# A new perspective on selenium’s impact on renal function: European population-based analysis of plasma proteome-mediated Mendelian randomization study

**DOI:** 10.3389/fendo.2024.1410463

**Published:** 2024-09-12

**Authors:** Shaojie Fu, Man Qian, Zishu Yuan, Sensen Su, Fuzhe Ma, Fan Li, Zhonggao Xu

**Affiliations:** ^1^ Department of Nephrology, The First Hospital of Jilin University, Changchun, China; ^2^ Department of Hepatology, The First Hospital of Jilin University, Changchun, China

**Keywords:** selenium, renal function, plasma proteins, Mendelian randomization analysis, mediation analysis

## Abstract

**Background:**

The relationship between selenium and renal function has always attracted widespread attention. Increased selenium level has been found to cause impaired renal function in our previous study, but the mechanism is not clear. In this study, we evaluate the potential mediating effects of plasma proteome in the association of selenium level and renal function to understand the mechanisms of selenium’s effect on renal function.

**Methods:**

Utilizing two-sample two-step mediating mendelian randomization (MR) methodology to investigate the genetically causal relationship between selenium level and renal function as well as the role of the plasma proteome in mediating them. Additionally, the mediating proteins were enriched and analyzed through bioinformatics to understand the potential mechanisms of selenium effects on renal function.

**Results:**

In the MR analysis, an increase in selenium level was found to decrease estimated glomerular filtration rate (eGFR). Specifically, for each standard deviation (SD) increase in selenium levels, eGFR levels are reduced by 0.003 SD [Beta (95% CI): -0.003 (-0.004 ~ -0.001), P=0.001, with no observed heterogeneity and pleiotropy]. Through mediation analysis, 35 proteins have been determined mediating the genetically causal effects of selenium on the levels of eGFR, including Fibroblast growth factor receptor 4 (FGFR4), Fibulin-1, Cilia- and flagella-associated protein 45, Mothers against decapentaplegic homolog 2 (SMAD2), and E3 ubiquitin-protein ligase ZNRF3, and the mediation effect rates of these proteins ranged from 1.59% to 23.70%. In the enrichment analysis, 13 signal transduction pathways, including FGFR4 mutant receptor activation and Defective SLC5A5 causing thyroid dyshormonogenesis 1, were involved in the effect of selenium on eGFR levels.

**Conclusion:**

Our finding has revealed the underlying mechanism by which increased selenium level lead to deterioration of renal function, effectively guiding the prevention of chronic kidney disease and paving the way for future studies.

## 1 Introduction

Chronic kidney disease (CKD) is defined as an abnormality in the structure or function of kidney, lasting more than 3 months, with specific implications for health (1). It could progress to end-stage renal disease (ESRD), which is fatal without renal replacement therapy (kidney transplantation or dialysis). Moreover, because of its effect on cardiovascular risk, CKD could directly affect the global burden of death caused by cardiovascular disease (2). Over 10% of the world’s population carries CKD and in the United States, more than 30 million people have CKD (3). In addition, with the rapid increase in the prevalence of risk factors such as hypertension, diabetes and obesity, the burden of CKD will become even heavier in the future. Therefore, CKD as a major public health problem has attracted more and more attention.

Selenium is an essential microelement for mammals and is widely distributed in all tissues in the body (4). Elemental selenium is biologically inactive by itself. It is involved in the synthesis of 25 selenoproteins in the form of selenocysteine and selenomethionine to perform its biological functions (5). Selenoproteins play important functions in the body such as combating oxidative stress, regulating immune system, regulating thyroxine metabolism and fertility (6). However, the biological activities of about 50% of selenoproteins are unknown and their roles in biology remain to be elucidated (7). Kidney is the organ with the highest selenium content (8). Many observational studies have found a strong association between selenium and CKD (9–11). Selenium levels are usually lower in CKD patients than normal people (12, 13), but whether selenium supplementation can benefit CKD patients is still controversial (14, 15).

Mendelian randomization (MR) is a research approach that utilizes genetic variation as an instrumental variables to examine whether exposure factor has a causal effect on health outcome (16). Compared to conventional methods, MR could avoid reverse causation bias and attenuates the interference of confounding factors, so it is becoming increasingly popular in epidemiologic studies (17). Using MR method, our team has previously found that increased selenium levels in the body could lead to a decrease of glomerular filtration rate and an increase in blood urea nitrogen levels, suggesting that selenium supplementation in CKD patients should be taken with great caution (18). However, the mechanism by which increased selenium levels in the body lead to the deterioration of renal function remains unclear. Genome-wide association studies (GWAS) have detected genetic variants associated with plasma proteome levels (19, 20), which provide an opportunity to utilize MR to reveal the underlying mechanisms behind the genetically causal relationship between exposure and outcome, as many circulating proteins always act as the principal regulators of molecular pathways (21).

In the current study, we further selected 3282 circulating plasma proteome levels to explore their potential mediating effect in the association of selenium levels and renal function. By evaluating these mediation effects, we can gain insights into the mechanistic pathways through which the increased selenium levels might influence the risk of renal failure, paving the way for potential therapeutic interventions.

## 2 Materials and methods

### 2.1 Overall study design

**Figure 1:**
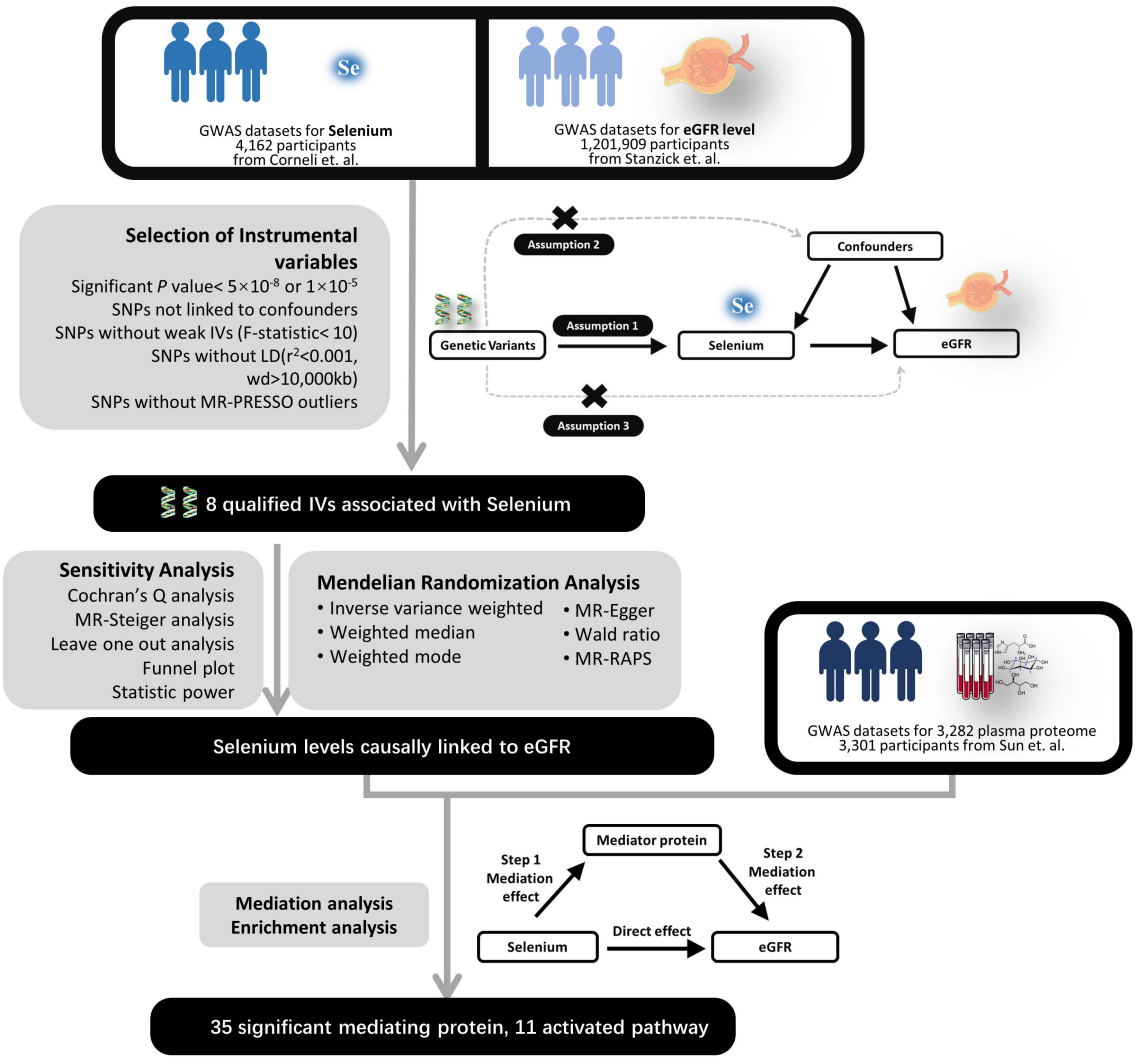
Experimental design flowchart. LD, Linkage Disequilibrium; SNP, Single Nucleotide Polymorphism; IV, Instrumental Variable; eGFR, Estimated Glomerular Filtration Rate; MR, Mendelian Randomization; RAPS, Robust Adjusted Profile Score.

This study utilized two-sample two-step mediating MR methodology to investigate the potential mediating effect of the plasma proteome in the genetically causal relationship between selenium levels and renal function (22), which was conducted in three stages (**Figure 1**). The first stage determined the genetically causal effects of selenium levels on renal function. The second stage focused on investigating the genetically causal effects of selenium levels on the plasma proteome and the genetically causal effects of the plasma proteome on renal function. The third stage aimed to investigate and quantify the potential mediating role of the plasma proteome in the genetically causal relationship between selenium levels and renal function, and perform enrichment analysis to the mediating proteins through bioinformatics for understanding the potential mechanisms involved in selenium’s influence on renal function. We reviewed our analytical process in accordance with the STROBE-MR Checklist to ensure the reliability of the results, with the checklist information available in **Supplementary Table 1**.

### 2.2 GWAS data sources

The instrumental variables closely associated with selenium levels were obtained from a large GWAS meta-analysis of toenail and blood selenium (23). Toenail selenium concentrations were collected from 4,162 individuals of European descent from four cohorts in the United States (adjusted for genetic associations based on sex, age, smoking status and study-specific covariates) (24–27). Blood selenium concentrations were collected from 2603 Australian twin pairs and their families and 2874 pregnant women from England (adjusted for genetic associations based on sex, age and within- family relatedness) (28). GWAS summary data for plasma proteome levels were gathered from the genomic atlas of the human plasma proteome published by Sun et al. in 2018 (19), which genome-wide tested 3282 plasma proteins of 10.6 million putative autosomal variants in 3301 healthy participants from 25 centers in England. GWAS summary data for kidney function (eGFR) were derived from the largest GWAS meta-analysis for kidney function to date (29), which included the data from UK Biobank (n = 436561) and Chronic Kidney Disease Genetics (CKDGen) Consortium (n = 765348). In UKB, GFR was estimated based on the CKD-EPI formula, whereas in CKDGen, GFR was estimated based on the CKD-EPI formula (for individuals > 18 years) and the Schwartz formula (for individuals ≤ 18 years). All participants have provided informed consent. **Table 1** details the characteristics of the involved GWAS datasets, and information on all GWAS datasets included in the study is presented in **Supplementary Table 2**. Based on the information from the data set sources, there is no sample overlap in the MR analysis of this study.

**Table 1:** Detailed information about the included GWAS studies.

Trait	Unit	Sample size	Year	Author	Gender	Population	NSNP	Reference (PMID)
eGFR	per 1 SD	1201909	2023	Stanzick	Males and Females	European	8844847	34272381
Selenium	per 1 SD	4162	2023	Corneli	Males and Females	European	21069470	25343990
Plasma proteomes	per 1 SD	3301	2018	Sun BB	Males and Females	European	10534735	29875488

Note: eGFR, estimated glomerular filtration rate; SD, standard deviation; GWAS, Genome-wide association studies; NSNP, Number of Single Nucleotide Polymorphism.

### 2.3 Selection of instrumental variables and mendelian randomization analysis

To ensure the reliability of MR analyses, three central assumptions must be met (30): (1) the instrumental variables are closely associated with exposure, (2) the instrumental variables independent of any confounders, (3) the instrumental variables affect the outcome only through exposure rather than any other causal pathway. Based on the above guidelines, we used the following steps to select instrumental variables: single nucleotide polymorphisms (SNPs) associated with exposure were selected at the genome-wide significant level (p-value < 5 × 10^-8^) or Genome-wide potential significance level (p-value < 1 × 10^-5^). SNPs exhibiting linkage disequilibrium, ambiguity, or being palindromic were excluded, and weak instruments were eliminated based on an F-statistic < 10 (31). The MR-PRESSO method was employed to detect and remove pleiotropic SNPs. The MR-Steiger test was utilized to identify and exclude SNPs with incorrect directionality of association. The web tool PhenoScanner and the R package “gwasrapidd” are utilized respectively to examine each included variation for its association with other traits, and SNPs associated with confounding factors are excluded. MR analyses were performed using inverse-variance weighting (IVW) (32), MR-Egger regression (33), weighted median (WM) (34), and MR-Robust Adjusted Profile Scores (MR-RAPS) (35).

### 2.4 Sensitivity analyses

Sensitivity analyses mainly include horizontal pleiotropy, heterogeneity tests and leave-one-out analysis. Horizontal pleiotropy was assessed using the MR-Egger method. When horizontal pleiotropy is detected, MR-Egger regression is preferred. Heterogeneity was assessed using Cochran’s Q test. According to MR operational guidelines, the random-effects IVW model is employed as the primary analysis method regardless of the presence of heterogeneity. We performed the leave-one-out analyses and plotted the funnel plots. In addition, MR-Steiger test was also performed to determine the overall causal direction was correct (36). Finally we calculated the statistical power to clarify the reliability of the negative results (18).

### 2.5 Mediation analyses of plasma proteome and enrichment analyses of pathways

In the mediation analyses, MR analysis of selenium levels on plasma proteome was performed in the first step, and MR analysis of plasma proteome on renal function was performed in the second step. Proteins that were significant in both steps of the MR analyses had a partial mediation effect. The mediation effect is calculated by multiplying the effect of first step and second step, while the mediation effect rate is the proportion of the mediation effect to the total effect. Then pathway enrichment of the discovered mediator proteins was performed in Reactome. Reactome is a peer-reviewed pathway database that provides intuitive bioinformatics tools for visualizing, interpreting and analyzing pathway information to support genome analyses (37).

### 2.6 Statistical analysis and graphing

For visualization, scatter plots were drawn for each SNP in this study, showing the relationship between exposure factors and outcome effects. Funnel plots were utilized to assess possible directional effects and pleiotropy. Forest plots were drawn for final causal estimation, presenting the results for each SNP and the results of the overall MR analysis. The full Mendelian randomization figure results can be found in **Supplementary Figures 1**-**4**. Statistical calculations and results visualization were conducted using R software (version 4.3.1) and R software packages “TwoSample MR”, “MR-PRESSO”, “mr.raps”.

## 3 Results

### 3.1 Dataset characteristics and screening for instrumental variables

**Figure 2:**
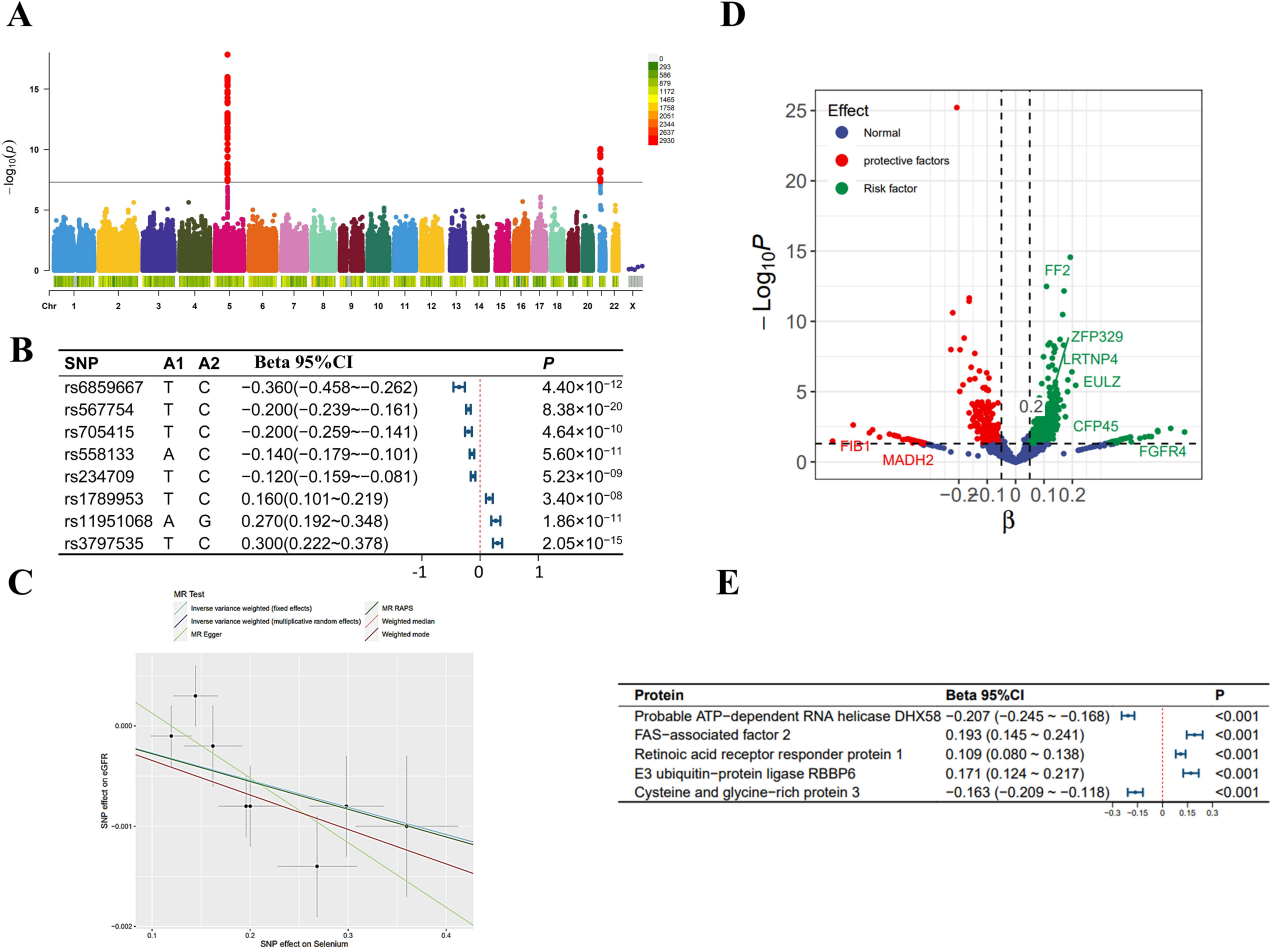
Selenium-related analysis diagram. **(A)** Manhattan plot of genome-wide association study summary data on selenium levels; **(B)** The Single Nucleotide Polymorphisms (SNPs) significantly associated with selenium levels involved as instrumental variables; **(C)** Scatter plot and regression curve of Mendelian Randomization exploring the association between selenium levels and eGFR; **(D)** Scatter plot of the effect-significance level of selenium levels on plasma proteins, with named proteins indicating mediating effects; **(E)** The top 5 plasma proteins most significantly influenced by selenium levels. Effects are scaled as “per 1 standard deviation”.

**Figure 3:**
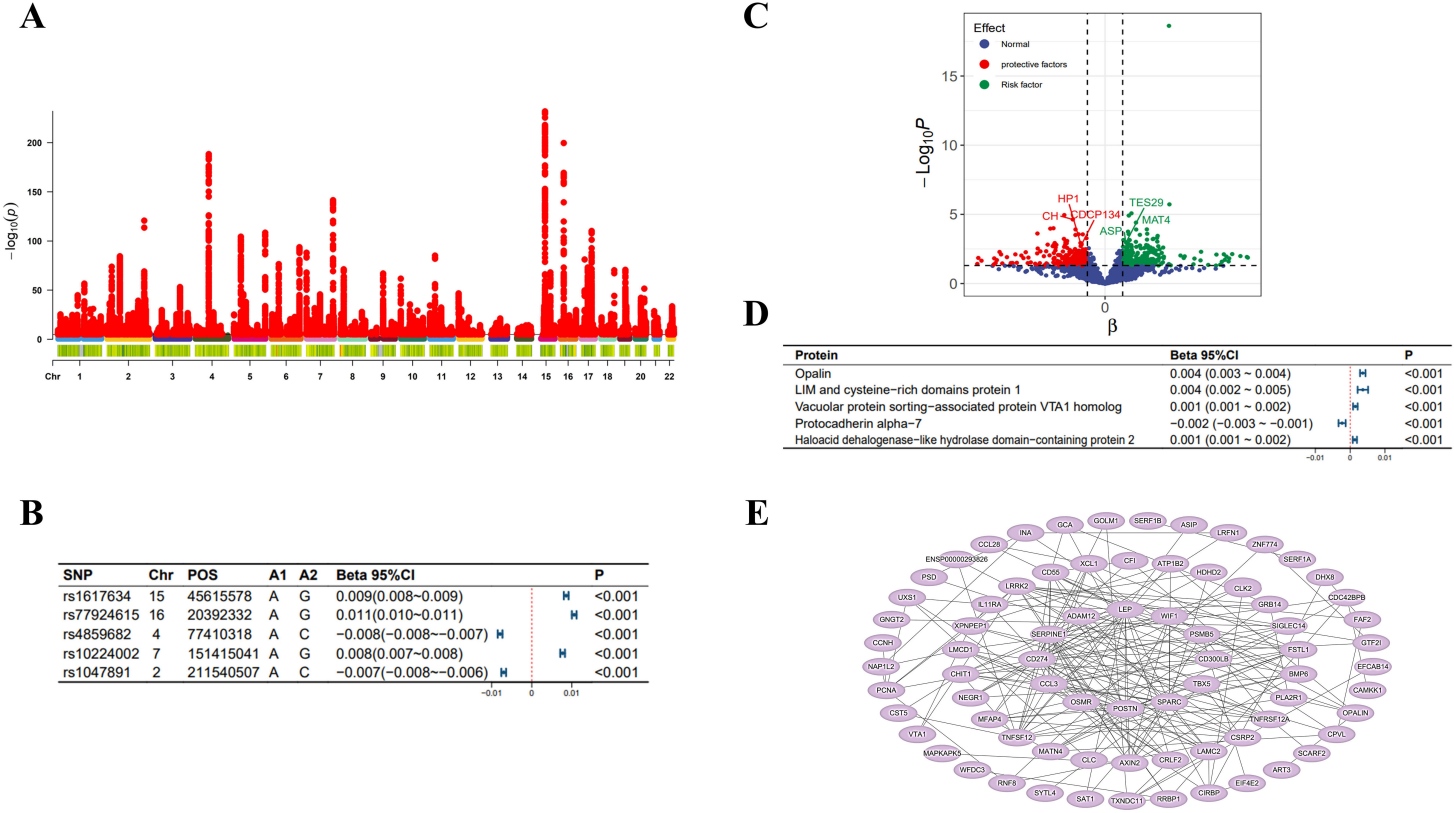
eGFR-related analysis diagram. **(A)** Manhattan plot of genome-wide association study summary data on eGFR levels; **(B)** The top 5 SNPs most significantly associated with eGFR levels; **(C)** Scatter plot of the effect-significance level of plasma proteins on eGFR levels, with named proteins indicating mediating effects; **(D)** The top 5 plasma proteins most significantly affecting eGFR levels; **(E)** Schematic diagram of plasma protein interactions affecting eGFR levels. Effects are scaled as “per 1 standard deviation”.

In the MR analysis, we initially screened 12 SNPs related to selenium levels, which primarily originate from chromosomes 5 and 21 (**Figure 2A**), and no weak instrumental variables were found; the MR-PRESSO test detected no SNPs with pleiotropy; the MR-Steiger test identified no SNPs with reverse causality. No SNPs associated with potential confounders (e.g., daily diet, occupational exposures, smoking, etc.) were found, but four SNPs potentially related to the outcomes were excluded. Ultimately, eight qualified SNPs were included in the study. Specifically, among these instrumental variables, there were three SNPs that upregulated selenium levels (rs3797535, rs11951068, and rs1789953), and five SNPs that downregulated selenium levels (rs234709, rs558133, rs567754, rs6859667 and rs705415). Details of these SNPs leading to changes in selenium body levels in are shown in **Figure 2B**. In the mediation MR analysis, we initially screened 249,286 SNPs related to the exposure, finding no weak instrumental variables. A total of 49,690 SNPs were absent in the outcome database and thus excluded; 23,526 ambiguous SNPs and palindromic SNPs were deleted when merging datasets. After Bonferroni correction, 3,203 SNPs directly related to the outcome were excluded. Ultimately, 172,870 qualified SNPs were included in the study. SNPs closely associated with eGFR levels are primarily distributed across multiple chromosomes (**Figure 3A**), with the top SNP being rs1617634. These SNPs mutations all contribute to the variations of eGFR (**Figure 3B**).

### 3.2 Mendelian randomization analysis

**Figure 4:**
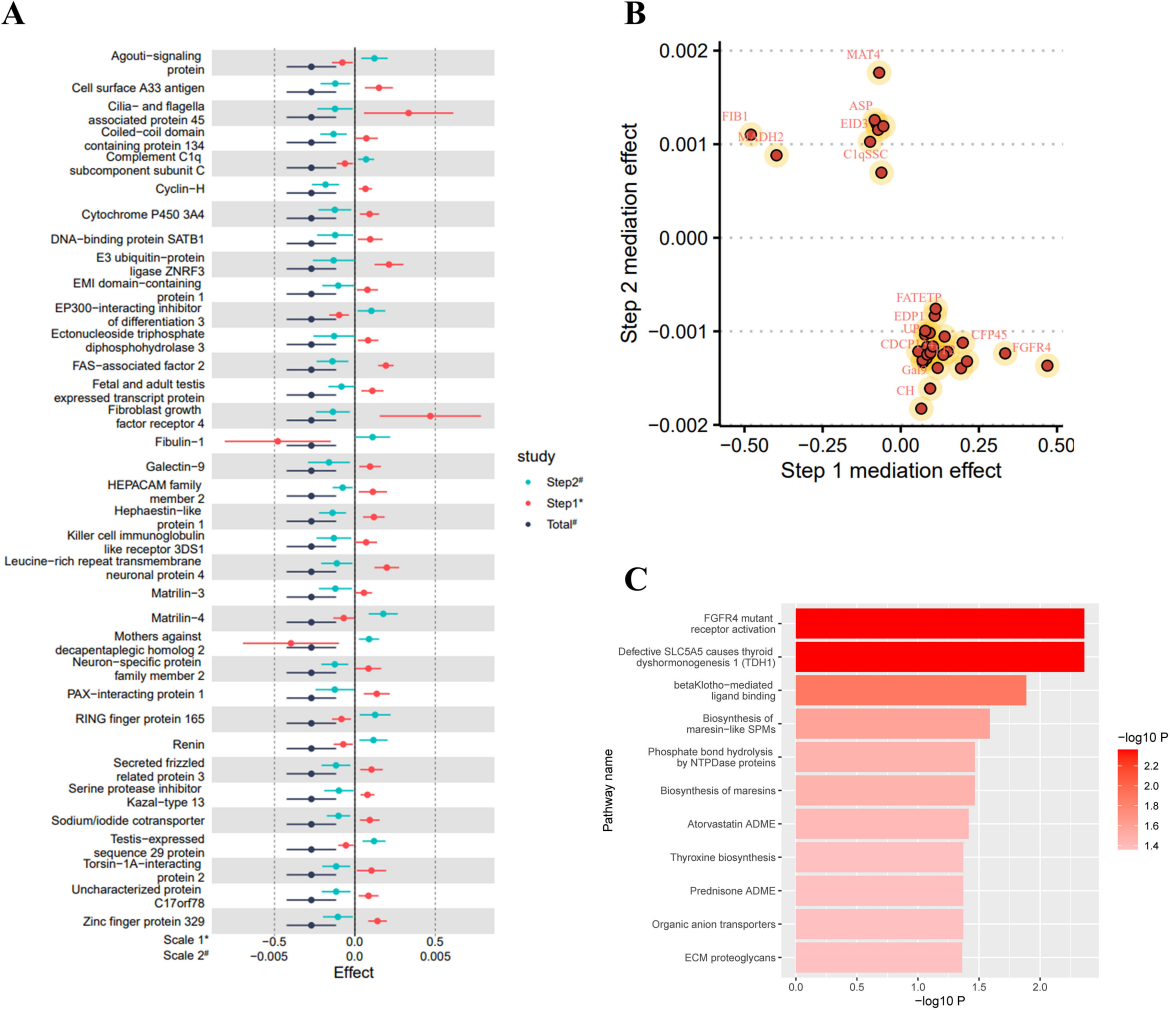
Mediation analysis and enrichment analysis results. **(A)** Forest plot of the total effect and two-step mediation effect; **(B)** Two-step effect scatter plot of mediating effect proteins; **(C)** Metabolic or signal transduction pathways mediating the effect of selenium levels on eGFR levels. Effects are scaled as “per 1 standard deviation”.

As shown in **Figure 2C**, in the MR analysis, for each standard deviation (SD) increase in selenium levels, eGFR levels are reduced by 0.003 SD [Beta (95% CI): -0.003 (-0.004 ~ -0.001), P=0.001, with no observed heterogeneity and pleiotropy]. Significant plasma protein effects mediated by selenium were shown in **Figure 2D**, among which the top 5 plasma proteins most significantly influenced by selenium levels were Probable ATP-dependent RNA helicase DHX58 (its level decreased by 0.207 SD for 1 SD increase in selenium level), FAS-associated factor 2 (its level increased by 0.193 SD for 1 SD increase in selenium level), Retinoic acid receptor responder protein 1 (its level increased by 0.109 SD for 1 SD increase in selenium level), E3 ubiqui-tin-protein ligase RBBP6 (its level increased by 0.171 SD for 1 SD increase in selenium level) and Cysteine and glycine-rich protein 3 (its level decreased by 0.163 SD for 1 SD increase in selenium level). Details of the effects of changes in selenium levels on them are shown in **Figure 2E**. The effect-significance level of plasma proteins on eGFR levels were shown in **Figure 3C**, among which the top 5 plasma proteins most significantly affecting eGFR levels were Opalin, LIM and cysteine-rich domains protein 1, Vacuolar protein sorting-associated protein VTA1 homolog, Protocadherin alpha-7 and Haloacid dehalogenase-like hydro-lase domain-containing protein 2 (**Figure 3D**). In addition, protein interaction analyses were performed and found sufficient interactions between the significant plasma proteins mediating eGFR alteration (**Figure 3E**). In the proteomic mediator analysis, 35 plasma proteins with mediating effects were identified, with mediation effect rates ranging from 1.59% to 23.70%. The full Mendelian randomization results and sensitivity analyses can be found in **Supplementary Tables 3**-**8**. Specifically, the top five proteins with the highest mediation effect rates were Fibroblast growth factor receptor 4, Fibu-lin-1, Cilia and flagella-associated protein 45, Mothers against decapentaplegic homolog 2, and E3 ubiquitin-protein ligase ZNRF3 (**Table 2**, **Figures 4A, B**).

**Table 2:** Significant results of two-step mediation analysis.

Mediator	X-Y		X-M		M-Y		Mediating direction	Mediating effect	Mediating ratio
	OR 95%CI	P	OR 95%CI	P	OR 95%CI	P			
**Agouti-signaling protein**	0.997(0.996~0.999)	0.001	0.925(0.867~0.987)	0.019	1.001(1.000~1.002)	0.004	TRUE	Partial	3.49%
**Uncharacterized protein C17orf78**	0.997(0.996~0.999)	0.001	1.088(1.023~1.158)	0.007	0.999(0.998~1.000)	0.011	TRUE	Partial	3.65%
**Complement C1q subcomponent subunit C**	0.997(0.996~0.999)	0.001	0.940(0.894~0.988)	0.015	1.001(1.000~1.001)	0.007	TRUE	Partial	1.59%
**Coiled-coil domain-containing protein 134**	0.997(0.996~0.999)	0.001	1.074(1.000~1.153)	0.049	0.999(0.998~0.999)	0.002	TRUE	Partial	3.51%
**Cyclin-H**	0.997(0.996~0.999)	0.001	1.068(1.025~1.112)	0.002	0.998(0.997~0.999)	<0.001	TRUE	Partial	4.44%
**Cilia- and flagella-associated protein 45**	0.997(0.996~0.999)	0.001	1.397(1.058~1.844)	0.04	0.999(0.998~1.000)	0.028	TRUE	Partial	15.26%
**Cytochrome P450 3A4**	0.997(0.996~0.999)	0.001	1.095(1.031~1.163)	0.003	0.999(0.998~1.000)	0.017	TRUE	Partial	4.19%
**EP300-interacting inhibitor of differentiation 3**	0.997(0.996~0.999)	0.001	0.907(0.852~0.964)	0.002	1.001(1.000~1.002)	0.02	TRUE	Partial	3.72%
**EMI domain-containing protein 1**	0.997(0.996~0.999)	0.001	1.081(1.012~1.155)	0.02	0.999(0.998~1.000)	0.041	TRUE	Partial	2.98%
**Ectonucleoside triphosphate diphosphohydrolase 3**	0.997(0.996~0.999)	0.001	1.085(1.018~1.157)	0.012	0.999(0.997~1.000)	0.049	TRUE	Partial	3.91%
**FAS-associated factor 2**	0.997(0.996~0.999)	0.001	1.213(1.156~1.272)	<0.001	0.999(0.998~1.000)	0.005	TRUE	Partial	9.94%
**Fetal and adult testis-expressed transcript protein**	0.997(0.996~0.999)	0.001	1.115(1.041~1.194)	0.002	0.999(0.998~1.000)	0.044	TRUE	Partial	3.35%
**Fibulin-1**	0.997(0.996~0.999)	0.001	0.619(0.445~0.861)	0.017	1.001(1.000~1.002)	0.048	TRUE	Partial	19.52%
**Fibroblast growth factor receptor 4**	0.997(0.996~0.999)	0.001	1.599(1.167~2.190)	0.015	0.999(0.998~1.000)	0.011	TRUE	Partial	23.70%
**Secreted frizzled-related protein 3**	0.997(0.996~0.999)	0.001	1.109(1.034~1.189)	0.004	0.999(0.998~1.000)	0.01	TRUE	Partial	4.48%
**Cell surface A33 antigen**	0.997(0.996~0.999)	0.001	1.161(1.064~1.267)	0.001	0.999(0.998~1.000)	0.01	TRUE	Partial	6.73%
**HEPACAM family member 2**	0.997(0.996~0.999)	0.001	1.118(1.024~1.221)	0.013	0.999(0.999~1.000)	0.017	TRUE	Partial	3.14%
**Hephaestin-like protein 1**	0.997(0.996~0.999)	0.001	1.125(1.052~1.204)	0.001	0.999(0.998~0.999)	0.001	TRUE	Partial	6.07%
**Killer cell immunoglobulin-like receptor 3DS1**	0.997(0.996~0.999)	0.001	1.073(1.003~1.147)	0.042	0.999(0.998~1.000)	0.017	TRUE	Partial	3.38%
**Galectin-9**	0.997(0.996~0.999)	0.001	1.099(1.028~1.175)	0.006	0.998(0.997~1.000)	0.015	TRUE	Partial	5.60%
**Leucine-rich repeat transmembrane neuronal protein 4**	0.997(0.996~0.999)	0.001	1.220(1.130~1.317)	<0.001	0.999(0.998~1.000)	0.024	TRUE	Partial	8.23%
**Matrilin-3**	0.997(0.996~0.999)	0.001	1.058(1.006~1.113)	0.029	0.999(0.998~1.000)	0.021	TRUE	Partial	2.52%
**Matrilin-4**	0.997(0.996~0.999)	0.001	0.933(0.874~0.996)	0.038	1.002(1.001~1.003)	<0.001	TRUE	Partial	4.50%
**Neuron-specific protein family member 2**	0.997(0.996~0.999)	0.001	1.088(1.006~1.177)	0.035	0.999(0.998~1.000)	0.003	TRUE	Partial	3.91%
**PAX-interacting protein 1**	0.997(0.996~0.999)	0.001	1.146(1.057~1.242)	0.001	0.999(0.998~1.000)	0.041	TRUE	Partial	6.28%
**Renin**	0.997(0.996~0.999)	0.001	0.930(0.876~0.988)	0.018	1.001(1.000~1.002)	0.01	TRUE	Partial	3.08%
**RING finger protein 165**	0.997(0.996~0.999)	0.001	0.920(0.867~0.976)	0.006	1.001(1.000~1.002)	0.01	TRUE	Partial	3.88%
**DNA-binding protein SATB1**	0.997(0.996~0.999)	0.001	1.100(1.018~1.188)	0.016	0.999(0.998~1.000)	0.032	TRUE	Partial	4.32%
**Sodium/iodide cotransporter**	0.997(0.996~0.999)	0.001	1.097(1.032~1.166)	0.003	0.999(0.998~1.000)	0.006	TRUE	Partial	3.48%
**Mothers against decapentaplegic homolog 2**	0.997(0.996~0.999)	0.001	0.672(0.498~0.906)	0.026	1.001(1.000~1.002)	0.006	TRUE	Partial	12.95%
**Serine protease inhibitor Kazal-type 13**	0.997(0.996~0.999)	0.001	1.081(1.036~1.129)	<0.001	0.999(0.998~1.000)	0.033	TRUE	Partial	2.88%
**Testis-expressed sequence 29 protein**	0.997(0.996~0.999)	0.001	0.946(0.901~0.992)	0.023	1.001(1.000~1.002)	0.001	TRUE	Partial	2.45%
**Torsin-1A-interacting protein 2**	0.997(0.996~0.999)	0.001	1.109(1.012~1.215)	0.027	0.999(0.998~1.000)	0.009	TRUE	Partial	4.42%
**Zinc finger protein 329**	0.997(0.996~0.999)	0.001	1.151(1.086~1.220)	<0.001	0.999(0.998~1.000)	0.026	TRUE	Partial	5.48%
**E3 ubiquitin-protein ligase ZNRF3**	0.997(0.996~0.999)	0.001	1.236(1.130~1.352)	<0.001	0.999(0.997~1.000)	0.045	TRUE	Partial	10.34%

OR, Odds ratio; CI, Confidence interval; X-Y, Total effect; X-M, step 1 mediating effect; M-Y, step 2 mediating effect.

### 3.3 Activation of metabolic and signaling pathways

In the enrichment analysis, 11 mediating pathways were identified that are implicated in the decrease of eGFR levels mediated by an increase in selenium levels. The five most significant pathways were FGFR4 mutant receptor activation (Reactions 5/5, Entities 1/1, pValue 0.004), Defective SLC5A5 causes thyroid dyshormonogenesis 1 (Reactions 1/1, Entities 1/1, pValue 0.004), betaKlotho-mediated ligand binding (Reactions 2/2, Entities 3/, pValue 0.013), Biosynthesis of maresin-like SPMs (Reactions 1/3, Entities 1/6, pValue 0.026), and Biosynthesis of maresins (Reactions 1/5, Entities 1/8, pValue 0.034) (**Figure 4C**; **Supplementary Table 9**).

## 4 Discussion

As a trace element, selenium is essential to the human body, and due to its important role in combating oxidative stress, regulating immune system, regulating thyroxine metabolism and fertility (6), there has been an increasing focus on selenium supplementation. As selenium levels in CKD patients are usually lower than normal (12, 13), they are more likely to favor selenium supplementation without professional guidance. Notably, it is important to note that the safe intake range for selenium is very narrow, and the optimal daily intake of selenium for the human body is only 30-400 *μ*g day^−1^ adult^−1^ (38), below which can lead to selenium deficiency, and above which can produce symptoms of toxicity, such as hair loss, thyroid dysfunction, and neurological damage (39). Therefore, selenium intake needs to be finely regulated. Moreover, selenium is not biologically active by itself, and it plays its biological roles mainly via selenoproteins (5). Currently, most of the functionally known selenoproteins of eukaryotes are oxidoreductases with antioxidant properties (40, 41). However, it is worth noting that taking GPX1 as an example, while it has anti-inflammatory and antioxidant effects, it has also been found to be associated with insulin resistance and type 2 diabetes (42), which fully illustrates the complexity of selenoproteins’ biological functions. Beneficial or harmful depending on the levels of selenoproteins in the body and other factors, and the biological effects of most selenoproteins, especially on kidney, remain unclear. Furthermore, the benefits and harms of selenium are dependent on its dose and form (43). With increasing selenium intake, selenoprotein expression tends to saturate and the excess selenium bind nonspecifically to human proteins through the substitution of methionine by selenomethionine (SeMet) resulting the production of non-specific selenium-containing proteins, and the non-specific selenium-containing proteins could cause damage to health (44, 45). Hence, lack or excess of selenium is a relative concept. CKD patients are characterized by decreased dietary intake, reduced intestinal resorption, increased urinary protein excretion, and reduced ability in synthesizing selenoproteins (46). Therefore, when supplemented with selenium, they are more susceptible to produce non-specific selenium-containing proteins and thus to toxic effects of selenium. Results of this study found that increased selenium levels could lead to decreased renal function, so supplementation of selenium must be taken with extreme caution, especially for patients with CKD.

In this study, we found that increased levels of selenium in body would lead to an increase of plasma protein levels such as FAS-associated factor 2 (FAF2), Retinoic acid receptor responder protein 1 (RARRES1). It may be related to the pro-oxidant effect of selenium compounds. As selenium levels increase, the selenium compounds, such as selenite, also increase, and selenite could lead to an excess of reactive oxygen species (ROS) in the cell, triggering oxidative stress (47). RARRES1 protein is involved in the regulation of signaling pathways in response to oxidative damage, so their up-regulation may reflect an adaptive reaction of the body to maintain cellular homeostasis facing oxidative challenges (48). The pro-oxidative effects of selenium compounds can also induce the activation of apoptosis and protein degradation pathways. FAF2, an apoptosis-associated protein involved in ubiquitination and protein degradation, may increase its expression in response to oxidative stress-induced protein damage (49). This reflects a complex mechanism of cellular regulation of oxidative damage and provides insight into the potential mechanisms of selenium toxicity.

Many circulating proteins are key regulators of molecular pathways, so exploring their potential mediating effect in the association of selenium levels and renal function could help to understand the underlying mechanisms of selenium’s influence on renal function. In this study, a total of 35 plasma proteins mediating between selenium levels and renal function were identified, and the three with the highest mediating effects were fibroblast growth factor receptor 4 (FGFR4), fibulin-1 (FBLN1), and cilia-and flagella-associated protein 45 (CFAP45). CKD patients have elevated serum levels of fibroblast growth factor (FGF), of which FGF23 has a strong dose-dependent association with cardiovascular morbidity, chronic inflammation and progression of kidney disease (50). FGFR4 is one of the receptors for FGF23. Activation of hepatic FGFR4 causes the production of inflammatory cytokines in the liver, thereby exacerbating the chronic inflammation *in vivo* and participating in the progression of CKD (51). In addition, it has been shown that FGF23 could participate in renal fibrosis by amplifying the activation of local renal fibroblast in injury and perpetuating pro-fibrotic signaling through FGFR4 (52). FBLN1 is an extracellular matrix (ECM) protein, which interacts with many ECM molecules (53). As a secreted plasma protein, FBLN1 has been found to be a good potential biomarker for renal impairment, significantly elevated in the plasma of CKD patients (54). The mechanism underlying the elevation of FBLN1 in patients with impaired renal function remains unknown. In the present study, we found that decreased plasma FBLN1 level could lead to the impairment of renal function, which is counterintuitive and suggests that, like BNP in heart failure, increased FBLN1 may be a compensatory result when renal function is impaired. However, the specific mechanisms behind this need to be studied further. CFAP45 protein is a static structural component of the ciliary axoneme and critical for motor cilia stability (55). Cilia is widely distributed in the proximal and distal tubules as well as the collecting ducts of kidney. Normal structure and function of cilia are essential for renal organogenesis and maintenance of epithelial cell differentiation and proliferation, and persistent ciliary dysfunction contributes to both early and progressive renal disease (56). However, the study on the relationship between CFAP45 and the kidney is still lacking, and its effect on renal cilia and renal function needs to be further explored in the future.

Eleven pathways, including thyroxine biosynthesis and defective SLC5A5 causes thyroid dyshormonogenesis were found to mediate the effects of selenium levels on renal function in the enrichment analysis. Selenium is important for thyroxine biosynthesis. The thyroid gland expresses selenoproteins such as glutathione peroxidase, selenoprotein P and thioredoxin reductase, which together form the antioxidant system to mitigate the damage to thyroid cells caused by reactive oxygen species generated during thyroxine biosynthesis, and thus maintains the normal function of thyroid tissue (57). In addition, iodothyronine deiodinases is also a selenoprotein. It converts the inactive precursor thyroxine to the active triiodothyronine. Abnormal selenium levels in the body affect the expression and activity of iodothyronine deiodinases, which can lead to disruption of thyroxine metabolism (58). The kidney is an important target organ for thyroxine action. Thyroxine affects protein synthesis and cell growth and therefore has an important role in the growth and development of kidney (59). Thyroxine regulates the activity of tubular ion transporters (Na+, K+, 2Cl− co-transport protein, etc.), which modulates tubuloglomerular feedback and affects glomerular filtration rate (60). It also regulates the expression of glomerular vasodilatory factors, such as vascular endothelial growth factor, and the synthesis and secretion of various components of the renin-angiotensin-aldosterone system, to modulate renal perfusion (61). Additionally, it has been found that abnormal increases or decreases of thyroxine are strongly associated with the development and progression of renal fibrosis (62, 63). These evidences support our finding that thyroxine disruption may be a potential mechanism by which selenium affects renal function.

Our two-sample MR analysis uses stringent criteria and provides compelling insights into the intricate relationship between the levels of selenium *in vivo* and renal function, suggesting that increased selenium levels are associated with decreased renal function. Furthermore, our mediation analysis revealed the important role of plasma proteome as mediators, depicting the causal trajectories from increased selenium levels to decreased renal function, which has profound significance in constructing a blueprint from the levels of micronutrients *in vivo* to the pathogenesis of CKD, thereby effectively guiding the prevention of CKD and delaying its progression.

However, it is crucial to acknowledge certain limitations in our study. First, the genetic analyses in this study were performed on individuals of European ancestry, so generalization of the findings to other ethnic populations is limited. Second, selenium levels in populations are closely related to geographic variation, thus also limiting the generalization of the findings to different geographic populations. Third, our data were derived from publicly available aggregated statistics and the raw clinical outcome data for each individual were not available, thus preventing further population stratification analyses. Fourth, this study used the relative scale of “per 1 SD” to determine the association of selenium with eGFR, and did not use an absolute scale due to limitations of the original data. Lastly, we did not perform experimental validations for the identified mediating proteins and potential mechanisms by which selenium affects renal function, and the role of environmental factors and the influence of genetic factors interacting with the environment on this causal effect should be further explored in future studies.

## 5 Conclusions

Our comprehensive MR analysis has revealed the complex relationship between selenium levels and renal function, and found that increased selenium level is one genetically causative factor for kidney function impairment, suggesting that supplementation of selenium must be taken with extreme caution, especially for patients with CKD. More importantly, we reveal the underlying mechanism by which increased selenium levels lead to deterioration of renal function, effectively guiding the prevention of CKD and paving the way for future studies.

## Data Availability

The original contributions presented in the study are included in the article/**Supplementary Material**. Further inquiries can be directed to the corresponding author.

## Supplementary material

Supplementary Figure 1 Forest plots of all the mendelian randomization analyses in this study.

Supplementary Figure 2 Scatter plots of all the mendelian randomization analyses in this study.

Supplementary Figure 3 Leave-one-out plots of all the mendelian randomization analyses in this study.

Supplementary Figure 4 Funnel plots of all the mendelian randomization analyses in this study.
