# Supplementary material for: A new perspective on selenium’s impact on renal function: European population-based analysis of plasma proteome-mediated Mendelian randomization study
Source: Front Endocrinol (Lausanne). 2024 Sep 12;15:1410463. doi: 10.3389/fendo.2024.1410463 (PMC11424436; doi:10.3389/fendo.2024.1410463)
Supplement: Supplementary Figure 1 — Forest plots of all the mendelian randomization analyses in this study. [file DataSheet1.pdf]

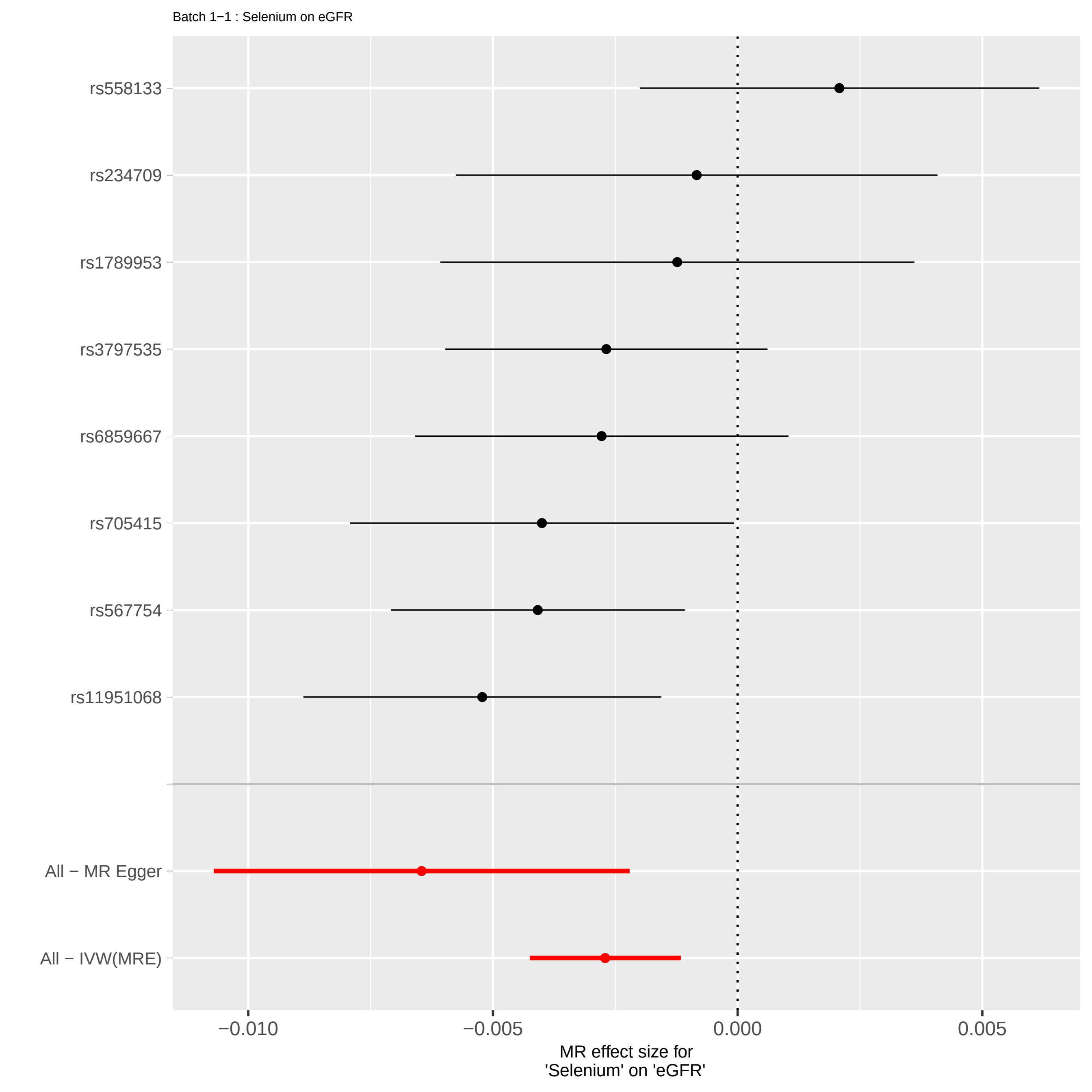
Batch 1−1 : Selenium on eGFR
rs558133
rs234709
rs1789953
rs3797535
rs6859667
rs705415
rs567754
rs11951068
All − MR Egger
All − IVW(MRE)
−0.010
−0.005
0.000
0.005
MR effect size for
'Selenium' on 'eGFR'

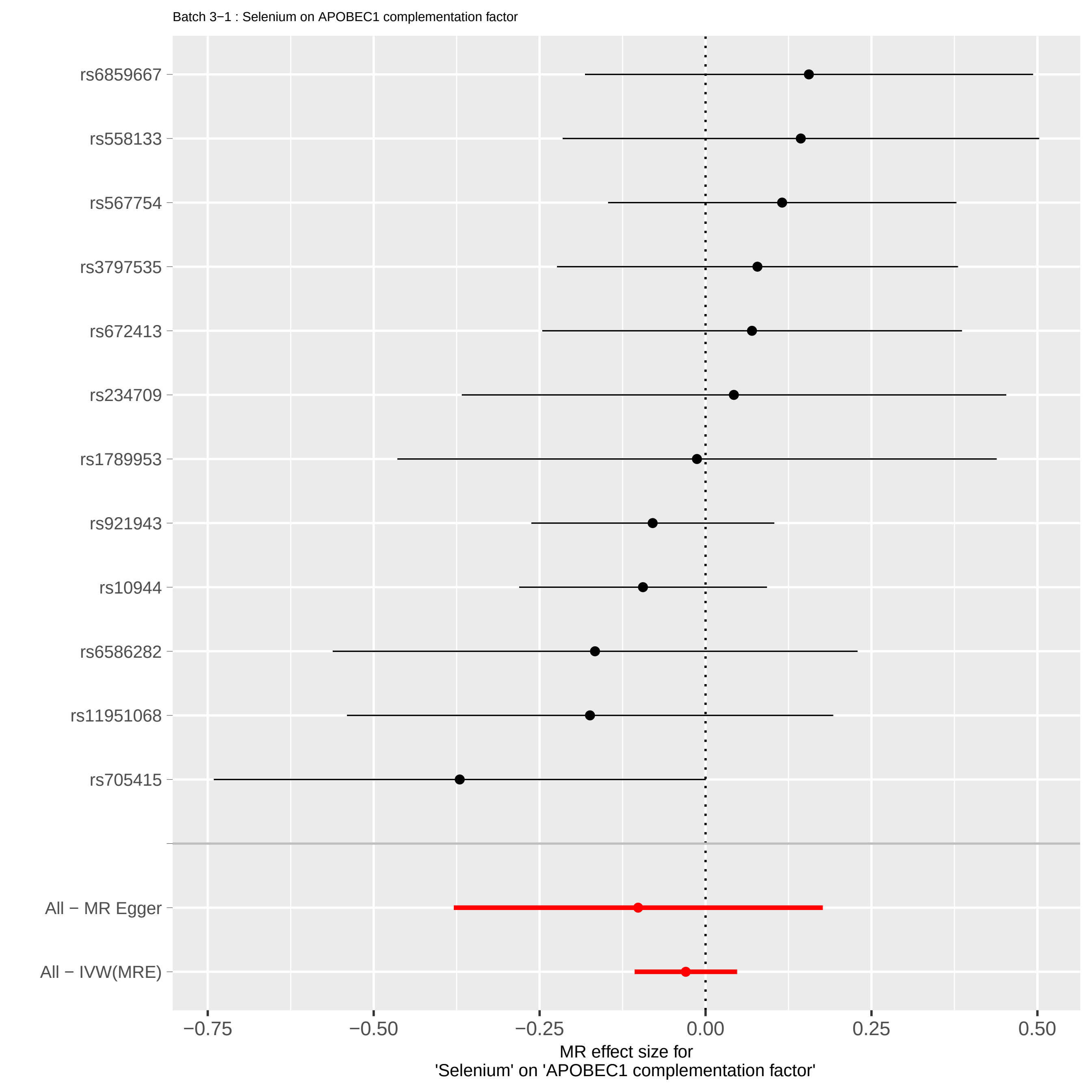

Batch 3−1 : Selenium on APOBEC1 complementation factor
rs6859667
rs558133
rs567754
rs3797535
rs672413
rs234709
rs1789953
rs921943
rs10944
rs6586282
rs11951068
rs705415
All − MR Egger
All − IVW(MRE)
−0.75
−0.50
−0.25
0.00
0.25
0.50
MR effect size for
'Selenium' on 'APOBEC1 complementation factor'

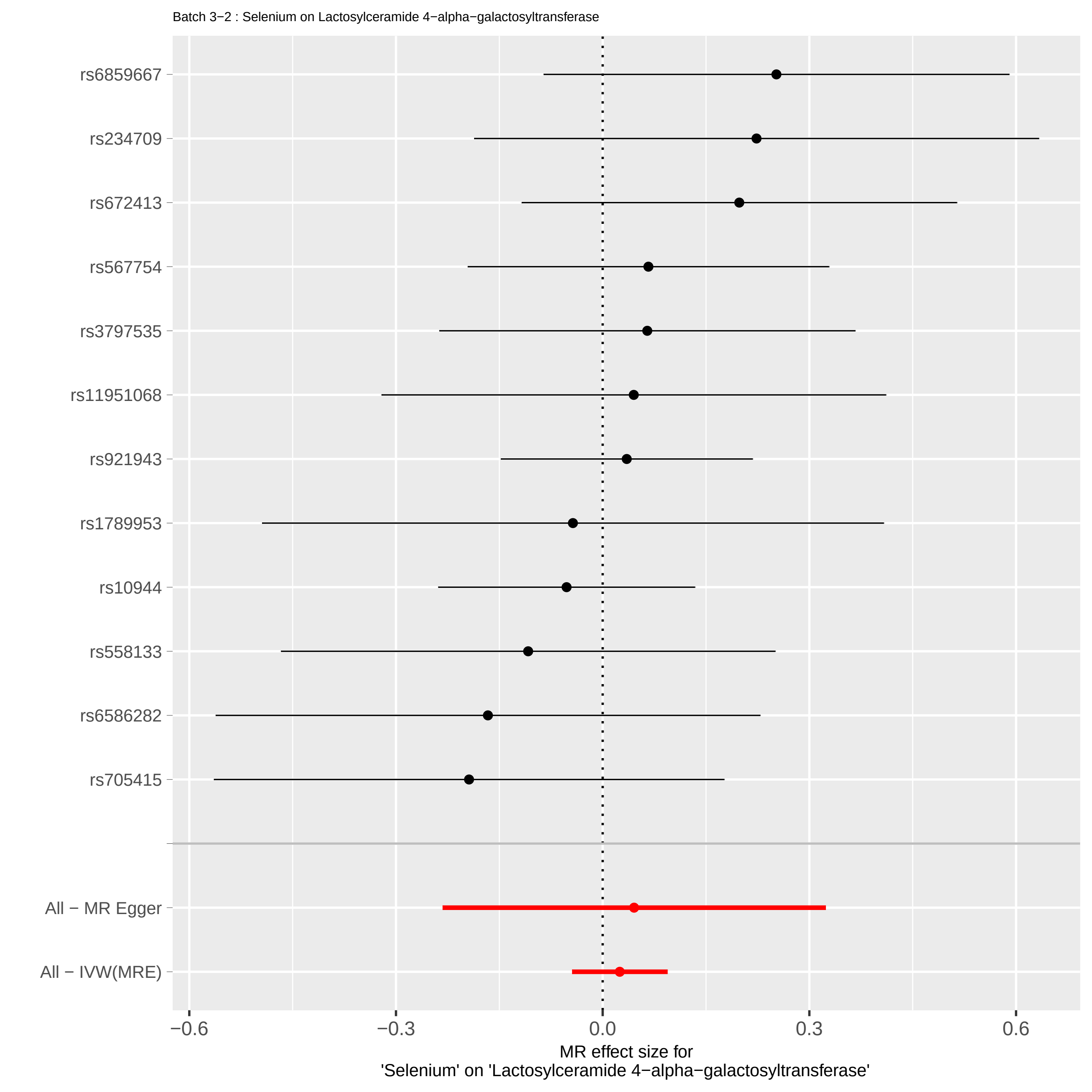

Batch 3−2 : Selenium on Lactosylceramide 4−alpha−galactosyltransferase
rs6859667
rs234709
rs672413
rs567754
rs3797535
rs11951068
rs921943
rs1789953
rs10944
rs558133
rs6586282
rs705415
All − MR Egger
All − IVW(MRE)
−0.6
−0.3
0.0
0.3
0.6
MR effect size for
'Selenium' on 'Lactosylceramide 4−alpha−galactosyltransferase'

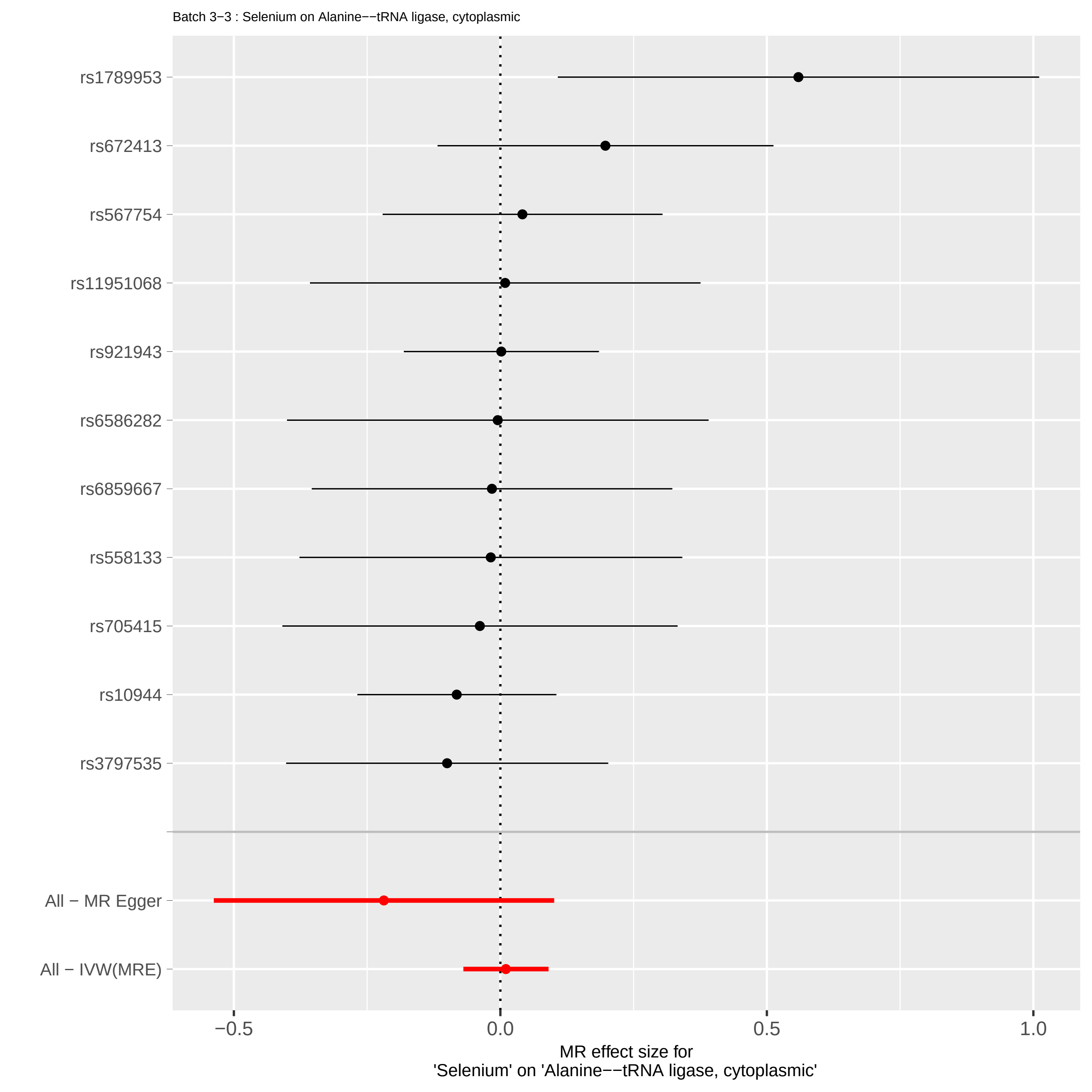
Batch 3−3 : Selenium on Alanine−−tRNA ligase, cytoplasmic
rs1789953
rs672413
rs567754
rs11951068
rs921943
rs6586282
rs6859667
rs558133
rs705415
rs10944
rs3797535
All − MR Egger
All − IVW(MRE)
−0.5
0.0
0.5
1.0
MR effect size for
'Selenium' on 'Alanine−−tRNA ligase, cytoplasmic'

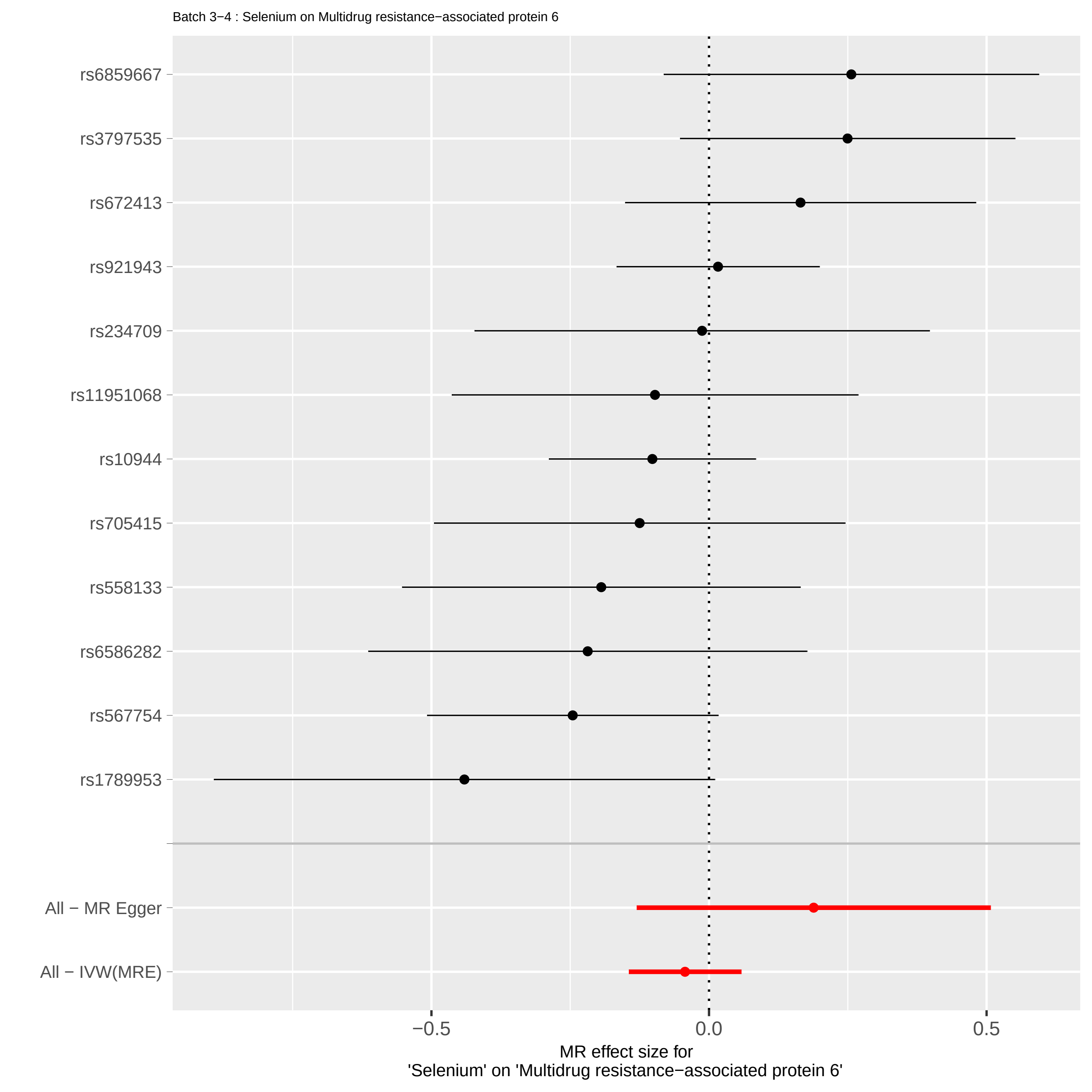

Batch 3−4 : Selenium on Multidrug resistance−associated protein 6
rs6859667
rs3797535
rs672413
rs921943
rs234709
rs11951068
rs10944
rs705415
rs558133
rs6586282
rs567754
rs1789953
All − MR Egger
All − IVW(MRE)
−0.5
0.0
0.5
MR effect size for
'Selenium' on 'Multidrug resistance−associated protein 6'

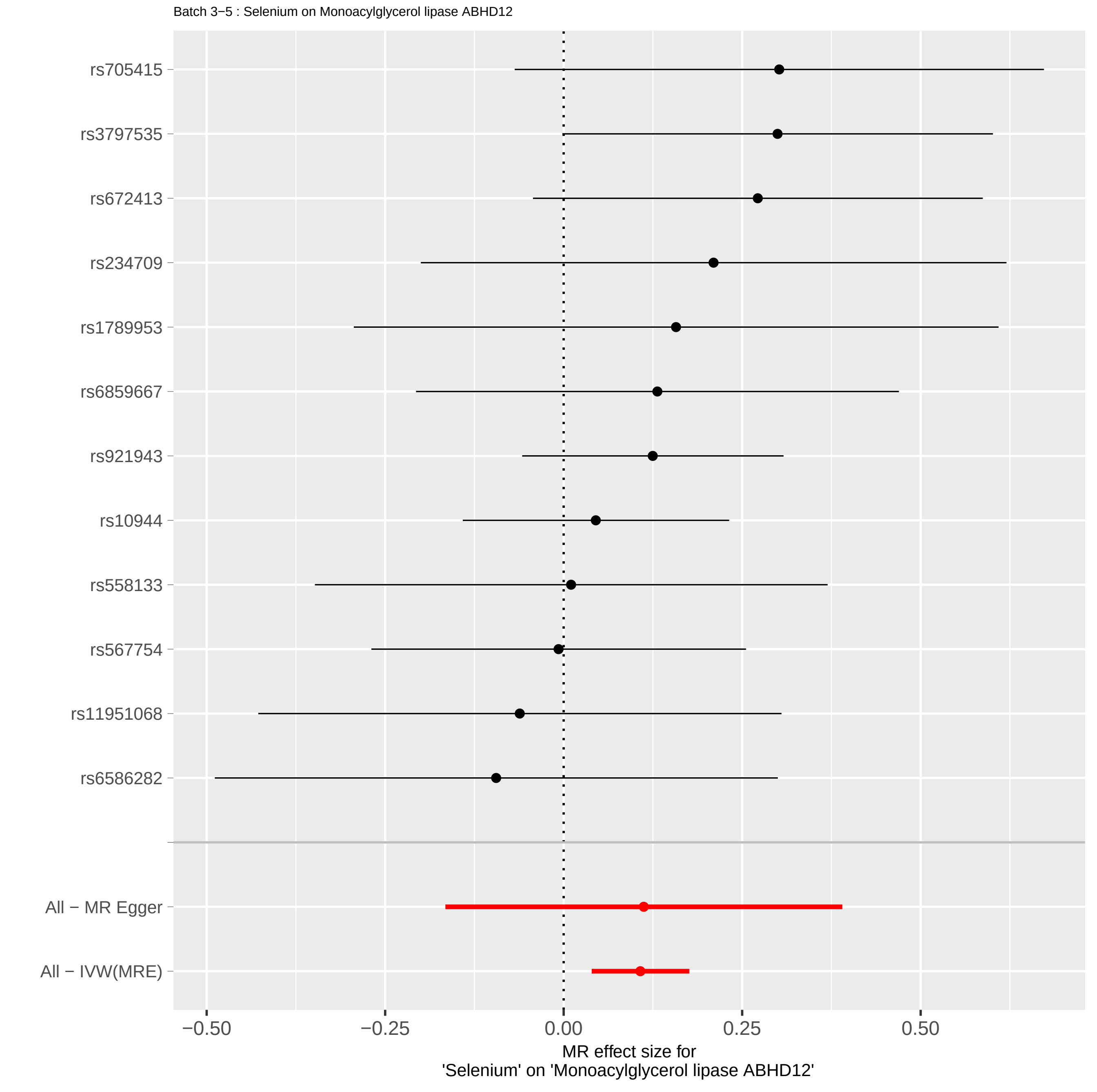
Batch 3−5 : Selenium on Monoacylglycerol lipase ABHD12
rs705415
rs3797535
rs672413
rs234709
rs1789953
rs6859667
rs921943
rs10944
rs558133
rs567754
rs11951068
rs6586282
All − MR Egger
All − IVW(MRE)
−0.50
−0.25
0.00
0.25
0.50
MR effect size for
'Selenium' on 'Monoacylglycerol lipase ABHD12'

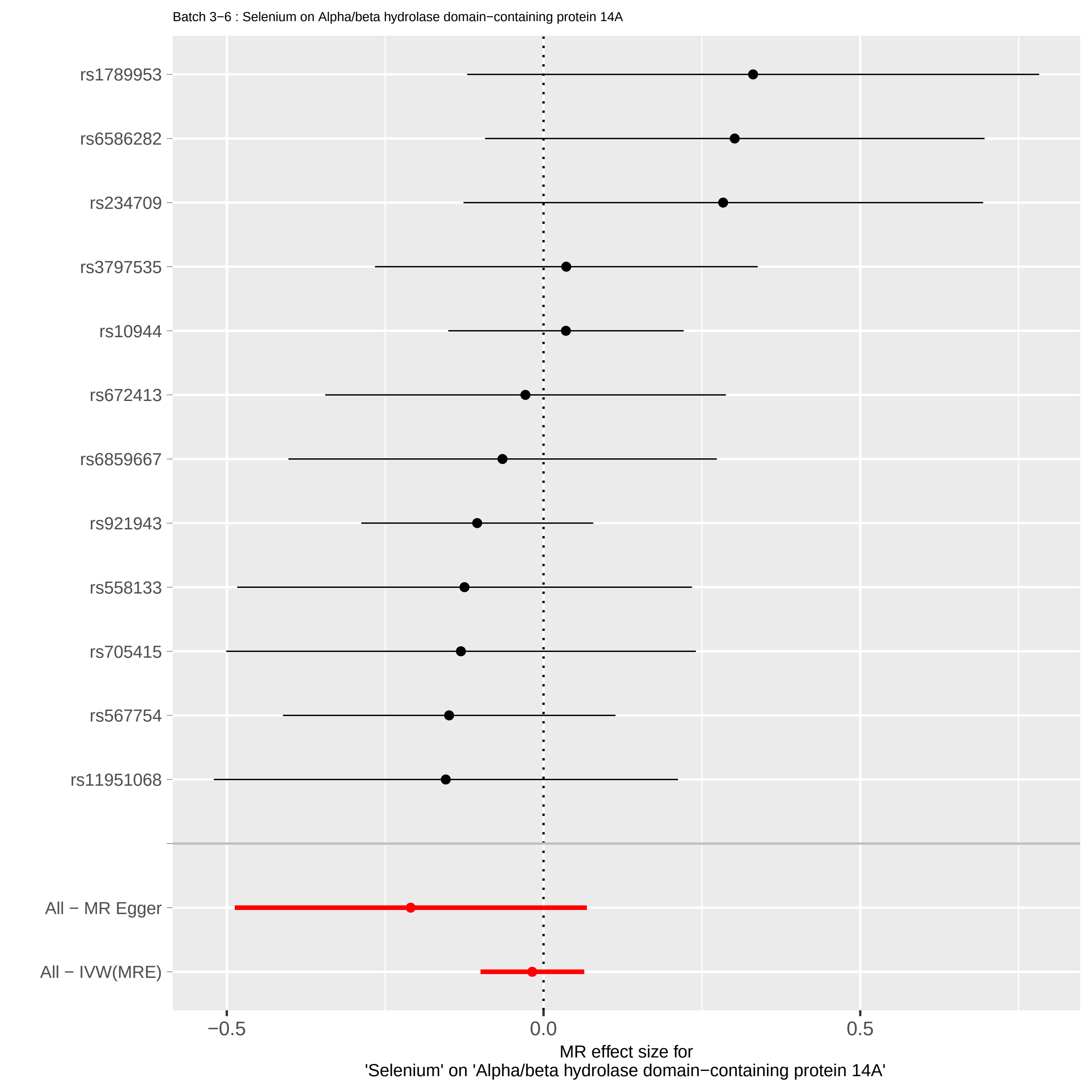

Batch 3−6 : Selenium on Alpha/beta hydrolase domain−containing protein 14A
rs1789953
rs6586282
rs234709
rs3797535
rs10944
rs672413
rs6859667
rs921943
rs558133
rs705415
rs567754
rs11951068
All − MR Egger
All − IVW(MRE)
−0.5
0.0
0.5
MR effect size for
'Selenium' on 'Alpha/beta hydrolase domain−containing protein 14A'

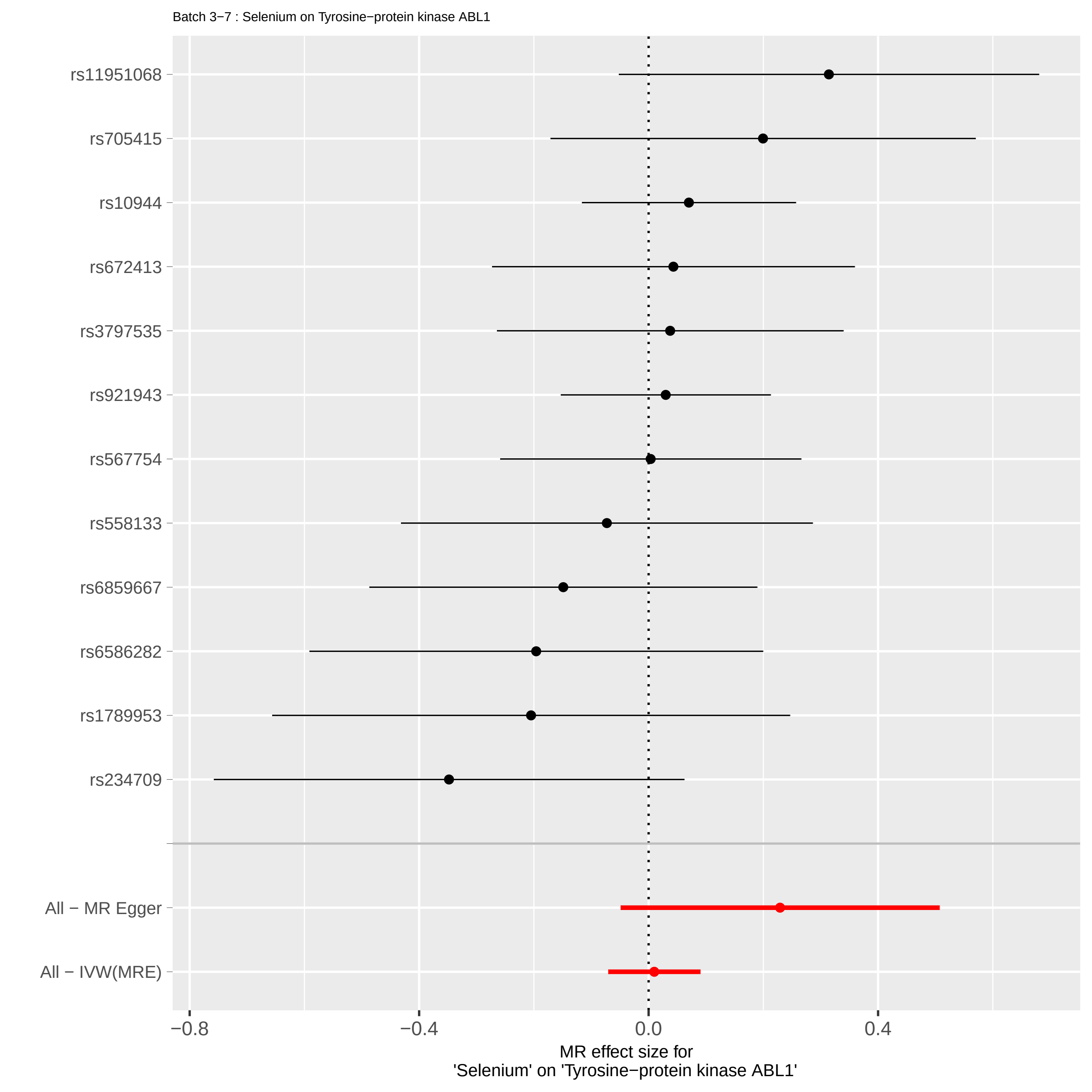
Batch 3−7 : Selenium on Tyrosine−protein kinase ABL1
rs11951068
rs705415
rs10944
rs672413
rs3797535
rs921943
rs567754
rs558133
rs6859667
rs6586282
rs1789953
rs234709
All − MR Egger
All − IVW(MRE)
−0.8
−0.4
0.0
0.4
MR effect size for
'Selenium' on 'Tyrosine−protein kinase ABL1'

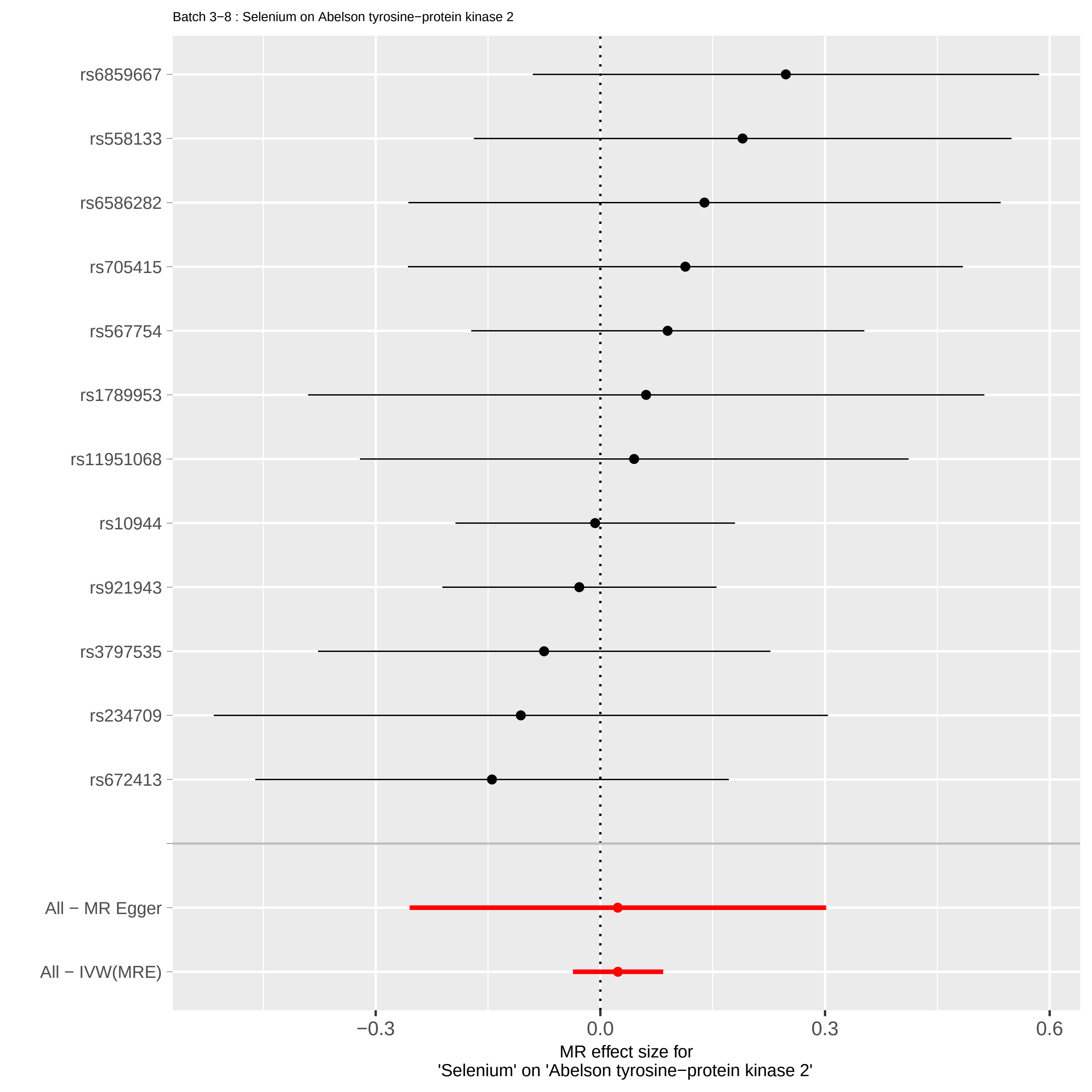
Batch 3−8 : Selenium on Abelson tyrosine−protein kinase 2
rs6859667
rs558133
rs6586282
rs705415
rs567754
rs1789953
rs11951068
rs10944
rs921943
rs3797535
rs234709
rs672413
All − MR Egger
All − IVW(MRE)
−0.3
0.0
0.3
0.6
MR effect size for
'Selenium' on 'Abelson tyrosine−protein kinase 2'

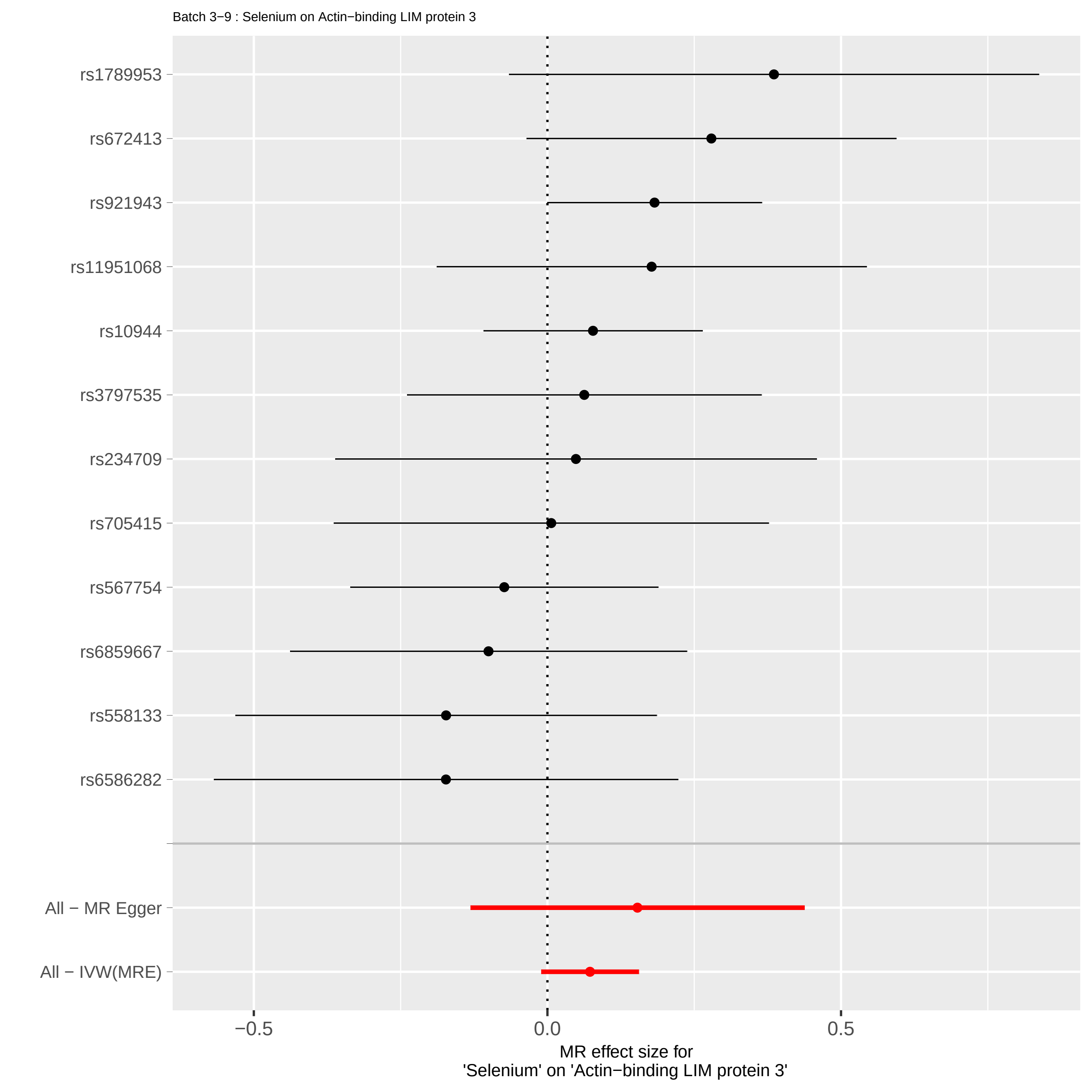

Batch 3−9 : Selenium on Actin−binding LIM protein 3
rs1789953
rs672413
rs921943
rs11951068
rs10944
rs3797535
rs234709
rs705415
rs567754
rs6859667
rs558133
rs6586282
All − MR Egger
All − IVW(MRE)
−0.5
0.0
0.5
MR effect size for
'Selenium' on 'Actin−binding LIM protein 3'

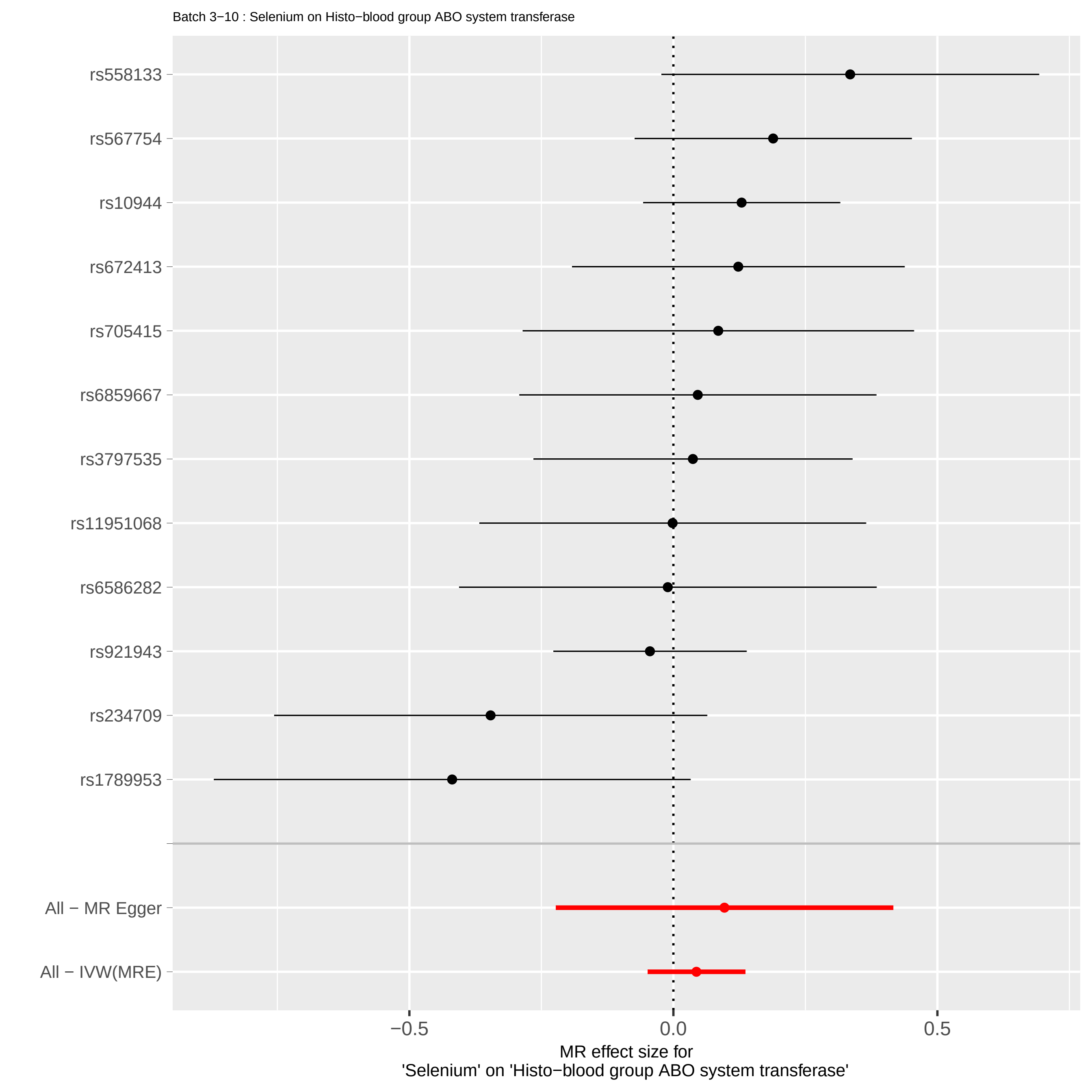
Batch 3−10 : Selenium on Histo−blood group ABO system transferase
rs558133
rs567754
rs10944
rs672413
rs705415
rs6859667
rs3797535
rs11951068
rs6586282
rs921943
rs234709
rs1789953
All − MR Egger
All − IVW(MRE)
−0.5
0.0
0.5
MR effect size for
'Selenium' on 'Histo−blood group ABO system transferase'

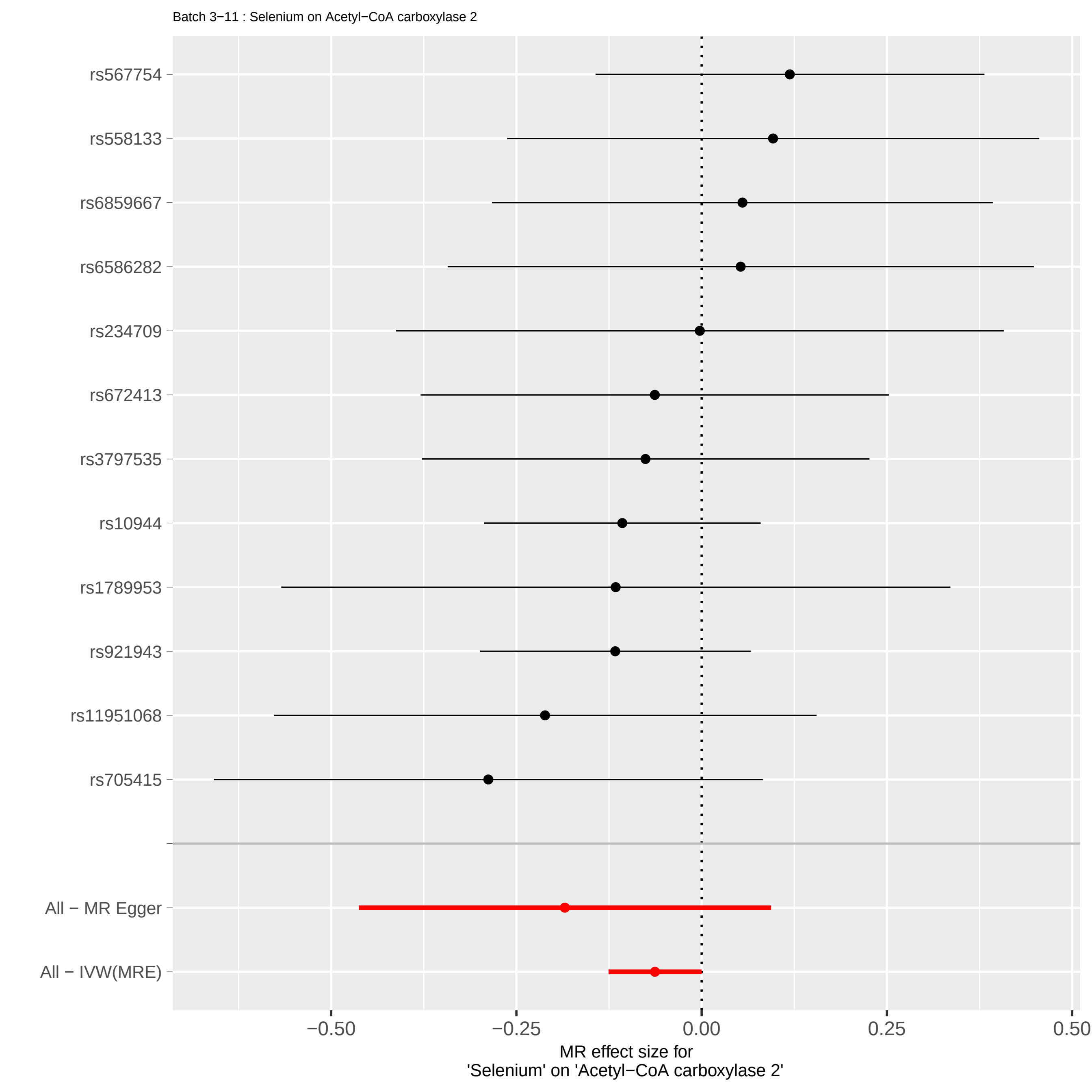
Batch 3−11 : Selenium on Acetyl−CoA carboxylase 2
rs567754
rs558133
rs6859667
rs6586282
rs234709
rs672413
rs3797535
rs10944
rs1789953
rs921943
rs11951068
rs705415
All − MR Egger
All − IVW(MRE)
−0.50
−0.25
0.00
0.25
0.50
MR effect size for
'Selenium' on 'Acetyl−CoA carboxylase 2'

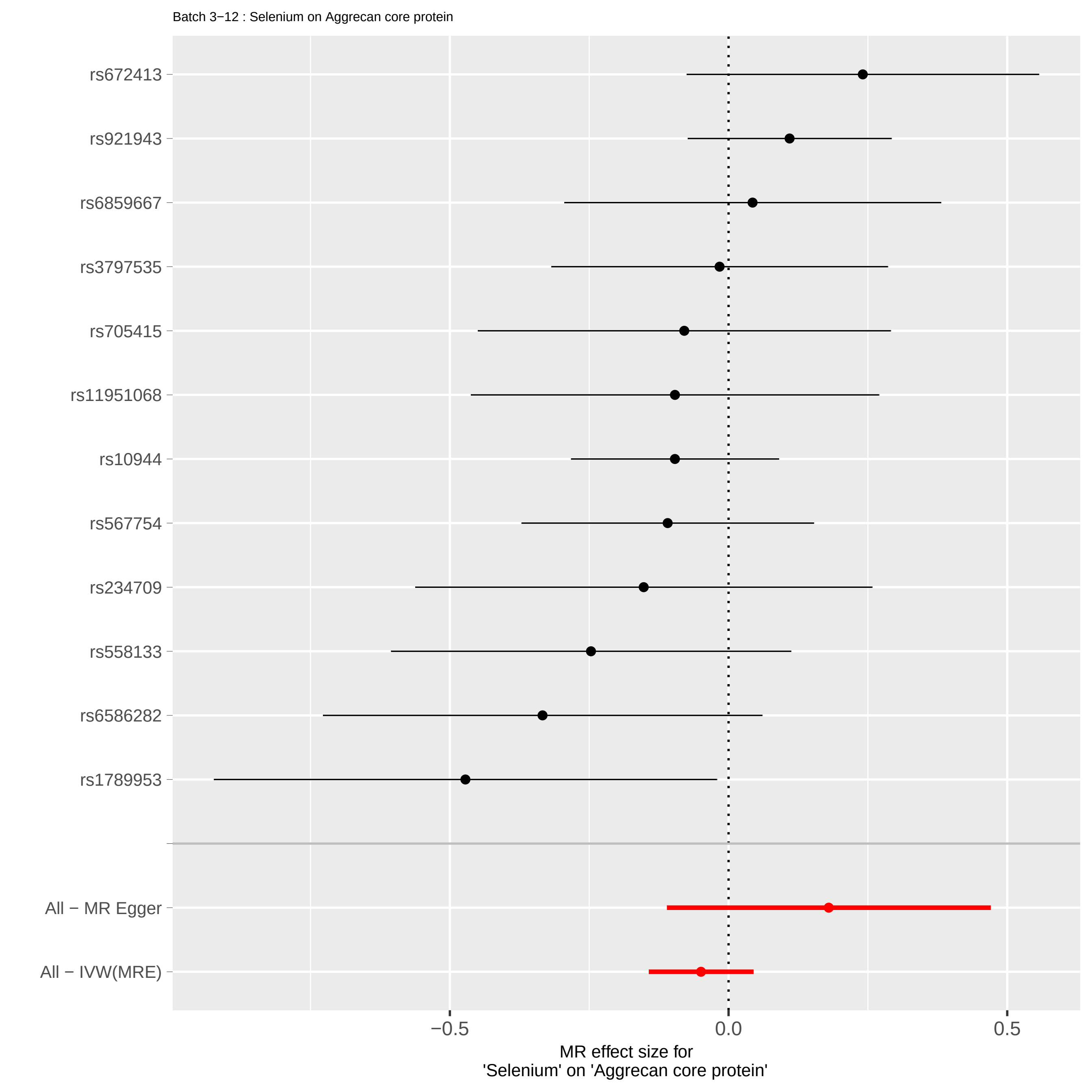

Batch 3−12 : Selenium on Aggrecan core protein
rs672413
rs921943
rs6859667
rs3797535
rs705415
rs11951068
rs10944
rs567754
rs234709
rs558133
rs6586282
rs1789953
All − MR Egger
All − IVW(MRE)
−0.5
0.0
0.5
MR effect size for
'Selenium' on 'Aggrecan core protein'

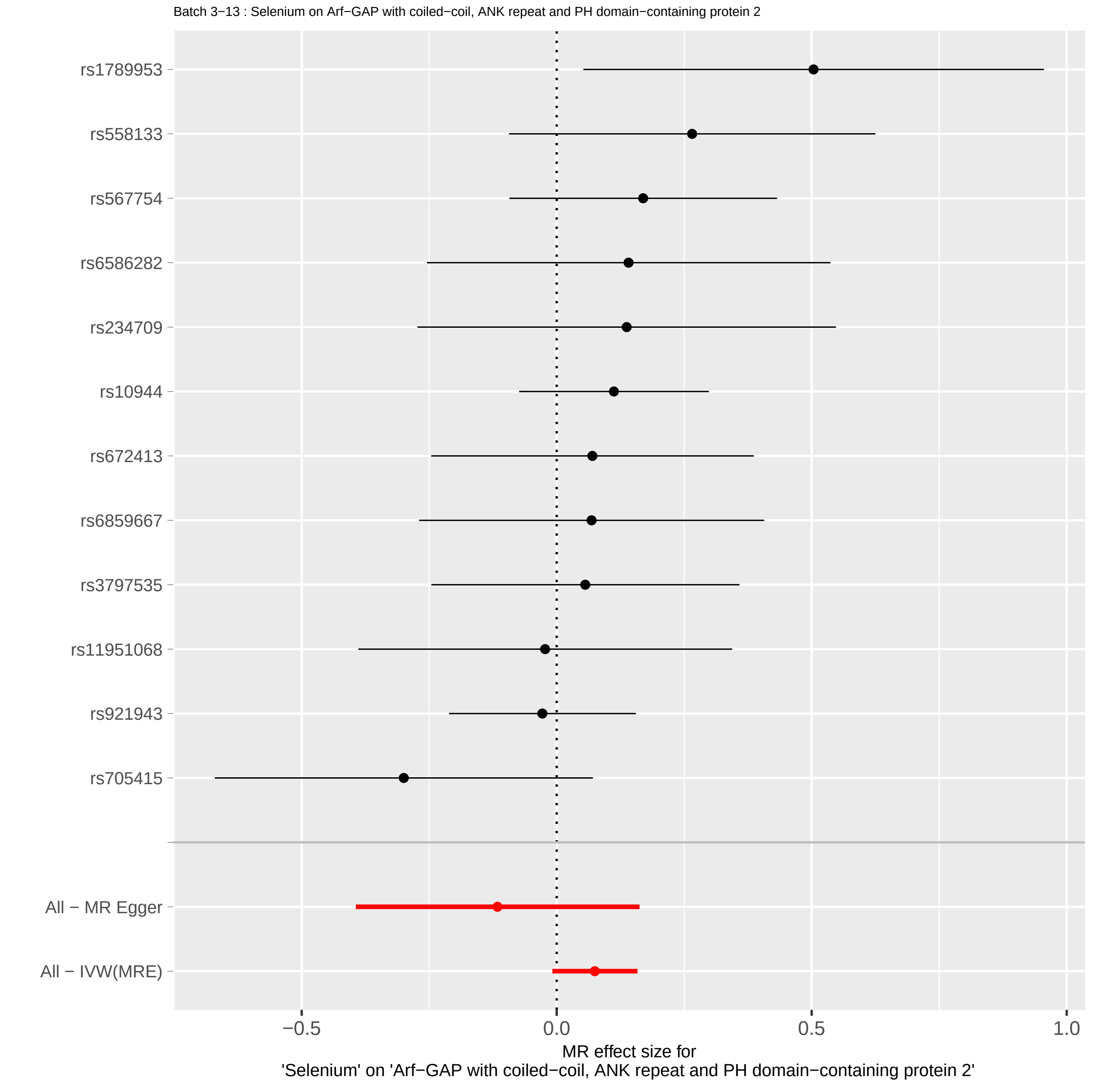

Batch 3−13 : Selenium on Arf−GAP with coiled−coil, ANK repeat and PH domain−containing protein 2
rs1789953
rs558133
rs567754
rs6586282
rs234709
rs10944
rs672413
rs6859667
rs3797535
rs11951068
rs921943
rs705415
All − MR Egger
All − IVW(MRE)
−0.5
0.0
0.5
1.0
MR effect size for
'Selenium' on 'Arf−GAP with coiled−coil, ANK repeat and PH domain−containing protein 2'

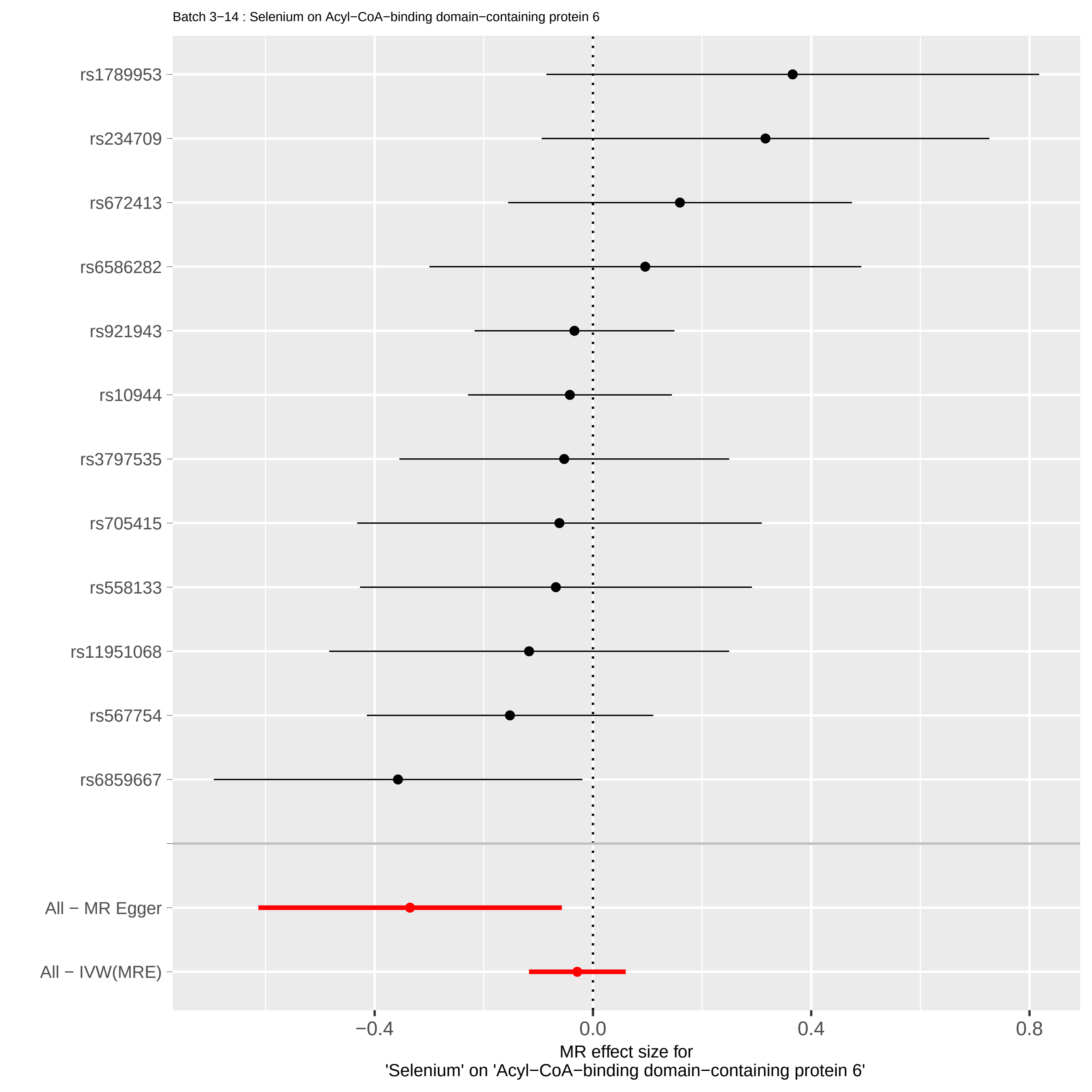

Batch 3−14 : Selenium on Acyl−CoA−binding domain−containing protein 6
rs1789953
rs234709
rs672413
rs6586282
rs921943
rs10944
rs3797535
rs705415
rs558133
rs11951068
rs567754
rs6859667
All − MR Egger
All − IVW(MRE)
−0.4
0.0
0.4
0.8
MR effect size for
'Selenium' on 'Acyl−CoA−binding domain−containing protein 6'

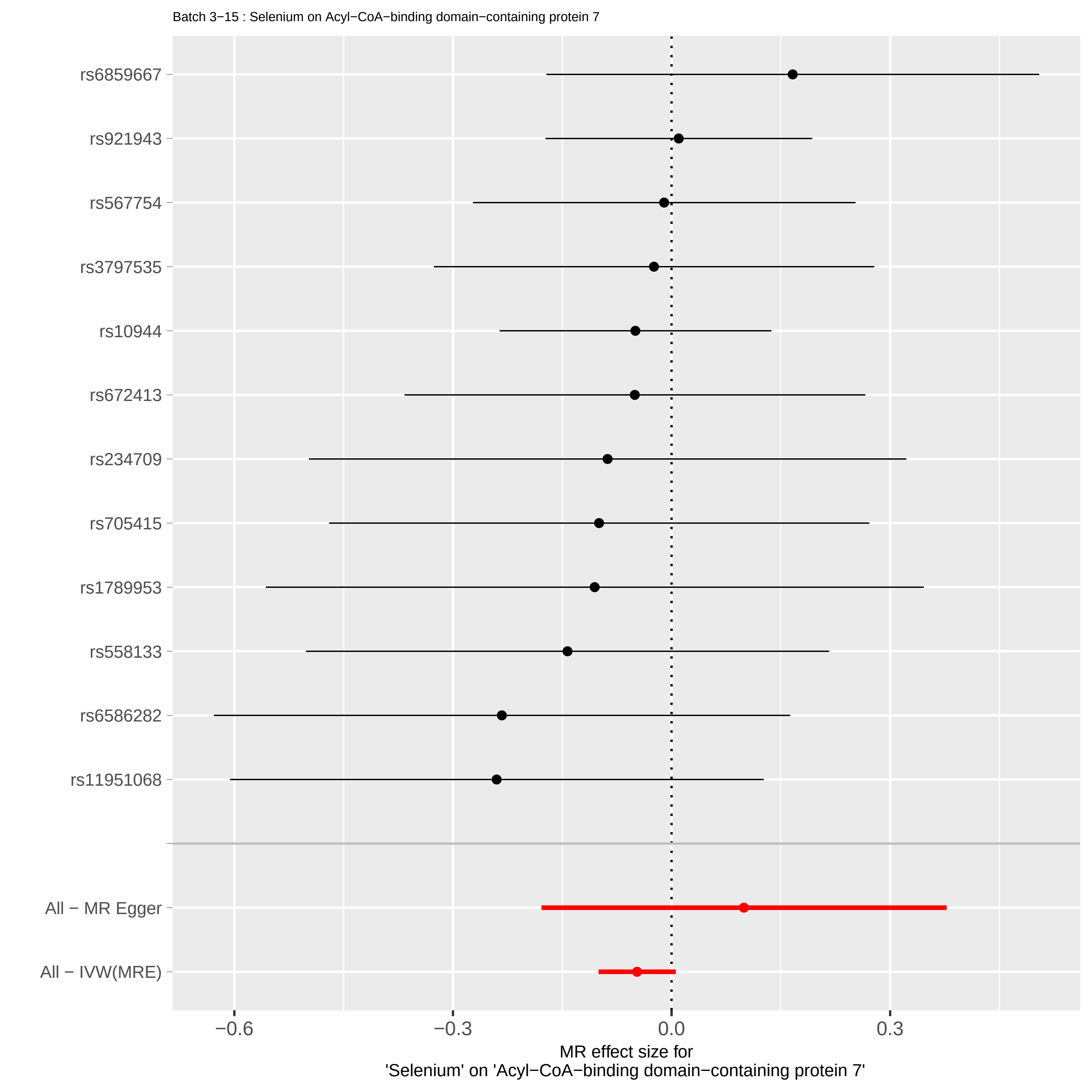
Batch 3−15 : Selenium on Acyl−CoA−binding domain−containing protein 7
rs6859667
rs921943
rs567754
rs3797535
rs10944
rs672413
rs234709
rs705415
rs1789953
rs558133
rs6586282
rs11951068
All − MR Egger
All − IVW(MRE)
−0.6
−0.3
0.0
0.3
MR effect size for
'Selenium' on 'Acyl−CoA−binding domain−containing protein 7'

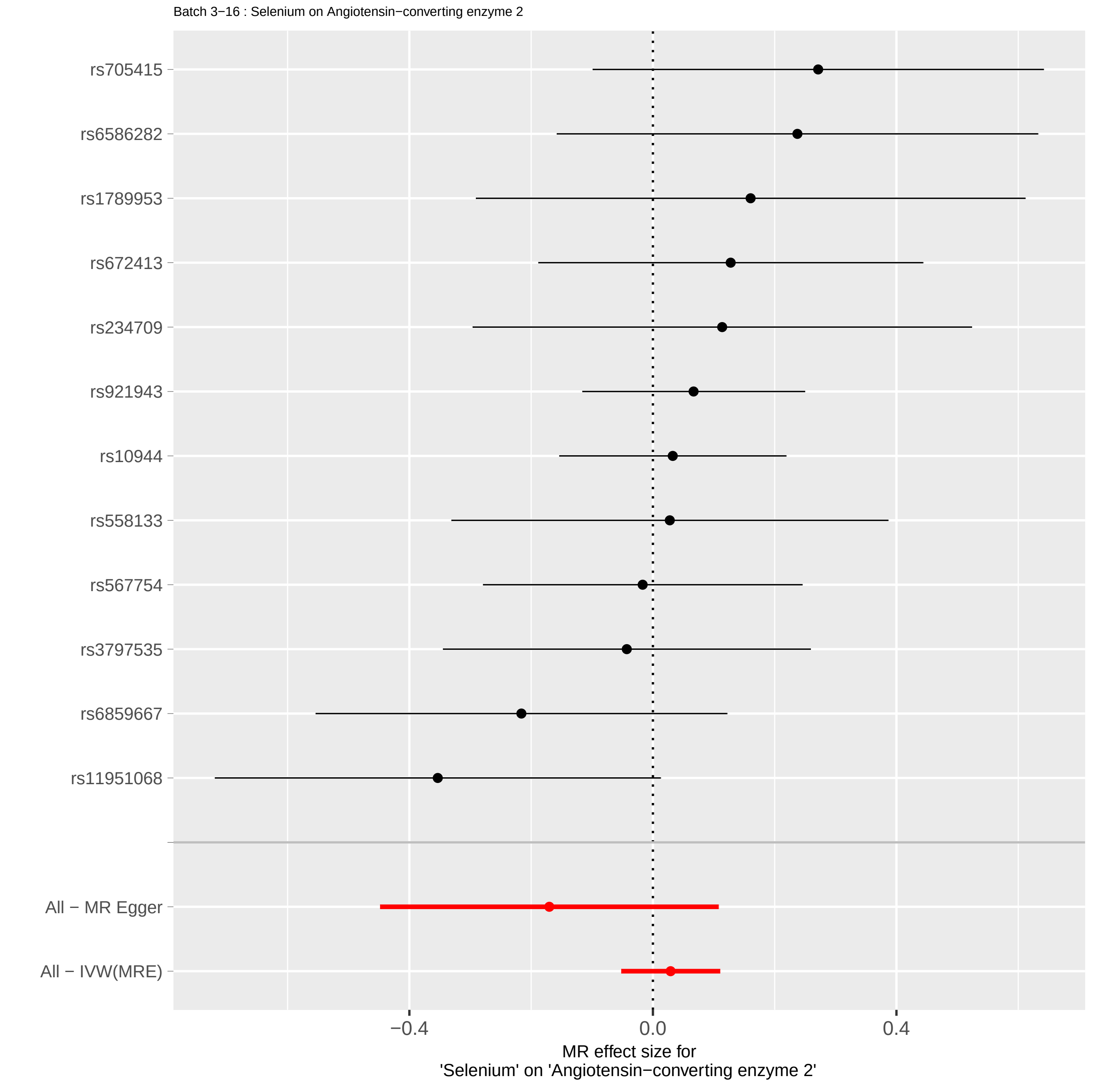

Batch 3−16 : Selenium on Angiotensin−converting enzyme 2
rs705415
rs6586282
rs1789953
rs672413
rs234709
rs921943
rs10944
rs558133
rs567754
rs3797535
rs6859667
rs11951068
All − MR Egger
All − IVW(MRE)
−0.4
0.0
0.4
MR effect size for
'Selenium' on 'Angiotensin−converting enzyme 2'

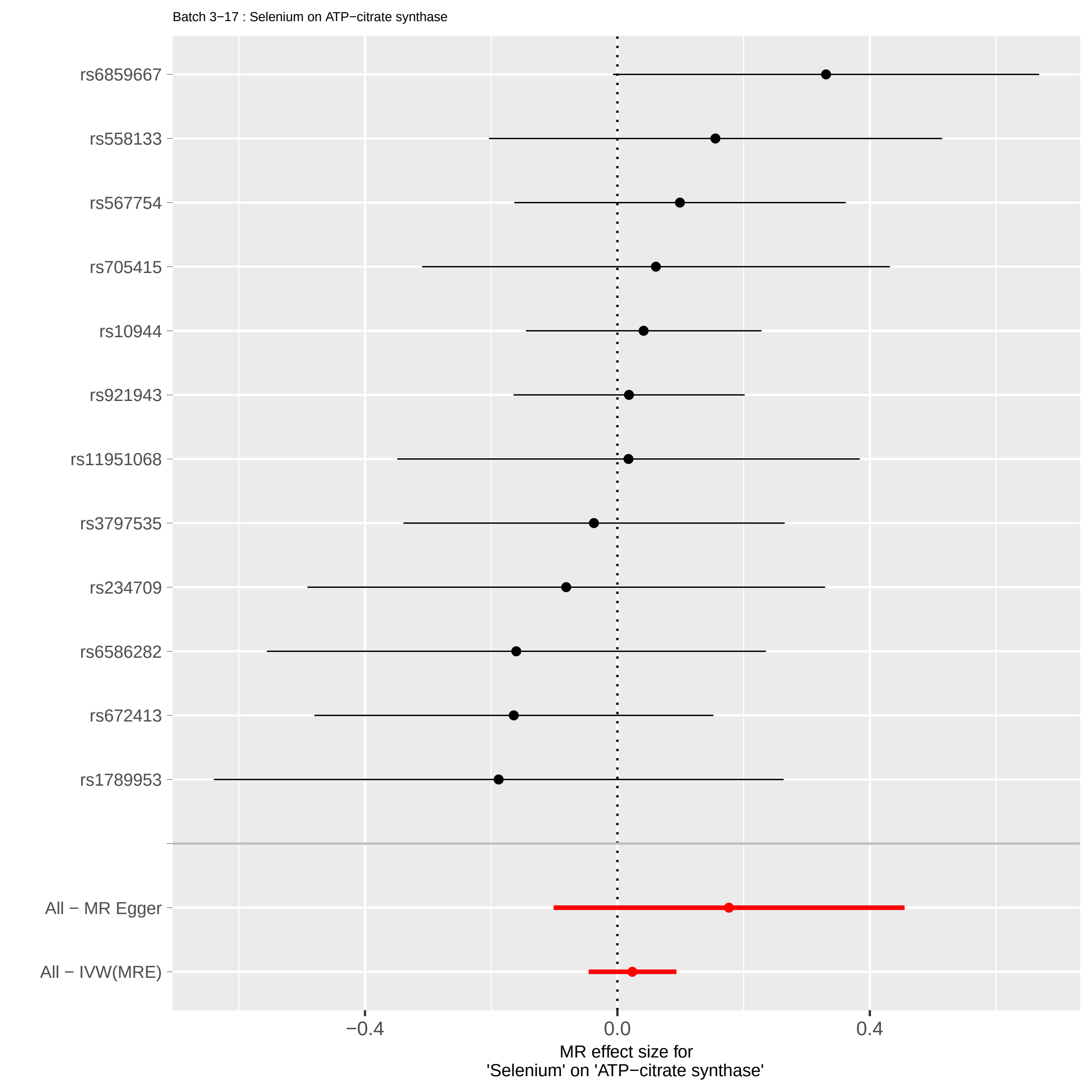
Batch 3−17 : Selenium on ATP−citrate synthase
rs6859667
rs558133
rs567754
rs705415
rs10944
rs921943
rs11951068
rs3797535
rs234709
rs6586282
rs672413
rs1789953
All − MR Egger
All − IVW(MRE)
−0.4
0.0
0.4
MR effect size for
'Selenium' on 'ATP−citrate synthase'

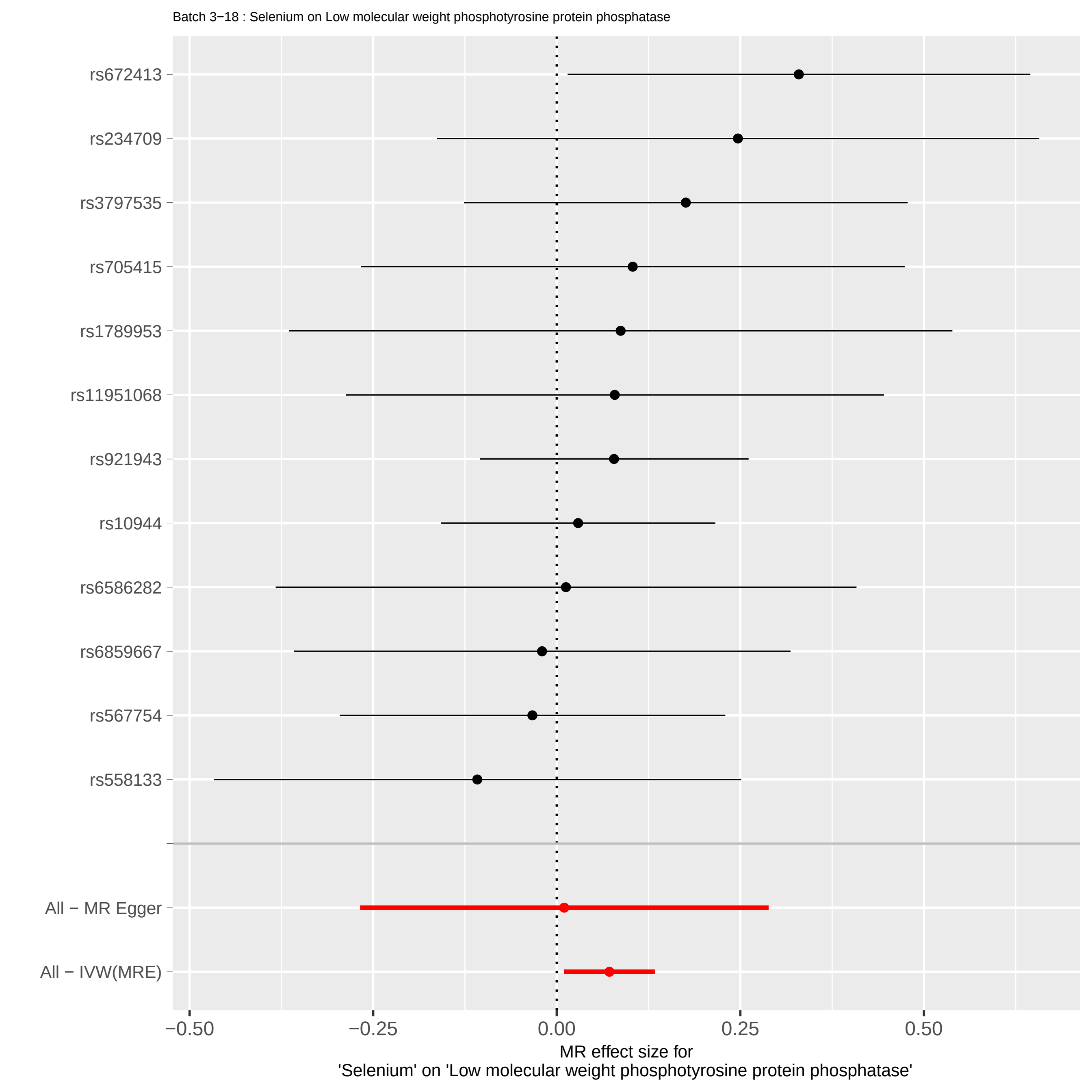
Batch 3−18 : Selenium on Low molecular weight phosphotyrosine protein phosphatase
rs672413
rs234709
rs3797535
rs705415
rs1789953
rs11951068
rs921943
rs10944
rs6586282
rs6859667
rs567754
rs558133
All − MR Egger
All − IVW(MRE)
−0.50
−0.25
0.00
0.25
0.50
MR effect size for
'Selenium' on 'Low molecular weight phosphotyrosine protein phosphatase'

Batch 3−19 : Selenium on Lysosomal acid phosphatase

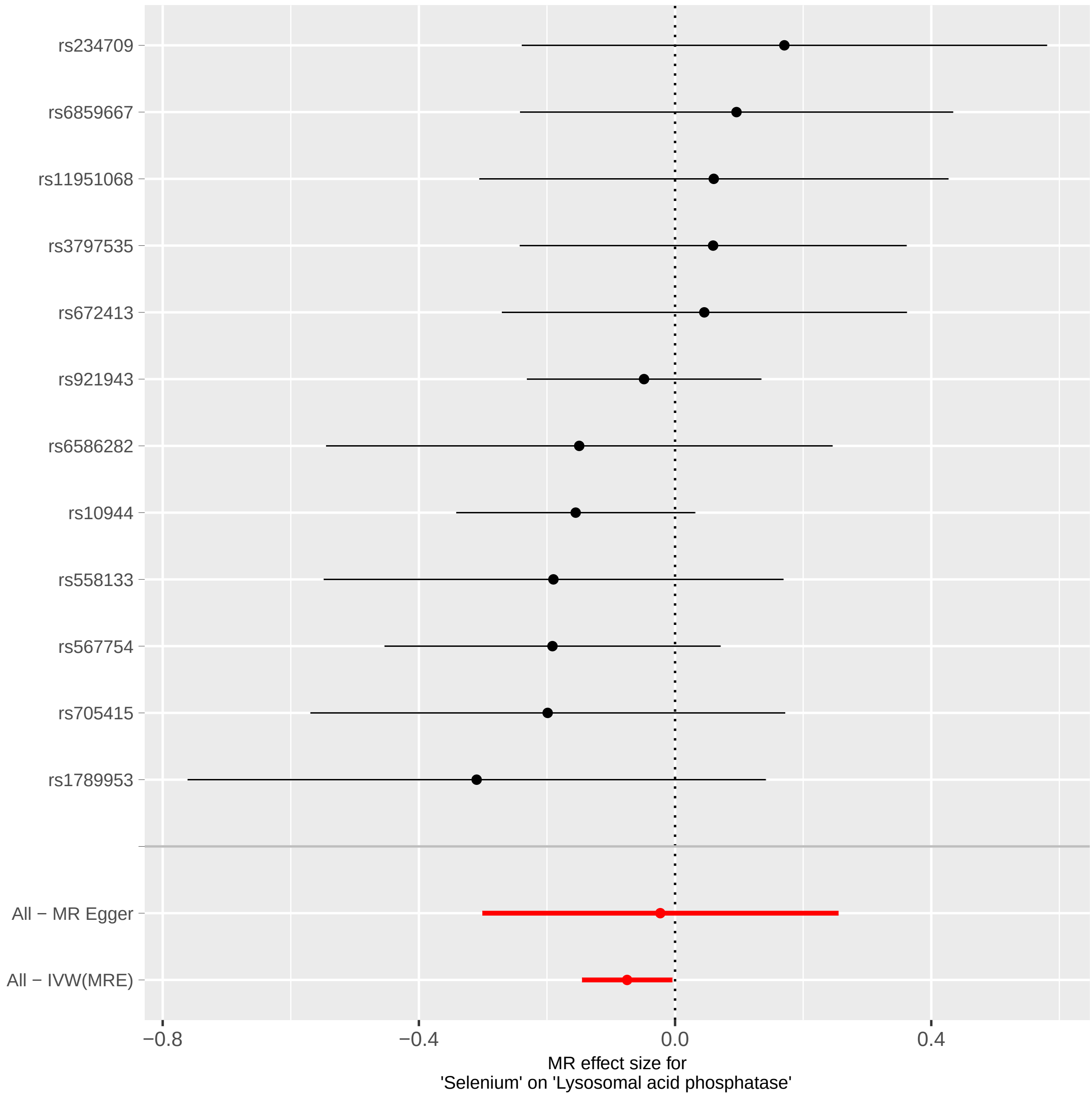

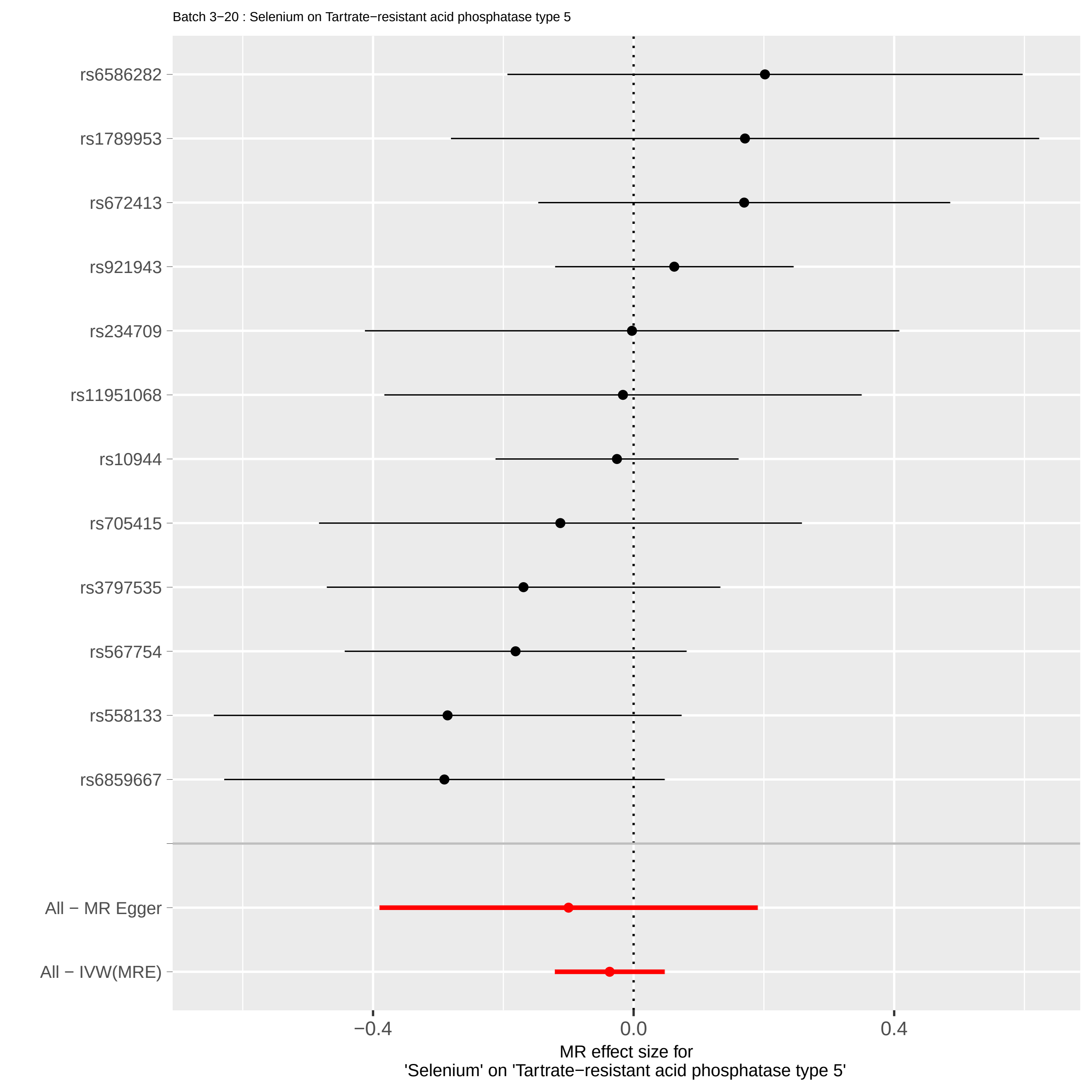
Batch 3−20 : Selenium on Tartrate−resistant acid phosphatase type 5
rs6586282
rs1789953
rs672413
rs921943
rs234709
rs11951068
rs10944
rs705415
rs3797535
rs567754
rs558133
rs6859667
All − MR Egger
All − IVW(MRE)
−0.4
0.0
0.4
MR effect size for
'Selenium' on 'Tartrate−resistant acid phosphatase type 5'

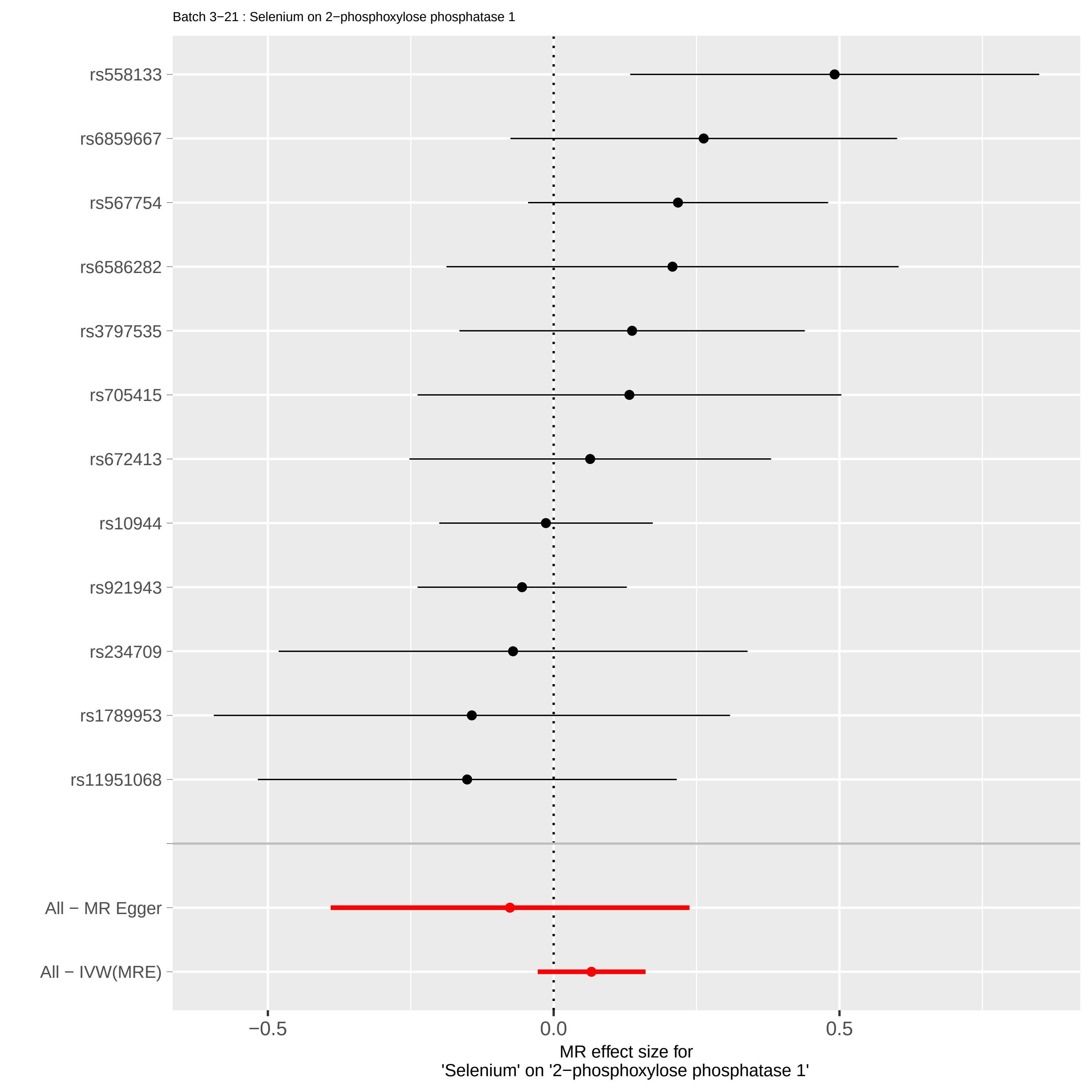

Batch 3−21 : Selenium on 2−phosphoxylose phosphatase 1
rs558133
rs6859667
rs567754
rs6586282
rs3797535
rs705415
rs672413
rs10944
rs921943
rs234709
rs1789953
rs11951068
All − MR Egger
All − IVW(MRE)
−0.5
0.0
0.5
MR effect size for
'Selenium' on '2−phosphoxylose phosphatase 1'

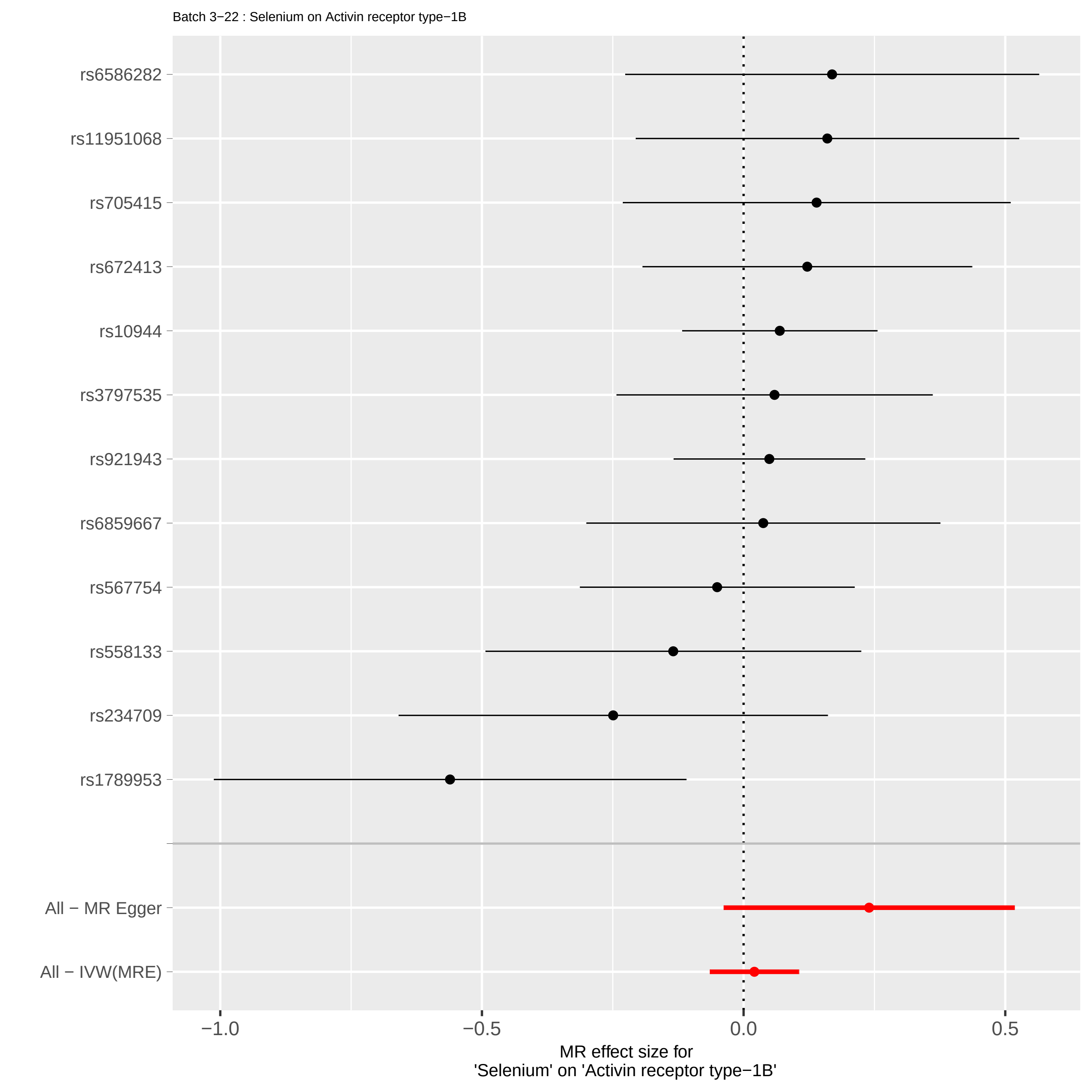
Batch 3−22 : Selenium on Activin receptor type−1B
rs6586282
rs11951068
rs705415
rs672413
rs10944
rs3797535
rs921943
rs6859667
rs567754
rs558133
rs234709
rs1789953
All − MR Egger
All − IVW(MRE)
−1.0
−0.5
0.0
0.5
MR effect size for
'Selenium' on 'Activin receptor type−1B'

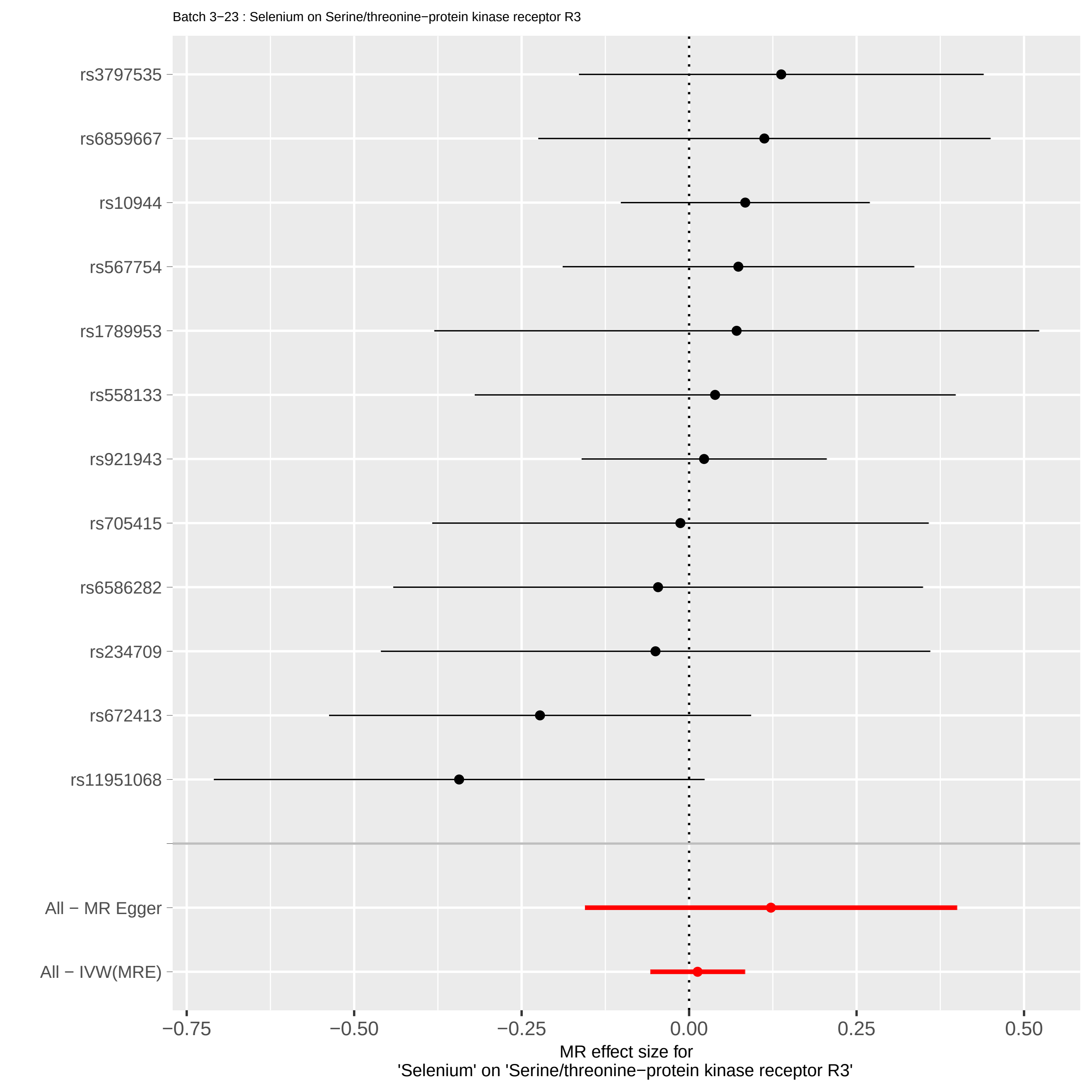
Batch 3−23 : Selenium on Serine/threonine−protein kinase receptor R3
rs3797535
rs6859667
rs10944
rs567754
rs1789953
rs558133
rs921943
rs705415
rs6586282
rs234709
rs672413
rs11951068
All − MR Egger
All − IVW(MRE)
−0.75
−0.50
−0.25
0.00
0.25
0.50
MR effect size for
'Selenium' on 'Serine/threonine−protein kinase receptor R3'

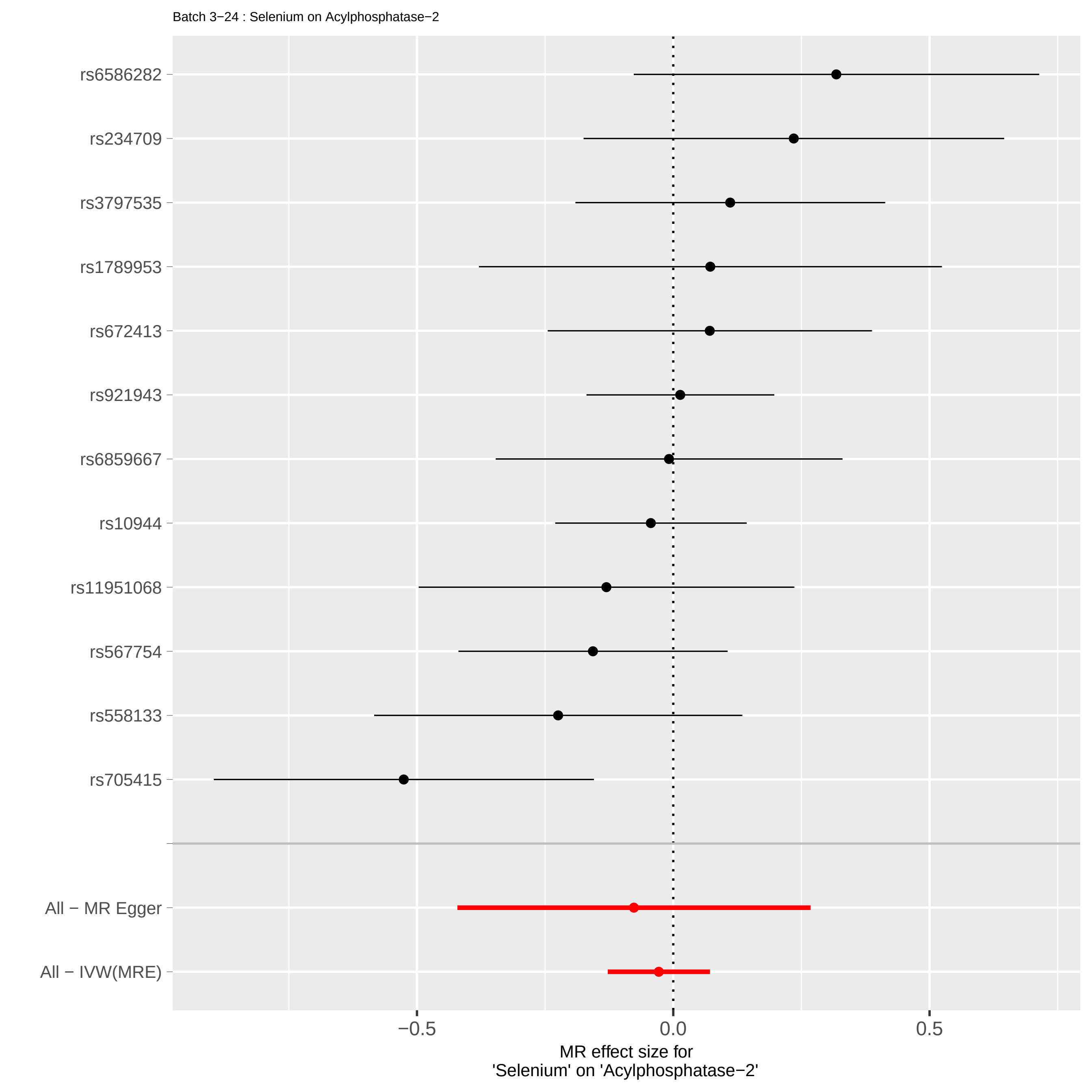
Batch 3−24 : Selenium on Acylphosphatase−2
rs6586282
rs234709
rs3797535
rs1789953
rs672413
rs921943
rs6859667
rs10944
rs11951068
rs567754
rs558133
rs705415
All − MR Egger
All − IVW(MRE)
−0.5
0.0
0.5
MR effect size for
'Selenium' on 'Acylphosphatase−2'

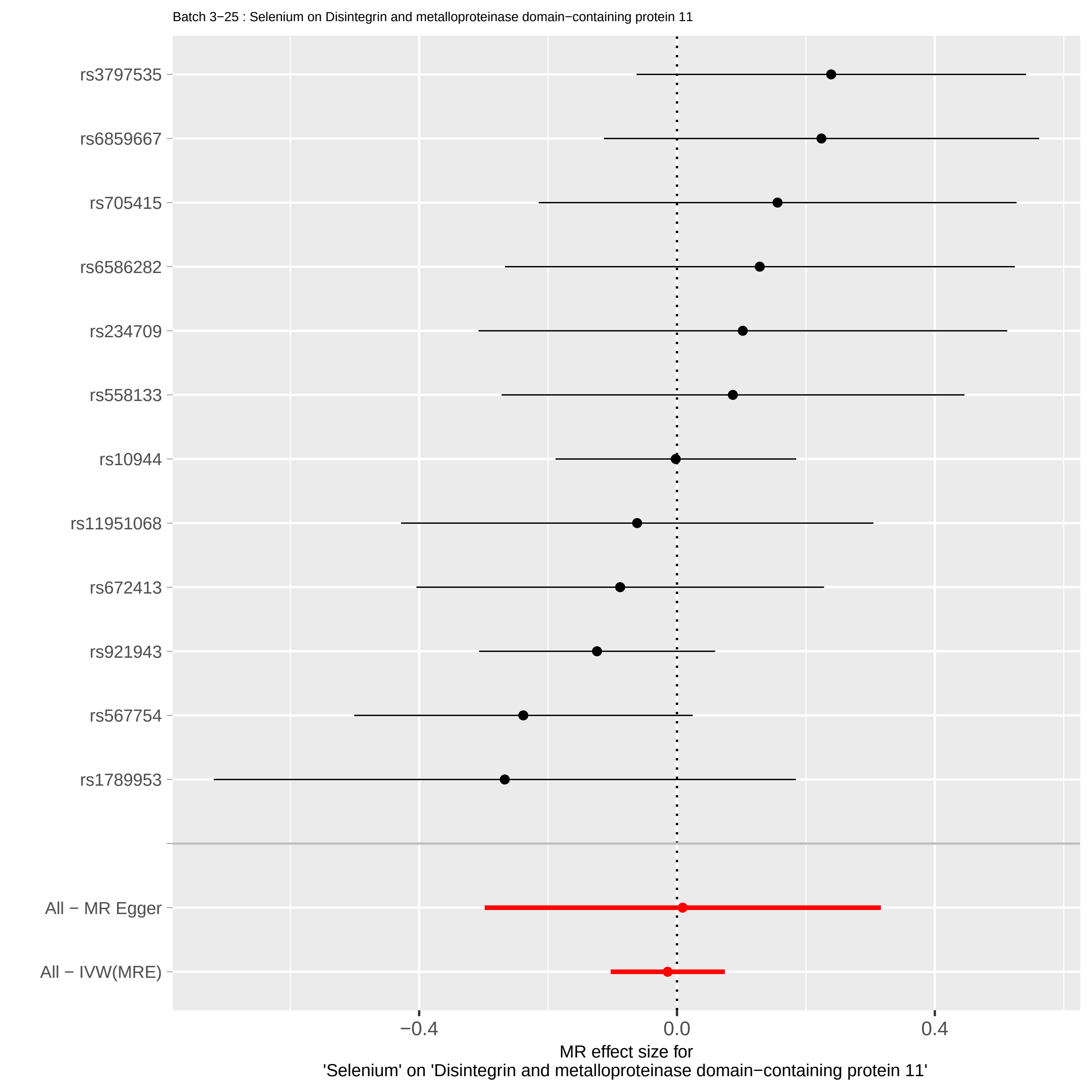
Batch 3−25 : Selenium on Disintegrin and metalloproteinase domain−containing protein 11
rs3797535
rs6859667
rs705415
rs6586282
rs234709
rs558133
rs10944
rs11951068
rs672413
rs921943
rs567754
rs1789953
All − MR Egger
All − IVW(MRE)
−0.4
0.0
0.4
MR effect size for
'Selenium' on 'Disintegrin and metalloproteinase domain−containing protein 11'

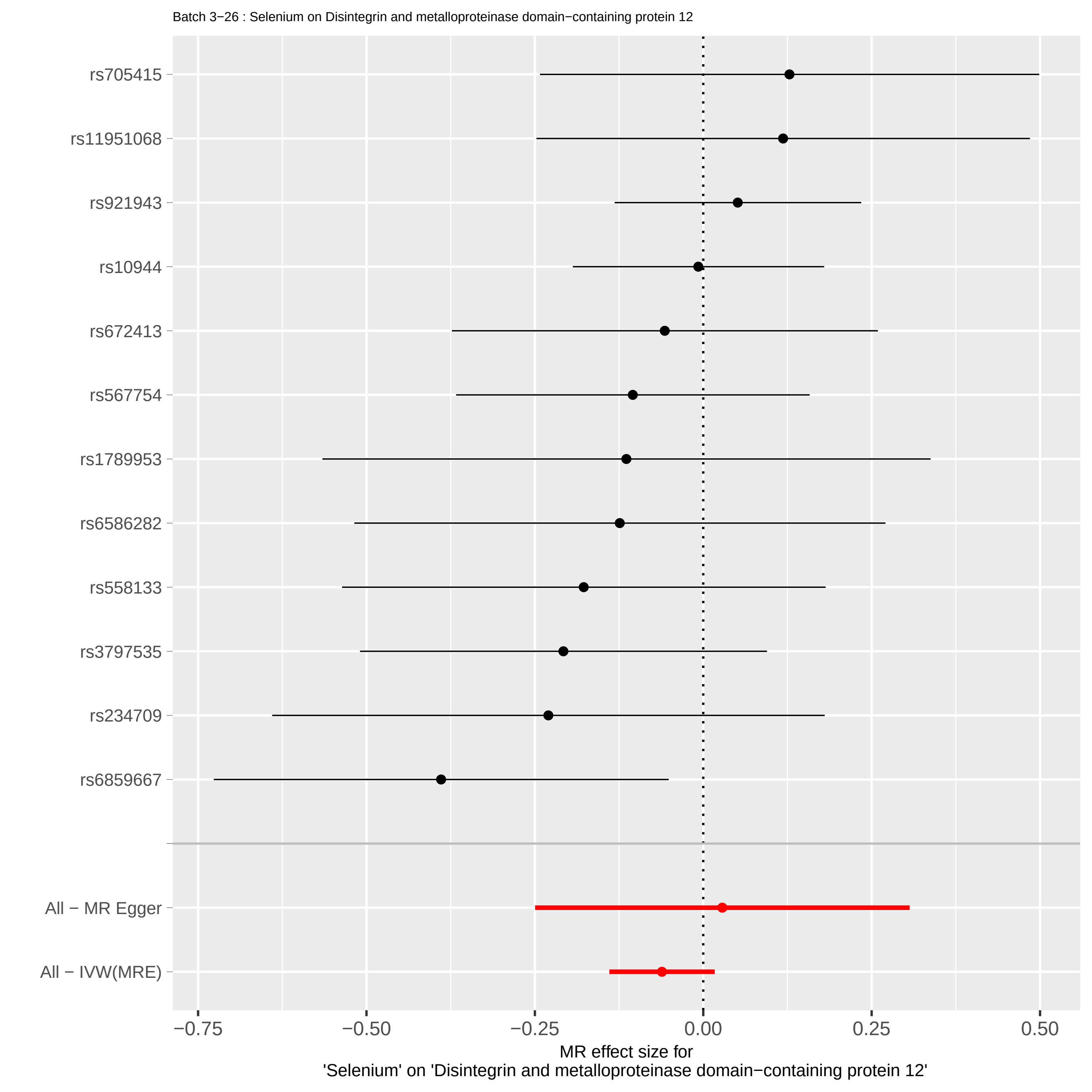

Batch 3−26 : Selenium on Disintegrin and metalloproteinase domain−containing protein 12
rs705415
rs11951068
rs921943
rs10944
rs672413
rs567754
rs1789953
rs6586282
rs558133
rs3797535
rs234709
rs6859667
All − MR Egger
All − IVW(MRE)
−0.75
−0.50
−0.25
0.00
0.25
0.50
MR effect size for
'Selenium' on 'Disintegrin and metalloproteinase domain−containing protein 12'

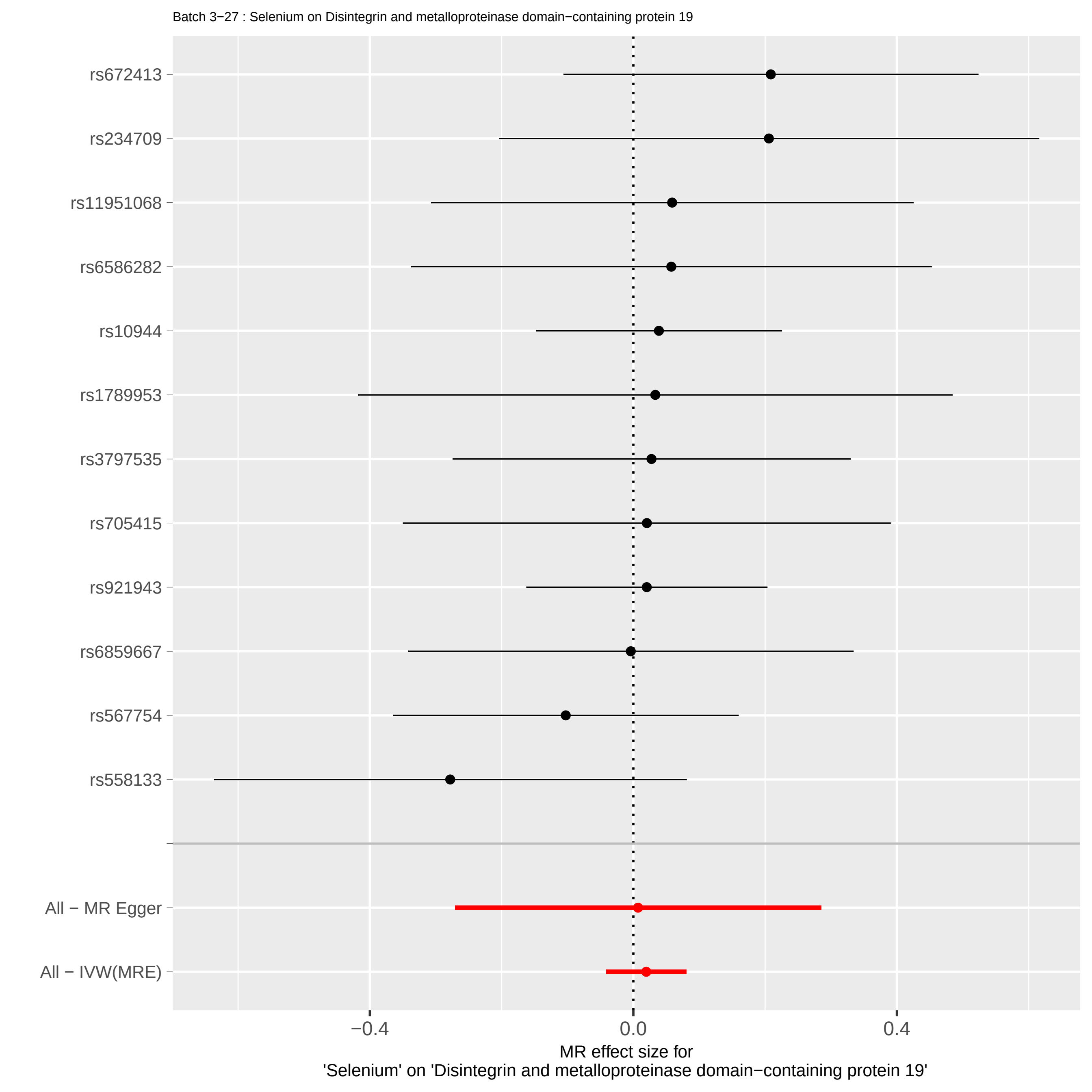

Batch 3−27 : Selenium on Disintegrin and metalloproteinase domain−containing protein 19
rs672413
rs234709
rs11951068
rs6586282
rs10944
rs1789953
rs3797535
rs705415
rs921943
rs6859667
rs567754
rs558133
All − MR Egger
All − IVW(MRE)
−0.4
0.0
0.4
MR effect size for
'Selenium' on 'Disintegrin and metalloproteinase domain−containing protein 19'

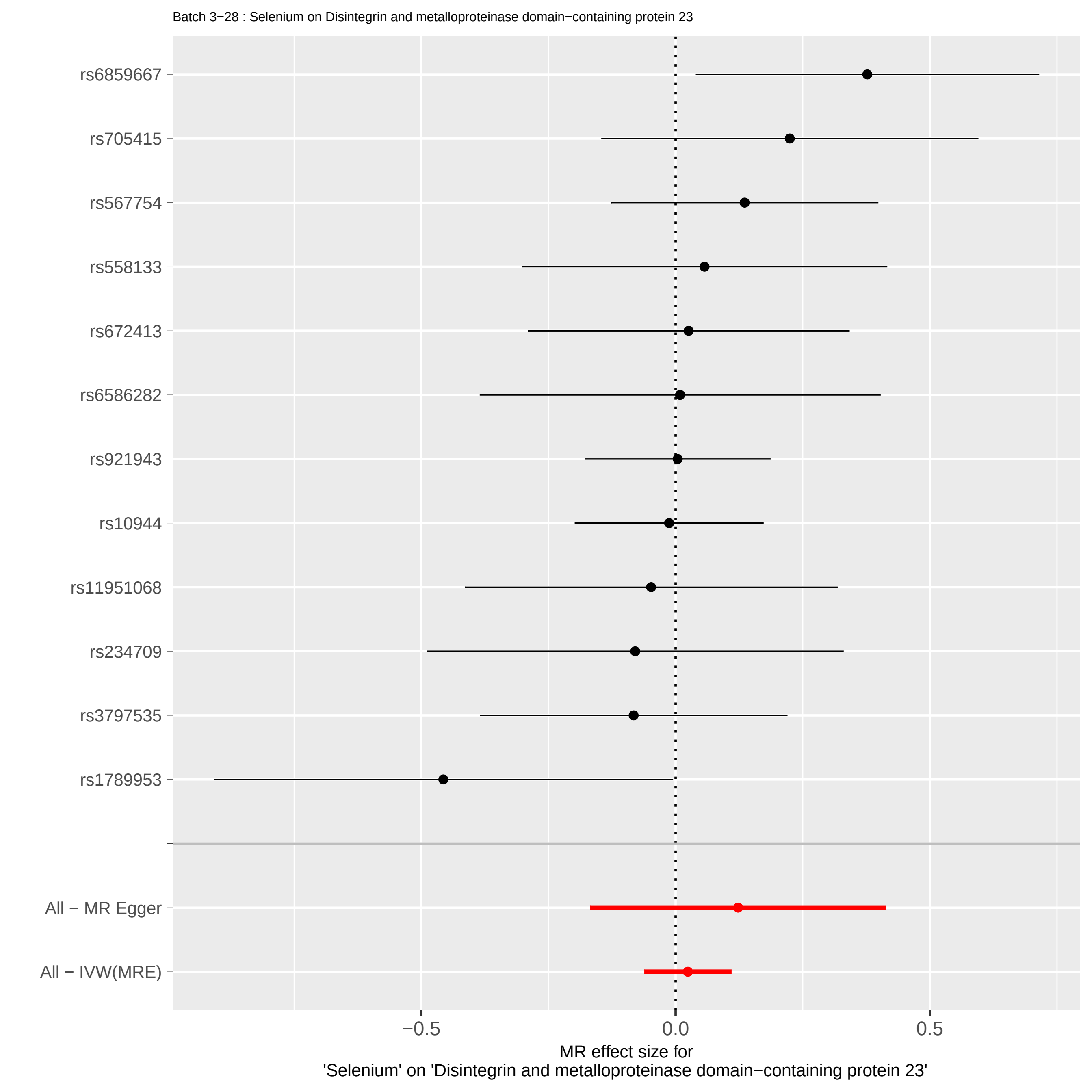

Batch 3−28 : Selenium on Disintegrin and metalloproteinase domain−containing protein 23
rs6859667
rs705415
rs567754
rs558133
rs672413
rs6586282
rs921943
rs10944
rs11951068
rs234709
rs3797535
rs1789953
All − MR Egger
All − IVW(MRE)
−0.5
0.0
0.5
MR effect size for
'Selenium' on 'Disintegrin and metalloproteinase domain−containing protein 23'

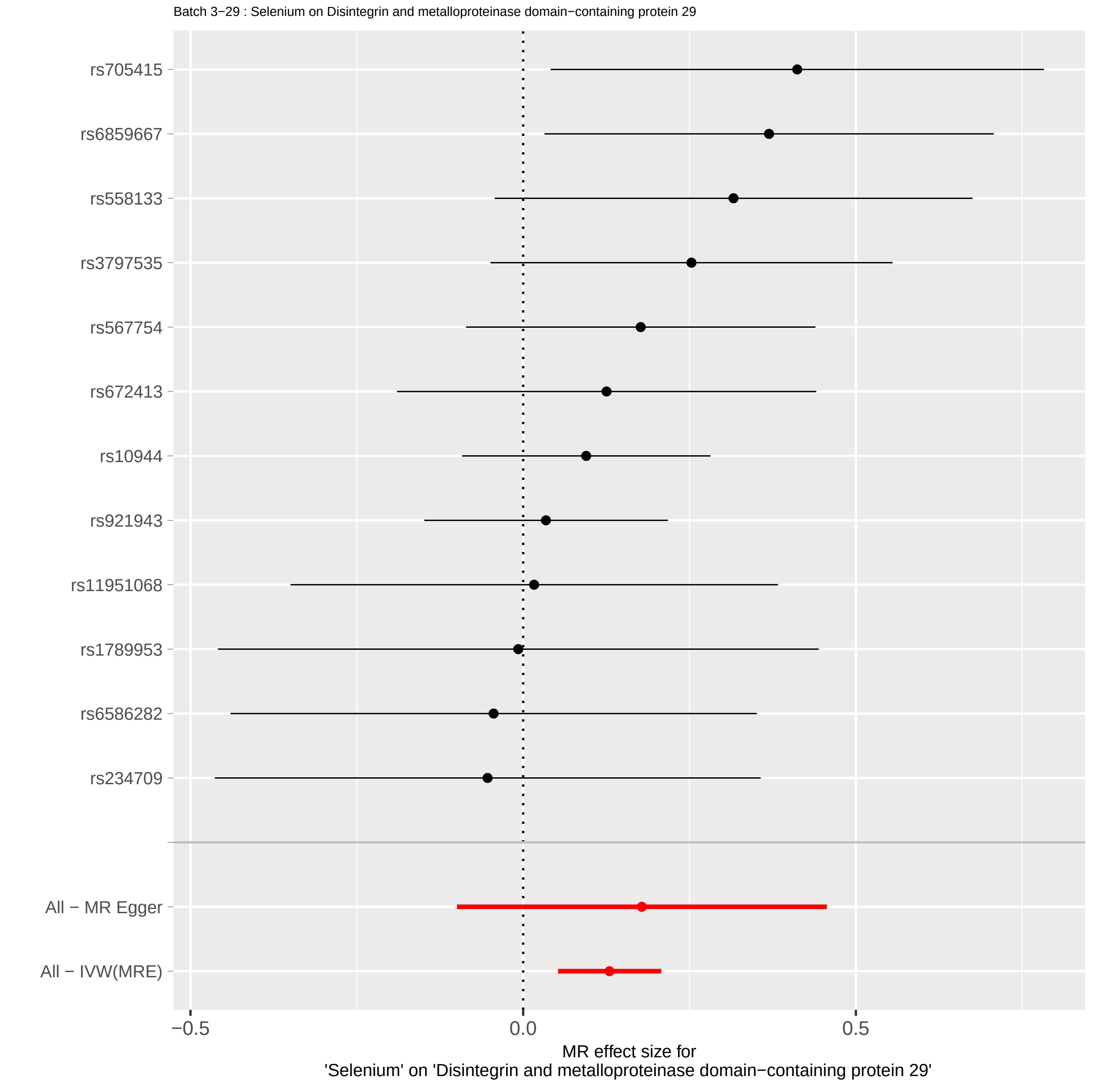

Batch 3−29 : Selenium on Disintegrin and metalloproteinase domain−containing protein 29
rs705415
rs6859667
rs558133
rs3797535
rs567754
rs672413
rs10944
rs921943
rs11951068
rs1789953
rs6586282
rs234709
All − MR Egger
All − IVW(MRE)
−0.5
0.0
0.5
MR effect size for
'Selenium' on 'Disintegrin and metalloproteinase domain−containing protein 29'

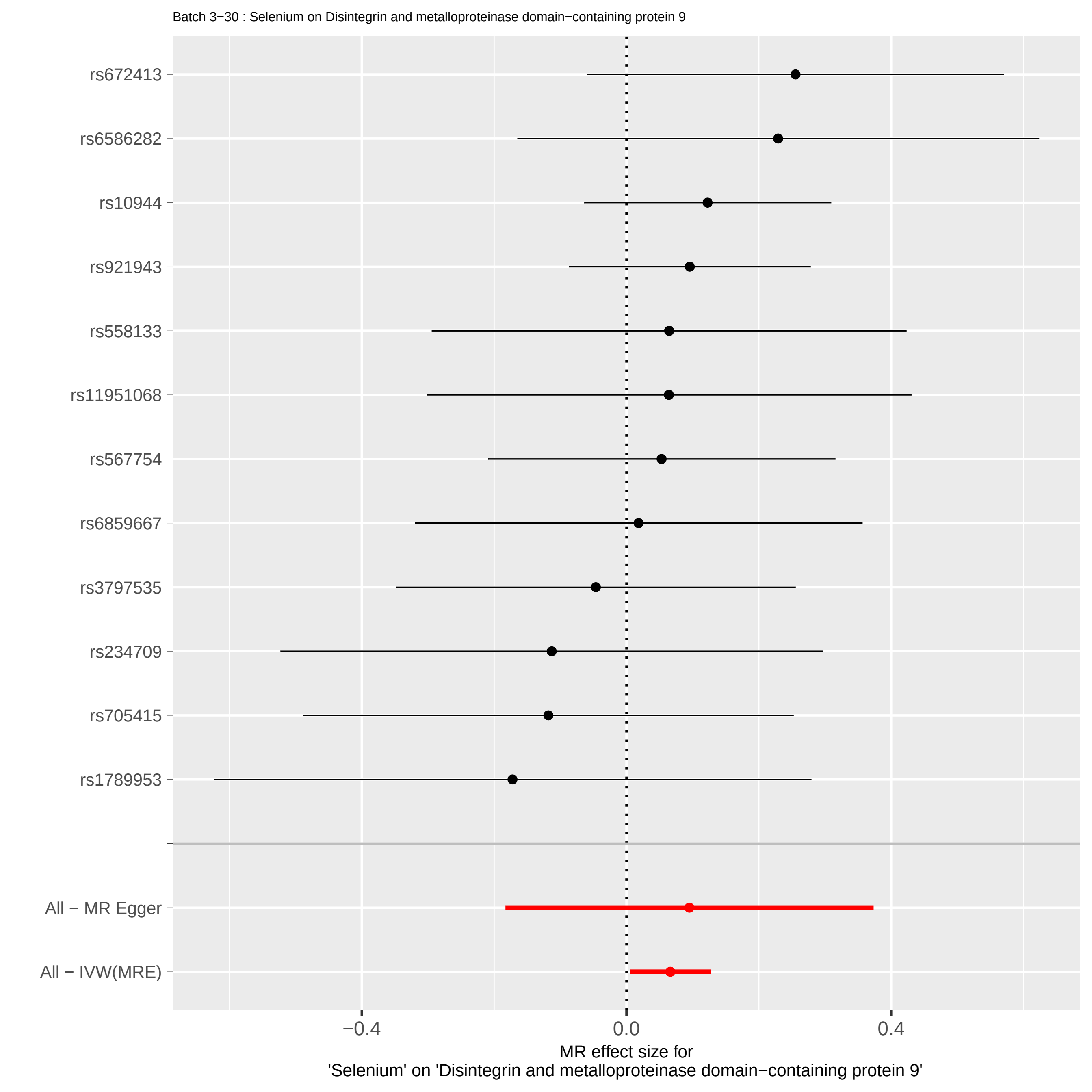

Batch 3−30 : Selenium on Disintegrin and metalloproteinase domain−containing protein 9
rs672413
rs6586282
rs10944
rs921943
rs558133
rs11951068
rs567754
rs6859667
rs3797535
rs234709
rs705415
rs1789953
All − MR Egger
All − IVW(MRE)
−0.4
0.0
0.4
MR effect size for
'Selenium' on 'Disintegrin and metalloproteinase domain−containing protein 9'

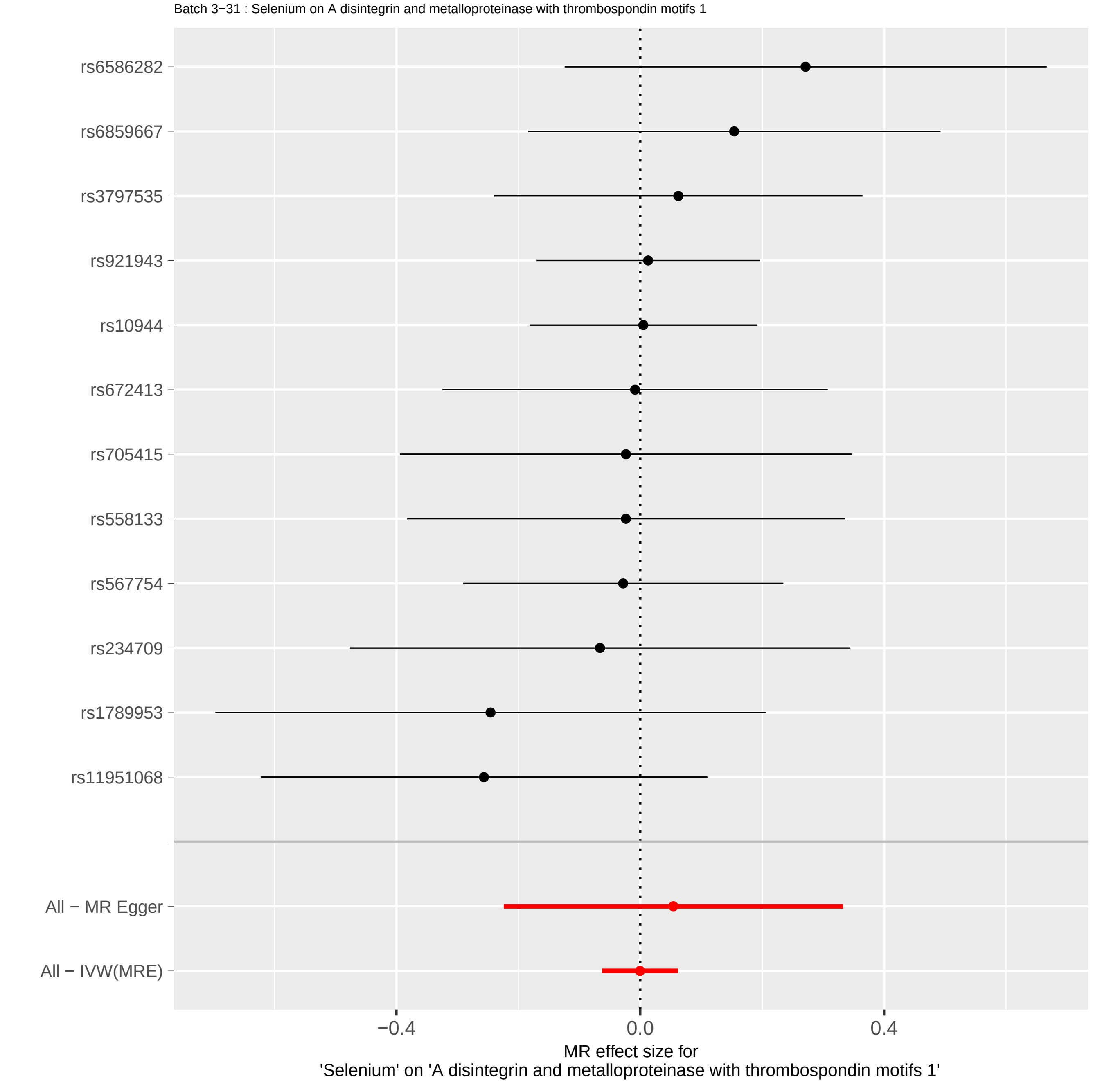

Batch 3−31 : Selenium on A disintegrin and metalloproteinase with thrombospondin motifs 1
rs6586282
rs6859667
rs3797535
rs921943
rs10944
rs672413
rs705415
rs558133
rs567754
rs234709
rs1789953
rs11951068
All − MR Egger
All − IVW(MRE)
−0.4
0.0
0.4
MR effect size for
'Selenium' on 'A disintegrin and metalloproteinase with thrombospondin motifs 1'

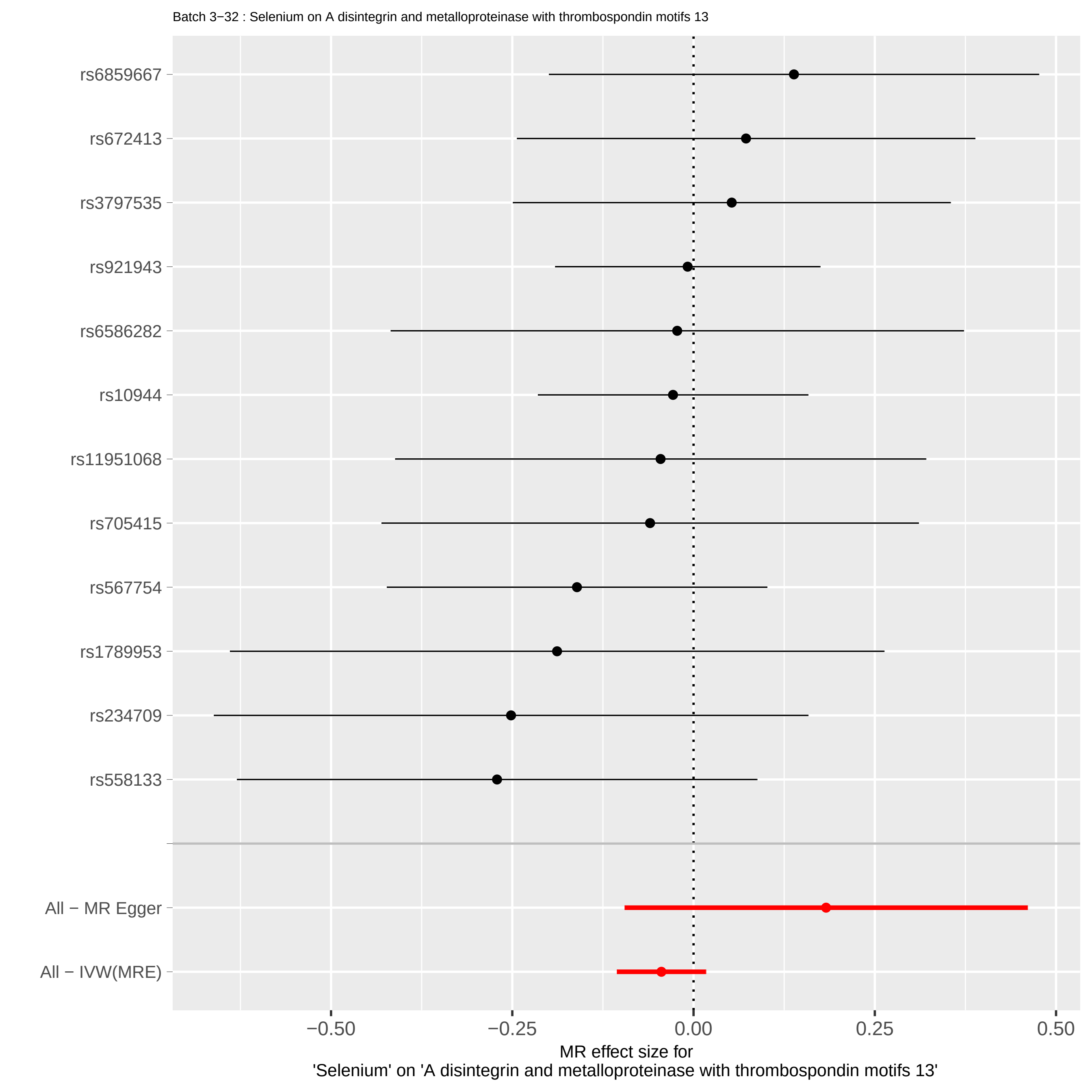
Batch 3−32 : Selenium on A disintegrin and metalloproteinase with thrombospondin motifs 13
rs6859667
rs672413
rs3797535
rs921943
rs6586282
rs10944
rs11951068
rs705415
rs567754
rs1789953
rs234709
rs558133
All − MR Egger
All − IVW(MRE)
−0.50
−0.25
0.00
0.25
0.50
MR effect size for
'Selenium' on 'A disintegrin and metalloproteinase with thrombospondin motifs 13'

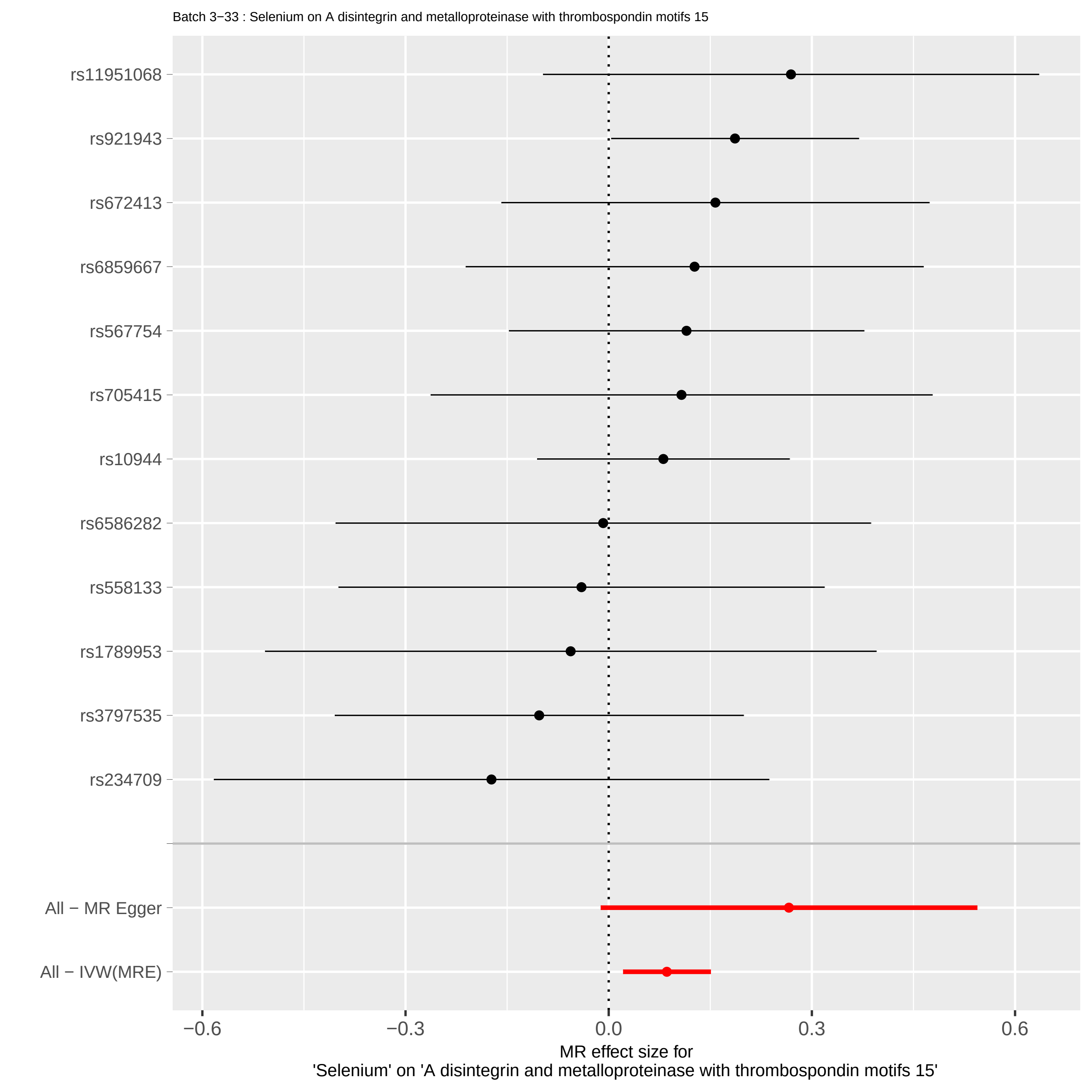

Batch 3−33 : Selenium on A disintegrin and metalloproteinase with thrombospondin motifs 15
rs11951068
rs921943
rs672413
rs6859667
rs567754
rs705415
rs10944
rs6586282
rs558133
rs1789953
rs3797535
rs234709
All − MR Egger
All − IVW(MRE)
−0.6
−0.3
0.0
0.3
0.6
MR effect size for
'Selenium' on 'A disintegrin and metalloproteinase with thrombospondin motifs 15'

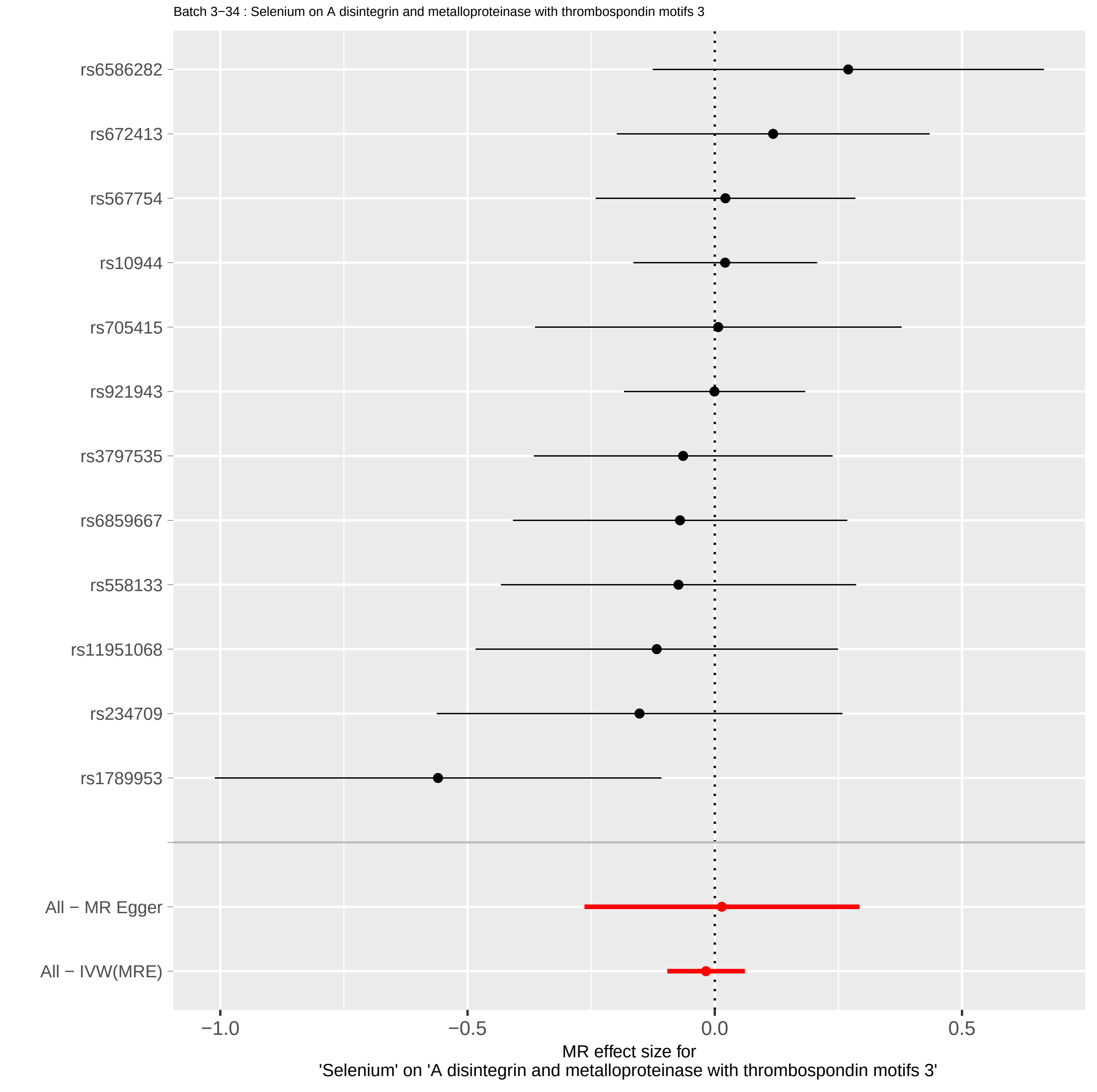

Batch 3−34 : Selenium on A disintegrin and metalloproteinase with thrombospondin motifs 3
rs6586282
rs672413
rs567754
rs10944
rs705415
rs921943
rs3797535
rs6859667
rs558133
rs11951068
rs234709
rs1789953
All − MR Egger
All − IVW(MRE)
−1.0
−0.5
0.0
0.5
MR effect size for
'Selenium' on 'A disintegrin and metalloproteinase with thrombospondin motifs 3'

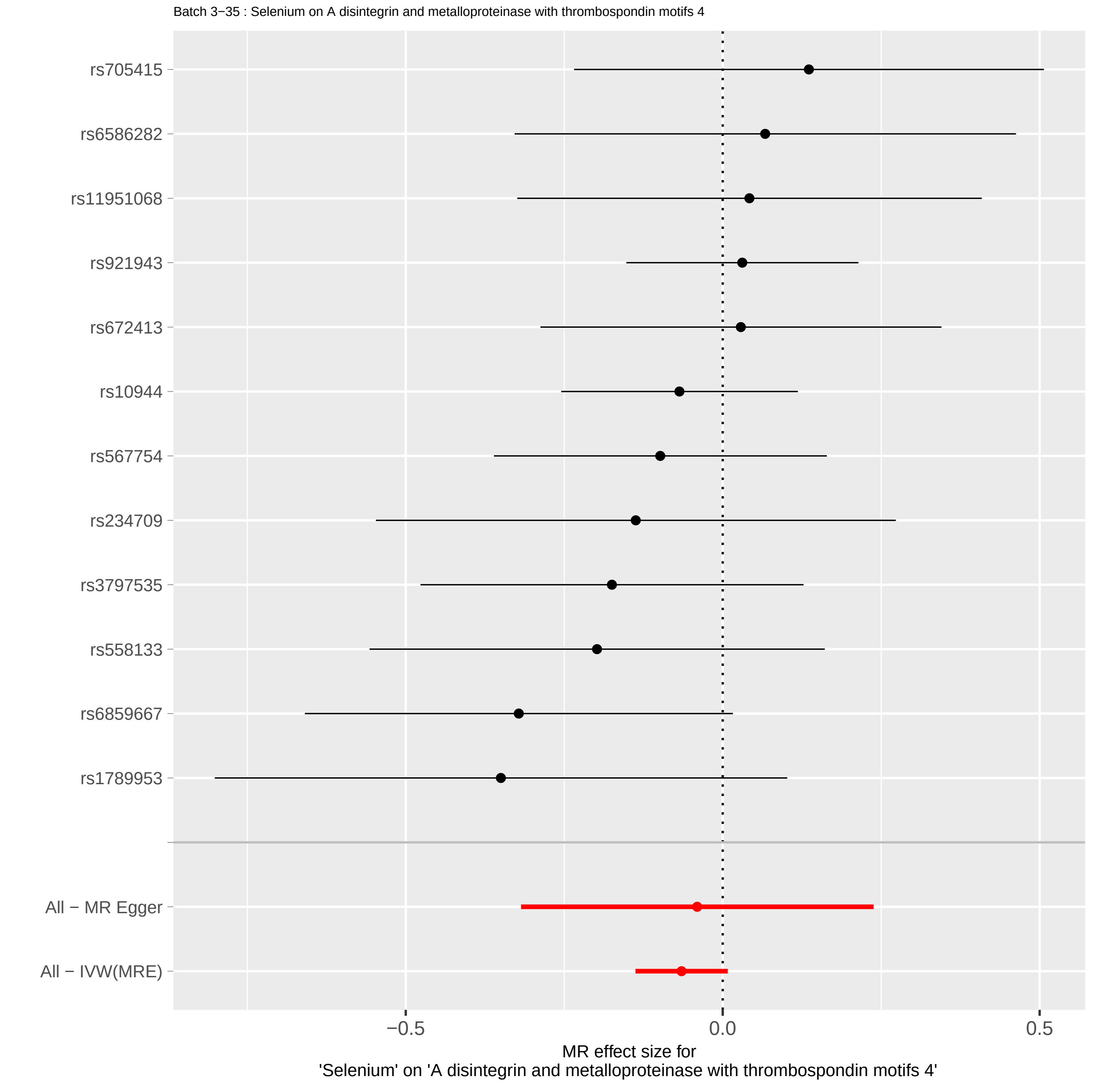

Batch 3−35 : Selenium on A disintegrin and metalloproteinase with thrombospondin motifs 4
rs705415
rs6586282
rs11951068
rs921943
rs672413
rs10944
rs567754
rs234709
rs3797535
rs558133
rs6859667
rs1789953
All − MR Egger
All − IVW(MRE)
−0.5
0.0
0.5
MR effect size for
'Selenium' on 'A disintegrin and metalloproteinase with thrombospondin motifs 4'

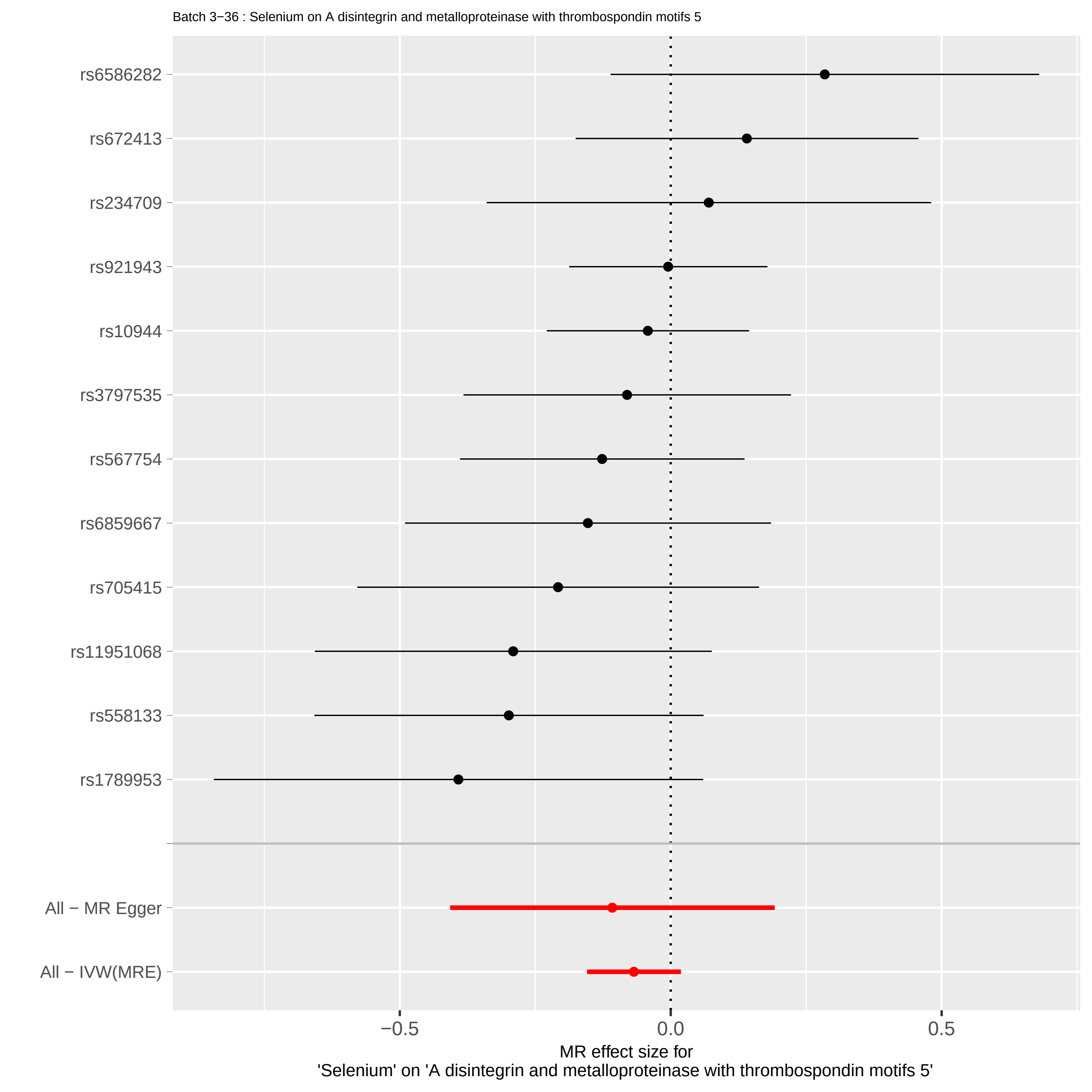
Batch 3−36 : Selenium on A disintegrin and metalloproteinase with thrombospondin motifs 5
rs6586282
rs672413
rs234709
rs921943
rs10944
rs3797535
rs567754
rs6859667
rs705415
rs11951068
rs558133
rs1789953
All − MR Egger
All − IVW(MRE)
−0.5
0.0
0.5
MR effect size for
'Selenium' on 'A disintegrin and metalloproteinase with thrombospondin motifs 5'

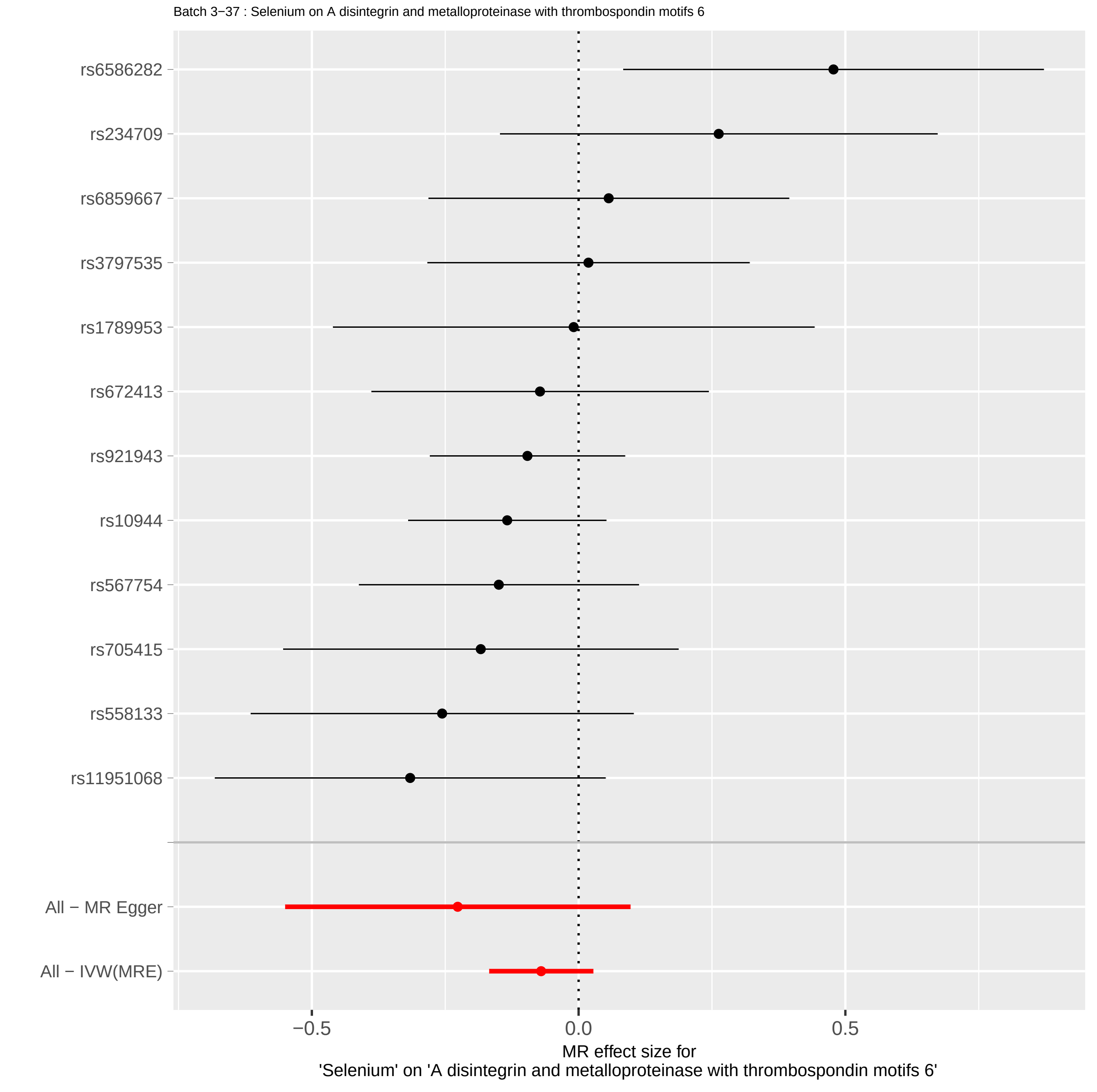
Batch 3−37 : Selenium on A disintegrin and metalloproteinase with thrombospondin motifs 6
rs6586282
rs234709
rs6859667
rs3797535
rs1789953
rs672413
rs921943
rs10944
rs567754
rs705415
rs558133
rs11951068
All − MR Egger
All − IVW(MRE)
−0.5
0.0
0.5
MR effect size for
'Selenium' on 'A disintegrin and metalloproteinase with thrombospondin motifs 6'

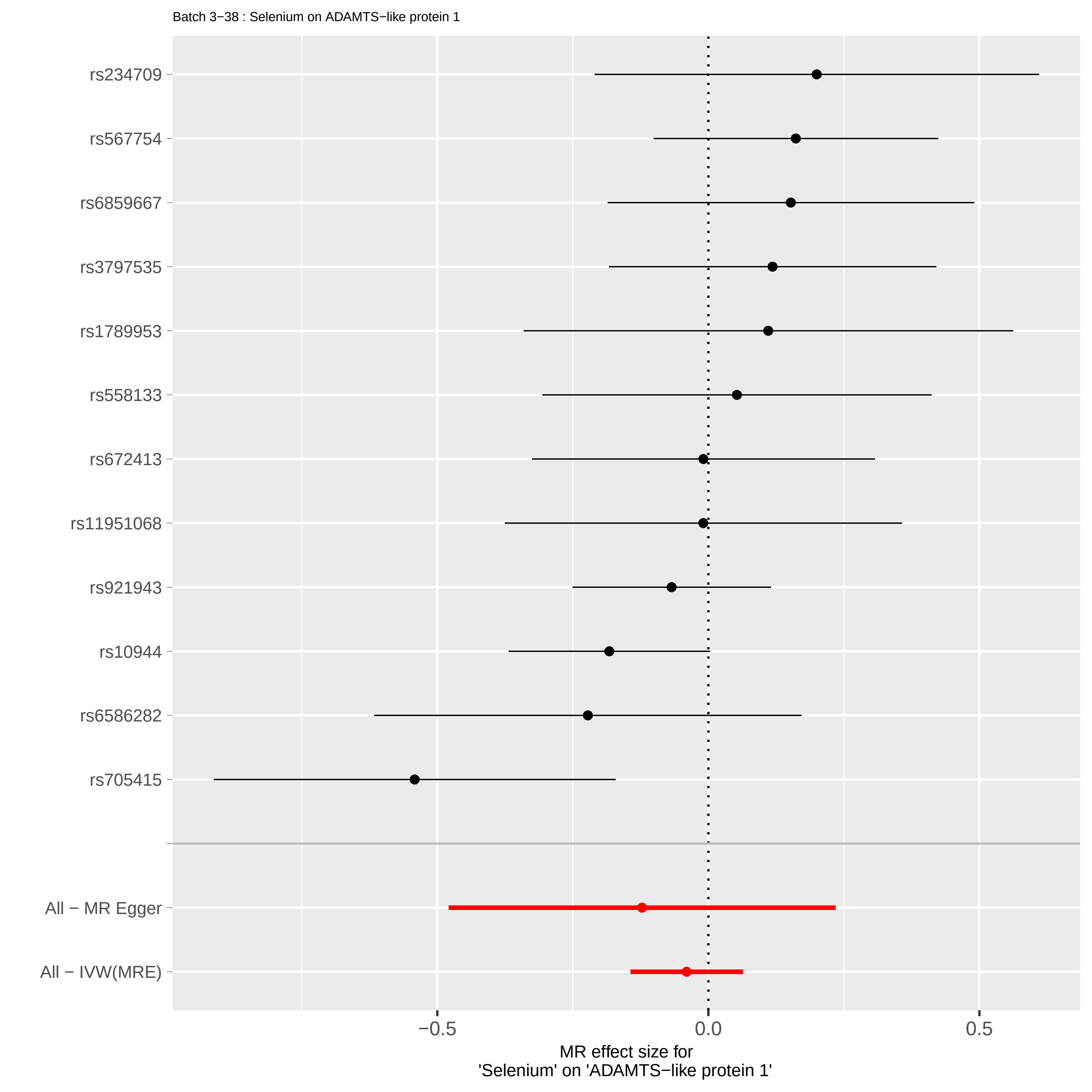

Batch 3−38 : Selenium on ADAMTS−like protein 1
rs234709
rs567754
rs6859667
rs3797535
rs1789953
rs558133
rs672413
rs11951068
rs921943
rs10944
rs6586282
rs705415
All − MR Egger
All − IVW(MRE)
−0.5
0.0
0.5
MR effect size for
'Selenium' on 'ADAMTS−like protein 1'

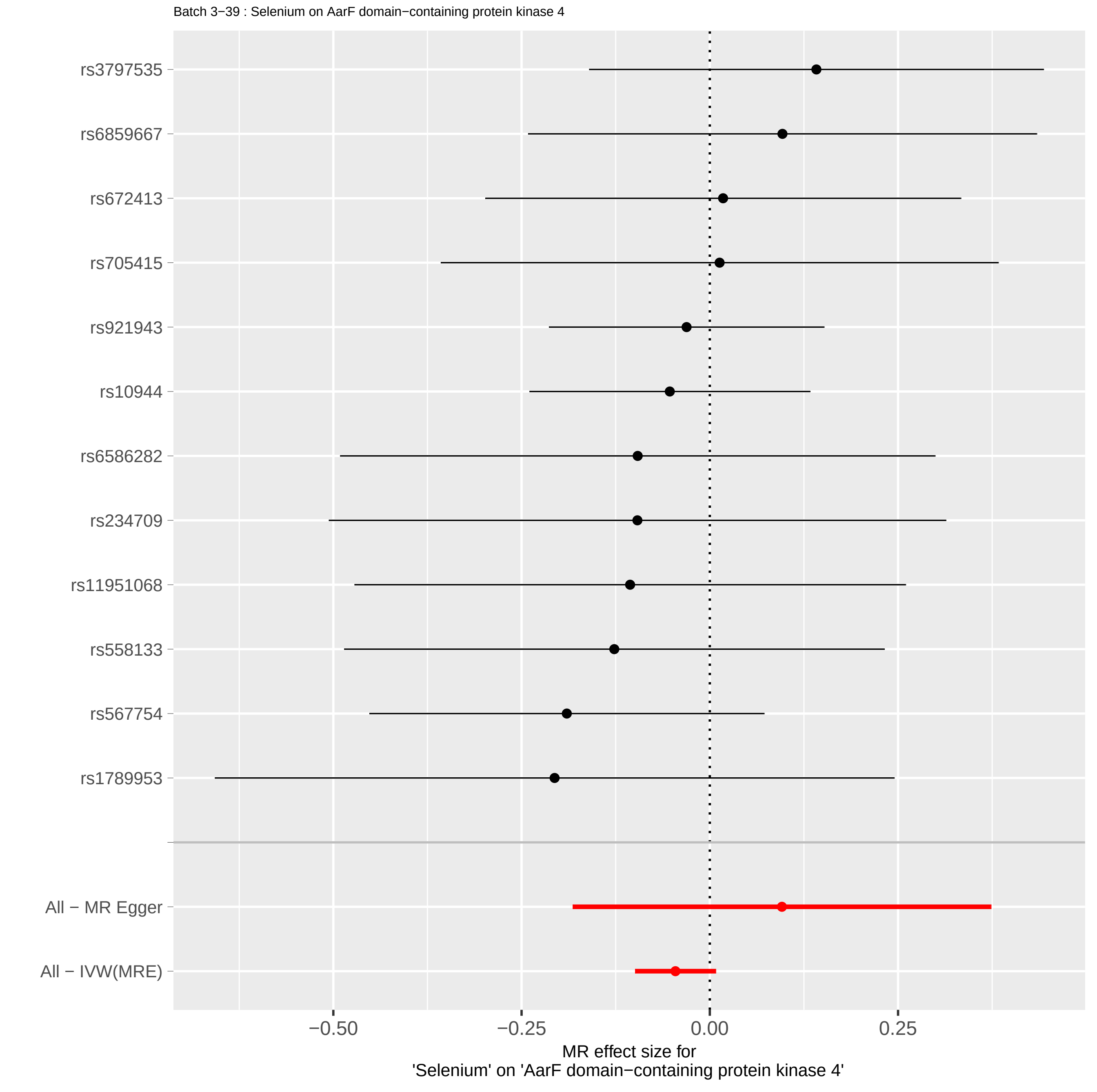
Batch 3−39 : Selenium on AarF domain−containing protein kinase 4
rs3797535
rs6859667
rs672413
rs705415
rs921943
rs10944
rs6586282
rs234709
rs11951068
rs558133
rs567754
rs1789953
All − MR Egger
All − IVW(MRE)
−0.50
−0.25
0.00
0.25
MR effect size for
'Selenium' on 'AarF domain−containing protein kinase 4'

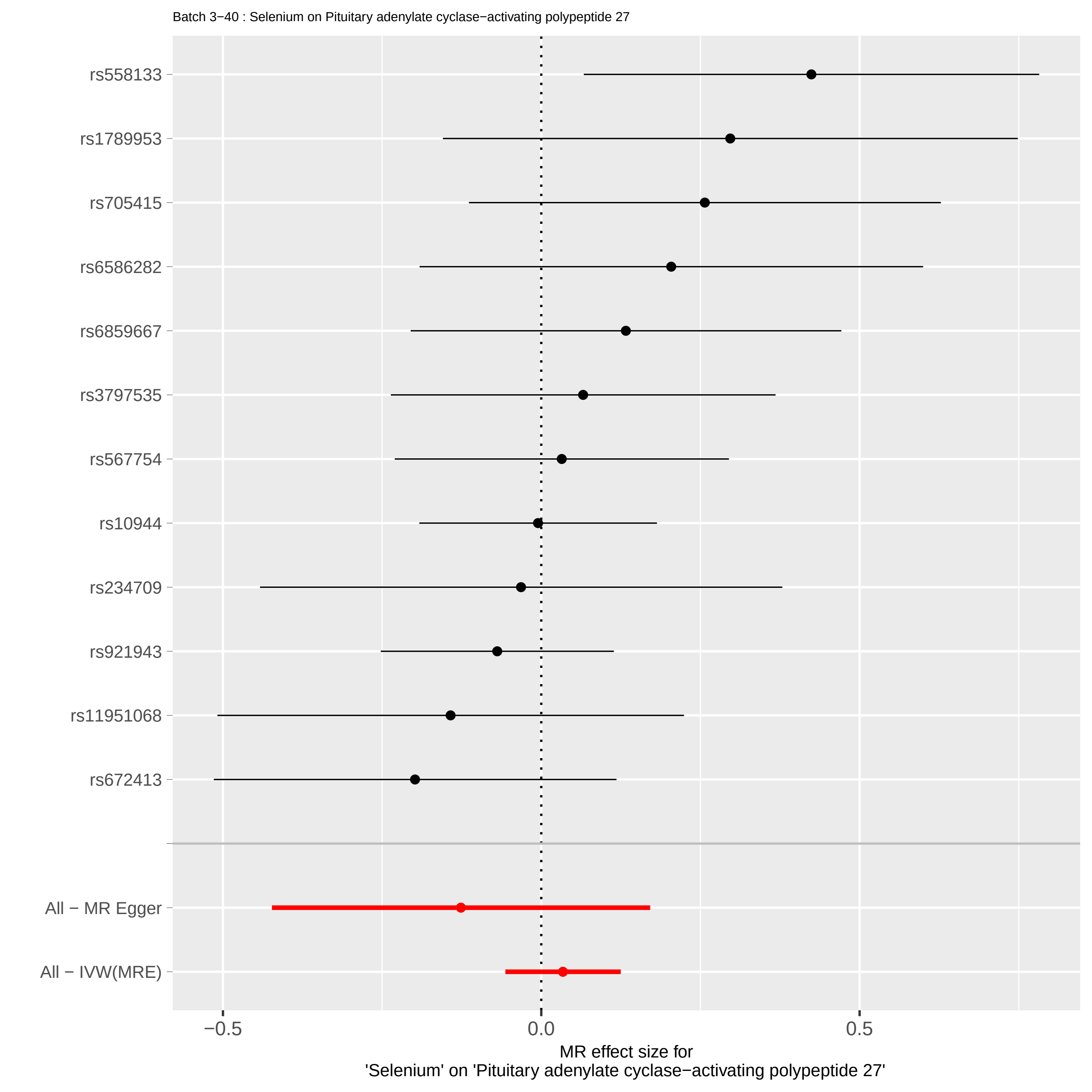
Batch 3−40 : Selenium on Pituitary adenylate cyclase−activating polypeptide 27
rs558133
rs1789953
rs705415
rs6586282
rs6859667
rs3797535
rs567754
rs10944
rs234709
rs921943
rs11951068
rs672413
All − MR Egger
All − IVW(MRE)
−0.5
0.0
0.5
MR effect size for
'Selenium' on 'Pituitary adenylate cyclase−activating polypeptide 27'

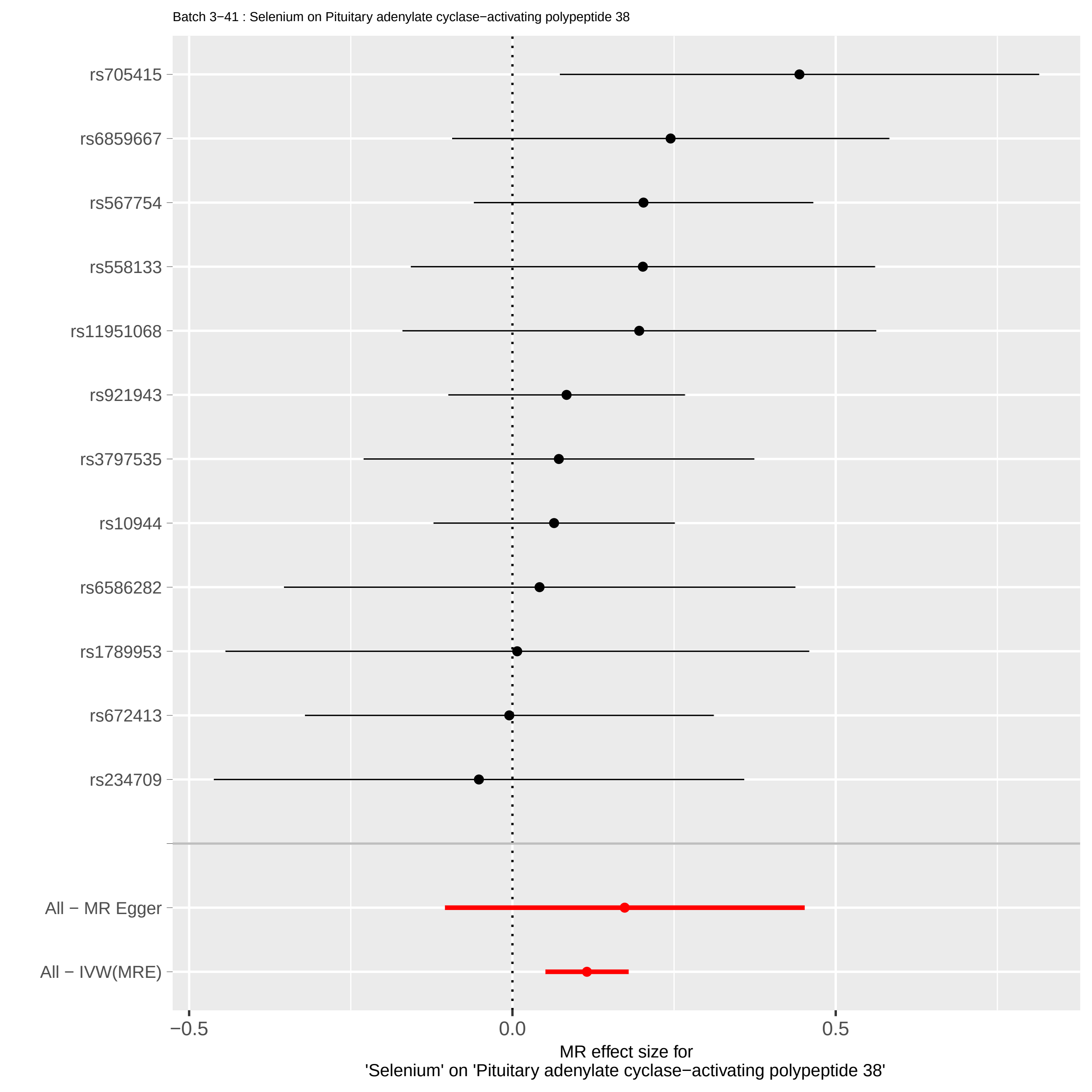

Batch 3−41 : Selenium on Pituitary adenylate cyclase−activating polypeptide 38
rs705415
rs6859667
rs567754
rs558133
rs11951068
rs921943
rs3797535
rs10944
rs6586282
rs1789953
rs672413
rs234709
All − MR Egger
All − IVW(MRE)
−0.5
0.0
0.5
MR effect size for
'Selenium' on 'Pituitary adenylate cyclase−activating polypeptide 38'

Batch 3−42 : Selenium on Pituitary adenylate cyclase−activating polypeptide

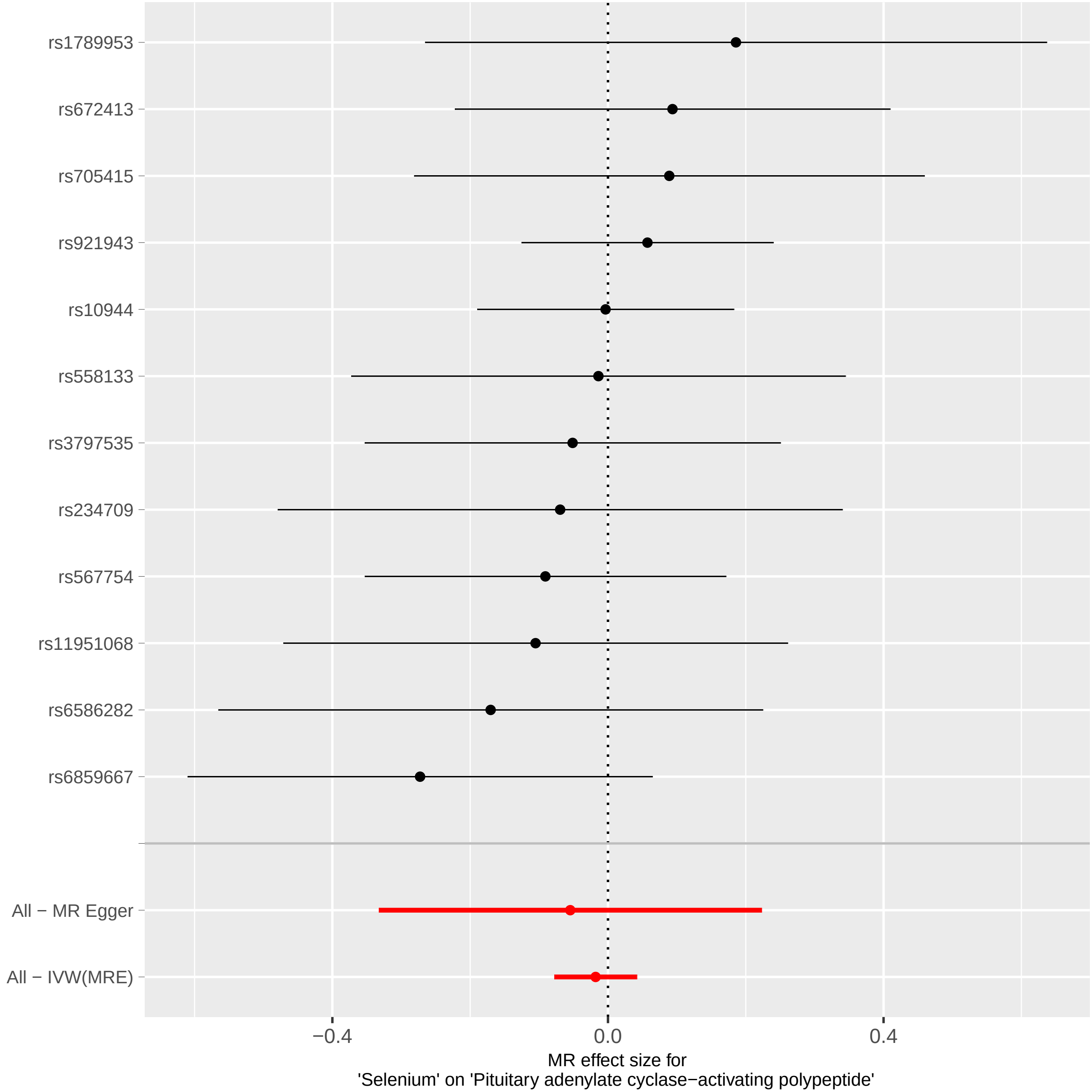

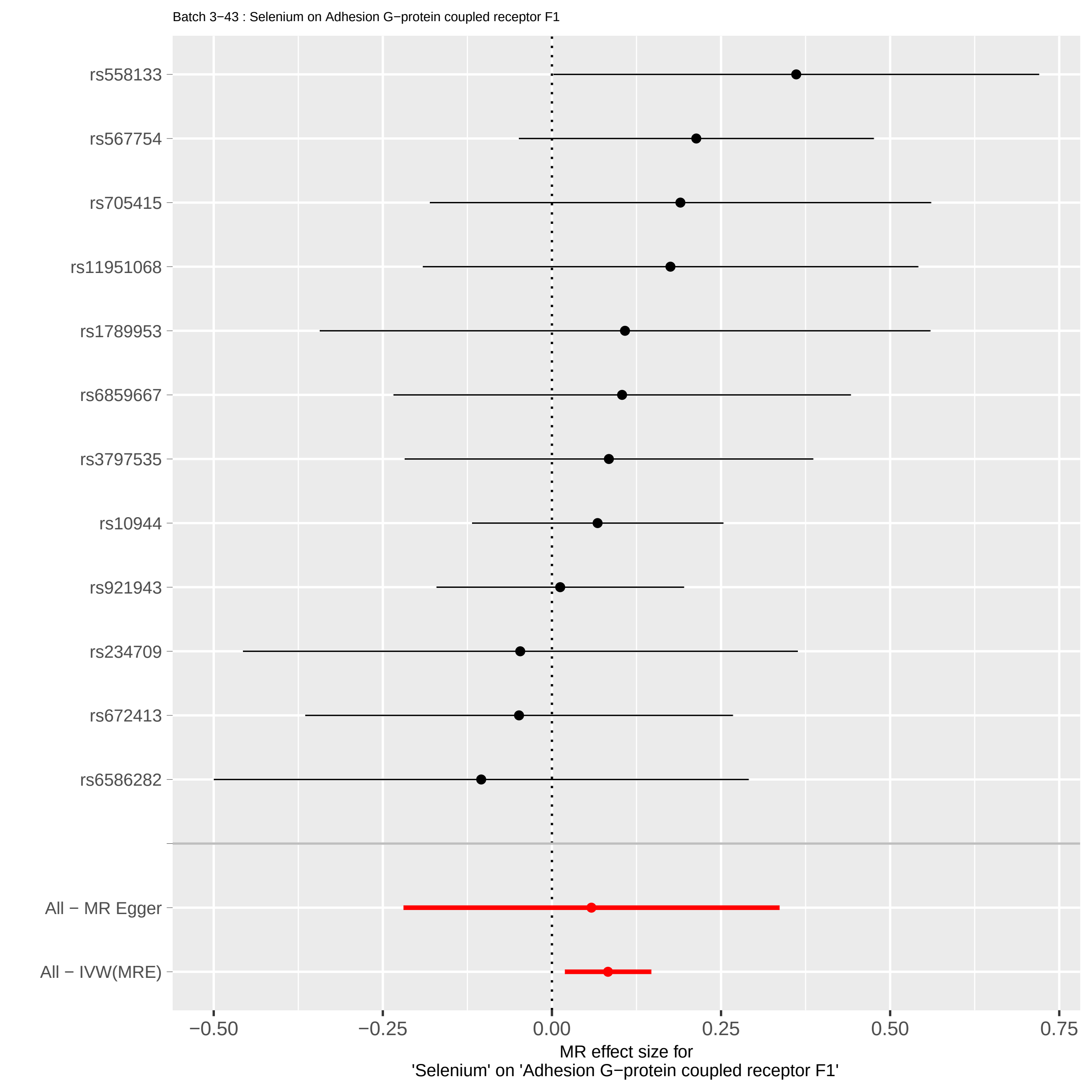

Batch 3−43 : Selenium on Adhesion G−protein coupled receptor F1
rs558133
rs567754
rs705415
rs11951068
rs1789953
rs6859667
rs3797535
rs10944
rs921943
rs234709
rs672413
rs6586282
All − MR Egger
All − IVW(MRE)
−0.50
−0.25
0.00
0.25
0.50
0.75
MR effect size for
'Selenium' on 'Adhesion G−protein coupled receptor F1'

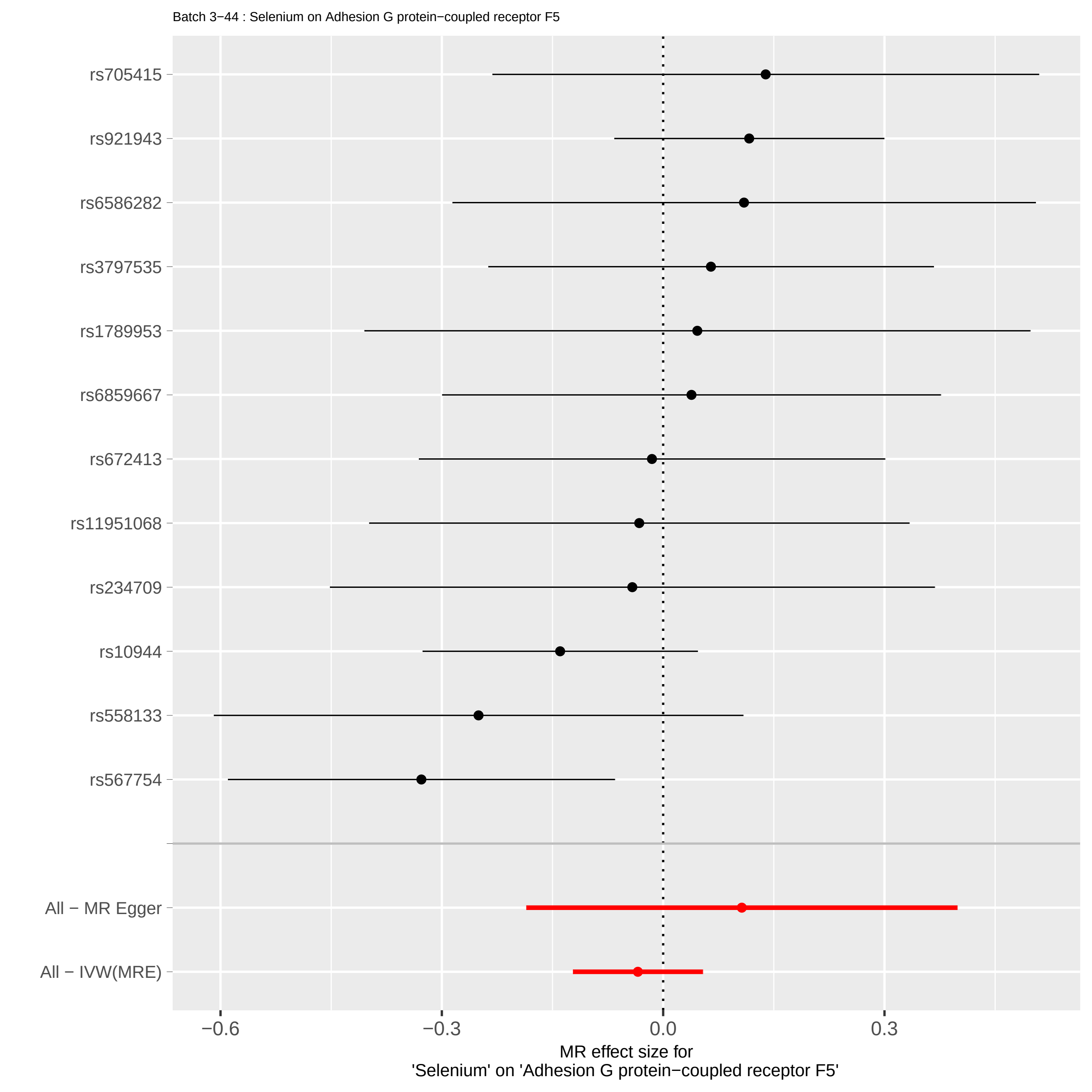

Batch 3−44 : Selenium on Adhesion G protein−coupled receptor F5
rs705415
rs921943
rs6586282
rs3797535
rs1789953
rs6859667
rs672413
rs11951068
rs234709
rs10944
rs558133
rs567754
All − MR Egger
All − IVW(MRE)
−0.6
−0.3
0.0
0.3
MR effect size for
'Selenium' on 'Adhesion G protein−coupled receptor F5'

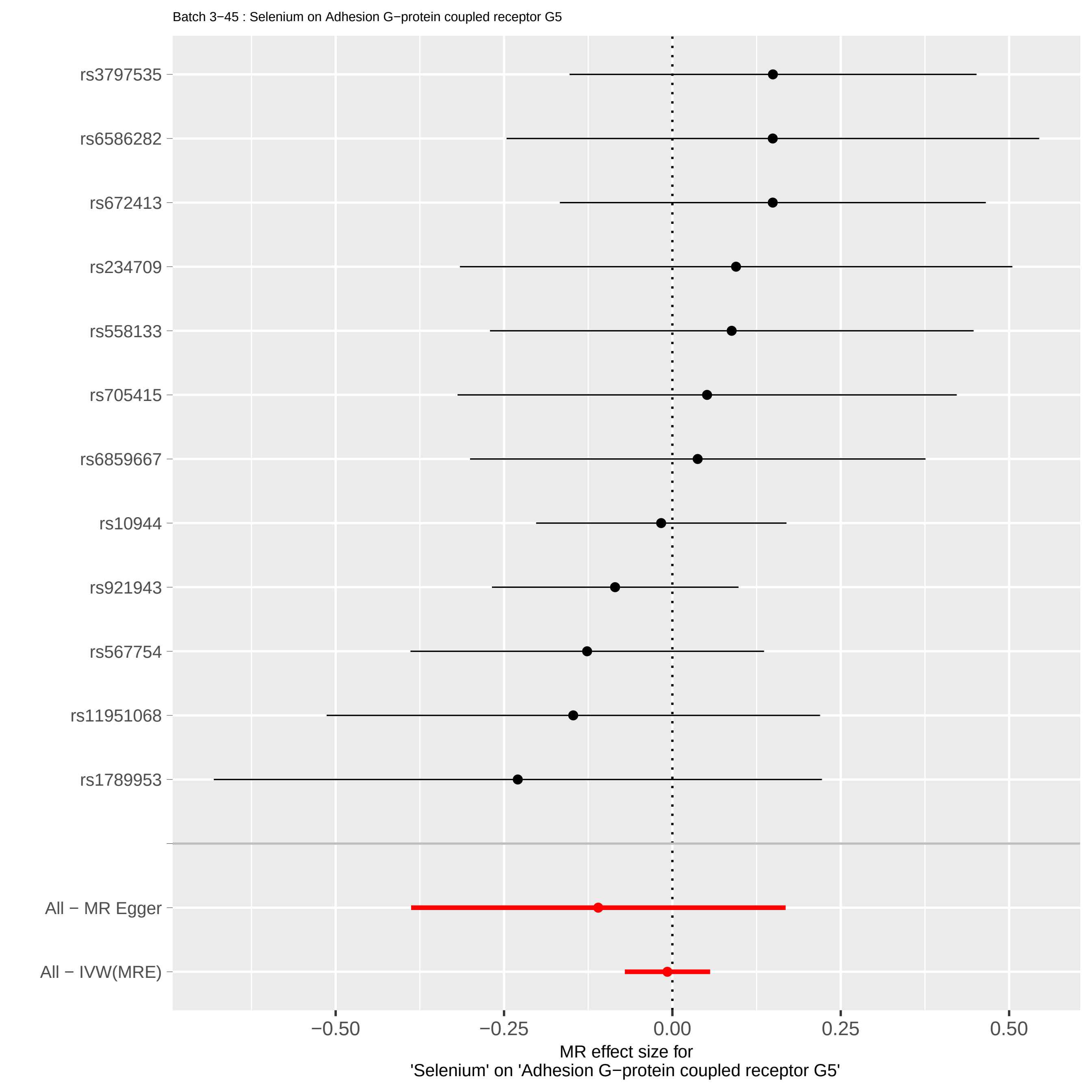
Batch 3−45 : Selenium on Adhesion G−protein coupled receptor G5
rs3797535
rs6586282
rs672413
rs234709
rs558133
rs705415
rs6859667
rs10944
rs921943
rs567754
rs11951068
rs1789953
All − MR Egger
All − IVW(MRE)
−0.50
−0.25
0.00
0.25
0.50
MR effect size for
'Selenium' on 'Adhesion G−protein coupled receptor G5'

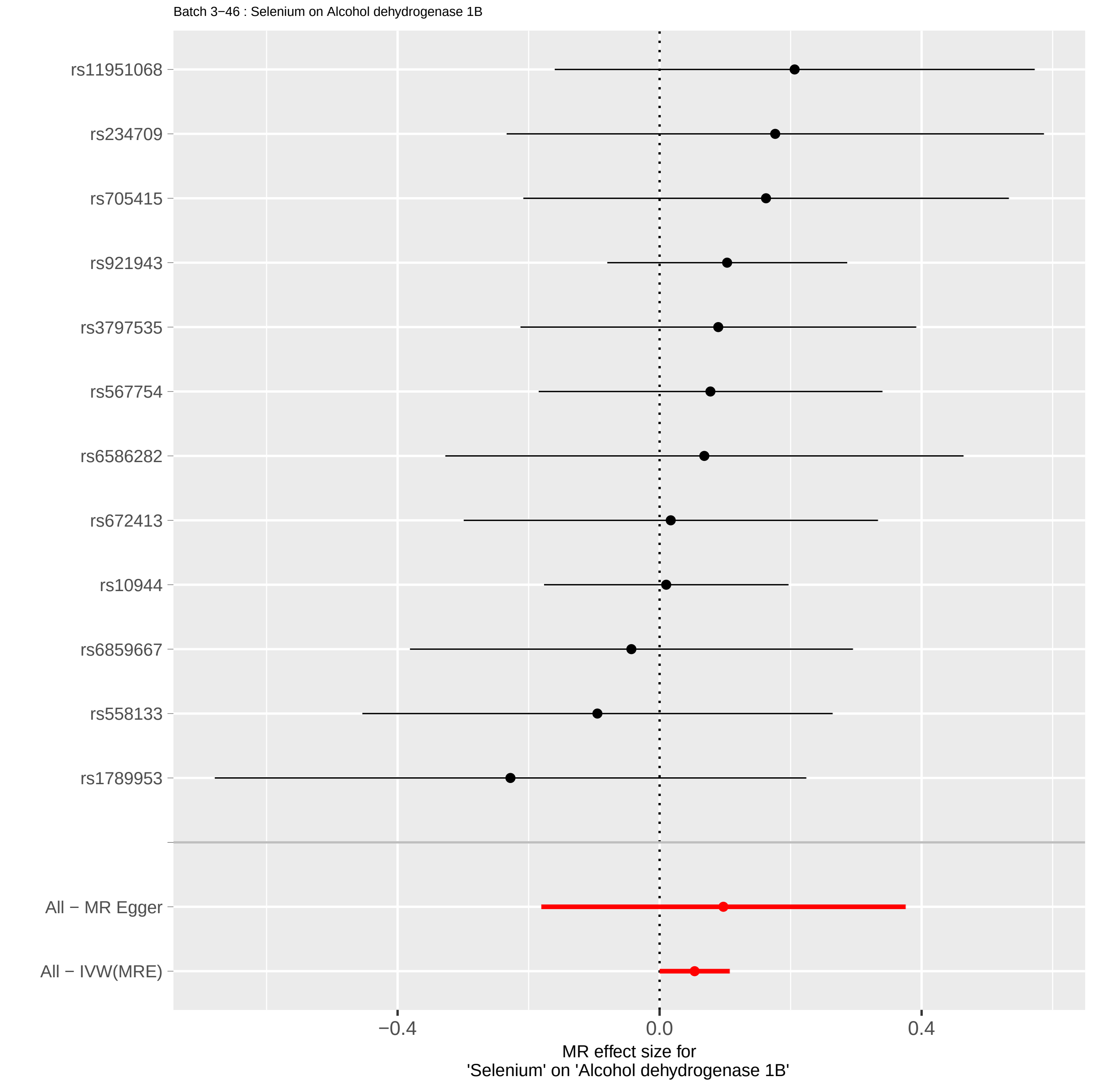
Batch 3−46 : Selenium on Alcohol dehydrogenase 1B
rs11951068
rs234709
rs705415
rs921943
rs3797535
rs567754
rs6586282
rs672413
rs10944
rs6859667
rs558133
rs1789953
All − MR Egger
All − IVW(MRE)
−0.4
0.0
0.4
MR effect size for
'Selenium' on 'Alcohol dehydrogenase 1B'

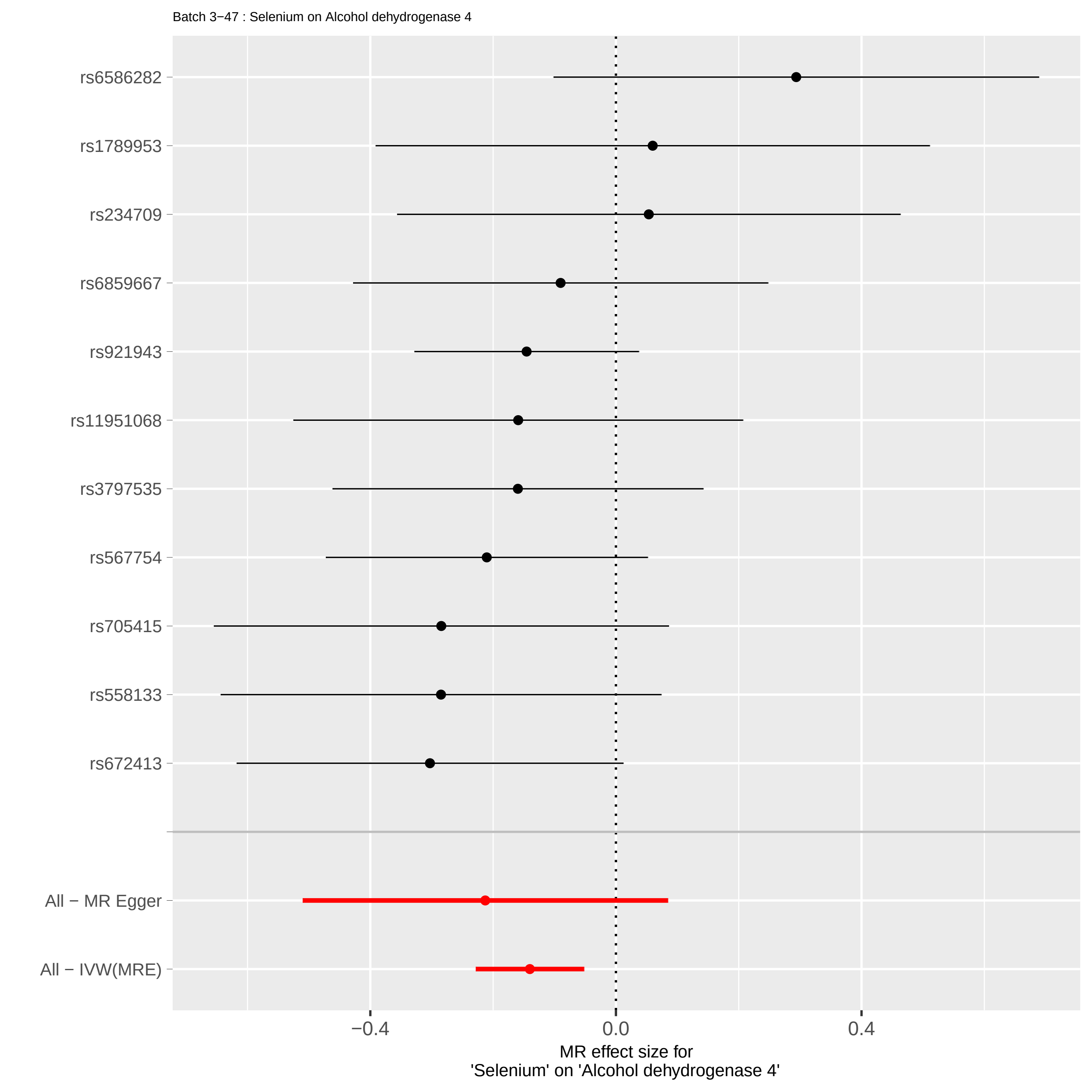
Batch 3−47 : Selenium on Alcohol dehydrogenase 4
rs6586282
rs1789953
rs234709
rs6859667
rs921943
rs11951068
rs3797535
rs567754
rs705415
rs558133
rs672413
All − MR Egger
All − IVW(MRE)
−0.4
0.0
0.4
MR effect size for
'Selenium' on 'Alcohol dehydrogenase 4'

Batch 3−48 : Selenium on Adrenomedullin

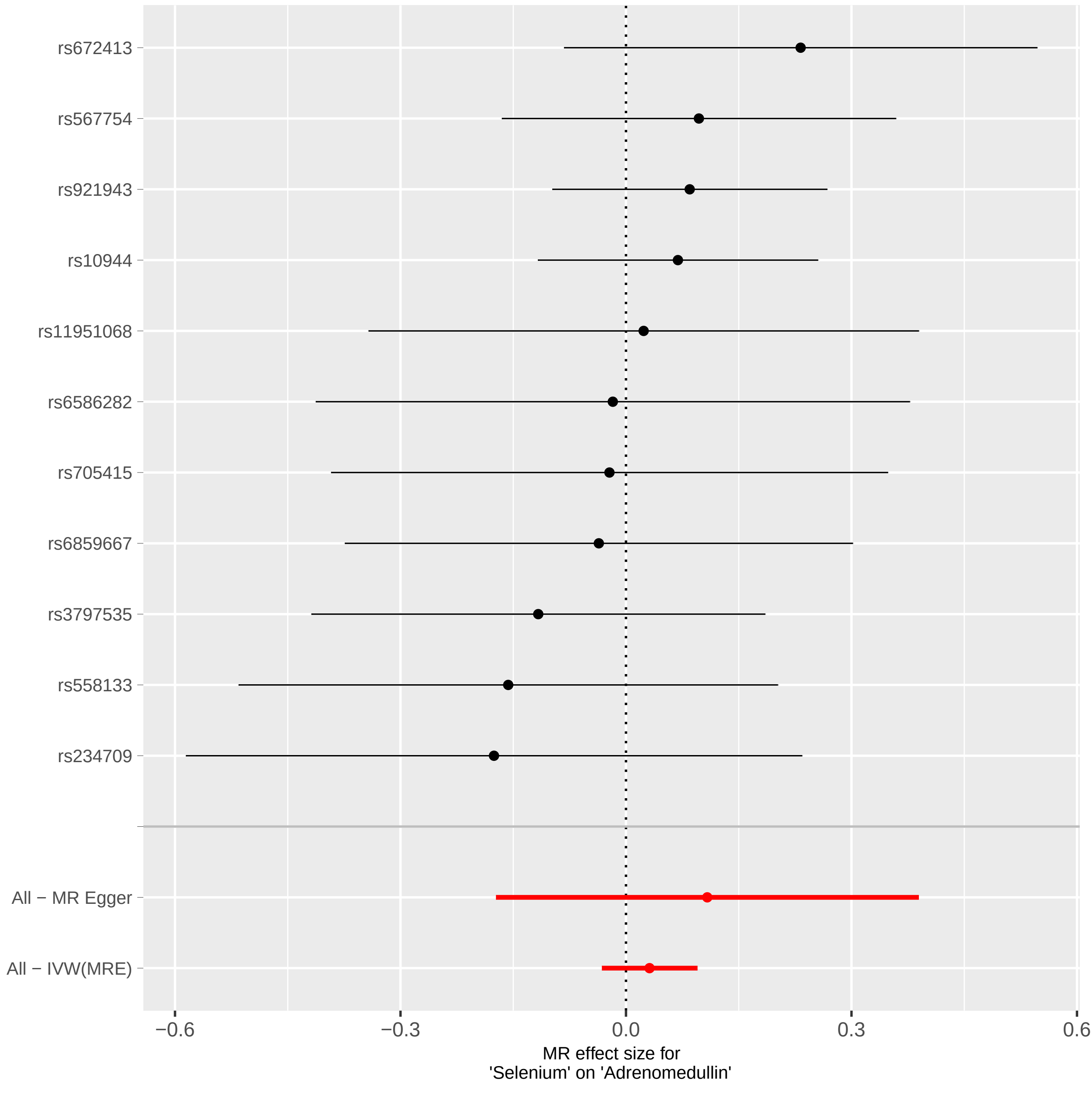

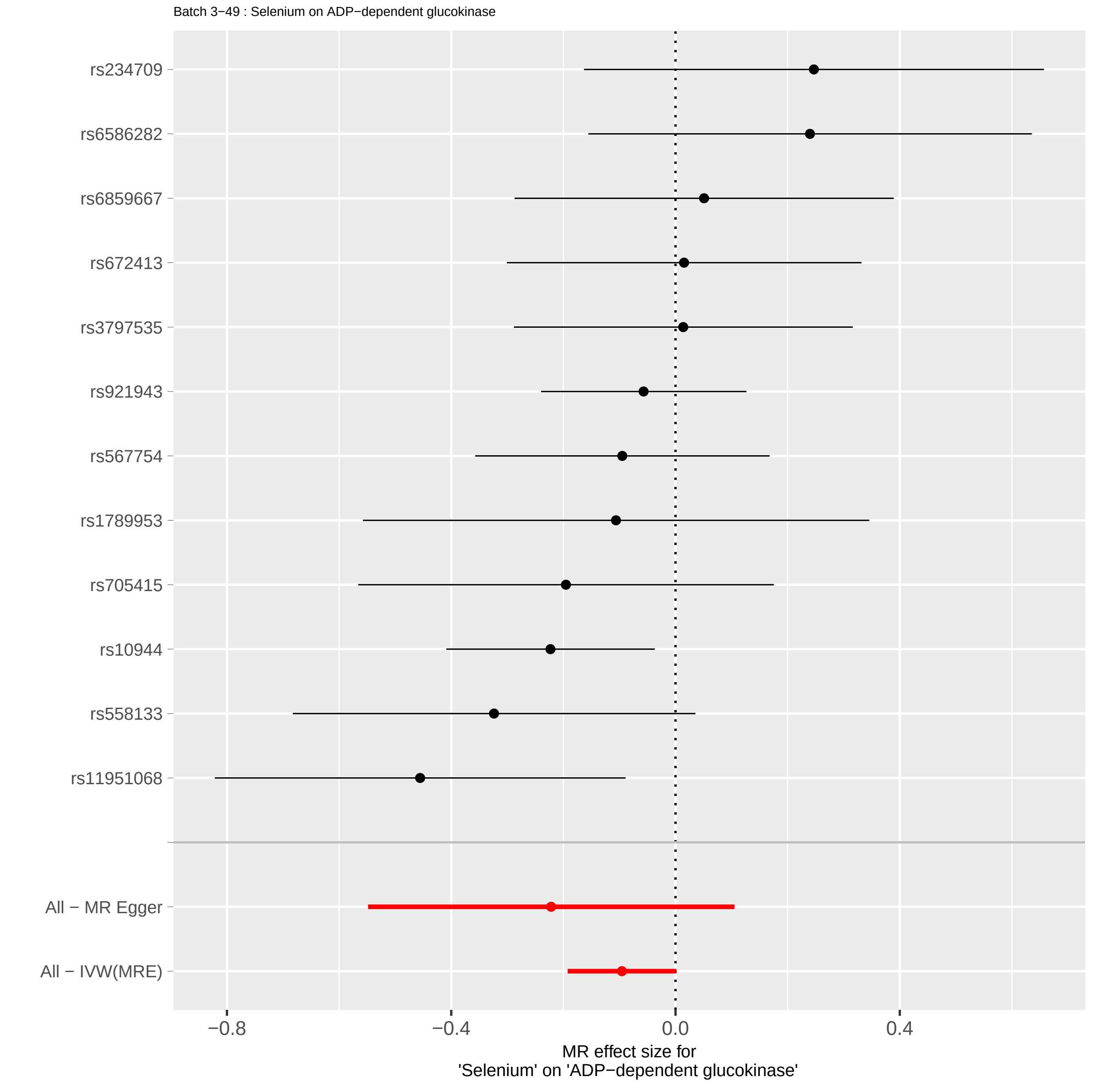

Batch 3−49 : Selenium on ADP−dependent glucokinase
rs234709
rs6586282
rs6859667
rs672413
rs3797535
rs921943
rs567754
rs1789953
rs705415
rs10944
rs558133
rs11951068
All − MR Egger
All − IVW(MRE)
−0.8
−0.4
0.0
0.4
MR effect size for
'Selenium' on 'ADP−dependent glucokinase'

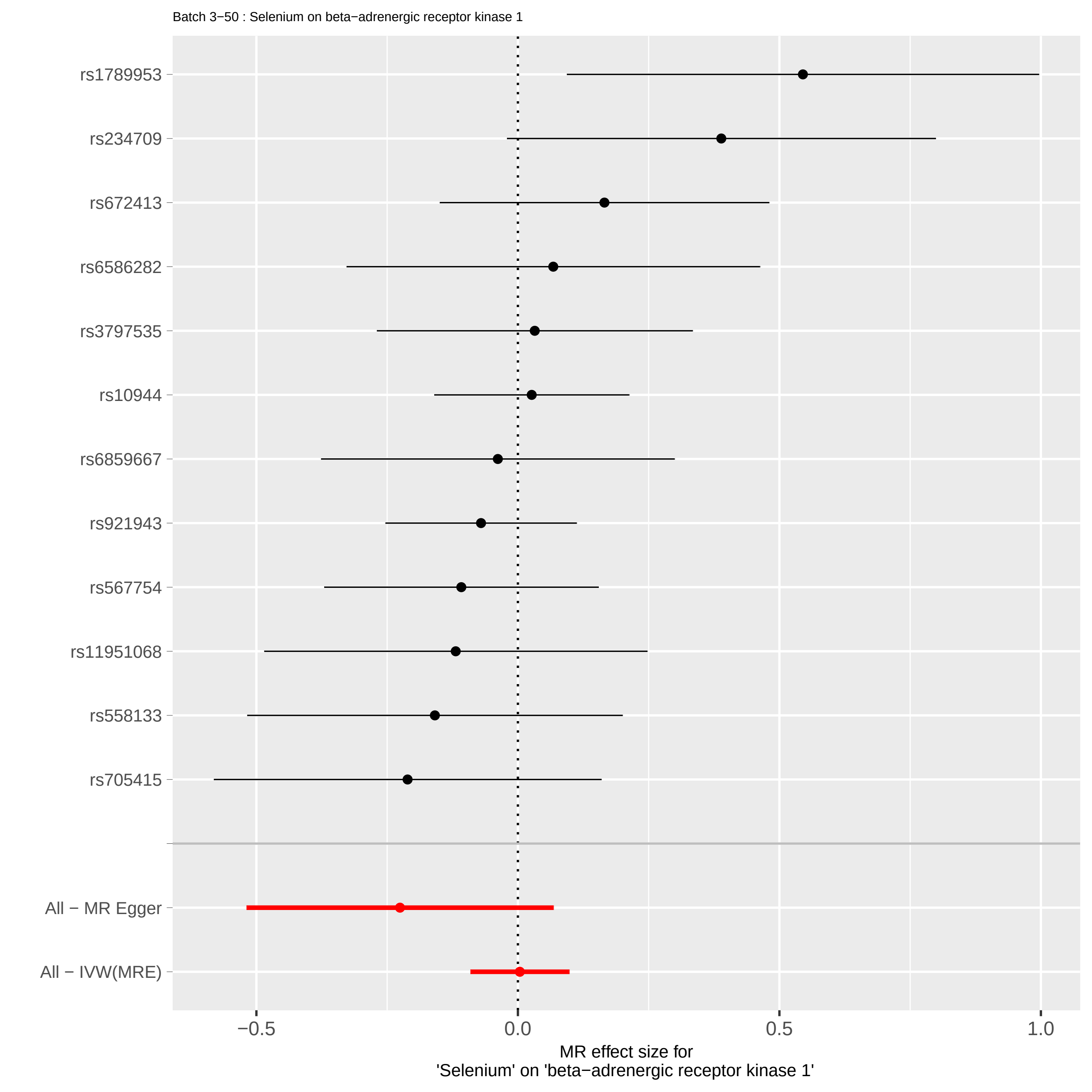

Batch 3−50 : Selenium on beta−adrenergic receptor kinase 1
rs1789953
rs234709
rs672413
rs6586282
rs3797535
rs10944
rs6859667
rs921943
rs567754
rs11951068
rs558133
rs705415
All − MR Egger
All − IVW(MRE)
−0.5
0.0
0.5
1.0
MR effect size for
'Selenium' on 'beta−adrenergic receptor kinase 1'

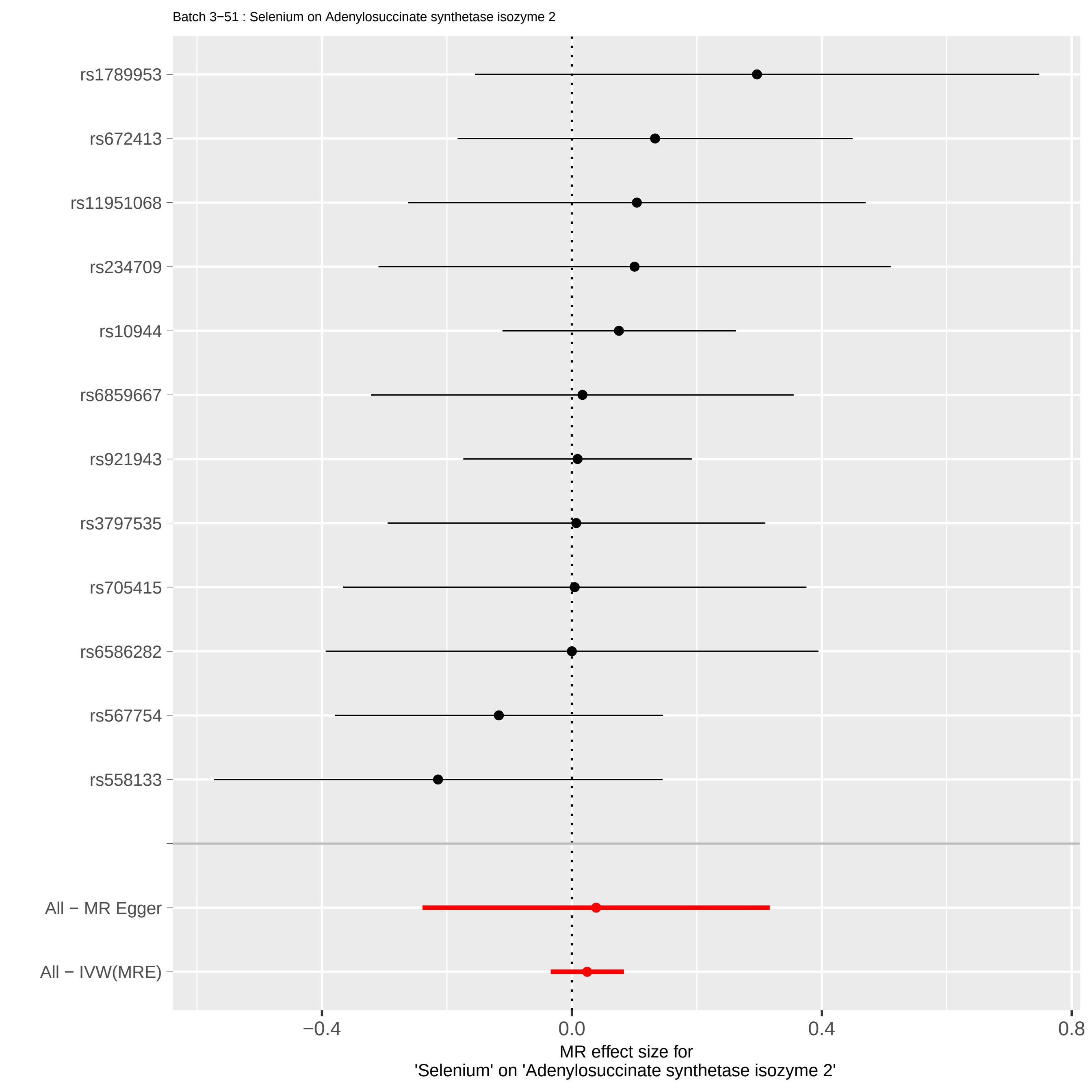
Batch 3−51 : Selenium on Adenylosuccinate synthetase isozyme 2
rs1789953
rs672413
rs11951068
rs234709
rs10944
rs6859667
rs921943
rs3797535
rs705415
rs6586282
rs567754
rs558133
All − MR Egger
All − IVW(MRE)
−0.4
0.0
0.4
0.8
MR effect size for
'Selenium' on 'Adenylosuccinate synthetase isozyme 2'

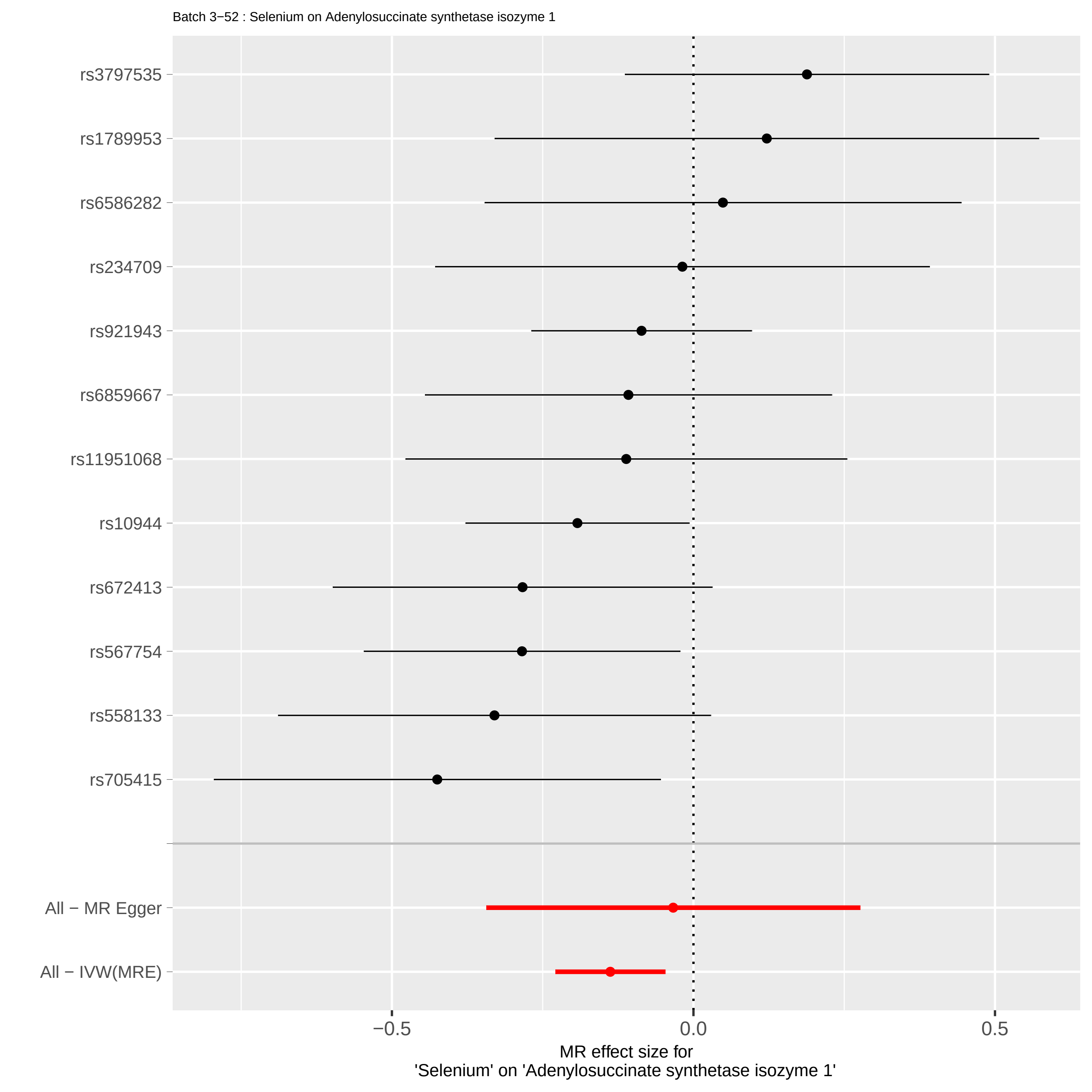

Batch 3−52 : Selenium on Adenylosuccinate synthetase isozyme 1
rs3797535
rs1789953
rs6586282
rs234709
rs921943
rs6859667
rs11951068
rs10944
rs672413
rs567754
rs558133
rs705415
All − MR Egger
All − IVW(MRE)
−0.5
0.0
0.5
MR effect size for
'Selenium' on 'Adenylosuccinate synthetase isozyme 1'

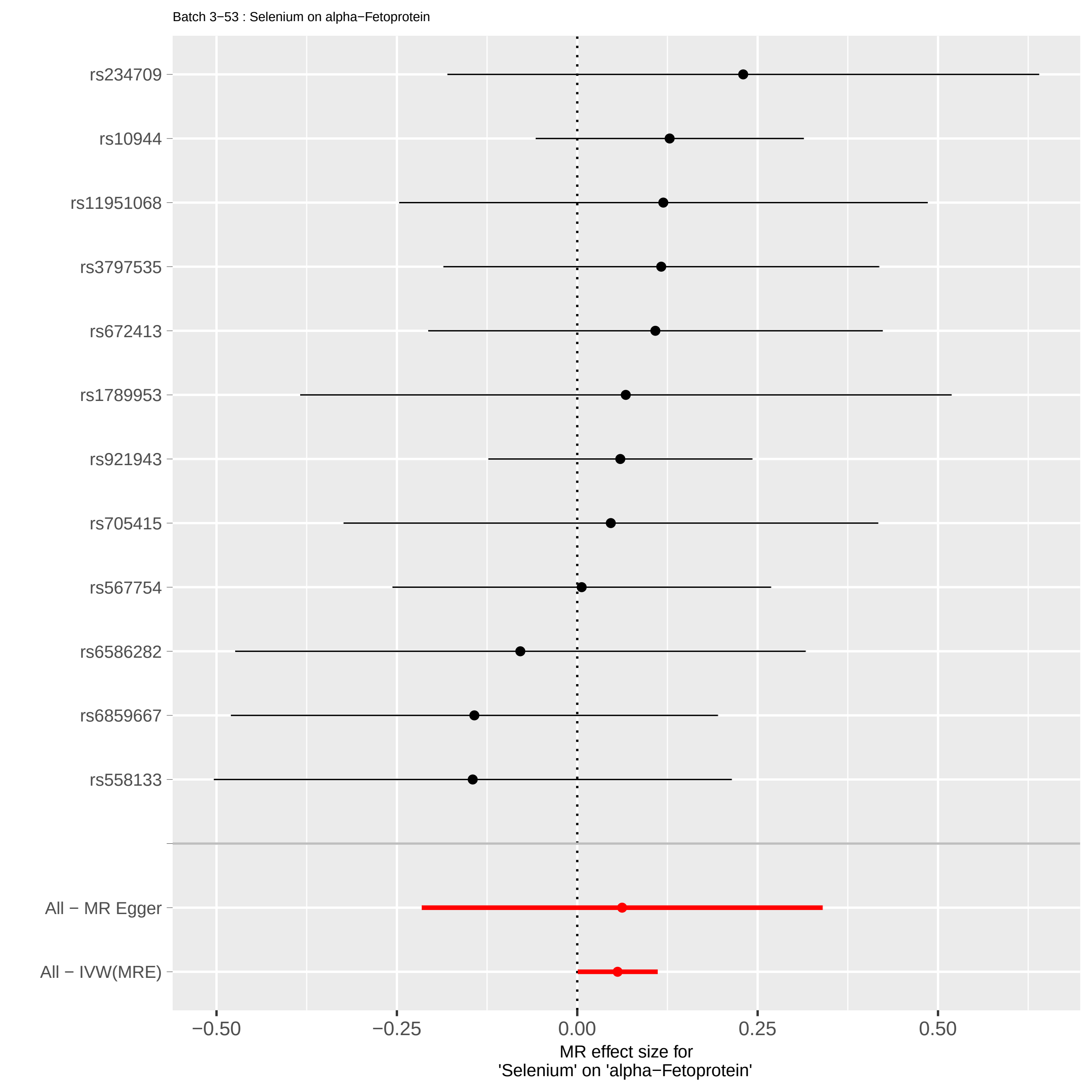

Batch 3−53 : Selenium on alpha−Fetoprotein
rs234709
rs10944
rs11951068
rs3797535
rs672413
rs1789953
rs921943
rs705415
rs567754
rs6586282
rs6859667
rs558133
All − MR Egger
All − IVW(MRE)
−0.50
−0.25
0.00
0.25
0.50
MR effect size for
'Selenium' on 'alpha−Fetoprotein'

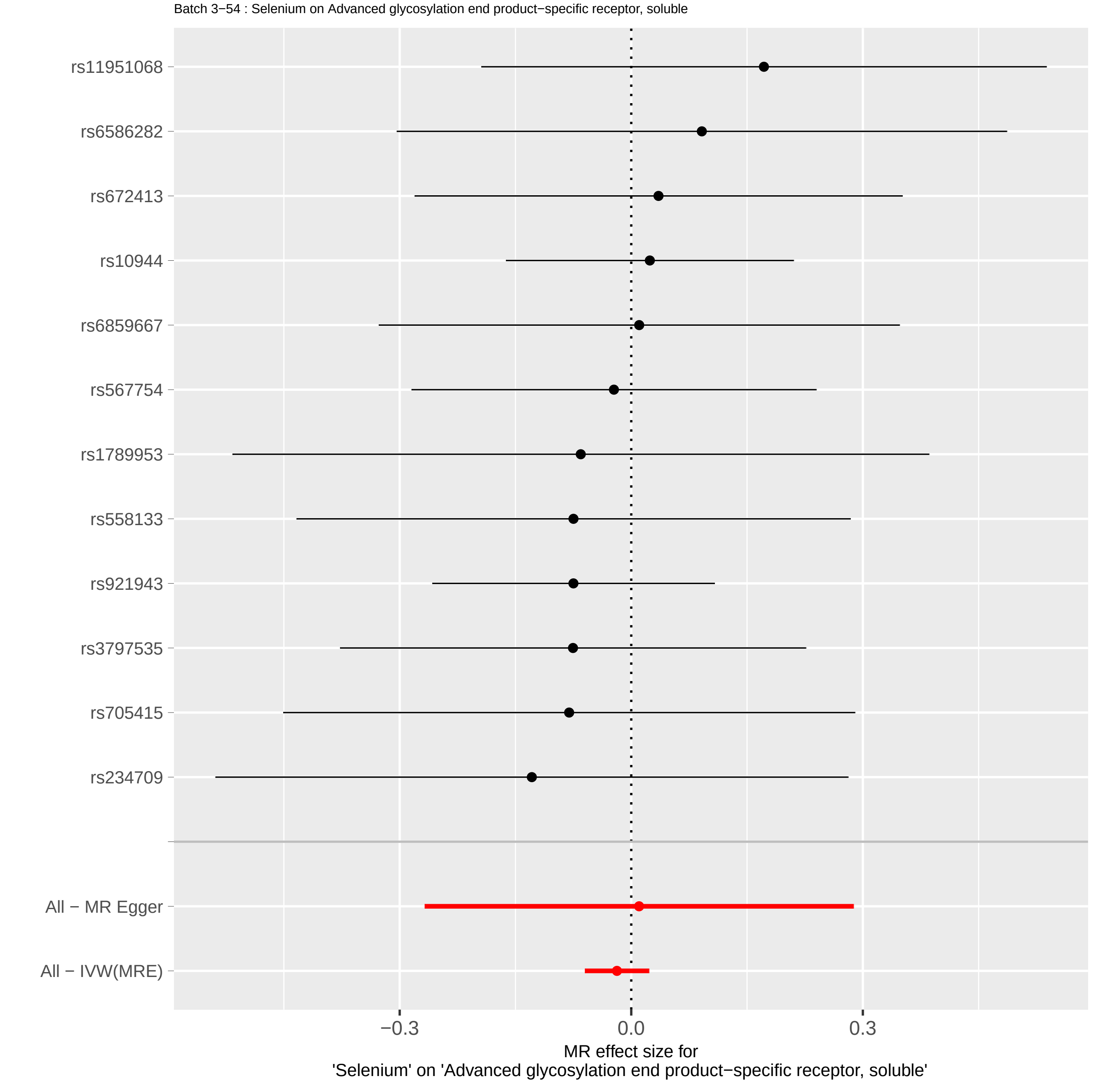

Batch 3−54 : Selenium on Advanced glycosylation end product−specific receptor, soluble
rs11951068
rs6586282
rs672413
rs10944
rs6859667
rs567754
rs1789953
rs558133
rs921943
rs3797535
rs705415
rs234709
All − MR Egger
All − IVW(MRE)
−0.3
0.0
0.3
MR effect size for
'Selenium' on 'Advanced glycosylation end product−specific receptor, soluble'

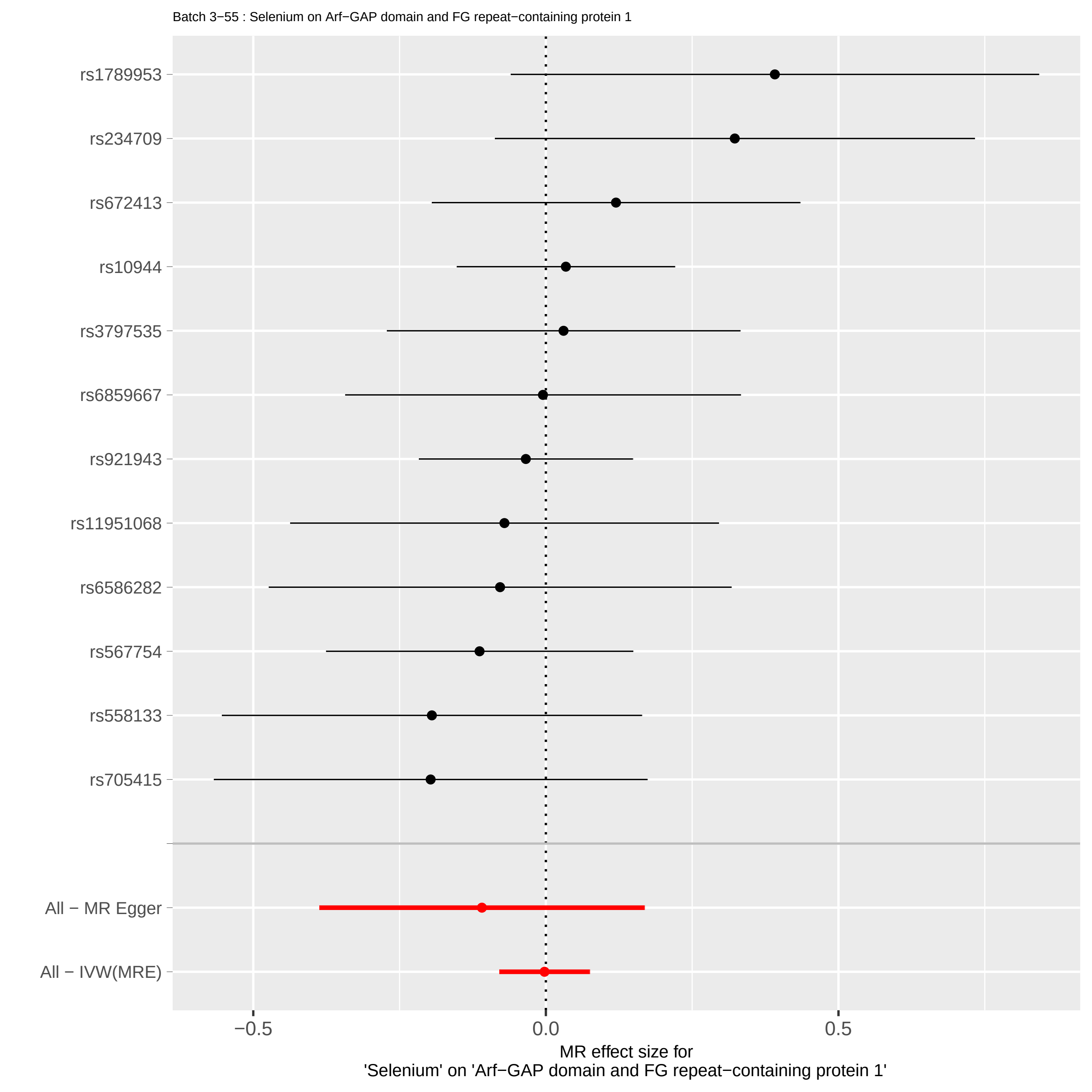

Batch 3−55 : Selenium on Arf−GAP domain and FG repeat−containing protein 1
rs1789953
rs234709
rs672413
rs10944
rs3797535
rs6859667
rs921943
rs11951068
rs6586282
rs567754
rs558133
rs705415
All − MR Egger
All − IVW(MRE)
−0.5
0.0
0.5
MR effect size for
'Selenium' on 'Arf−GAP domain and FG repeat−containing protein 1'

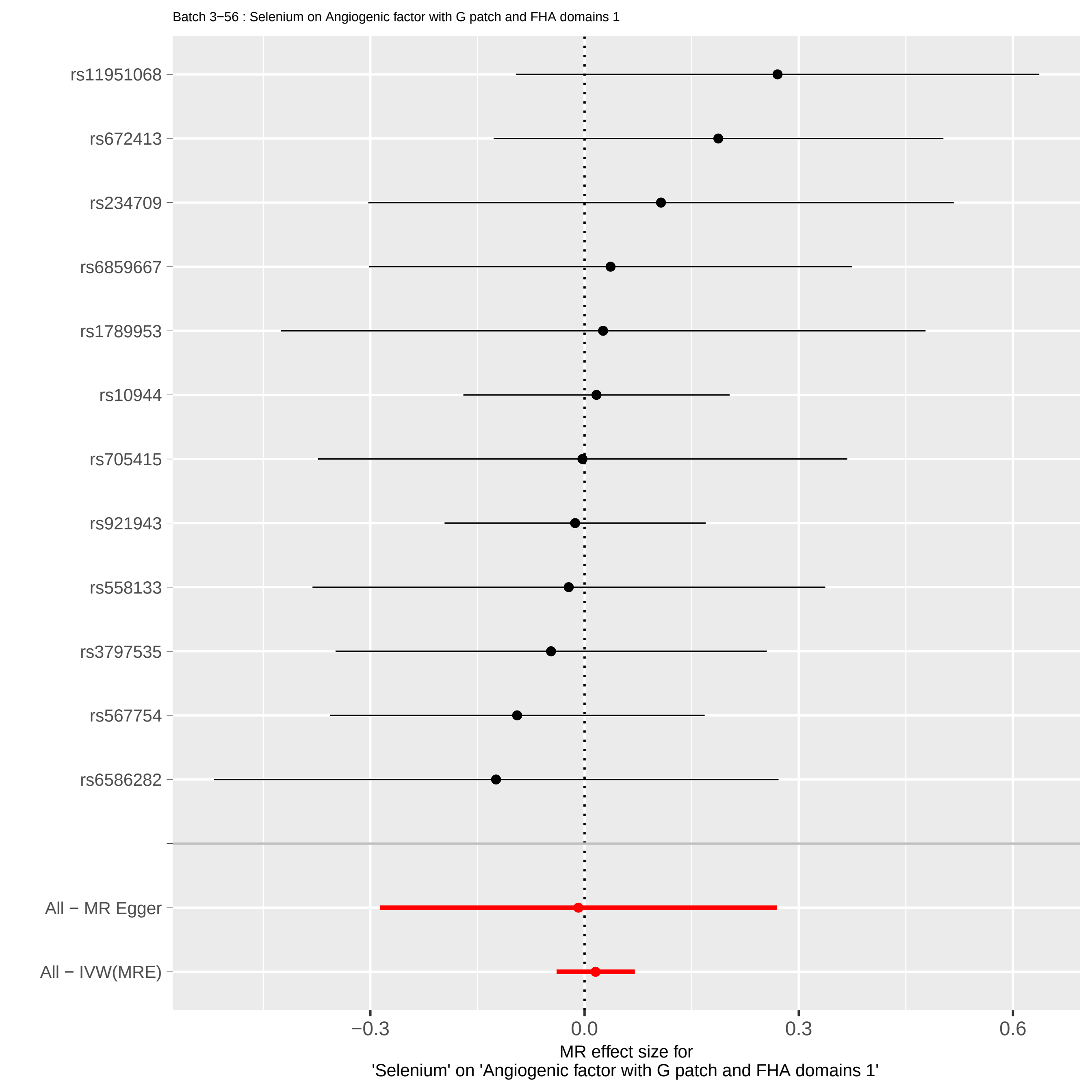
Batch 3−56 : Selenium on Angiogenic factor with G patch and FHA domains 1
rs11951068
rs672413
rs234709
rs6859667
rs1789953
rs10944
rs705415
rs921943
rs558133
rs3797535
rs567754
rs6586282
All − MR Egger
All − IVW(MRE)
−0.3
0.0
0.3
0.6
MR effect size for
'Selenium' on 'Angiogenic factor with G patch and FHA domains 1'

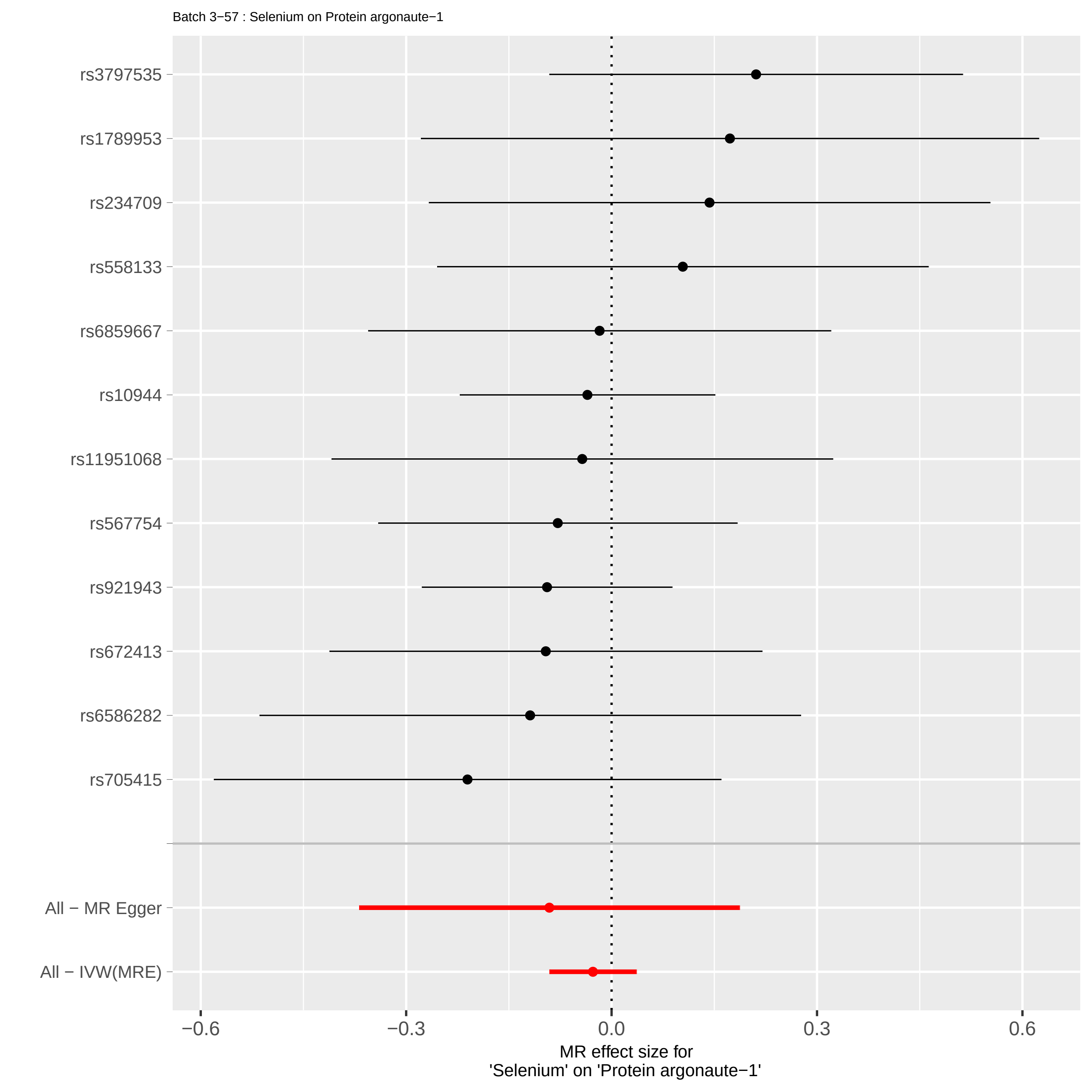
Batch 3−57 : Selenium on Protein argonaute−1
rs3797535
rs1789953
rs234709
rs558133
rs6859667
rs10944
rs11951068
rs567754
rs921943
rs672413
rs6586282
rs705415
All − MR Egger
All − IVW(MRE)
−0.6
−0.3
0.0
0.3
0.6
MR effect size for
'Selenium' on 'Protein argonaute−1'

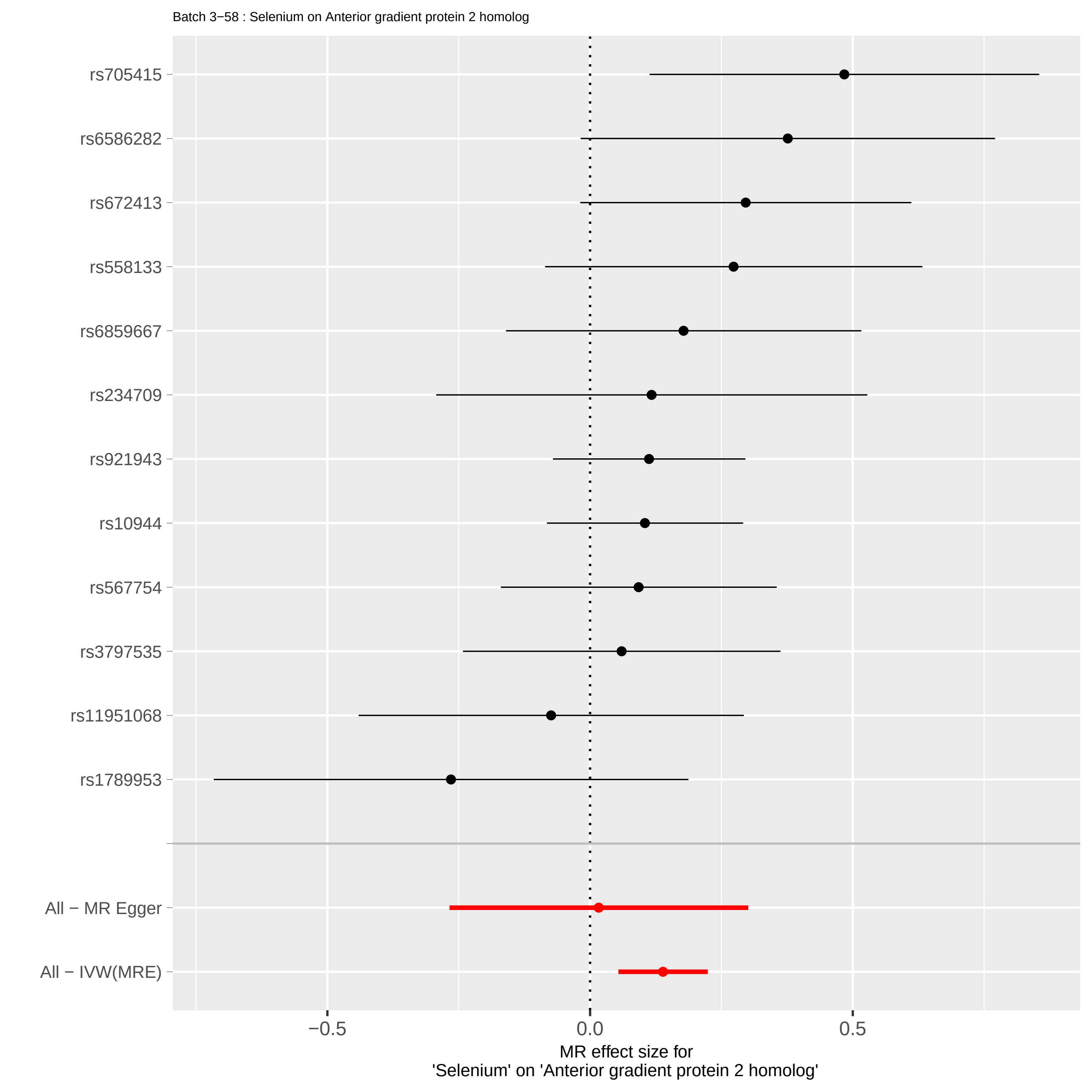
Batch 3−58 : Selenium on Anterior gradient protein 2 homolog
rs705415
rs6586282
rs672413
rs558133
rs6859667
rs234709
rs921943
rs10944
rs567754
rs3797535
rs11951068
rs1789953
All − MR Egger
All − IVW(MRE)
−0.5
0.0
0.5
MR effect size for
'Selenium' on 'Anterior gradient protein 2 homolog'

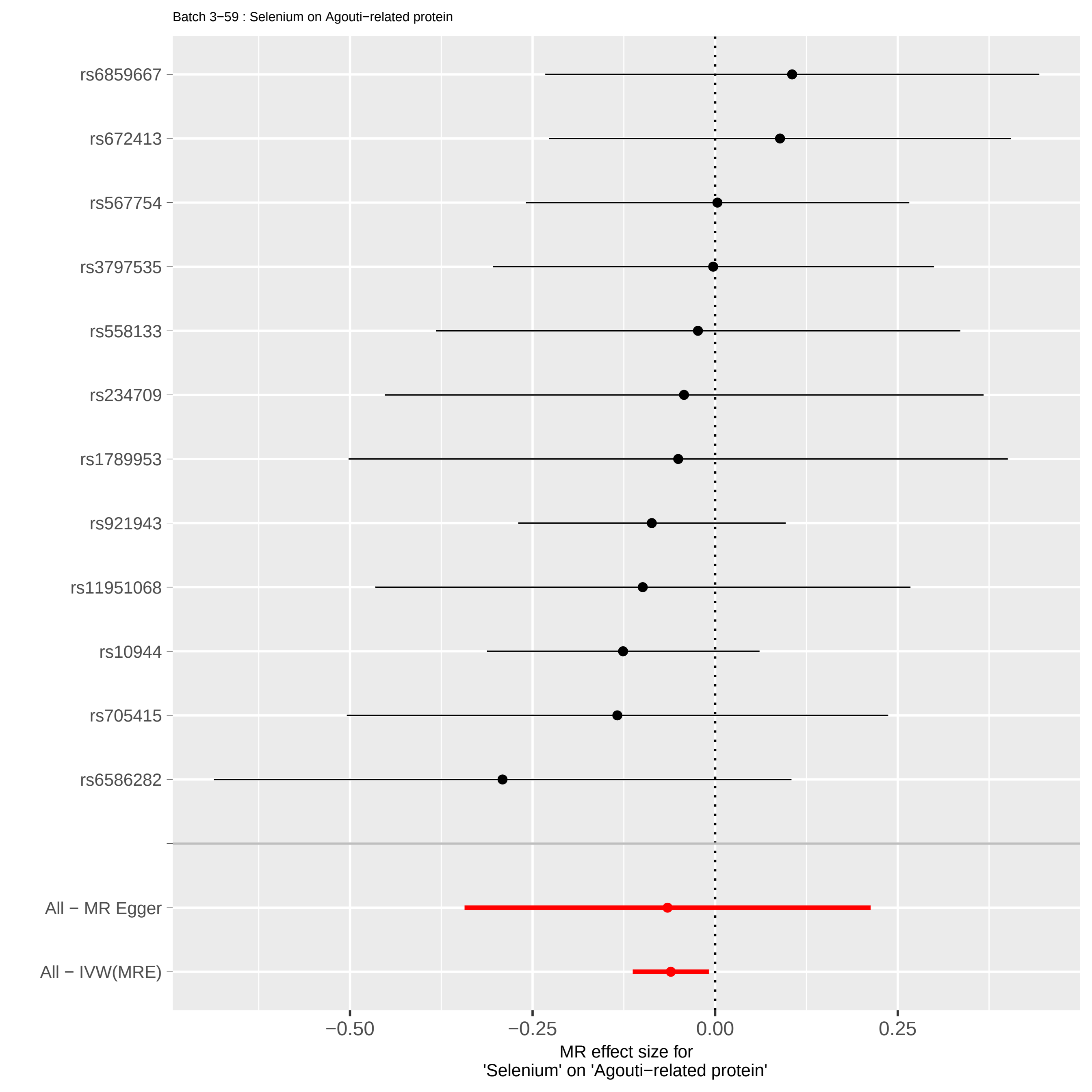
Batch 3−59 : Selenium on Agouti−related protein
rs6859667
rs672413
rs567754
rs3797535
rs558133
rs234709
rs1789953
rs921943
rs11951068
rs10944
rs705415
rs6586282
All − MR Egger
All − IVW(MRE)
−0.50
−0.25
0.00
0.25
MR effect size for
'Selenium' on 'Agouti−related protein'

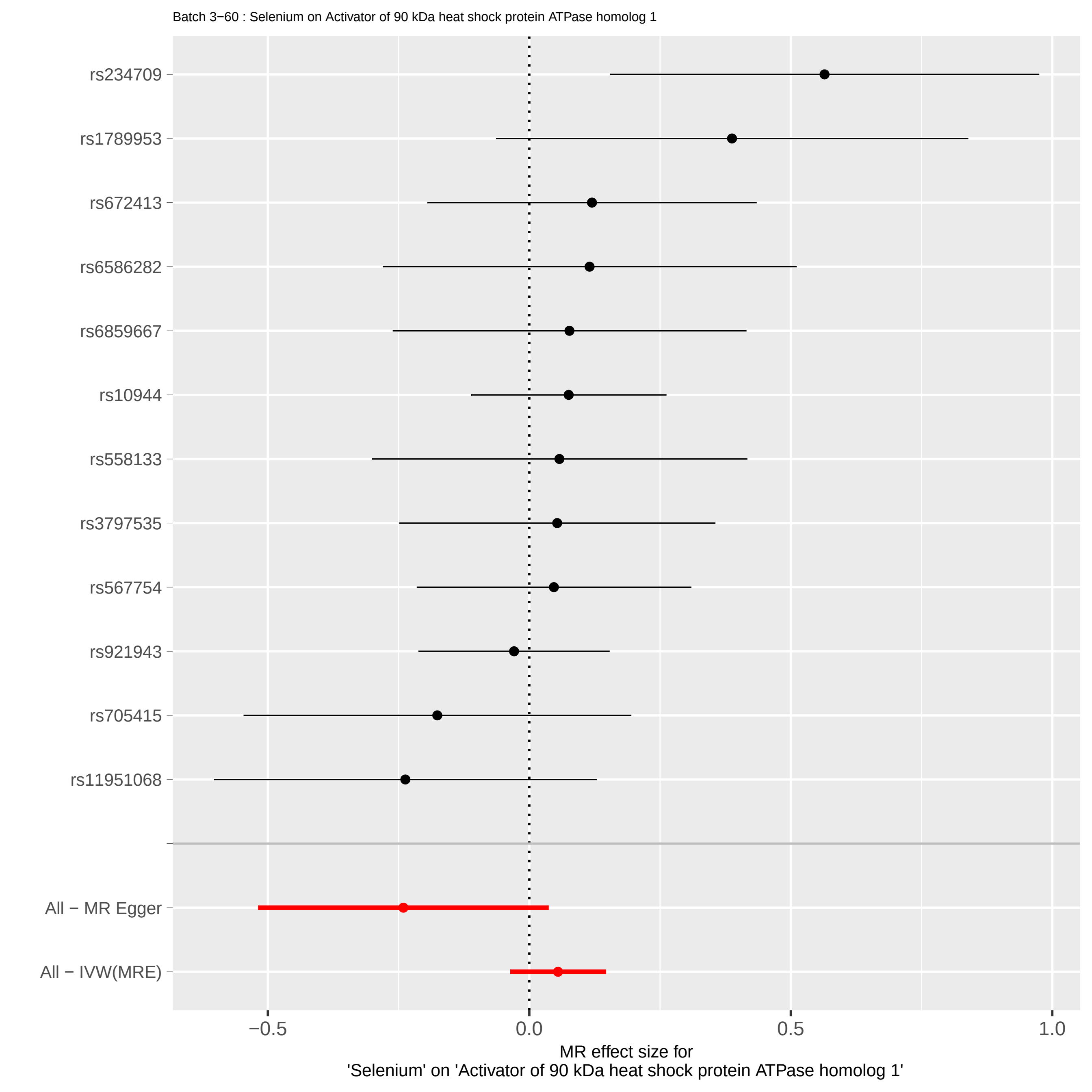

Batch 3−60 : Selenium on Activator of 90 kDa heat shock protein ATPase homolog 1
rs234709
rs1789953
rs672413
rs6586282
rs6859667
rs10944
rs558133
rs3797535
rs567754
rs921943
rs705415
rs11951068
All − MR Egger
All − IVW(MRE)
−0.5
0.0
0.5
1.0
MR effect size for
'Selenium' on 'Activator of 90 kDa heat shock protein ATPase homolog 1'

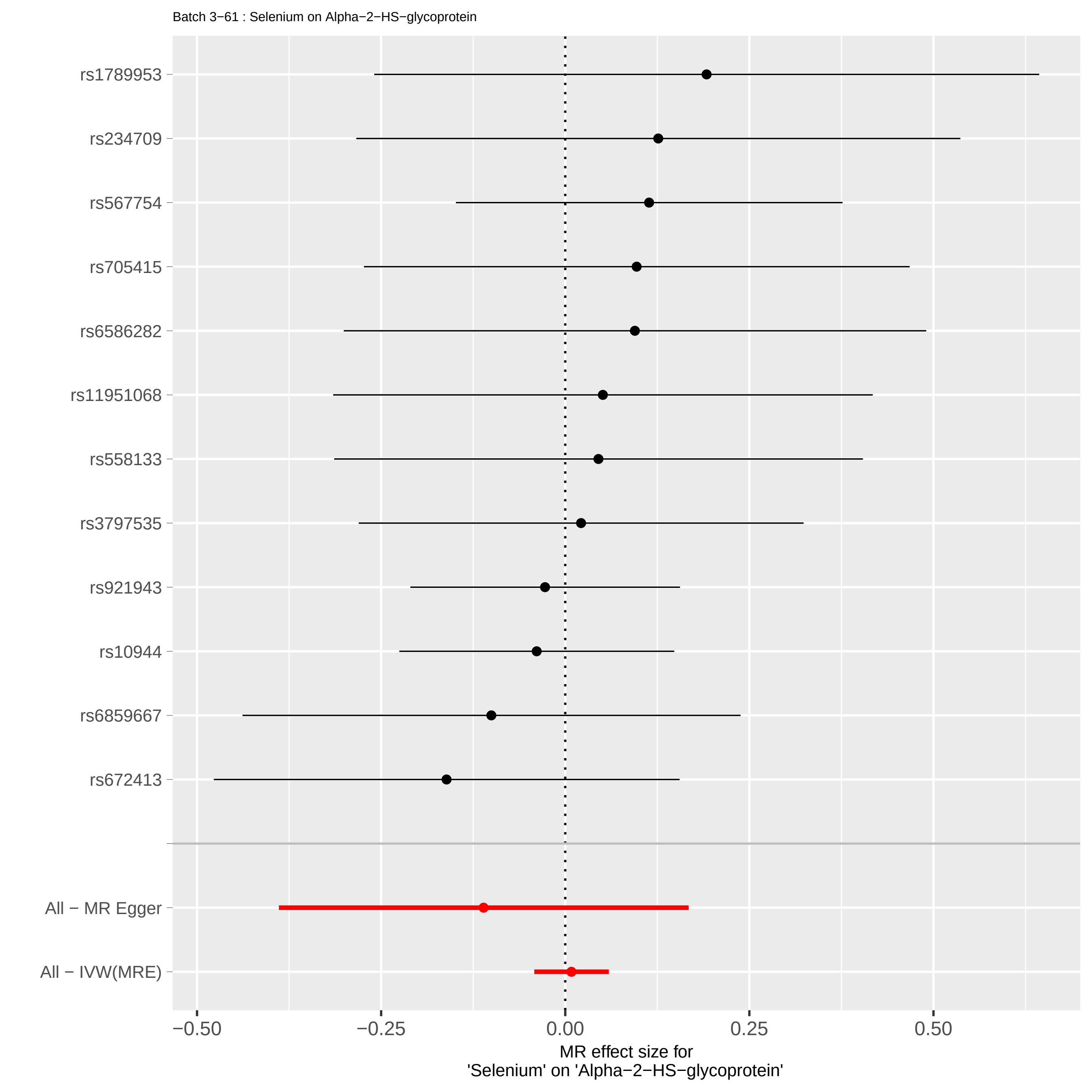

Batch 3−61 : Selenium on Alpha−2−HS−glycoprotein
rs1789953
rs234709
rs567754
rs705415
rs6586282
rs11951068
rs558133
rs3797535
rs921943
rs10944
rs6859667
rs672413
All − MR Egger
All − IVW(MRE)
−0.50
−0.25
0.00
0.25
0.50
MR effect size for
'Selenium' on 'Alpha−2−HS−glycoprotein'

Batch 3−62 : Selenium on Allograft inflammatory factor 1

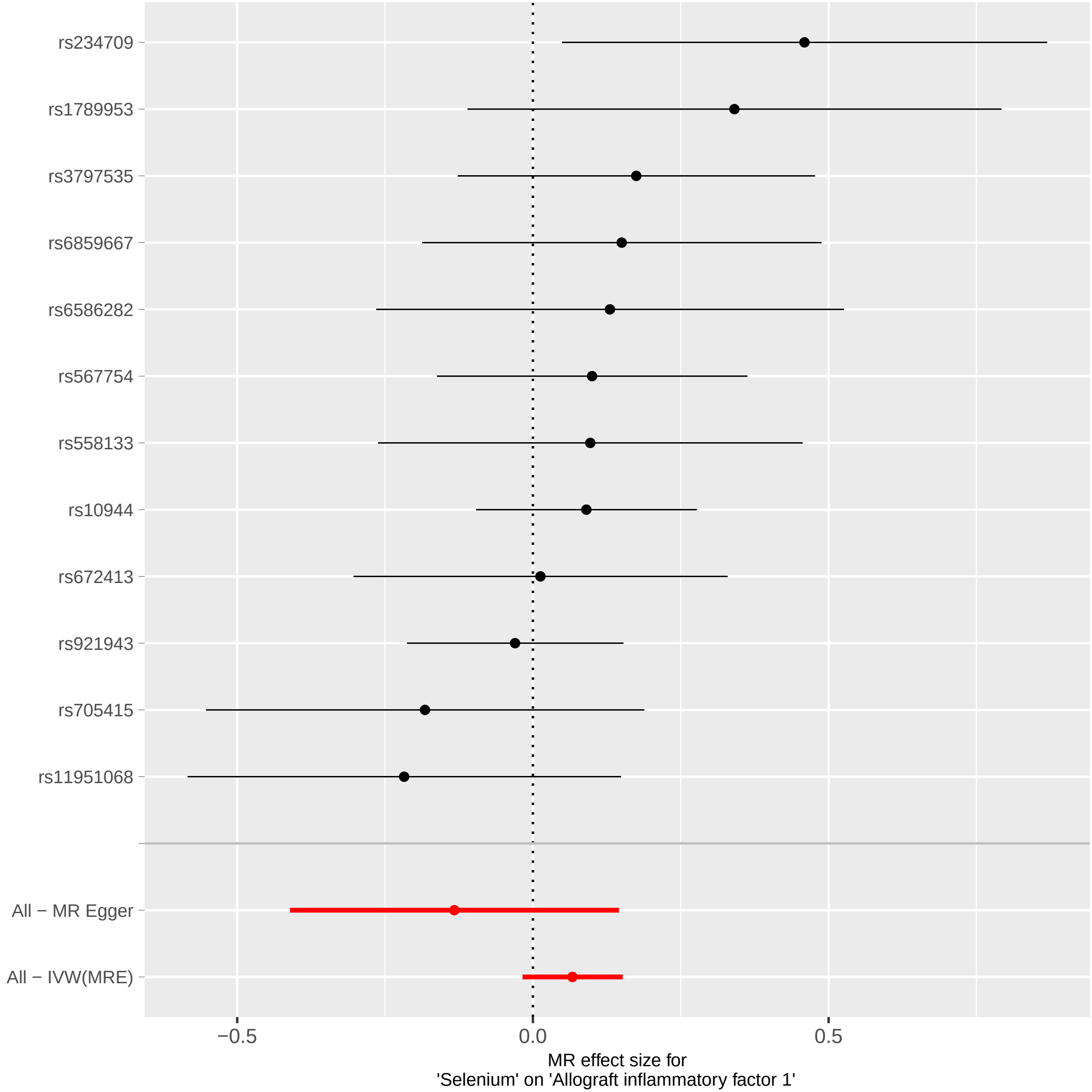

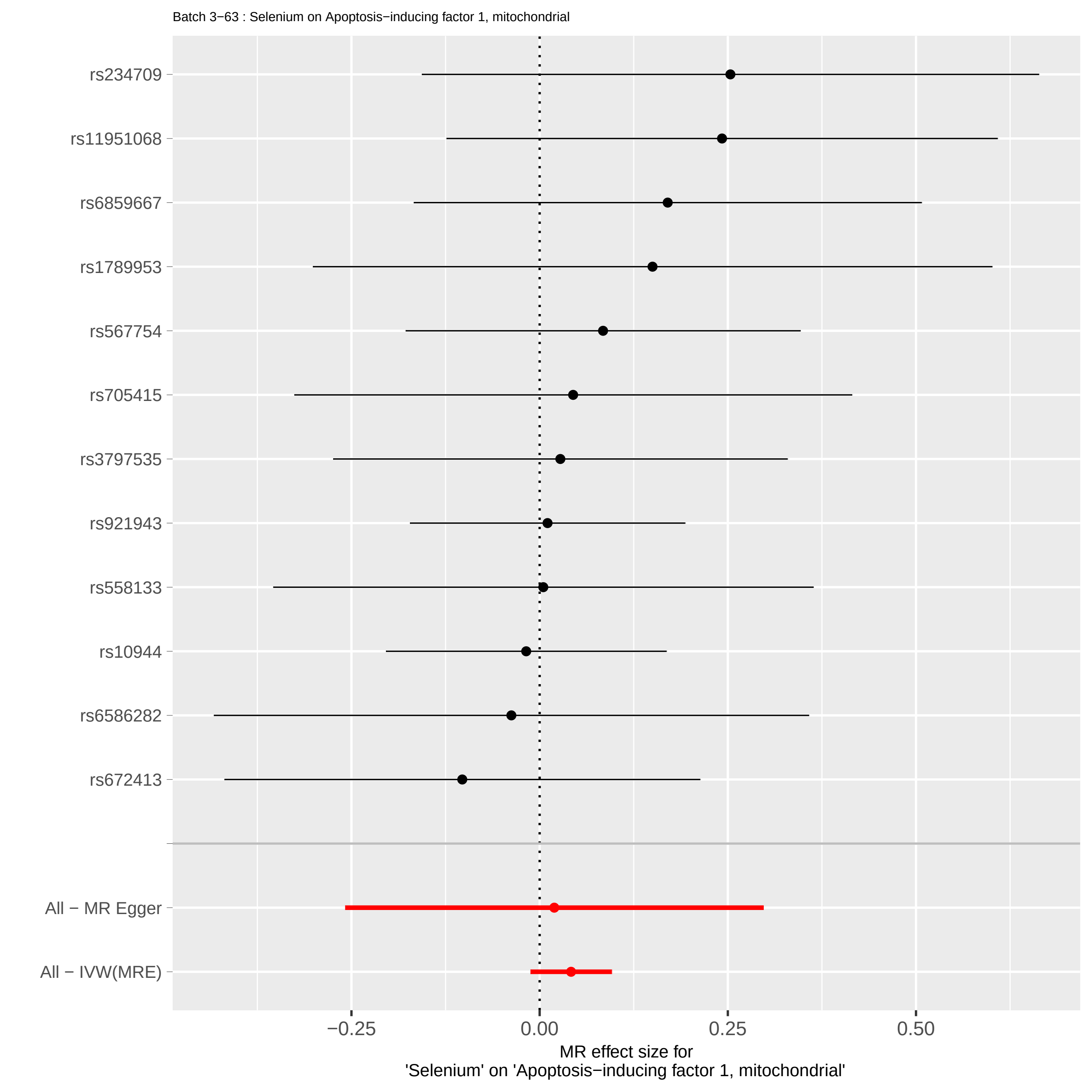
Batch 3−63 : Selenium on Apoptosis−inducing factor 1, mitochondrial
rs234709
rs11951068
rs6859667
rs1789953
rs567754
rs705415
rs3797535
rs921943
rs558133
rs10944
rs6586282
rs672413
All − MR Egger
All − IVW(MRE)
−0.25
0.00
0.25
0.50
MR effect size for
'Selenium' on 'Apoptosis−inducing factor 1, mitochondrial'

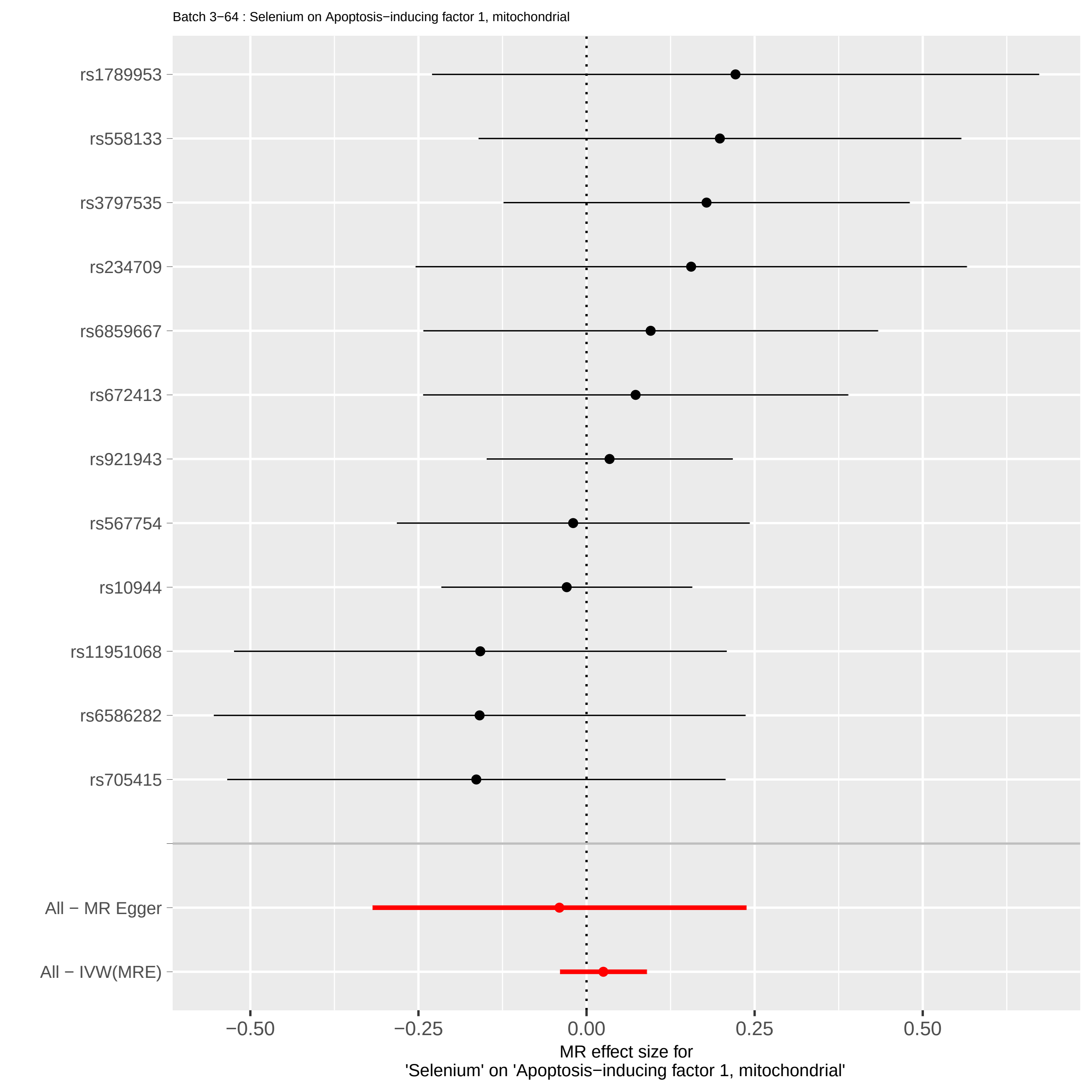

Batch 3−64 : Selenium on Apoptosis−inducing factor 1, mitochondrial
rs1789953
rs558133
rs3797535
rs234709
rs6859667
rs672413
rs921943
rs567754
rs10944
rs11951068
rs6586282
rs705415
All − MR Egger
All − IVW(MRE)
−0.50
−0.25
0.00
0.25
0.50
MR effect size for
'Selenium' on 'Apoptosis−inducing factor 1, mitochondrial'

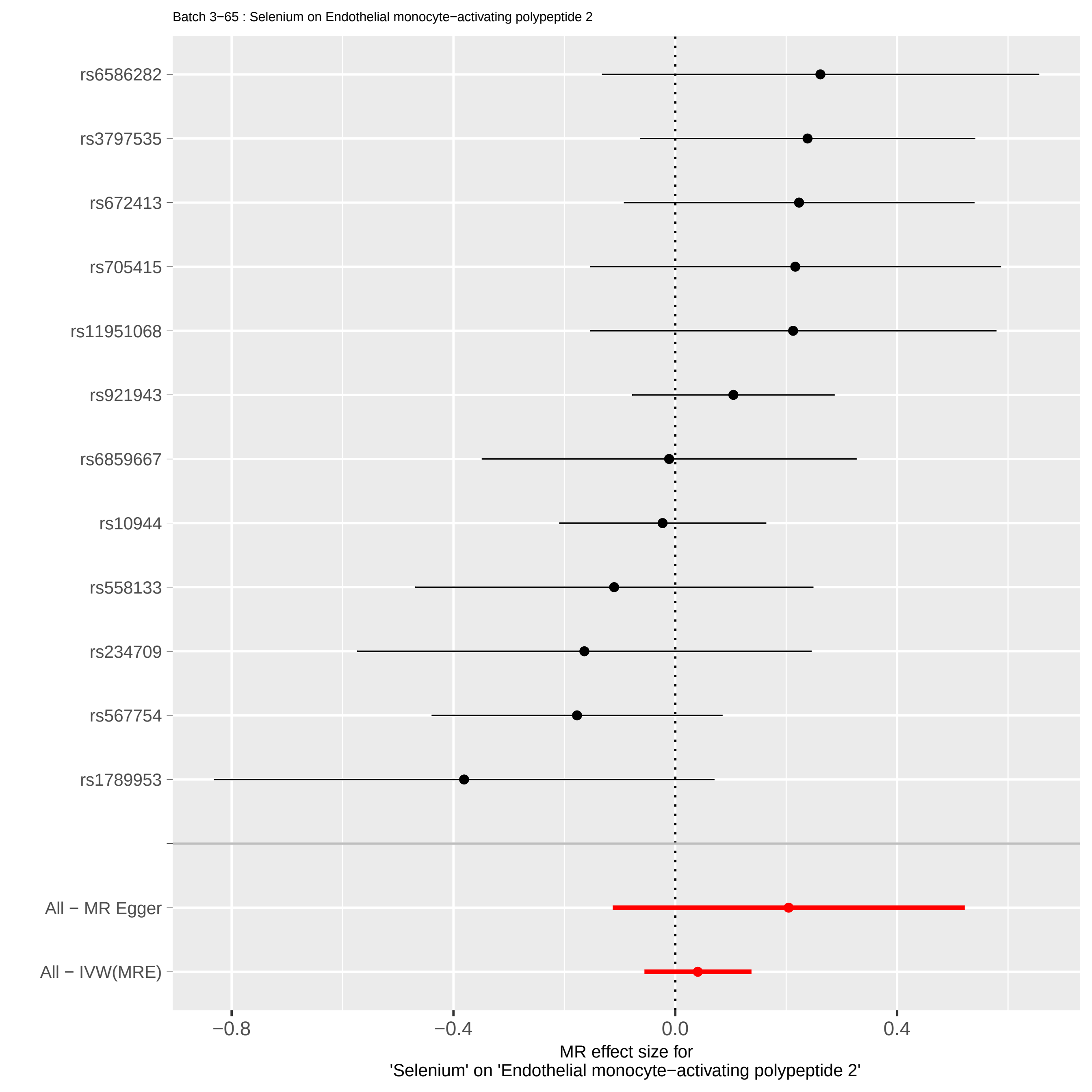

Batch 3−65 : Selenium on Endothelial monocyte−activating polypeptide 2
rs6586282
rs3797535
rs672413
rs705415
rs11951068
rs921943
rs6859667
rs10944
rs558133
rs234709
rs567754
rs1789953
All − MR Egger
All − IVW(MRE)
−0.8
−0.4
0.0
0.4
MR effect size for
'Selenium' on 'Endothelial monocyte−activating polypeptide 2'

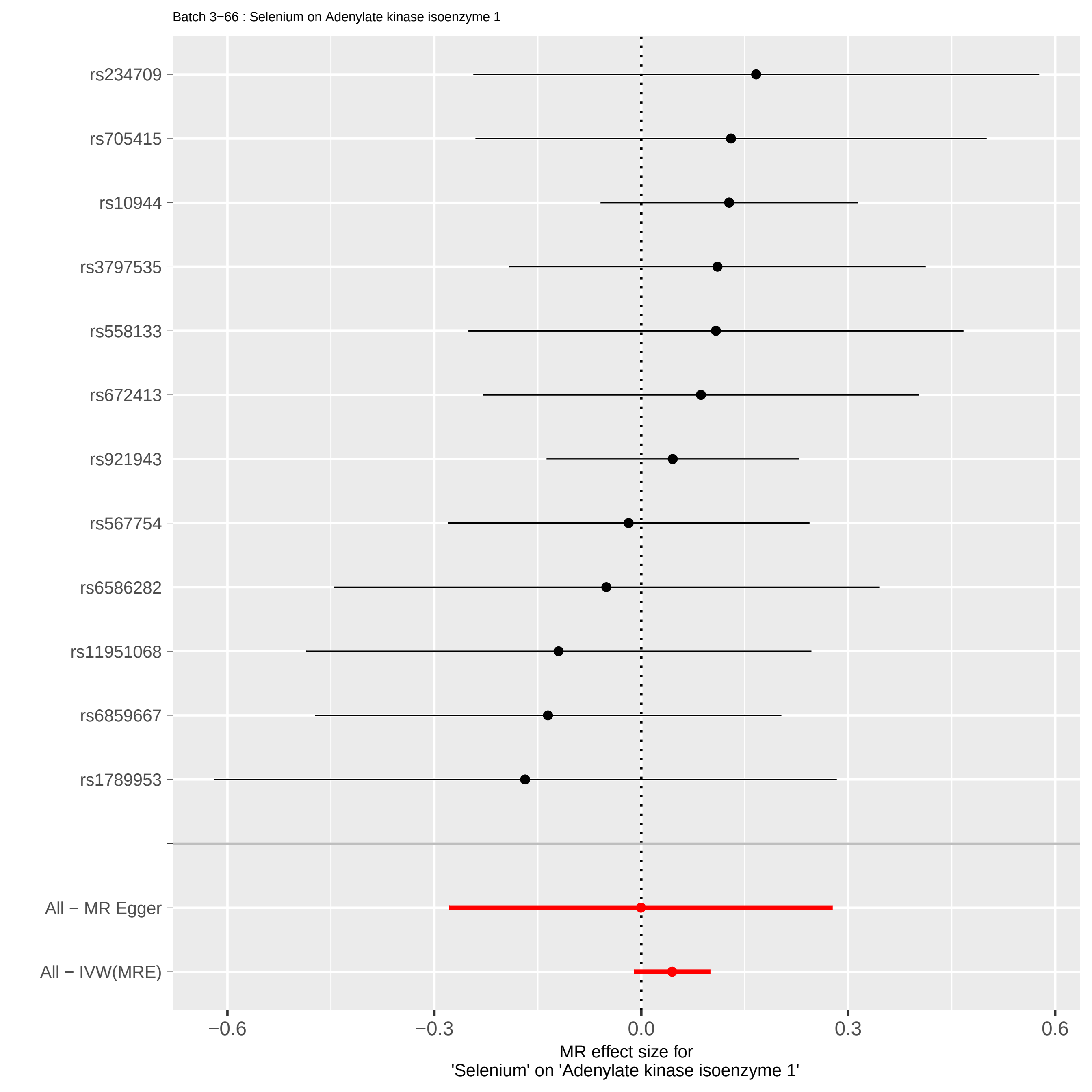
Batch 3−66 : Selenium on Adenylate kinase isoenzyme 1
rs234709
rs705415
rs10944
rs3797535
rs558133
rs672413
rs921943
rs567754
rs6586282
rs11951068
rs6859667
rs1789953
All − MR Egger
All − IVW(MRE)
−0.6
−0.3
0.0
0.3
0.6
MR effect size for
'Selenium' on 'Adenylate kinase isoenzyme 1'

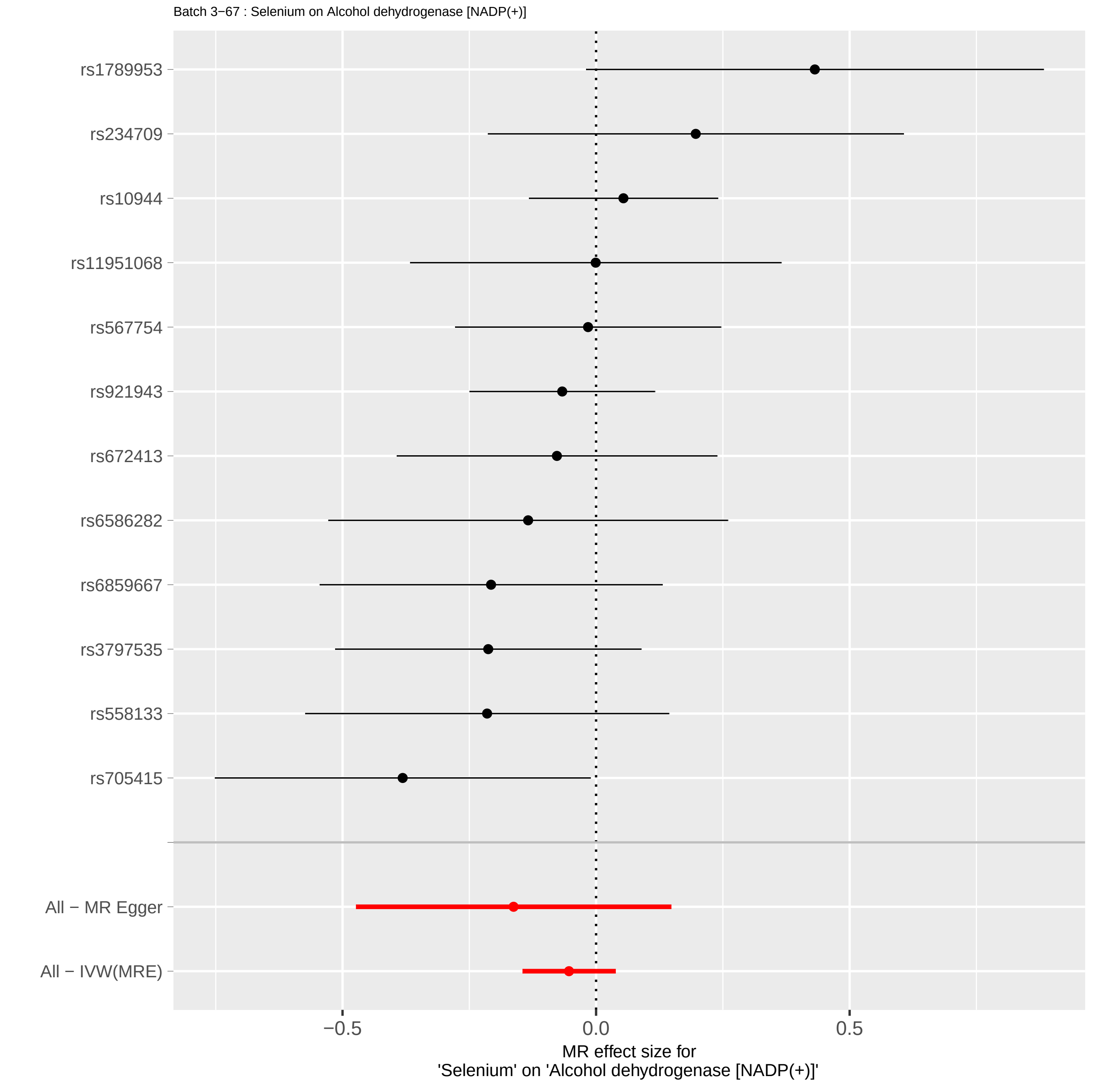

Batch 3−67 : Selenium on Alcohol dehydrogenase [NADP(+)]
rs1789953
rs234709
rs10944
rs11951068
rs567754
rs921943
rs672413
rs6586282
rs6859667
rs3797535
rs558133
rs705415
All − MR Egger
All − IVW(MRE)
−0.5
0.0
0.5
MR effect size for
'Selenium' on 'Alcohol dehydrogenase [NADP(+)]'

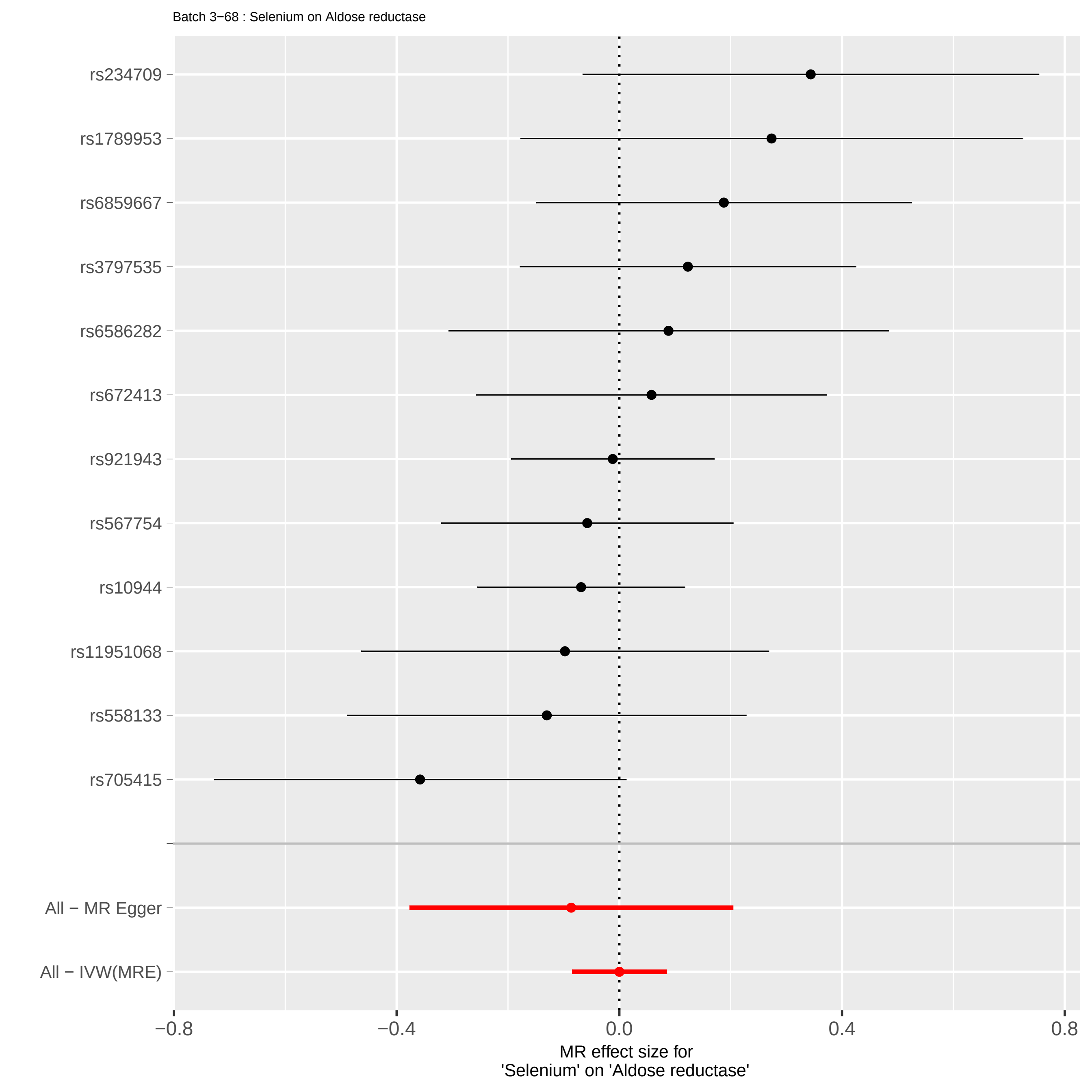
Batch 3−68 : Selenium on Aldose reductase
rs234709
rs1789953
rs6859667
rs3797535
rs6586282
rs672413
rs921943
rs567754
rs10944
rs11951068
rs558133
rs705415
All − MR Egger
All − IVW(MRE)
−0.8
−0.4
0.0
0.4
0.8
MR effect size for
'Selenium' on 'Aldose reductase'

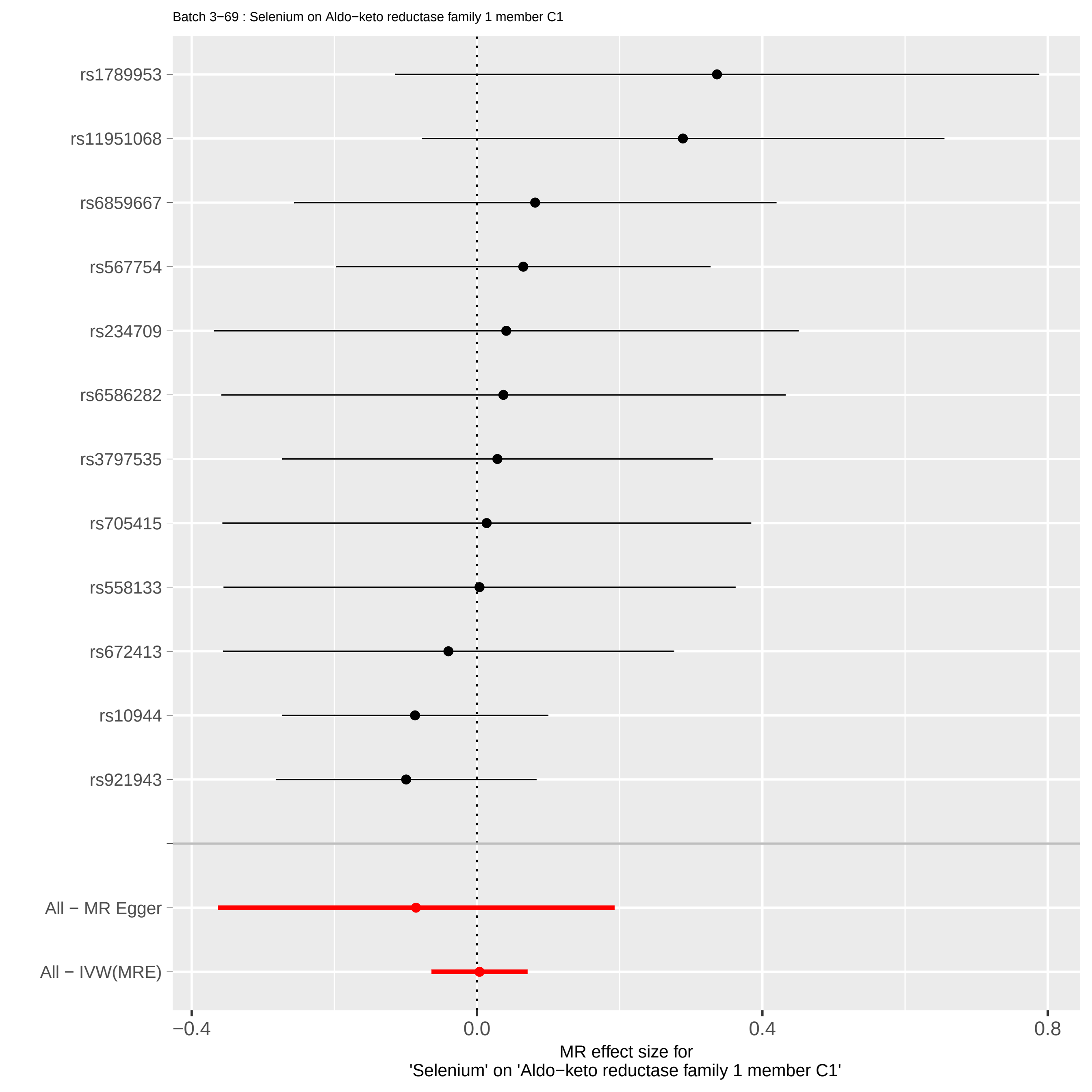

Batch 3−69 : Selenium on Aldo−keto reductase family 1 member C1
rs1789953
rs11951068
rs6859667
rs567754
rs234709
rs6586282
rs3797535
rs705415
rs558133
rs672413
rs10944
rs921943
All − MR Egger
All − IVW(MRE)
−0.4
0.0
0.4
0.8
MR effect size for
'Selenium' on 'Aldo−keto reductase family 1 member C1'

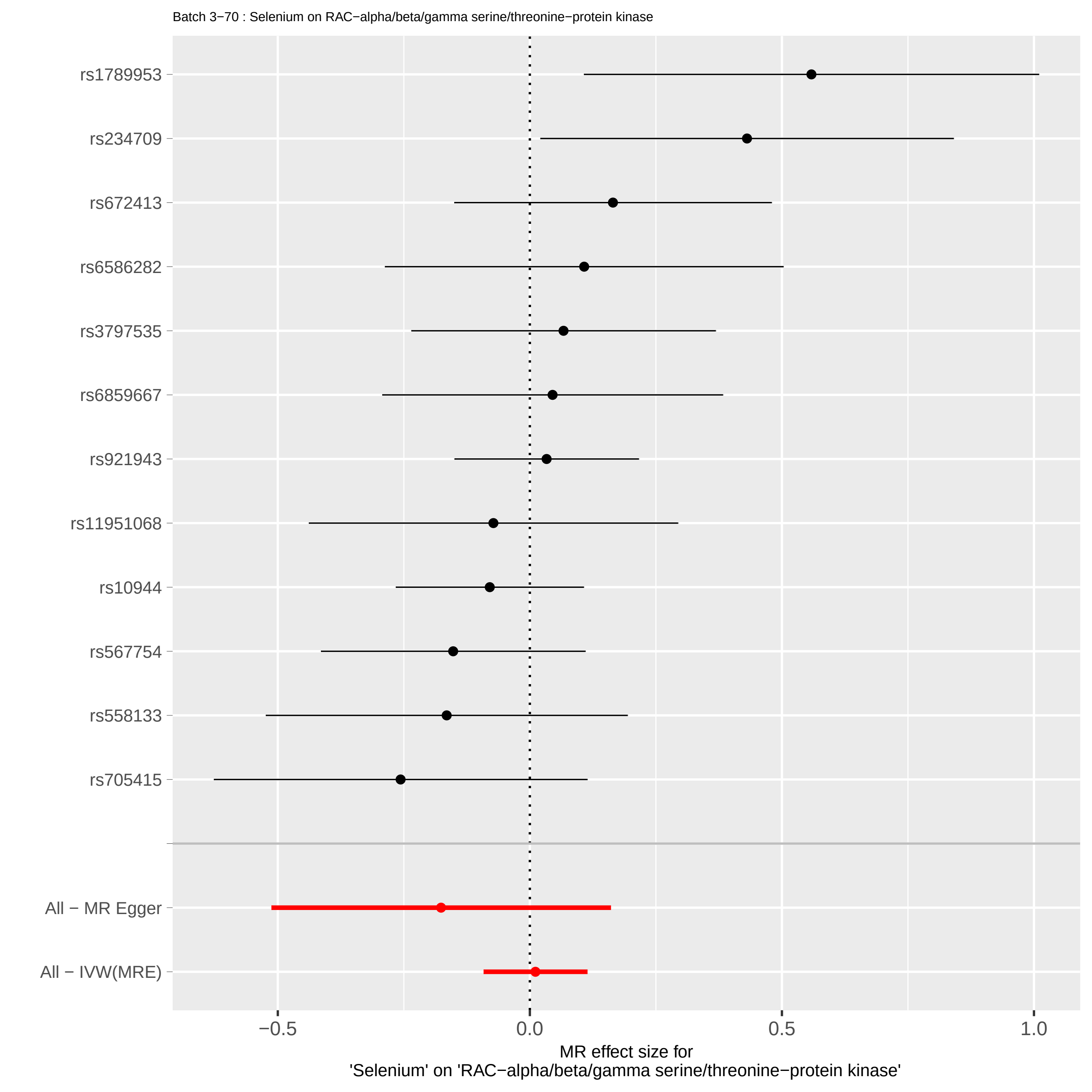
Batch 3−70 : Selenium on RAC−alpha/beta/gamma serine/threonine−protein kinase
rs1789953
rs234709
rs672413
rs6586282
rs3797535
rs6859667
rs921943
rs11951068
rs10944
rs567754
rs558133
rs705415
All − MR Egger
All − IVW(MRE)
−0.5
0.0
0.5
1.0
MR effect size for
'Selenium' on 'RAC−alpha/beta/gamma serine/threonine−protein kinase'

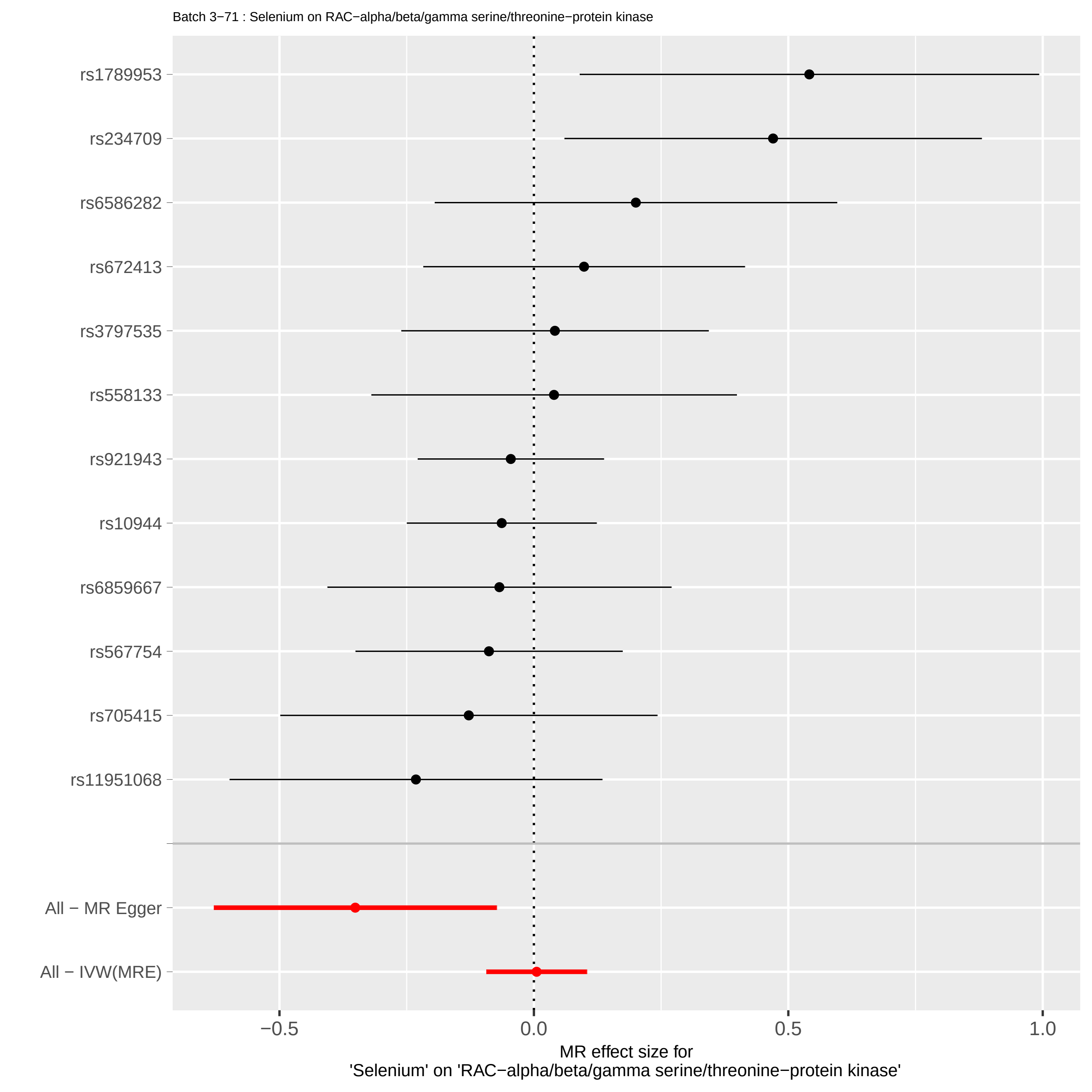
Batch 3−71 : Selenium on RAC−alpha/beta/gamma serine/threonine−protein kinase
rs1789953
rs234709
rs6586282
rs672413
rs3797535
rs558133
rs921943
rs10944
rs6859667
rs567754
rs705415
rs11951068
All − MR Egger
All − IVW(MRE)
−0.5
0.0
0.5
1.0
MR effect size for
'Selenium' on 'RAC−alpha/beta/gamma serine/threonine−protein kinase'

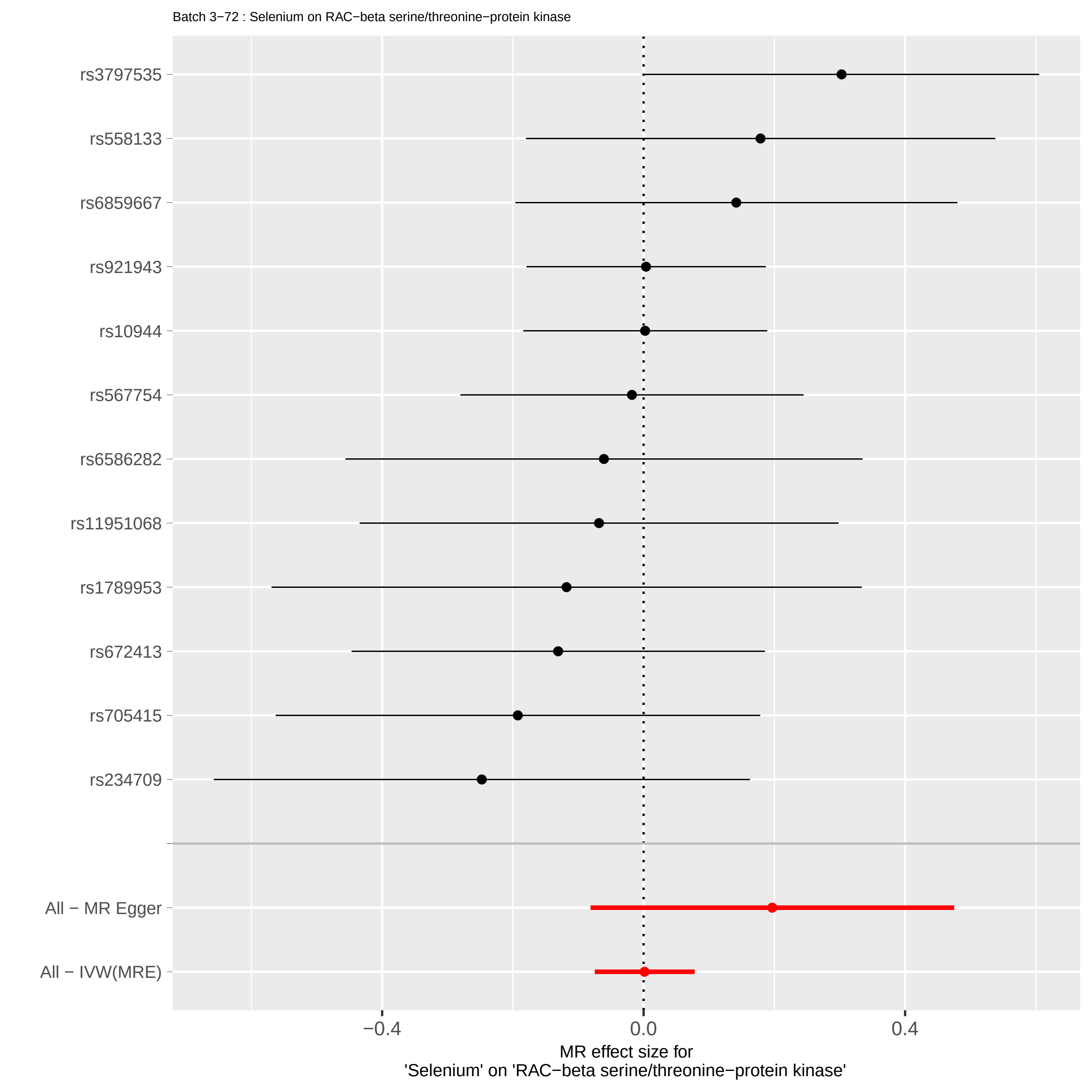
Batch 3−72 : Selenium on RAC−beta serine/threonine−protein kinase
rs3797535
rs558133
rs6859667
rs921943
rs10944
rs567754
rs6586282
rs11951068
rs1789953
rs672413
rs705415
rs234709
All − MR Egger
All − IVW(MRE)
−0.4
0.0
0.4
MR effect size for
'Selenium' on 'RAC−beta serine/threonine−protein kinase'

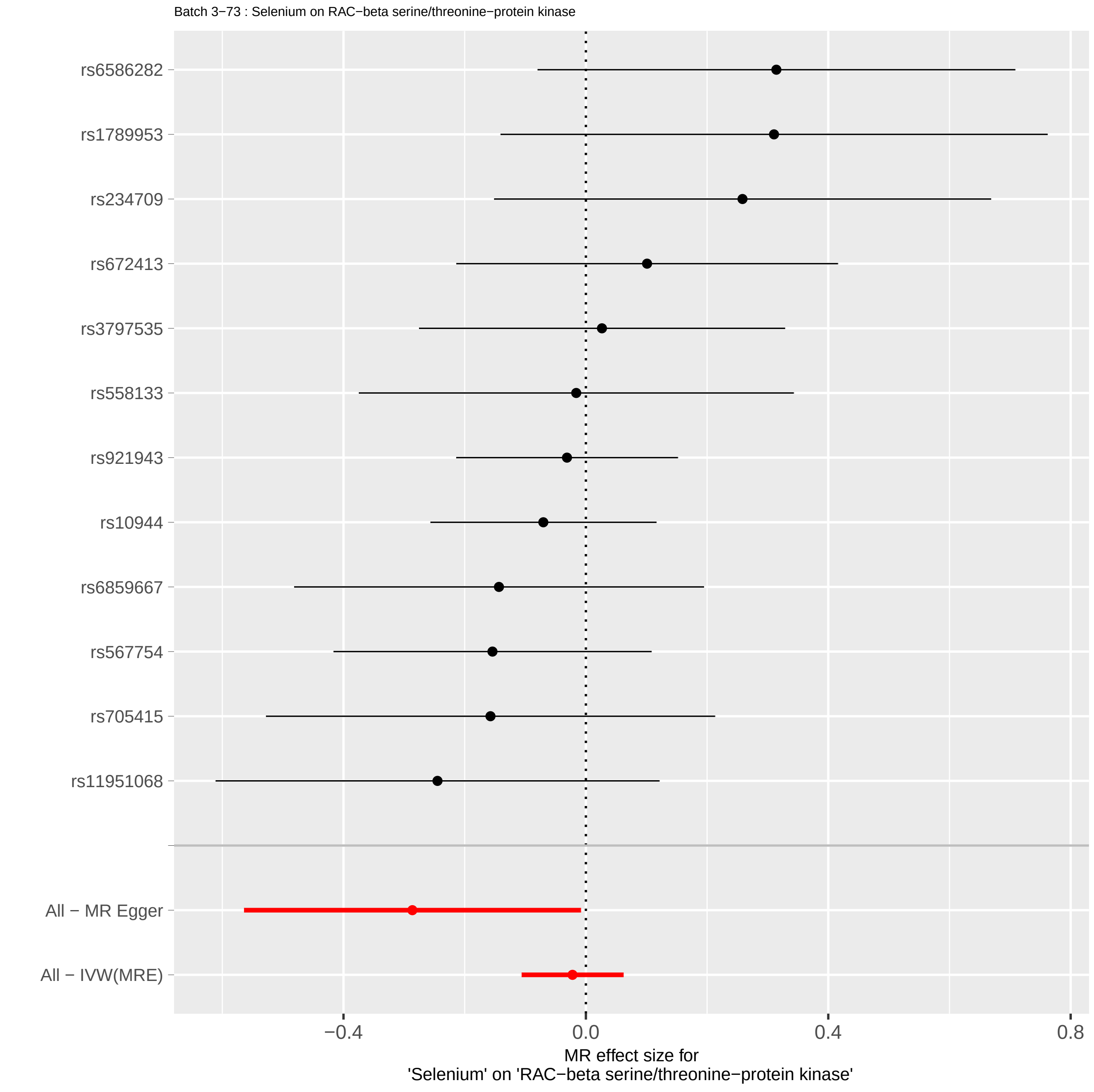

Batch 3−73 : Selenium on RAC−beta serine/threonine−protein kinase
rs6586282
rs1789953
rs234709
rs672413
rs3797535
rs558133
rs921943
rs10944
rs6859667
rs567754
rs705415
rs11951068
All − MR Egger
All − IVW(MRE)
−0.4
0.0
0.4
0.8
MR effect size for
'Selenium' on 'RAC−beta serine/threonine−protein kinase'

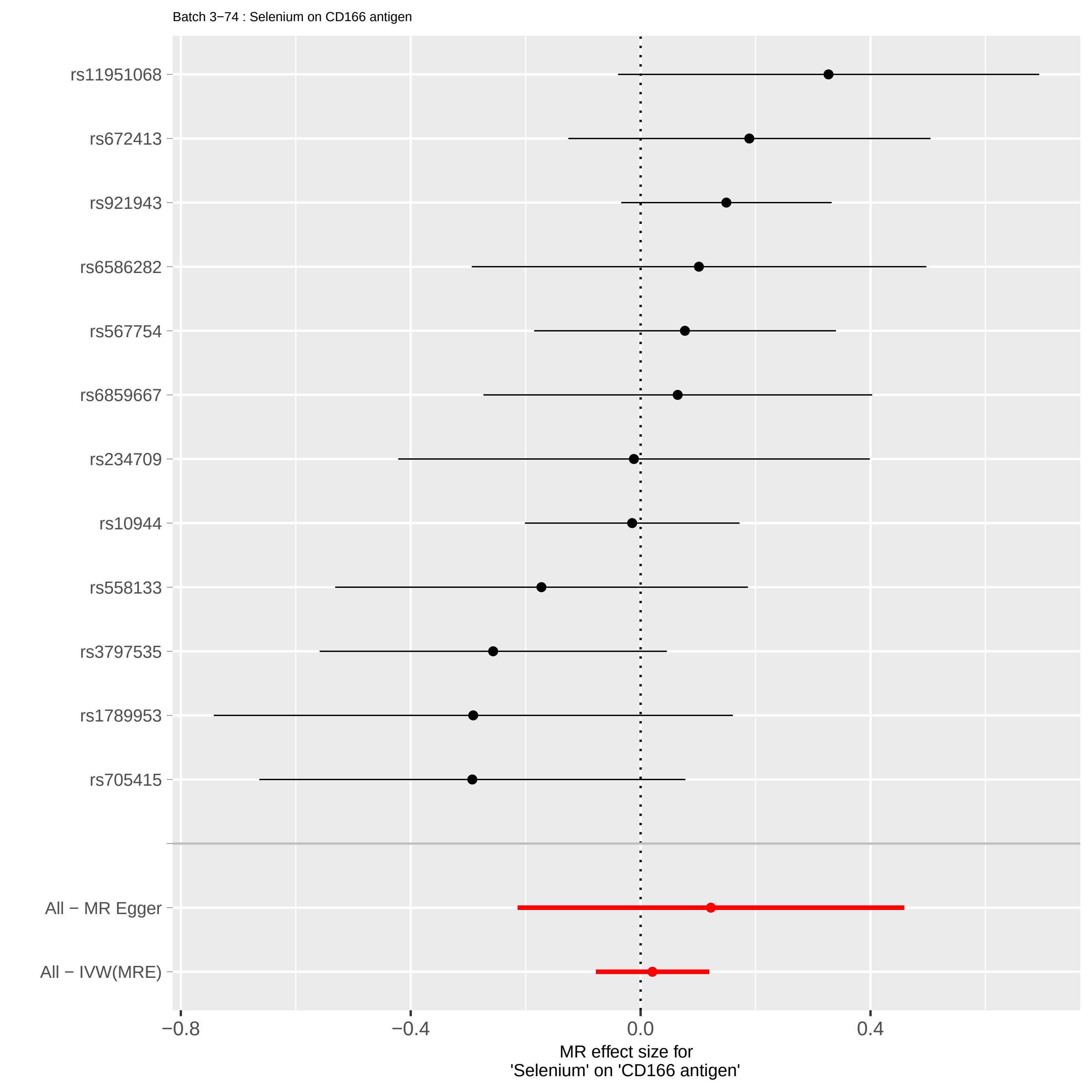
Batch 3−74 : Selenium on CD166 antigen
rs11951068
rs672413
rs921943
rs6586282
rs567754
rs6859667
rs234709
rs10944
rs558133
rs3797535
rs1789953
rs705415
All − MR Egger
All − IVW(MRE)
−0.8
−0.4
0.0
0.4
MR effect size for
'Selenium' on 'CD166 antigen'

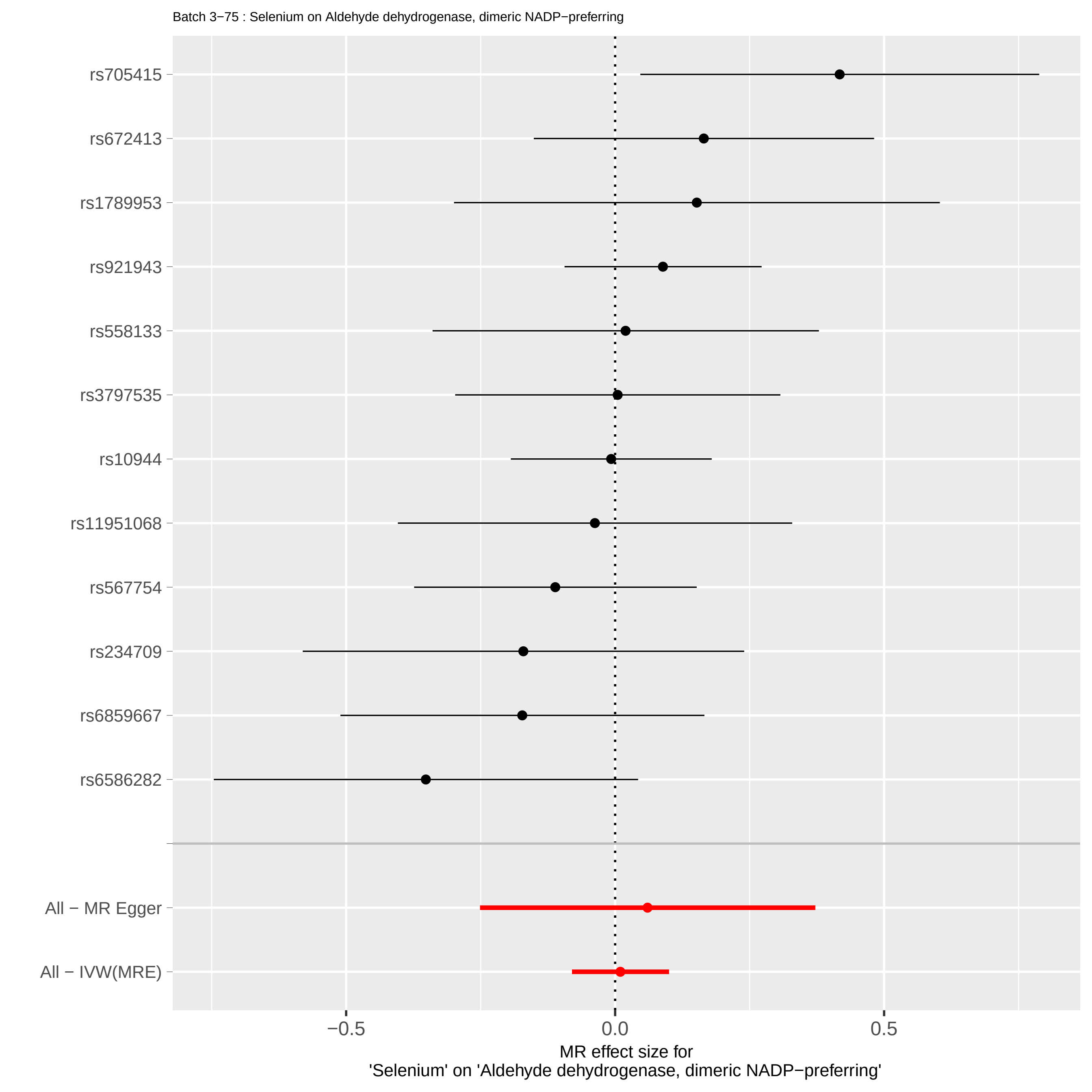
Batch 3−75 : Selenium on Aldehyde dehydrogenase, dimeric NADP−preferring
rs705415
rs672413
rs1789953
rs921943
rs558133
rs3797535
rs10944
rs11951068
rs567754
rs234709
rs6859667
rs6586282
All − MR Egger
All − IVW(MRE)
−0.5
0.0
0.5
MR effect size for
'Selenium' on 'Aldehyde dehydrogenase, dimeric NADP−preferring'

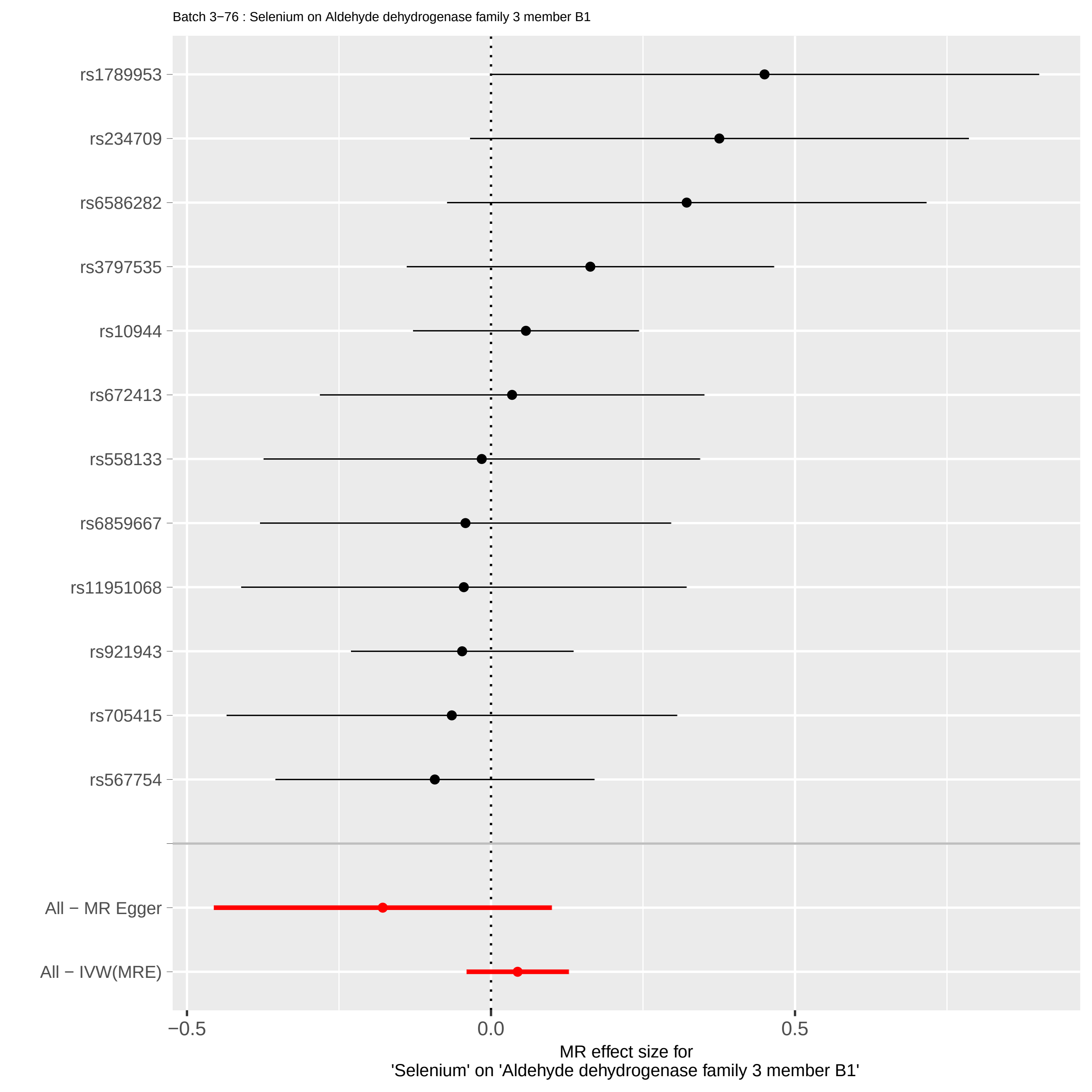

Batch 3−76 : Selenium on Aldehyde dehydrogenase family 3 member B1
rs1789953
rs234709
rs6586282
rs3797535
rs10944
rs672413
rs558133
rs6859667
rs11951068
rs921943
rs705415
rs567754
All − MR Egger
All − IVW(MRE)
−0.5
0.0
0.5
MR effect size for
'Selenium' on 'Aldehyde dehydrogenase family 3 member B1'

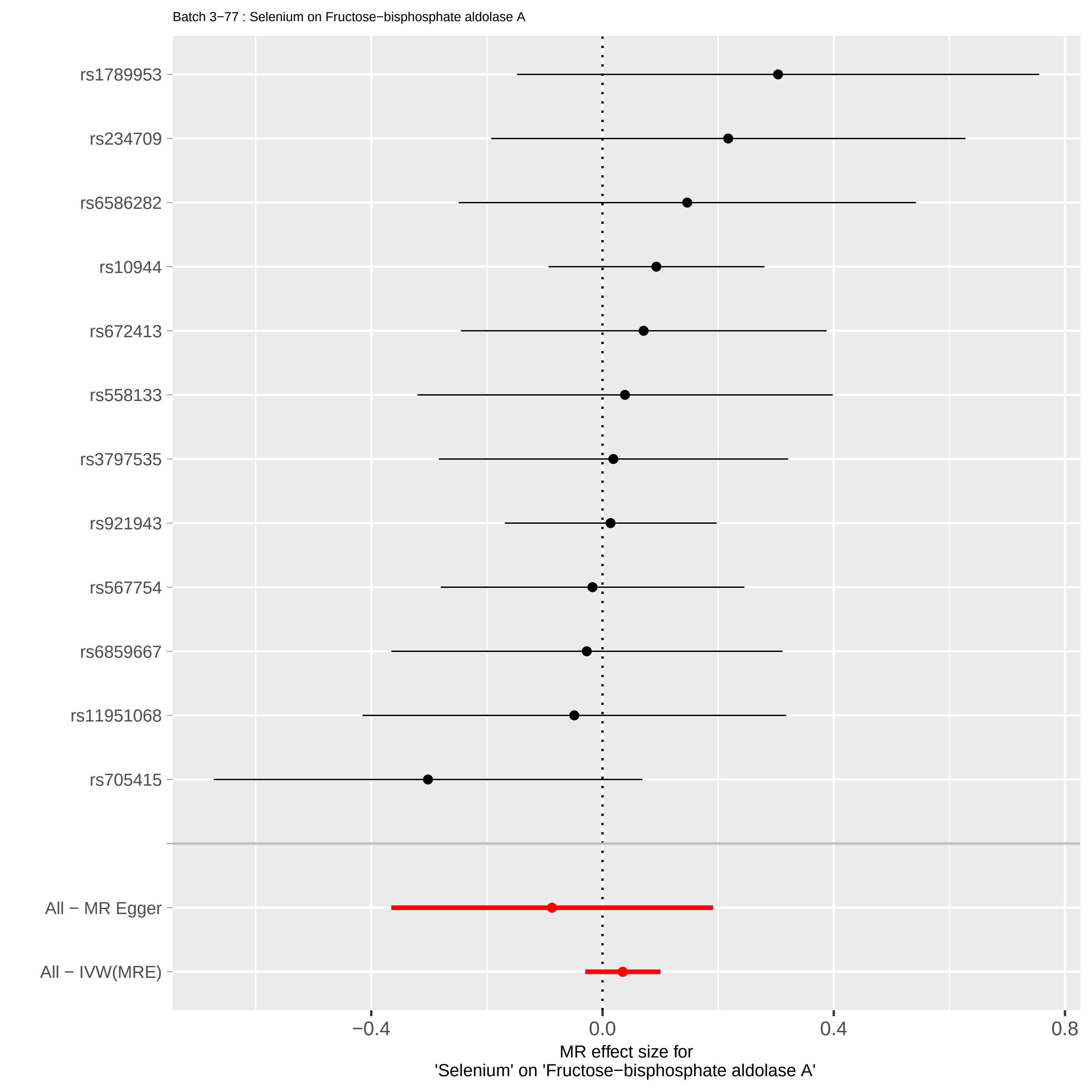
Batch 3−77 : Selenium on Fructose−bisphosphate aldolase A
rs1789953
rs234709
rs6586282
rs10944
rs672413
rs558133
rs3797535
rs921943
rs567754
rs6859667
rs11951068
rs705415
All − MR Egger
All − IVW(MRE)
−0.4
0.0
0.4
0.8
MR effect size for
'Selenium' on 'Fructose−bisphosphate aldolase A'

Batch 3−78 : Selenium on Fructose−bisphosphate aldolase C

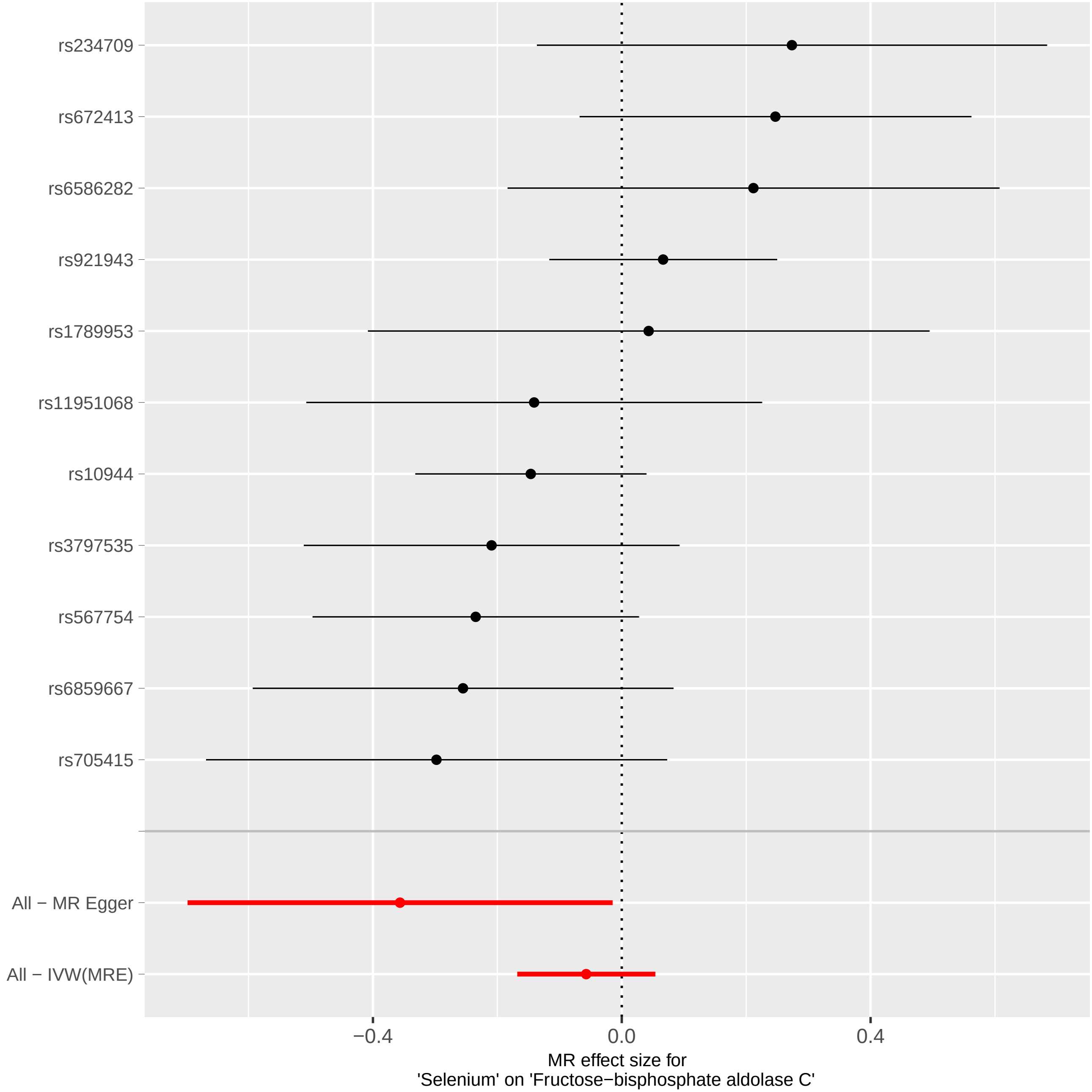

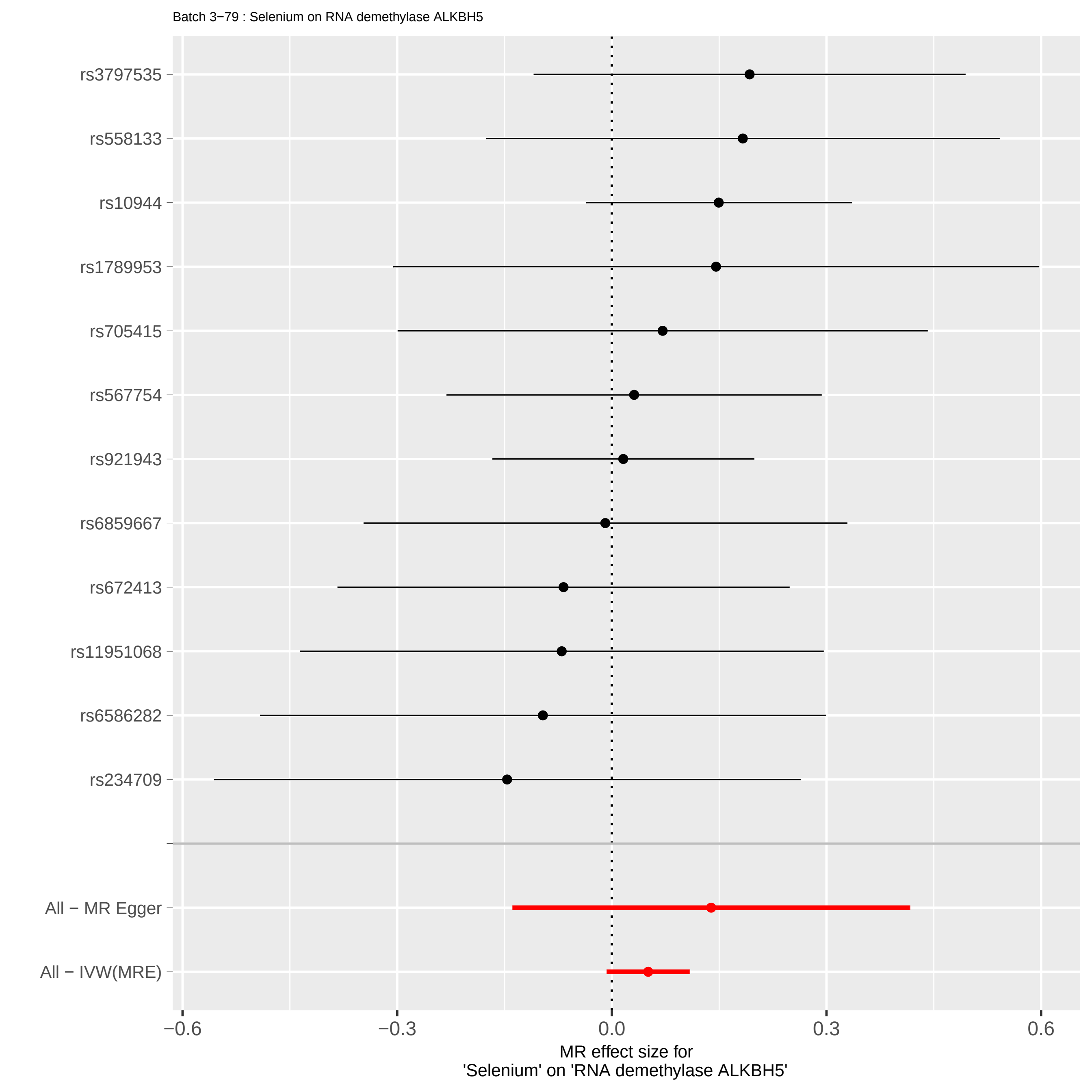

Batch 3−79 : Selenium on RNA demethylase ALKBH5
rs3797535
rs558133
rs10944
rs1789953
rs705415
rs567754
rs921943
rs6859667
rs672413
rs11951068
rs6586282
rs234709
All − MR Egger
All − IVW(MRE)
−0.6
−0.3
0.0
0.3
0.6
MR effect size for
'Selenium' on 'RNA demethylase ALKBH5'

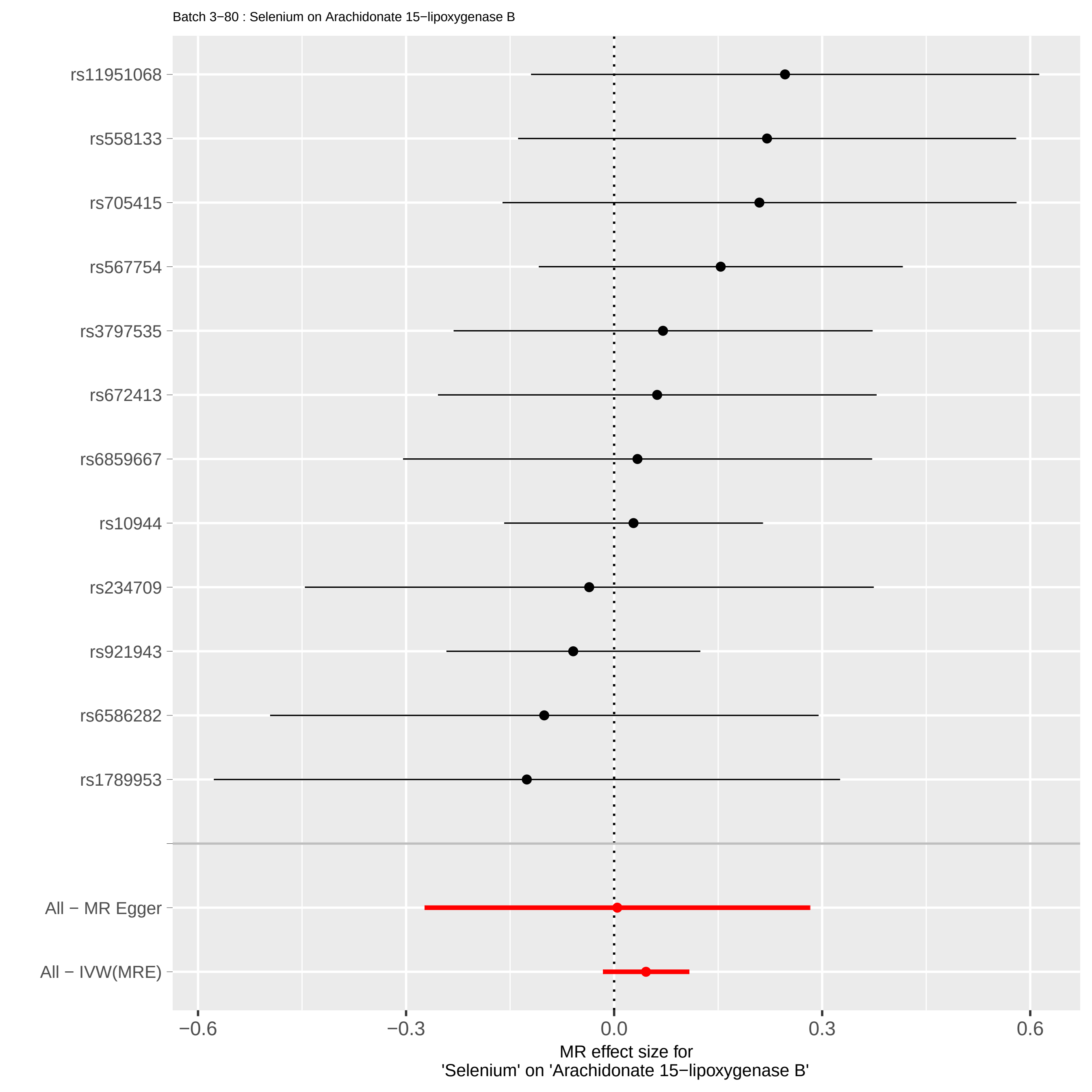
Batch 3−80 : Selenium on Arachidonate 15−lipoxygenase B
rs11951068
rs558133
rs705415
rs567754
rs3797535
rs672413
rs6859667
rs10944
rs234709
rs921943
rs6586282
rs1789953
All − MR Egger
All − IVW(MRE)
−0.6
−0.3
0.0
0.3
0.6
MR effect size for
'Selenium' on 'Arachidonate 15−lipoxygenase B'

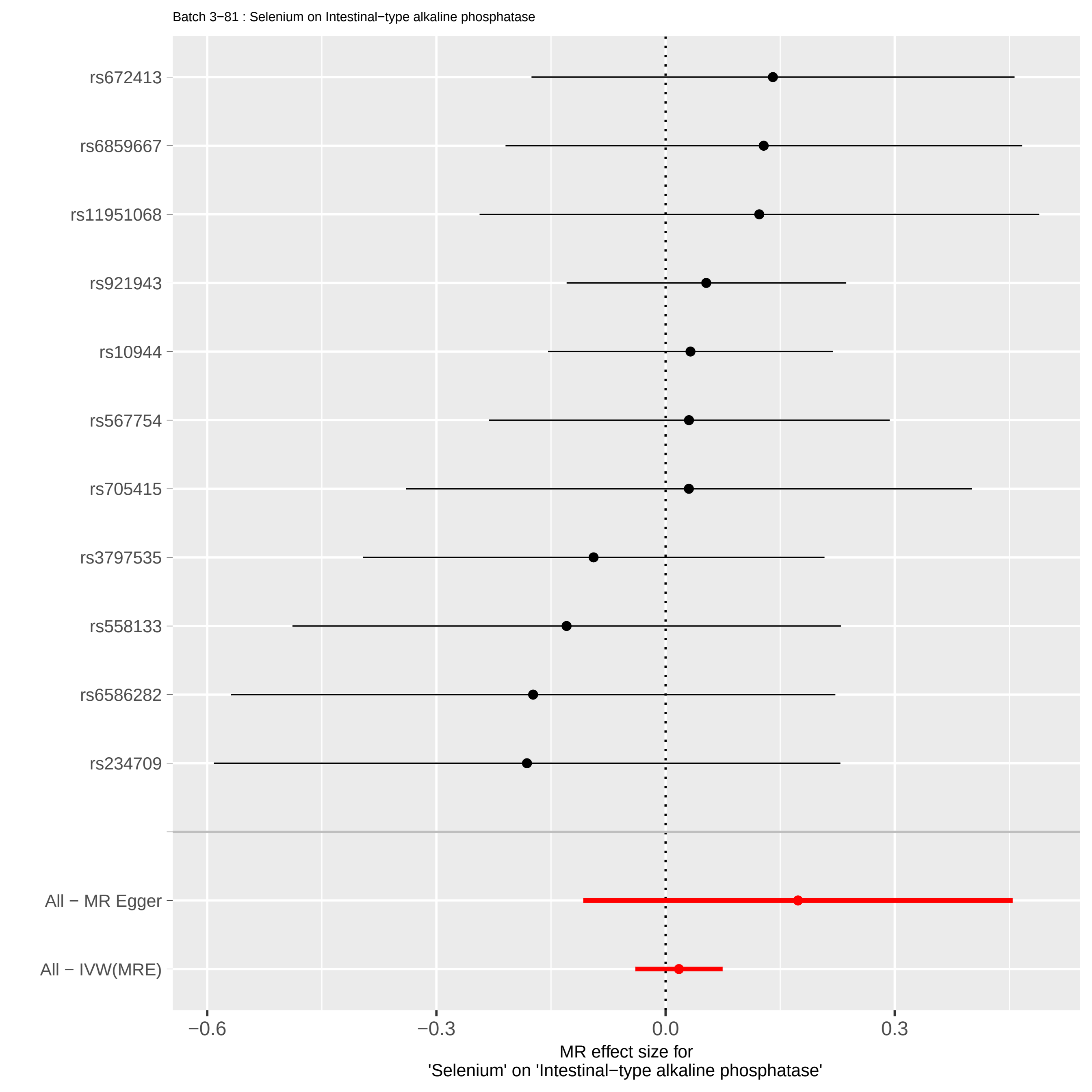

Batch 3−81 : Selenium on Intestinal−type alkaline phosphatase
rs672413
rs6859667
rs11951068
rs921943
rs10944
rs567754
rs705415
rs3797535
rs558133
rs6586282
rs234709
All − MR Egger
All − IVW(MRE)
−0.6
−0.3
0.0
0.3
MR effect size for
'Selenium' on 'Intestinal−type alkaline phosphatase'

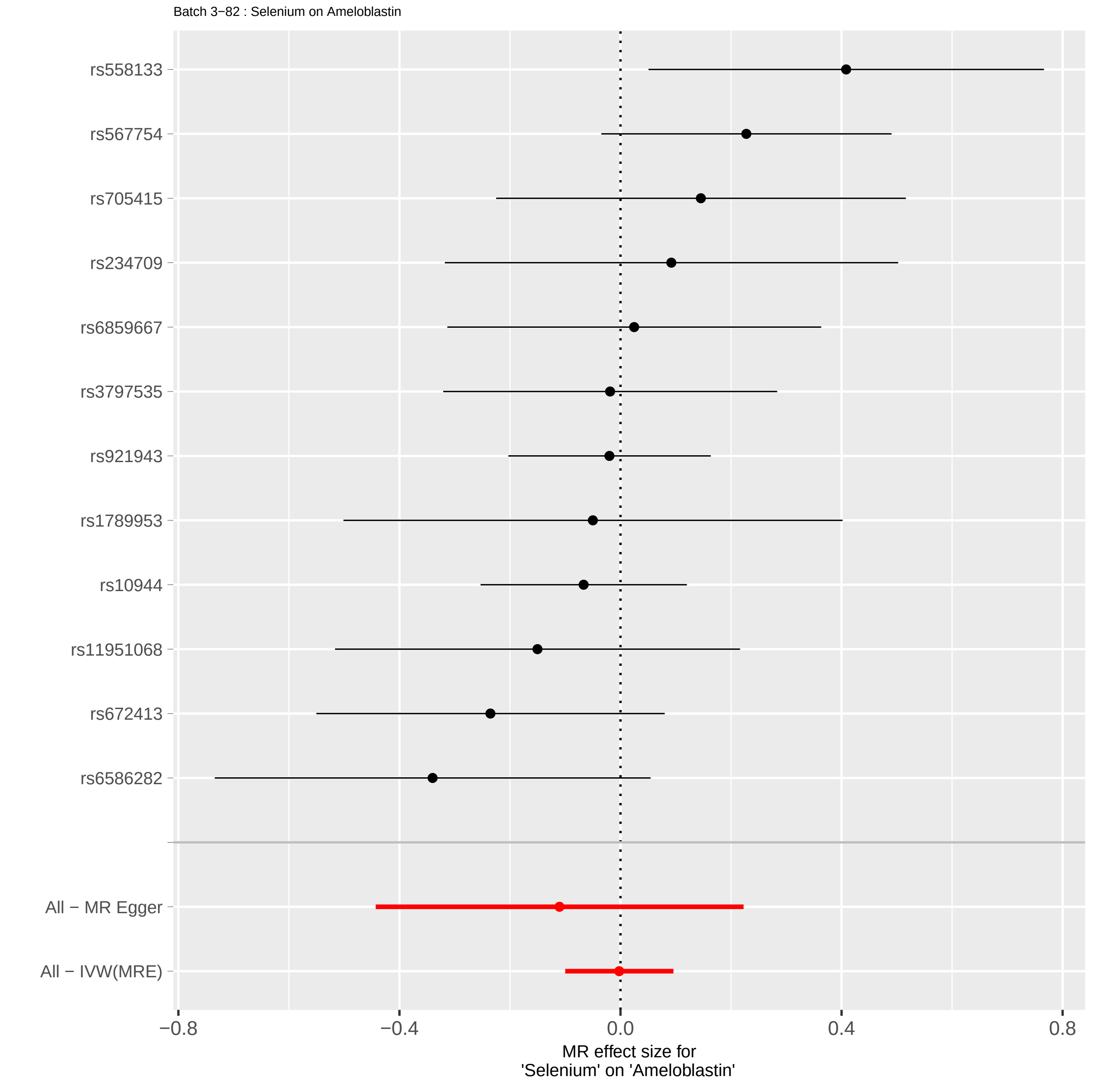
Batch 3−82 : Selenium on Ameloblastin
rs558133
rs567754
rs705415
rs234709
rs6859667
rs3797535
rs921943
rs1789953
rs10944
rs11951068
rs672413
rs6586282
All − MR Egger
All − IVW(MRE)
−0.8
−0.4
0.0
0.4
0.8
MR effect size for
'Selenium' on 'Ameloblastin'

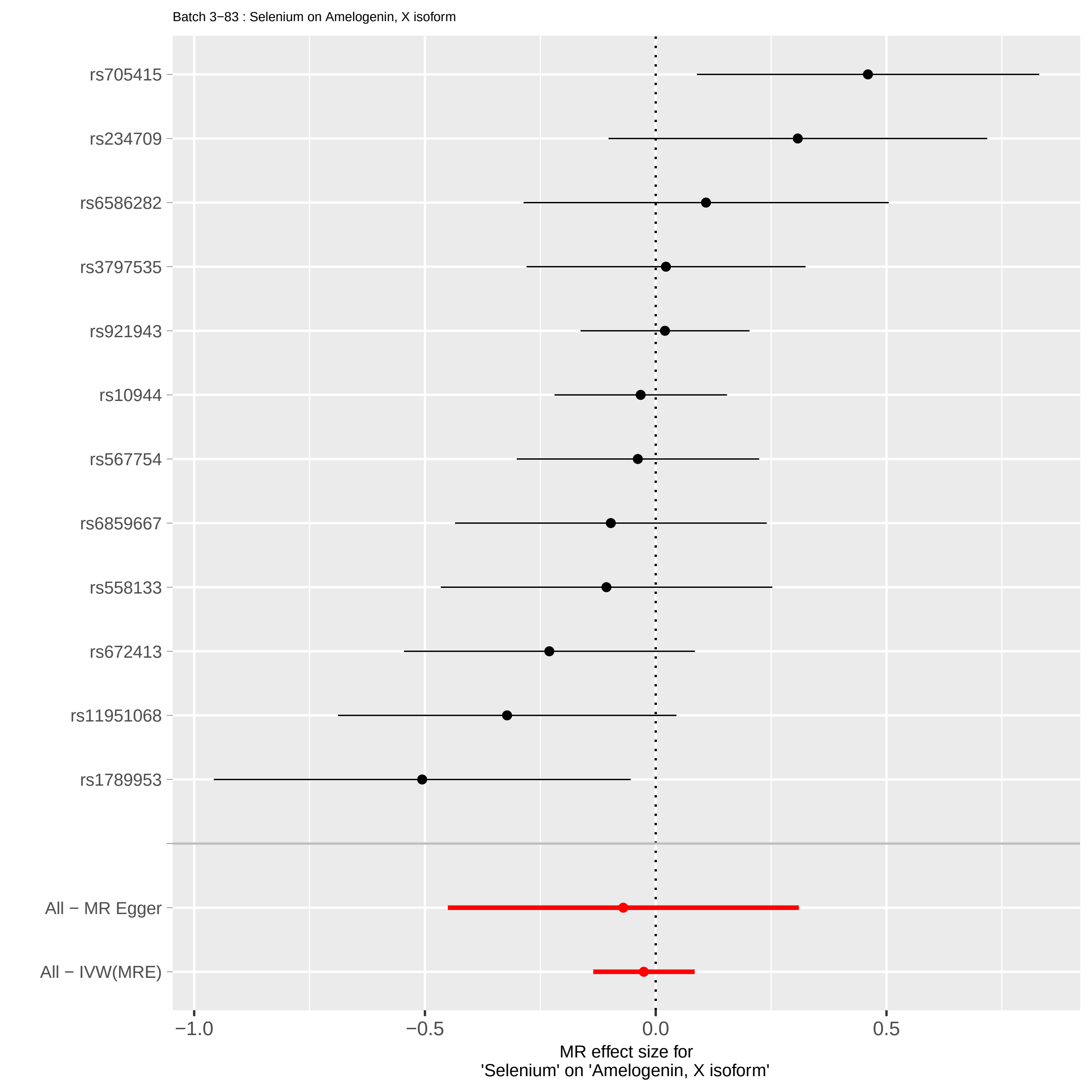
Batch 3−83 : Selenium on Amelogenin, X isoform
rs705415
rs234709
rs6586282
rs3797535
rs921943
rs10944
rs567754
rs6859667
rs558133
rs672413
rs11951068
rs1789953
All − MR Egger
All − IVW(MRE)
−1.0
−0.5
0.0
0.5
MR effect size for
'Selenium' on 'Amelogenin, X isoform'

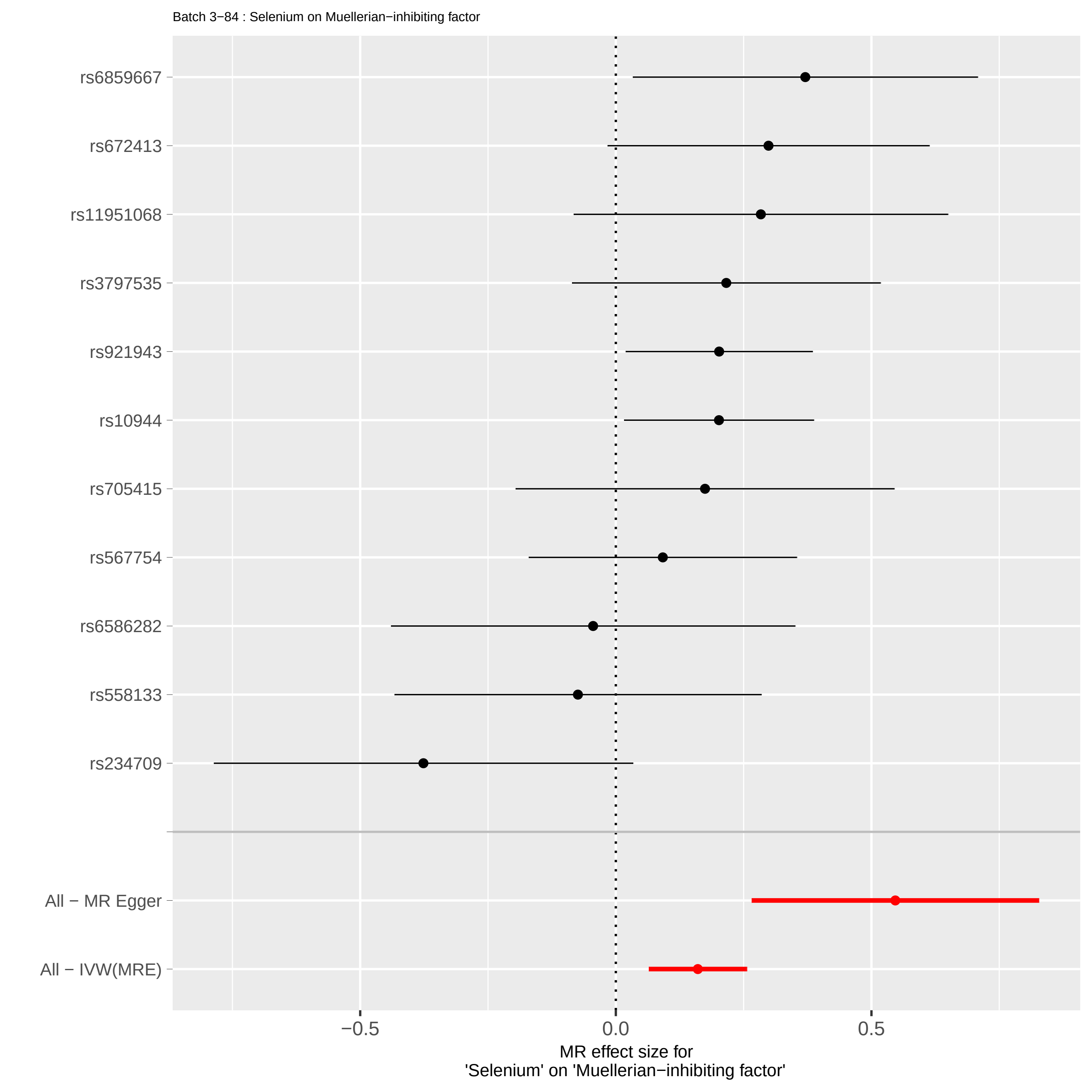
Batch 3−84 : Selenium on Muellerian−inhibiting factor
rs6859667
rs672413
rs11951068
rs3797535
rs921943
rs10944
rs705415
rs567754
rs6586282
rs558133
rs234709
All − MR Egger
All − IVW(MRE)
−0.5
0.0
0.5
MR effect size for
'Selenium' on 'Muellerian−inhibiting factor'

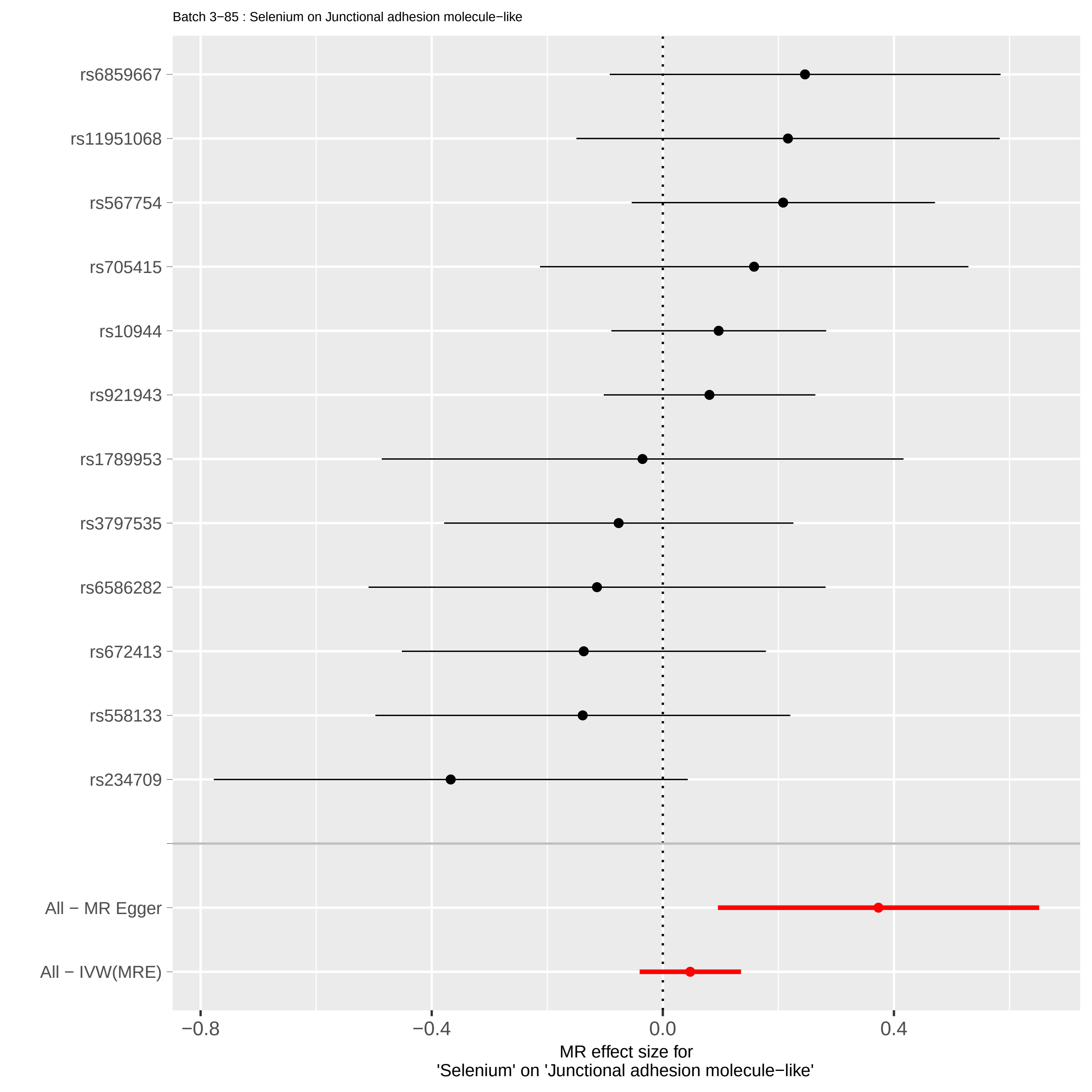
Batch 3−85 : Selenium on Junctional adhesion molecule−like
rs6859667
rs11951068
rs567754
rs705415
rs10944
rs921943
rs1789953
rs3797535
rs6586282
rs672413
rs558133
rs234709
All − MR Egger
All − IVW(MRE)
−0.8
−0.4
0.0
0.4
MR effect size for
'Selenium' on 'Junctional adhesion molecule−like'

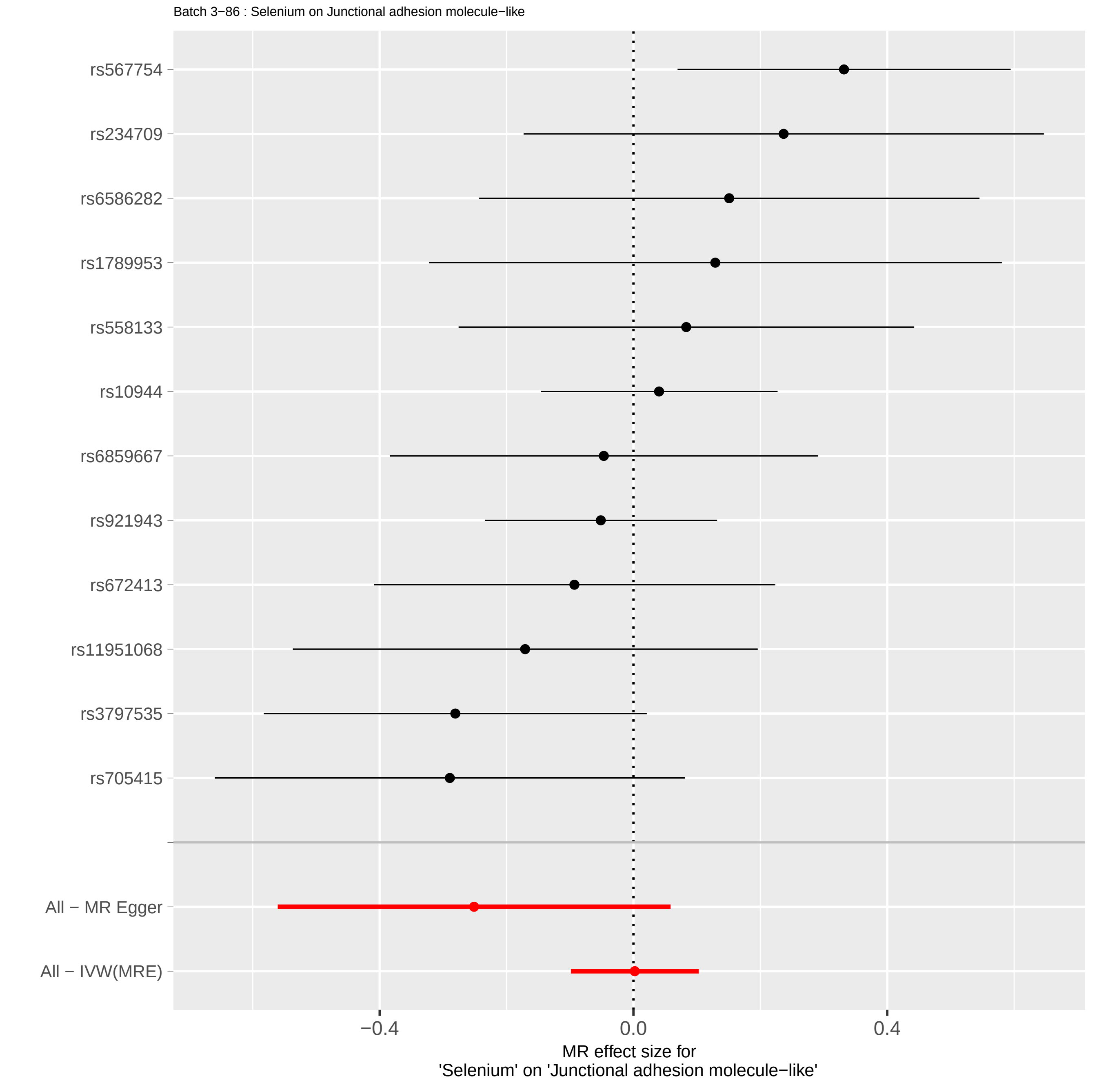

Batch 3−86 : Selenium on Junctional adhesion molecule−like
rs567754
rs234709
rs6586282
rs1789953
rs558133
rs10944
rs6859667
rs921943
rs672413
rs11951068
rs3797535
rs705415
All − MR Egger
All − IVW(MRE)
−0.4
0.0
0.4
MR effect size for
'Selenium' on 'Junctional adhesion molecule−like'

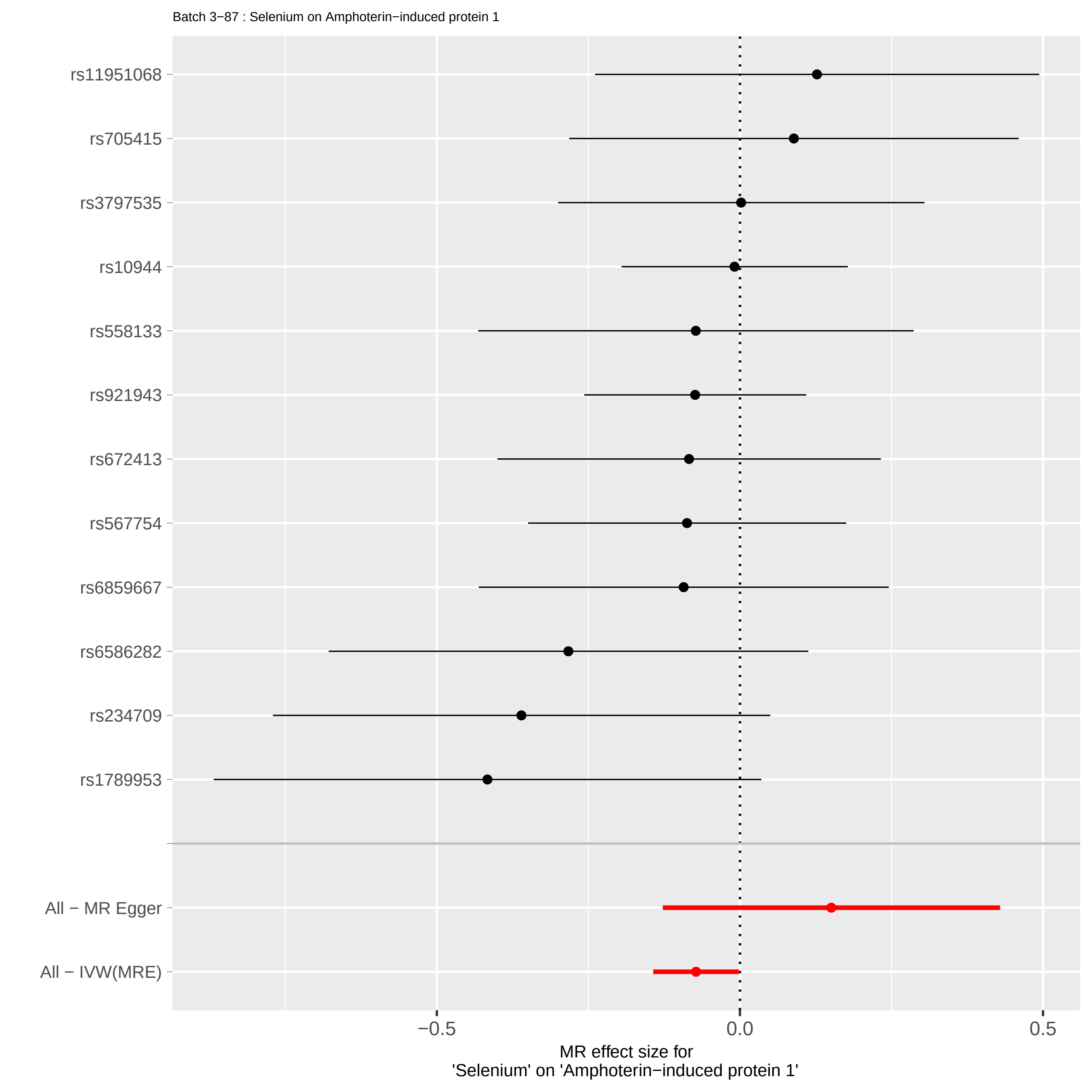

Batch 3−87 : Selenium on Amphoterin−induced protein 1
rs11951068
rs705415
rs3797535
rs10944
rs558133
rs921943
rs672413
rs567754
rs6859667
rs6586282
rs234709
rs1789953
All − MR Egger
All − IVW(MRE)
−0.5
0.0
0.5
MR effect size for
'Selenium' on 'Amphoterin−induced protein 1'

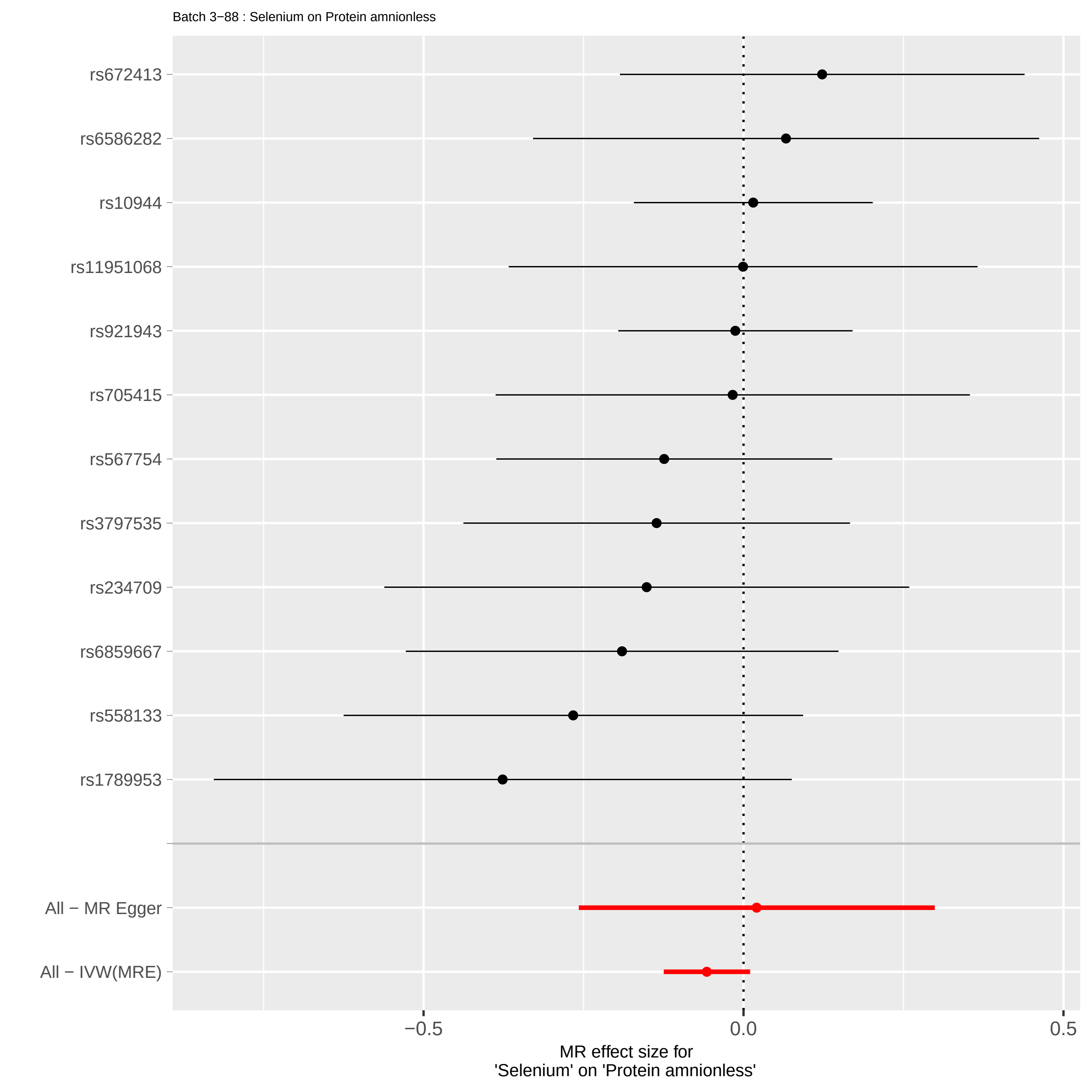
Batch 3−88 : Selenium on Protein amnionless
rs672413
rs6586282
rs10944
rs11951068
rs921943
rs705415
rs567754
rs3797535
rs234709
rs6859667
rs558133
rs1789953
All − MR Egger
All − IVW(MRE)
−0.5
0.0
0.5
MR effect size for
'Selenium' on 'Protein amnionless'

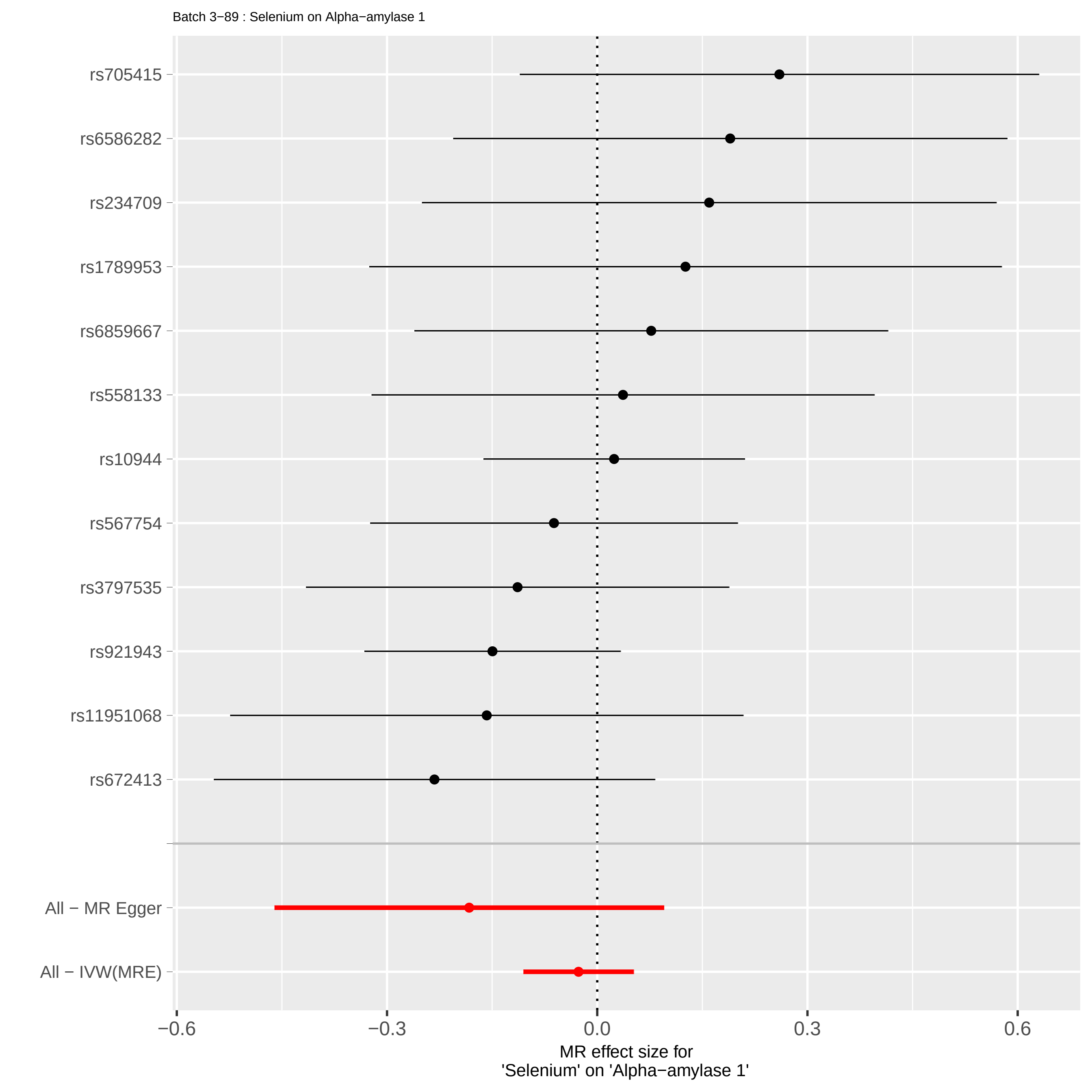
Batch 3−89 : Selenium on Alpha−amylase 1
rs705415
rs6586282
rs234709
rs1789953
rs6859667
rs558133
rs10944
rs567754
rs3797535
rs921943
rs11951068
rs672413
All − MR Egger
All − IVW(MRE)
−0.6
−0.3
0.0
0.3
0.6
MR effect size for
'Selenium' on 'Alpha−amylase 1'

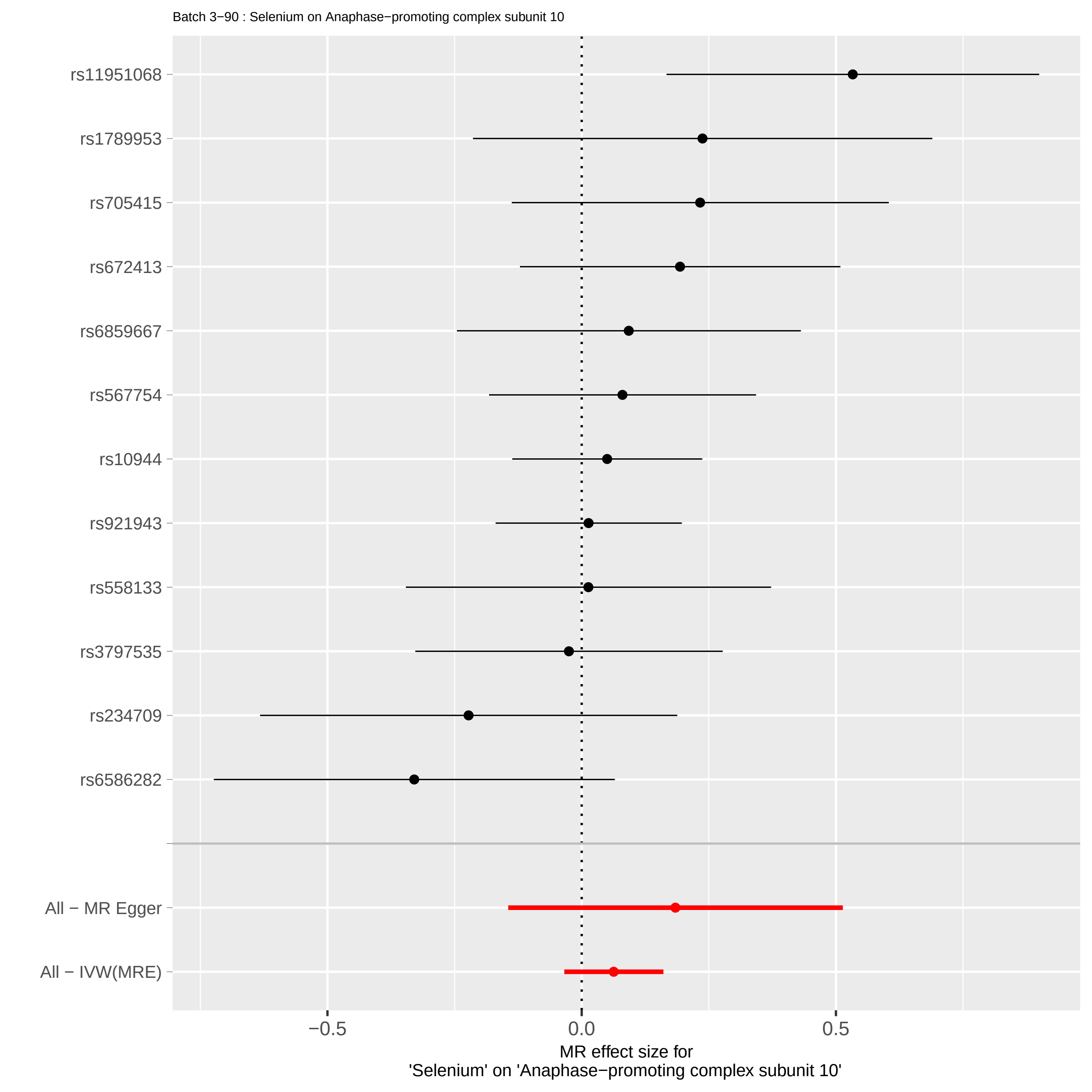

Batch 3−90 : Selenium on Anaphase−promoting complex subunit 10
rs11951068
rs1789953
rs705415
rs672413
rs6859667
rs567754
rs10944
rs921943
rs558133
rs3797535
rs234709
rs6586282
All − MR Egger
All − IVW(MRE)
−0.5
0.0
0.5
MR effect size for
'Selenium' on 'Anaphase−promoting complex subunit 10'

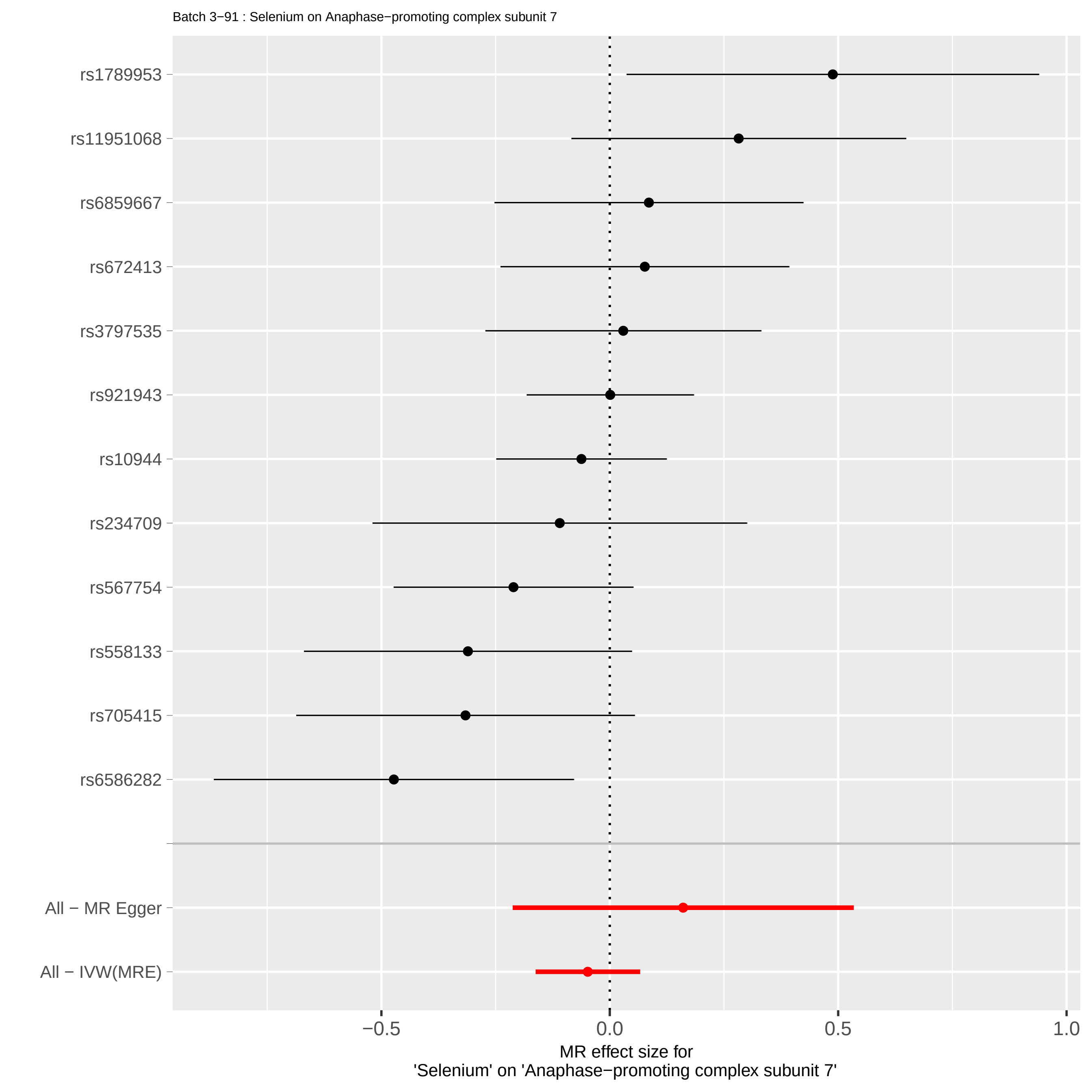

Batch 3−91 : Selenium on Anaphase−promoting complex subunit 7
rs1789953
rs11951068
rs6859667
rs672413
rs3797535
rs921943
rs10944
rs234709
rs567754
rs558133
rs705415
rs6586282
All − MR Egger
All − IVW(MRE)
−0.5
0.0
0.5
1.0
MR effect size for
'Selenium' on 'Anaphase−promoting complex subunit 7'

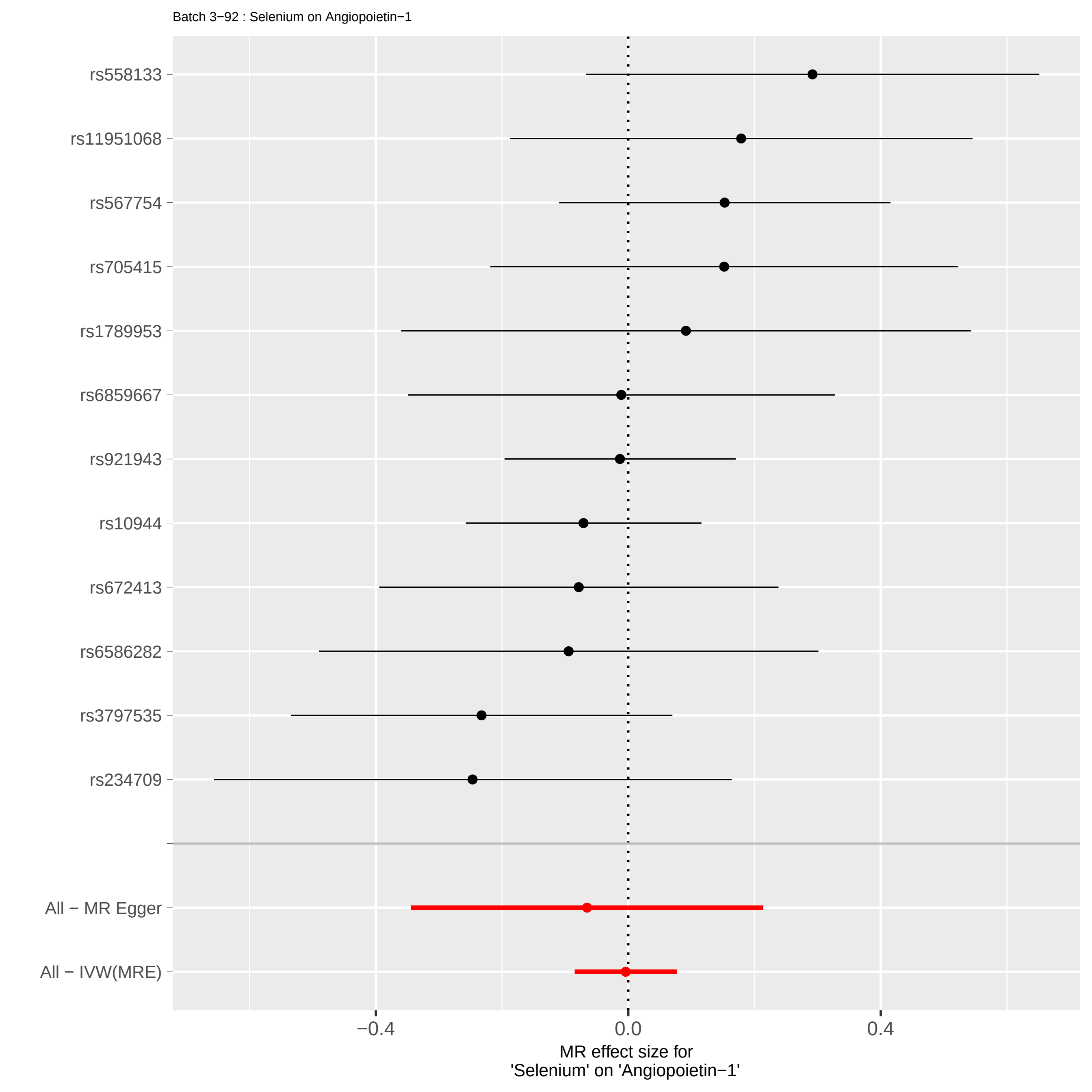
Batch 3−92 : Selenium on Angiopoietin−1
rs558133
rs11951068
rs567754
rs705415
rs1789953
rs6859667
rs921943
rs10944
rs672413
rs6586282
rs3797535
rs234709
All − MR Egger
All − IVW(MRE)
−0.4
0.0
0.4
MR effect size for
'Selenium' on 'Angiopoietin−1'

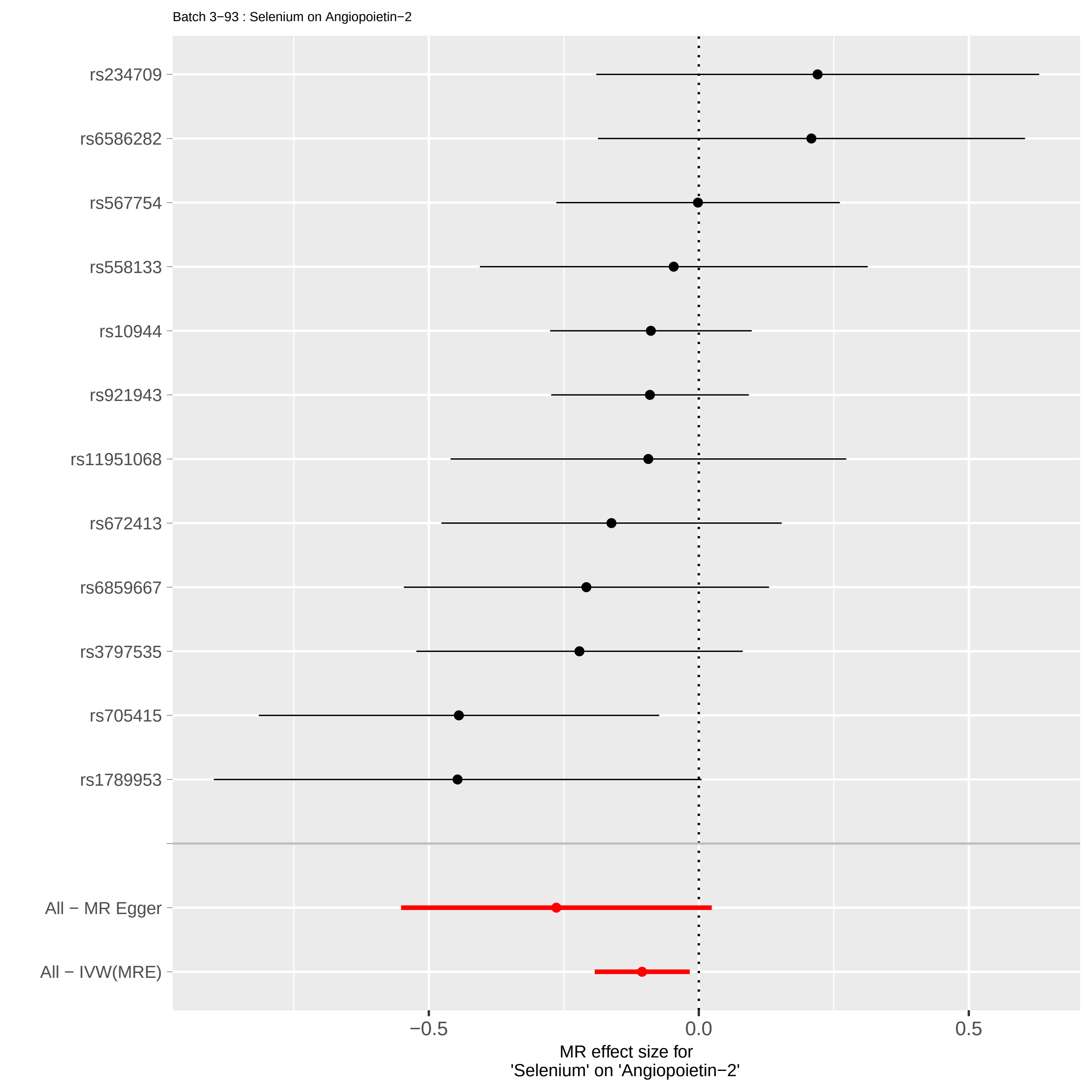
Batch 3−93 : Selenium on Angiopoietin−2
rs234709
rs6586282
rs567754
rs558133
rs10944
rs921943
rs11951068
rs672413
rs6859667
rs3797535
rs705415
rs1789953
All − MR Egger
All − IVW(MRE)
−0.5
0.0
0.5
MR effect size for
'Selenium' on 'Angiopoietin−2'

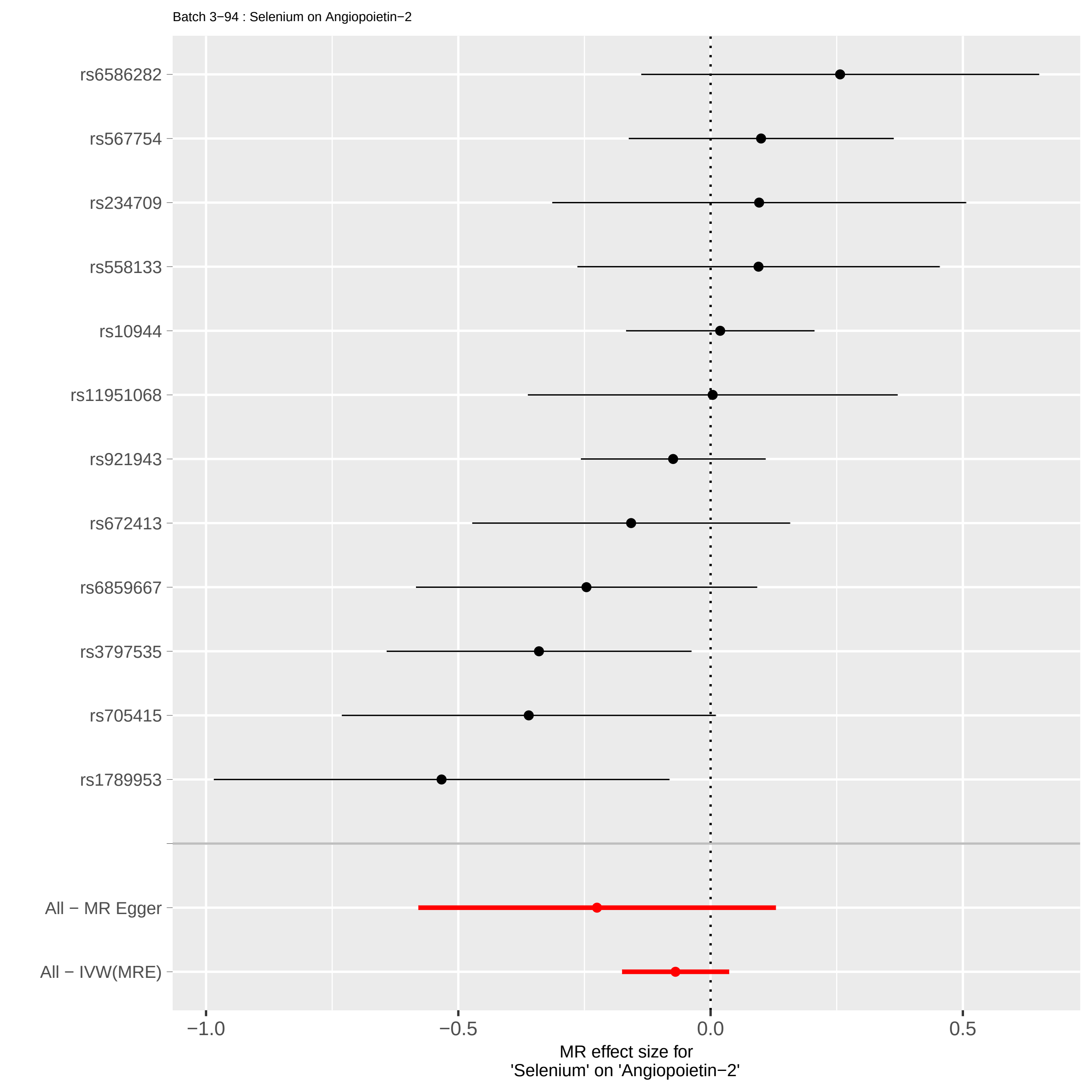

Batch 3−94 : Selenium on Angiopoietin−2
rs6586282
rs567754
rs234709
rs558133
rs10944
rs11951068
rs921943
rs672413
rs6859667
rs3797535
rs705415
rs1789953
All − MR Egger
All − IVW(MRE)
−1.0
−0.5
0.0
0.5
MR effect size for
'Selenium' on 'Angiopoietin−2'

Batch 3−95 : Selenium on Angiopoietin−4

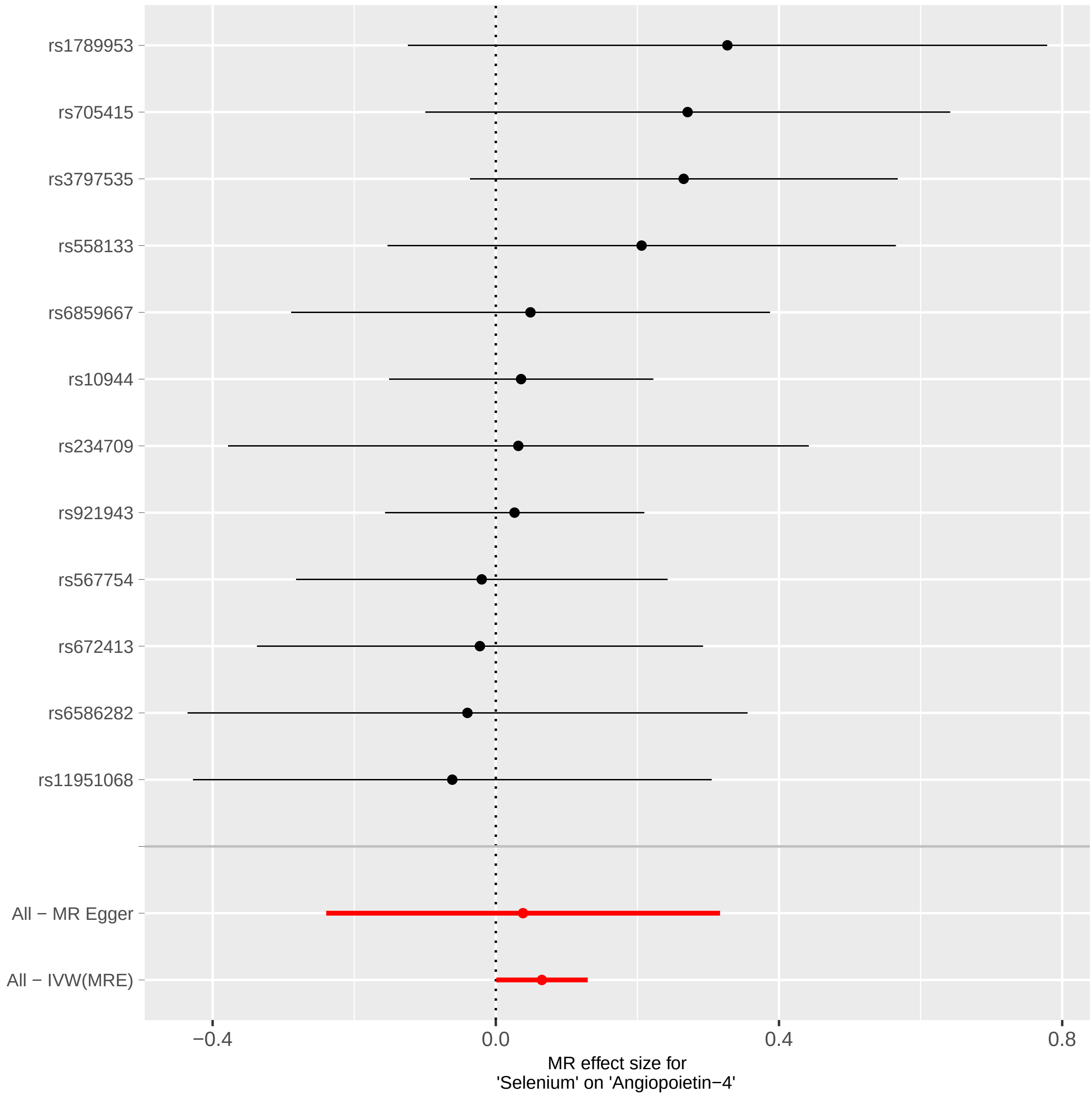

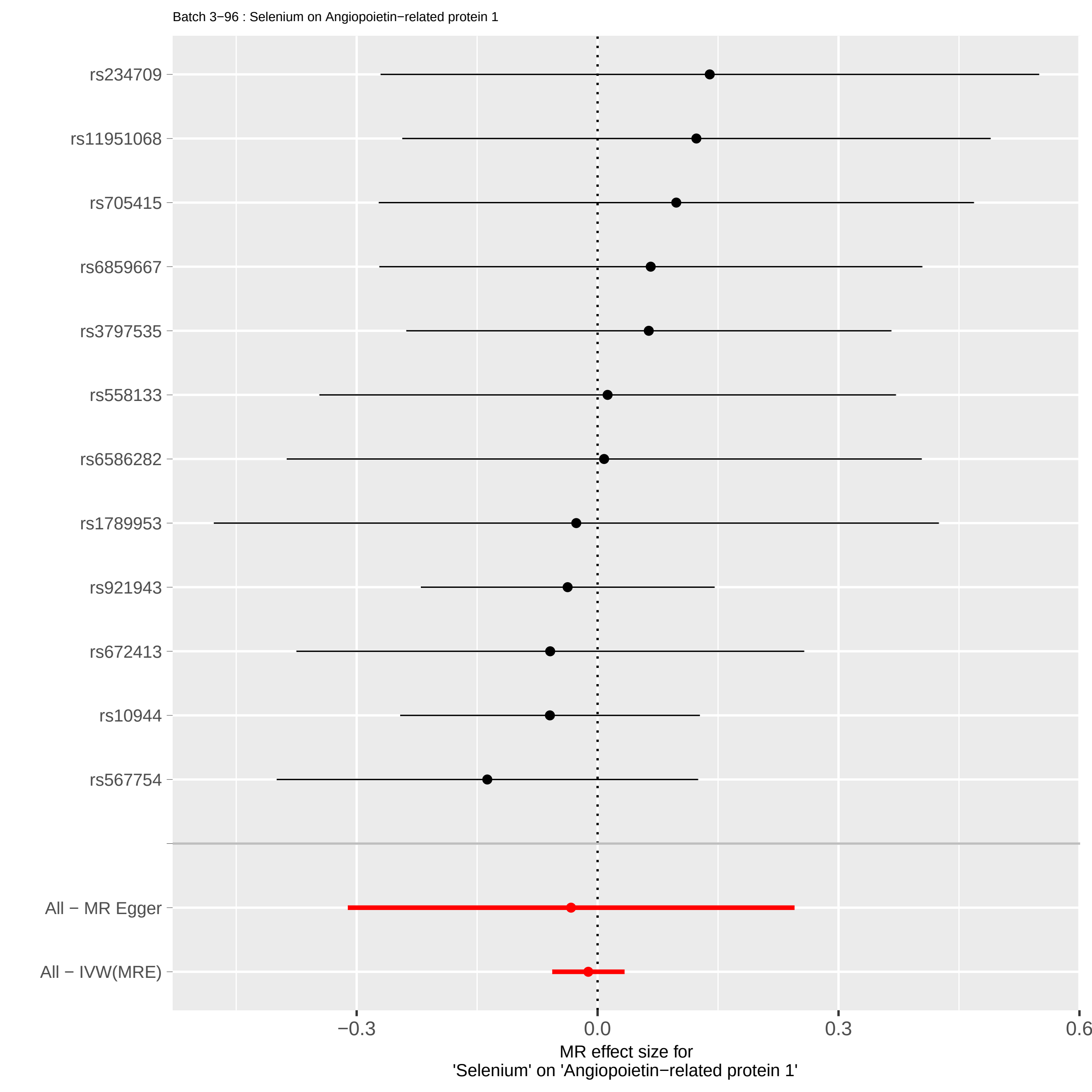
Batch 3−96 : Selenium on Angiopoietin−related protein 1
rs234709
rs11951068
rs705415
rs6859667
rs3797535
rs558133
rs6586282
rs1789953
rs921943
rs672413
rs10944
rs567754
All − MR Egger
All − IVW(MRE)
−0.3
0.0
0.3
0.6
MR effect size for
'Selenium' on 'Angiopoietin−related protein 1'

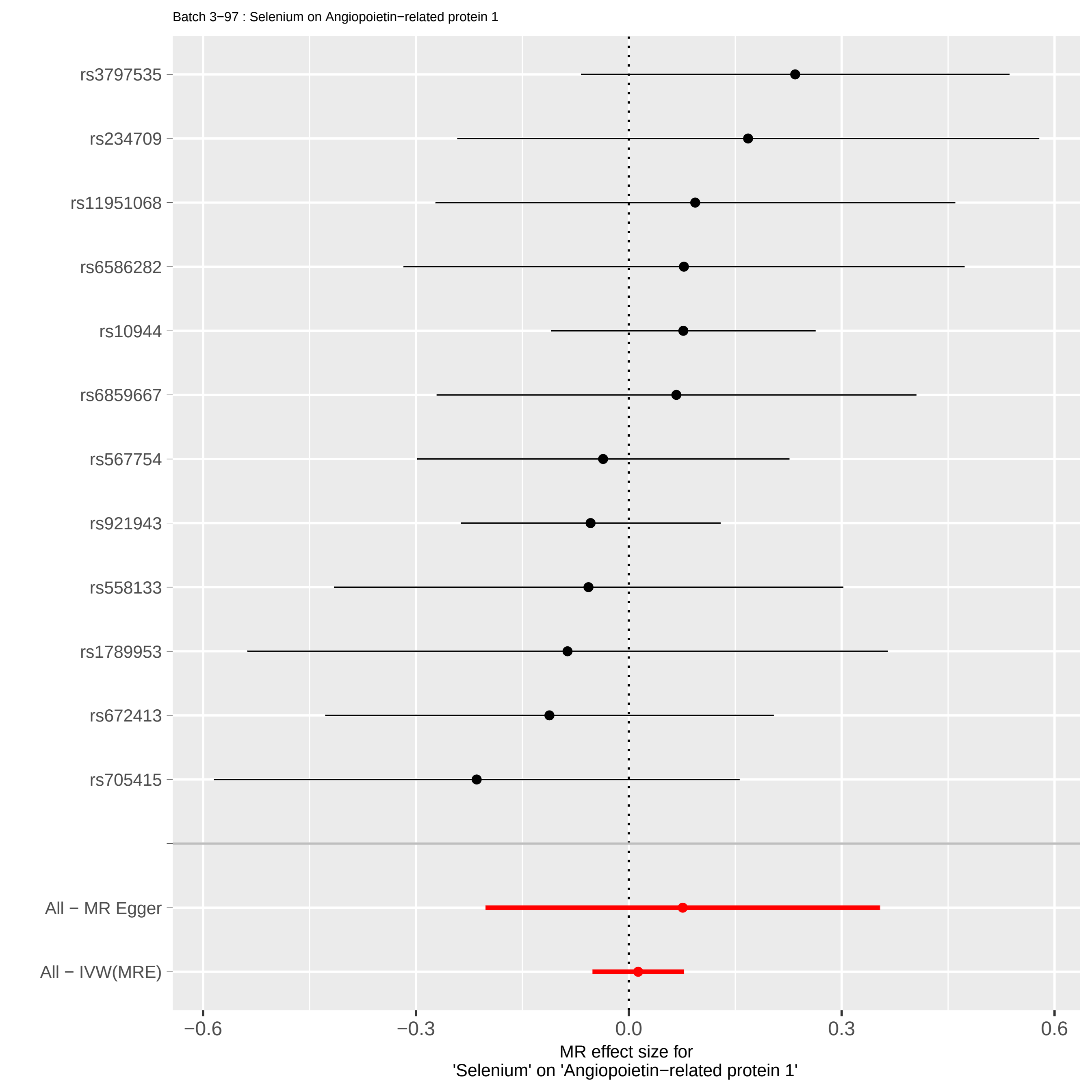

Batch 3−97 : Selenium on Angiopoietin−related protein 1
rs3797535
rs234709
rs11951068
rs6586282
rs10944
rs6859667
rs567754
rs921943
rs558133
rs1789953
rs672413
rs705415
All − MR Egger
All − IVW(MRE)
−0.6
−0.3
0.0
0.3
0.6
MR effect size for
'Selenium' on 'Angiopoietin−related protein 1'

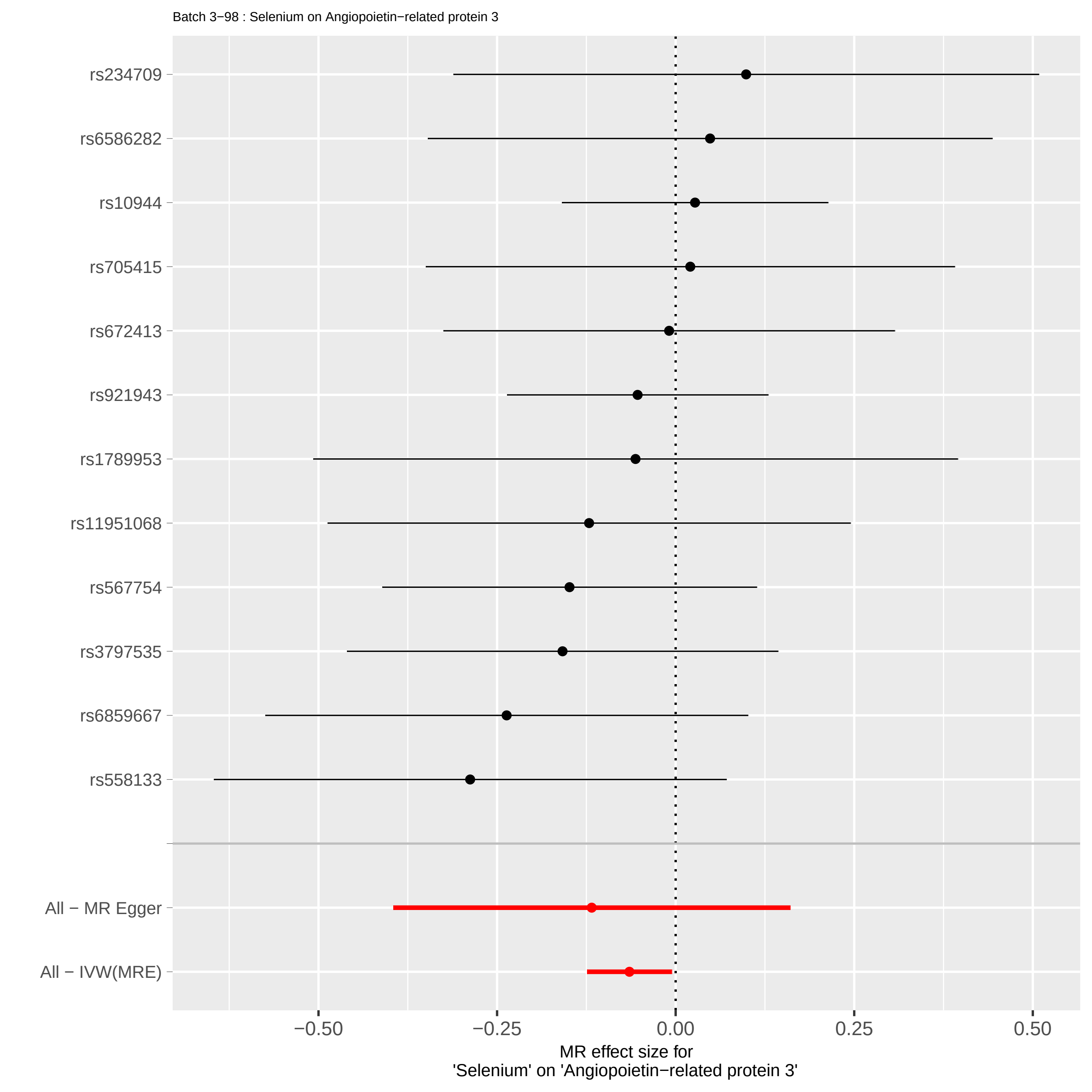

Batch 3−98 : Selenium on Angiopoietin−related protein 3
rs234709
rs6586282
rs10944
rs705415
rs672413
rs921943
rs1789953
rs11951068
rs567754
rs3797535
rs6859667
rs558133
All − MR Egger
All − IVW(MRE)
−0.50
−0.25
0.00
0.25
0.50
MR effect size for
'Selenium' on 'Angiopoietin−related protein 3'

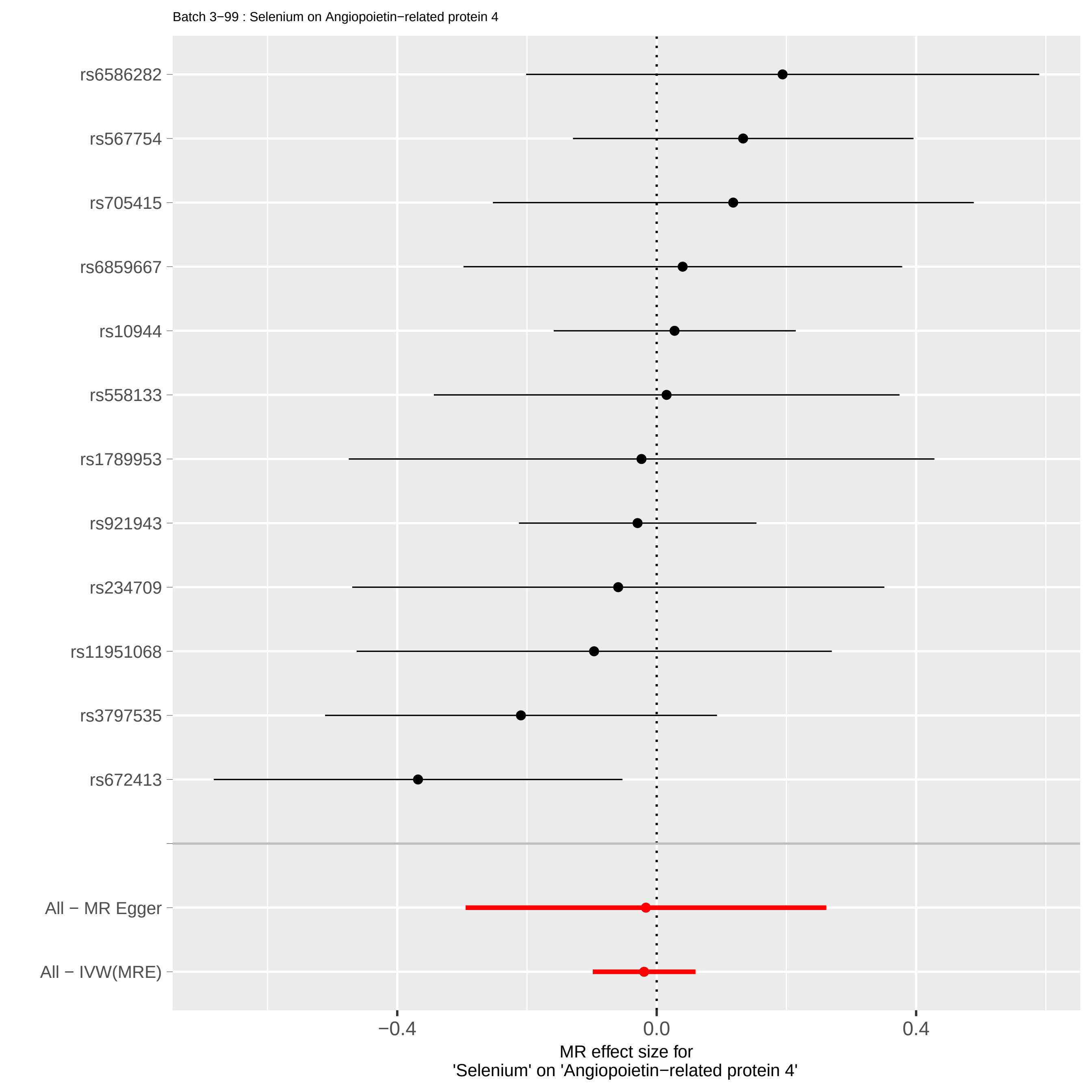
Batch 3−99 : Selenium on Angiopoietin−related protein 4
rs6586282
rs567754
rs705415
rs6859667
rs10944
rs558133
rs1789953
rs921943
rs234709
rs11951068
rs3797535
rs672413
All − MR Egger
All − IVW(MRE)
−0.4
0.0
0.4
MR effect size for
'Selenium' on 'Angiopoietin−related protein 4'

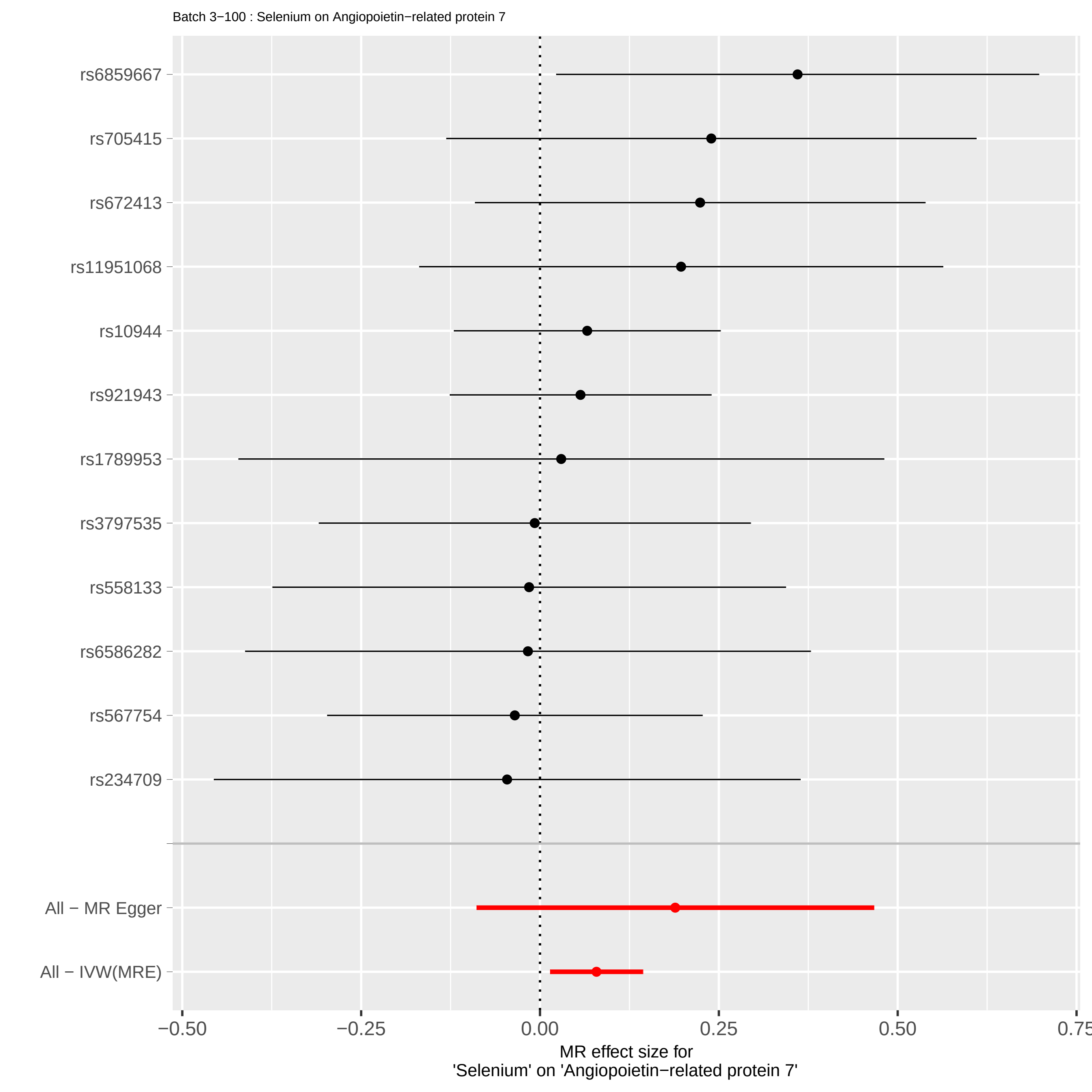

Batch 3−100 : Selenium on Angiopoietin−related protein 7
rs6859667
rs705415
rs672413
rs11951068
rs10944
rs921943
rs1789953
rs3797535
rs558133
rs6586282
rs567754
rs234709
All − MR Egger
All − IVW(MRE)
−0.50
−0.25
0.00
0.25
0.50
0.75
MR effect size for
'Selenium' on 'Angiopoietin−related protein 7'

Batch 3−101 : Selenium on Ankyrin−2

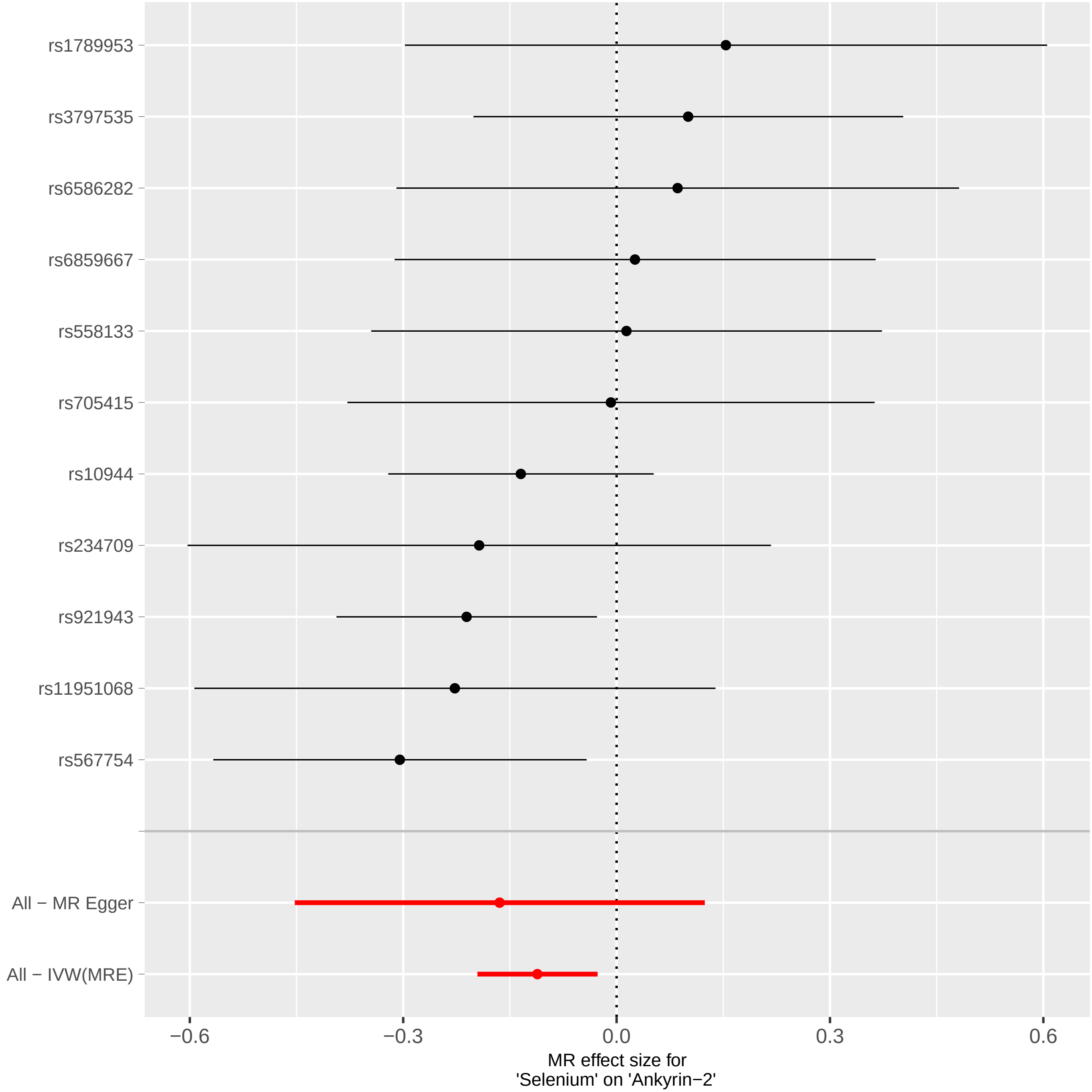

Batch 3−102 : Selenium on Ankyrin repeat domain−containing protein 27

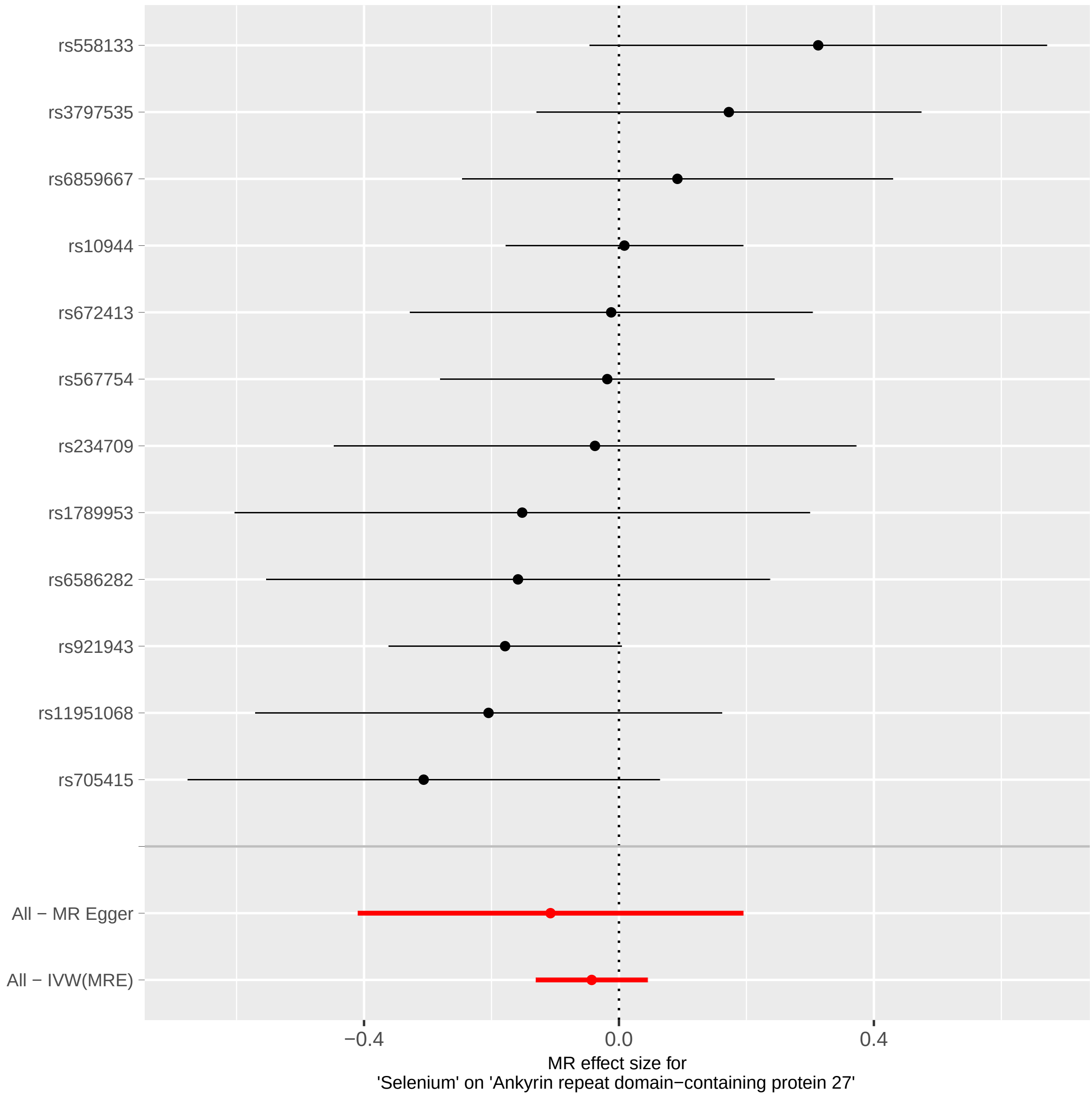

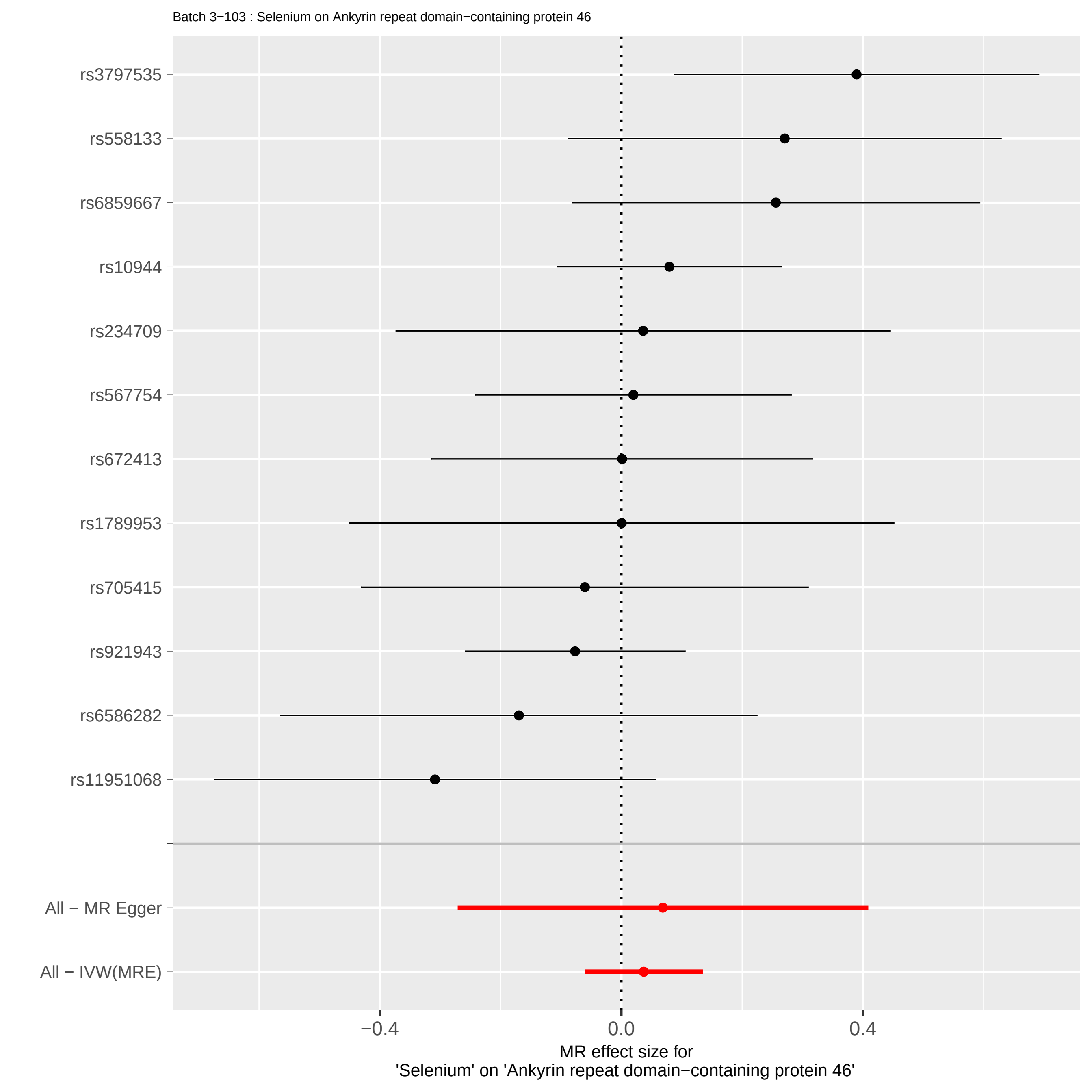
Batch 3−103 : Selenium on Ankyrin repeat domain−containing protein 46
rs3797535
rs558133
rs6859667
rs10944
rs234709
rs567754
rs672413
rs1789953
rs705415
rs921943
rs6586282
rs11951068
All − MR Egger
All − IVW(MRE)
−0.4
0.0
0.4
MR effect size for
'Selenium' on 'Ankyrin repeat domain−containing protein 46'

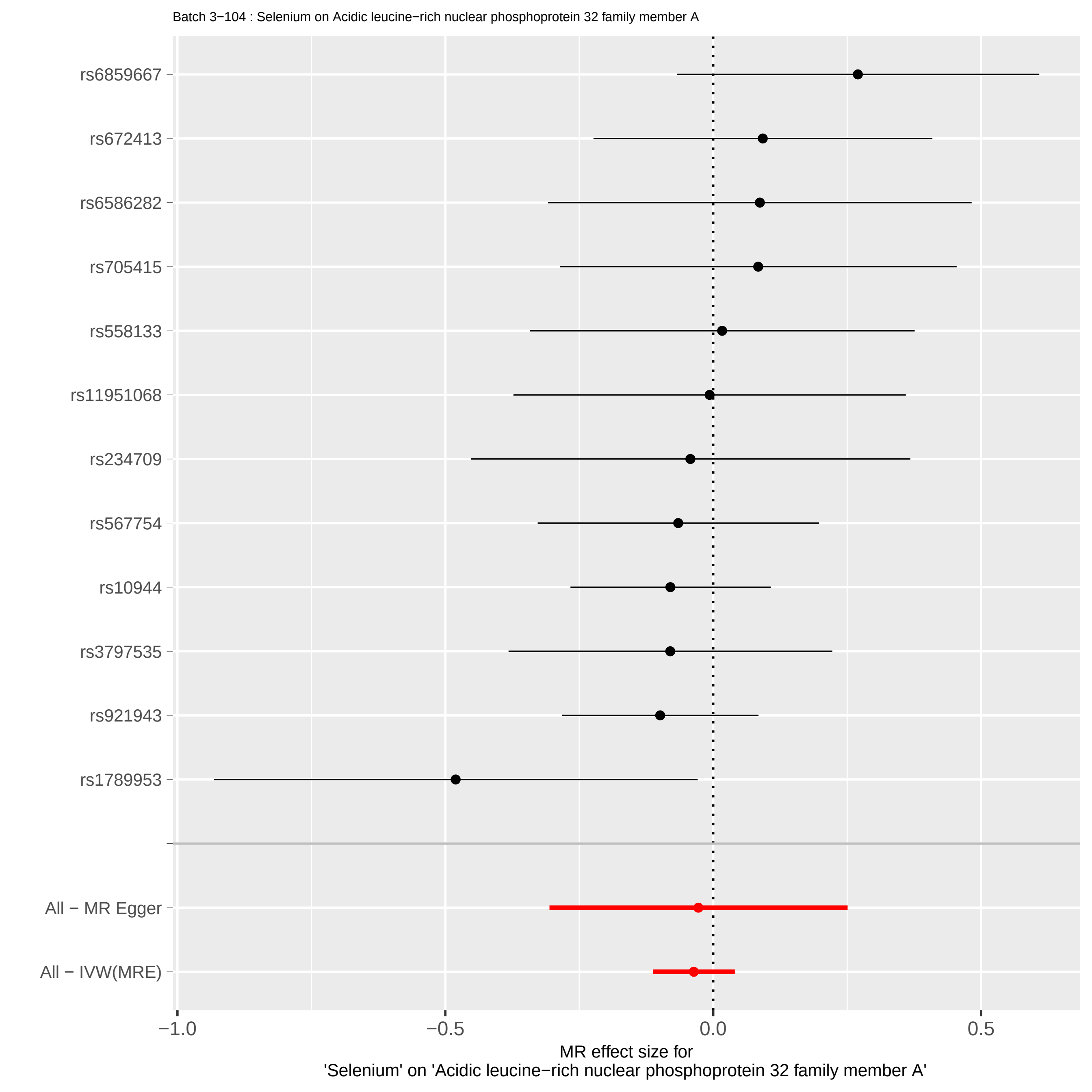

Batch 3−104 : Selenium on Acidic leucine−rich nuclear phosphoprotein 32 family member A
rs6859667
rs672413
rs6586282
rs705415
rs558133
rs11951068
rs234709
rs567754
rs10944
rs3797535
rs921943
rs1789953
All − MR Egger
All − IVW(MRE)
−1.0
−0.5
0.0
0.5
MR effect size for
'Selenium' on 'Acidic leucine−rich nuclear phosphoprotein 32 family member A'

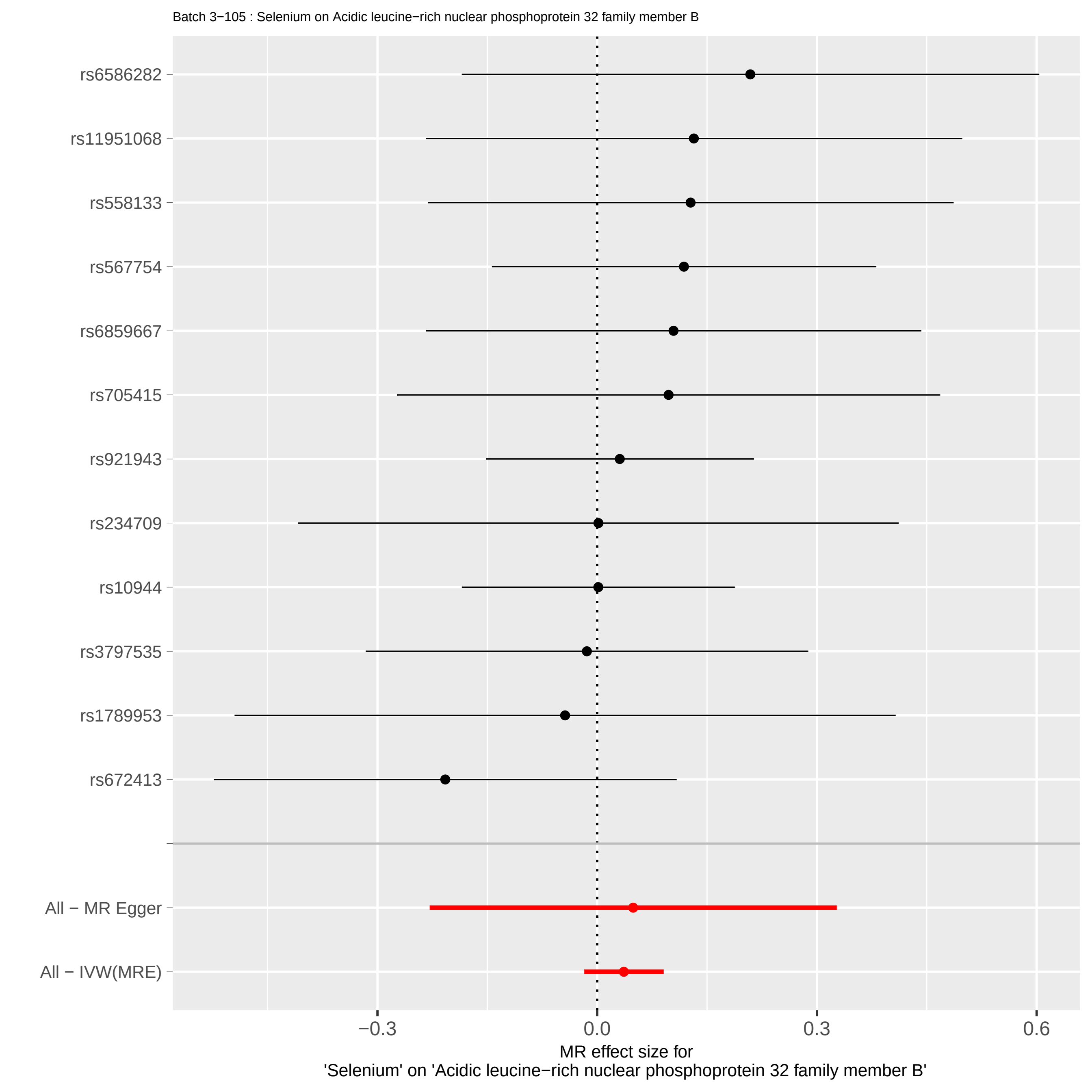
Batch 3−105 : Selenium on Acidic leucine−rich nuclear phosphoprotein 32 family member B
rs6586282
rs11951068
rs558133
rs567754
rs6859667
rs705415
rs921943
rs234709
rs10944
rs3797535
rs1789953
rs672413
All − MR Egger
All − IVW(MRE)
−0.3
0.0
0.3
0.6
MR effect size for
'Selenium' on 'Acidic leucine−rich nuclear phosphoprotein 32 family member B'

Batch 3−106 : Selenium on Annexin A10

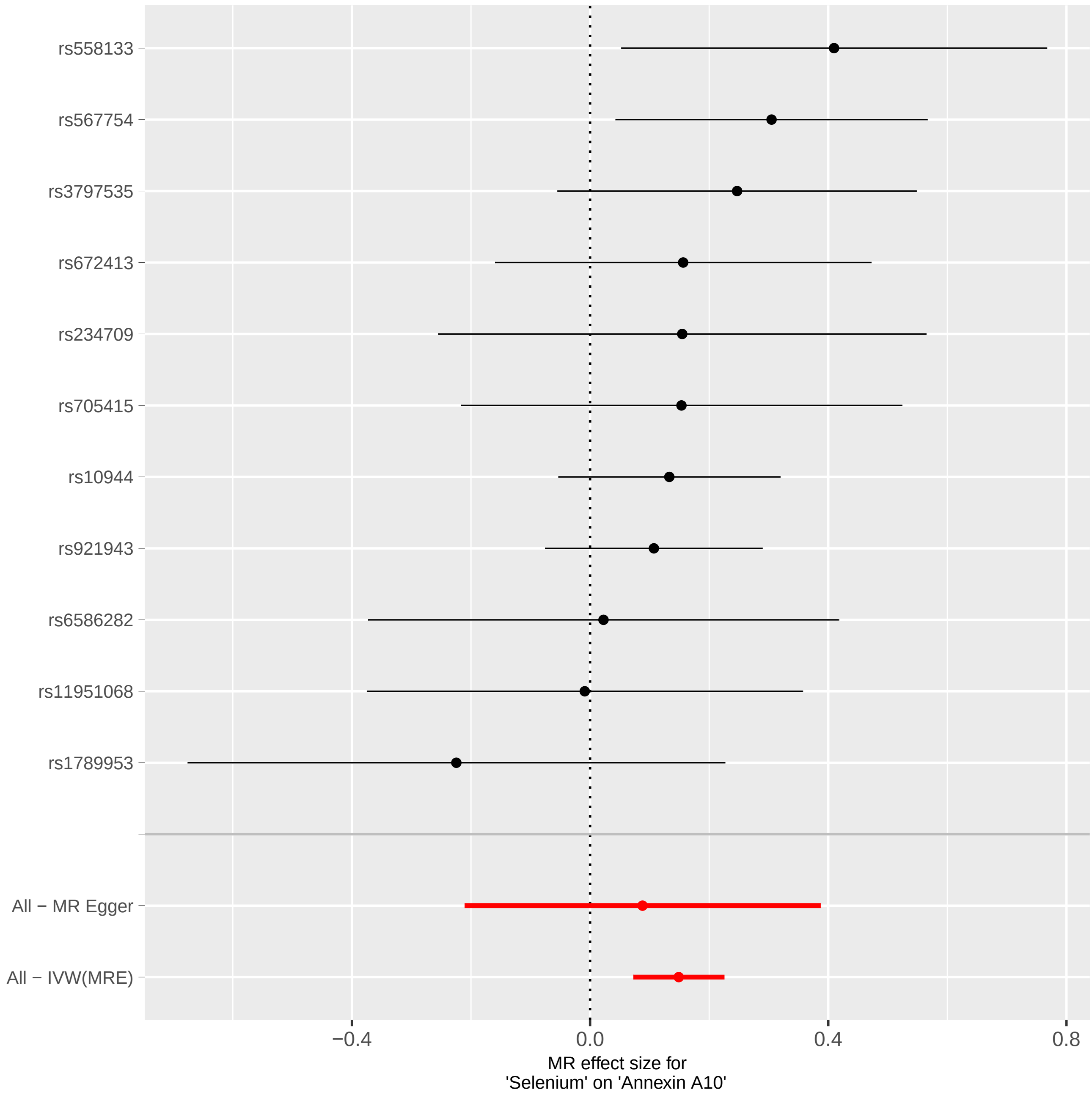

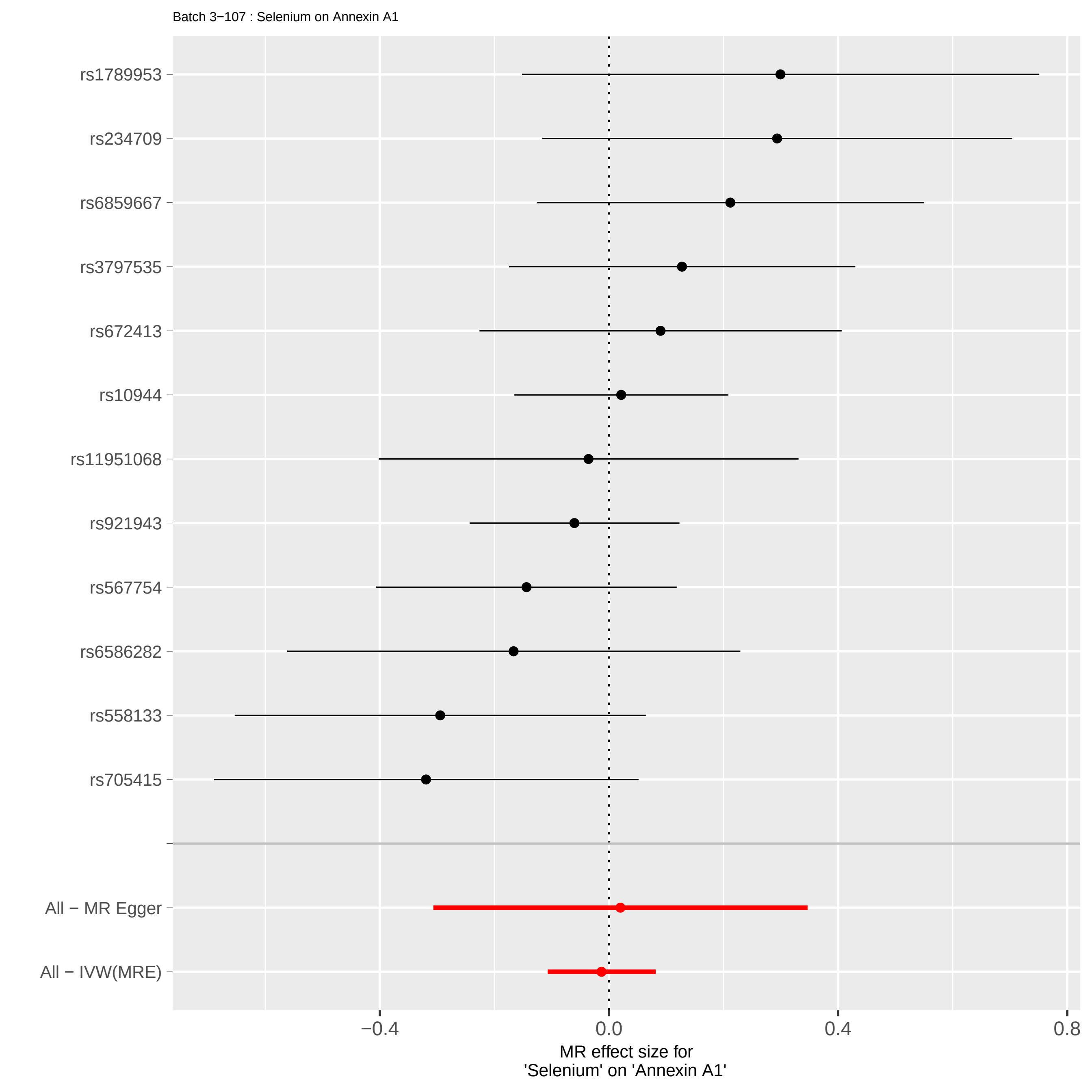
Batch 3−107 : Selenium on Annexin A1
rs1789953
rs234709
rs6859667
rs3797535
rs672413
rs10944
rs11951068
rs921943
rs567754
rs6586282
rs558133
rs705415
All − MR Egger
All − IVW(MRE)
−0.4
0.0
0.4
0.8
MR effect size for
'Selenium' on 'Annexin A1'

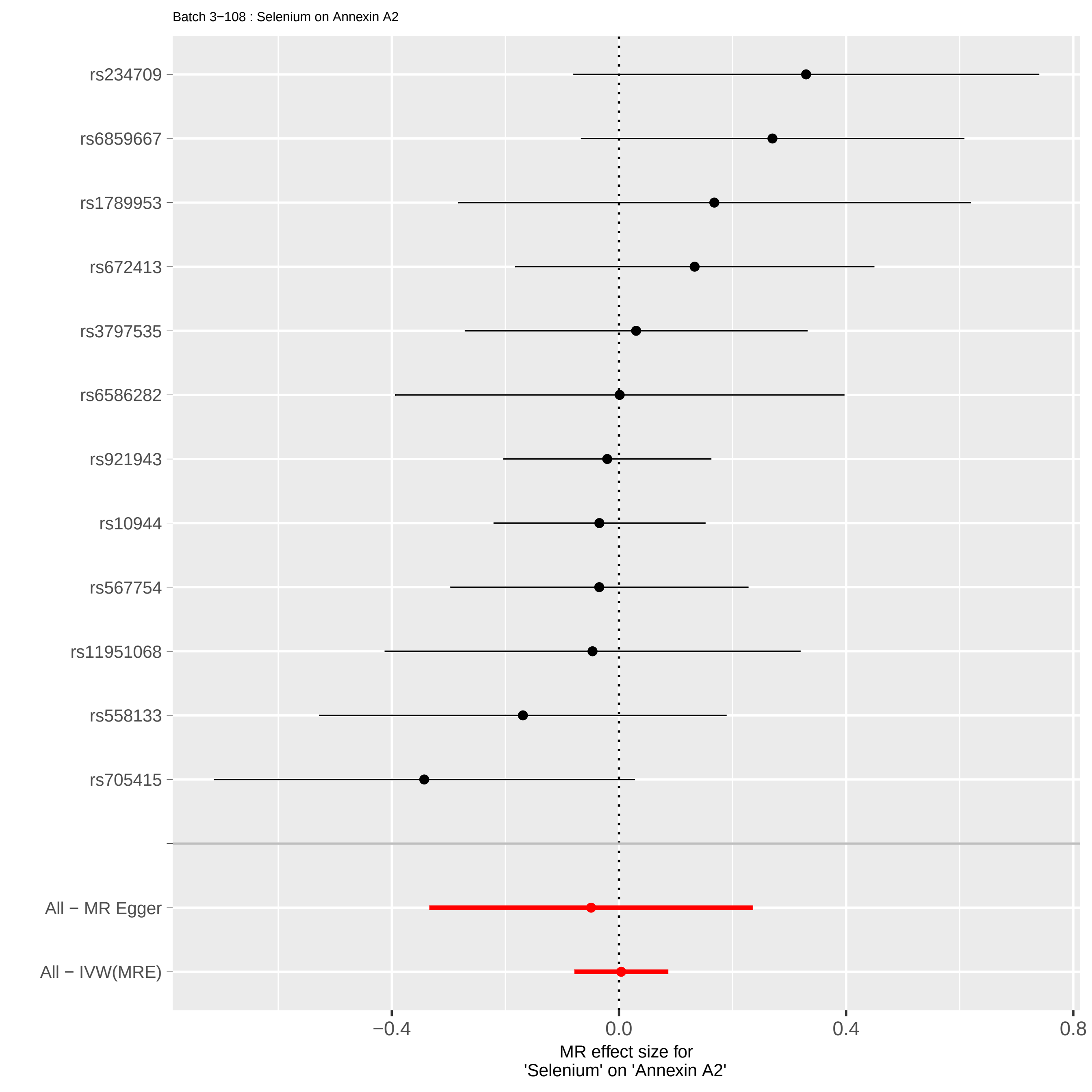

Batch 3−108 : Selenium on Annexin A2
rs234709
rs6859667
rs1789953
rs672413
rs3797535
rs6586282
rs921943
rs10944
rs567754
rs11951068
rs558133
rs705415
All − MR Egger
All − IVW(MRE)
−0.4
0.0
0.4
0.8
MR effect size for
'Selenium' on 'Annexin A2'

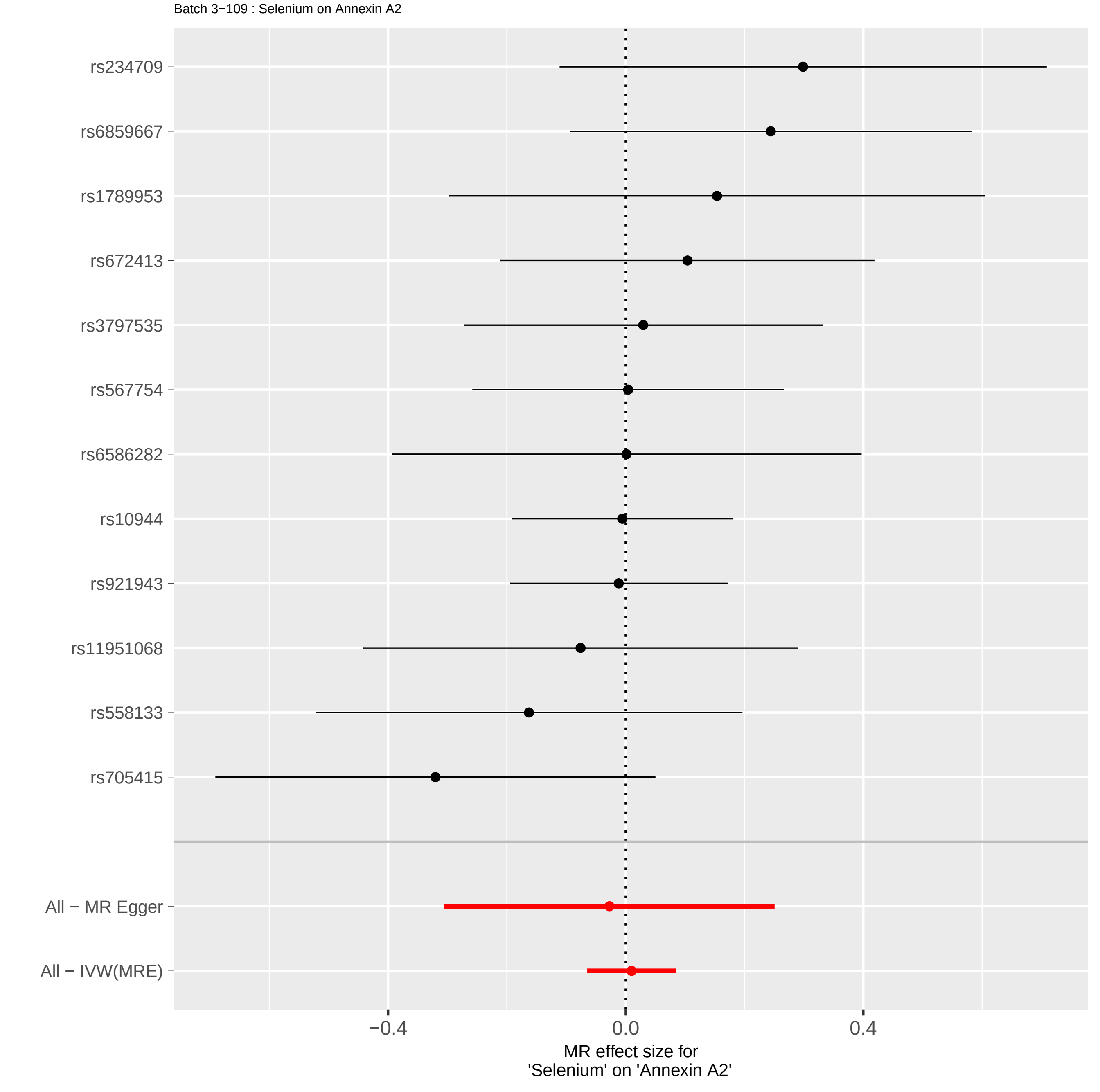
Batch 3−109 : Selenium on Annexin A2
rs234709
rs6859667
rs1789953
rs672413
rs3797535
rs567754
rs6586282
rs10944
rs921943
rs11951068
rs558133
rs705415
All − MR Egger
All − IVW(MRE)
−0.4
0.0
0.4
MR effect size for
'Selenium' on 'Annexin A2'

Batch 3−110 : Selenium on Annexin A9

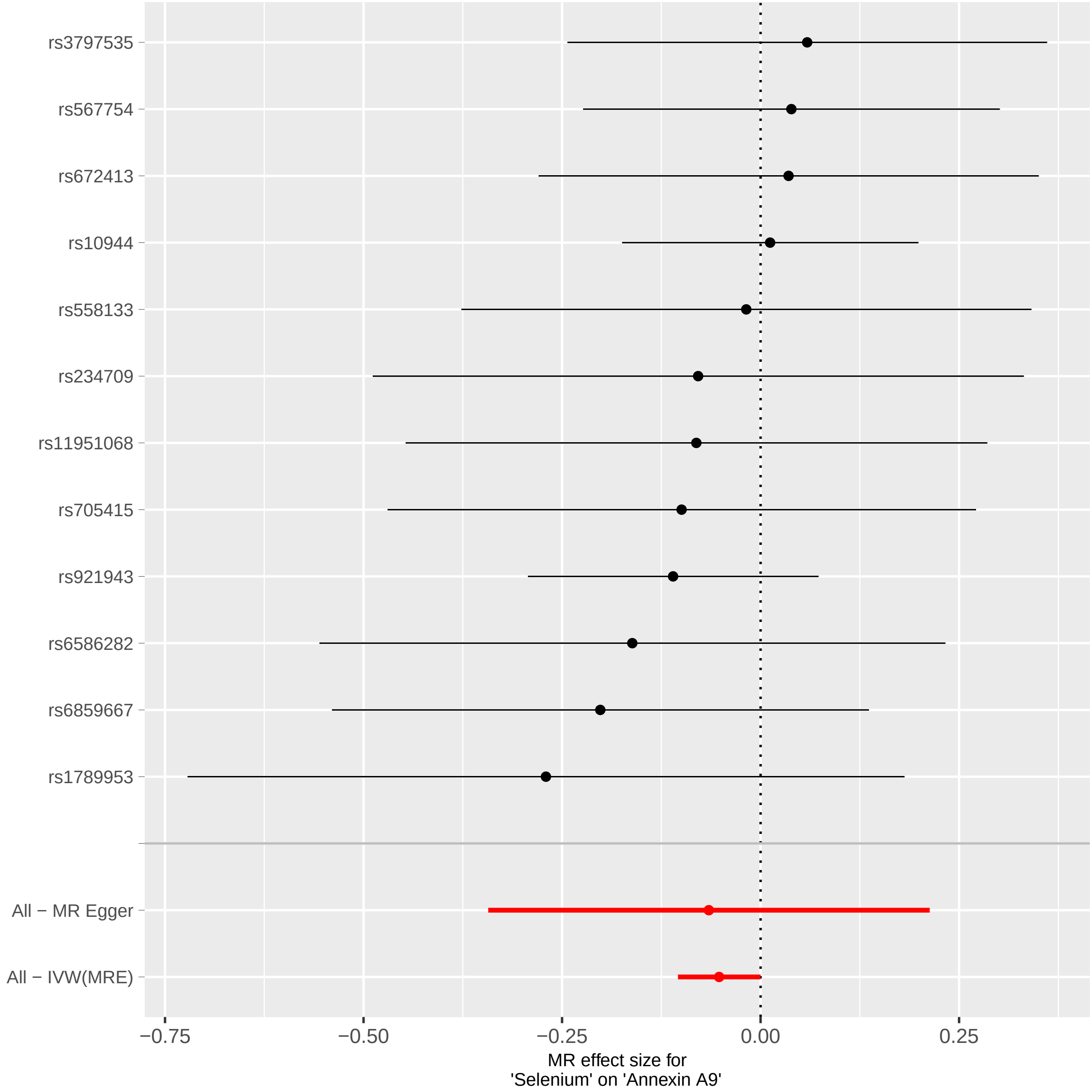

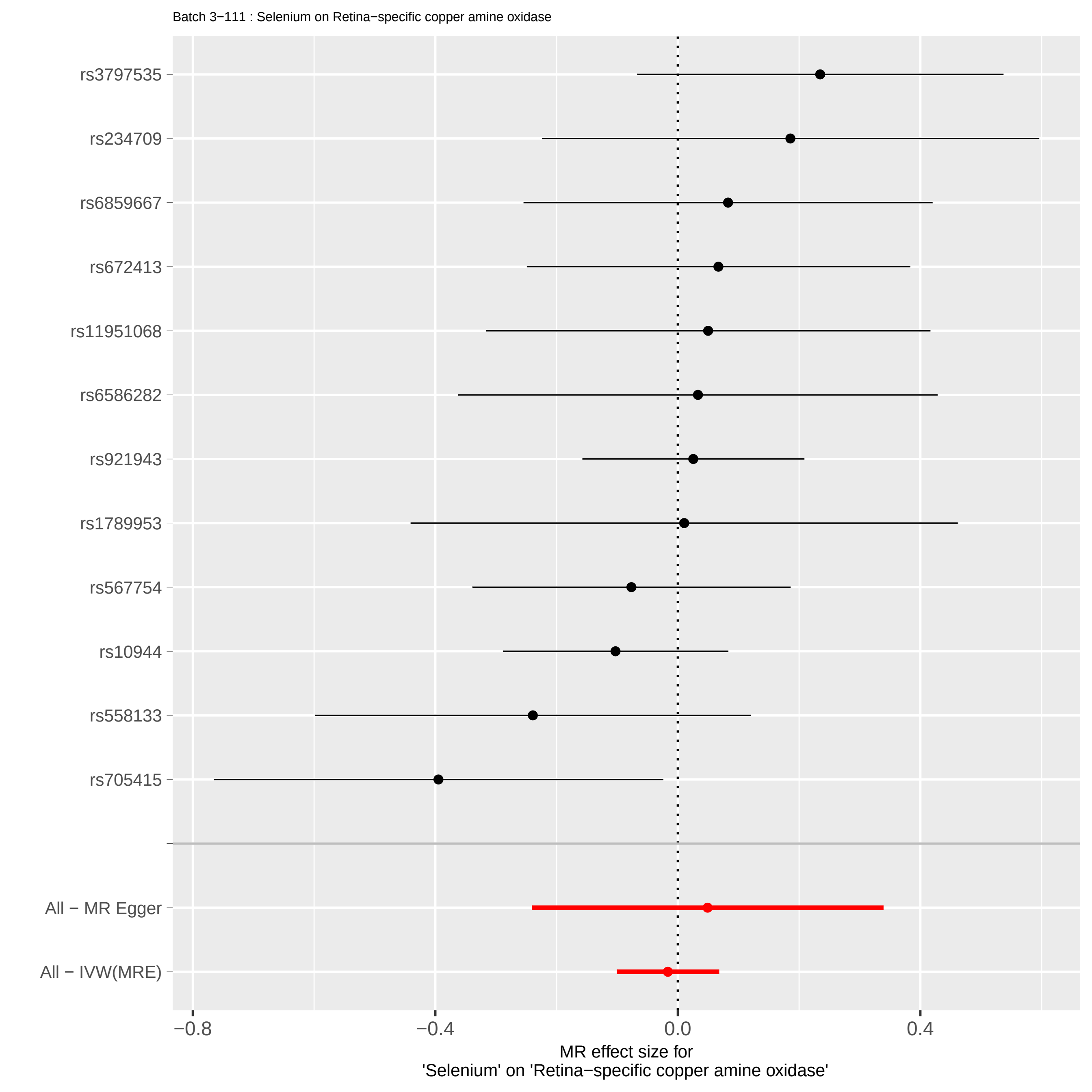

Batch 3−111 : Selenium on Retina−specific copper amine oxidase
rs3797535
rs234709
rs6859667
rs672413
rs11951068
rs6586282
rs921943
rs1789953
rs567754
rs10944
rs558133
rs705415
All − MR Egger
All − IVW(MRE)
−0.8
−0.4
0.0
0.4
MR effect size for
'Selenium' on 'Retina−specific copper amine oxidase'

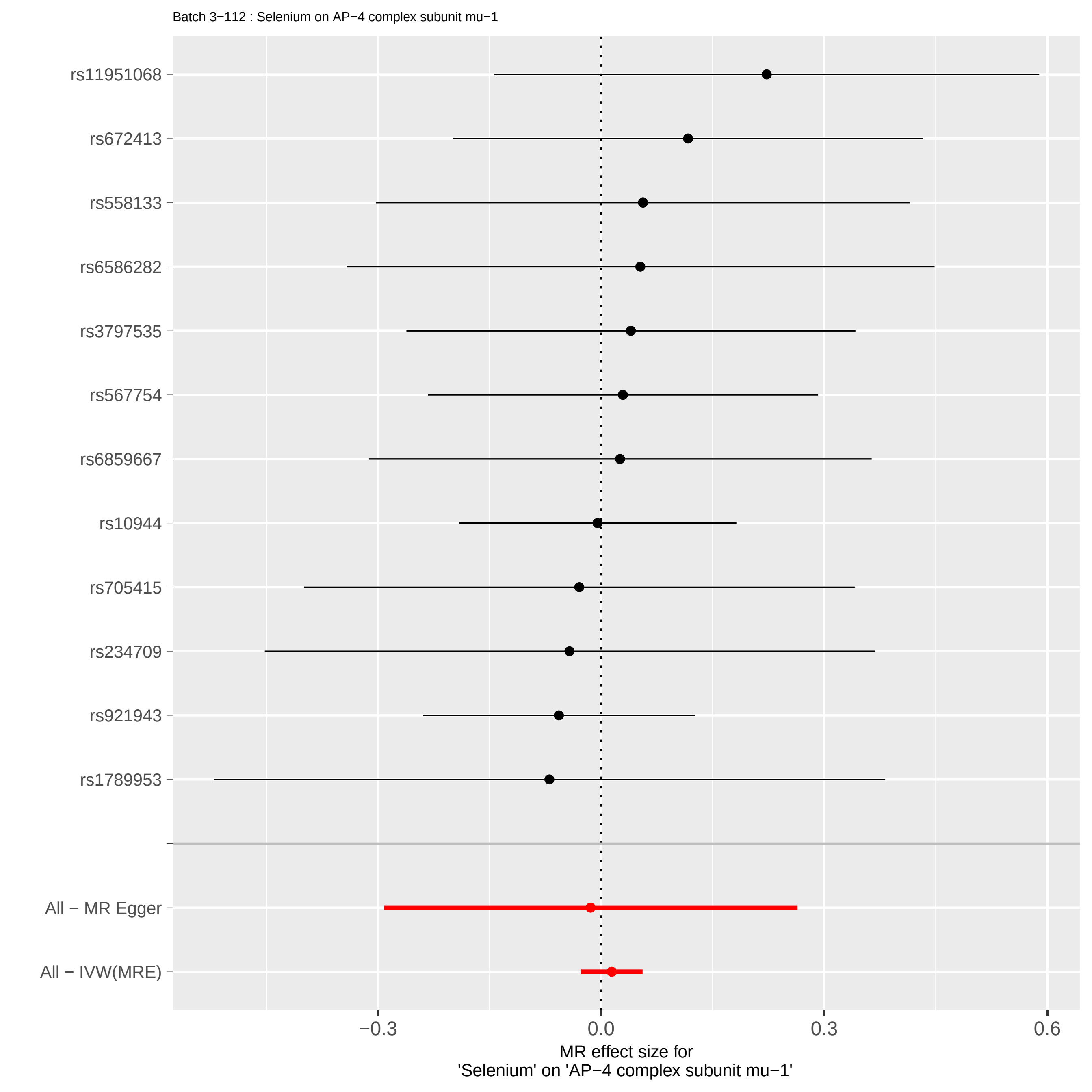
Batch 3−112 : Selenium on AP−4 complex subunit mu−1
rs11951068
rs672413
rs558133
rs6586282
rs3797535
rs567754
rs6859667
rs10944
rs705415
rs234709
rs921943
rs1789953
All − MR Egger
All − IVW(MRE)
−0.3
0.0
0.3
0.6
MR effect size for
'Selenium' on 'AP−4 complex subunit mu−1'

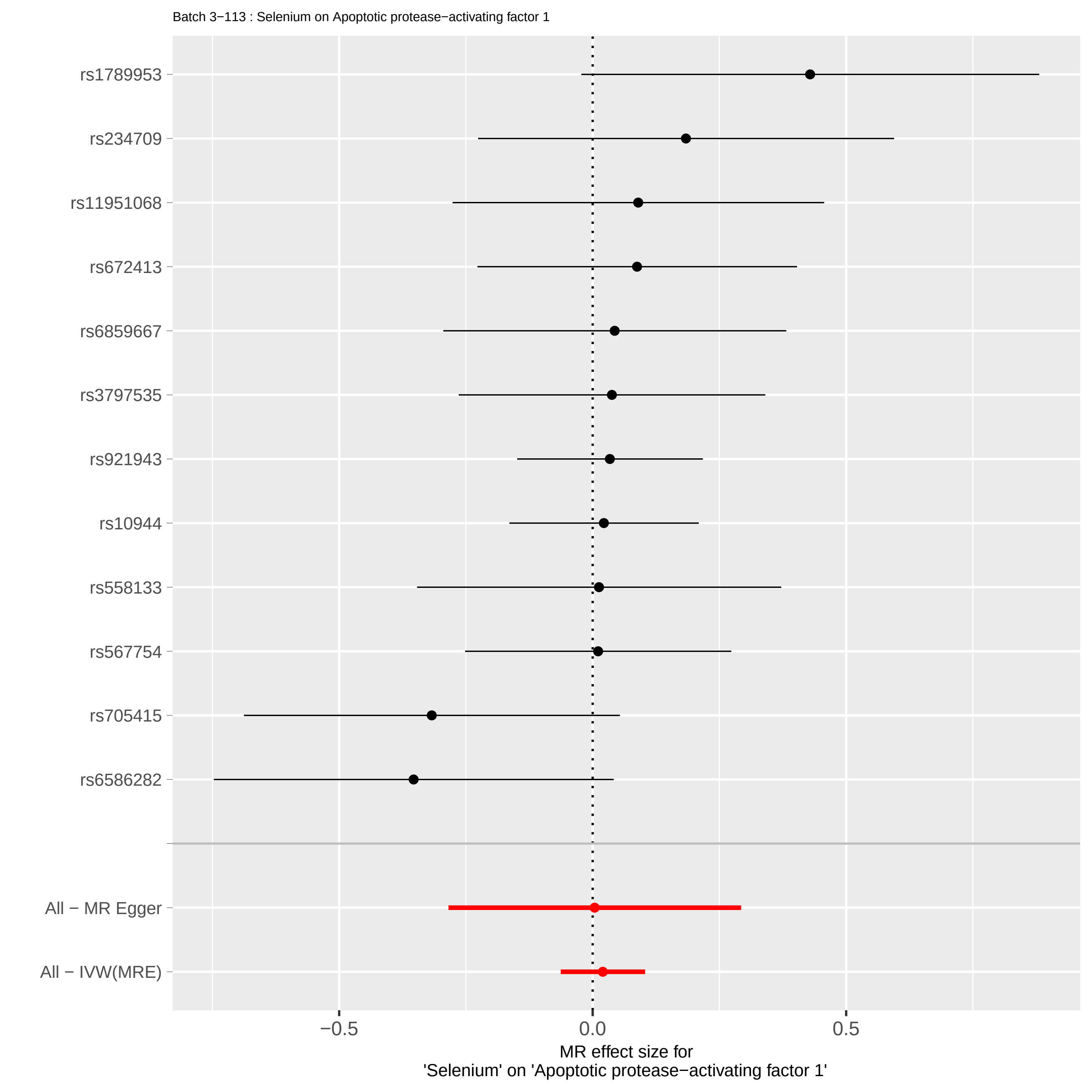

Batch 3−113 : Selenium on Apoptotic protease−activating factor 1
rs1789953
rs234709
rs11951068
rs672413
rs6859667
rs3797535
rs921943
rs10944
rs558133
rs567754
rs705415
rs6586282
All − MR Egger
All − IVW(MRE)
−0.5
0.0
0.5
MR effect size for
'Selenium' on 'Apoptotic protease−activating factor 1'

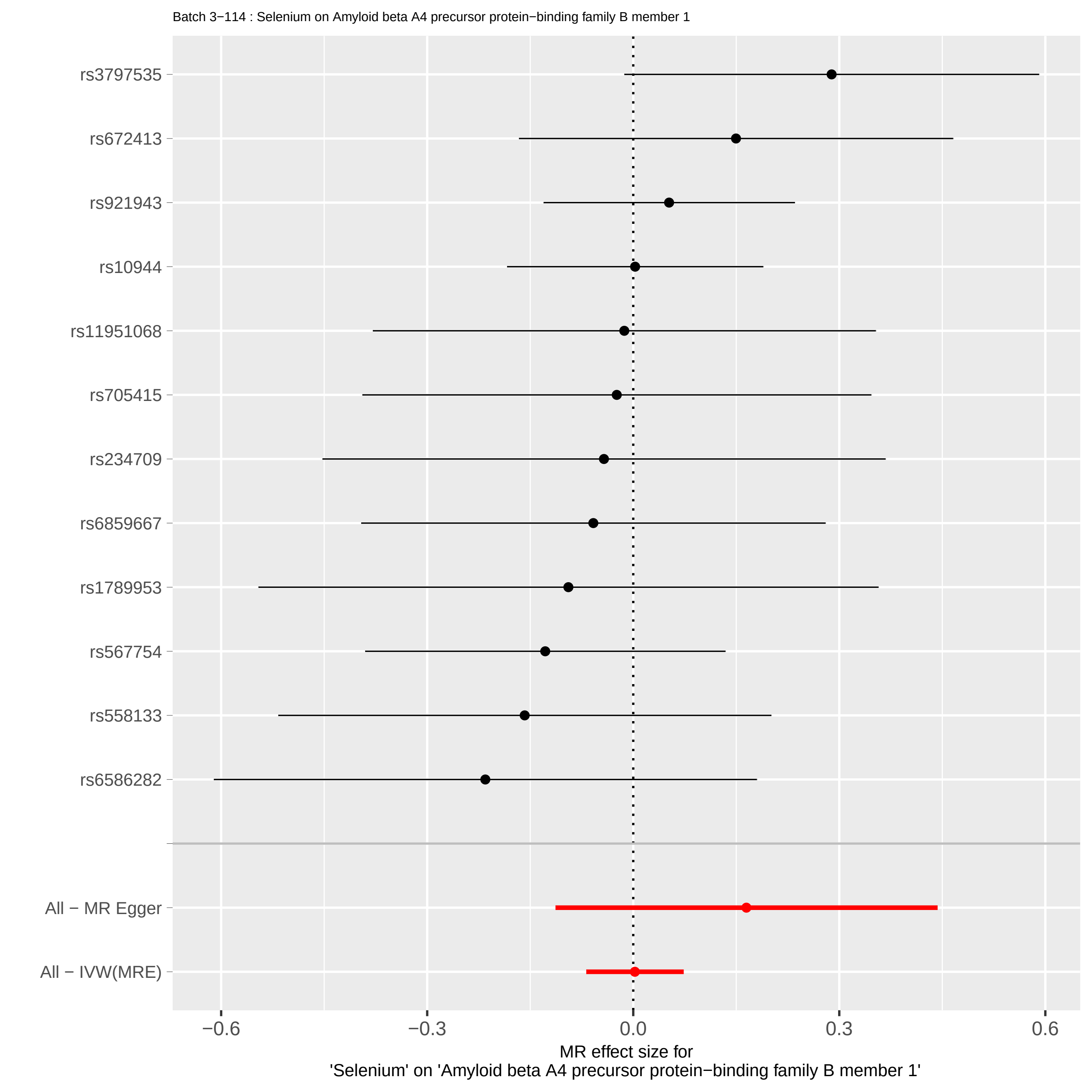

Batch 3−114 : Selenium on Amyloid beta A4 precursor protein−binding family B member 1
rs3797535
rs672413
rs921943
rs10944
rs11951068
rs705415
rs234709
rs6859667
rs1789953
rs567754
rs558133
rs6586282
All − MR Egger
All − IVW(MRE)
−0.6
−0.3
0.0
0.3
0.6
MR effect size for
'Selenium' on 'Amyloid beta A4 precursor protein−binding family B member 1'

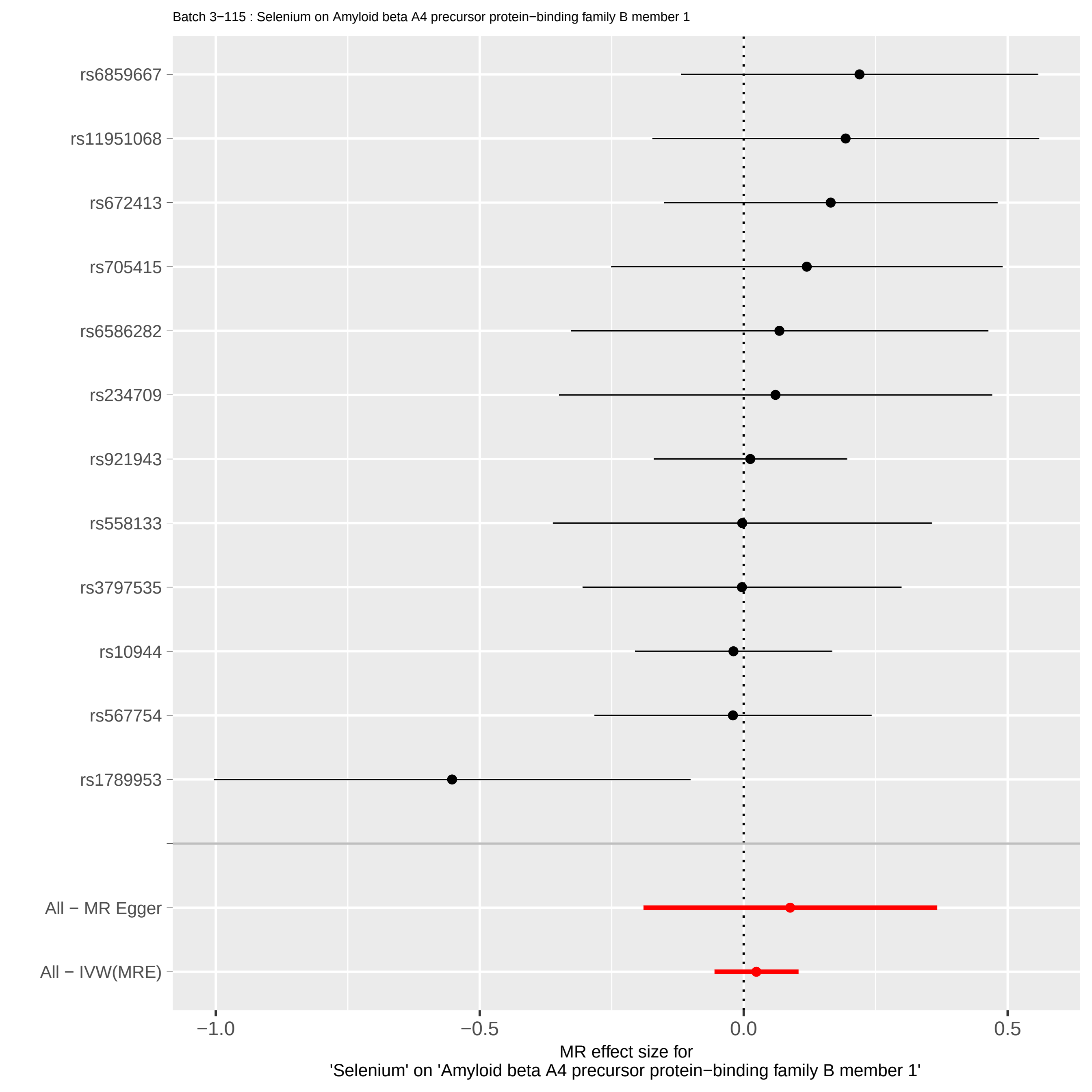

Batch 3−115 : Selenium on Amyloid beta A4 precursor protein−binding family B member 1
rs6859667
rs11951068
rs672413
rs705415
rs6586282
rs234709
rs921943
rs558133
rs3797535
rs10944
rs567754
rs1789953
All − MR Egger
All − IVW(MRE)
−1.0
−0.5
0.0
0.5
MR effect size for
'Selenium' on 'Amyloid beta A4 precursor protein−binding family B member 1'

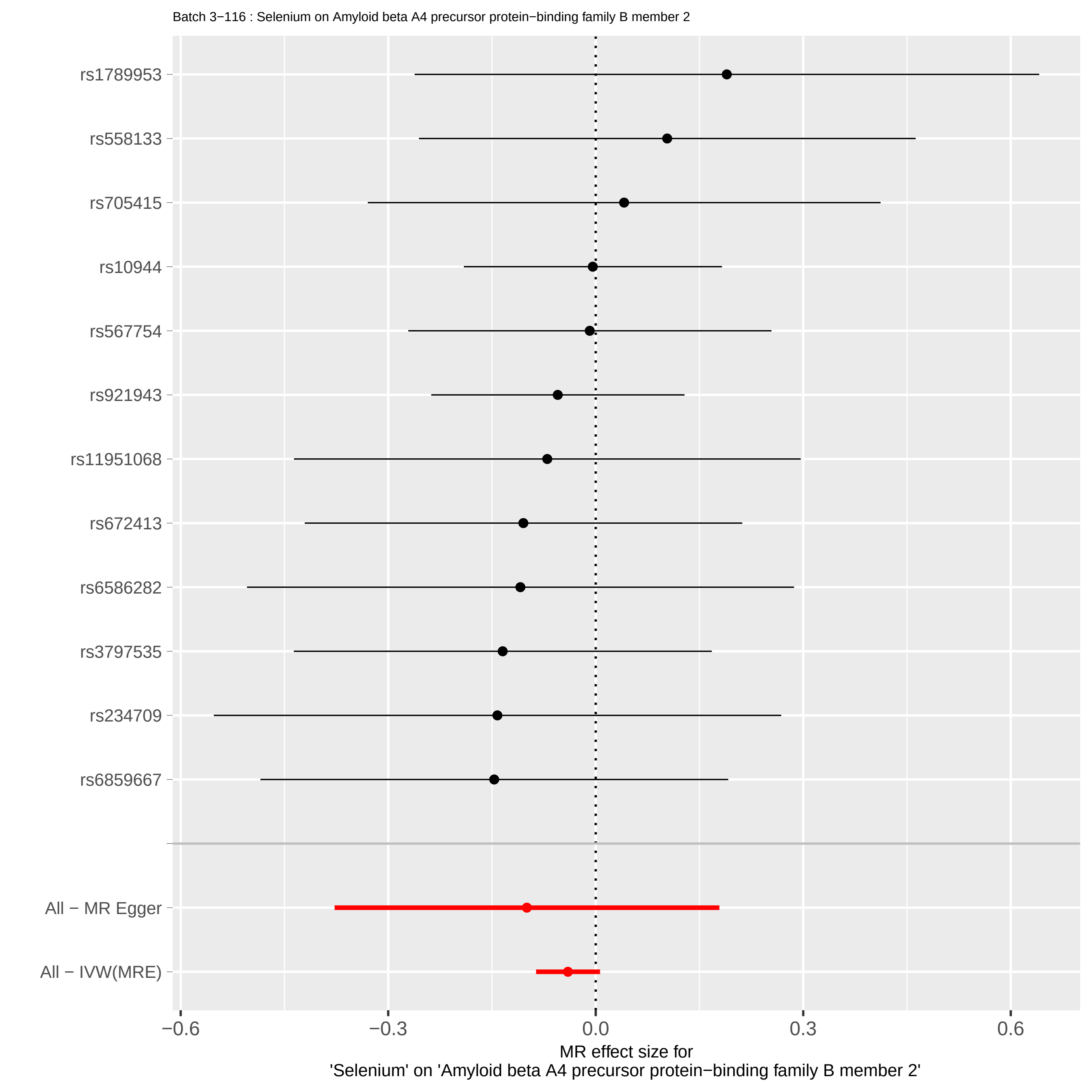

Batch 3−116 : Selenium on Amyloid beta A4 precursor protein−binding family B member 2
rs1789953
rs558133
rs705415
rs10944
rs567754
rs921943
rs11951068
rs672413
rs6586282
rs3797535
rs234709
rs6859667
All − MR Egger
All − IVW(MRE)
−0.6
−0.3
0.0
0.3
0.6
MR effect size for
'Selenium' on 'Amyloid beta A4 precursor protein−binding family B member 2'

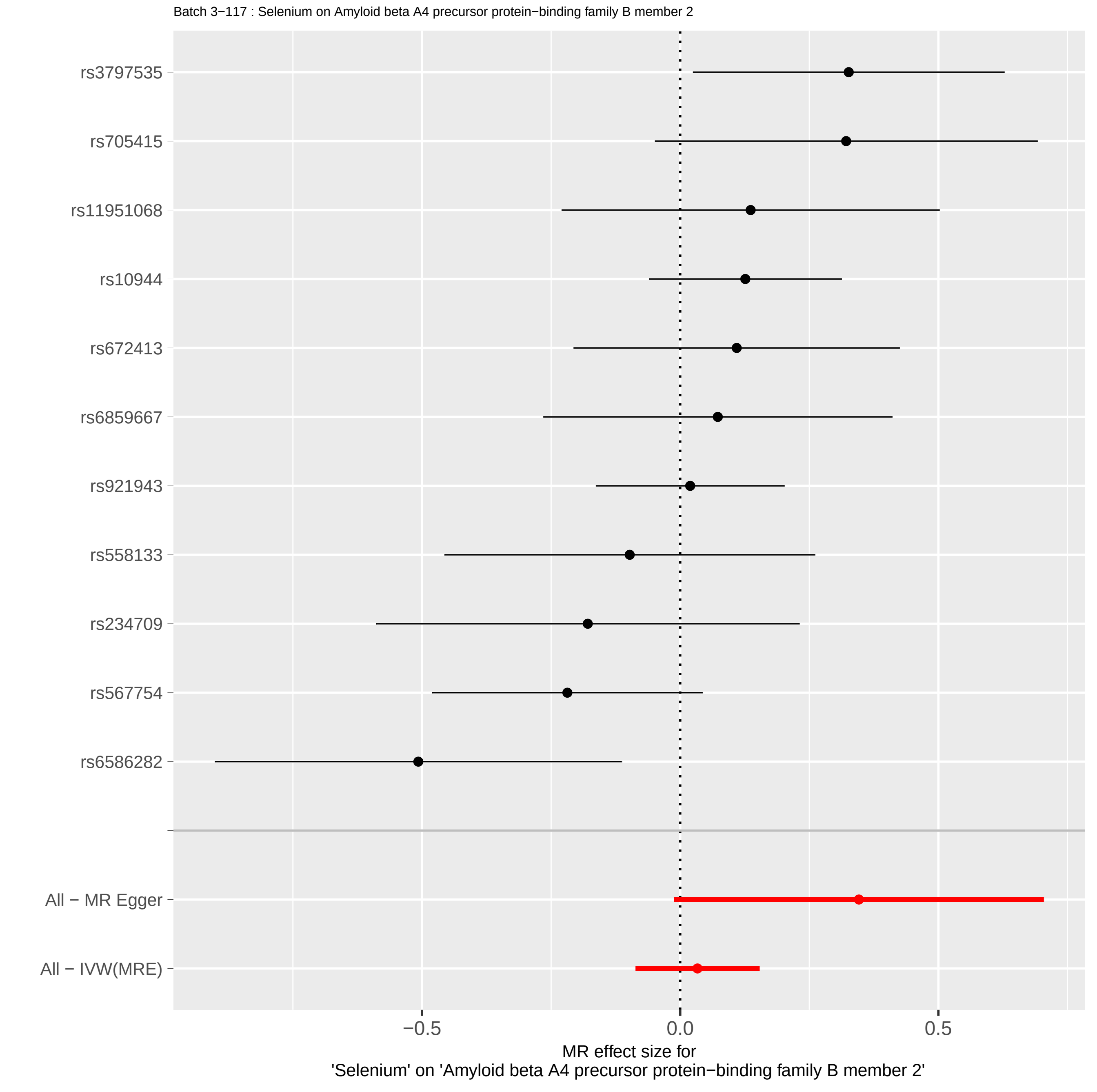

Batch 3−117 : Selenium on Amyloid beta A4 precursor protein−binding family B member 2
rs3797535
rs705415
rs11951068
rs10944
rs672413
rs6859667
rs921943
rs558133
rs234709
rs567754
rs6586282
All − MR Egger
All − IVW(MRE)
−0.5
0.0
0.5
MR effect size for
'Selenium' on 'Amyloid beta A4 precursor protein−binding family B member 2'

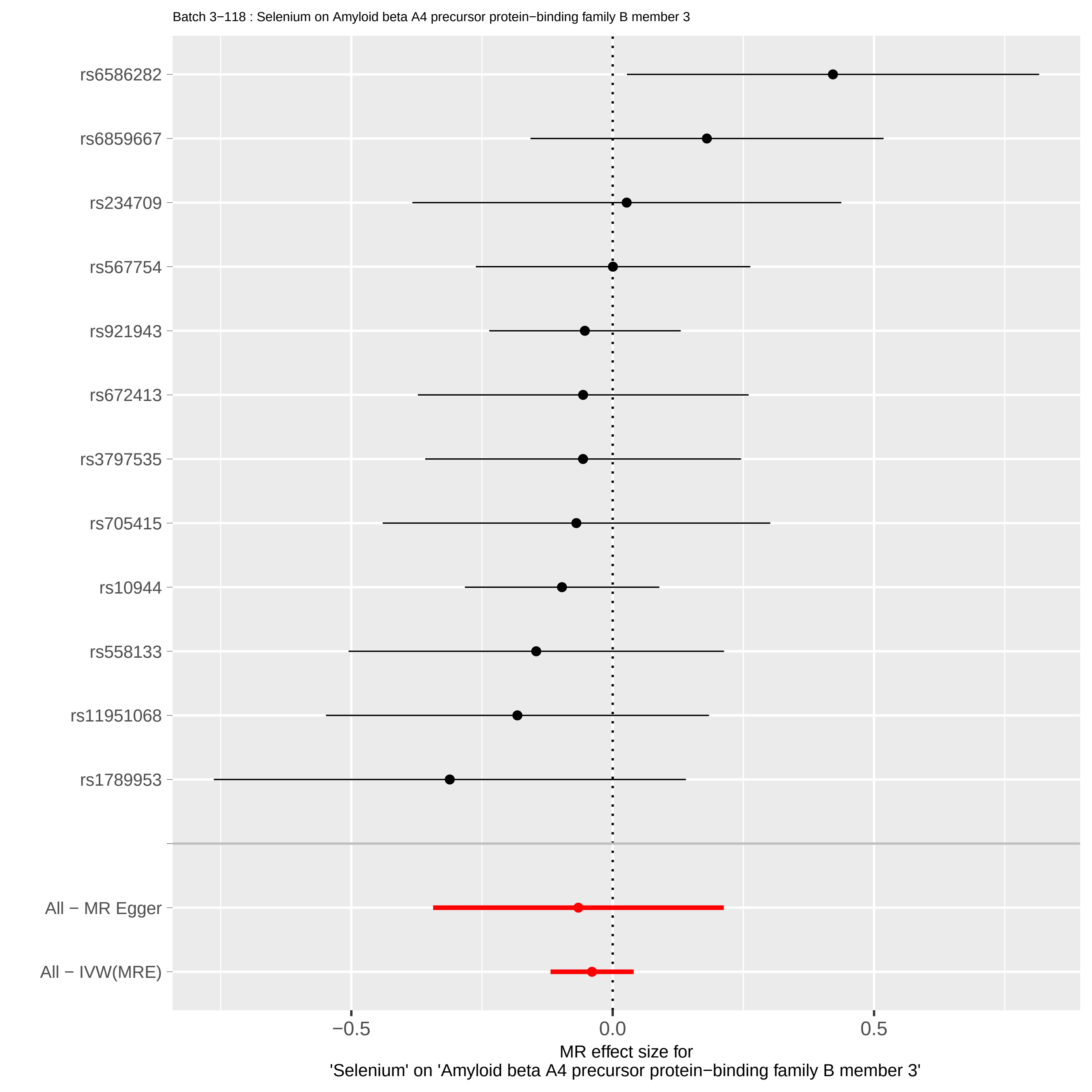
Batch 3−118 : Selenium on Amyloid beta A4 precursor protein−binding family B member 3
rs6586282
rs6859667
rs234709
rs567754
rs921943
rs672413
rs3797535
rs705415
rs10944
rs558133
rs11951068
rs1789953
All − MR Egger
All − IVW(MRE)
−0.5
0.0
0.5
MR effect size for
'Selenium' on 'Amyloid beta A4 precursor protein−binding family B member 3'

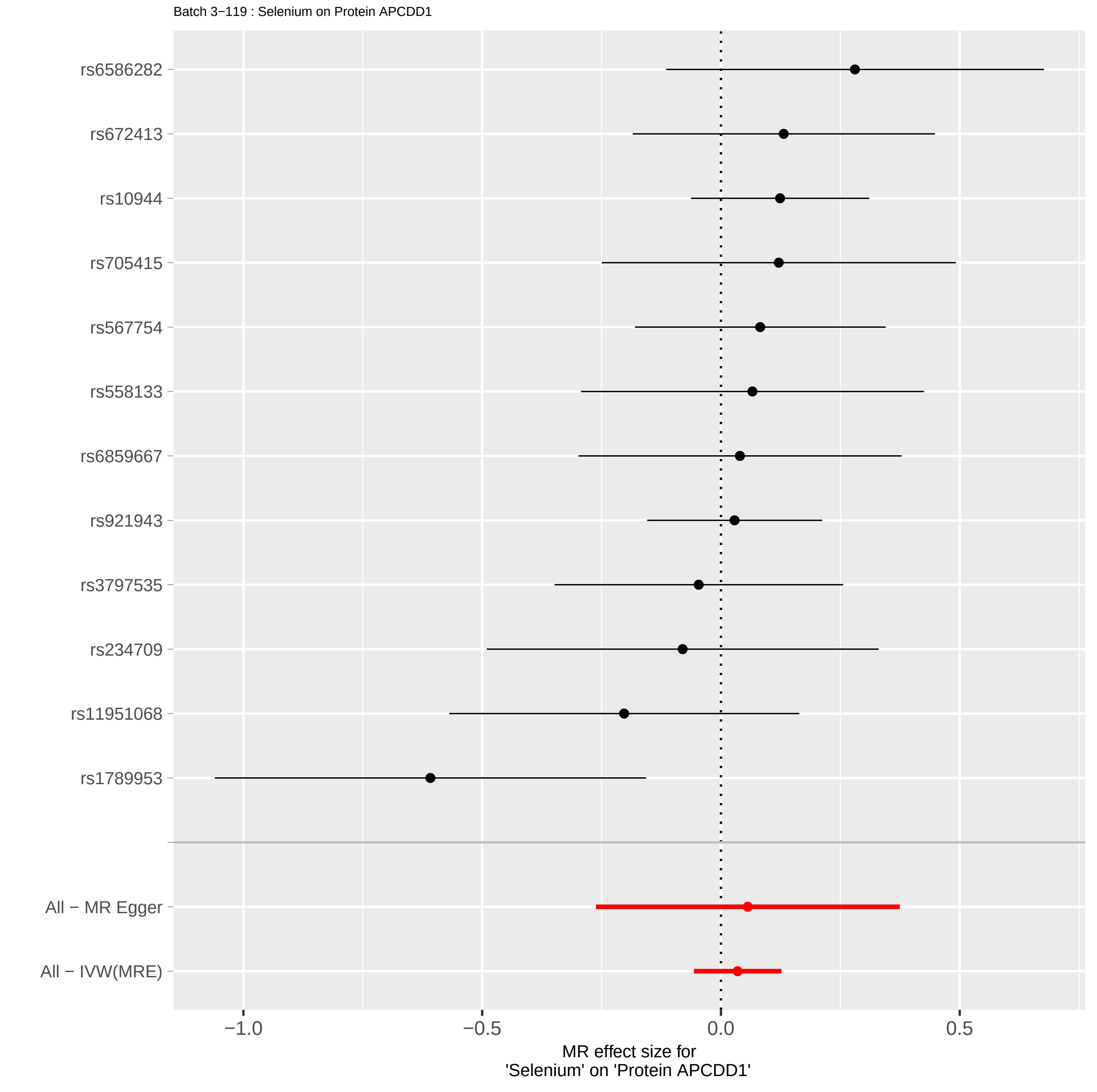

Batch 3−119 : Selenium on Protein APCDD1
rs6586282
rs672413
rs10944
rs705415
rs567754
rs558133
rs6859667
rs921943
rs3797535
rs234709
rs11951068
rs1789953
All − MR Egger
All − IVW(MRE)
−1.0
−0.5
0.0
0.5
MR effect size for
'Selenium' on 'Protein APCDD1'

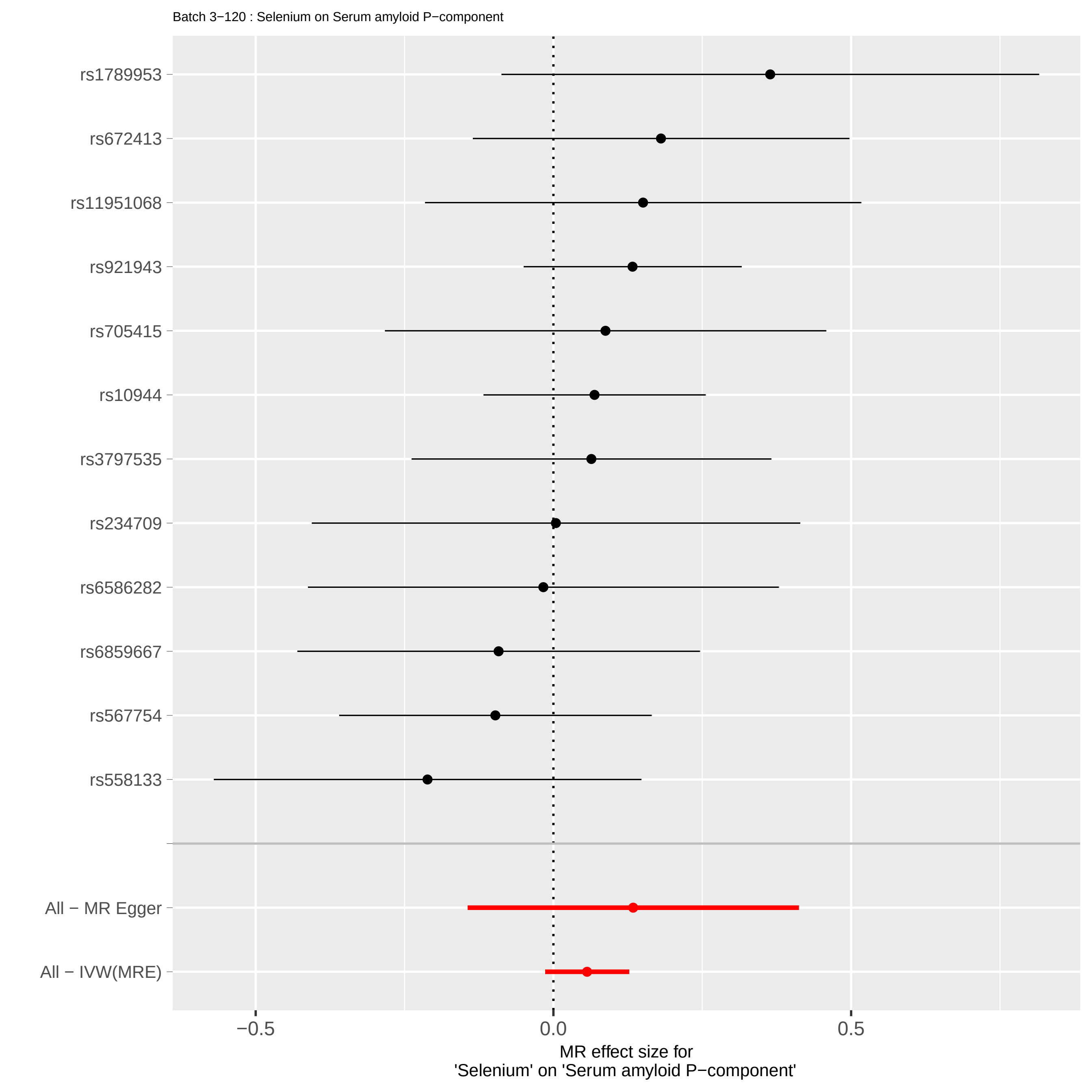
Batch 3−120 : Selenium on Serum amyloid P−component
rs1789953
rs672413
rs11951068
rs921943
rs705415
rs10944
rs3797535
rs234709
rs6586282
rs6859667
rs567754
rs558133
All − MR Egger
All − IVW(MRE)
−0.5
0.0
0.5
MR effect size for
'Selenium' on 'Serum amyloid P−component'

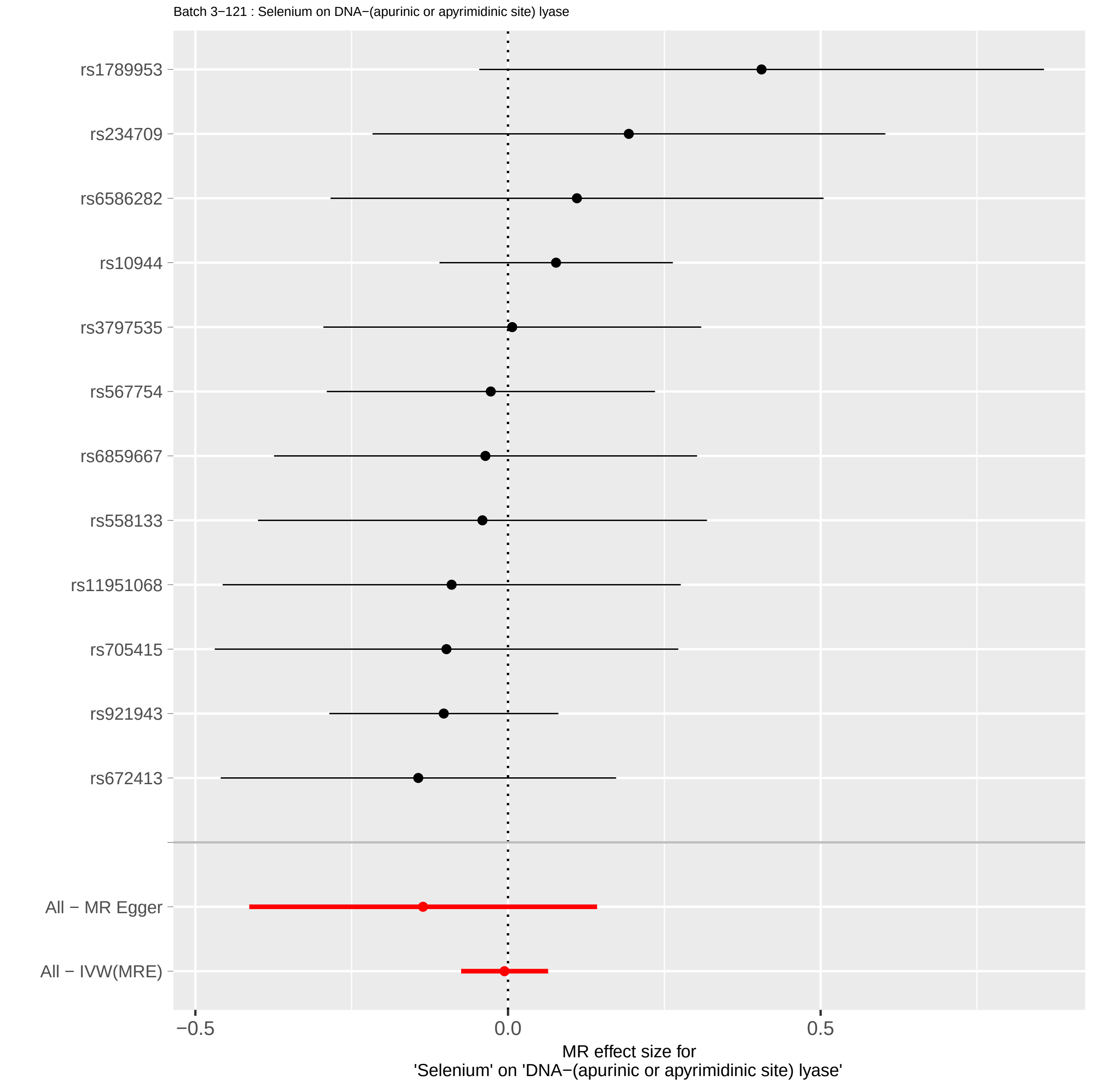
Batch 3−121 : Selenium on DNA−(apurinic or apyrimidinic site) lyase
rs1789953
rs234709
rs6586282
rs10944
rs3797535
rs567754
rs6859667
rs558133
rs11951068
rs705415
rs921943
rs672413
All − MR Egger
All − IVW(MRE)
−0.5
0.0
0.5
MR effect size for
'Selenium' on 'DNA−(apurinic or apyrimidinic site) lyase'

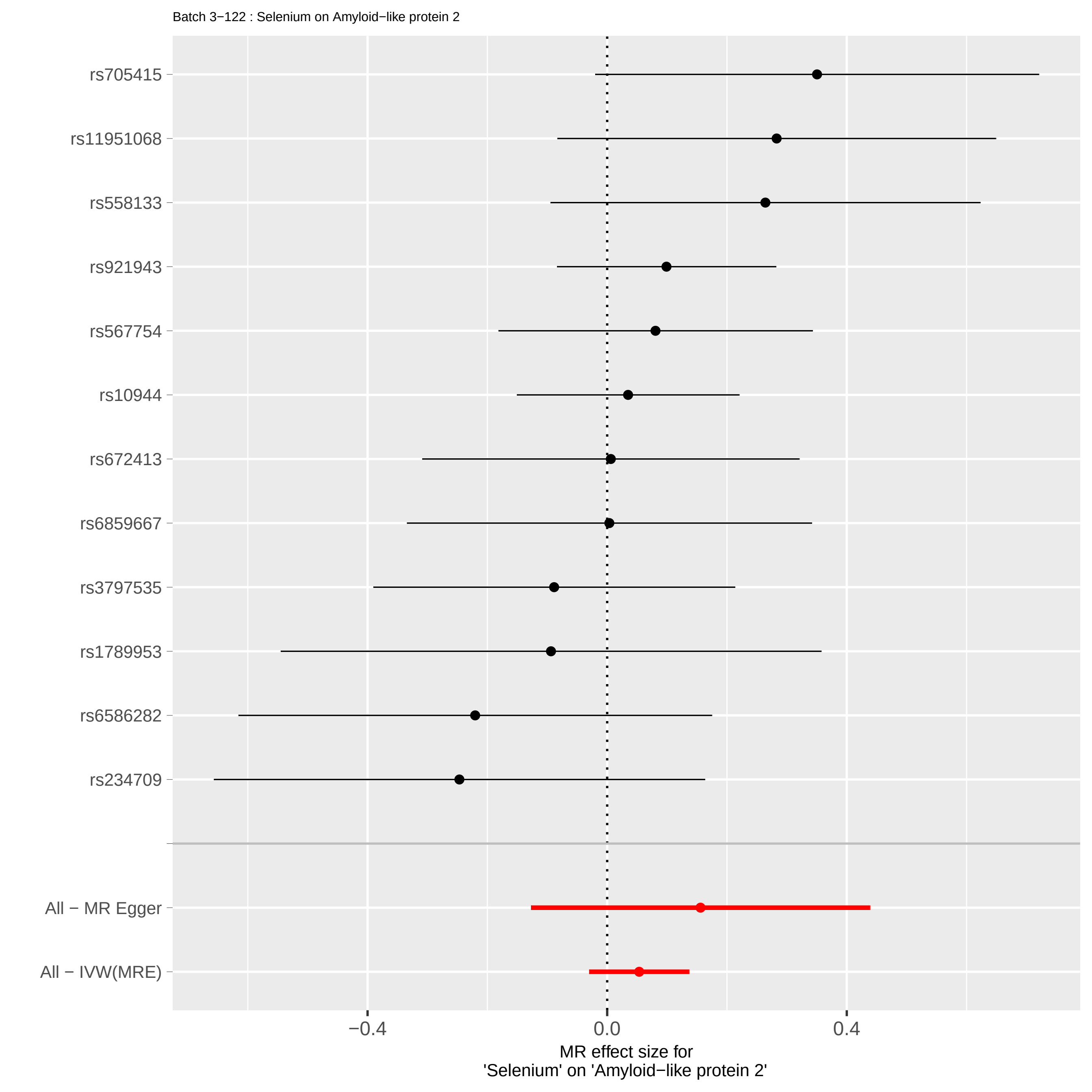
Batch 3−122 : Selenium on Amyloid−like protein 2
rs705415
rs11951068
rs558133
rs921943
rs567754
rs10944
rs672413
rs6859667
rs3797535
rs1789953
rs6586282
rs234709
All − MR Egger
All − IVW(MRE)
−0.4
0.0
0.4
MR effect size for
'Selenium' on 'Amyloid−like protein 2'

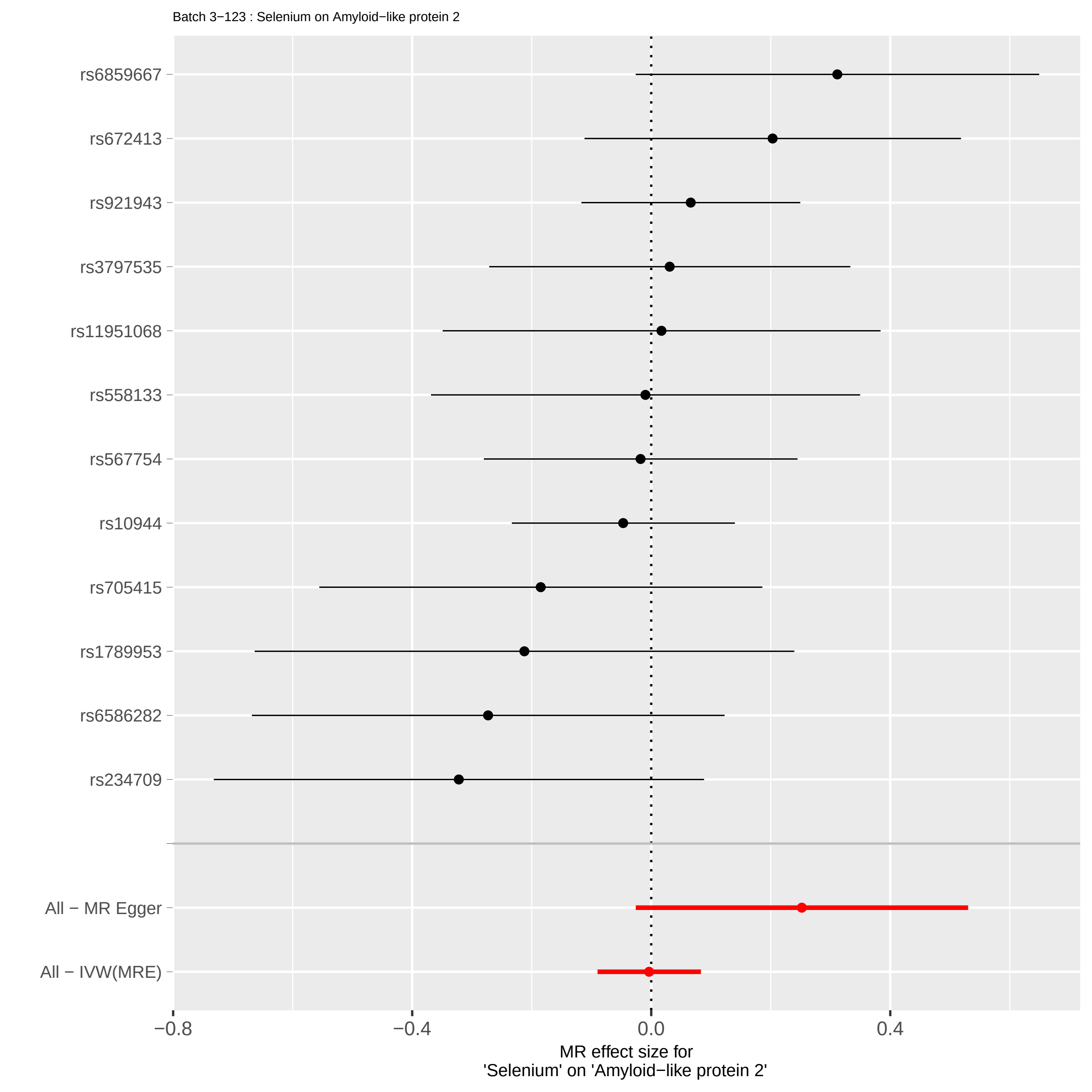

Batch 3−123 : Selenium on Amyloid−like protein 2
rs6859667
rs672413
rs921943
rs3797535
rs11951068
rs558133
rs567754
rs10944
rs705415
rs1789953
rs6586282
rs234709
All − MR Egger
All − IVW(MRE)
−0.8
−0.4
0.0
0.4
MR effect size for
'Selenium' on 'Amyloid−like protein 2'

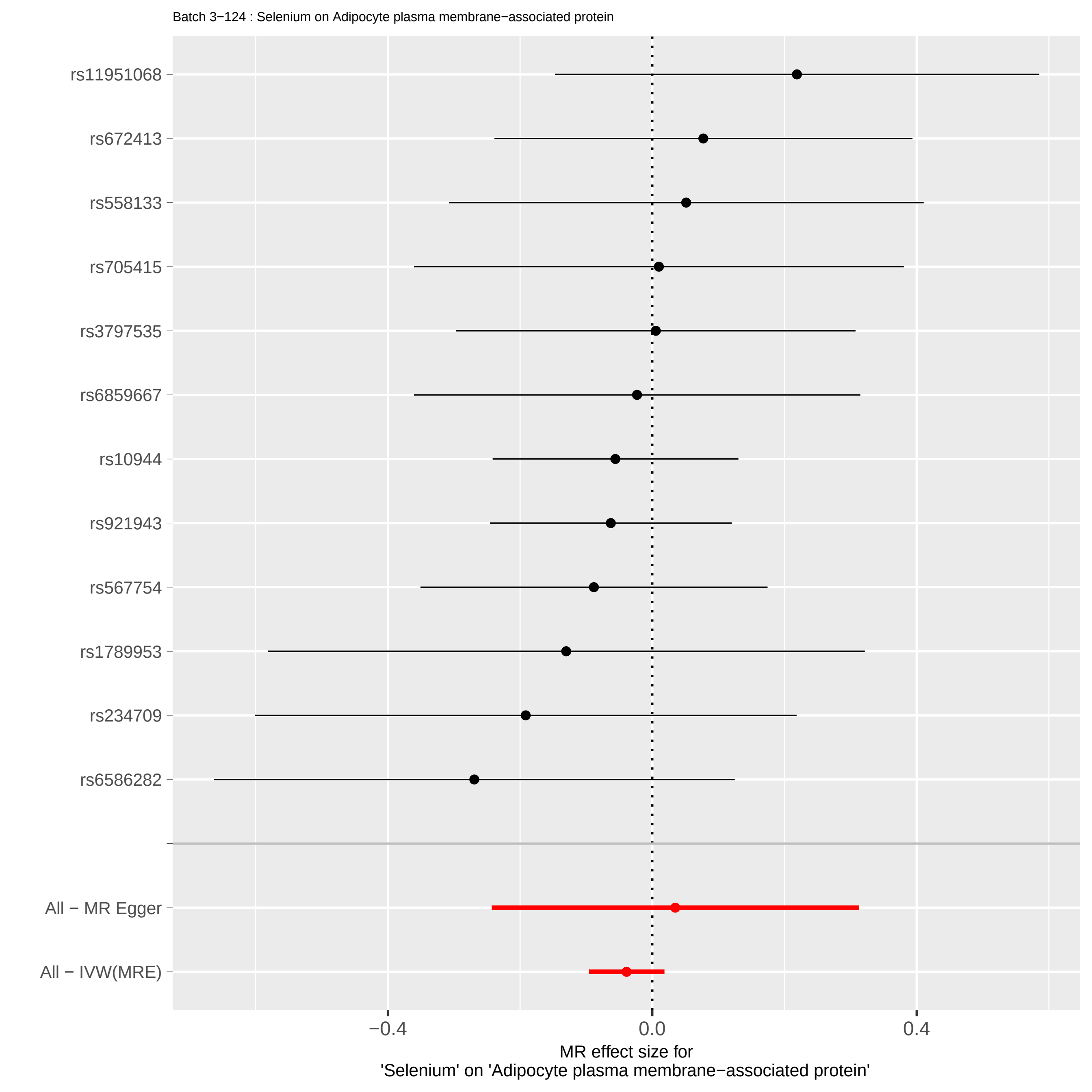

Batch 3−124 : Selenium on Adipocyte plasma membrane−associated protein
rs11951068
rs672413
rs558133
rs705415
rs3797535
rs6859667
rs10944
rs921943
rs567754
rs1789953
rs234709
rs6586282
All − MR Egger
All − IVW(MRE)
−0.4
0.0
0.4
MR effect size for
'Selenium' on 'Adipocyte plasma membrane−associated protein'

Batch 3−125 : Selenium on Apolipoprotein A−V

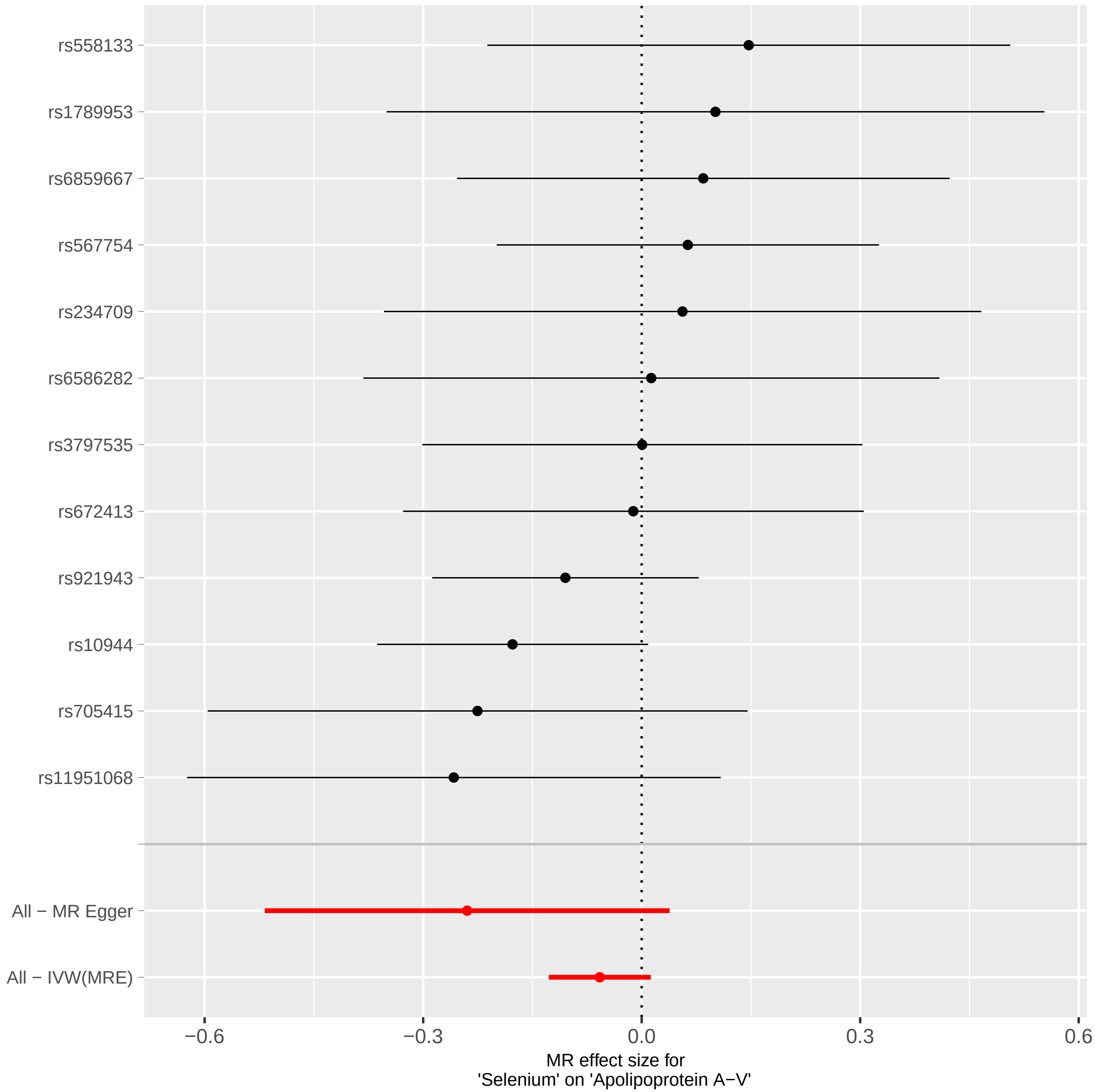

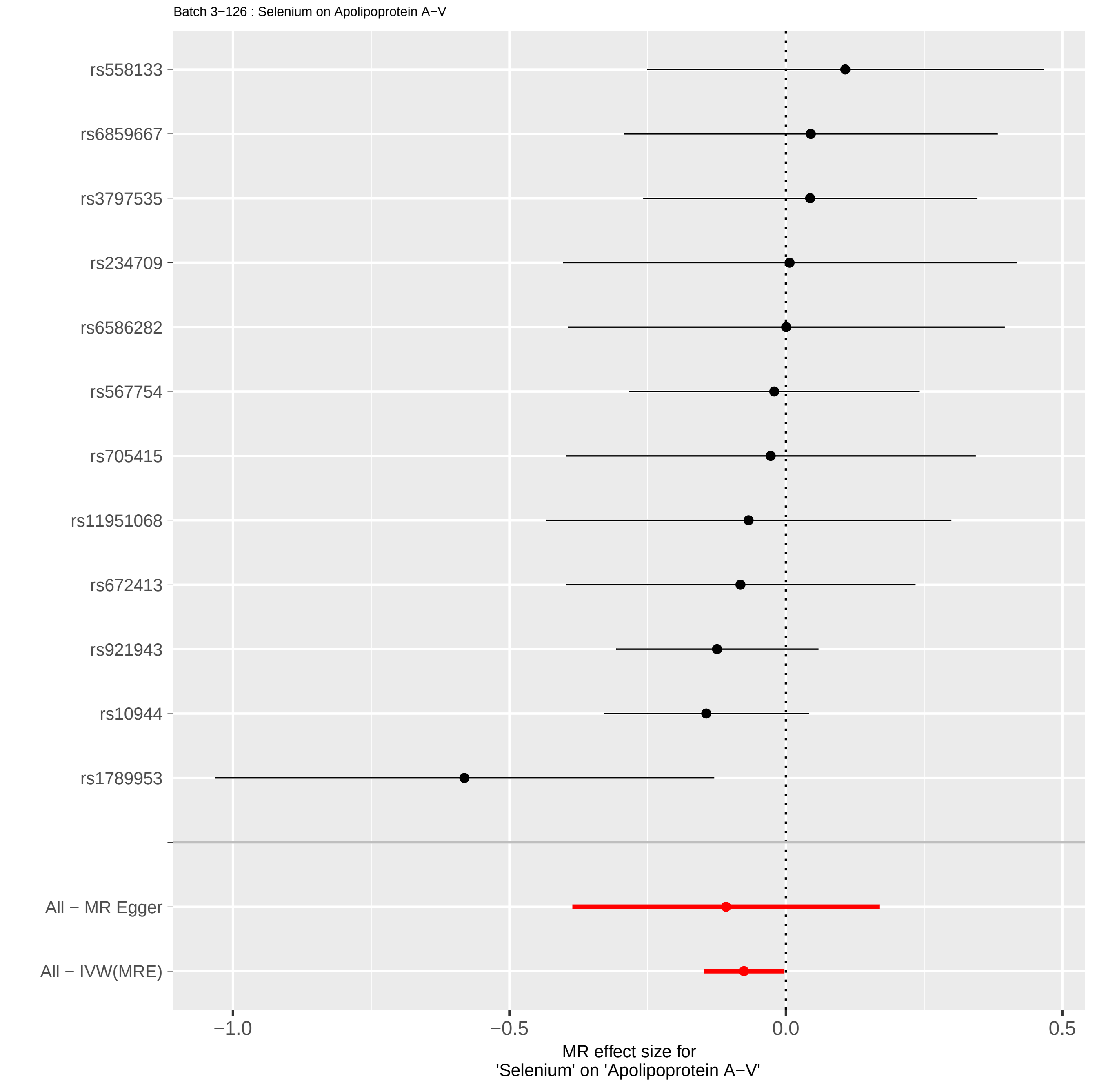

Batch 3−126 : Selenium on Apolipoprotein A−V
rs558133
rs6859667
rs3797535
rs234709
rs6586282
rs567754
rs705415
rs11951068
rs672413
rs921943
rs10944
rs1789953
All − MR Egger
All − IVW(MRE)
−1.0
−0.5
0.0
0.5
MR effect size for
'Selenium' on 'Apolipoprotein A−V'

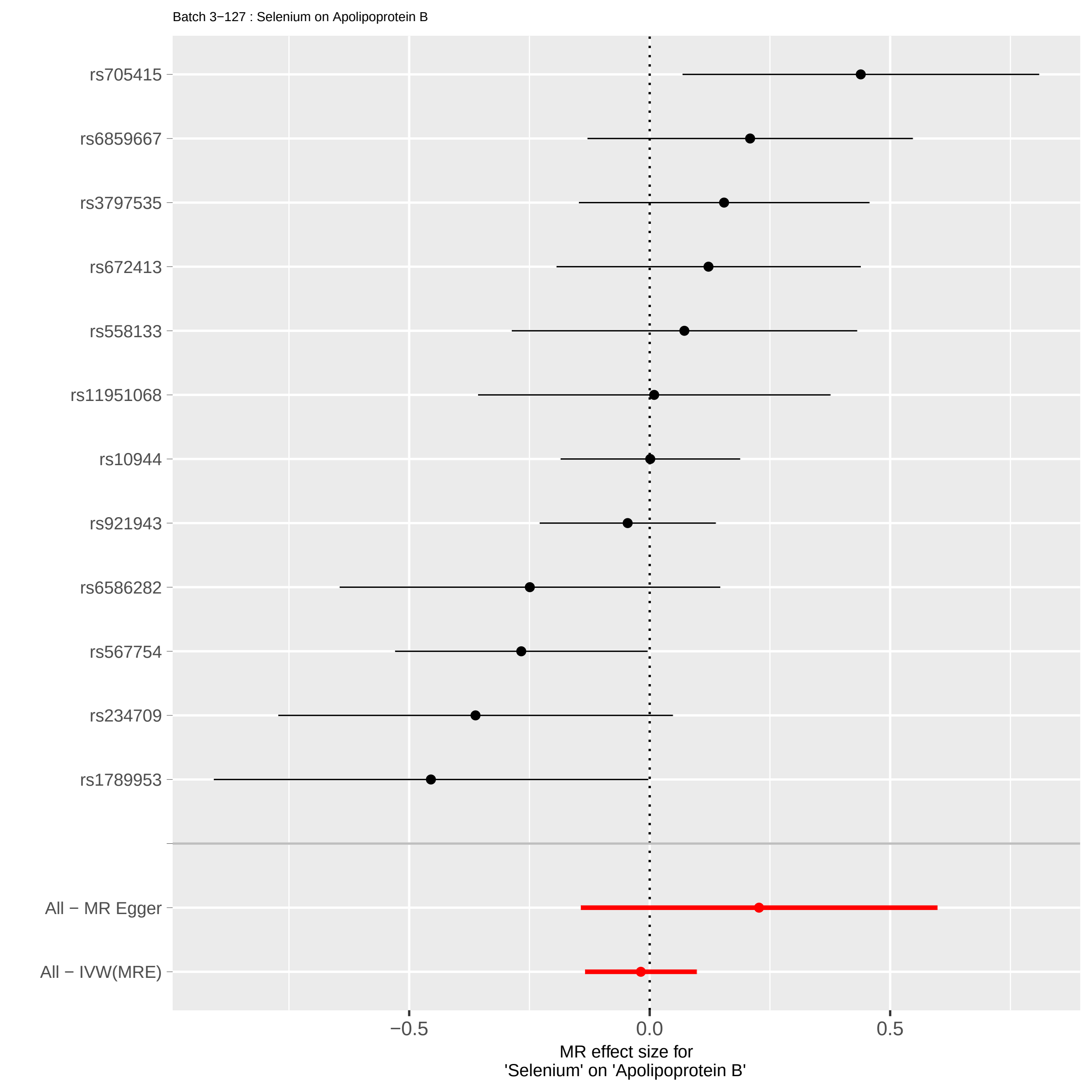
Batch 3−127 : Selenium on Apolipoprotein B
rs705415
rs6859667
rs3797535
rs672413
rs558133
rs11951068
rs10944
rs921943
rs6586282
rs567754
rs234709
rs1789953
All − MR Egger
All − IVW(MRE)
−0.5
0.0
0.5
MR effect size for
'Selenium' on 'Apolipoprotein B'

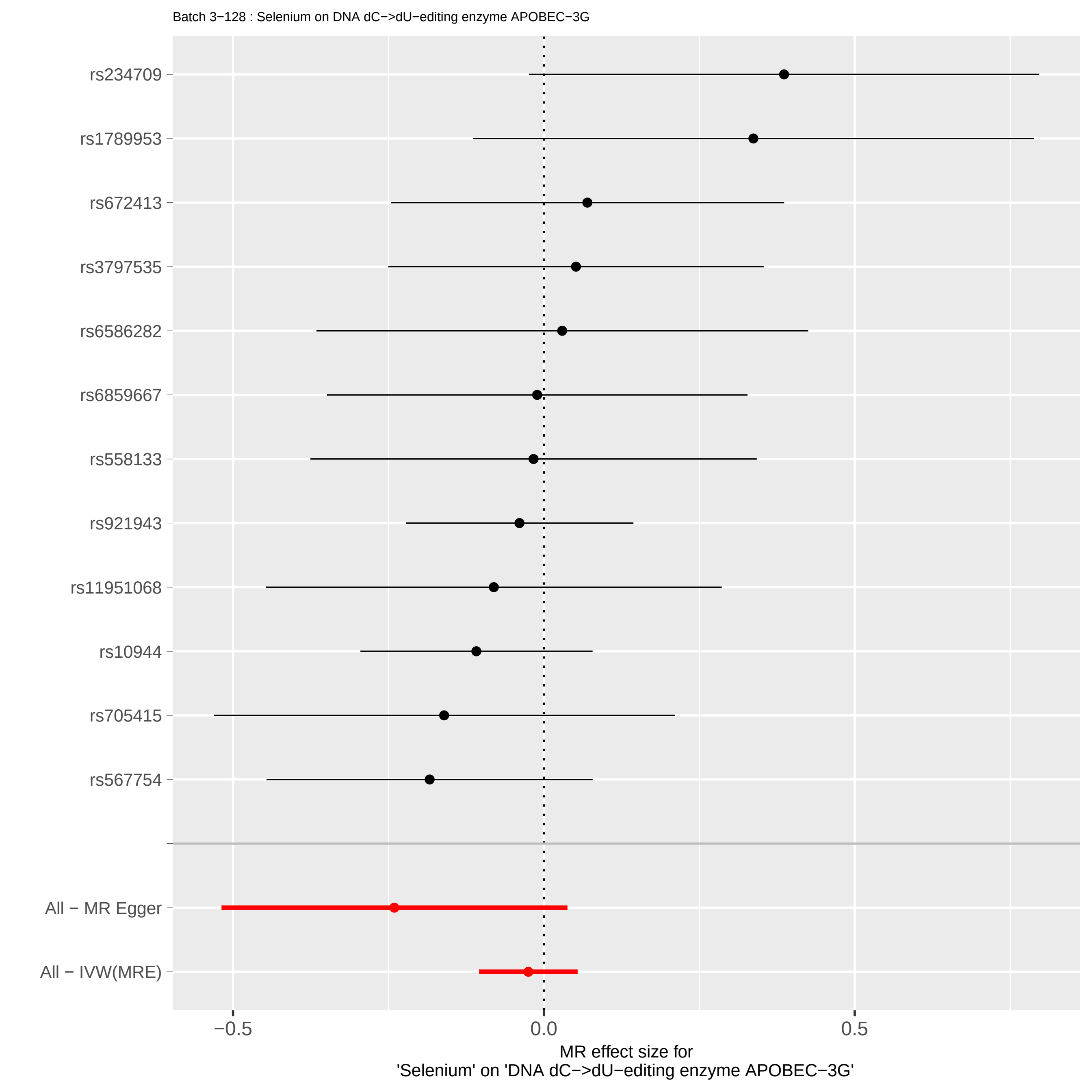

Batch 3−128 : Selenium on DNA dC−>dU−editing enzyme APOBEC−3G
rs234709
rs1789953
rs672413
rs3797535
rs6586282
rs6859667
rs558133
rs921943
rs11951068
rs10944
rs705415
rs567754
All − MR Egger
All − IVW(MRE)
−0.5
0.0
0.5
MR effect size for
'Selenium' on 'DNA dC−>dU−editing enzyme APOBEC−3G'

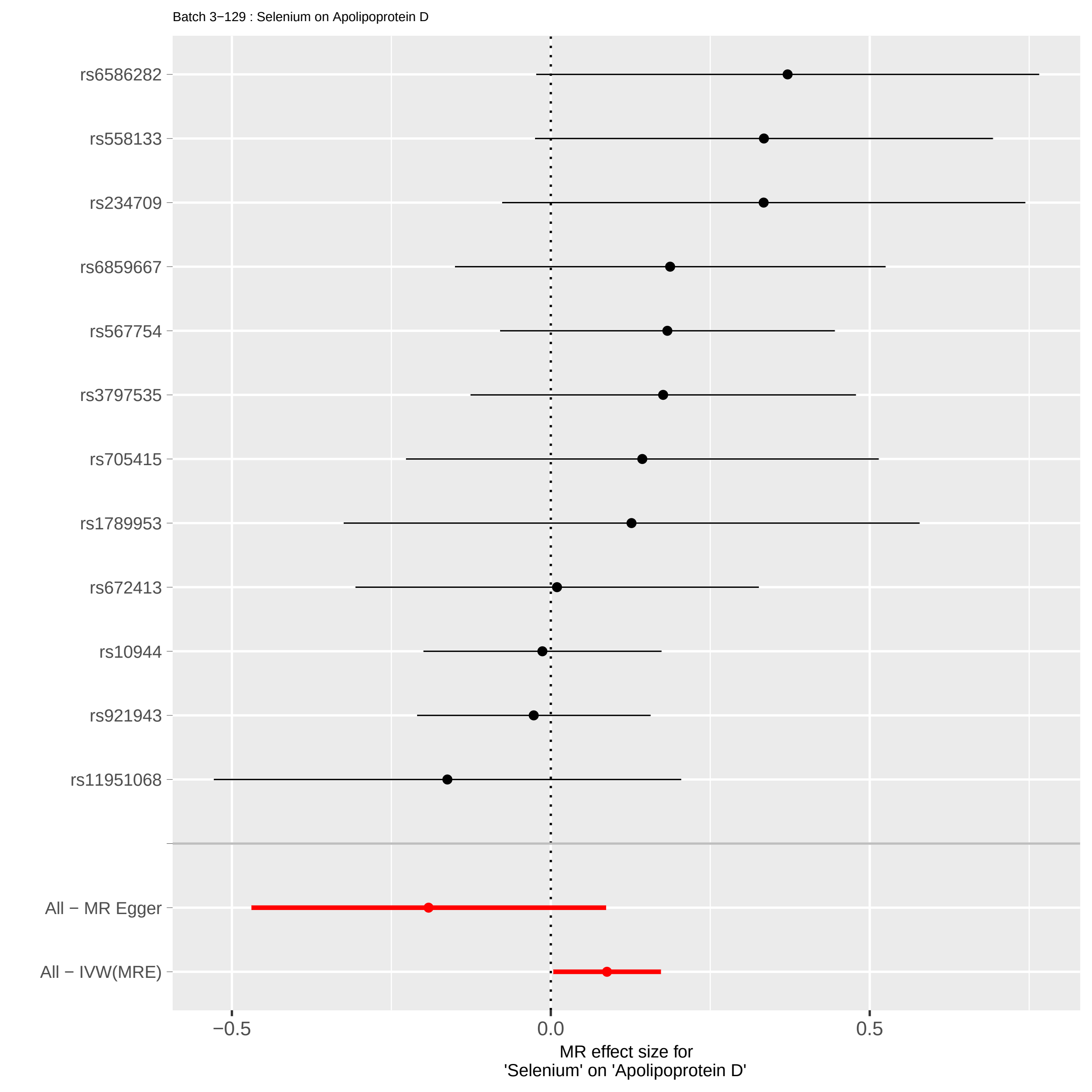
Batch 3−129 : Selenium on Apolipoprotein D
rs6586282
rs558133
rs234709
rs6859667
rs567754
rs3797535
rs705415
rs1789953
rs672413
rs10944
rs921943
rs11951068
All − MR Egger
All − IVW(MRE)
−0.5
0.0
0.5
MR effect size for
'Selenium' on 'Apolipoprotein D'

Batch 3−130 : Selenium on Apolipoprotein D

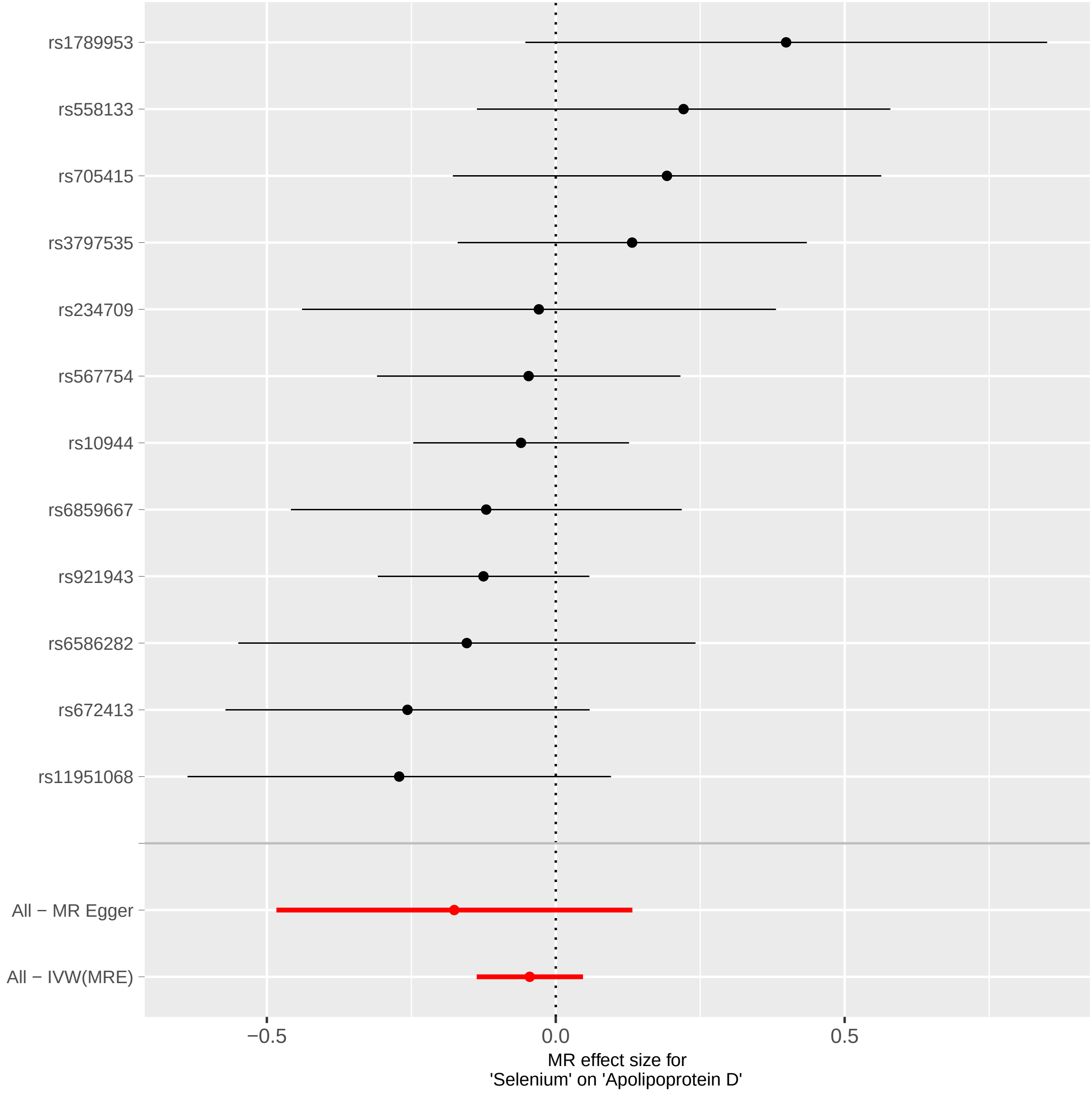

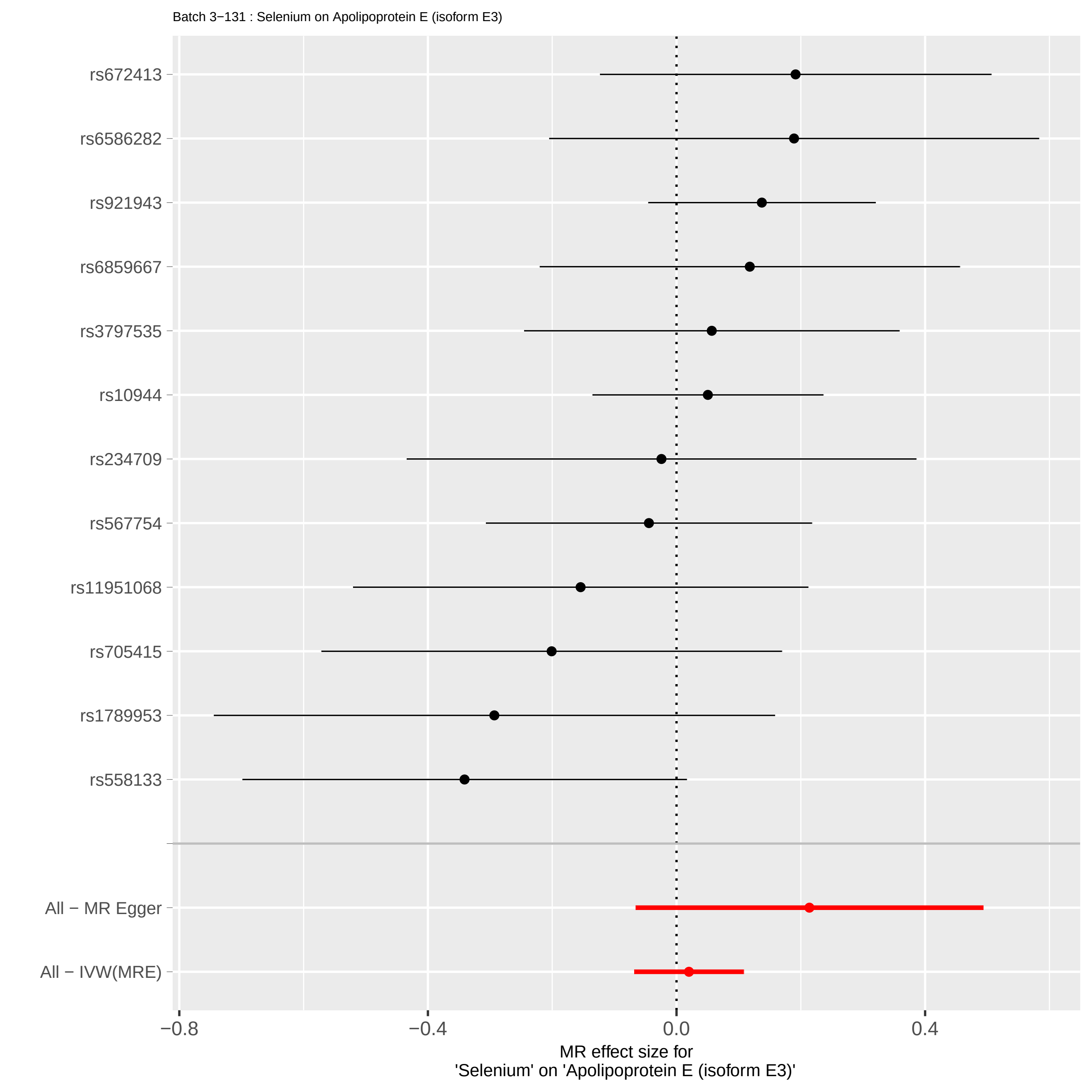
Batch 3−131 : Selenium on Apolipoprotein E (isoform E3)
rs672413
rs6586282
rs921943
rs6859667
rs3797535
rs10944
rs234709
rs567754
rs11951068
rs705415
rs1789953
rs558133
All − MR Egger
All − IVW(MRE)
−0.8
−0.4
0.0
0.4
MR effect size for
'Selenium' on 'Apolipoprotein E (isoform E3)'

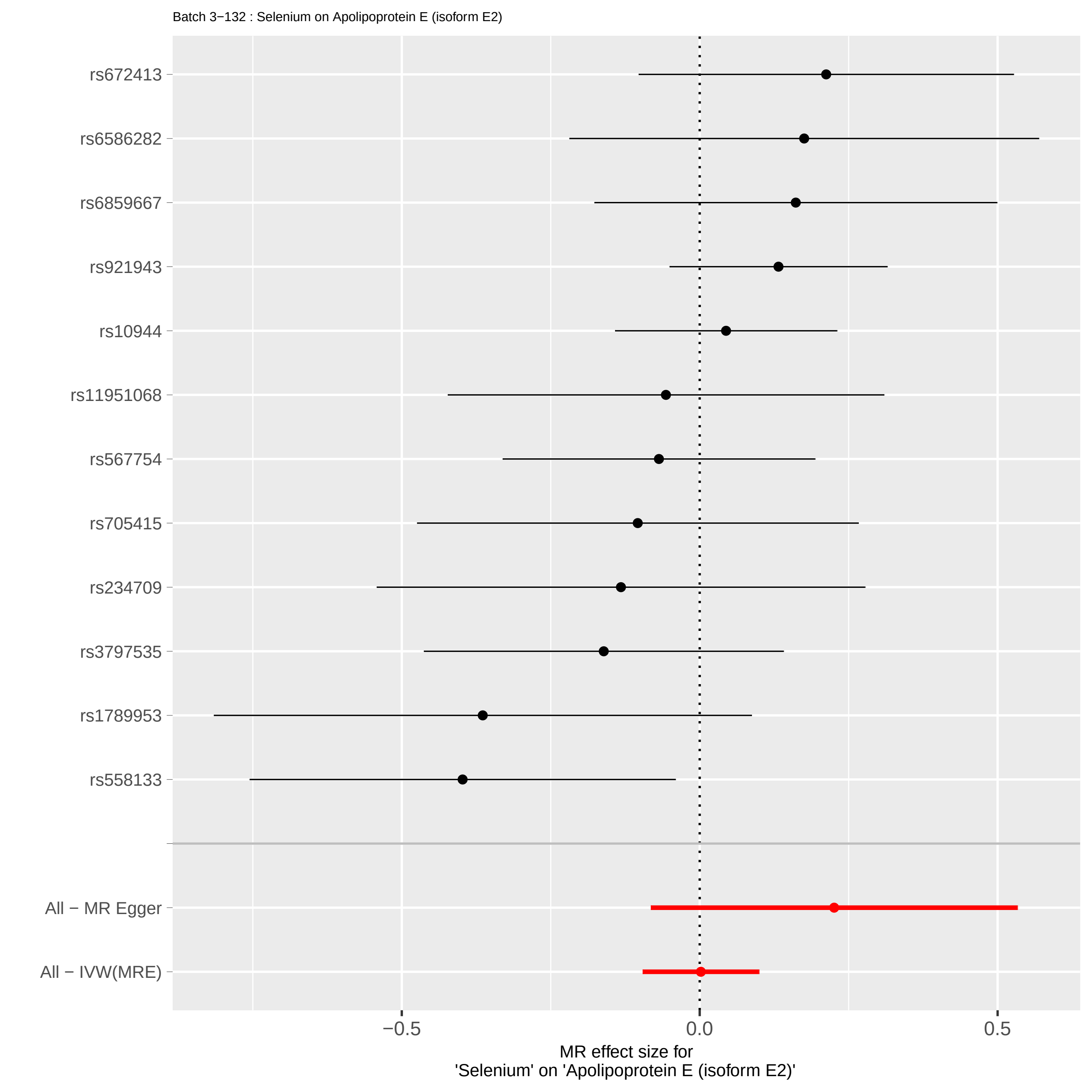

Batch 3−132 : Selenium on Apolipoprotein E (isoform E2)
rs672413
rs6586282
rs6859667
rs921943
rs10944
rs11951068
rs567754
rs705415
rs234709
rs3797535
rs1789953
rs558133
All − MR Egger
All − IVW(MRE)
−0.5
0.0
0.5
MR effect size for
'Selenium' on 'Apolipoprotein E (isoform E2)'

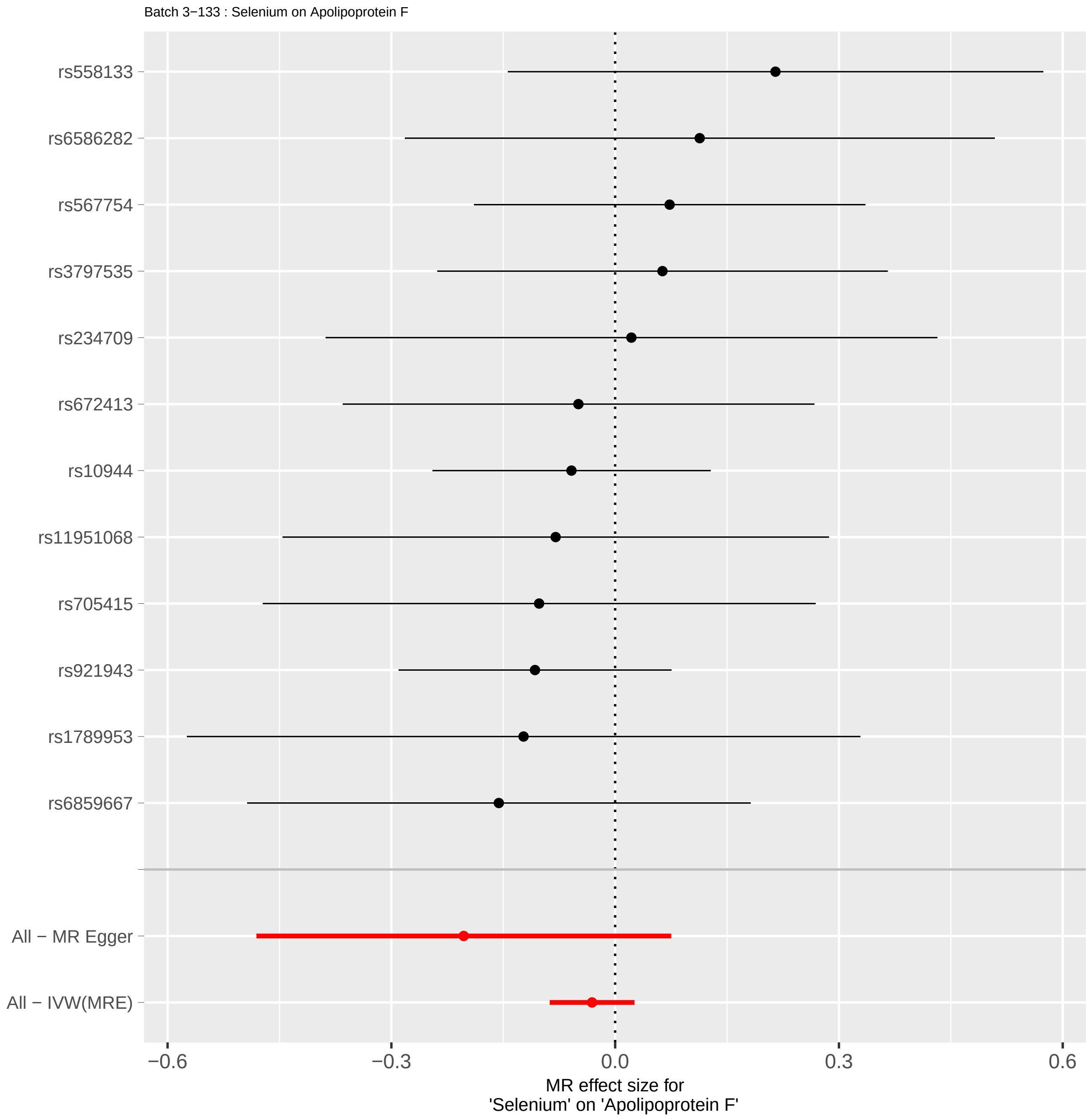
Batch 3−133 : Selenium on Apolipoprotein F
rs558133
rs6586282
rs567754
rs3797535
rs234709
rs672413
rs10944
rs11951068
rs705415
rs921943
rs1789953
rs6859667
All − MR Egger
All − IVW(MRE)
−0.6
−0.3
0.0
0.3
0.6
MR effect size for
'Selenium' on 'Apolipoprotein F'

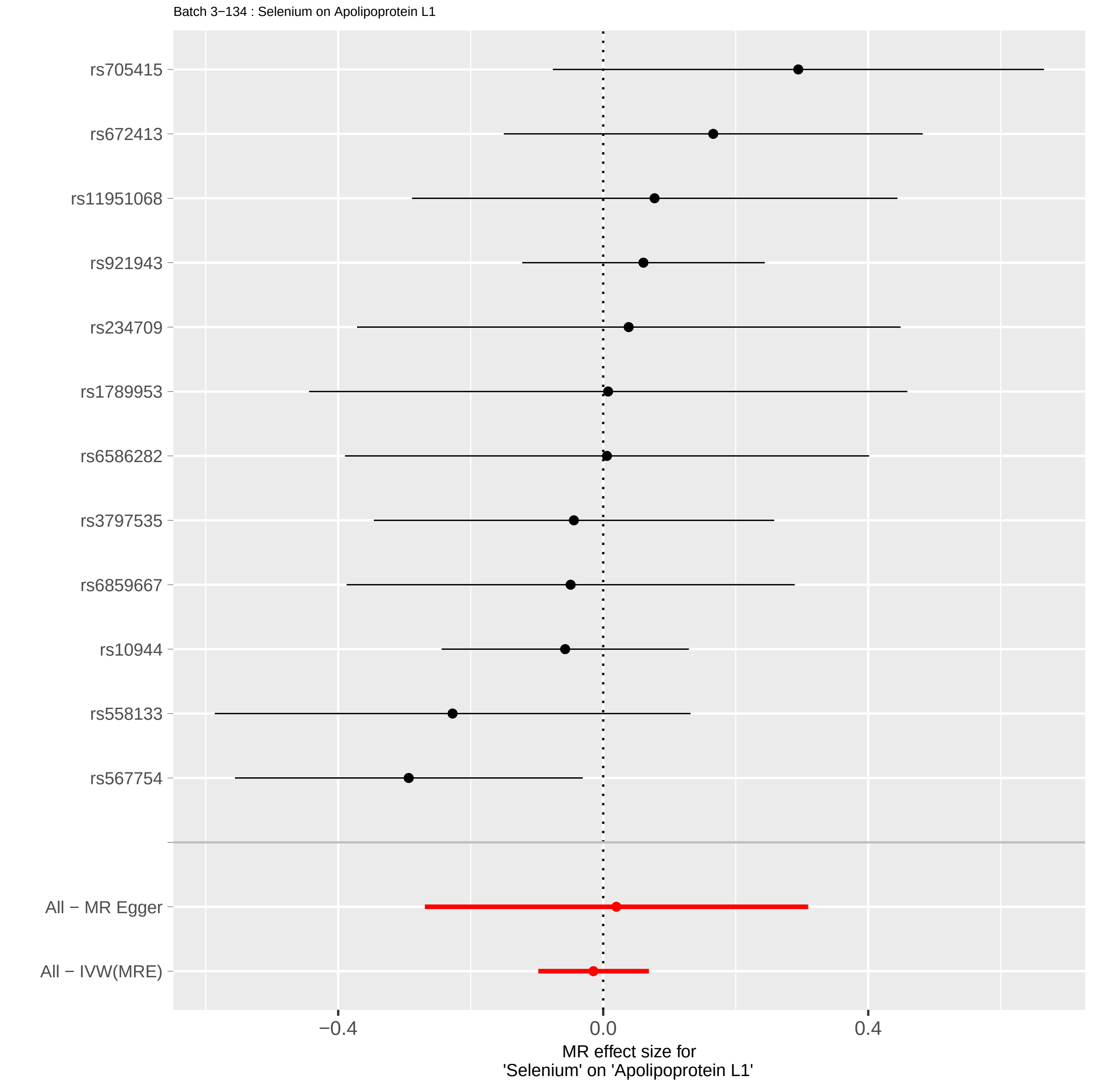

Batch 3−134 : Selenium on Apolipoprotein L1
rs705415
rs672413
rs11951068
rs921943
rs234709
rs1789953
rs6586282
rs3797535
rs6859667
rs10944
rs558133
rs567754
All − MR Egger
All − IVW(MRE)
−0.4
0.0
0.4
MR effect size for
'Selenium' on 'Apolipoprotein L1'

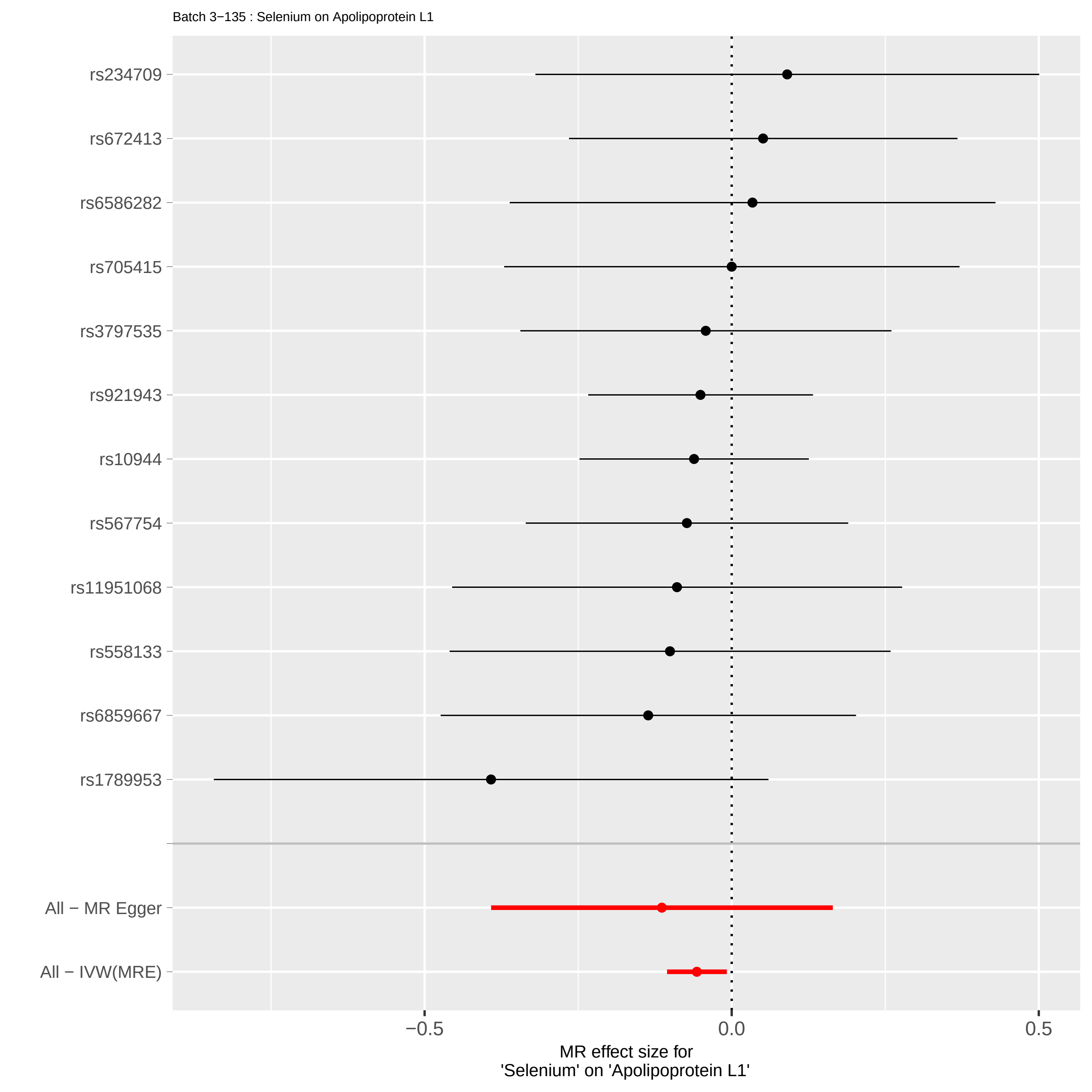
Batch 3−135 : Selenium on Apolipoprotein L1
rs234709
rs672413
rs6586282
rs705415
rs3797535
rs921943
rs10944
rs567754
rs11951068
rs558133
rs6859667
rs1789953
All − MR Egger
All − IVW(MRE)
−0.5
0.0
0.5
MR effect size for
'Selenium' on 'Apolipoprotein L1'

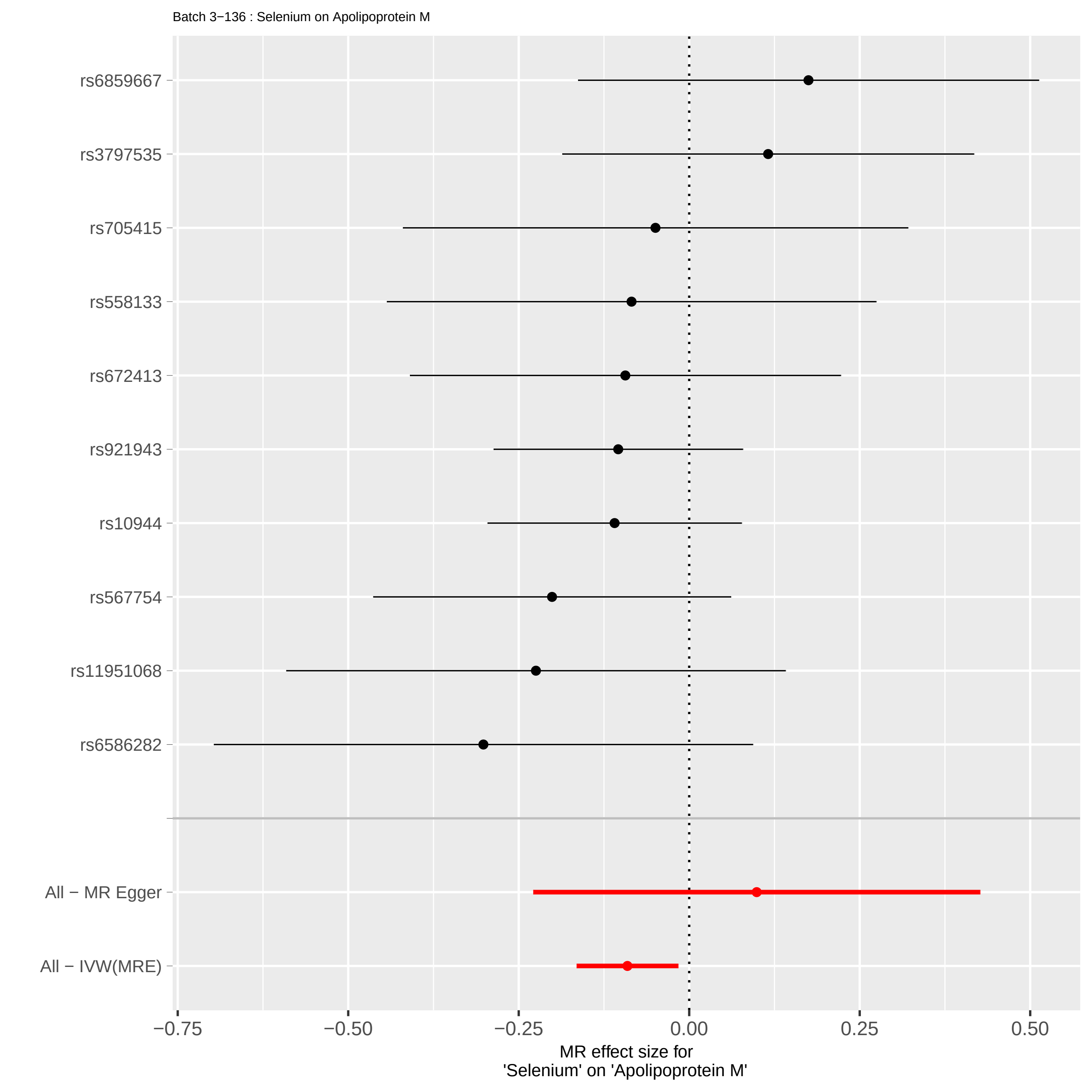

Batch 3−136 : Selenium on Apolipoprotein M
rs6859667
rs3797535
rs705415
rs558133
rs672413
rs921943
rs10944
rs567754
rs11951068
rs6586282
All − MR Egger
All − IVW(MRE)
−0.75
−0.50
−0.25
0.00
0.25
0.50
MR effect size for
'Selenium' on 'Apolipoprotein M'

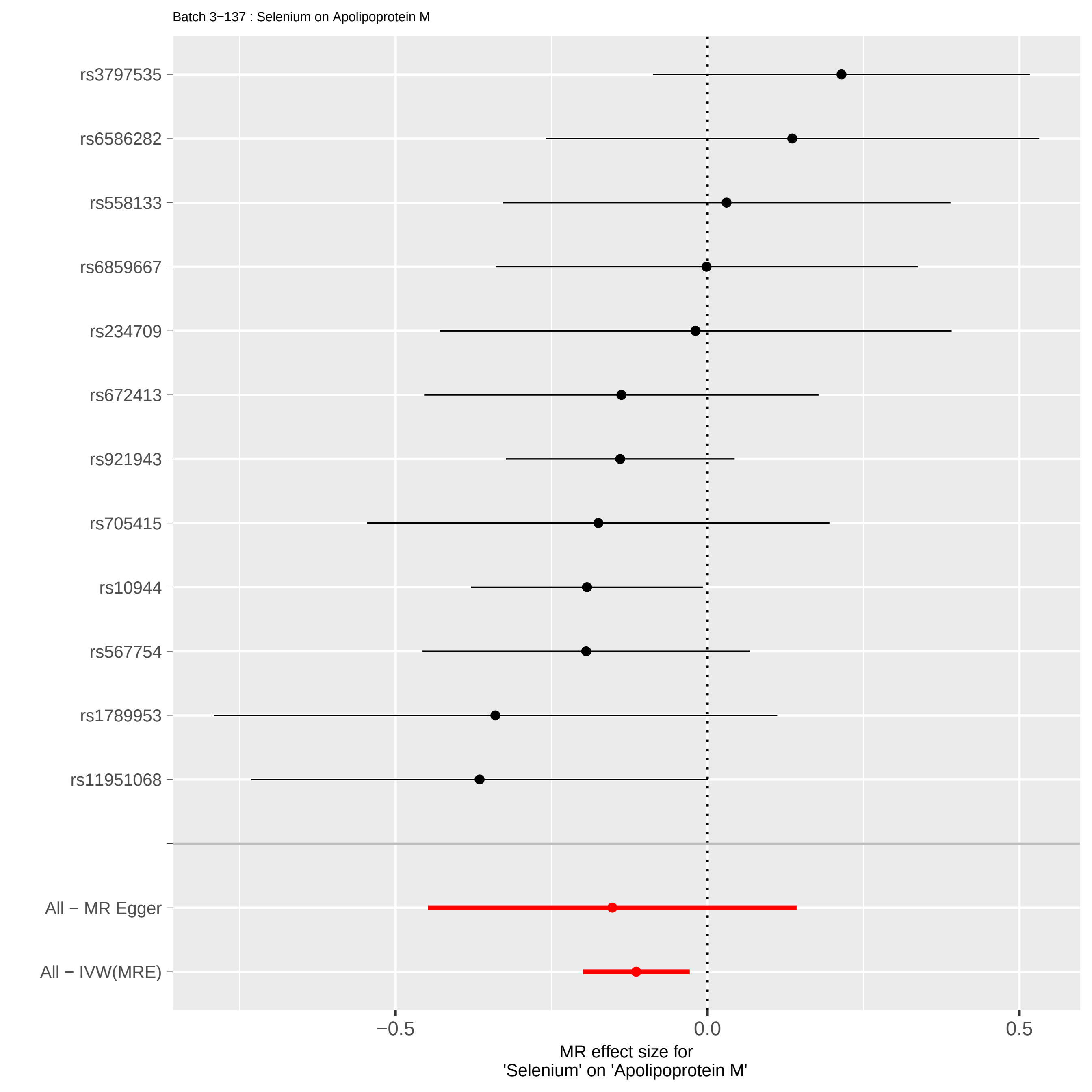

Batch 3−137 : Selenium on Apolipoprotein M
rs3797535
rs6586282
rs558133
rs6859667
rs234709
rs672413
rs921943
rs705415
rs10944
rs567754
rs1789953
rs11951068
All − MR Egger
All − IVW(MRE)
−0.5
0.0
0.5
MR effect size for
'Selenium' on 'Apolipoprotein M'

Batch 3−138 : Selenium on MICOS complex subunit MIC26

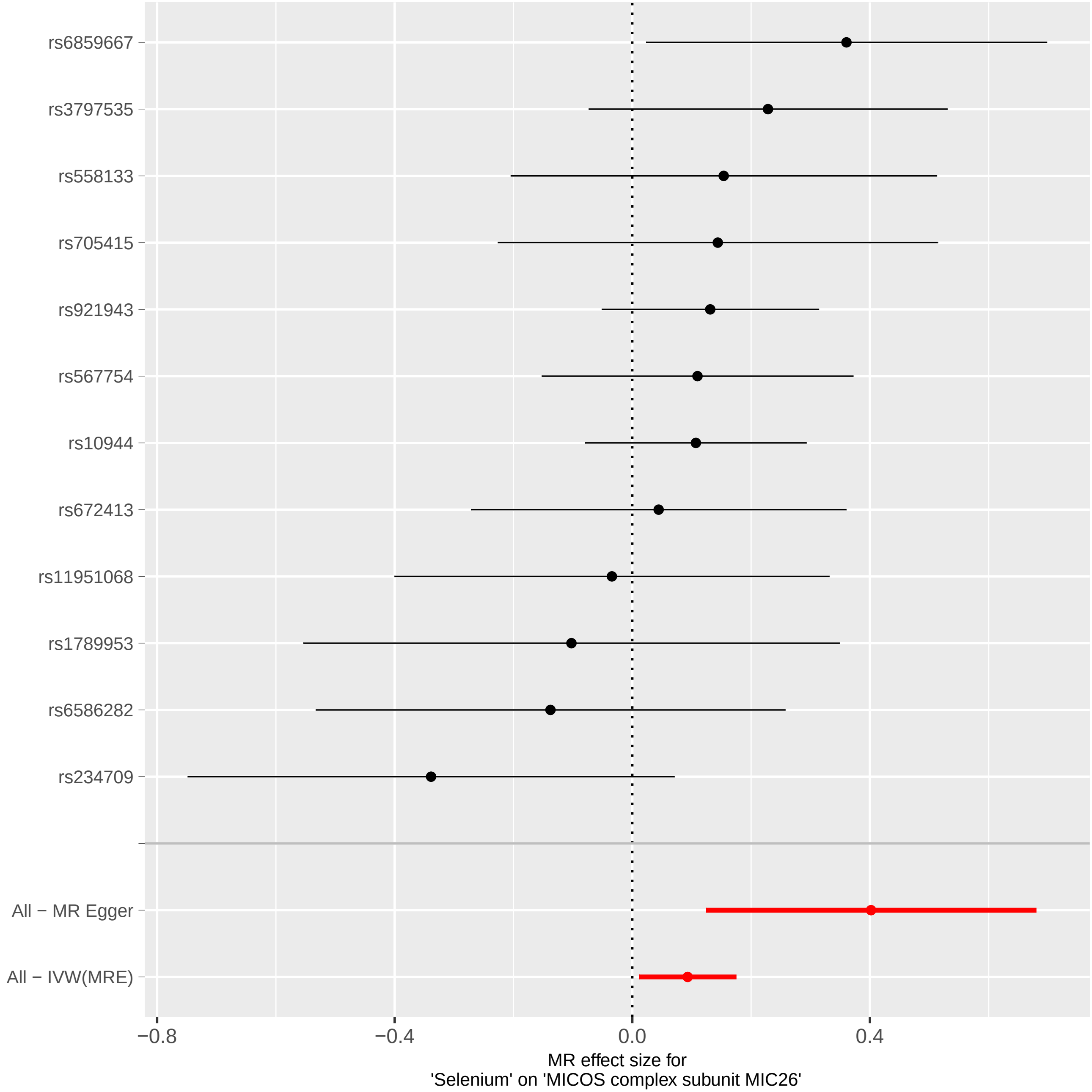

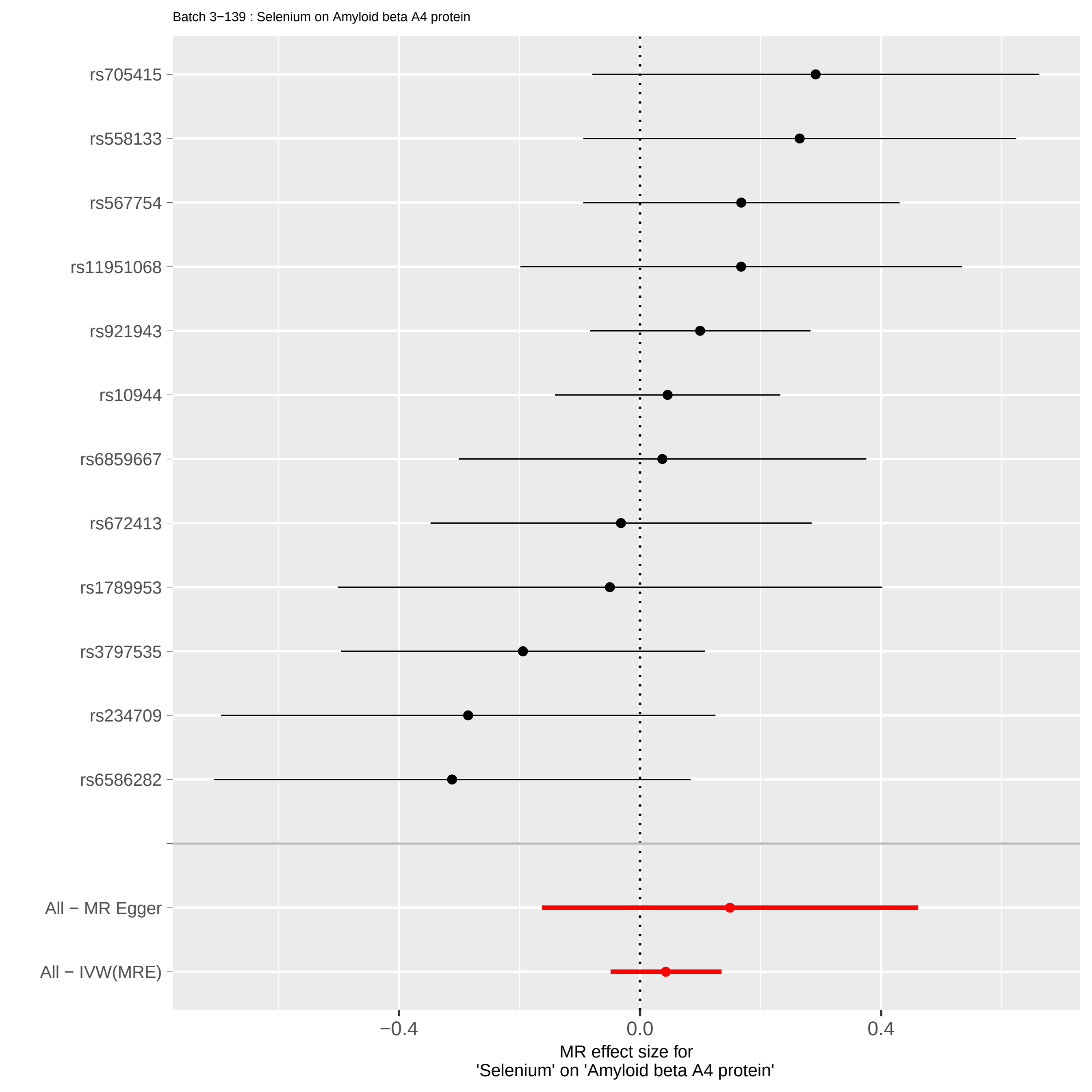
Batch 3−139 : Selenium on Amyloid beta A4 protein
rs705415
rs558133
rs567754
rs11951068
rs921943
rs10944
rs6859667
rs672413
rs1789953
rs3797535
rs234709
rs6586282
All − MR Egger
All − IVW(MRE)
−0.4
0.0
0.4
MR effect size for
'Selenium' on 'Amyloid beta A4 protein'

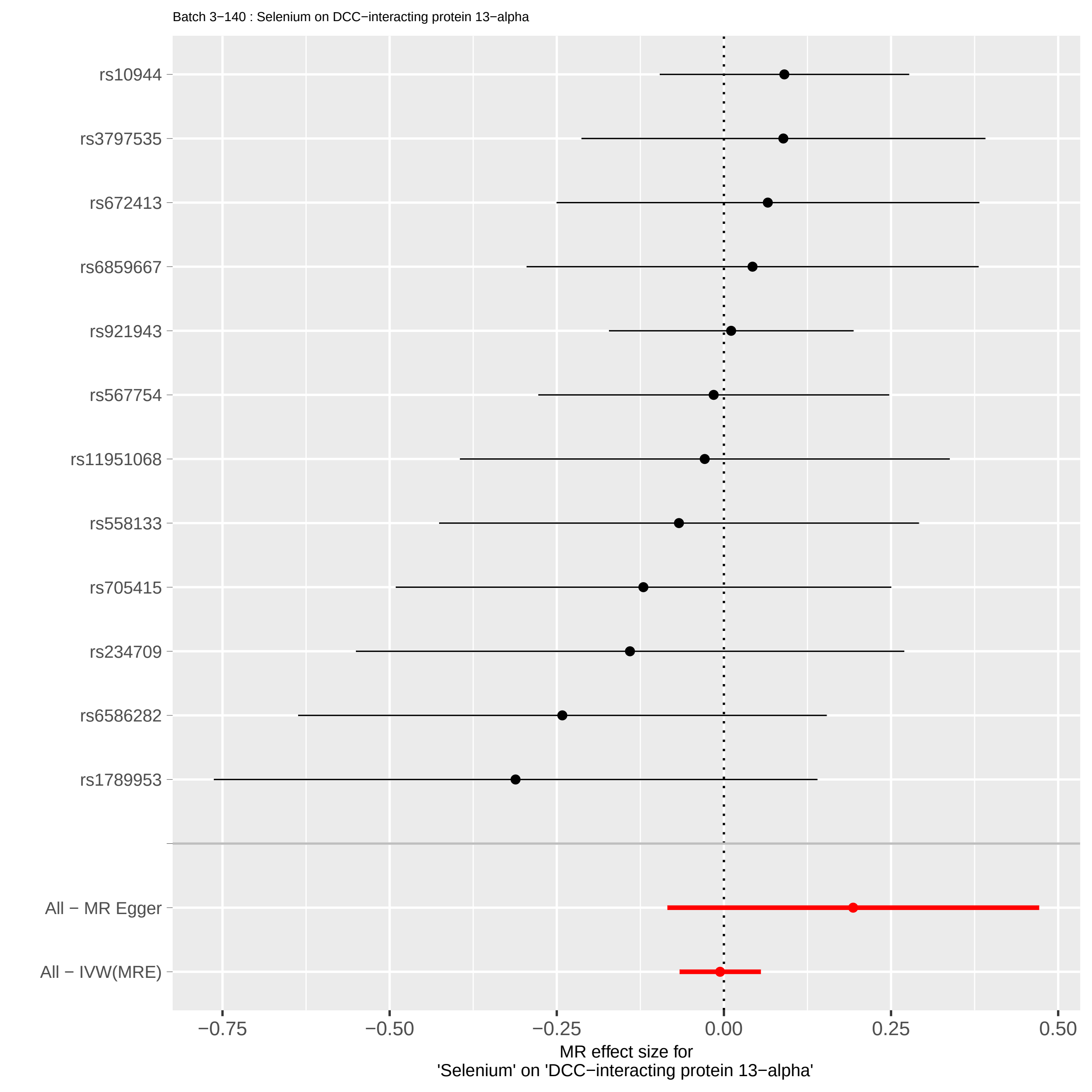

Batch 3−140 : Selenium on DCC−interacting protein 13−alpha
rs10944
rs3797535
rs672413
rs6859667
rs921943
rs567754
rs11951068
rs558133
rs705415
rs234709
rs6586282
rs1789953
All − MR Egger
All − IVW(MRE)
−0.75
−0.50
−0.25
0.00
0.25
0.50
MR effect size for
'Selenium' on 'DCC−interacting protein 13−alpha'

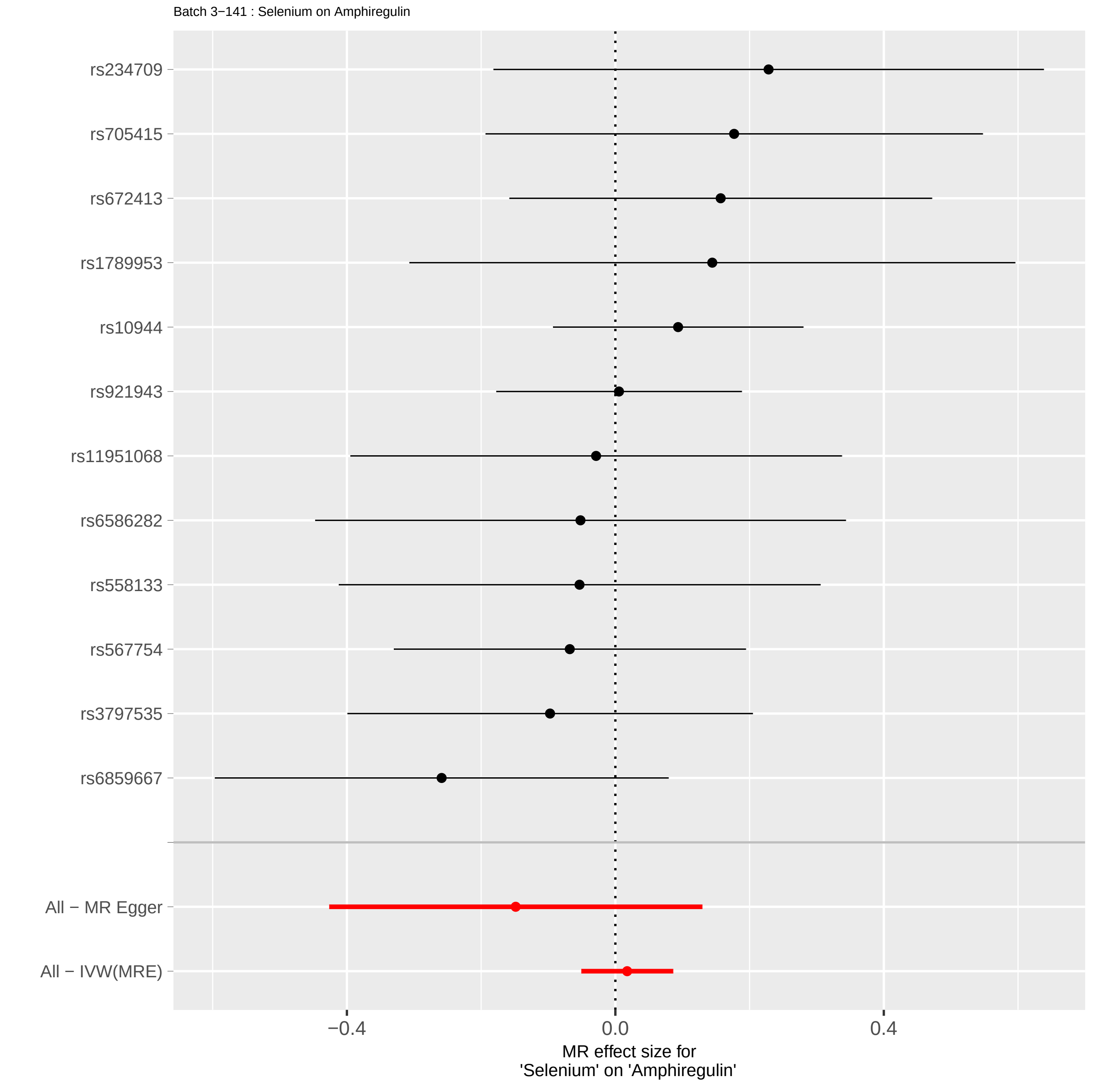
Batch 3−141 : Selenium on Amphiregulin
rs234709
rs705415
rs672413
rs1789953
rs10944
rs921943
rs11951068
rs6586282
rs558133
rs567754
rs3797535
rs6859667
All − MR Egger
All − IVW(MRE)
−0.4
0.0
0.4
MR effect size for
'Selenium' on 'Amphiregulin'

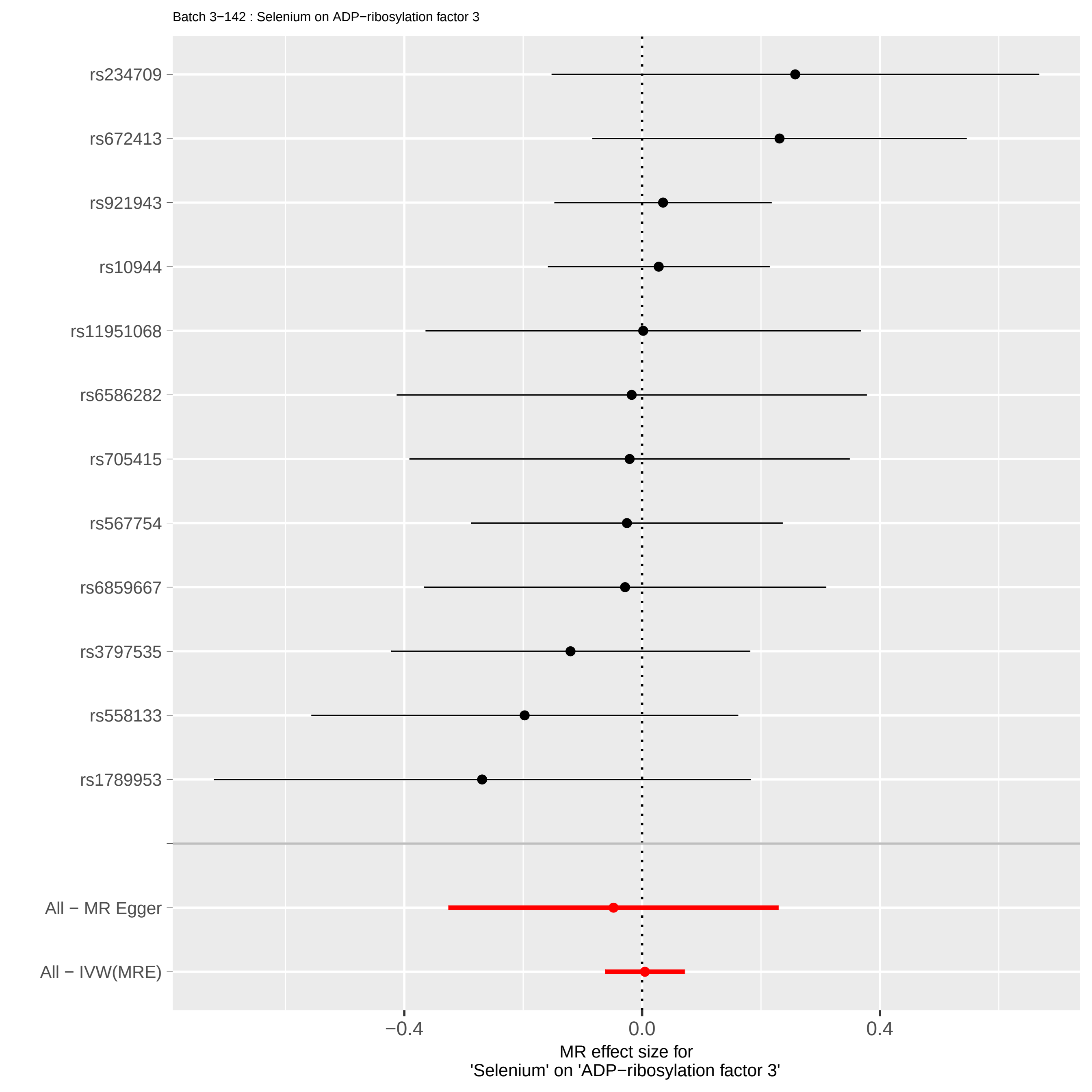
Batch 3−142 : Selenium on ADP−ribosylation factor 3
rs234709
rs672413
rs921943
rs10944
rs11951068
rs6586282
rs705415
rs567754
rs6859667
rs3797535
rs558133
rs1789953
All − MR Egger
All − IVW(MRE)
−0.4
0.0
0.4
MR effect size for
'Selenium' on 'ADP−ribosylation factor 3'

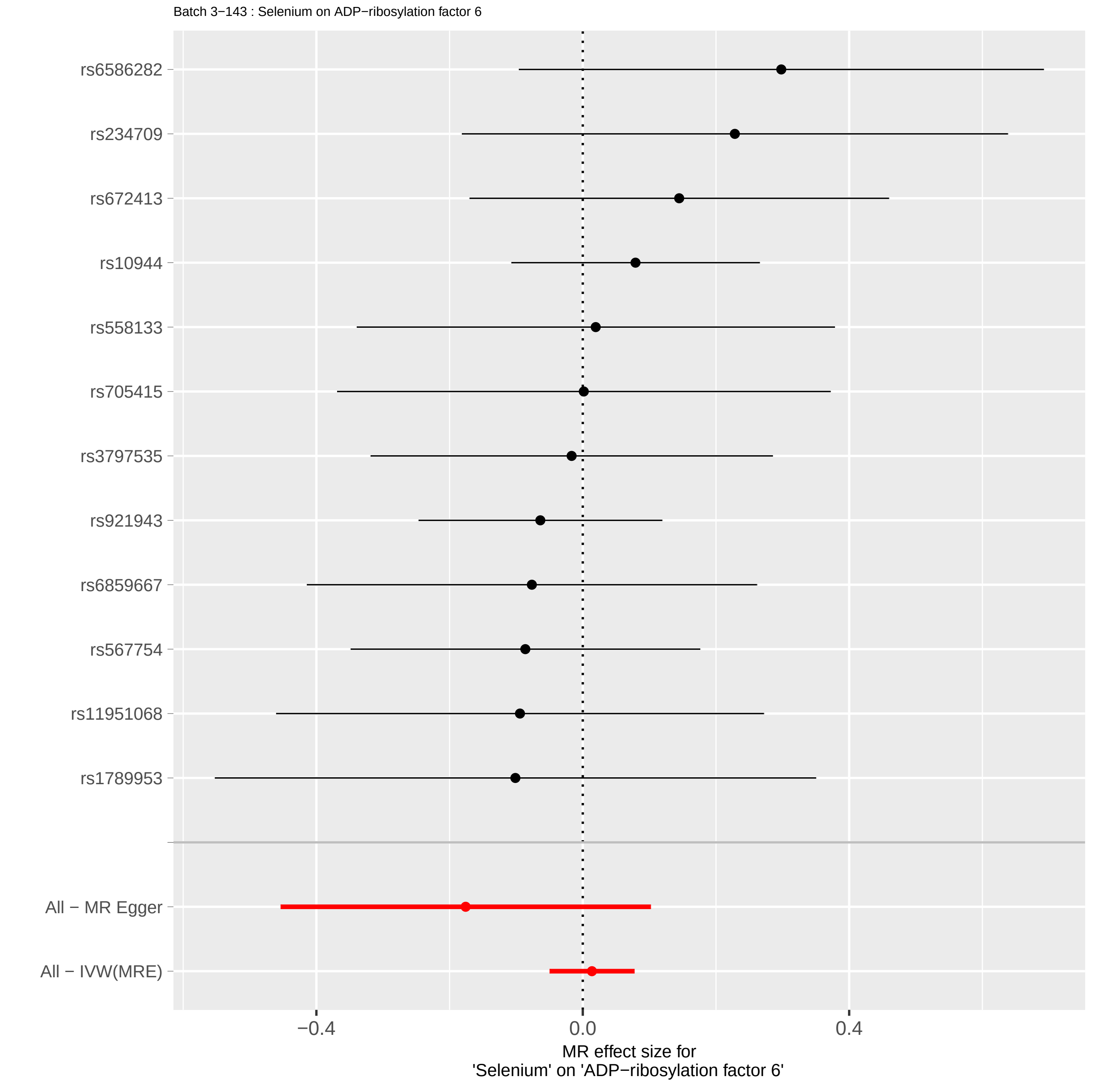

Batch 3−143 : Selenium on ADP−ribosylation factor 6
rs6586282
rs234709
rs672413
rs10944
rs558133
rs705415
rs3797535
rs921943
rs6859667
rs567754
rs11951068
rs1789953
All − MR Egger
All − IVW(MRE)
−0.4
0.0
0.4
MR effect size for
'Selenium' on 'ADP−ribosylation factor 6'

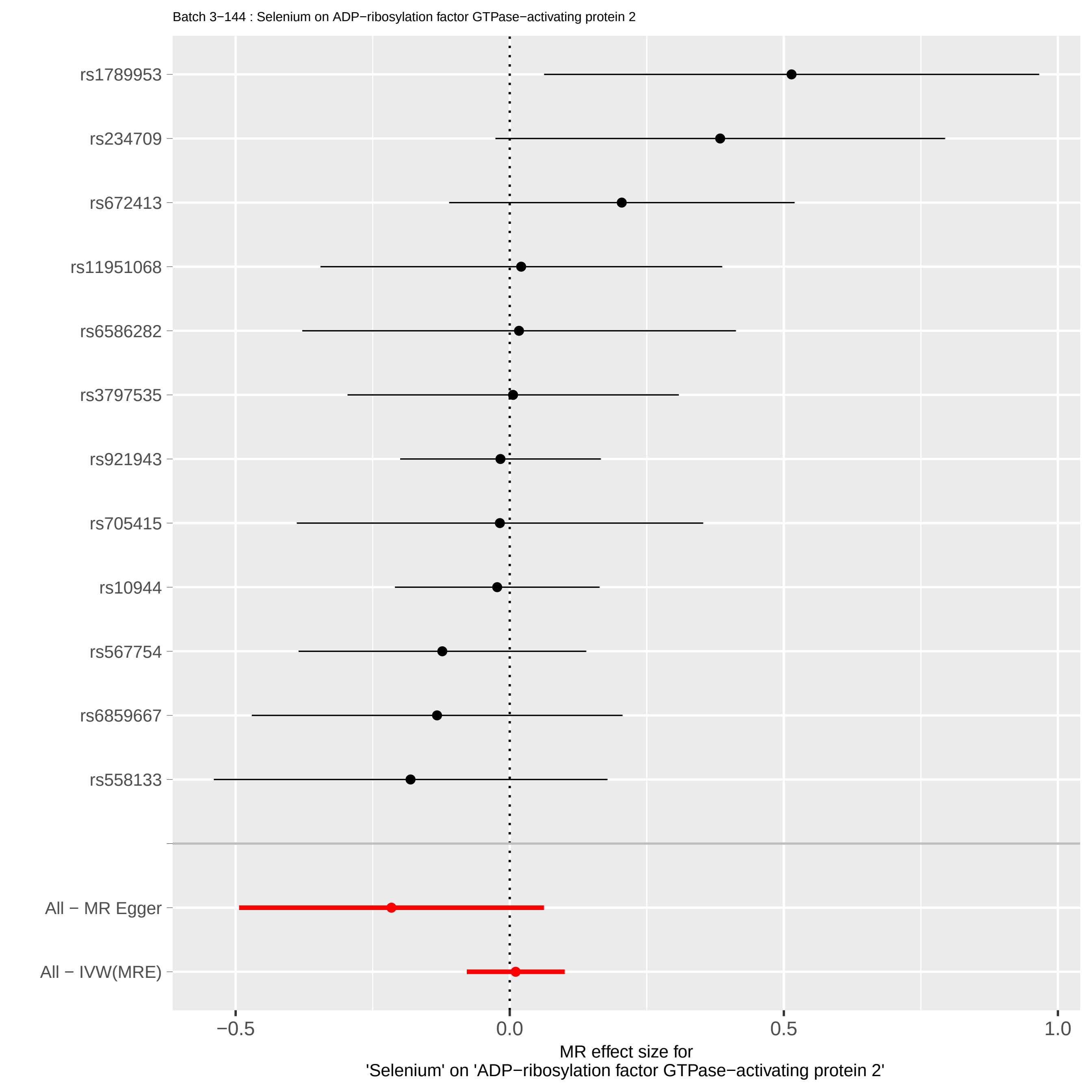

Batch 3−144 : Selenium on ADP−ribosylation factor GTPase−activating protein 2
rs1789953
rs234709
rs672413
rs11951068
rs6586282
rs3797535
rs921943
rs705415
rs10944
rs567754
rs6859667
rs558133
All − MR Egger
All − IVW(MRE)
−0.5
0.0
0.5
1.0
MR effect size for
'Selenium' on 'ADP−ribosylation factor GTPase−activating protein 2'

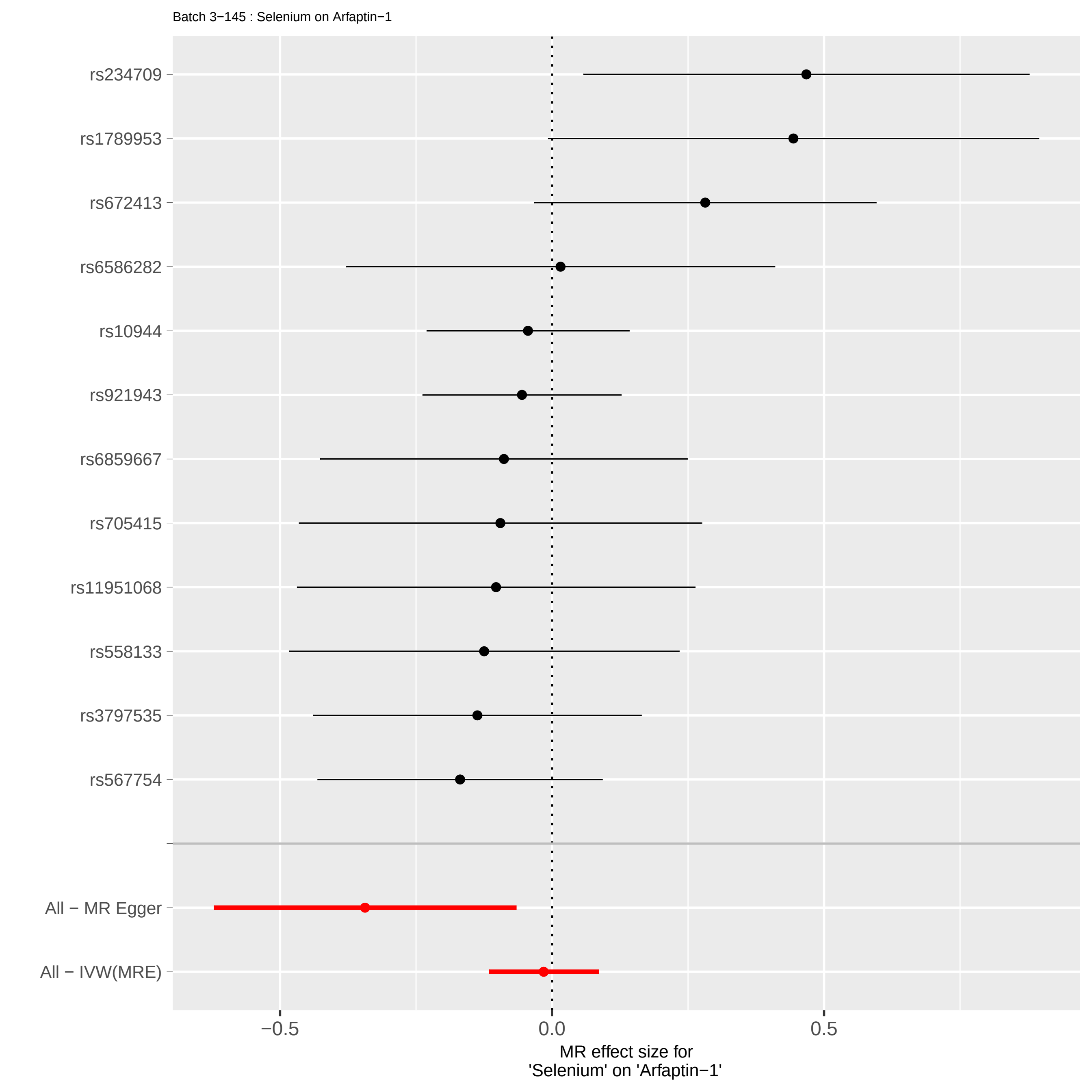

Batch 3−145 : Selenium on Arfaptin−1
rs234709
rs1789953
rs672413
rs6586282
rs10944
rs921943
rs6859667
rs705415
rs11951068
rs558133
rs3797535
rs567754
All − MR Egger
All − IVW(MRE)
−0.5
0.0
0.5
MR effect size for
'Selenium' on 'Arfaptin−1'

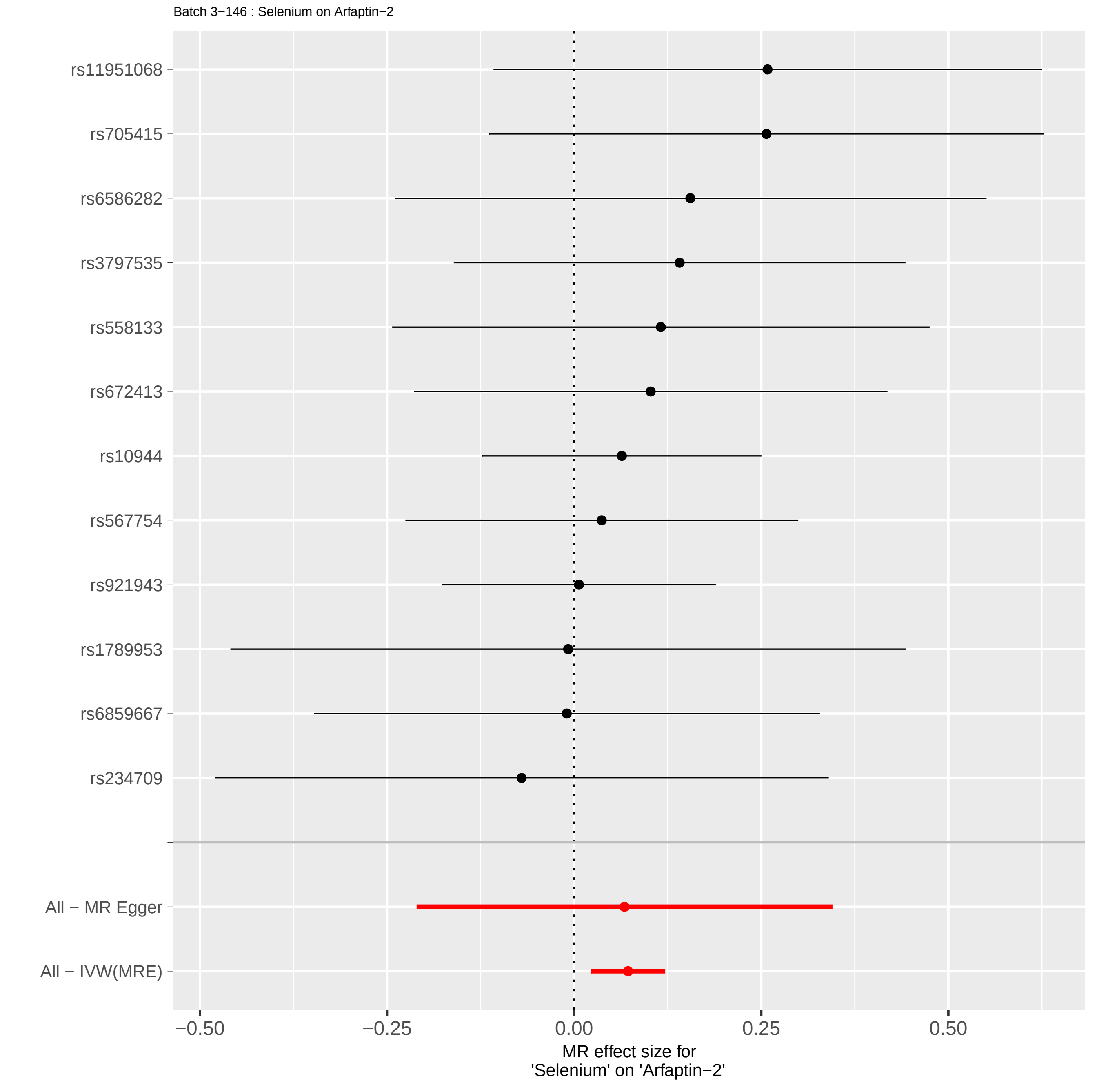
Batch 3−146 : Selenium on Arfaptin−2
rs11951068
rs705415
rs6586282
rs3797535
rs558133
rs672413
rs10944
rs567754
rs921943
rs1789953
rs6859667
rs234709
All − MR Egger
All − IVW(MRE)
−0.50
−0.25
0.00
0.25
0.50
MR effect size for
'Selenium' on 'Arfaptin−2'

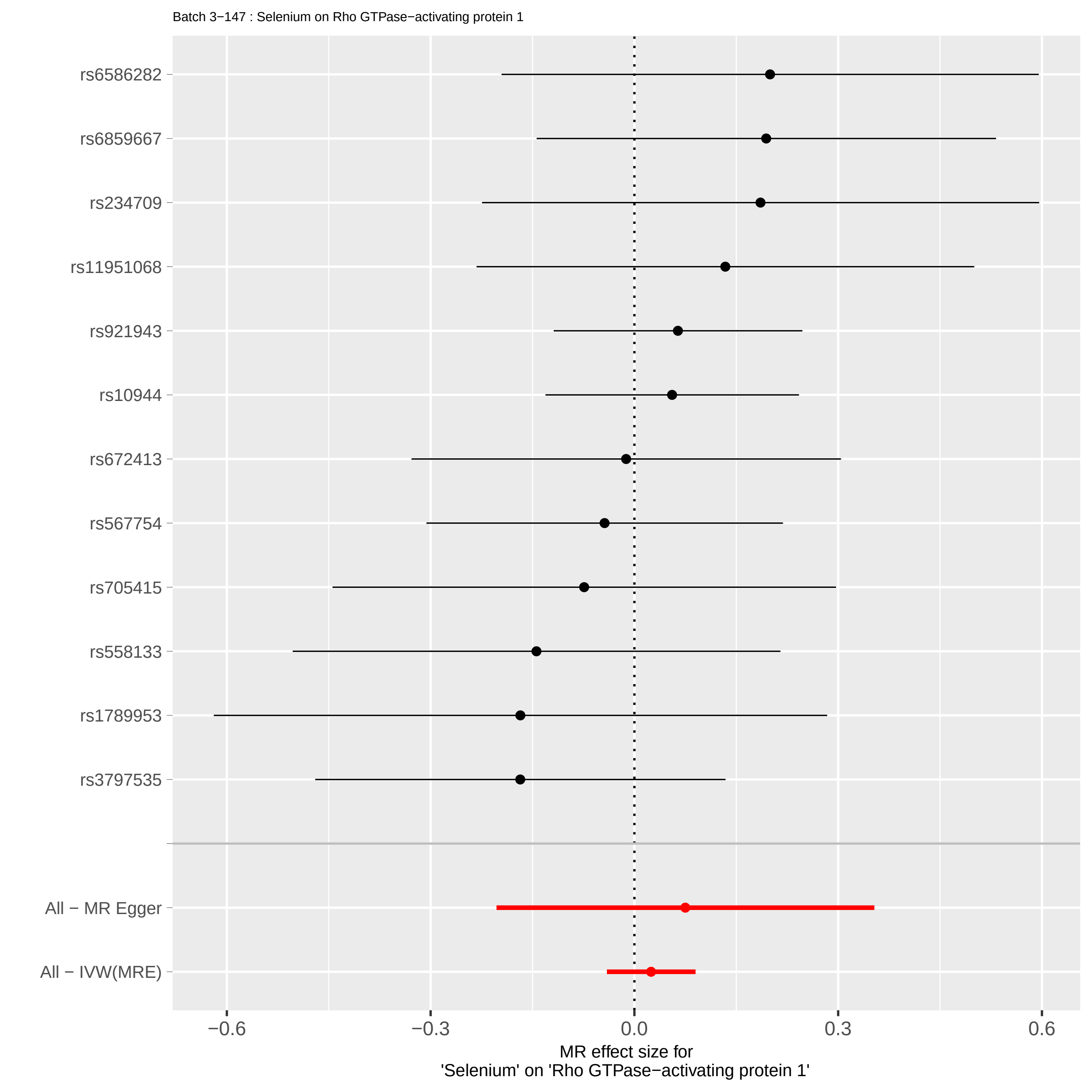
Batch 3−147 : Selenium on Rho GTPase−activating protein 1
rs6586282
rs6859667
rs234709
rs11951068
rs921943
rs10944
rs672413
rs567754
rs705415
rs558133
rs1789953
rs3797535
All − MR Egger
All − IVW(MRE)
−0.6
−0.3
0.0
0.3
0.6
MR effect size for
'Selenium' on 'Rho GTPase−activating protein 1'

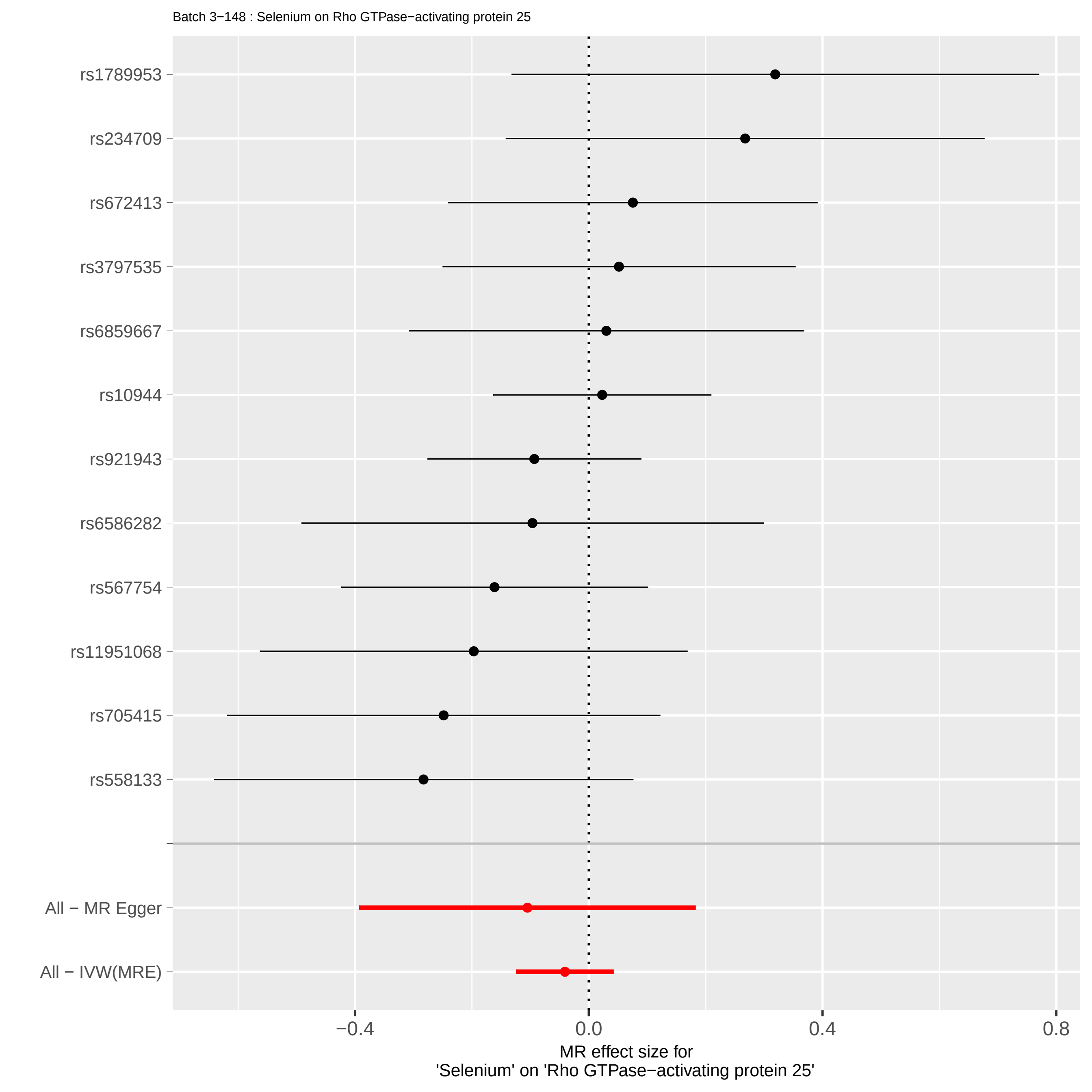

Batch 3−148 : Selenium on Rho GTPase−activating protein 25
rs1789953
rs234709
rs672413
rs3797535
rs6859667
rs10944
rs921943
rs6586282
rs567754
rs11951068
rs705415
rs558133
All − MR Egger
All − IVW(MRE)
−0.4
0.0
0.4
0.8
MR effect size for
'Selenium' on 'Rho GTPase−activating protein 25'

Batch 3−149 : Selenium on Rho GTPase−activating protein 30

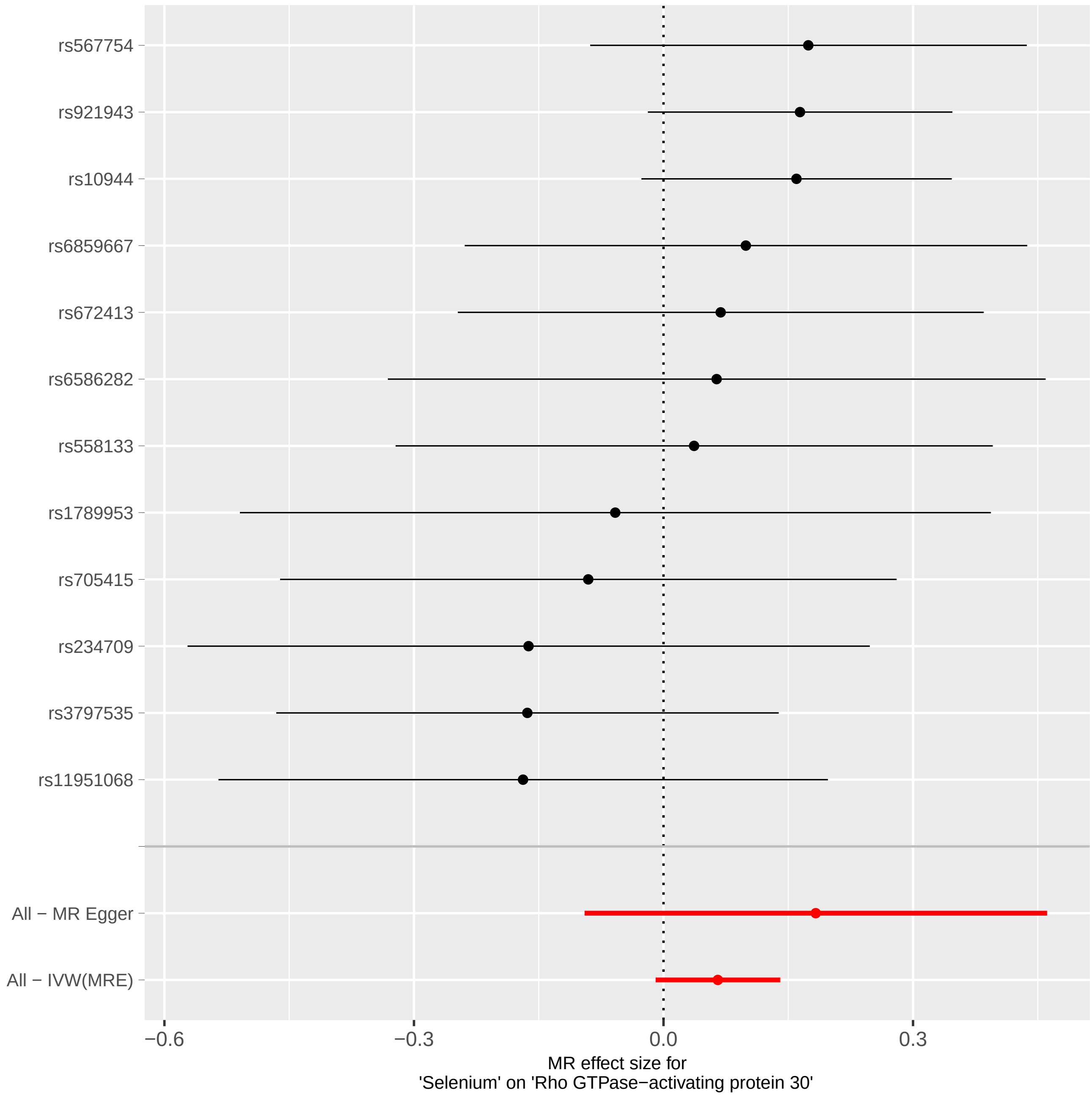

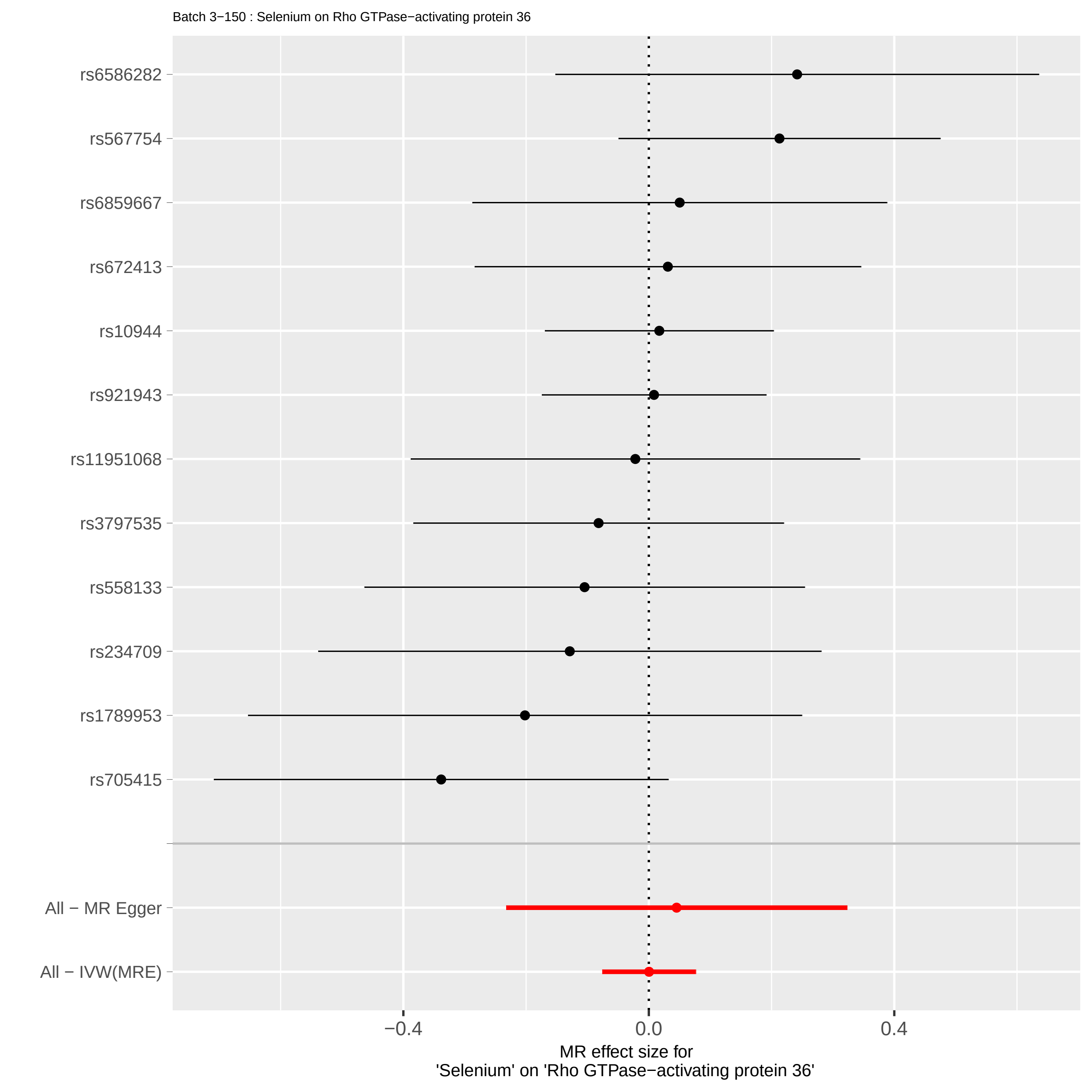

Batch 3−150 : Selenium on Rho GTPase−activating protein 36
rs6586282
rs567754
rs6859667
rs672413
rs10944
rs921943
rs11951068
rs3797535
rs558133
rs234709
rs1789953
rs705415
All − MR Egger
All − IVW(MRE)
−0.4
0.0
0.4
MR effect size for
'Selenium' on 'Rho GTPase−activating protein 36'

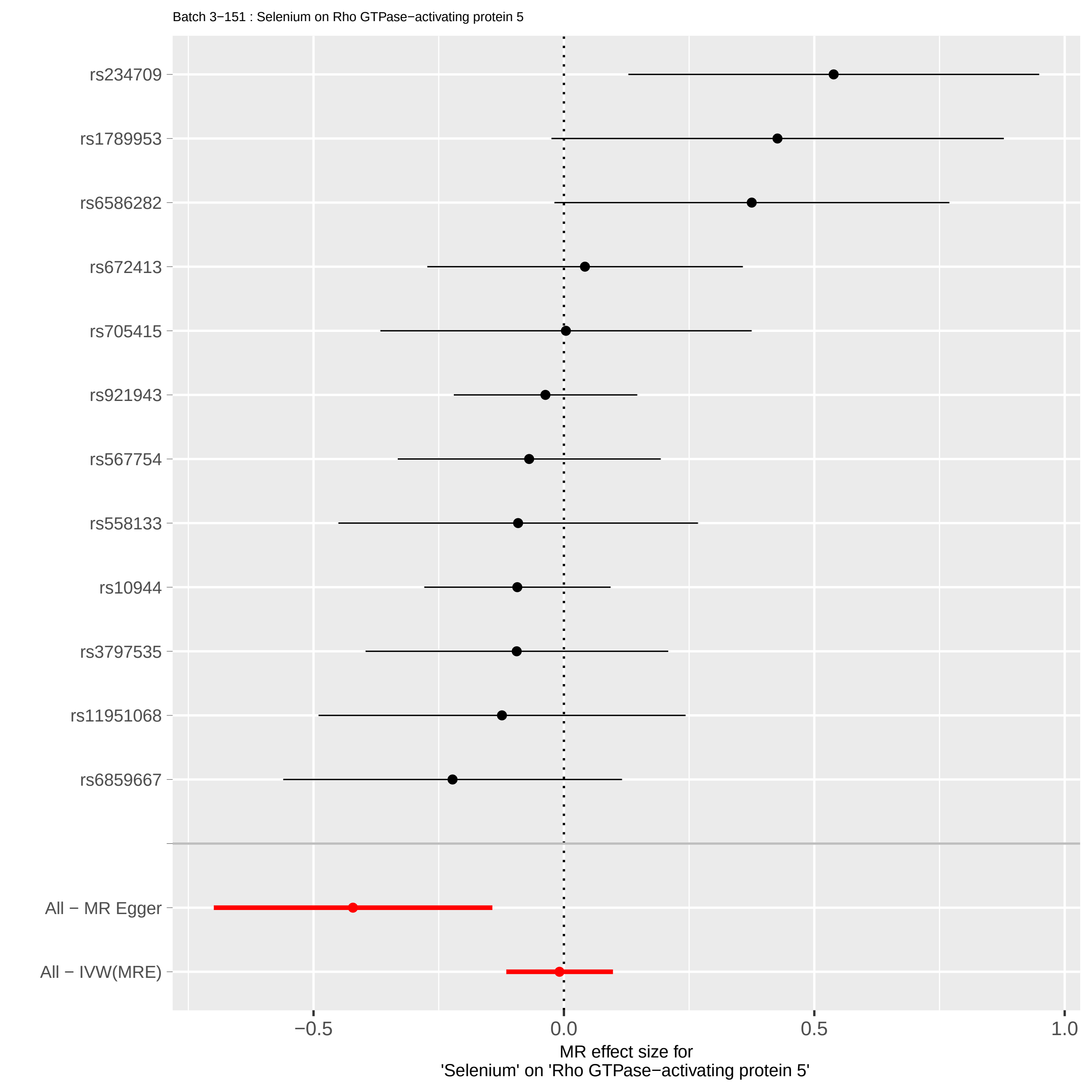
Batch 3−151 : Selenium on Rho GTPase−activating protein 5
rs234709
rs1789953
rs6586282
rs672413
rs705415
rs921943
rs567754
rs558133
rs10944
rs3797535
rs11951068
rs6859667
All − MR Egger
All − IVW(MRE)
−0.5
0.0
0.5
1.0
MR effect size for
'Selenium' on 'Rho GTPase−activating protein 5'

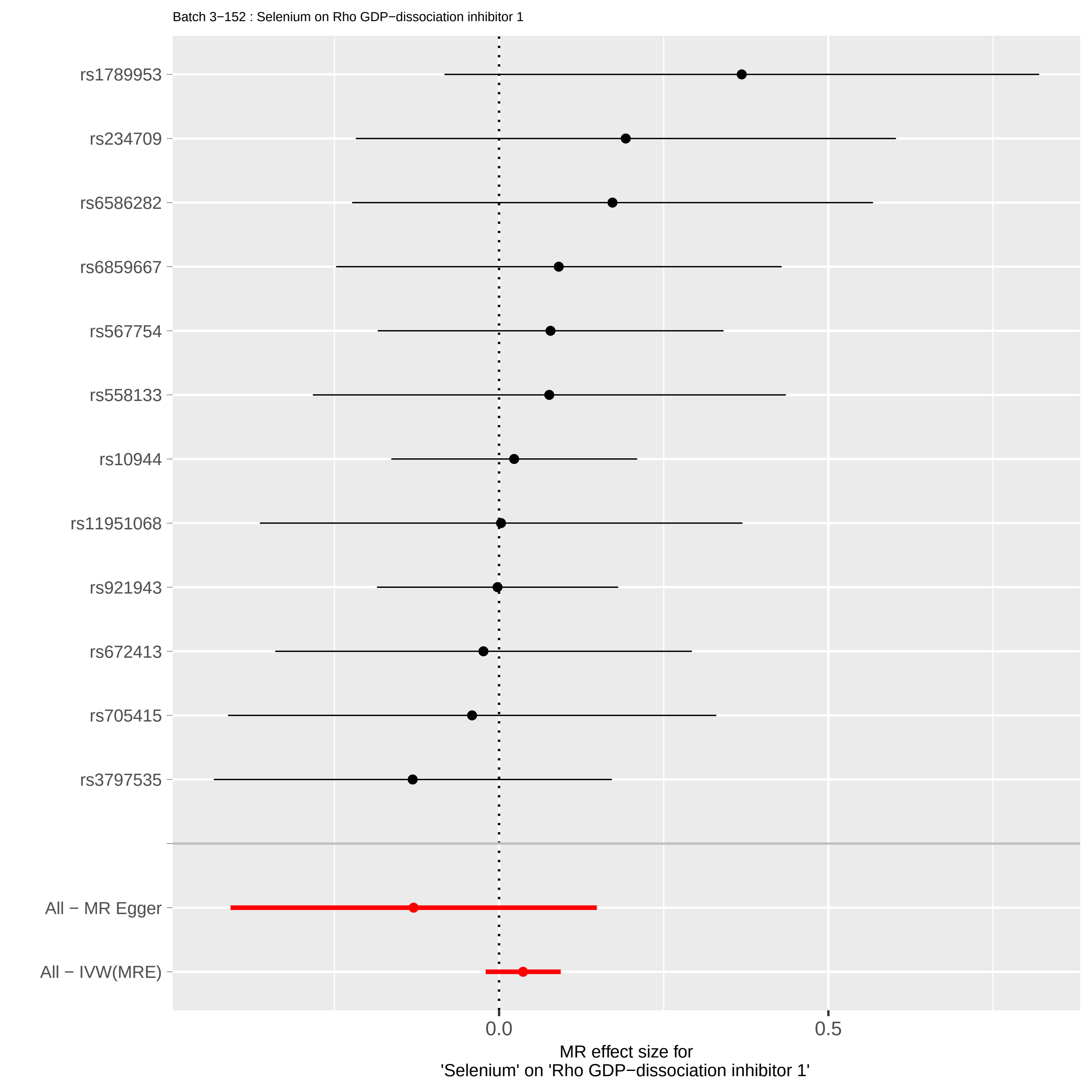

Batch 3−152 : Selenium on Rho GDP−dissociation inhibitor 1
rs1789953
rs234709
rs6586282
rs6859667
rs567754
rs558133
rs10944
rs11951068
rs921943
rs672413
rs705415
rs3797535
All − MR Egger
All − IVW(MRE)
0.0
0.5
MR effect size for
'Selenium' on 'Rho GDP−dissociation inhibitor 1'

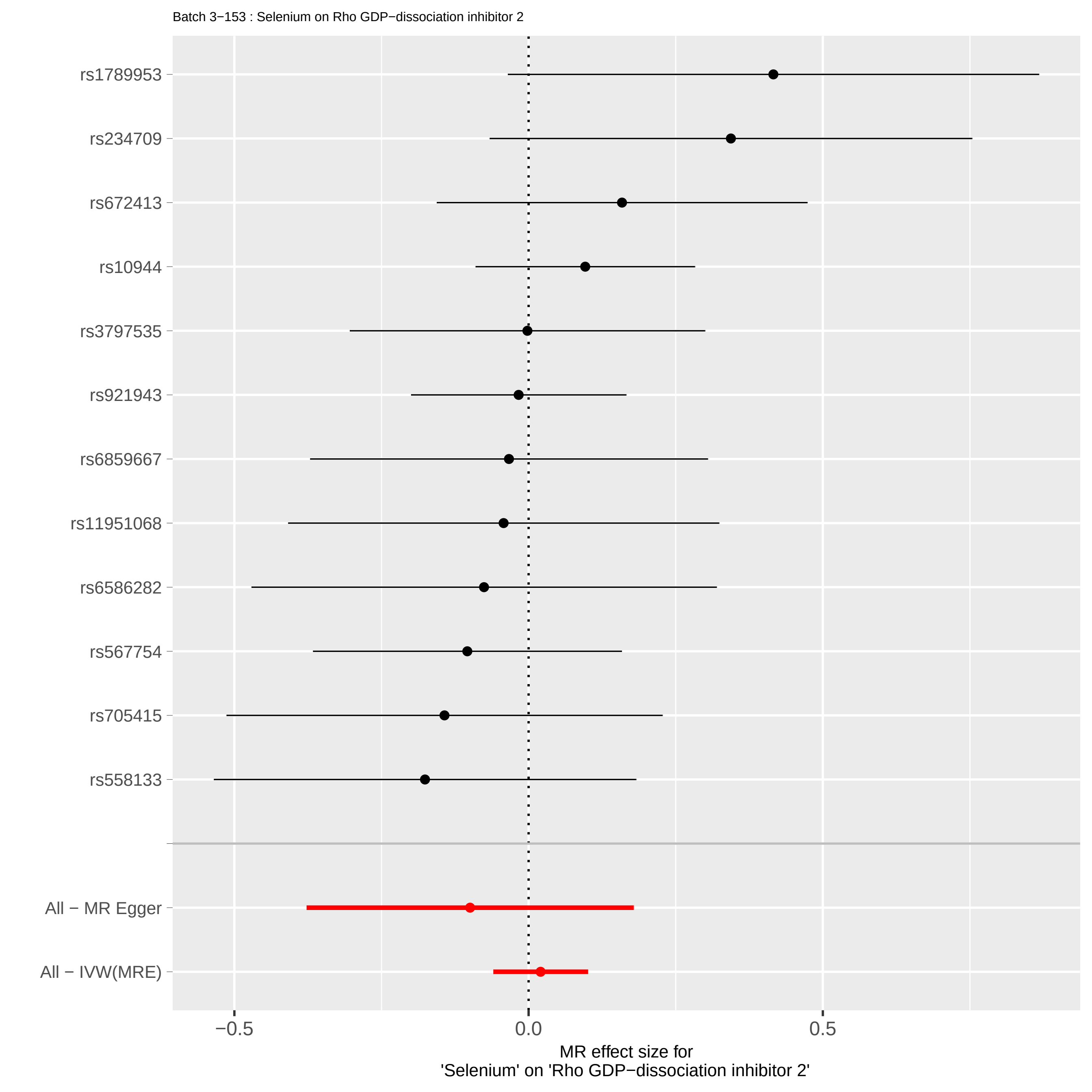

Batch 3−153 : Selenium on Rho GDP−dissociation inhibitor 2
rs1789953
rs234709
rs672413
rs10944
rs3797535
rs921943
rs6859667
rs11951068
rs6586282
rs567754
rs705415
rs558133
All − MR Egger
All − IVW(MRE)
−0.5
0.0
0.5
MR effect size for
'Selenium' on 'Rho GDP−dissociation inhibitor 2'

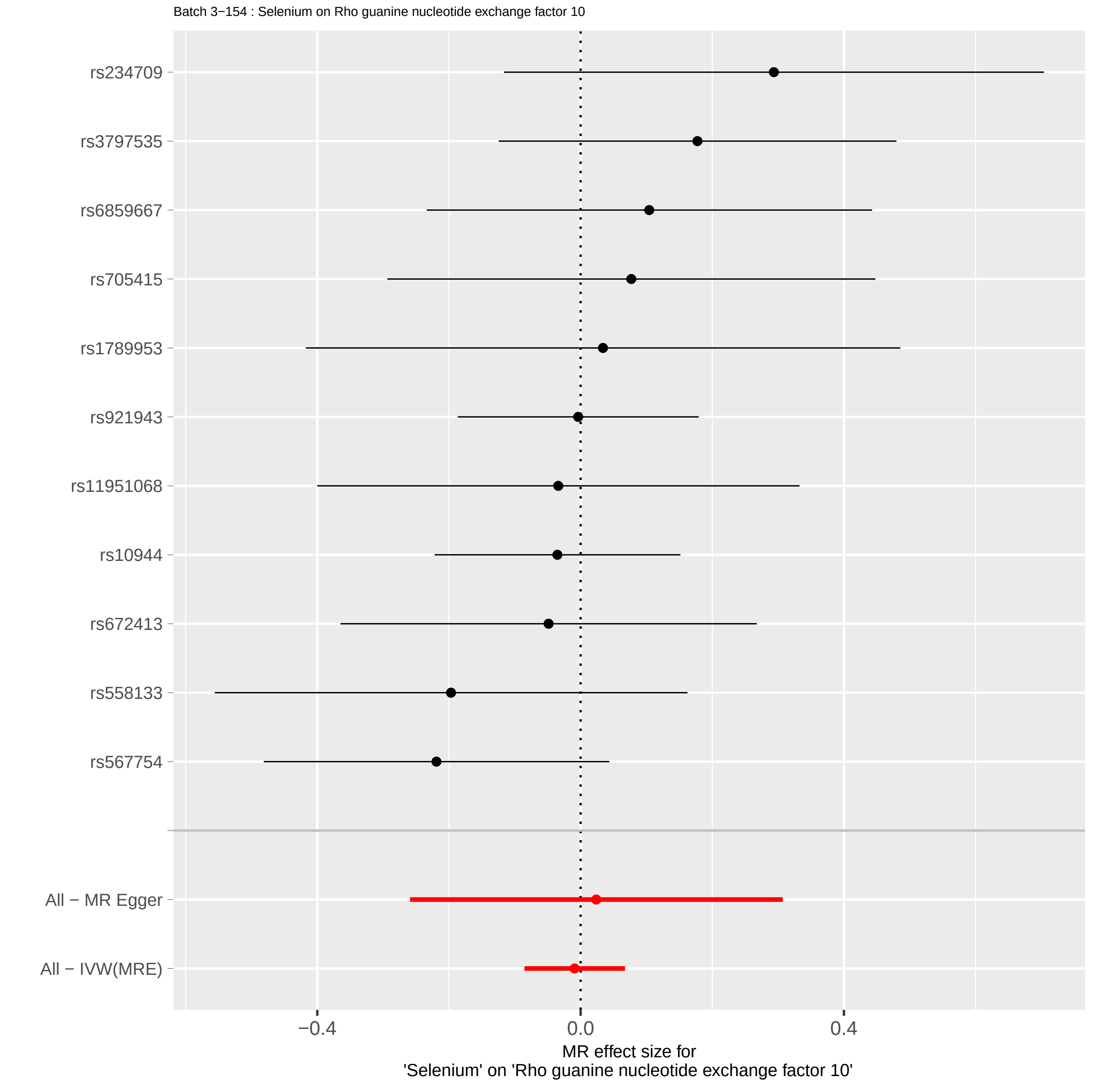
Batch 3−154 : Selenium on Rho guanine nucleotide exchange factor 10
rs234709
rs3797535
rs6859667
rs705415
rs1789953
rs921943
rs11951068
rs10944
rs672413
rs558133
rs567754
All − MR Egger
All − IVW(MRE)
−0.4
0.0
0.4
MR effect size for
'Selenium' on 'Rho guanine nucleotide exchange factor 10'

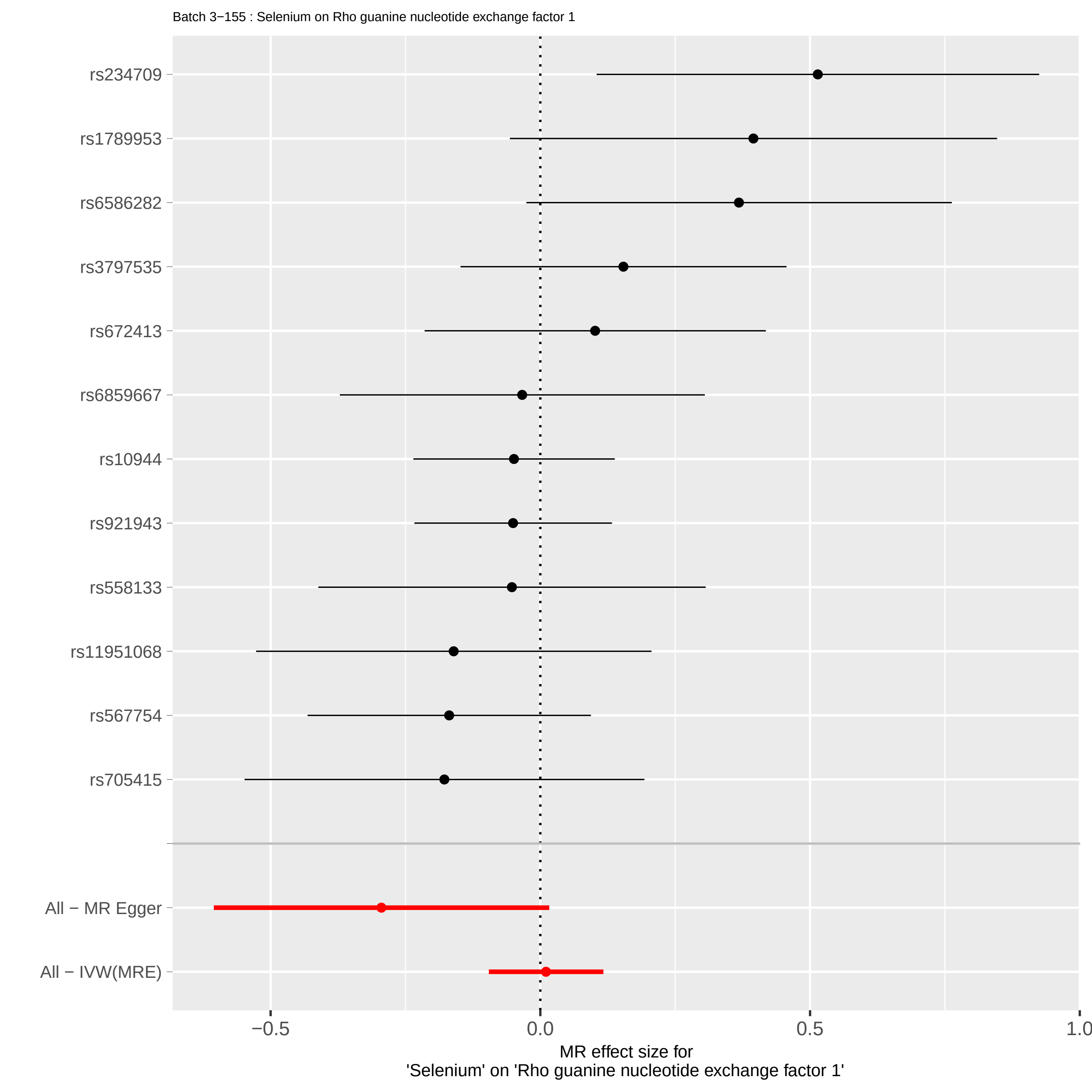

Batch 3−155 : Selenium on Rho guanine nucleotide exchange factor 1
rs234709
rs1789953
rs6586282
rs3797535
rs672413
rs6859667
rs10944
rs921943
rs558133
rs11951068
rs567754
rs705415
All − MR Egger
All − IVW(MRE)
−0.5
0.0
0.5
1.0
MR effect size for
'Selenium' on 'Rho guanine nucleotide exchange factor 1'

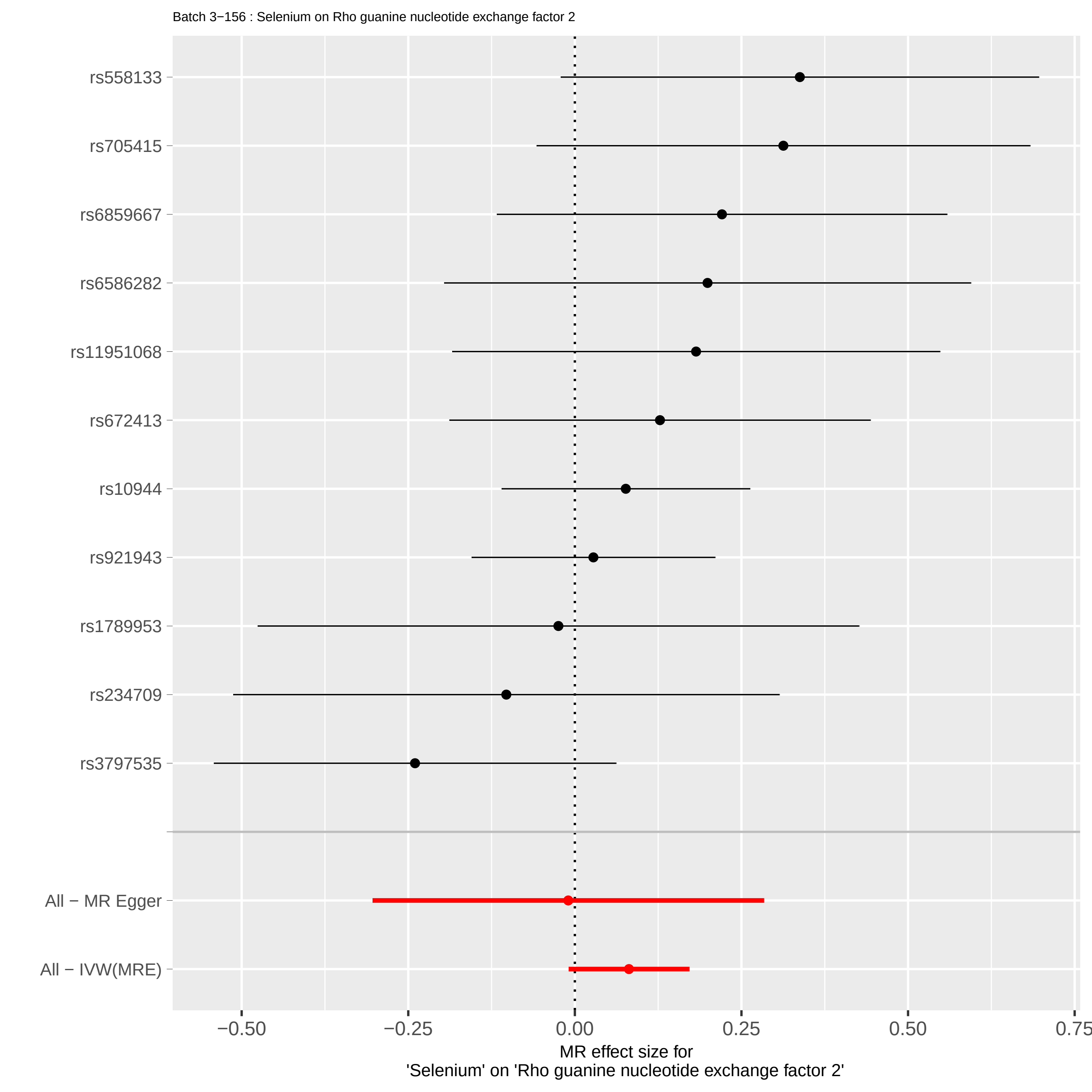
Batch 3−156 : Selenium on Rho guanine nucleotide exchange factor 2
rs558133
rs705415
rs6859667
rs6586282
rs11951068
rs672413
rs10944
rs921943
rs1789953
rs234709
rs3797535
All − MR Egger
All − IVW(MRE)
−0.50
−0.25
0.00
0.25
0.50
0.75
MR effect size for
'Selenium' on 'Rho guanine nucleotide exchange factor 2'

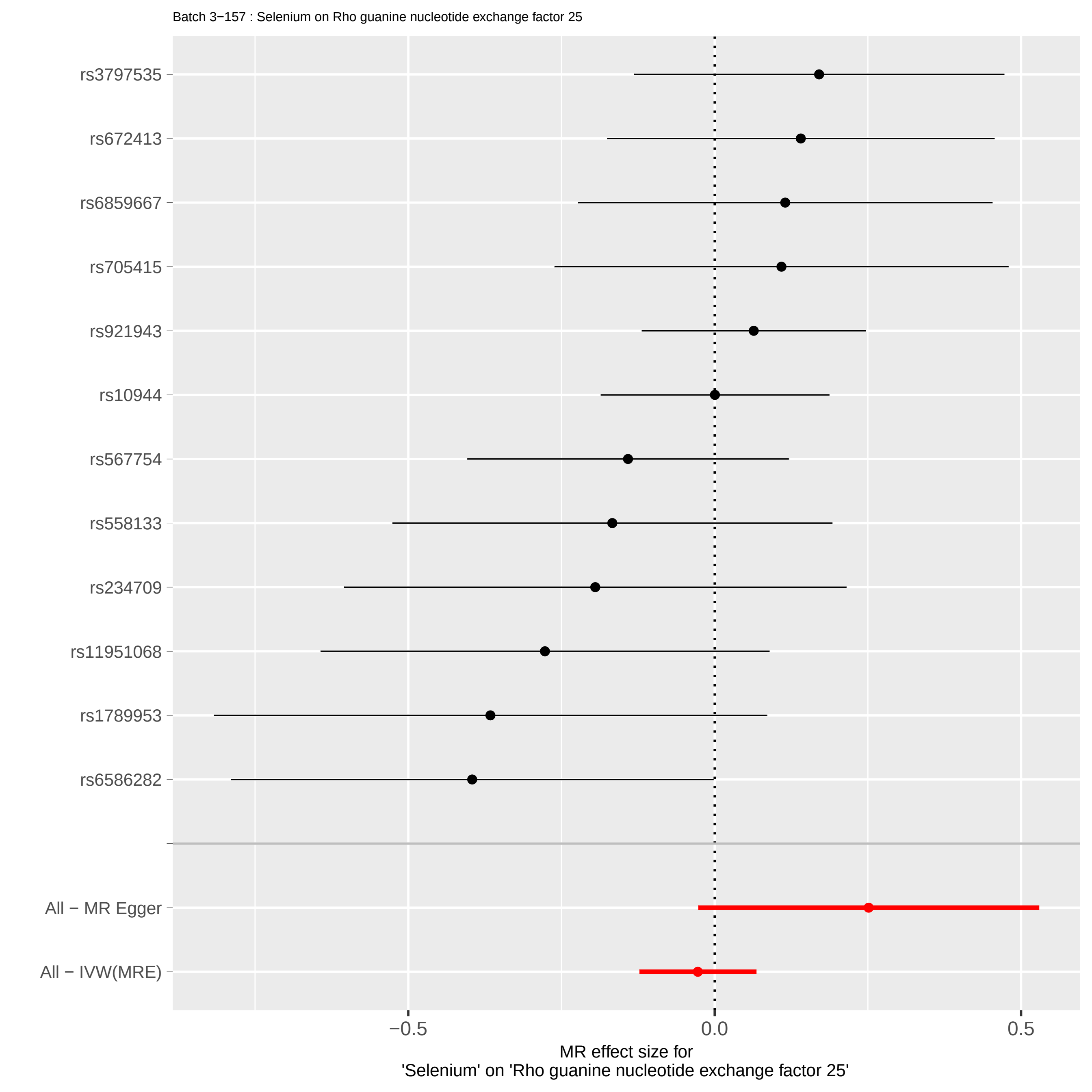

Batch 3−157 : Selenium on Rho guanine nucleotide exchange factor 25
rs3797535
rs672413
rs6859667
rs705415
rs921943
rs10944
rs567754
rs558133
rs234709
rs11951068
rs1789953
rs6586282
All − MR Egger
All − IVW(MRE)
−0.5
0.0
0.5
MR effect size for
'Selenium' on 'Rho guanine nucleotide exchange factor 25'

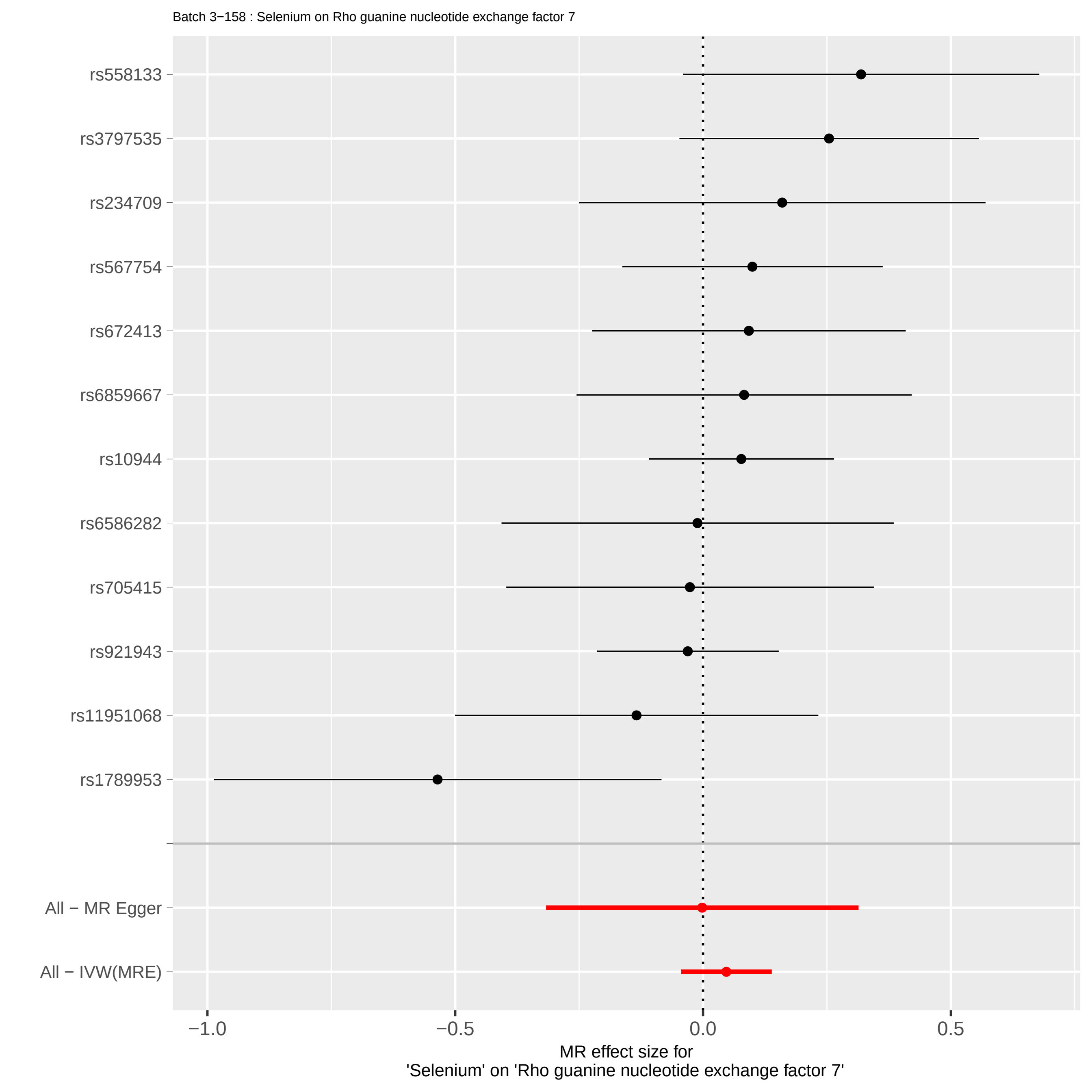

Batch 3−158 : Selenium on Rho guanine nucleotide exchange factor 7
rs558133
rs3797535
rs234709
rs567754
rs672413
rs6859667
rs10944
rs6586282
rs705415
rs921943
rs11951068
rs1789953
All − MR Egger
All − IVW(MRE)
−1.0
−0.5
0.0
0.5
MR effect size for
'Selenium' on 'Rho guanine nucleotide exchange factor 7'

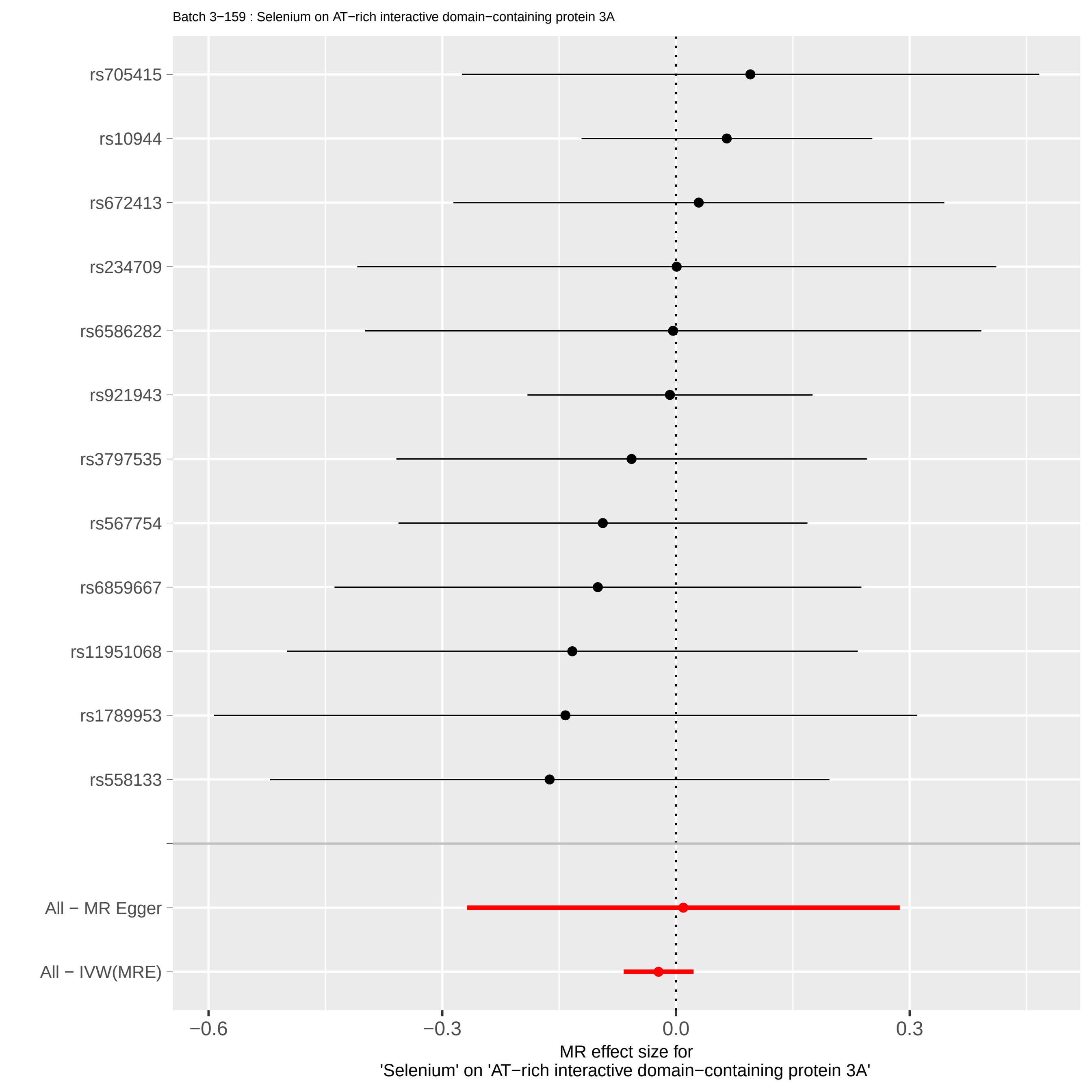
Batch 3−159 : Selenium on AT−rich interactive domain−containing protein 3A
rs705415
rs10944
rs672413
rs234709
rs6586282
rs921943
rs3797535
rs567754
rs6859667
rs11951068
rs1789953
rs558133
All − MR Egger
All − IVW(MRE)
−0.6
−0.3
0.0
0.3
MR effect size for
'Selenium' on 'AT−rich interactive domain−containing protein 3A'

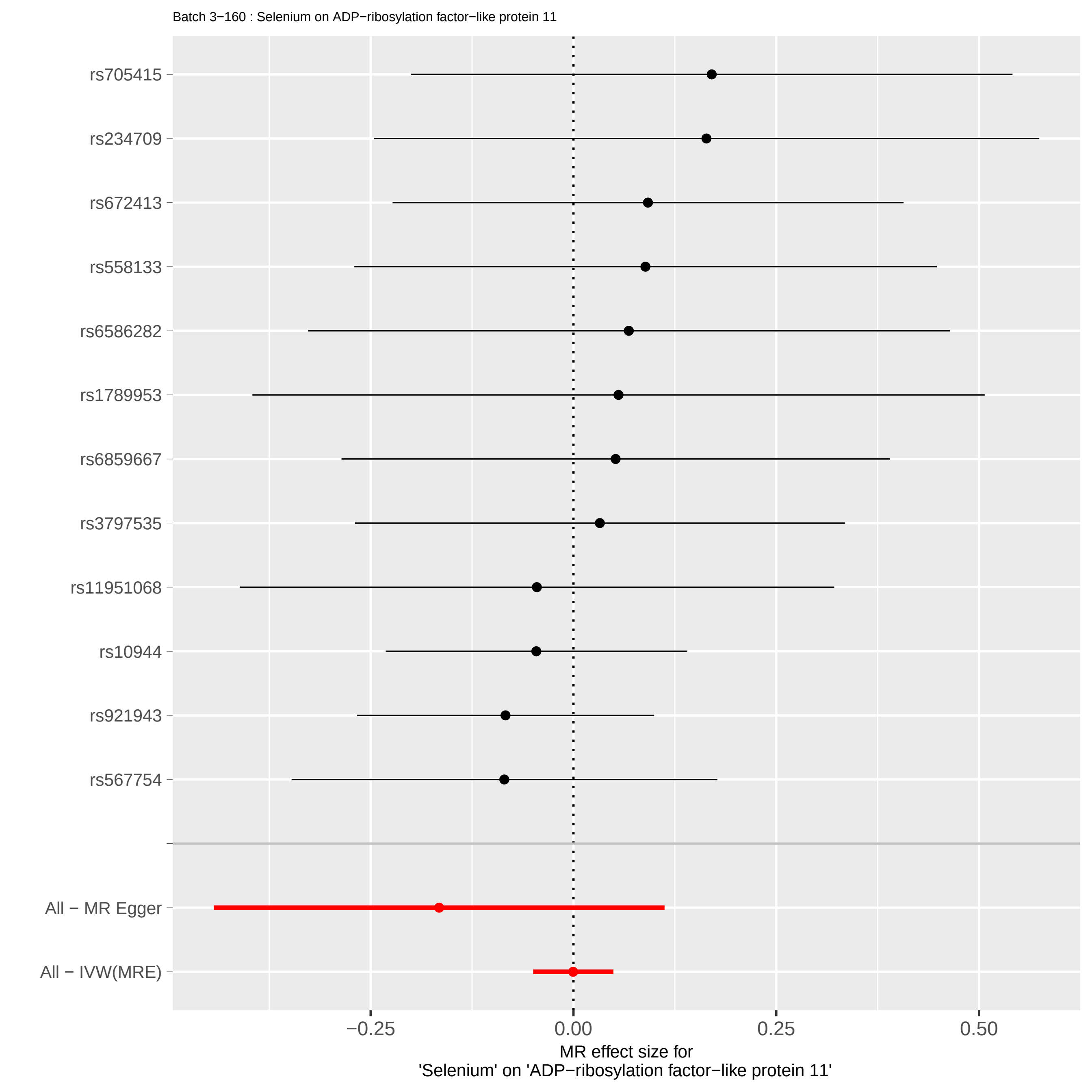
Batch 3−160 : Selenium on ADP−ribosylation factor−like protein 11
rs705415
rs234709
rs672413
rs558133
rs6586282
rs1789953
rs6859667
rs3797535
rs11951068
rs10944
rs921943
rs567754
All − MR Egger
All − IVW(MRE)
−0.25
0.00
0.25
0.50
MR effect size for
'Selenium' on 'ADP−ribosylation factor−like protein 11'

Batch 3−161 : Selenium on ADP−ribosylation factor−like protein 1

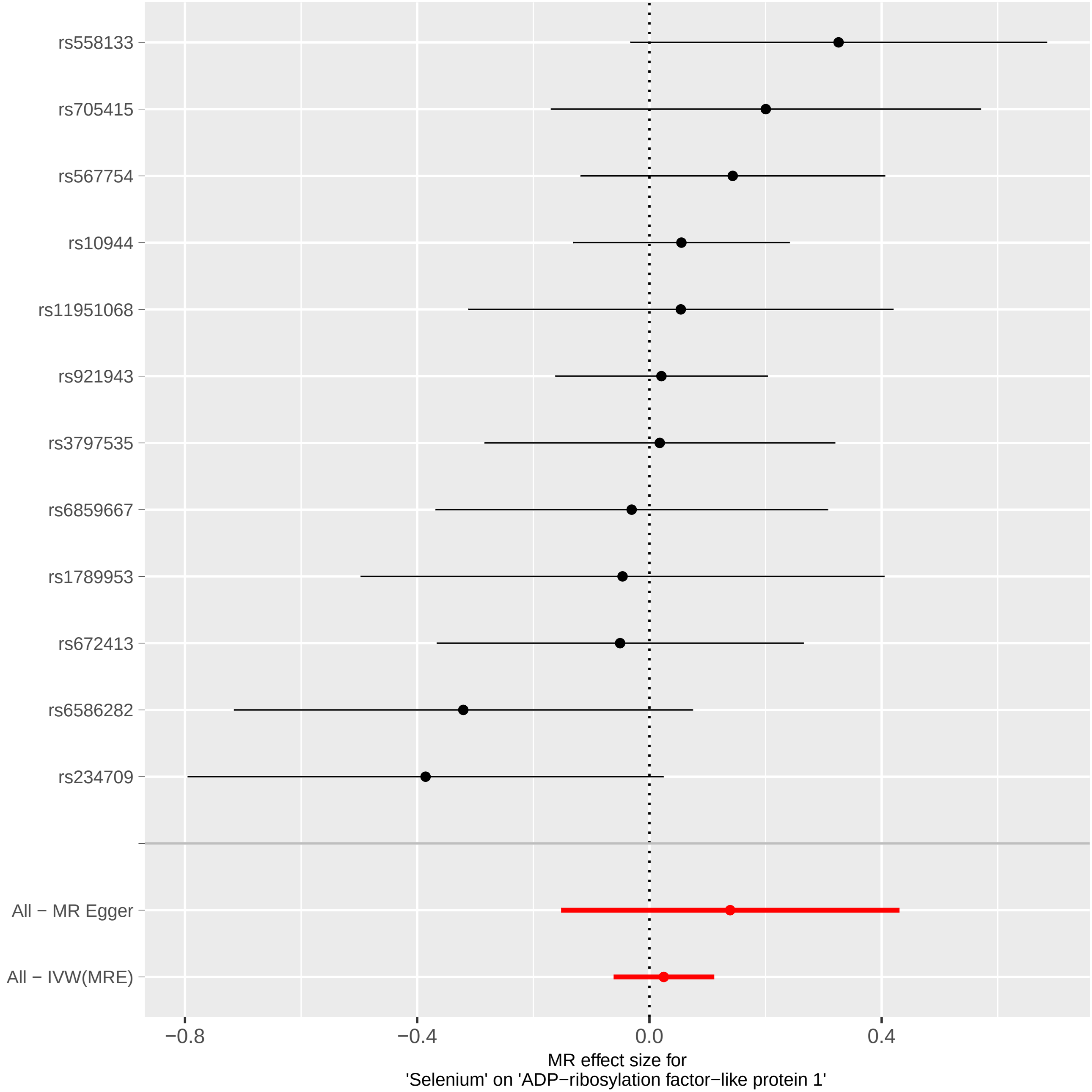

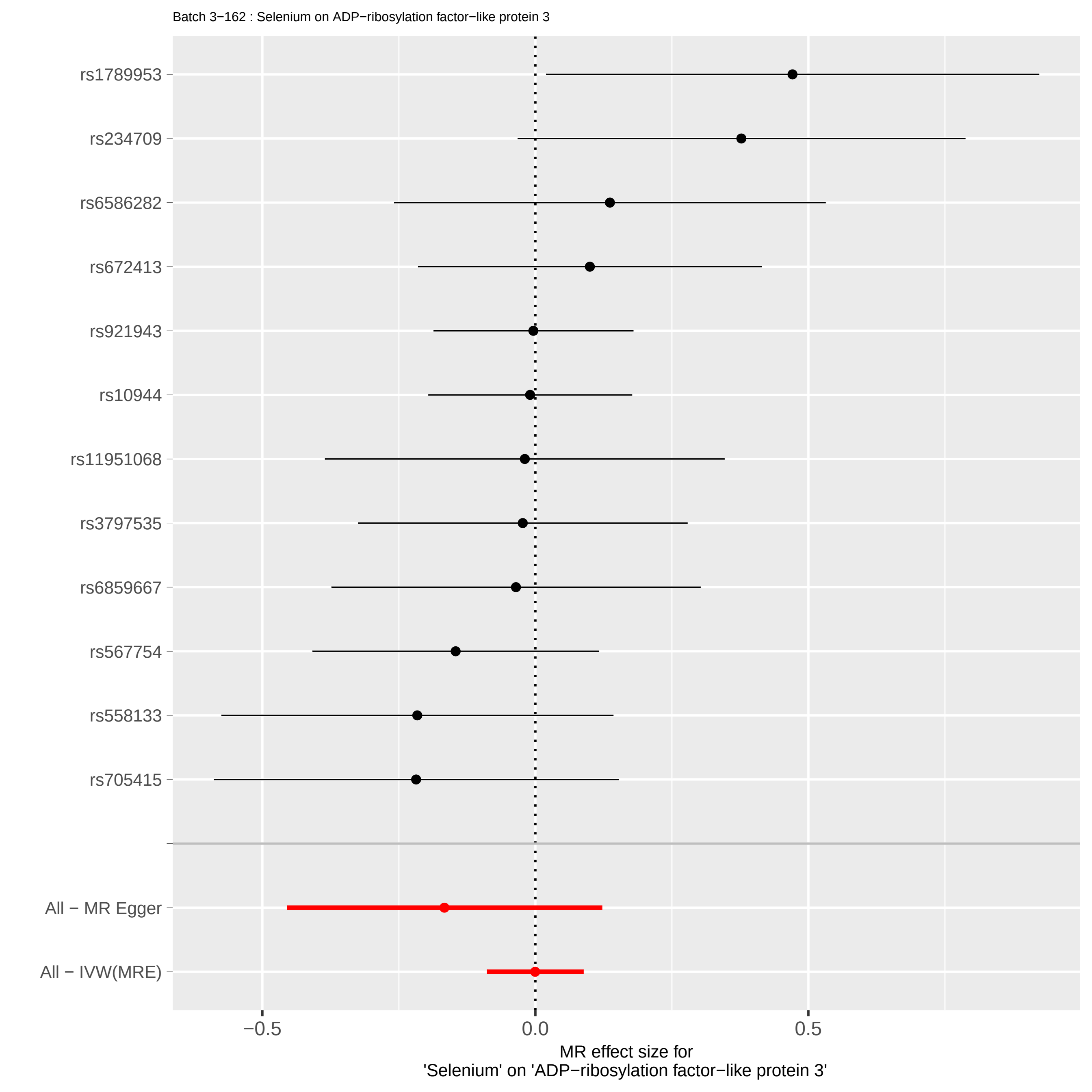

Batch 3−162 : Selenium on ADP−ribosylation factor−like protein 3
rs1789953
rs234709
rs6586282
rs672413
rs921943
rs10944
rs11951068
rs3797535
rs6859667
rs567754
rs558133
rs705415
All − MR Egger
All − IVW(MRE)
−0.5
0.0
0.5
MR effect size for
'Selenium' on 'ADP−ribosylation factor−like protein 3'

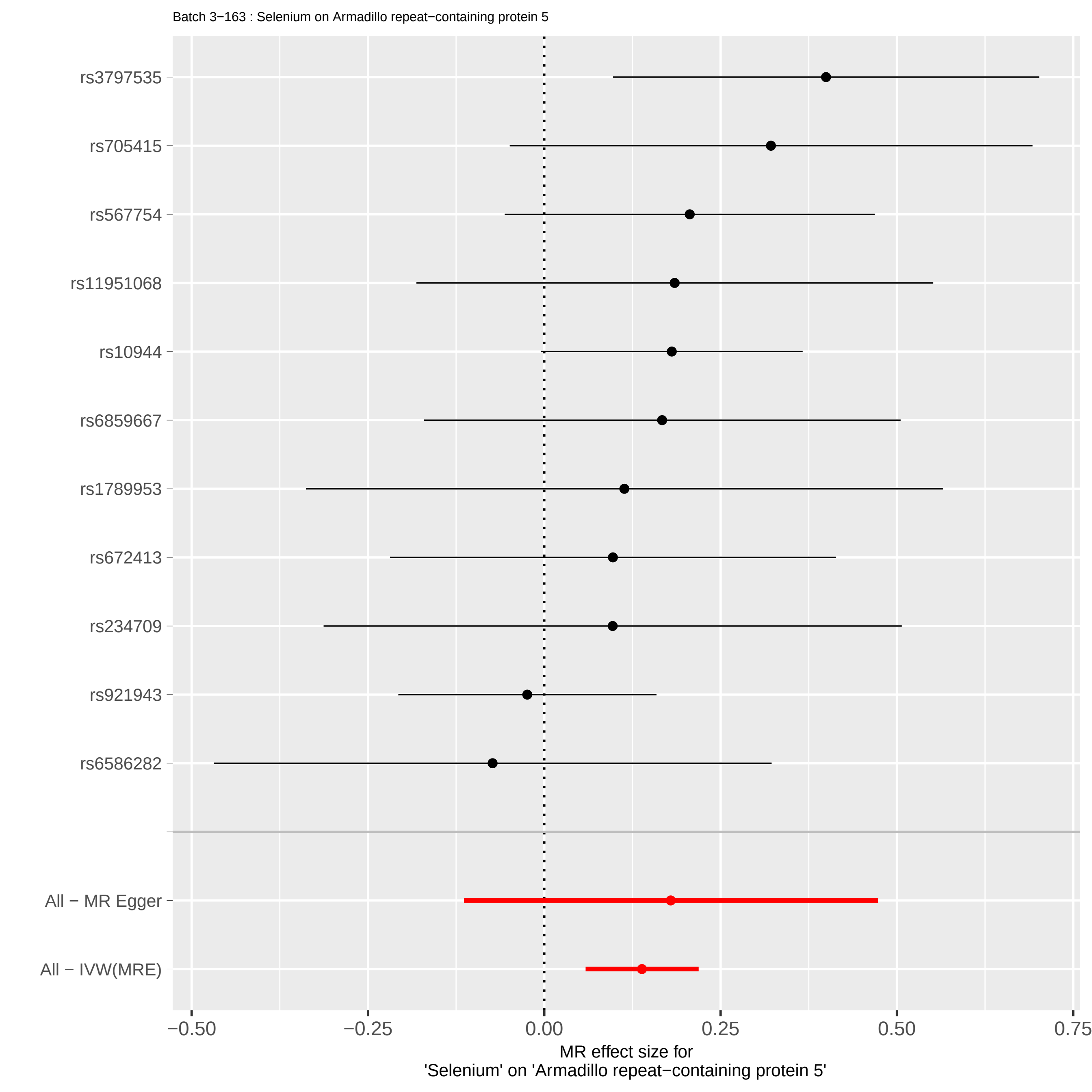
Batch 3−163 : Selenium on Armadillo repeat−containing protein 5
rs3797535
rs705415
rs567754
rs11951068
rs10944
rs6859667
rs1789953
rs672413
rs234709
rs921943
rs6586282
All − MR Egger
All − IVW(MRE)
−0.50
−0.25
0.00
0.25
0.50
0.75
MR effect size for
'Selenium' on 'Armadillo repeat−containing protein 5'

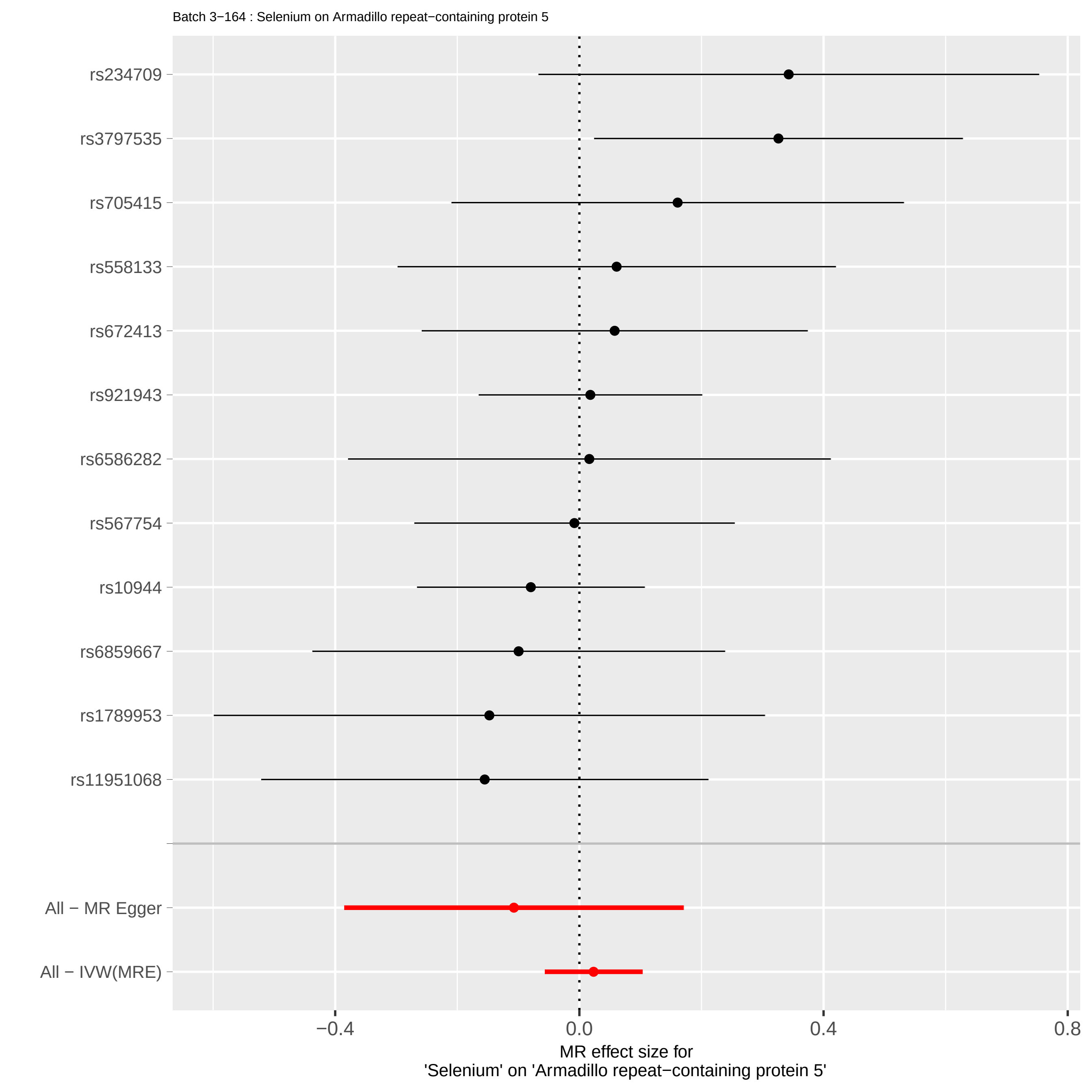
Batch 3−164 : Selenium on Armadillo repeat−containing protein 5
rs234709
rs3797535
rs705415
rs558133
rs672413
rs921943
rs6586282
rs567754
rs10944
rs6859667
rs1789953
rs11951068
All − MR Egger
All − IVW(MRE)
−0.4
0.0
0.4
0.8
MR effect size for
'Selenium' on 'Armadillo repeat−containing protein 5'

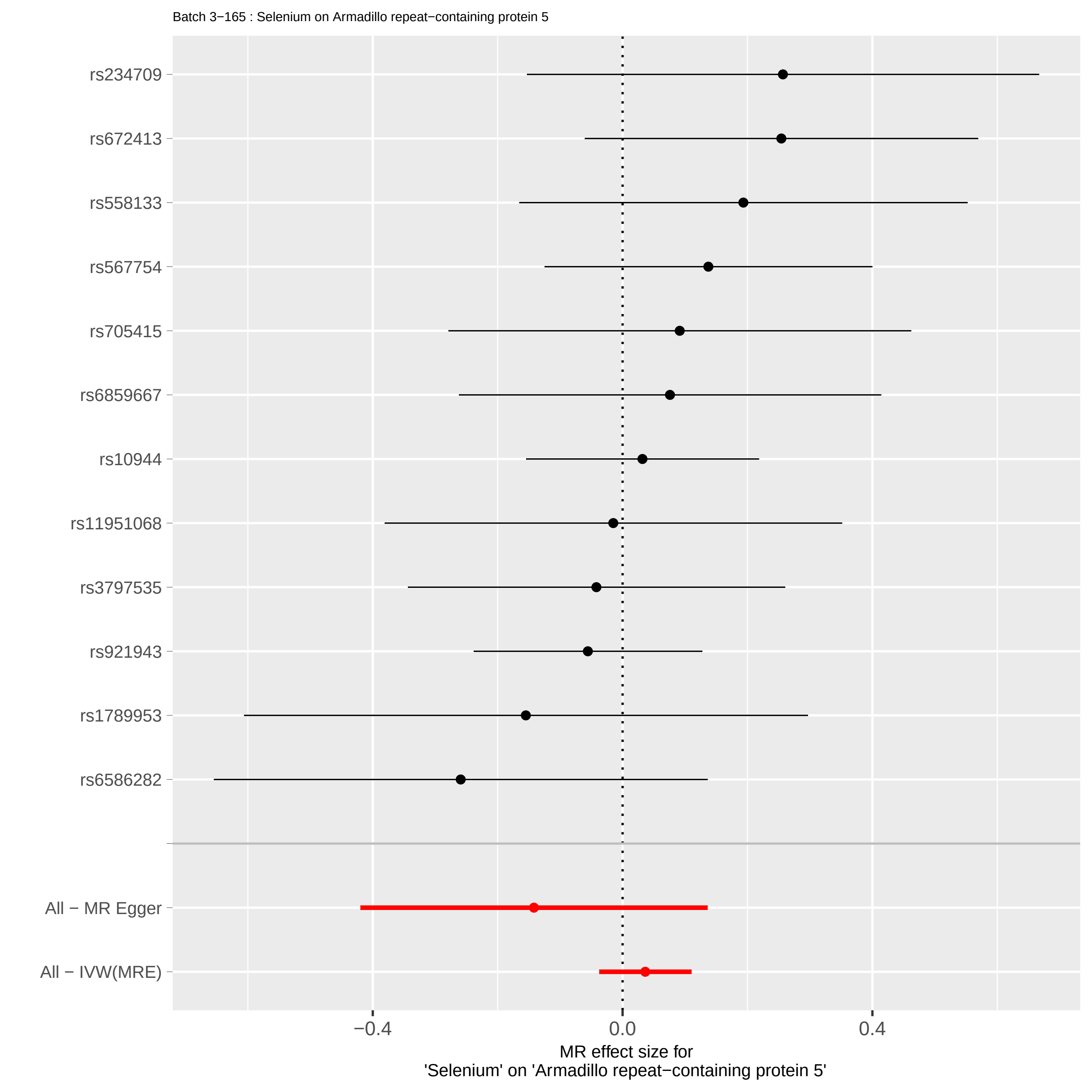

Batch 3−165 : Selenium on Armadillo repeat−containing protein 5
rs234709
rs672413
rs558133
rs567754
rs705415
rs6859667
rs10944
rs11951068
rs3797535
rs921943
rs1789953
rs6586282
All − MR Egger
All − IVW(MRE)
−0.4
0.0
0.4
MR effect size for
'Selenium' on 'Armadillo repeat−containing protein 5'

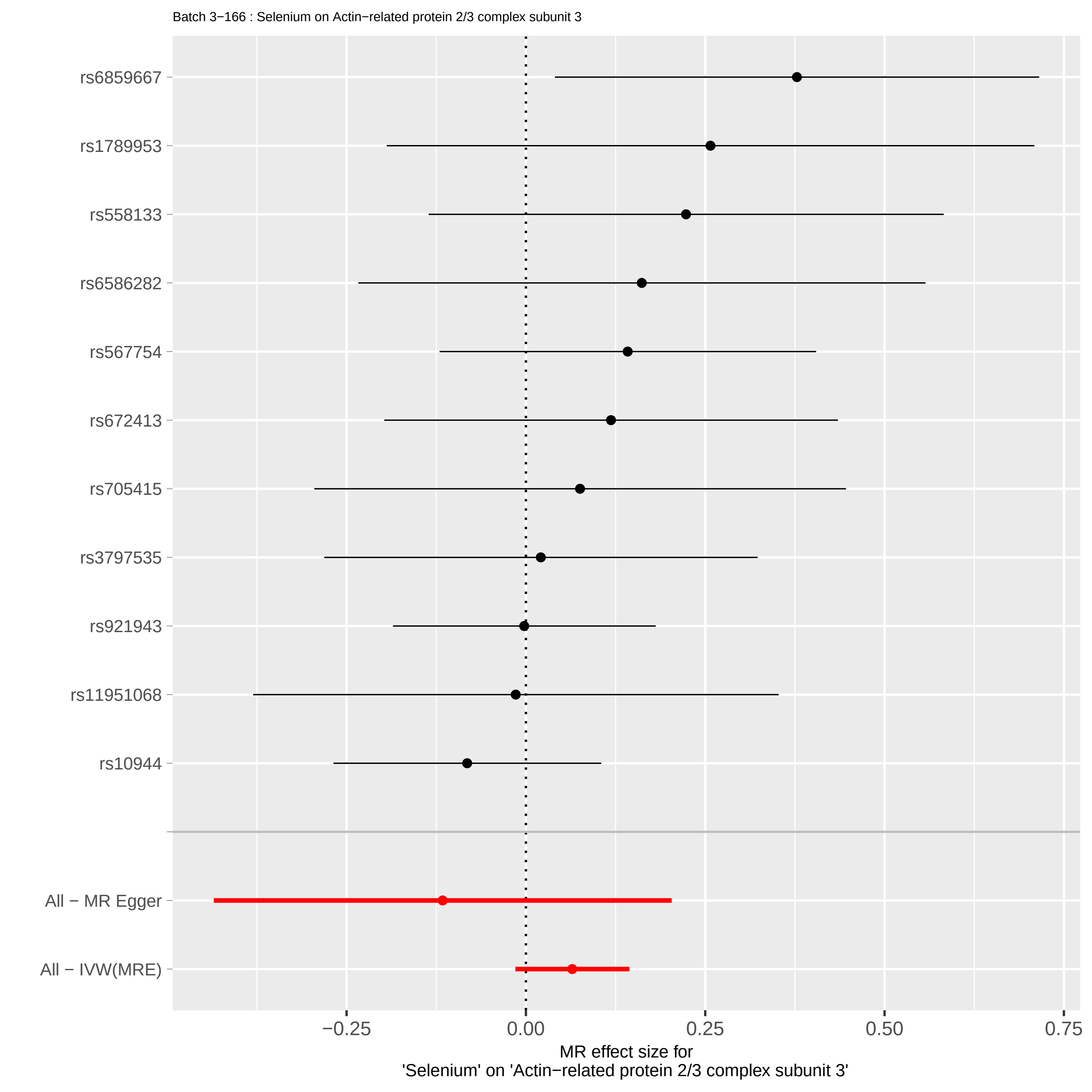
Batch 3−166 : Selenium on Actin−related protein 2/3 complex subunit 3
rs6859667
rs1789953
rs558133
rs6586282
rs567754
rs672413
rs705415
rs3797535
rs921943
rs11951068
rs10944
All − MR Egger
All − IVW(MRE)
−0.25
0.00
0.25
0.50
0.75
MR effect size for
'Selenium' on 'Actin−related protein 2/3 complex subunit 3'

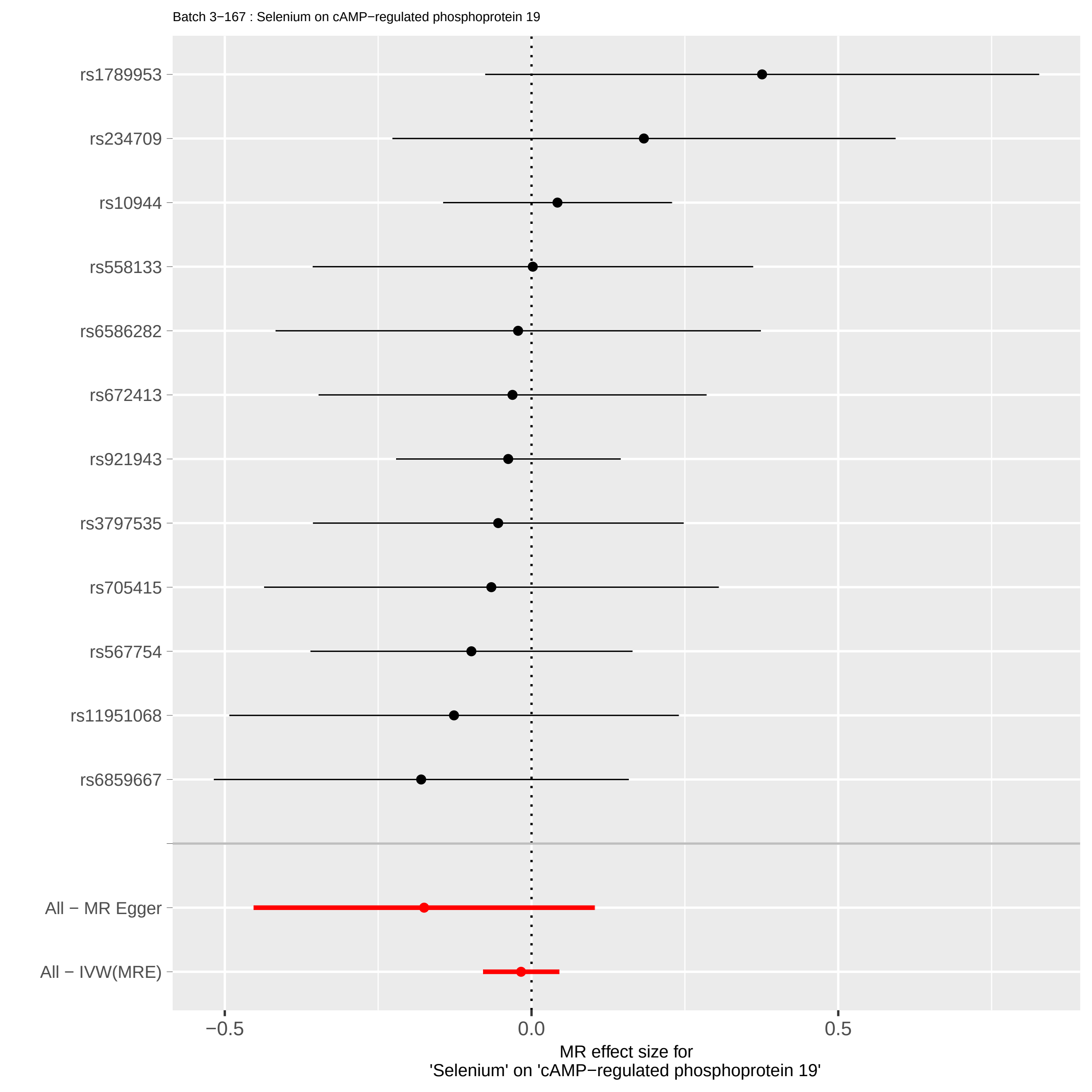

Batch 3−167 : Selenium on cAMP−regulated phosphoprotein 19
rs1789953
rs234709
rs10944
rs558133
rs6586282
rs672413
rs921943
rs3797535
rs705415
rs567754
rs11951068
rs6859667
All − MR Egger
All − IVW(MRE)
−0.5
0.0
0.5
MR effect size for
'Selenium' on 'cAMP−regulated phosphoprotein 19'

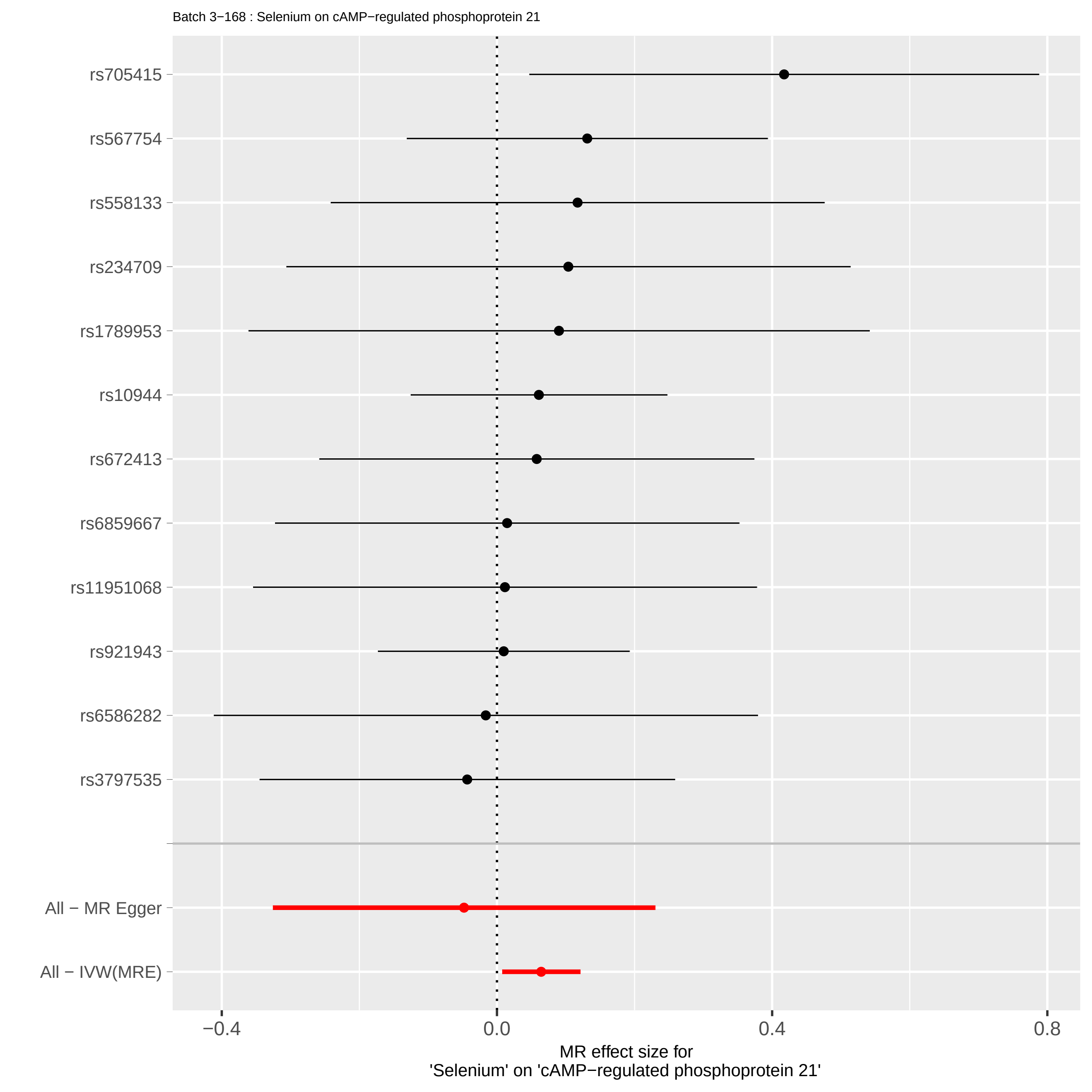

Batch 3−168 : Selenium on cAMP−regulated phosphoprotein 21
rs705415
rs567754
rs558133
rs234709
rs1789953
rs10944
rs672413
rs6859667
rs11951068
rs921943
rs6586282
rs3797535
All − MR Egger
All − IVW(MRE)
−0.4
0.0
0.4
0.8
MR effect size for
'Selenium' on 'cAMP−regulated phosphoprotein 21'

Batch 3−169 : Selenium on Beta−arrestin−1

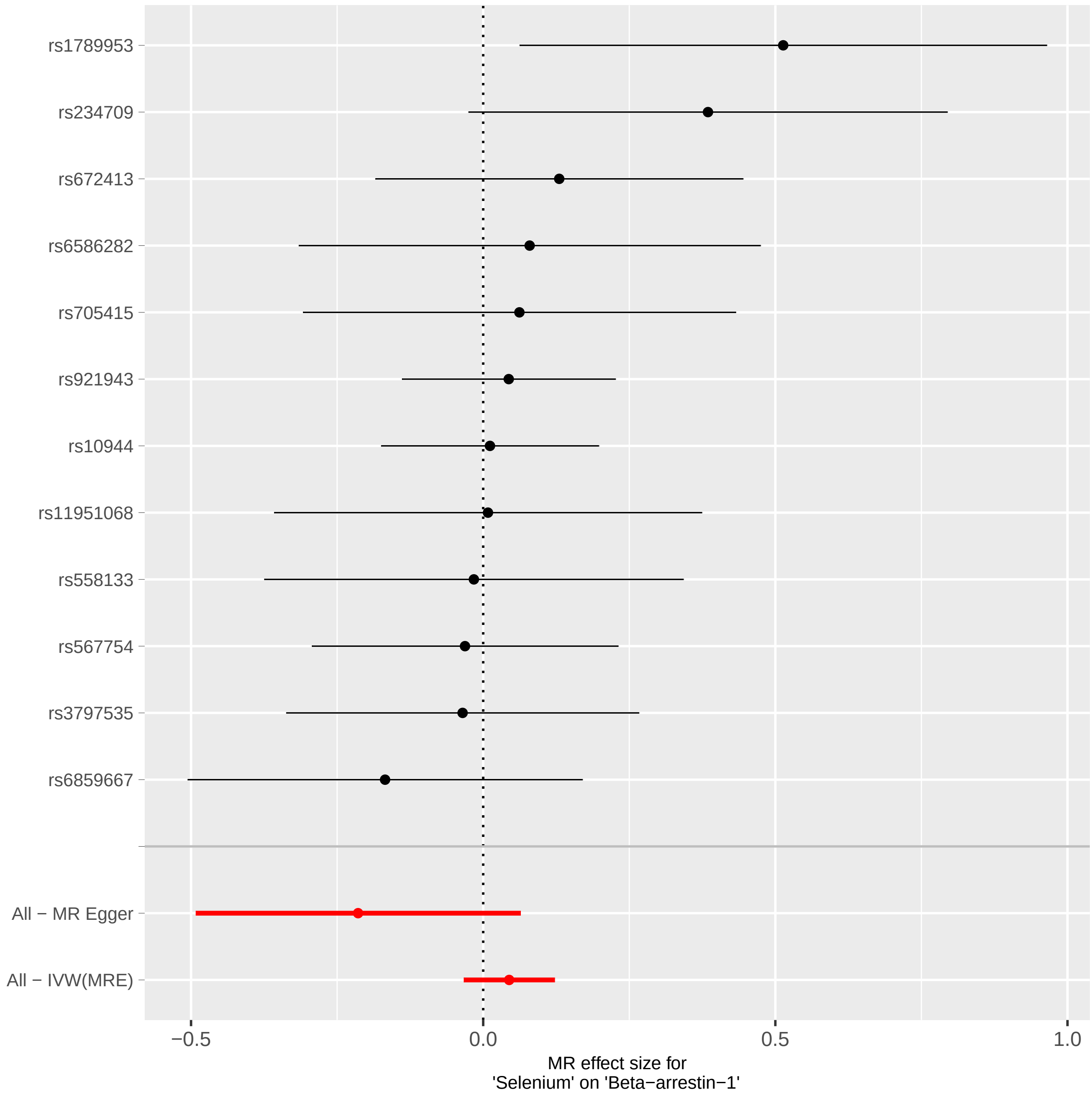

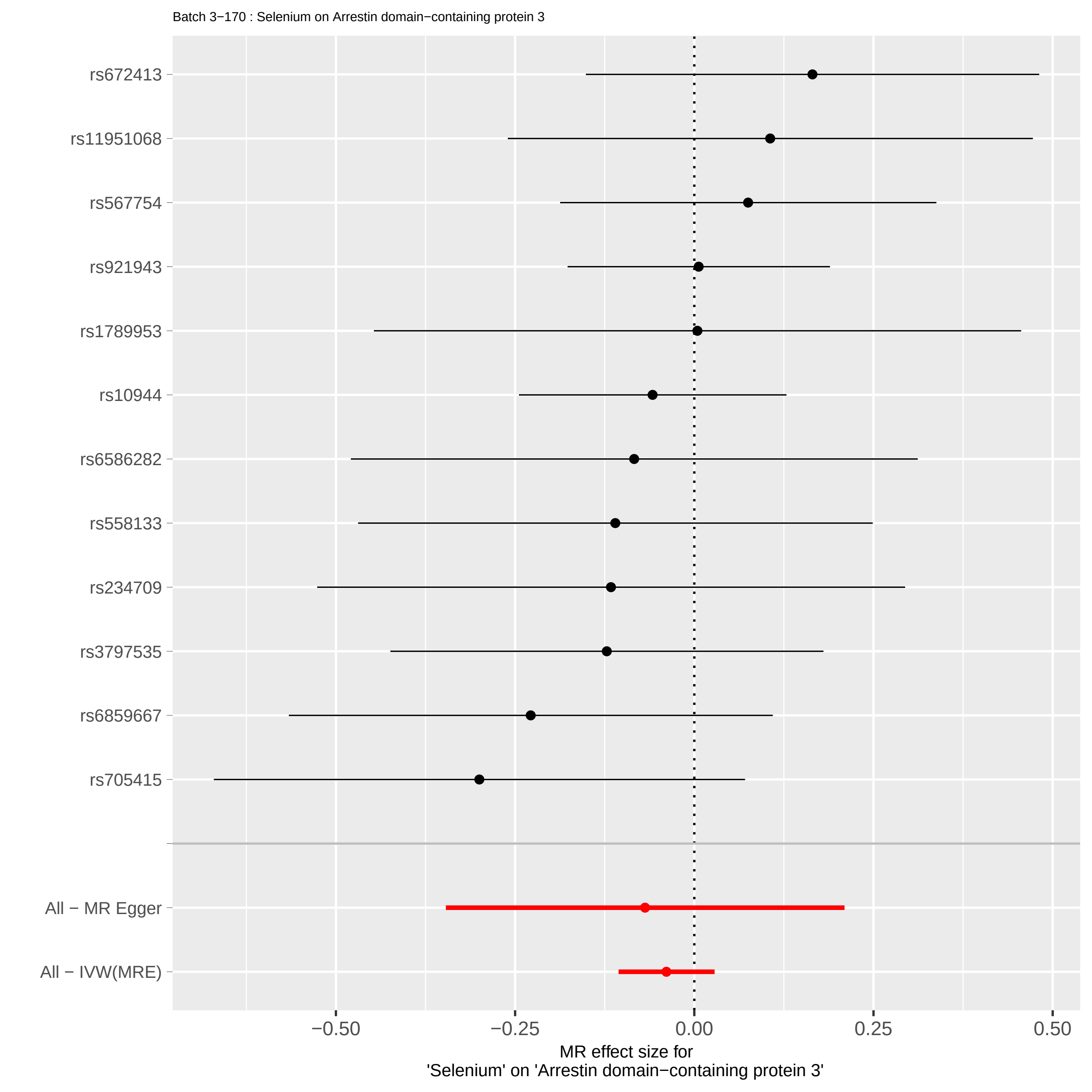

Batch 3−170 : Selenium on Arrestin domain−containing protein 3
rs672413
rs11951068
rs567754
rs921943
rs1789953
rs10944
rs6586282
rs558133
rs234709
rs3797535
rs6859667
rs705415
All − MR Egger
All − IVW(MRE)
−0.50
−0.25
0.00
0.25
0.50
MR effect size for
'Selenium' on 'Arrestin domain−containing protein 3'

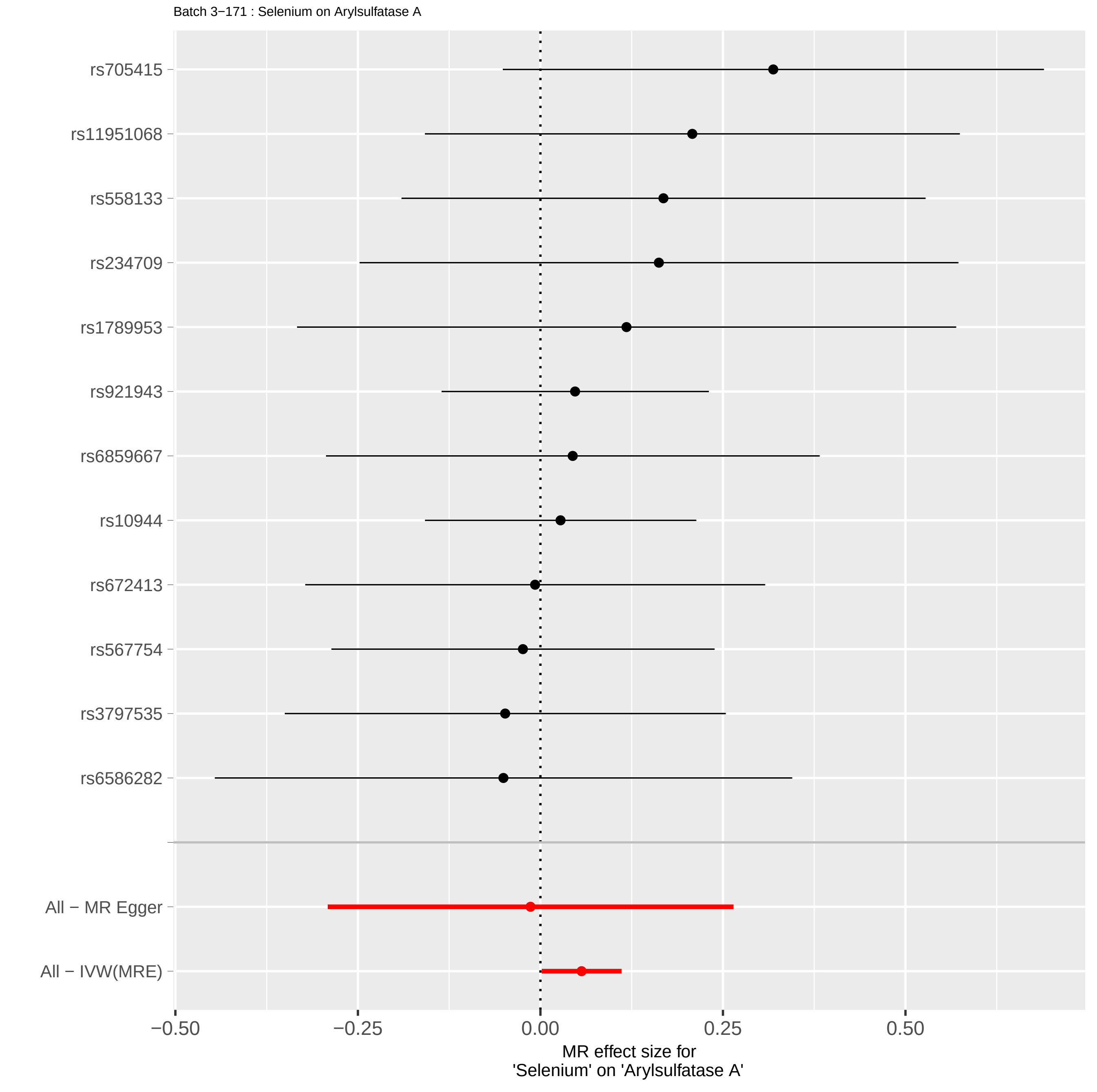
Batch 3−171 : Selenium on Arylsulfatase A
rs705415
rs11951068
rs558133
rs234709
rs1789953
rs921943
rs6859667
rs10944
rs672413
rs567754
rs3797535
rs6586282
All − MR Egger
All − IVW(MRE)
−0.50
−0.25
0.00
0.25
0.50
MR effect size for
'Selenium' on 'Arylsulfatase A'

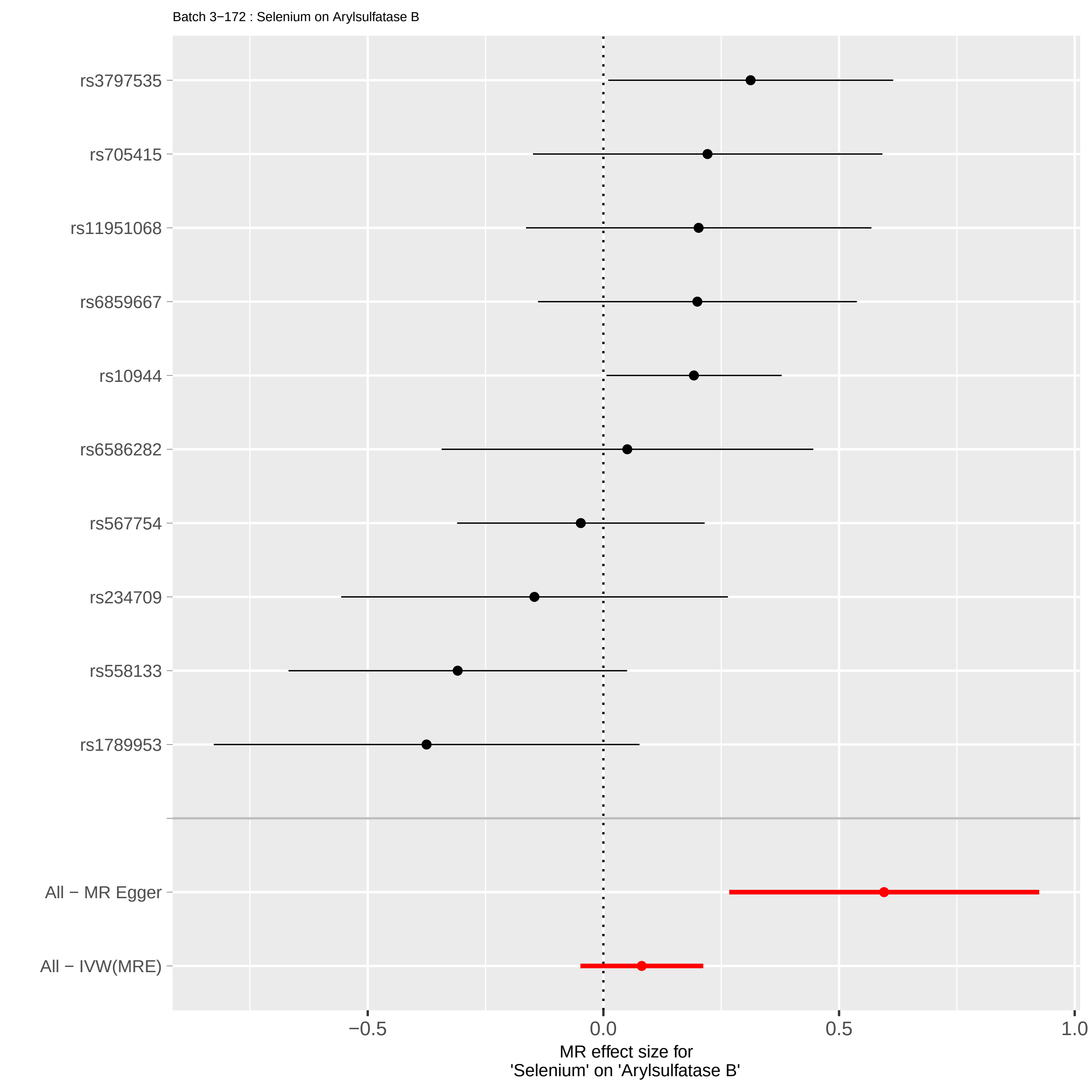

Batch 3−172 : Selenium on Arylsulfatase B
rs3797535
rs705415
rs11951068
rs6859667
rs10944
rs6586282
rs567754
rs234709
rs558133
rs1789953
All − MR Egger
All − IVW(MRE)
−0.5
0.0
0.5
1.0
MR effect size for
'Selenium' on 'Arylsulfatase B'

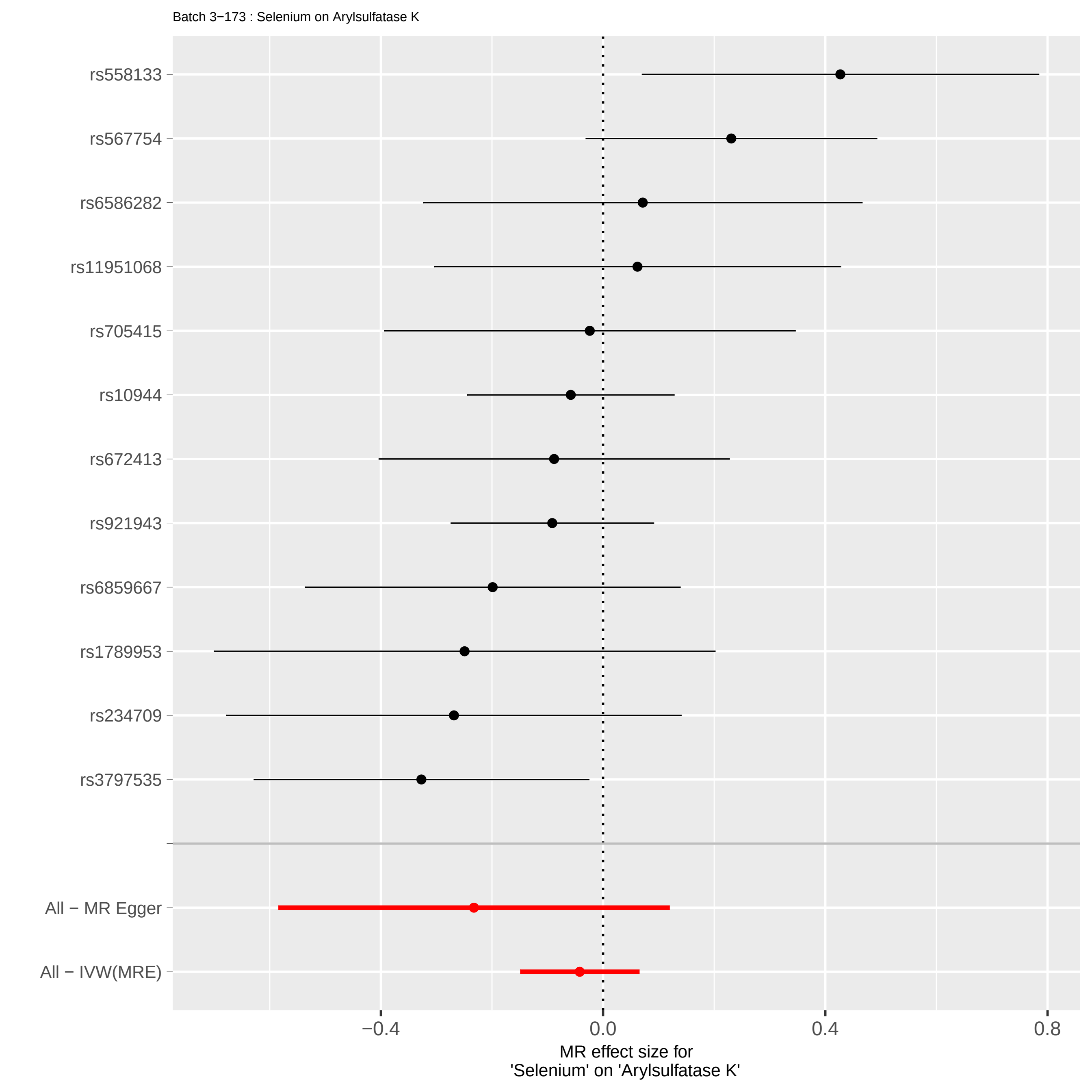

Batch 3−173 : Selenium on Arylsulfatase K
rs558133
rs567754
rs6586282
rs11951068
rs705415
rs10944
rs672413
rs921943
rs6859667
rs1789953
rs234709
rs3797535
All − MR Egger
All − IVW(MRE)
−0.4
0.0
0.4
0.8
MR effect size for
'Selenium' on 'Arylsulfatase K'

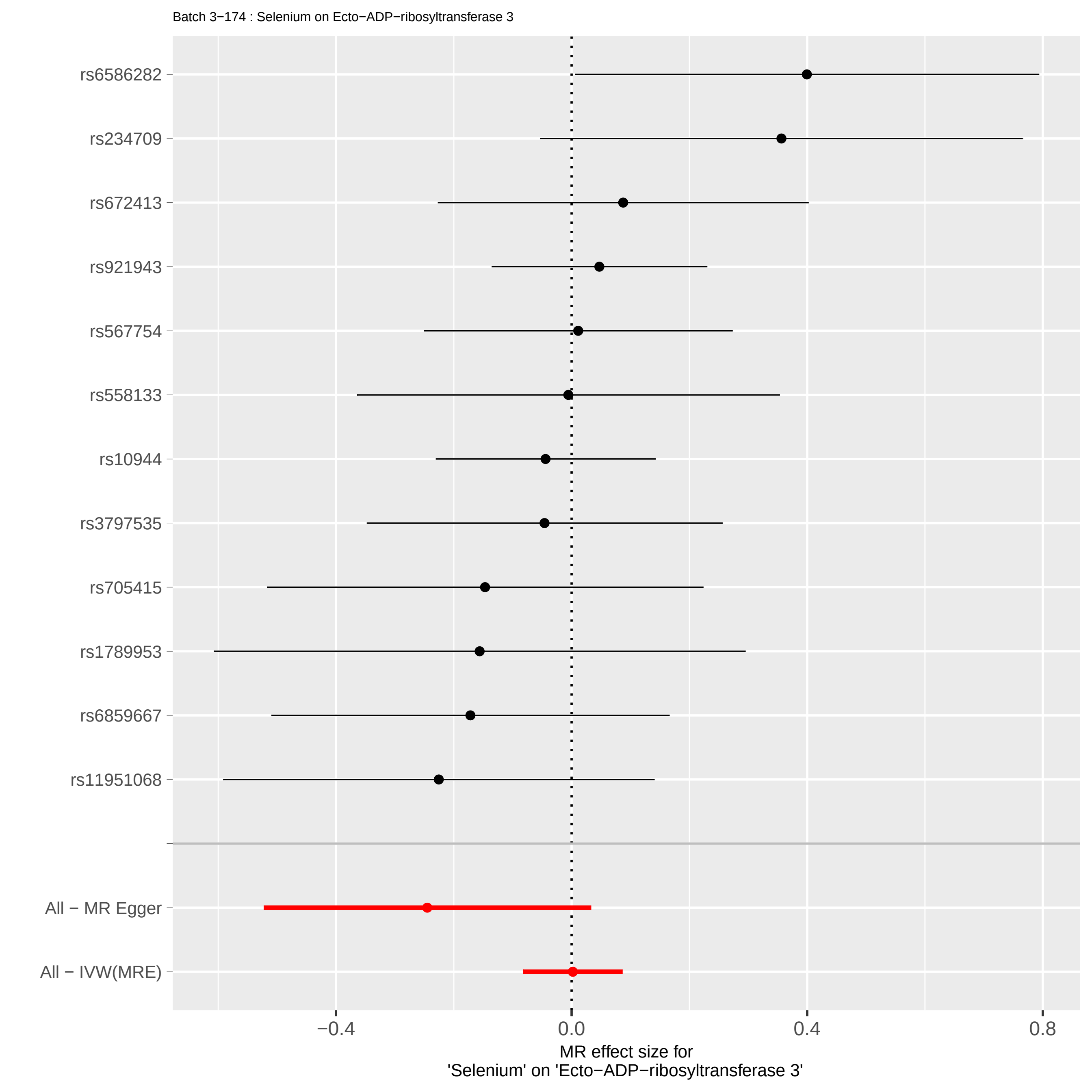

Batch 3−174 : Selenium on Ecto−ADP−ribosyltransferase 3
rs6586282
rs234709
rs672413
rs921943
rs567754
rs558133
rs10944
rs3797535
rs705415
rs1789953
rs6859667
rs11951068
All − MR Egger
All − IVW(MRE)
−0.4
0.0
0.4
0.8
MR effect size for
'Selenium' on 'Ecto−ADP−ribosyltransferase 3'

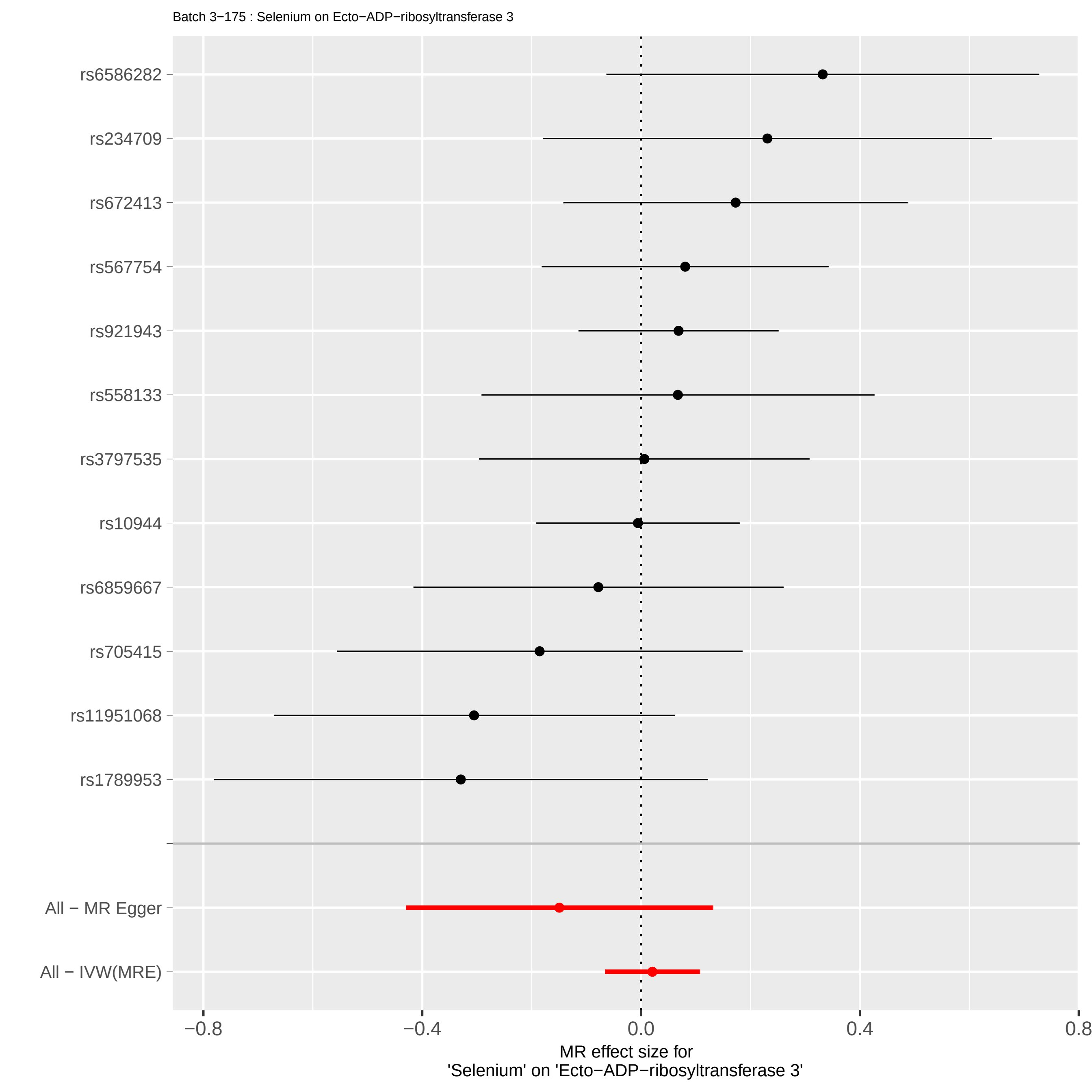
Batch 3−175 : Selenium on Ecto−ADP−ribosyltransferase 3
rs6586282
rs234709
rs672413
rs567754
rs921943
rs558133
rs3797535
rs10944
rs6859667
rs705415
rs11951068
rs1789953
All − MR Egger
All − IVW(MRE)
−0.8
−0.4
0.0
0.4
0.8
MR effect size for
'Selenium' on 'Ecto−ADP−ribosyltransferase 3'

Batch 3−176 : Selenium on Ecto−ADP−ribosyltransferase 4

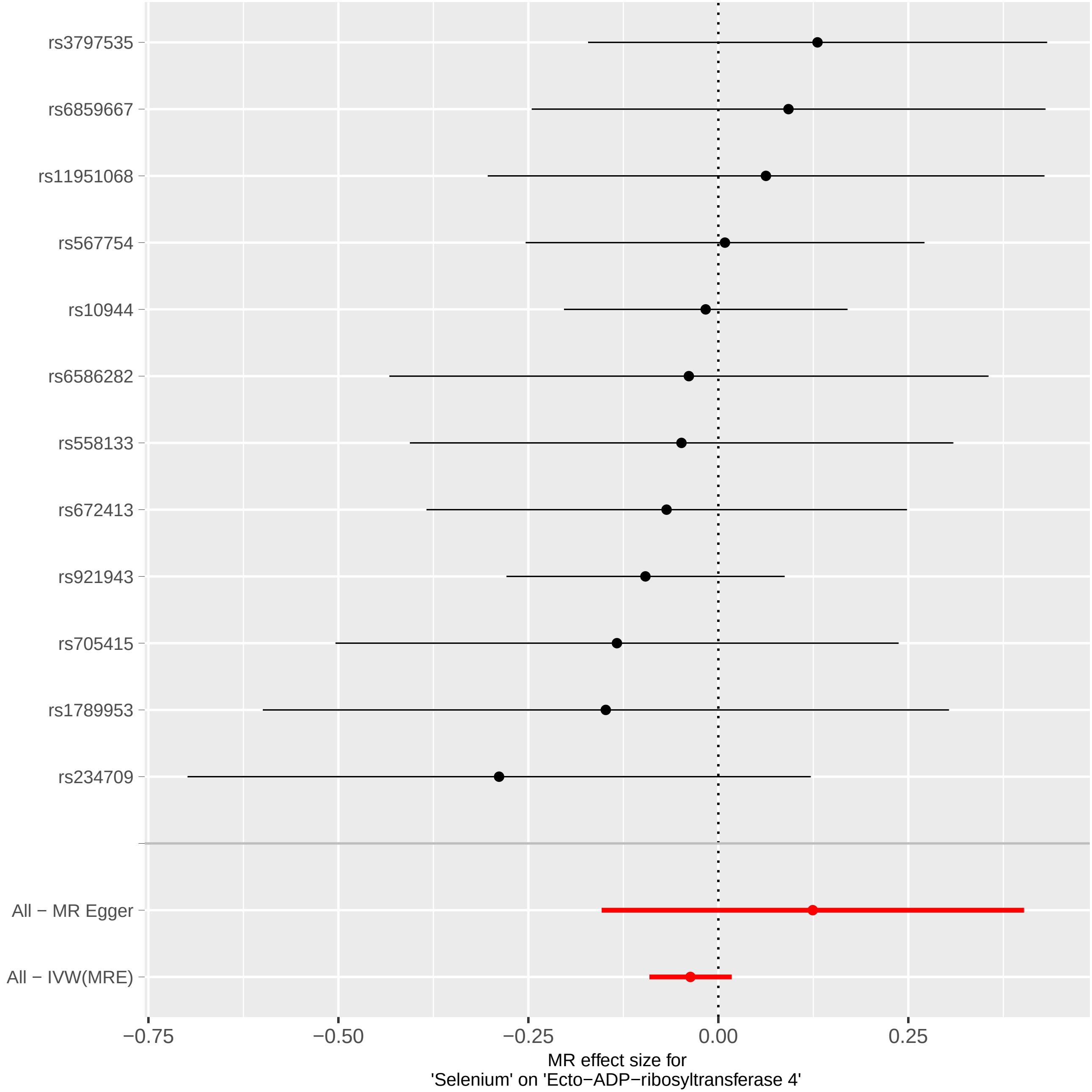

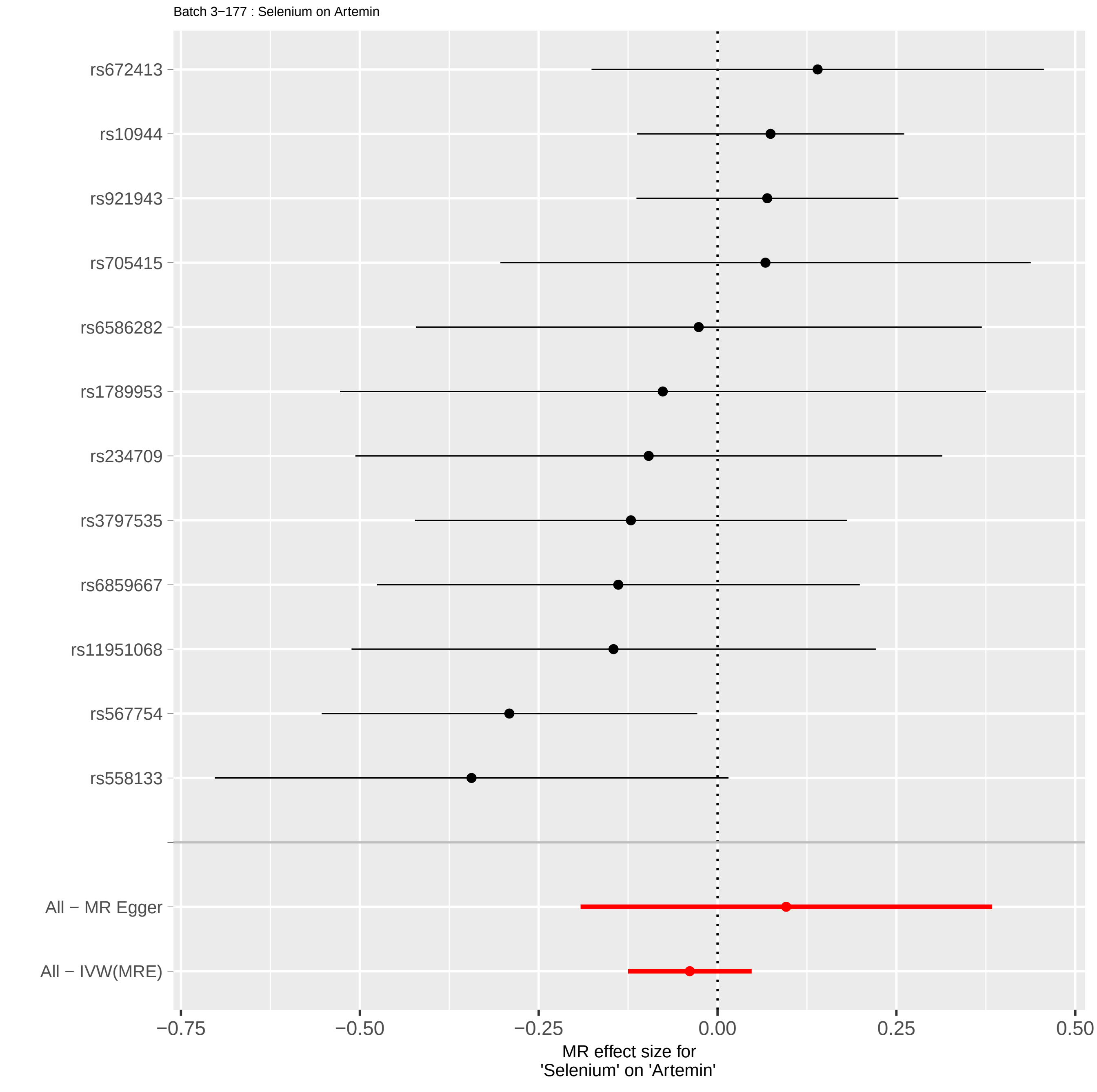

Batch 3−177 : Selenium on Artemin
rs672413
rs10944
rs921943
rs705415
rs6586282
rs1789953
rs234709
rs3797535
rs6859667
rs11951068
rs567754
rs558133
All − MR Egger
All − IVW(MRE)
−0.75
−0.50
−0.25
0.00
0.25
0.50
MR effect size for
'Selenium' on 'Artemin'

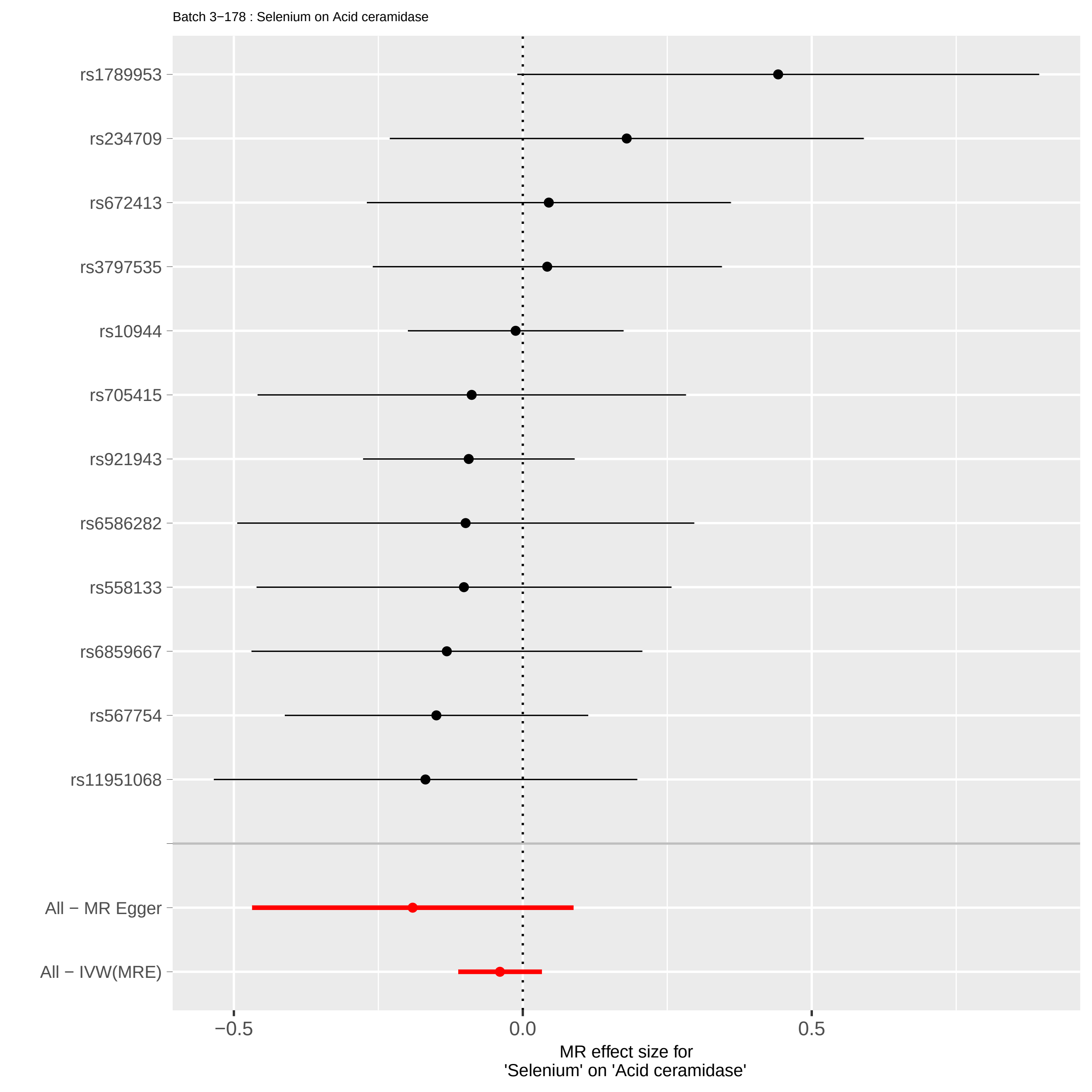

Batch 3−178 : Selenium on Acid ceramidase
rs1789953
rs234709
rs672413
rs3797535
rs10944
rs705415
rs921943
rs6586282
rs558133
rs6859667
rs567754
rs11951068
All − MR Egger
All − IVW(MRE)
−0.5
0.0
0.5
MR effect size for
'Selenium' on 'Acid ceramidase'

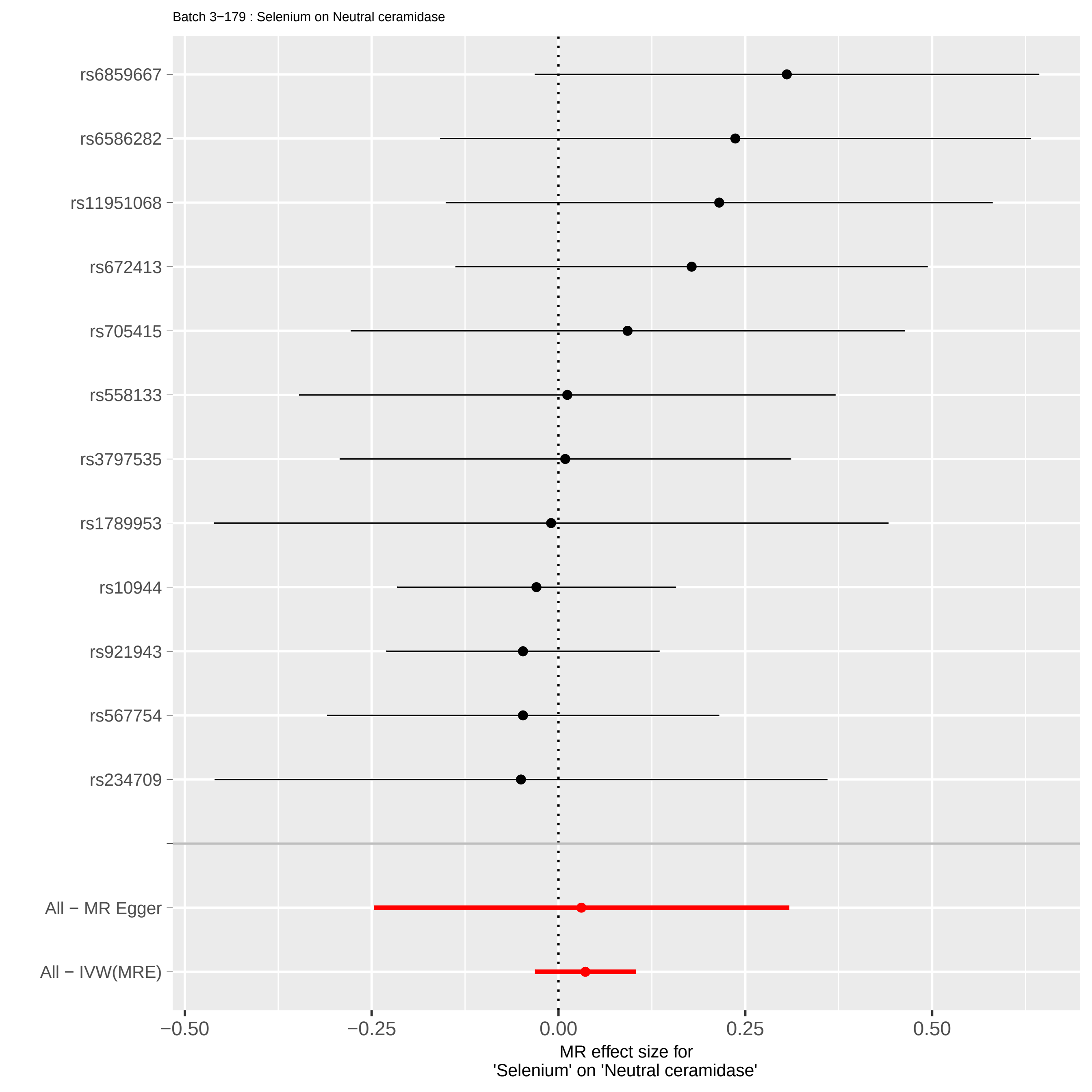

Batch 3−179 : Selenium on Neutral ceramidase
rs6859667
rs6586282
rs11951068
rs672413
rs705415
rs558133
rs3797535
rs1789953
rs10944
rs921943
rs567754
rs234709
All − MR Egger
All − IVW(MRE)
−0.50
−0.25
0.00
0.25
0.50
MR effect size for
'Selenium' on 'Neutral ceramidase'

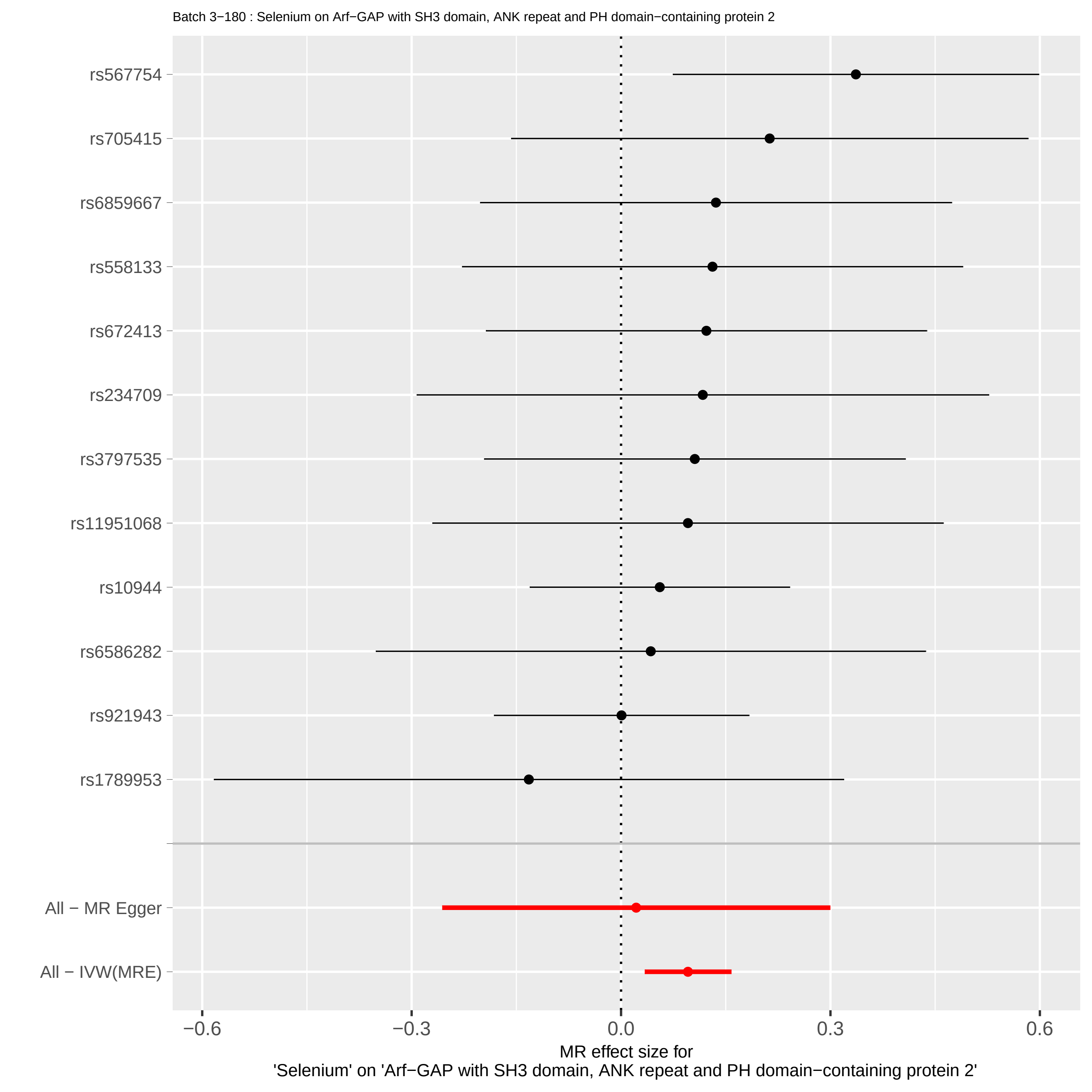
Batch 3−180 : Selenium on Arf−GAP with SH3 domain, ANK repeat and PH domain−containing protein 2
rs567754
rs705415
rs6859667
rs558133
rs672413
rs234709
rs3797535
rs11951068
rs10944
rs6586282
rs921943
rs1789953
All − MR Egger
All − IVW(MRE)
−0.6
−0.3
0.0
0.3
0.6
MR effect size for
'Selenium' on 'Arf−GAP with SH3 domain, ANK repeat and PH domain−containing protein 2'

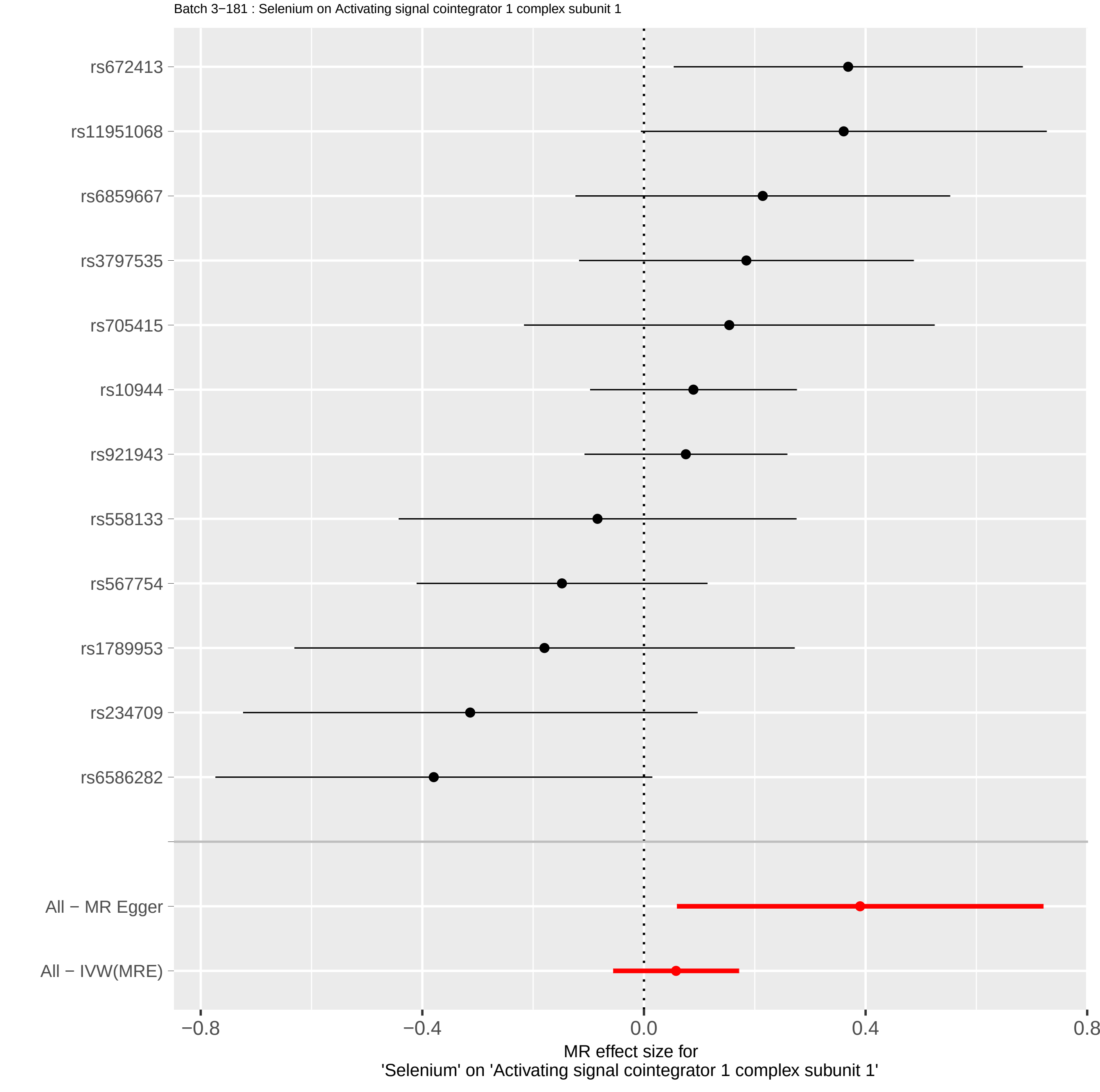
Batch 3−181 : Selenium on Activating signal cointegrator 1 complex subunit 1
rs672413
rs11951068
rs6859667
rs3797535
rs705415
rs10944
rs921943
rs558133
rs567754
rs1789953
rs234709
rs6586282
All − MR Egger
All − IVW(MRE)
−0.8
−0.4
0.0
0.4
0.8
MR effect size for
'Selenium' on 'Activating signal cointegrator 1 complex subunit 1'

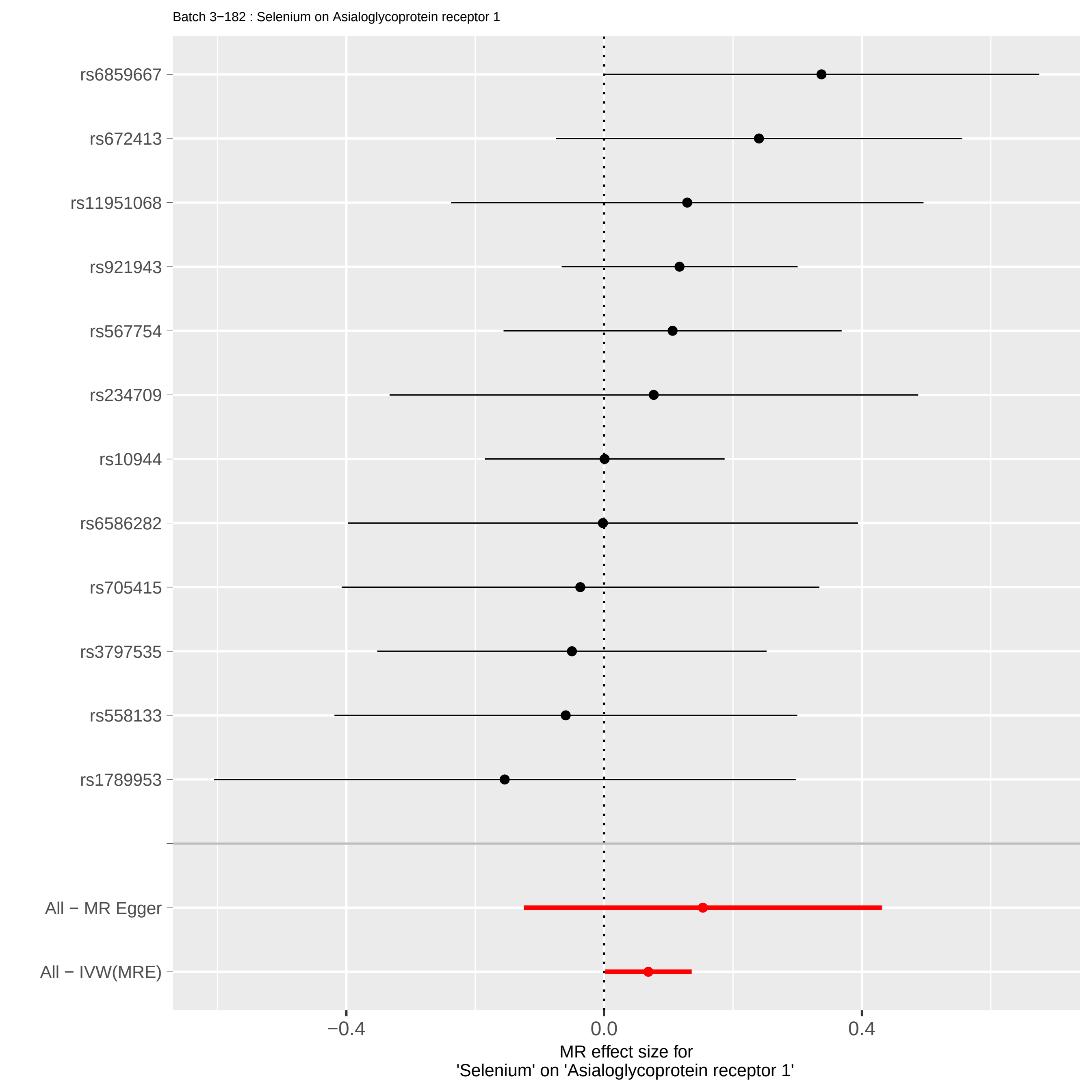
Batch 3−182 : Selenium on Asialoglycoprotein receptor 1
rs6859667
rs672413
rs11951068
rs921943
rs567754
rs234709
rs10944
rs6586282
rs705415
rs3797535
rs558133
rs1789953
All − MR Egger
All − IVW(MRE)
−0.4
0.0
0.4
MR effect size for
'Selenium' on 'Asialoglycoprotein receptor 1'

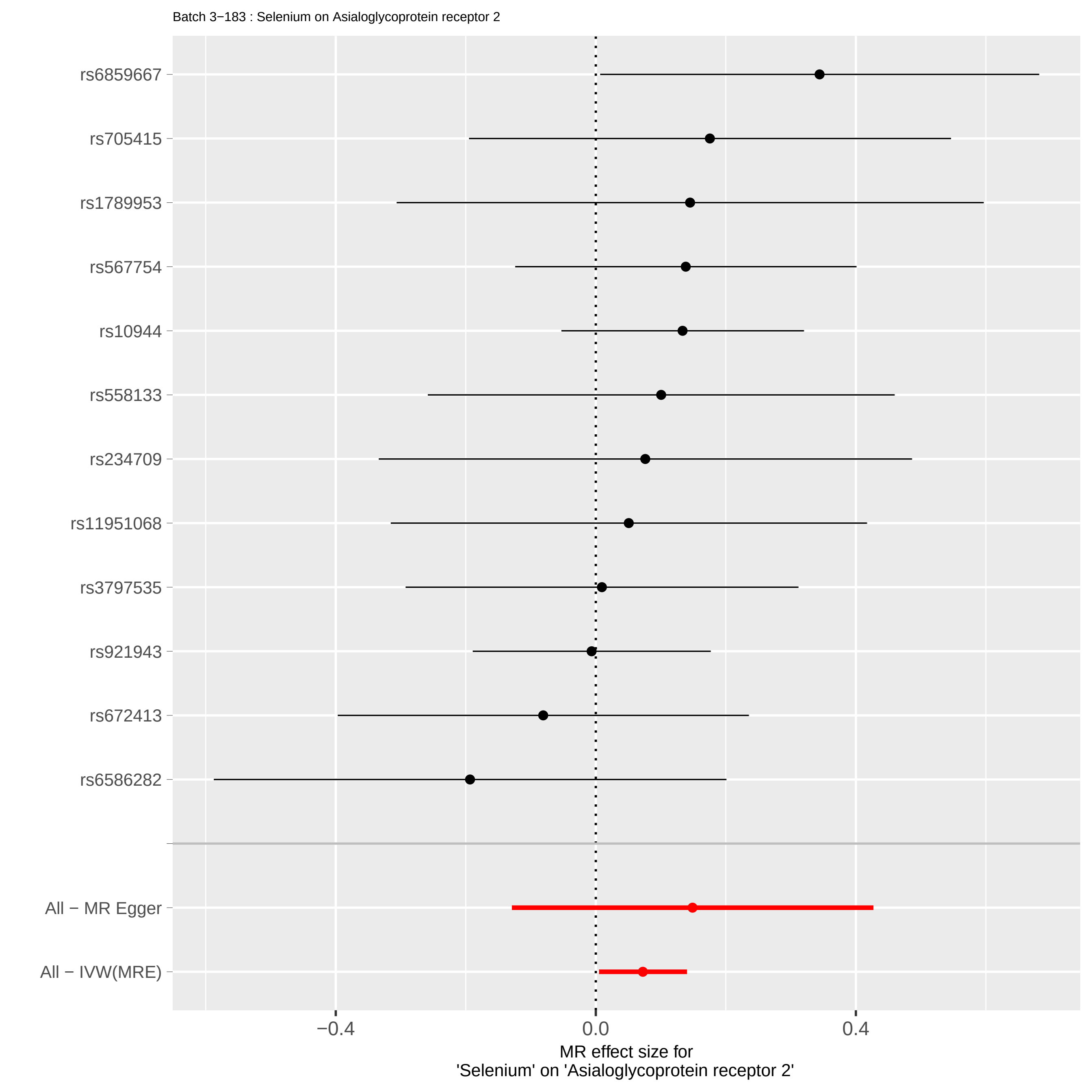

Batch 3−183 : Selenium on Asialoglycoprotein receptor 2
rs6859667
rs705415
rs1789953
rs567754
rs10944
rs558133
rs234709
rs11951068
rs3797535
rs921943
rs672413
rs6586282
All − MR Egger
All − IVW(MRE)
−0.4
0.0
0.4
MR effect size for
'Selenium' on 'Asialoglycoprotein receptor 2'

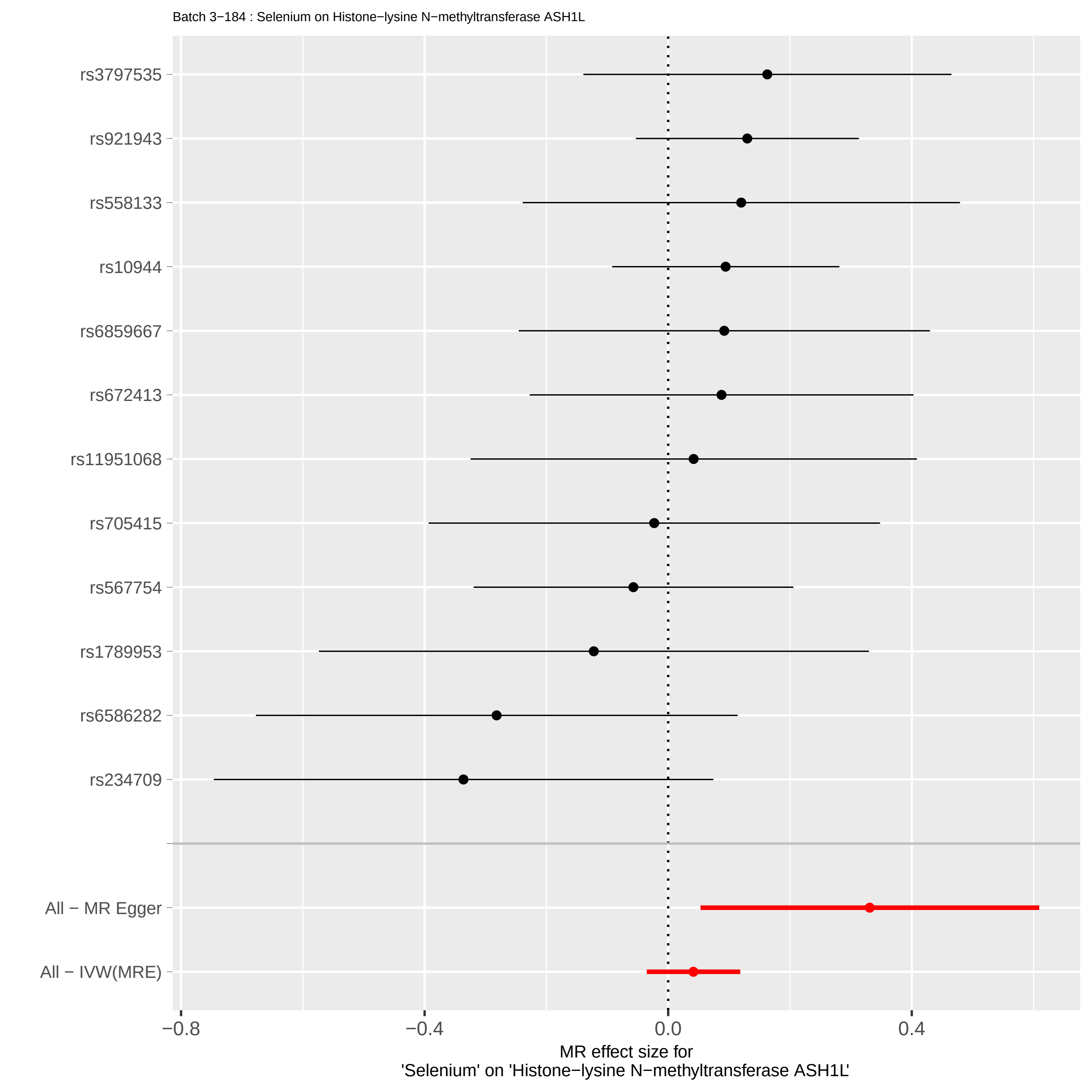
Batch 3−184 : Selenium on Histone−lysine N−methyltransferase ASH1L
rs3797535
rs921943
rs558133
rs10944
rs6859667
rs672413
rs11951068
rs705415
rs567754
rs1789953
rs6586282
rs234709
All − MR Egger
All − IVW(MRE)
−0.8
−0.4
0.0
0.4
MR effect size for
'Selenium' on 'Histone−lysine N−methyltransferase ASH1L'

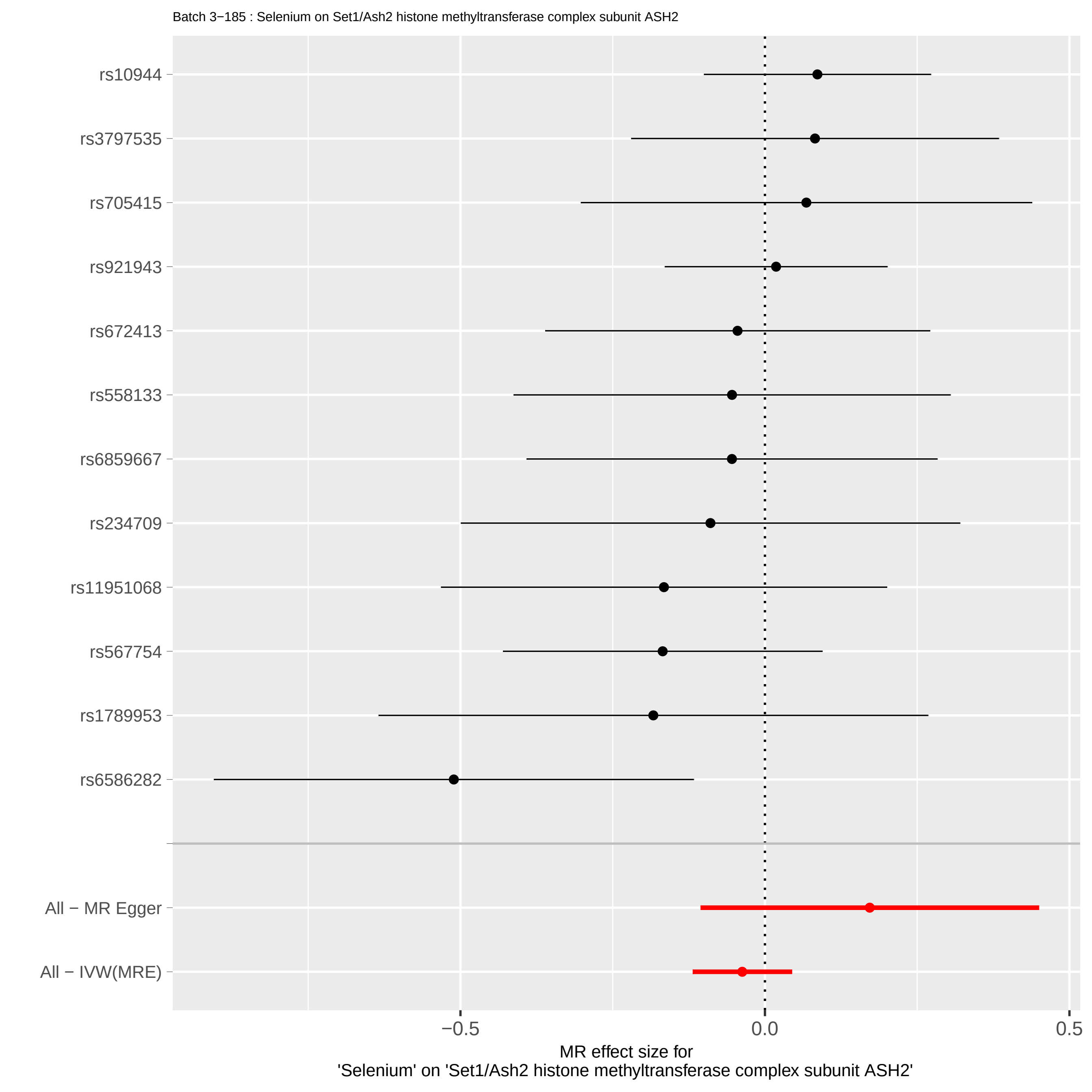

Batch 3−185 : Selenium on Set1/Ash2 histone methyltransferase complex subunit ASH2
rs10944
rs3797535
rs705415
rs921943
rs672413
rs558133
rs6859667
rs234709
rs11951068
rs567754
rs1789953
rs6586282
All − MR Egger
All − IVW(MRE)
−0.5
0.0
0.5
MR effect size for
'Selenium' on 'Set1/Ash2 histone methyltransferase complex subunit ASH2'

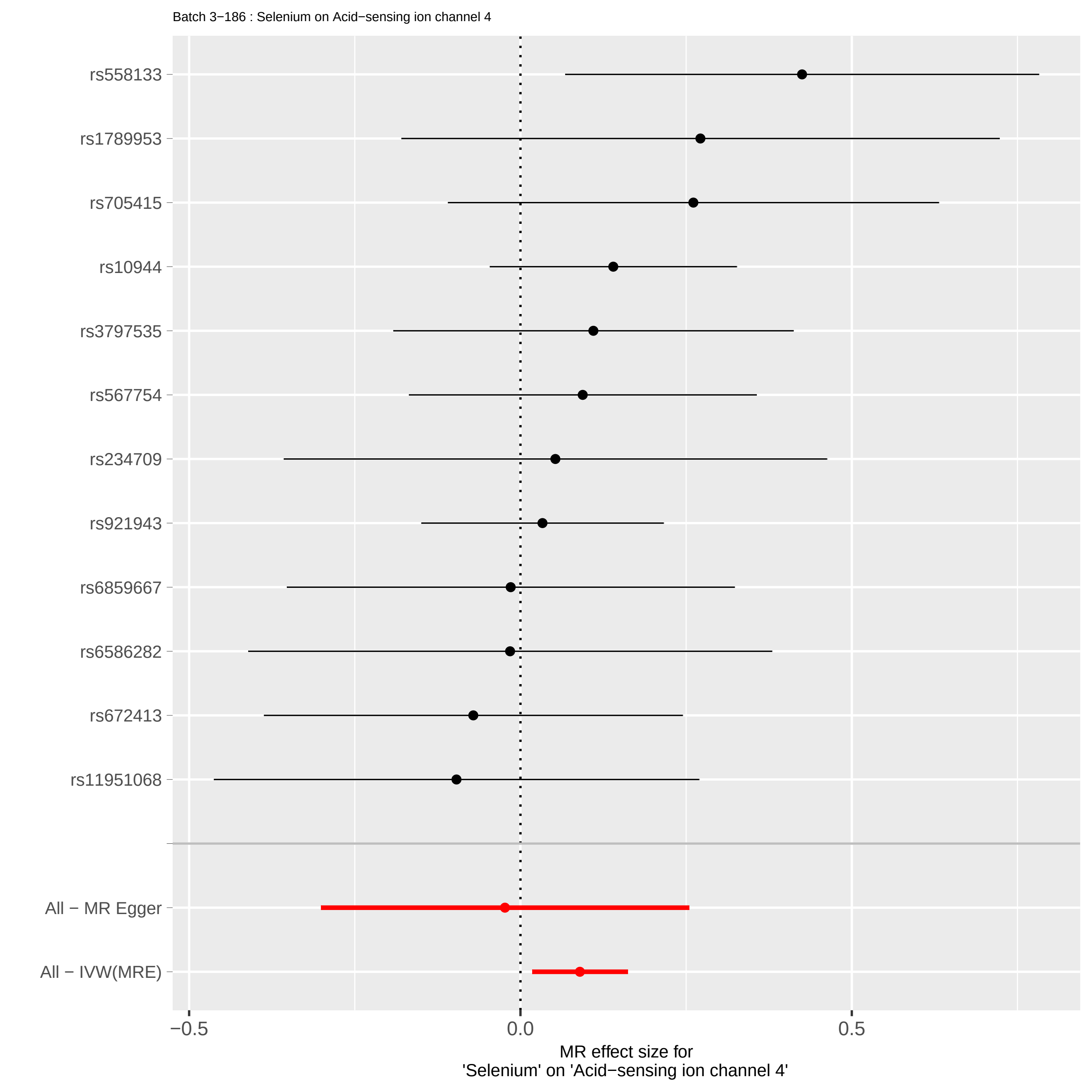
Batch 3−186 : Selenium on Acid−sensing ion channel 4
rs558133
rs1789953
rs705415
rs10944
rs3797535
rs567754
rs234709
rs921943
rs6859667
rs6586282
rs672413
rs11951068
All − MR Egger
All − IVW(MRE)
−0.5
0.0
0.5
MR effect size for
'Selenium' on 'Acid−sensing ion channel 4'

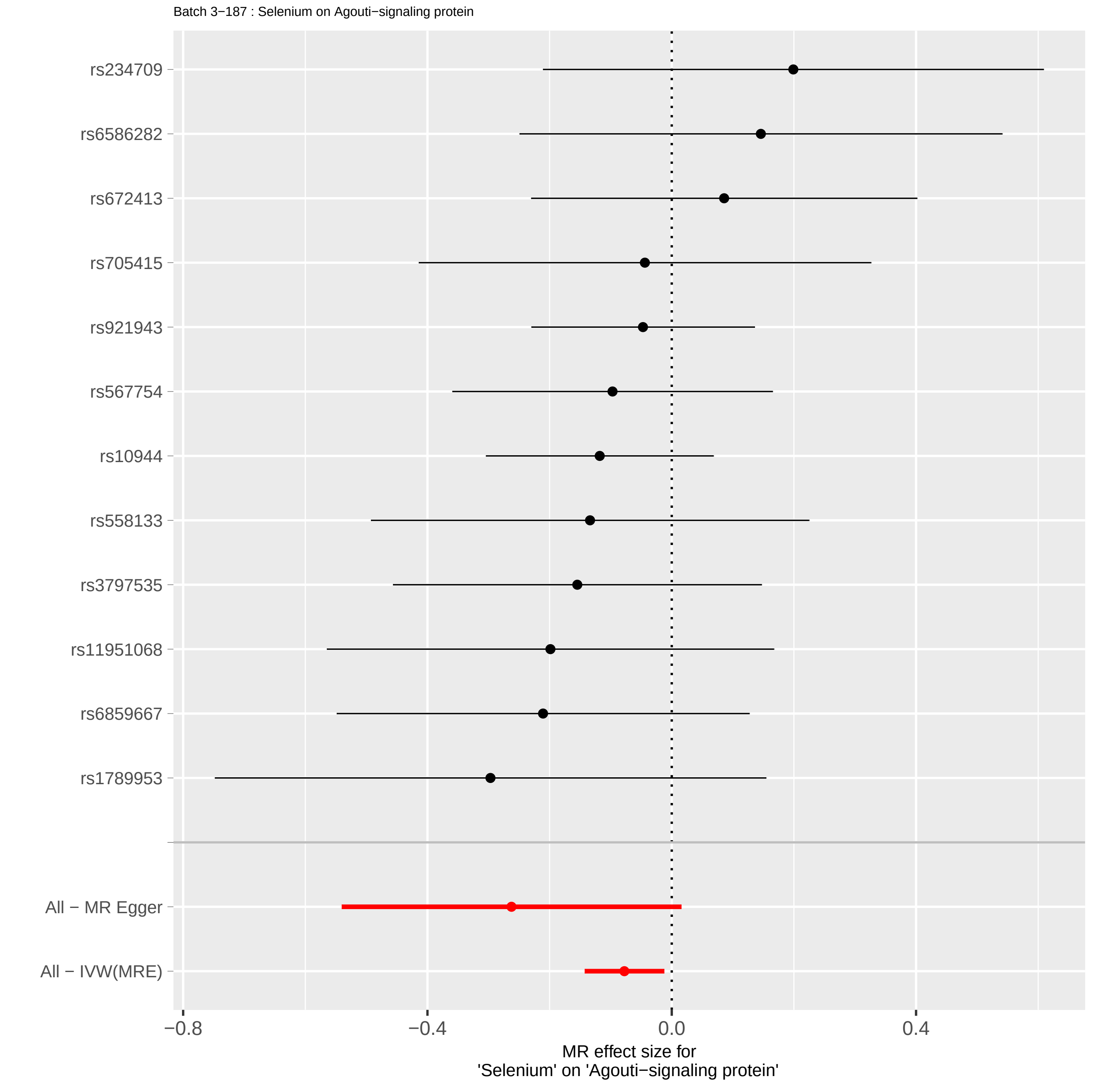

Batch 3−187 : Selenium on Agouti−signaling protein
rs234709
rs6586282
rs672413
rs705415
rs921943
rs567754
rs10944
rs558133
rs3797535
rs11951068
rs6859667
rs1789953
All − MR Egger
All − IVW(MRE)
−0.8
−0.4
0.0
0.4
MR effect size for
'Selenium' on 'Agouti−signaling protein'

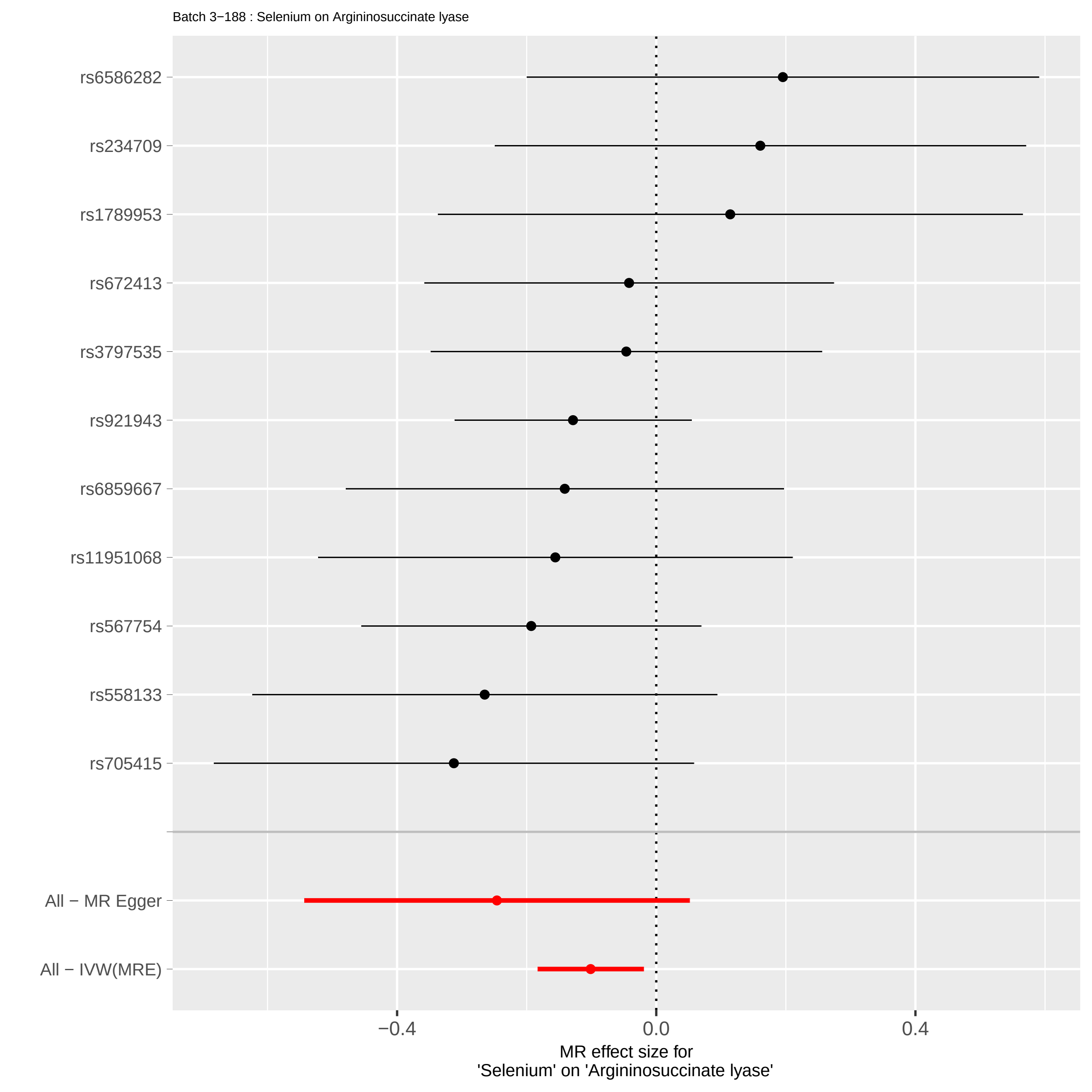

Batch 3−188 : Selenium on Argininosuccinate lyase
rs6586282
rs234709
rs1789953
rs672413
rs3797535
rs921943
rs6859667
rs11951068
rs567754
rs558133
rs705415
All − MR Egger
All − IVW(MRE)
−0.4
0.0
0.4
MR effect size for
'Selenium' on 'Argininosuccinate lyase'

Batch 3−189 : Selenium on N−acetylserotonin O−methyltransferase−like protein

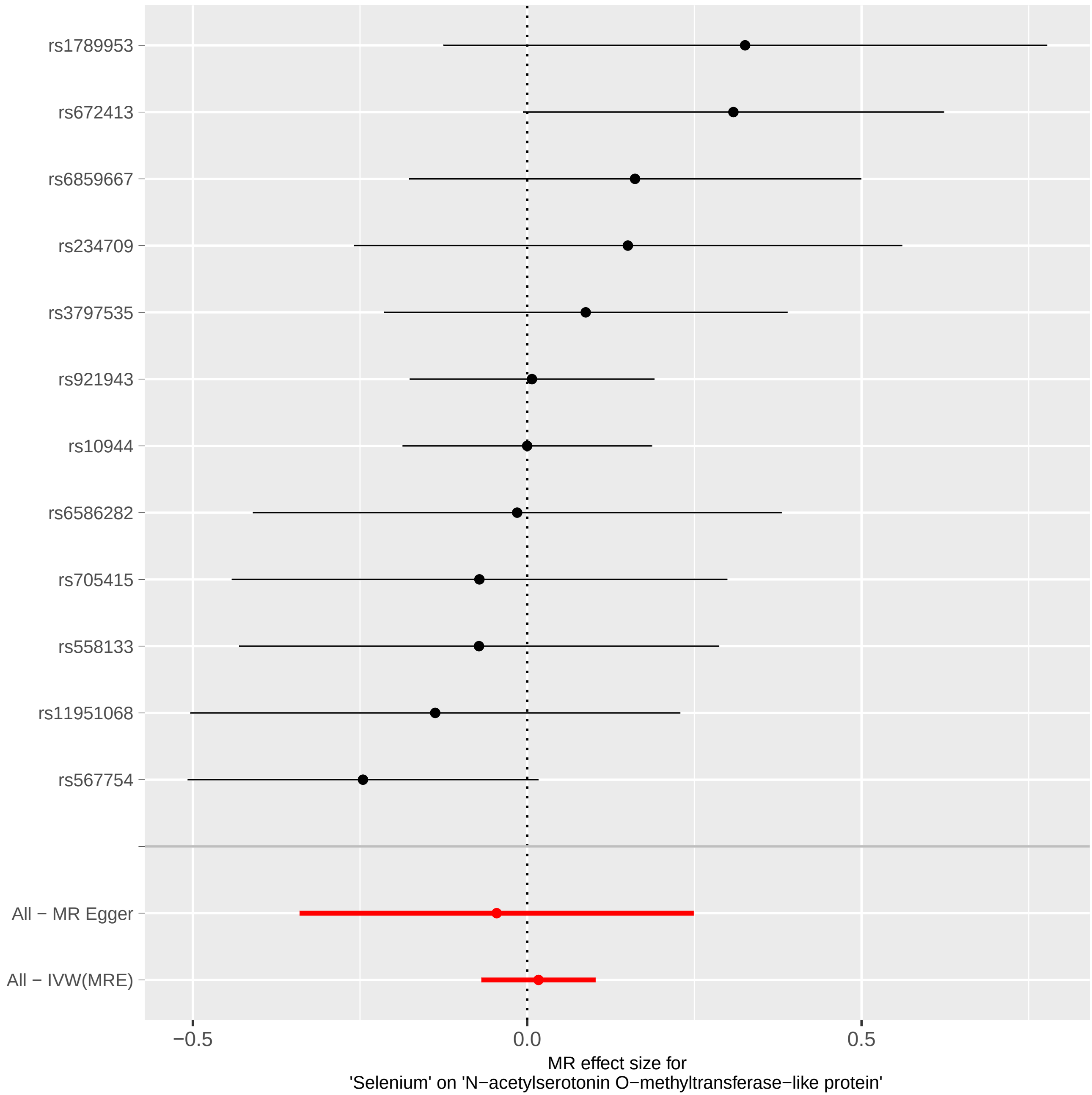

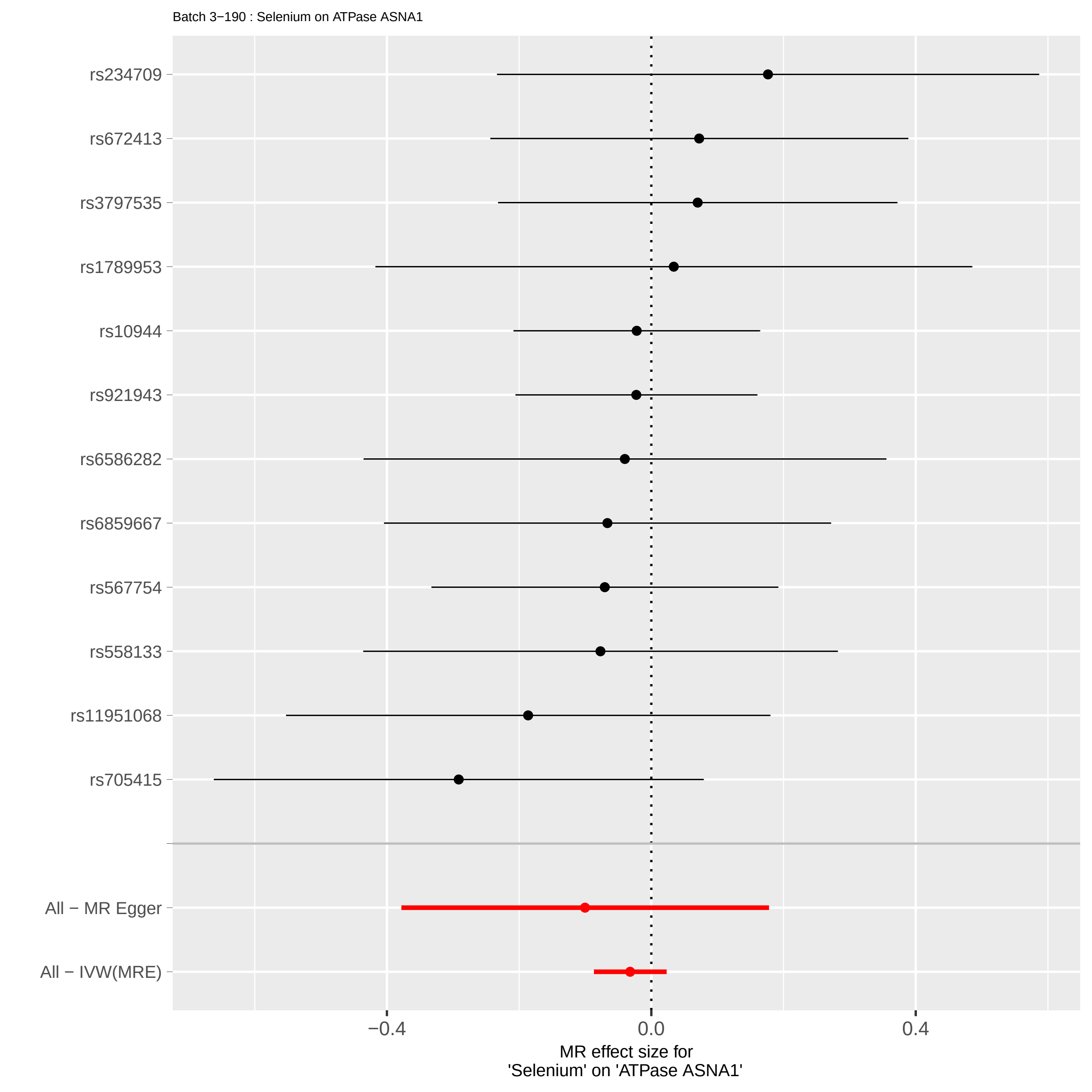

Batch 3−190 : Selenium on ATPase ASNA1
rs234709
rs672413
rs3797535
rs1789953
rs10944
rs921943
rs6586282
rs6859667
rs567754
rs558133
rs11951068
rs705415
All − MR Egger
All − IVW(MRE)
−0.4
0.0
0.4
MR effect size for
'Selenium' on 'ATPase ASNA1'

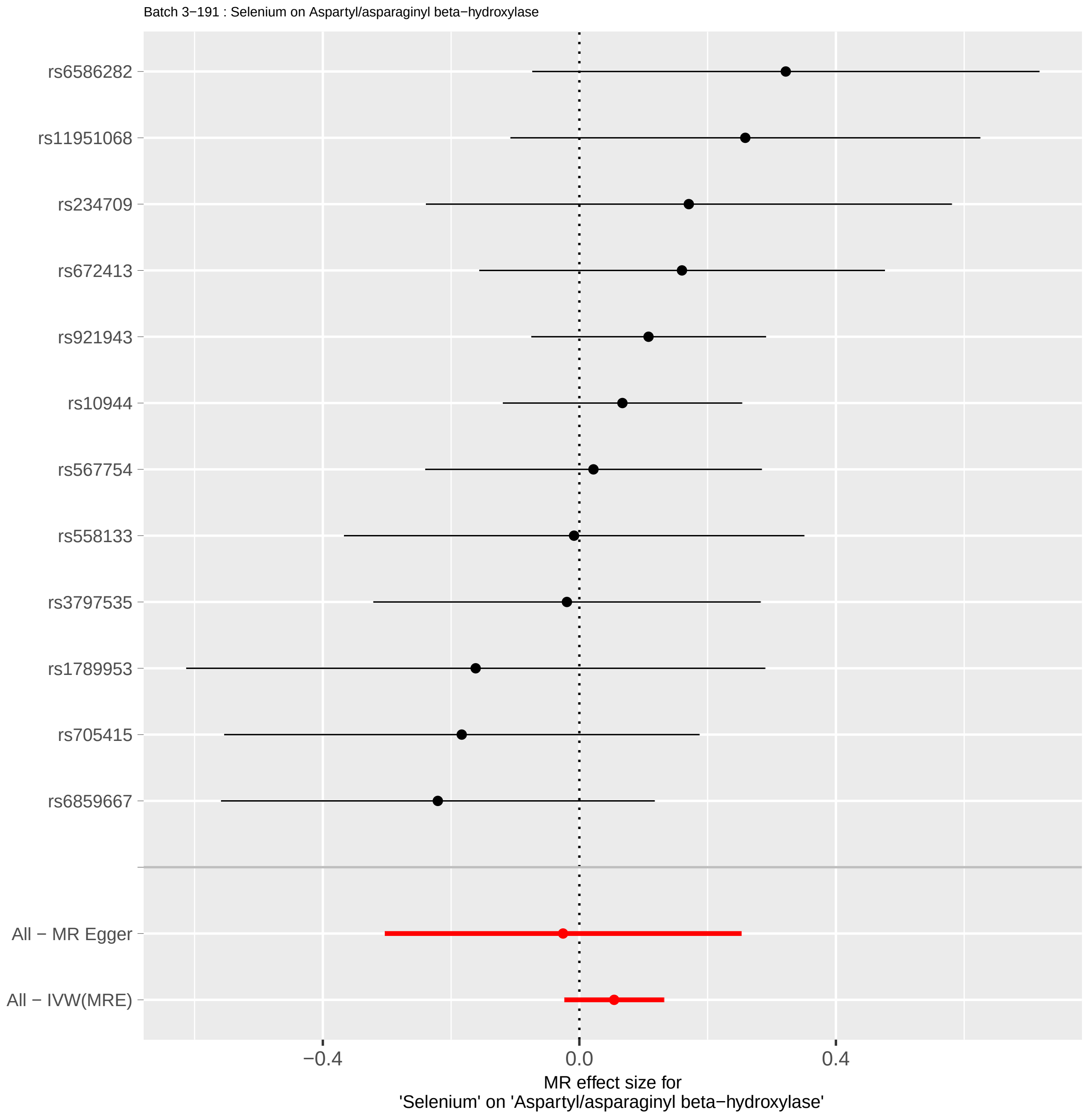
Batch 3−191 : Selenium on Aspartyl/asparaginyl beta−hydroxylase
rs6586282
rs11951068
rs234709
rs672413
rs921943
rs10944
rs567754
rs558133
rs3797535
rs1789953
rs705415
rs6859667
All − MR Egger
All − IVW(MRE)
−0.4
0.0
0.4
MR effect size for
'Selenium' on 'Aspartyl/asparaginyl beta−hydroxylase'

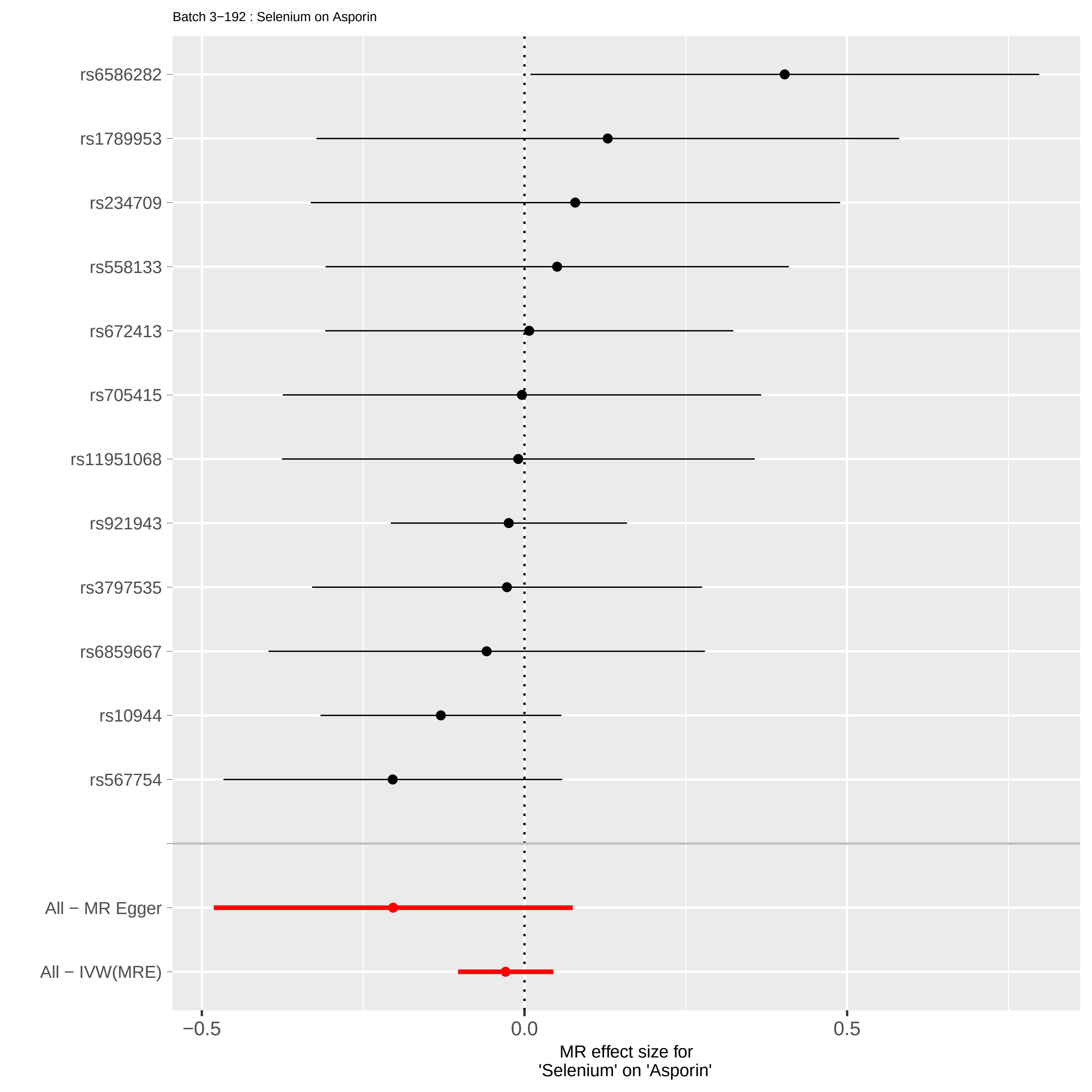
Batch 3−192 : Selenium on Asporin
rs6586282
rs1789953
rs234709
rs558133
rs672413
rs705415
rs11951068
rs921943
rs3797535
rs6859667
rs10944
rs567754
All − MR Egger
All − IVW(MRE)
−0.5
0.0
0.5
MR effect size for
'Selenium' on 'Asporin'

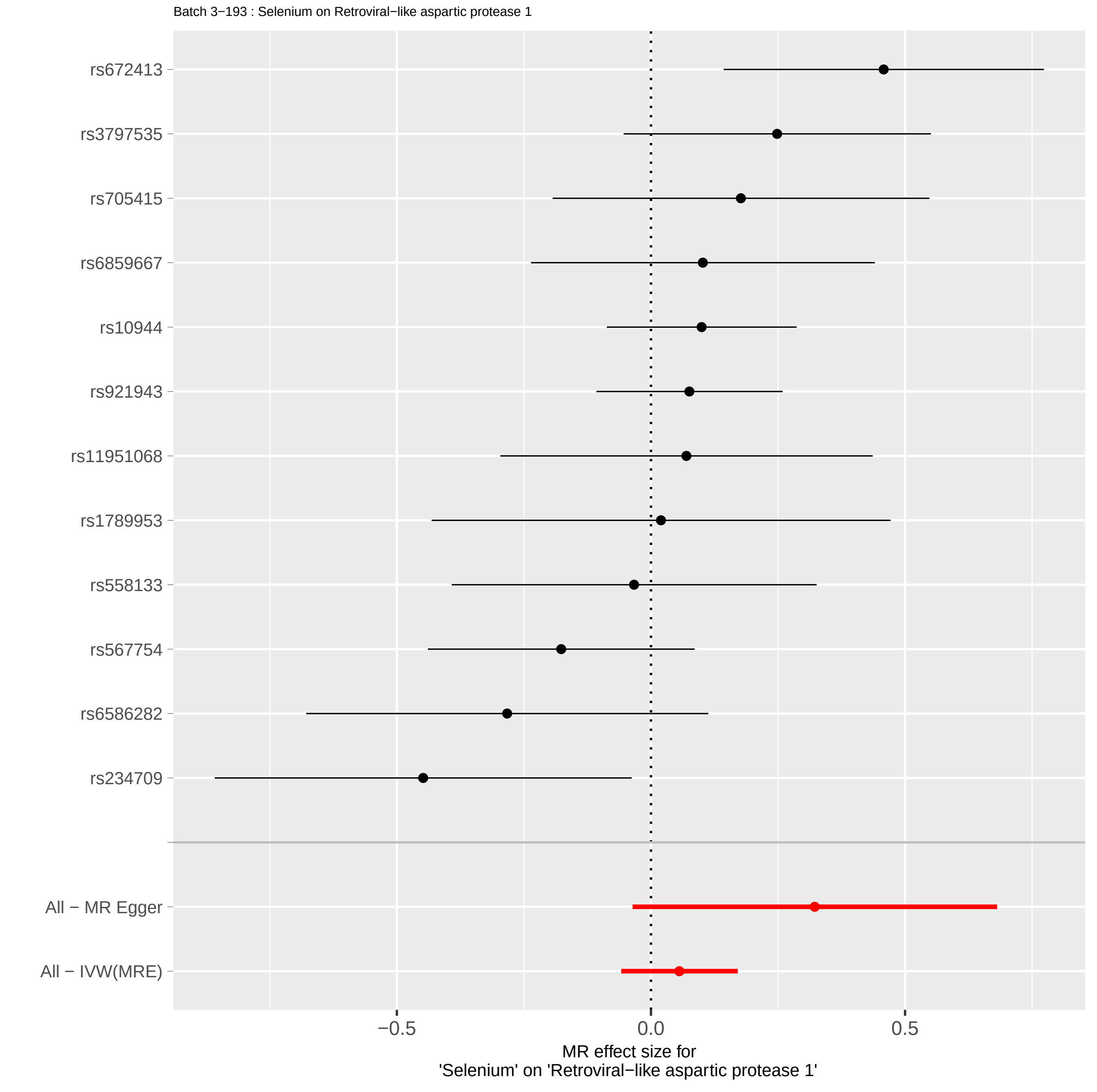

Batch 3−193 : Selenium on Retroviral−like aspartic protease 1
rs672413
rs3797535
rs705415
rs6859667
rs10944
rs921943
rs11951068
rs1789953
rs558133
rs567754
rs6586282
rs234709
All − MR Egger
All − IVW(MRE)
−0.5
0.0
0.5
MR effect size for
'Selenium' on 'Retroviral−like aspartic protease 1'

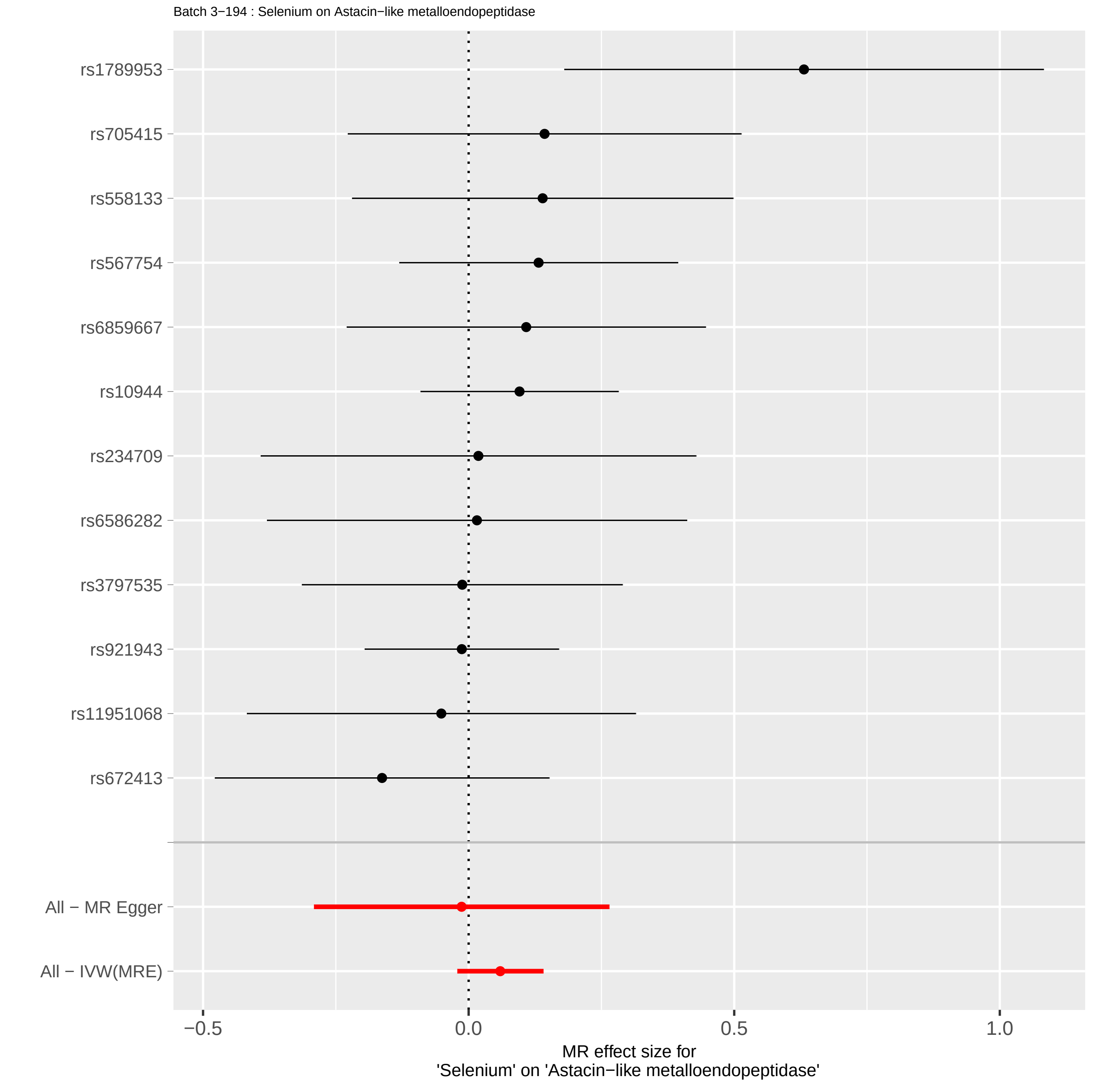

Batch 3−194 : Selenium on Astacin−like metalloendopeptidase
rs1789953
rs705415
rs558133
rs567754
rs6859667
rs10944
rs234709
rs6586282
rs3797535
rs921943
rs11951068
rs672413
All − MR Egger
All − IVW(MRE)
−0.5
0.0
0.5
1.0
MR effect size for
'Selenium' on 'Astacin−like metalloendopeptidase'

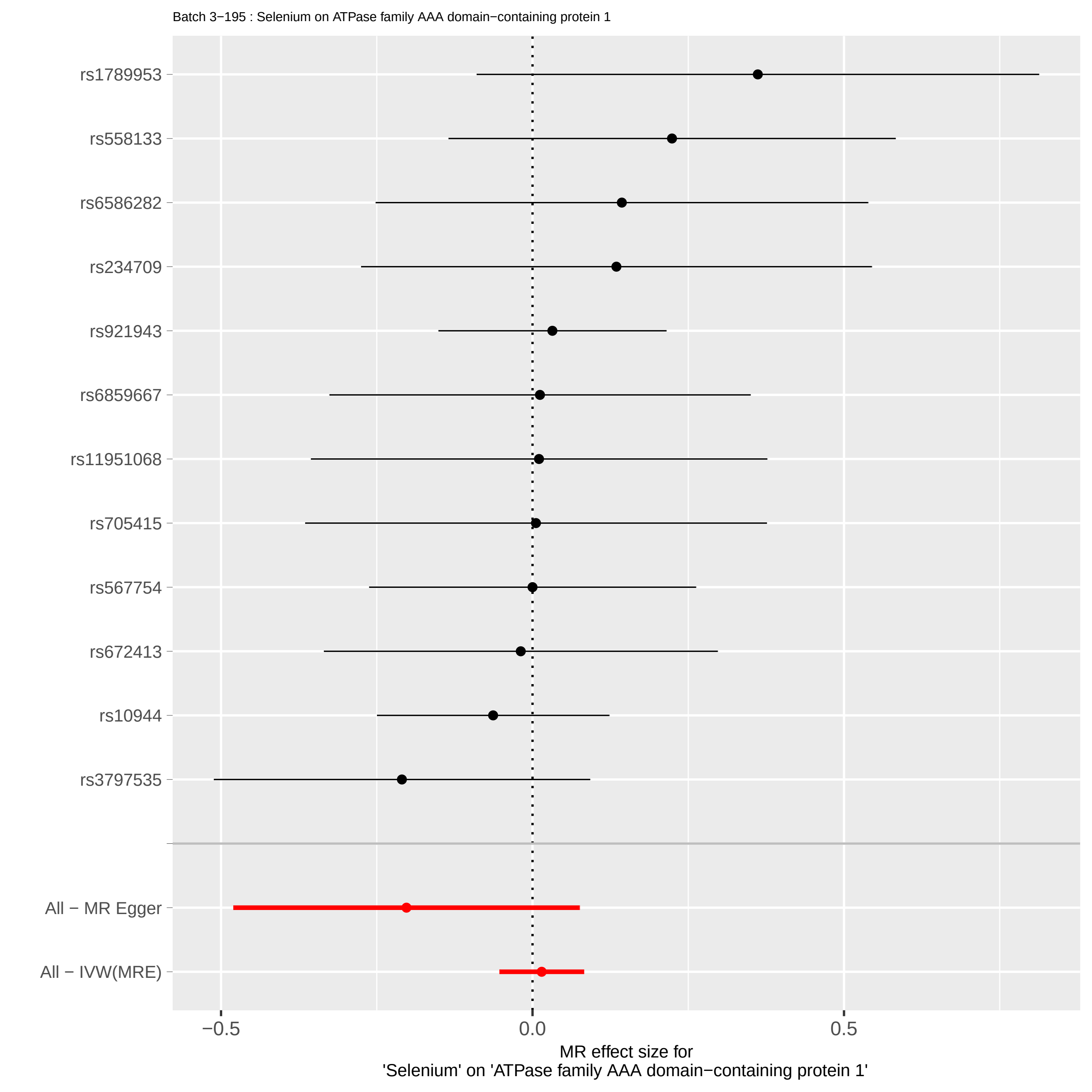

Batch 3−195 : Selenium on ATPase family AAA domain−containing protein 1
rs1789953
rs558133
rs6586282
rs234709
rs921943
rs6859667
rs11951068
rs705415
rs567754
rs672413
rs10944
rs3797535
All − MR Egger
All − IVW(MRE)
−0.5
0.0
0.5
MR effect size for
'Selenium' on 'ATPase family AAA domain−containing protein 1'

Batch 3−196 : Selenium on ATPase family AAA domain−containing protein 2

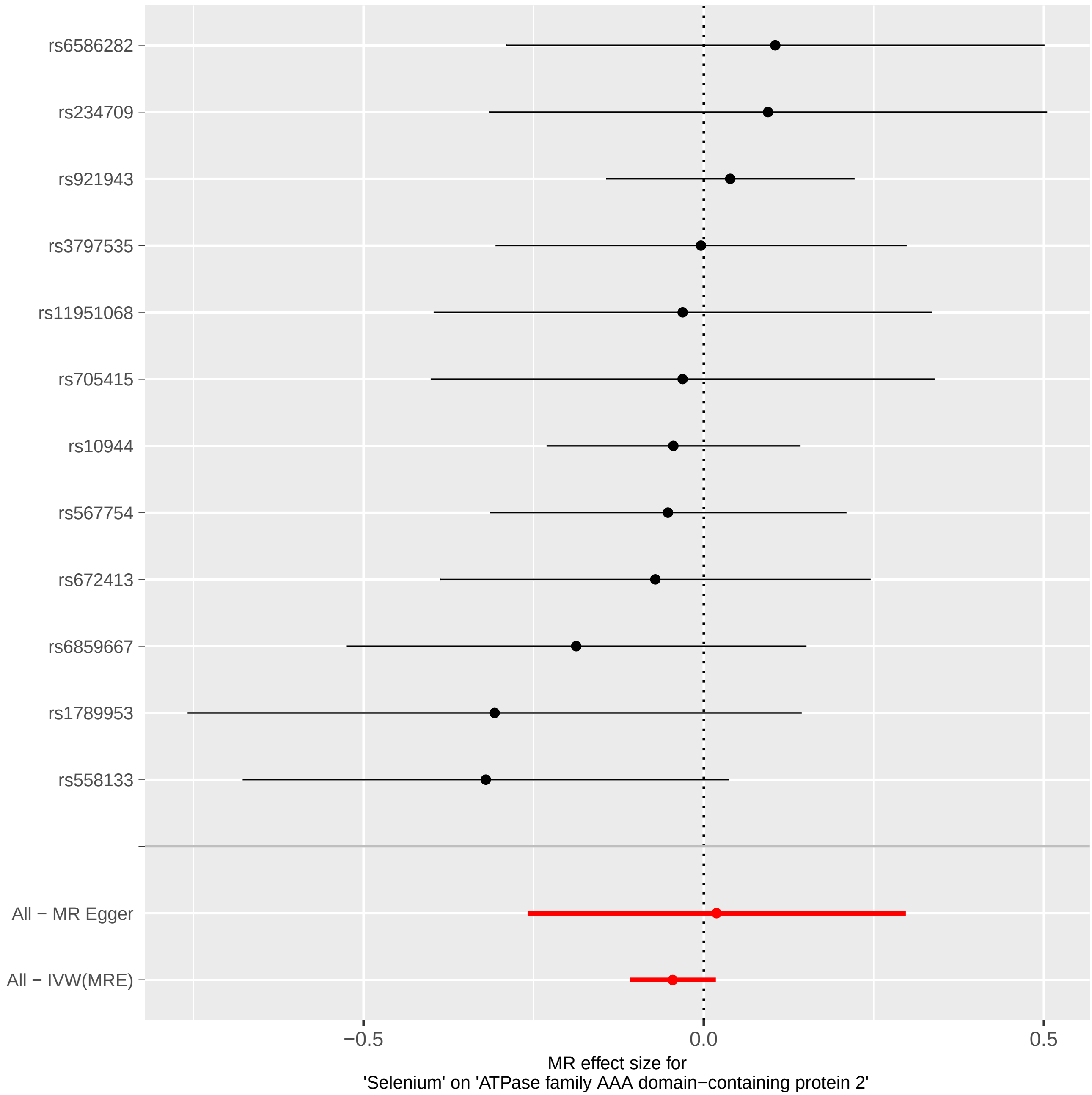

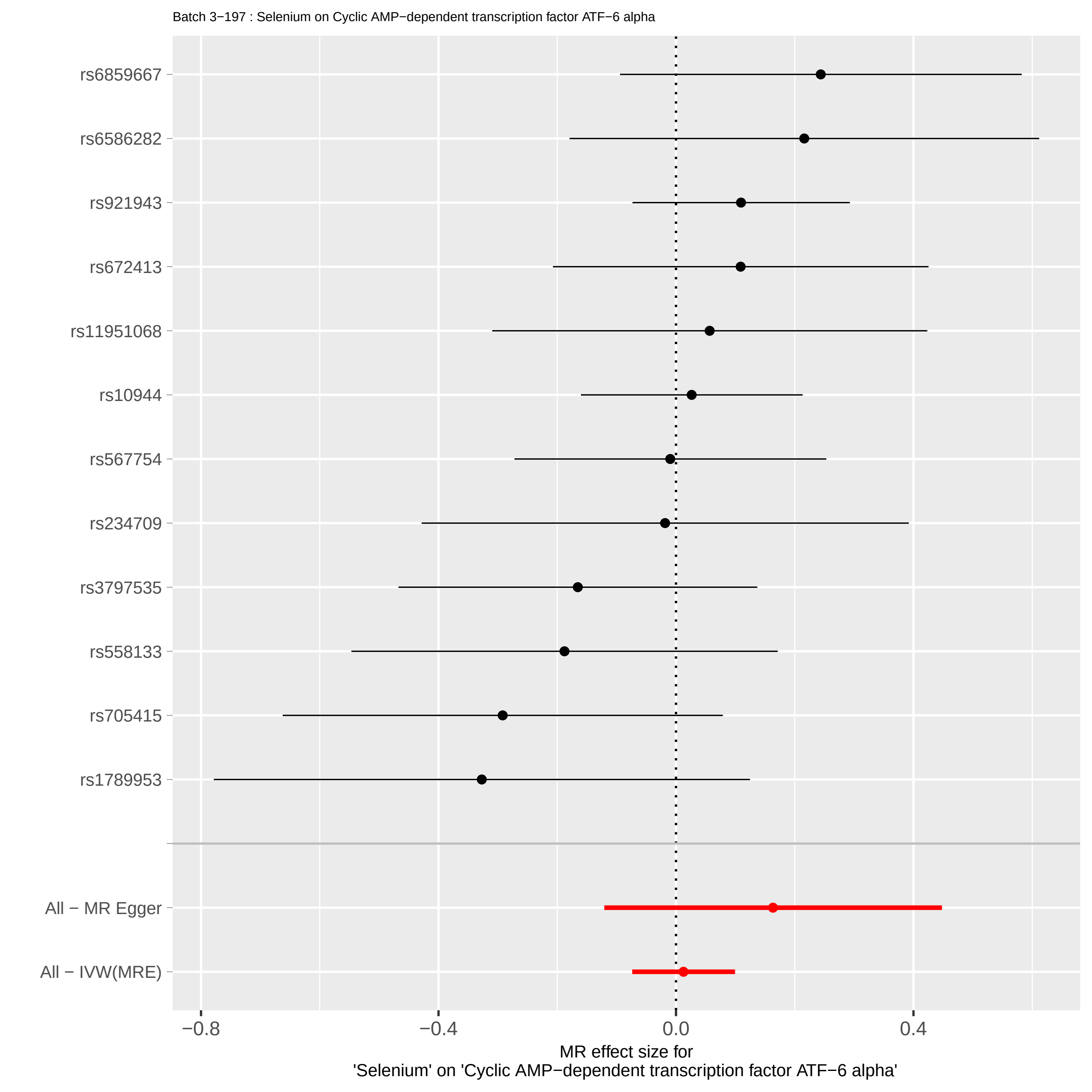
Batch 3−197 : Selenium on Cyclic AMP−dependent transcription factor ATF−6 alpha
rs6859667
rs6586282
rs921943
rs672413
rs11951068
rs10944
rs567754
rs234709
rs3797535
rs558133
rs705415
rs1789953
All − MR Egger
All − IVW(MRE)
−0.8
−0.4
0.0
0.4
MR effect size for
'Selenium' on 'Cyclic AMP−dependent transcription factor ATF−6 alpha'

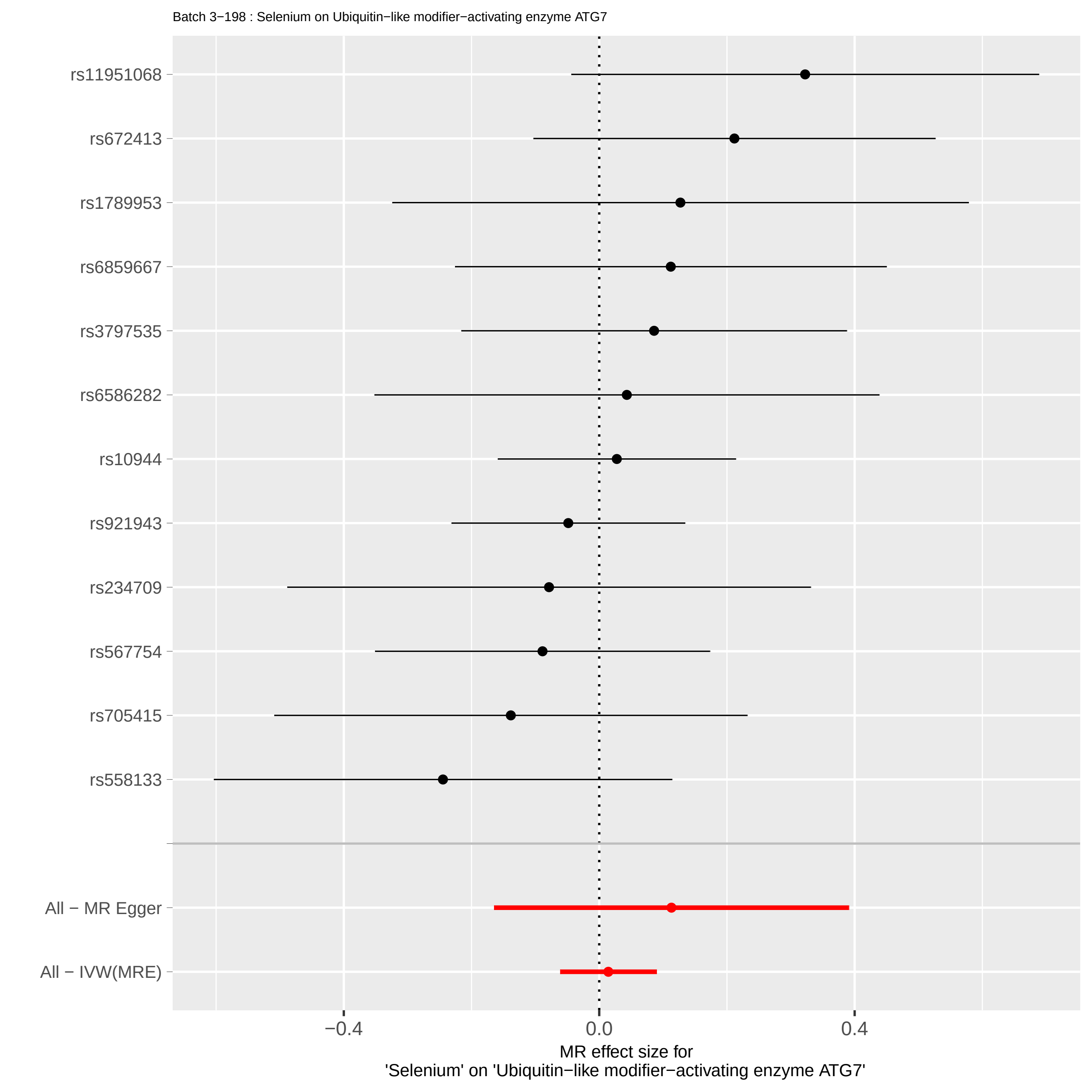
Batch 3−198 : Selenium on Ubiquitin−like modifier−activating enzyme ATG7
rs11951068
rs672413
rs1789953
rs6859667
rs3797535
rs6586282
rs10944
rs921943
rs234709
rs567754
rs705415
rs558133
All − MR Egger
All − IVW(MRE)
−0.4
0.0
0.4
MR effect size for
'Selenium' on 'Ubiquitin−like modifier−activating enzyme ATG7'

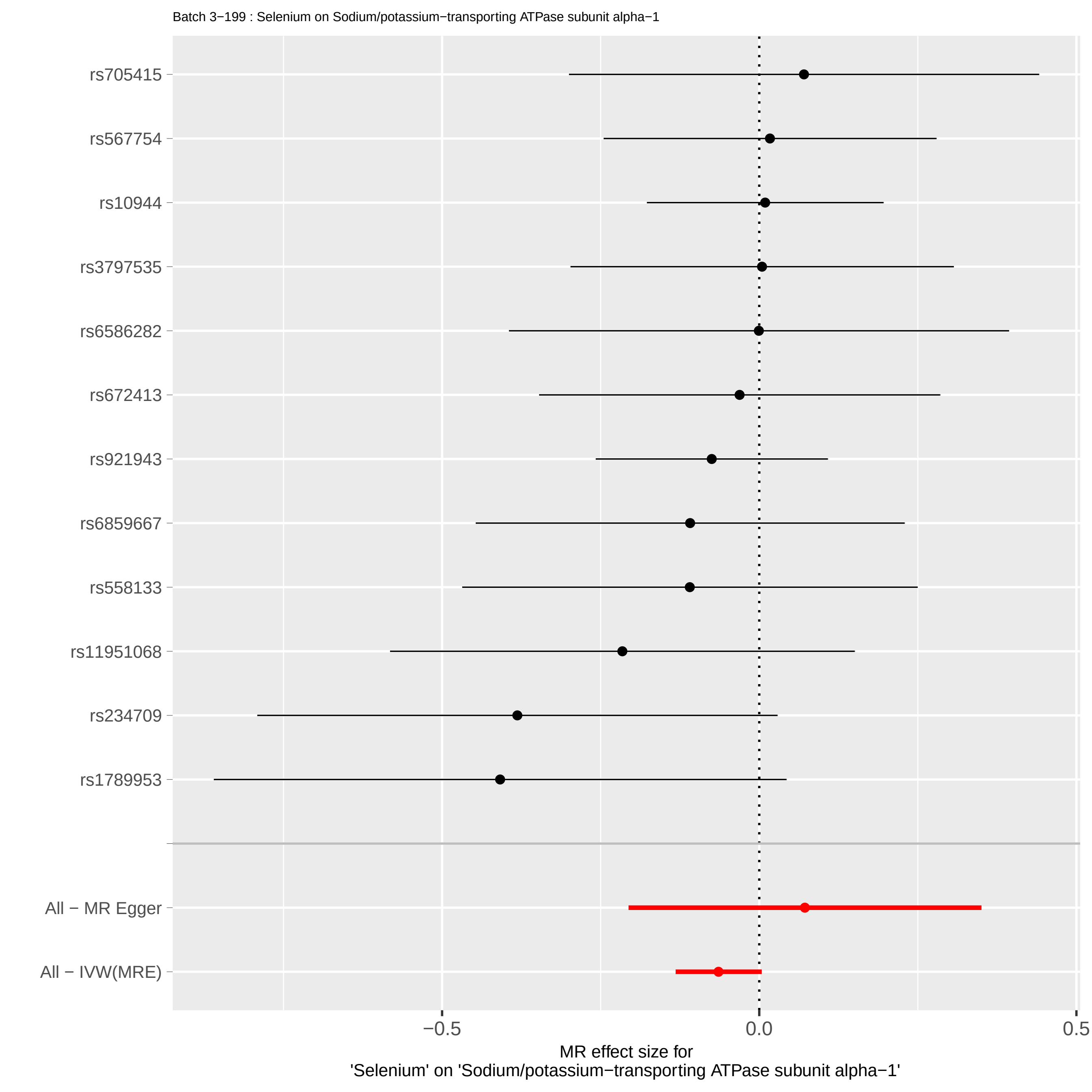

Batch 3−199 : Selenium on Sodium/potassium−transporting ATPase subunit alpha−1
rs705415
rs567754
rs10944
rs3797535
rs6586282
rs672413
rs921943
rs6859667
rs558133
rs11951068
rs234709
rs1789953
All − MR Egger
All − IVW(MRE)
−0.5
0.0
0.5
MR effect size for
'Selenium' on 'Sodium/potassium−transporting ATPase subunit alpha−1'

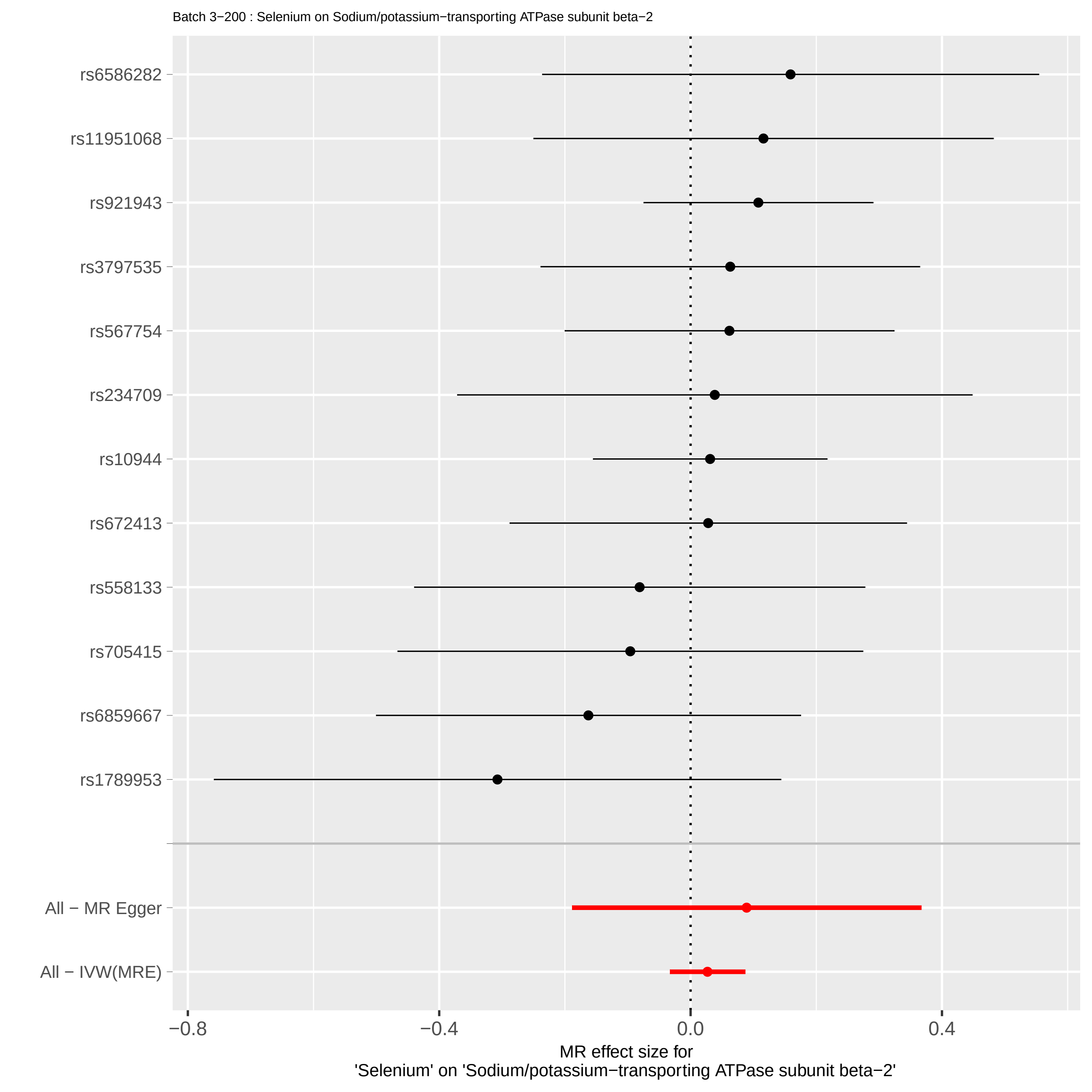
Batch 3−200 : Selenium on Sodium/potassium−transporting ATPase subunit beta−2
rs6586282
rs11951068
rs921943
rs3797535
rs567754
rs234709
rs10944
rs672413
rs558133
rs705415
rs6859667
rs1789953
All − MR Egger
All − IVW(MRE)
−0.8
−0.4
0.0
0.4
MR effect size for
'Selenium' on 'Sodium/potassium−transporting ATPase subunit beta−2'

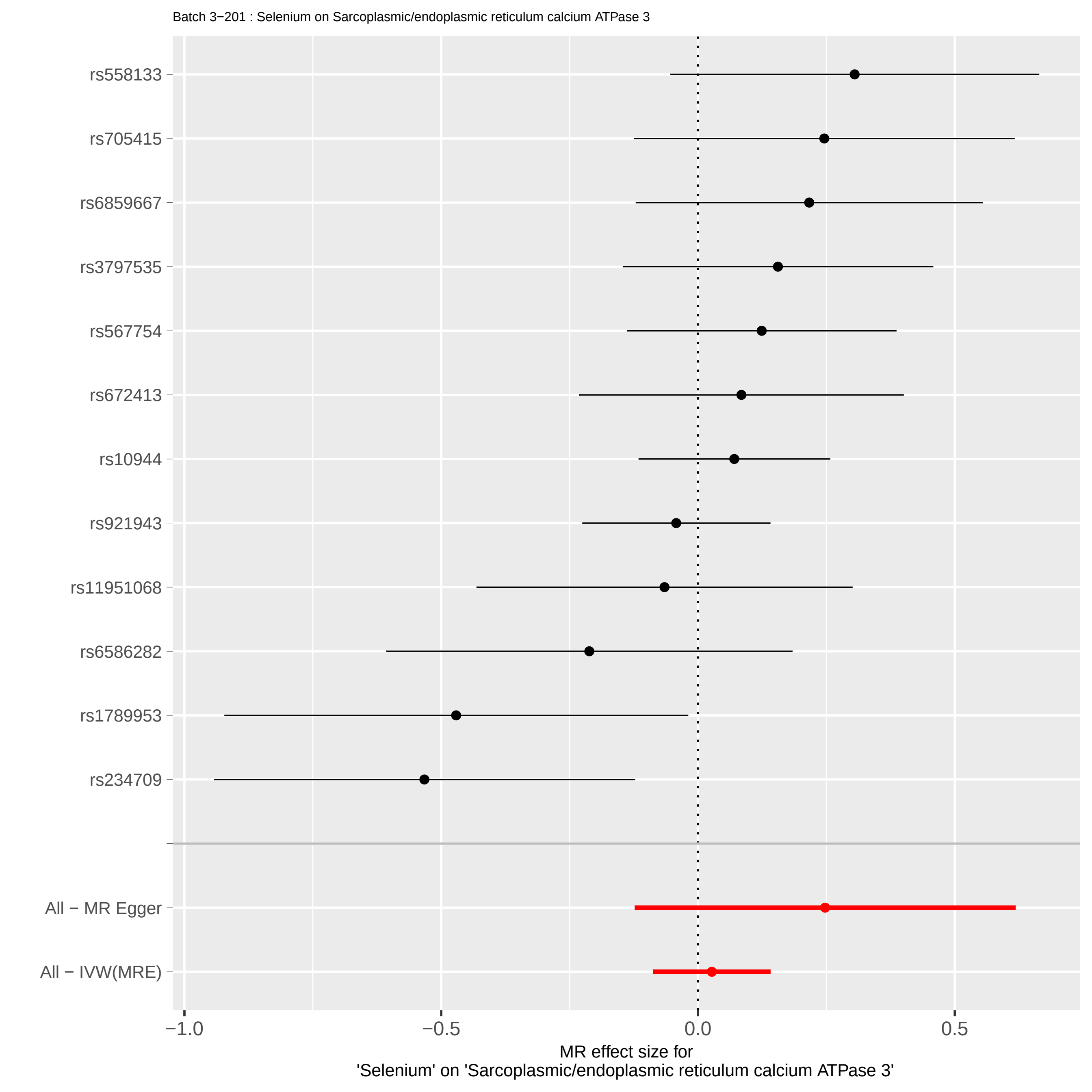
Batch 3−201 : Selenium on Sarcoplasmic/endoplasmic reticulum calcium ATPase 3
rs558133
rs705415
rs6859667
rs3797535
rs567754
rs672413
rs10944
rs921943
rs11951068
rs6586282
rs1789953
rs234709
All − MR Egger
All − IVW(MRE)
−1.0
−0.5
0.0
0.5
MR effect size for
'Selenium' on 'Sarcoplasmic/endoplasmic reticulum calcium ATPase 3'

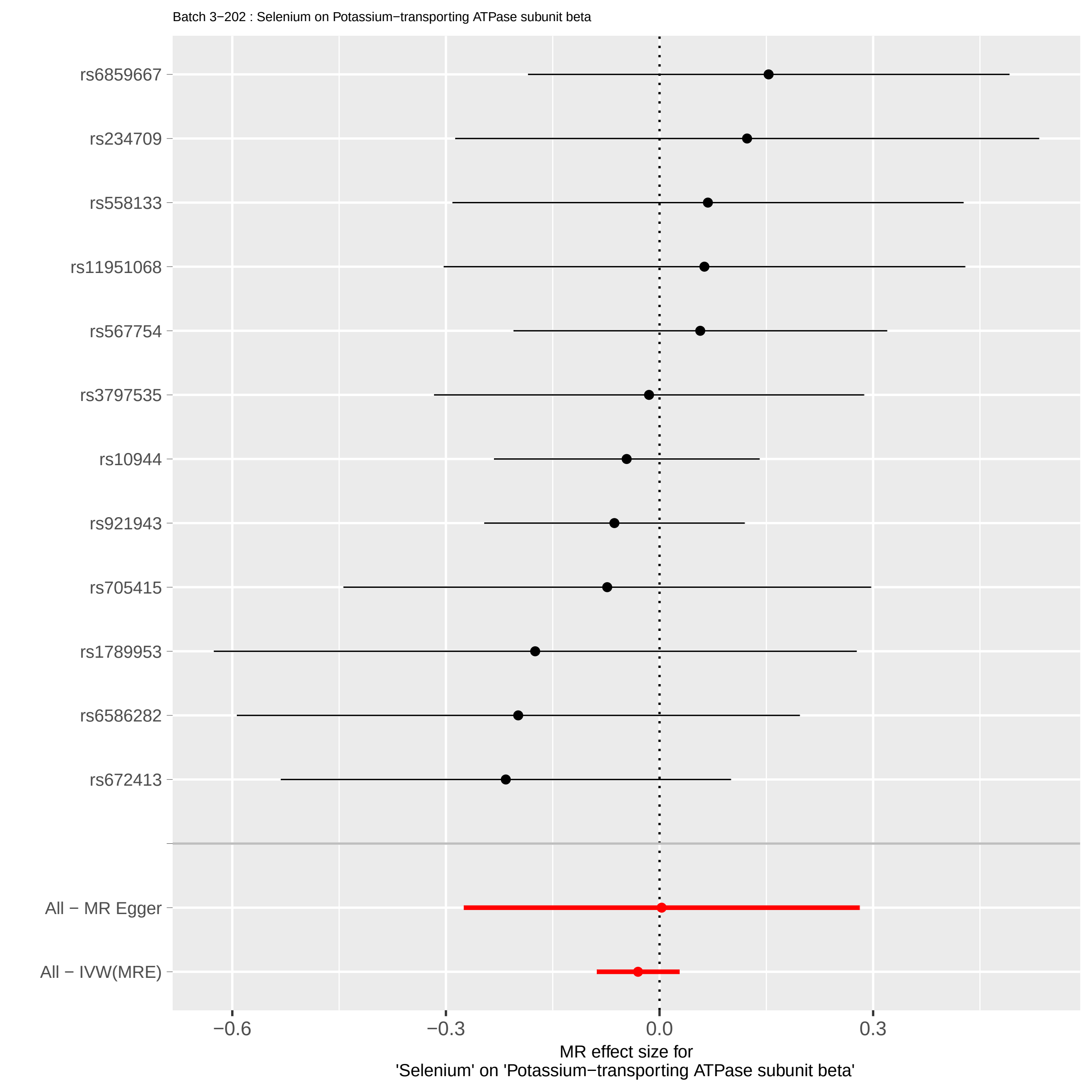

Batch 3−202 : Selenium on Potassium−transporting ATPase subunit beta
rs6859667
rs234709
rs558133
rs11951068
rs567754
rs3797535
rs10944
rs921943
rs705415
rs1789953
rs6586282
rs672413
All − MR Egger
All − IVW(MRE)
−0.6
−0.3
0.0
0.3
MR effect size for
'Selenium' on 'Potassium−transporting ATPase subunit beta'

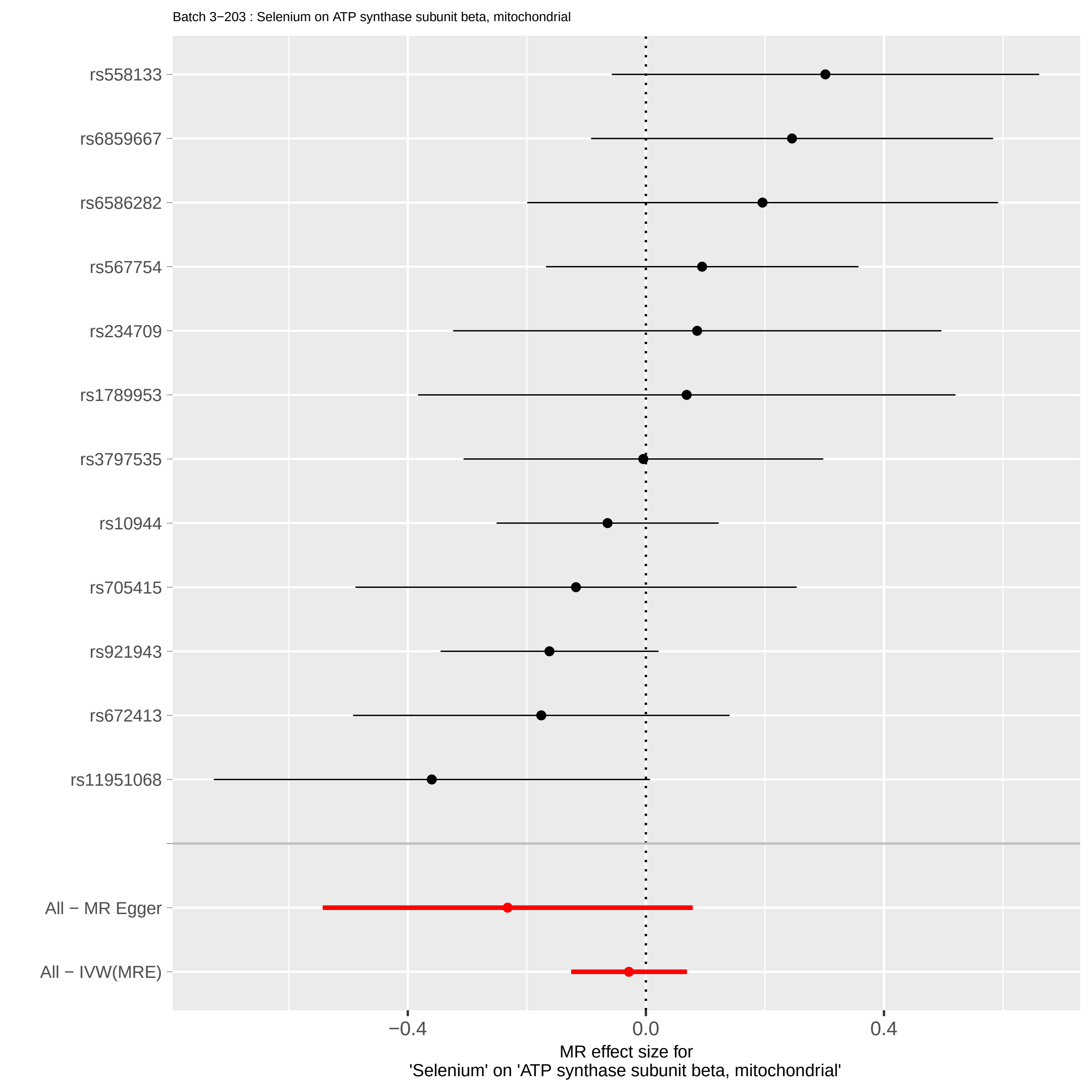

Batch 3−203 : Selenium on ATP synthase subunit beta, mitochondrial
rs558133
rs6859667
rs6586282
rs567754
rs234709
rs1789953
rs3797535
rs10944
rs705415
rs921943
rs672413
rs11951068
All − MR Egger
All − IVW(MRE)
−0.4
0.0
0.4
MR effect size for
'Selenium' on 'ATP synthase subunit beta, mitochondrial'

Batch 3−204 : Selenium on Aurora kinase A

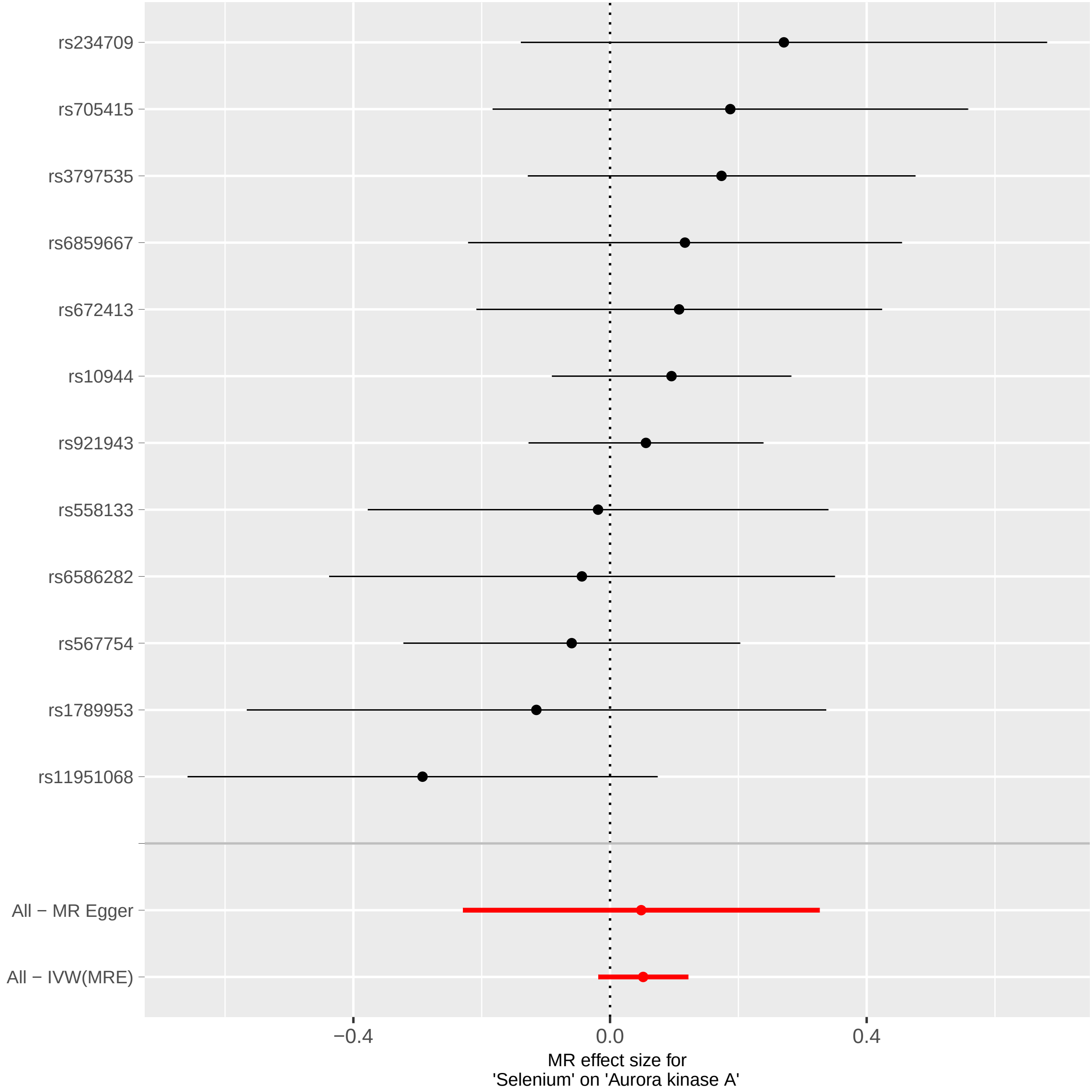

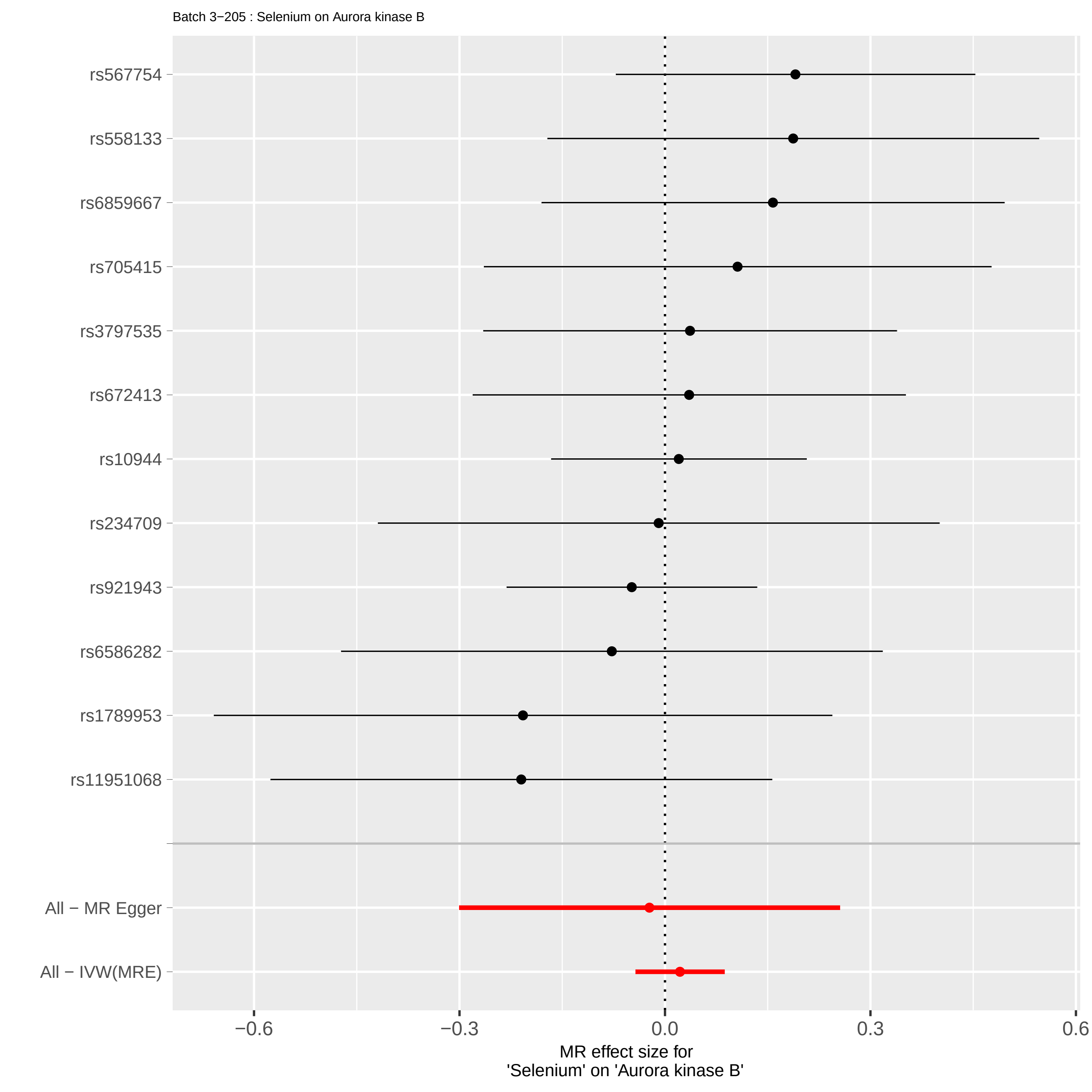

Batch 3−205 : Selenium on Aurora kinase B
rs567754
rs558133
rs6859667
rs705415
rs3797535
rs672413
rs10944
rs234709
rs921943
rs6586282
rs1789953
rs11951068
All − MR Egger
All − IVW(MRE)
−0.6
−0.3
0.0
0.3
0.6
MR effect size for
'Selenium' on 'Aurora kinase B'

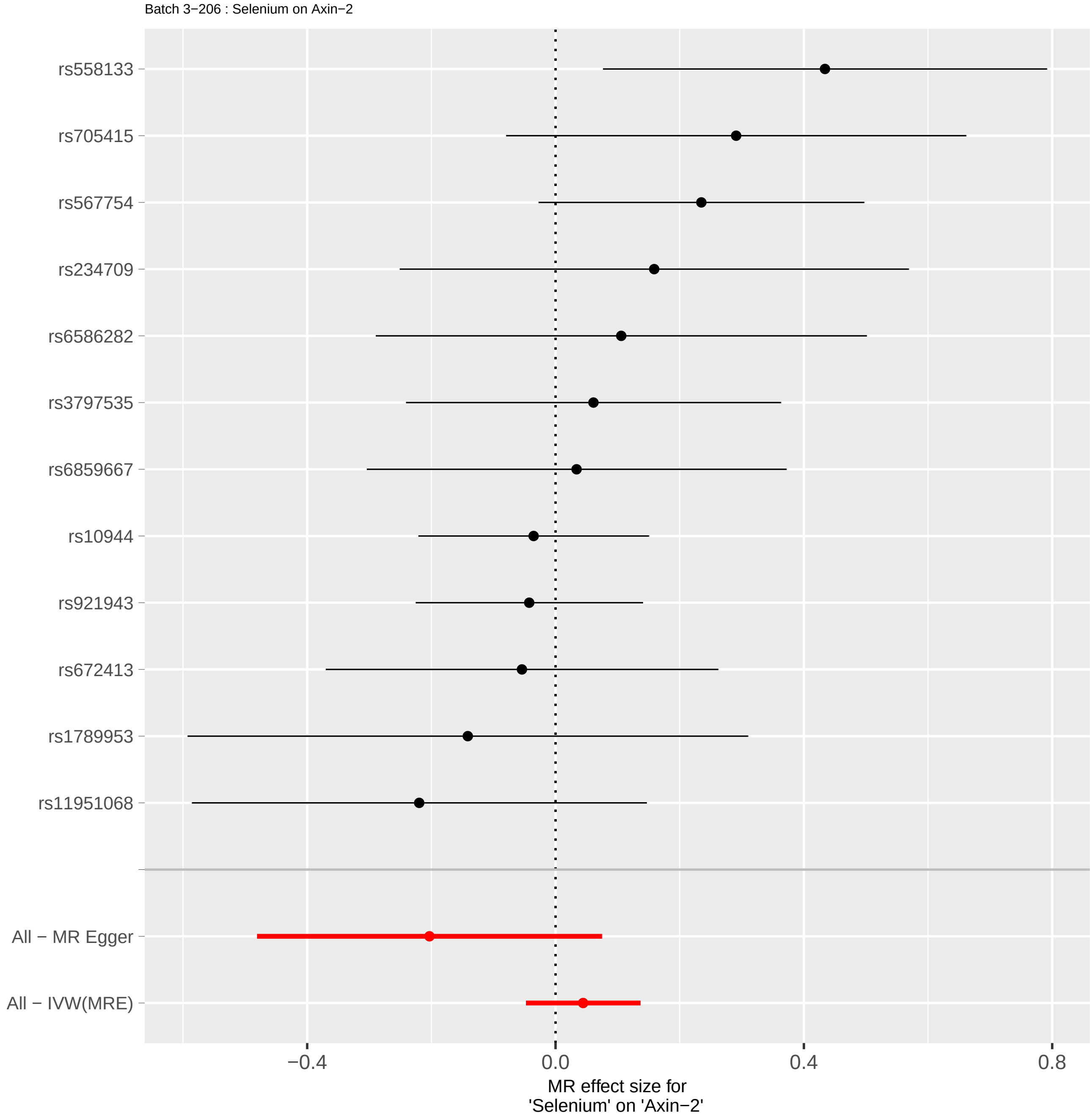

Batch 3−206 : Selenium on Axin−2
rs558133
rs705415
rs567754
rs234709
rs6586282
rs3797535
rs6859667
rs10944
rs921943
rs672413
rs1789953
rs11951068
All − MR Egger
All − IVW(MRE)
−0.4
0.0
0.4
0.8
MR effect size for
'Selenium' on 'Axin−2'

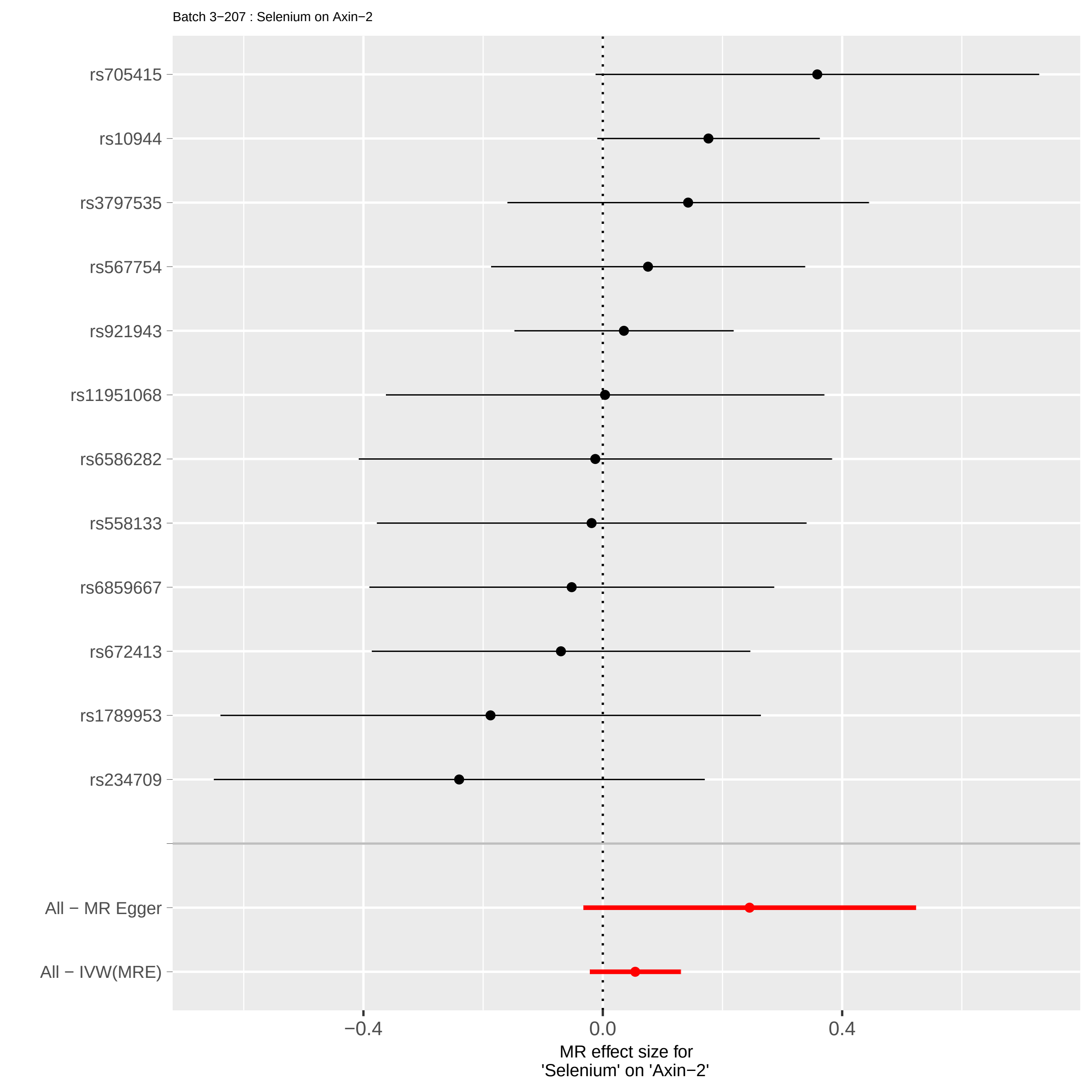

Batch 3−207 : Selenium on Axin−2
rs705415
rs10944
rs3797535
rs567754
rs921943
rs11951068
rs6586282
rs558133
rs6859667
rs672413
rs1789953
rs234709
All − MR Egger
All − IVW(MRE)
−0.4
0.0
0.4
MR effect size for
'Selenium' on 'Axin−2'

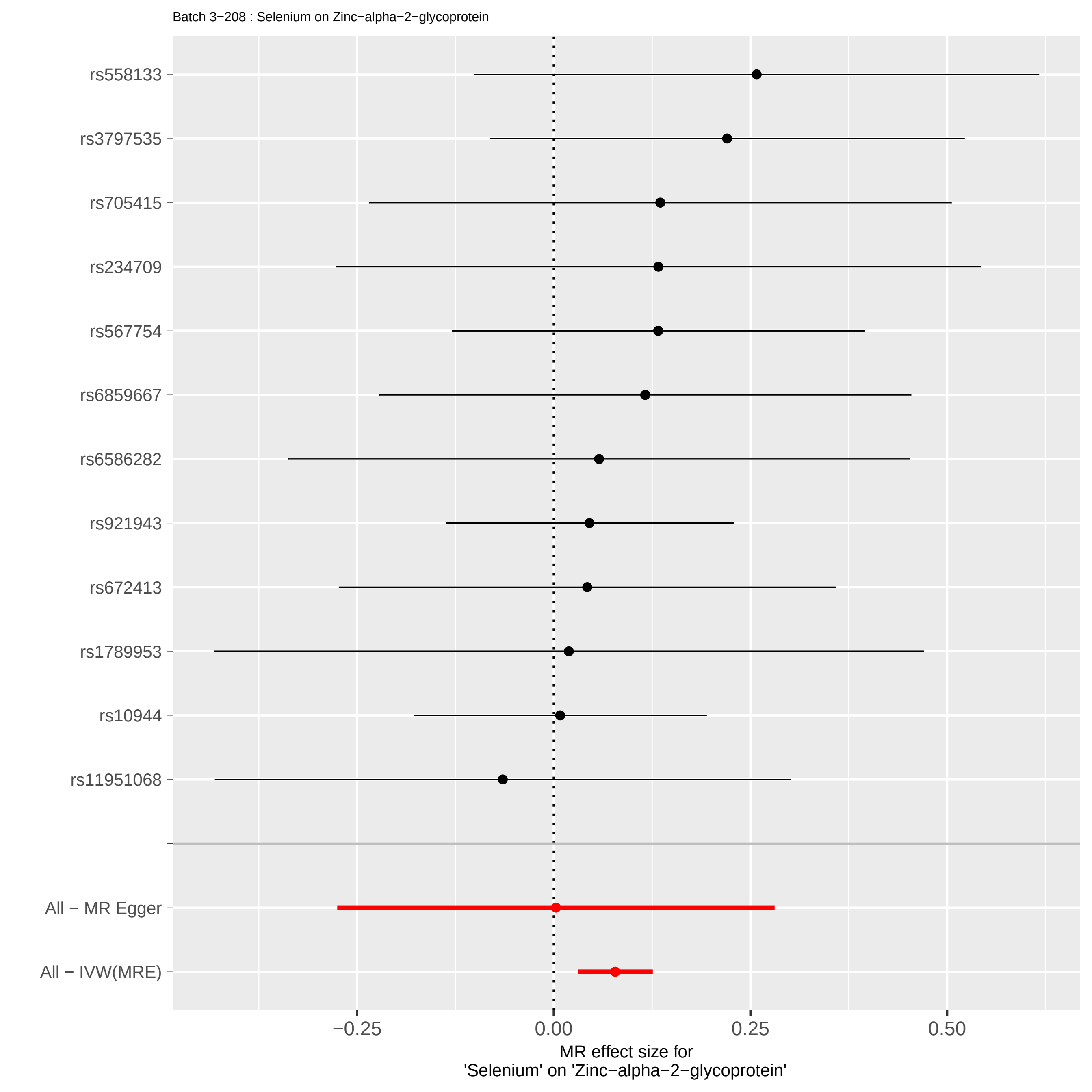
Batch 3−208 : Selenium on Zinc−alpha−2−glycoprotein
rs558133
rs3797535
rs705415
rs234709
rs567754
rs6859667
rs6586282
rs921943
rs672413
rs1789953
rs10944
rs11951068
All − MR Egger
All − IVW(MRE)
−0.25
0.00
0.25
0.50
MR effect size for
'Selenium' on 'Zinc−alpha−2−glycoprotein'

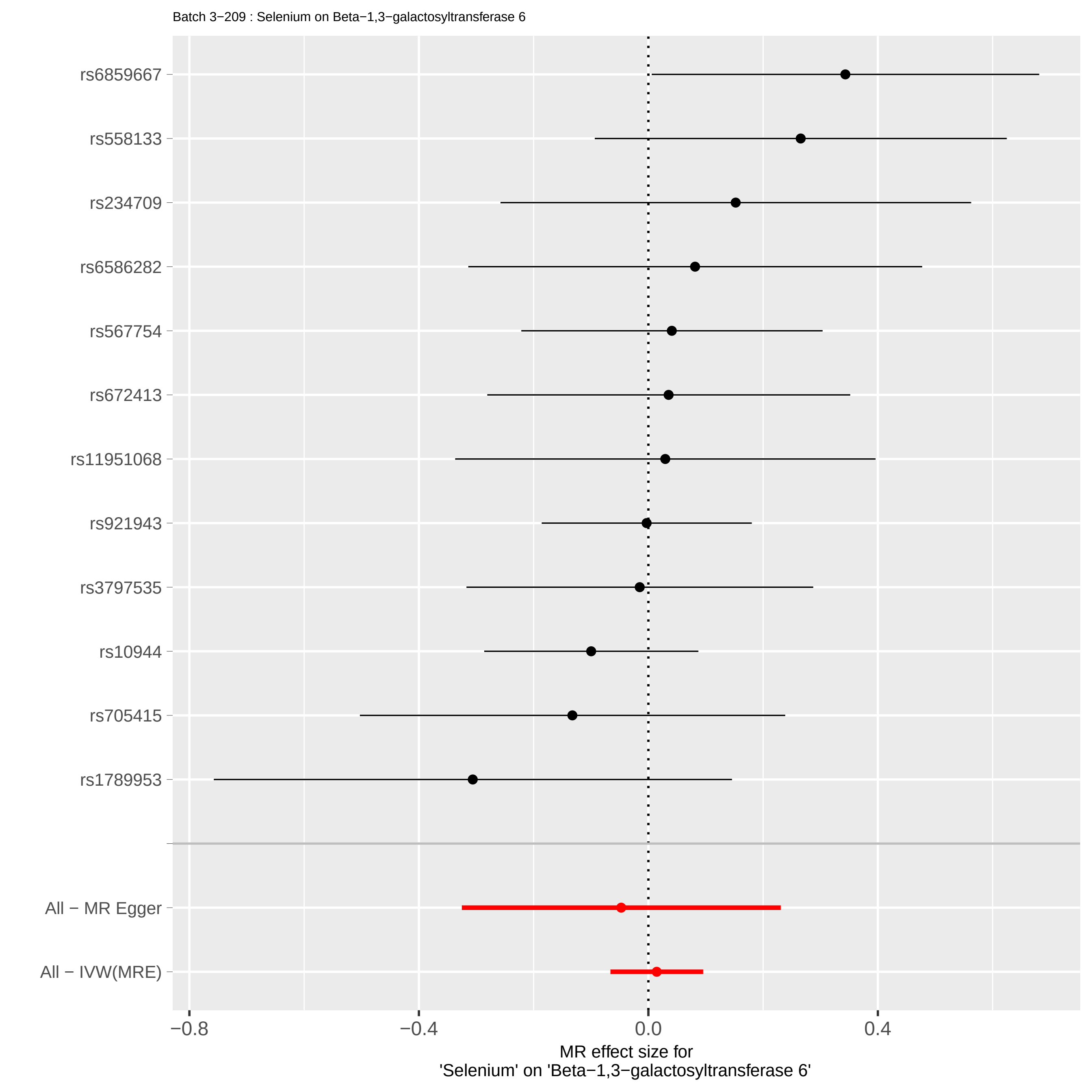
Batch 3−209 : Selenium on Beta−1,3−galactosyltransferase 6
rs6859667
rs558133
rs234709
rs6586282
rs567754
rs672413
rs11951068
rs921943
rs3797535
rs10944
rs705415
rs1789953
All − MR Egger
All − IVW(MRE)
−0.8
−0.4
0.0
0.4
MR effect size for
'Selenium' on 'Beta−1,3−galactosyltransferase 6'

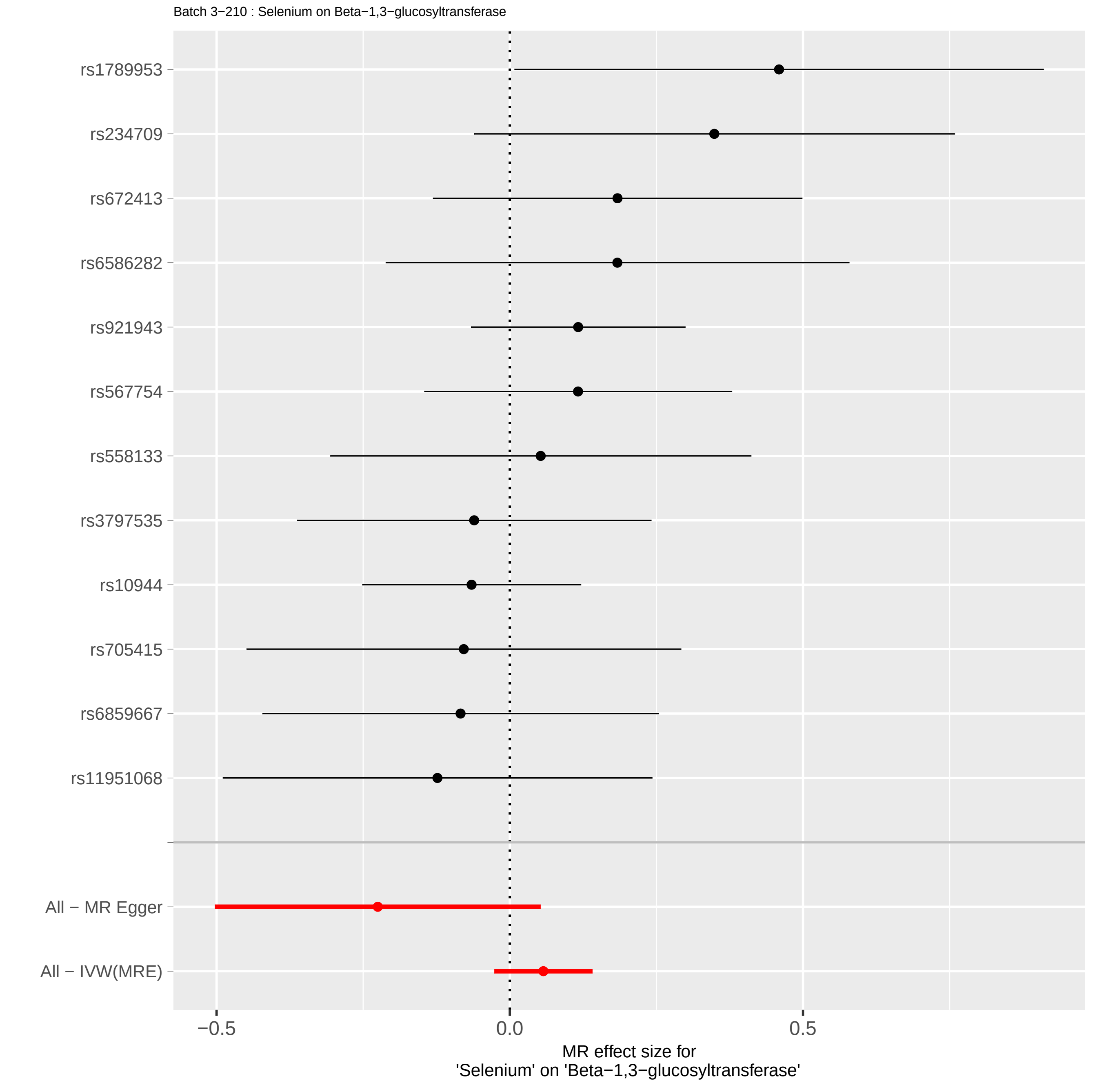

Batch 3−210 : Selenium on Beta−1,3−glucosyltransferase
rs1789953
rs234709
rs672413
rs6586282
rs921943
rs567754
rs558133
rs3797535
rs10944
rs705415
rs6859667
rs11951068
All − MR Egger
All − IVW(MRE)
−0.5
0.0
0.5
MR effect size for
'Selenium' on 'Beta−1,3−glucosyltransferase'

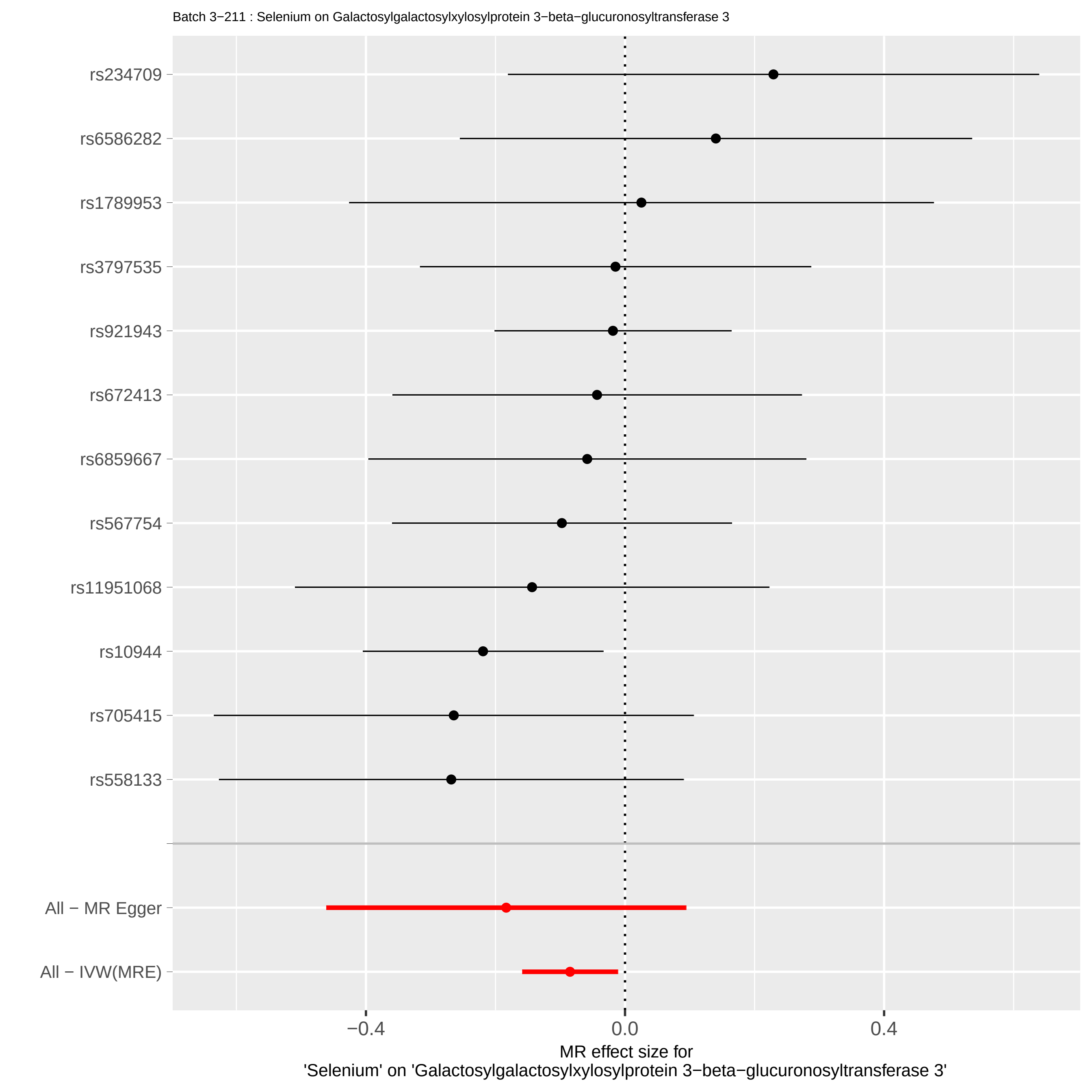
Batch 3−211 : Selenium on Galactosylgalactosylxylosylprotein 3−beta−glucuronosyltransferase 3
rs234709
rs6586282
rs1789953
rs3797535
rs921943
rs672413
rs6859667
rs567754
rs11951068
rs10944
rs705415
rs558133
All − MR Egger
All − IVW(MRE)
−0.4
0.0
0.4
MR effect size for
'Selenium' on 'Galactosylgalactosylxylosylprotein 3−beta−glucuronosyltransferase 3'

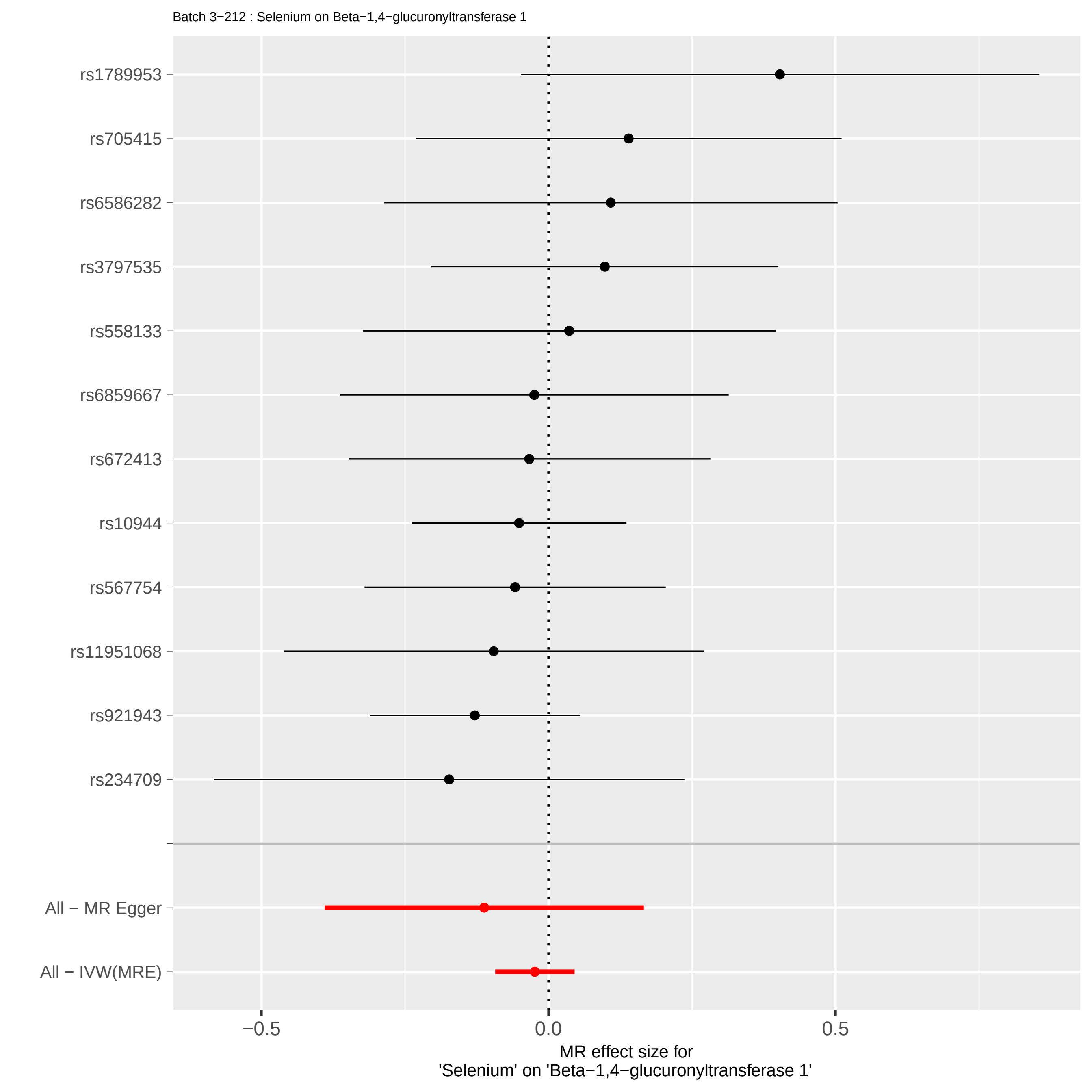

Batch 3−212 : Selenium on Beta−1,4−glucuronyltransferase 1
rs1789953
rs705415
rs6586282
rs3797535
rs558133
rs6859667
rs672413
rs10944
rs567754
rs11951068
rs921943
rs234709
All − MR Egger
All − IVW(MRE)
−0.5
0.0
0.5
MR effect size for
'Selenium' on 'Beta−1,4−glucuronyltransferase 1'

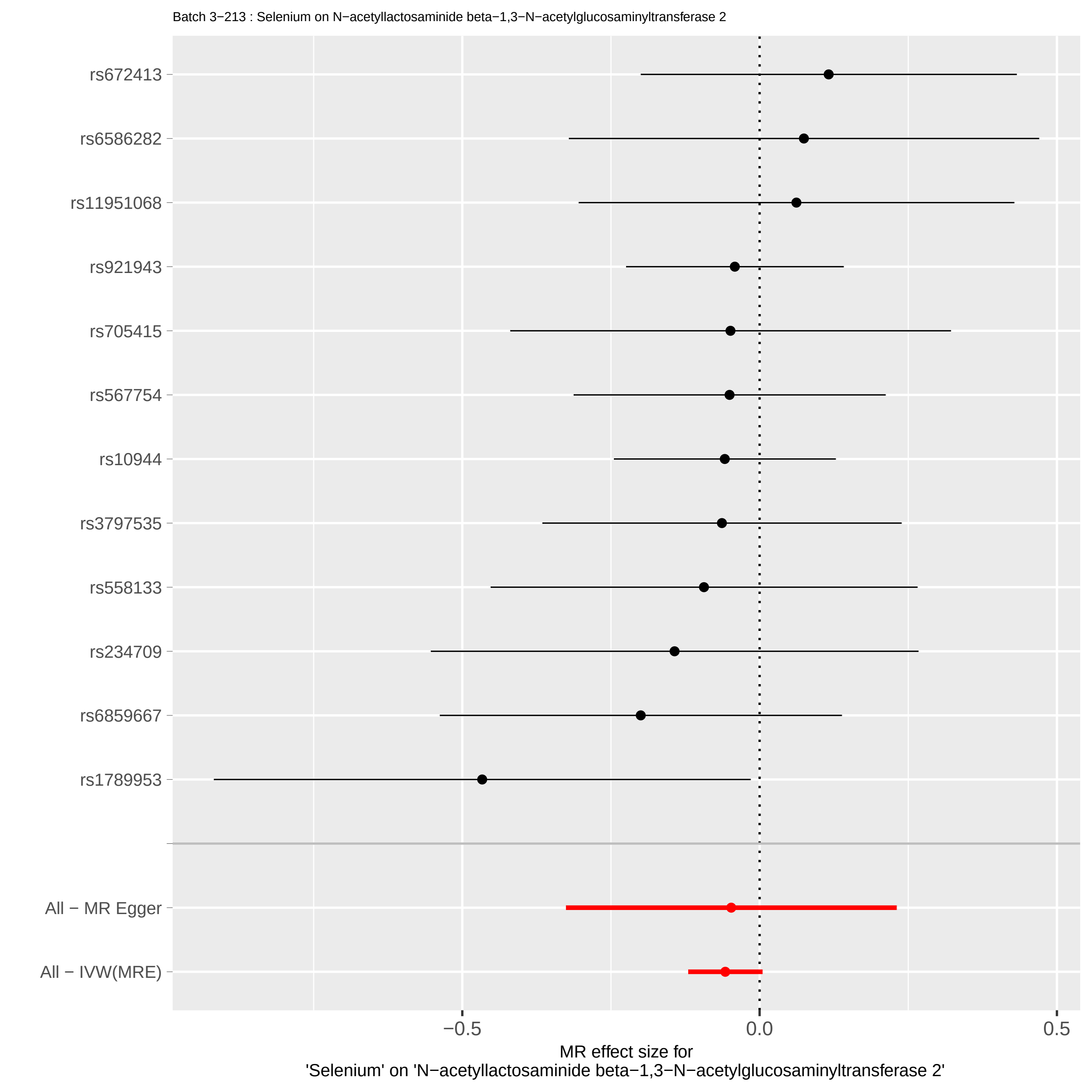
Batch 3−213 : Selenium on N−acetyllactosaminide beta−1,3−N−acetylglucosaminyltransferase 2
rs672413
rs6586282
rs11951068
rs921943
rs705415
rs567754
rs10944
rs3797535
rs558133
rs234709
rs6859667
rs1789953
All − MR Egger
All − IVW(MRE)
−0.5
0.0
0.5
MR effect size for
'Selenium' on 'N−acetyllactosaminide beta−1,3−N−acetylglucosaminyltransferase 2'

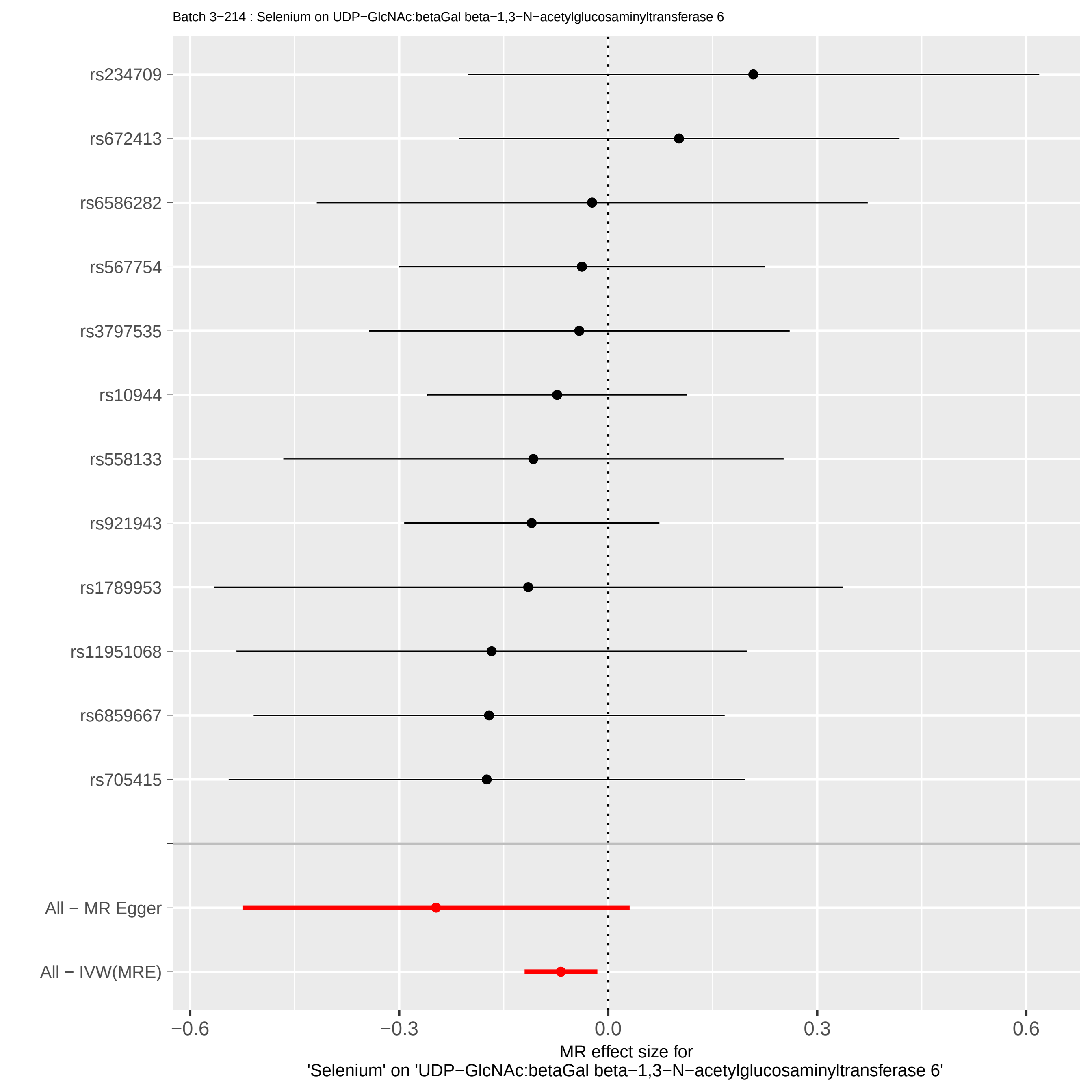

Batch 3−214 : Selenium on UDP−GlcNAc:betaGal beta−1,3−N−acetylglucosaminyltransferase 6
rs234709
rs672413
rs6586282
rs567754
rs3797535
rs10944
rs558133
rs921943
rs1789953
rs11951068
rs6859667
rs705415
All − MR Egger
All − IVW(MRE)
−0.6
−0.3
0.0
0.3
0.6
MR effect size for
'Selenium' on 'UDP−GlcNAc:betaGal beta−1,3−N−acetylglucosaminyltransferase 6'

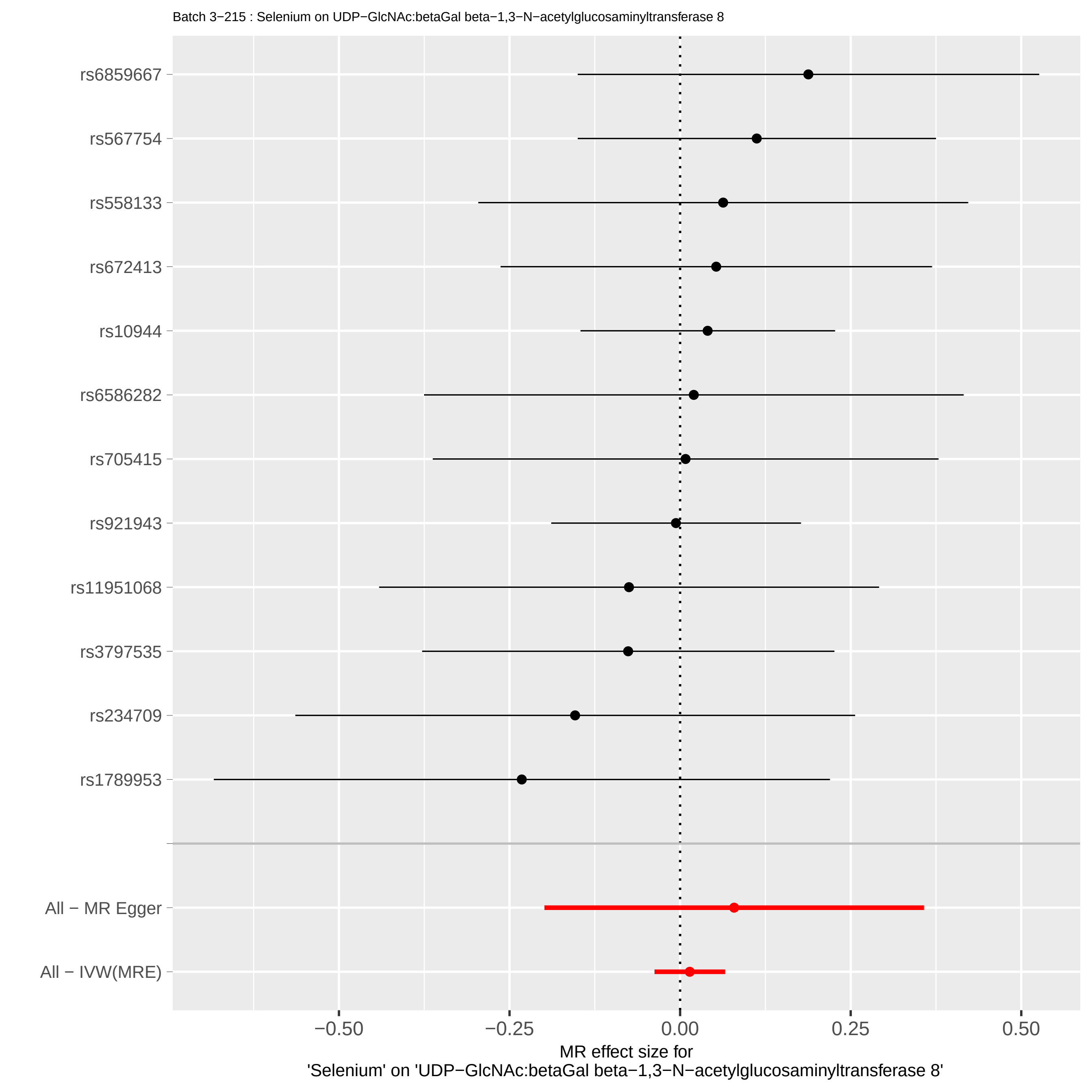

Batch 3−215 : Selenium on UDP−GlcNAc:betaGal beta−1,3−N−acetylglucosaminyltransferase 8
rs6859667
rs567754
rs558133
rs672413
rs10944
rs6586282
rs705415
rs921943
rs11951068
rs3797535
rs234709
rs1789953
All − MR Egger
All − IVW(MRE)
−0.50
−0.25
0.00
0.25
0.50
MR effect size for
'Selenium' on 'UDP−GlcNAc:betaGal beta−1,3−N−acetylglucosaminyltransferase 8'

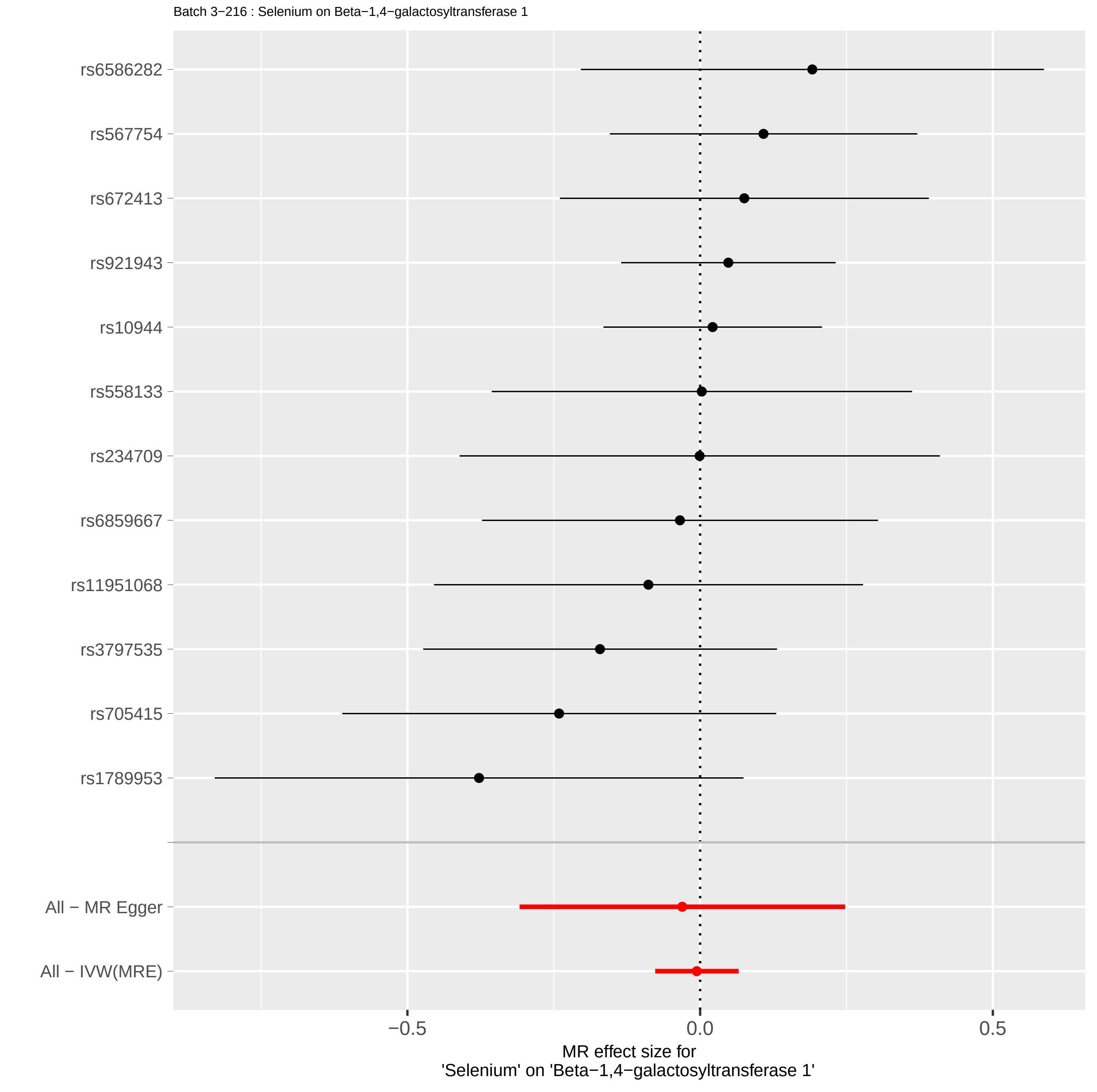

Batch 3−216 : Selenium on Beta−1,4−galactosyltransferase 1
rs6586282
rs567754
rs672413
rs921943
rs10944
rs558133
rs234709
rs6859667
rs11951068
rs3797535
rs705415
rs1789953
All − MR Egger
All − IVW(MRE)
−0.5
0.0
0.5
MR effect size for
'Selenium' on 'Beta−1,4−galactosyltransferase 1'

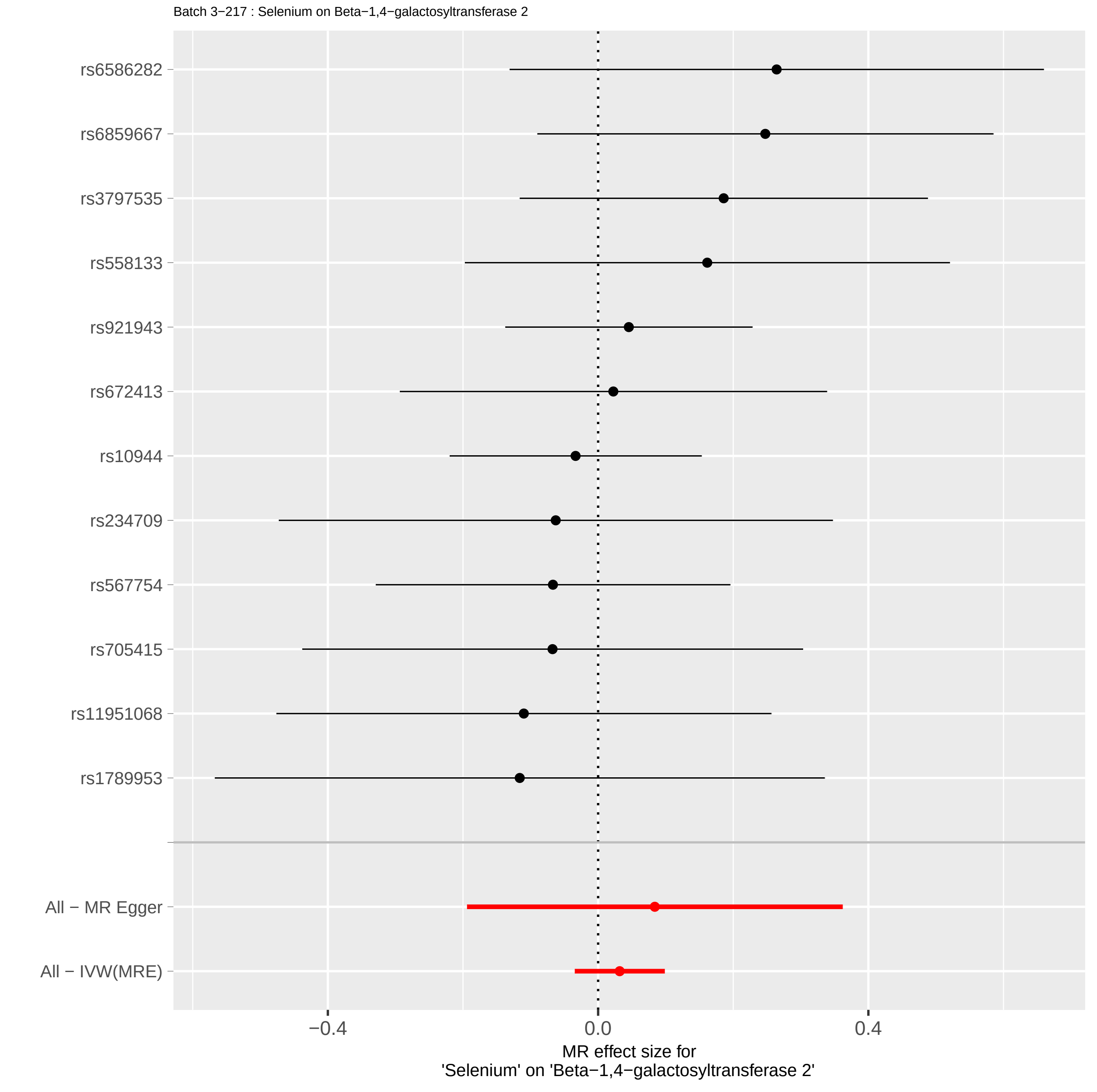

Batch 3−217 : Selenium on Beta−1,4−galactosyltransferase 2
rs6586282
rs6859667
rs3797535
rs558133
rs921943
rs672413
rs10944
rs234709
rs567754
rs705415
rs11951068
rs1789953
All − MR Egger
All − IVW(MRE)
−0.4
0.0
0.4
MR effect size for
'Selenium' on 'Beta−1,4−galactosyltransferase 2'

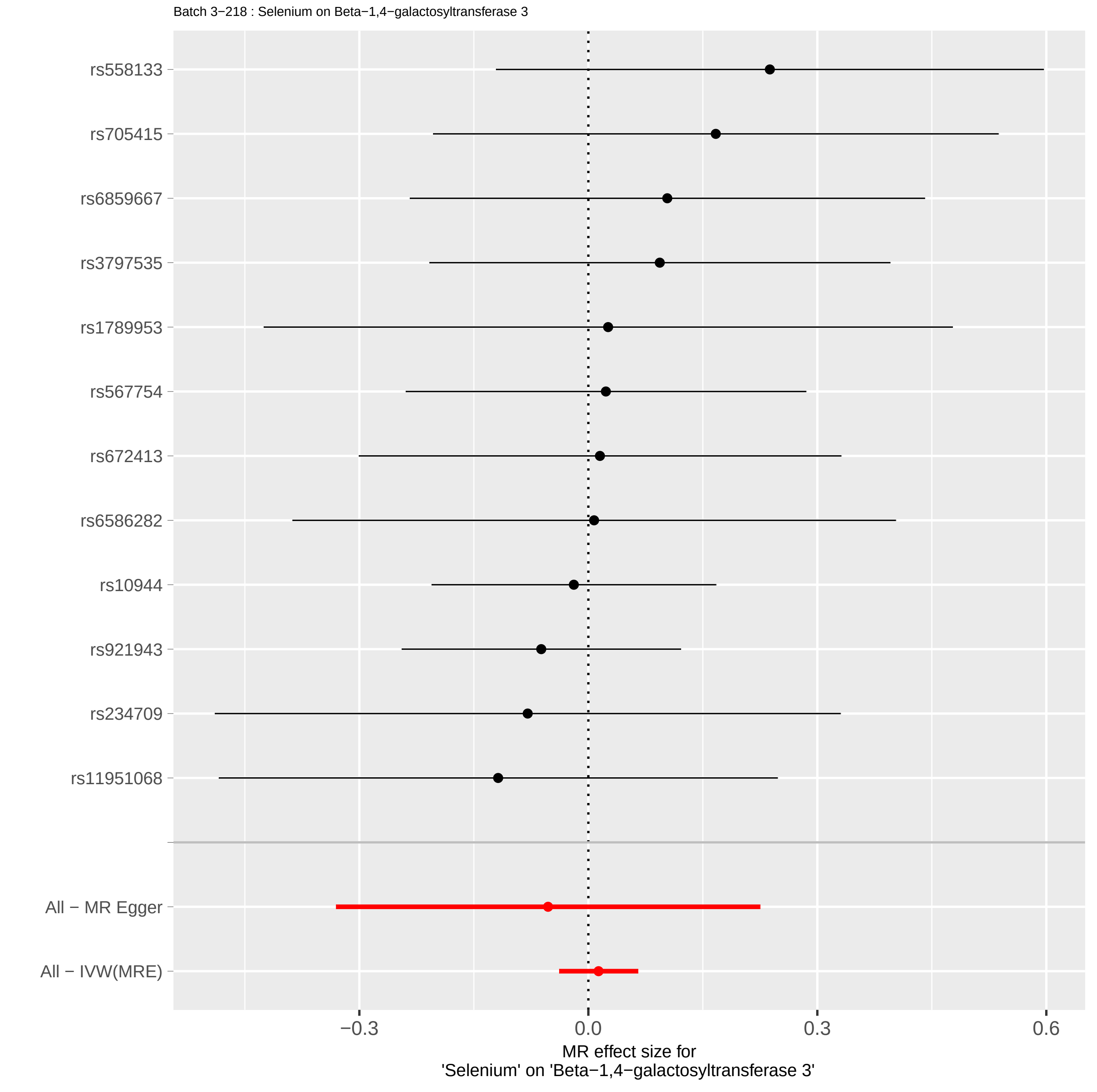
Batch 3−218 : Selenium on Beta−1,4−galactosyltransferase 3
rs558133
rs705415
rs6859667
rs3797535
rs1789953
rs567754
rs672413
rs6586282
rs10944
rs921943
rs234709
rs11951068
All − MR Egger
All − IVW(MRE)
−0.3
0.0
0.3
0.6
MR effect size for
'Selenium' on 'Beta−1,4−galactosyltransferase 3'

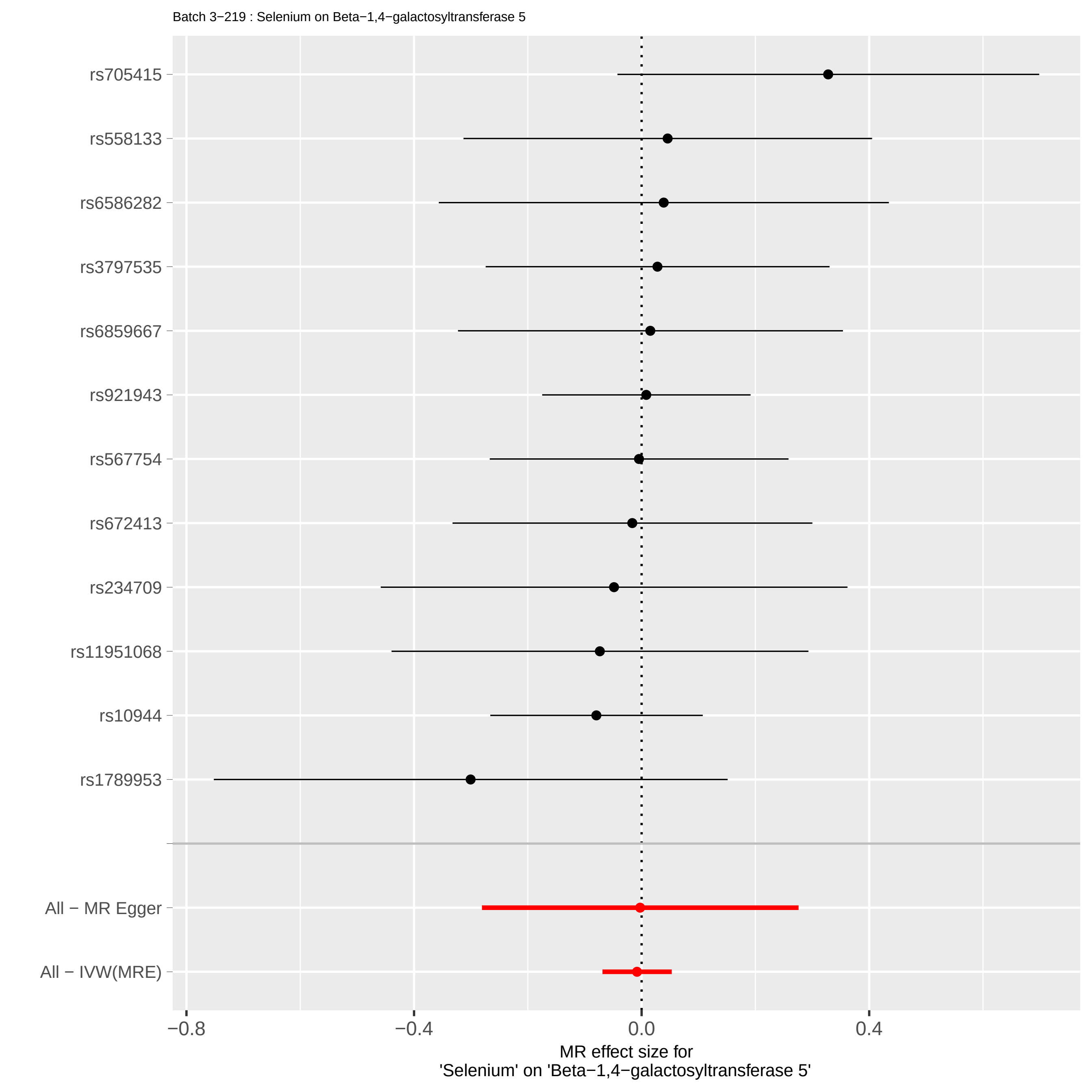
Batch 3−219 : Selenium on Beta−1,4−galactosyltransferase 5
rs705415
rs558133
rs6586282
rs3797535
rs6859667
rs921943
rs567754
rs672413
rs234709
rs11951068
rs10944
rs1789953
All − MR Egger
All − IVW(MRE)
−0.8
−0.4
0.0
0.4
MR effect size for
'Selenium' on 'Beta−1,4−galactosyltransferase 5'

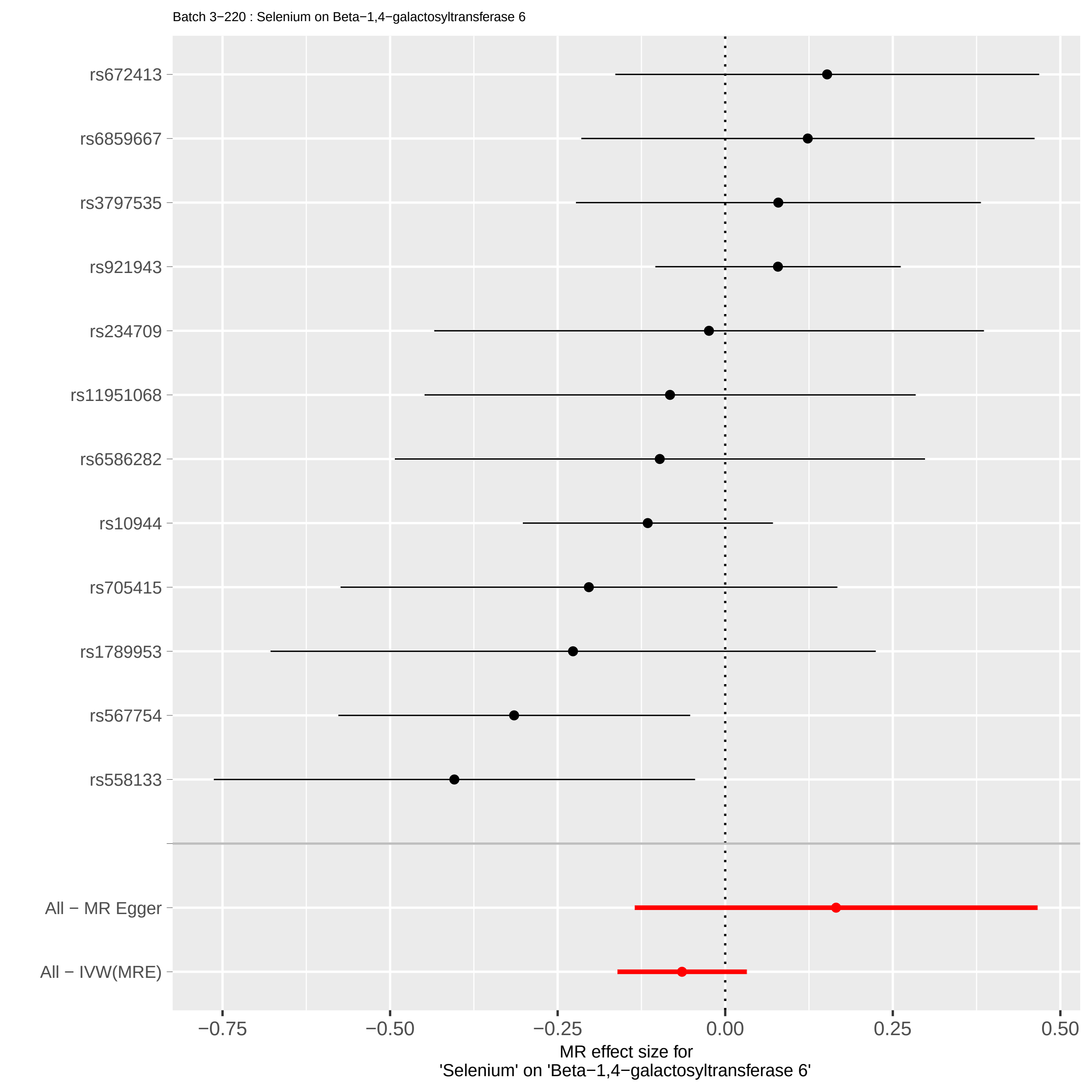

Batch 3−220 : Selenium on Beta−1,4−galactosyltransferase 6
rs672413
rs6859667
rs3797535
rs921943
rs234709
rs11951068
rs6586282
rs10944
rs705415
rs1789953
rs567754
rs558133
All − MR Egger
All − IVW(MRE)
−0.75
−0.50
−0.25
0.00
0.25
0.50
MR effect size for
'Selenium' on 'Beta−1,4−galactosyltransferase 6'

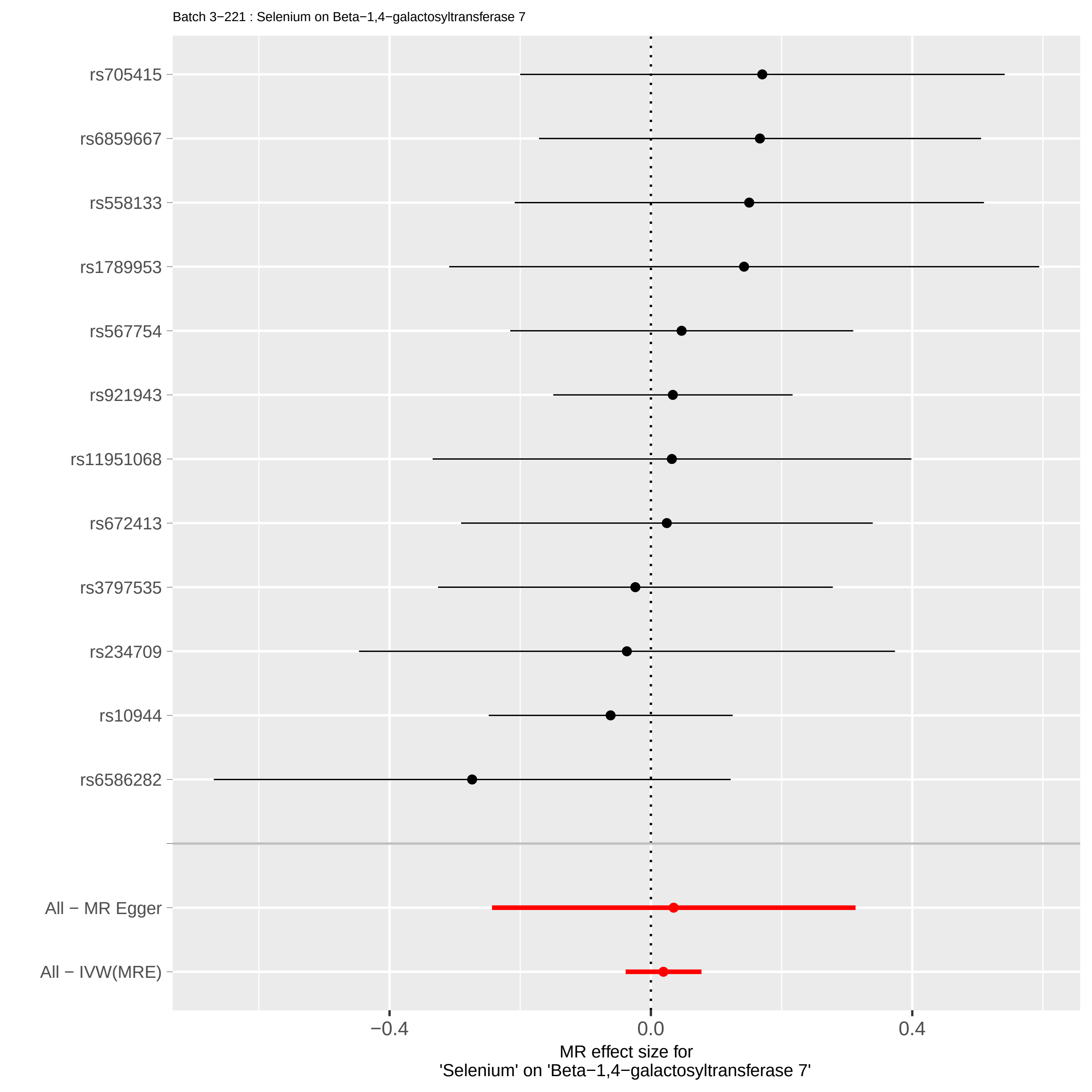

Batch 3−221 : Selenium on Beta−1,4−galactosyltransferase 7
rs705415
rs6859667
rs558133
rs1789953
rs567754
rs921943
rs11951068
rs672413
rs3797535
rs234709
rs10944
rs6586282
All − MR Egger
All − IVW(MRE)
−0.4
0.0
0.4
MR effect size for
'Selenium' on 'Beta−1,4−galactosyltransferase 7'

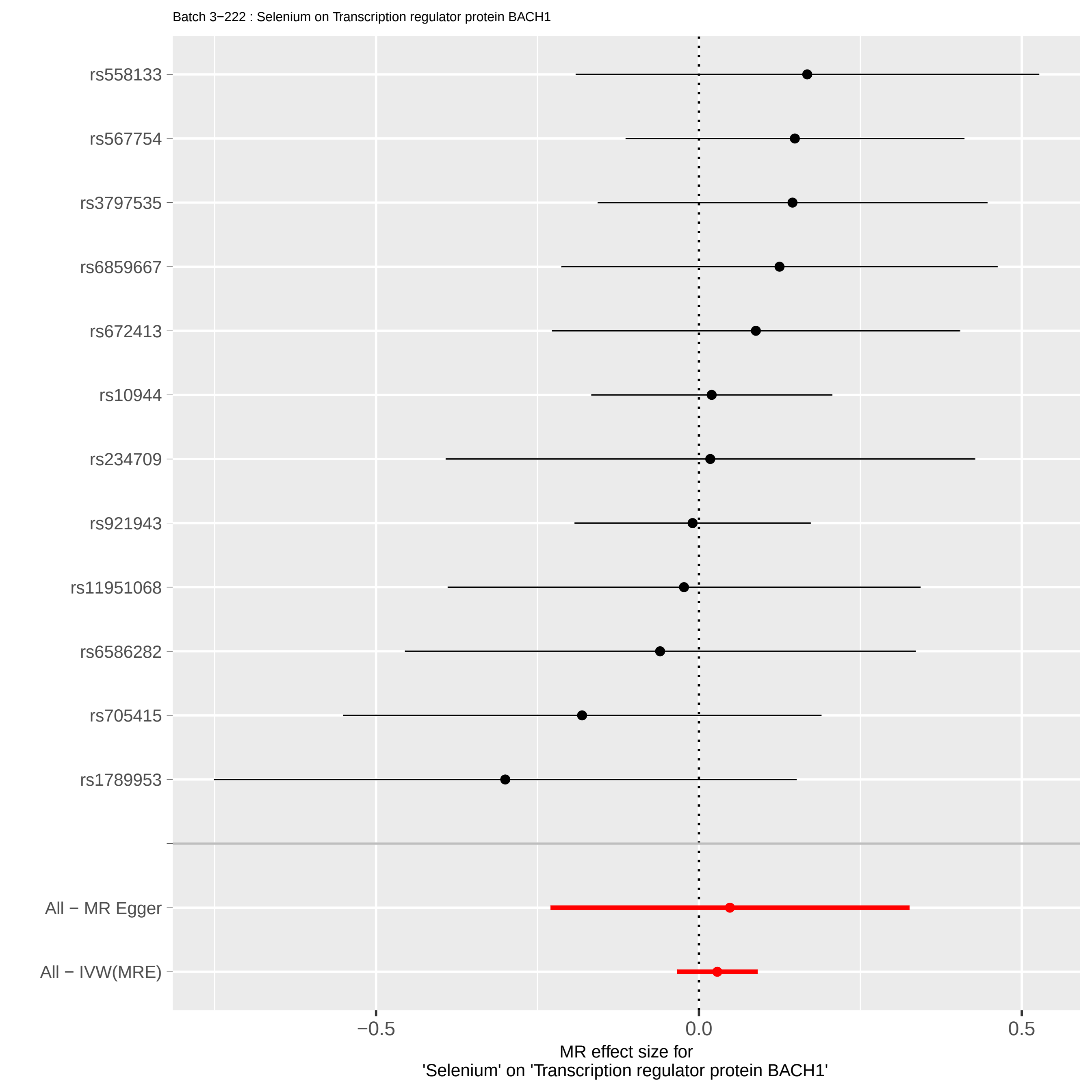
Batch 3−222 : Selenium on Transcription regulator protein BACH1
rs558133
rs567754
rs3797535
rs6859667
rs672413
rs10944
rs234709
rs921943
rs11951068
rs6586282
rs705415
rs1789953
All − MR Egger
All − IVW(MRE)
−0.5
0.0
0.5
MR effect size for
'Selenium' on 'Transcription regulator protein BACH1'

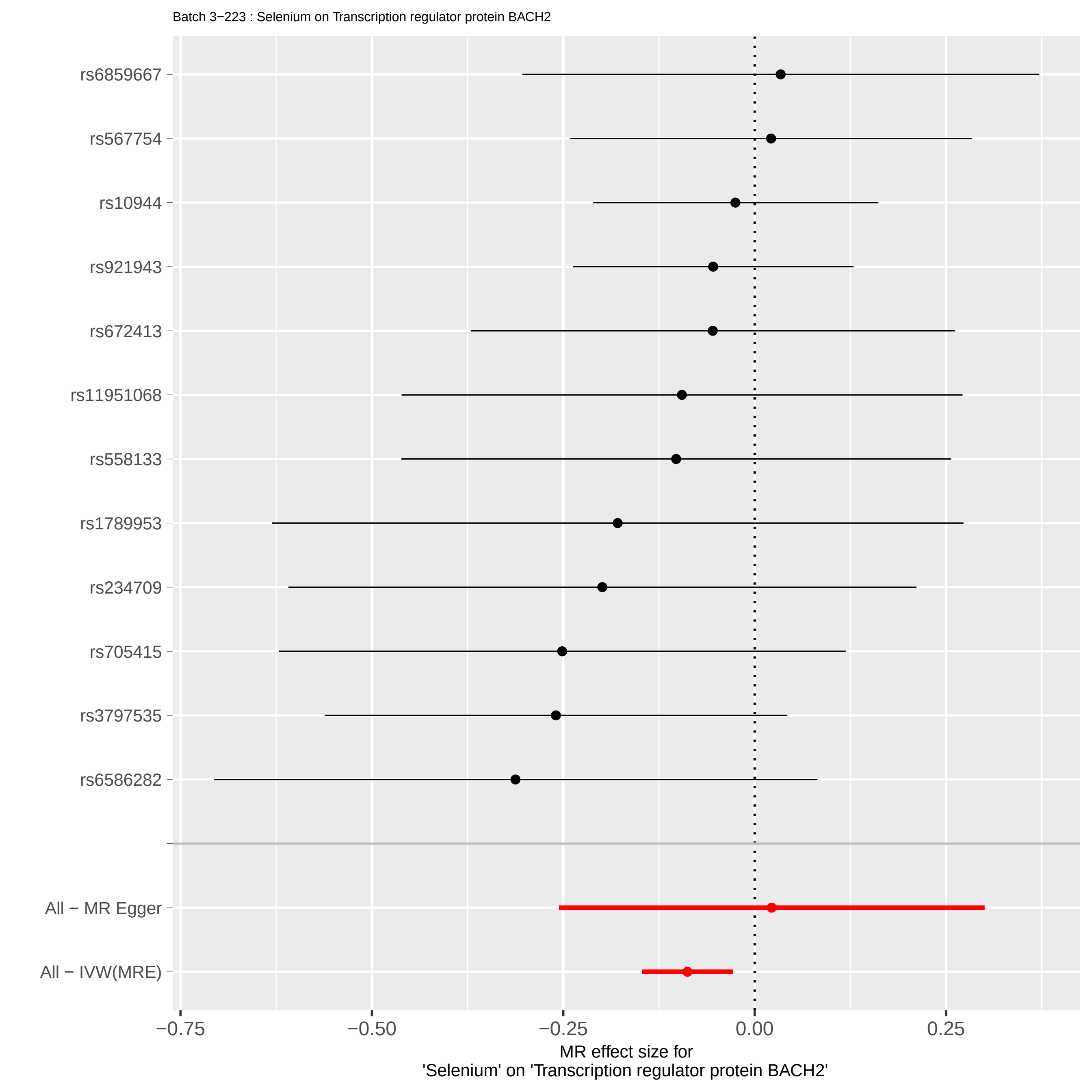

Batch 3−223 : Selenium on Transcription regulator protein BACH2
rs6859667
rs567754
rs10944
rs921943
rs672413
rs11951068
rs558133
rs1789953
rs234709
rs705415
rs3797535
rs6586282
All − MR Egger
All − IVW(MRE)
−0.75
−0.50
−0.25
0.00
0.25
MR effect size for
'Selenium' on 'Transcription regulator protein BACH2'

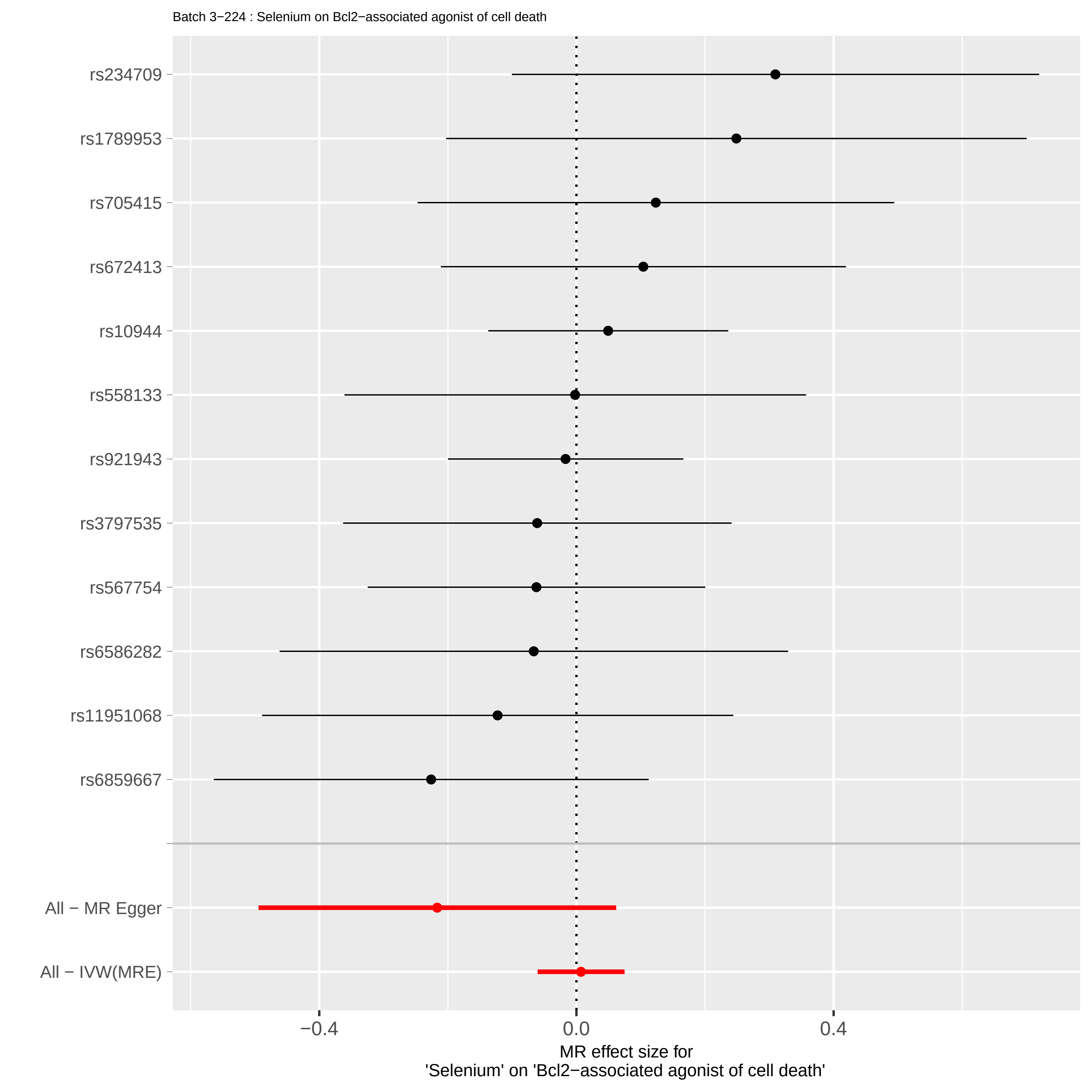
Batch 3−224 : Selenium on Bcl2−associated agonist of cell death
rs234709
rs1789953
rs705415
rs672413
rs10944
rs558133
rs921943
rs3797535
rs567754
rs6586282
rs11951068
rs6859667
All − MR Egger
All − IVW(MRE)
−0.4
0.0
0.4
MR effect size for
'Selenium' on 'Bcl2−associated agonist of cell death'

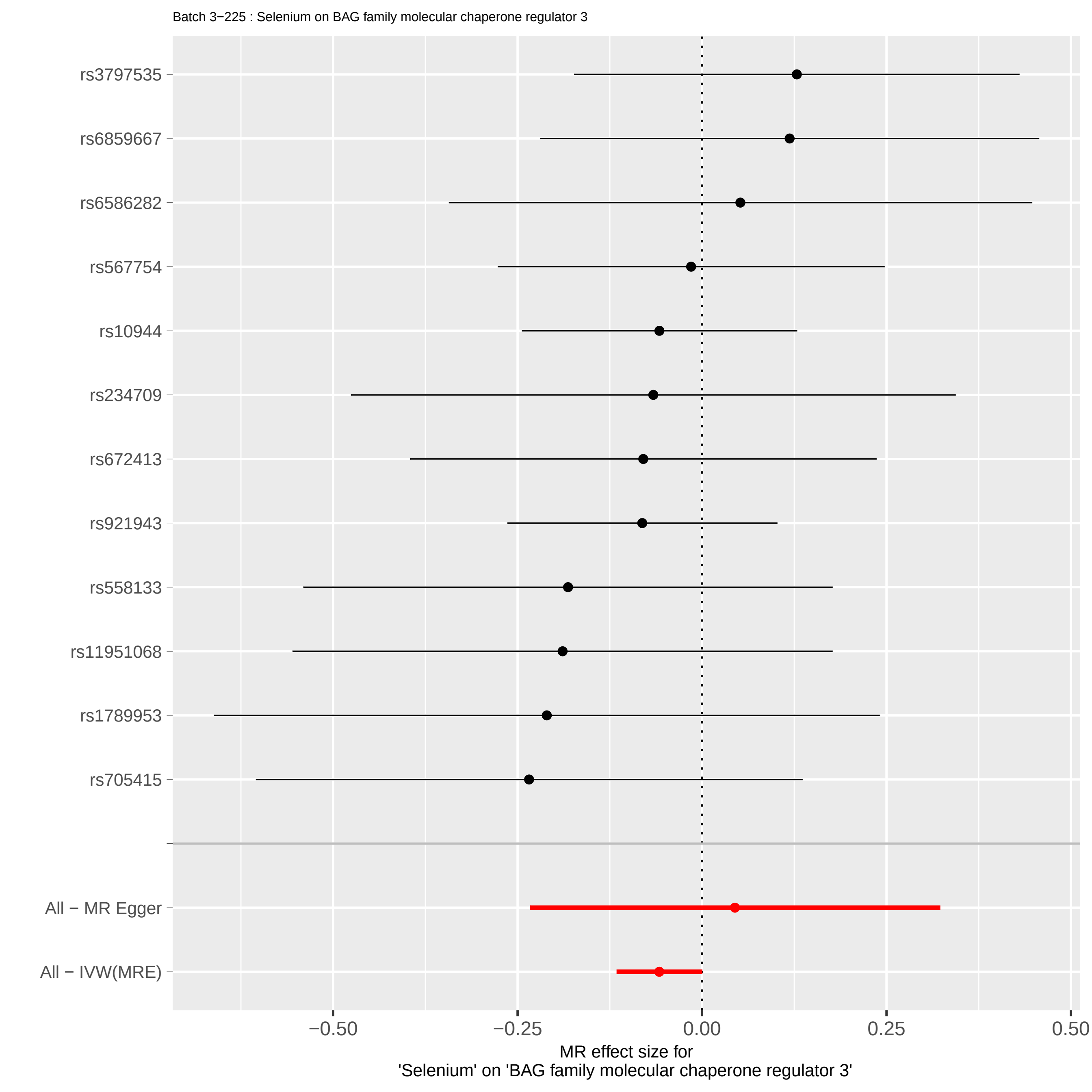

Batch 3−225 : Selenium on BAG family molecular chaperone regulator 3
rs3797535
rs6859667
rs6586282
rs567754
rs10944
rs234709
rs672413
rs921943
rs558133
rs11951068
rs1789953
rs705415
All − MR Egger
All − IVW(MRE)
−0.50
−0.25
0.00
0.25
0.50
MR effect size for
'Selenium' on 'BAG family molecular chaperone regulator 3'

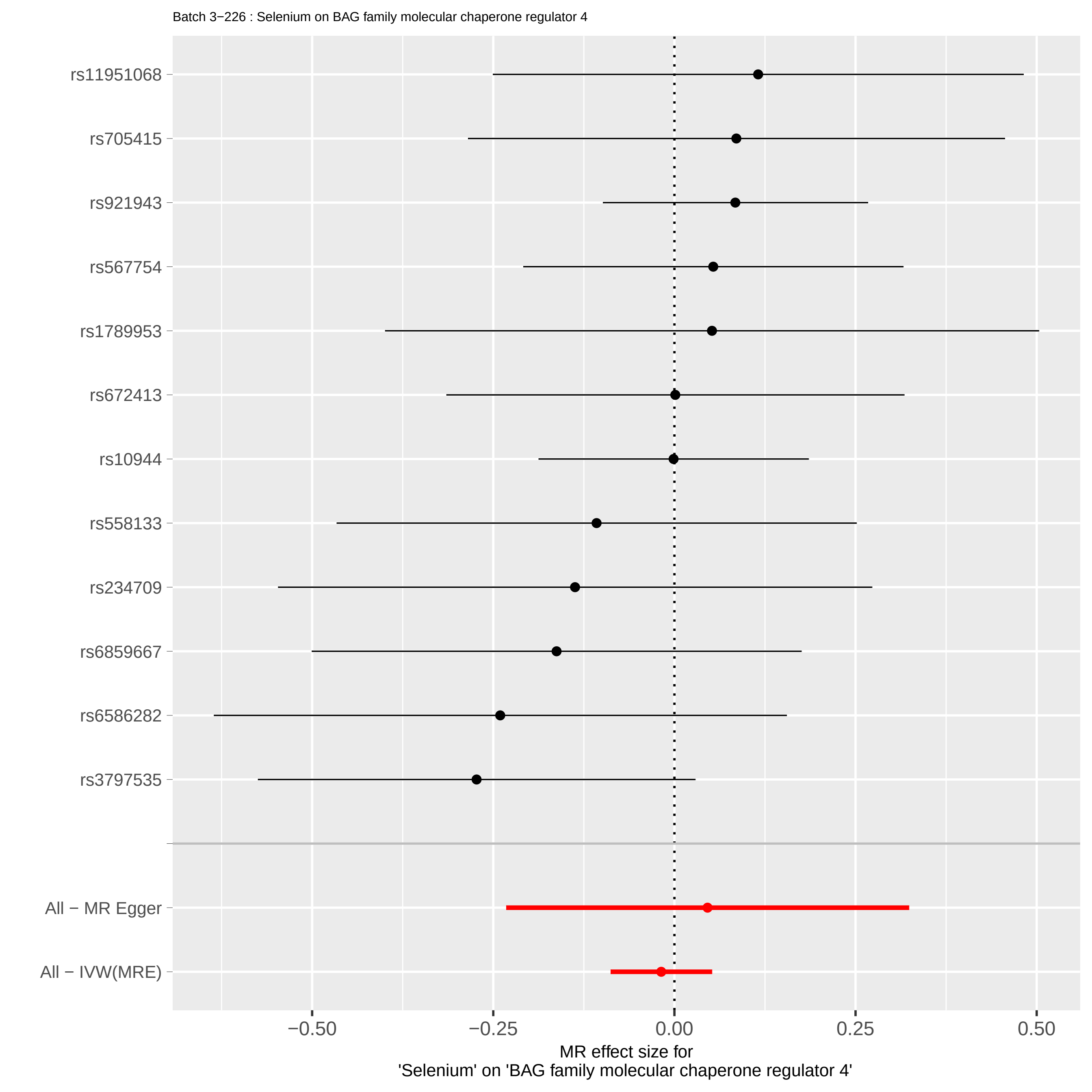
Batch 3−226 : Selenium on BAG family molecular chaperone regulator 4
rs11951068
rs705415
rs921943
rs567754
rs1789953
rs672413
rs10944
rs558133
rs234709
rs6859667
rs6586282
rs3797535
All − MR Egger
All − IVW(MRE)
−0.50
−0.25
0.00
0.25
0.50
MR effect size for
'Selenium' on 'BAG family molecular chaperone regulator 4'

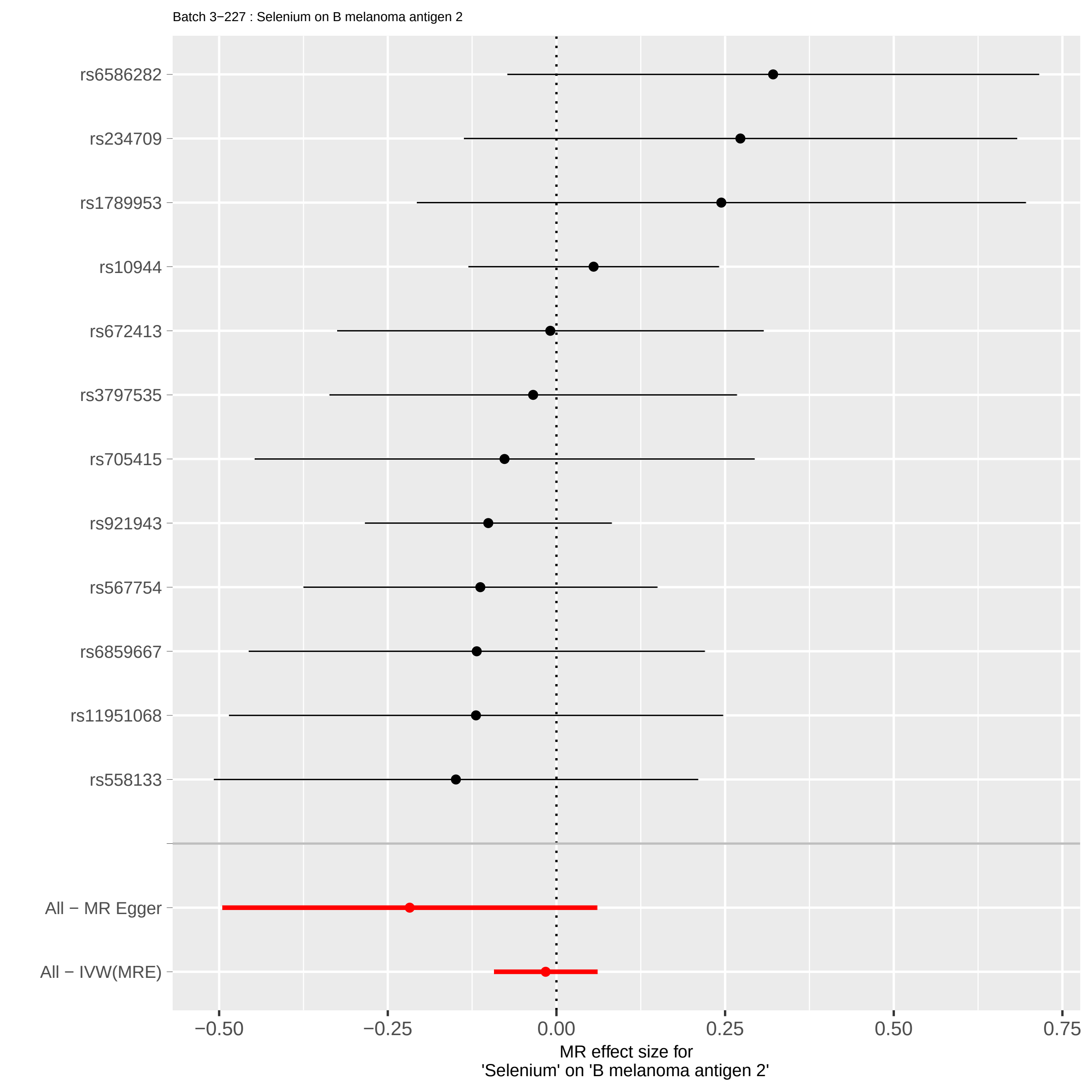

Batch 3−227 : Selenium on B melanoma antigen 2
rs6586282
rs234709
rs1789953
rs10944
rs672413
rs3797535
rs705415
rs921943
rs567754
rs6859667
rs11951068
rs558133
All − MR Egger
All − IVW(MRE)
−0.50
−0.25
0.00
0.25
0.50
0.75
MR effect size for
'Selenium' on 'B melanoma antigen 2'

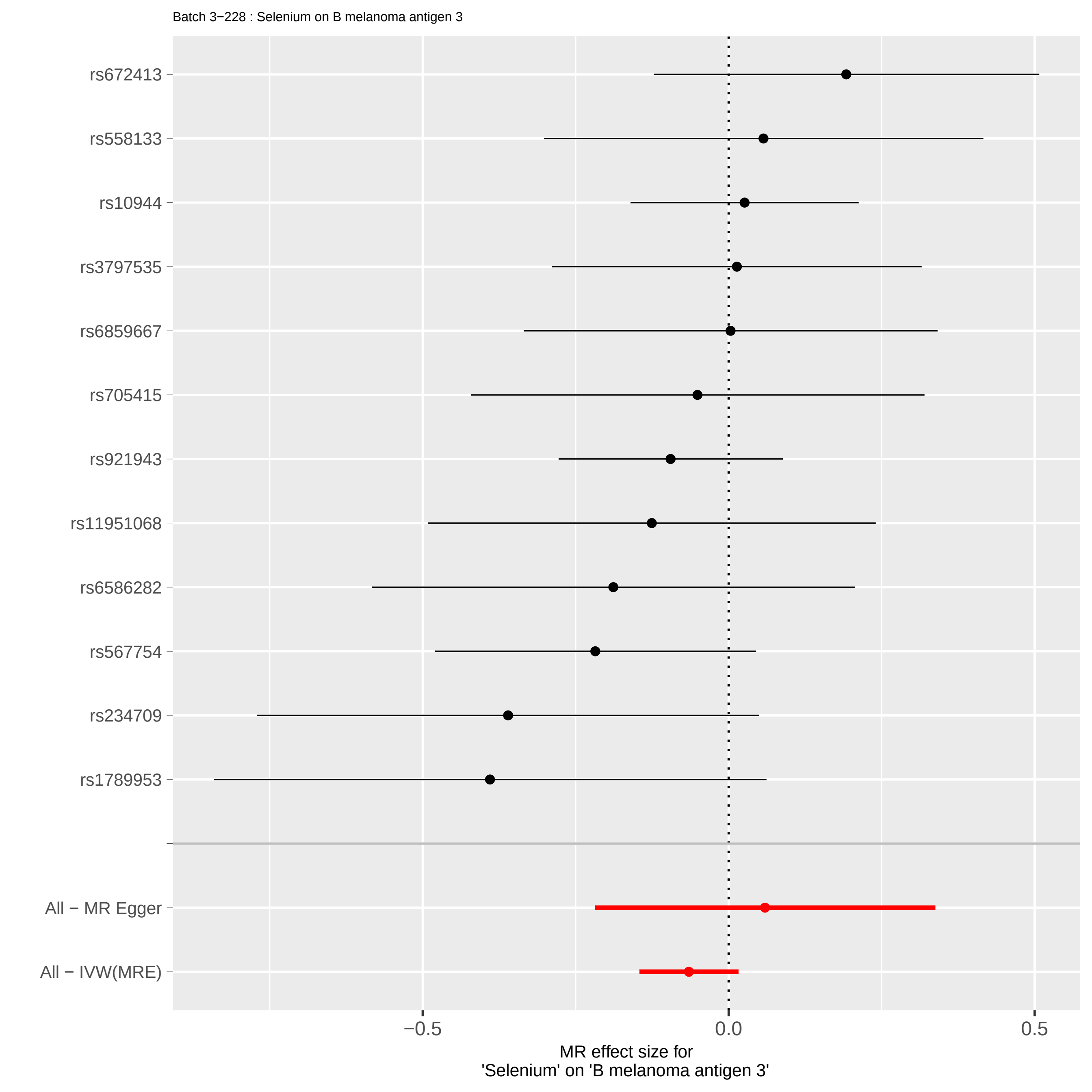
Batch 3−228 : Selenium on B melanoma antigen 3
rs672413
rs558133
rs10944
rs3797535
rs6859667
rs705415
rs921943
rs11951068
rs6586282
rs567754
rs234709
rs1789953
All − MR Egger
All − IVW(MRE)
−0.5
0.0
0.5
MR effect size for
'Selenium' on 'B melanoma antigen 3'

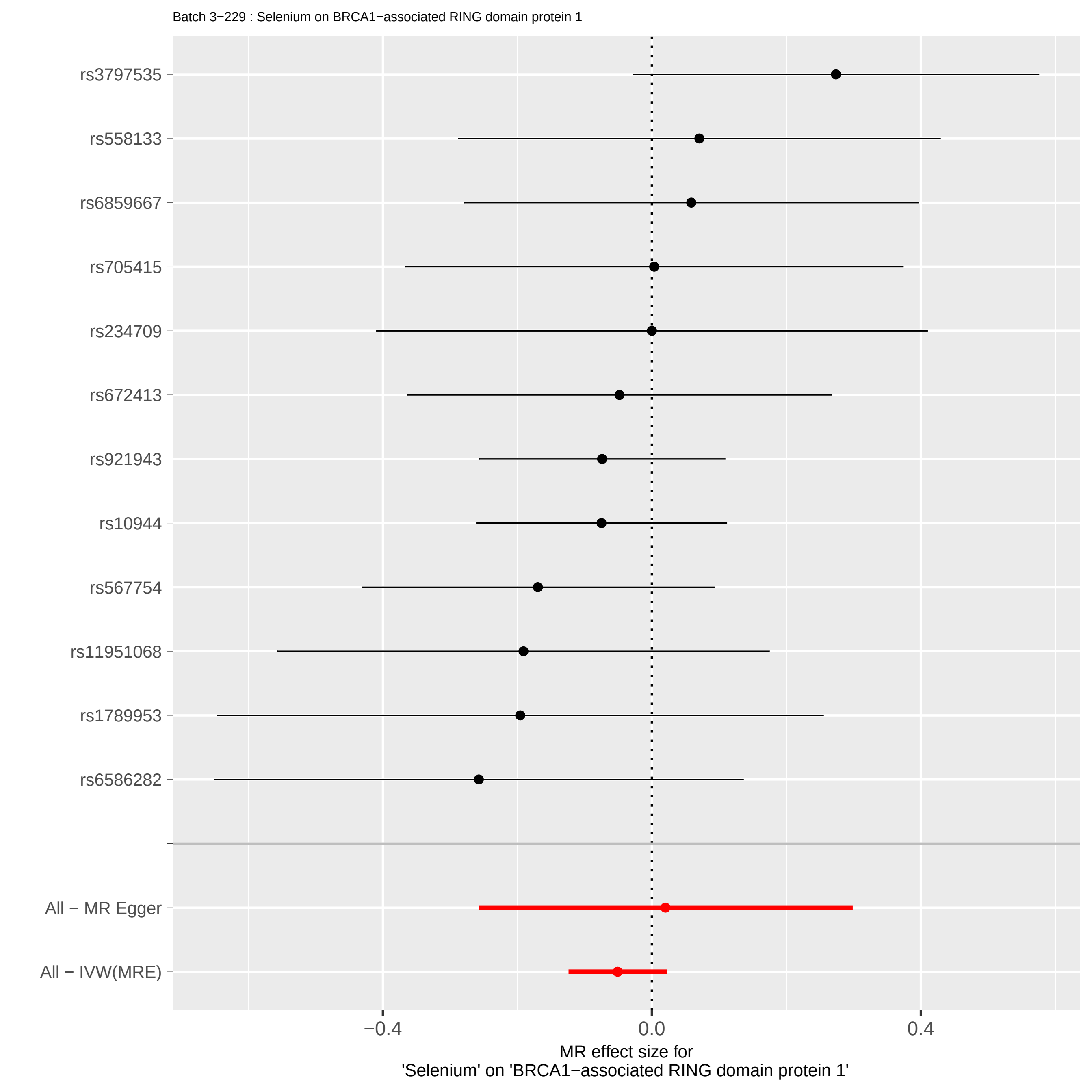

Batch 3−229 : Selenium on BRCA1−associated RING domain protein 1
rs3797535
rs558133
rs6859667
rs705415
rs234709
rs672413
rs921943
rs10944
rs567754
rs11951068
rs1789953
rs6586282
All − MR Egger
All − IVW(MRE)
−0.4
0.0
0.4
MR effect size for
'Selenium' on 'BRCA1−associated RING domain protein 1'

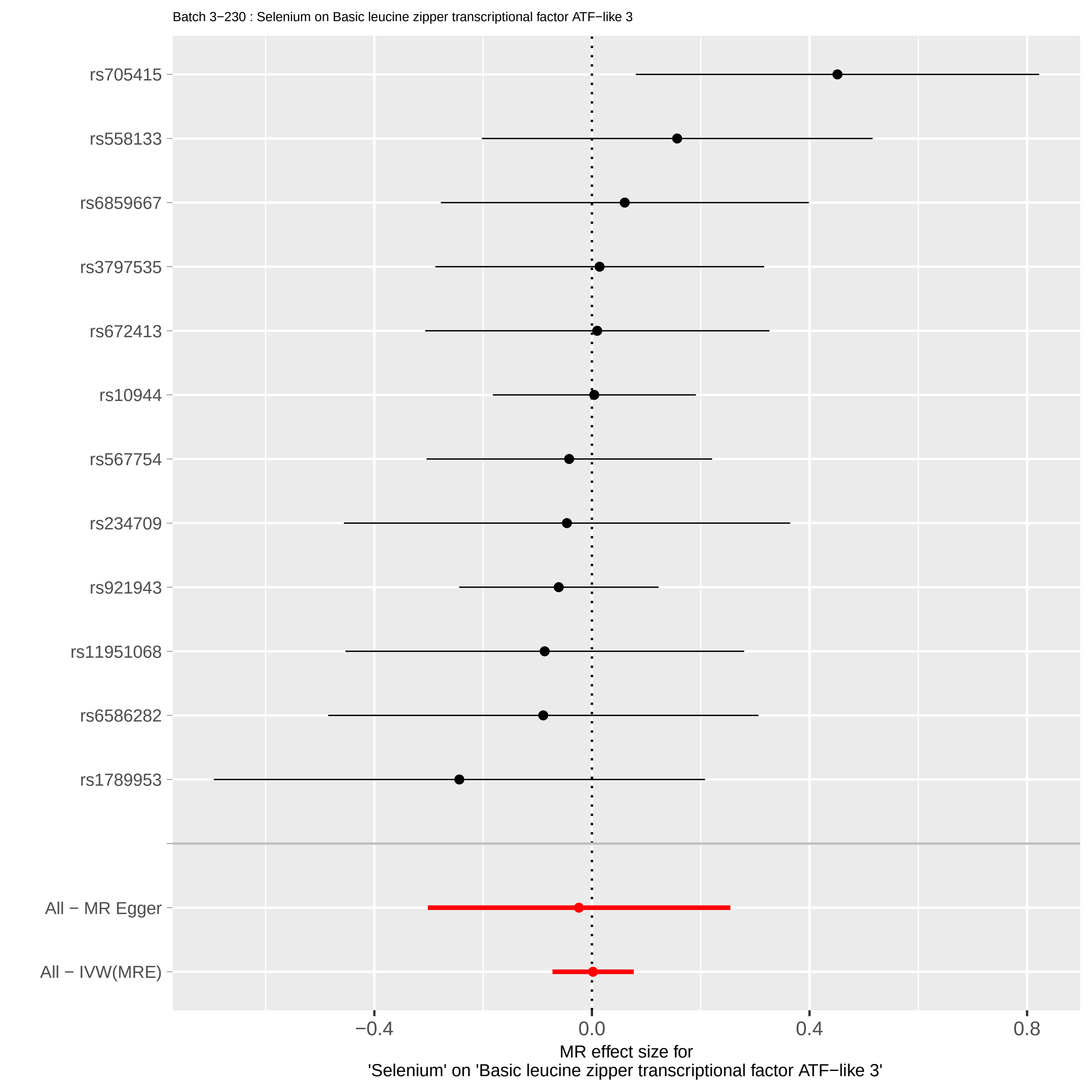
Batch 3−230 : Selenium on Basic leucine zipper transcriptional factor ATF−like 3
rs705415
rs558133
rs6859667
rs3797535
rs672413
rs10944
rs567754
rs234709
rs921943
rs11951068
rs6586282
rs1789953
All − MR Egger
All − IVW(MRE)
−0.4
0.0
0.4
0.8
MR effect size for
'Selenium' on 'Basic leucine zipper transcriptional factor ATF−like 3'

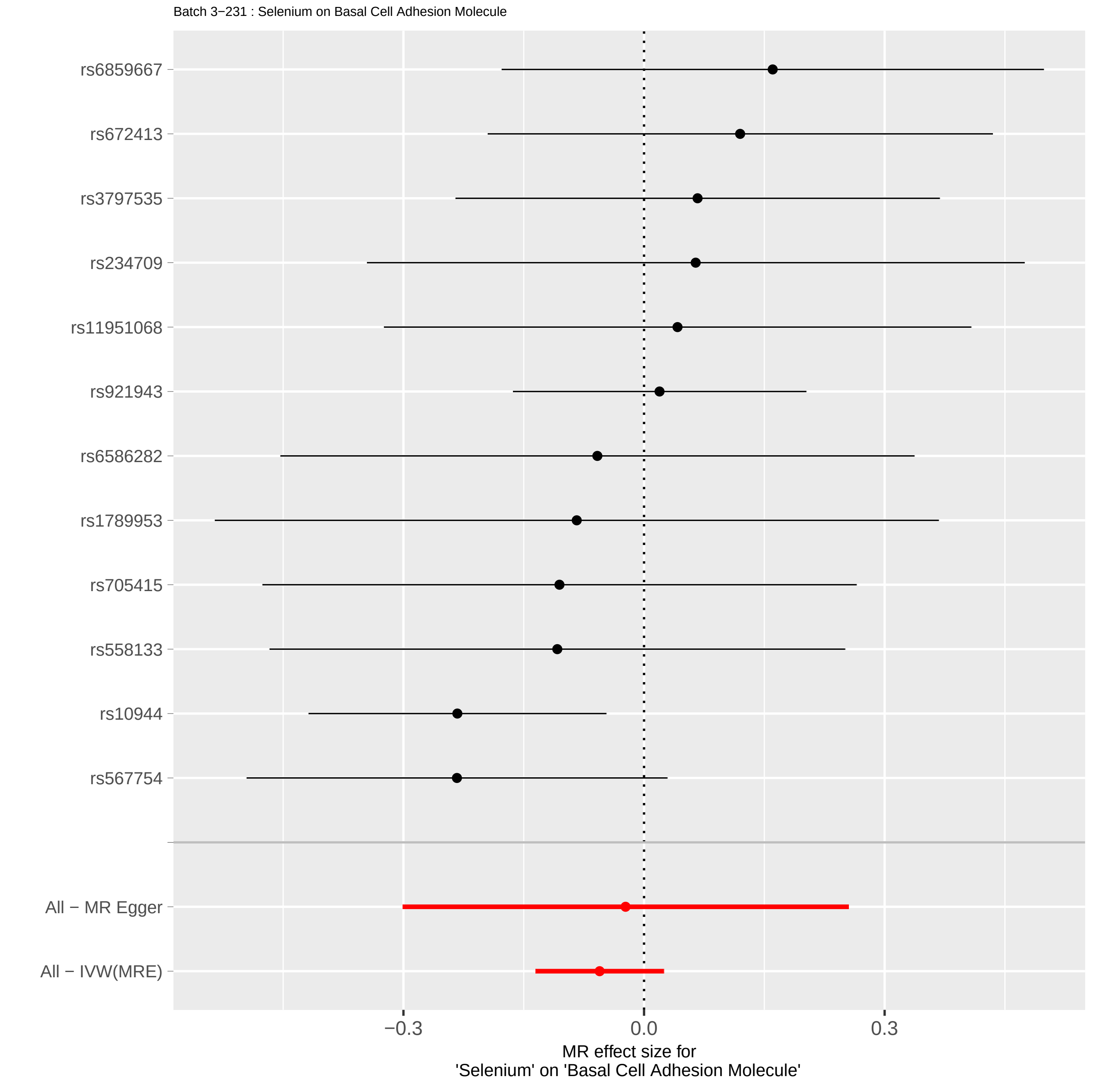

Batch 3−231 : Selenium on Basal Cell Adhesion Molecule
rs6859667
rs672413
rs3797535
rs234709
rs11951068
rs921943
rs6586282
rs1789953
rs705415
rs558133
rs10944
rs567754
All − MR Egger
All − IVW(MRE)
−0.3
0.0
0.3
MR effect size for
'Selenium' on 'Basal Cell Adhesion Molecule'

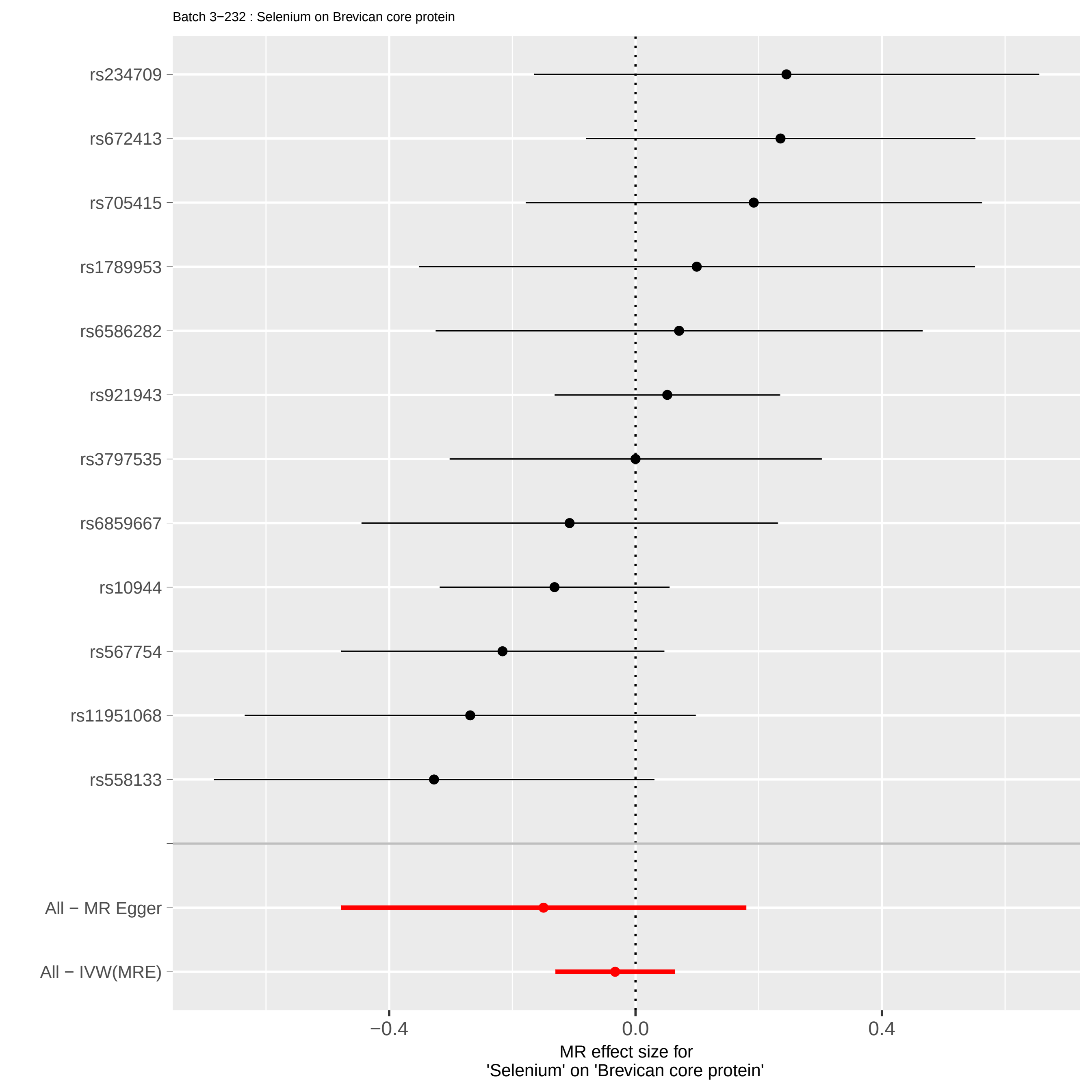
Batch 3−232 : Selenium on Brevican core protein
rs234709
rs672413
rs705415
rs1789953
rs6586282
rs921943
rs3797535
rs6859667
rs10944
rs567754
rs11951068
rs558133
All − MR Egger
All − IVW(MRE)
−0.4
0.0
0.4
MR effect size for
'Selenium' on 'Brevican core protein'

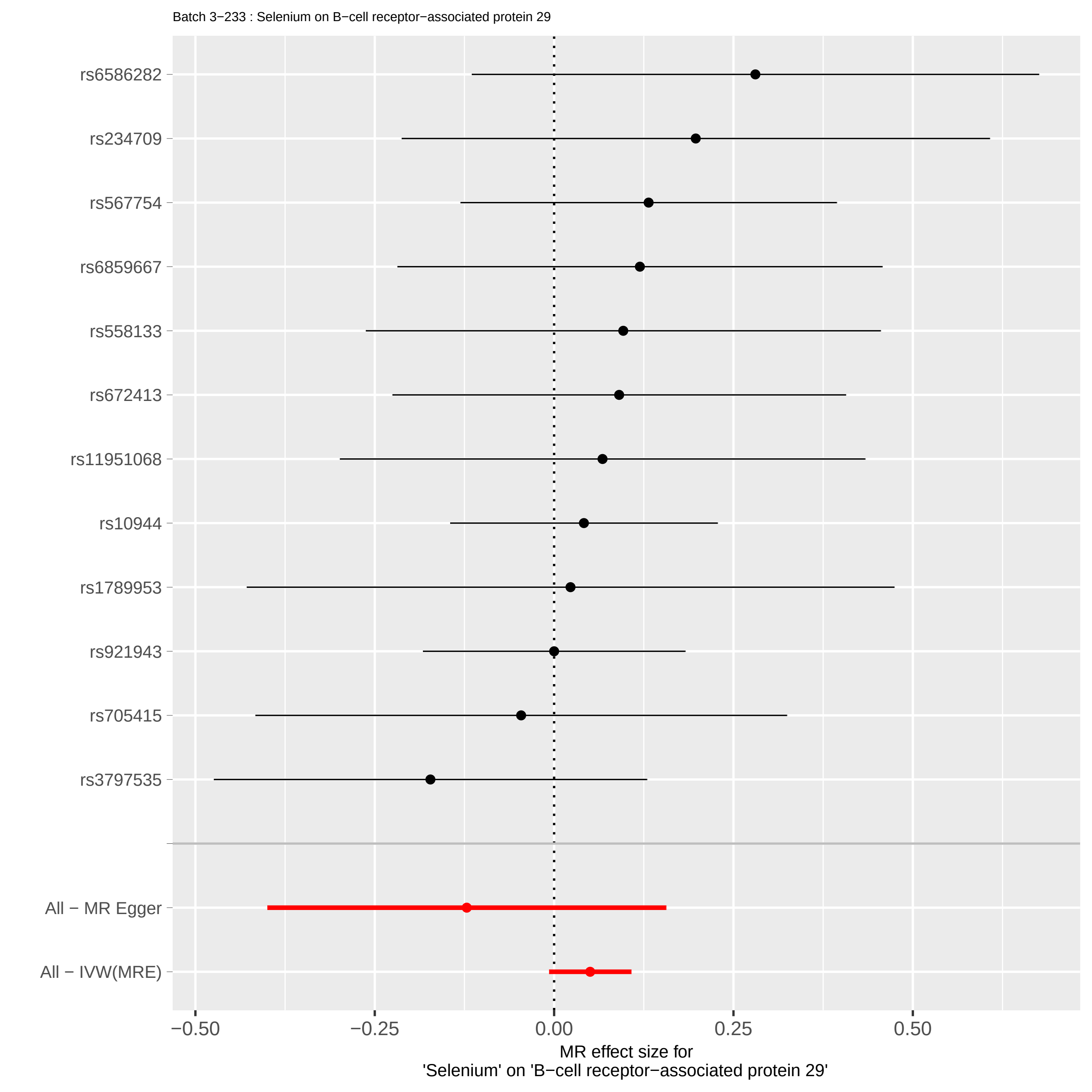

Batch 3−233 : Selenium on B−cell receptor−associated protein 29
rs6586282
rs234709
rs567754
rs6859667
rs558133
rs672413
rs11951068
rs10944
rs1789953
rs921943
rs705415
rs3797535
All − MR Egger
All − IVW(MRE)
−0.50
−0.25
0.00
0.25
0.50
MR effect size for
'Selenium' on 'B−cell receptor−associated protein 29'

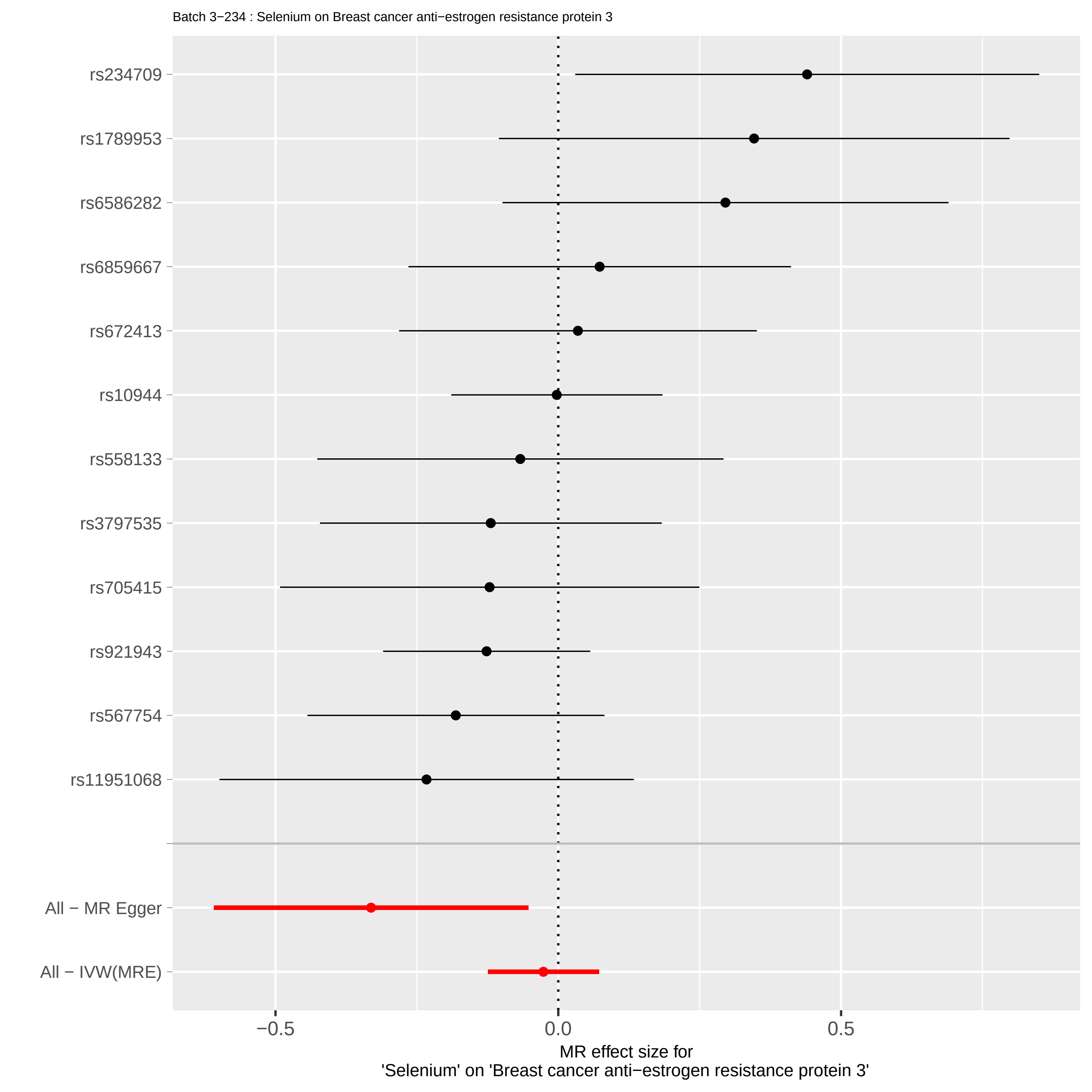
Batch 3−234 : Selenium on Breast cancer anti−estrogen resistance protein 3
rs234709
rs1789953
rs6586282
rs6859667
rs672413
rs10944
rs558133
rs3797535
rs705415
rs921943
rs567754
rs11951068
All − MR Egger
All − IVW(MRE)
−0.5
0.0
0.5
MR effect size for
'Selenium' on 'Breast cancer anti−estrogen resistance protein 3'

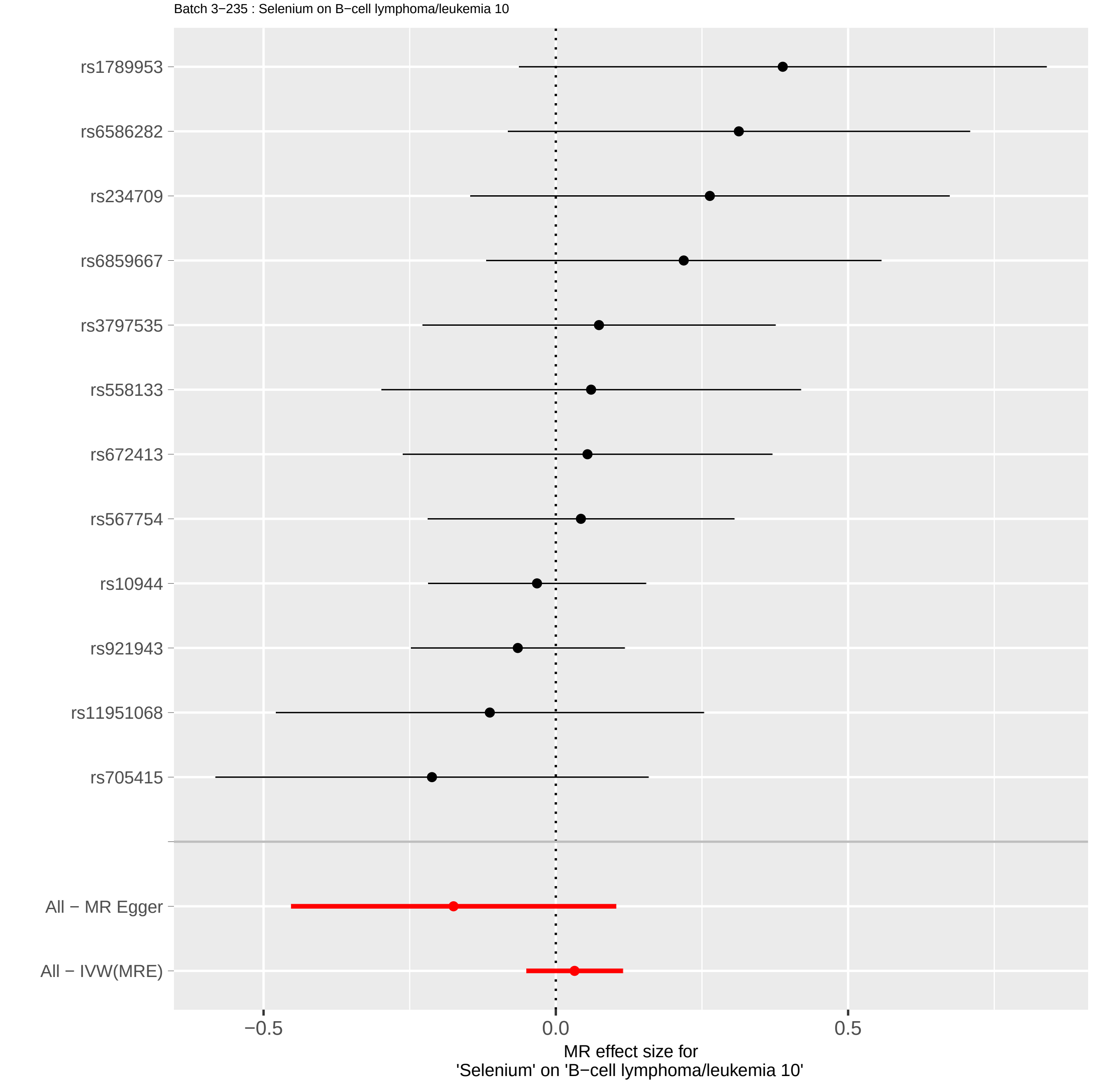

Batch 3−235 : Selenium on B−cell lymphoma/leukemia 10
rs1789953
rs6586282
rs234709
rs6859667
rs3797535
rs558133
rs672413
rs567754
rs10944
rs921943
rs11951068
rs705415
All − MR Egger
All − IVW(MRE)
−0.5
0.0
0.5
MR effect size for
'Selenium' on 'B−cell lymphoma/leukemia 10'

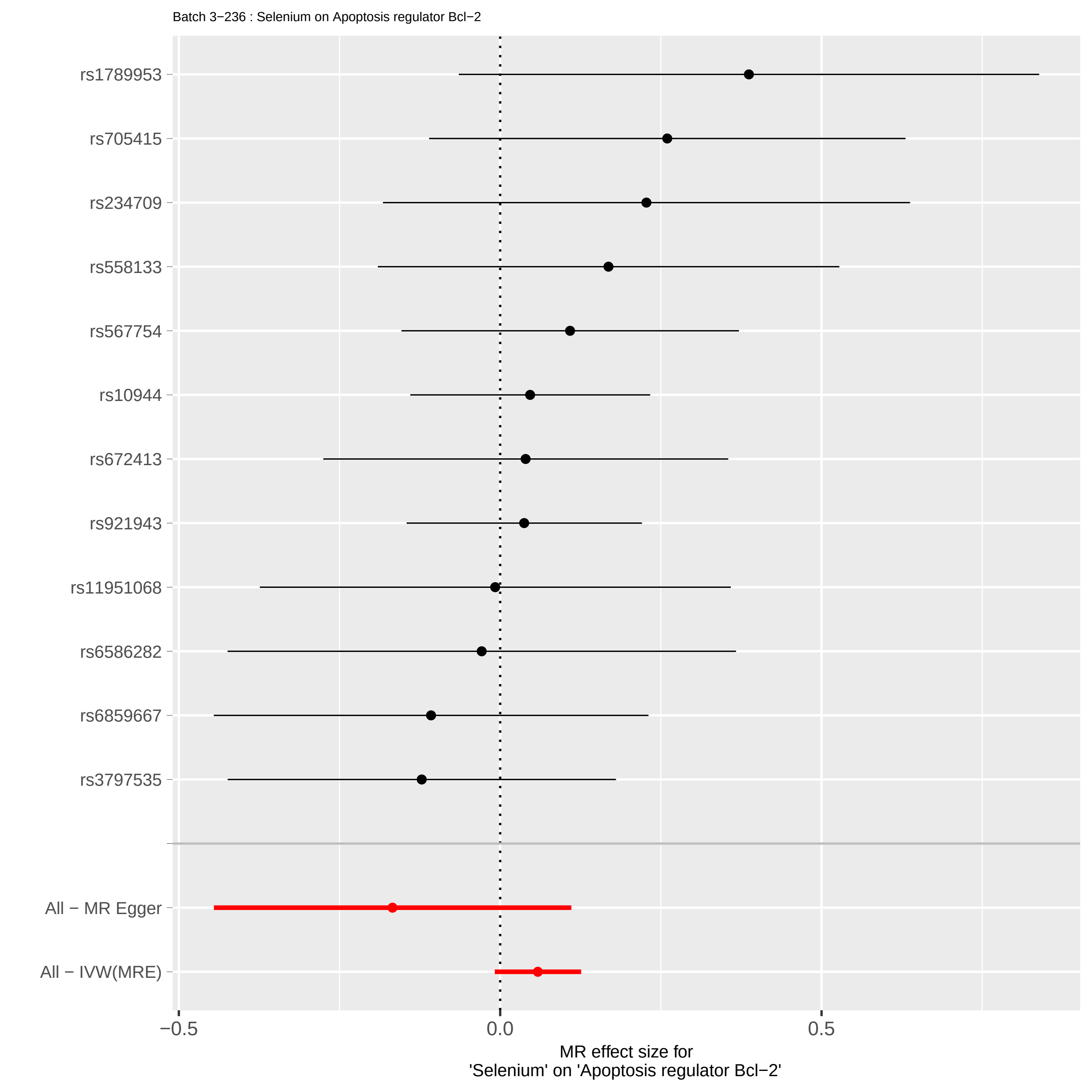

Batch 3−236 : Selenium on Apoptosis regulator Bcl−2
rs1789953
rs705415
rs234709
rs558133
rs567754
rs10944
rs672413
rs921943
rs11951068
rs6586282
rs6859667
rs3797535
All − MR Egger
All − IVW(MRE)
−0.5
0.0
0.5
MR effect size for
'Selenium' on 'Apoptosis regulator Bcl−2'

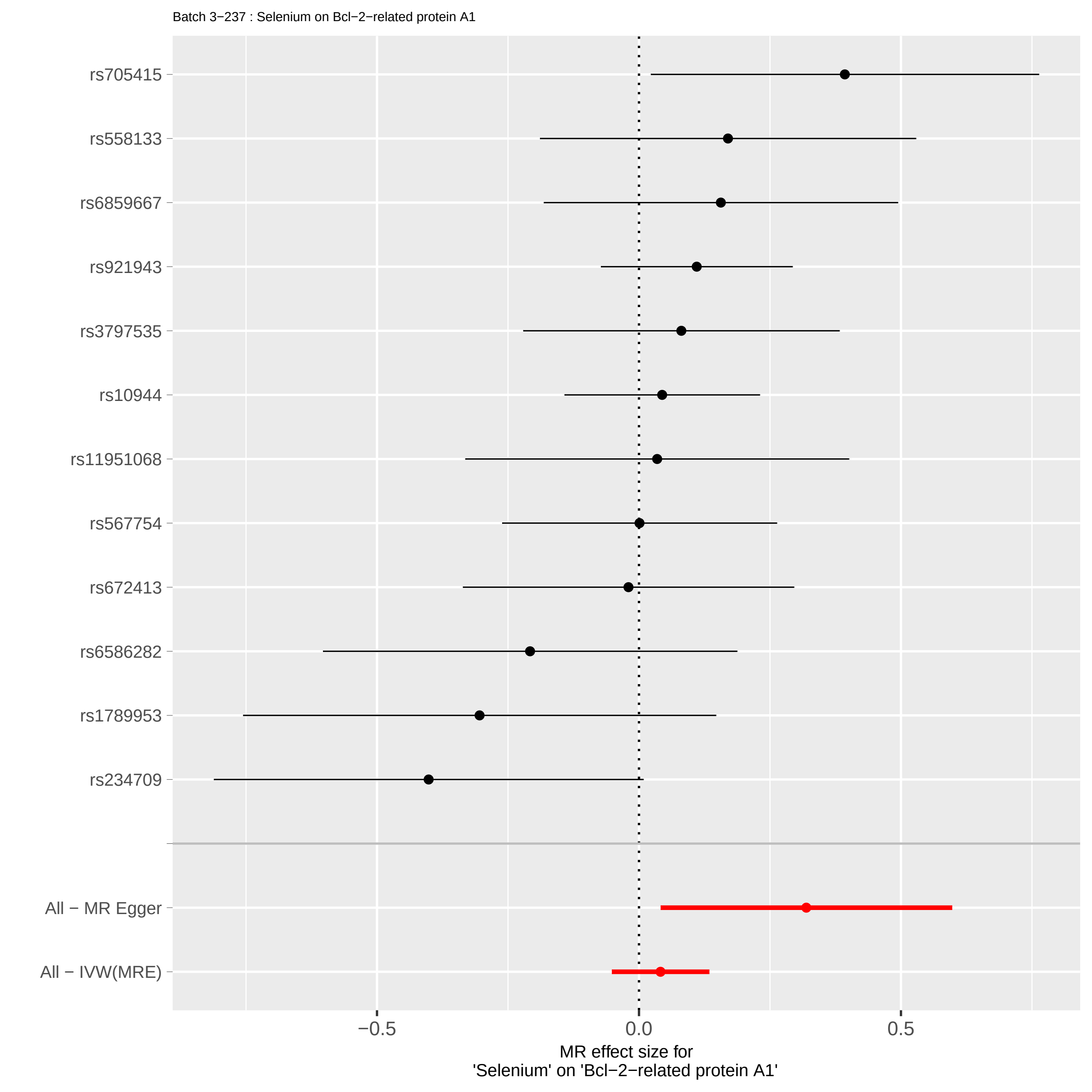
Batch 3−237 : Selenium on Bcl−2−related protein A1
rs705415
rs558133
rs6859667
rs921943
rs3797535
rs10944
rs11951068
rs567754
rs672413
rs6586282
rs1789953
rs234709
All − MR Egger
All − IVW(MRE)
−0.5
0.0
0.5
MR effect size for
'Selenium' on 'Bcl−2−related protein A1'

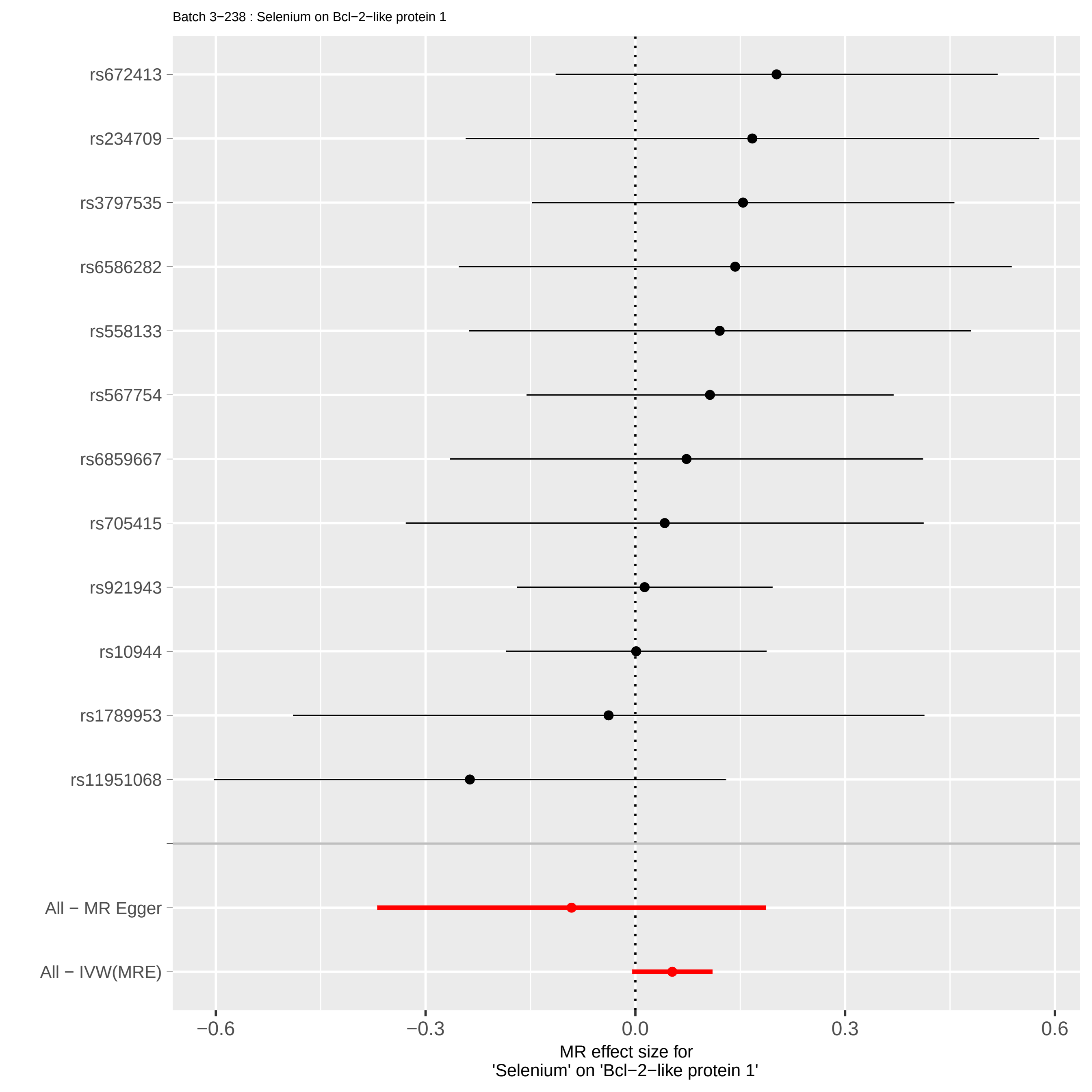
Batch 3−238 : Selenium on Bcl−2−like protein 1
rs672413
rs234709
rs3797535
rs6586282
rs558133
rs567754
rs6859667
rs705415
rs921943
rs10944
rs1789953
rs11951068
All − MR Egger
All − IVW(MRE)
−0.6
−0.3
0.0
0.3
0.6
MR effect size for
'Selenium' on 'Bcl−2−like protein 1'

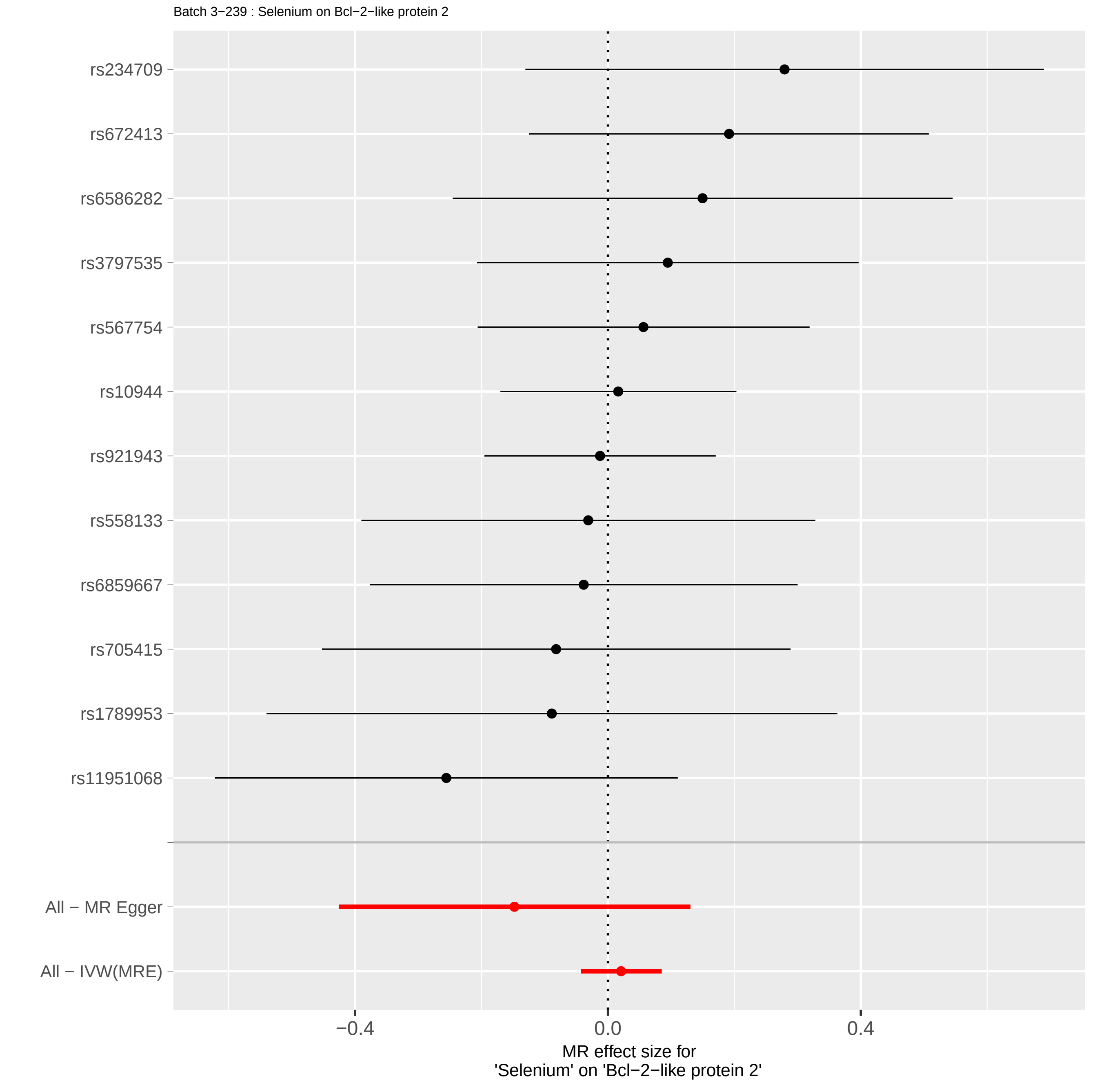
Batch 3−239 : Selenium on Bcl−2−like protein 2
rs234709
rs672413
rs6586282
rs3797535
rs567754
rs10944
rs921943
rs558133
rs6859667
rs705415
rs1789953
rs11951068
All − MR Egger
All − IVW(MRE)
−0.4
0.0
0.4
MR effect size for
'Selenium' on 'Bcl−2−like protein 2'

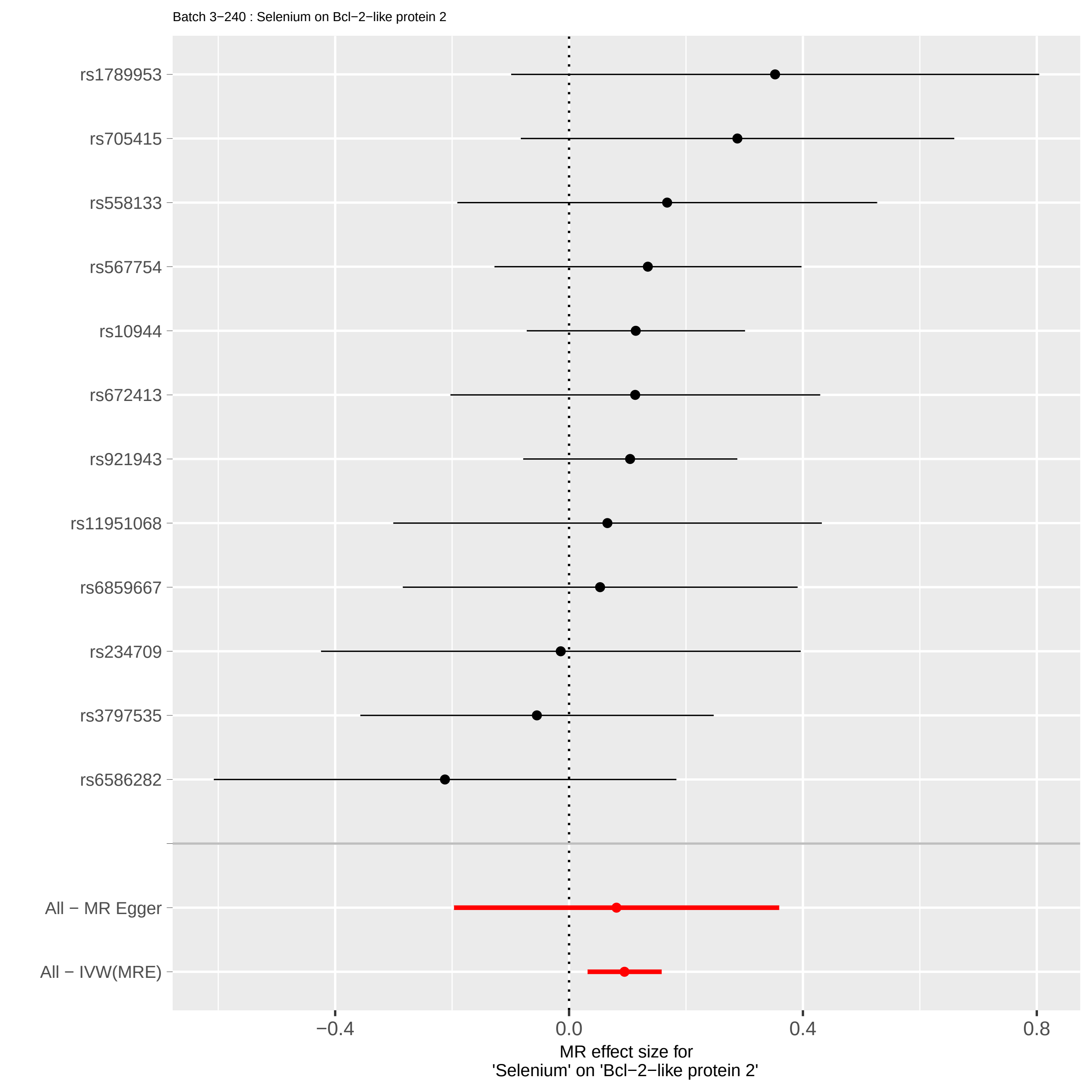
Batch 3−240 : Selenium on Bcl−2−like protein 2
rs1789953
rs705415
rs558133
rs567754
rs10944
rs672413
rs921943
rs11951068
rs6859667
rs234709
rs3797535
rs6586282
All − MR Egger
All − IVW(MRE)
−0.4
0.0
0.4
0.8
MR effect size for
'Selenium' on 'Bcl−2−like protein 2'

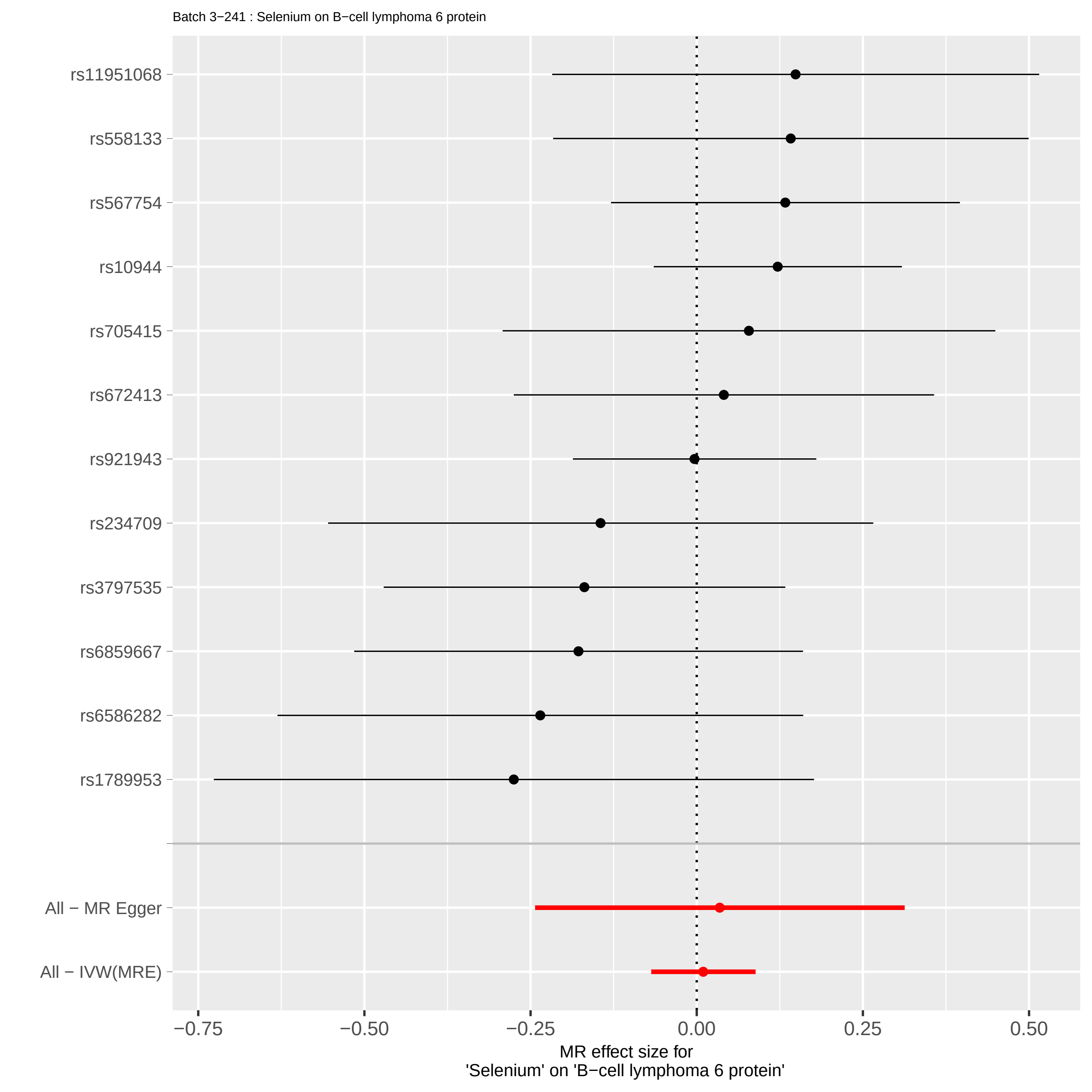

Batch 3−241 : Selenium on B−cell lymphoma 6 protein
rs11951068
rs558133
rs567754
rs10944
rs705415
rs672413
rs921943
rs234709
rs3797535
rs6859667
rs6586282
rs1789953
All − MR Egger
All − IVW(MRE)
−0.75
−0.50
−0.25
0.00
0.25
0.50
MR effect size for
'Selenium' on 'B−cell lymphoma 6 protein'

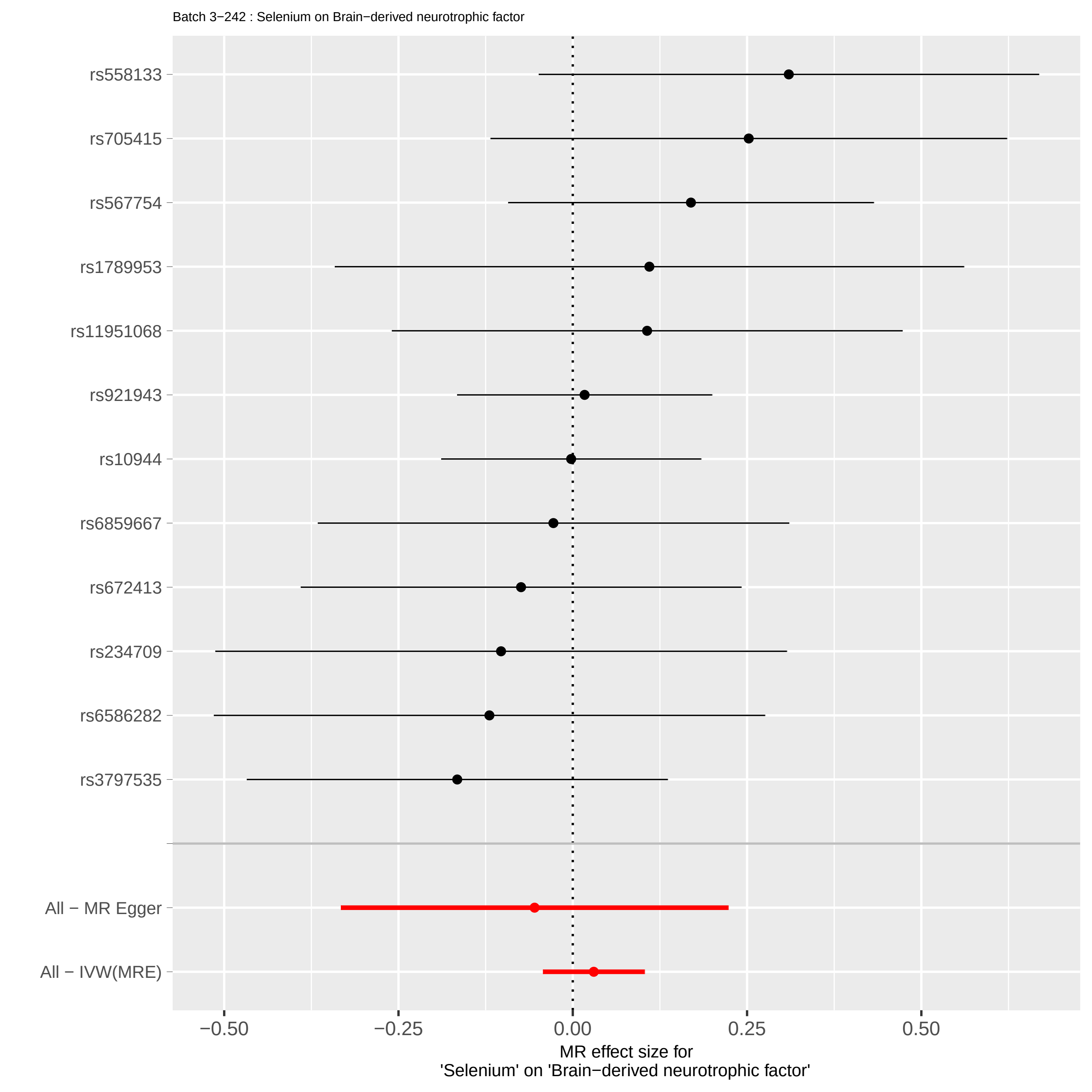
Batch 3−242 : Selenium on Brain−derived neurotrophic factor
rs558133
rs705415
rs567754
rs1789953
rs11951068
rs921943
rs10944
rs6859667
rs672413
rs234709
rs6586282
rs3797535
All − MR Egger
All − IVW(MRE)
−0.50
−0.25
0.00
0.25
0.50
MR effect size for
'Selenium' on 'Brain−derived neurotrophic factor'

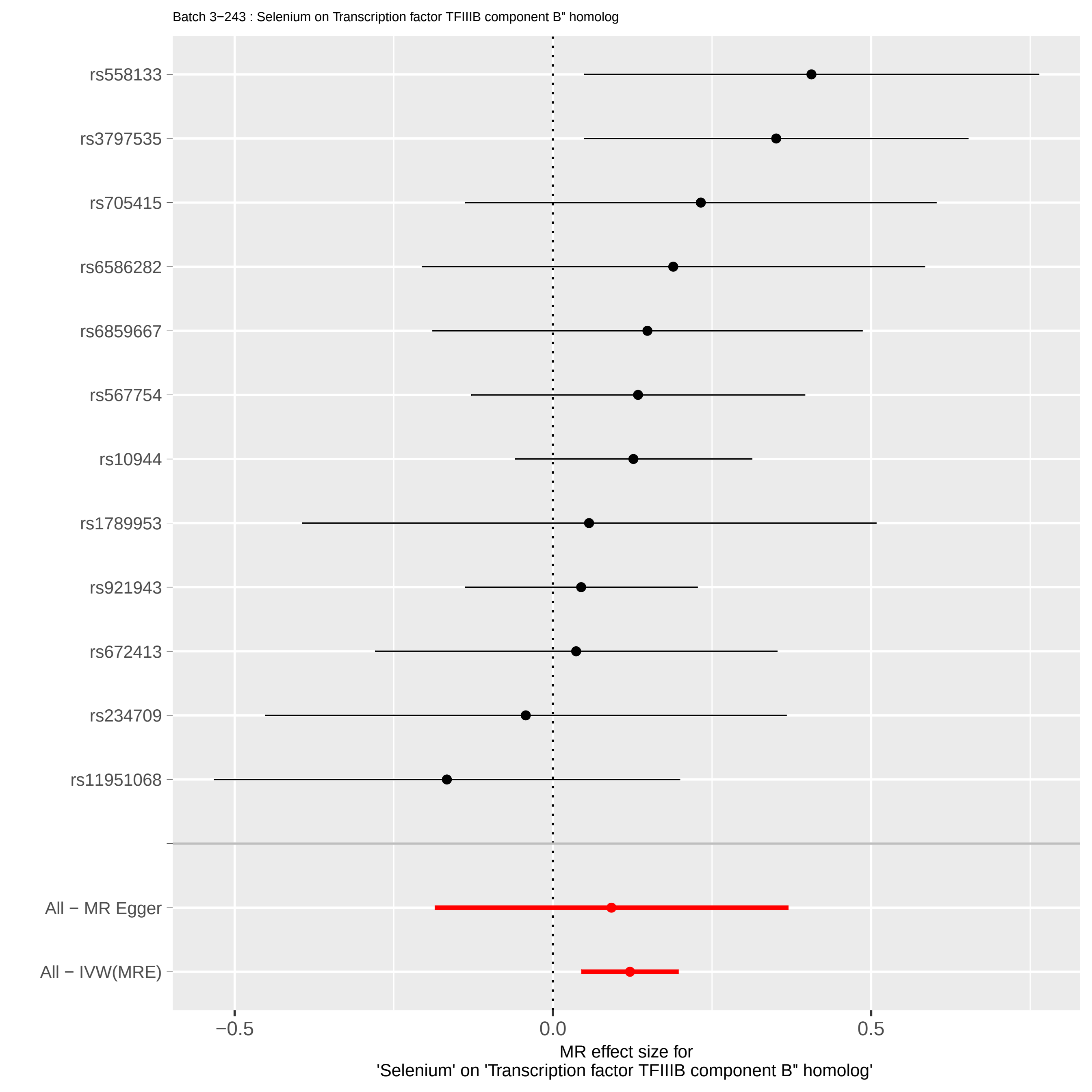

Batch 3−243 : Selenium on Transcription factor TFIIIB component B" homolog
rs558133
rs3797535
rs705415
rs6586282
rs6859667
rs567754
rs10944
rs1789953
rs921943
rs672413
rs234709
rs11951068
All − MR Egger
All − IVW(MRE)
−0.5
0.0
0.5
MR effect size for
'Selenium' on 'Transcription factor TFIIIB component B" homolog'

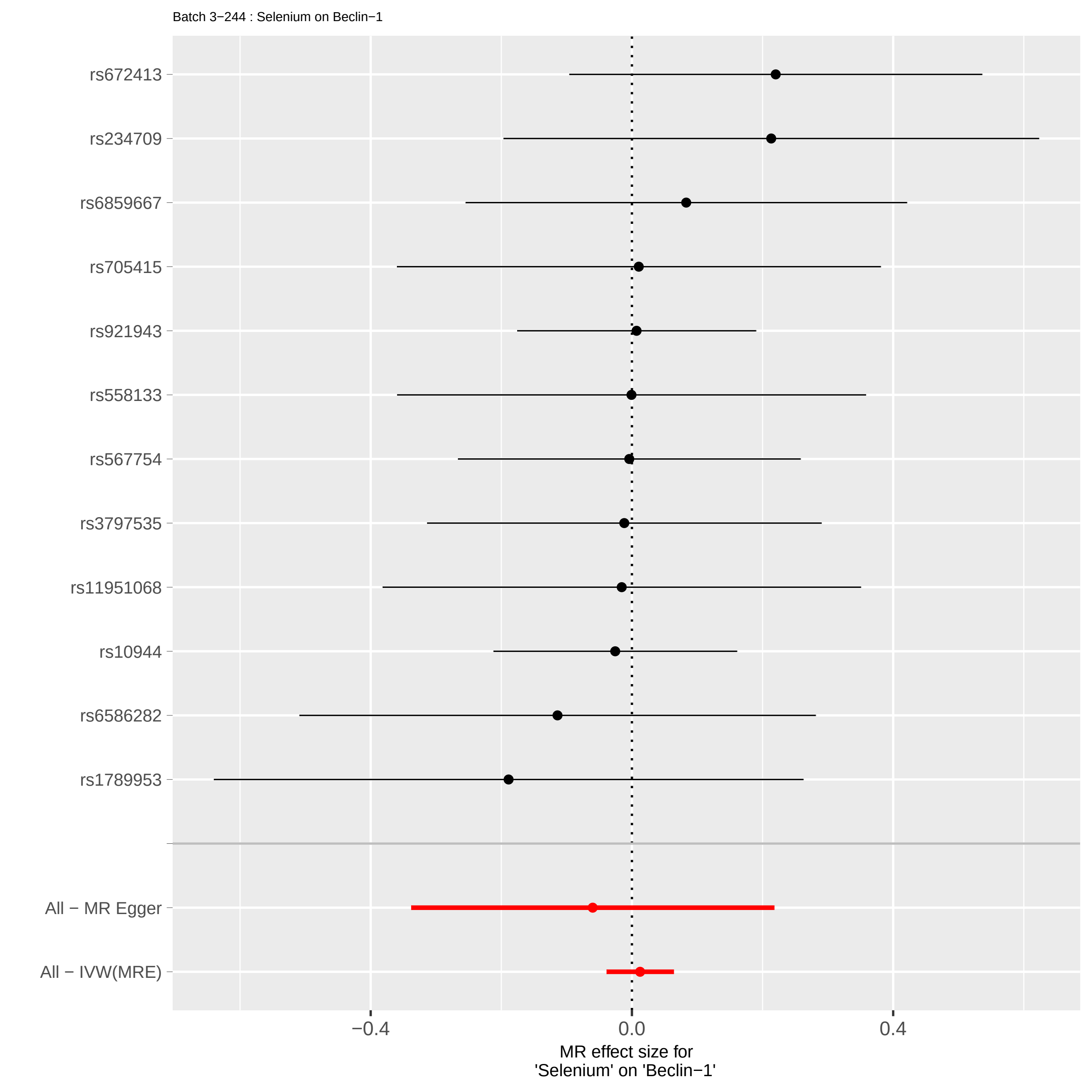

Batch 3−244 : Selenium on Beclin−1
rs672413
rs234709
rs6859667
rs705415
rs921943
rs558133
rs567754
rs3797535
rs11951068
rs10944
rs6586282
rs1789953
All − MR Egger
All − IVW(MRE)
−0.4
0.0
0.4
MR effect size for
'Selenium' on 'Beclin−1'

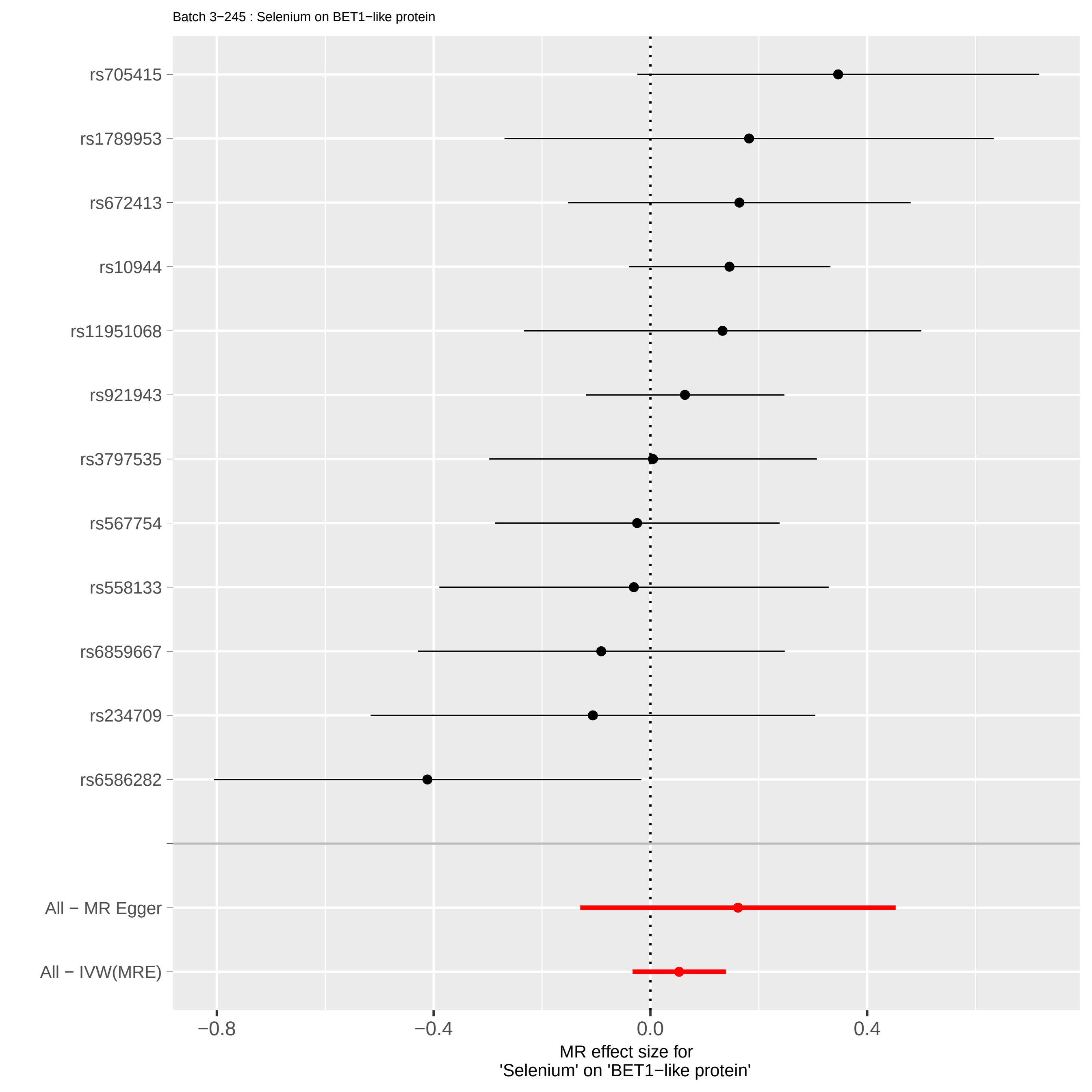

Batch 3−245 : Selenium on BET1−like protein
rs705415
rs1789953
rs672413
rs10944
rs11951068
rs921943
rs3797535
rs567754
rs558133
rs6859667
rs234709
rs6586282
All − MR Egger
All − IVW(MRE)
−0.8
−0.4
0.0
0.4
MR effect size for
'Selenium' on 'BET1−like protein'

Batch 3−246 : Selenium on Osteocalcin

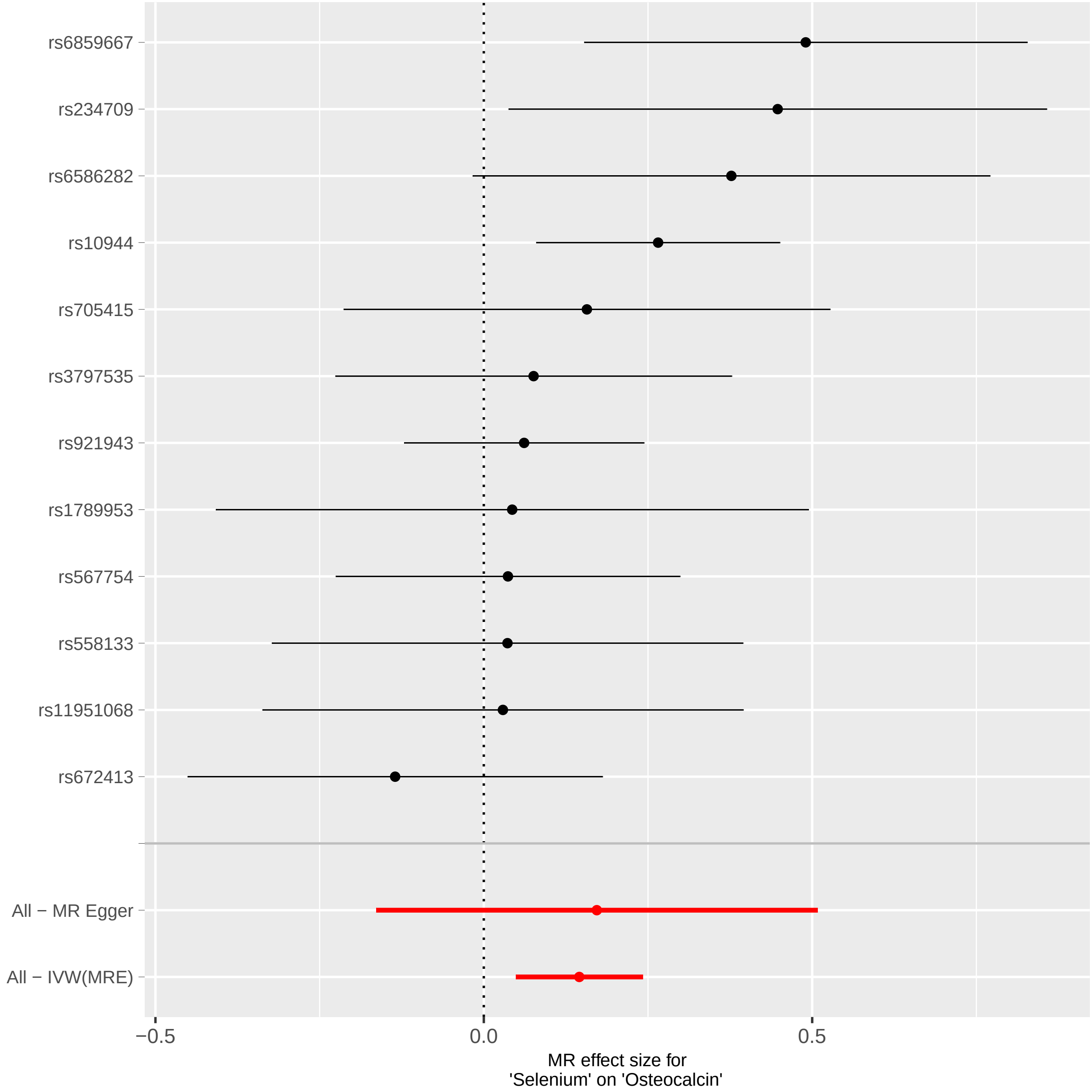

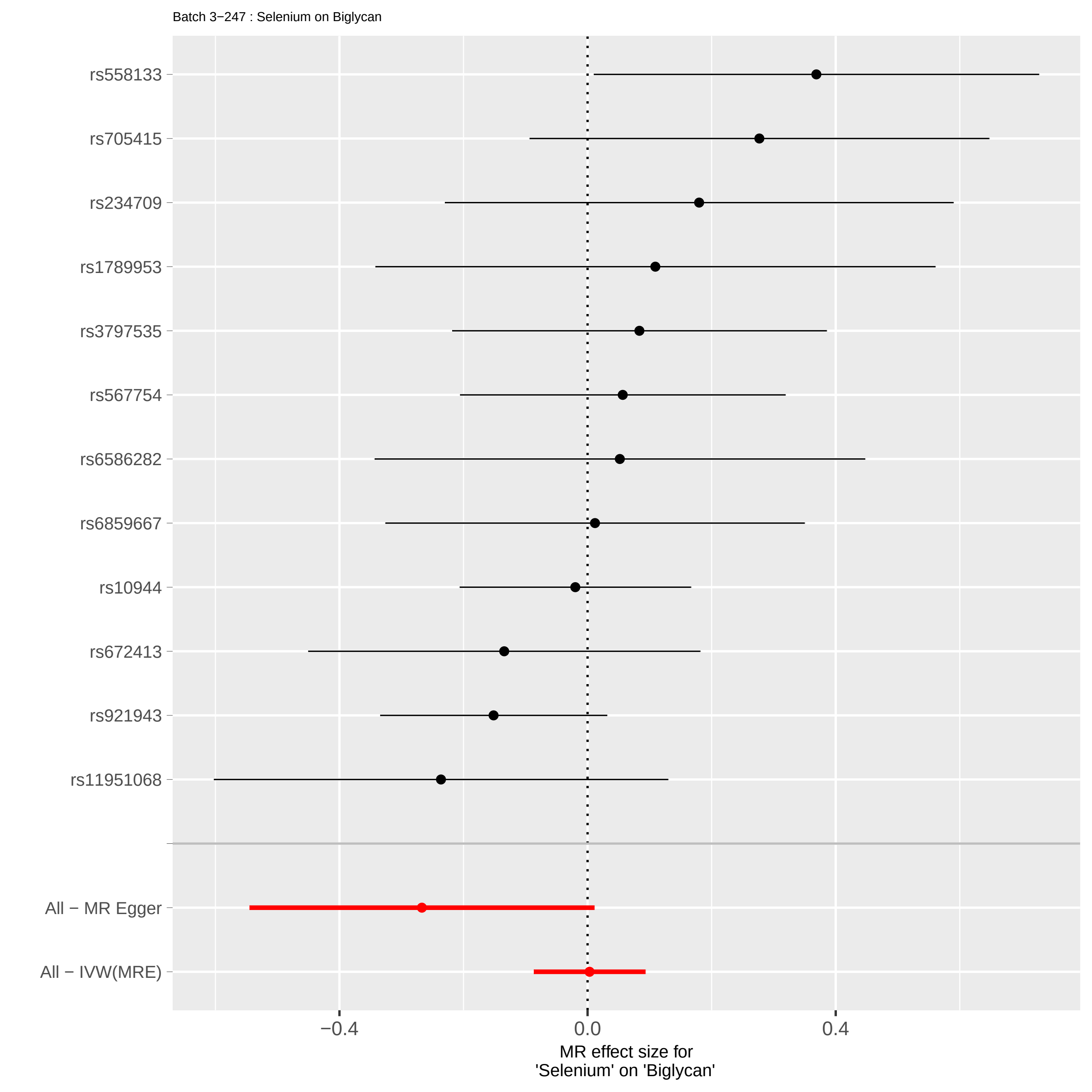
Batch 3−247 : Selenium on Biglycan
rs558133
rs705415
rs234709
rs1789953
rs3797535
rs567754
rs6586282
rs6859667
rs10944
rs672413
rs921943
rs11951068
All − MR Egger
All − IVW(MRE)
−0.4
0.0
0.4
MR effect size for
'Selenium' on 'Biglycan'

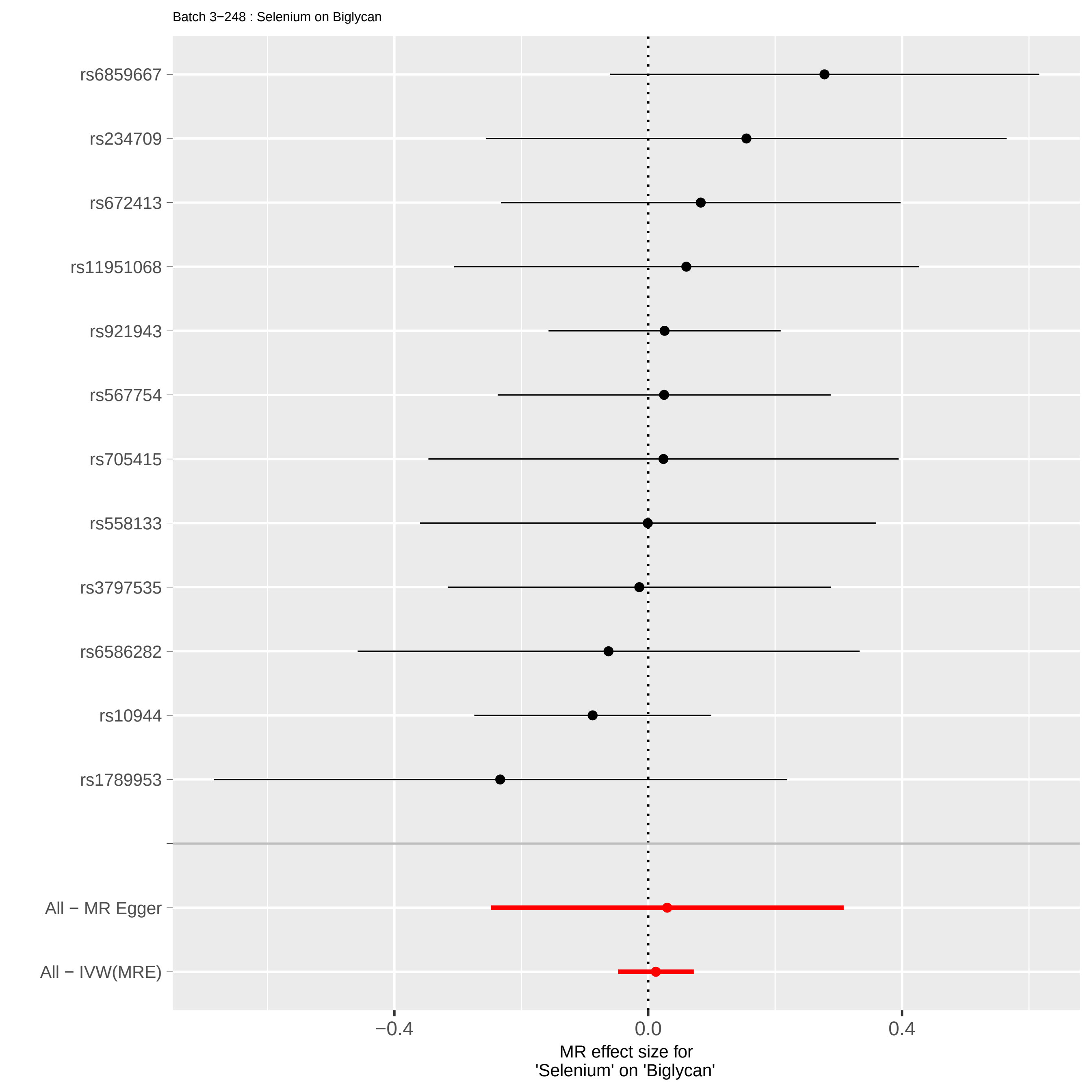
Batch 3−248 : Selenium on Biglycan
rs6859667
rs234709
rs672413
rs11951068
rs921943
rs567754
rs705415
rs558133
rs3797535
rs6586282
rs10944
rs1789953
All − MR Egger
All − IVW(MRE)
−0.4
0.0
0.4
MR effect size for
'Selenium' on 'Biglycan'

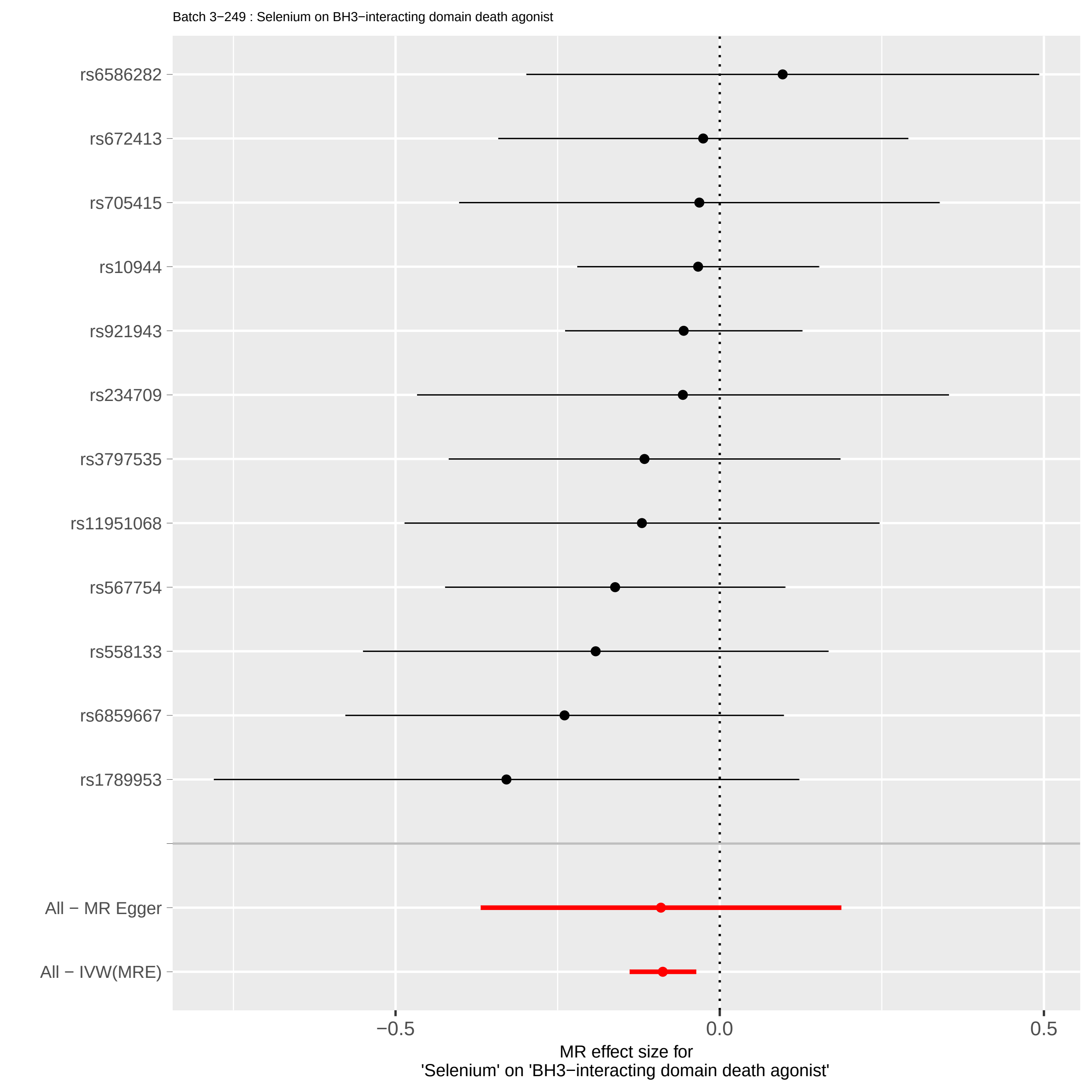

Batch 3−249 : Selenium on BH3−interacting domain death agonist
rs6586282
rs672413
rs705415
rs10944
rs921943
rs234709
rs3797535
rs11951068
rs567754
rs558133
rs6859667
rs1789953
All − MR Egger
All − IVW(MRE)
−0.5
0.0
0.5
MR effect size for
'Selenium' on 'BH3−interacting domain death agonist'

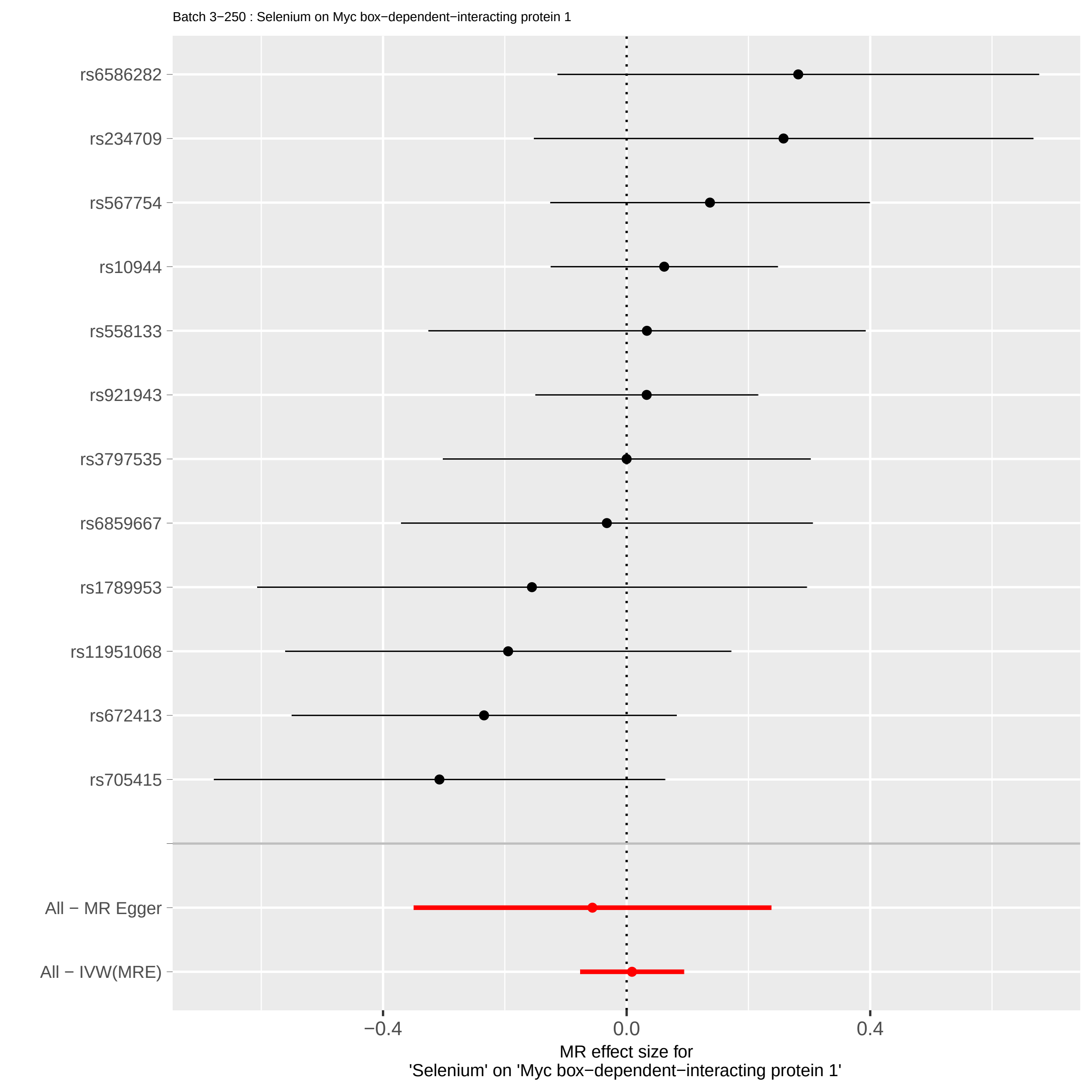
Batch 3−250 : Selenium on Myc box−dependent−interacting protein 1
rs6586282
rs234709
rs567754
rs10944
rs558133
rs921943
rs3797535
rs6859667
rs1789953
rs11951068
rs672413
rs705415
All − MR Egger
All − IVW(MRE)
−0.4
0.0
0.4
MR effect size for
'Selenium' on 'Myc box−dependent−interacting protein 1'

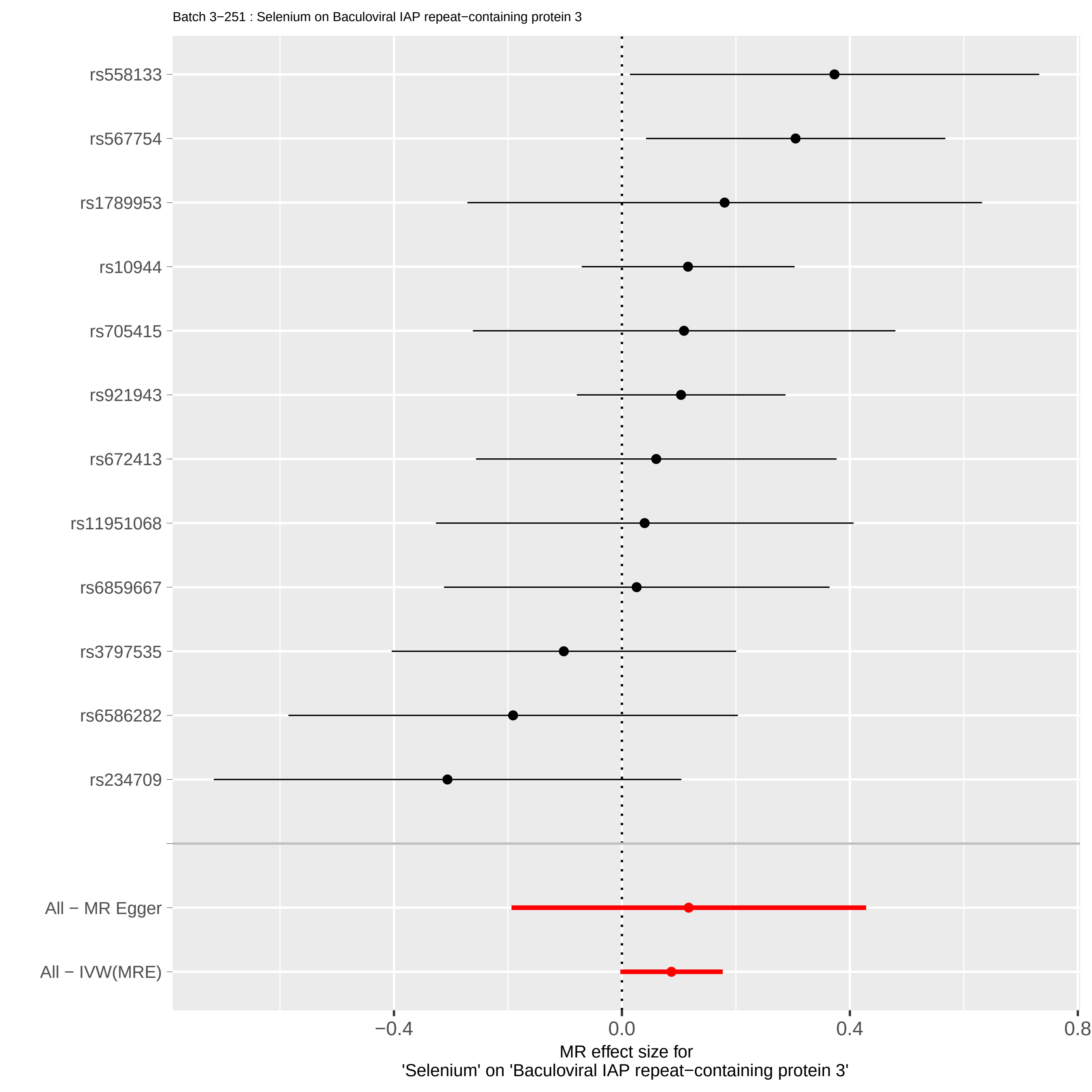

Batch 3−251 : Selenium on Baculoviral IAP repeat−containing protein 3
rs558133
rs567754
rs1789953
rs10944
rs705415
rs921943
rs672413
rs11951068
rs6859667
rs3797535
rs6586282
rs234709
All − MR Egger
All − IVW(MRE)
−0.4
0.0
0.4
0.8
MR effect size for
'Selenium' on 'Baculoviral IAP repeat−containing protein 3'

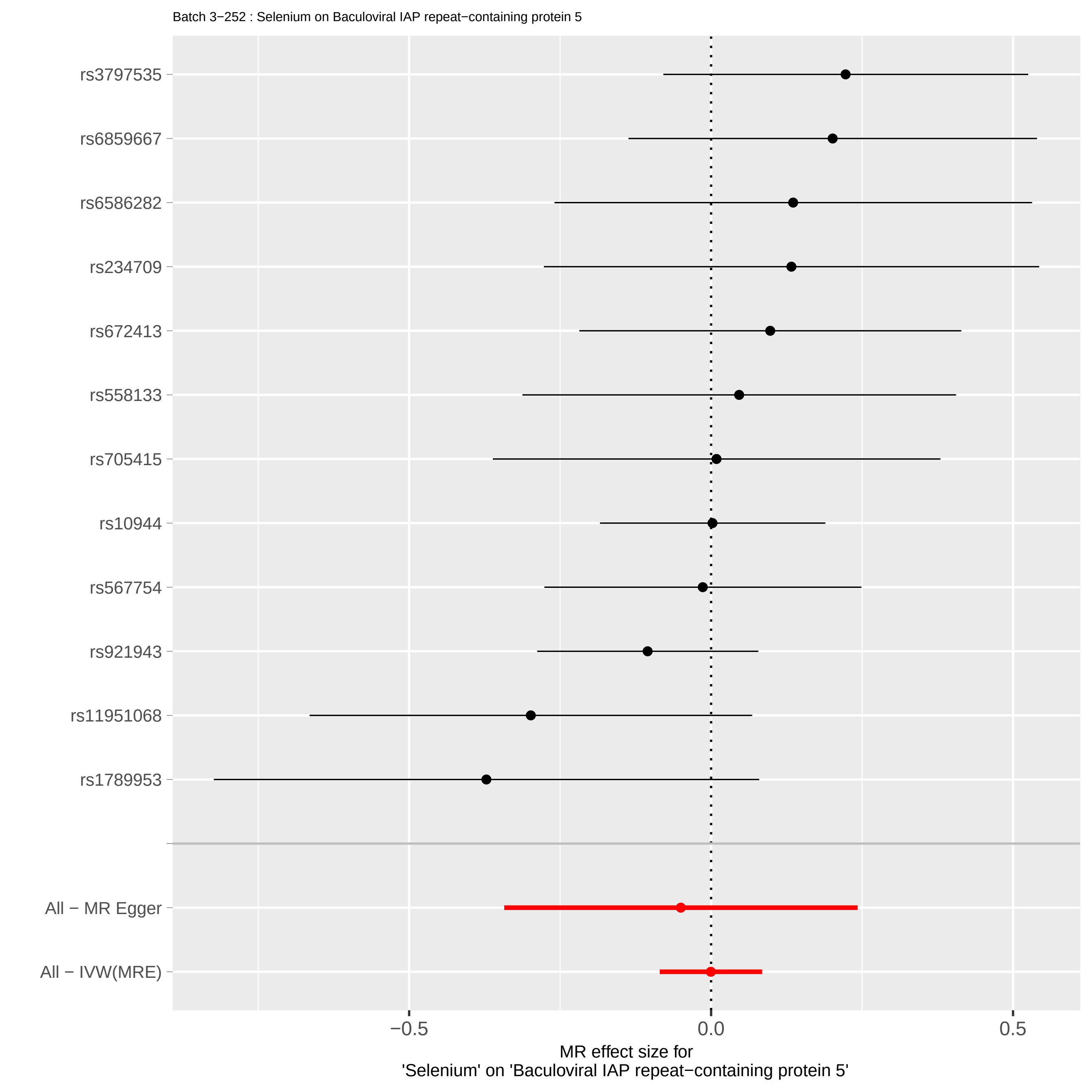

Batch 3−252 : Selenium on Baculoviral IAP repeat−containing protein 5
rs3797535
rs6859667
rs6586282
rs234709
rs672413
rs558133
rs705415
rs10944
rs567754
rs921943
rs11951068
rs1789953
All − MR Egger
All − IVW(MRE)
−0.5
0.0
0.5
MR effect size for
'Selenium' on 'Baculoviral IAP repeat−containing protein 5'

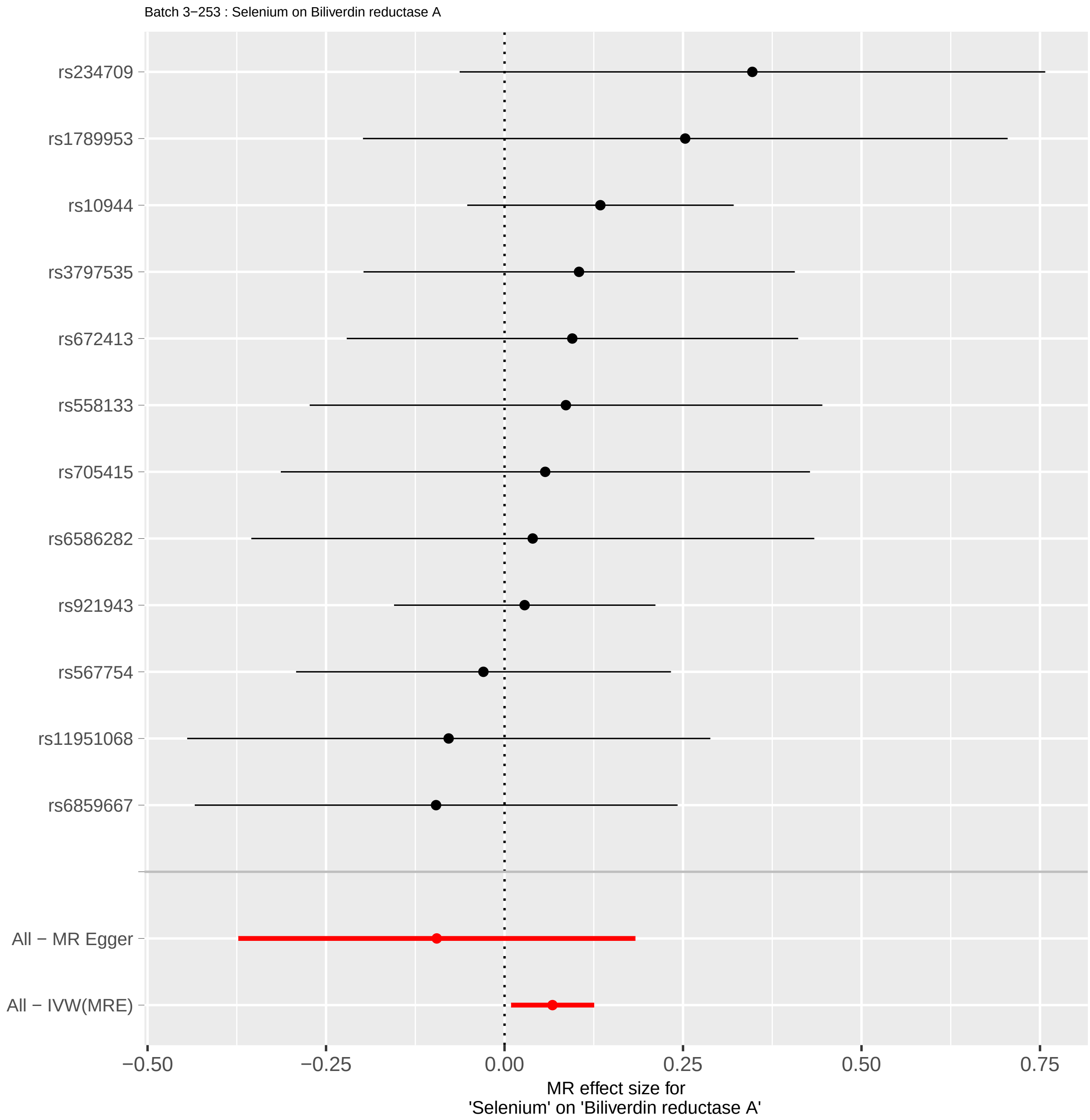

Batch 3−253 : Selenium on Biliverdin reductase A
rs234709
rs1789953
rs10944
rs3797535
rs672413
rs558133
rs705415
rs6586282
rs921943
rs567754
rs11951068
rs6859667
All − MR Egger
All − IVW(MRE)
−0.50
−0.25
0.00
0.25
0.50
0.75
MR effect size for
'Selenium' on 'Biliverdin reductase A'

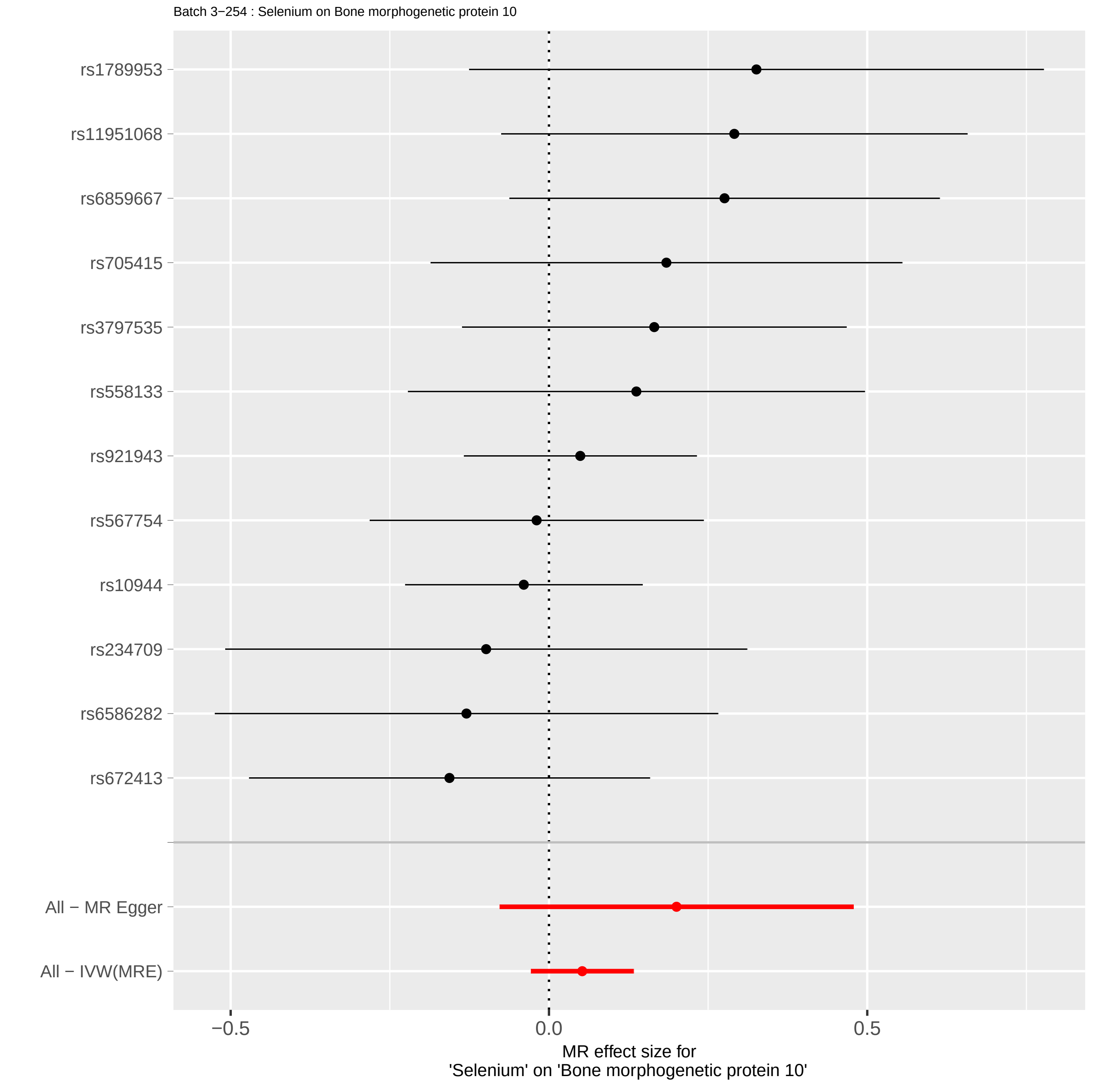

Batch 3−254 : Selenium on Bone morphogenetic protein 10
rs1789953
rs11951068
rs6859667
rs705415
rs3797535
rs558133
rs921943
rs567754
rs10944
rs234709
rs6586282
rs672413
All − MR Egger
All − IVW(MRE)
−0.5
0.0
0.5
MR effect size for
'Selenium' on 'Bone morphogenetic protein 10'

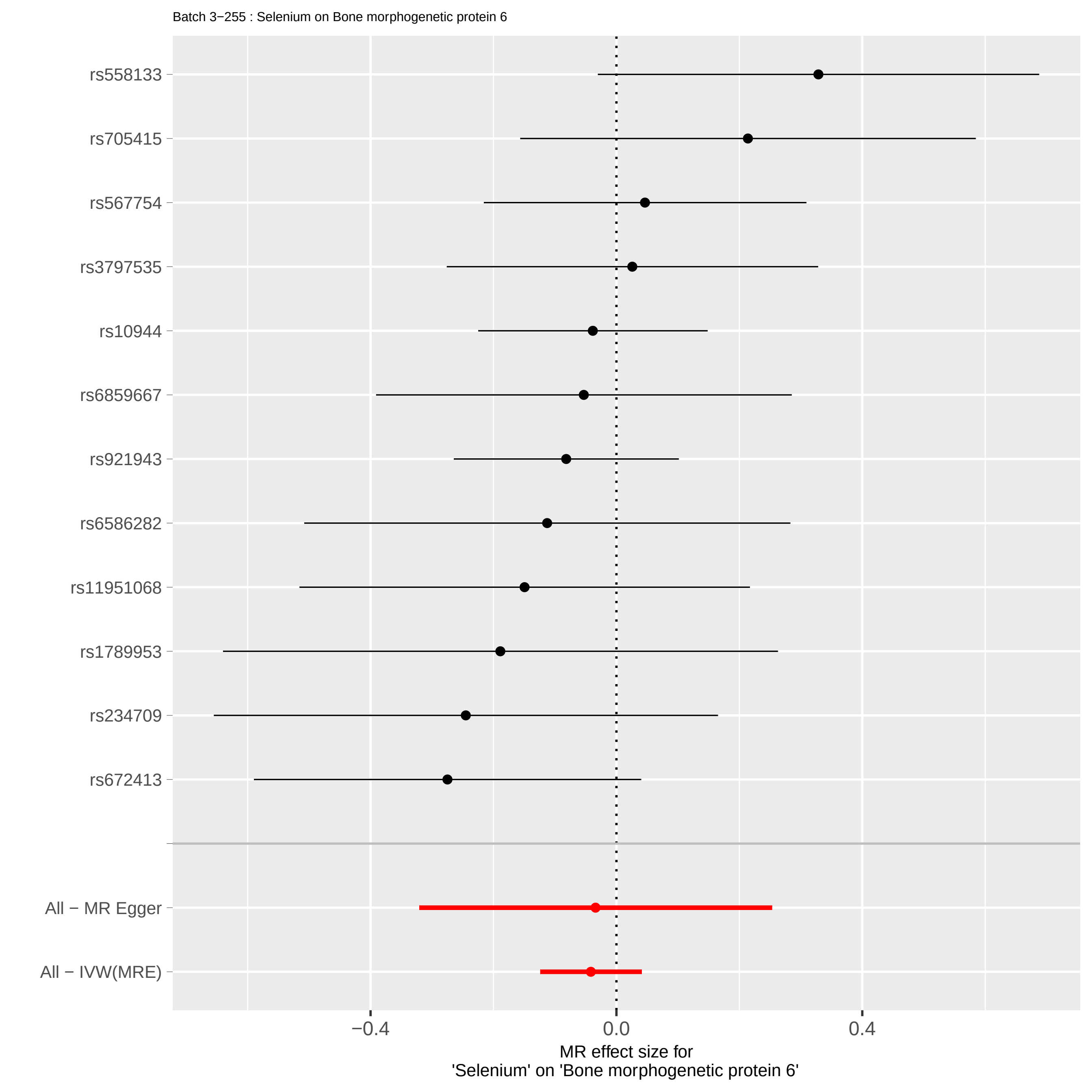
Batch 3−255 : Selenium on Bone morphogenetic protein 6
rs558133
rs705415
rs567754
rs3797535
rs10944
rs6859667
rs921943
rs6586282
rs11951068
rs1789953
rs234709
rs672413
All − MR Egger
All − IVW(MRE)
−0.4
0.0
0.4
MR effect size for
'Selenium' on 'Bone morphogenetic protein 6'

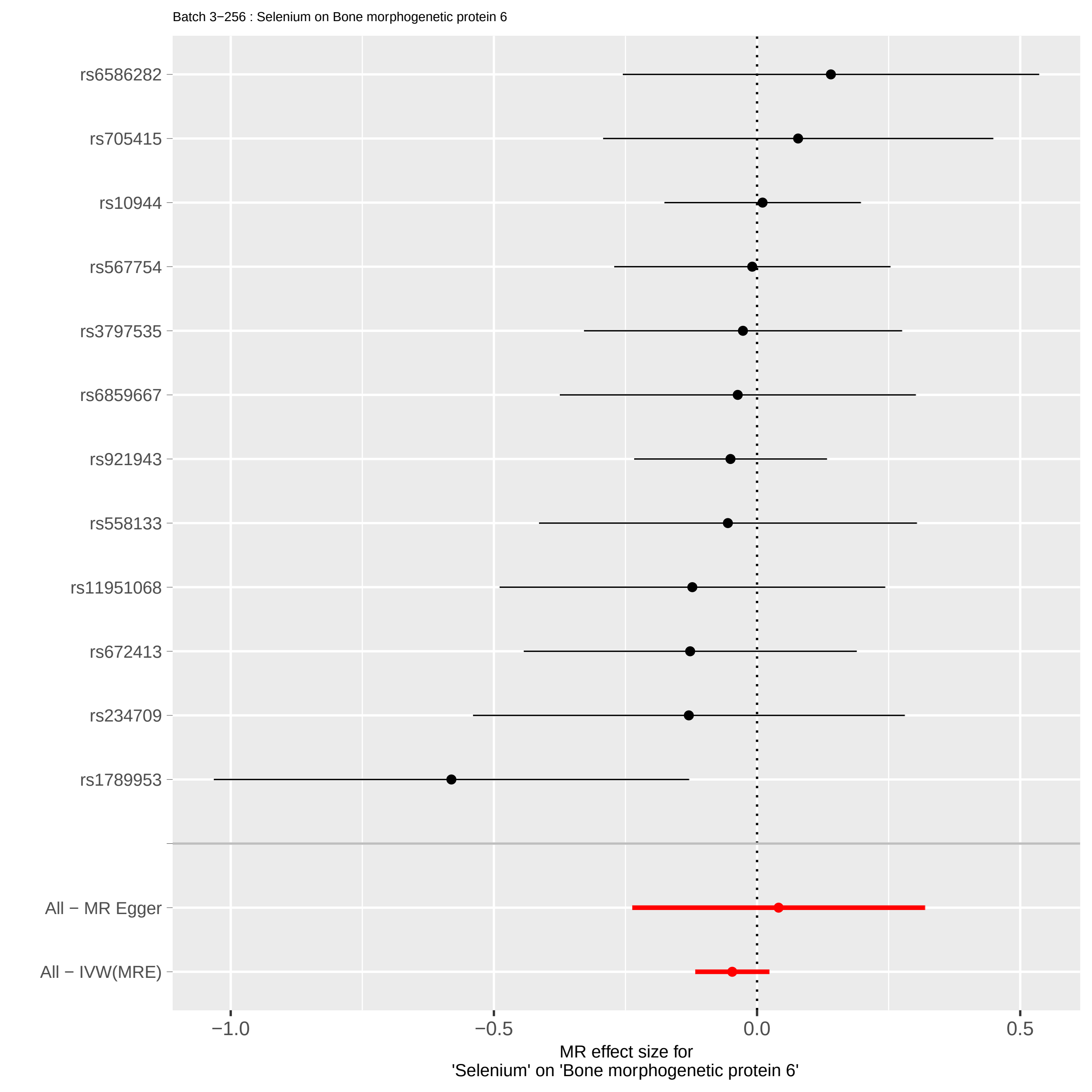

Batch 3−256 : Selenium on Bone morphogenetic protein 6
rs6586282
rs705415
rs10944
rs567754
rs3797535
rs6859667
rs921943
rs558133
rs11951068
rs672413
rs234709
rs1789953
All − MR Egger
All − IVW(MRE)
−1.0
−0.5
0.0
0.5
MR effect size for
'Selenium' on 'Bone morphogenetic protein 6'

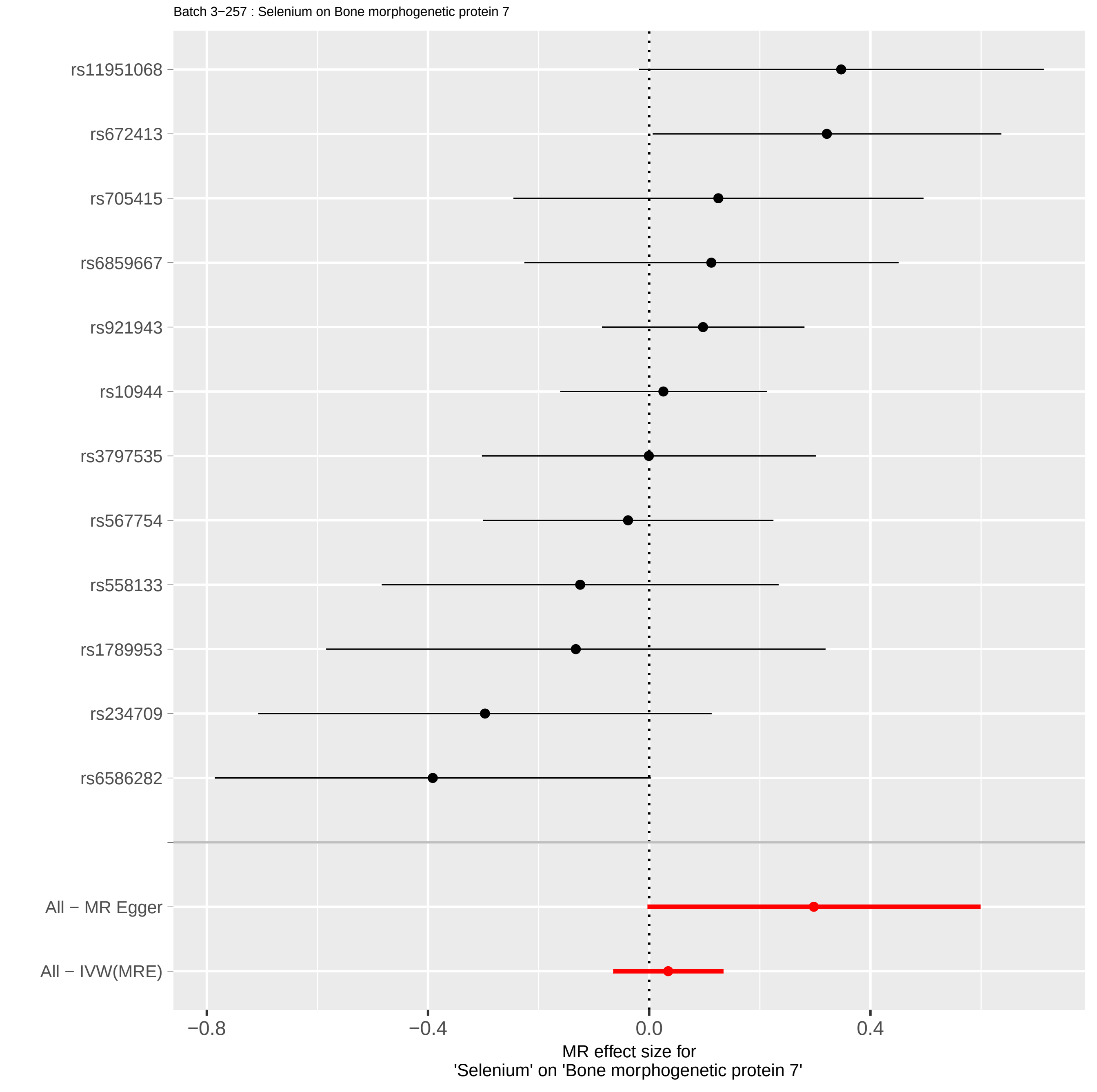
Batch 3−257 : Selenium on Bone morphogenetic protein 7
rs11951068
rs672413
rs705415
rs6859667
rs921943
rs10944
rs3797535
rs567754
rs558133
rs1789953
rs234709
rs6586282
All − MR Egger
All − IVW(MRE)
−0.8
−0.4
0.0
0.4
MR effect size for
'Selenium' on 'Bone morphogenetic protein 7'

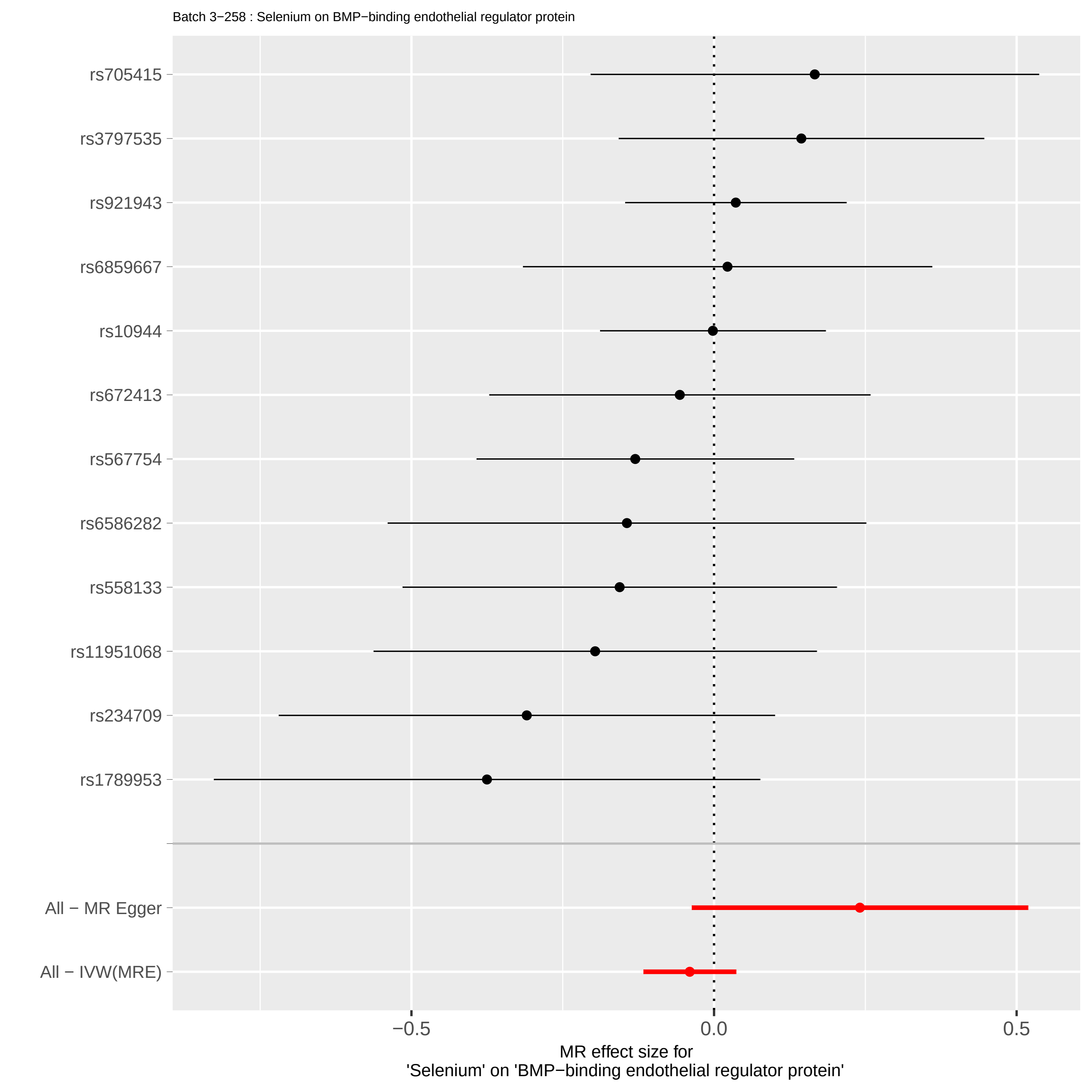

Batch 3−258 : Selenium on BMP−binding endothelial regulator protein
rs705415
rs3797535
rs921943
rs6859667
rs10944
rs672413
rs567754
rs6586282
rs558133
rs11951068
rs234709
rs1789953
All − MR Egger
All − IVW(MRE)
−0.5
0.0
0.5
MR effect size for
'Selenium' on 'BMP−binding endothelial regulator protein'

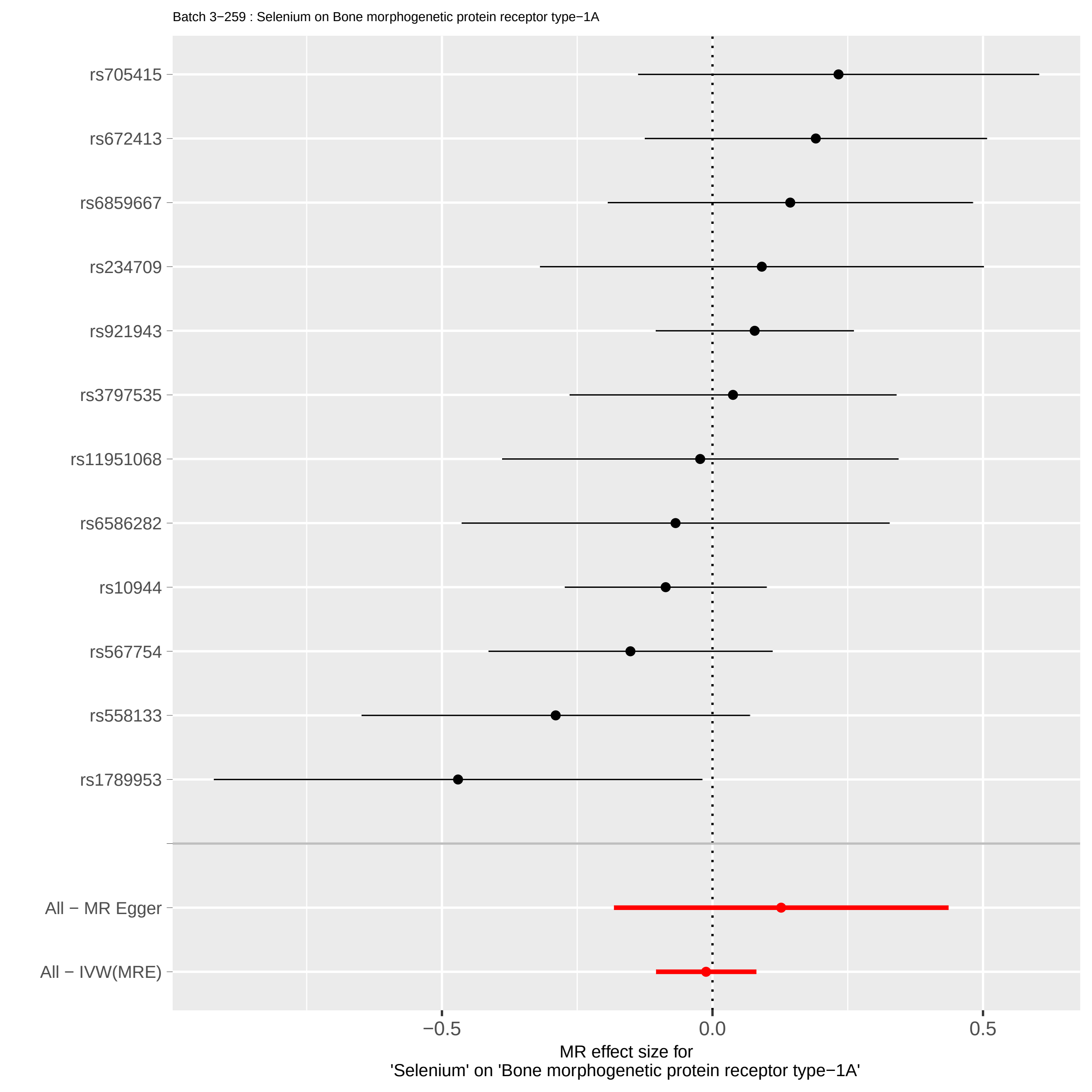
Batch 3−259 : Selenium on Bone morphogenetic protein receptor type−1A
rs705415
rs672413
rs6859667
rs234709
rs921943
rs3797535
rs11951068
rs6586282
rs10944
rs567754
rs558133
rs1789953
All − MR Egger
All − IVW(MRE)
−0.5
0.0
0.5
MR effect size for
'Selenium' on 'Bone morphogenetic protein receptor type−1A'

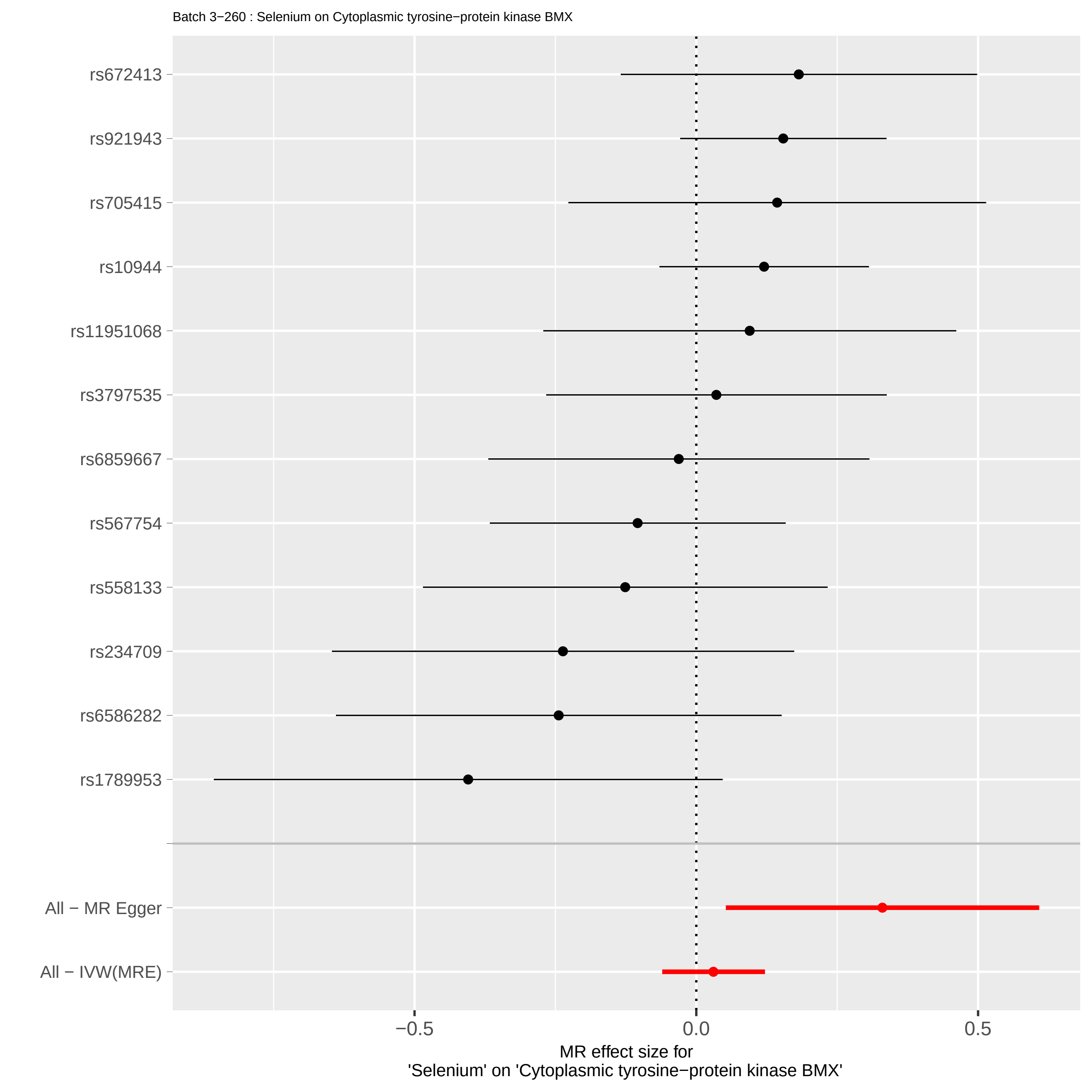
Batch 3−260 : Selenium on Cytoplasmic tyrosine−protein kinase BMX
rs672413
rs921943
rs705415
rs10944
rs11951068
rs3797535
rs6859667
rs567754
rs558133
rs234709
rs6586282
rs1789953
All − MR Egger
All − IVW(MRE)
−0.5
0.0
0.5
MR effect size for
'Selenium' on 'Cytoplasmic tyrosine−protein kinase BMX'

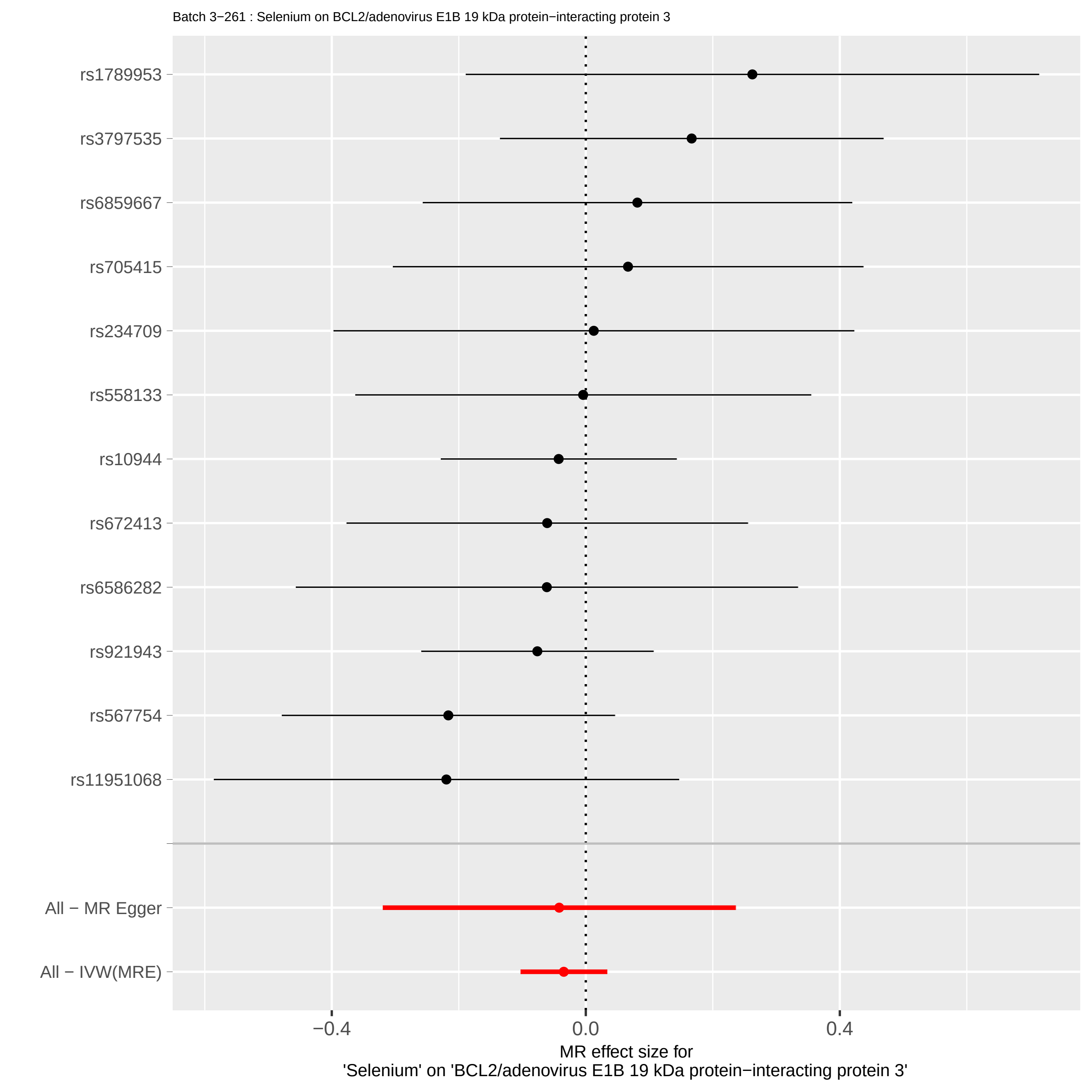

Batch 3−261 : Selenium on BCL2/adenovirus E1B 19 kDa protein−interacting protein 3
rs1789953
rs3797535
rs6859667
rs705415
rs234709
rs558133
rs10944
rs672413
rs6586282
rs921943
rs567754
rs11951068
All − MR Egger
All − IVW(MRE)
−0.4
0.0
0.4
MR effect size for
'Selenium' on 'BCL2/adenovirus E1B 19 kDa protein−interacting protein 3'

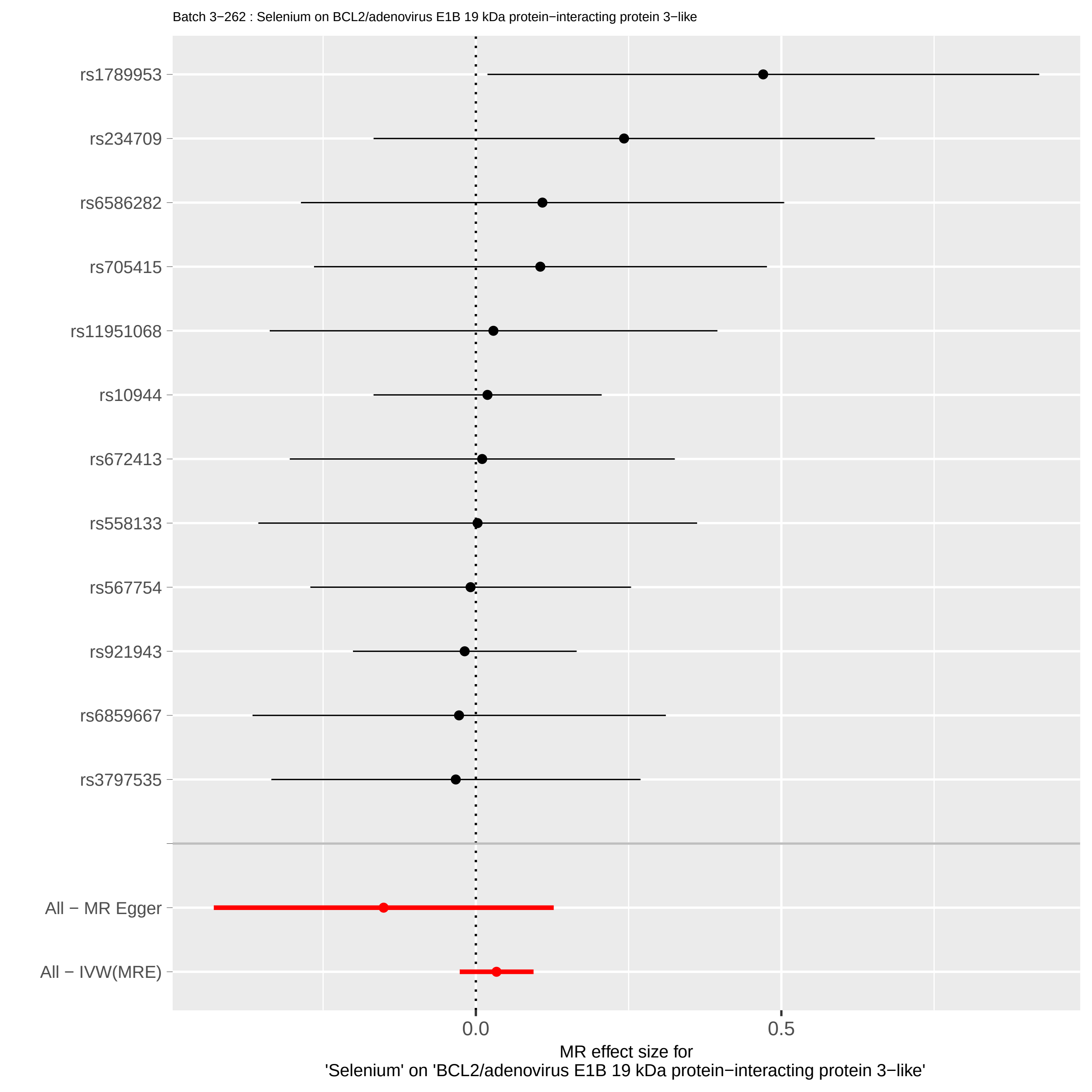
Batch 3−262 : Selenium on BCL2/adenovirus E1B 19 kDa protein−interacting protein 3−like
rs1789953
rs234709
rs6586282
rs705415
rs11951068
rs10944
rs672413
rs558133
rs567754
rs921943
rs6859667
rs3797535
All − MR Egger
All − IVW(MRE)
0.0
0.5
MR effect size for
'Selenium' on 'BCL2/adenovirus E1B 19 kDa protein−interacting protein 3−like'

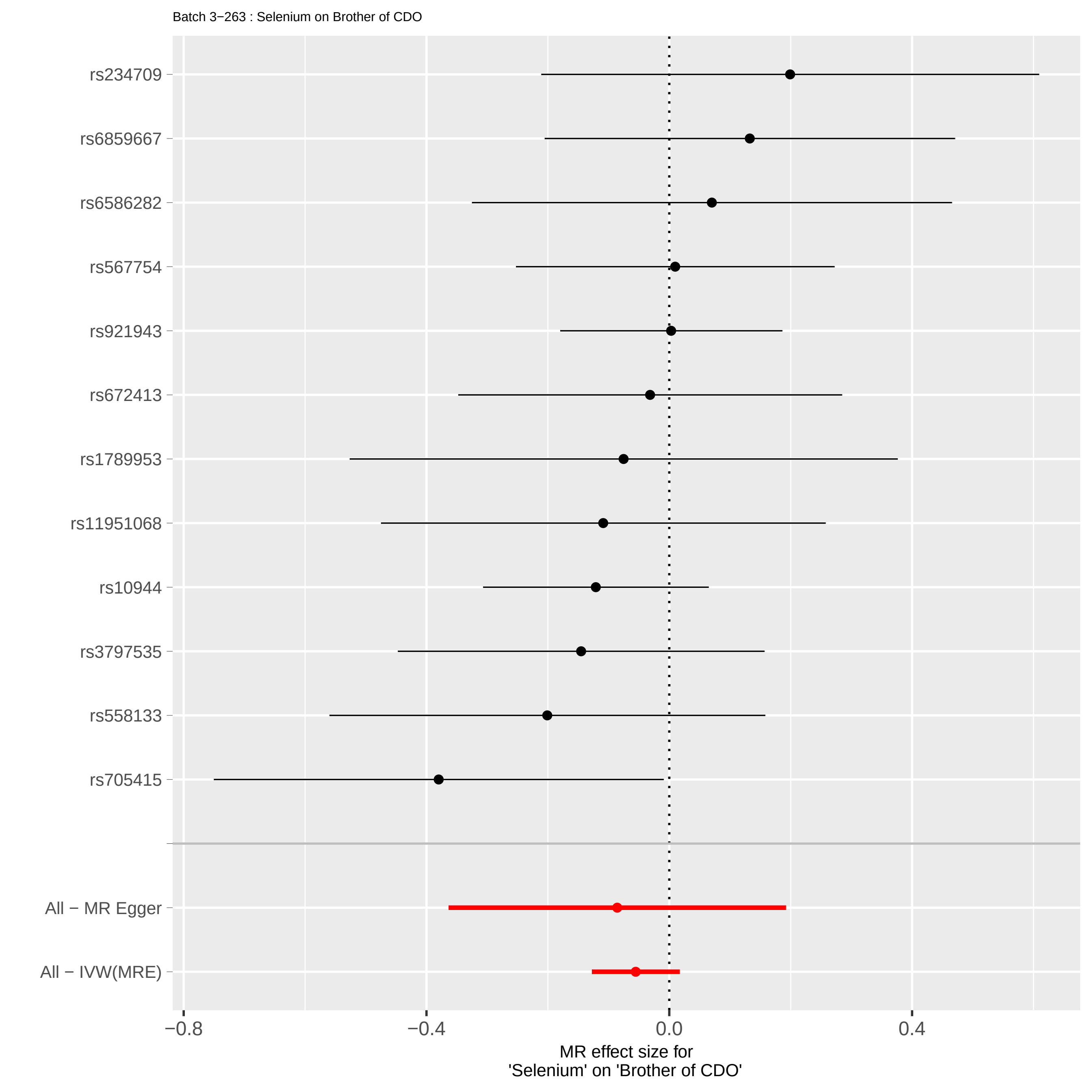
Batch 3−263 : Selenium on Brother of CDO
rs234709
rs6859667
rs6586282
rs567754
rs921943
rs672413
rs1789953
rs11951068
rs10944
rs3797535
rs558133
rs705415
All − MR Egger
All − IVW(MRE)
−0.8
−0.4
0.0
0.4
MR effect size for
'Selenium' on 'Brother of CDO'

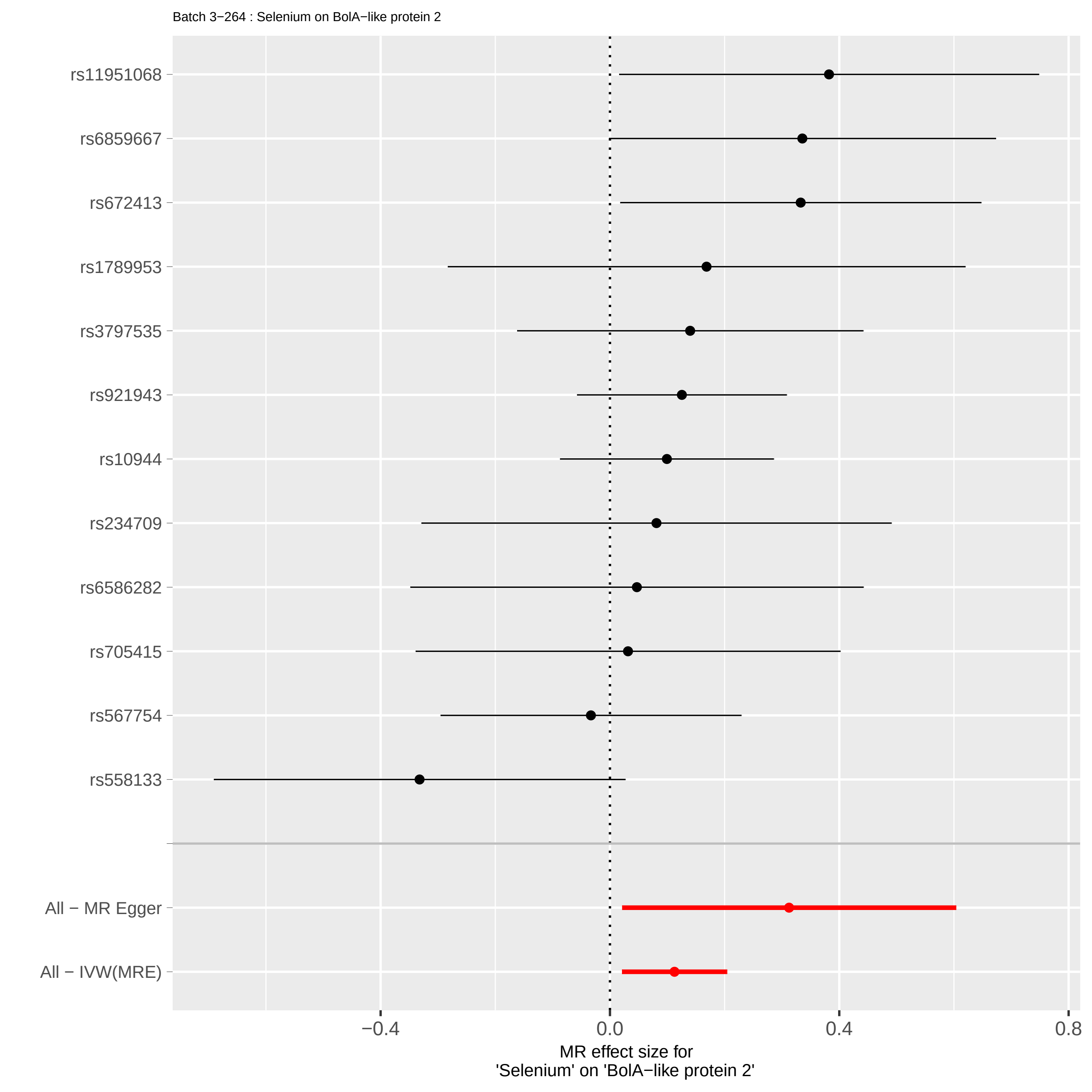

Batch 3−264 : Selenium on BolA−like protein 2
rs11951068
rs6859667
rs672413
rs1789953
rs3797535
rs921943
rs10944
rs234709
rs6586282
rs705415
rs567754
rs558133
All − MR Egger
All − IVW(MRE)
−0.4
0.0
0.4
0.8
MR effect size for
'Selenium' on 'BolA−like protein 2'

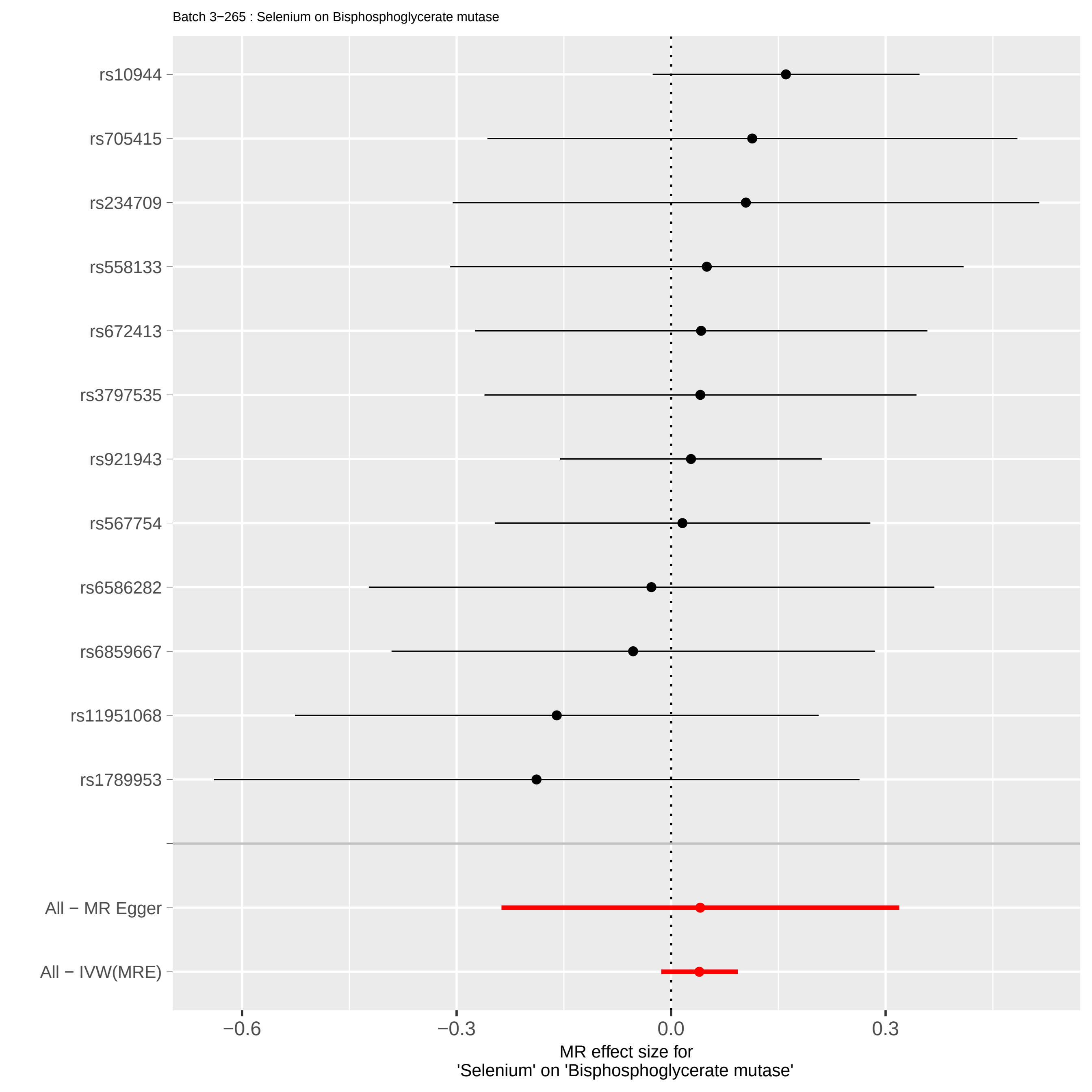

Batch 3−265 : Selenium on Bisphosphoglycerate mutase
rs10944
rs705415
rs234709
rs558133
rs672413
rs3797535
rs921943
rs567754
rs6586282
rs6859667
rs11951068
rs1789953
All − MR Egger
All − IVW(MRE)
−0.6
−0.3
0.0
0.3
MR effect size for
'Selenium' on 'Bisphosphoglycerate mutase'

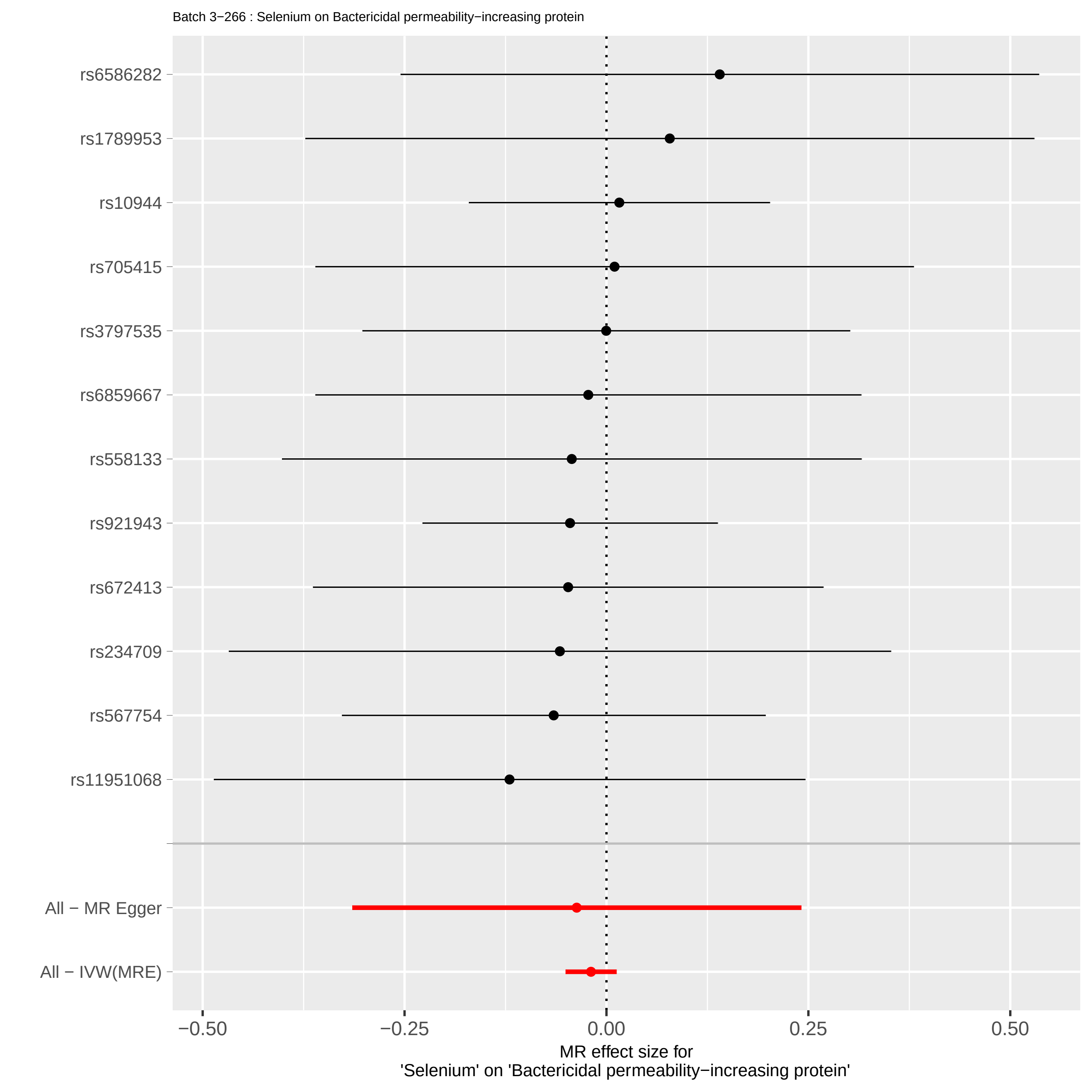

Batch 3−266 : Selenium on Bactericidal permeability−increasing protein
rs6586282
rs1789953
rs10944
rs705415
rs3797535
rs6859667
rs558133
rs921943
rs672413
rs234709
rs567754
rs11951068
All − MR Egger
All − IVW(MRE)
−0.50
−0.25
0.00
0.25
0.50
MR effect size for
'Selenium' on 'Bactericidal permeability−increasing protein'

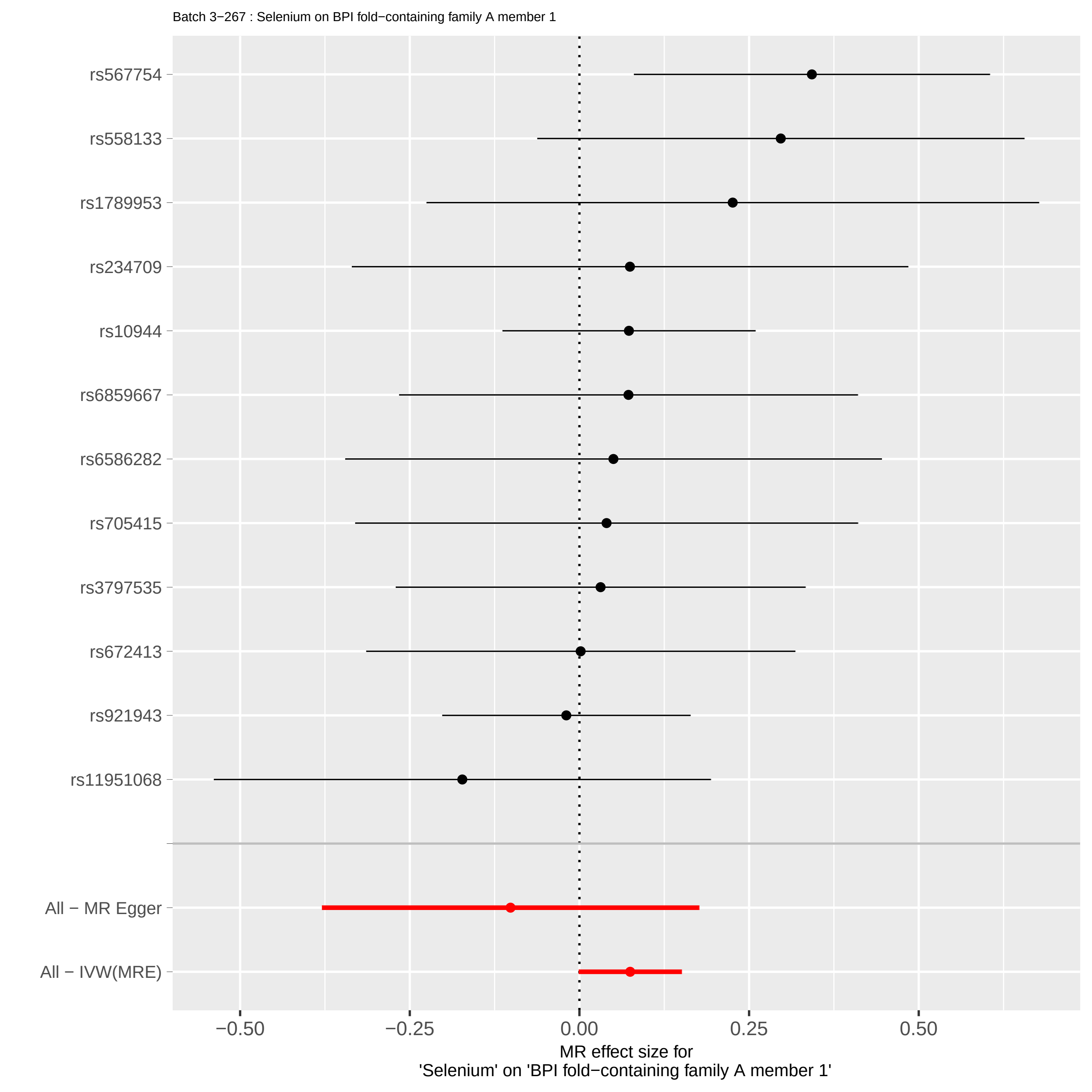

Batch 3−267 : Selenium on BPI fold−containing family A member 1
rs567754
rs558133
rs1789953
rs234709
rs10944
rs6859667
rs6586282
rs705415
rs3797535
rs672413
rs921943
rs11951068
All − MR Egger
All − IVW(MRE)
−0.50
−0.25
0.00
0.25
0.50
MR effect size for
'Selenium' on 'BPI fold−containing family A member 1'

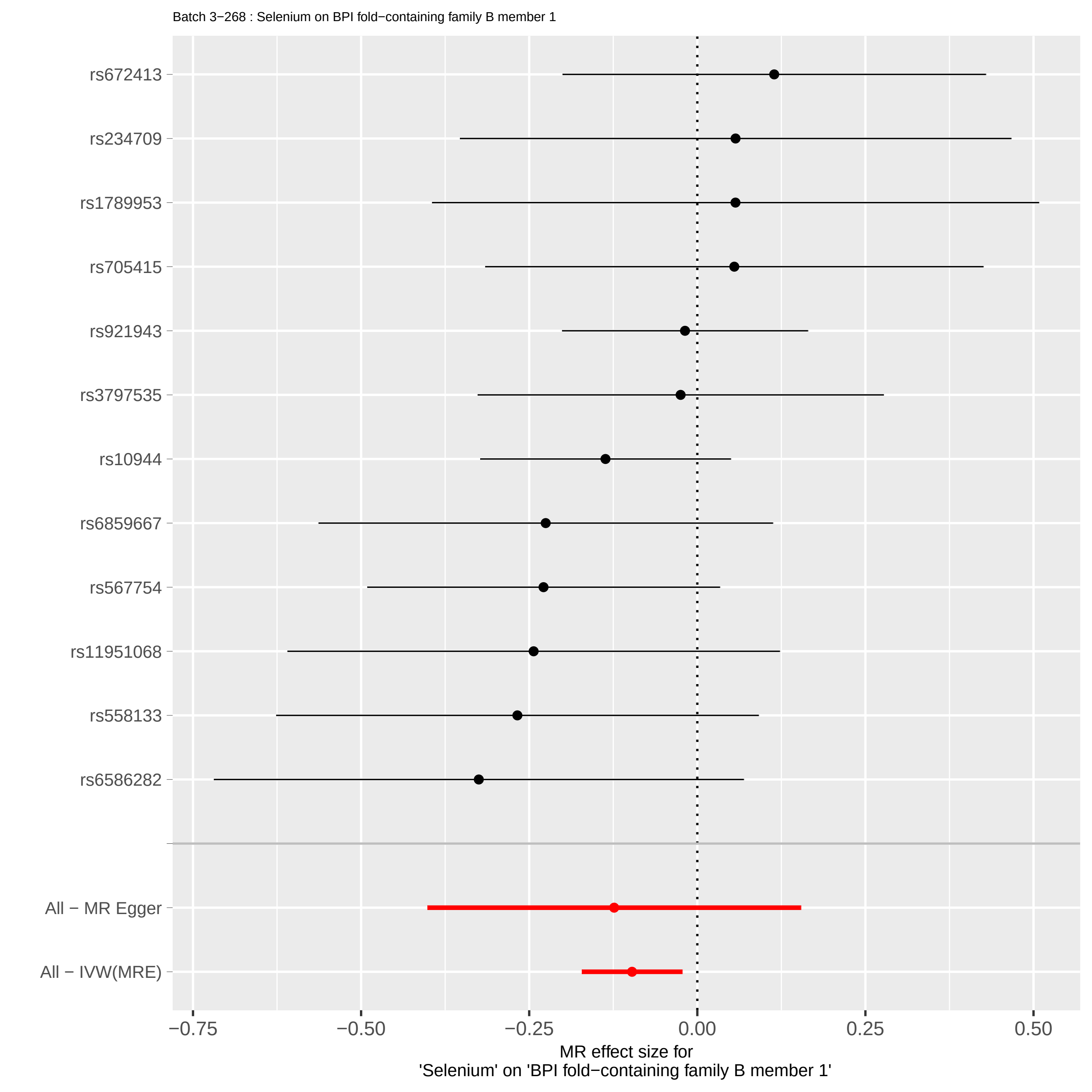

Batch 3−268 : Selenium on BPI fold−containing family B member 1
rs672413
rs234709
rs1789953
rs705415
rs921943
rs3797535
rs10944
rs6859667
rs567754
rs11951068
rs558133
rs6586282
All − MR Egger
All − IVW(MRE)
−0.75
−0.50
−0.25
0.00
0.25
0.50
MR effect size for
'Selenium' on 'BPI fold−containing family B member 1'

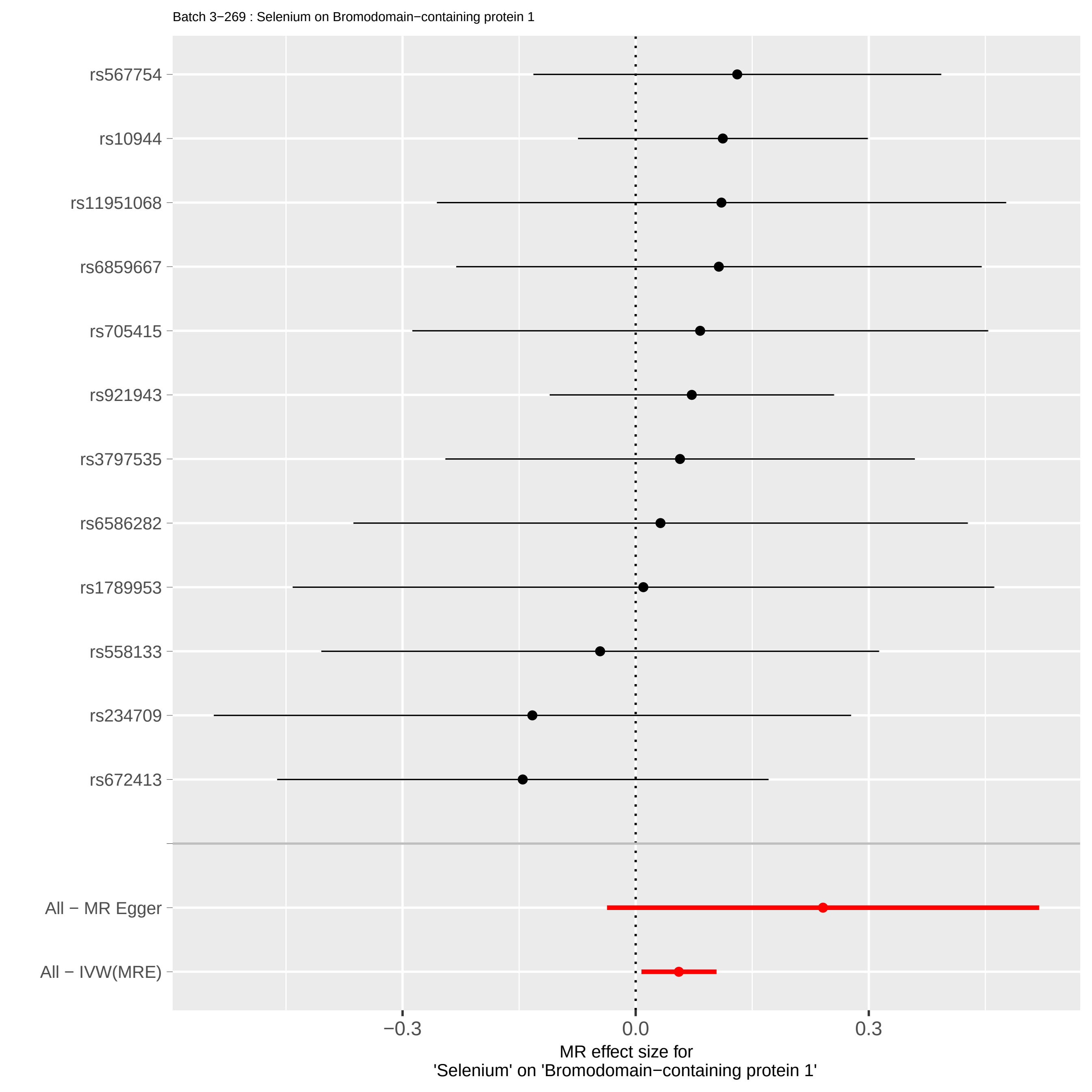

Batch 3−269 : Selenium on Bromodomain−containing protein 1
rs567754
rs10944
rs11951068
rs6859667
rs705415
rs921943
rs3797535
rs6586282
rs1789953
rs558133
rs234709
rs672413
All − MR Egger
All − IVW(MRE)
−0.3
0.0
0.3
MR effect size for
'Selenium' on 'Bromodomain−containing protein 1'

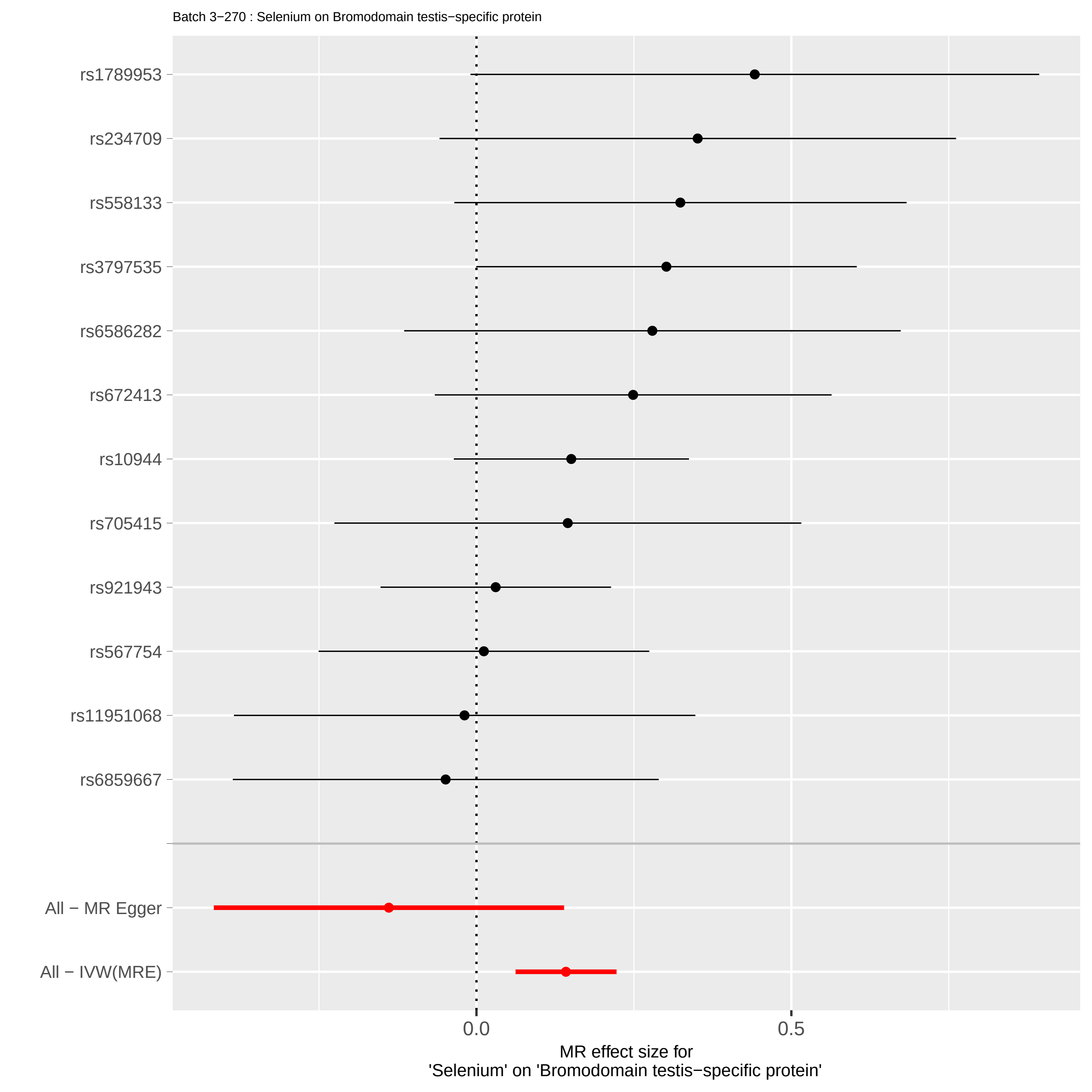
Batch 3−270 : Selenium on Bromodomain testis−specific protein
rs1789953
rs234709
rs558133
rs3797535
rs6586282
rs672413
rs10944
rs705415
rs921943
rs567754
rs11951068
rs6859667
All − MR Egger
All − IVW(MRE)
0.0
0.5
MR effect size for
'Selenium' on 'Bromodomain testis−specific protein'

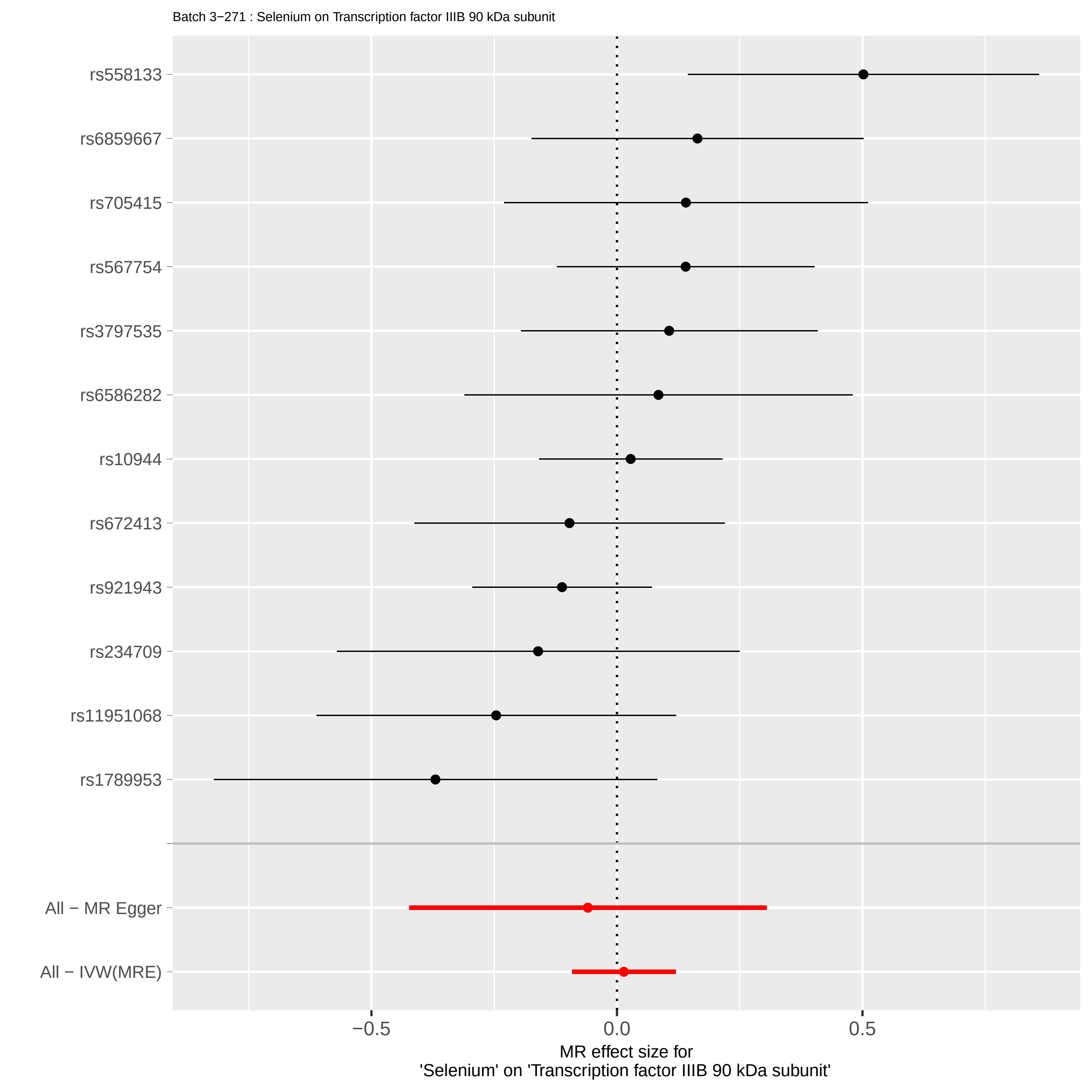
Batch 3−271 : Selenium on Transcription factor IIIB 90 kDa subunit
rs558133
rs6859667
rs705415
rs567754
rs3797535
rs6586282
rs10944
rs672413
rs921943
rs234709
rs11951068
rs1789953
All − MR Egger
All − IVW(MRE)
−0.5
0.0
0.5
MR effect size for
'Selenium' on 'Transcription factor IIIB 90 kDa subunit'

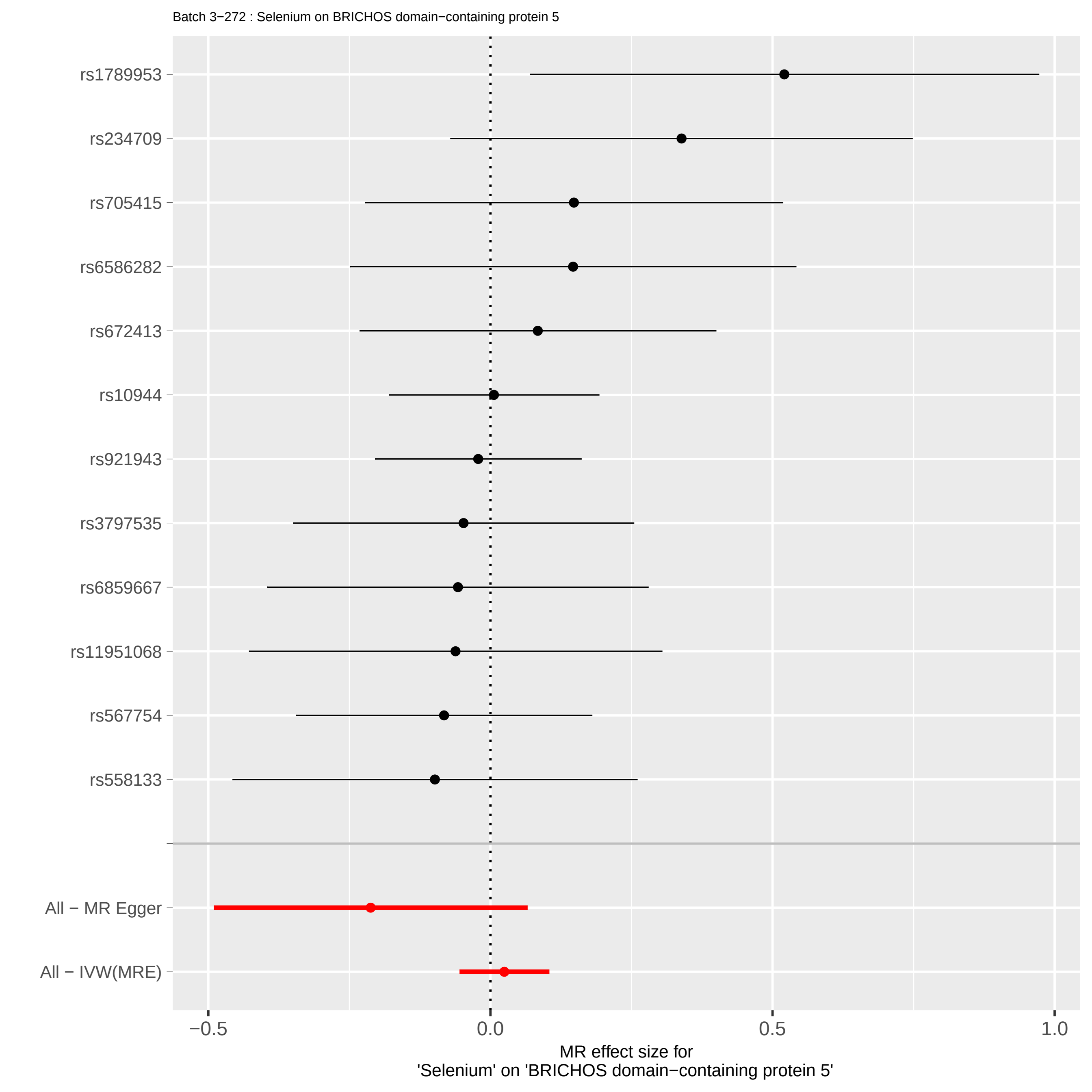

Batch 3−272 : Selenium on BRICHOS domain−containing protein 5
rs1789953
rs234709
rs705415
rs6586282
rs672413
rs10944
rs921943
rs3797535
rs6859667
rs11951068
rs567754
rs558133
All − MR Egger
All − IVW(MRE)
−0.5
0.0
0.5
1.0
MR effect size for
'Selenium' on 'BRICHOS domain−containing protein 5'

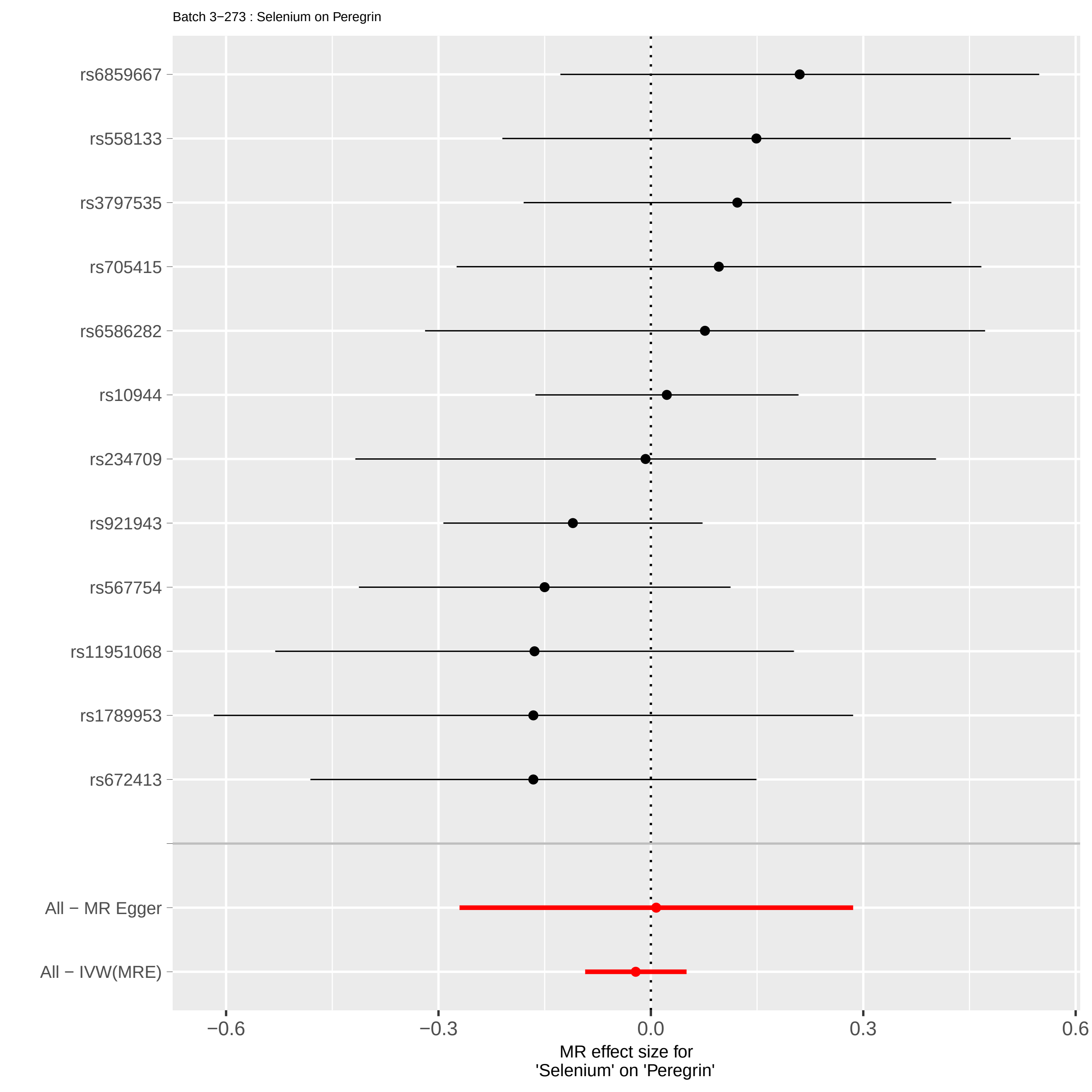
Batch 3−273 : Selenium on Peregrin
rs6859667
rs558133
rs3797535
rs705415
rs6586282
rs10944
rs234709
rs921943
rs567754
rs11951068
rs1789953
rs672413
All − MR Egger
All − IVW(MRE)
−0.6
−0.3
0.0
0.3
0.6
MR effect size for
'Selenium' on 'Peregrin'

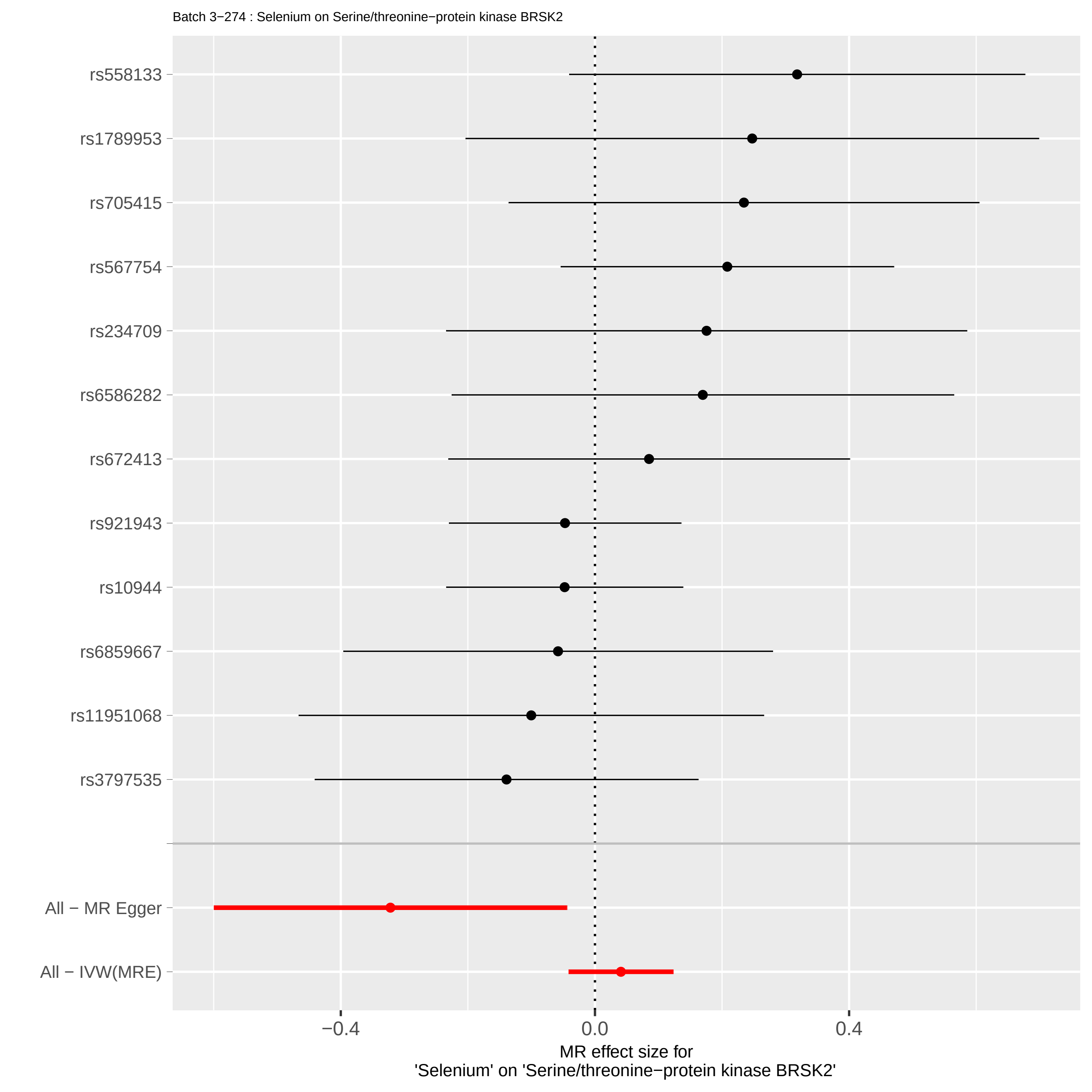
Batch 3−274 : Selenium on Serine/threonine−protein kinase BRSK2
rs558133
rs1789953
rs705415
rs567754
rs234709
rs6586282
rs672413
rs921943
rs10944
rs6859667
rs11951068
rs3797535
All − MR Egger
All − IVW(MRE)
−0.4
0.0
0.4
MR effect size for
'Selenium' on 'Serine/threonine−protein kinase BRSK2'

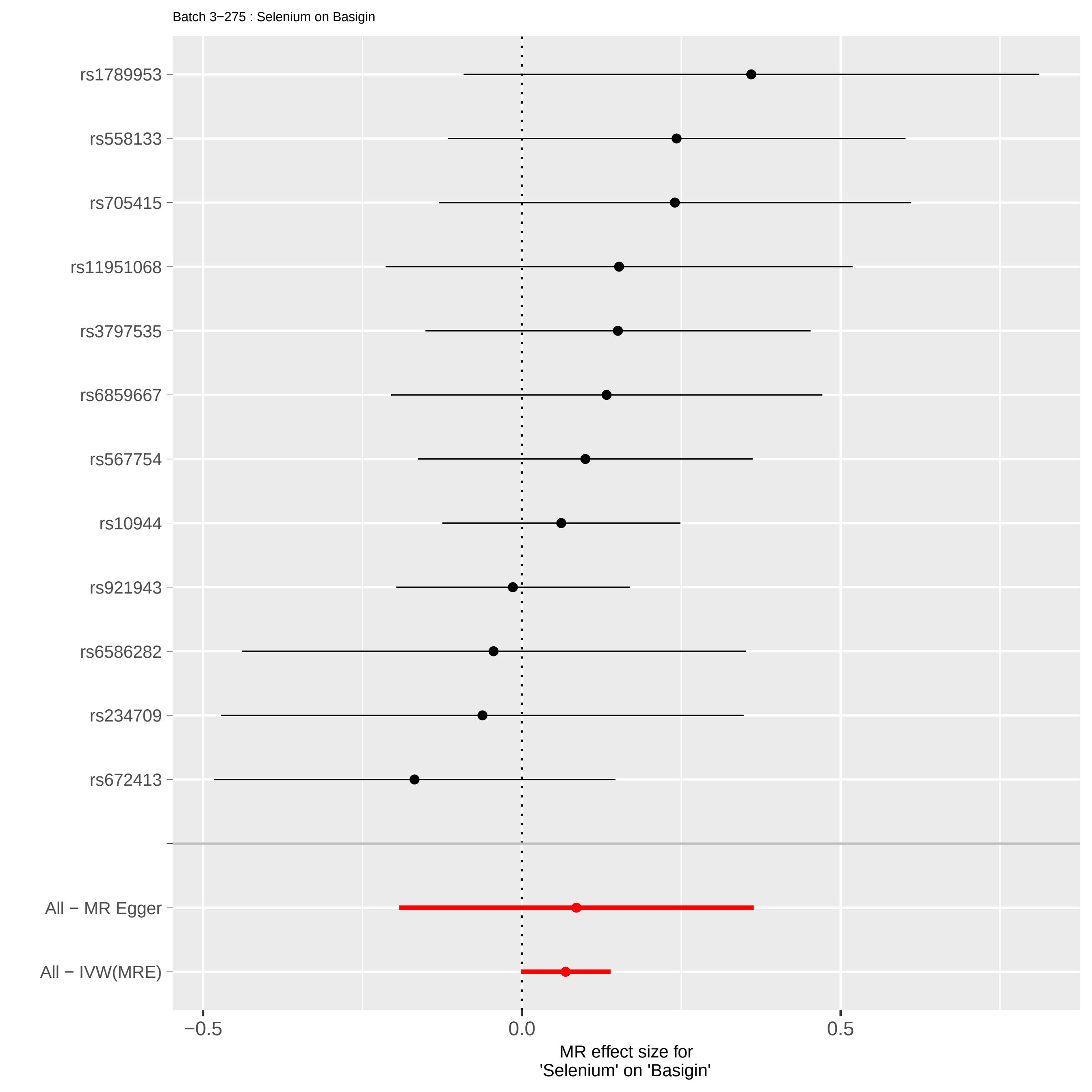
Batch 3−275 : Selenium on Basigin
rs1789953
rs558133
rs705415
rs11951068
rs3797535
rs6859667
rs567754
rs10944
rs921943
rs6586282
rs234709
rs672413
All − MR Egger
All − IVW(MRE)
−0.5
0.0
0.5
MR effect size for
'Selenium' on 'Basigin'

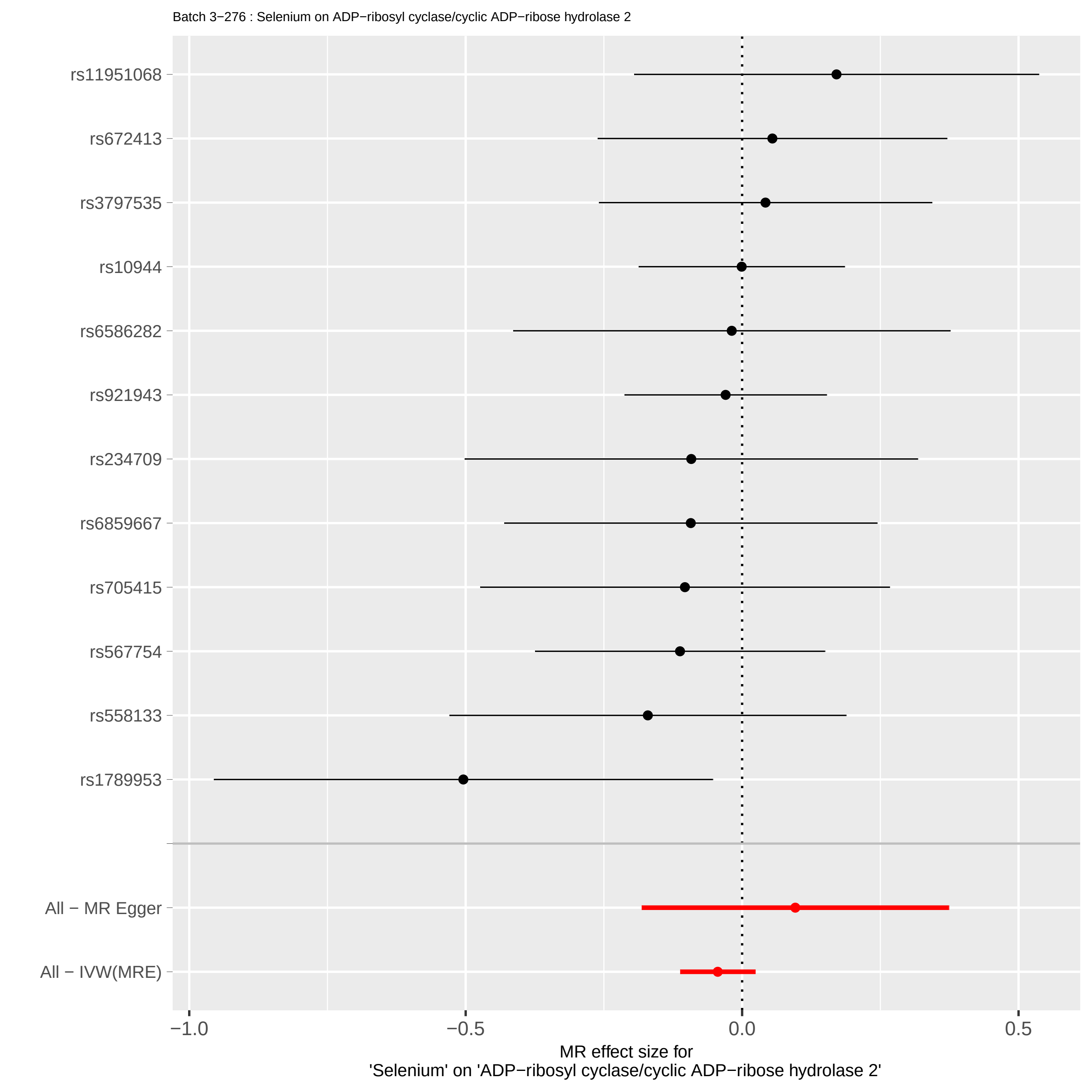
Batch 3−276 : Selenium on ADP−ribosyl cyclase/cyclic ADP−ribose hydrolase 2
rs11951068
rs672413
rs3797535
rs10944
rs6586282
rs921943
rs234709
rs6859667
rs705415
rs567754
rs558133
rs1789953
All − MR Egger
All − IVW(MRE)
−1.0
−0.5
0.0
0.5
MR effect size for
'Selenium' on 'ADP−ribosyl cyclase/cyclic ADP−ribose hydrolase 2'

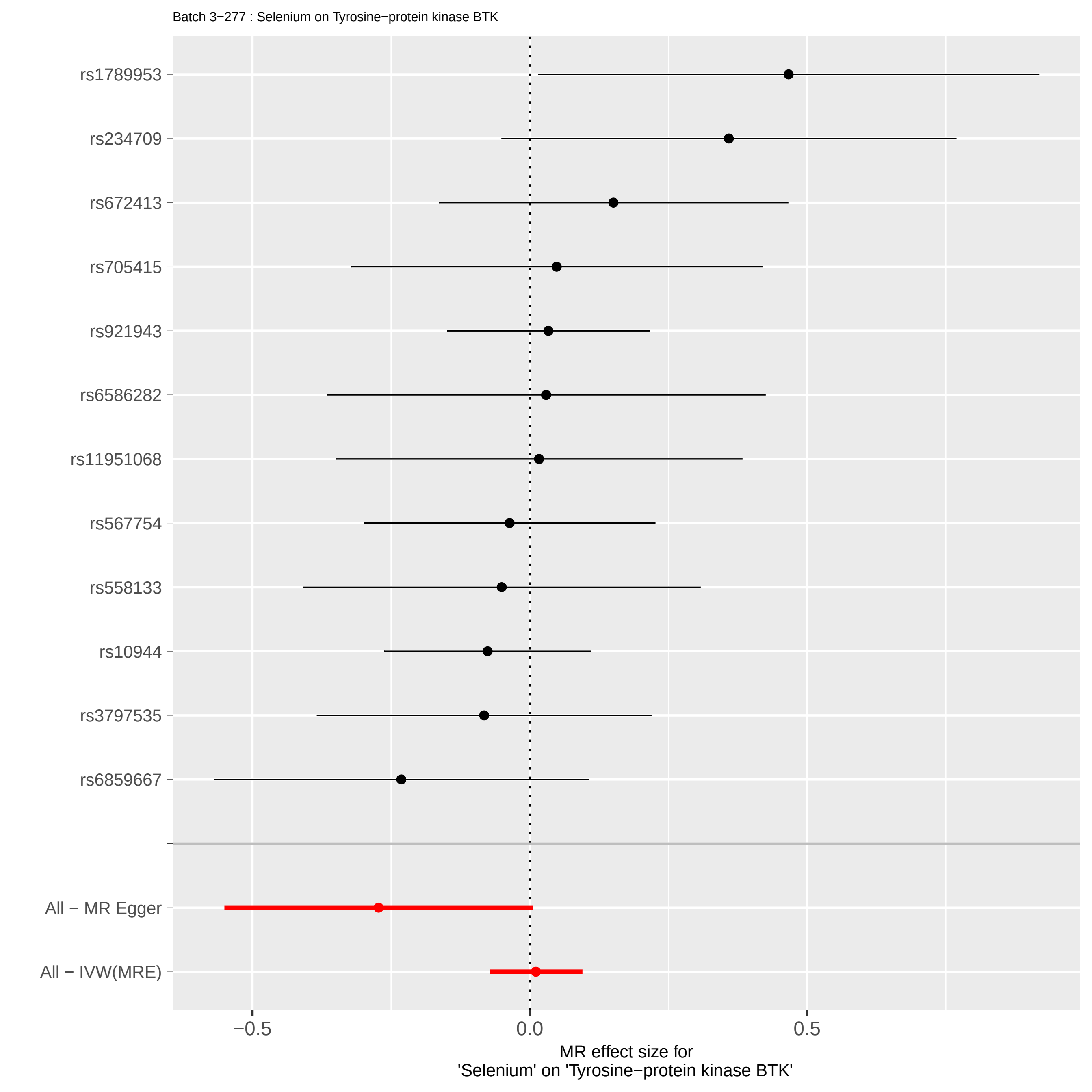
Batch 3−277 : Selenium on Tyrosine−protein kinase BTK
rs1789953
rs234709
rs672413
rs705415
rs921943
rs6586282
rs11951068
rs567754
rs558133
rs10944
rs3797535
rs6859667
All − MR Egger
All − IVW(MRE)
−0.5
0.0
0.5
MR effect size for
'Selenium' on 'Tyrosine−protein kinase BTK'

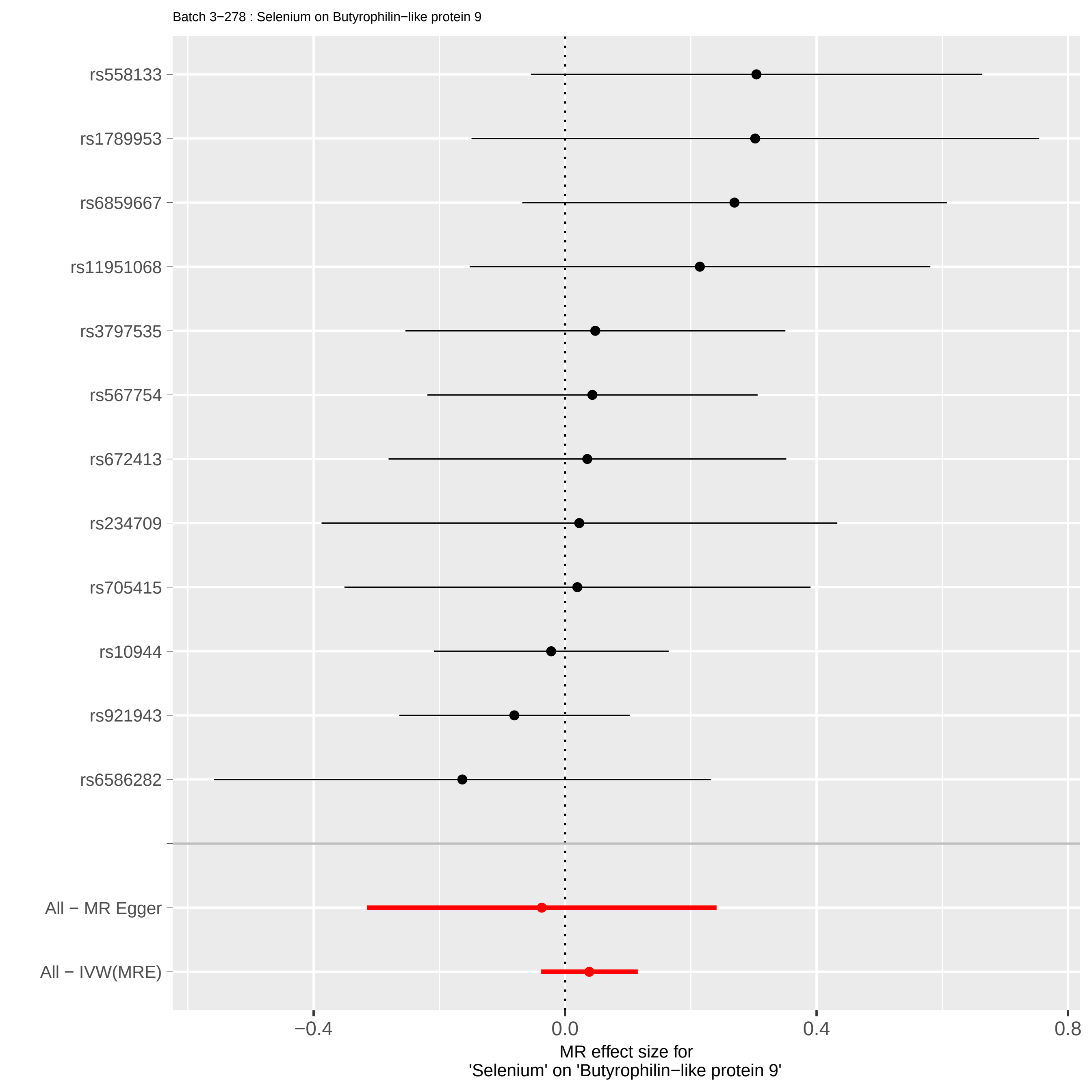
Batch 3−278 : Selenium on Butyrophilin−like protein 9
rs558133
rs1789953
rs6859667
rs11951068
rs3797535
rs567754
rs672413
rs234709
rs705415
rs10944
rs921943
rs6586282
All − MR Egger
All − IVW(MRE)
−0.4
0.0
0.4
0.8
MR effect size for
'Selenium' on 'Butyrophilin−like protein 9'

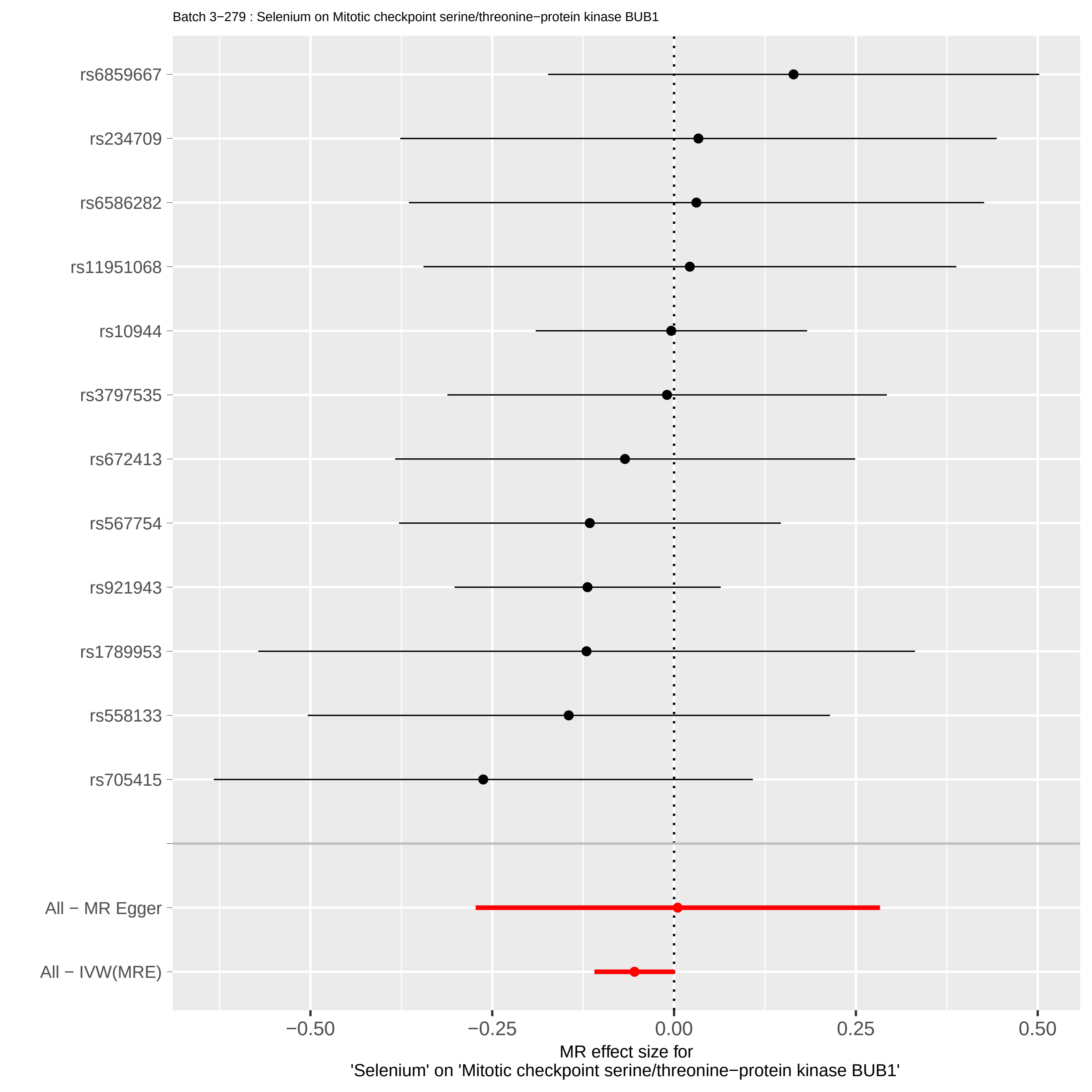

Batch 3−279 : Selenium on Mitotic checkpoint serine/threonine−protein kinase BUB1
rs6859667
rs234709
rs6586282
rs11951068
rs10944
rs3797535
rs672413
rs567754
rs921943
rs1789953
rs558133
rs705415
All − MR Egger
All − IVW(MRE)
−0.50
−0.25
0.00
0.25
0.50
MR effect size for
'Selenium' on 'Mitotic checkpoint serine/threonine−protein kinase BUB1'

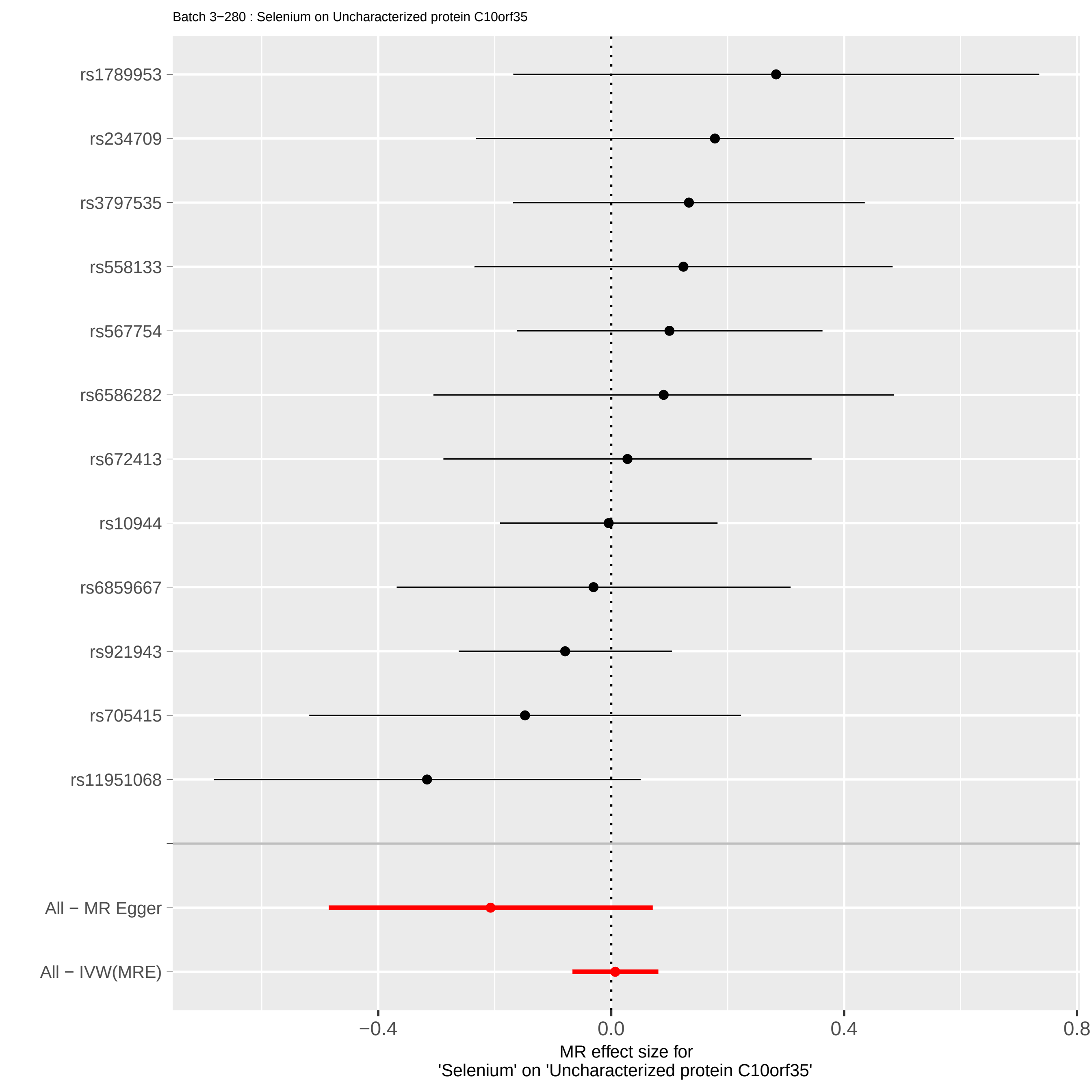
Batch 3−280 : Selenium on Uncharacterized protein C10orf35
rs1789953
rs234709
rs3797535
rs558133
rs567754
rs6586282
rs672413
rs10944
rs6859667
rs921943
rs705415
rs11951068
All − MR Egger
All − IVW(MRE)
−0.4
0.0
0.4
0.8
MR effect size for
'Selenium' on 'Uncharacterized protein C10orf35'

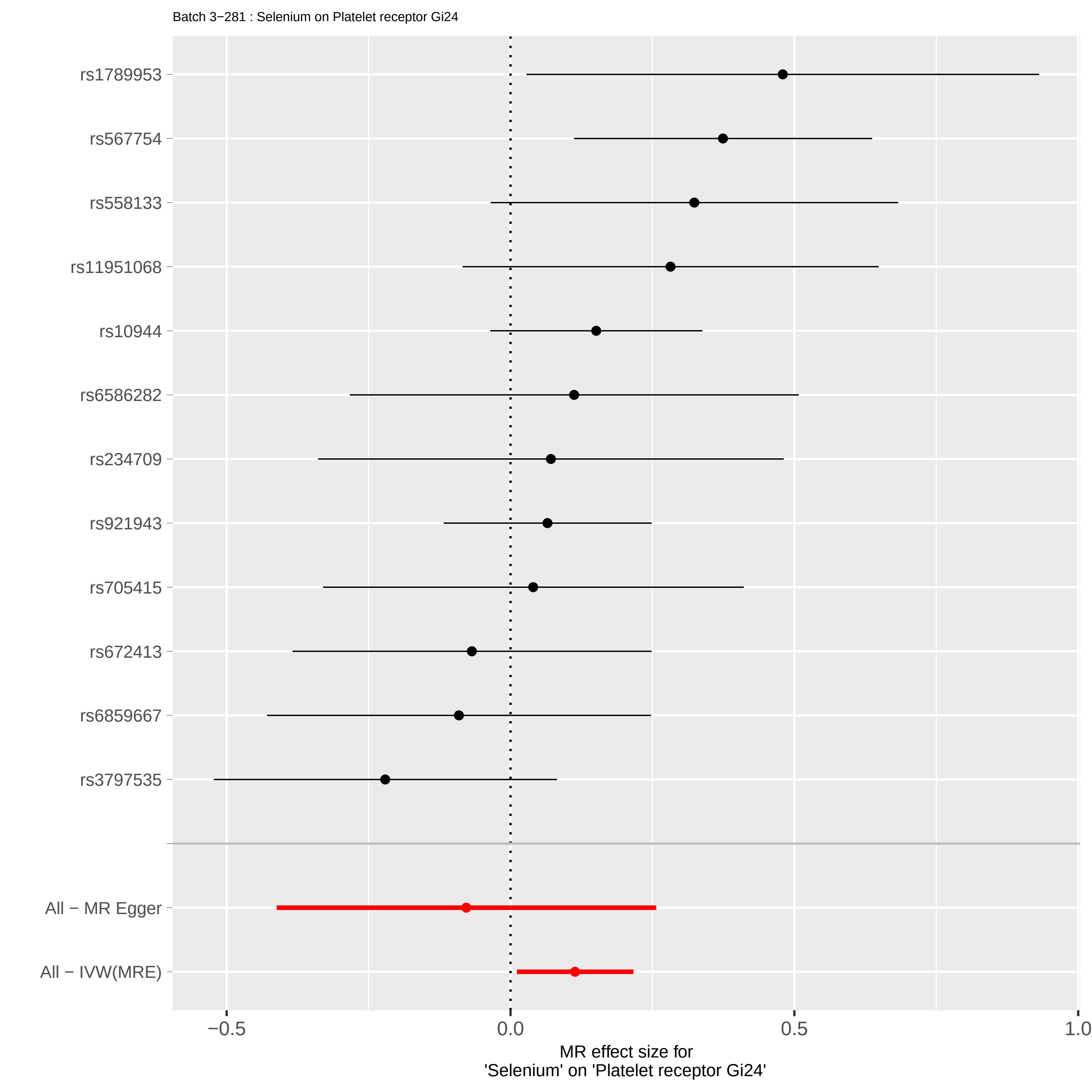

Batch 3−281 : Selenium on Platelet receptor Gi24
rs1789953
rs567754
rs558133
rs11951068
rs10944
rs6586282
rs234709
rs921943
rs705415
rs672413
rs6859667
rs3797535
All − MR Egger
All − IVW(MRE)
−0.5
0.0
0.5
1.0
MR effect size for
'Selenium' on 'Platelet receptor Gi24'

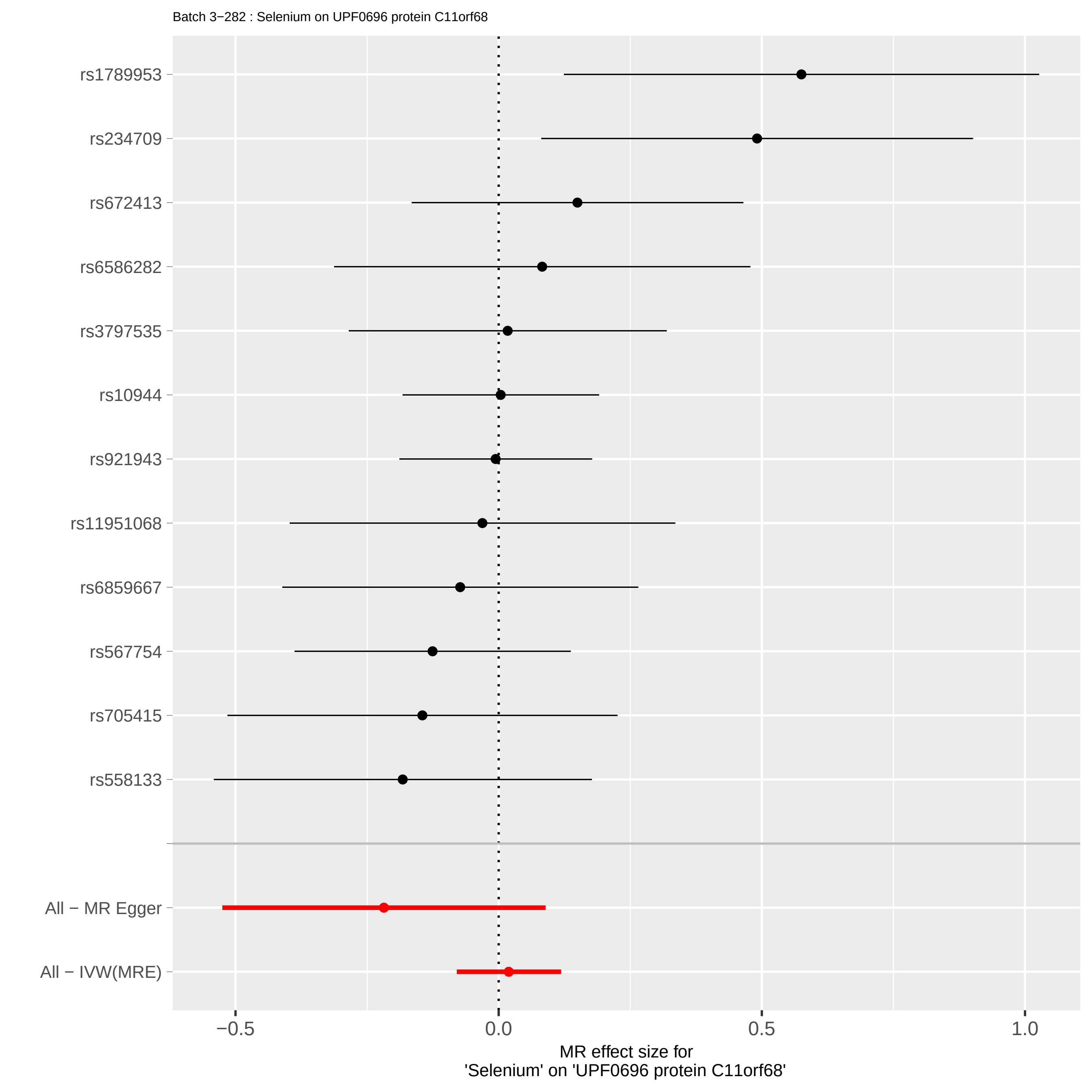
Batch 3−282 : Selenium on UPF0696 protein C11orf68
rs1789953
rs234709
rs672413
rs6586282
rs3797535
rs10944
rs921943
rs11951068
rs6859667
rs567754
rs705415
rs558133
All − MR Egger
All − IVW(MRE)
−0.5
0.0
0.5
1.0
MR effect size for
'Selenium' on 'UPF0696 protein C11orf68'

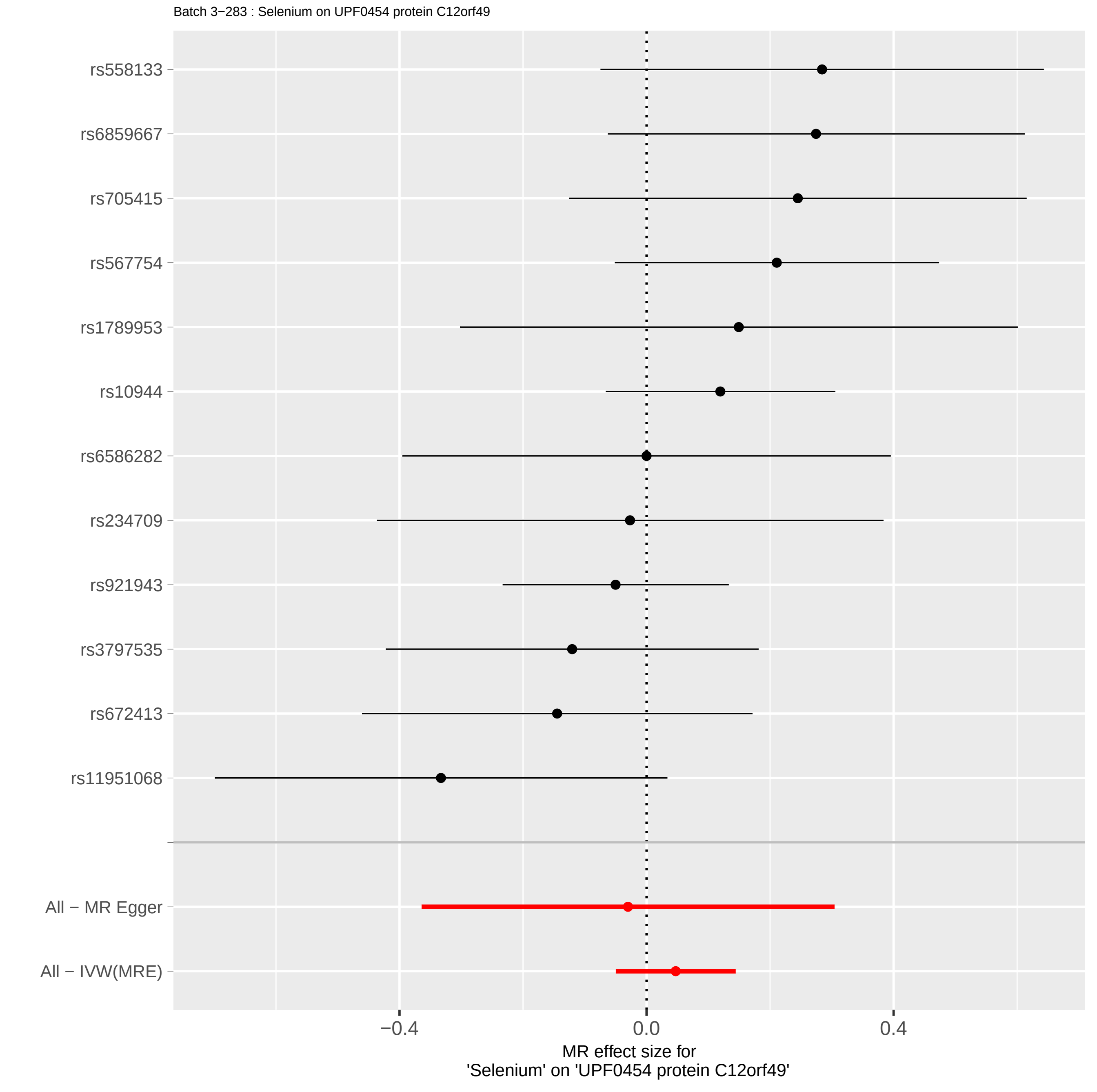
Batch 3−283 : Selenium on UPF0454 protein C12orf49
rs558133
rs6859667
rs705415
rs567754
rs1789953
rs10944
rs6586282
rs234709
rs921943
rs3797535
rs672413
rs11951068
All − MR Egger
All − IVW(MRE)
−0.4
0.0
0.4
MR effect size for
'Selenium' on 'UPF0454 protein C12orf49'

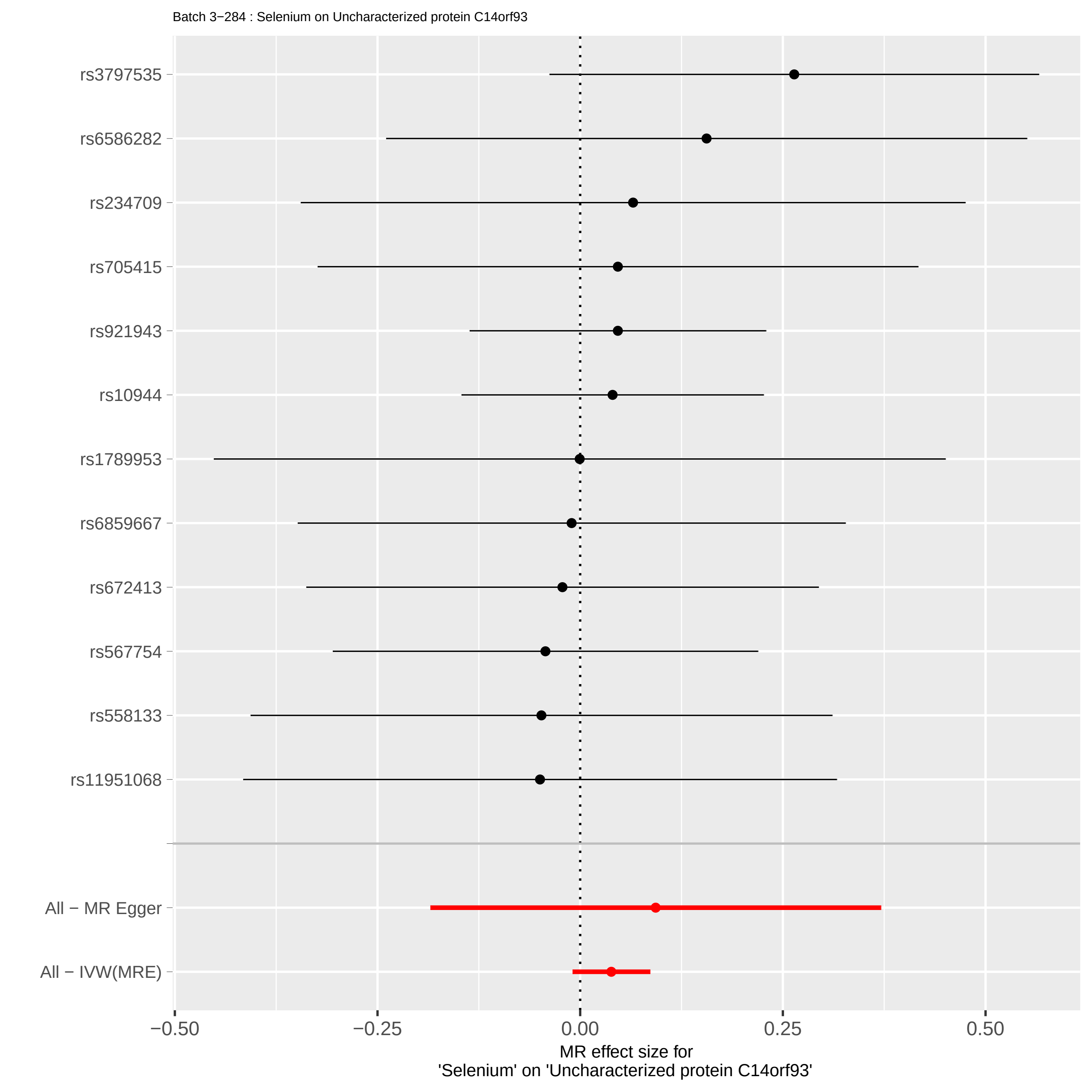
Batch 3−284 : Selenium on Uncharacterized protein C14orf93
rs3797535
rs6586282
rs234709
rs705415
rs921943
rs10944
rs1789953
rs6859667
rs672413
rs567754
rs558133
rs11951068
All − MR Egger
All − IVW(MRE)
−0.50
−0.25
0.00
0.25
0.50
MR effect size for
'Selenium' on 'Uncharacterized protein C14orf93'

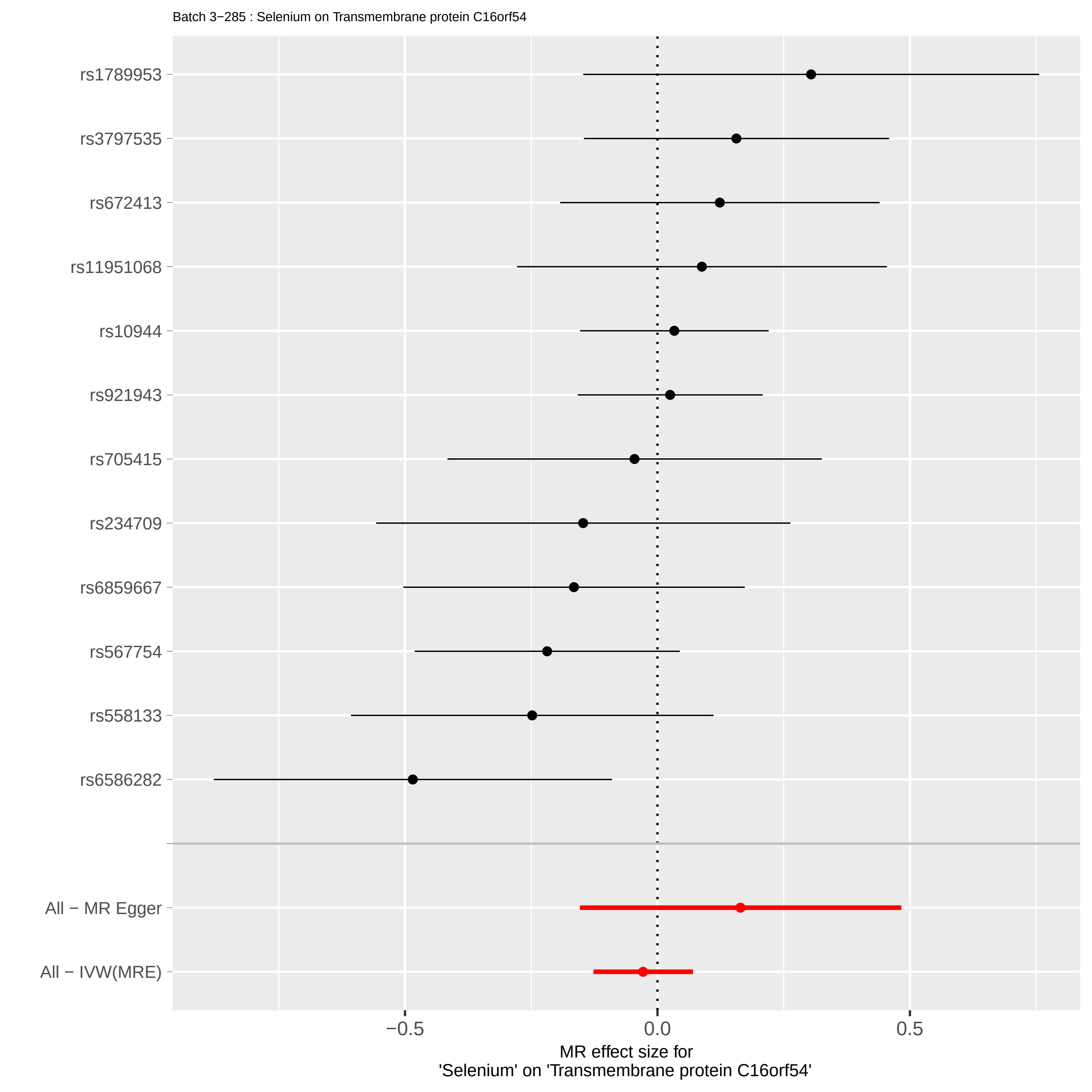
Batch 3−285 : Selenium on Transmembrane protein C16orf54
rs1789953
rs3797535
rs672413
rs11951068
rs10944
rs921943
rs705415
rs234709
rs6859667
rs567754
rs558133
rs6586282
All − MR Egger
All − IVW(MRE)
−0.5
0.0
0.5
MR effect size for
'Selenium' on 'Transmembrane protein C16orf54'

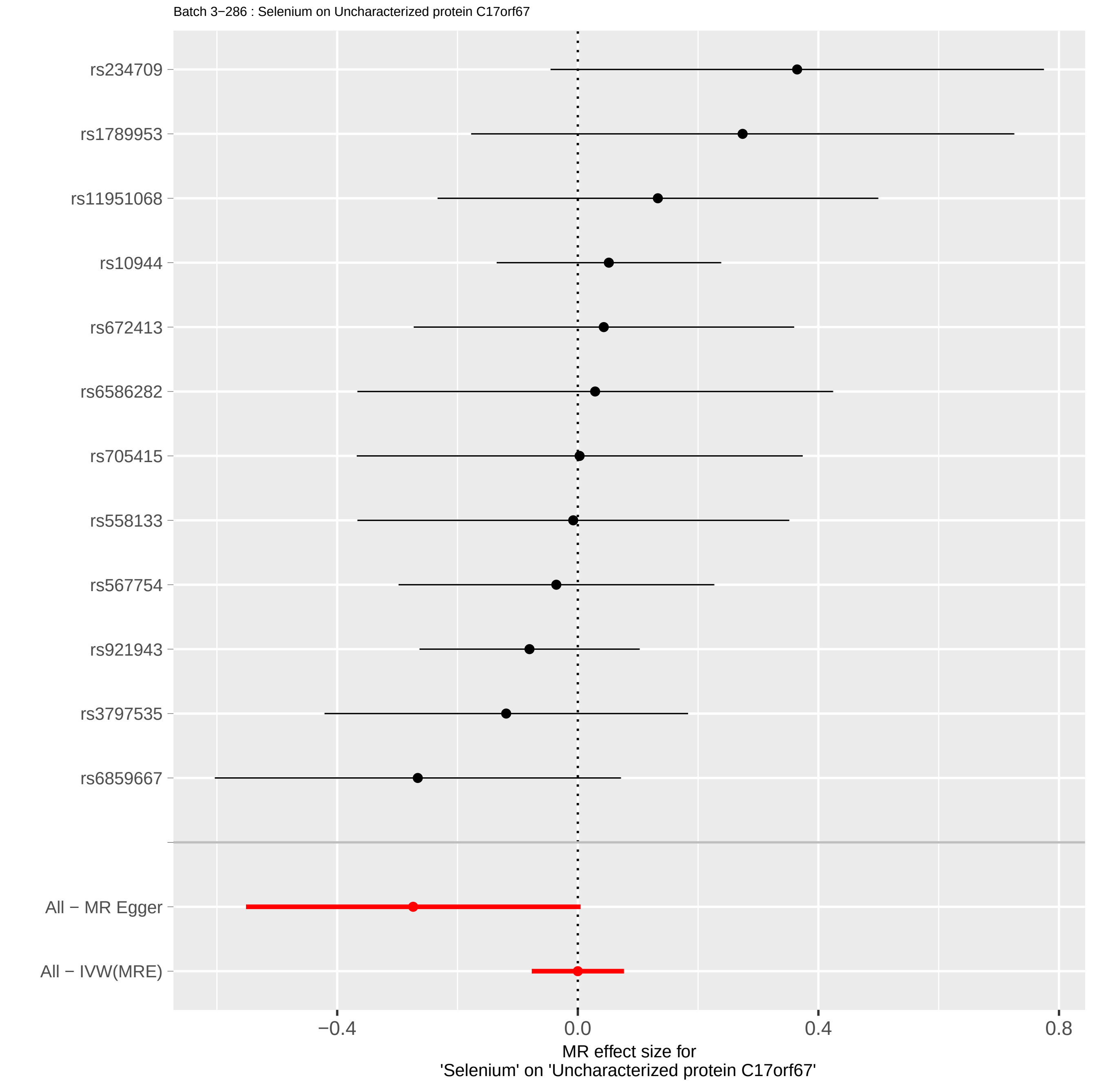

Batch 3−286 : Selenium on Uncharacterized protein C17orf67
rs234709
rs1789953
rs11951068
rs10944
rs672413
rs6586282
rs705415
rs558133
rs567754
rs921943
rs3797535
rs6859667
All − MR Egger
All − IVW(MRE)
−0.4
0.0
0.4
0.8
MR effect size for
'Selenium' on 'Uncharacterized protein C17orf67'

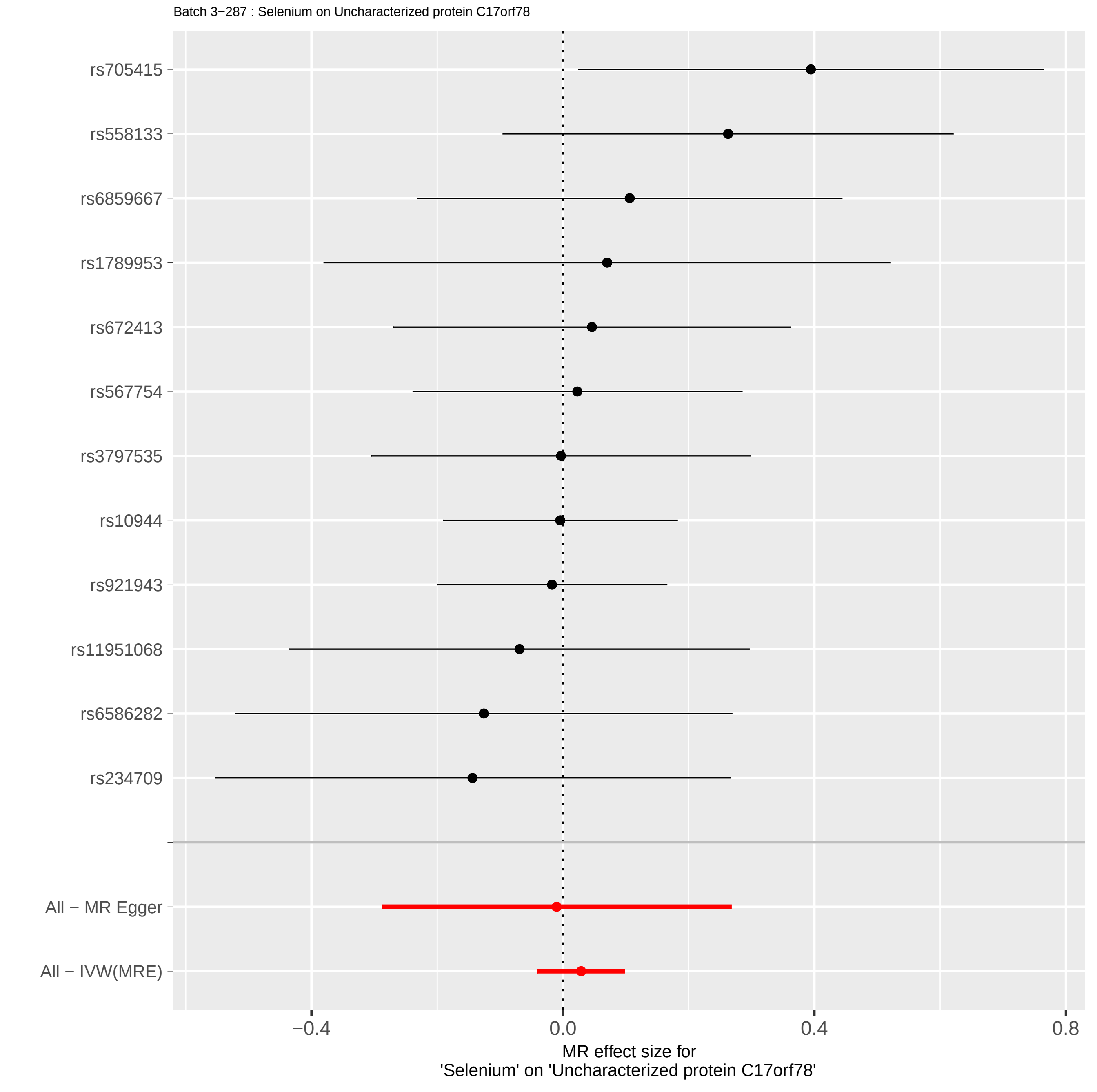

Batch 3−287 : Selenium on Uncharacterized protein C17orf78
rs705415
rs558133
rs6859667
rs1789953
rs672413
rs567754
rs3797535
rs10944
rs921943
rs11951068
rs6586282
rs234709
All − MR Egger
All − IVW(MRE)
−0.4
0.0
0.4
0.8
MR effect size for
'Selenium' on 'Uncharacterized protein C17orf78'

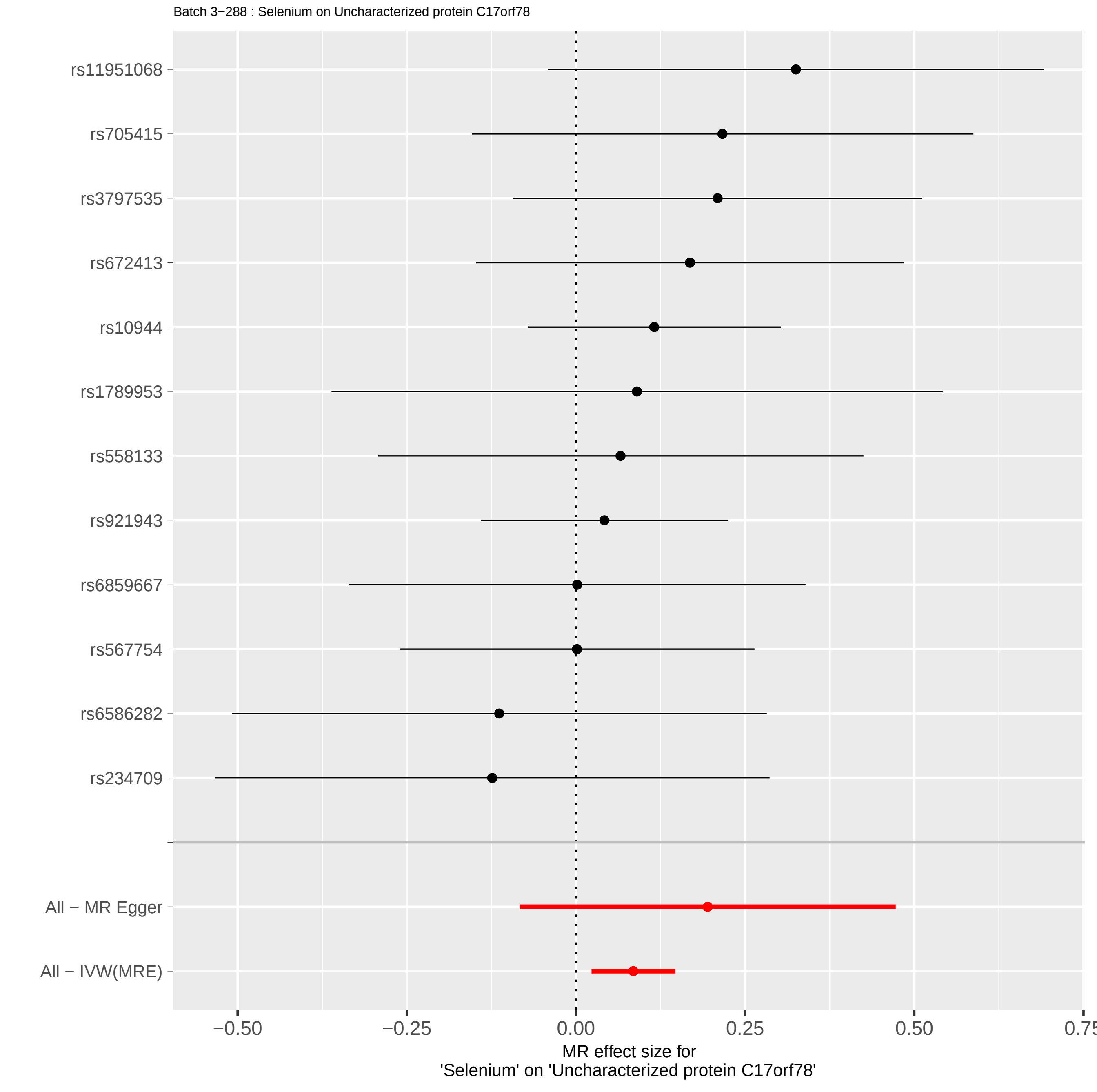

Batch 3−288 : Selenium on Uncharacterized protein C17orf78
rs11951068
rs705415
rs3797535
rs672413
rs10944
rs1789953
rs558133
rs921943
rs6859667
rs567754
rs6586282
rs234709
All − MR Egger
All − IVW(MRE)
−0.50
−0.25
0.00
0.25
0.50
0.75
MR effect size for
'Selenium' on 'Uncharacterized protein C17orf78'

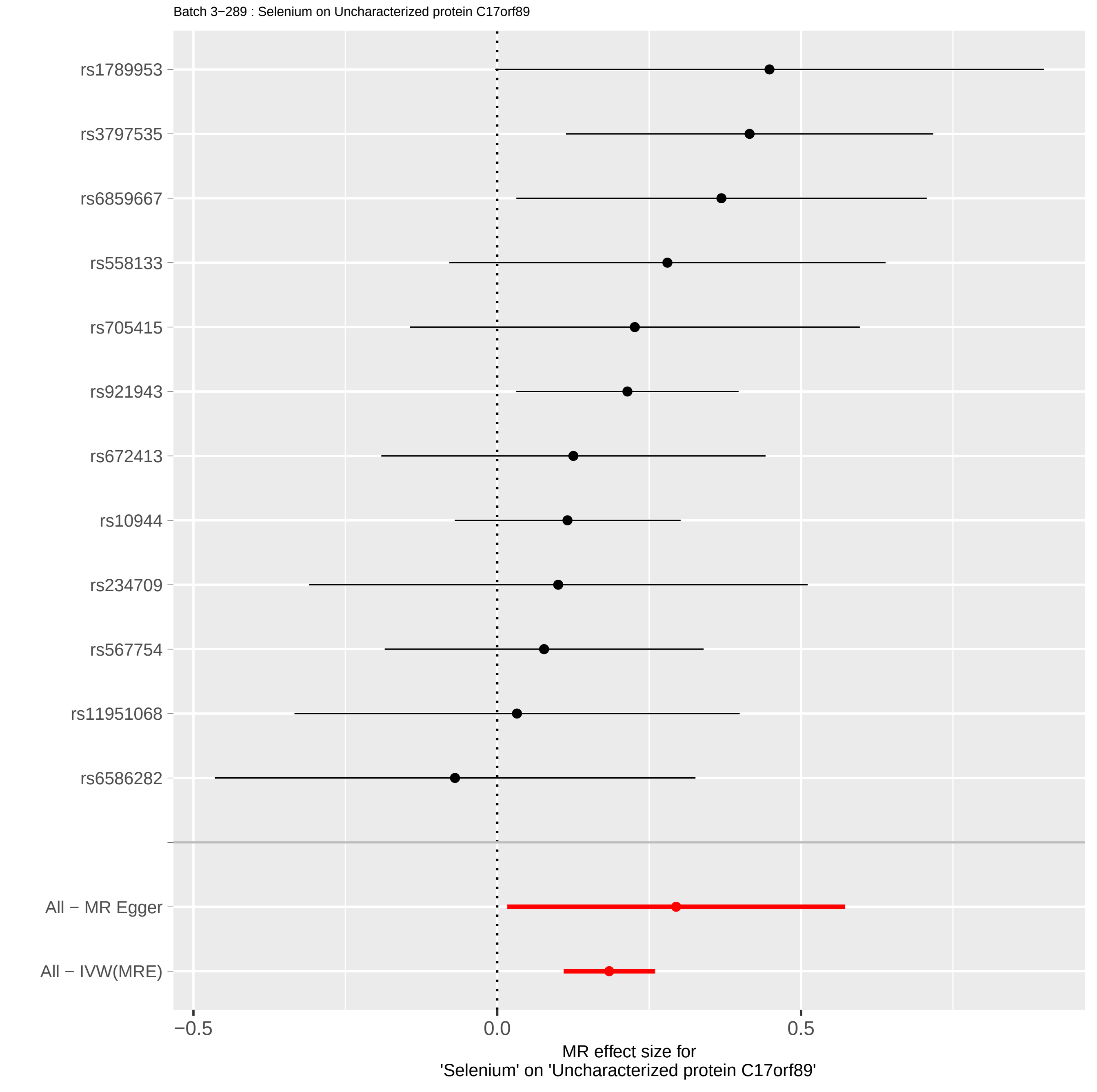
Batch 3−289 : Selenium on Uncharacterized protein C17orf89
rs1789953
rs3797535
rs6859667
rs558133
rs705415
rs921943
rs672413
rs10944
rs234709
rs567754
rs11951068
rs6586282
All − MR Egger
All − IVW(MRE)
−0.5
0.0
0.5
MR effect size for
'Selenium' on 'Uncharacterized protein C17orf89'

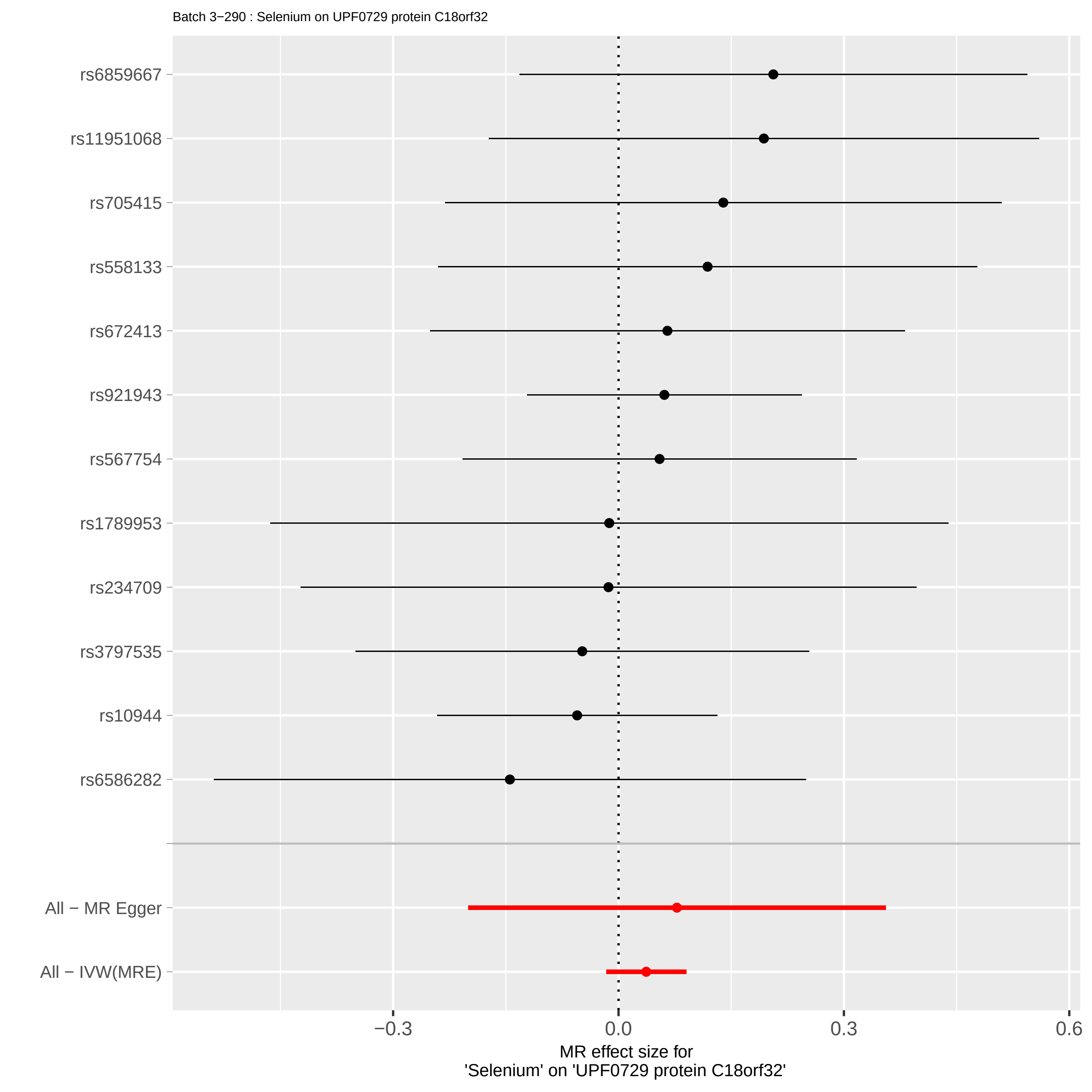

Batch 3−290 : Selenium on UPF0729 protein C18orf32
rs6859667
rs11951068
rs705415
rs558133
rs672413
rs921943
rs567754
rs1789953
rs234709
rs3797535
rs10944
rs6586282
All − MR Egger
All − IVW(MRE)
−0.3
0.0
0.3
0.6
MR effect size for
'Selenium' on 'UPF0729 protein C18orf32'

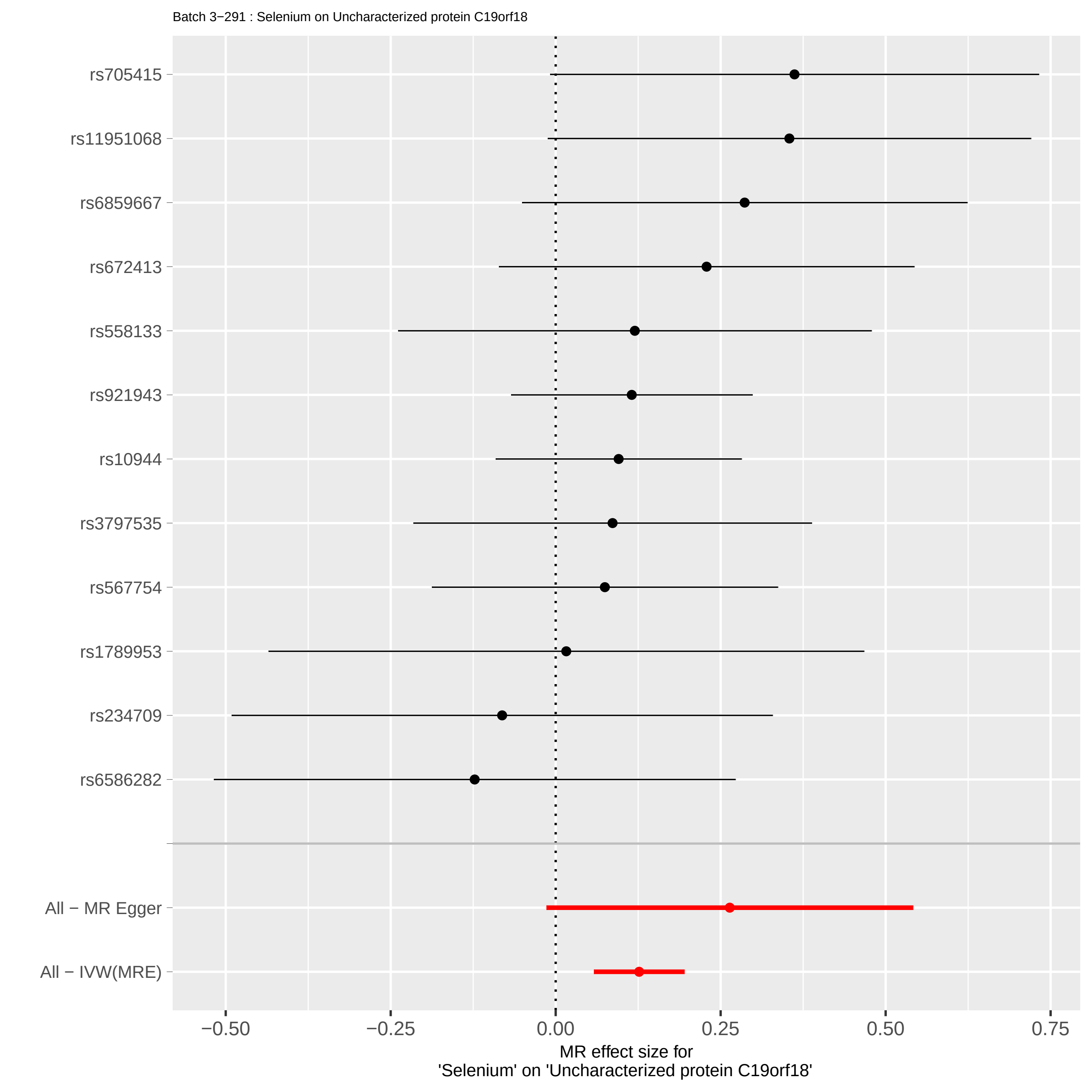

Batch 3−291 : Selenium on Uncharacterized protein C19orf18
rs705415
rs11951068
rs6859667
rs672413
rs558133
rs921943
rs10944
rs3797535
rs567754
rs1789953
rs234709
rs6586282
All − MR Egger
All − IVW(MRE)
−0.50
−0.25
0.00
0.25
0.50
0.75
MR effect size for
'Selenium' on 'Uncharacterized protein C19orf18'

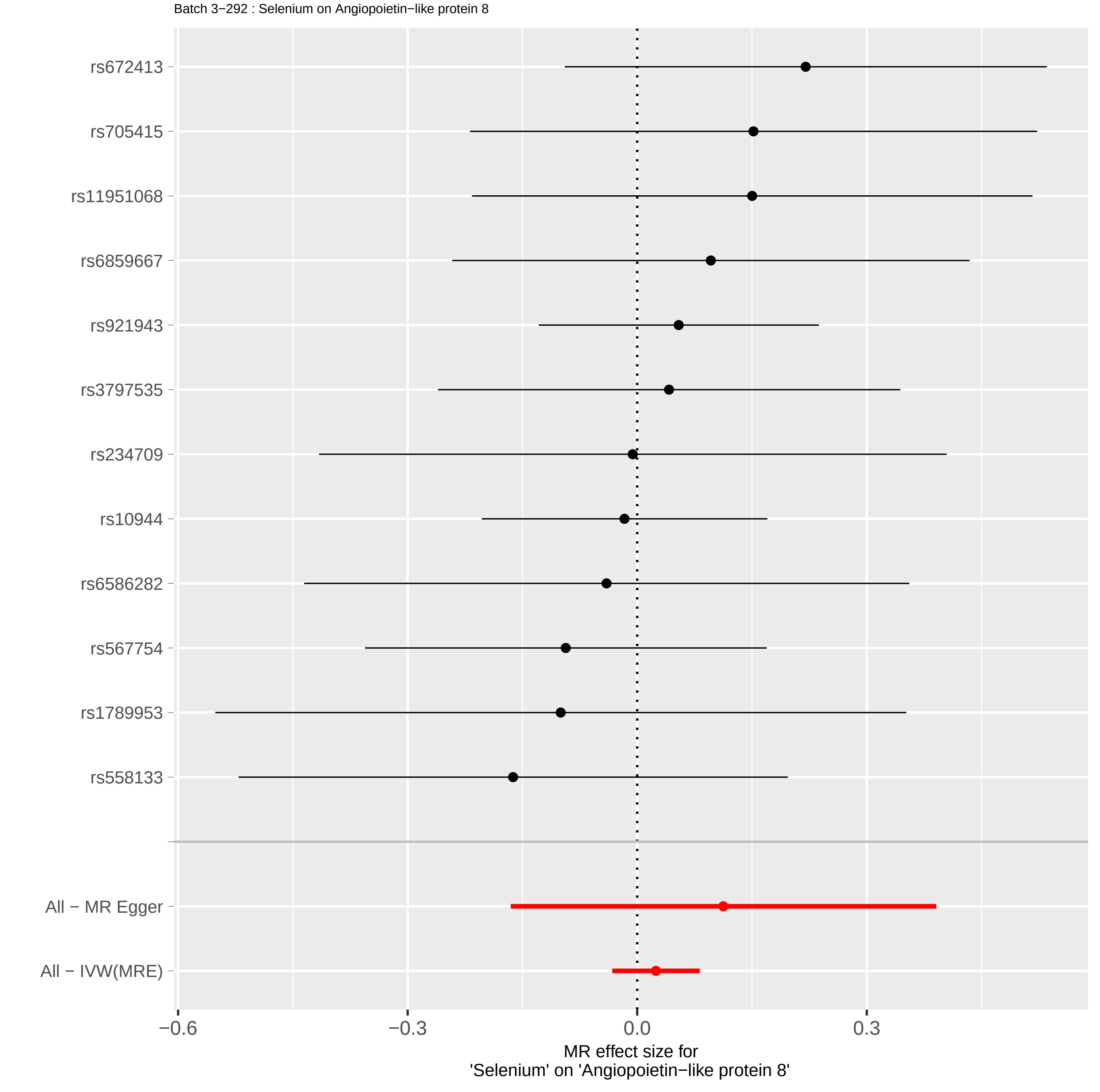
Batch 3−292 : Selenium on Angiopoietin−like protein 8
rs672413
rs705415
rs11951068
rs6859667
rs921943
rs3797535
rs234709
rs10944
rs6586282
rs567754
rs1789953
rs558133
All − MR Egger
All − IVW(MRE)
−0.6
−0.3
0.0
0.3
MR effect size for
'Selenium' on 'Angiopoietin−like protein 8'

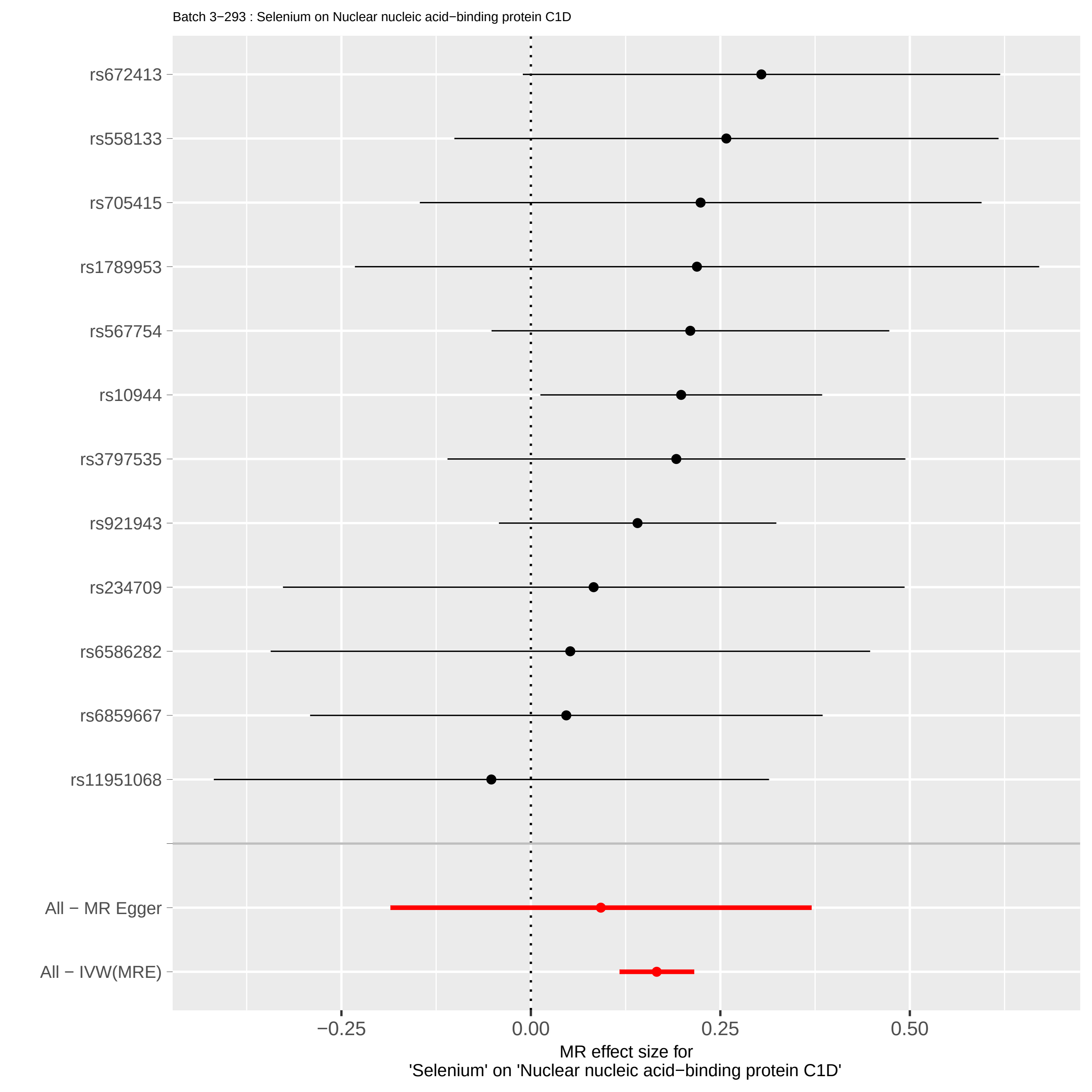

Batch 3−293 : Selenium on Nuclear nucleic acid−binding protein C1D
rs672413
rs558133
rs705415
rs1789953
rs567754
rs10944
rs3797535
rs921943
rs234709
rs6586282
rs6859667
rs11951068
All − MR Egger
All − IVW(MRE)
−0.25
0.00
0.25
0.50
MR effect size for
'Selenium' on 'Nuclear nucleic acid−binding protein C1D'

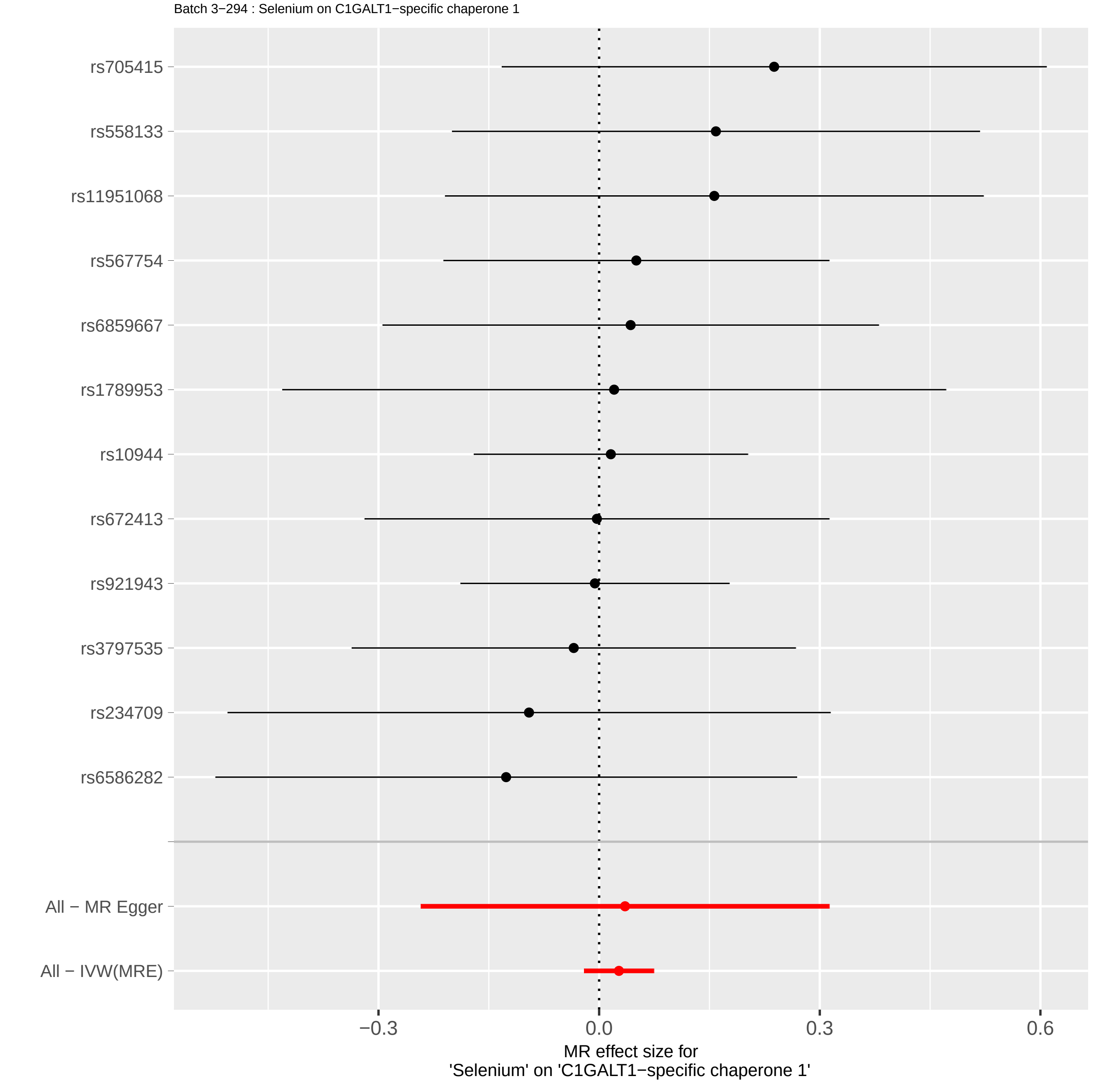
Batch 3−294 : Selenium on C1GALT1−specific chaperone 1
rs705415
rs558133
rs11951068
rs567754
rs6859667
rs1789953
rs10944
rs672413
rs921943
rs3797535
rs234709
rs6586282
All − MR Egger
All − IVW(MRE)
−0.3
0.0
0.3
0.6
MR effect size for
'Selenium' on 'C1GALT1−specific chaperone 1'

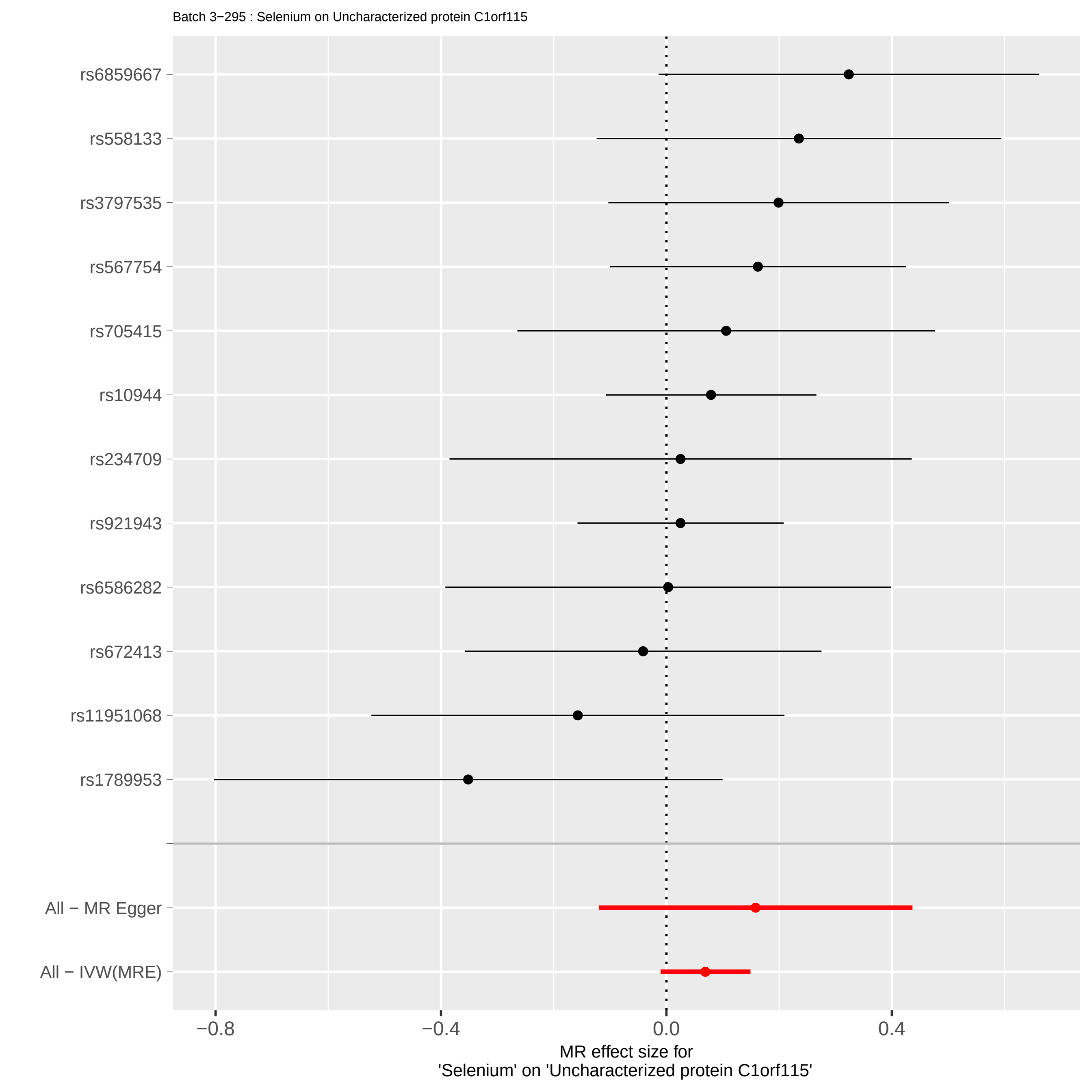

Batch 3−295 : Selenium on Uncharacterized protein C1orf115
rs6859667
rs558133
rs3797535
rs567754
rs705415
rs10944
rs234709
rs921943
rs6586282
rs672413
rs11951068
rs1789953
All − MR Egger
All − IVW(MRE)
−0.8
−0.4
0.0
0.4
MR effect size for
'Selenium' on 'Uncharacterized protein C1orf115'

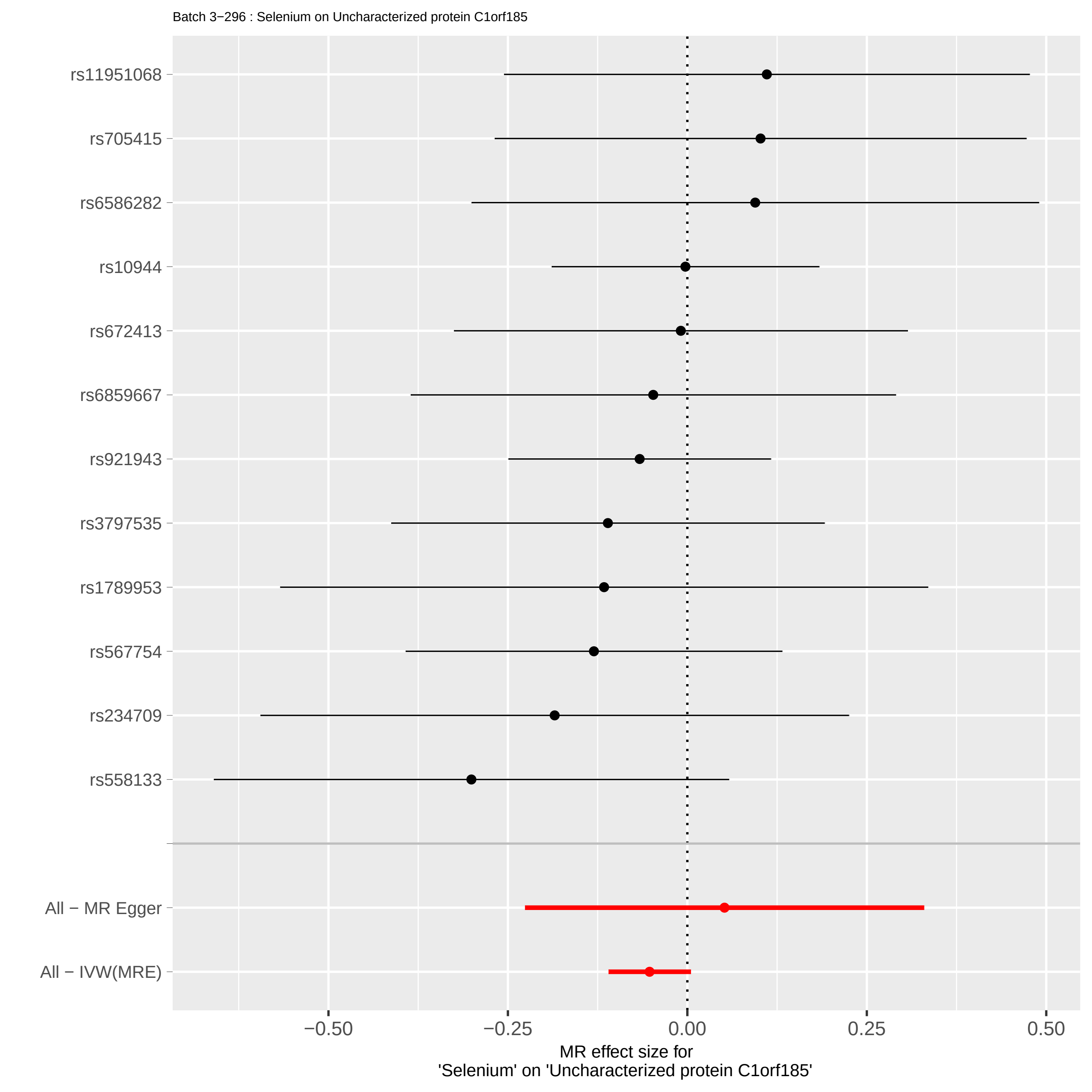

Batch 3−296 : Selenium on Uncharacterized protein C1orf185
rs11951068
rs705415
rs6586282
rs10944
rs672413
rs6859667
rs921943
rs3797535
rs1789953
rs567754
rs234709
rs558133
All − MR Egger
All − IVW(MRE)
−0.50
−0.25
0.00
0.25
0.50
MR effect size for
'Selenium' on 'Uncharacterized protein C1orf185'

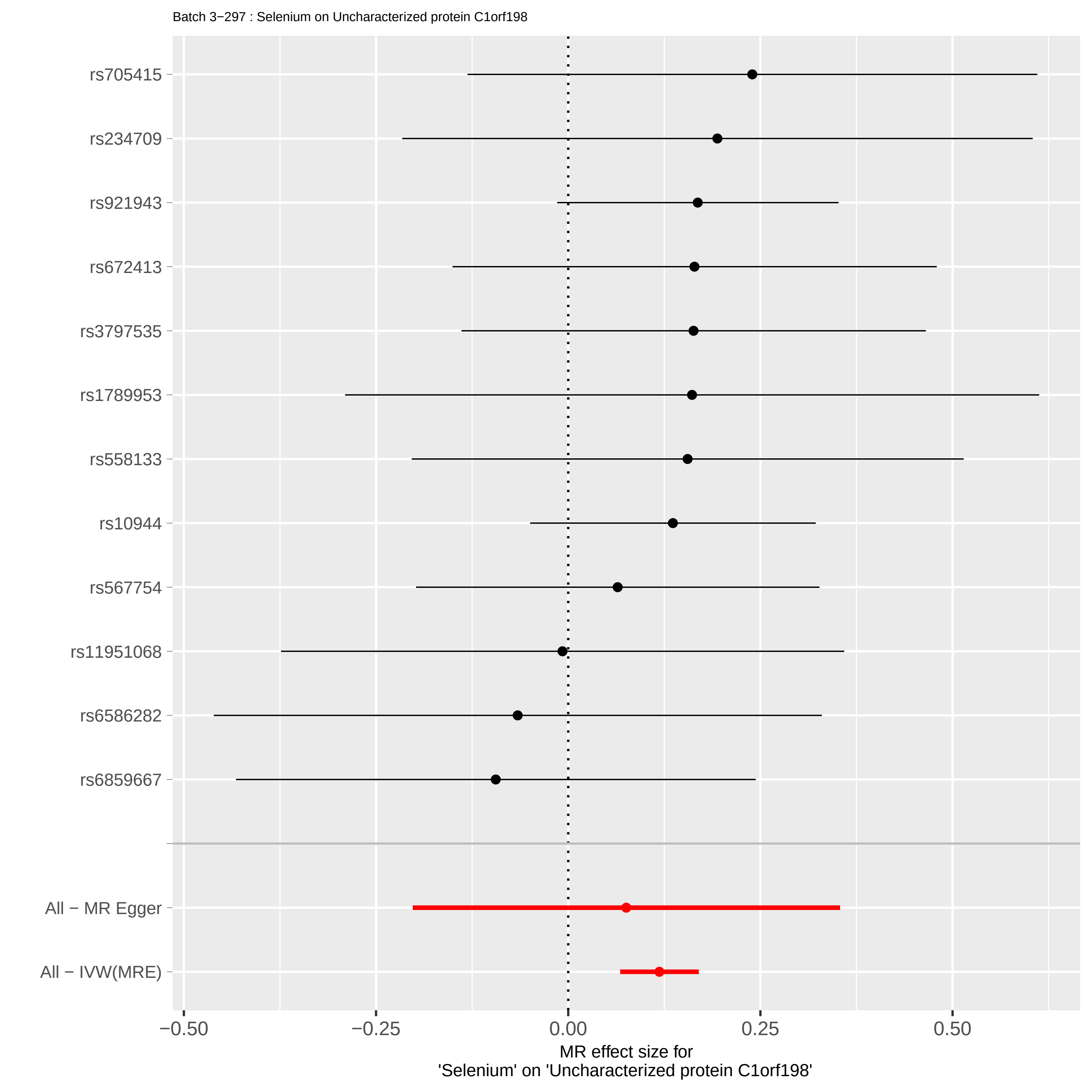

Batch 3−297 : Selenium on Uncharacterized protein C1orf198
rs705415
rs234709
rs921943
rs672413
rs3797535
rs1789953
rs558133
rs10944
rs567754
rs11951068
rs6586282
rs6859667
All − MR Egger
All − IVW(MRE)
−0.50
−0.25
0.00
0.25
0.50
MR effect size for
'Selenium' on 'Uncharacterized protein C1orf198'

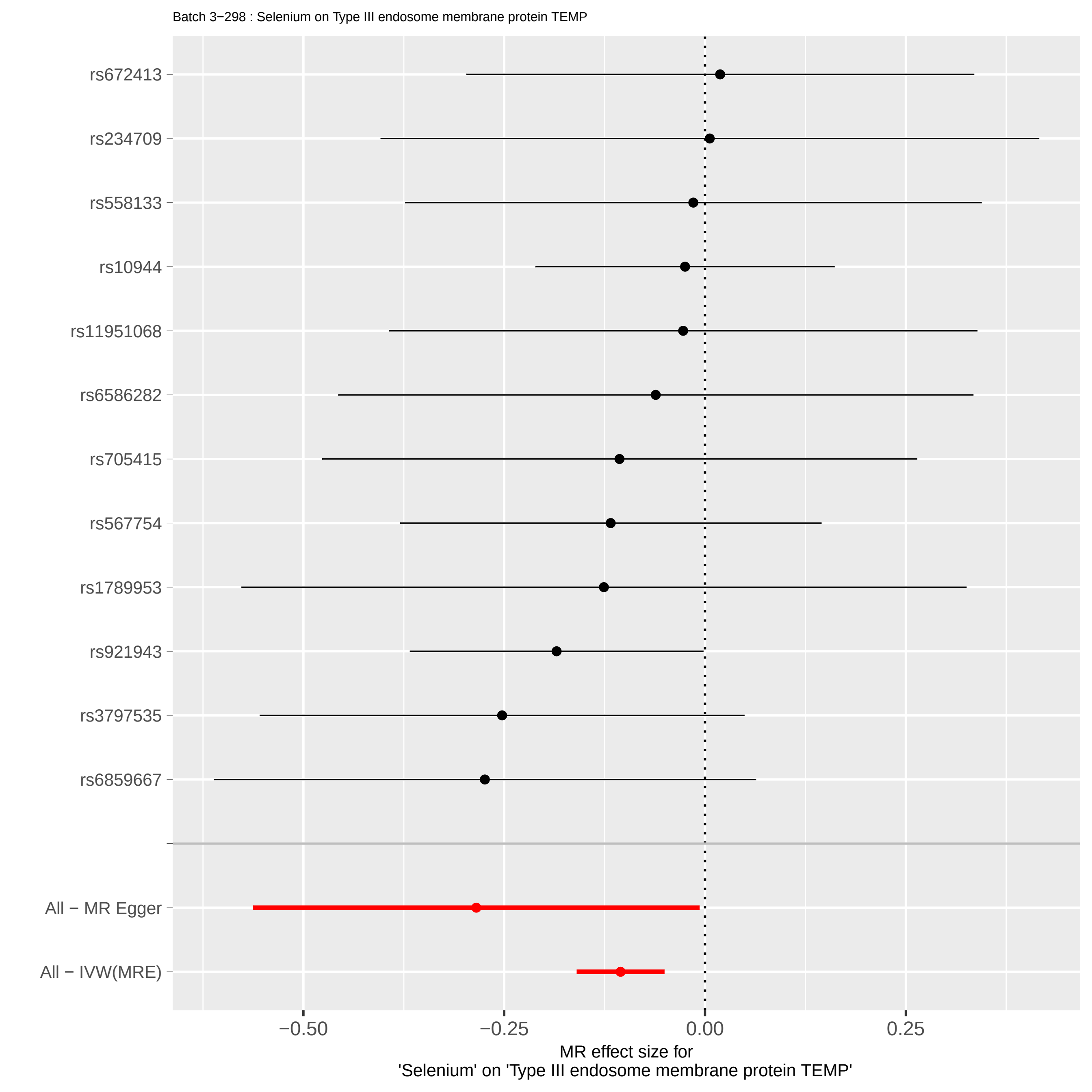

Batch 3−298 : Selenium on Type III endosome membrane protein TEMP
rs672413
rs234709
rs558133
rs10944
rs11951068
rs6586282
rs705415
rs567754
rs1789953
rs921943
rs3797535
rs6859667
All − MR Egger
All − IVW(MRE)
−0.50
−0.25
0.00
0.25
MR effect size for
'Selenium' on 'Type III endosome membrane protein TEMP'

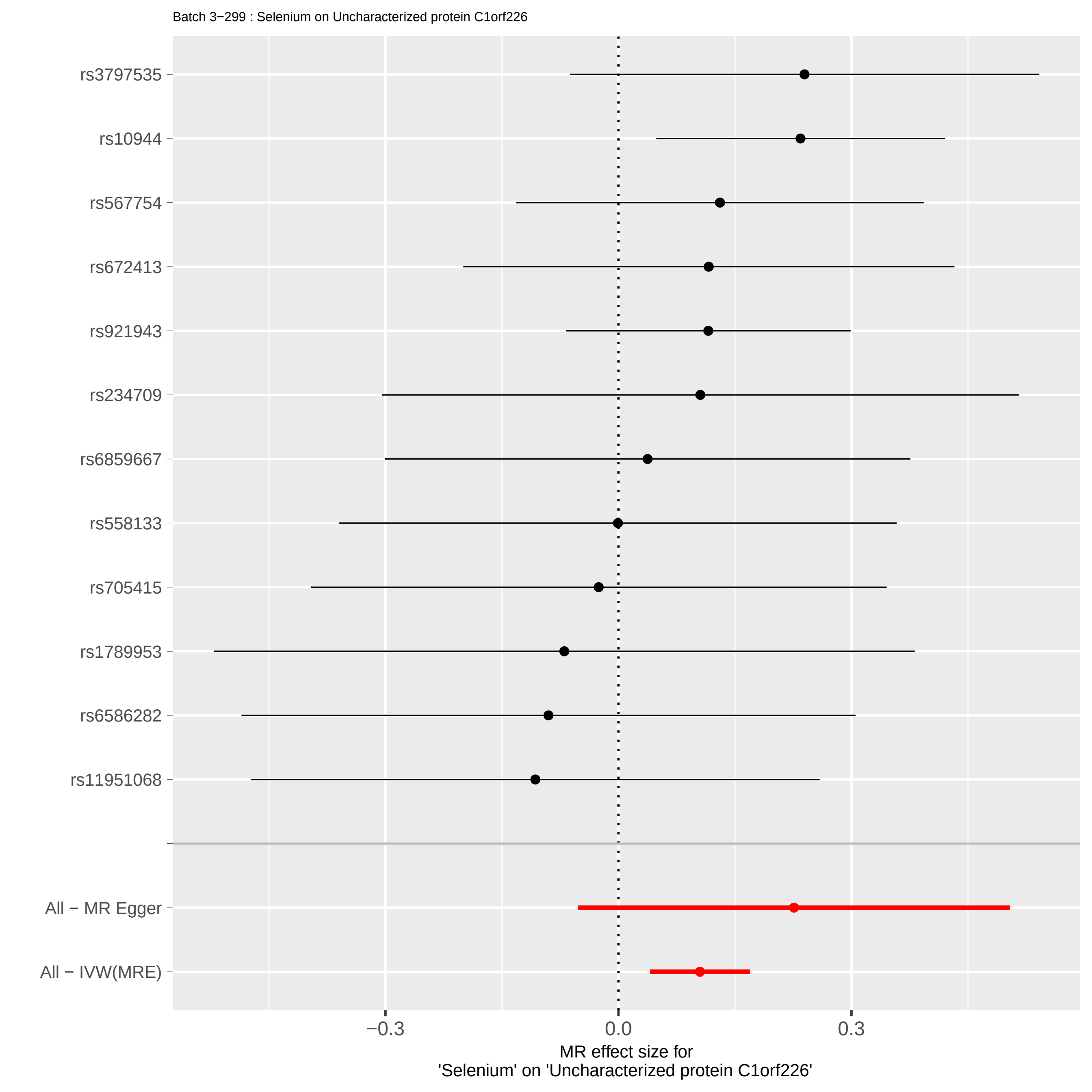

Batch 3−299 : Selenium on Uncharacterized protein C1orf226
rs3797535
rs10944
rs567754
rs672413
rs921943
rs234709
rs6859667
rs558133
rs705415
rs1789953
rs6586282
rs11951068
All − MR Egger
All − IVW(MRE)
−0.3
0.0
0.3
MR effect size for
'Selenium' on 'Uncharacterized protein C1orf226'

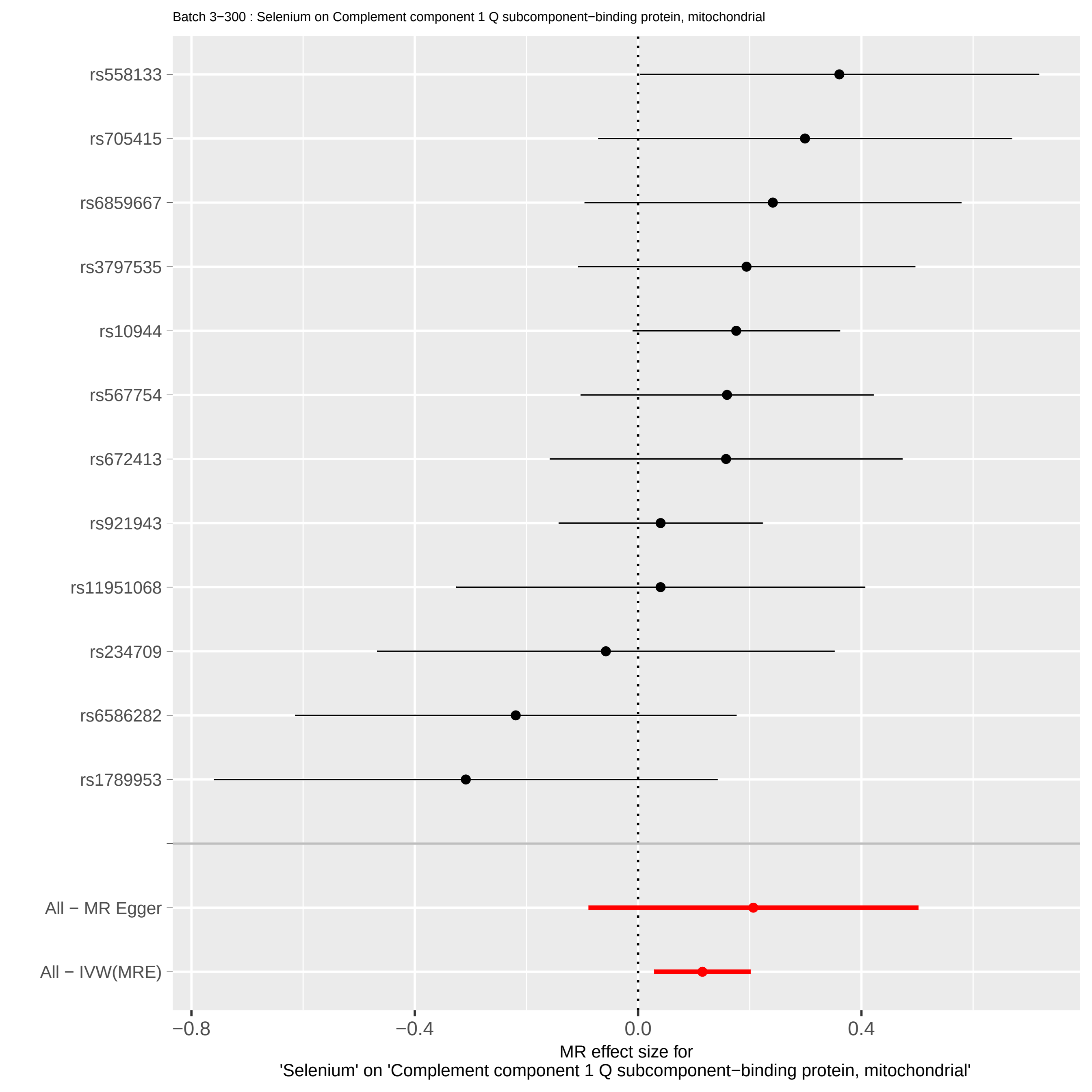
Batch 3−300 : Selenium on Complement component 1 Q subcomponent−binding protein, mitochondrial
rs558133
rs705415
rs6859667
rs3797535
rs10944
rs567754
rs672413
rs921943
rs11951068
rs234709
rs6586282
rs1789953
All − MR Egger
All − IVW(MRE)
−0.8
−0.4
0.0
0.4
MR effect size for
'Selenium' on 'Complement component 1 Q subcomponent−binding protein, mitochondrial'

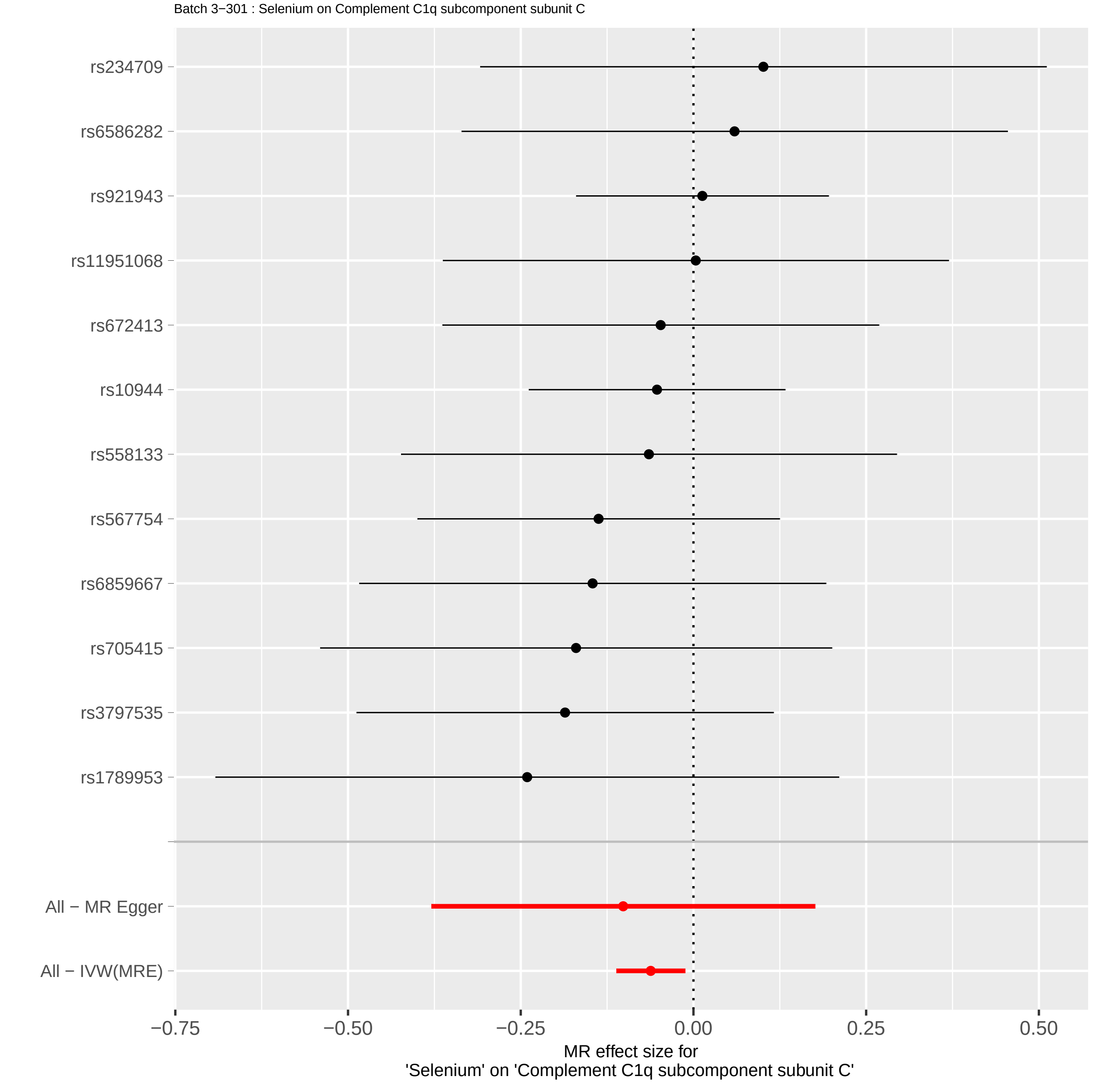

Batch 3−301 : Selenium on Complement C1q subcomponent subunit C
rs234709
rs6586282
rs921943
rs11951068
rs672413
rs10944
rs558133
rs567754
rs6859667
rs705415
rs3797535
rs1789953
All − MR Egger
All − IVW(MRE)
−0.75
−0.50
−0.25
0.00
0.25
0.50
MR effect size for
'Selenium' on 'Complement C1q subcomponent subunit C'

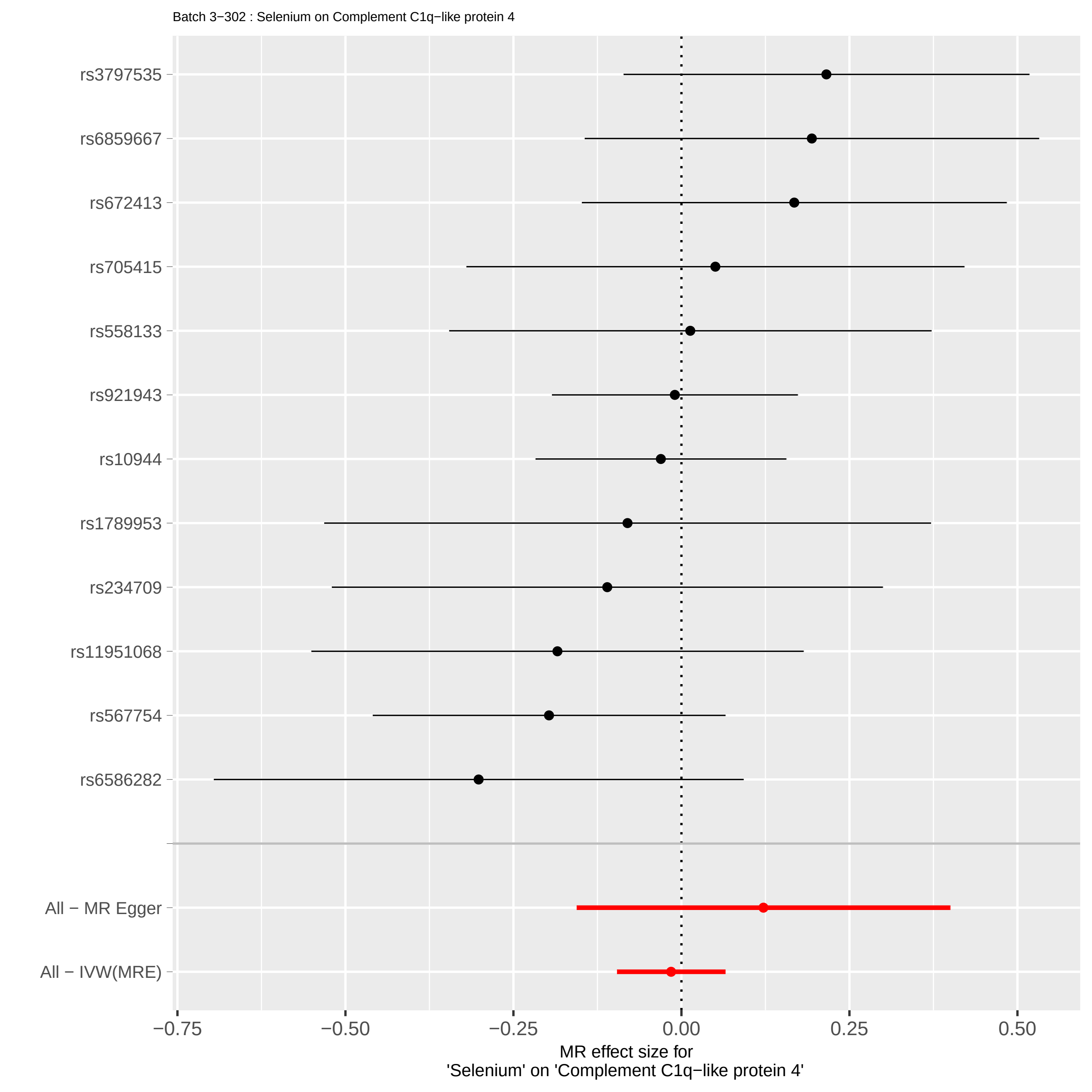

Batch 3−302 : Selenium on Complement C1q−like protein 4
rs3797535
rs6859667
rs672413
rs705415
rs558133
rs921943
rs10944
rs1789953
rs234709
rs11951068
rs567754
rs6586282
All − MR Egger
All − IVW(MRE)
−0.75
−0.50
−0.25
0.00
0.25
0.50
MR effect size for
'Selenium' on 'Complement C1q−like protein 4'

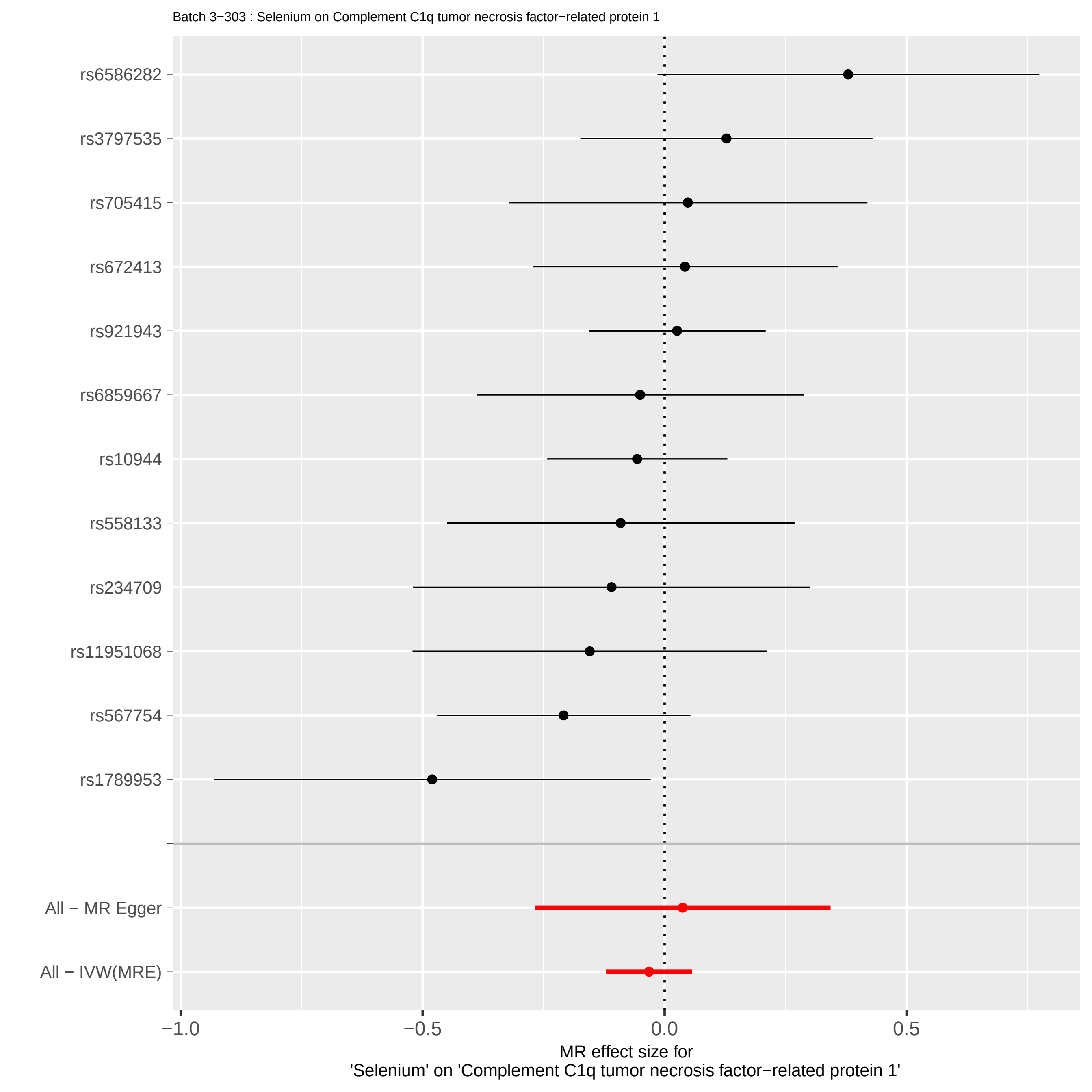
Batch 3−303 : Selenium on Complement C1q tumor necrosis factor−related protein 1
rs6586282
rs3797535
rs705415
rs672413
rs921943
rs6859667
rs10944
rs558133
rs234709
rs11951068
rs567754
rs1789953
All − MR Egger
All − IVW(MRE)
−1.0
−0.5
0.0
0.5
MR effect size for
'Selenium' on 'Complement C1q tumor necrosis factor−related protein 1'

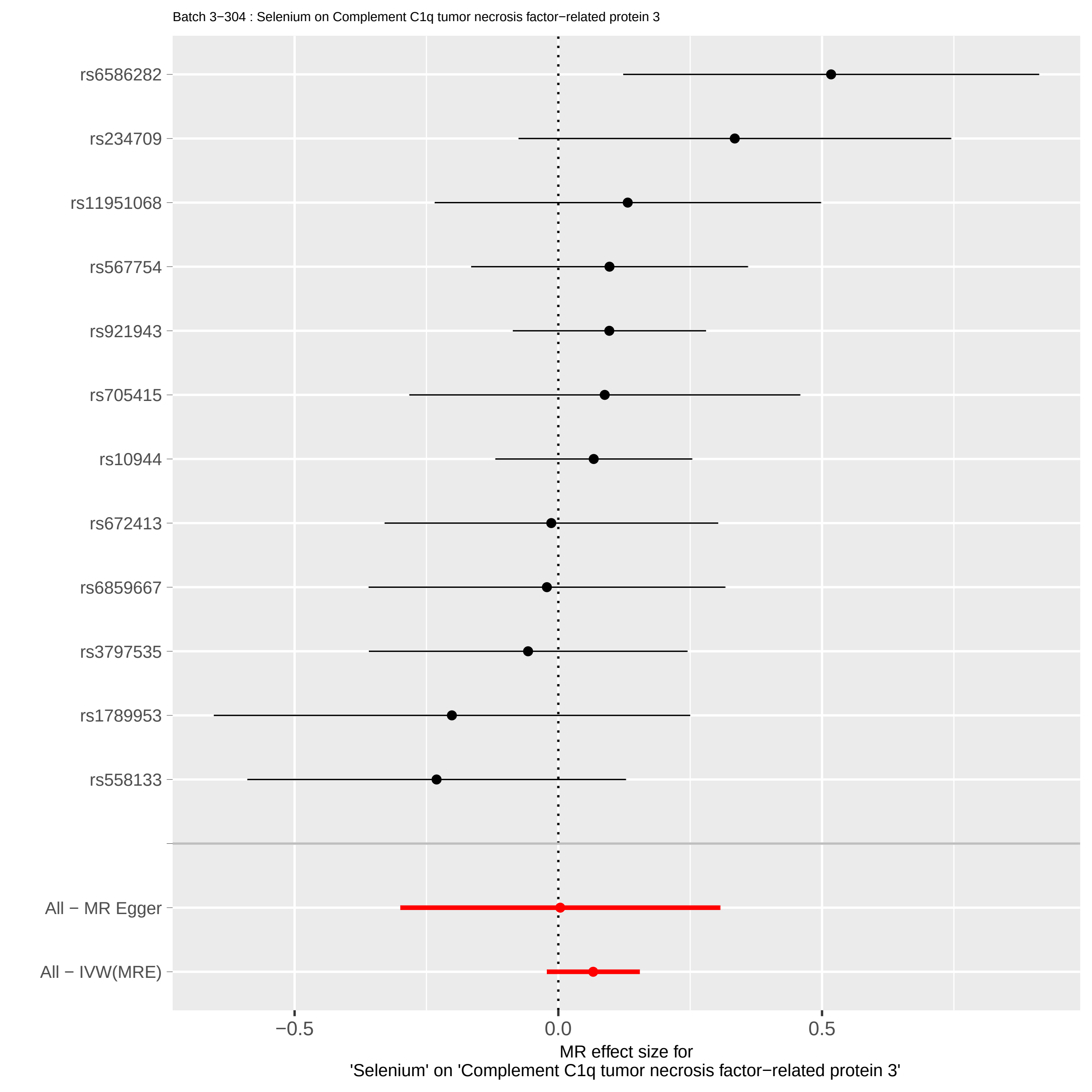
Batch 3−304 : Selenium on Complement C1q tumor necrosis factor−related protein 3
rs6586282
rs234709
rs11951068
rs567754
rs921943
rs705415
rs10944
rs672413
rs6859667
rs3797535
rs1789953
rs558133
All − MR Egger
All − IVW(MRE)
−0.5
0.0
0.5
MR effect size for
'Selenium' on 'Complement C1q tumor necrosis factor−related protein 3'

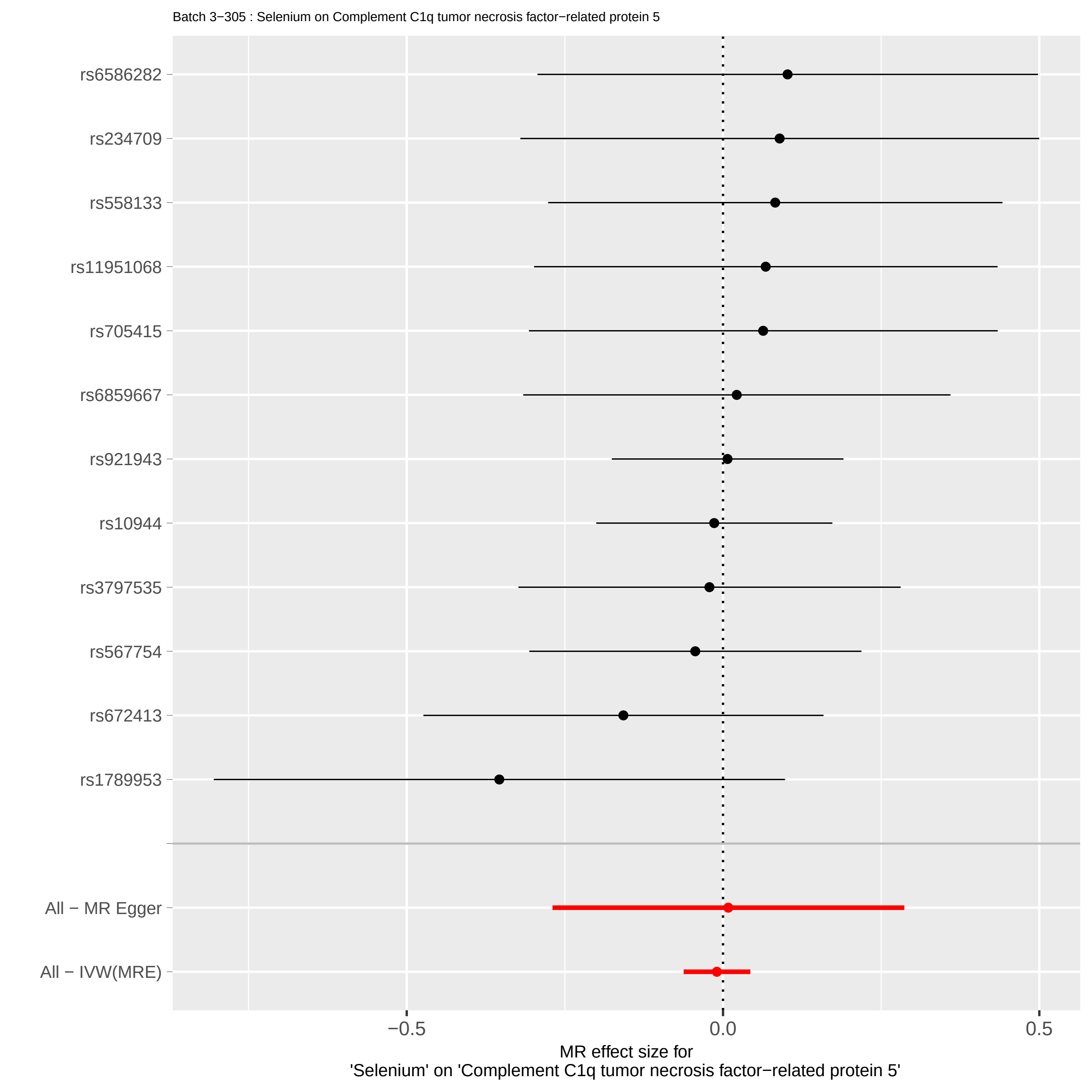

Batch 3−305 : Selenium on Complement C1q tumor necrosis factor−related protein 5
rs6586282
rs234709
rs558133
rs11951068
rs705415
rs6859667
rs921943
rs10944
rs3797535
rs567754
rs672413
rs1789953
All − MR Egger
All − IVW(MRE)
−0.5
0.0
0.5
MR effect size for
'Selenium' on 'Complement C1q tumor necrosis factor−related protein 5'

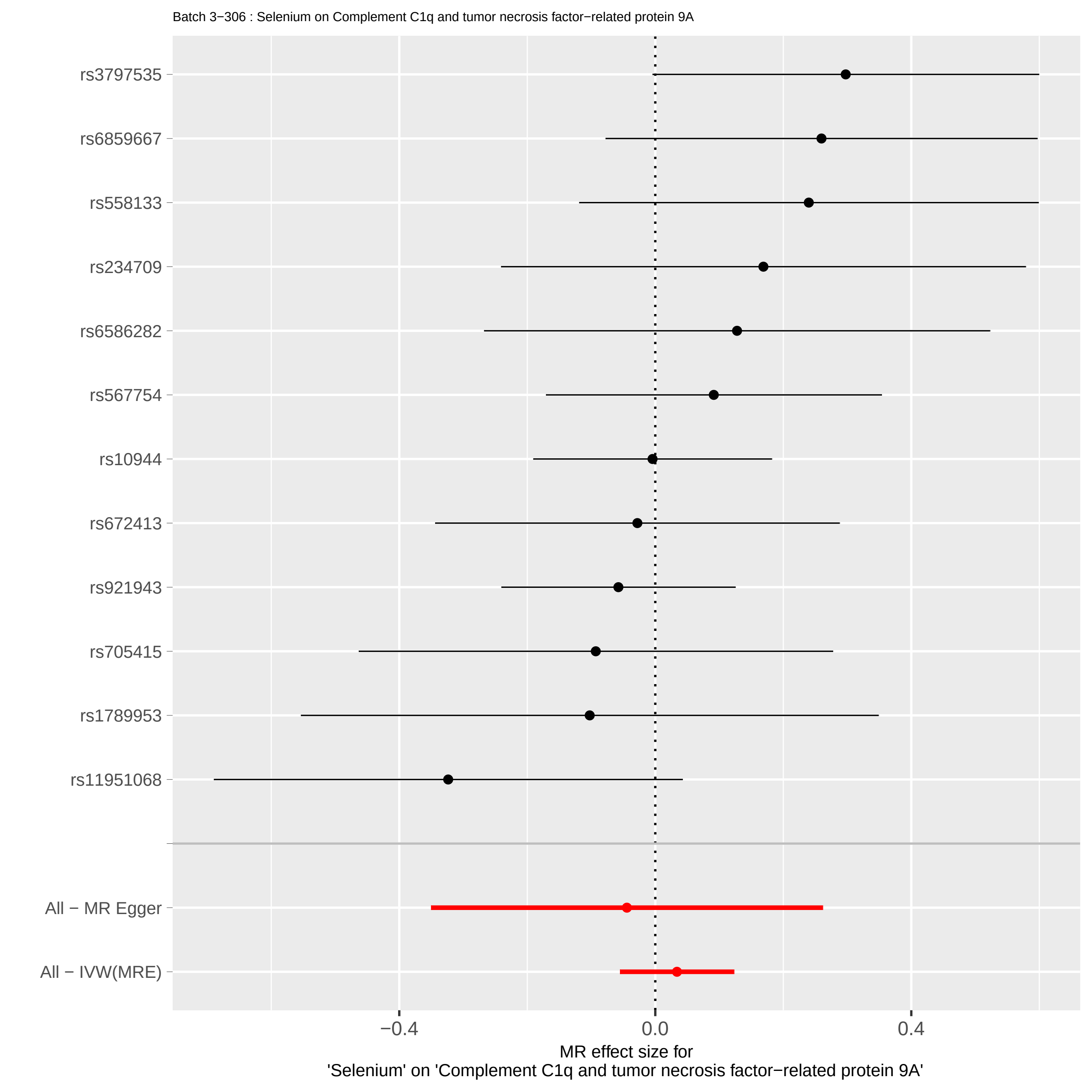

Batch 3−306 : Selenium on Complement C1q and tumor necrosis factor−related protein 9A
rs3797535
rs6859667
rs558133
rs234709
rs6586282
rs567754
rs10944
rs672413
rs921943
rs705415
rs1789953
rs11951068
All − MR Egger
All − IVW(MRE)
−0.4
0.0
0.4
MR effect size for
'Selenium' on 'Complement C1q and tumor necrosis factor−related protein 9A'

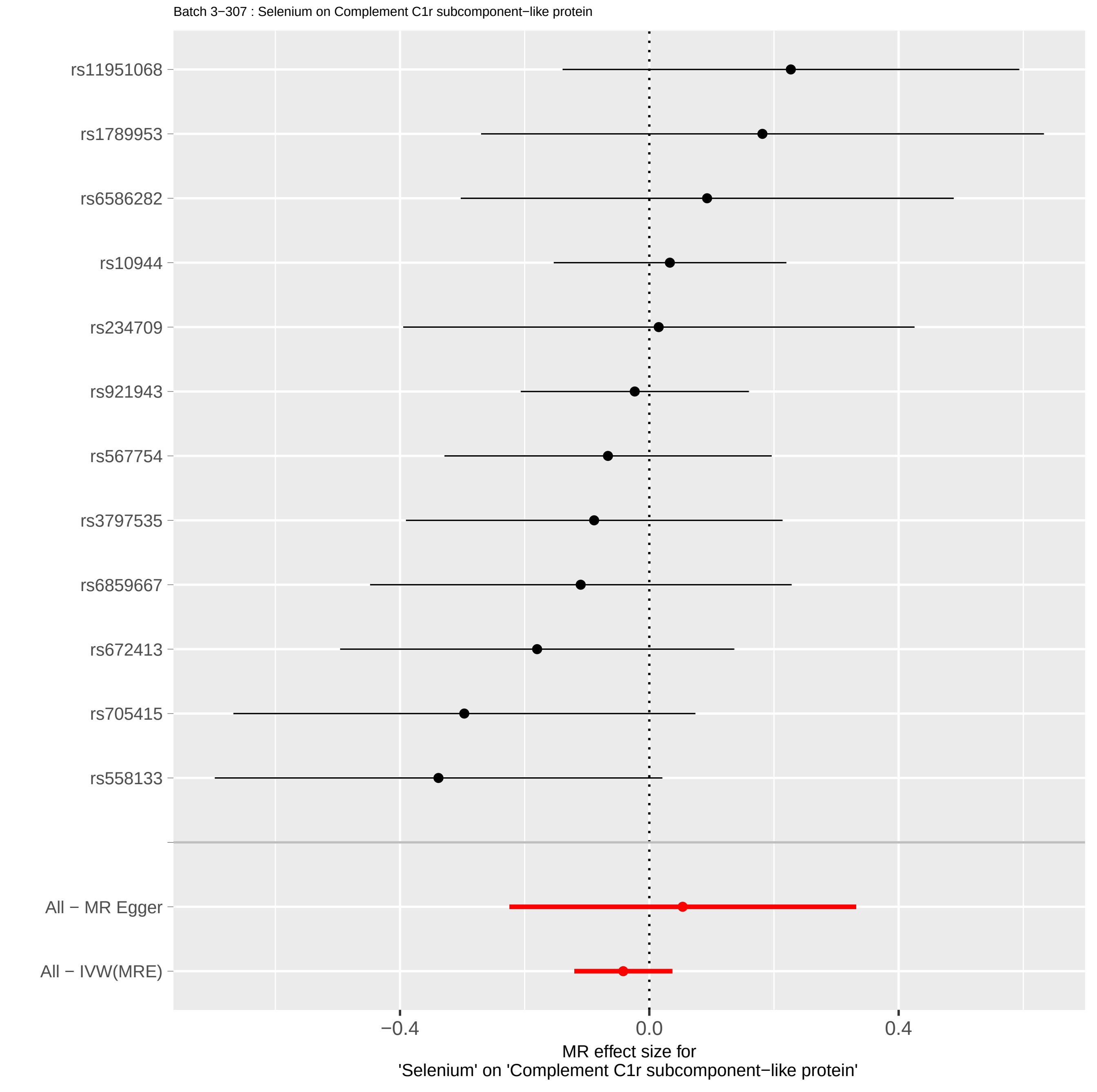

Batch 3−307 : Selenium on Complement C1r subcomponent−like protein
rs11951068
rs1789953
rs6586282
rs10944
rs234709
rs921943
rs567754
rs3797535
rs6859667
rs672413
rs705415
rs558133
All − MR Egger
All − IVW(MRE)
−0.4
0.0
0.4
MR effect size for
'Selenium' on 'Complement C1r subcomponent−like protein'

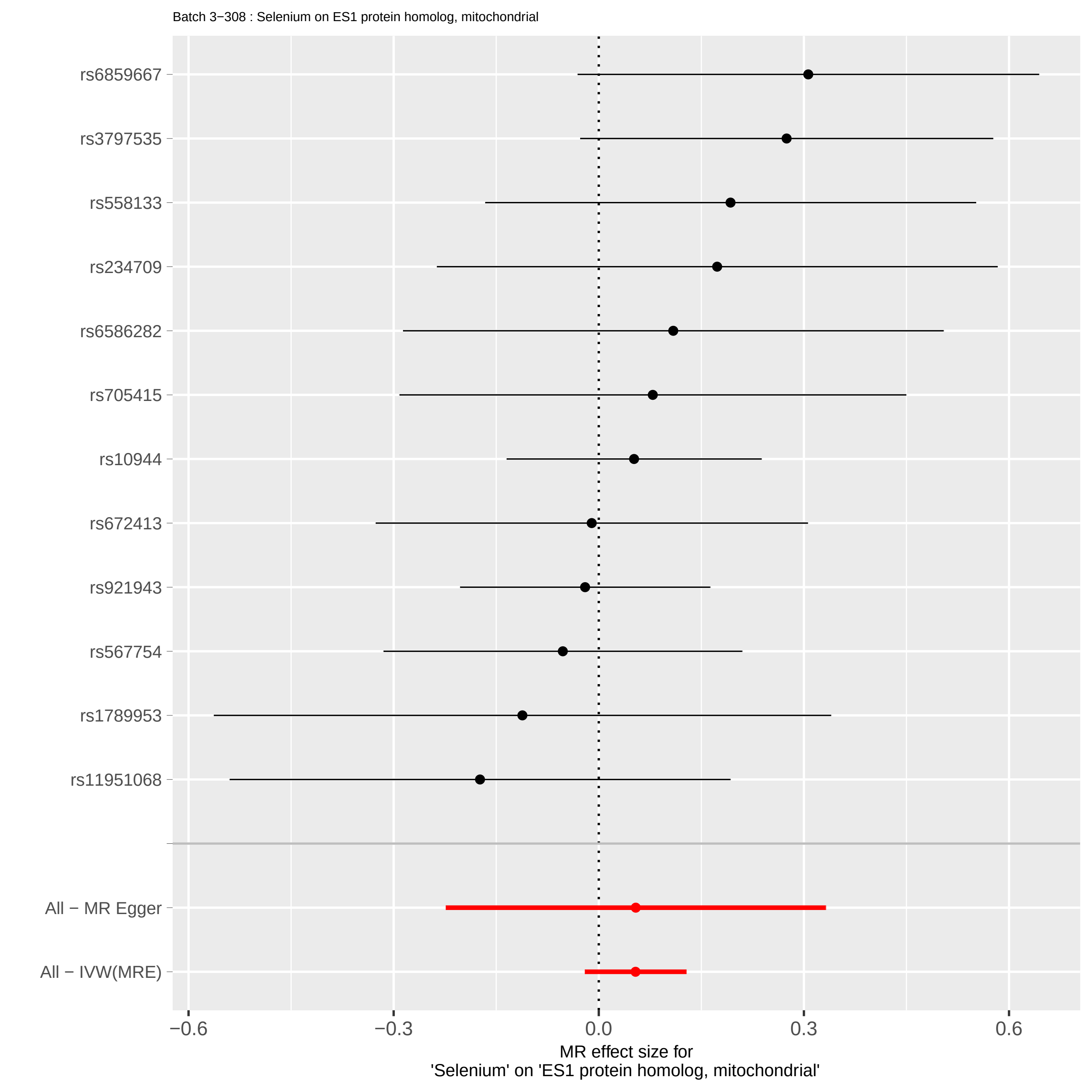

Batch 3−308 : Selenium on ES1 protein homolog, mitochondrial
rs6859667
rs3797535
rs558133
rs234709
rs6586282
rs705415
rs10944
rs672413
rs921943
rs567754
rs1789953
rs11951068
All − MR Egger
All − IVW(MRE)
−0.6
−0.3
0.0
0.3
0.6
MR effect size for
'Selenium' on 'ES1 protein homolog, mitochondrial'

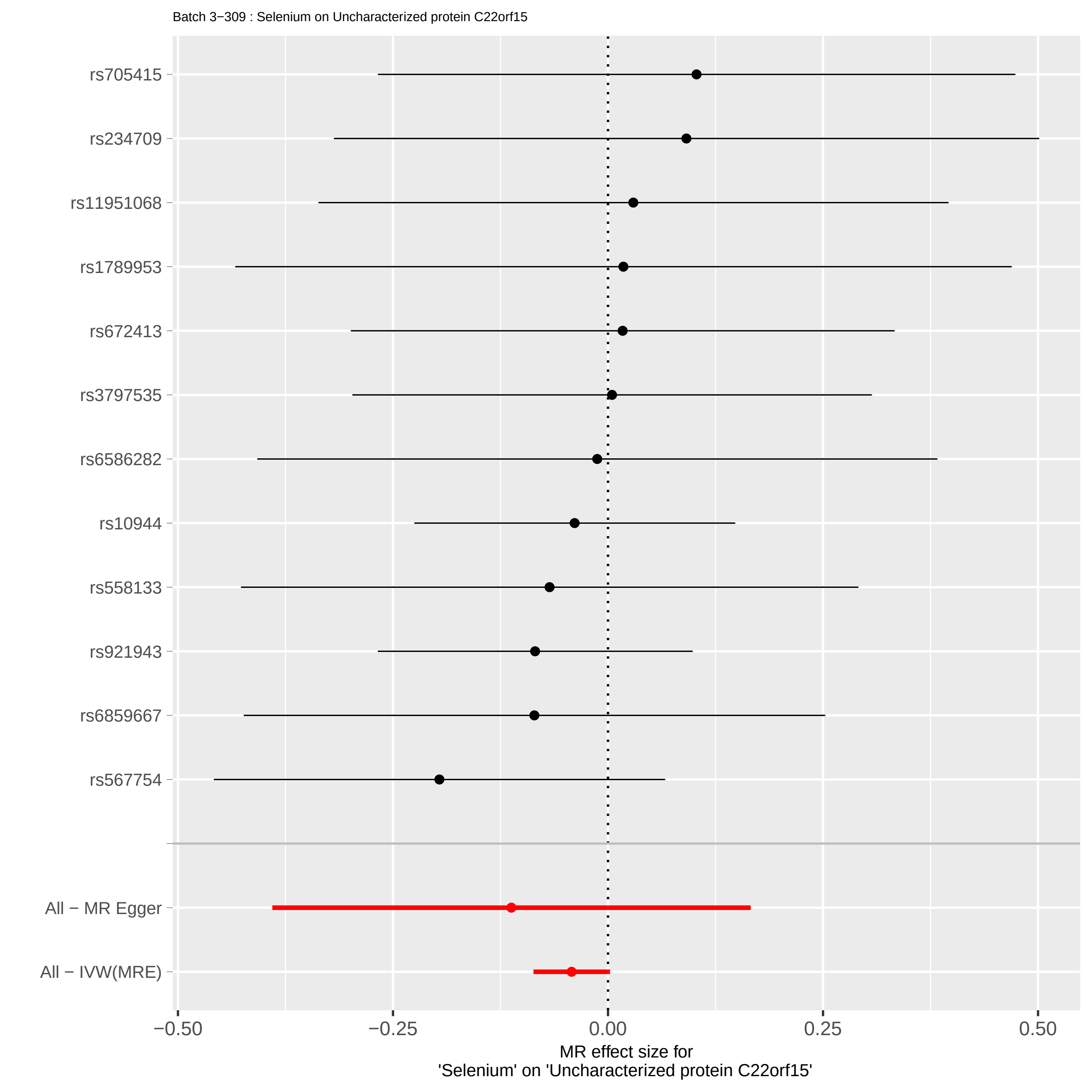
Batch 3−309 : Selenium on Uncharacterized protein C22orf15
rs705415
rs234709
rs11951068
rs1789953
rs672413
rs3797535
rs6586282
rs10944
rs558133
rs921943
rs6859667
rs567754
All − MR Egger
All − IVW(MRE)
−0.50
−0.25
0.00
0.25
0.50
MR effect size for
'Selenium' on 'Uncharacterized protein C22orf15'

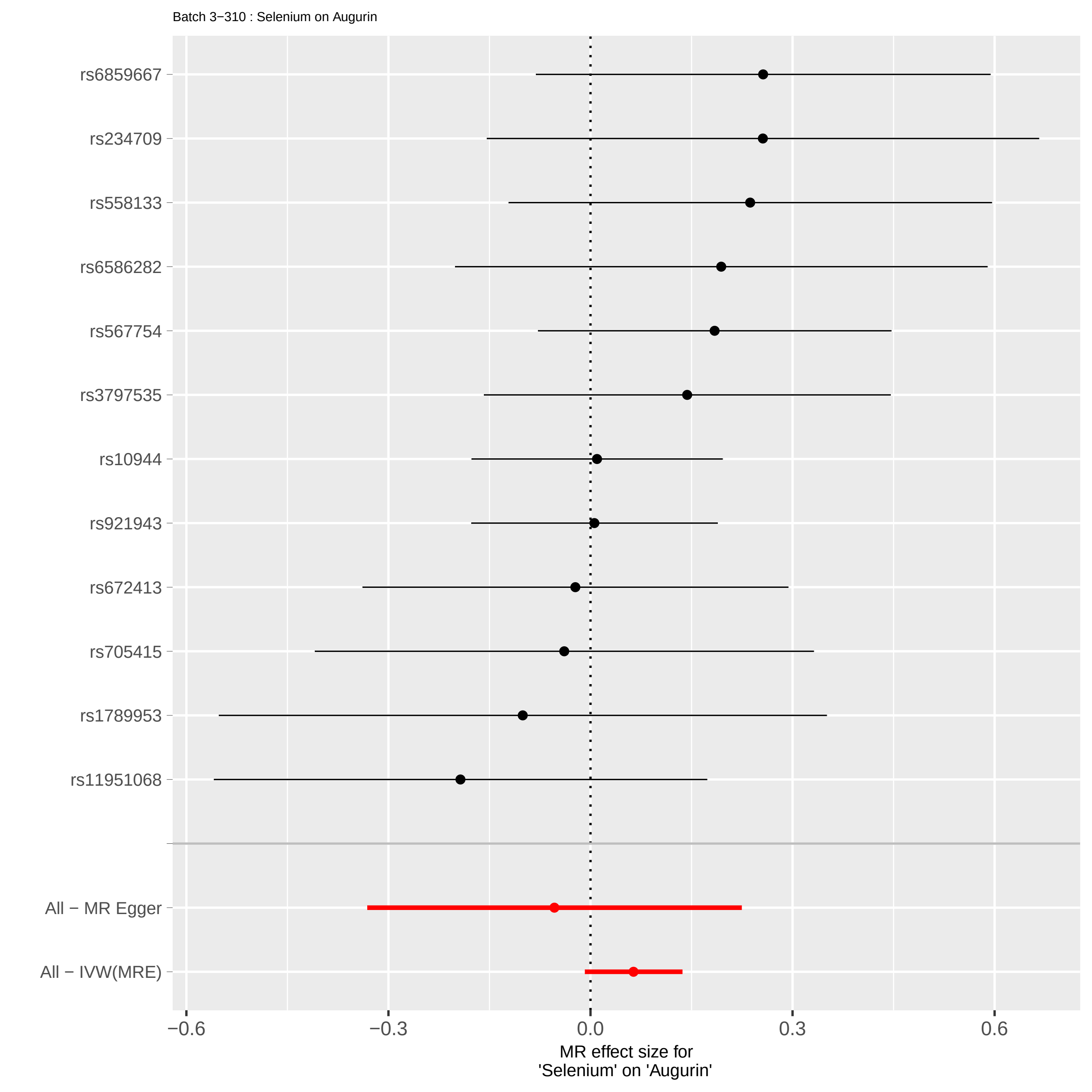

Batch 3−310 : Selenium on Augurin
rs6859667
rs234709
rs558133
rs6586282
rs567754
rs3797535
rs10944
rs921943
rs672413
rs705415
rs1789953
rs11951068
All − MR Egger
All − IVW(MRE)
−0.6
−0.3
0.0
0.3
0.6
MR effect size for
'Selenium' on 'Augurin'

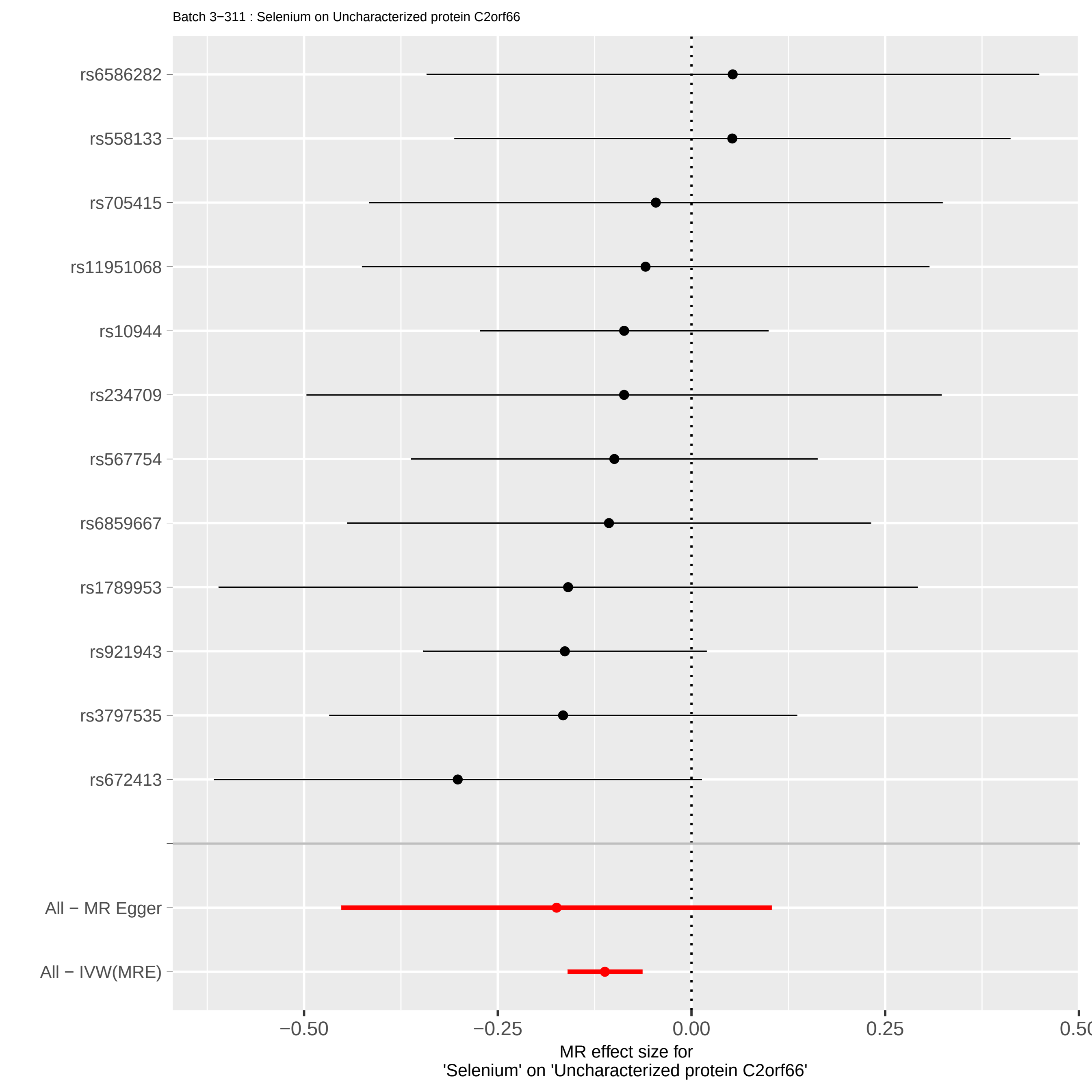
Batch 3−311 : Selenium on Uncharacterized protein C2orf66
rs6586282
rs558133
rs705415
rs11951068
rs10944
rs234709
rs567754
rs6859667
rs1789953
rs921943
rs3797535
rs672413
All − MR Egger
All − IVW(MRE)
−0.50
−0.25
0.00
0.25
0.50
MR effect size for
'Selenium' on 'Uncharacterized protein C2orf66'

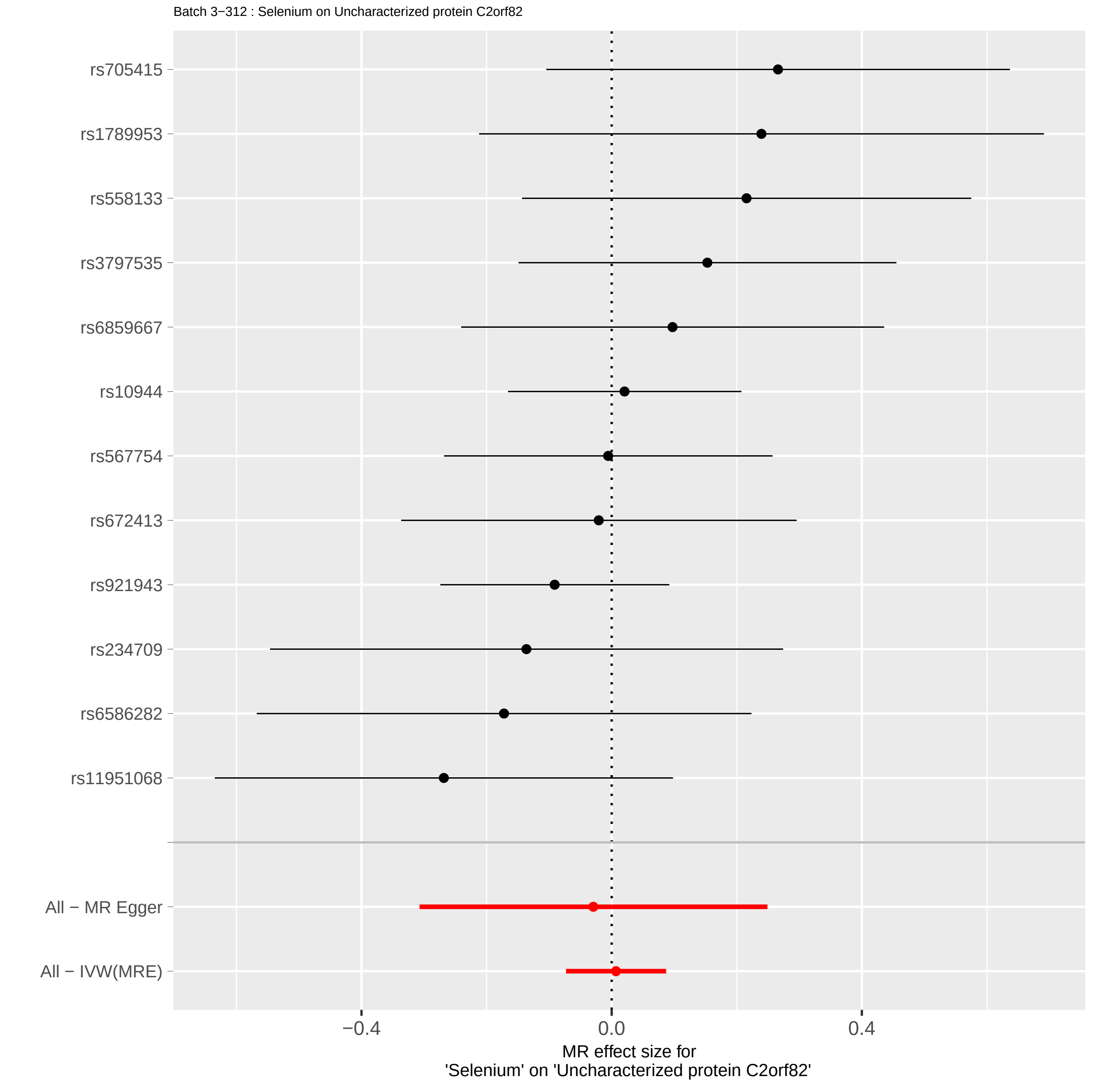
Batch 3−312 : Selenium on Uncharacterized protein C2orf82
rs705415
rs1789953
rs558133
rs3797535
rs6859667
rs10944
rs567754
rs672413
rs921943
rs234709
rs6586282
rs11951068
All − MR Egger
All − IVW(MRE)
−0.4
0.0
0.4
MR effect size for
'Selenium' on 'Uncharacterized protein C2orf82'

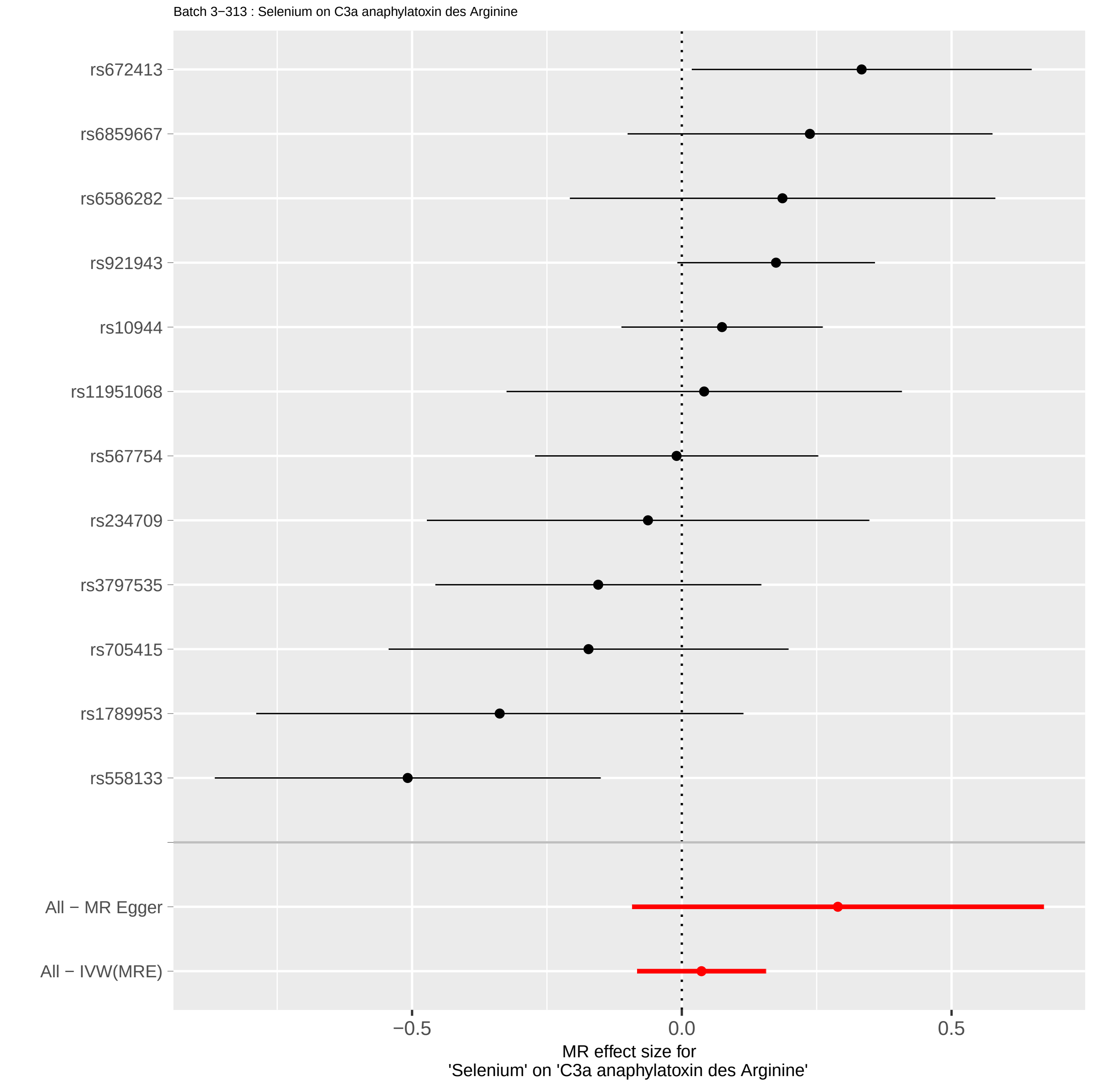

Batch 3−313 : Selenium on C3a anaphylatoxin des Arginine
rs672413
rs6859667
rs6586282
rs921943
rs10944
rs11951068
rs567754
rs234709
rs3797535
rs705415
rs1789953
rs558133
All − MR Egger
All − IVW(MRE)
−0.5
0.0
0.5
MR effect size for
'Selenium' on 'C3a anaphylatoxin des Arginine'

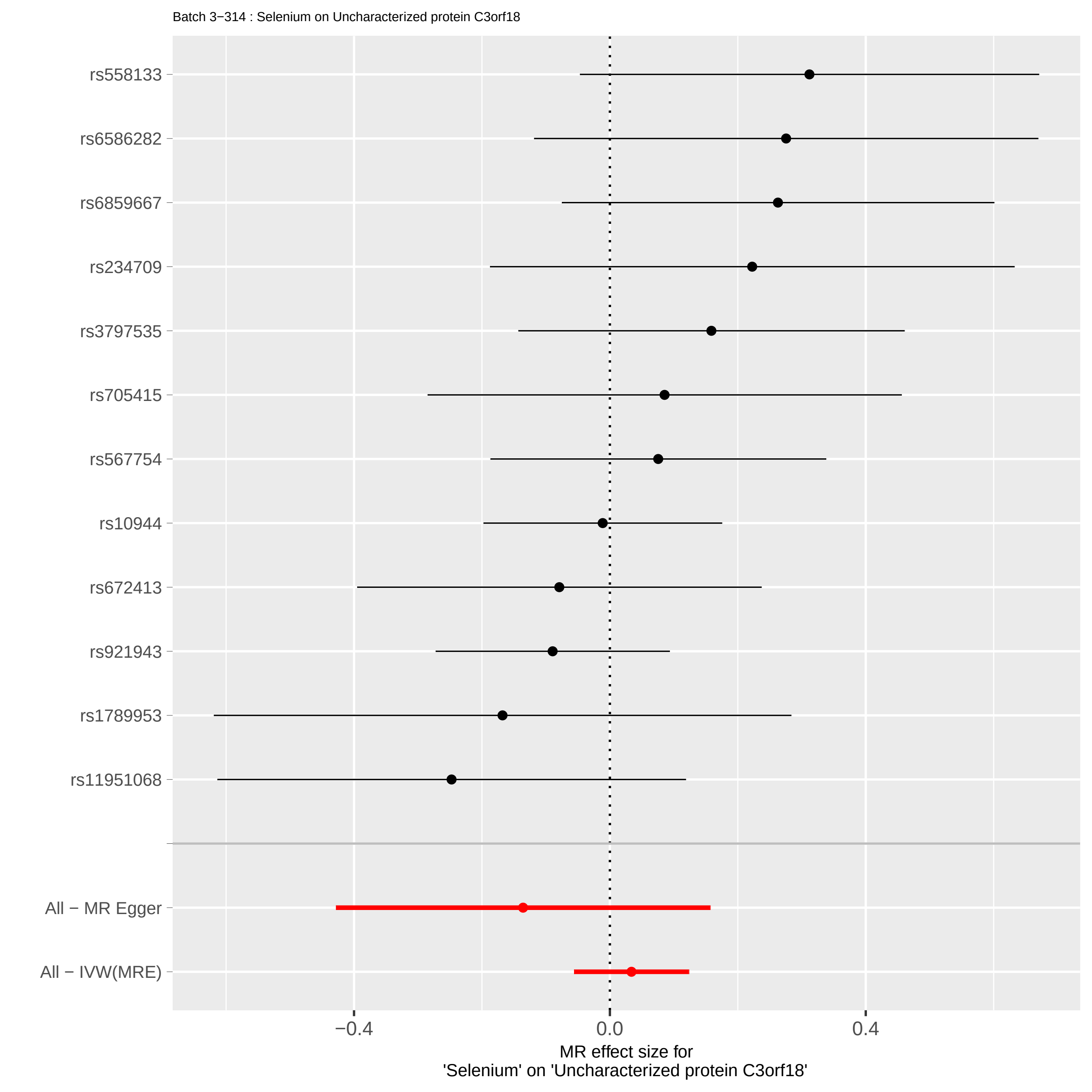

Batch 3−314 : Selenium on Uncharacterized protein C3orf18
rs558133
rs6586282
rs6859667
rs234709
rs3797535
rs705415
rs567754
rs10944
rs672413
rs921943
rs1789953
rs11951068
All − MR Egger
All − IVW(MRE)
−0.4
0.0
0.4
MR effect size for
'Selenium' on 'Uncharacterized protein C3orf18'

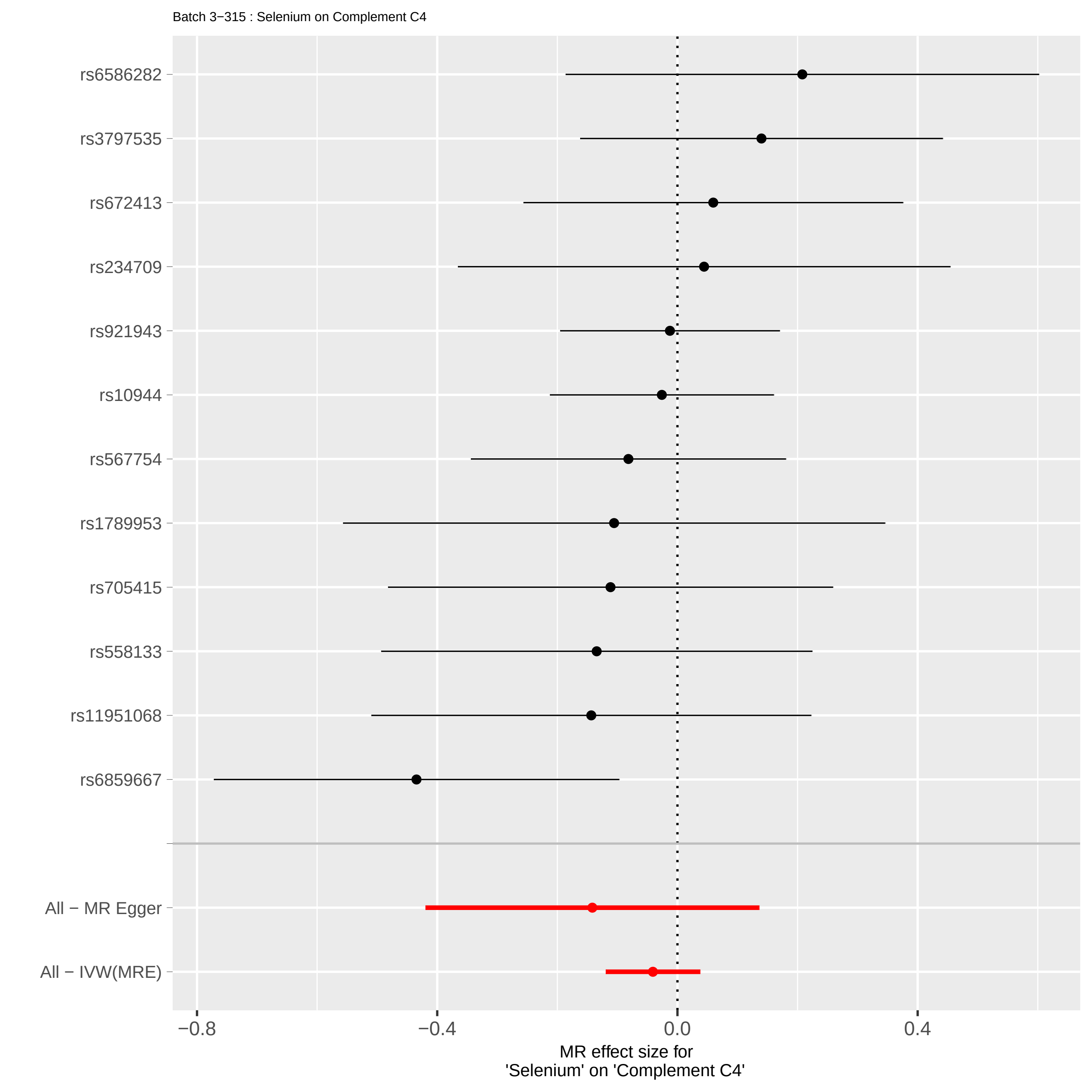

Batch 3−315 : Selenium on Complement C4
rs6586282
rs3797535
rs672413
rs234709
rs921943
rs10944
rs567754
rs1789953
rs705415
rs558133
rs11951068
rs6859667
All − MR Egger
All − IVW(MRE)
−0.8
−0.4
0.0
0.4
MR effect size for
'Selenium' on 'Complement C4'

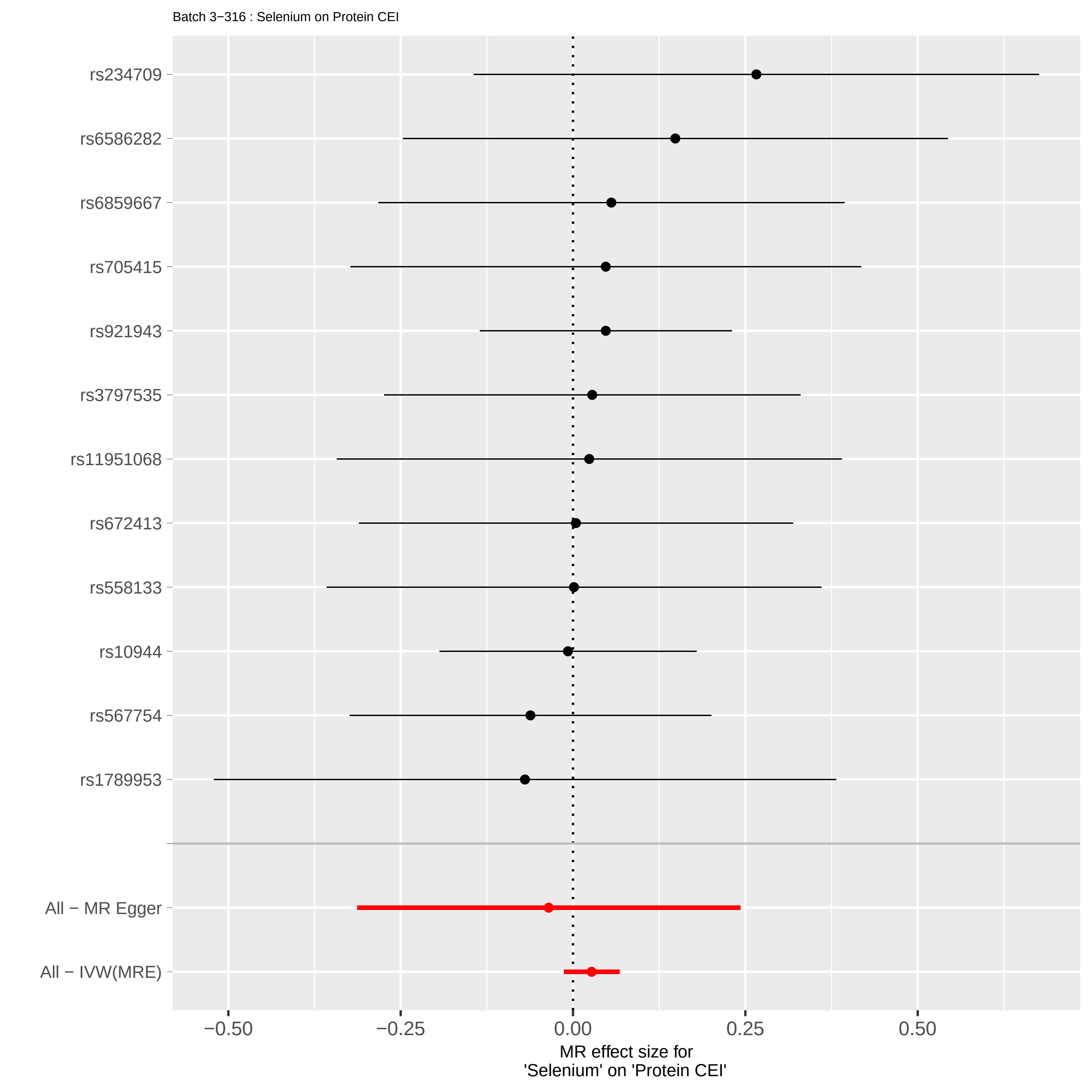

Batch 3−316 : Selenium on Protein CEI
rs234709
rs6586282
rs6859667
rs705415
rs921943
rs3797535
rs11951068
rs672413
rs558133
rs10944
rs567754
rs1789953
All − MR Egger
All − IVW(MRE)
−0.50
−0.25
0.00
0.25
0.50
MR effect size for
'Selenium' on 'Protein CEI'

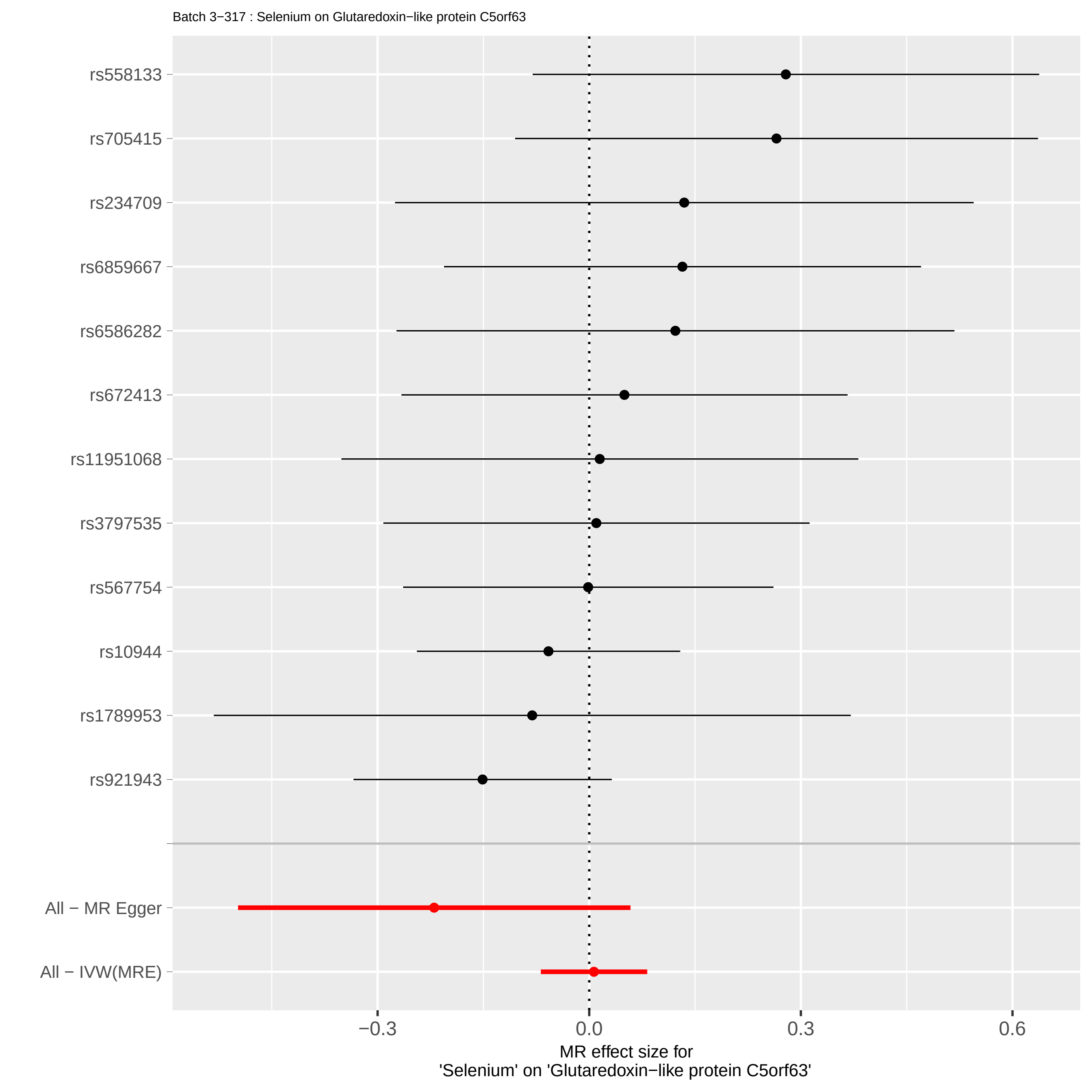
Batch 3−317 : Selenium on Glutaredoxin−like protein C5orf63
rs558133
rs705415
rs234709
rs6859667
rs6586282
rs672413
rs11951068
rs3797535
rs567754
rs10944
rs1789953
rs921943
All − MR Egger
All − IVW(MRE)
−0.3
0.0
0.3
0.6
MR effect size for
'Selenium' on 'Glutaredoxin−like protein C5orf63'

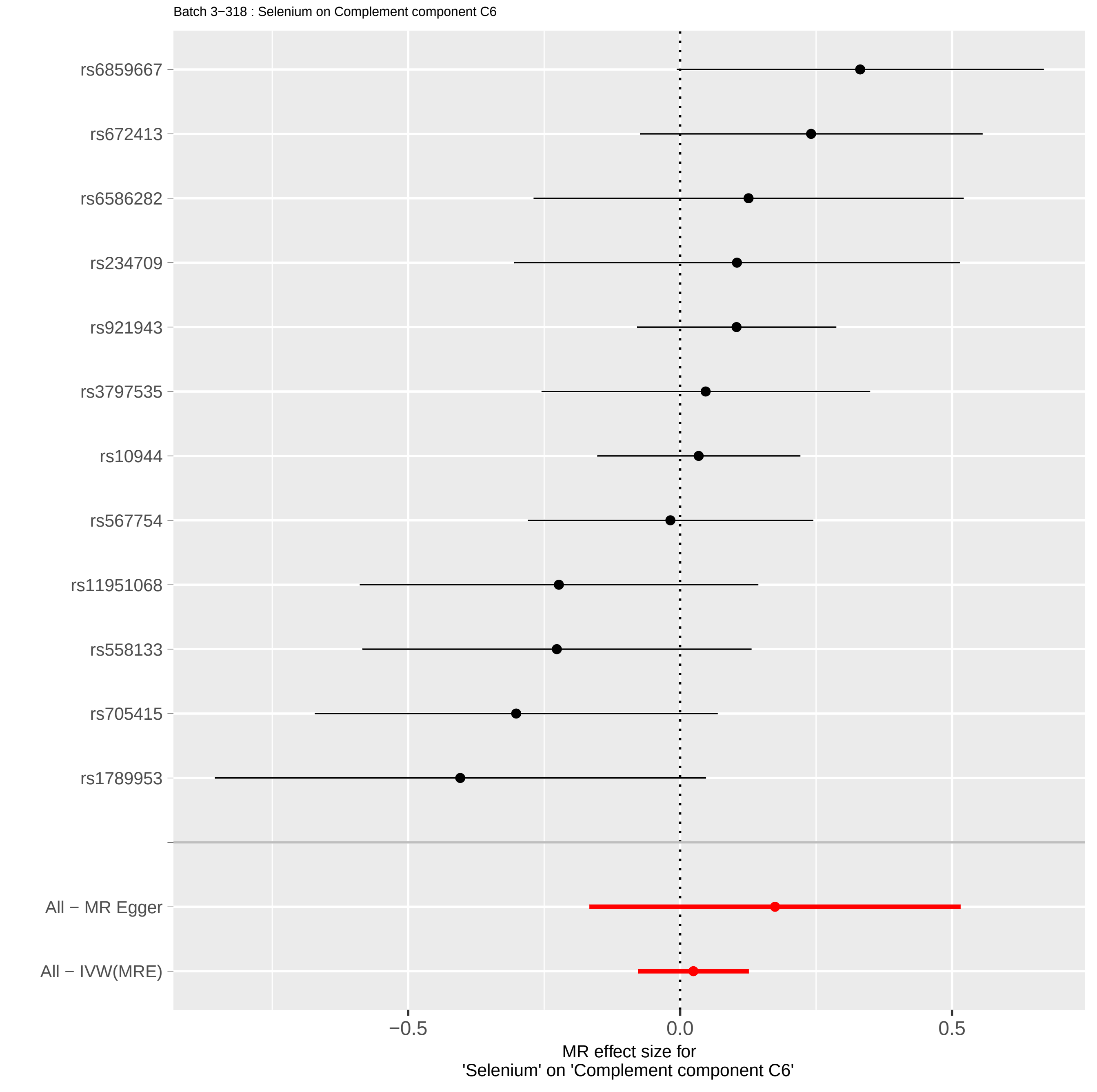

Batch 3−318 : Selenium on Complement component C6
rs6859667
rs672413
rs6586282
rs234709
rs921943
rs3797535
rs10944
rs567754
rs11951068
rs558133
rs705415
rs1789953
All − MR Egger
All − IVW(MRE)
−0.5
0.0
0.5
MR effect size for
'Selenium' on 'Complement component C6'

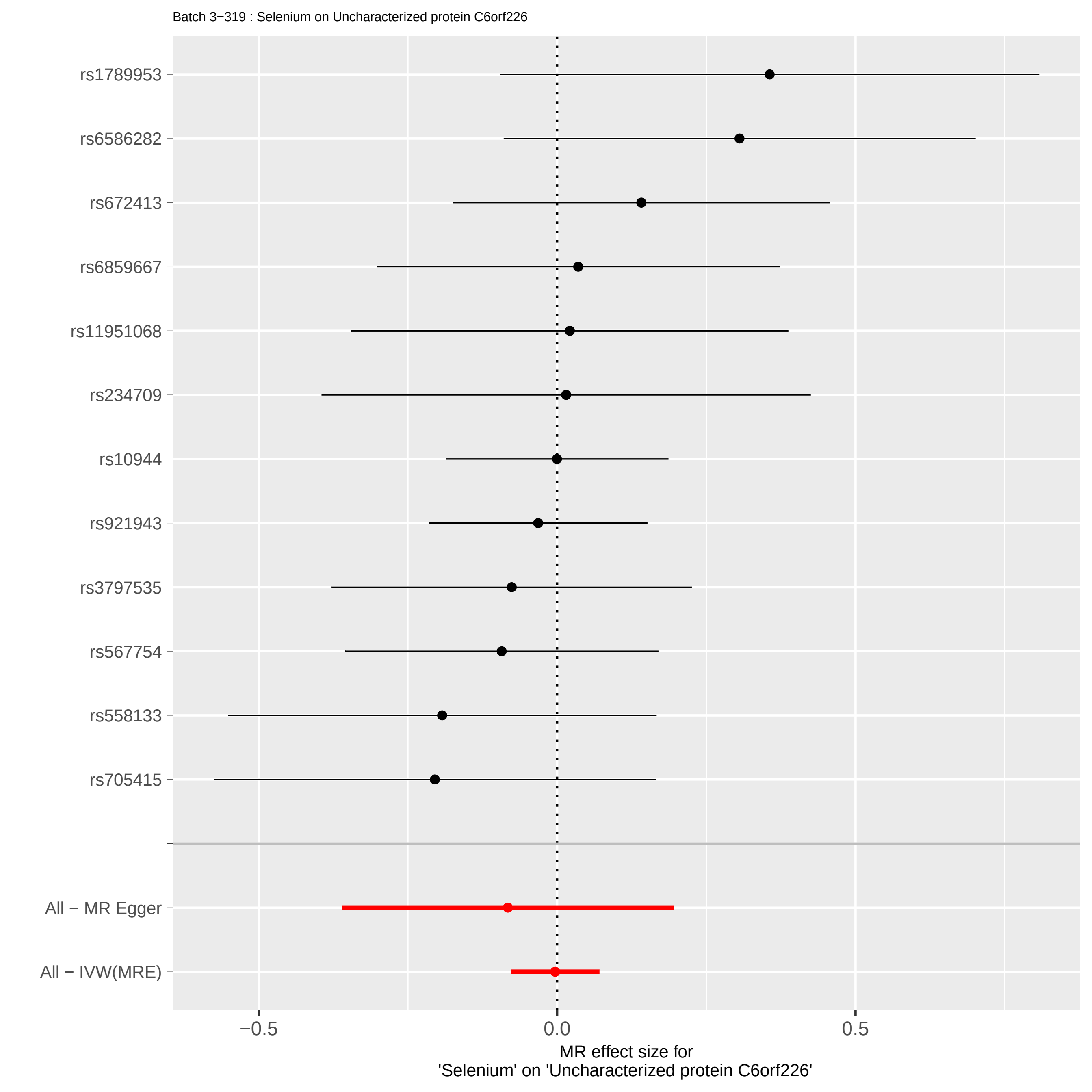

Batch 3−319 : Selenium on Uncharacterized protein C6orf226
rs1789953
rs6586282
rs672413
rs6859667
rs11951068
rs234709
rs10944
rs921943
rs3797535
rs567754
rs558133
rs705415
All − MR Egger
All − IVW(MRE)
−0.5
0.0
0.5
MR effect size for
'Selenium' on 'Uncharacterized protein C6orf226'

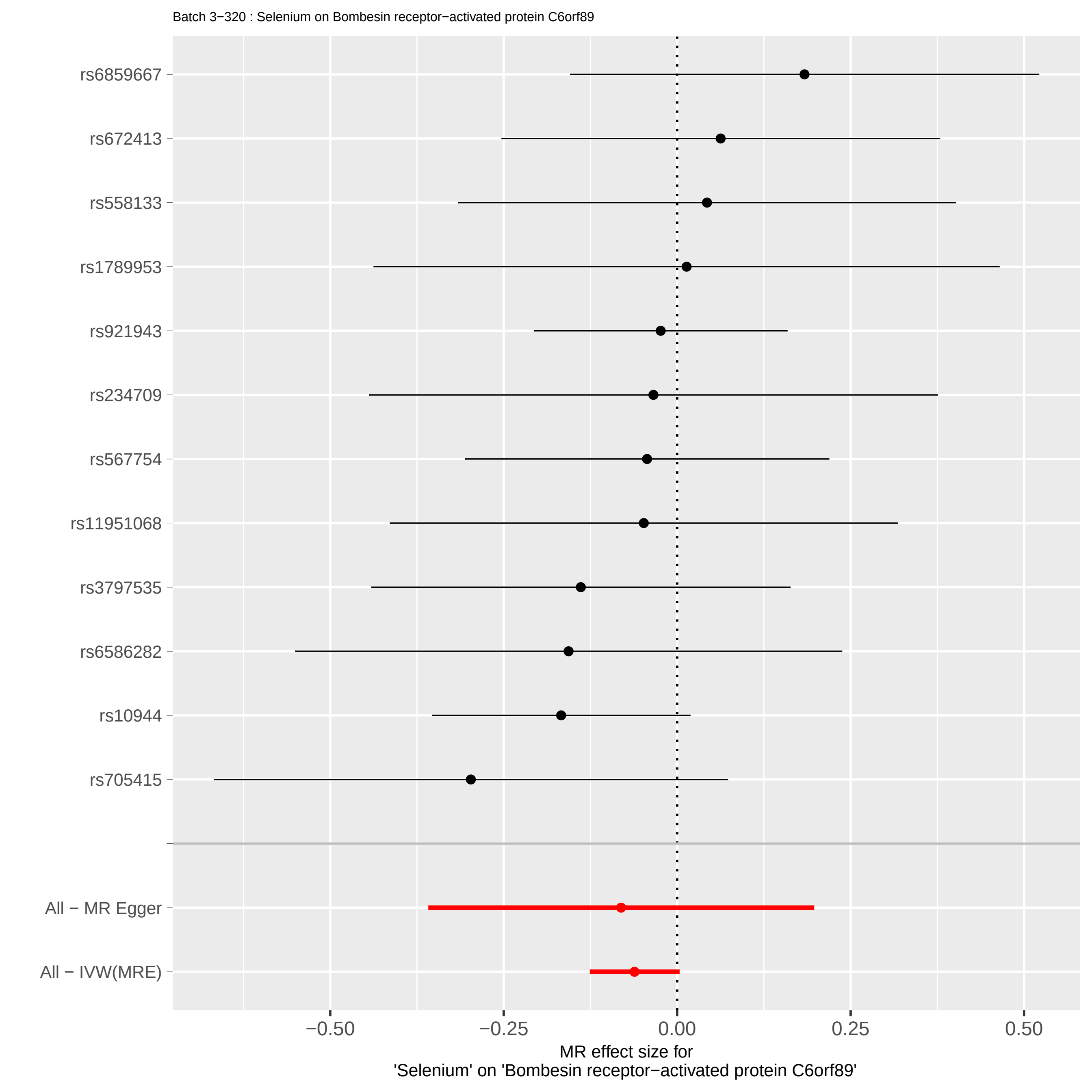

Batch 3−320 : Selenium on Bombesin receptor−activated protein C6orf89
rs6859667
rs672413
rs558133
rs1789953
rs921943
rs234709
rs567754
rs11951068
rs3797535
rs6586282
rs10944
rs705415
All − MR Egger
All − IVW(MRE)
−0.50
−0.25
0.00
0.25
0.50
MR effect size for
'Selenium' on 'Bombesin receptor−activated protein C6orf89'

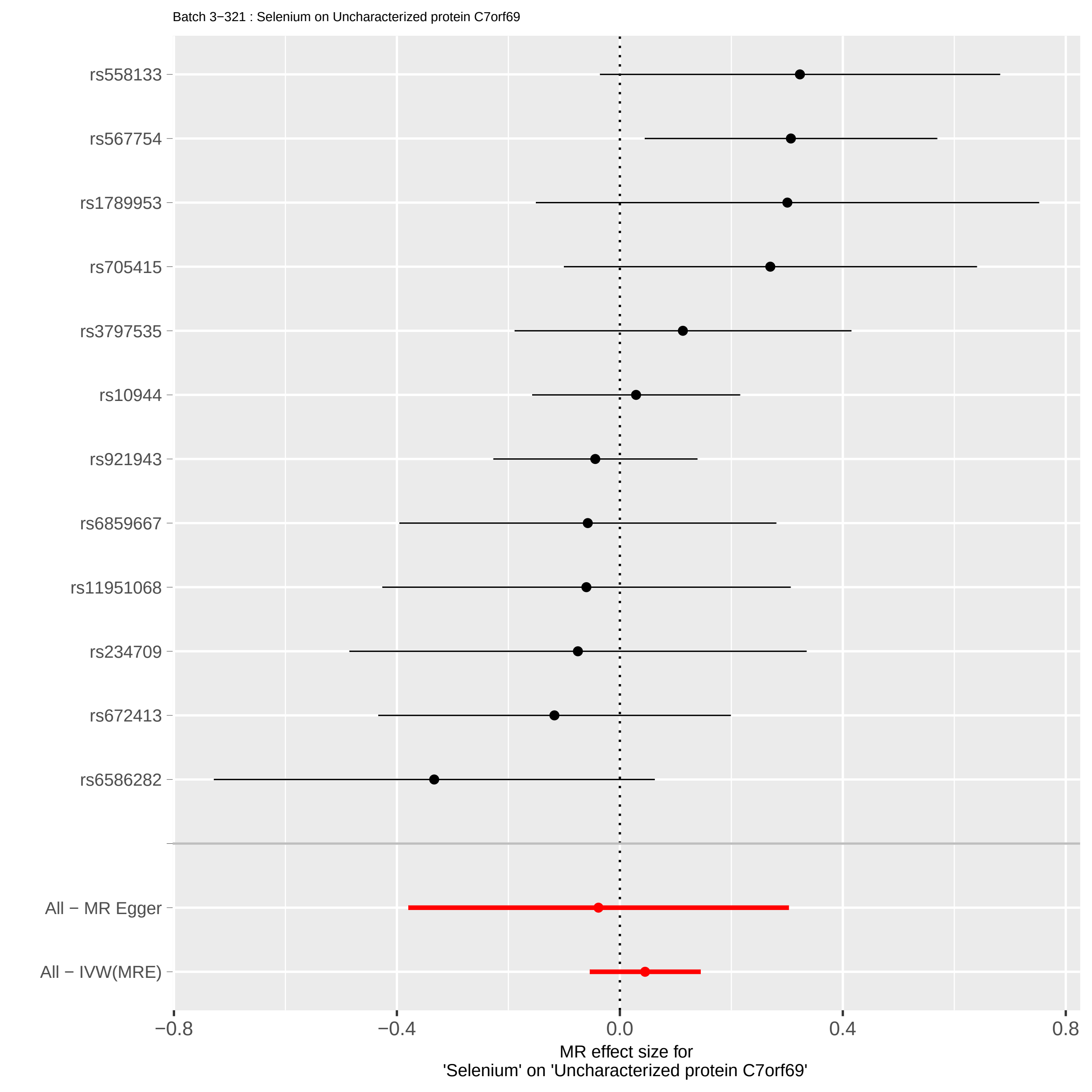

Batch 3−321 : Selenium on Uncharacterized protein C7orf69
rs558133
rs567754
rs1789953
rs705415
rs3797535
rs10944
rs921943
rs6859667
rs11951068
rs234709
rs672413
rs6586282
All − MR Egger
All − IVW(MRE)
−0.8
−0.4
0.0
0.4
0.8
MR effect size for
'Selenium' on 'Uncharacterized protein C7orf69'

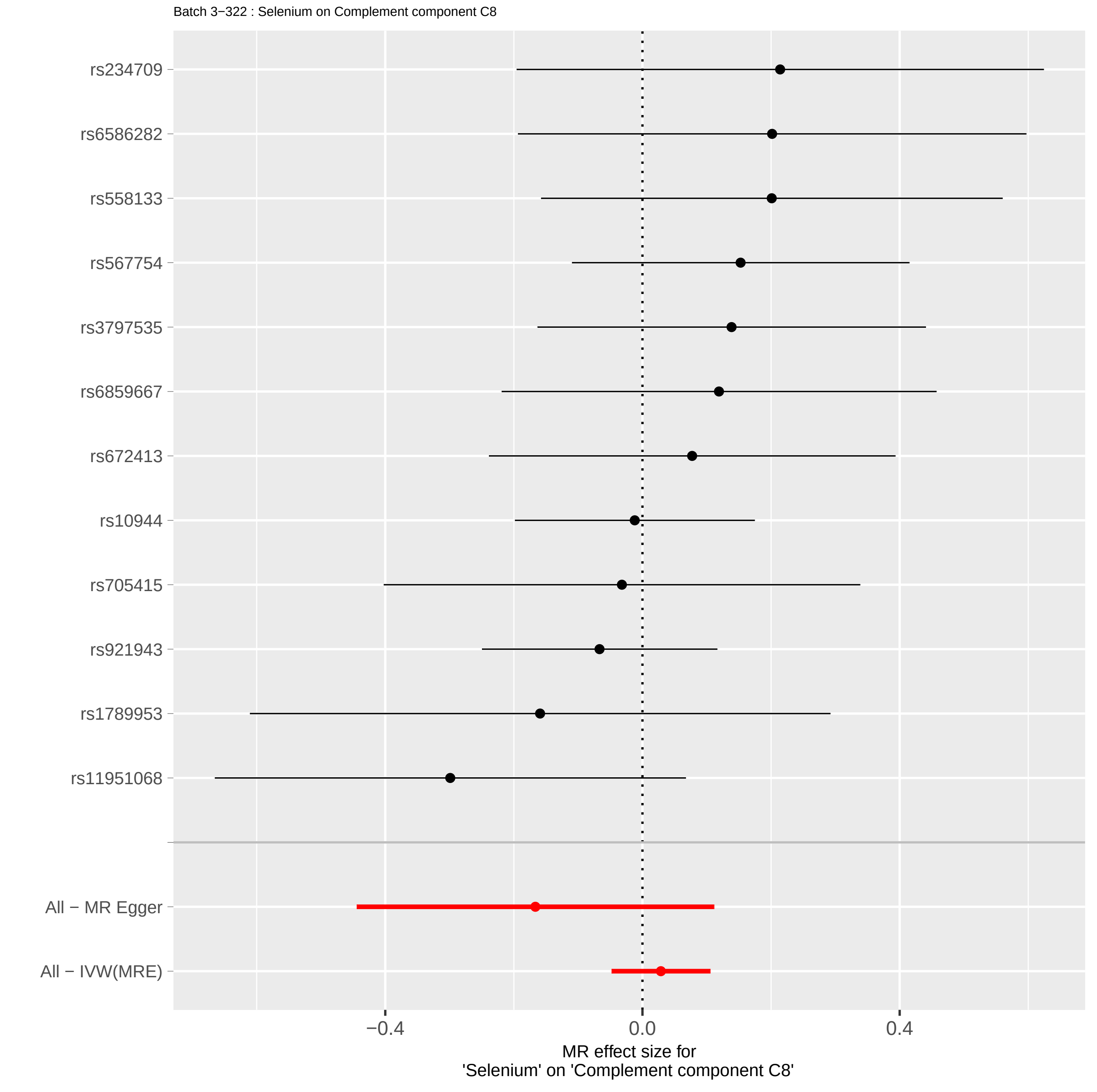

Batch 3−322 : Selenium on Complement component C8
rs234709
rs6586282
rs558133
rs567754
rs3797535
rs6859667
rs672413
rs10944
rs705415
rs921943
rs1789953
rs11951068
All − MR Egger
All − IVW(MRE)
−0.4
0.0
0.4
MR effect size for
'Selenium' on 'Complement component C8'

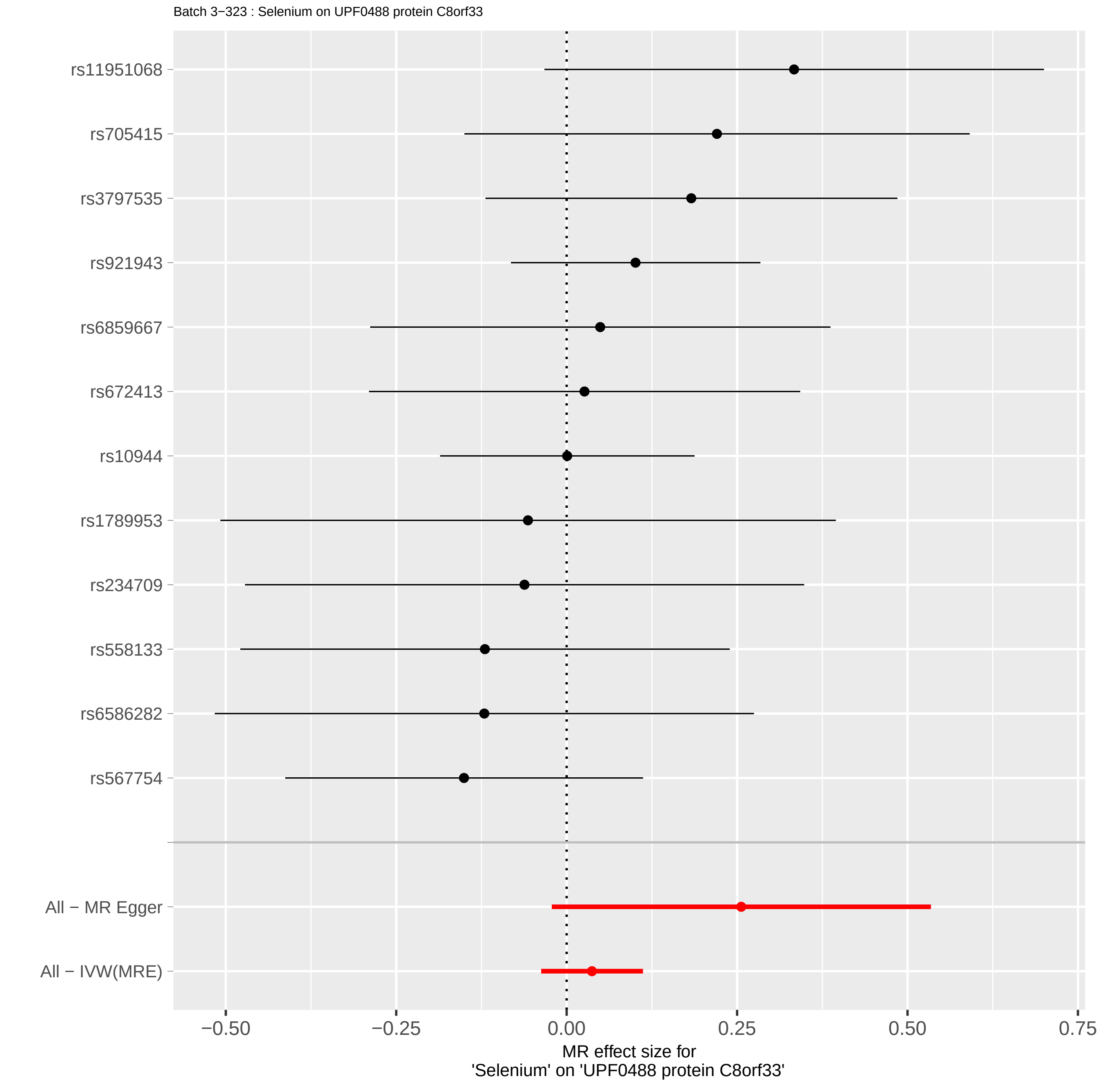
Batch 3−323 : Selenium on UPF0488 protein C8orf33
rs11951068
rs705415
rs3797535
rs921943
rs6859667
rs672413
rs10944
rs1789953
rs234709
rs558133
rs6586282
rs567754
All − MR Egger
All − IVW(MRE)
−0.50
−0.25
0.00
0.25
0.50
0.75
MR effect size for
'Selenium' on 'UPF0488 protein C8orf33'

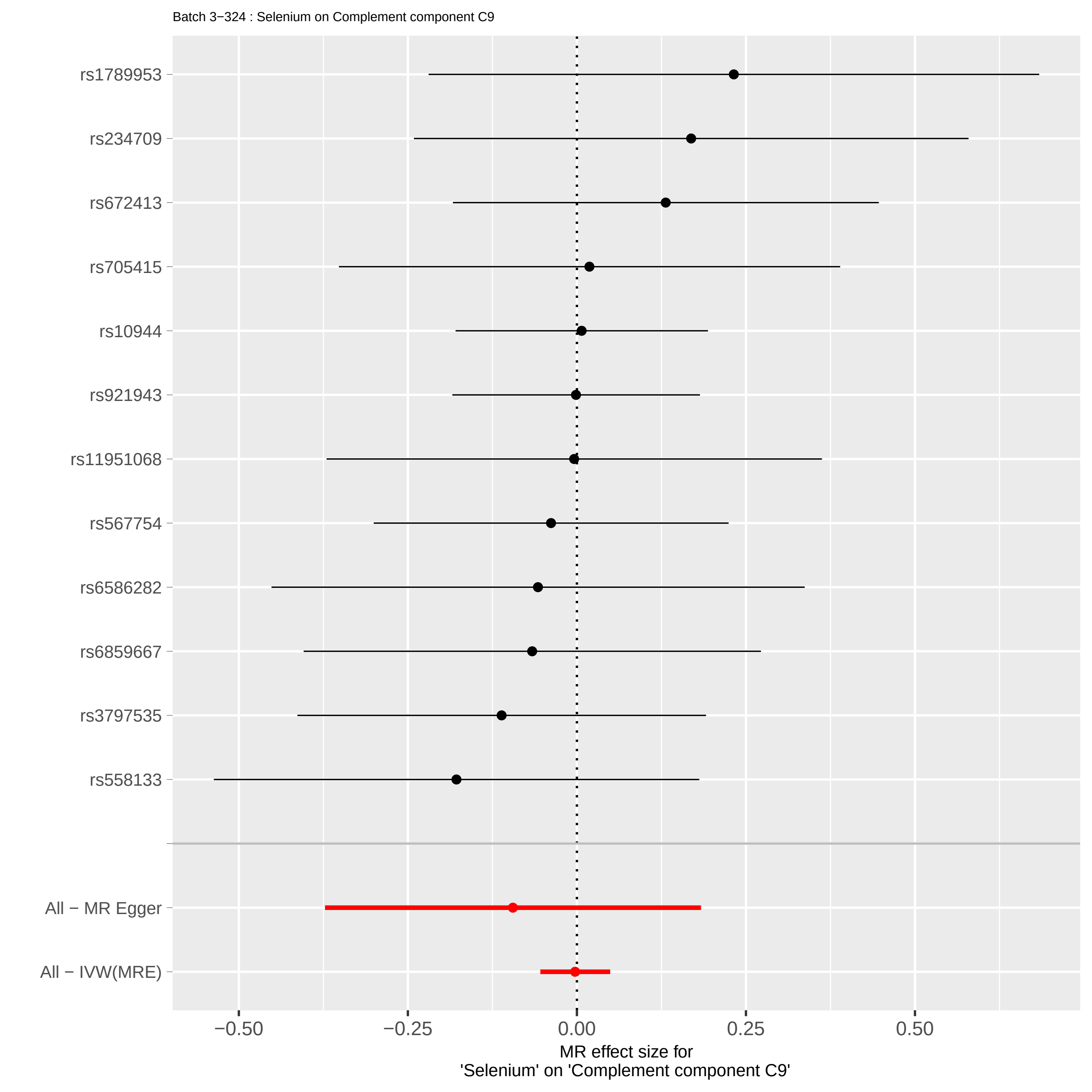

Batch 3−324 : Selenium on Complement component C9
rs1789953
rs234709
rs672413
rs705415
rs10944
rs921943
rs11951068
rs567754
rs6586282
rs6859667
rs3797535
rs558133
All − MR Egger
All − IVW(MRE)
−0.50
−0.25
0.00
0.25
0.50
MR effect size for
'Selenium' on 'Complement component C9'

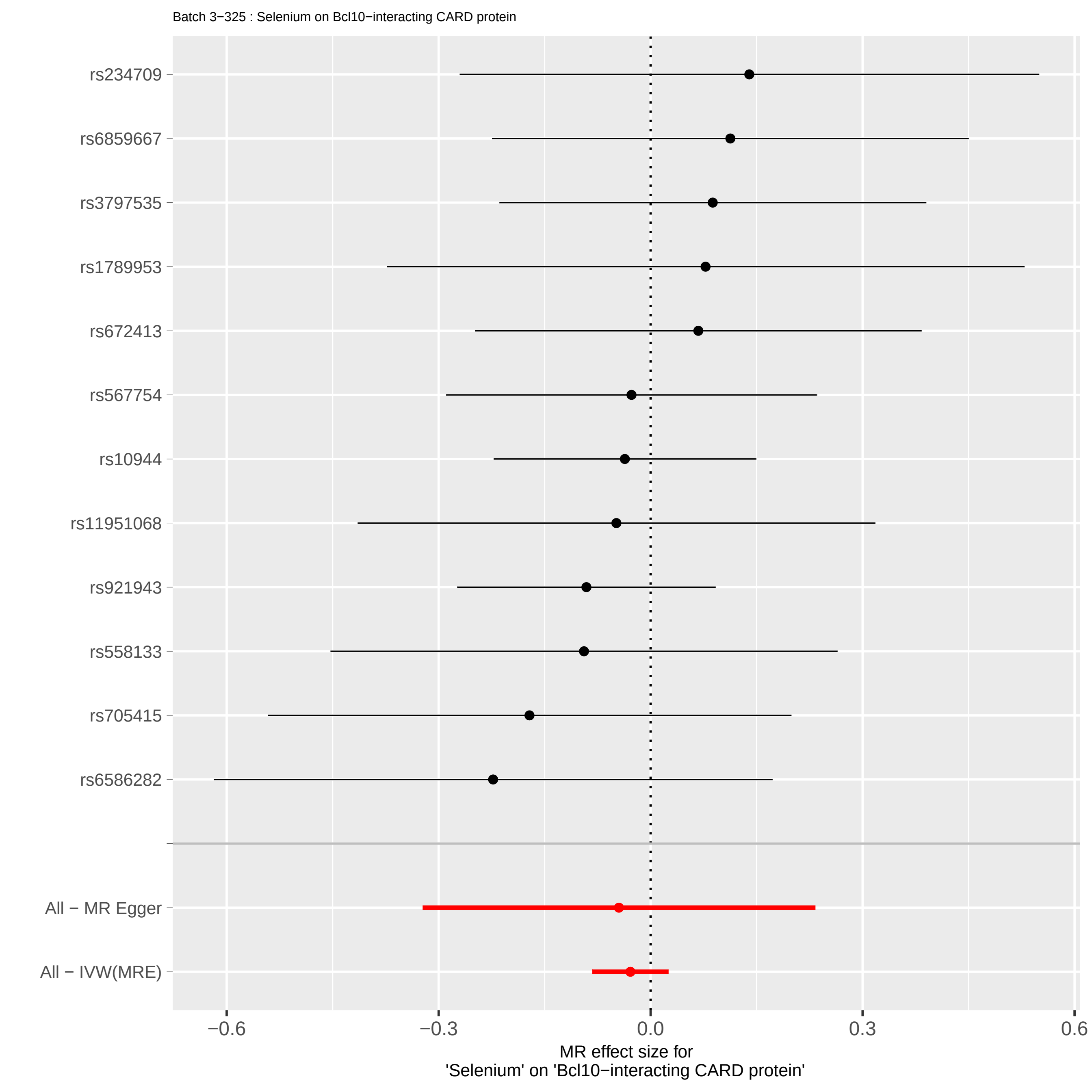
Batch 3−325 : Selenium on Bcl10−interacting CARD protein
rs234709
rs6859667
rs3797535
rs1789953
rs672413
rs567754
rs10944
rs11951068
rs921943
rs558133
rs705415
rs6586282
All − MR Egger
All − IVW(MRE)
−0.6
−0.3
0.0
0.3
0.6
MR effect size for
'Selenium' on 'Bcl10−interacting CARD protein'

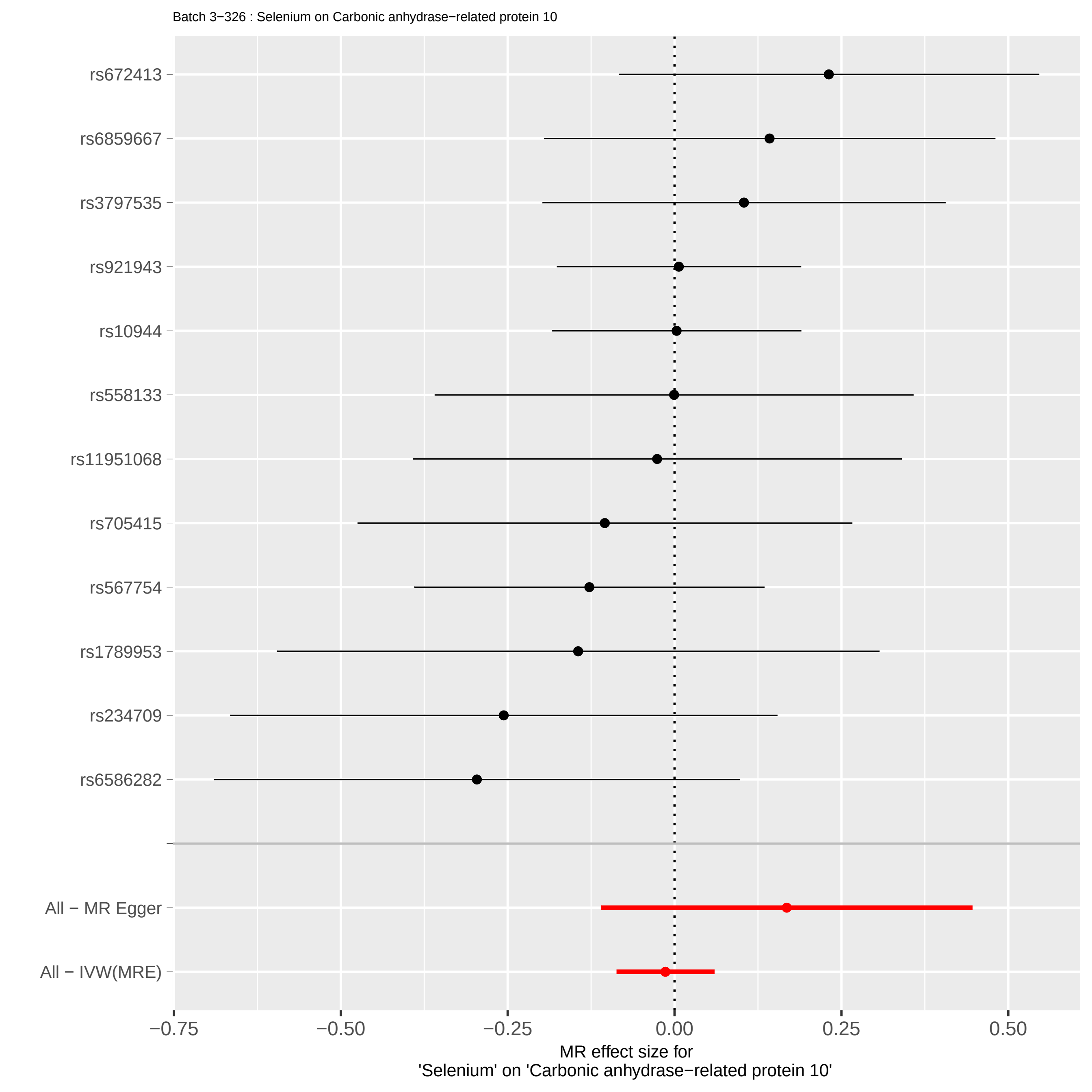

Batch 3−326 : Selenium on Carbonic anhydrase−related protein 10
rs672413
rs6859667
rs3797535
rs921943
rs10944
rs558133
rs11951068
rs705415
rs567754
rs1789953
rs234709
rs6586282
All − MR Egger
All − IVW(MRE)
−0.75
−0.50
−0.25
0.00
0.25
0.50
MR effect size for
'Selenium' on 'Carbonic anhydrase−related protein 10'

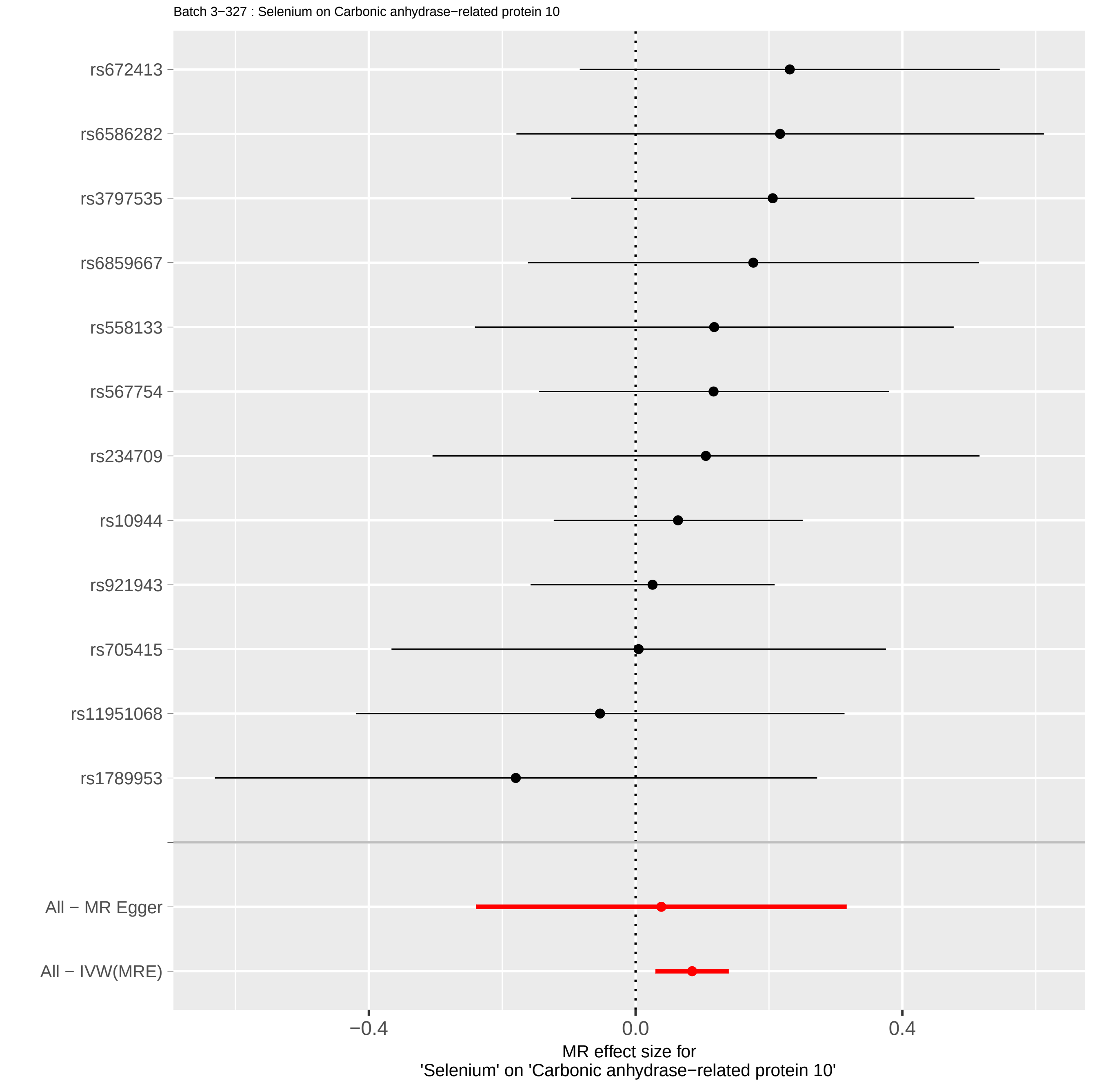
Batch 3−327 : Selenium on Carbonic anhydrase−related protein 10
rs672413
rs6586282
rs3797535
rs6859667
rs558133
rs567754
rs234709
rs10944
rs921943
rs705415
rs11951068
rs1789953
All − MR Egger
All − IVW(MRE)
−0.4
0.0
0.4
MR effect size for
'Selenium' on 'Carbonic anhydrase−related protein 10'

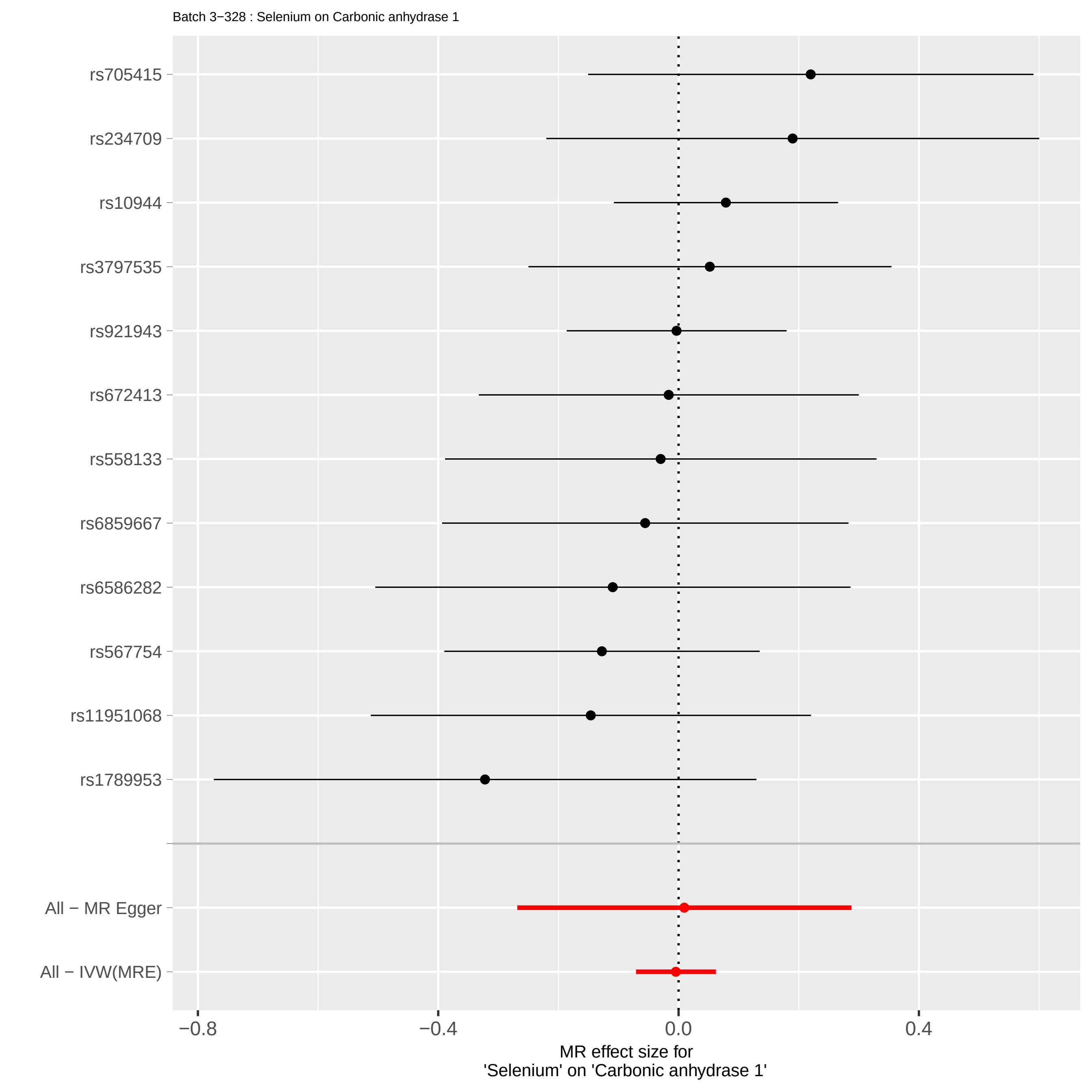

Batch 3−328 : Selenium on Carbonic anhydrase 1
rs705415
rs234709
rs10944
rs3797535
rs921943
rs672413
rs558133
rs6859667
rs6586282
rs567754
rs11951068
rs1789953
All − MR Egger
All − IVW(MRE)
−0.8
−0.4
0.0
0.4
MR effect size for
'Selenium' on 'Carbonic anhydrase 1'

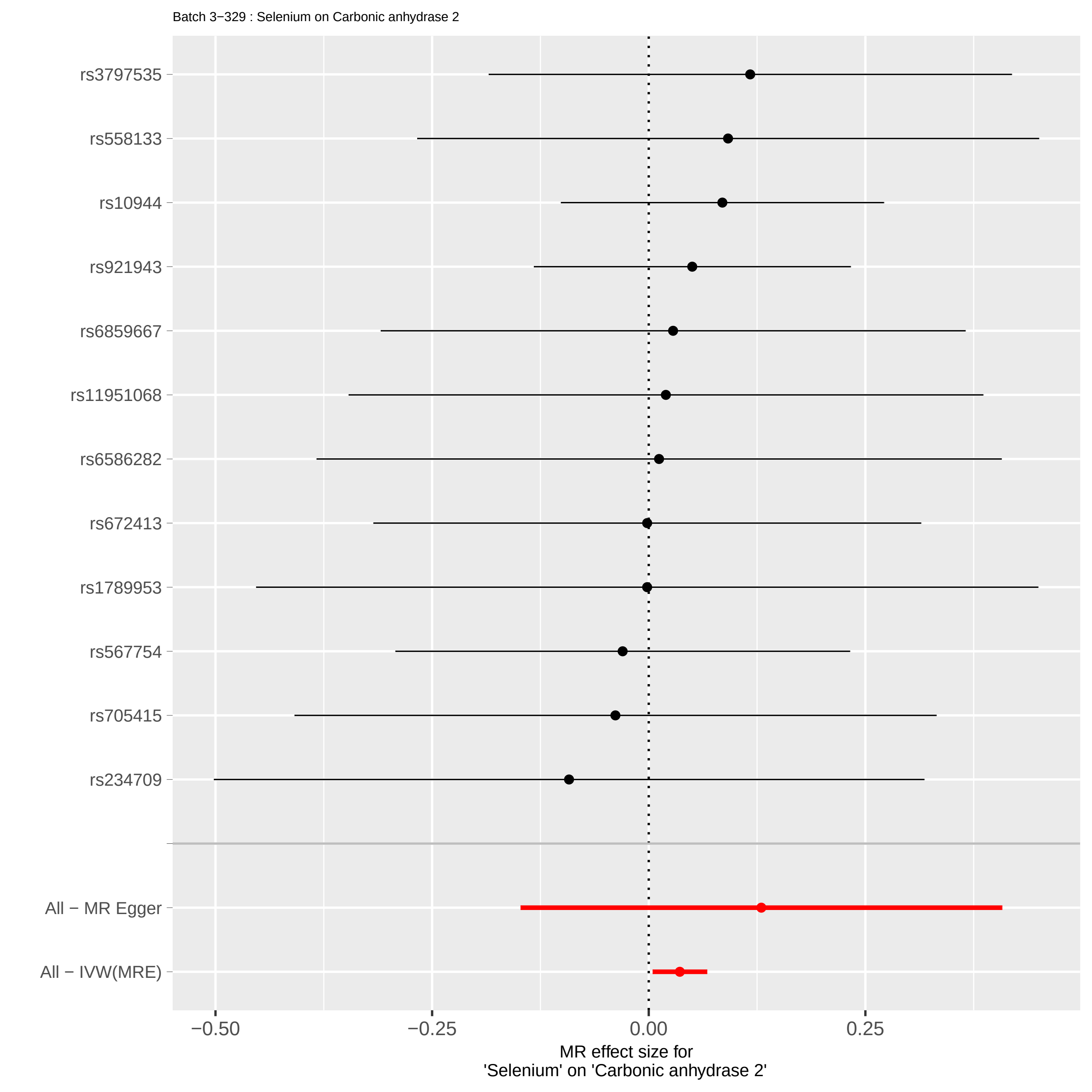
Batch 3−329 : Selenium on Carbonic anhydrase 2
rs3797535
rs558133
rs10944
rs921943
rs6859667
rs11951068
rs6586282
rs672413
rs1789953
rs567754
rs705415
rs234709
All − MR Egger
All − IVW(MRE)
−0.50
−0.25
0.00
0.25
MR effect size for
'Selenium' on 'Carbonic anhydrase 2'

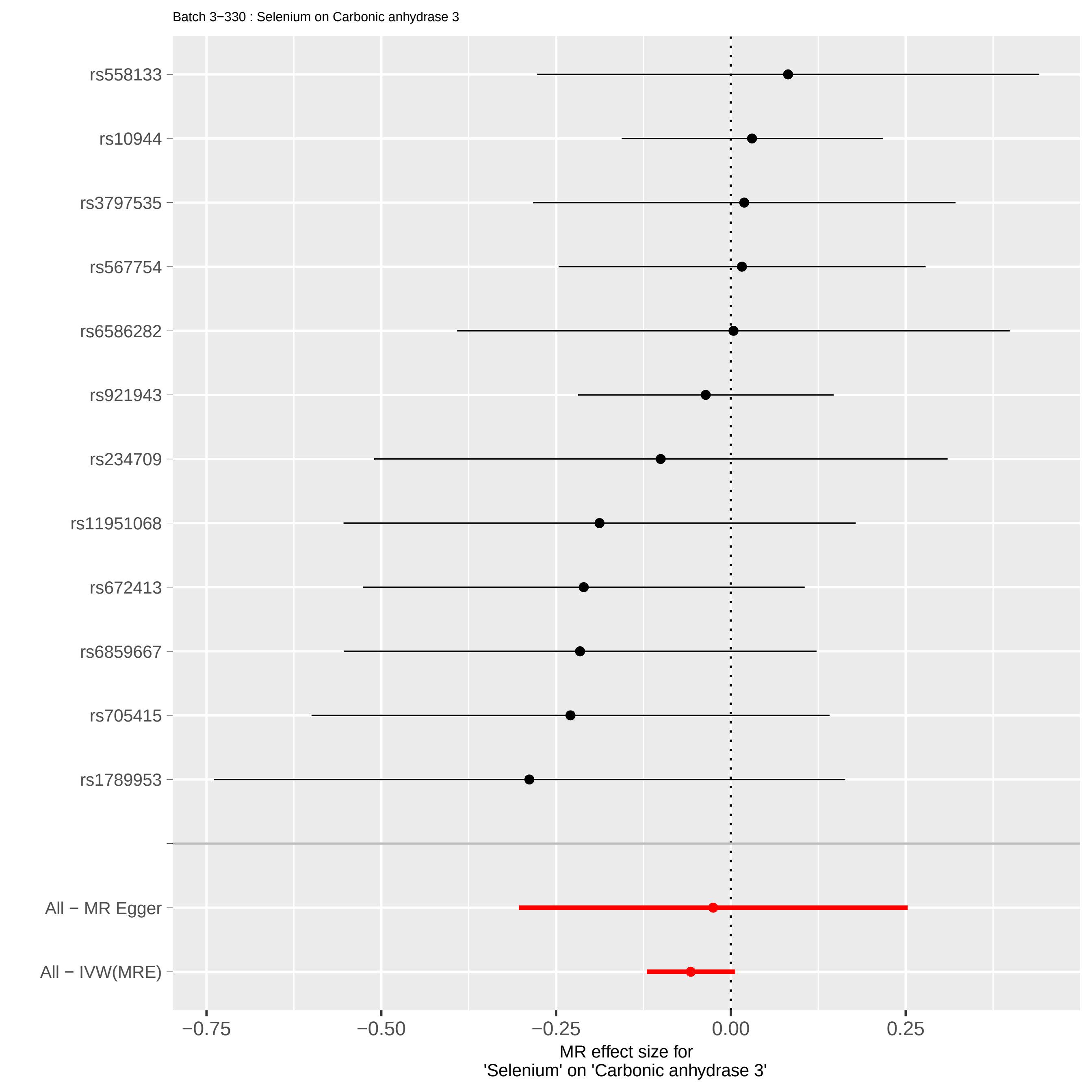

Batch 3−330 : Selenium on Carbonic anhydrase 3
rs558133
rs10944
rs3797535
rs567754
rs6586282
rs921943
rs234709
rs11951068
rs672413
rs6859667
rs705415
rs1789953
All − MR Egger
All − IVW(MRE)
−0.75
−0.50
−0.25
0.00
0.25
MR effect size for
'Selenium' on 'Carbonic anhydrase 3'

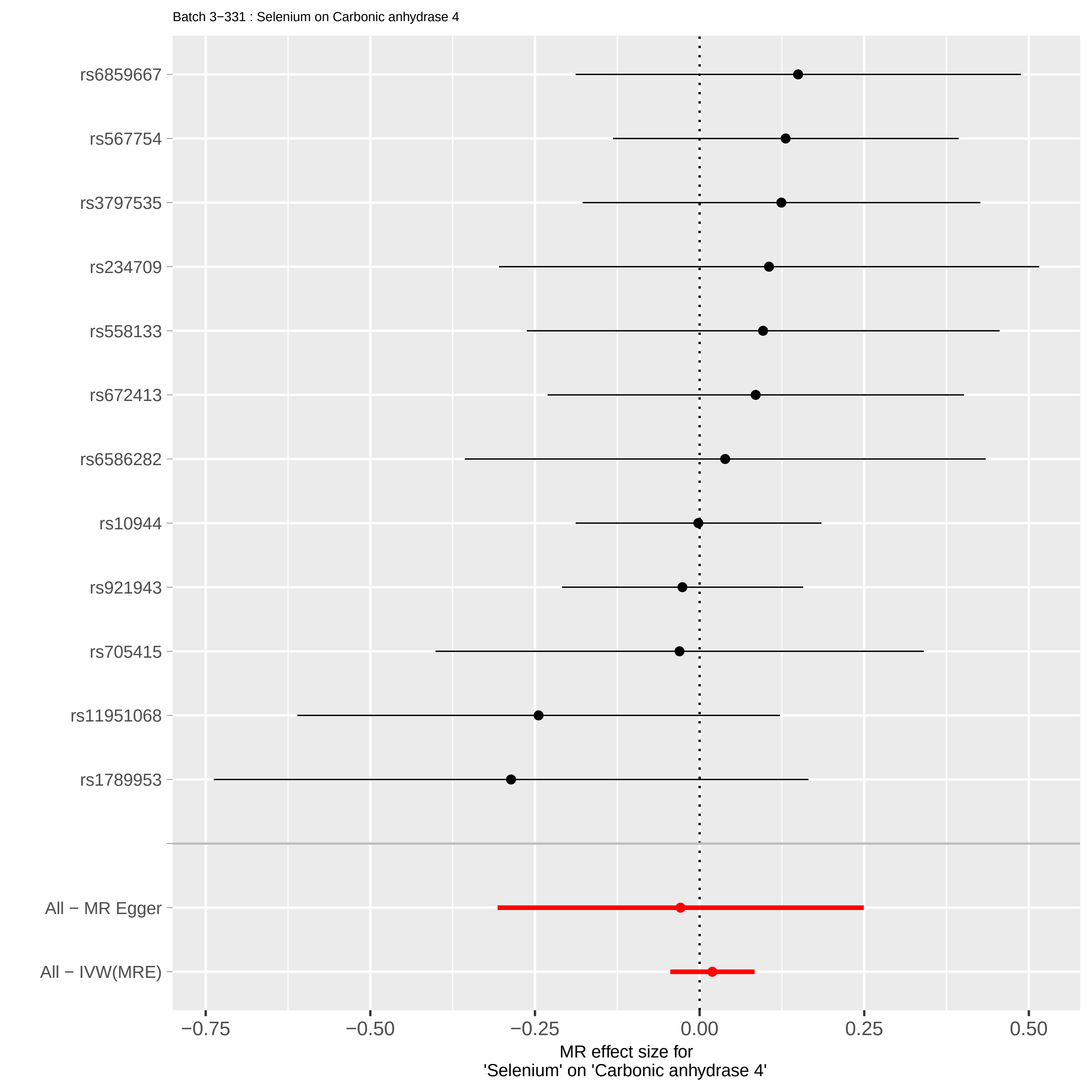

Batch 3−331 : Selenium on Carbonic anhydrase 4
rs6859667
rs567754
rs3797535
rs234709
rs558133
rs672413
rs6586282
rs10944
rs921943
rs705415
rs11951068
rs1789953
All − MR Egger
All − IVW(MRE)
−0.75
−0.50
−0.25
0.00
0.25
0.50
MR effect size for
'Selenium' on 'Carbonic anhydrase 4'

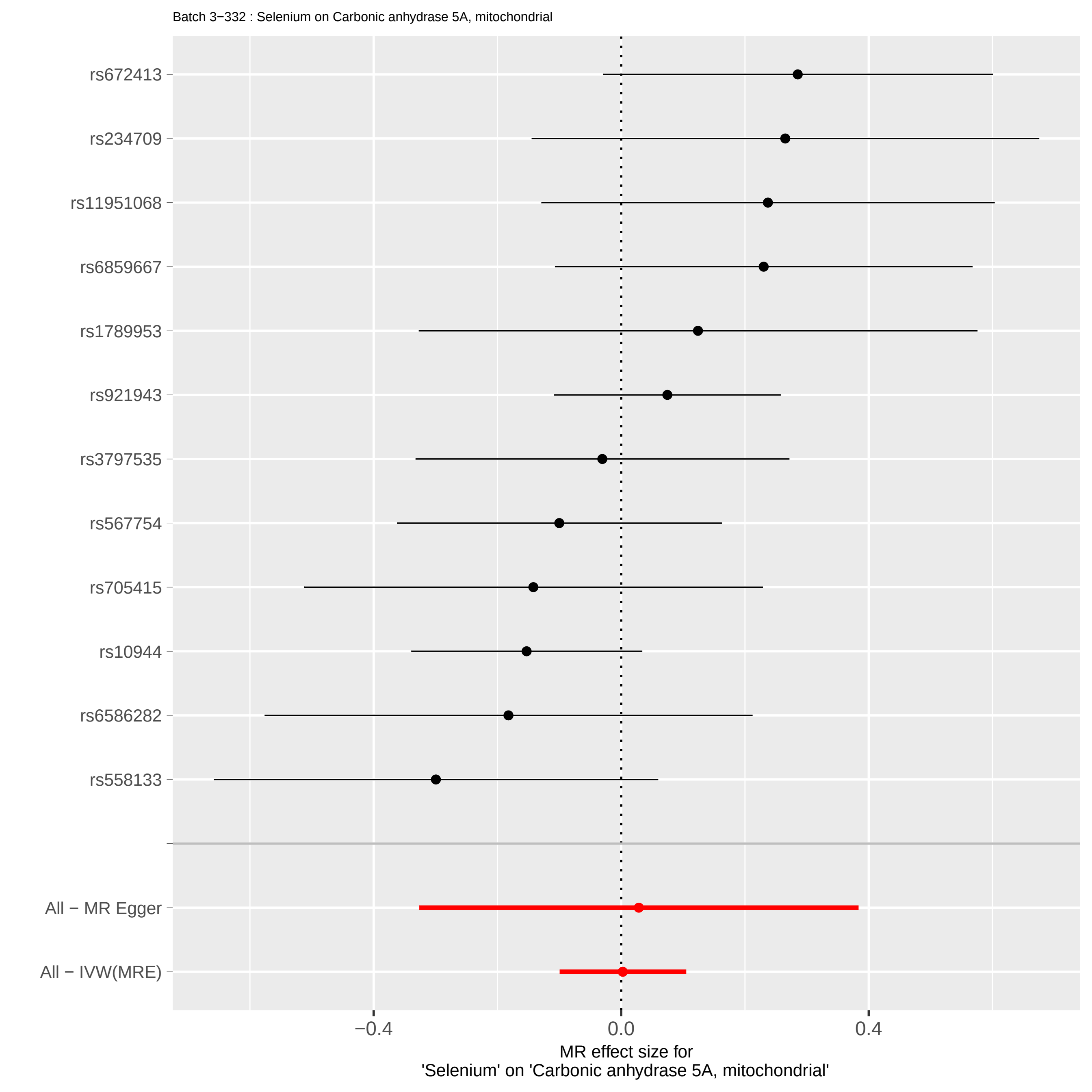
Batch 3−332 : Selenium on Carbonic anhydrase 5A, mitochondrial
rs672413
rs234709
rs11951068
rs6859667
rs1789953
rs921943
rs3797535
rs567754
rs705415
rs10944
rs6586282
rs558133
All − MR Egger
All − IVW(MRE)
−0.4
0.0
0.4
MR effect size for
'Selenium' on 'Carbonic anhydrase 5A, mitochondrial'

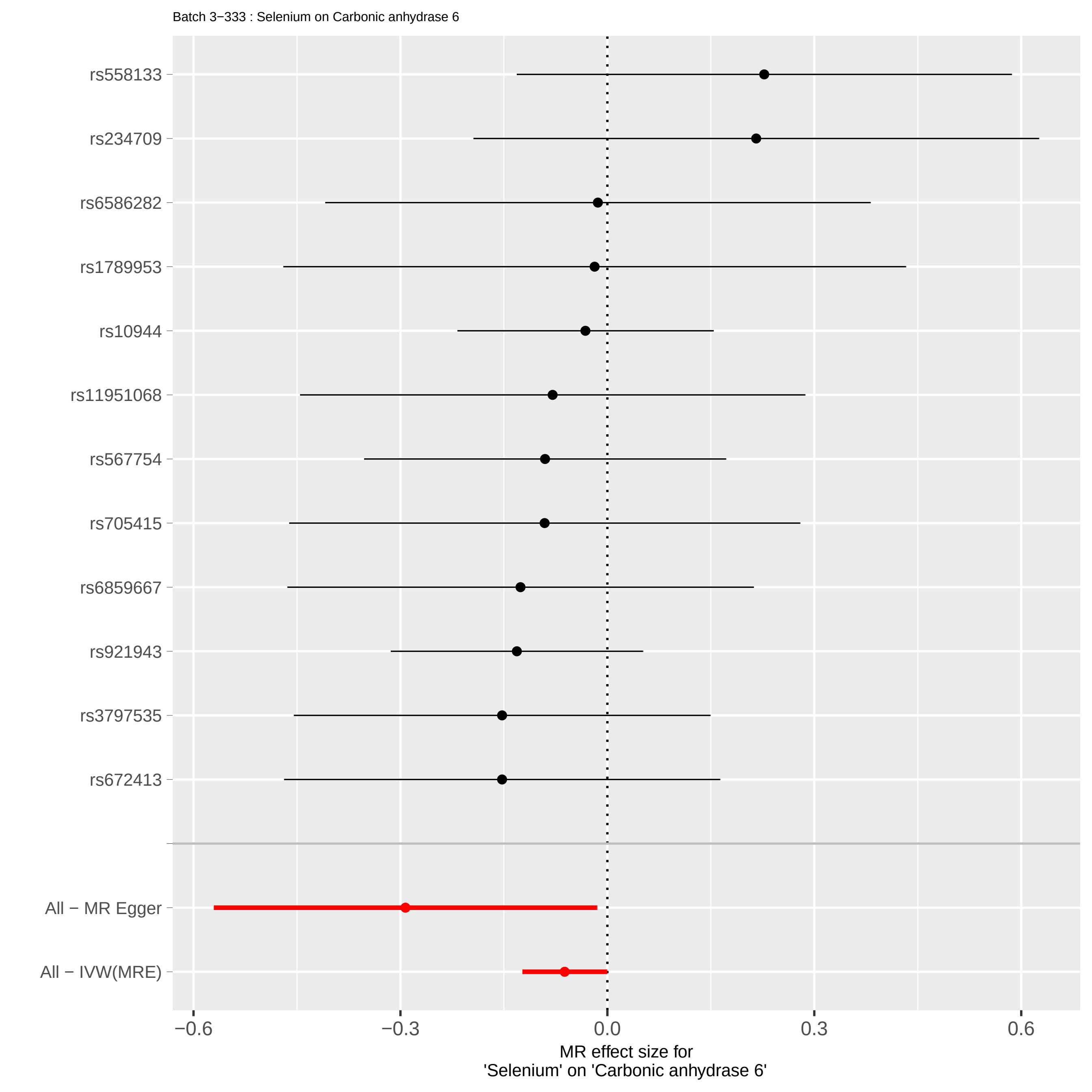

Batch 3−333 : Selenium on Carbonic anhydrase 6
rs558133
rs234709
rs6586282
rs1789953
rs10944
rs11951068
rs567754
rs705415
rs6859667
rs921943
rs3797535
rs672413
All − MR Egger
All − IVW(MRE)
−0.6
−0.3
0.0
0.3
0.6
MR effect size for
'Selenium' on 'Carbonic anhydrase 6'

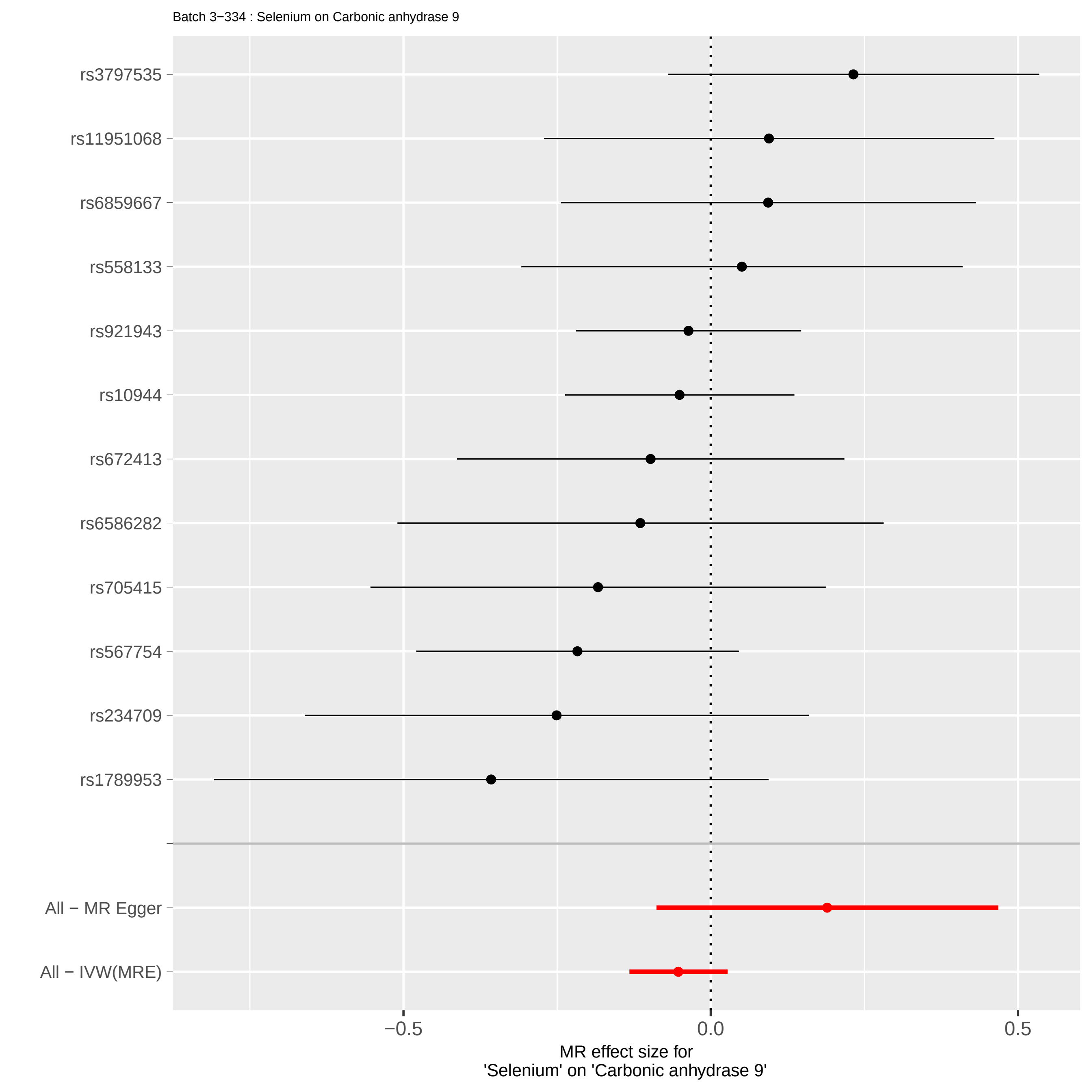

Batch 3−334 : Selenium on Carbonic anhydrase 9
rs3797535
rs11951068
rs6859667
rs558133
rs921943
rs10944
rs672413
rs6586282
rs705415
rs567754
rs234709
rs1789953
All − MR Egger
All − IVW(MRE)
−0.5
0.0
0.5
MR effect size for
'Selenium' on 'Carbonic anhydrase 9'

Batch 3−335 : Selenium on Voltage−dependent calcium channel subunit alpha−2/delta−3

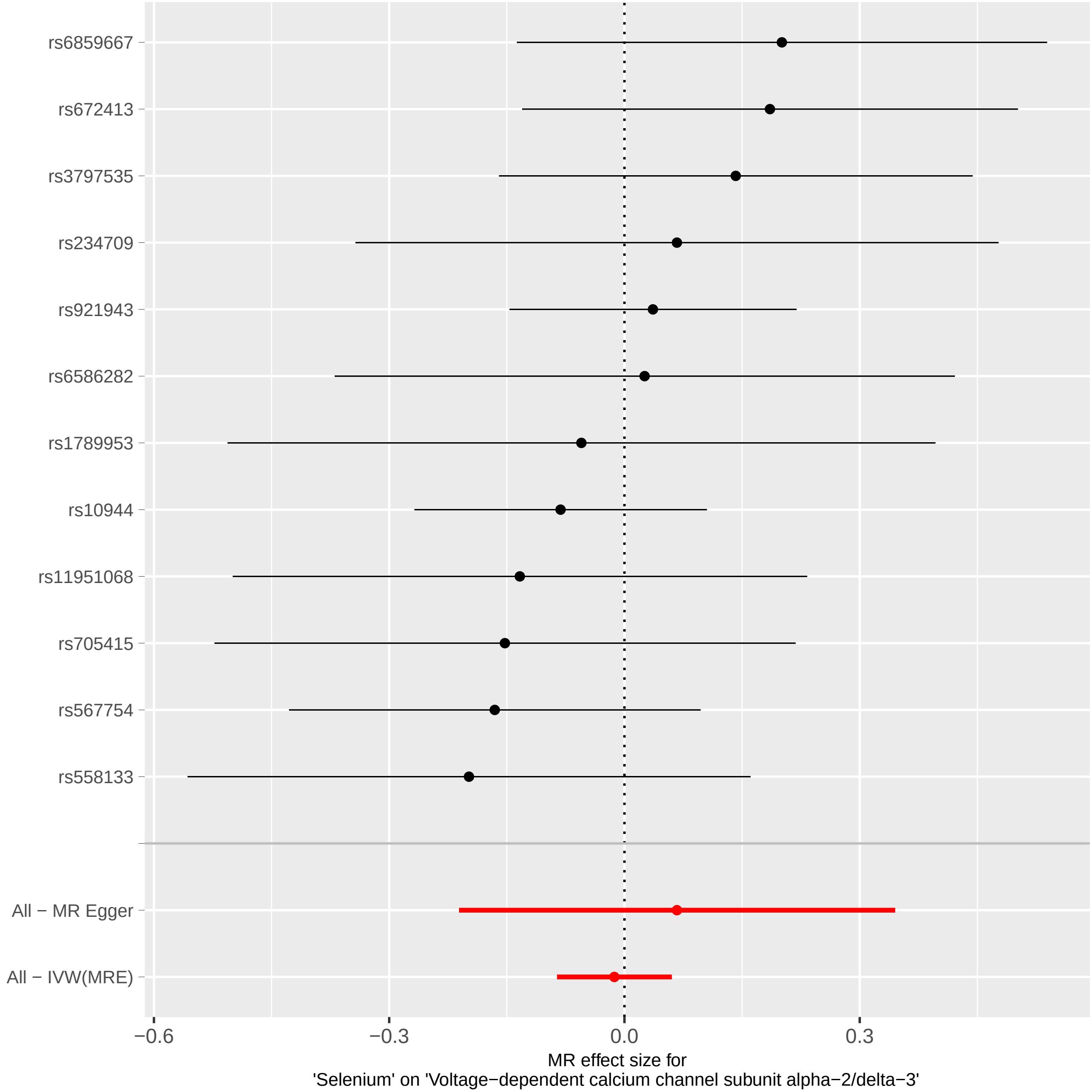

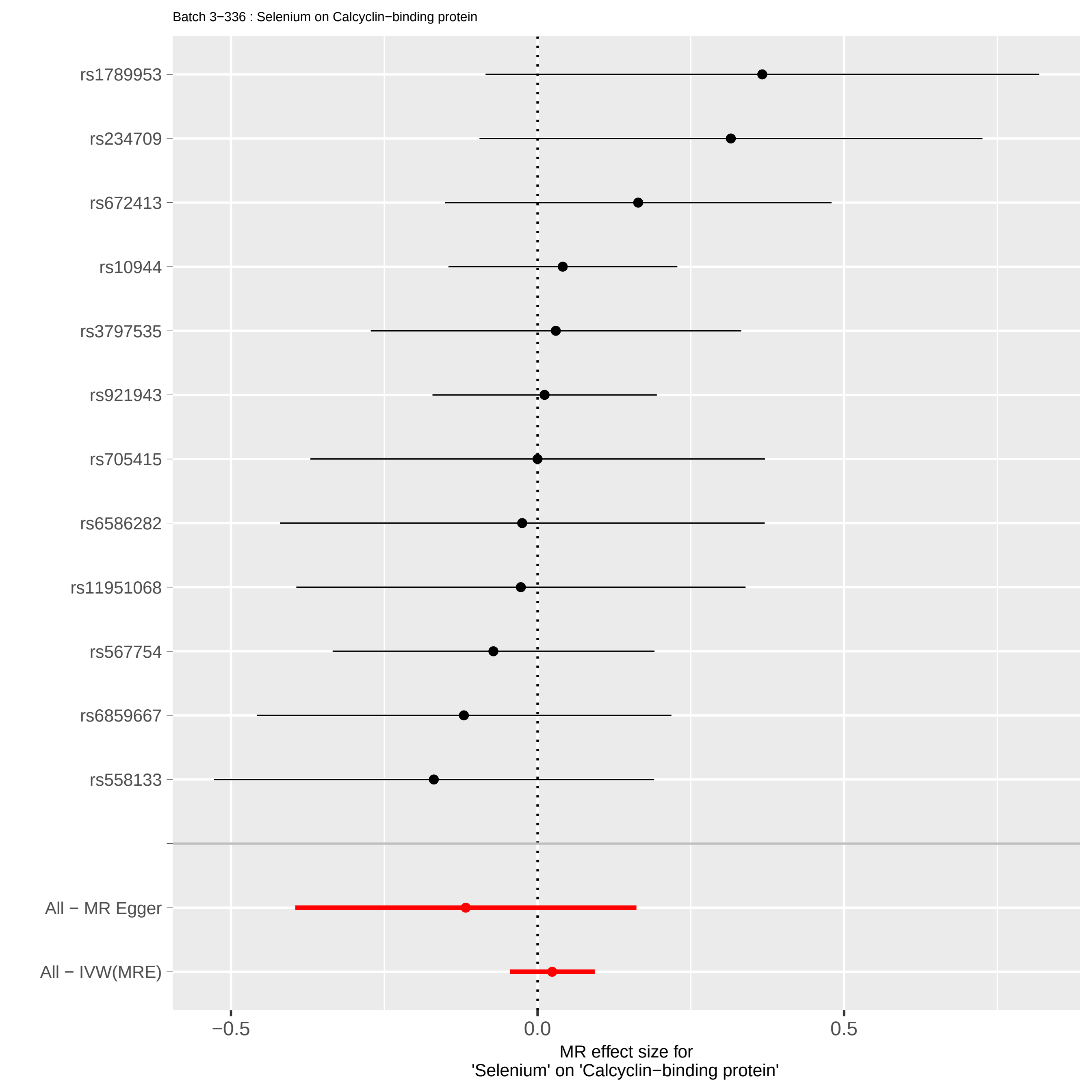

Batch 3−336 : Selenium on Calcyclin−binding protein
rs1789953
rs234709
rs672413
rs10944
rs3797535
rs921943
rs705415
rs6586282
rs11951068
rs567754
rs6859667
rs558133
All − MR Egger
All − IVW(MRE)
−0.5
0.0
0.5
MR effect size for
'Selenium' on 'Calcyclin−binding protein'

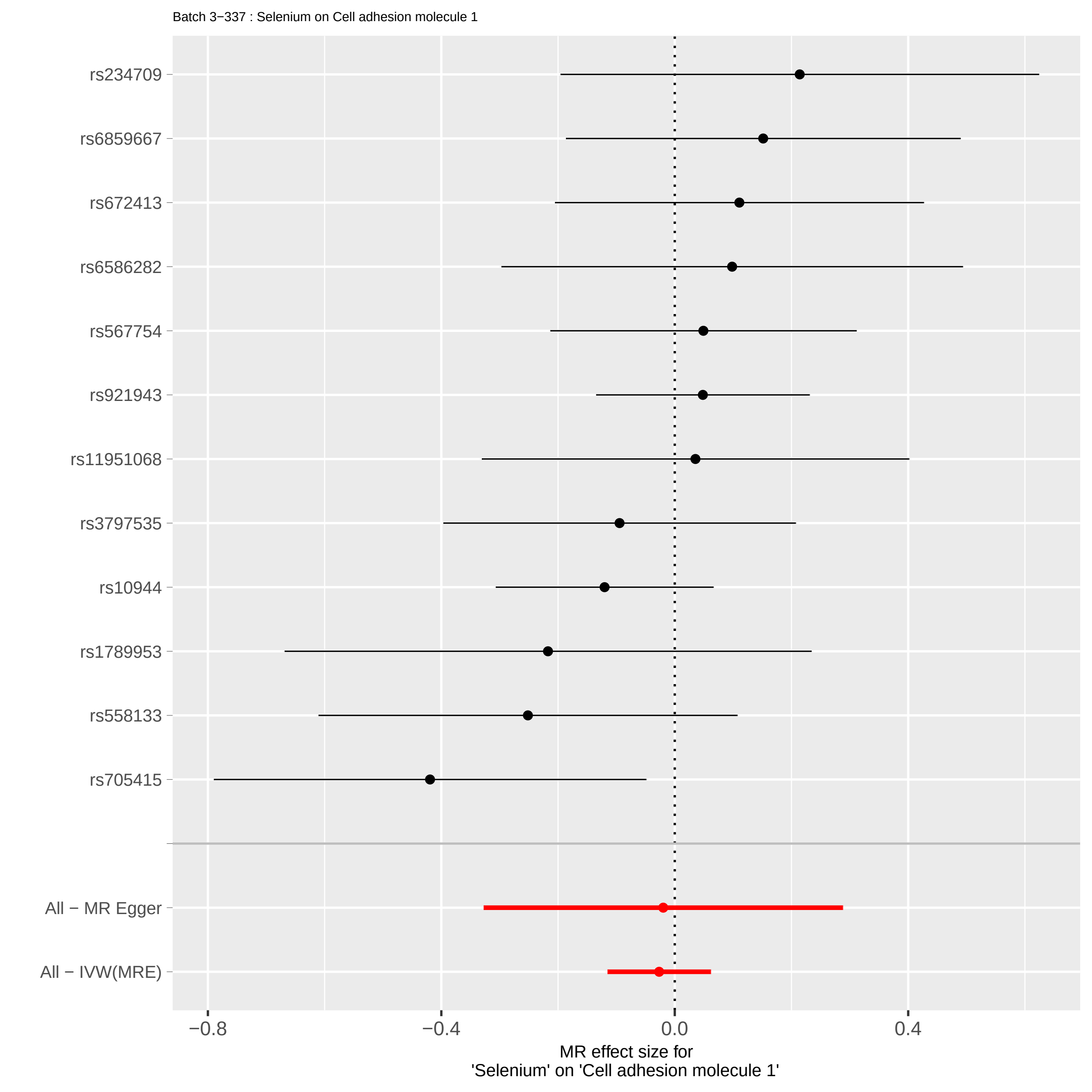

Batch 3−337 : Selenium on Cell adhesion molecule 1
rs234709
rs6859667
rs672413
rs6586282
rs567754
rs921943
rs11951068
rs3797535
rs10944
rs1789953
rs558133
rs705415
All − MR Egger
All − IVW(MRE)
−0.8
−0.4
0.0
0.4
MR effect size for
'Selenium' on 'Cell adhesion molecule 1'

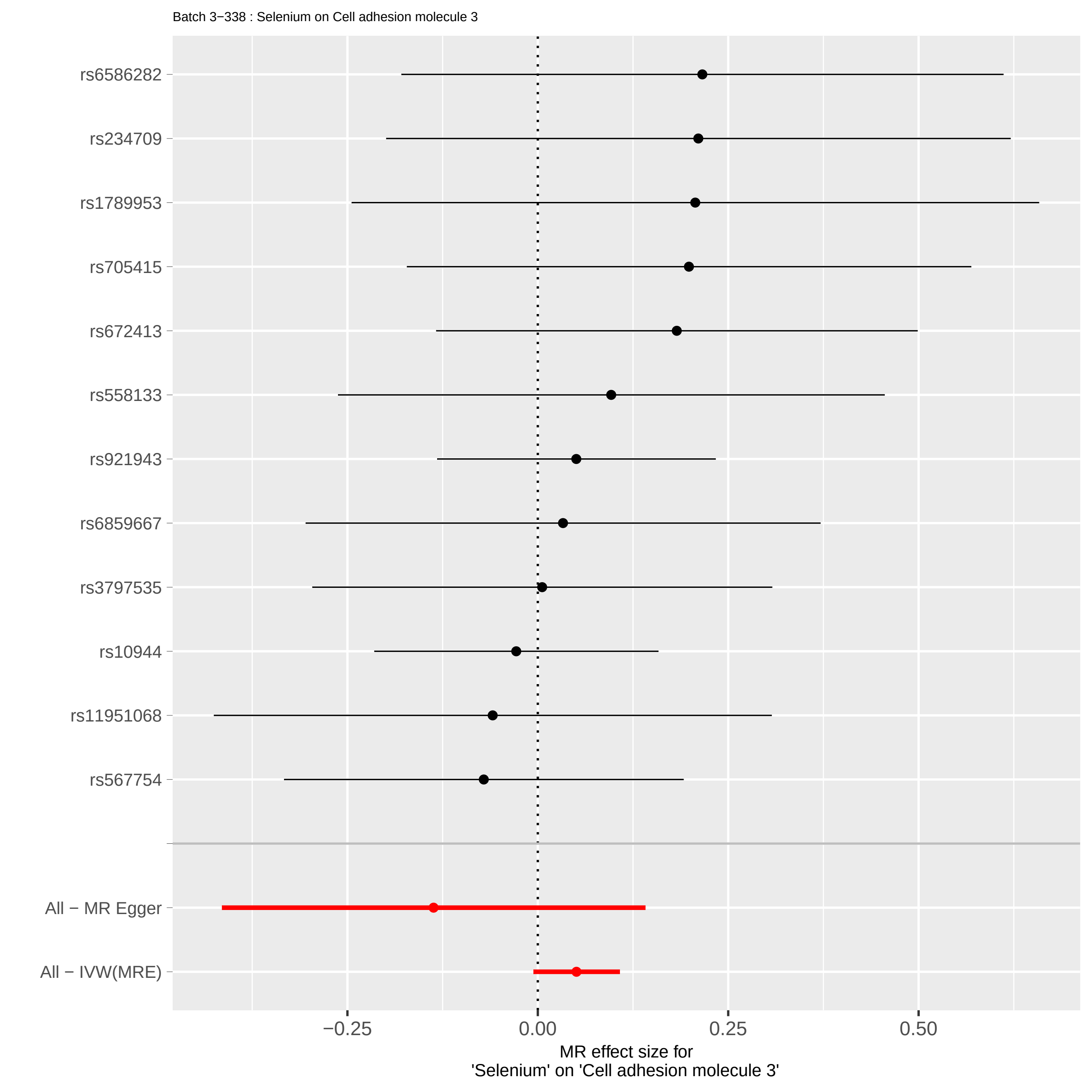
Batch 3−338 : Selenium on Cell adhesion molecule 3
rs6586282
rs234709
rs1789953
rs705415
rs672413
rs558133
rs921943
rs6859667
rs3797535
rs10944
rs11951068
rs567754
All − MR Egger
All − IVW(MRE)
−0.25
0.00
0.25
0.50
MR effect size for
'Selenium' on 'Cell adhesion molecule 3'

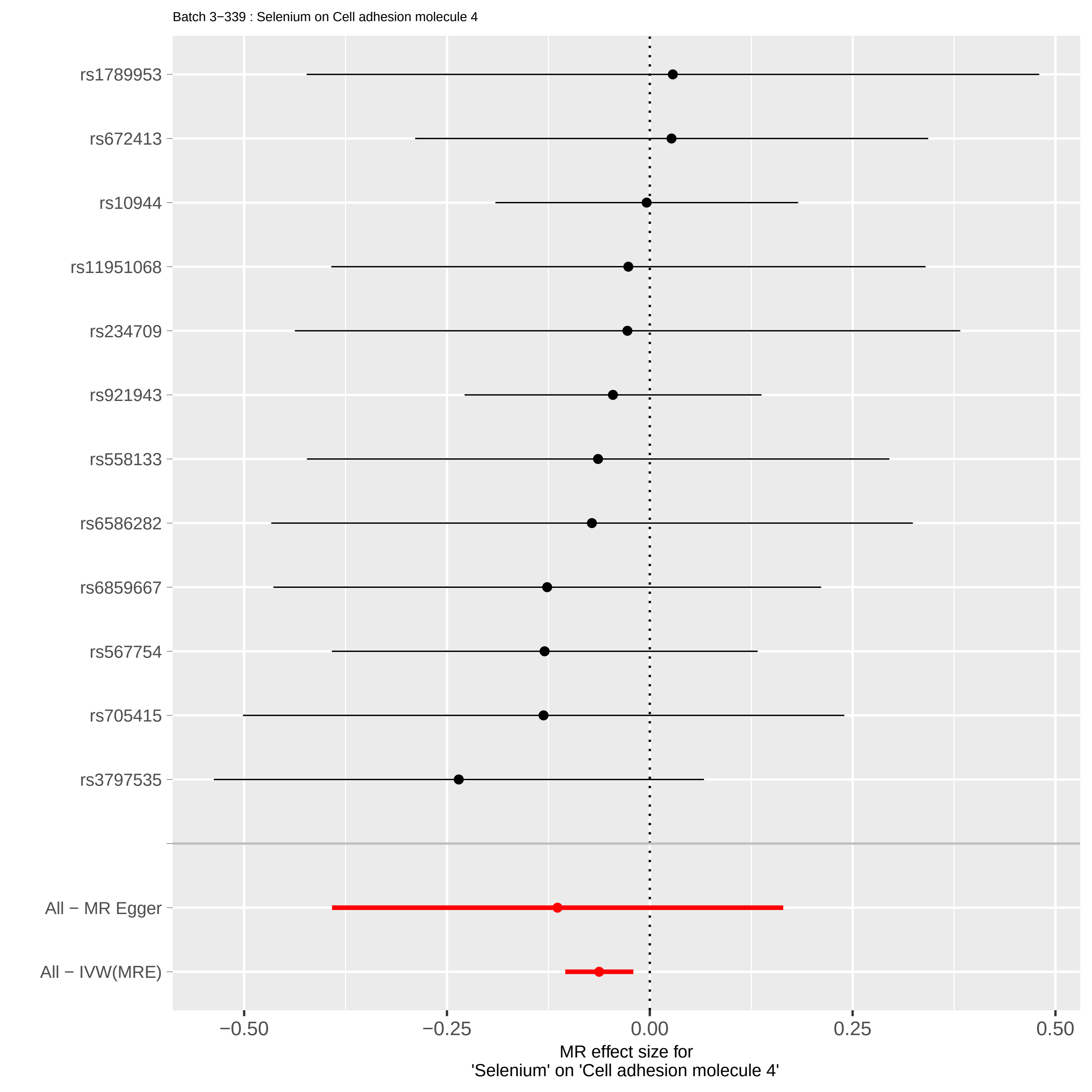
Batch 3−339 : Selenium on Cell adhesion molecule 4
rs1789953
rs672413
rs10944
rs11951068
rs234709
rs921943
rs558133
rs6586282
rs6859667
rs567754
rs705415
rs3797535
All − MR Egger
All − IVW(MRE)
−0.50
−0.25
0.00
0.25
0.50
MR effect size for
'Selenium' on 'Cell adhesion molecule 4'

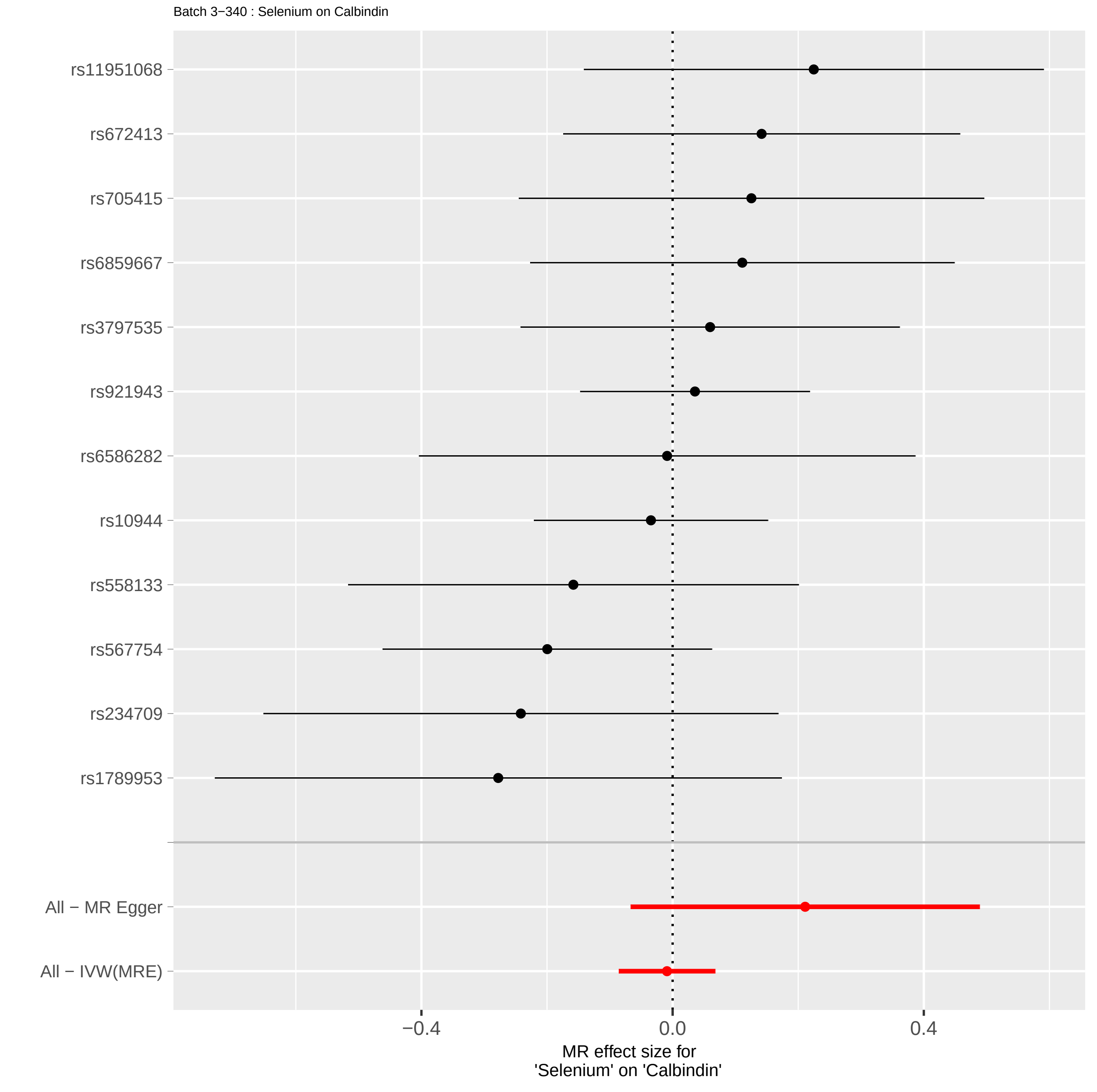

Batch 3−340 : Selenium on Calbindin
rs11951068
rs672413
rs705415
rs6859667
rs3797535
rs921943
rs6586282
rs10944
rs558133
rs567754
rs234709
rs1789953
All − MR Egger
All − IVW(MRE)
−0.4
0.0
0.4
MR effect size for
'Selenium' on 'Calbindin'

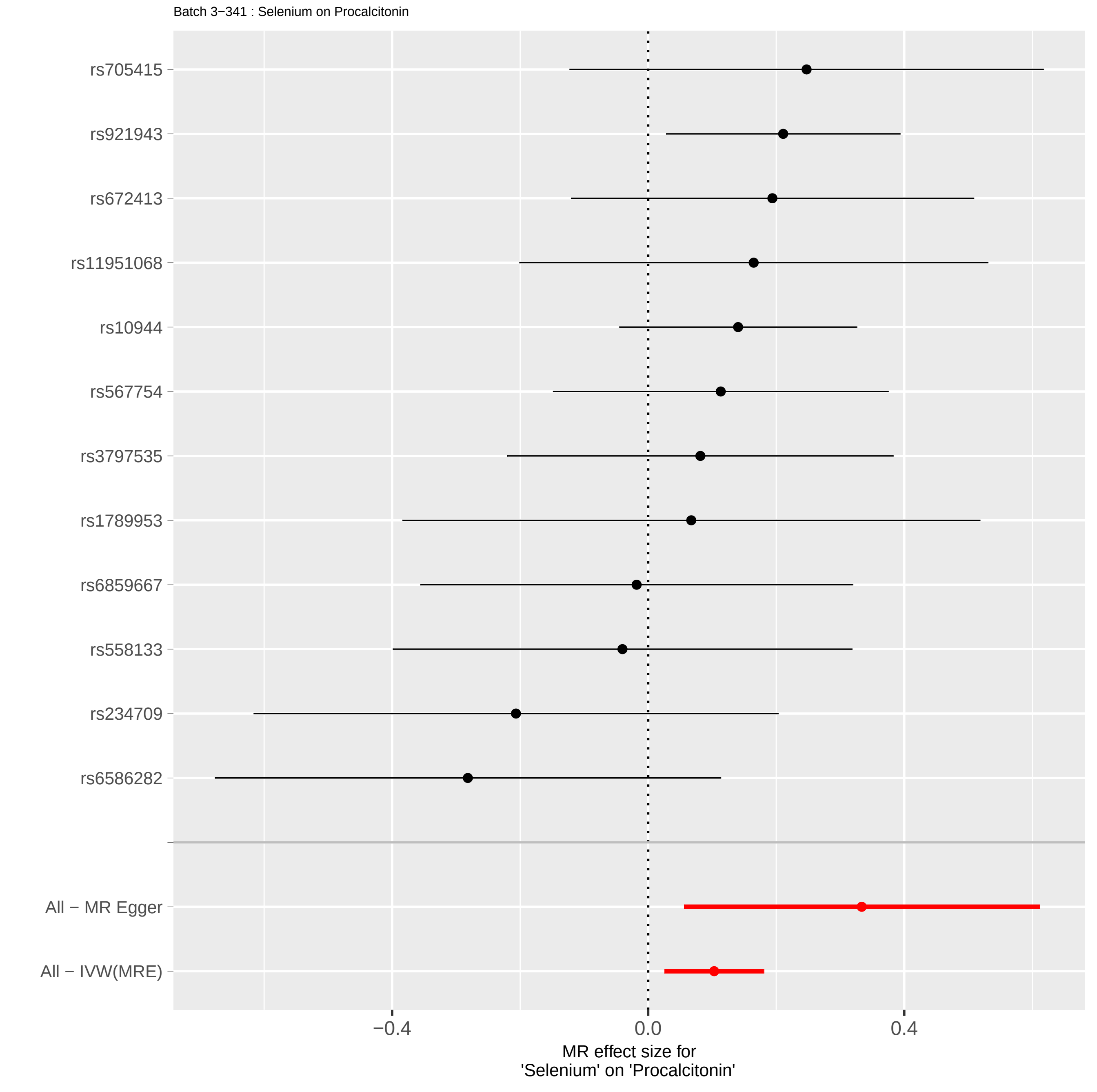

Batch 3−341 : Selenium on Procalcitonin
rs705415
rs921943
rs672413
rs11951068
rs10944
rs567754
rs3797535
rs1789953
rs6859667
rs558133
rs234709
rs6586282
All − MR Egger
All − IVW(MRE)
−0.4
0.0
0.4
MR effect size for
'Selenium' on 'Procalcitonin'

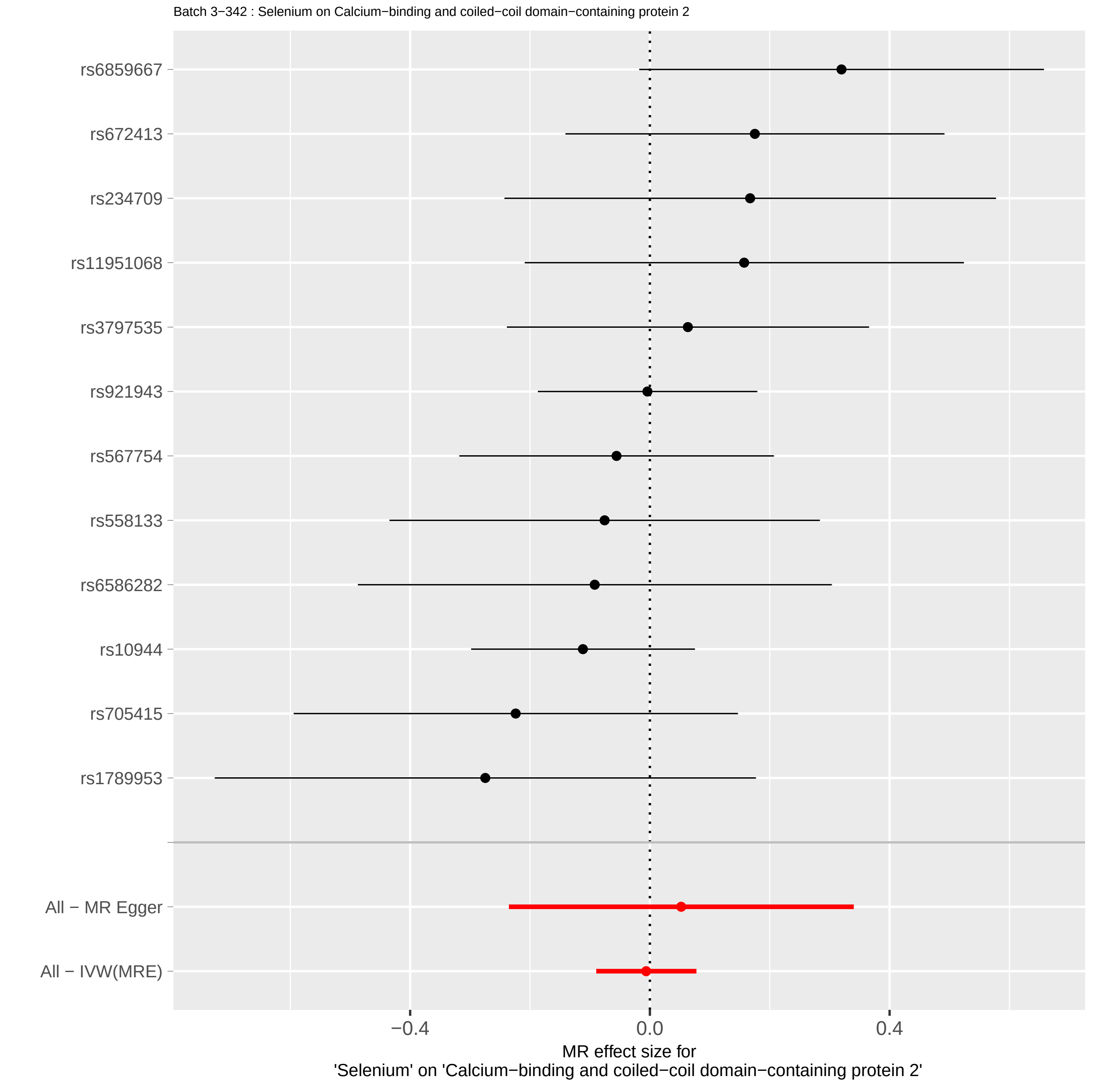
Batch 3−342 : Selenium on Calcium−binding and coiled−coil domain−containing protein 2
rs6859667
rs672413
rs234709
rs11951068
rs3797535
rs921943
rs567754
rs558133
rs6586282
rs10944
rs705415
rs1789953
All − MR Egger
All − IVW(MRE)
−0.4
0.0
0.4
MR effect size for
'Selenium' on 'Calcium−binding and coiled−coil domain−containing protein 2'

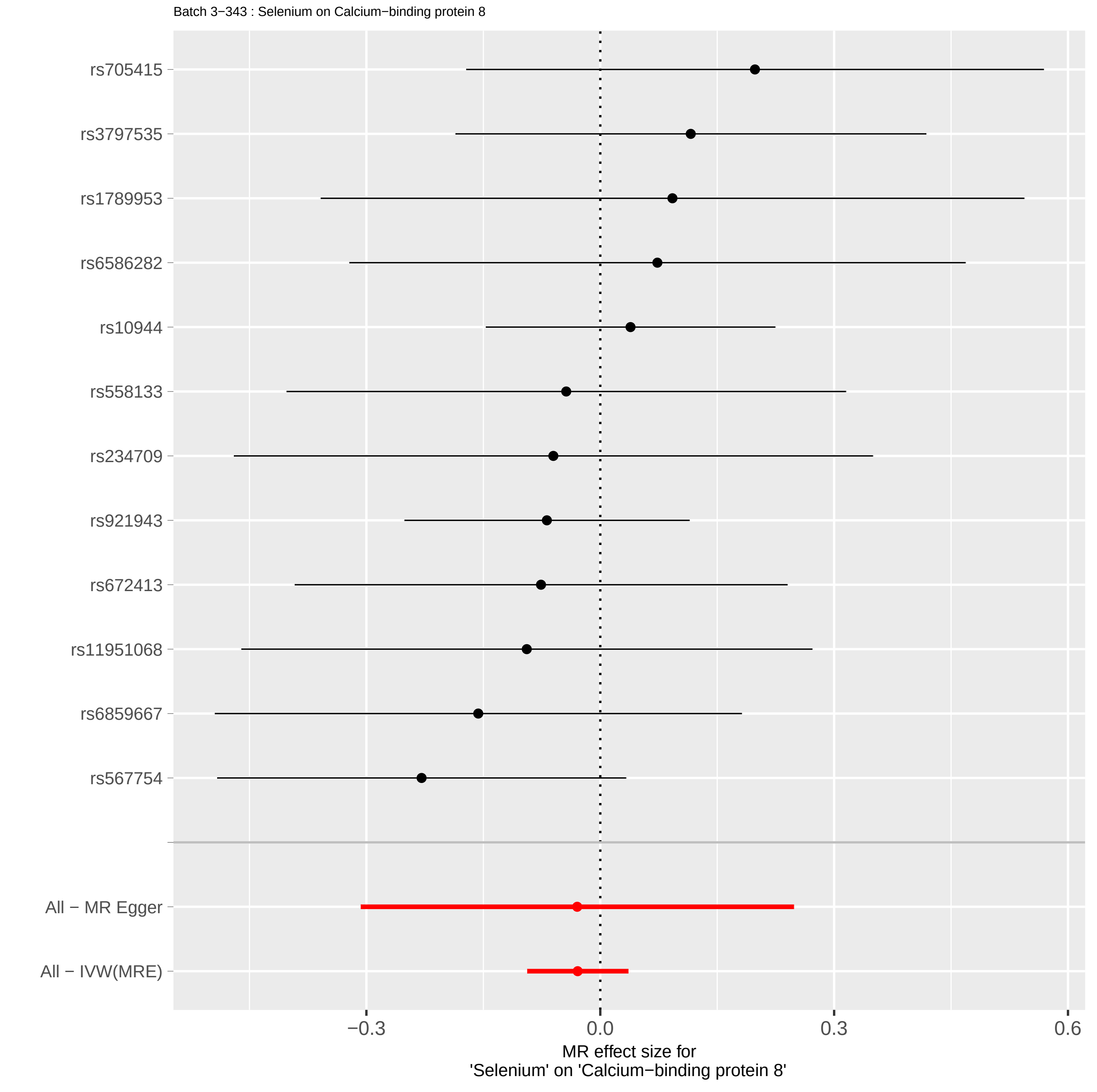
Batch 3−343 : Selenium on Calcium−binding protein 8
rs705415
rs3797535
rs1789953
rs6586282
rs10944
rs558133
rs234709
rs921943
rs672413
rs11951068
rs6859667
rs567754
All − MR Egger
All − IVW(MRE)
−0.3
0.0
0.3
0.6
MR effect size for
'Selenium' on 'Calcium−binding protein 8'

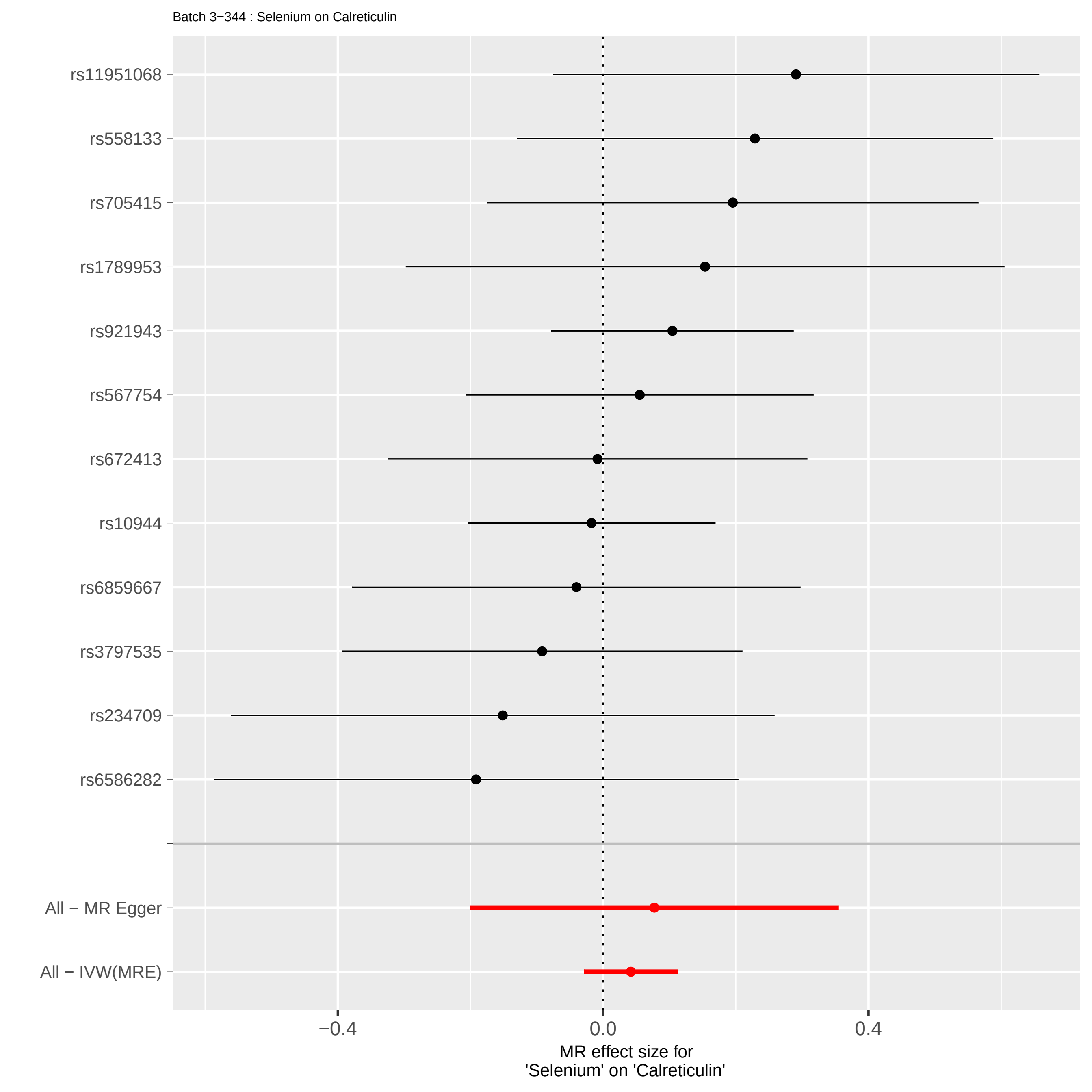

Batch 3−344 : Selenium on Calreticulin
rs11951068
rs558133
rs705415
rs1789953
rs921943
rs567754
rs672413
rs10944
rs6859667
rs3797535
rs234709
rs6586282
All − MR Egger
All − IVW(MRE)
−0.4
0.0
0.4
MR effect size for
'Selenium' on 'Calreticulin'

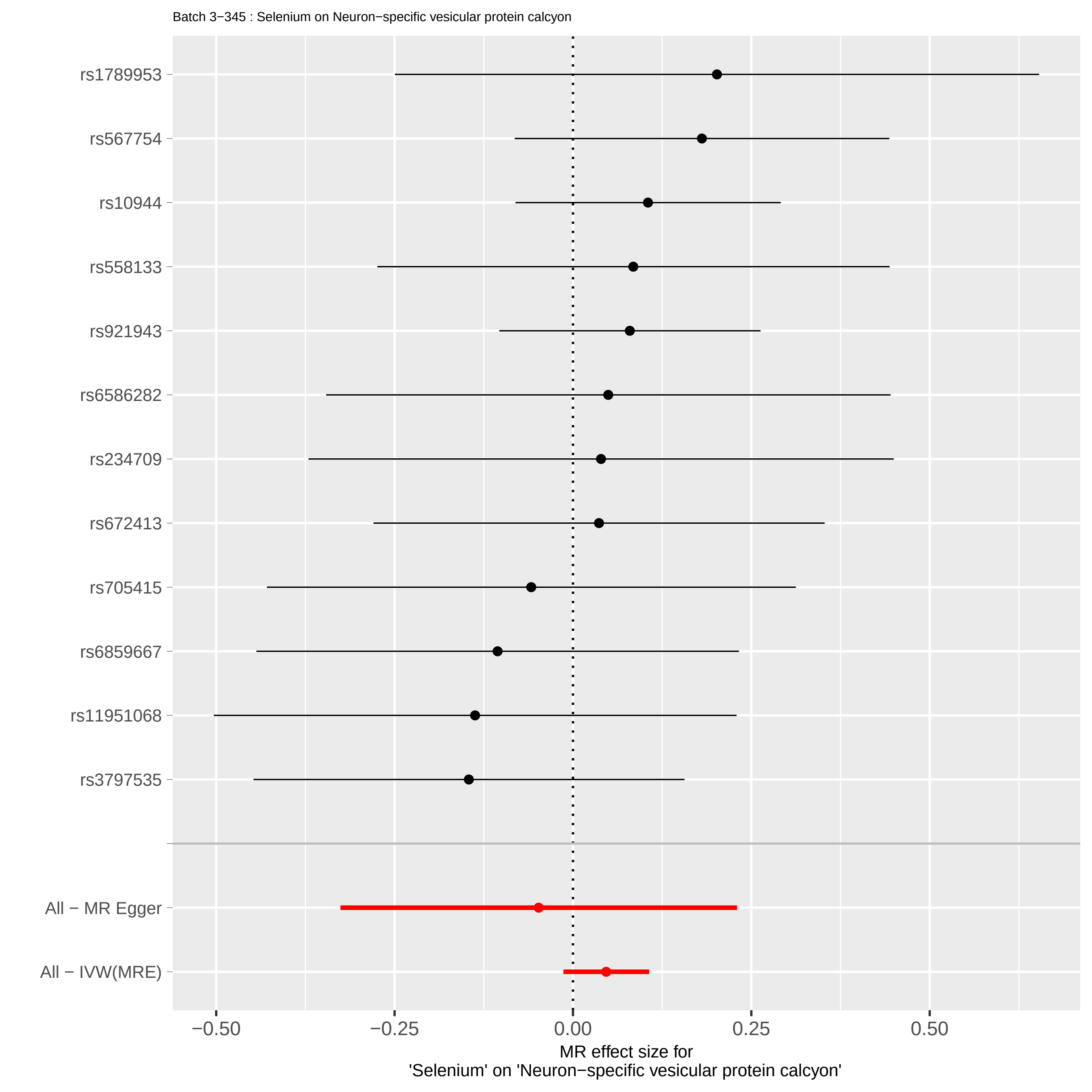

Batch 3−345 : Selenium on Neuron−specific vesicular protein calcyon
rs1789953
rs567754
rs10944
rs558133
rs921943
rs6586282
rs234709
rs672413
rs705415
rs6859667
rs11951068
rs3797535
All − MR Egger
All − IVW(MRE)
−0.50
−0.25
0.00
0.25
0.50
MR effect size for
'Selenium' on 'Neuron−specific vesicular protein calcyon'

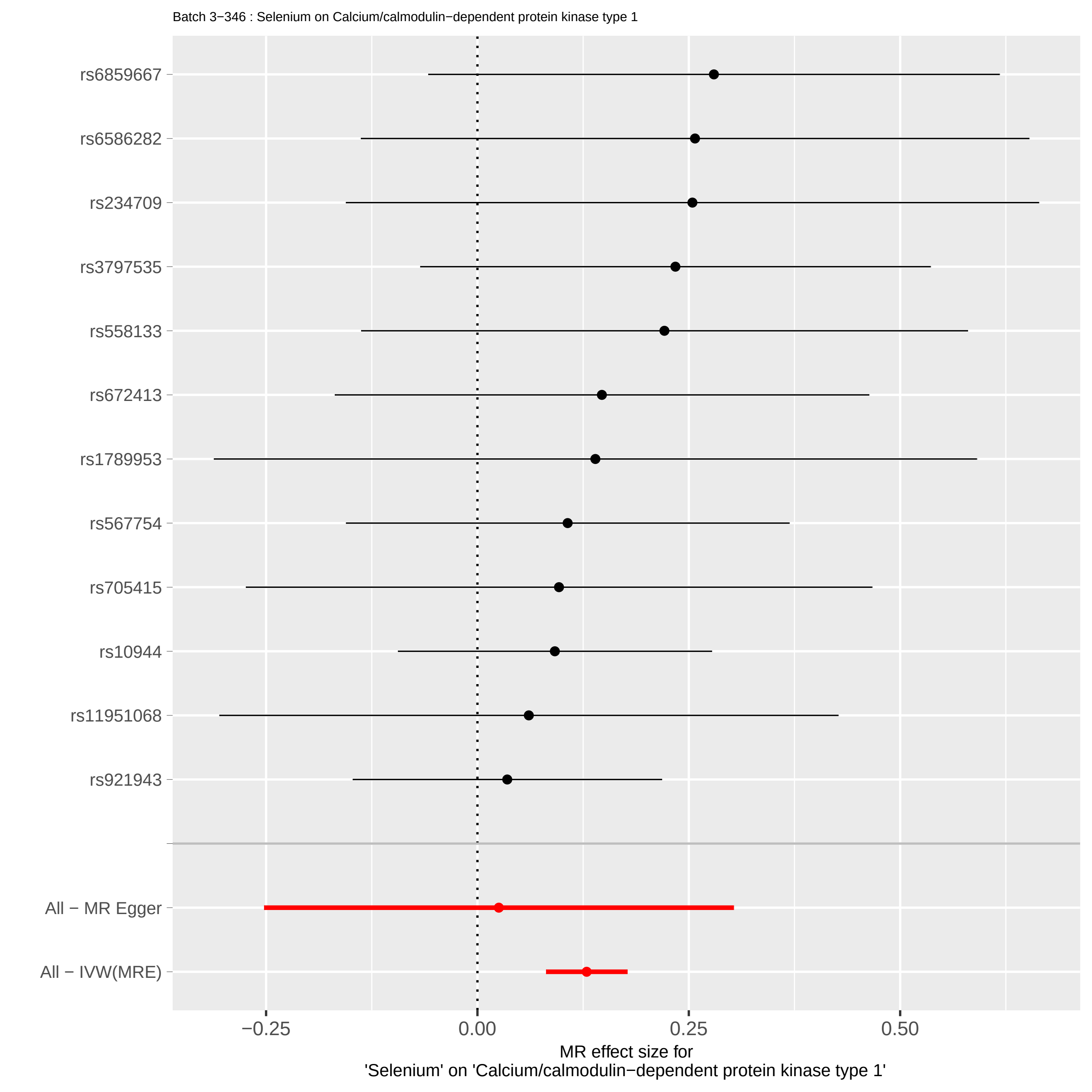

Batch 3−346 : Selenium on Calcium/calmodulin−dependent protein kinase type 1
rs6859667
rs6586282
rs234709
rs3797535
rs558133
rs672413
rs1789953
rs567754
rs705415
rs10944
rs11951068
rs921943
All − MR Egger
All − IVW(MRE)
−0.25
0.00
0.25
0.50
MR effect size for
'Selenium' on 'Calcium/calmodulin−dependent protein kinase type 1'

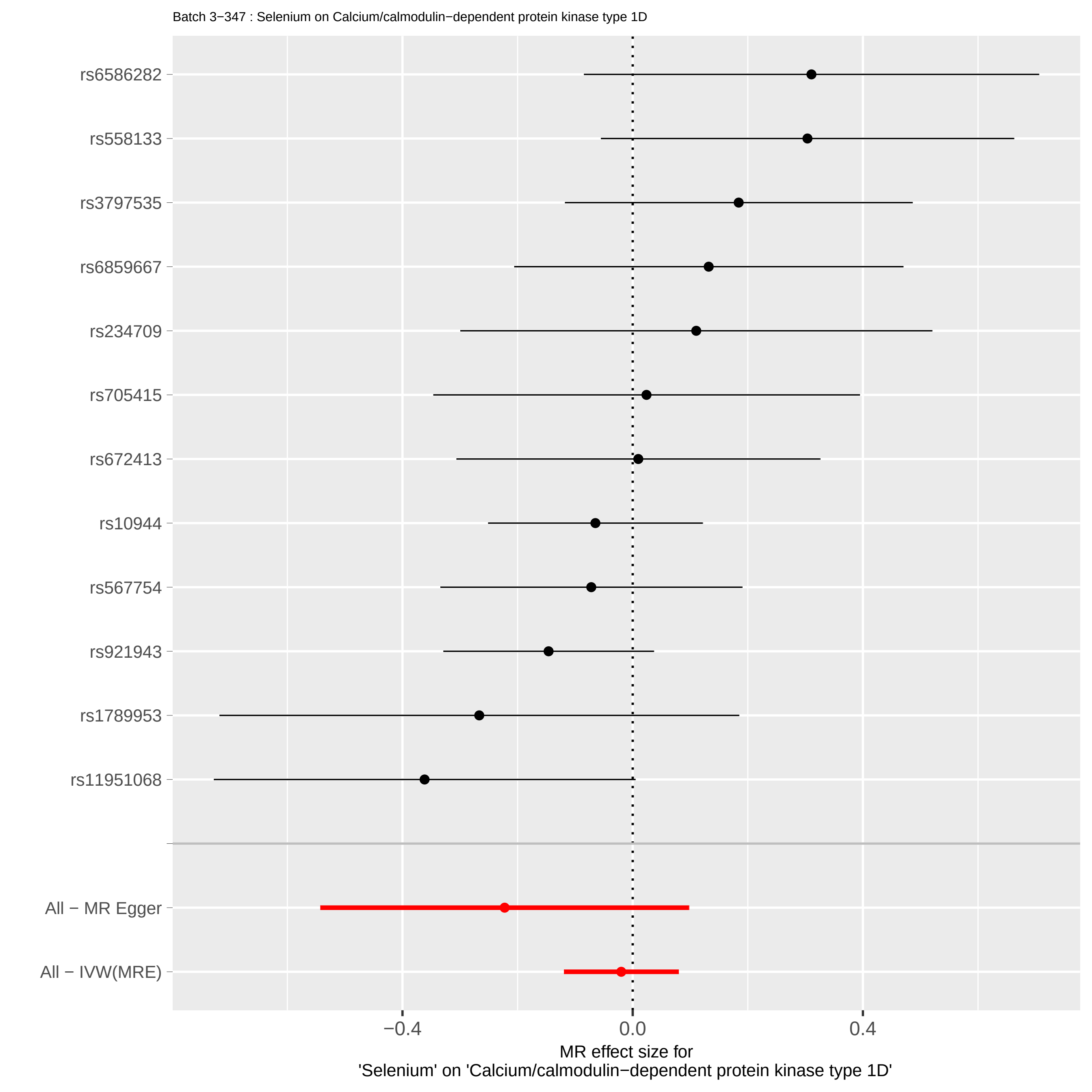

Batch 3−347 : Selenium on Calcium/calmodulin−dependent protein kinase type 1D
rs6586282
rs558133
rs3797535
rs6859667
rs234709
rs705415
rs672413
rs10944
rs567754
rs921943
rs1789953
rs11951068
All − MR Egger
All − IVW(MRE)
−0.4
0.0
0.4
MR effect size for
'Selenium' on 'Calcium/calmodulin−dependent protein kinase type 1D'

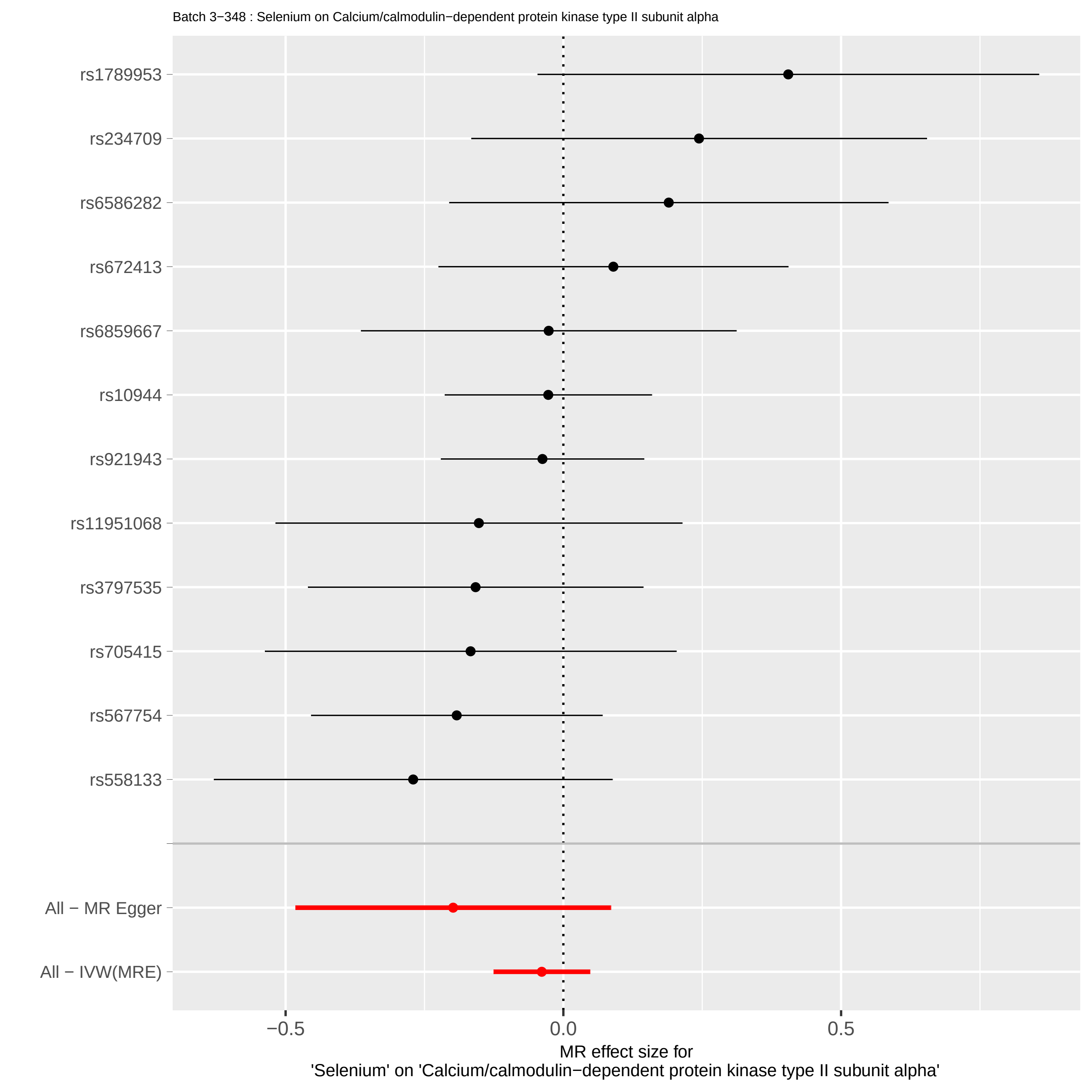
Batch 3−348 : Selenium on Calcium/calmodulin−dependent protein kinase type II subunit alpha
rs1789953
rs234709
rs6586282
rs672413
rs6859667
rs10944
rs921943
rs11951068
rs3797535
rs705415
rs567754
rs558133
All − MR Egger
All − IVW(MRE)
−0.5
0.0
0.5
MR effect size for
'Selenium' on 'Calcium/calmodulin−dependent protein kinase type II subunit alpha'

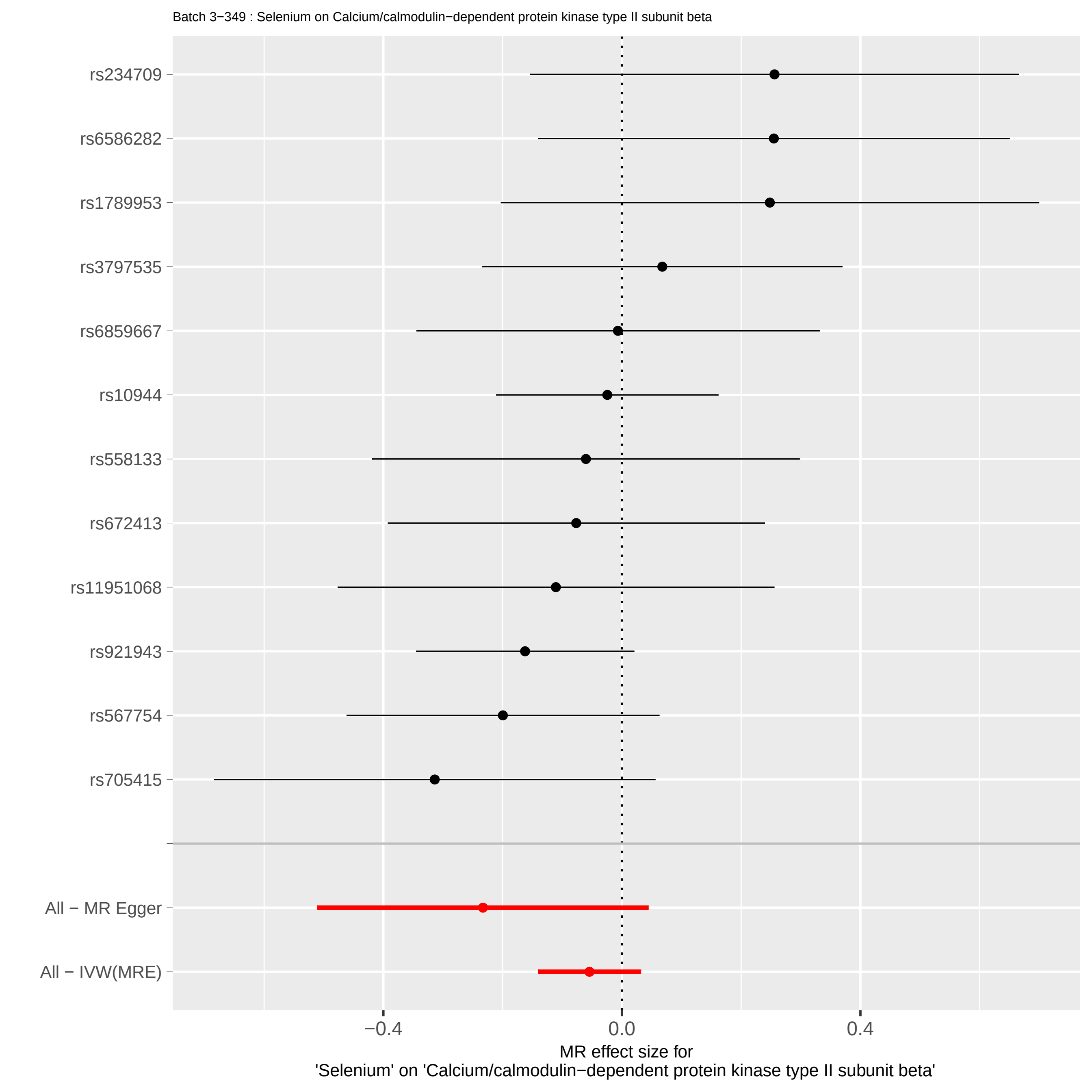

Batch 3−349 : Selenium on Calcium/calmodulin−dependent protein kinase type II subunit beta
rs234709
rs6586282
rs1789953
rs3797535
rs6859667
rs10944
rs558133
rs672413
rs11951068
rs921943
rs567754
rs705415
All − MR Egger
All − IVW(MRE)
−0.4
0.0
0.4
MR effect size for
'Selenium' on 'Calcium/calmodulin−dependent protein kinase type II subunit beta'

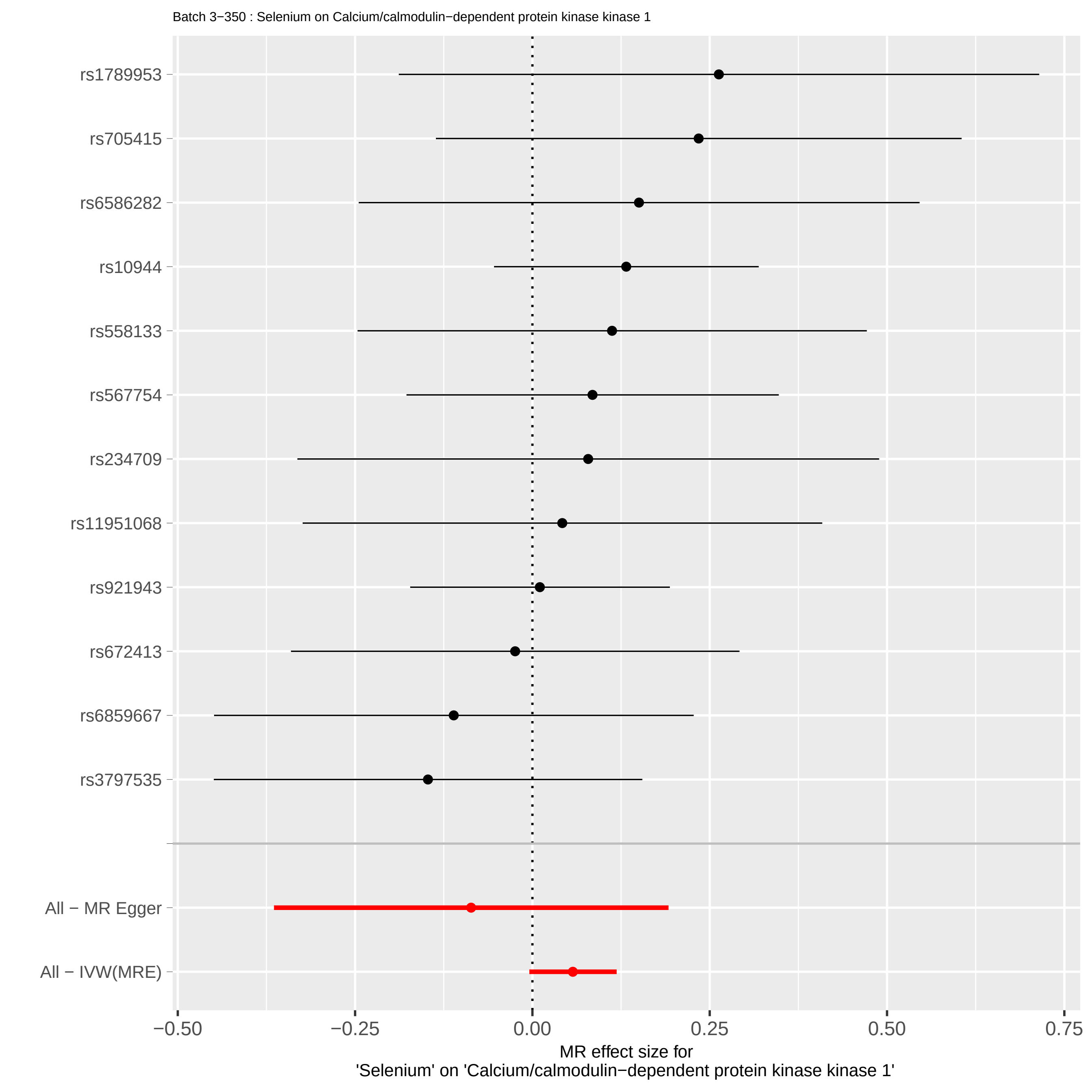
Batch 3−350 : Selenium on Calcium/calmodulin−dependent protein kinase kinase 1
rs1789953
rs705415
rs6586282
rs10944
rs558133
rs567754
rs234709
rs11951068
rs921943
rs672413
rs6859667
rs3797535
All − MR Egger
All − IVW(MRE)
−0.50
−0.25
0.00
0.25
0.50
0.75
MR effect size for
'Selenium' on 'Calcium/calmodulin−dependent protein kinase kinase 1'

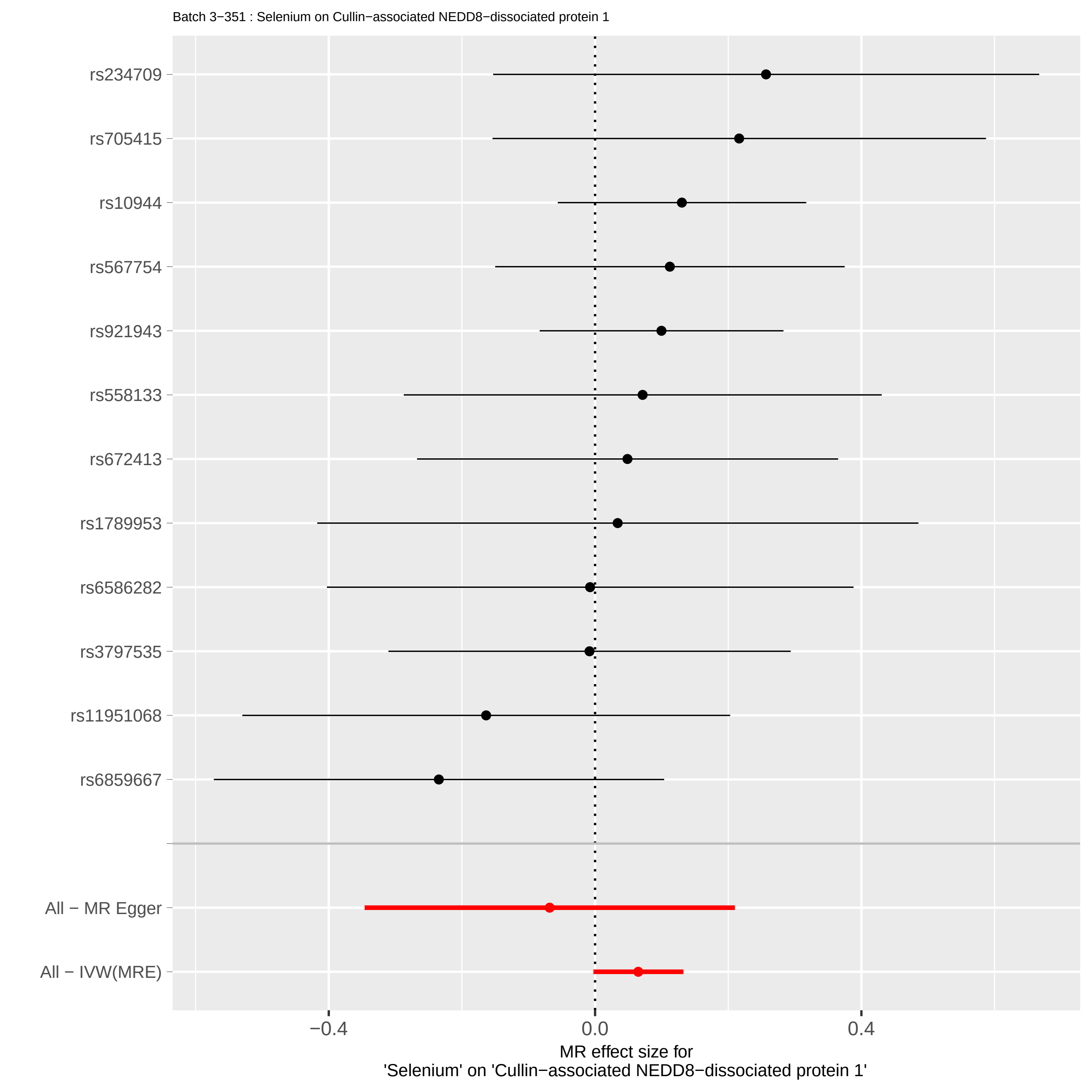

Batch 3−351 : Selenium on Cullin−associated NEDD8−dissociated protein 1
rs234709
rs705415
rs10944
rs567754
rs921943
rs558133
rs672413
rs1789953
rs6586282
rs3797535
rs11951068
rs6859667
All − MR Egger
All − IVW(MRE)
−0.4
0.0
0.4
MR effect size for
'Selenium' on 'Cullin−associated NEDD8−dissociated protein 1'

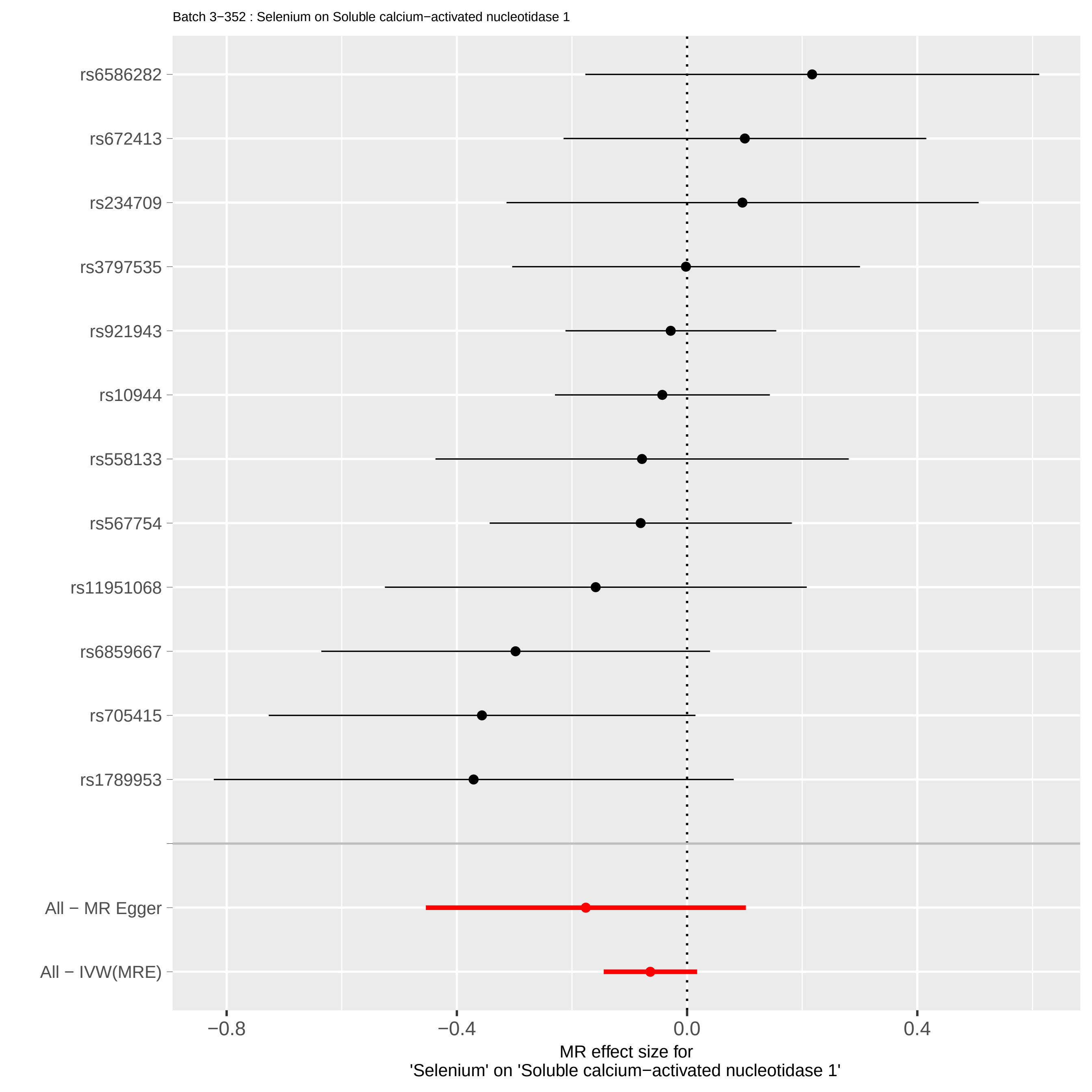
Batch 3−352 : Selenium on Soluble calcium−activated nucleotidase 1
rs6586282
rs672413
rs234709
rs3797535
rs921943
rs10944
rs558133
rs567754
rs11951068
rs6859667
rs705415
rs1789953
All − MR Egger
All − IVW(MRE)
−0.8
−0.4
0.0
0.4
MR effect size for
'Selenium' on 'Soluble calcium−activated nucleotidase 1'

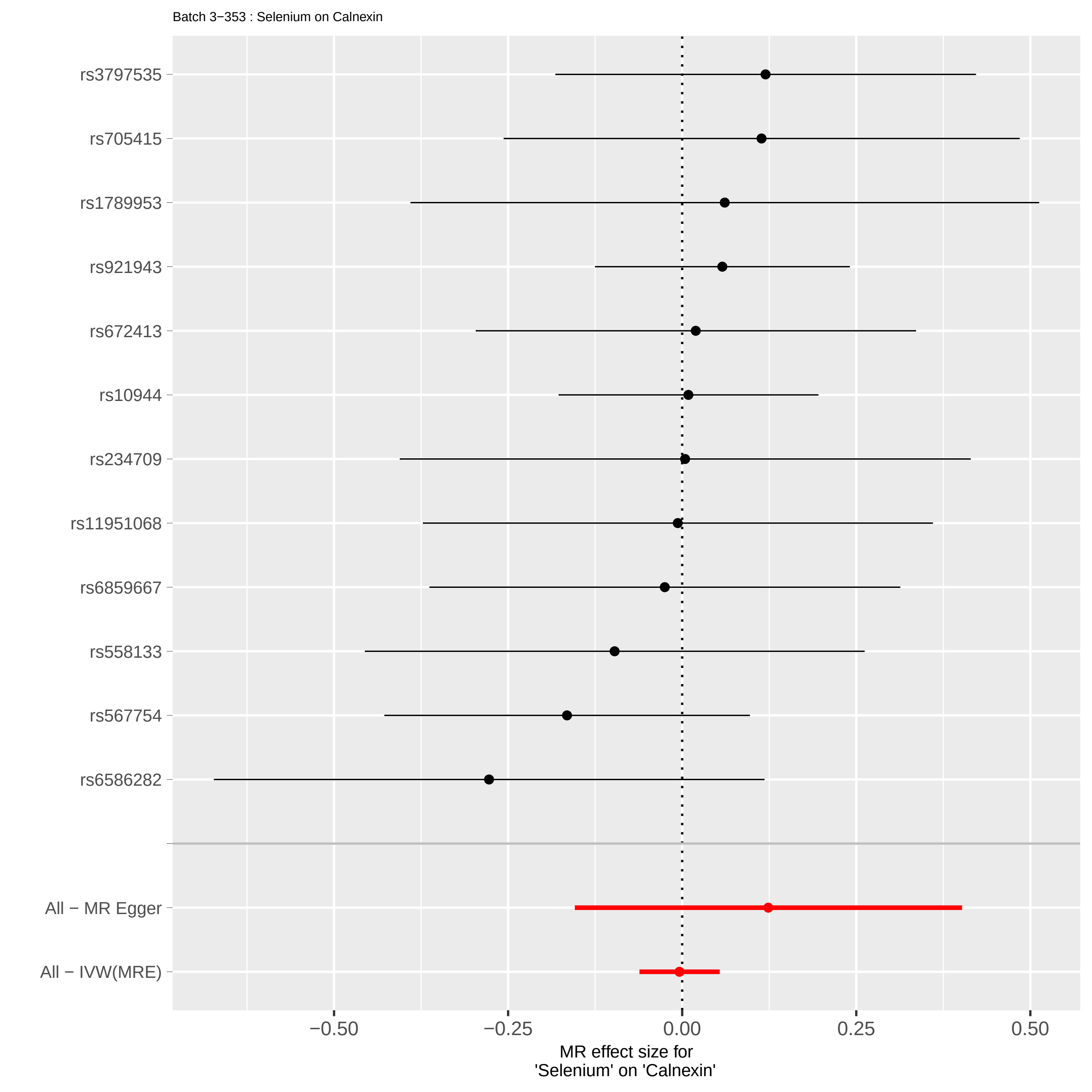

Batch 3−353 : Selenium on Calnexin
rs3797535
rs705415
rs1789953
rs921943
rs672413
rs10944
rs234709
rs11951068
rs6859667
rs558133
rs567754
rs6586282
All − MR Egger
All − IVW(MRE)
−0.50
−0.25
0.00
0.25
0.50
MR effect size for
'Selenium' on 'Calnexin'

Batch 3−354 : Selenium on Calpain−2 catalytic subunit

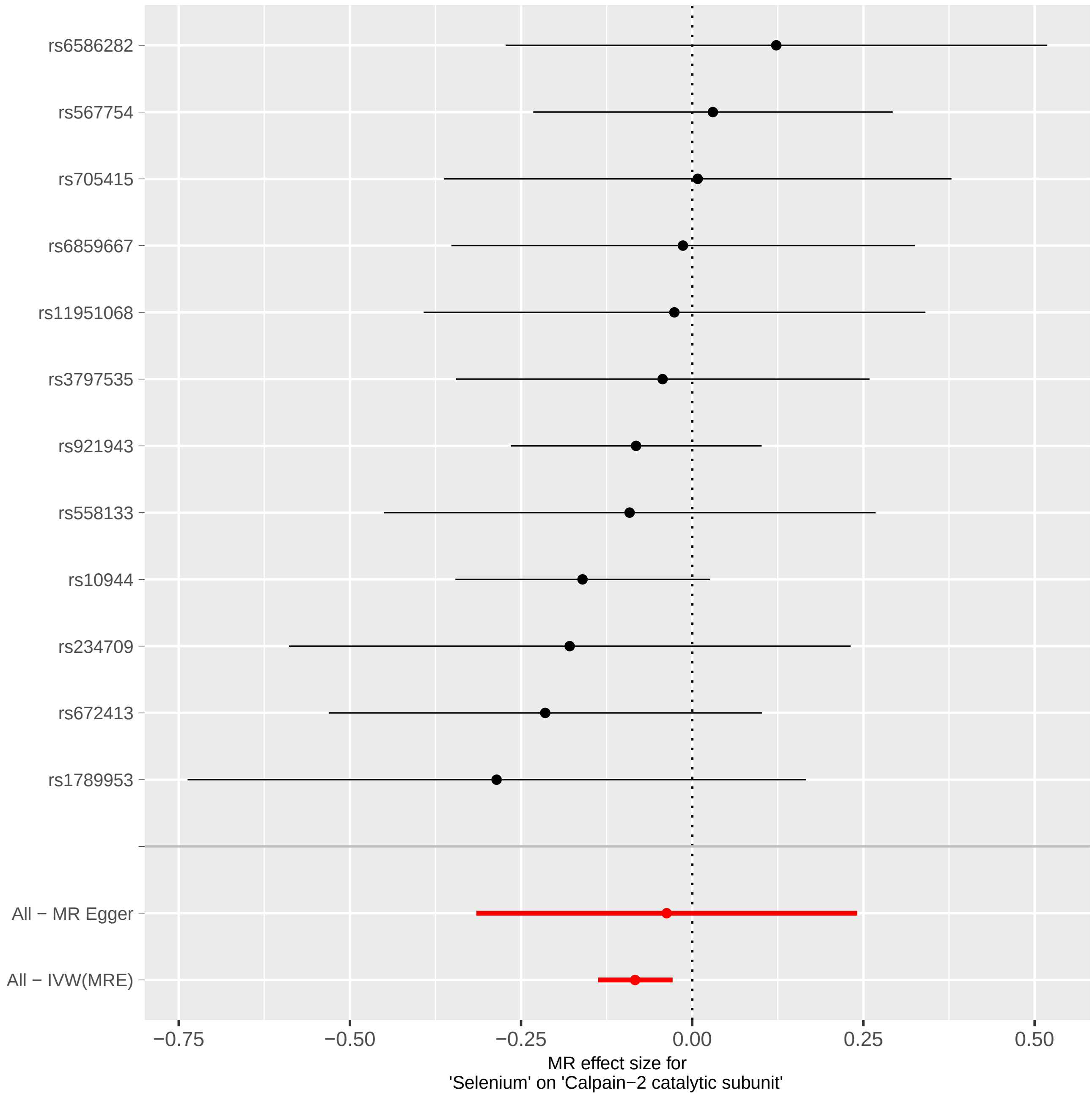

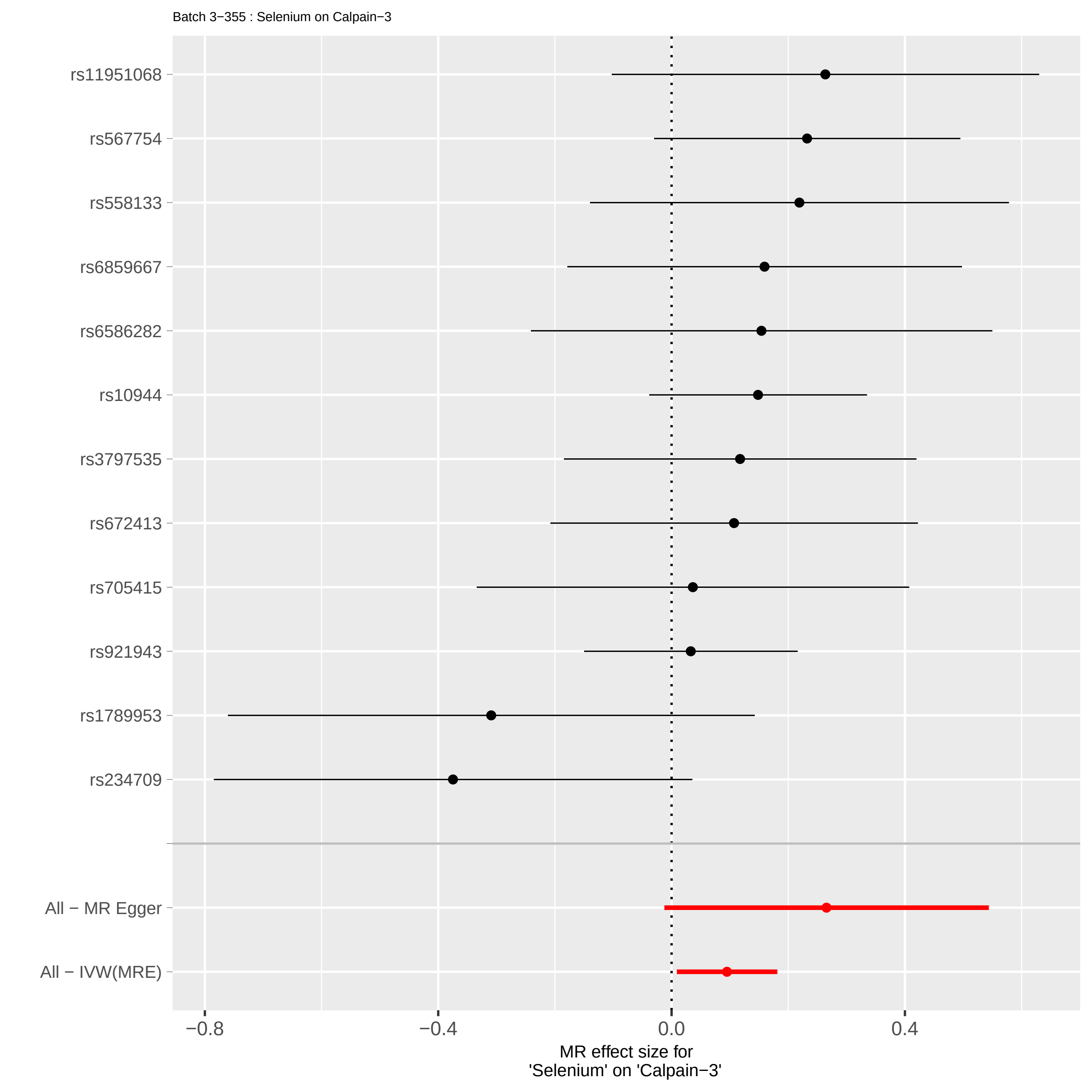

Batch 3−355 : Selenium on Calpain−3
rs11951068
rs567754
rs558133
rs6859667
rs6586282
rs10944
rs3797535
rs672413
rs705415
rs921943
rs1789953
rs234709
All − MR Egger
All − IVW(MRE)
−0.8
−0.4
0.0
0.4
MR effect size for
'Selenium' on 'Calpain−3'

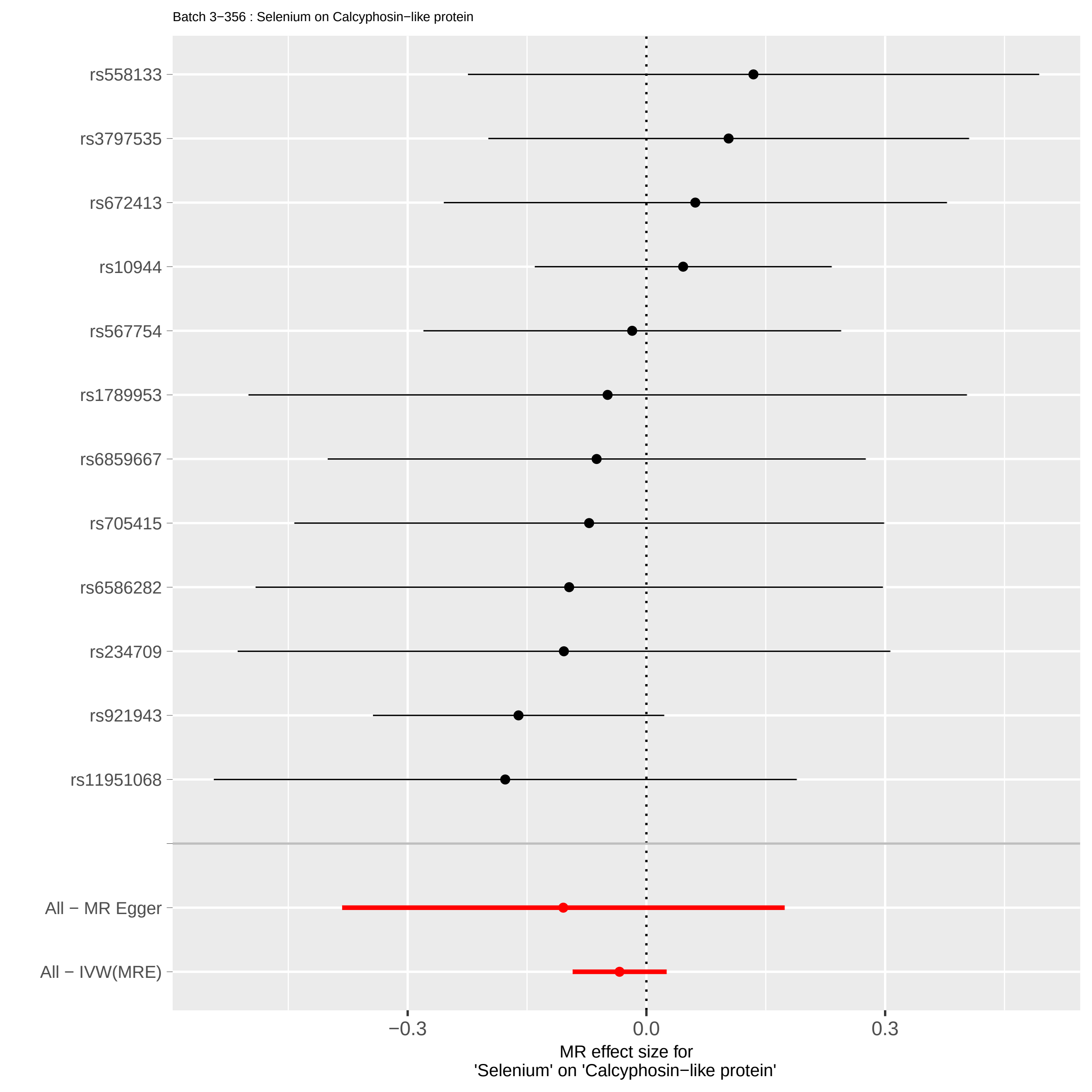

Batch 3−356 : Selenium on Calcyphosin−like protein
rs558133
rs3797535
rs672413
rs10944
rs567754
rs1789953
rs6859667
rs705415
rs6586282
rs234709
rs921943
rs11951068
All − MR Egger
All − IVW(MRE)
−0.3
0.0
0.3
MR effect size for
'Selenium' on 'Calcyphosin−like protein'

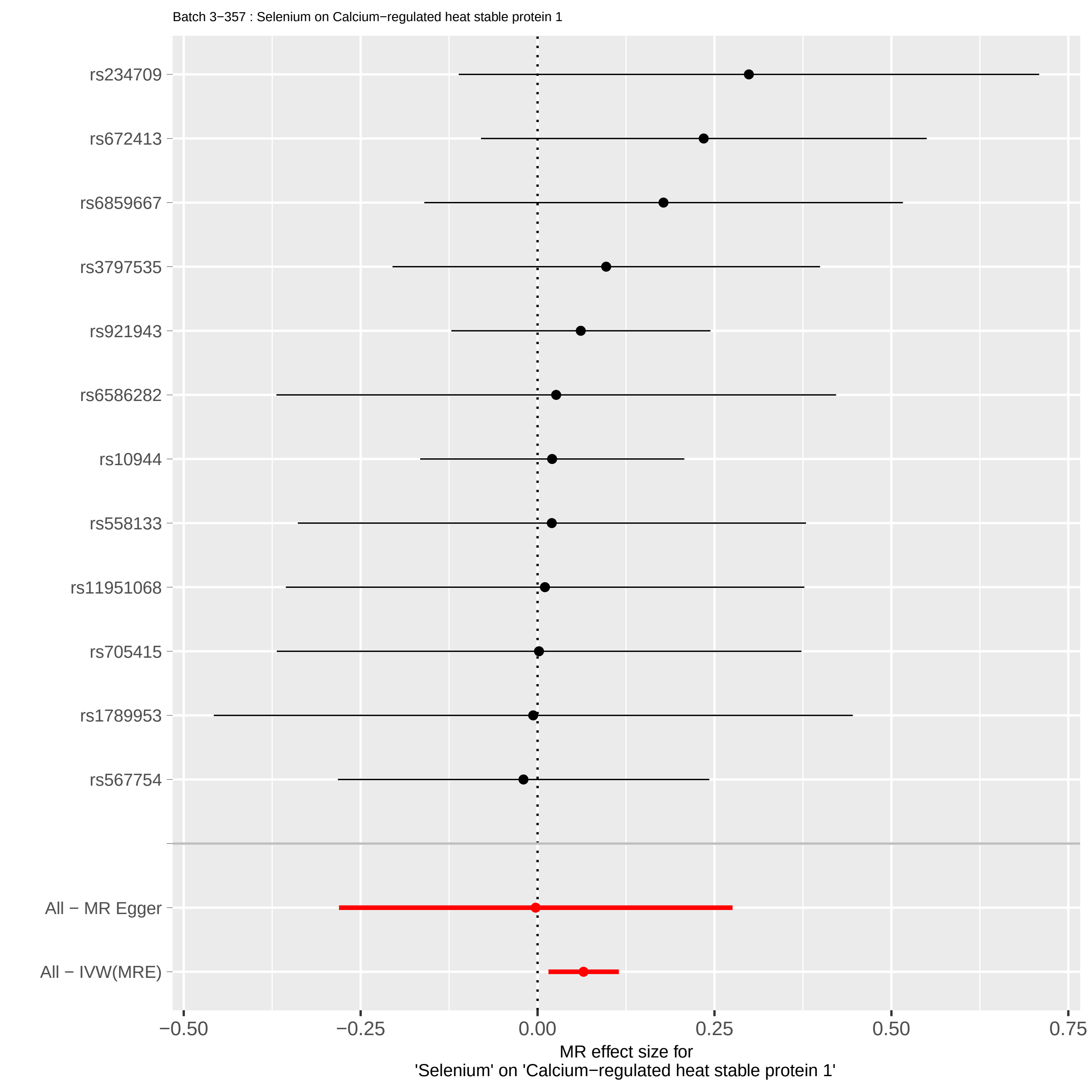
Batch 3−357 : Selenium on Calcium−regulated heat stable protein 1
rs234709
rs672413
rs6859667
rs3797535
rs921943
rs6586282
rs10944
rs558133
rs11951068
rs705415
rs1789953
rs567754
All − MR Egger
All − IVW(MRE)
−0.50
−0.25
0.00
0.25
0.50
0.75
MR effect size for
'Selenium' on 'Calcium−regulated heat stable protein 1'

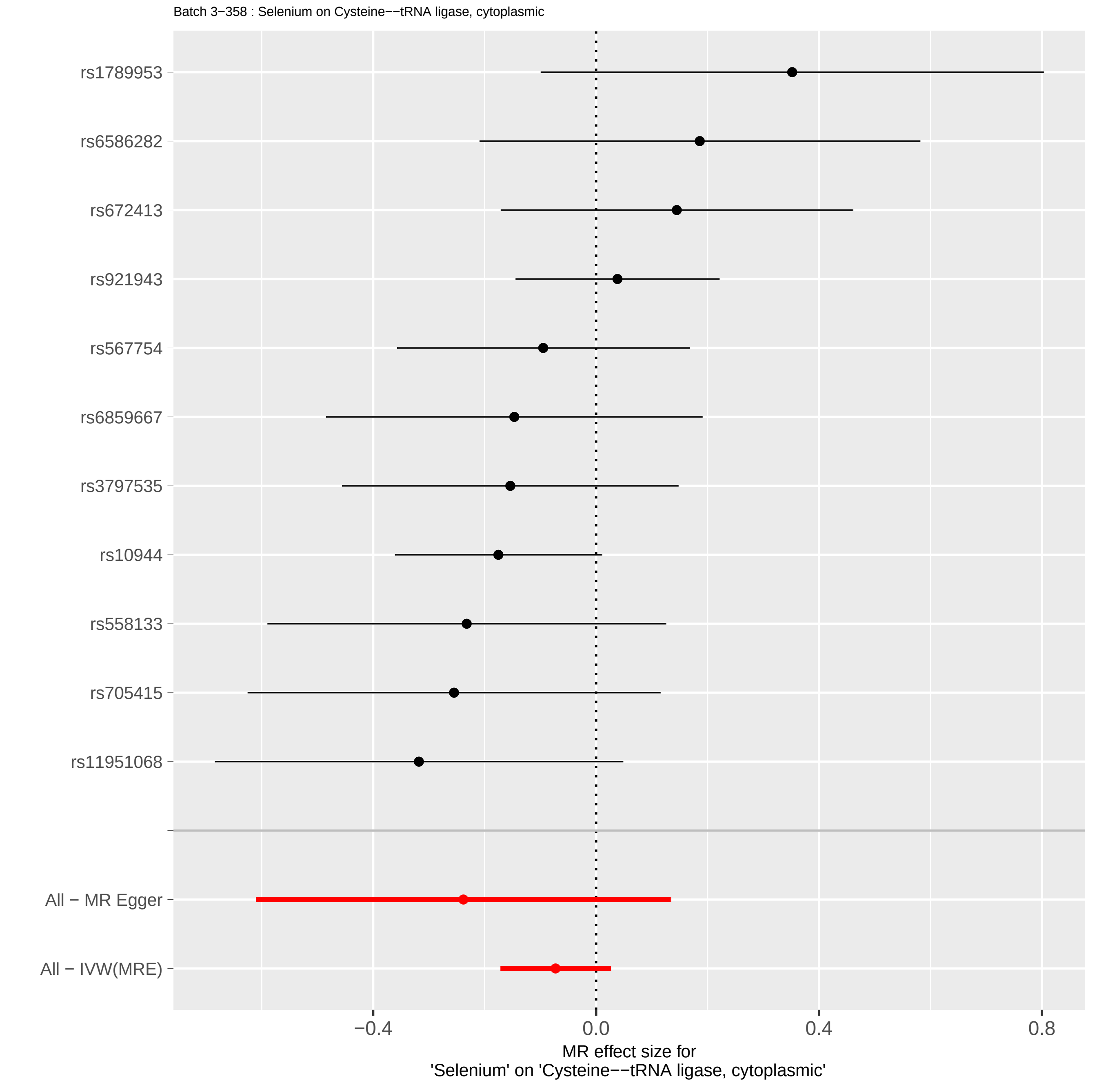

Batch 3−358 : Selenium on Cysteine−−tRNA ligase, cytoplasmic
rs1789953
rs6586282
rs672413
rs921943
rs567754
rs6859667
rs3797535
rs10944
rs558133
rs705415
rs11951068
All − MR Egger
All − IVW(MRE)
−0.4
0.0
0.4
0.8
MR effect size for
'Selenium' on 'Cysteine−−tRNA ligase, cytoplasmic'

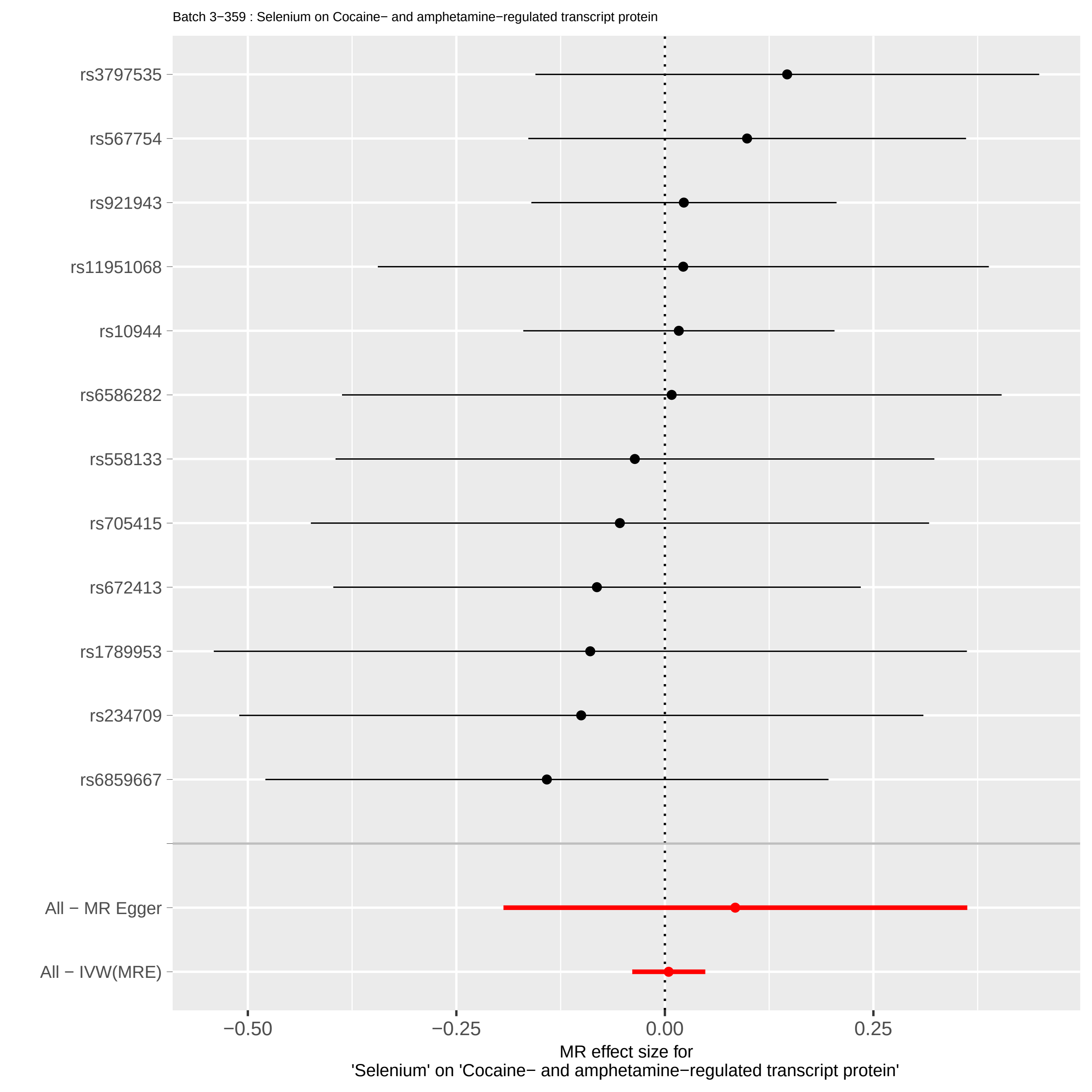
Batch 3−359 : Selenium on Cocaine− and amphetamine−regulated transcript protein
rs3797535
rs567754
rs921943
rs11951068
rs10944
rs6586282
rs558133
rs705415
rs672413
rs1789953
rs234709
rs6859667
All − MR Egger
All − IVW(MRE)
−0.50
−0.25
0.00
0.25
MR effect size for
'Selenium' on 'Cocaine− and amphetamine−regulated transcript protein'

Batch 3−360 : Selenium on Protein CASC4

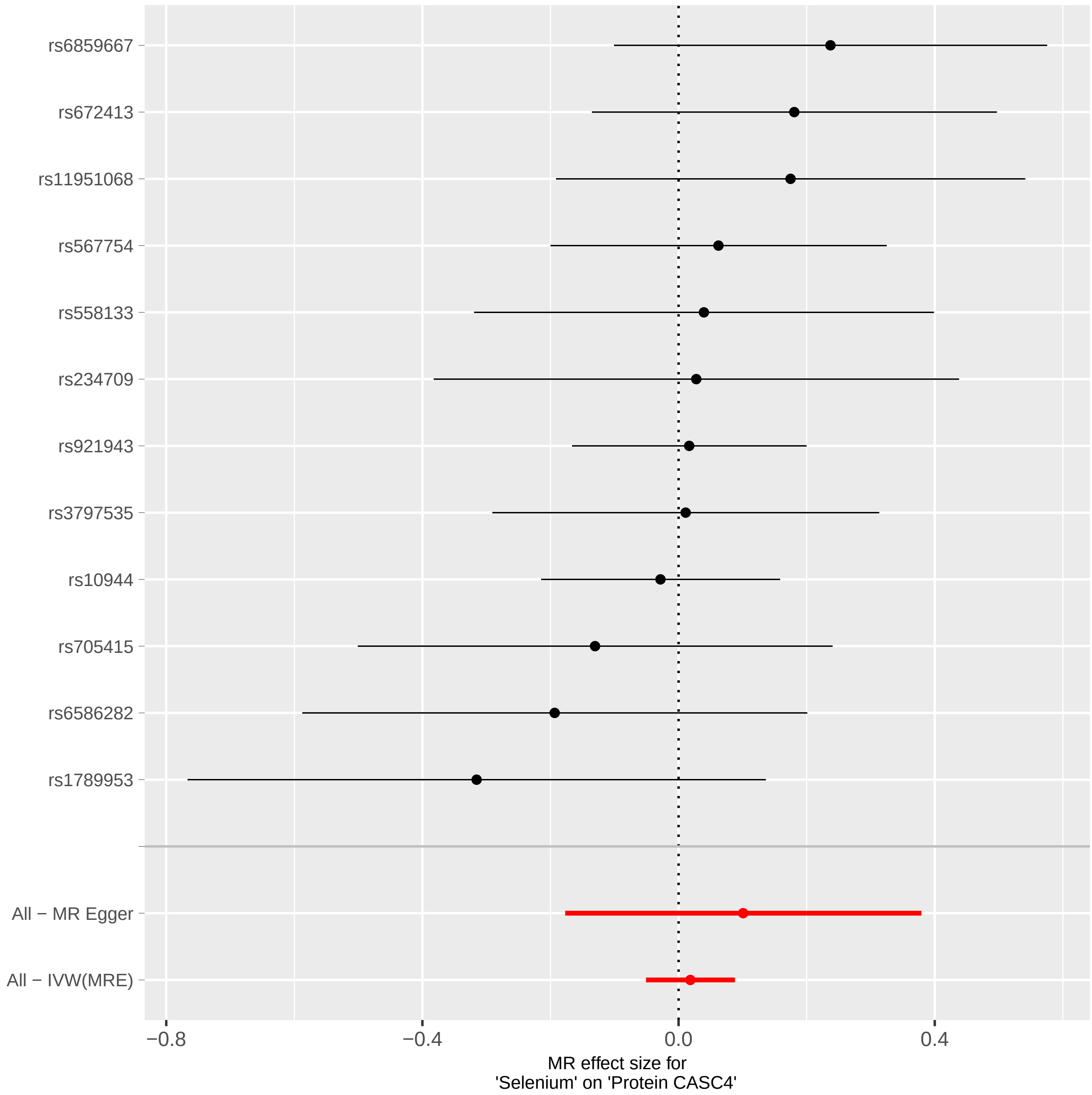

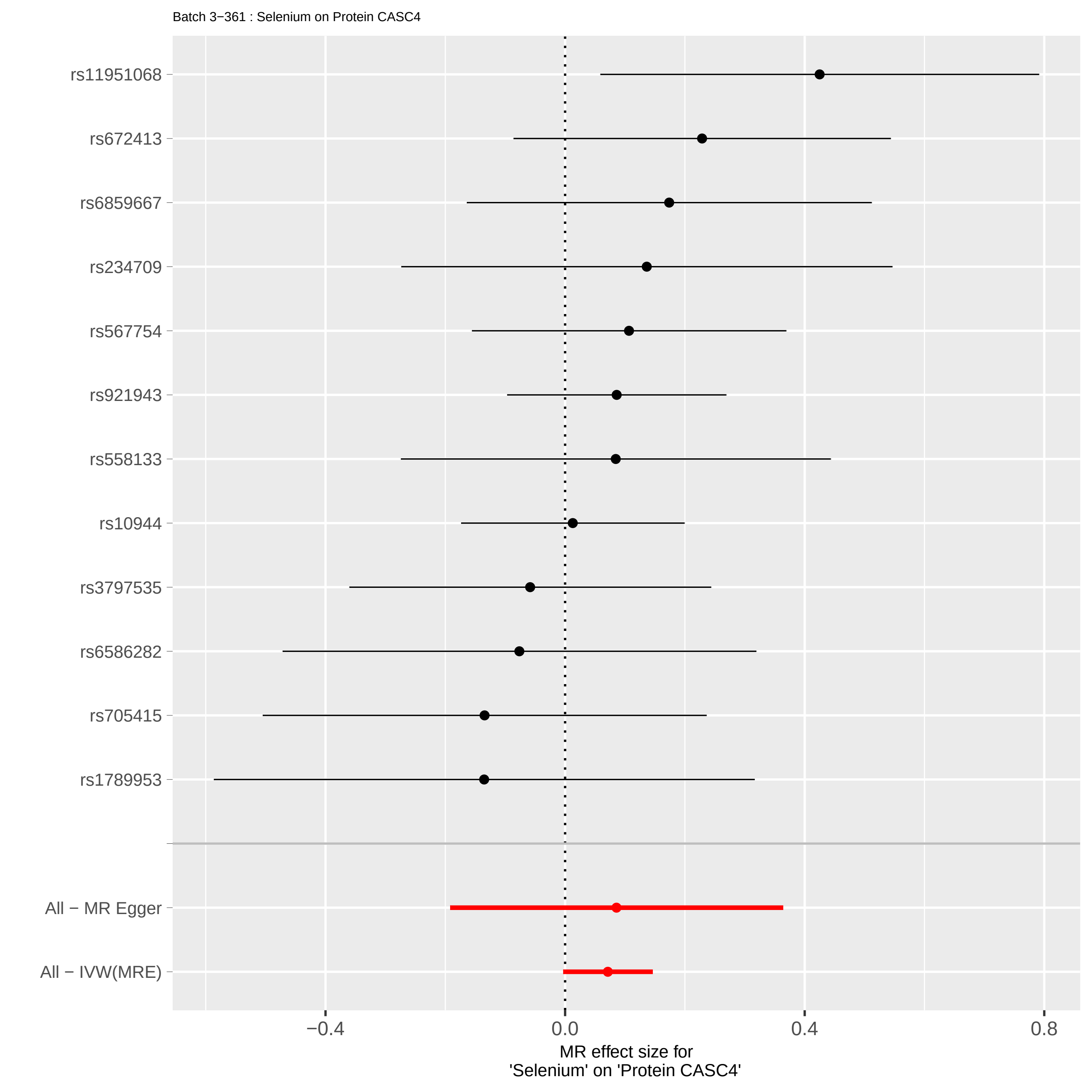

Batch 3−361 : Selenium on Protein CASC4
rs11951068
rs672413
rs6859667
rs234709
rs567754
rs921943
rs558133
rs10944
rs3797535
rs6586282
rs705415
rs1789953
All − MR Egger
All − IVW(MRE)
−0.4
0.0
0.4
0.8
MR effect size for
'Selenium' on 'Protein CASC4'

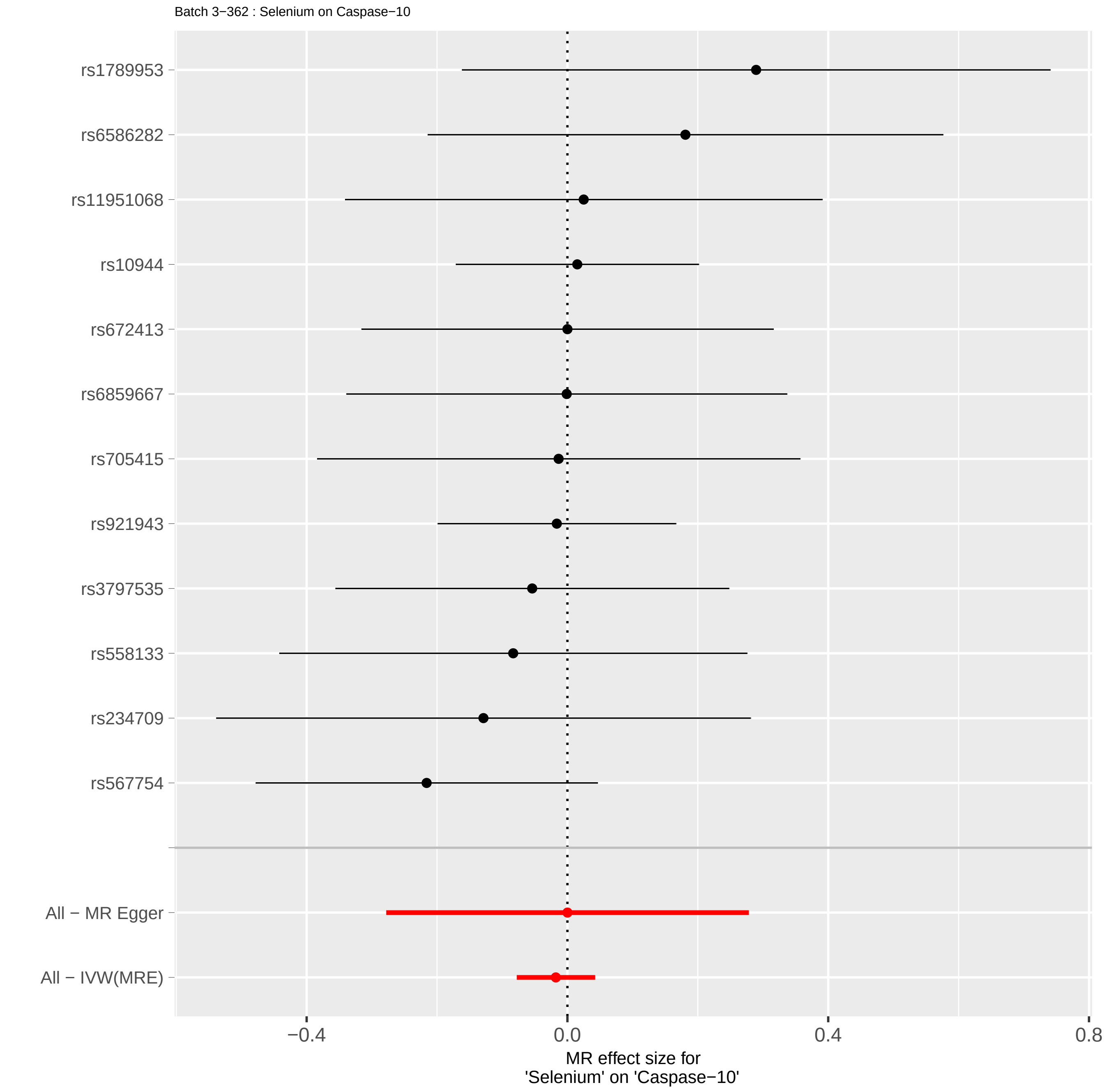
Batch 3−362 : Selenium on Caspase−10
rs1789953
rs6586282
rs11951068
rs10944
rs672413
rs6859667
rs705415
rs921943
rs3797535
rs558133
rs234709
rs567754
All − MR Egger
All − IVW(MRE)
−0.4
0.0
0.4
0.8
MR effect size for
'Selenium' on 'Caspase−10'

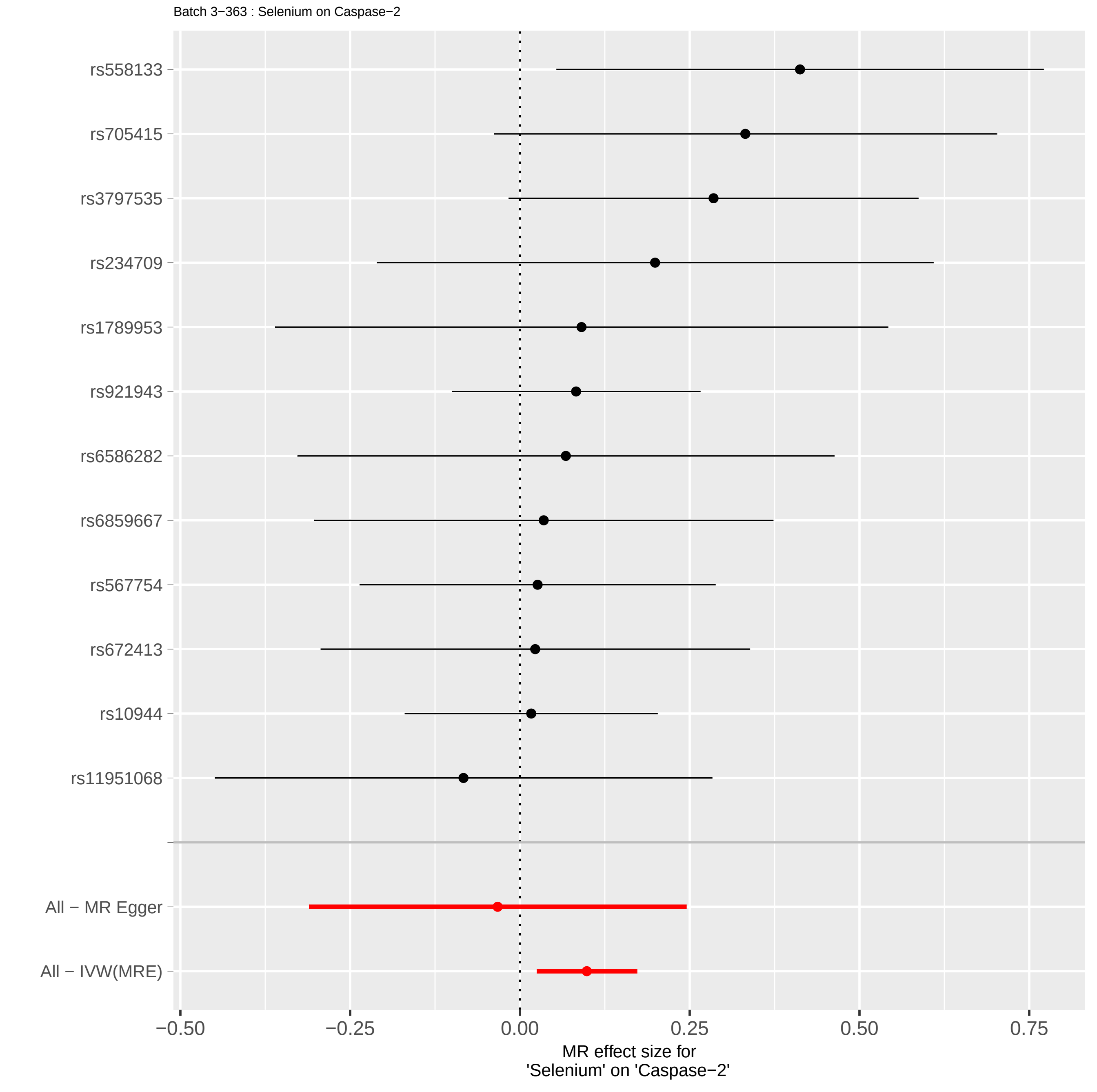
Batch 3−363 : Selenium on Caspase−2
rs558133
rs705415
rs3797535
rs234709
rs1789953
rs921943
rs6586282
rs6859667
rs567754
rs672413
rs10944
rs11951068
All − MR Egger
All − IVW(MRE)
−0.50
−0.25
0.00
0.25
0.50
0.75
MR effect size for
'Selenium' on 'Caspase−2'

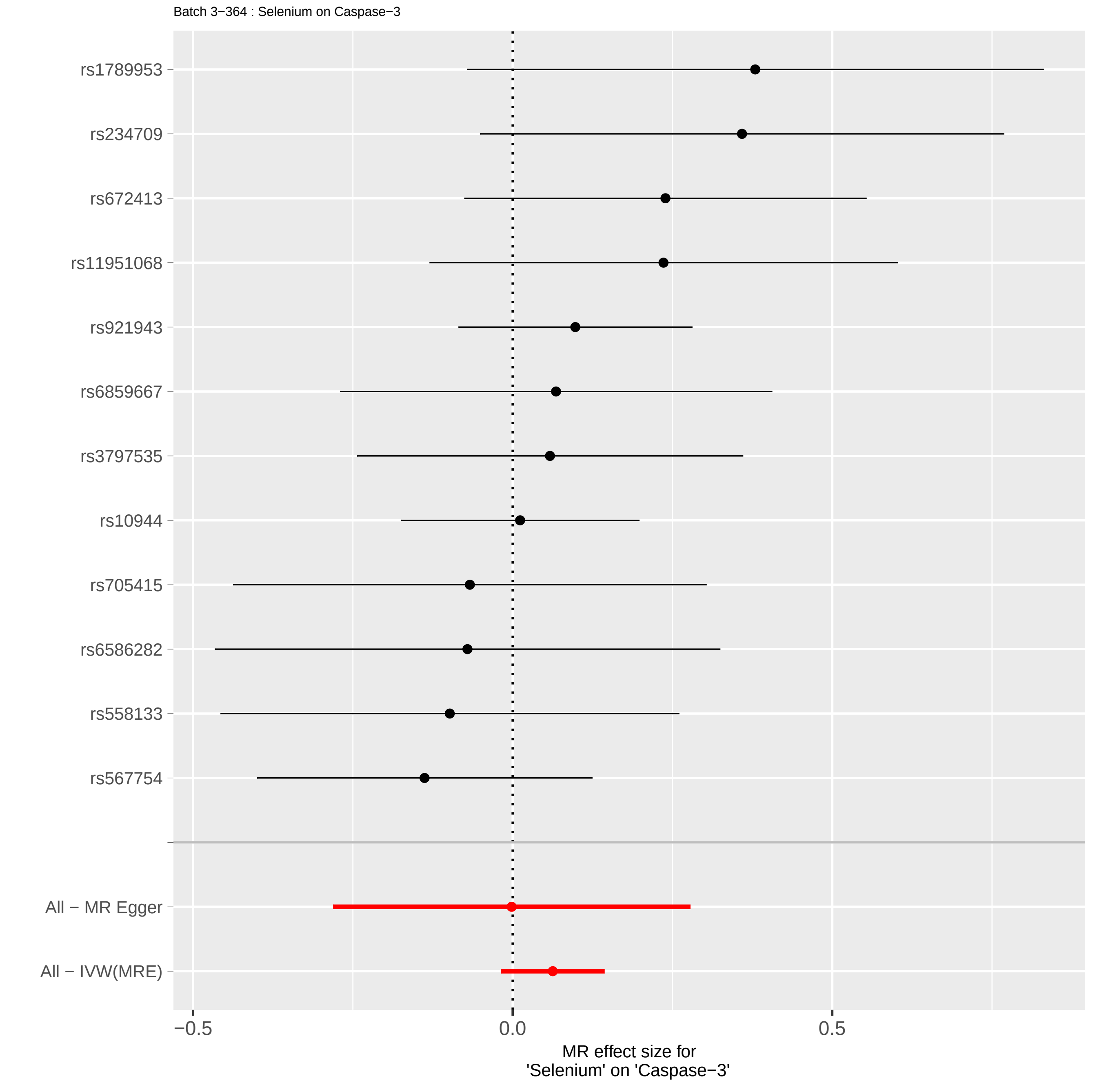

Batch 3−364 : Selenium on Caspase−3
rs1789953
rs234709
rs672413
rs11951068
rs921943
rs6859667
rs3797535
rs10944
rs705415
rs6586282
rs558133
rs567754
All − MR Egger
All − IVW(MRE)
−0.5
0.0
0.5
MR effect size for
'Selenium' on 'Caspase−3'

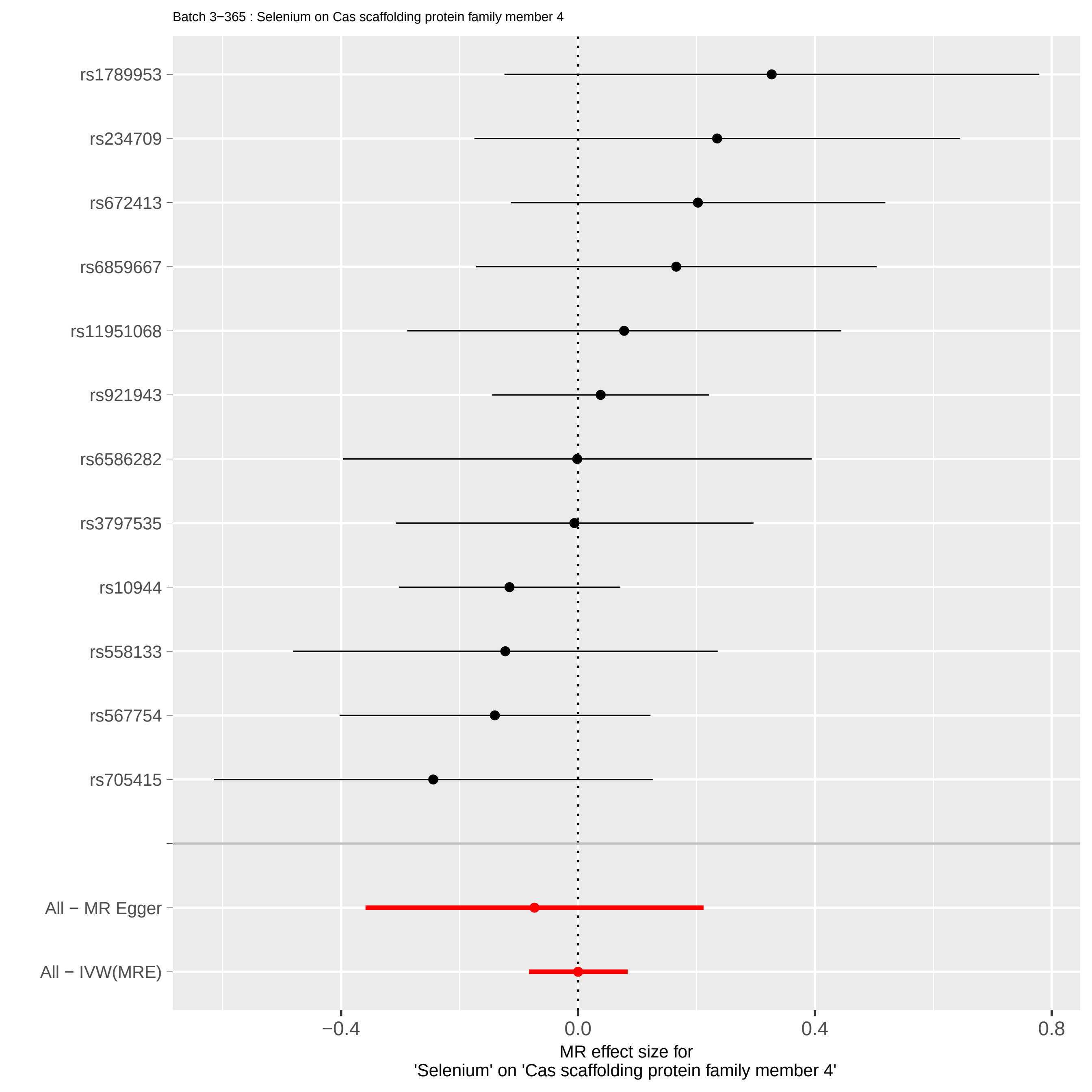
Batch 3−365 : Selenium on Cas scaffolding protein family member 4
rs1789953
rs234709
rs672413
rs6859667
rs11951068
rs921943
rs6586282
rs3797535
rs10944
rs558133
rs567754
rs705415
All − MR Egger
All − IVW(MRE)
−0.4
0.0
0.4
0.8
MR effect size for
'Selenium' on 'Cas scaffolding protein family member 4'

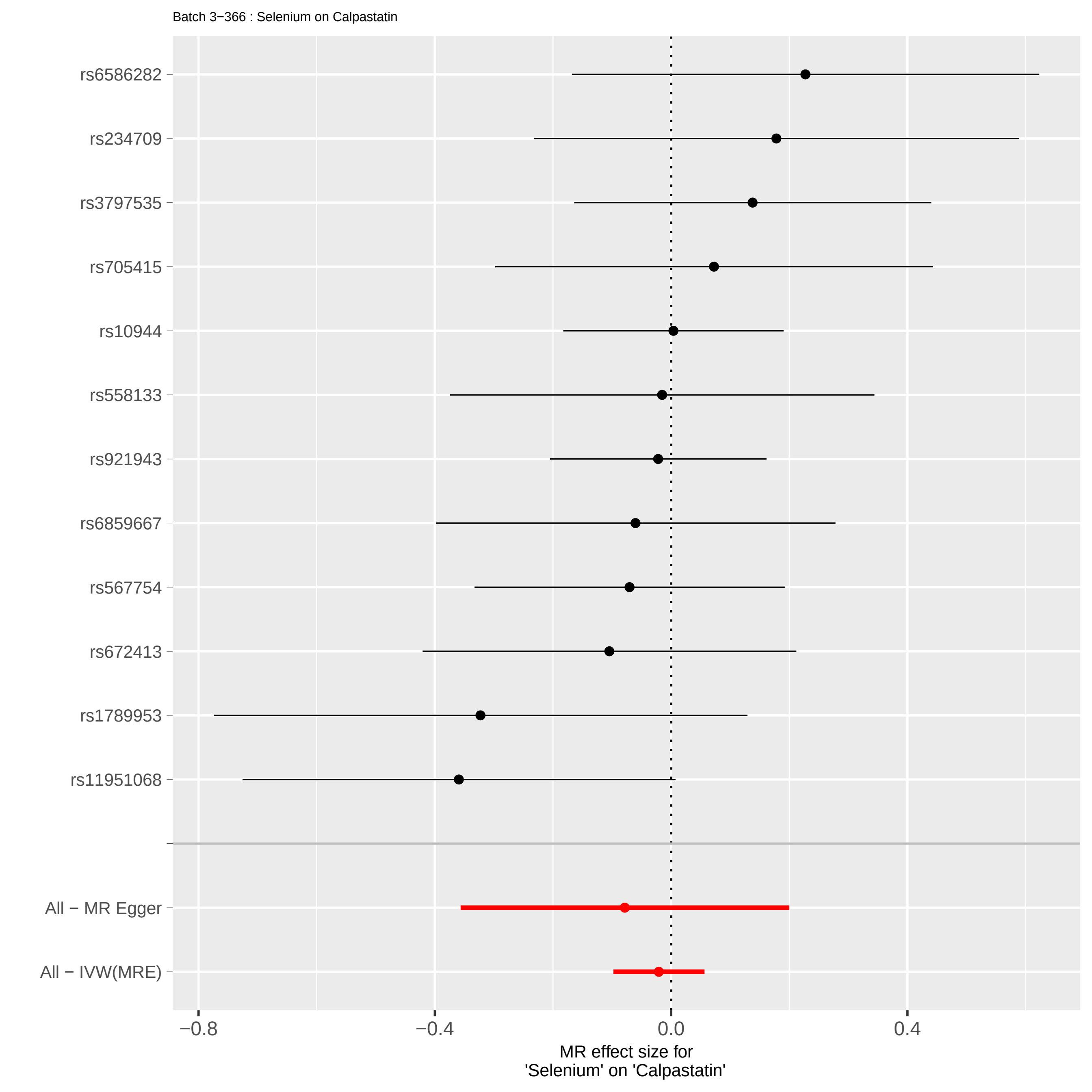

Batch 3−366 : Selenium on Calpastatin
rs6586282
rs234709
rs3797535
rs705415
rs10944
rs558133
rs921943
rs6859667
rs567754
rs672413
rs1789953
rs11951068
All − MR Egger
All − IVW(MRE)
−0.8
−0.4
0.0
0.4
MR effect size for
'Selenium' on 'Calpastatin'

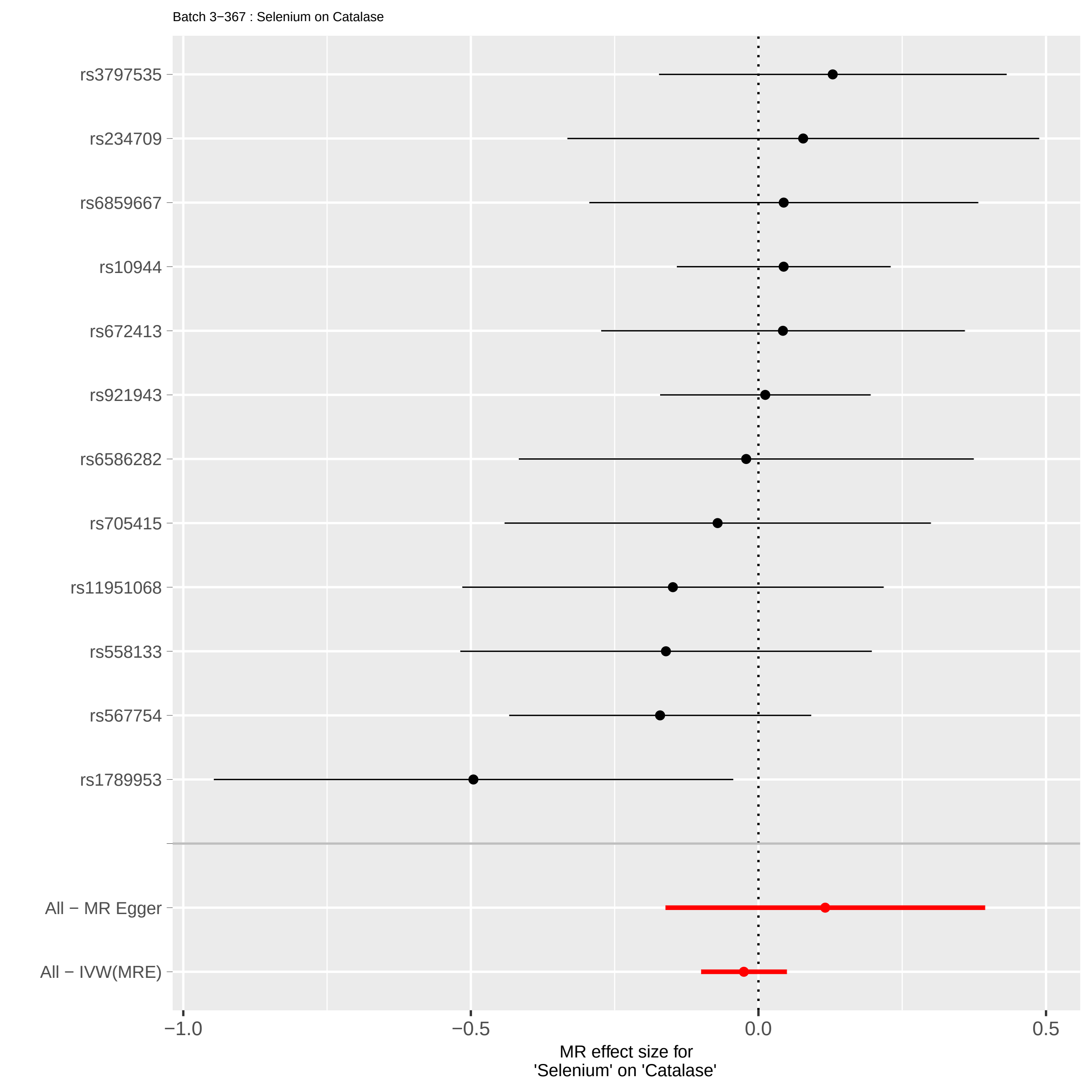
Batch 3−367 : Selenium on Catalase
rs3797535
rs234709
rs6859667
rs10944
rs672413
rs921943
rs6586282
rs705415
rs11951068
rs558133
rs567754
rs1789953
All − MR Egger
All − IVW(MRE)
−1.0
−0.5
0.0
0.5
MR effect size for
'Selenium' on 'Catalase'

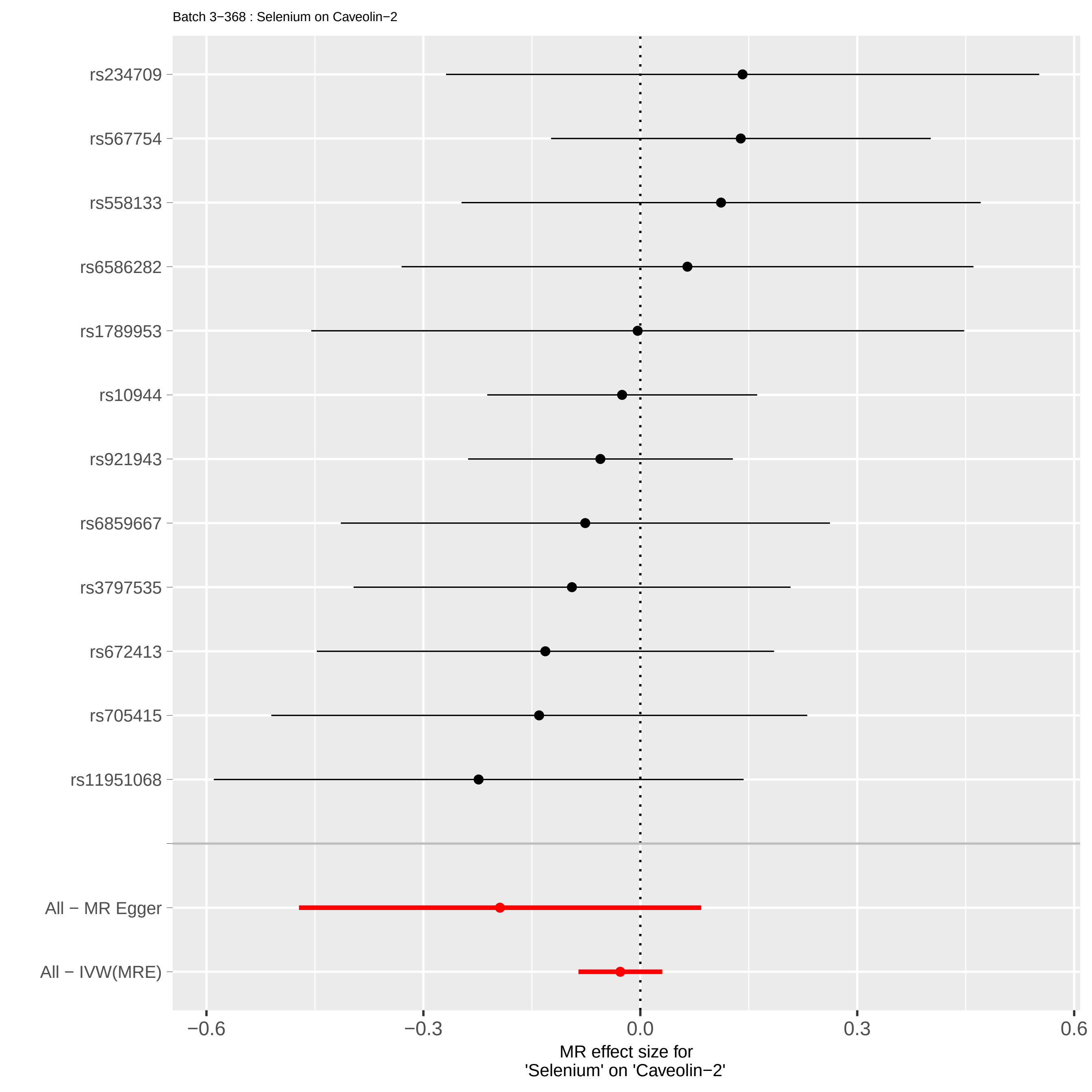

Batch 3−368 : Selenium on Caveolin−2
rs234709
rs567754
rs558133
rs6586282
rs1789953
rs10944
rs921943
rs6859667
rs3797535
rs672413
rs705415
rs11951068
All − MR Egger
All − IVW(MRE)
−0.6
−0.3
0.0
0.3
0.6
MR effect size for
'Selenium' on 'Caveolin−2'

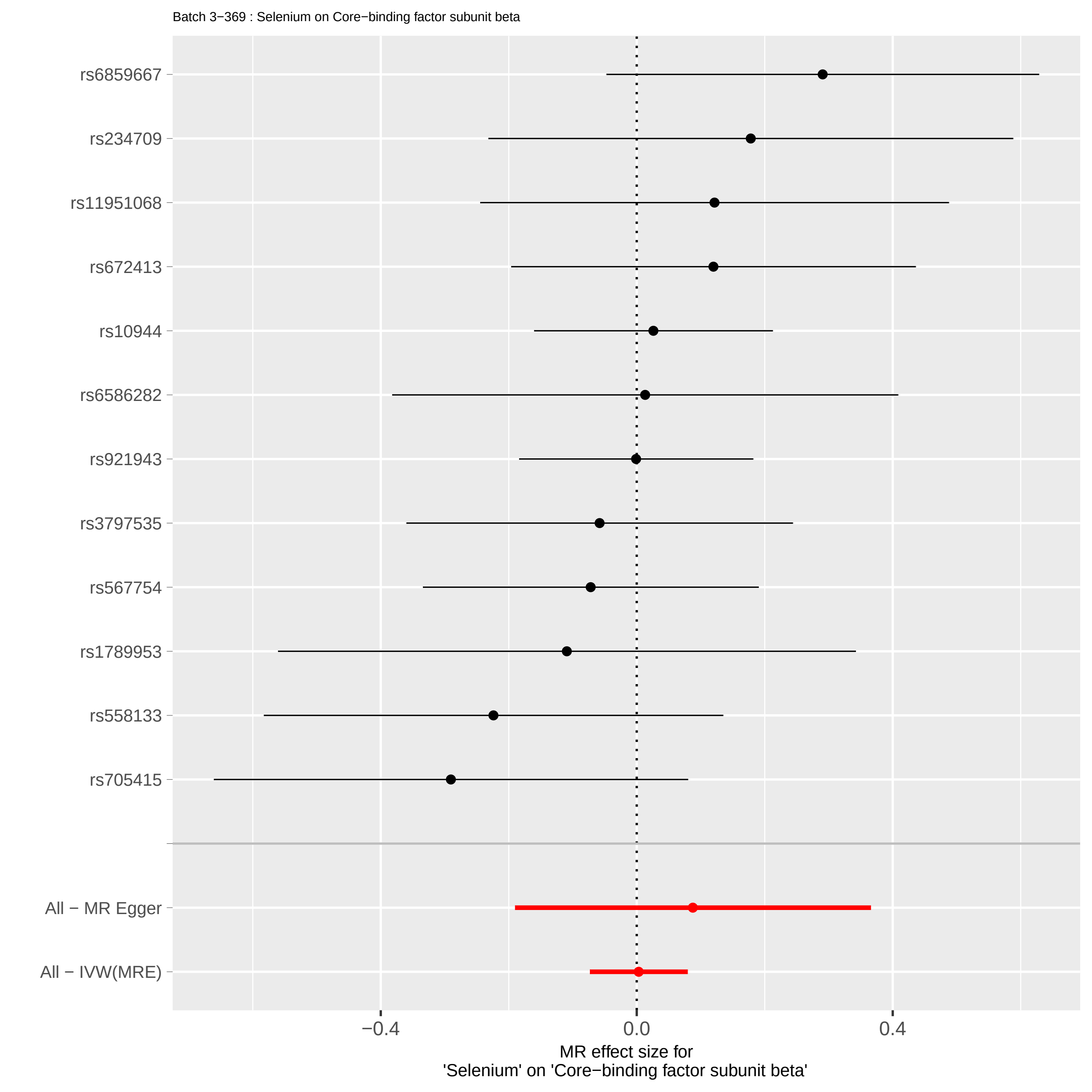

Batch 3−369 : Selenium on Core−binding factor subunit beta
rs6859667
rs234709
rs11951068
rs672413
rs10944
rs6586282
rs921943
rs3797535
rs567754
rs1789953
rs558133
rs705415
All − MR Egger
All − IVW(MRE)
−0.4
0.0
0.4
MR effect size for
'Selenium' on 'Core−binding factor subunit beta'

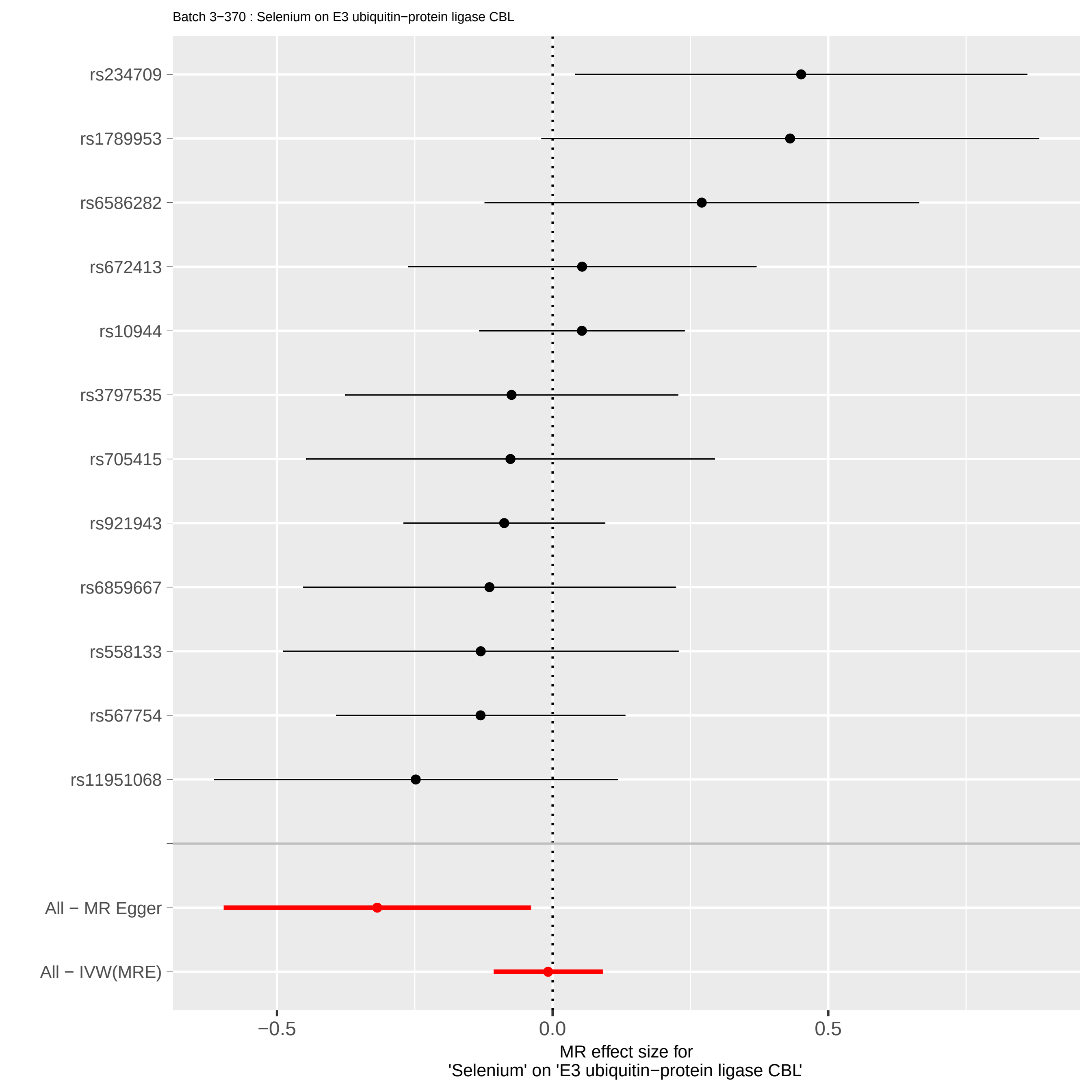
Batch 3−370 : Selenium on E3 ubiquitin−protein ligase CBL
rs234709
rs1789953
rs6586282
rs672413
rs10944
rs3797535
rs705415
rs921943
rs6859667
rs558133
rs567754
rs11951068
All − MR Egger
All − IVW(MRE)
−0.5
0.0
0.5
MR effect size for
'Selenium' on 'E3 ubiquitin−protein ligase CBL'

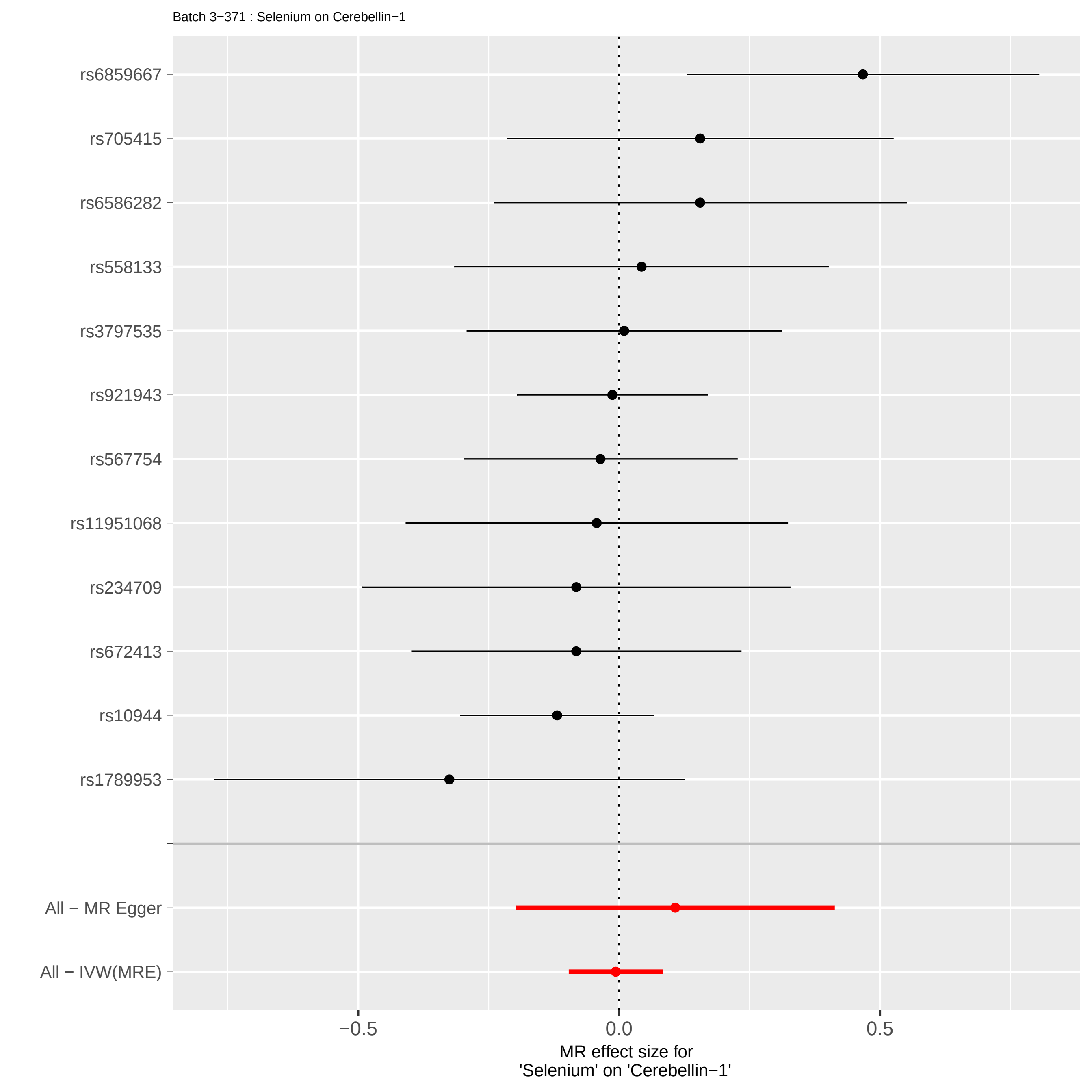
Batch 3−371 : Selenium on Cerebellin−1
rs6859667
rs705415
rs6586282
rs558133
rs3797535
rs921943
rs567754
rs11951068
rs234709
rs672413
rs10944
rs1789953
All − MR Egger
All − IVW(MRE)
−0.5
0.0
0.5
MR effect size for
'Selenium' on 'Cerebellin−1'

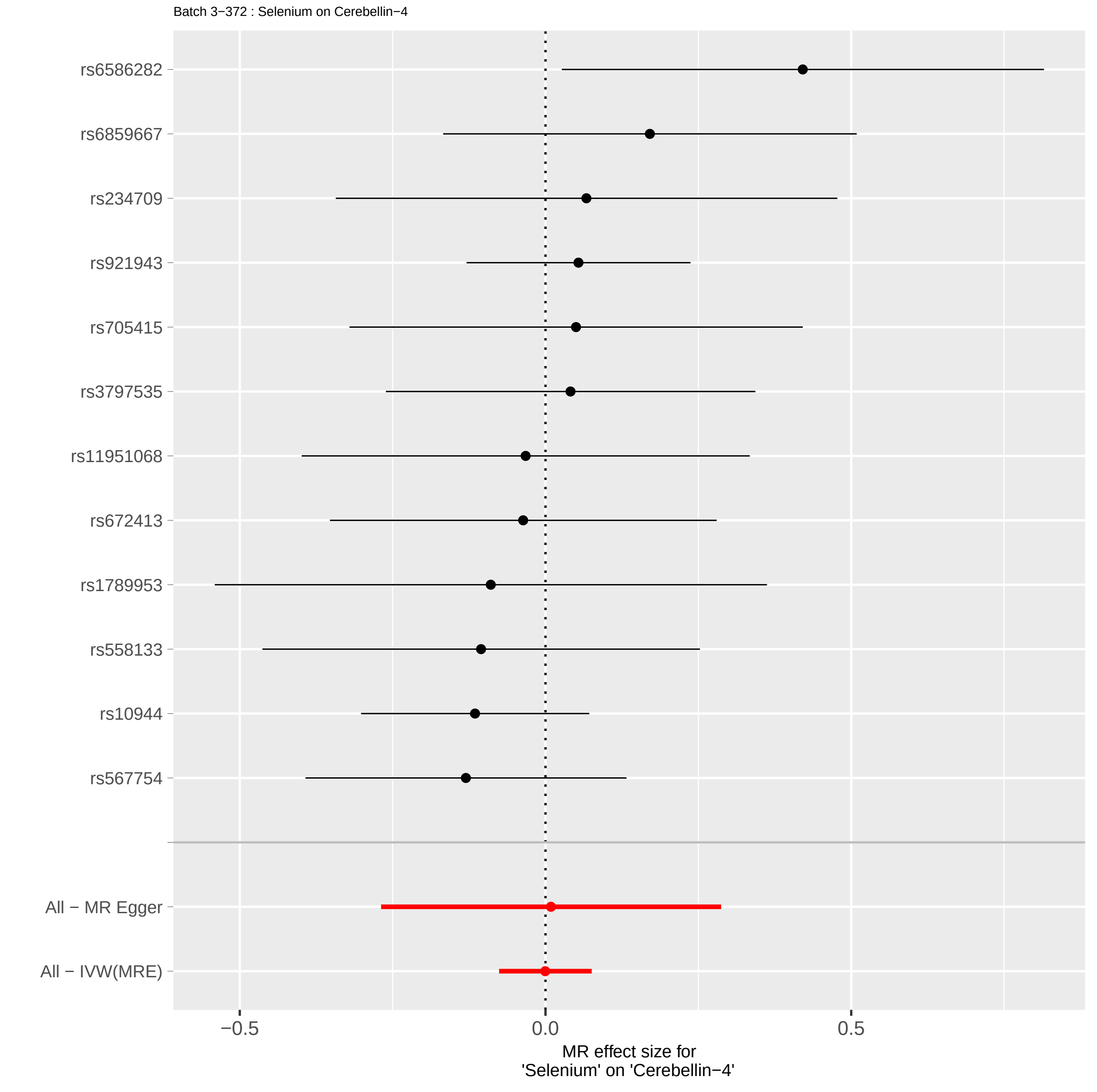

Batch 3−372 : Selenium on Cerebellin−4
rs6586282
rs6859667
rs234709
rs921943
rs705415
rs3797535
rs11951068
rs672413
rs1789953
rs558133
rs10944
rs567754
All − MR Egger
All − IVW(MRE)
−0.5
0.0
0.5
MR effect size for
'Selenium' on 'Cerebellin−4'

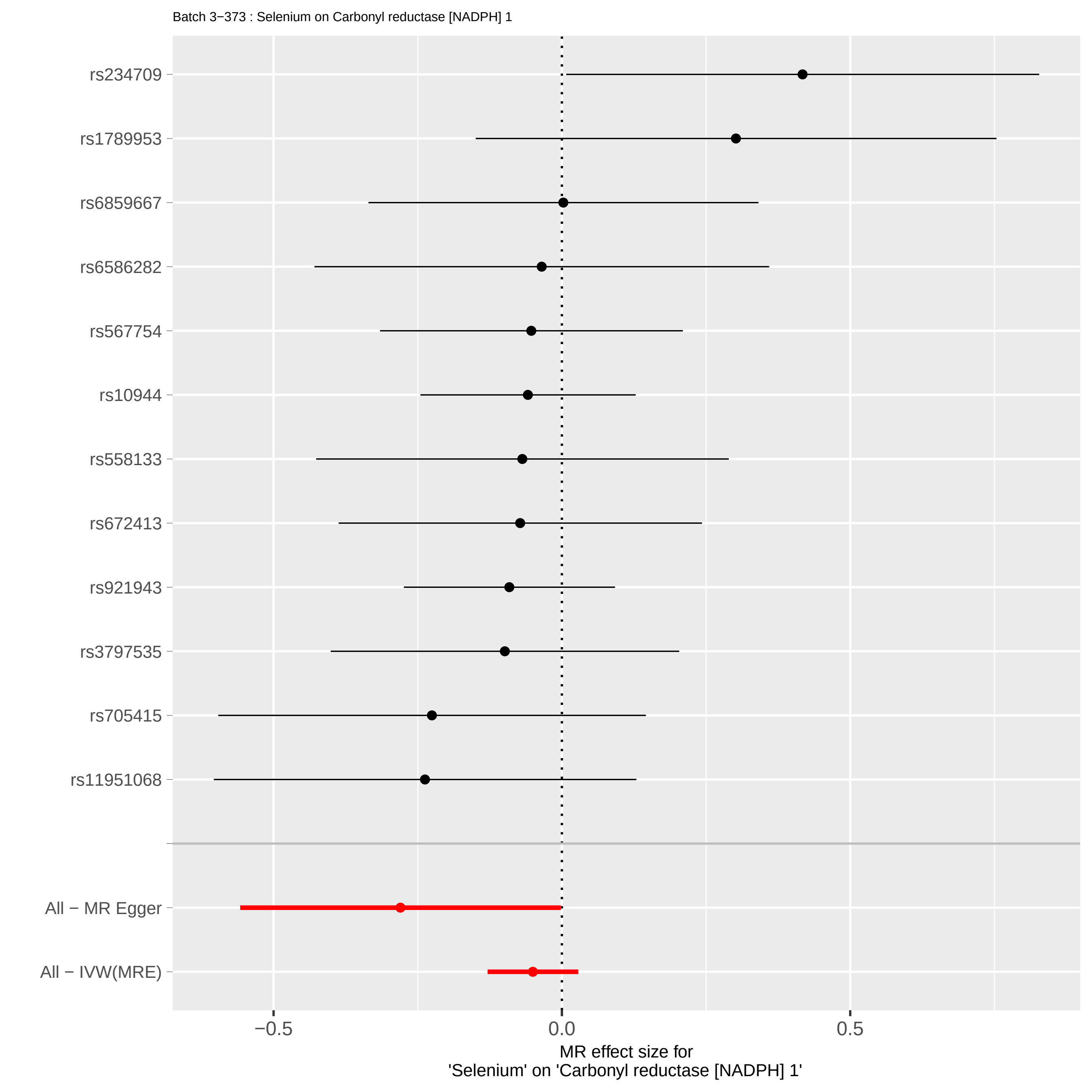
Batch 3−373 : Selenium on Carbonyl reductase [NADPH] 1
rs234709
rs1789953
rs6859667
rs6586282
rs567754
rs10944
rs558133
rs672413
rs921943
rs3797535
rs705415
rs11951068
All − MR Egger
All − IVW(MRE)
−0.5
0.0
0.5
MR effect size for
'Selenium' on 'Carbonyl reductase [NADPH] 1'

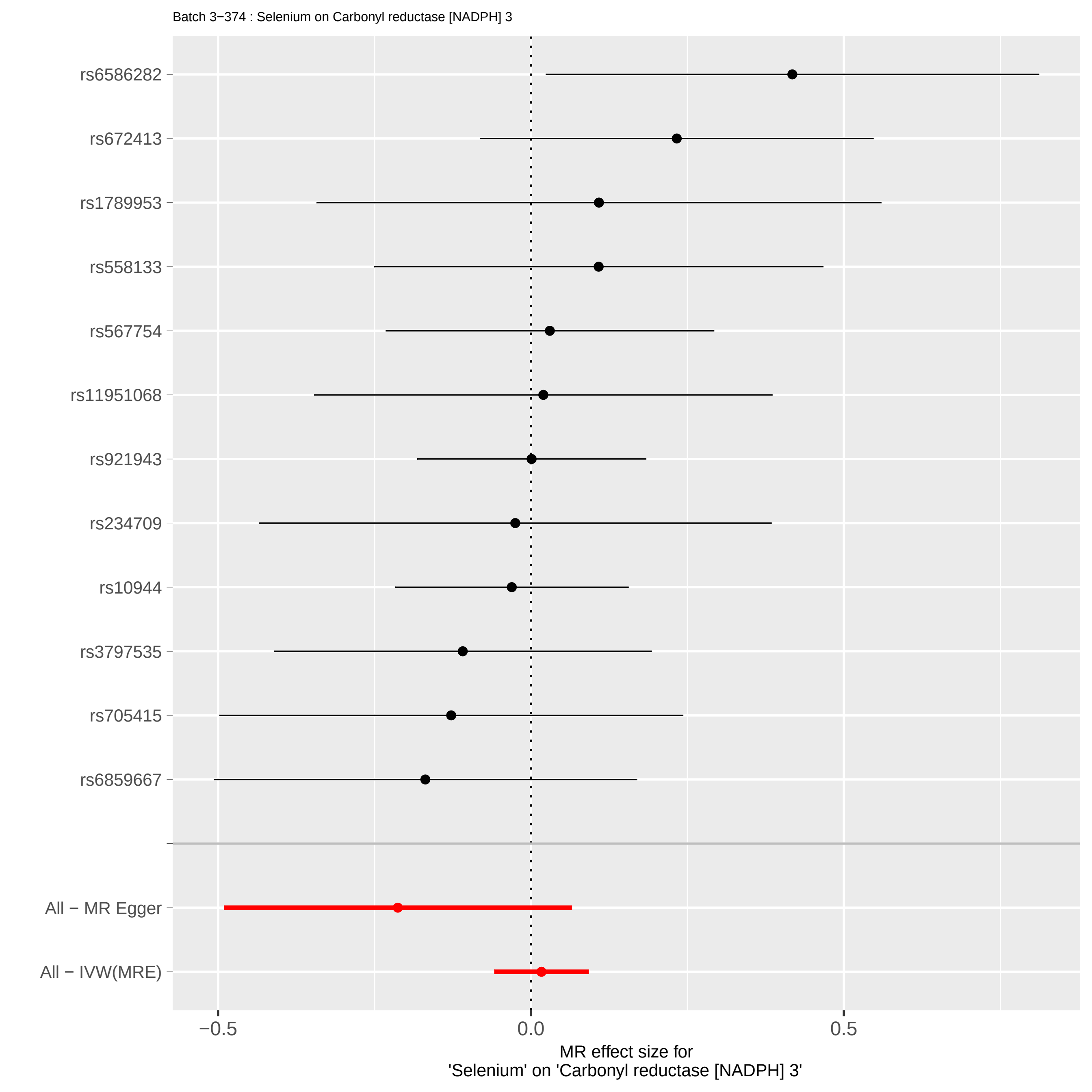
Batch 3−374 : Selenium on Carbonyl reductase [NADPH] 3
rs6586282
rs672413
rs1789953
rs558133
rs567754
rs11951068
rs921943
rs234709
rs10944
rs3797535
rs705415
rs6859667
All − MR Egger
All − IVW(MRE)
−0.5
0.0
0.5
MR effect size for
'Selenium' on 'Carbonyl reductase [NADPH] 3'

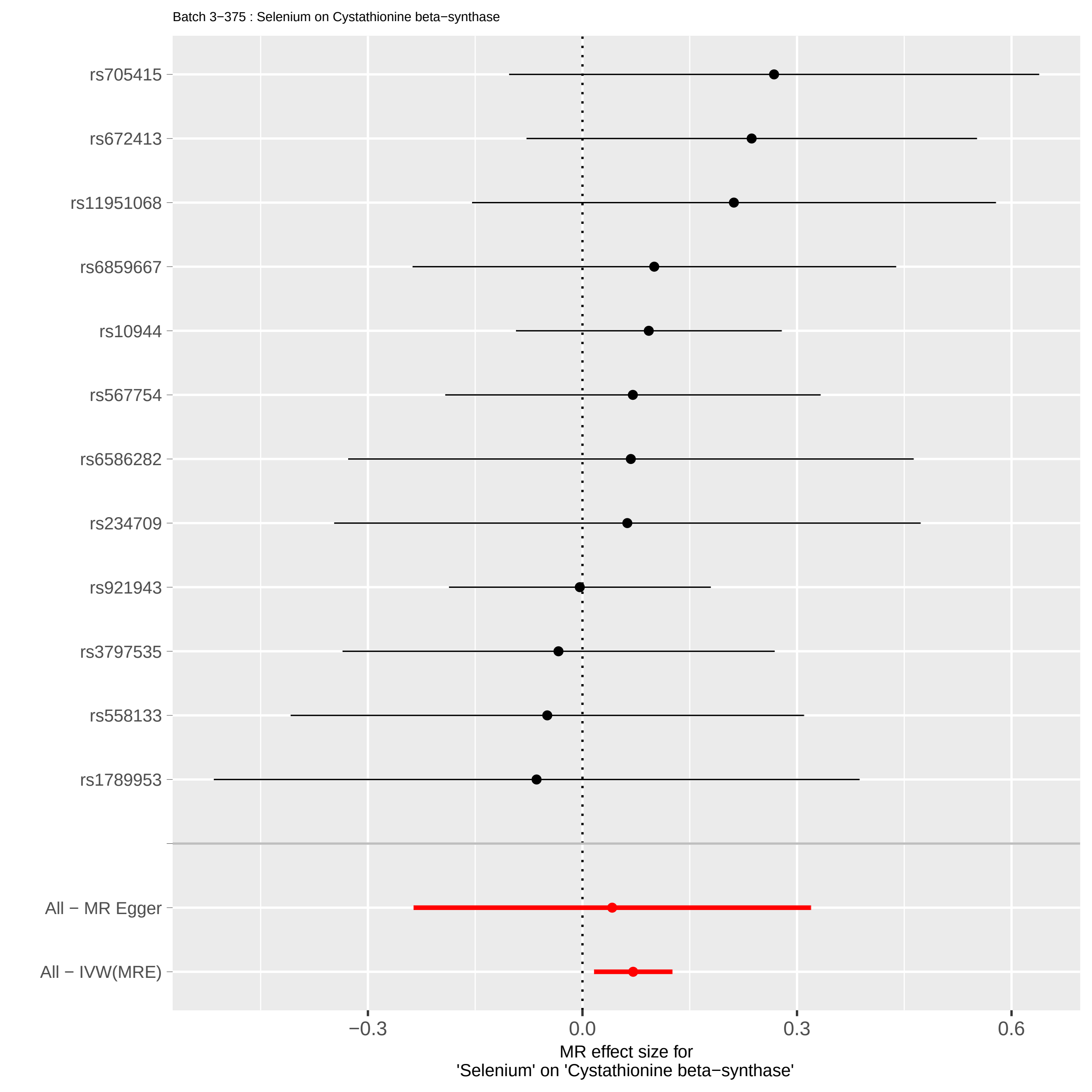

Batch 3−375 : Selenium on Cystathionine beta−synthase
rs705415
rs672413
rs11951068
rs6859667
rs10944
rs567754
rs6586282
rs234709
rs921943
rs3797535
rs558133
rs1789953
All − MR Egger
All − IVW(MRE)
−0.3
0.0
0.3
0.6
MR effect size for
'Selenium' on 'Cystathionine beta−synthase'

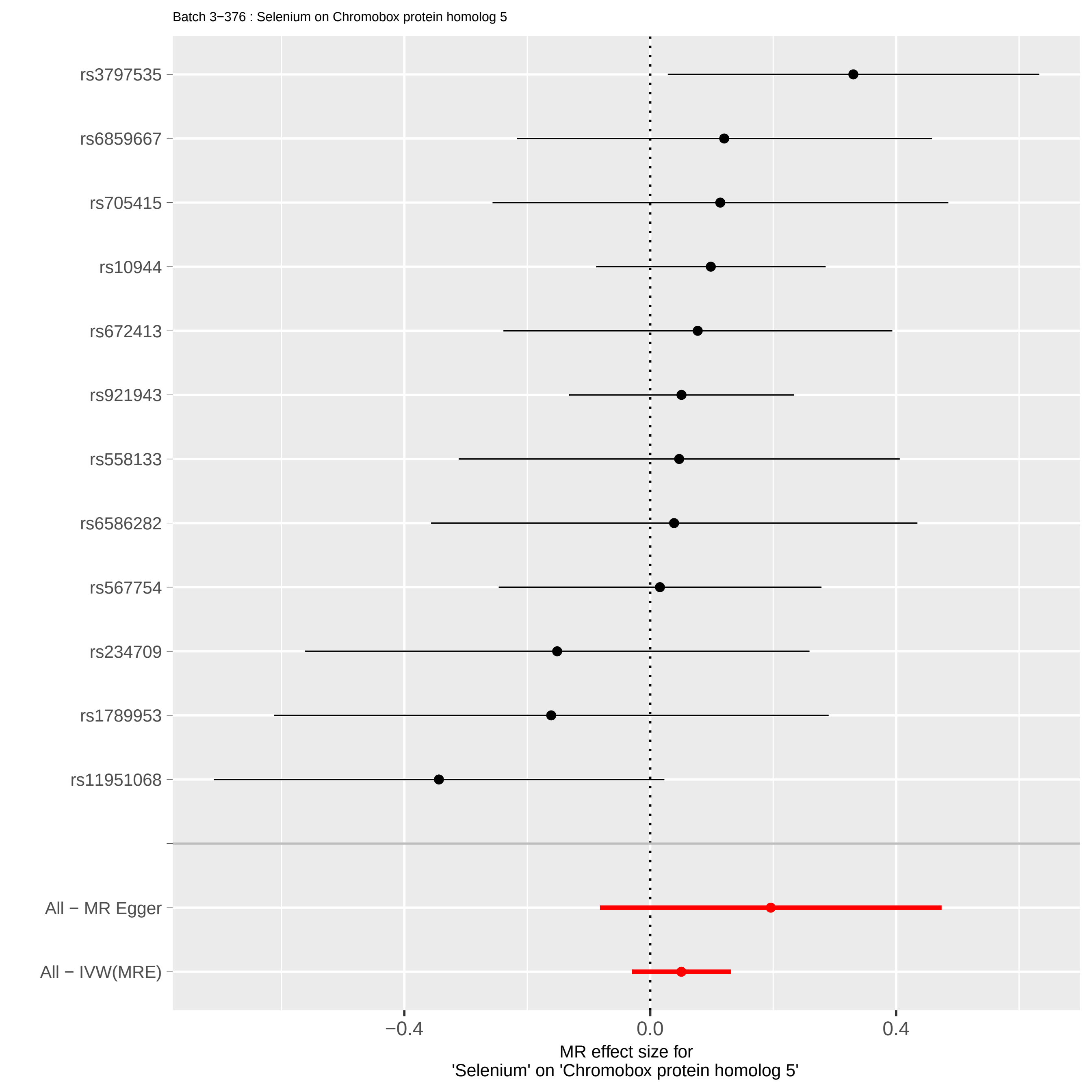
Batch 3−376 : Selenium on Chromobox protein homolog 5
rs3797535
rs6859667
rs705415
rs10944
rs672413
rs921943
rs558133
rs6586282
rs567754
rs234709
rs1789953
rs11951068
All − MR Egger
All − IVW(MRE)
−0.4
0.0
0.4
MR effect size for
'Selenium' on 'Chromobox protein homolog 5'

Batch 3−377 : Selenium on Chromobox protein homolog 7

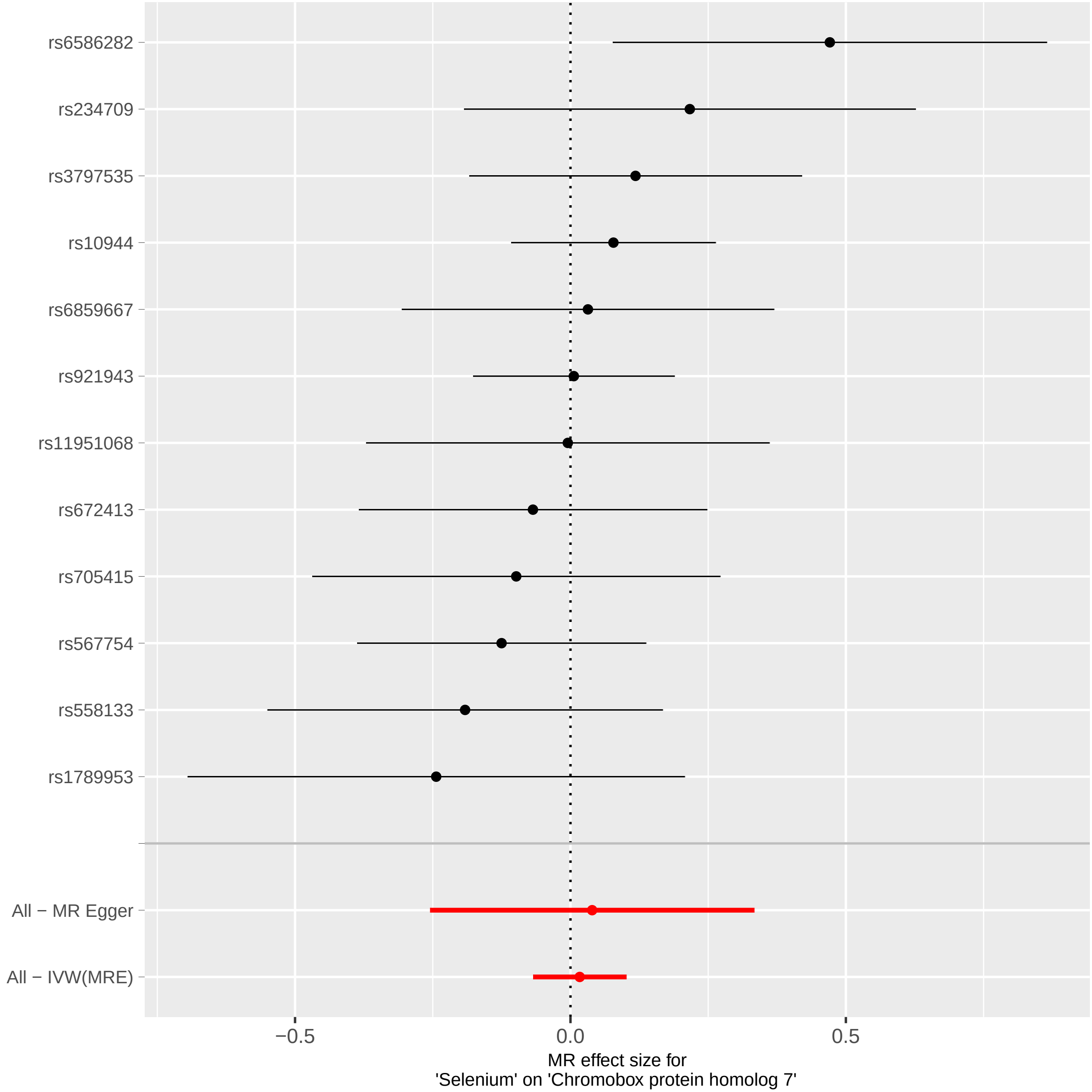

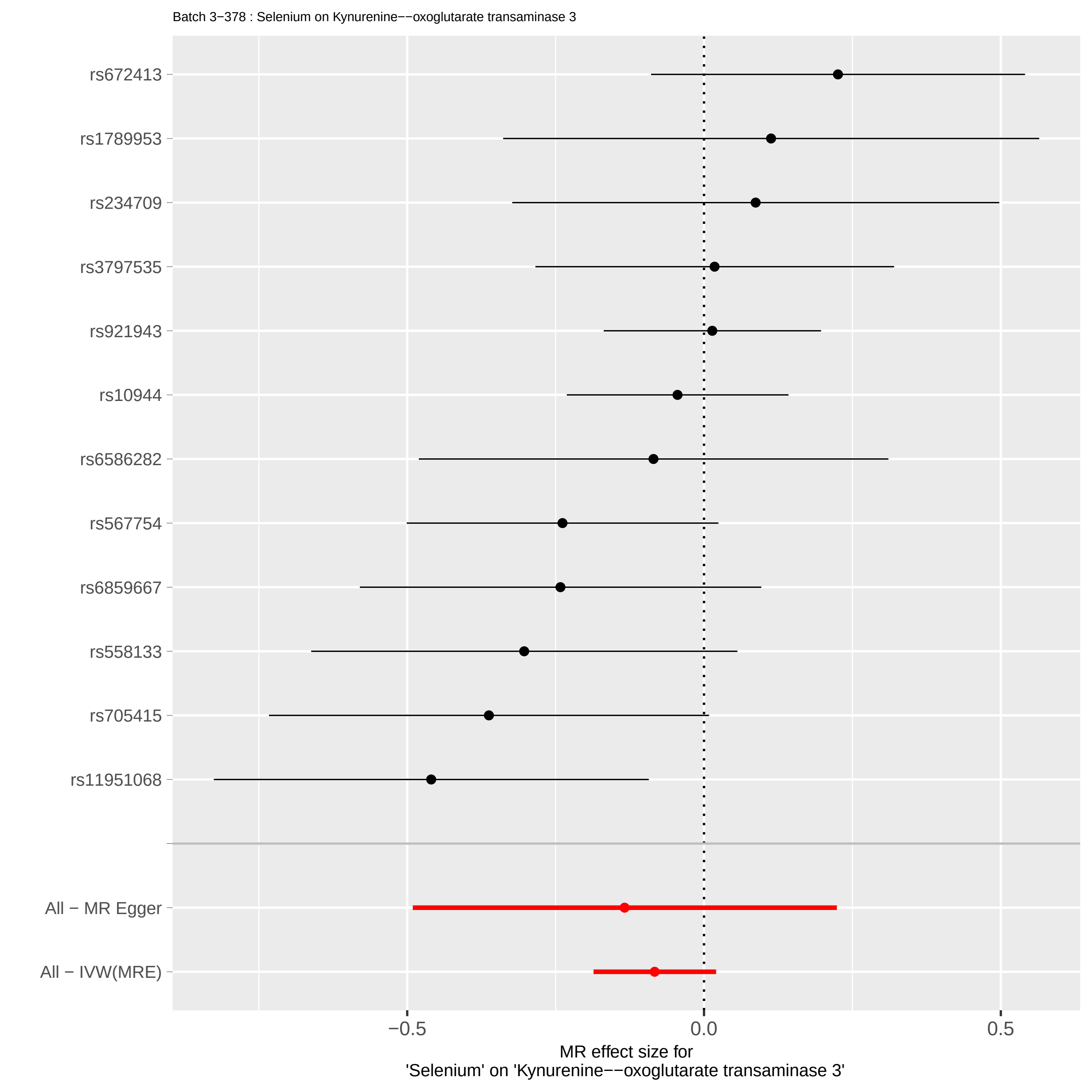
Batch 3−378 : Selenium on Kynurenine−−oxoglutarate transaminase 3
rs672413
rs1789953
rs234709
rs3797535
rs921943
rs10944
rs6586282
rs567754
rs6859667
rs558133
rs705415
rs11951068
All − MR Egger
All − IVW(MRE)
−0.5
0.0
0.5
MR effect size for
'Selenium' on 'Kynurenine−−oxoglutarate transaminase 3'

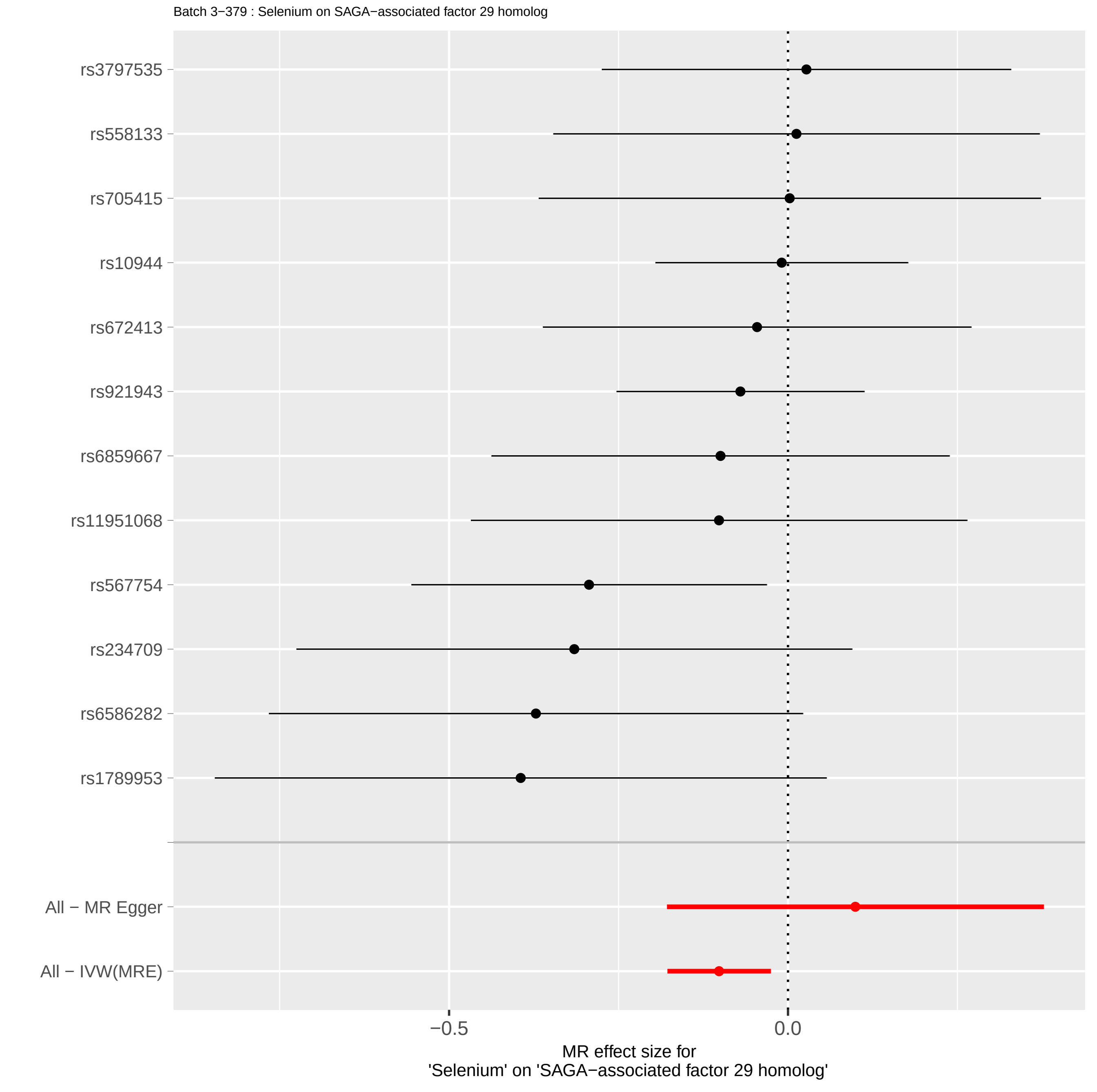
Batch 3−379 : Selenium on SAGA−associated factor 29 homolog
rs3797535
rs558133
rs705415
rs10944
rs672413
rs921943
rs6859667
rs11951068
rs567754
rs234709
rs6586282
rs1789953
All − MR Egger
All − IVW(MRE)
−0.5
0.0
MR effect size for
'Selenium' on 'SAGA−associated factor 29 homolog'

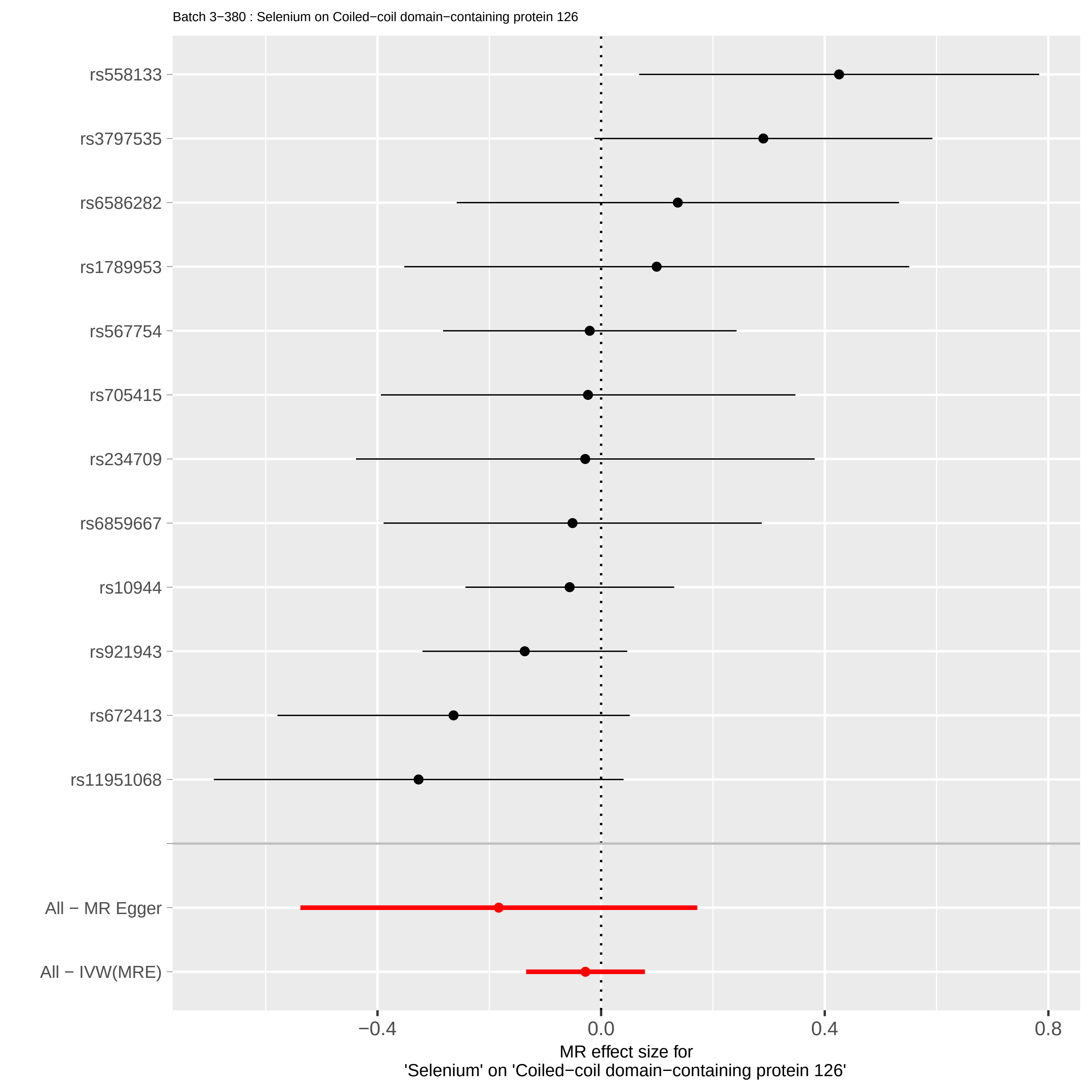

Batch 3−380 : Selenium on Coiled−coil domain−containing protein 126
rs558133
rs3797535
rs6586282
rs1789953
rs567754
rs705415
rs234709
rs6859667
rs10944
rs921943
rs672413
rs11951068
All − MR Egger
All − IVW(MRE)
−0.4
0.0
0.4
0.8
MR effect size for
'Selenium' on 'Coiled−coil domain−containing protein 126'

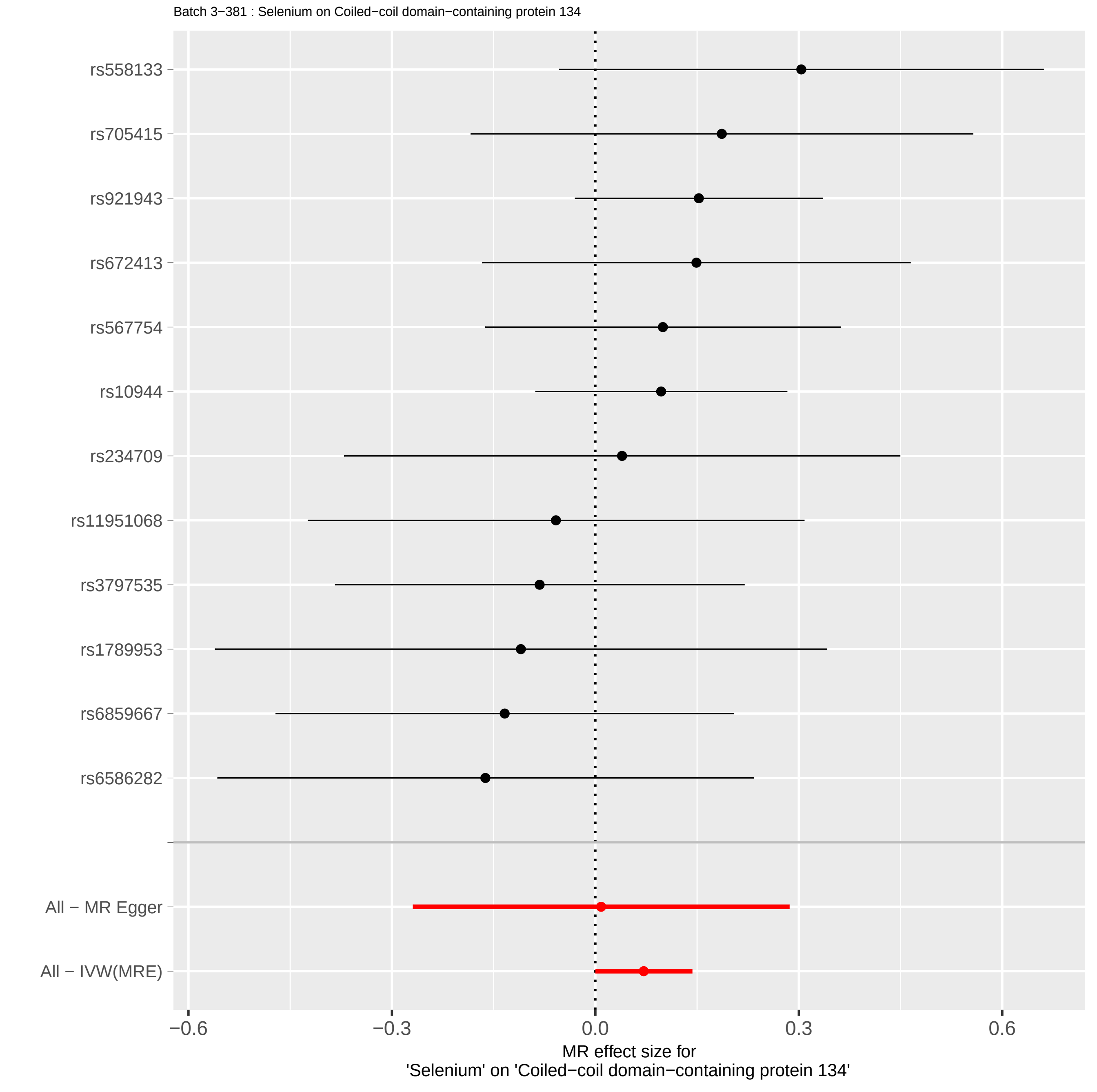
Batch 3−381 : Selenium on Coiled−coil domain−containing protein 134
rs558133
rs705415
rs921943
rs672413
rs567754
rs10944
rs234709
rs11951068
rs3797535
rs1789953
rs6859667
rs6586282
All − MR Egger
All − IVW(MRE)
−0.6
−0.3
0.0
0.3
0.6
MR effect size for
'Selenium' on 'Coiled−coil domain−containing protein 134'

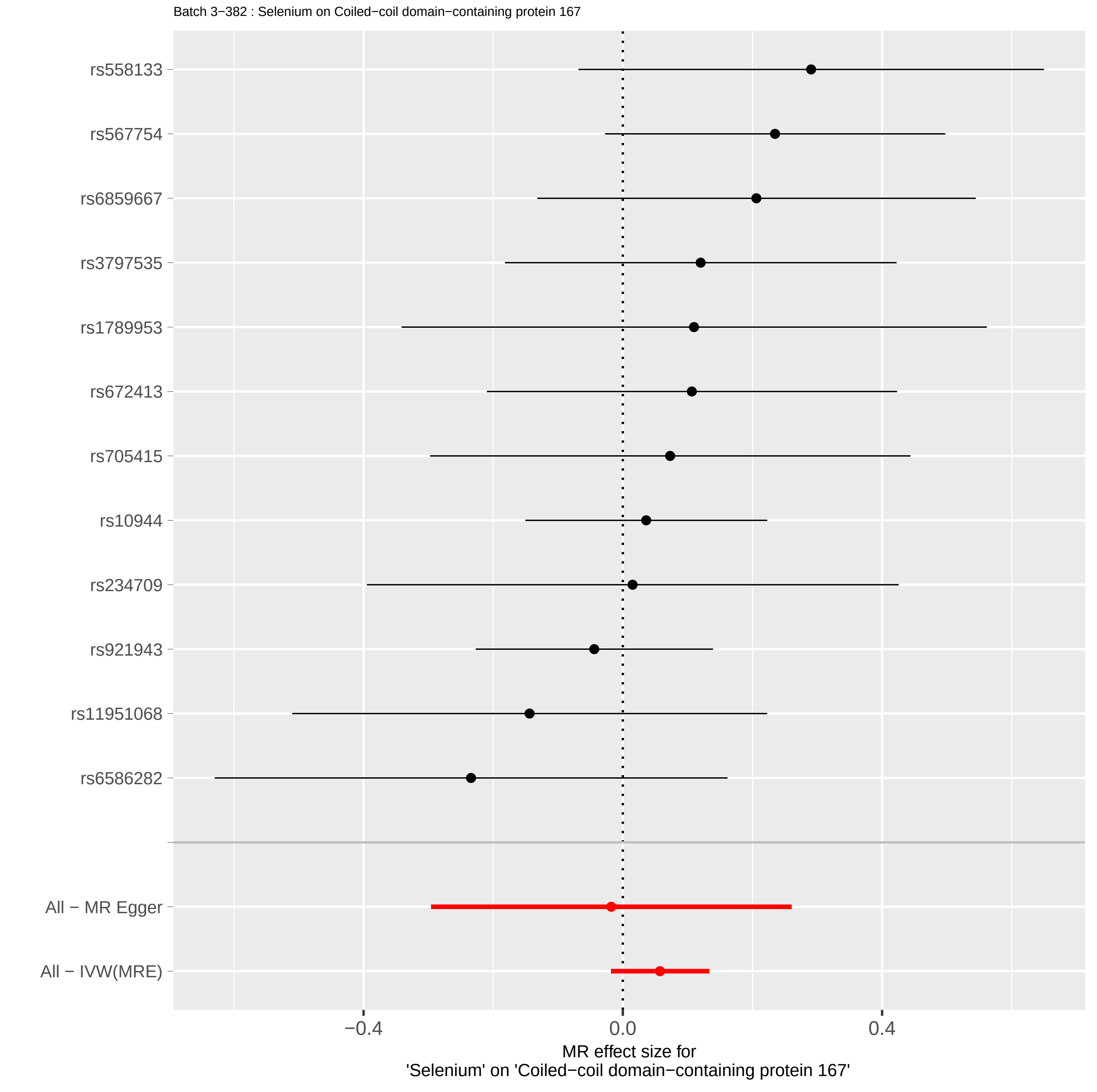

Batch 3−382 : Selenium on Coiled−coil domain−containing protein 167
rs558133
rs567754
rs6859667
rs3797535
rs1789953
rs672413
rs705415
rs10944
rs234709
rs921943
rs11951068
rs6586282
All − MR Egger
All − IVW(MRE)
−0.4
0.0
0.4
MR effect size for
'Selenium' on 'Coiled−coil domain−containing protein 167'

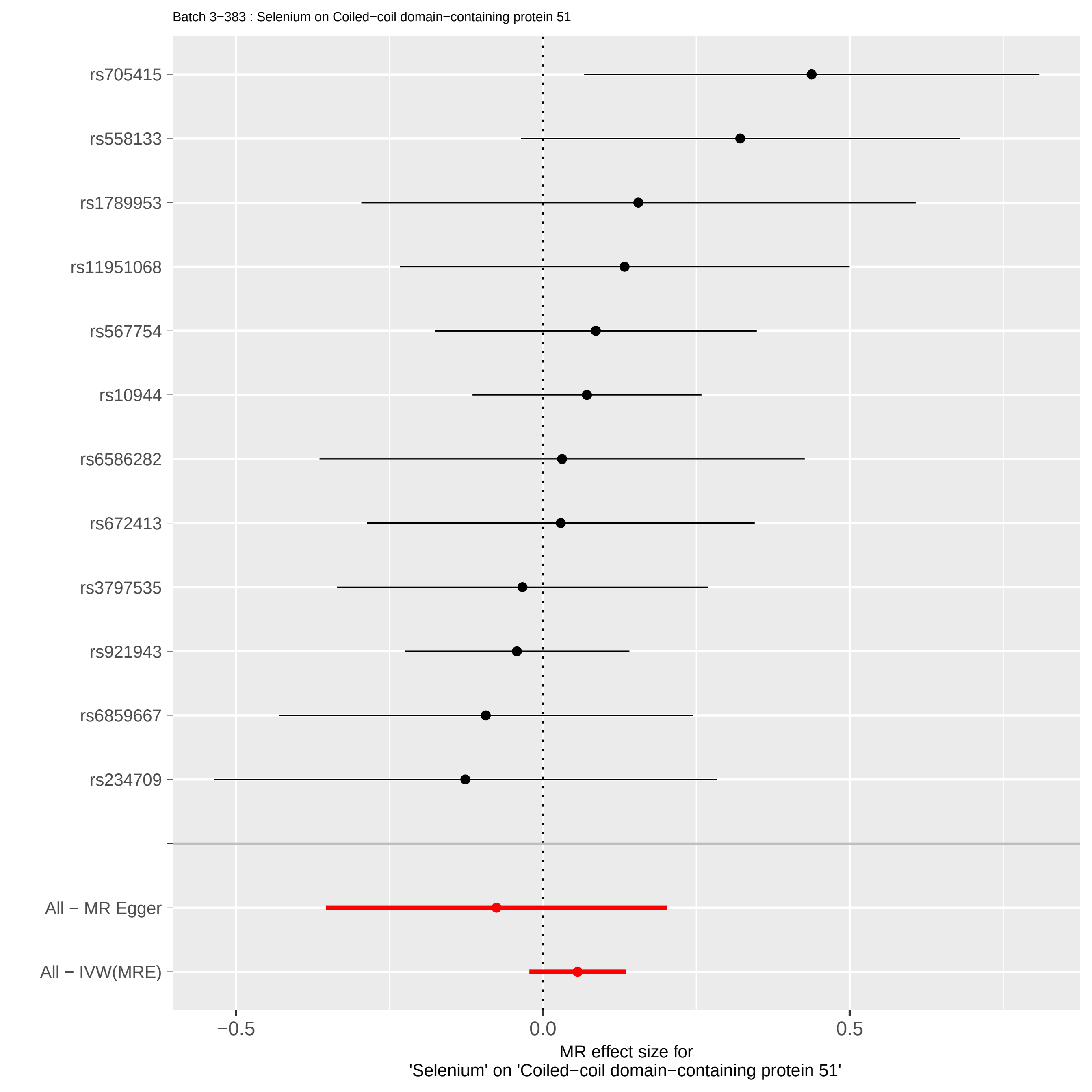
Batch 3−383 : Selenium on Coiled−coil domain−containing protein 51
rs705415
rs558133
rs1789953
rs11951068
rs567754
rs10944
rs6586282
rs672413
rs3797535
rs921943
rs6859667
rs234709
All − MR Egger
All − IVW(MRE)
−0.5
0.0
0.5
MR effect size for
'Selenium' on 'Coiled−coil domain−containing protein 51'

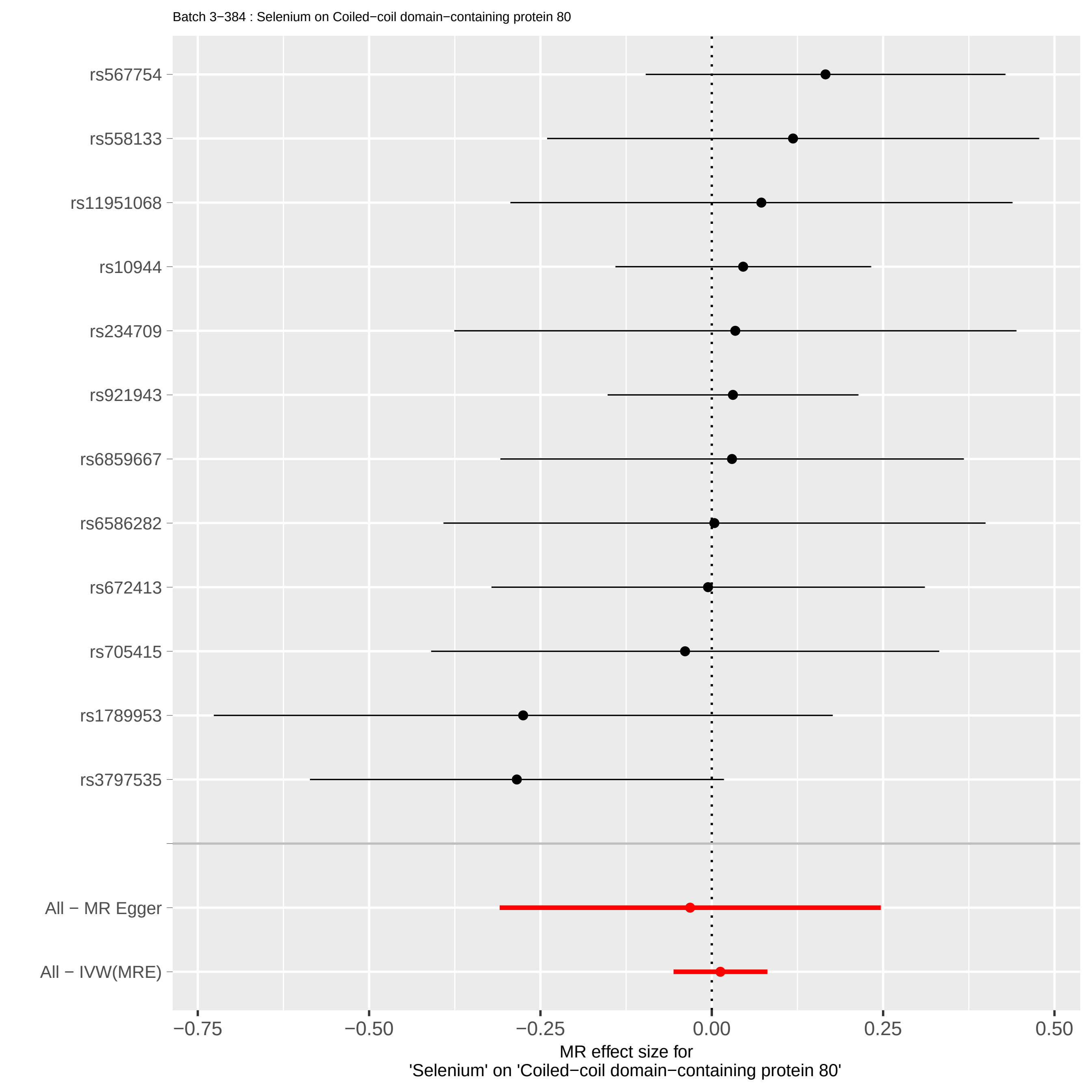

Batch 3−384 : Selenium on Coiled−coil domain−containing protein 80
rs567754
rs558133
rs11951068
rs10944
rs234709
rs921943
rs6859667
rs6586282
rs672413
rs705415
rs1789953
rs3797535
All − MR Egger
All − IVW(MRE)
−0.75
−0.50
−0.25
0.00
0.25
0.50
MR effect size for
'Selenium' on 'Coiled−coil domain−containing protein 80'

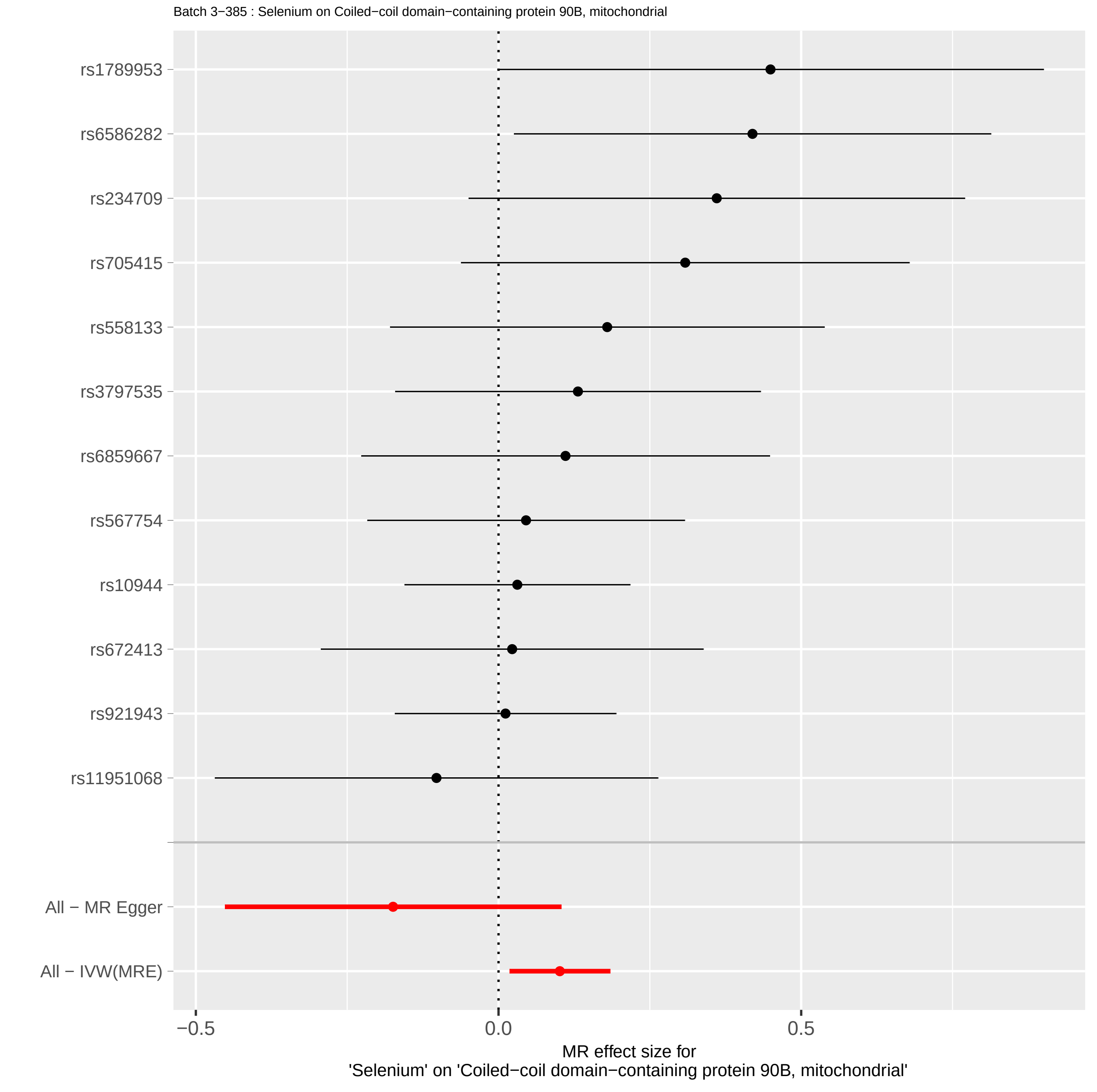

Batch 3−385 : Selenium on Coiled−coil domain−containing protein 90B, mitochondrial
rs1789953
rs6586282
rs234709
rs705415
rs558133
rs3797535
rs6859667
rs567754
rs10944
rs672413
rs921943
rs11951068
All − MR Egger
All − IVW(MRE)
−0.5
0.0
0.5
MR effect size for
'Selenium' on 'Coiled−coil domain−containing protein 90B, mitochondrial'

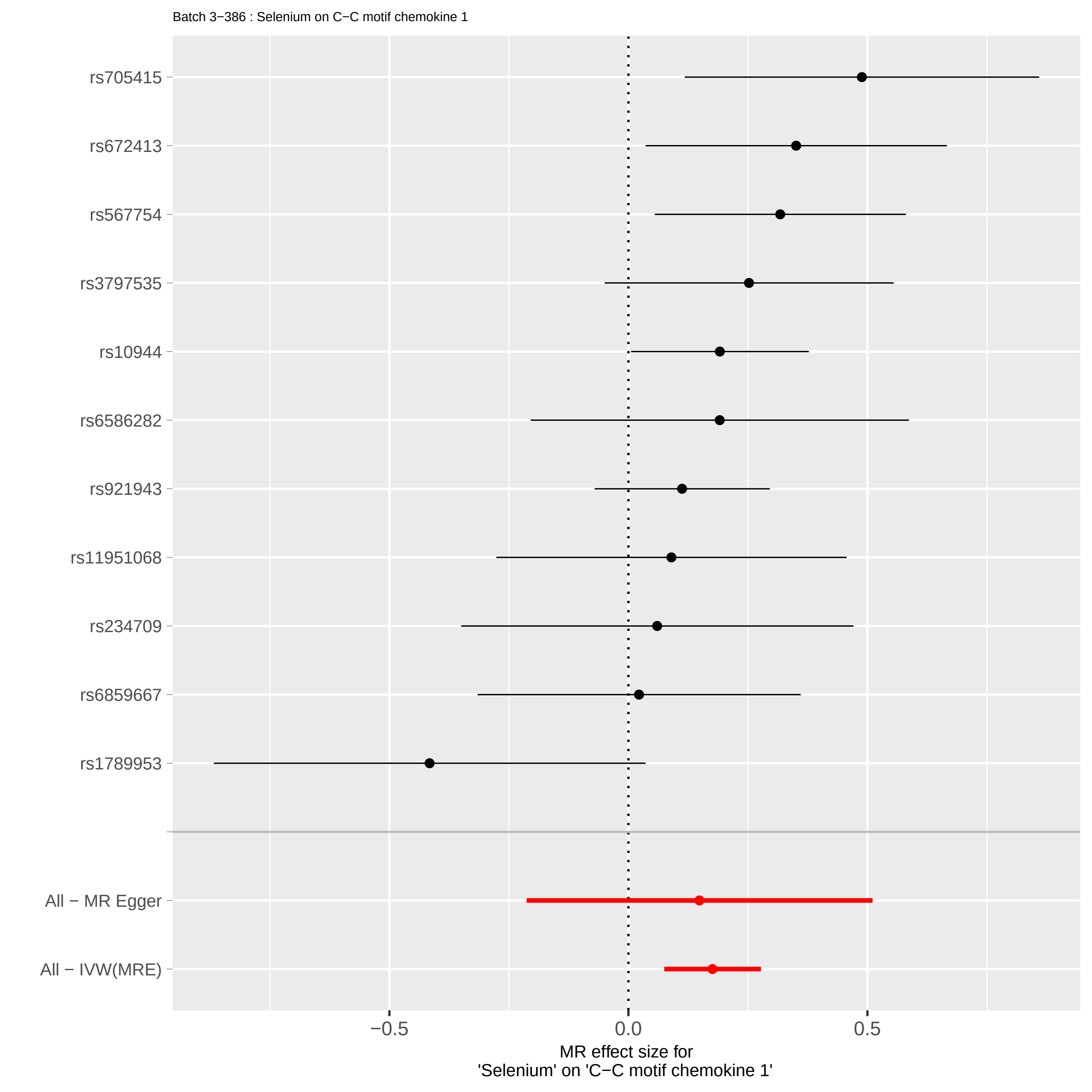
Batch 3−386 : Selenium on C−C motif chemokine 1
rs705415
rs672413
rs567754
rs3797535
rs10944
rs6586282
rs921943
rs11951068
rs234709
rs6859667
rs1789953
All − MR Egger
All − IVW(MRE)
−0.5
0.0
0.5
MR effect size for
'Selenium' on 'C−C motif chemokine 1'

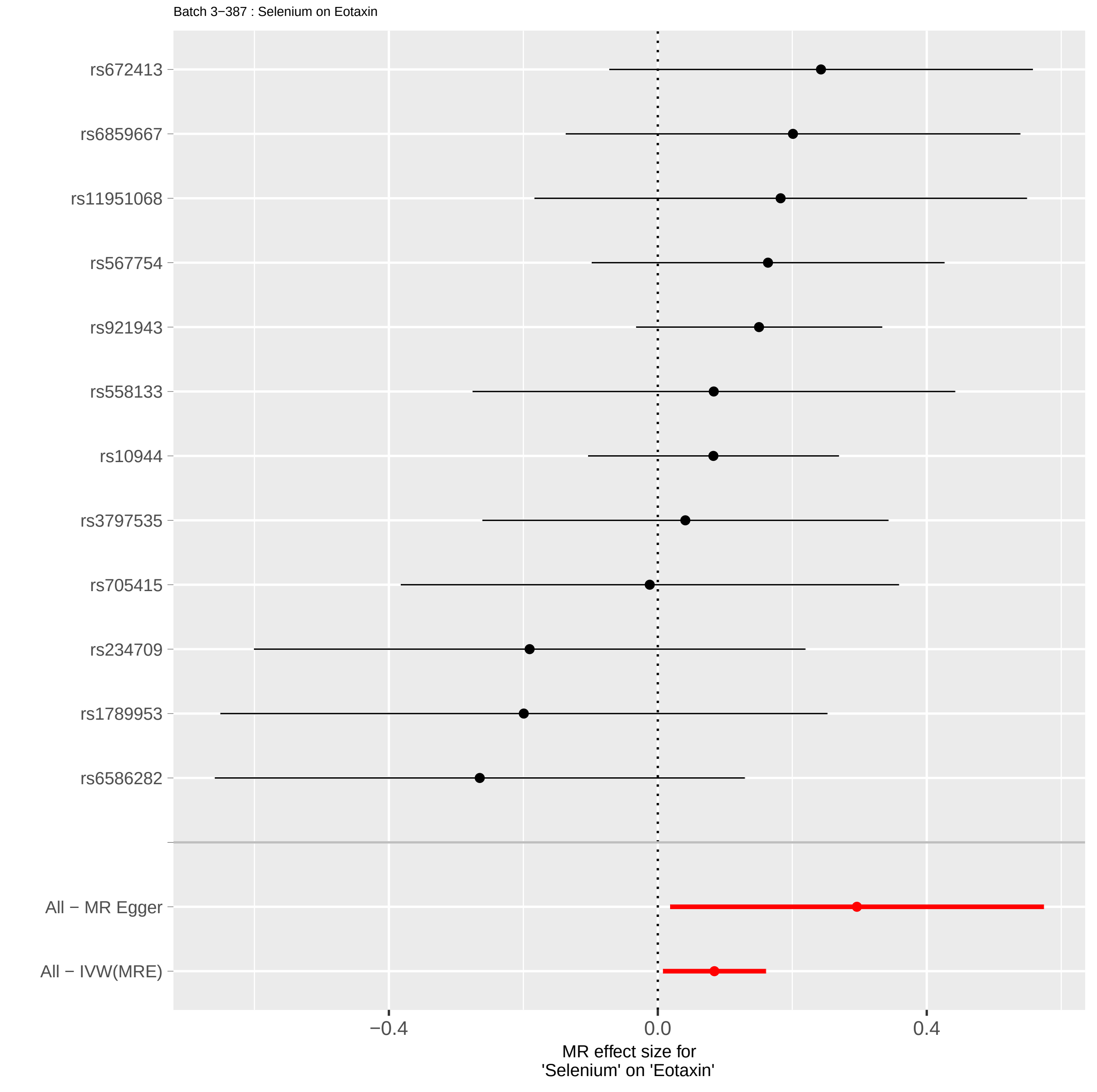

Batch 3−387 : Selenium on Eotaxin
rs672413
rs6859667
rs11951068
rs567754
rs921943
rs558133
rs10944
rs3797535
rs705415
rs234709
rs1789953
rs6586282
All − MR Egger
All − IVW(MRE)
−0.4
0.0
0.4
MR effect size for
'Selenium' on 'Eotaxin'

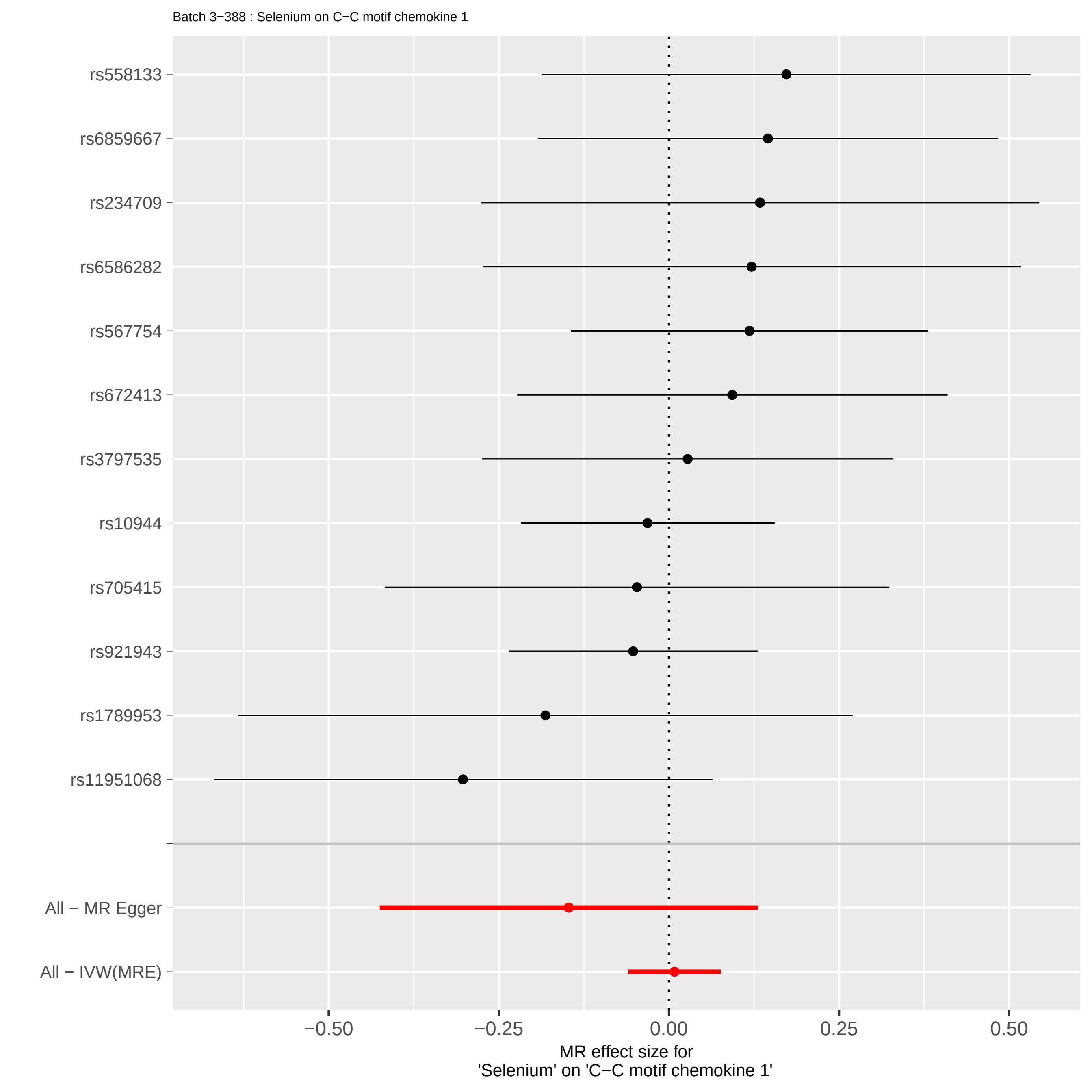

Batch 3−388 : Selenium on C−C motif chemokine 1
rs558133
rs6859667
rs234709
rs6586282
rs567754
rs672413
rs3797535
rs10944
rs705415
rs921943
rs1789953
rs11951068
All − MR Egger
All − IVW(MRE)
−0.50
−0.25
0.00
0.25
0.50
MR effect size for
'Selenium' on 'C−C motif chemokine 1'

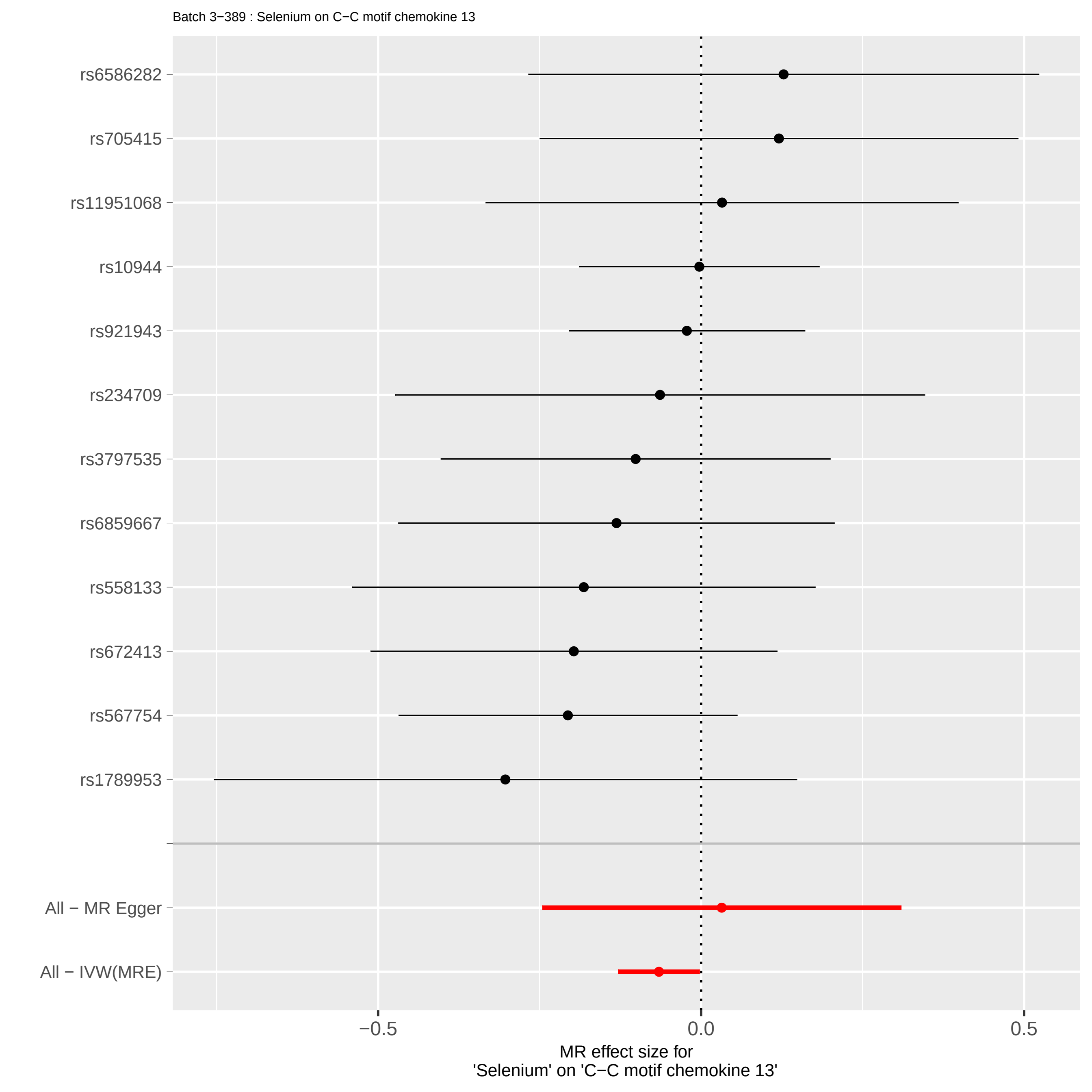
Batch 3−389 : Selenium on C−C motif chemokine 13
rs6586282
rs705415
rs11951068
rs10944
rs921943
rs234709
rs3797535
rs6859667
rs558133
rs672413
rs567754
rs1789953
All − MR Egger
All − IVW(MRE)
−0.5
0.0
0.5
MR effect size for
'Selenium' on 'C−C motif chemokine 13'

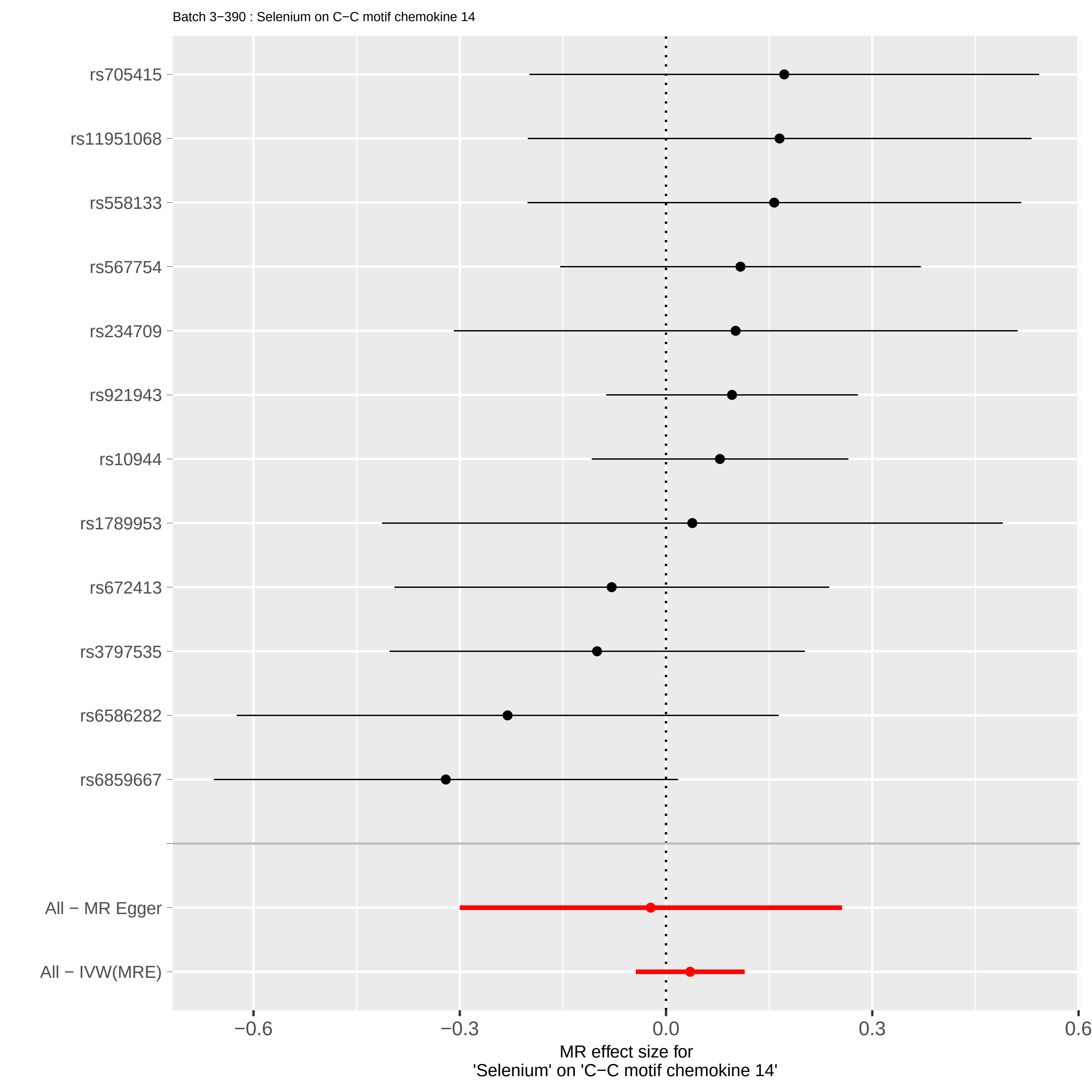
Batch 3−390 : Selenium on C−C motif chemokine 14
rs705415
rs11951068
rs558133
rs567754
rs234709
rs921943
rs10944
rs1789953
rs672413
rs3797535
rs6586282
rs6859667
All − MR Egger
All − IVW(MRE)
−0.6
−0.3
0.0
0.3
0.6
MR effect size for
'Selenium' on 'C−C motif chemokine 14'

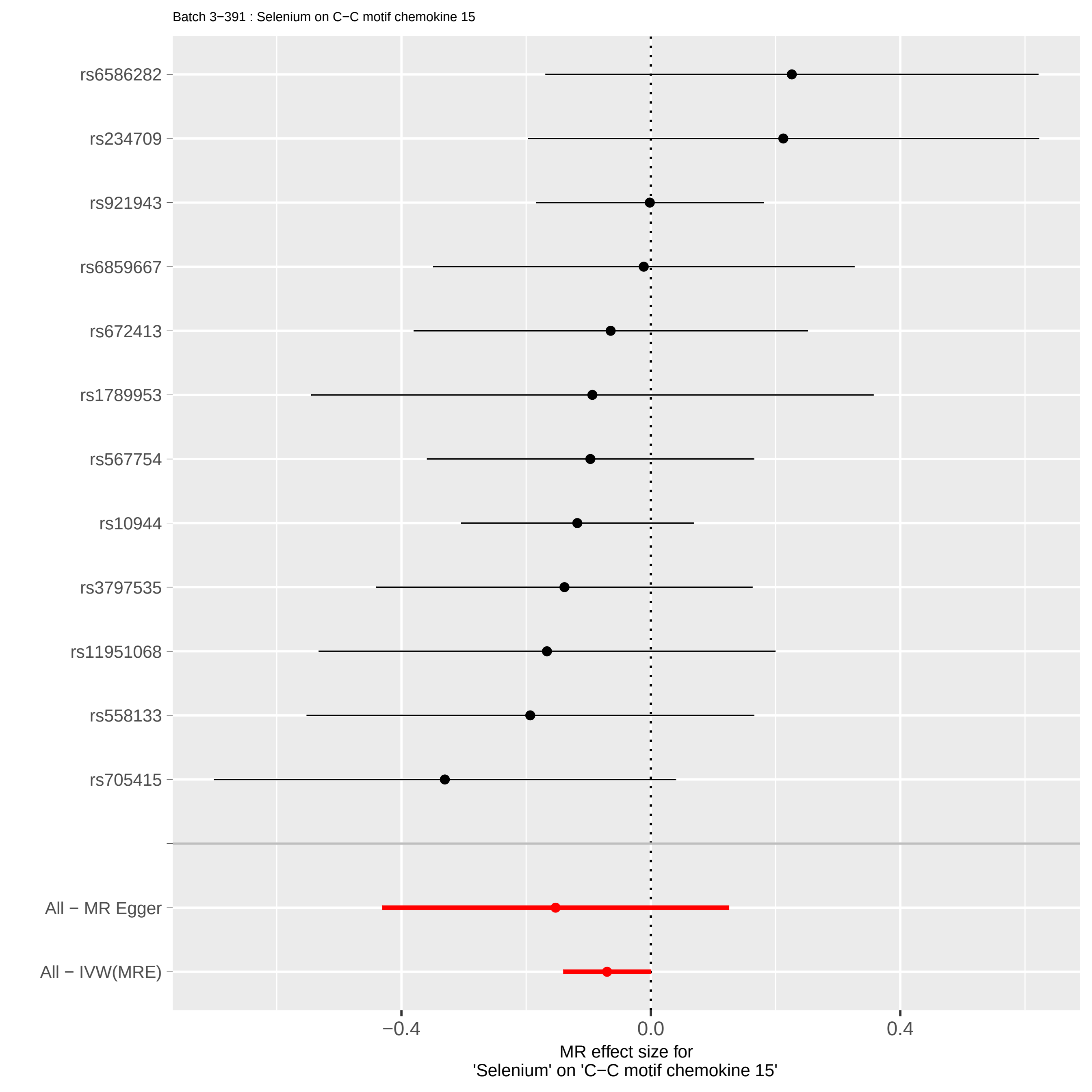

Batch 3−391 : Selenium on C−C motif chemokine 15
rs6586282
rs234709
rs921943
rs6859667
rs672413
rs1789953
rs567754
rs10944
rs3797535
rs11951068
rs558133
rs705415
All − MR Egger
All − IVW(MRE)
−0.4
0.0
0.4
MR effect size for
'Selenium' on 'C−C motif chemokine 15'

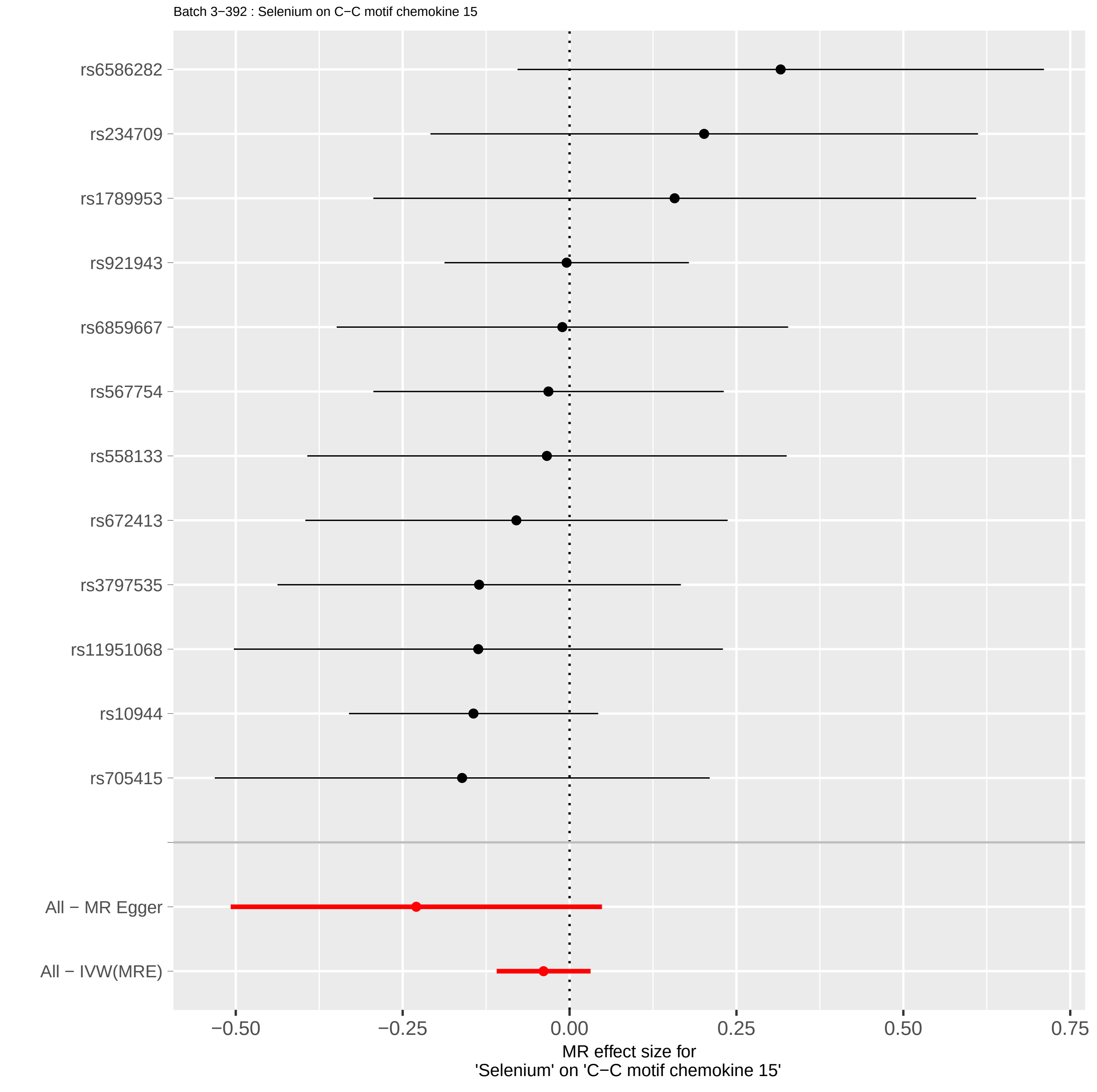

Batch 3−392 : Selenium on C−C motif chemokine 15
rs6586282
rs234709
rs1789953
rs921943
rs6859667
rs567754
rs558133
rs672413
rs3797535
rs11951068
rs10944
rs705415
All − MR Egger
All − IVW(MRE)
−0.50
−0.25
0.00
0.25
0.50
0.75
MR effect size for
'Selenium' on 'C−C motif chemokine 15'

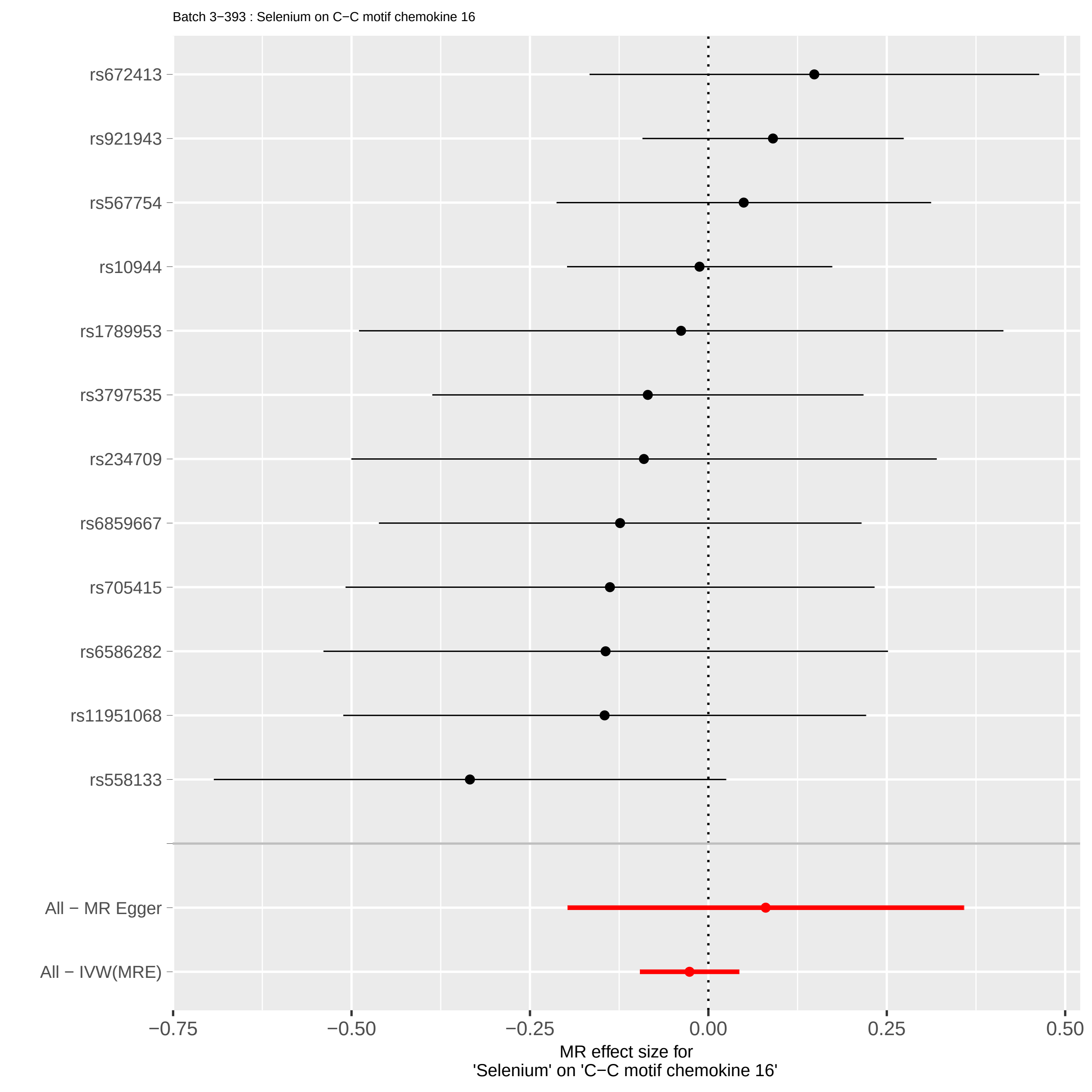
Batch 3−393 : Selenium on C−C motif chemokine 16
rs672413
rs921943
rs567754
rs10944
rs1789953
rs3797535
rs234709
rs6859667
rs705415
rs6586282
rs11951068
rs558133
All − MR Egger
All − IVW(MRE)
−0.75
−0.50
−0.25
0.00
0.25
0.50
MR effect size for
'Selenium' on 'C−C motif chemokine 16'

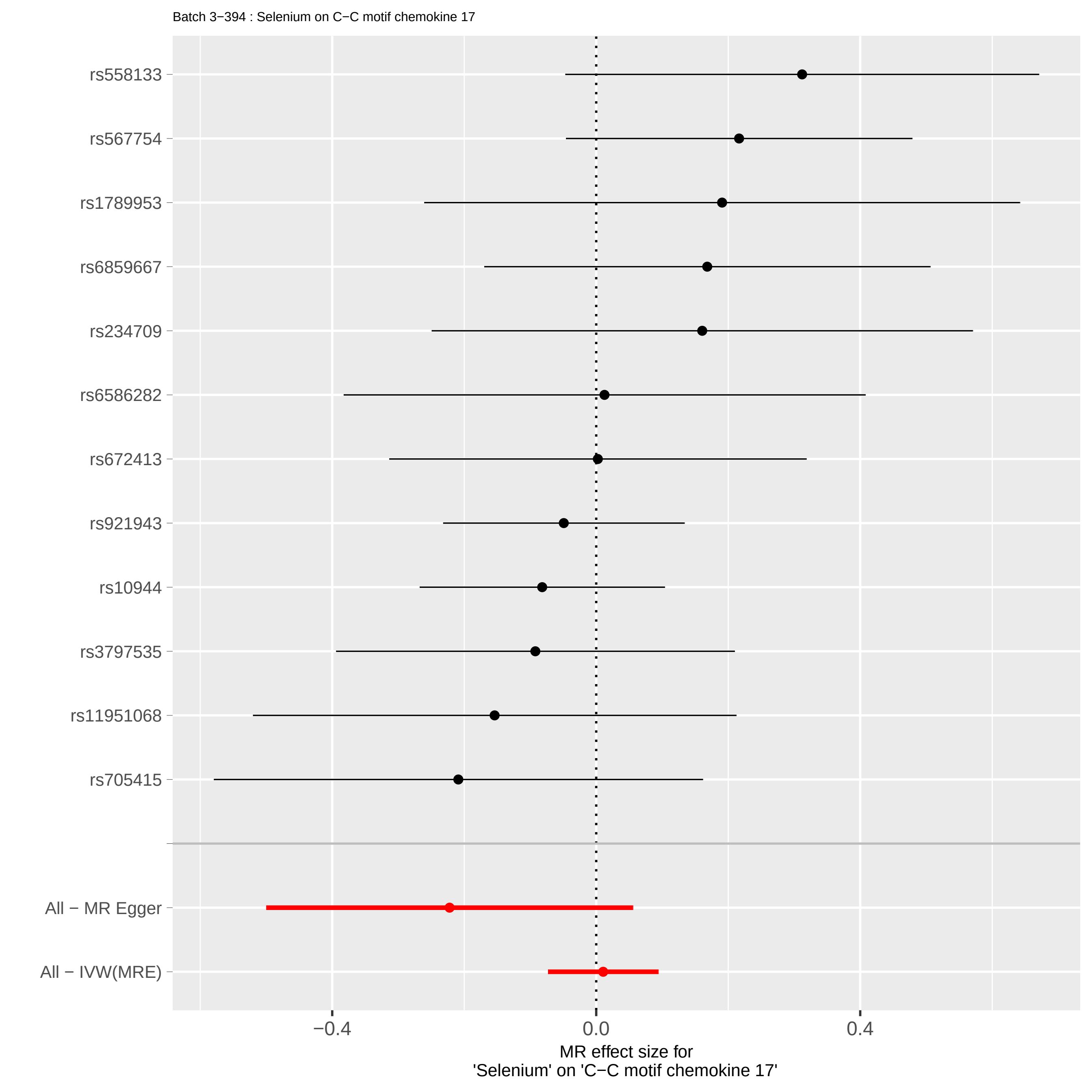

Batch 3−394 : Selenium on C−C motif chemokine 17
rs558133
rs567754
rs1789953
rs6859667
rs234709
rs6586282
rs672413
rs921943
rs10944
rs3797535
rs11951068
rs705415
All − MR Egger
All − IVW(MRE)
−0.4
0.0
0.4
MR effect size for
'Selenium' on 'C−C motif chemokine 17'

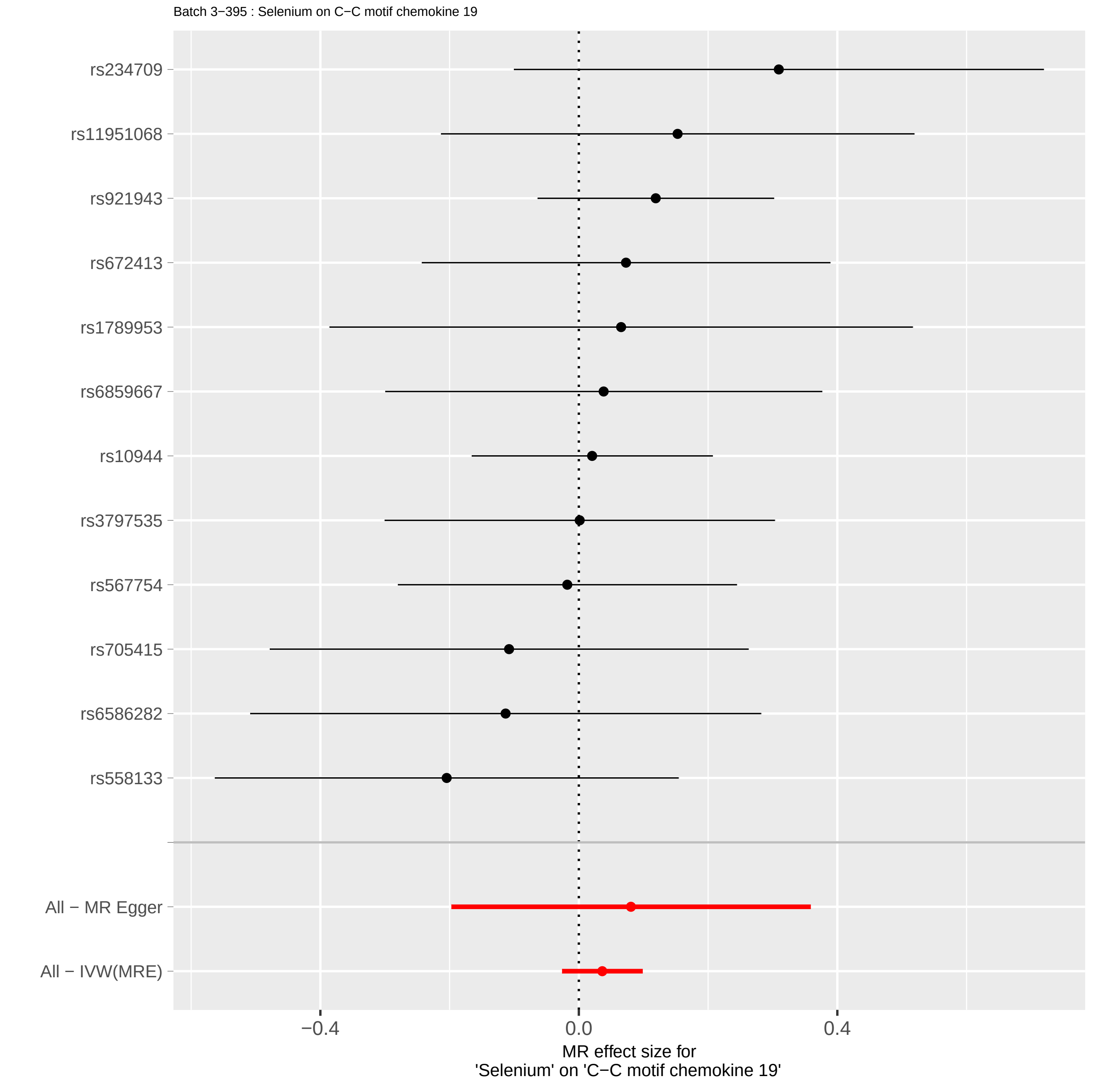
Batch 3−395 : Selenium on C−C motif chemokine 19
rs234709
rs11951068
rs921943
rs672413
rs1789953
rs6859667
rs10944
rs3797535
rs567754
rs705415
rs6586282
rs558133
All − MR Egger
All − IVW(MRE)
−0.4
0.0
0.4
MR effect size for
'Selenium' on 'C−C motif chemokine 19'

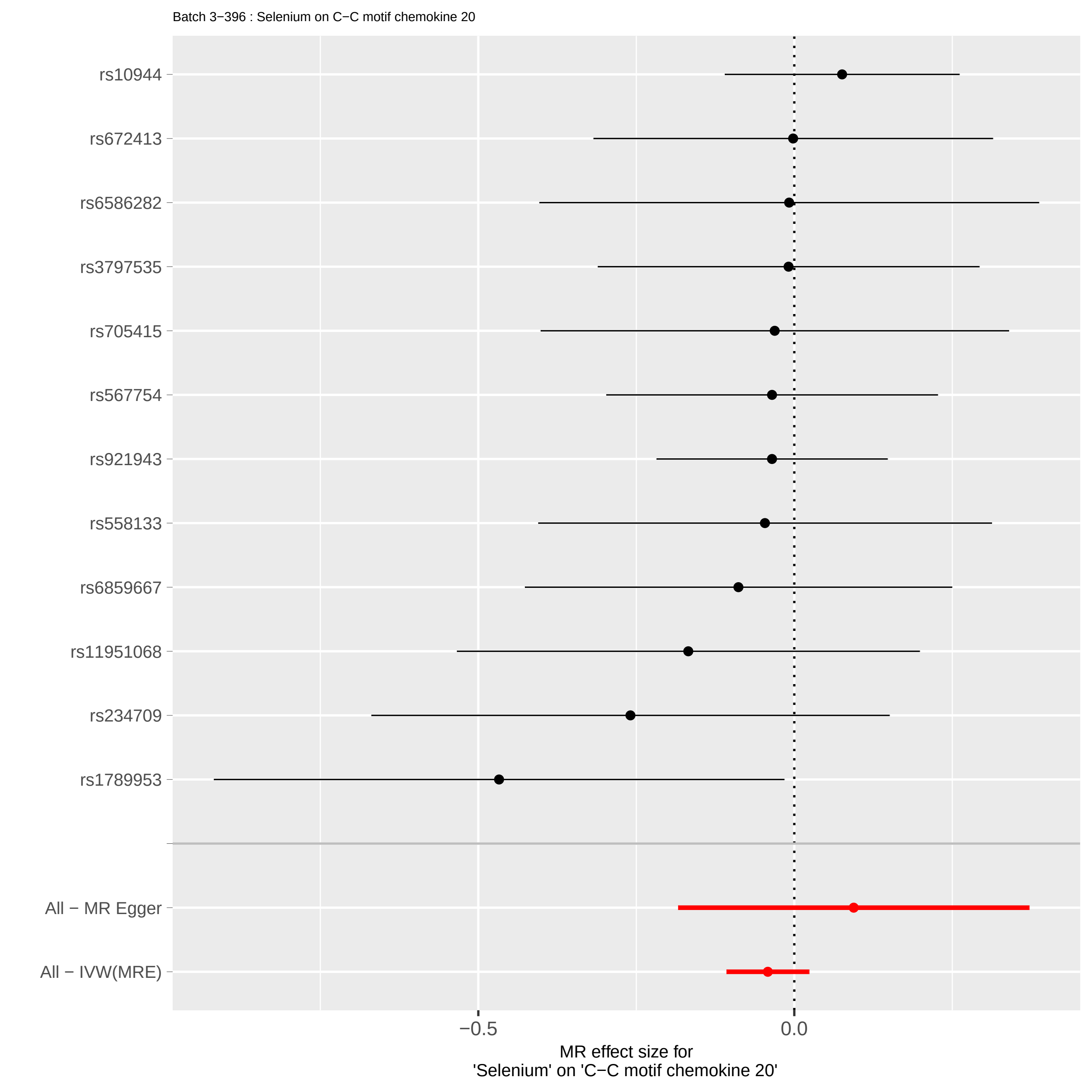
Batch 3−396 : Selenium on C−C motif chemokine 20
rs10944
rs672413
rs6586282
rs3797535
rs705415
rs567754
rs921943
rs558133
rs6859667
rs11951068
rs234709
rs1789953
All − MR Egger
All − IVW(MRE)
−0.5
0.0
MR effect size for
'Selenium' on 'C−C motif chemokine 20'

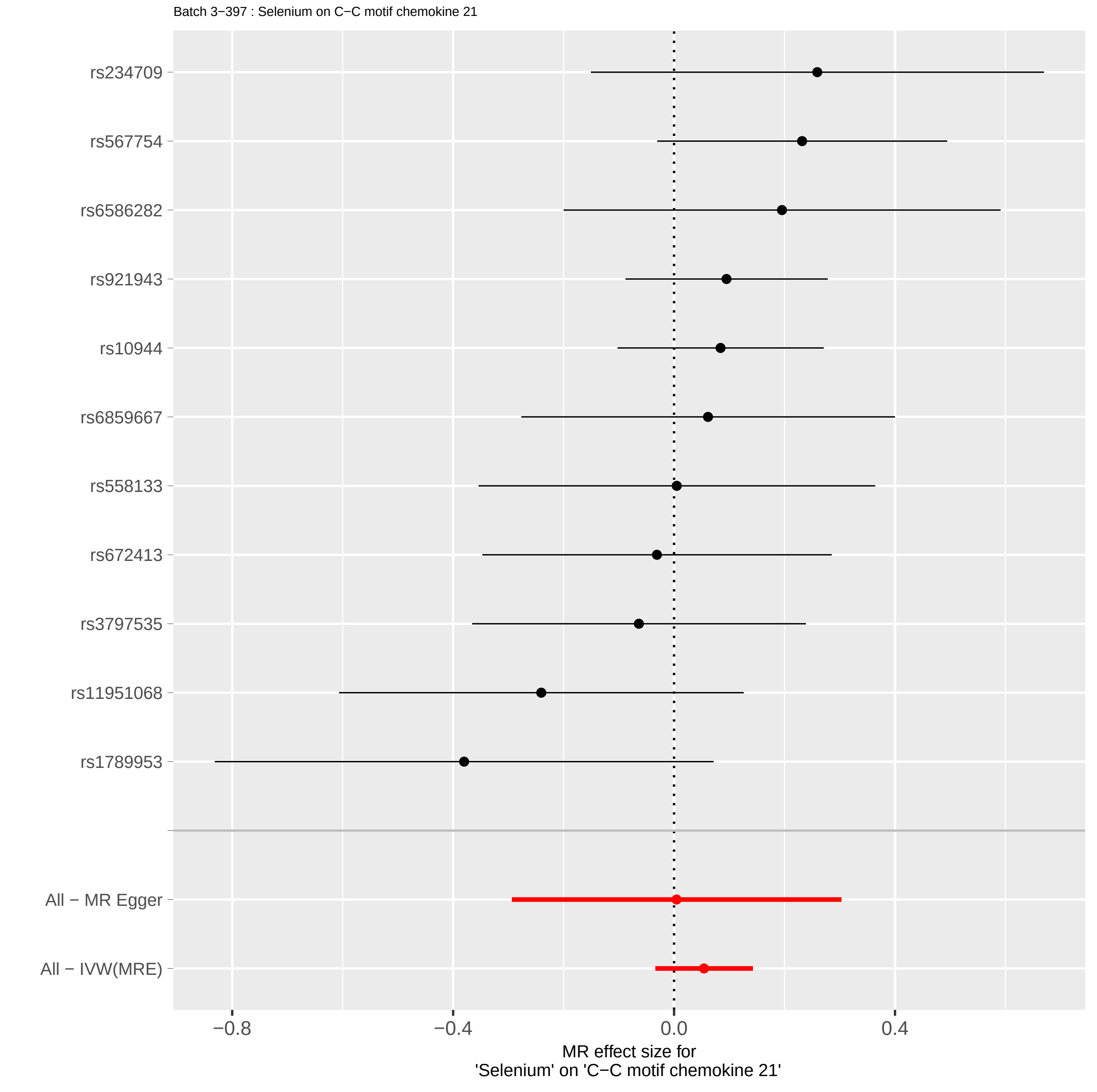
Batch 3−397 : Selenium on C−C motif chemokine 21
rs234709
rs567754
rs6586282
rs921943
rs10944
rs6859667
rs558133
rs672413
rs3797535
rs11951068
rs1789953
All − MR Egger
All − IVW(MRE)
−0.8
−0.4
0.0
0.4
MR effect size for
'Selenium' on 'C−C motif chemokine 21'

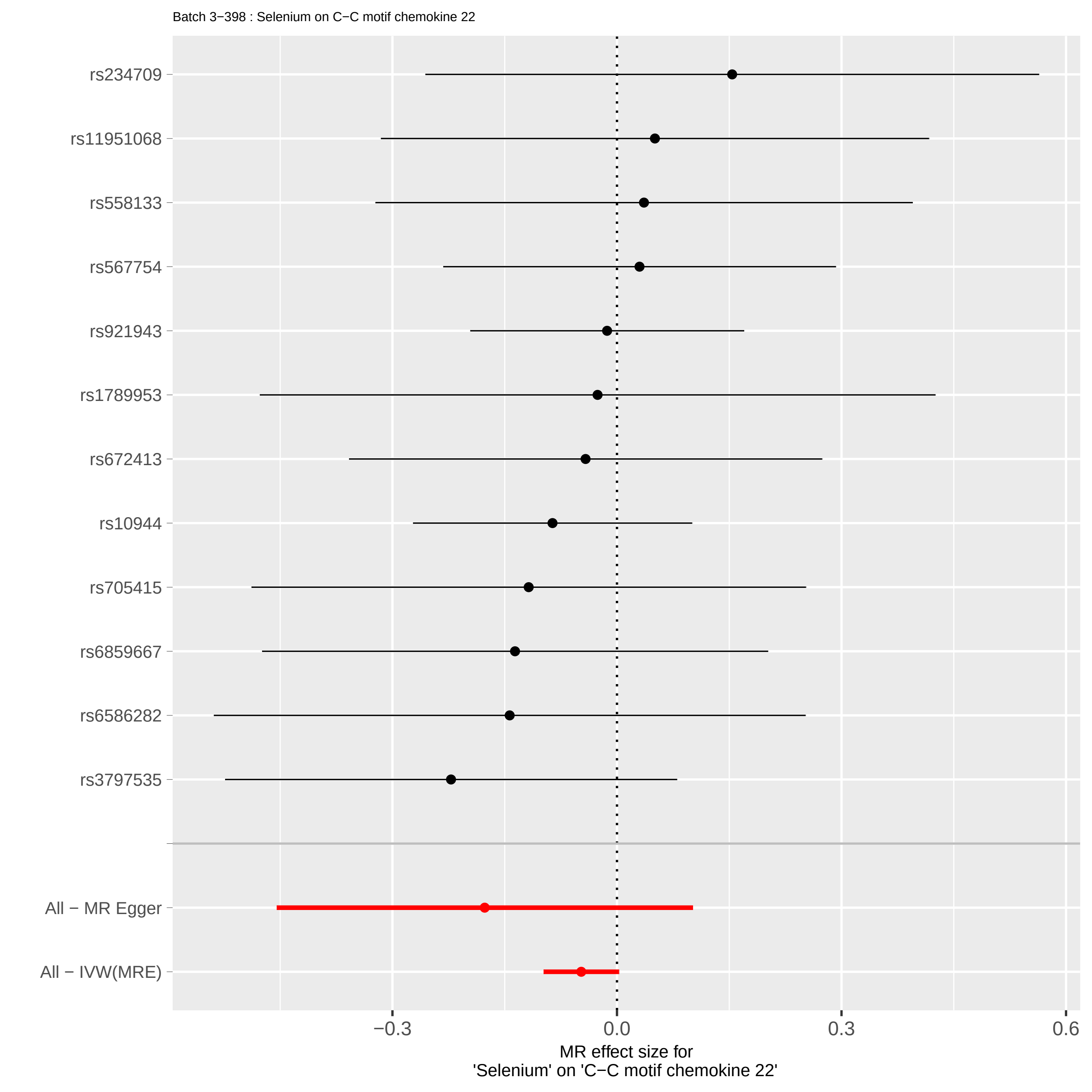
Batch 3−398 : Selenium on C−C motif chemokine 22
rs234709
rs11951068
rs558133
rs567754
rs921943
rs1789953
rs672413
rs10944
rs705415
rs6859667
rs6586282
rs3797535
All − MR Egger
All − IVW(MRE)
−0.3
0.0
0.3
0.6
MR effect size for
'Selenium' on 'C−C motif chemokine 22'

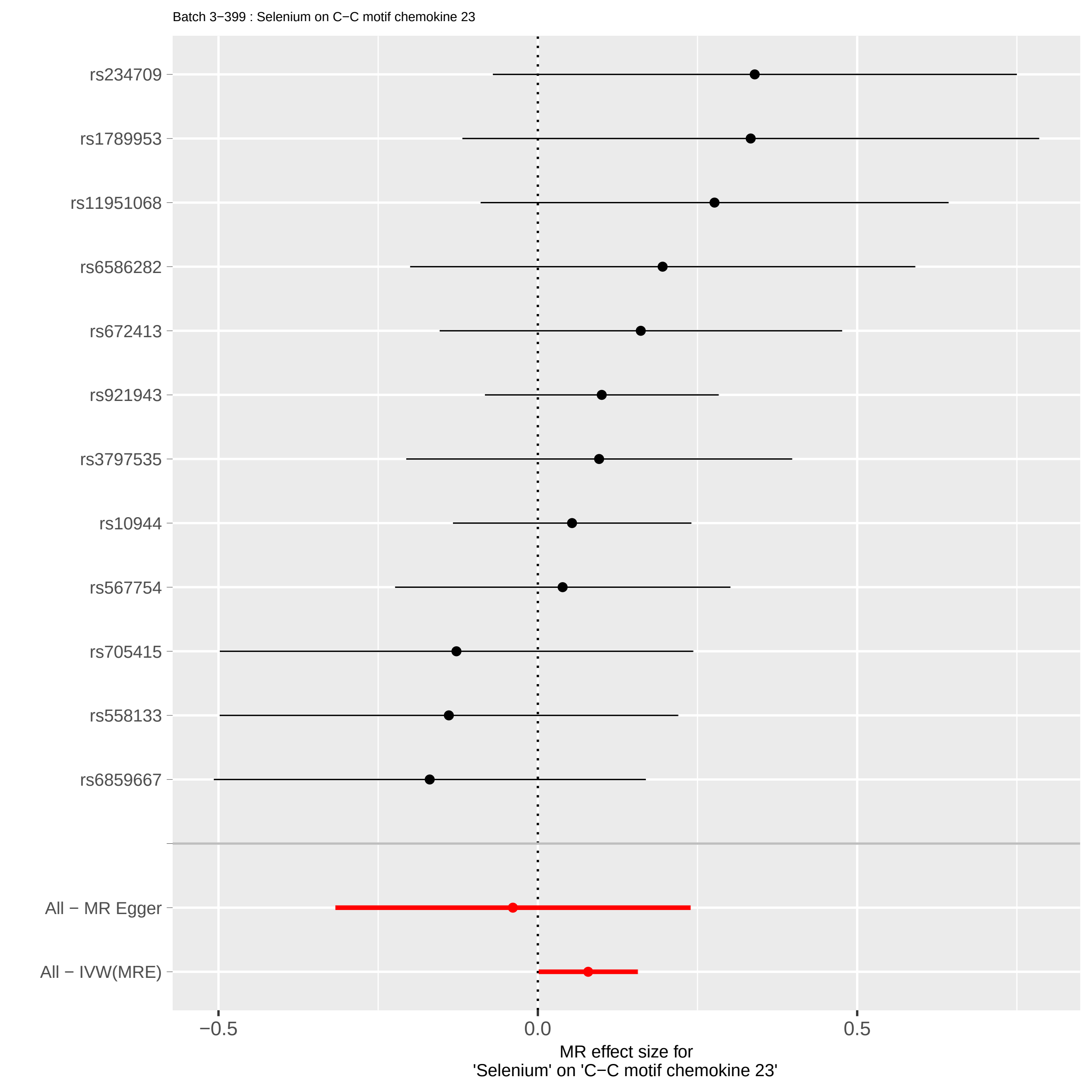
Batch 3−399 : Selenium on C−C motif chemokine 23
rs234709
rs1789953
rs11951068
rs6586282
rs672413
rs921943
rs3797535
rs10944
rs567754
rs705415
rs558133
rs6859667
All − MR Egger
All − IVW(MRE)
−0.5
0.0
0.5
MR effect size for
'Selenium' on 'C−C motif chemokine 23'

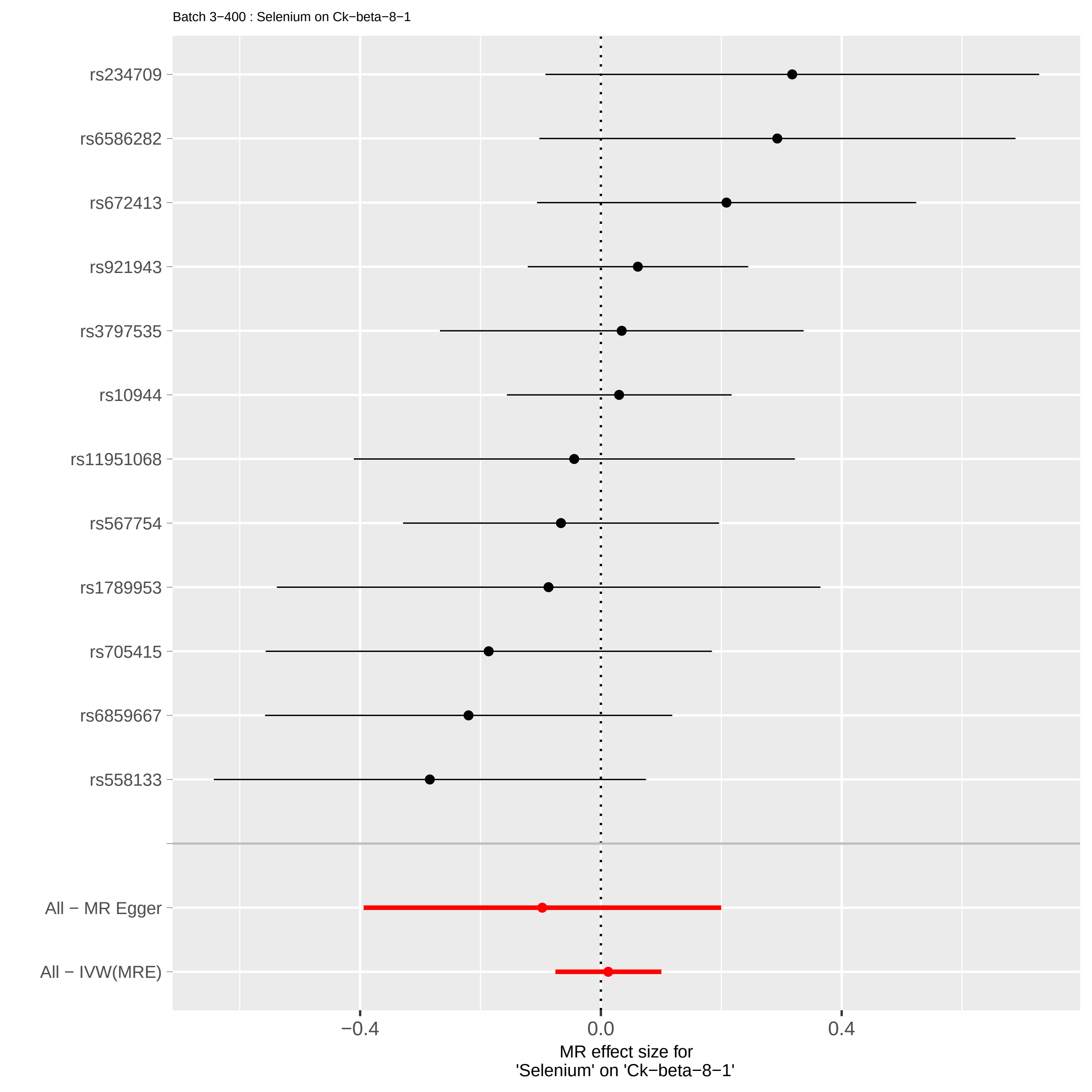

Batch 3−400 : Selenium on Ck−beta−8−1
rs234709
rs6586282
rs672413
rs921943
rs3797535
rs10944
rs11951068
rs567754
rs1789953
rs705415
rs6859667
rs558133
All − MR Egger
All − IVW(MRE)
−0.4
0.0
0.4
MR effect size for
'Selenium' on 'Ck−beta−8−1'

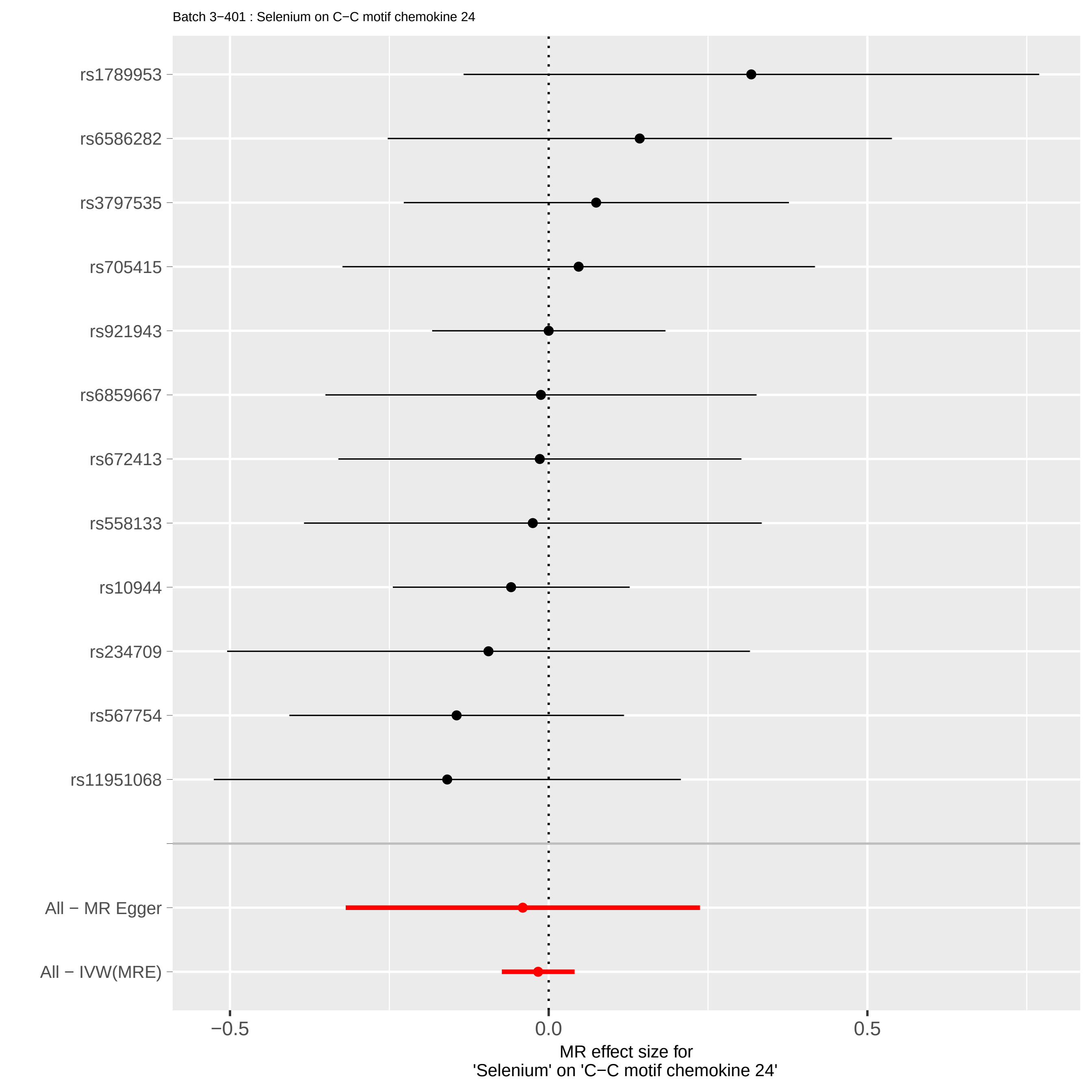
Batch 3−401 : Selenium on C−C motif chemokine 24
rs1789953
rs6586282
rs3797535
rs705415
rs921943
rs6859667
rs672413
rs558133
rs10944
rs234709
rs567754
rs11951068
All − MR Egger
All − IVW(MRE)
−0.5
0.0
0.5
MR effect size for
'Selenium' on 'C−C motif chemokine 24'

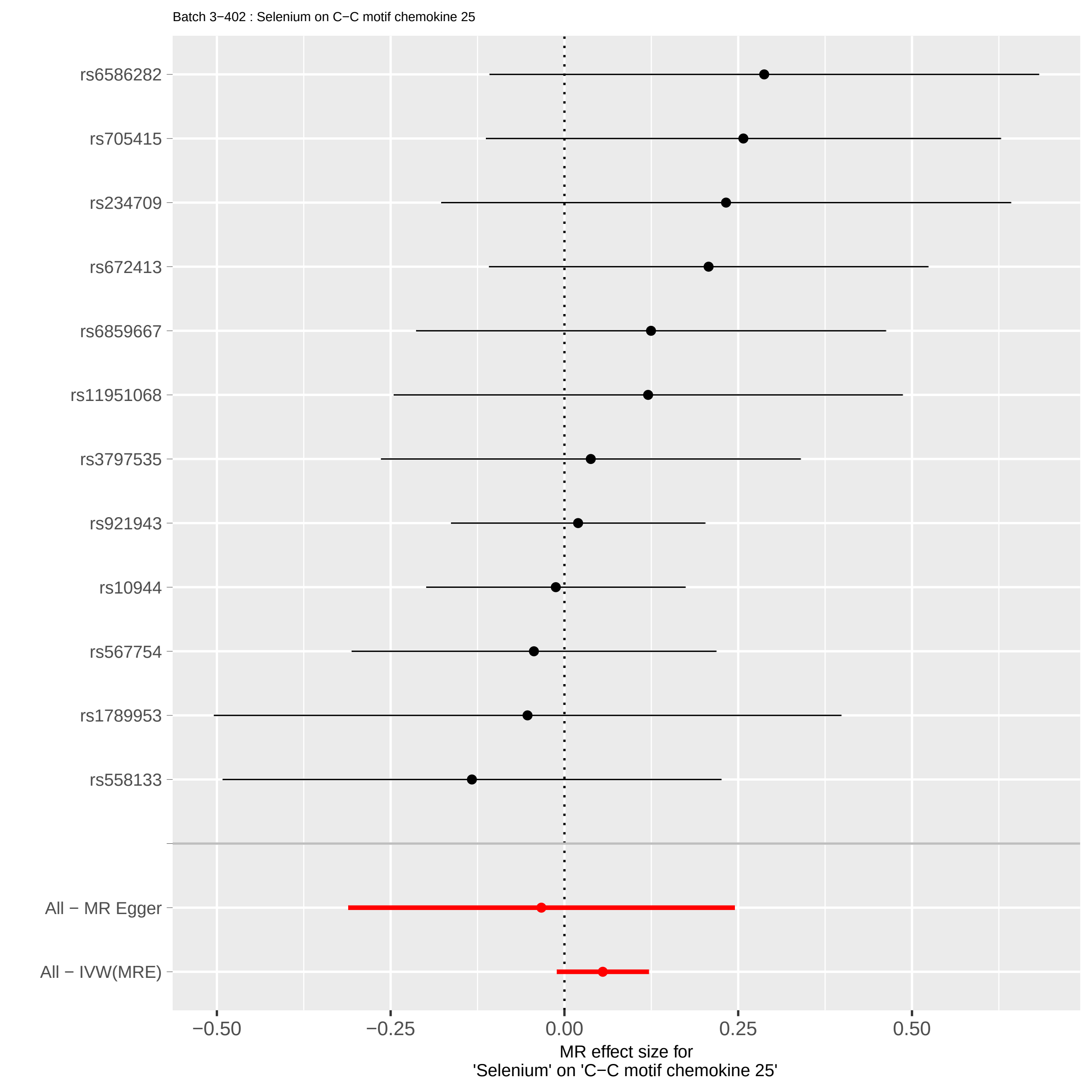

Batch 3−402 : Selenium on C−C motif chemokine 25
rs6586282
rs705415
rs234709
rs672413
rs6859667
rs11951068
rs3797535
rs921943
rs10944
rs567754
rs1789953
rs558133
All − MR Egger
All − IVW(MRE)
−0.50
−0.25
0.00
0.25
0.50
MR effect size for
'Selenium' on 'C−C motif chemokine 25'

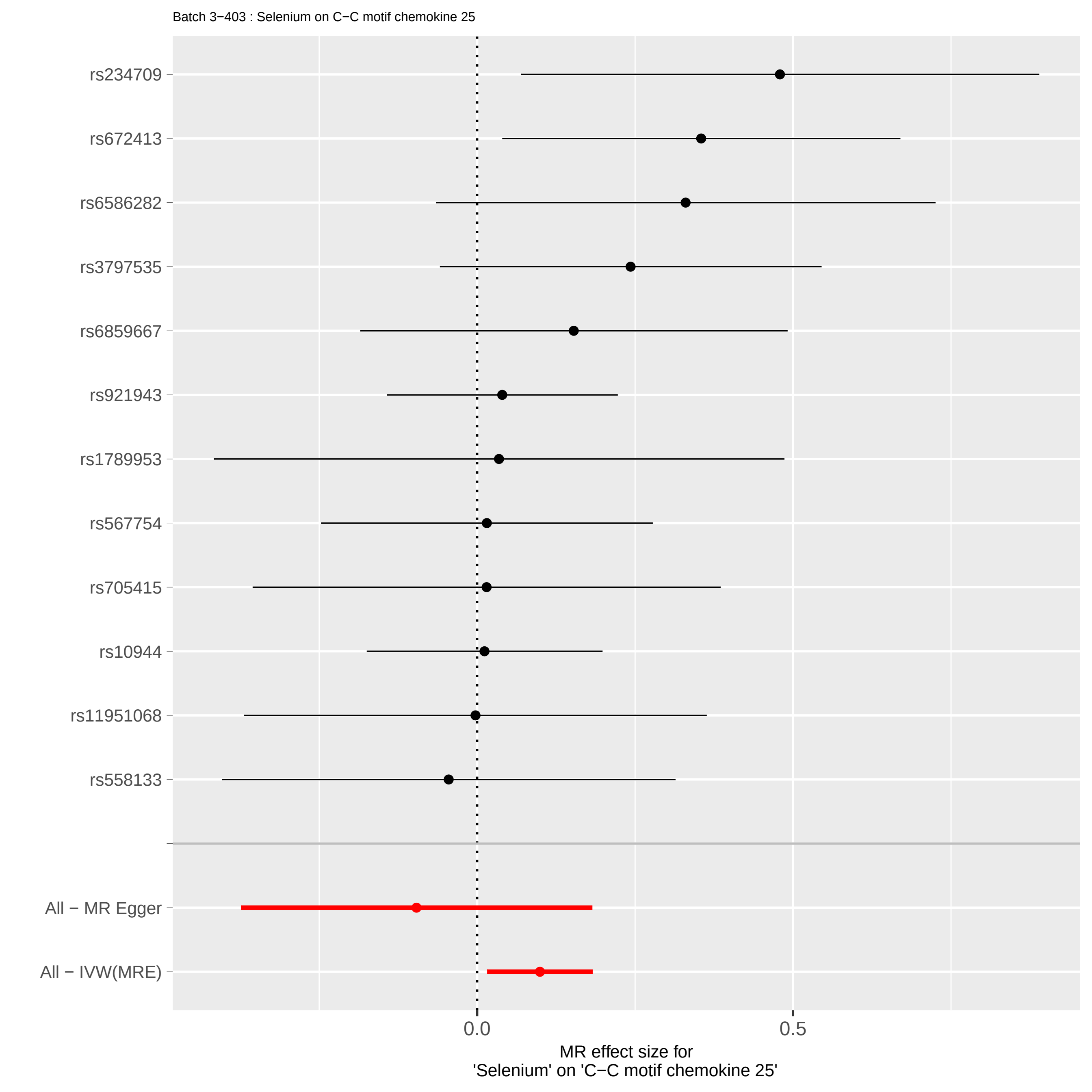
Batch 3−403 : Selenium on C−C motif chemokine 25
rs234709
rs672413
rs6586282
rs3797535
rs6859667
rs921943
rs1789953
rs567754
rs705415
rs10944
rs11951068
rs558133
All − MR Egger
All − IVW(MRE)
0.0
0.5
MR effect size for
'Selenium' on 'C−C motif chemokine 25'

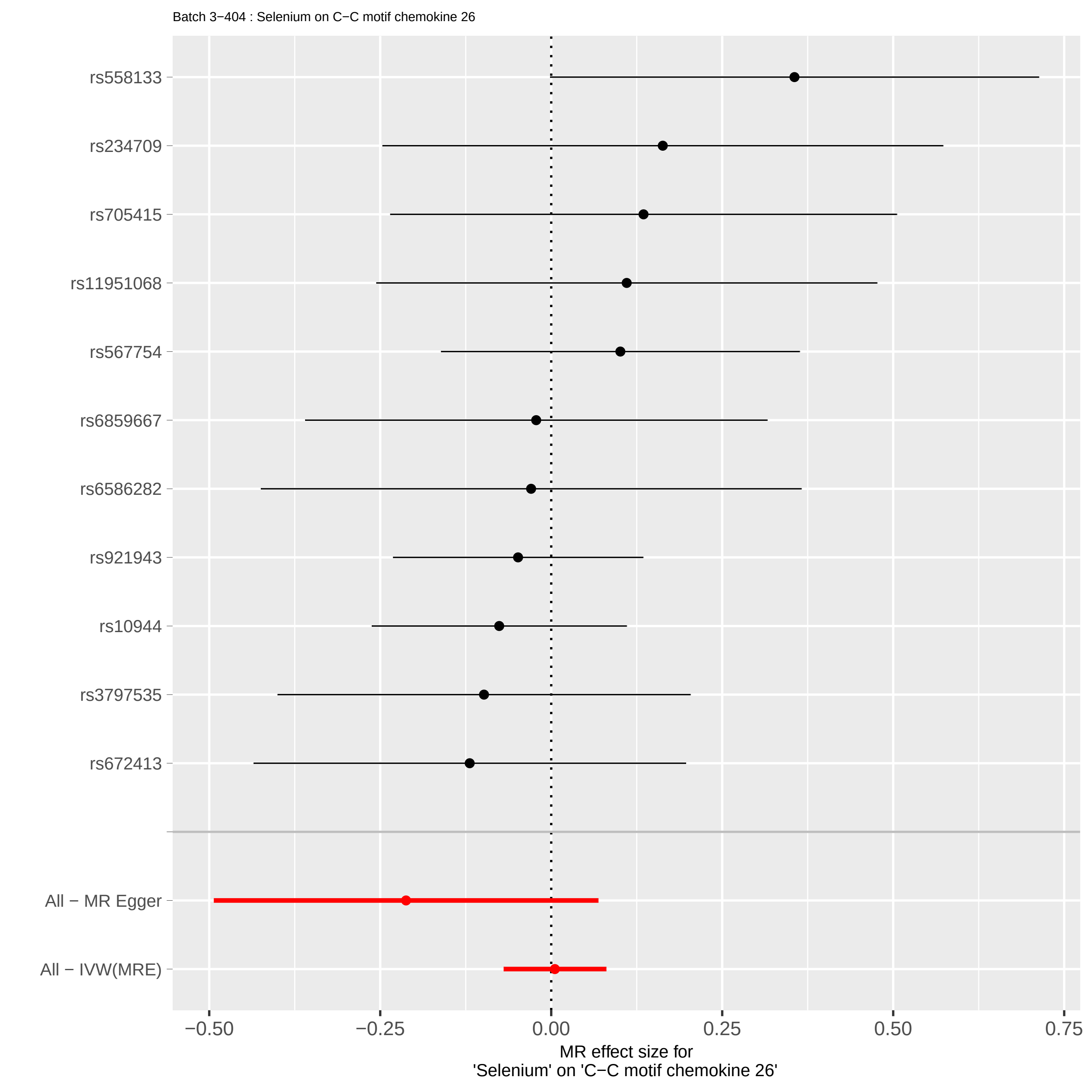
Batch 3−404 : Selenium on C−C motif chemokine 26
rs558133
rs234709
rs705415
rs11951068
rs567754
rs6859667
rs6586282
rs921943
rs10944
rs3797535
rs672413
All − MR Egger
All − IVW(MRE)
−0.50
−0.25
0.00
0.25
0.50
0.75
MR effect size for
'Selenium' on 'C−C motif chemokine 26'

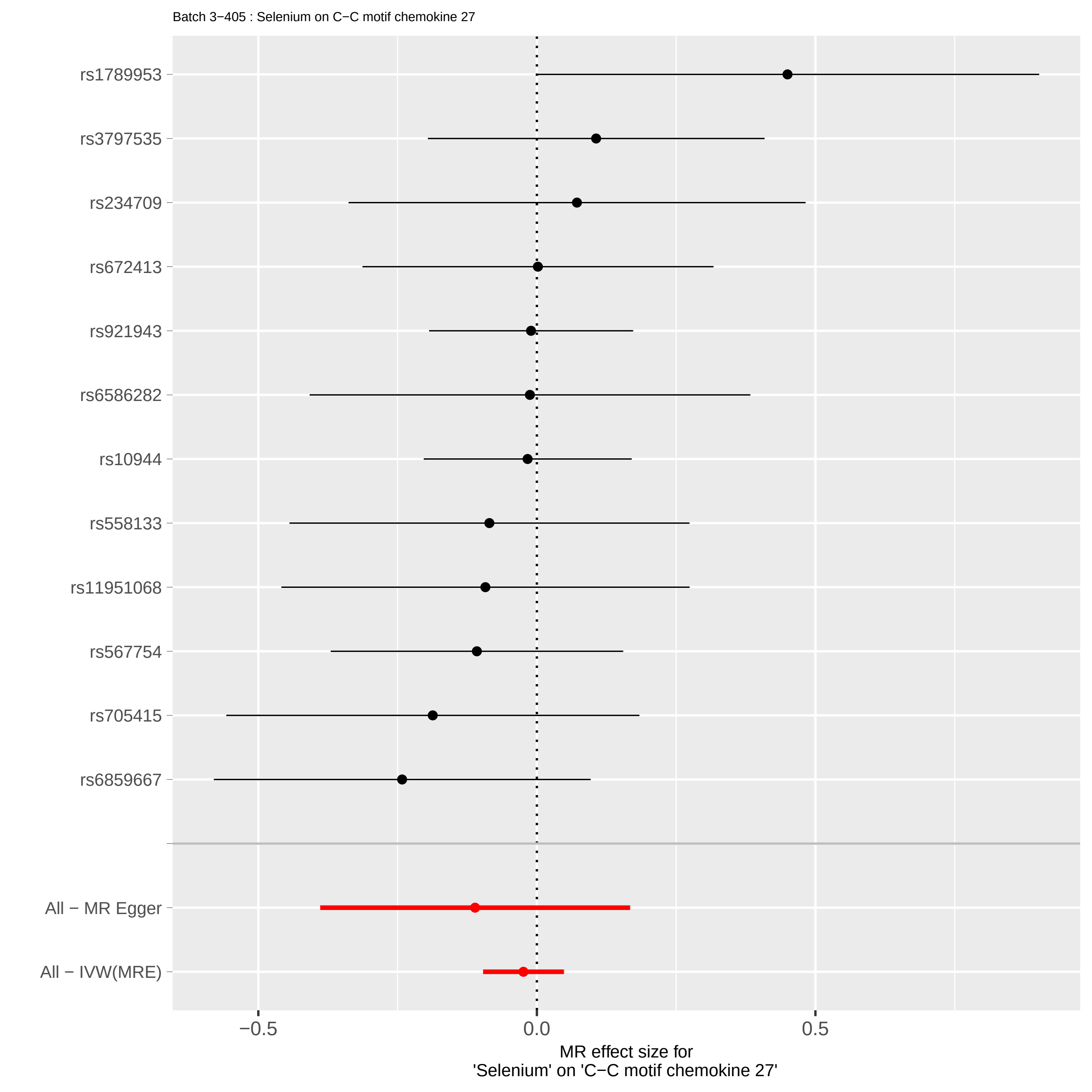

Batch 3−405 : Selenium on C−C motif chemokine 27
rs1789953
rs3797535
rs234709
rs672413
rs921943
rs6586282
rs10944
rs558133
rs11951068
rs567754
rs705415
rs6859667
All − MR Egger
All − IVW(MRE)
−0.5
0.0
0.5
MR effect size for
'Selenium' on 'C−C motif chemokine 27'

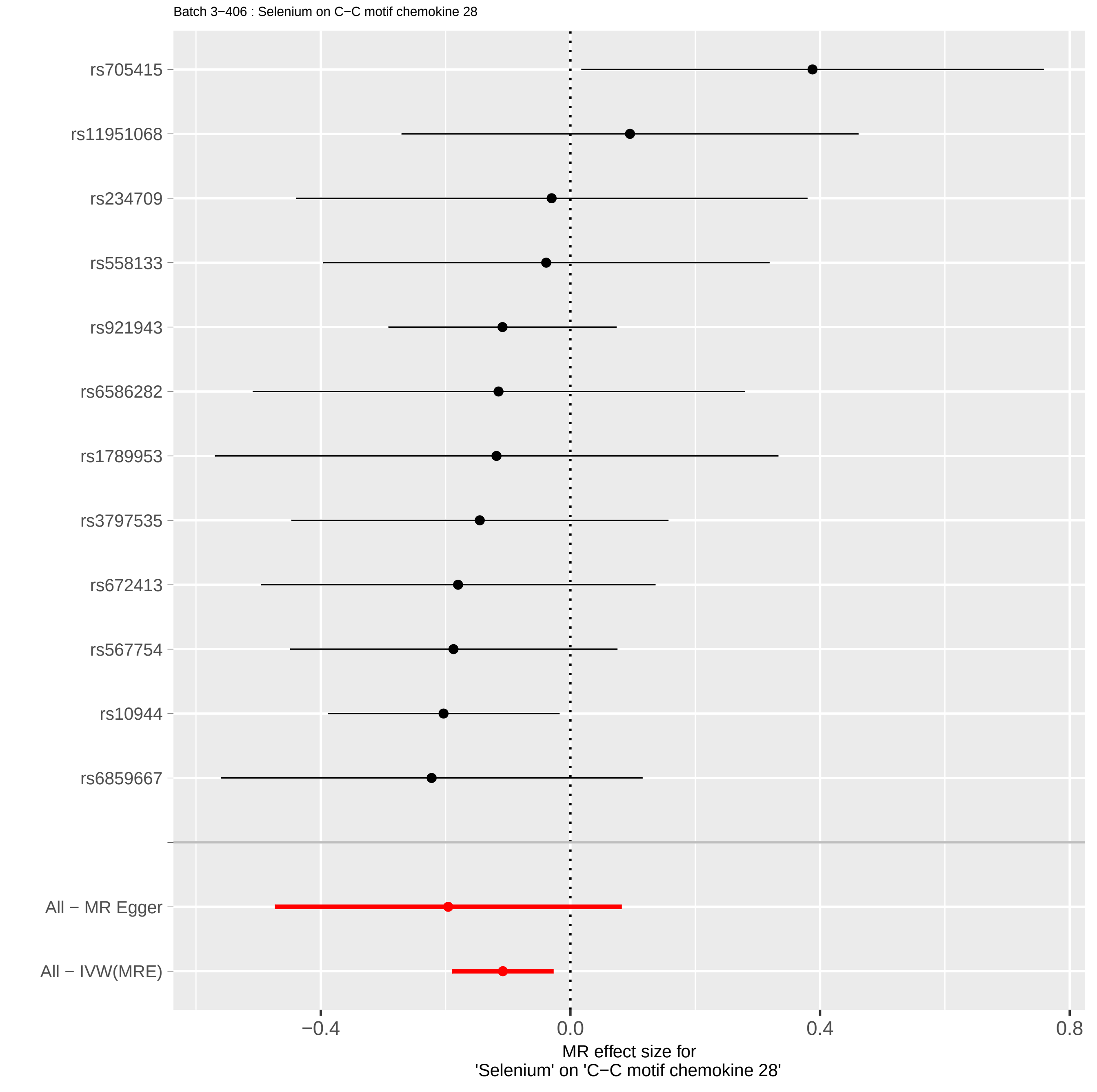
Batch 3−406 : Selenium on C−C motif chemokine 28
rs705415
rs11951068
rs234709
rs558133
rs921943
rs6586282
rs1789953
rs3797535
rs672413
rs567754
rs10944
rs6859667
All − MR Egger
All − IVW(MRE)
−0.4
0.0
0.4
0.8
MR effect size for
'Selenium' on 'C−C motif chemokine 28'

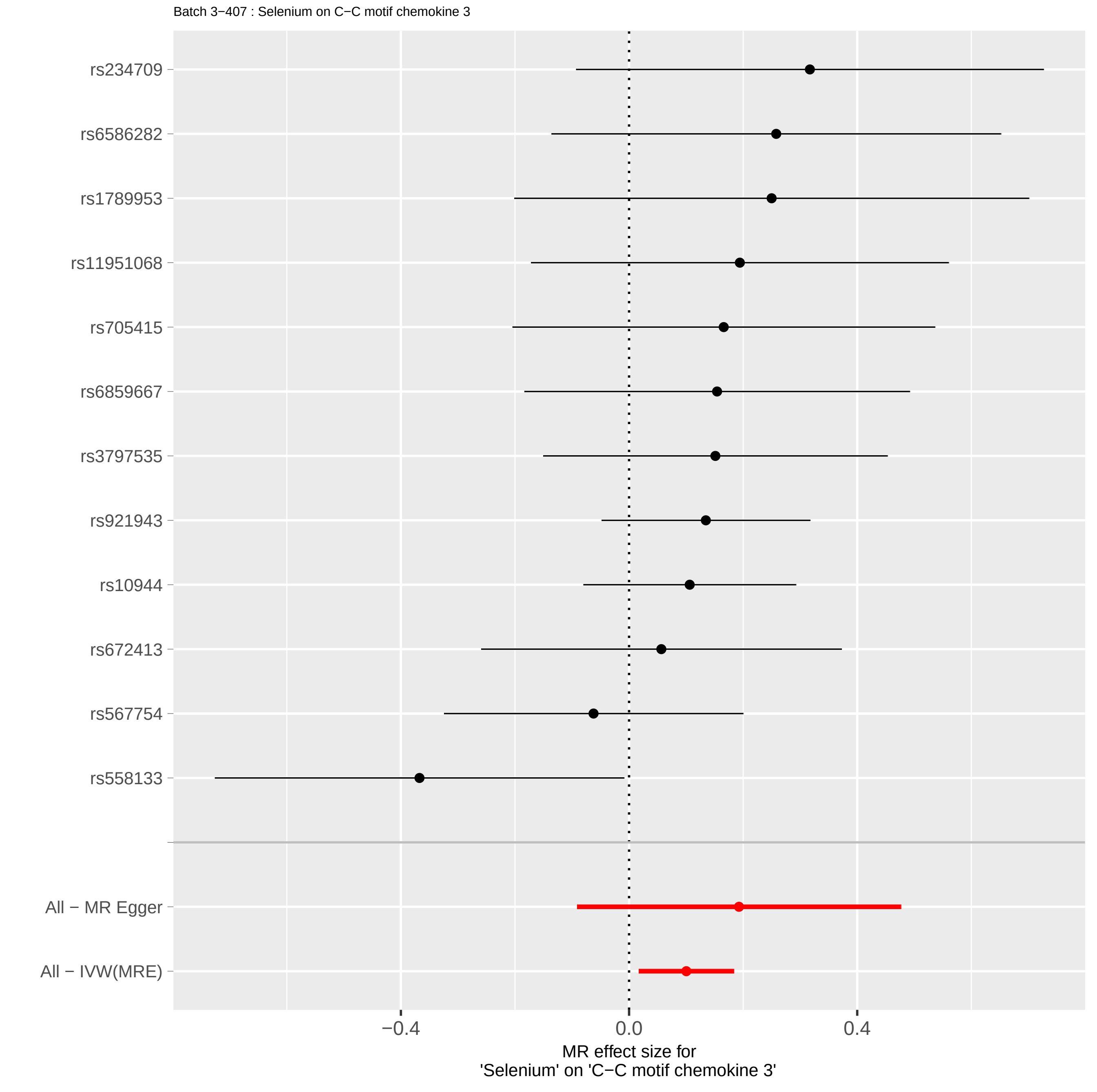

Batch 3−407 : Selenium on C−C motif chemokine 3
rs234709
rs6586282
rs1789953
rs11951068
rs705415
rs6859667
rs3797535
rs921943
rs10944
rs672413
rs567754
rs558133
All − MR Egger
All − IVW(MRE)
−0.4
0.0
0.4
MR effect size for
'Selenium' on 'C−C motif chemokine 3'

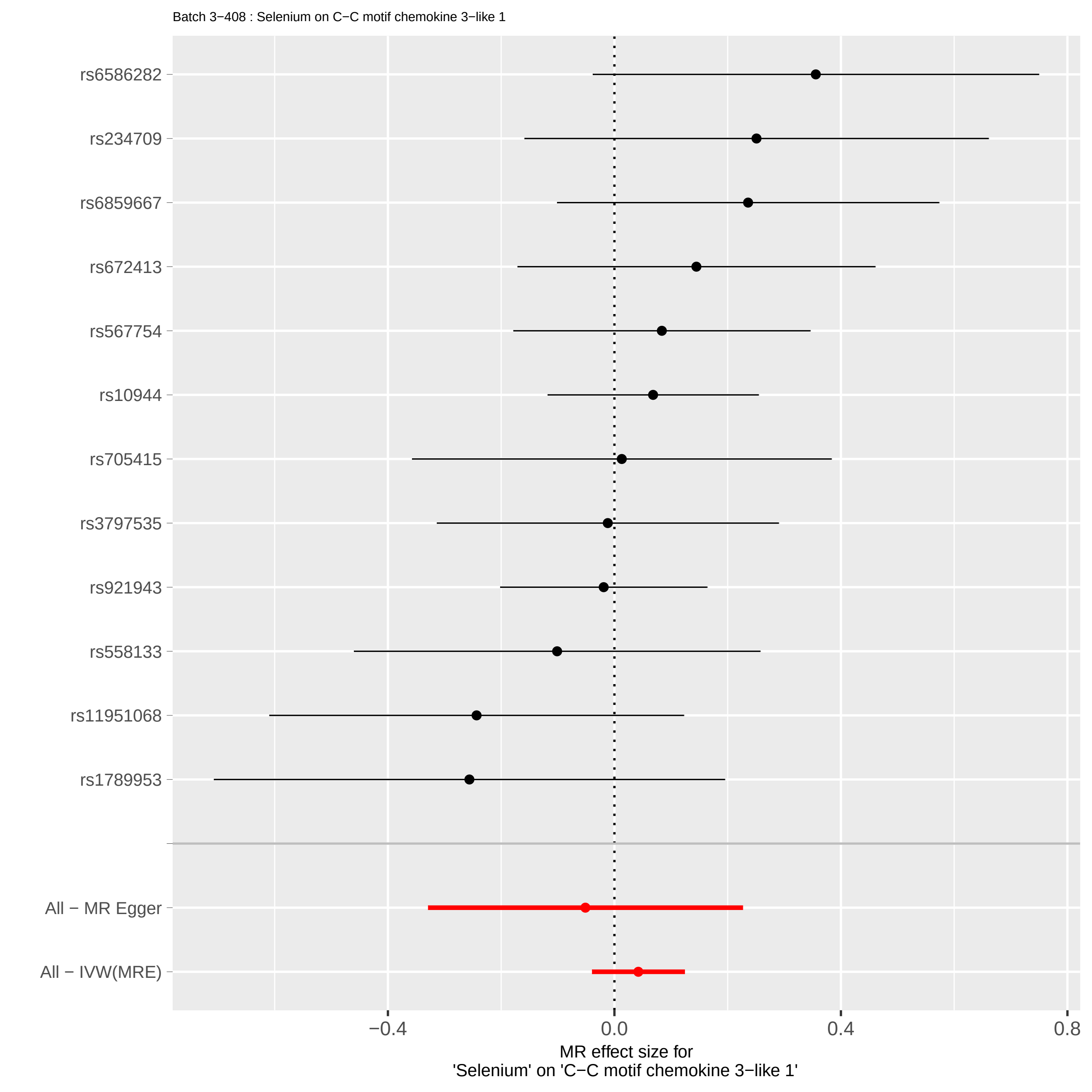

Batch 3−408 : Selenium on C−C motif chemokine 3−like 1
rs6586282
rs234709
rs6859667
rs672413
rs567754
rs10944
rs705415
rs3797535
rs921943
rs558133
rs11951068
rs1789953
All − MR Egger
All − IVW(MRE)
−0.4
0.0
0.4
0.8
MR effect size for
'Selenium' on 'C−C motif chemokine 3−like 1'

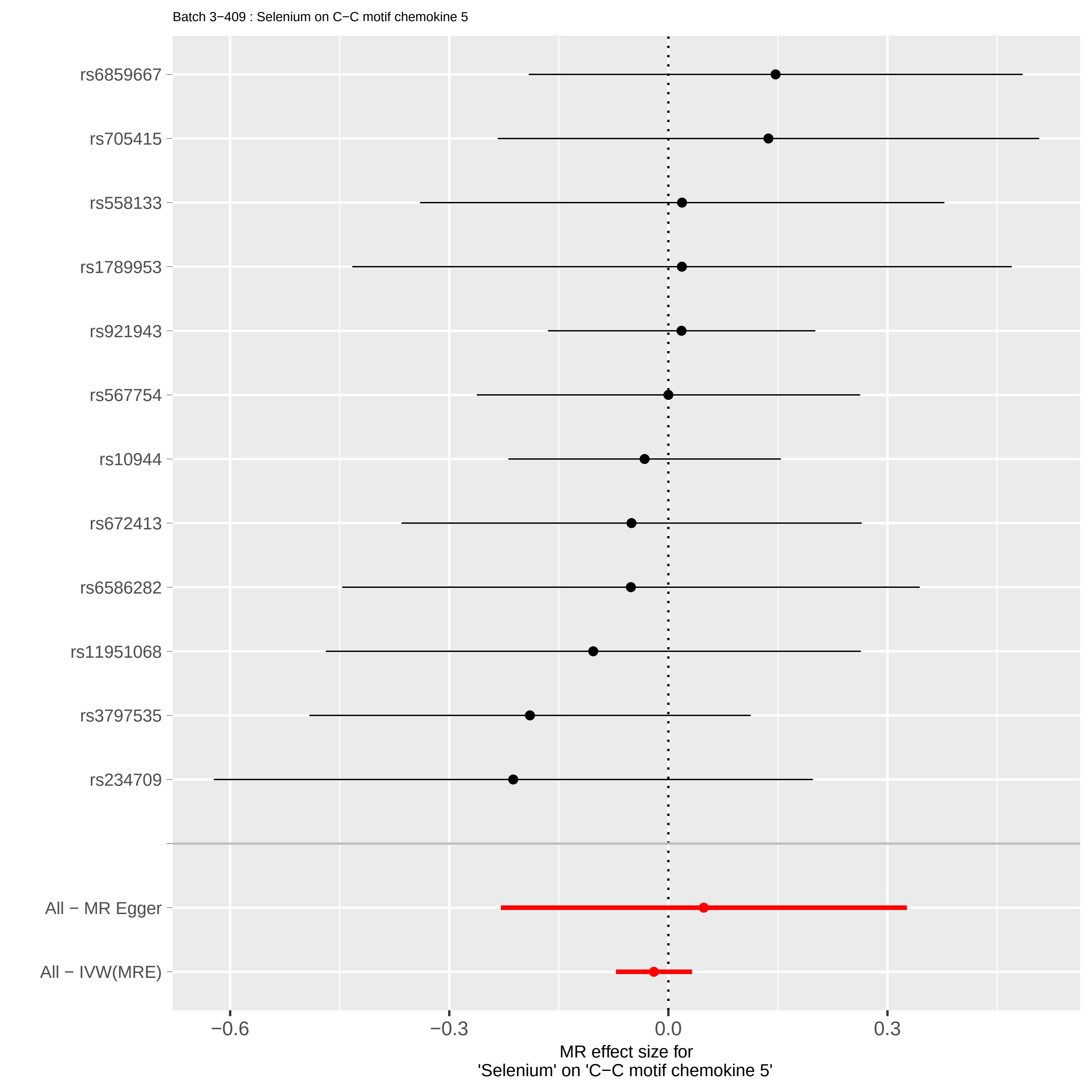

Batch 3−409 : Selenium on C−C motif chemokine 5
rs6859667
rs705415
rs558133
rs1789953
rs921943
rs567754
rs10944
rs672413
rs6586282
rs11951068
rs3797535
rs234709
All − MR Egger
All − IVW(MRE)
−0.6
−0.3
0.0
0.3
MR effect size for
'Selenium' on 'C−C motif chemokine 5'

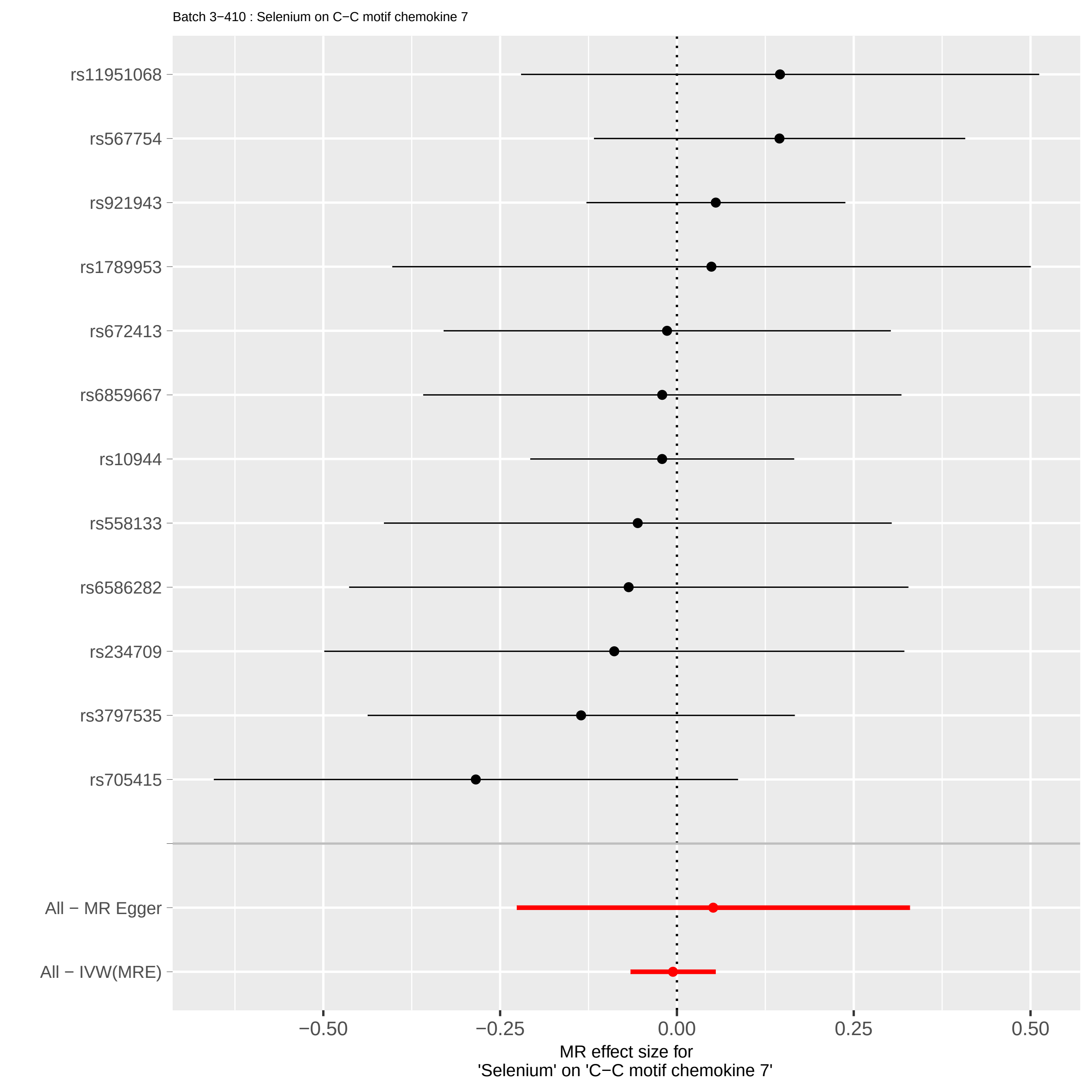

Batch 3−410 : Selenium on C−C motif chemokine 7
rs11951068
rs567754
rs921943
rs1789953
rs672413
rs6859667
rs10944
rs558133
rs6586282
rs234709
rs3797535
rs705415
All − MR Egger
All − IVW(MRE)
−0.50
−0.25
0.00
0.25
0.50
MR effect size for
'Selenium' on 'C−C motif chemokine 7'

Batch 3−411 : Selenium on C−C motif chemokine 8

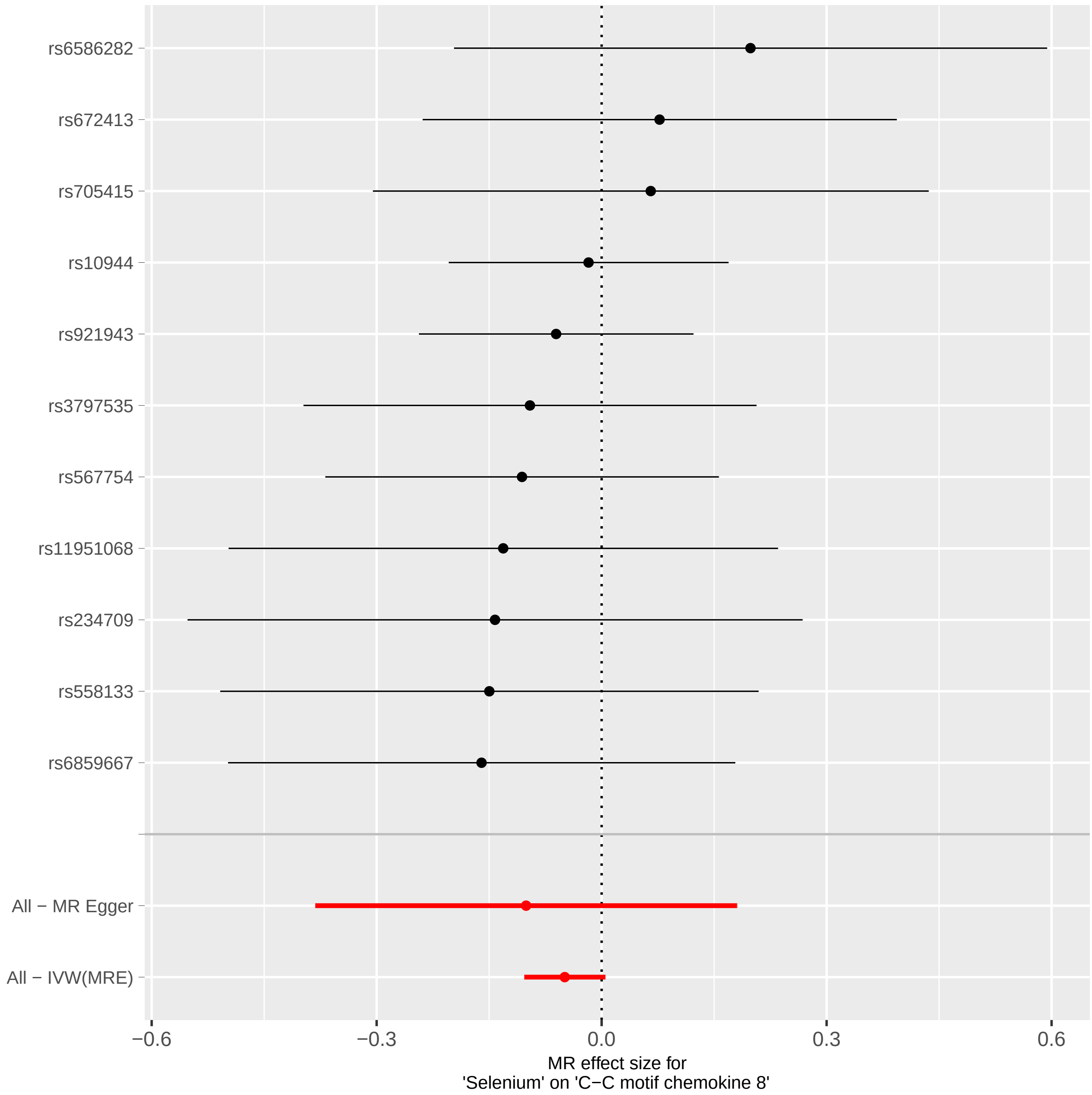

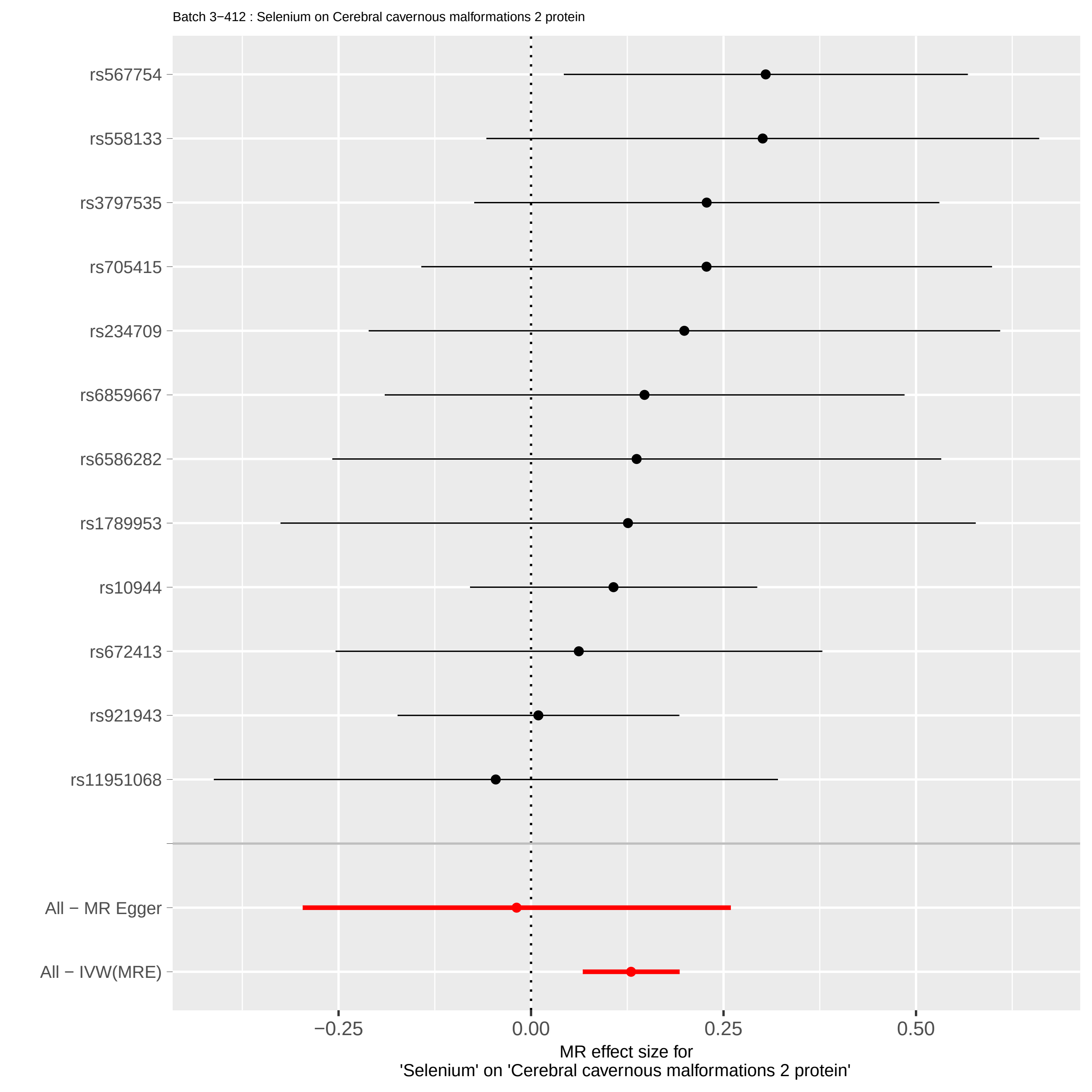
Batch 3−412 : Selenium on Cerebral cavernous malformations 2 protein
rs567754
rs558133
rs3797535
rs705415
rs234709
rs6859667
rs6586282
rs1789953
rs10944
rs672413
rs921943
rs11951068
All − MR Egger
All − IVW(MRE)
−0.25
0.00
0.25
0.50
MR effect size for
'Selenium' on 'Cerebral cavernous malformations 2 protein'

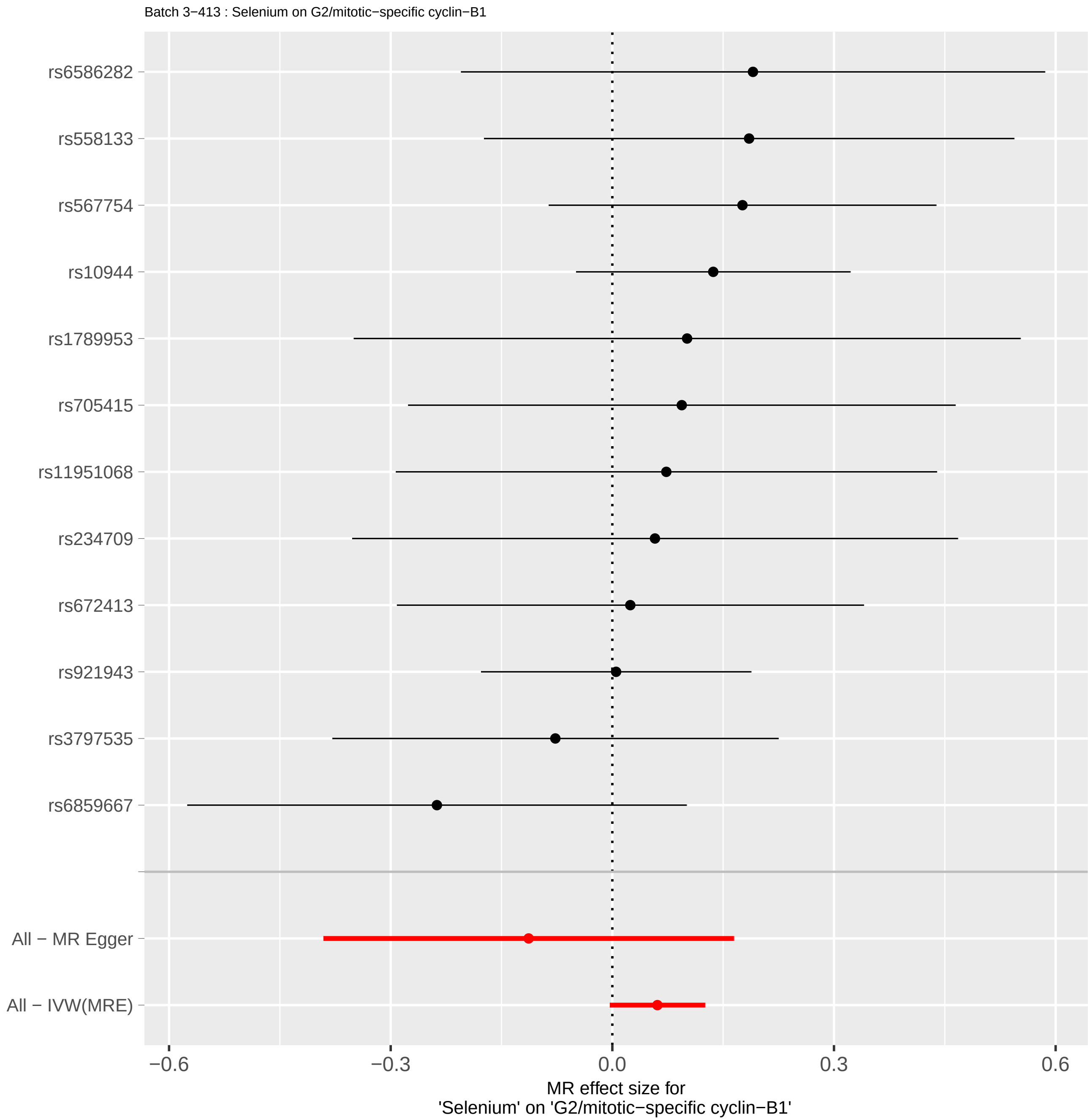
Batch 3−413 : Selenium on G2/mitotic−specific cyclin−B1
rs6586282
rs558133
rs567754
rs10944
rs1789953
rs705415
rs11951068
rs234709
rs672413
rs921943
rs3797535
rs6859667
All − MR Egger
All − IVW(MRE)
−0.6
−0.3
0.0
0.3
0.6
MR effect size for
'Selenium' on 'G2/mitotic−specific cyclin−B1'

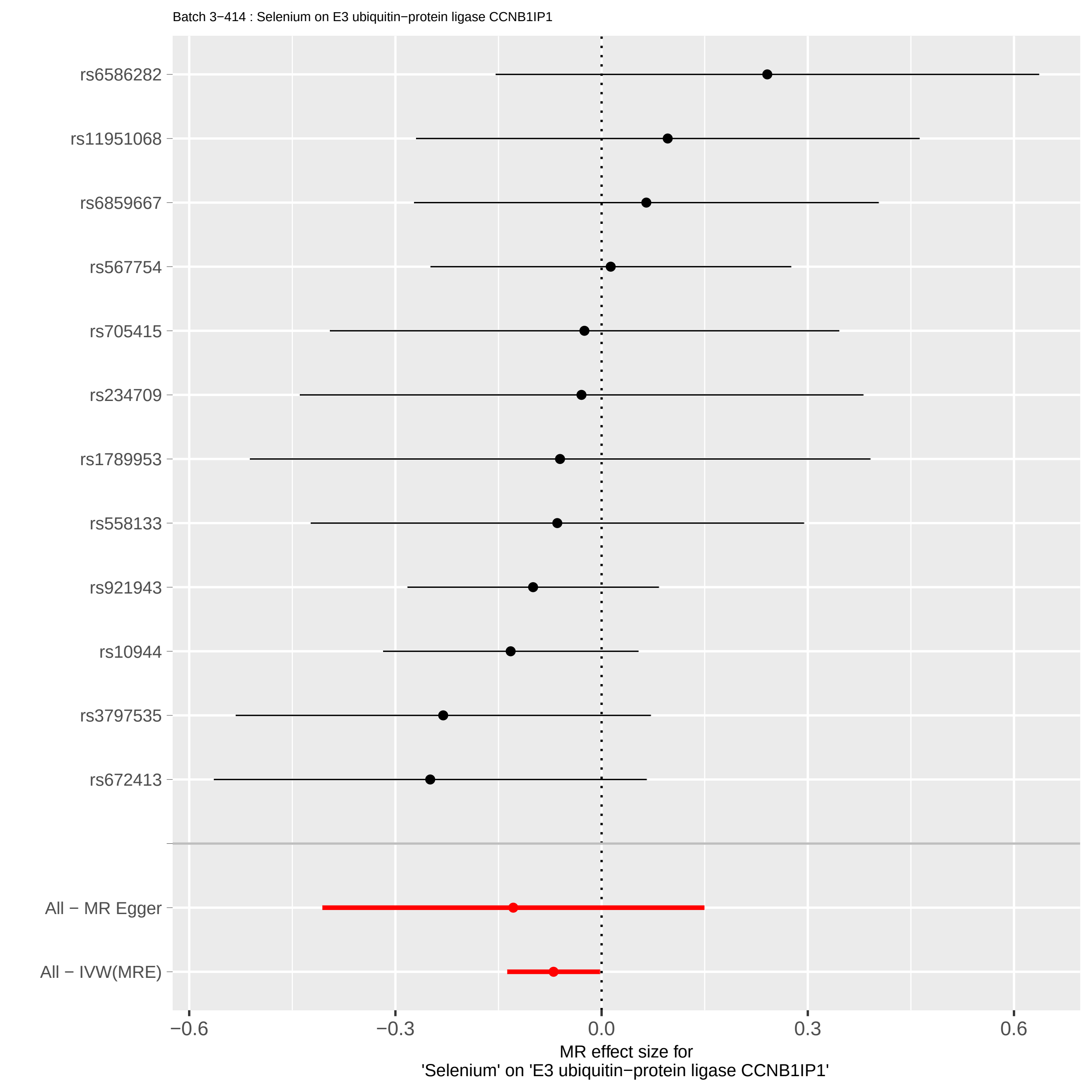
Batch 3−414 : Selenium on E3 ubiquitin−protein ligase CCNB1IP1
rs6586282
rs11951068
rs6859667
rs567754
rs705415
rs234709
rs1789953
rs558133
rs921943
rs10944
rs3797535
rs672413
All − MR Egger
All − IVW(MRE)
−0.6
−0.3
0.0
0.3
0.6
MR effect size for
'Selenium' on 'E3 ubiquitin−protein ligase CCNB1IP1'

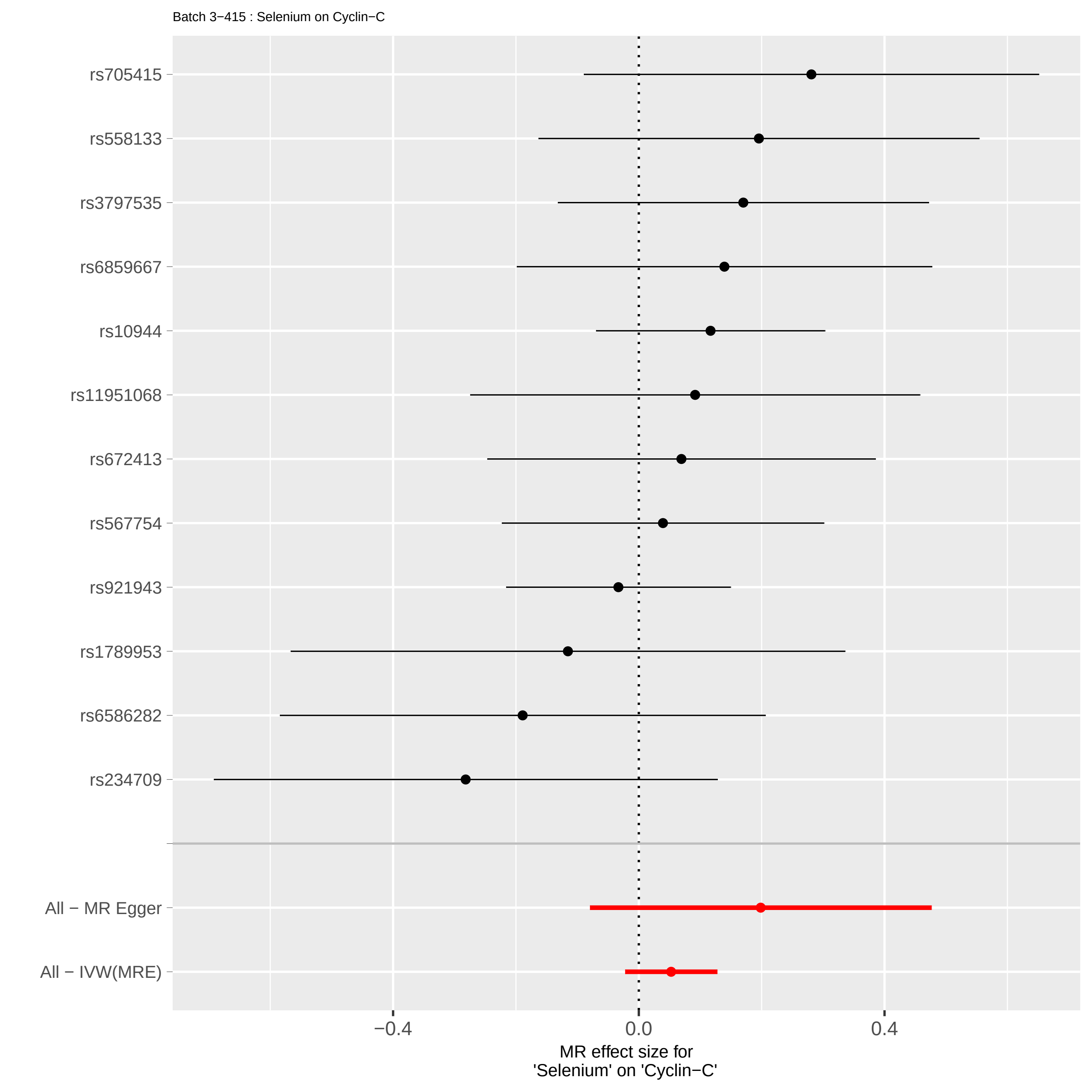

Batch 3−415 : Selenium on Cyclin−C
rs705415
rs558133
rs3797535
rs6859667
rs10944
rs11951068
rs672413
rs567754
rs921943
rs1789953
rs6586282
rs234709
All − MR Egger
All − IVW(MRE)
−0.4
0.0
0.4
MR effect size for
'Selenium' on 'Cyclin−C'

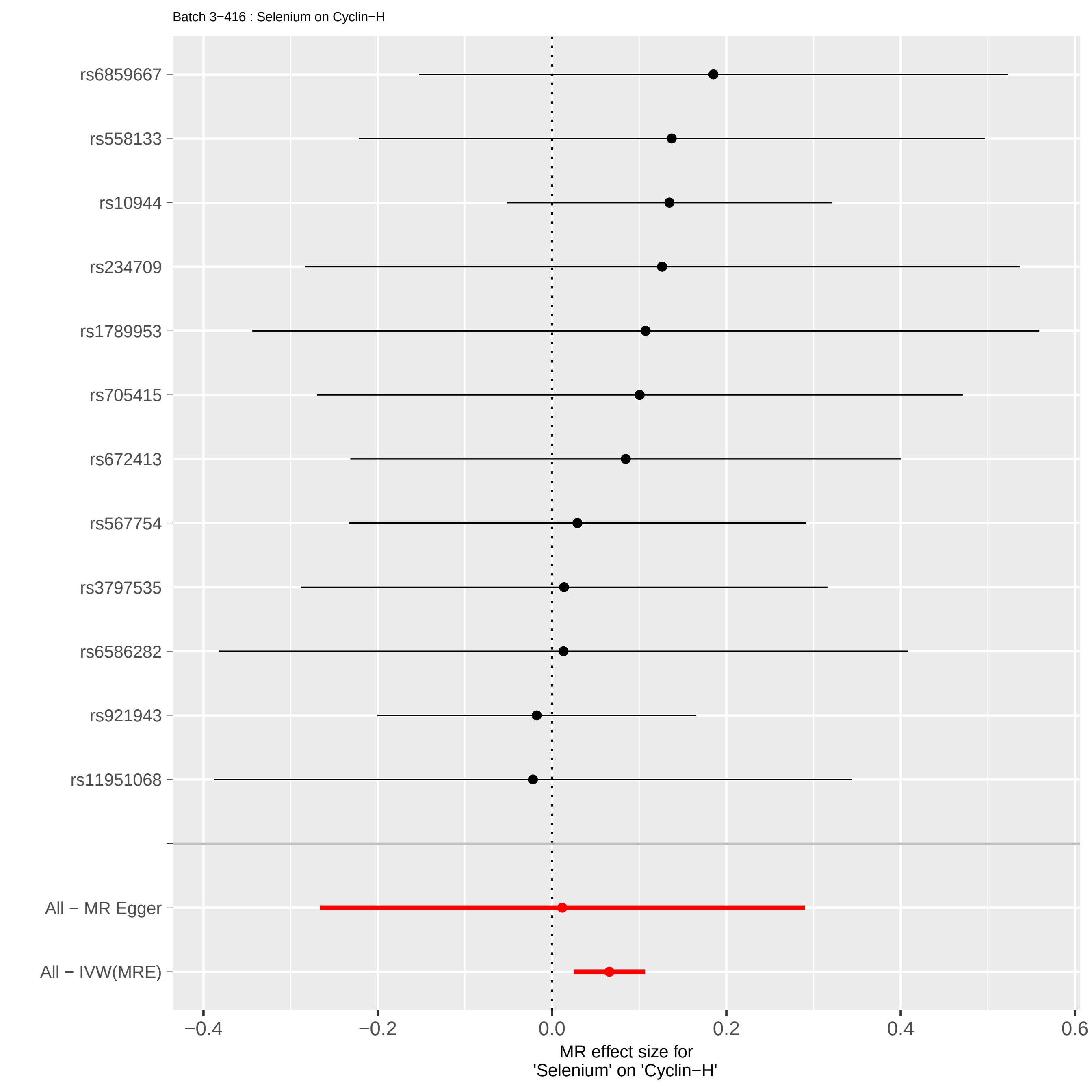

Batch 3−416 : Selenium on Cyclin−H
rs6859667
rs558133
rs10944
rs234709
rs1789953
rs705415
rs672413
rs567754
rs3797535
rs6586282
rs921943
rs11951068
All − MR Egger
All − IVW(MRE)
−0.4
−0.2
0.0
0.2
0.4
0.6
MR effect size for
'Selenium' on 'Cyclin−H'

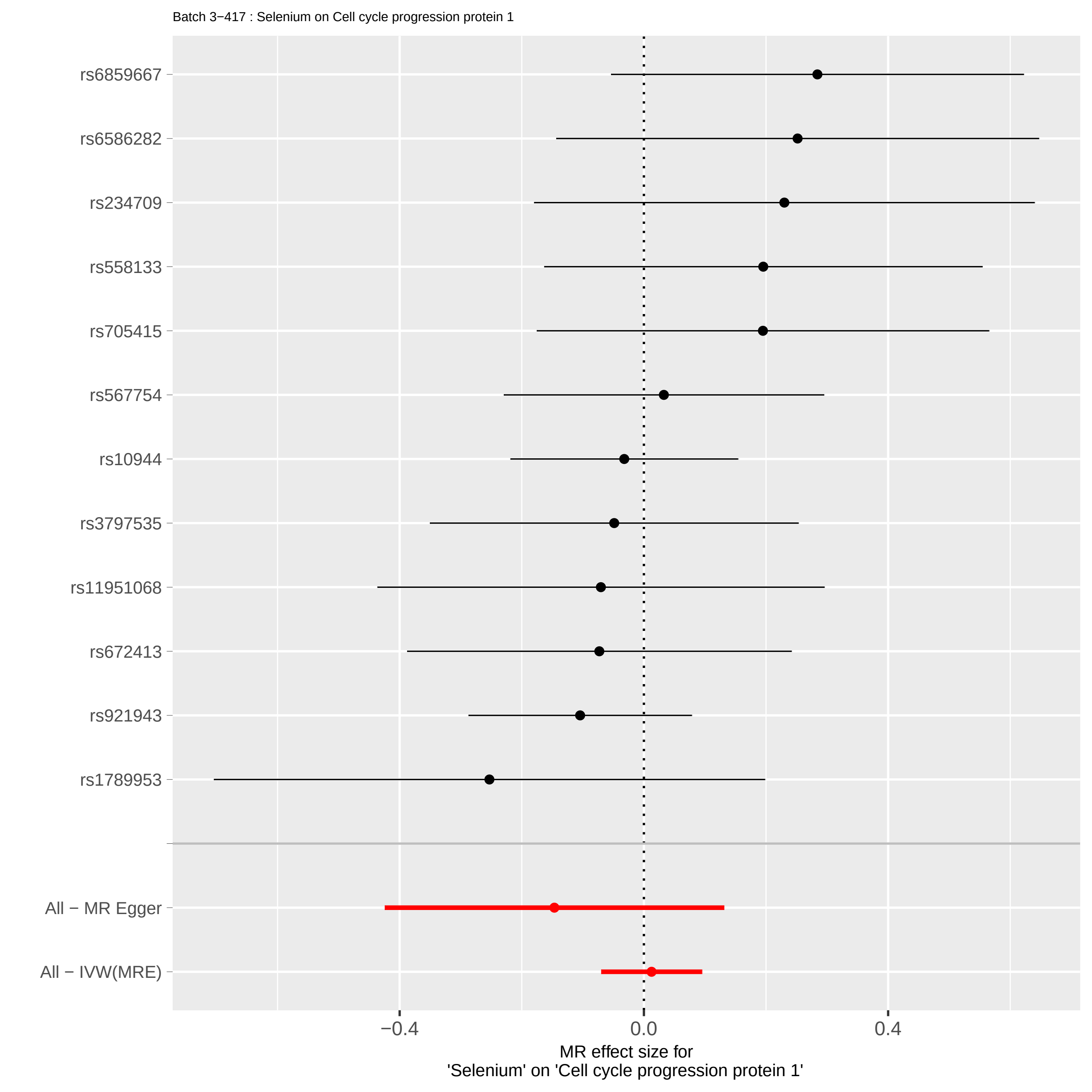
Batch 3−417 : Selenium on Cell cycle progression protein 1
rs6859667
rs6586282
rs234709
rs558133
rs705415
rs567754
rs10944
rs3797535
rs11951068
rs672413
rs921943
rs1789953
All − MR Egger
All − IVW(MRE)
−0.4
0.0
0.4
MR effect size for
'Selenium' on 'Cell cycle progression protein 1'

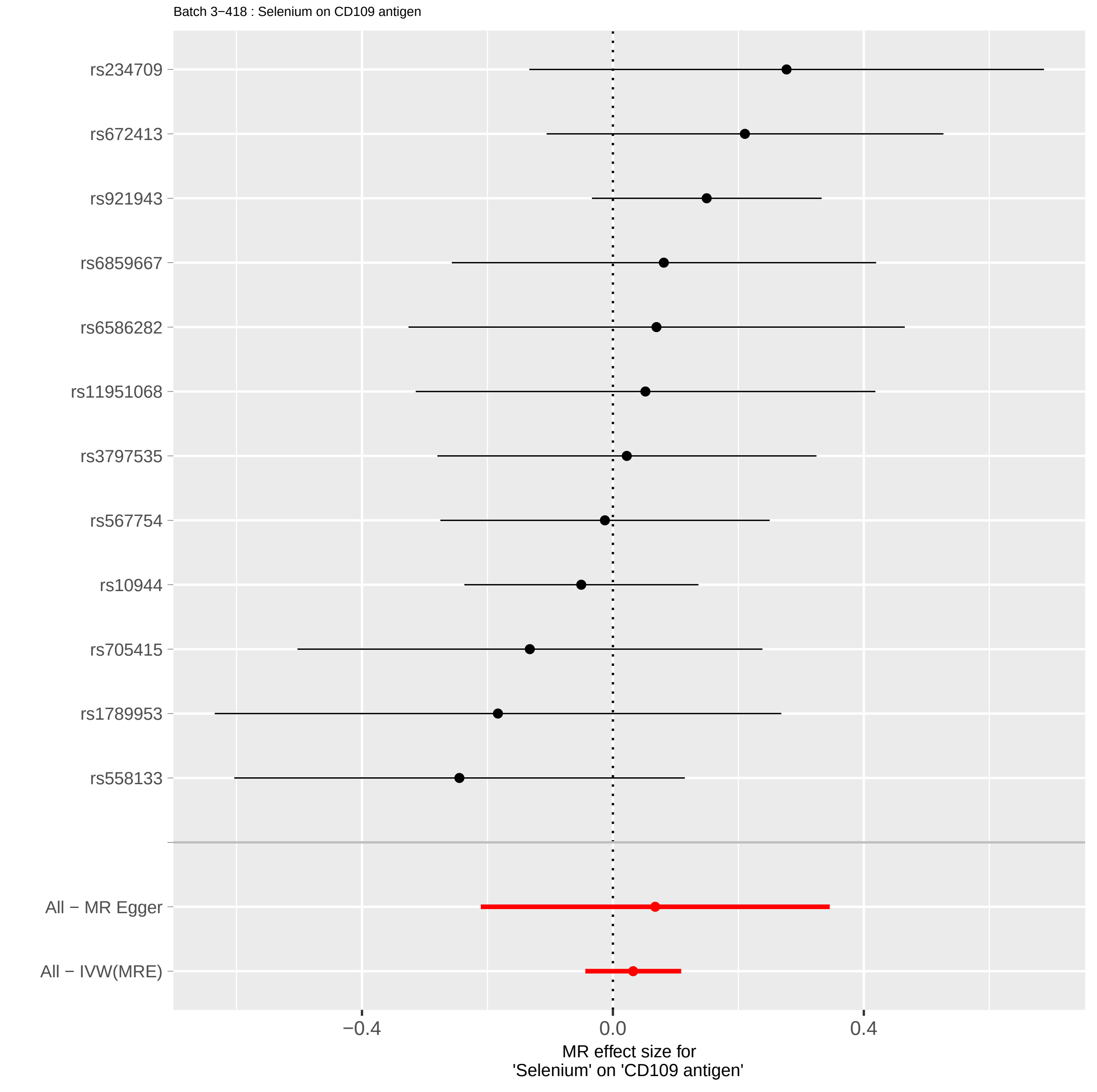

Batch 3−418 : Selenium on CD109 antigen
rs234709
rs672413
rs921943
rs6859667
rs6586282
rs11951068
rs3797535
rs567754
rs10944
rs705415
rs1789953
rs558133
All − MR Egger
All − IVW(MRE)
−0.4
0.0
0.4
MR effect size for
'Selenium' on 'CD109 antigen'

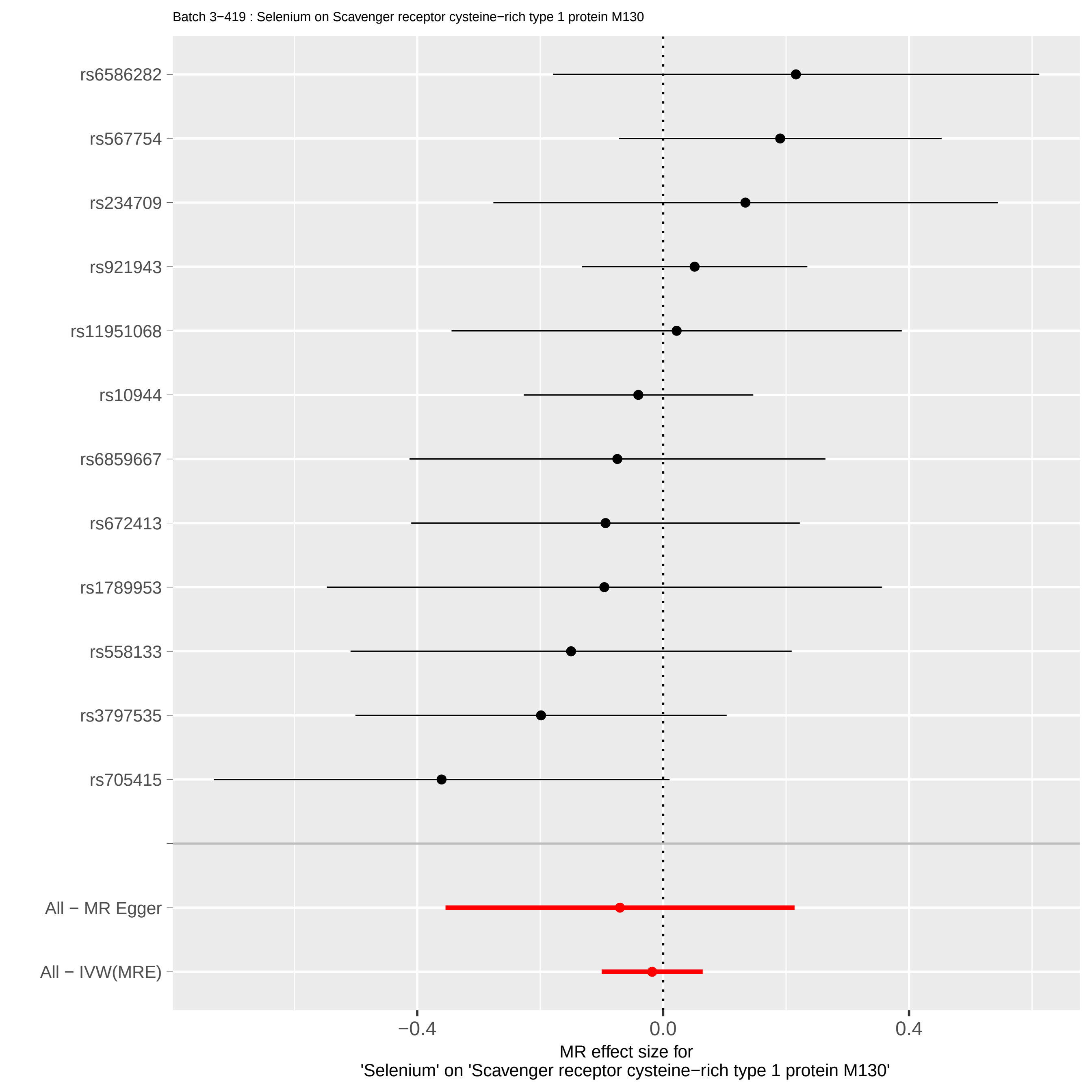

Batch 3−419 : Selenium on Scavenger receptor cysteine−rich type 1 protein M130
rs6586282
rs567754
rs234709
rs921943
rs11951068
rs10944
rs6859667
rs672413
rs1789953
rs558133
rs3797535
rs705415
All − MR Egger
All − IVW(MRE)
−0.4
0.0
0.4
MR effect size for
'Selenium' on 'Scavenger receptor cysteine−rich type 1 protein M130'

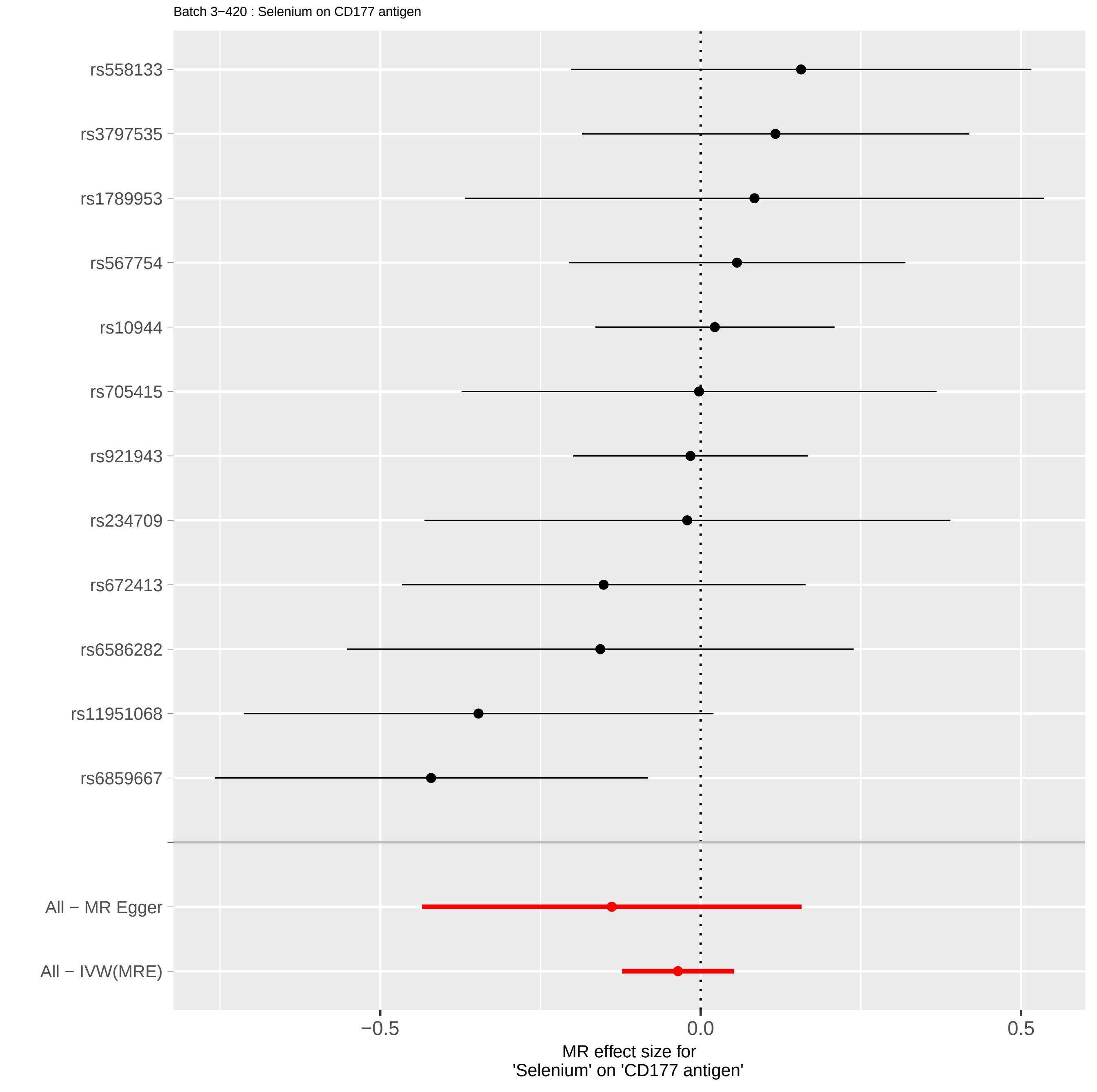

Batch 3−420 : Selenium on CD177 antigen
rs558133
rs3797535
rs1789953
rs567754
rs10944
rs705415
rs921943
rs234709
rs672413
rs6586282
rs11951068
rs6859667
All − MR Egger
All − IVW(MRE)
−0.5
0.0
0.5
MR effect size for
'Selenium' on 'CD177 antigen'

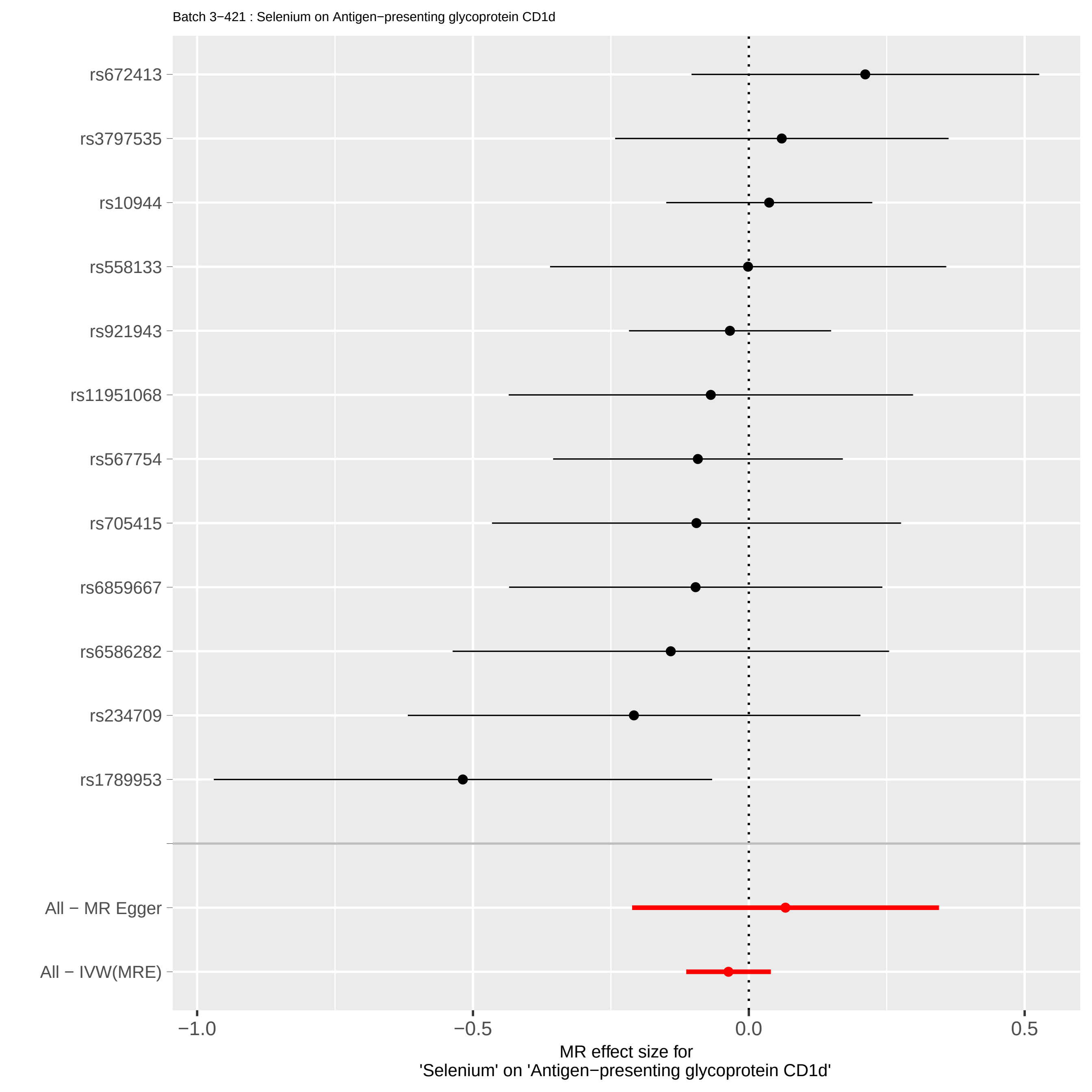
Batch 3−421 : Selenium on Antigen−presenting glycoprotein CD1d
rs672413
rs3797535
rs10944
rs558133
rs921943
rs11951068
rs567754
rs705415
rs6859667
rs6586282
rs234709
rs1789953
All − MR Egger
All − IVW(MRE)
−1.0
−0.5
0.0
0.5
MR effect size for
'Selenium' on 'Antigen−presenting glycoprotein CD1d'

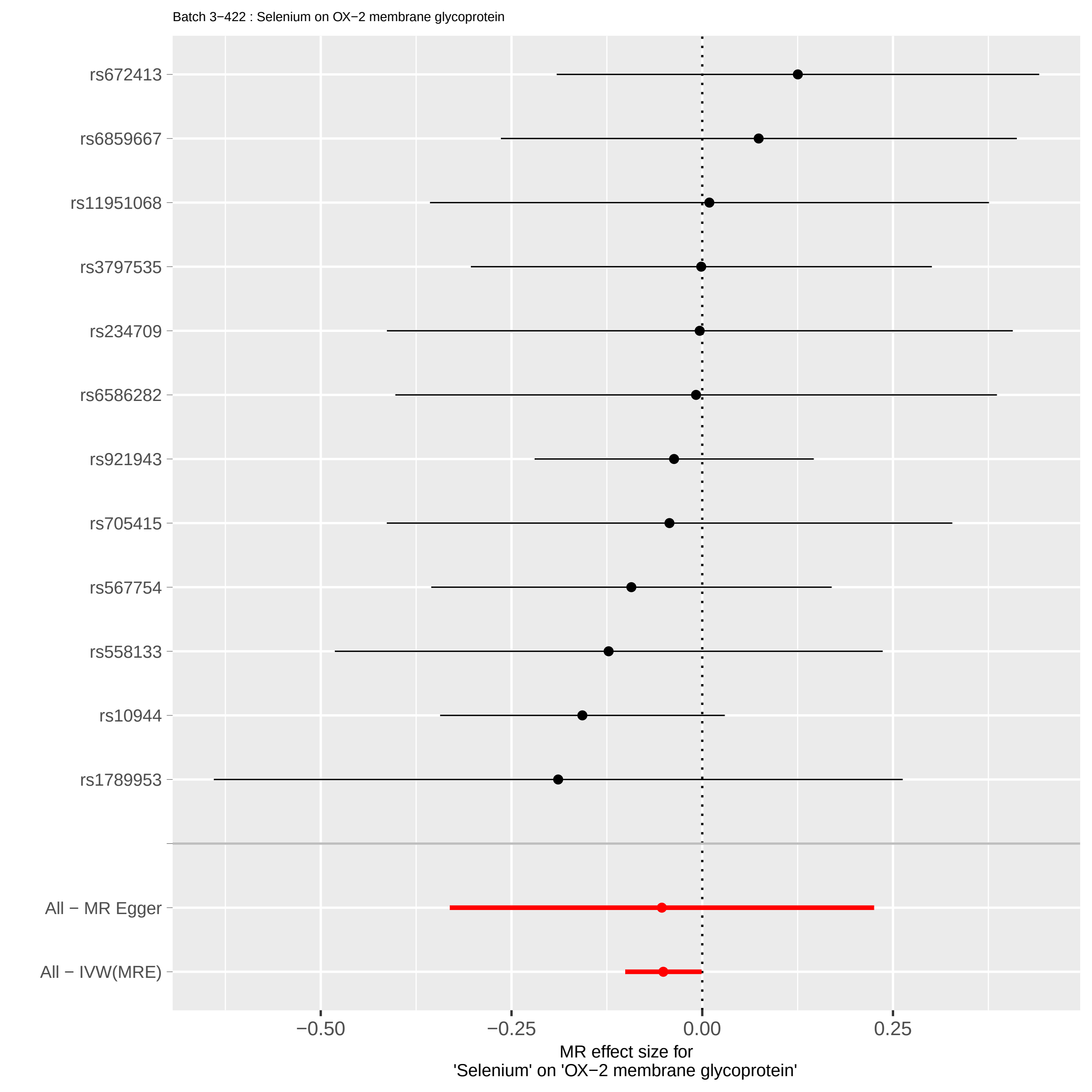

Batch 3−422 : Selenium on OX−2 membrane glycoprotein
rs672413
rs6859667
rs11951068
rs3797535
rs234709
rs6586282
rs921943
rs705415
rs567754
rs558133
rs10944
rs1789953
All − MR Egger
All − IVW(MRE)
−0.50
−0.25
0.00
0.25
MR effect size for
'Selenium' on 'OX−2 membrane glycoprotein'

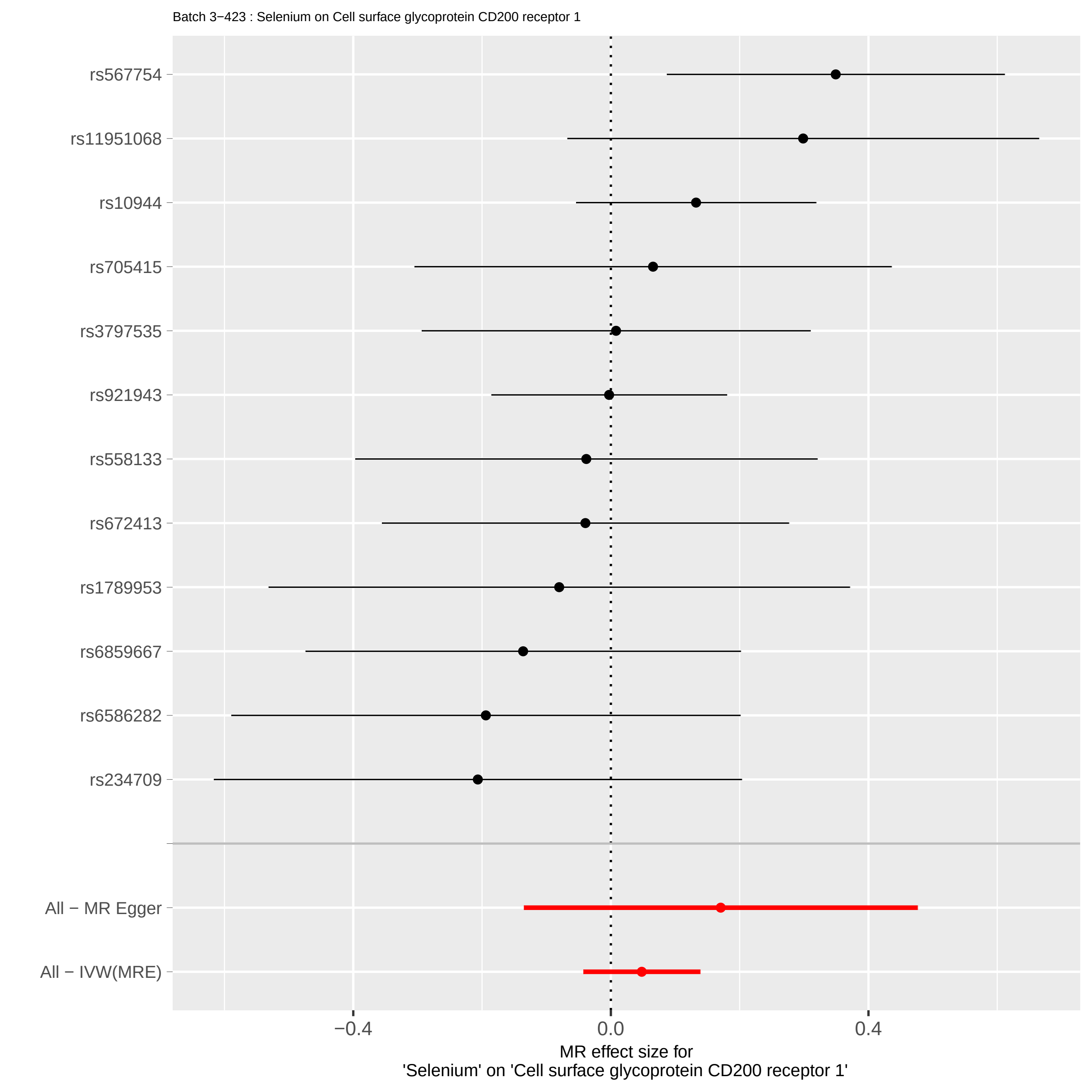

Batch 3−423 : Selenium on Cell surface glycoprotein CD200 receptor 1
rs567754
rs11951068
rs10944
rs705415
rs3797535
rs921943
rs558133
rs672413
rs1789953
rs6859667
rs6586282
rs234709
All − MR Egger
All − IVW(MRE)
−0.4
0.0
0.4
MR effect size for
'Selenium' on 'Cell surface glycoprotein CD200 receptor 1'

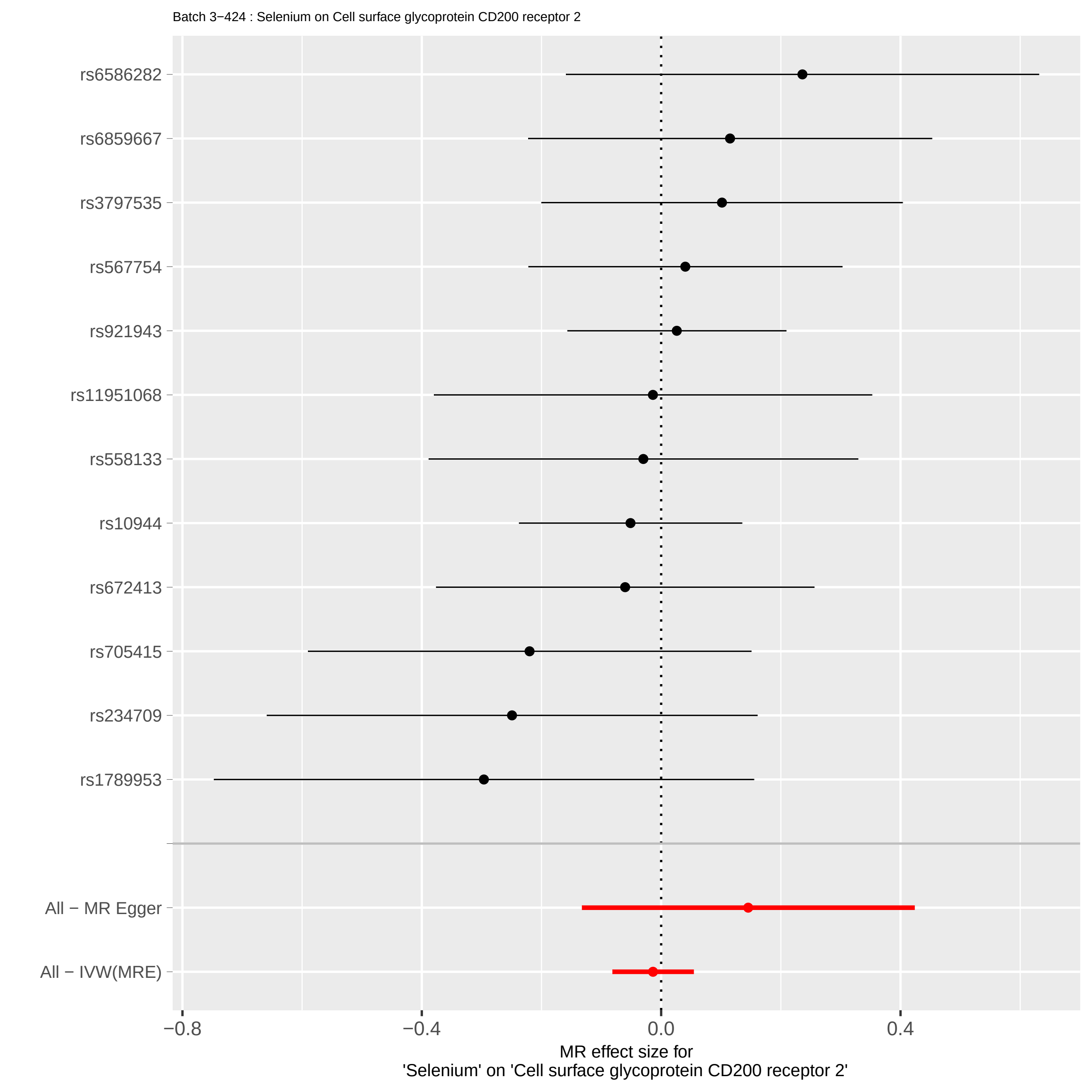

Batch 3−424 : Selenium on Cell surface glycoprotein CD200 receptor 2
rs6586282
rs6859667
rs3797535
rs567754
rs921943
rs11951068
rs558133
rs10944
rs672413
rs705415
rs234709
rs1789953
All − MR Egger
All − IVW(MRE)
−0.8
−0.4
0.0
0.4
MR effect size for
'Selenium' on 'Cell surface glycoprotein CD200 receptor 2'

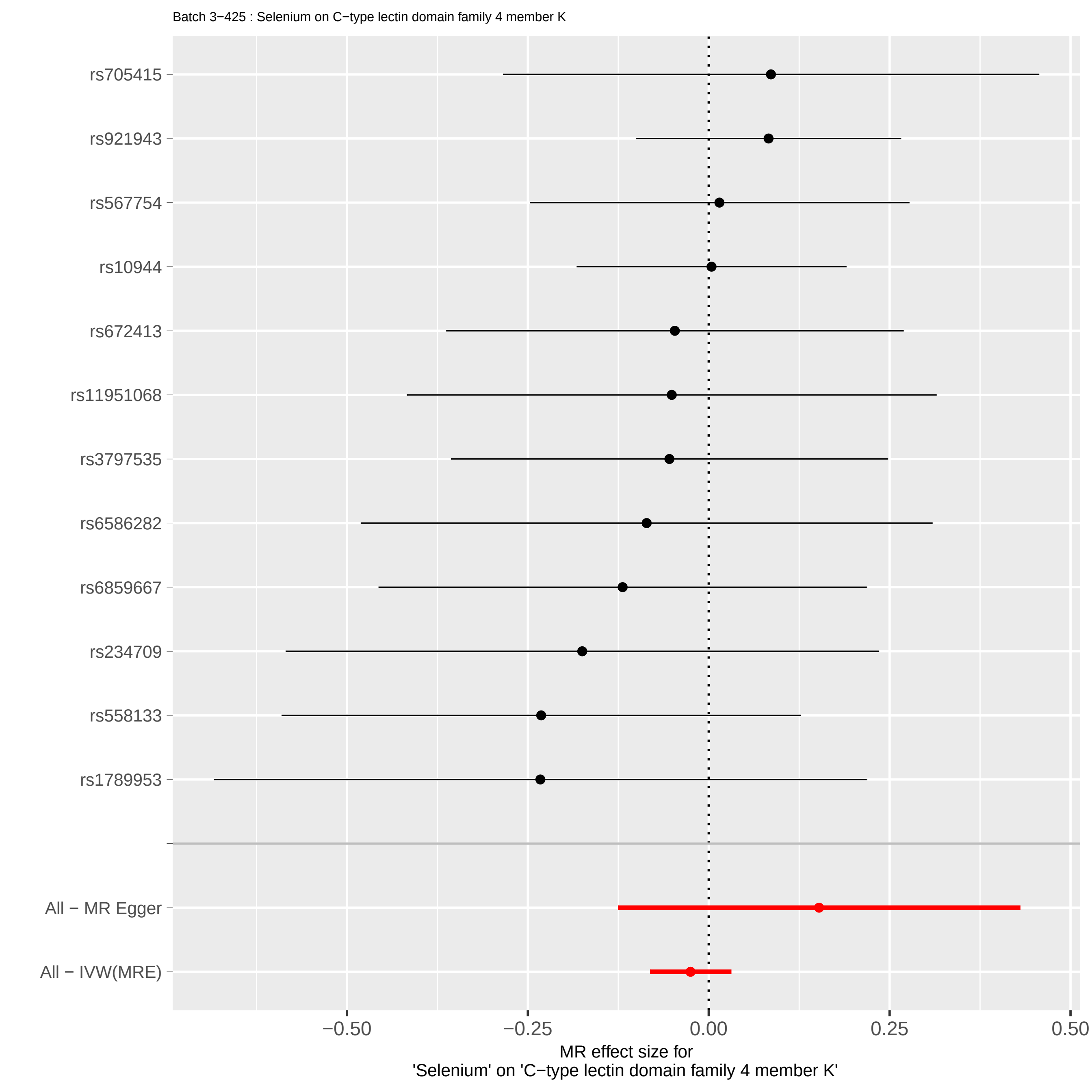
Batch 3−425 : Selenium on C−type lectin domain family 4 member K
rs705415
rs921943
rs567754
rs10944
rs672413
rs11951068
rs3797535
rs6586282
rs6859667
rs234709
rs558133
rs1789953
All − MR Egger
All − IVW(MRE)
−0.50
−0.25
0.00
0.25
0.50
MR effect size for
'Selenium' on 'C−type lectin domain family 4 member K'

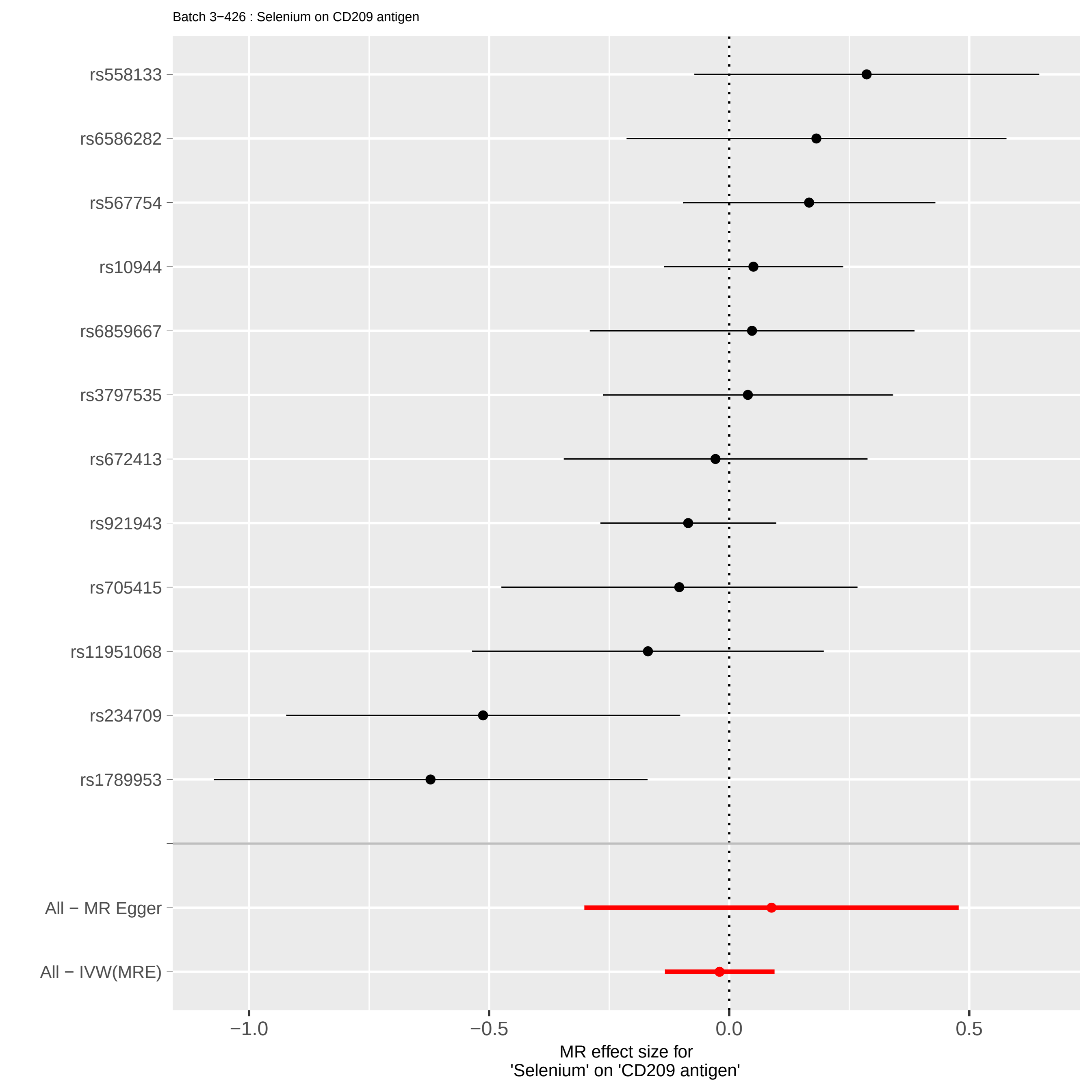

Batch 3−426 : Selenium on CD209 antigen
rs558133
rs6586282
rs567754
rs10944
rs6859667
rs3797535
rs672413
rs921943
rs705415
rs11951068
rs234709
rs1789953
All − MR Egger
All − IVW(MRE)
−1.0
−0.5
0.0
0.5
MR effect size for
'Selenium' on 'CD209 antigen'

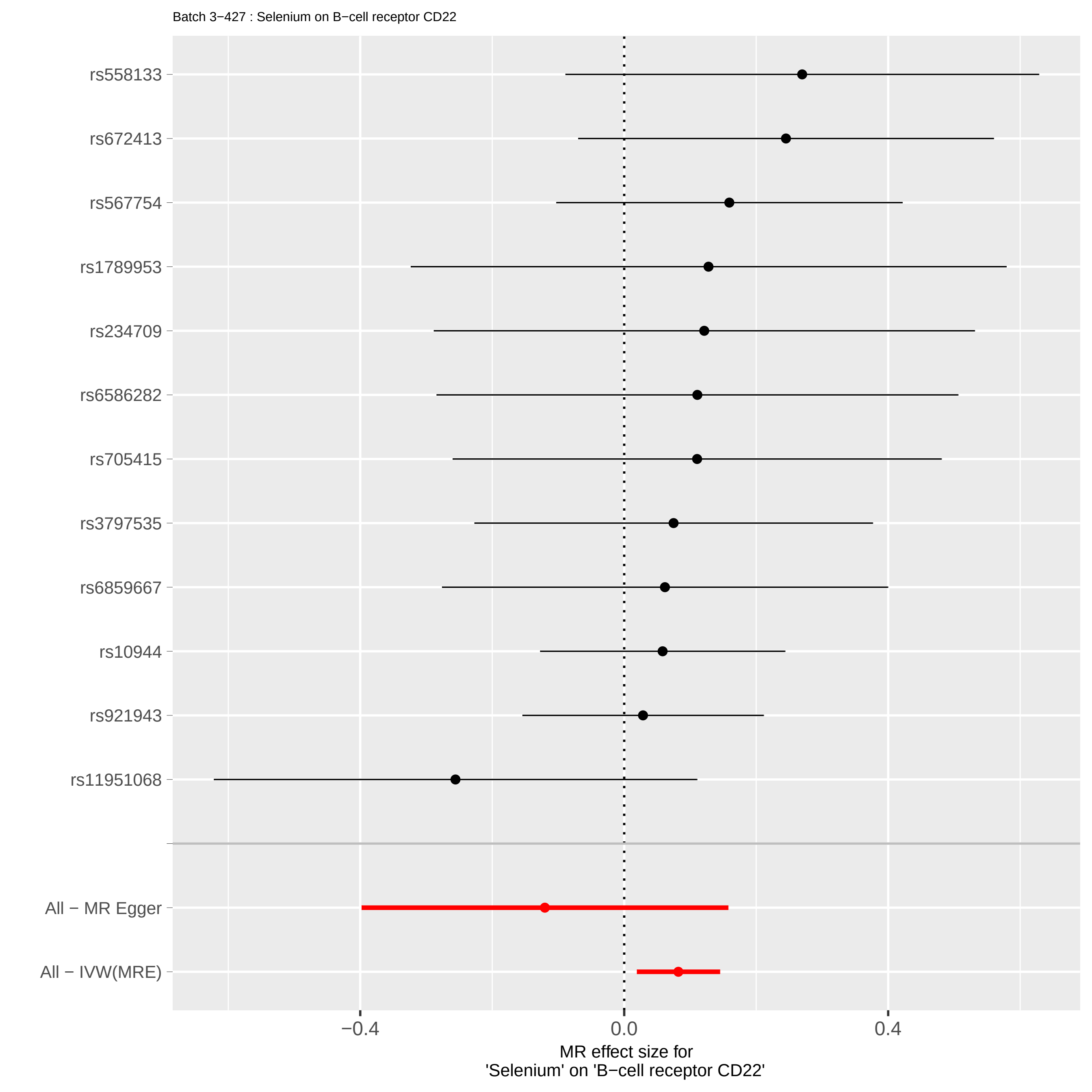

Batch 3−427 : Selenium on B−cell receptor CD22
rs558133
rs672413
rs567754
rs1789953
rs234709
rs6586282
rs705415
rs3797535
rs6859667
rs10944
rs921943
rs11951068
All − MR Egger
All − IVW(MRE)
−0.4
0.0
0.4
MR effect size for
'Selenium' on 'B−cell receptor CD22'

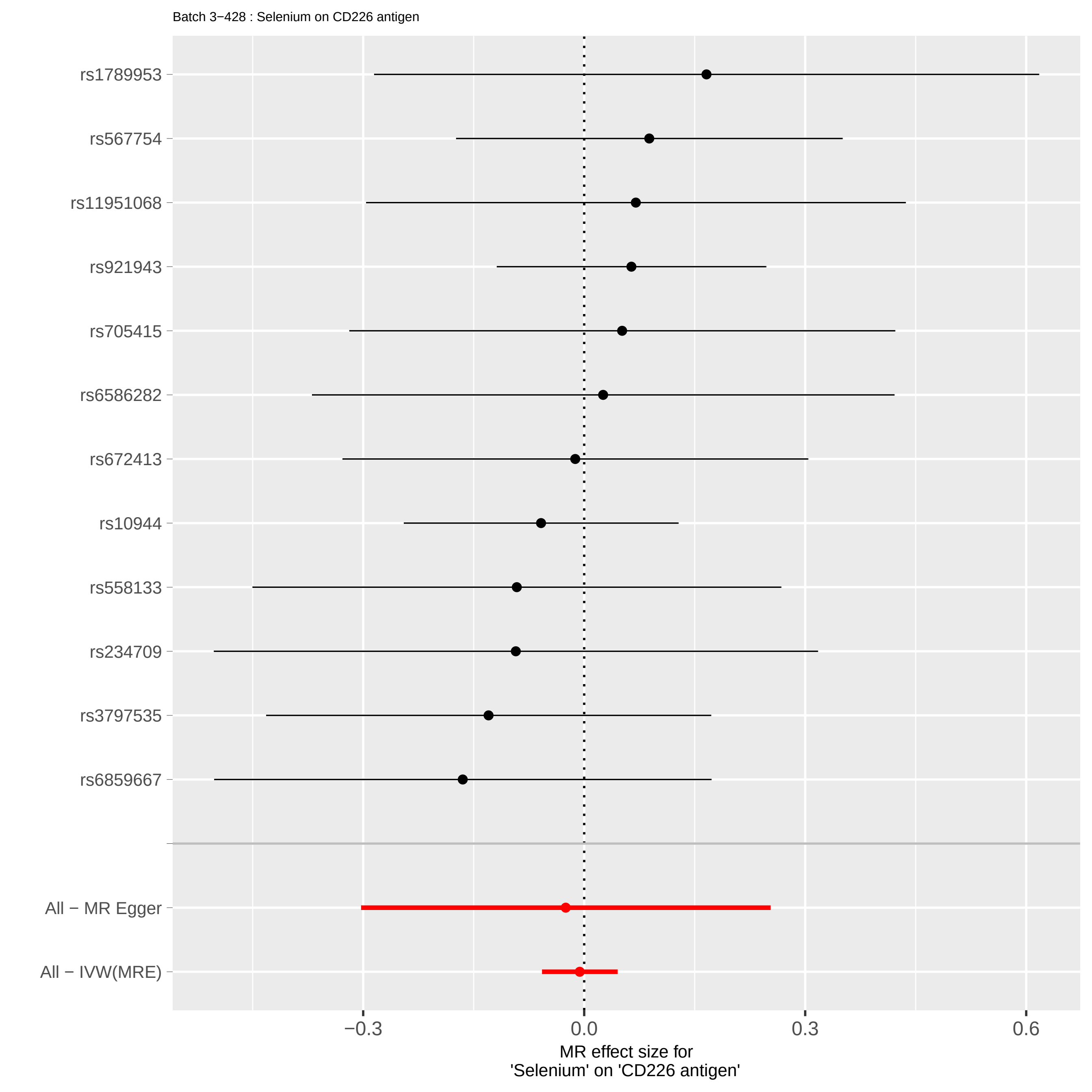
Batch 3−428 : Selenium on CD226 antigen
rs1789953
rs567754
rs11951068
rs921943
rs705415
rs6586282
rs672413
rs10944
rs558133
rs234709
rs3797535
rs6859667
All − MR Egger
All − IVW(MRE)
−0.3
0.0
0.3
0.6
MR effect size for
'Selenium' on 'CD226 antigen'

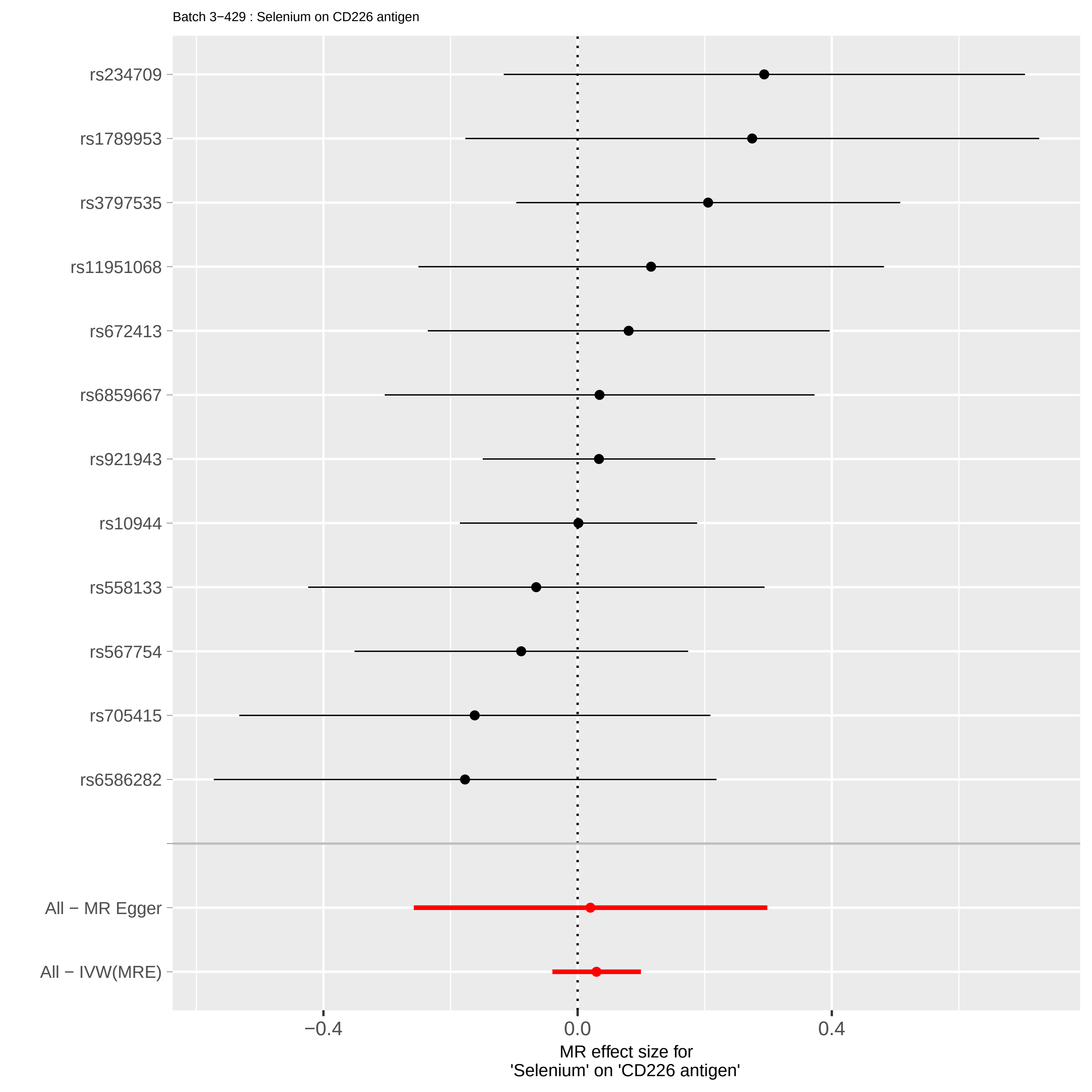
Batch 3−429 : Selenium on CD226 antigen
rs234709
rs1789953
rs3797535
rs11951068
rs672413
rs6859667
rs921943
rs10944
rs558133
rs567754
rs705415
rs6586282
All − MR Egger
All − IVW(MRE)
−0.4
0.0
0.4
MR effect size for
'Selenium' on 'CD226 antigen'

Batch 3−430 : Selenium on T−cell surface antigen CD2

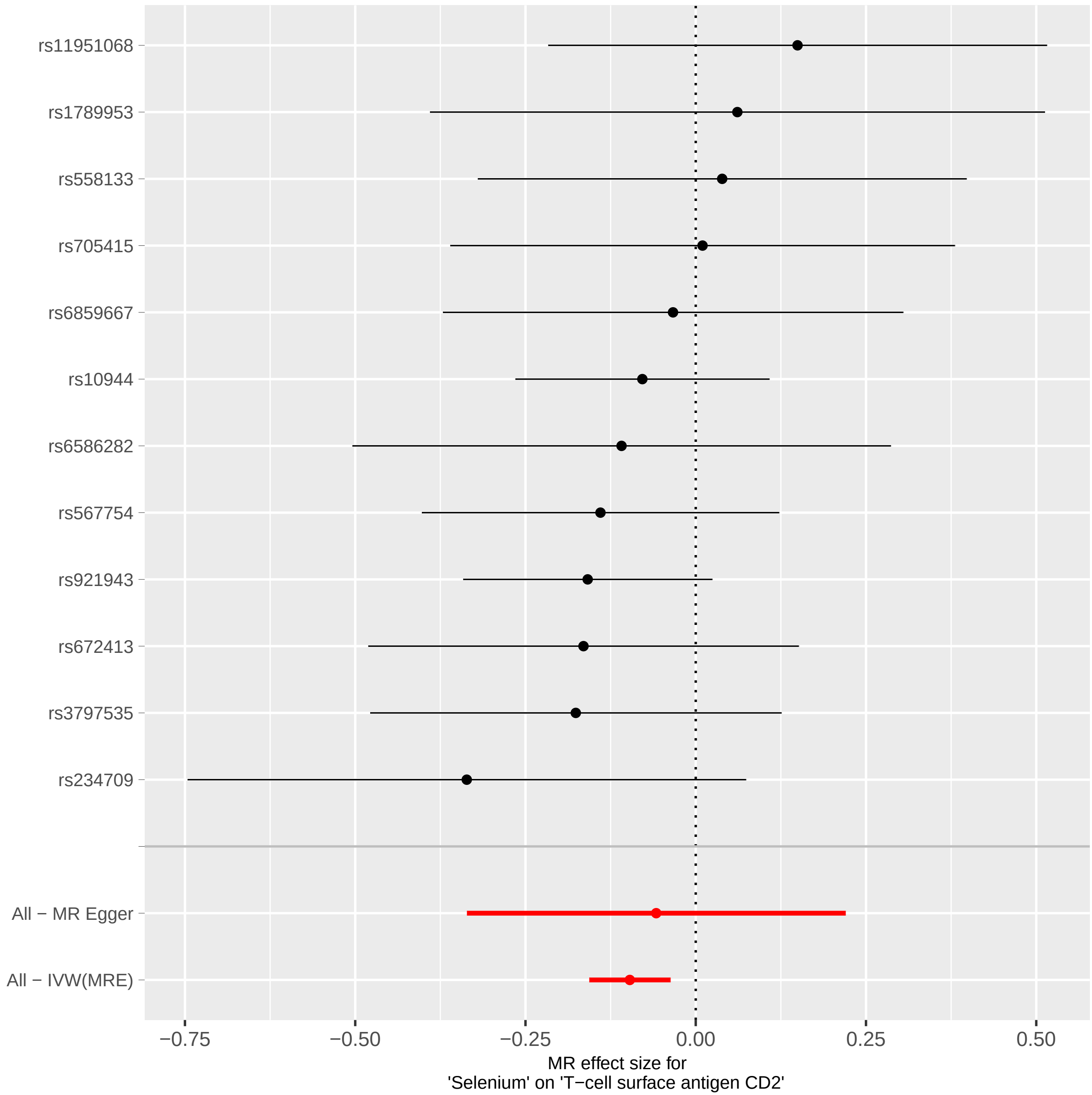

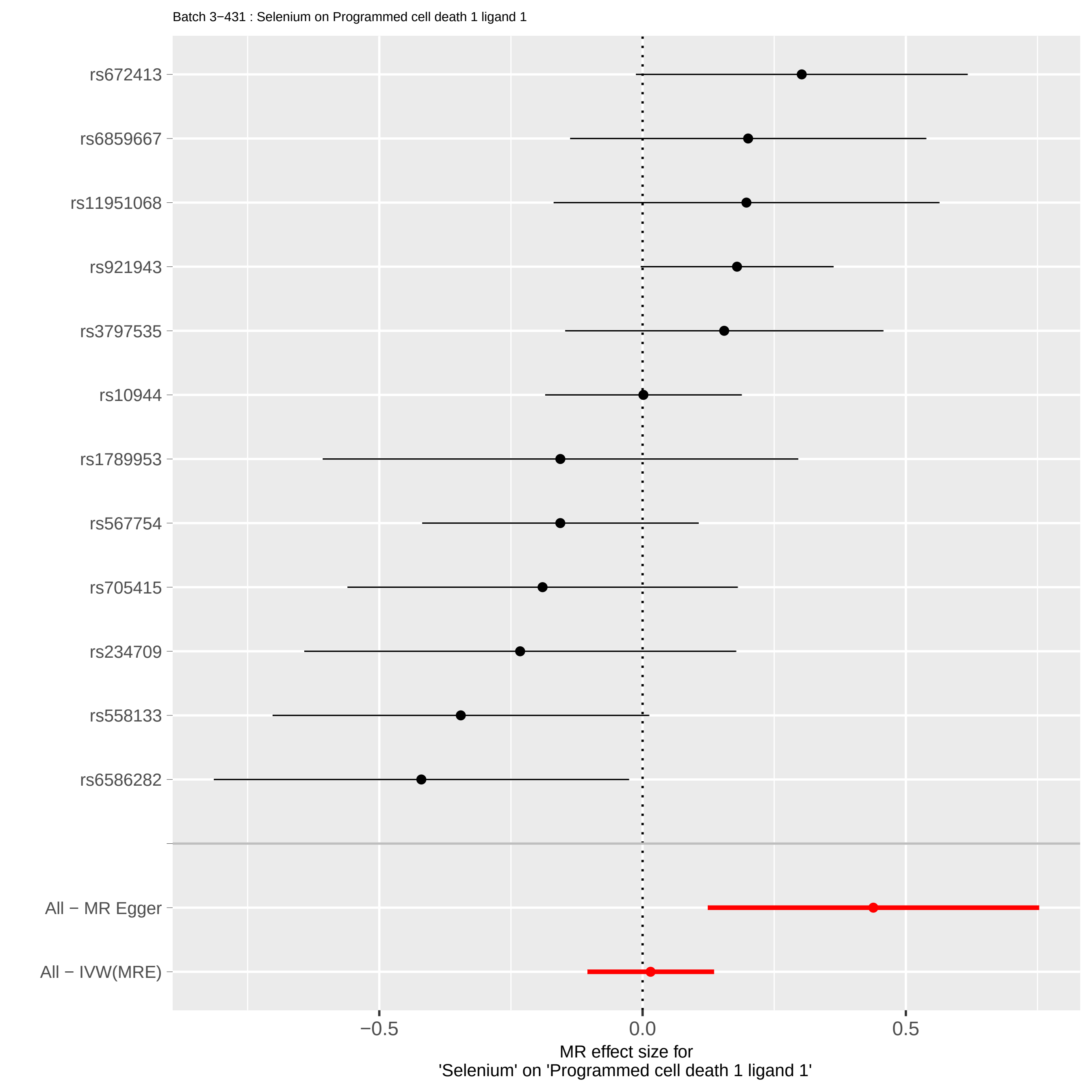

Batch 3−431 : Selenium on Programmed cell death 1 ligand 1
rs672413
rs6859667
rs11951068
rs921943
rs3797535
rs10944
rs1789953
rs567754
rs705415
rs234709
rs558133
rs6586282
All − MR Egger
All − IVW(MRE)
−0.5
0.0
0.5
MR effect size for
'Selenium' on 'Programmed cell death 1 ligand 1'

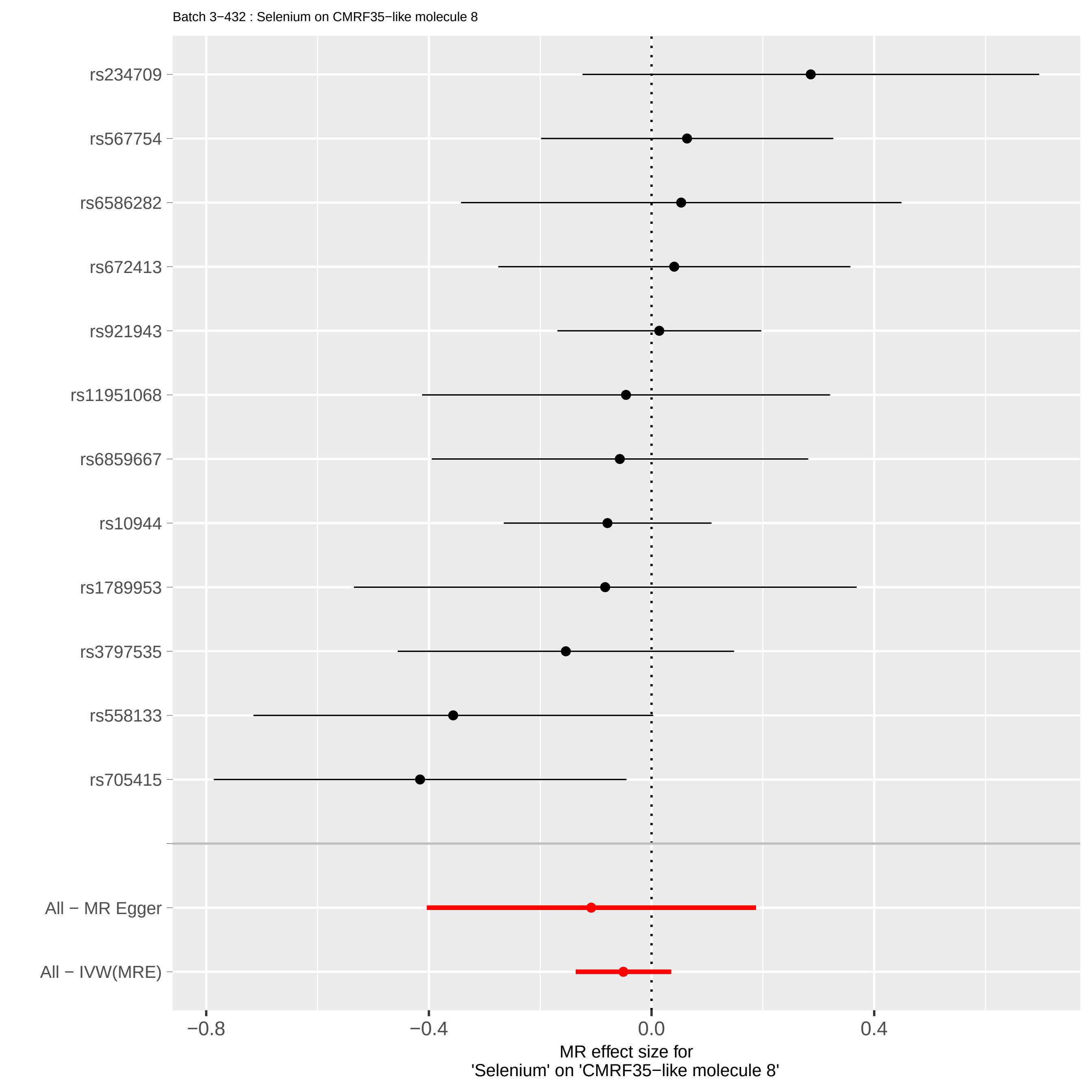

Batch 3−432 : Selenium on CMRF35−like molecule 8
rs234709
rs567754
rs6586282
rs672413
rs921943
rs11951068
rs6859667
rs10944
rs1789953
rs3797535
rs558133
rs705415
All − MR Egger
All − IVW(MRE)
−0.8
−0.4
0.0
0.4
MR effect size for
'Selenium' on 'CMRF35−like molecule 8'

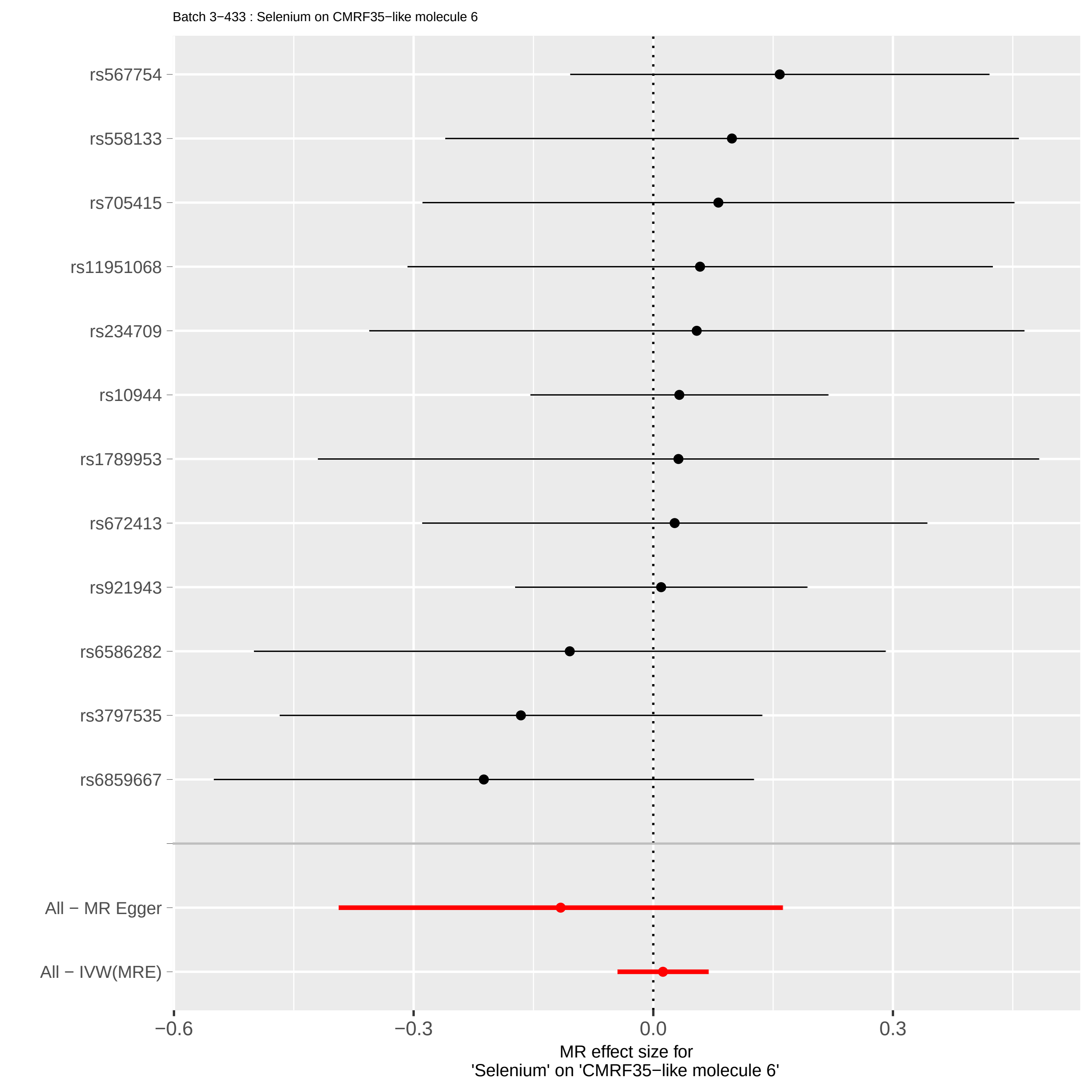
Batch 3−433 : Selenium on CMRF35−like molecule 6
rs567754
rs558133
rs705415
rs11951068
rs234709
rs10944
rs1789953
rs672413
rs921943
rs6586282
rs3797535
rs6859667
All − MR Egger
All − IVW(MRE)
−0.6
−0.3
0.0
0.3
MR effect size for
'Selenium' on 'CMRF35−like molecule 6'

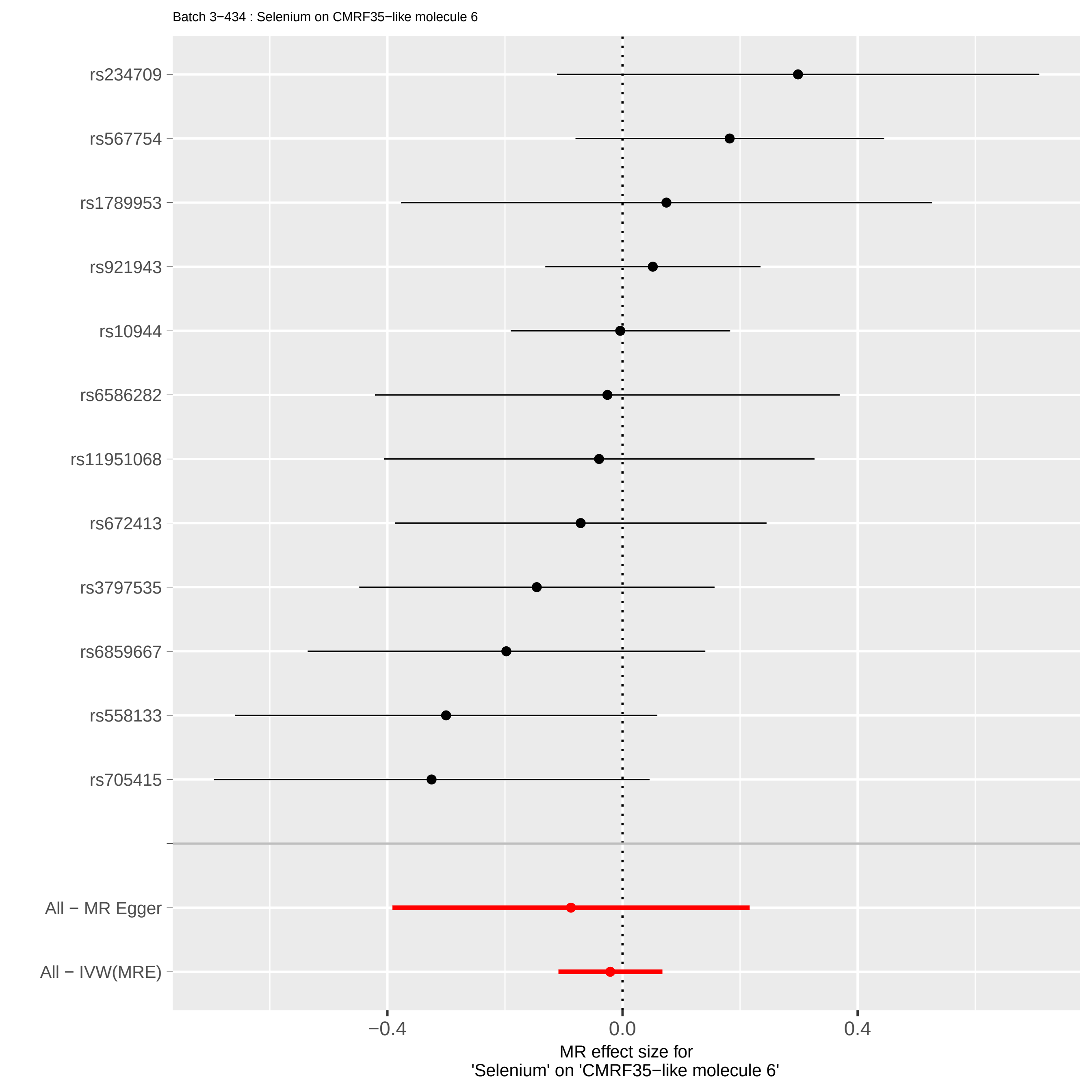
Batch 3−434 : Selenium on CMRF35−like molecule 6
rs234709
rs567754
rs1789953
rs921943
rs10944
rs6586282
rs11951068
rs672413
rs3797535
rs6859667
rs558133
rs705415
All − MR Egger
All − IVW(MRE)
−0.4
0.0
0.4
MR effect size for
'Selenium' on 'CMRF35−like molecule 6'

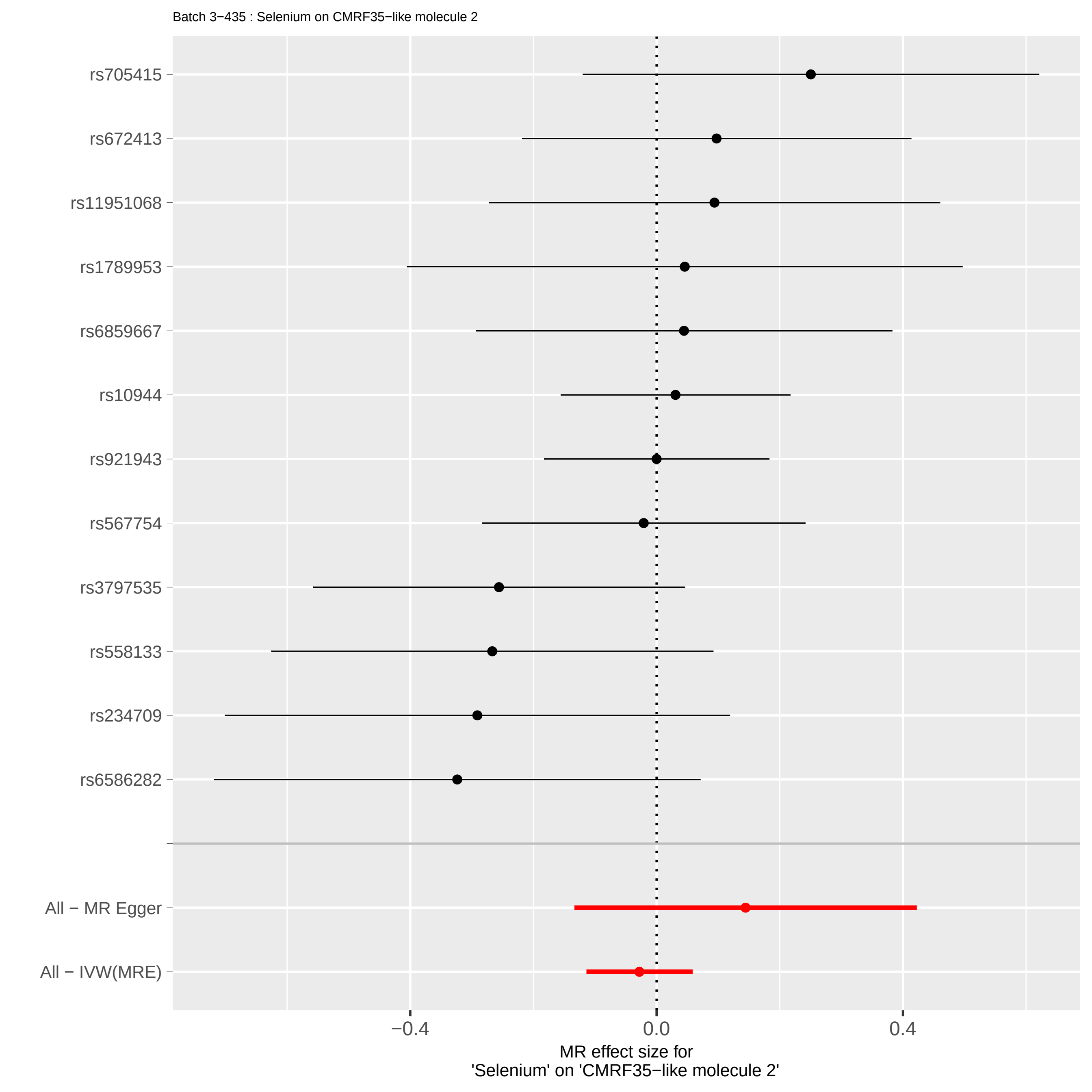
Batch 3−435 : Selenium on CMRF35−like molecule 2
rs705415
rs672413
rs11951068
rs1789953
rs6859667
rs10944
rs921943
rs567754
rs3797535
rs558133
rs234709
rs6586282
All − MR Egger
All − IVW(MRE)
−0.4
0.0
0.4
MR effect size for
'Selenium' on 'CMRF35−like molecule 2'

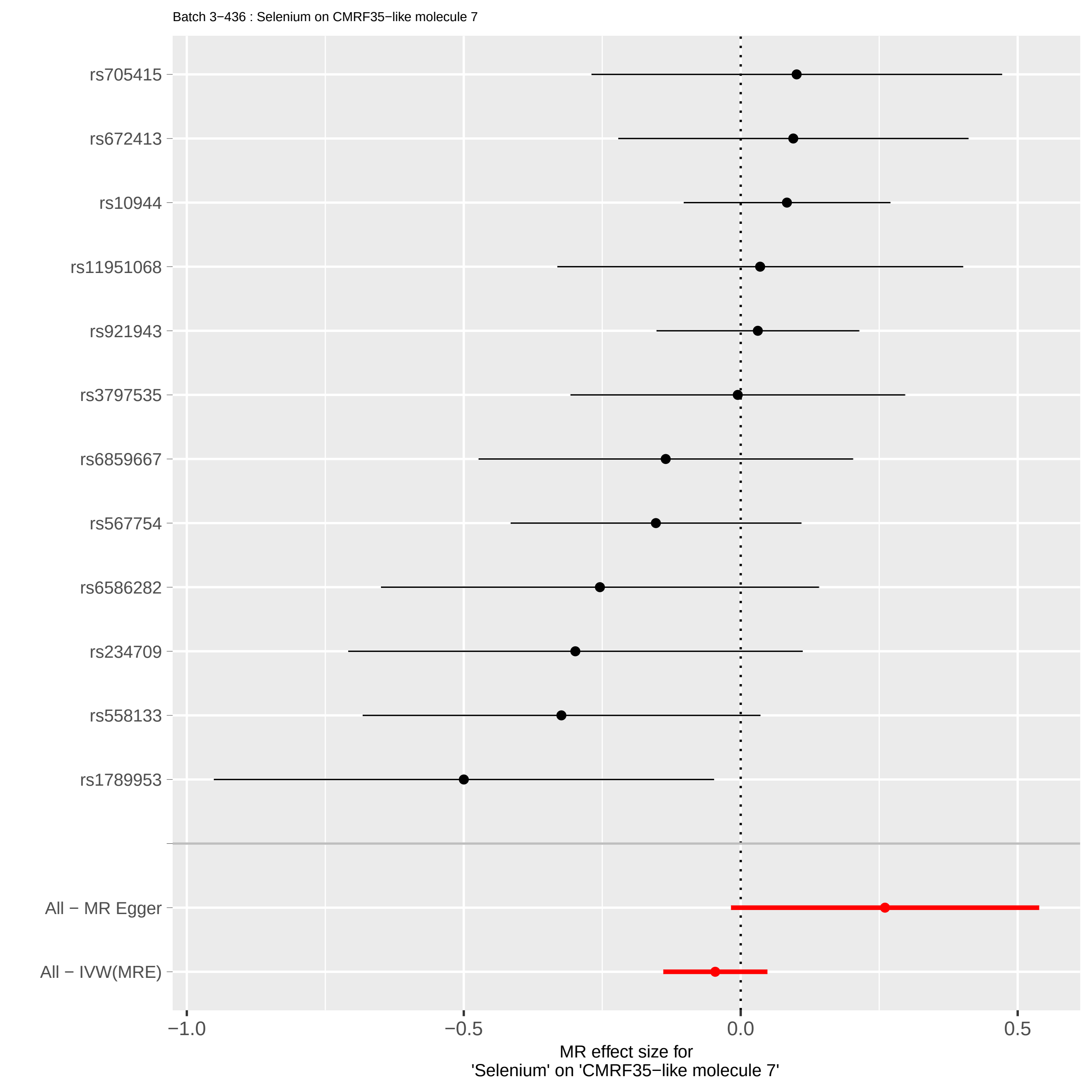
Batch 3−436 : Selenium on CMRF35−like molecule 7
rs705415
rs672413
rs10944
rs11951068
rs921943
rs3797535
rs6859667
rs567754
rs6586282
rs234709
rs558133
rs1789953
All − MR Egger
All − IVW(MRE)
−1.0
−0.5
0.0
0.5
MR effect size for
'Selenium' on 'CMRF35−like molecule 7'

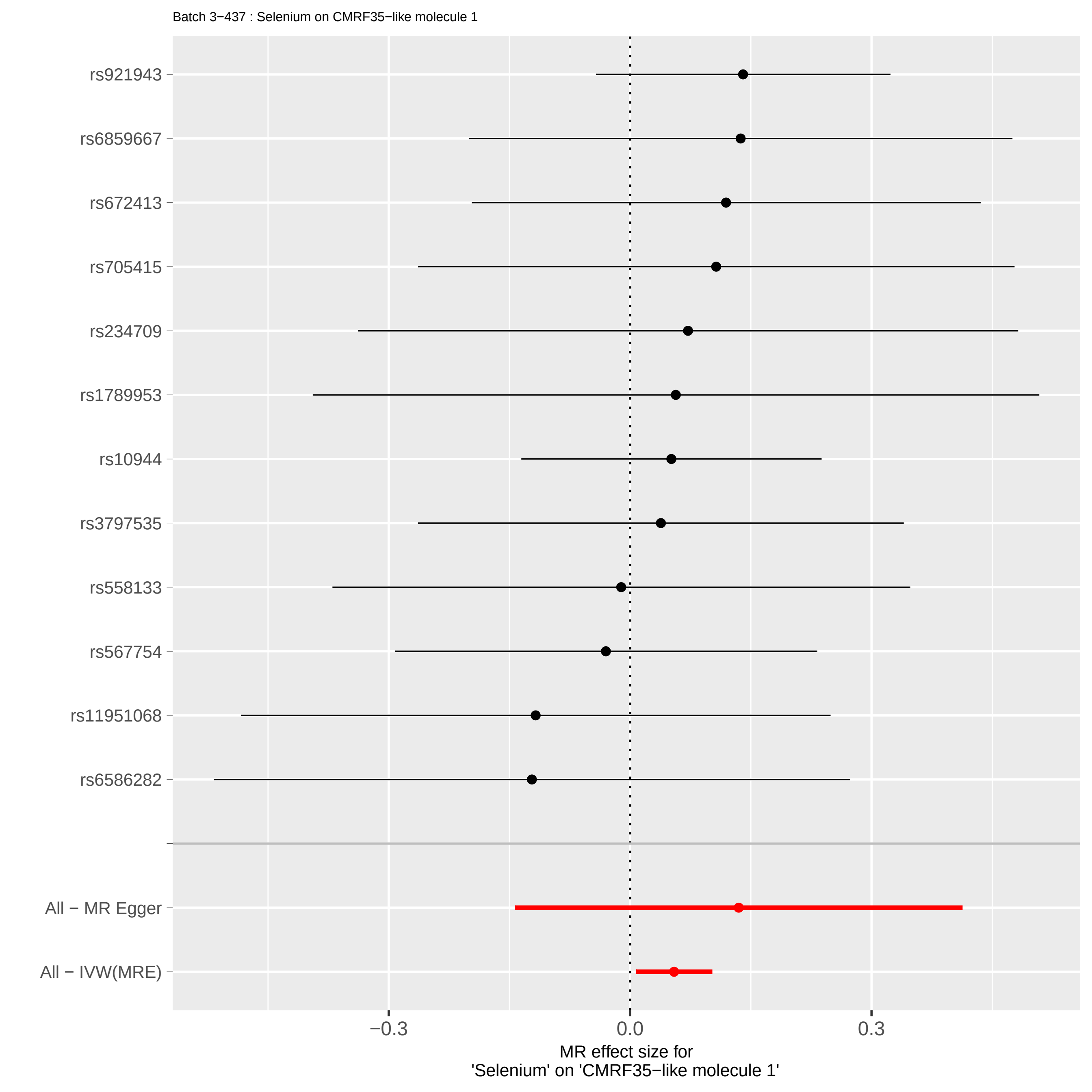

Batch 3−437 : Selenium on CMRF35−like molecule 1
rs921943
rs6859667
rs672413
rs705415
rs234709
rs1789953
rs10944
rs3797535
rs558133
rs567754
rs11951068
rs6586282
All − MR Egger
All − IVW(MRE)
−0.3
0.0
0.3
MR effect size for
'Selenium' on 'CMRF35−like molecule 1'

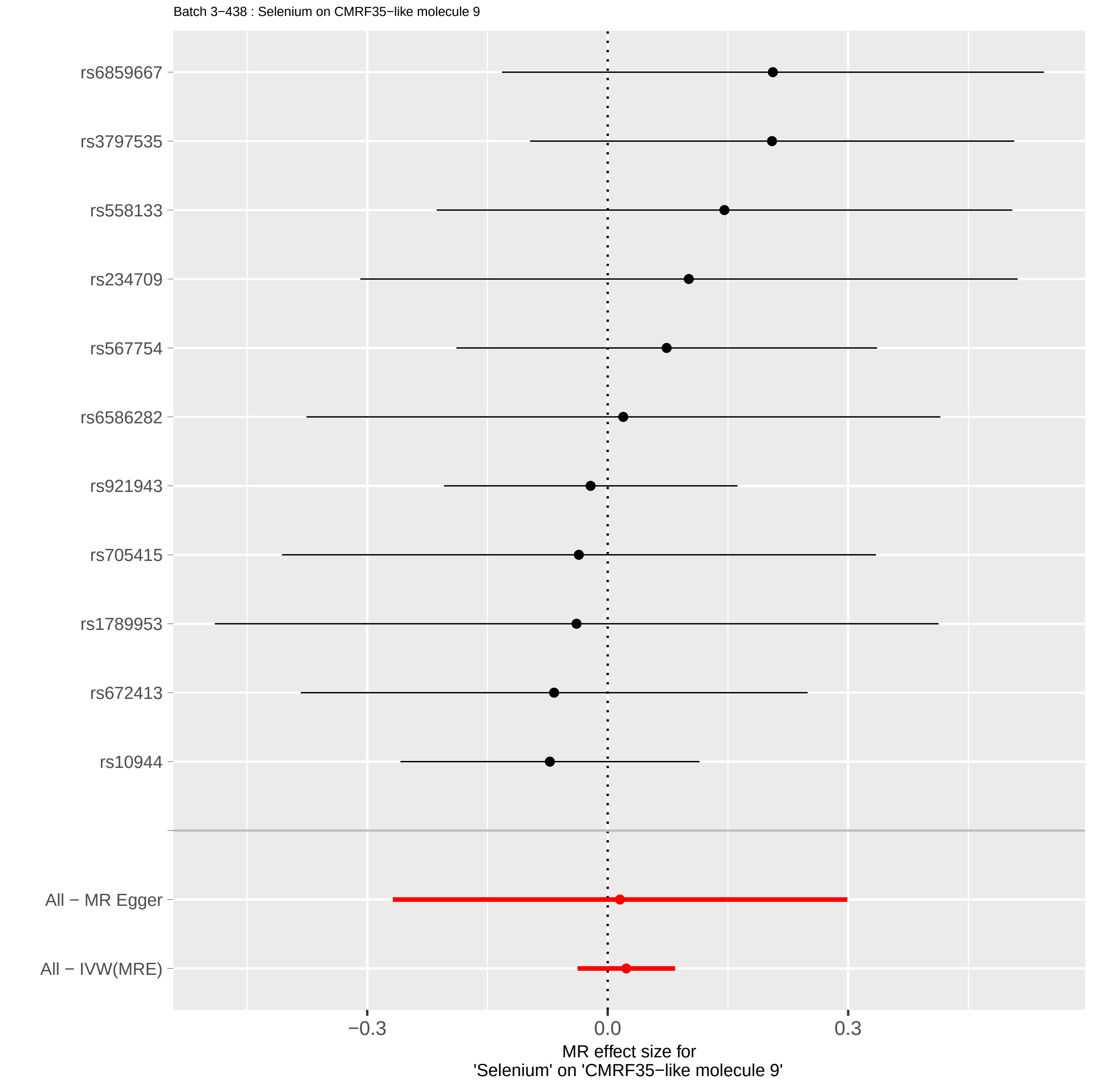

Batch 3−438 : Selenium on CMRF35−like molecule 9
rs6859667
rs3797535
rs558133
rs234709
rs567754
rs6586282
rs921943
rs705415
rs1789953
rs672413
rs10944
All − MR Egger
All − IVW(MRE)
−0.3
0.0
0.3
MR effect size for
'Selenium' on 'CMRF35−like molecule 9'

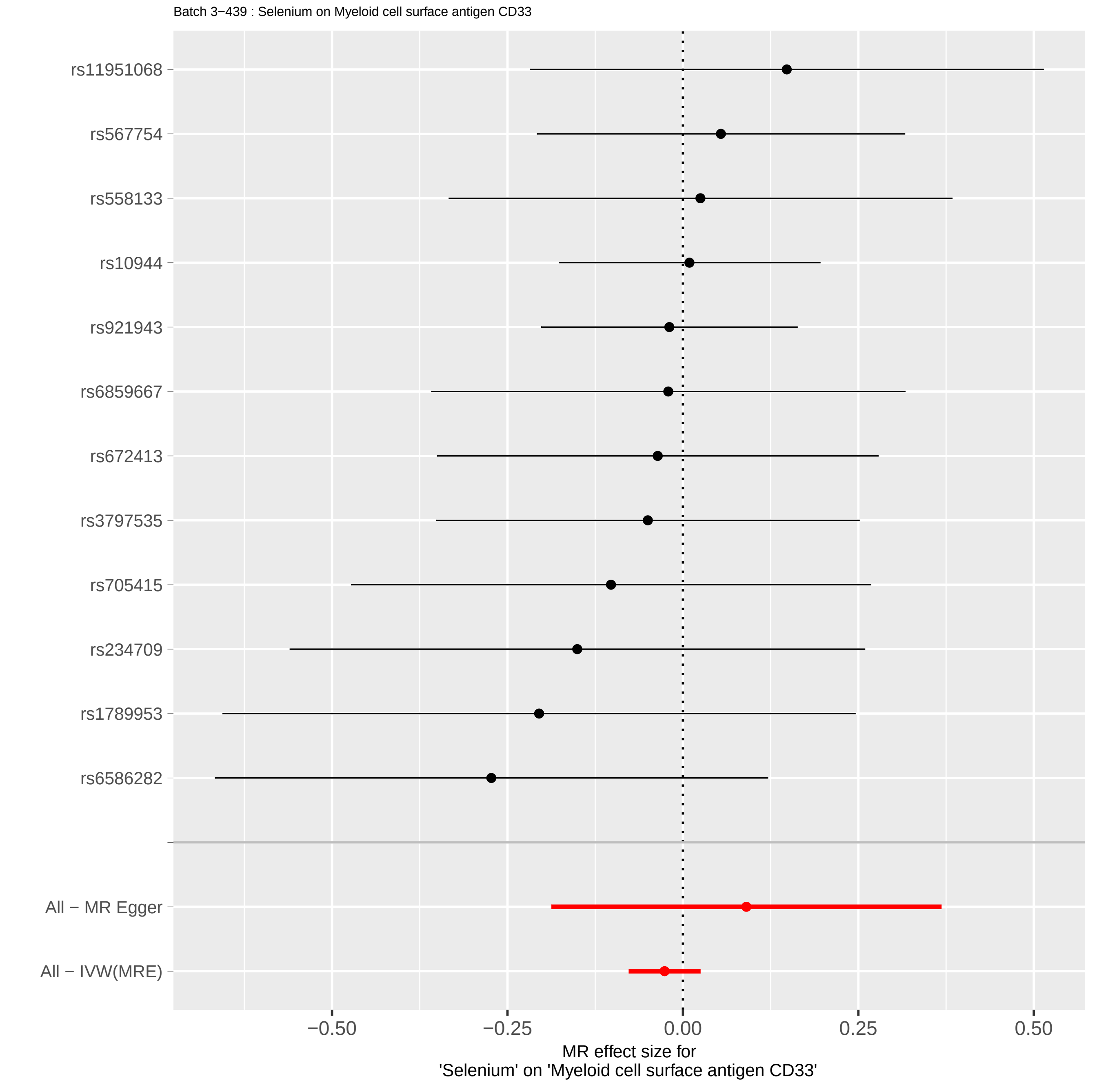

Batch 3−439 : Selenium on Myeloid cell surface antigen CD33
rs11951068
rs567754
rs558133
rs10944
rs921943
rs6859667
rs672413
rs3797535
rs705415
rs234709
rs1789953
rs6586282
All − MR Egger
All − IVW(MRE)
−0.50
−0.25
0.00
0.25
0.50
MR effect size for
'Selenium' on 'Myeloid cell surface antigen CD33'

Batch 3−440 : Selenium on Hematopoietic progenitor cell antigen CD34

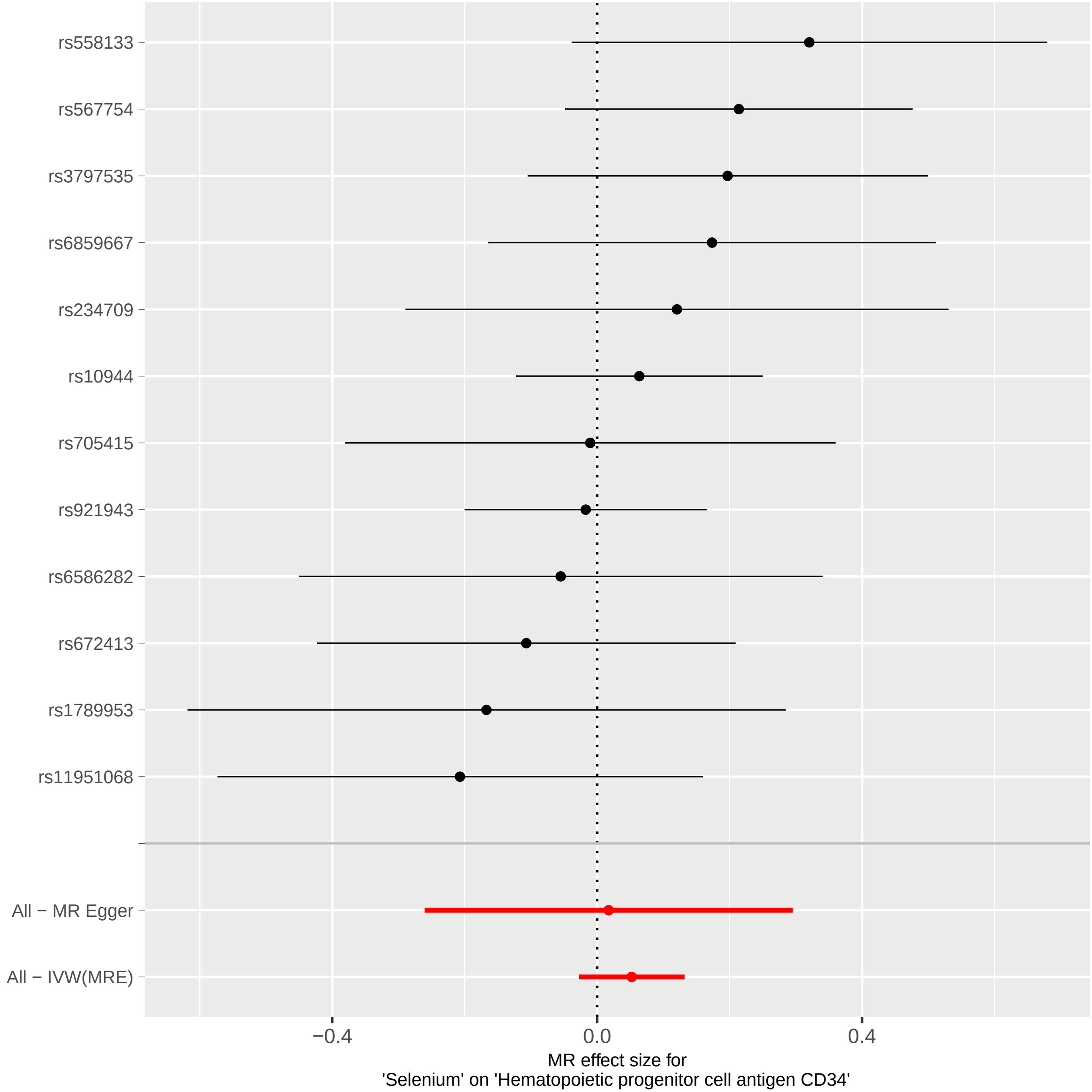

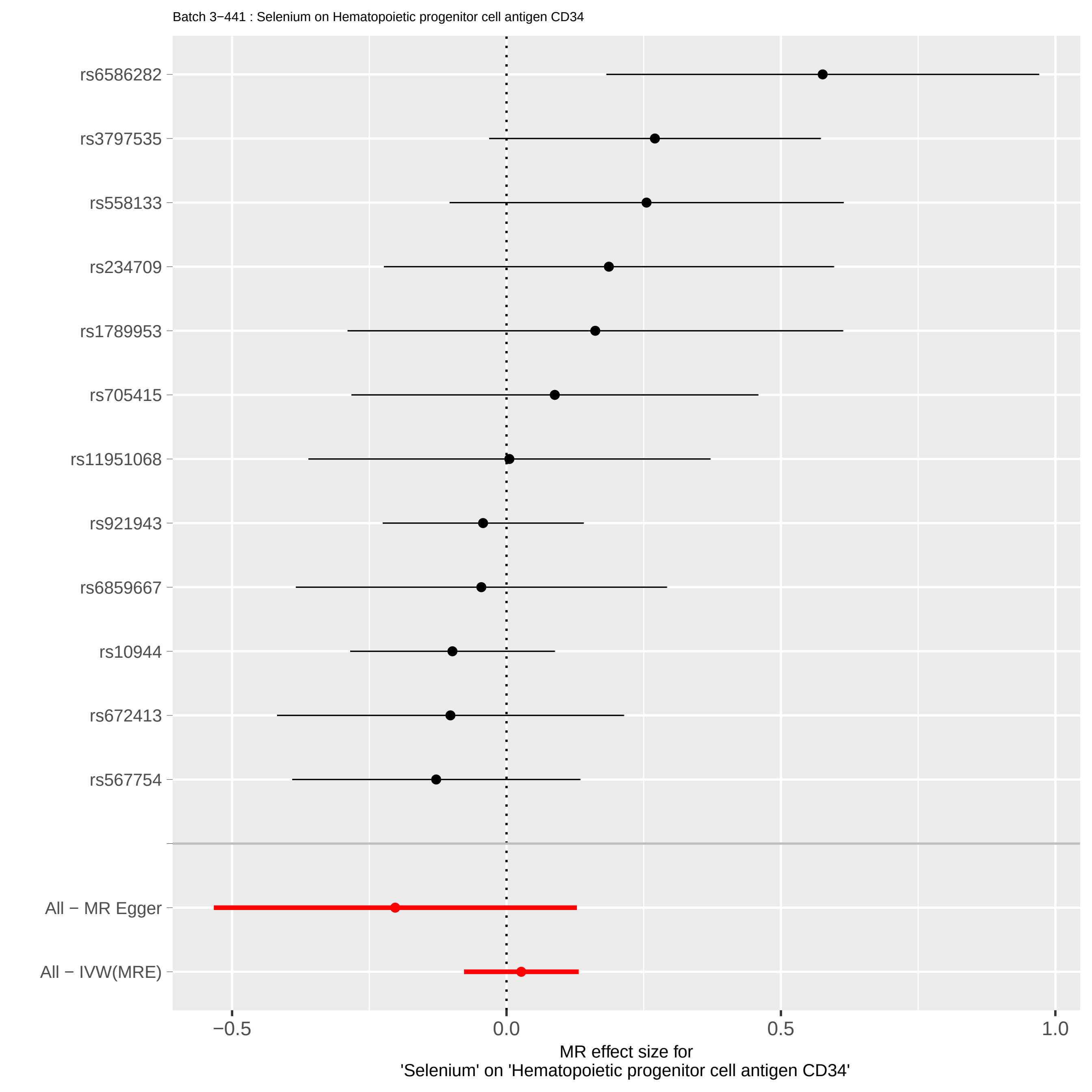
Batch 3−441 : Selenium on Hematopoietic progenitor cell antigen CD34
rs6586282
rs3797535
rs558133
rs234709
rs1789953
rs705415
rs11951068
rs921943
rs6859667
rs10944
rs672413
rs567754
All − MR Egger
All − IVW(MRE)
−0.5
0.0
0.5
1.0
MR effect size for
'Selenium' on 'Hematopoietic progenitor cell antigen CD34'

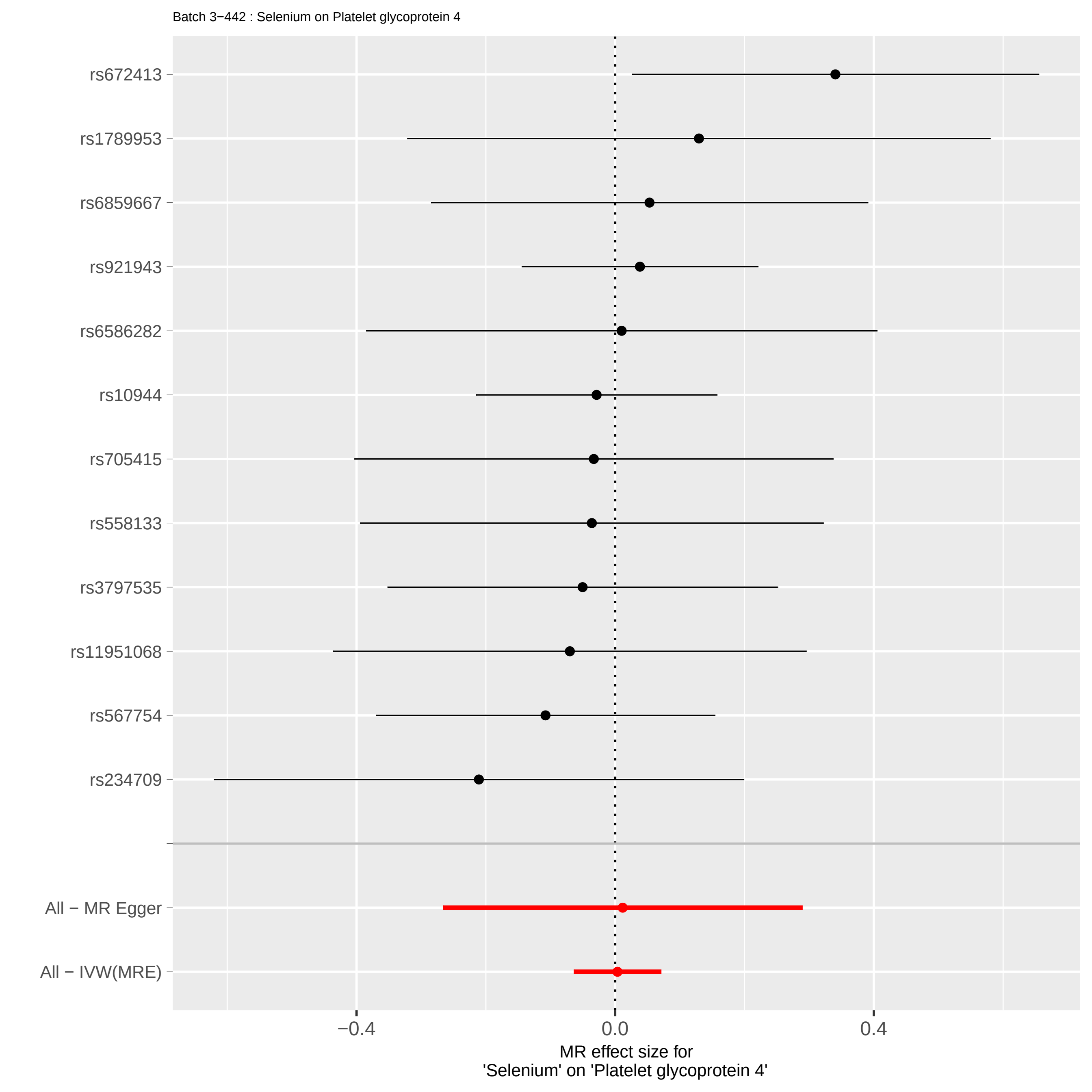

Batch 3−442 : Selenium on Platelet glycoprotein 4
rs672413
rs1789953
rs6859667
rs921943
rs6586282
rs10944
rs705415
rs558133
rs3797535
rs11951068
rs567754
rs234709
All − MR Egger
All − IVW(MRE)
−0.4
0.0
0.4
MR effect size for
'Selenium' on 'Platelet glycoprotein 4'

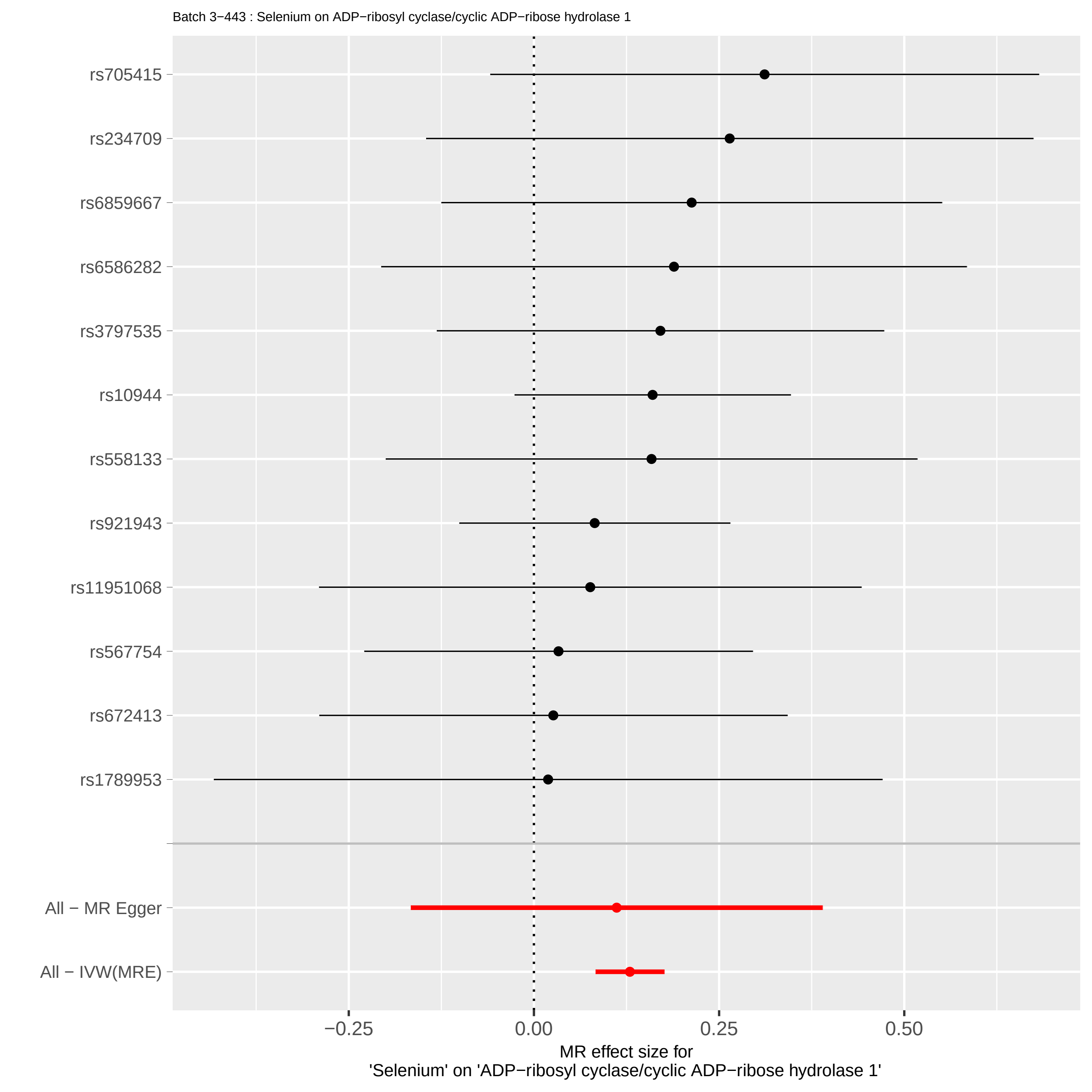

Batch 3−443 : Selenium on ADP−ribosyl cyclase/cyclic ADP−ribose hydrolase 1
rs705415
rs234709
rs6859667
rs6586282
rs3797535
rs10944
rs558133
rs921943
rs11951068
rs567754
rs672413
rs1789953
All − MR Egger
All − IVW(MRE)
−0.25
0.00
0.25
0.50
MR effect size for
'Selenium' on 'ADP−ribosyl cyclase/cyclic ADP−ribose hydrolase 1'

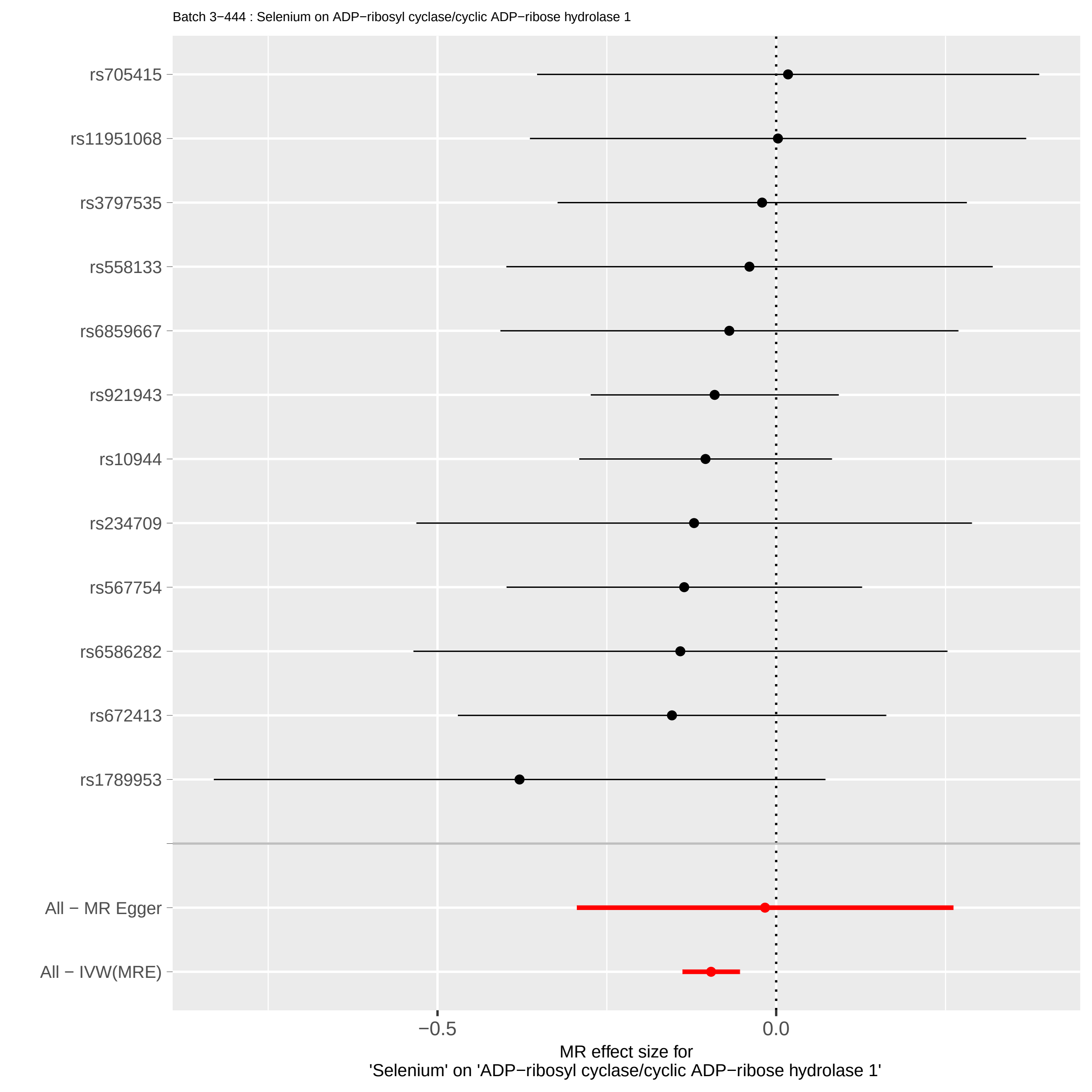
Batch 3−444 : Selenium on ADP−ribosyl cyclase/cyclic ADP−ribose hydrolase 1
rs705415
rs11951068
rs3797535
rs558133
rs6859667
rs921943
rs10944
rs234709
rs567754
rs6586282
rs672413
rs1789953
All − MR Egger
All − IVW(MRE)
−0.5
0.0
MR effect size for
'Selenium' on 'ADP−ribosyl cyclase/cyclic ADP−ribose hydrolase 1'

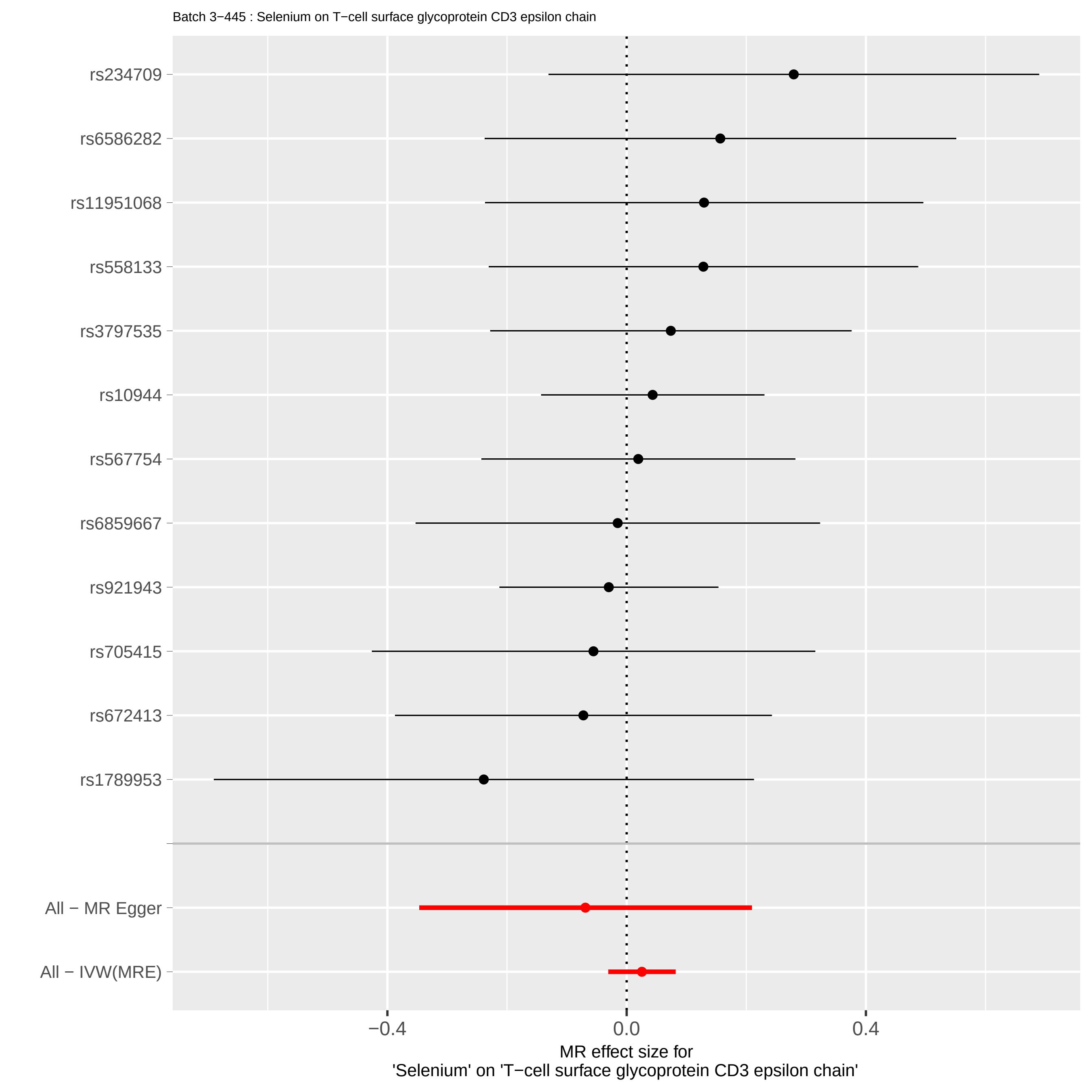

Batch 3−445 : Selenium on T−cell surface glycoprotein CD3 epsilon chain
rs234709
rs6586282
rs11951068
rs558133
rs3797535
rs10944
rs567754
rs6859667
rs921943
rs705415
rs672413
rs1789953
All − MR Egger
All − IVW(MRE)
−0.4
0.0
0.4
MR effect size for
'Selenium' on 'T−cell surface glycoprotein CD3 epsilon chain'

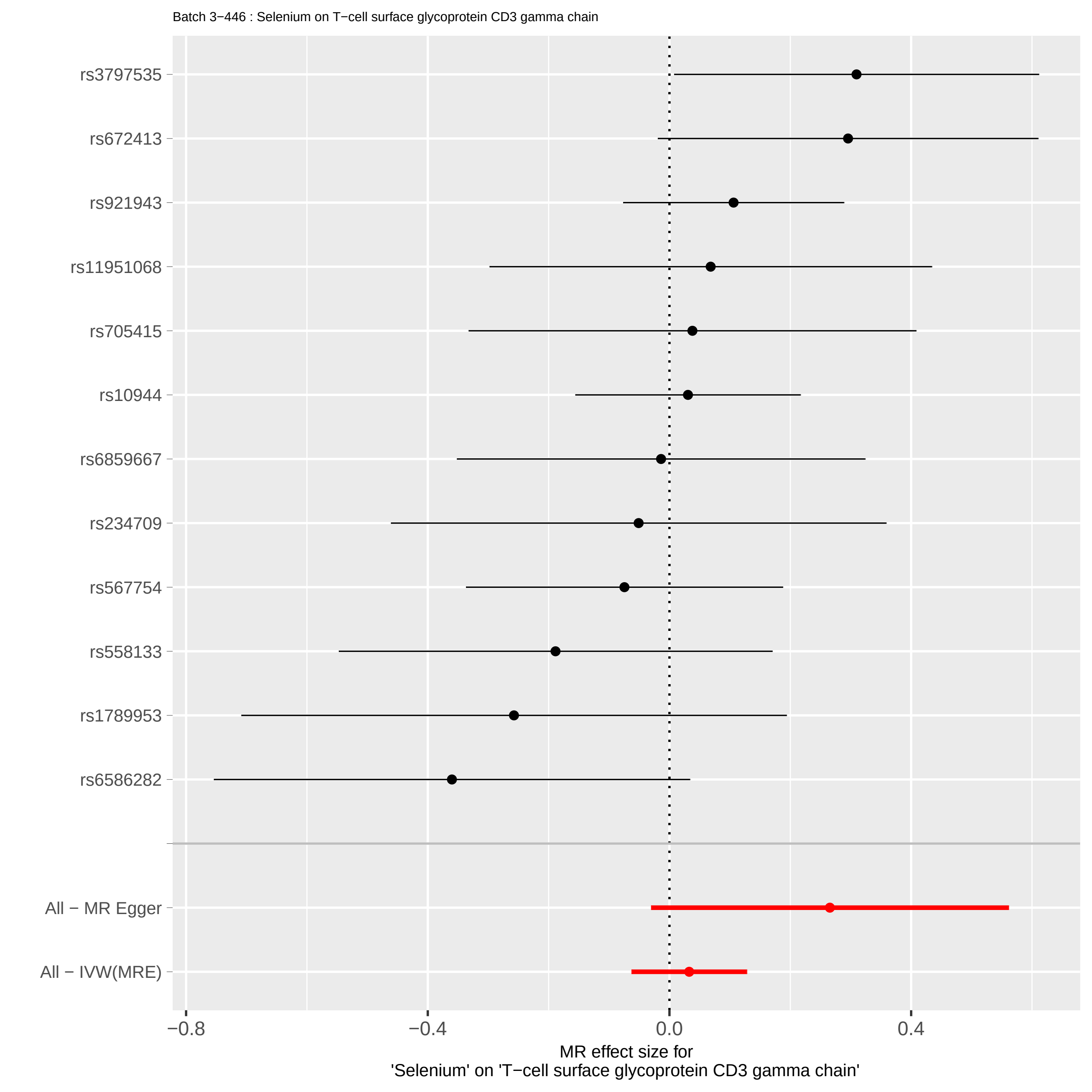
Batch 3−446 : Selenium on T−cell surface glycoprotein CD3 gamma chain
rs3797535
rs672413
rs921943
rs11951068
rs705415
rs10944
rs6859667
rs234709
rs567754
rs558133
rs1789953
rs6586282
All − MR Egger
All − IVW(MRE)
−0.8
−0.4
0.0
0.4
MR effect size for
'Selenium' on 'T−cell surface glycoprotein CD3 gamma chain'

Batch 3−447 : Selenium on CD40 ligand

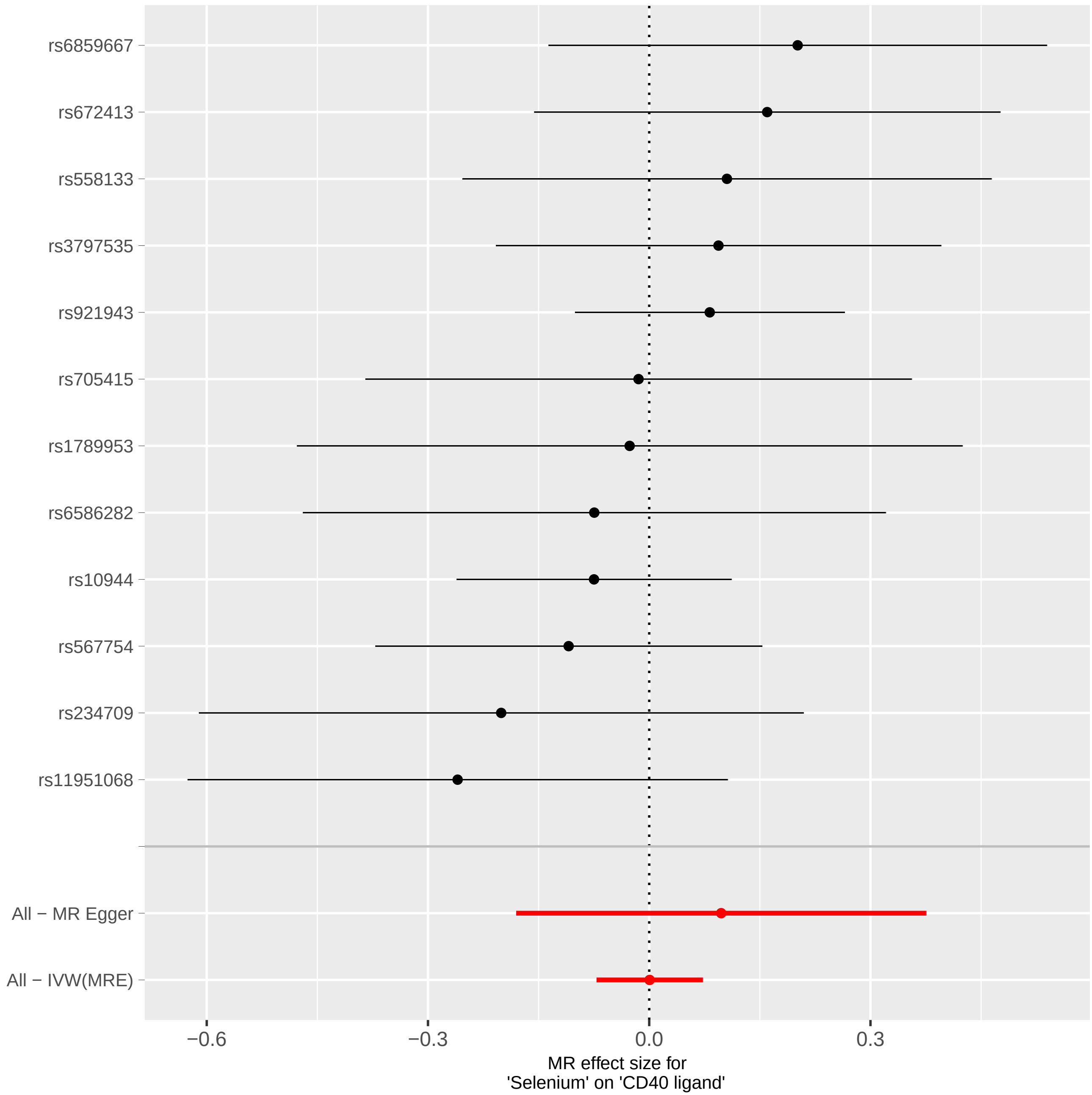

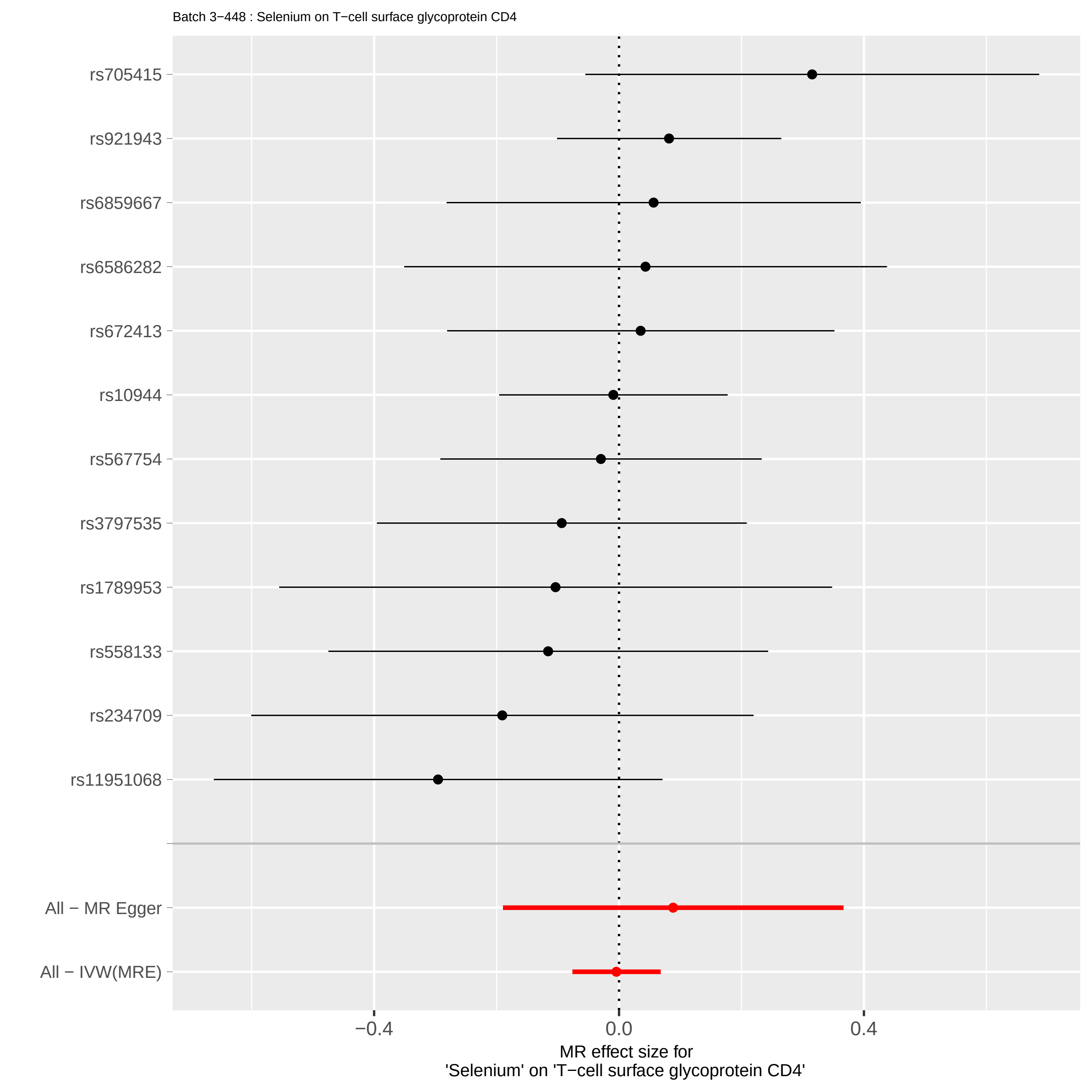
Batch 3−448 : Selenium on T−cell surface glycoprotein CD4
rs705415
rs921943
rs6859667
rs6586282
rs672413
rs10944
rs567754
rs3797535
rs1789953
rs558133
rs234709
rs11951068
All − MR Egger
All − IVW(MRE)
−0.4
0.0
0.4
MR effect size for
'Selenium' on 'T−cell surface glycoprotein CD4'

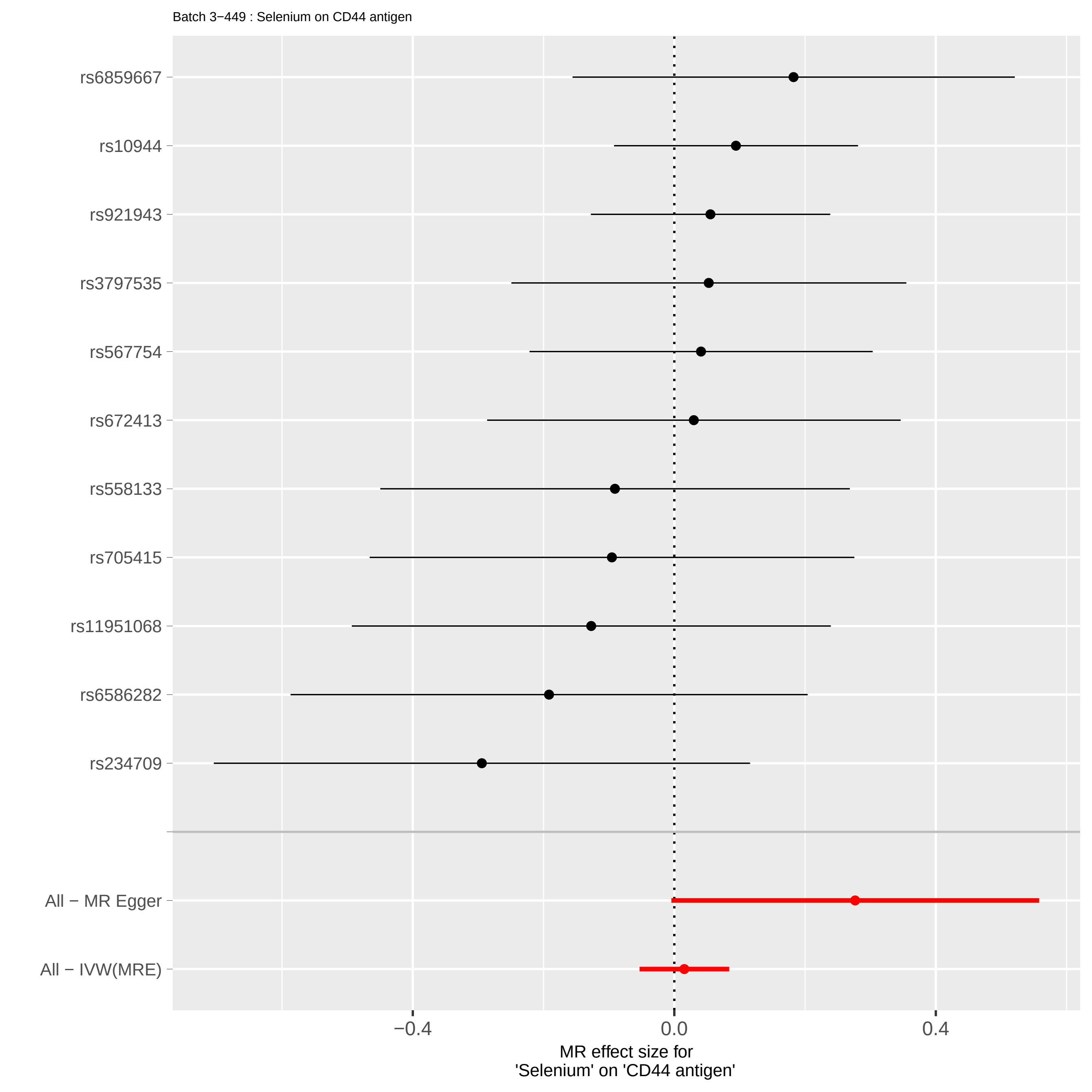

Batch 3−449 : Selenium on CD44 antigen
rs6859667
rs10944
rs921943
rs3797535
rs567754
rs672413
rs558133
rs705415
rs11951068
rs6586282
rs234709
All − MR Egger
All − IVW(MRE)
−0.4
0.0
0.4
MR effect size for
'Selenium' on 'CD44 antigen'

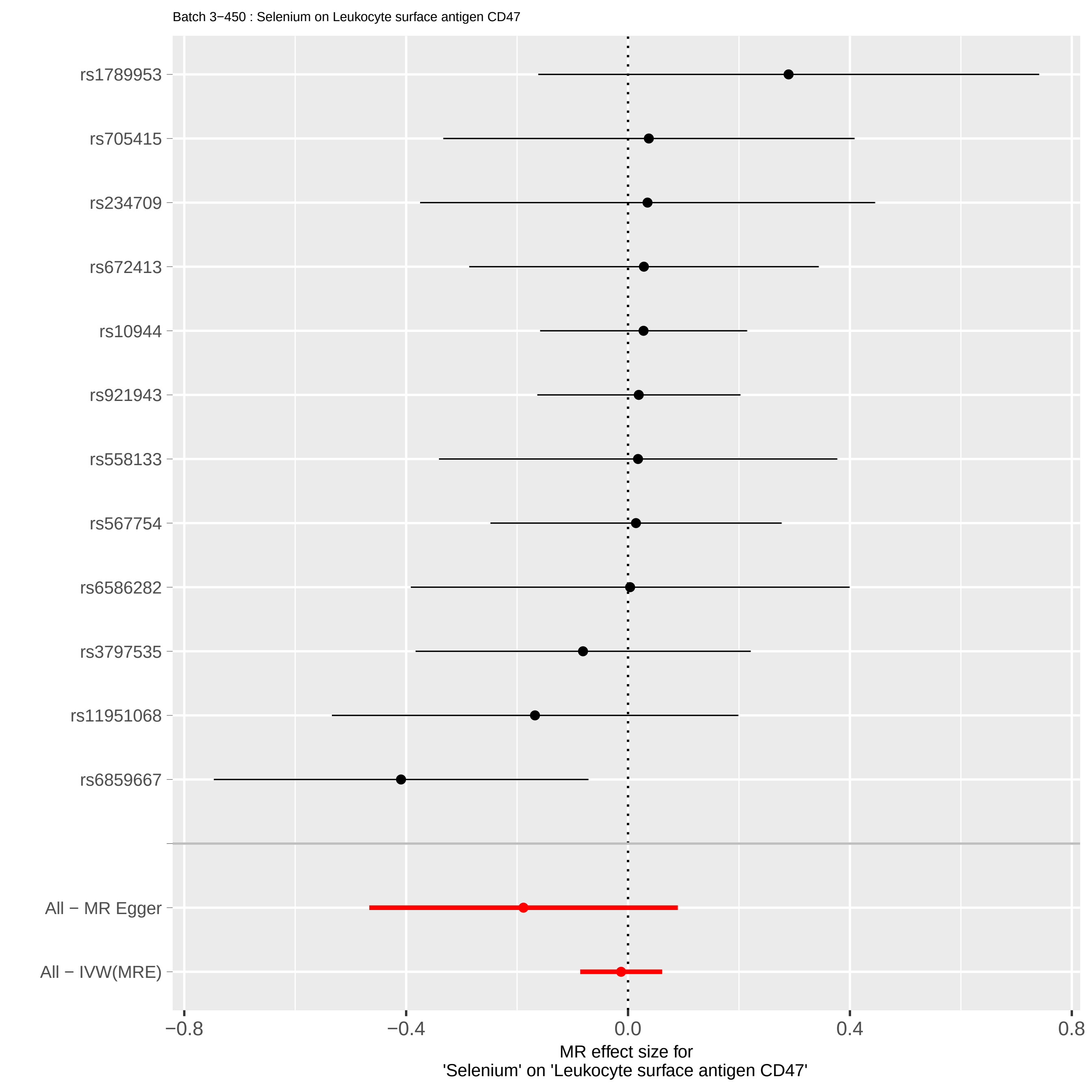
Batch 3−450 : Selenium on Leukocyte surface antigen CD47
rs1789953
rs705415
rs234709
rs672413
rs10944
rs921943
rs558133
rs567754
rs6586282
rs3797535
rs11951068
rs6859667
All − MR Egger
All − IVW(MRE)
−0.8
−0.4
0.0
0.4
0.8
MR effect size for
'Selenium' on 'Leukocyte surface antigen CD47'

Batch 3−451 : Selenium on CD48 antigen

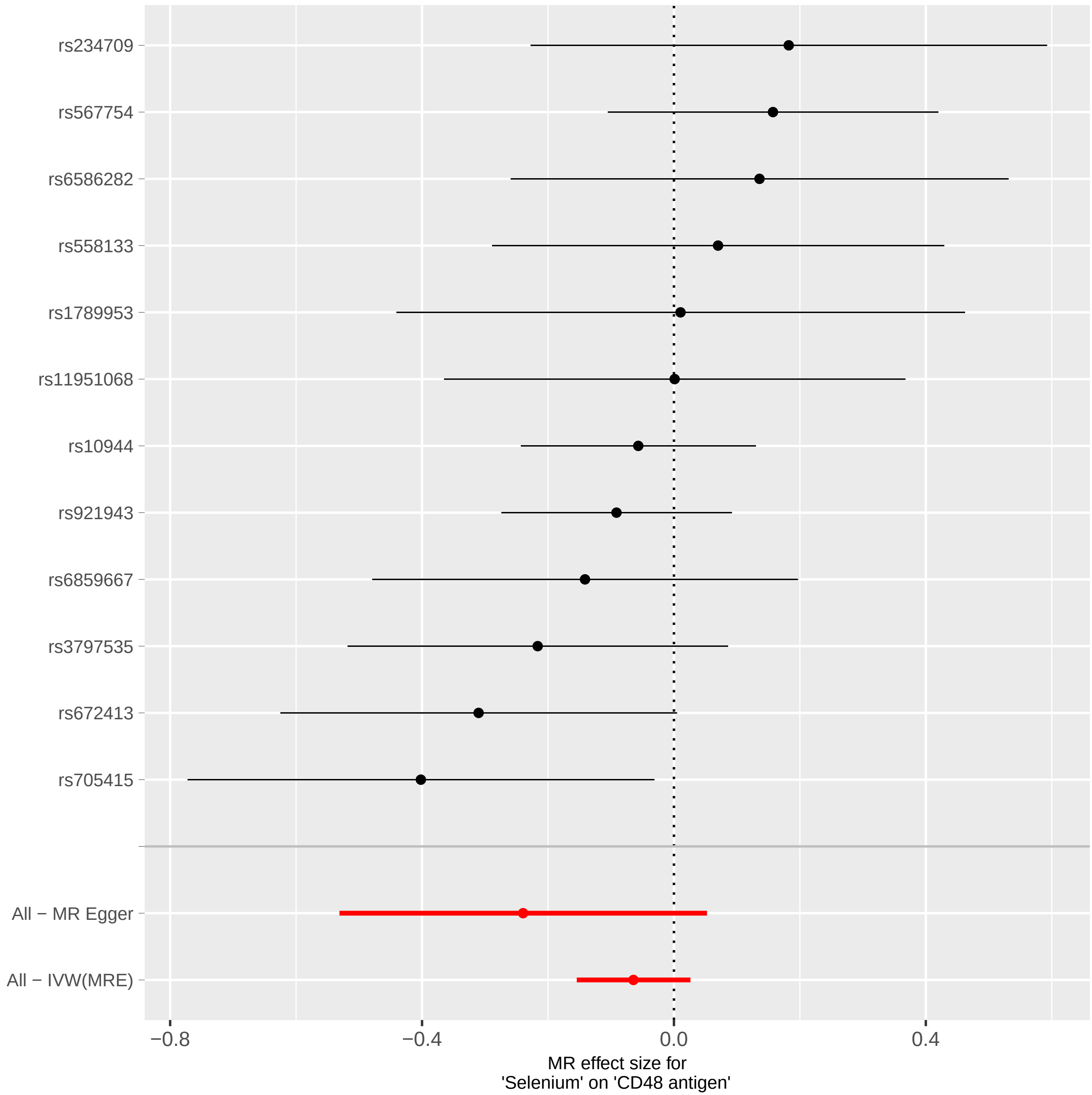

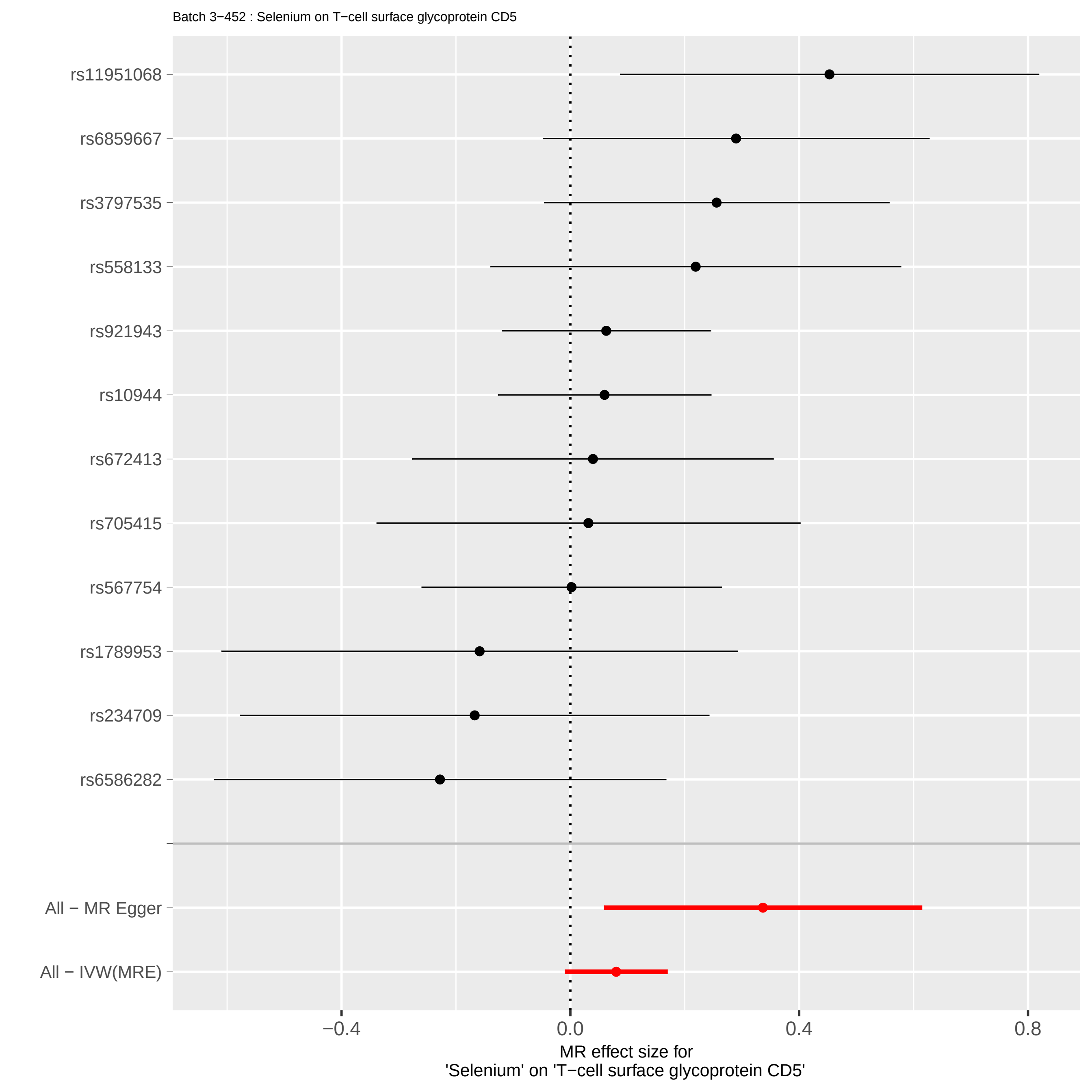

Batch 3−452 : Selenium on T−cell surface glycoprotein CD5
rs11951068
rs6859667
rs3797535
rs558133
rs921943
rs10944
rs672413
rs705415
rs567754
rs1789953
rs234709
rs6586282
All − MR Egger
All − IVW(MRE)
−0.4
0.0
0.4
0.8
MR effect size for
'Selenium' on 'T−cell surface glycoprotein CD5'

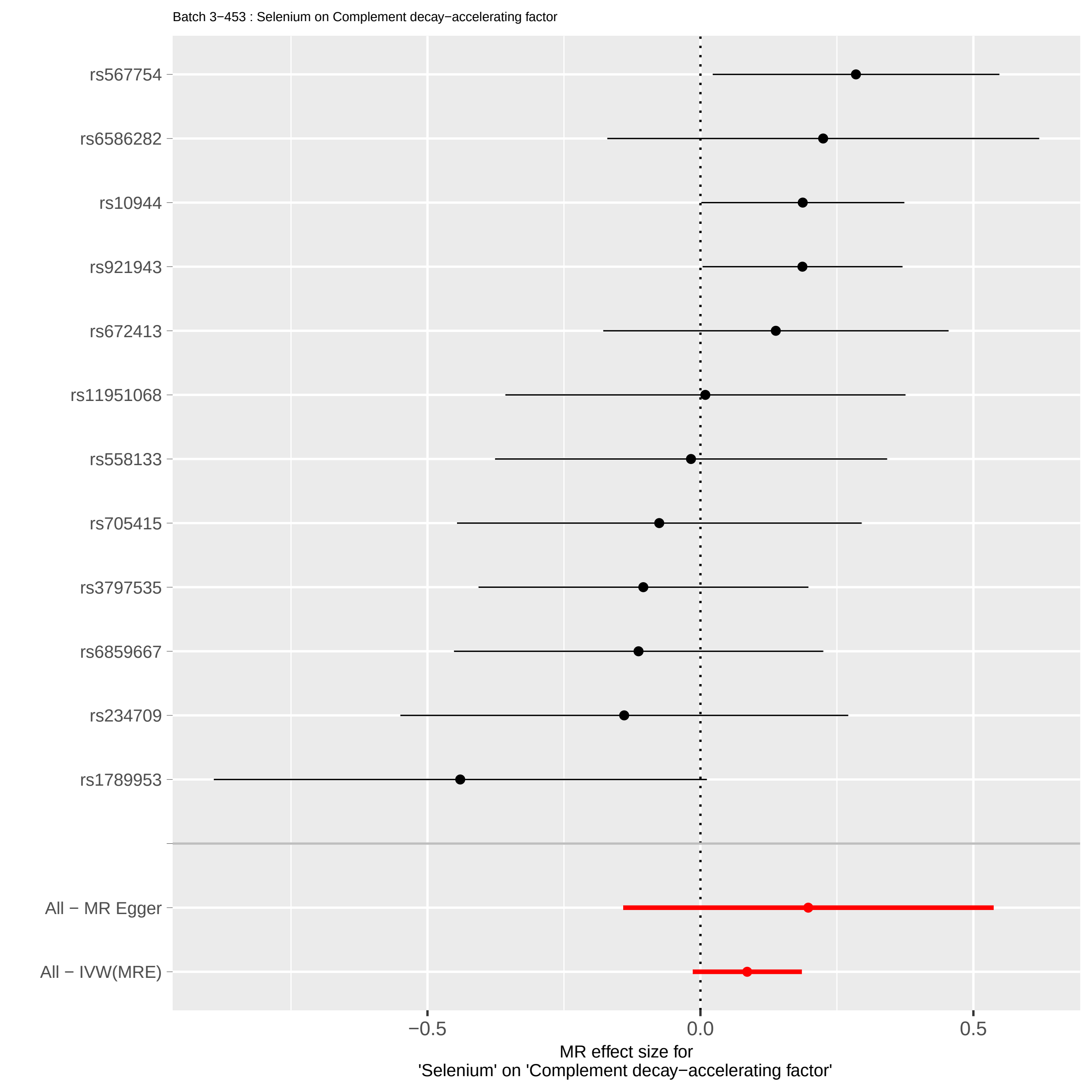

Batch 3−453 : Selenium on Complement decay−accelerating factor
rs567754
rs6586282
rs10944
rs921943
rs672413
rs11951068
rs558133
rs705415
rs3797535
rs6859667
rs234709
rs1789953
All − MR Egger
All − IVW(MRE)
−0.5
0.0
0.5
MR effect size for
'Selenium' on 'Complement decay−accelerating factor'

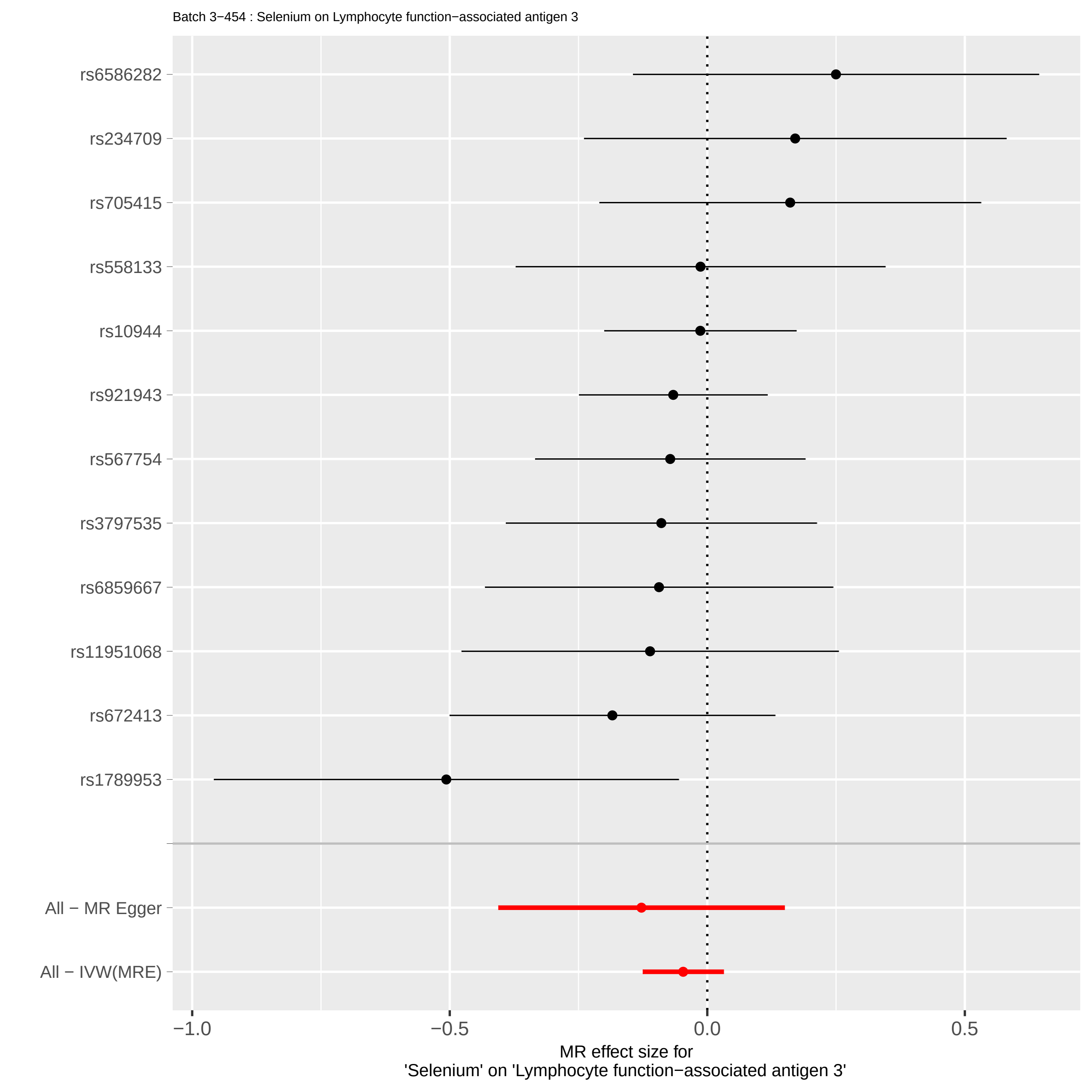

Batch 3−454 : Selenium on Lymphocyte function−associated antigen 3
rs6586282
rs234709
rs705415
rs558133
rs10944
rs921943
rs567754
rs3797535
rs6859667
rs11951068
rs672413
rs1789953
All − MR Egger
All − IVW(MRE)
−1.0
−0.5
0.0
0.5
MR effect size for
'Selenium' on 'Lymphocyte function−associated antigen 3'

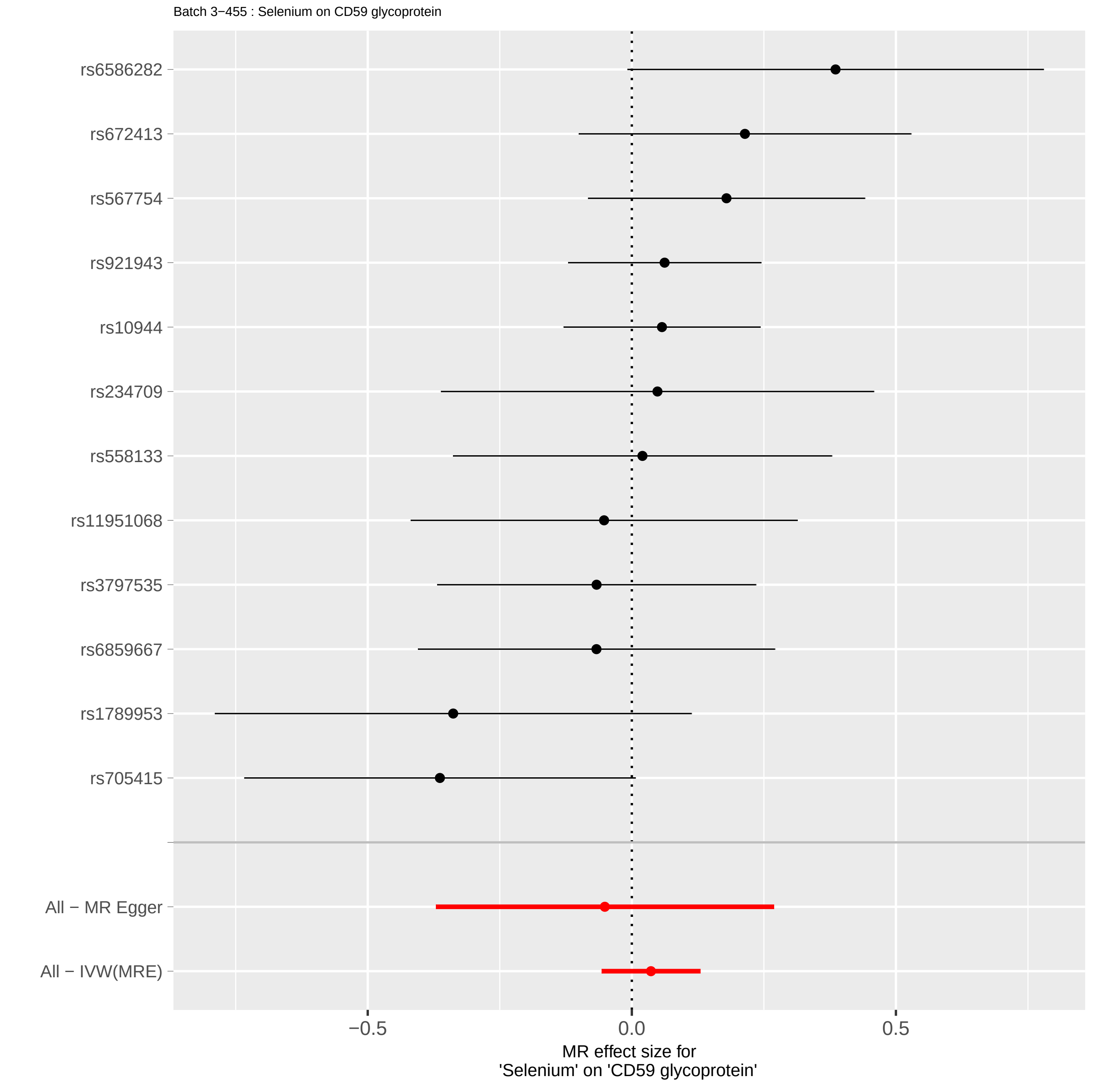

Batch 3−455 : Selenium on CD59 glycoprotein
rs6586282
rs672413
rs567754
rs921943
rs10944
rs234709
rs558133
rs11951068
rs3797535
rs6859667
rs1789953
rs705415
All − MR Egger
All − IVW(MRE)
−0.5
0.0
0.5
MR effect size for
'Selenium' on 'CD59 glycoprotein'

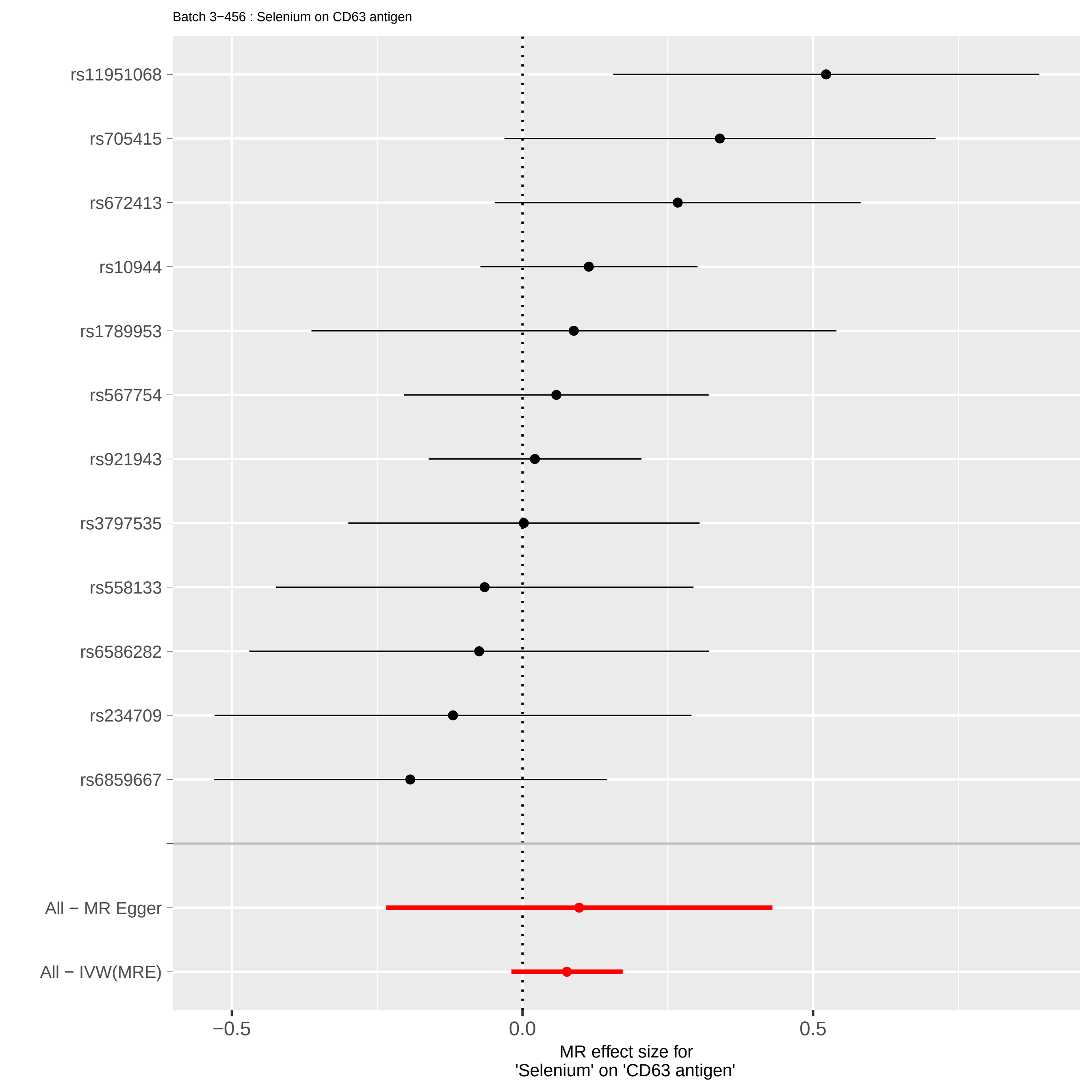

Batch 3−456 : Selenium on CD63 antigen
rs11951068
rs705415
rs672413
rs10944
rs1789953
rs567754
rs921943
rs3797535
rs558133
rs6586282
rs234709
rs6859667
All − MR Egger
All − IVW(MRE)
−0.5
0.0
0.5
MR effect size for
'Selenium' on 'CD63 antigen'

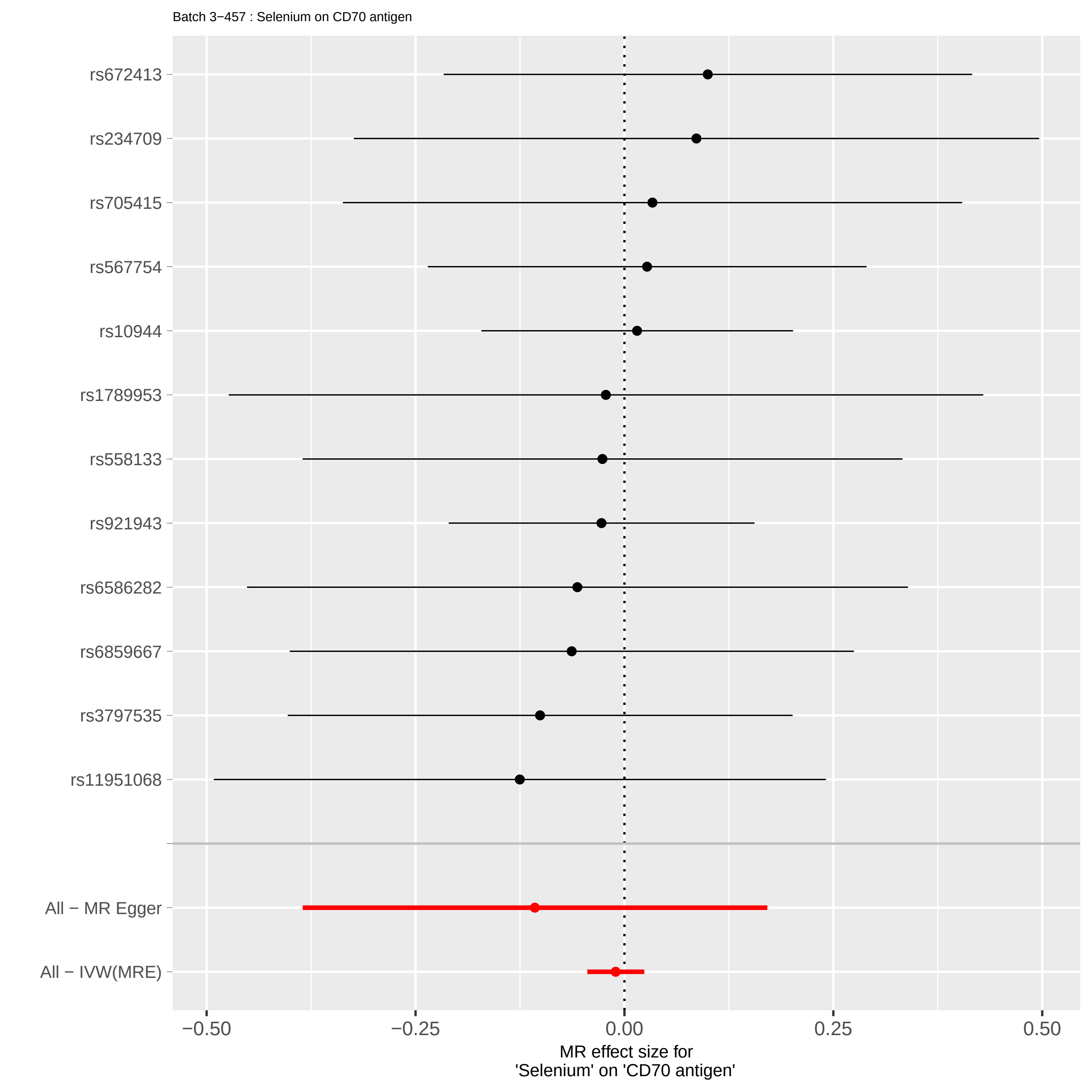
Batch 3−457 : Selenium on CD70 antigen
rs672413
rs234709
rs705415
rs567754
rs10944
rs1789953
rs558133
rs921943
rs6586282
rs6859667
rs3797535
rs11951068
All − MR Egger
All − IVW(MRE)
−0.50
−0.25
0.00
0.25
0.50
MR effect size for
'Selenium' on 'CD70 antigen'

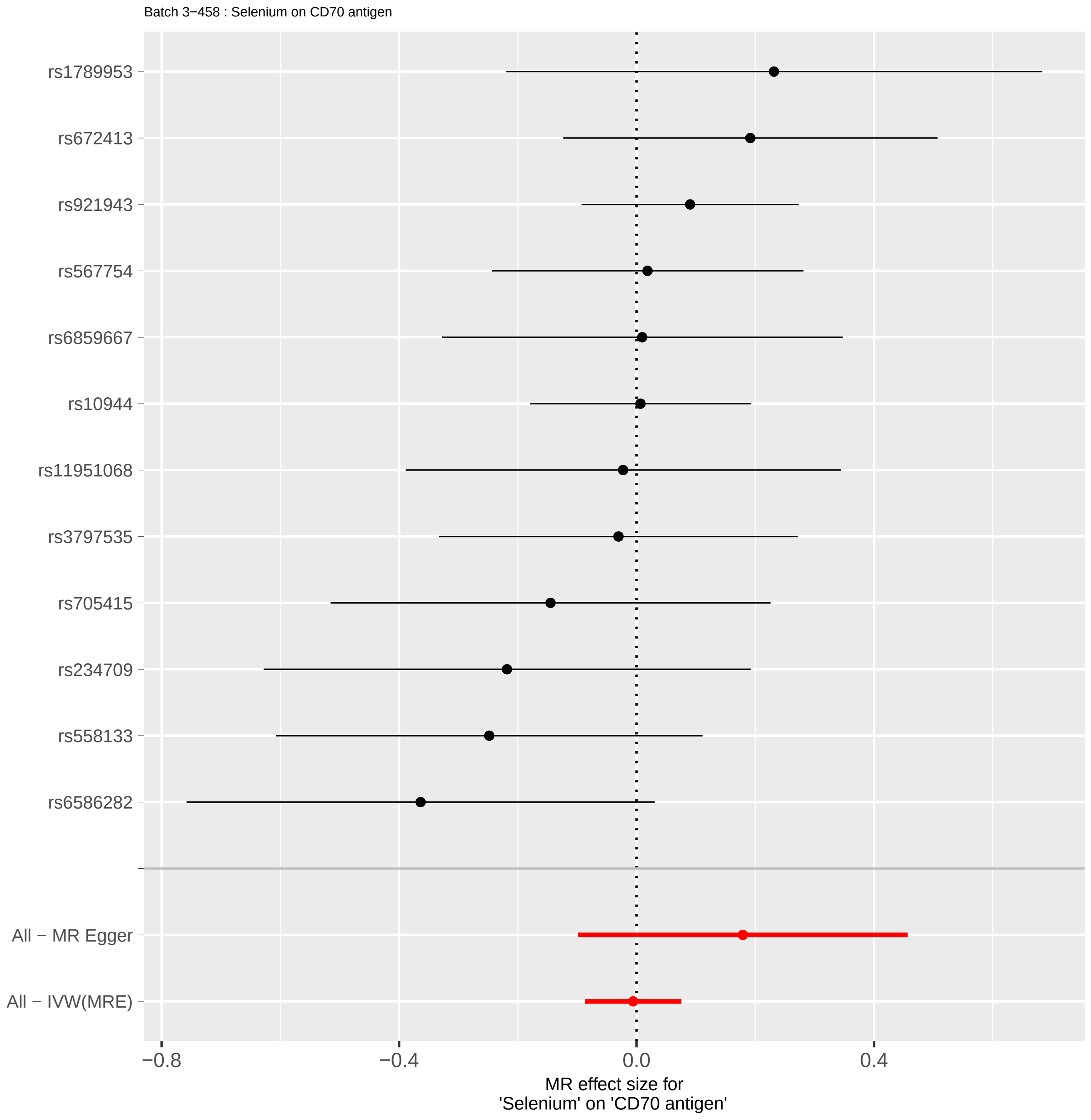

Batch 3−458 : Selenium on CD70 antigen
rs1789953
rs672413
rs921943
rs567754
rs6859667
rs10944
rs11951068
rs3797535
rs705415
rs234709
rs558133
rs6586282
All − MR Egger
All − IVW(MRE)
−0.8
−0.4
0.0
0.4
MR effect size for
'Selenium' on 'CD70 antigen'

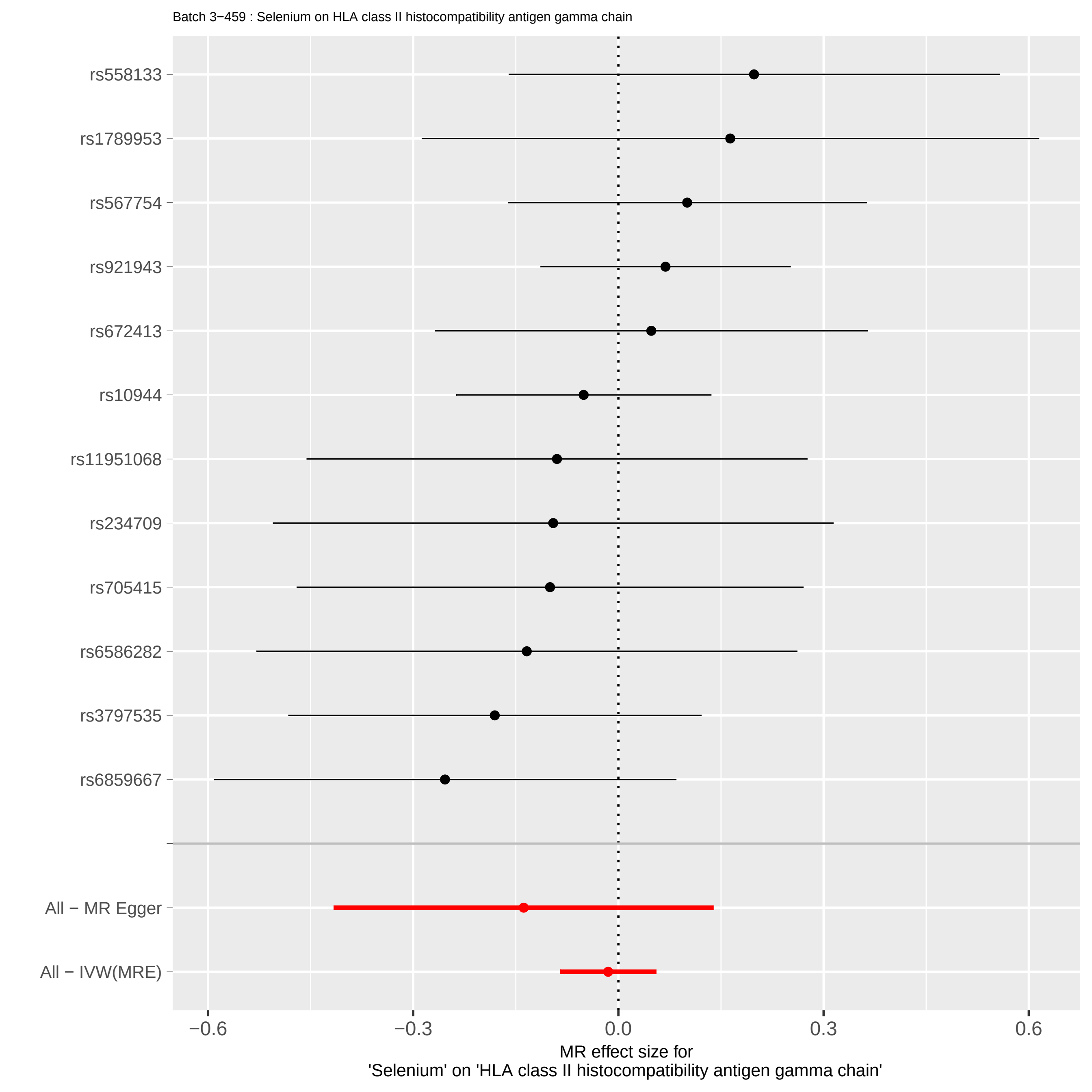
Batch 3−459 : Selenium on HLA class II histocompatibility antigen gamma chain
rs558133
rs1789953
rs567754
rs921943
rs672413
rs10944
rs11951068
rs234709
rs705415
rs6586282
rs3797535
rs6859667
All − MR Egger
All − IVW(MRE)
−0.6
−0.3
0.0
0.3
0.6
MR effect size for
'Selenium' on 'HLA class II histocompatibility antigen gamma chain'

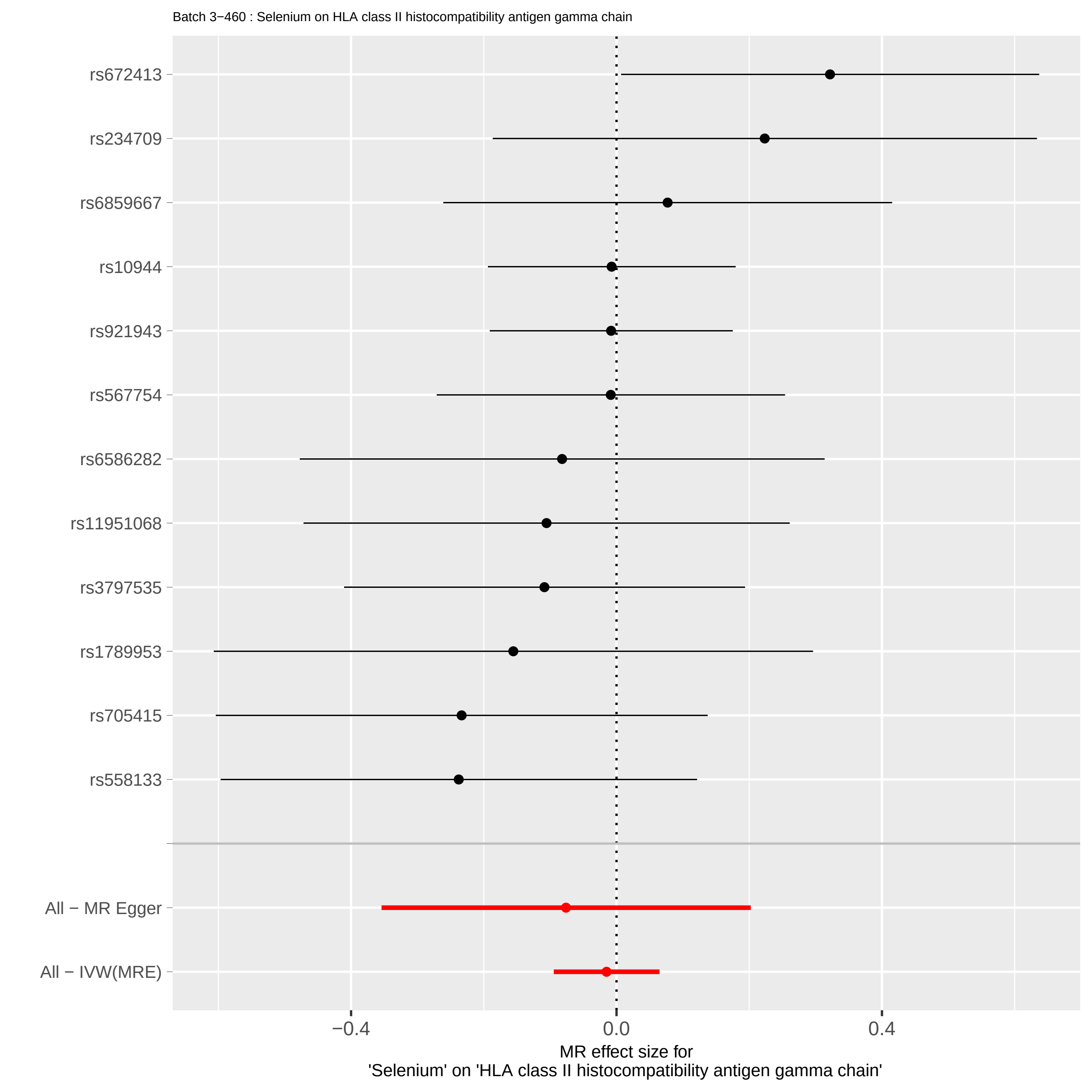
Batch 3−460 : Selenium on HLA class II histocompatibility antigen gamma chain
rs672413
rs234709
rs6859667
rs10944
rs921943
rs567754
rs6586282
rs11951068
rs3797535
rs1789953
rs705415
rs558133
All − MR Egger
All − IVW(MRE)
−0.4
0.0
0.4
MR effect size for
'Selenium' on 'HLA class II histocompatibility antigen gamma chain'

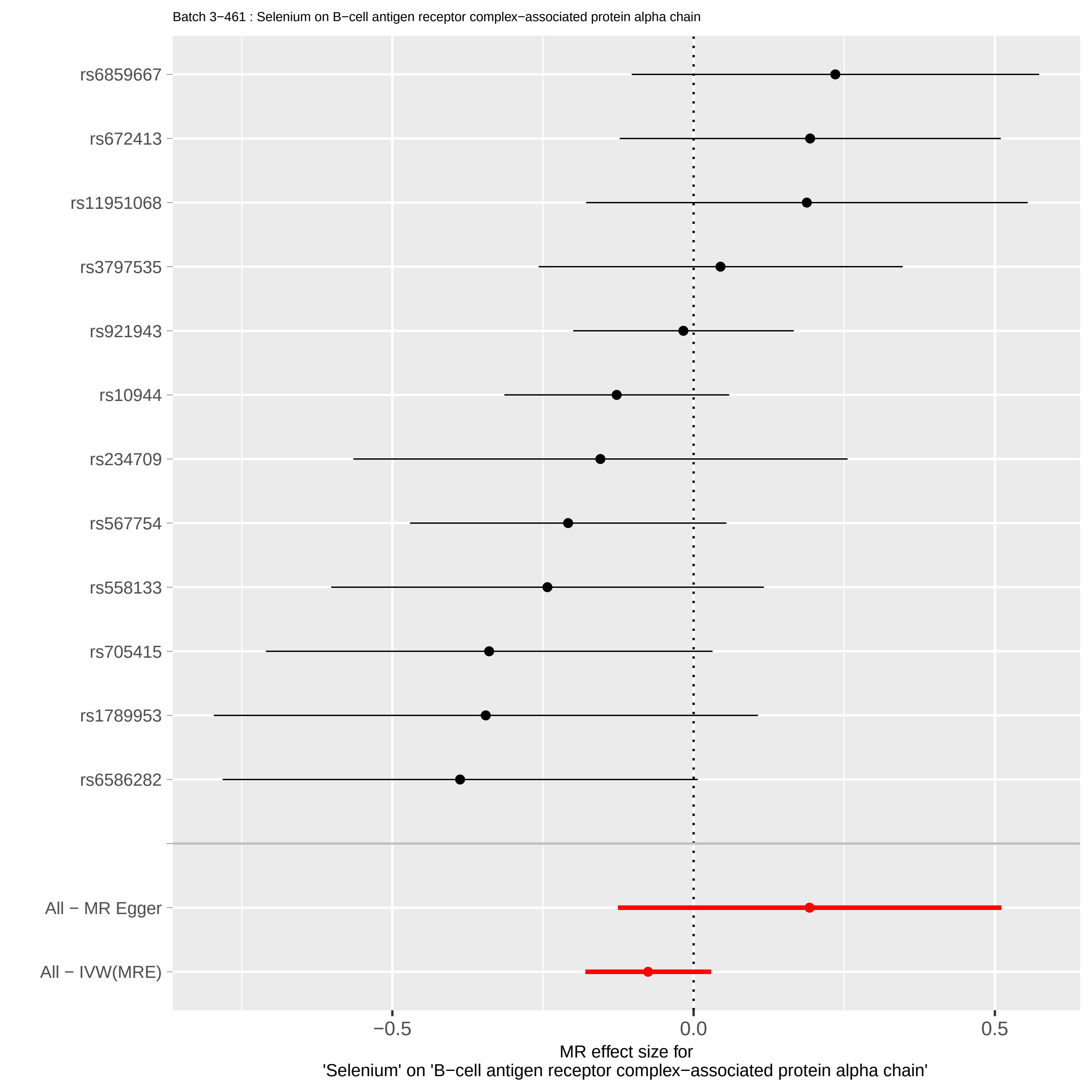

Batch 3−461 : Selenium on B−cell antigen receptor complex−associated protein alpha chain
rs6859667
rs672413
rs11951068
rs3797535
rs921943
rs10944
rs234709
rs567754
rs558133
rs705415
rs1789953
rs6586282
All − MR Egger
All − IVW(MRE)
−0.5
0.0
0.5
MR effect size for
'Selenium' on 'B−cell antigen receptor complex−associated protein alpha chain'

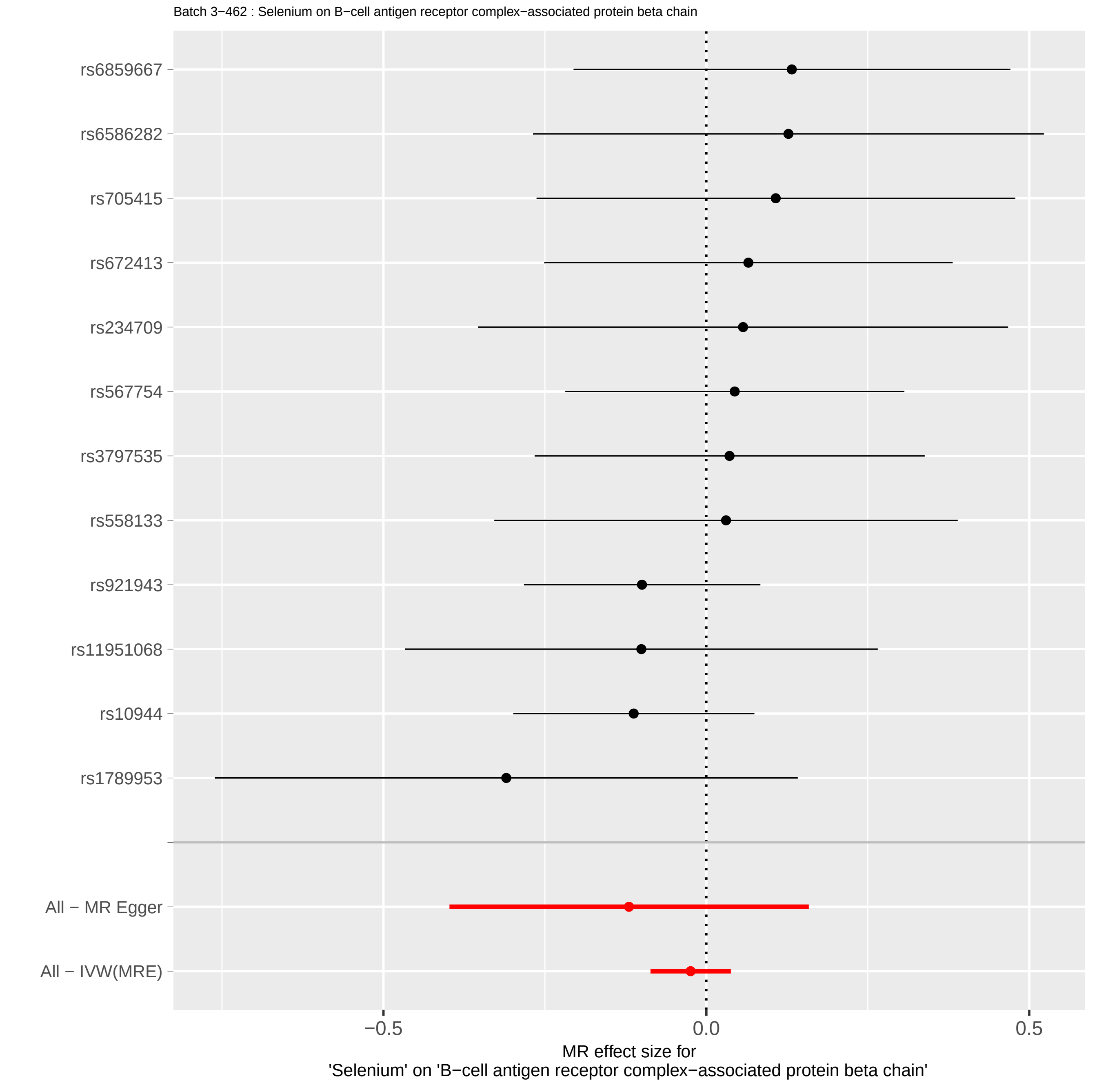
Batch 3−462 : Selenium on B−cell antigen receptor complex−associated protein beta chain
rs6859667
rs6586282
rs705415
rs672413
rs234709
rs567754
rs3797535
rs558133
rs921943
rs11951068
rs10944
rs1789953
All − MR Egger
All − IVW(MRE)
−0.5
0.0
0.5
MR effect size for
'Selenium' on 'B−cell antigen receptor complex−associated protein beta chain'

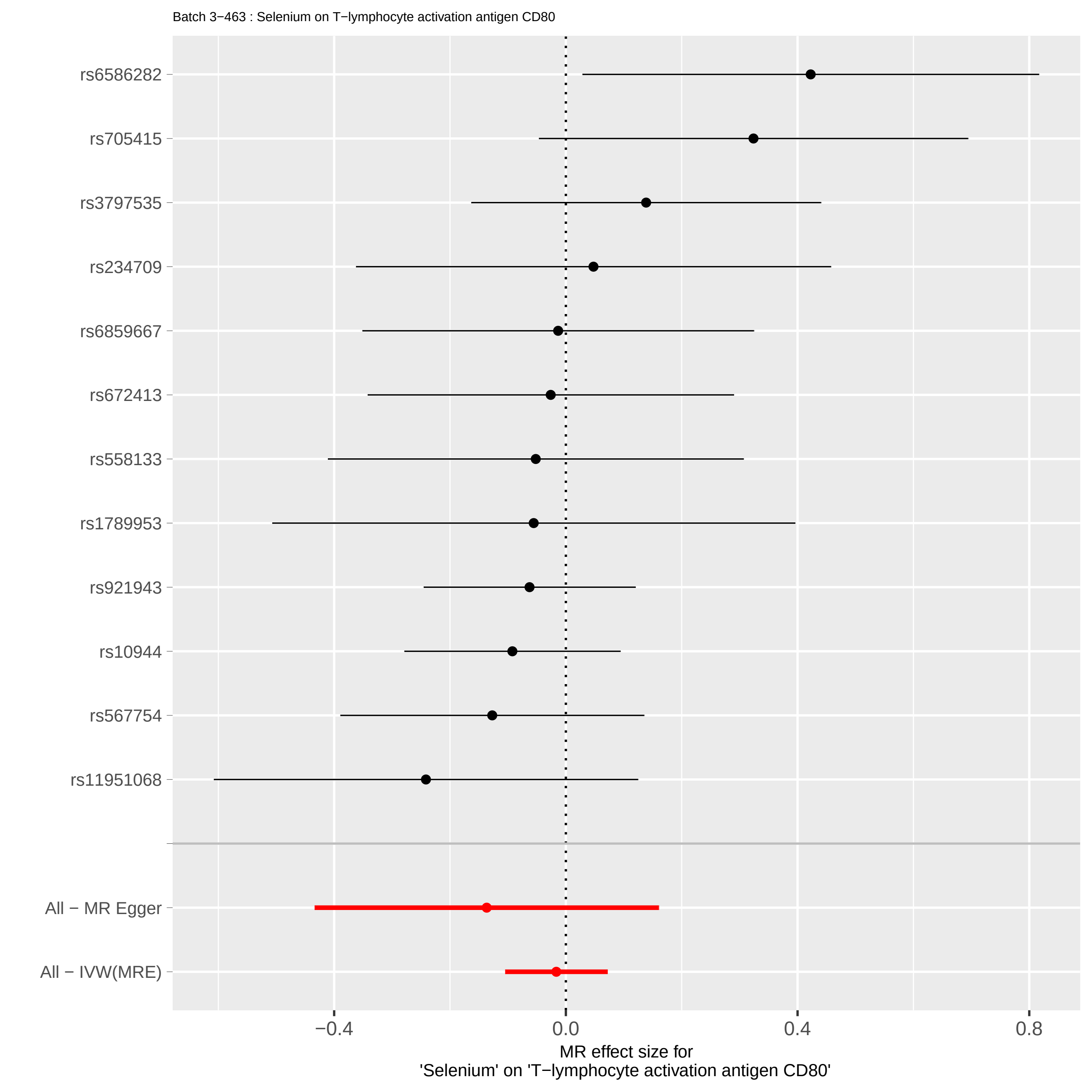

Batch 3−463 : Selenium on T−lymphocyte activation antigen CD80
rs6586282
rs705415
rs3797535
rs234709
rs6859667
rs672413
rs558133
rs1789953
rs921943
rs10944
rs567754
rs11951068
All − MR Egger
All − IVW(MRE)
−0.4
0.0
0.4
0.8
MR effect size for
'Selenium' on 'T−lymphocyte activation antigen CD80'

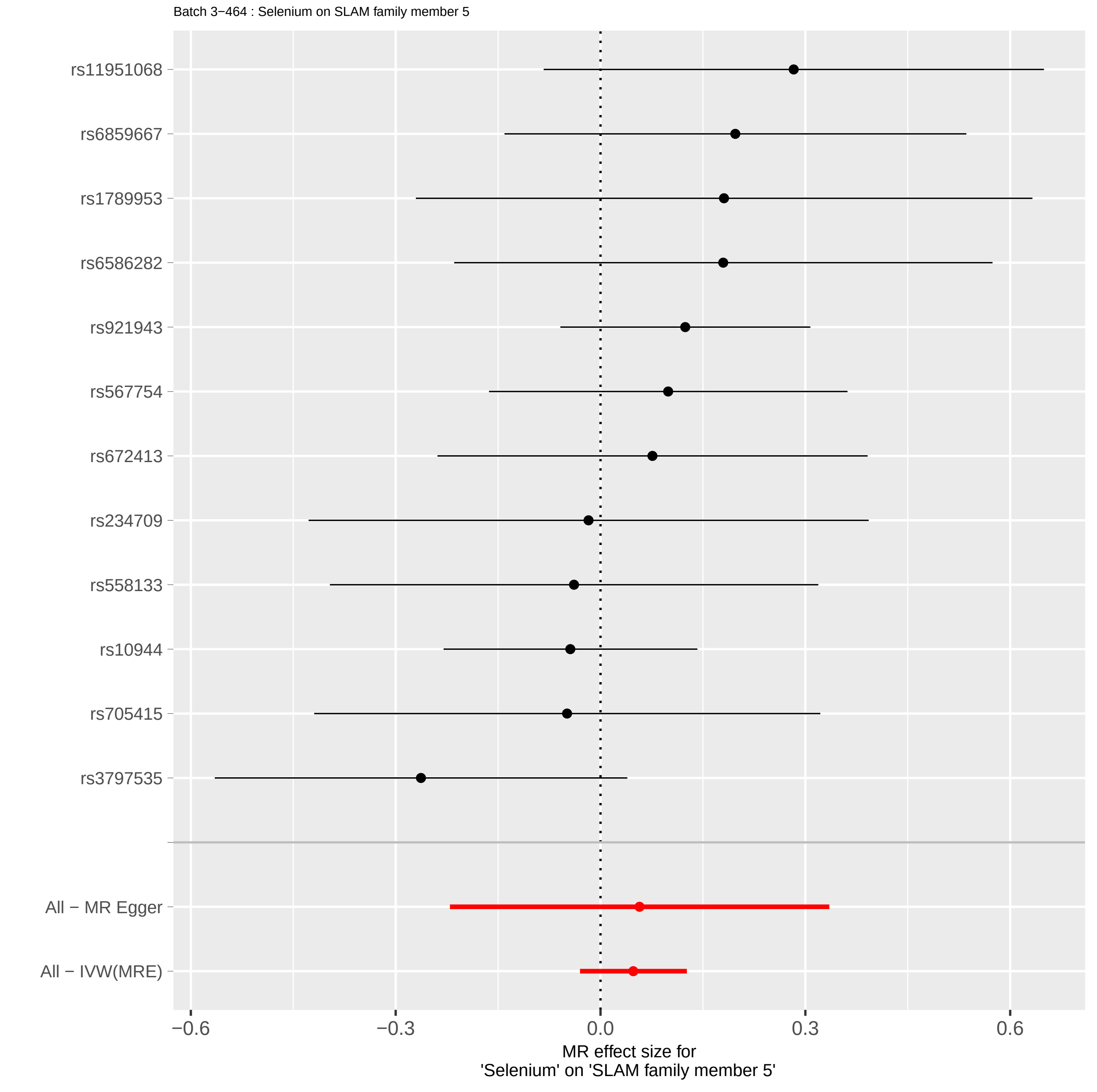

Batch 3−464 : Selenium on SLAM family member 5
rs11951068
rs6859667
rs1789953
rs6586282
rs921943
rs567754
rs672413
rs234709
rs558133
rs10944
rs705415
rs3797535
All − MR Egger
All − IVW(MRE)
−0.6
−0.3
0.0
0.3
0.6
MR effect size for
'Selenium' on 'SLAM family member 5'

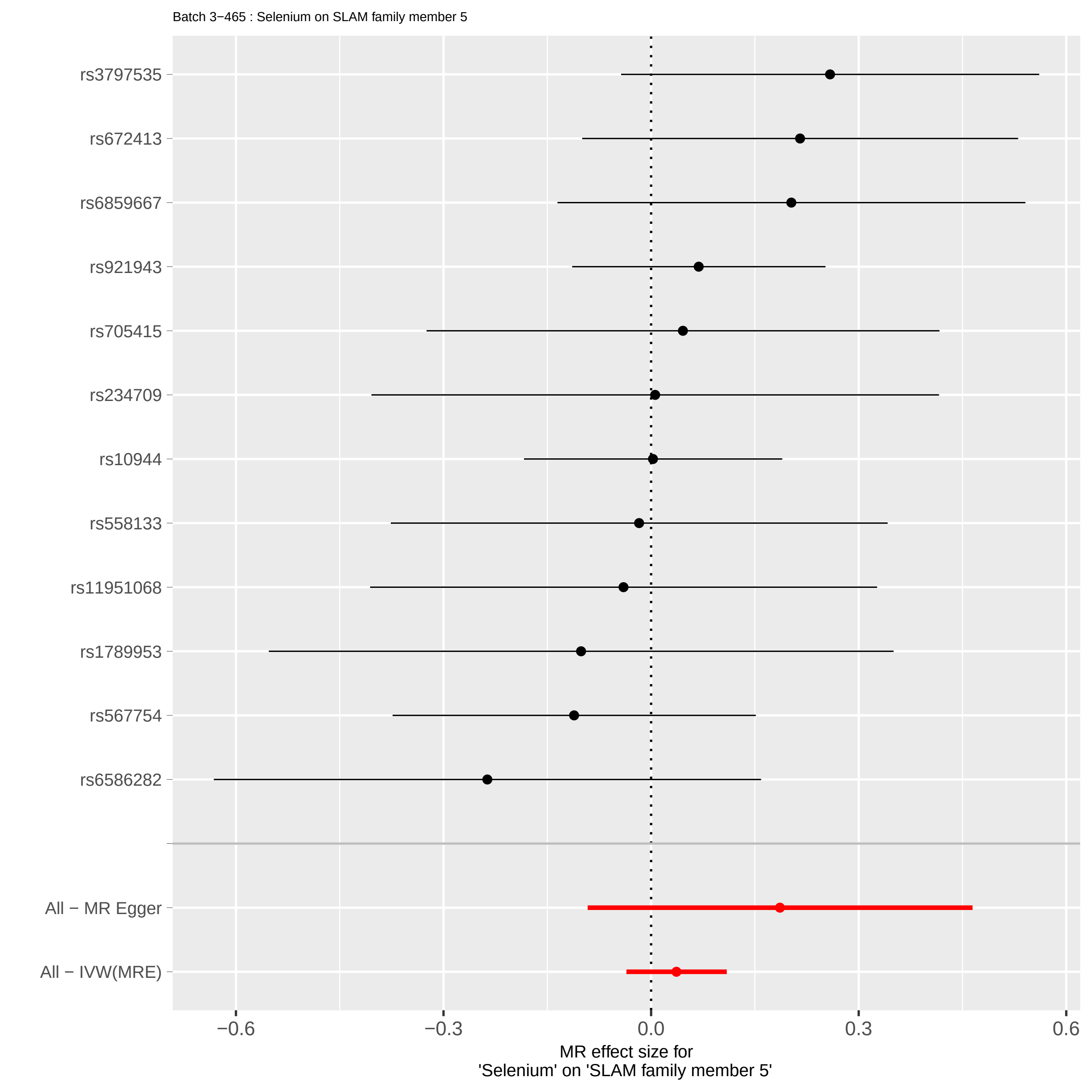

Batch 3−465 : Selenium on SLAM family member 5
rs3797535
rs672413
rs6859667
rs921943
rs705415
rs234709
rs10944
rs558133
rs11951068
rs1789953
rs567754
rs6586282
All − MR Egger
All − IVW(MRE)
−0.6
−0.3
0.0
0.3
0.6
MR effect size for
'Selenium' on 'SLAM family member 5'

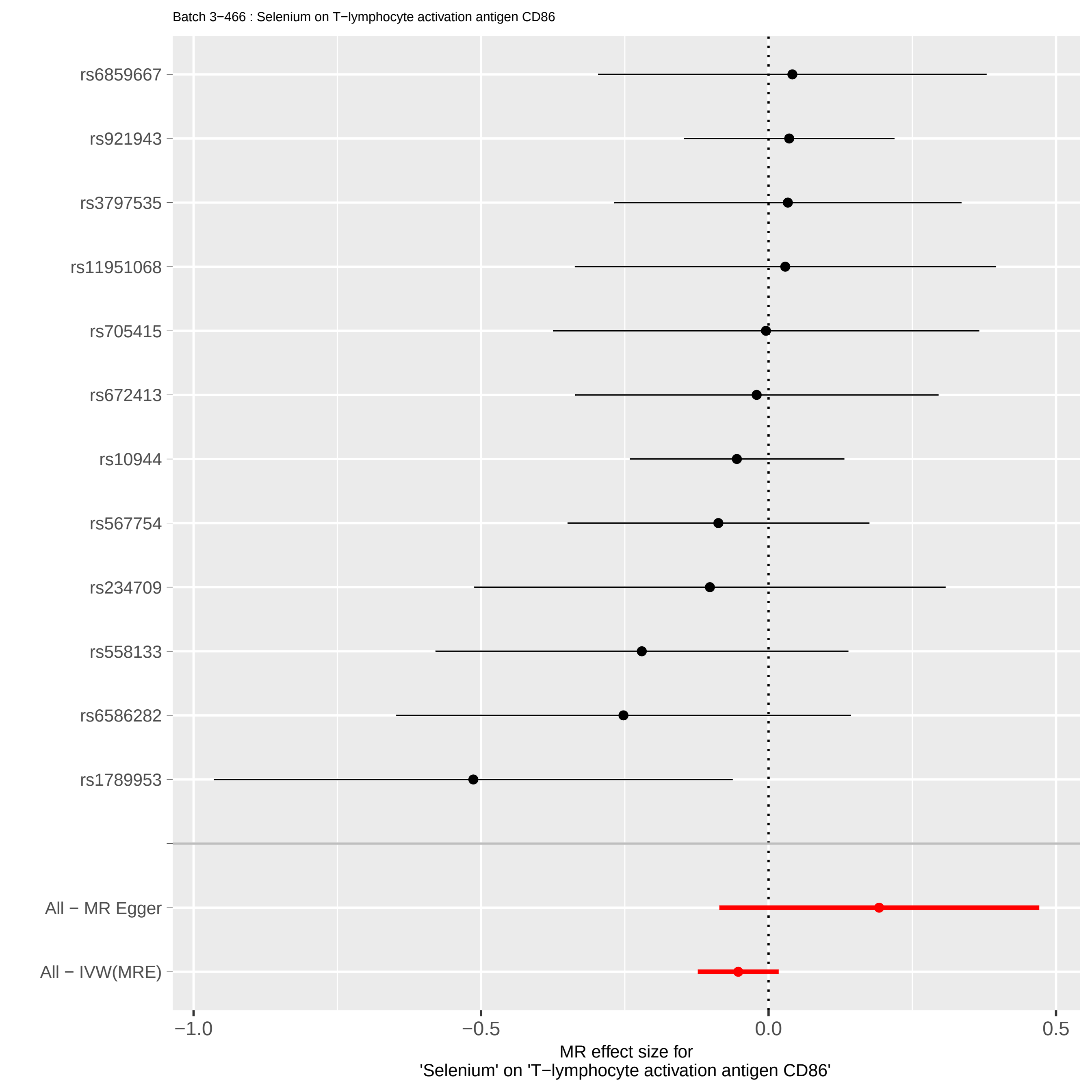

Batch 3−466 : Selenium on T−lymphocyte activation antigen CD86
rs6859667
rs921943
rs3797535
rs11951068
rs705415
rs672413
rs10944
rs567754
rs234709
rs558133
rs6586282
rs1789953
All − MR Egger
All − IVW(MRE)
−1.0
−0.5
0.0
0.5
MR effect size for
'Selenium' on 'T−lymphocyte activation antigen CD86'

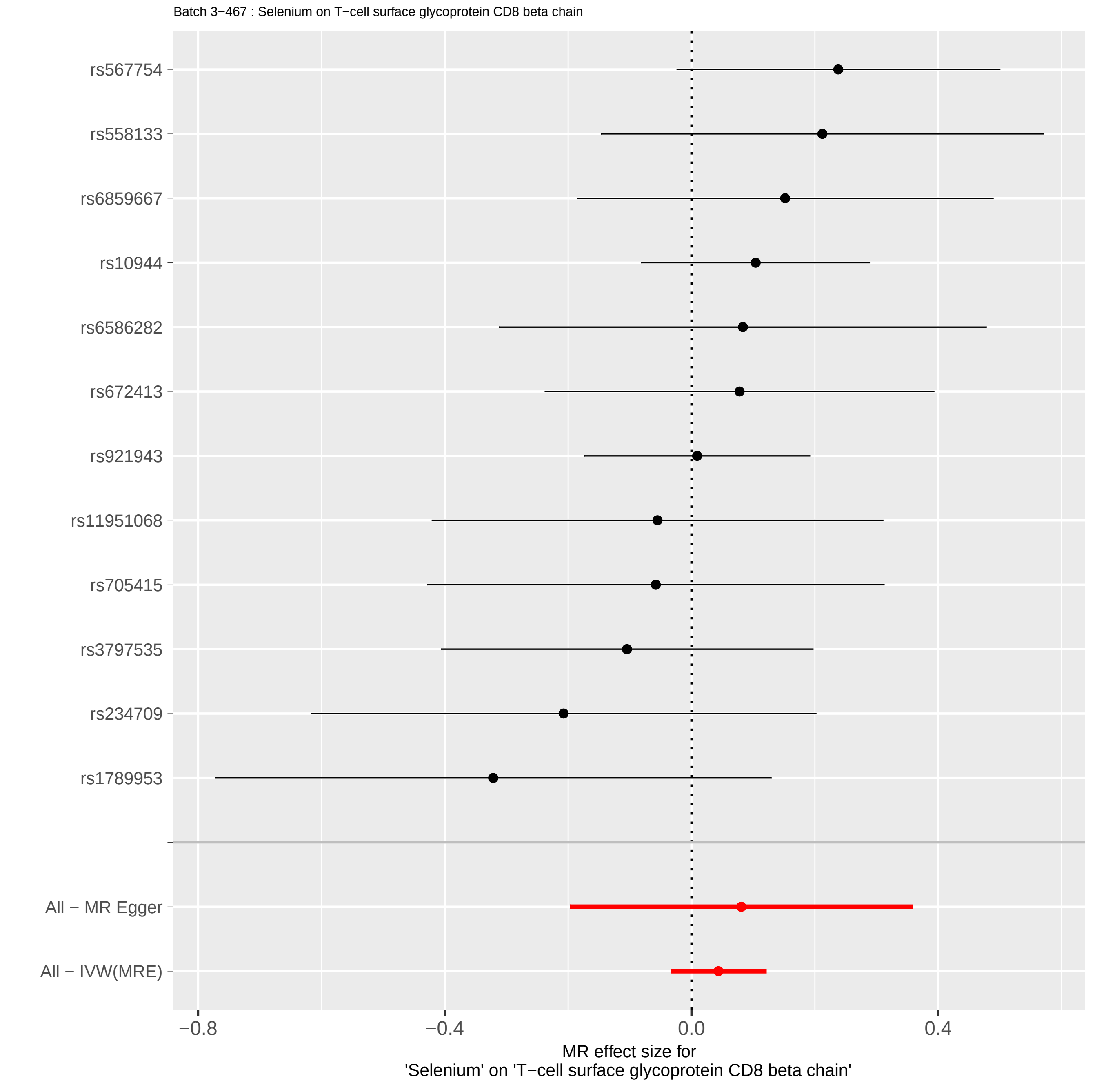

Batch 3−467 : Selenium on T−cell surface glycoprotein CD8 beta chain
rs567754
rs558133
rs6859667
rs10944
rs6586282
rs672413
rs921943
rs11951068
rs705415
rs3797535
rs234709
rs1789953
All − MR Egger
All − IVW(MRE)
−0.8
−0.4
0.0
0.4
MR effect size for
'Selenium' on 'T−cell surface glycoprotein CD8 beta chain'

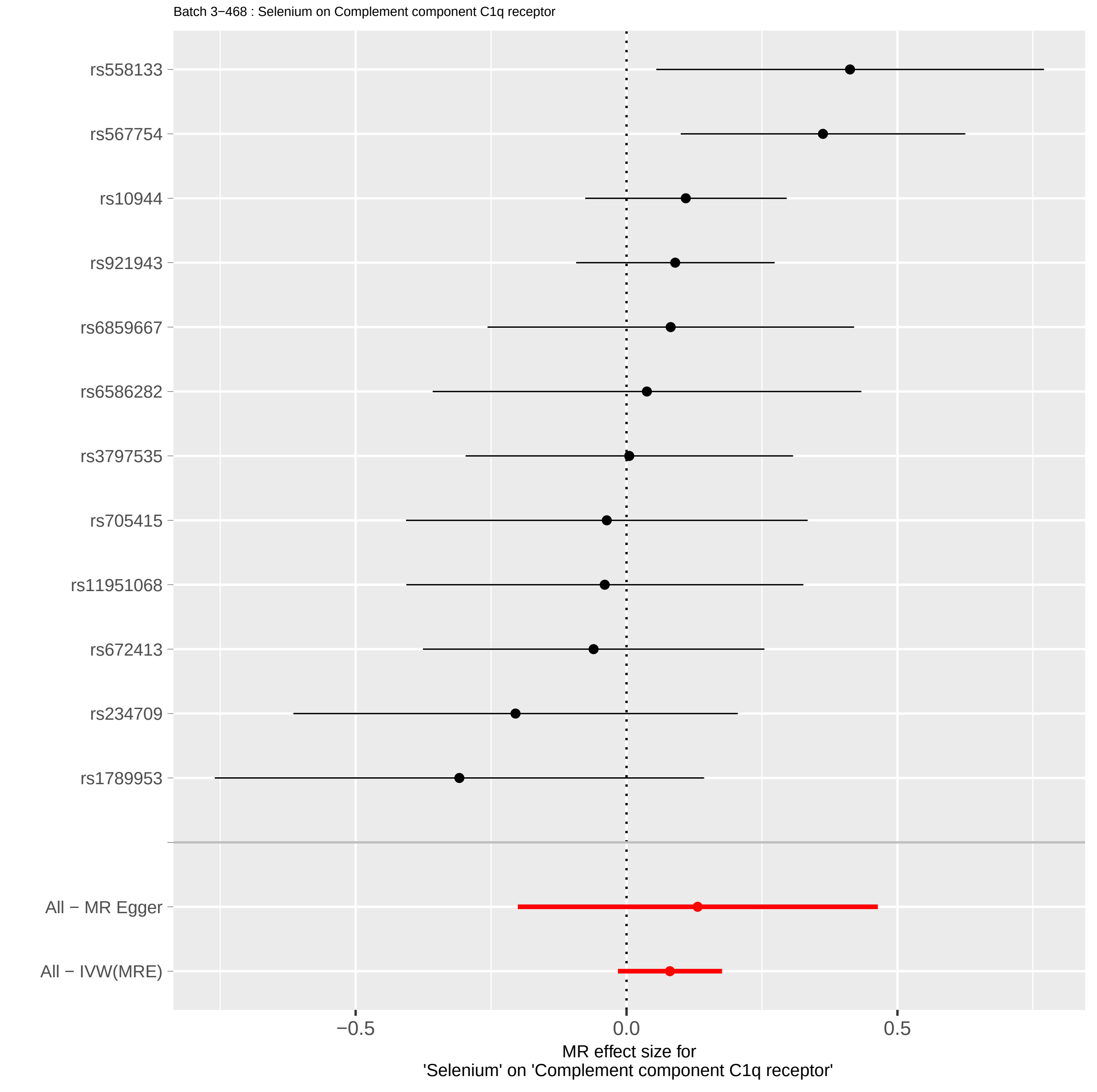

Batch 3−468 : Selenium on Complement component C1q receptor
rs558133
rs567754
rs10944
rs921943
rs6859667
rs6586282
rs3797535
rs705415
rs11951068
rs672413
rs234709
rs1789953
All − MR Egger
All − IVW(MRE)
−0.5
0.0
0.5
MR effect size for
'Selenium' on 'Complement component C1q receptor'

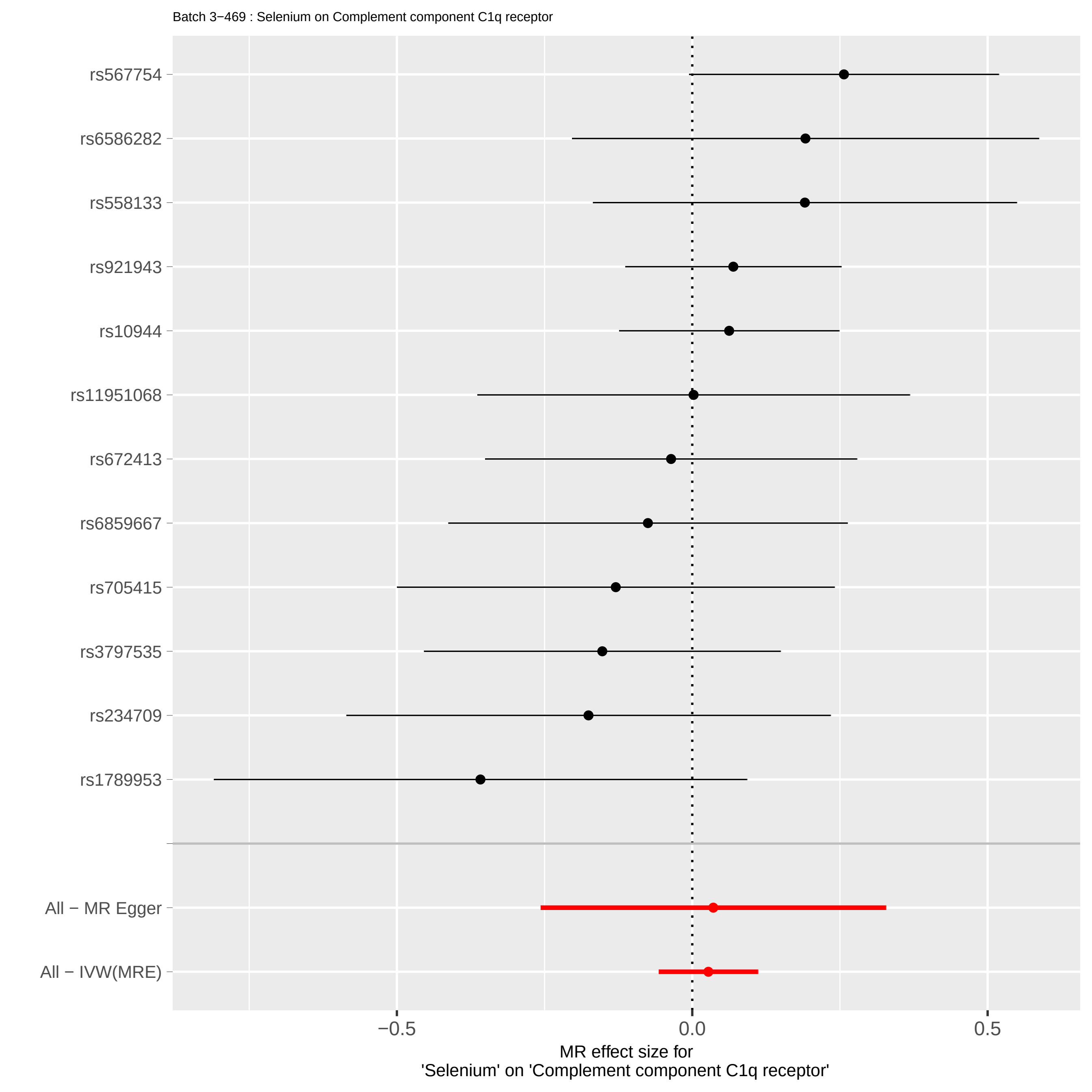

Batch 3−469 : Selenium on Complement component C1q receptor
rs567754
rs6586282
rs558133
rs921943
rs10944
rs11951068
rs672413
rs6859667
rs705415
rs3797535
rs234709
rs1789953
All − MR Egger
All − IVW(MRE)
−0.5
0.0
0.5
MR effect size for
'Selenium' on 'Complement component C1q receptor'

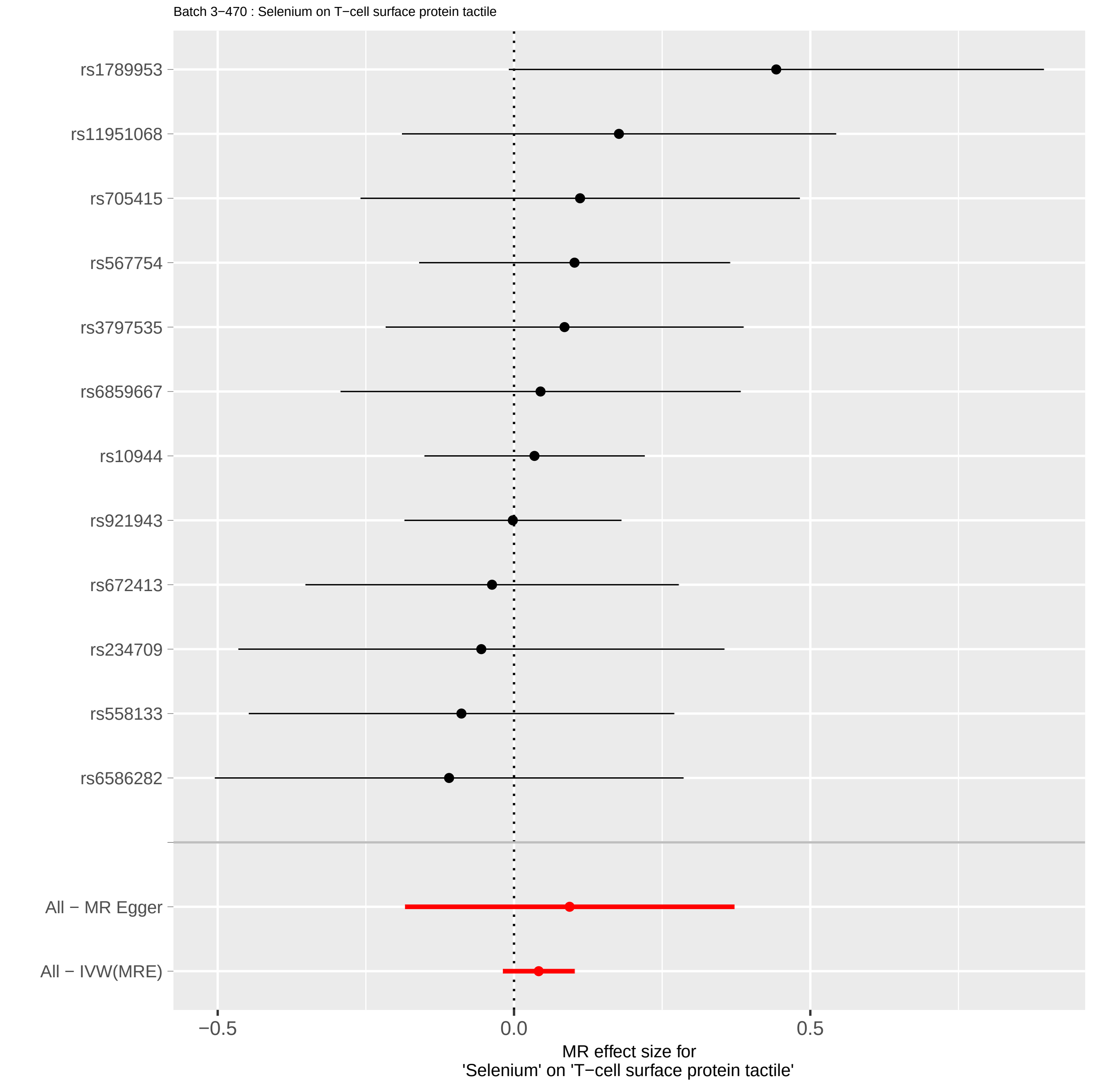

Batch 3−470 : Selenium on T−cell surface protein tactile
rs1789953
rs11951068
rs705415
rs567754
rs3797535
rs6859667
rs10944
rs921943
rs672413
rs234709
rs558133
rs6586282
All − MR Egger
All − IVW(MRE)
−0.5
0.0
0.5
MR effect size for
'Selenium' on 'T−cell surface protein tactile'

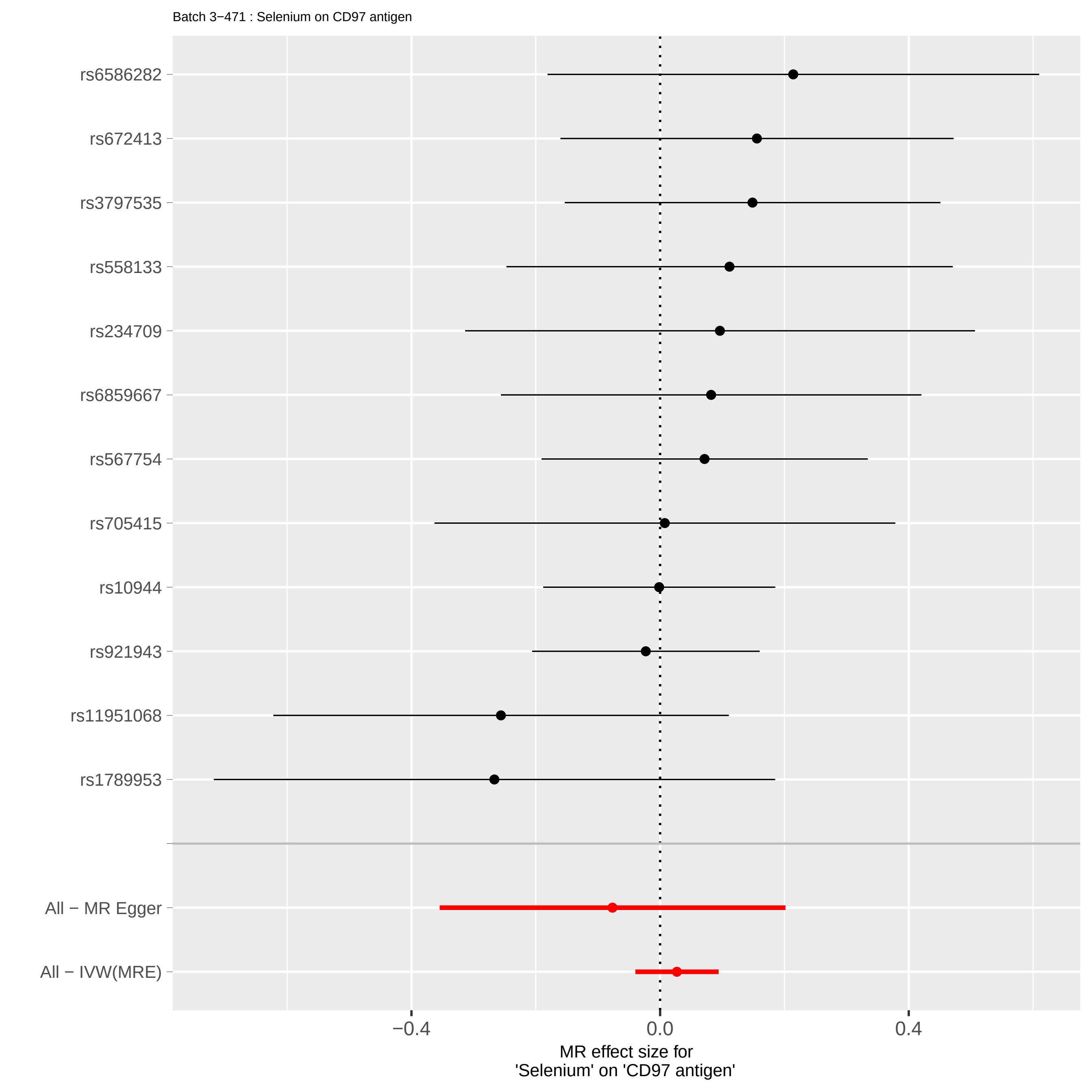

Batch 3−471 : Selenium on CD97 antigen
rs6586282
rs672413
rs3797535
rs558133
rs234709
rs6859667
rs567754
rs705415
rs10944
rs921943
rs11951068
rs1789953
All − MR Egger
All − IVW(MRE)
−0.4
0.0
0.4
MR effect size for
'Selenium' on 'CD97 antigen'

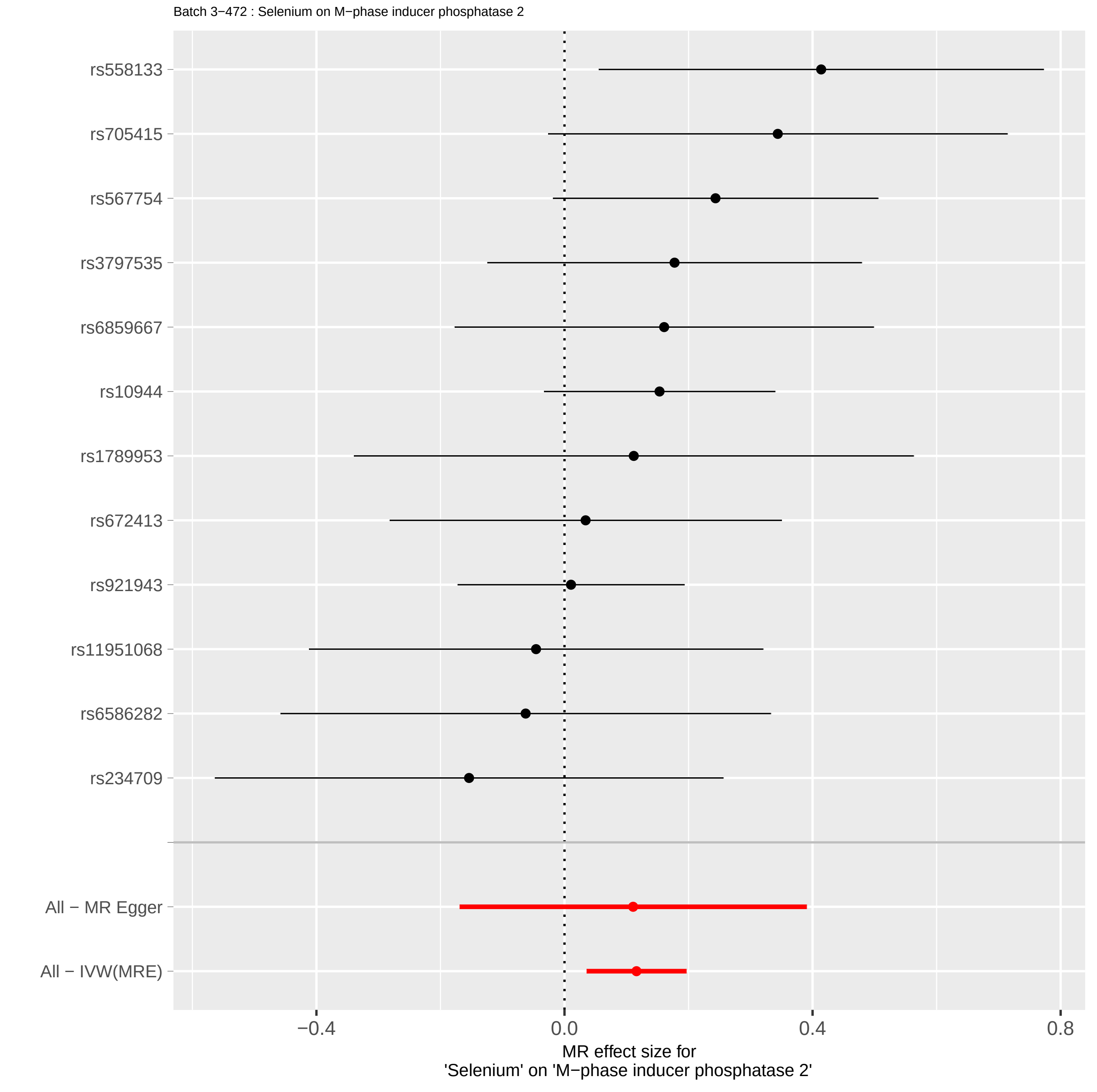
Batch 3−472 : Selenium on M−phase inducer phosphatase 2
rs558133
rs705415
rs567754
rs3797535
rs6859667
rs10944
rs1789953
rs672413
rs921943
rs11951068
rs6586282
rs234709
All − MR Egger
All − IVW(MRE)
−0.4
0.0
0.4
0.8
MR effect size for
'Selenium' on 'M−phase inducer phosphatase 2'

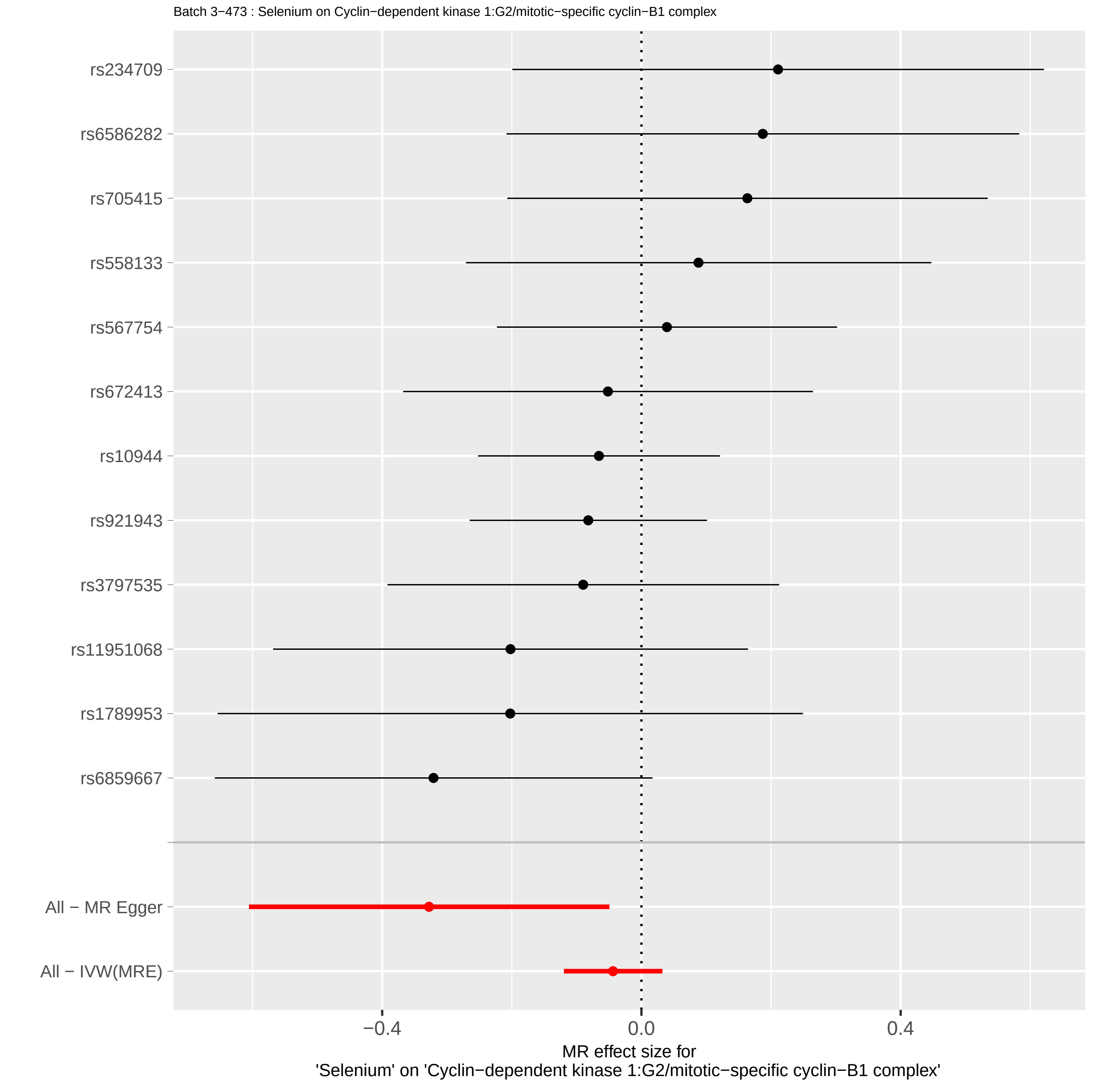

Batch 3−473 : Selenium on Cyclin−dependent kinase 1:G2/mitotic−specific cyclin−B1 complex
rs234709
rs6586282
rs705415
rs558133
rs567754
rs672413
rs10944
rs921943
rs3797535
rs11951068
rs1789953
rs6859667
All − MR Egger
All − IVW(MRE)
−0.4
0.0
0.4
MR effect size for
'Selenium' on 'Cyclin−dependent kinase 1:G2/mitotic−specific cyclin−B1 complex'

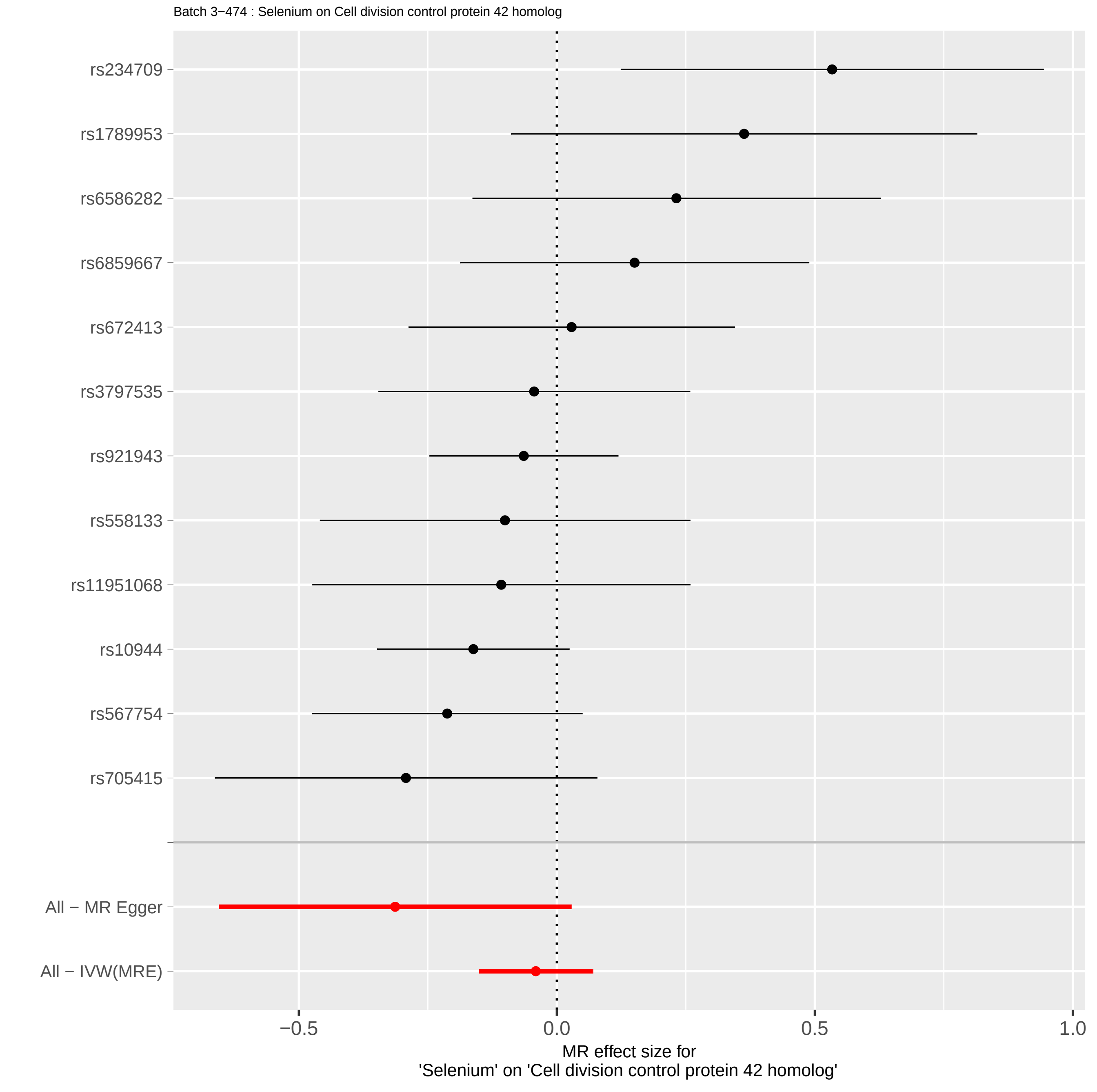

Batch 3−474 : Selenium on Cell division control protein 42 homolog
rs234709
rs1789953
rs6586282
rs6859667
rs672413
rs3797535
rs921943
rs558133
rs11951068
rs10944
rs567754
rs705415
All − MR Egger
All − IVW(MRE)
−0.5
0.0
0.5
1.0
MR effect size for
'Selenium' on 'Cell division control protein 42 homolog'

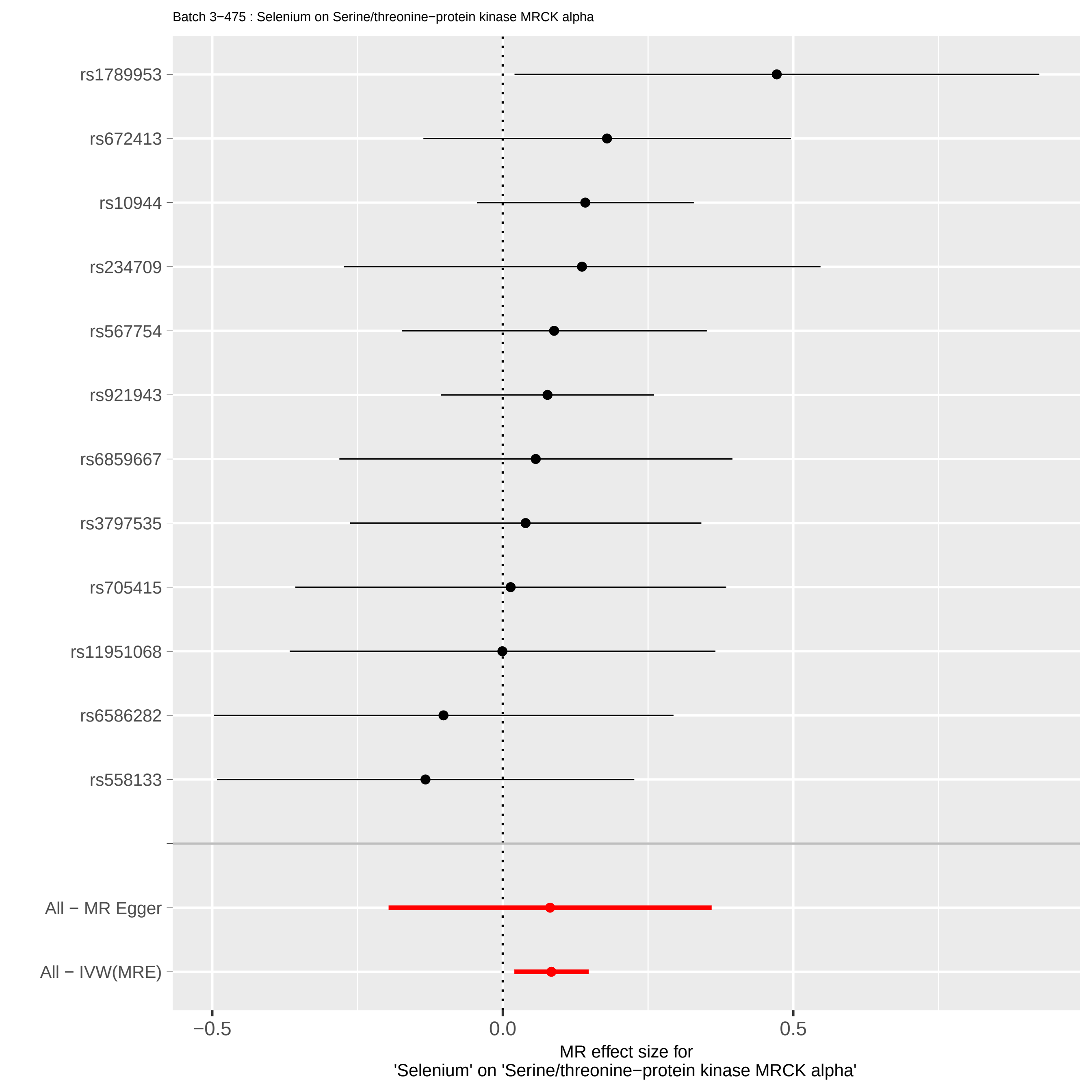
Batch 3−475 : Selenium on Serine/threonine−protein kinase MRCK alpha
rs1789953
rs672413
rs10944
rs234709
rs567754
rs921943
rs6859667
rs3797535
rs705415
rs11951068
rs6586282
rs558133
All − MR Egger
All − IVW(MRE)
−0.5
0.0
0.5
MR effect size for
'Selenium' on 'Serine/threonine−protein kinase MRCK alpha'

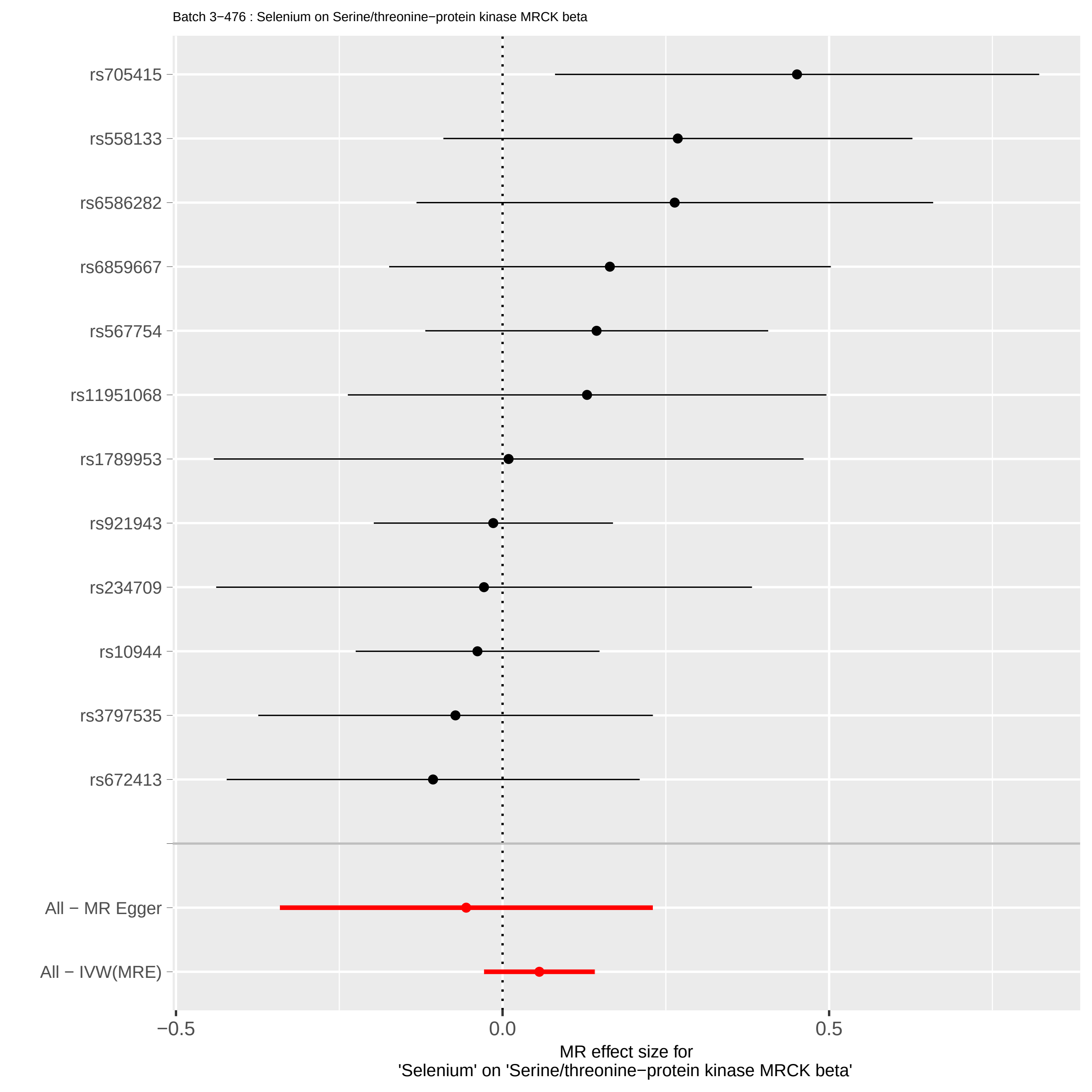

Batch 3−476 : Selenium on Serine/threonine−protein kinase MRCK beta
rs705415
rs558133
rs6586282
rs6859667
rs567754
rs11951068
rs1789953
rs921943
rs234709
rs10944
rs3797535
rs672413
All − MR Egger
All − IVW(MRE)
−0.5
0.0
0.5
MR effect size for
'Selenium' on 'Serine/threonine−protein kinase MRCK beta'

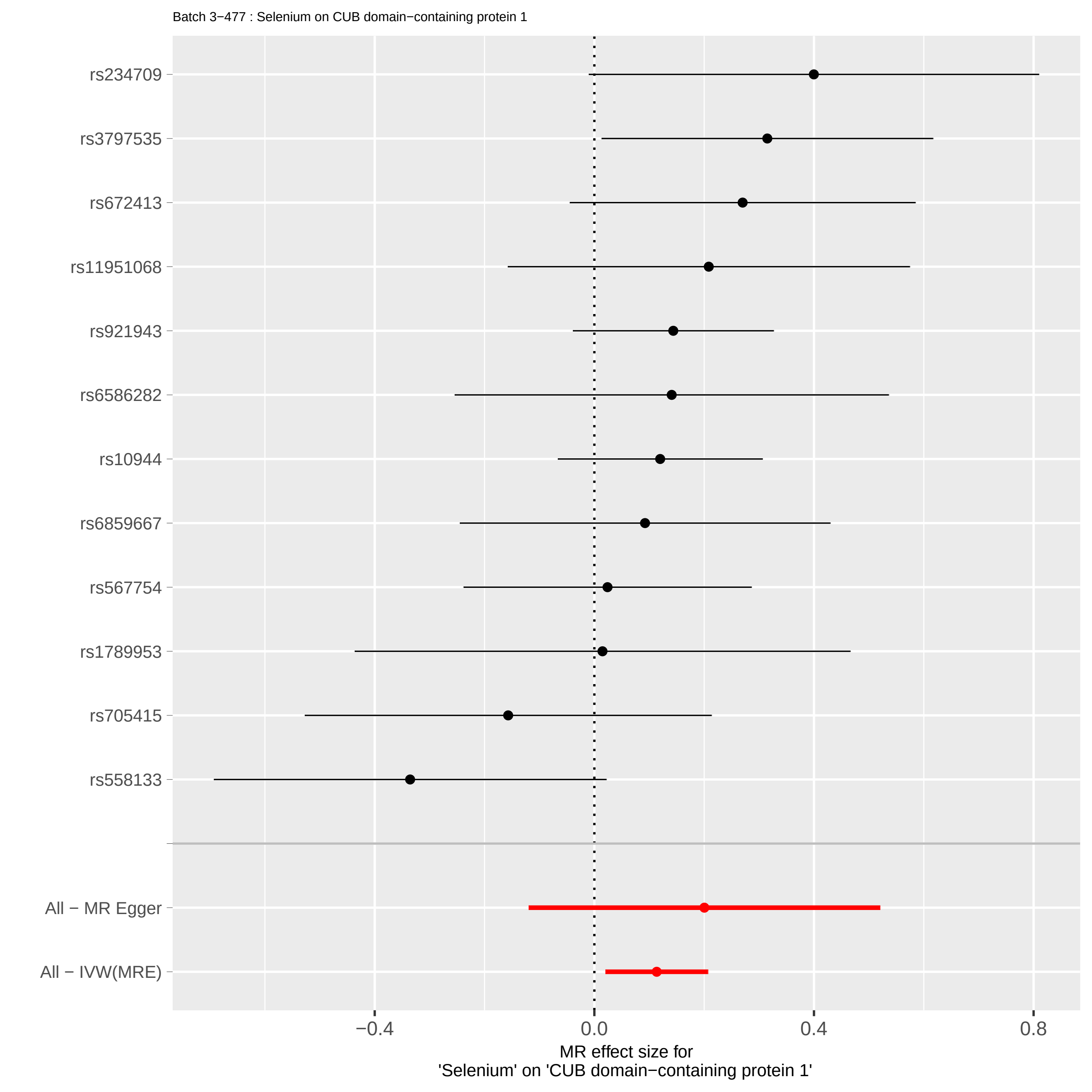

Batch 3−477 : Selenium on CUB domain−containing protein 1
rs234709
rs3797535
rs672413
rs11951068
rs921943
rs6586282
rs10944
rs6859667
rs567754
rs1789953
rs705415
rs558133
All − MR Egger
All − IVW(MRE)
−0.4
0.0
0.4
0.8
MR effect size for
'Selenium' on 'CUB domain−containing protein 1'

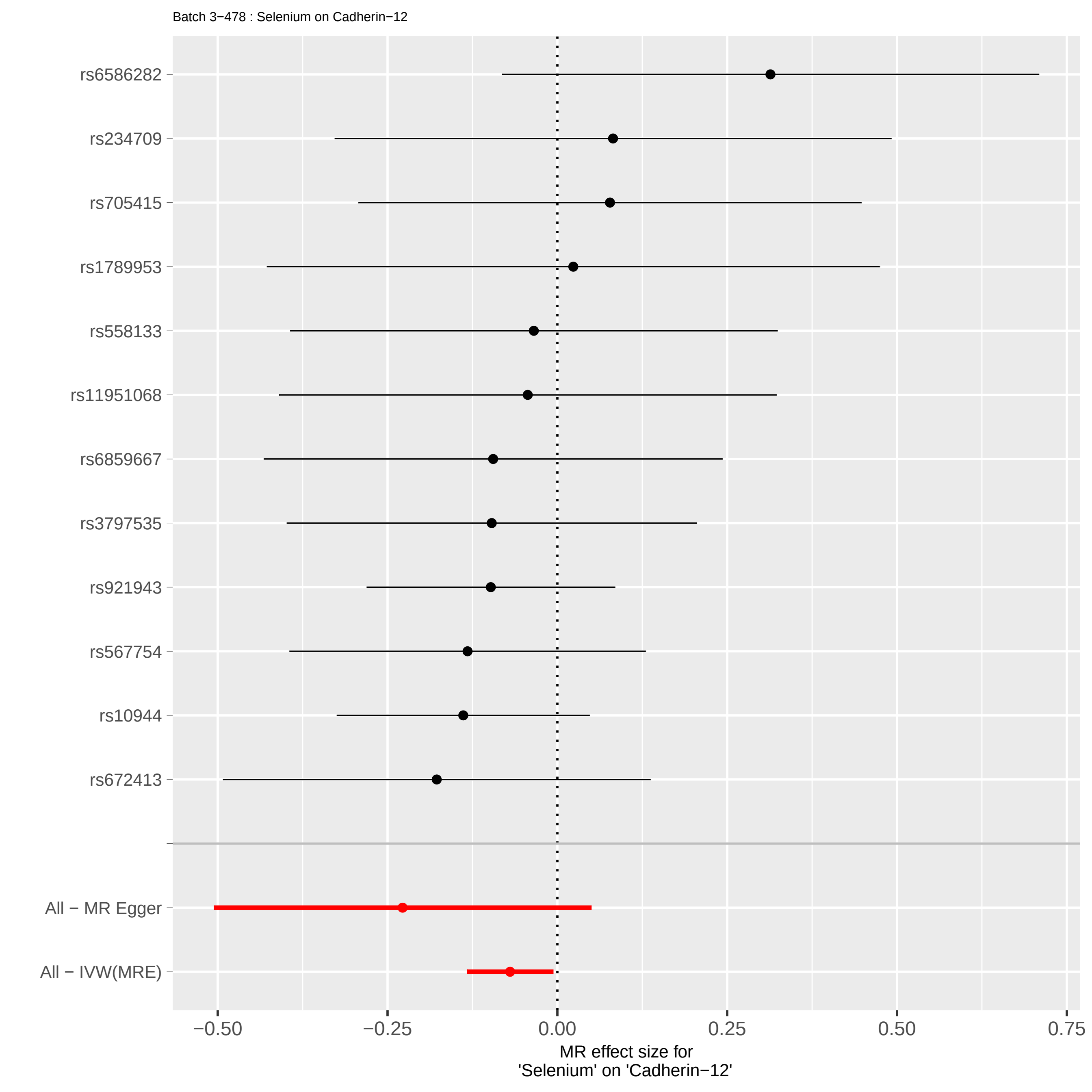

Batch 3−478 : Selenium on Cadherin−12
rs6586282
rs234709
rs705415
rs1789953
rs558133
rs11951068
rs6859667
rs3797535
rs921943
rs567754
rs10944
rs672413
All − MR Egger
All − IVW(MRE)
−0.50
−0.25
0.00
0.25
0.50
0.75
MR effect size for
'Selenium' on 'Cadherin−12'

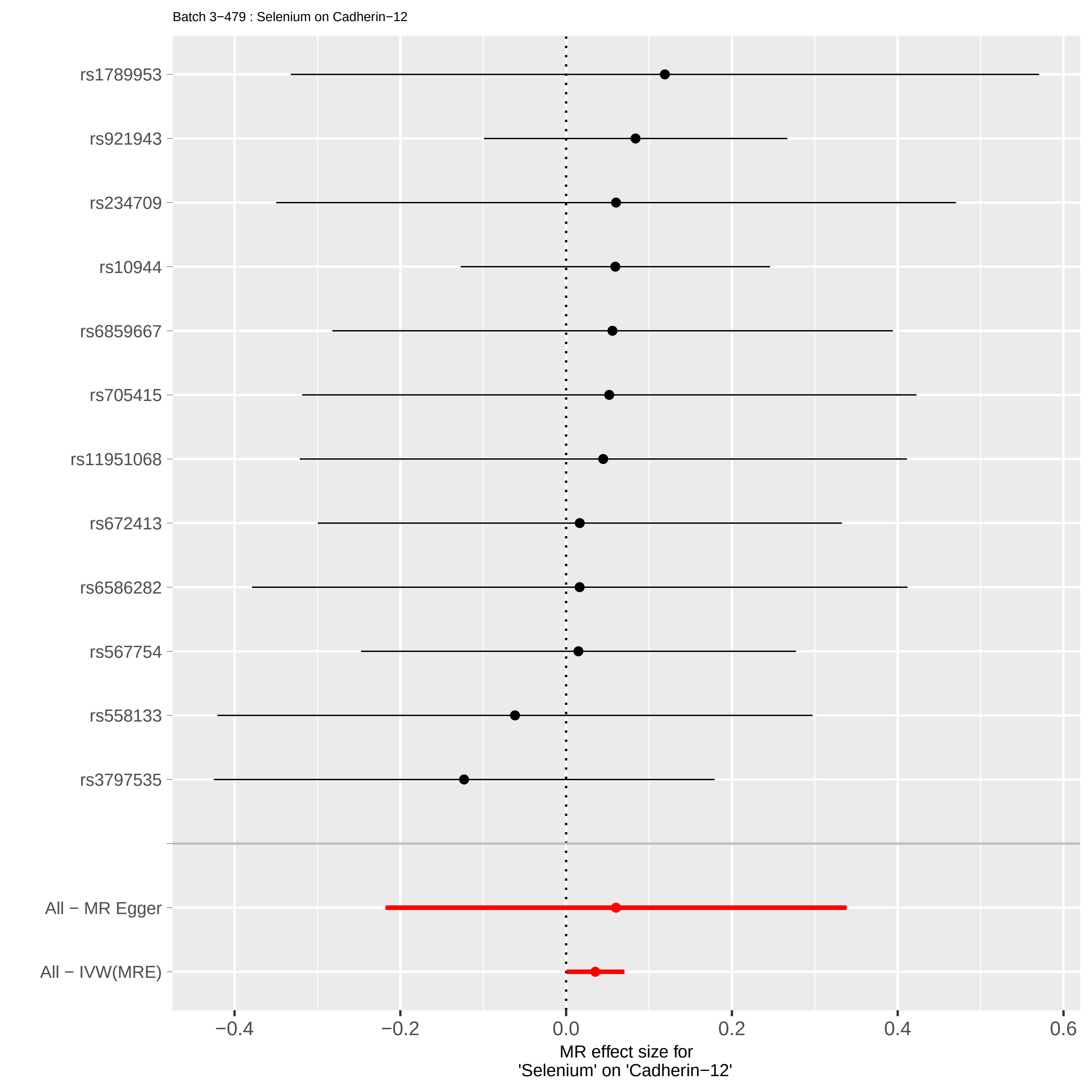
Batch 3−479 : Selenium on Cadherin−12
rs1789953
rs921943
rs234709
rs10944
rs6859667
rs705415
rs11951068
rs672413
rs6586282
rs567754
rs558133
rs3797535
All − MR Egger
All − IVW(MRE)
−0.4
−0.2
0.0
0.2
0.4
0.6
MR effect size for
'Selenium' on 'Cadherin−12'

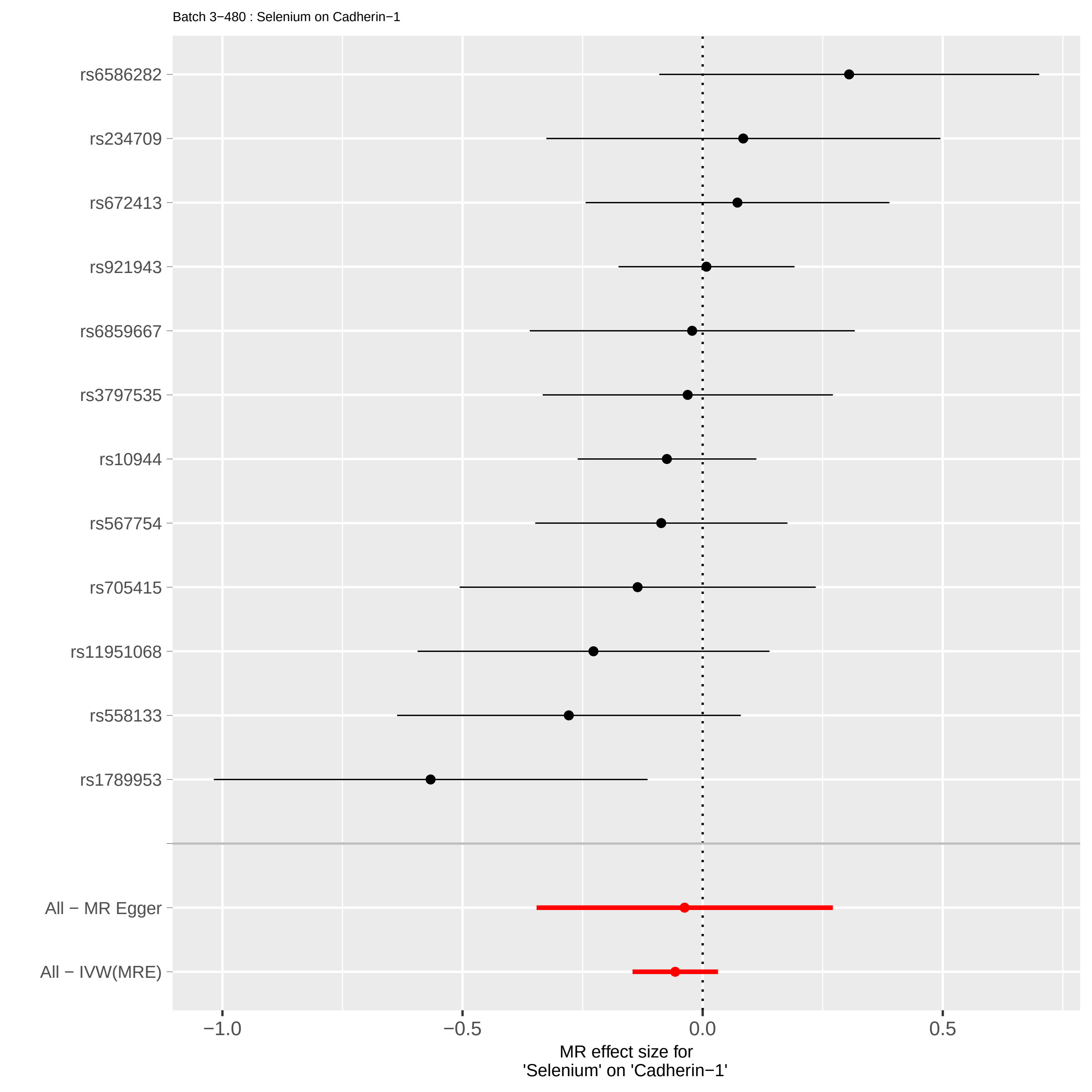

Batch 3−480 : Selenium on Cadherin−1
rs6586282
rs234709
rs672413
rs921943
rs6859667
rs3797535
rs10944
rs567754
rs705415
rs11951068
rs558133
rs1789953
All − MR Egger
All − IVW(MRE)
−1.0
−0.5
0.0
0.5
MR effect size for
'Selenium' on 'Cadherin−1'

Batch 3−481 : Selenium on Cadherin−15

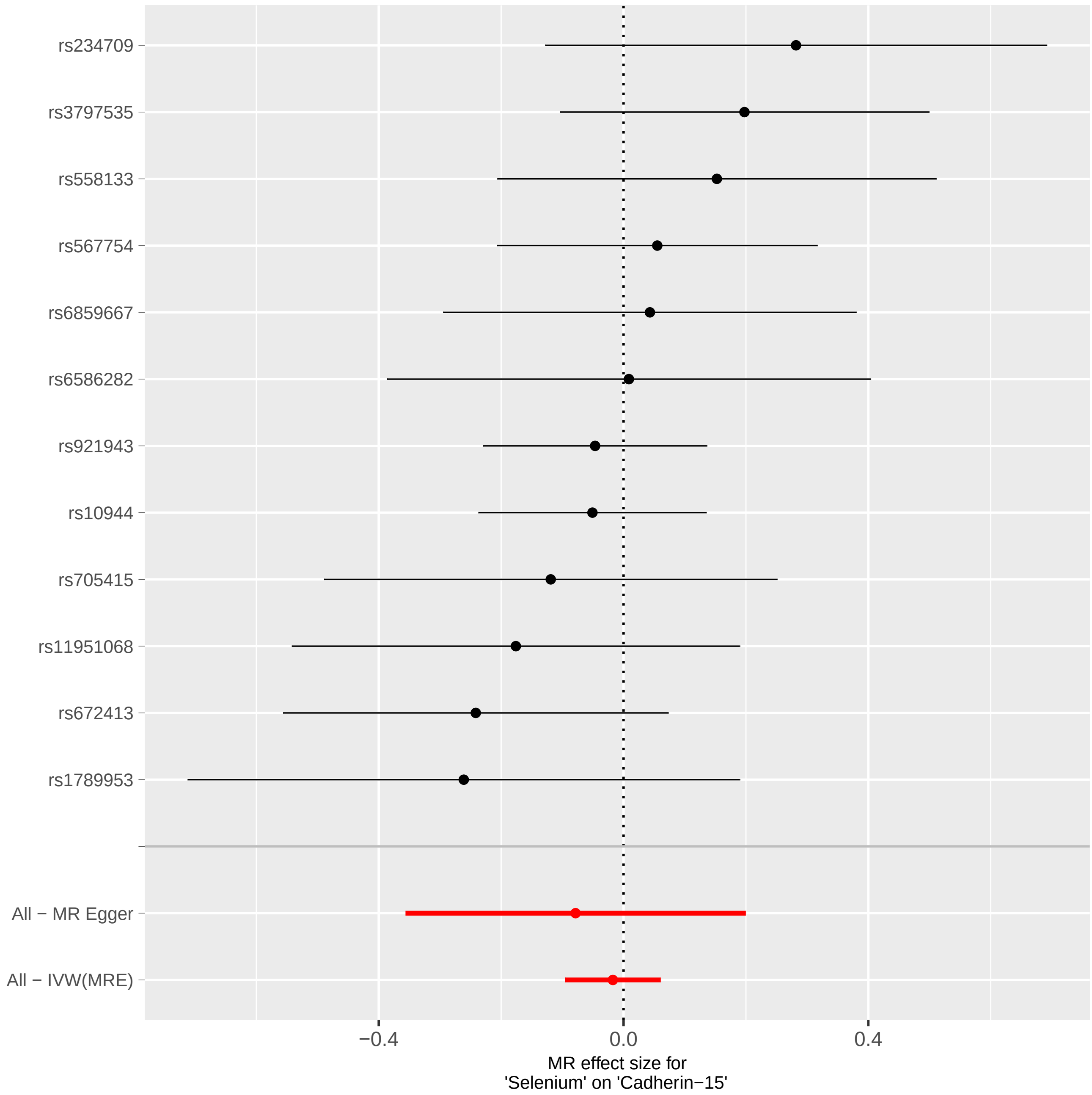

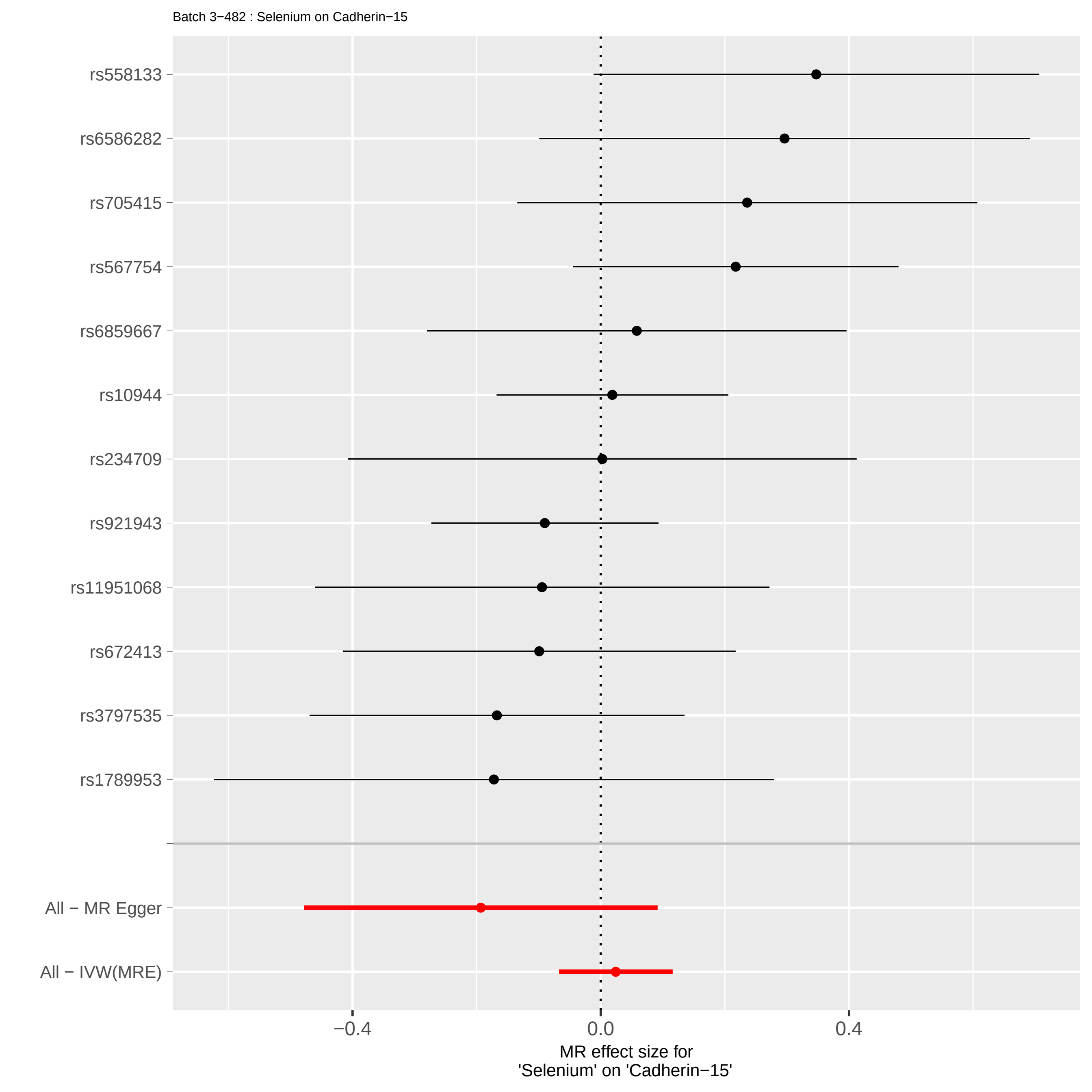
Batch 3−482 : Selenium on Cadherin−15
rs558133
rs6586282
rs705415
rs567754
rs6859667
rs10944
rs234709
rs921943
rs11951068
rs672413
rs3797535
rs1789953
All − MR Egger
All − IVW(MRE)
−0.4
0.0
0.4
MR effect size for
'Selenium' on 'Cadherin−15'

Batch 3−483 : Selenium on Cadherin−2

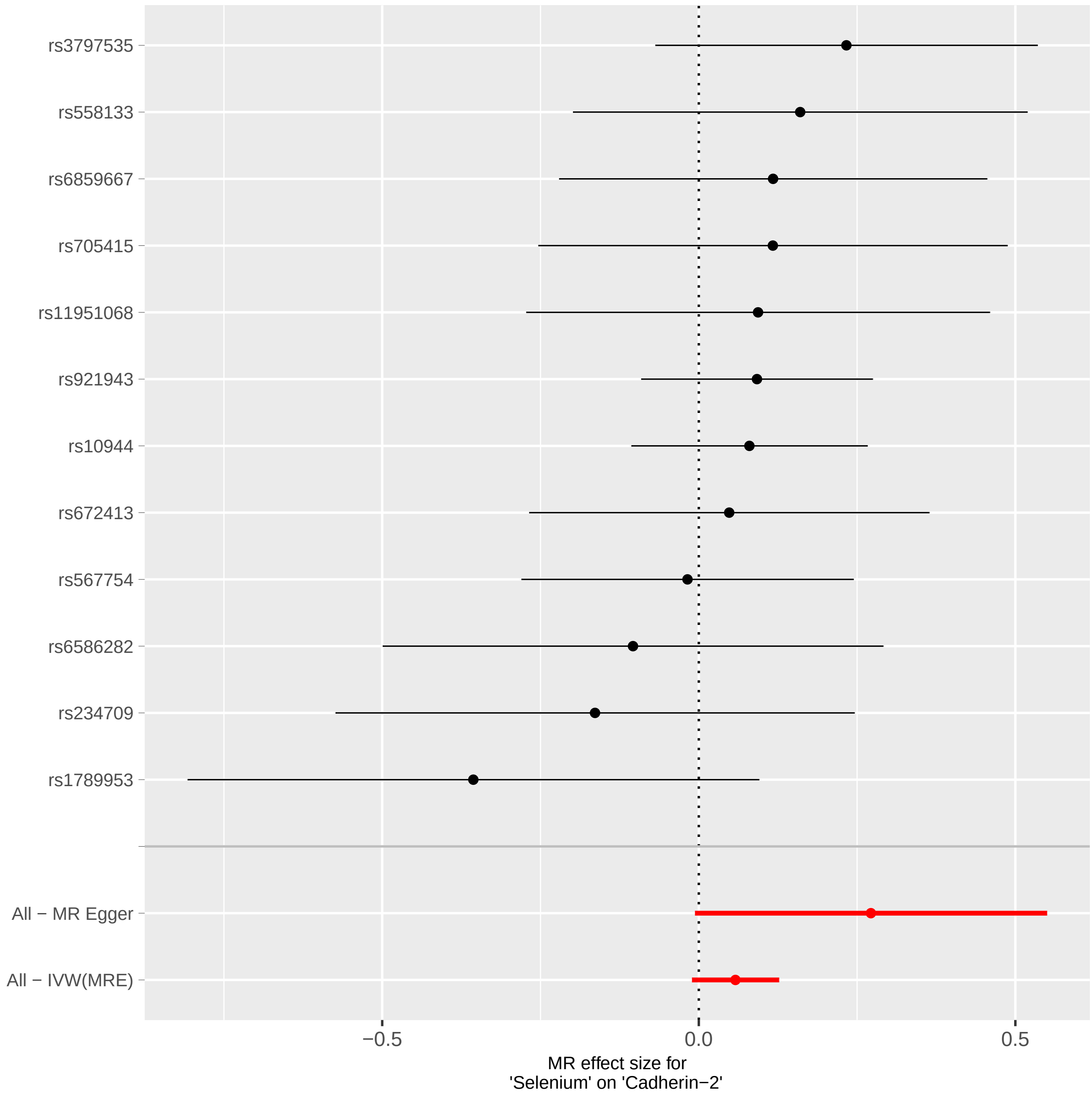

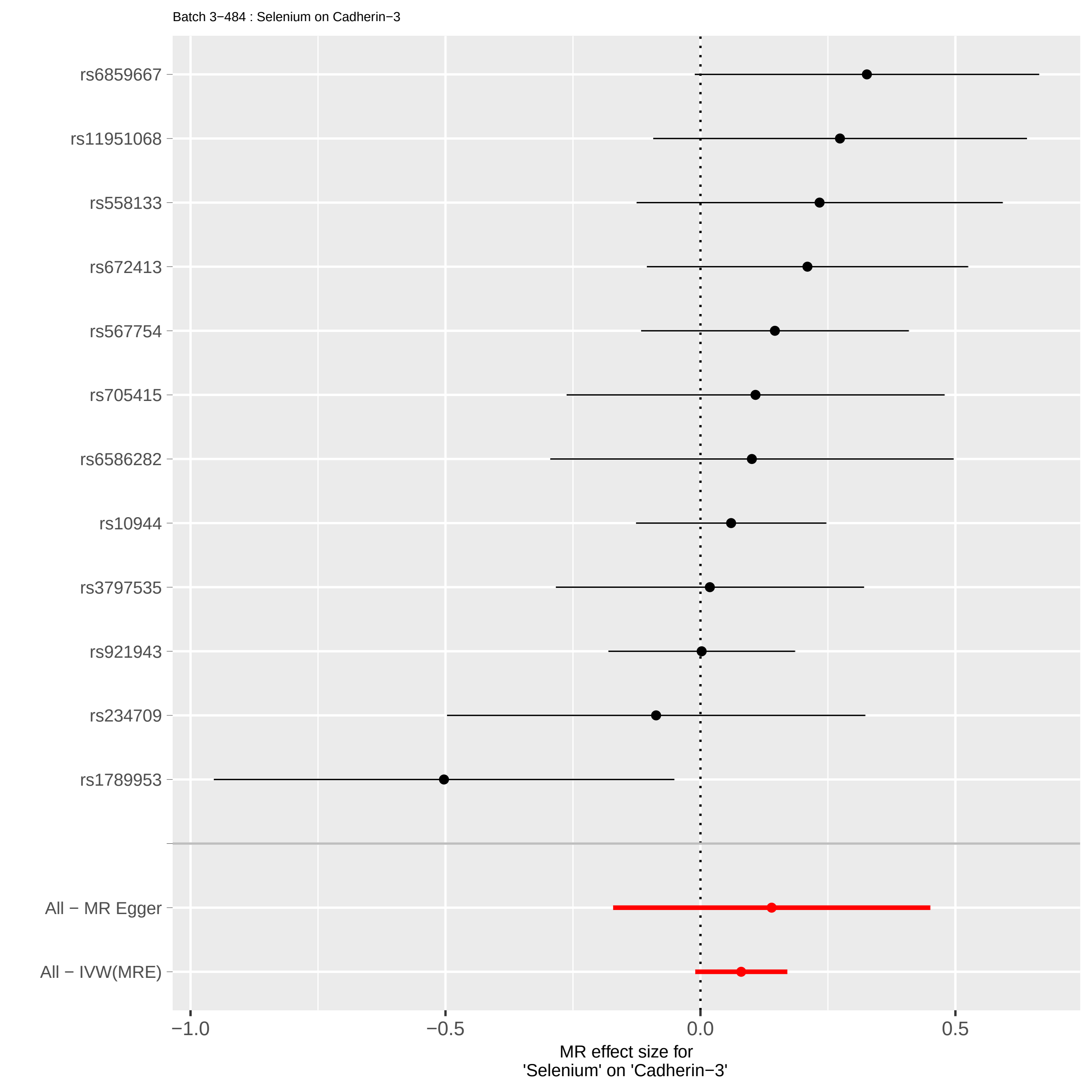

Batch 3−484 : Selenium on Cadherin−3
rs6859667
rs11951068
rs558133
rs672413
rs567754
rs705415
rs6586282
rs10944
rs3797535
rs921943
rs234709
rs1789953
All − MR Egger
All − IVW(MRE)
−1.0
−0.5
0.0
0.5
MR effect size for
'Selenium' on 'Cadherin−3'

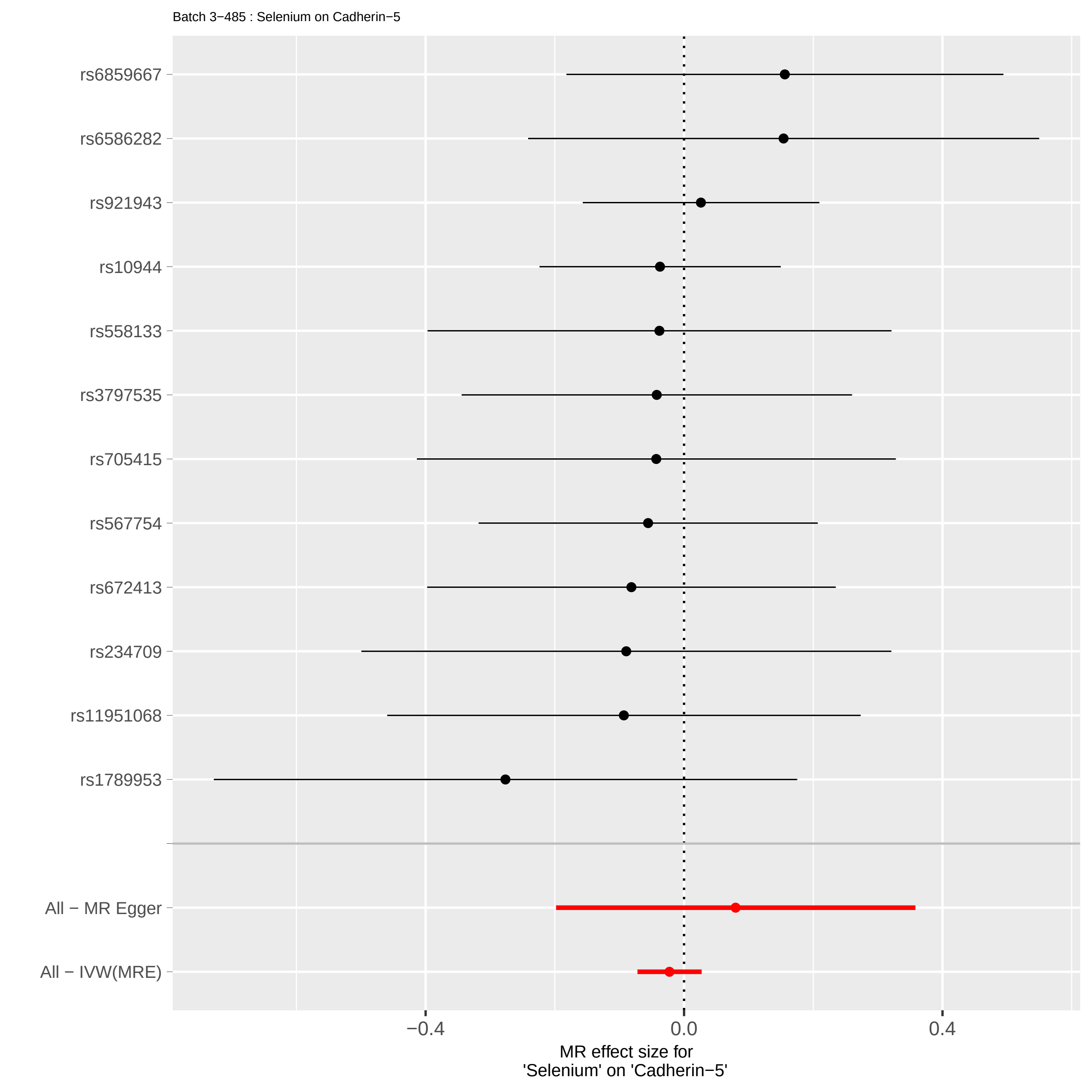
Batch 3−485 : Selenium on Cadherin−5
rs6859667
rs6586282
rs921943
rs10944
rs558133
rs3797535
rs705415
rs567754
rs672413
rs234709
rs11951068
rs1789953
All − MR Egger
All − IVW(MRE)
−0.4
0.0
0.4
MR effect size for
'Selenium' on 'Cadherin−5'

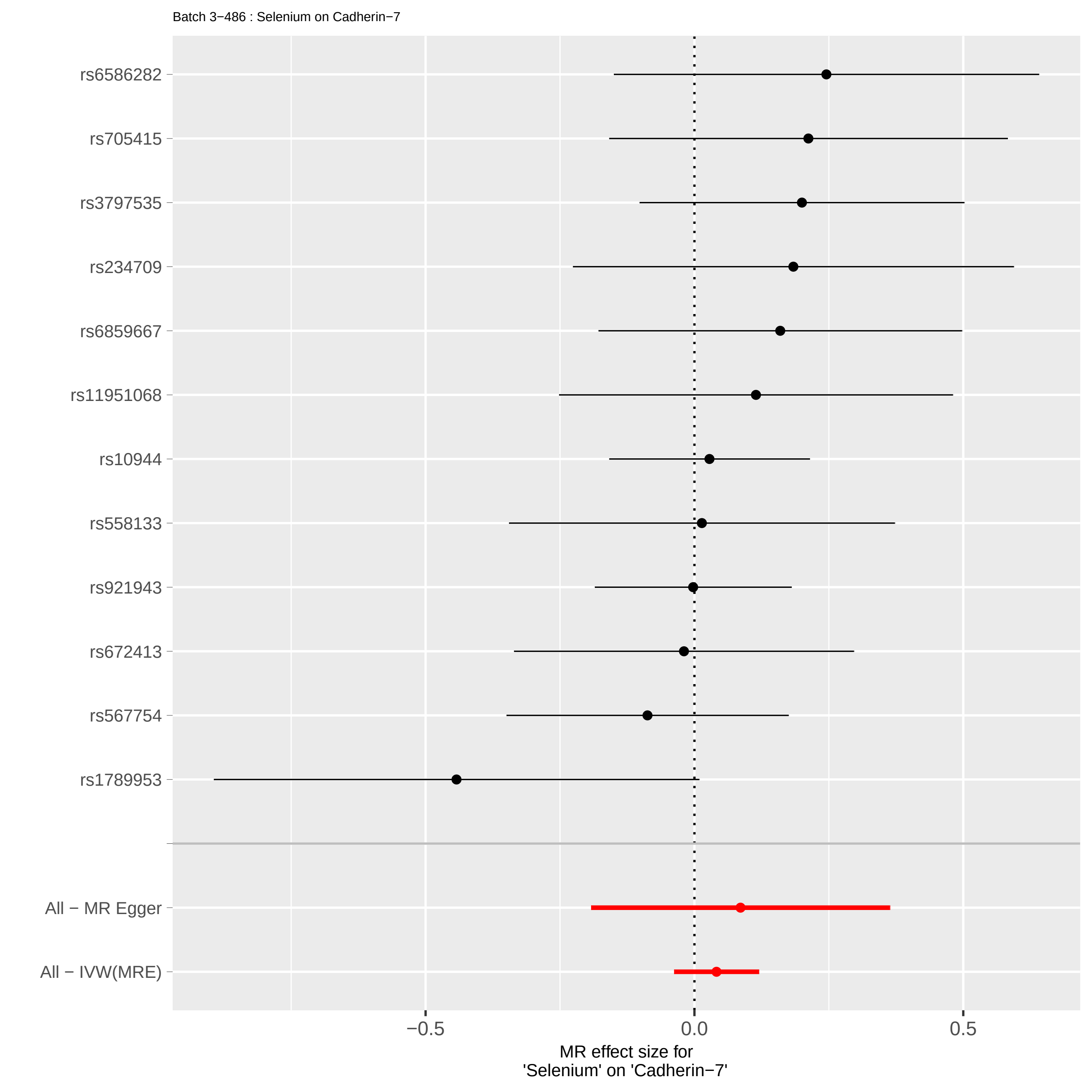
Batch 3−486 : Selenium on Cadherin−7
rs6586282
rs705415
rs3797535
rs234709
rs6859667
rs11951068
rs10944
rs558133
rs921943
rs672413
rs567754
rs1789953
All − MR Egger
All − IVW(MRE)
−0.5
0.0
0.5
MR effect size for
'Selenium' on 'Cadherin−7'

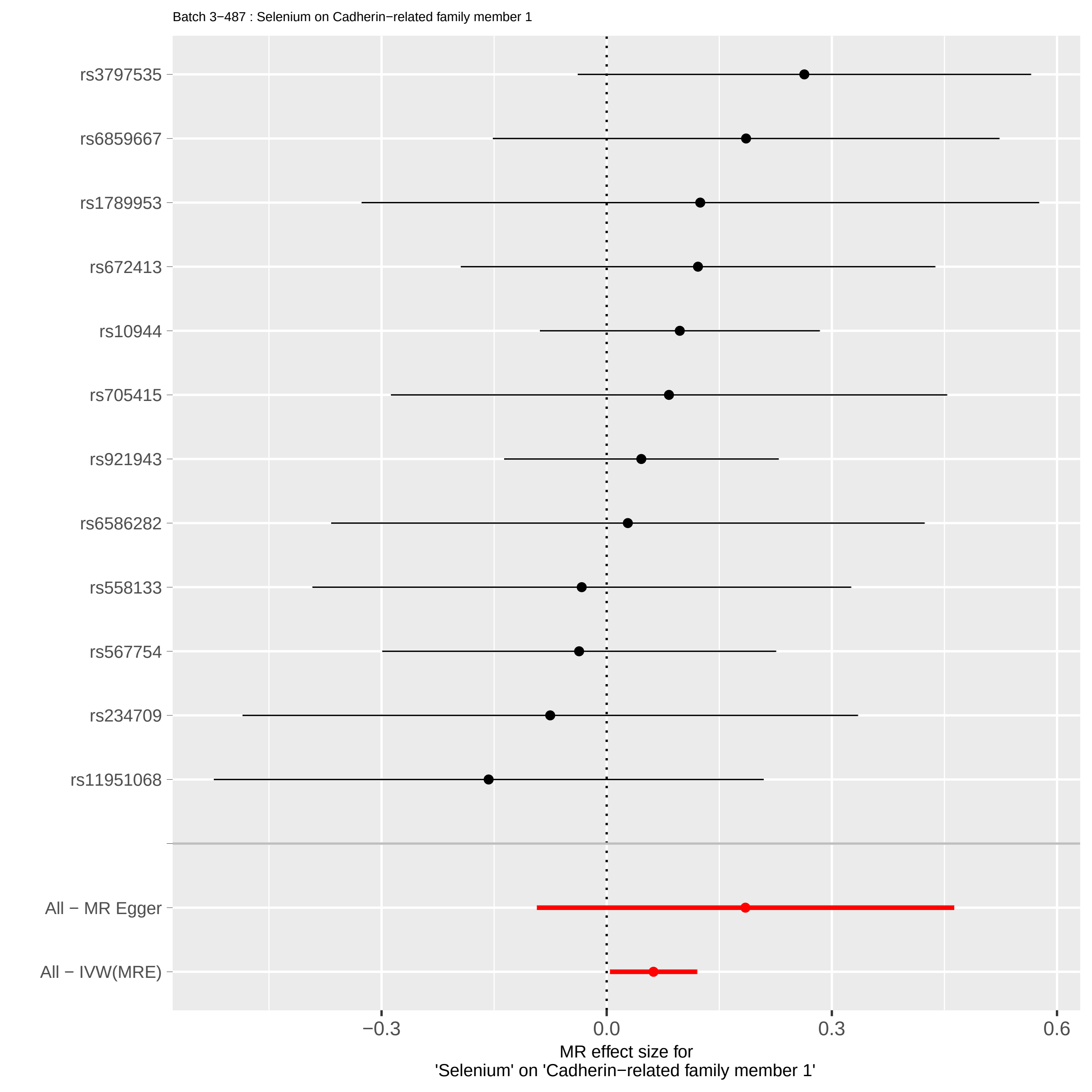
Batch 3−487 : Selenium on Cadherin−related family member 1
rs3797535
rs6859667
rs1789953
rs672413
rs10944
rs705415
rs921943
rs6586282
rs558133
rs567754
rs234709
rs11951068
All − MR Egger
All − IVW(MRE)
−0.3
0.0
0.3
0.6
MR effect size for
'Selenium' on 'Cadherin−related family member 1'

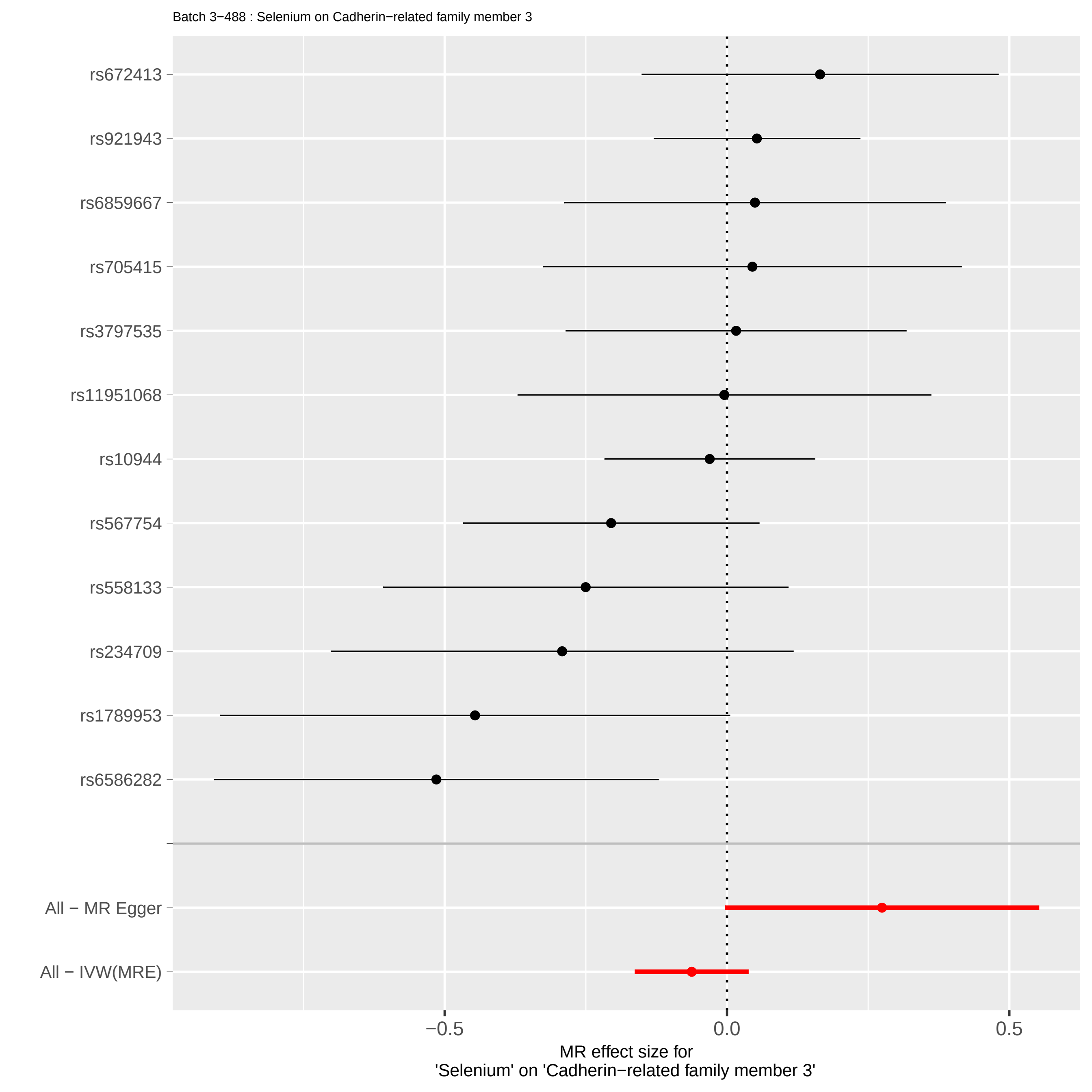
Batch 3−488 : Selenium on Cadherin−related family member 3
rs672413
rs921943
rs6859667
rs705415
rs3797535
rs11951068
rs10944
rs567754
rs558133
rs234709
rs1789953
rs6586282
All − MR Egger
All − IVW(MRE)
−0.5
0.0
0.5
MR effect size for
'Selenium' on 'Cadherin−related family member 3'

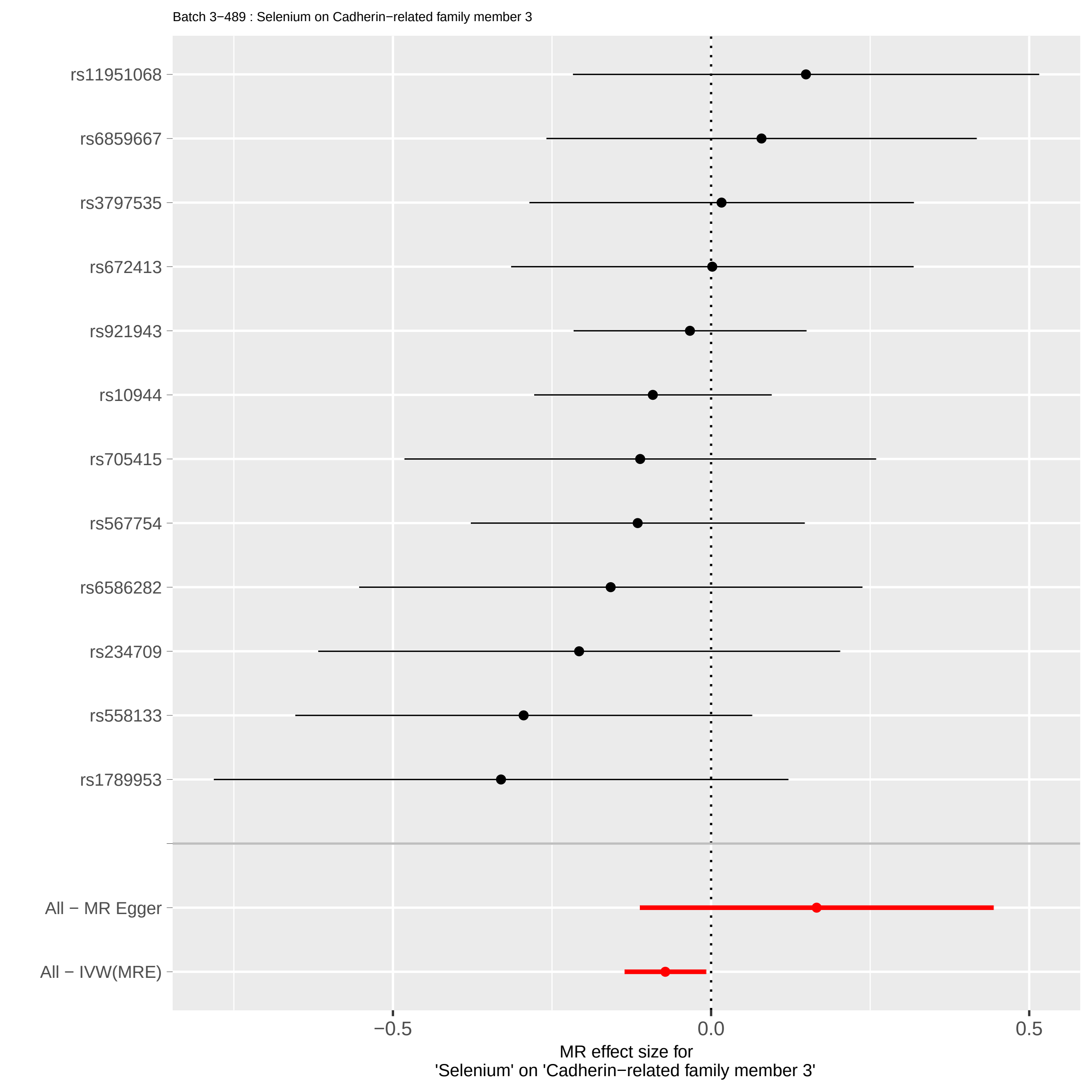
Batch 3−489 : Selenium on Cadherin−related family member 3
rs11951068
rs6859667
rs3797535
rs672413
rs921943
rs10944
rs705415
rs567754
rs6586282
rs234709
rs558133
rs1789953
All − MR Egger
All − IVW(MRE)
−0.5
0.0
0.5
MR effect size for
'Selenium' on 'Cadherin−related family member 3'

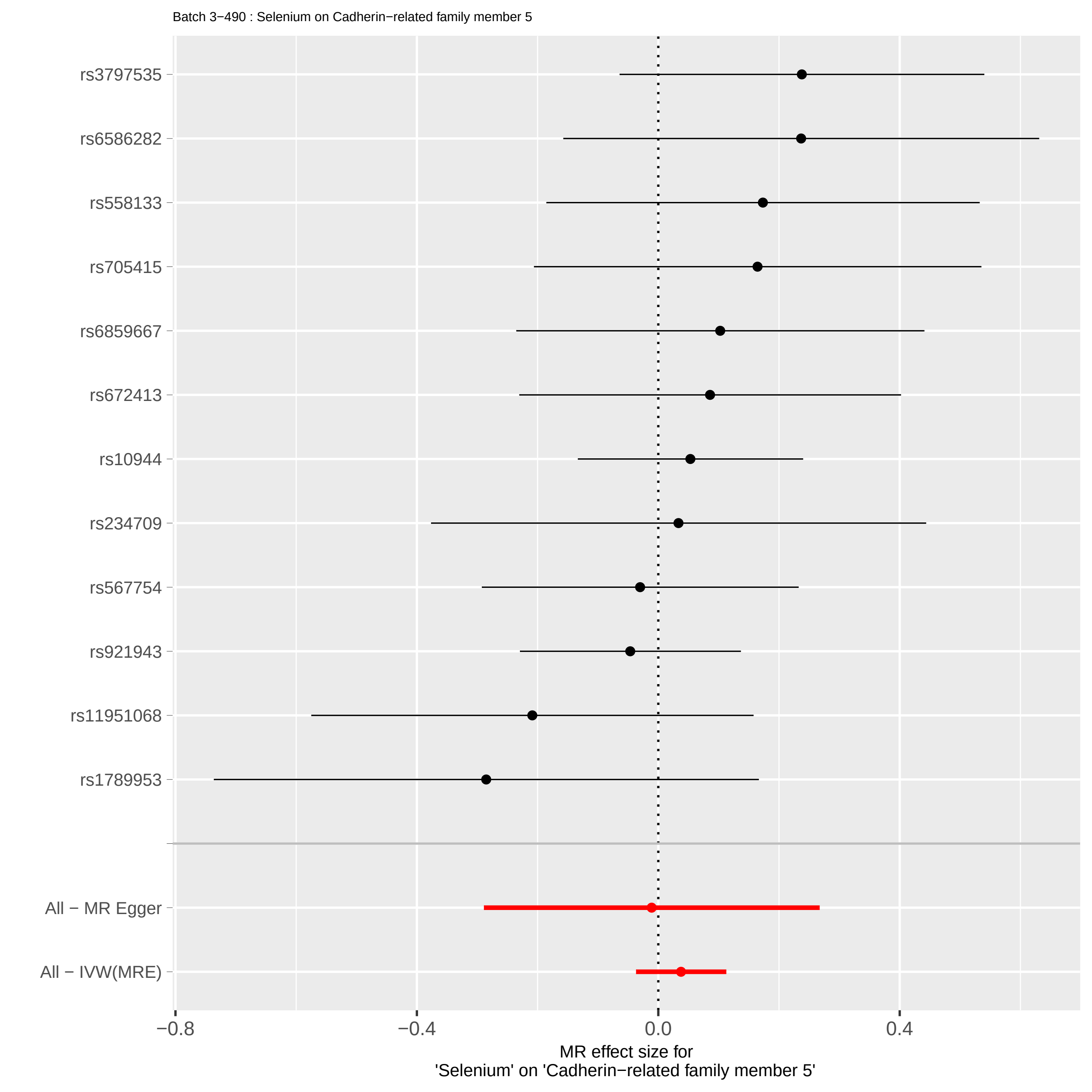
Batch 3−490 : Selenium on Cadherin−related family member 5
rs3797535
rs6586282
rs558133
rs705415
rs6859667
rs672413
rs10944
rs234709
rs567754
rs921943
rs11951068
rs1789953
All − MR Egger
All − IVW(MRE)
−0.8
−0.4
0.0
0.4
MR effect size for
'Selenium' on 'Cadherin−related family member 5'

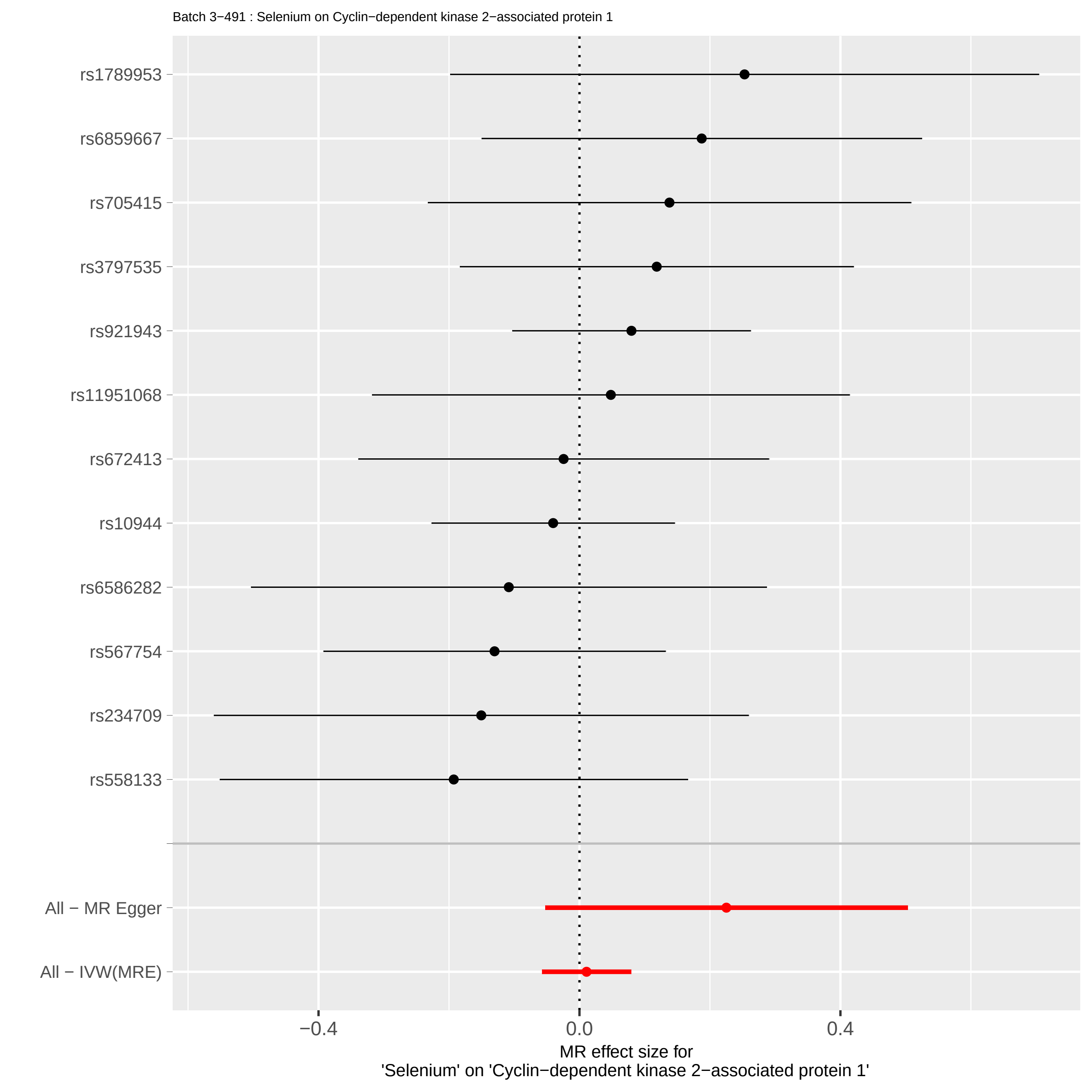

Batch 3−491 : Selenium on Cyclin−dependent kinase 2−associated protein 1
rs1789953
rs6859667
rs705415
rs3797535
rs921943
rs11951068
rs672413
rs10944
rs6586282
rs567754
rs234709
rs558133
All − MR Egger
All − IVW(MRE)
−0.4
0.0
0.4
MR effect size for
'Selenium' on 'Cyclin−dependent kinase 2−associated protein 1'

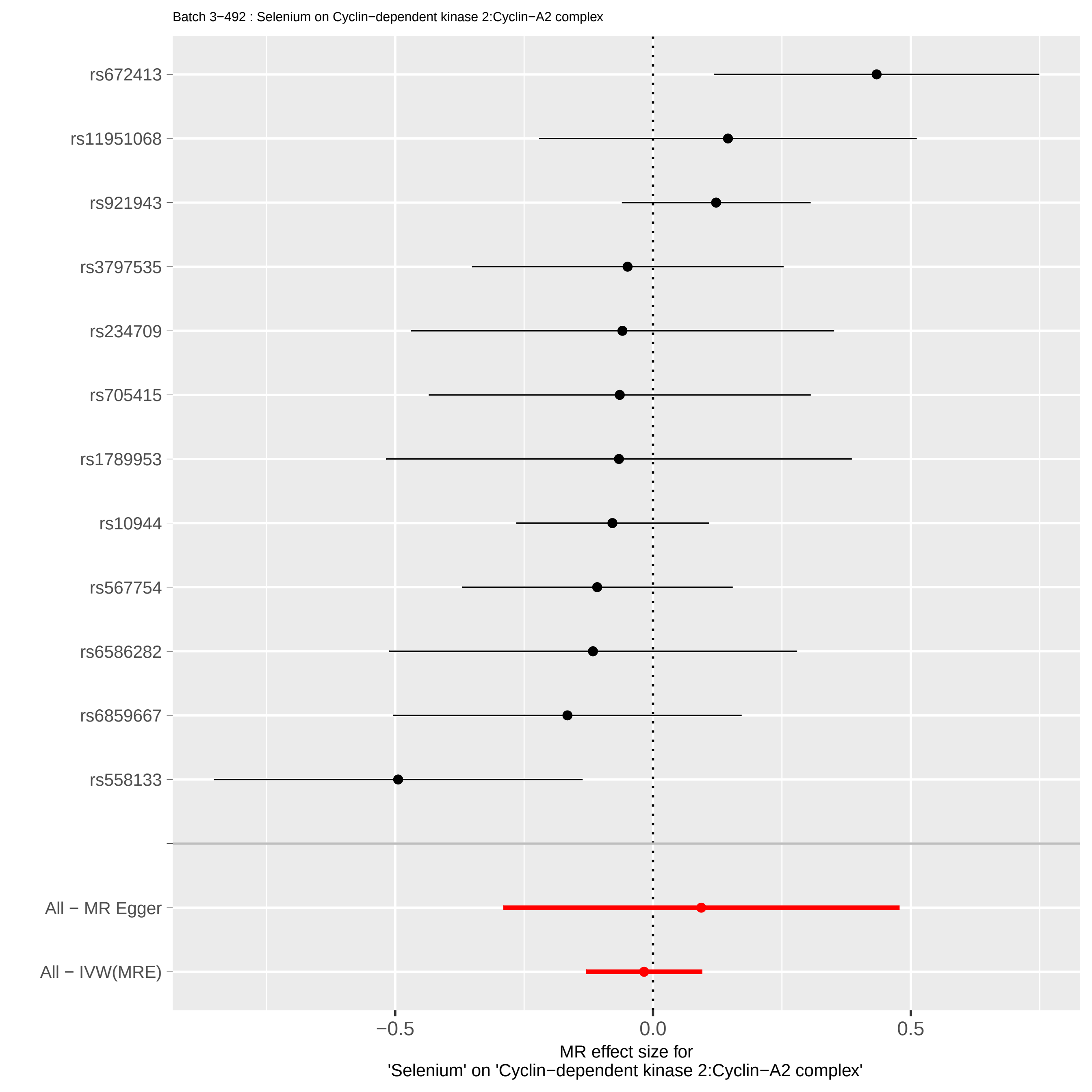

Batch 3−492 : Selenium on Cyclin−dependent kinase 2:Cyclin−A2 complex
rs672413
rs11951068
rs921943
rs3797535
rs234709
rs705415
rs1789953
rs10944
rs567754
rs6586282
rs6859667
rs558133
All − MR Egger
All − IVW(MRE)
−0.5
0.0
0.5
MR effect size for
'Selenium' on 'Cyclin−dependent kinase 2:Cyclin−A2 complex'

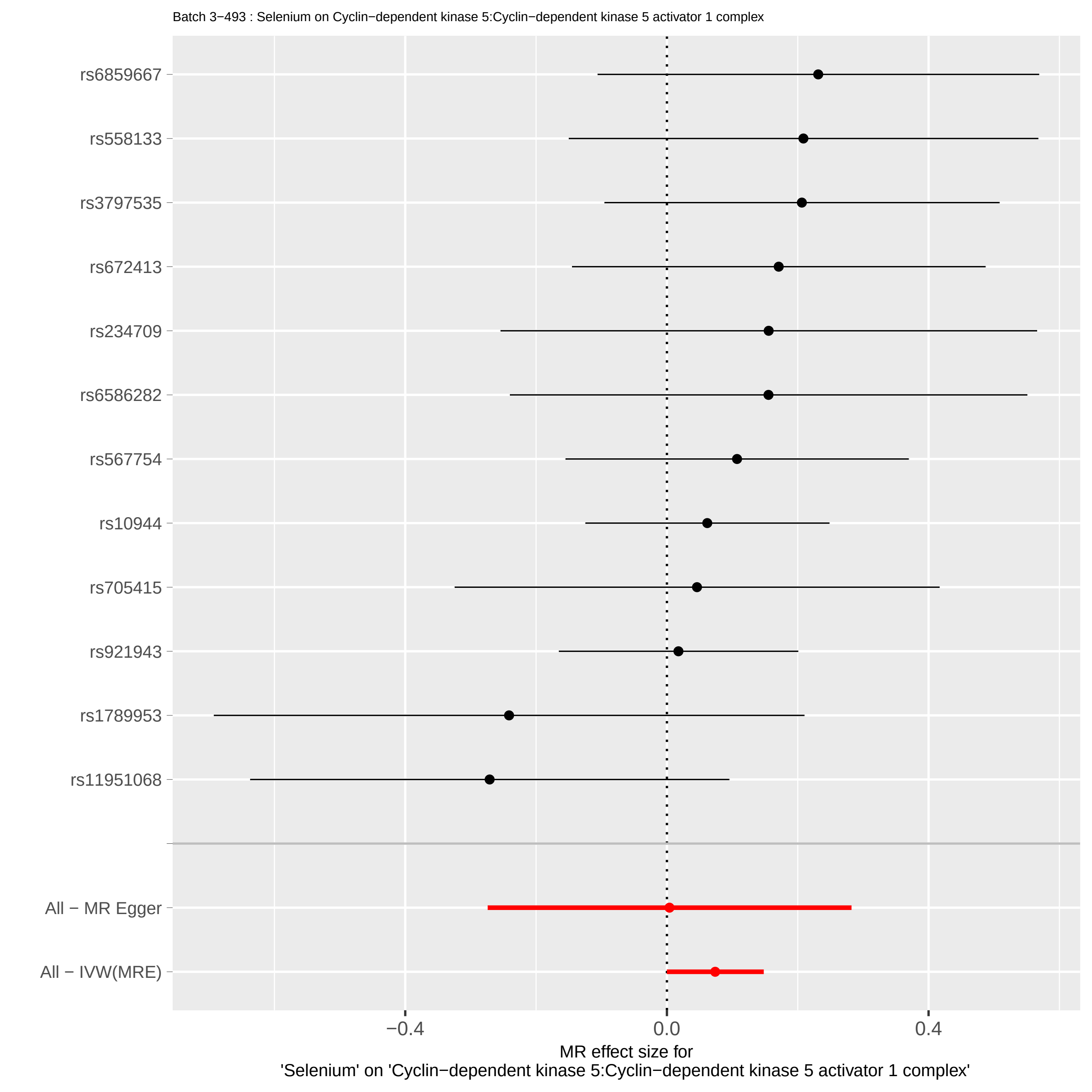

Batch 3−493 : Selenium on Cyclin−dependent kinase 5:Cyclin−dependent kinase 5 activator 1 complex
rs6859667
rs558133
rs3797535
rs672413
rs234709
rs6586282
rs567754
rs10944
rs705415
rs921943
rs1789953
rs11951068
All − MR Egger
All − IVW(MRE)
−0.4
0.0
0.4
MR effect size for
'Selenium' on 'Cyclin−dependent kinase 5:Cyclin−dependent kinase 5 activator 1 complex'

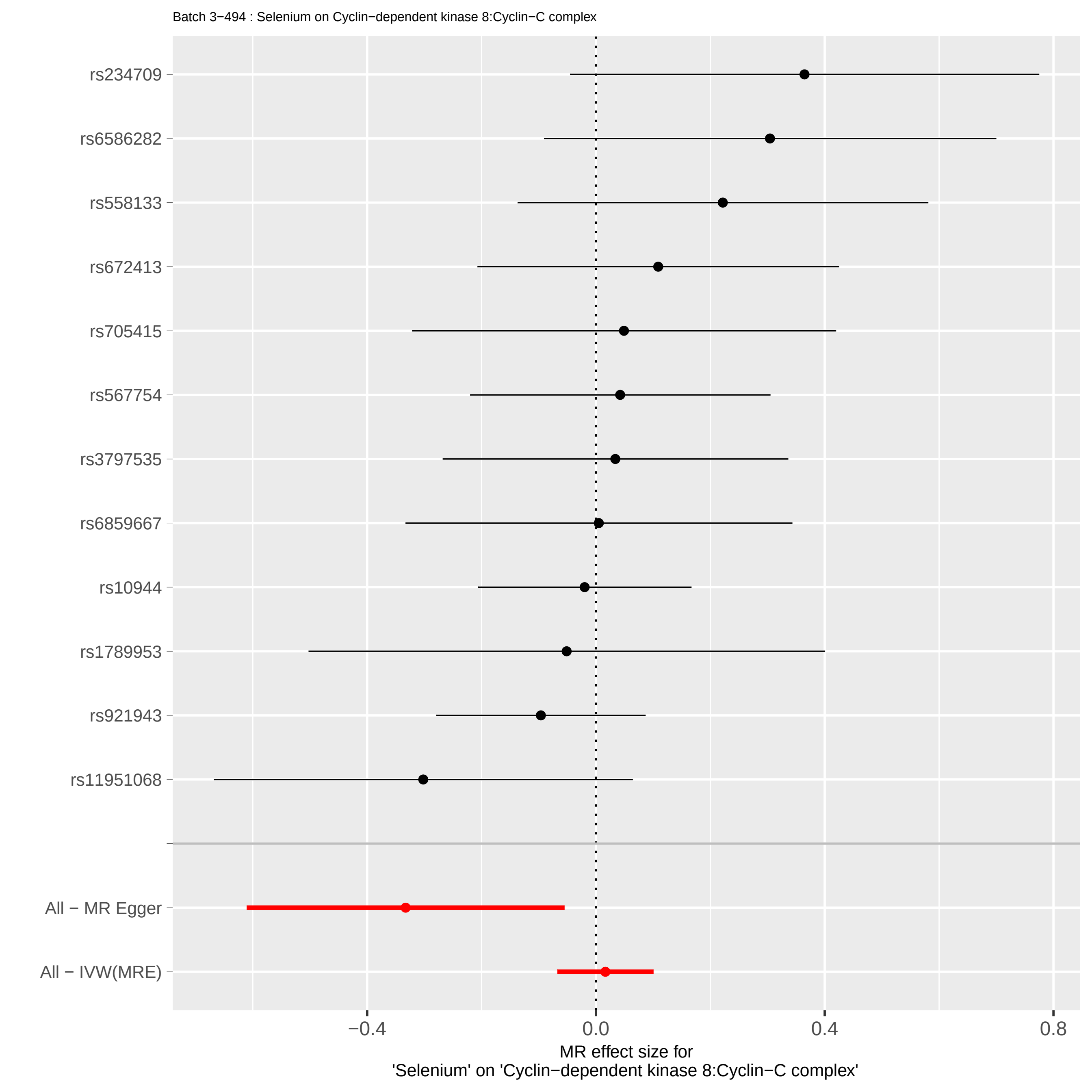

Batch 3−494 : Selenium on Cyclin−dependent kinase 8:Cyclin−C complex
rs234709
rs6586282
rs558133
rs672413
rs705415
rs567754
rs3797535
rs6859667
rs10944
rs1789953
rs921943
rs11951068
All − MR Egger
All − IVW(MRE)
−0.4
0.0
0.4
0.8
MR effect size for
'Selenium' on 'Cyclin−dependent kinase 8:Cyclin−C complex'

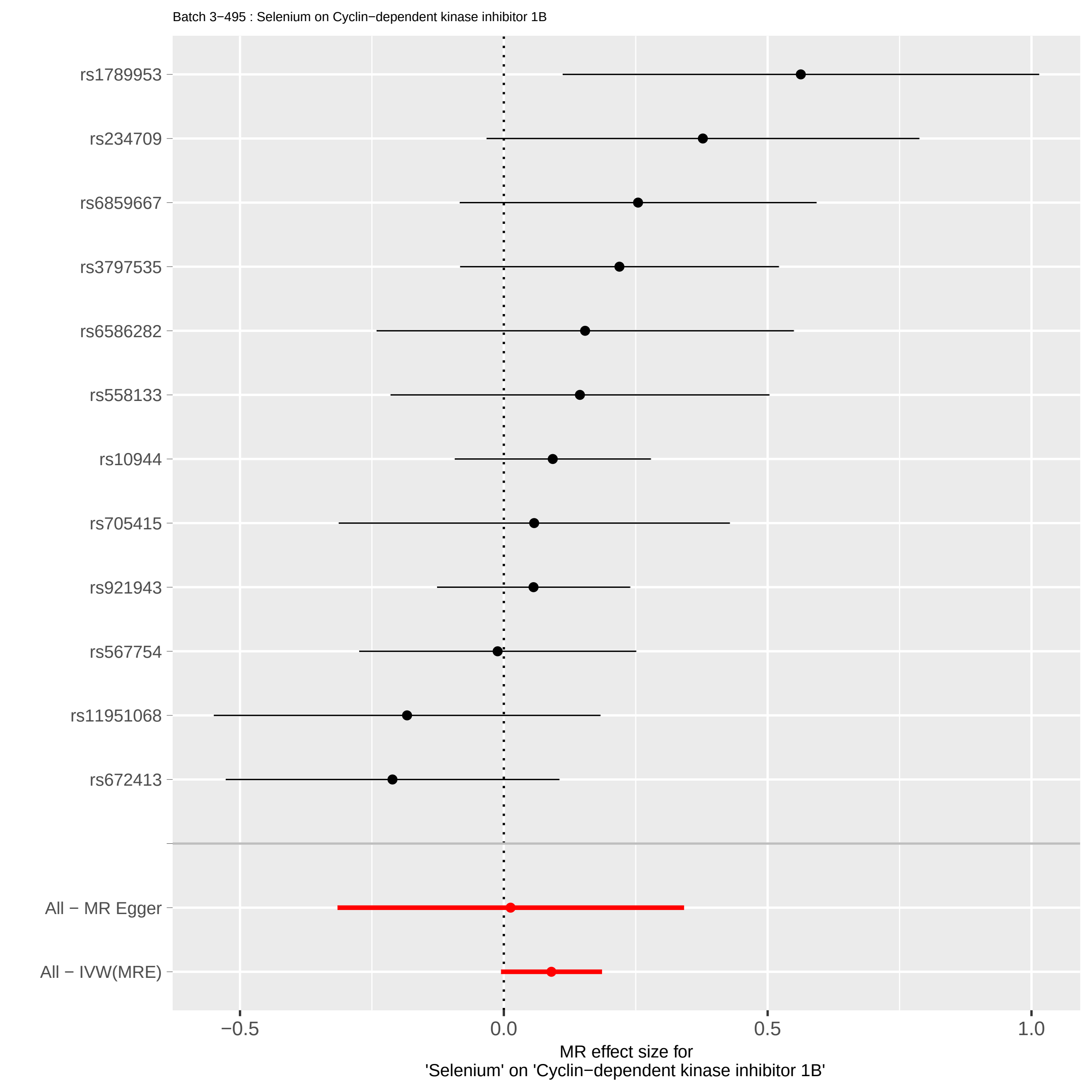

Batch 3−495 : Selenium on Cyclin−dependent kinase inhibitor 1B
rs1789953
rs234709
rs6859667
rs3797535
rs6586282
rs558133
rs10944
rs705415
rs921943
rs567754
rs11951068
rs672413
All − MR Egger
All − IVW(MRE)
−0.5
0.0
0.5
1.0
MR effect size for
'Selenium' on 'Cyclin−dependent kinase inhibitor 1B'

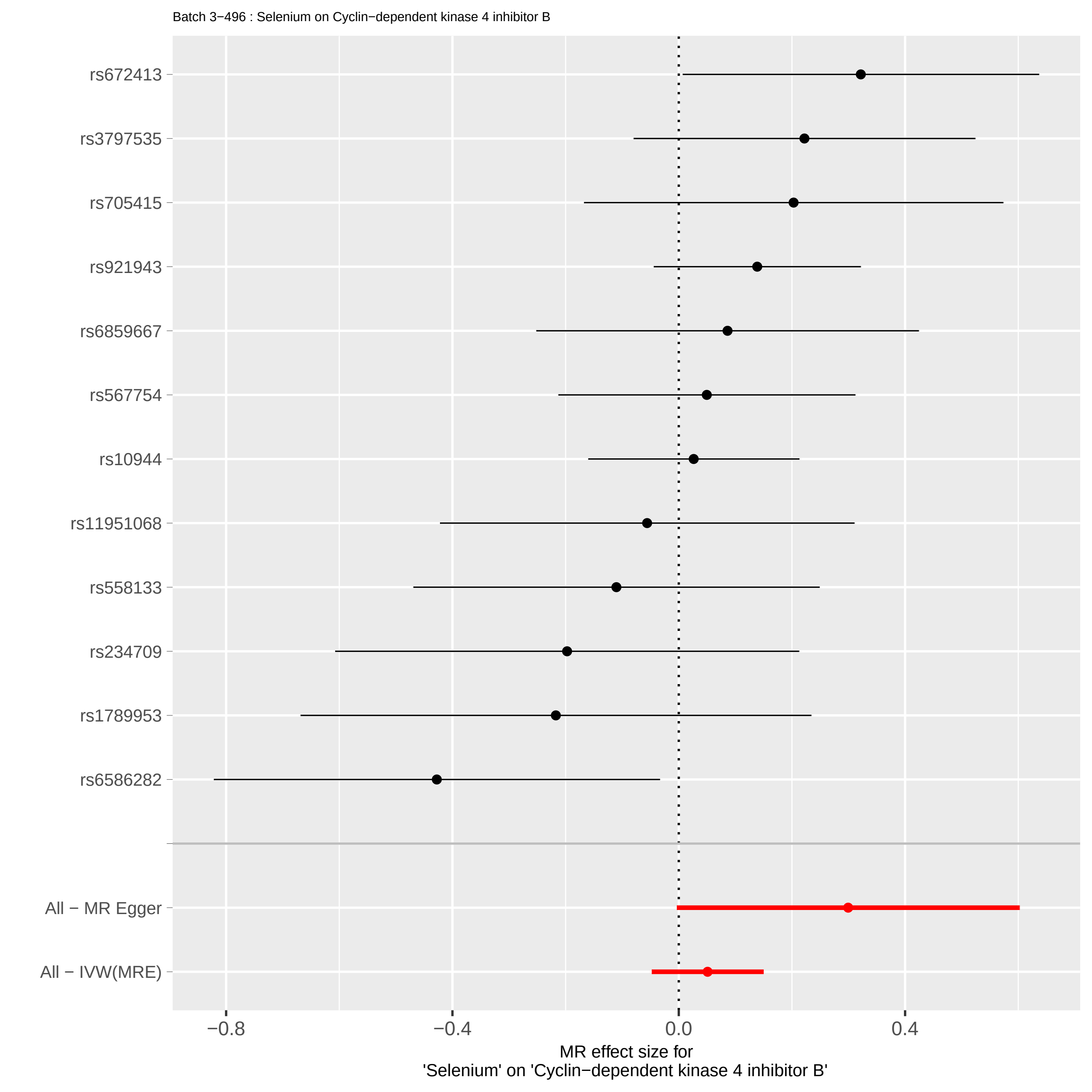

Batch 3−496 : Selenium on Cyclin−dependent kinase 4 inhibitor B
rs672413
rs3797535
rs705415
rs921943
rs6859667
rs567754
rs10944
rs11951068
rs558133
rs234709
rs1789953
rs6586282
All − MR Egger
All − IVW(MRE)
−0.8
−0.4
0.0
0.4
MR effect size for
'Selenium' on 'Cyclin−dependent kinase 4 inhibitor B'

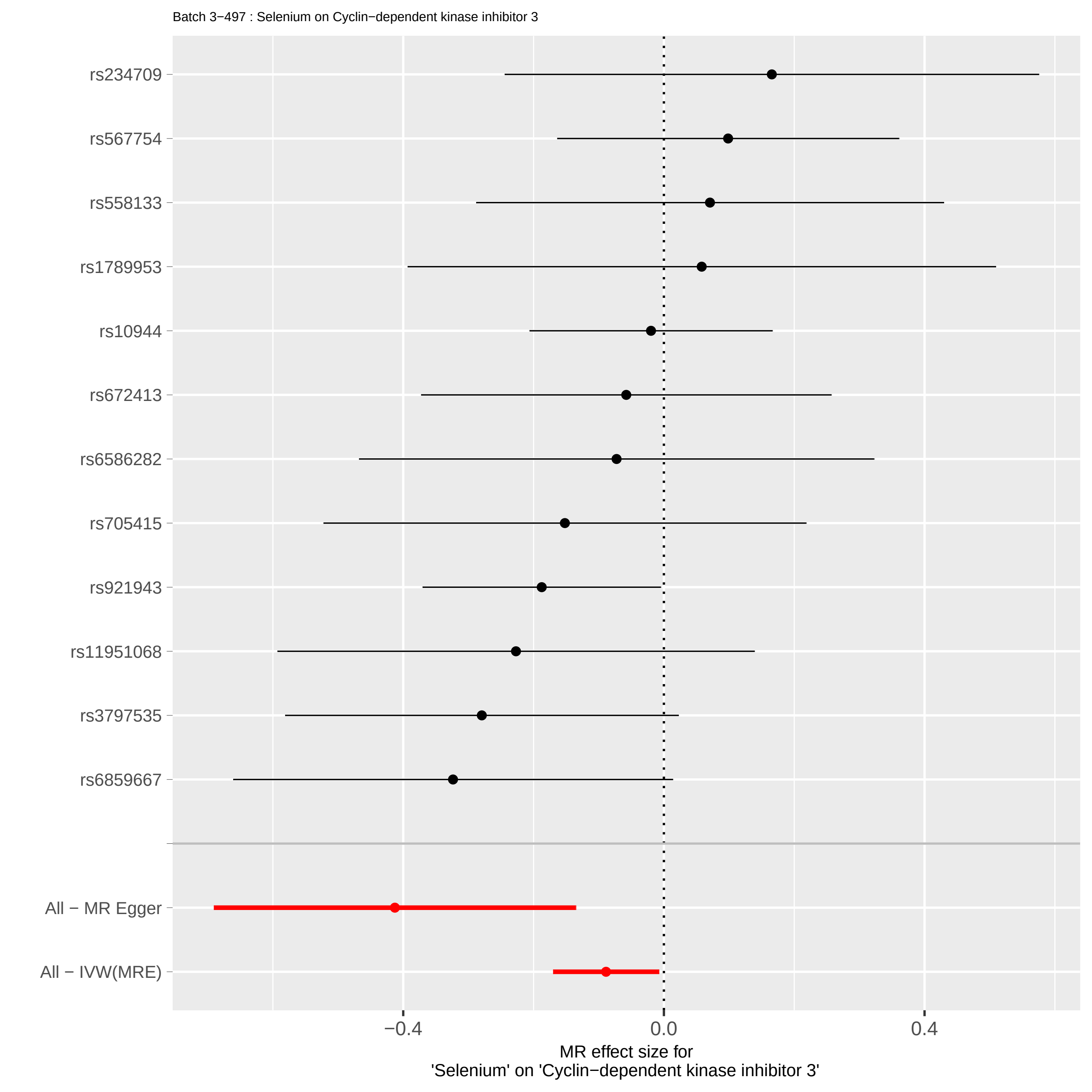

Batch 3−497 : Selenium on Cyclin−dependent kinase inhibitor 3
rs234709
rs567754
rs558133
rs1789953
rs10944
rs672413
rs6586282
rs705415
rs921943
rs11951068
rs3797535
rs6859667
All − MR Egger
All − IVW(MRE)
−0.4
0.0
0.4
MR effect size for
'Selenium' on 'Cyclin−dependent kinase inhibitor 3'

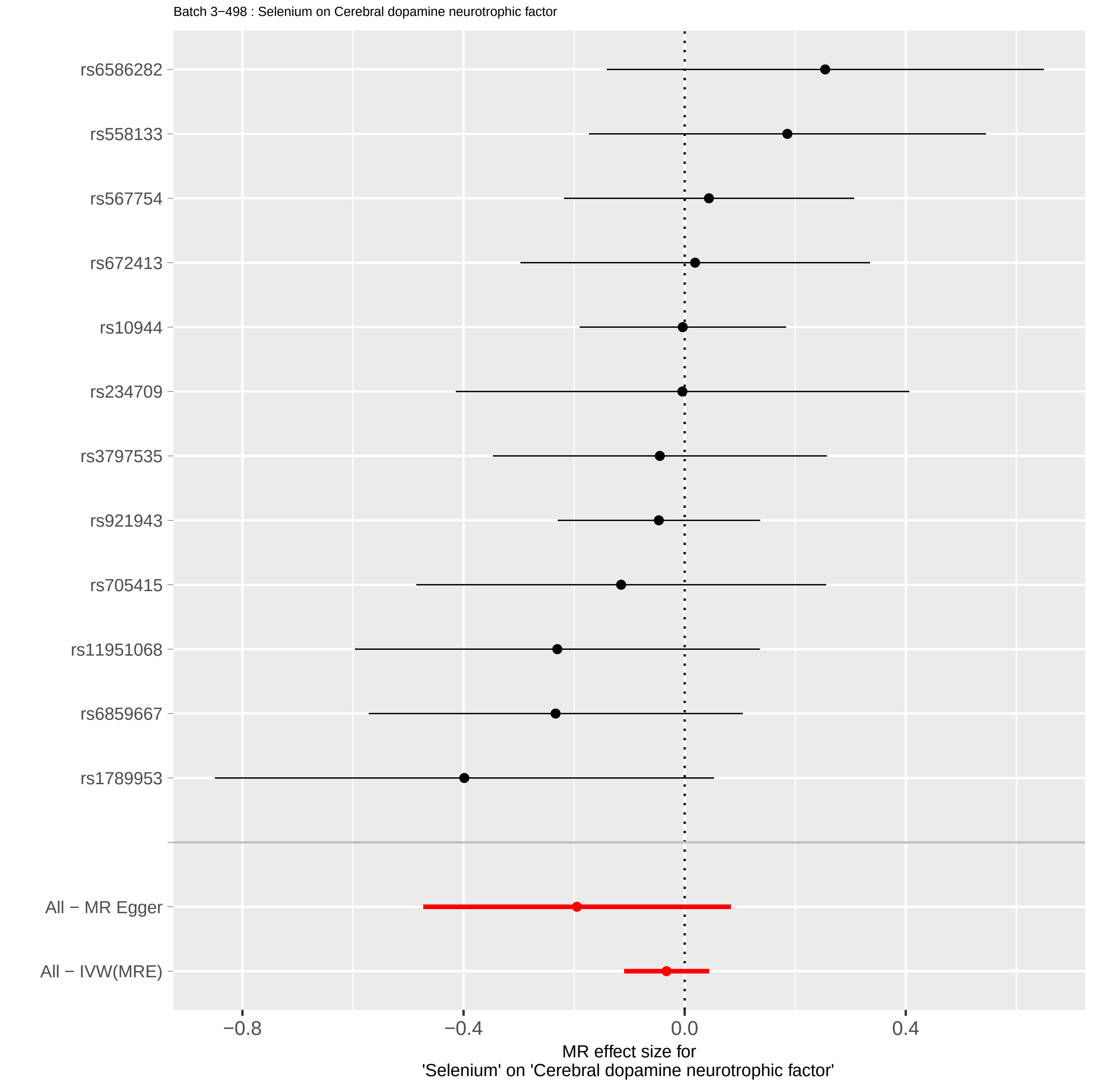

Batch 3−498 : Selenium on Cerebral dopamine neurotrophic factor
rs6586282
rs558133
rs567754
rs672413
rs10944
rs234709
rs3797535
rs921943
rs705415
rs11951068
rs6859667
rs1789953
All − MR Egger
All − IVW(MRE)
−0.8
−0.4
0.0
0.4
MR effect size for
'Selenium' on 'Cerebral dopamine neurotrophic factor'

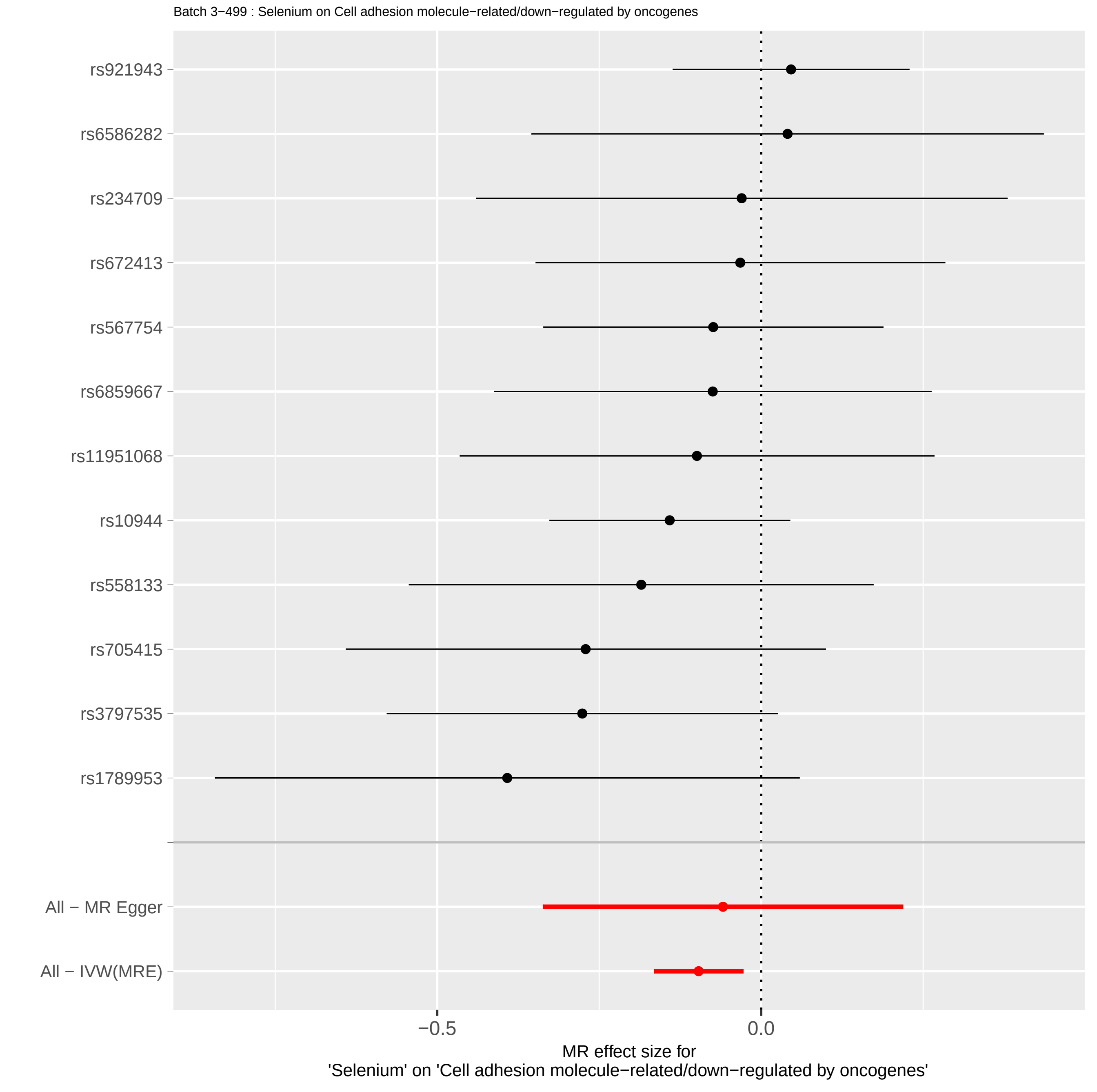
Batch 3−499 : Selenium on Cell adhesion molecule−related/down−regulated by oncogenes
rs921943
rs6586282
rs234709
rs672413
rs567754
rs6859667
rs11951068
rs10944
rs558133
rs705415
rs3797535
rs1789953
All − MR Egger
All − IVW(MRE)
−0.5
0.0
MR effect size for
'Selenium' on 'Cell adhesion molecule−related/down−regulated by oncogenes'

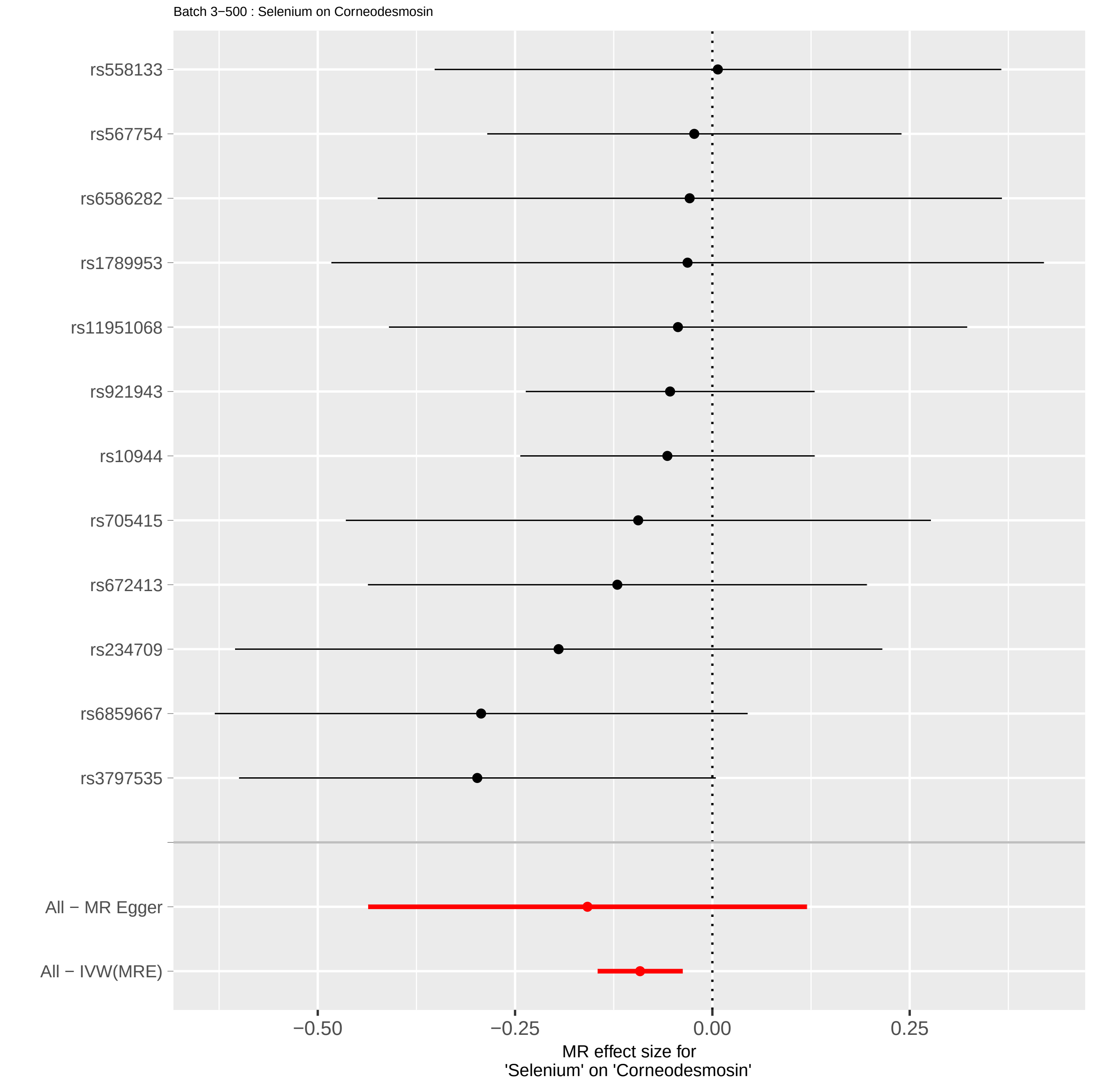

Batch 3−500 : Selenium on Corneodesmosin
rs558133
rs567754
rs6586282
rs1789953
rs11951068
rs921943
rs10944
rs705415
rs672413
rs234709
rs6859667
rs3797535
All − MR Egger
All − IVW(MRE)
−0.50
−0.25
0.00
0.25
MR effect size for
'Selenium' on 'Corneodesmosin'

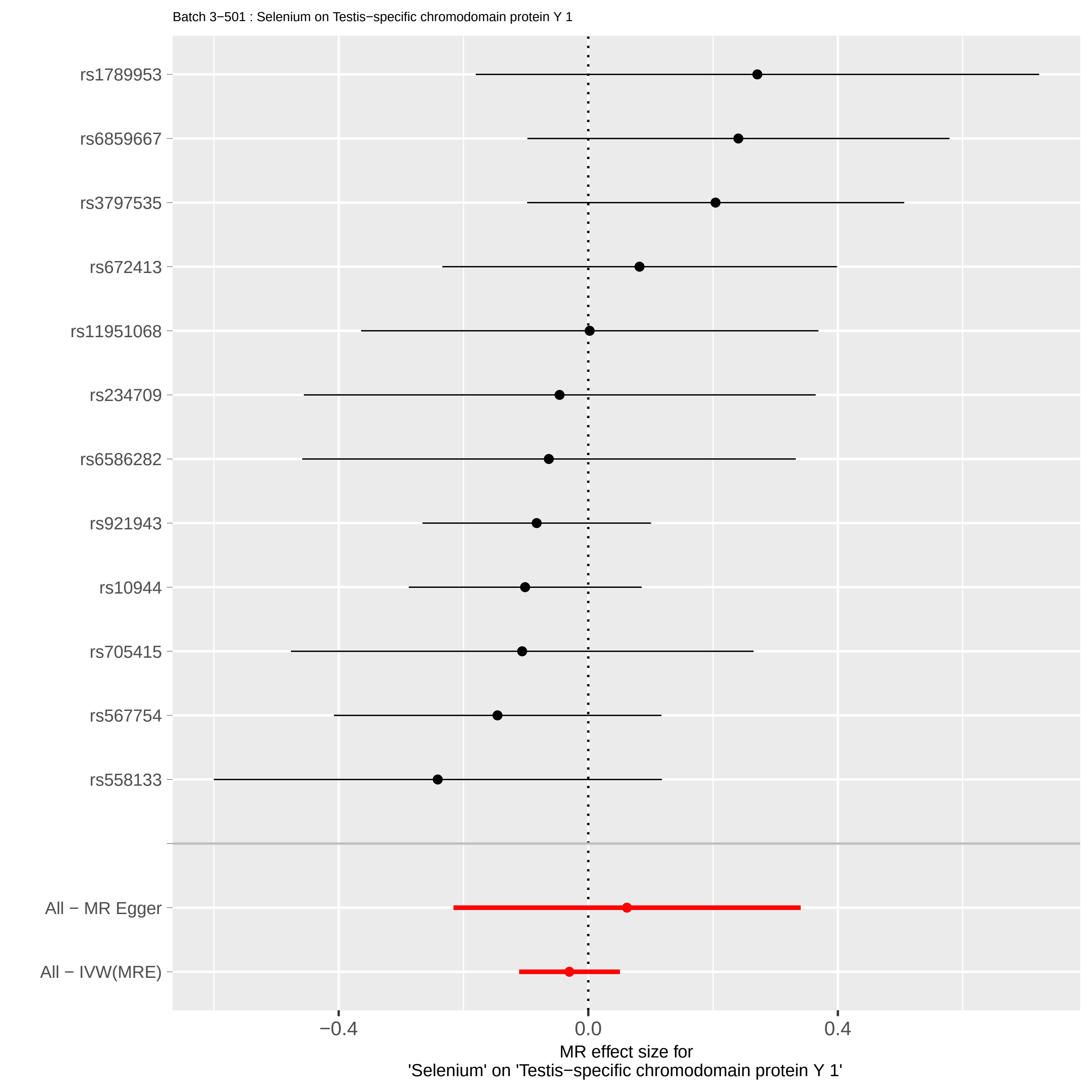
Batch 3−501 : Selenium on Testis−specific chromodomain protein Y 1
rs1789953
rs6859667
rs3797535
rs672413
rs11951068
rs234709
rs6586282
rs921943
rs10944
rs705415
rs567754
rs558133
All − MR Egger
All − IVW(MRE)
−0.4
0.0
0.4
MR effect size for
'Selenium' on 'Testis−specific chromodomain protein Y 1'

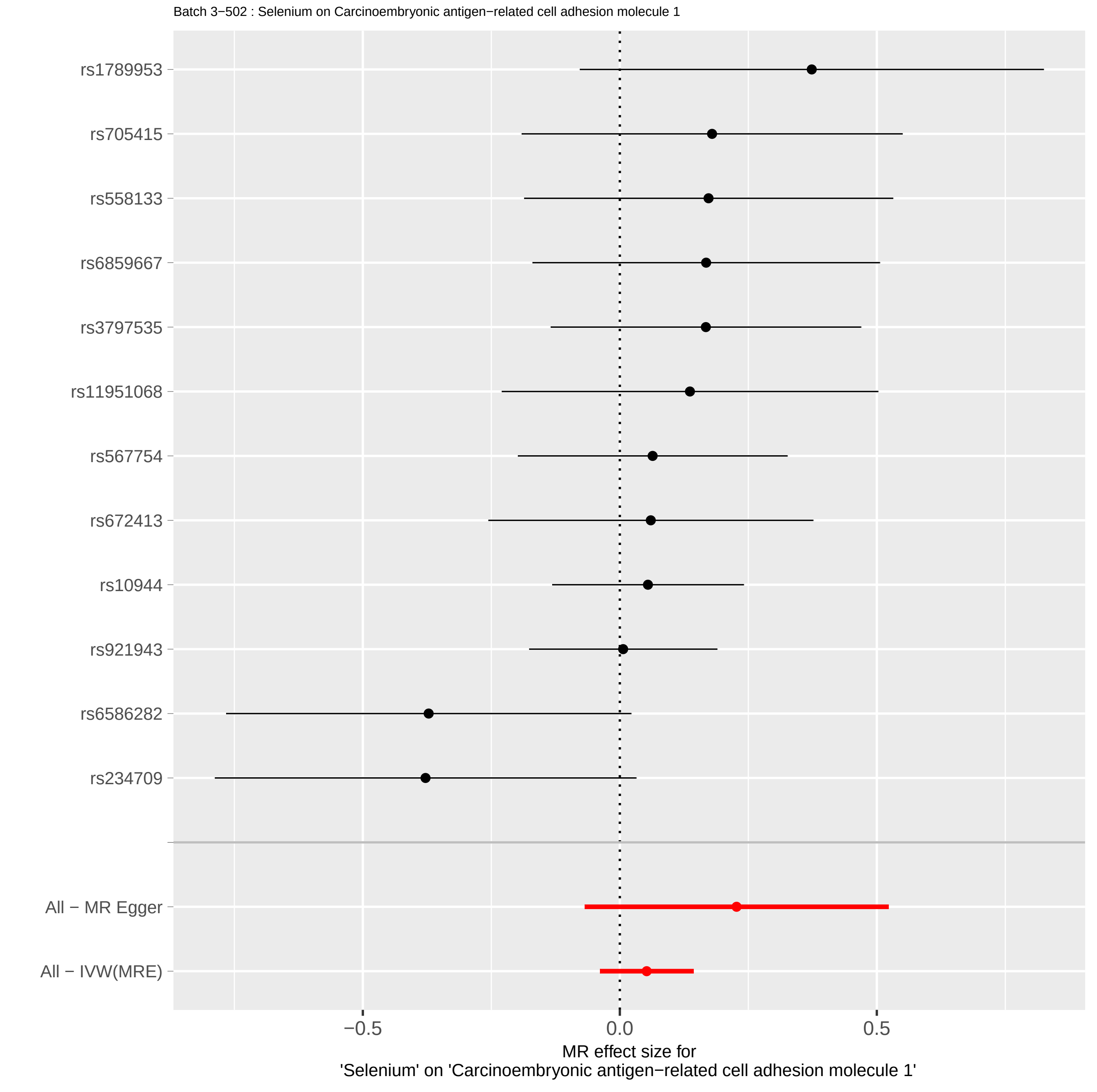

Batch 3−502 : Selenium on Carcinoembryonic antigen−related cell adhesion molecule 1
rs1789953
rs705415
rs558133
rs6859667
rs3797535
rs11951068
rs567754
rs672413
rs10944
rs921943
rs6586282
rs234709
All − MR Egger
All − IVW(MRE)
−0.5
0.0
0.5
MR effect size for
'Selenium' on 'Carcinoembryonic antigen−related cell adhesion molecule 1'

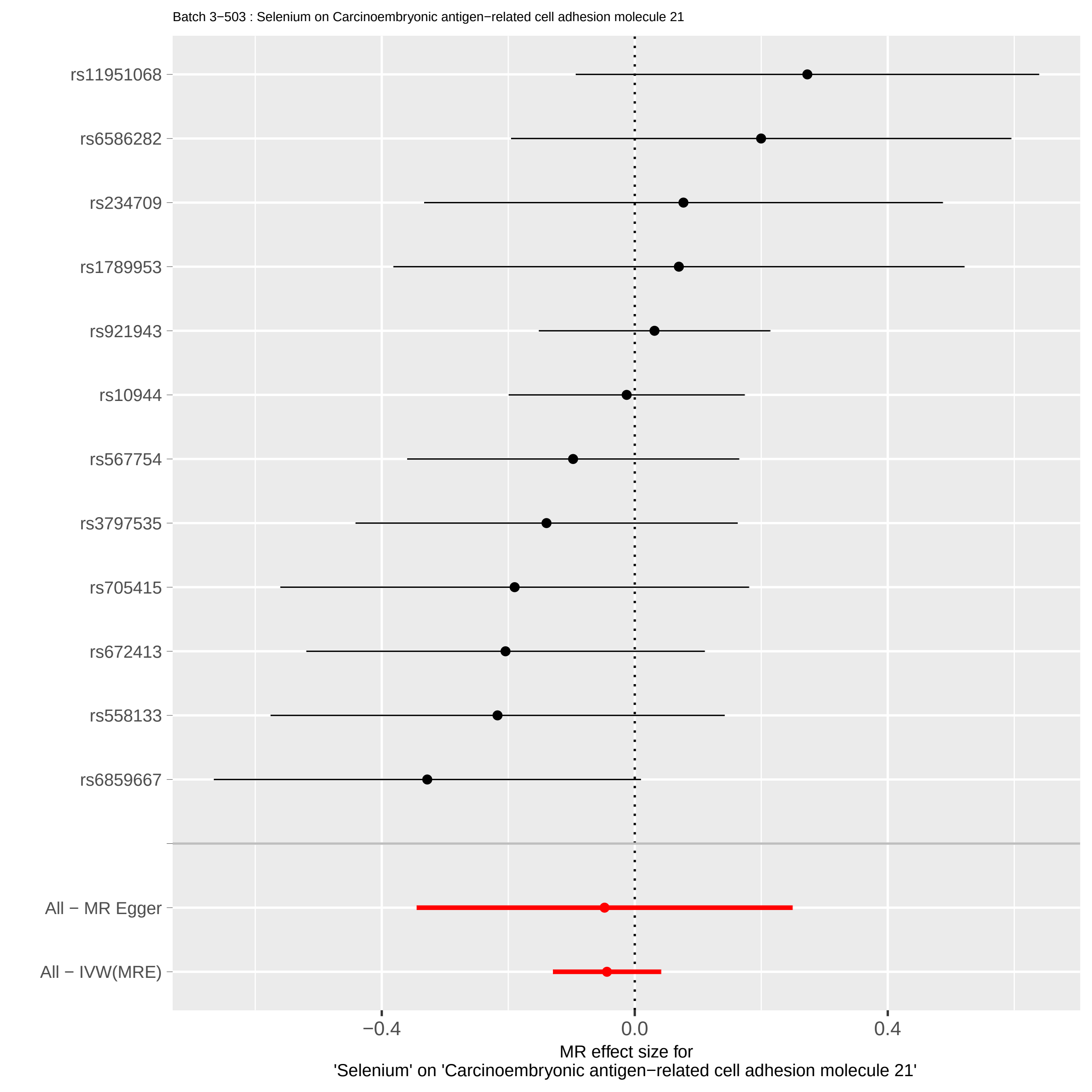

Batch 3−503 : Selenium on Carcinoembryonic antigen−related cell adhesion molecule 21
rs11951068
rs6586282
rs234709
rs1789953
rs921943
rs10944
rs567754
rs3797535
rs705415
rs672413
rs558133
rs6859667
All − MR Egger
All − IVW(MRE)
−0.4
0.0
0.4
MR effect size for
'Selenium' on 'Carcinoembryonic antigen−related cell adhesion molecule 21'

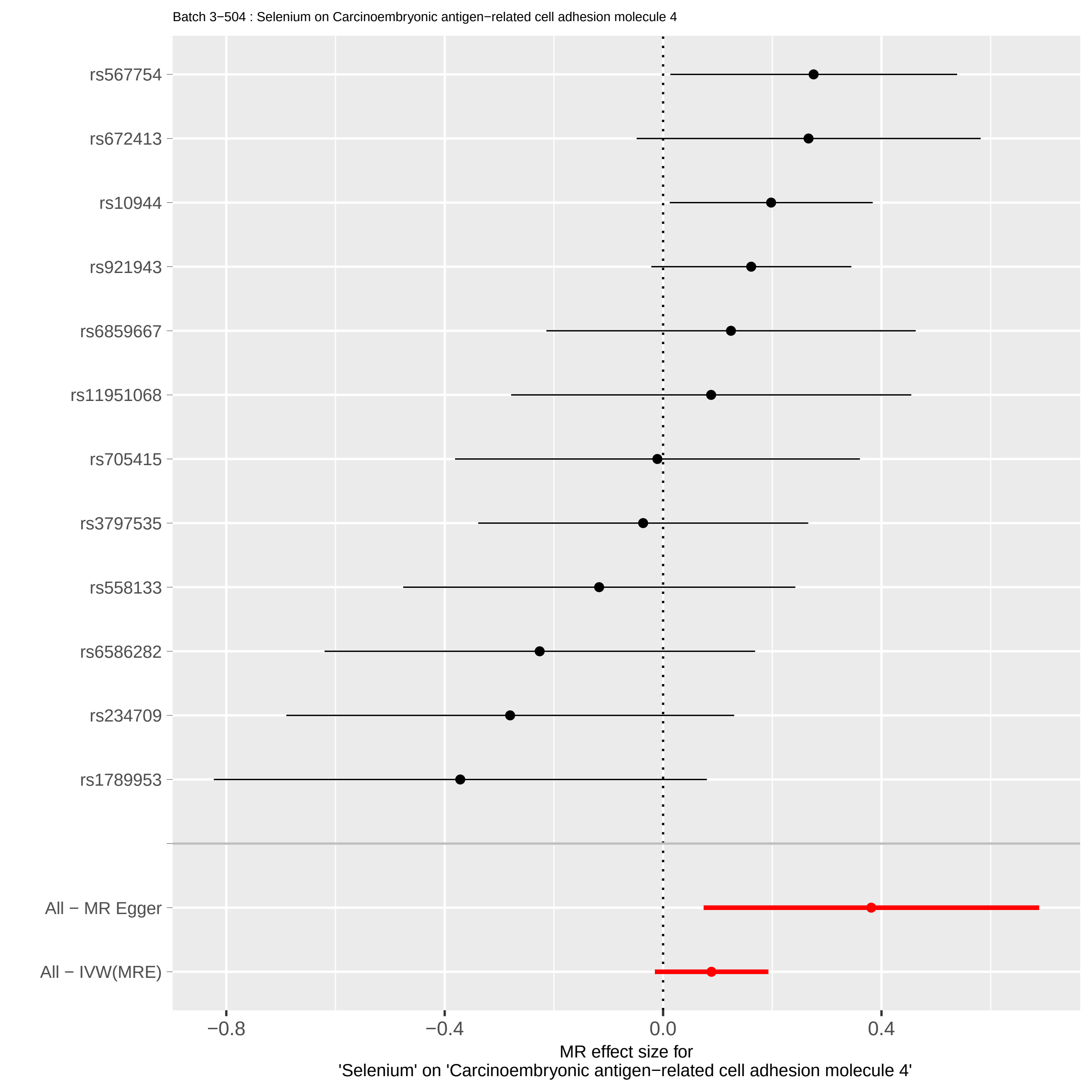

Batch 3−504 : Selenium on Carcinoembryonic antigen−related cell adhesion molecule 4
rs567754
rs672413
rs10944
rs921943
rs6859667
rs11951068
rs705415
rs3797535
rs558133
rs6586282
rs234709
rs1789953
All − MR Egger
All − IVW(MRE)
−0.8
−0.4
0.0
0.4
MR effect size for
'Selenium' on 'Carcinoembryonic antigen−related cell adhesion molecule 4'

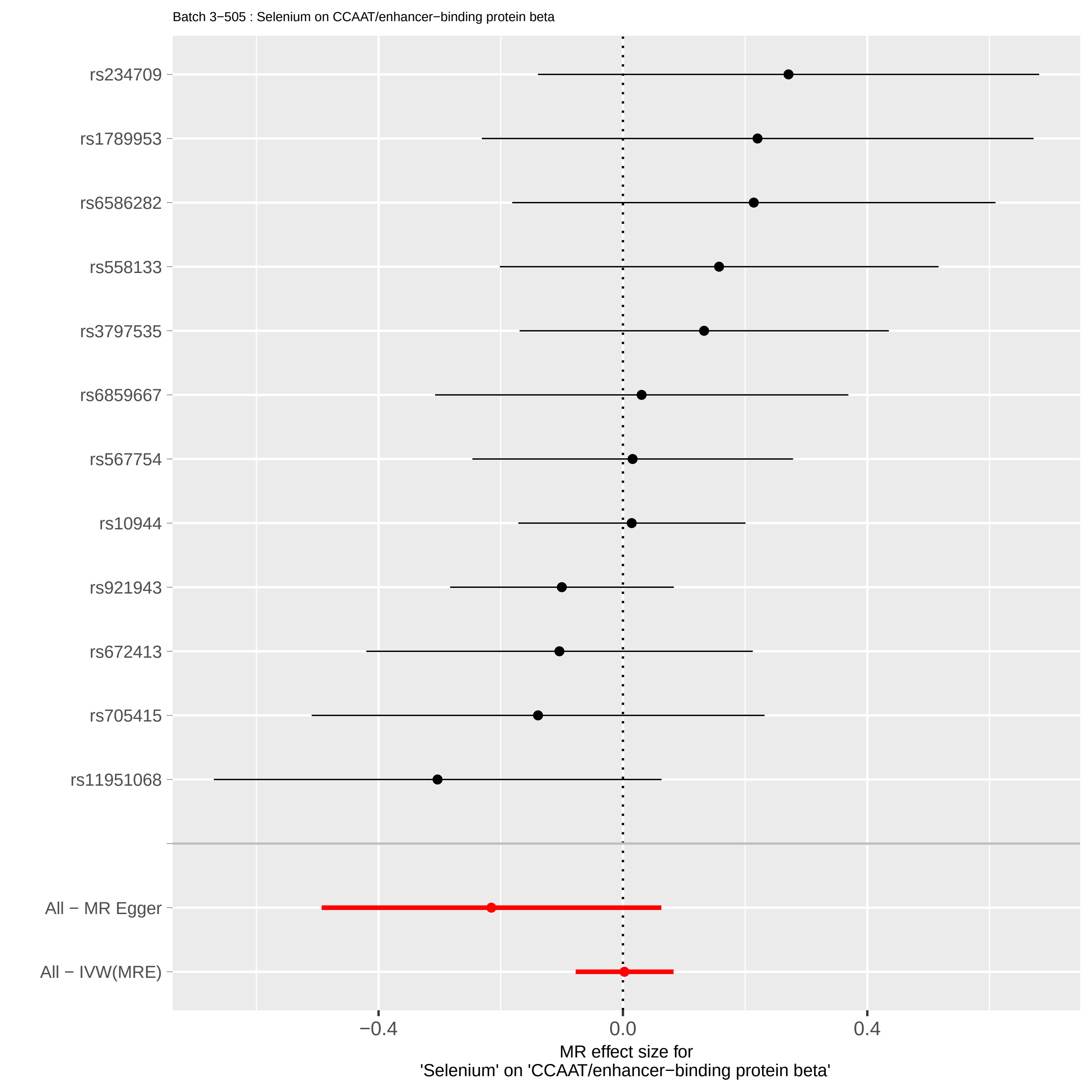
Batch 3−505 : Selenium on CCAAT/enhancer−binding protein beta
rs234709
rs1789953
rs6586282
rs558133
rs3797535
rs6859667
rs567754
rs10944
rs921943
rs672413
rs705415
rs11951068
All − MR Egger
All − IVW(MRE)
−0.4
0.0
0.4
MR effect size for
'Selenium' on 'CCAAT/enhancer−binding protein beta'

Batch 3−506 : Selenium on Adenosine deaminase CECR1

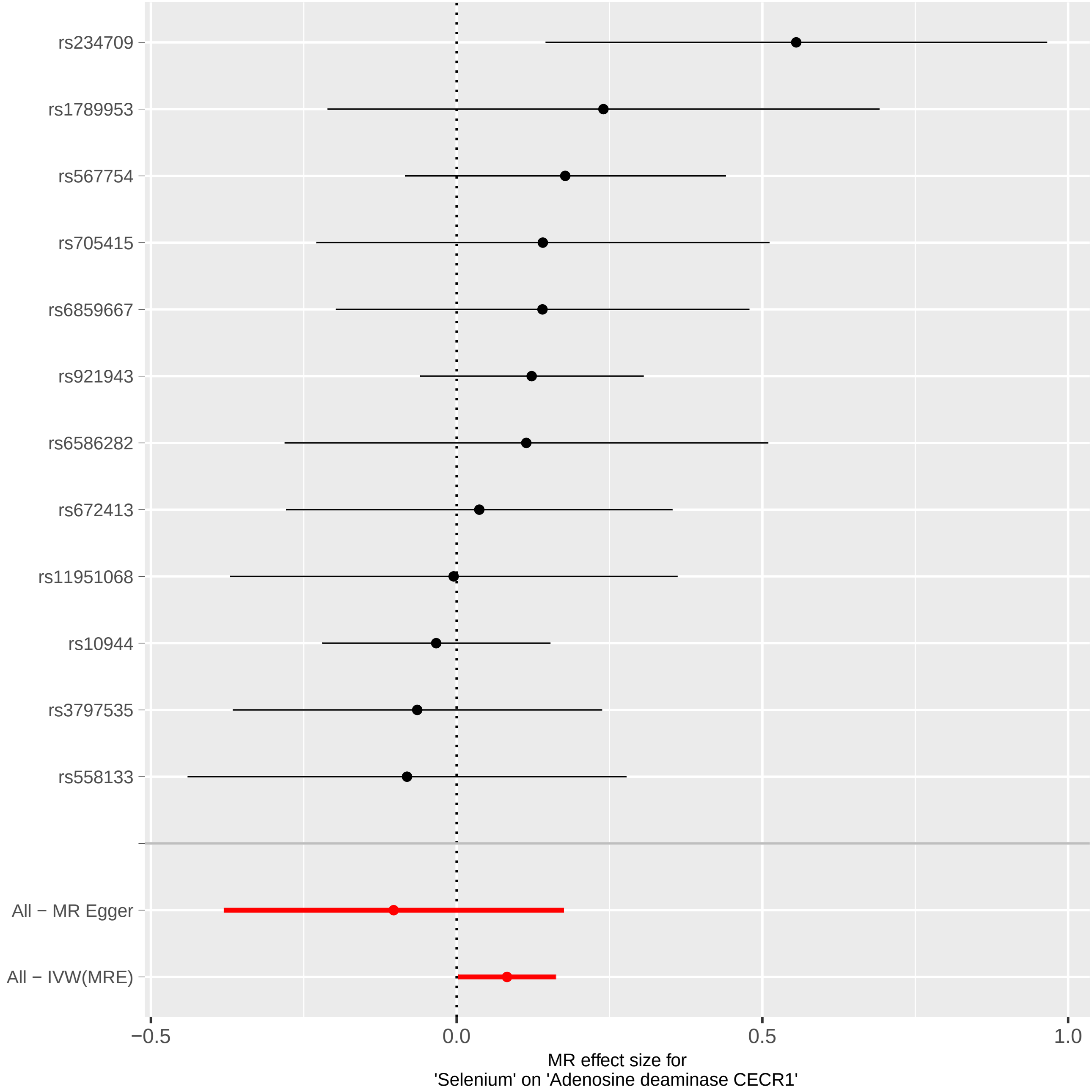

Batch 3−507 : Selenium on Bile salt−activated lipase

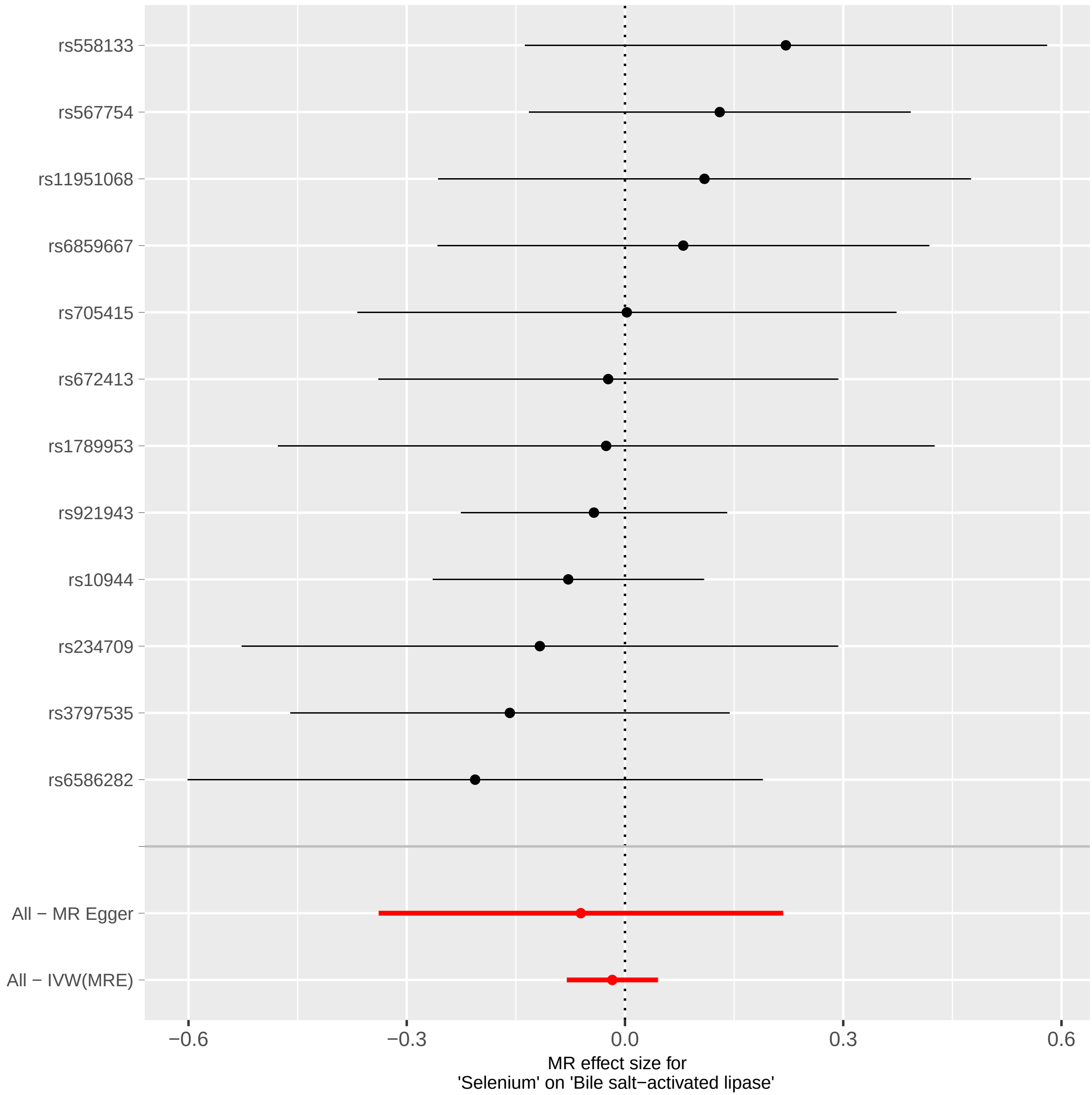

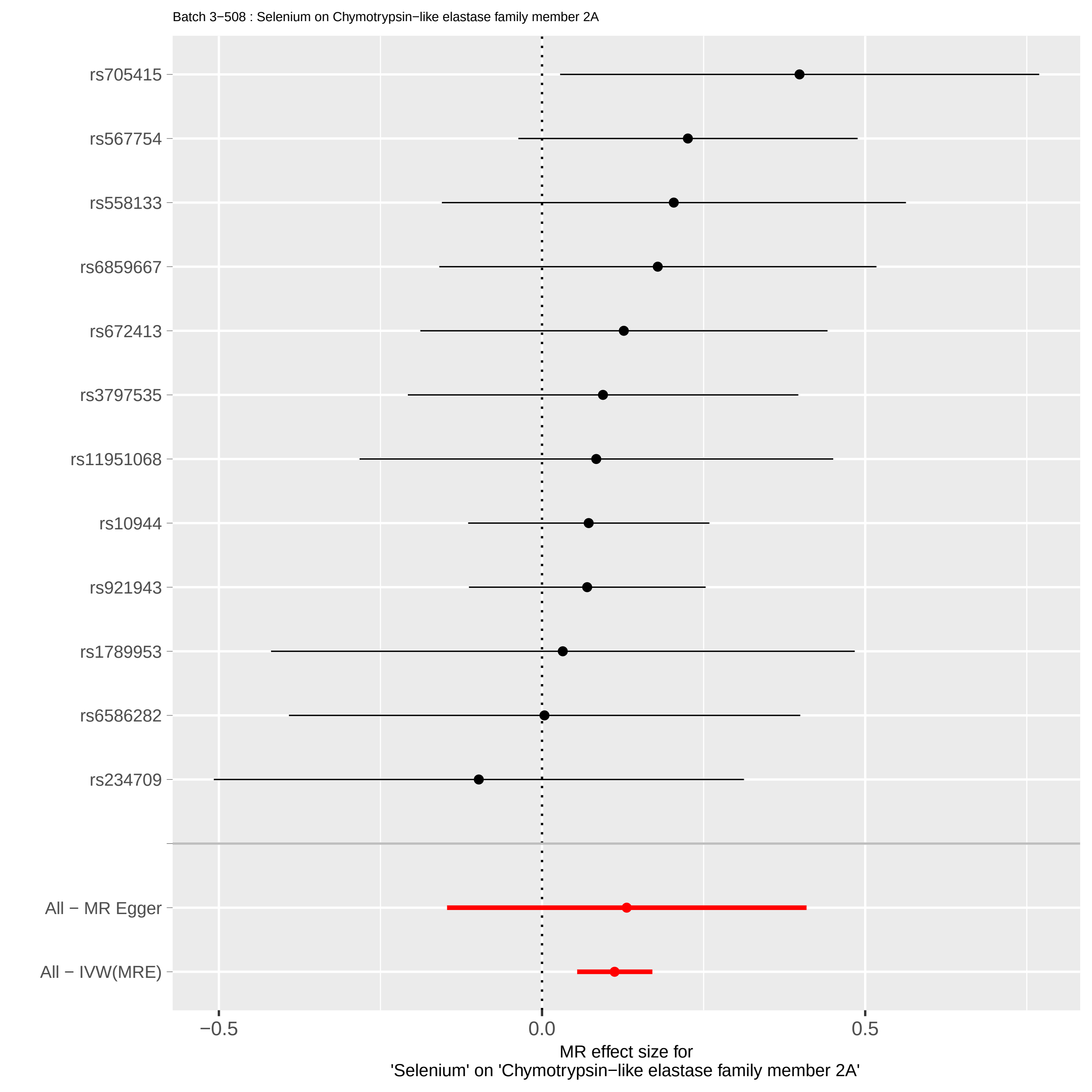
Batch 3−508 : Selenium on Chymotrypsin−like elastase family member 2A
rs705415
rs567754
rs558133
rs6859667
rs672413
rs3797535
rs11951068
rs10944
rs921943
rs1789953
rs6586282
rs234709
All − MR Egger
All − IVW(MRE)
−0.5
0.0
0.5
MR effect size for
'Selenium' on 'Chymotrypsin−like elastase family member 2A'

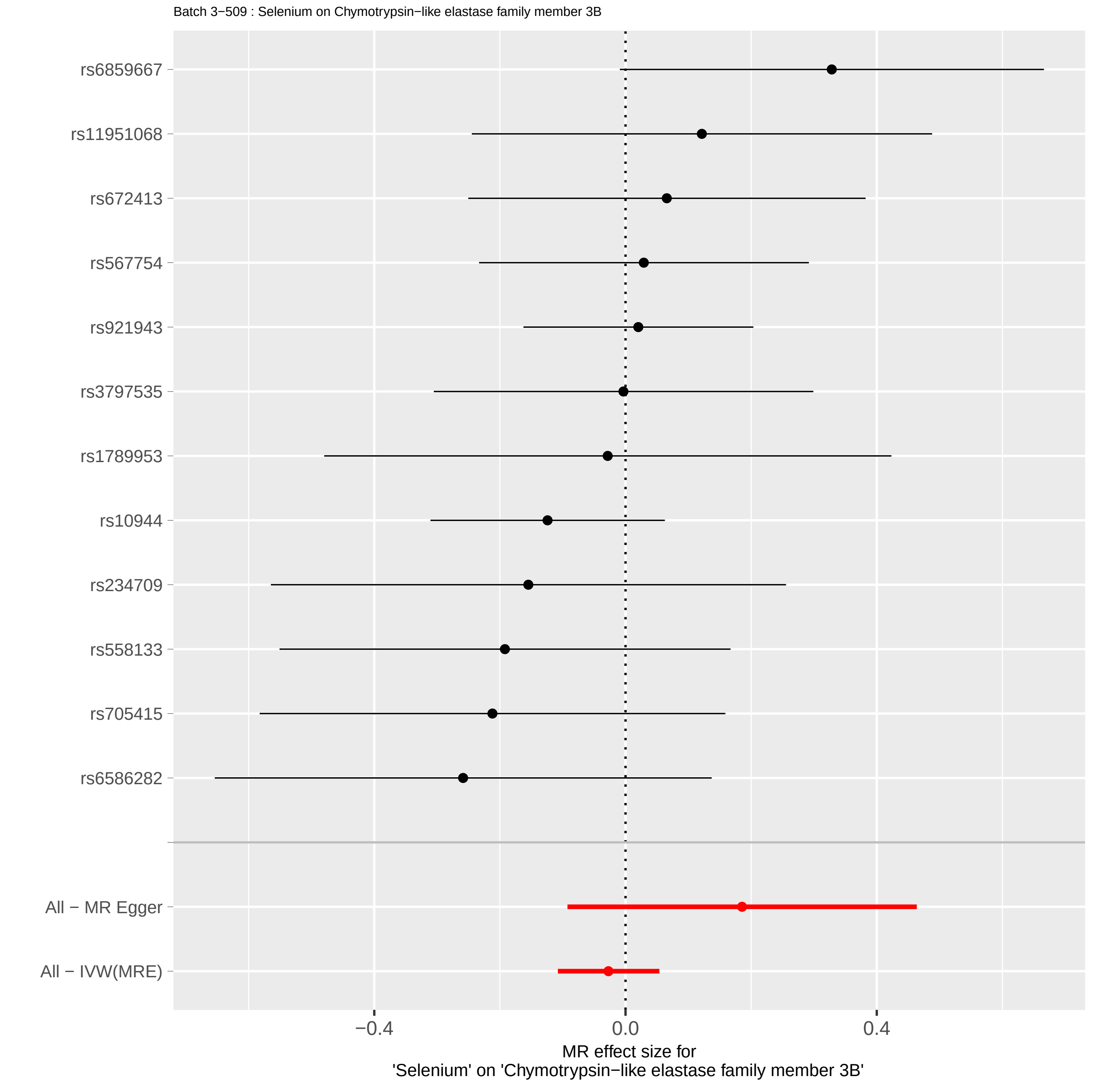

Batch 3−509 : Selenium on Chymotrypsin−like elastase family member 3B
rs6859667
rs11951068
rs672413
rs567754
rs921943
rs3797535
rs1789953
rs10944
rs234709
rs558133
rs705415
rs6586282
All − MR Egger
All − IVW(MRE)
−0.4
0.0
0.4
MR effect size for
'Selenium' on 'Chymotrypsin−like elastase family member 3B'

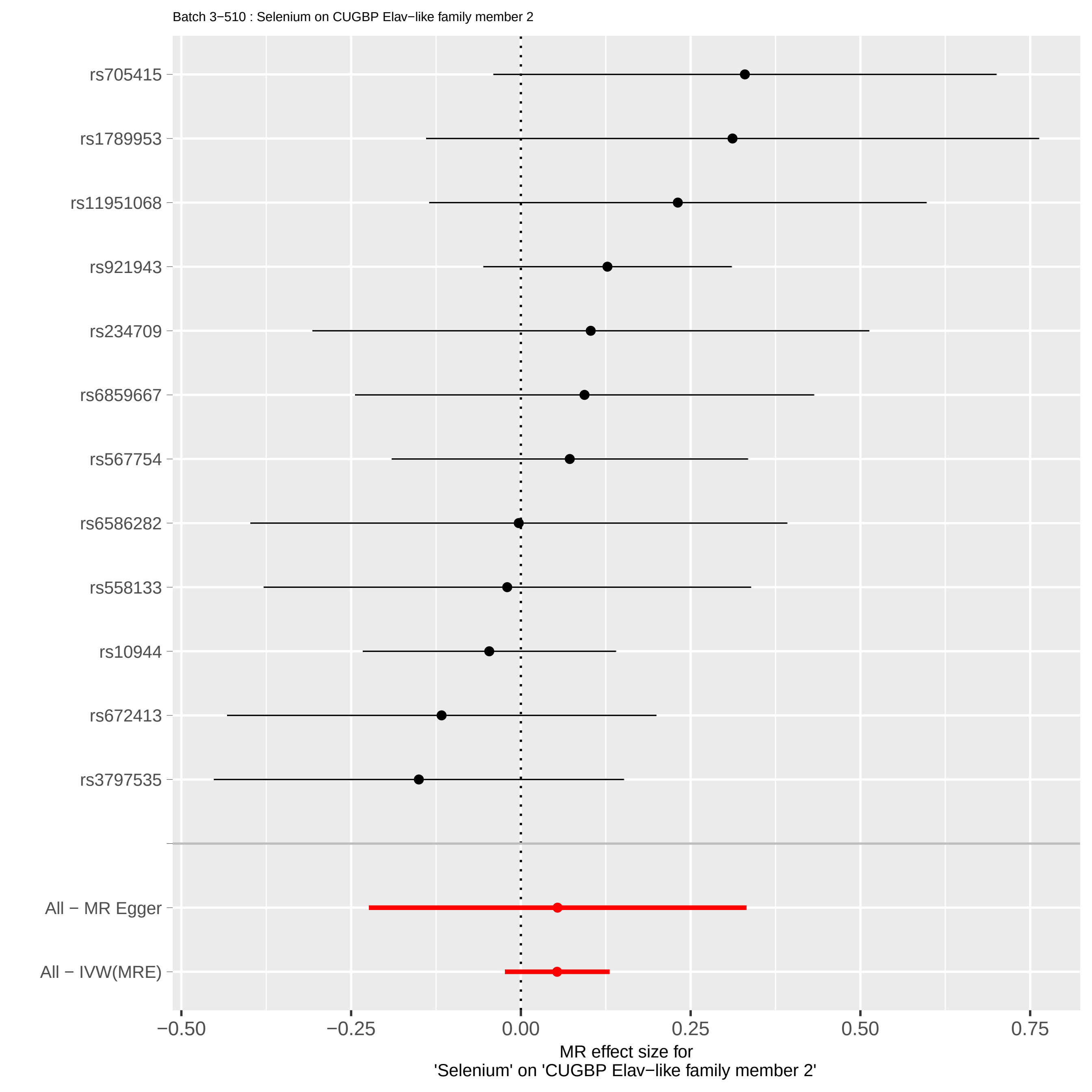

Batch 3−510 : Selenium on CUGBP Elav−like family member 2
rs705415
rs1789953
rs11951068
rs921943
rs234709
rs6859667
rs567754
rs6586282
rs558133
rs10944
rs672413
rs3797535
All − MR Egger
All − IVW(MRE)
−0.50
−0.25
0.00
0.25
0.50
0.75
MR effect size for
'Selenium' on 'CUGBP Elav−like family member 2'

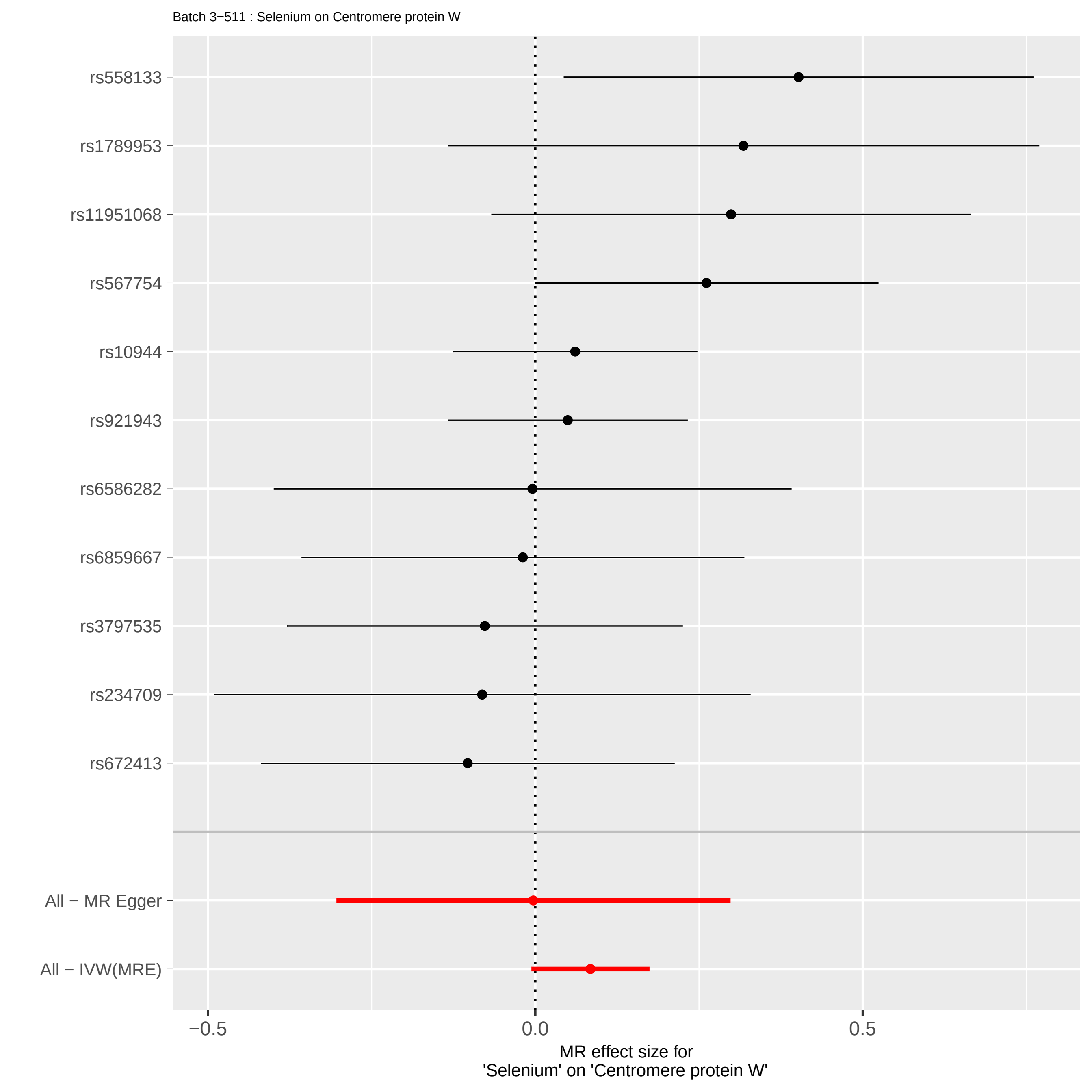

Batch 3−511 : Selenium on Centromere protein W
rs558133
rs1789953
rs11951068
rs567754
rs10944
rs921943
rs6586282
rs6859667
rs3797535
rs234709
rs672413
All − MR Egger
All − IVW(MRE)
−0.5
0.0
0.5
MR effect size for
'Selenium' on 'Centromere protein W'

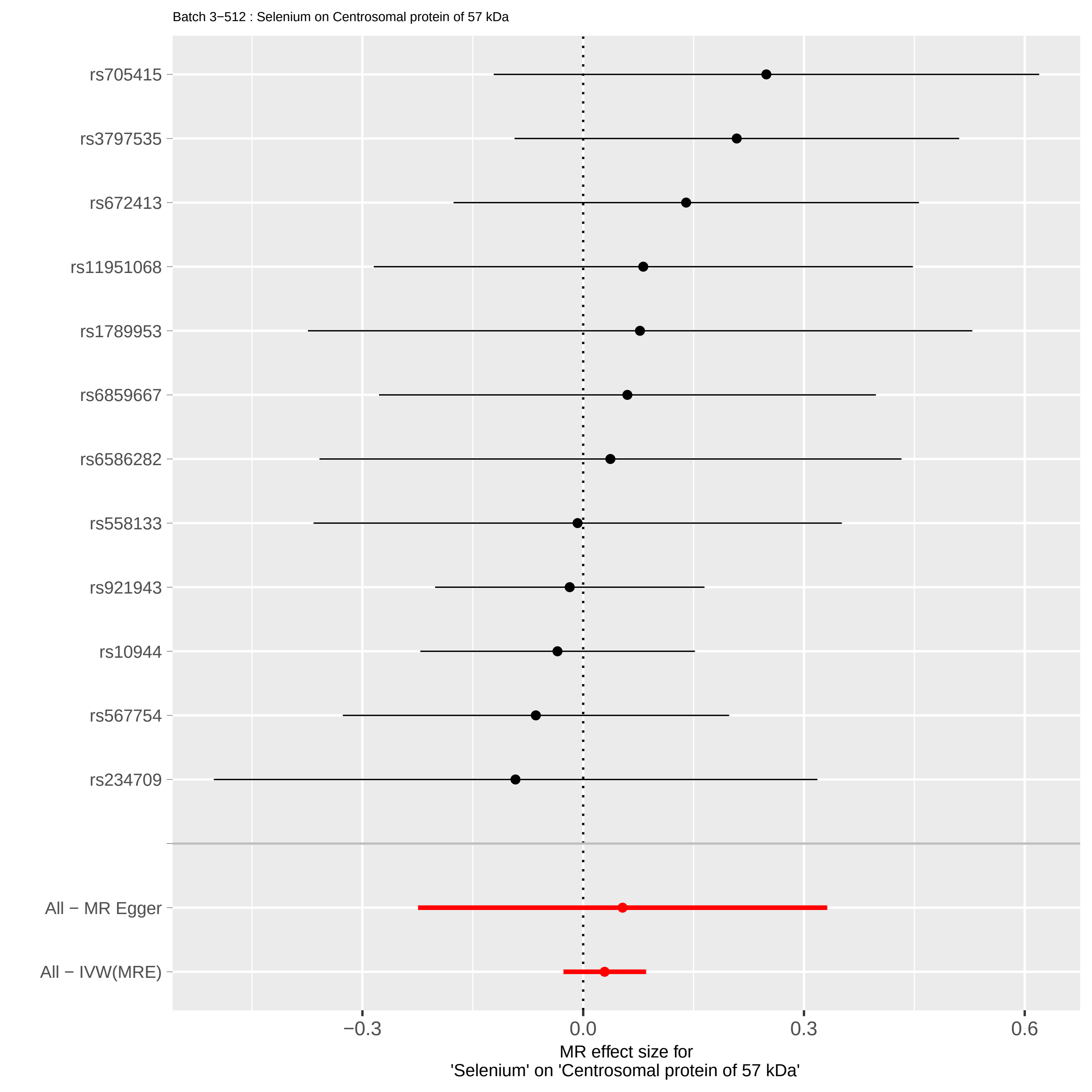

Batch 3−512 : Selenium on Centrosomal protein of 57 kDa
rs705415
rs3797535
rs672413
rs11951068
rs1789953
rs6859667
rs6586282
rs558133
rs921943
rs10944
rs567754
rs234709
All − MR Egger
All − IVW(MRE)
−0.3
0.0
0.3
0.6
MR effect size for
'Selenium' on 'Centrosomal protein of 57 kDa'

Batch 3−513 : Selenium on Centrosomal protein of 57 kDa

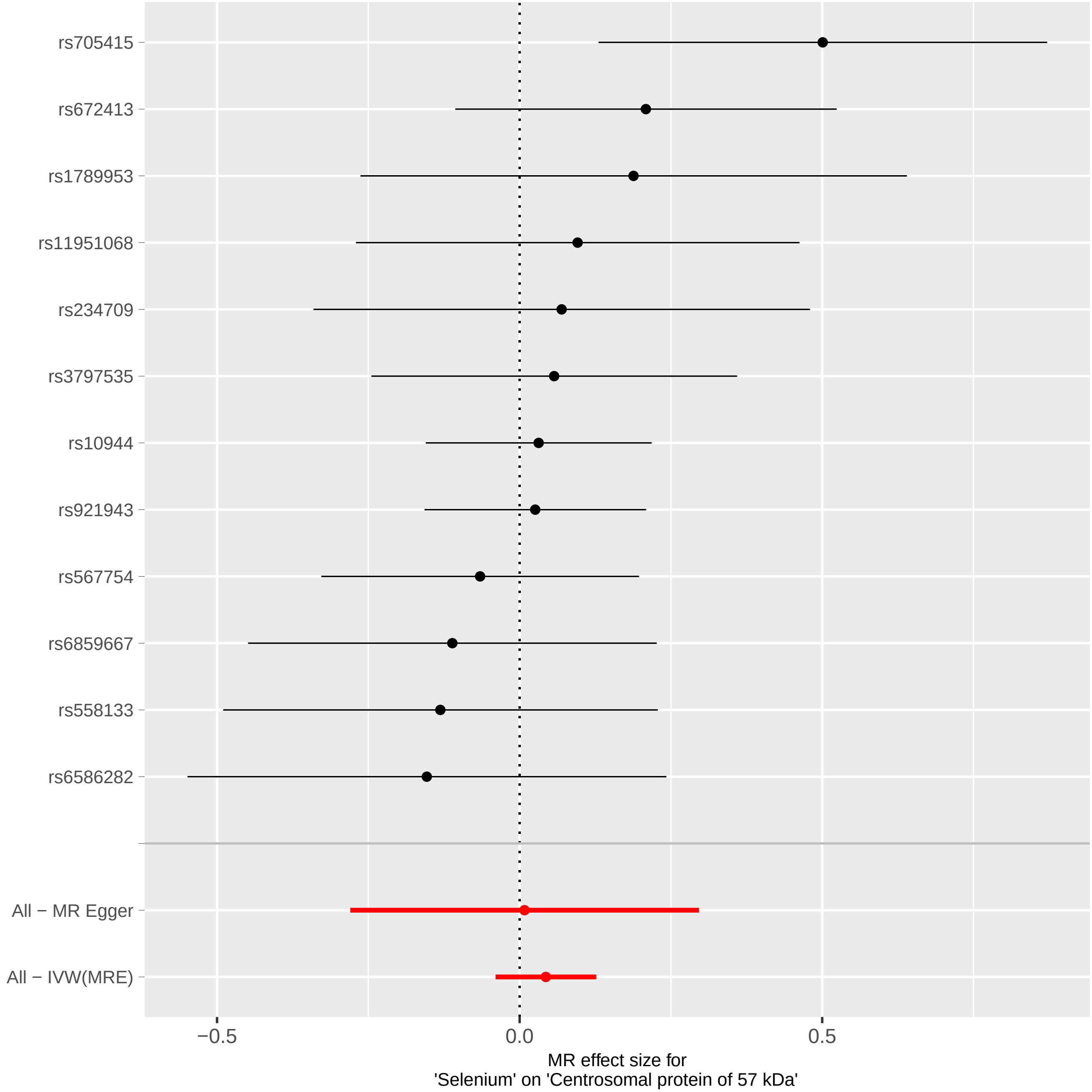

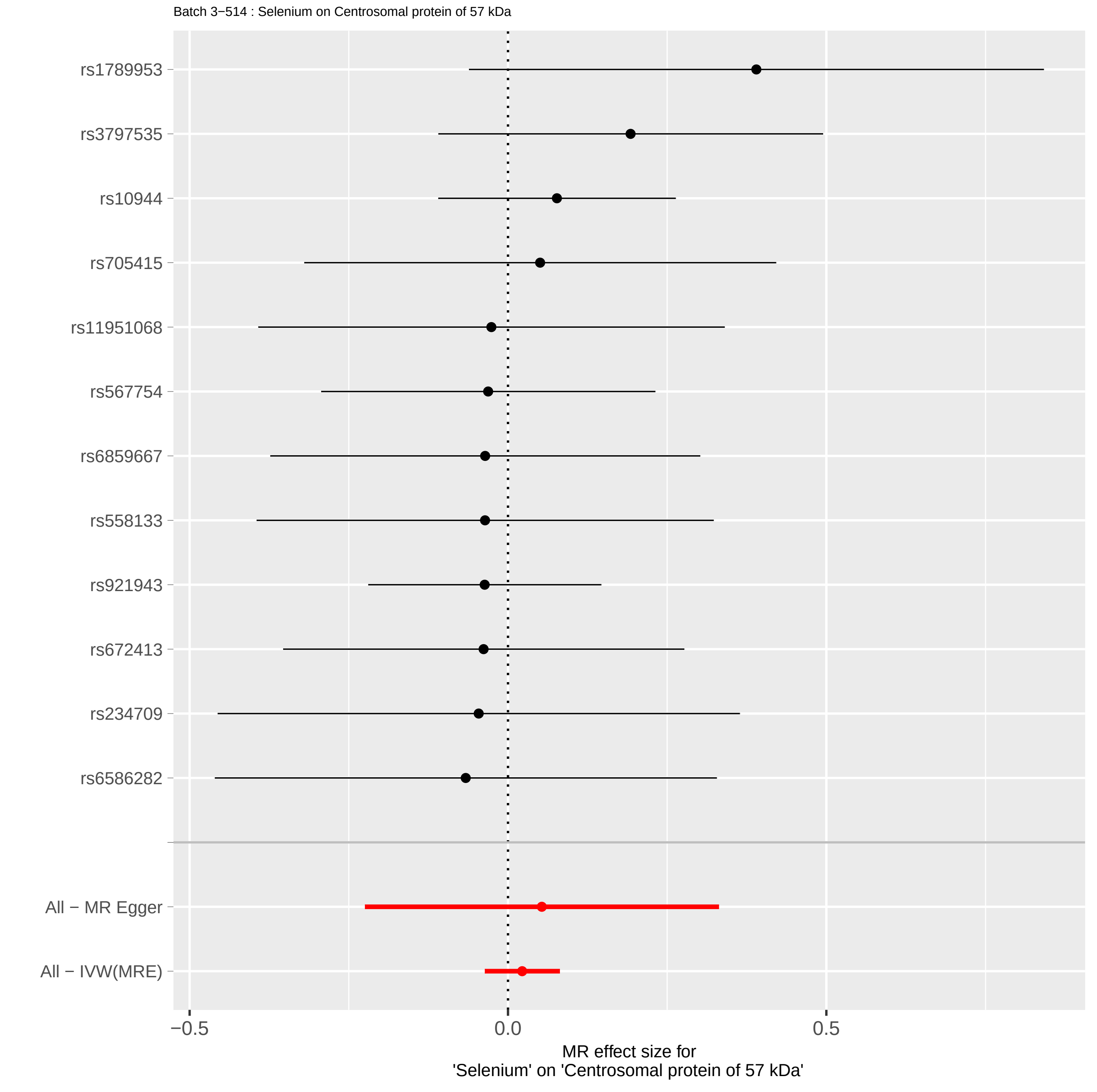

Batch 3−514 : Selenium on Centrosomal protein of 57 kDa
rs1789953
rs3797535
rs10944
rs705415
rs11951068
rs567754
rs6859667
rs558133
rs921943
rs672413
rs234709
rs6586282
All − MR Egger
All − IVW(MRE)
−0.5
0.0
0.5
MR effect size for
'Selenium' on 'Centrosomal protein of 57 kDa'

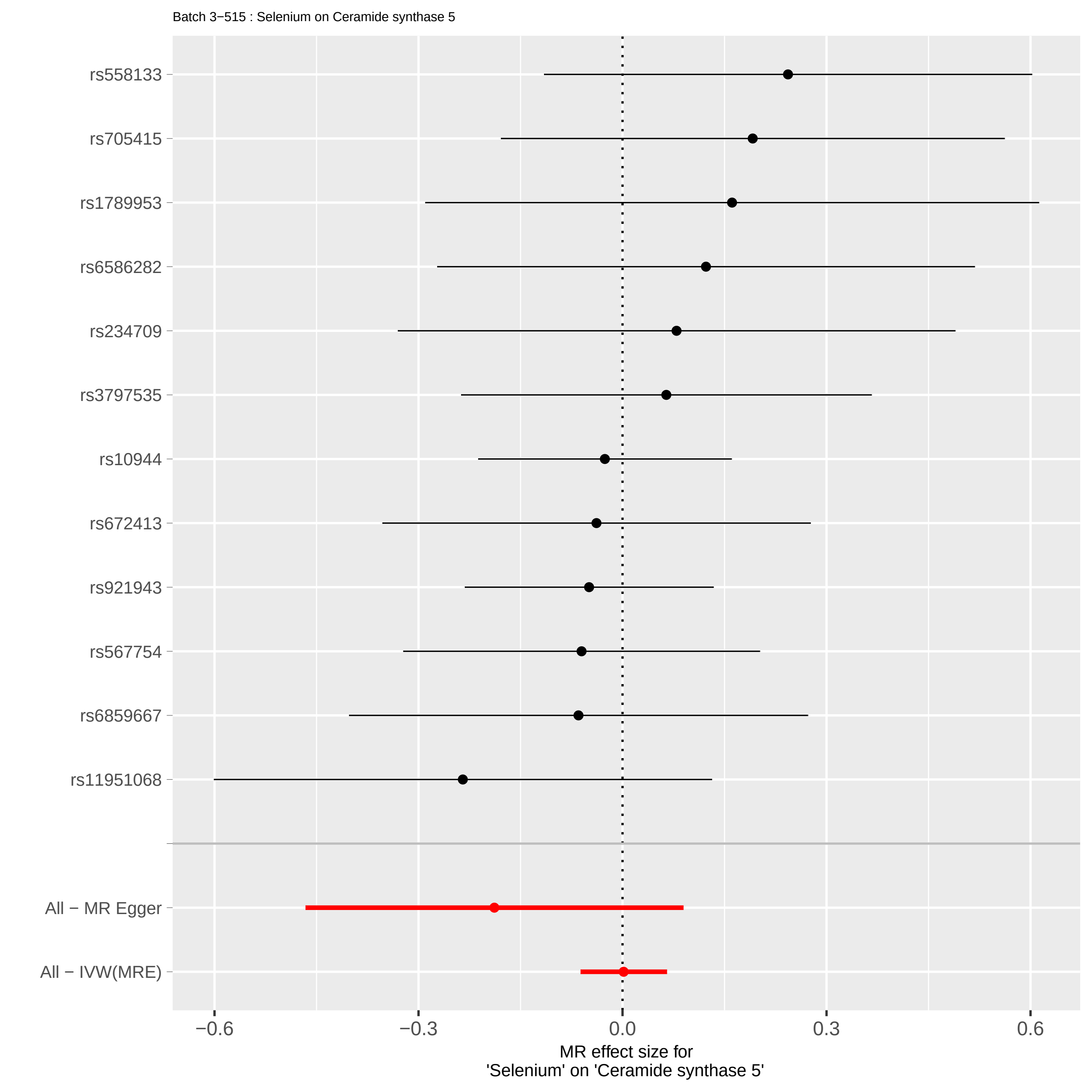

Batch 3−515 : Selenium on Ceramide synthase 5
rs558133
rs705415
rs1789953
rs6586282
rs234709
rs3797535
rs10944
rs672413
rs921943
rs567754
rs6859667
rs11951068
All − MR Egger
All − IVW(MRE)
−0.6
−0.3
0.0
0.3
0.6
MR effect size for
'Selenium' on 'Ceramide synthase 5'

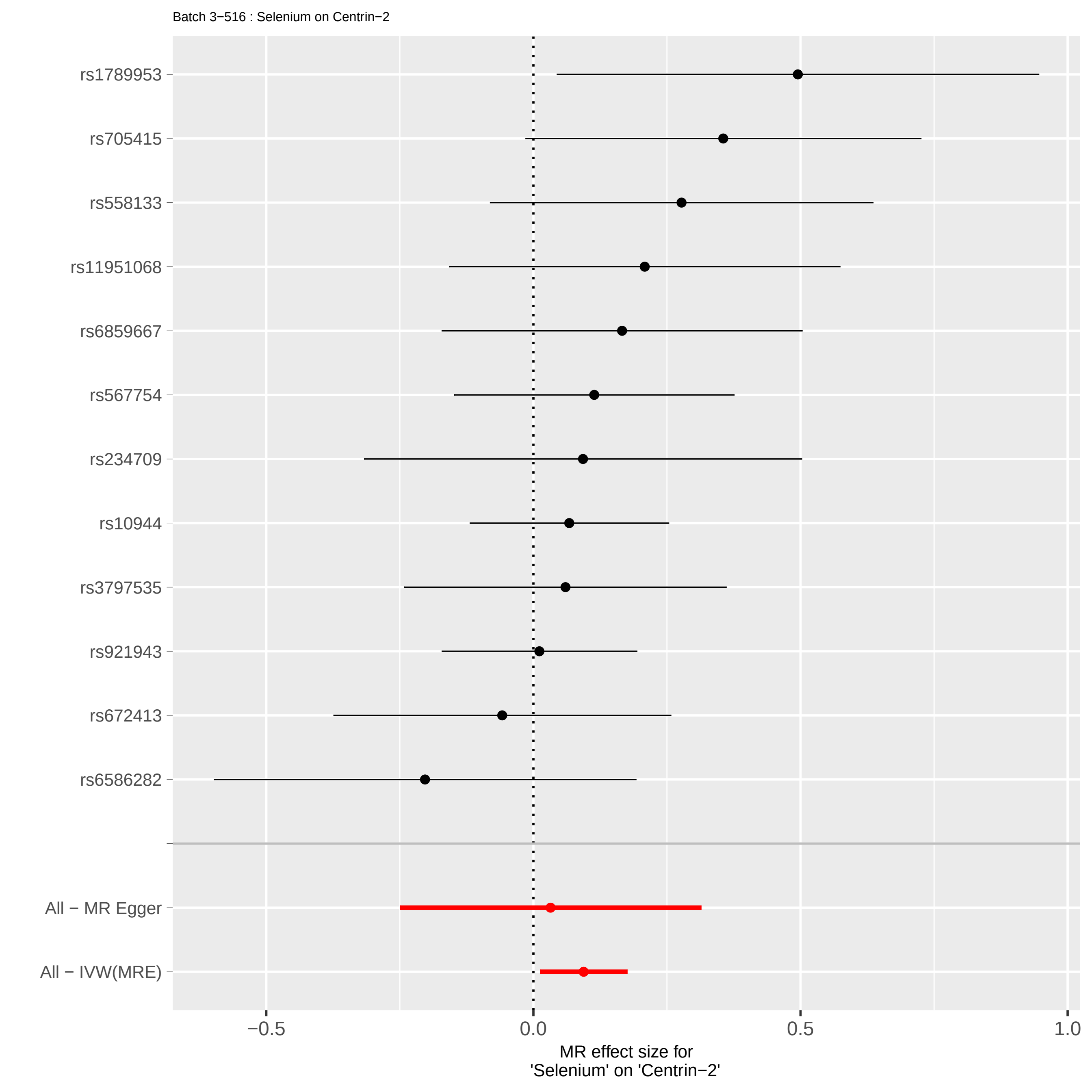

Batch 3−516 : Selenium on Centrin−2
rs1789953
rs705415
rs558133
rs11951068
rs6859667
rs567754
rs234709
rs10944
rs3797535
rs921943
rs672413
rs6586282
All − MR Egger
All − IVW(MRE)
−0.5
0.0
0.5
1.0
MR effect size for
'Selenium' on 'Centrin−2'

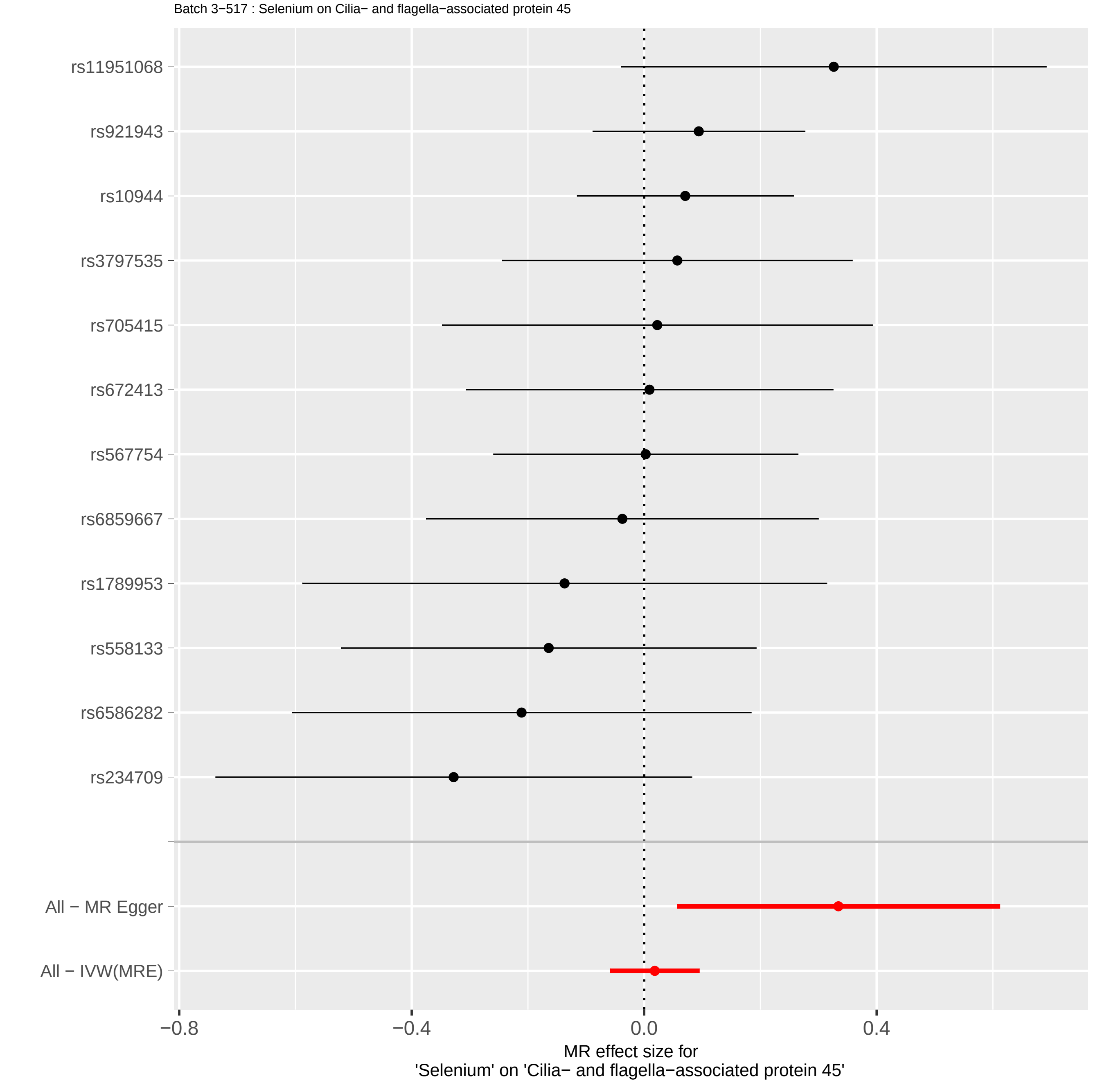

Batch 3−517 : Selenium on Cilia− and flagella−associated protein 45
rs11951068
rs921943
rs10944
rs3797535
rs705415
rs672413
rs567754
rs6859667
rs1789953
rs558133
rs6586282
rs234709
All − MR Egger
All − IVW(MRE)
−0.8
−0.4
0.0
0.4
MR effect size for
'Selenium' on 'Cilia− and flagella−associated protein 45'

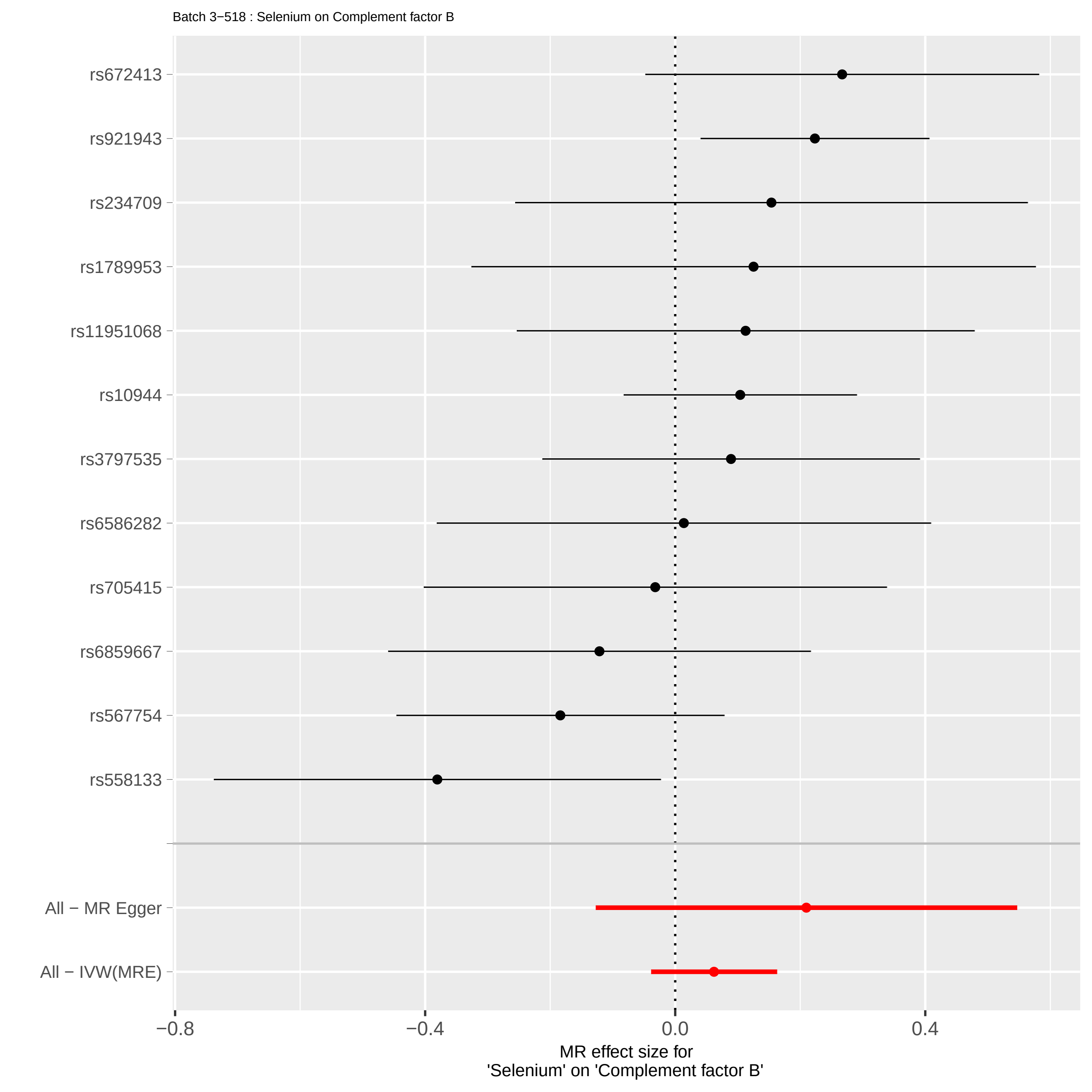

Batch 3−518 : Selenium on Complement factor B
rs672413
rs921943
rs234709
rs1789953
rs11951068
rs10944
rs3797535
rs6586282
rs705415
rs6859667
rs567754
rs558133
All − MR Egger
All − IVW(MRE)
−0.8
−0.4
0.0
0.4
MR effect size for
'Selenium' on 'Complement factor B'

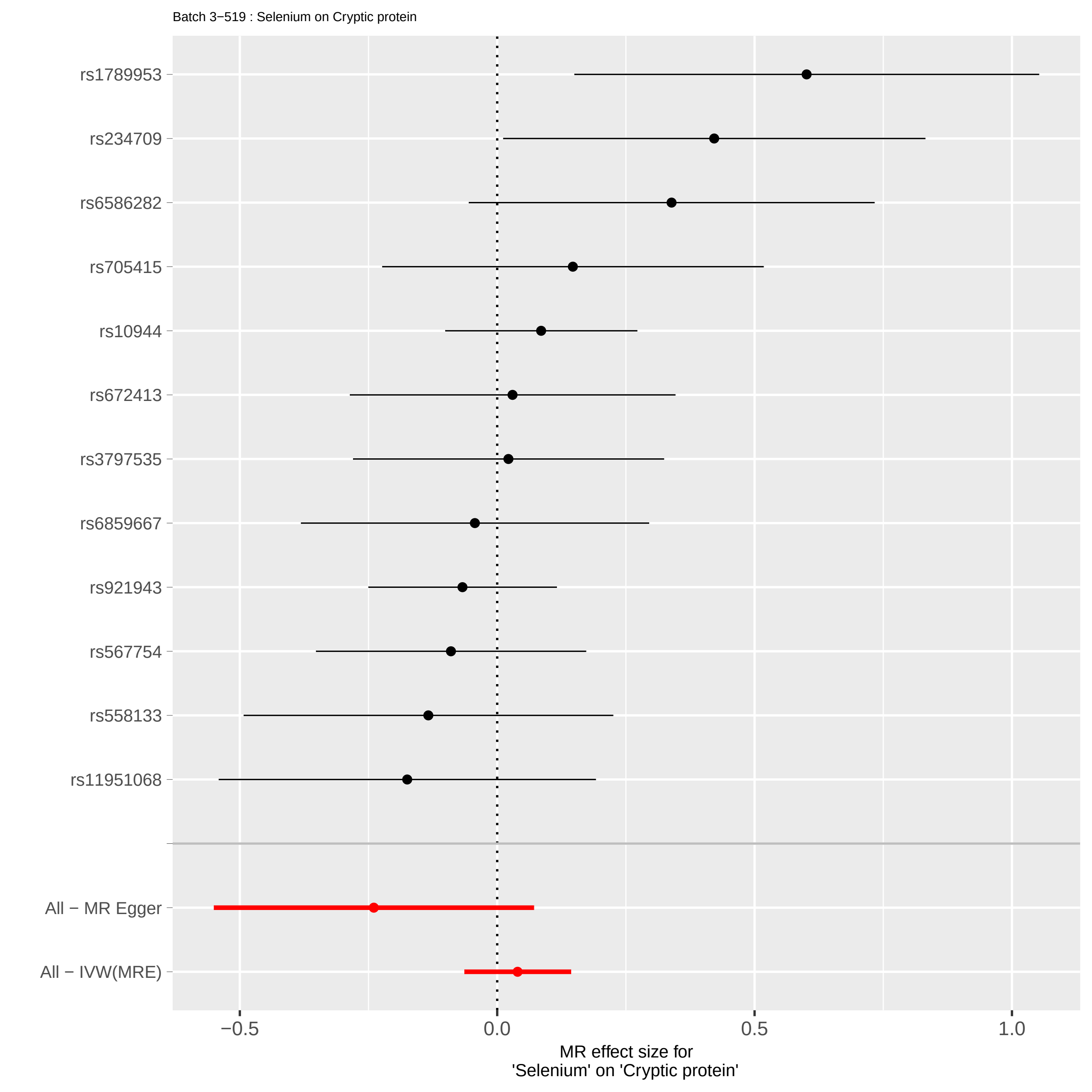
Batch 3−519 : Selenium on Cryptic protein
rs1789953
rs234709
rs6586282
rs705415
rs10944
rs672413
rs3797535
rs6859667
rs921943
rs567754
rs558133
rs11951068
All − MR Egger
All − IVW(MRE)
−0.5
0.0
0.5
1.0
MR effect size for
'Selenium' on 'Cryptic protein'

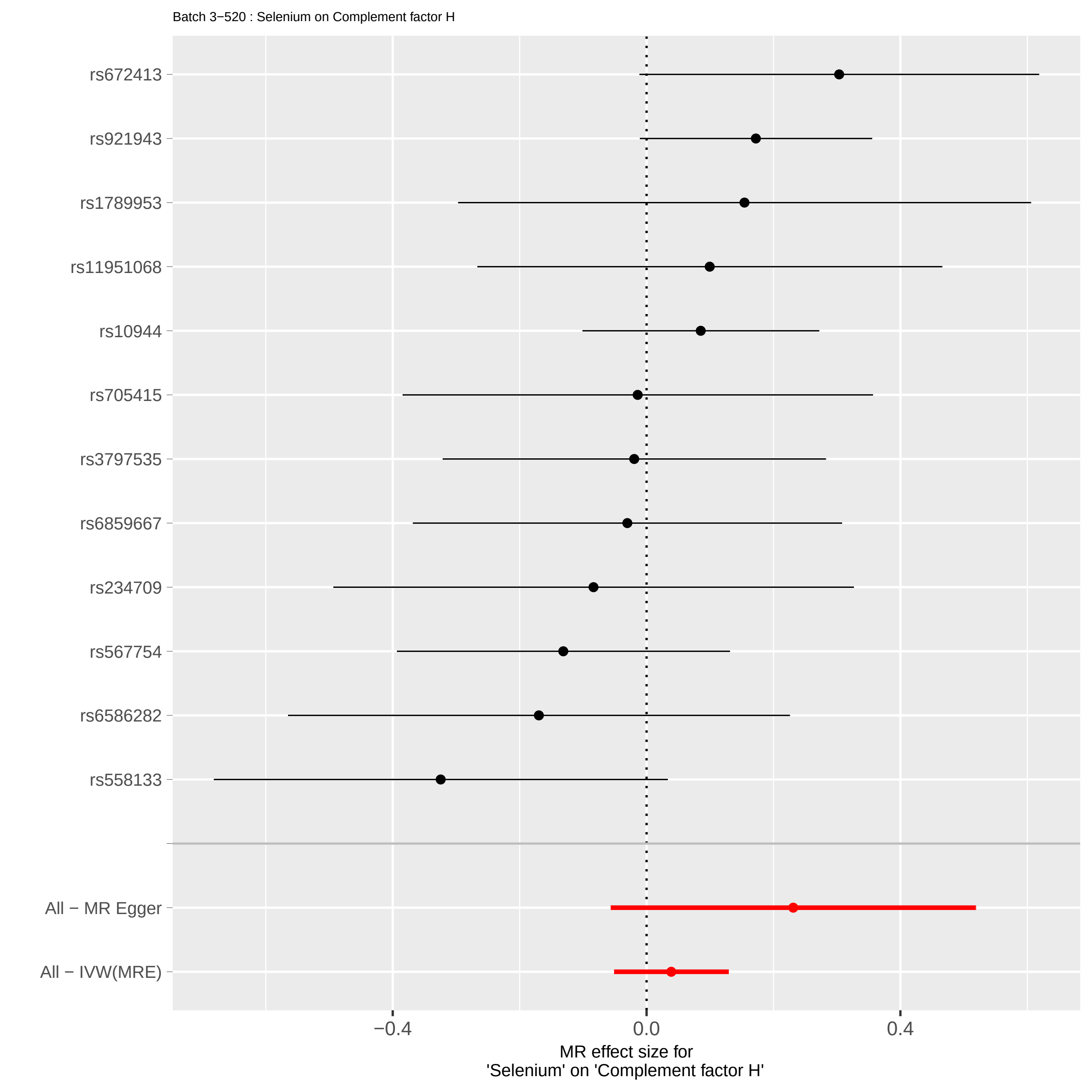
Batch 3−520 : Selenium on Complement factor H
rs672413
rs921943
rs1789953
rs11951068
rs10944
rs705415
rs3797535
rs6859667
rs234709
rs567754
rs6586282
rs558133
All − MR Egger
All − IVW(MRE)
−0.4
0.0
0.4
MR effect size for
'Selenium' on 'Complement factor H'

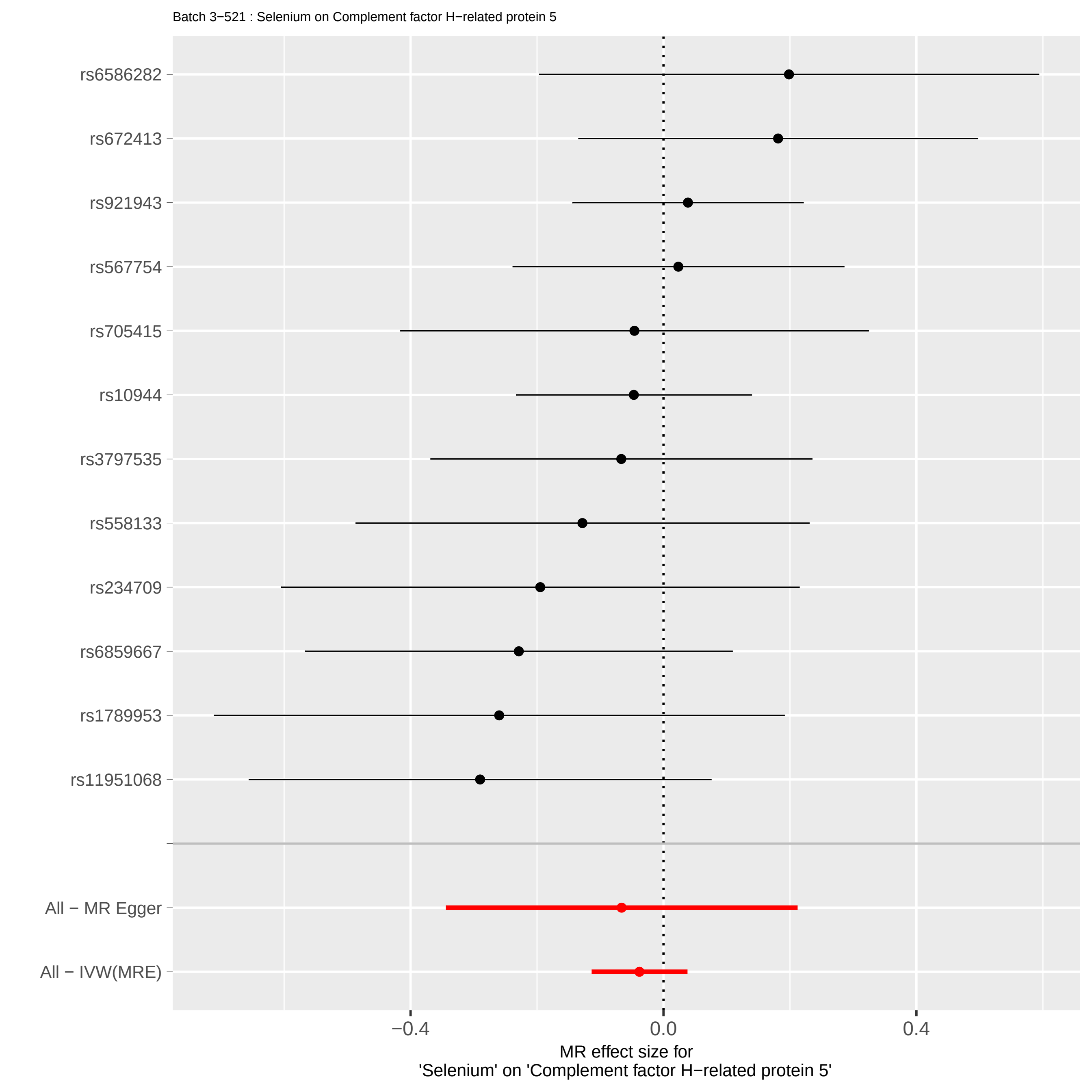

Batch 3−521 : Selenium on Complement factor H−related protein 5
rs6586282
rs672413
rs921943
rs567754
rs705415
rs10944
rs3797535
rs558133
rs234709
rs6859667
rs1789953
rs11951068
All − MR Egger
All − IVW(MRE)
−0.4
0.0
0.4
MR effect size for
'Selenium' on 'Complement factor H−related protein 5'

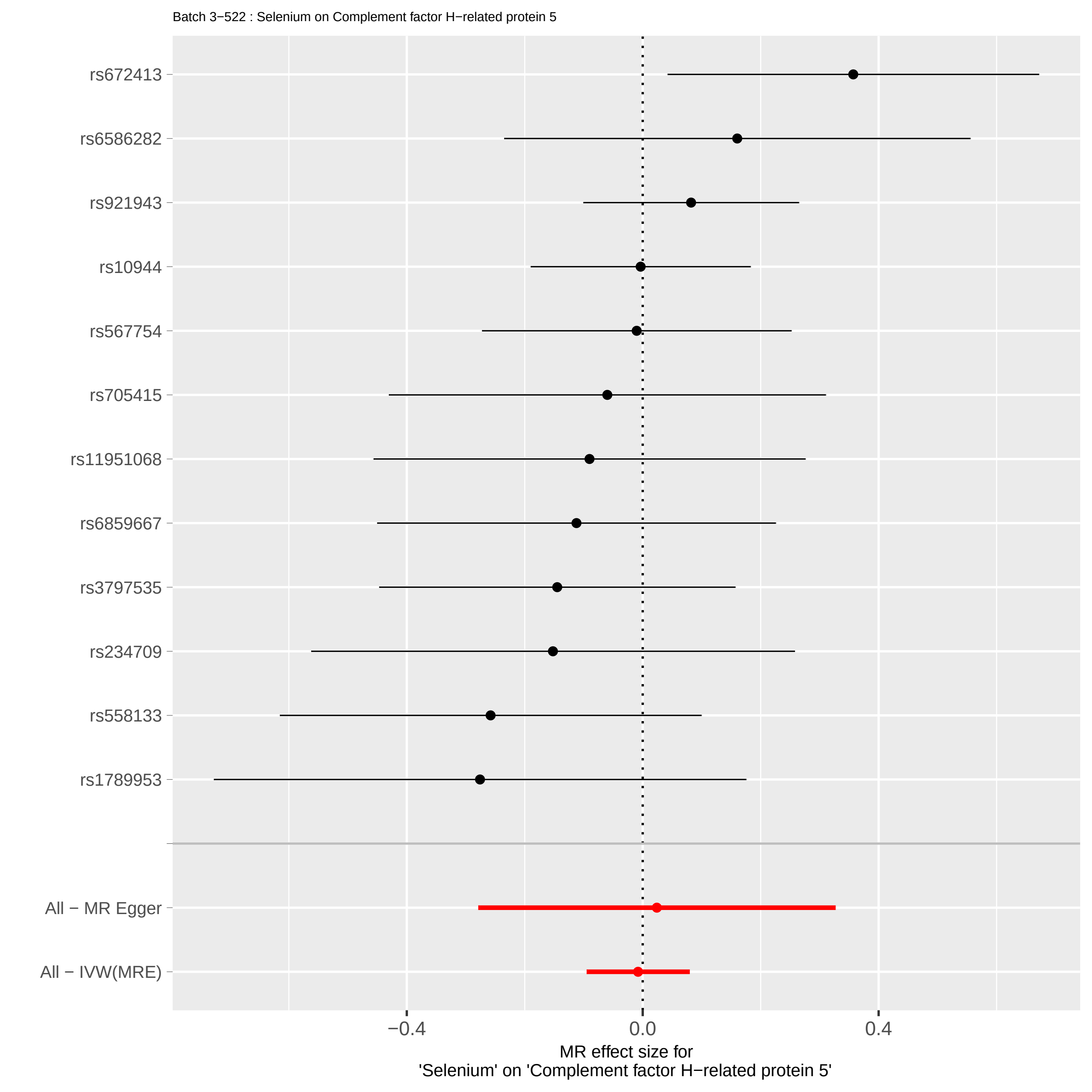
Batch 3−522 : Selenium on Complement factor H−related protein 5
rs672413
rs6586282
rs921943
rs10944
rs567754
rs705415
rs11951068
rs6859667
rs3797535
rs234709
rs558133
rs1789953
All − MR Egger
All − IVW(MRE)
−0.4
0.0
0.4
MR effect size for
'Selenium' on 'Complement factor H−related protein 5'

Batch 3−523 : Selenium on Complement factor I

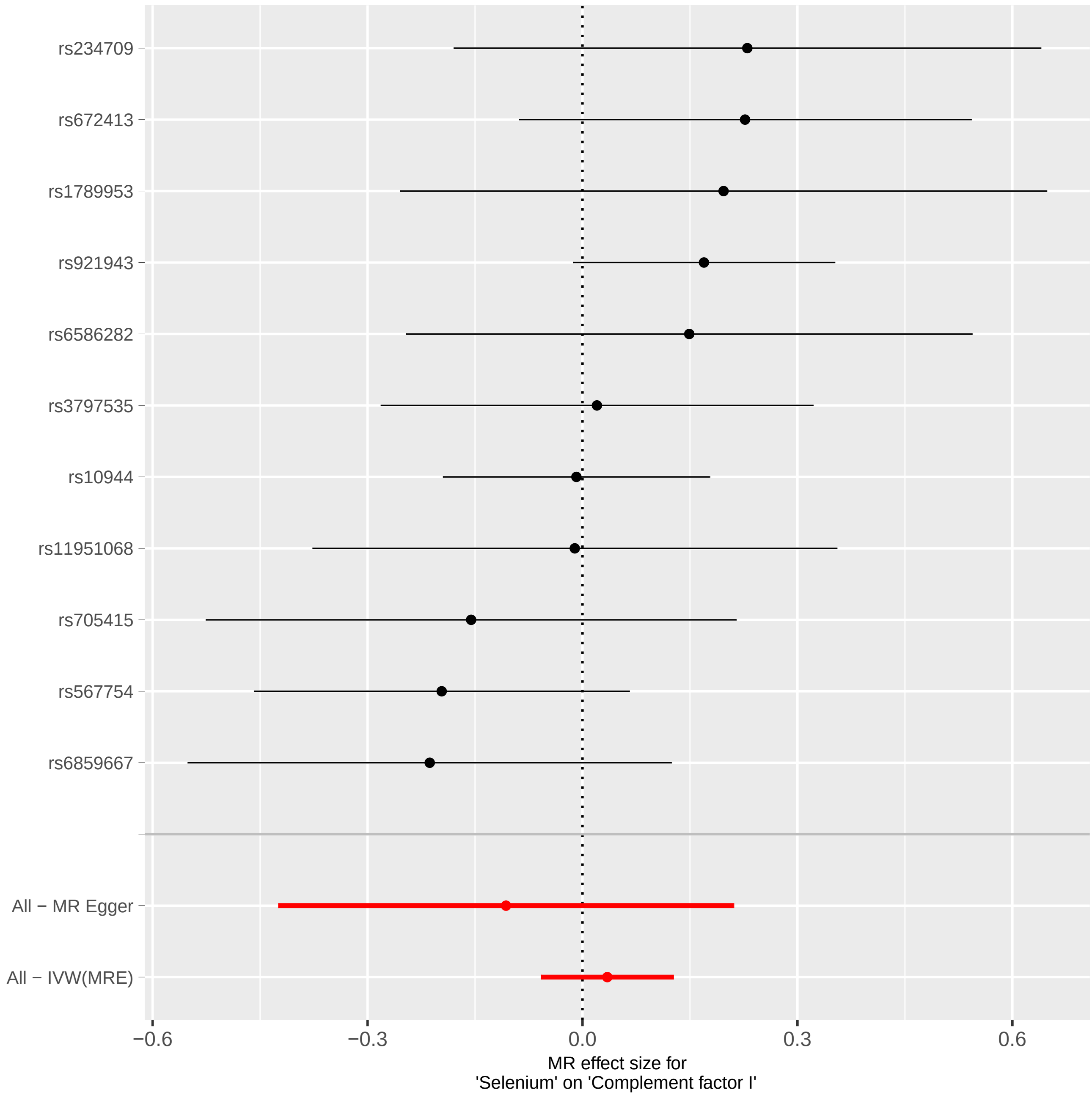

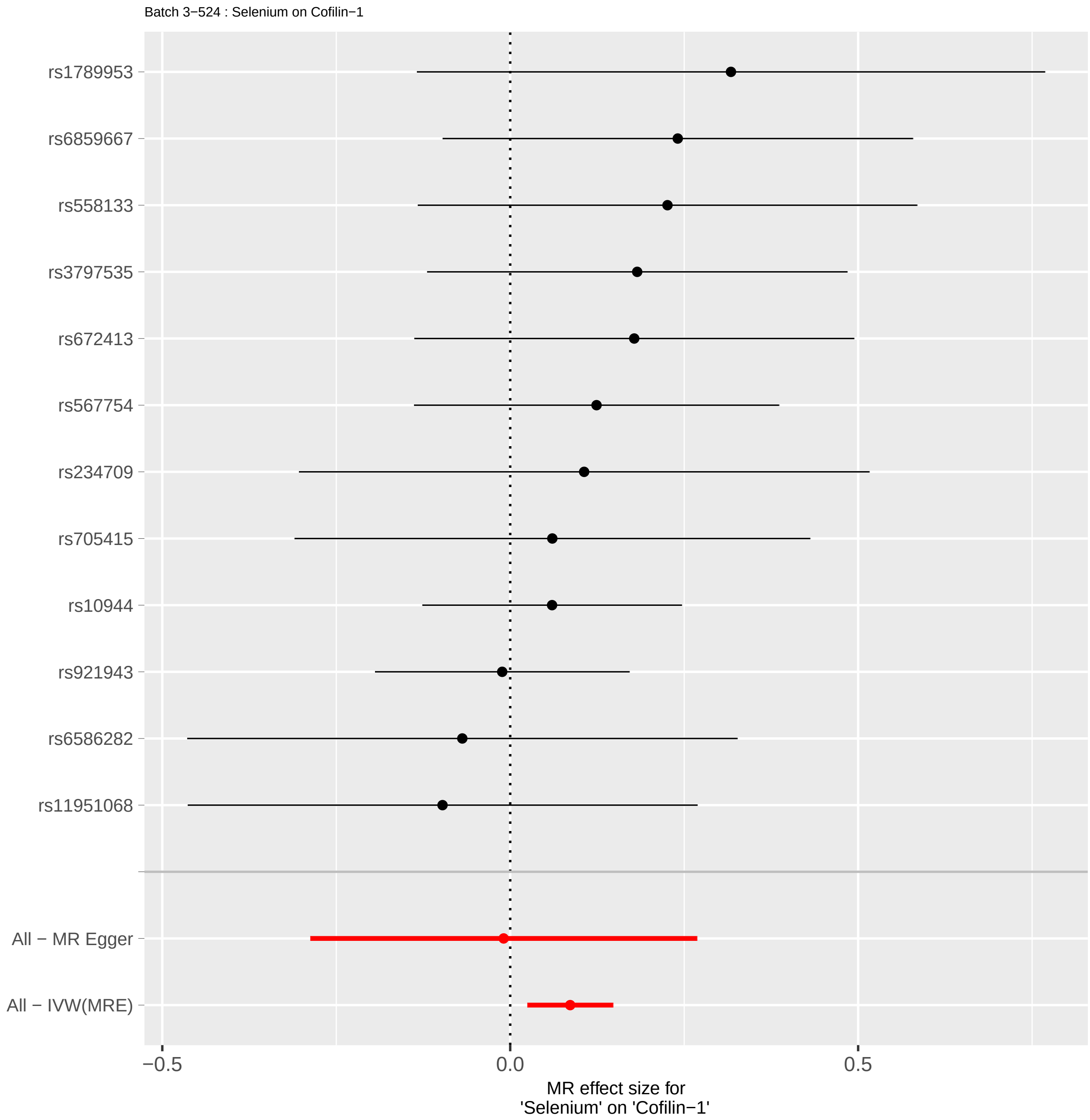

Batch 3−524 : Selenium on Cofilin−1
rs1789953
rs6859667
rs558133
rs3797535
rs672413
rs567754
rs234709
rs705415
rs10944
rs921943
rs6586282
rs11951068
All − MR Egger
All − IVW(MRE)
−0.5
0.0
0.5
MR effect size for
'Selenium' on 'Cofilin−1'

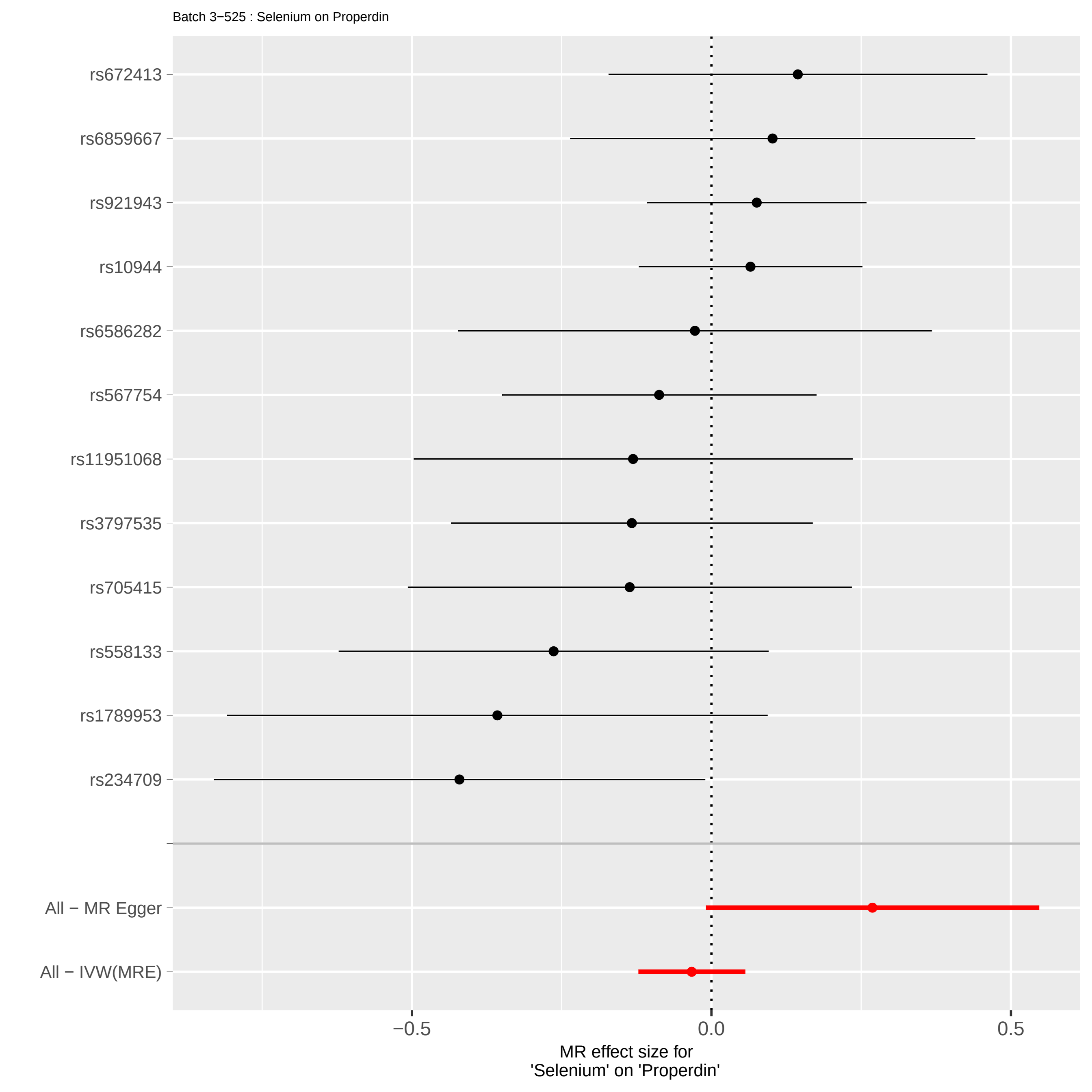

Batch 3−525 : Selenium on Properdin
rs672413
rs6859667
rs921943
rs10944
rs6586282
rs567754
rs11951068
rs3797535
rs705415
rs558133
rs1789953
rs234709
All − MR Egger
All − IVW(MRE)
−0.5
0.0
0.5
MR effect size for
'Selenium' on 'Properdin'

Batch 3−526 : Selenium on Glycoprotein hormones alpha chain

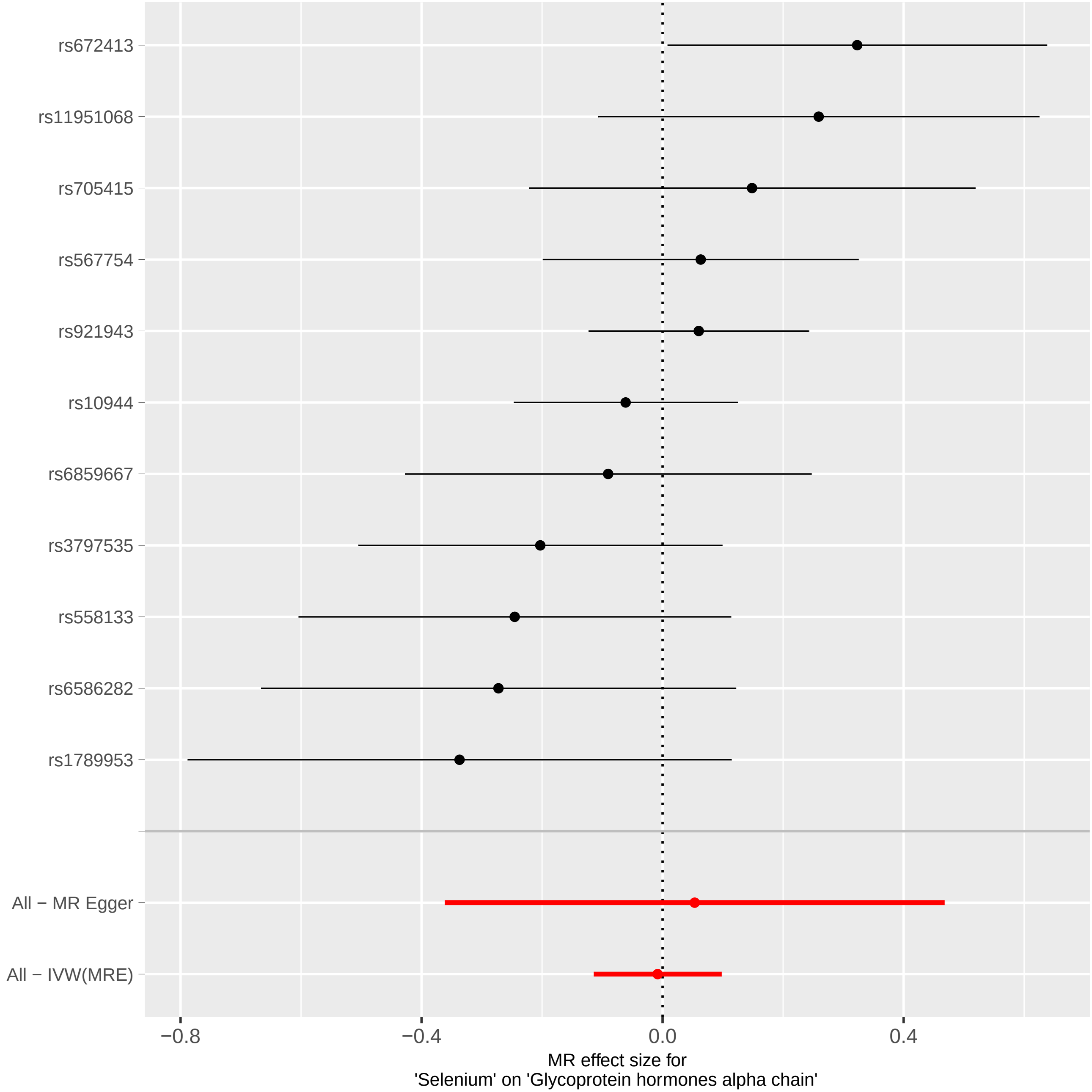

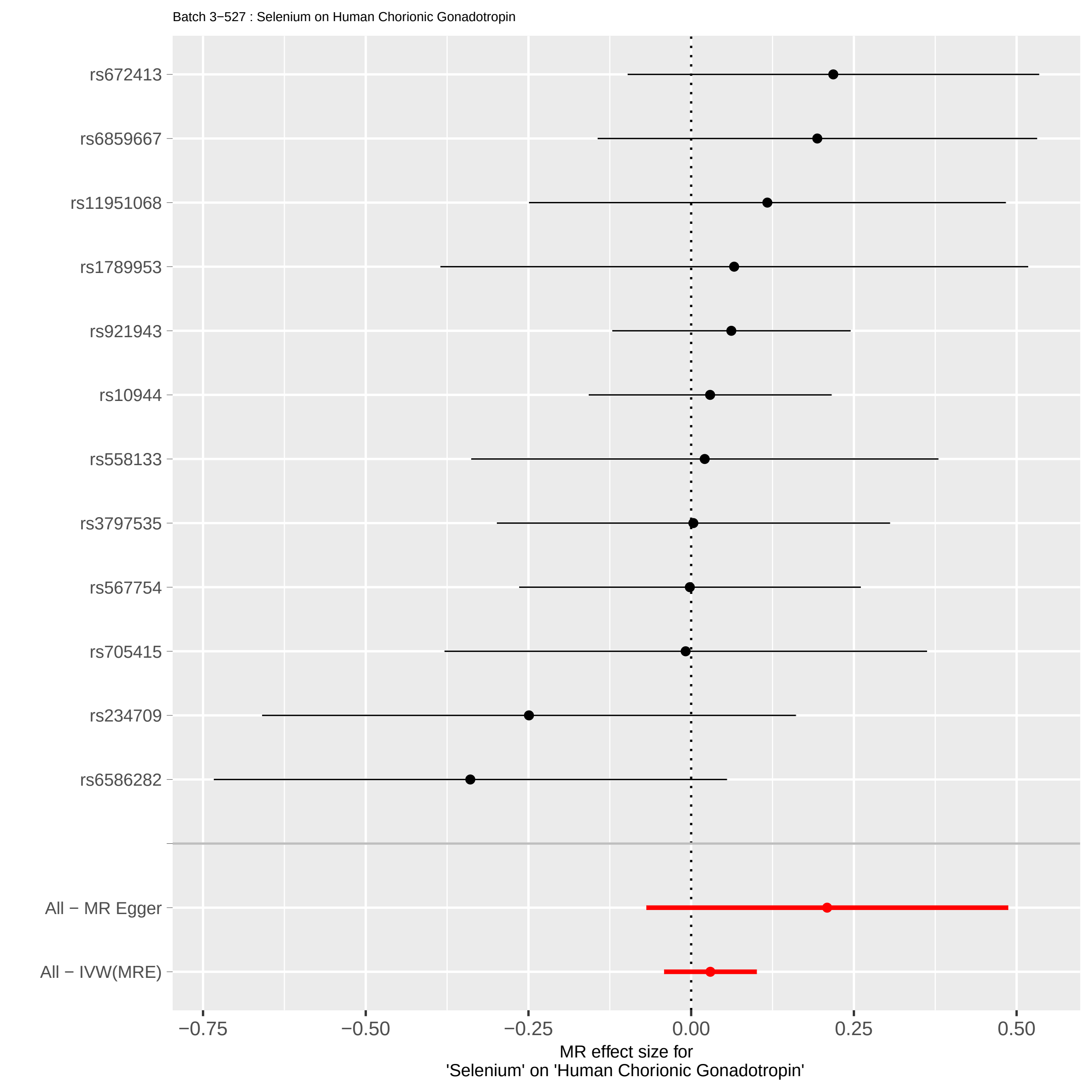
Batch 3−527 : Selenium on Human Chorionic Gonadotropin
rs672413
rs6859667
rs11951068
rs1789953
rs921943
rs10944
rs558133
rs3797535
rs567754
rs705415
rs234709
rs6586282
All − MR Egger
All − IVW(MRE)
−0.75
−0.50
−0.25
0.00
0.25
0.50
MR effect size for
'Selenium' on 'Human Chorionic Gonadotropin'

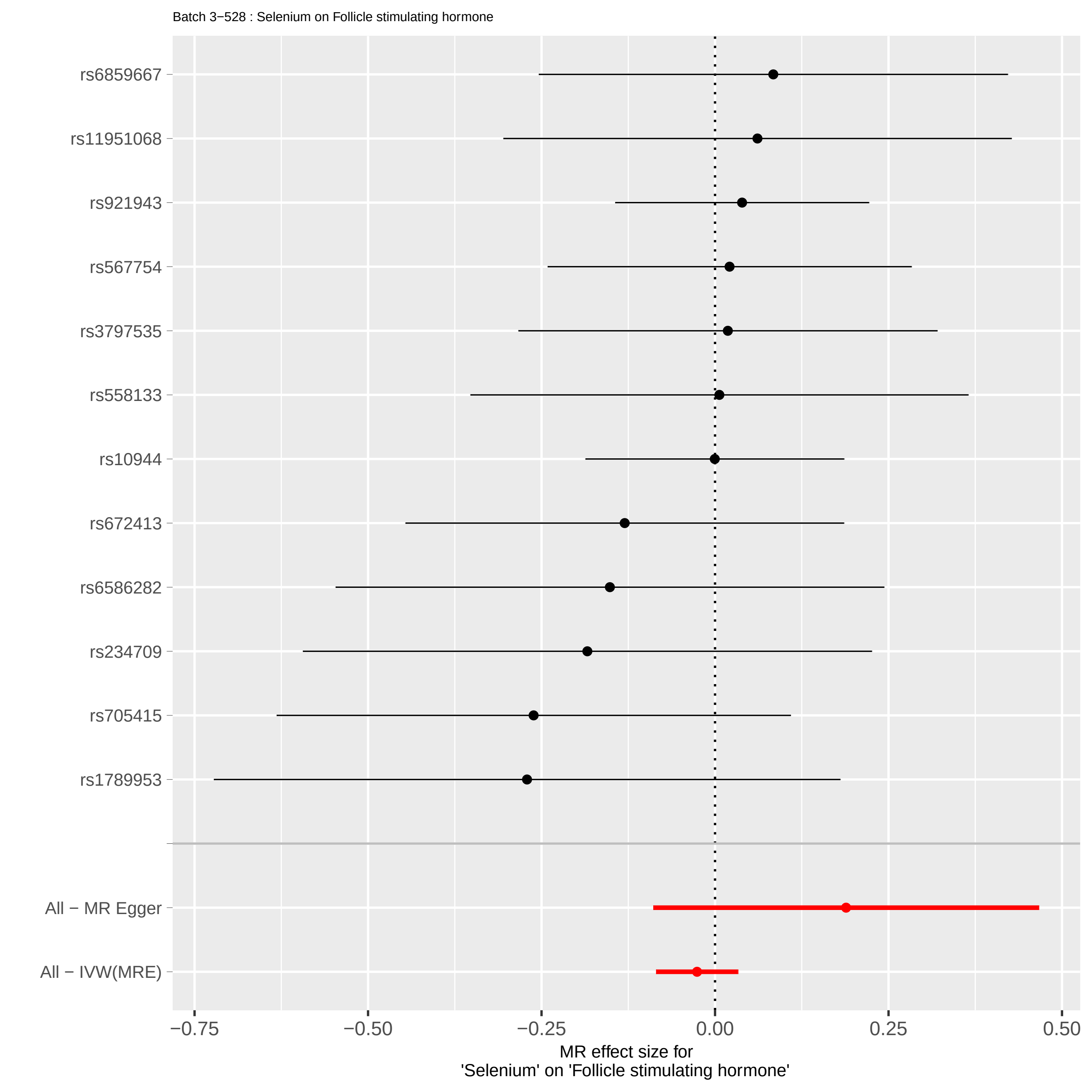

Batch 3−528 : Selenium on Follicle stimulating hormone
rs6859667
rs11951068
rs921943
rs567754
rs3797535
rs558133
rs10944
rs672413
rs6586282
rs234709
rs705415
rs1789953
All − MR Egger
All − IVW(MRE)
−0.75
−0.50
−0.25
0.00
0.25
0.50
MR effect size for
'Selenium' on 'Follicle stimulating hormone'

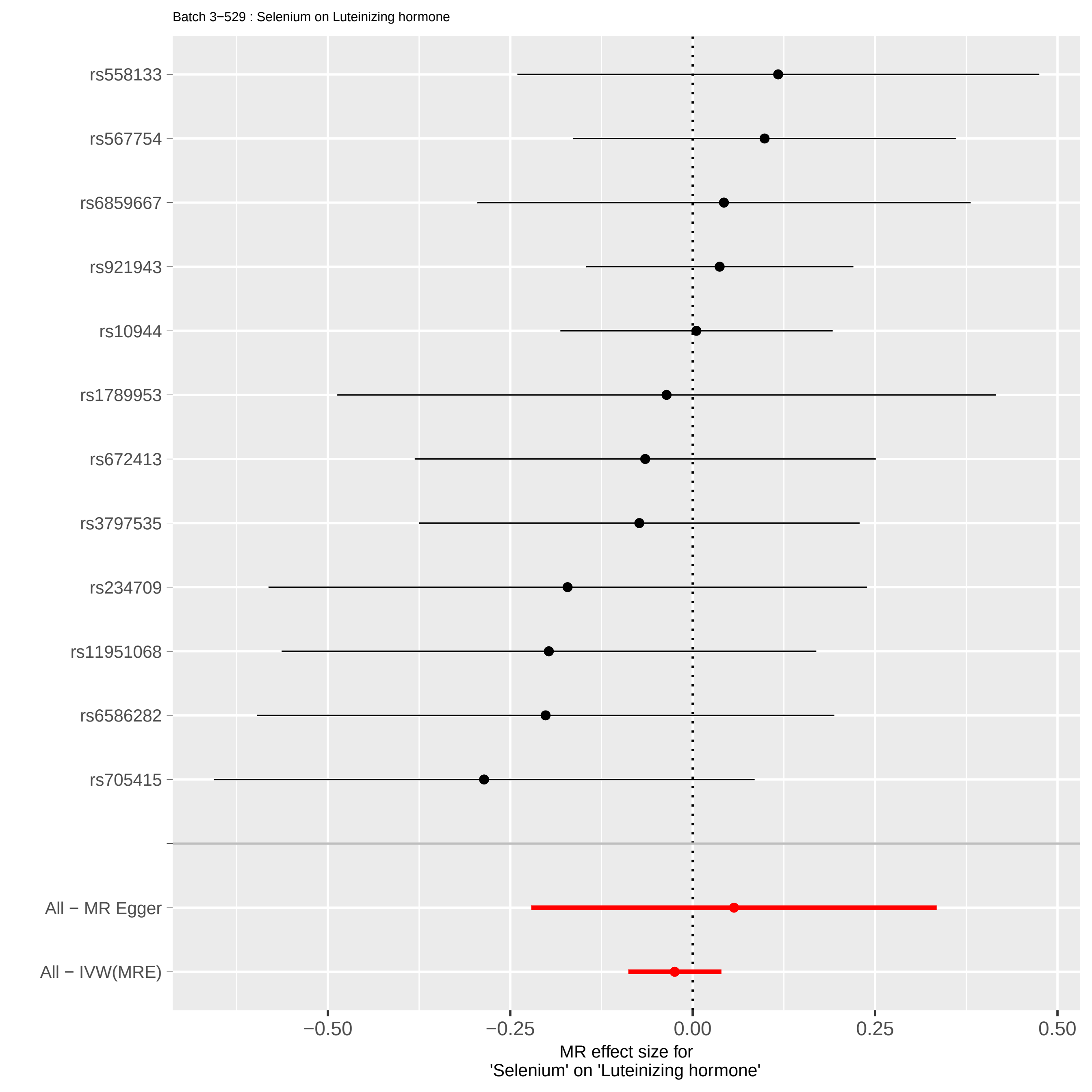
Batch 3−529 : Selenium on Luteinizing hormone
rs558133
rs567754
rs6859667
rs921943
rs10944
rs1789953
rs672413
rs3797535
rs234709
rs11951068
rs6586282
rs705415
All − MR Egger
All − IVW(MRE)
−0.50
−0.25
0.00
0.25
0.50
MR effect size for
'Selenium' on 'Luteinizing hormone'

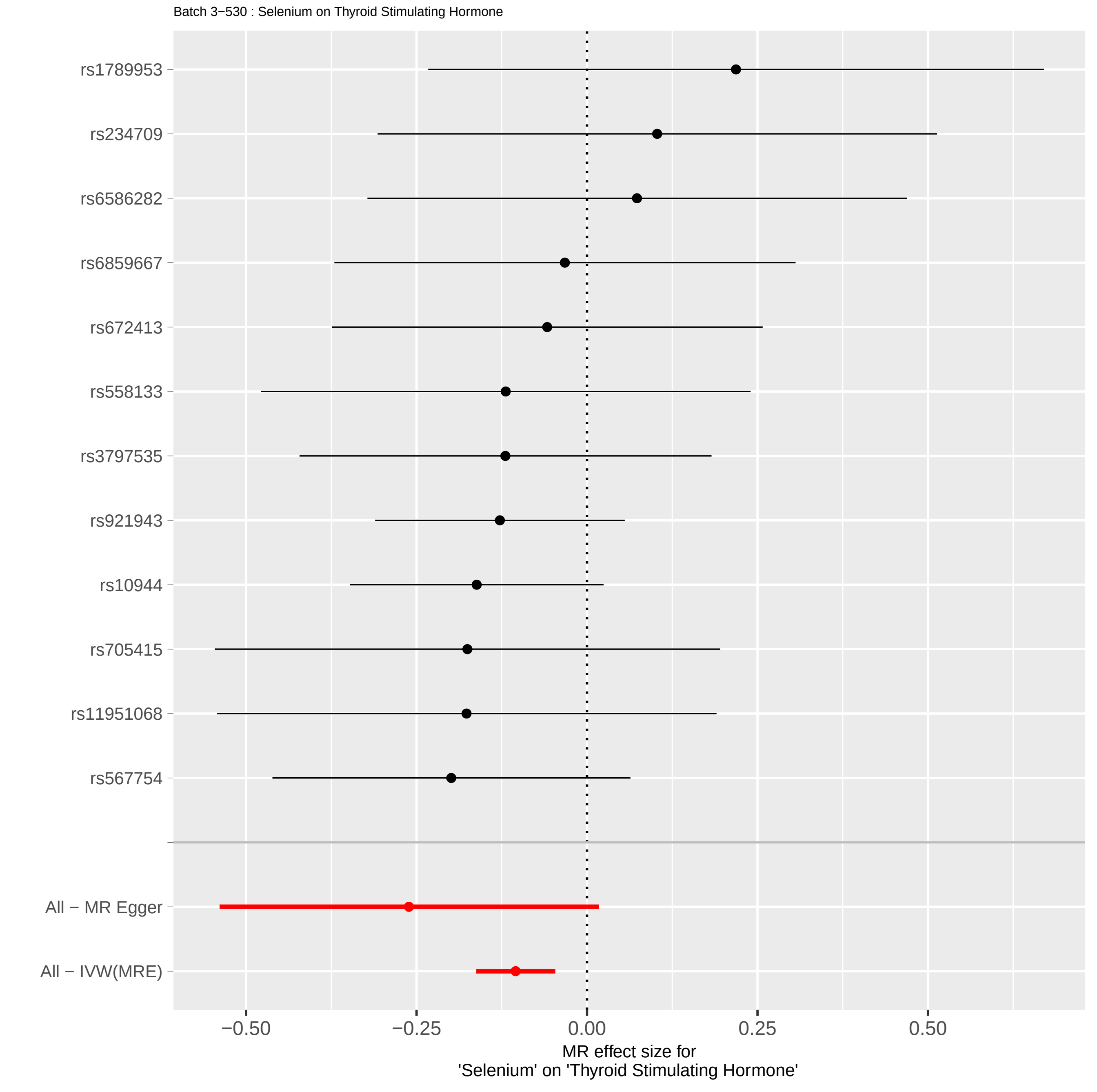
Batch 3−530 : Selenium on Thyroid Stimulating Hormone
rs1789953
rs234709
rs6586282
rs6859667
rs672413
rs558133
rs3797535
rs921943
rs10944
rs705415
rs11951068
rs567754
All − MR Egger
All − IVW(MRE)
−0.50
−0.25
0.00
0.25
0.50
MR effect size for
'Selenium' on 'Thyroid Stimulating Hormone'

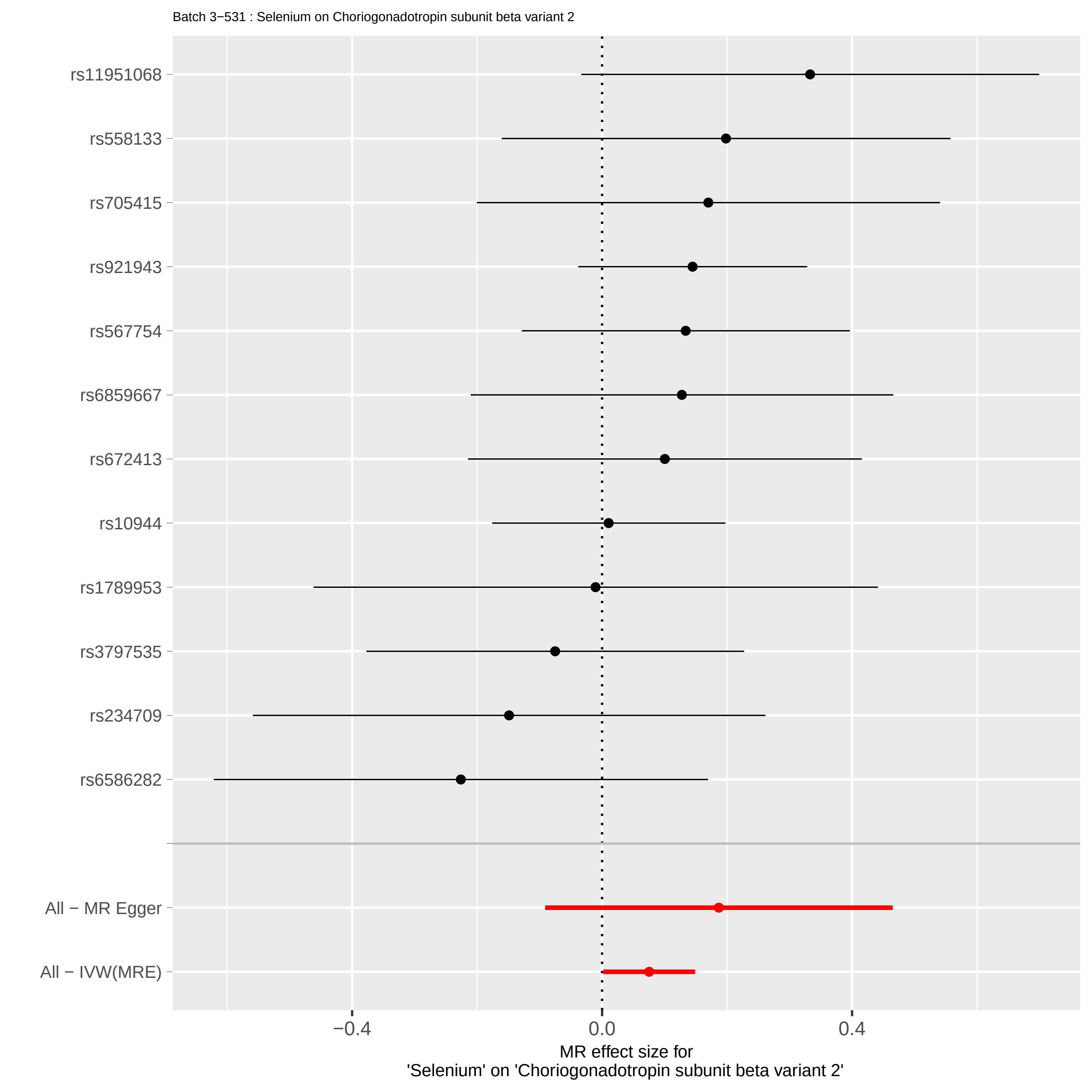

Batch 3−531 : Selenium on Choriogonadotropin subunit beta variant 2
rs11951068
rs558133
rs705415
rs921943
rs567754
rs6859667
rs672413
rs10944
rs1789953
rs3797535
rs234709
rs6586282
All − MR Egger
All − IVW(MRE)
−0.4
0.0
0.4
MR effect size for
'Selenium' on 'Choriogonadotropin subunit beta variant 2'

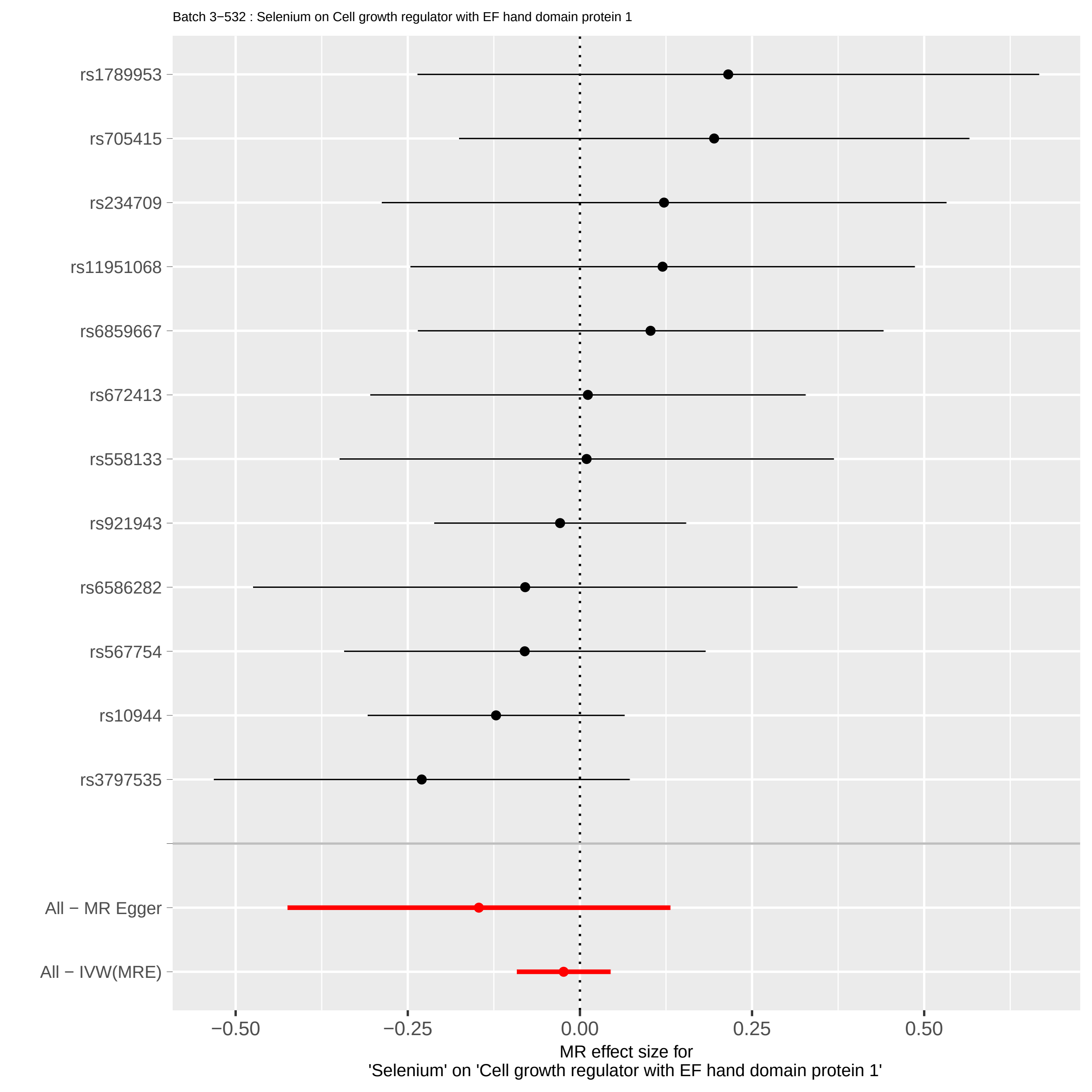
Batch 3−532 : Selenium on Cell growth regulator with EF hand domain protein 1
rs1789953
rs705415
rs234709
rs11951068
rs6859667
rs672413
rs558133
rs921943
rs6586282
rs567754
rs10944
rs3797535
All − MR Egger
All − IVW(MRE)
−0.50
−0.25
0.00
0.25
0.50
MR effect size for
'Selenium' on 'Cell growth regulator with EF hand domain protein 1'

Batch 3−533 : Selenium on Chondroadherin

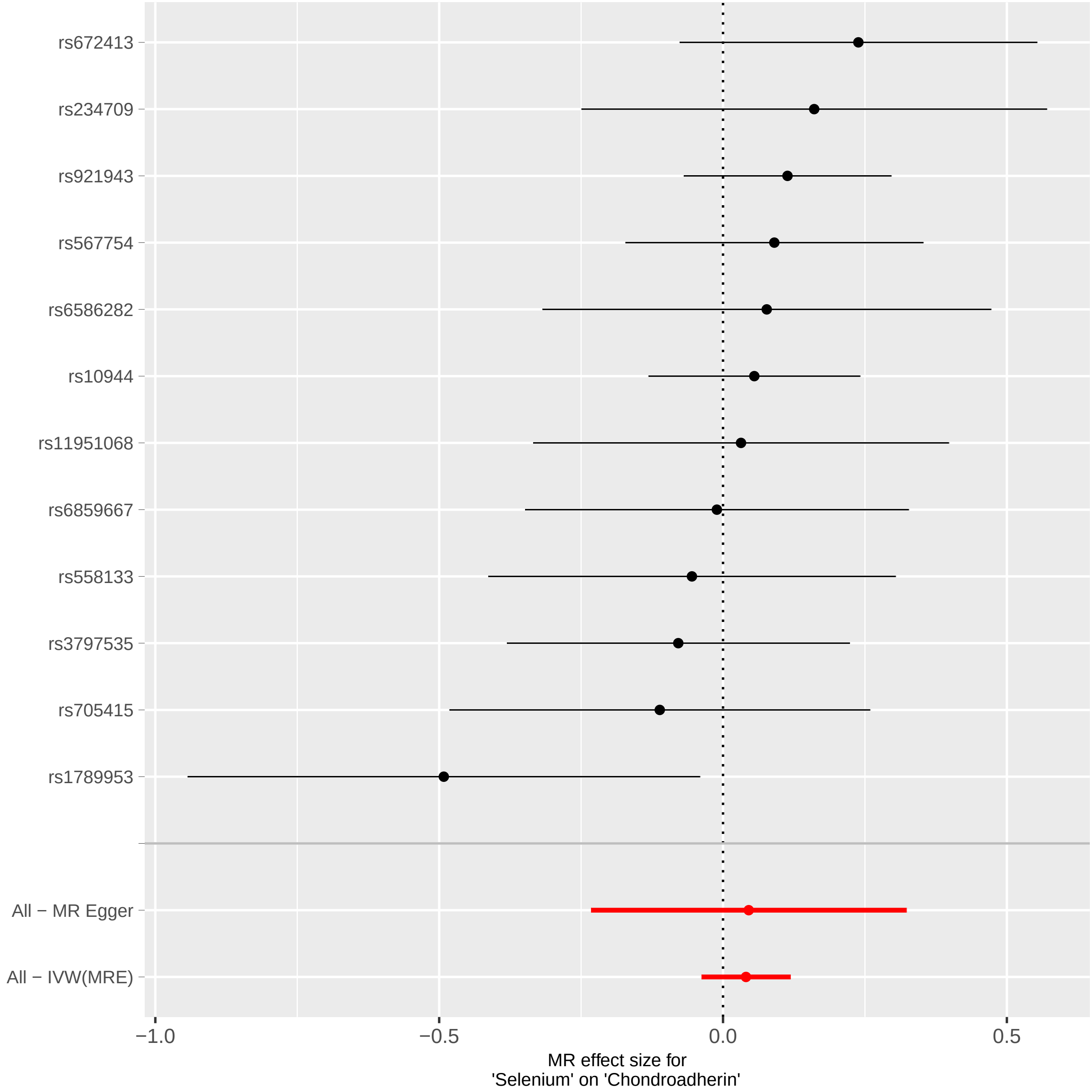

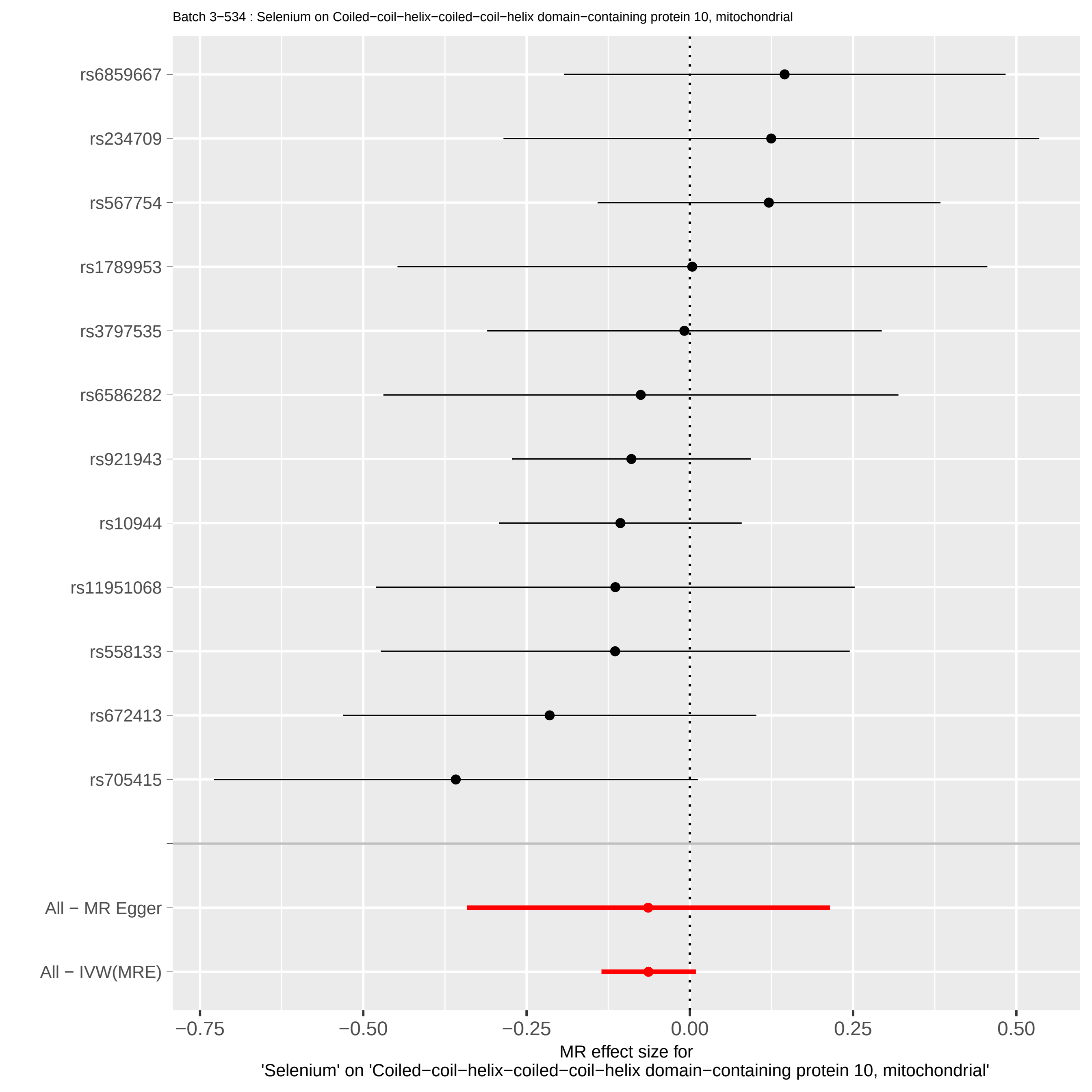

Batch 3−534 : Selenium on Coiled−coil−helix−coiled−coil−helix domain−containing protein 10, mitochondrial
rs6859667
rs234709
rs567754
rs1789953
rs3797535
rs6586282
rs921943
rs10944
rs11951068
rs558133
rs672413
rs705415
All − MR Egger
All − IVW(MRE)
−0.75
−0.50
−0.25
0.00
0.25
0.50
MR effect size for
'Selenium' on 'Coiled−coil−helix−coiled−coil−helix domain−containing protein 10, mitochondrial'

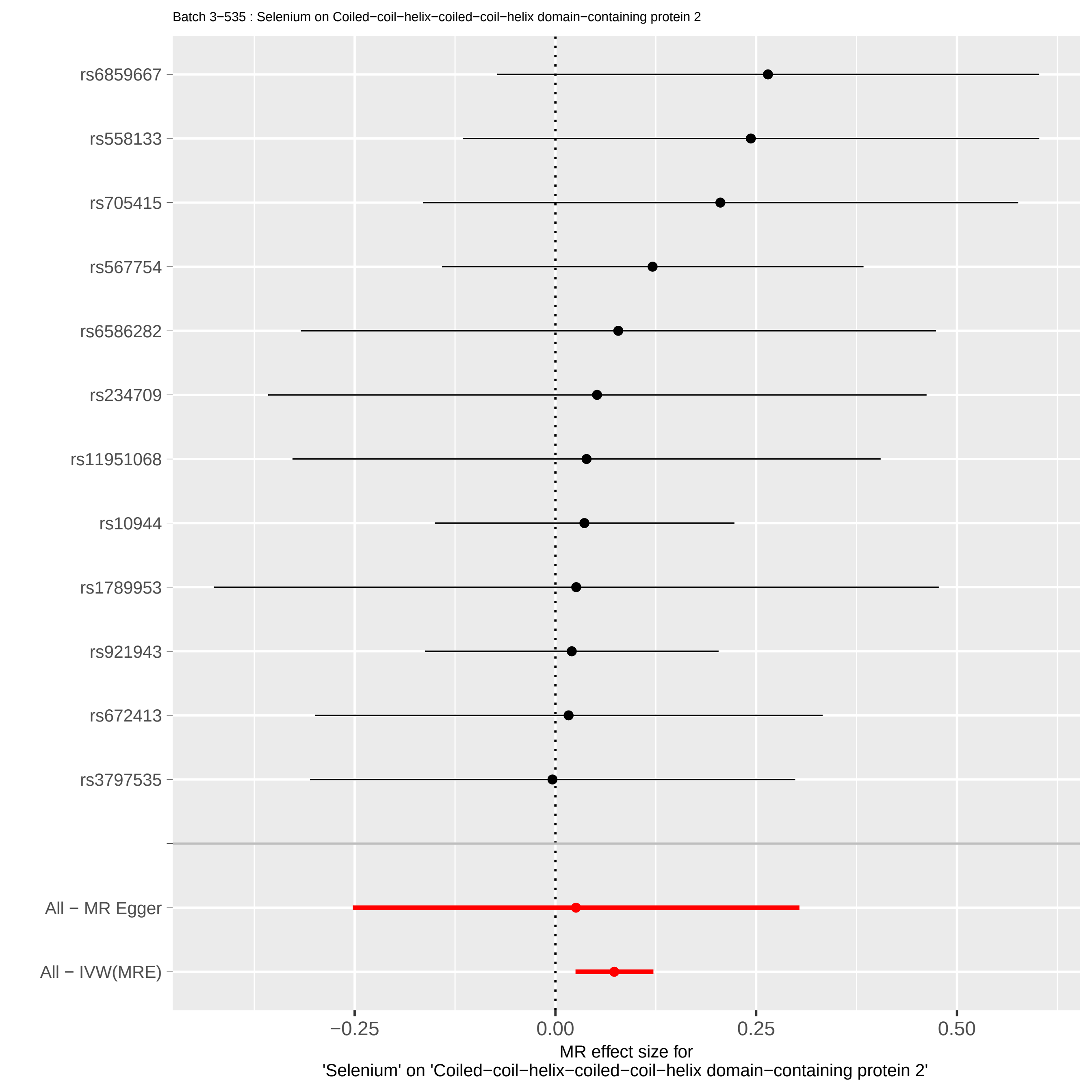
Batch 3−535 : Selenium on Coiled−coil−helix−coiled−coil−helix domain−containing protein 2
rs6859667
rs558133
rs705415
rs567754
rs6586282
rs234709
rs11951068
rs10944
rs1789953
rs921943
rs672413
rs3797535
All − MR Egger
All − IVW(MRE)
−0.25
0.00
0.25
0.50
MR effect size for
'Selenium' on 'Coiled−coil−helix−coiled−coil−helix domain−containing protein 2'

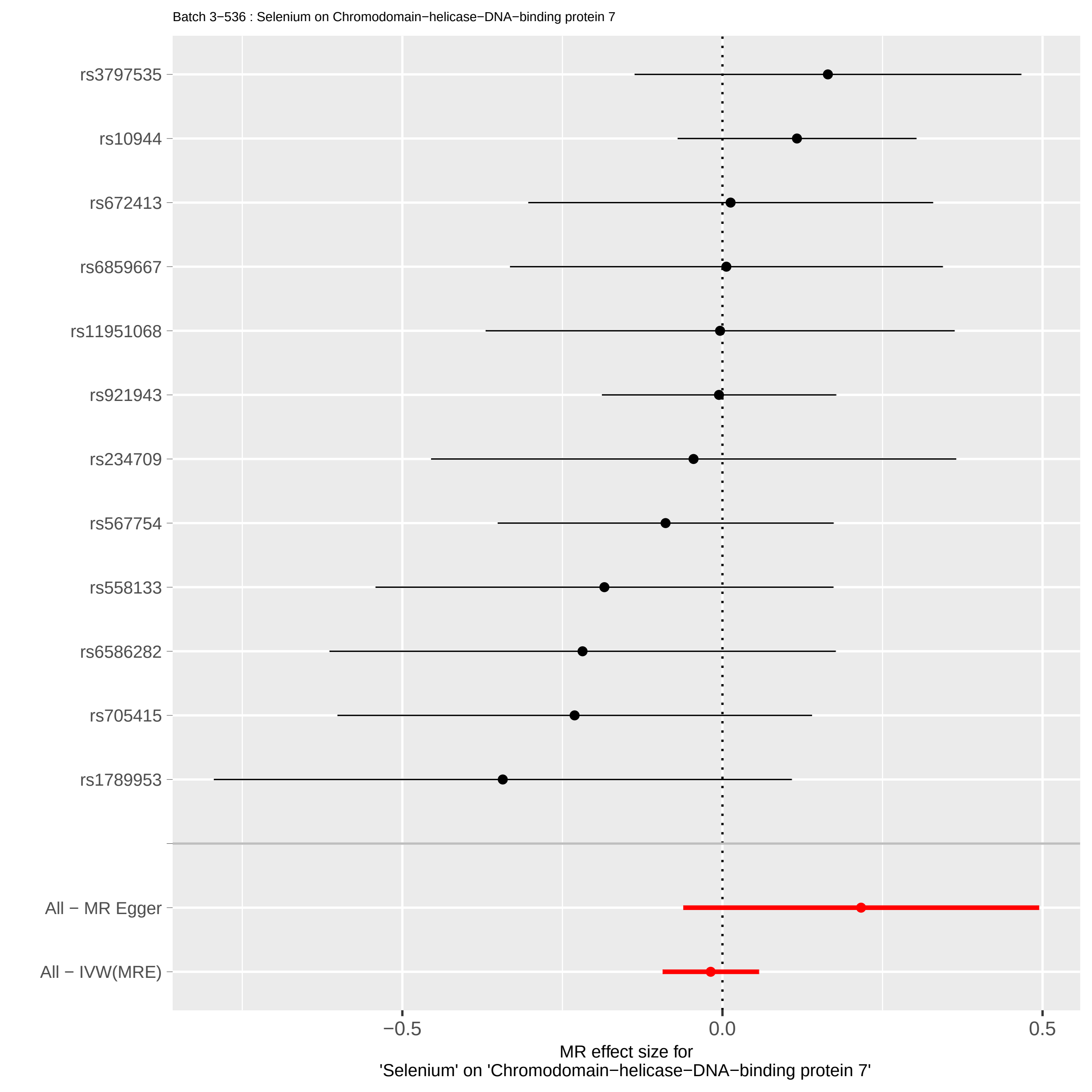

Batch 3−536 : Selenium on Chromodomain−helicase−DNA−binding protein 7
rs3797535
rs10944
rs672413
rs6859667
rs11951068
rs921943
rs234709
rs567754
rs558133
rs6586282
rs705415
rs1789953
All − MR Egger
All − IVW(MRE)
−0.5
0.0
0.5
MR effect size for
'Selenium' on 'Chromodomain−helicase−DNA−binding protein 7'

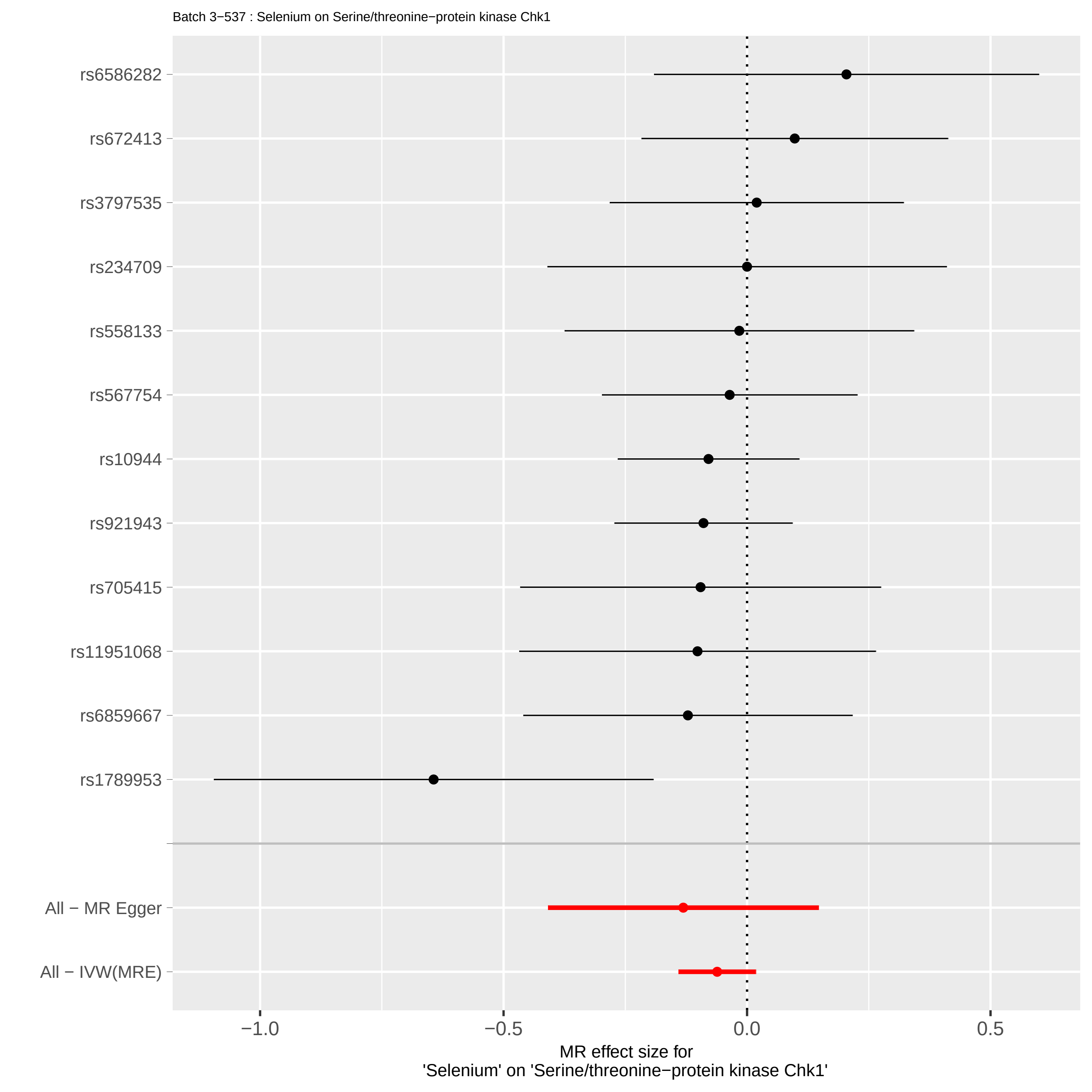

Batch 3−537 : Selenium on Serine/threonine−protein kinase Chk1
rs6586282
rs672413
rs3797535
rs234709
rs558133
rs567754
rs10944
rs921943
rs705415
rs11951068
rs6859667
rs1789953
All − MR Egger
All − IVW(MRE)
−1.0
−0.5
0.0
0.5
MR effect size for
'Selenium' on 'Serine/threonine−protein kinase Chk1'

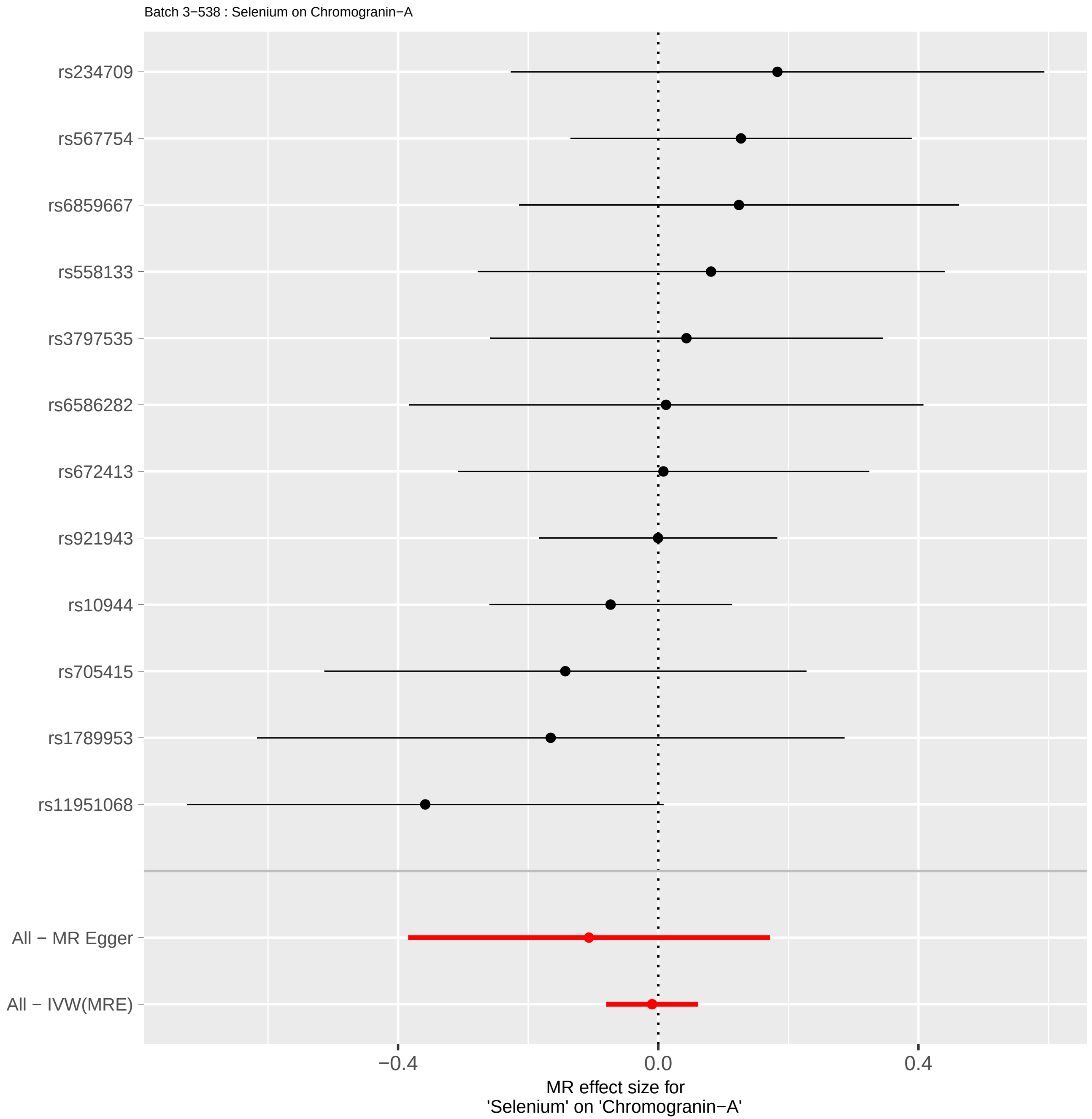

Batch 3−538 : Selenium on Chromogranin−A
rs234709
rs567754
rs6859667
rs558133
rs3797535
rs6586282
rs672413
rs921943
rs10944
rs705415
rs1789953
rs11951068
All − MR Egger
All − IVW(MRE)
−0.4
0.0
0.4
MR effect size for
'Selenium' on 'Chromogranin−A'

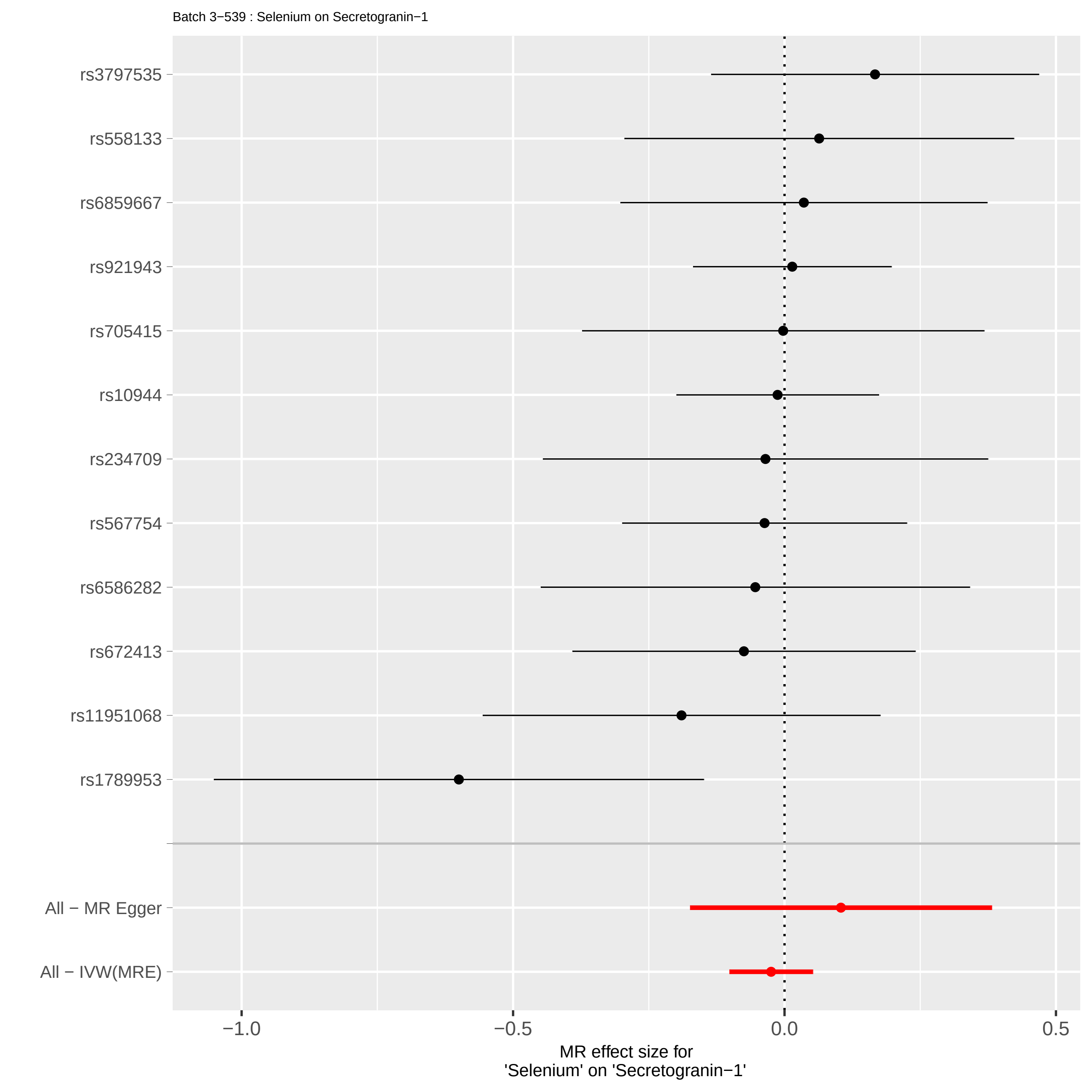
Batch 3−539 : Selenium on Secretogranin−1
rs3797535
rs558133
rs6859667
rs921943
rs705415
rs10944
rs234709
rs567754
rs6586282
rs672413
rs11951068
rs1789953
All − MR Egger
All − IVW(MRE)
−1.0
−0.5
0.0
0.5
MR effect size for
'Selenium' on 'Secretogranin−1'

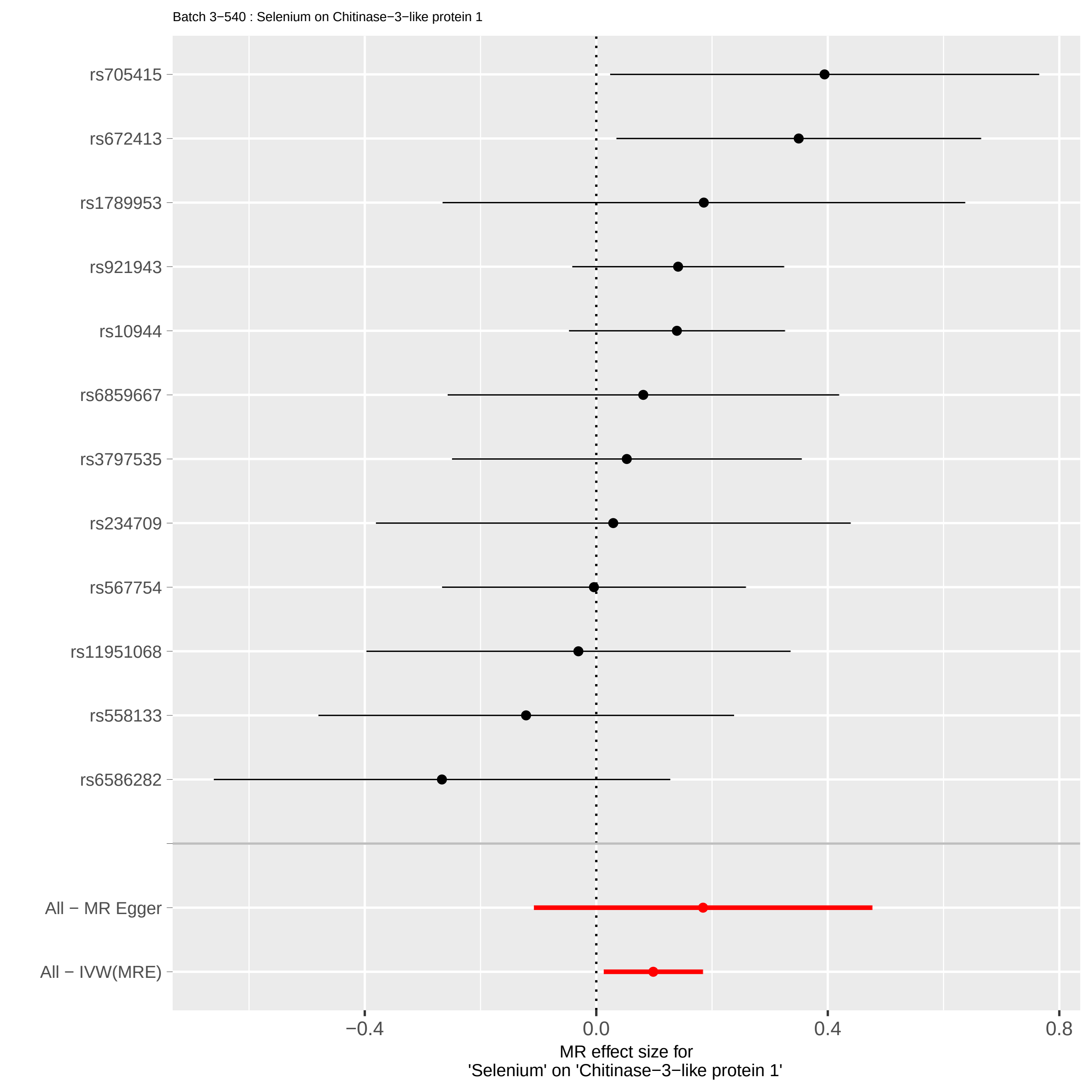

Batch 3−540 : Selenium on Chitinase−3−like protein 1
rs705415
rs672413
rs1789953
rs921943
rs10944
rs6859667
rs3797535
rs234709
rs567754
rs11951068
rs558133
rs6586282
All − MR Egger
All − IVW(MRE)
−0.4
0.0
0.4
0.8
MR effect size for
'Selenium' on 'Chitinase−3−like protein 1'

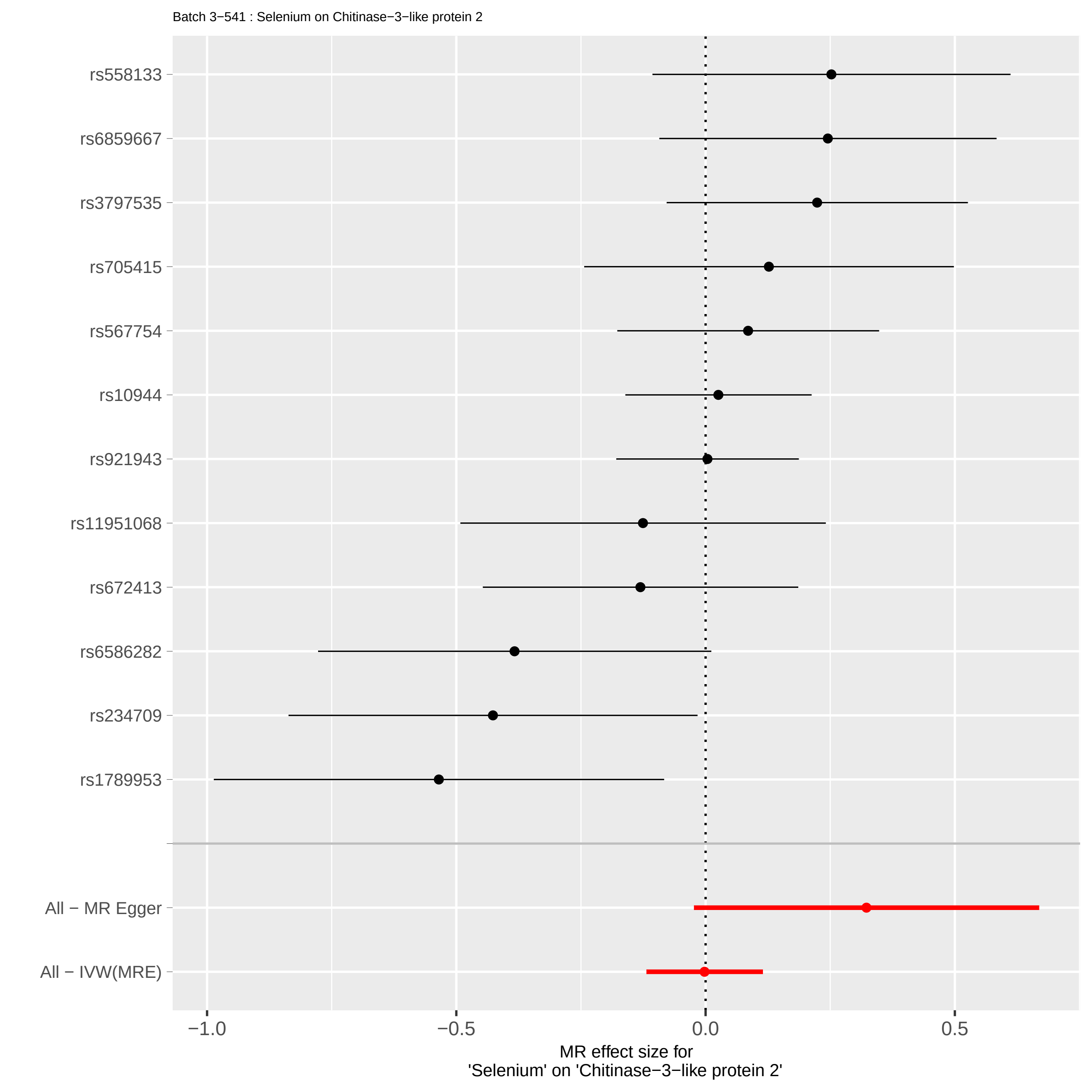

Batch 3−541 : Selenium on Chitinase−3−like protein 2
rs558133
rs6859667
rs3797535
rs705415
rs567754
rs10944
rs921943
rs11951068
rs672413
rs6586282
rs234709
rs1789953
All − MR Egger
All − IVW(MRE)
−1.0
−0.5
0.0
0.5
MR effect size for
'Selenium' on 'Chitinase−3−like protein 2'

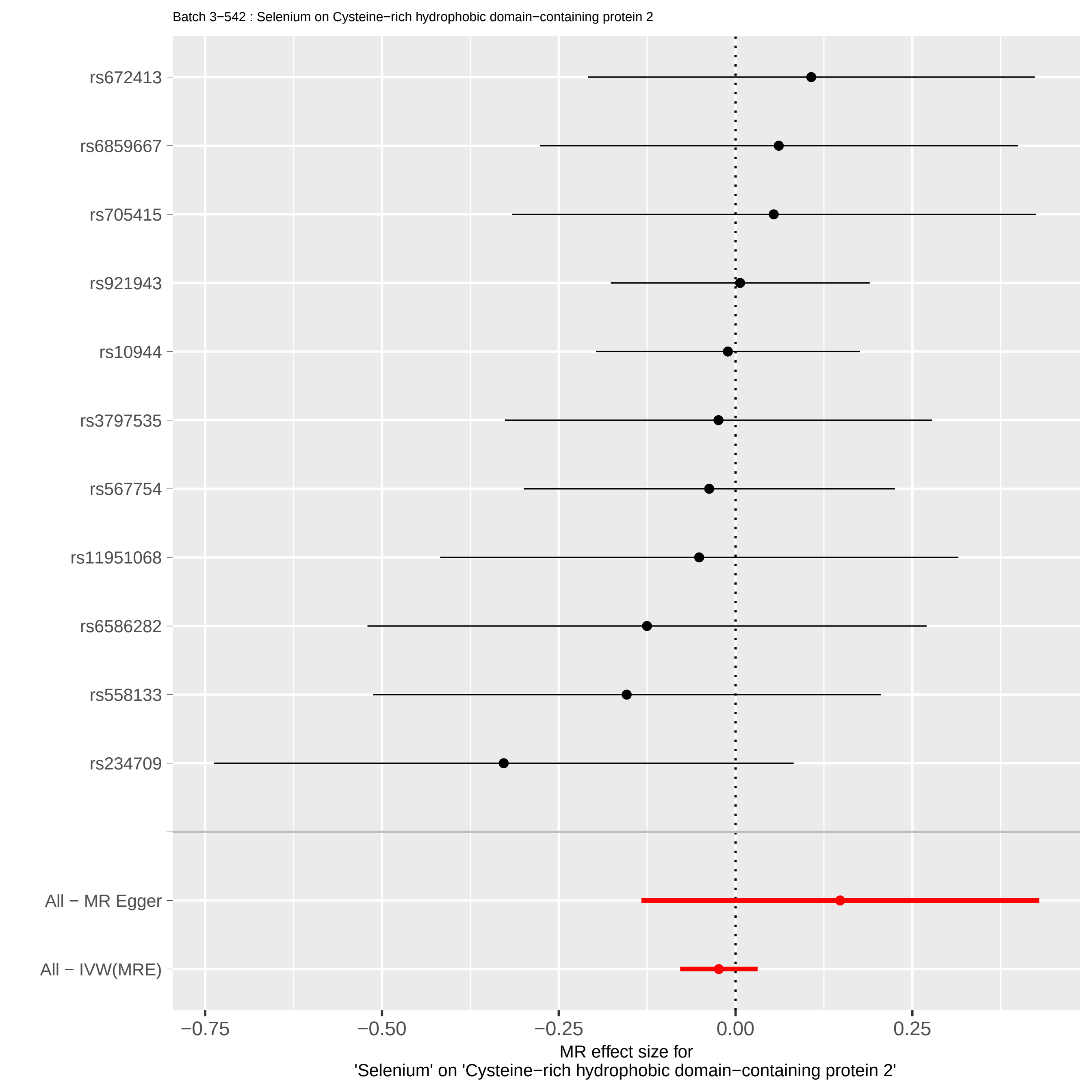

Batch 3−542 : Selenium on Cysteine−rich hydrophobic domain−containing protein 2
rs672413
rs6859667
rs705415
rs921943
rs10944
rs3797535
rs567754
rs11951068
rs6586282
rs558133
rs234709
All − MR Egger
All − IVW(MRE)
−0.75
−0.50
−0.25
0.00
0.25
MR effect size for
'Selenium' on 'Cysteine−rich hydrophobic domain−containing protein 2'

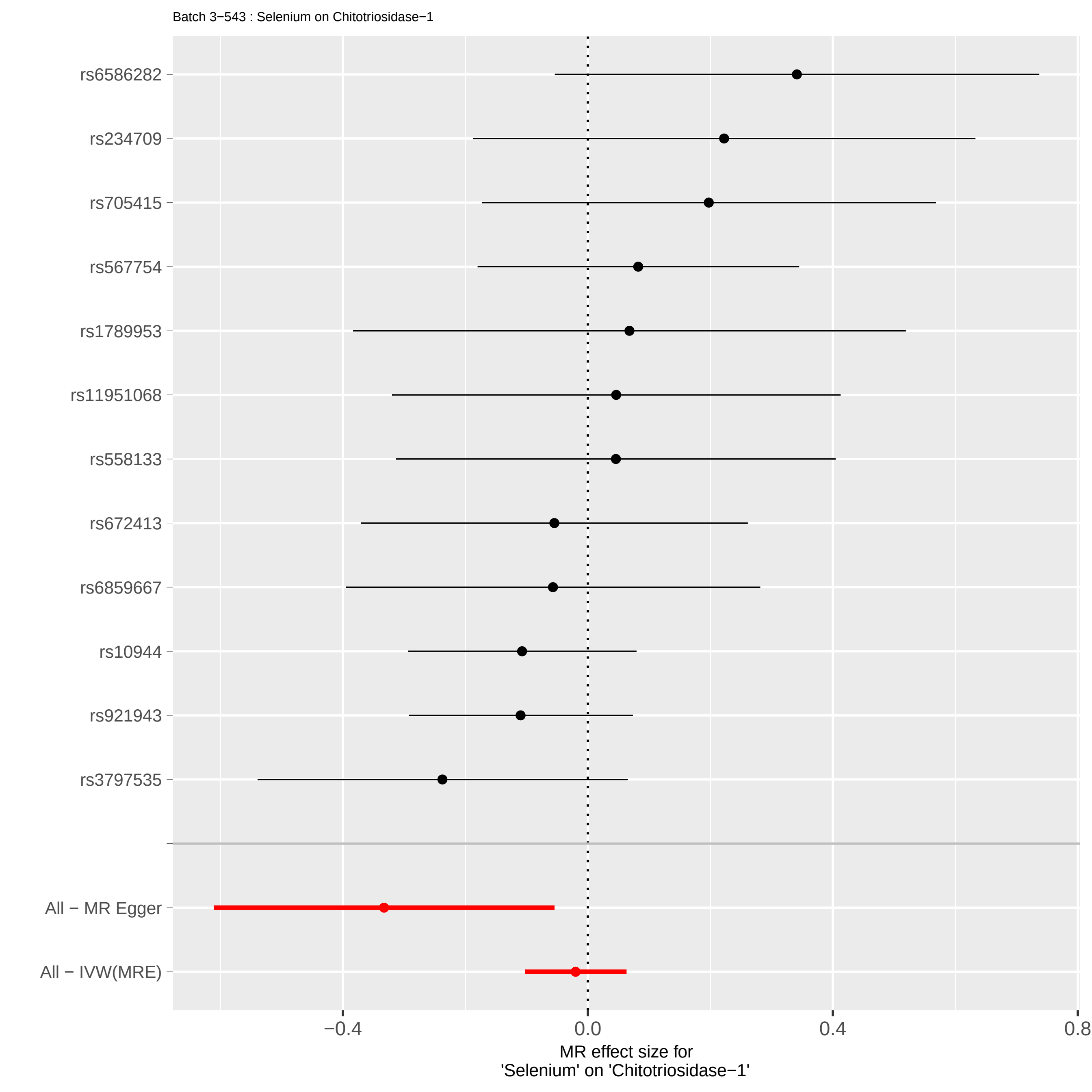
Batch 3−543 : Selenium on Chitotriosidase−1
rs6586282
rs234709
rs705415
rs567754
rs1789953
rs11951068
rs558133
rs672413
rs6859667
rs10944
rs921943
rs3797535
All − MR Egger
All − IVW(MRE)
−0.4
0.0
0.4
0.8
MR effect size for
'Selenium' on 'Chitotriosidase−1'

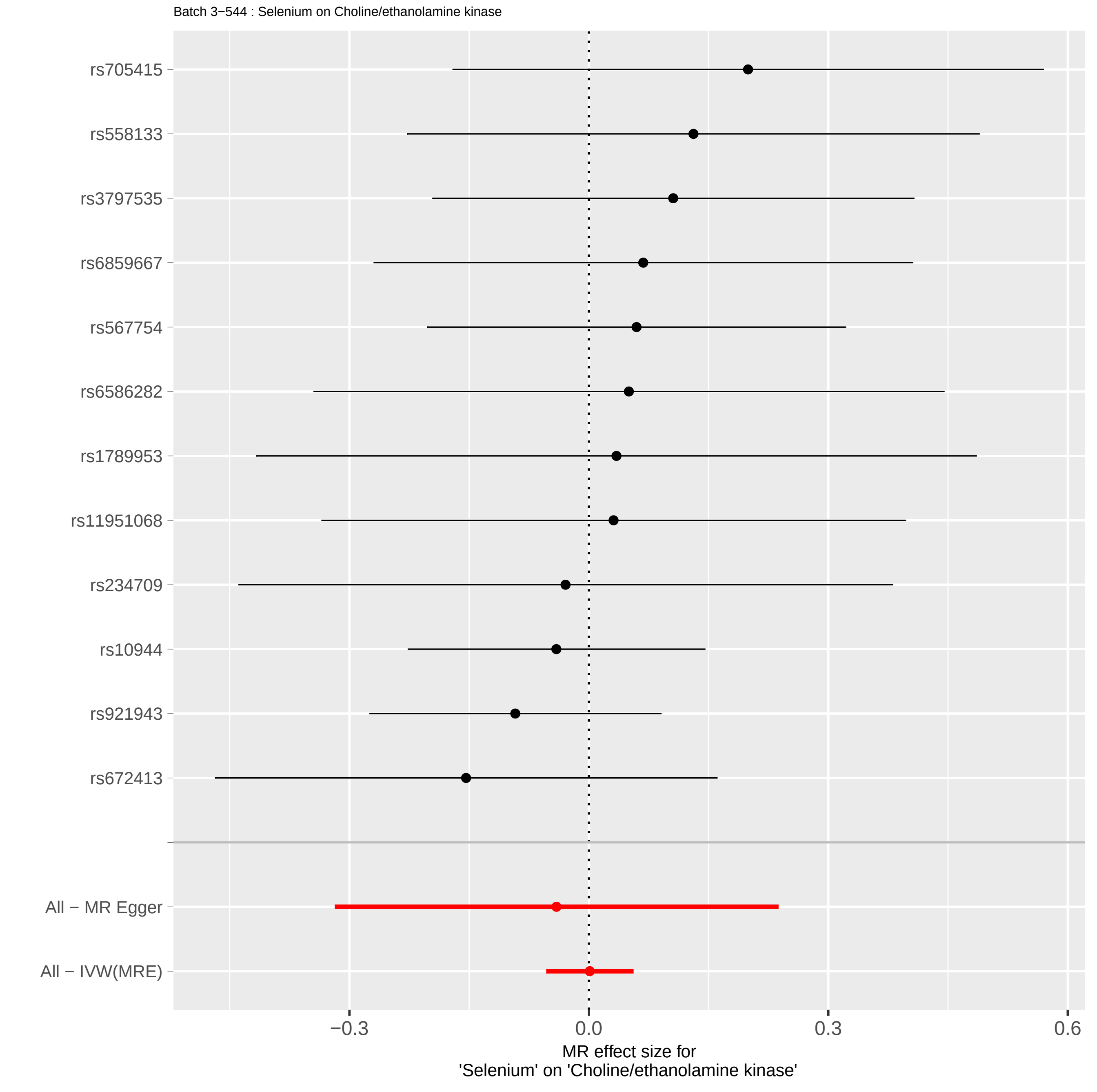

Batch 3−544 : Selenium on Choline/ethanolamine kinase
rs705415
rs558133
rs3797535
rs6859667
rs567754
rs6586282
rs1789953
rs11951068
rs234709
rs10944
rs921943
rs672413
All − MR Egger
All − IVW(MRE)
−0.3
0.0
0.3
0.6
MR effect size for
'Selenium' on 'Choline/ethanolamine kinase'

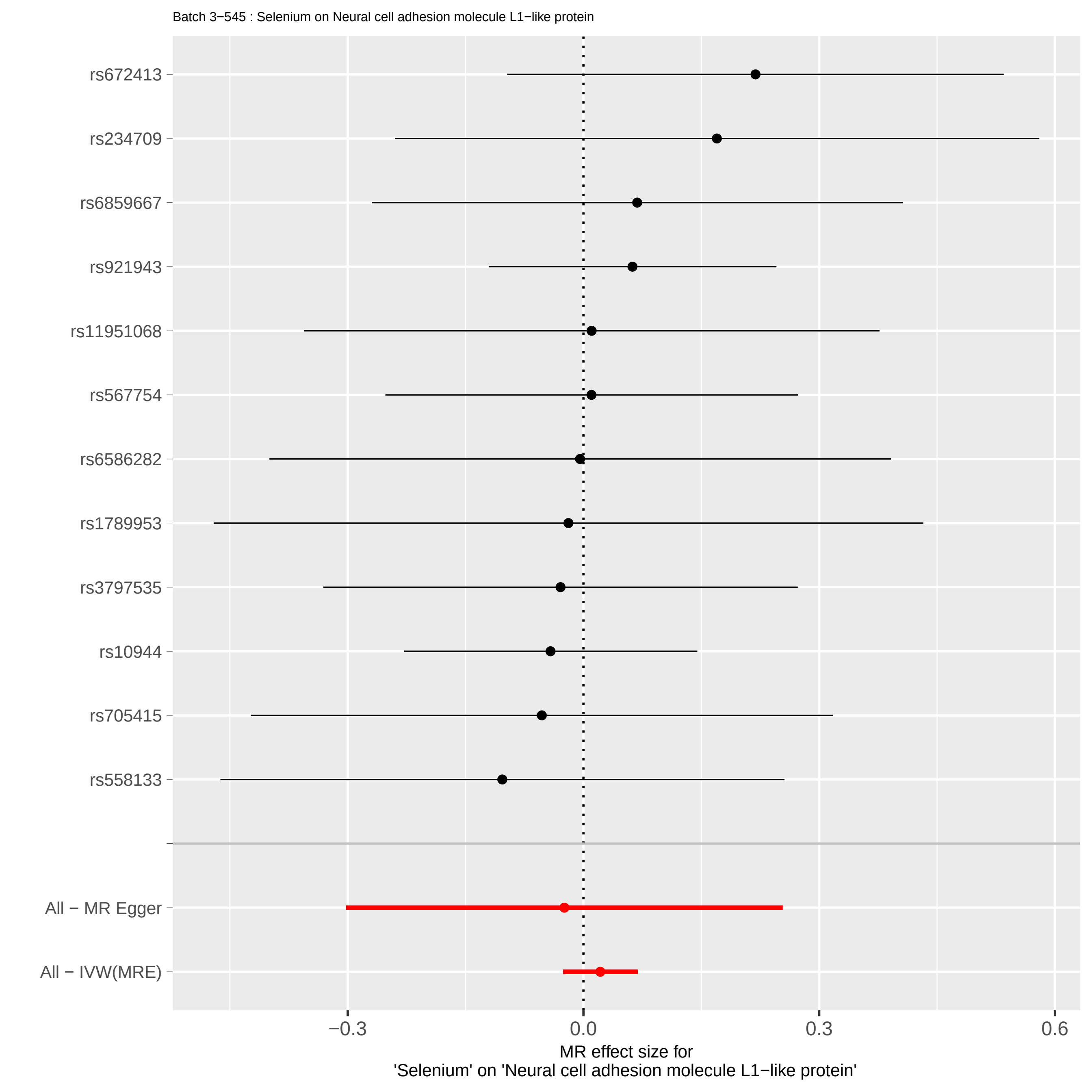
Batch 3−545 : Selenium on Neural cell adhesion molecule L1−like protein
rs672413
rs234709
rs6859667
rs921943
rs11951068
rs567754
rs6586282
rs1789953
rs3797535
rs10944
rs705415
rs558133
All − MR Egger
All − IVW(MRE)
−0.3
0.0
0.3
0.6
MR effect size for
'Selenium' on 'Neural cell adhesion molecule L1−like protein'

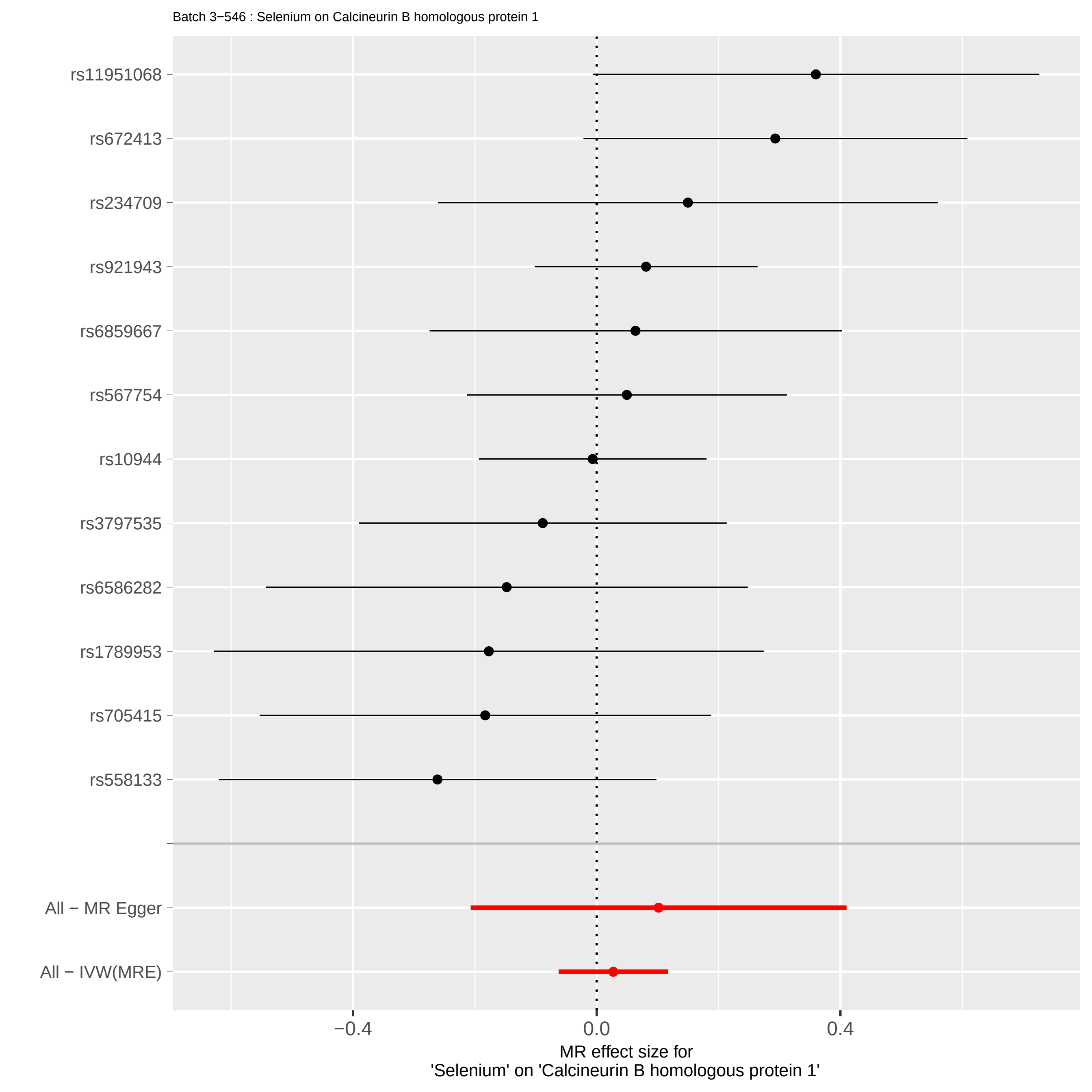

Batch 3−546 : Selenium on Calcineurin B homologous protein 1
rs11951068
rs672413
rs234709
rs921943
rs6859667
rs567754
rs10944
rs3797535
rs6586282
rs1789953
rs705415
rs558133
All − MR Egger
All − IVW(MRE)
−0.4
0.0
0.4
MR effect size for
'Selenium' on 'Calcineurin B homologous protein 1'

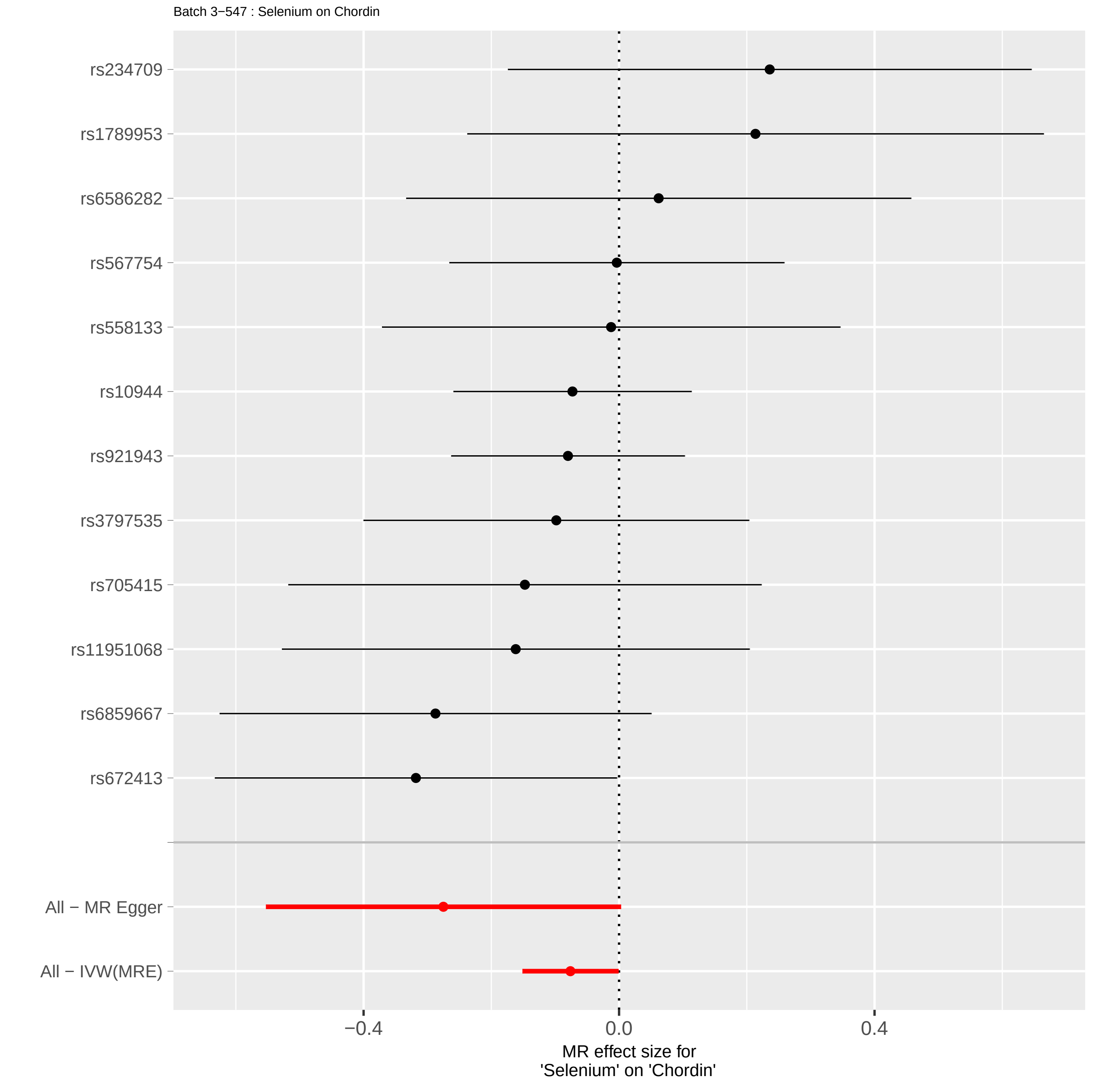

Batch 3−547 : Selenium on Chordin
rs234709
rs1789953
rs6586282
rs567754
rs558133
rs10944
rs921943
rs3797535
rs705415
rs11951068
rs6859667
rs672413
All − MR Egger
All − IVW(MRE)
−0.4
0.0
0.4
MR effect size for
'Selenium' on 'Chordin'

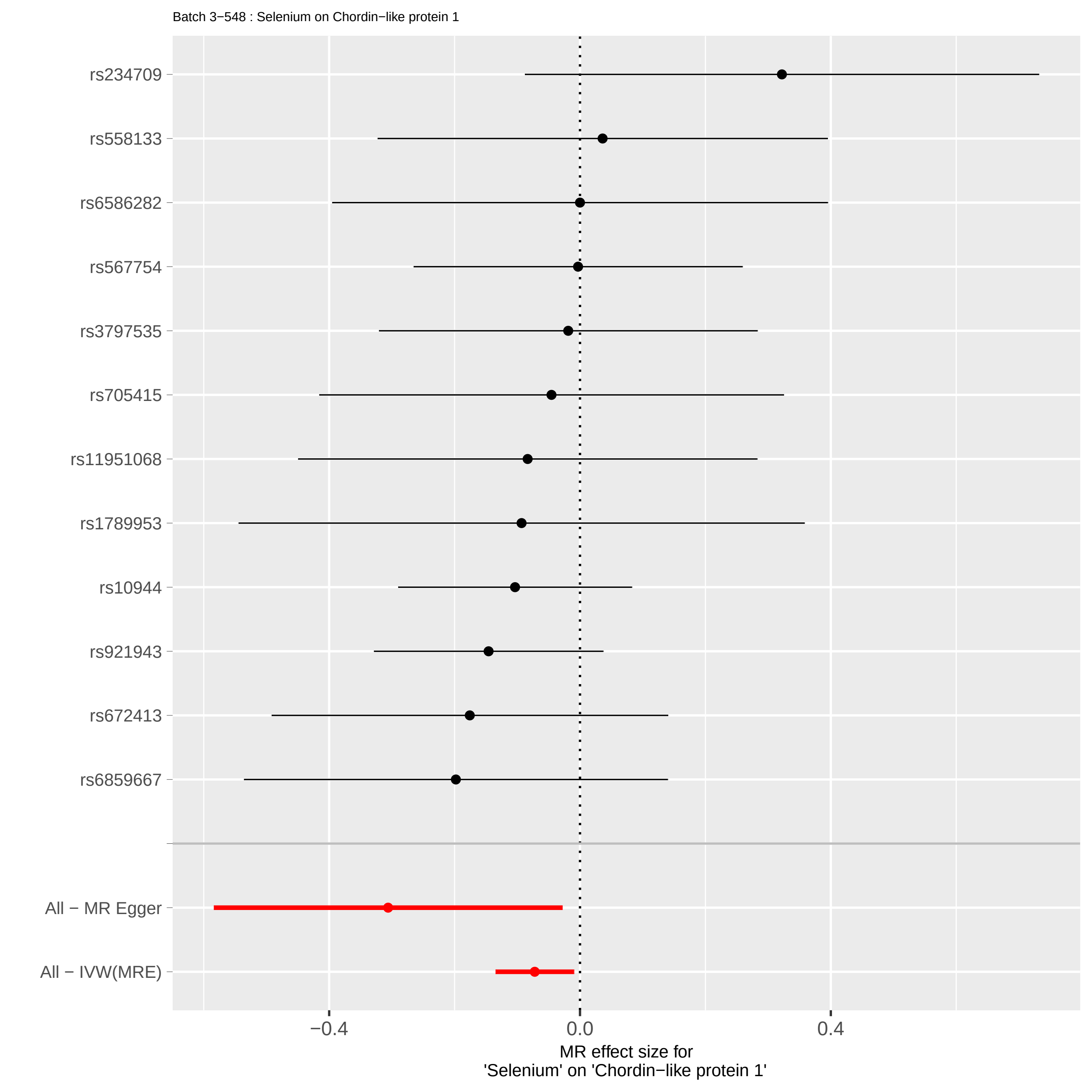

Batch 3−548 : Selenium on Chordin−like protein 1
rs234709
rs558133
rs6586282
rs567754
rs3797535
rs705415
rs11951068
rs1789953
rs10944
rs921943
rs672413
rs6859667
All − MR Egger
All − IVW(MRE)
−0.4
0.0
0.4
MR effect size for
'Selenium' on 'Chordin−like protein 1'

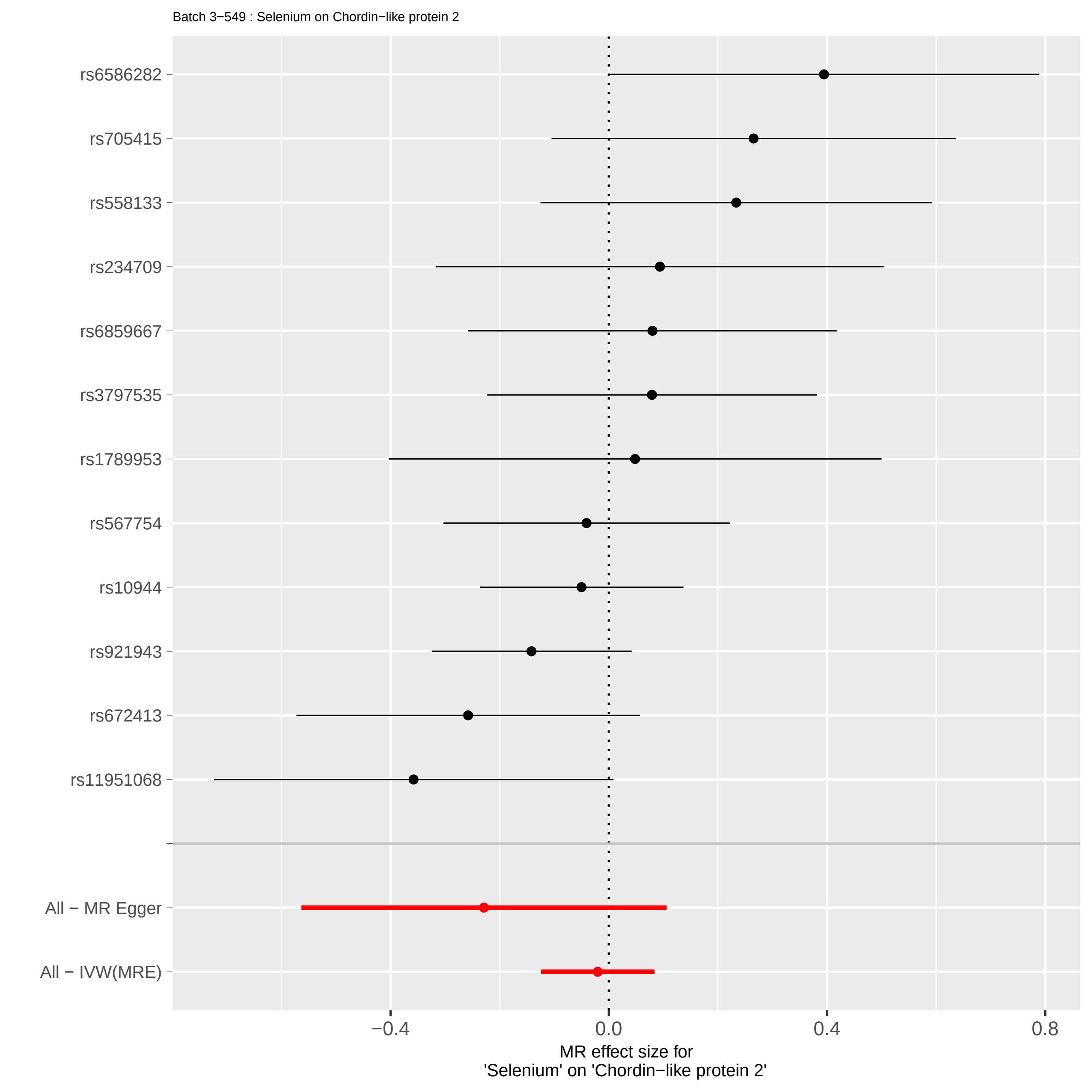
Batch 3−549 : Selenium on Chordin−like protein 2
rs6586282
rs705415
rs558133
rs234709
rs6859667
rs3797535
rs1789953
rs567754
rs10944
rs921943
rs672413
rs11951068
All − MR Egger
All − IVW(MRE)
−0.4
0.0
0.4
0.8
MR effect size for
'Selenium' on 'Chordin−like protein 2'

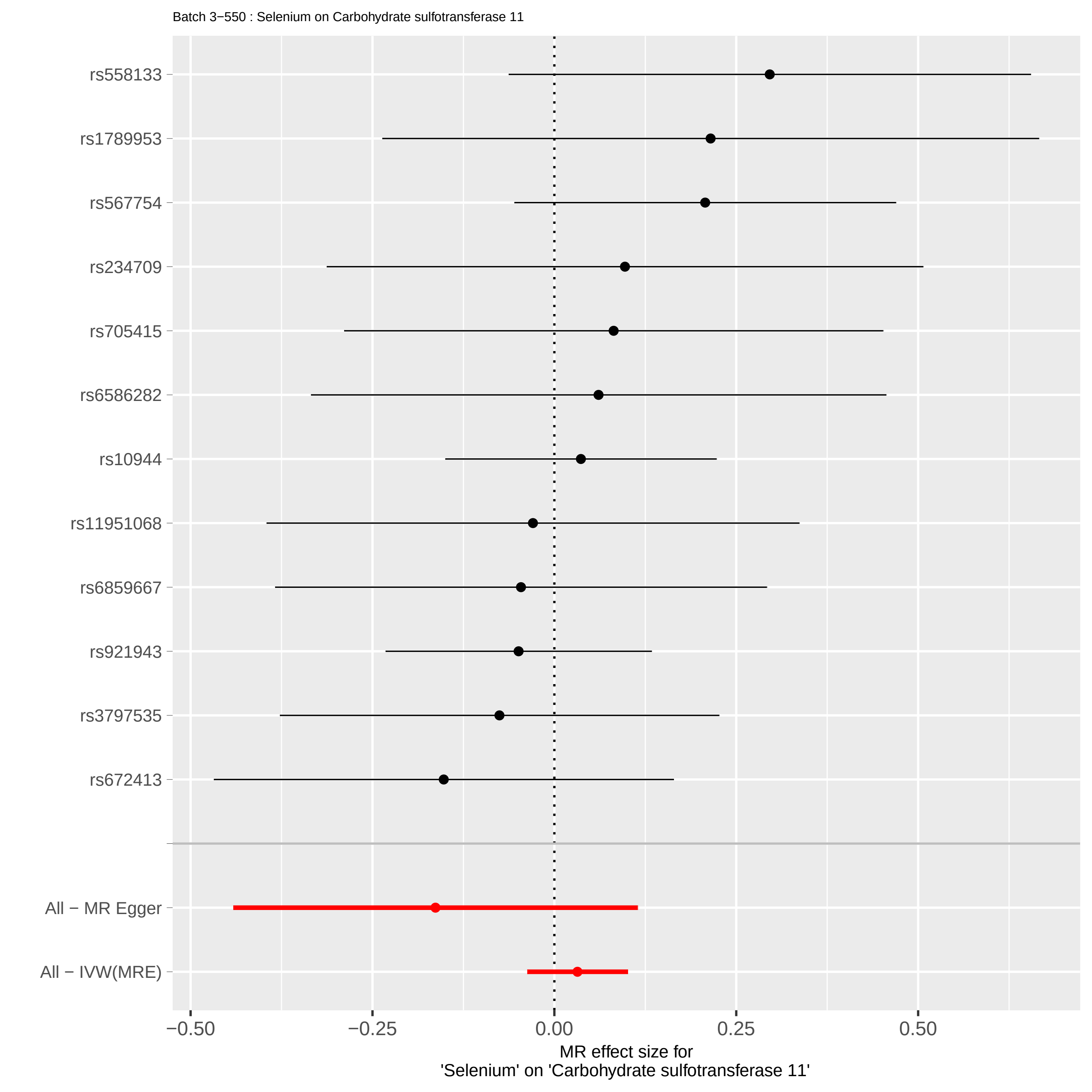
Batch 3−550 : Selenium on Carbohydrate sulfotransferase 11
rs558133
rs1789953
rs567754
rs234709
rs705415
rs6586282
rs10944
rs11951068
rs6859667
rs921943
rs3797535
rs672413
All − MR Egger
All − IVW(MRE)
−0.50
−0.25
0.00
0.25
0.50
MR effect size for
'Selenium' on 'Carbohydrate sulfotransferase 11'

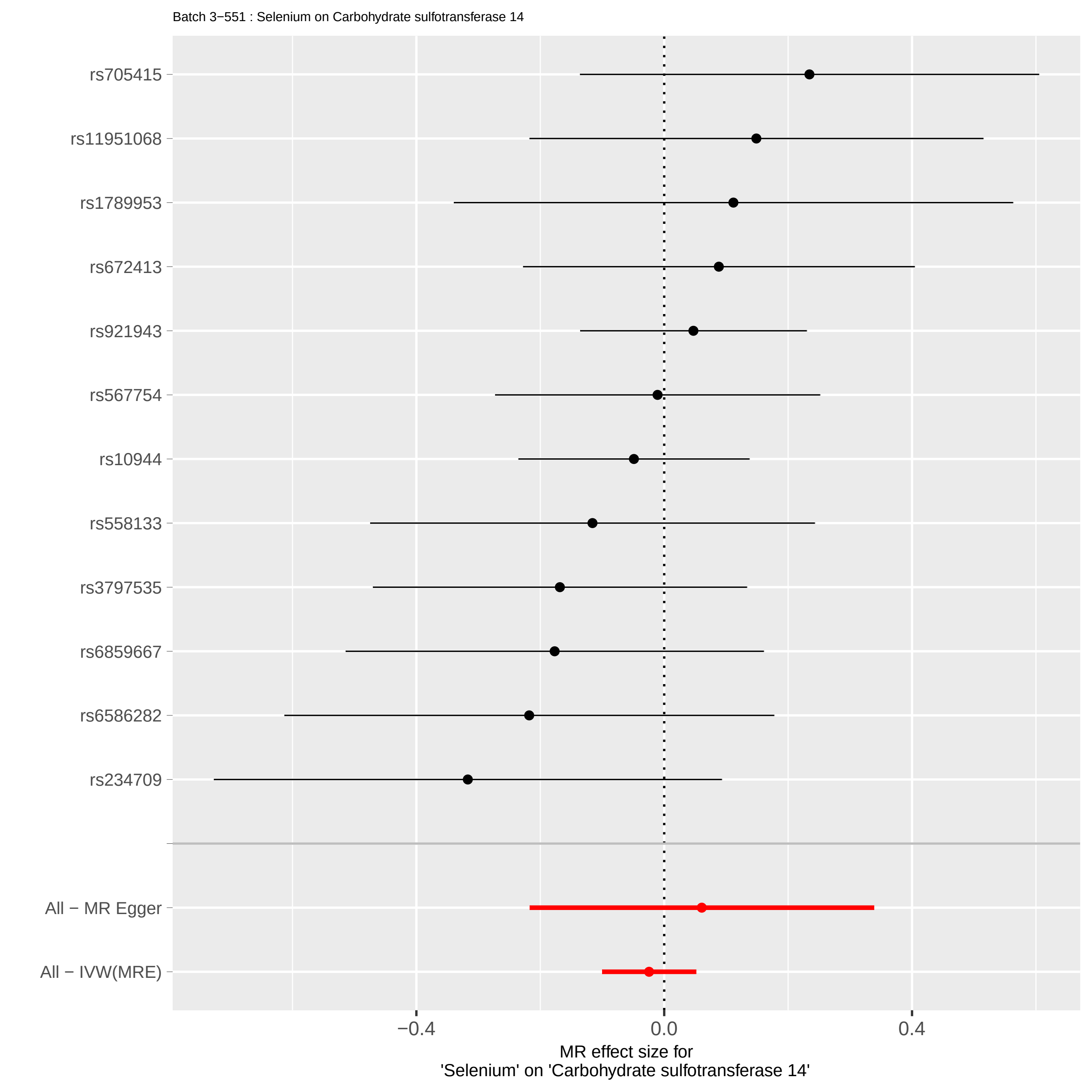

Batch 3−551 : Selenium on Carbohydrate sulfotransferase 14
rs705415
rs11951068
rs1789953
rs672413
rs921943
rs567754
rs10944
rs558133
rs3797535
rs6859667
rs6586282
rs234709
All − MR Egger
All − IVW(MRE)
−0.4
0.0
0.4
MR effect size for
'Selenium' on 'Carbohydrate sulfotransferase 14'

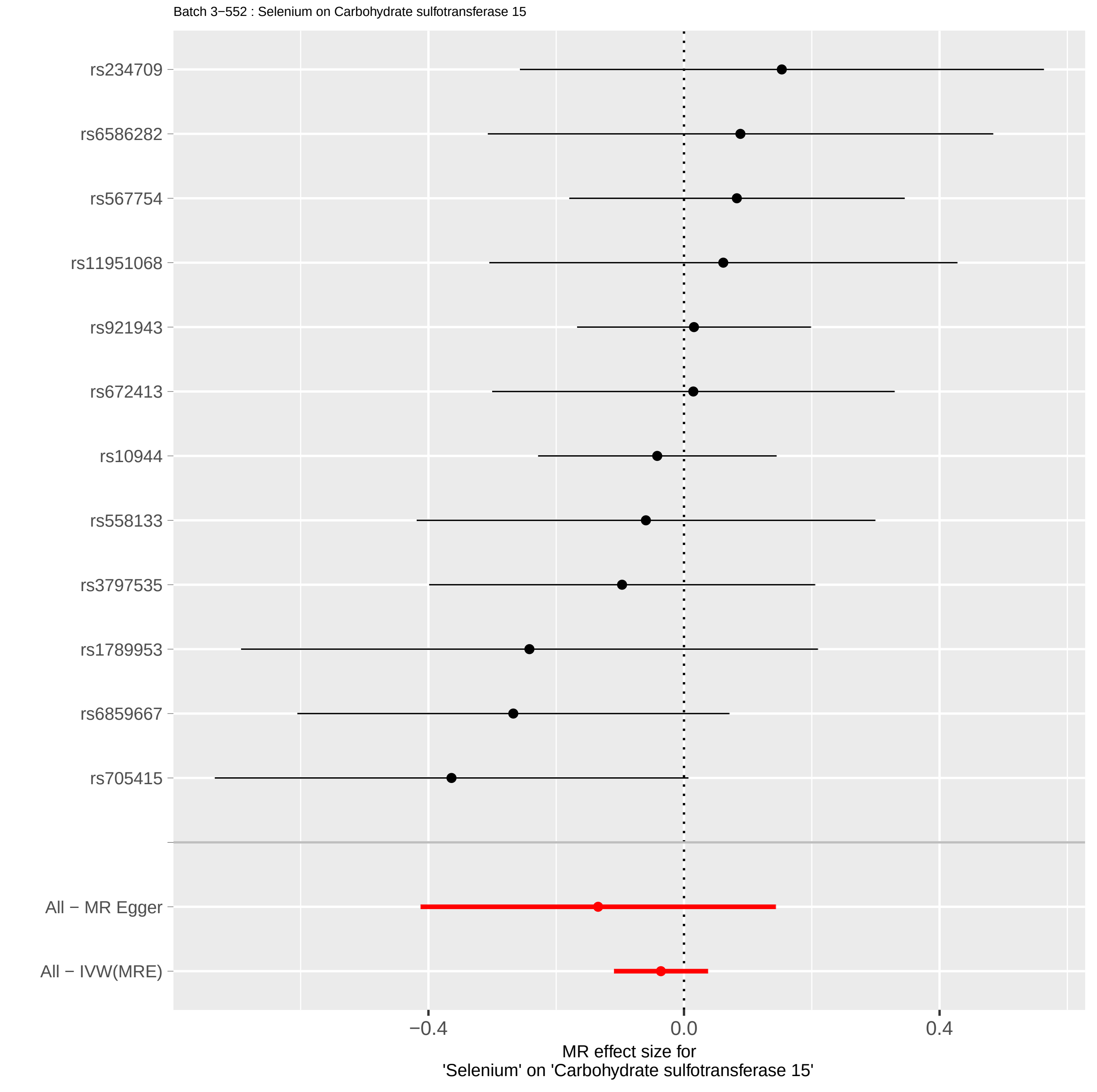
Batch 3−552 : Selenium on Carbohydrate sulfotransferase 15
rs234709
rs6586282
rs567754
rs11951068
rs921943
rs672413
rs10944
rs558133
rs3797535
rs1789953
rs6859667
rs705415
All − MR Egger
All − IVW(MRE)
−0.4
0.0
0.4
MR effect size for
'Selenium' on 'Carbohydrate sulfotransferase 15'

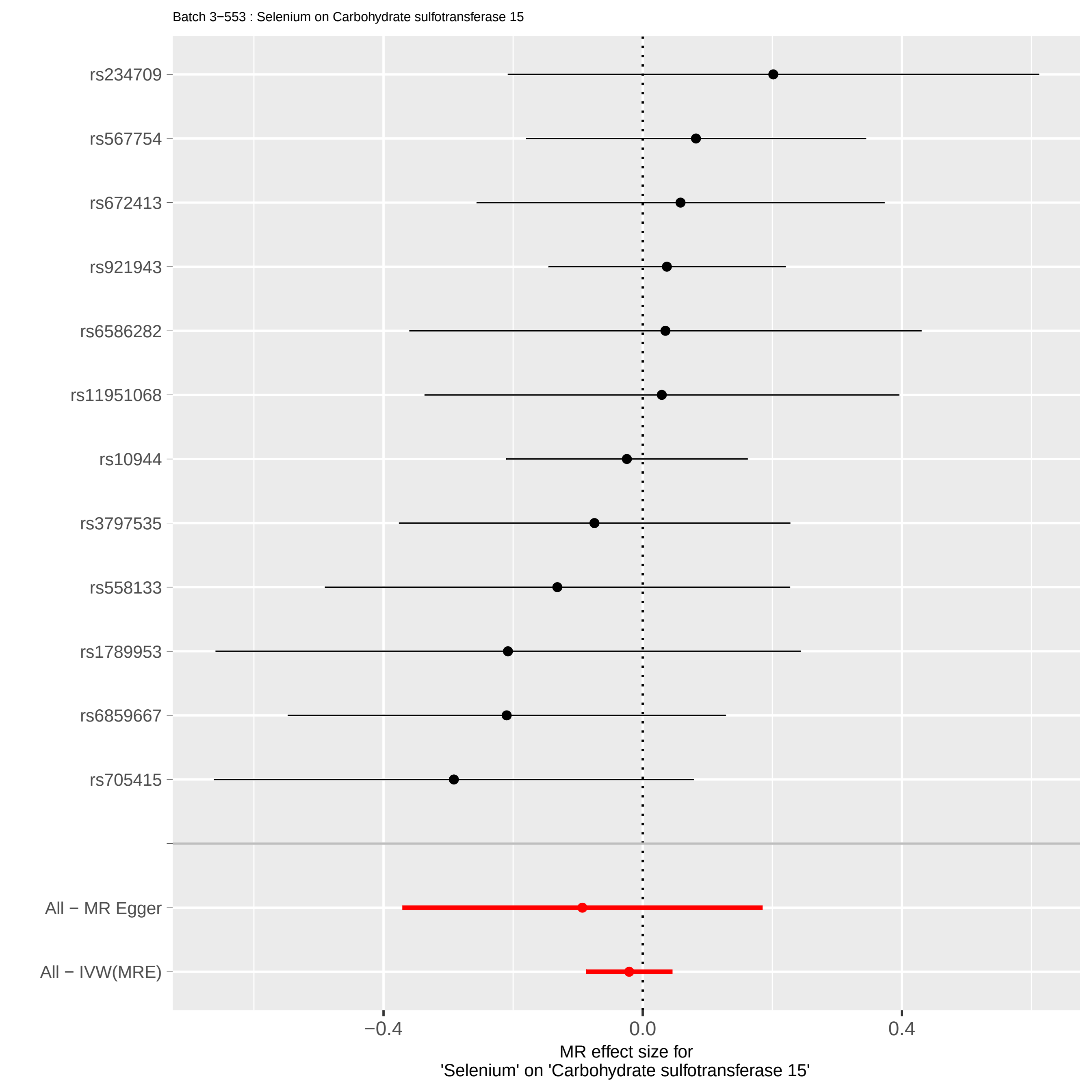

Batch 3−553 : Selenium on Carbohydrate sulfotransferase 15
rs234709
rs567754
rs672413
rs921943
rs6586282
rs11951068
rs10944
rs3797535
rs558133
rs1789953
rs6859667
rs705415
All − MR Egger
All − IVW(MRE)
−0.4
0.0
0.4
MR effect size for
'Selenium' on 'Carbohydrate sulfotransferase 15'

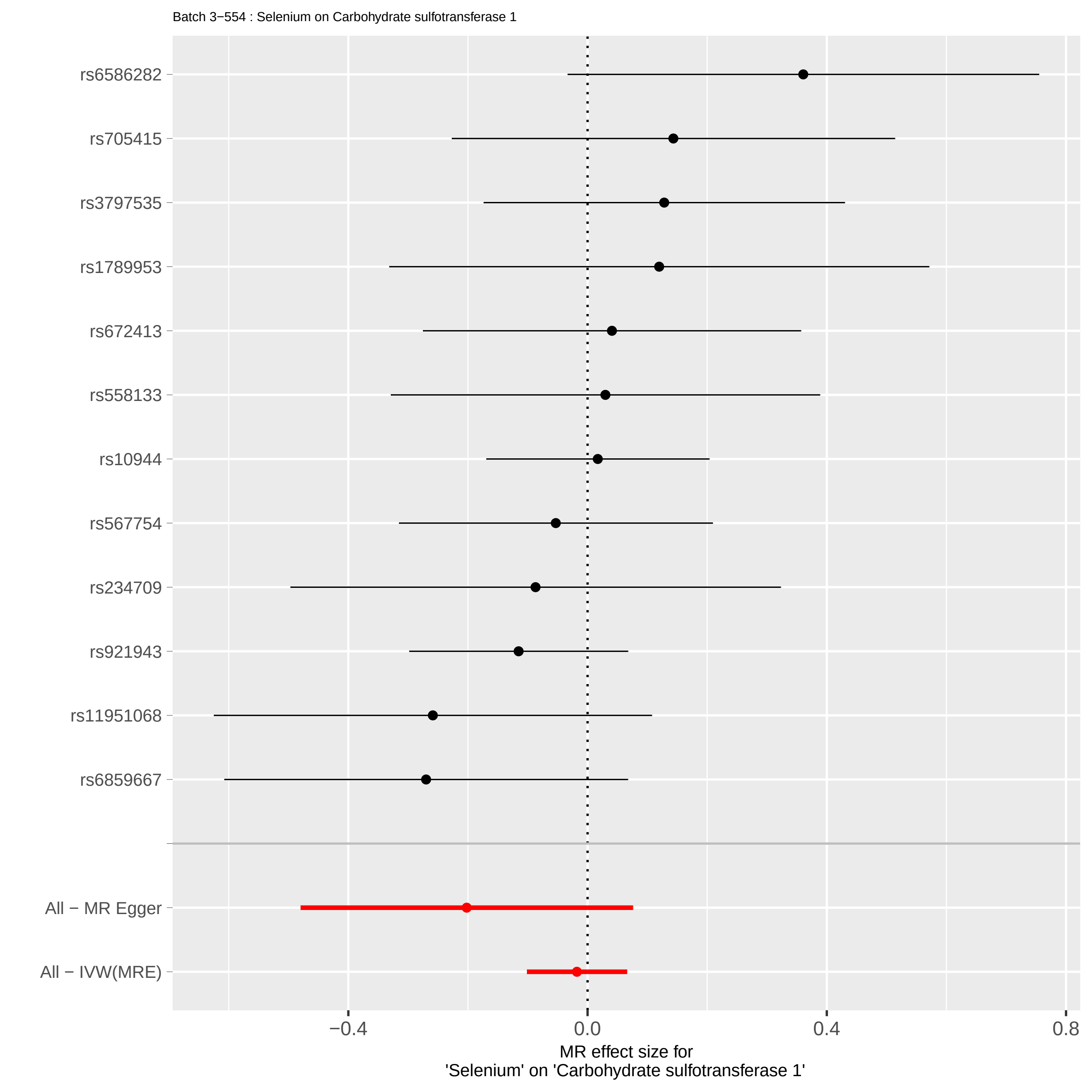

Batch 3−554 : Selenium on Carbohydrate sulfotransferase 1
rs6586282
rs705415
rs3797535
rs1789953
rs672413
rs558133
rs10944
rs567754
rs234709
rs921943
rs11951068
rs6859667
All − MR Egger
All − IVW(MRE)
−0.4
0.0
0.4
0.8
MR effect size for
'Selenium' on 'Carbohydrate sulfotransferase 1'

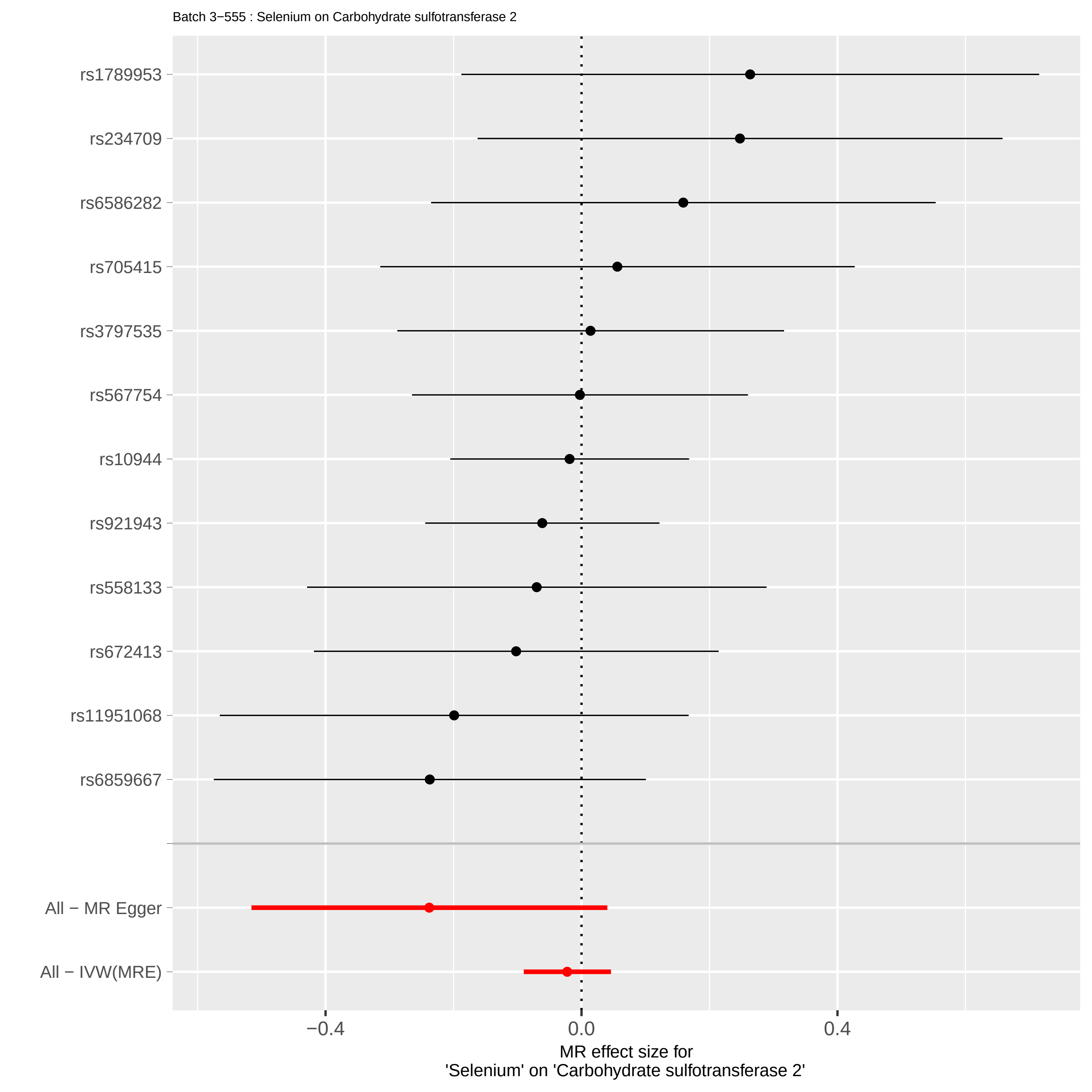

Batch 3−555 : Selenium on Carbohydrate sulfotransferase 2
rs1789953
rs234709
rs6586282
rs705415
rs3797535
rs567754
rs10944
rs921943
rs558133
rs672413
rs11951068
rs6859667
All − MR Egger
All − IVW(MRE)
−0.4
0.0
0.4
MR effect size for
'Selenium' on 'Carbohydrate sulfotransferase 2'

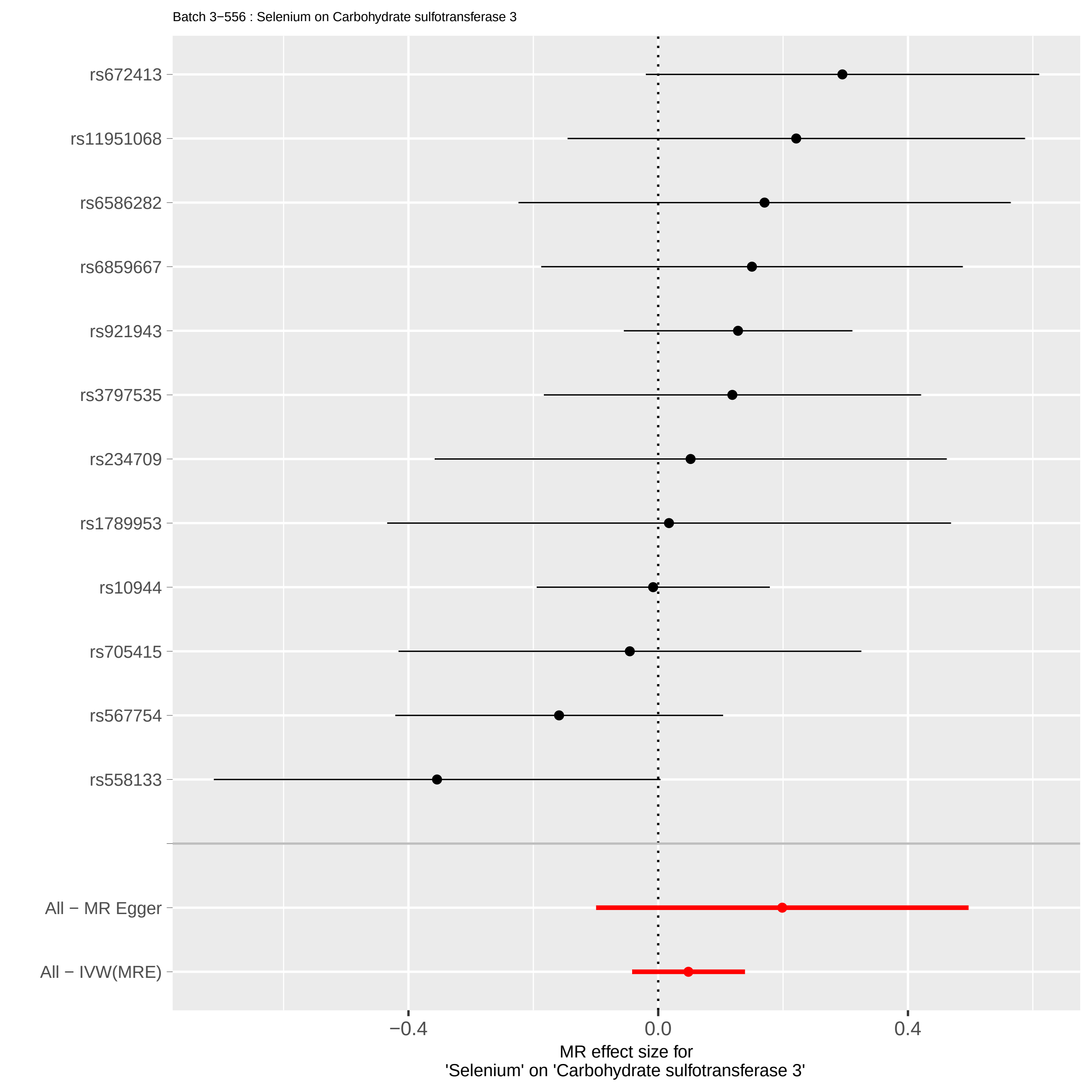
Batch 3−556 : Selenium on Carbohydrate sulfotransferase 3
rs672413
rs11951068
rs6586282
rs6859667
rs921943
rs3797535
rs234709
rs1789953
rs10944
rs705415
rs567754
rs558133
All − MR Egger
All − IVW(MRE)
−0.4
0.0
0.4
MR effect size for
'Selenium' on 'Carbohydrate sulfotransferase 3'

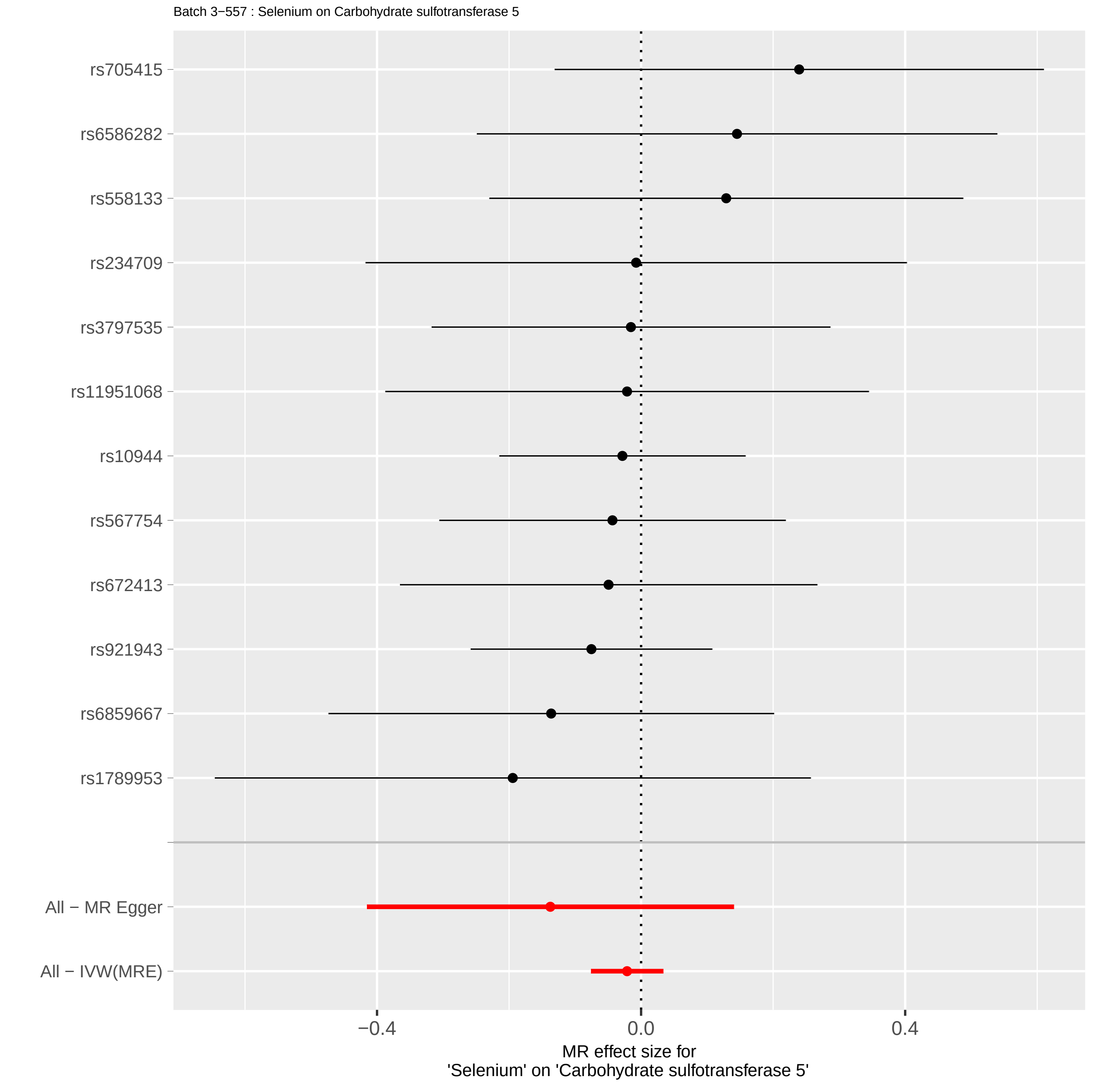
Batch 3−557 : Selenium on Carbohydrate sulfotransferase 5
rs705415
rs6586282
rs558133
rs234709
rs3797535
rs11951068
rs10944
rs567754
rs672413
rs921943
rs6859667
rs1789953
All − MR Egger
All − IVW(MRE)
−0.4
0.0
0.4
MR effect size for
'Selenium' on 'Carbohydrate sulfotransferase 5'

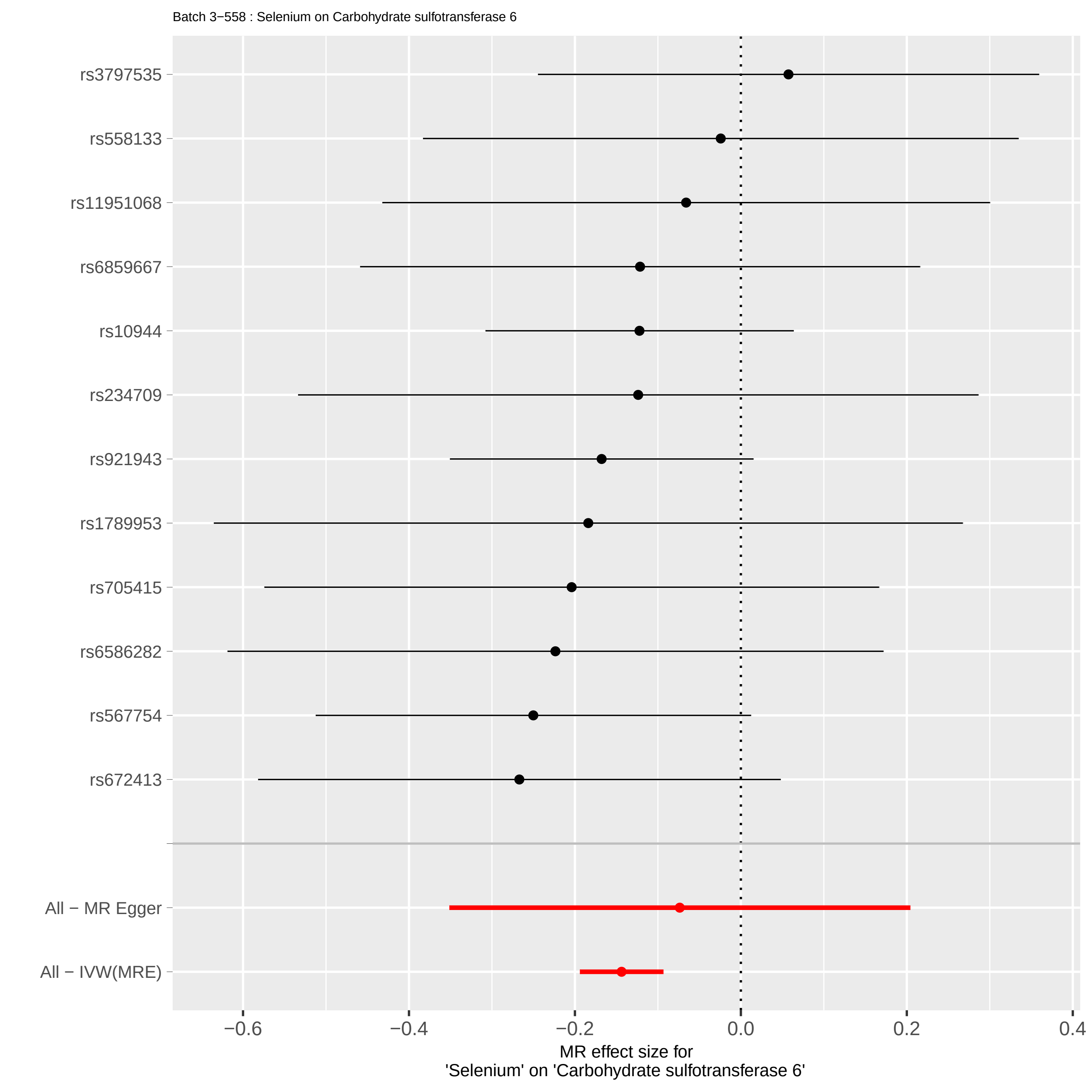

Batch 3−558 : Selenium on Carbohydrate sulfotransferase 6
rs3797535
rs558133
rs11951068
rs6859667
rs10944
rs234709
rs921943
rs1789953
rs705415
rs6586282
rs567754
rs672413
All − MR Egger
All − IVW(MRE)
−0.6
−0.4
−0.2
0.0
0.2
0.4
MR effect size for
'Selenium' on 'Carbohydrate sulfotransferase 6'

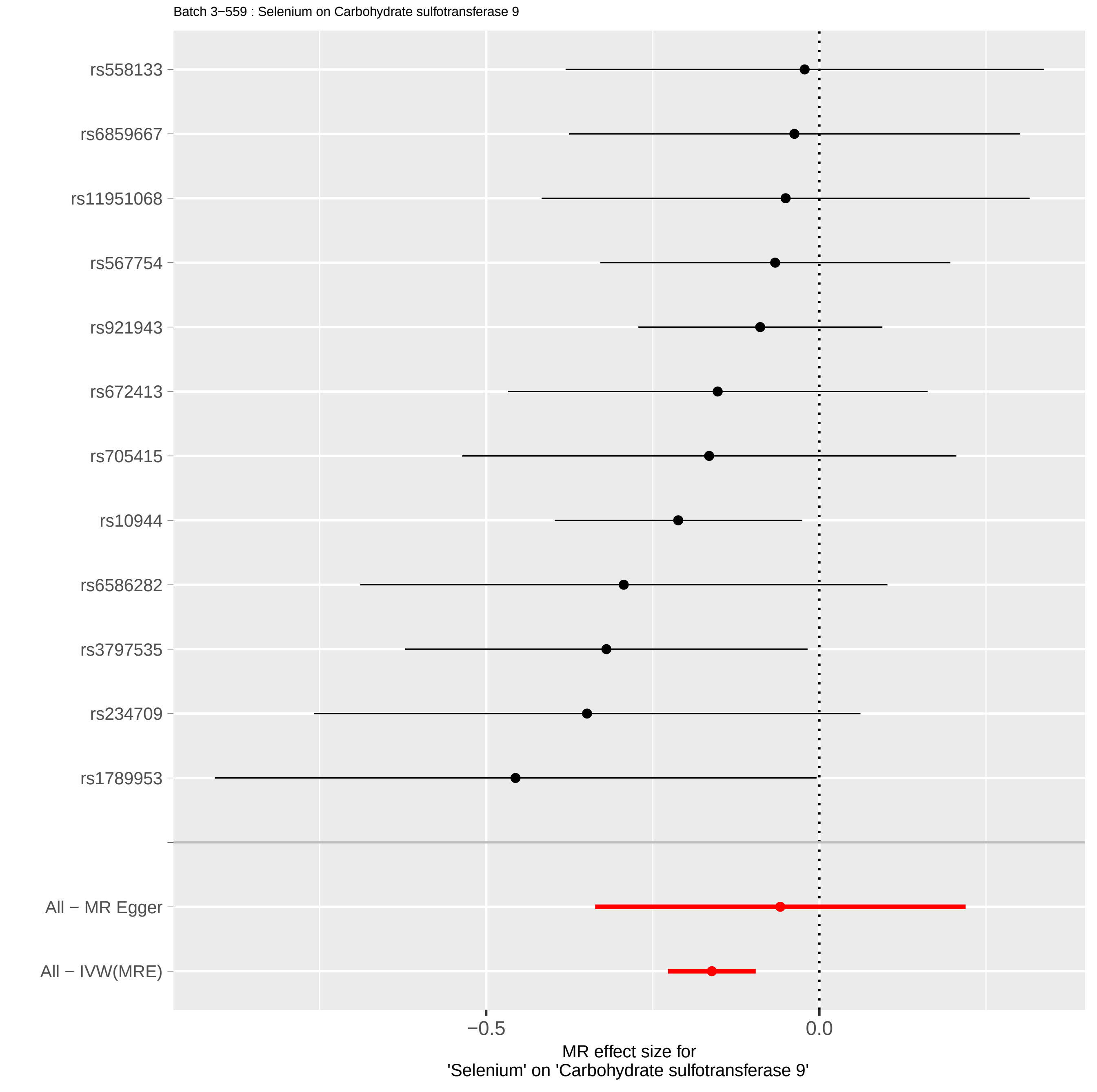

Batch 3−559 : Selenium on Carbohydrate sulfotransferase 9
rs558133
rs6859667
rs11951068
rs567754
rs921943
rs672413
rs705415
rs10944
rs6586282
rs3797535
rs234709
rs1789953
All − MR Egger
All − IVW(MRE)
−0.5
0.0
MR effect size for
'Selenium' on 'Carbohydrate sulfotransferase 9'

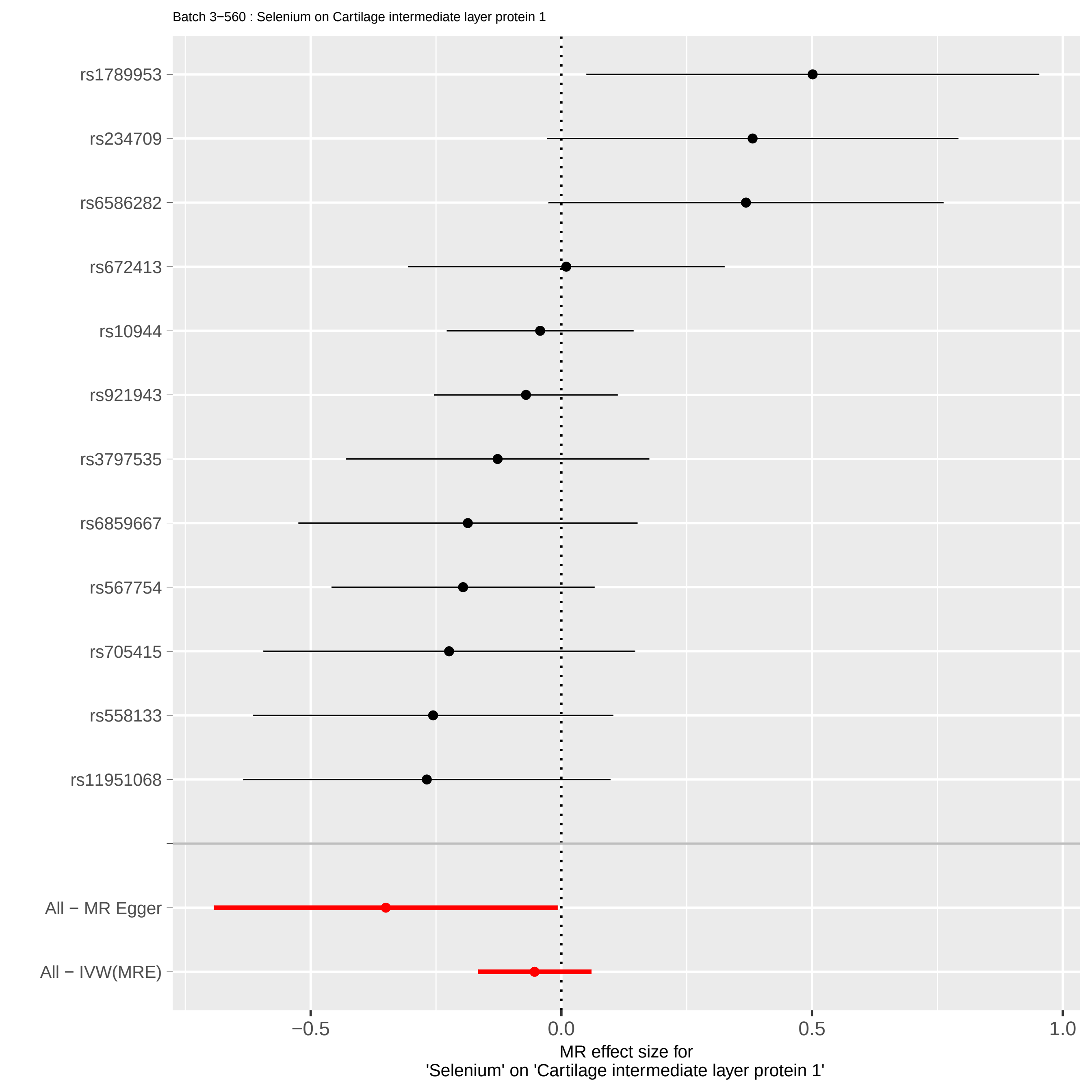

Batch 3−560 : Selenium on Cartilage intermediate layer protein 1
rs1789953
rs234709
rs6586282
rs672413
rs10944
rs921943
rs3797535
rs6859667
rs567754
rs705415
rs558133
rs11951068
All − MR Egger
All − IVW(MRE)
−0.5
0.0
0.5
1.0
MR effect size for
'Selenium' on 'Cartilage intermediate layer protein 1'

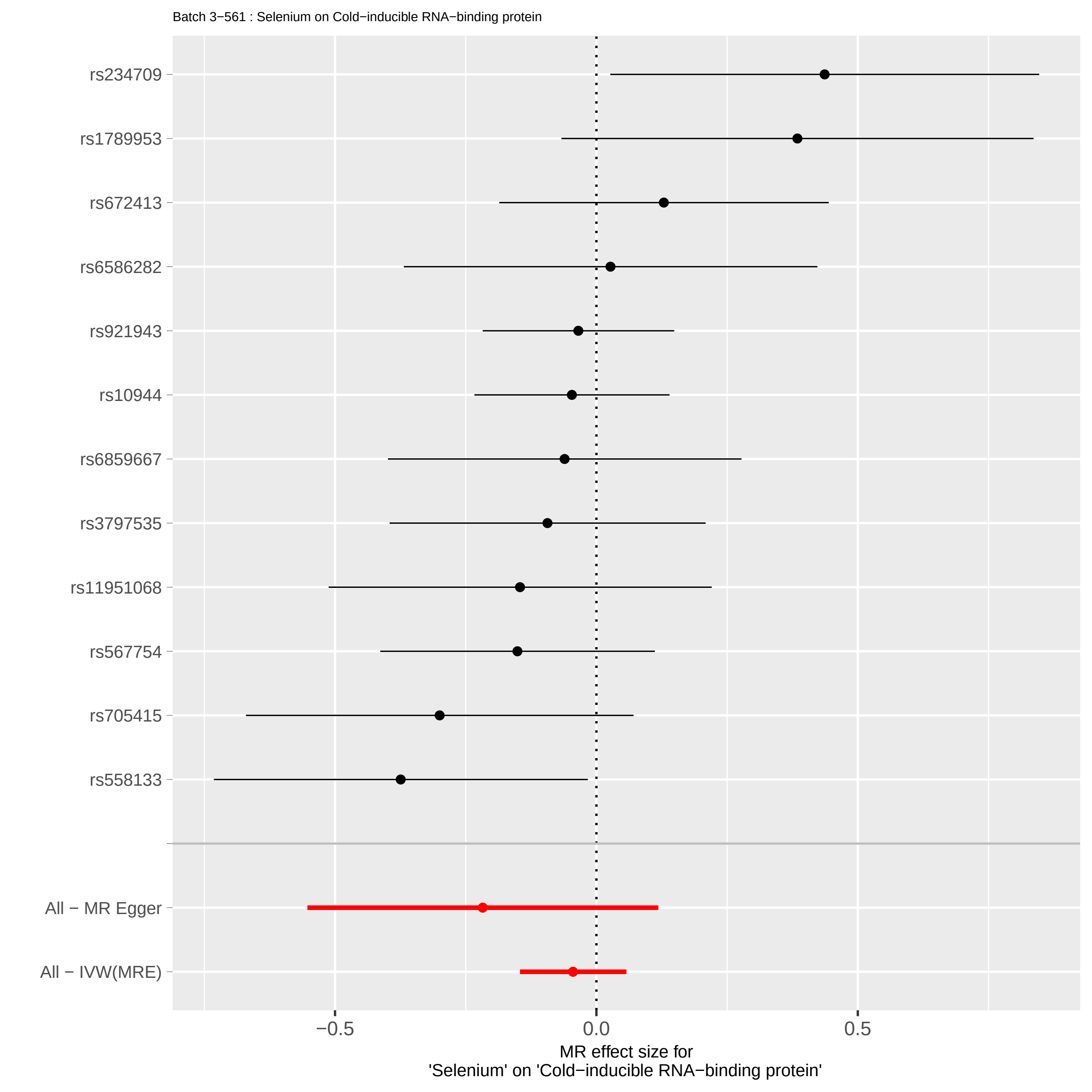

Batch 3−561 : Selenium on Cold−inducible RNA−binding protein
rs234709
rs1789953
rs672413
rs6586282
rs921943
rs10944
rs6859667
rs3797535
rs11951068
rs567754
rs705415
rs558133
All − MR Egger
All − IVW(MRE)
−0.5
0.0
0.5
MR effect size for
'Selenium' on 'Cold−inducible RNA−binding protein'

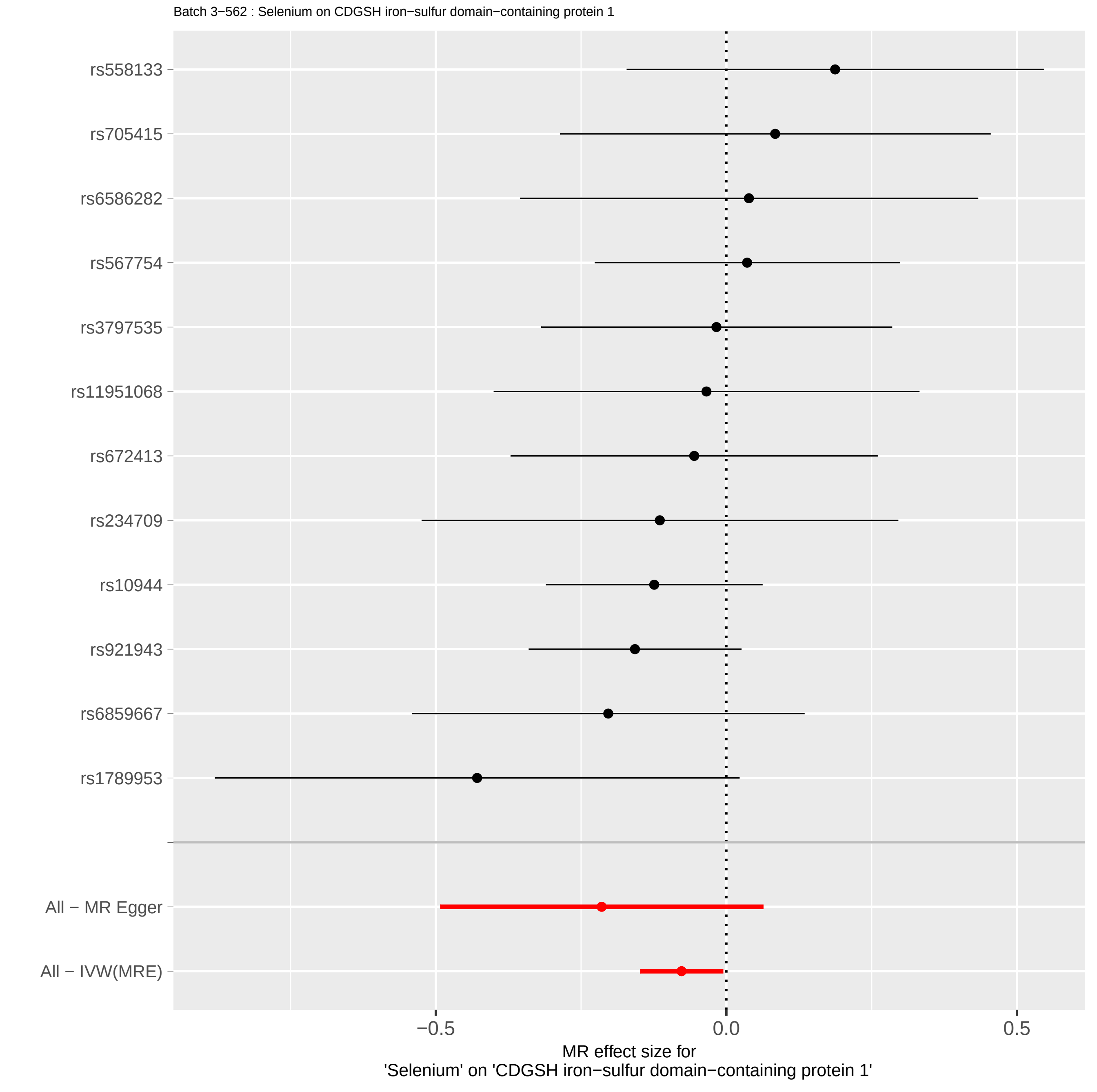

Batch 3−562 : Selenium on CDGSH iron−sulfur domain−containing protein 1
rs558133
rs705415
rs6586282
rs567754
rs3797535
rs11951068
rs672413
rs234709
rs10944
rs921943
rs6859667
rs1789953
All − MR Egger
All − IVW(MRE)
−0.5
0.0
0.5
MR effect size for
'Selenium' on 'CDGSH iron−sulfur domain−containing protein 1'

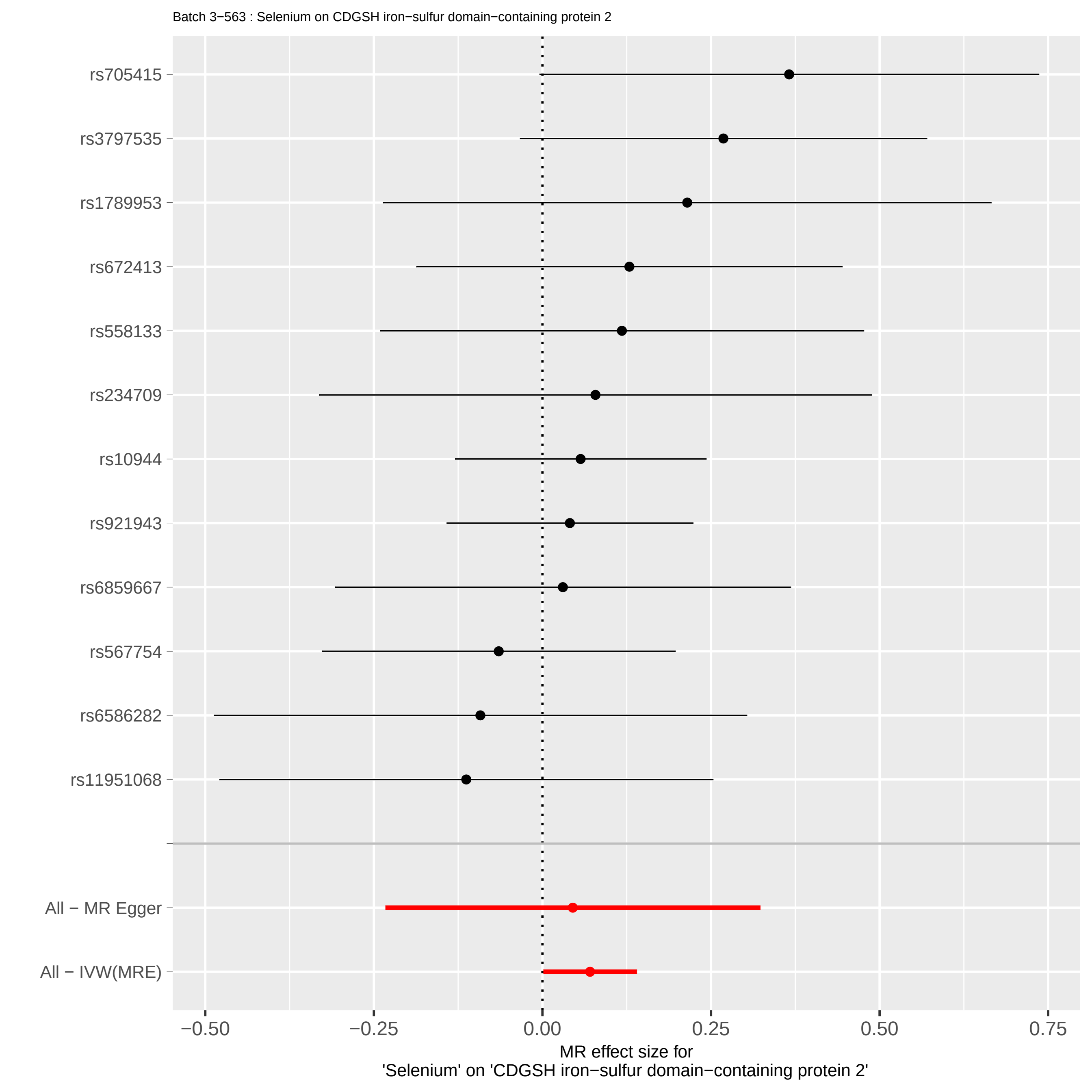
Batch 3−563 : Selenium on CDGSH iron−sulfur domain−containing protein 2
rs705415
rs3797535
rs1789953
rs672413
rs558133
rs234709
rs10944
rs921943
rs6859667
rs567754
rs6586282
rs11951068
All − MR Egger
All − IVW(MRE)
−0.50
−0.25
0.00
0.25
0.50
0.75
MR effect size for
'Selenium' on 'CDGSH iron−sulfur domain−containing protein 2'

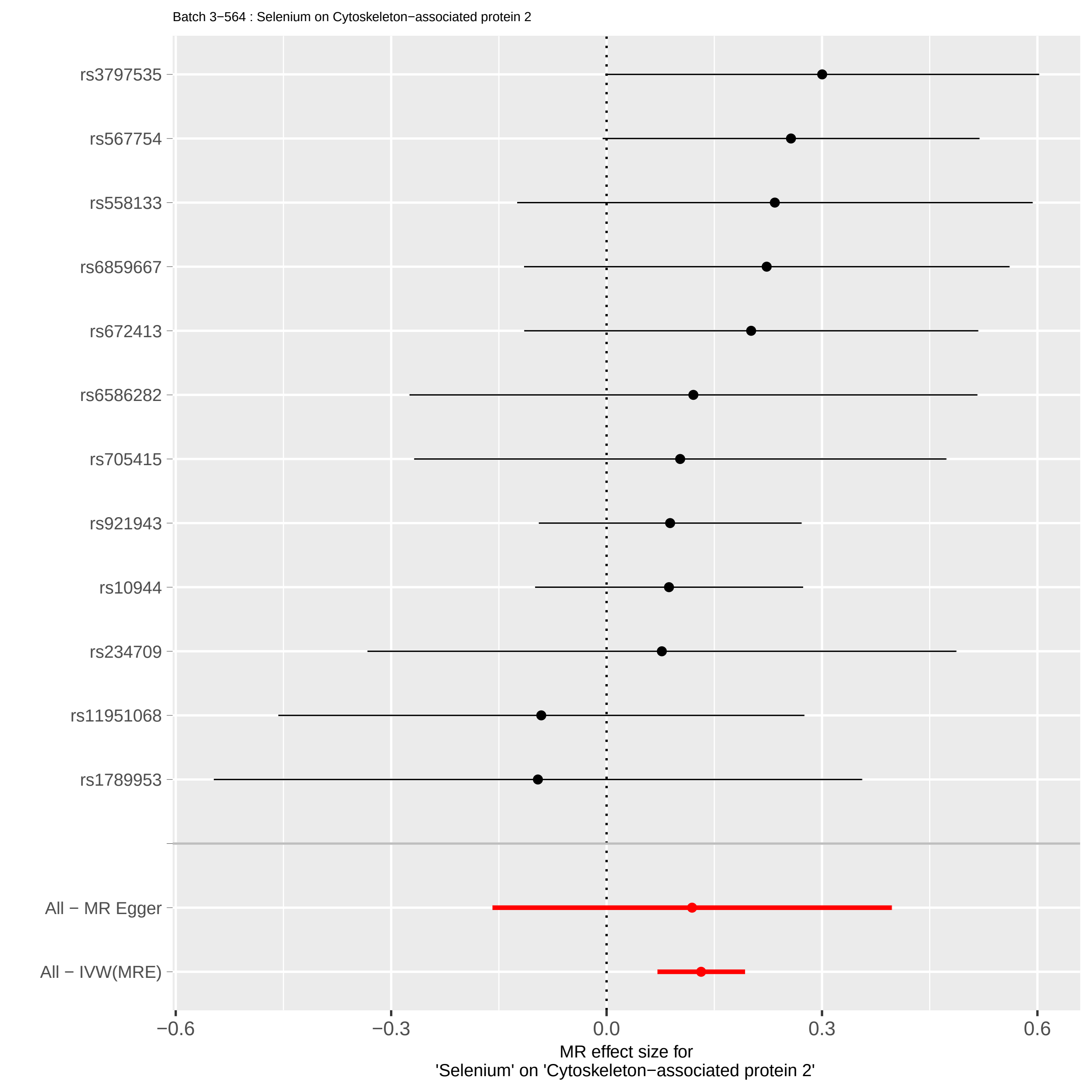

Batch 3−564 : Selenium on Cytoskeleton−associated protein 2
rs3797535
rs567754
rs558133
rs6859667
rs672413
rs6586282
rs705415
rs921943
rs10944
rs234709
rs11951068
rs1789953
All − MR Egger
All − IVW(MRE)
−0.6
−0.3
0.0
0.3
0.6
MR effect size for
'Selenium' on 'Cytoskeleton−associated protein 2'

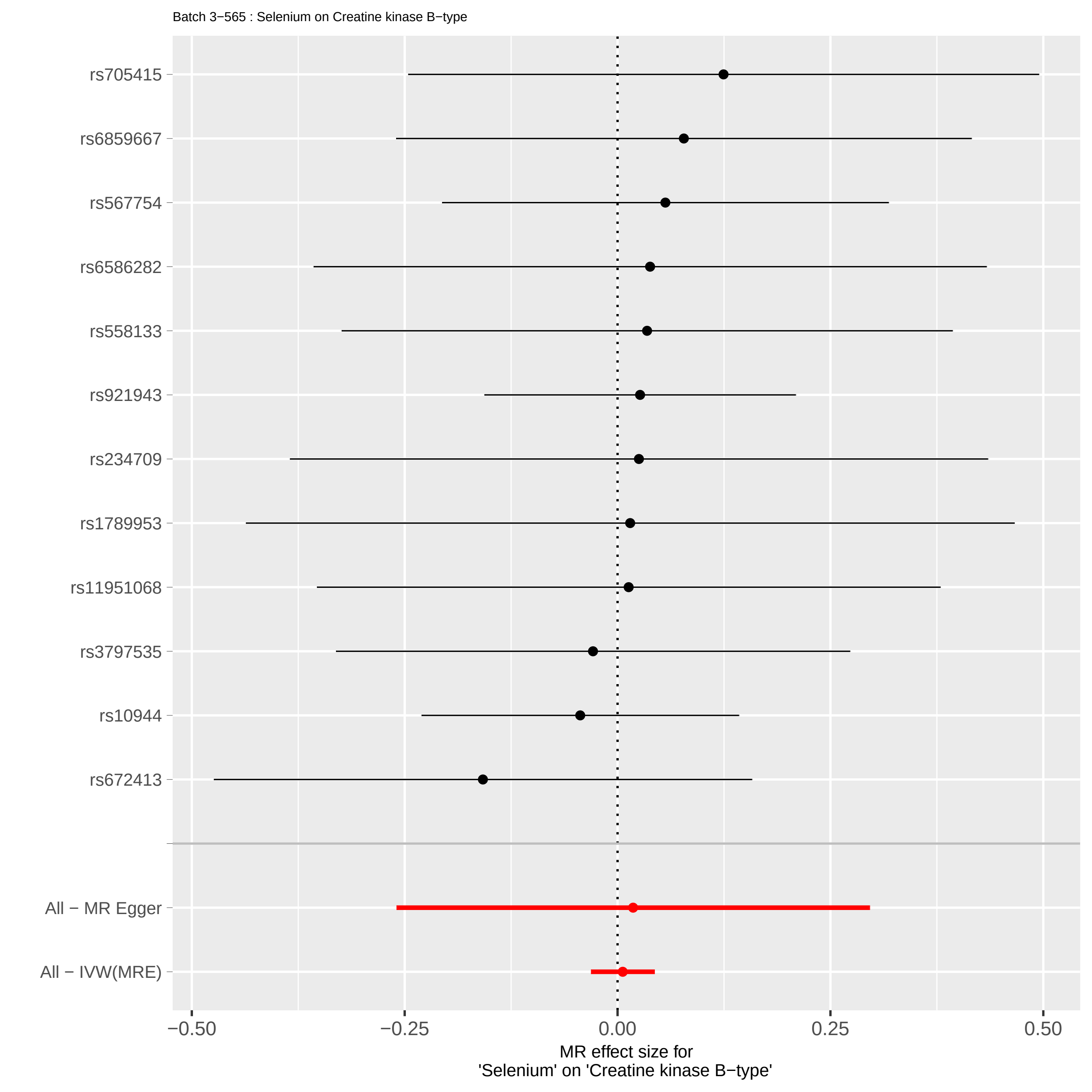
Batch 3−565 : Selenium on Creatine kinase B−type
rs705415
rs6859667
rs567754
rs6586282
rs558133
rs921943
rs234709
rs1789953
rs11951068
rs3797535
rs10944
rs672413
All − MR Egger
All − IVW(MRE)
−0.50
−0.25
0.00
0.25
0.50
MR effect size for
'Selenium' on 'Creatine kinase B−type'

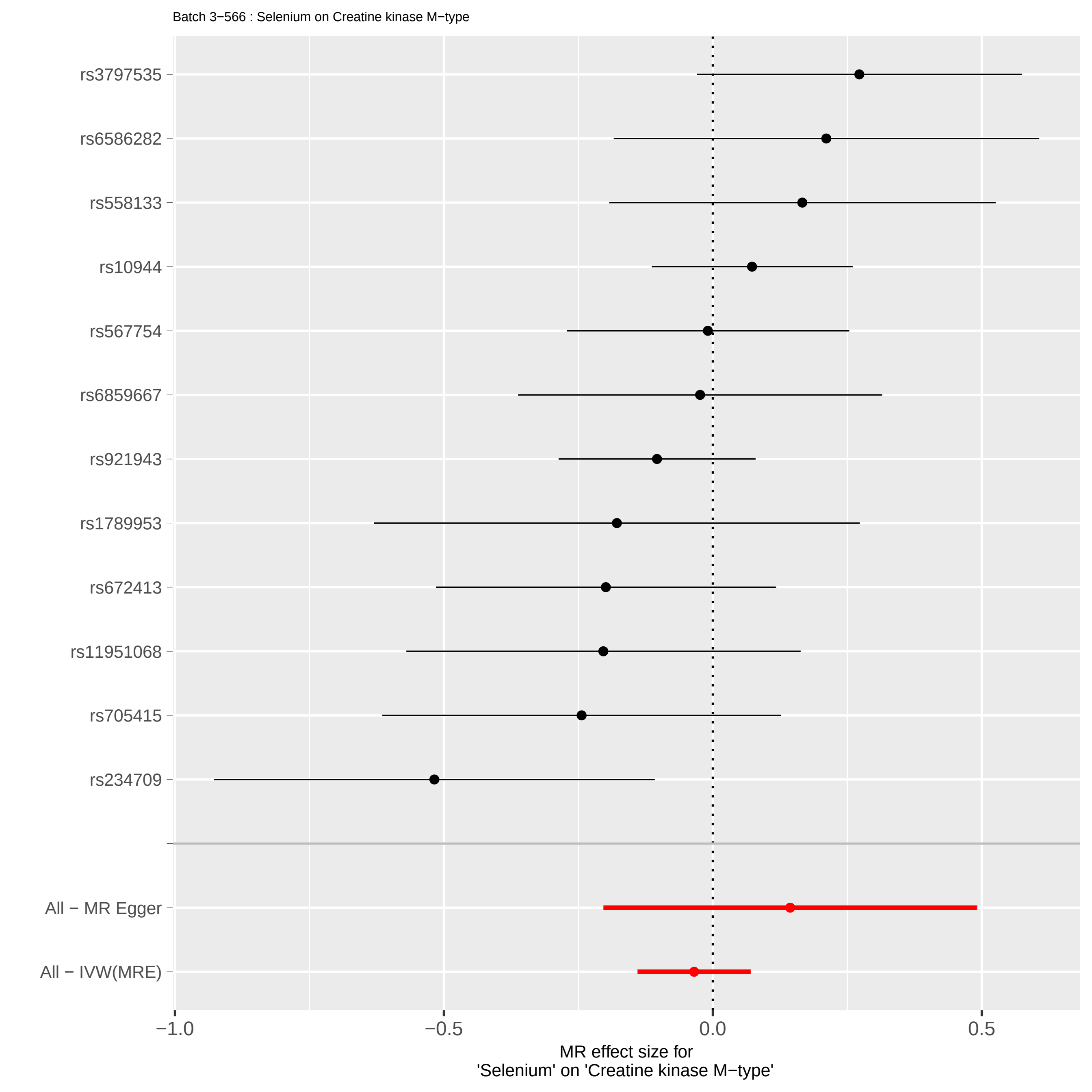

Batch 3−566 : Selenium on Creatine kinase M−type
rs3797535
rs6586282
rs558133
rs10944
rs567754
rs6859667
rs921943
rs1789953
rs672413
rs11951068
rs705415
rs234709
All − MR Egger
All − IVW(MRE)
−1.0
−0.5
0.0
0.5
MR effect size for
'Selenium' on 'Creatine kinase M−type'

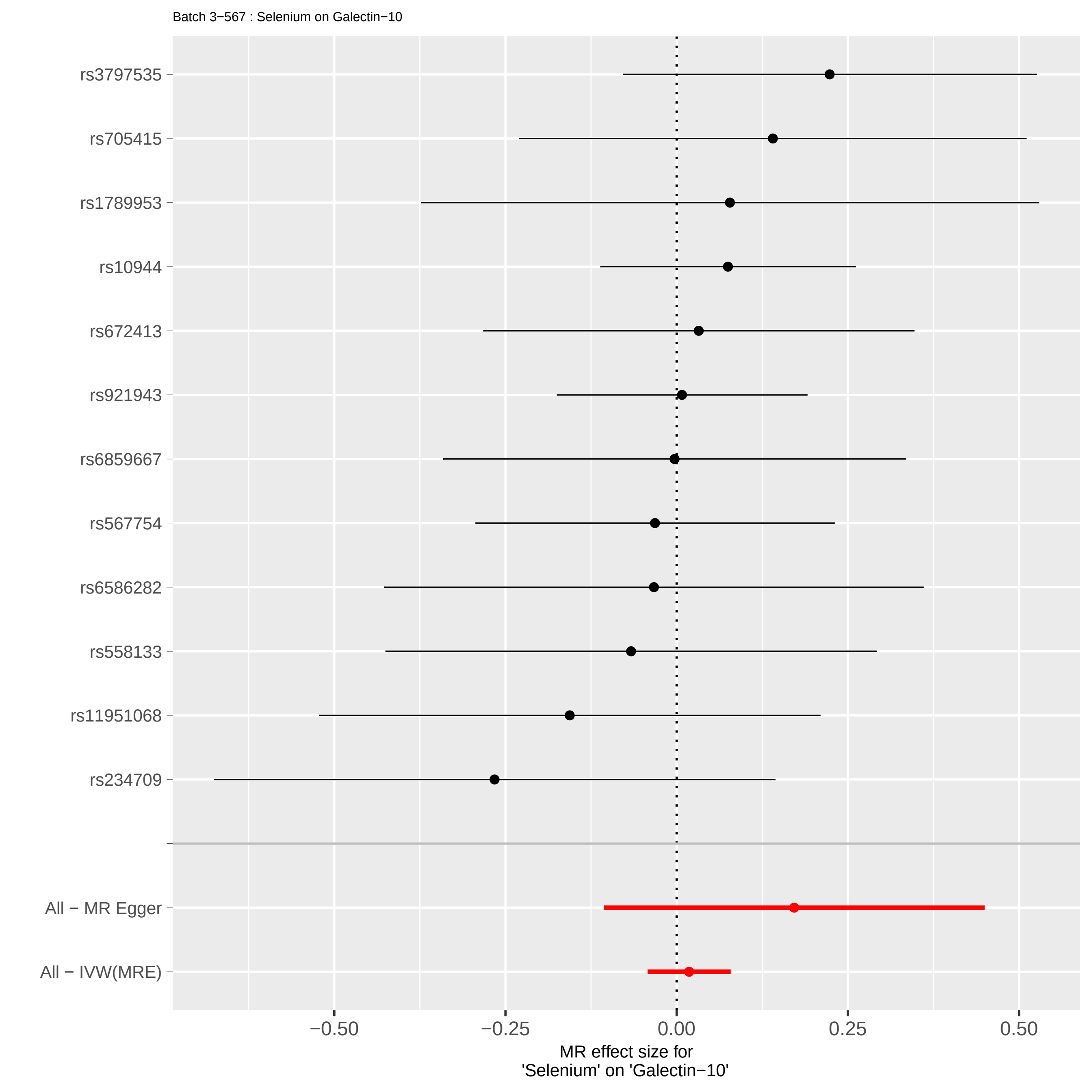

Batch 3−567 : Selenium on Galectin−10
rs3797535
rs705415
rs1789953
rs10944
rs672413
rs921943
rs6859667
rs567754
rs6586282
rs558133
rs11951068
rs234709
All − MR Egger
All − IVW(MRE)
−0.50
−0.25
0.00
0.25
0.50
MR effect size for
'Selenium' on 'Galectin−10'

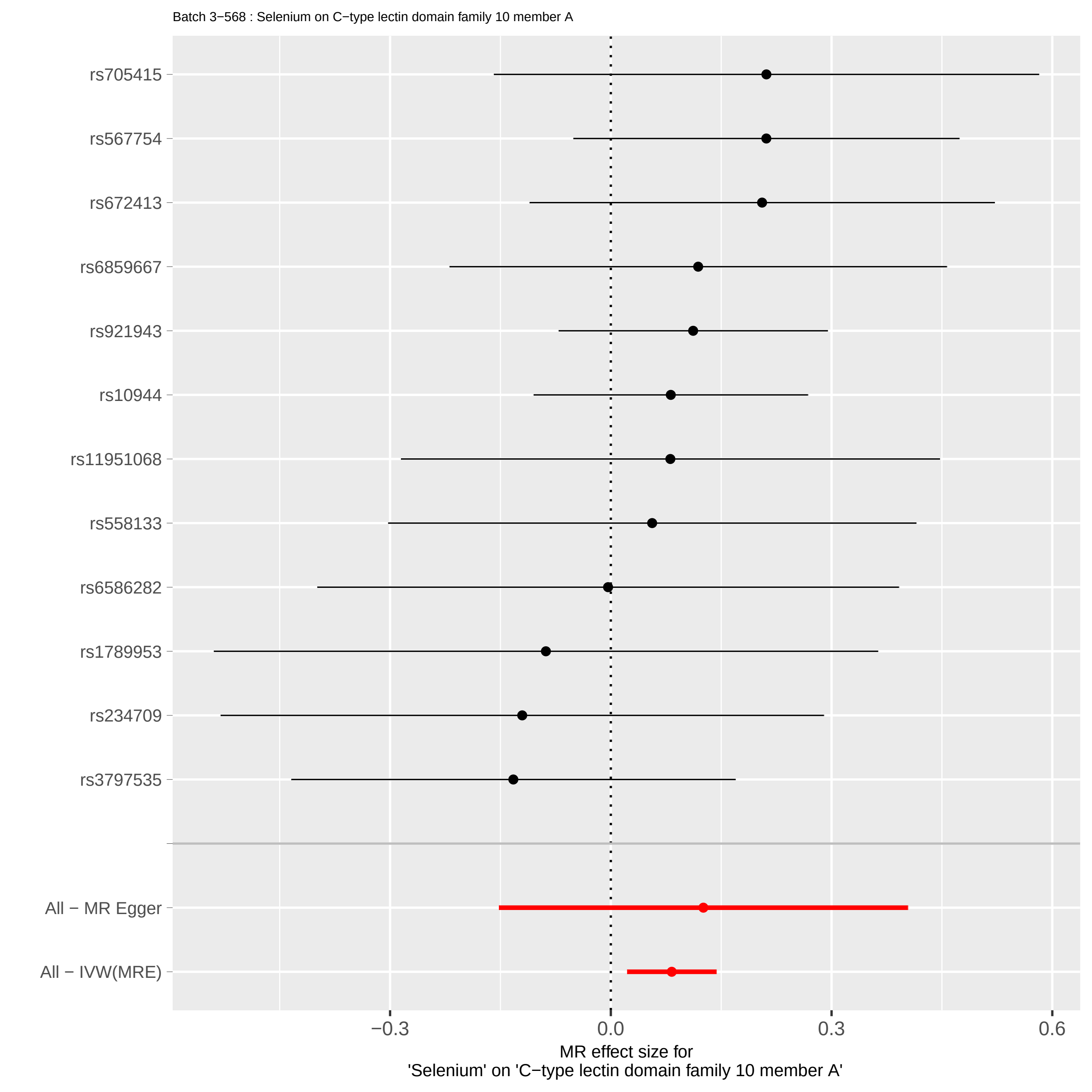

Batch 3−568 : Selenium on C−type lectin domain family 10 member A
rs705415
rs567754
rs672413
rs6859667
rs921943
rs10944
rs11951068
rs558133
rs6586282
rs1789953
rs234709
rs3797535
All − MR Egger
All − IVW(MRE)
−0.3
0.0
0.3
0.6
MR effect size for
'Selenium' on 'C−type lectin domain family 10 member A'

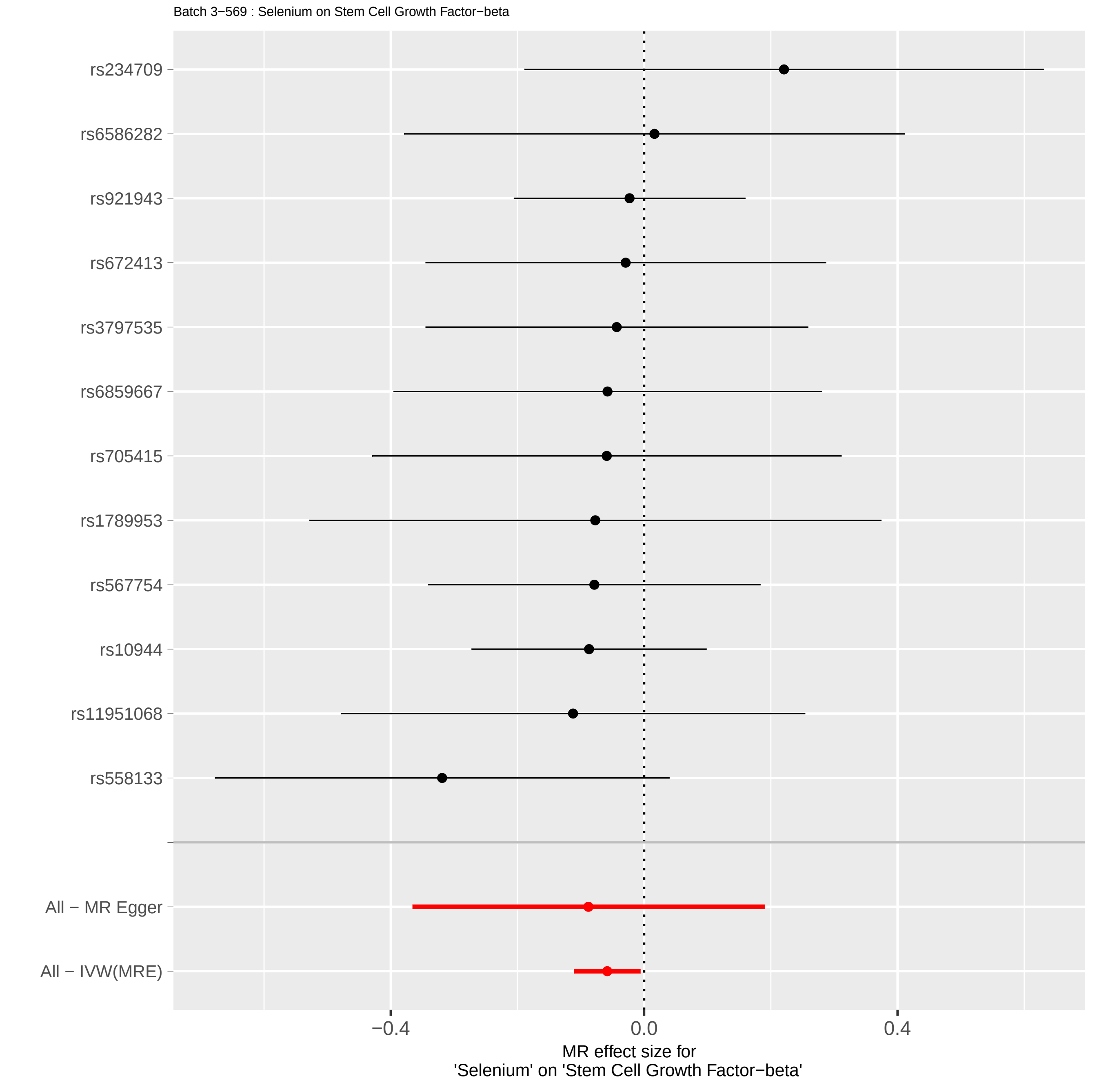
Batch 3−569 : Selenium on Stem Cell Growth Factor−beta
rs234709
rs6586282
rs921943
rs672413
rs3797535
rs6859667
rs705415
rs1789953
rs567754
rs10944
rs11951068
rs558133
All − MR Egger
All − IVW(MRE)
−0.4
0.0
0.4
MR effect size for
'Selenium' on 'Stem Cell Growth Factor−beta'

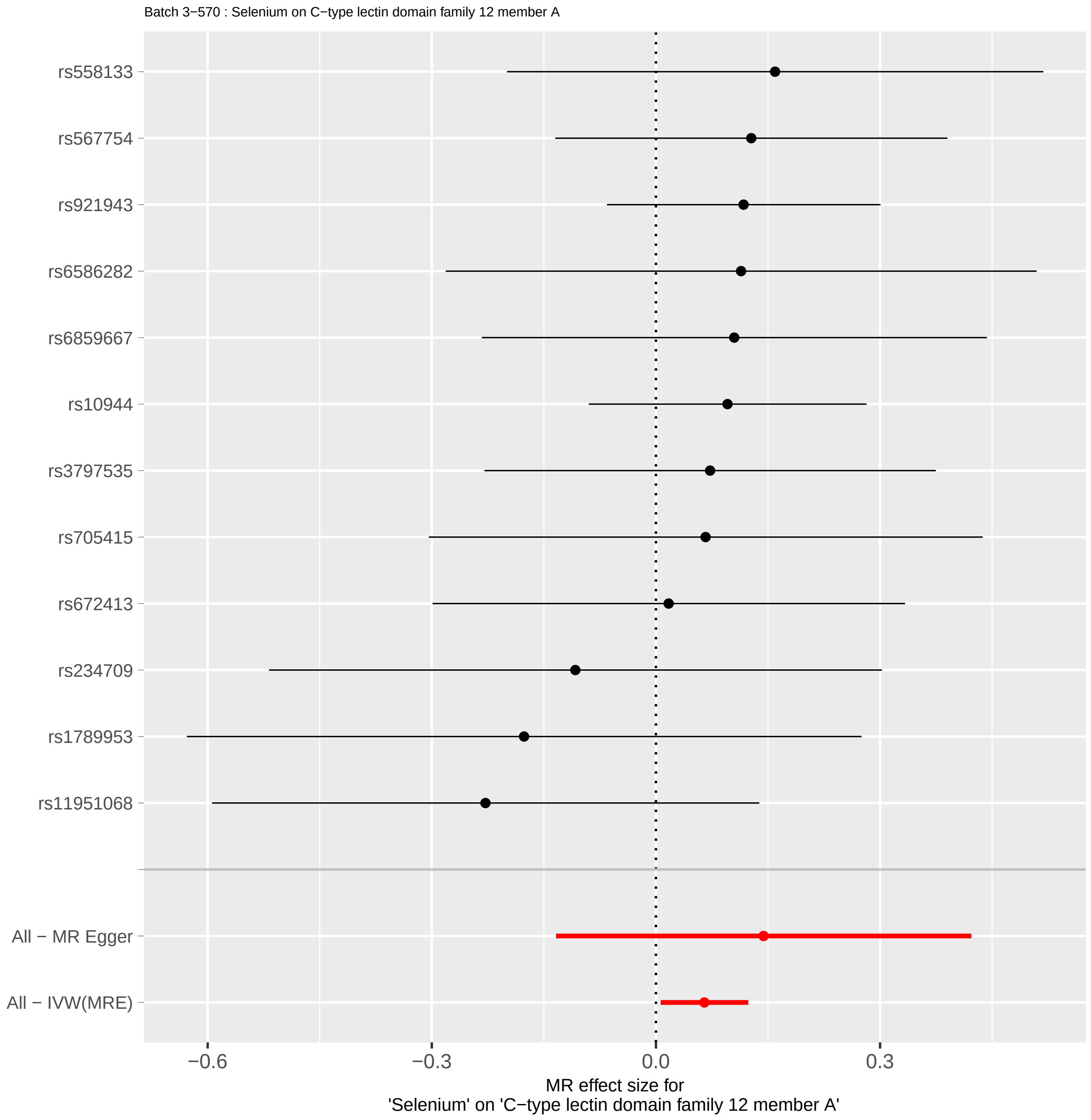

Batch 3−570 : Selenium on C−type lectin domain family 12 member A
rs558133
rs567754
rs921943
rs6586282
rs6859667
rs10944
rs3797535
rs705415
rs672413
rs234709
rs1789953
rs11951068
All − MR Egger
All − IVW(MRE)
−0.6
−0.3
0.0
0.3
MR effect size for
'Selenium' on 'C−type lectin domain family 12 member A'

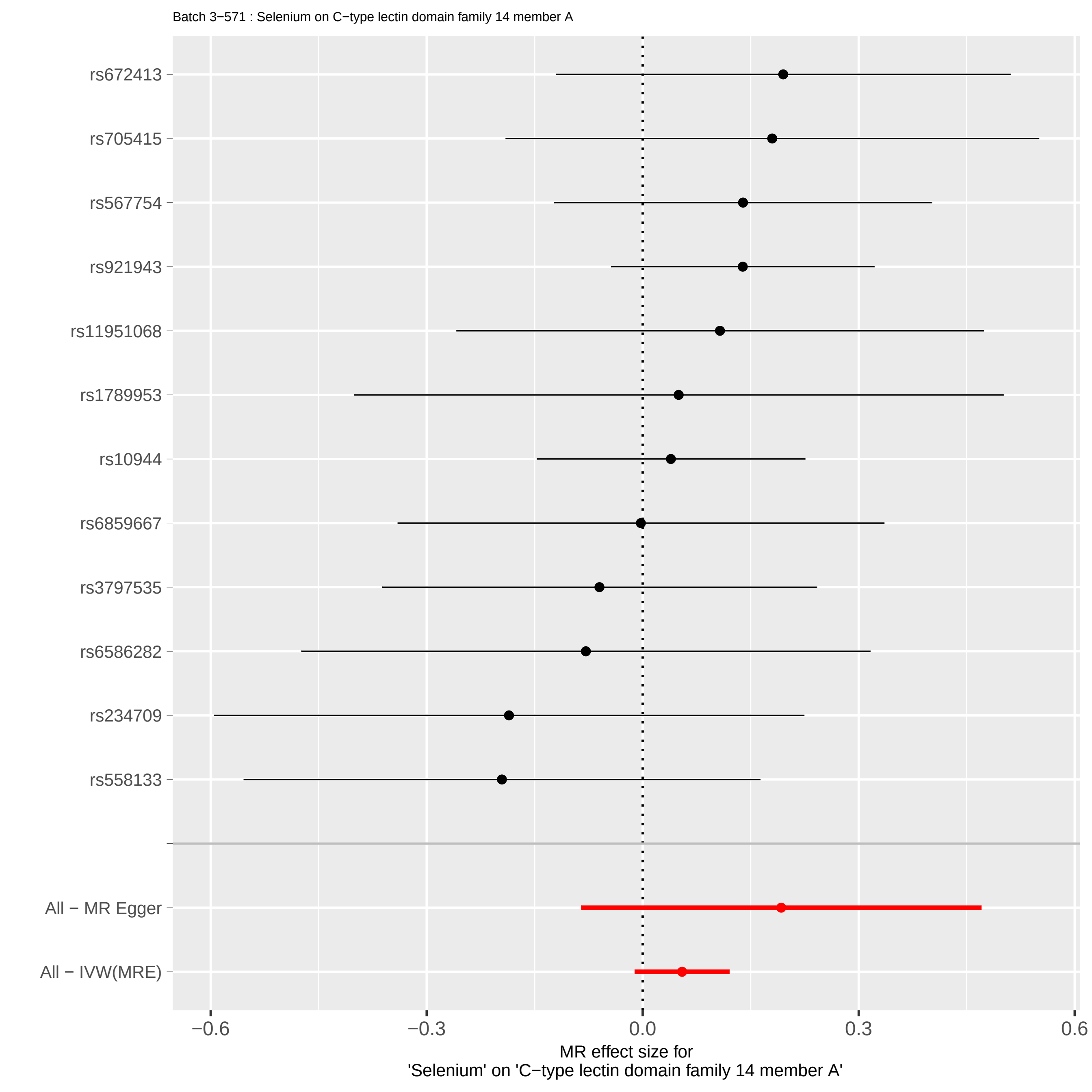

Batch 3−571 : Selenium on C−type lectin domain family 14 member A
rs672413
rs705415
rs567754
rs921943
rs11951068
rs1789953
rs10944
rs6859667
rs3797535
rs6586282
rs234709
rs558133
All − MR Egger
All − IVW(MRE)
−0.6
−0.3
0.0
0.3
0.6
MR effect size for
'Selenium' on 'C−type lectin domain family 14 member A'

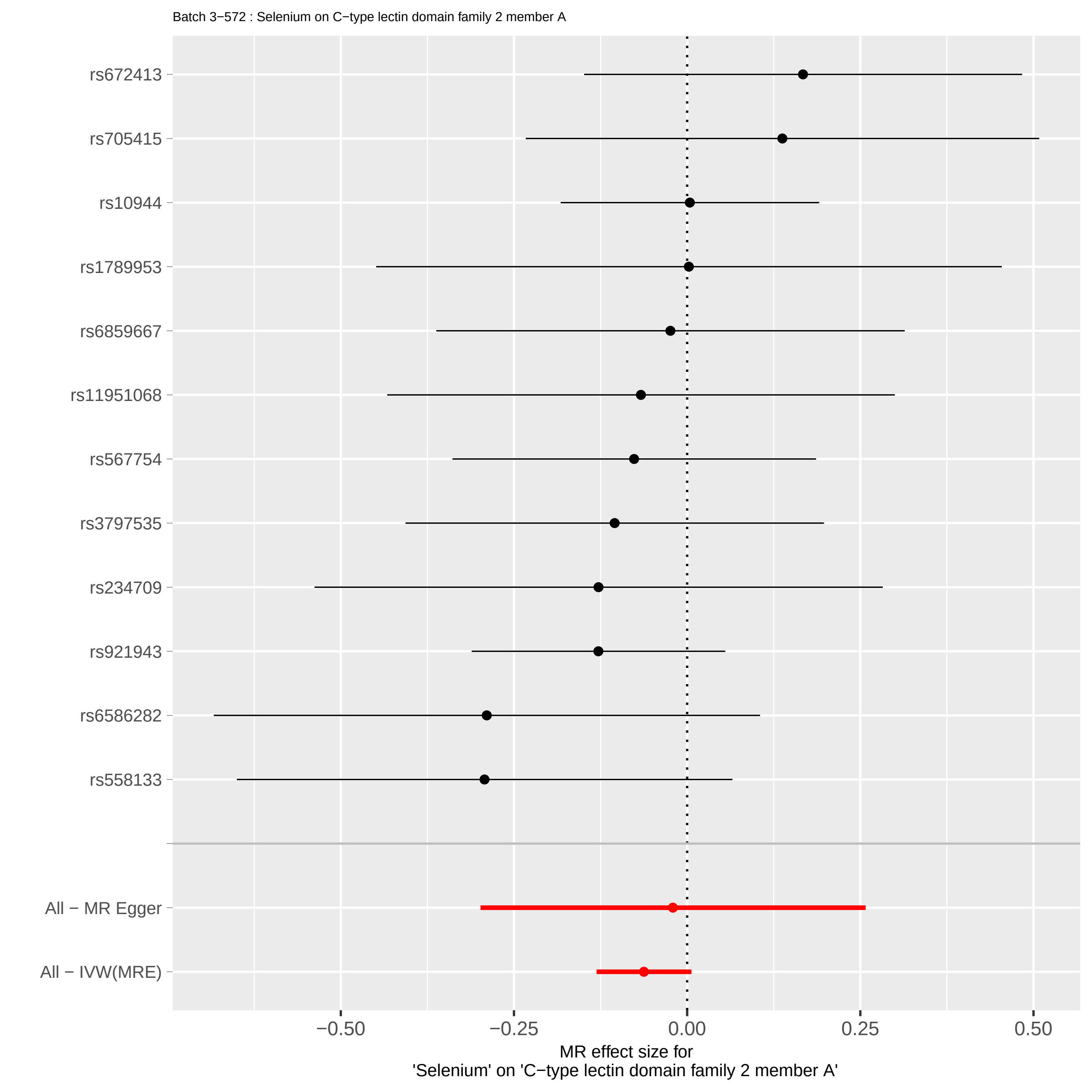

Batch 3−572 : Selenium on C−type lectin domain family 2 member A
rs672413
rs705415
rs10944
rs1789953
rs6859667
rs11951068
rs567754
rs3797535
rs234709
rs921943
rs6586282
rs558133
All − MR Egger
All − IVW(MRE)
−0.50
−0.25
0.00
0.25
0.50
MR effect size for
'Selenium' on 'C−type lectin domain family 2 member A'

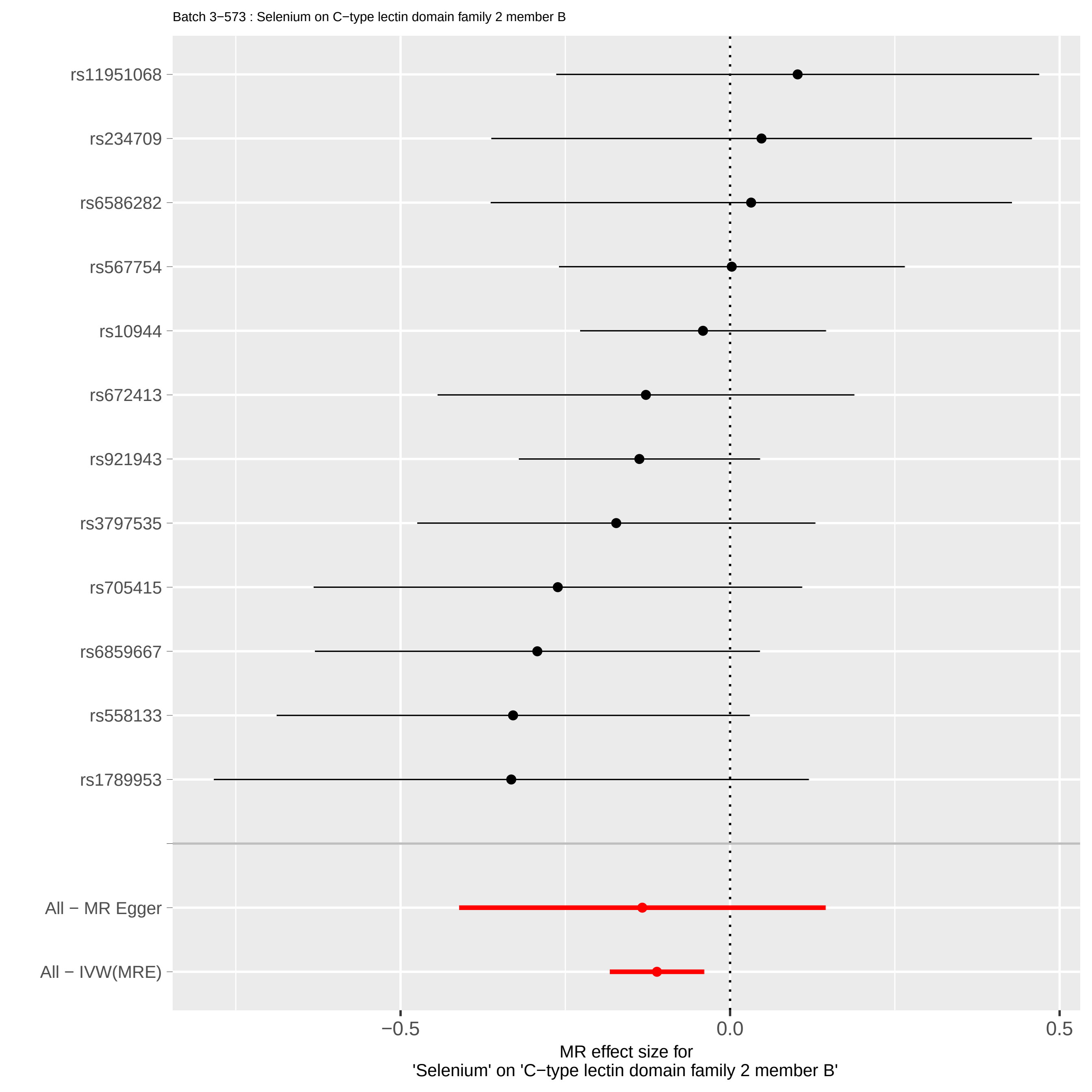
Batch 3−573 : Selenium on C−type lectin domain family 2 member B
rs11951068
rs234709
rs6586282
rs567754
rs10944
rs672413
rs921943
rs3797535
rs705415
rs6859667
rs558133
rs1789953
All − MR Egger
All − IVW(MRE)
−0.5
0.0
0.5
MR effect size for
'Selenium' on 'C−type lectin domain family 2 member B'

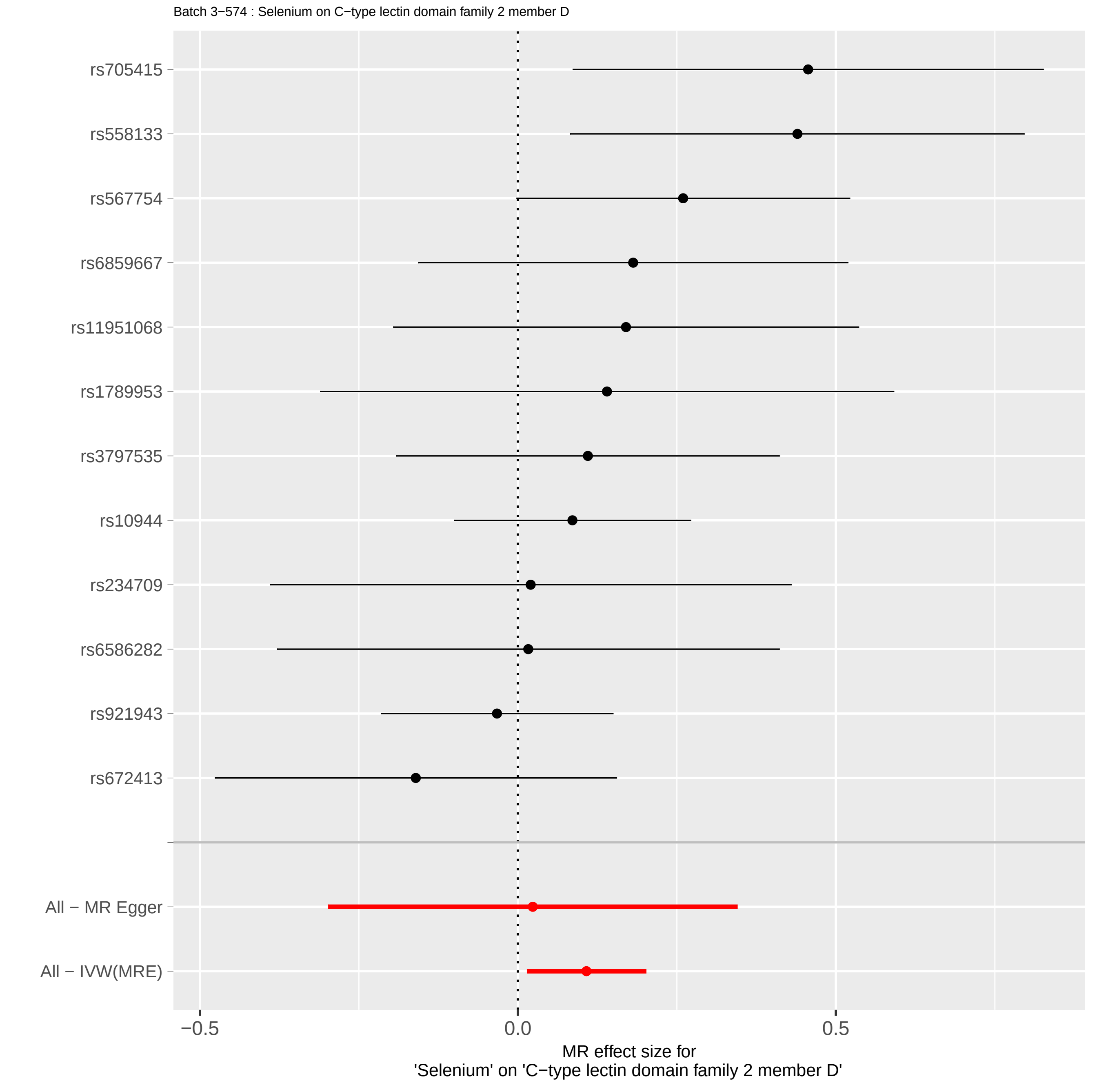

Batch 3−574 : Selenium on C−type lectin domain family 2 member D
rs705415
rs558133
rs567754
rs6859667
rs11951068
rs1789953
rs3797535
rs10944
rs234709
rs6586282
rs921943
rs672413
All − MR Egger
All − IVW(MRE)
−0.5
0.0
0.5
MR effect size for
'Selenium' on 'C−type lectin domain family 2 member D'

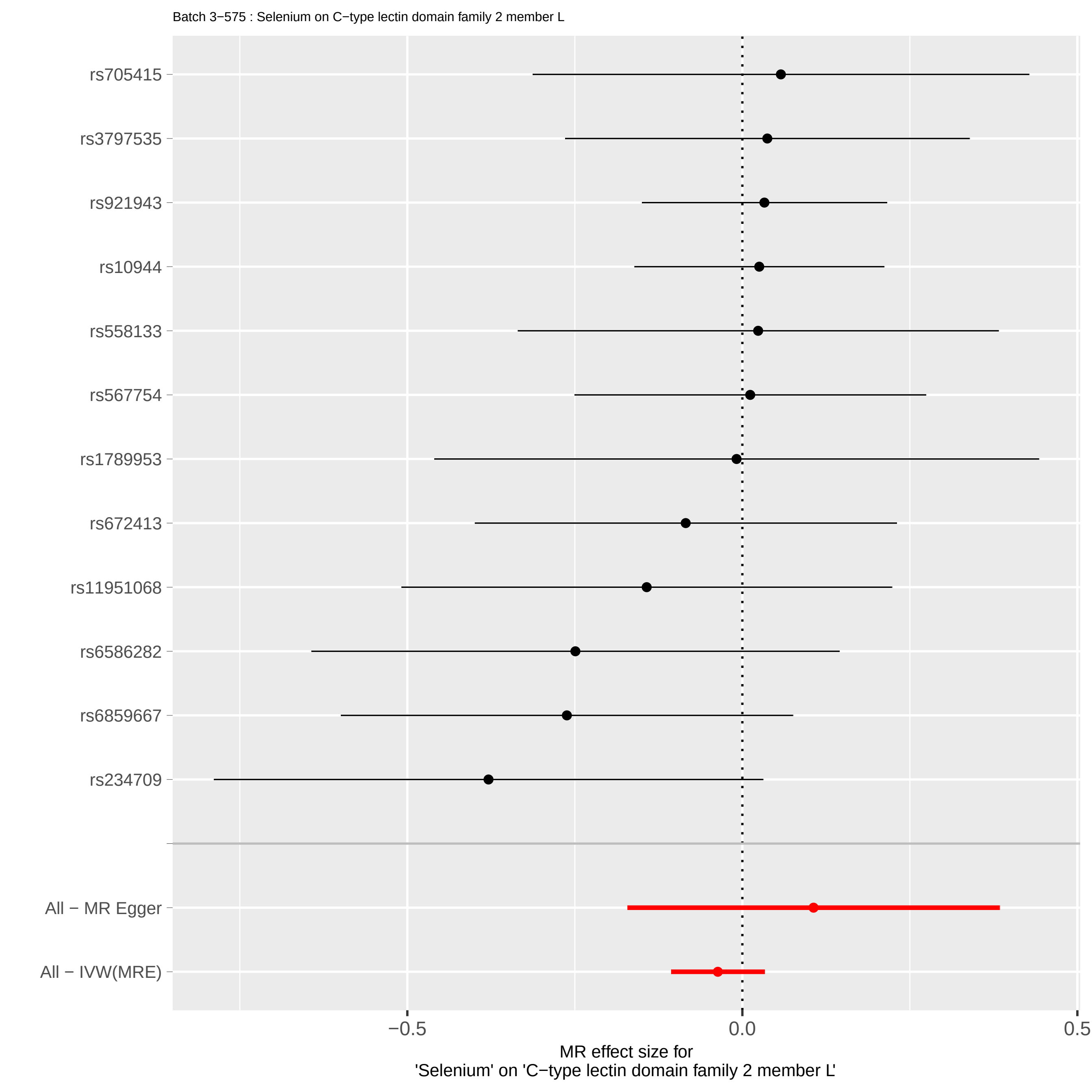

Batch 3−575 : Selenium on C−type lectin domain family 2 member L
rs705415
rs3797535
rs921943
rs10944
rs558133
rs567754
rs1789953
rs672413
rs11951068
rs6586282
rs6859667
rs234709
All − MR Egger
All − IVW(MRE)
−0.5
0.0
0.5
MR effect size for
'Selenium' on 'C−type lectin domain family 2 member L'

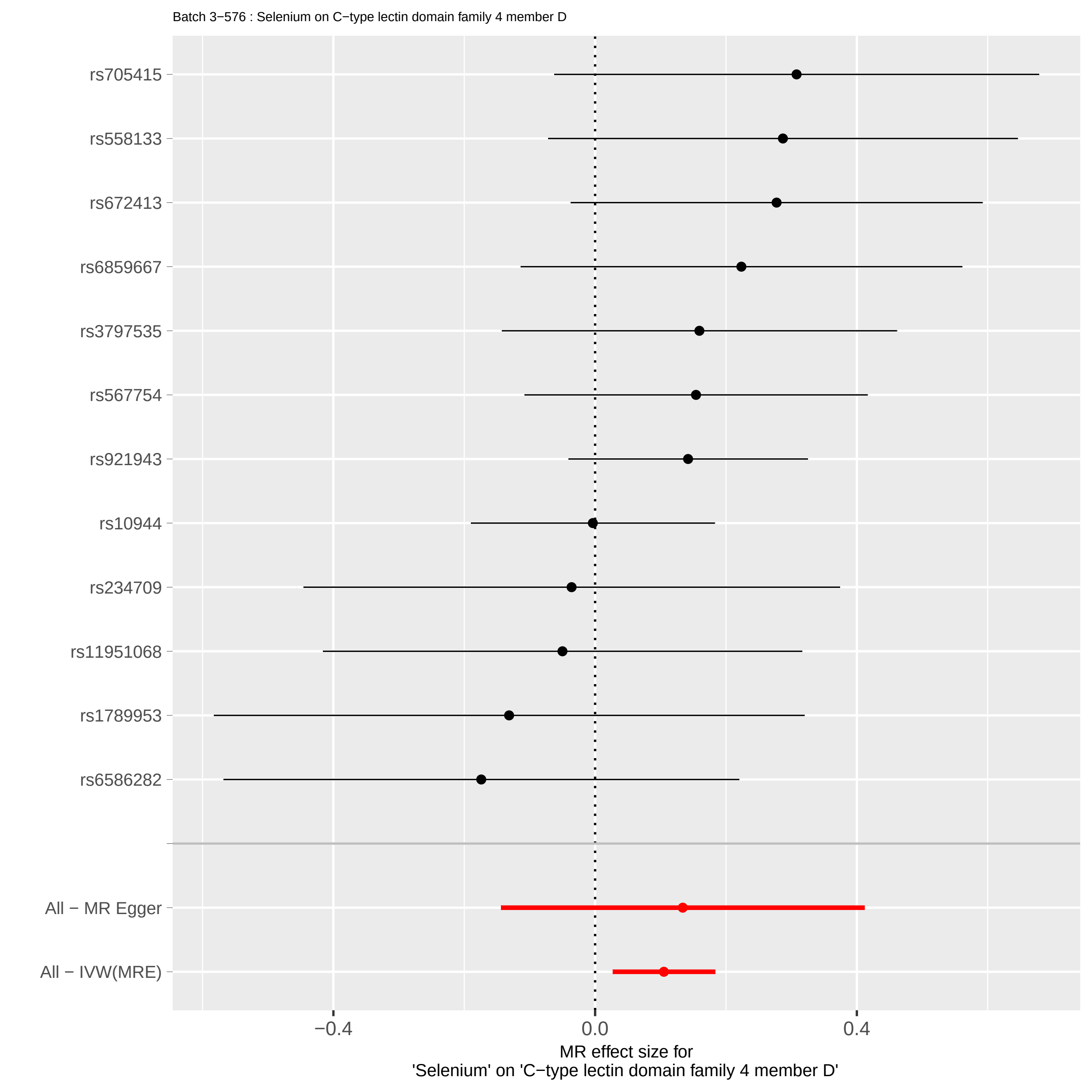
Batch 3−576 : Selenium on C−type lectin domain family 4 member D
rs705415
rs558133
rs672413
rs6859667
rs3797535
rs567754
rs921943
rs10944
rs234709
rs11951068
rs1789953
rs6586282
All − MR Egger
All − IVW(MRE)
−0.4
0.0
0.4
MR effect size for
'Selenium' on 'C−type lectin domain family 4 member D'

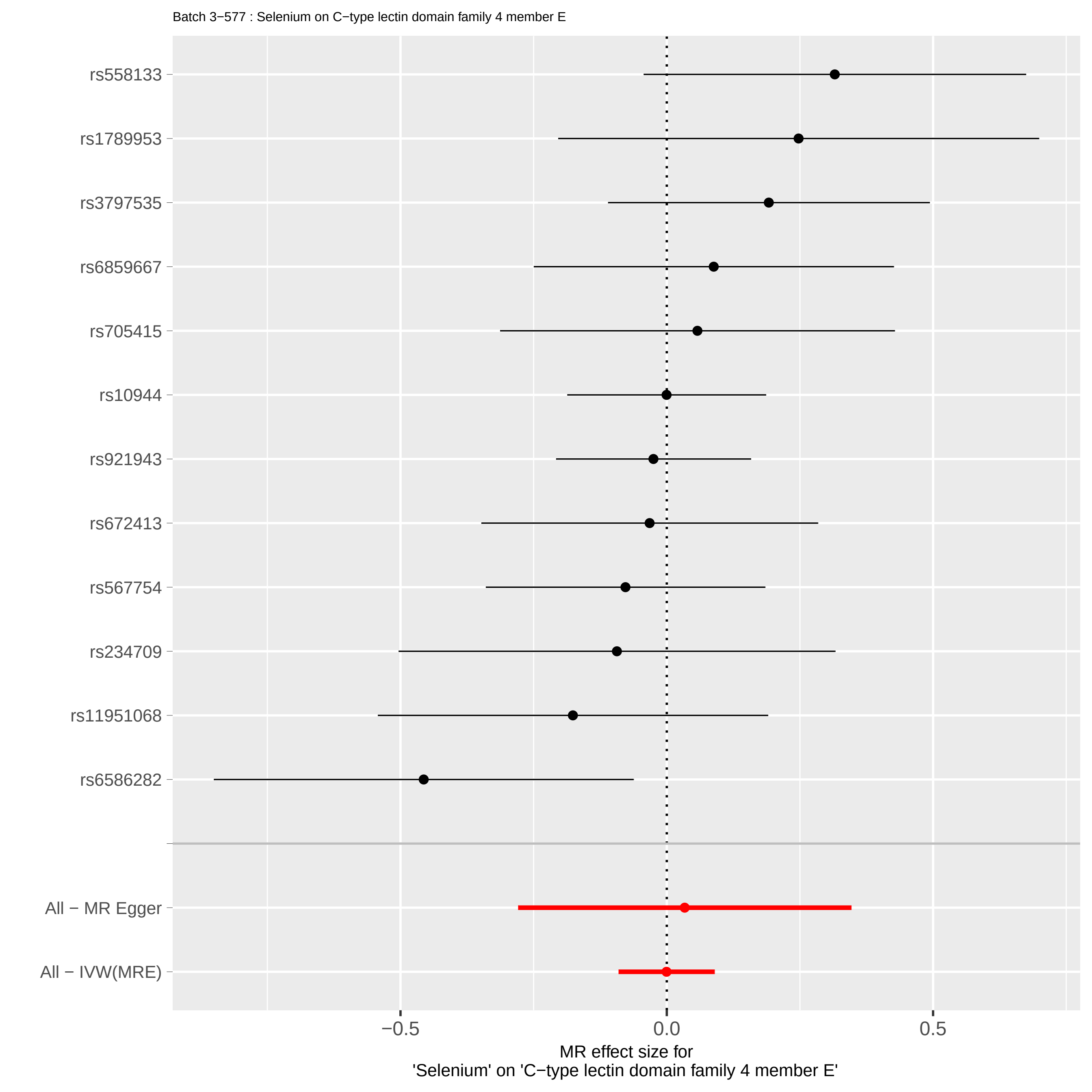
Batch 3−577 : Selenium on C−type lectin domain family 4 member E
rs558133
rs1789953
rs3797535
rs6859667
rs705415
rs10944
rs921943
rs672413
rs567754
rs234709
rs11951068
rs6586282
All − MR Egger
All − IVW(MRE)
−0.5
0.0
0.5
MR effect size for
'Selenium' on 'C−type lectin domain family 4 member E'

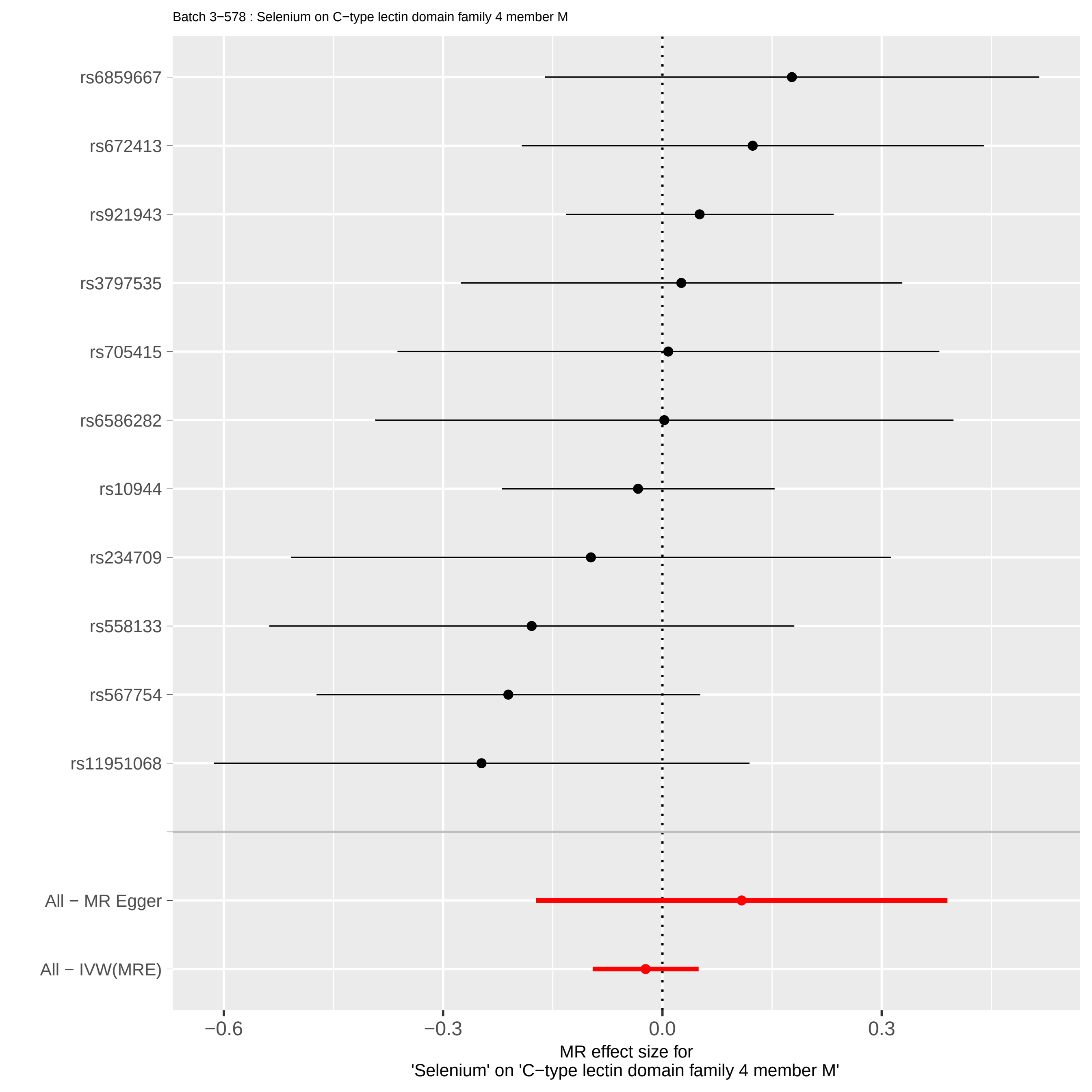
Batch 3−578 : Selenium on C−type lectin domain family 4 member M
rs6859667
rs672413
rs921943
rs3797535
rs705415
rs6586282
rs10944
rs234709
rs558133
rs567754
rs11951068
All − MR Egger
All − IVW(MRE)
−0.6
−0.3
0.0
0.3
MR effect size for
'Selenium' on 'C−type lectin domain family 4 member M'

Batch 3−579 : Selenium on C−type lectin domain family 5 member A

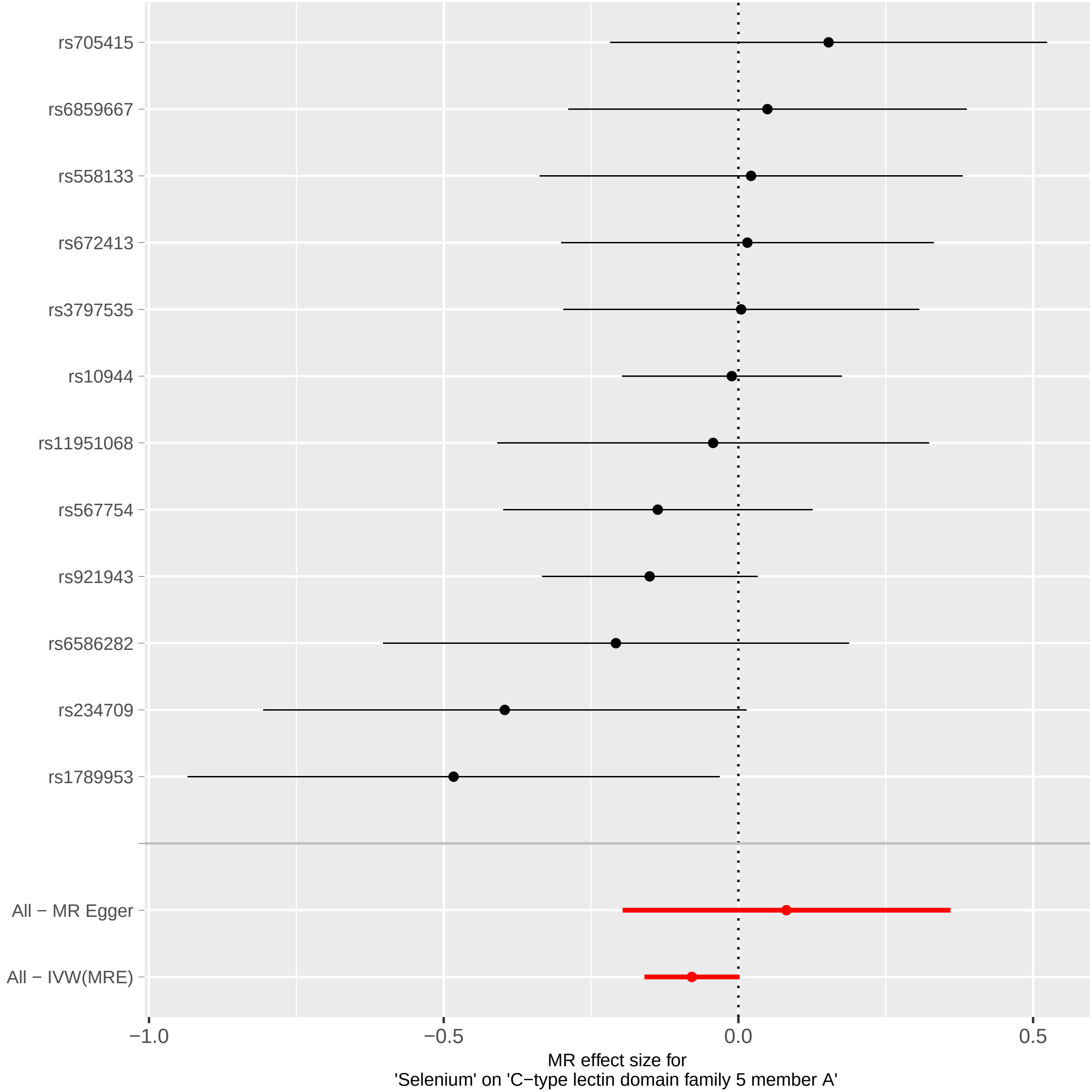

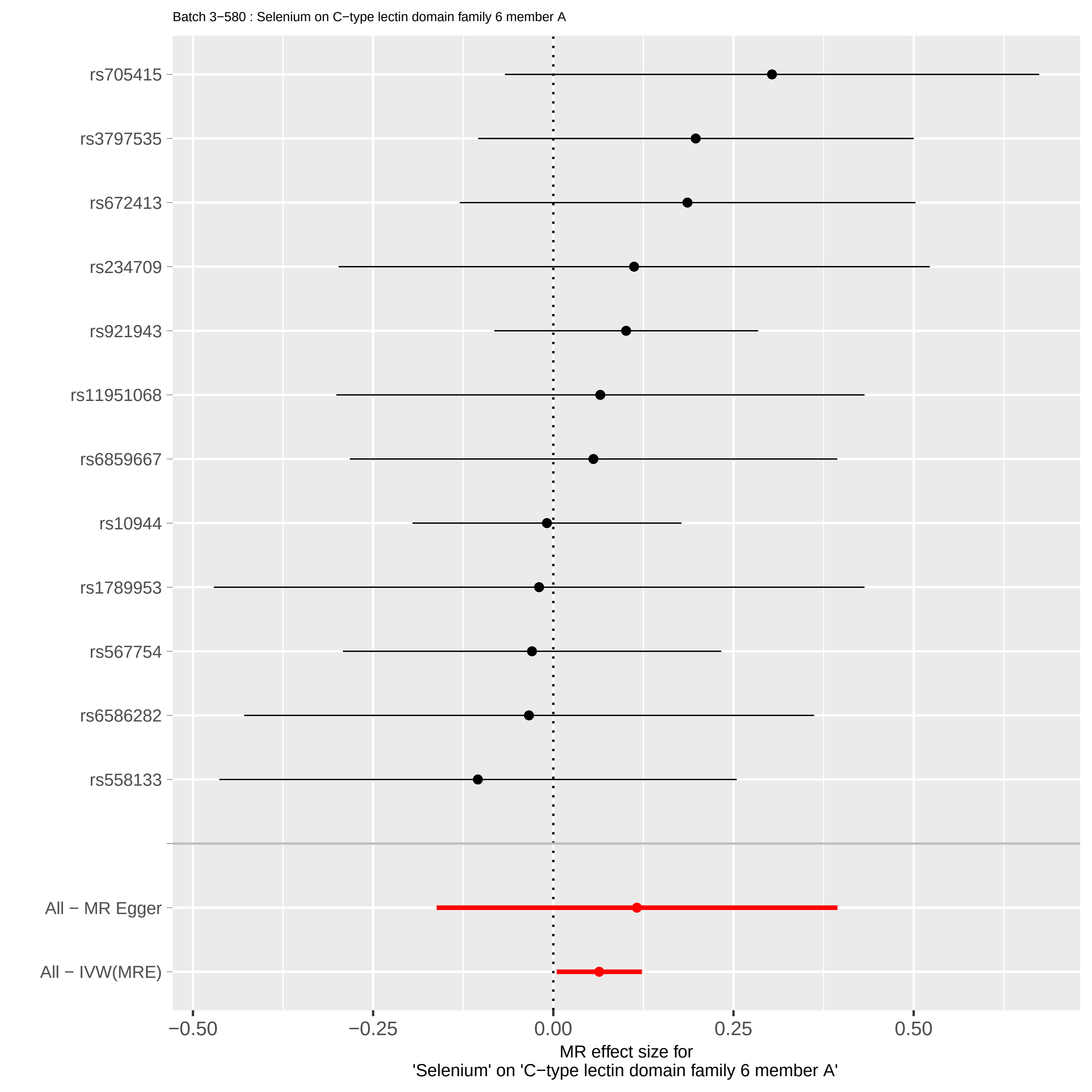
Batch 3−580 : Selenium on C−type lectin domain family 6 member A
rs705415
rs3797535
rs672413
rs234709
rs921943
rs11951068
rs6859667
rs10944
rs1789953
rs567754
rs6586282
rs558133
All − MR Egger
All − IVW(MRE)
−0.50
−0.25
0.00
0.25
0.50
MR effect size for
'Selenium' on 'C−type lectin domain family 6 member A'

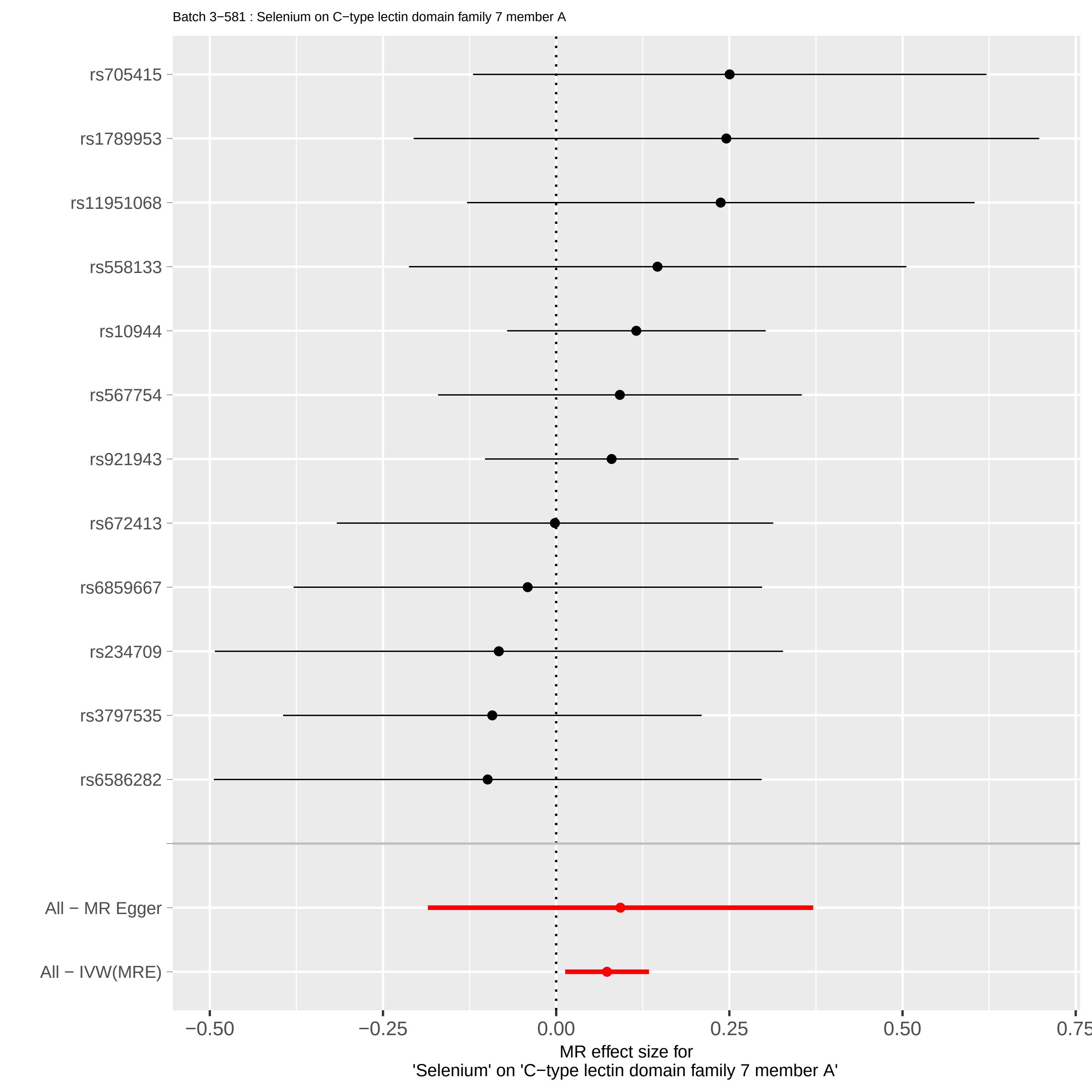

Batch 3−581 : Selenium on C−type lectin domain family 7 member A
rs705415
rs1789953
rs11951068
rs558133
rs10944
rs567754
rs921943
rs672413
rs6859667
rs234709
rs3797535
rs6586282
All − MR Egger
All − IVW(MRE)
−0.50
−0.25
0.00
0.25
0.50
0.75
MR effect size for
'Selenium' on 'C−type lectin domain family 7 member A'

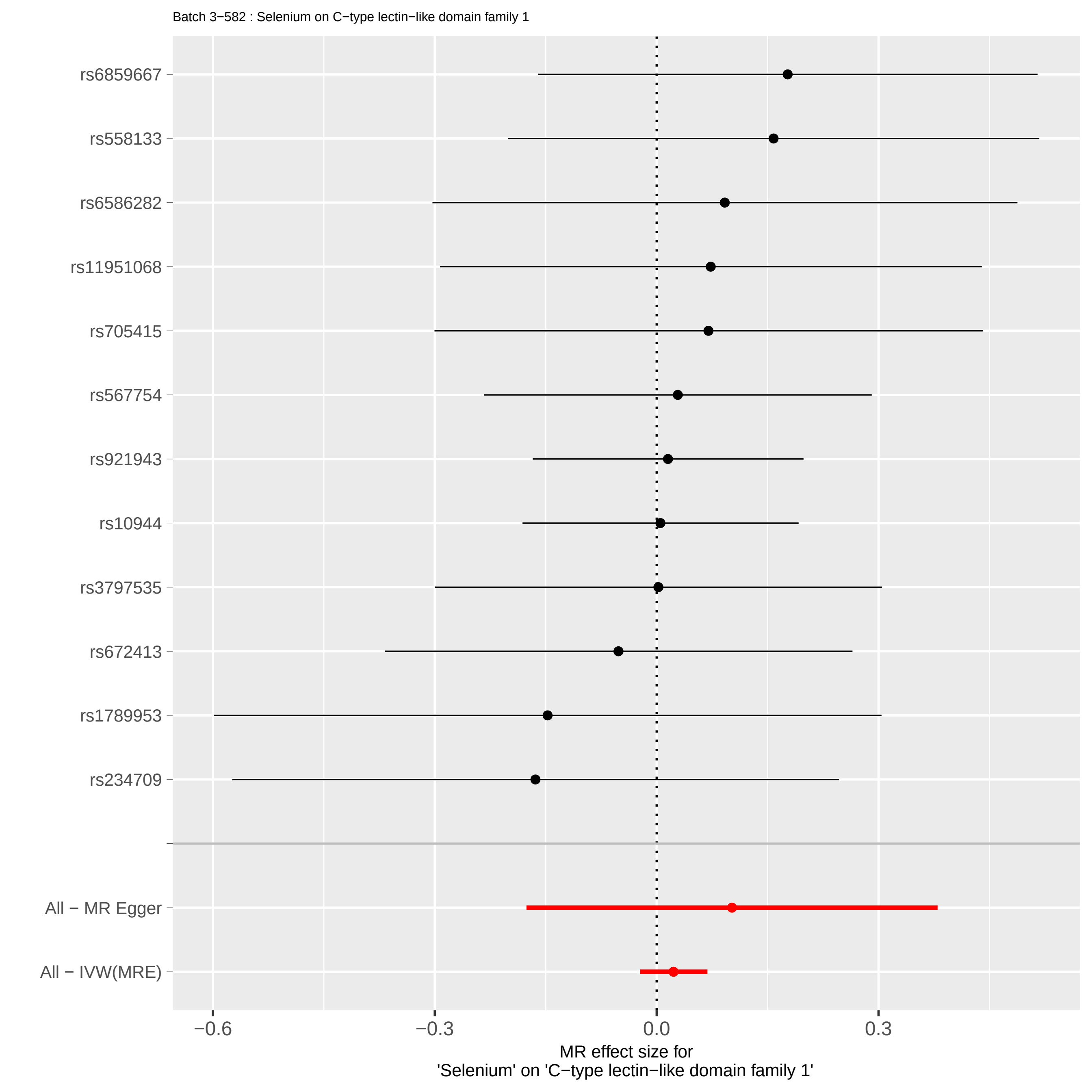
Batch 3−582 : Selenium on C−type lectin−like domain family 1
rs6859667
rs558133
rs6586282
rs11951068
rs705415
rs567754
rs921943
rs10944
rs3797535
rs672413
rs1789953
rs234709
All − MR Egger
All − IVW(MRE)
−0.6
−0.3
0.0
0.3
MR effect size for
'Selenium' on 'C−type lectin−like domain family 1'

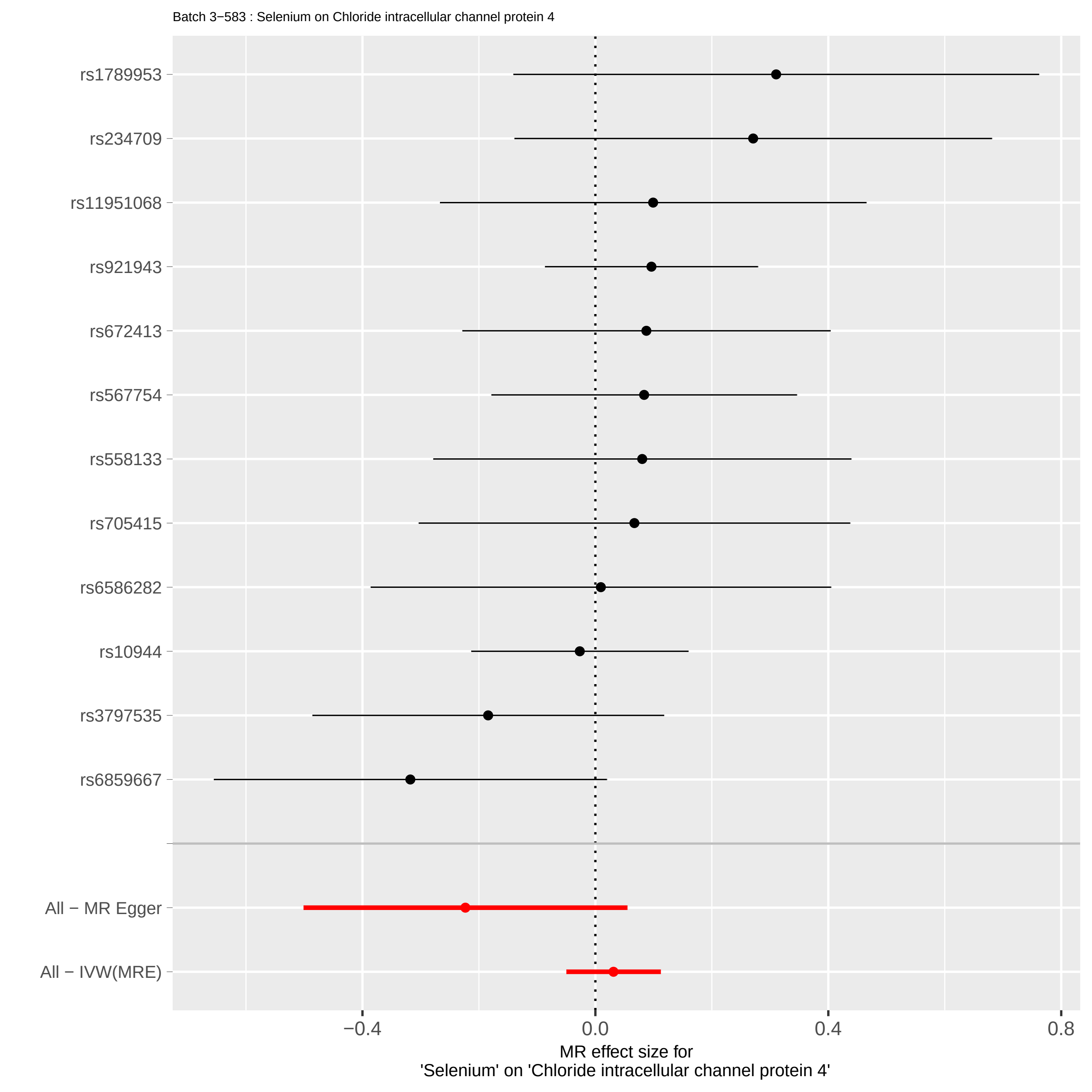
Batch 3−583 : Selenium on Chloride intracellular channel protein 4
rs1789953
rs234709
rs11951068
rs921943
rs672413
rs567754
rs558133
rs705415
rs6586282
rs10944
rs3797535
rs6859667
All − MR Egger
All − IVW(MRE)
−0.4
0.0
0.4
0.8
MR effect size for
'Selenium' on 'Chloride intracellular channel protein 4'

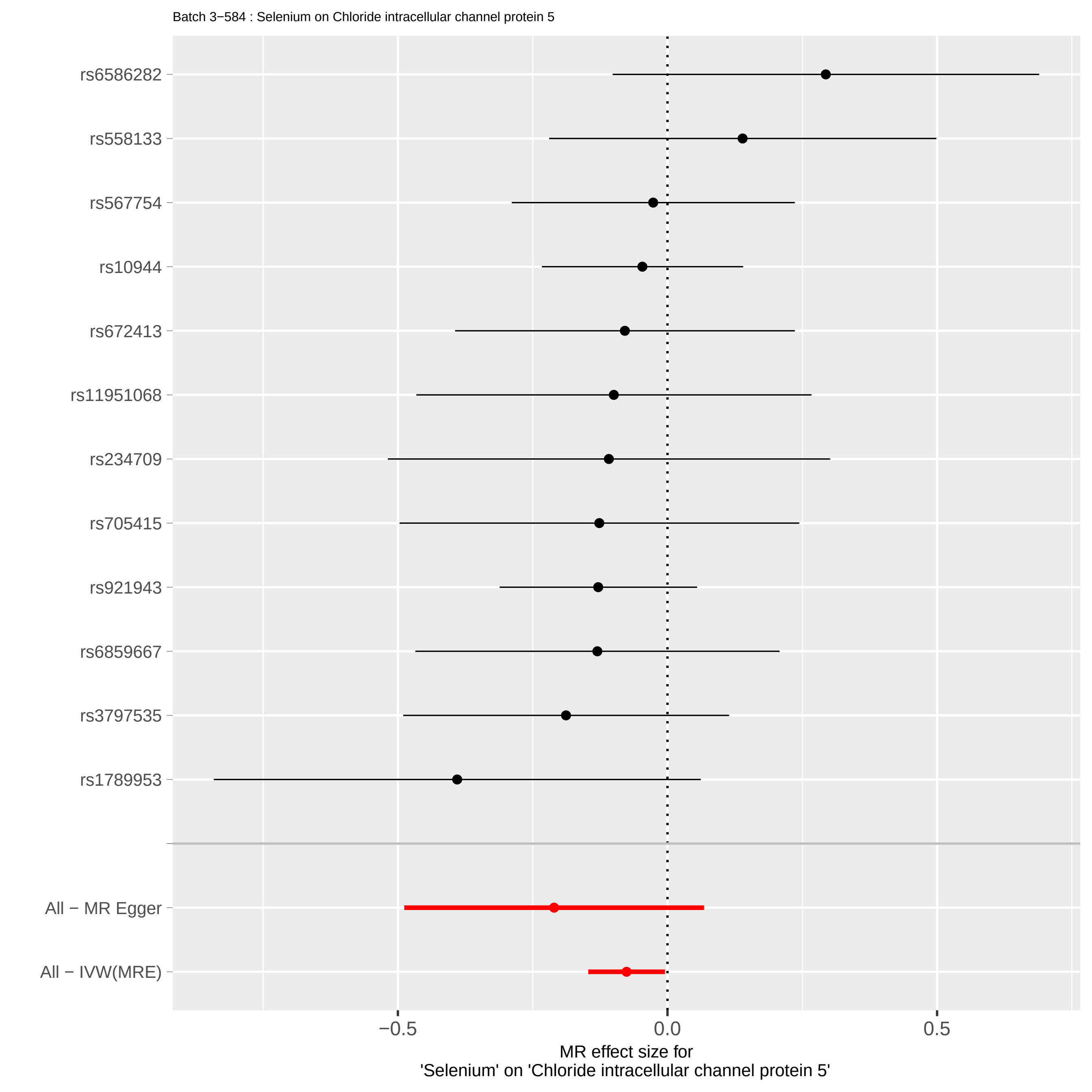
Batch 3−584 : Selenium on Chloride intracellular channel protein 5
rs6586282
rs558133
rs567754
rs10944
rs672413
rs11951068
rs234709
rs705415
rs921943
rs6859667
rs3797535
rs1789953
All − MR Egger
All − IVW(MRE)
−0.5
0.0
0.5
MR effect size for
'Selenium' on 'Chloride intracellular channel protein 5'

Batch 3−585 : Selenium on Clathrin interactor 1

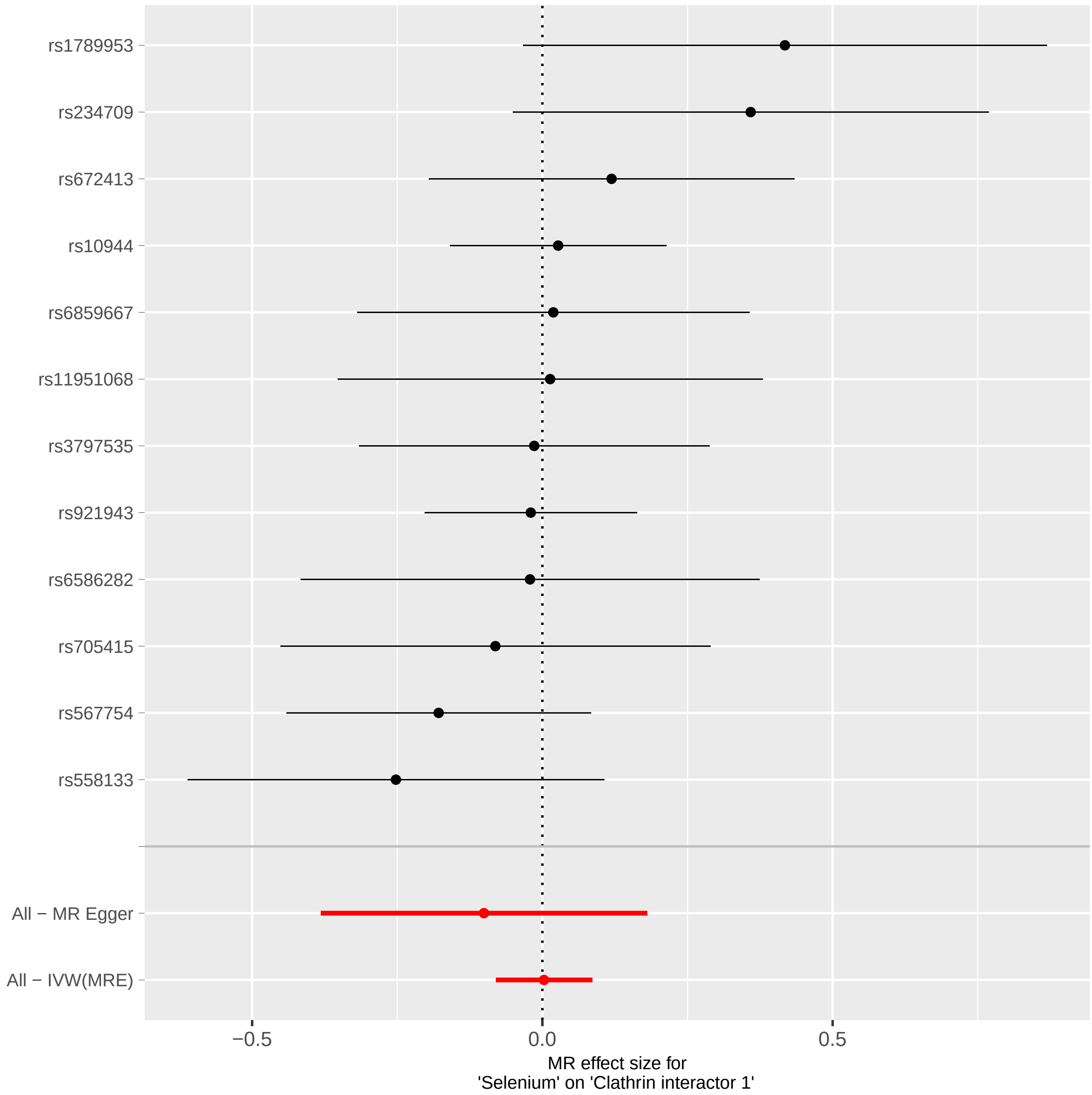

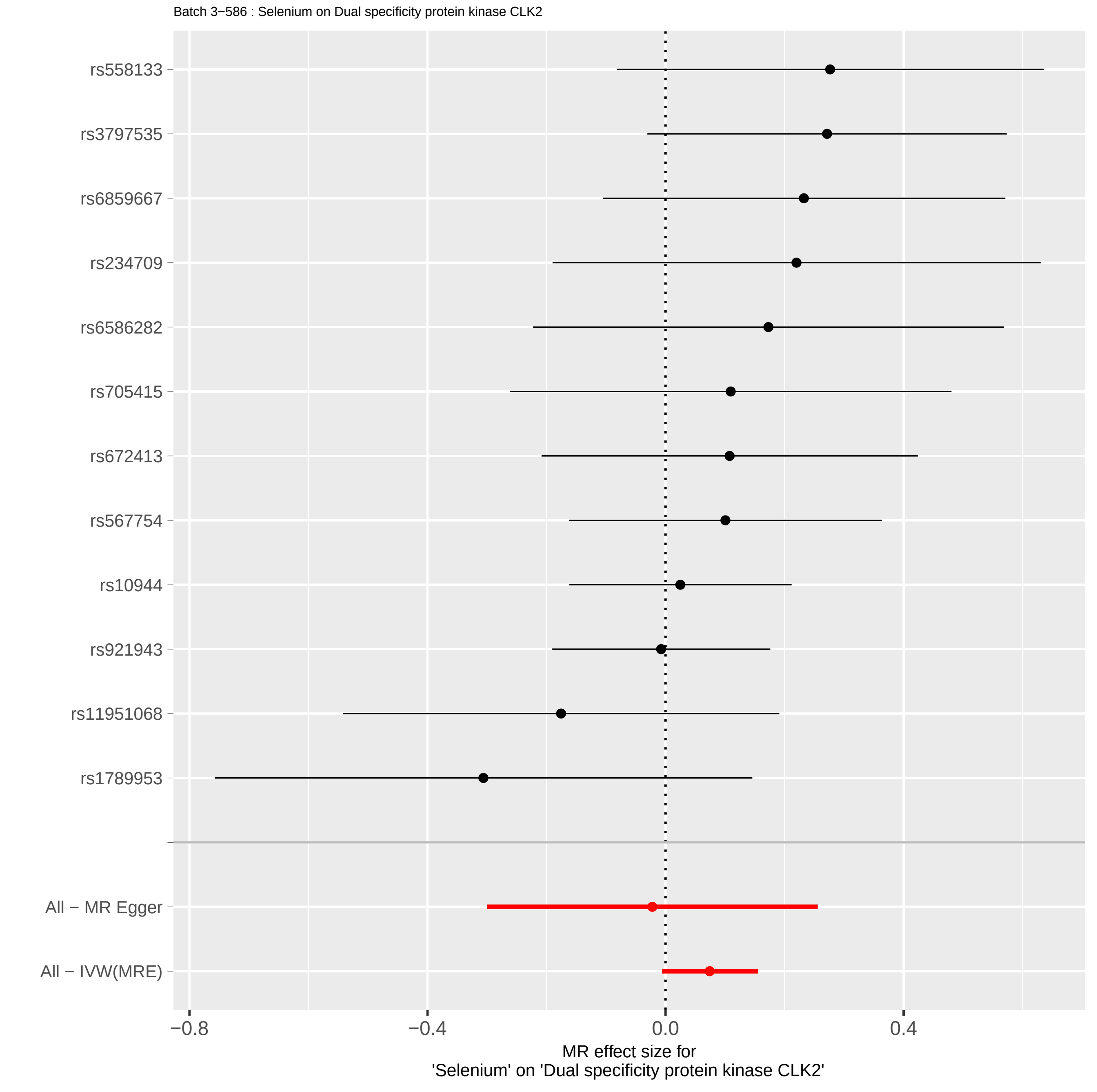

Batch 3−586 : Selenium on Dual specificity protein kinase CLK2
rs558133
rs3797535
rs6859667
rs234709
rs6586282
rs705415
rs672413
rs567754
rs10944
rs921943
rs11951068
rs1789953
All − MR Egger
All − IVW(MRE)
−0.8
−0.4
0.0
0.4
MR effect size for
'Selenium' on 'Dual specificity protein kinase CLK2'

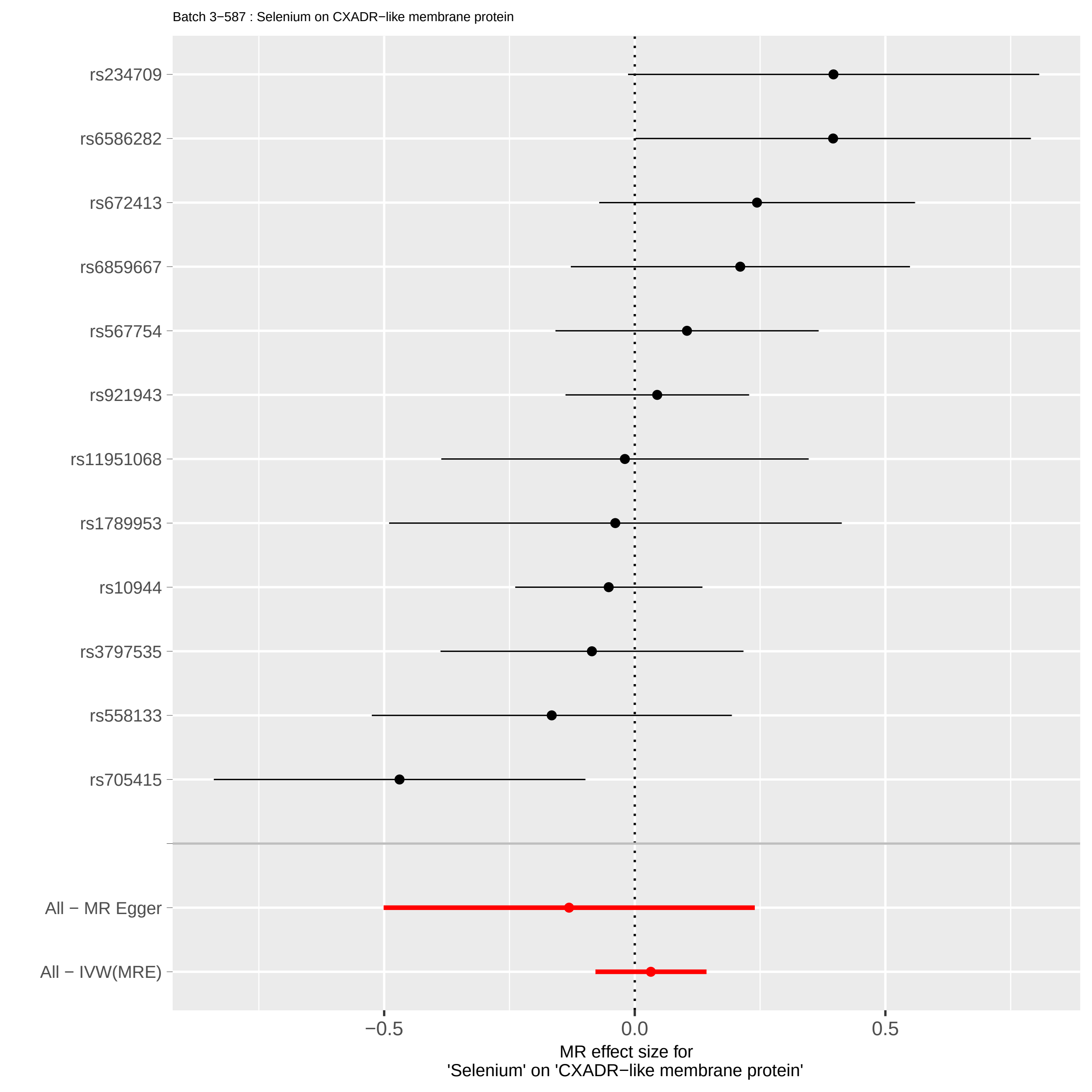

Batch 3−587 : Selenium on CXADR−like membrane protein
rs234709
rs6586282
rs672413
rs6859667
rs567754
rs921943
rs11951068
rs1789953
rs10944
rs3797535
rs558133
rs705415
All − MR Egger
All − IVW(MRE)
−0.5
0.0
0.5
MR effect size for
'Selenium' on 'CXADR−like membrane protein'

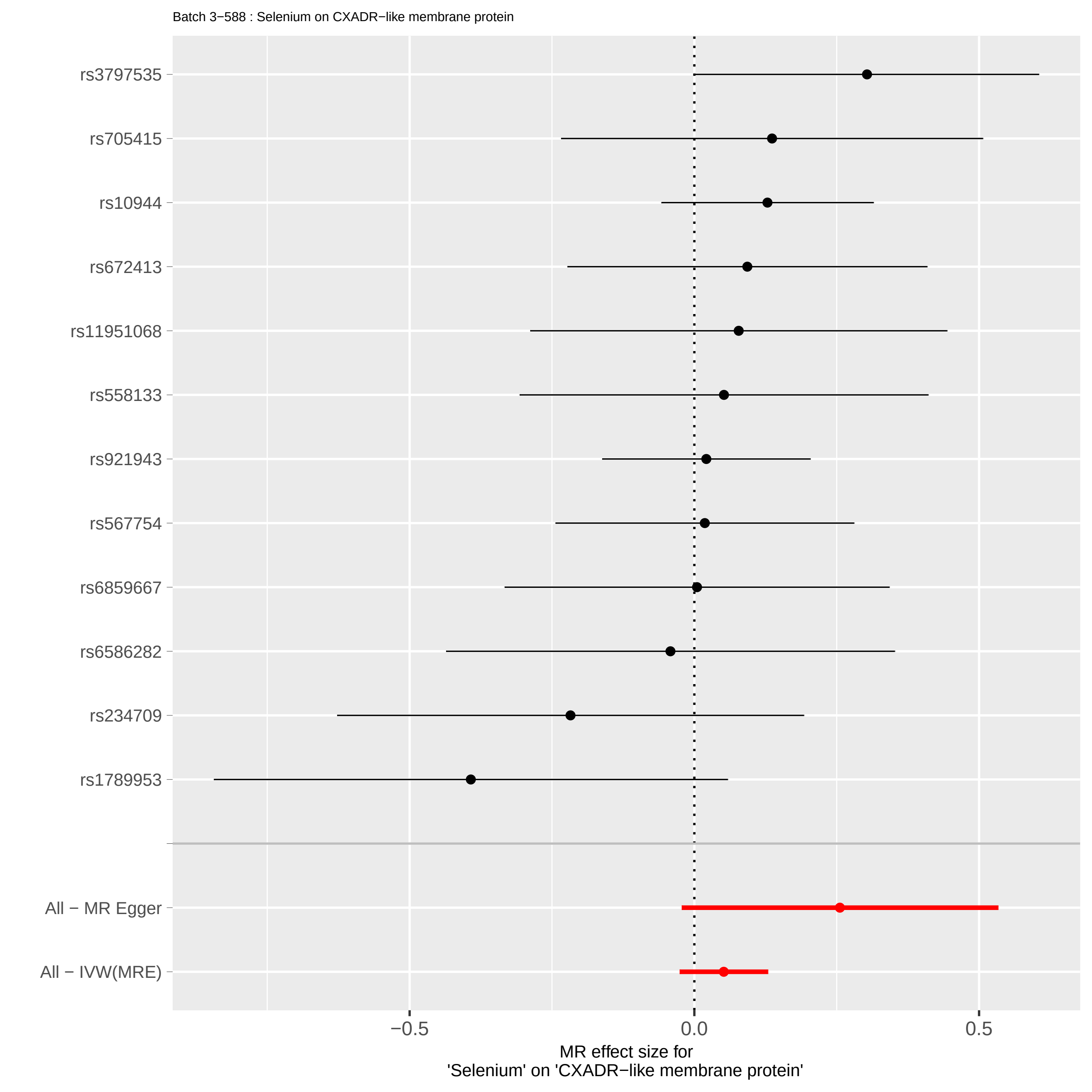

Batch 3−588 : Selenium on CXADR−like membrane protein
rs3797535
rs705415
rs10944
rs672413
rs11951068
rs558133
rs921943
rs567754
rs6859667
rs6586282
rs234709
rs1789953
All − MR Egger
All − IVW(MRE)
−0.5
0.0
0.5
MR effect size for
'Selenium' on 'CXADR−like membrane protein'

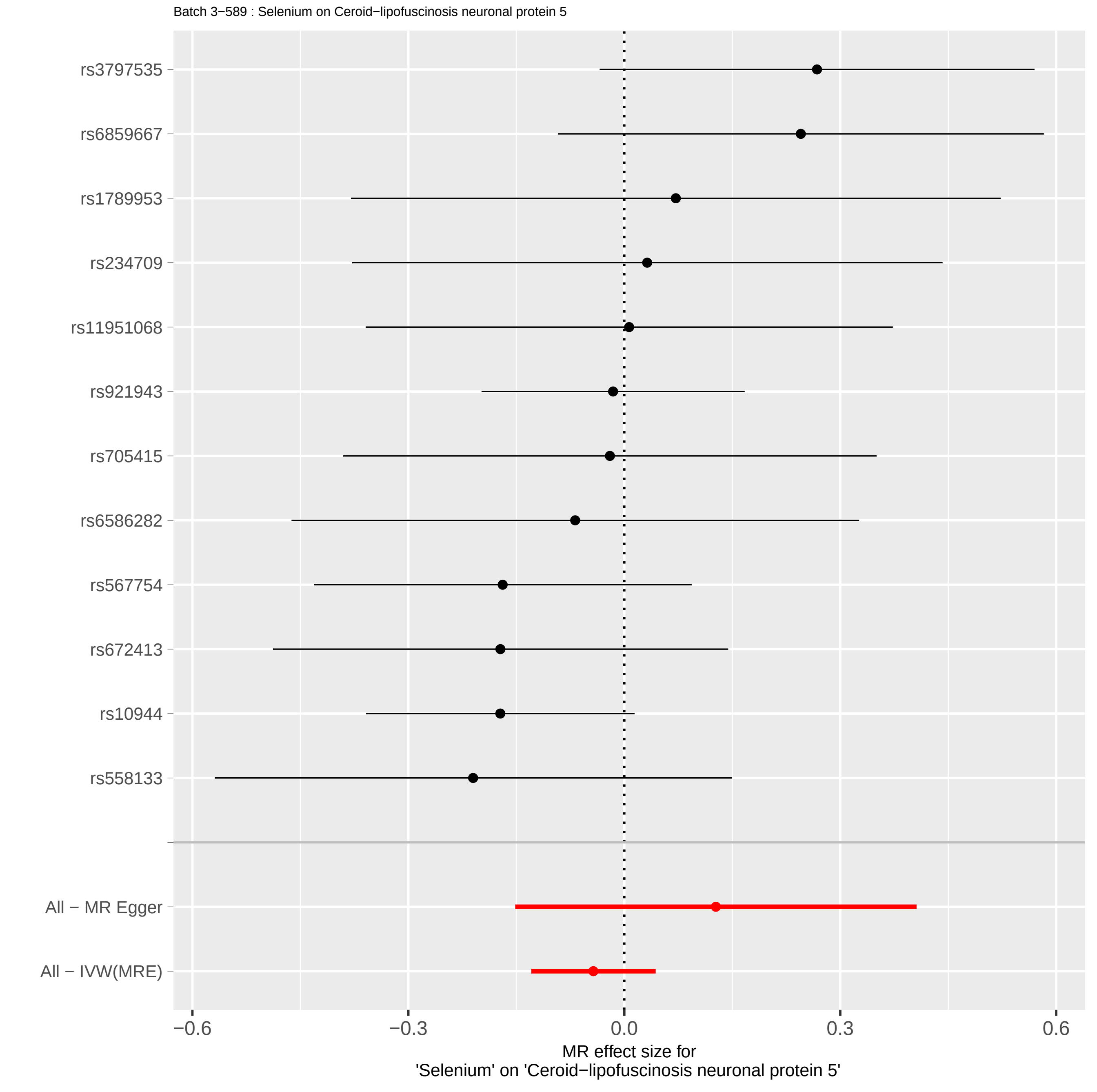

Batch 3−589 : Selenium on Ceroid−lipofuscinosis neuronal protein 5
rs3797535
rs6859667
rs1789953
rs234709
rs11951068
rs921943
rs705415
rs6586282
rs567754
rs672413
rs10944
rs558133
All − MR Egger
All − IVW(MRE)
−0.6
−0.3
0.0
0.3
0.6
MR effect size for
'Selenium' on 'Ceroid−lipofuscinosis neuronal protein 5'

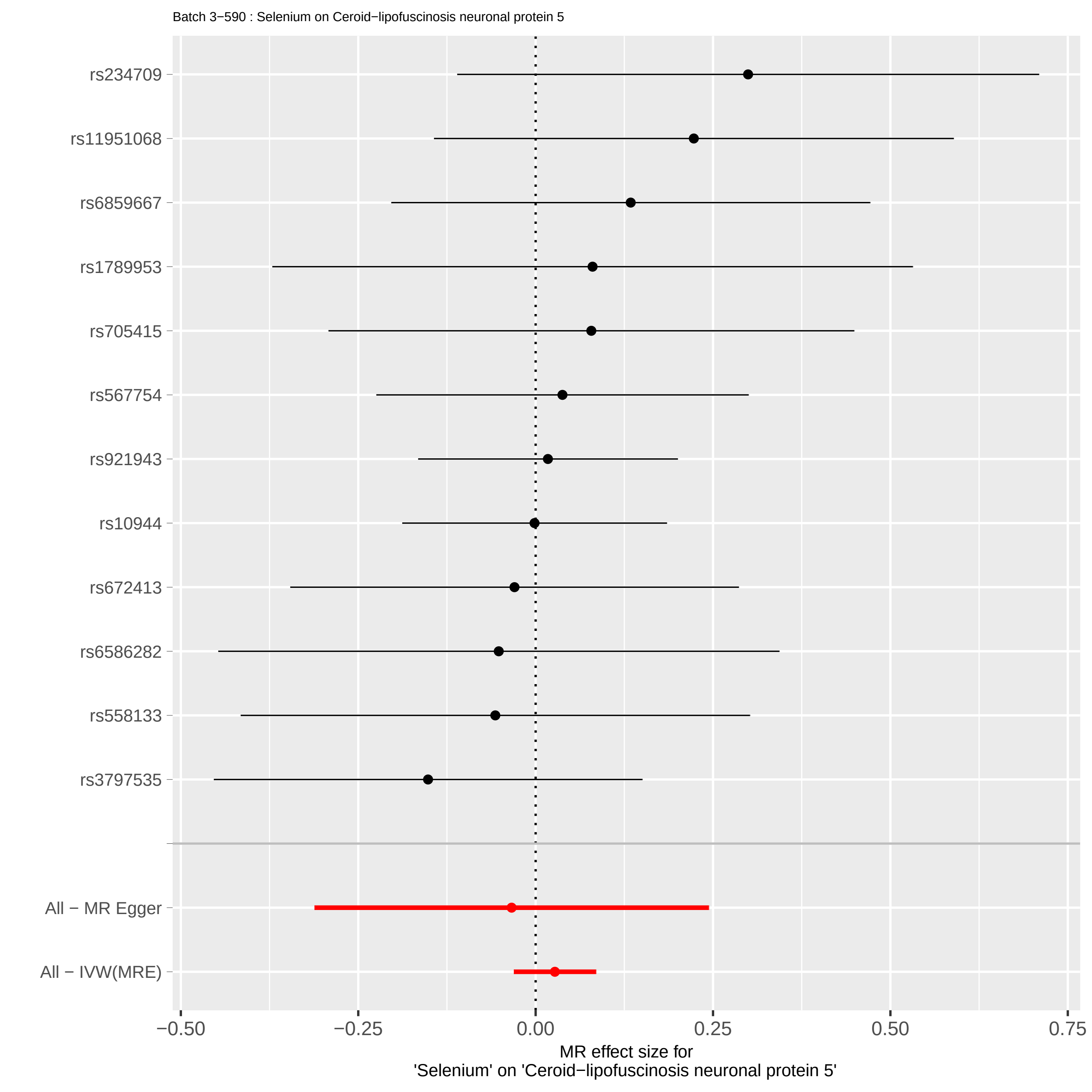

Batch 3−590 : Selenium on Ceroid−lipofuscinosis neuronal protein 5
rs234709
rs11951068
rs6859667
rs1789953
rs705415
rs567754
rs921943
rs10944
rs672413
rs6586282
rs558133
rs3797535
All − MR Egger
All − IVW(MRE)
−0.50
−0.25
0.00
0.25
0.50
0.75
MR effect size for
'Selenium' on 'Ceroid−lipofuscinosis neuronal protein 5'

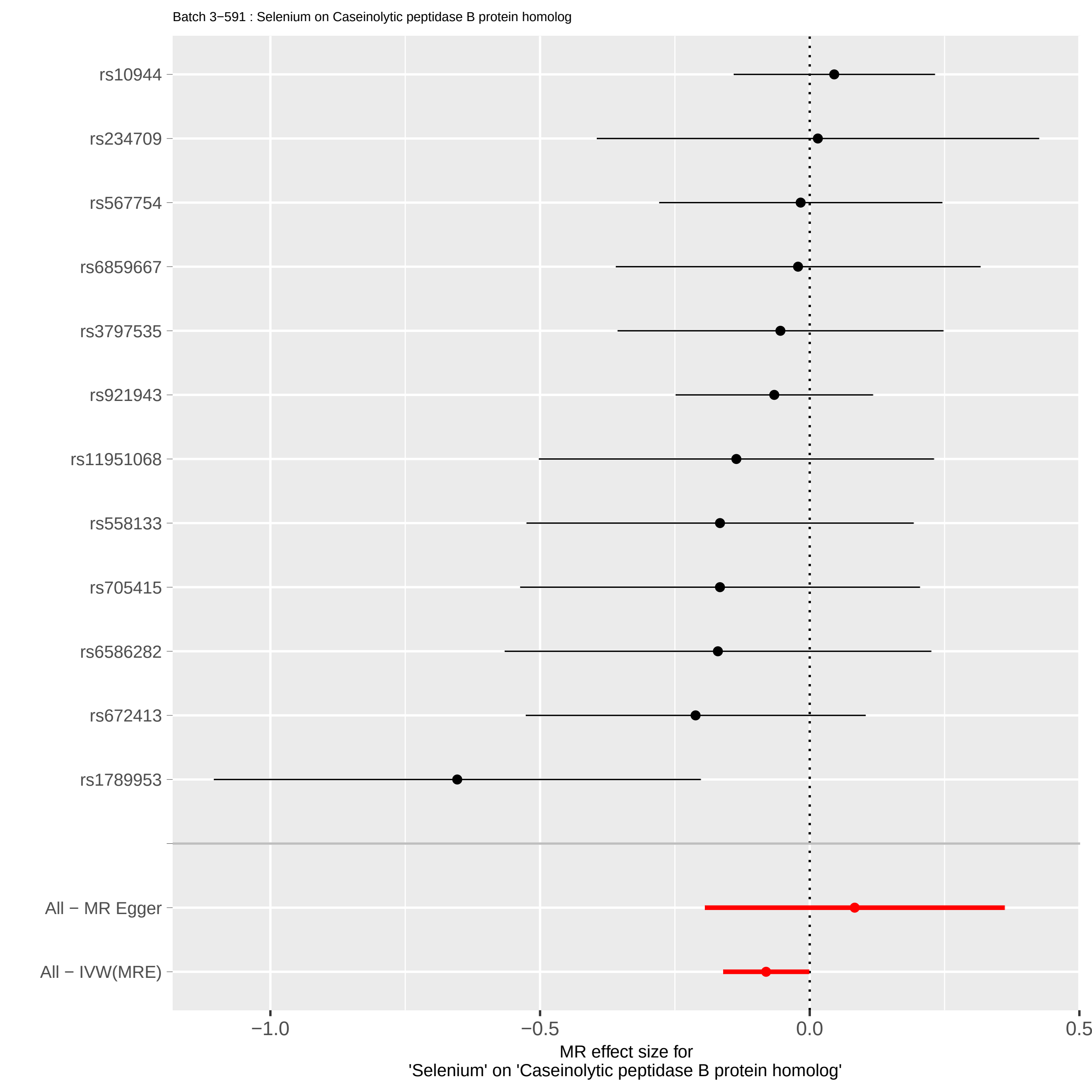

Batch 3−591 : Selenium on Caseinolytic peptidase B protein homolog
rs10944
rs234709
rs567754
rs6859667
rs3797535
rs921943
rs11951068
rs558133
rs705415
rs6586282
rs672413
rs1789953
All − MR Egger
All − IVW(MRE)
−1.0
−0.5
0.0
0.5
MR effect size for
'Selenium' on 'Caseinolytic peptidase B protein homolog'

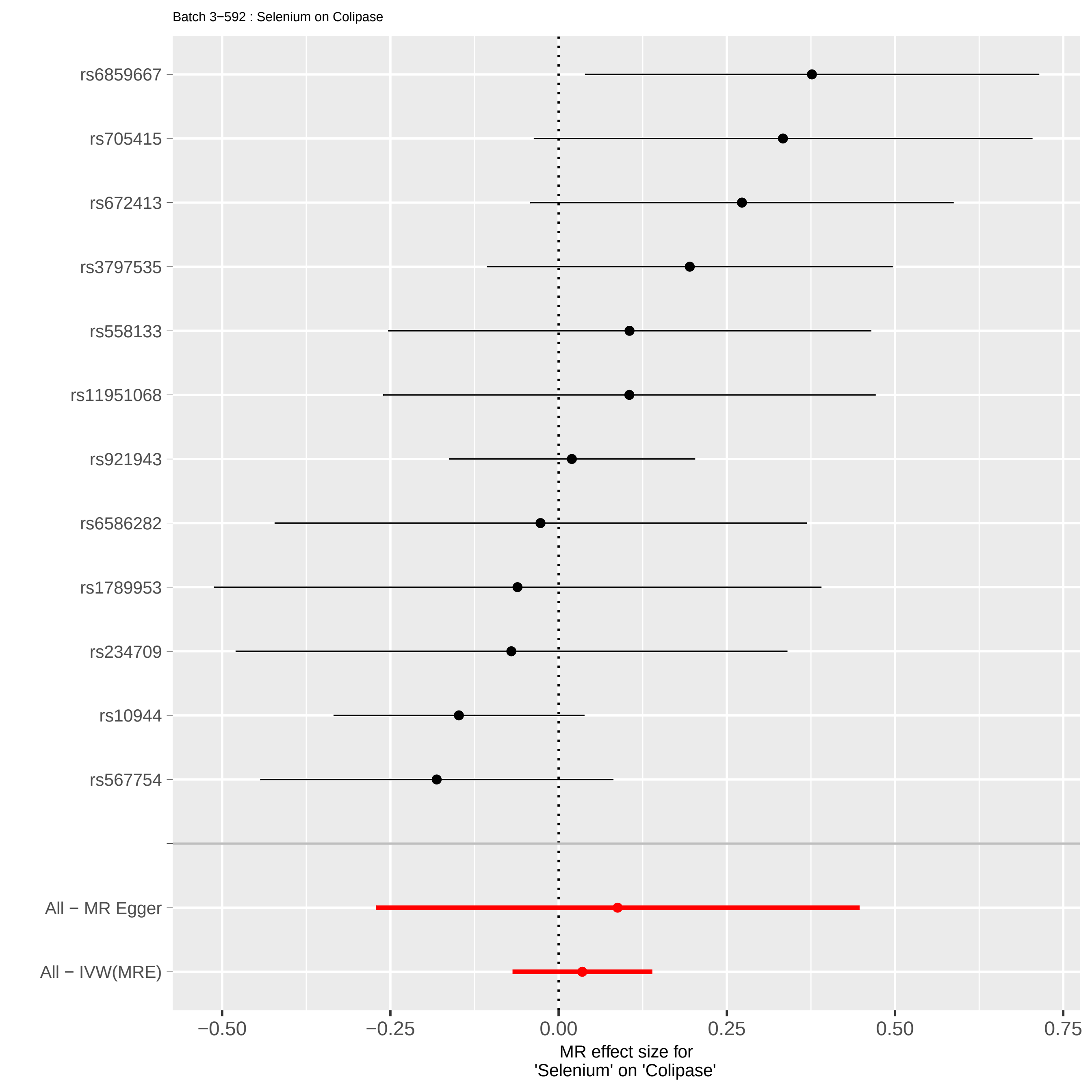
Batch 3−592 : Selenium on Colipase
rs6859667
rs705415
rs672413
rs3797535
rs558133
rs11951068
rs921943
rs6586282
rs1789953
rs234709
rs10944
rs567754
All − MR Egger
All − IVW(MRE)
−0.50
−0.25
0.00
0.25
0.50
0.75
MR effect size for
'Selenium' on 'Colipase'

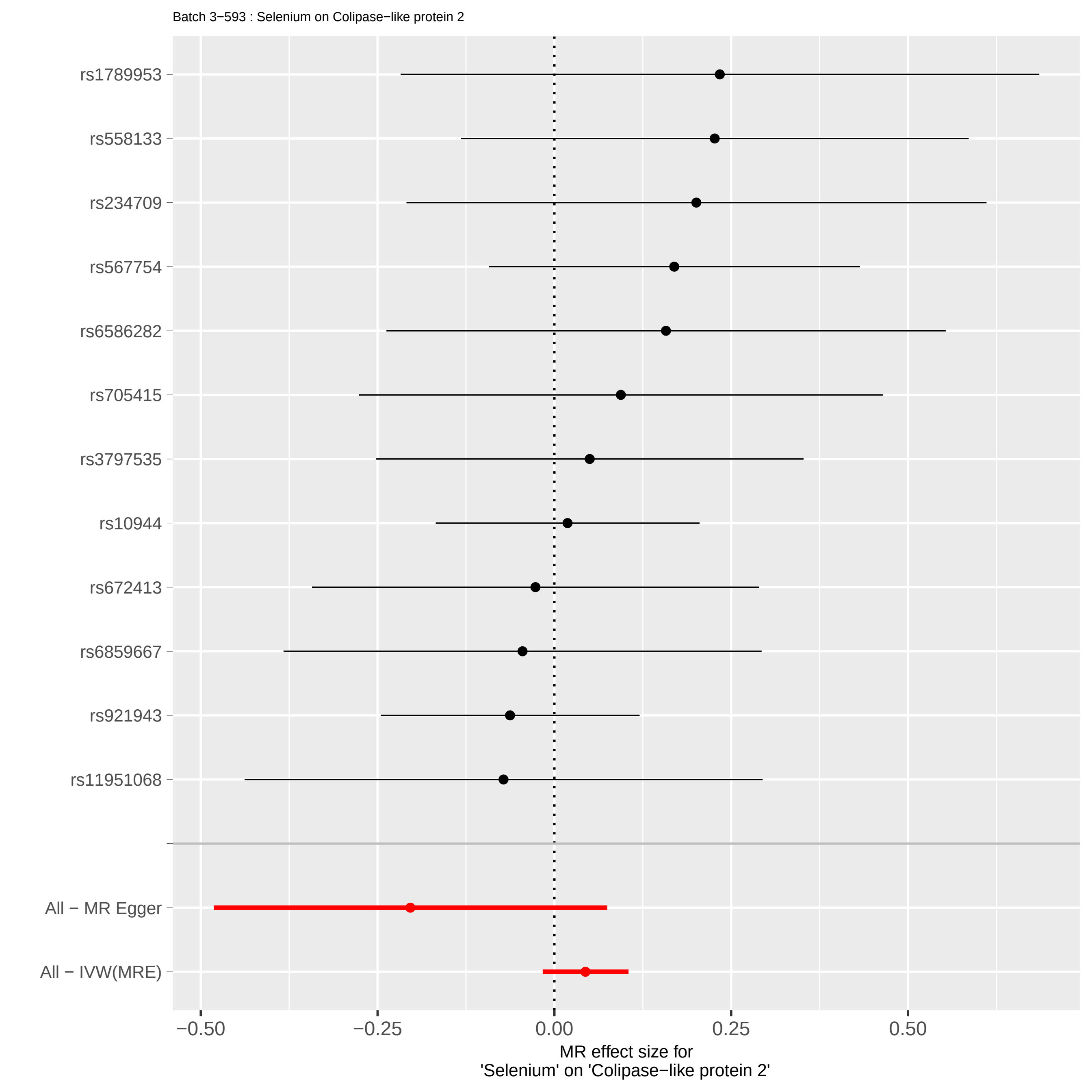
Batch 3−593 : Selenium on Colipase−like protein 2
rs1789953
rs558133
rs234709
rs567754
rs6586282
rs705415
rs3797535
rs10944
rs672413
rs6859667
rs921943
rs11951068
All − MR Egger
All − IVW(MRE)
−0.50
−0.25
0.00
0.25
0.50
MR effect size for
'Selenium' on 'Colipase−like protein 2'

Batch 3−594 : Selenium on Calsyntenin−3

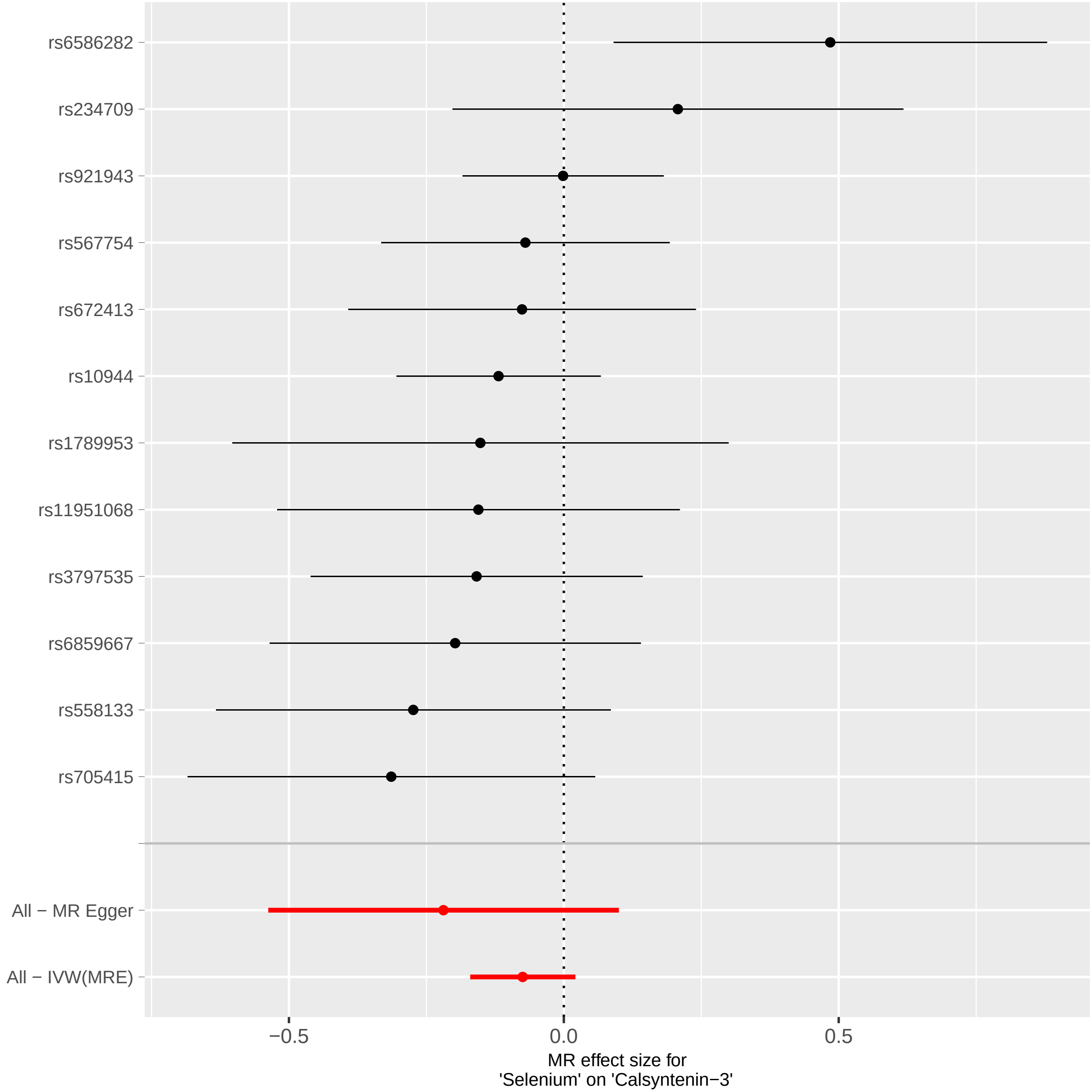

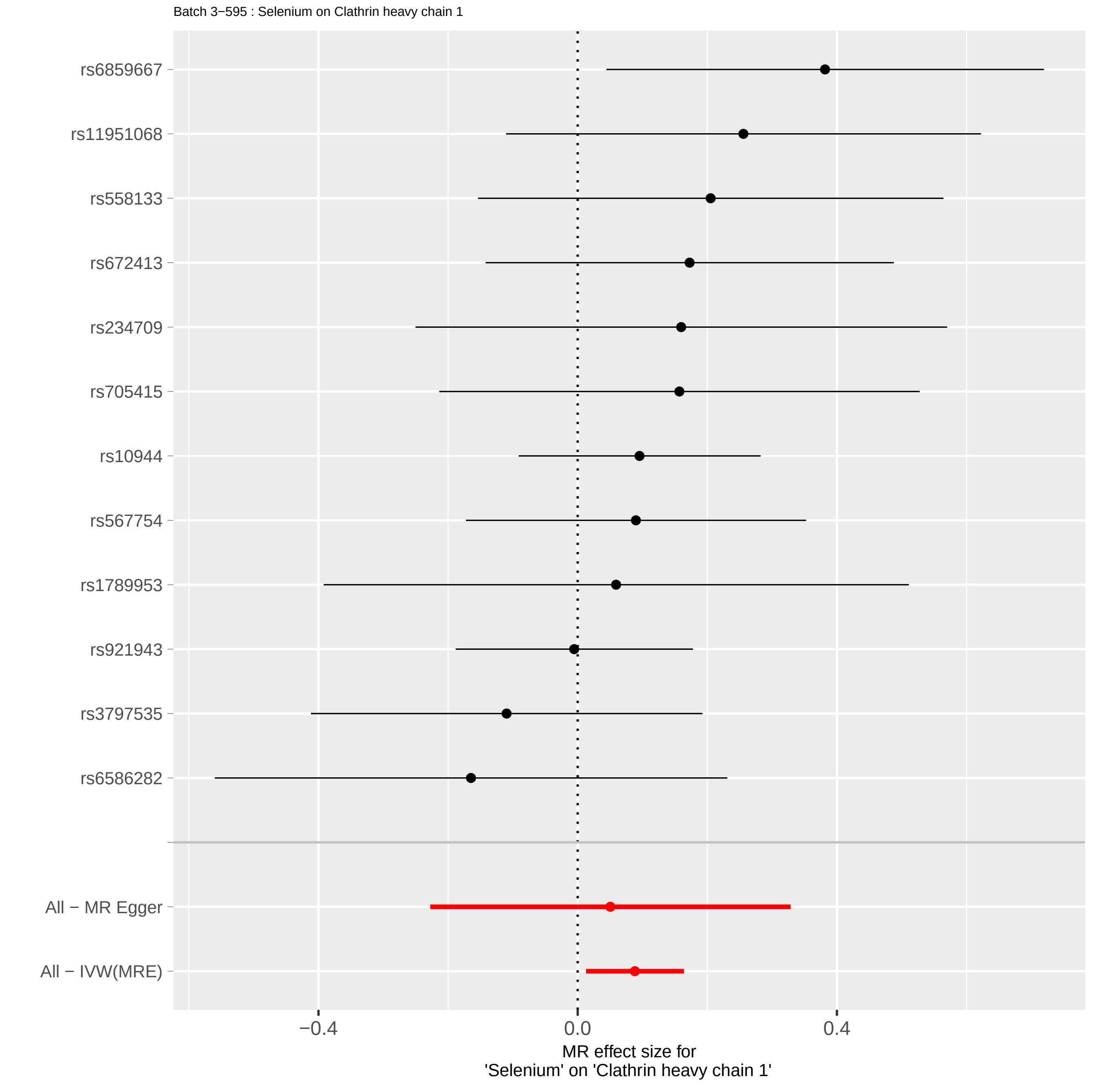

Batch 3−595 : Selenium on Clathrin heavy chain 1
rs6859667
rs11951068
rs558133
rs672413
rs234709
rs705415
rs10944
rs567754
rs1789953
rs921943
rs3797535
rs6586282
All − MR Egger
All − IVW(MRE)
−0.4
0.0
0.4
MR effect size for
'Selenium' on 'Clathrin heavy chain 1'

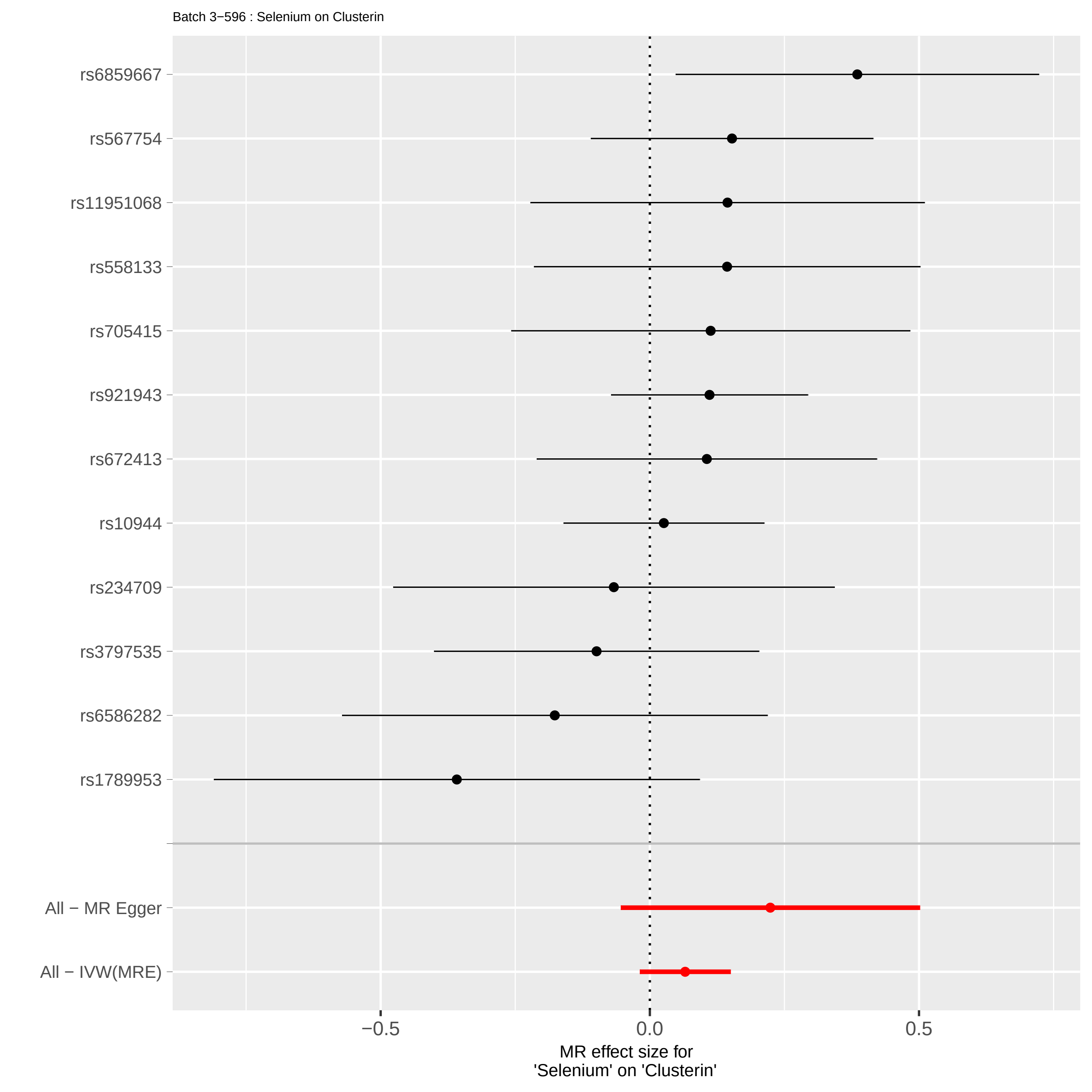
Batch 3−596 : Selenium on Clusterin
rs6859667
rs567754
rs11951068
rs558133
rs705415
rs921943
rs672413
rs10944
rs234709
rs3797535
rs6586282
rs1789953
All − MR Egger
All − IVW(MRE)
−0.5
0.0
0.5
MR effect size for
'Selenium' on 'Clusterin'

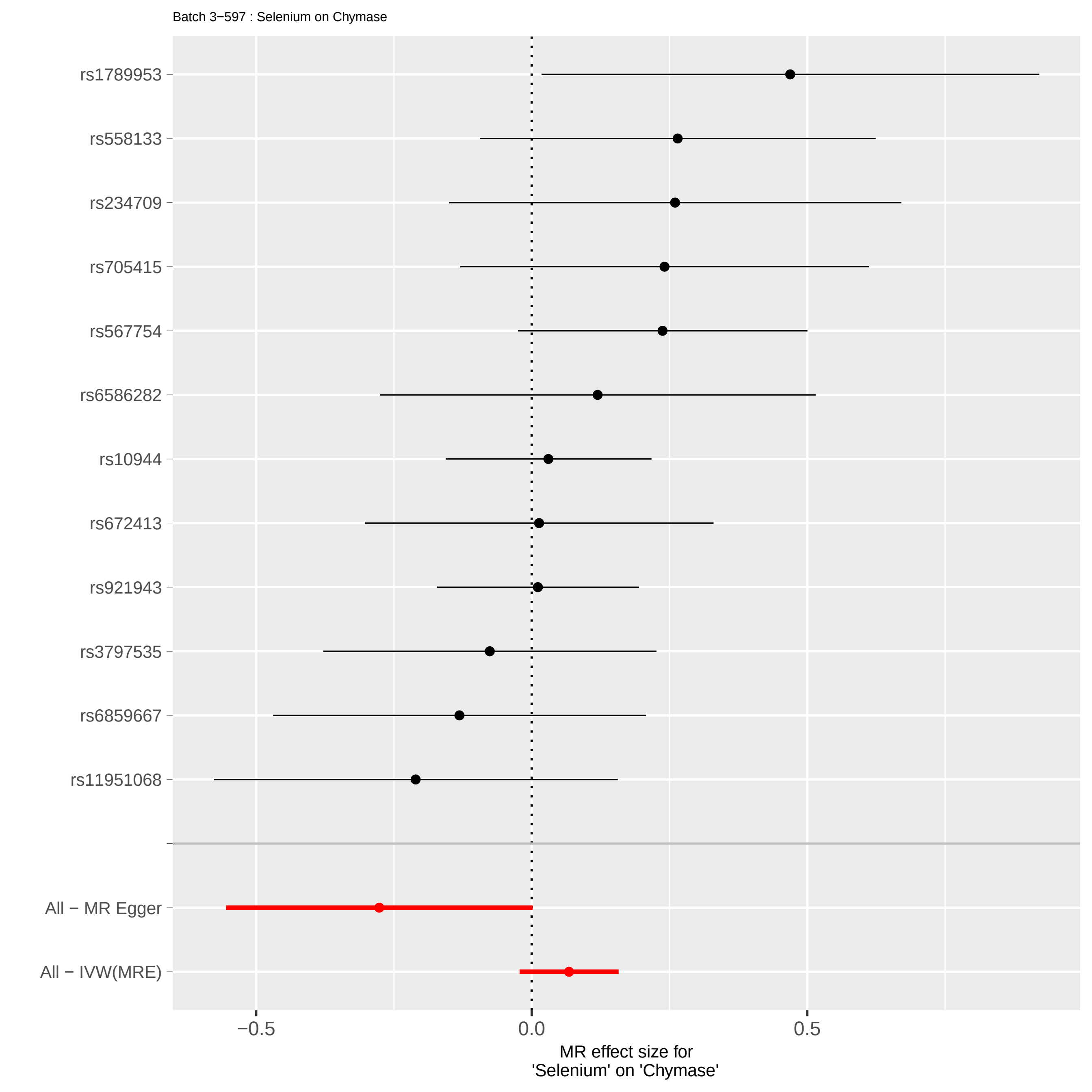

Batch 3−597 : Selenium on Chymase
rs1789953
rs558133
rs234709
rs705415
rs567754
rs6586282
rs10944
rs672413
rs921943
rs3797535
rs6859667
rs11951068
All − MR Egger
All − IVW(MRE)
−0.5
0.0
0.5
MR effect size for
'Selenium' on 'Chymase'

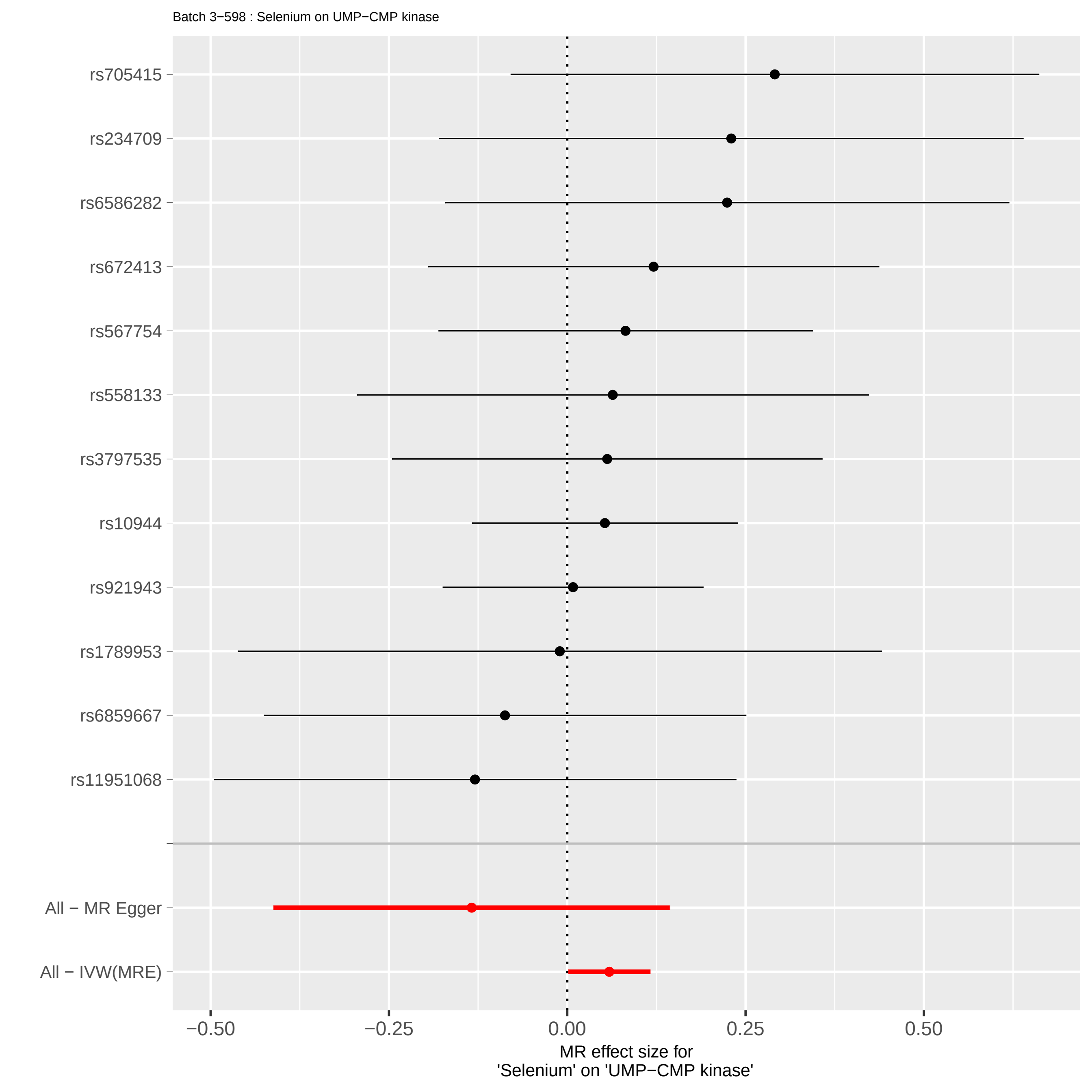

Batch 3−598 : Selenium on UMP−CMP kinase
rs705415
rs234709
rs6586282
rs672413
rs567754
rs558133
rs3797535
rs10944
rs921943
rs1789953
rs6859667
rs11951068
All − MR Egger
All − IVW(MRE)
−0.50
−0.25
0.00
0.25
0.50
MR effect size for
'Selenium' on 'UMP−CMP kinase'

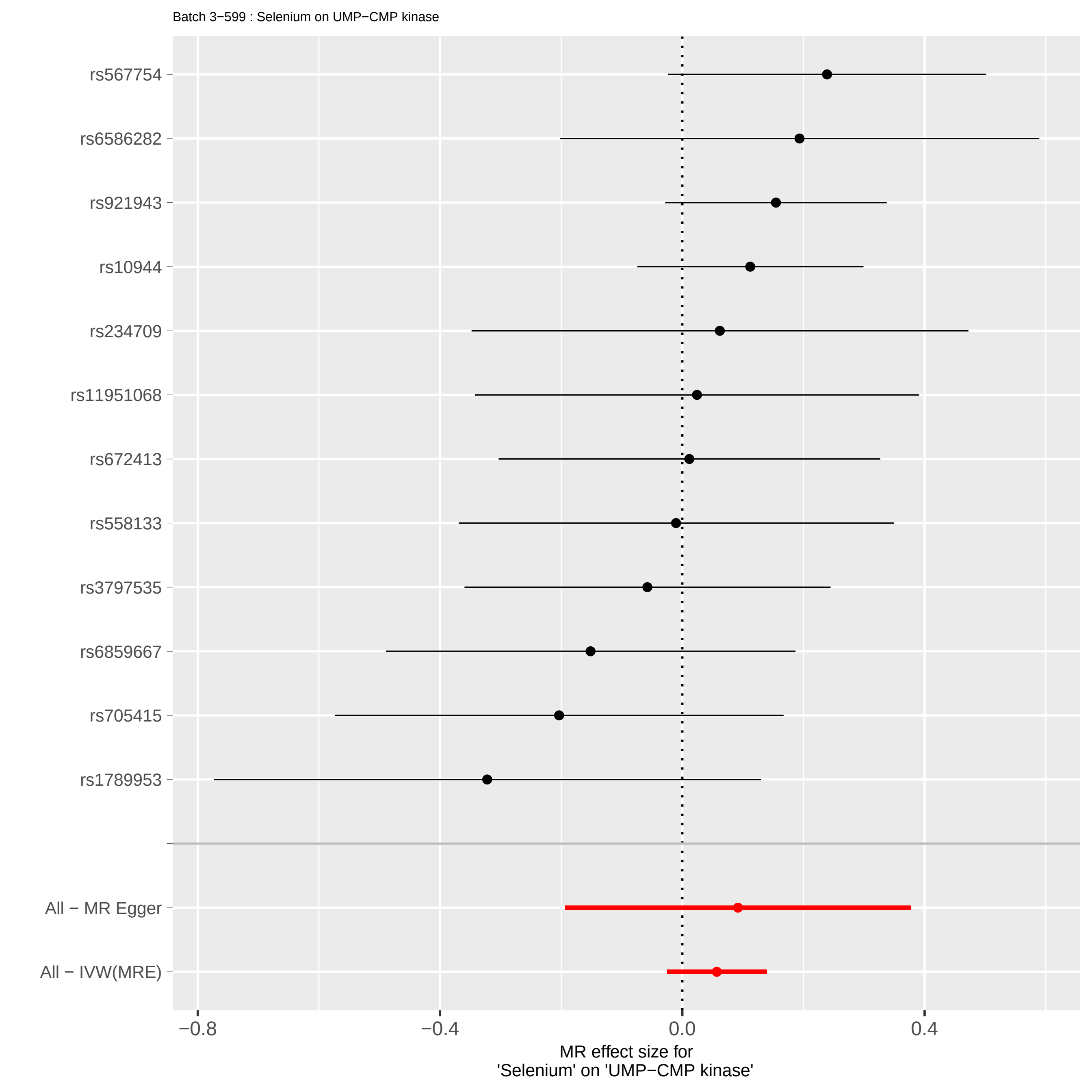
Batch 3−599 : Selenium on UMP−CMP kinase
rs567754
rs6586282
rs921943
rs10944
rs234709
rs11951068
rs672413
rs558133
rs3797535
rs6859667
rs705415
rs1789953
All − MR Egger
All − IVW(MRE)
−0.8
−0.4
0.0
0.4
MR effect size for
'Selenium' on 'UMP−CMP kinase'

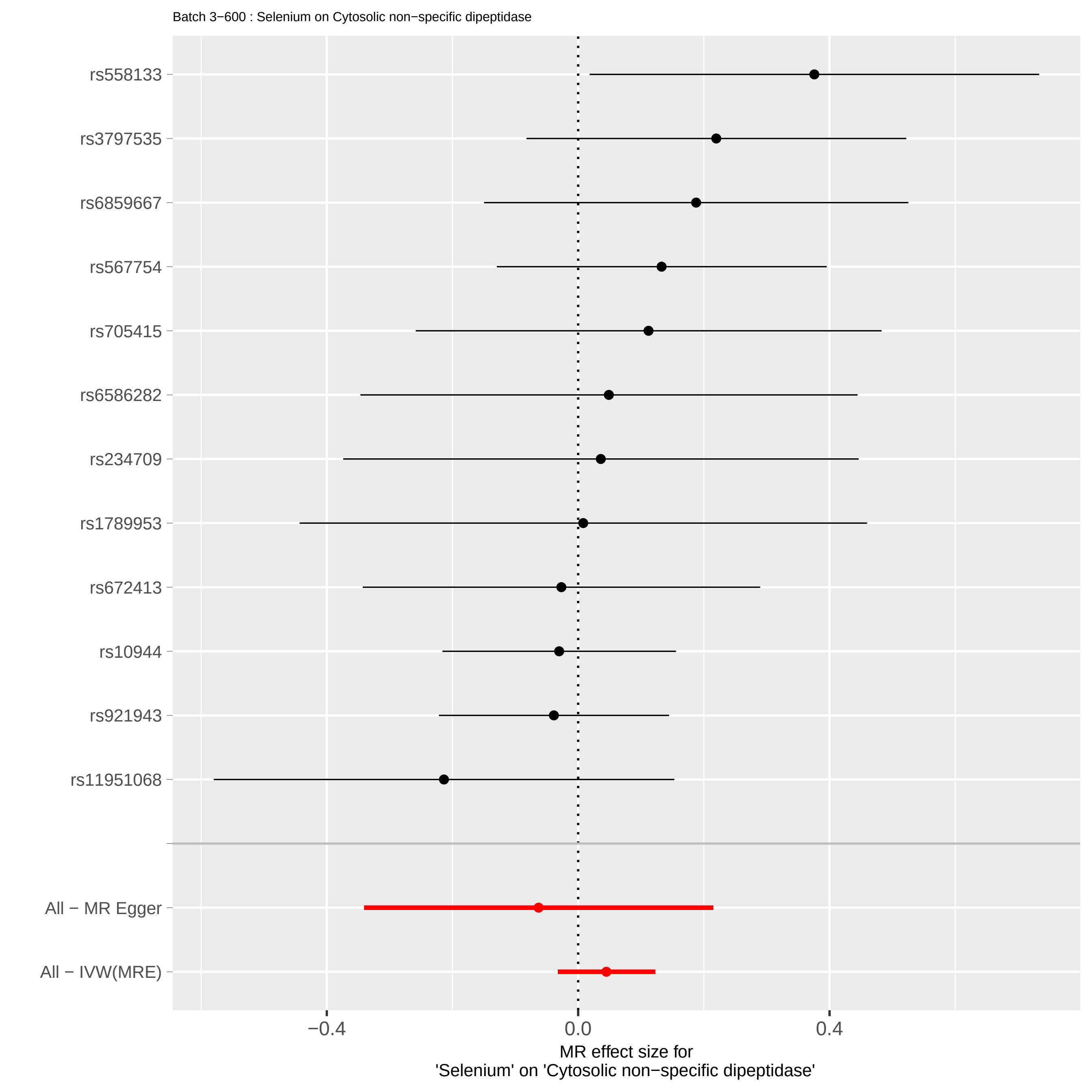
Batch 3−600 : Selenium on Cytosolic non−specific dipeptidase
rs558133
rs3797535
rs6859667
rs567754
rs705415
rs6586282
rs234709
rs1789953
rs672413
rs10944
rs921943
rs11951068
All − MR Egger
All − IVW(MRE)
−0.4
0.0
0.4
MR effect size for
'Selenium' on 'Cytosolic non−specific dipeptidase'

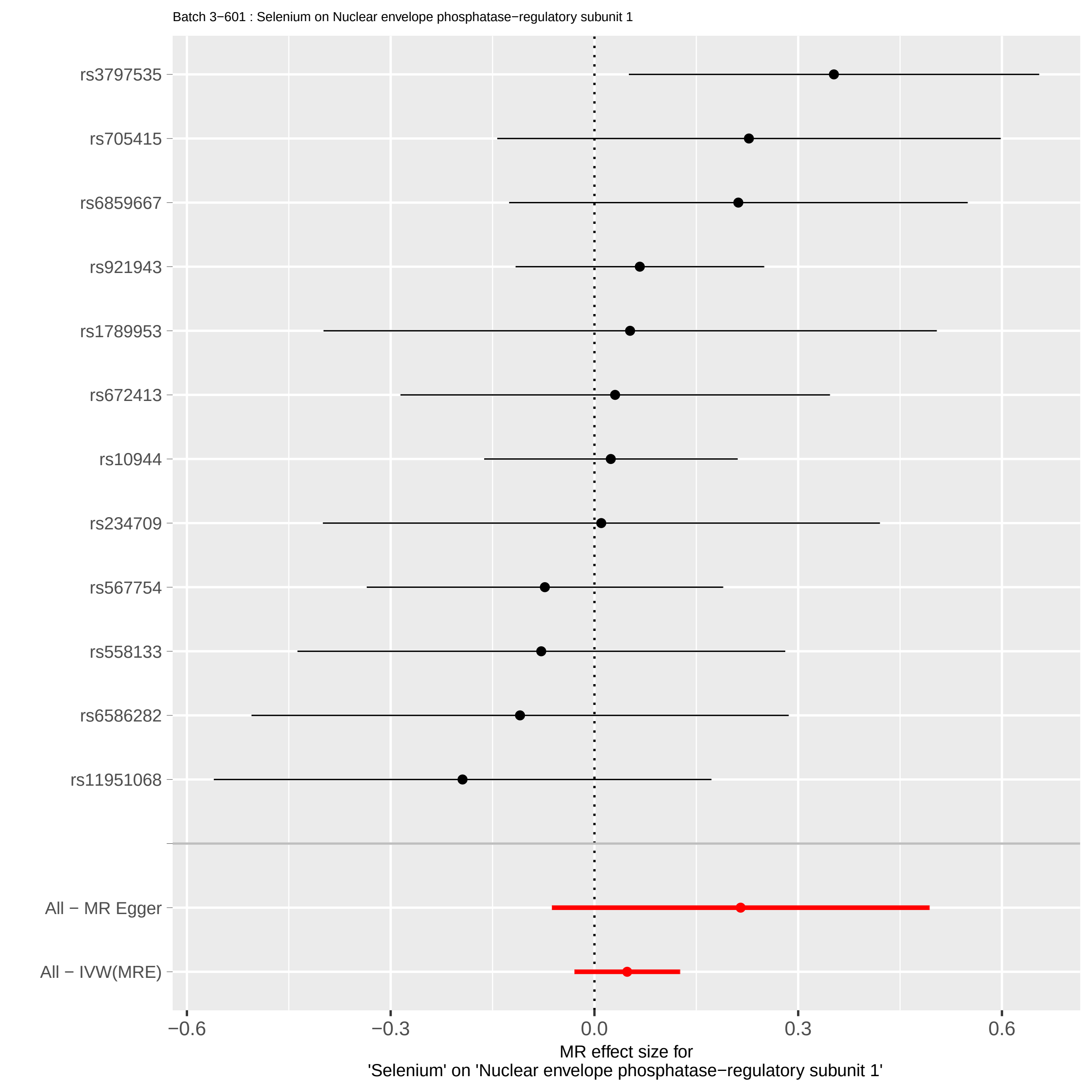

Batch 3−601 : Selenium on Nuclear envelope phosphatase−regulatory subunit 1
rs3797535
rs705415
rs6859667
rs921943
rs1789953
rs672413
rs10944
rs234709
rs567754
rs558133
rs6586282
rs11951068
All − MR Egger
All − IVW(MRE)
−0.6
−0.3
0.0
0.3
0.6
MR effect size for
'Selenium' on 'Nuclear envelope phosphatase−regulatory subunit 1'

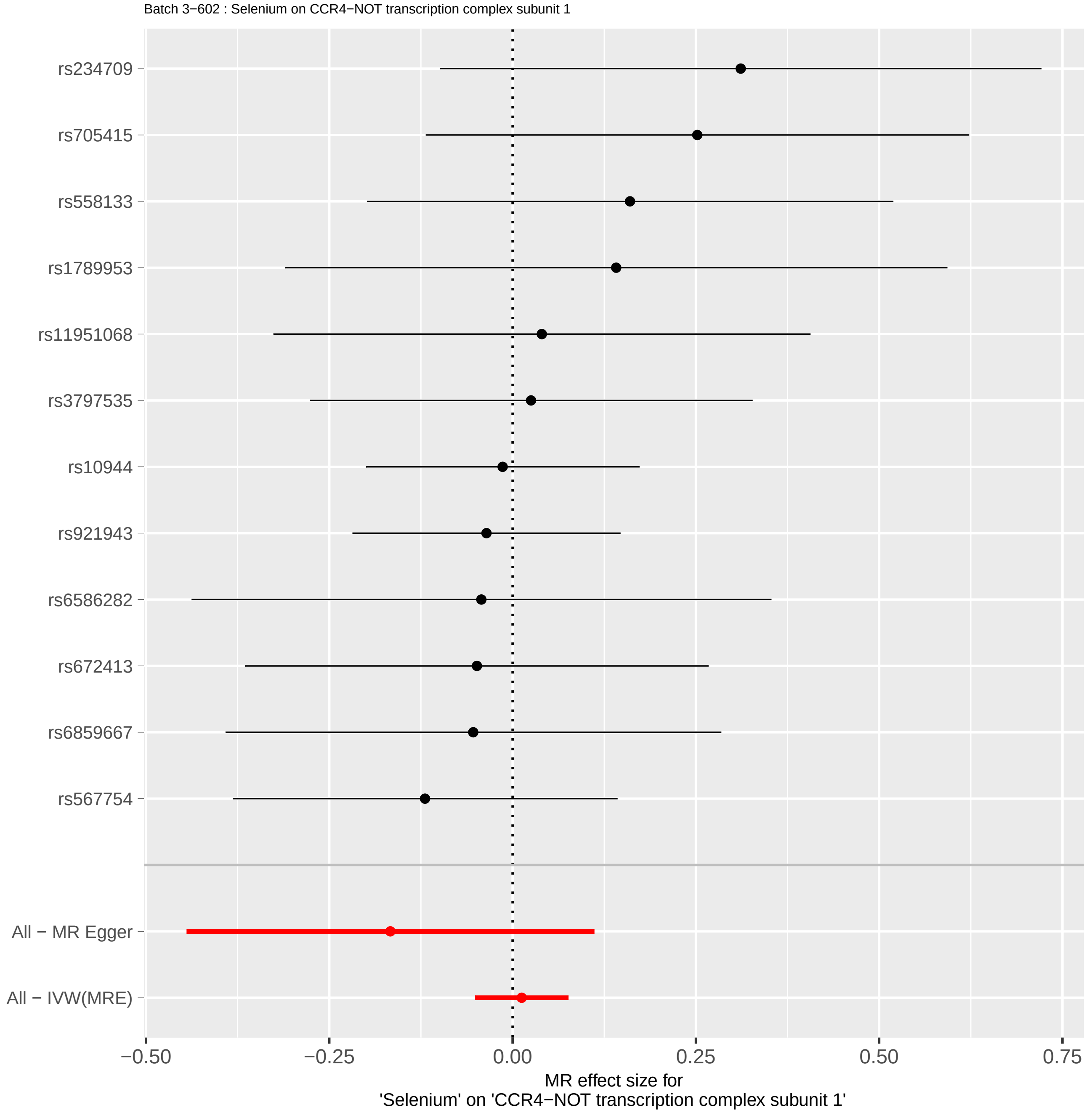

Batch 3−602 : Selenium on CCR4−NOT transcription complex subunit 1
rs234709
rs705415
rs558133
rs1789953
rs11951068
rs3797535
rs10944
rs921943
rs6586282
rs672413
rs6859667
rs567754
All − MR Egger
All − IVW(MRE)
−0.50
−0.25
0.00
0.25
0.50
0.75
MR effect size for
'Selenium' on 'CCR4−NOT transcription complex subunit 1'

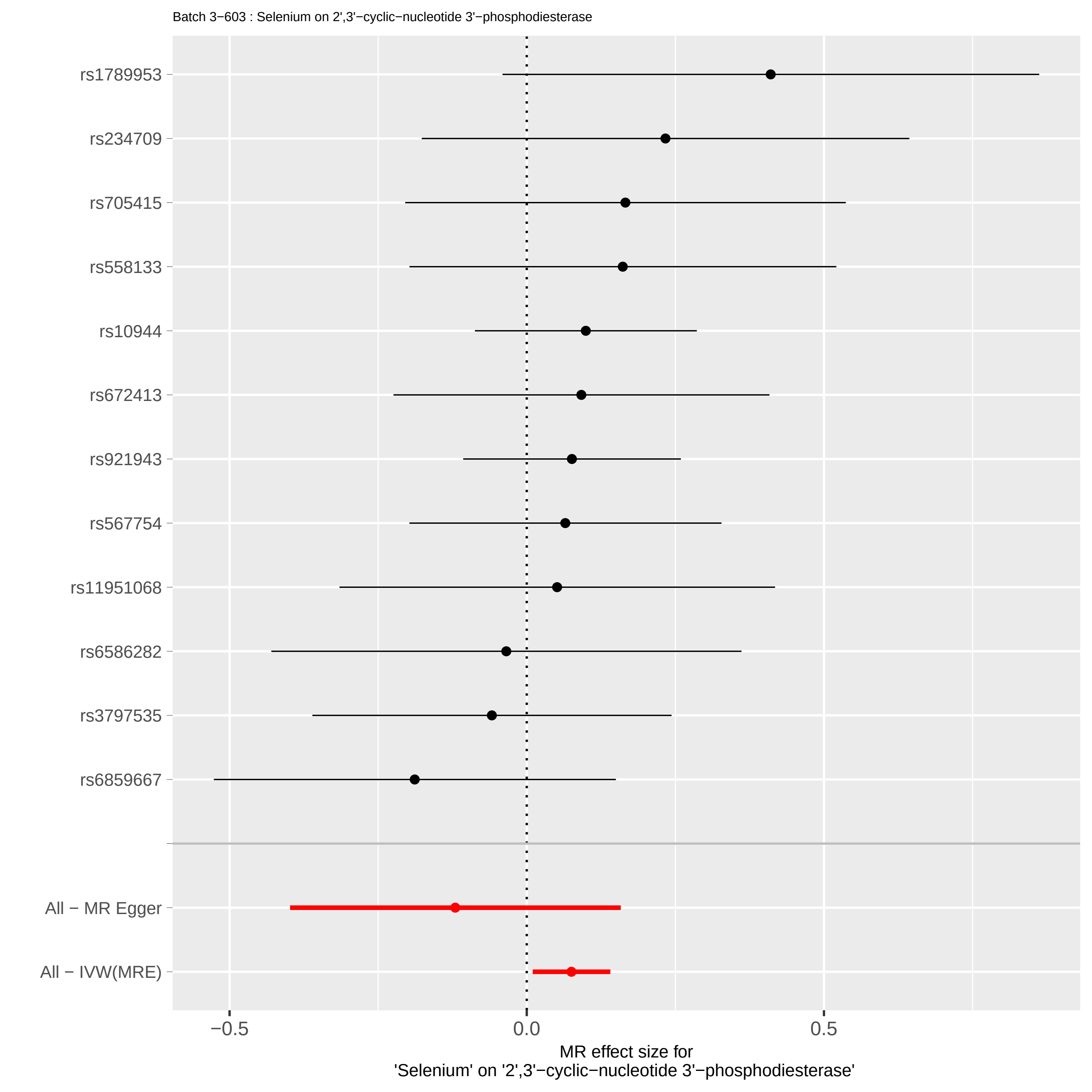

Batch 3−603 : Selenium on 2',3'−cyclic−nucleotide 3'−phosphodiesterase
rs1789953
rs234709
rs705415
rs558133
rs10944
rs672413
rs921943
rs567754
rs11951068
rs6586282
rs3797535
rs6859667
All − MR Egger
All − IVW(MRE)
−0.5
0.0
0.5
MR effect size for
'Selenium' on '2',3'−cyclic−nucleotide 3'−phosphodiesterase'

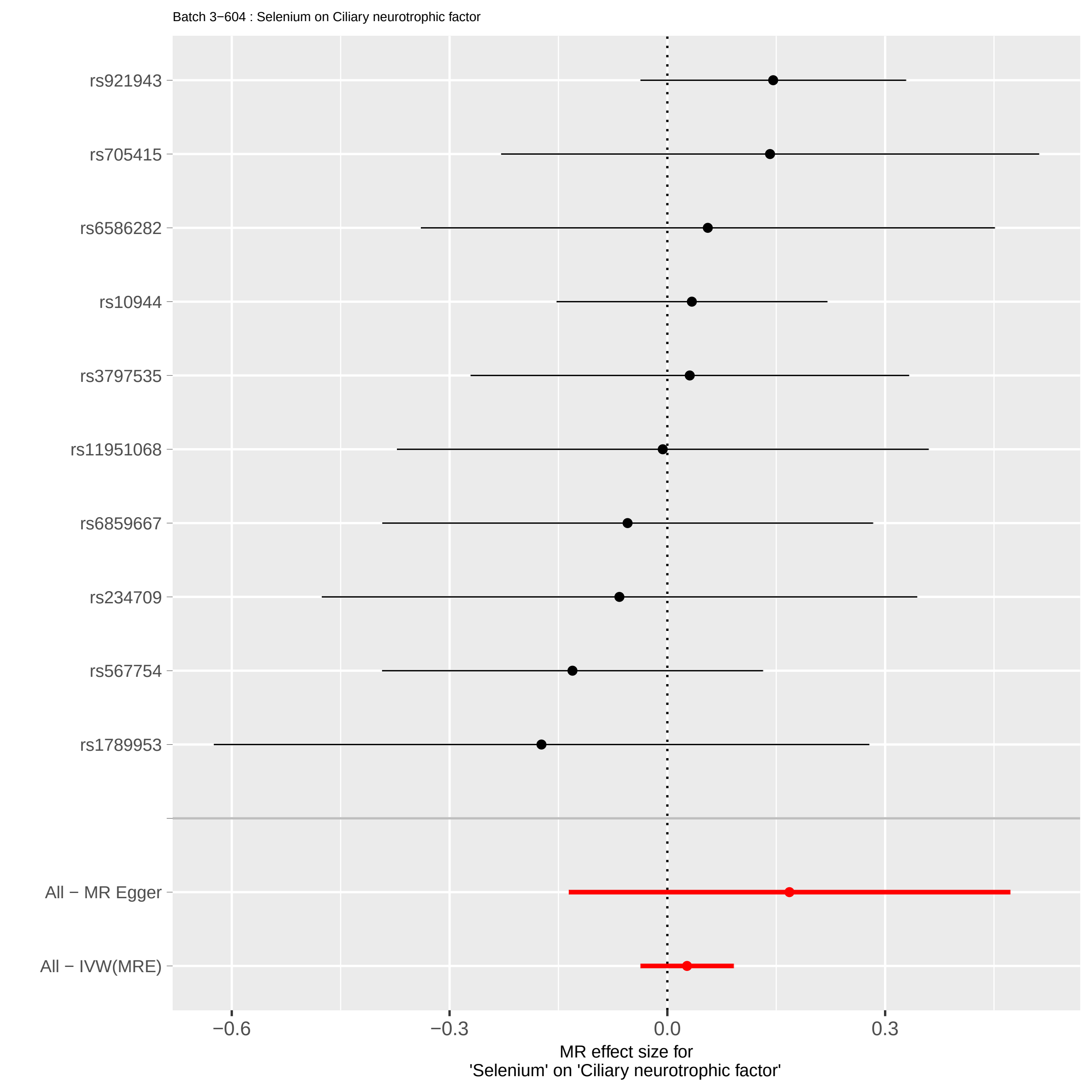
Batch 3−604 : Selenium on Ciliary neurotrophic factor
rs921943
rs705415
rs6586282
rs10944
rs3797535
rs11951068
rs6859667
rs234709
rs567754
rs1789953
All − MR Egger
All − IVW(MRE)
−0.6
−0.3
0.0
0.3
MR effect size for
'Selenium' on 'Ciliary neurotrophic factor'

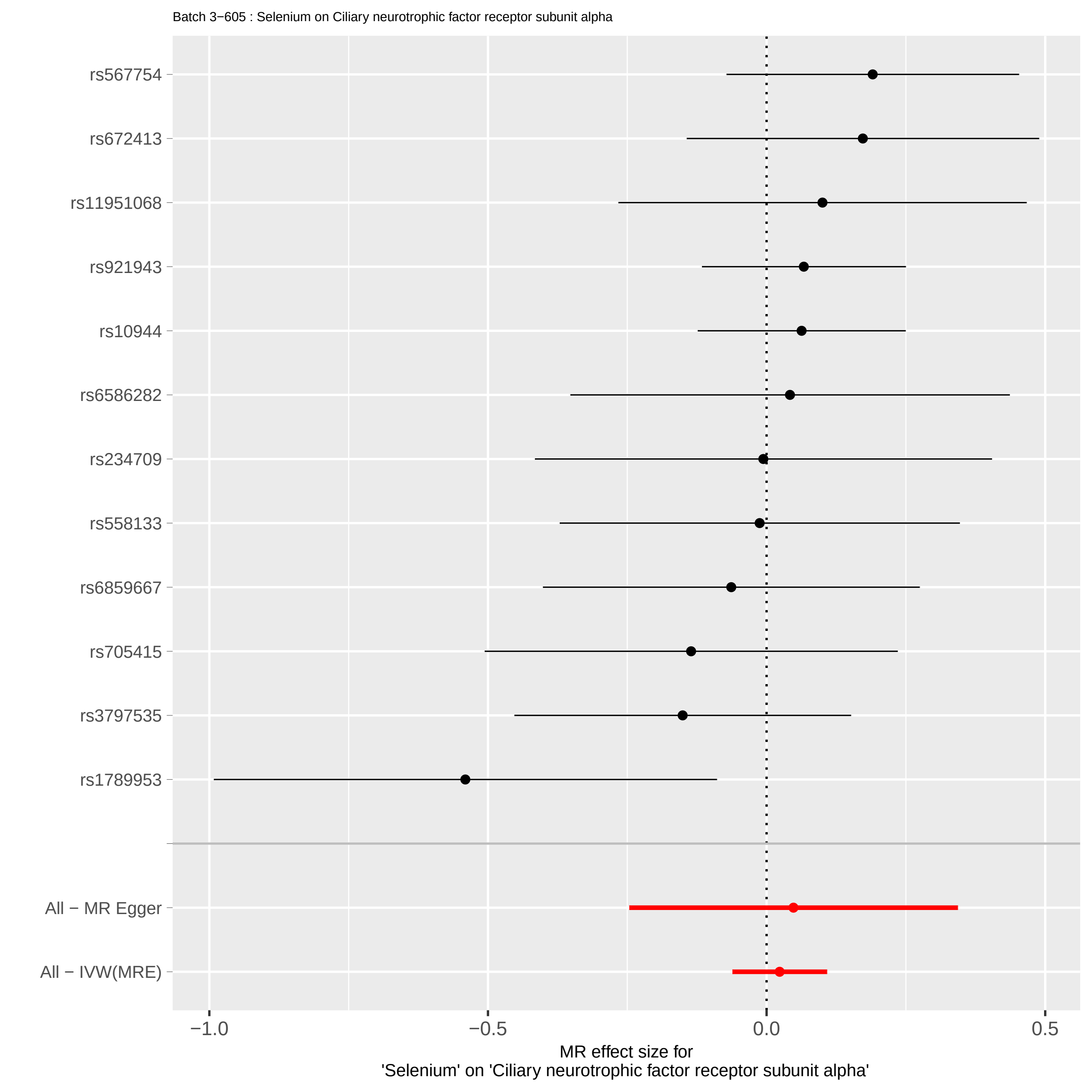

Batch 3−605 : Selenium on Ciliary neurotrophic factor receptor subunit alpha
rs567754
rs672413
rs11951068
rs921943
rs10944
rs6586282
rs234709
rs558133
rs6859667
rs705415
rs3797535
rs1789953
All − MR Egger
All − IVW(MRE)
−1.0
−0.5
0.0
0.5
MR effect size for
'Selenium' on 'Ciliary neurotrophic factor receptor subunit alpha'

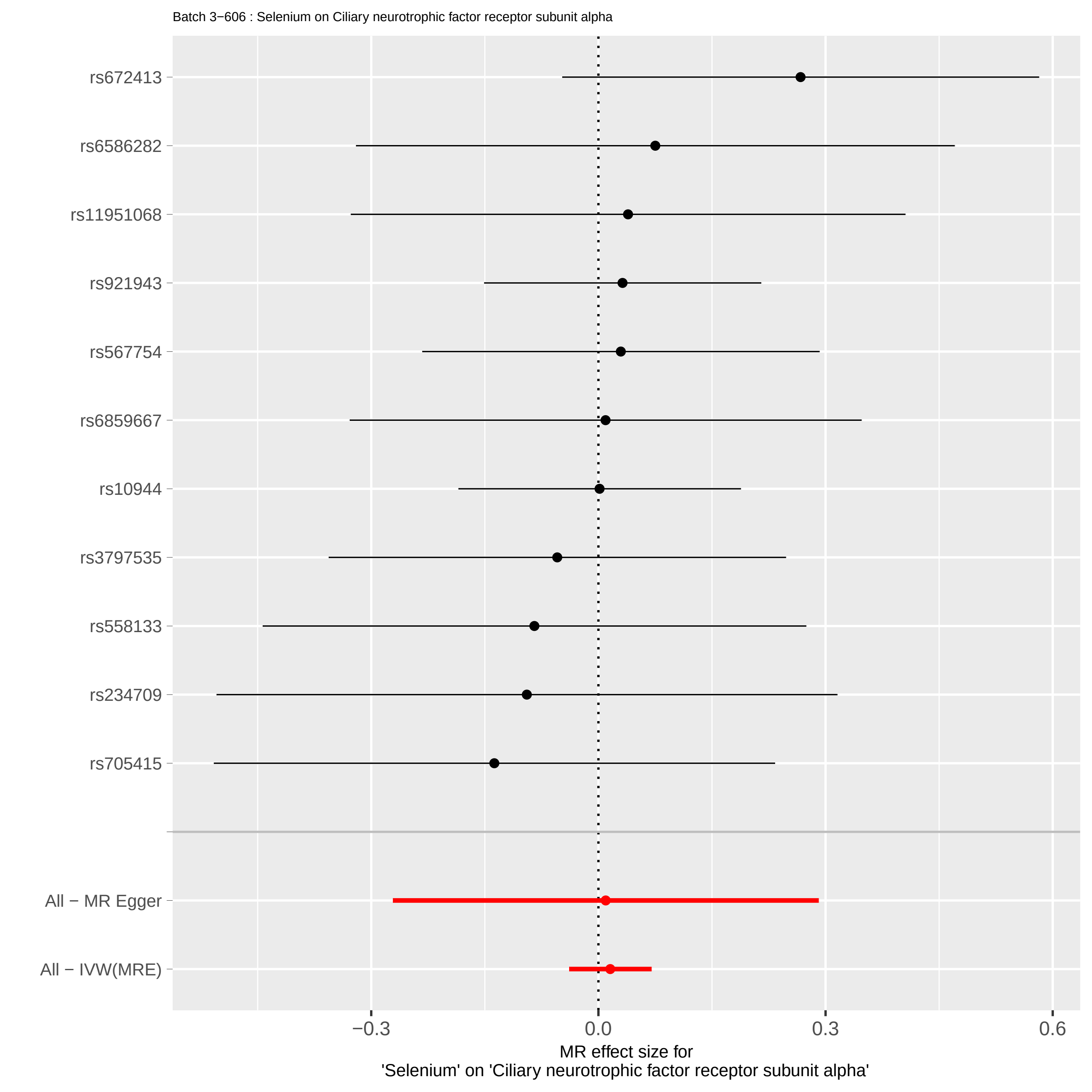
Batch 3−606 : Selenium on Ciliary neurotrophic factor receptor subunit alpha
rs672413
rs6586282
rs11951068
rs921943
rs567754
rs6859667
rs10944
rs3797535
rs558133
rs234709
rs705415
All − MR Egger
All − IVW(MRE)
−0.3
0.0
0.3
0.6
MR effect size for
'Selenium' on 'Ciliary neurotrophic factor receptor subunit alpha'

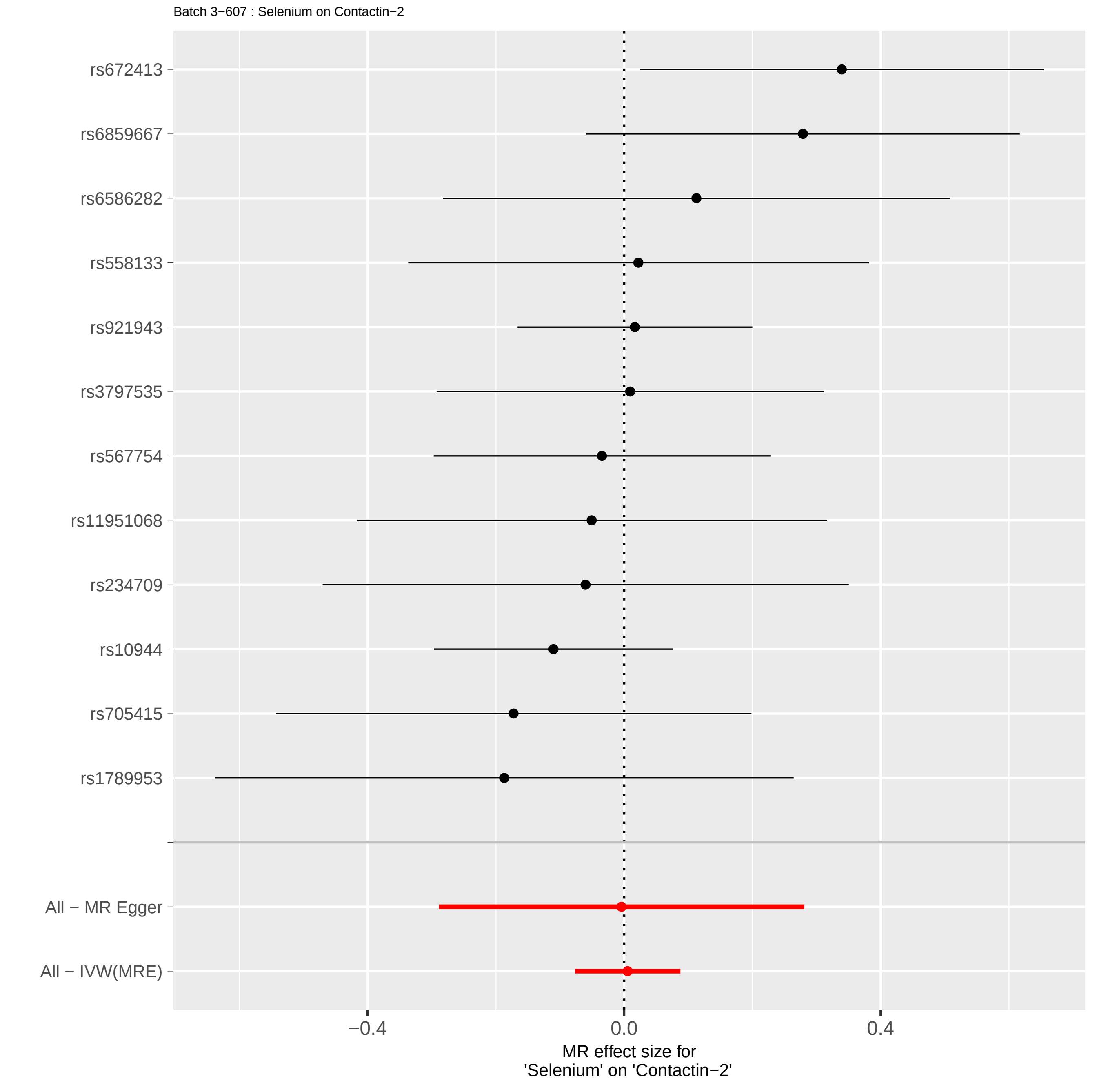
Batch 3−607 : Selenium on Contactin−2
rs672413
rs6859667
rs6586282
rs558133
rs921943
rs3797535
rs567754
rs11951068
rs234709
rs10944
rs705415
rs1789953
All − MR Egger
All − IVW(MRE)
−0.4
0.0
0.4
MR effect size for
'Selenium' on 'Contactin−2'

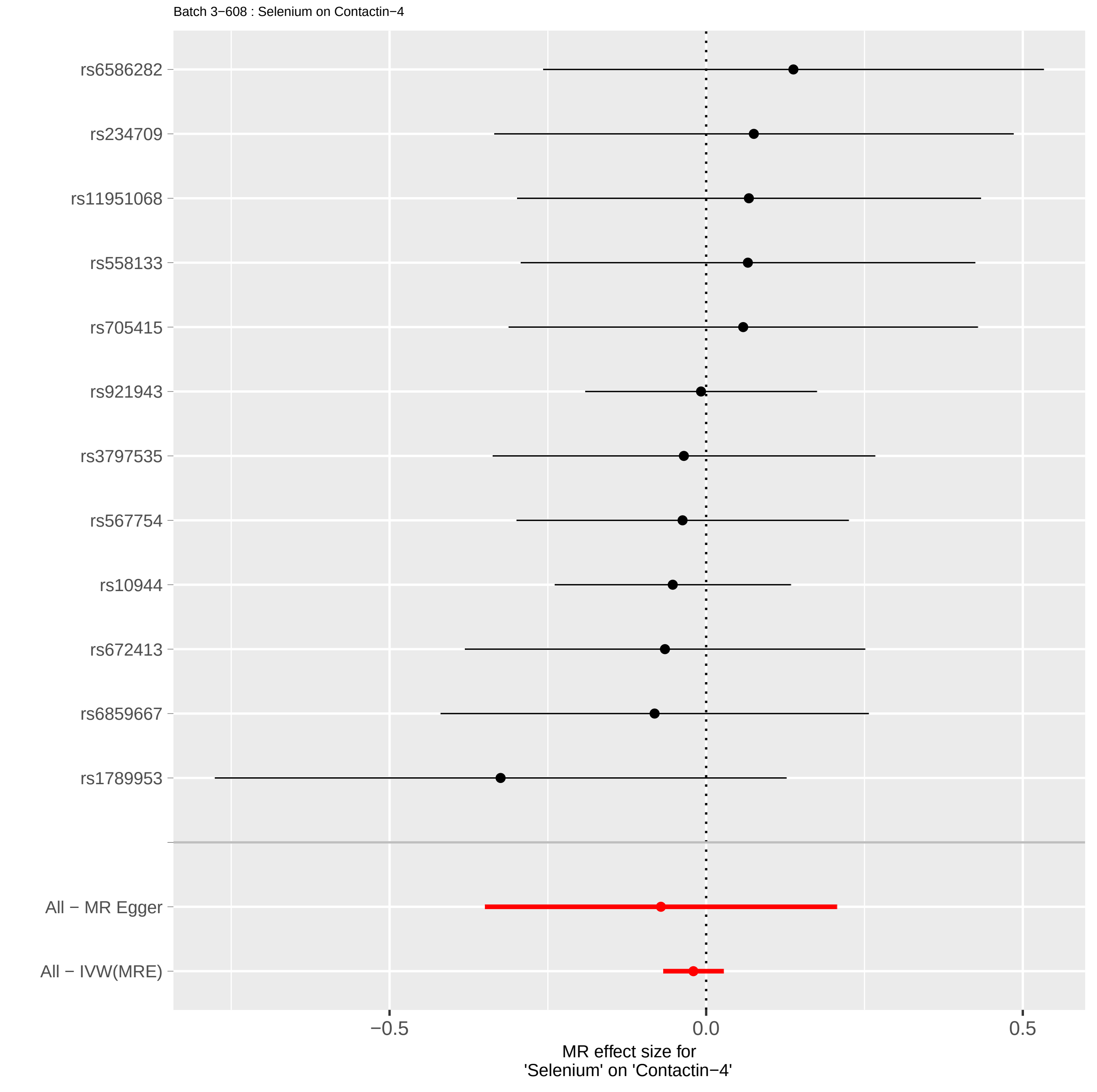

Batch 3−608 : Selenium on Contactin−4
rs6586282
rs234709
rs11951068
rs558133
rs705415
rs921943
rs3797535
rs567754
rs10944
rs672413
rs6859667
rs1789953
All − MR Egger
All − IVW(MRE)
−0.5
0.0
0.5
MR effect size for
'Selenium' on 'Contactin−4'

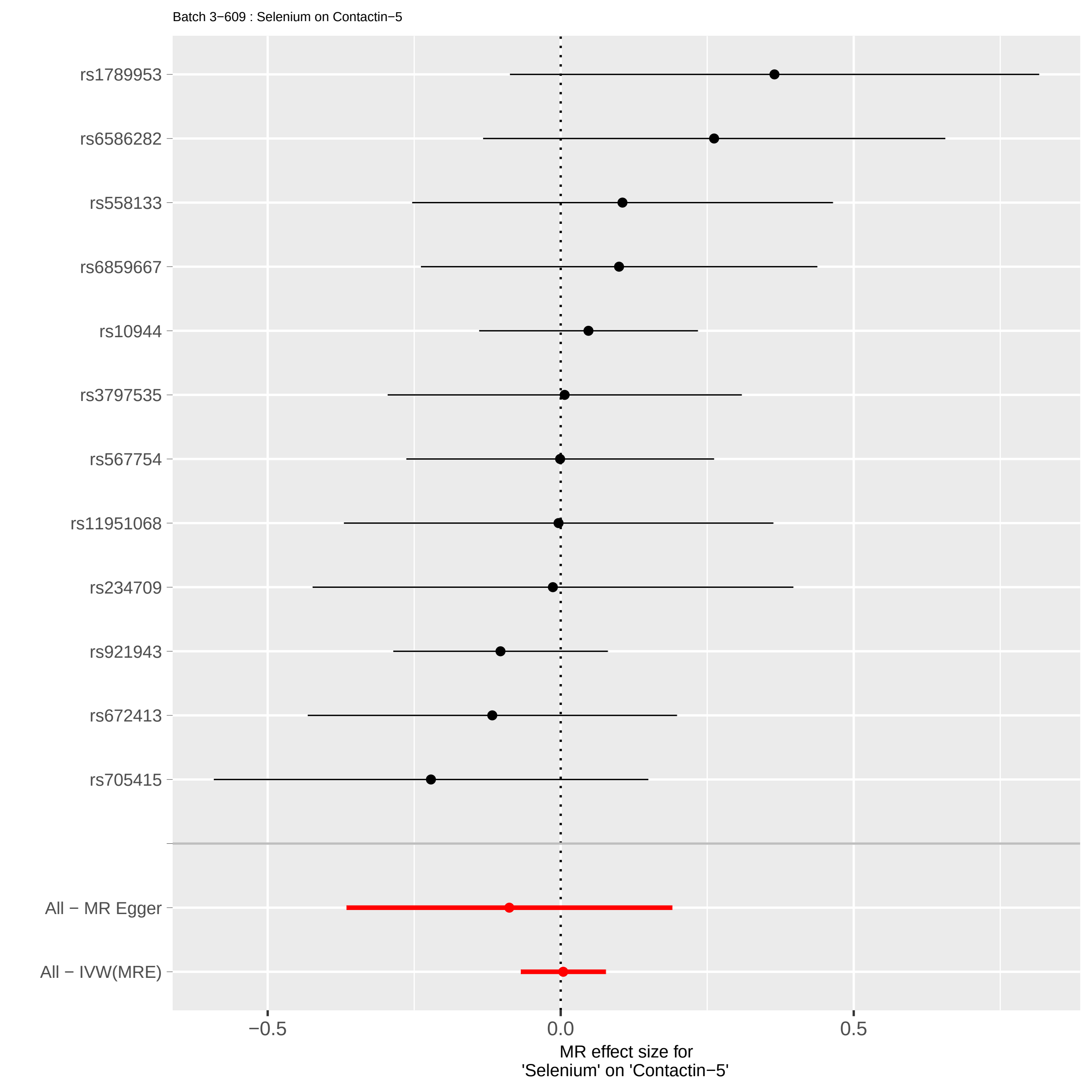
Batch 3−609 : Selenium on Contactin−5
rs1789953
rs6586282
rs558133
rs6859667
rs10944
rs3797535
rs567754
rs11951068
rs234709
rs921943
rs672413
rs705415
All − MR Egger
All − IVW(MRE)
−0.5
0.0
0.5
MR effect size for
'Selenium' on 'Contactin−5'

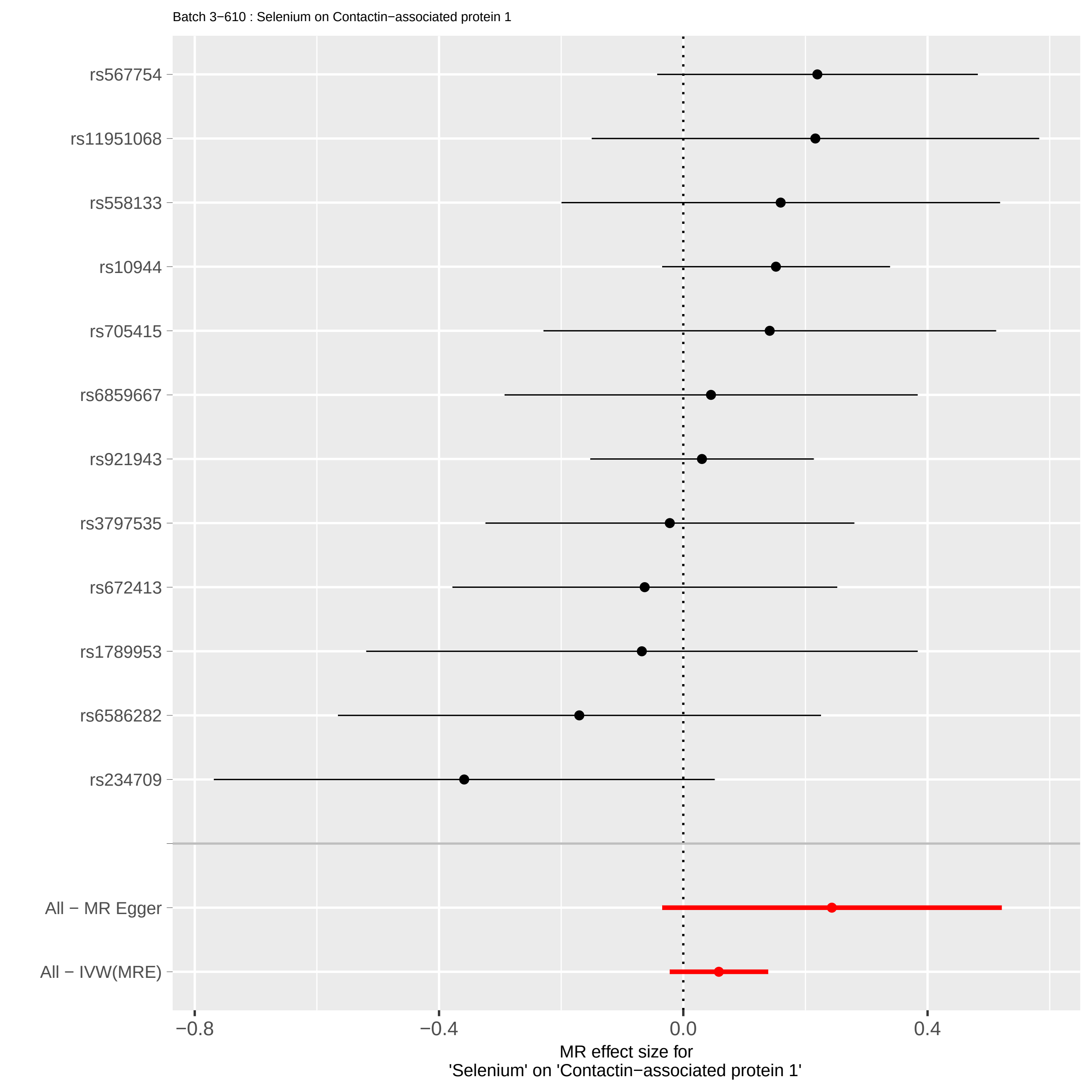

Batch 3−610 : Selenium on Contactin−associated protein 1
rs567754
rs11951068
rs558133
rs10944
rs705415
rs6859667
rs921943
rs3797535
rs672413
rs1789953
rs6586282
rs234709
All − MR Egger
All − IVW(MRE)
−0.8
−0.4
0.0
0.4
MR effect size for
'Selenium' on 'Contactin−associated protein 1'

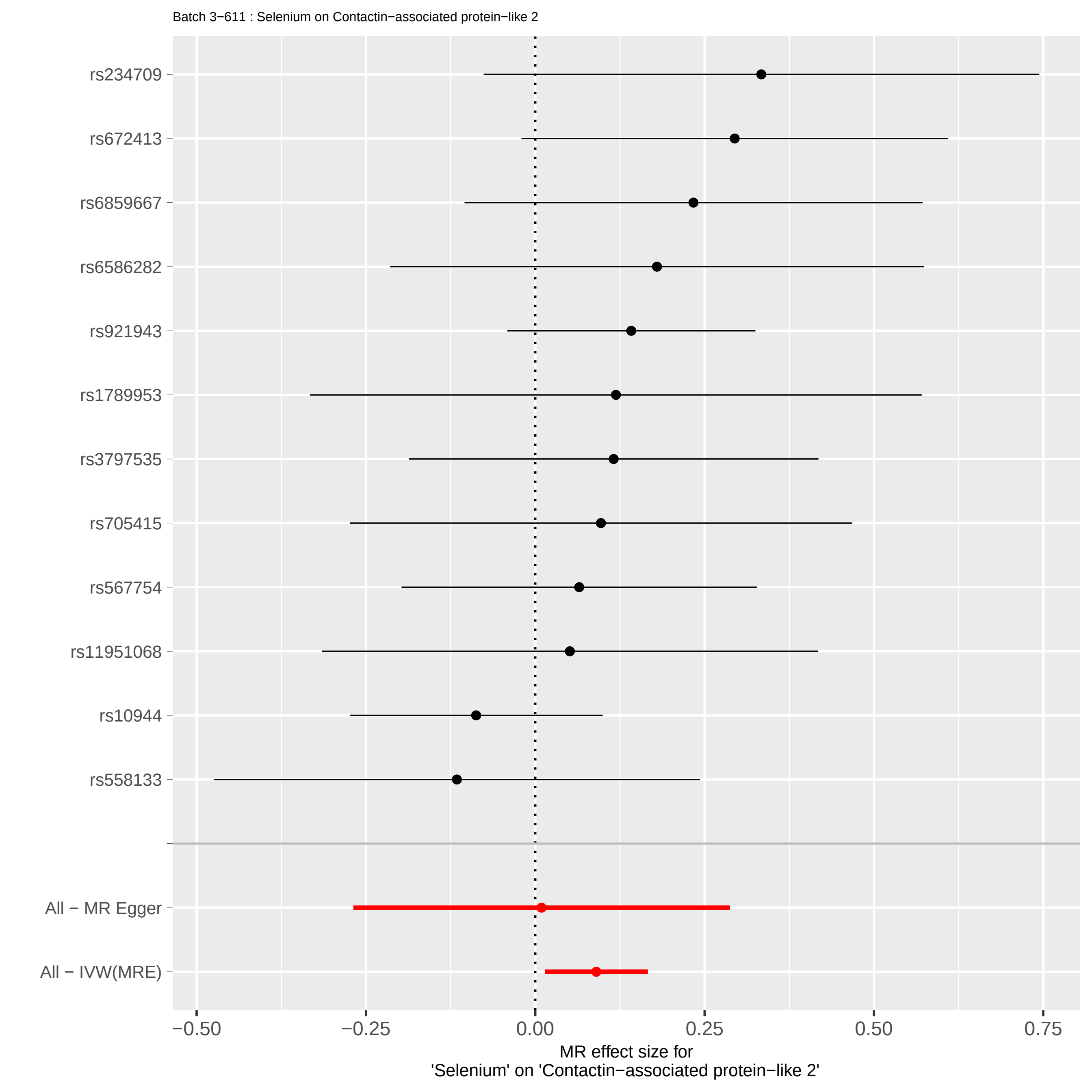
Batch 3−611 : Selenium on Contactin−associated protein−like 2
rs234709
rs672413
rs6859667
rs6586282
rs921943
rs1789953
rs3797535
rs705415
rs567754
rs11951068
rs10944
rs558133
All − MR Egger
All − IVW(MRE)
−0.50
−0.25
0.00
0.25
0.50
0.75
MR effect size for
'Selenium' on 'Contactin−associated protein−like 2'

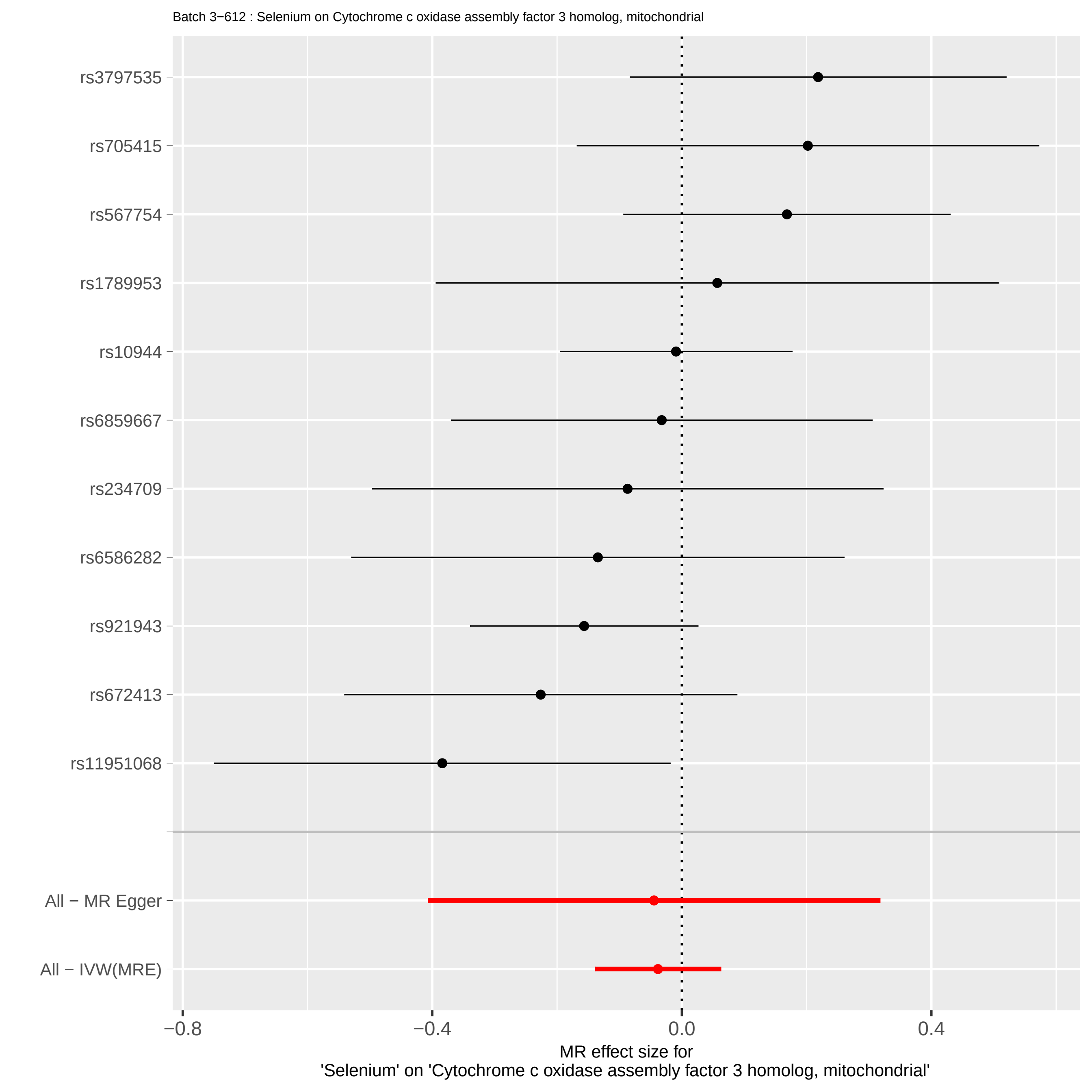
Batch 3−612 : Selenium on Cytochrome c oxidase assembly factor 3 homolog, mitochondrial
rs3797535
rs705415
rs567754
rs1789953
rs10944
rs6859667
rs234709
rs6586282
rs921943
rs672413
rs11951068
All − MR Egger
All − IVW(MRE)
−0.8
−0.4
0.0
0.4
MR effect size for
'Selenium' on 'Cytochrome c oxidase assembly factor 3 homolog, mitochondrial'

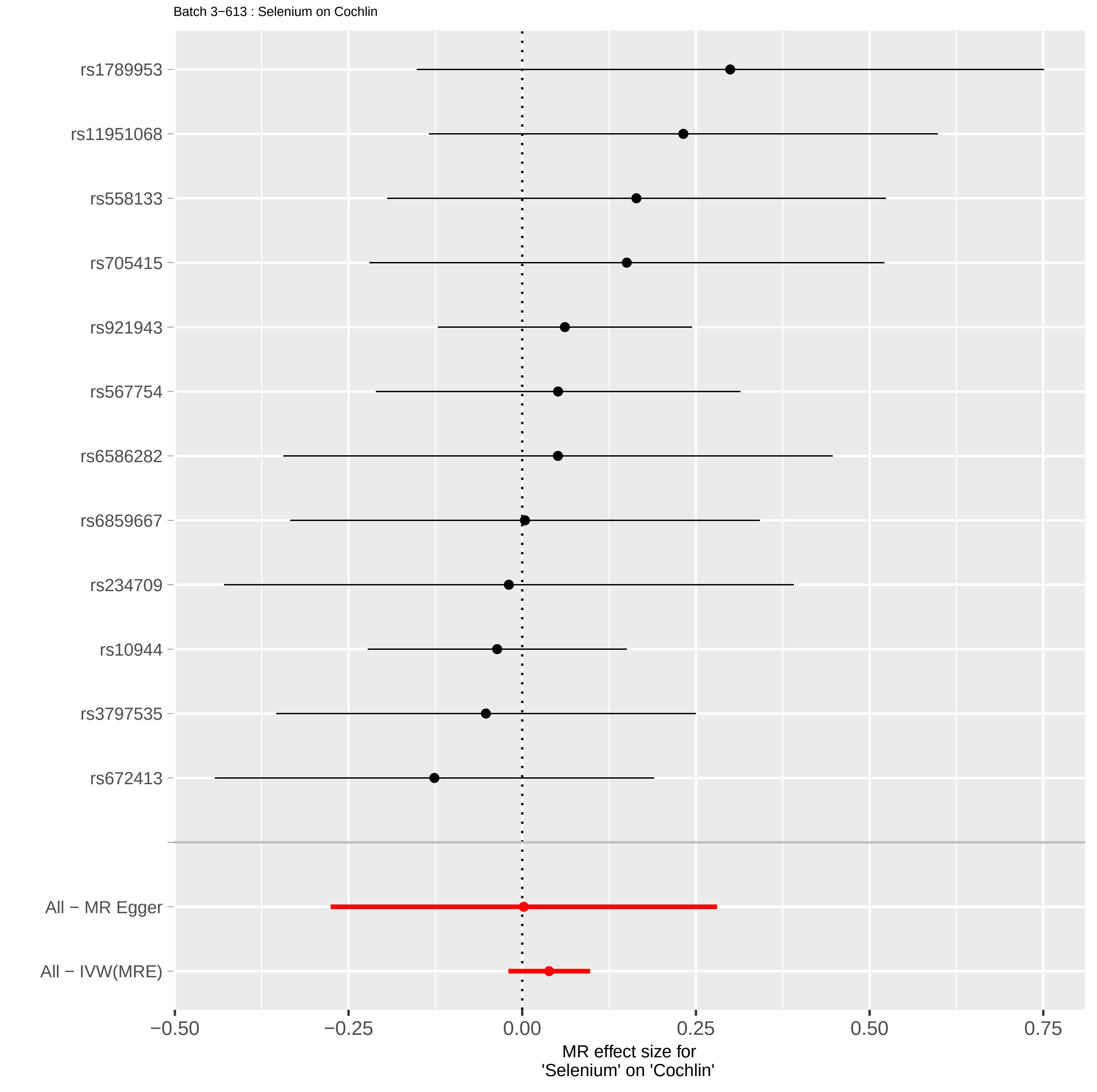

Batch 3−613 : Selenium on Cochlin
rs1789953
rs11951068
rs558133
rs705415
rs921943
rs567754
rs6586282
rs6859667
rs234709
rs10944
rs3797535
rs672413
All − MR Egger
All − IVW(MRE)
−0.50
−0.25
0.00
0.25
0.50
0.75
MR effect size for
'Selenium' on 'Cochlin'

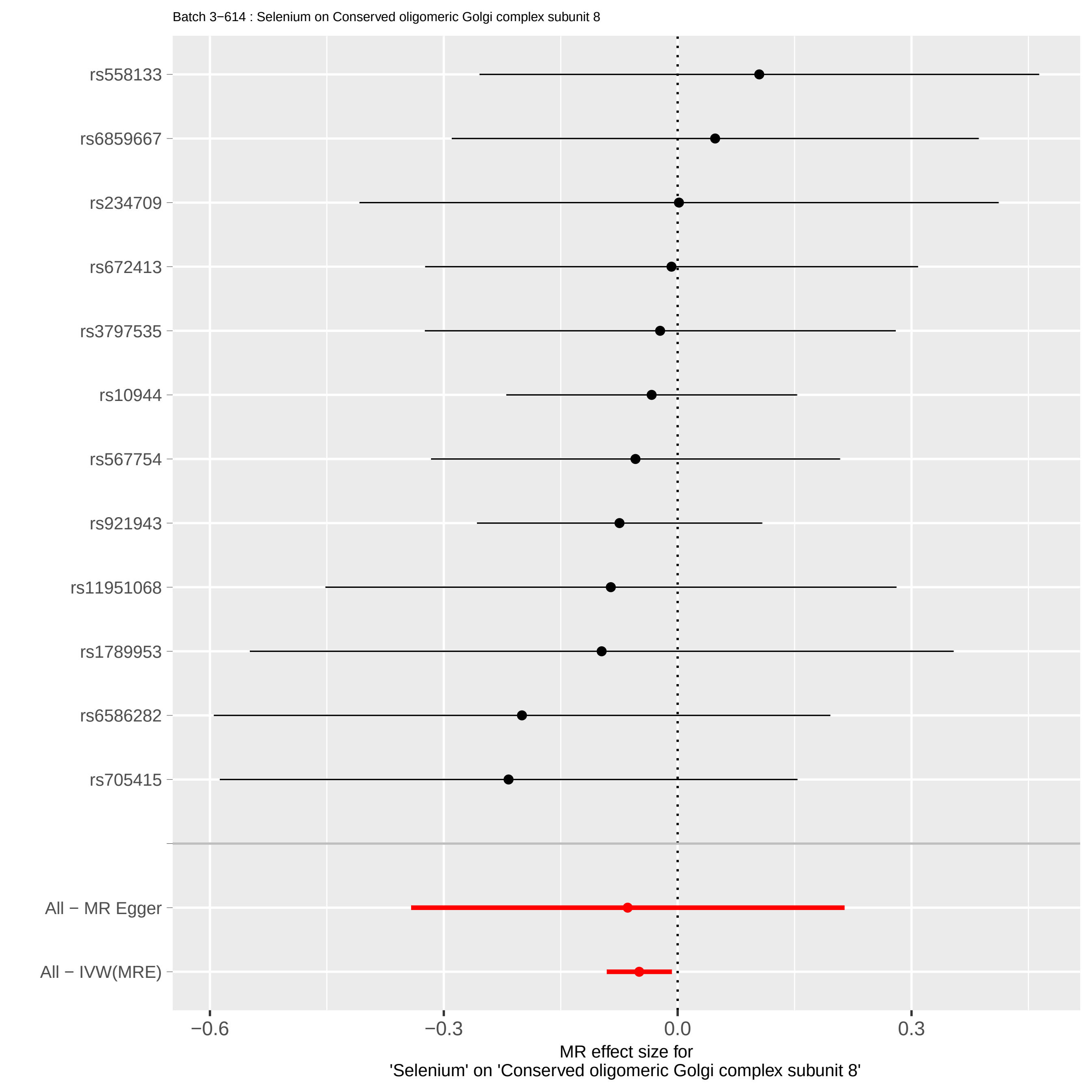

Batch 3−614 : Selenium on Conserved oligomeric Golgi complex subunit 8
rs558133
rs6859667
rs234709
rs672413
rs3797535
rs10944
rs567754
rs921943
rs11951068
rs1789953
rs6586282
rs705415
All − MR Egger
All − IVW(MRE)
−0.6
−0.3
0.0
0.3
MR effect size for
'Selenium' on 'Conserved oligomeric Golgi complex subunit 8'

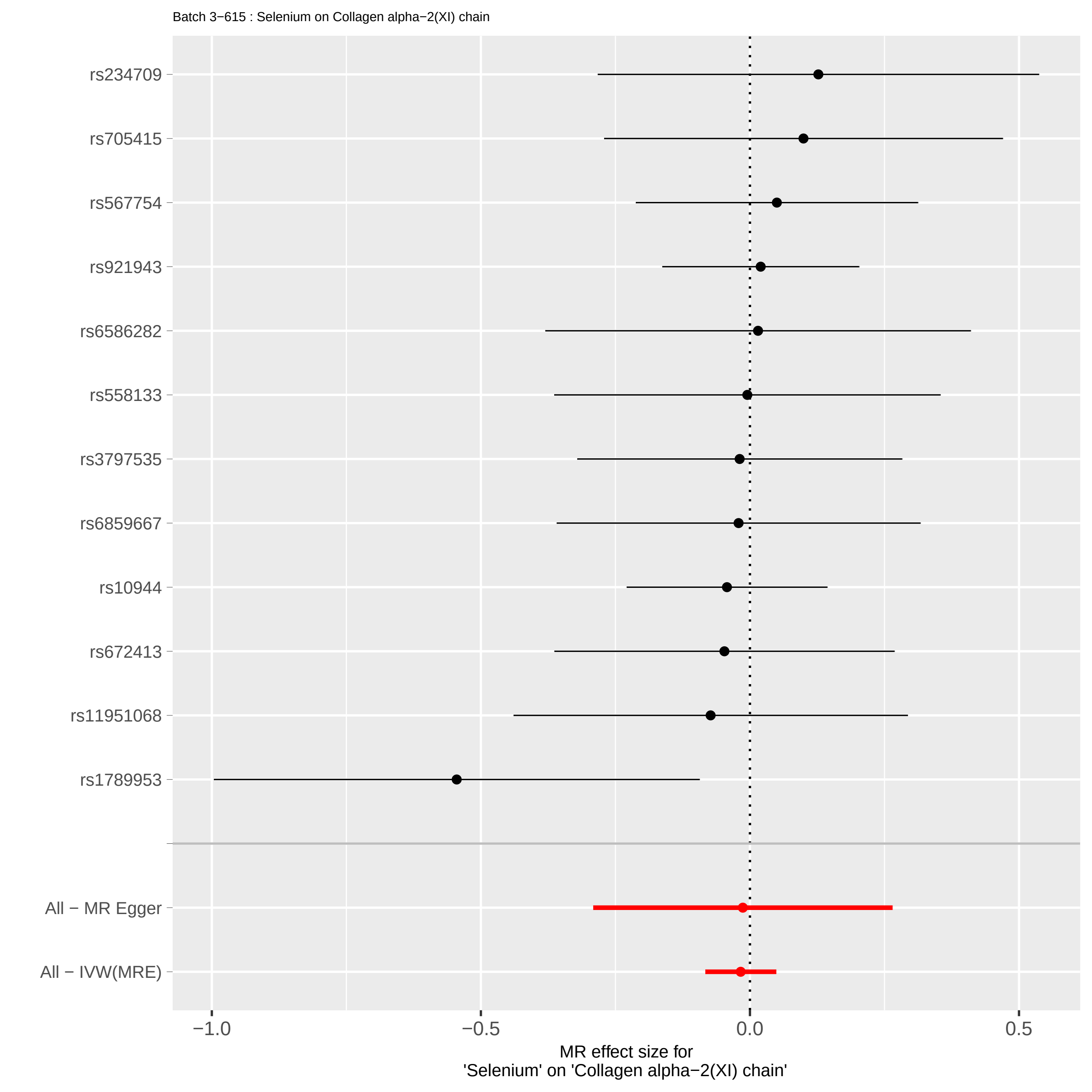

Batch 3−615 : Selenium on Collagen alpha−2(XI) chain
rs234709
rs705415
rs567754
rs921943
rs6586282
rs558133
rs3797535
rs6859667
rs10944
rs672413
rs11951068
rs1789953
All − MR Egger
All − IVW(MRE)
−1.0
−0.5
0.0
0.5
MR effect size for
'Selenium' on 'Collagen alpha−2(XI) chain'

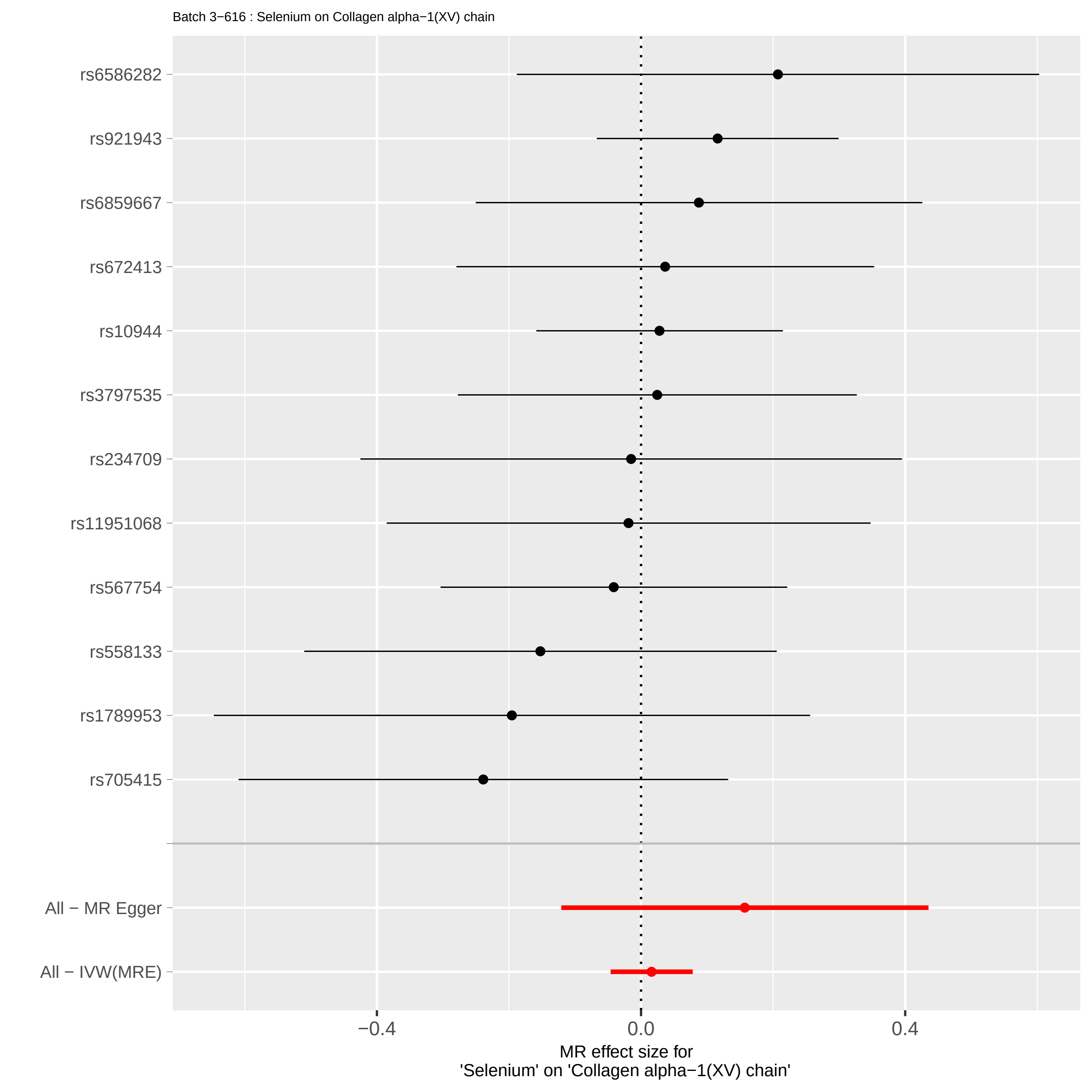
Batch 3−616 : Selenium on Collagen alpha−1(XV) chain
rs6586282
rs921943
rs6859667
rs672413
rs10944
rs3797535
rs234709
rs11951068
rs567754
rs558133
rs1789953
rs705415
All − MR Egger
All − IVW(MRE)
−0.4
0.0
0.4
MR effect size for
'Selenium' on 'Collagen alpha−1(XV) chain'

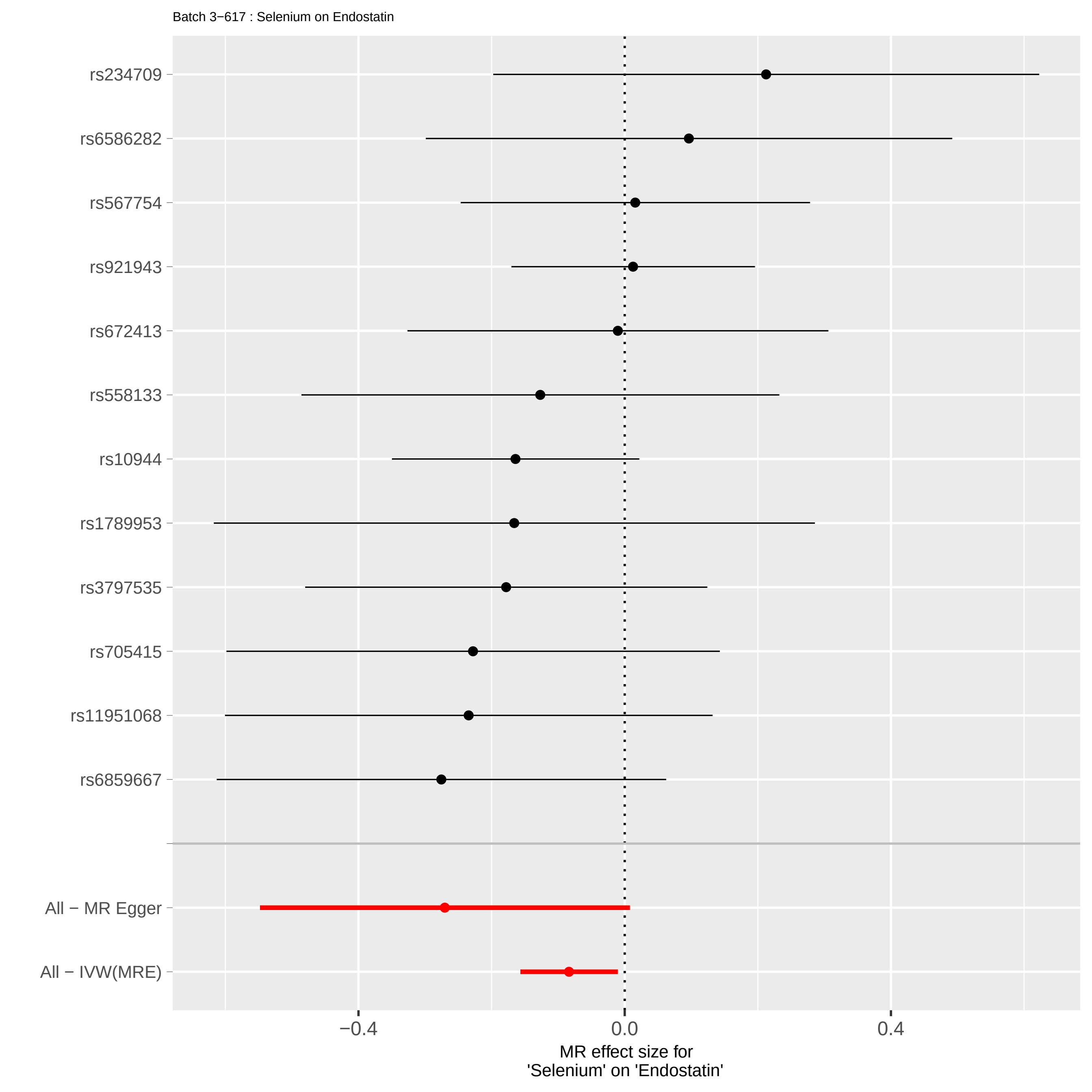

Batch 3−617 : Selenium on Endostatin
rs234709
rs6586282
rs567754
rs921943
rs672413
rs558133
rs10944
rs1789953
rs3797535
rs705415
rs11951068
rs6859667
All − MR Egger
All − IVW(MRE)
−0.4
0.0
0.4
MR effect size for
'Selenium' on 'Endostatin'

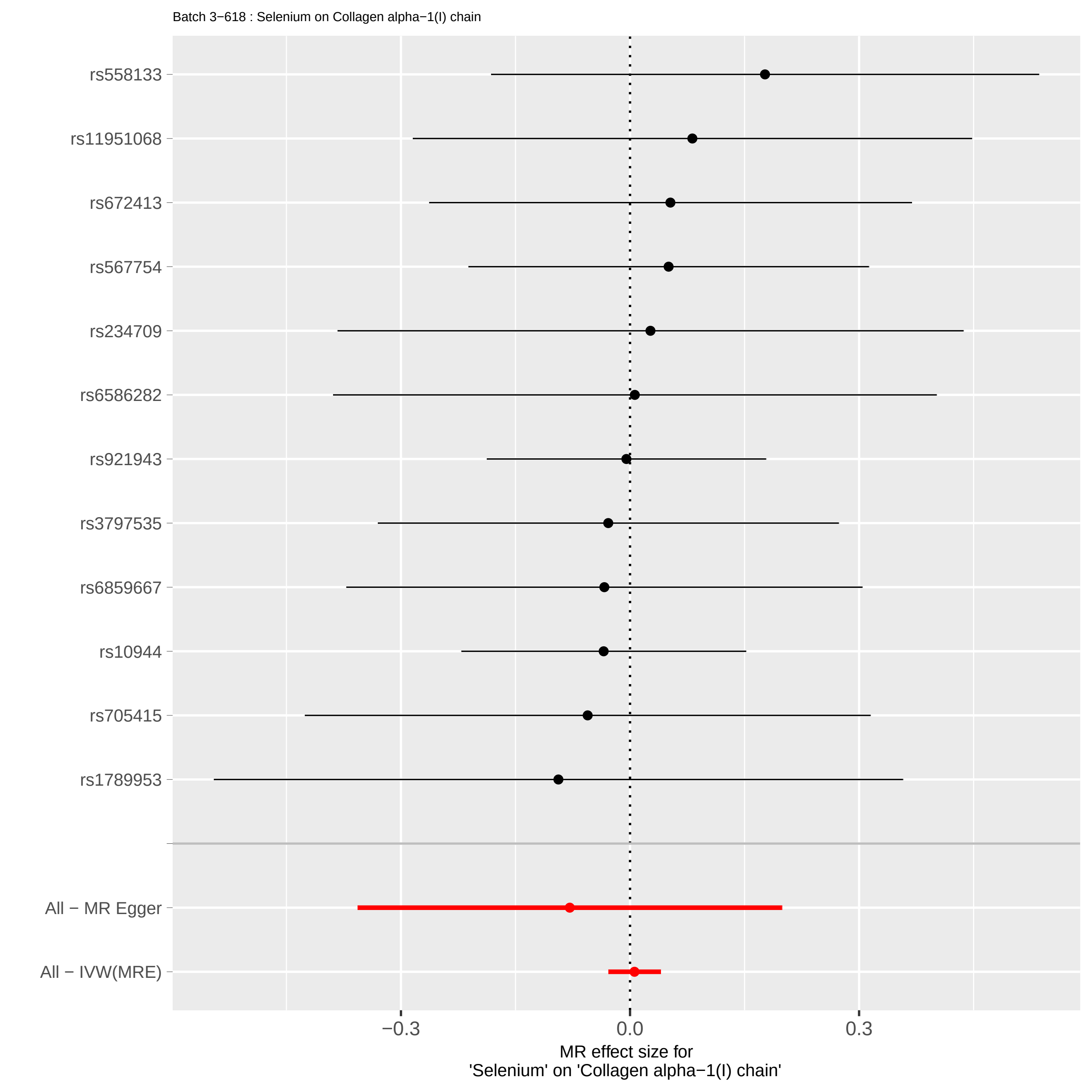
Batch 3−618 : Selenium on Collagen alpha−1(I) chain
rs558133
rs11951068
rs672413
rs567754
rs234709
rs6586282
rs921943
rs3797535
rs6859667
rs10944
rs705415
rs1789953
All − MR Egger
All − IVW(MRE)
−0.3
0.0
0.3
MR effect size for
'Selenium' on 'Collagen alpha−1(I) chain'

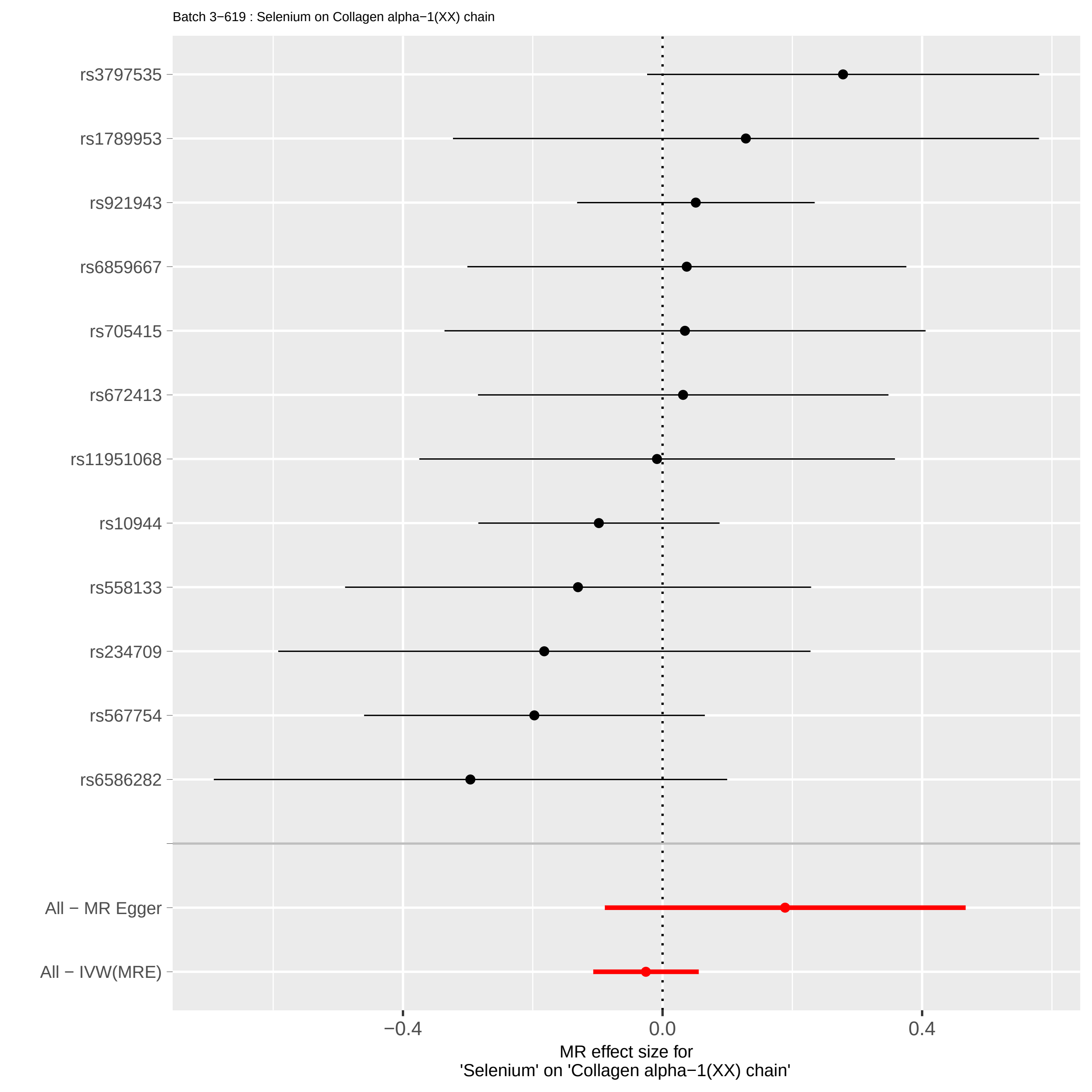

Batch 3−619 : Selenium on Collagen alpha−1(XX) chain
rs3797535
rs1789953
rs921943
rs6859667
rs705415
rs672413
rs11951068
rs10944
rs558133
rs234709
rs567754
rs6586282
All − MR Egger
All − IVW(MRE)
−0.4
0.0
0.4
MR effect size for
'Selenium' on 'Collagen alpha−1(XX) chain'

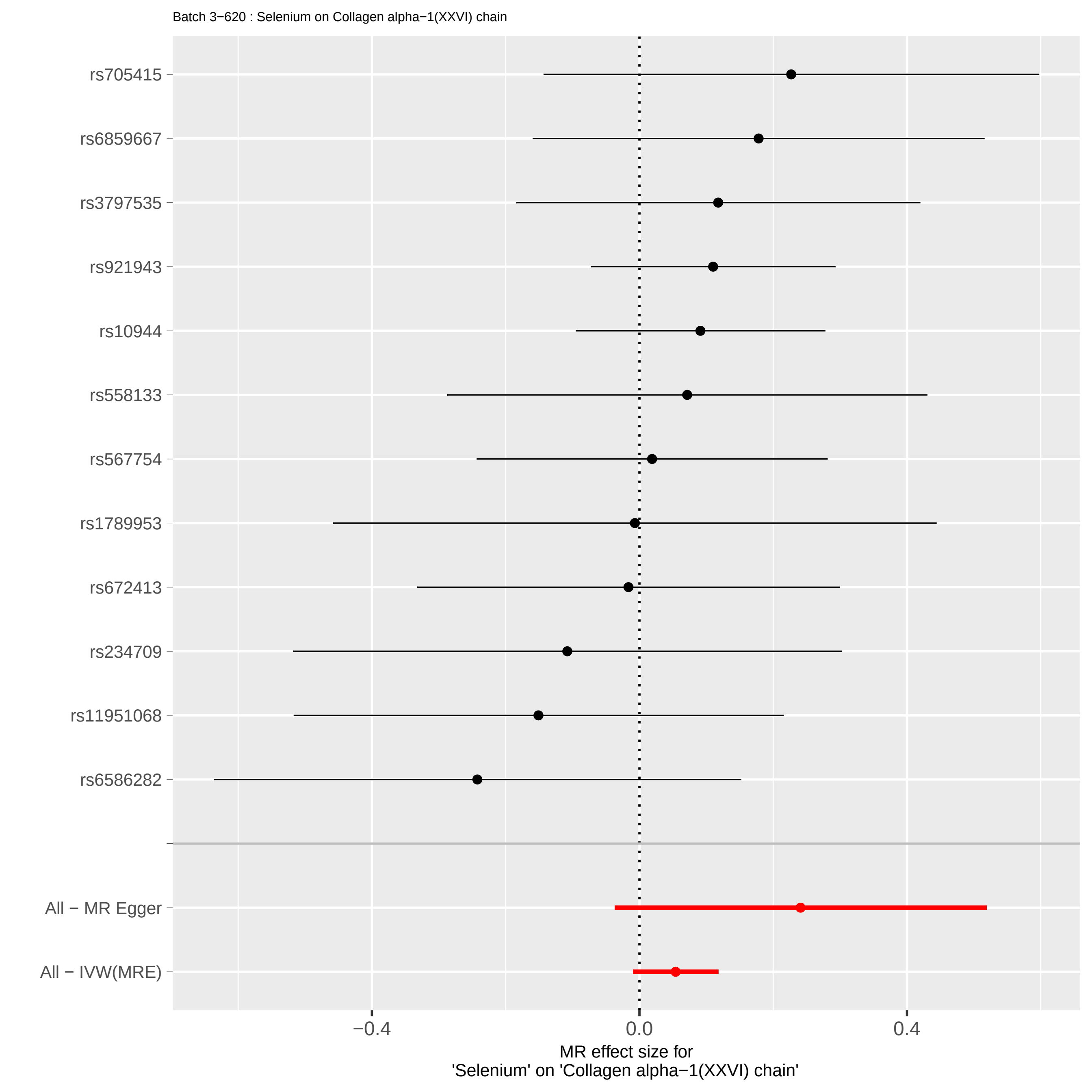

Batch 3−620 : Selenium on Collagen alpha−1(XXVI) chain
rs705415
rs6859667
rs3797535
rs921943
rs10944
rs558133
rs567754
rs1789953
rs672413
rs234709
rs11951068
rs6586282
All − MR Egger
All − IVW(MRE)
−0.4
0.0
0.4
MR effect size for
'Selenium' on 'Collagen alpha−1(XXVI) chain'

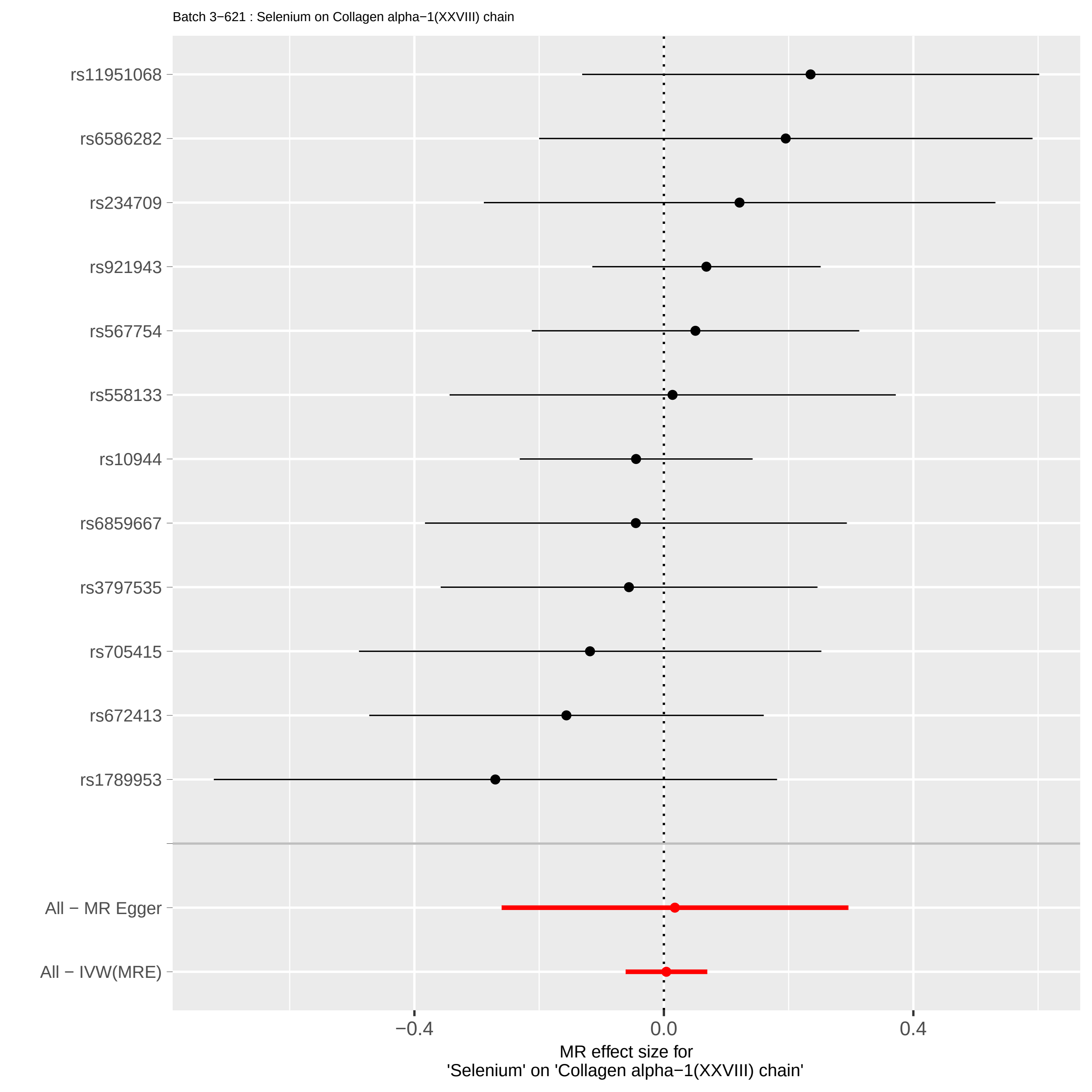

Batch 3−621 : Selenium on Collagen alpha−1(XXVIII) chain
rs11951068
rs6586282
rs234709
rs921943
rs567754
rs558133
rs10944
rs6859667
rs3797535
rs705415
rs672413
rs1789953
All − MR Egger
All − IVW(MRE)
−0.4
0.0
0.4
MR effect size for
'Selenium' on 'Collagen alpha−1(XXVIII) chain'

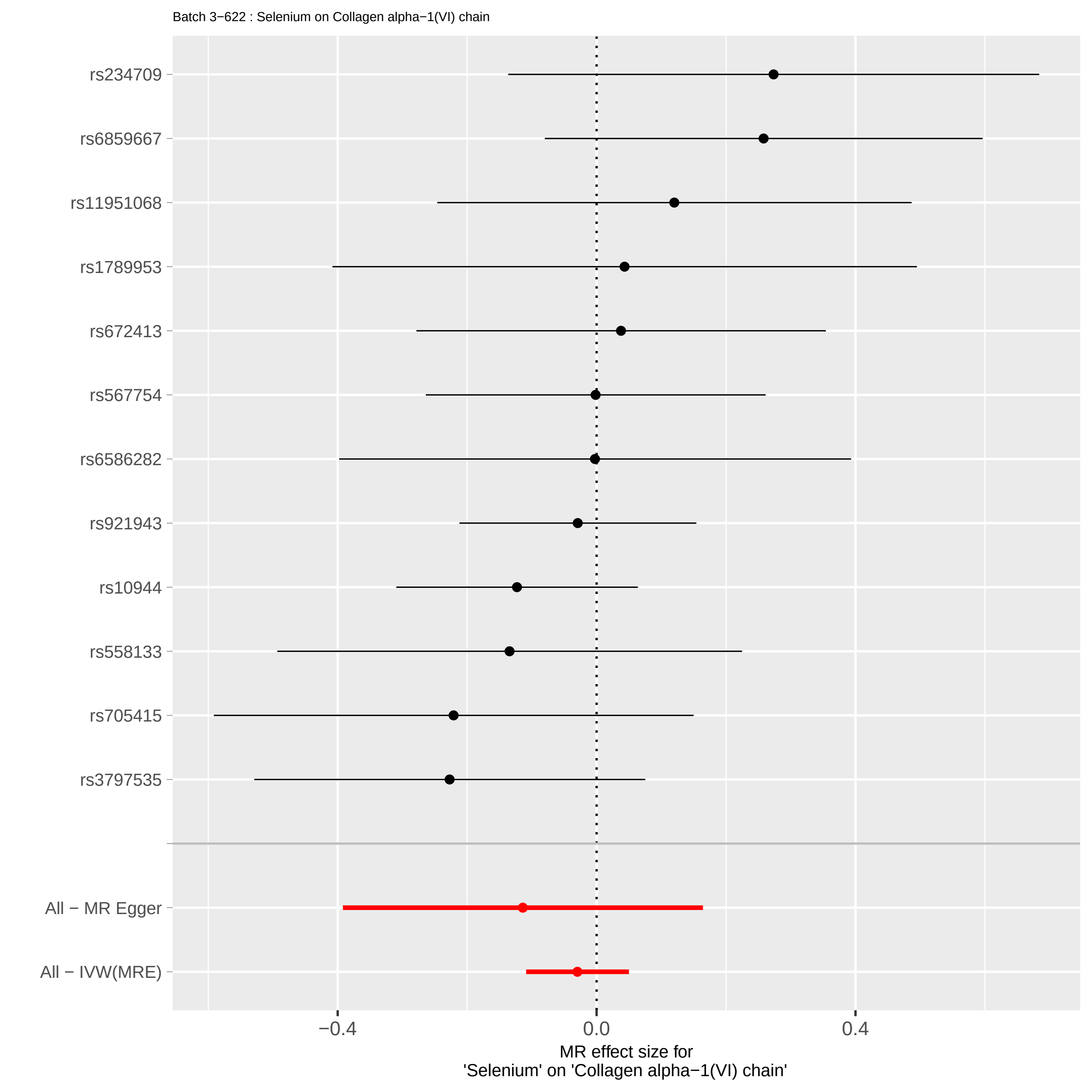

Batch 3−622 : Selenium on Collagen alpha−1(VI) chain
rs234709
rs6859667
rs11951068
rs1789953
rs672413
rs567754
rs6586282
rs921943
rs10944
rs558133
rs705415
rs3797535
All − MR Egger
All − IVW(MRE)
−0.4
0.0
0.4
MR effect size for
'Selenium' on 'Collagen alpha−1(VI) chain'

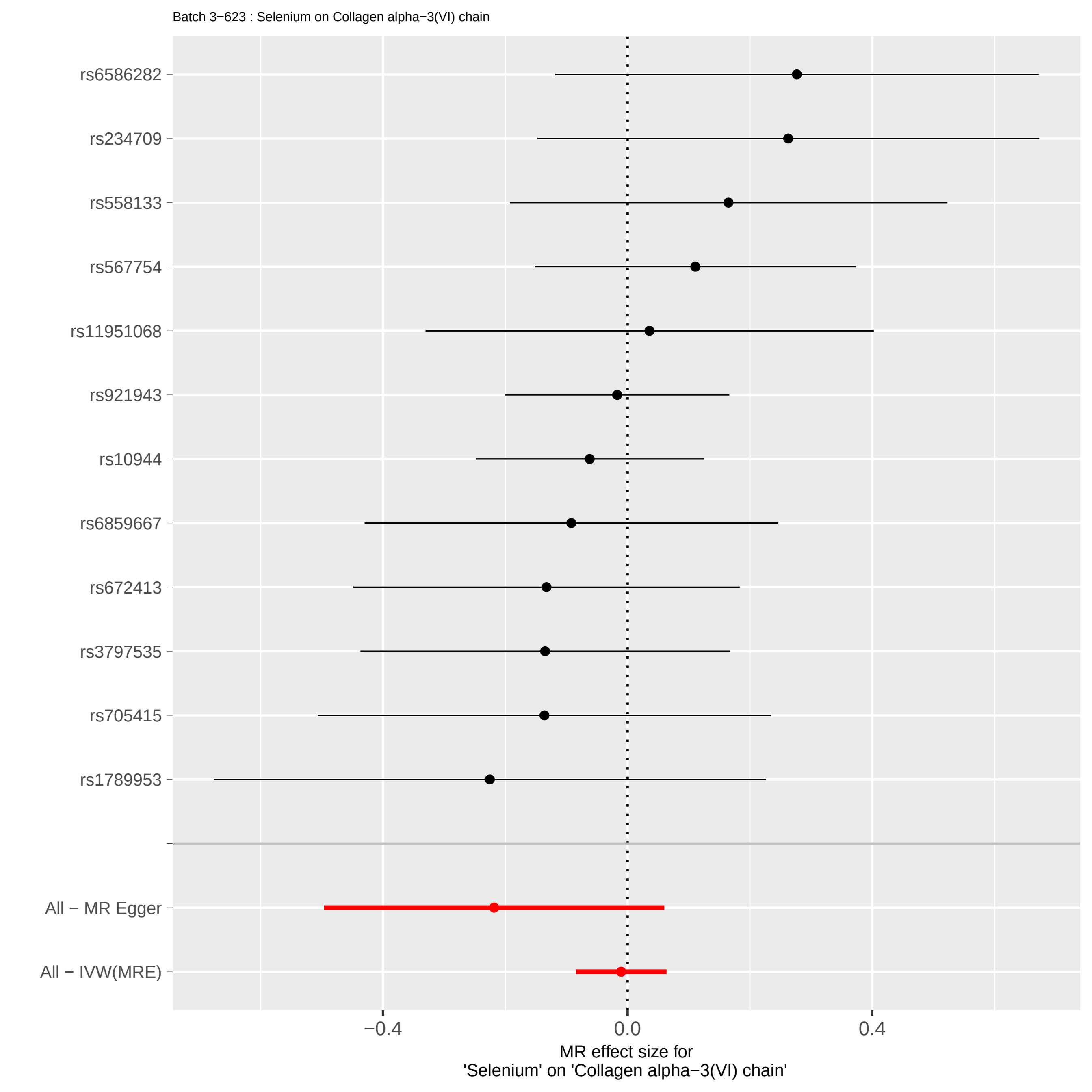
Batch 3−623 : Selenium on Collagen alpha−3(VI) chain
rs6586282
rs234709
rs558133
rs567754
rs11951068
rs921943
rs10944
rs6859667
rs672413
rs3797535
rs705415
rs1789953
All − MR Egger
All − IVW(MRE)
−0.4
0.0
0.4
MR effect size for
'Selenium' on 'Collagen alpha−3(VI) chain'

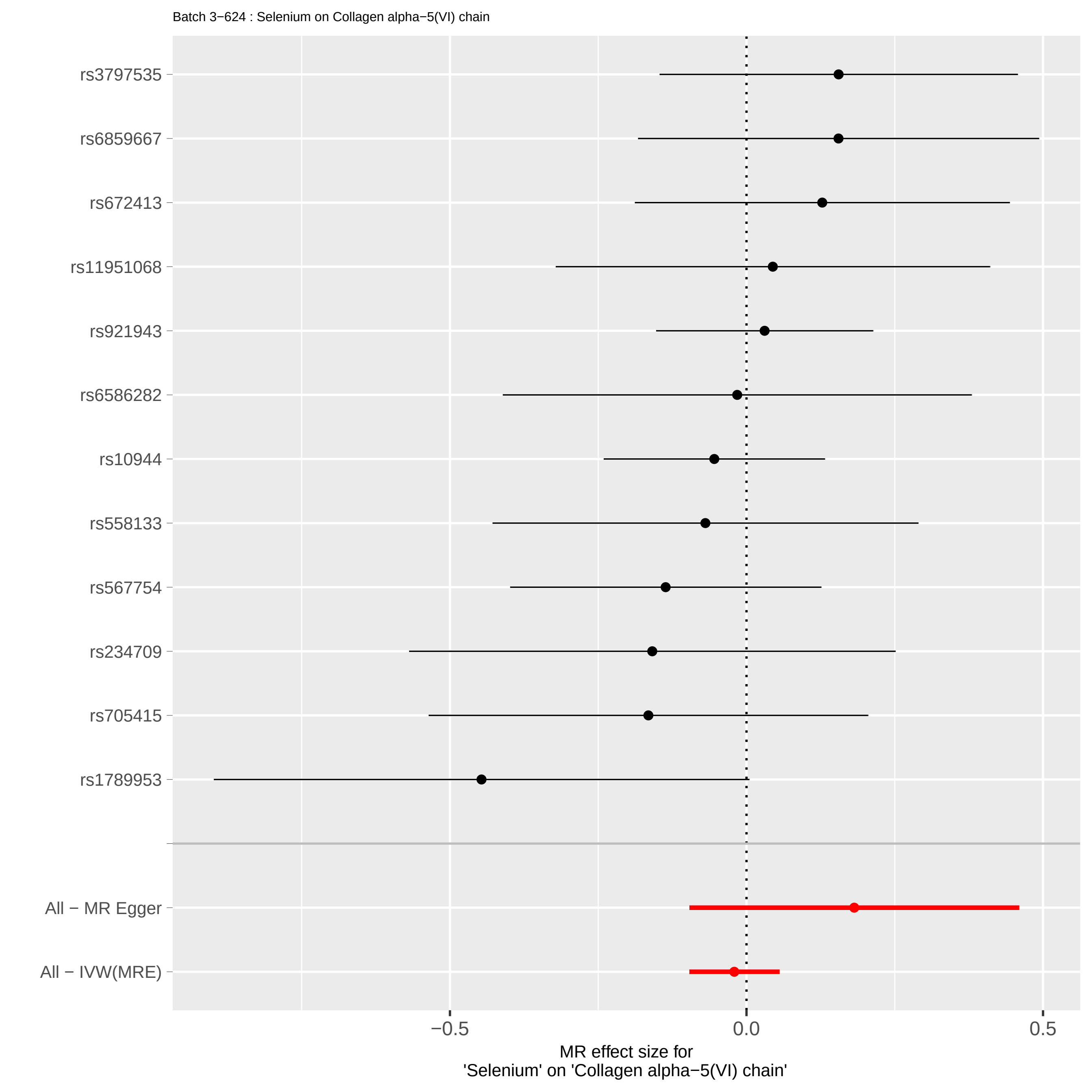

Batch 3−624 : Selenium on Collagen alpha−5(VI) chain
rs3797535
rs6859667
rs672413
rs11951068
rs921943
rs6586282
rs10944
rs558133
rs567754
rs234709
rs705415
rs1789953
All − MR Egger
All − IVW(MRE)
−0.5
0.0
0.5
MR effect size for
'Selenium' on 'Collagen alpha−5(VI) chain'

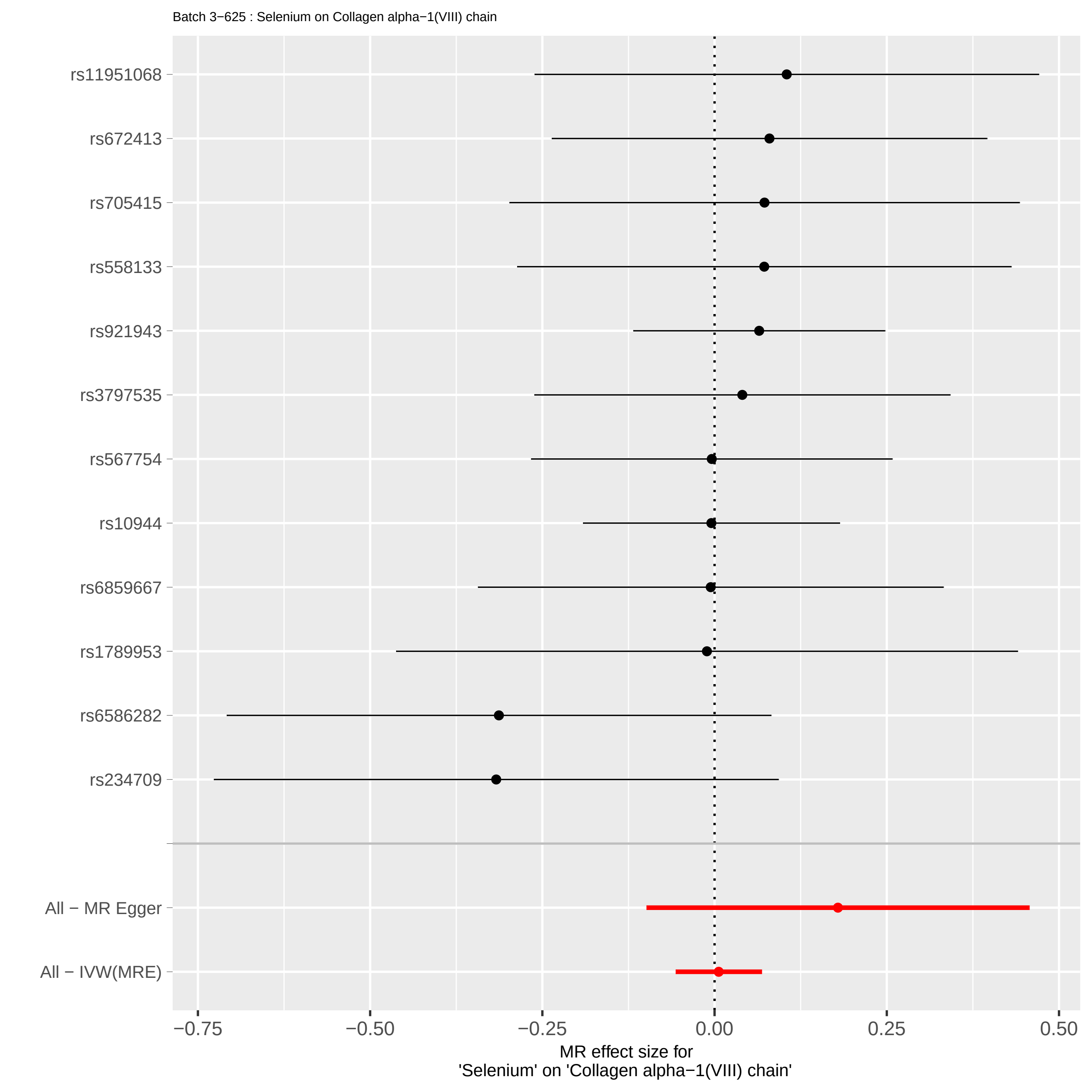

Batch 3−625 : Selenium on Collagen alpha−1(VIII) chain
rs11951068
rs672413
rs705415
rs558133
rs921943
rs3797535
rs567754
rs10944
rs6859667
rs1789953
rs6586282
rs234709
All − MR Egger
All − IVW(MRE)
−0.75
−0.50
−0.25
0.00
0.25
0.50
MR effect size for
'Selenium' on 'Collagen alpha−1(VIII) chain'

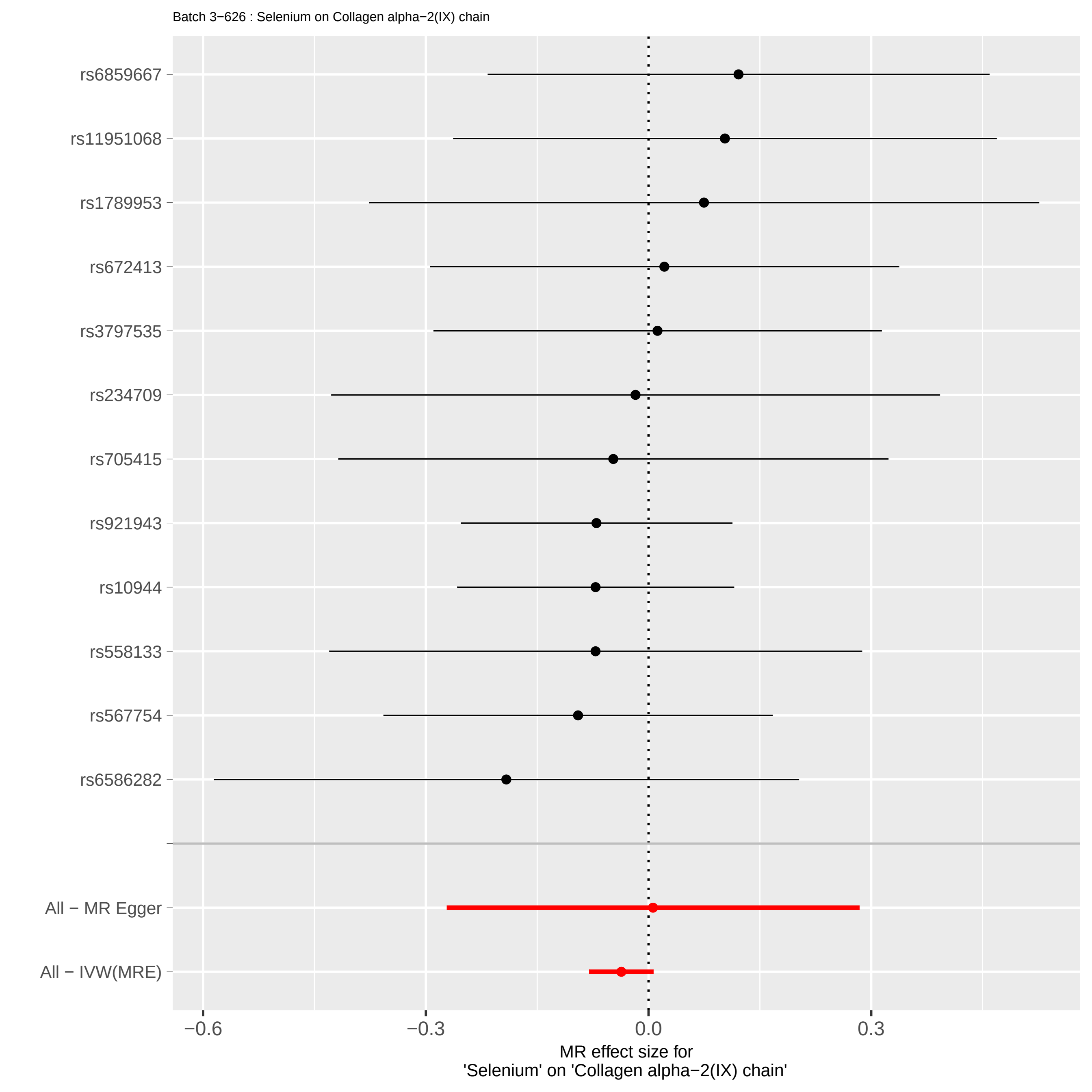

Batch 3−626 : Selenium on Collagen alpha−2(IX) chain
rs6859667
rs11951068
rs1789953
rs672413
rs3797535
rs234709
rs705415
rs921943
rs10944
rs558133
rs567754
rs6586282
All − MR Egger
All − IVW(MRE)
−0.6
−0.3
0.0
0.3
MR effect size for
'Selenium' on 'Collagen alpha−2(IX) chain'

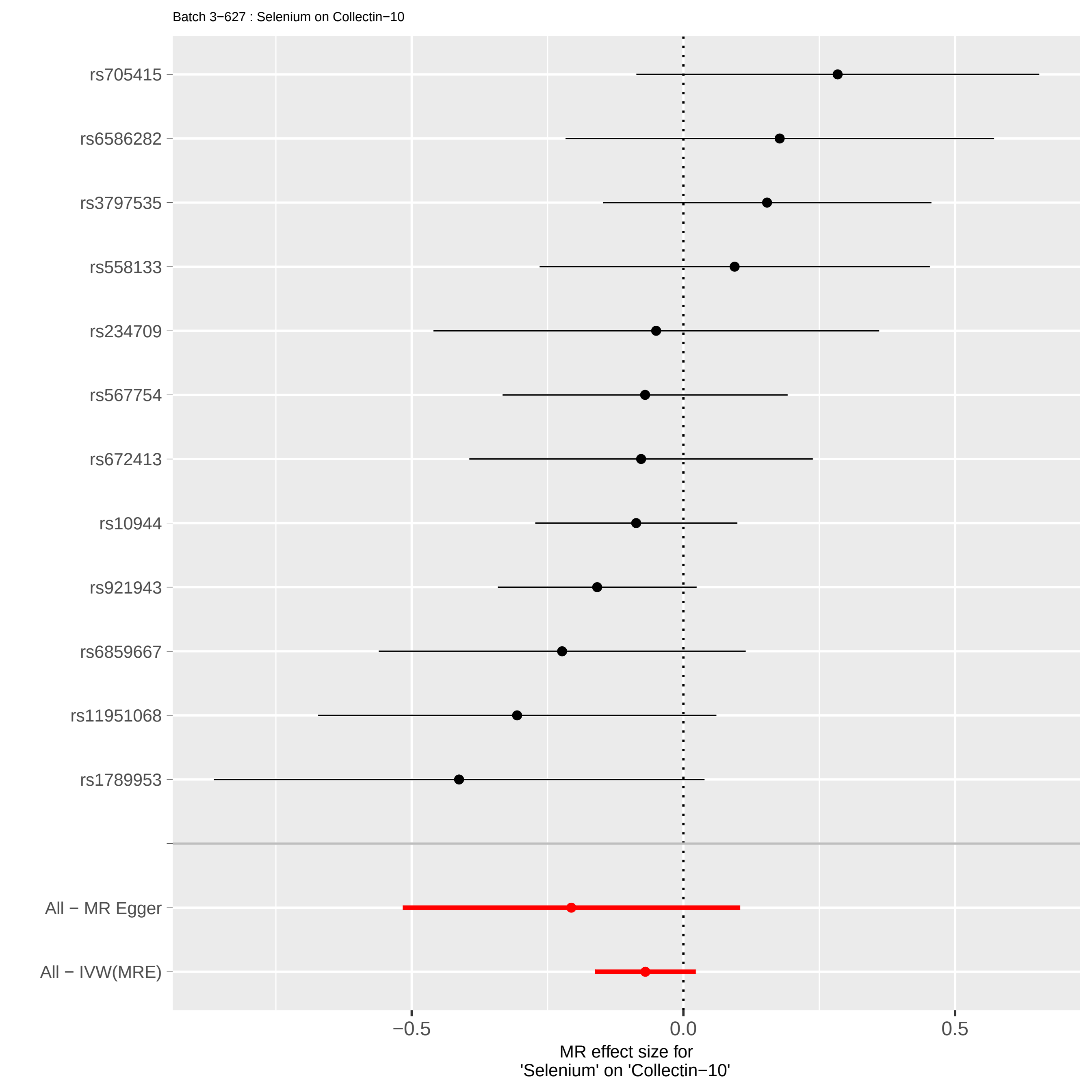
Batch 3−627 : Selenium on Collectin−10
rs705415
rs6586282
rs3797535
rs558133
rs234709
rs567754
rs672413
rs10944
rs921943
rs6859667
rs11951068
rs1789953
All − MR Egger
All − IVW(MRE)
−0.5
0.0
0.5
MR effect size for
'Selenium' on 'Collectin−10'

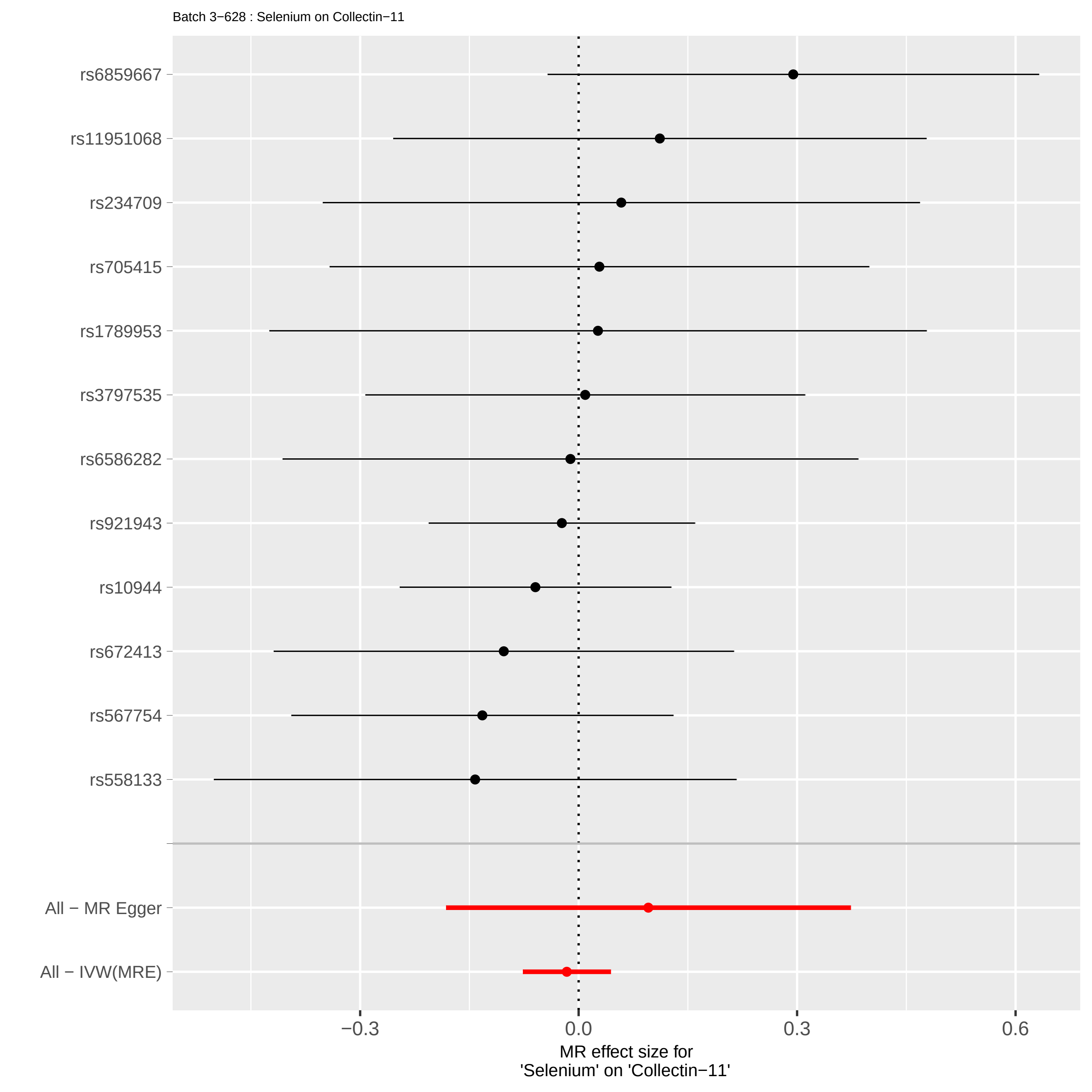

Batch 3−628 : Selenium on Collectin−11
rs6859667
rs11951068
rs234709
rs705415
rs1789953
rs3797535
rs6586282
rs921943
rs10944
rs672413
rs567754
rs558133
All − MR Egger
All − IVW(MRE)
−0.3
0.0
0.3
0.6
MR effect size for
'Selenium' on 'Collectin−11'

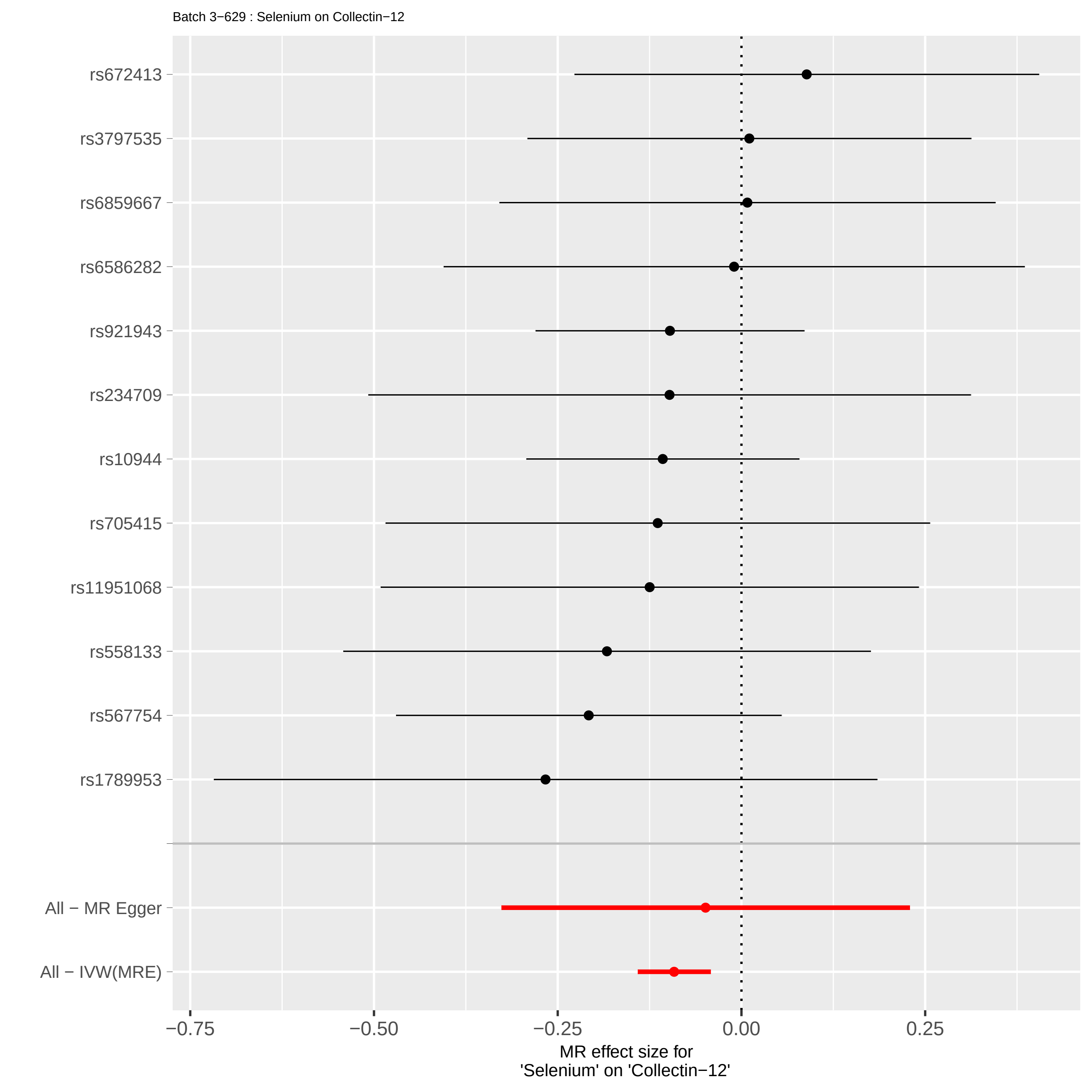

Batch 3−629 : Selenium on Collectin−12
rs672413
rs3797535
rs6859667
rs6586282
rs921943
rs234709
rs10944
rs705415
rs11951068
rs558133
rs567754
rs1789953
All − MR Egger
All − IVW(MRE)
−0.75
−0.50
−0.25
0.00
0.25
MR effect size for
'Selenium' on 'Collectin−12'

Batch 3−630 : Selenium on Collectin−12

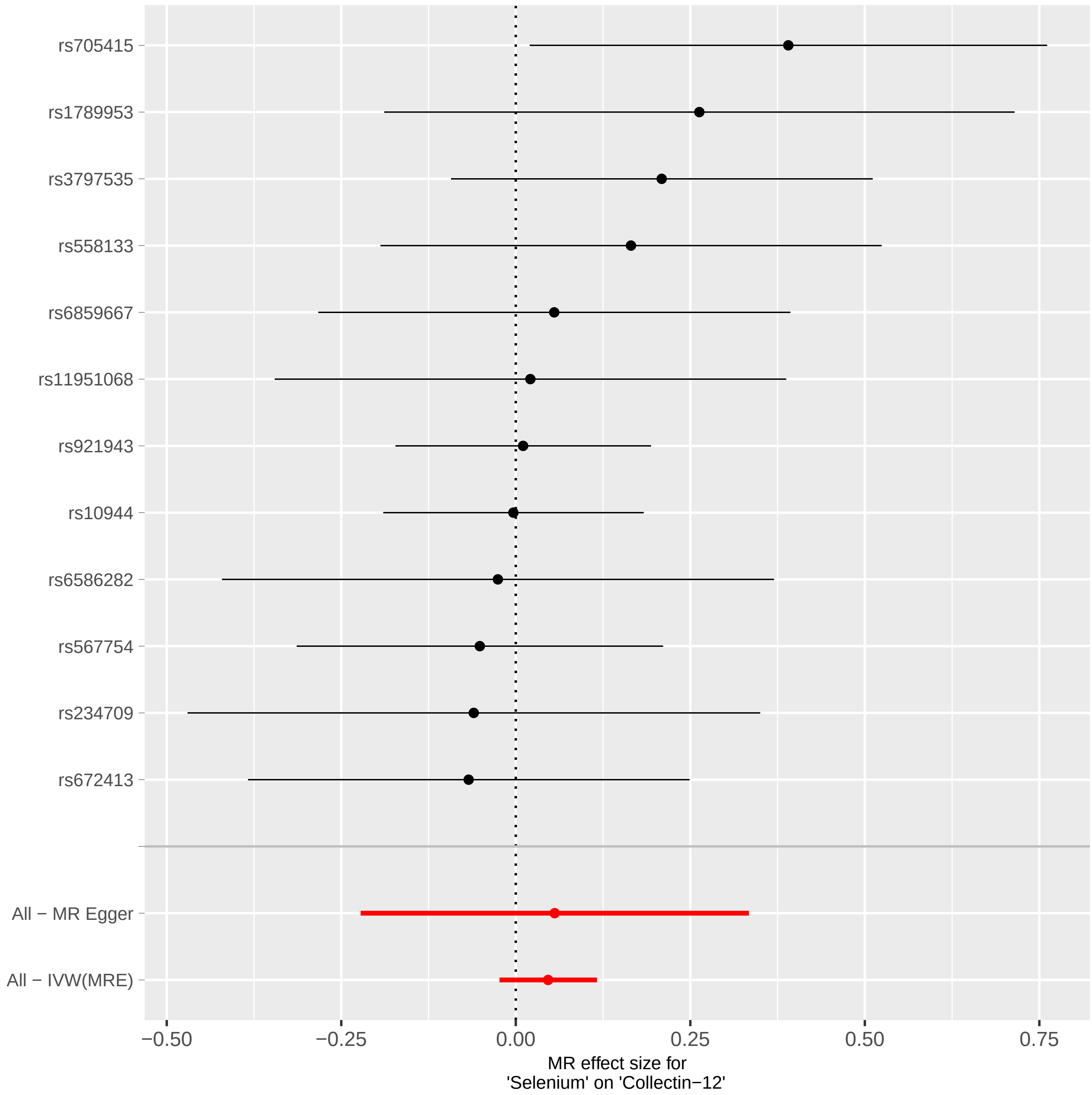

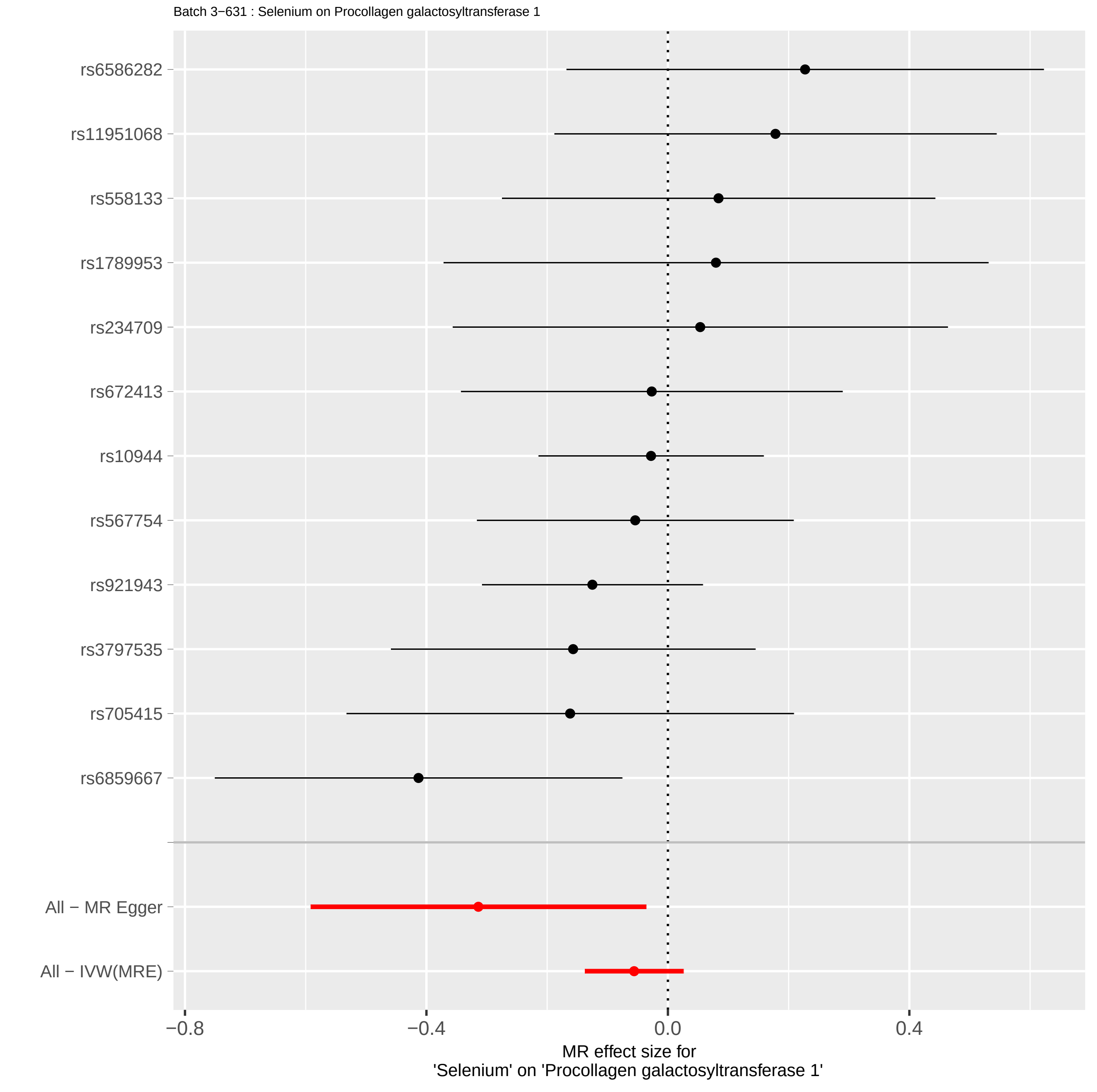

Batch 3−631 : Selenium on Procollagen galactosyltransferase 1
rs6586282
rs11951068
rs558133
rs1789953
rs234709
rs672413
rs10944
rs567754
rs921943
rs3797535
rs705415
rs6859667
All − MR Egger
All − IVW(MRE)
−0.8
−0.4
0.0
0.4
MR effect size for
'Selenium' on 'Procollagen galactosyltransferase 1'

Batch 3−632 : Selenium on Procollagen galactosyltransferase 2

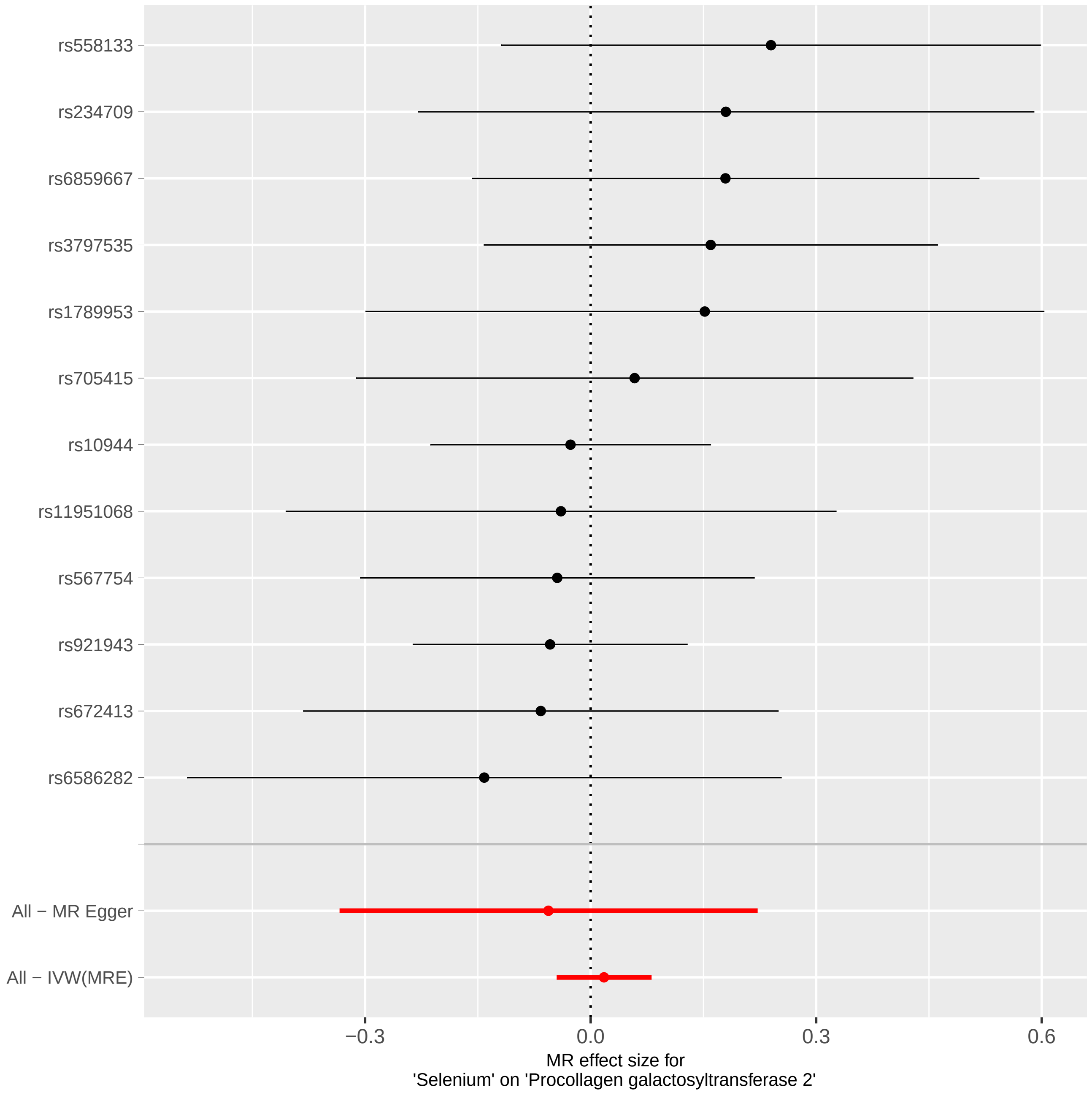

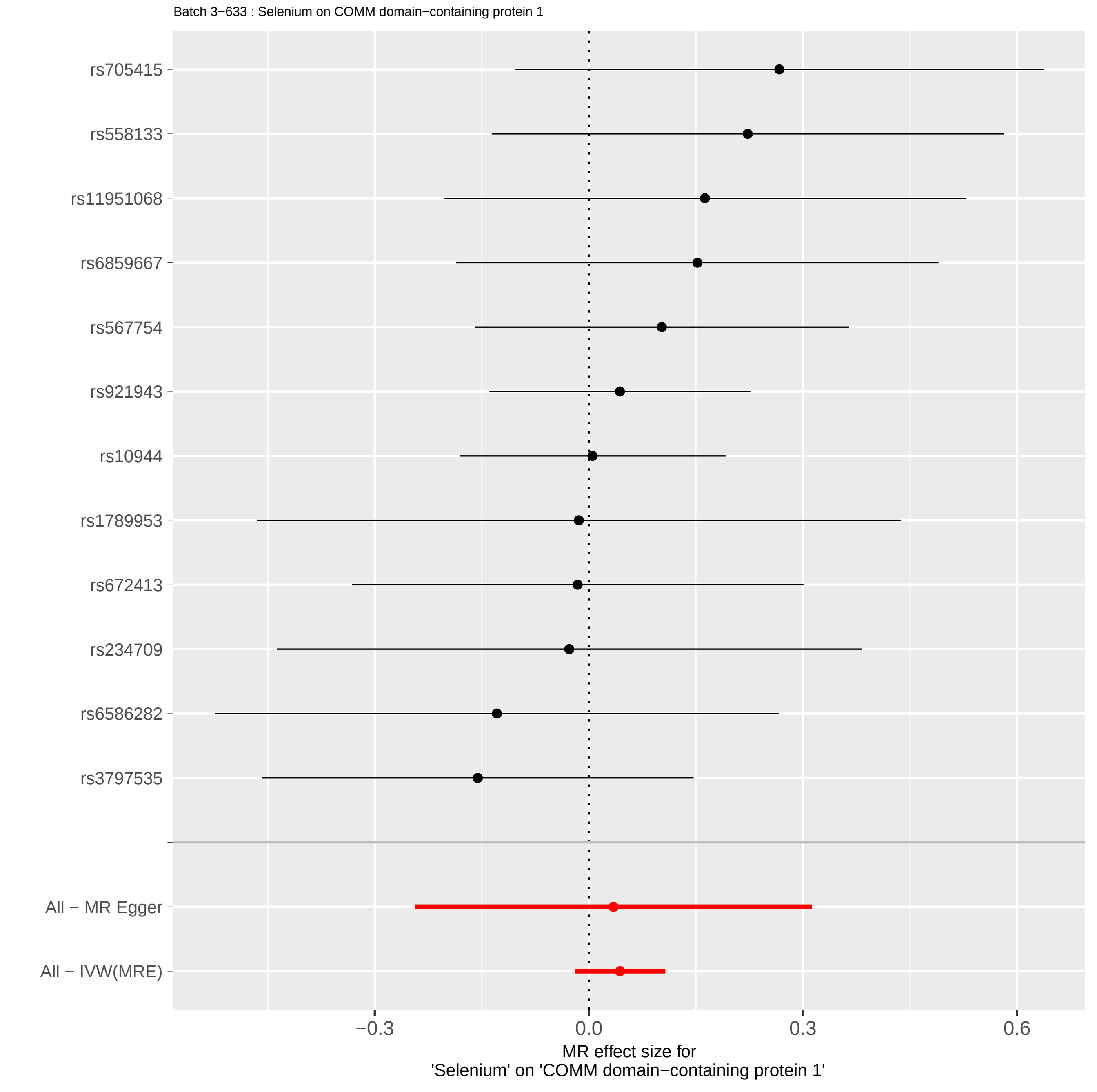

Batch 3−633 : Selenium on COMM domain−containing protein 1
rs705415
rs558133
rs11951068
rs6859667
rs567754
rs921943
rs10944
rs1789953
rs672413
rs234709
rs6586282
rs3797535
All − MR Egger
All − IVW(MRE)
−0.3
0.0
0.3
0.6
MR effect size for
'Selenium' on 'COMM domain−containing protein 1'

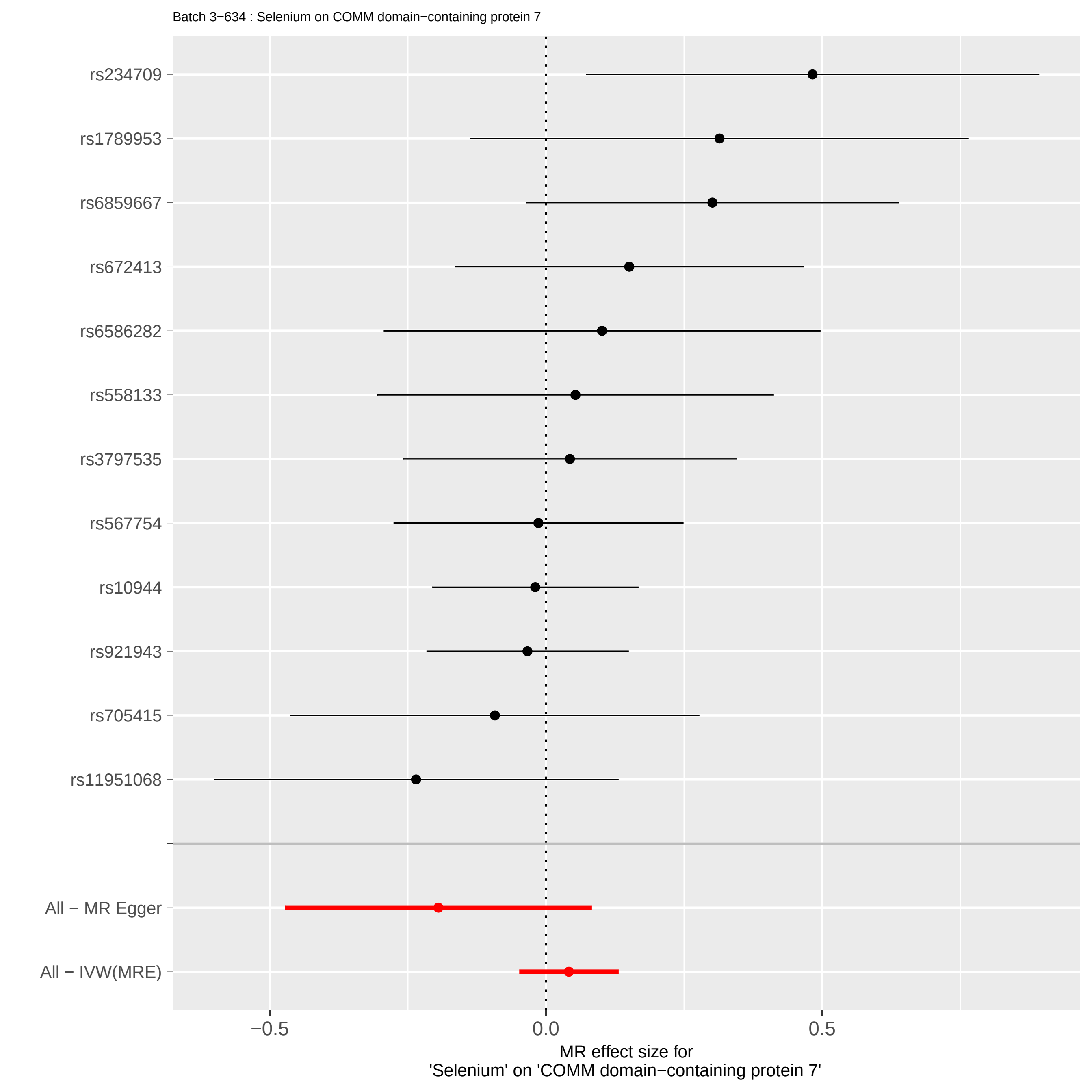

Batch 3−634 : Selenium on COMM domain−containing protein 7
rs234709
rs1789953
rs6859667
rs672413
rs6586282
rs558133
rs3797535
rs567754
rs10944
rs921943
rs705415
rs11951068
All − MR Egger
All − IVW(MRE)
−0.5
0.0
0.5
MR effect size for
'Selenium' on 'COMM domain−containing protein 7'

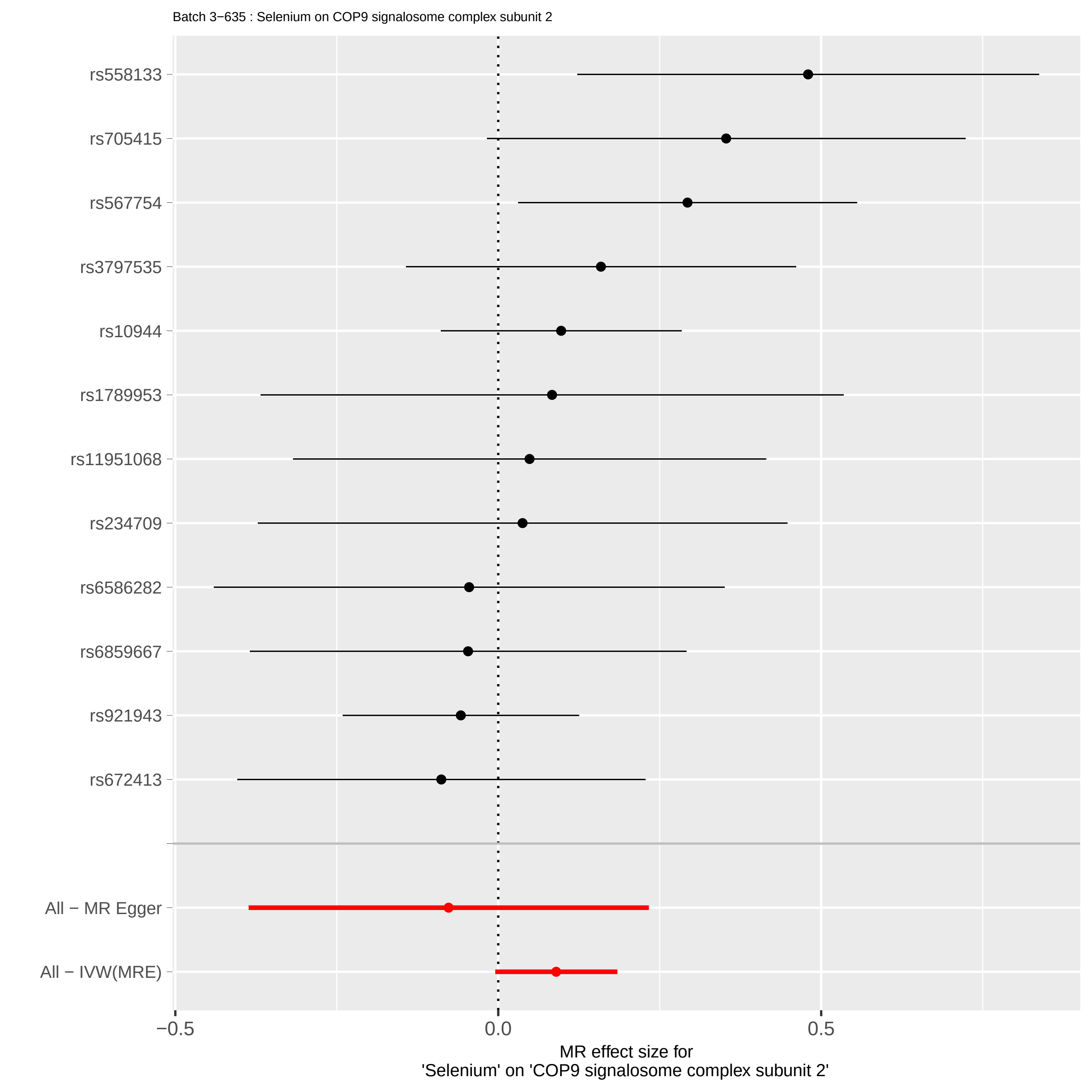

Batch 3−635 : Selenium on COP9 signalosome complex subunit 2
rs558133
rs705415
rs567754
rs3797535
rs10944
rs1789953
rs11951068
rs234709
rs6586282
rs6859667
rs921943
rs672413
All − MR Egger
All − IVW(MRE)
−0.5
0.0
0.5
MR effect size for
'Selenium' on 'COP9 signalosome complex subunit 2'

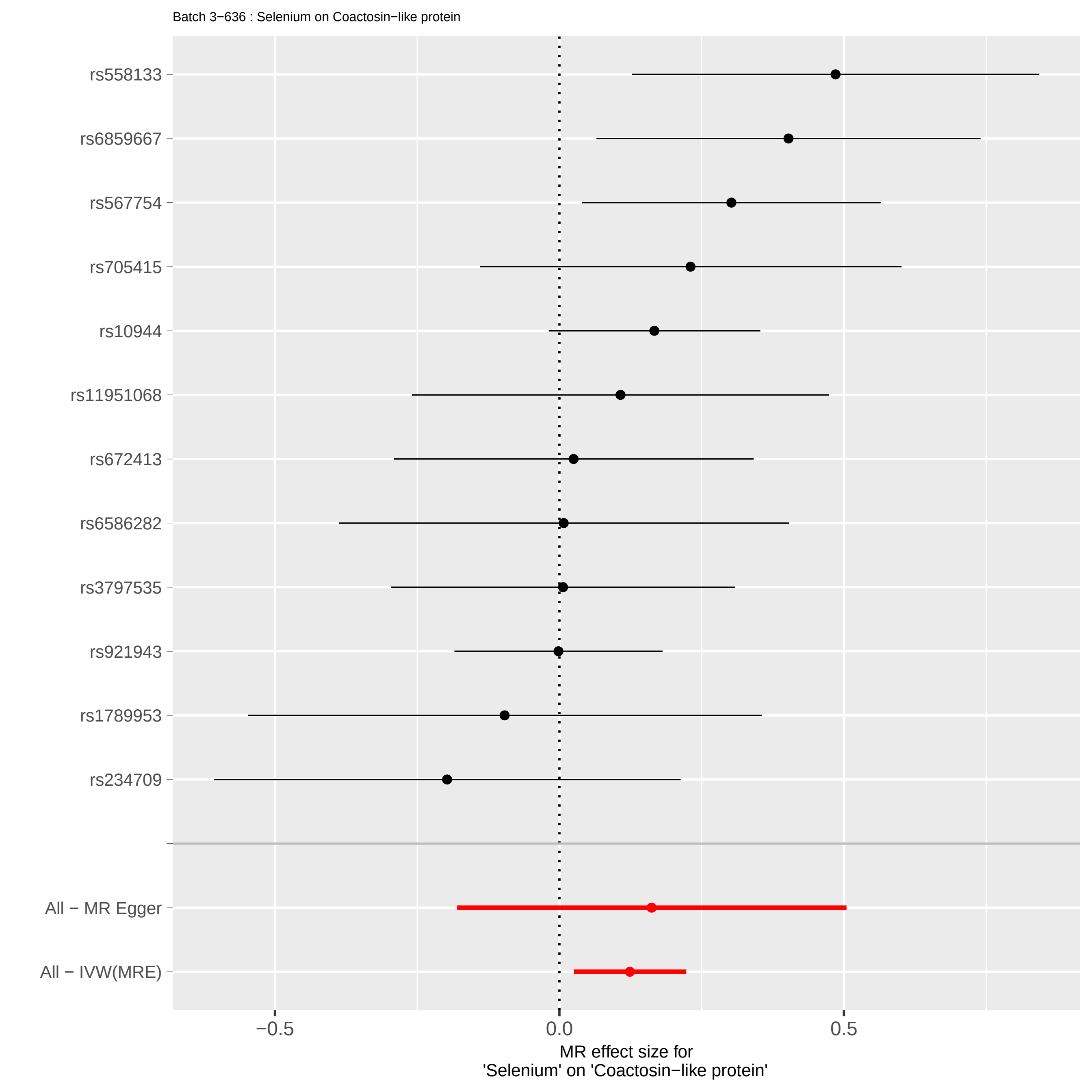
Batch 3−636 : Selenium on Coactosin−like protein
rs558133
rs6859667
rs567754
rs705415
rs10944
rs11951068
rs672413
rs6586282
rs3797535
rs921943
rs1789953
rs234709
All − MR Egger
All − IVW(MRE)
−0.5
0.0
0.5
MR effect size for
'Selenium' on 'Coactosin−like protein'

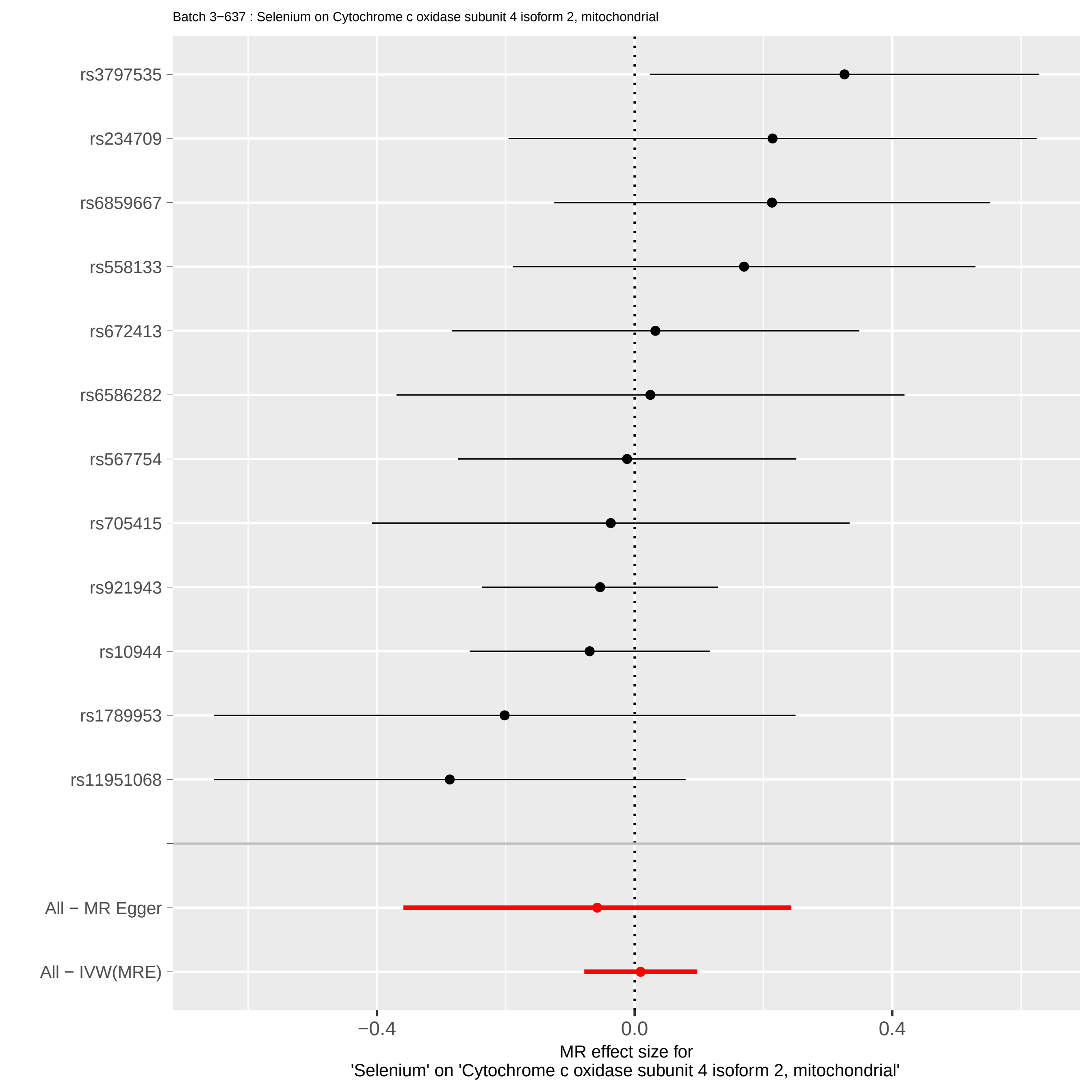

Batch 3−637 : Selenium on Cytochrome c oxidase subunit 4 isoform 2, mitochondrial
rs3797535
rs234709
rs6859667
rs558133
rs672413
rs6586282
rs567754
rs705415
rs921943
rs10944
rs1789953
rs11951068
All − MR Egger
All − IVW(MRE)
−0.4
0.0
0.4
MR effect size for
'Selenium' on 'Cytochrome c oxidase subunit 4 isoform 2, mitochondrial'

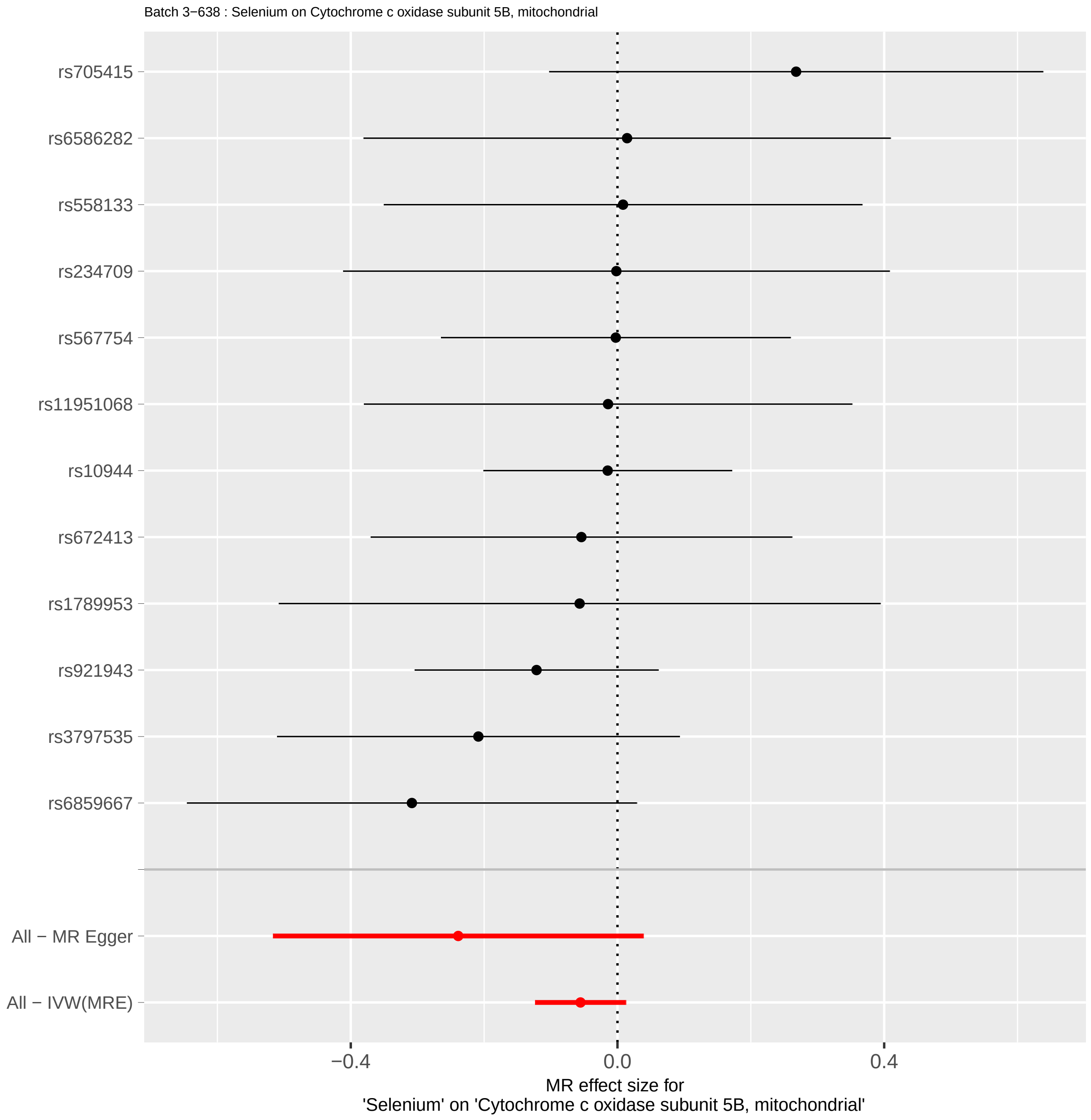

Batch 3−638 : Selenium on Cytochrome c oxidase subunit 5B, mitochondrial
rs705415
rs6586282
rs558133
rs234709
rs567754
rs11951068
rs10944
rs672413
rs1789953
rs921943
rs3797535
rs6859667
All − MR Egger
All − IVW(MRE)
−0.4
0.0
0.4
MR effect size for
'Selenium' on 'Cytochrome c oxidase subunit 5B, mitochondrial'

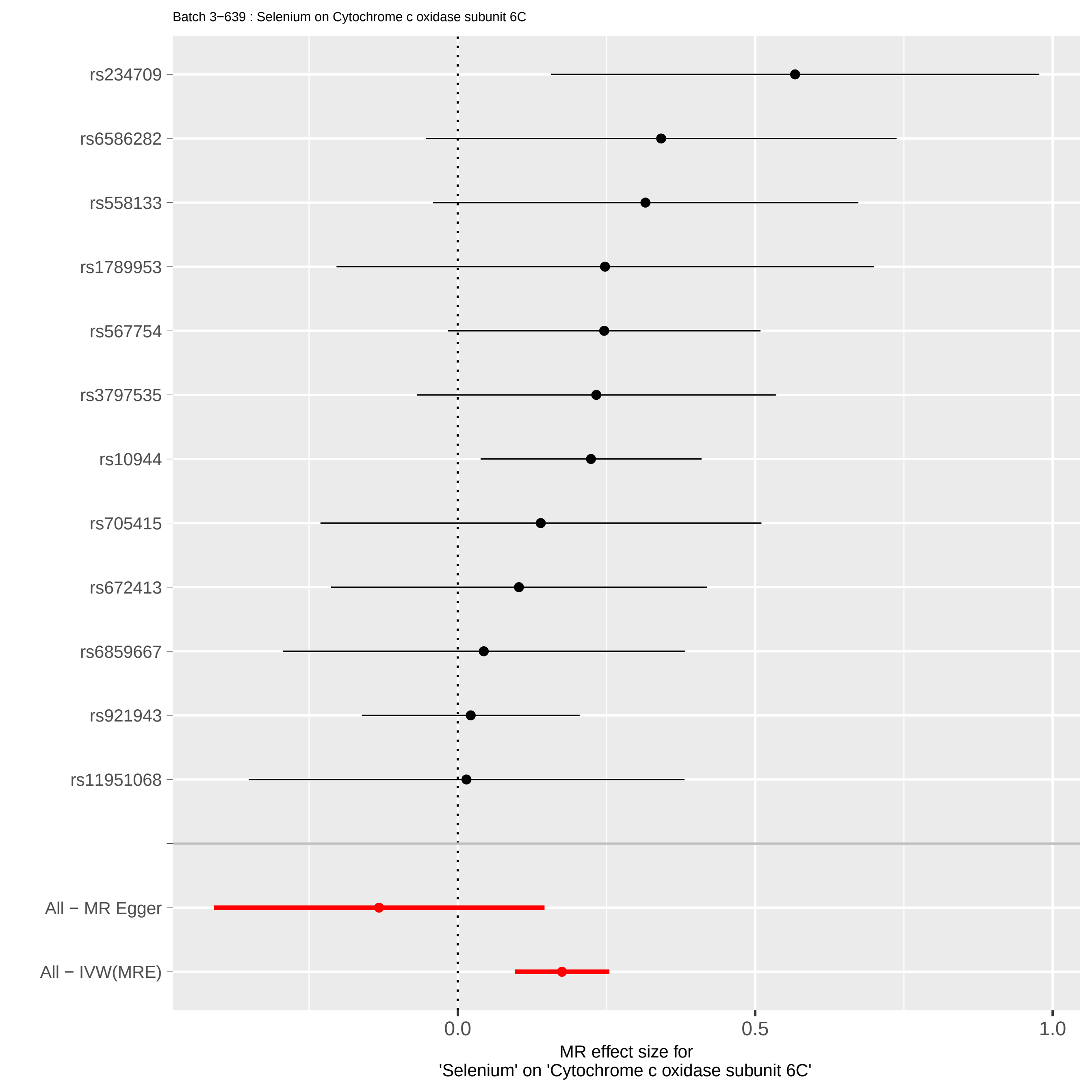

Batch 3−639 : Selenium on Cytochrome c oxidase subunit 6C
rs234709
rs6586282
rs558133
rs1789953
rs567754
rs3797535
rs10944
rs705415
rs672413
rs6859667
rs921943
rs11951068
All − MR Egger
All − IVW(MRE)
0.0
0.5
1.0
MR effect size for
'Selenium' on 'Cytochrome c oxidase subunit 6C'

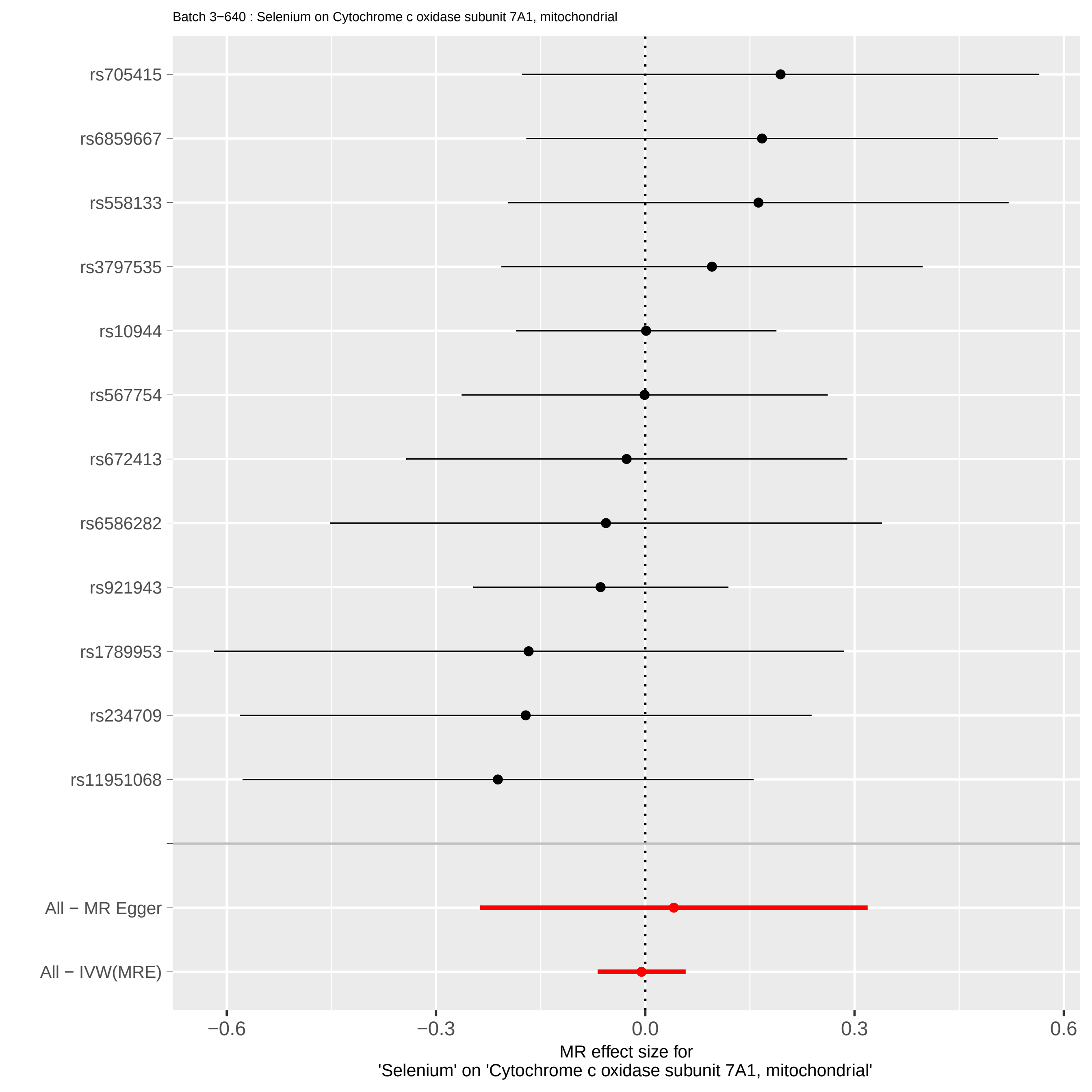

Batch 3−640 : Selenium on Cytochrome c oxidase subunit 7A1, mitochondrial
rs705415
rs6859667
rs558133
rs3797535
rs10944
rs567754
rs672413
rs6586282
rs921943
rs1789953
rs234709
rs11951068
All − MR Egger
All − IVW(MRE)
−0.6
−0.3
0.0
0.3
0.6
MR effect size for
'Selenium' on 'Cytochrome c oxidase subunit 7A1, mitochondrial'

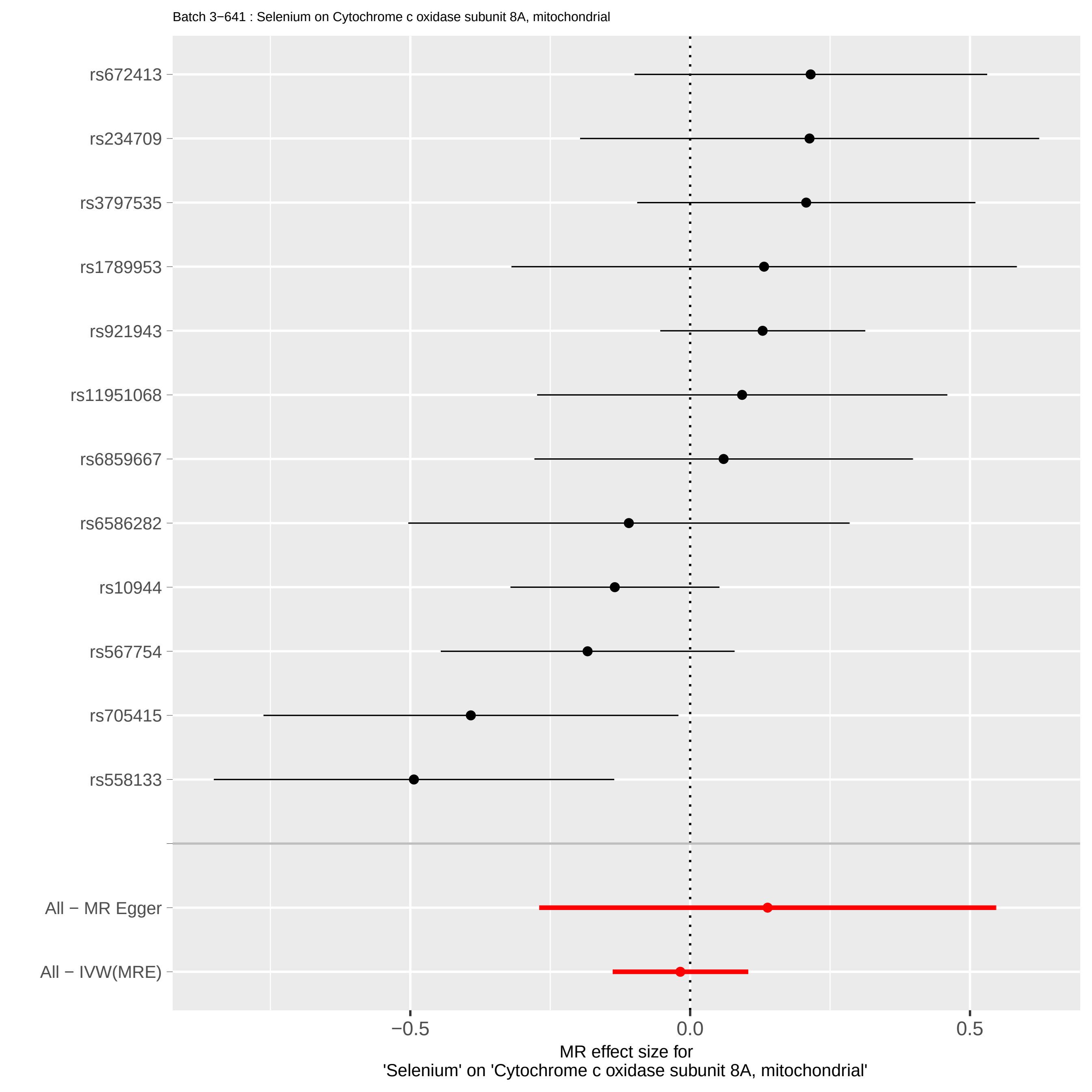
Batch 3−641 : Selenium on Cytochrome c oxidase subunit 8A, mitochondrial
rs672413
rs234709
rs3797535
rs1789953
rs921943
rs11951068
rs6859667
rs6586282
rs10944
rs567754
rs705415
rs558133
All − MR Egger
All − IVW(MRE)
−0.5
0.0
0.5
MR effect size for
'Selenium' on 'Cytochrome c oxidase subunit 8A, mitochondrial'

Batch 3−642 : Selenium on Carboxypeptidase A2

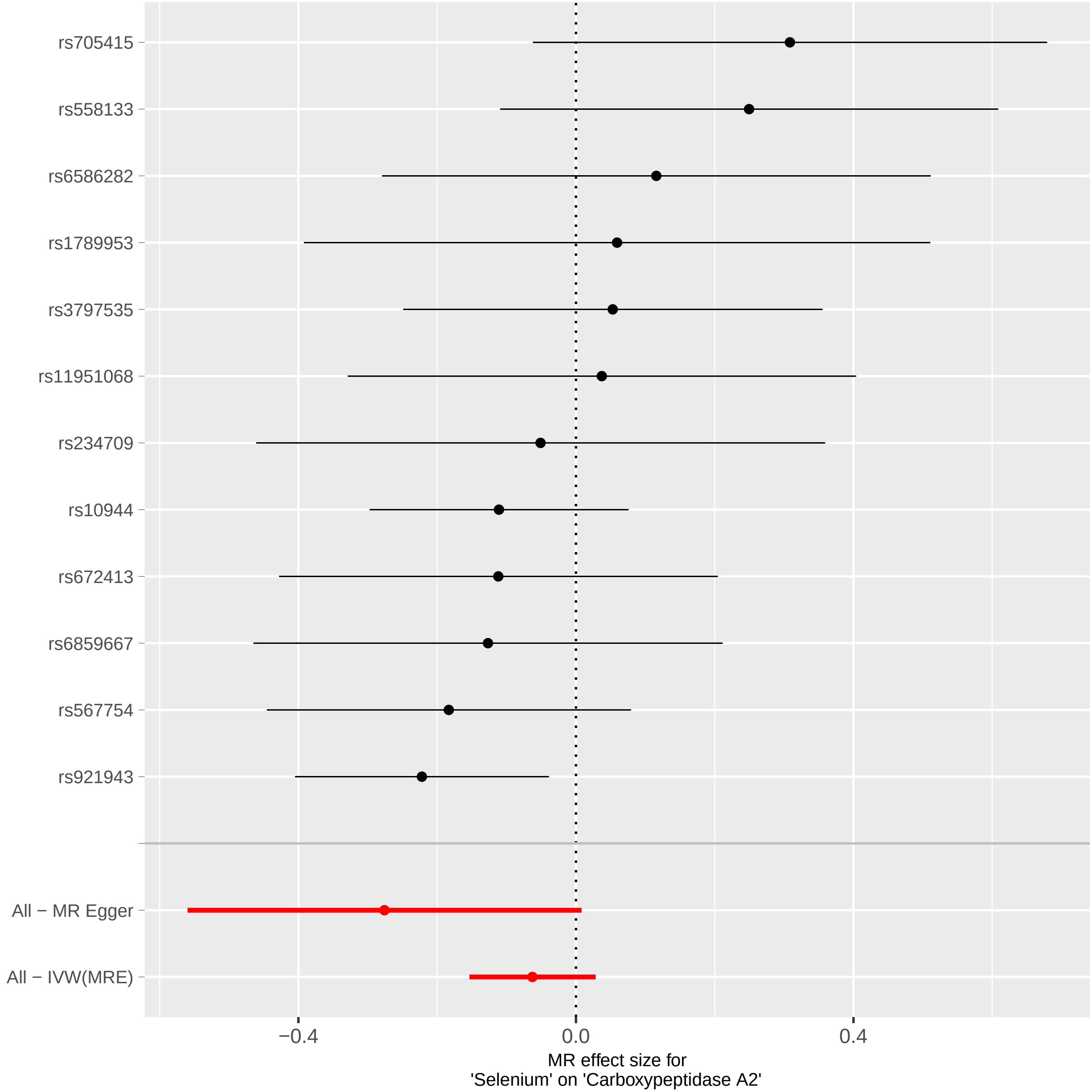

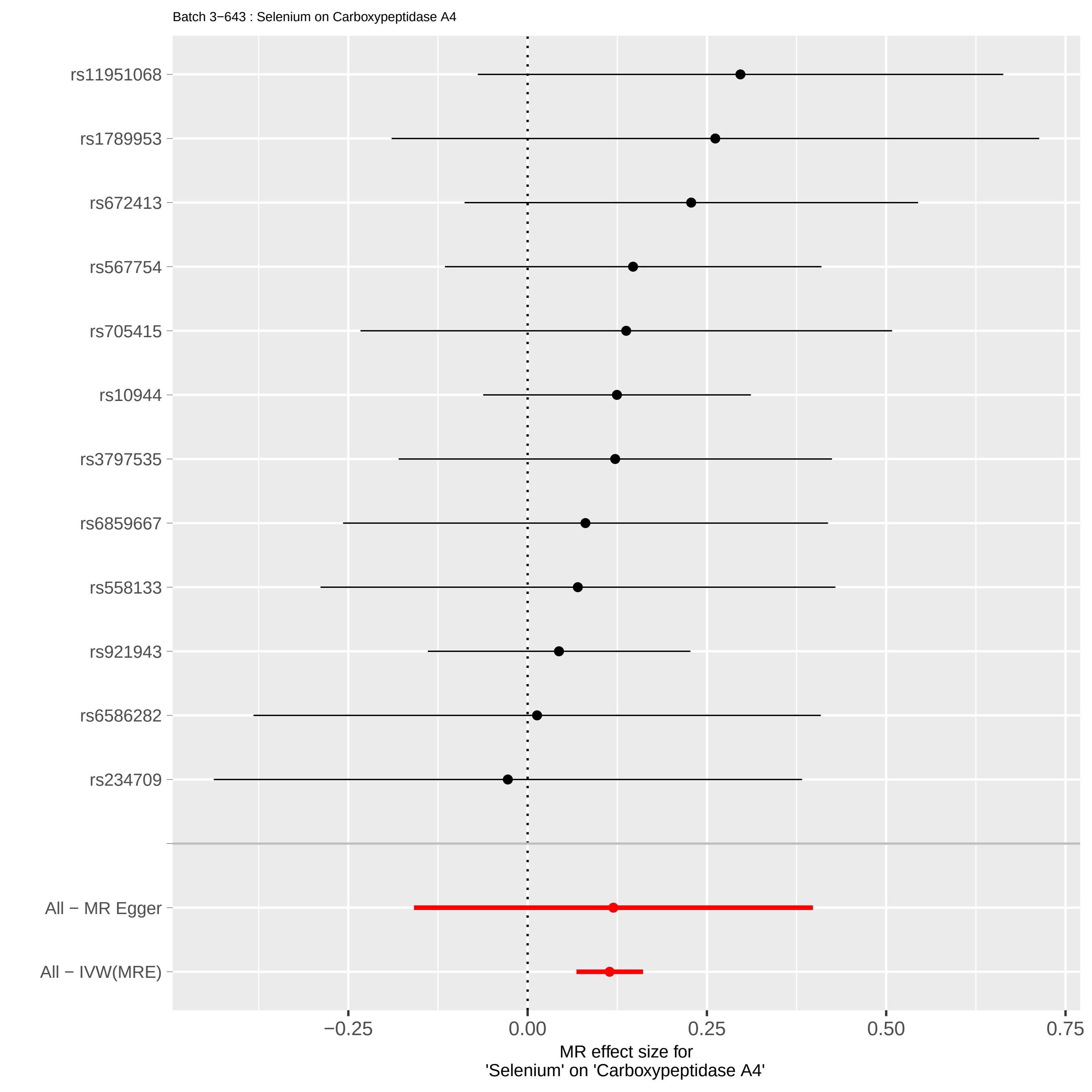
Batch 3−643 : Selenium on Carboxypeptidase A4
rs11951068
rs1789953
rs672413
rs567754
rs705415
rs10944
rs3797535
rs6859667
rs558133
rs921943
rs6586282
rs234709
All − MR Egger
All − IVW(MRE)
−0.25
0.00
0.25
0.50
0.75
MR effect size for
'Selenium' on 'Carboxypeptidase A4'

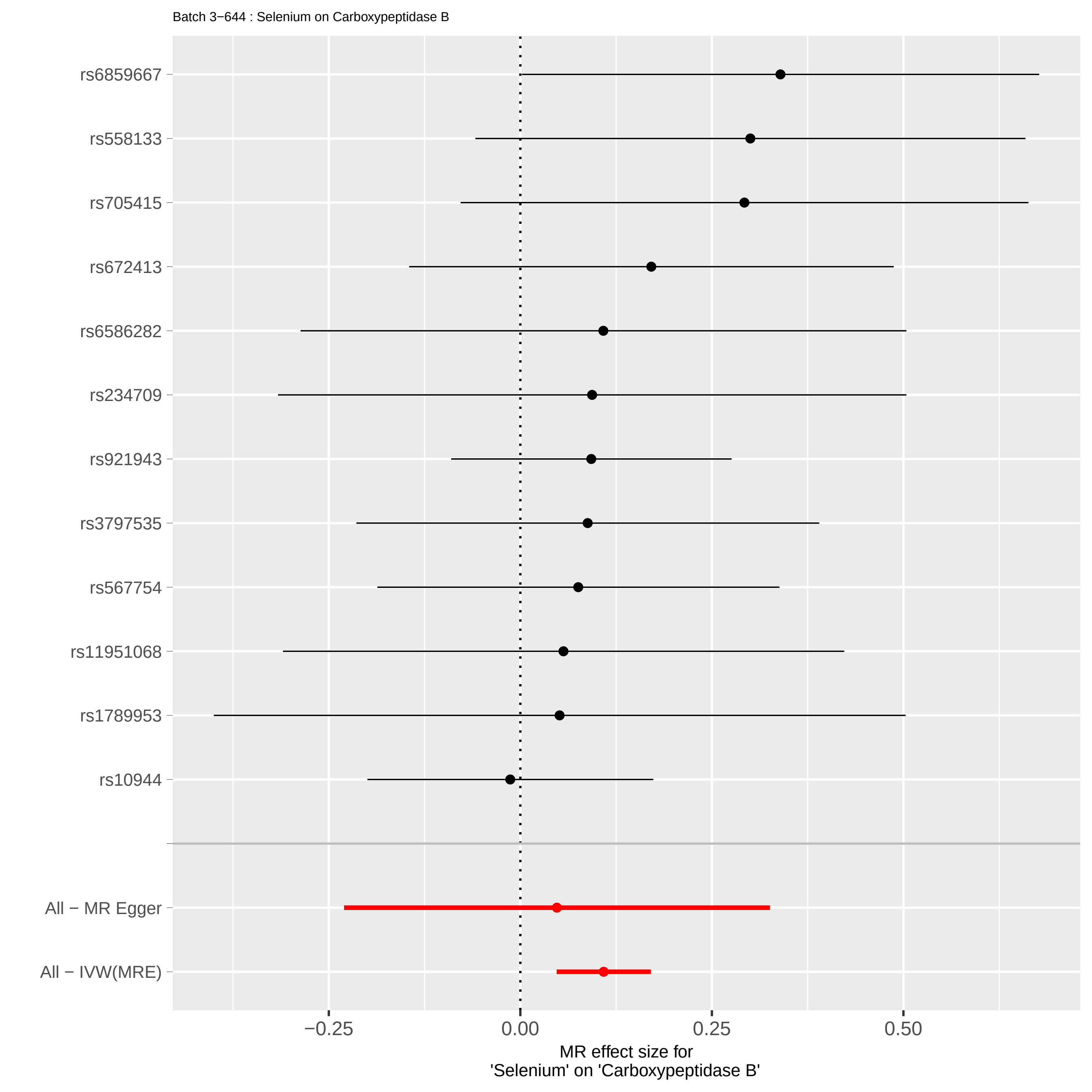
Batch 3−644 : Selenium on Carboxypeptidase B
rs6859667
rs558133
rs705415
rs672413
rs6586282
rs234709
rs921943
rs3797535
rs567754
rs11951068
rs1789953
rs10944
All − MR Egger
All − IVW(MRE)
−0.25
0.00
0.25
0.50
MR effect size for
'Selenium' on 'Carboxypeptidase B'

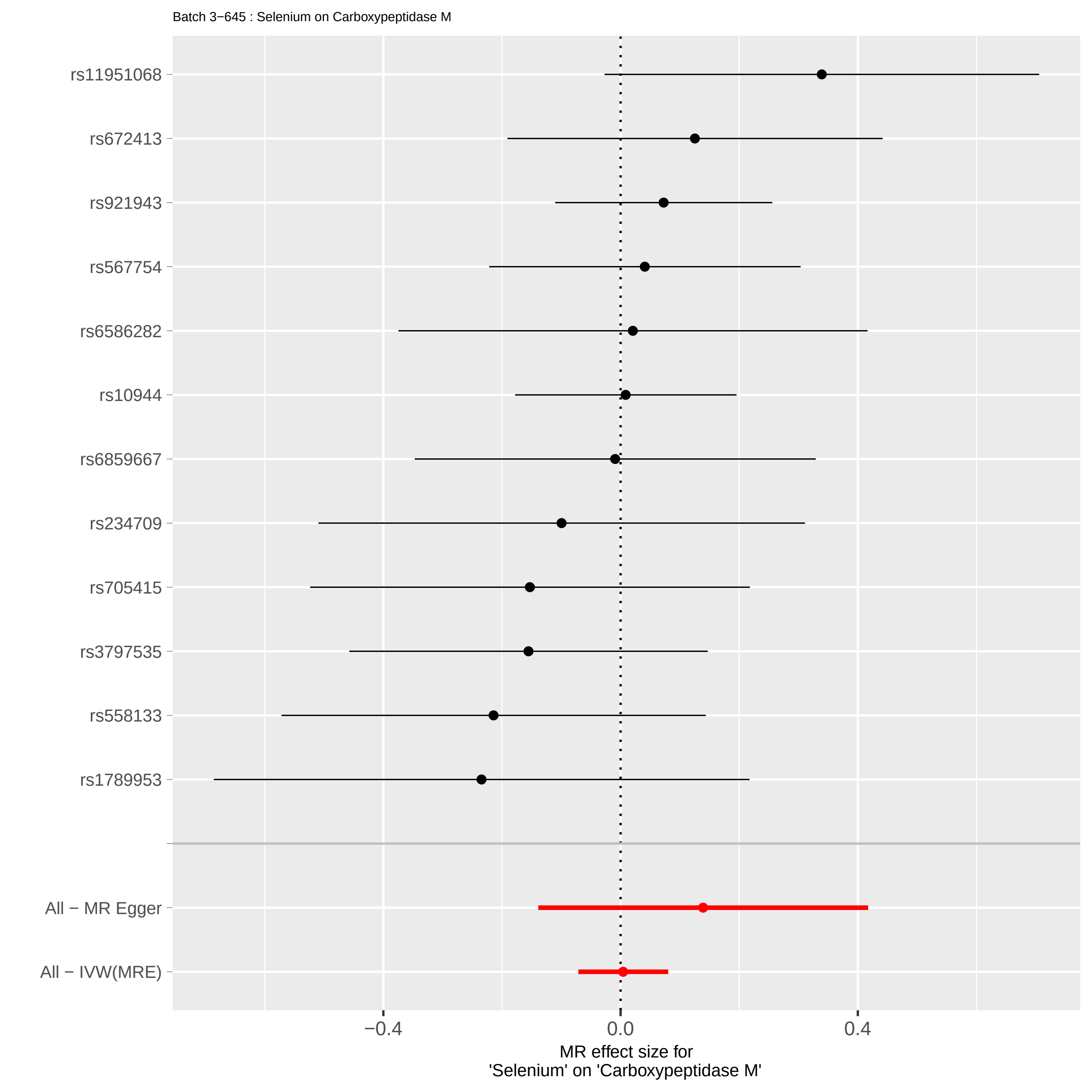

Batch 3−645 : Selenium on Carboxypeptidase M
rs11951068
rs672413
rs921943
rs567754
rs6586282
rs10944
rs6859667
rs234709
rs705415
rs3797535
rs558133
rs1789953
All − MR Egger
All − IVW(MRE)
−0.4
0.0
0.4
MR effect size for
'Selenium' on 'Carboxypeptidase M'

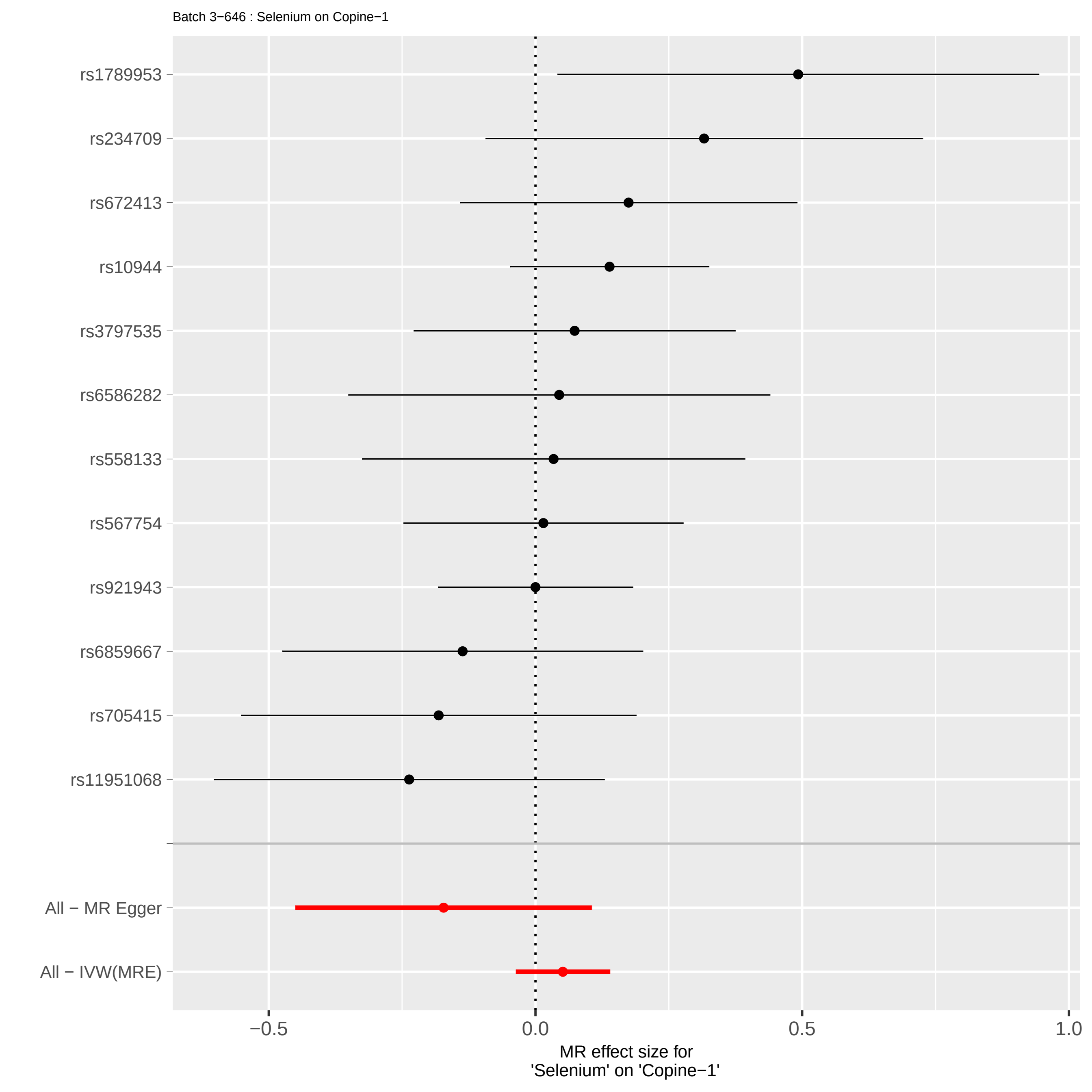

Batch 3−646 : Selenium on Copine−1
rs1789953
rs234709
rs672413
rs10944
rs3797535
rs6586282
rs558133
rs567754
rs921943
rs6859667
rs705415
rs11951068
All − MR Egger
All − IVW(MRE)
−0.5
0.0
0.5
1.0
MR effect size for
'Selenium' on 'Copine−1'

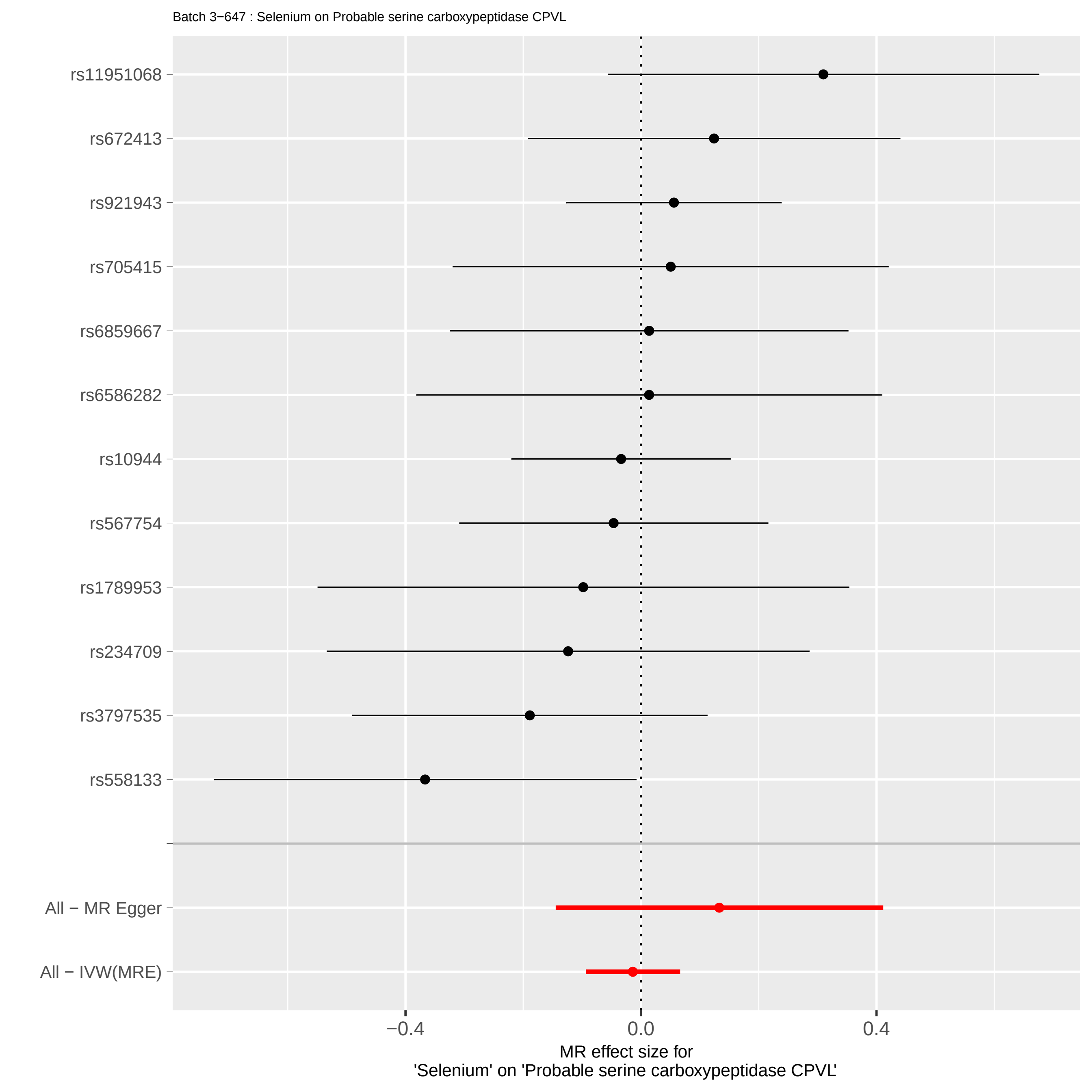
Batch 3−647 : Selenium on Probable serine carboxypeptidase CPVL
rs11951068
rs672413
rs921943
rs705415
rs6859667
rs6586282
rs10944
rs567754
rs1789953
rs234709
rs3797535
rs558133
All − MR Egger
All − IVW(MRE)
−0.4
0.0
0.4
MR effect size for
'Selenium' on 'Probable serine carboxypeptidase CPVL'

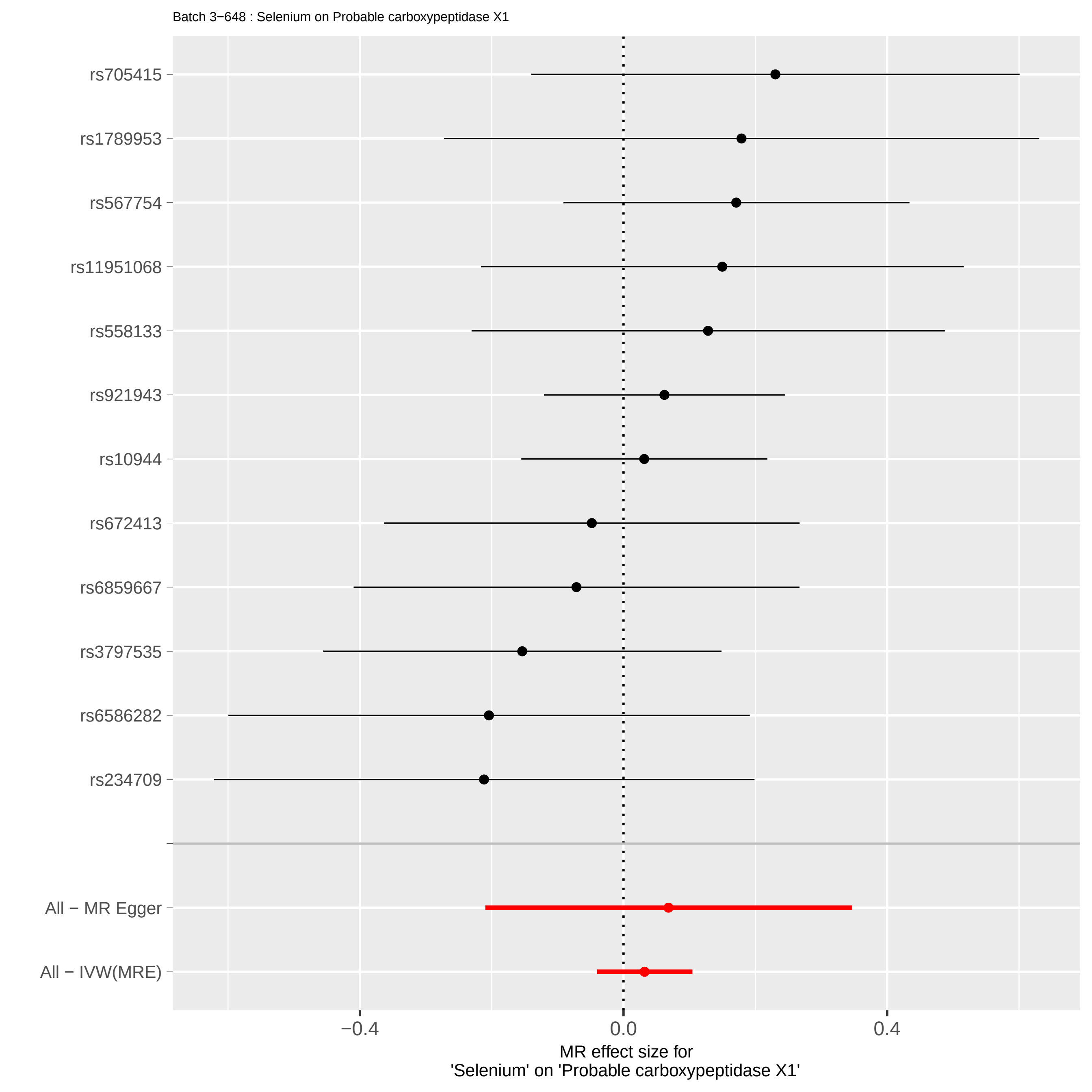

Batch 3−648 : Selenium on Probable carboxypeptidase X1
rs705415
rs1789953
rs567754
rs11951068
rs558133
rs921943
rs10944
rs672413
rs6859667
rs3797535
rs6586282
rs234709
All − MR Egger
All − IVW(MRE)
−0.4
0.0
0.4
MR effect size for
'Selenium' on 'Probable carboxypeptidase X1'

Batch 3−649 : Selenium on Carboxypeptidase Z

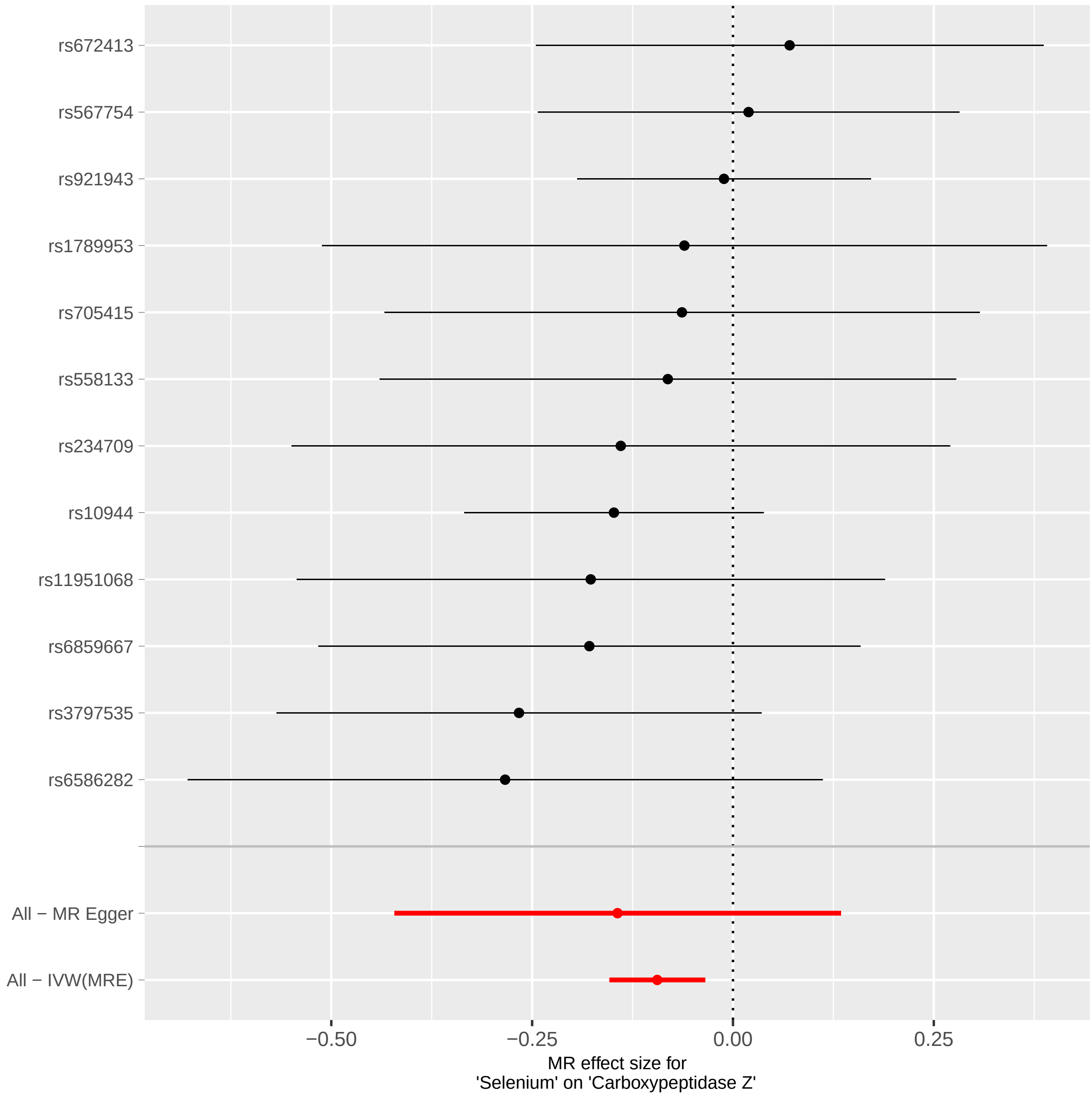

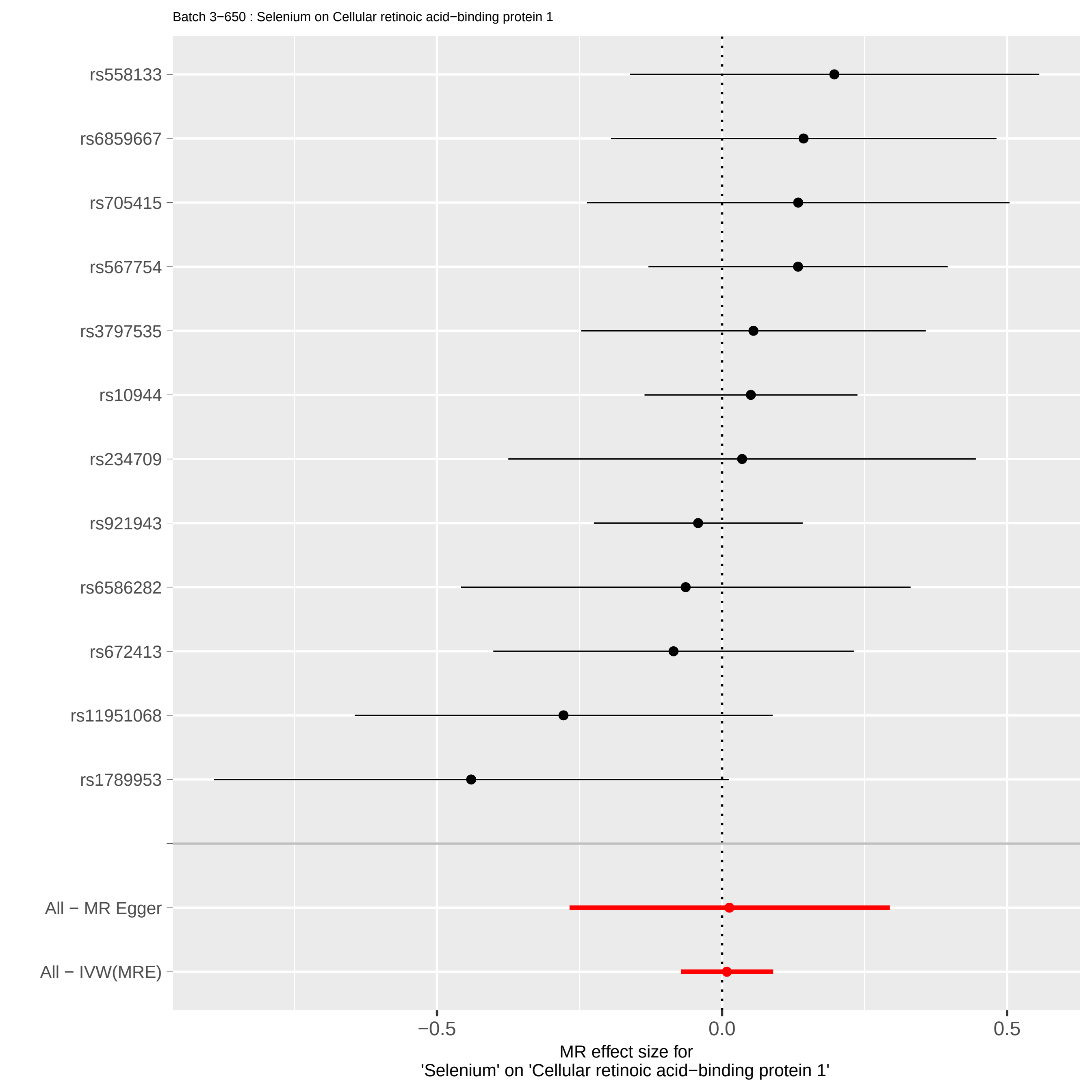

Batch 3−650 : Selenium on Cellular retinoic acid−binding protein 1
rs558133
rs6859667
rs705415
rs567754
rs3797535
rs10944
rs234709
rs921943
rs6586282
rs672413
rs11951068
rs1789953
All − MR Egger
All − IVW(MRE)
−0.5
0.0
0.5
MR effect size for
'Selenium' on 'Cellular retinoic acid−binding protein 1'

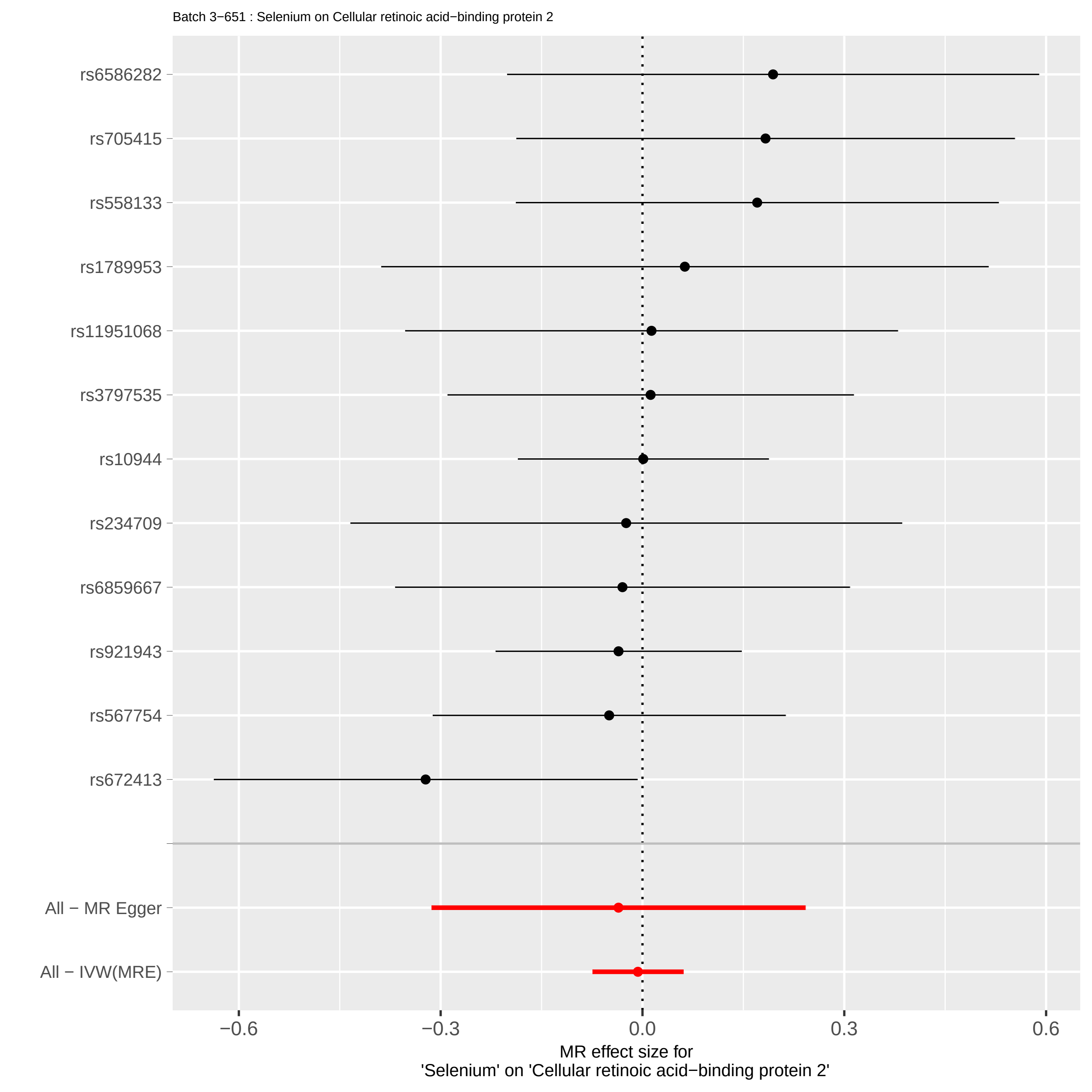
Batch 3−651 : Selenium on Cellular retinoic acid−binding protein 2
rs6586282
rs705415
rs558133
rs1789953
rs11951068
rs3797535
rs10944
rs234709
rs6859667
rs921943
rs567754
rs672413
All − MR Egger
All − IVW(MRE)
−0.6
−0.3
0.0
0.3
0.6
MR effect size for
'Selenium' on 'Cellular retinoic acid−binding protein 2'

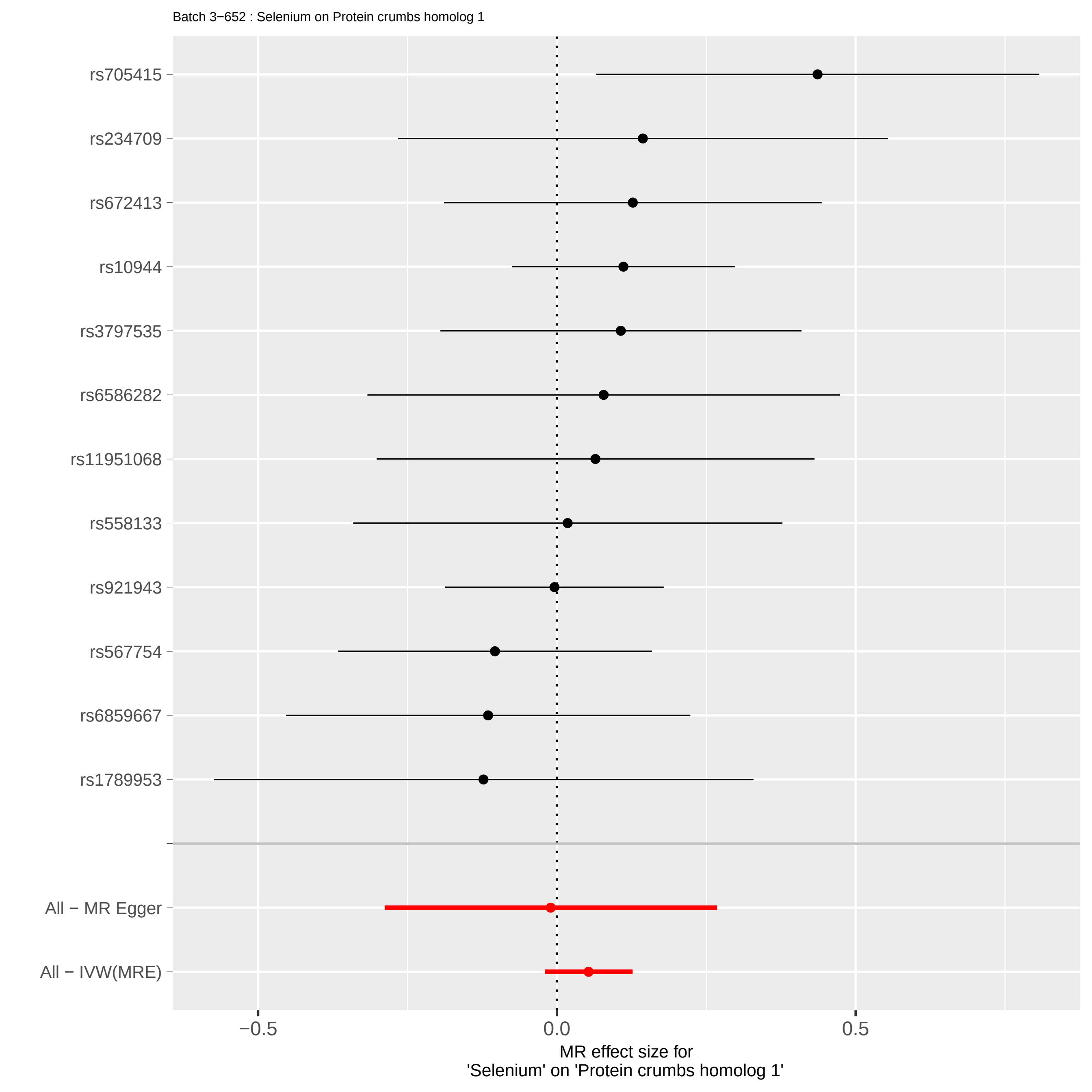

Batch 3−652 : Selenium on Protein crumbs homolog 1
rs705415
rs234709
rs672413
rs10944
rs3797535
rs6586282
rs11951068
rs558133
rs921943
rs567754
rs6859667
rs1789953
All − MR Egger
All − IVW(MRE)
−0.5
0.0
0.5
MR effect size for
'Selenium' on 'Protein crumbs homolog 1'

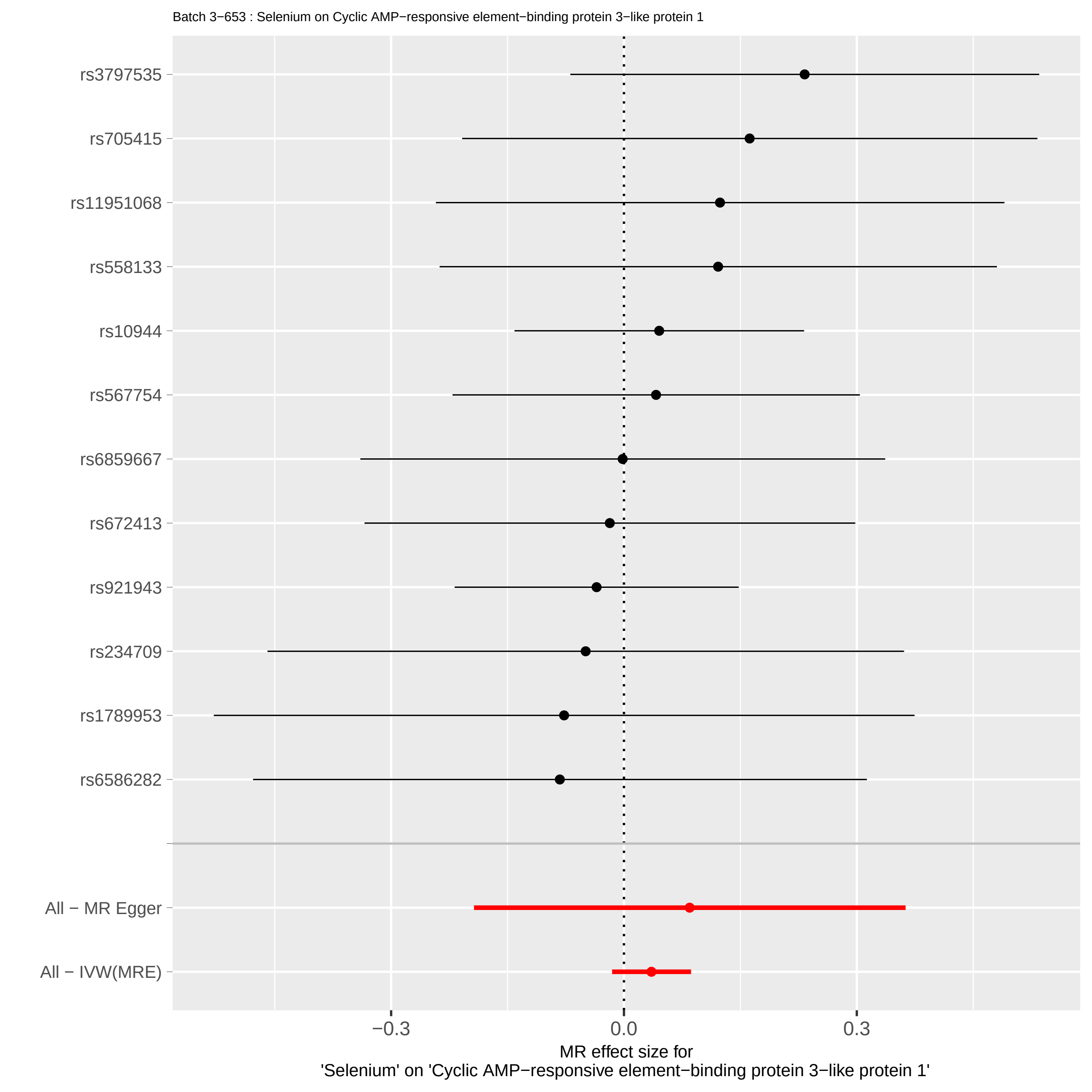
Batch 3−653 : Selenium on Cyclic AMP−responsive element−binding protein 3−like protein 1
rs3797535
rs705415
rs11951068
rs558133
rs10944
rs567754
rs6859667
rs672413
rs921943
rs234709
rs1789953
rs6586282
All − MR Egger
All − IVW(MRE)
−0.3
0.0
0.3
MR effect size for
'Selenium' on 'Cyclic AMP−responsive element−binding protein 3−like protein 1'

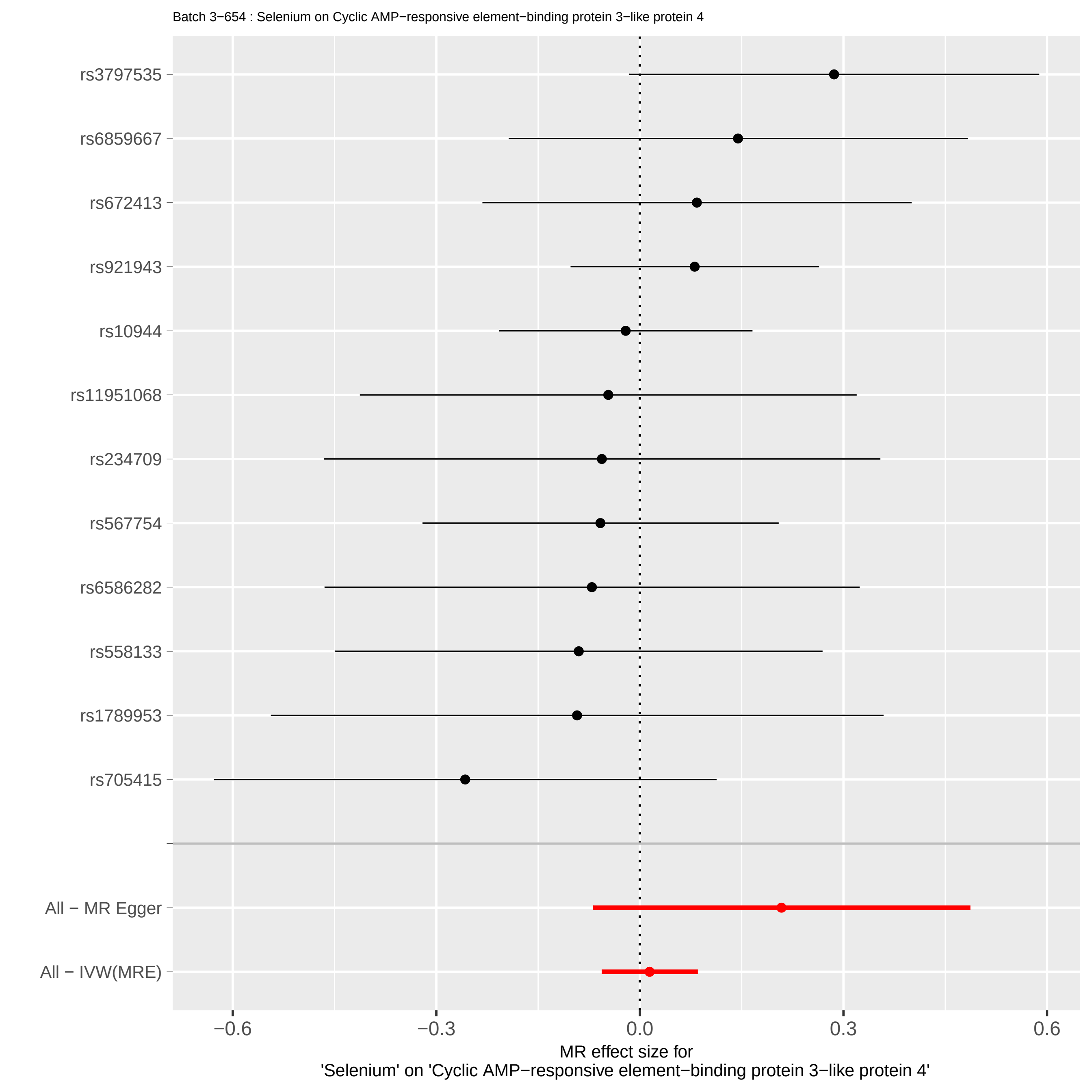
Batch 3−654 : Selenium on Cyclic AMP−responsive element−binding protein 3−like protein 4
rs3797535
rs6859667
rs672413
rs921943
rs10944
rs11951068
rs234709
rs567754
rs6586282
rs558133
rs1789953
rs705415
All − MR Egger
All − IVW(MRE)
−0.6
−0.3
0.0
0.3
0.6
MR effect size for
'Selenium' on 'Cyclic AMP−responsive element−binding protein 3−like protein 4'

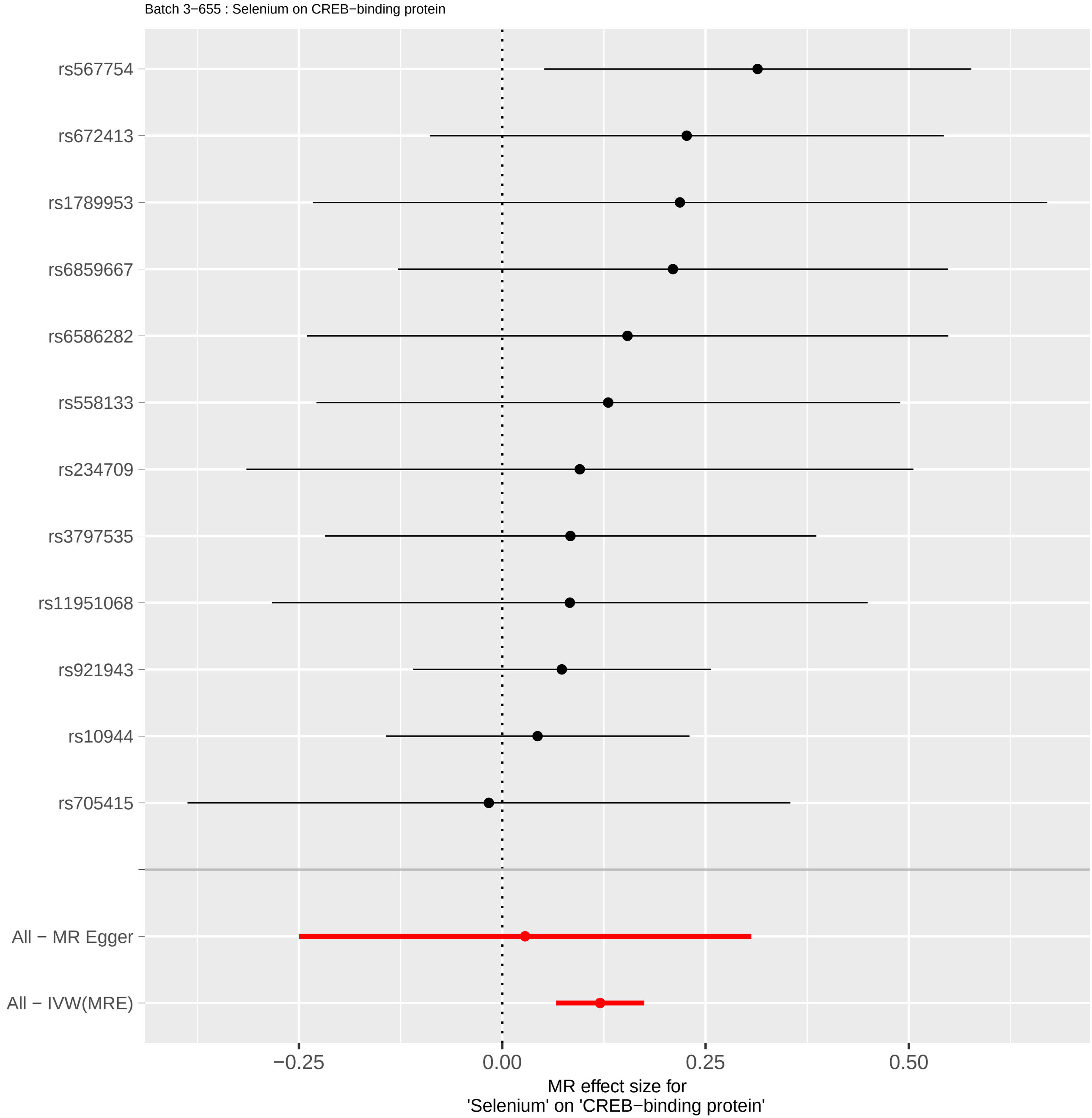

Batch 3−655 : Selenium on CREB−binding protein
rs567754
rs672413
rs1789953
rs6859667
rs6586282
rs558133
rs234709
rs3797535
rs11951068
rs921943
rs10944
rs705415
All − MR Egger
All − IVW(MRE)
−0.25
0.00
0.25
0.50
MR effect size for
'Selenium' on 'CREB−binding protein'

Batch 3−656 : Selenium on Protein CREG1

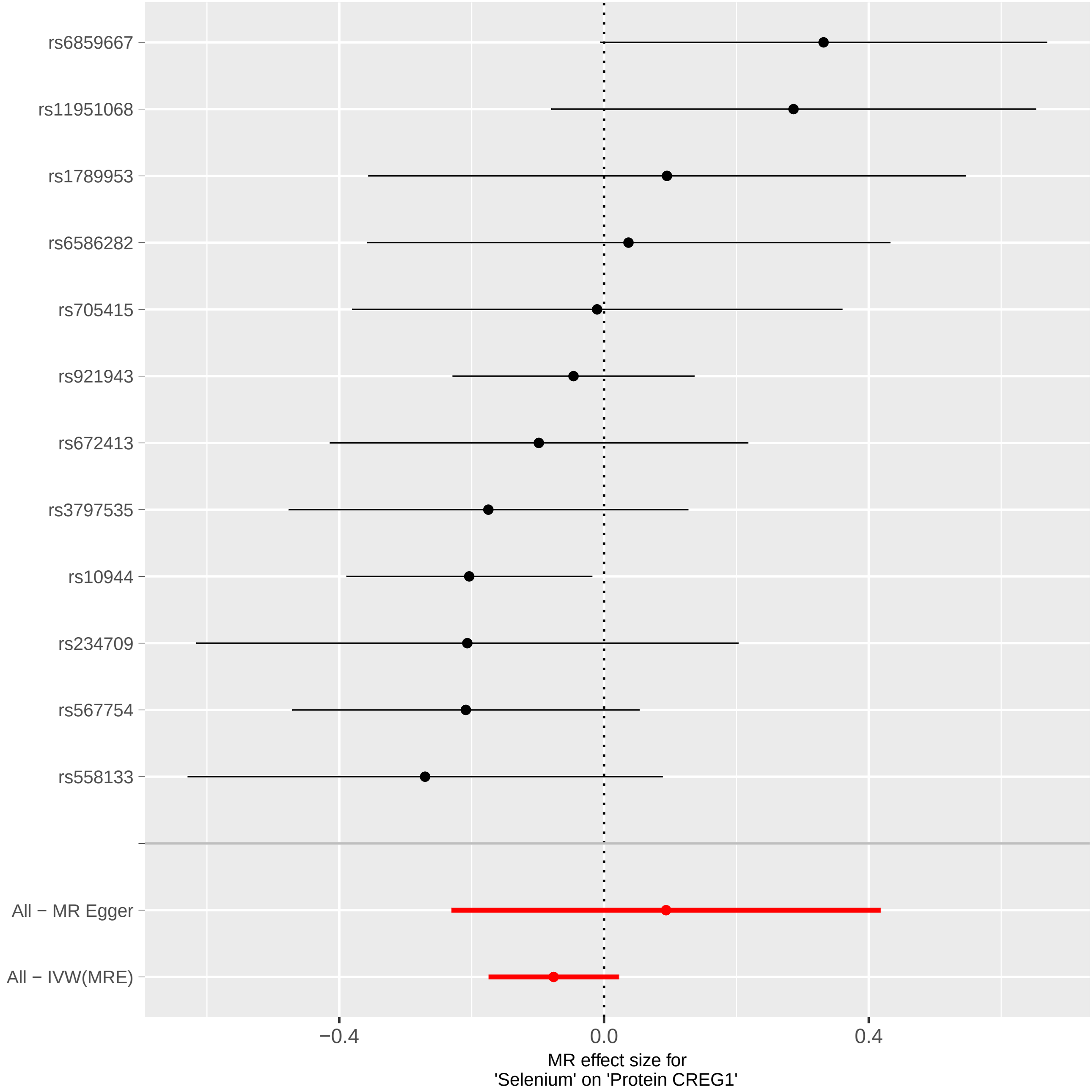

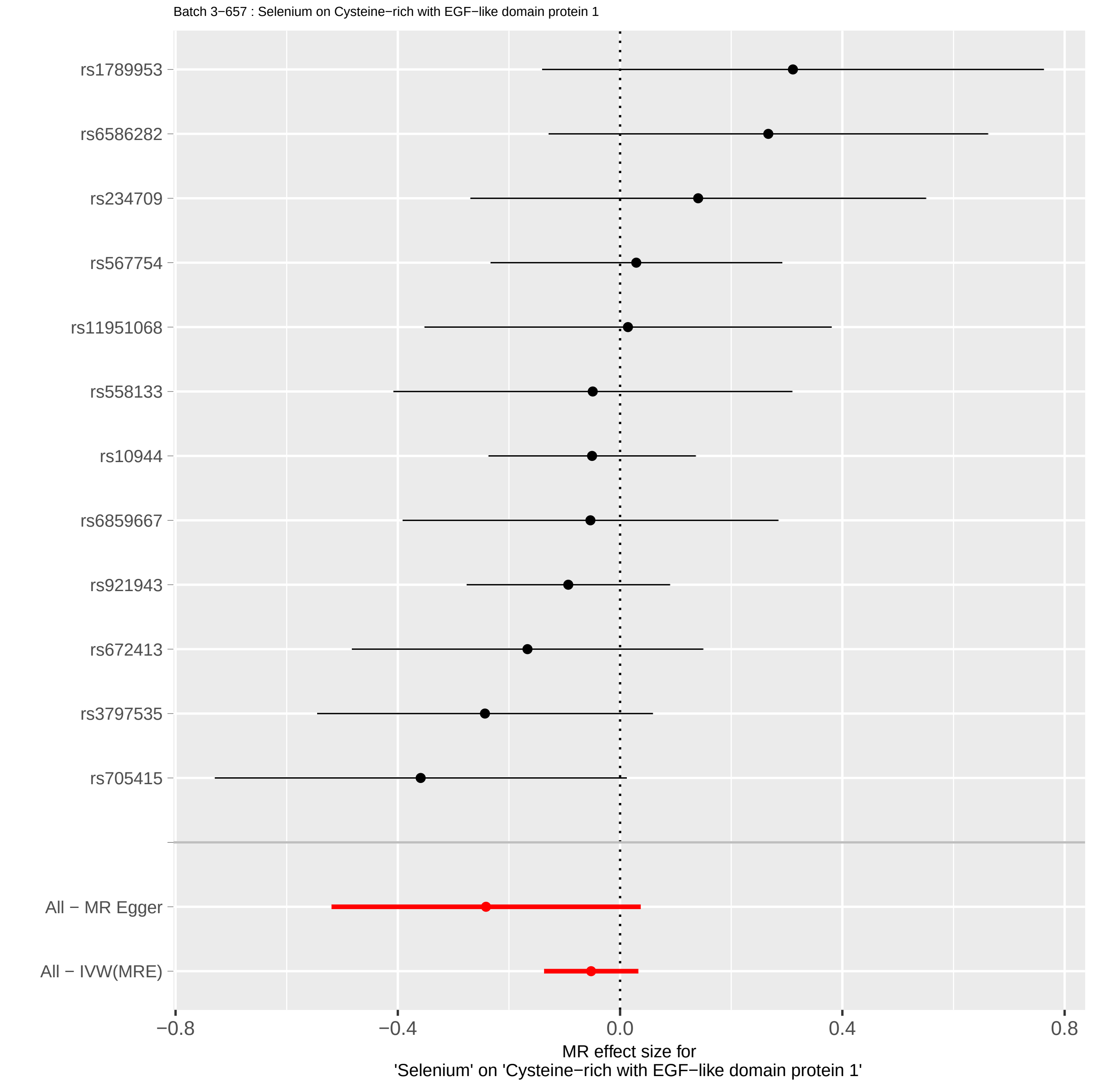

Batch 3−657 : Selenium on Cysteine−rich with EGF−like domain protein 1
rs1789953
rs6586282
rs234709
rs567754
rs11951068
rs558133
rs10944
rs6859667
rs921943
rs672413
rs3797535
rs705415
All − MR Egger
All − IVW(MRE)
−0.8
−0.4
0.0
0.4
0.8
MR effect size for
'Selenium' on 'Cysteine−rich with EGF−like domain protein 1'

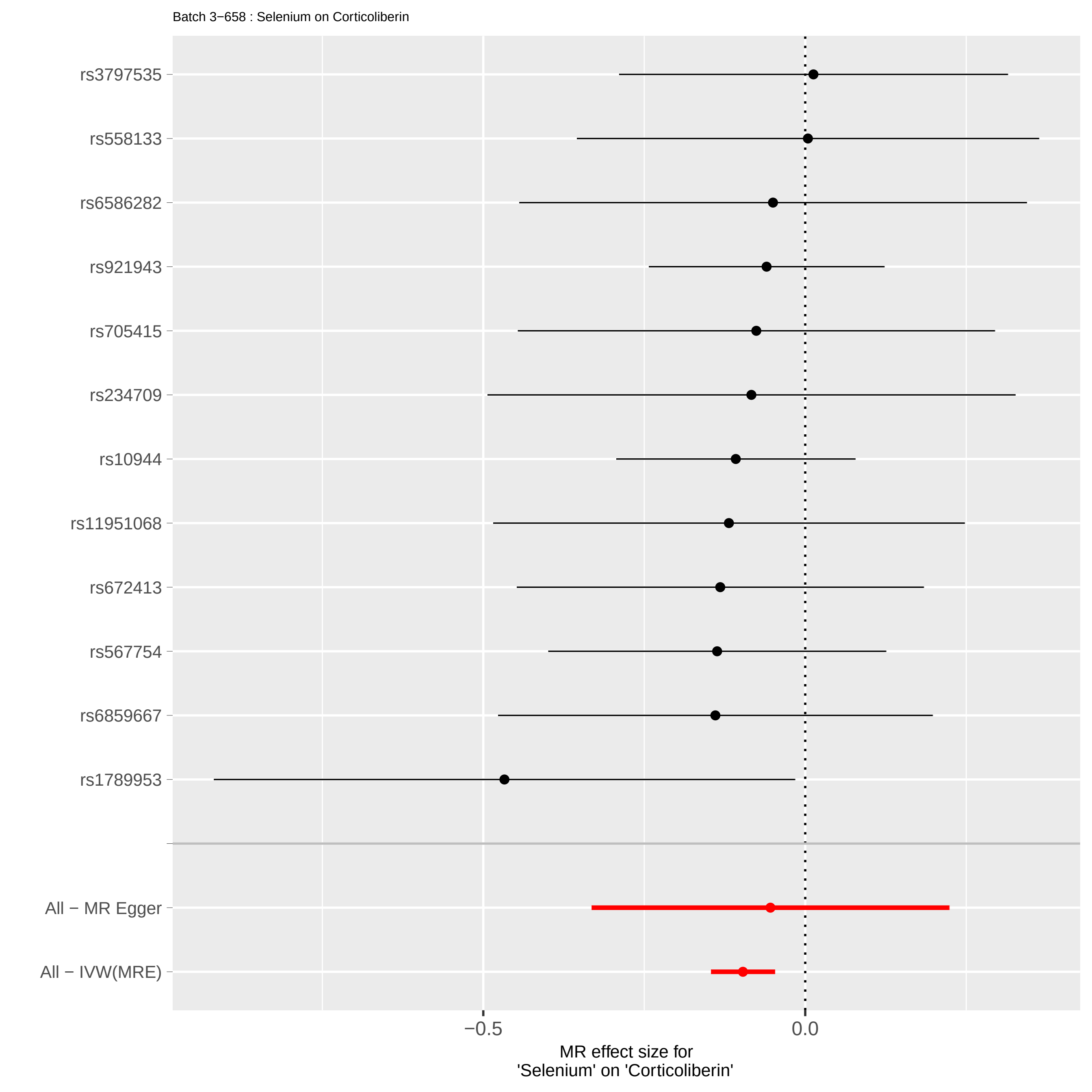

Batch 3−658 : Selenium on Corticoliberin
rs3797535
rs558133
rs6586282
rs921943
rs705415
rs234709
rs10944
rs11951068
rs672413
rs567754
rs6859667
rs1789953
All − MR Egger
All − IVW(MRE)
−0.5
0.0
MR effect size for
'Selenium' on 'Corticoliberin'

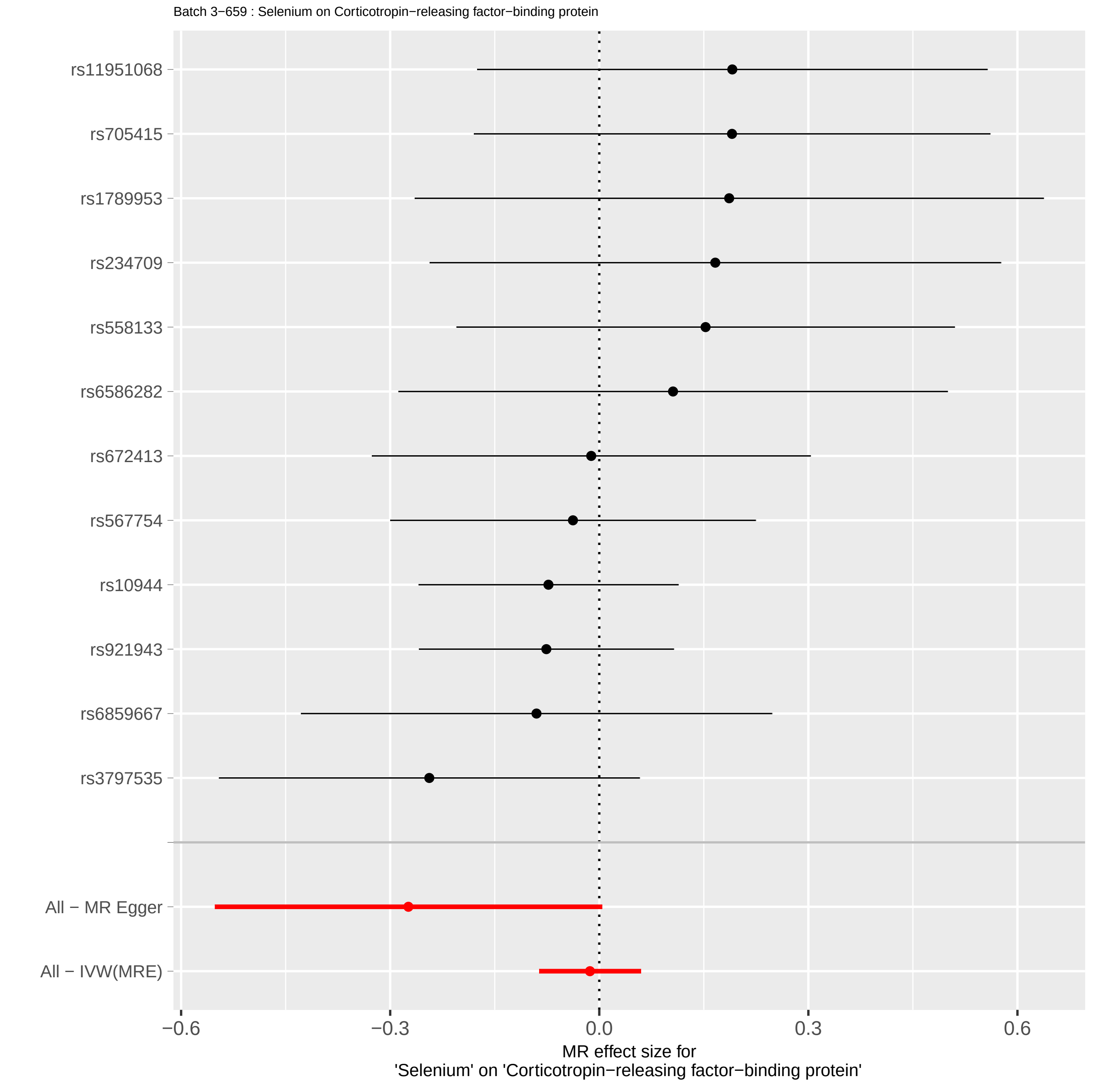

Batch 3−659 : Selenium on Corticotropin−releasing factor−binding protein
rs11951068
rs705415
rs1789953
rs234709
rs558133
rs6586282
rs672413
rs567754
rs10944
rs921943
rs6859667
rs3797535
All − MR Egger
All − IVW(MRE)
−0.6
−0.3
0.0
0.3
0.6
MR effect size for
'Selenium' on 'Corticotropin−releasing factor−binding protein'

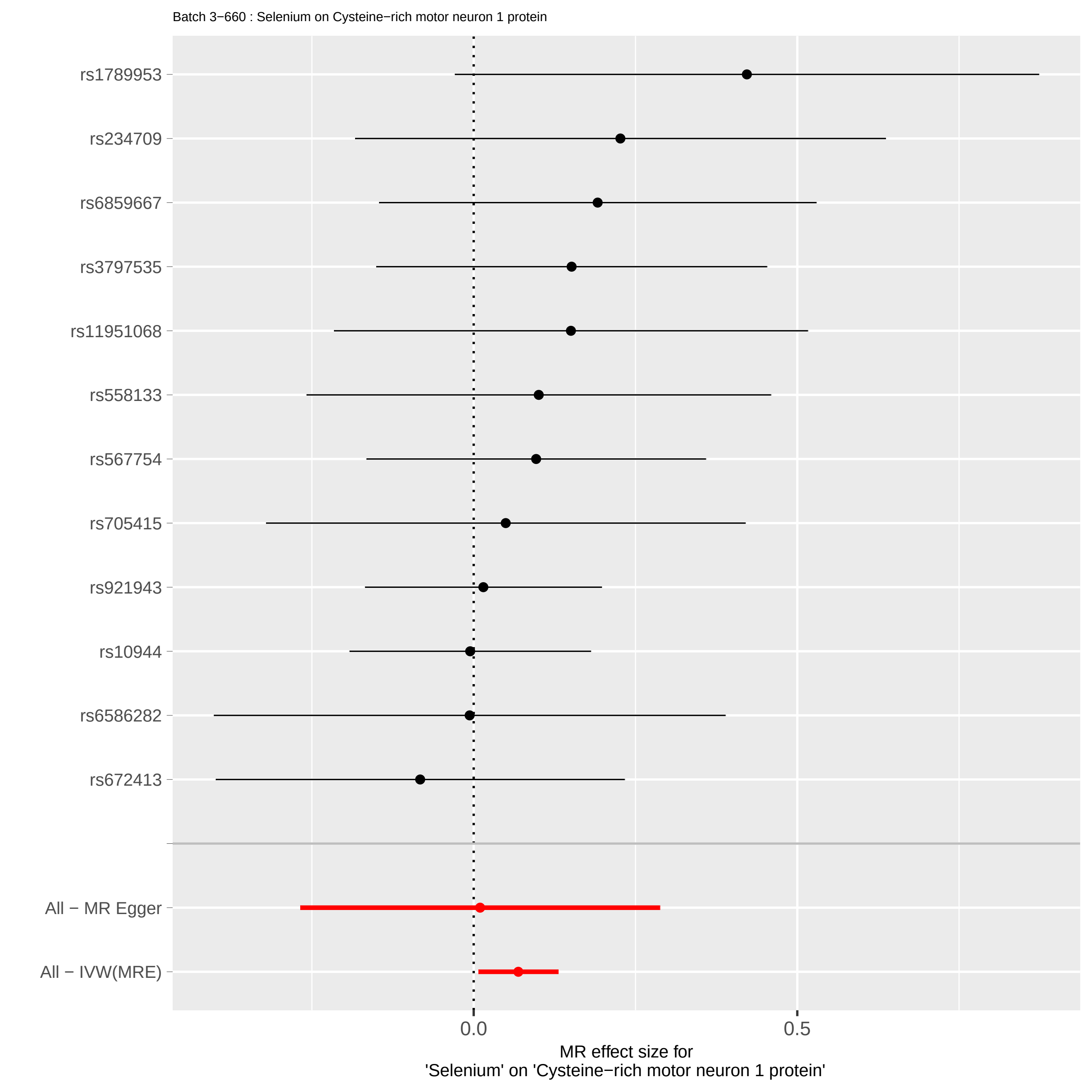

Batch 3−660 : Selenium on Cysteine−rich motor neuron 1 protein
rs1789953
rs234709
rs6859667
rs3797535
rs11951068
rs558133
rs567754
rs705415
rs921943
rs10944
rs6586282
rs672413
All − MR Egger
All − IVW(MRE)
0.0
0.5
MR effect size for
'Selenium' on 'Cysteine−rich motor neuron 1 protein'

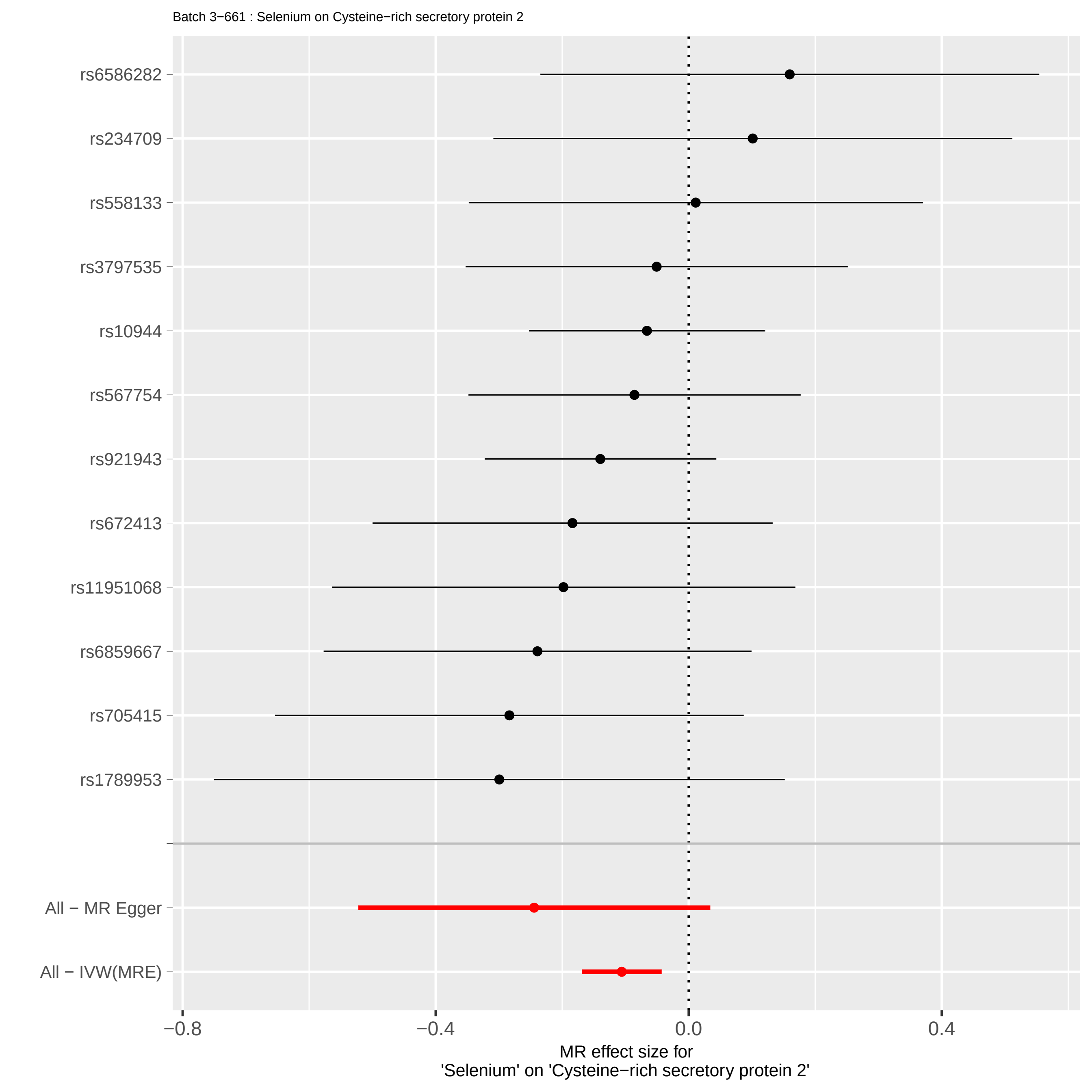
Batch 3−661 : Selenium on Cysteine−rich secretory protein 2
rs6586282
rs234709
rs558133
rs3797535
rs10944
rs567754
rs921943
rs672413
rs11951068
rs6859667
rs705415
rs1789953
All − MR Egger
All − IVW(MRE)
−0.8
−0.4
0.0
0.4
MR effect size for
'Selenium' on 'Cysteine−rich secretory protein 2'

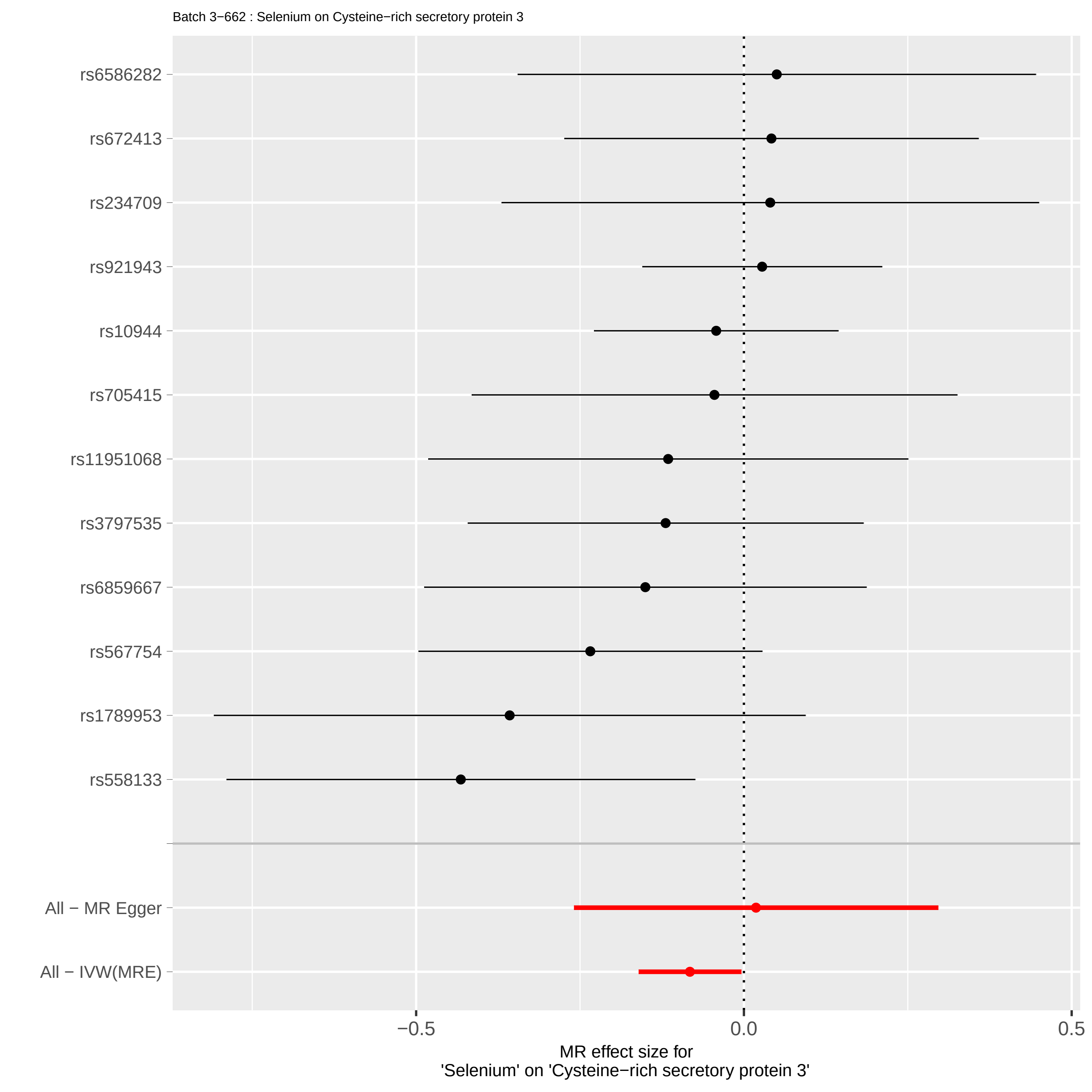

Batch 3−662 : Selenium on Cysteine−rich secretory protein 3
rs6586282
rs672413
rs234709
rs921943
rs10944
rs705415
rs11951068
rs3797535
rs6859667
rs567754
rs1789953
rs558133
All − MR Egger
All − IVW(MRE)
−0.5
0.0
0.5
MR effect size for
'Selenium' on 'Cysteine−rich secretory protein 3'

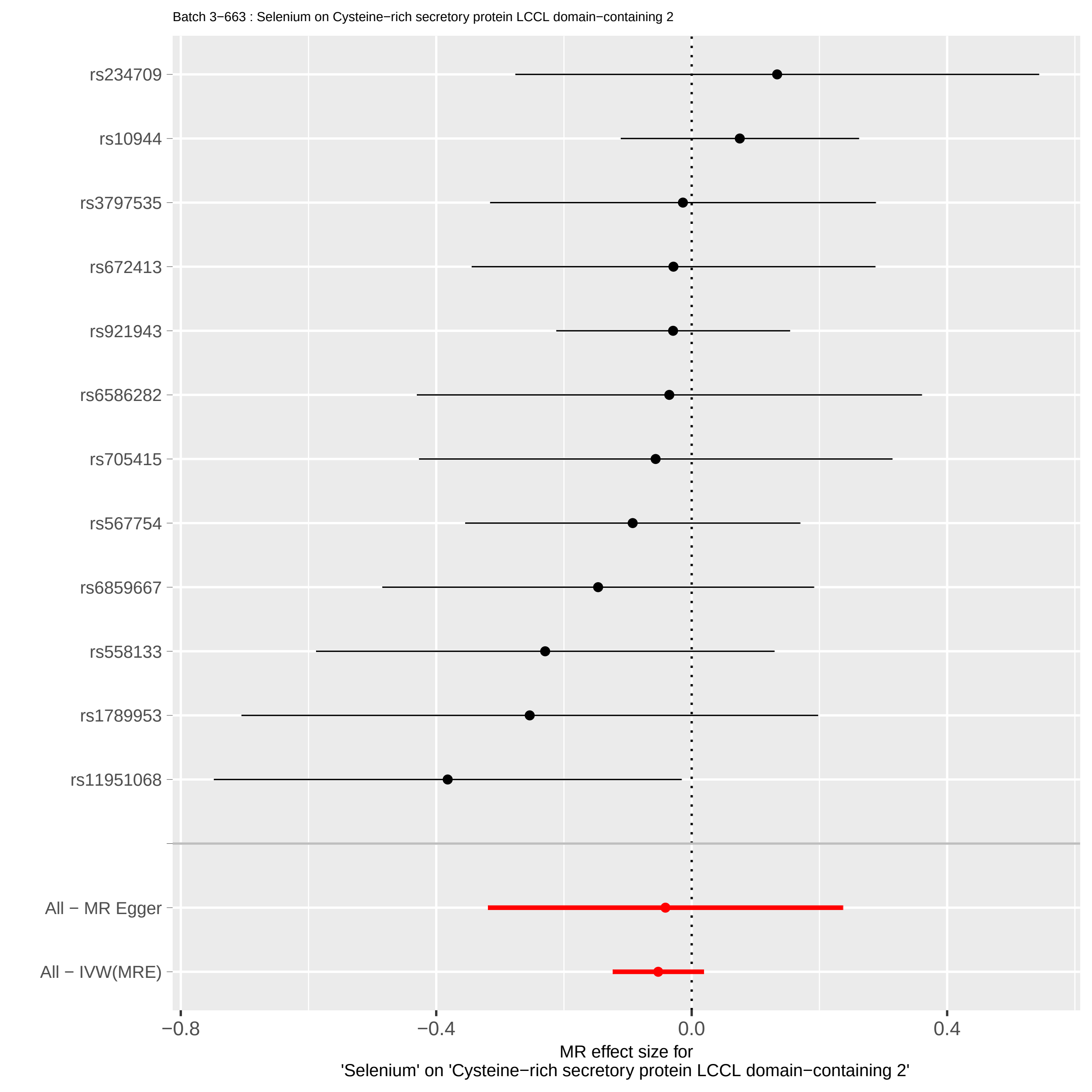
Batch 3−663 : Selenium on Cysteine−rich secretory protein LCCL domain−containing 2
rs234709
rs10944
rs3797535
rs672413
rs921943
rs6586282
rs705415
rs567754
rs6859667
rs558133
rs1789953
rs11951068
All − MR Egger
All − IVW(MRE)
−0.8
−0.4
0.0
0.4
MR effect size for
'Selenium' on 'Cysteine−rich secretory protein LCCL domain−containing 2'

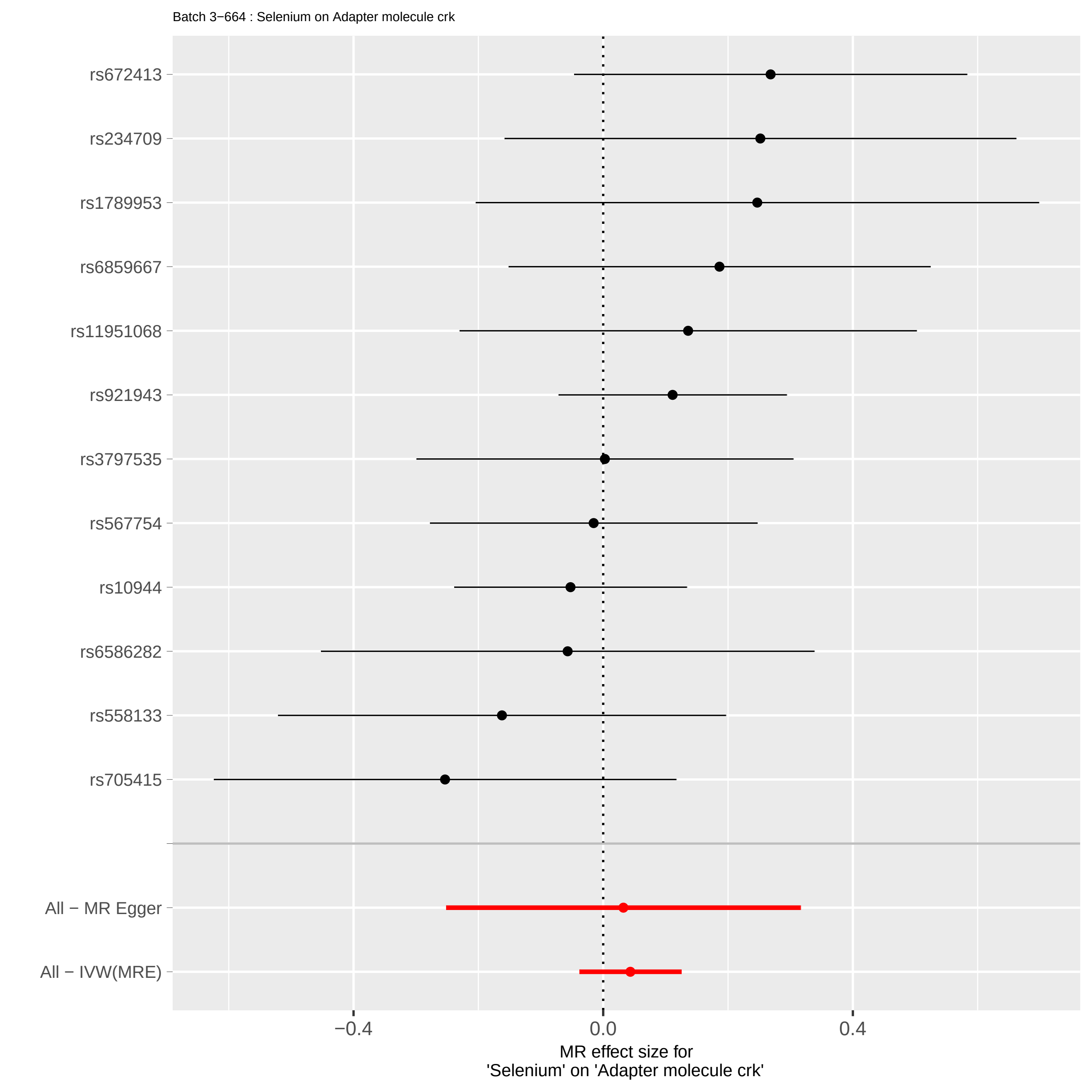

Batch 3−664 : Selenium on Adapter molecule crk
rs672413
rs234709
rs1789953
rs6859667
rs11951068
rs921943
rs3797535
rs567754
rs10944
rs6586282
rs558133
rs705415
All − MR Egger
All − IVW(MRE)
−0.4
0.0
0.4
MR effect size for
'Selenium' on 'Adapter molecule crk'

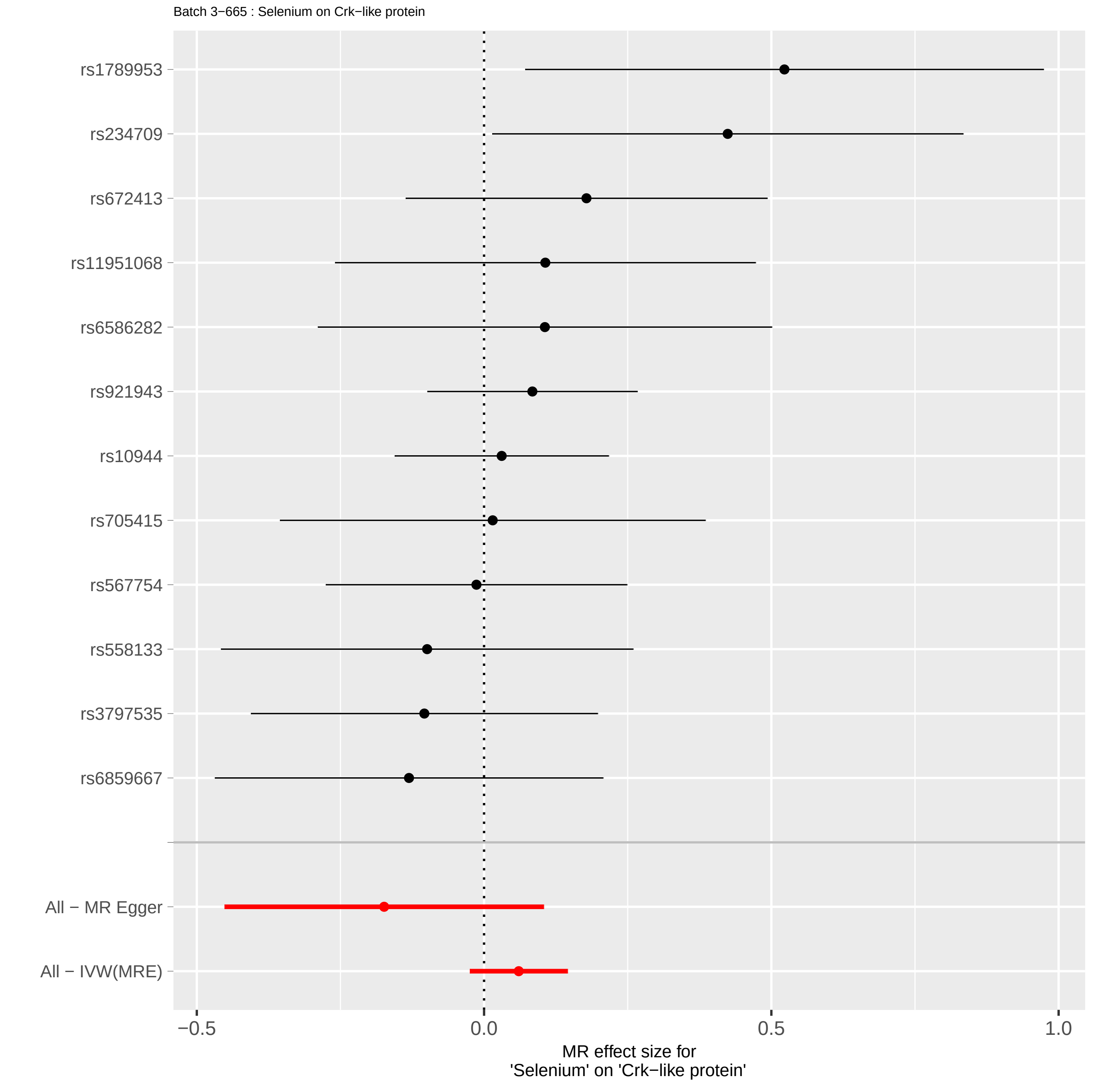

Batch 3−665 : Selenium on Crk−like protein
rs1789953
rs234709
rs672413
rs11951068
rs6586282
rs921943
rs10944
rs705415
rs567754
rs558133
rs3797535
rs6859667
All − MR Egger
All − IVW(MRE)
−0.5
0.0
0.5
1.0
MR effect size for
'Selenium' on 'Crk−like protein'

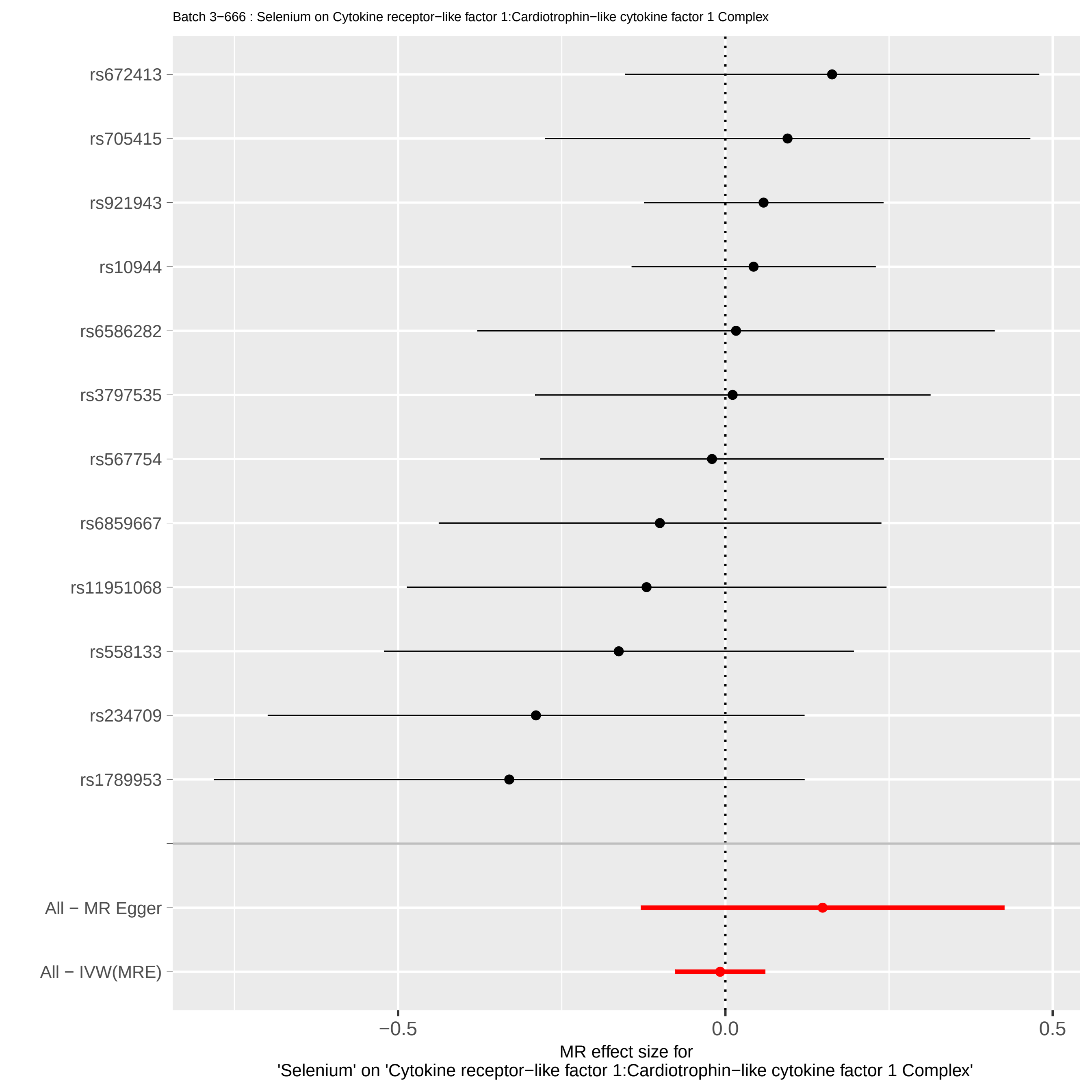

Batch 3−666 : Selenium on Cytokine receptor−like factor 1:Cardiotrophin−like cytokine factor 1 Complex
rs672413
rs705415
rs921943
rs10944
rs6586282
rs3797535
rs567754
rs6859667
rs11951068
rs558133
rs234709
rs1789953
All − MR Egger
All − IVW(MRE)
−0.5
0.0
0.5
MR effect size for
'Selenium' on 'Cytokine receptor−like factor 1:Cardiotrophin−like cytokine factor 1 Complex'

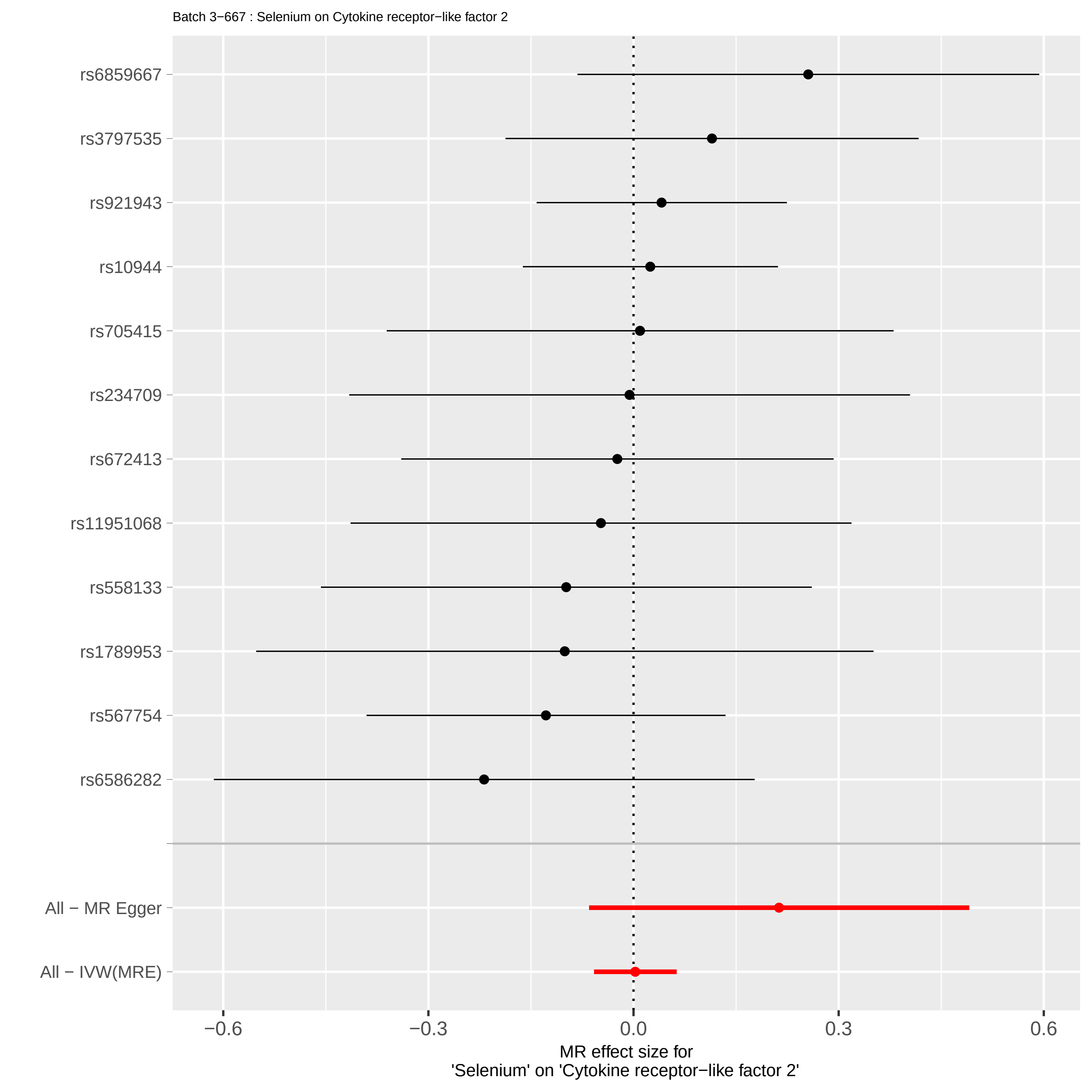
Batch 3−667 : Selenium on Cytokine receptor−like factor 2
rs6859667
rs3797535
rs921943
rs10944
rs705415
rs234709
rs672413
rs11951068
rs558133
rs1789953
rs567754
rs6586282
All − MR Egger
All − IVW(MRE)
−0.6
−0.3
0.0
0.3
0.6
MR effect size for
'Selenium' on 'Cytokine receptor−like factor 2'

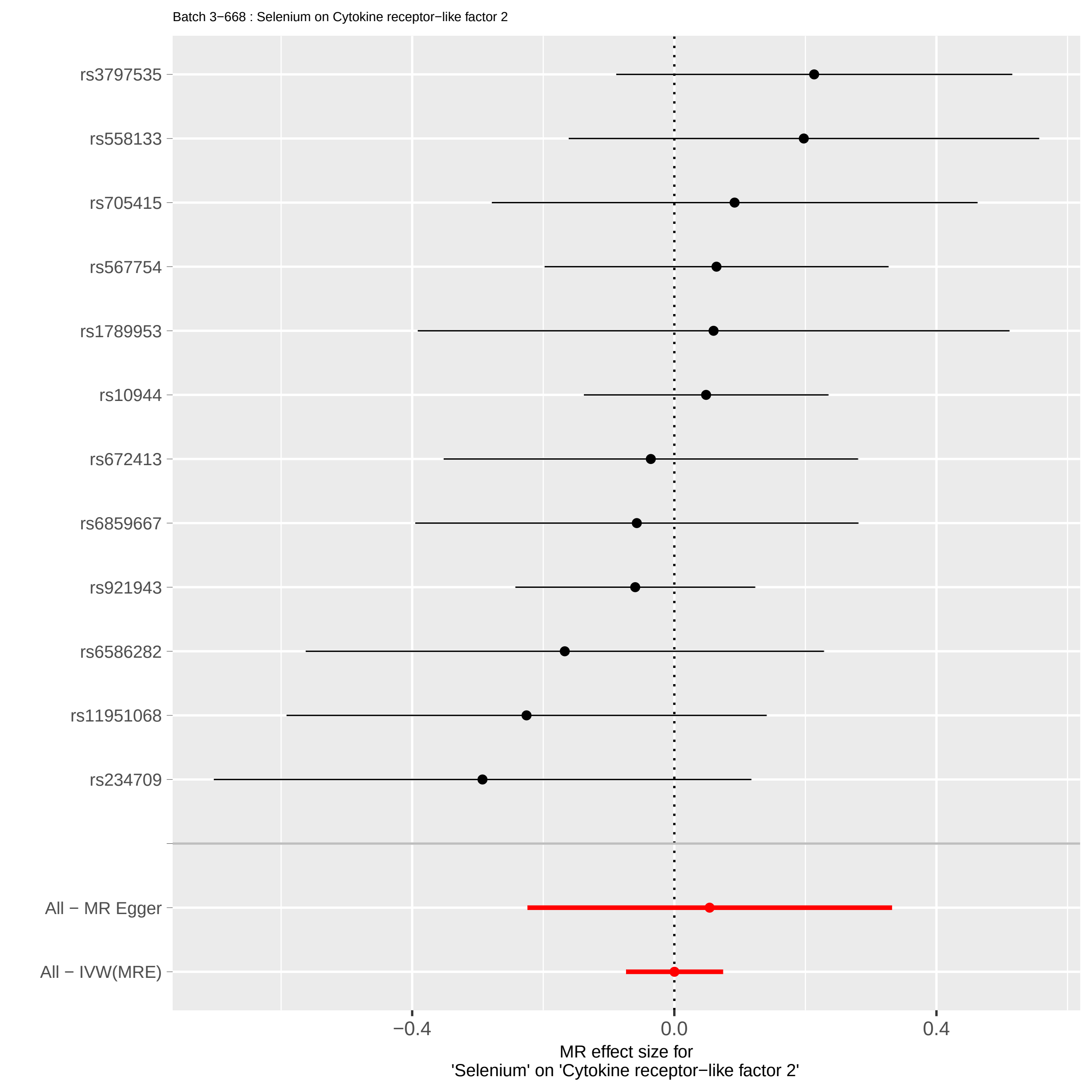
Batch 3−668 : Selenium on Cytokine receptor−like factor 2
rs3797535
rs558133
rs705415
rs567754
rs1789953
rs10944
rs672413
rs6859667
rs921943
rs6586282
rs11951068
rs234709
All − MR Egger
All − IVW(MRE)
−0.4
0.0
0.4
MR effect size for
'Selenium' on 'Cytokine receptor−like factor 2'

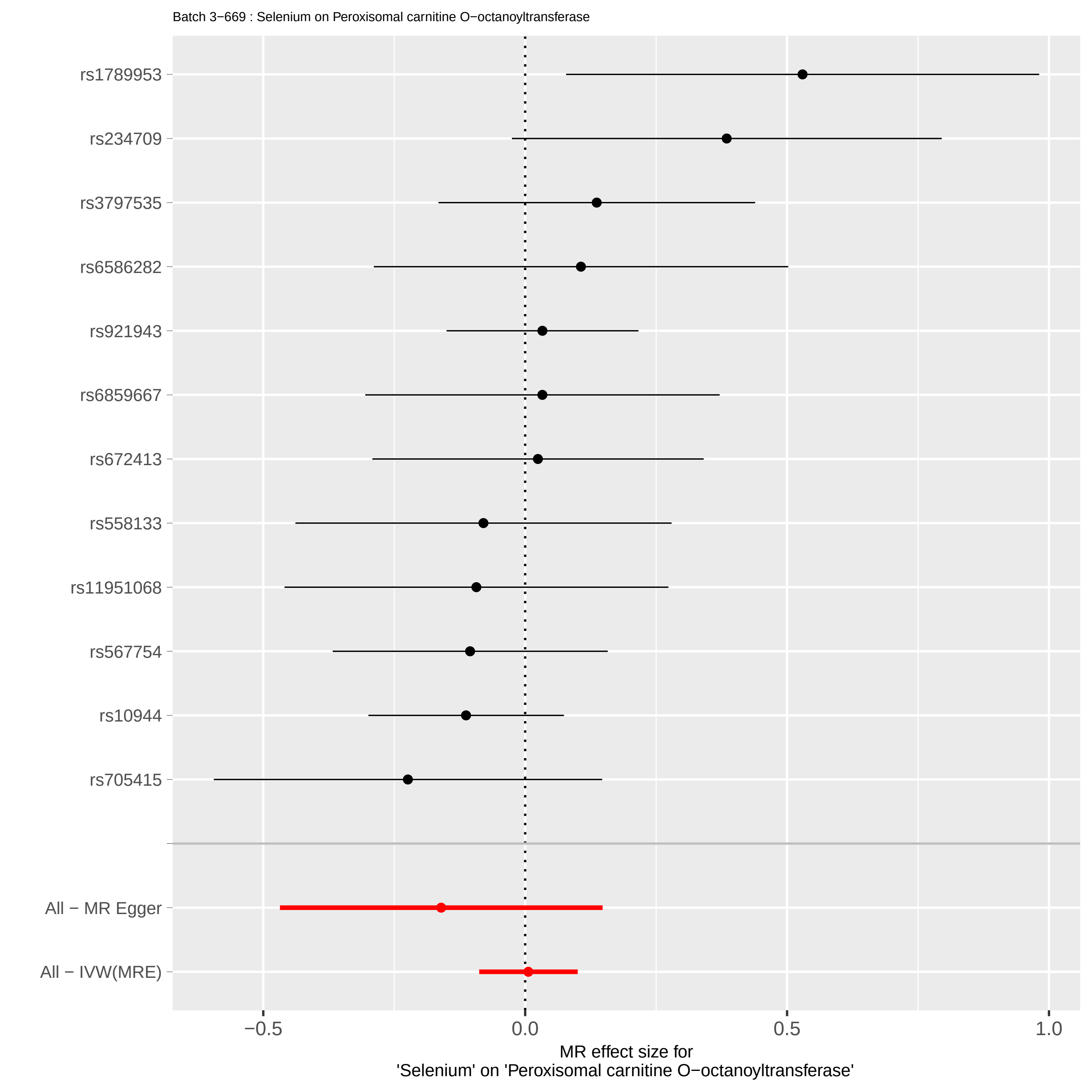

Batch 3−669 : Selenium on Peroxisomal carnitine O−octanoyltransferase
rs1789953
rs234709
rs3797535
rs6586282
rs921943
rs6859667
rs672413
rs558133
rs11951068
rs567754
rs10944
rs705415
All − MR Egger
All − IVW(MRE)
−0.5
0.0
0.5
1.0
MR effect size for
'Selenium' on 'Peroxisomal carnitine O−octanoyltransferase'

Batch 3−670 : Selenium on C−reactive protein

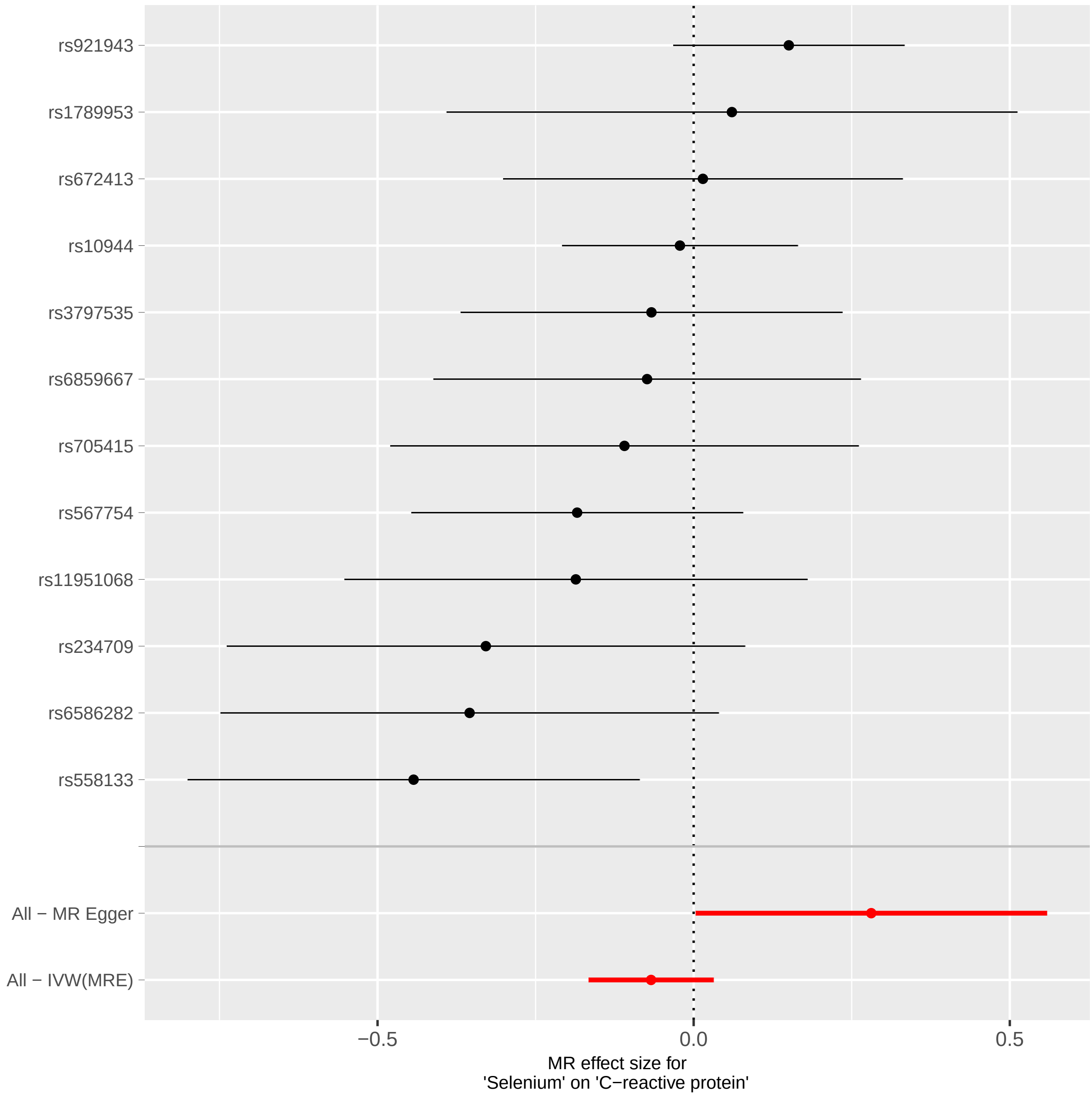

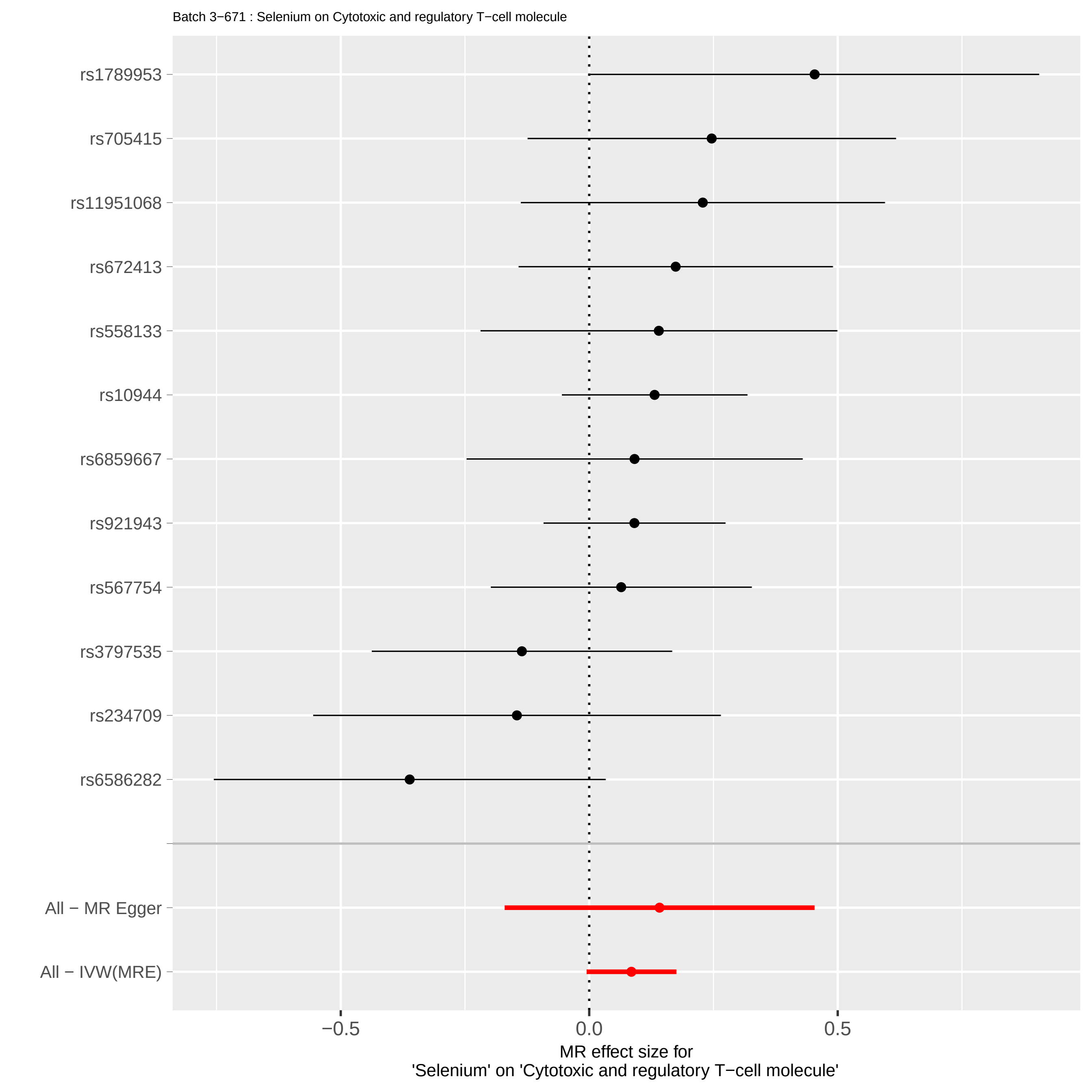
Batch 3−671 : Selenium on Cytotoxic and regulatory T−cell molecule
rs1789953
rs705415
rs11951068
rs672413
rs558133
rs10944
rs6859667
rs921943
rs567754
rs3797535
rs234709
rs6586282
All − MR Egger
All − IVW(MRE)
−0.5
0.0
0.5
MR effect size for
'Selenium' on 'Cytotoxic and regulatory T−cell molecule'

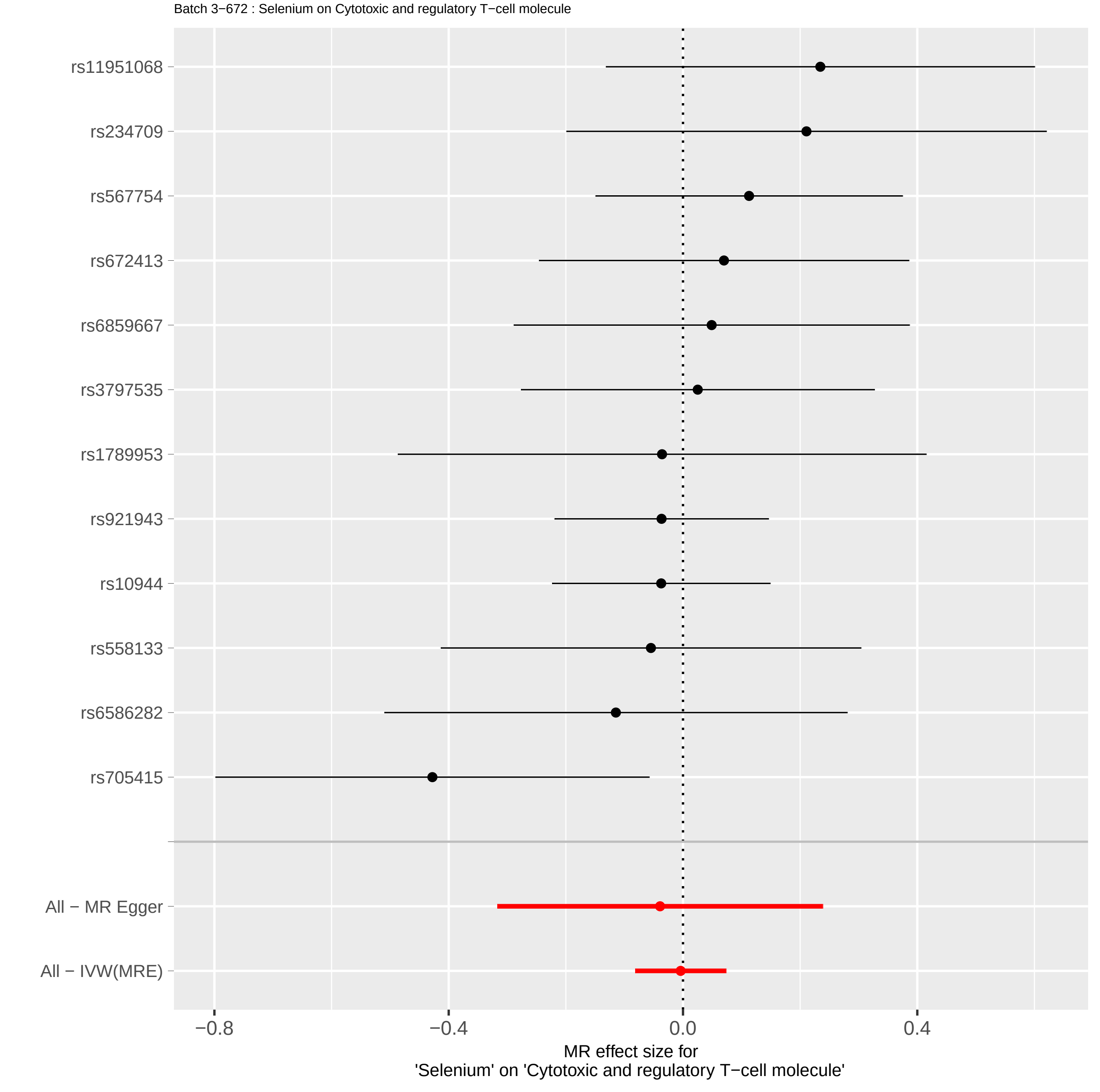
Batch 3−672 : Selenium on Cytotoxic and regulatory T−cell molecule
rs11951068
rs234709
rs567754
rs672413
rs6859667
rs3797535
rs1789953
rs921943
rs10944
rs558133
rs6586282
rs705415
All − MR Egger
All − IVW(MRE)
−0.8
−0.4
0.0
0.4
MR effect size for
'Selenium' on 'Cytotoxic and regulatory T−cell molecule'

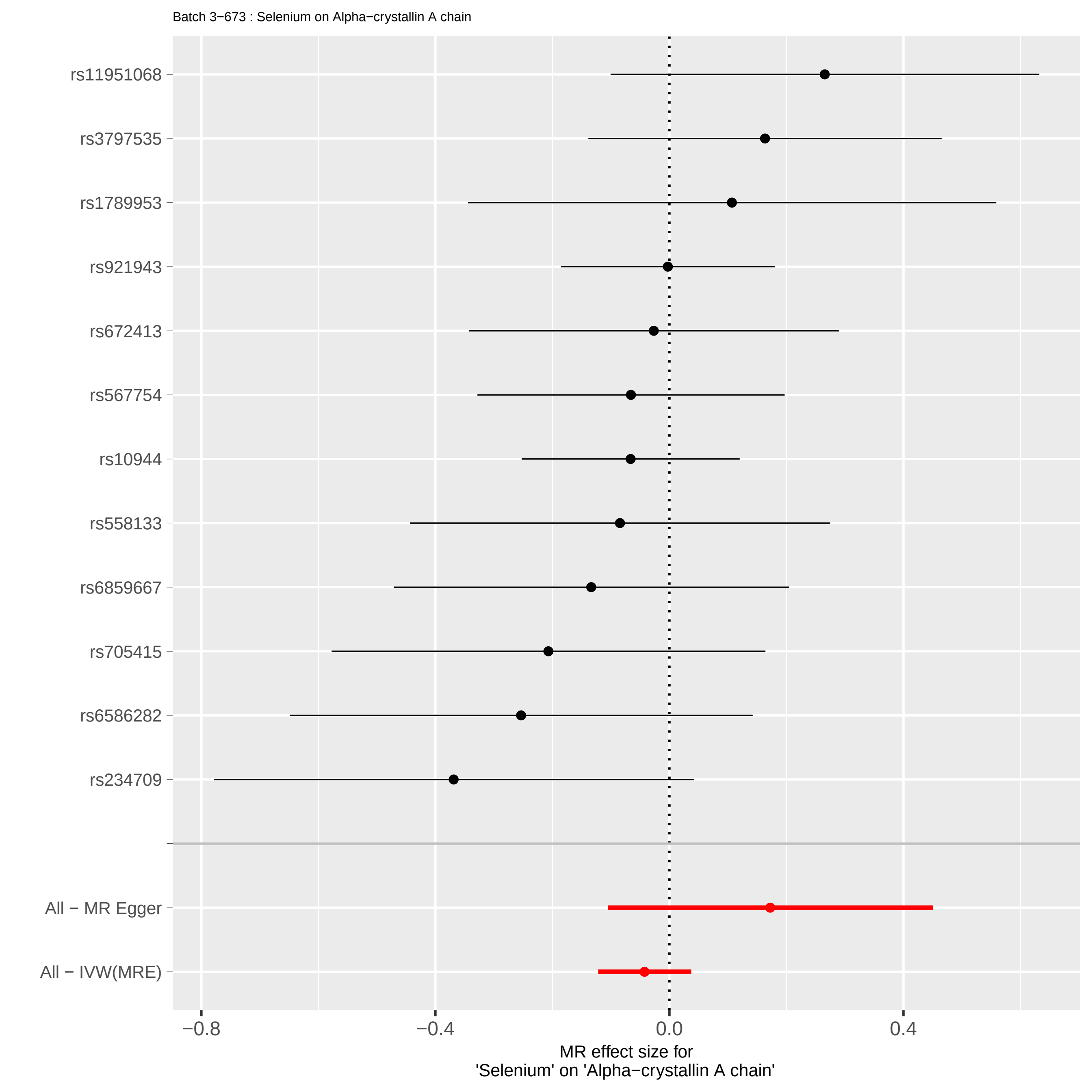

Batch 3−673 : Selenium on Alpha−crystallin A chain
rs11951068
rs3797535
rs1789953
rs921943
rs672413
rs567754
rs10944
rs558133
rs6859667
rs705415
rs6586282
rs234709
All − MR Egger
All − IVW(MRE)
−0.8
−0.4
0.0
0.4
MR effect size for
'Selenium' on 'Alpha−crystallin A chain'

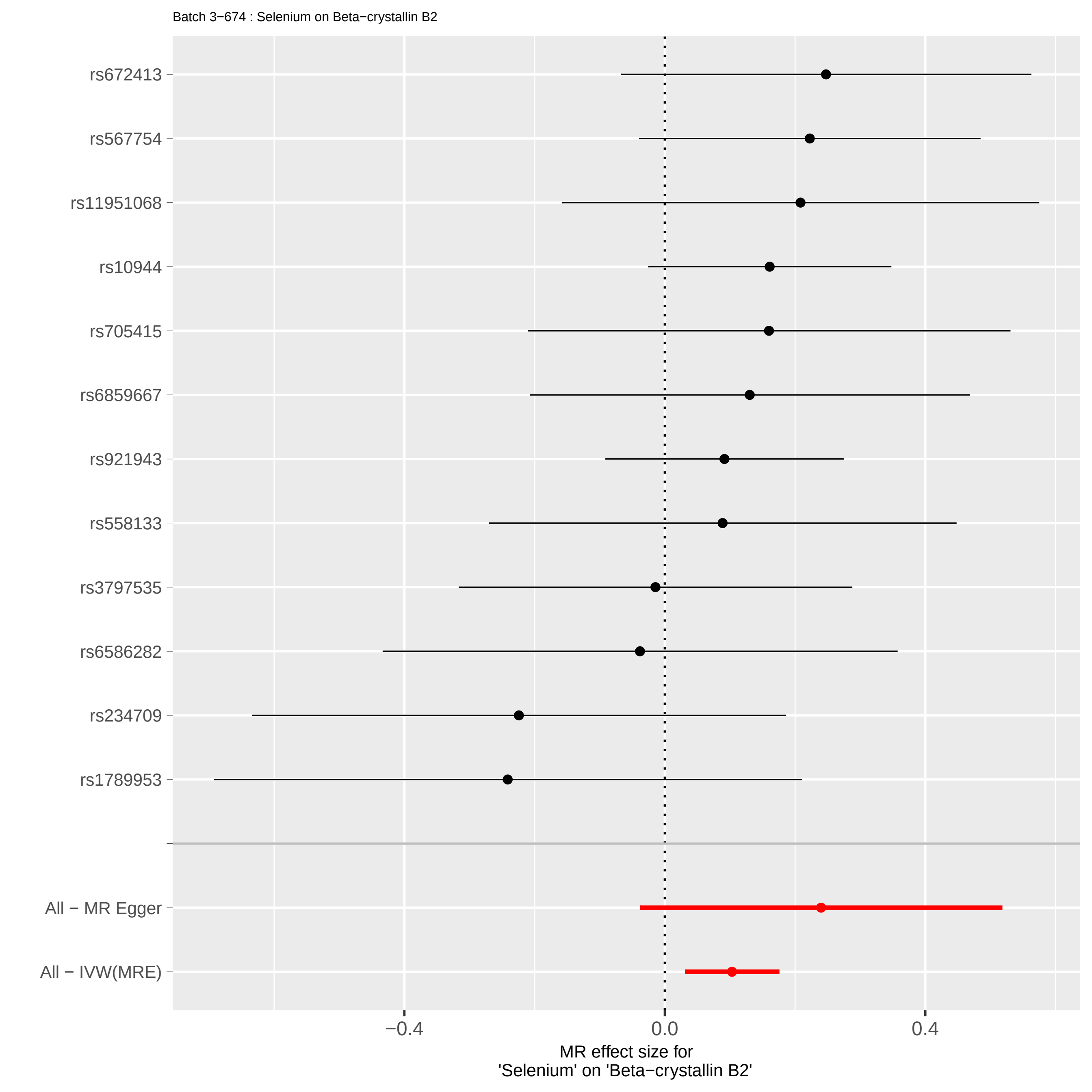

Batch 3−674 : Selenium on Beta−crystallin B2
rs672413
rs567754
rs11951068
rs10944
rs705415
rs6859667
rs921943
rs558133
rs3797535
rs6586282
rs234709
rs1789953
All − MR Egger
All − IVW(MRE)
−0.4
0.0
0.4
MR effect size for
'Selenium' on 'Beta−crystallin B2'

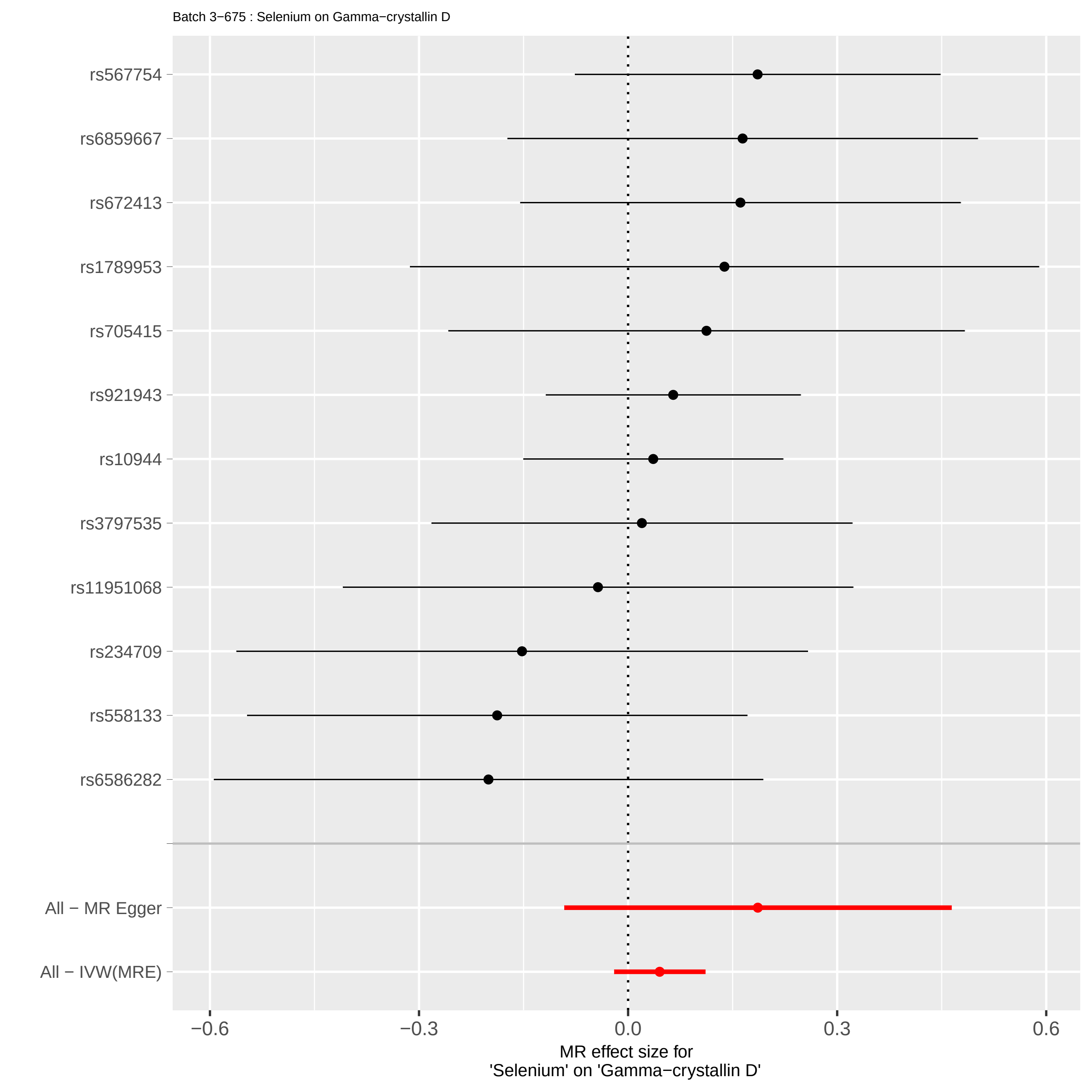
Batch 3−675 : Selenium on Gamma−crystallin D
rs567754
rs6859667
rs672413
rs1789953
rs705415
rs921943
rs10944
rs3797535
rs11951068
rs234709
rs558133
rs6586282
All − MR Egger
All − IVW(MRE)
−0.6
−0.3
0.0
0.3
0.6
MR effect size for
'Selenium' on 'Gamma−crystallin D'

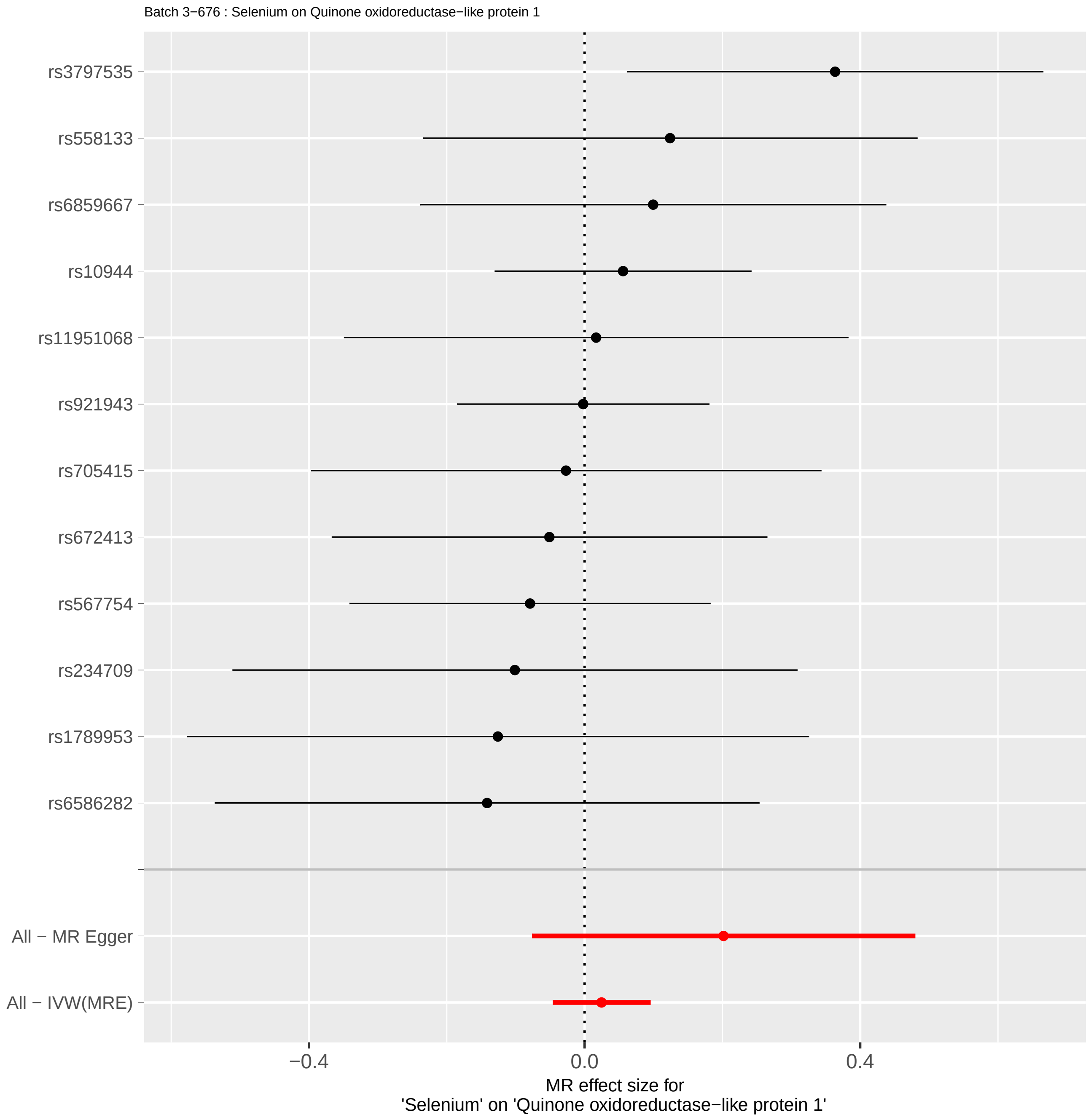

Batch 3−676 : Selenium on Quinone oxidoreductase−like protein 1
rs3797535
rs558133
rs6859667
rs10944
rs11951068
rs921943
rs705415
rs672413
rs567754
rs234709
rs1789953
rs6586282
All − MR Egger
All − IVW(MRE)
−0.4
0.0
0.4
MR effect size for
'Selenium' on 'Quinone oxidoreductase−like protein 1'

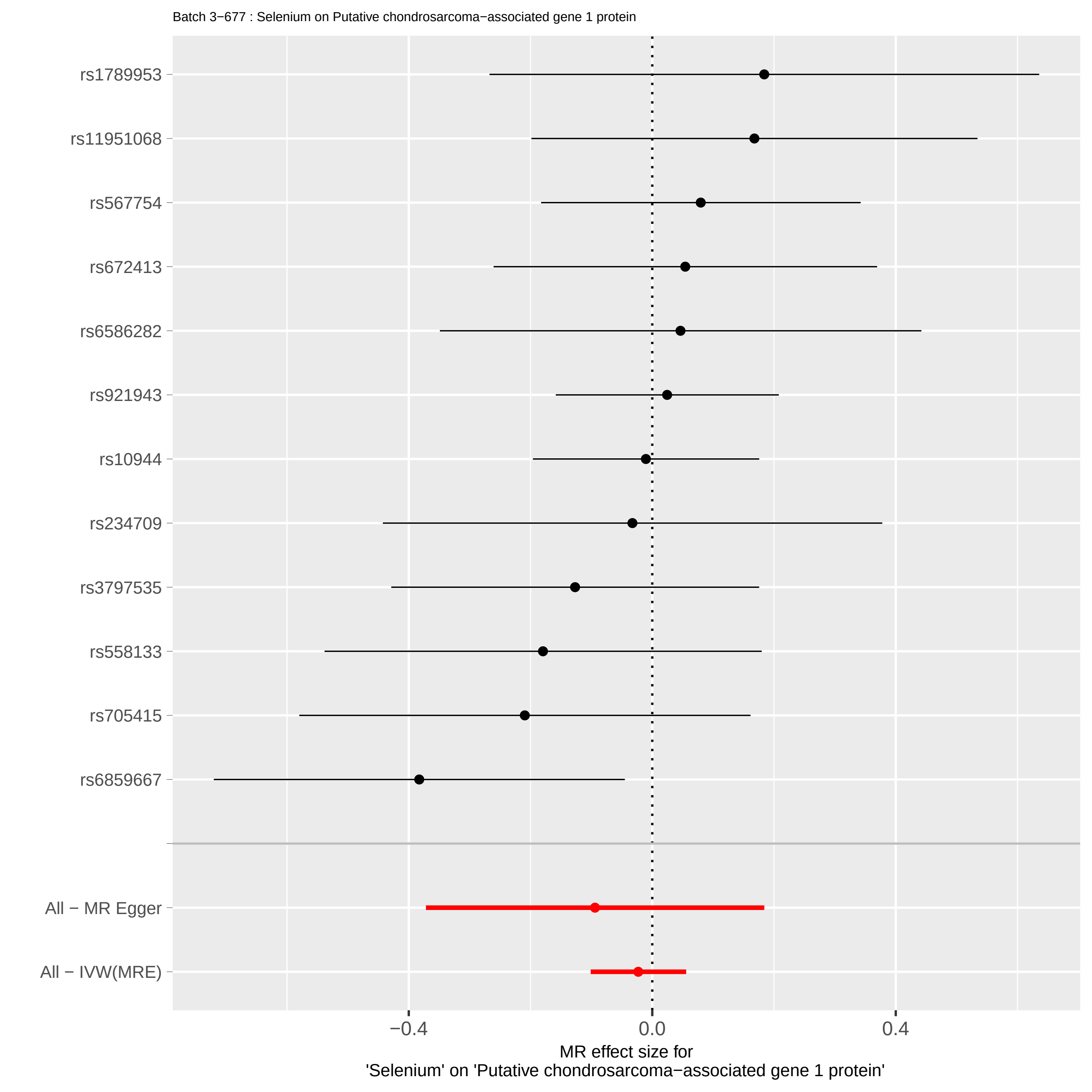

Batch 3−677 : Selenium on Putative chondrosarcoma−associated gene 1 protein
rs1789953
rs11951068
rs567754
rs672413
rs6586282
rs921943
rs10944
rs234709
rs3797535
rs558133
rs705415
rs6859667
All − MR Egger
All − IVW(MRE)
−0.4
0.0
0.4
MR effect size for
'Selenium' on 'Putative chondrosarcoma−associated gene 1 protein'

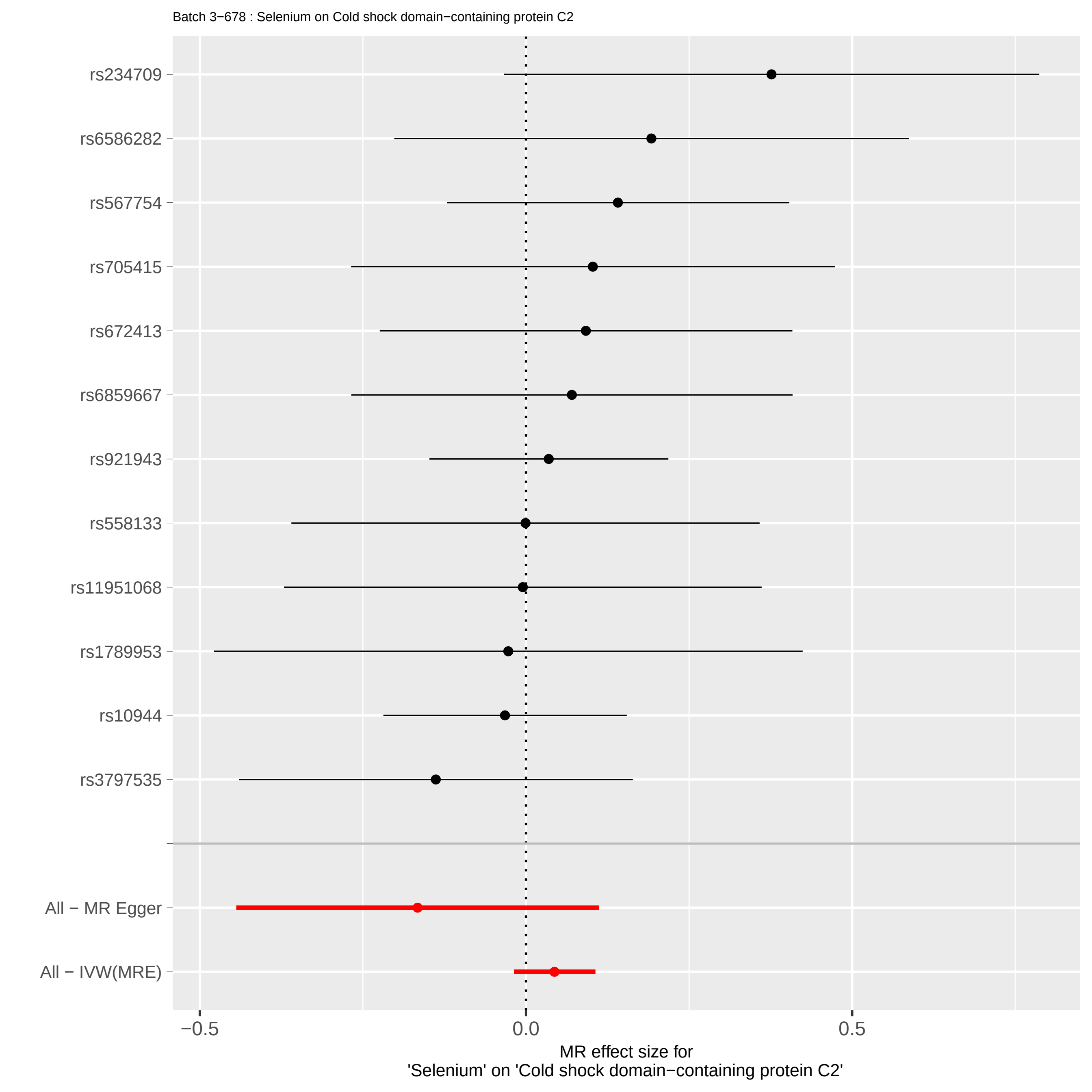

Batch 3−678 : Selenium on Cold shock domain−containing protein C2
rs234709
rs6586282
rs567754
rs705415
rs672413
rs6859667
rs921943
rs558133
rs11951068
rs1789953
rs10944
rs3797535
All − MR Egger
All − IVW(MRE)
−0.5
0.0
0.5
MR effect size for
'Selenium' on 'Cold shock domain−containing protein C2'

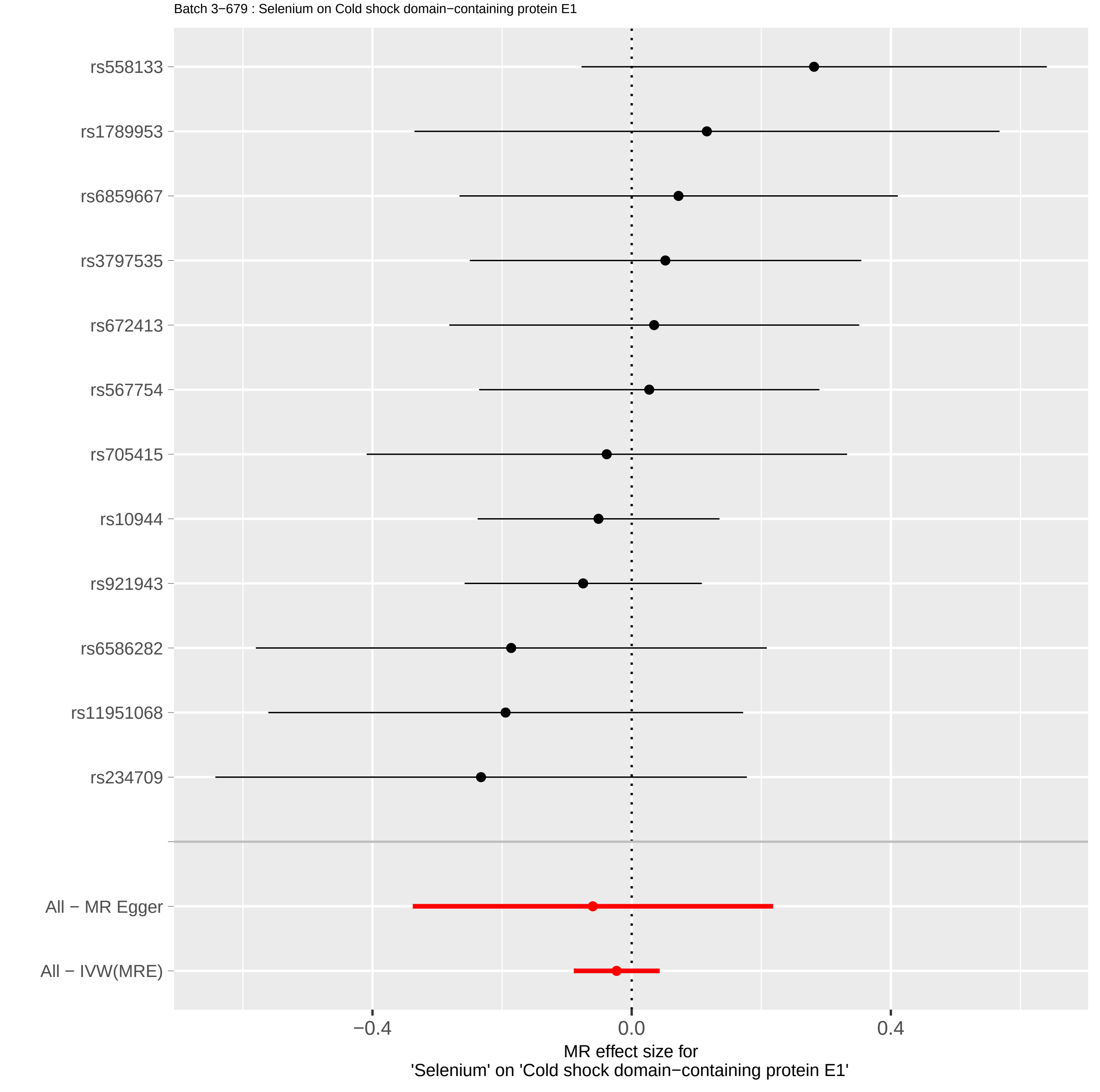

Batch 3−679 : Selenium on Cold shock domain−containing protein E1
rs558133
rs1789953
rs6859667
rs3797535
rs672413
rs567754
rs705415
rs10944
rs921943
rs6586282
rs11951068
rs234709
All − MR Egger
All − IVW(MRE)
−0.4
0.0
0.4
MR effect size for
'Selenium' on 'Cold shock domain−containing protein E1'

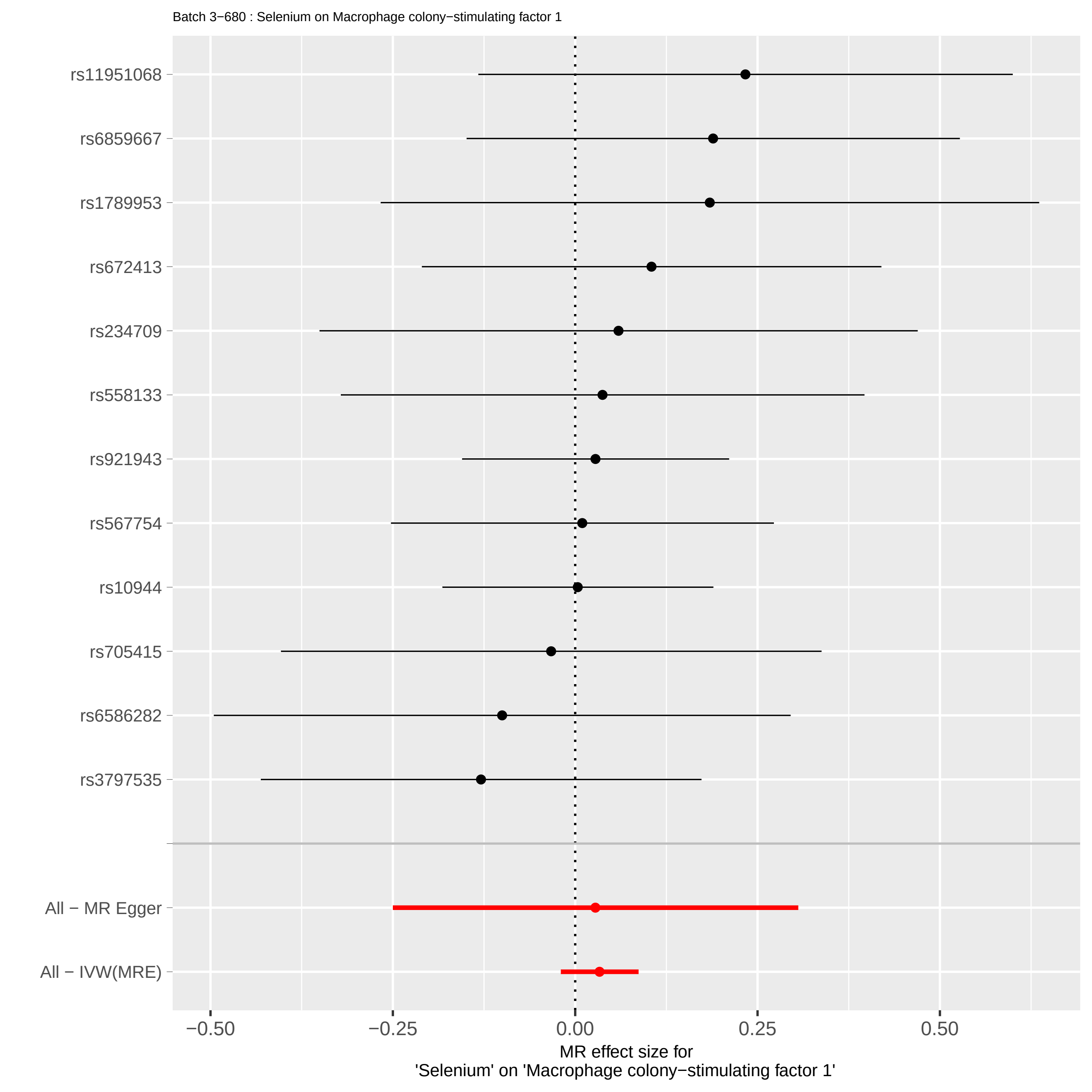
Batch 3−680 : Selenium on Macrophage colony−stimulating factor 1
rs11951068
rs6859667
rs1789953
rs672413
rs234709
rs558133
rs921943
rs567754
rs10944
rs705415
rs6586282
rs3797535
All − MR Egger
All − IVW(MRE)
−0.50
−0.25
0.00
0.25
0.50
MR effect size for
'Selenium' on 'Macrophage colony−stimulating factor 1'

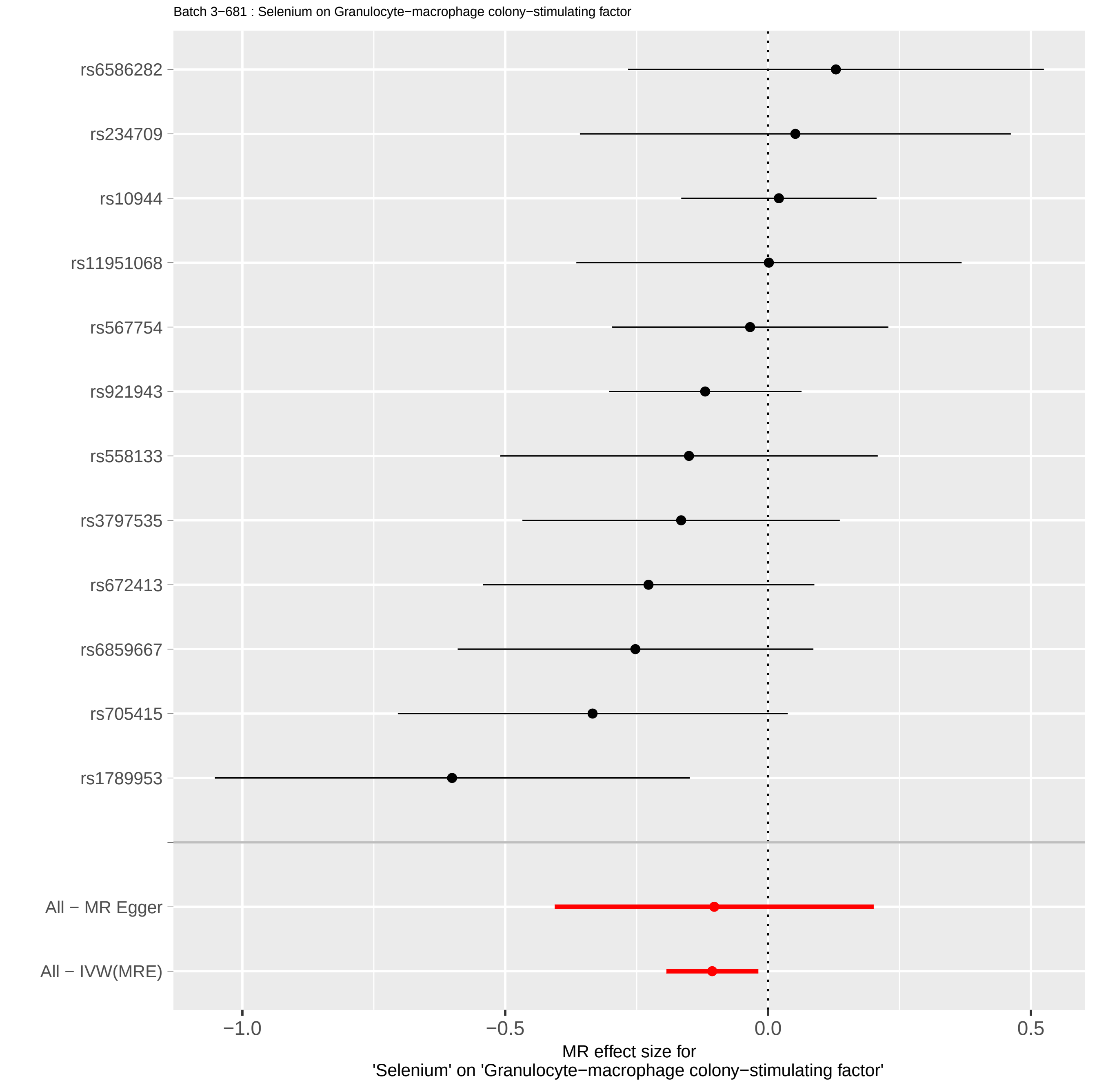

Batch 3−681 : Selenium on Granulocyte−macrophage colony−stimulating factor
rs6586282
rs234709
rs10944
rs11951068
rs567754
rs921943
rs558133
rs3797535
rs672413
rs6859667
rs705415
rs1789953
All − MR Egger
All − IVW(MRE)
−1.0
−0.5
0.0
0.5
MR effect size for
'Selenium' on 'Granulocyte−macrophage colony−stimulating factor'

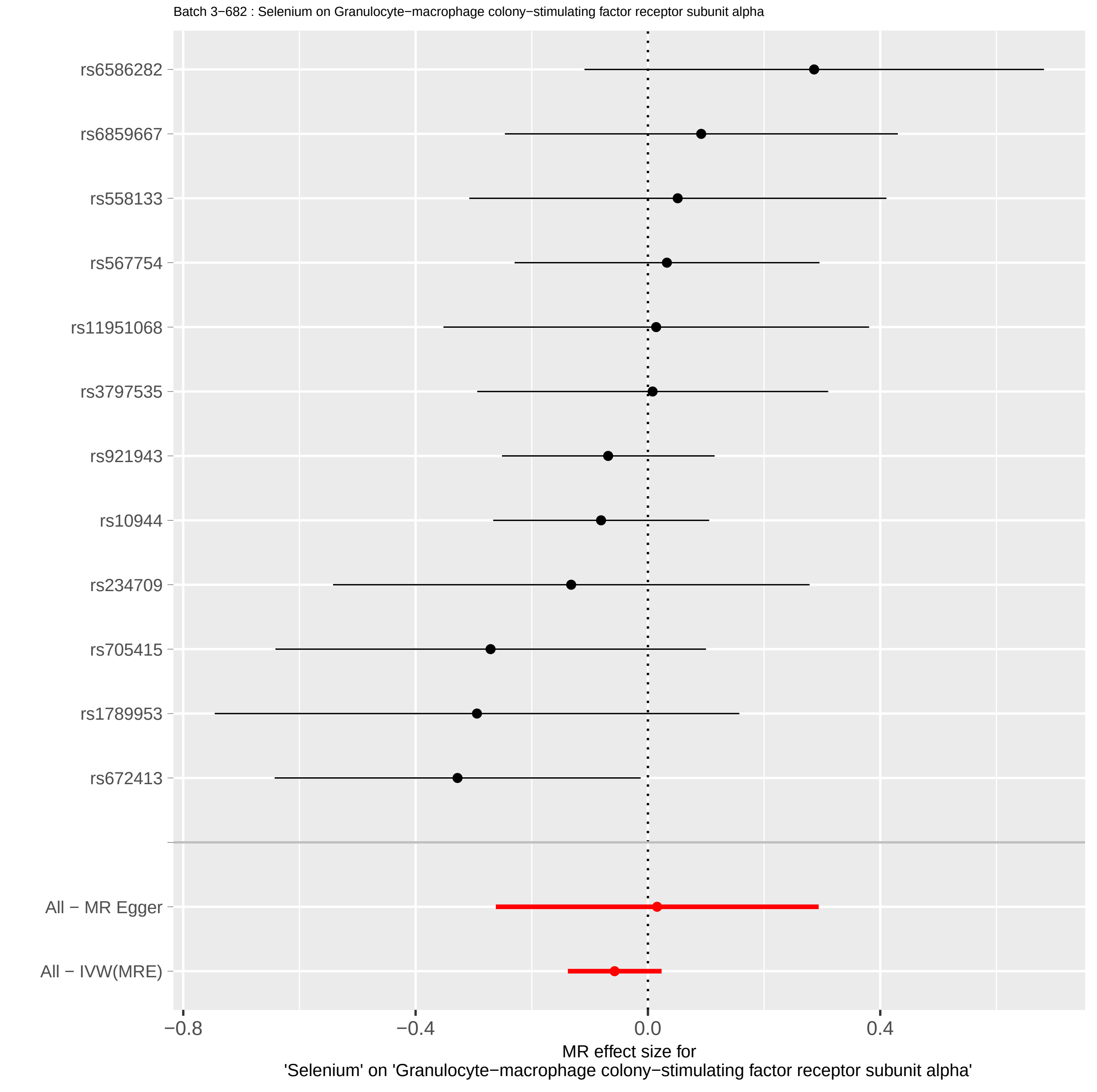

Batch 3−682 : Selenium on Granulocyte−macrophage colony−stimulating factor receptor subunit alpha
rs6586282
rs6859667
rs558133
rs567754
rs11951068
rs3797535
rs921943
rs10944
rs234709
rs705415
rs1789953
rs672413
All − MR Egger
All − IVW(MRE)
−0.8
−0.4
0.0
0.4
MR effect size for
'Selenium' on 'Granulocyte−macrophage colony−stimulating factor receptor subunit alpha'

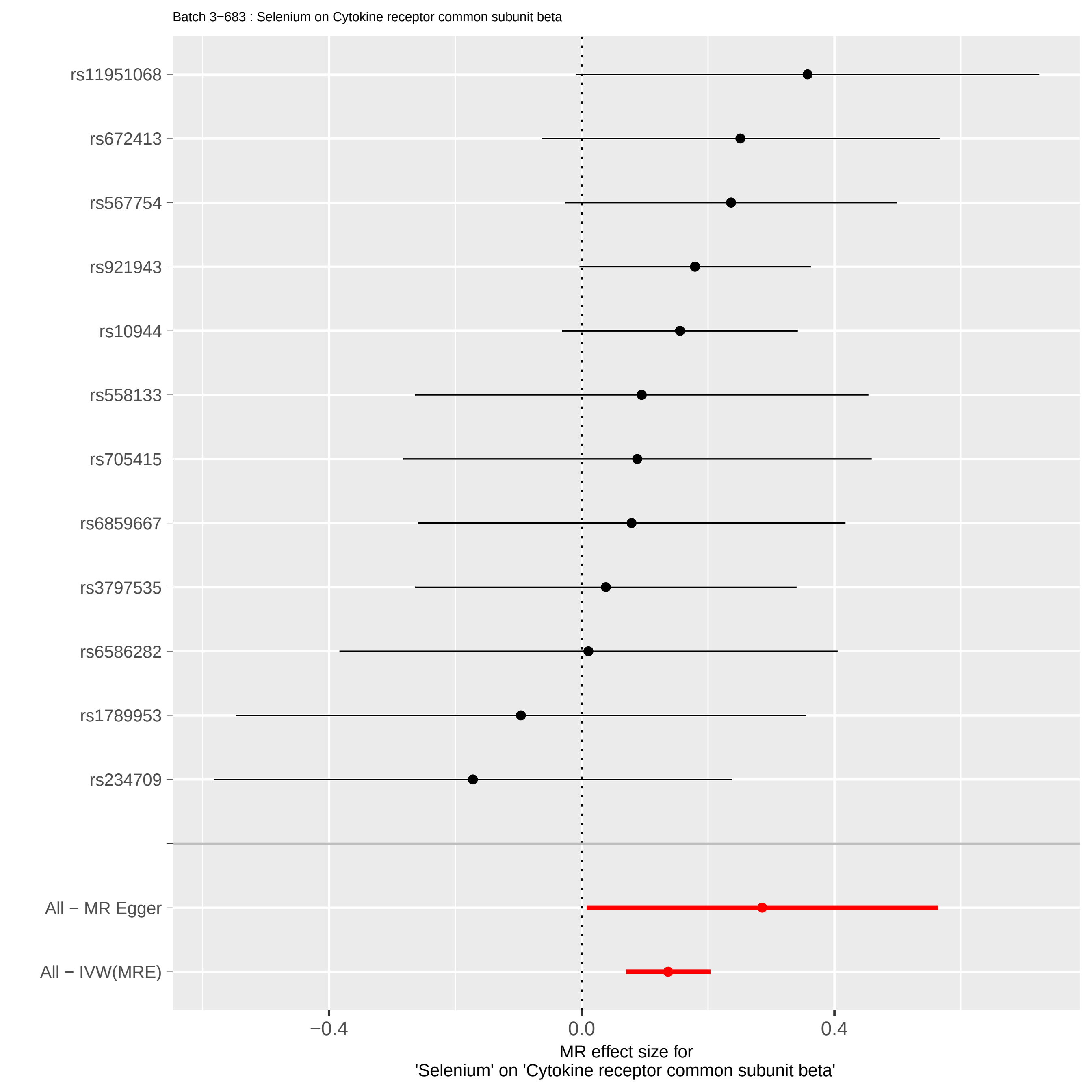

Batch 3−683 : Selenium on Cytokine receptor common subunit beta
rs11951068
rs672413
rs567754
rs921943
rs10944
rs558133
rs705415
rs6859667
rs3797535
rs6586282
rs1789953
rs234709
All − MR Egger
All − IVW(MRE)
−0.4
0.0
0.4
MR effect size for
'Selenium' on 'Cytokine receptor common subunit beta'

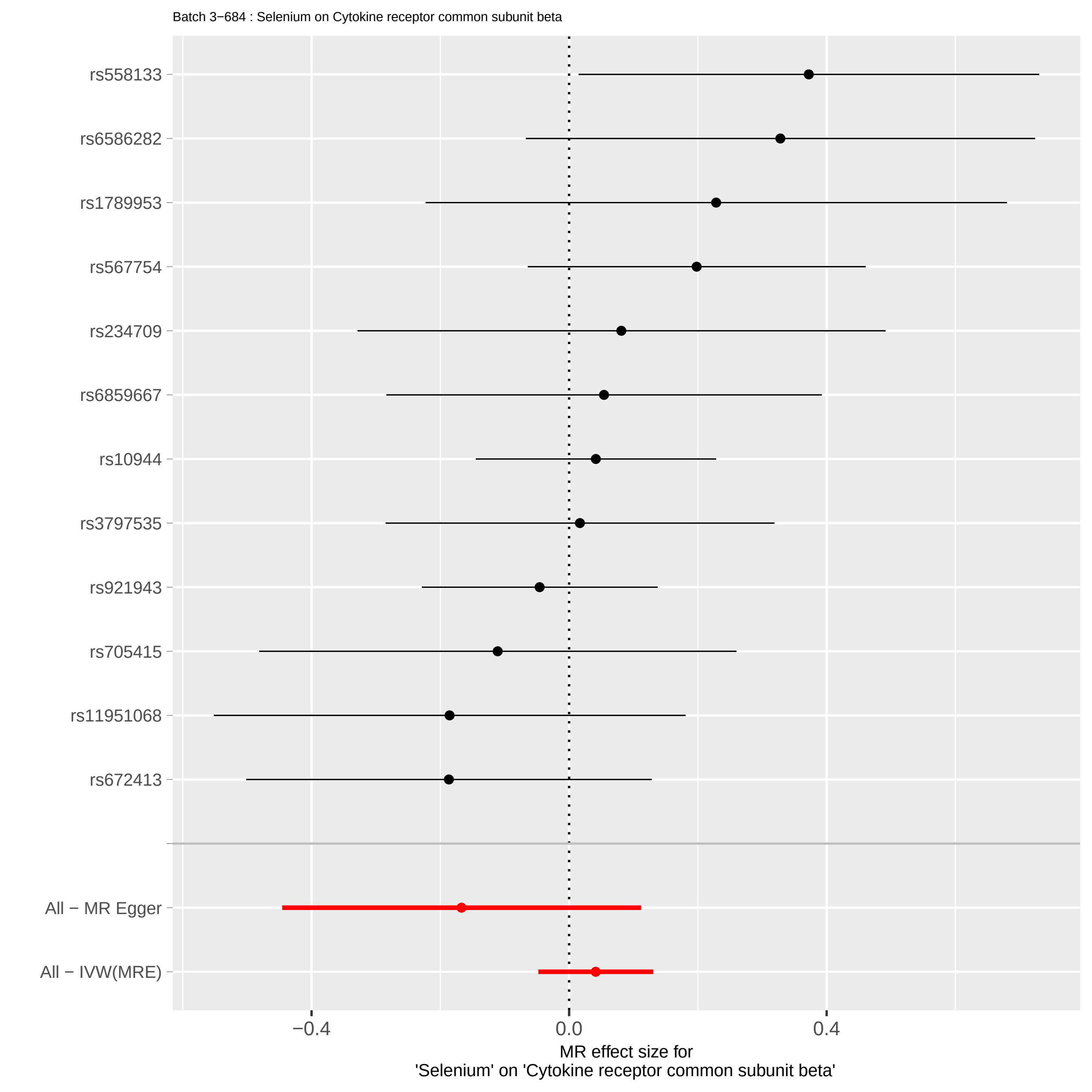
Batch 3−684 : Selenium on Cytokine receptor common subunit beta
rs558133
rs6586282
rs1789953
rs567754
rs234709
rs6859667
rs10944
rs3797535
rs921943
rs705415
rs11951068
rs672413
All − MR Egger
All − IVW(MRE)
−0.4
0.0
0.4
MR effect size for
'Selenium' on 'Cytokine receptor common subunit beta'

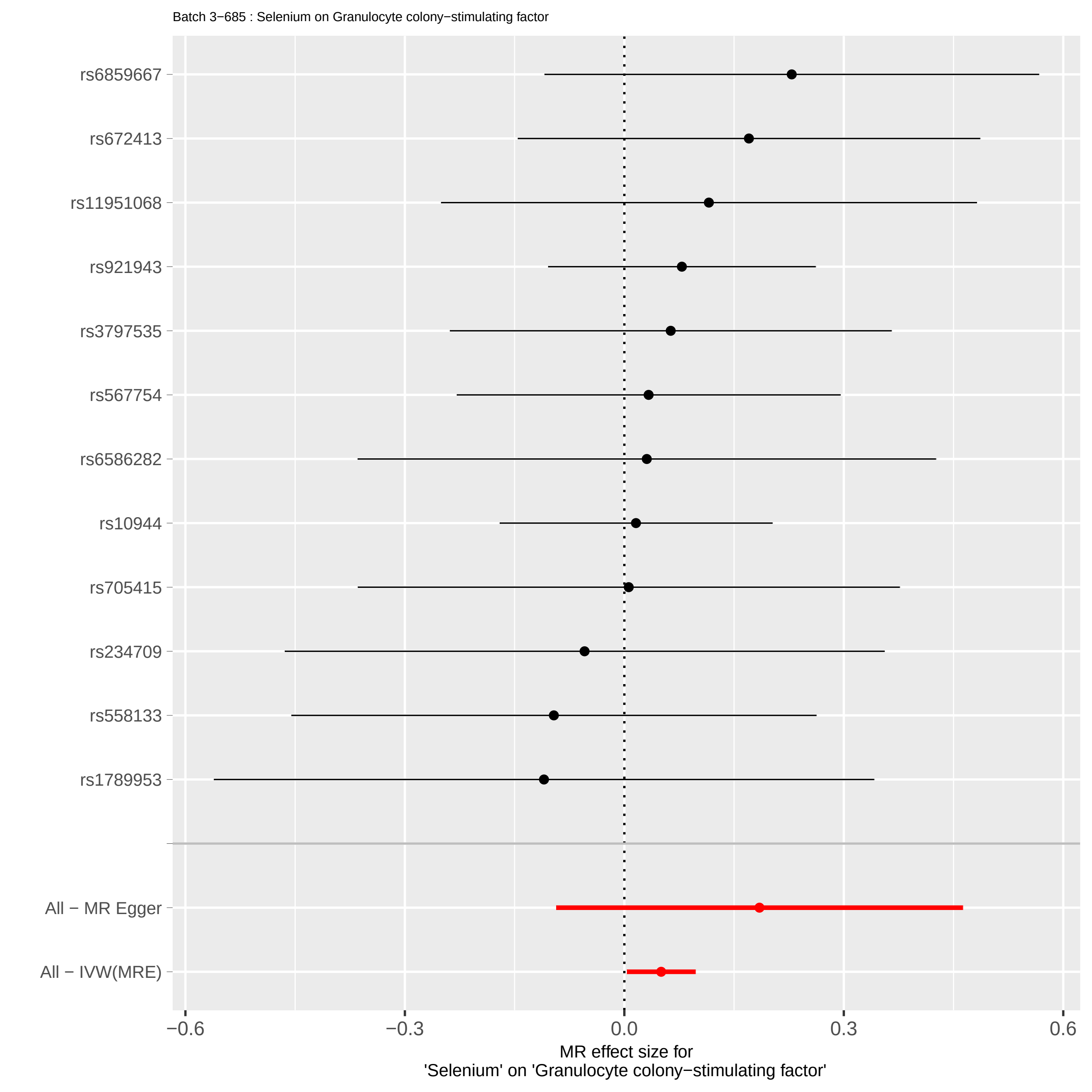
Batch 3−685 : Selenium on Granulocyte colony−stimulating factor
rs6859667
rs672413
rs11951068
rs921943
rs3797535
rs567754
rs6586282
rs10944
rs705415
rs234709
rs558133
rs1789953
All − MR Egger
All − IVW(MRE)
−0.6
−0.3
0.0
0.3
0.6
MR effect size for
'Selenium' on 'Granulocyte colony−stimulating factor'

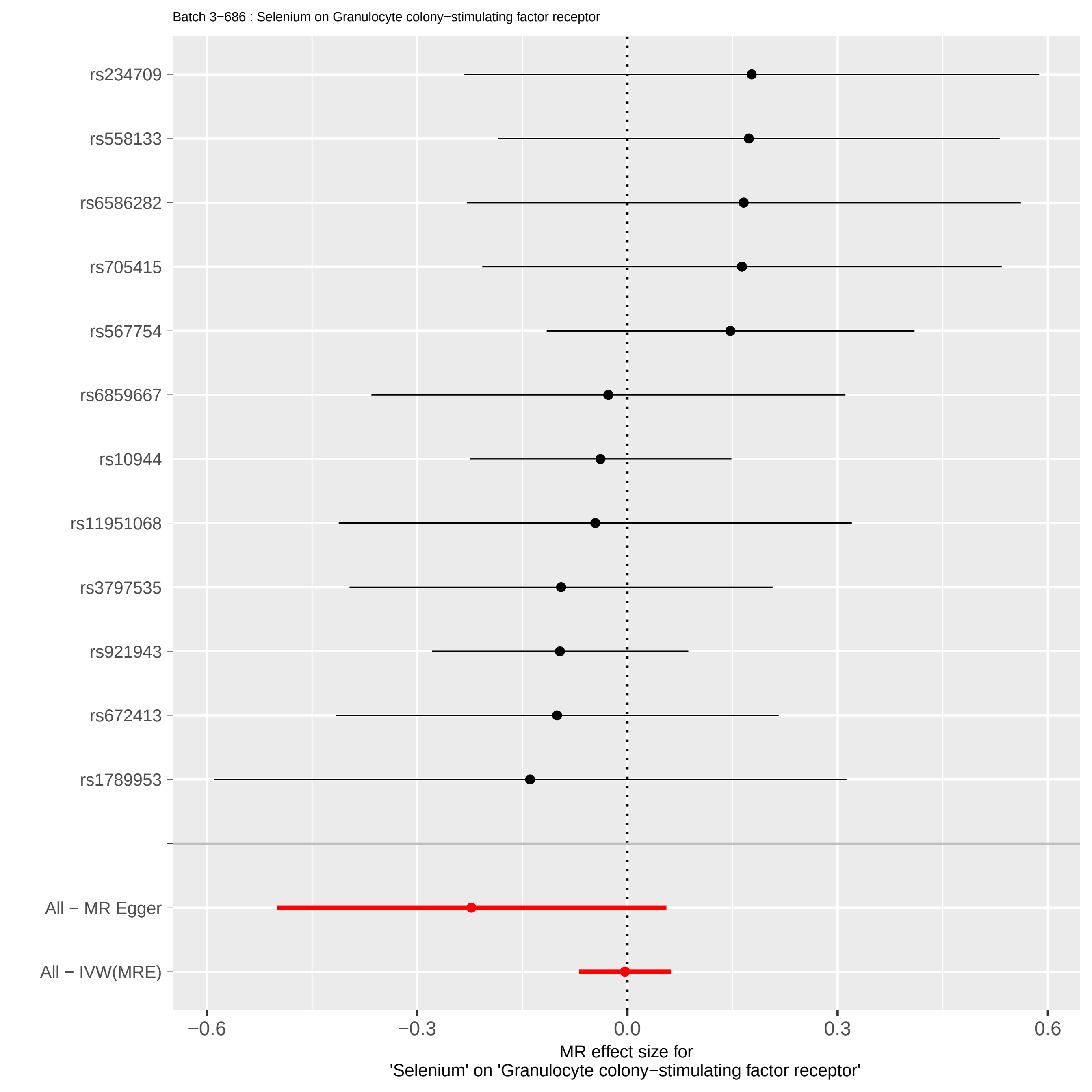
Batch 3−686 : Selenium on Granulocyte colony−stimulating factor receptor
rs234709
rs558133
rs6586282
rs705415
rs567754
rs6859667
rs10944
rs11951068
rs3797535
rs921943
rs672413
rs1789953
All − MR Egger
All − IVW(MRE)
−0.6
−0.3
0.0
0.3
0.6
MR effect size for
'Selenium' on 'Granulocyte colony−stimulating factor receptor'

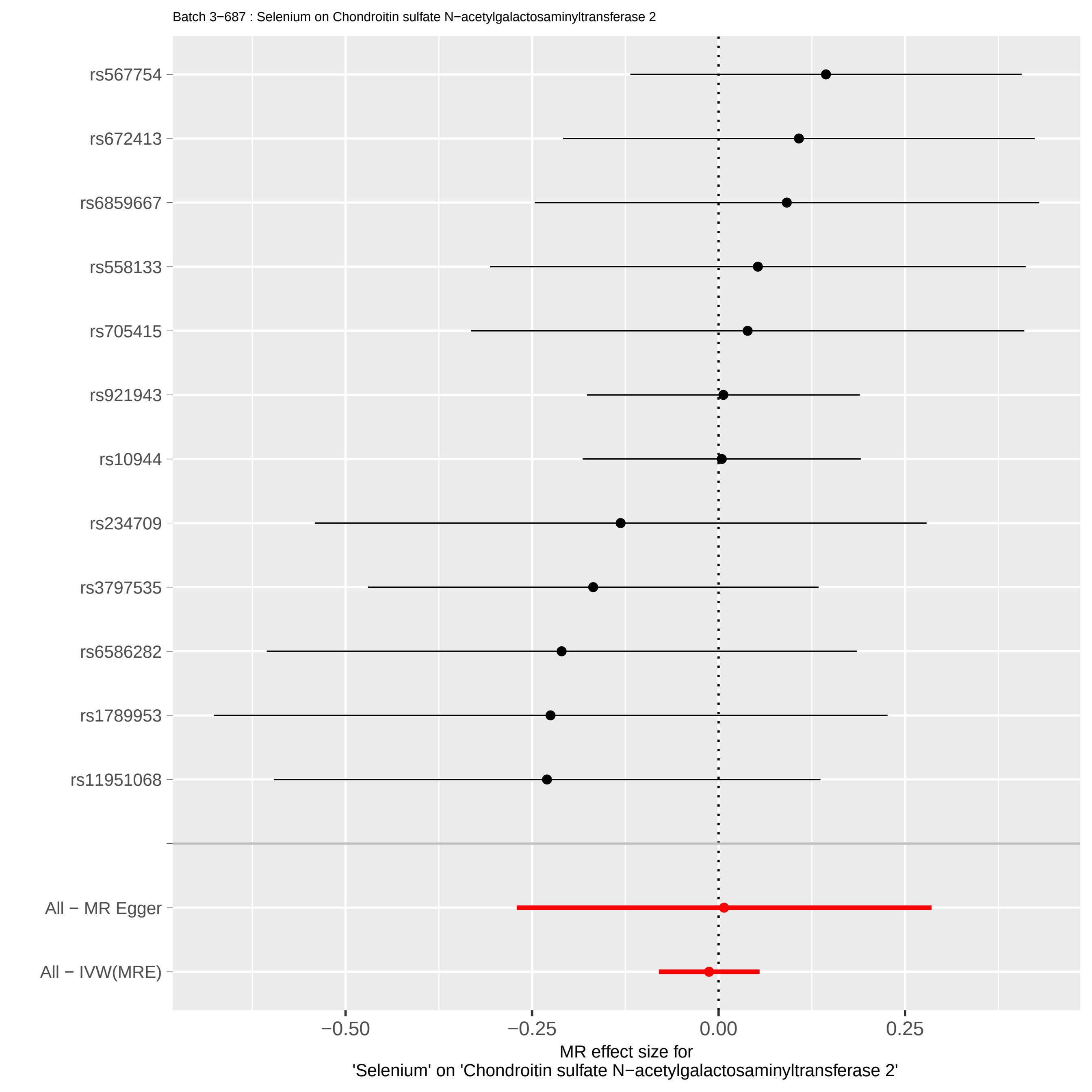

Batch 3−687 : Selenium on Chondroitin sulfate N−acetylgalactosaminyltransferase 2
rs567754
rs672413
rs6859667
rs558133
rs705415
rs921943
rs10944
rs234709
rs3797535
rs6586282
rs1789953
rs11951068
All − MR Egger
All − IVW(MRE)
−0.50
−0.25
0.00
0.25
MR effect size for
'Selenium' on 'Chondroitin sulfate N−acetylgalactosaminyltransferase 2'

Batch 3−688 : Selenium on Chorionic somatomammotropin hormone

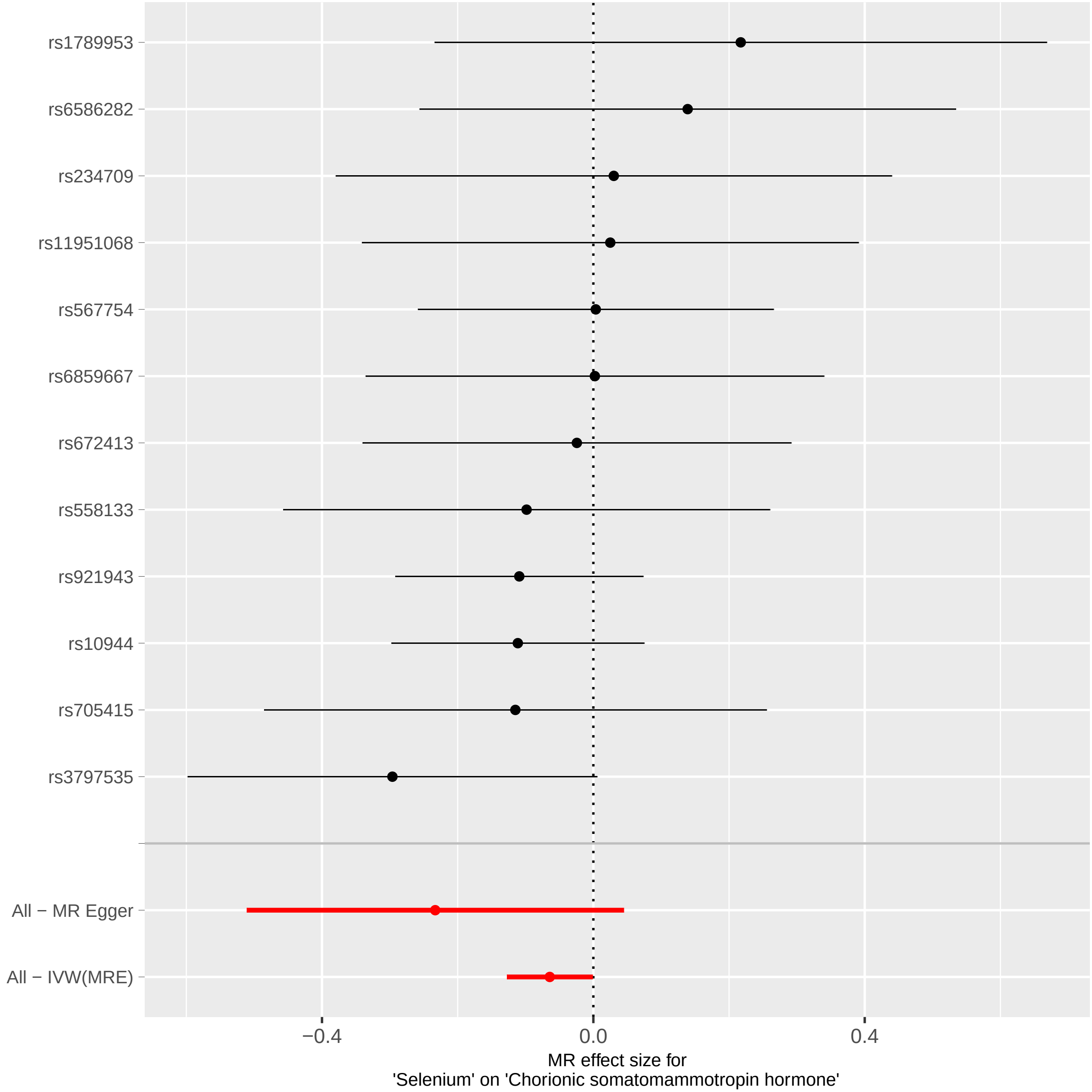

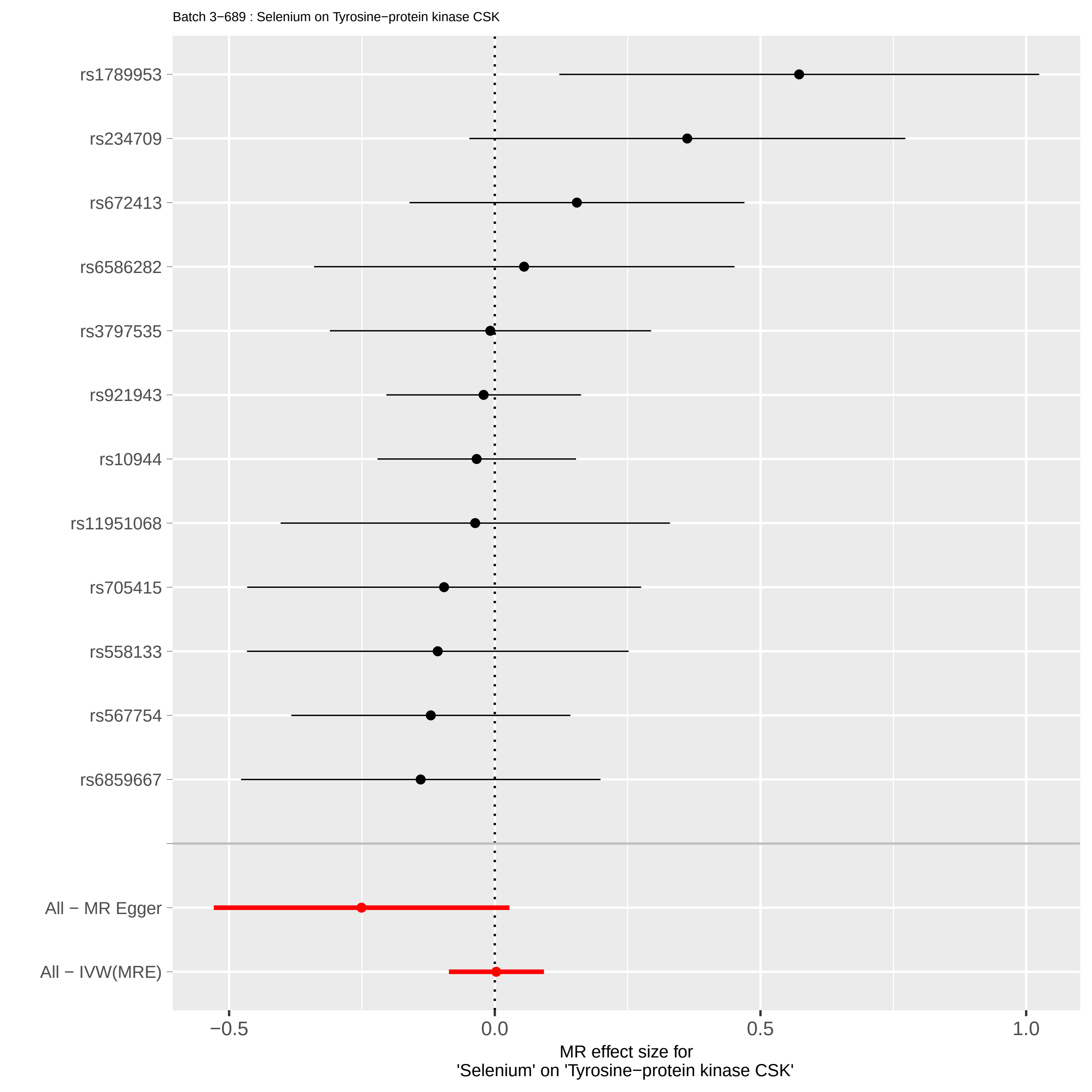

Batch 3−689 : Selenium on Tyrosine−protein kinase CSK
rs1789953
rs234709
rs672413
rs6586282
rs3797535
rs921943
rs10944
rs11951068
rs705415
rs558133
rs567754
rs6859667
All − MR Egger
All − IVW(MRE)
−0.5
0.0
0.5
1.0
MR effect size for
'Selenium' on 'Tyrosine−protein kinase CSK'

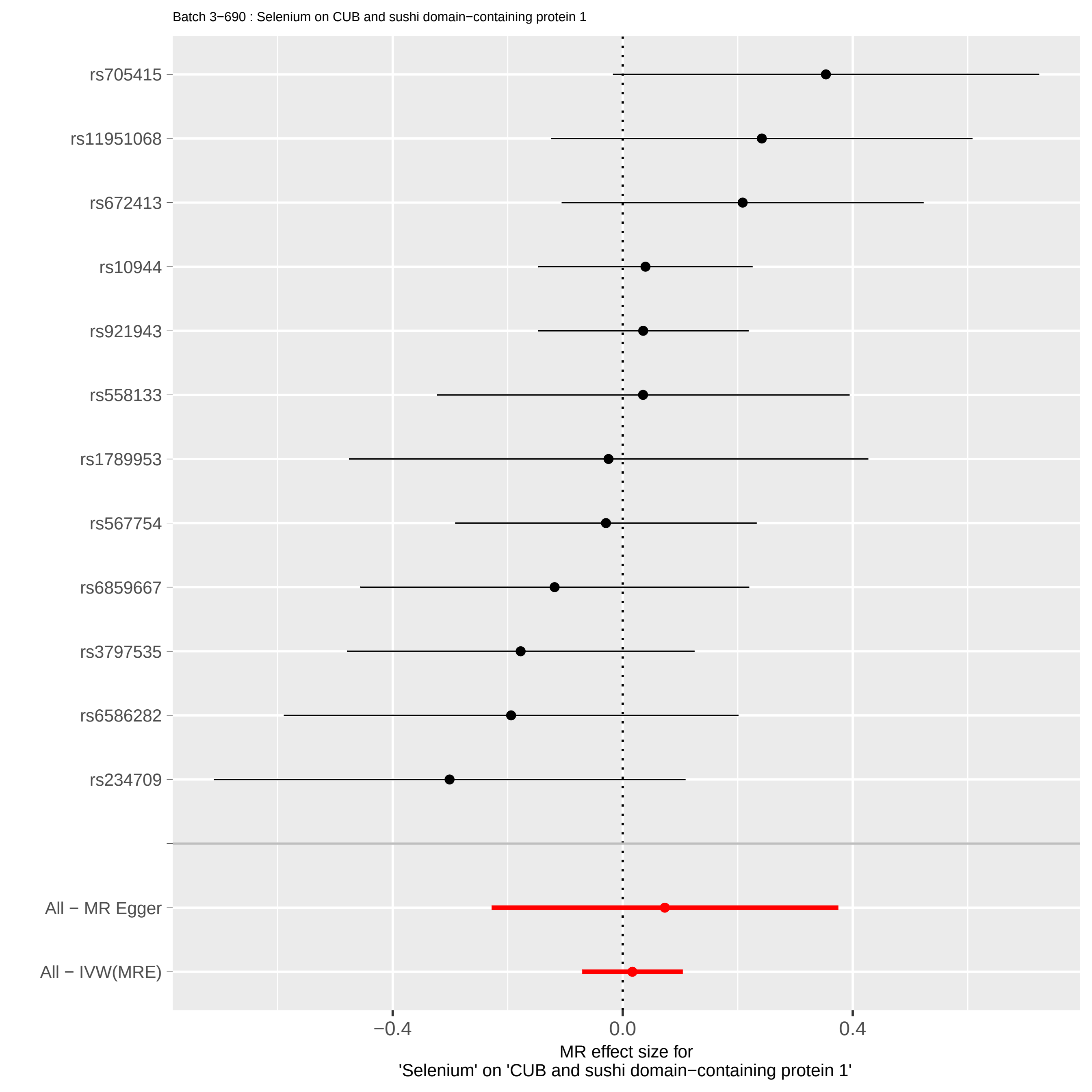
Batch 3−690 : Selenium on CUB and sushi domain−containing protein 1
rs705415
rs11951068
rs672413
rs10944
rs921943
rs558133
rs1789953
rs567754
rs6859667
rs3797535
rs6586282
rs234709
All − MR Egger
All − IVW(MRE)
−0.4
0.0
0.4
MR effect size for
'Selenium' on 'CUB and sushi domain−containing protein 1'

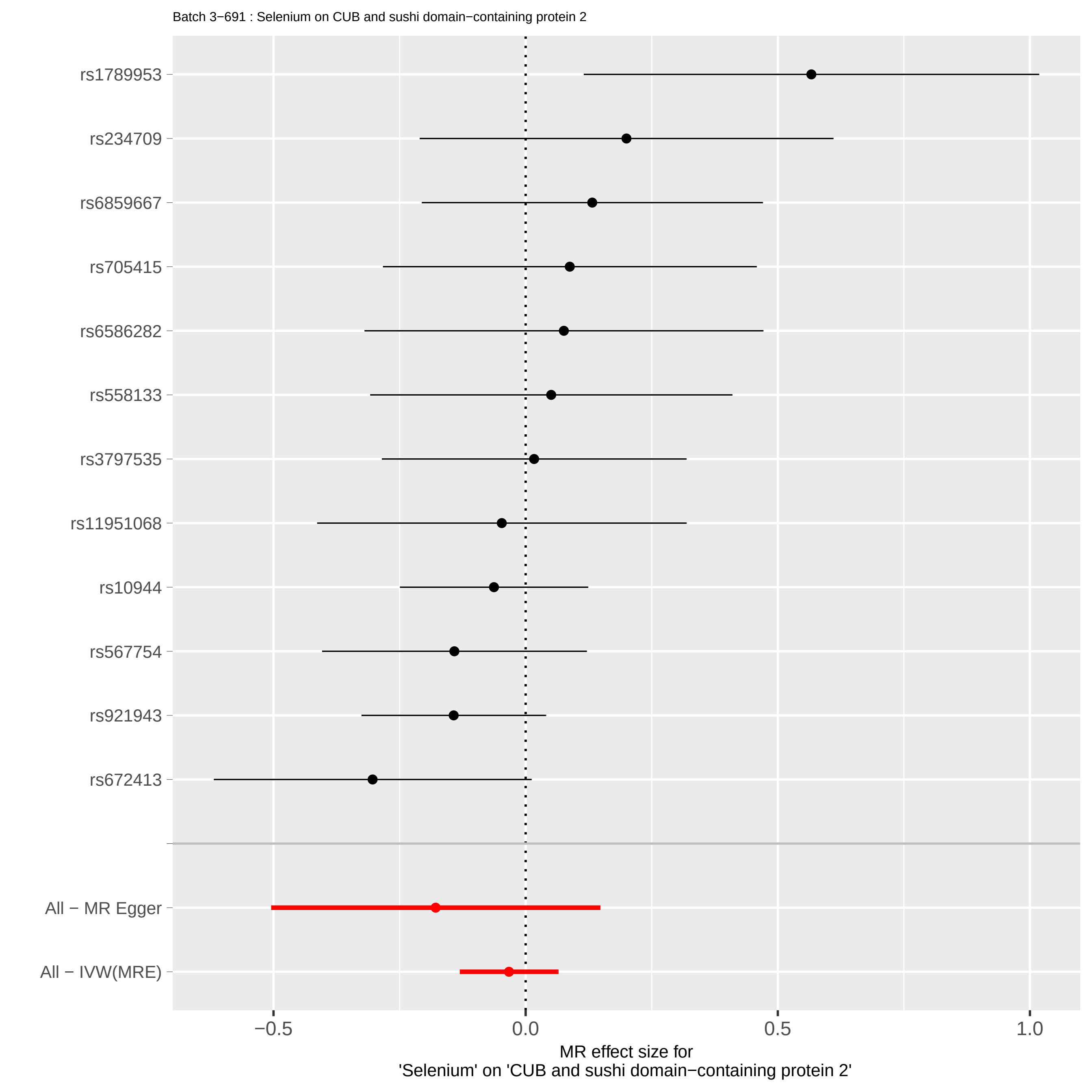
Batch 3−691 : Selenium on CUB and sushi domain−containing protein 2
rs1789953
rs234709
rs6859667
rs705415
rs6586282
rs558133
rs3797535
rs11951068
rs10944
rs567754
rs921943
rs672413
All − MR Egger
All − IVW(MRE)
−0.5
0.0
0.5
1.0
MR effect size for
'Selenium' on 'CUB and sushi domain−containing protein 2'

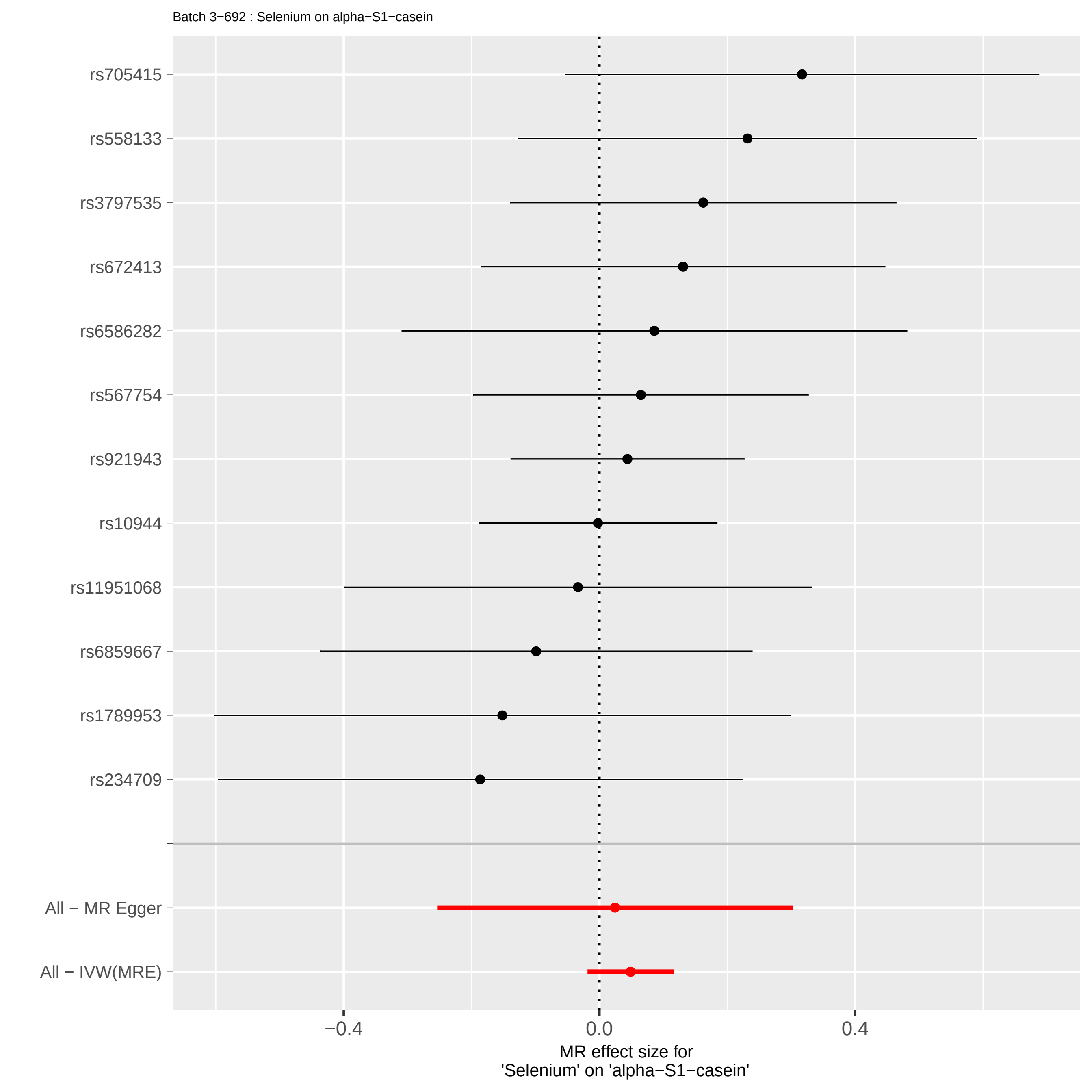

Batch 3−692 : Selenium on alpha−S1−casein
rs705415
rs558133
rs3797535
rs672413
rs6586282
rs567754
rs921943
rs10944
rs11951068
rs6859667
rs1789953
rs234709
All − MR Egger
All − IVW(MRE)
−0.4
0.0
0.4
MR effect size for
'Selenium' on 'alpha−S1−casein'

Batch 3−693 : Selenium on Casein kinase I isoform delta

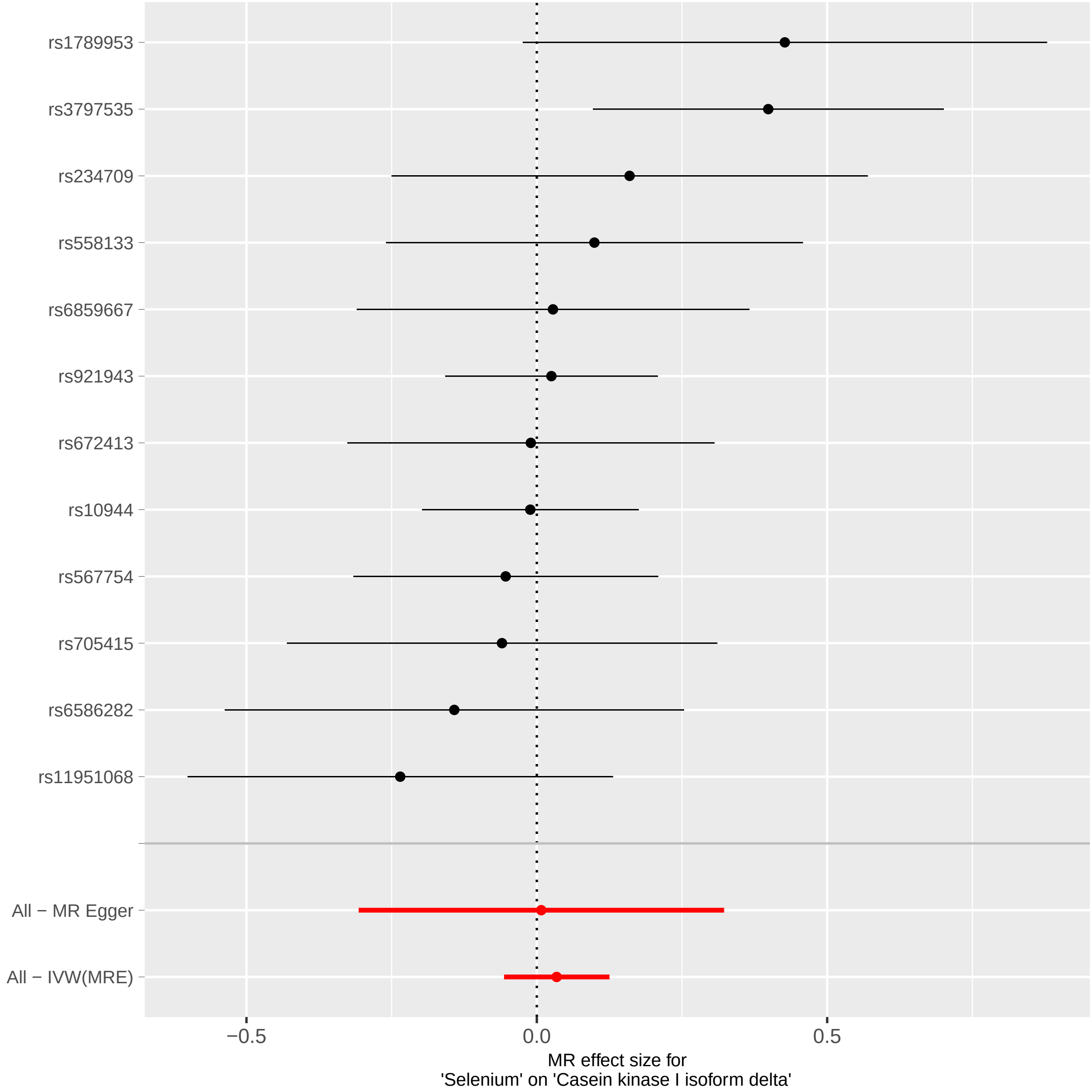

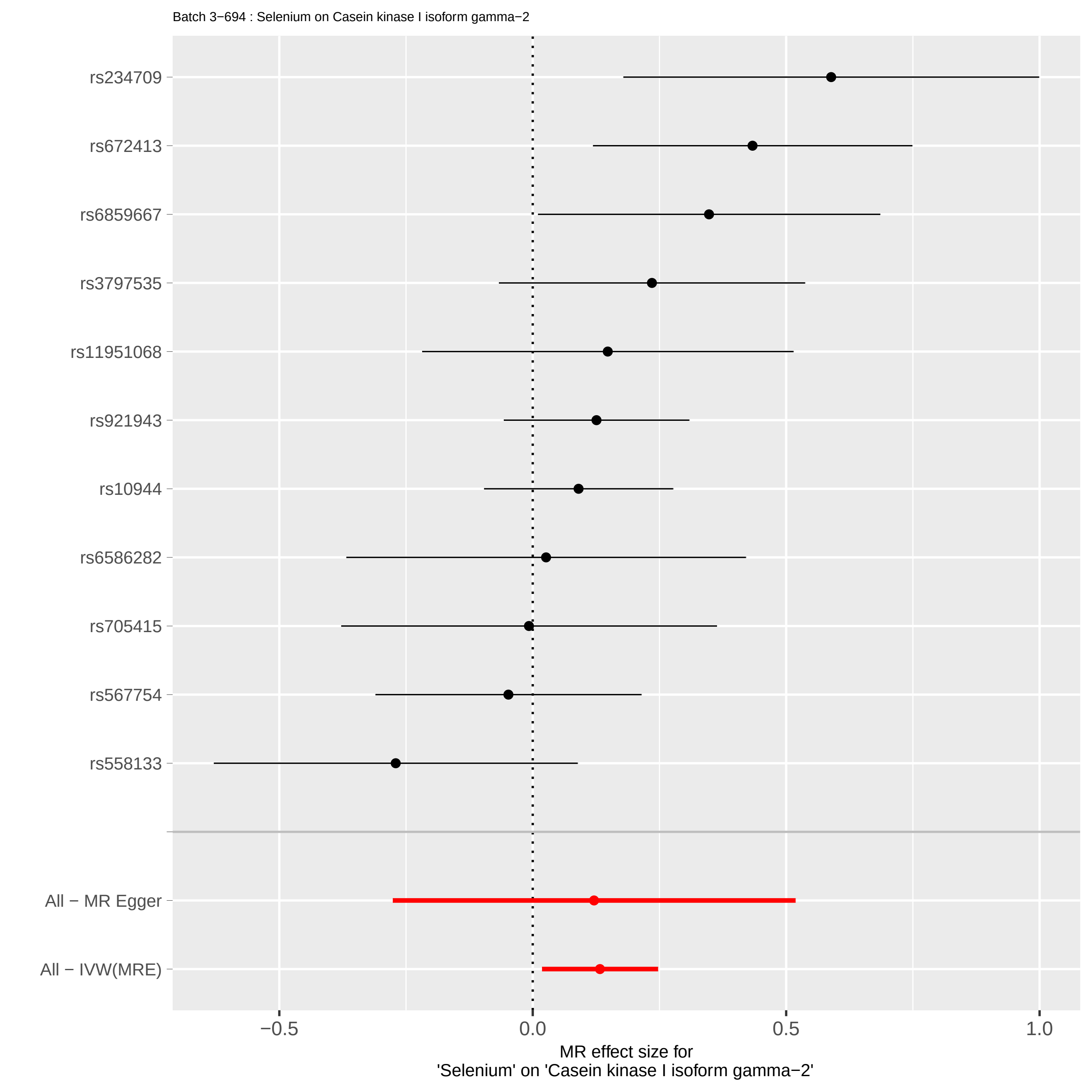

Batch 3−694 : Selenium on Casein kinase I isoform gamma−2
rs234709
rs672413
rs6859667
rs3797535
rs11951068
rs921943
rs10944
rs6586282
rs705415
rs567754
rs558133
All − MR Egger
All − IVW(MRE)
−0.5
0.0
0.5
1.0
MR effect size for
'Selenium' on 'Casein kinase I isoform gamma−2'

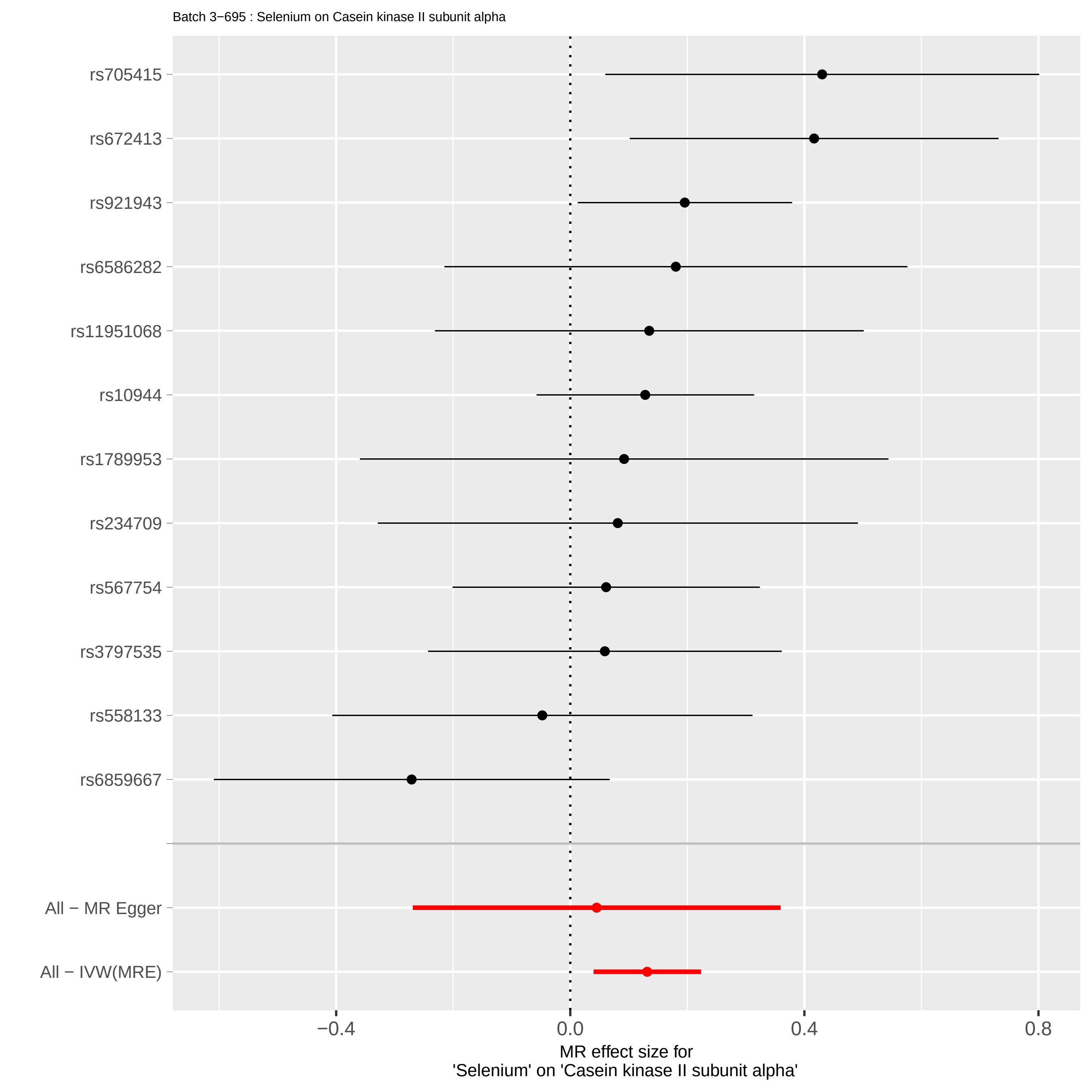

Batch 3−695 : Selenium on Casein kinase II subunit alpha
rs705415
rs672413
rs921943
rs6586282
rs11951068
rs10944
rs1789953
rs234709
rs567754
rs3797535
rs558133
rs6859667
All − MR Egger
All − IVW(MRE)
−0.4
0.0
0.4
0.8
MR effect size for
'Selenium' on 'Casein kinase II subunit alpha'

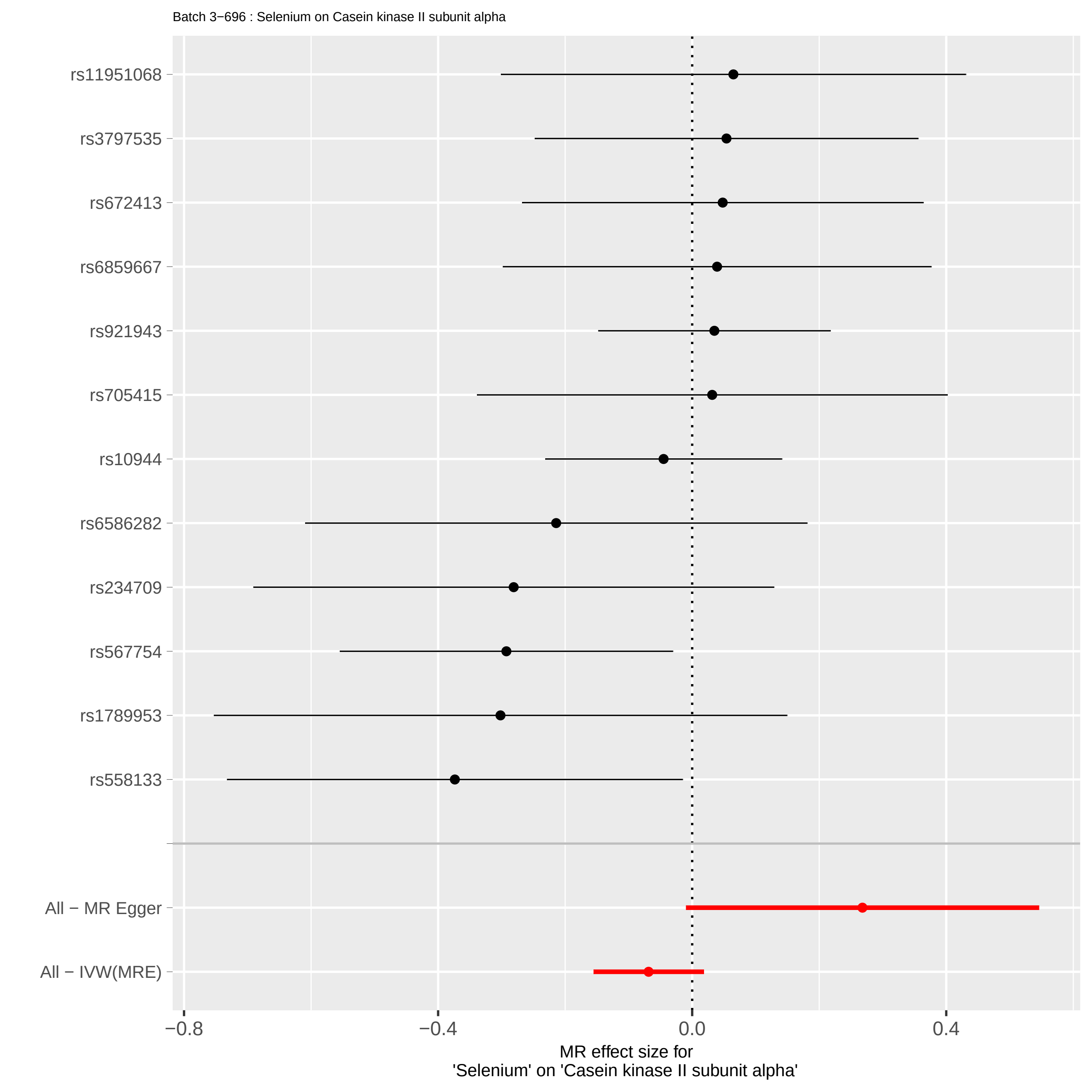

Batch 3−696 : Selenium on Casein kinase II subunit alpha
rs11951068
rs3797535
rs672413
rs6859667
rs921943
rs705415
rs10944
rs6586282
rs234709
rs567754
rs1789953
rs558133
All − MR Egger
All − IVW(MRE)
−0.8
−0.4
0.0
0.4
MR effect size for
'Selenium' on 'Casein kinase II subunit alpha'

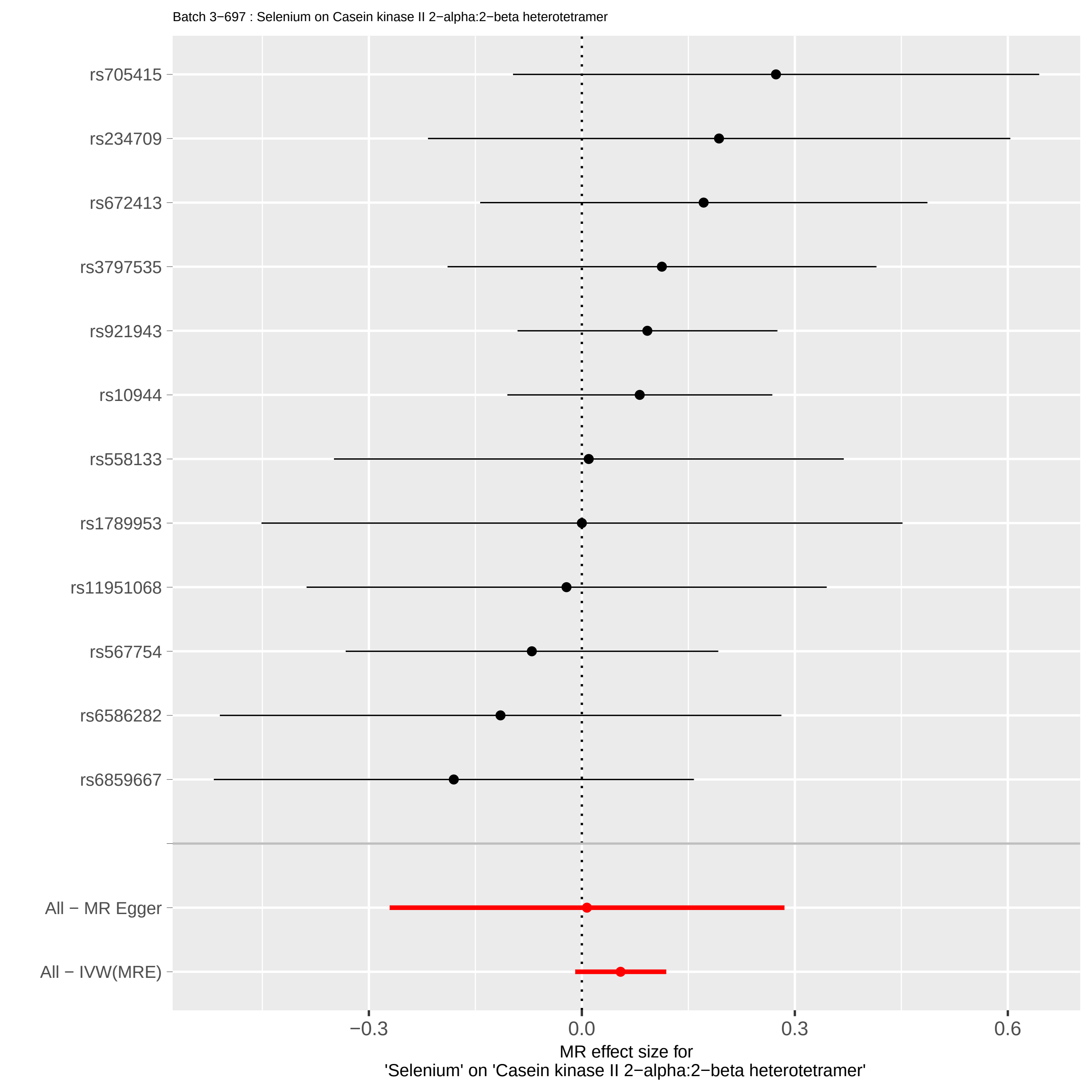

Batch 3−697 : Selenium on Casein kinase II 2−alpha:2−beta heterotetramer
rs705415
rs234709
rs672413
rs3797535
rs921943
rs10944
rs558133
rs1789953
rs11951068
rs567754
rs6586282
rs6859667
All − MR Egger
All − IVW(MRE)
−0.3
0.0
0.3
0.6
MR effect size for
'Selenium' on 'Casein kinase II 2−alpha:2−beta heterotetramer'

Batch 3−698 : Selenium on Casein kinase II 2−alpha':2−beta heterotetramer

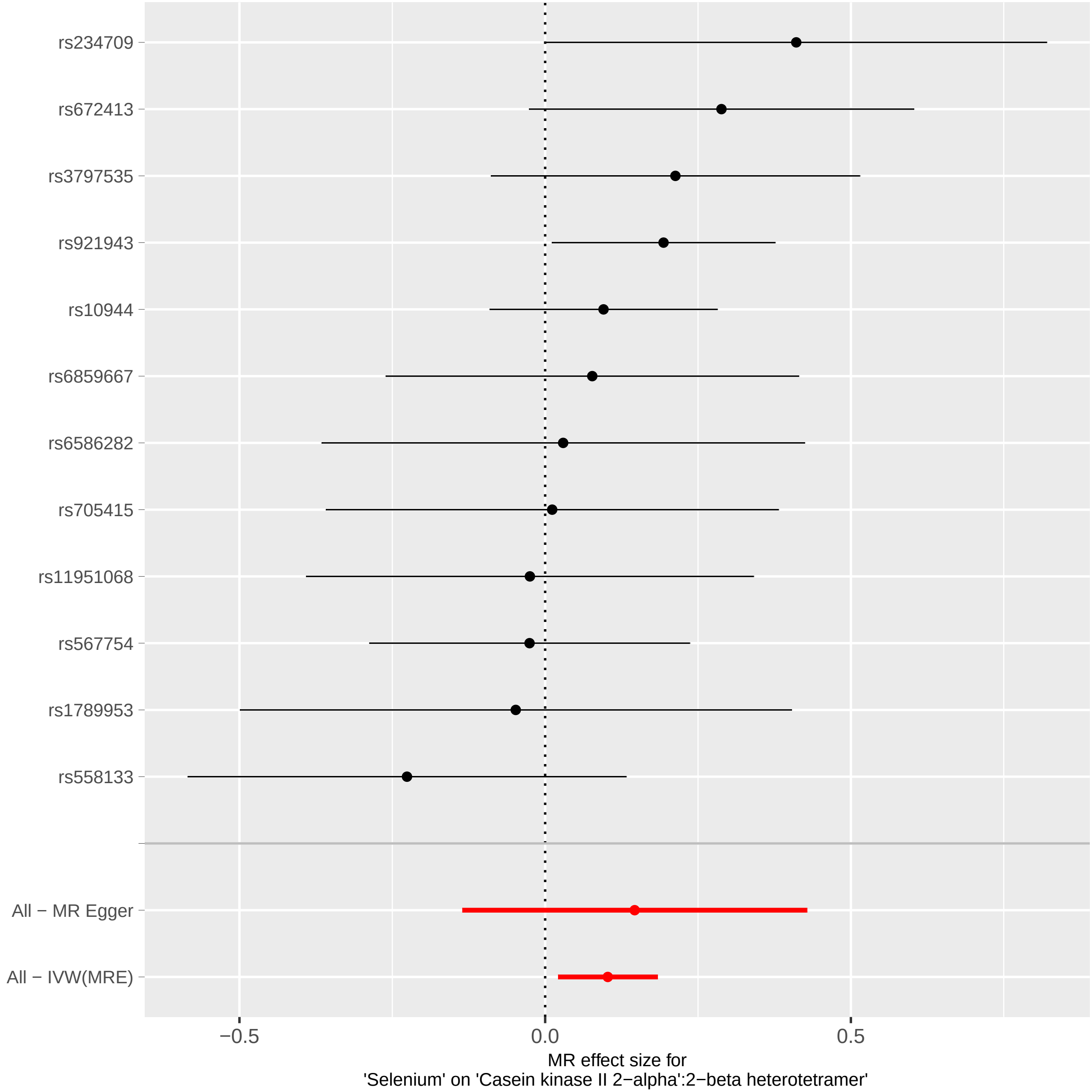

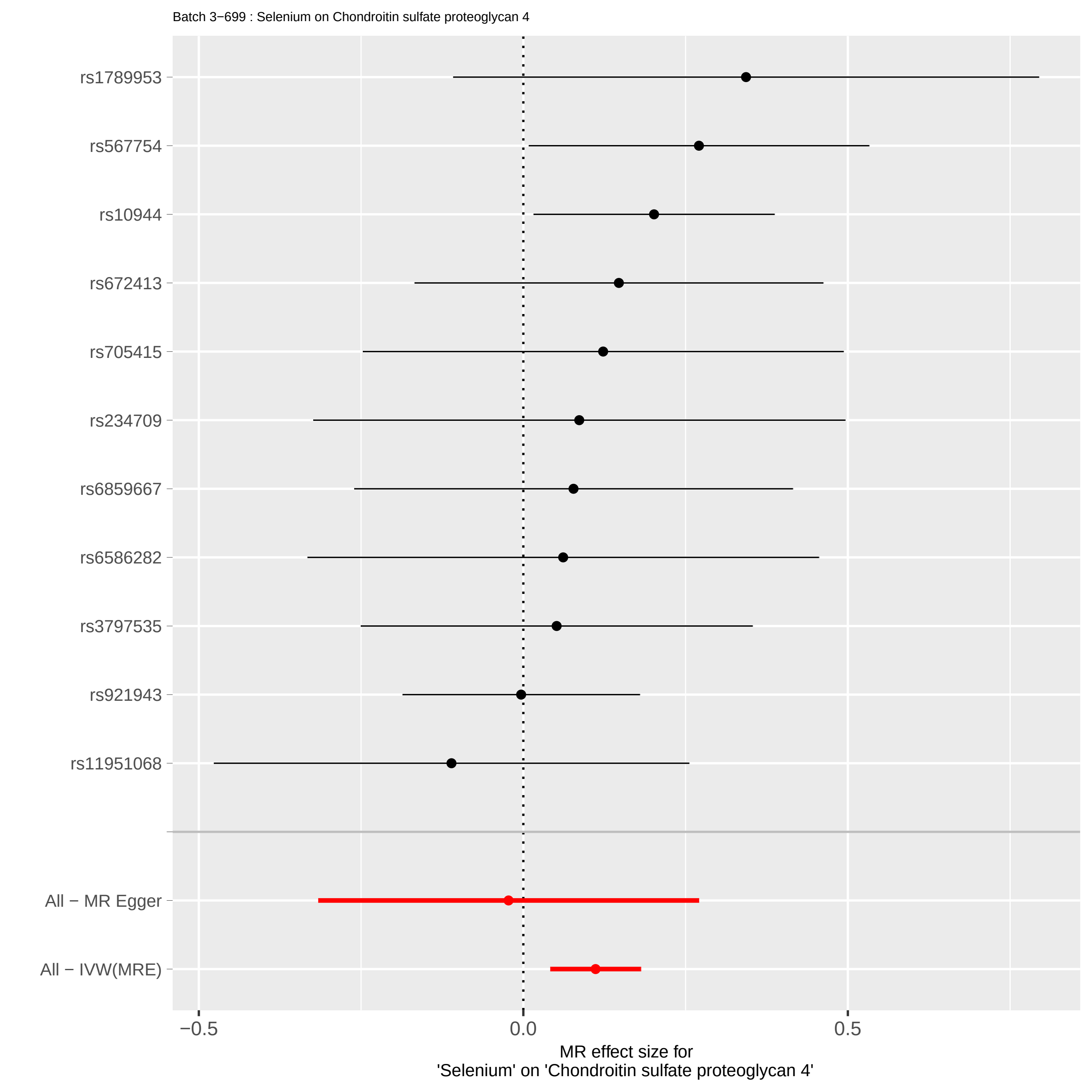
Batch 3−699 : Selenium on Chondroitin sulfate proteoglycan 4
rs1789953
rs567754
rs10944
rs672413
rs705415
rs234709
rs6859667
rs6586282
rs3797535
rs921943
rs11951068
All − MR Egger
All − IVW(MRE)
−0.5
0.0
0.5
MR effect size for
'Selenium' on 'Chondroitin sulfate proteoglycan 4'

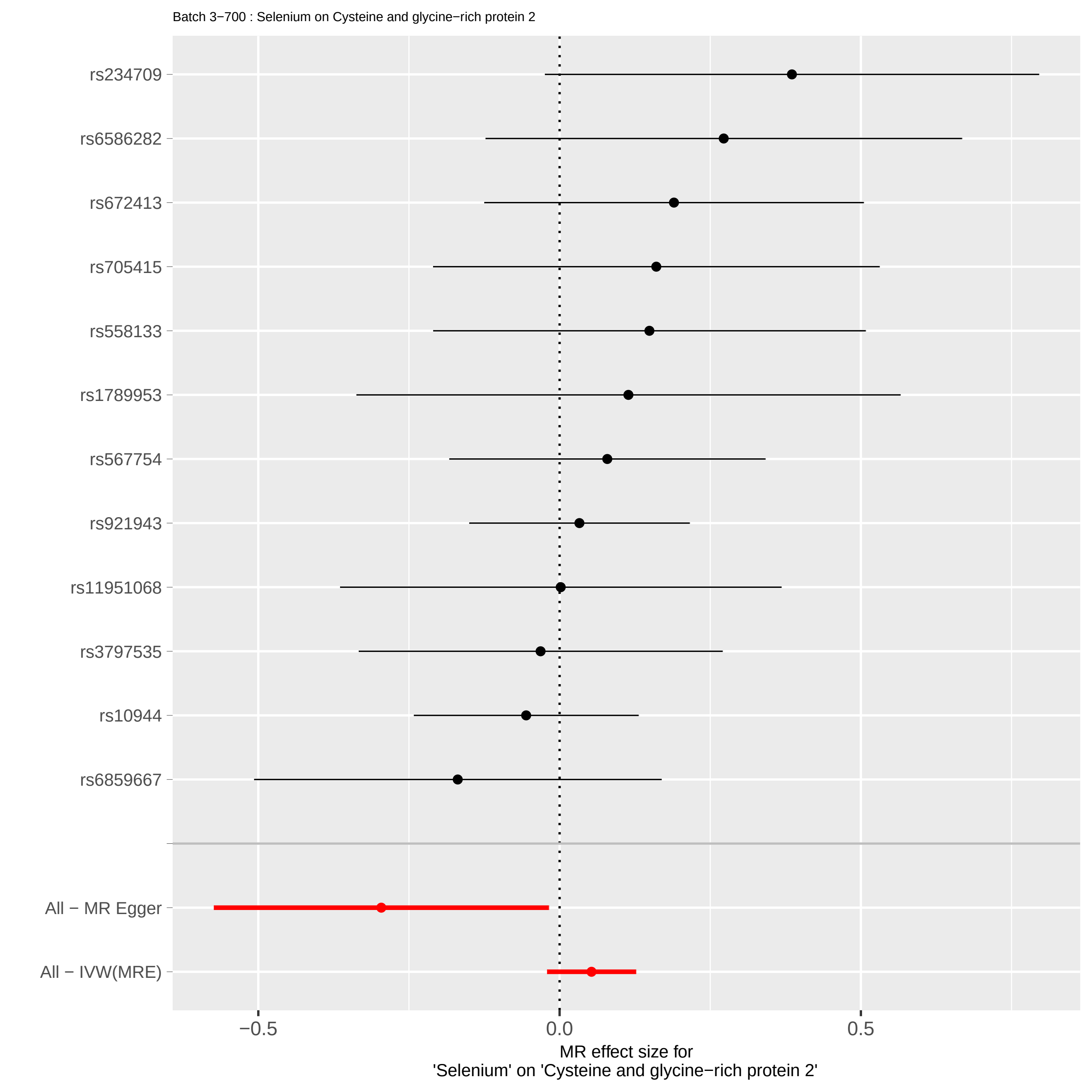
Batch 3−700 : Selenium on Cysteine and glycine−rich protein 2
rs234709
rs6586282
rs672413
rs705415
rs558133
rs1789953
rs567754
rs921943
rs11951068
rs3797535
rs10944
rs6859667
All − MR Egger
All − IVW(MRE)
−0.5
0.0
0.5
MR effect size for
'Selenium' on 'Cysteine and glycine−rich protein 2'

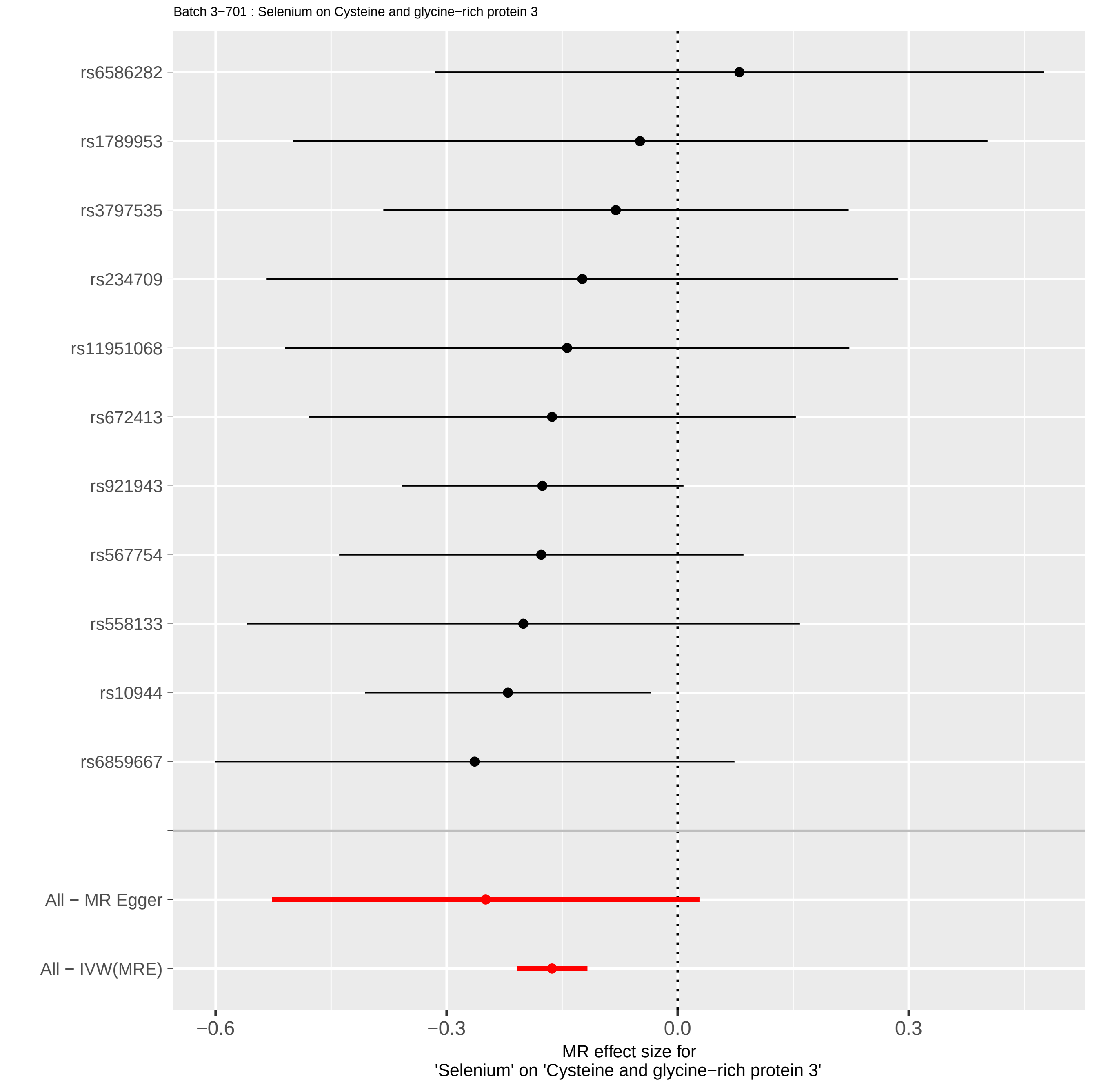

Batch 3−701 : Selenium on Cysteine and glycine−rich protein 3
rs6586282
rs1789953
rs3797535
rs234709
rs11951068
rs672413
rs921943
rs567754
rs558133
rs10944
rs6859667
All − MR Egger
All − IVW(MRE)
−0.6
−0.3
0.0
0.3
MR effect size for
'Selenium' on 'Cysteine and glycine−rich protein 3'

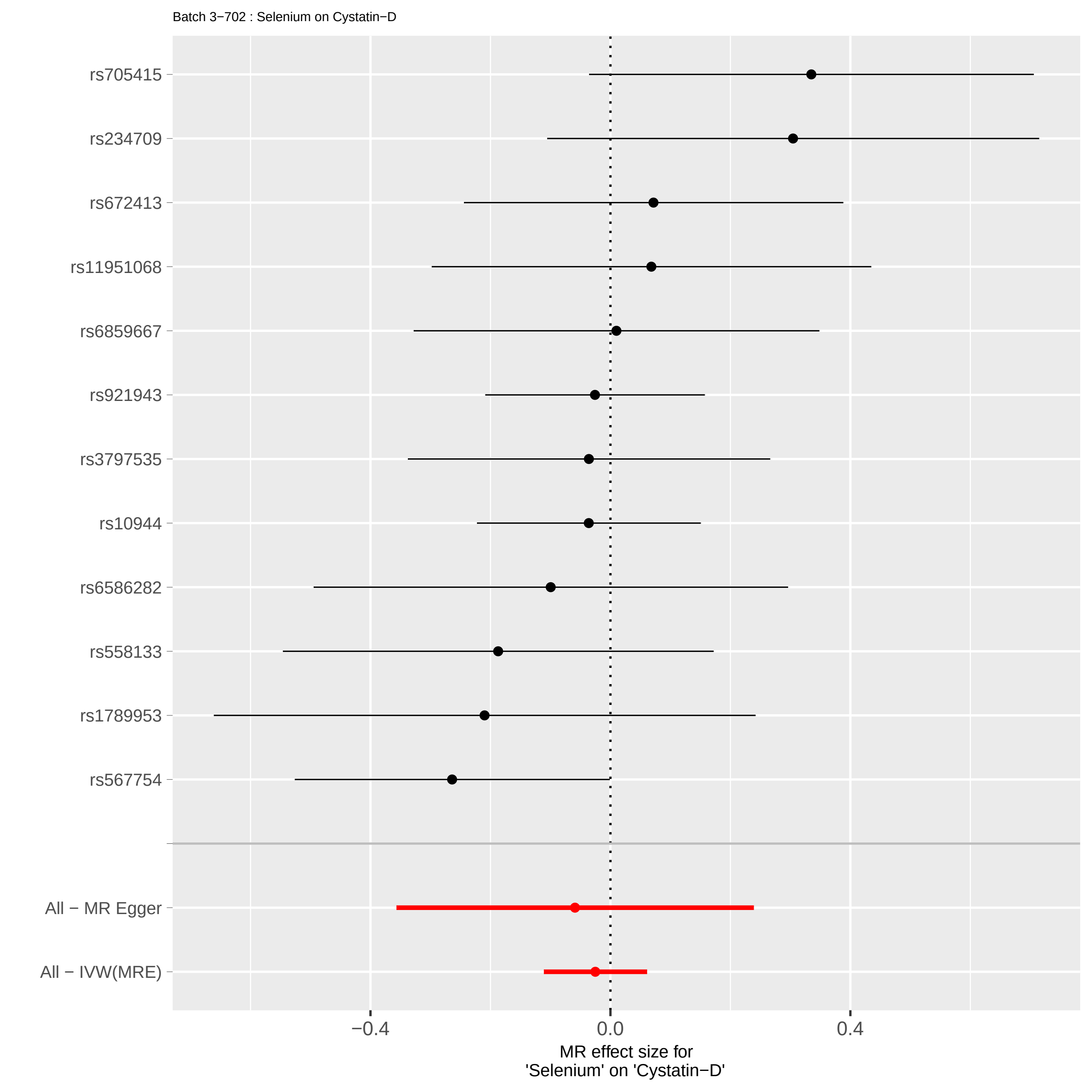

Batch 3−702 : Selenium on Cystatin−D
rs705415
rs234709
rs672413
rs11951068
rs6859667
rs921943
rs3797535
rs10944
rs6586282
rs558133
rs1789953
rs567754
All − MR Egger
All − IVW(MRE)
−0.4
0.0
0.4
MR effect size for
'Selenium' on 'Cystatin−D'

Batch 3−703 : Selenium on Cystatin−M

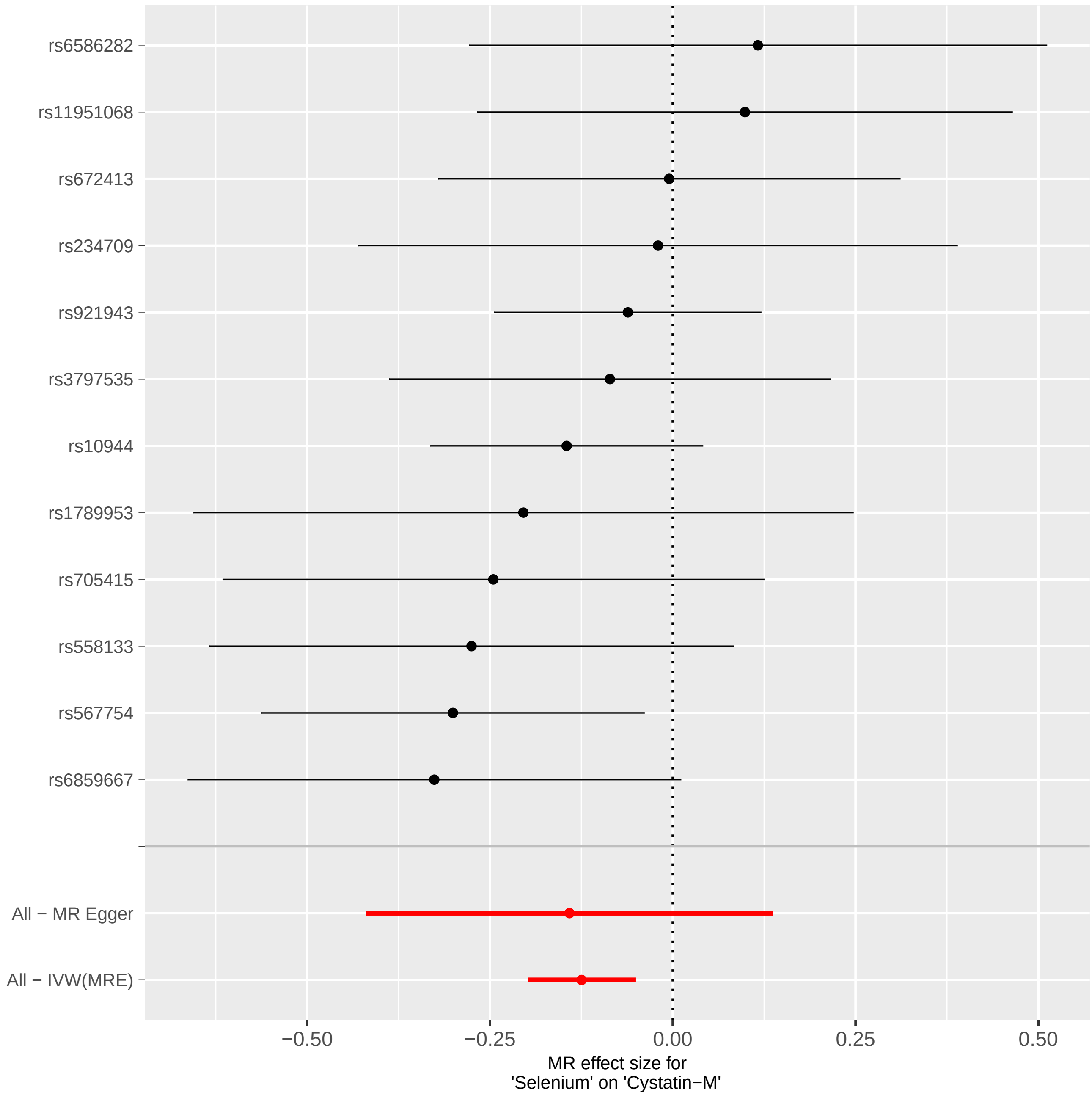

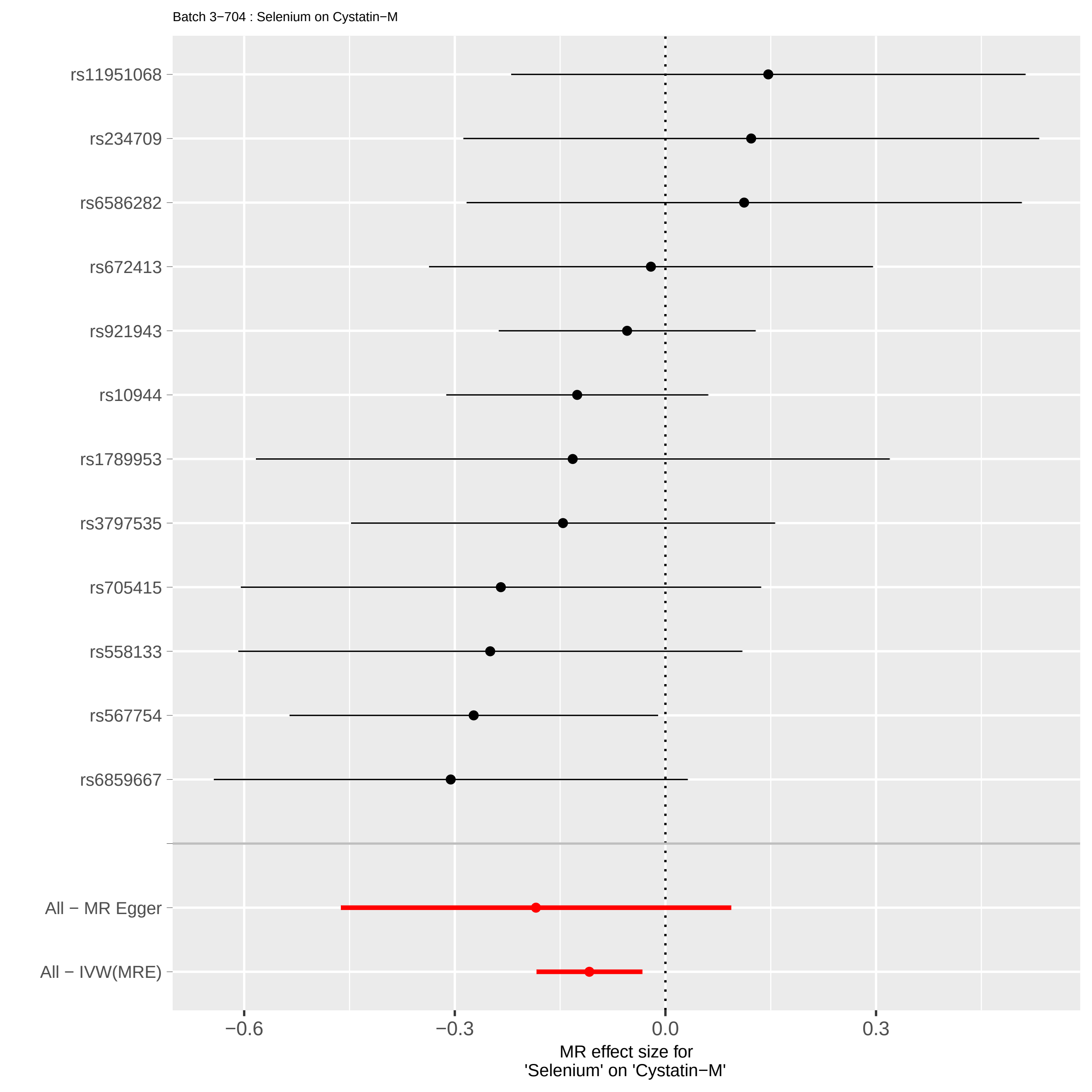

Batch 3−704 : Selenium on Cystatin−M
rs11951068
rs234709
rs6586282
rs672413
rs921943
rs10944
rs1789953
rs3797535
rs705415
rs558133
rs567754
rs6859667
All − MR Egger
All − IVW(MRE)
−0.6
−0.3
0.0
0.3
MR effect size for
'Selenium' on 'Cystatin−M'

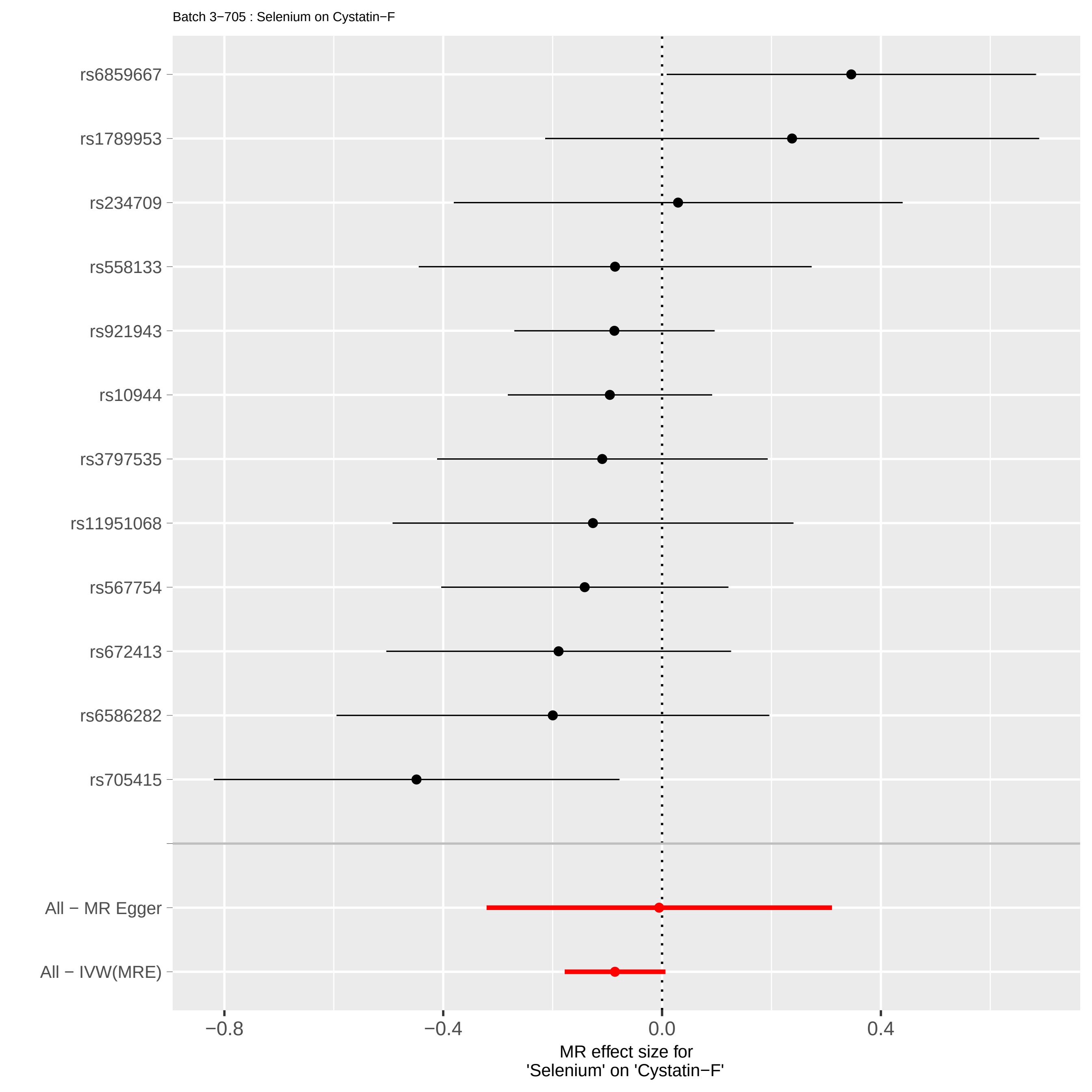
Batch 3−705 : Selenium on Cystatin−F
rs6859667
rs1789953
rs234709
rs558133
rs921943
rs10944
rs3797535
rs11951068
rs567754
rs672413
rs6586282
rs705415
All − MR Egger
All − IVW(MRE)
−0.8
−0.4
0.0
0.4
MR effect size for
'Selenium' on 'Cystatin−F'

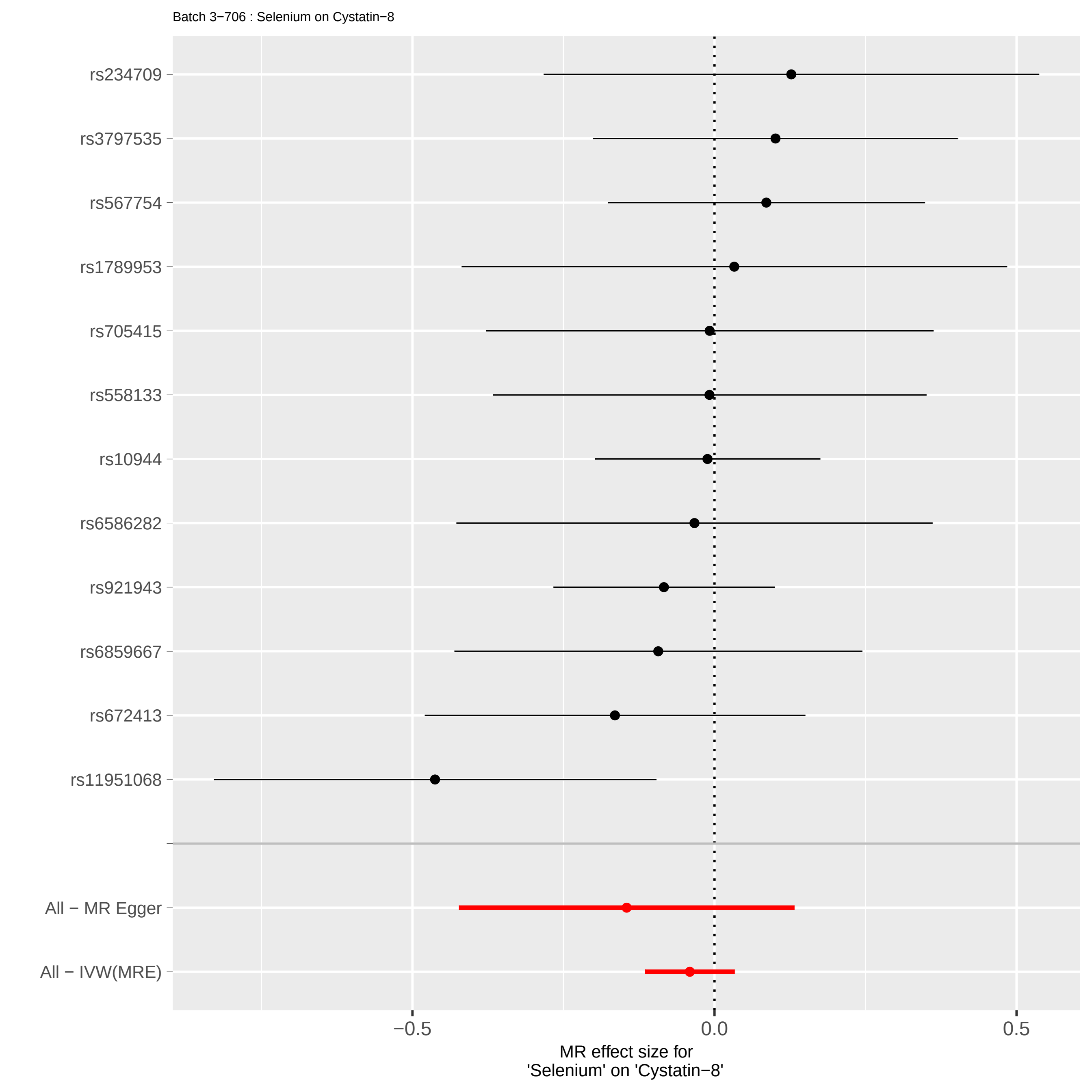
Batch 3−706 : Selenium on Cystatin−8
rs234709
rs3797535
rs567754
rs1789953
rs705415
rs558133
rs10944
rs6586282
rs921943
rs6859667
rs672413
rs11951068
All − MR Egger
All − IVW(MRE)
−0.5
0.0
0.5
MR effect size for
'Selenium' on 'Cystatin−8'

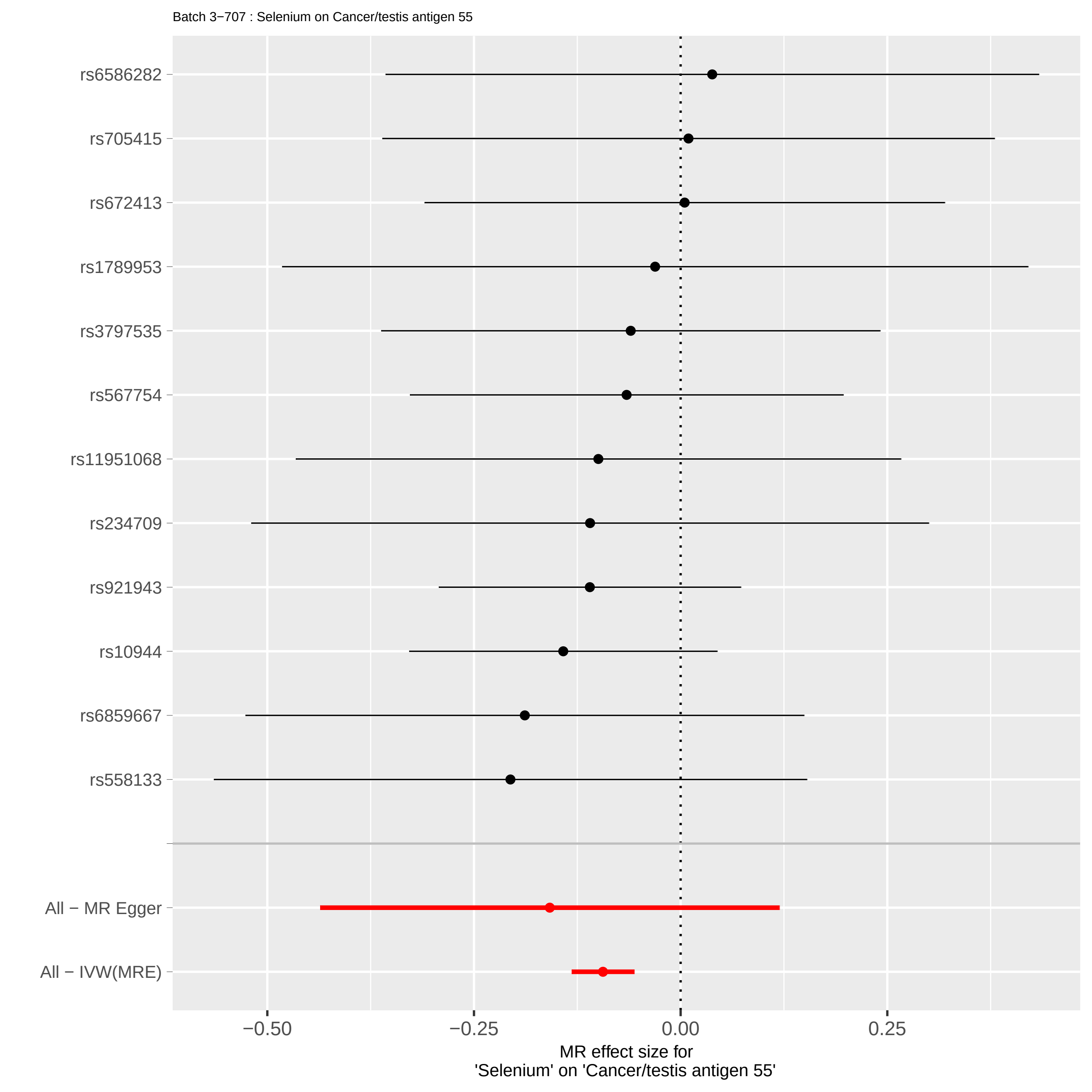

Batch 3−707 : Selenium on Cancer/testis antigen 55
rs6586282
rs705415
rs672413
rs1789953
rs3797535
rs567754
rs11951068
rs234709
rs921943
rs10944
rs6859667
rs558133
All − MR Egger
All − IVW(MRE)
−0.50
−0.25
0.00
0.25
MR effect size for
'Selenium' on 'Cancer/testis antigen 55'

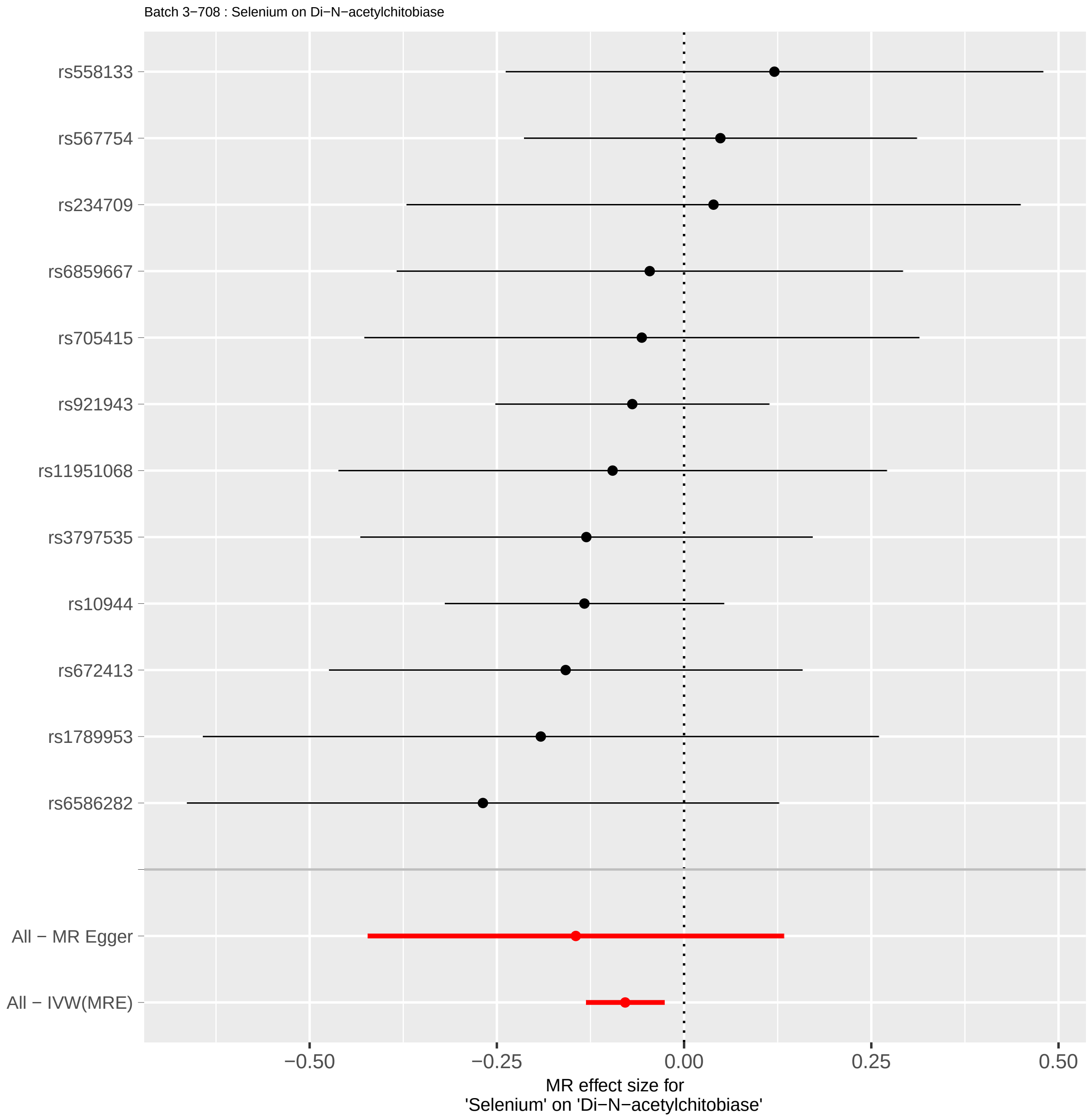

Batch 3−708 : Selenium on Di−N−acetylchitobiase
rs558133
rs567754
rs234709
rs6859667
rs705415
rs921943
rs11951068
rs3797535
rs10944
rs672413
rs1789953
rs6586282
All − MR Egger
All − IVW(MRE)
−0.50
−0.25
0.00
0.25
0.50
MR effect size for
'Selenium' on 'Di−N−acetylchitobiase'

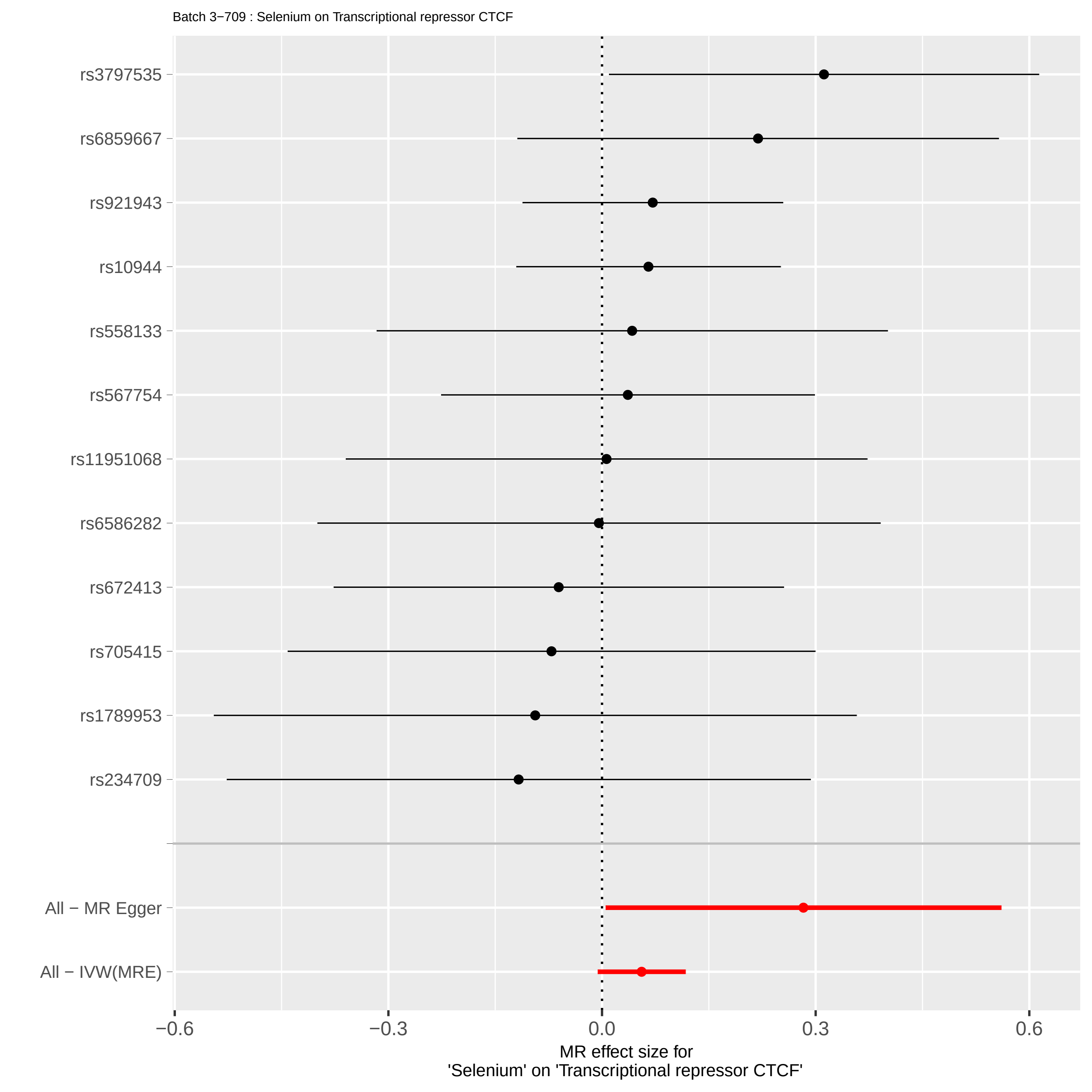

Batch 3−709 : Selenium on Transcriptional repressor CTCF
rs3797535
rs6859667
rs921943
rs10944
rs558133
rs567754
rs11951068
rs6586282
rs672413
rs705415
rs1789953
rs234709
All − MR Egger
All − IVW(MRE)
−0.6
−0.3
0.0
0.3
0.6
MR effect size for
'Selenium' on 'Transcriptional repressor CTCF'

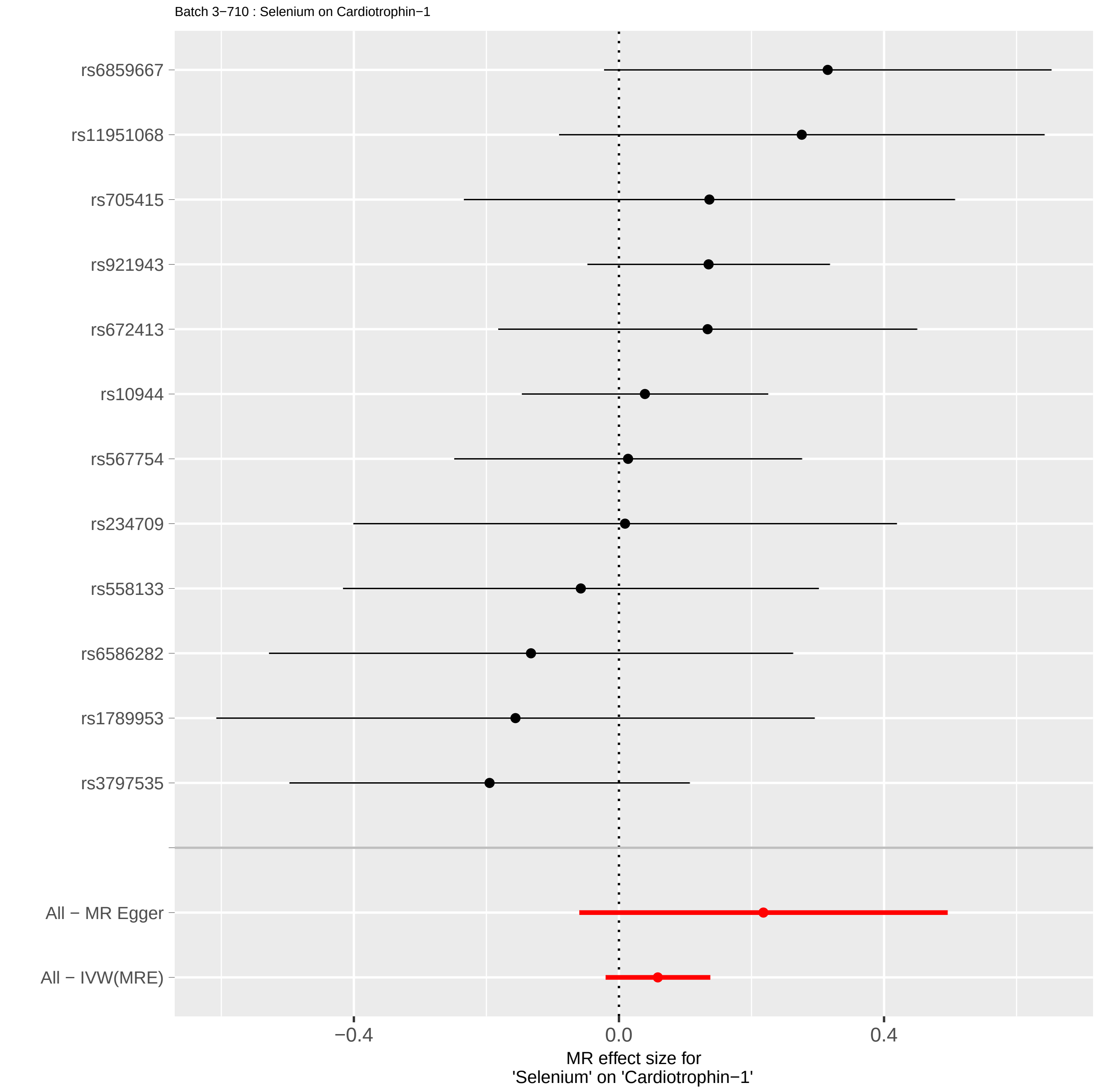
Batch 3−710 : Selenium on Cardiotrophin−1
rs6859667
rs11951068
rs705415
rs921943
rs672413
rs10944
rs567754
rs234709
rs558133
rs6586282
rs1789953
rs3797535
All − MR Egger
All − IVW(MRE)
−0.4
0.0
0.4
MR effect size for
'Selenium' on 'Cardiotrophin−1'

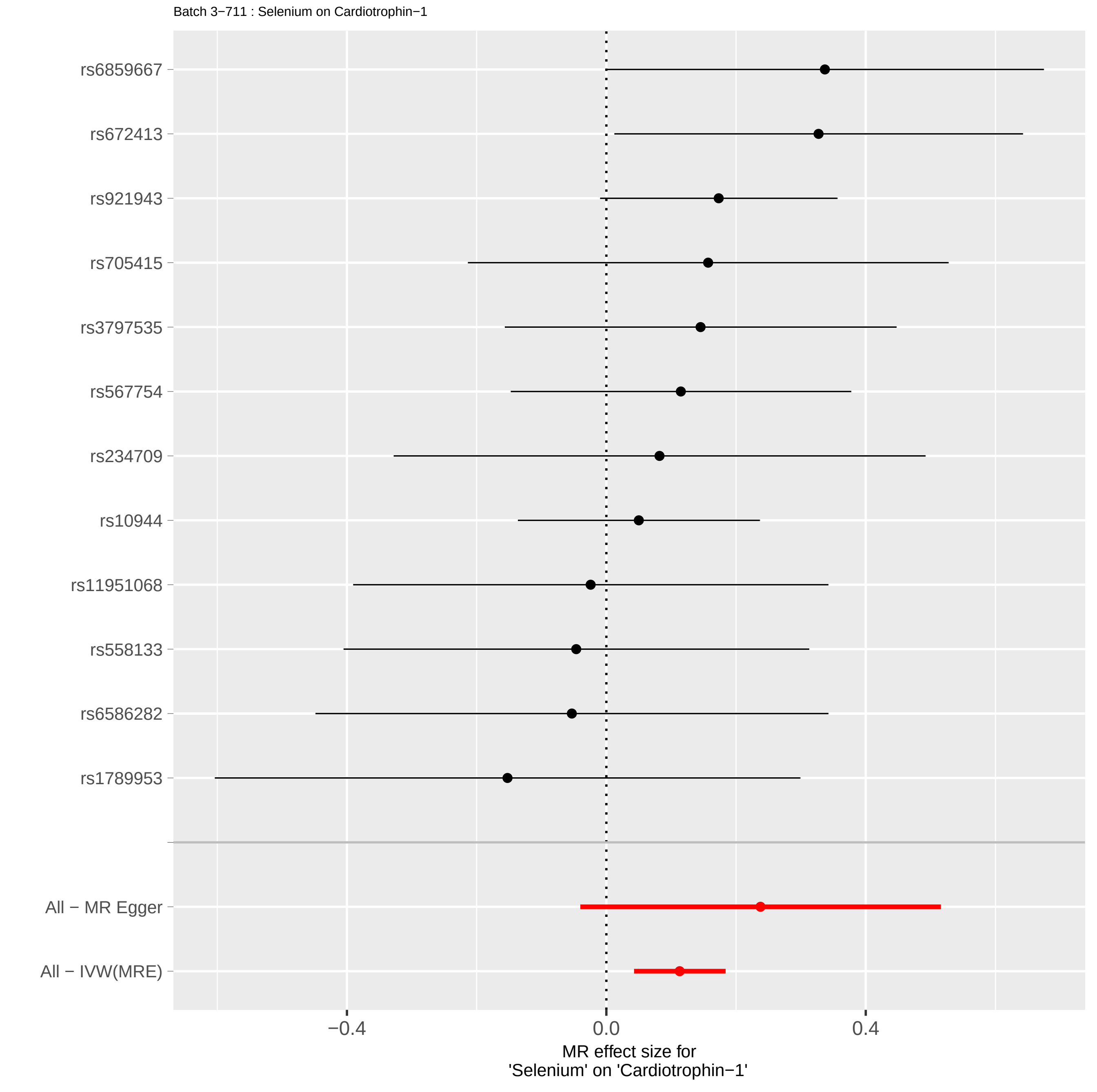
Batch 3−711 : Selenium on Cardiotrophin−1
rs6859667
rs672413
rs921943
rs705415
rs3797535
rs567754
rs234709
rs10944
rs11951068
rs558133
rs6586282
rs1789953
All − MR Egger
All − IVW(MRE)
−0.4
0.0
0.4
MR effect size for
'Selenium' on 'Cardiotrophin−1'

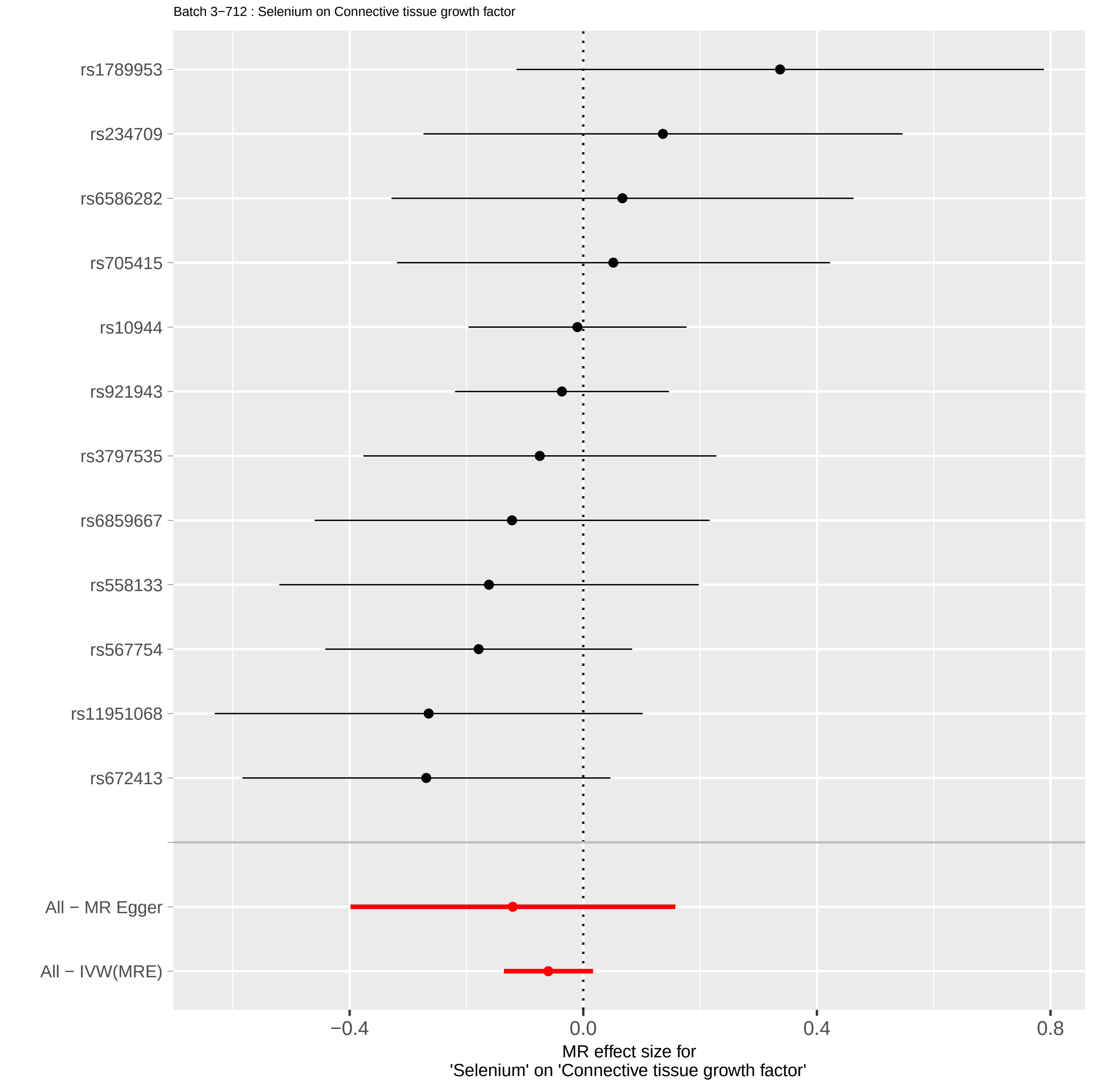

Batch 3−712 : Selenium on Connective tissue growth factor
rs1789953
rs234709
rs6586282
rs705415
rs10944
rs921943
rs3797535
rs6859667
rs558133
rs567754
rs11951068
rs672413
All − MR Egger
All − IVW(MRE)
−0.4
0.0
0.4
0.8
MR effect size for
'Selenium' on 'Connective tissue growth factor'

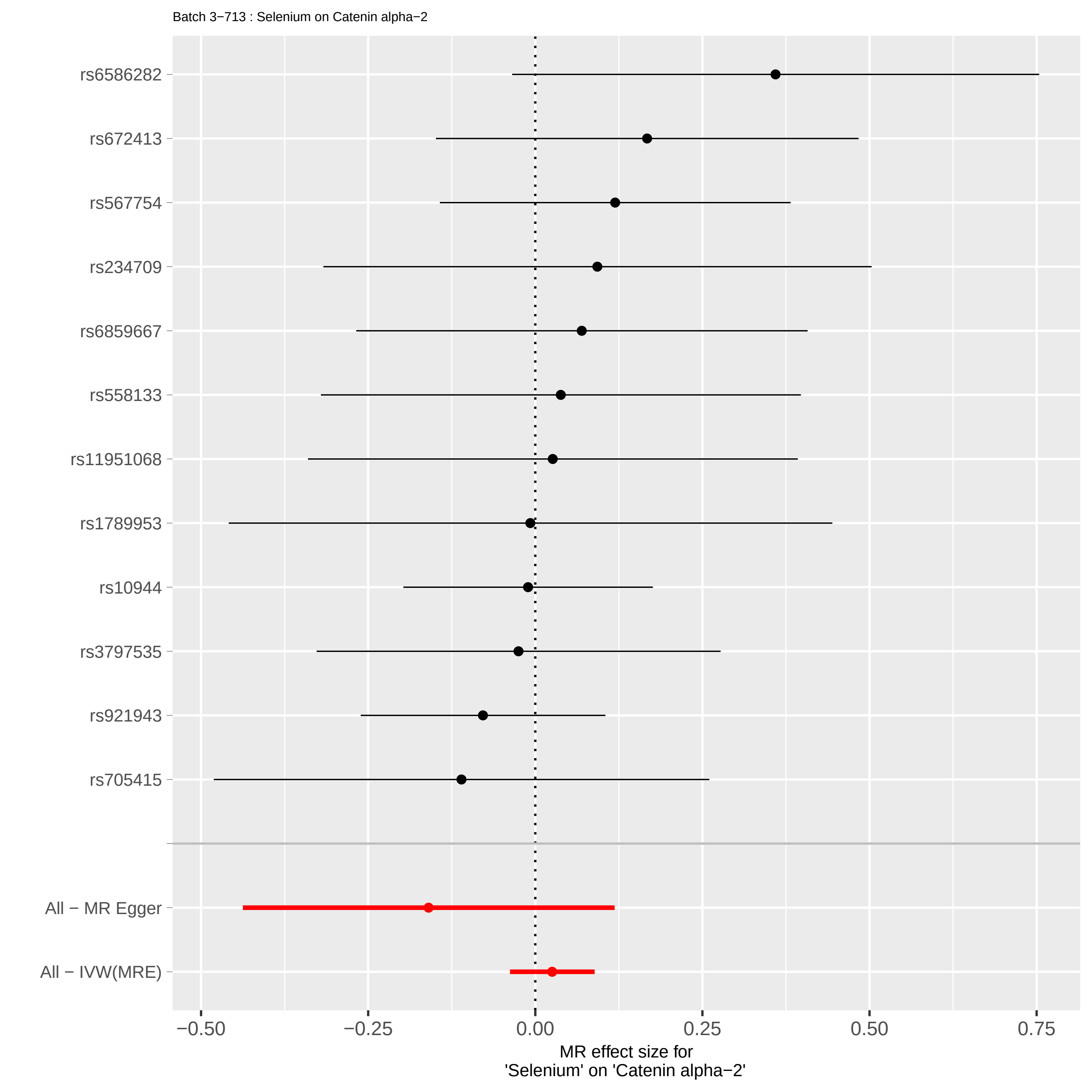
Batch 3−713 : Selenium on Catenin alpha−2
rs6586282
rs672413
rs567754
rs234709
rs6859667
rs558133
rs11951068
rs1789953
rs10944
rs3797535
rs921943
rs705415
All − MR Egger
All − IVW(MRE)
−0.50
−0.25
0.00
0.25
0.50
0.75
MR effect size for
'Selenium' on 'Catenin alpha−2'

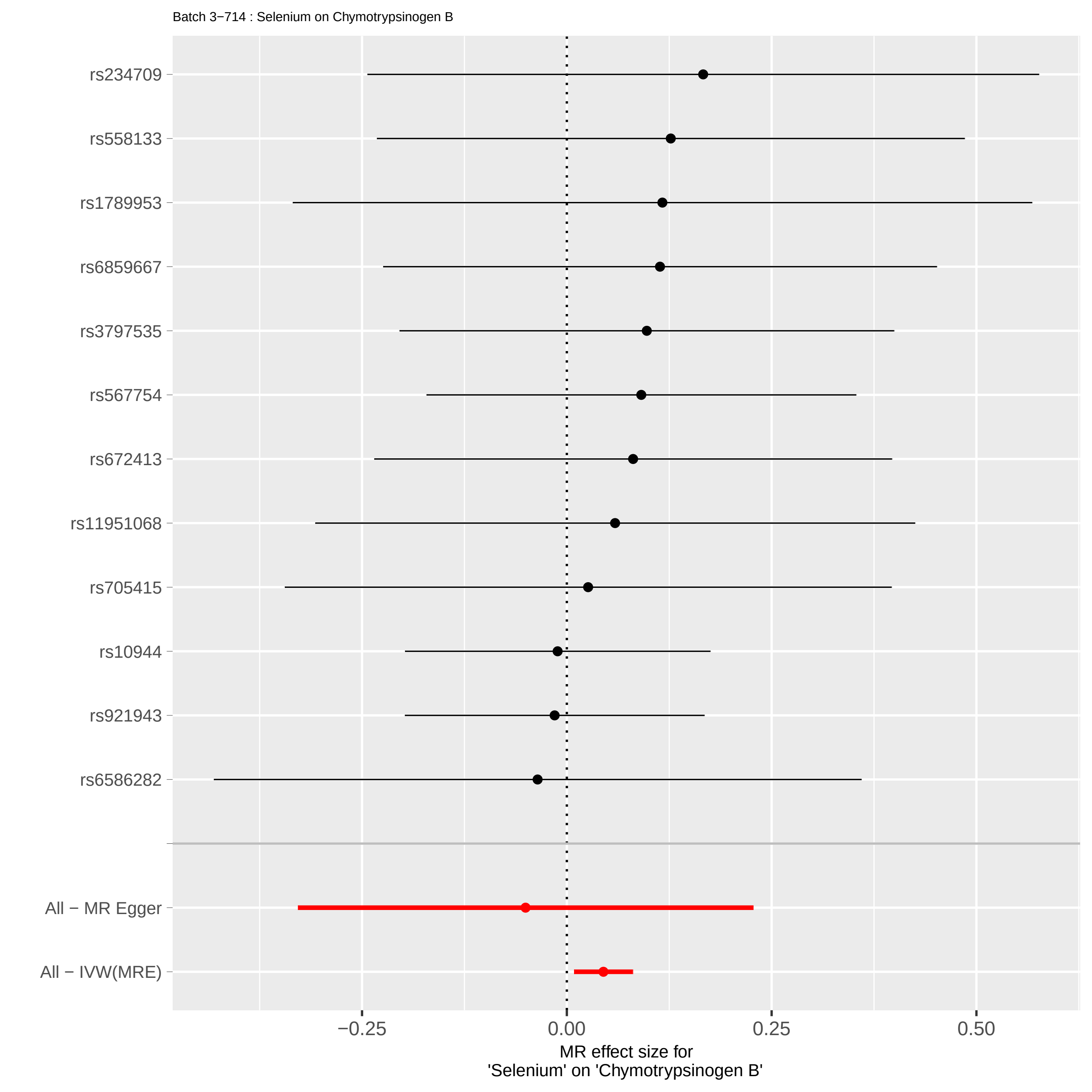

Batch 3−714 : Selenium on Chymotrypsinogen B
rs234709
rs558133
rs1789953
rs6859667
rs3797535
rs567754
rs672413
rs11951068
rs705415
rs10944
rs921943
rs6586282
All − MR Egger
All − IVW(MRE)
−0.25
0.00
0.25
0.50
MR effect size for
'Selenium' on 'Chymotrypsinogen B'

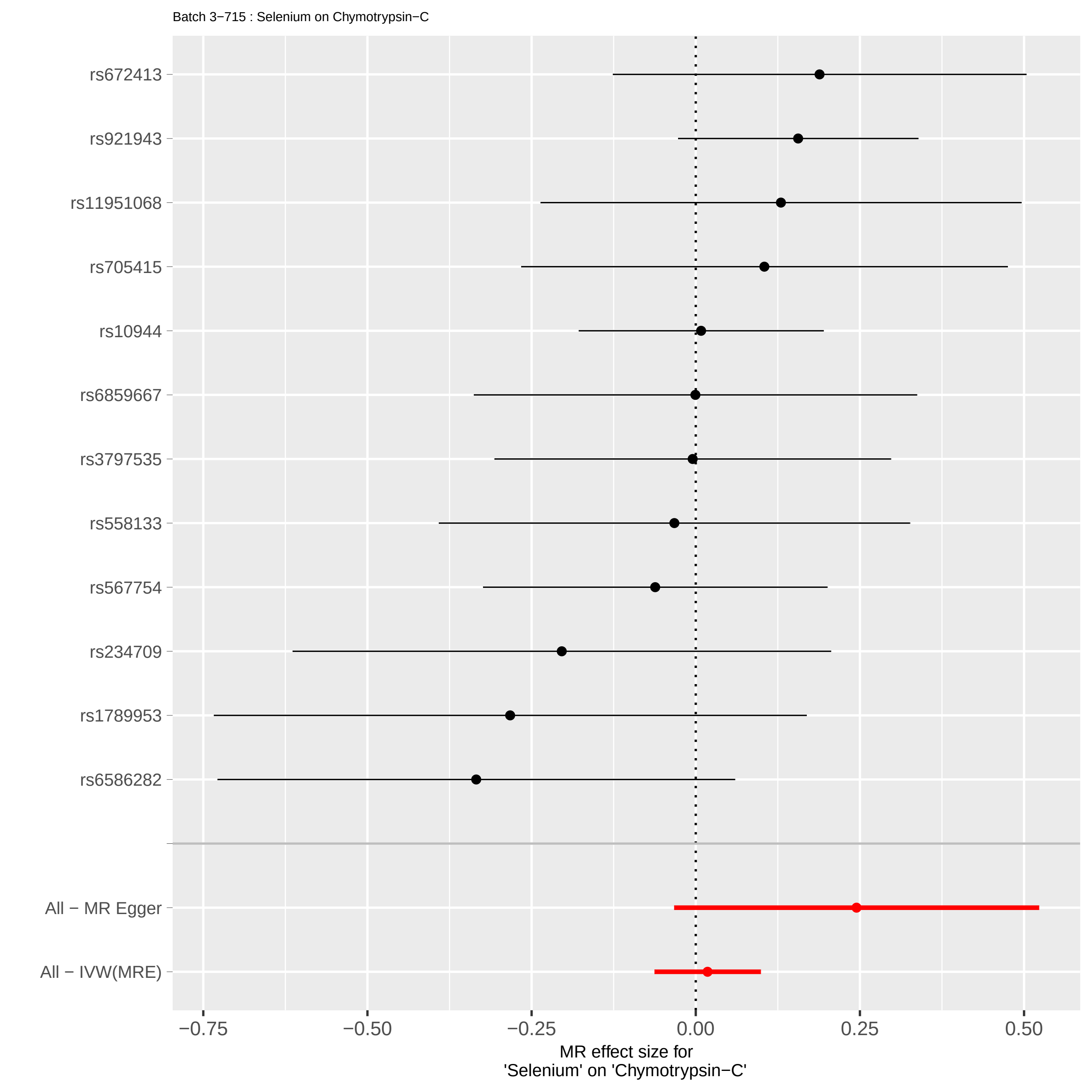

Batch 3−715 : Selenium on Chymotrypsin−C
rs672413
rs921943
rs11951068
rs705415
rs10944
rs6859667
rs3797535
rs558133
rs567754
rs234709
rs1789953
rs6586282
All − MR Egger
All − IVW(MRE)
−0.75
−0.50
−0.25
0.00
0.25
0.50
MR effect size for
'Selenium' on 'Chymotrypsin−C'

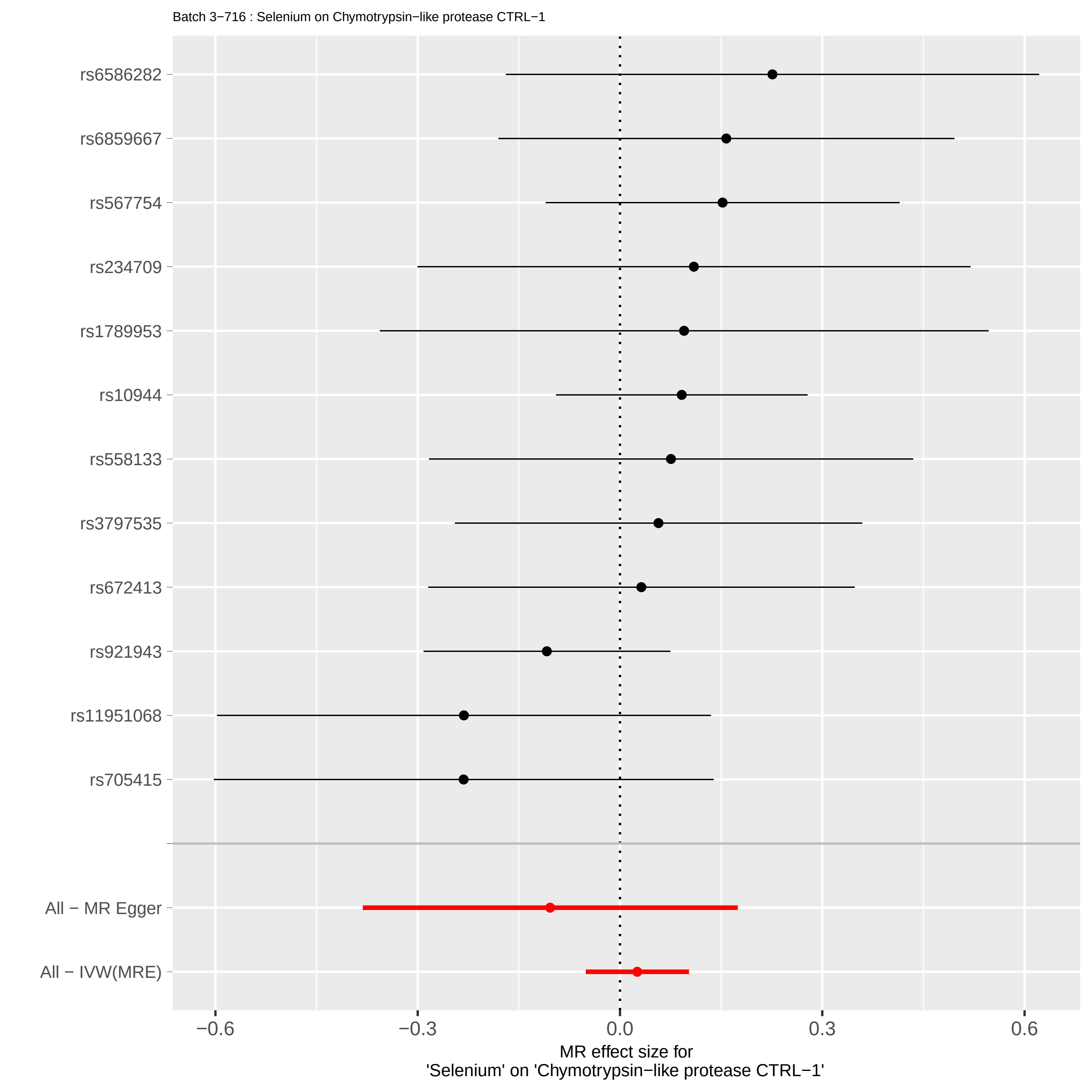
Batch 3−716 : Selenium on Chymotrypsin−like protease CTRL−1
rs6586282
rs6859667
rs567754
rs234709
rs1789953
rs10944
rs558133
rs3797535
rs672413
rs921943
rs11951068
rs705415
All − MR Egger
All − IVW(MRE)
−0.6
−0.3
0.0
0.3
0.6
MR effect size for
'Selenium' on 'Chymotrypsin−like protease CTRL−1'

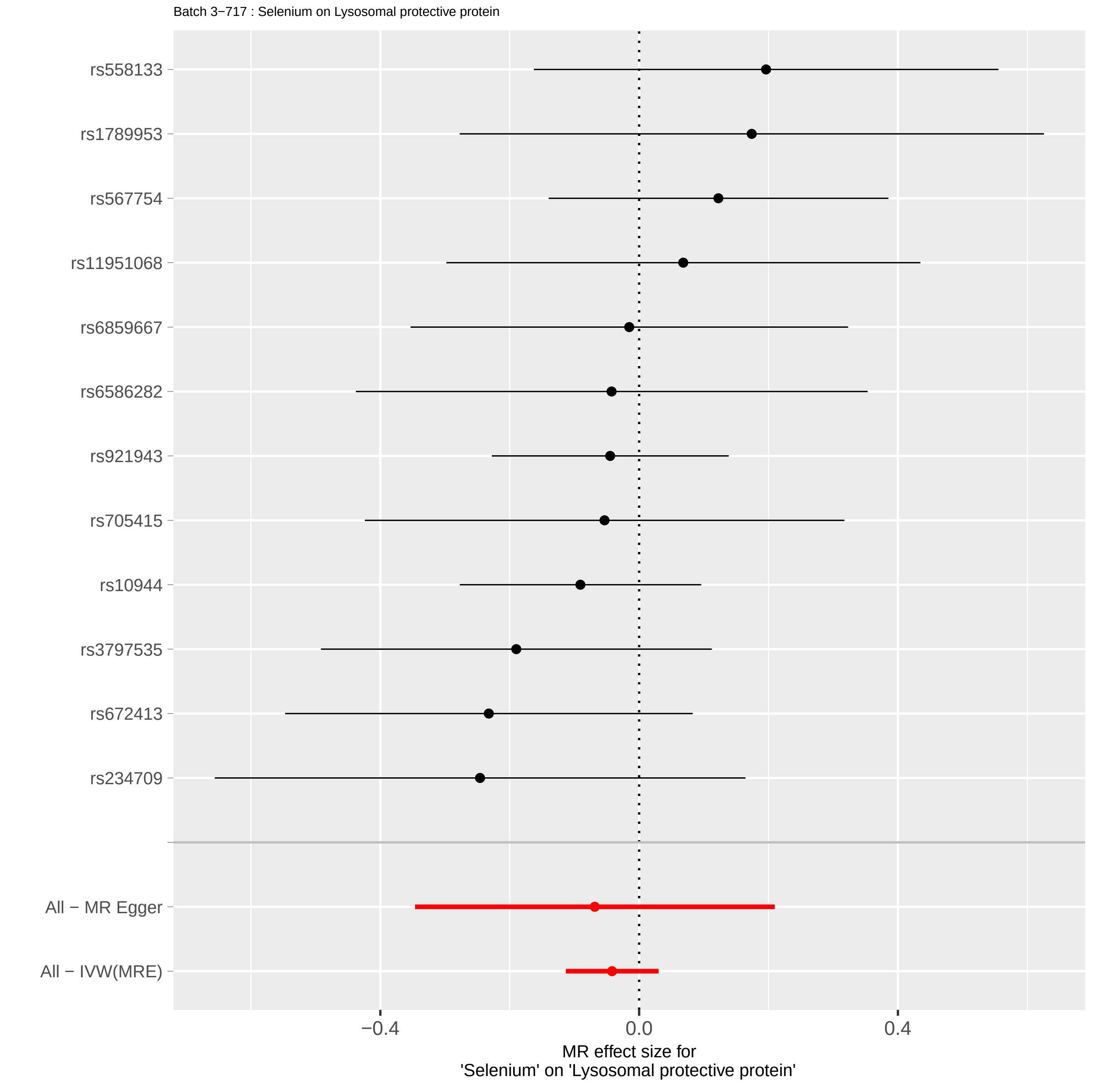
Batch 3−717 : Selenium on Lysosomal protective protein
rs558133
rs1789953
rs567754
rs11951068
rs6859667
rs6586282
rs921943
rs705415
rs10944
rs3797535
rs672413
rs234709
All − MR Egger
All − IVW(MRE)
−0.4
0.0
0.4
MR effect size for
'Selenium' on 'Lysosomal protective protein'

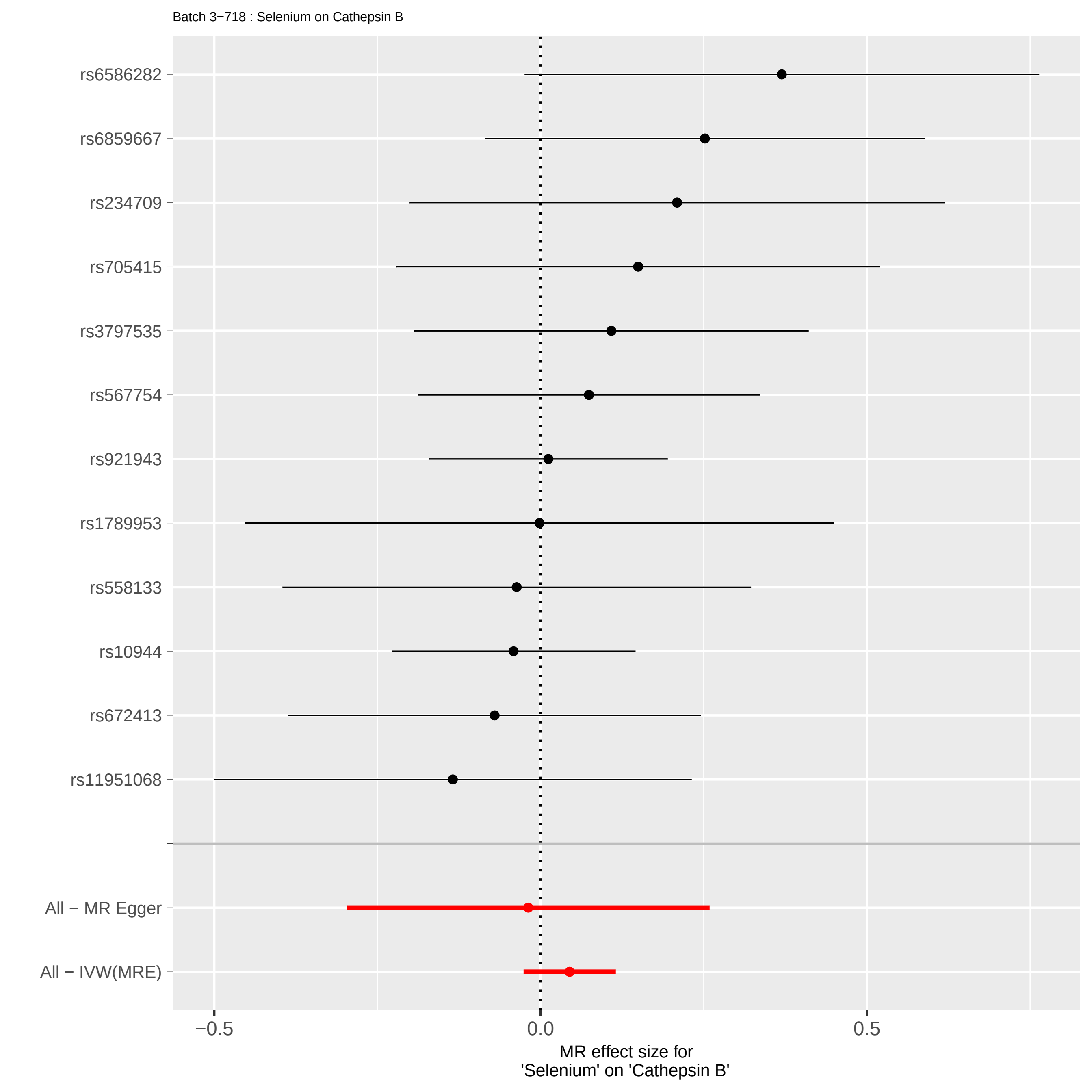
Batch 3−718 : Selenium on Cathepsin B
rs6586282
rs6859667
rs234709
rs705415
rs3797535
rs567754
rs921943
rs1789953
rs558133
rs10944
rs672413
rs11951068
All − MR Egger
All − IVW(MRE)
−0.5
0.0
0.5
MR effect size for
'Selenium' on 'Cathepsin B'

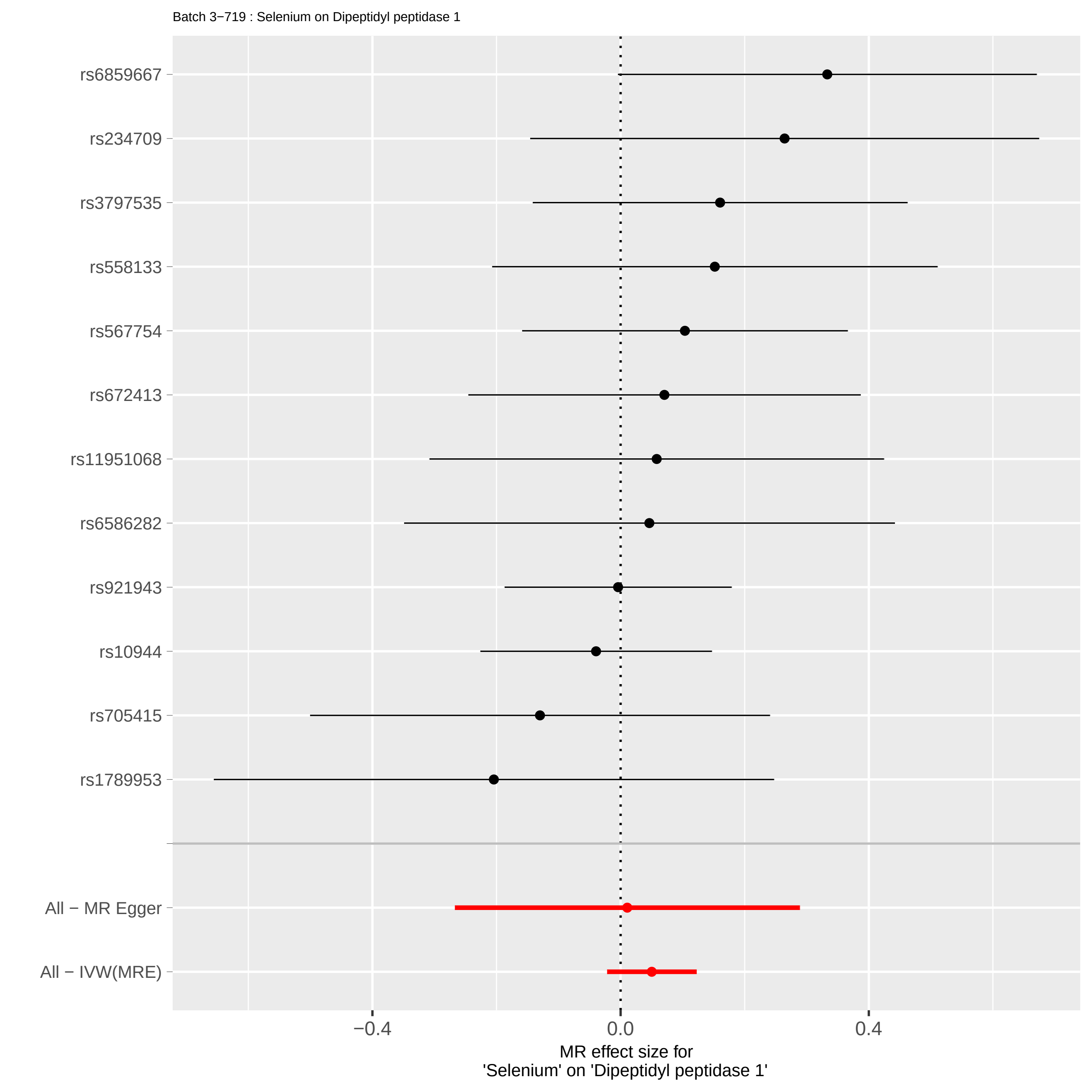

Batch 3−719 : Selenium on Dipeptidyl peptidase 1
rs6859667
rs234709
rs3797535
rs558133
rs567754
rs672413
rs11951068
rs6586282
rs921943
rs10944
rs705415
rs1789953
All − MR Egger
All − IVW(MRE)
−0.4
0.0
0.4
MR effect size for
'Selenium' on 'Dipeptidyl peptidase 1'

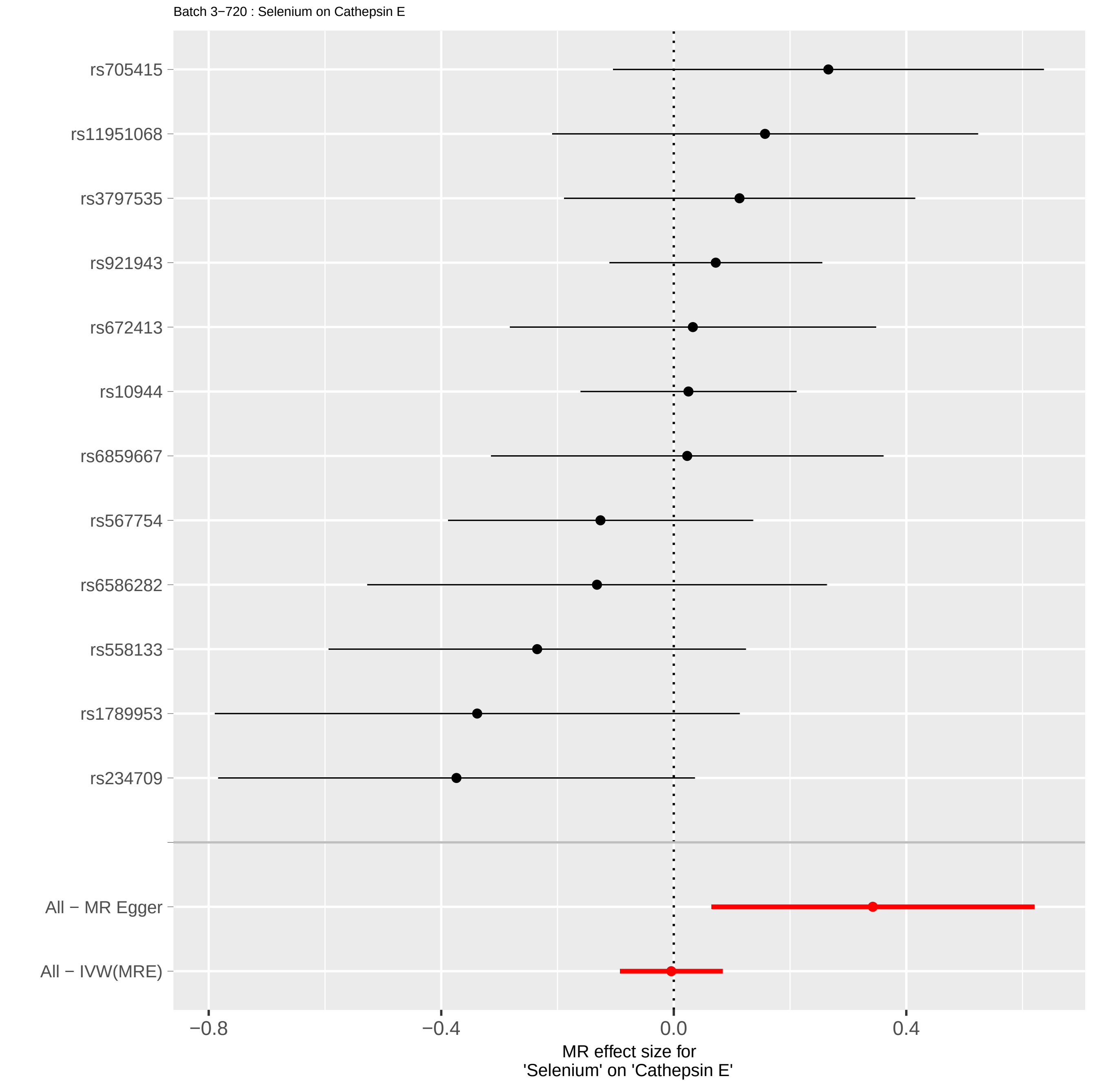
Batch 3−720 : Selenium on Cathepsin E
rs705415
rs11951068
rs3797535
rs921943
rs672413
rs10944
rs6859667
rs567754
rs6586282
rs558133
rs1789953
rs234709
All − MR Egger
All − IVW(MRE)
−0.8
−0.4
0.0
0.4
MR effect size for
'Selenium' on 'Cathepsin E'

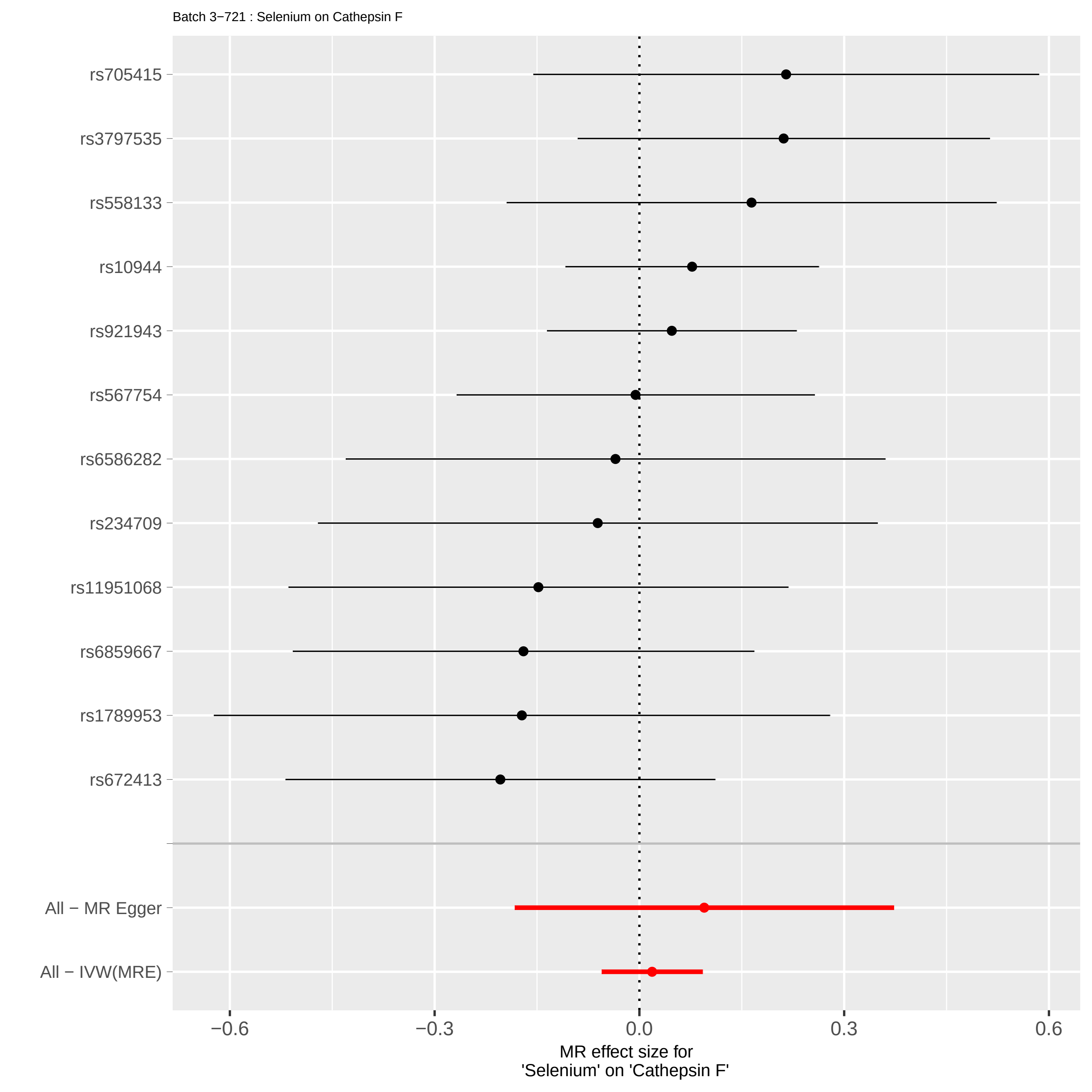

Batch 3−721 : Selenium on Cathepsin F
rs705415
rs3797535
rs558133
rs10944
rs921943
rs567754
rs6586282
rs234709
rs11951068
rs6859667
rs1789953
rs672413
All − MR Egger
All − IVW(MRE)
−0.6
−0.3
0.0
0.3
0.6
MR effect size for
'Selenium' on 'Cathepsin F'

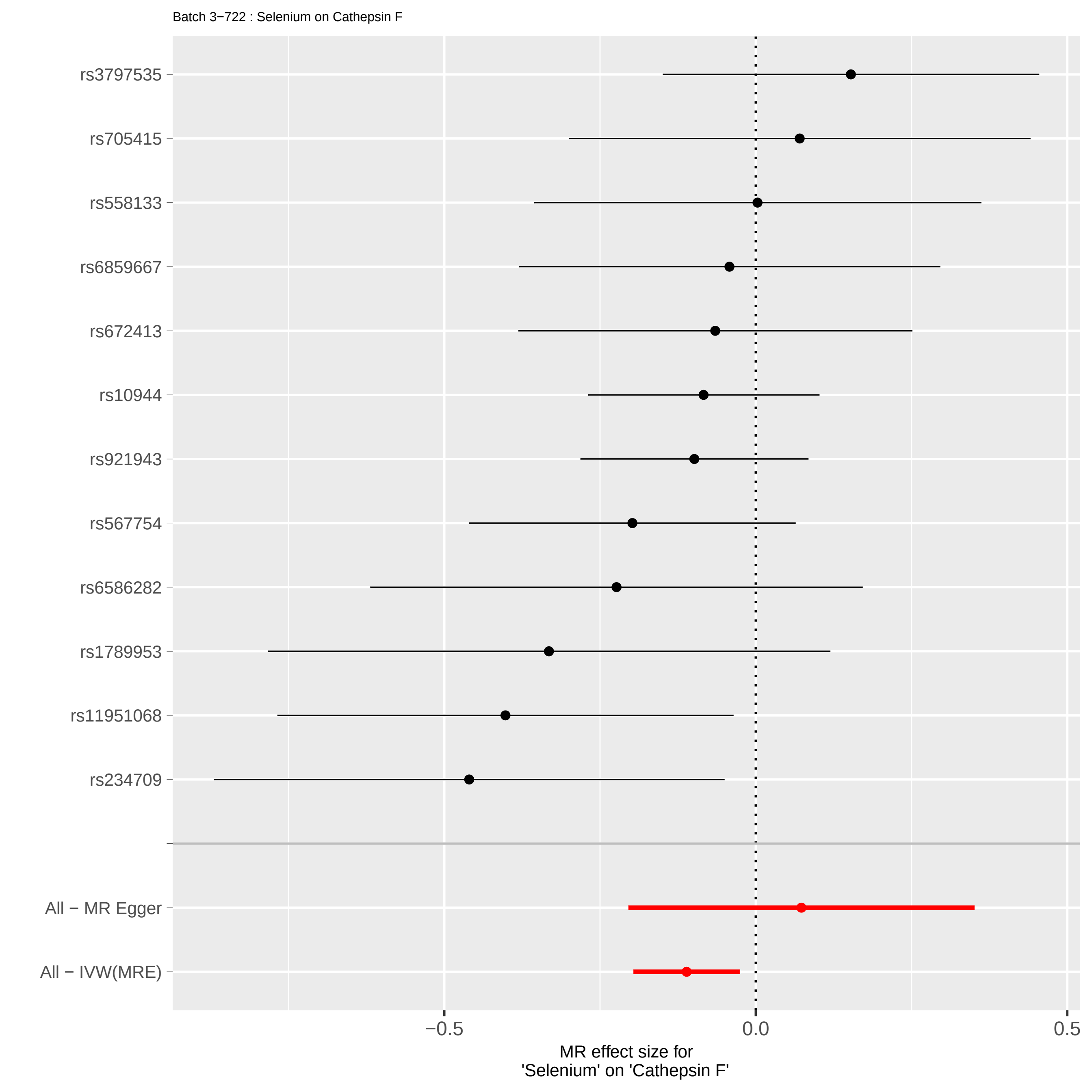

Batch 3−722 : Selenium on Cathepsin F
rs3797535
rs705415
rs558133
rs6859667
rs672413
rs10944
rs921943
rs567754
rs6586282
rs1789953
rs11951068
rs234709
All − MR Egger
All − IVW(MRE)
−0.5
0.0
0.5
MR effect size for
'Selenium' on 'Cathepsin F'

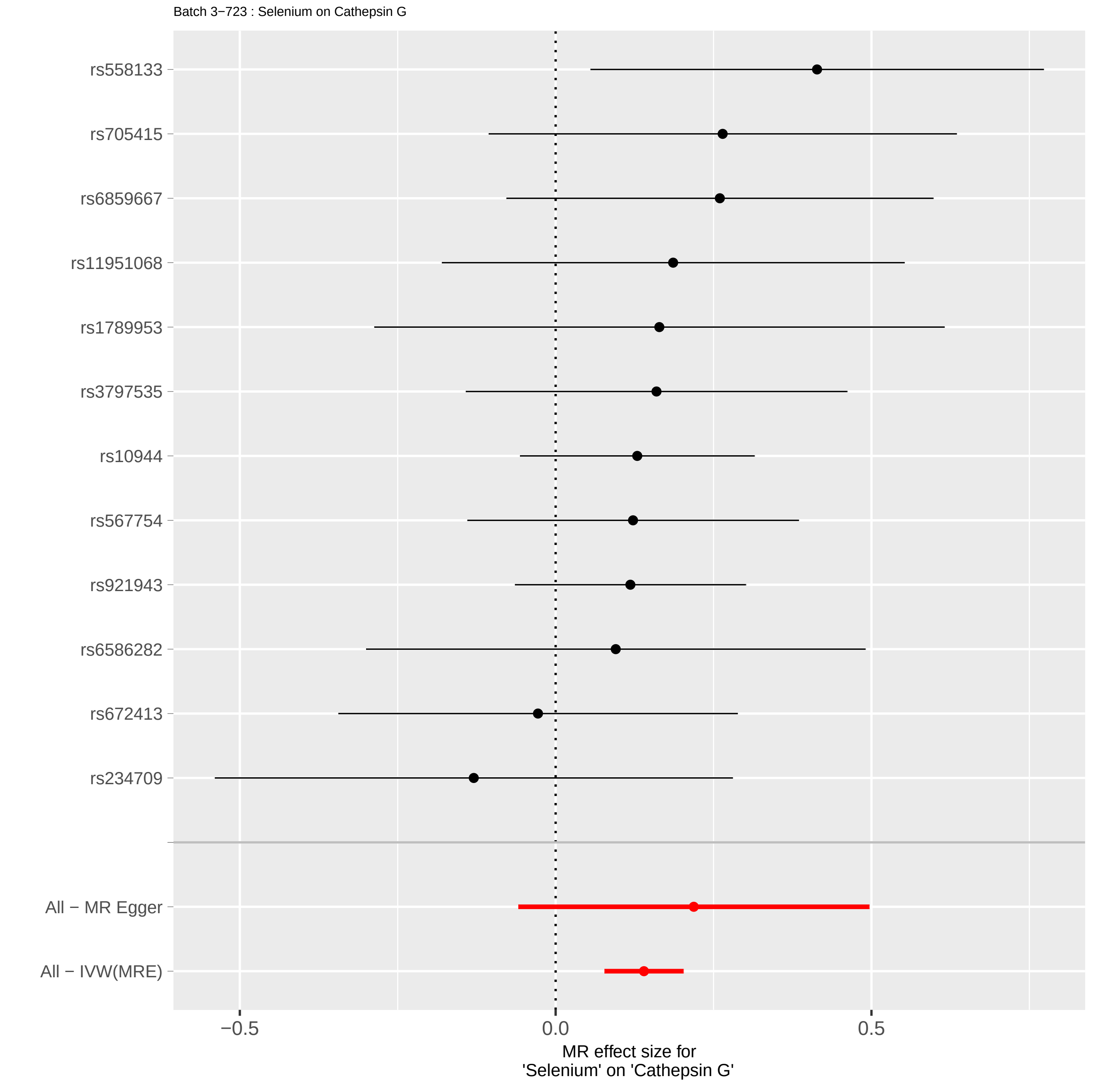

Batch 3−723 : Selenium on Cathepsin G
rs558133
rs705415
rs6859667
rs11951068
rs1789953
rs3797535
rs10944
rs567754
rs921943
rs6586282
rs672413
rs234709
All − MR Egger
All − IVW(MRE)
−0.5
0.0
0.5
MR effect size for
'Selenium' on 'Cathepsin G'

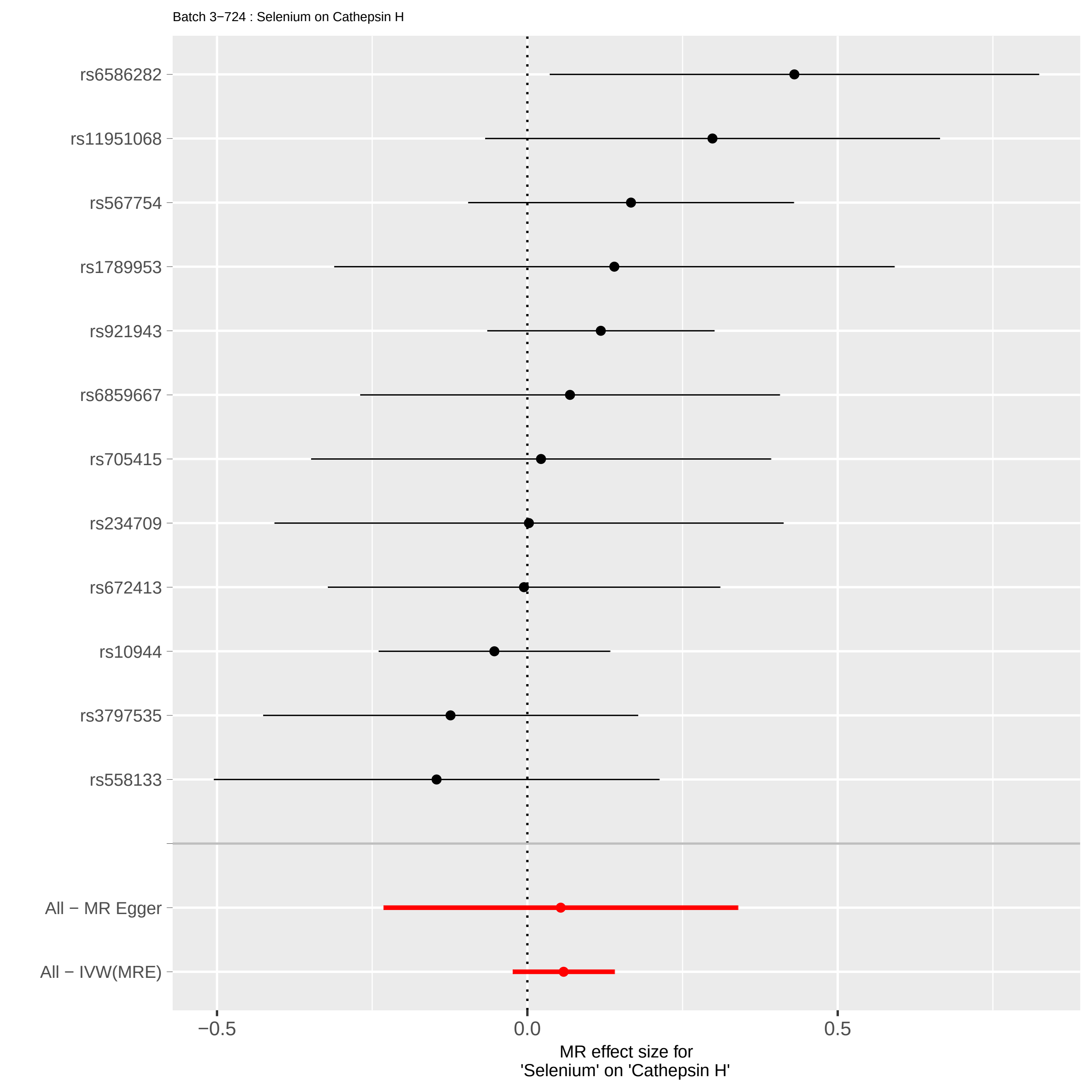

Batch 3−724 : Selenium on Cathepsin H
rs6586282
rs11951068
rs567754
rs1789953
rs921943
rs6859667
rs705415
rs234709
rs672413
rs10944
rs3797535
rs558133
All − MR Egger
All − IVW(MRE)
−0.5
0.0
0.5
MR effect size for
'Selenium' on 'Cathepsin H'

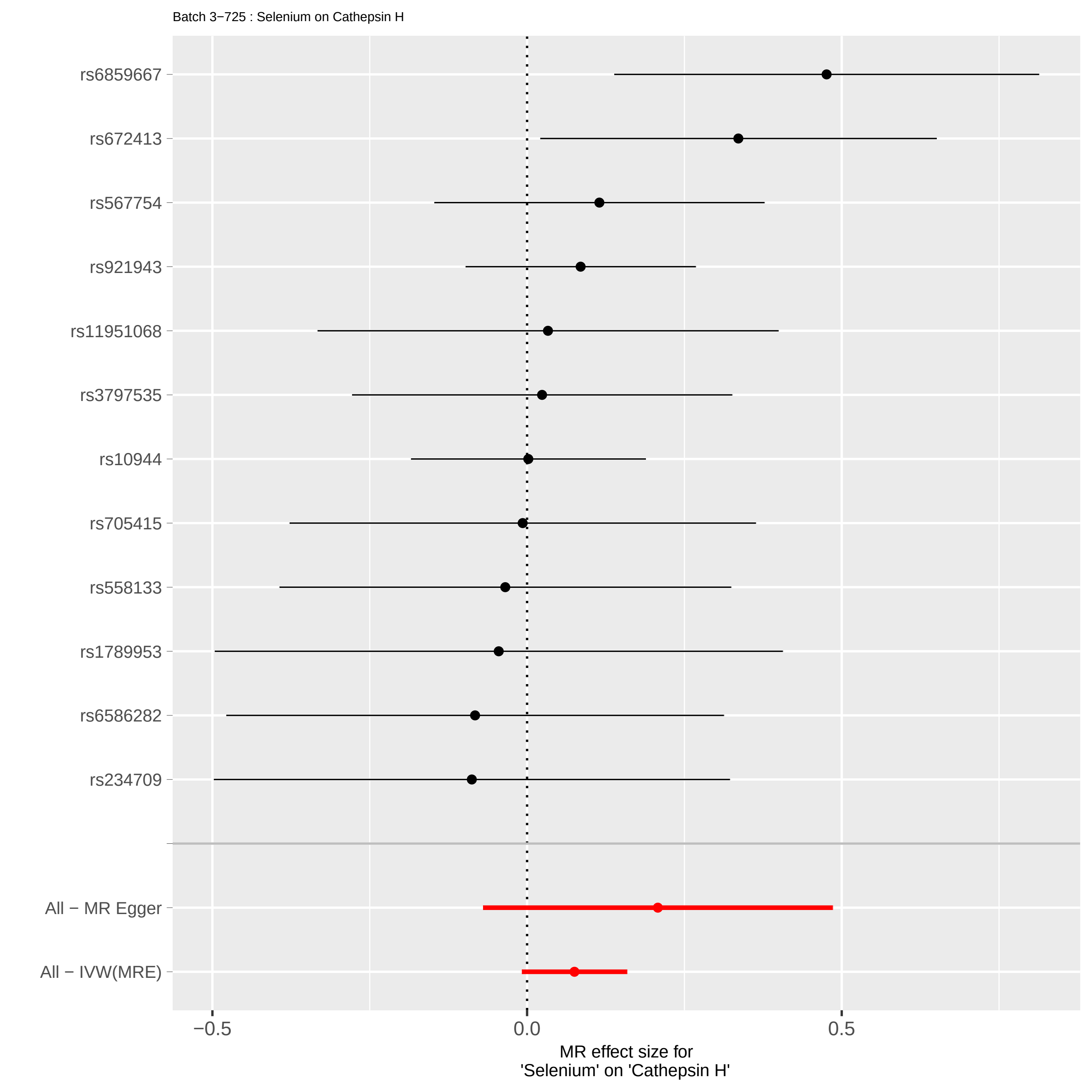

Batch 3−725 : Selenium on Cathepsin H
rs6859667
rs672413
rs567754
rs921943
rs11951068
rs3797535
rs10944
rs705415
rs558133
rs1789953
rs6586282
rs234709
All − MR Egger
All − IVW(MRE)
−0.5
0.0
0.5
MR effect size for
'Selenium' on 'Cathepsin H'

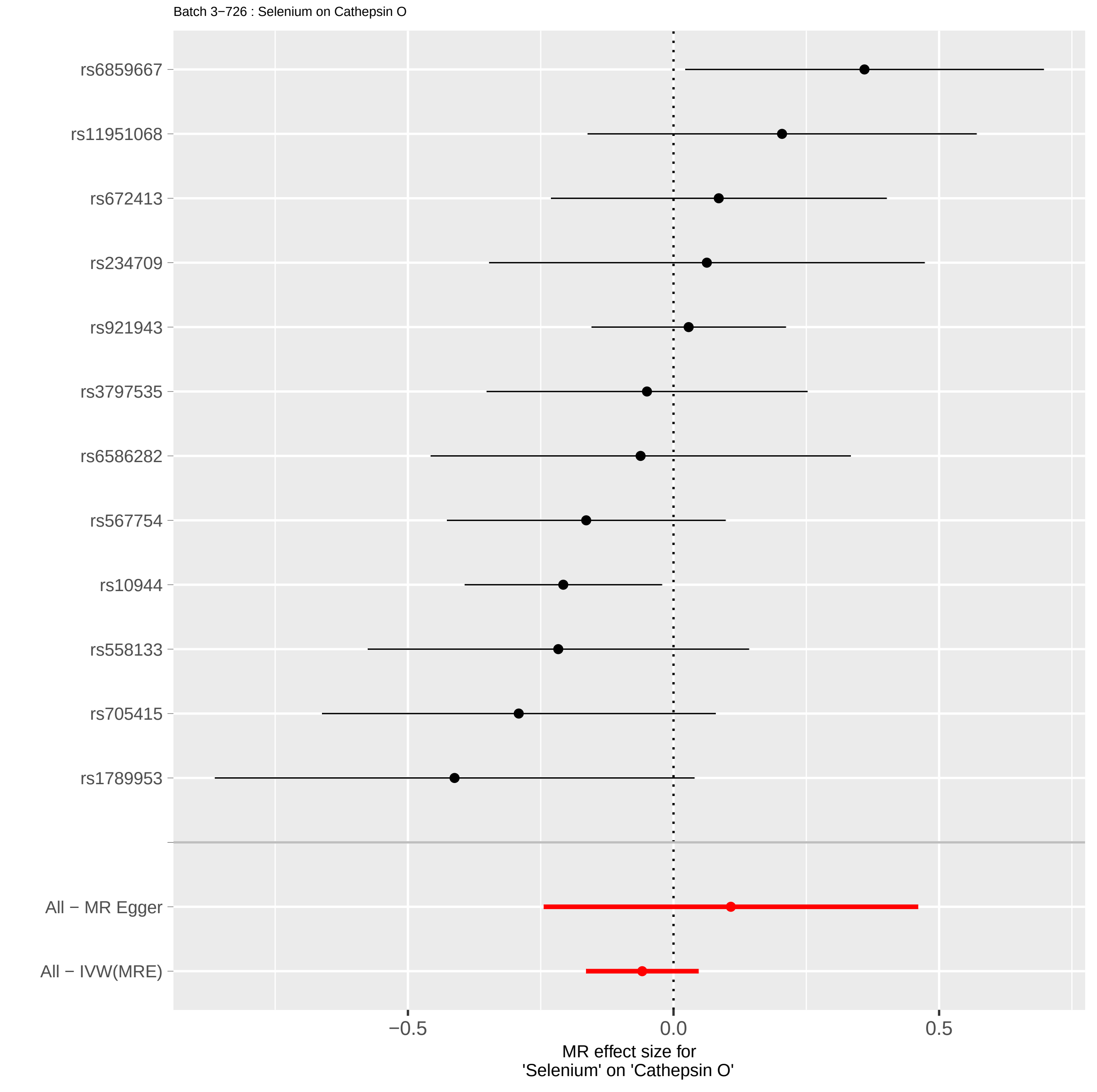

Batch 3−726 : Selenium on Cathepsin O
rs6859667
rs11951068
rs672413
rs234709
rs921943
rs3797535
rs6586282
rs567754
rs10944
rs558133
rs705415
rs1789953
All − MR Egger
All − IVW(MRE)
−0.5
0.0
0.5
MR effect size for
'Selenium' on 'Cathepsin O'

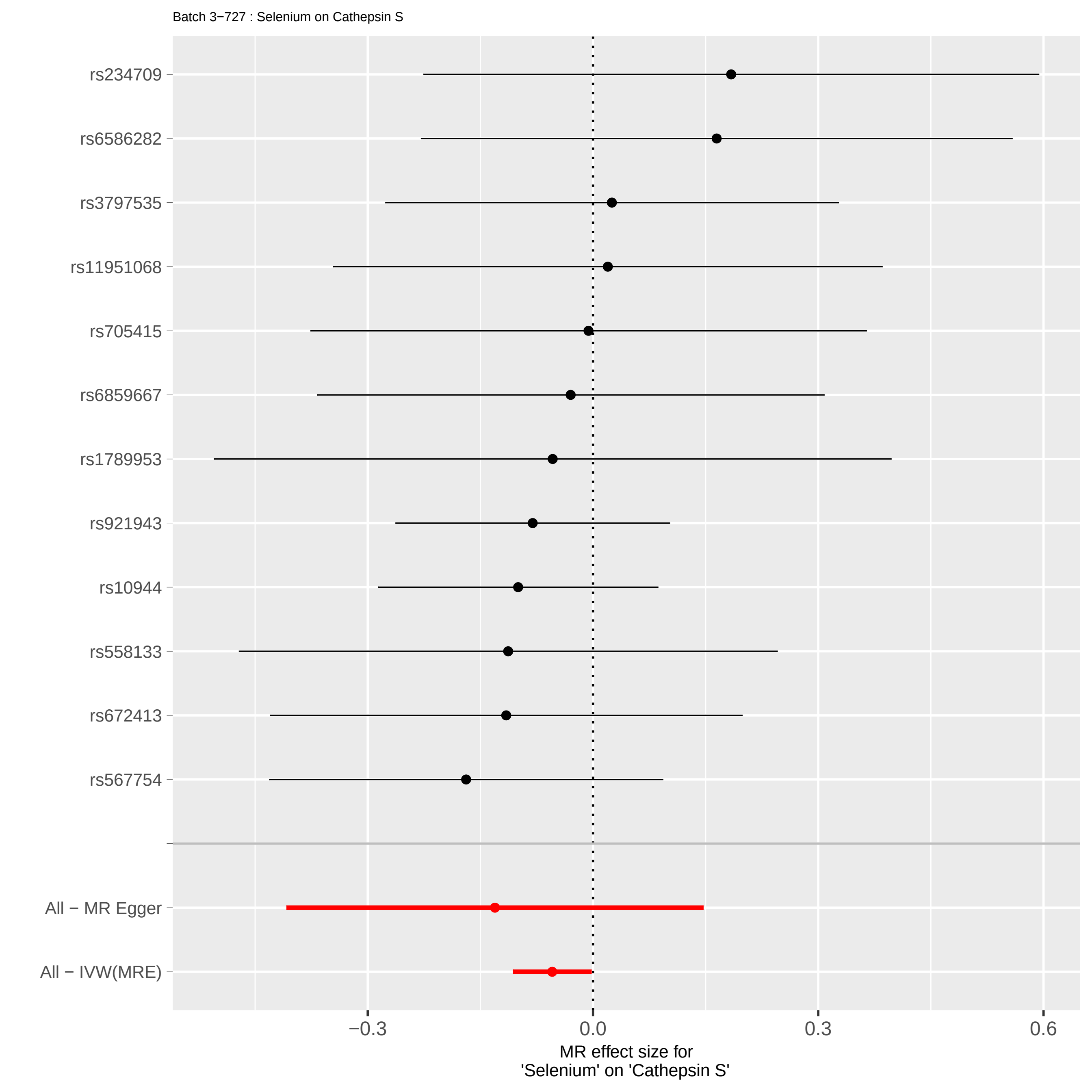
Batch 3−727 : Selenium on Cathepsin S
rs234709
rs6586282
rs3797535
rs11951068
rs705415
rs6859667
rs1789953
rs921943
rs10944
rs558133
rs672413
rs567754
All − MR Egger
All − IVW(MRE)
−0.3
0.0
0.3
0.6
MR effect size for
'Selenium' on 'Cathepsin S'

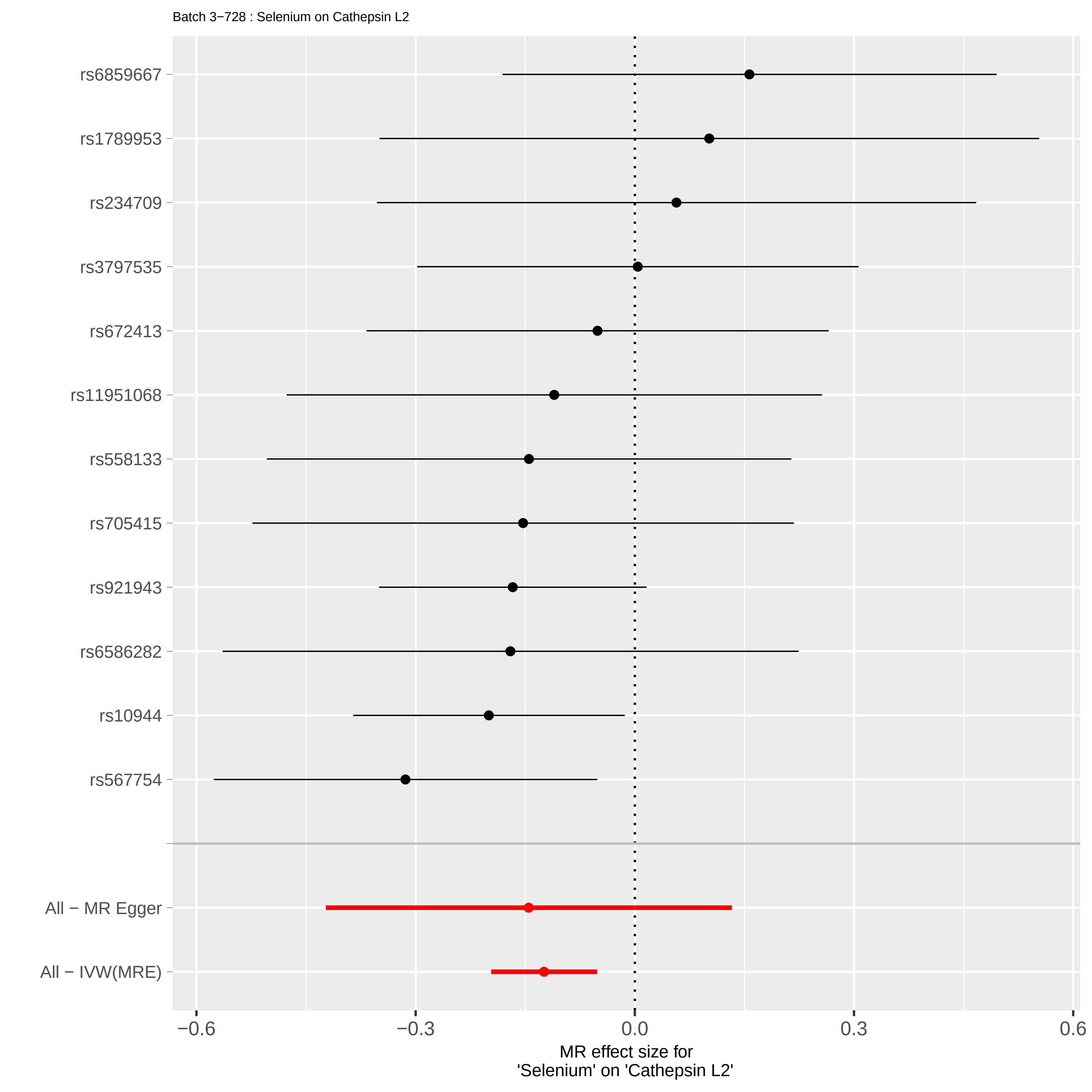

Batch 3−728 : Selenium on Cathepsin L2
rs6859667
rs1789953
rs234709
rs3797535
rs672413
rs11951068
rs558133
rs705415
rs921943
rs6586282
rs10944
rs567754
All − MR Egger
All − IVW(MRE)
−0.6
−0.3
0.0
0.3
0.6
MR effect size for
'Selenium' on 'Cathepsin L2'

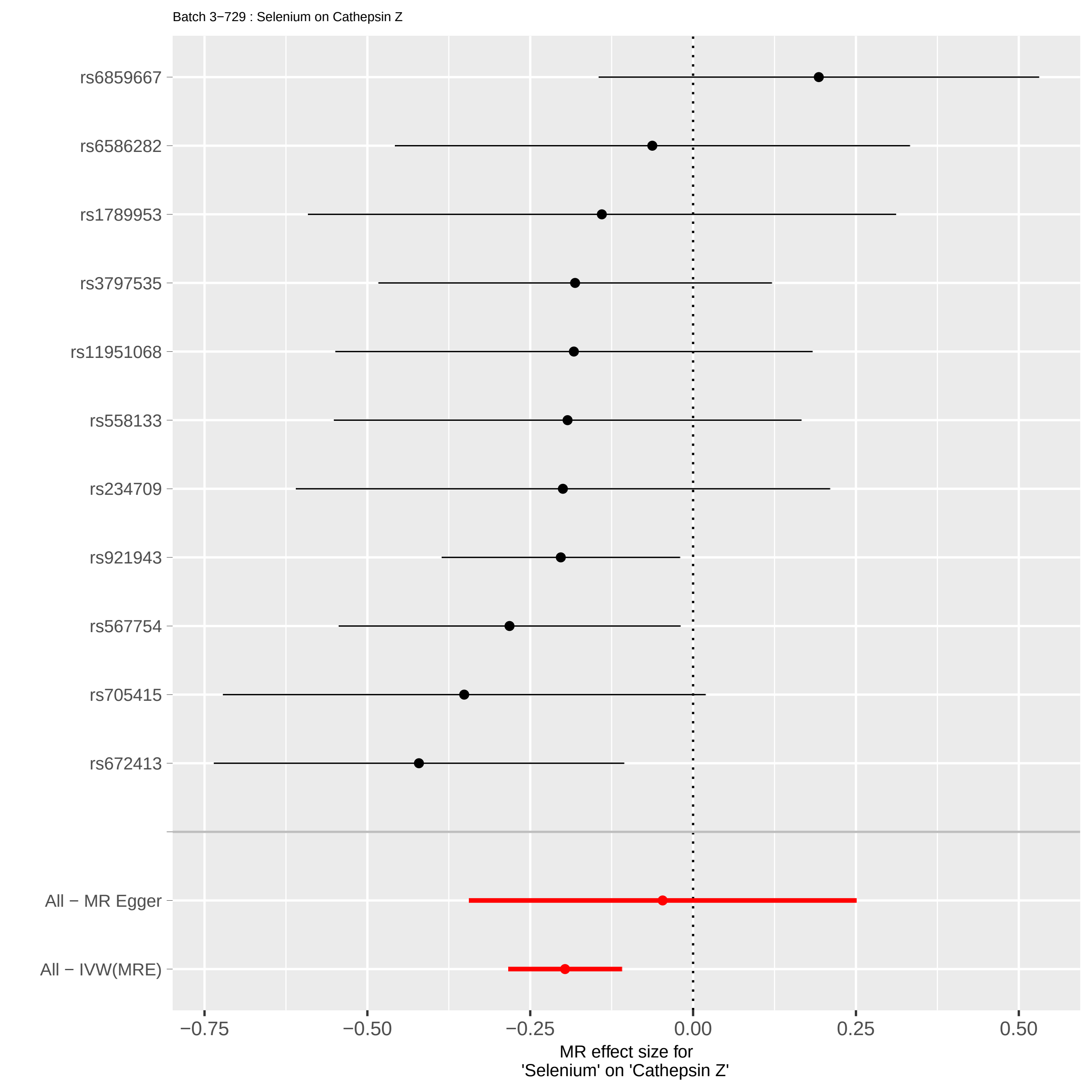
Batch 3−729 : Selenium on Cathepsin Z
rs6859667
rs6586282
rs1789953
rs3797535
rs11951068
rs558133
rs234709
rs921943
rs567754
rs705415
rs672413
All − MR Egger
All − IVW(MRE)
−0.75
−0.50
−0.25
0.00
0.25
0.50
MR effect size for
'Selenium' on 'Cathepsin Z'

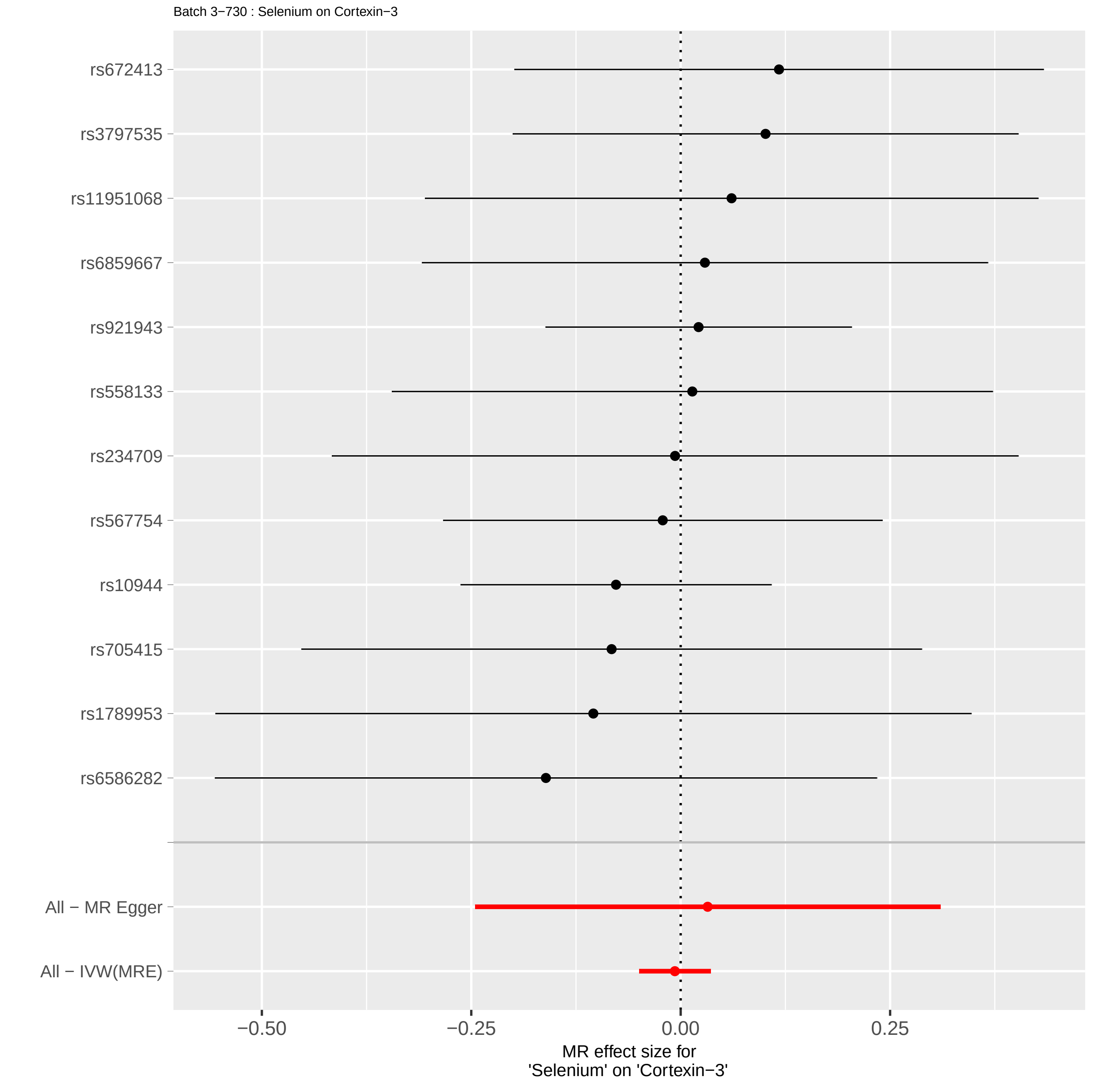
Batch 3−730 : Selenium on Cortexin−3
rs672413
rs3797535
rs11951068
rs6859667
rs921943
rs558133
rs234709
rs567754
rs10944
rs705415
rs1789953
rs6586282
All − MR Egger
All − IVW(MRE)
−0.50
−0.25
0.00
0.25
MR effect size for
'Selenium' on 'Cortexin−3'

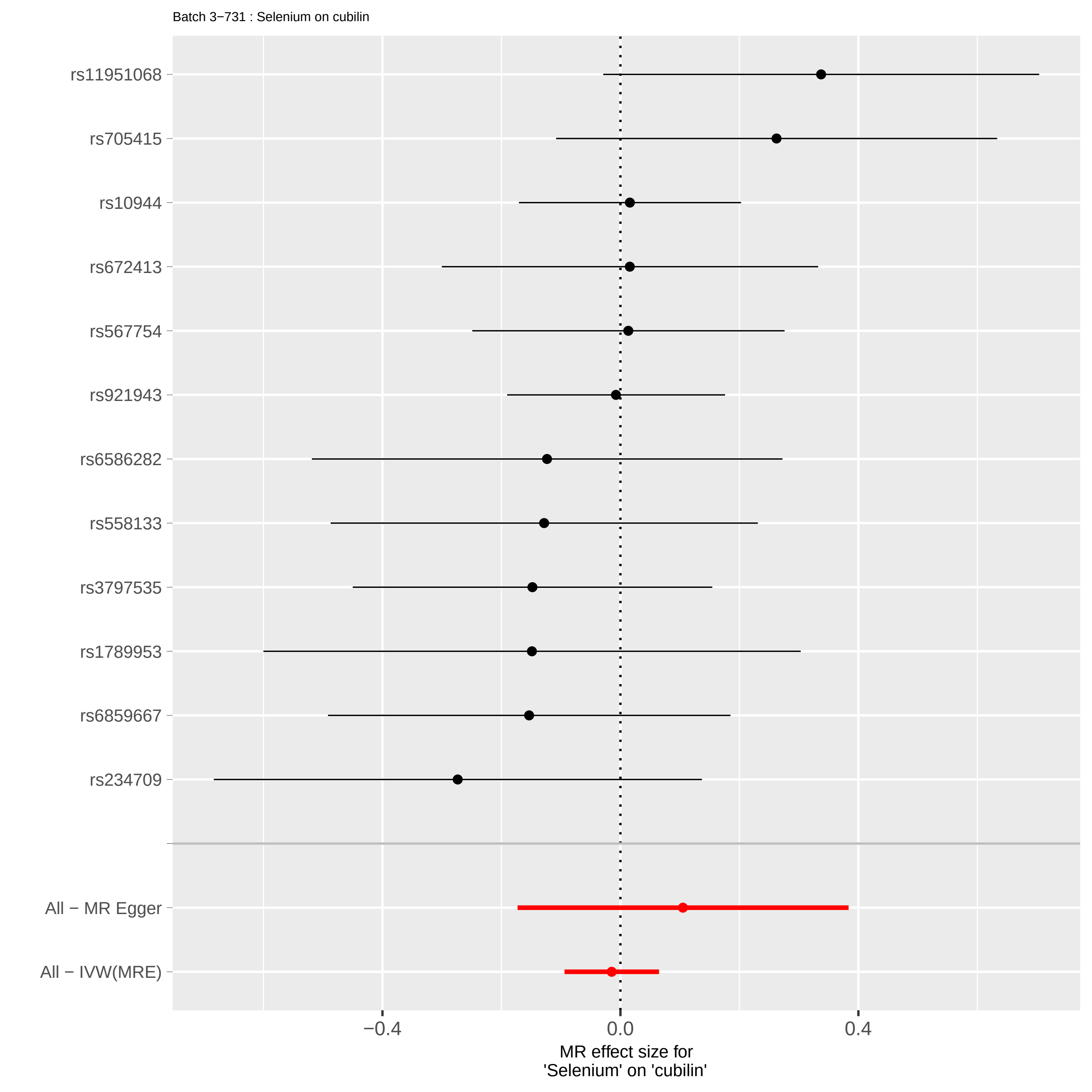
Batch 3−731 : Selenium on cubilin
rs11951068
rs705415
rs10944
rs672413
rs567754
rs921943
rs6586282
rs558133
rs3797535
rs1789953
rs6859667
rs234709
All − MR Egger
All − IVW(MRE)
−0.4
0.0
0.4
MR effect size for
'Selenium' on 'cubilin'

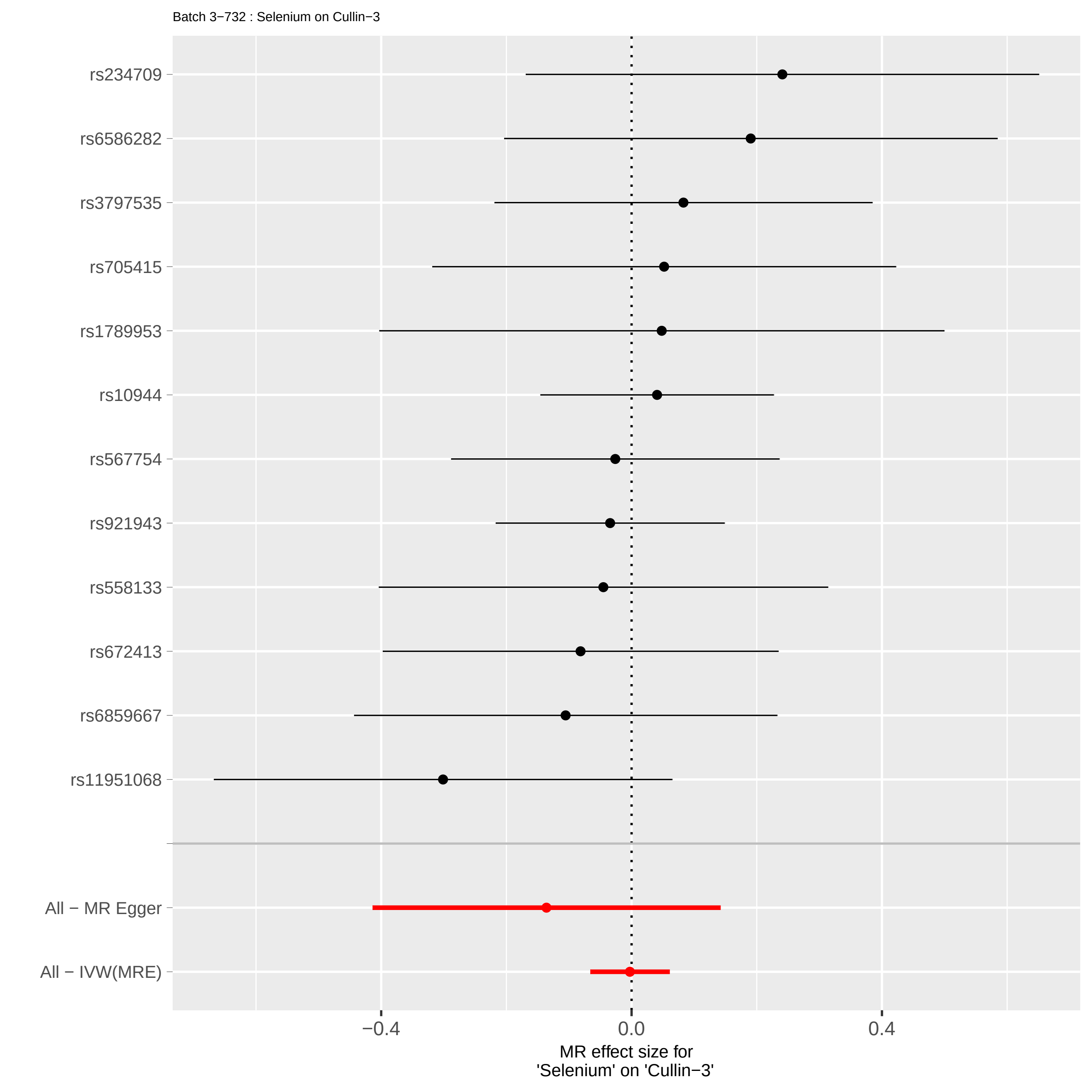

Batch 3−732 : Selenium on Cullin−3
rs234709
rs6586282
rs3797535
rs705415
rs1789953
rs10944
rs567754
rs921943
rs558133
rs672413
rs6859667
rs11951068
All − MR Egger
All − IVW(MRE)
−0.4
0.0
0.4
MR effect size for
'Selenium' on 'Cullin−3'

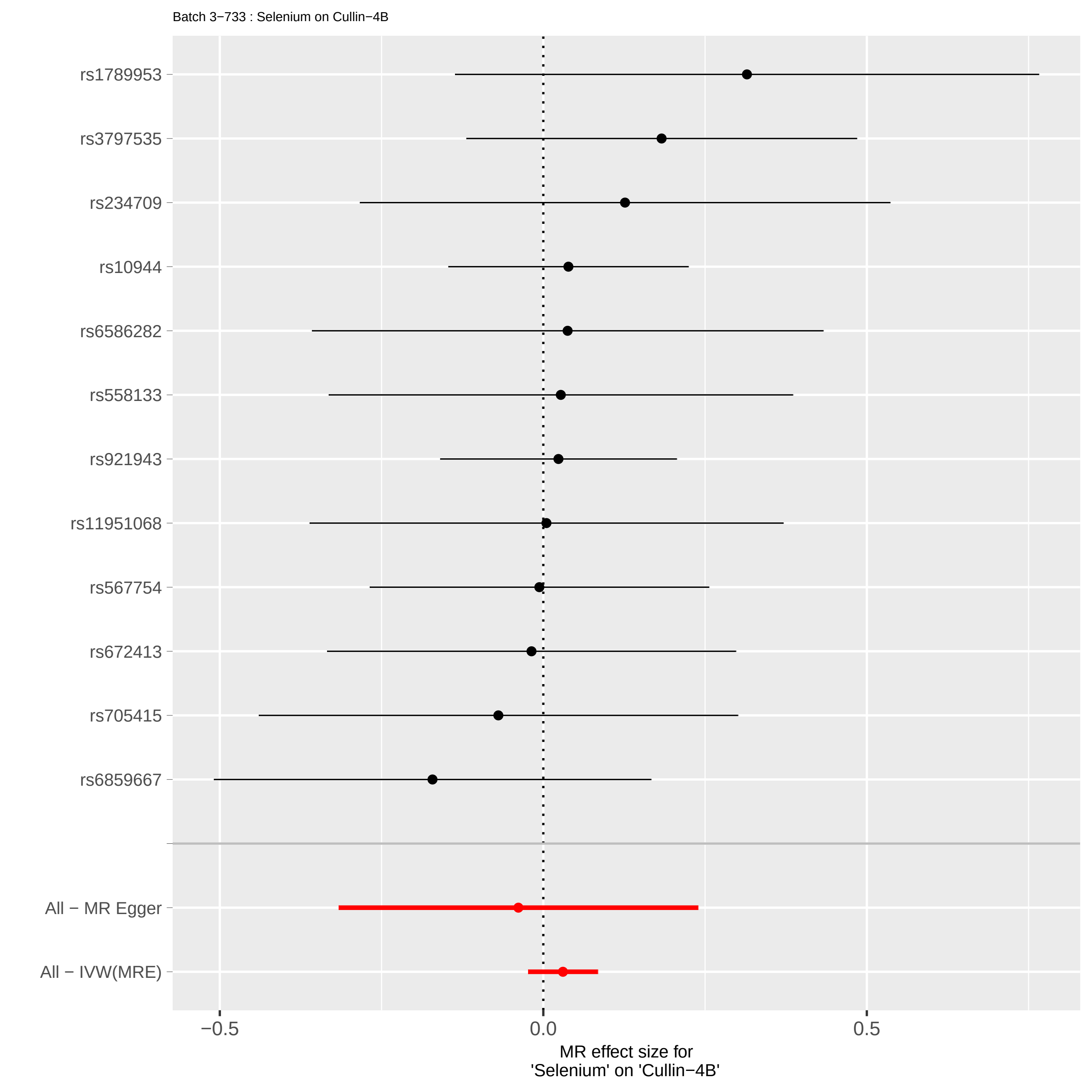
Batch 3−733 : Selenium on Cullin−4B
rs1789953
rs3797535
rs234709
rs10944
rs6586282
rs558133
rs921943
rs11951068
rs567754
rs672413
rs705415
rs6859667
All − MR Egger
All − IVW(MRE)
−0.5
0.0
0.5
MR effect size for
'Selenium' on 'Cullin−4B'

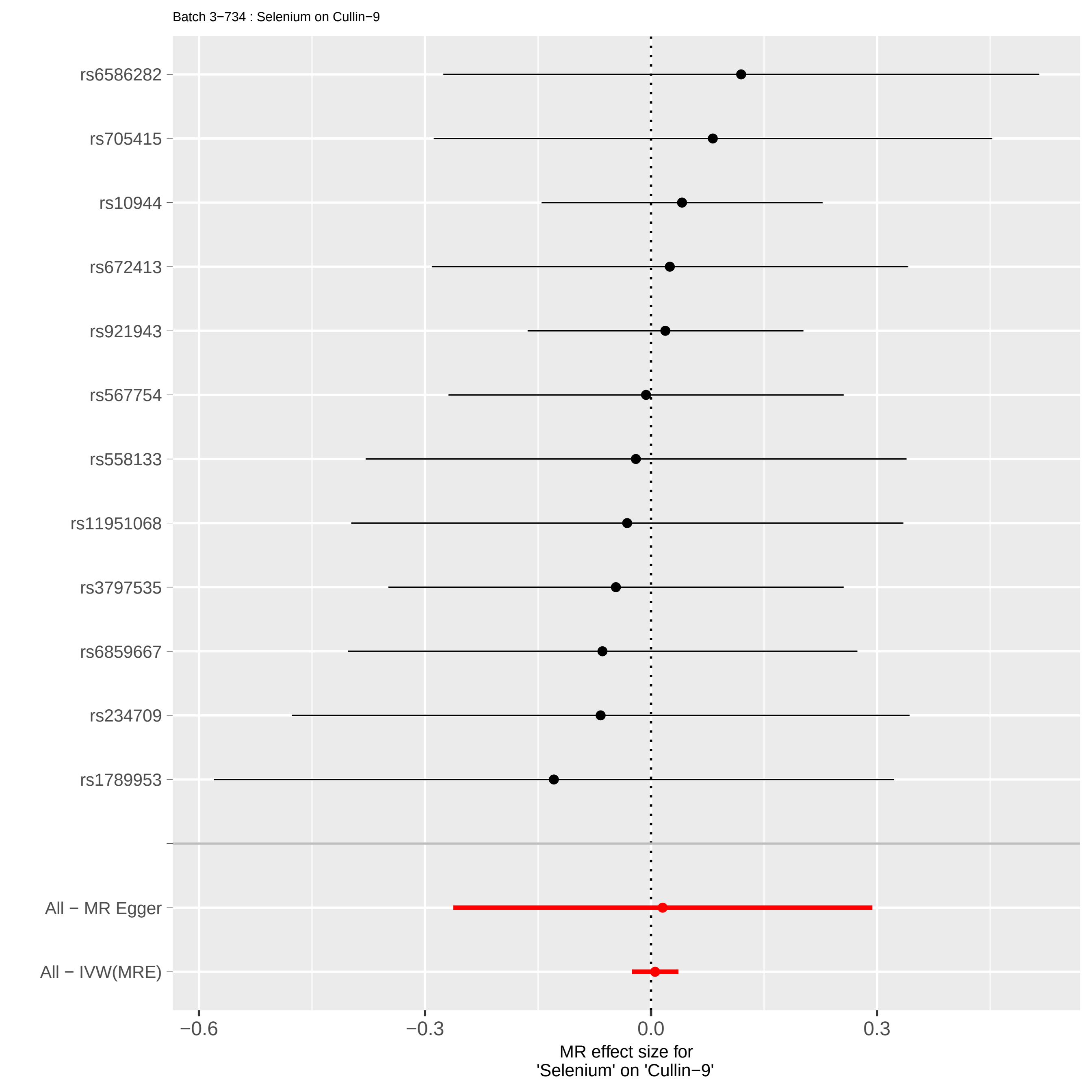
Batch 3−734 : Selenium on Cullin−9
rs6586282
rs705415
rs10944
rs672413
rs921943
rs567754
rs558133
rs11951068
rs3797535
rs6859667
rs234709
rs1789953
All − MR Egger
All − IVW(MRE)
−0.6
−0.3
0.0
0.3
MR effect size for
'Selenium' on 'Cullin−9'

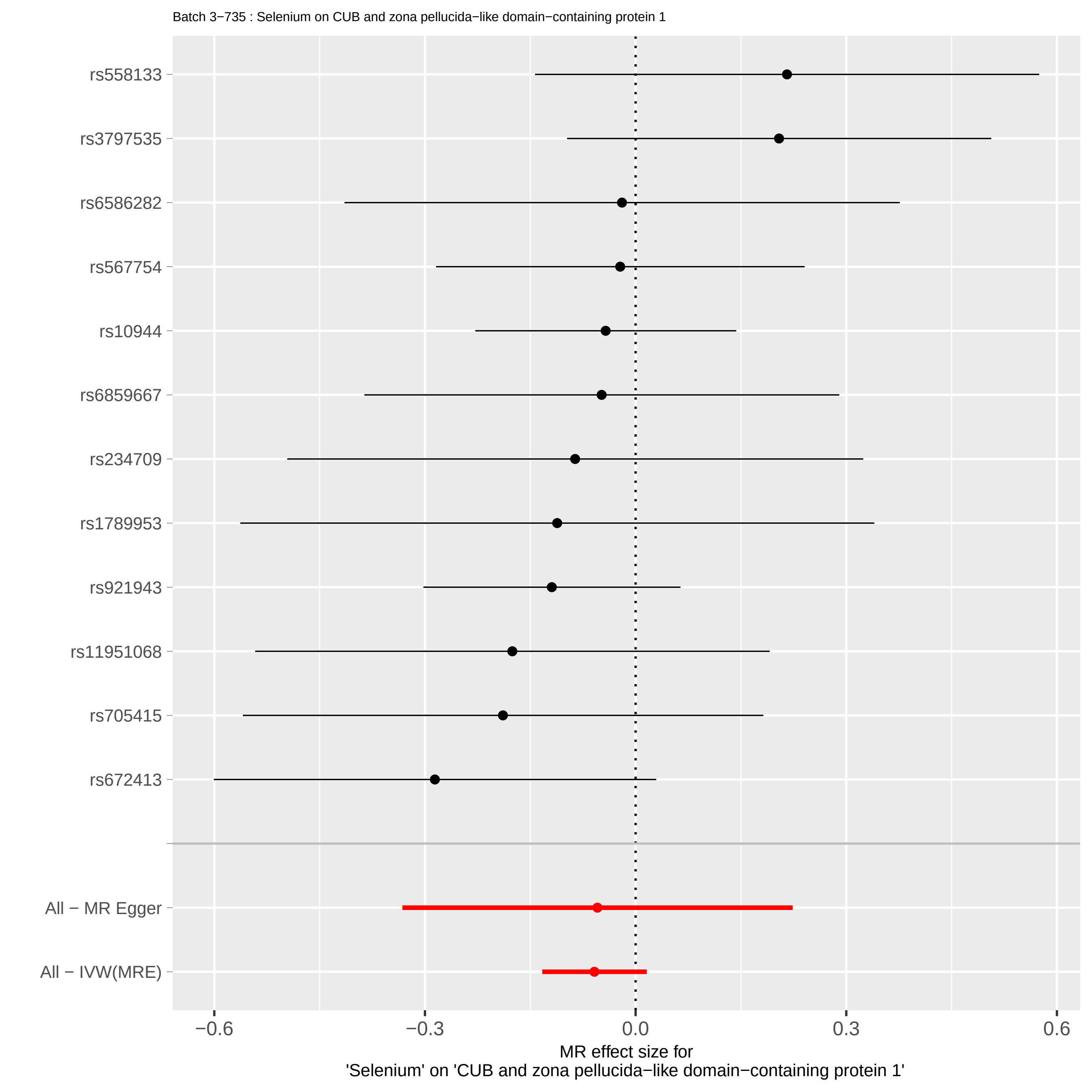

Batch 3−735 : Selenium on CUB and zona pellucida−like domain−containing protein 1
rs558133
rs3797535
rs6586282
rs567754
rs10944
rs6859667
rs234709
rs1789953
rs921943
rs11951068
rs705415
rs672413
All − MR Egger
All − IVW(MRE)
−0.6
−0.3
0.0
0.3
0.6
MR effect size for
'Selenium' on 'CUB and zona pellucida−like domain−containing protein 1'

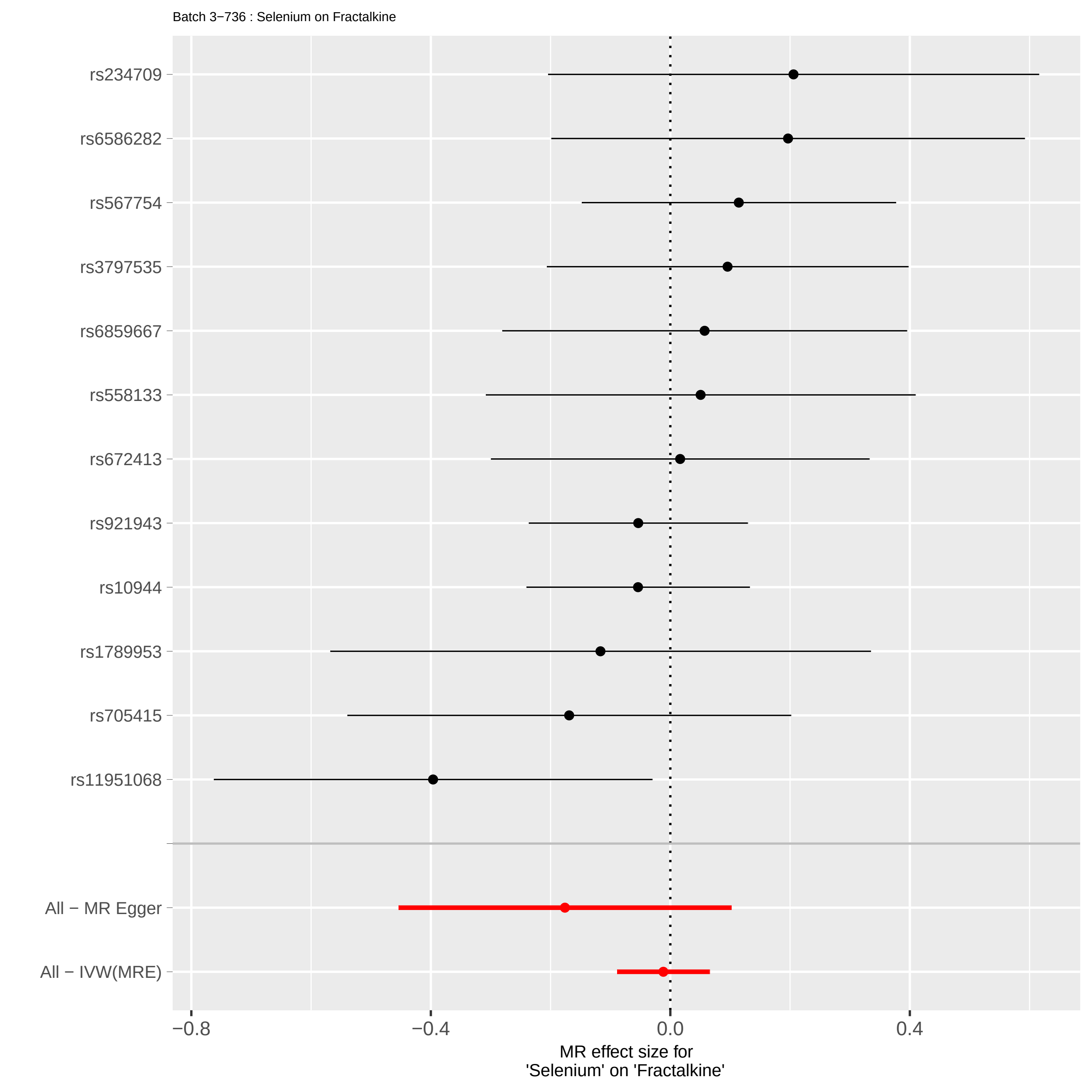

Batch 3−736 : Selenium on Fractalkine
rs234709
rs6586282
rs567754
rs3797535
rs6859667
rs558133
rs672413
rs921943
rs10944
rs1789953
rs705415
rs11951068
All − MR Egger
All − IVW(MRE)
−0.8
−0.4
0.0
0.4
MR effect size for
'Selenium' on 'Fractalkine'

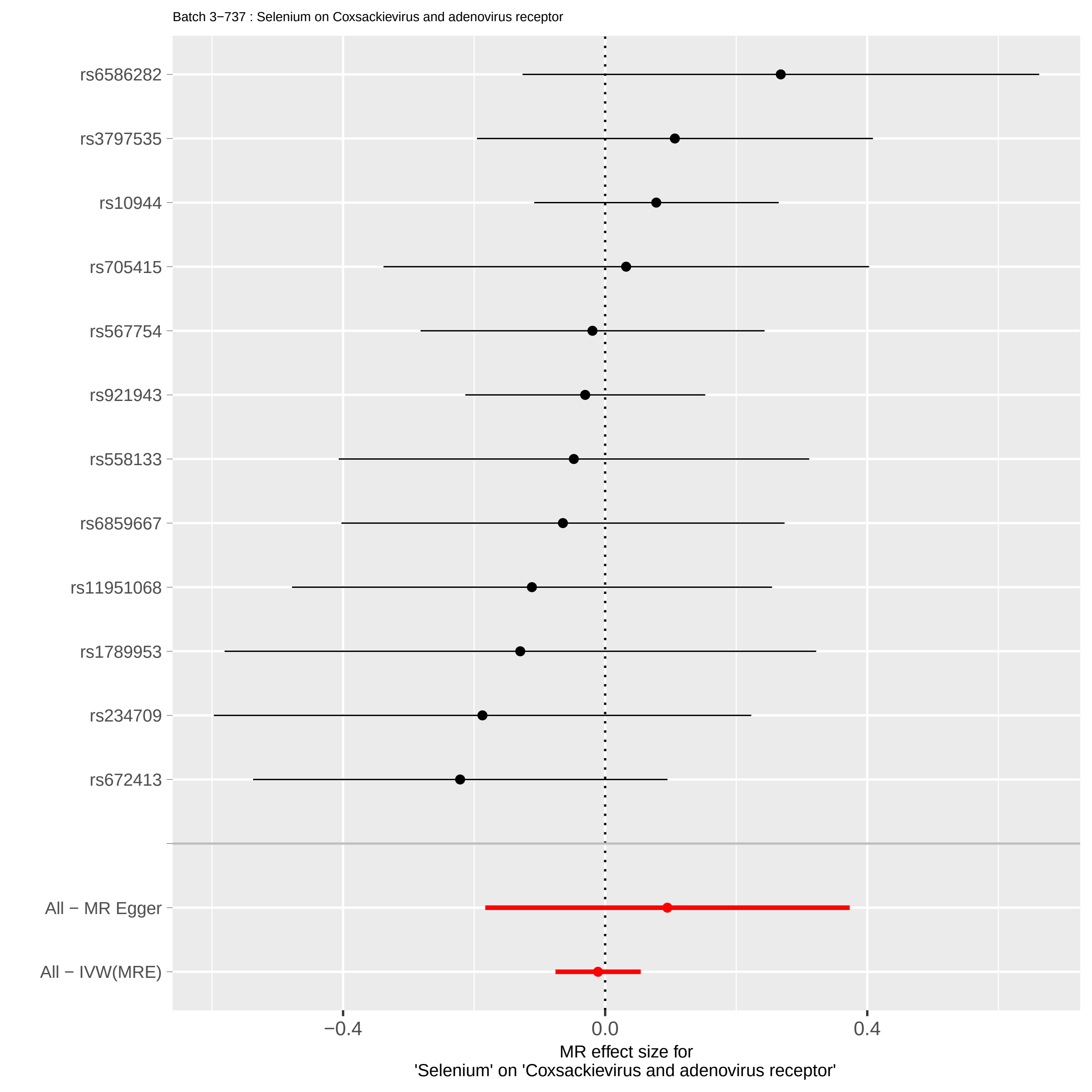
Batch 3−737 : Selenium on Coxsackievirus and adenovirus receptor
rs6586282
rs3797535
rs10944
rs705415
rs567754
rs921943
rs558133
rs6859667
rs11951068
rs1789953
rs234709
rs672413
All − MR Egger
All − IVW(MRE)
−0.4
0.0
0.4
MR effect size for
'Selenium' on 'Coxsackievirus and adenovirus receptor'

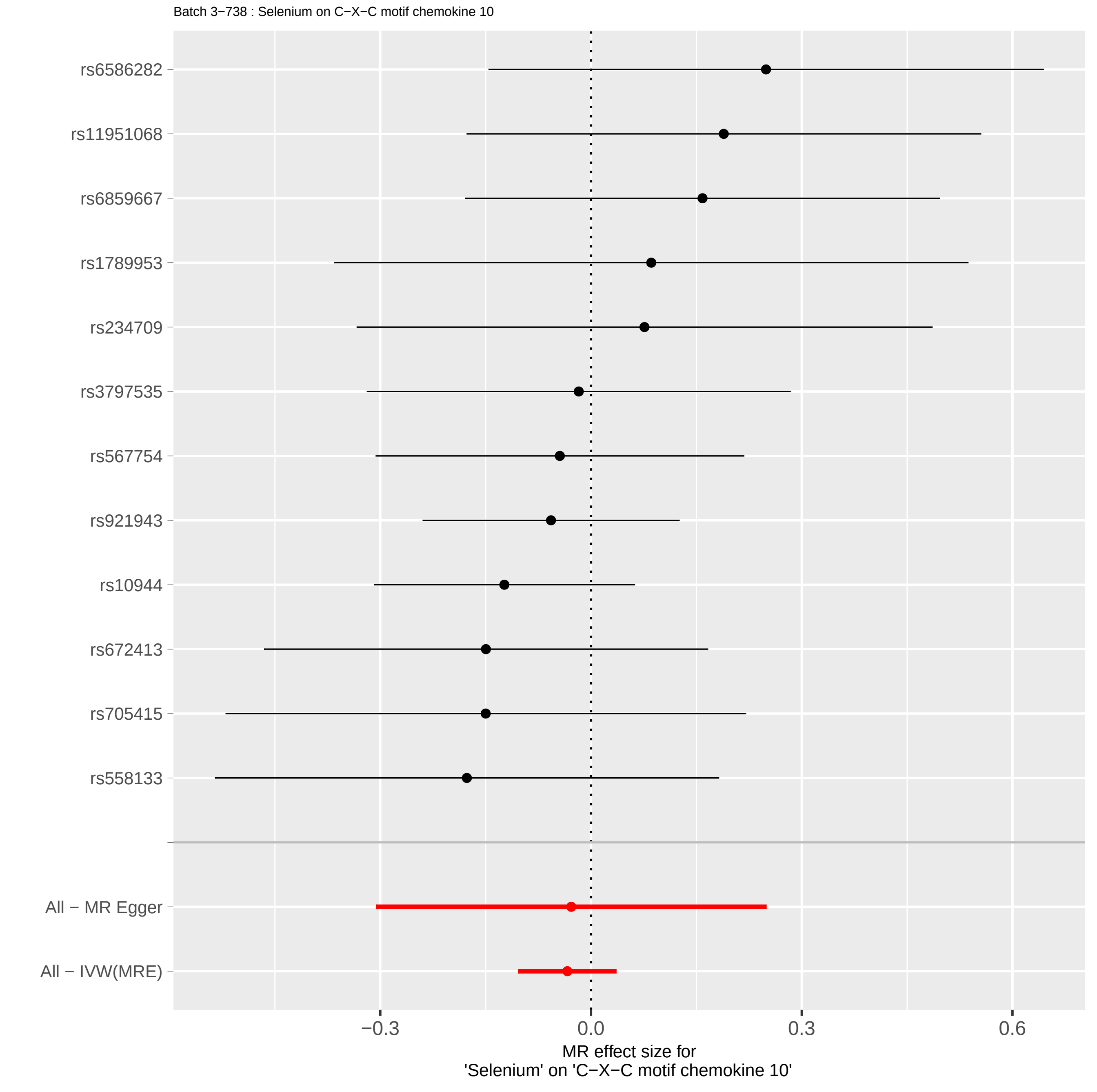
Batch 3−738 : Selenium on C−X−C motif chemokine 10
rs6586282
rs11951068
rs6859667
rs1789953
rs234709
rs3797535
rs567754
rs921943
rs10944
rs672413
rs705415
rs558133
All − MR Egger
All − IVW(MRE)
−0.3
0.0
0.3
0.6
MR effect size for
'Selenium' on 'C−X−C motif chemokine 10'

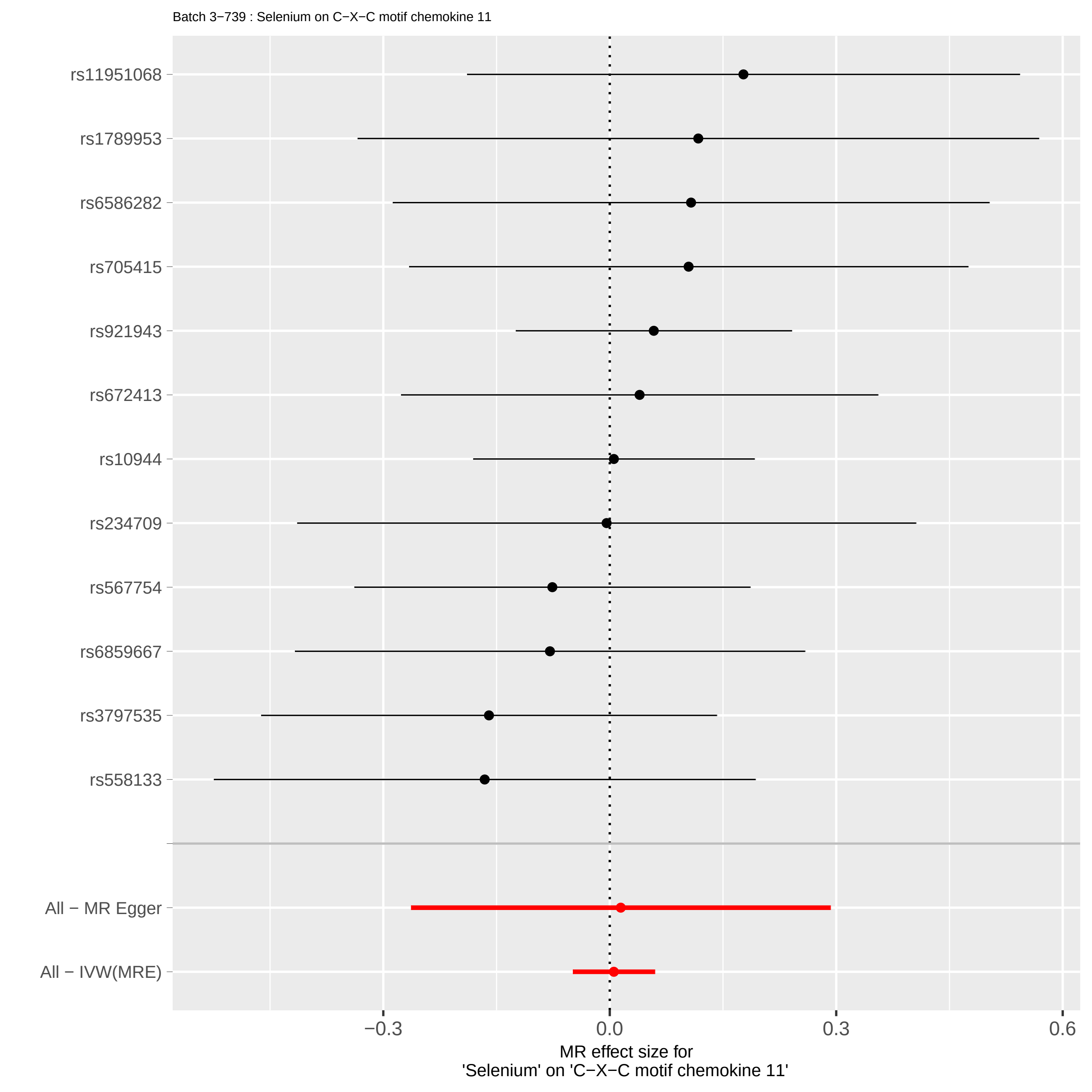

Batch 3−739 : Selenium on C−X−C motif chemokine 11
rs11951068
rs1789953
rs6586282
rs705415
rs921943
rs672413
rs10944
rs234709
rs567754
rs6859667
rs3797535
rs558133
All − MR Egger
All − IVW(MRE)
−0.3
0.0
0.3
0.6
MR effect size for
'Selenium' on 'C−X−C motif chemokine 11'

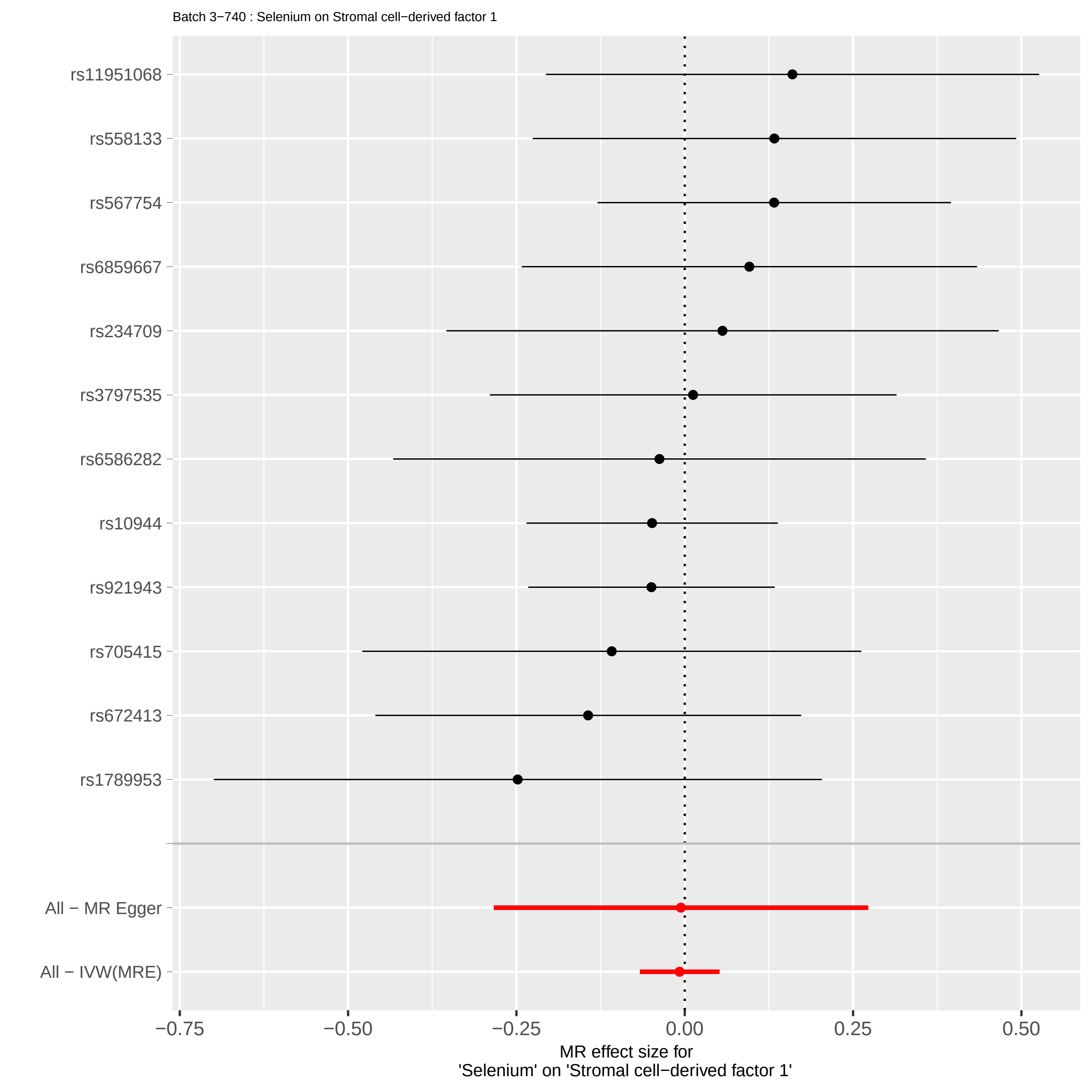
Batch 3−740 : Selenium on Stromal cell−derived factor 1
rs11951068
rs558133
rs567754
rs6859667
rs234709
rs3797535
rs6586282
rs10944
rs921943
rs705415
rs672413
rs1789953
All − MR Egger
All − IVW(MRE)
−0.75
−0.50
−0.25
0.00
0.25
0.50
MR effect size for
'Selenium' on 'Stromal cell−derived factor 1'

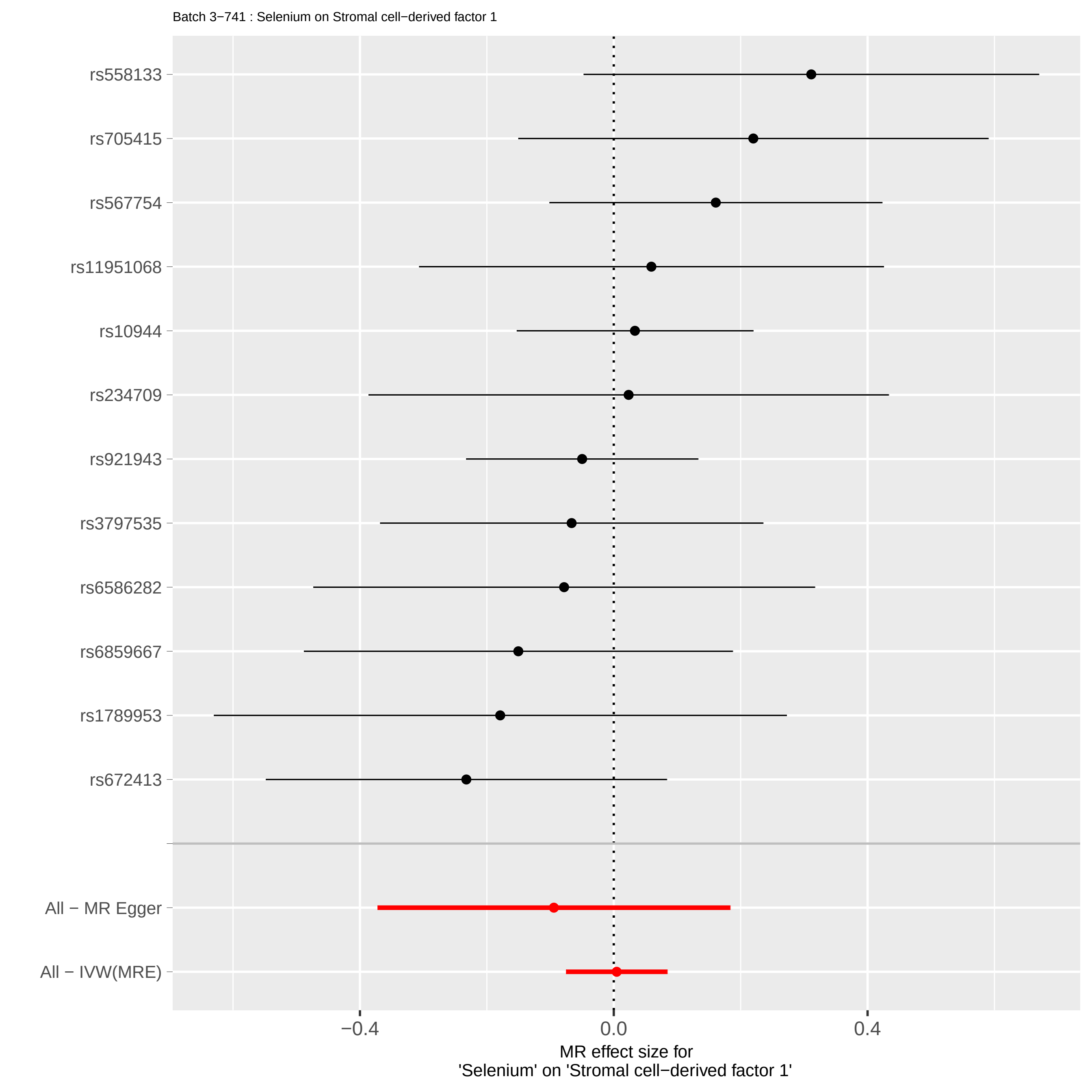
Batch 3−741 : Selenium on Stromal cell−derived factor 1
rs558133
rs705415
rs567754
rs11951068
rs10944
rs234709
rs921943
rs3797535
rs6586282
rs6859667
rs1789953
rs672413
All − MR Egger
All − IVW(MRE)
−0.4
0.0
0.4
MR effect size for
'Selenium' on 'Stromal cell−derived factor 1'

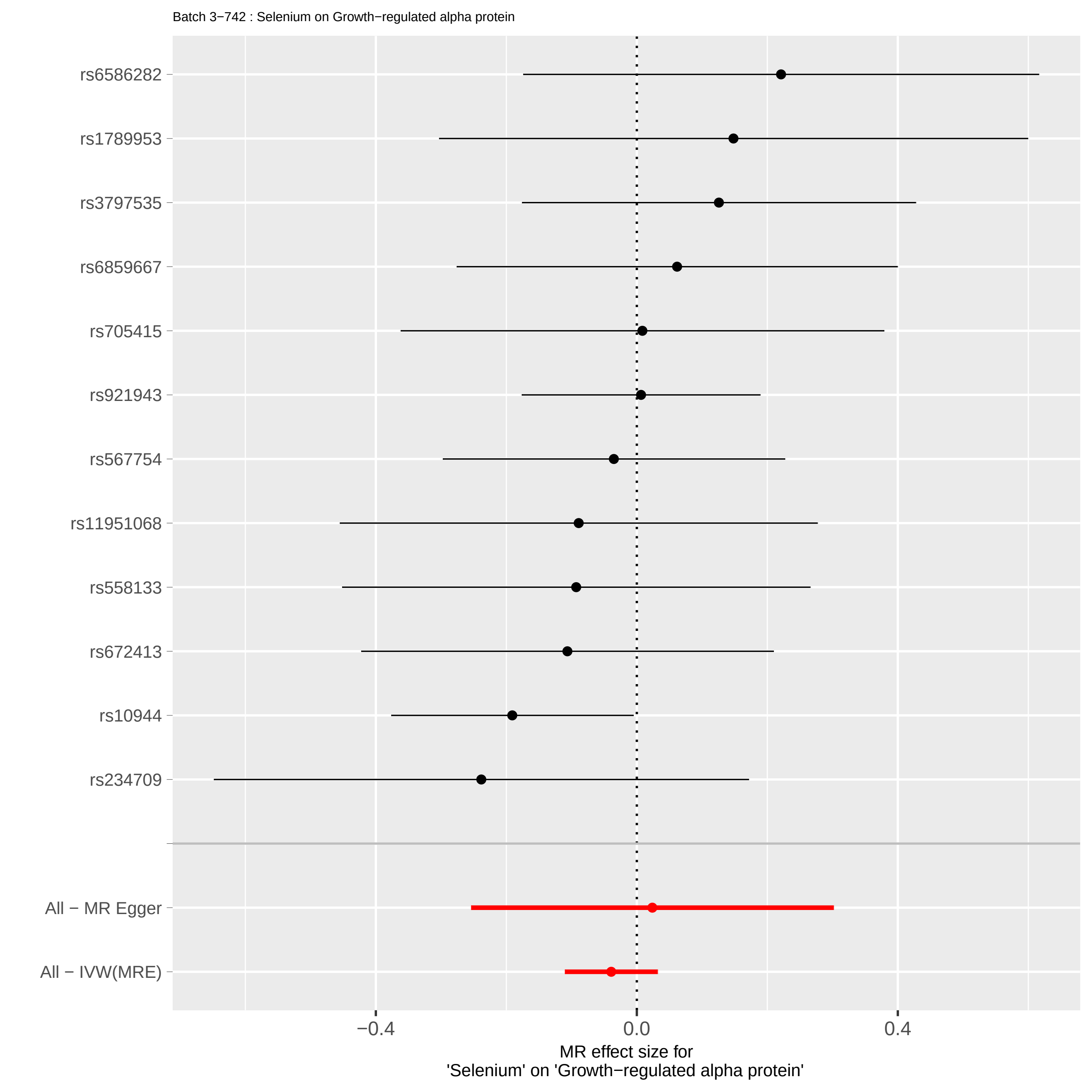
Batch 3−742 : Selenium on Growth−regulated alpha protein
rs6586282
rs1789953
rs3797535
rs6859667
rs705415
rs921943
rs567754
rs11951068
rs558133
rs672413
rs10944
rs234709
All − MR Egger
All − IVW(MRE)
−0.4
0.0
0.4
MR effect size for
'Selenium' on 'Growth−regulated alpha protein'

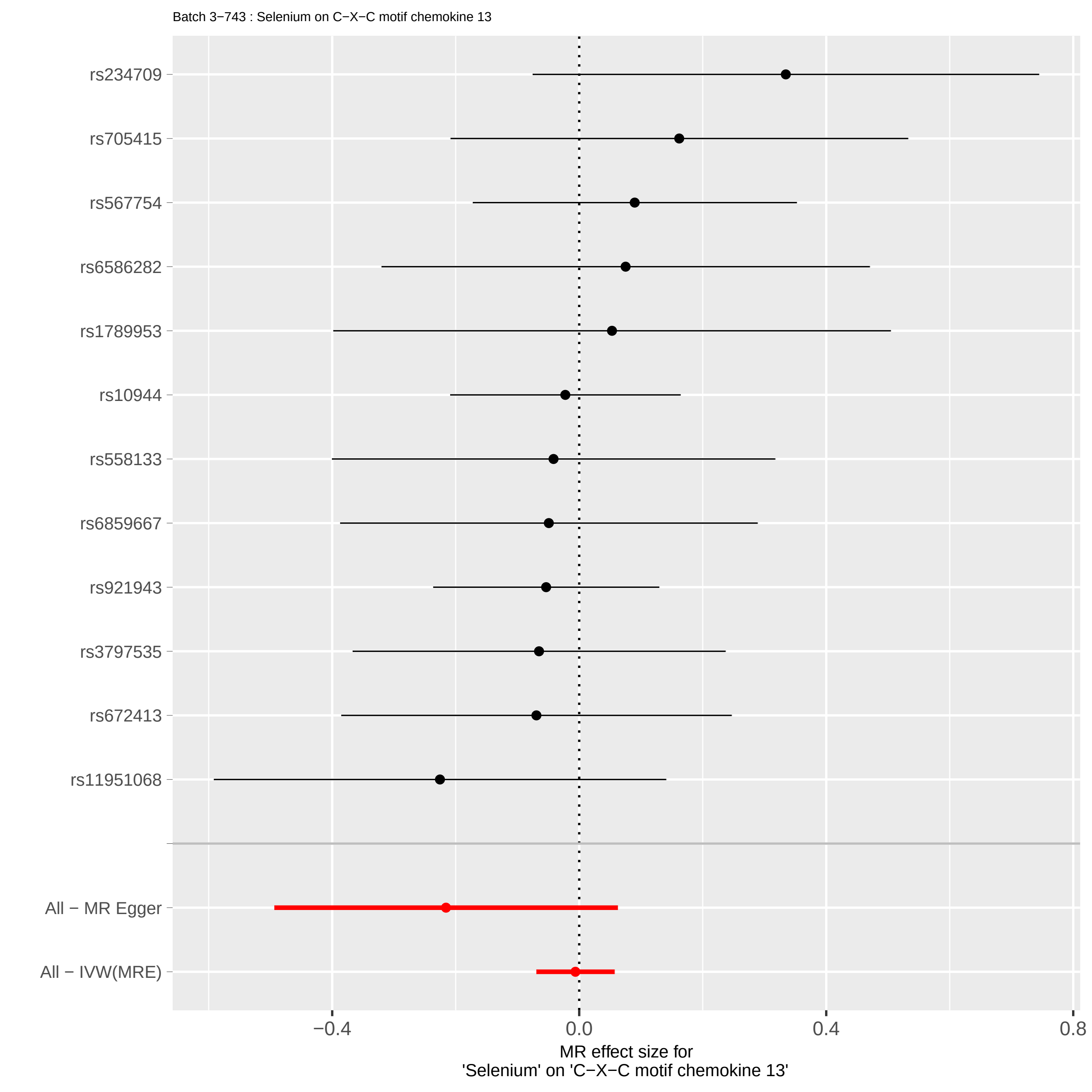

Batch 3−743 : Selenium on C−X−C motif chemokine 13
rs234709
rs705415
rs567754
rs6586282
rs1789953
rs10944
rs558133
rs6859667
rs921943
rs3797535
rs672413
rs11951068
All − MR Egger
All − IVW(MRE)
−0.4
0.0
0.4
0.8
MR effect size for
'Selenium' on 'C−X−C motif chemokine 13'

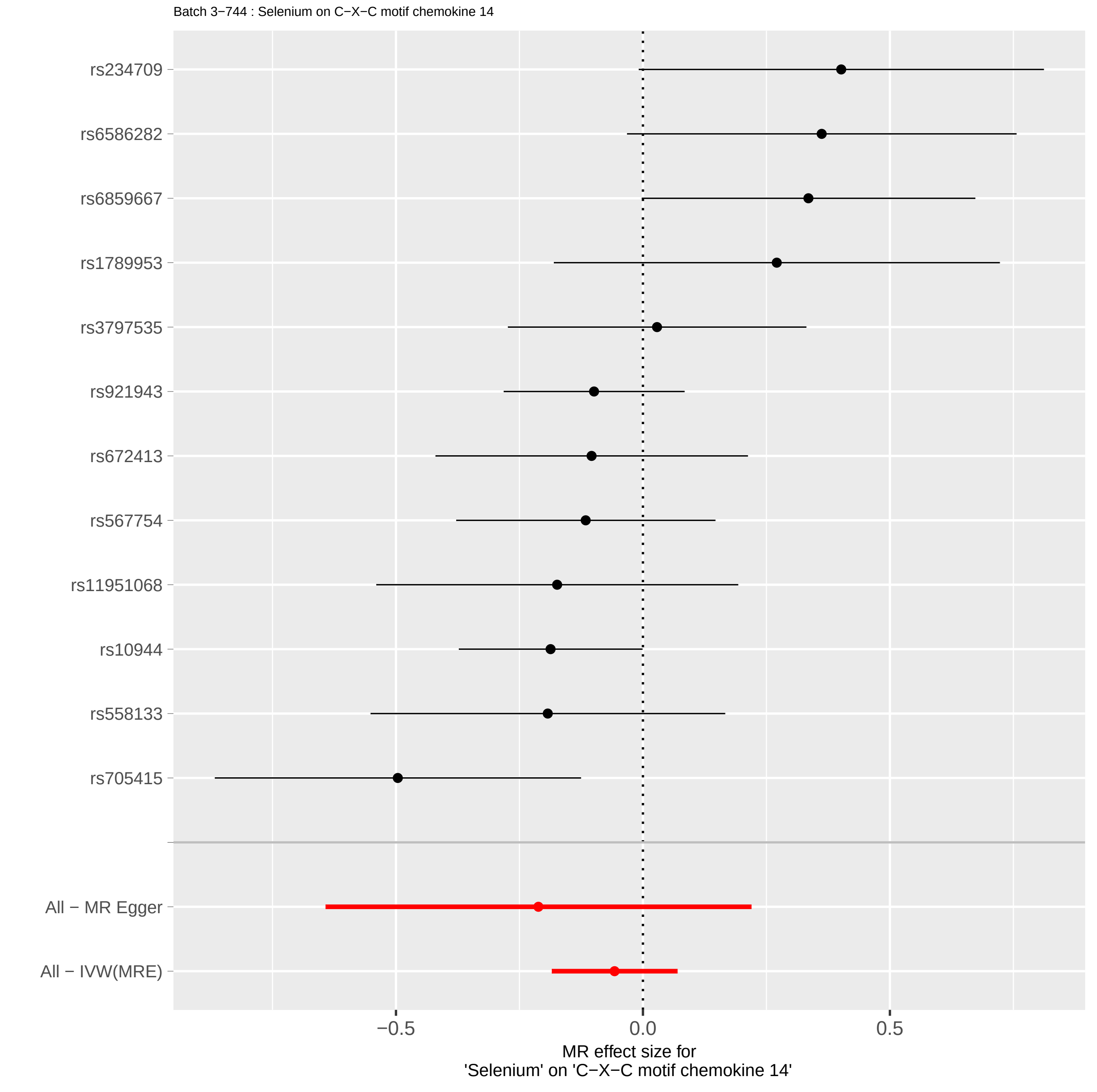
Batch 3−744 : Selenium on C−X−C motif chemokine 14
rs234709
rs6586282
rs6859667
rs1789953
rs3797535
rs921943
rs672413
rs567754
rs11951068
rs10944
rs558133
rs705415
All − MR Egger
All − IVW(MRE)
−0.5
0.0
0.5
MR effect size for
'Selenium' on 'C−X−C motif chemokine 14'

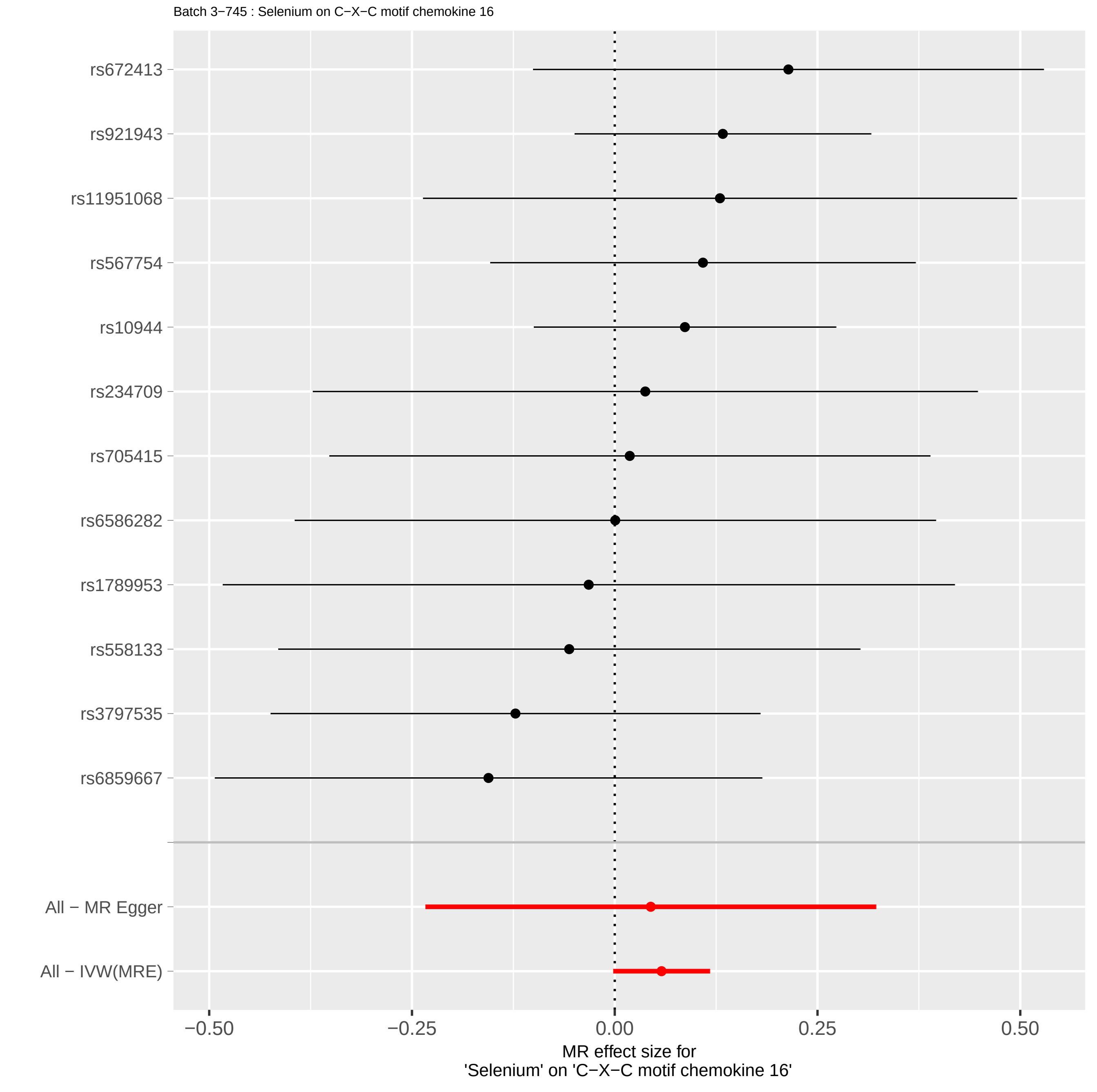

Batch 3−745 : Selenium on C−X−C motif chemokine 16
rs672413
rs921943
rs11951068
rs567754
rs10944
rs234709
rs705415
rs6586282
rs1789953
rs558133
rs3797535
rs6859667
All − MR Egger
All − IVW(MRE)
−0.50
−0.25
0.00
0.25
0.50
MR effect size for
'Selenium' on 'C−X−C motif chemokine 16'

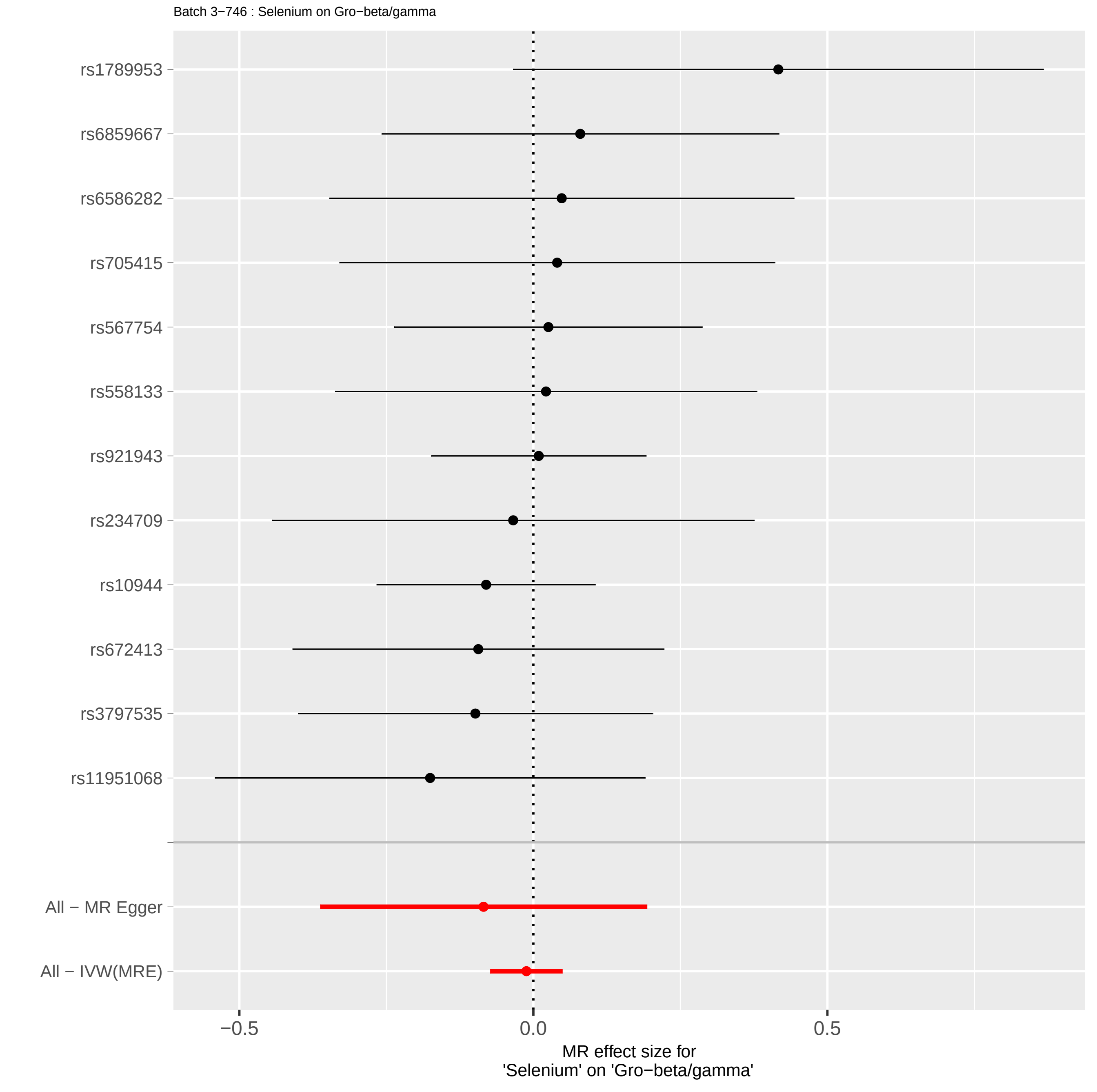

Batch 3−746 : Selenium on Gro−beta/gamma
rs1789953
rs6859667
rs6586282
rs705415
rs567754
rs558133
rs921943
rs234709
rs10944
rs672413
rs3797535
rs11951068
All − MR Egger
All − IVW(MRE)
−0.5
0.0
0.5
MR effect size for
'Selenium' on 'Gro−beta/gamma'

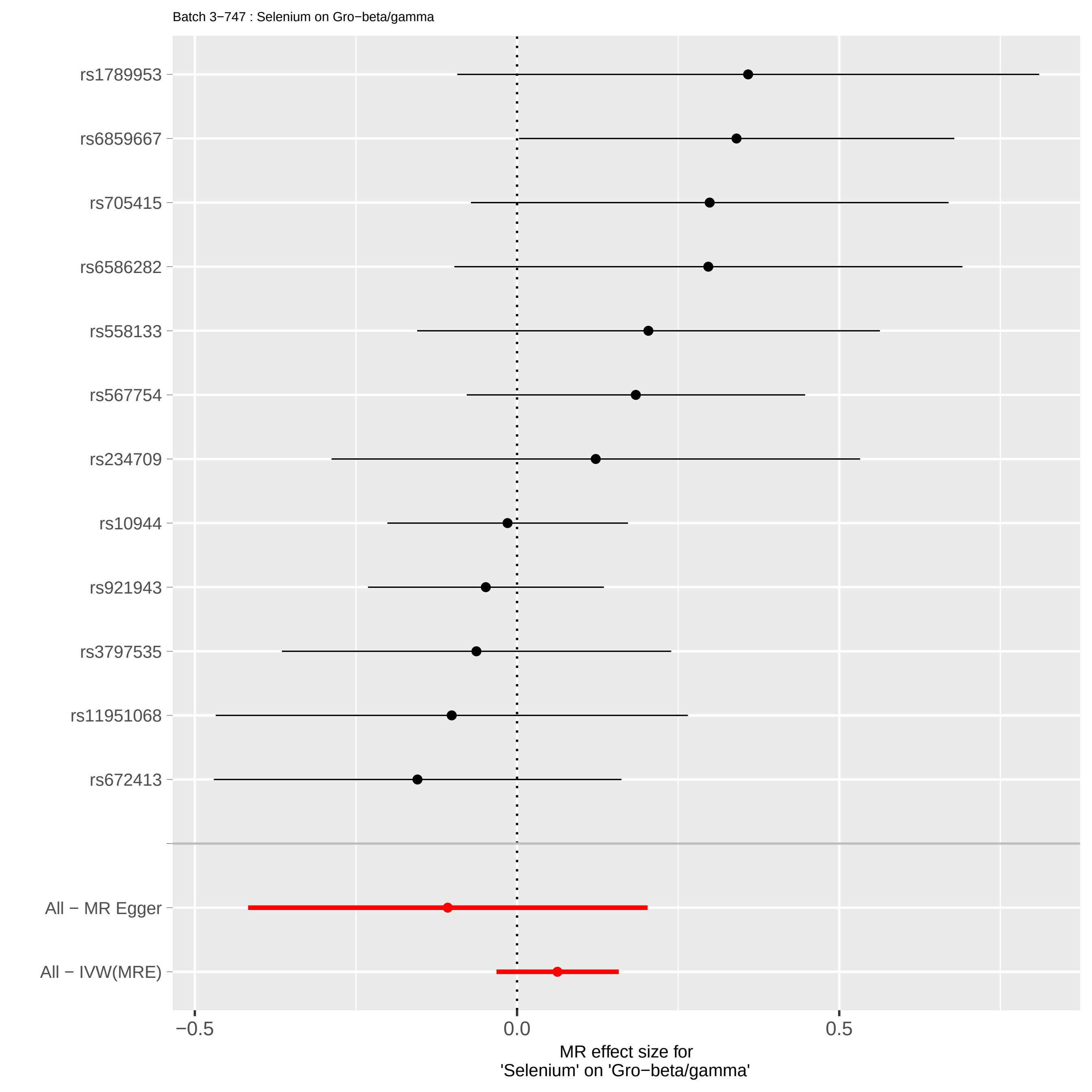
Batch 3−747 : Selenium on Gro−beta/gamma
rs1789953
rs6859667
rs705415
rs6586282
rs558133
rs567754
rs234709
rs10944
rs921943
rs3797535
rs11951068
rs672413
All − MR Egger
All − IVW(MRE)
−0.5
0.0
0.5
MR effect size for
'Selenium' on 'Gro−beta/gamma'

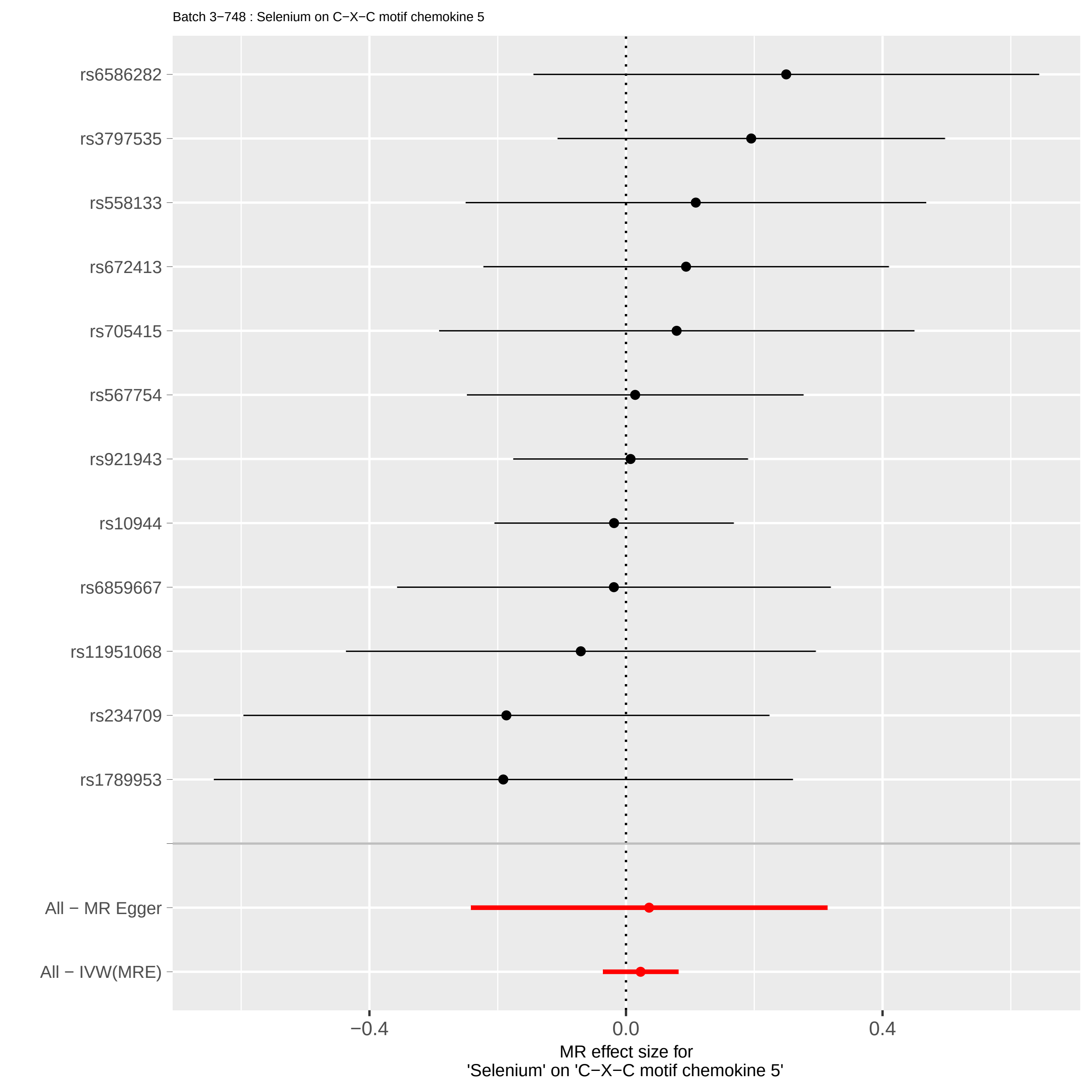

Batch 3−748 : Selenium on C−X−C motif chemokine 5
rs6586282
rs3797535
rs558133
rs672413
rs705415
rs567754
rs921943
rs10944
rs6859667
rs11951068
rs234709
rs1789953
All − MR Egger
All − IVW(MRE)
−0.4
0.0
0.4
MR effect size for
'Selenium' on 'C−X−C motif chemokine 5'

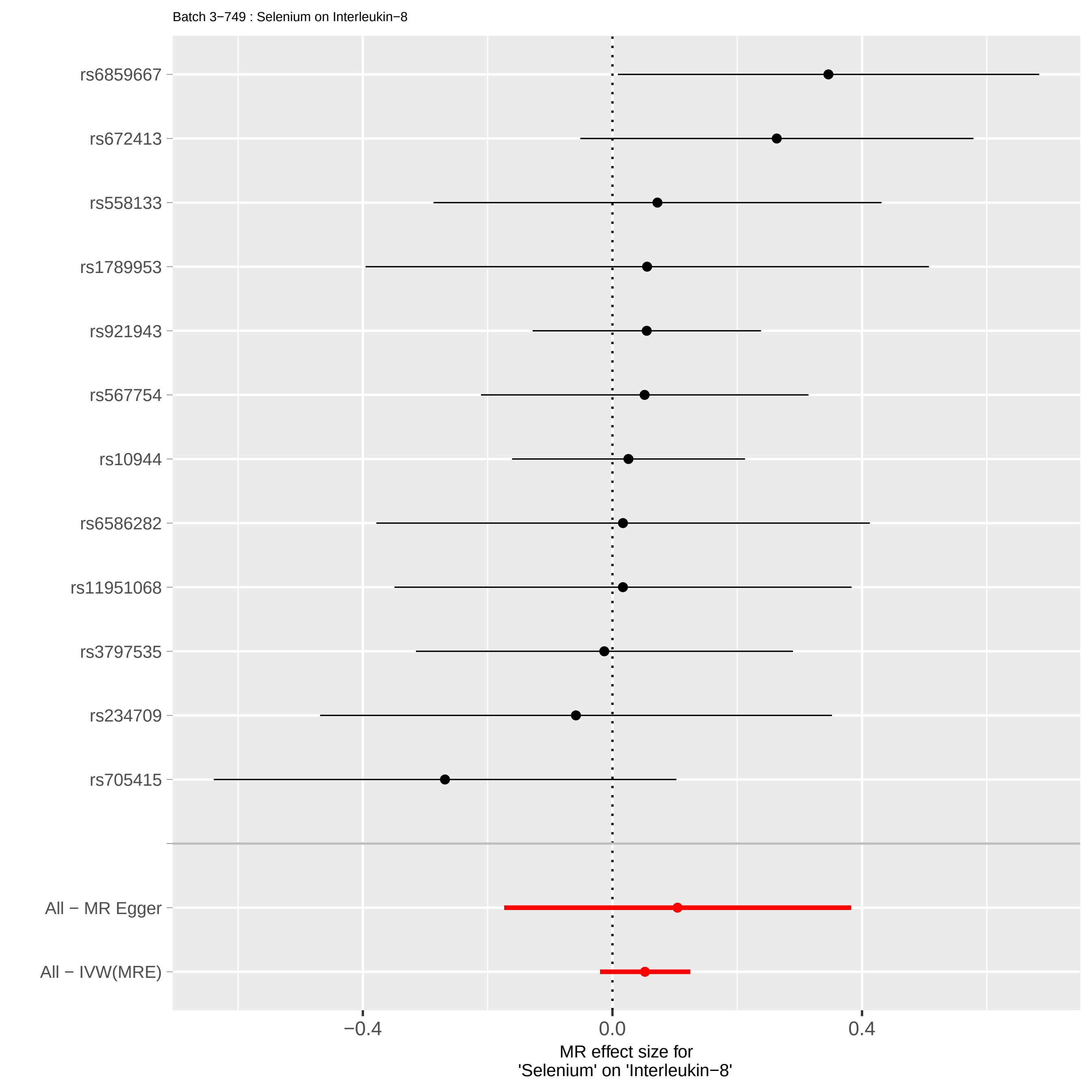
Batch 3−749 : Selenium on Interleukin−8
rs6859667
rs672413
rs558133
rs1789953
rs921943
rs567754
rs10944
rs6586282
rs11951068
rs3797535
rs234709
rs705415
All − MR Egger
All − IVW(MRE)
−0.4
0.0
0.4
MR effect size for
'Selenium' on 'Interleukin−8'

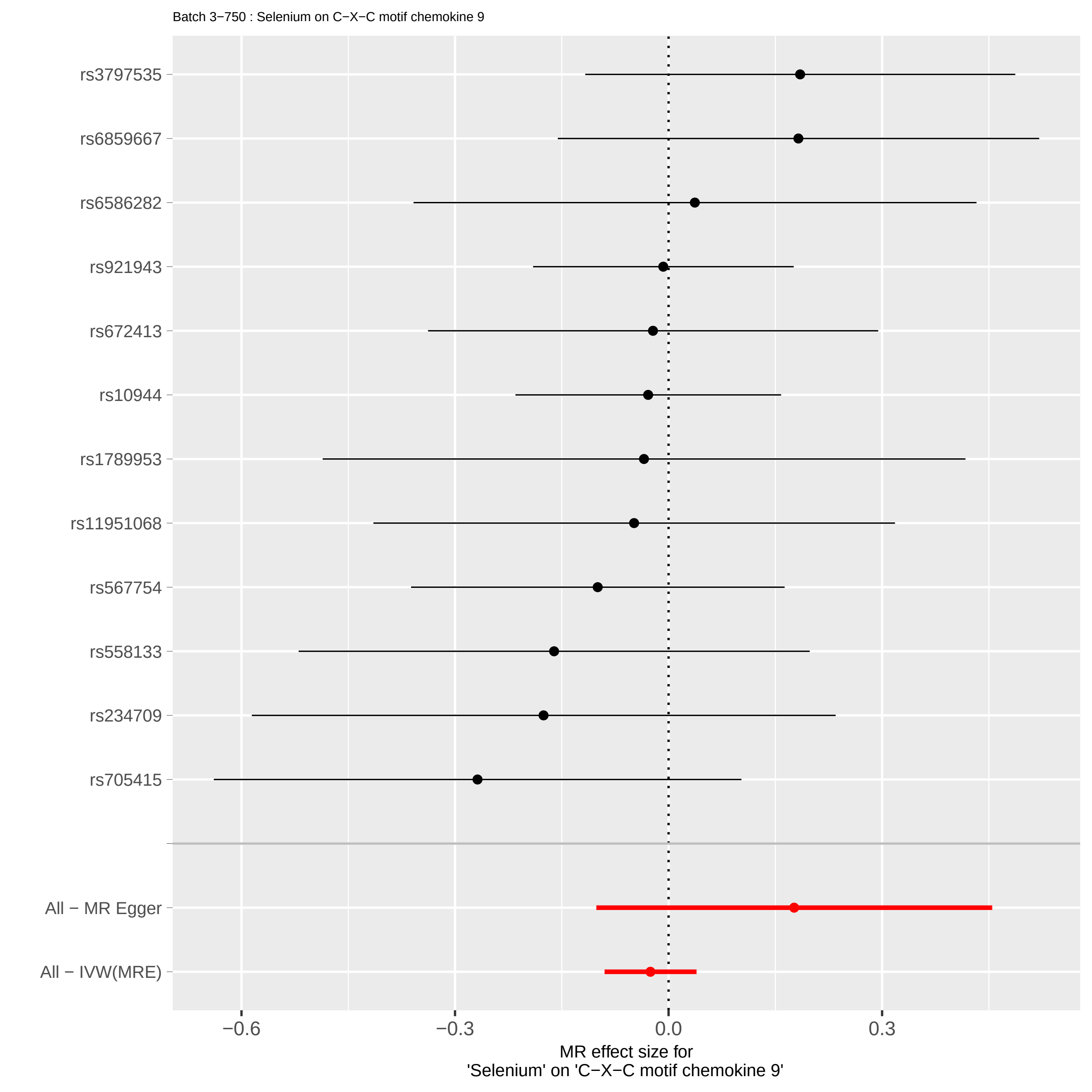
Batch 3−750 : Selenium on C−X−C motif chemokine 9
rs3797535
rs6859667
rs6586282
rs921943
rs672413
rs10944
rs1789953
rs11951068
rs567754
rs558133
rs234709
rs705415
All − MR Egger
All − IVW(MRE)
−0.6
−0.3
0.0
0.3
MR effect size for
'Selenium' on 'C−X−C motif chemokine 9'

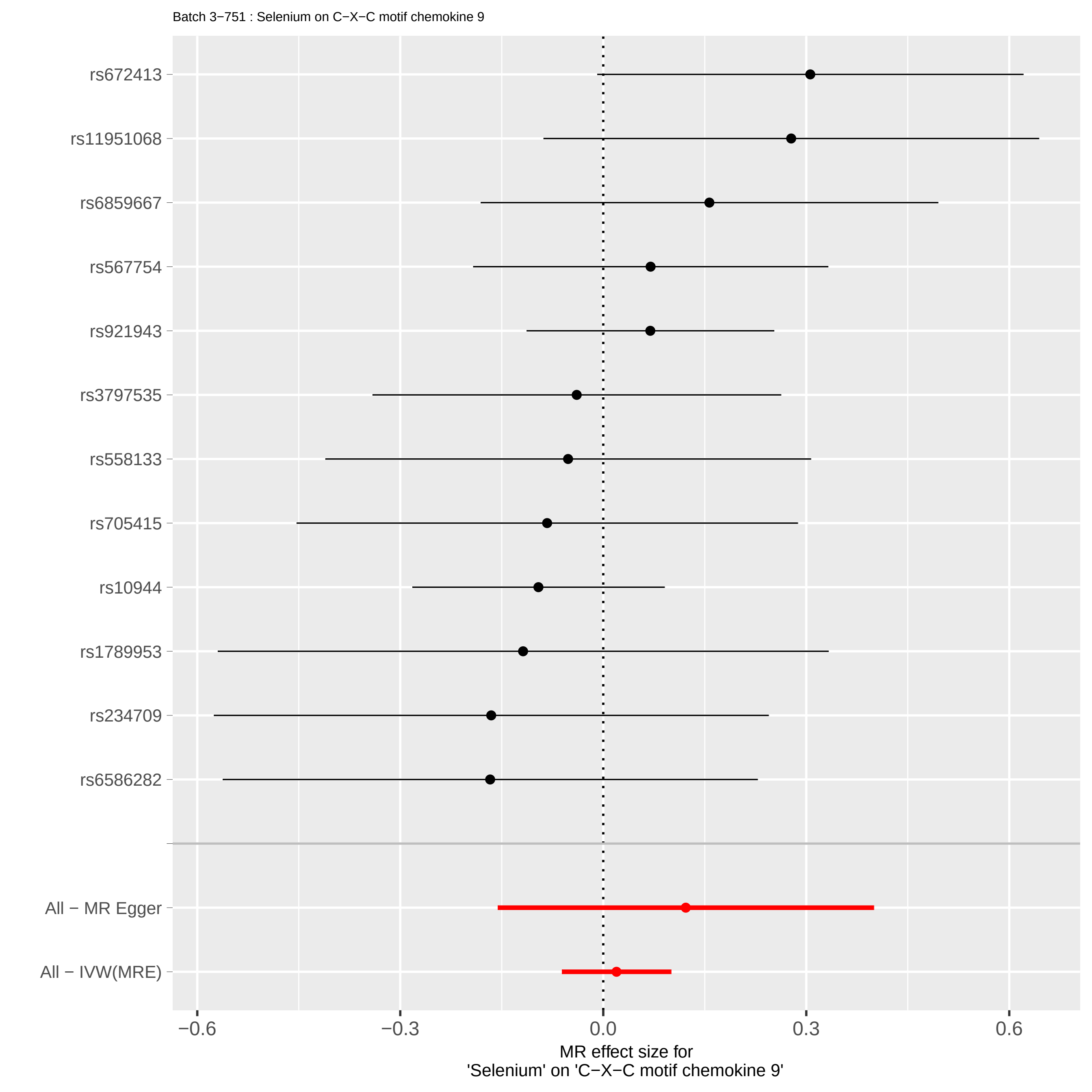
Batch 3−751 : Selenium on C−X−C motif chemokine 9
rs672413
rs11951068
rs6859667
rs567754
rs921943
rs3797535
rs558133
rs705415
rs10944
rs1789953
rs234709
rs6586282
All − MR Egger
All − IVW(MRE)
−0.6
−0.3
0.0
0.3
0.6
MR effect size for
'Selenium' on 'C−X−C motif chemokine 9'

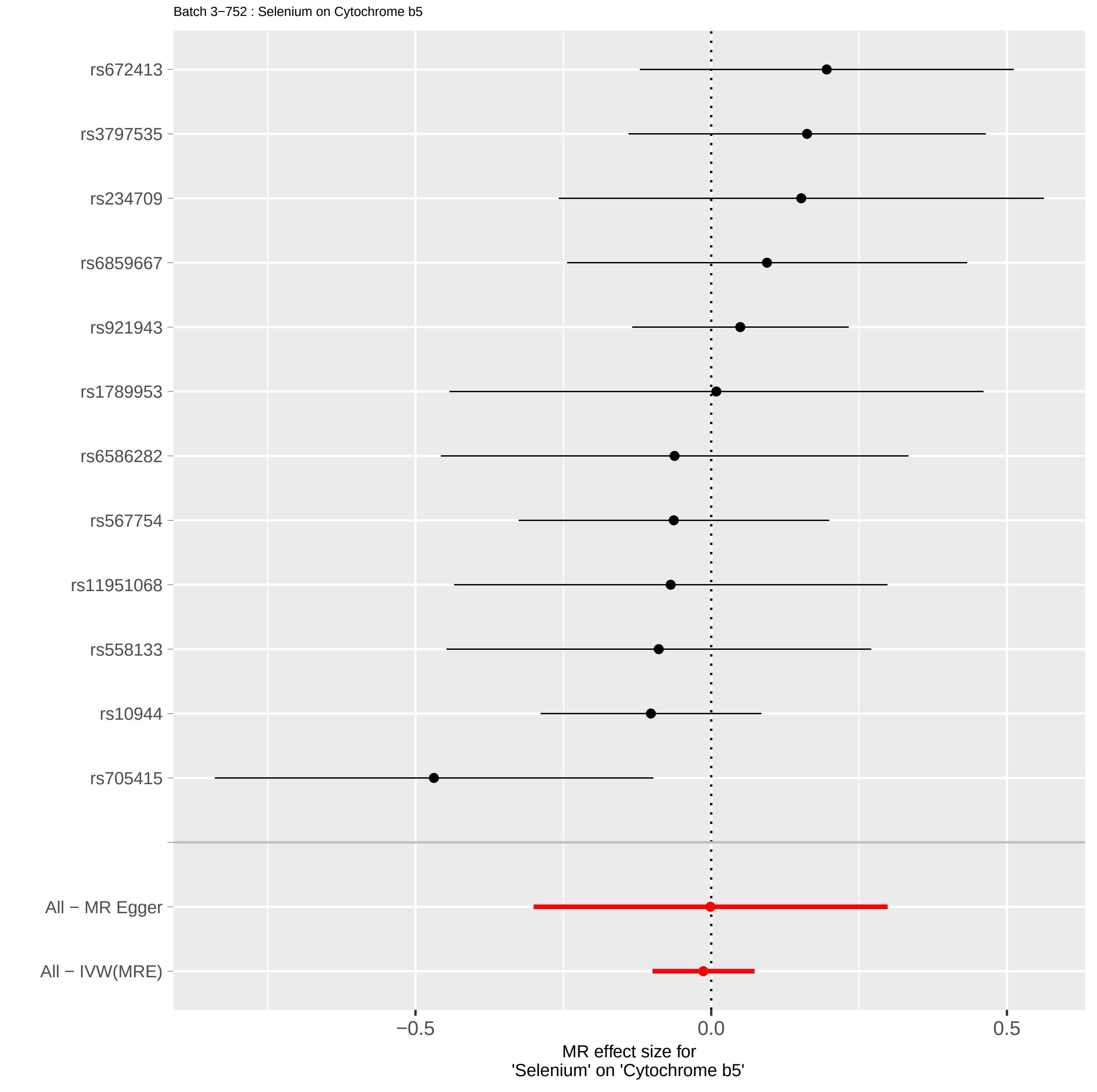

Batch 3−752 : Selenium on Cytochrome b5
rs672413
rs3797535
rs234709
rs6859667
rs921943
rs1789953
rs6586282
rs567754
rs11951068
rs558133
rs10944
rs705415
All − MR Egger
All − IVW(MRE)
−0.5
0.0
0.5
MR effect size for
'Selenium' on 'Cytochrome b5'

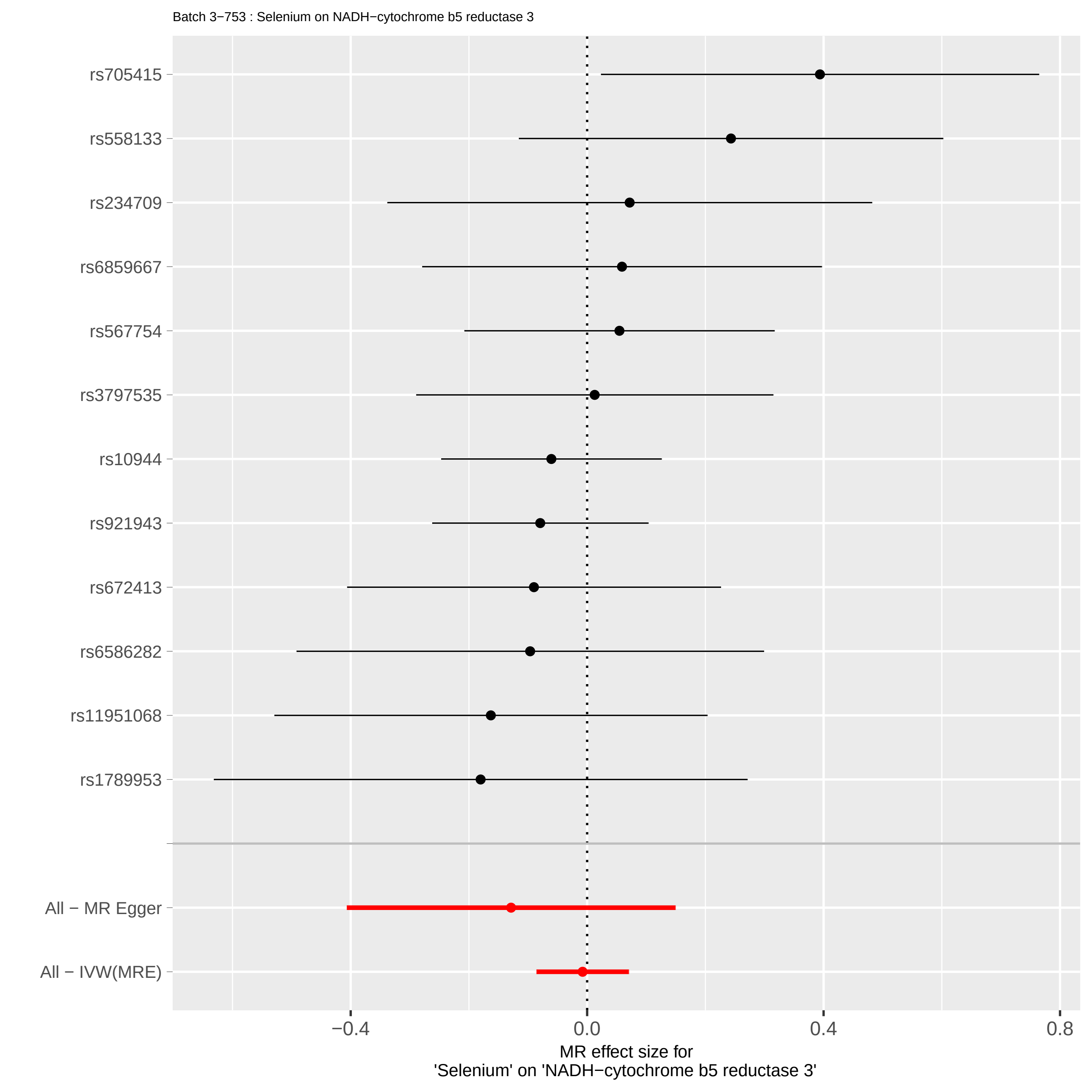

Batch 3−753 : Selenium on NADH−cytochrome b5 reductase 3
rs705415
rs558133
rs234709
rs6859667
rs567754
rs3797535
rs10944
rs921943
rs672413
rs6586282
rs11951068
rs1789953
All − MR Egger
All − IVW(MRE)
−0.4
0.0
0.4
0.8
MR effect size for
'Selenium' on 'NADH−cytochrome b5 reductase 3'

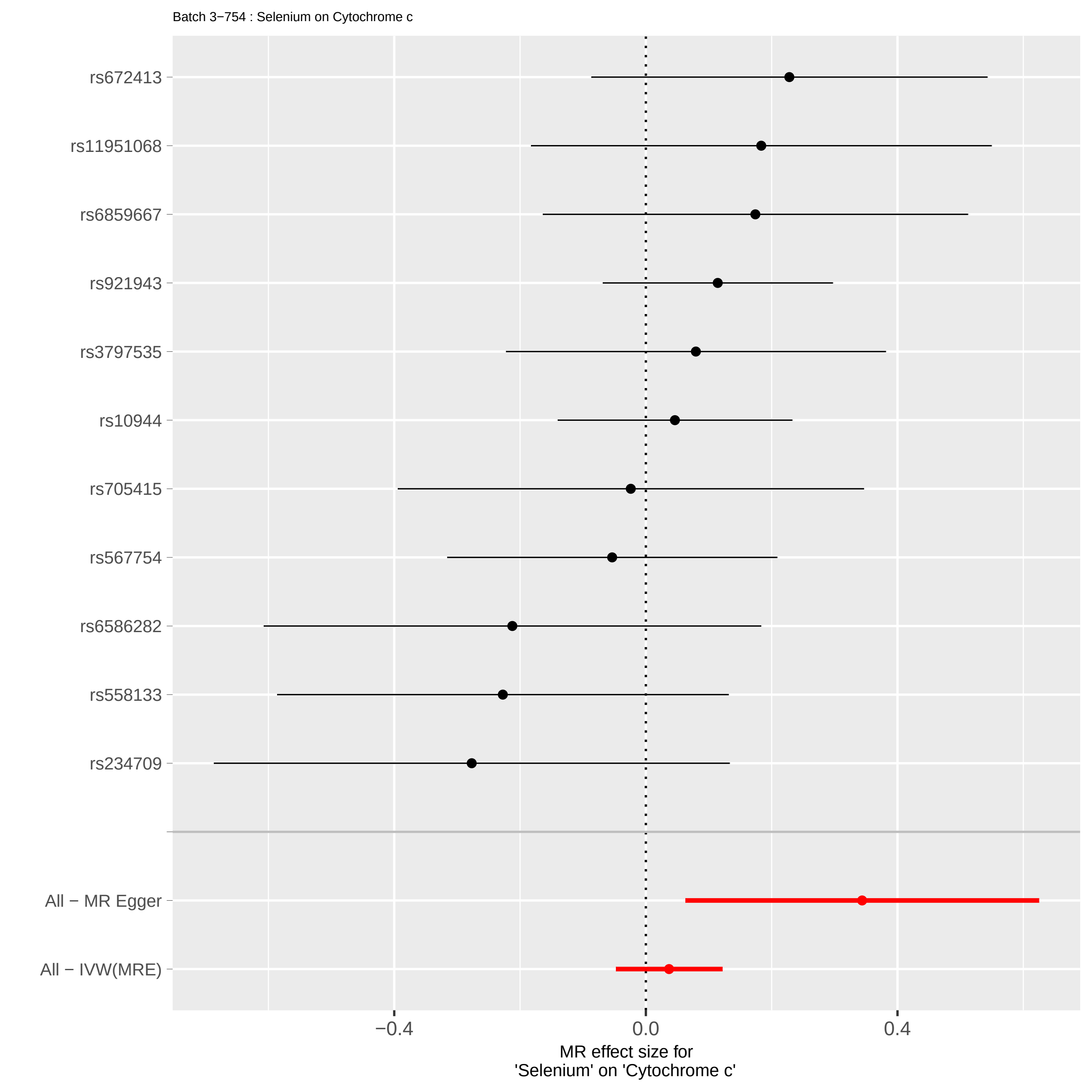

Batch 3−754 : Selenium on Cytochrome c
rs672413
rs11951068
rs6859667
rs921943
rs3797535
rs10944
rs705415
rs567754
rs6586282
rs558133
rs234709
All − MR Egger
All − IVW(MRE)
−0.4
0.0
0.4
MR effect size for
'Selenium' on 'Cytochrome c'

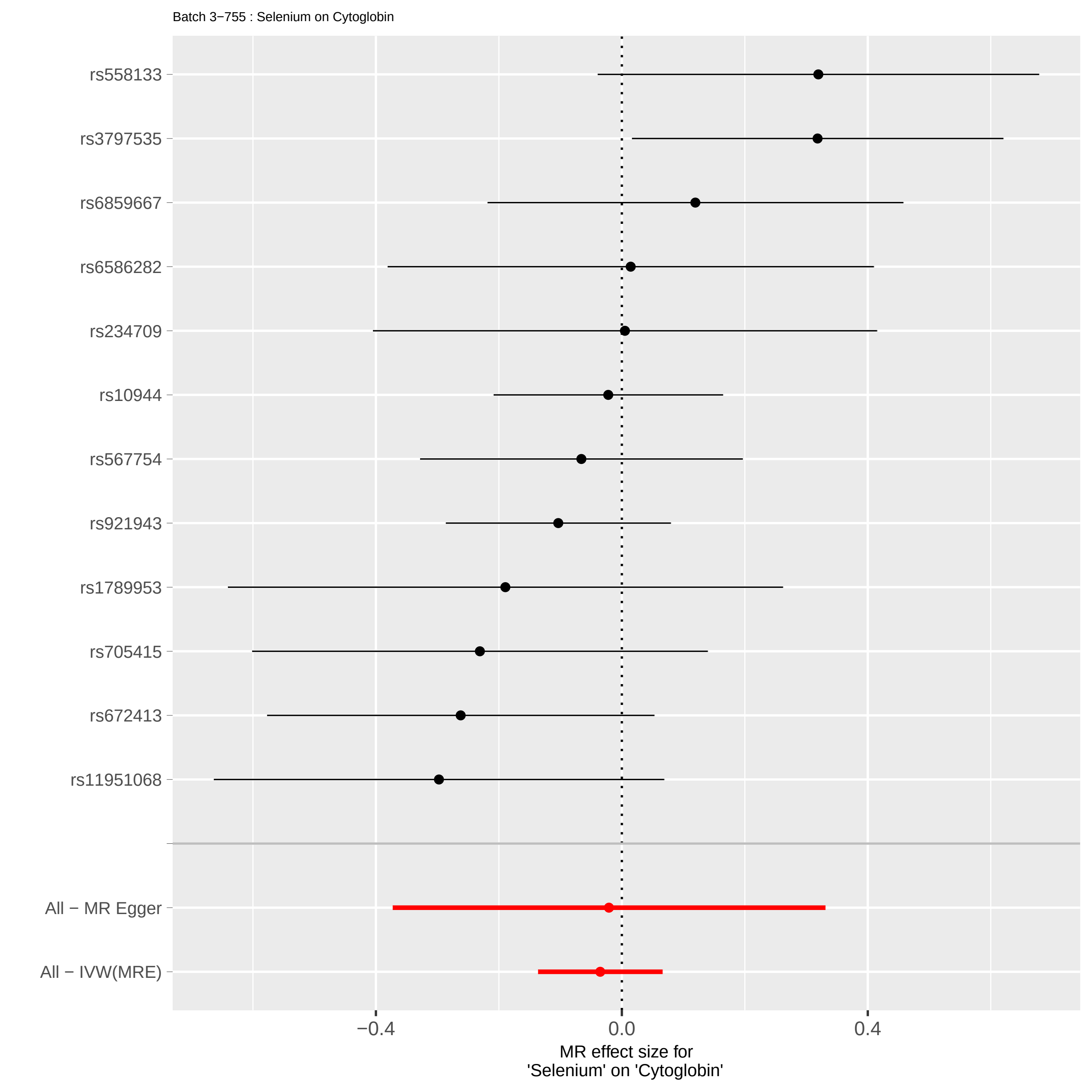

Batch 3−755 : Selenium on Cytoglobin
rs558133
rs3797535
rs6859667
rs6586282
rs234709
rs10944
rs567754
rs921943
rs1789953
rs705415
rs672413
rs11951068
All − MR Egger
All − IVW(MRE)
−0.4
0.0
0.4
MR effect size for
'Selenium' on 'Cytoglobin'

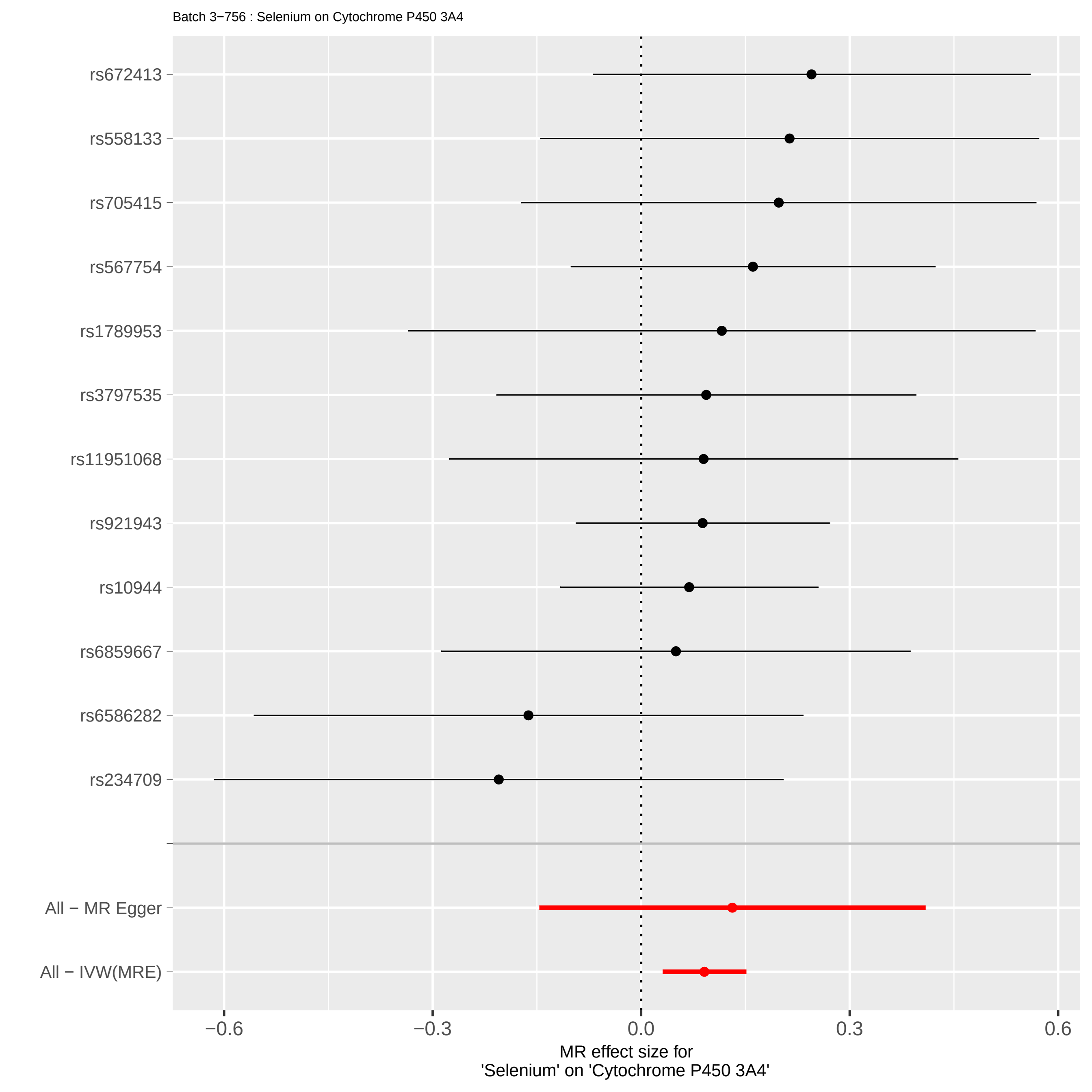
Batch 3−756 : Selenium on Cytochrome P450 3A4
rs672413
rs558133
rs705415
rs567754
rs1789953
rs3797535
rs11951068
rs921943
rs10944
rs6859667
rs6586282
rs234709
All − MR Egger
All − IVW(MRE)
−0.6
−0.3
0.0
0.3
0.6
MR effect size for
'Selenium' on 'Cytochrome P450 3A4'

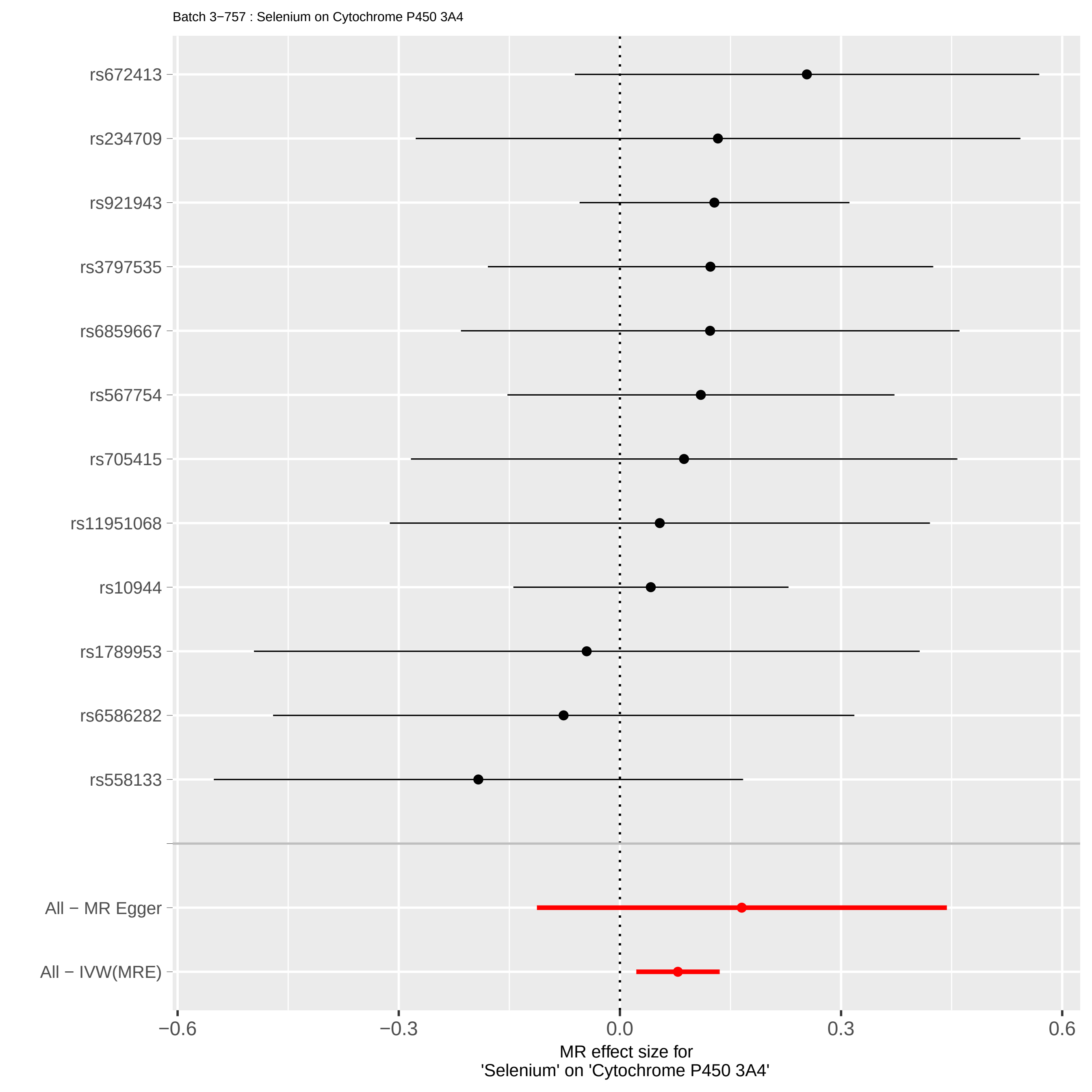

Batch 3−757 : Selenium on Cytochrome P450 3A4
rs672413
rs234709
rs921943
rs3797535
rs6859667
rs567754
rs705415
rs11951068
rs10944
rs1789953
rs6586282
rs558133
All − MR Egger
All − IVW(MRE)
−0.6
−0.3
0.0
0.3
0.6
MR effect size for
'Selenium' on 'Cytochrome P450 3A4'

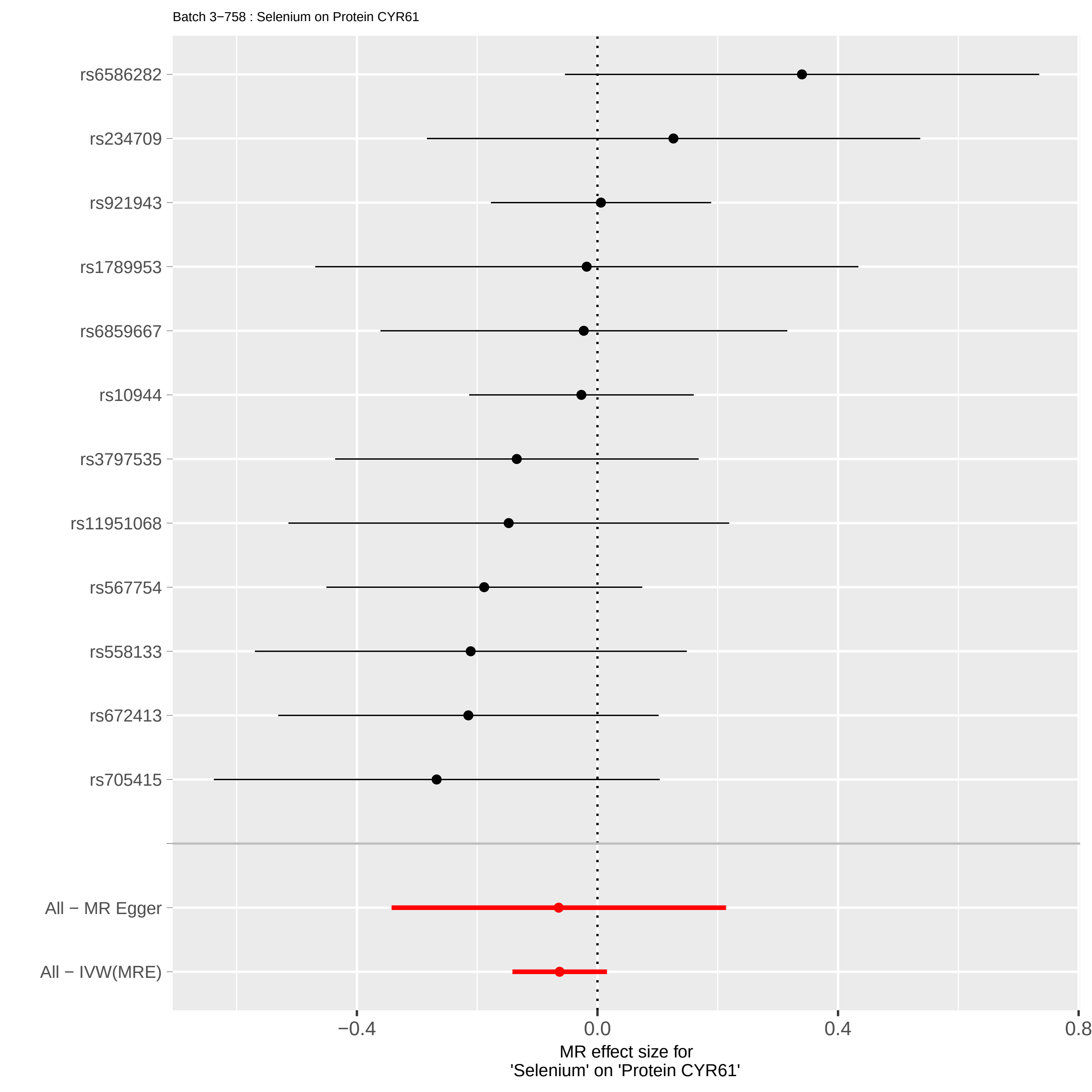
Batch 3−758 : Selenium on Protein CYR61
rs6586282
rs234709
rs921943
rs1789953
rs6859667
rs10944
rs3797535
rs11951068
rs567754
rs558133
rs672413
rs705415
All − MR Egger
All − IVW(MRE)
−0.4
0.0
0.4
0.8
MR effect size for
'Selenium' on 'Protein CYR61'

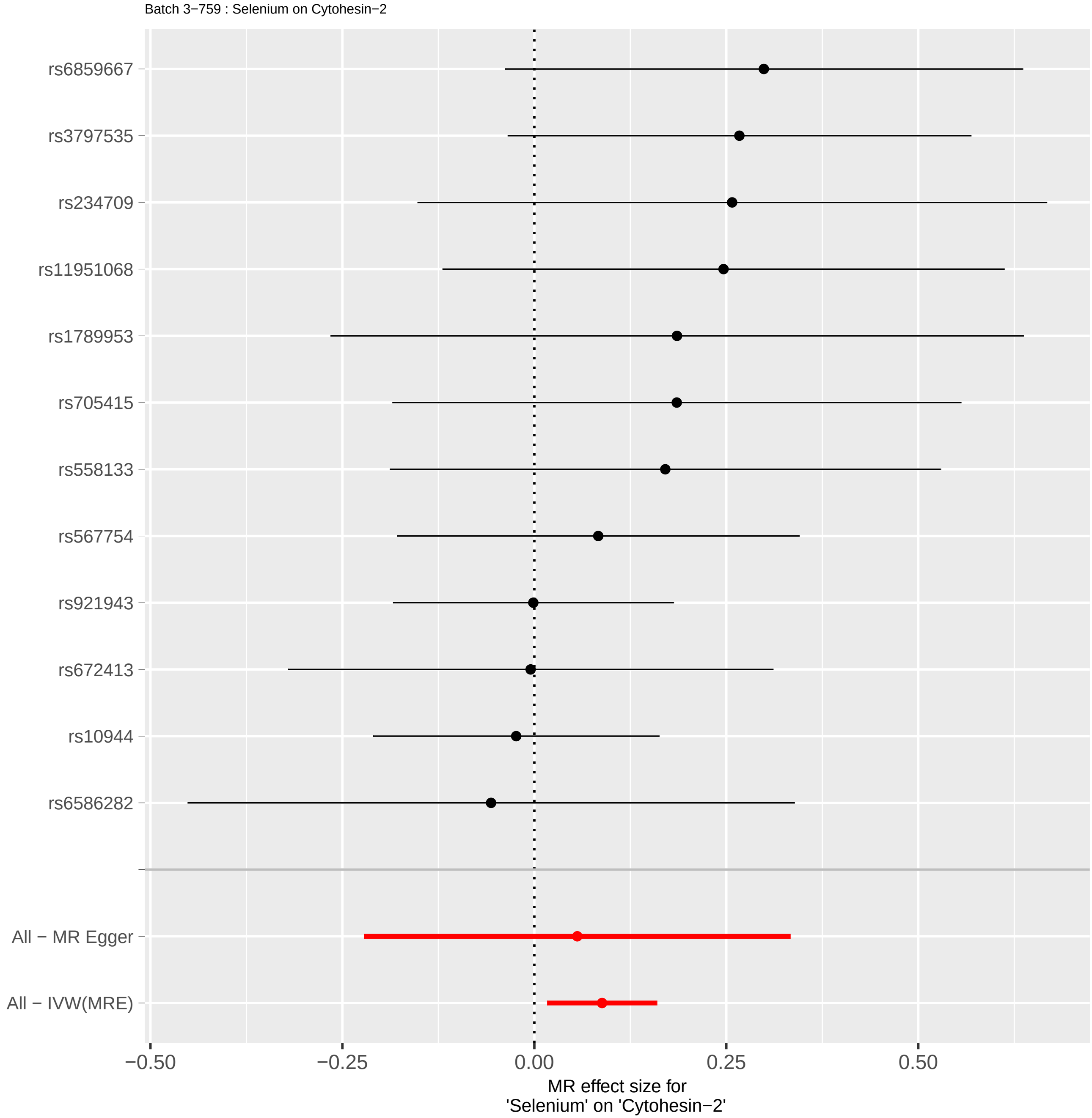

Batch 3−759 : Selenium on Cytohesin−2
rs6859667
rs3797535
rs234709
rs11951068
rs1789953
rs705415
rs558133
rs567754
rs921943
rs672413
rs10944
rs6586282
All − MR Egger
All − IVW(MRE)
−0.50
−0.25
0.00
0.25
0.50
MR effect size for
'Selenium' on 'Cytohesin−2'

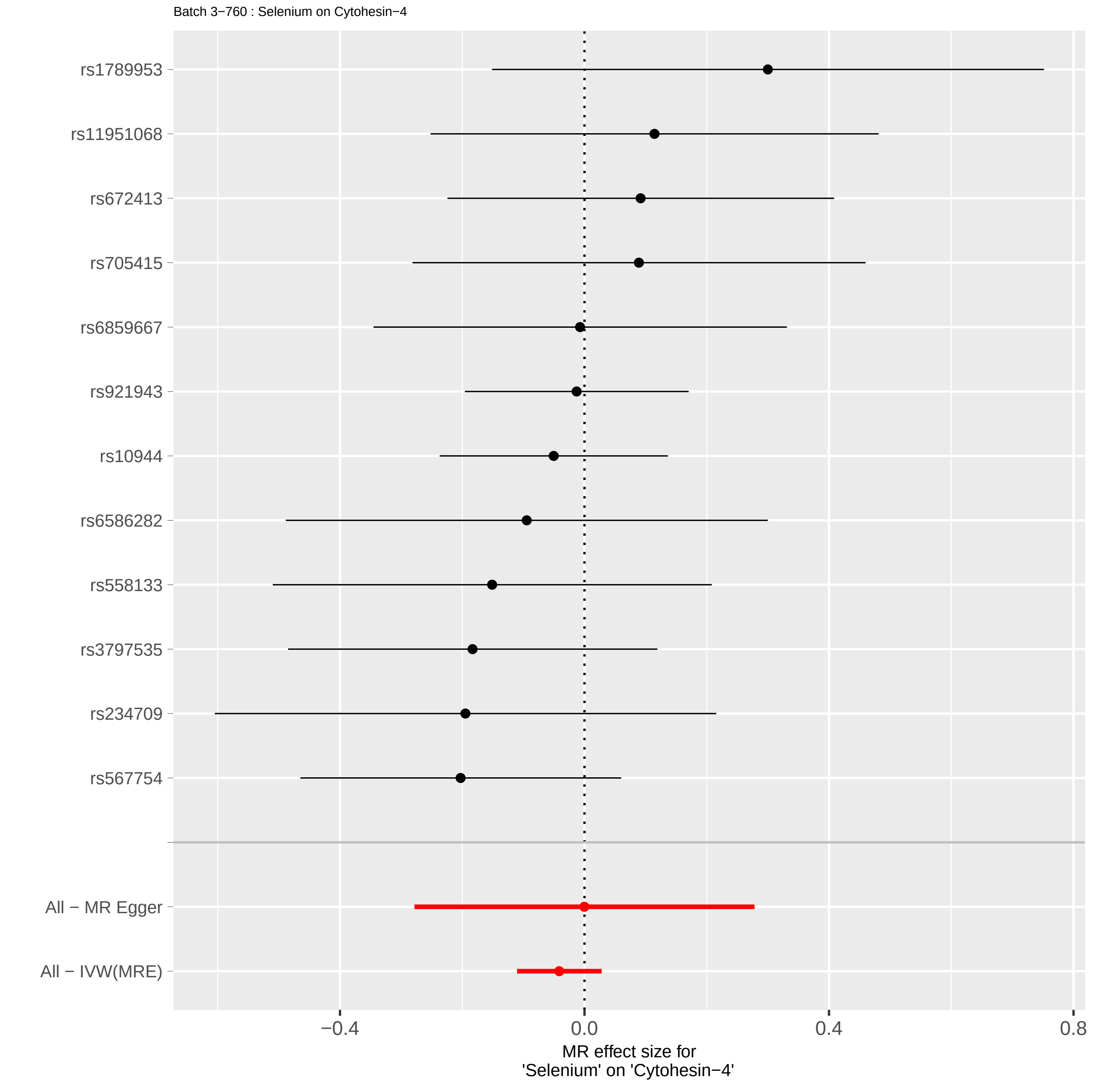

Batch 3−760 : Selenium on Cytohesin−4
rs1789953
rs11951068
rs672413
rs705415
rs6859667
rs921943
rs10944
rs6586282
rs558133
rs3797535
rs234709
rs567754
All − MR Egger
All − IVW(MRE)
−0.4
0.0
0.4
0.8
MR effect size for
'Selenium' on 'Cytohesin−4'

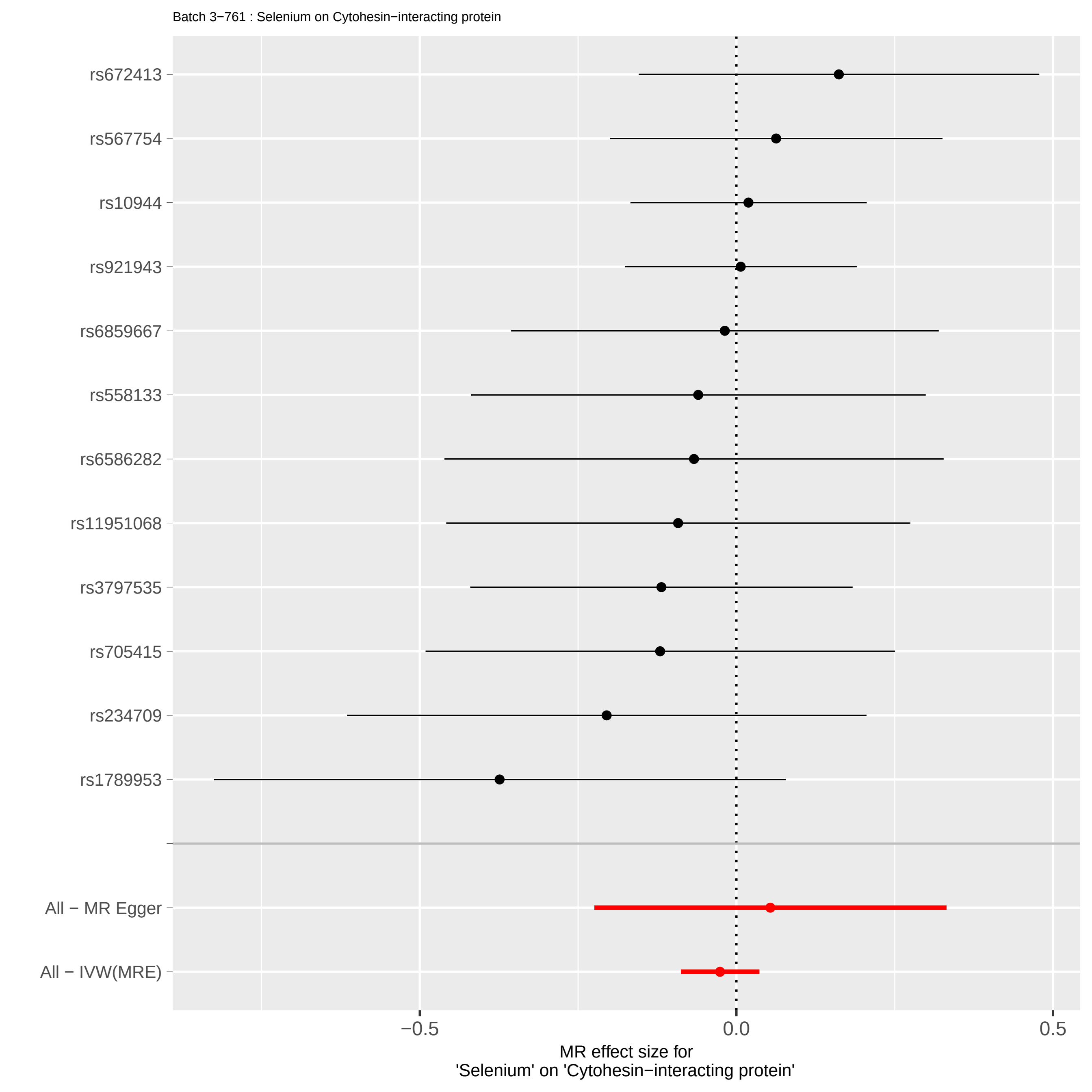
Batch 3−761 : Selenium on Cytohesin−interacting protein
rs672413
rs567754
rs10944
rs921943
rs6859667
rs558133
rs6586282
rs11951068
rs3797535
rs705415
rs234709
rs1789953
All − MR Egger
All − IVW(MRE)
−0.5
0.0
0.5
MR effect size for
'Selenium' on 'Cytohesin−interacting protein'

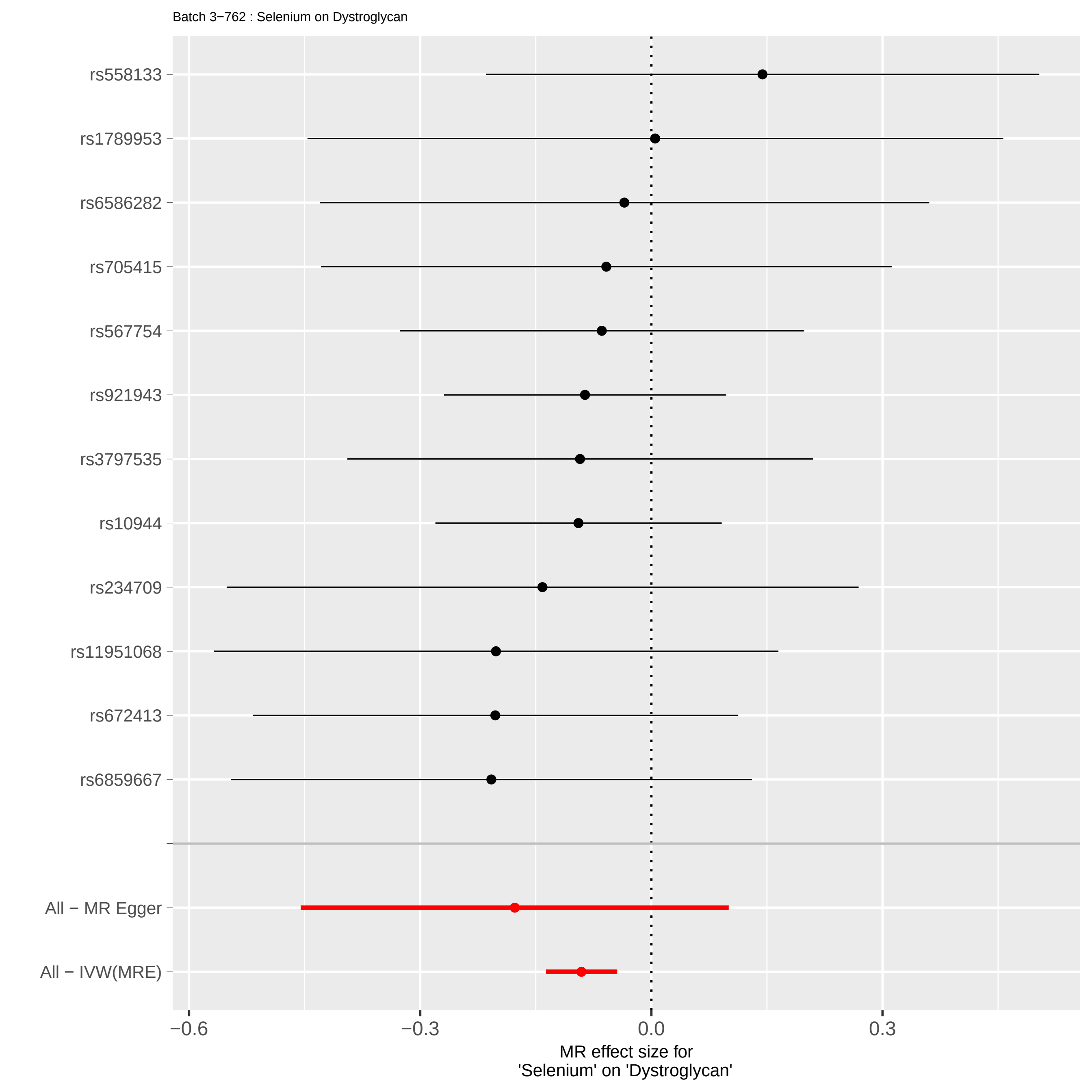

Batch 3−762 : Selenium on Dystroglycan
rs558133
rs1789953
rs6586282
rs705415
rs567754
rs921943
rs3797535
rs10944
rs234709
rs11951068
rs672413
rs6859667
All − MR Egger
All − IVW(MRE)
−0.6
−0.3
0.0
0.3
MR effect size for
'Selenium' on 'Dystroglycan'

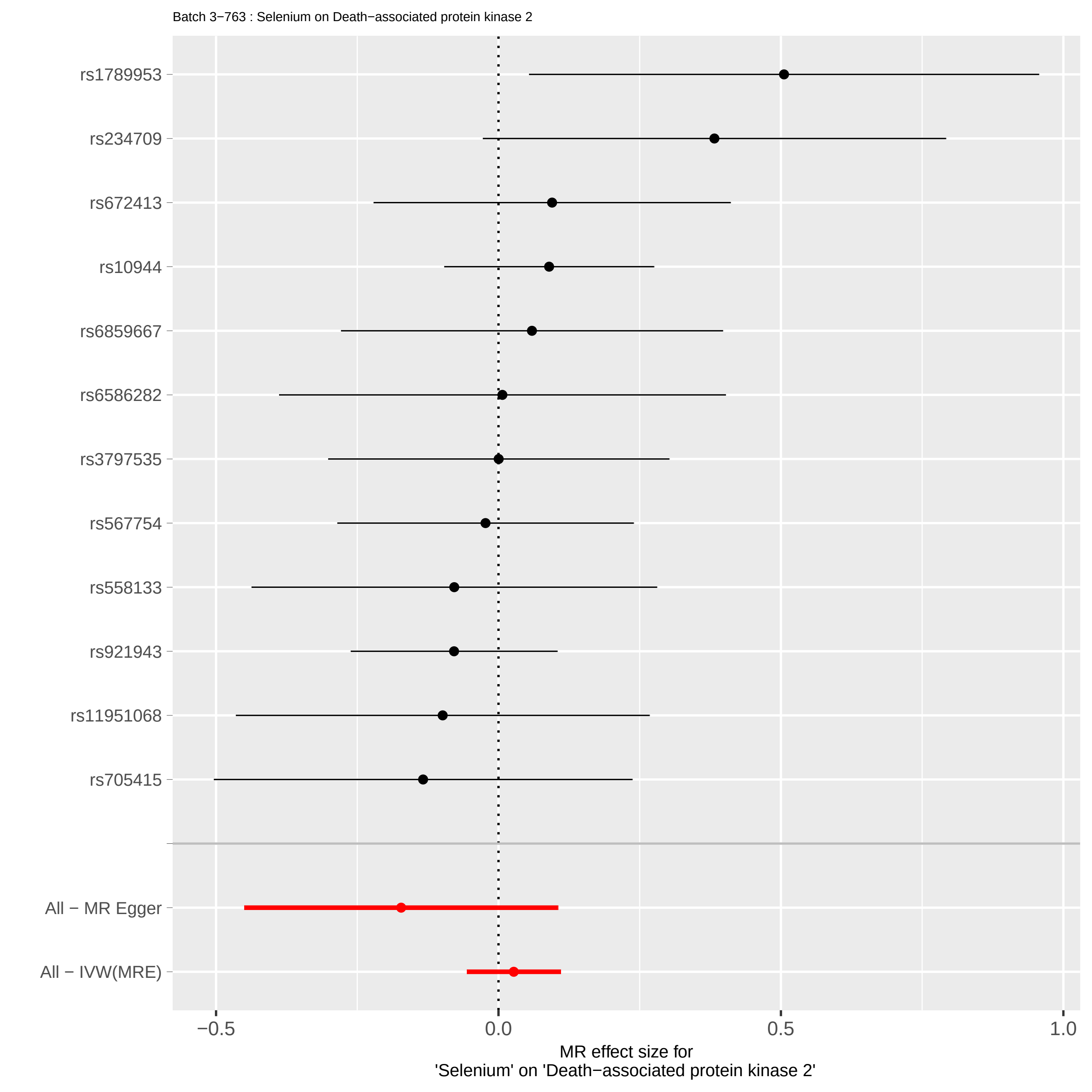

Batch 3−763 : Selenium on Death−associated protein kinase 2
rs1789953
rs234709
rs672413
rs10944
rs6859667
rs6586282
rs3797535
rs567754
rs558133
rs921943
rs11951068
rs705415
All − MR Egger
All − IVW(MRE)
−0.5
0.0
0.5
1.0
MR effect size for
'Selenium' on 'Death−associated protein kinase 2'

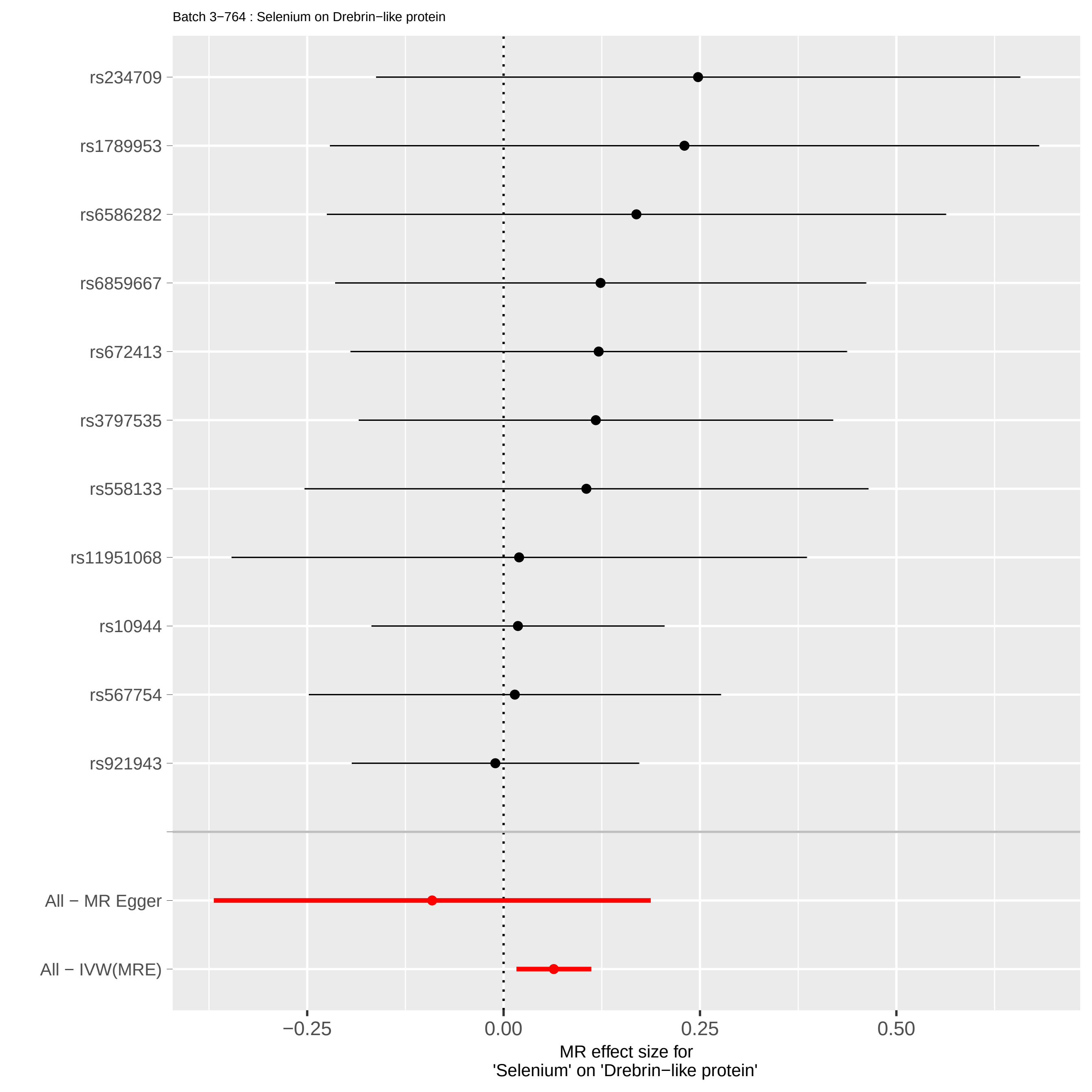
Batch 3−764 : Selenium on Drebrin−like protein
rs234709
rs1789953
rs6586282
rs6859667
rs672413
rs3797535
rs558133
rs11951068
rs10944
rs567754
rs921943
All − MR Egger
All − IVW(MRE)
−0.25
0.00
0.25
0.50
MR effect size for
'Selenium' on 'Drebrin−like protein'

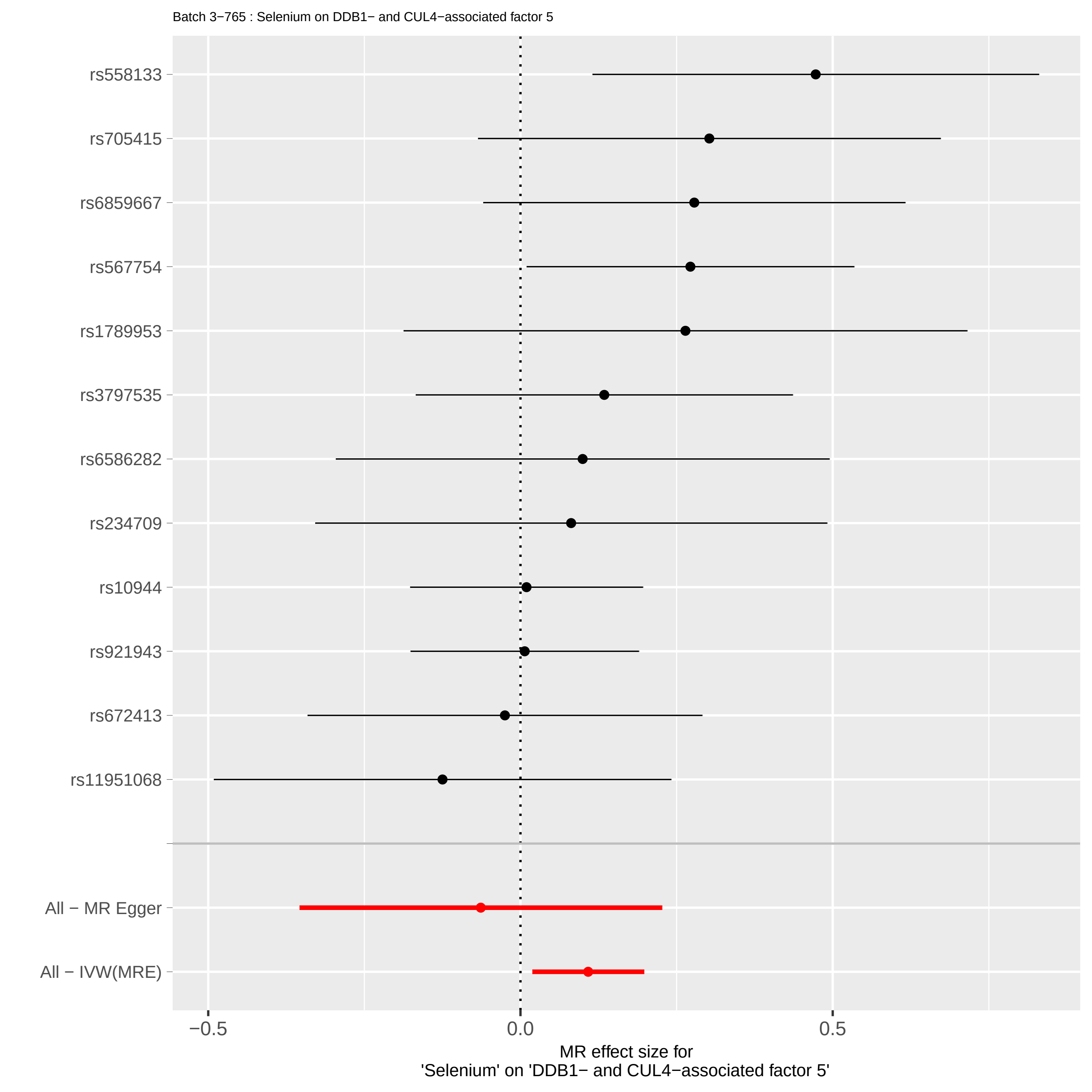
Batch 3−765 : Selenium on DDB1− and CUL4−associated factor 5
rs558133
rs705415
rs6859667
rs567754
rs1789953
rs3797535
rs6586282
rs234709
rs10944
rs921943
rs672413
rs11951068
All − MR Egger
All − IVW(MRE)
−0.5
0.0
0.5
MR effect size for
'Selenium' on 'DDB1− and CUL4−associated factor 5'

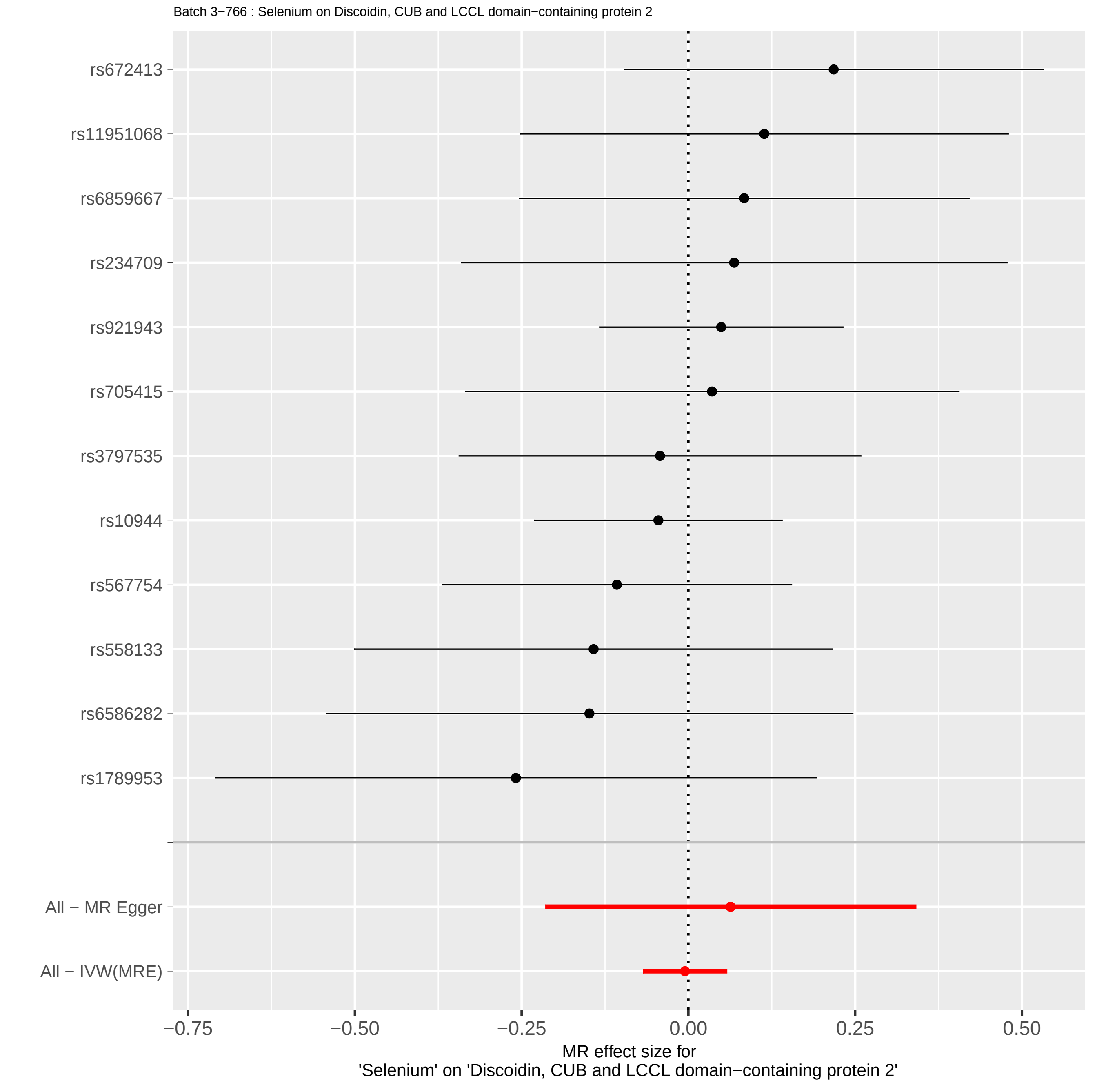
Batch 3−766 : Selenium on Discoidin, CUB and LCCL domain−containing protein 2
rs672413
rs11951068
rs6859667
rs234709
rs921943
rs705415
rs3797535
rs10944
rs567754
rs558133
rs6586282
rs1789953
All − MR Egger
All − IVW(MRE)
−0.75
−0.50
−0.25
0.00
0.25
0.50
MR effect size for
'Selenium' on 'Discoidin, CUB and LCCL domain−containing protein 2'

Batch 3−767 : Selenium on Deoxycytidine kinase

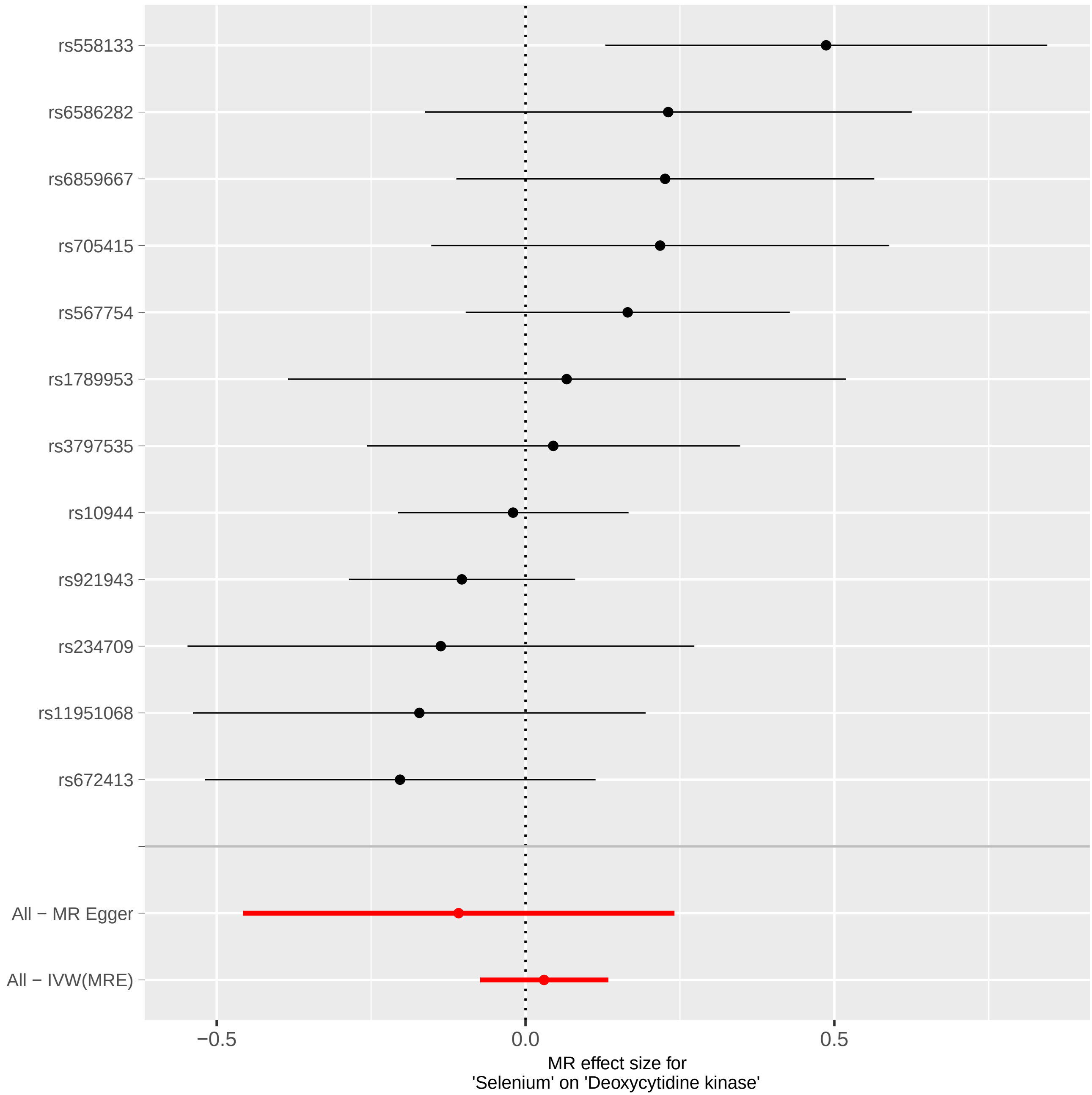

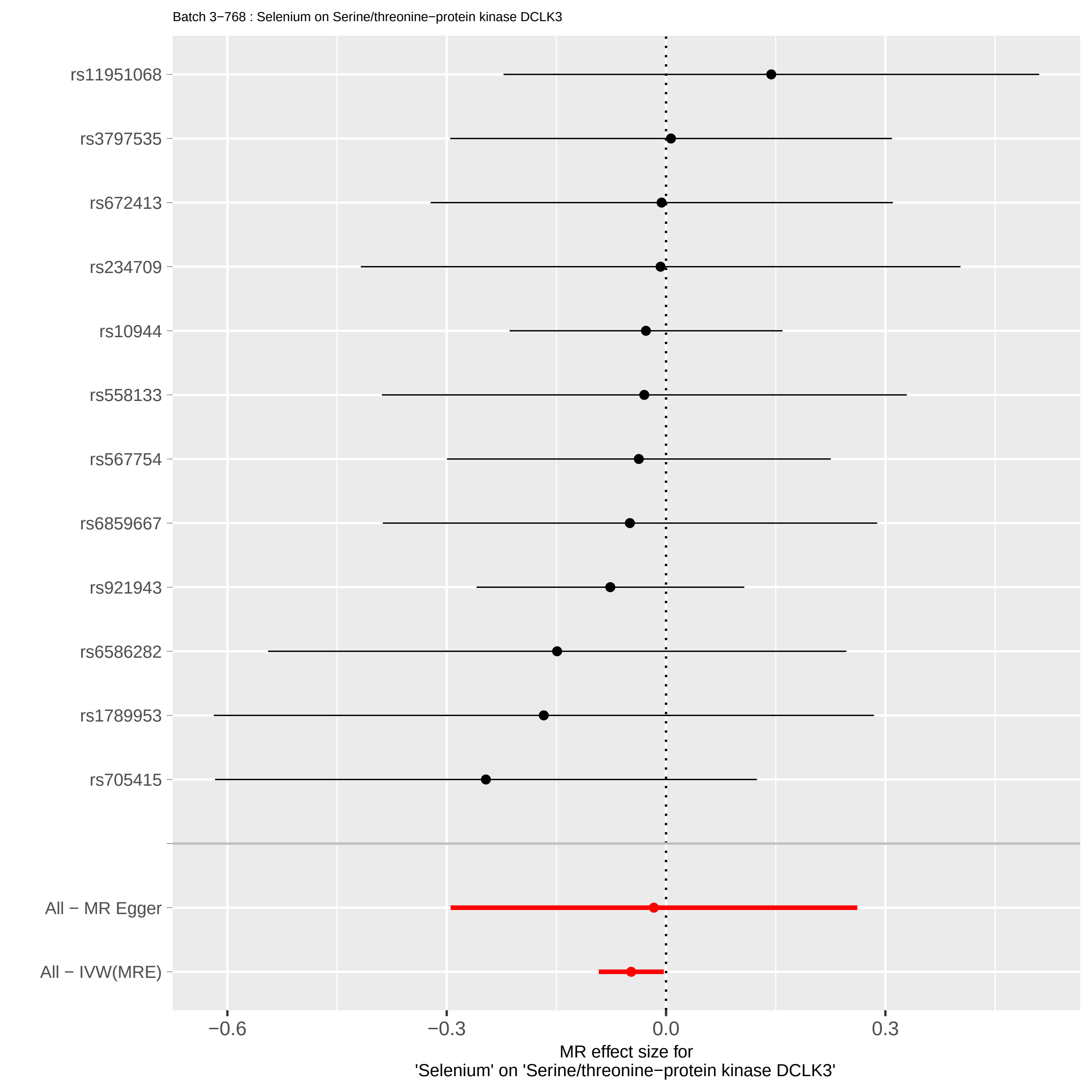
Batch 3−768 : Selenium on Serine/threonine−protein kinase DCLK3
rs11951068
rs3797535
rs672413
rs234709
rs10944
rs558133
rs567754
rs6859667
rs921943
rs6586282
rs1789953
rs705415
All − MR Egger
All − IVW(MRE)
−0.6
−0.3
0.0
0.3
MR effect size for
'Selenium' on 'Serine/threonine−protein kinase DCLK3'

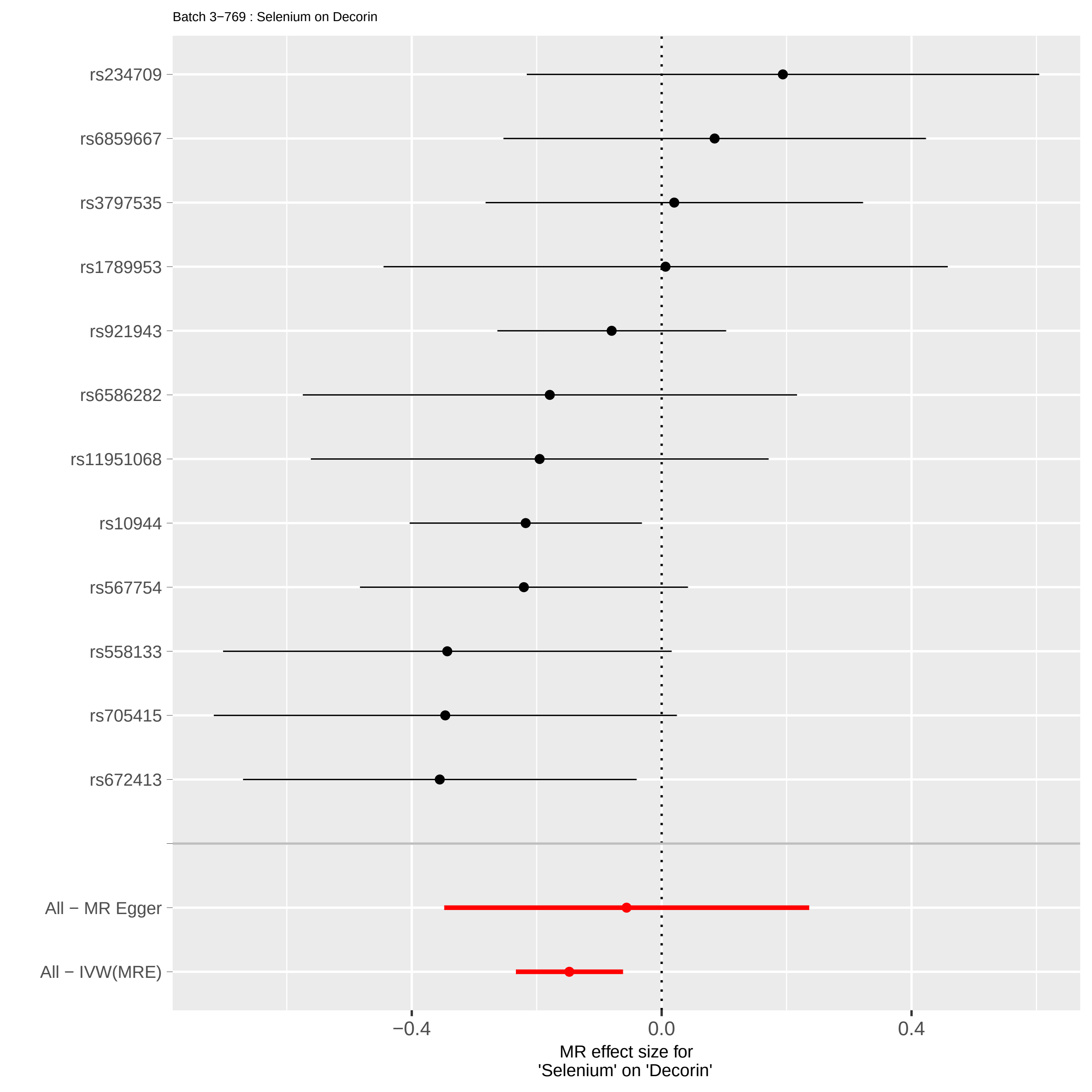
Batch 3−769 : Selenium on Decorin
rs234709
rs6859667
rs3797535
rs1789953
rs921943
rs6586282
rs11951068
rs10944
rs567754
rs558133
rs705415
rs672413
All − MR Egger
All − IVW(MRE)
−0.4
0.0
0.4
MR effect size for
'Selenium' on 'Decorin'

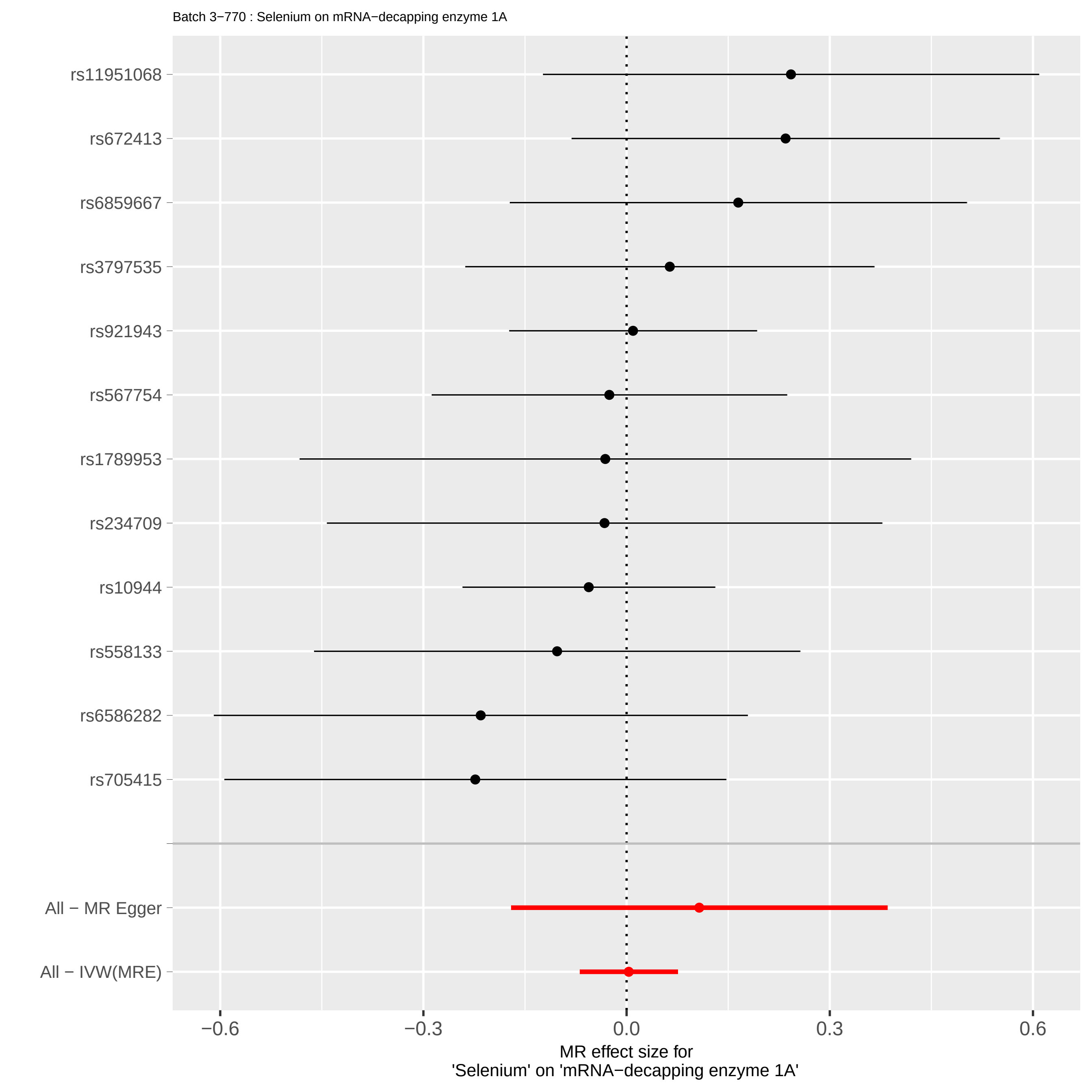

Batch 3−770 : Selenium on mRNA−decapping enzyme 1A
rs11951068
rs672413
rs6859667
rs3797535
rs921943
rs567754
rs1789953
rs234709
rs10944
rs558133
rs6586282
rs705415
All − MR Egger
All − IVW(MRE)
−0.6
−0.3
0.0
0.3
0.6
MR effect size for
'Selenium' on 'mRNA−decapping enzyme 1A'

Batch 3−771 : Selenium on Dynactin subunit 2

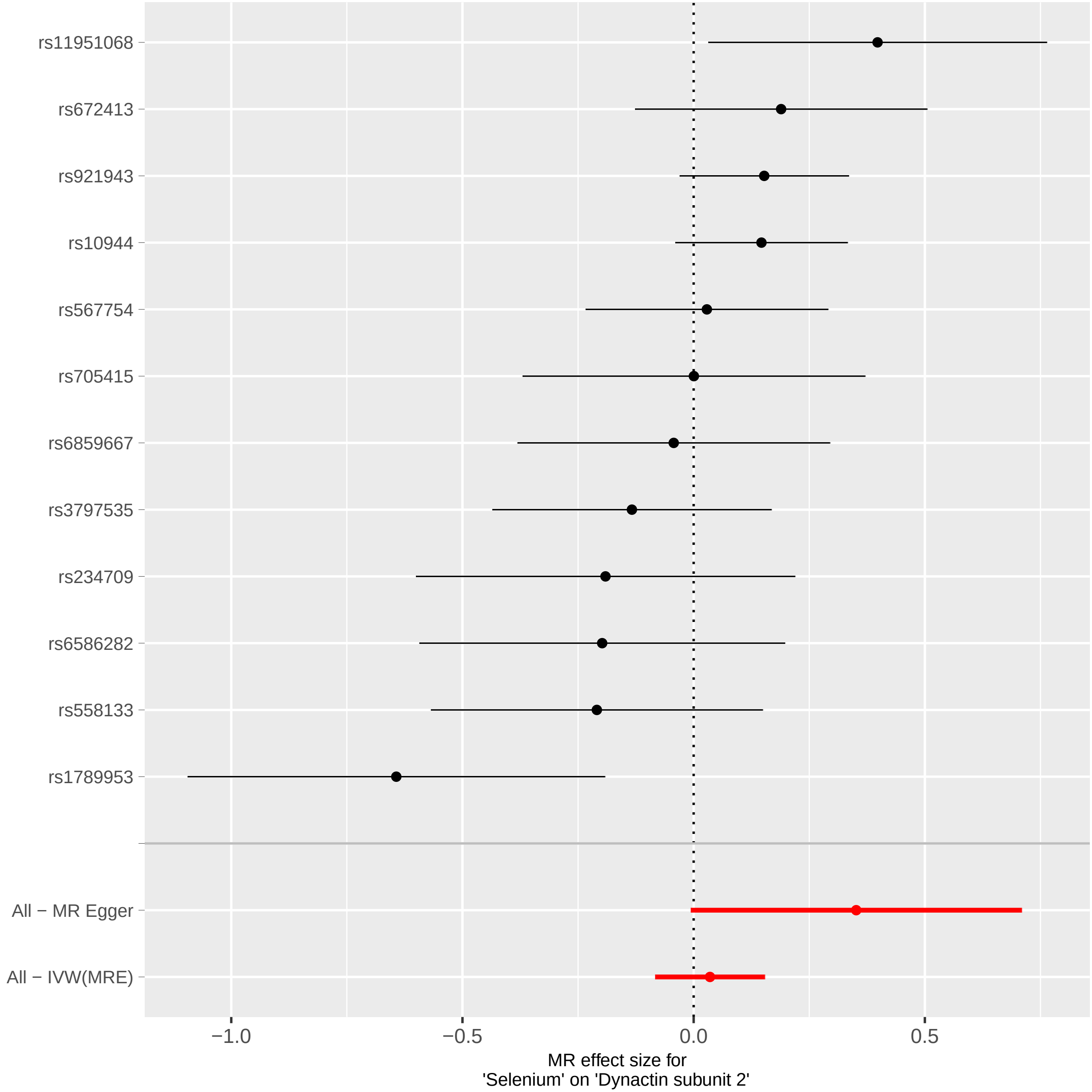

Batch 3−772 : Selenium on dCTP pyrophosphatase 1

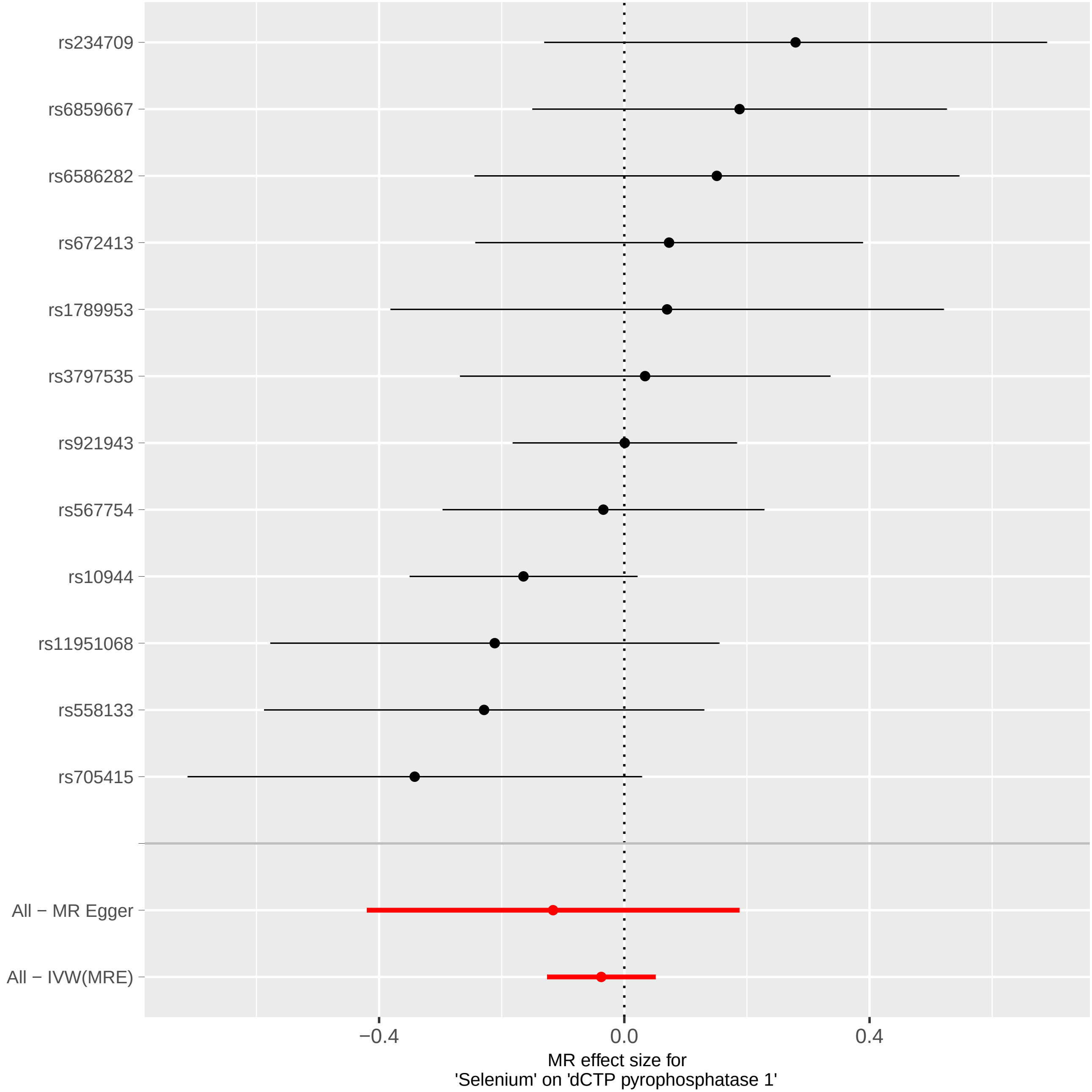

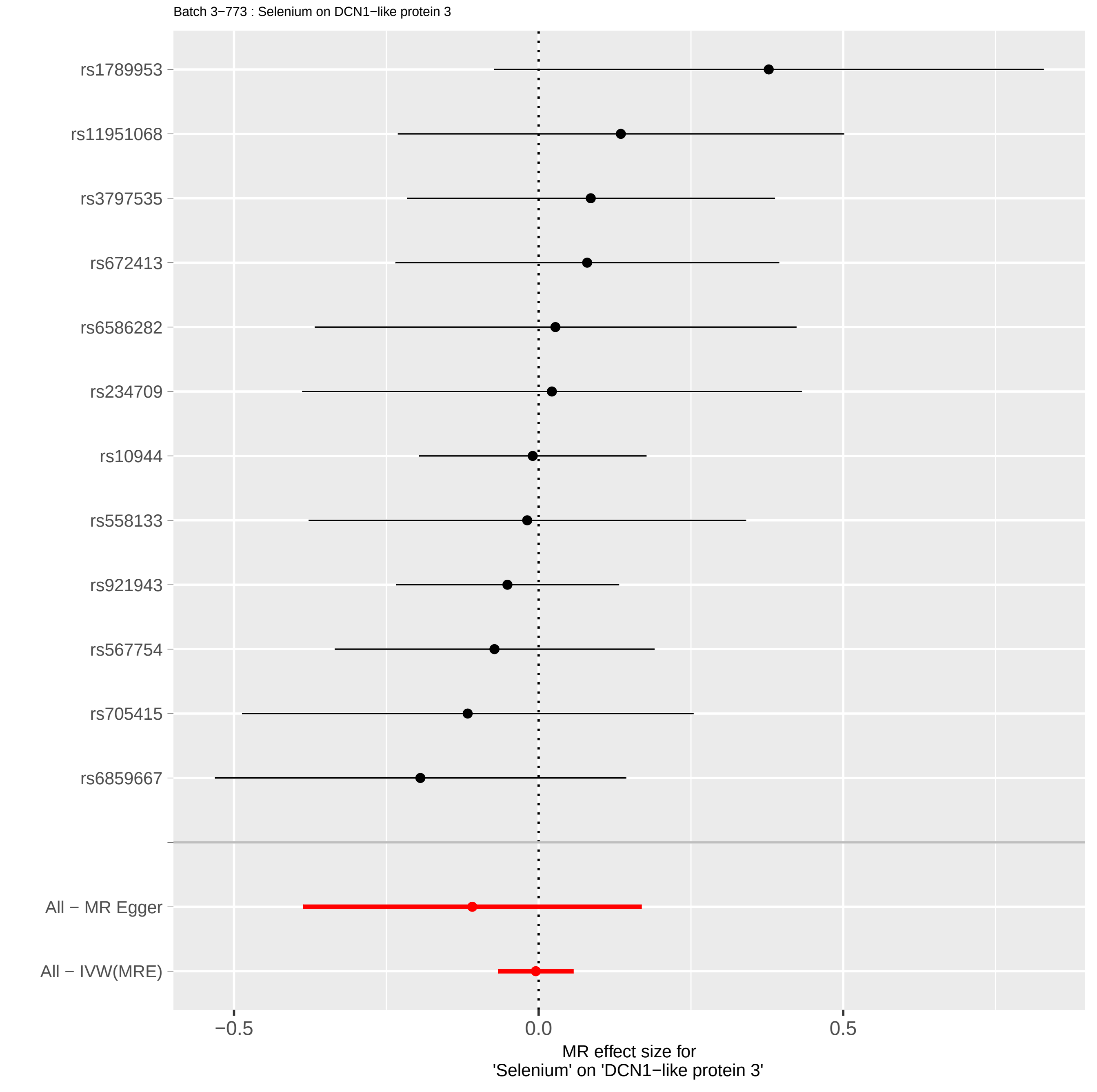

Batch 3−773 : Selenium on DCN1−like protein 3
rs1789953
rs11951068
rs3797535
rs672413
rs6586282
rs234709
rs10944
rs558133
rs921943
rs567754
rs705415
rs6859667
All − MR Egger
All − IVW(MRE)
−0.5
0.0
0.5
MR effect size for
'Selenium' on 'DCN1−like protein 3'

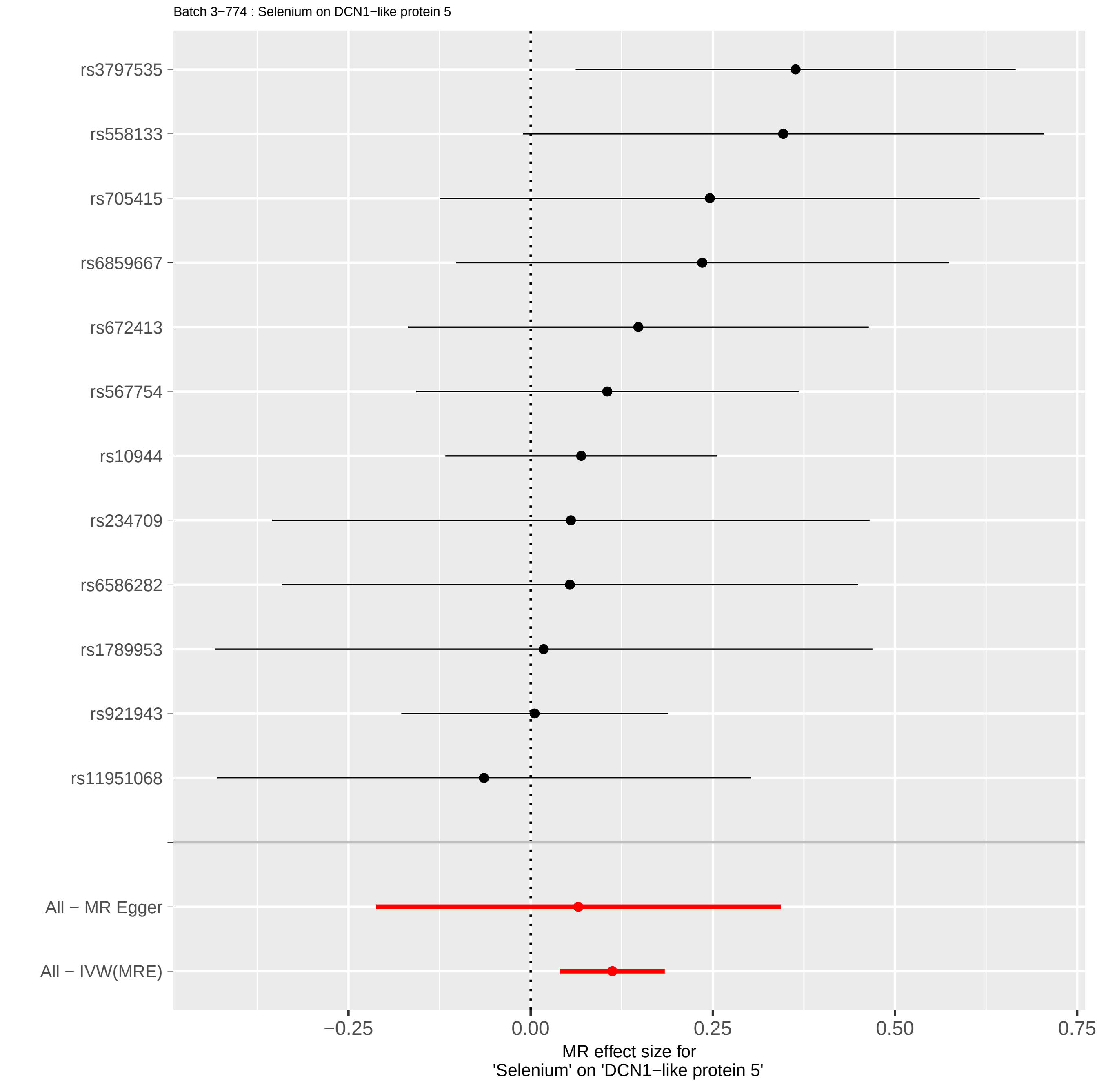

Batch 3−774 : Selenium on DCN1−like protein 5
rs3797535
rs558133
rs705415
rs6859667
rs672413
rs567754
rs10944
rs234709
rs6586282
rs1789953
rs921943
rs11951068
All − MR Egger
All − IVW(MRE)
−0.25
0.00
0.25
0.50
0.75
MR effect size for
'Selenium' on 'DCN1−like protein 5'

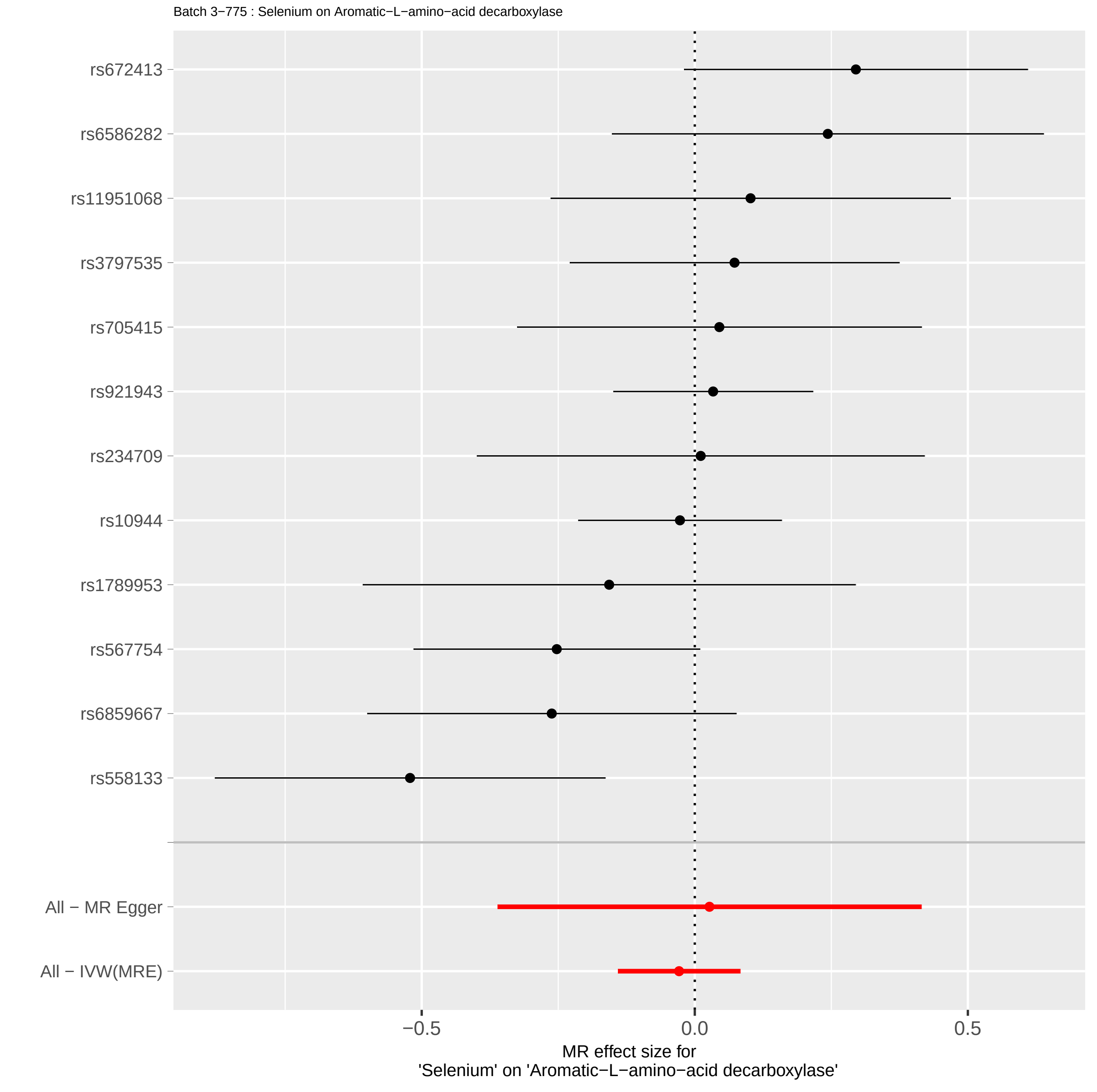
Batch 3−775 : Selenium on Aromatic−L−amino−acid decarboxylase
rs672413
rs6586282
rs11951068
rs3797535
rs705415
rs921943
rs234709
rs10944
rs1789953
rs567754
rs6859667
rs558133
All − MR Egger
All − IVW(MRE)
−0.5
0.0
0.5
MR effect size for
'Selenium' on 'Aromatic−L−amino−acid decarboxylase'

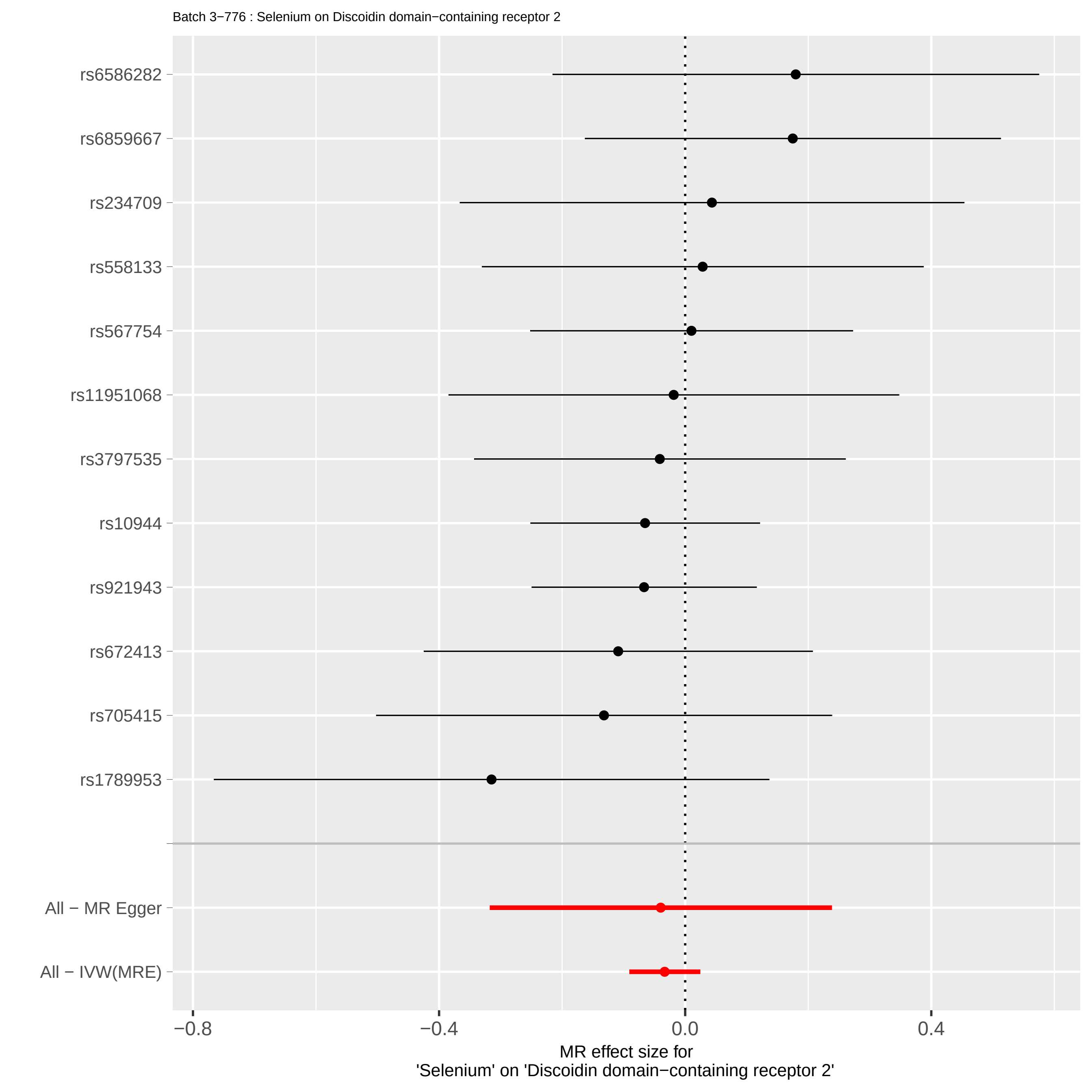
Batch 3−776 : Selenium on Discoidin domain−containing receptor 2
rs6586282
rs6859667
rs234709
rs558133
rs567754
rs11951068
rs3797535
rs10944
rs921943
rs672413
rs705415
rs1789953
All − MR Egger
All − IVW(MRE)
−0.8
−0.4
0.0
0.4
MR effect size for
'Selenium' on 'Discoidin domain−containing receptor 2'

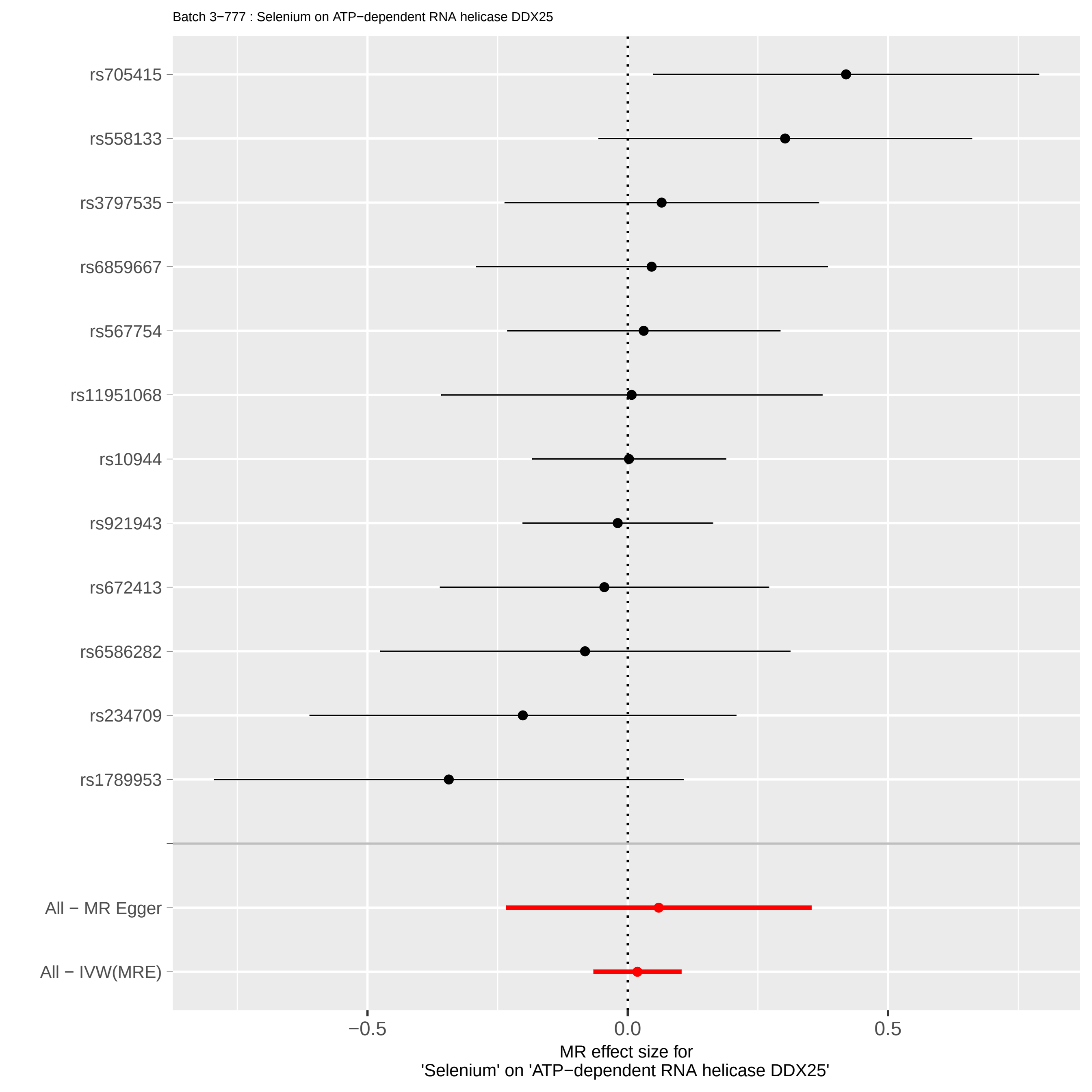

Batch 3−777 : Selenium on ATP−dependent RNA helicase DDX25
rs705415
rs558133
rs3797535
rs6859667
rs567754
rs11951068
rs10944
rs921943
rs672413
rs6586282
rs234709
rs1789953
All − MR Egger
All − IVW(MRE)
−0.5
0.0
0.5
MR effect size for
'Selenium' on 'ATP−dependent RNA helicase DDX25'

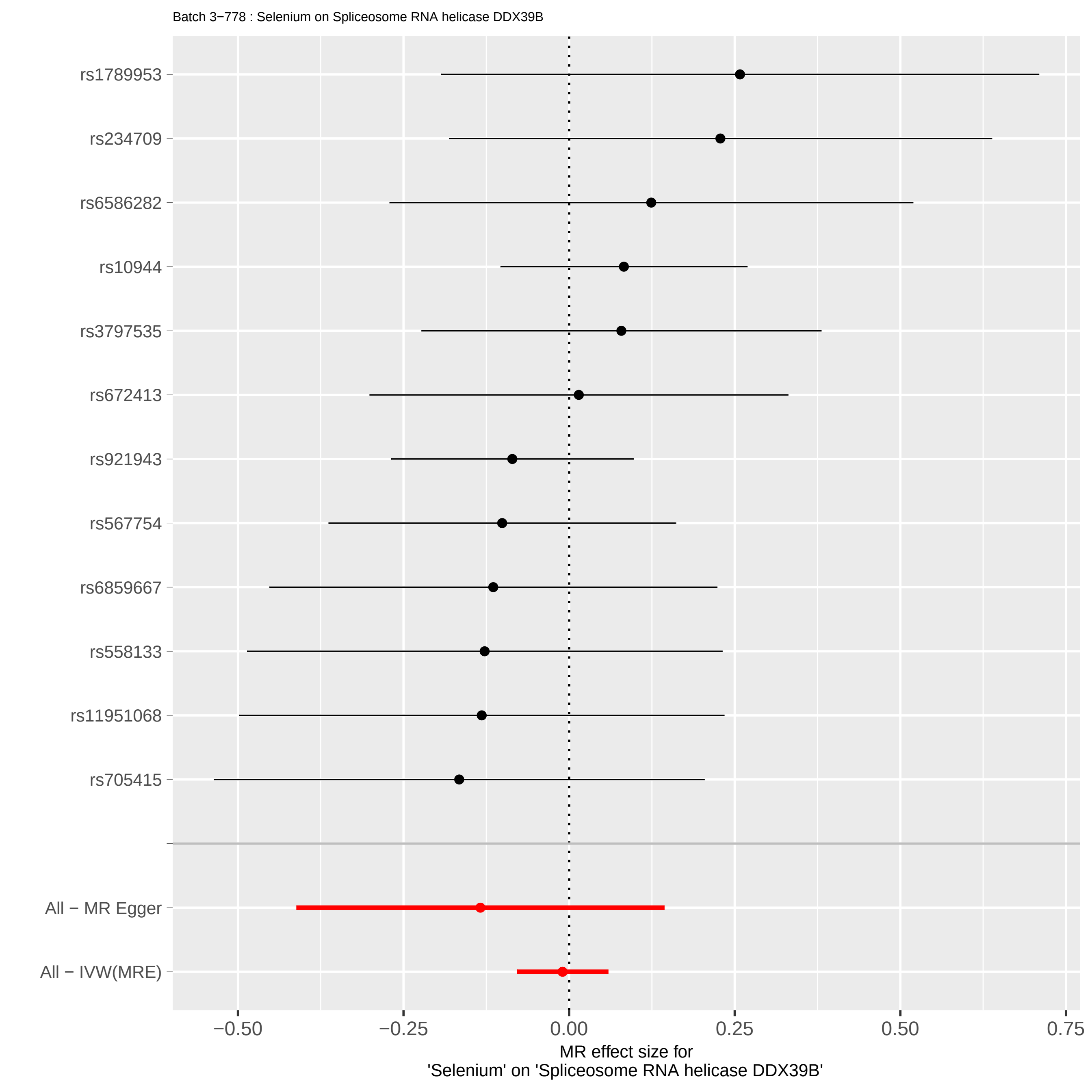
Batch 3−778 : Selenium on Spliceosome RNA helicase DDX39B
rs1789953
rs234709
rs6586282
rs10944
rs3797535
rs672413
rs921943
rs567754
rs6859667
rs558133
rs11951068
rs705415
All − MR Egger
All − IVW(MRE)
−0.50
−0.25
0.00
0.25
0.50
0.75
MR effect size for
'Selenium' on 'Spliceosome RNA helicase DDX39B'

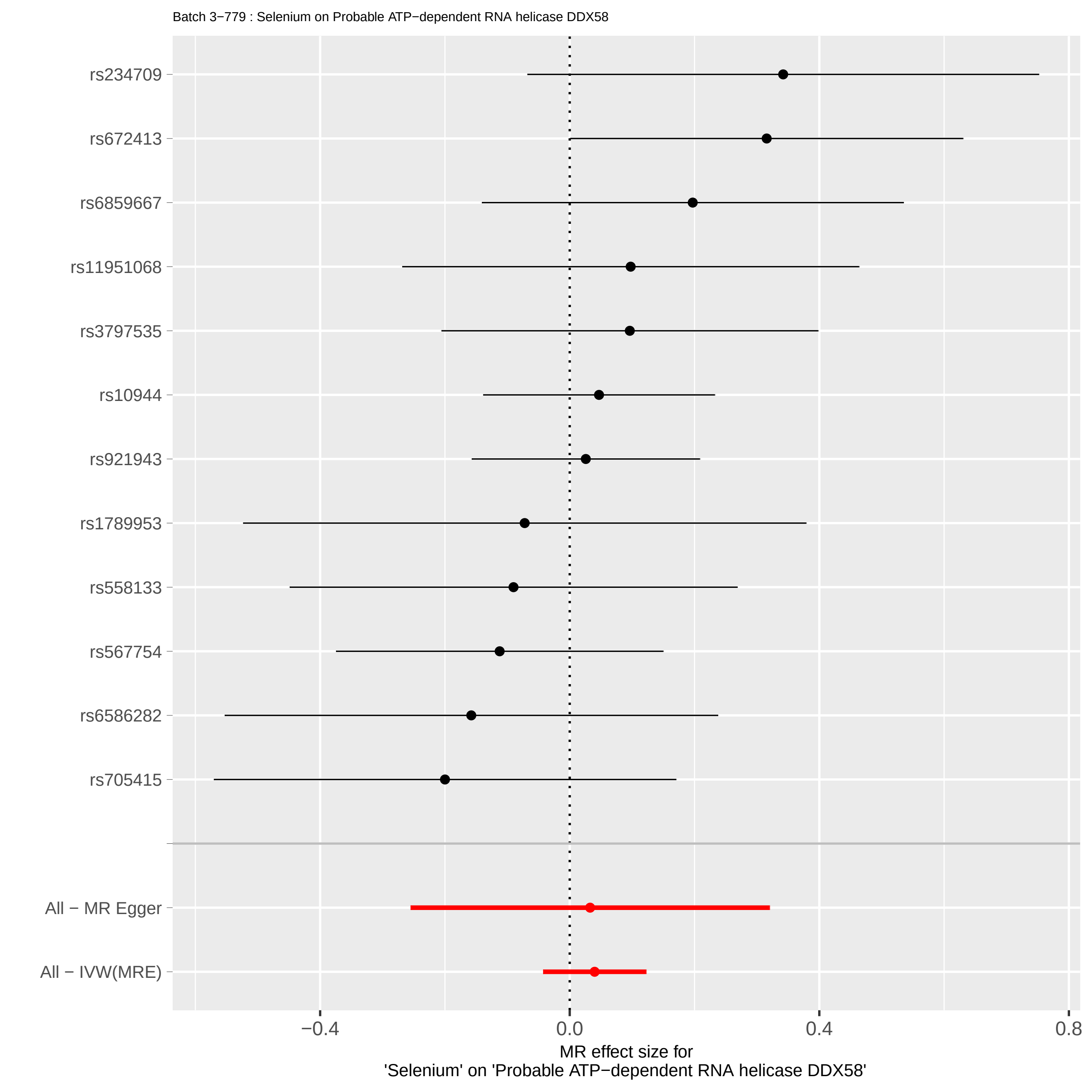

Batch 3−779 : Selenium on Probable ATP−dependent RNA helicase DDX58
rs234709
rs672413
rs6859667
rs11951068
rs3797535
rs10944
rs921943
rs1789953
rs558133
rs567754
rs6586282
rs705415
All − MR Egger
All − IVW(MRE)
−0.4
0.0
0.4
0.8
MR effect size for
'Selenium' on 'Probable ATP−dependent RNA helicase DDX58'

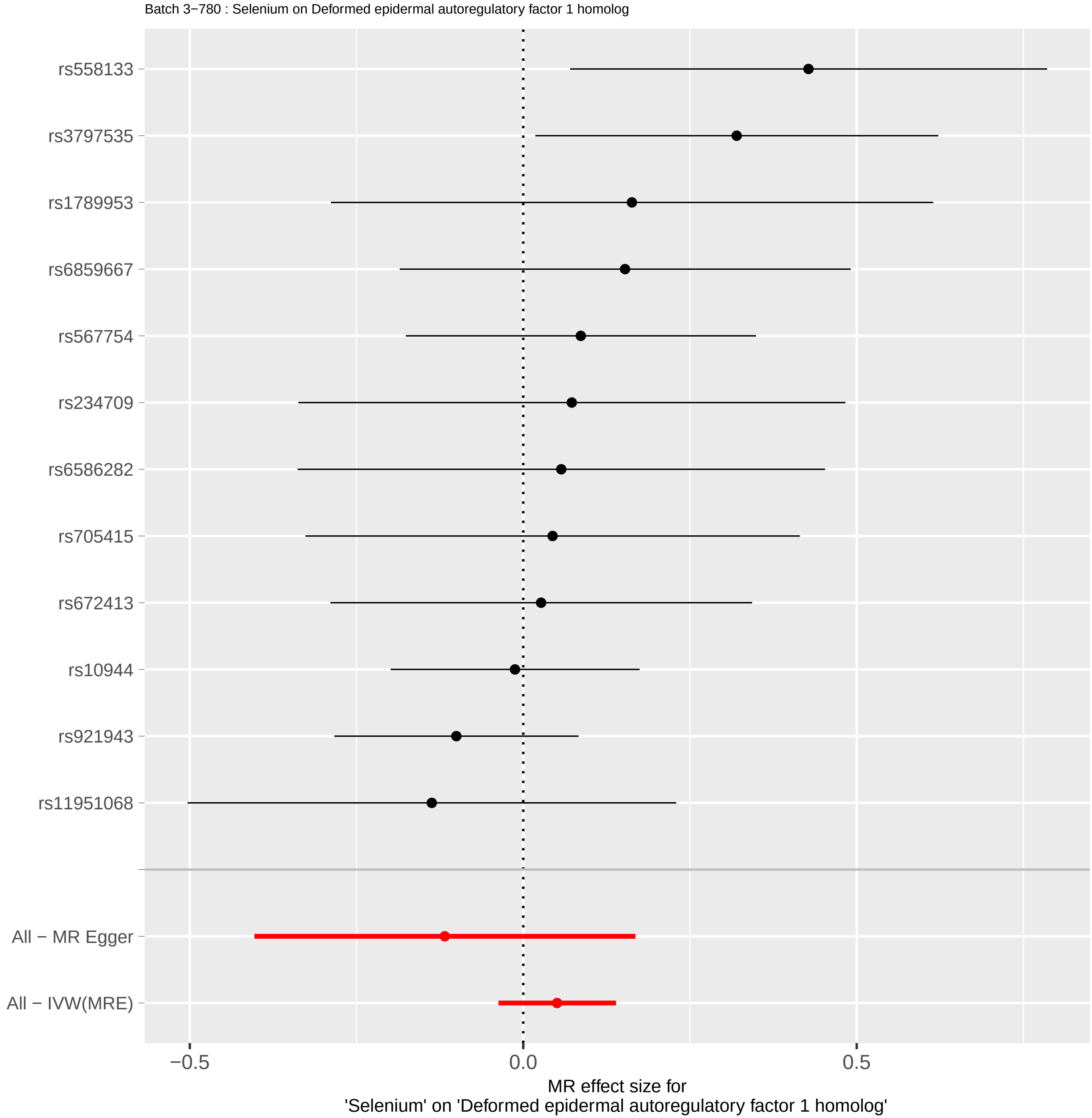

Batch 3−780 : Selenium on Deformed epidermal autoregulatory factor 1 homolog
rs558133
rs3797535
rs1789953
rs6859667
rs567754
rs234709
rs6586282
rs705415
rs672413
rs10944
rs921943
rs11951068
All − MR Egger
All − IVW(MRE)
−0.5
0.0
0.5
MR effect size for
'Selenium' on 'Deformed epidermal autoregulatory factor 1 homolog'

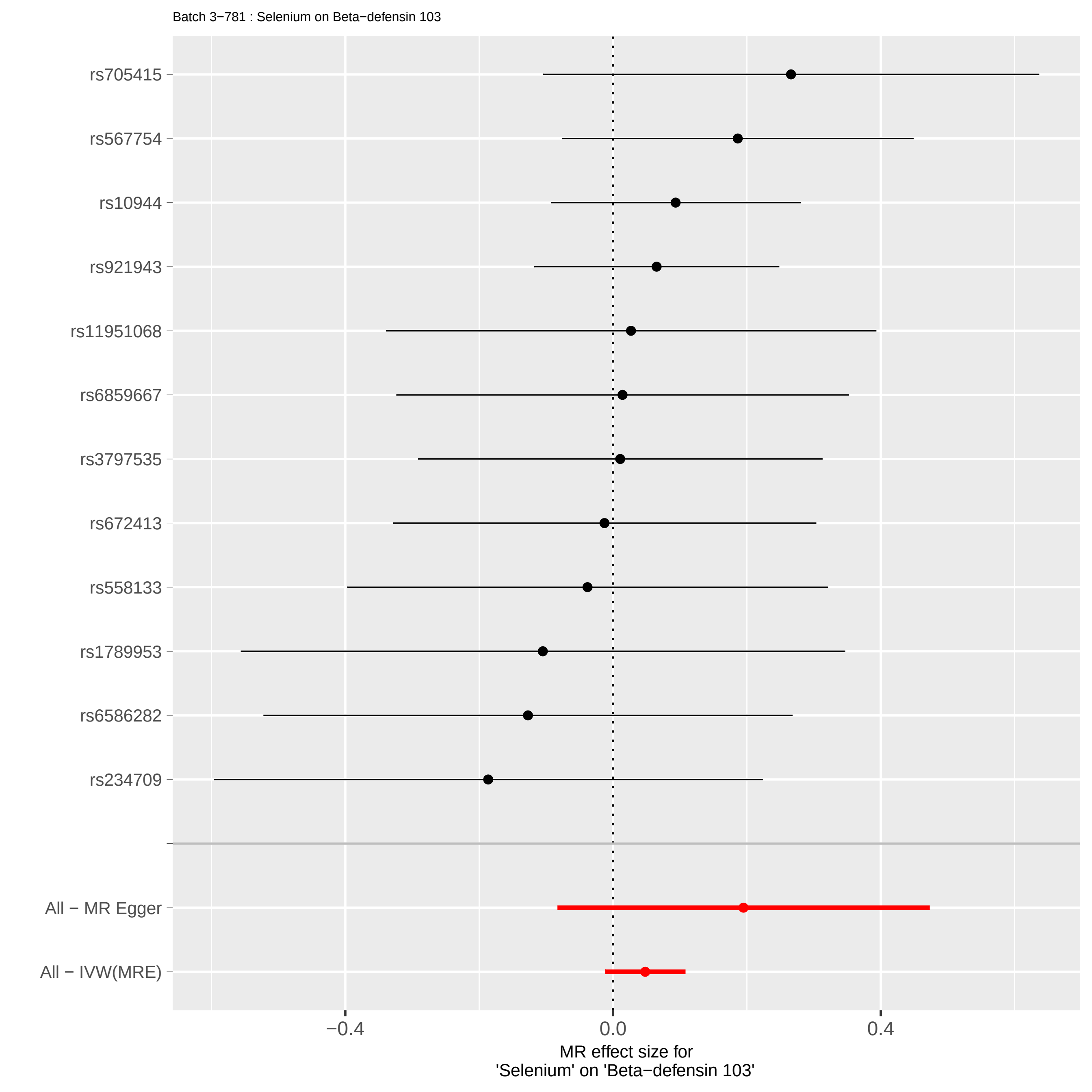
Batch 3−781 : Selenium on Beta−defensin 103
rs705415
rs567754
rs10944
rs921943
rs11951068
rs6859667
rs3797535
rs672413
rs558133
rs1789953
rs6586282
rs234709
All − MR Egger
All − IVW(MRE)
−0.4
0.0
0.4
MR effect size for
'Selenium' on 'Beta−defensin 103'

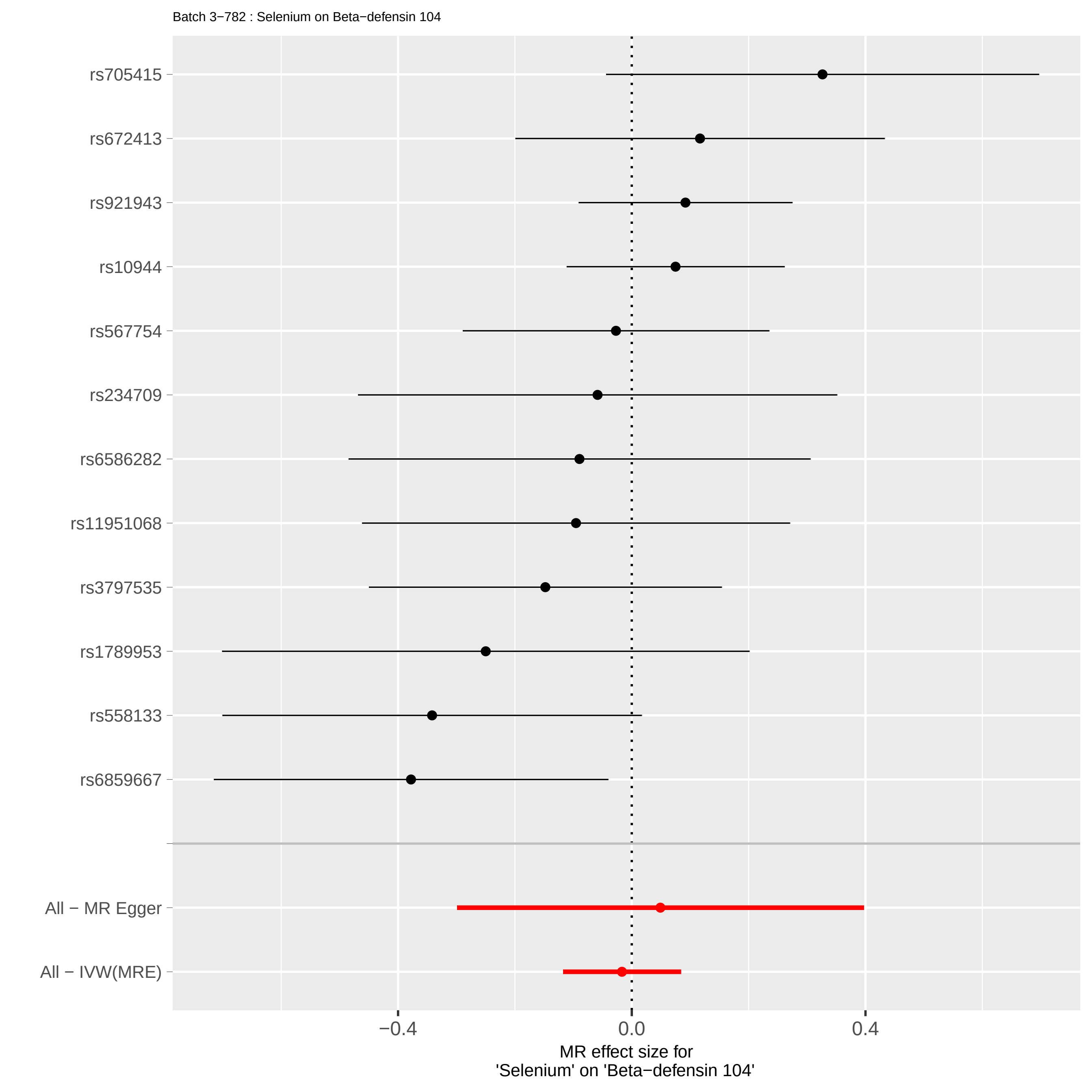

Batch 3−782 : Selenium on Beta−defensin 104
rs705415
rs672413
rs921943
rs10944
rs567754
rs234709
rs6586282
rs11951068
rs3797535
rs1789953
rs558133
rs6859667
All − MR Egger
All − IVW(MRE)
−0.4
0.0
0.4
MR effect size for
'Selenium' on 'Beta−defensin 104'

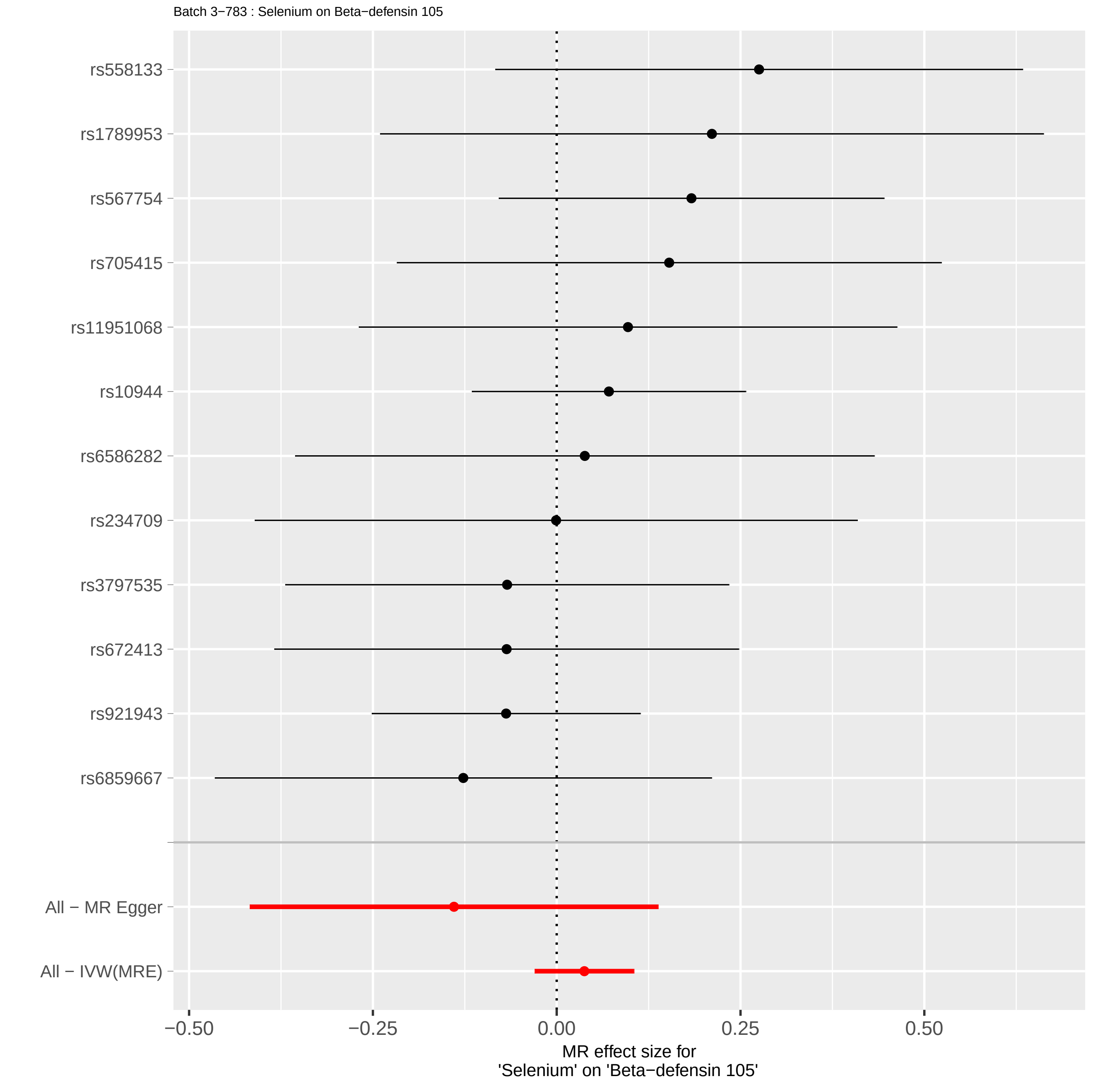
Batch 3−783 : Selenium on Beta−defensin 105
rs558133
rs1789953
rs567754
rs705415
rs11951068
rs10944
rs6586282
rs234709
rs3797535
rs672413
rs921943
rs6859667
All − MR Egger
All − IVW(MRE)
−0.50
−0.25
0.00
0.25
0.50
MR effect size for
'Selenium' on 'Beta−defensin 105'

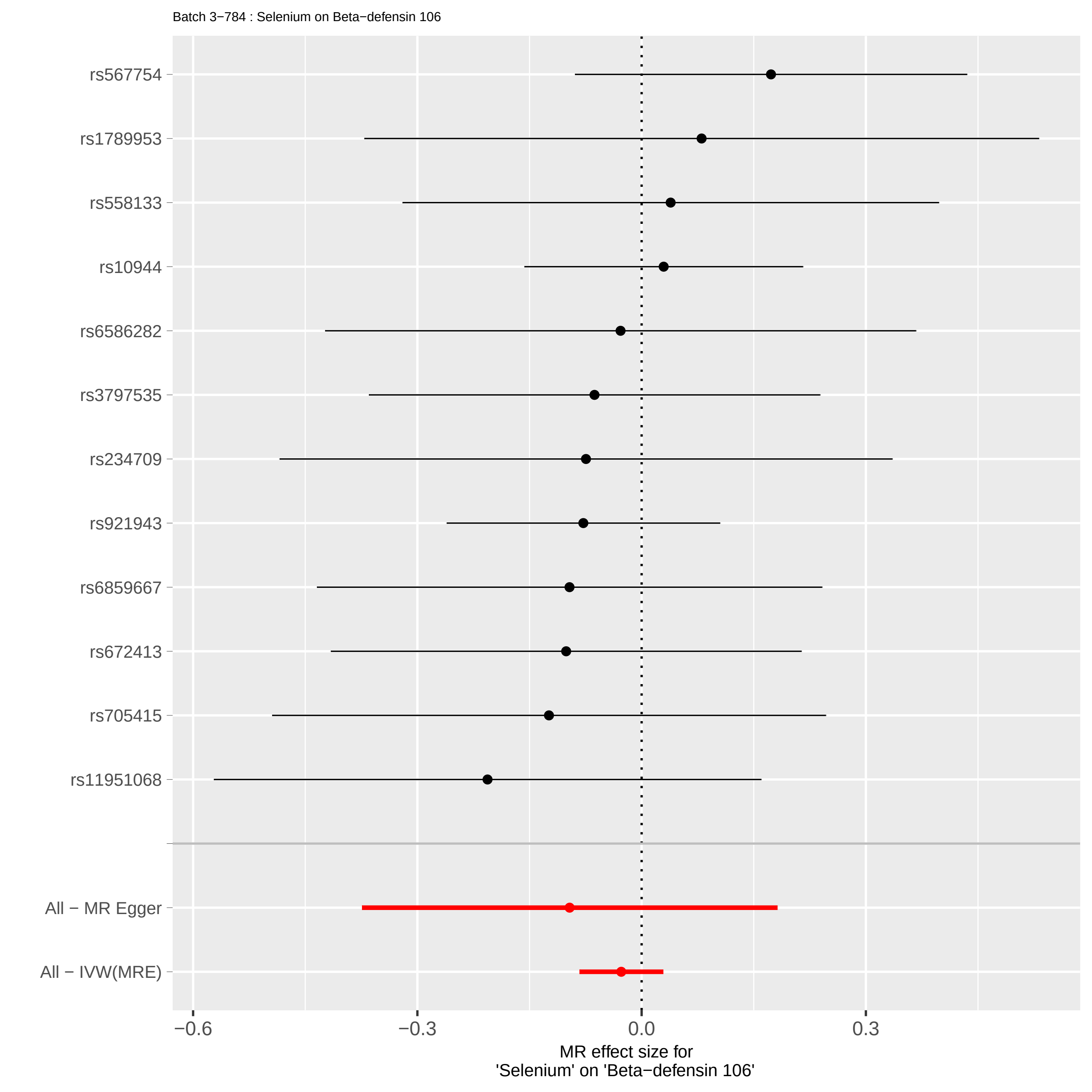

Batch 3−784 : Selenium on Beta−defensin 106
rs567754
rs1789953
rs558133
rs10944
rs6586282
rs3797535
rs234709
rs921943
rs6859667
rs672413
rs705415
rs11951068
All − MR Egger
All − IVW(MRE)
−0.6
−0.3
0.0
0.3
MR effect size for
'Selenium' on 'Beta−defensin 106'

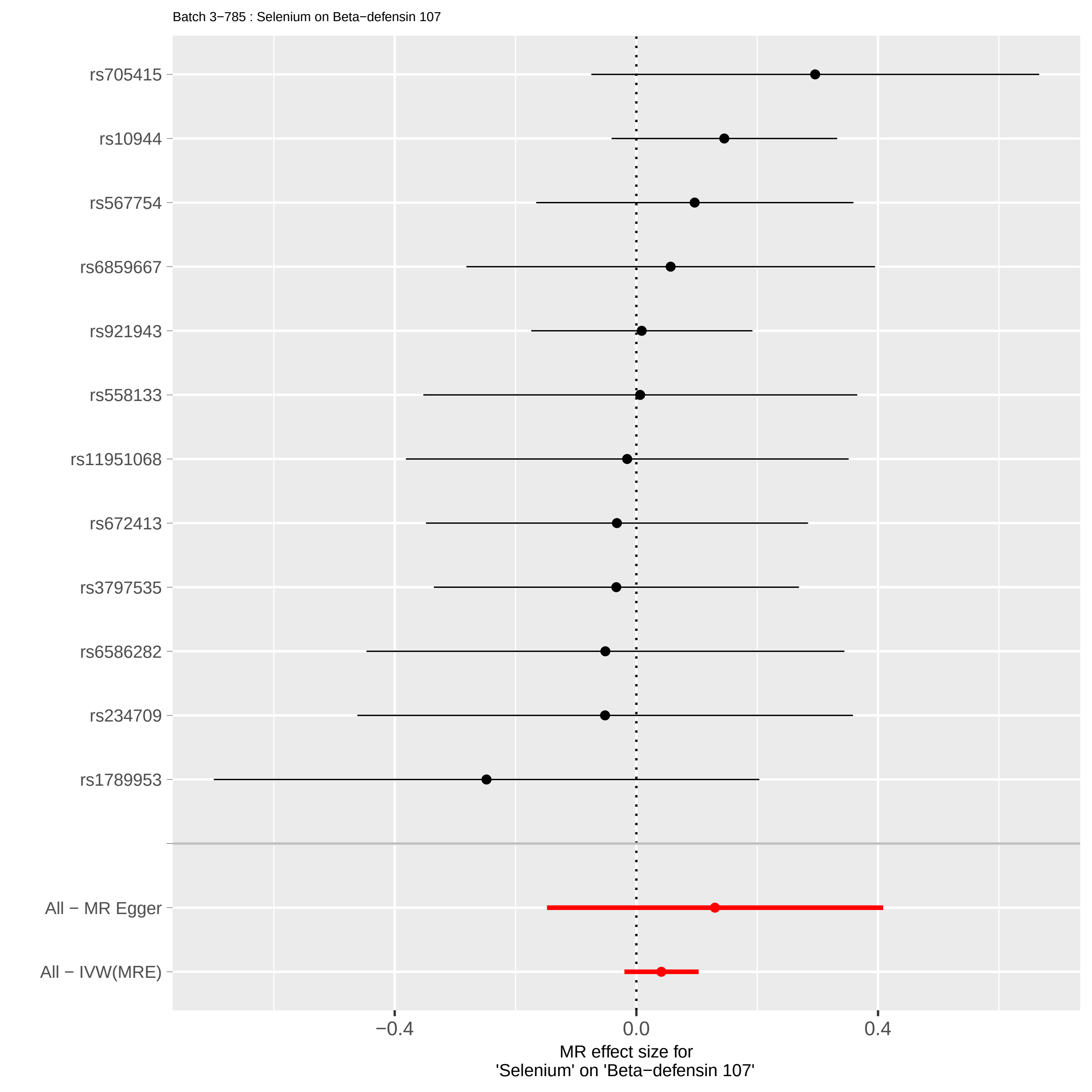

Batch 3−785 : Selenium on Beta−defensin 107
rs705415
rs10944
rs567754
rs6859667
rs921943
rs558133
rs11951068
rs672413
rs3797535
rs6586282
rs234709
rs1789953
All − MR Egger
All − IVW(MRE)
−0.4
0.0
0.4
MR effect size for
'Selenium' on 'Beta−defensin 107'

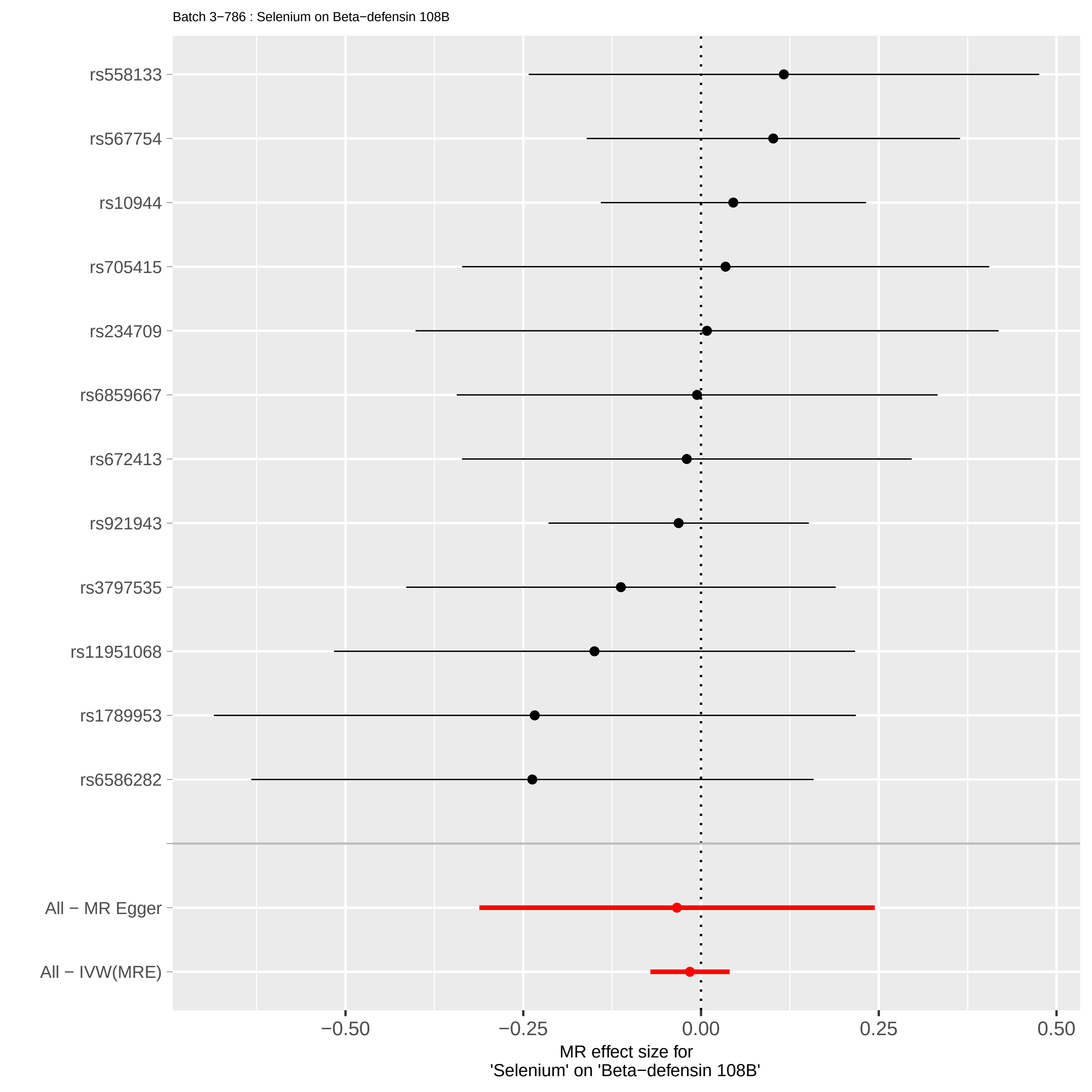

Batch 3−786 : Selenium on Beta−defensin 108B
rs558133
rs567754
rs10944
rs705415
rs234709
rs6859667
rs672413
rs921943
rs3797535
rs11951068
rs1789953
rs6586282
All − MR Egger
All − IVW(MRE)
−0.50
−0.25
0.00
0.25
0.50
MR effect size for
'Selenium' on 'Beta−defensin 108B'

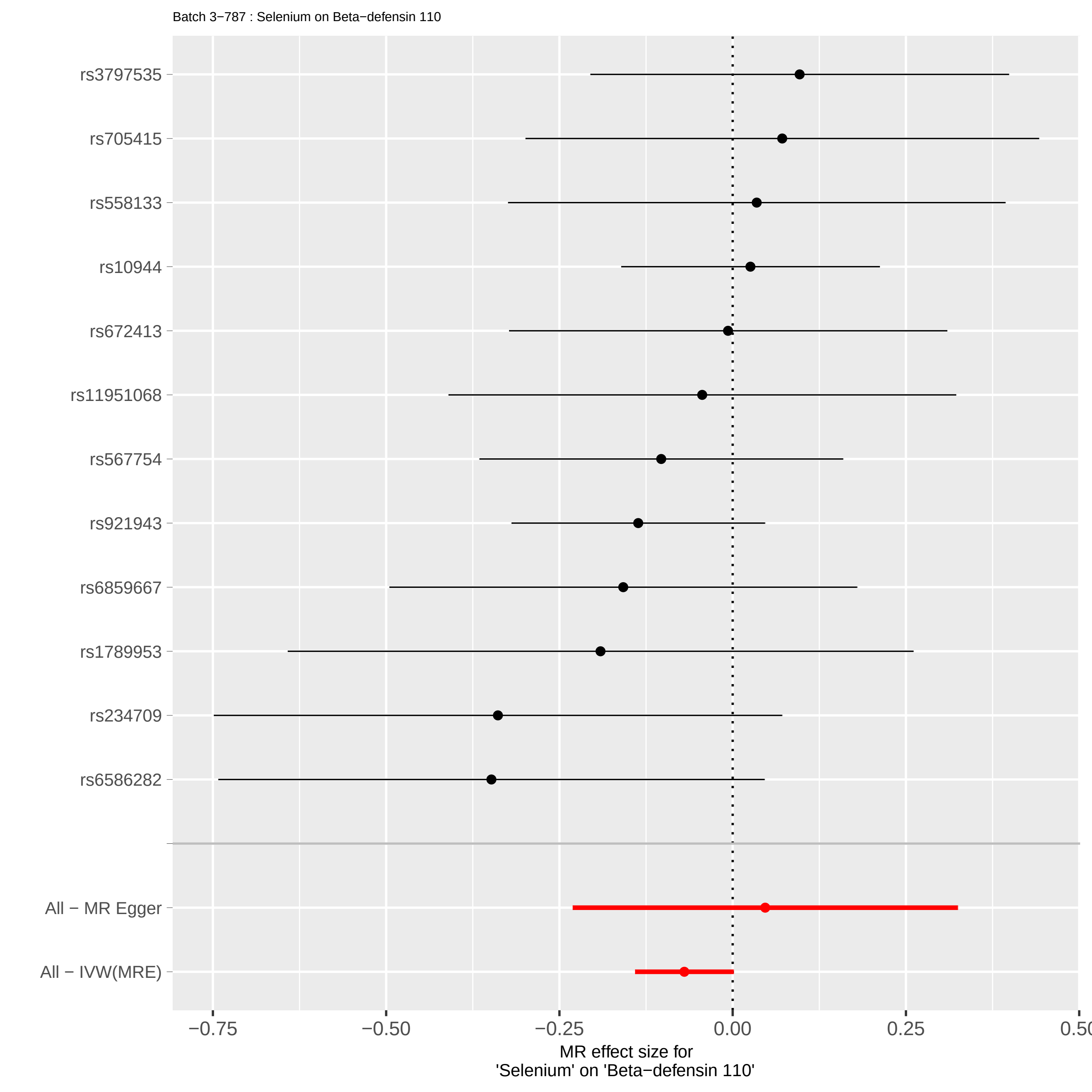

Batch 3−787 : Selenium on Beta−defensin 110
rs3797535
rs705415
rs558133
rs10944
rs672413
rs11951068
rs567754
rs921943
rs6859667
rs1789953
rs234709
rs6586282
All − MR Egger
All − IVW(MRE)
−0.75
−0.50
−0.25
0.00
0.25
0.50
MR effect size for
'Selenium' on 'Beta−defensin 110'

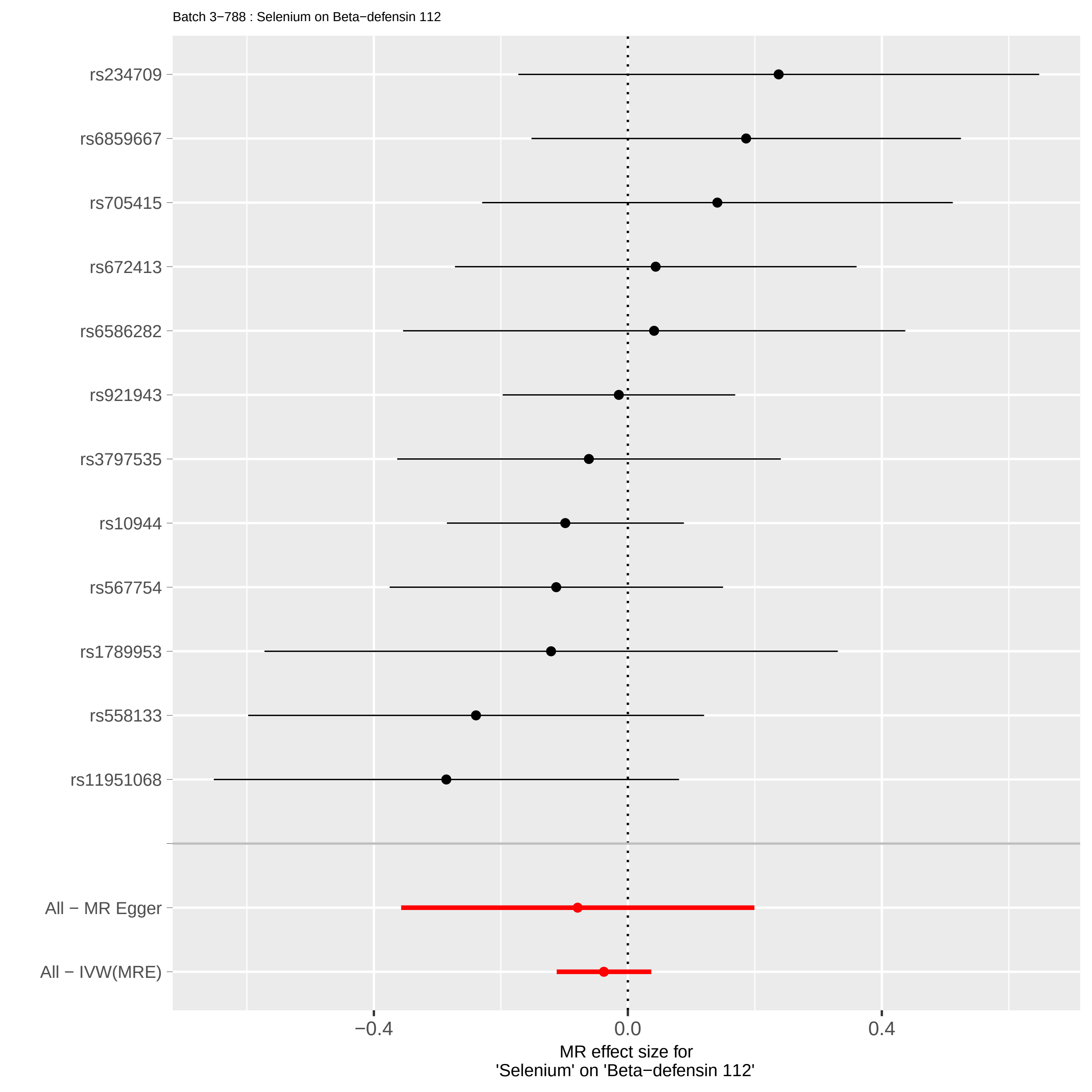
Batch 3−788 : Selenium on Beta−defensin 112
rs234709
rs6859667
rs705415
rs672413
rs6586282
rs921943
rs3797535
rs10944
rs567754
rs1789953
rs558133
rs11951068
All − MR Egger
All − IVW(MRE)
−0.4
0.0
0.4
MR effect size for
'Selenium' on 'Beta−defensin 112'

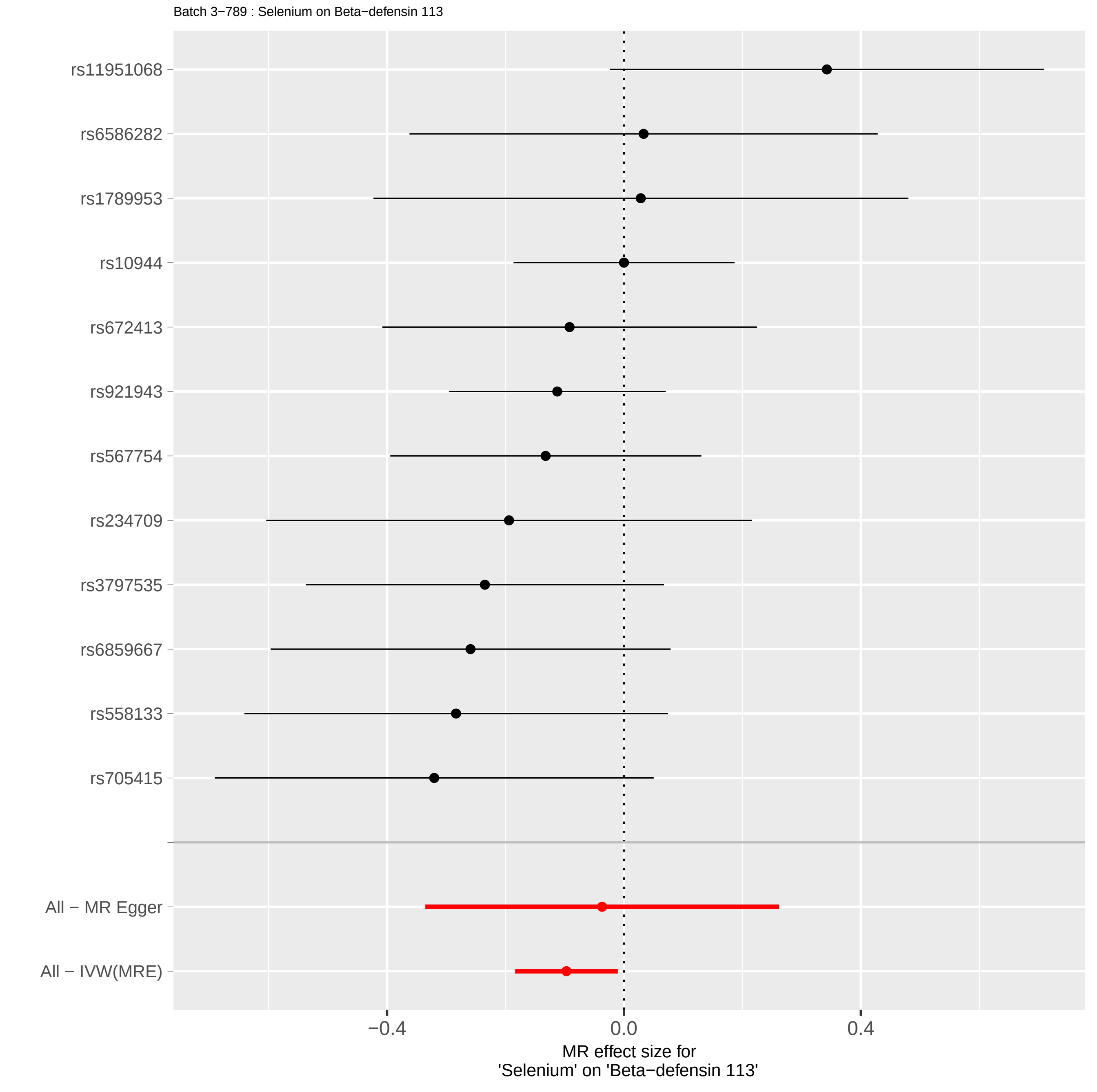
Batch 3−789 : Selenium on Beta−defensin 113
rs11951068
rs6586282
rs1789953
rs10944
rs672413
rs921943
rs567754
rs234709
rs3797535
rs6859667
rs558133
rs705415
All − MR Egger
All − IVW(MRE)
−0.4
0.0
0.4
MR effect size for
'Selenium' on 'Beta−defensin 113'

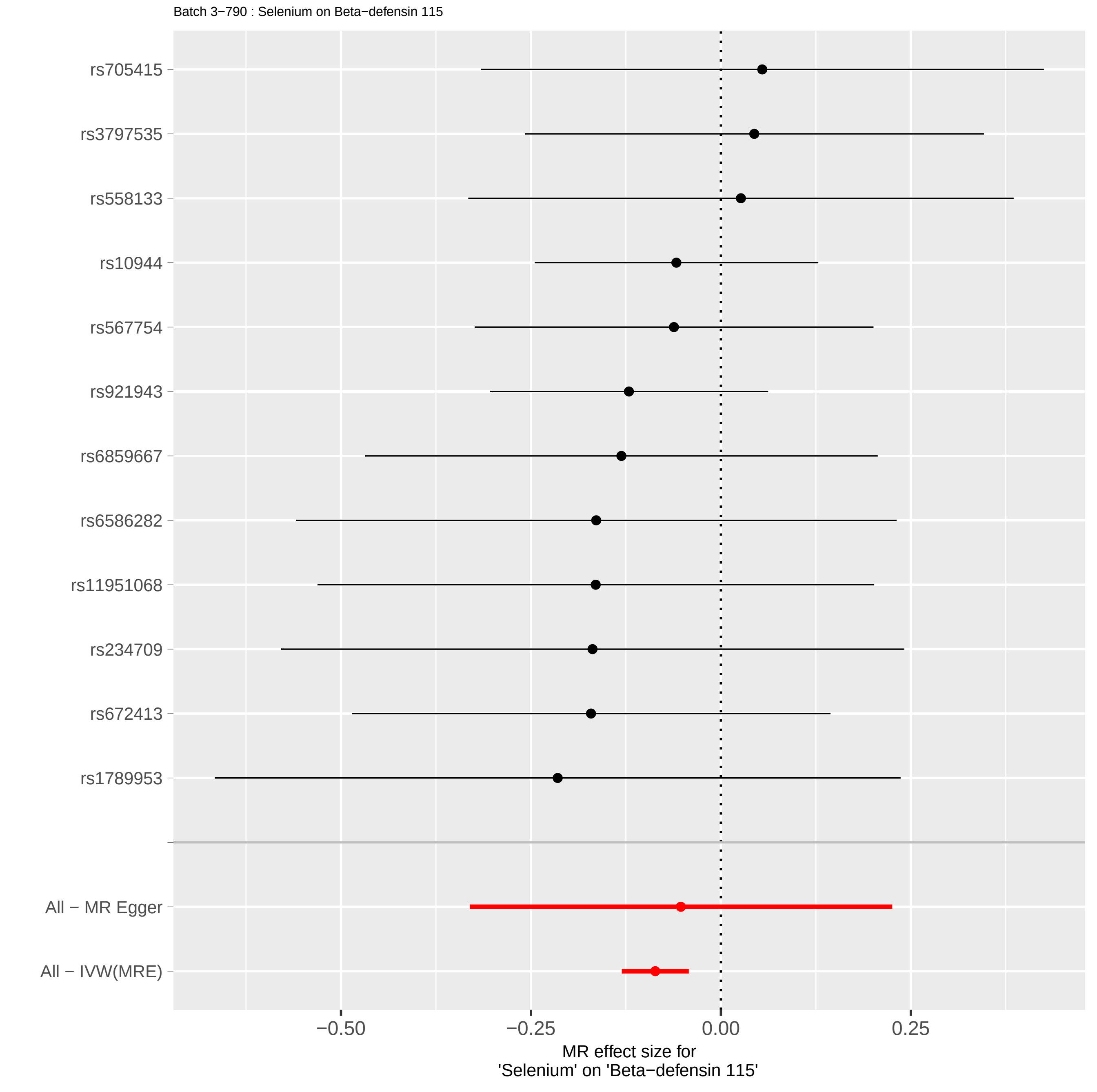
Batch 3−790 : Selenium on Beta−defensin 115
rs705415
rs3797535
rs558133
rs10944
rs567754
rs921943
rs6859667
rs6586282
rs11951068
rs234709
rs672413
rs1789953
All − MR Egger
All − IVW(MRE)
−0.50
−0.25
0.00
0.25
MR effect size for
'Selenium' on 'Beta−defensin 115'

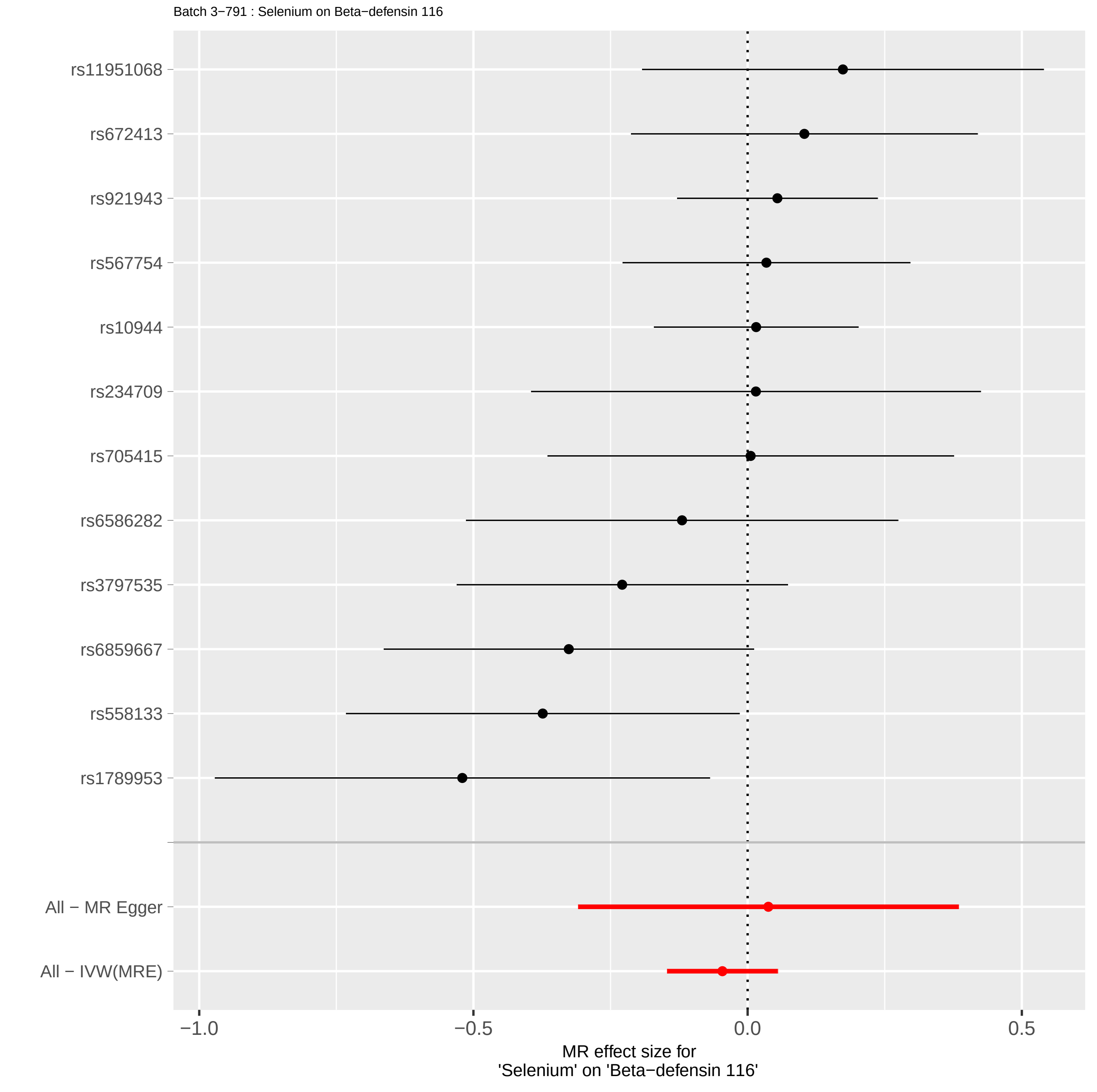

Batch 3−791 : Selenium on Beta−defensin 116
rs11951068
rs672413
rs921943
rs567754
rs10944
rs234709
rs705415
rs6586282
rs3797535
rs6859667
rs558133
rs1789953
All − MR Egger
All − IVW(MRE)
−1.0
−0.5
0.0
0.5
MR effect size for
'Selenium' on 'Beta−defensin 116'

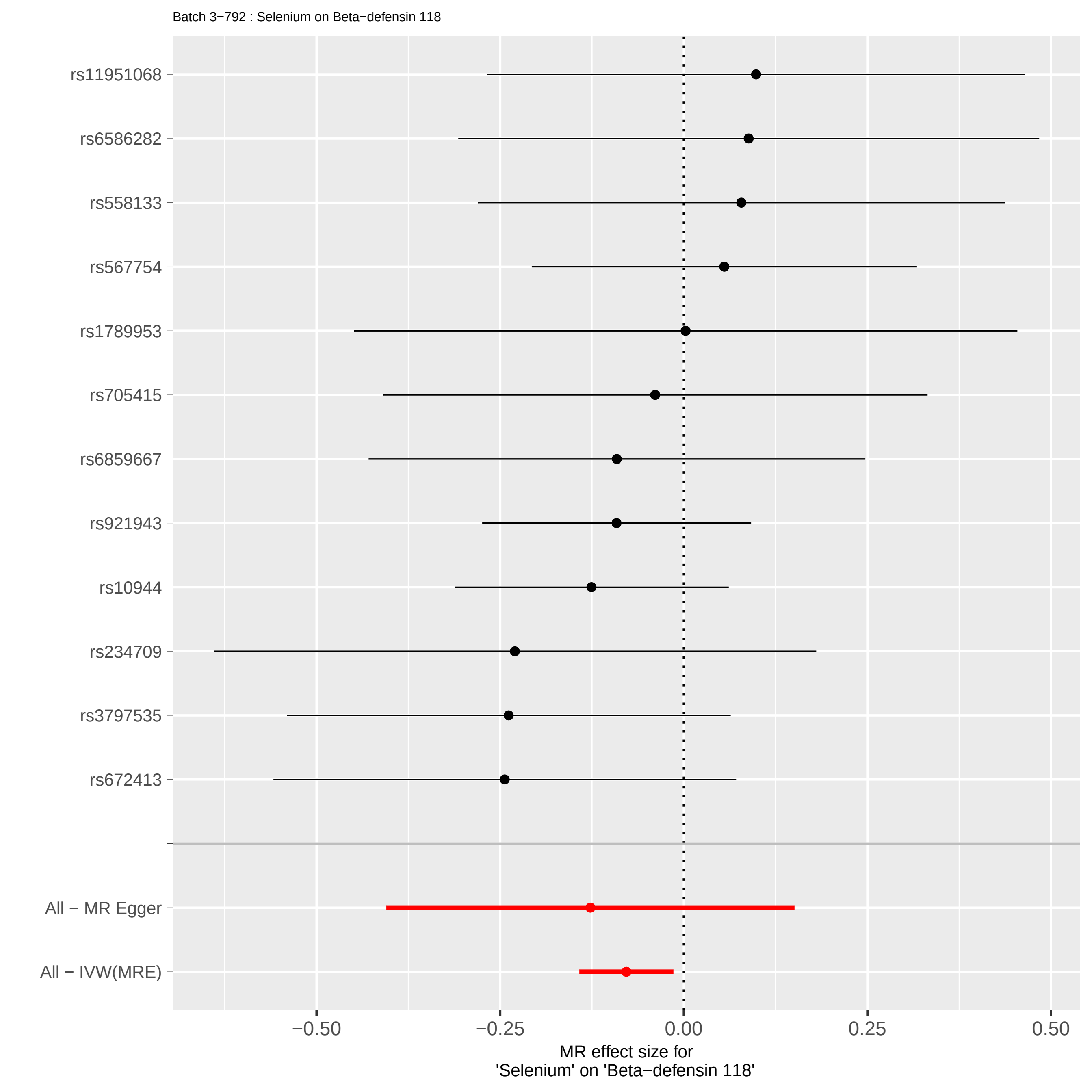

Batch 3−792 : Selenium on Beta−defensin 118
rs11951068
rs6586282
rs558133
rs567754
rs1789953
rs705415
rs6859667
rs921943
rs10944
rs234709
rs3797535
rs672413
All − MR Egger
All − IVW(MRE)
−0.50
−0.25
0.00
0.25
0.50
MR effect size for
'Selenium' on 'Beta−defensin 118'

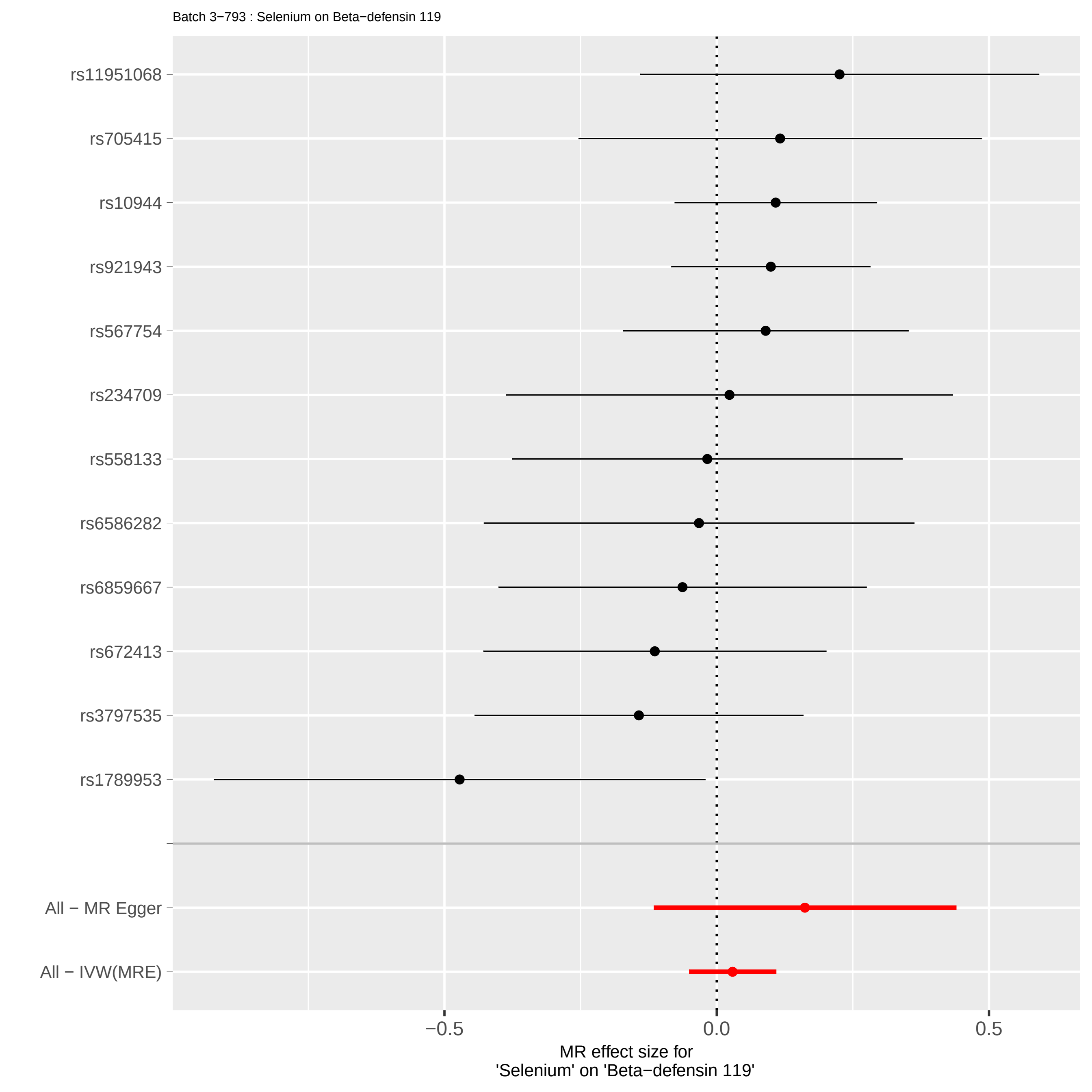
Batch 3−793 : Selenium on Beta−defensin 119
rs11951068
rs705415
rs10944
rs921943
rs567754
rs234709
rs558133
rs6586282
rs6859667
rs672413
rs3797535
rs1789953
All − MR Egger
All − IVW(MRE)
−0.5
0.0
0.5
MR effect size for
'Selenium' on 'Beta−defensin 119'

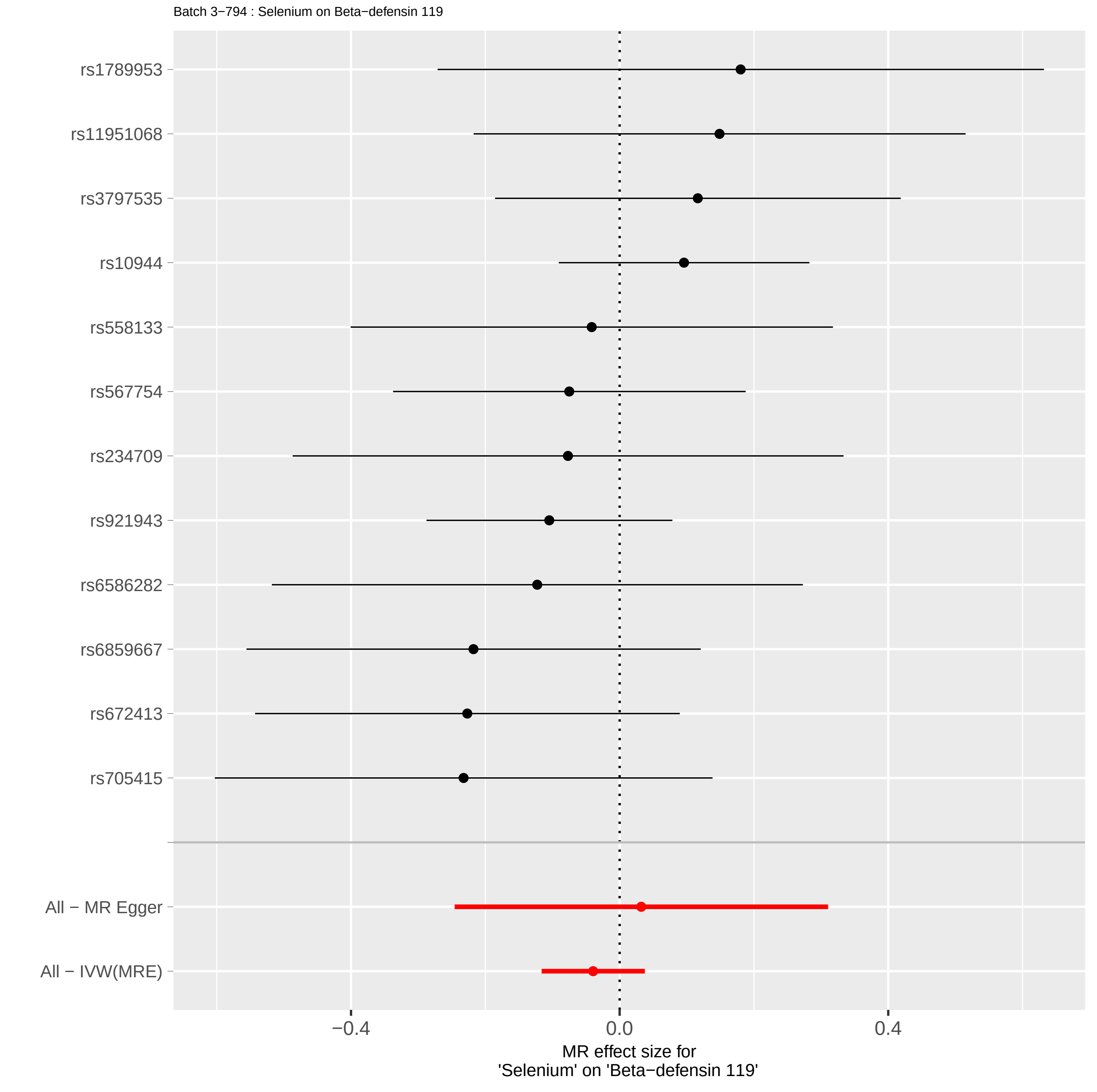

Batch 3−794 : Selenium on Beta−defensin 119
rs1789953
rs11951068
rs3797535
rs10944
rs558133
rs567754
rs234709
rs921943
rs6586282
rs6859667
rs672413
rs705415
All − MR Egger
All − IVW(MRE)
−0.4
0.0
0.4
MR effect size for
'Selenium' on 'Beta−defensin 119'

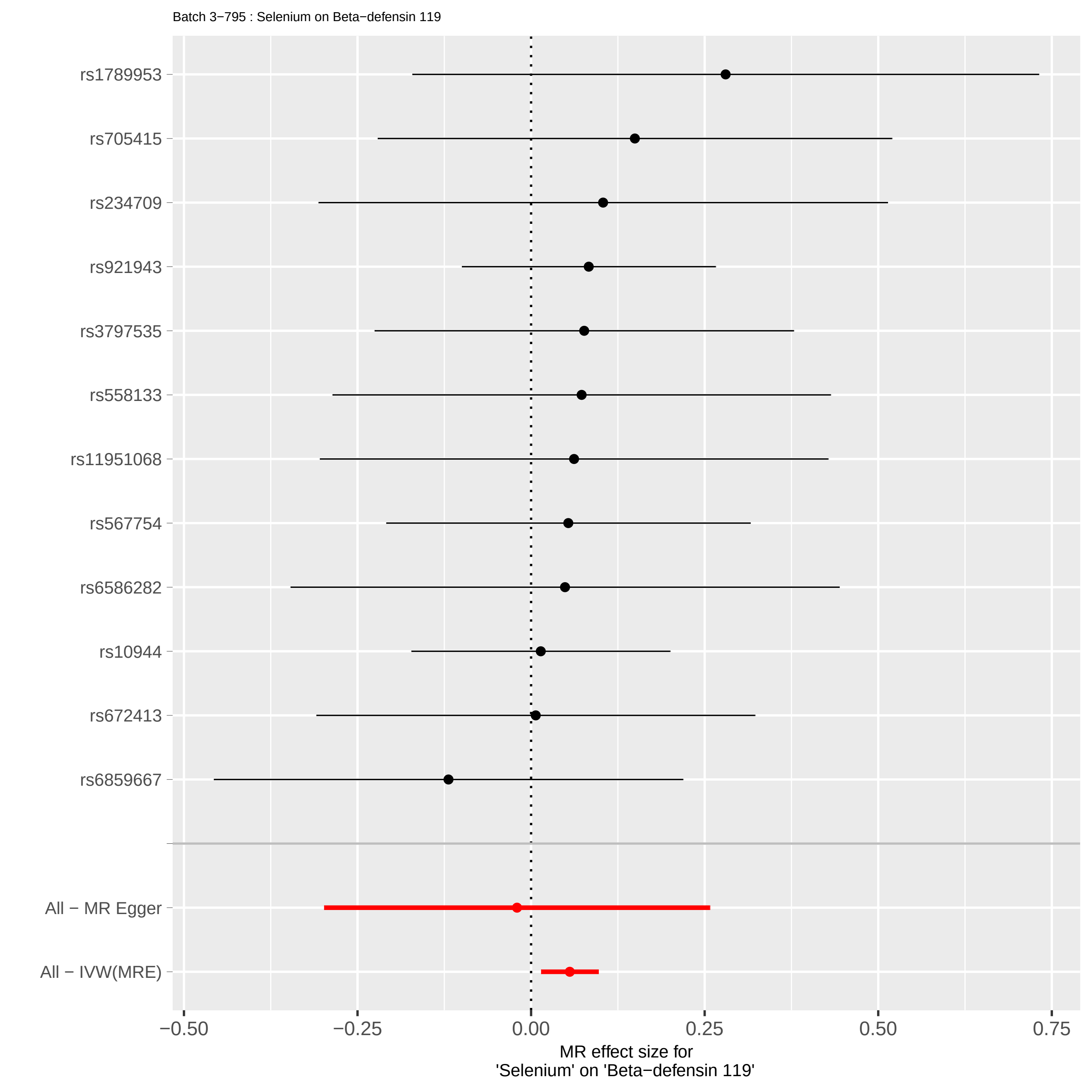

Batch 3−795 : Selenium on Beta−defensin 119
rs1789953
rs705415
rs234709
rs921943
rs3797535
rs558133
rs11951068
rs567754
rs6586282
rs10944
rs672413
rs6859667
All − MR Egger
All − IVW(MRE)
−0.50
−0.25
0.00
0.25
0.50
0.75
MR effect size for
'Selenium' on 'Beta−defensin 119'

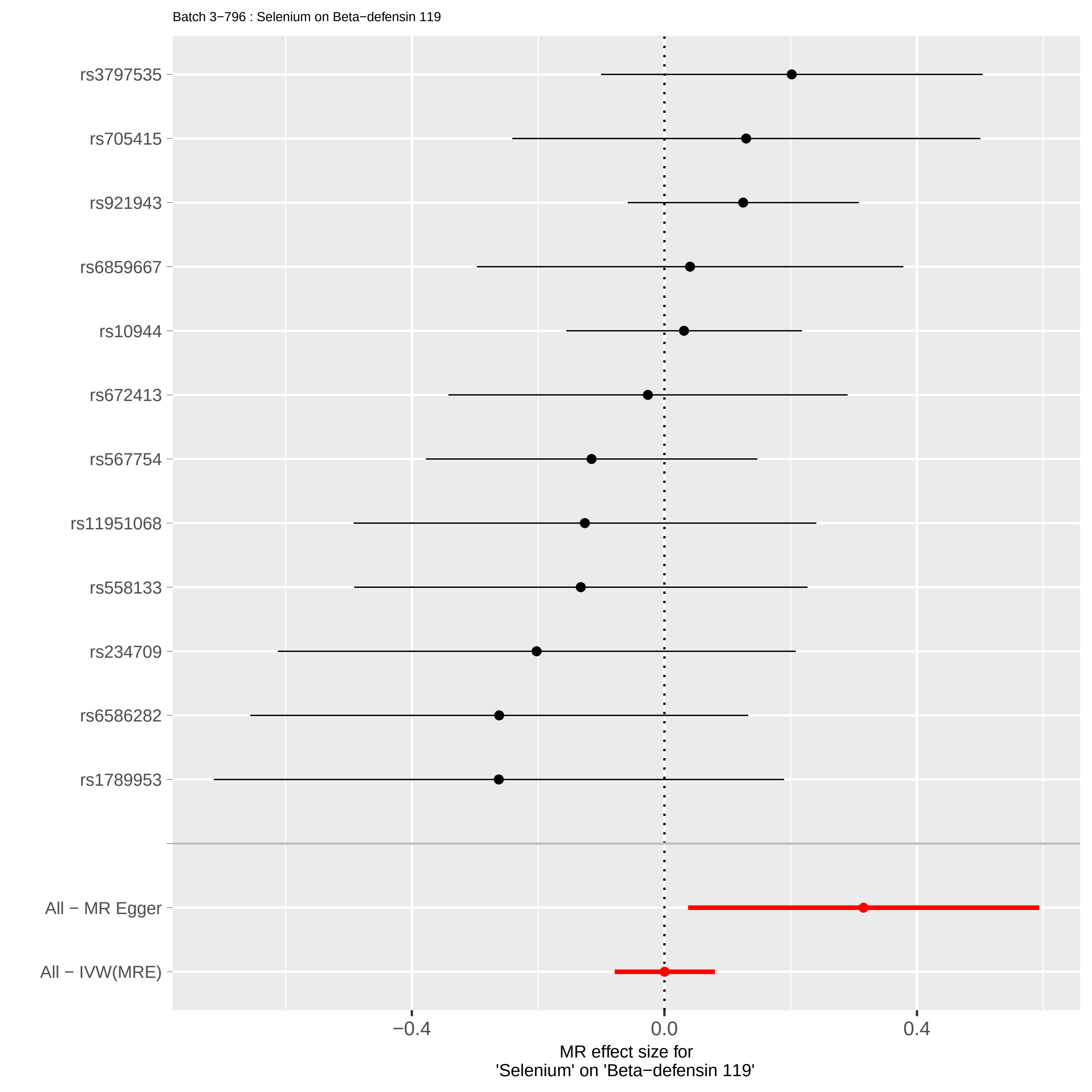
Batch 3−796 : Selenium on Beta−defensin 119
rs3797535
rs705415
rs921943
rs6859667
rs10944
rs672413
rs567754
rs11951068
rs558133
rs234709
rs6586282
rs1789953
All − MR Egger
All − IVW(MRE)
−0.4
0.0
0.4
MR effect size for
'Selenium' on 'Beta−defensin 119'

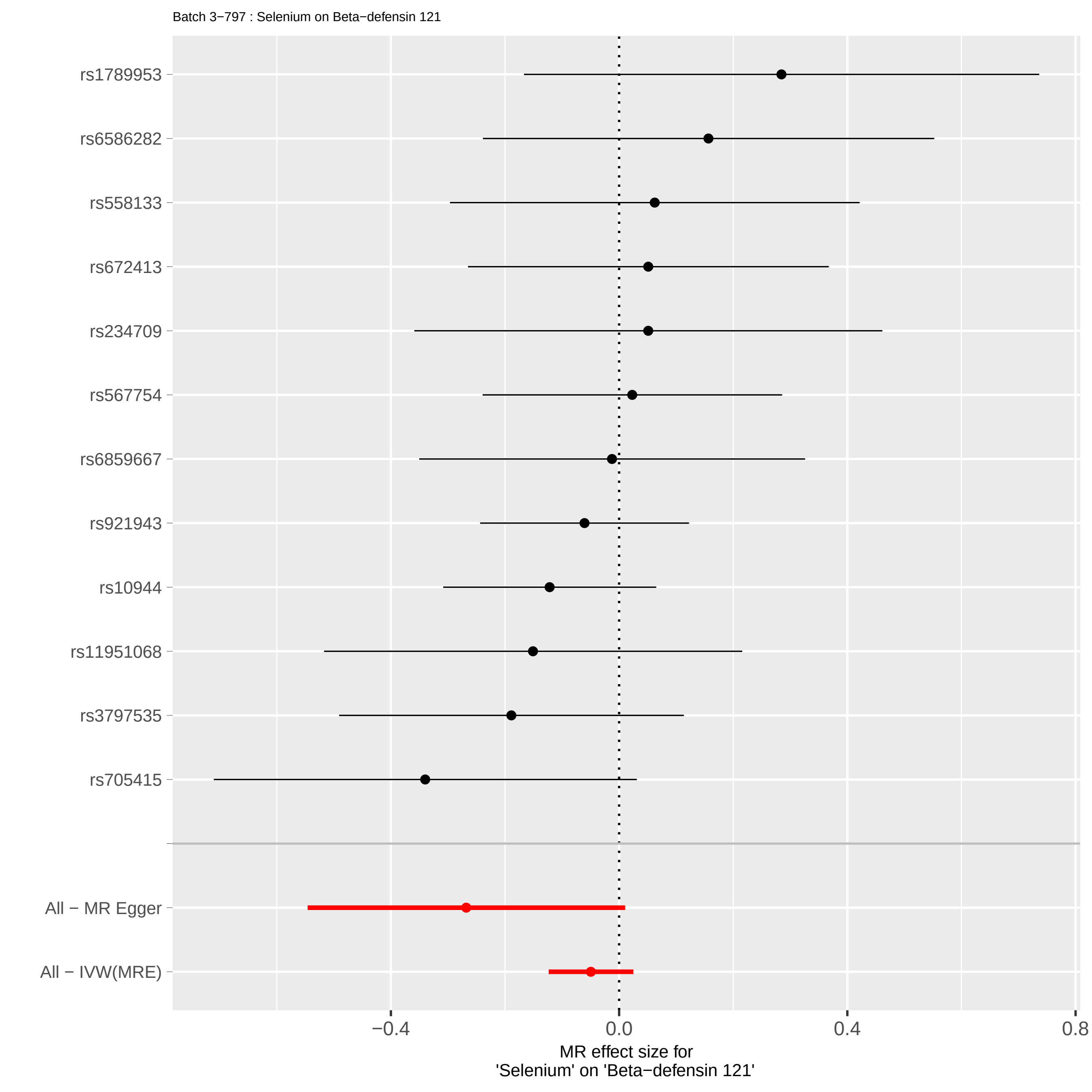
Batch 3−797 : Selenium on Beta−defensin 121
rs1789953
rs6586282
rs558133
rs672413
rs234709
rs567754
rs6859667
rs921943
rs10944
rs11951068
rs3797535
rs705415
All − MR Egger
All − IVW(MRE)
−0.4
0.0
0.4
0.8
MR effect size for
'Selenium' on 'Beta−defensin 121'

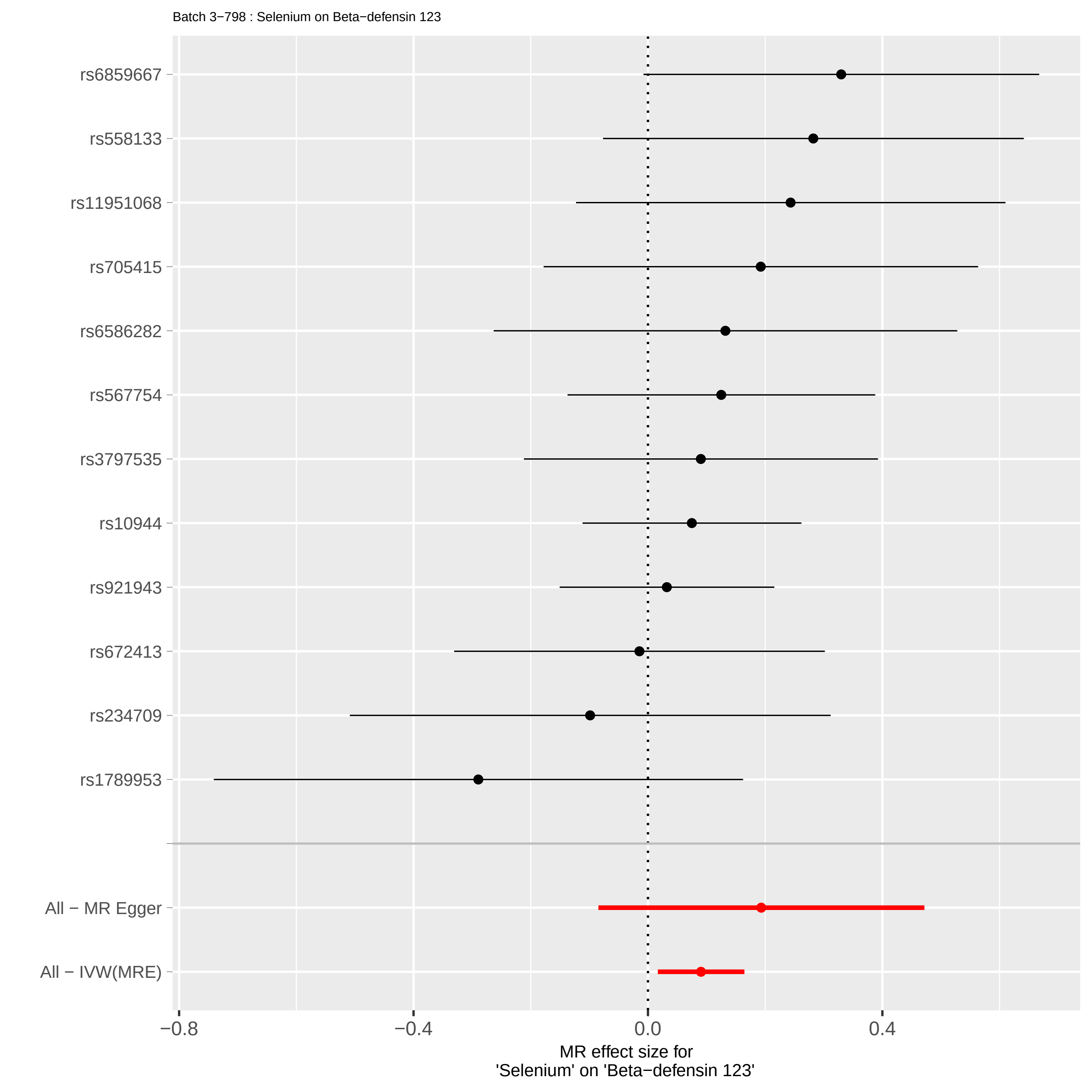
Batch 3−798 : Selenium on Beta−defensin 123
rs6859667
rs558133
rs11951068
rs705415
rs6586282
rs567754
rs3797535
rs10944
rs921943
rs672413
rs234709
rs1789953
All − MR Egger
All − IVW(MRE)
−0.8
−0.4
0.0
0.4
MR effect size for
'Selenium' on 'Beta−defensin 123'

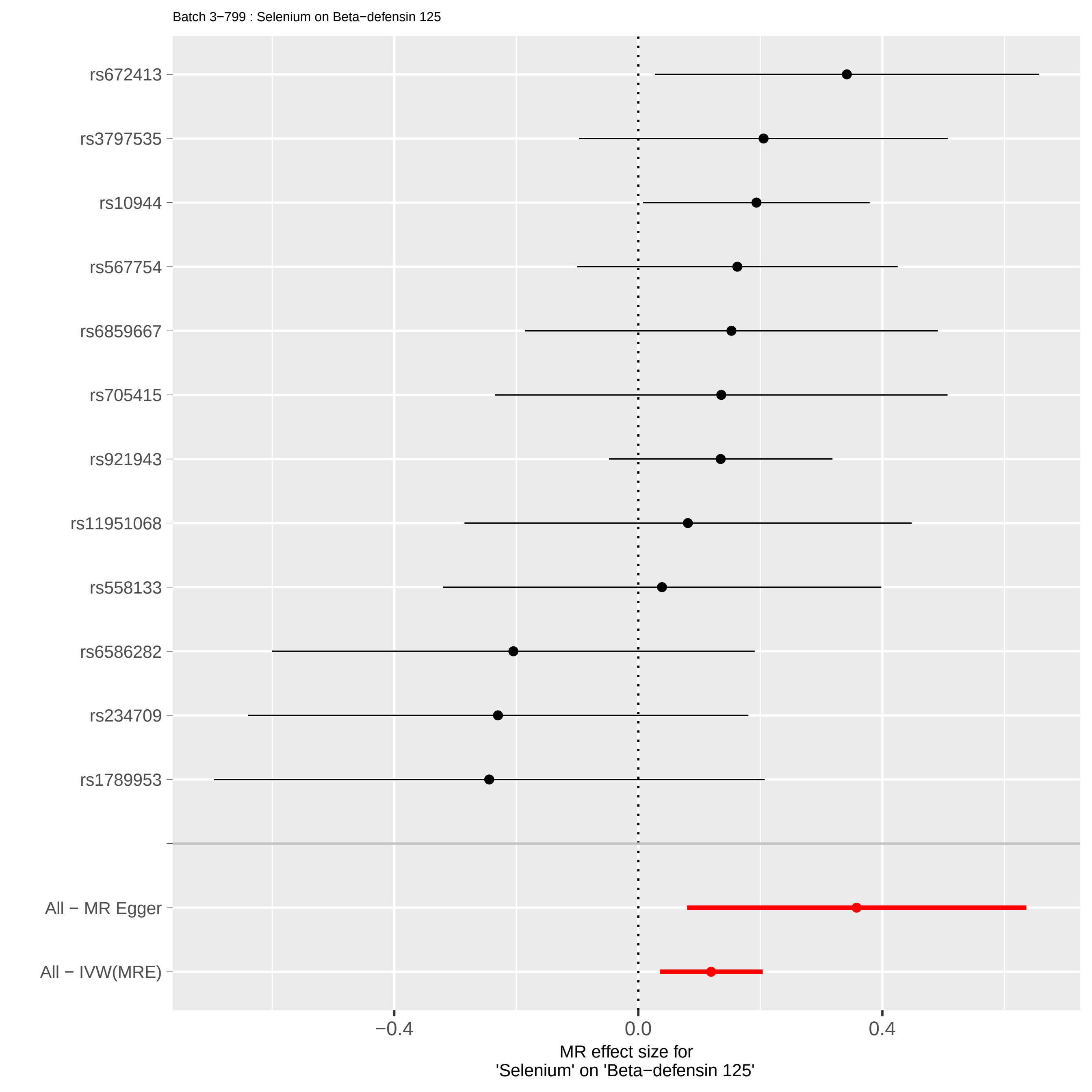
Batch 3−799 : Selenium on Beta−defensin 125
rs672413
rs3797535
rs10944
rs567754
rs6859667
rs705415
rs921943
rs11951068
rs558133
rs6586282
rs234709
rs1789953
All − MR Egger
All − IVW(MRE)
−0.4
0.0
0.4
MR effect size for
'Selenium' on 'Beta−defensin 125'

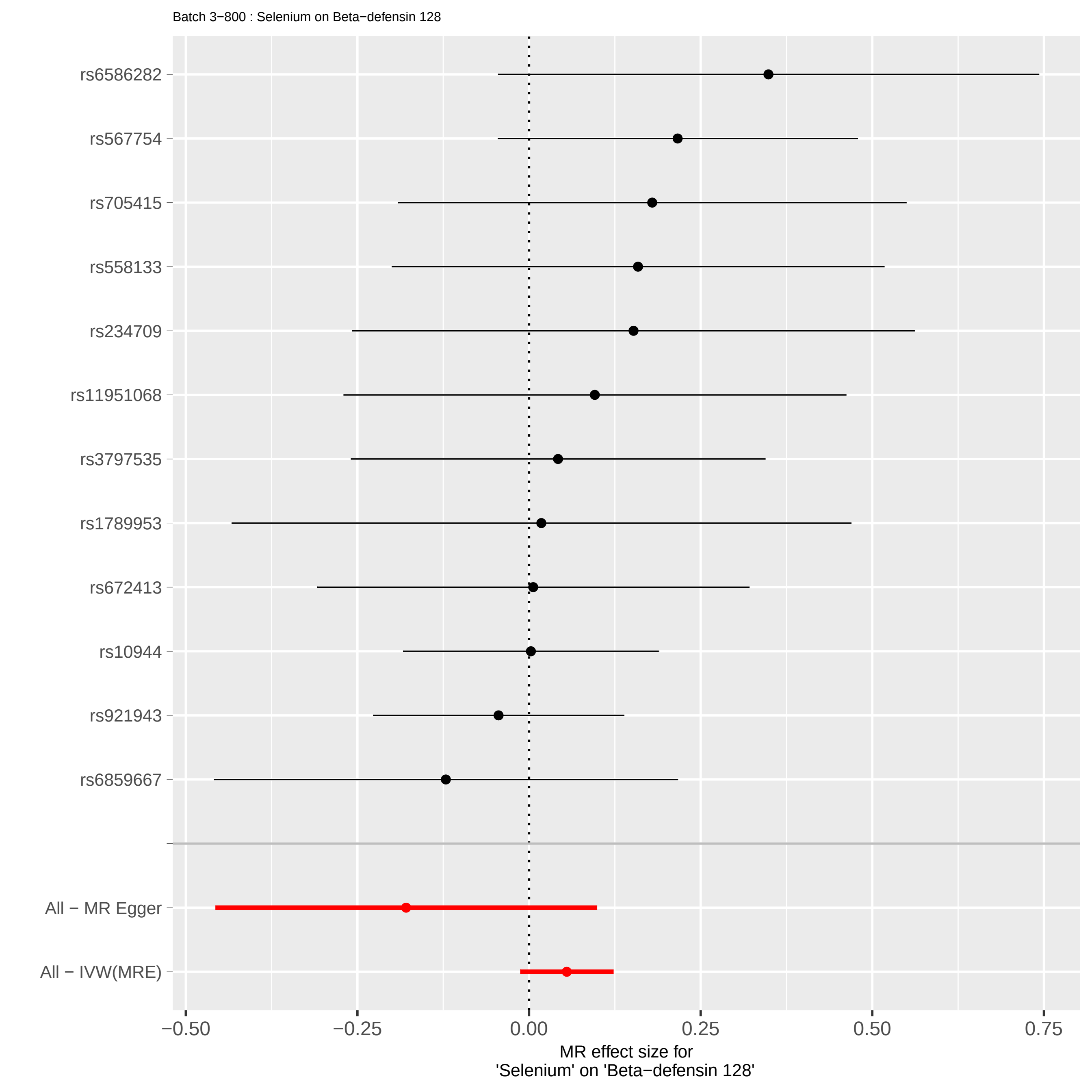

Batch 3−800 : Selenium on Beta−defensin 128
rs6586282
rs567754
rs705415
rs558133
rs234709
rs11951068
rs3797535
rs1789953
rs672413
rs10944
rs921943
rs6859667
All − MR Egger
All − IVW(MRE)
−0.50
−0.25
0.00
0.25
0.50
0.75
MR effect size for
'Selenium' on 'Beta−defensin 128'

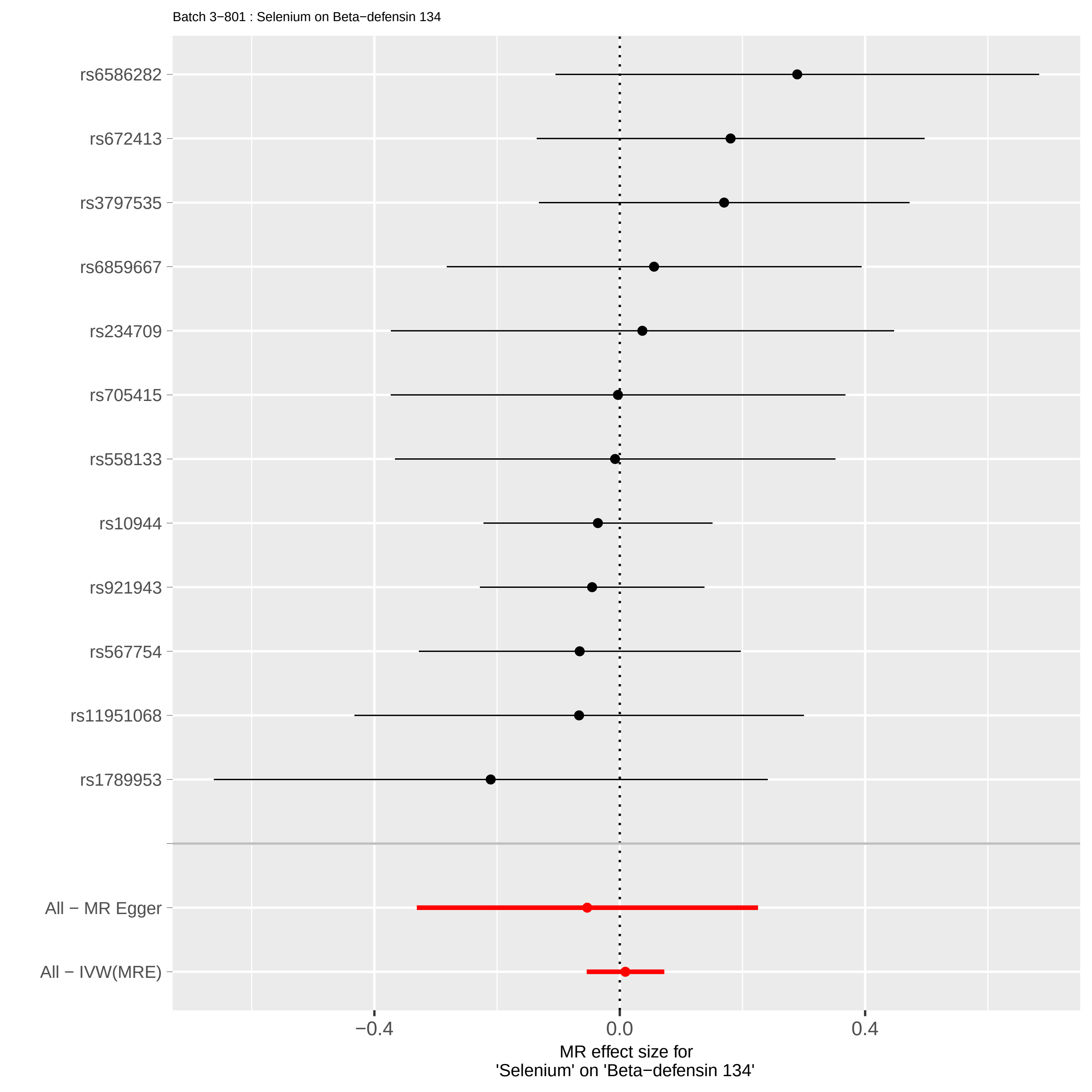
Batch 3−801 : Selenium on Beta−defensin 134
rs6586282
rs672413
rs3797535
rs6859667
rs234709
rs705415
rs558133
rs10944
rs921943
rs567754
rs11951068
rs1789953
All − MR Egger
All − IVW(MRE)
−0.4
0.0
0.4
MR effect size for
'Selenium' on 'Beta−defensin 134'

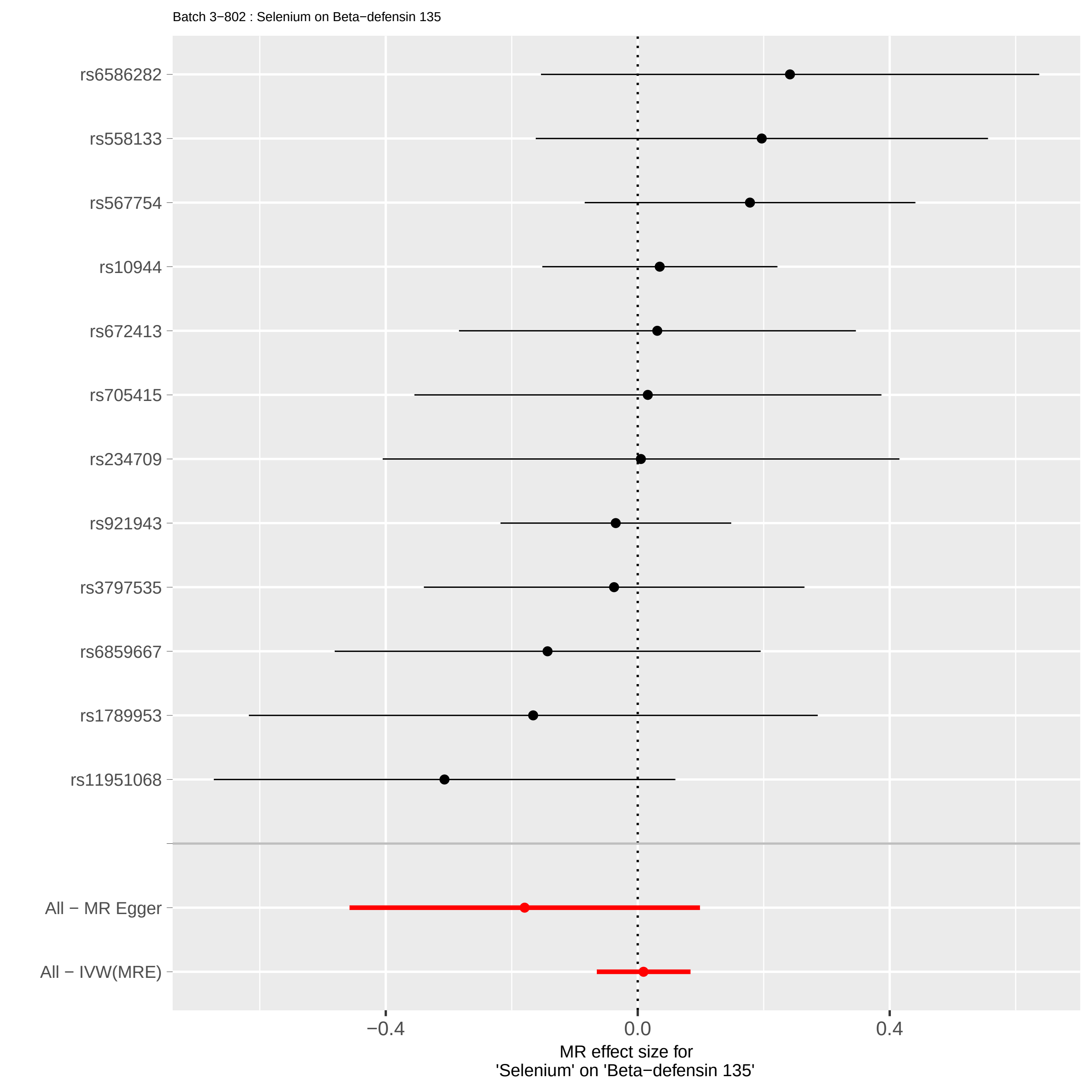

Batch 3−802 : Selenium on Beta−defensin 135
rs6586282
rs558133
rs567754
rs10944
rs672413
rs705415
rs234709
rs921943
rs3797535
rs6859667
rs1789953
rs11951068
All − MR Egger
All − IVW(MRE)
−0.4
0.0
0.4
MR effect size for
'Selenium' on 'Beta−defensin 135'

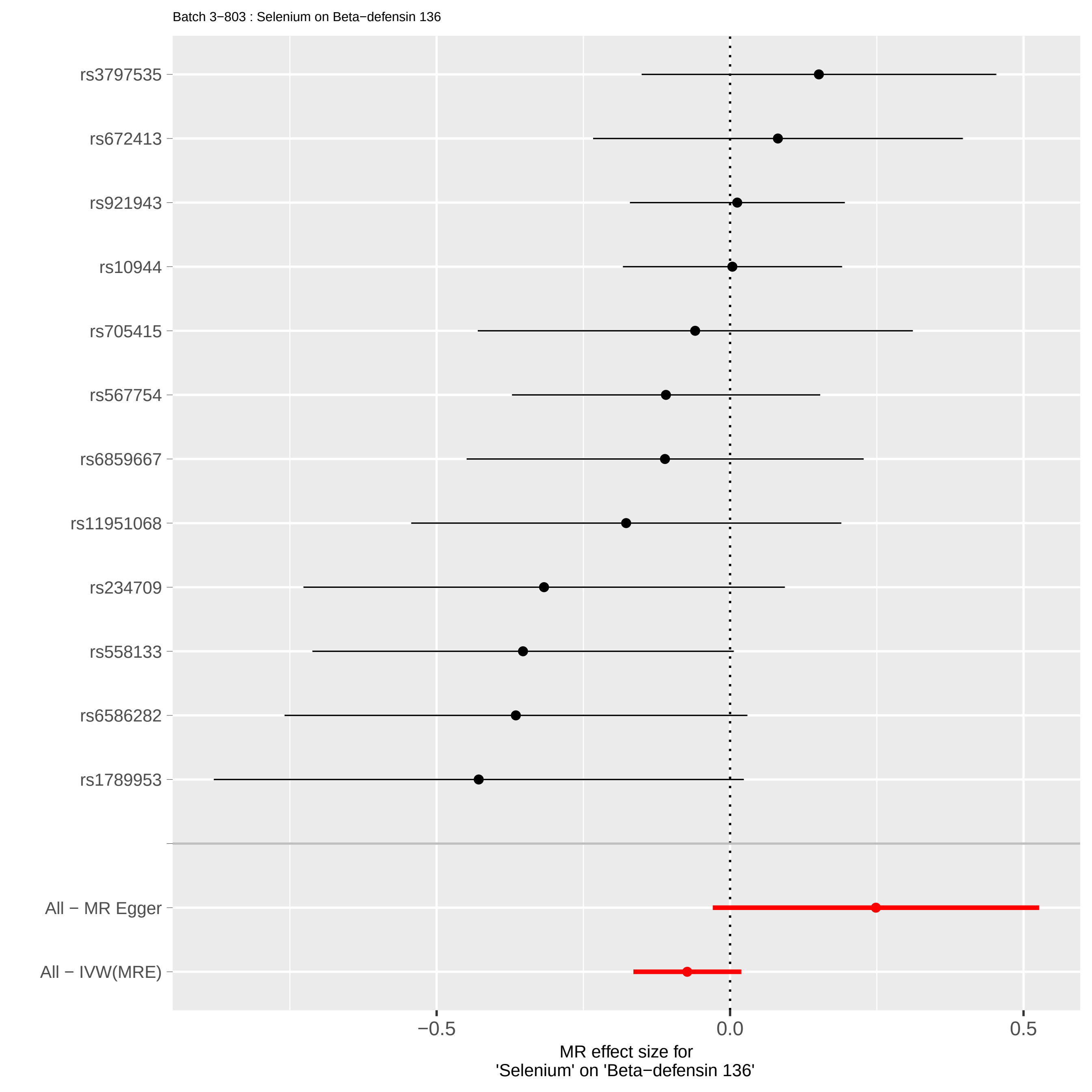
Batch 3−803 : Selenium on Beta−defensin 136
rs3797535
rs672413
rs921943
rs10944
rs705415
rs567754
rs6859667
rs11951068
rs234709
rs558133
rs6586282
rs1789953
All − MR Egger
All − IVW(MRE)
−0.5
0.0
0.5
MR effect size for
'Selenium' on 'Beta−defensin 136'

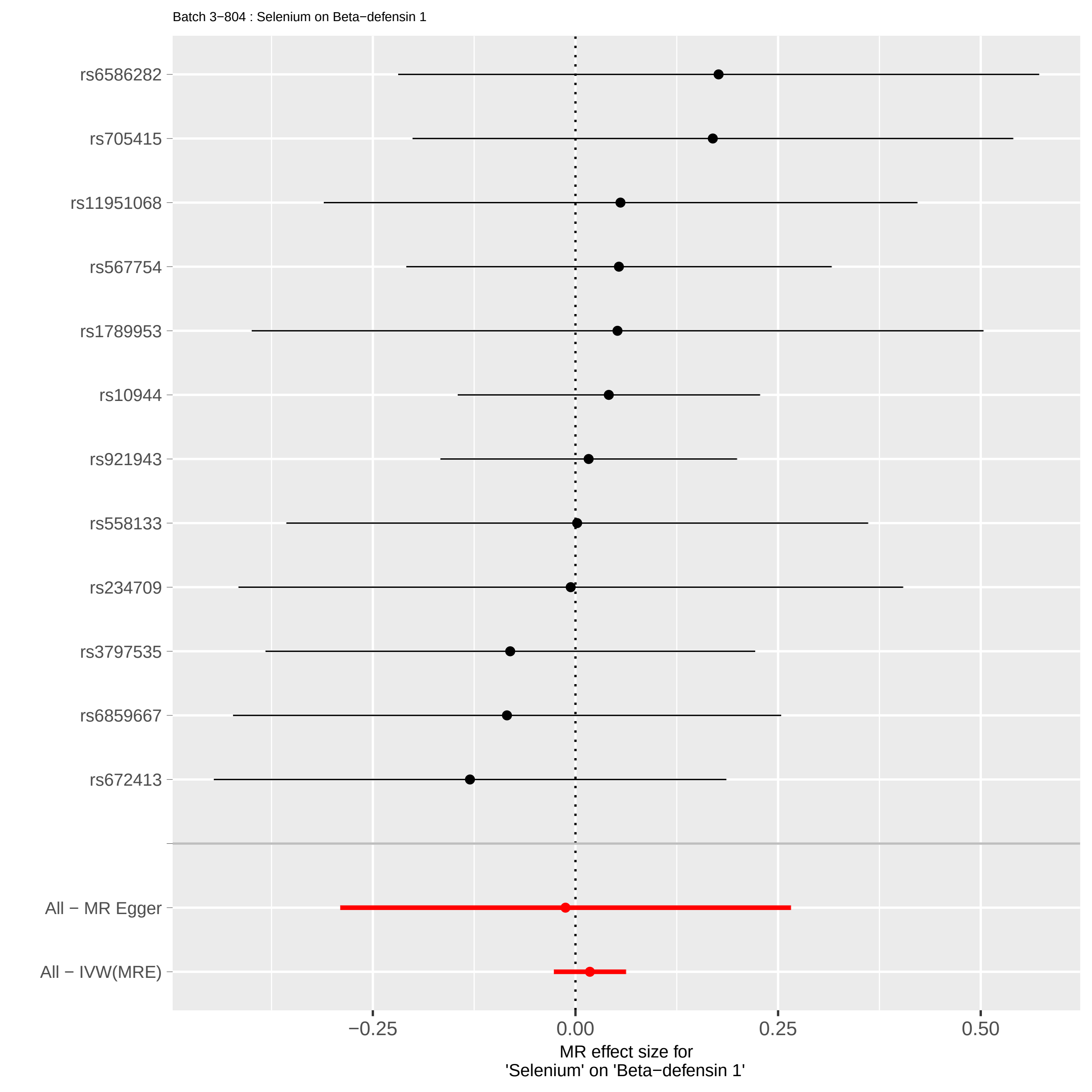
Batch 3−804 : Selenium on Beta−defensin 1
rs6586282
rs705415
rs11951068
rs567754
rs1789953
rs10944
rs921943
rs558133
rs234709
rs3797535
rs6859667
rs672413
All − MR Egger
All − IVW(MRE)
−0.25
0.00
0.25
0.50
MR effect size for
'Selenium' on 'Beta−defensin 1'

Batch 3−805 : Selenium on Protein DEPP

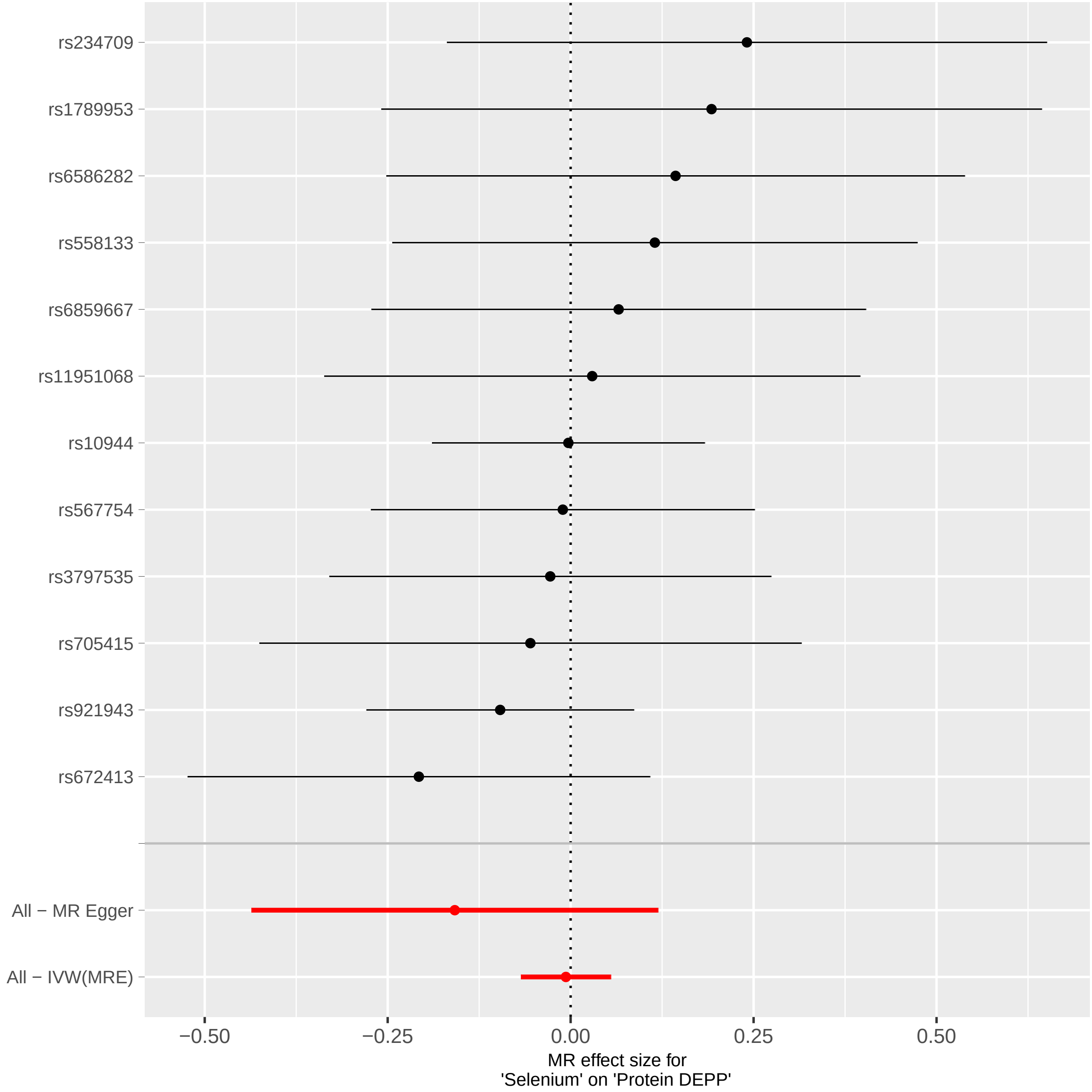

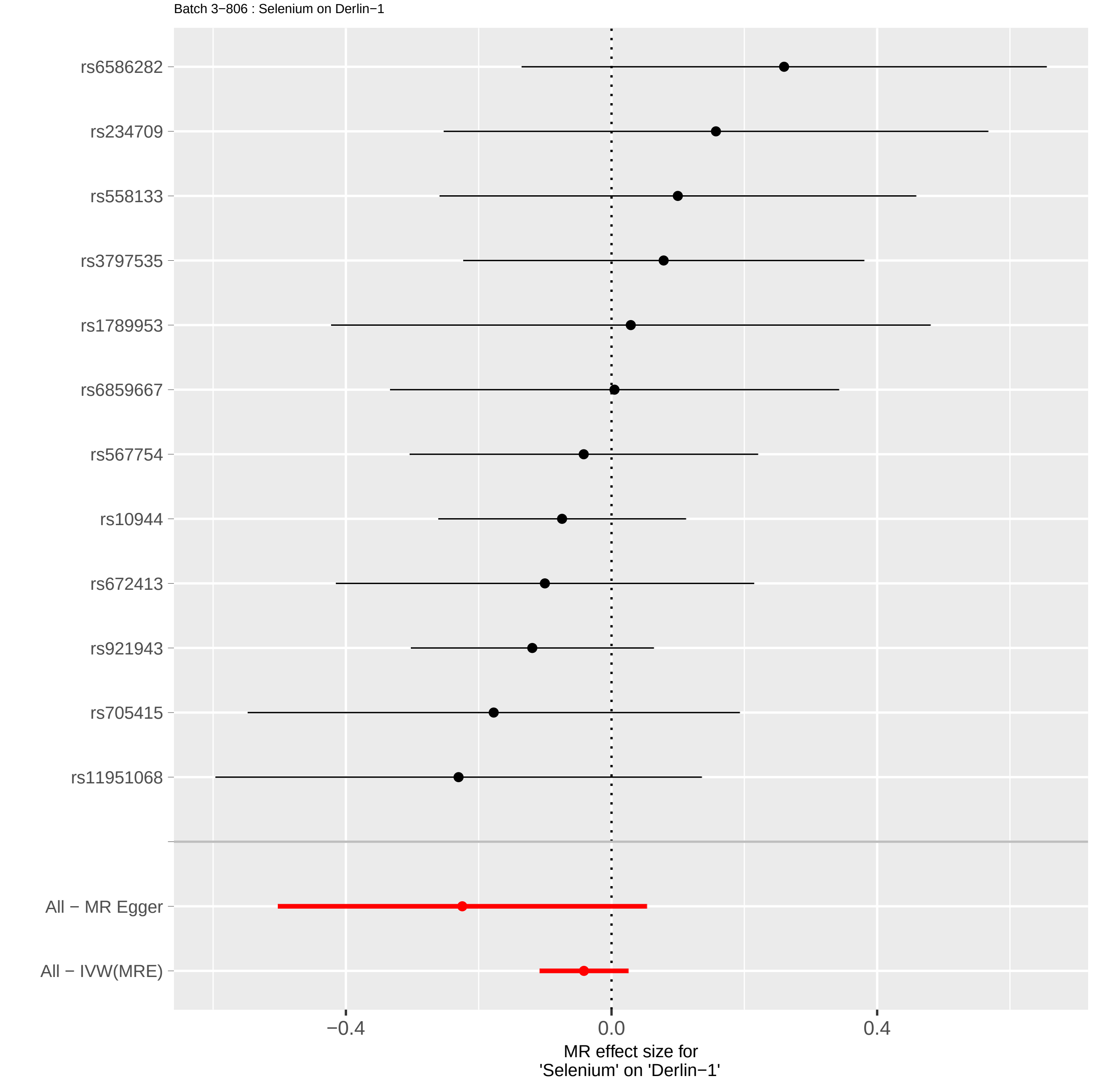

Batch 3−806 : Selenium on Derlin−1
rs6586282
rs234709
rs558133
rs3797535
rs1789953
rs6859667
rs567754
rs10944
rs672413
rs921943
rs705415
rs11951068
All − MR Egger
All − IVW(MRE)
−0.4
0.0
0.4
MR effect size for
'Selenium' on 'Derlin−1'

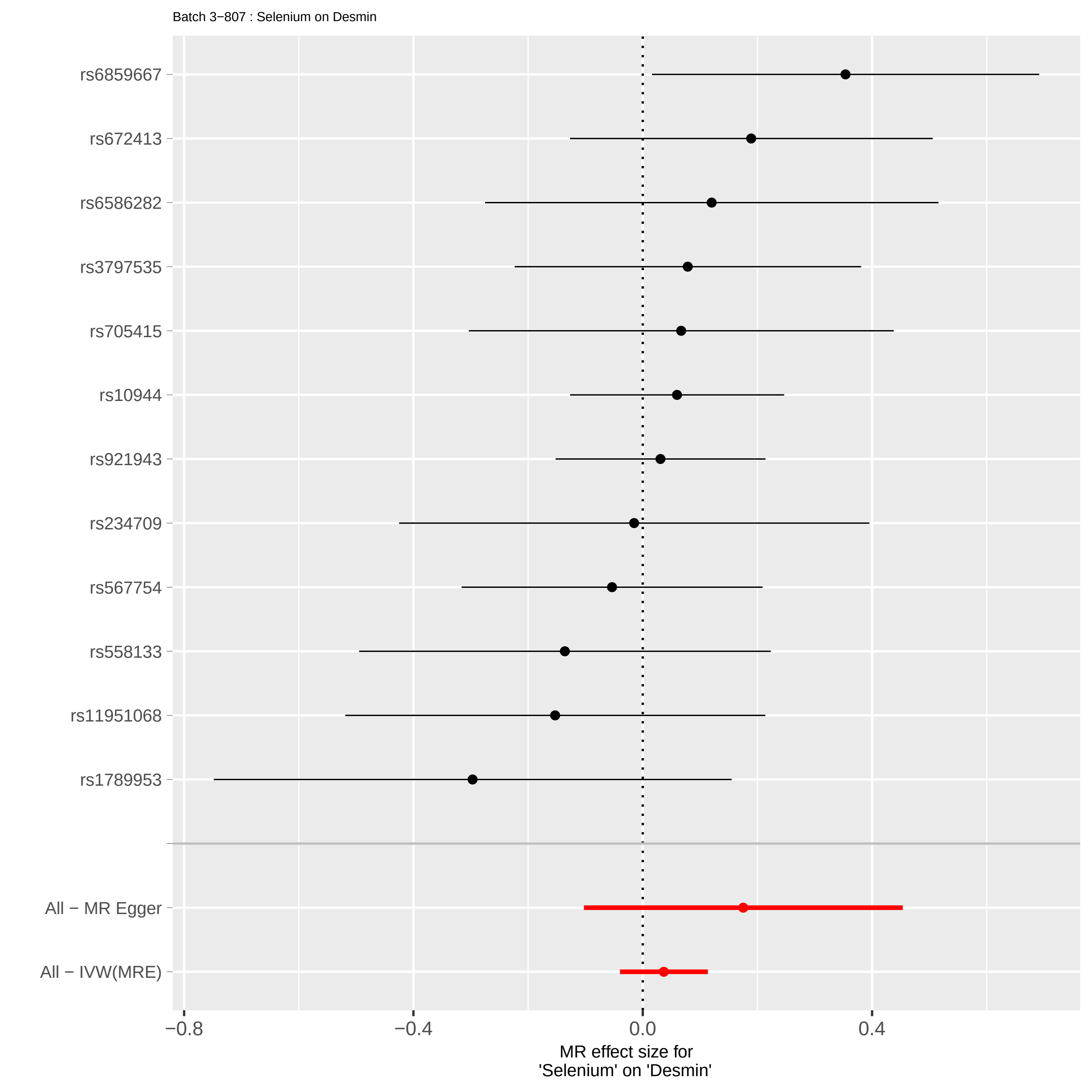

Batch 3−807 : Selenium on Desmin
rs6859667
rs672413
rs6586282
rs3797535
rs705415
rs10944
rs921943
rs234709
rs567754
rs558133
rs11951068
rs1789953
All − MR Egger
All − IVW(MRE)
−0.8
−0.4
0.0
0.4
MR effect size for
'Selenium' on 'Desmin'

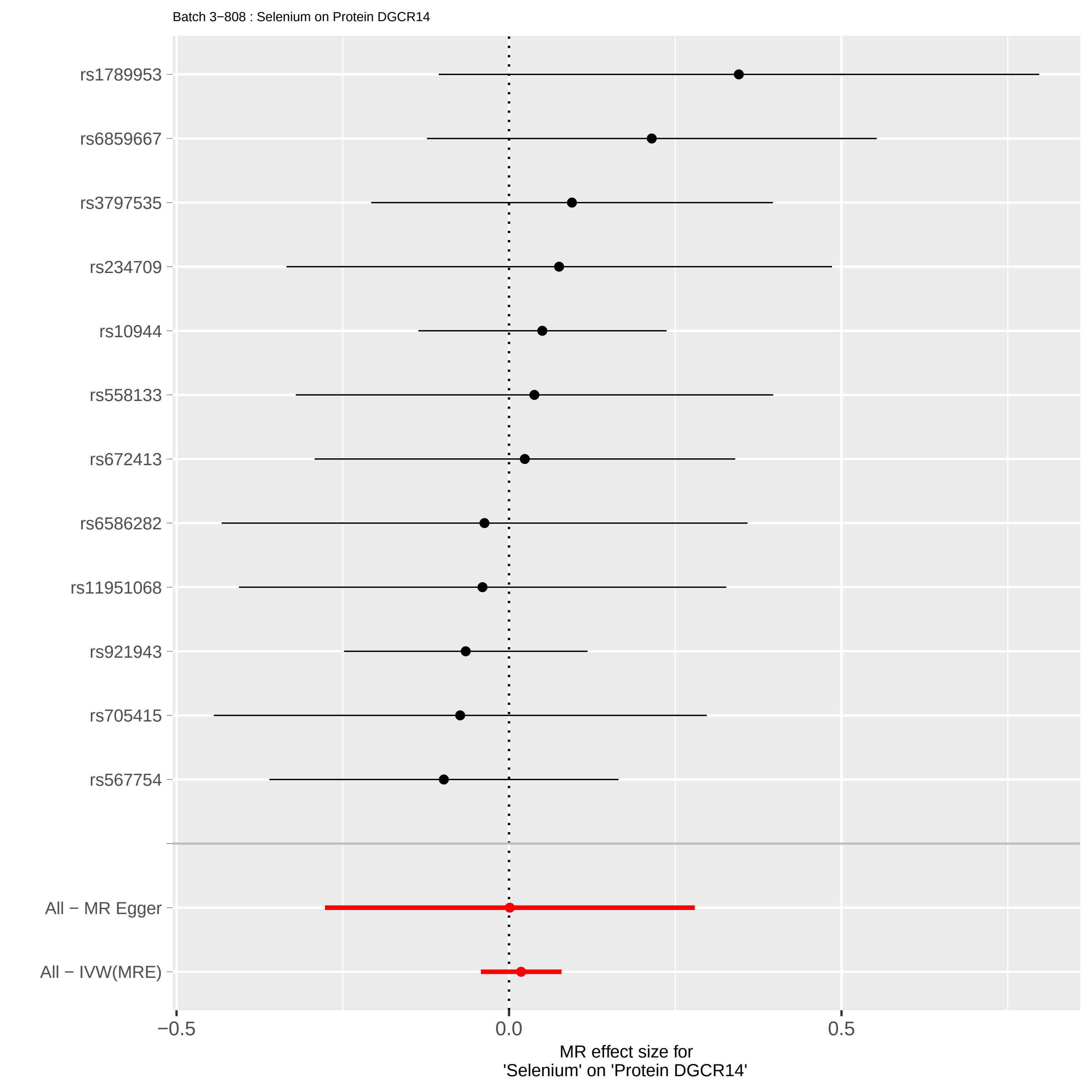
Batch 3−808 : Selenium on Protein DGCR14
rs1789953
rs6859667
rs3797535
rs234709
rs10944
rs558133
rs672413
rs6586282
rs11951068
rs921943
rs705415
rs567754
All − MR Egger
All − IVW(MRE)
−0.5
0.0
0.5
MR effect size for
'Selenium' on 'Protein DGCR14'

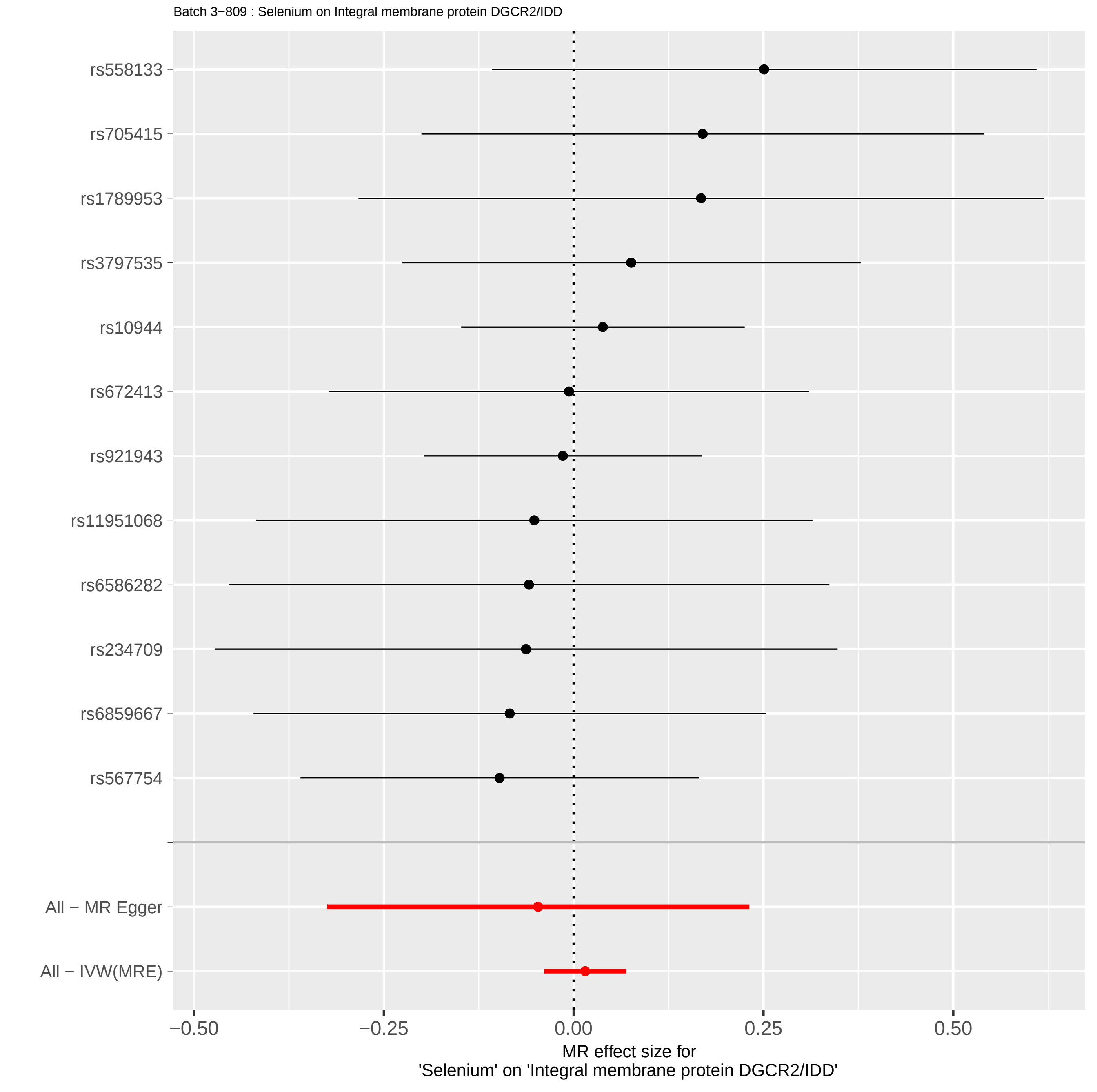

Batch 3−809 : Selenium on Integral membrane protein DGCR2/IDD
rs558133
rs705415
rs1789953
rs3797535
rs10944
rs672413
rs921943
rs11951068
rs6586282
rs234709
rs6859667
rs567754
All − MR Egger
All − IVW(MRE)
−0.50
−0.25
0.00
0.25
0.50
MR effect size for
'Selenium' on 'Integral membrane protein DGCR2/IDD'

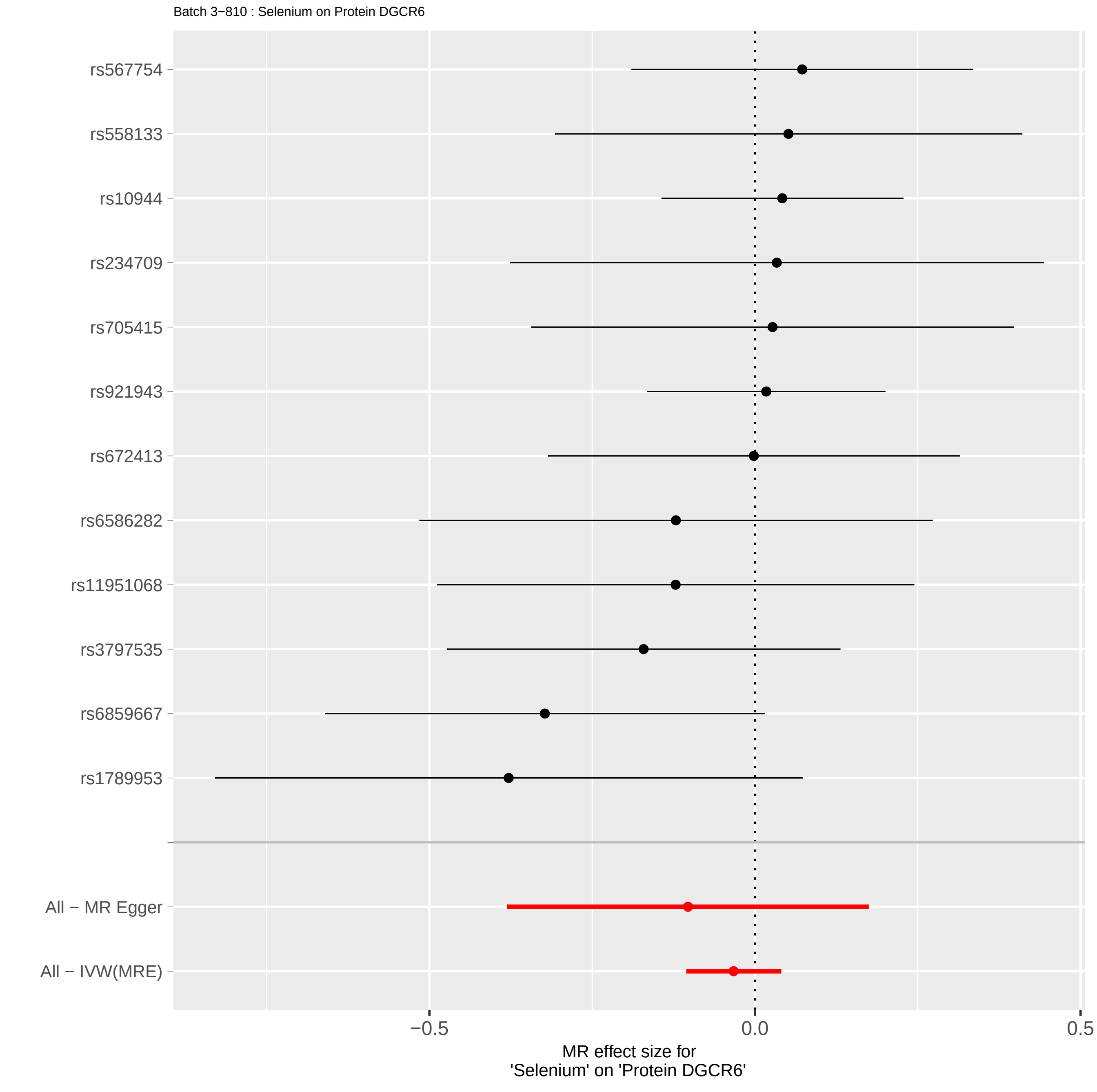

Batch 3−810 : Selenium on Protein DGCR6
rs567754
rs558133
rs10944
rs234709
rs705415
rs921943
rs672413
rs6586282
rs11951068
rs3797535
rs6859667
rs1789953
All − MR Egger
All − IVW(MRE)
−0.5
0.0
0.5
MR effect size for
'Selenium' on 'Protein DGCR6'

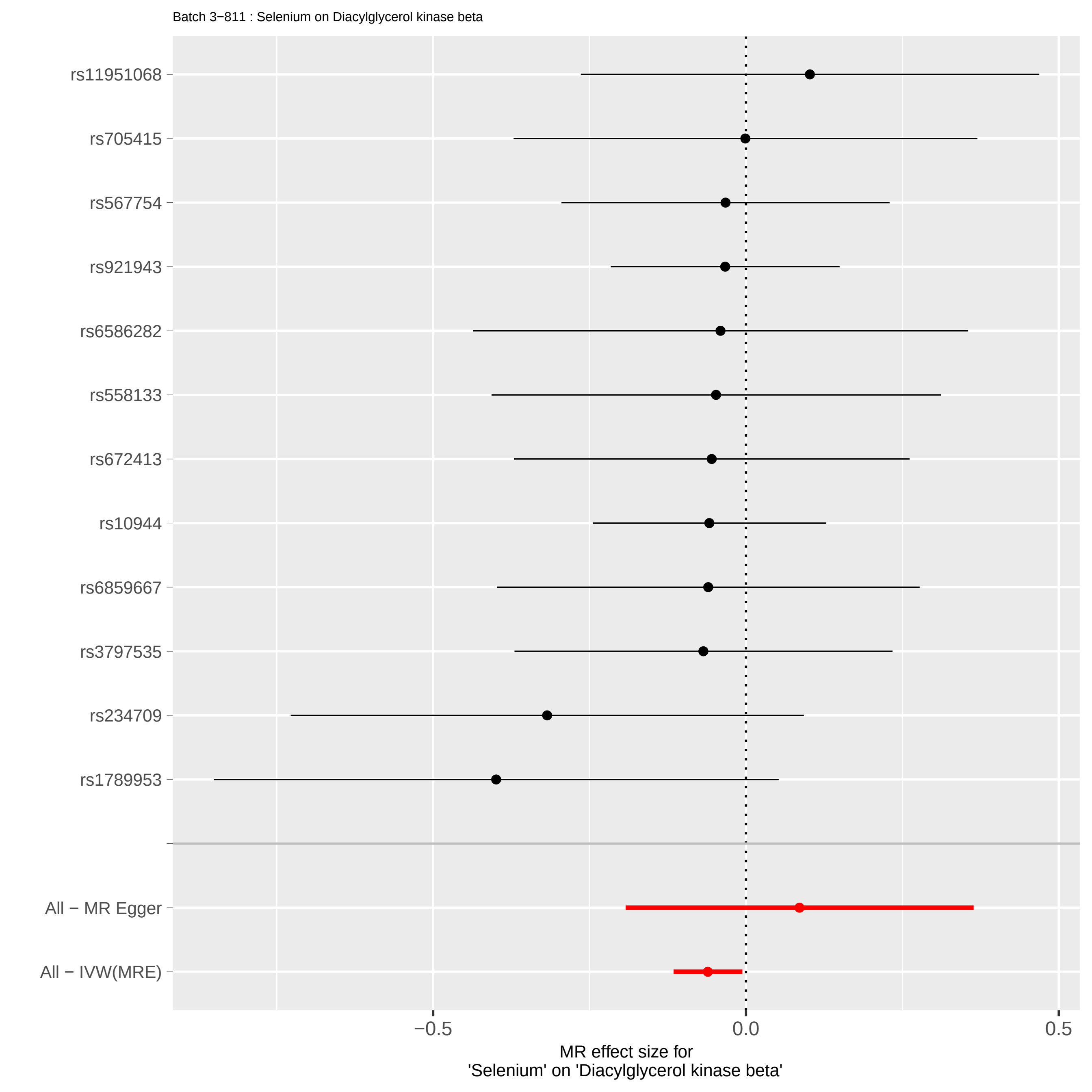
Batch 3−811 : Selenium on Diacylglycerol kinase beta
rs11951068
rs705415
rs567754
rs921943
rs6586282
rs558133
rs672413
rs10944
rs6859667
rs3797535
rs234709
rs1789953
All − MR Egger
All − IVW(MRE)
−0.5
0.0
0.5
MR effect size for
'Selenium' on 'Diacylglycerol kinase beta'

Batch 3−812 : Selenium on Dihydrofolate reductase

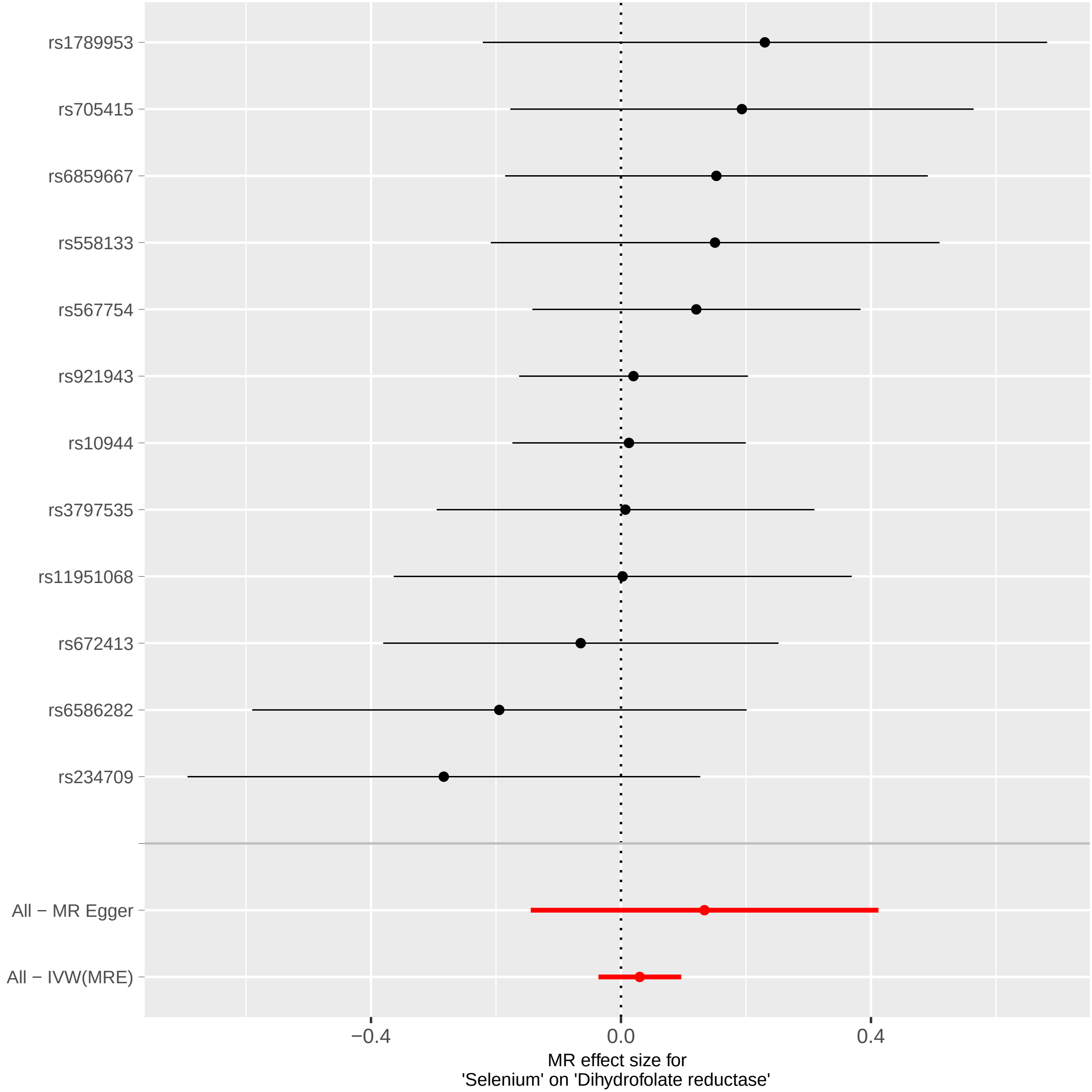

Batch 3−813 : Selenium on Desert hedgehog protein N−product

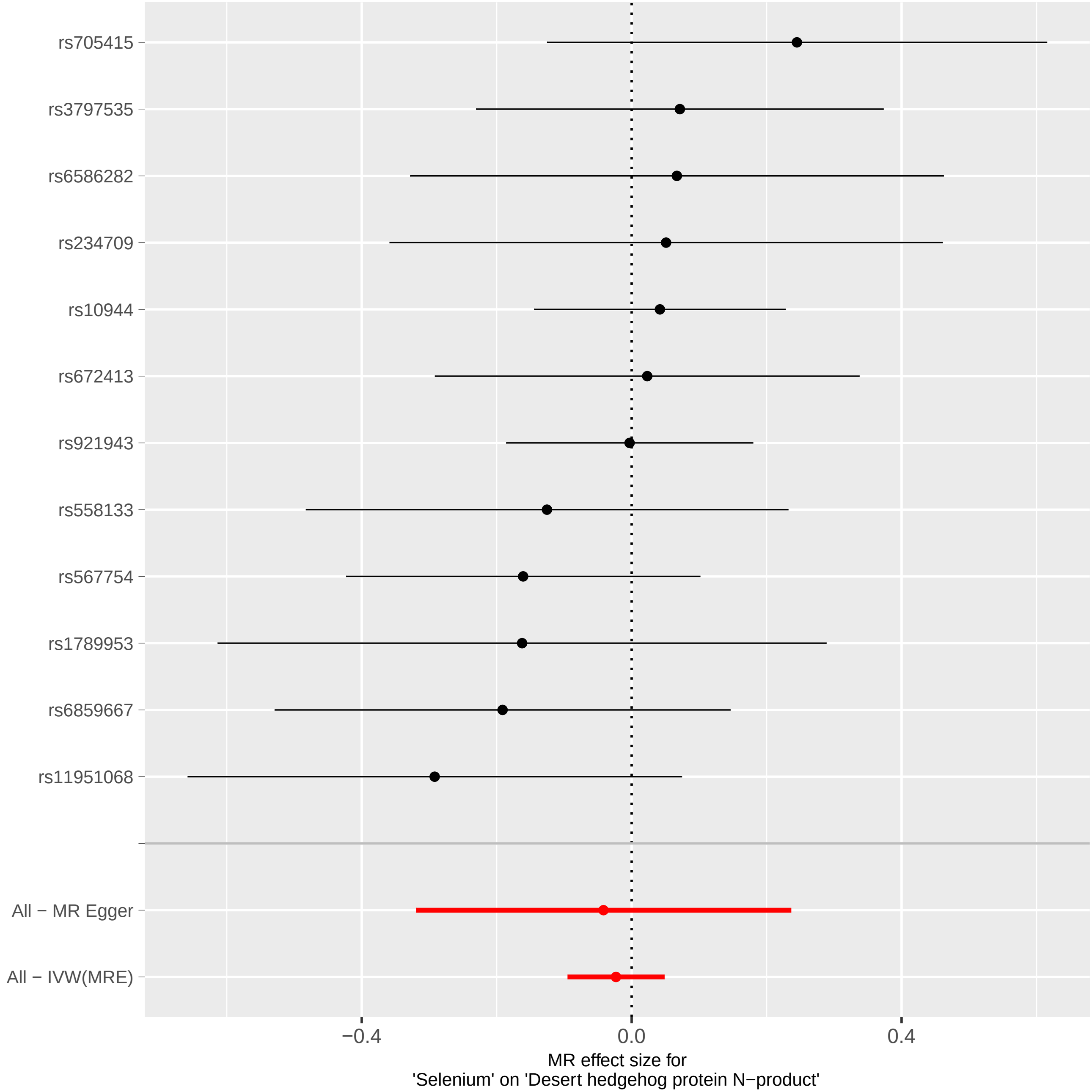

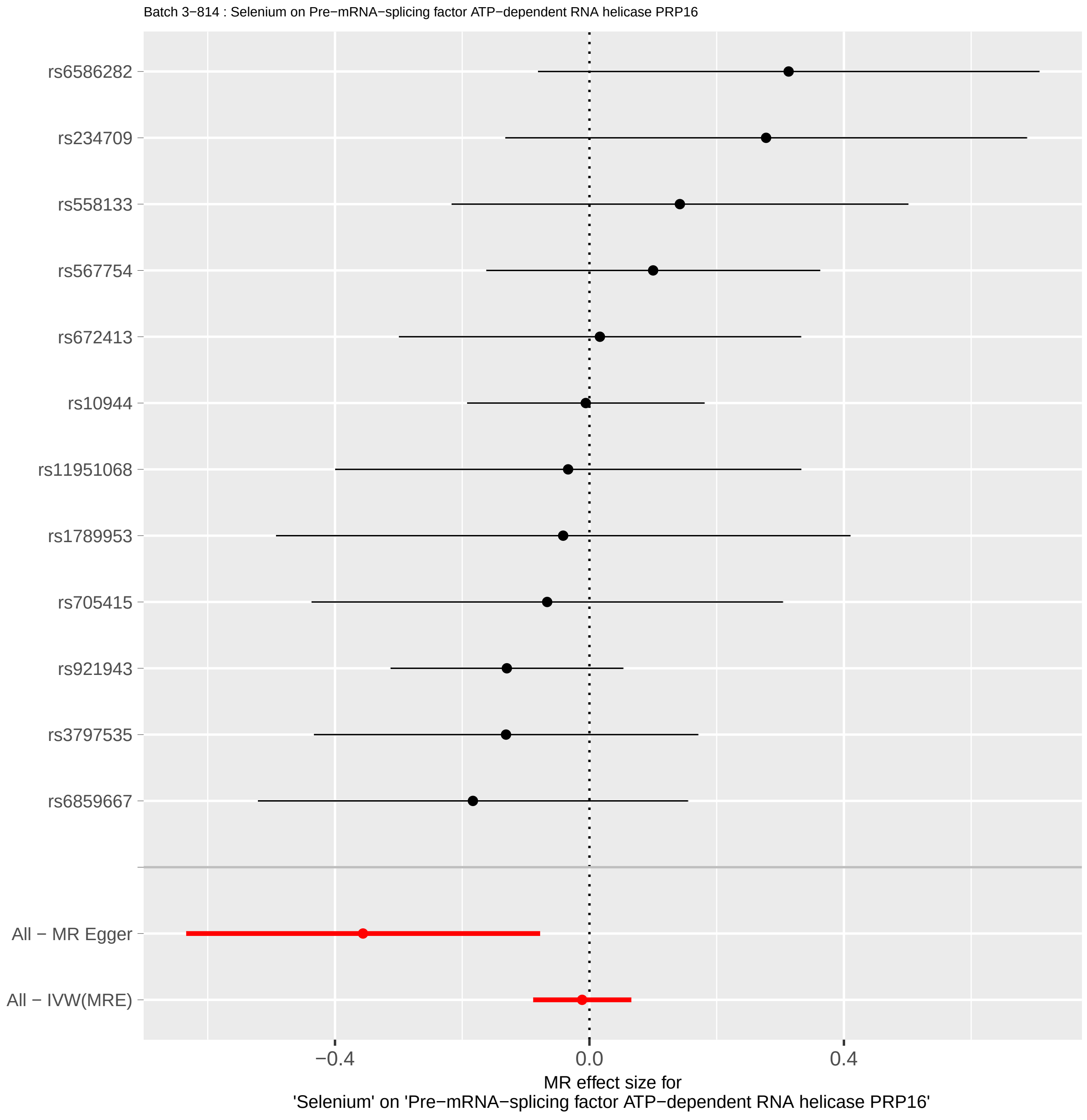
Batch 3−814 : Selenium on Pre−mRNA−splicing factor ATP−dependent RNA helicase PRP16
rs6586282
rs234709
rs558133
rs567754
rs672413
rs10944
rs11951068
rs1789953
rs705415
rs921943
rs3797535
rs6859667
All − MR Egger
All − IVW(MRE)
−0.4
0.0
0.4
MR effect size for
'Selenium' on 'Pre−mRNA−splicing factor ATP−dependent RNA helicase PRP16'

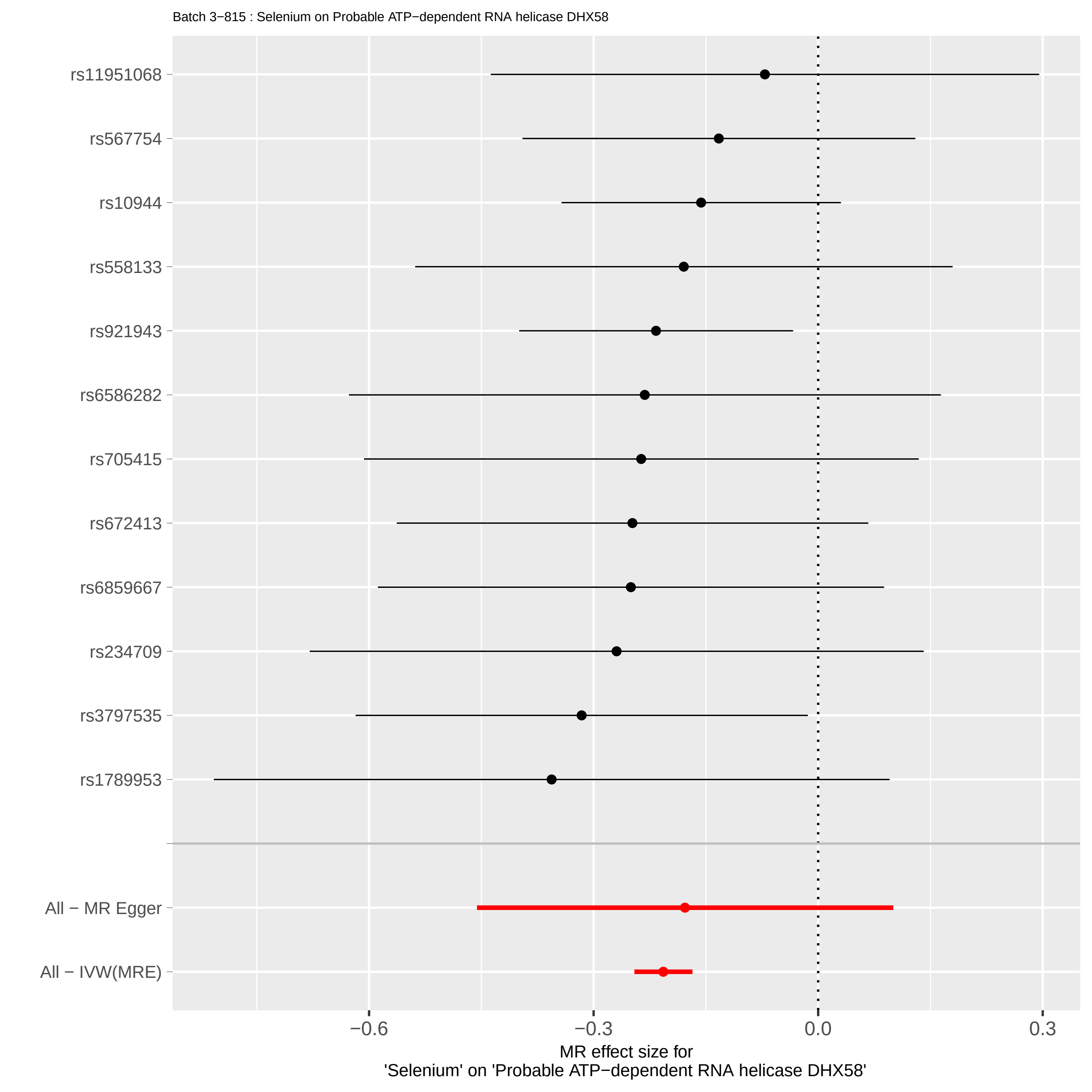

Batch 3−815 : Selenium on Probable ATP−dependent RNA helicase DHX58
rs11951068
rs567754
rs10944
rs558133
rs921943
rs6586282
rs705415
rs672413
rs6859667
rs234709
rs3797535
rs1789953
All − MR Egger
All − IVW(MRE)
−0.6
−0.3
0.0
0.3
MR effect size for
'Selenium' on 'Probable ATP−dependent RNA helicase DHX58'

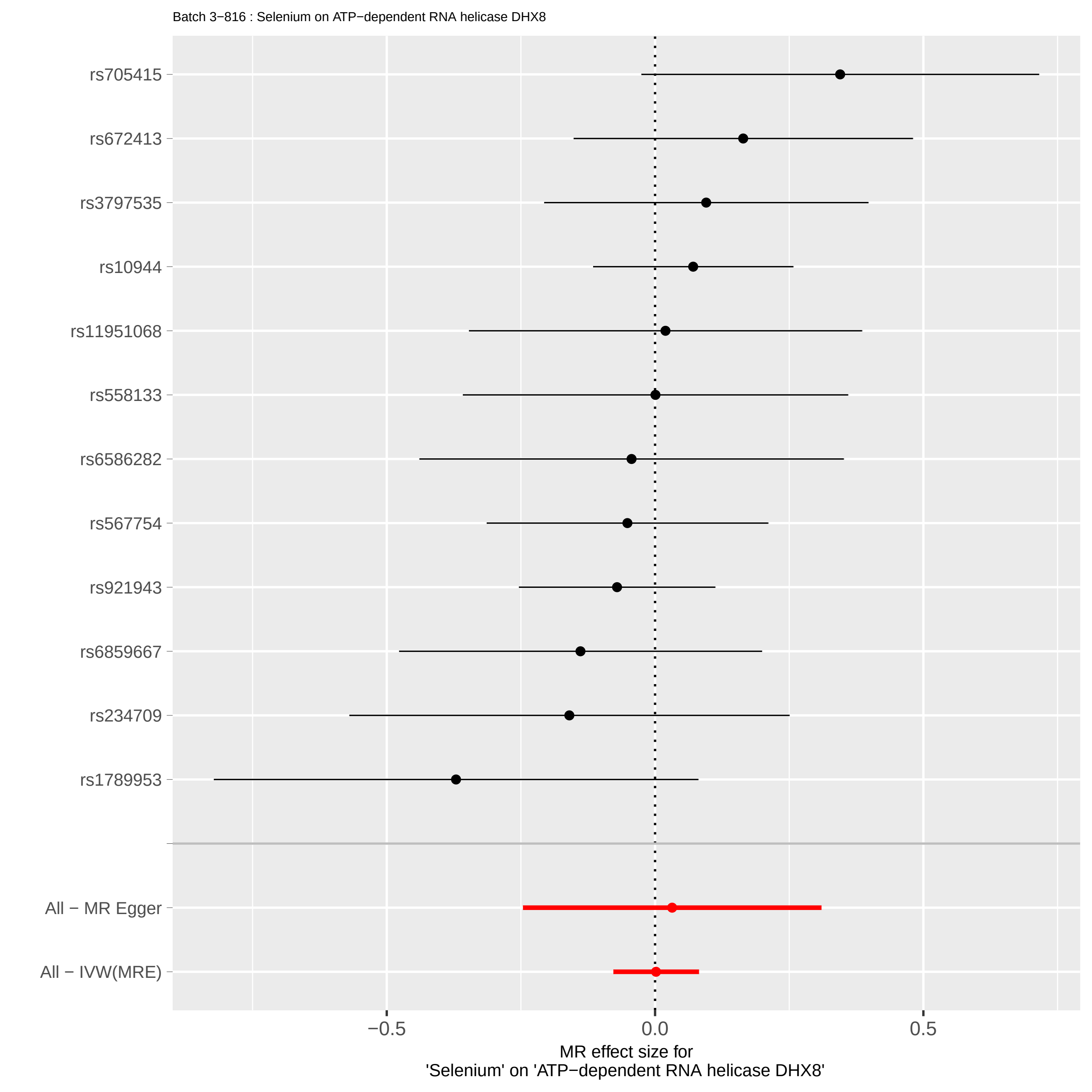

Batch 3−816 : Selenium on ATP−dependent RNA helicase DHX8
rs705415
rs672413
rs3797535
rs10944
rs11951068
rs558133
rs6586282
rs567754
rs921943
rs6859667
rs234709
rs1789953
All − MR Egger
All − IVW(MRE)
−0.5
0.0
0.5
MR effect size for
'Selenium' on 'ATP−dependent RNA helicase DHX8'

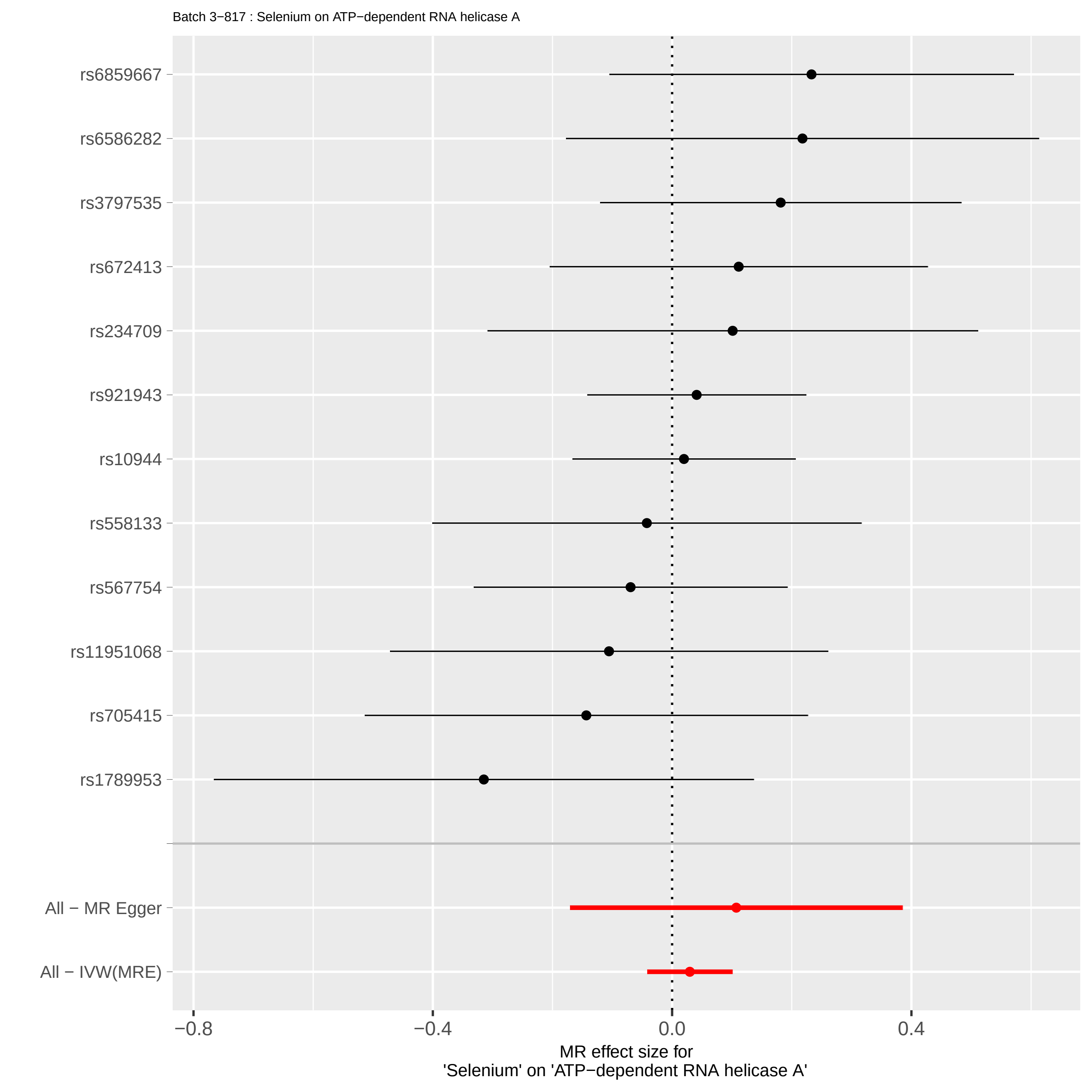
Batch 3−817 : Selenium on ATP−dependent RNA helicase A
rs6859667
rs6586282
rs3797535
rs672413
rs234709
rs921943
rs10944
rs558133
rs567754
rs11951068
rs705415
rs1789953
All − MR Egger
All − IVW(MRE)
−0.8
−0.4
0.0
0.4
MR effect size for
'Selenium' on 'ATP−dependent RNA helicase A'

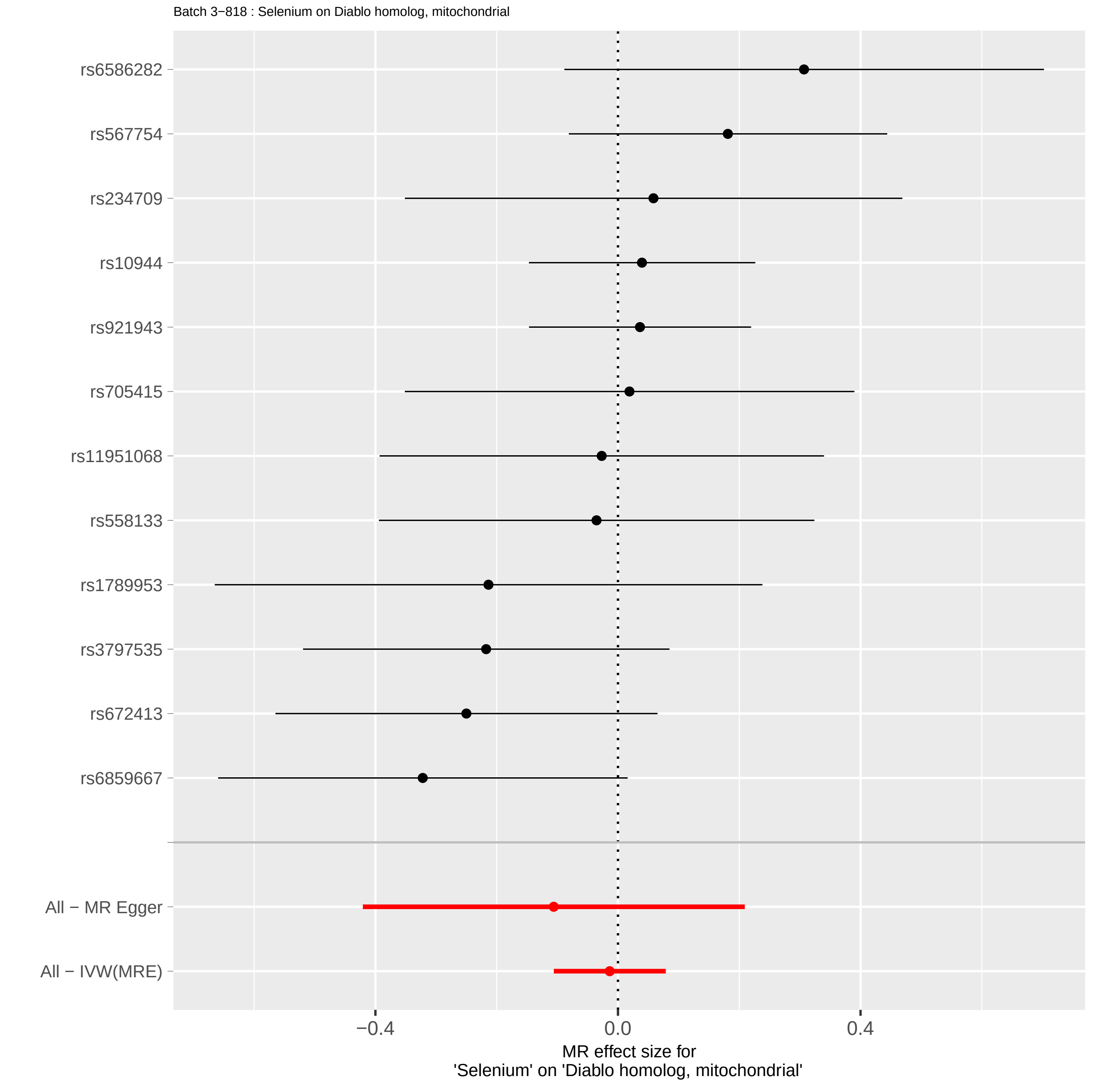
Batch 3−818 : Selenium on Diablo homolog, mitochondrial
rs6586282
rs567754
rs234709
rs10944
rs921943
rs705415
rs11951068
rs558133
rs1789953
rs3797535
rs672413
rs6859667
All − MR Egger
All − IVW(MRE)
−0.4
0.0
0.4
MR effect size for
'Selenium' on 'Diablo homolog, mitochondrial'

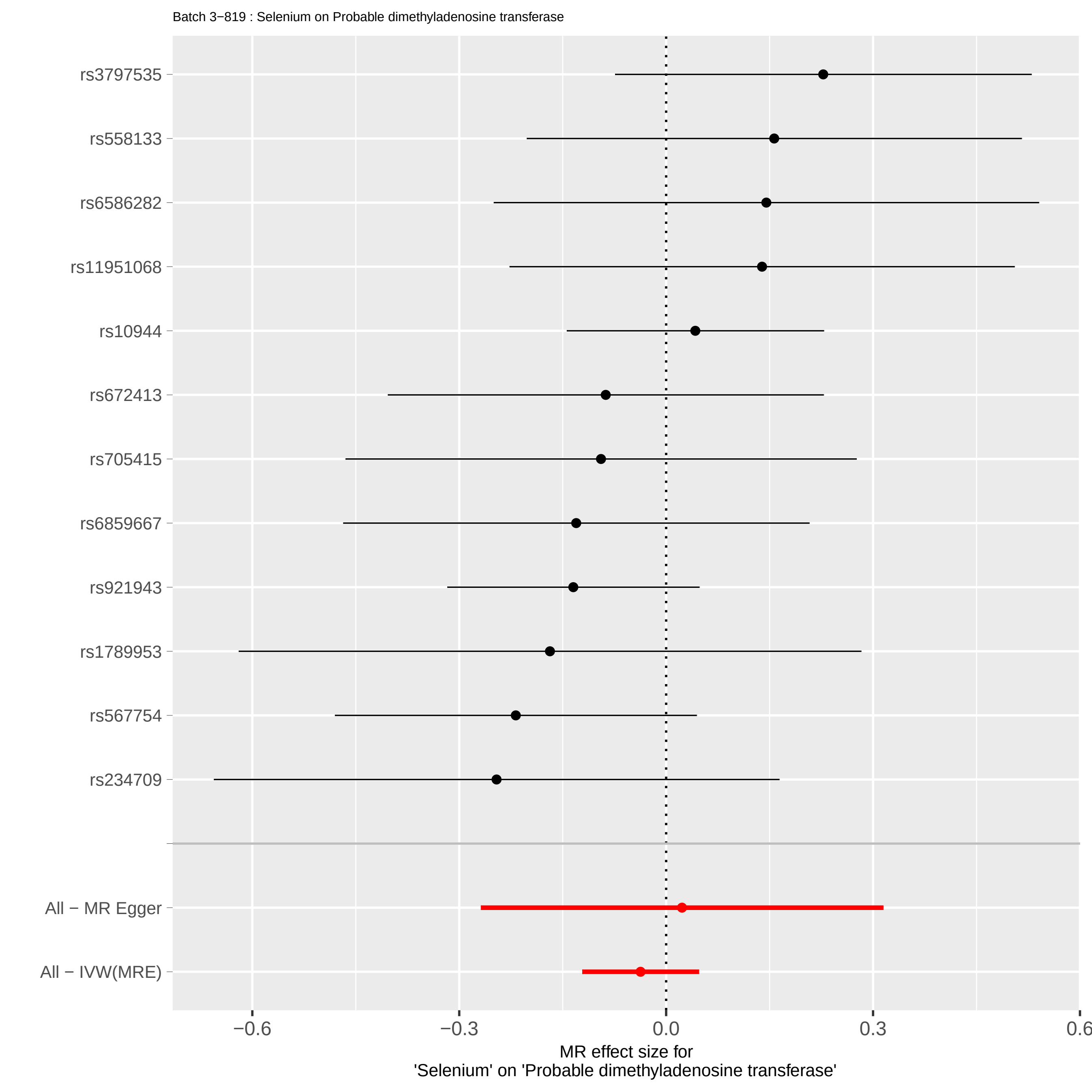

Batch 3−819 : Selenium on Probable dimethyladenosine transferase
rs3797535
rs558133
rs6586282
rs11951068
rs10944
rs672413
rs705415
rs6859667
rs921943
rs1789953
rs567754
rs234709
All − MR Egger
All − IVW(MRE)
−0.6
−0.3
0.0
0.3
0.6
MR effect size for
'Selenium' on 'Probable dimethyladenosine transferase'

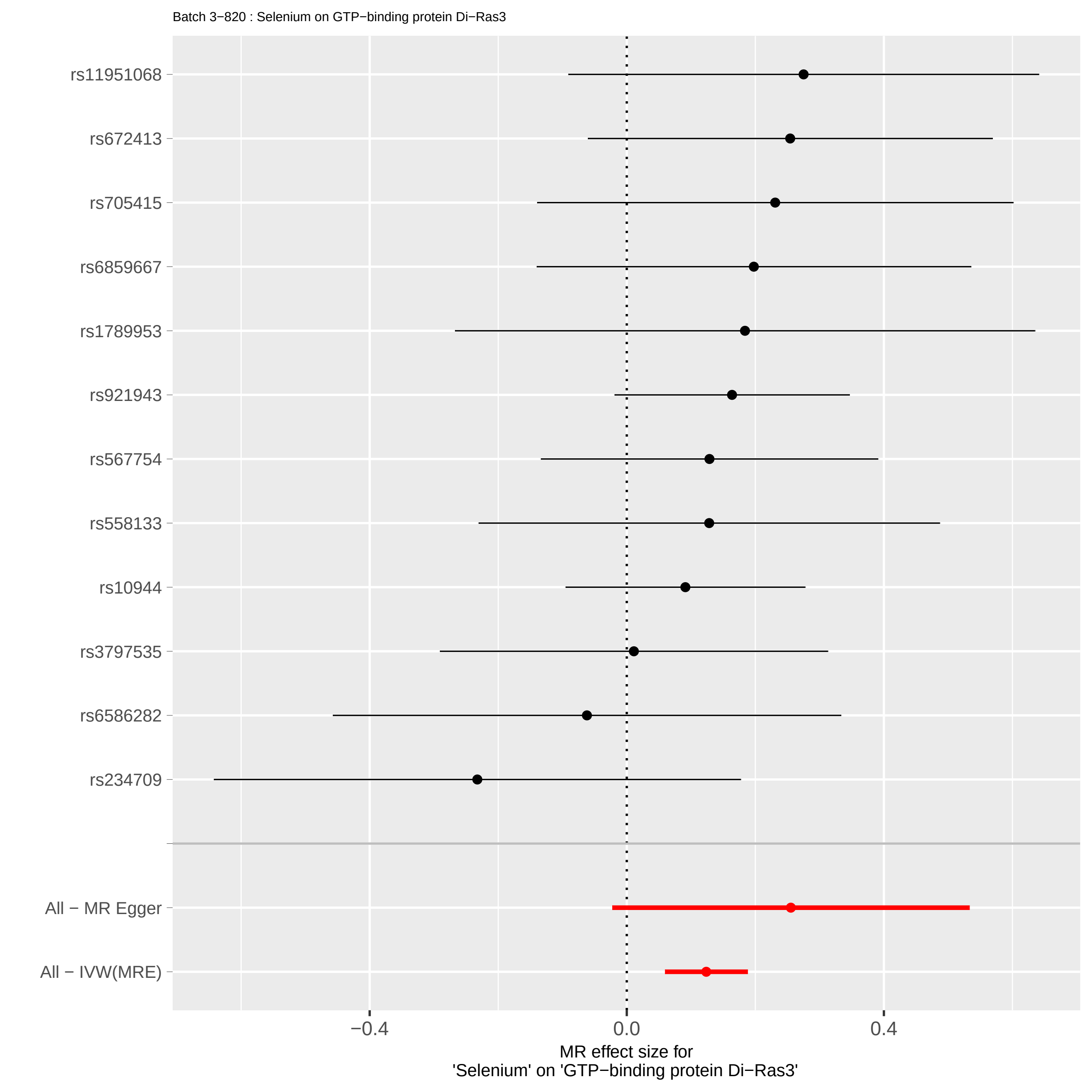

Batch 3−820 : Selenium on GTP−binding protein Di−Ras3
rs11951068
rs672413
rs705415
rs6859667
rs1789953
rs921943
rs567754
rs558133
rs10944
rs3797535
rs6586282
rs234709
All − MR Egger
All − IVW(MRE)
−0.4
0.0
0.4
MR effect size for
'Selenium' on 'GTP−binding protein Di−Ras3'

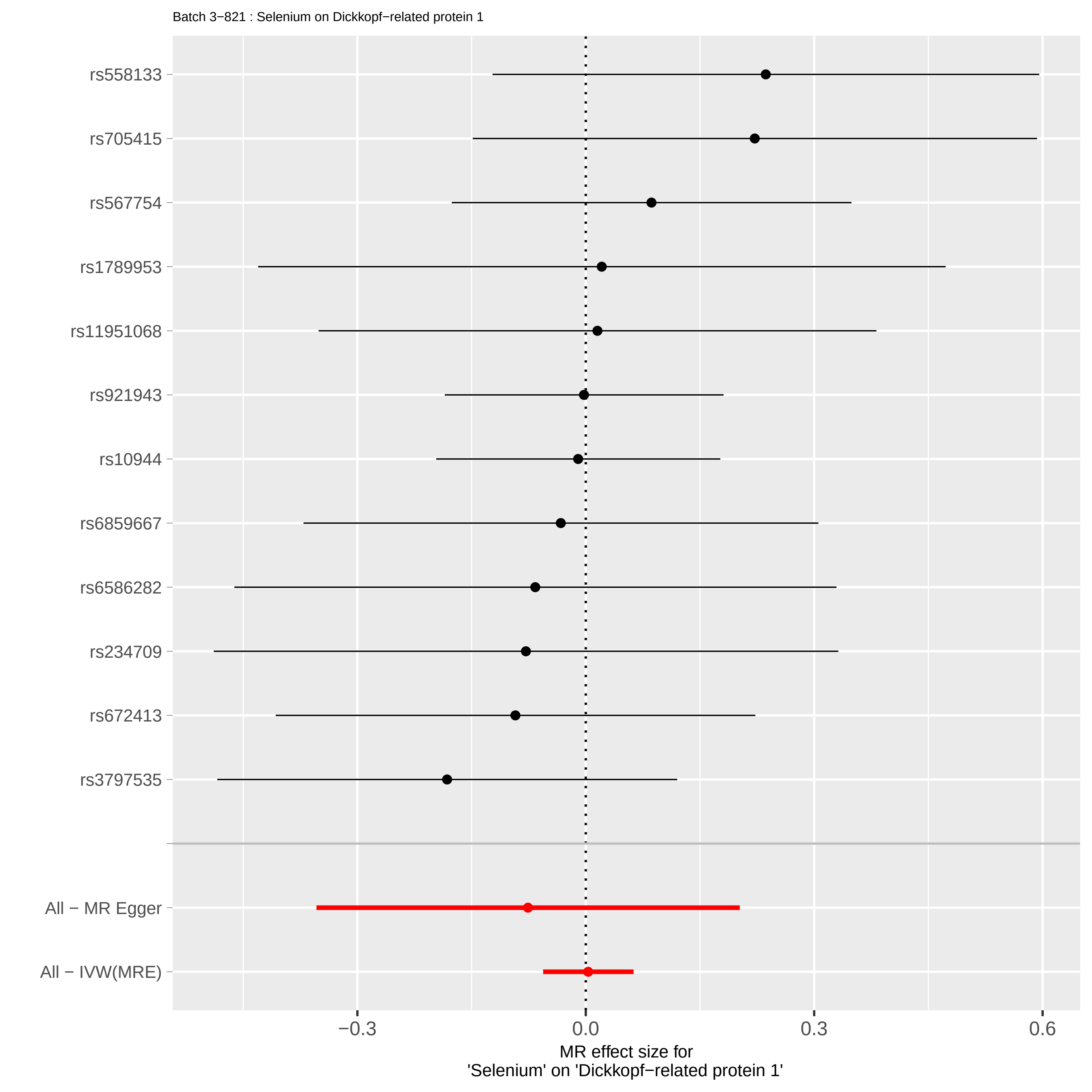

Batch 3−821 : Selenium on Dickkopf−related protein 1
rs558133
rs705415
rs567754
rs1789953
rs11951068
rs921943
rs10944
rs6859667
rs6586282
rs234709
rs672413
rs3797535
All − MR Egger
All − IVW(MRE)
−0.3
0.0
0.3
0.6
MR effect size for
'Selenium' on 'Dickkopf−related protein 1'

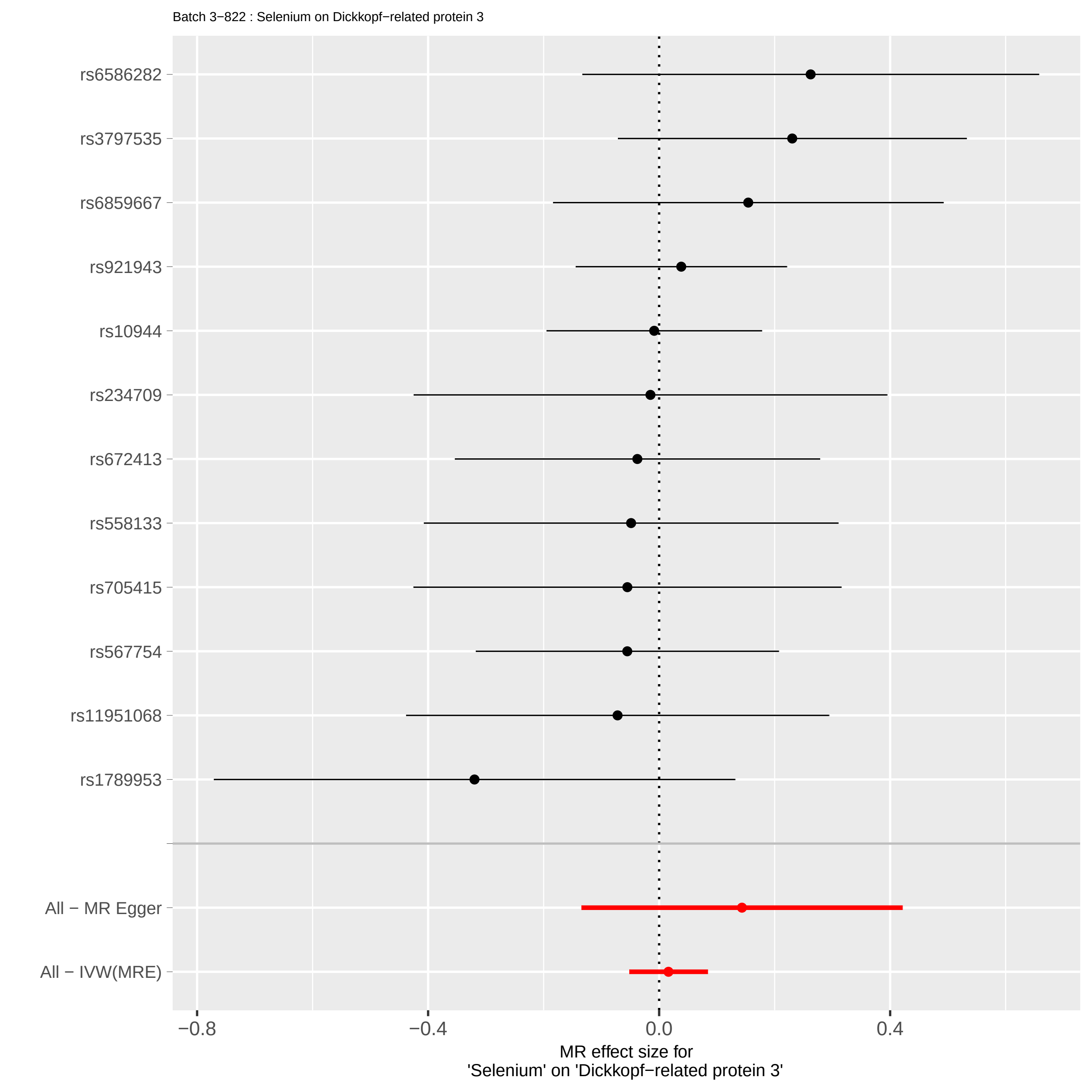

Batch 3−822 : Selenium on Dickkopf−related protein 3
rs6586282
rs3797535
rs6859667
rs921943
rs10944
rs234709
rs672413
rs558133
rs705415
rs567754
rs11951068
rs1789953
All − MR Egger
All − IVW(MRE)
−0.8
−0.4
0.0
0.4
MR effect size for
'Selenium' on 'Dickkopf−related protein 3'

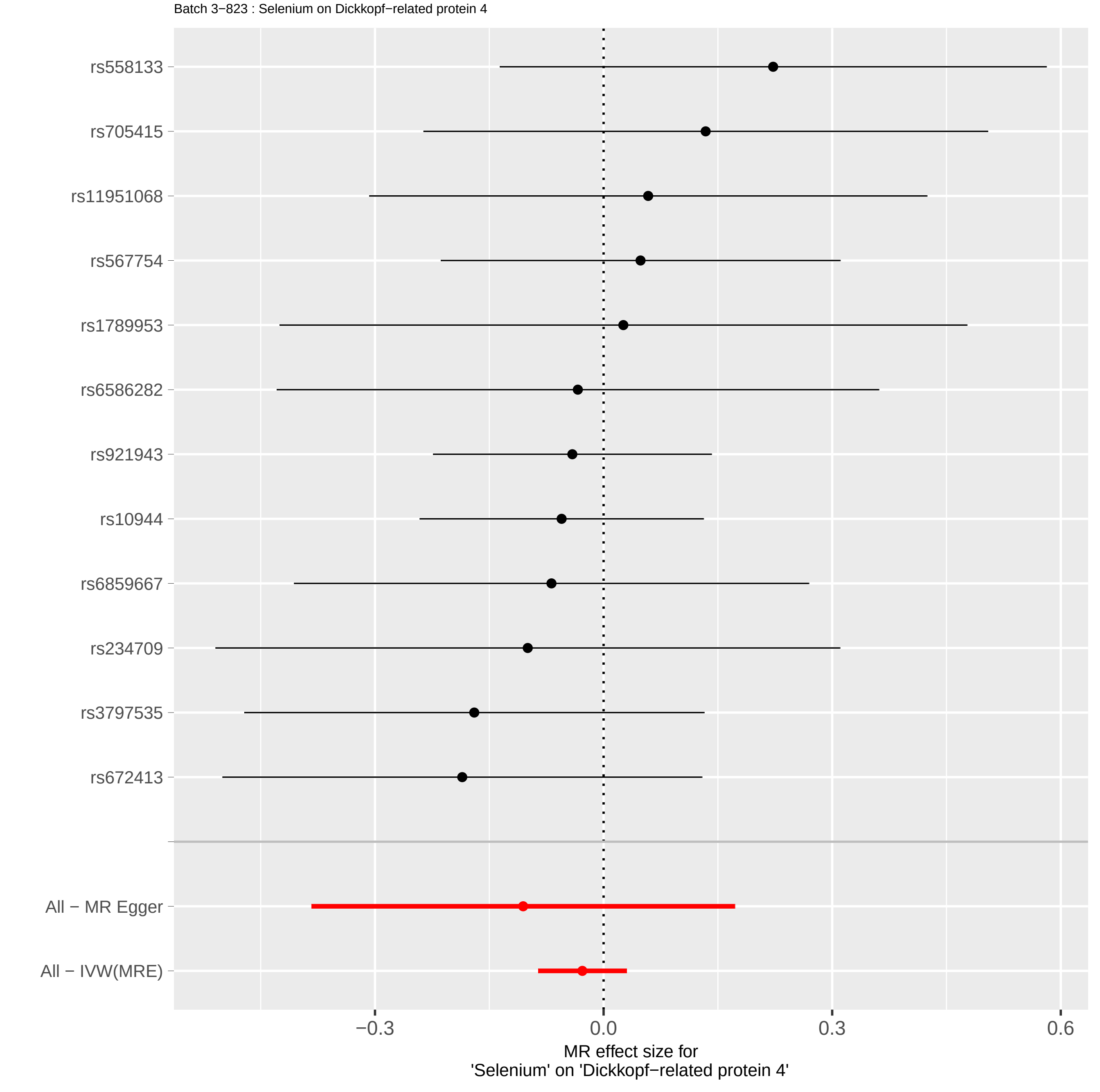

Batch 3−823 : Selenium on Dickkopf−related protein 4
rs558133
rs705415
rs11951068
rs567754
rs1789953
rs6586282
rs921943
rs10944
rs6859667
rs234709
rs3797535
rs672413
All − MR Egger
All − IVW(MRE)
−0.3
0.0
0.3
0.6
MR effect size for
'Selenium' on 'Dickkopf−related protein 4'

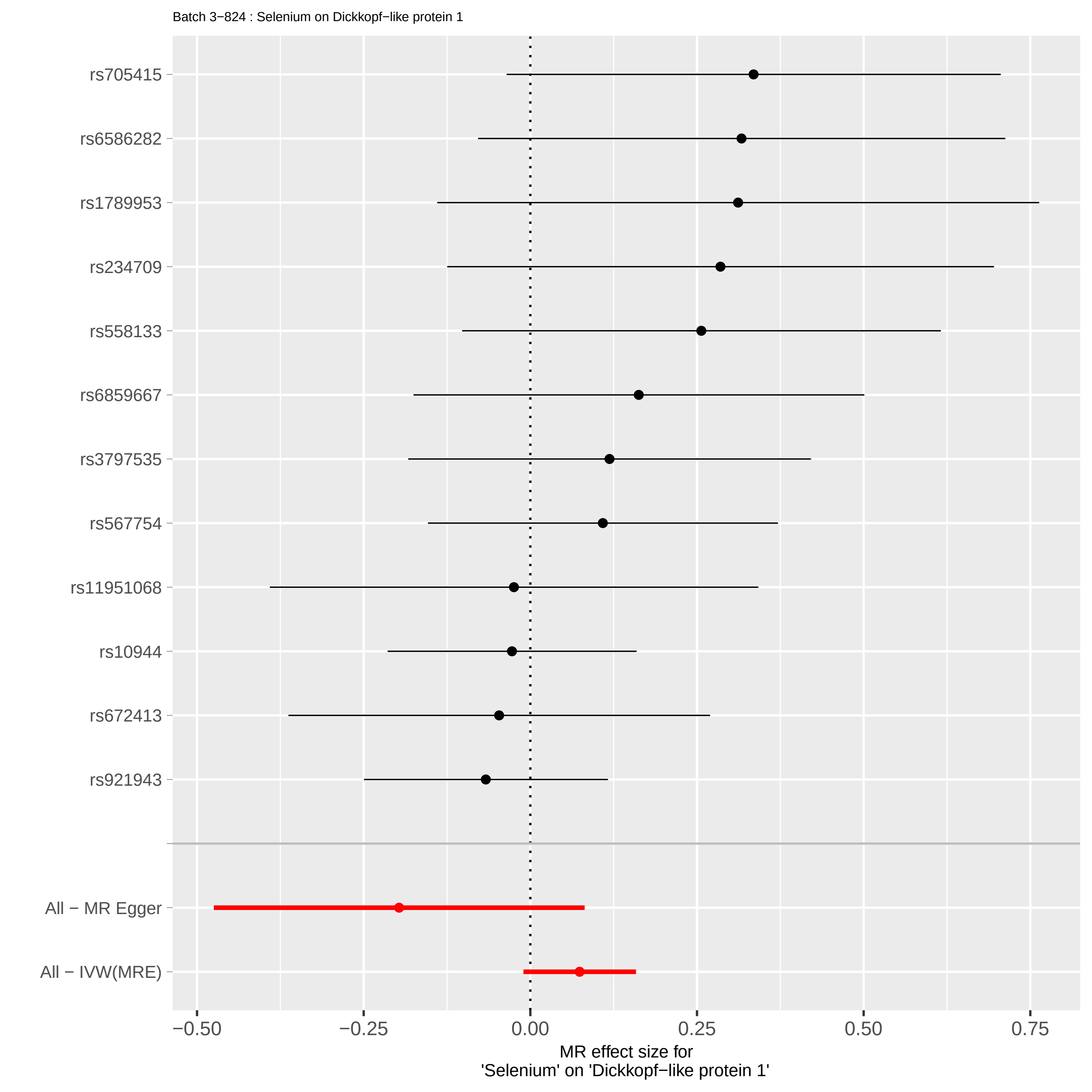

Batch 3−824 : Selenium on Dickkopf−like protein 1
rs705415
rs6586282
rs1789953
rs234709
rs558133
rs6859667
rs3797535
rs567754
rs11951068
rs10944
rs672413
rs921943
All − MR Egger
All − IVW(MRE)
−0.50
−0.25
0.00
0.25
0.50
0.75
MR effect size for
'Selenium' on 'Dickkopf−like protein 1'

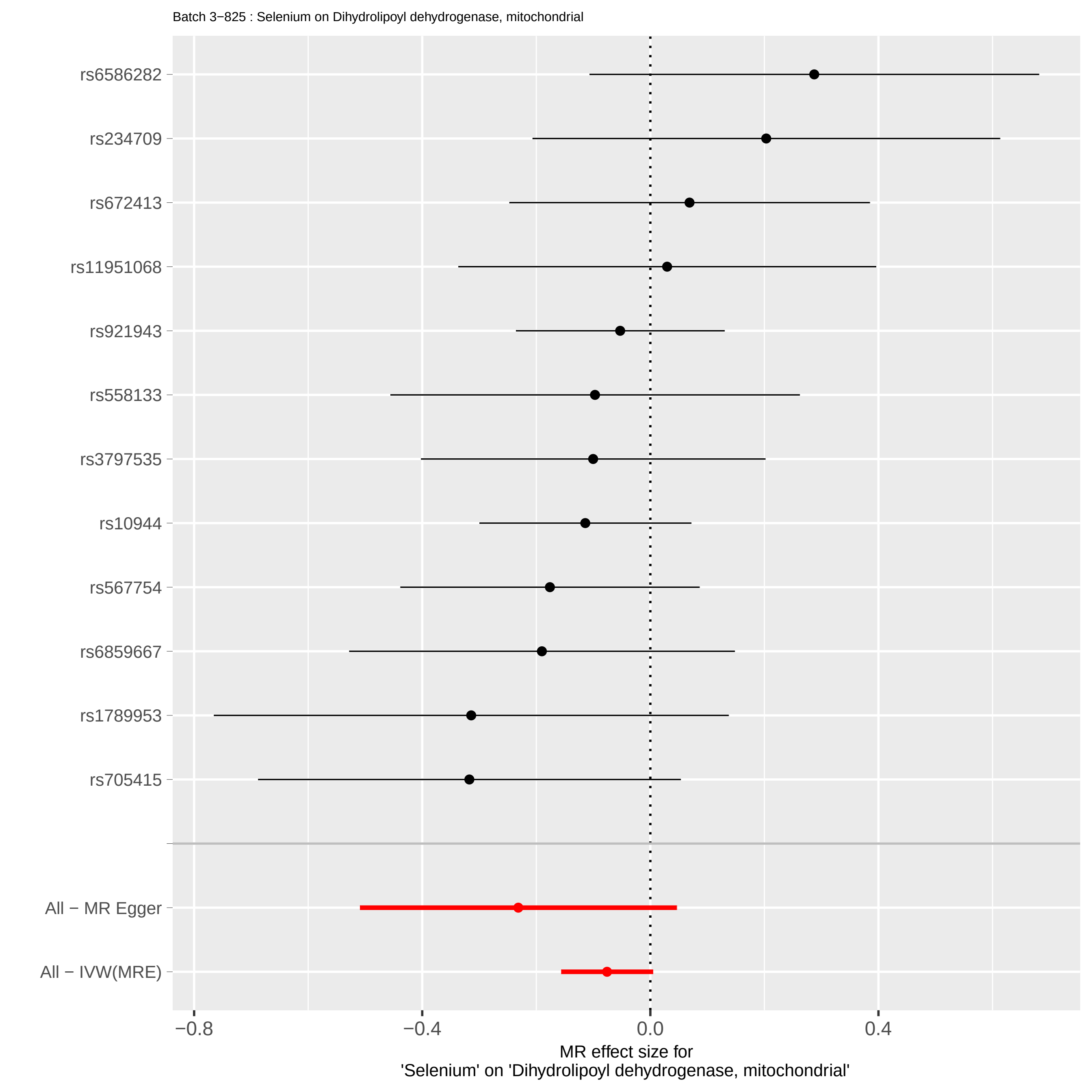

Batch 3−825 : Selenium on Dihydrolipoyl dehydrogenase, mitochondrial
rs6586282
rs234709
rs672413
rs11951068
rs921943
rs558133
rs3797535
rs10944
rs567754
rs6859667
rs1789953
rs705415
All − MR Egger
All − IVW(MRE)
−0.8
−0.4
0.0
0.4
MR effect size for
'Selenium' on 'Dihydrolipoyl dehydrogenase, mitochondrial'

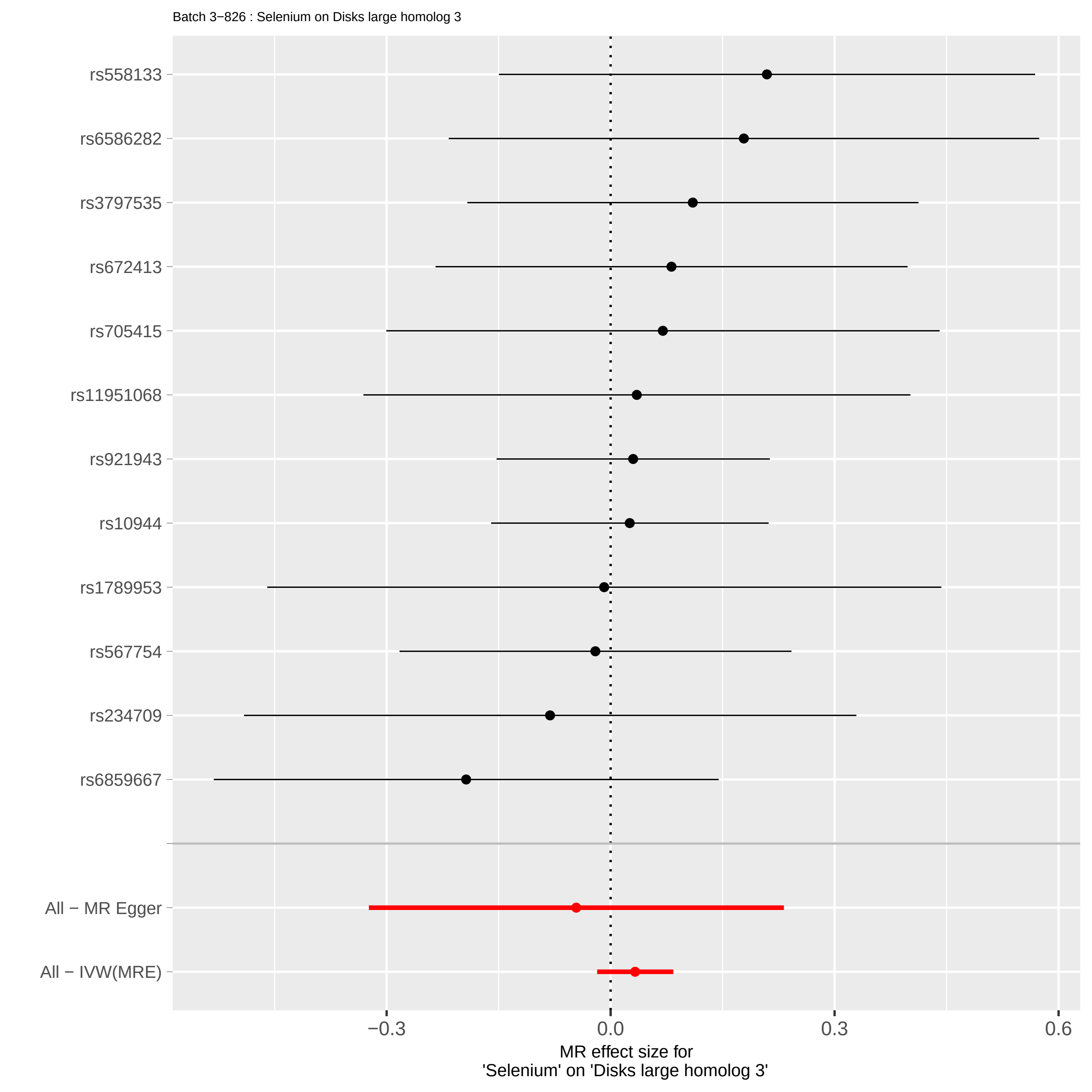
Batch 3−826 : Selenium on Disks large homolog 3
rs558133
rs6586282
rs3797535
rs672413
rs705415
rs11951068
rs921943
rs10944
rs1789953
rs567754
rs234709
rs6859667
All − MR Egger
All − IVW(MRE)
−0.3
0.0
0.3
0.6
MR effect size for
'Selenium' on 'Disks large homolog 3'

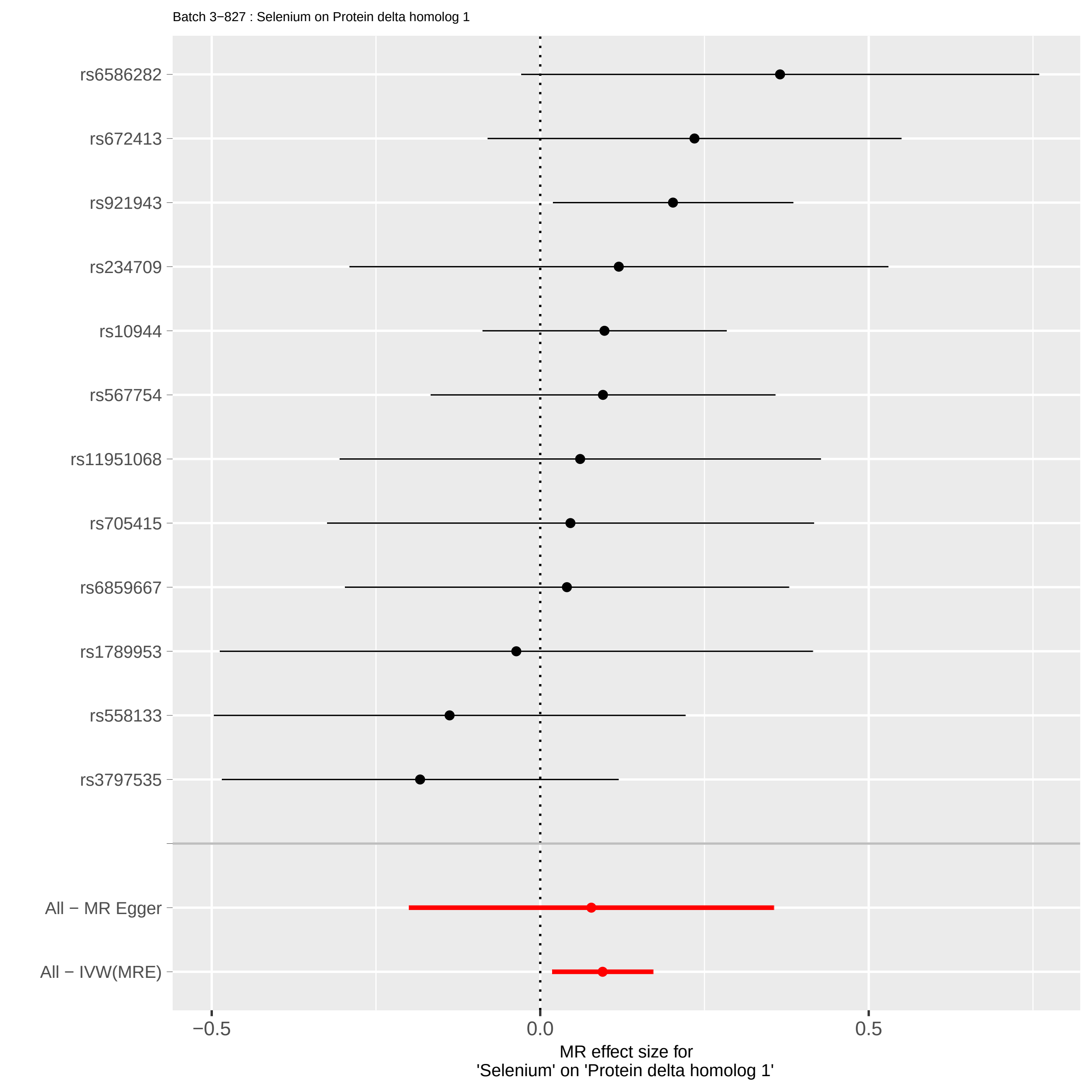

Batch 3−827 : Selenium on Protein delta homolog 1
rs6586282
rs672413
rs921943
rs234709
rs10944
rs567754
rs11951068
rs705415
rs6859667
rs1789953
rs558133
rs3797535
All − MR Egger
All − IVW(MRE)
−0.5
0.0
0.5
MR effect size for
'Selenium' on 'Protein delta homolog 1'

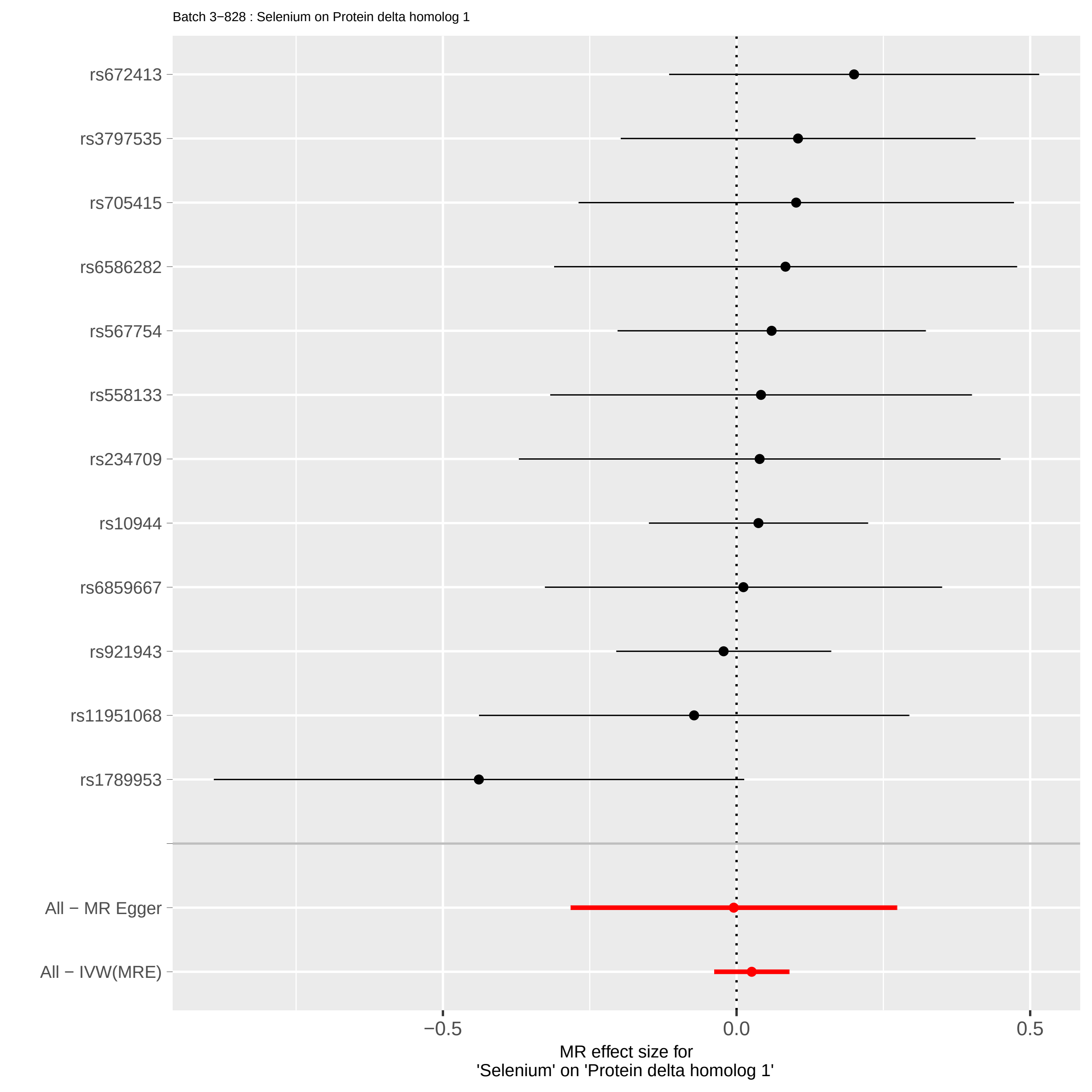
Batch 3−828 : Selenium on Protein delta homolog 1
rs672413
rs3797535
rs705415
rs6586282
rs567754
rs558133
rs234709
rs10944
rs6859667
rs921943
rs11951068
rs1789953
All − MR Egger
All − IVW(MRE)
−0.5
0.0
0.5
MR effect size for
'Selenium' on 'Protein delta homolog 1'

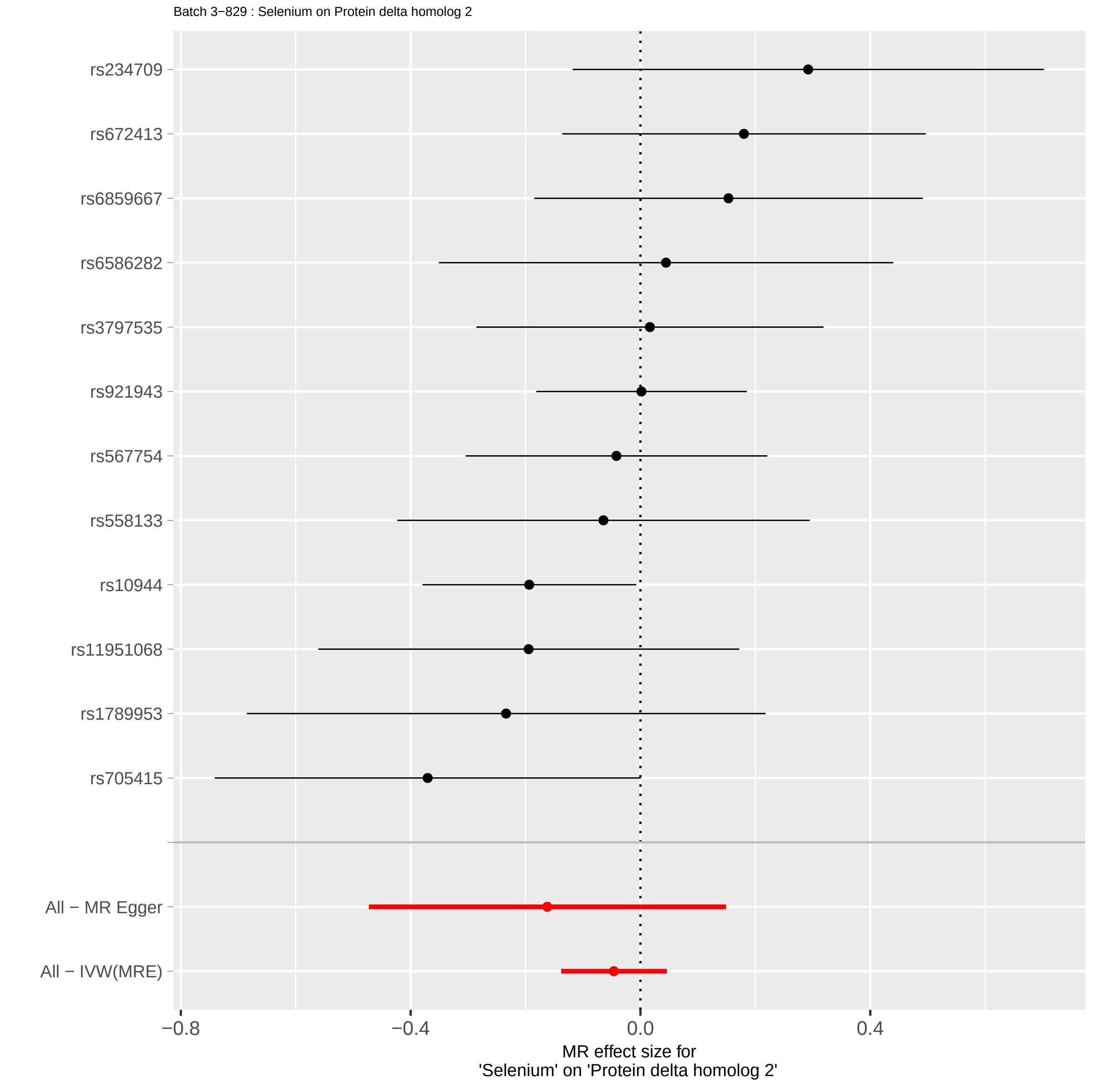

Batch 3−829 : Selenium on Protein delta homolog 2
rs234709
rs672413
rs6859667
rs6586282
rs3797535
rs921943
rs567754
rs558133
rs10944
rs11951068
rs1789953
rs705415
All − MR Egger
All − IVW(MRE)
−0.8
−0.4
0.0
0.4
MR effect size for
'Selenium' on 'Protein delta homolog 2'

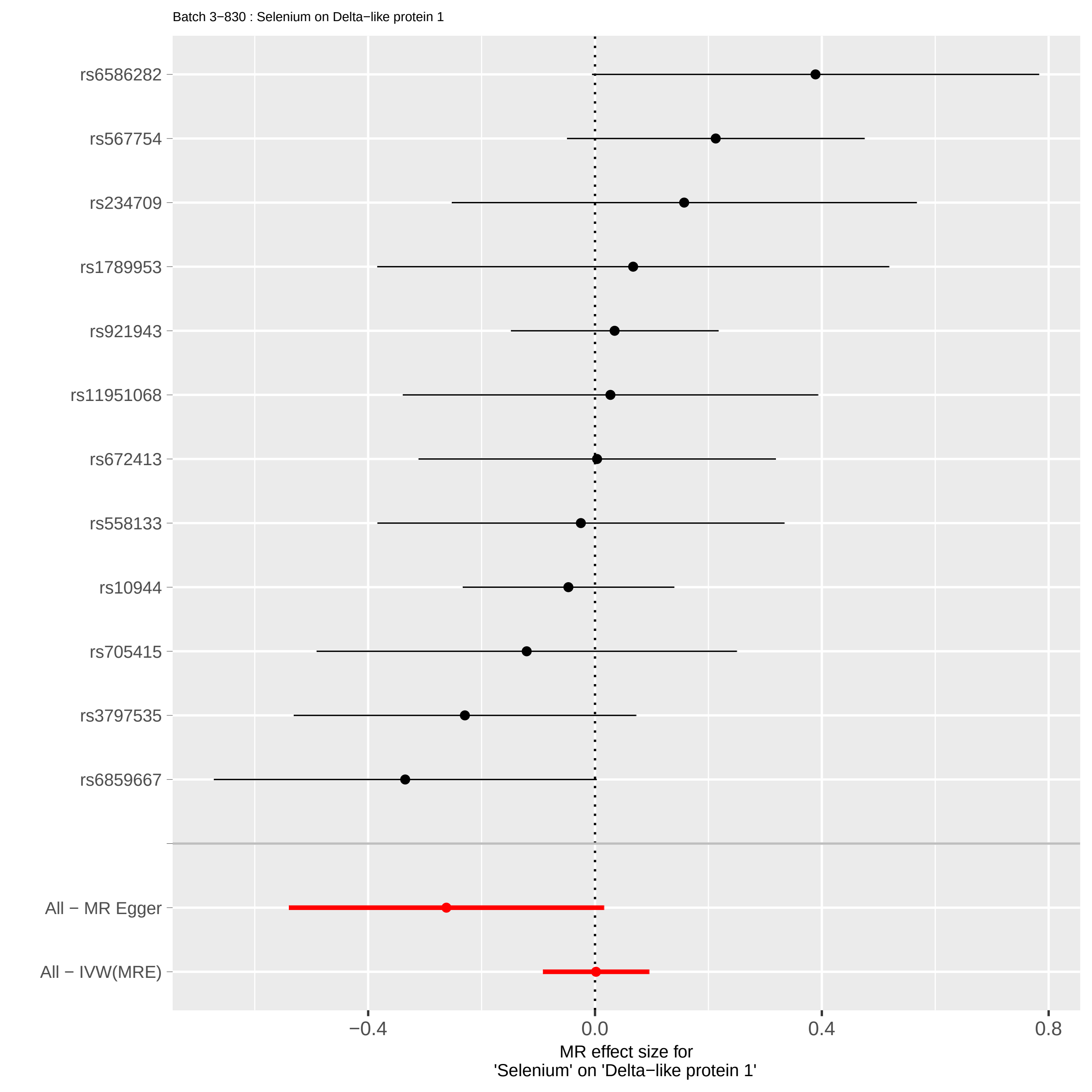
Batch 3−830 : Selenium on Delta−like protein 1
rs6586282
rs567754
rs234709
rs1789953
rs921943
rs11951068
rs672413
rs558133
rs10944
rs705415
rs3797535
rs6859667
All − MR Egger
All − IVW(MRE)
−0.4
0.0
0.4
0.8
MR effect size for
'Selenium' on 'Delta−like protein 1'

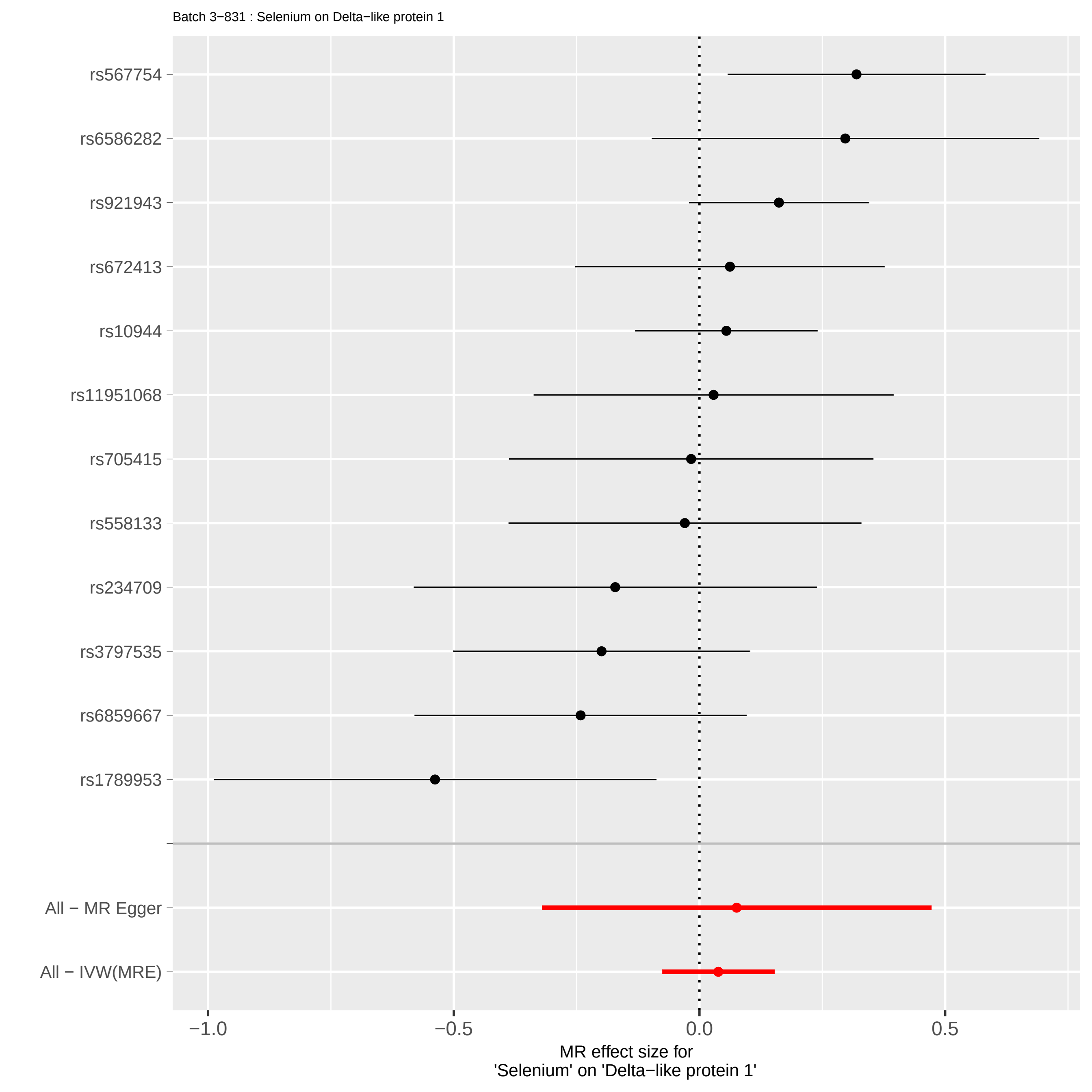

Batch 3−831 : Selenium on Delta−like protein 1
rs567754
rs6586282
rs921943
rs672413
rs10944
rs11951068
rs705415
rs558133
rs234709
rs3797535
rs6859667
rs1789953
All − MR Egger
All − IVW(MRE)
−1.0
−0.5
0.0
0.5
MR effect size for
'Selenium' on 'Delta−like protein 1'

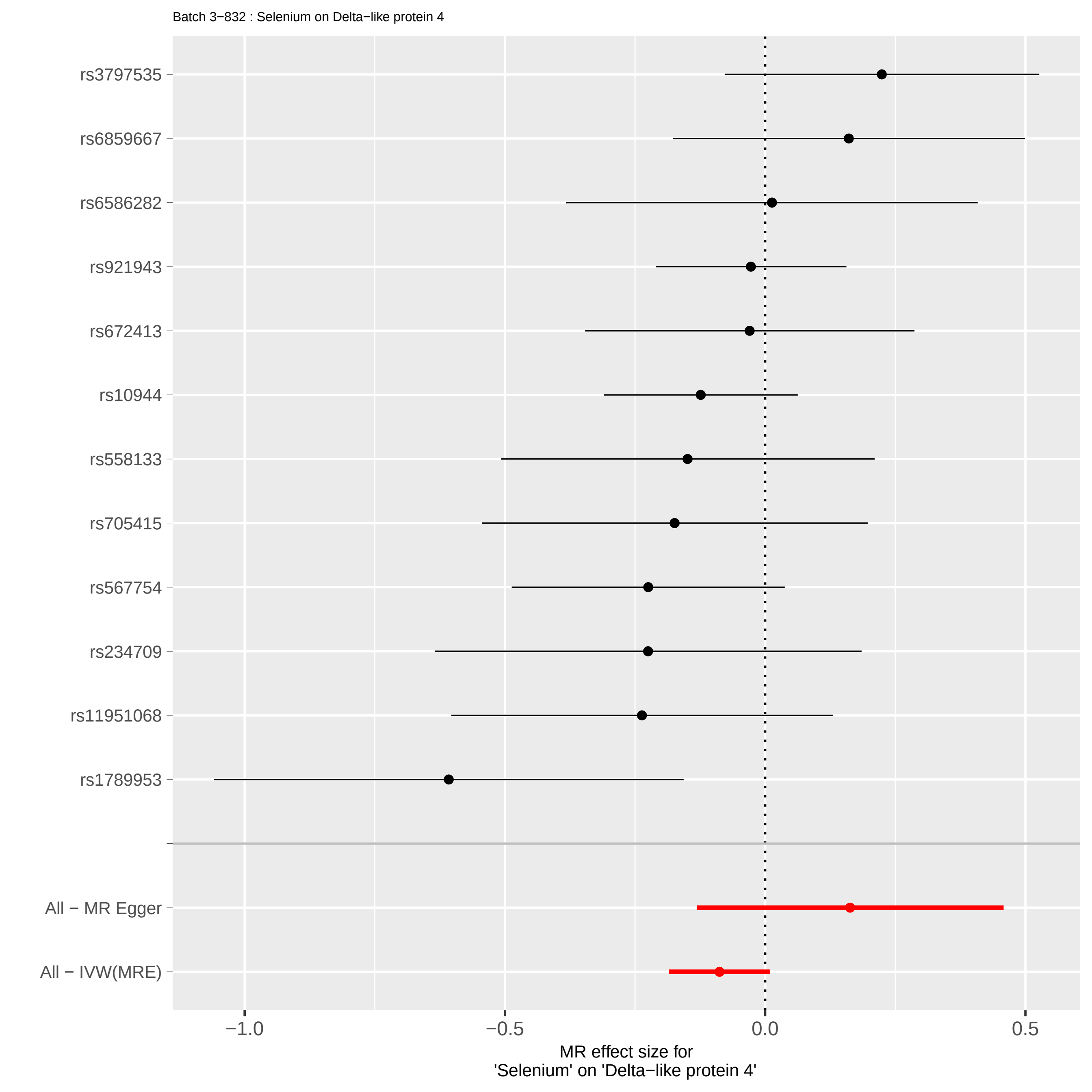
Batch 3−832 : Selenium on Delta−like protein 4
rs3797535
rs6859667
rs6586282
rs921943
rs672413
rs10944
rs558133
rs705415
rs567754
rs234709
rs11951068
rs1789953
All − MR Egger
All − IVW(MRE)
−1.0
−0.5
0.0
0.5
MR effect size for
'Selenium' on 'Delta−like protein 4'

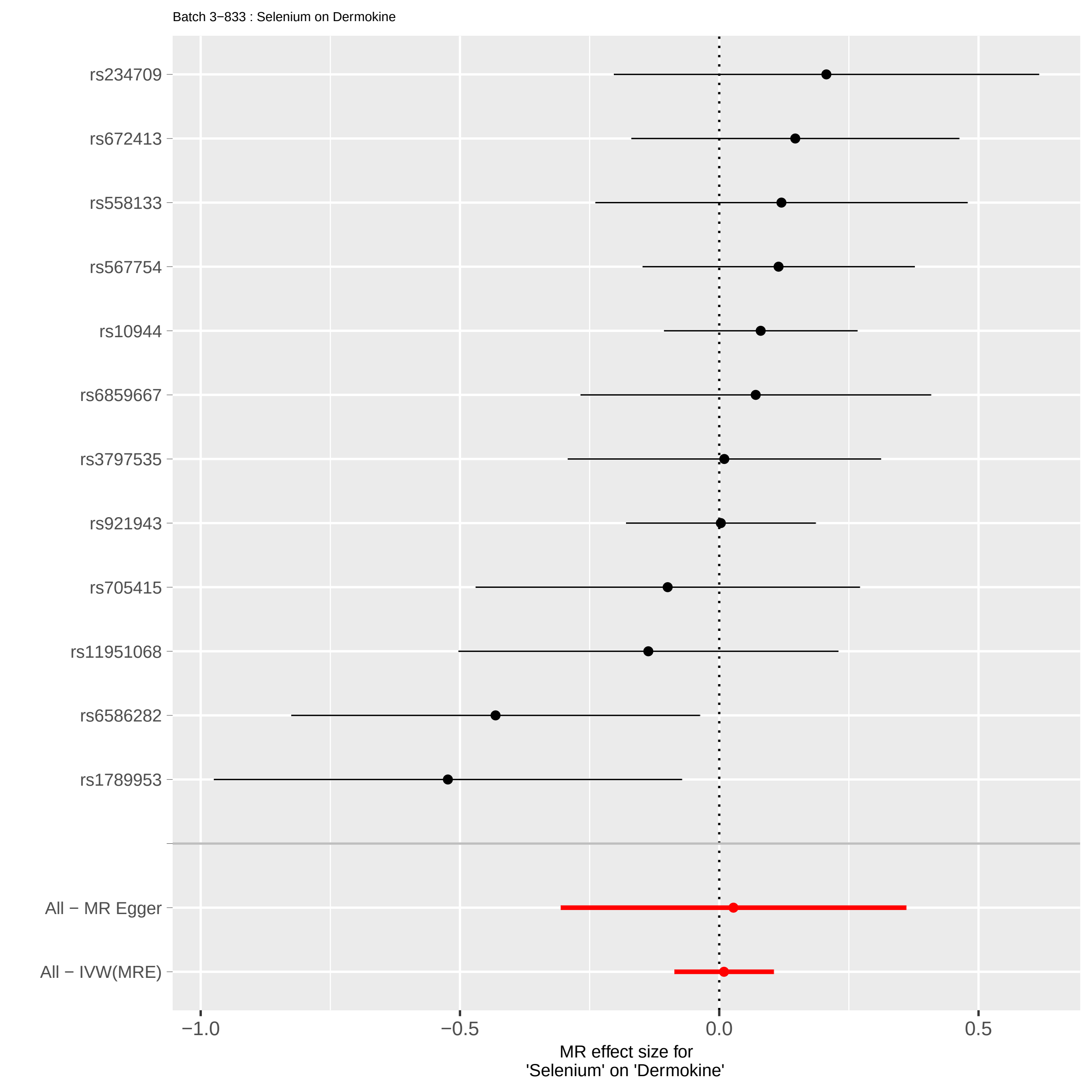
Batch 3−833 : Selenium on Dermokine
rs234709
rs672413
rs558133
rs567754
rs10944
rs6859667
rs3797535
rs921943
rs705415
rs11951068
rs6586282
rs1789953
All − MR Egger
All − IVW(MRE)
−1.0
−0.5
0.0
0.5
MR effect size for
'Selenium' on 'Dermokine'

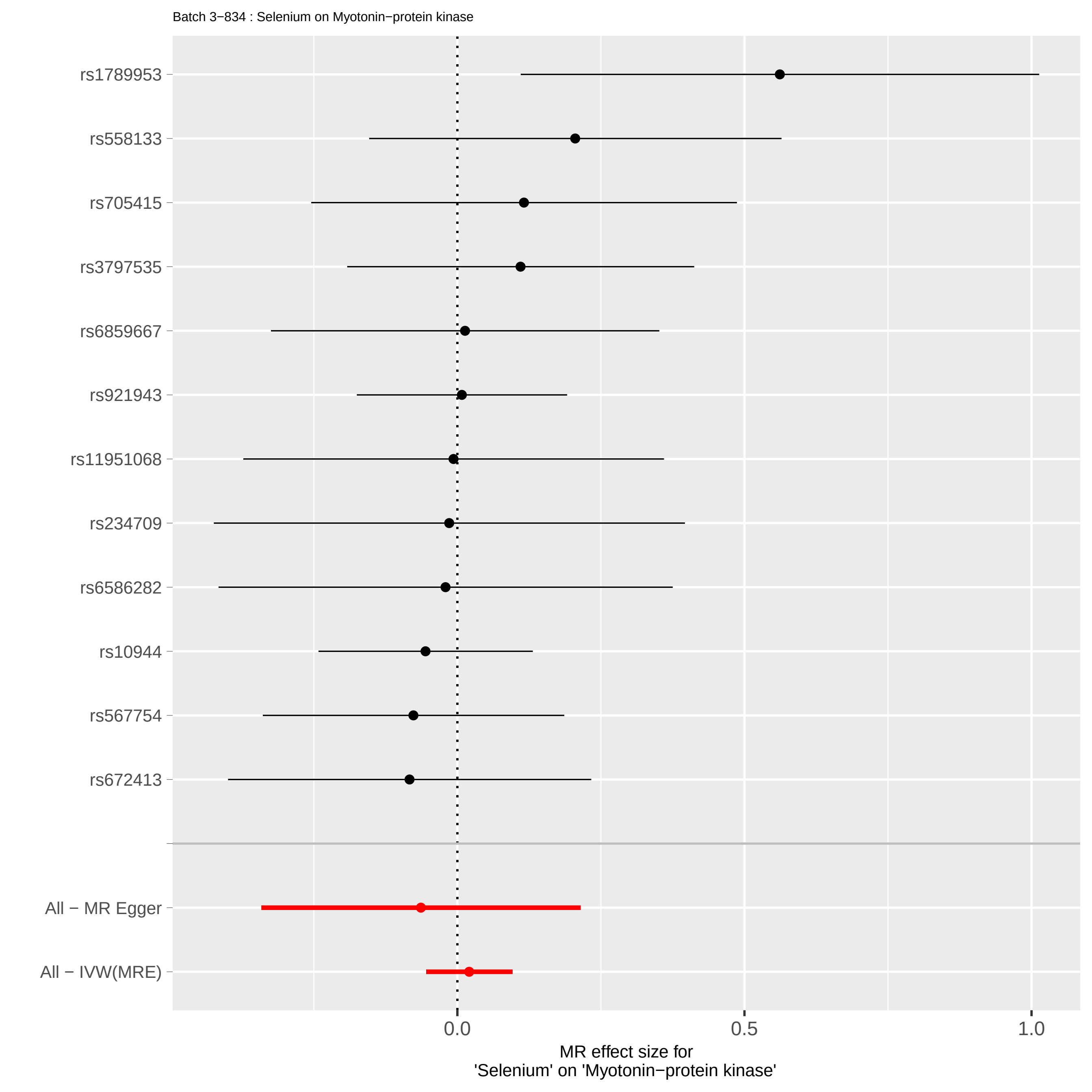
Batch 3−834 : Selenium on Myotonin−protein kinase
rs1789953
rs558133
rs705415
rs3797535
rs6859667
rs921943
rs11951068
rs234709
rs6586282
rs10944
rs567754
rs672413
All − MR Egger
All − IVW(MRE)
0.0
0.5
1.0
MR effect size for
'Selenium' on 'Myotonin−protein kinase'

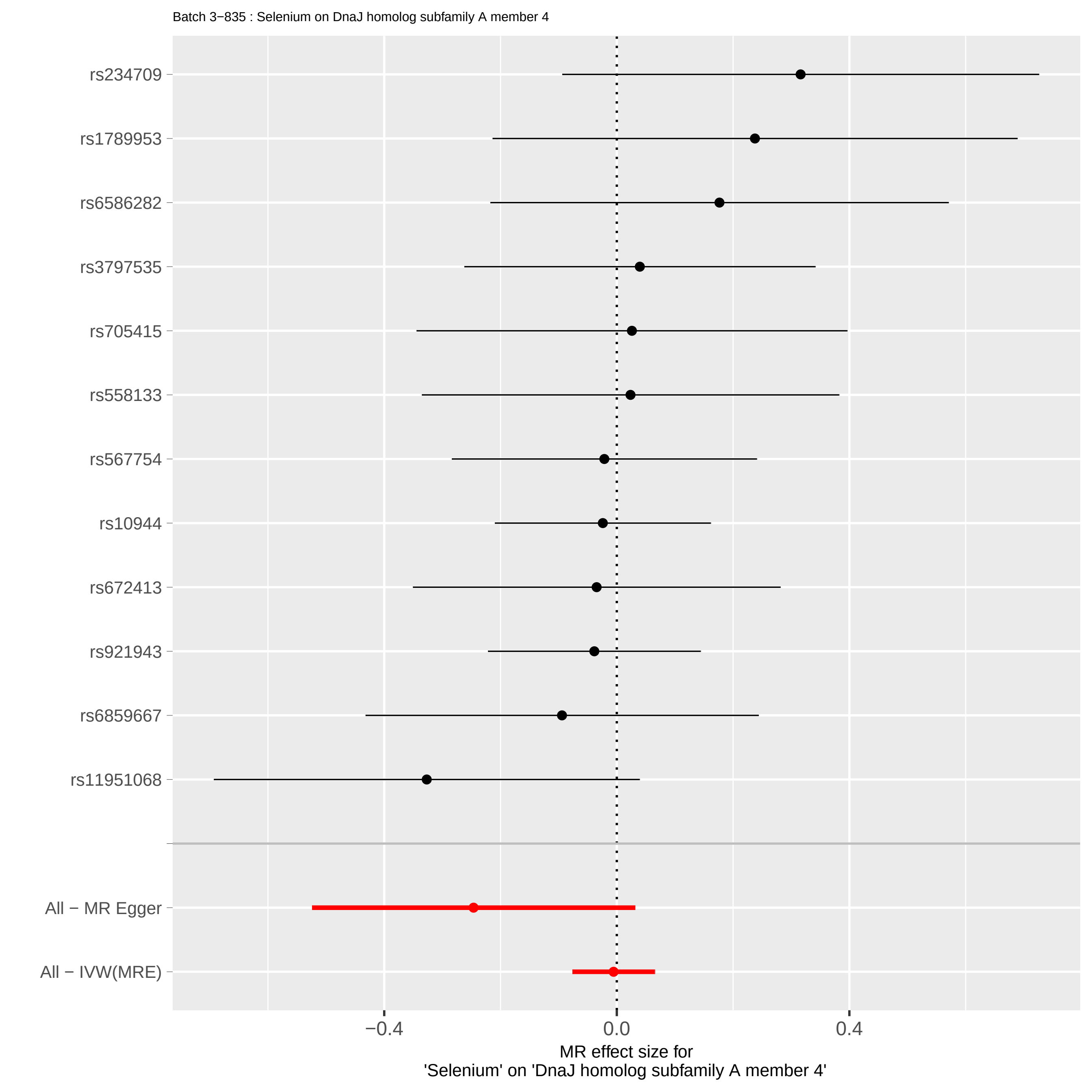

Batch 3−835 : Selenium on DnaJ homolog subfamily A member 4
rs234709
rs1789953
rs6586282
rs3797535
rs705415
rs558133
rs567754
rs10944
rs672413
rs921943
rs6859667
rs11951068
All − MR Egger
All − IVW(MRE)
−0.4
0.0
0.4
MR effect size for
'Selenium' on 'DnaJ homolog subfamily A member 4'

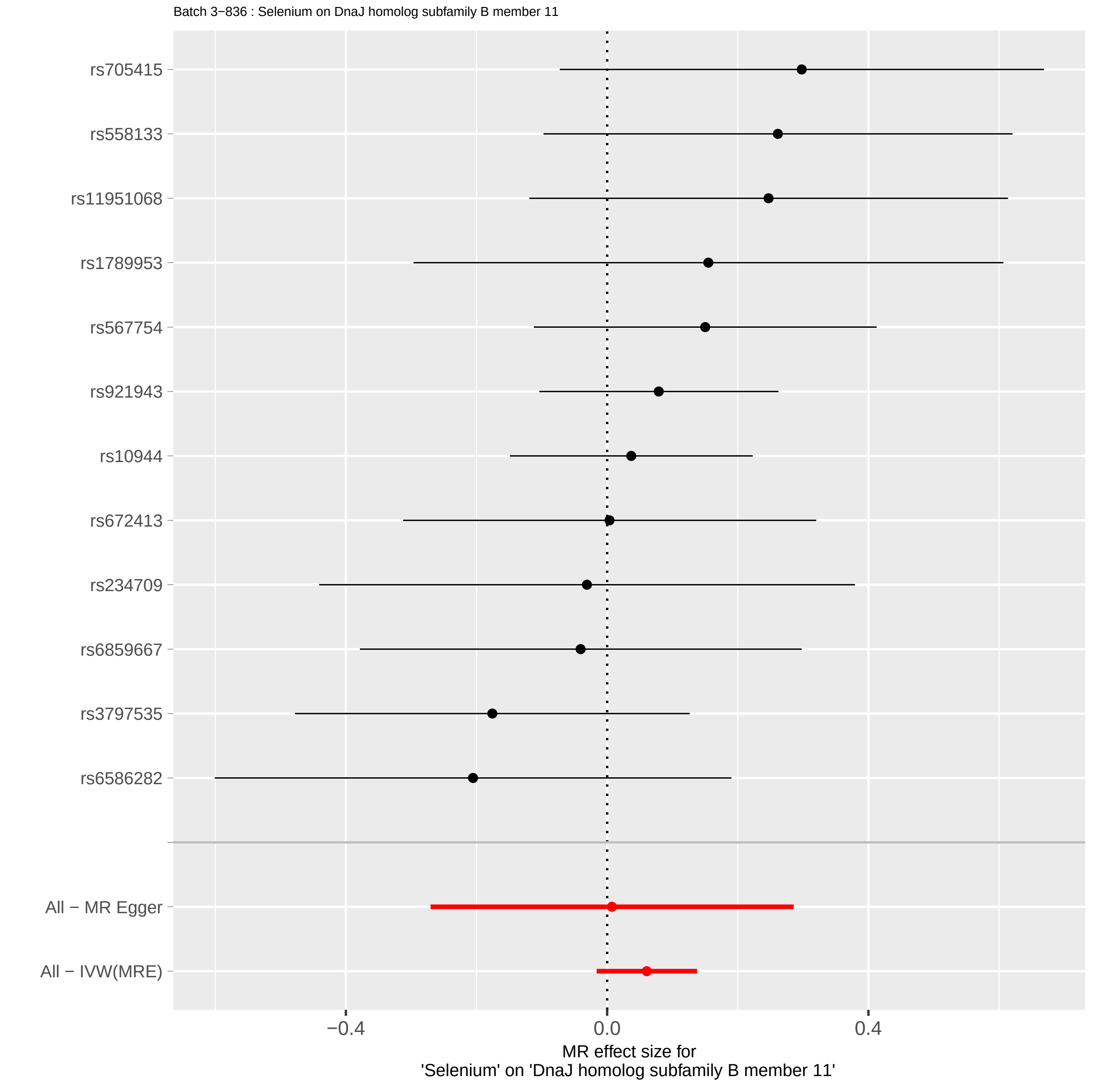

Batch 3−836 : Selenium on DnaJ homolog subfamily B member 11
rs705415
rs558133
rs11951068
rs1789953
rs567754
rs921943
rs10944
rs672413
rs234709
rs6859667
rs3797535
rs6586282
All − MR Egger
All − IVW(MRE)
−0.4
0.0
0.4
MR effect size for
'Selenium' on 'DnaJ homolog subfamily B member 11'

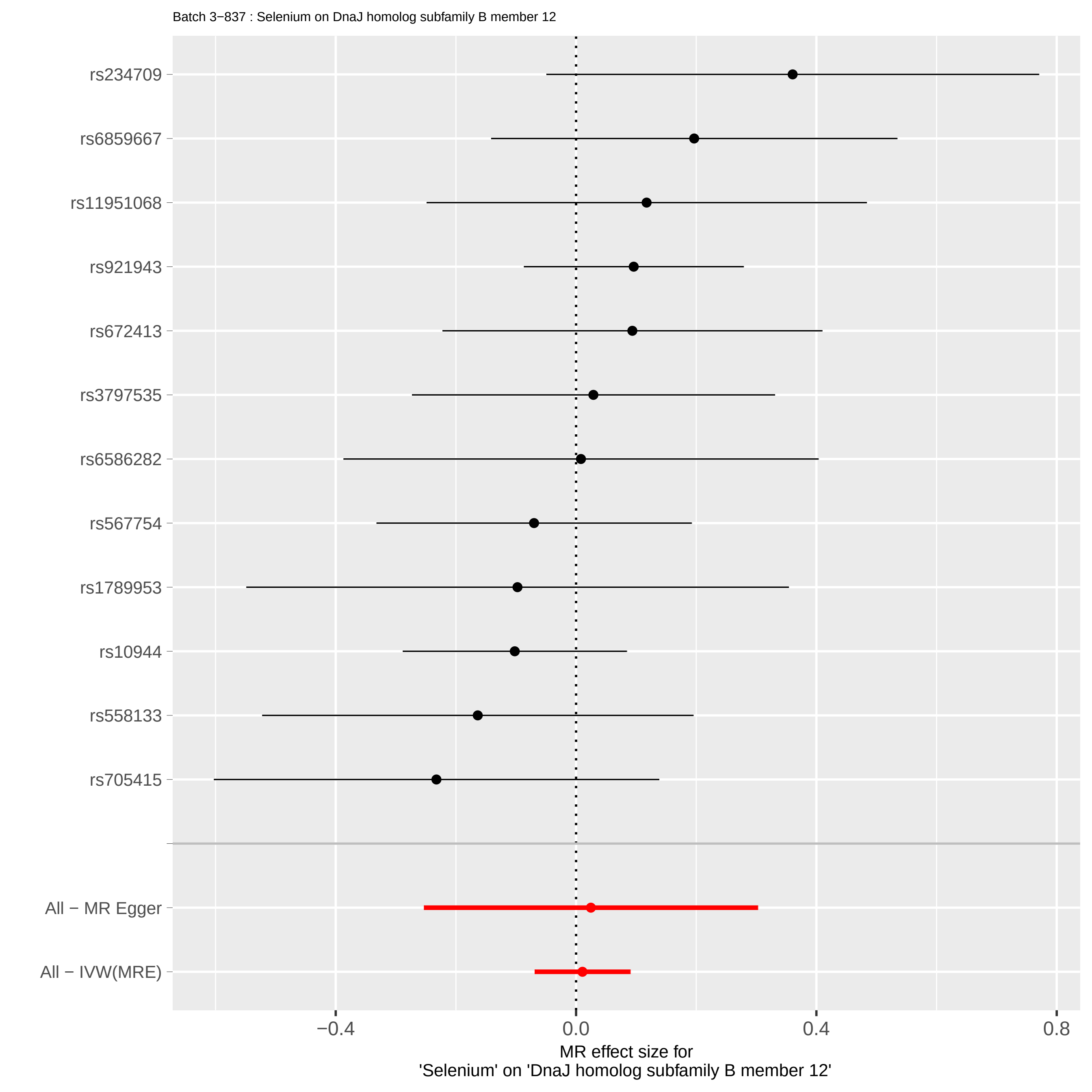

Batch 3−837 : Selenium on DnaJ homolog subfamily B member 12
rs234709
rs6859667
rs11951068
rs921943
rs672413
rs3797535
rs6586282
rs567754
rs1789953
rs10944
rs558133
rs705415
All − MR Egger
All − IVW(MRE)
−0.4
0.0
0.4
0.8
MR effect size for
'Selenium' on 'DnaJ homolog subfamily B member 12'

Batch 3−838 : Selenium on DnaJ homolog subfamily B member 1

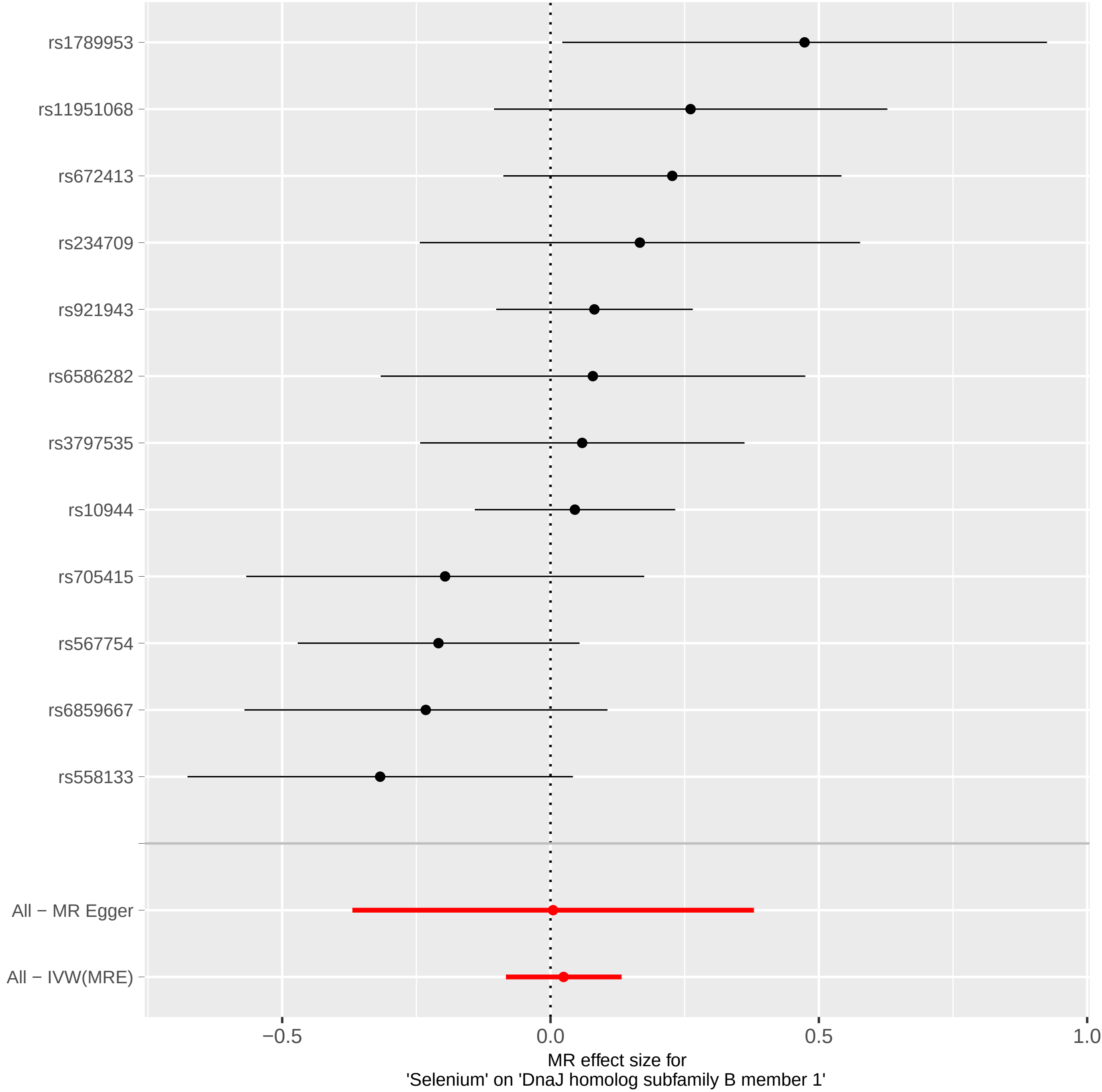

Batch 3−839 : Selenium on DnaJ homolog subfamily B member 14

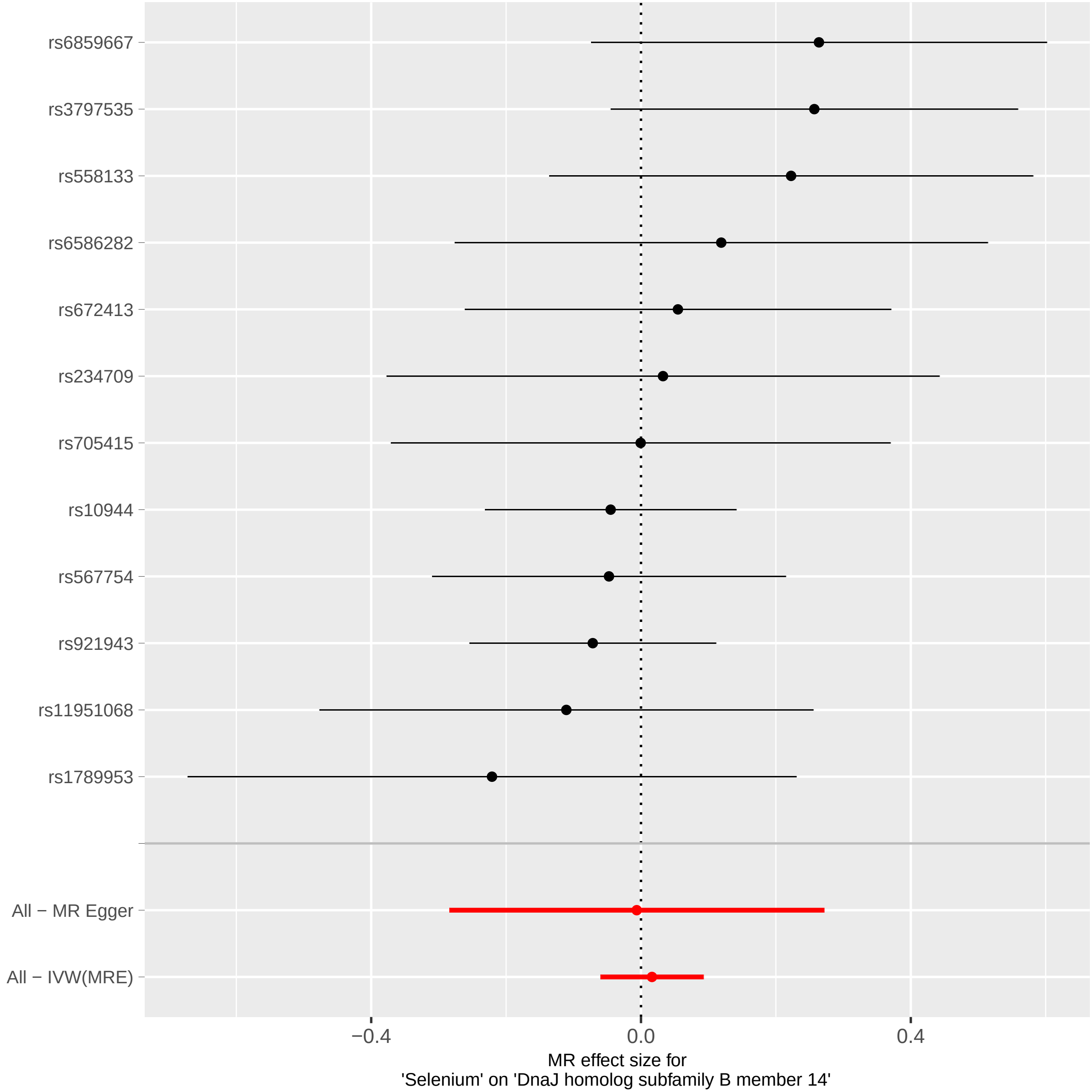

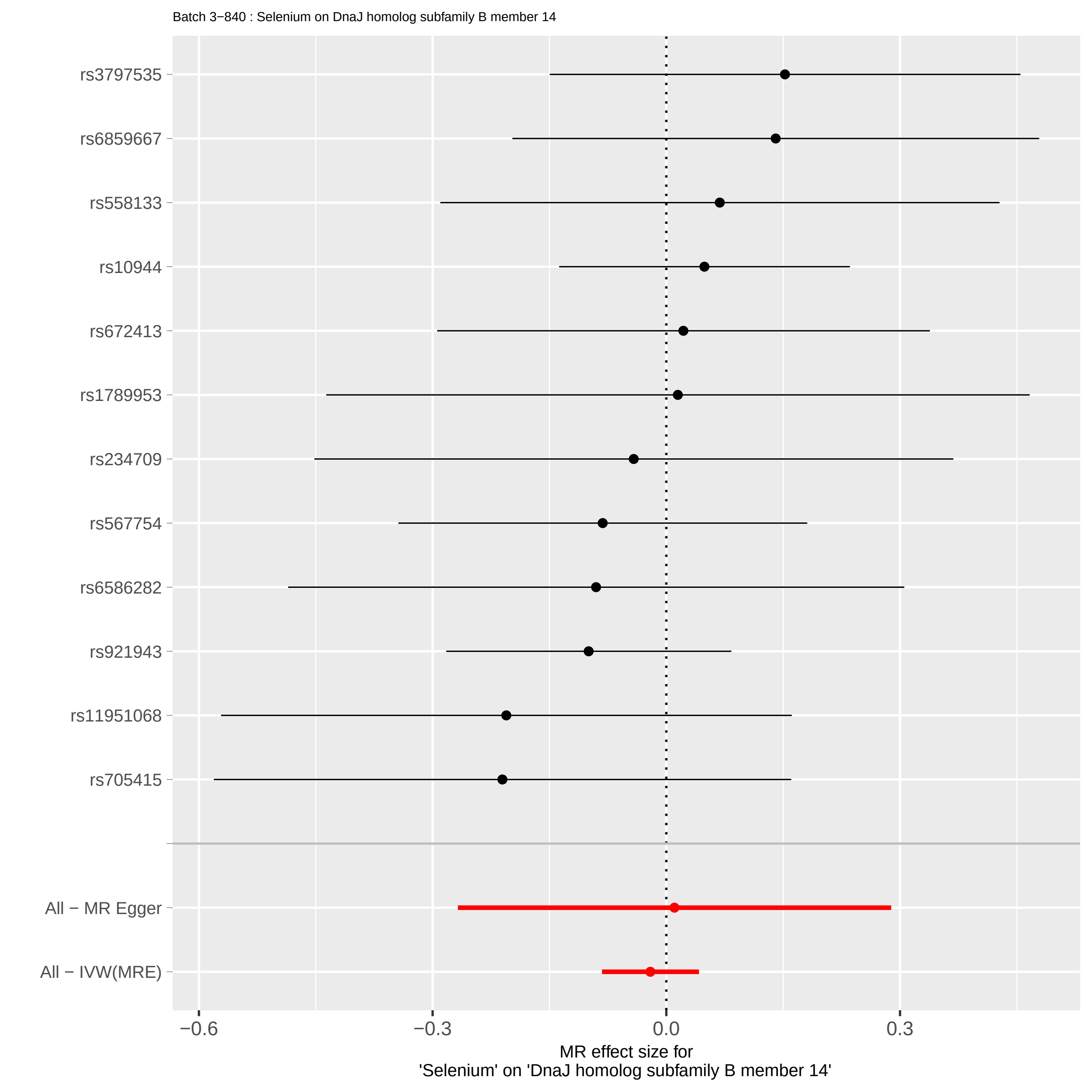
Batch 3−840 : Selenium on DnaJ homolog subfamily B member 14
rs3797535
rs6859667
rs558133
rs10944
rs672413
rs1789953
rs234709
rs567754
rs6586282
rs921943
rs11951068
rs705415
All − MR Egger
All − IVW(MRE)
−0.6
−0.3
0.0
0.3
MR effect size for
'Selenium' on 'DnaJ homolog subfamily B member 14'

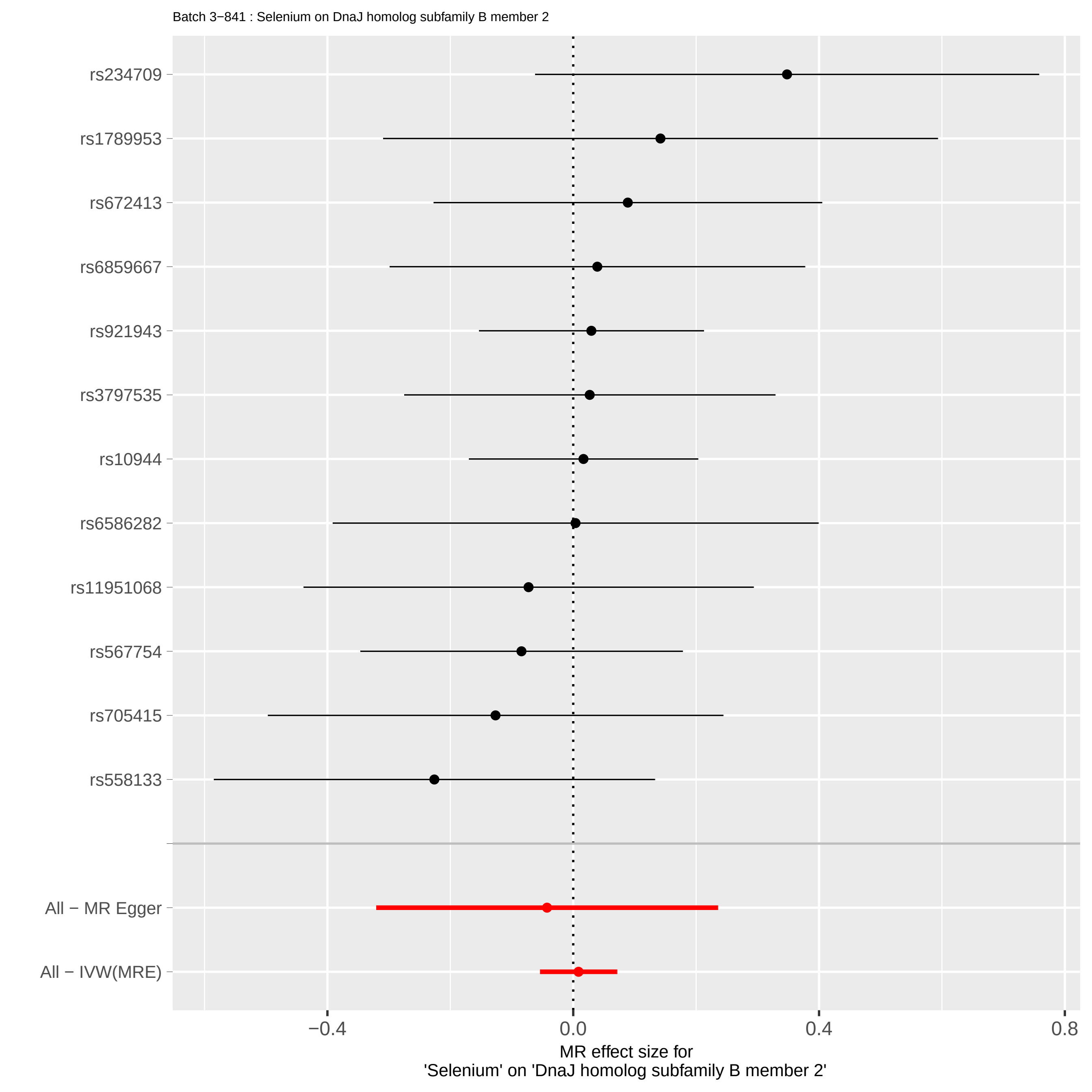
Batch 3−841 : Selenium on DnaJ homolog subfamily B member 2
rs234709
rs1789953
rs672413
rs6859667
rs921943
rs3797535
rs10944
rs6586282
rs11951068
rs567754
rs705415
rs558133
All − MR Egger
All − IVW(MRE)
−0.4
0.0
0.4
0.8
MR effect size for
'Selenium' on 'DnaJ homolog subfamily B member 2'

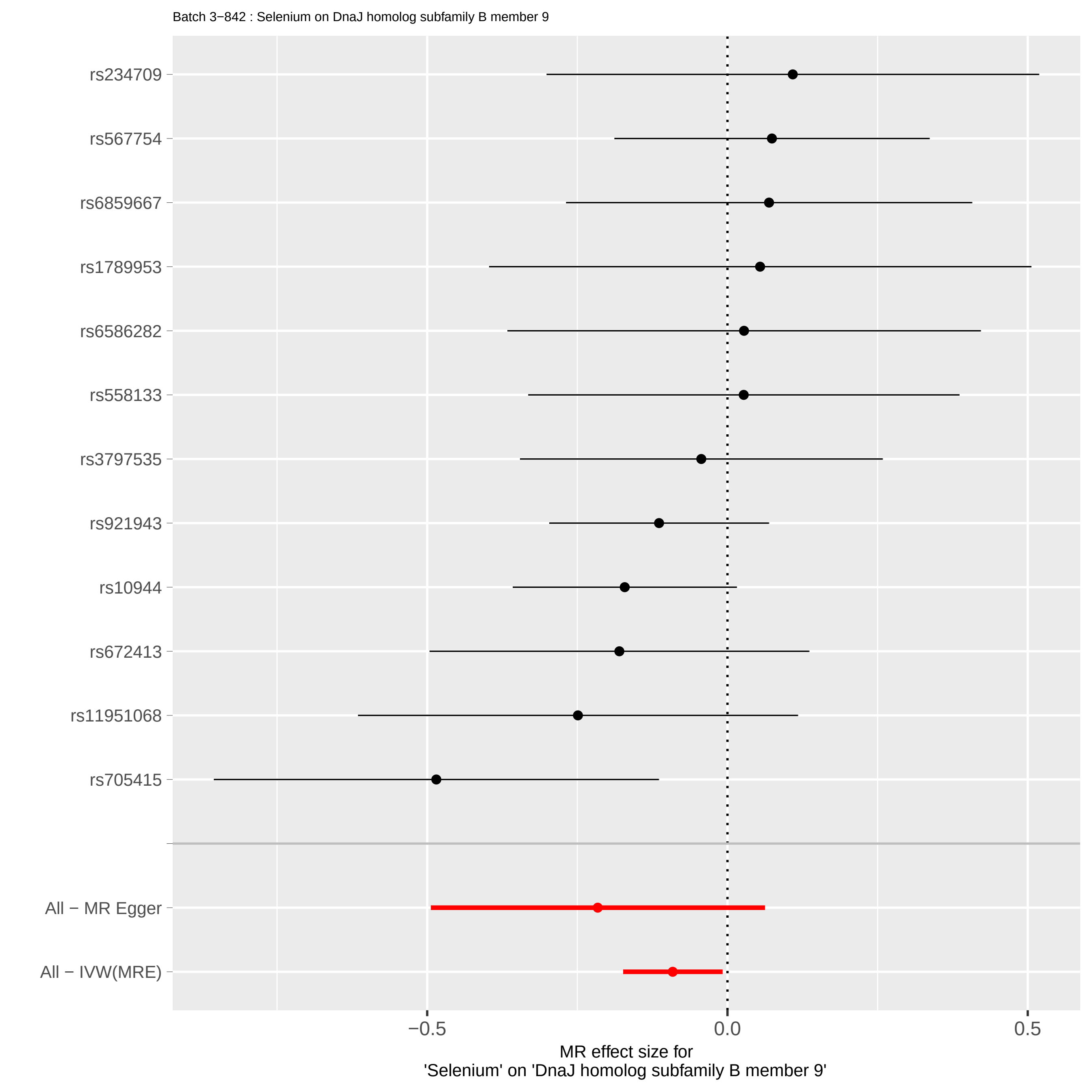

Batch 3−842 : Selenium on DnaJ homolog subfamily B member 9
rs234709
rs567754
rs6859667
rs1789953
rs6586282
rs558133
rs3797535
rs921943
rs10944
rs672413
rs11951068
rs705415
All − MR Egger
All − IVW(MRE)
−0.5
0.0
0.5
MR effect size for
'Selenium' on 'DnaJ homolog subfamily B member 9'

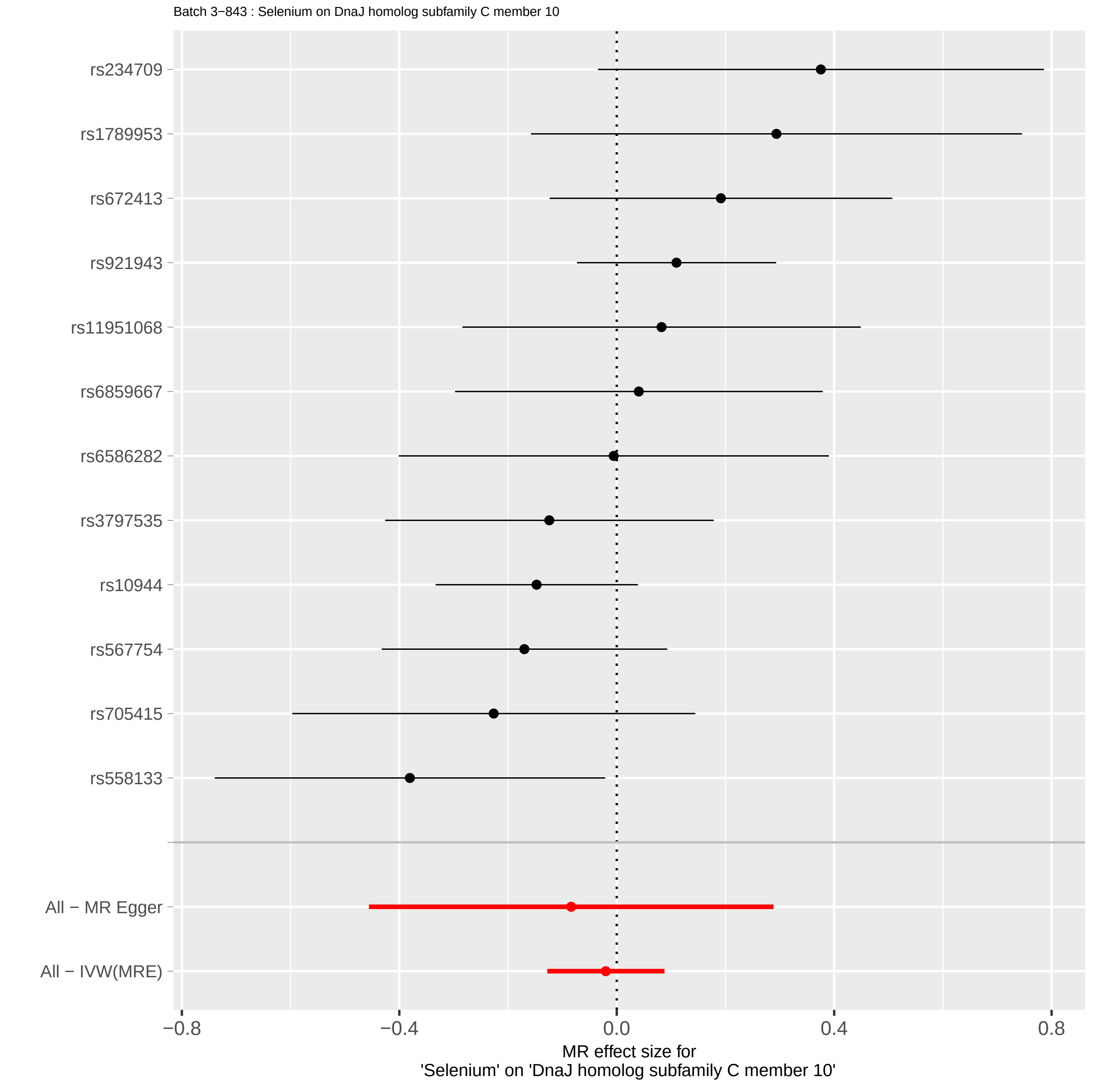

Batch 3−843 : Selenium on DnaJ homolog subfamily C member 10
rs234709
rs1789953
rs672413
rs921943
rs11951068
rs6859667
rs6586282
rs3797535
rs10944
rs567754
rs705415
rs558133
All − MR Egger
All − IVW(MRE)
−0.8
−0.4
0.0
0.4
0.8
MR effect size for
'Selenium' on 'DnaJ homolog subfamily C member 10'

Batch 3−844 : Selenium on DnaJ homolog subfamily C member 15

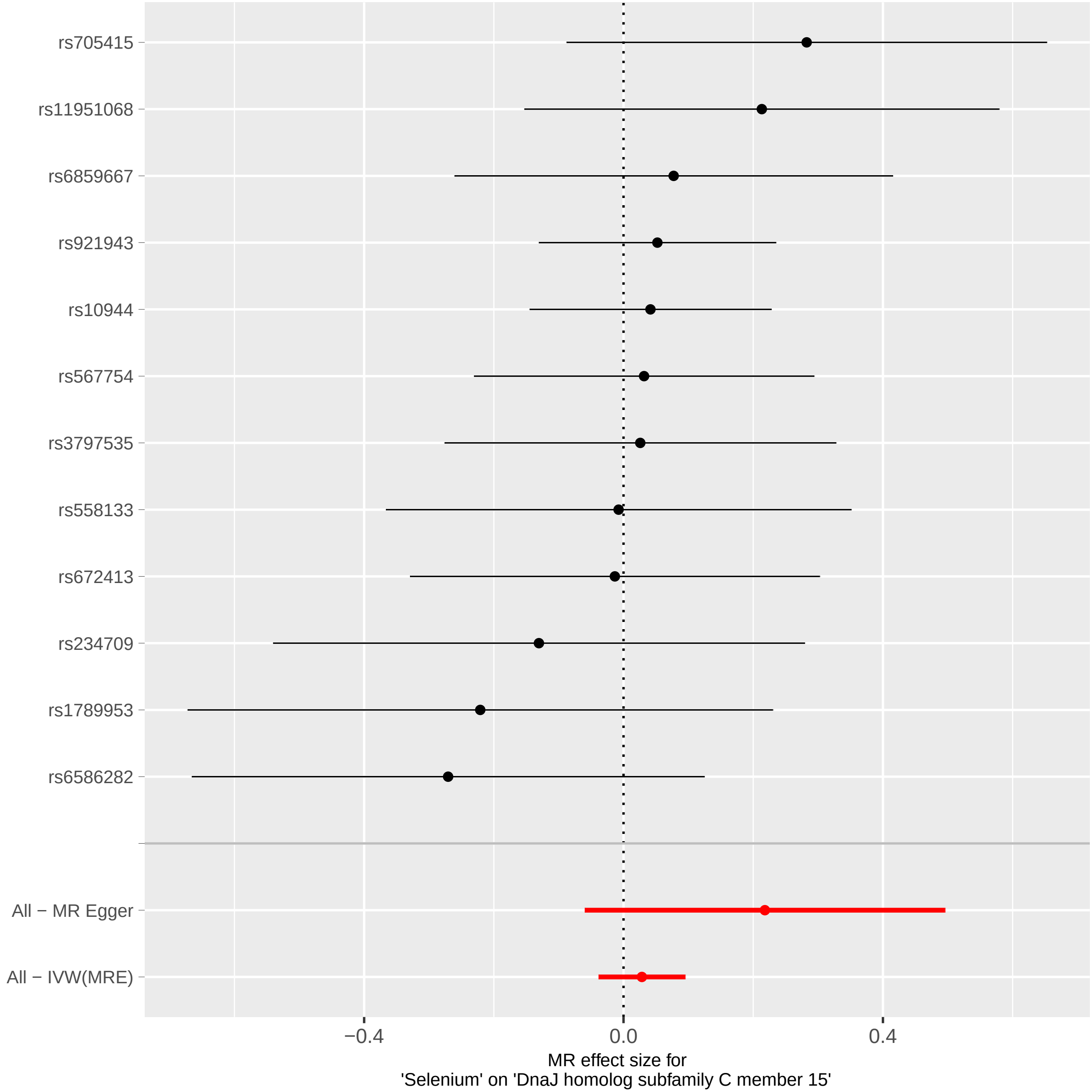

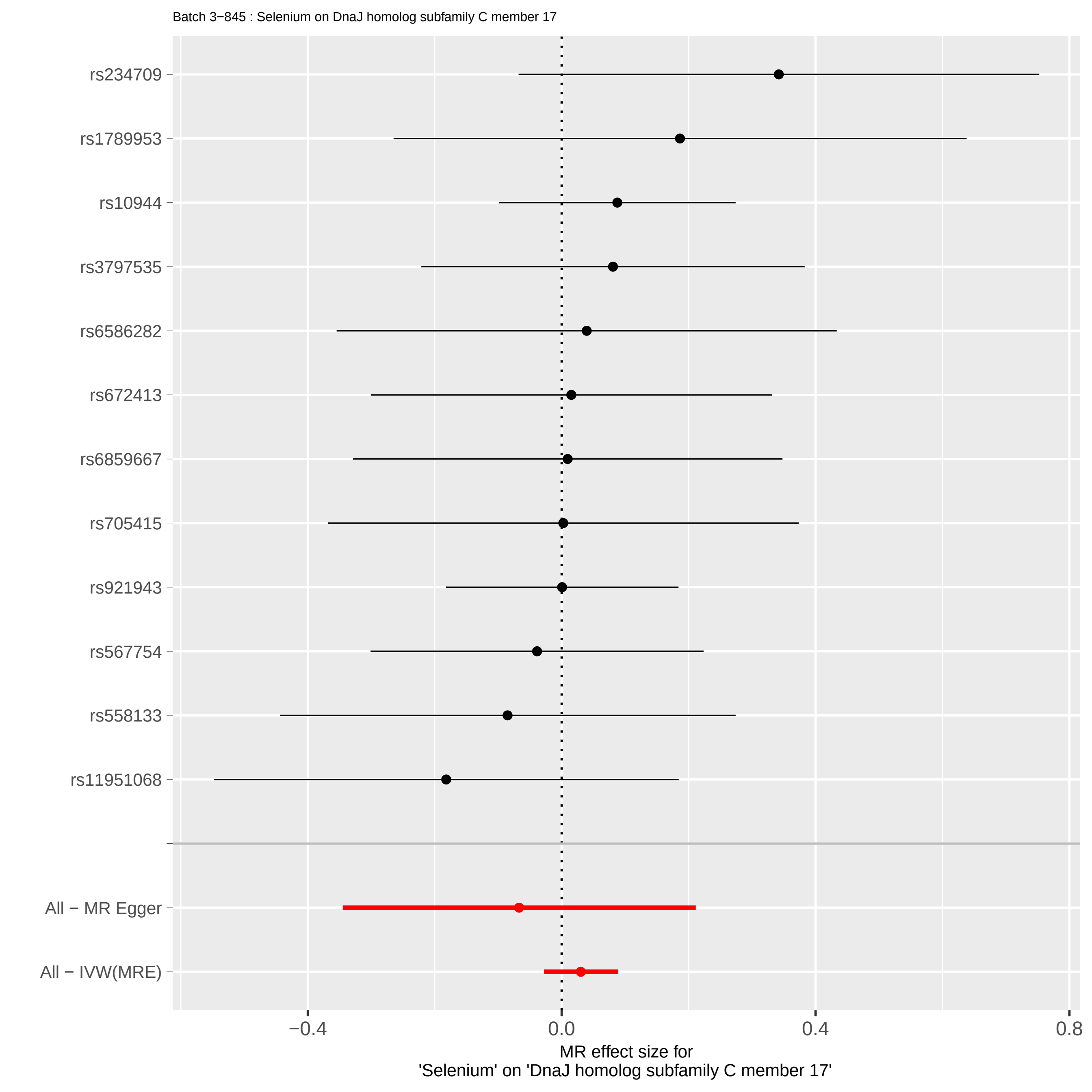

Batch 3−845 : Selenium on DnaJ homolog subfamily C member 17
rs234709
rs1789953
rs10944
rs3797535
rs6586282
rs672413
rs6859667
rs705415
rs921943
rs567754
rs558133
rs11951068
All − MR Egger
All − IVW(MRE)
−0.4
0.0
0.4
0.8
MR effect size for
'Selenium' on 'DnaJ homolog subfamily C member 17'

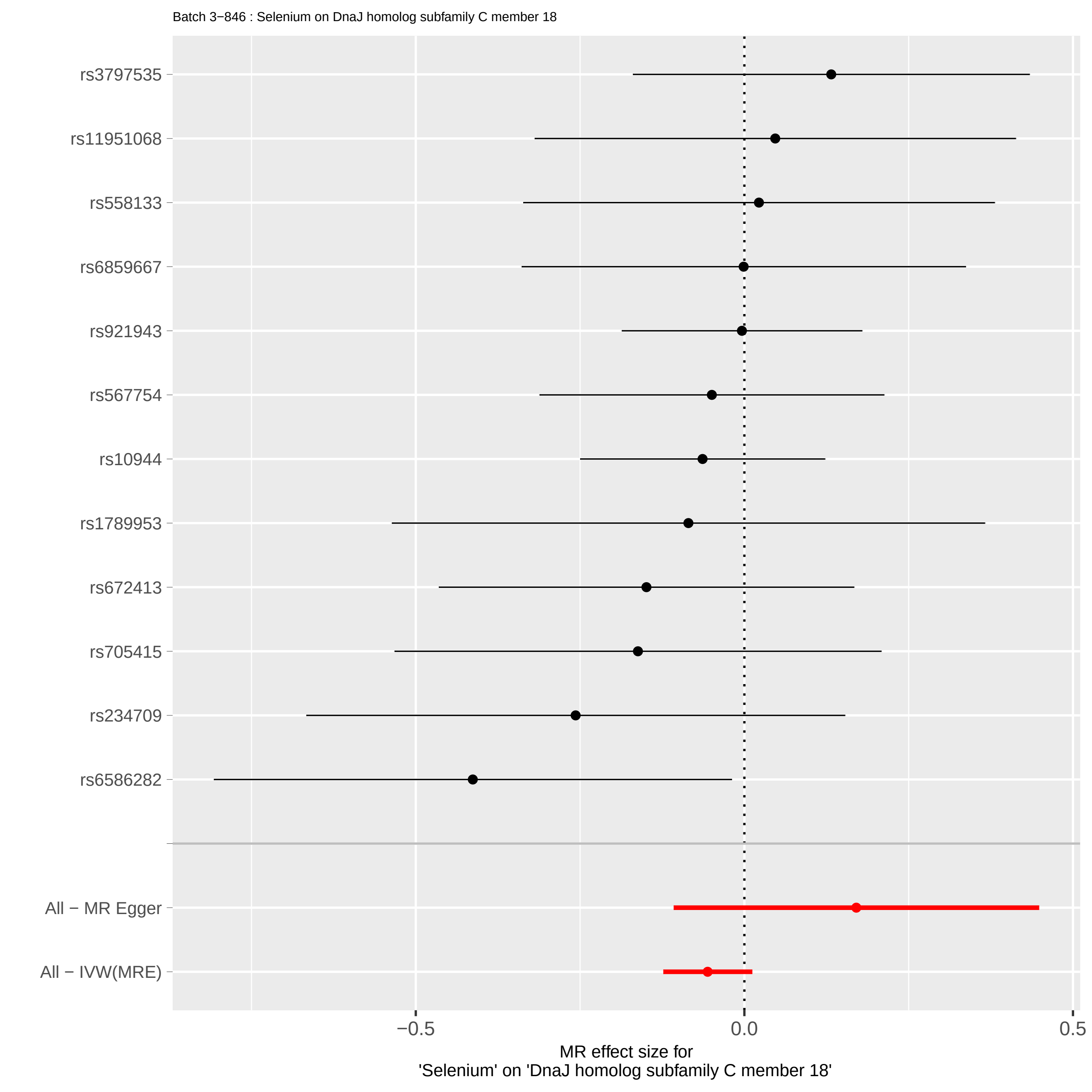
Batch 3−846 : Selenium on DnaJ homolog subfamily C member 18
rs3797535
rs11951068
rs558133
rs6859667
rs921943
rs567754
rs10944
rs1789953
rs672413
rs705415
rs234709
rs6586282
All − MR Egger
All − IVW(MRE)
−0.5
0.0
0.5
MR effect size for
'Selenium' on 'DnaJ homolog subfamily C member 18'

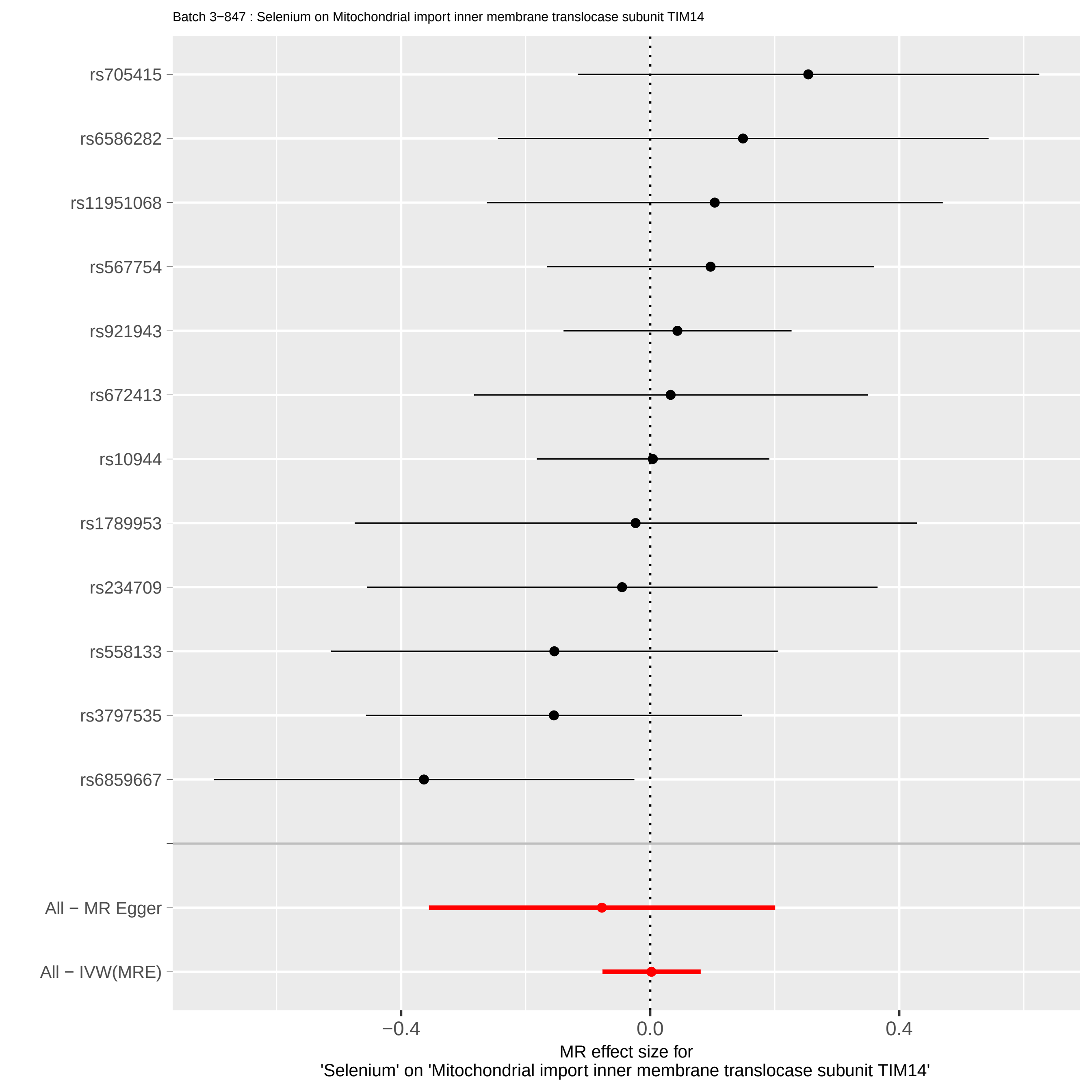
Batch 3−847 : Selenium on Mitochondrial import inner membrane translocase subunit TIM14
rs705415
rs6586282
rs11951068
rs567754
rs921943
rs672413
rs10944
rs1789953
rs234709
rs558133
rs3797535
rs6859667
All − MR Egger
All − IVW(MRE)
−0.4
0.0
0.4
MR effect size for
'Selenium' on 'Mitochondrial import inner membrane translocase subunit TIM14'

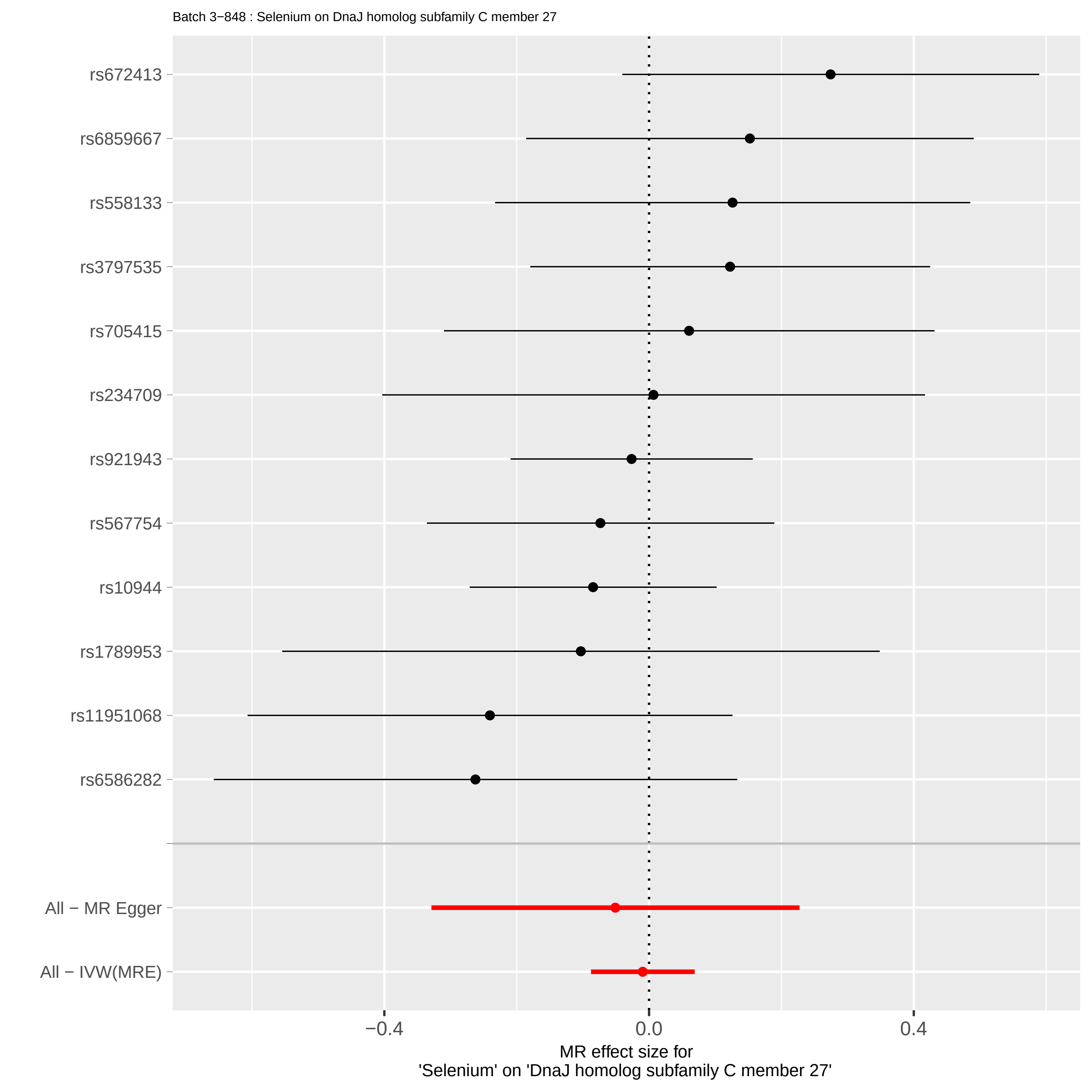

Batch 3−848 : Selenium on DnaJ homolog subfamily C member 27
rs672413
rs6859667
rs558133
rs3797535
rs705415
rs234709
rs921943
rs567754
rs10944
rs1789953
rs11951068
rs6586282
All − MR Egger
All − IVW(MRE)
−0.4
0.0
0.4
MR effect size for
'Selenium' on 'DnaJ homolog subfamily C member 27'

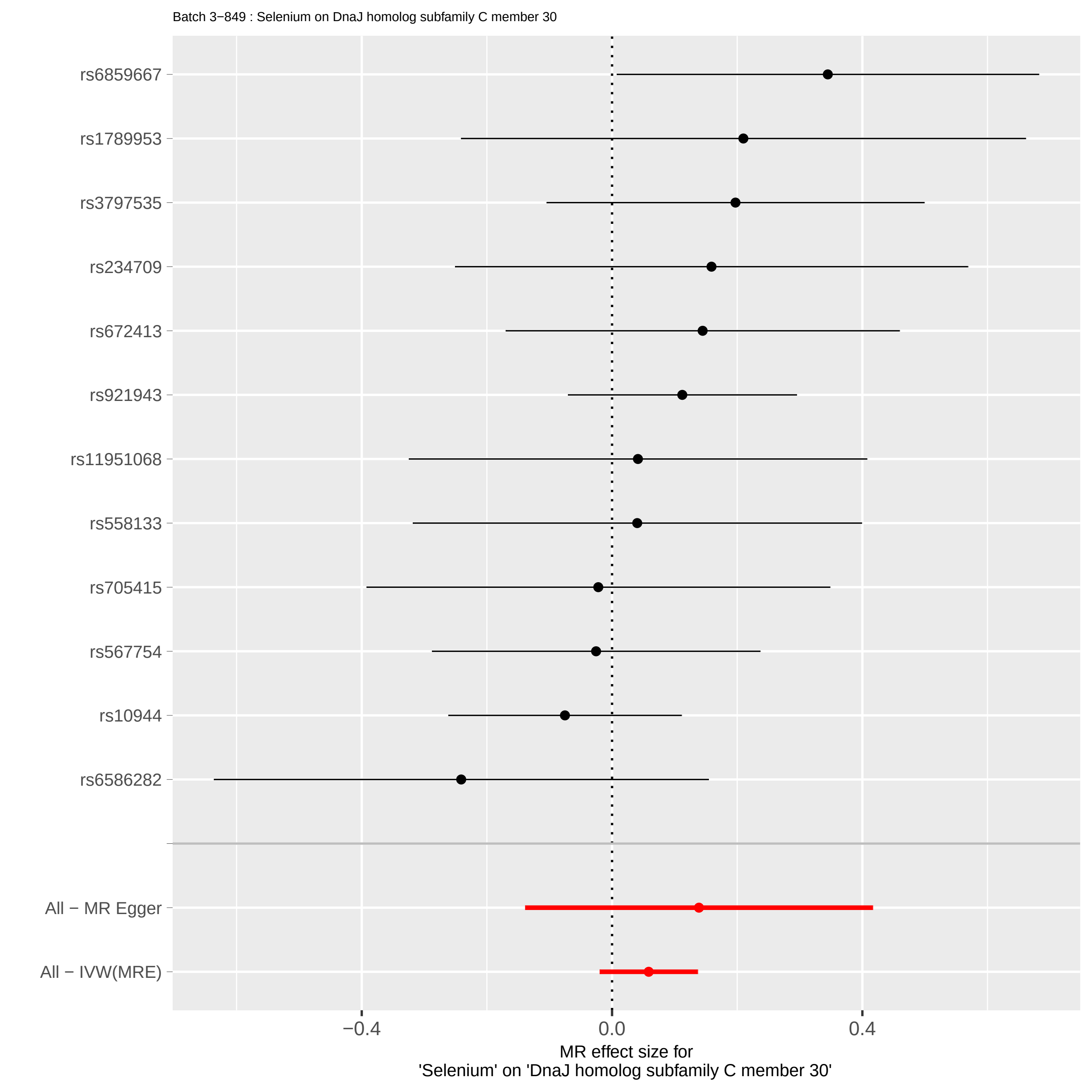

Batch 3−849 : Selenium on DnaJ homolog subfamily C member 30
rs6859667
rs1789953
rs3797535
rs234709
rs672413
rs921943
rs11951068
rs558133
rs705415
rs567754
rs10944
rs6586282
All − MR Egger
All − IVW(MRE)
−0.4
0.0
0.4
MR effect size for
'Selenium' on 'DnaJ homolog subfamily C member 30'

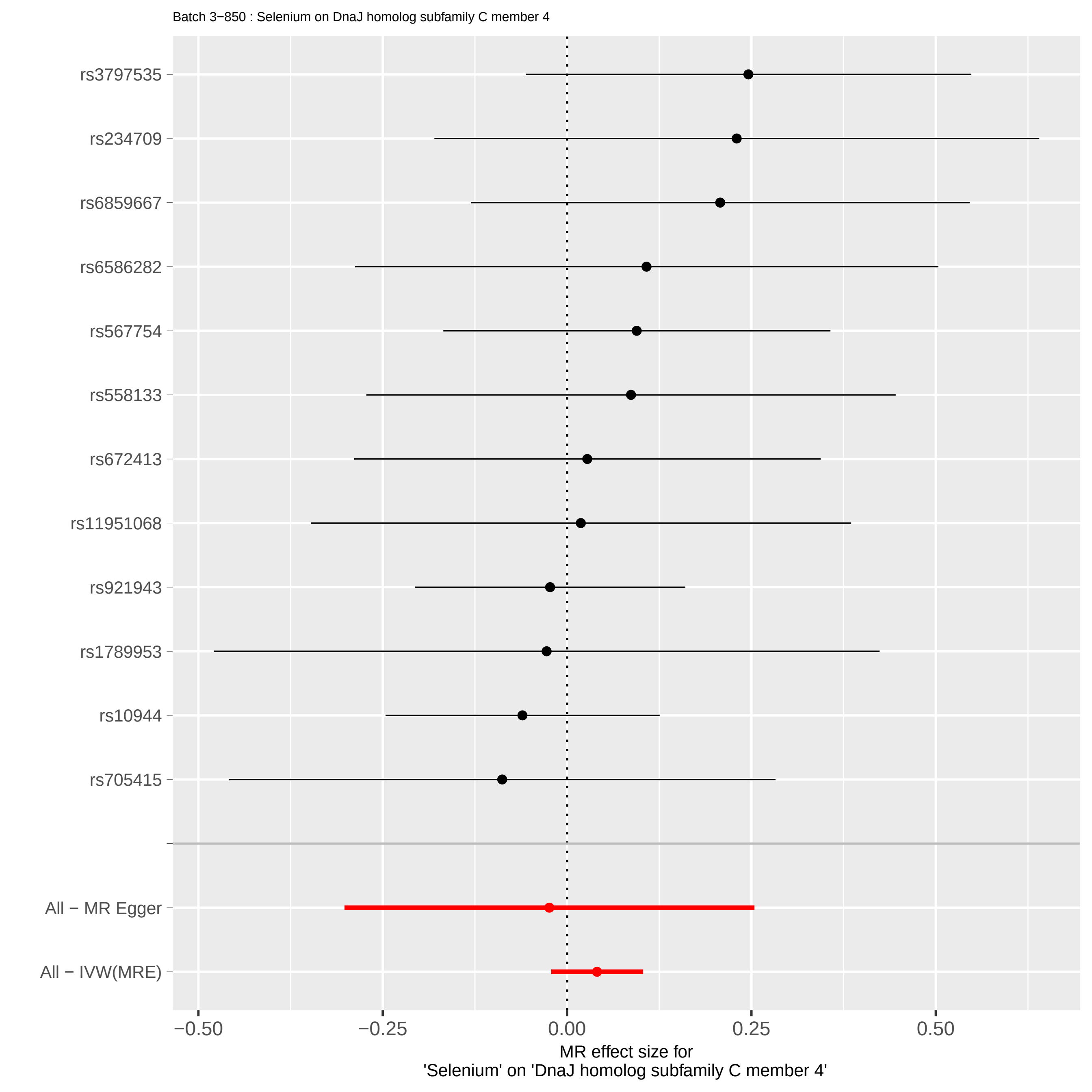
Batch 3−850 : Selenium on DnaJ homolog subfamily C member 4
rs3797535
rs234709
rs6859667
rs6586282
rs567754
rs558133
rs672413
rs11951068
rs921943
rs1789953
rs10944
rs705415
All − MR Egger
All − IVW(MRE)
−0.50
−0.25
0.00
0.25
0.50
MR effect size for
'Selenium' on 'DnaJ homolog subfamily C member 4'

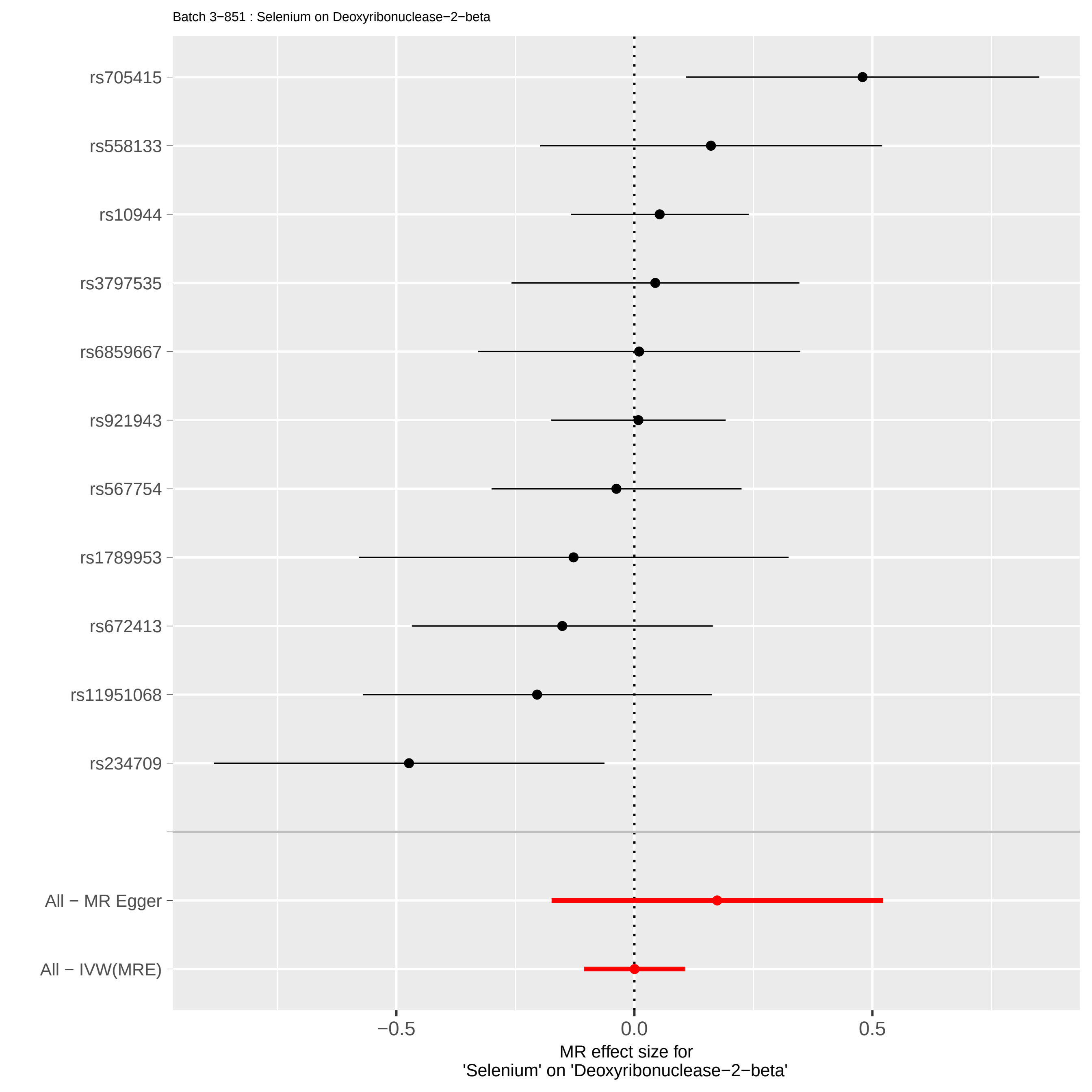

Batch 3−851 : Selenium on Deoxyribonuclease−2−beta
rs705415
rs558133
rs10944
rs3797535
rs6859667
rs921943
rs567754
rs1789953
rs672413
rs11951068
rs234709
All − MR Egger
All − IVW(MRE)
−0.5
0.0
0.5
MR effect size for
'Selenium' on 'Deoxyribonuclease−2−beta'

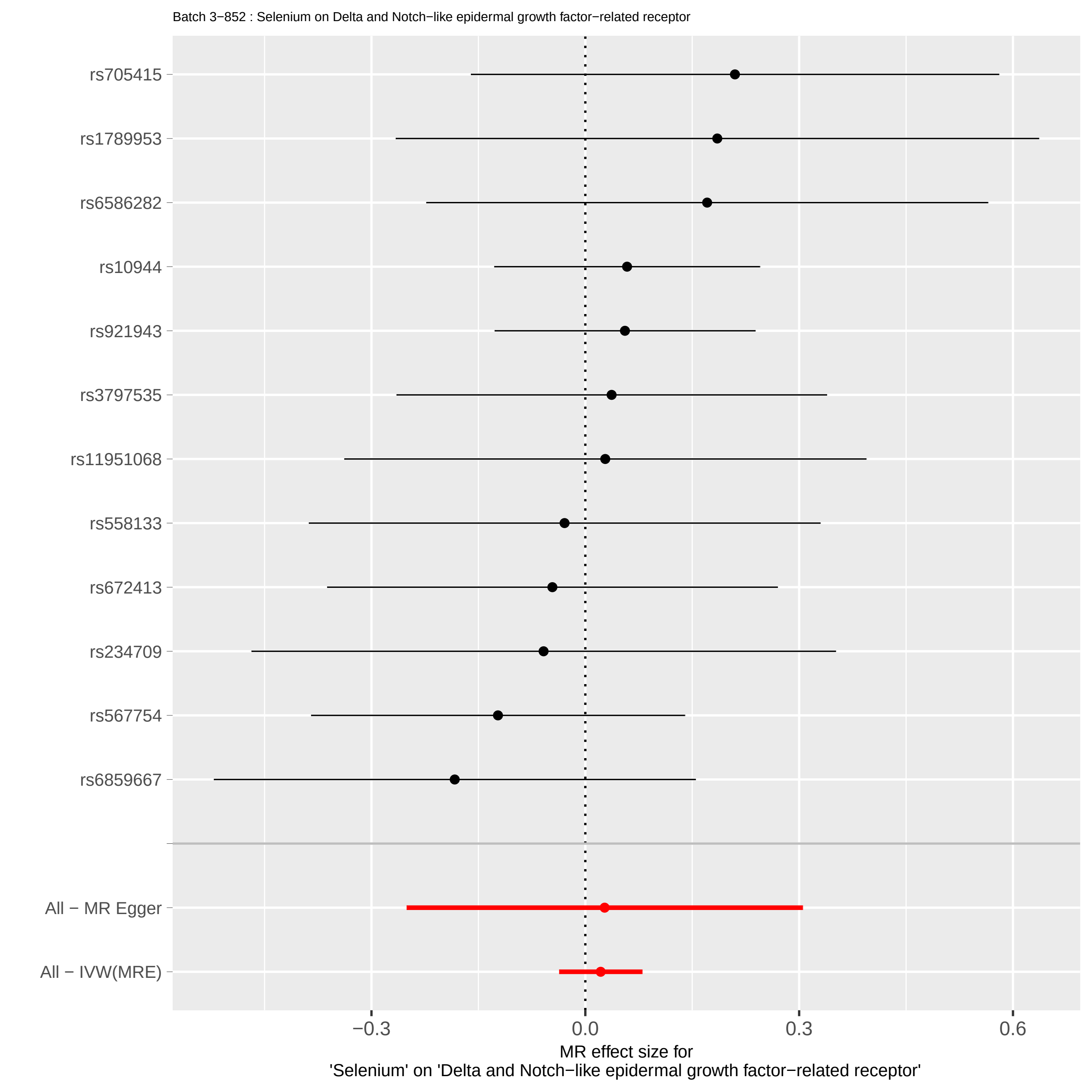

Batch 3−852 : Selenium on Delta and Notch−like epidermal growth factor−related receptor
rs705415
rs1789953
rs6586282
rs10944
rs921943
rs3797535
rs11951068
rs558133
rs672413
rs234709
rs567754
rs6859667
All − MR Egger
All − IVW(MRE)
−0.3
0.0
0.3
0.6
MR effect size for
'Selenium' on 'Delta and Notch−like epidermal growth factor−related receptor'

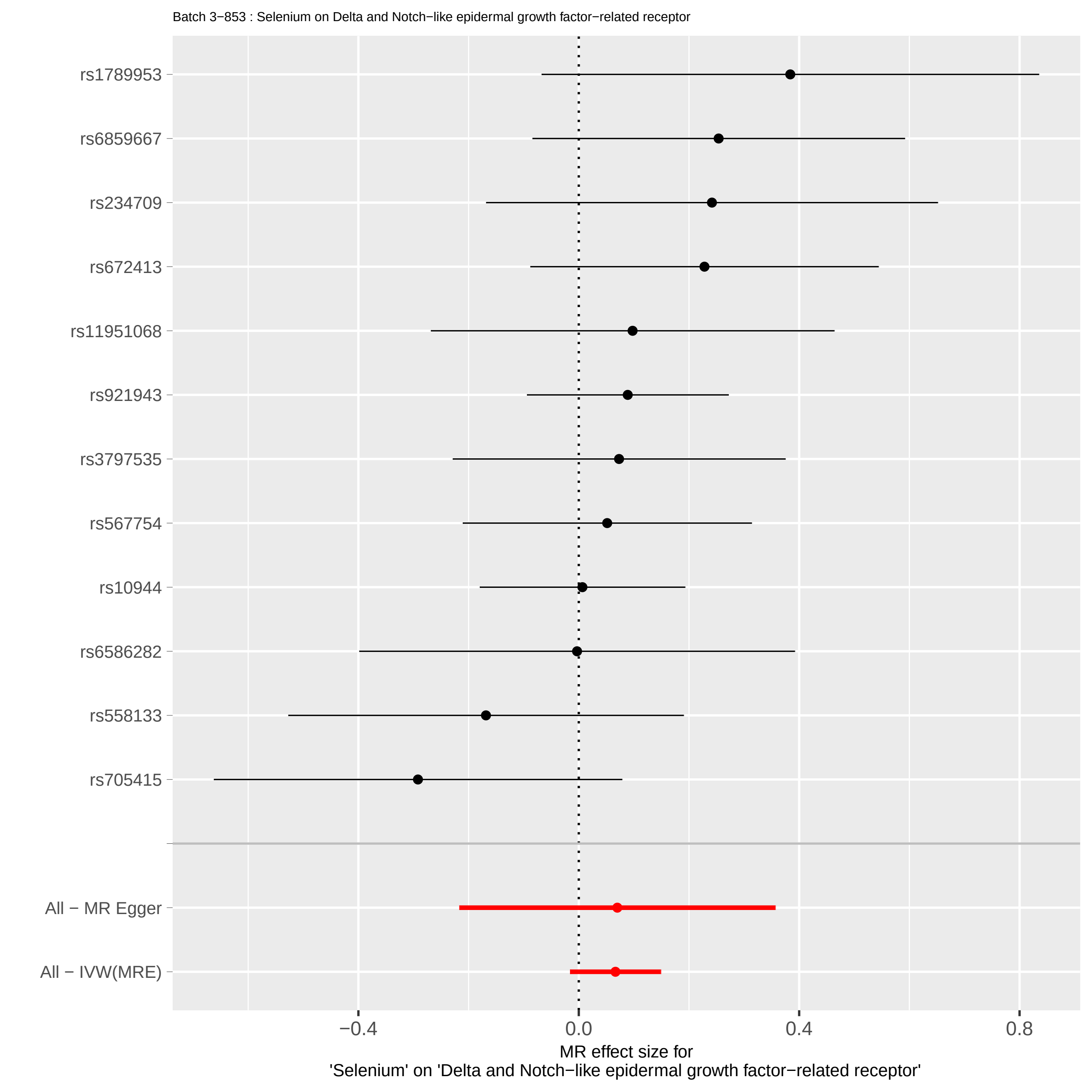

Batch 3−853 : Selenium on Delta and Notch−like epidermal growth factor−related receptor
rs1789953
rs6859667
rs234709
rs672413
rs11951068
rs921943
rs3797535
rs567754
rs10944
rs6586282
rs558133
rs705415
All − MR Egger
All − IVW(MRE)
−0.4
0.0
0.4
0.8
MR effect size for
'Selenium' on 'Delta and Notch−like epidermal growth factor−related receptor'

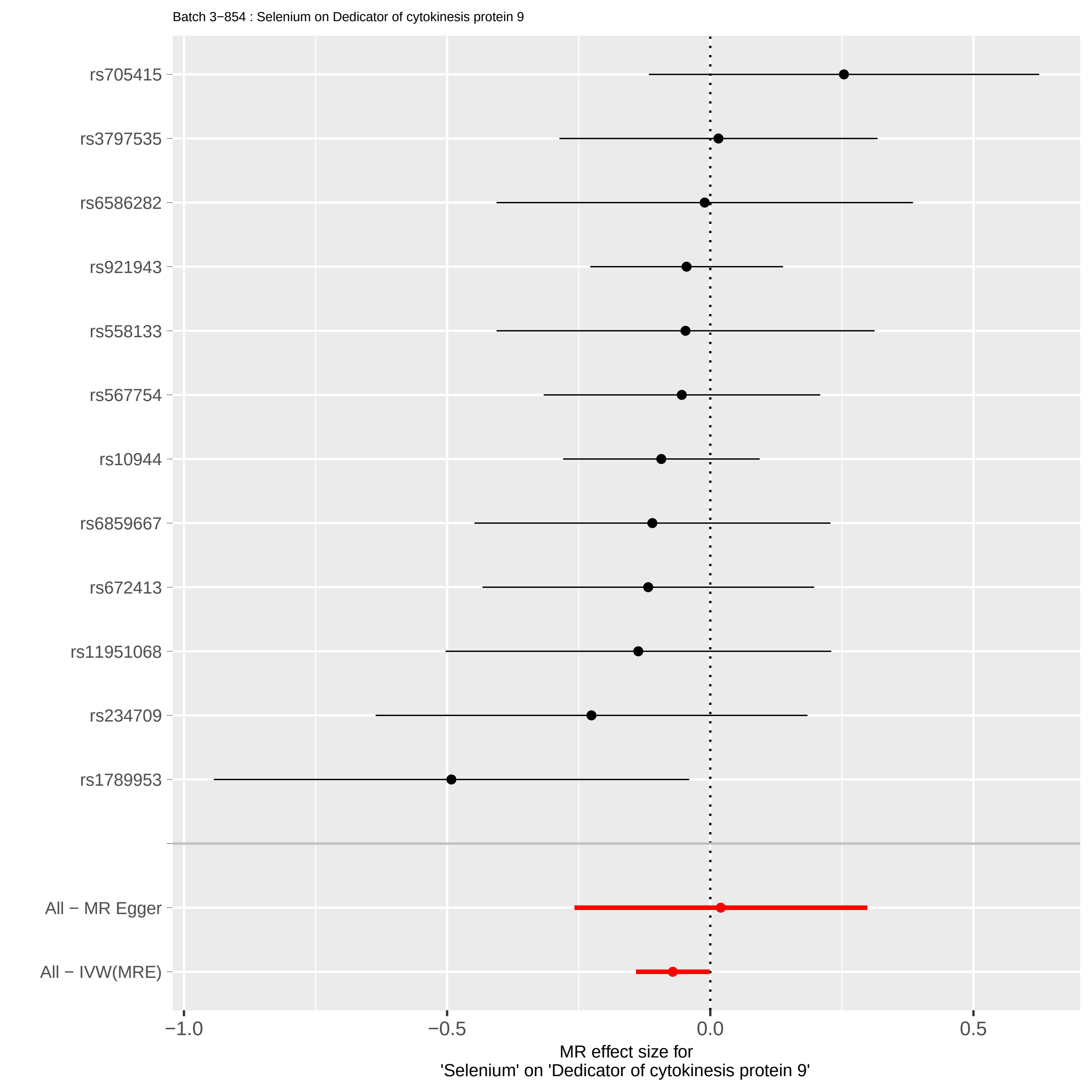

Batch 3−854 : Selenium on Dedicator of cytokinesis protein 9
rs705415
rs3797535
rs6586282
rs921943
rs558133
rs567754
rs10944
rs6859667
rs672413
rs11951068
rs234709
rs1789953
All − MR Egger
All − IVW(MRE)
−1.0
−0.5
0.0
0.5
MR effect size for
'Selenium' on 'Dedicator of cytokinesis protein 9'

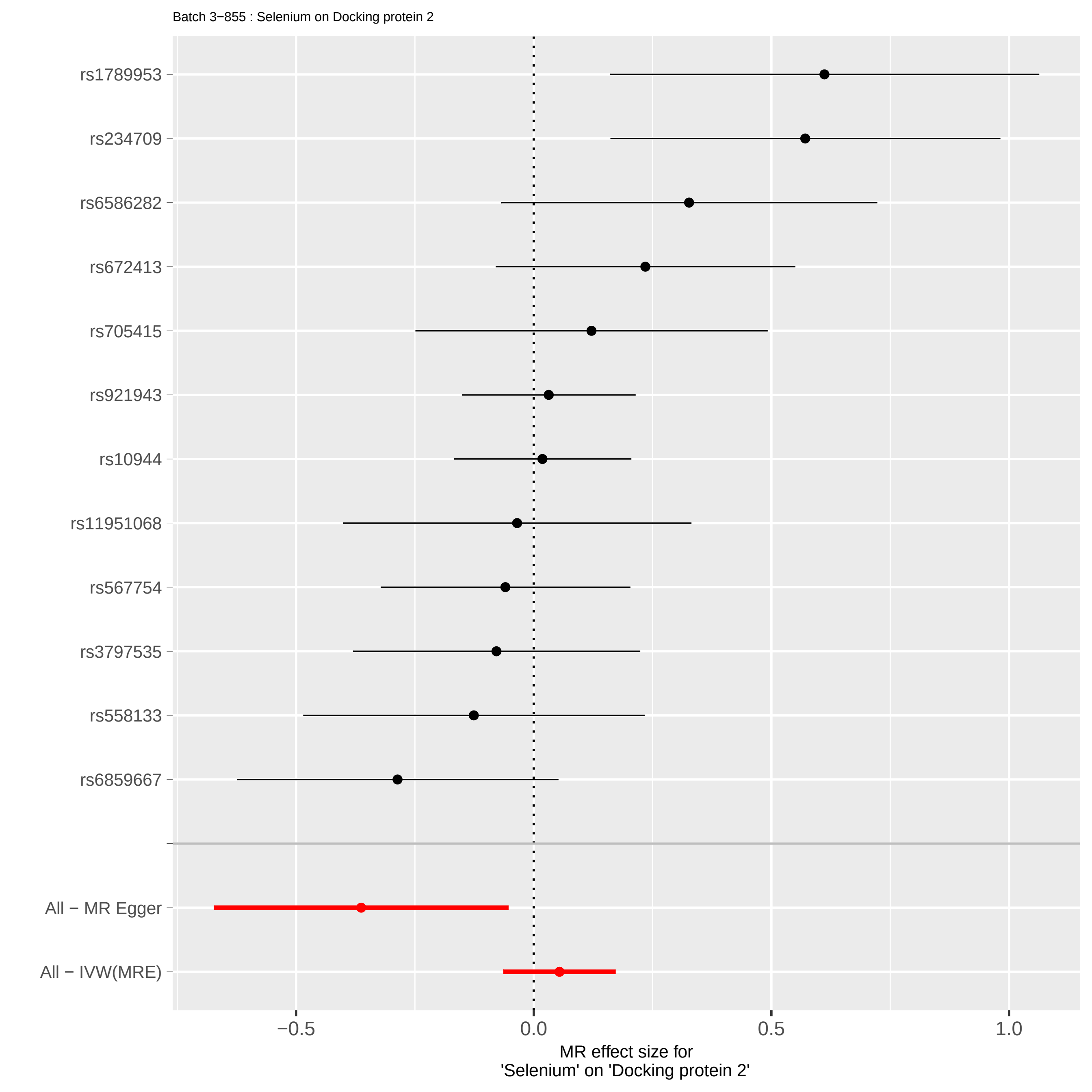

Batch 3−855 : Selenium on Docking protein 2
rs1789953
rs234709
rs6586282
rs672413
rs705415
rs921943
rs10944
rs11951068
rs567754
rs3797535
rs558133
rs6859667
All − MR Egger
All − IVW(MRE)
−0.5
0.0
0.5
1.0
MR effect size for
'Selenium' on 'Docking protein 2'

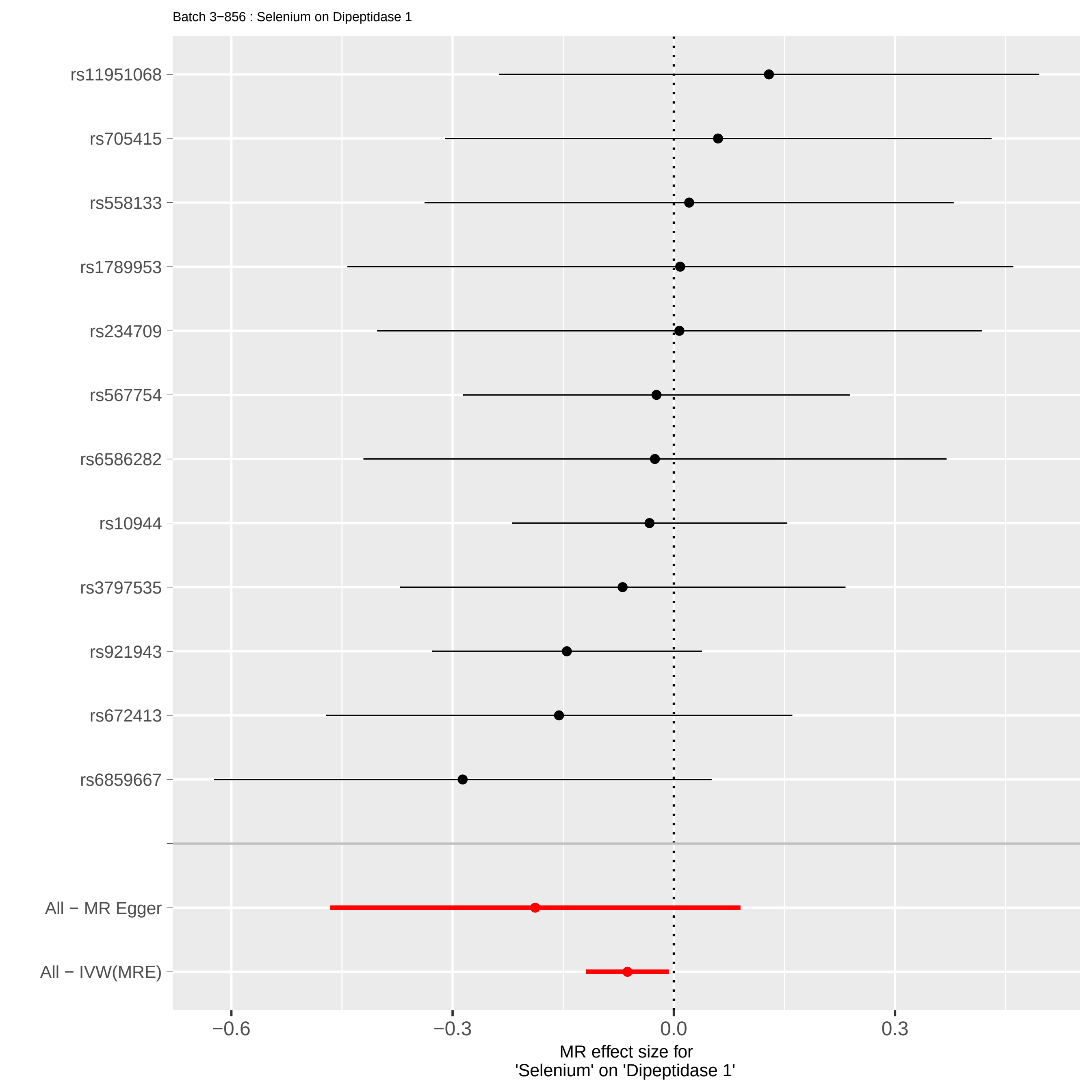

Batch 3−856 : Selenium on Dipeptidase 1
rs11951068
rs705415
rs558133
rs1789953
rs234709
rs567754
rs6586282
rs10944
rs3797535
rs921943
rs672413
rs6859667
All − MR Egger
All − IVW(MRE)
−0.6
−0.3
0.0
0.3
MR effect size for
'Selenium' on 'Dipeptidase 1'

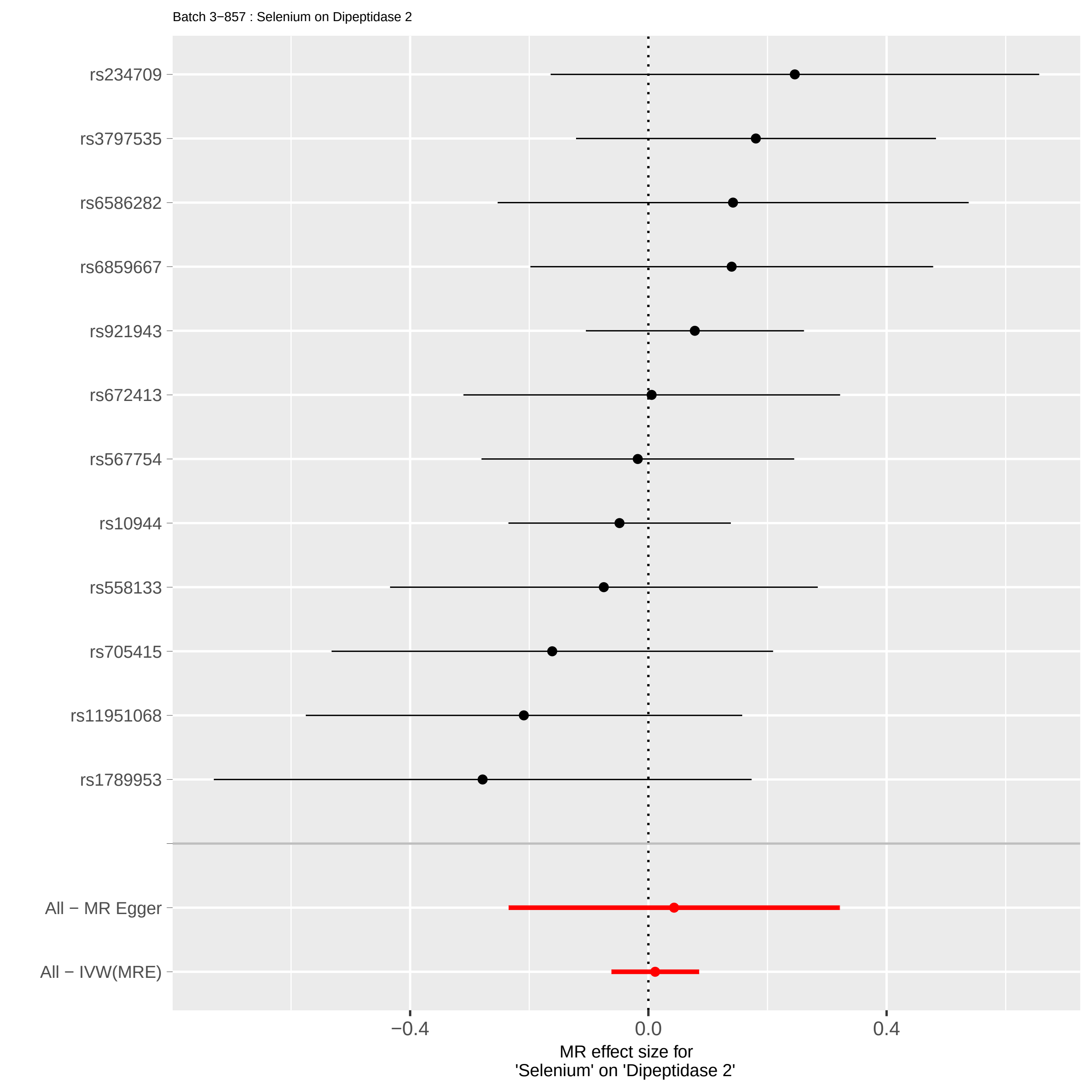

Batch 3−857 : Selenium on Dipeptidase 2
rs234709
rs3797535
rs6586282
rs6859667
rs921943
rs672413
rs567754
rs10944
rs558133
rs705415
rs11951068
rs1789953
All − MR Egger
All − IVW(MRE)
−0.4
0.0
0.4
MR effect size for
'Selenium' on 'Dipeptidase 2'

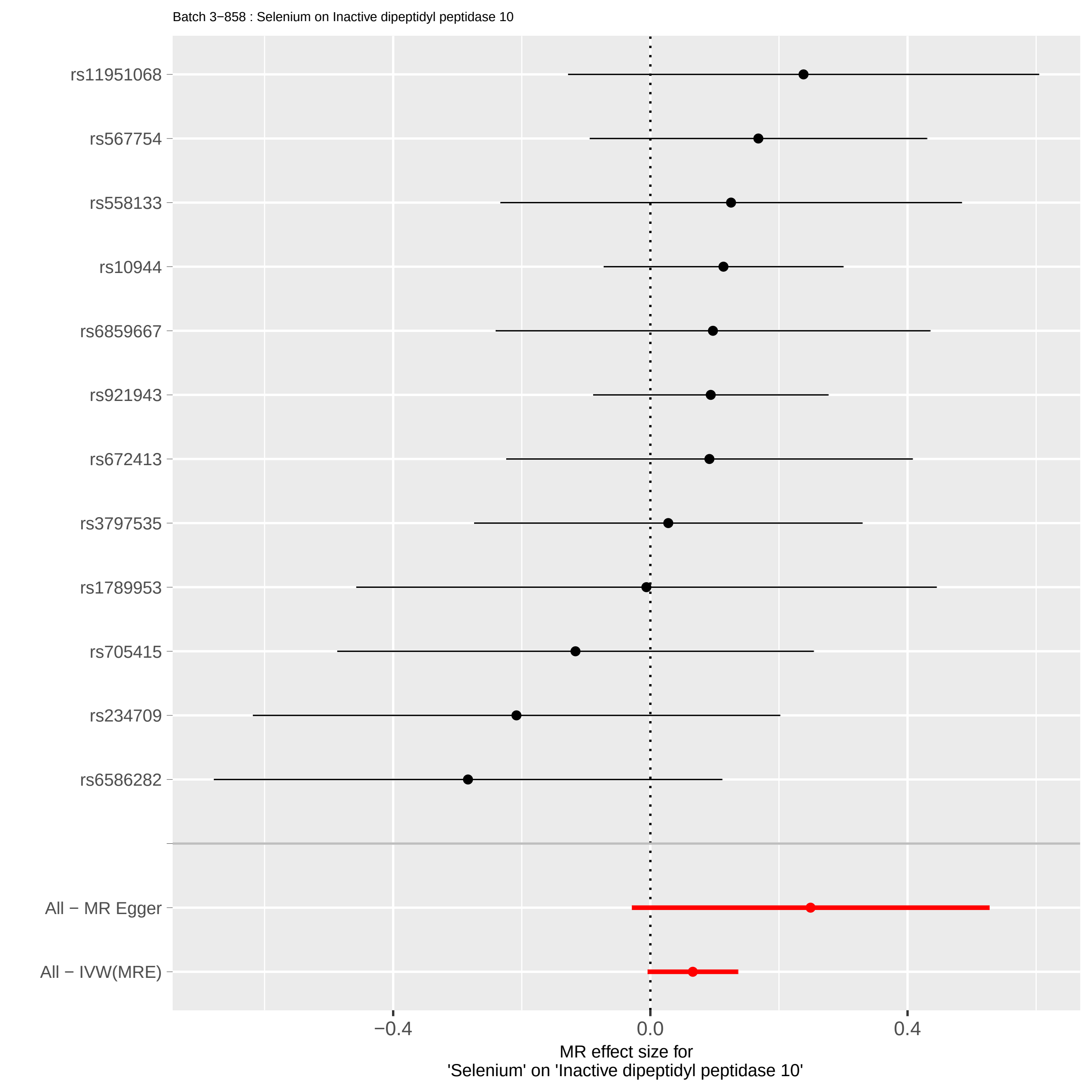
Batch 3−858 : Selenium on Inactive dipeptidyl peptidase 10
rs11951068
rs567754
rs558133
rs10944
rs6859667
rs921943
rs672413
rs3797535
rs1789953
rs705415
rs234709
rs6586282
All − MR Egger
All − IVW(MRE)
−0.4
0.0
0.4
MR effect size for
'Selenium' on 'Inactive dipeptidyl peptidase 10'

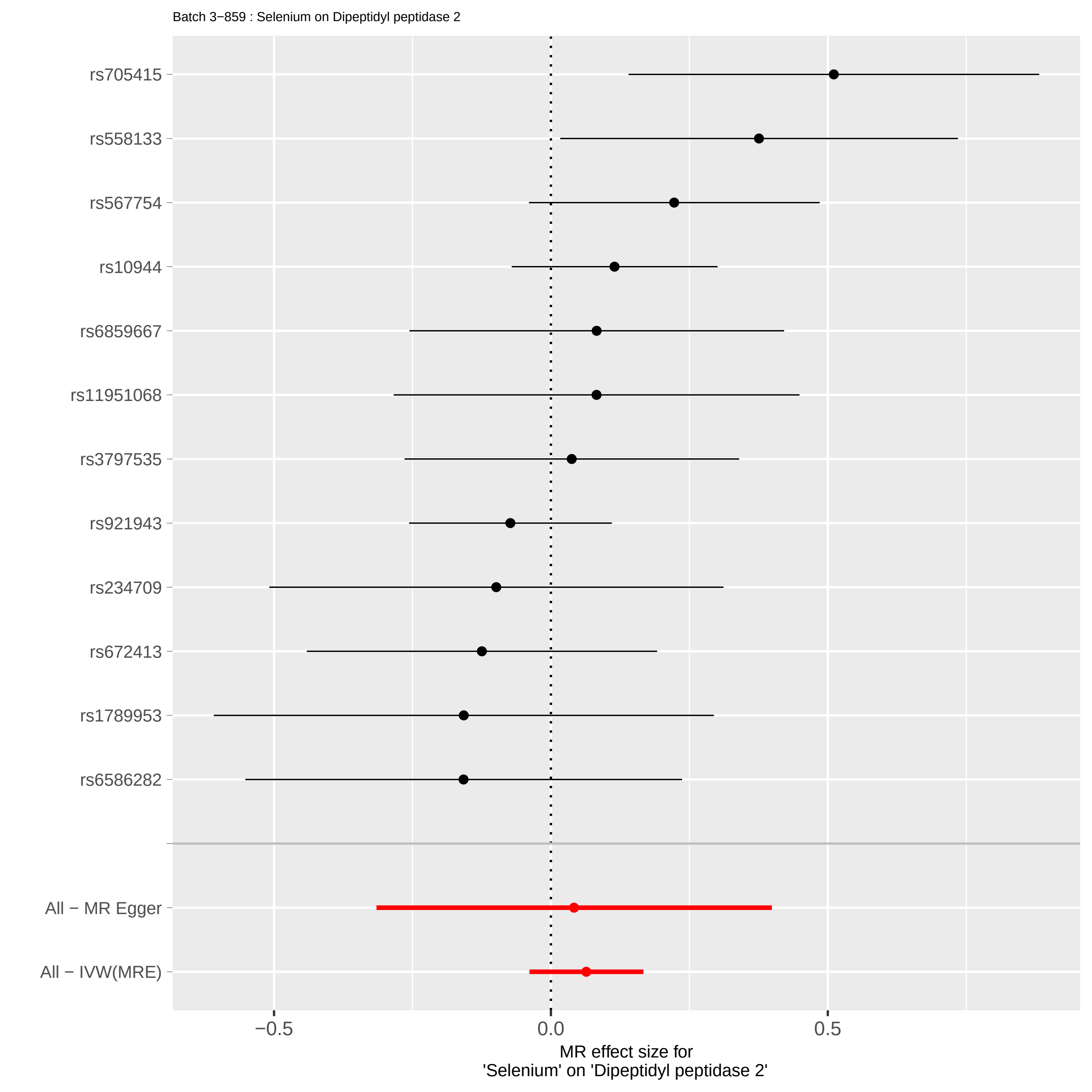
Batch 3−859 : Selenium on Dipeptidyl peptidase 2
rs705415
rs558133
rs567754
rs10944
rs6859667
rs11951068
rs3797535
rs921943
rs234709
rs672413
rs1789953
rs6586282
All − MR Egger
All − IVW(MRE)
−0.5
0.0
0.5
MR effect size for
'Selenium' on 'Dipeptidyl peptidase 2'

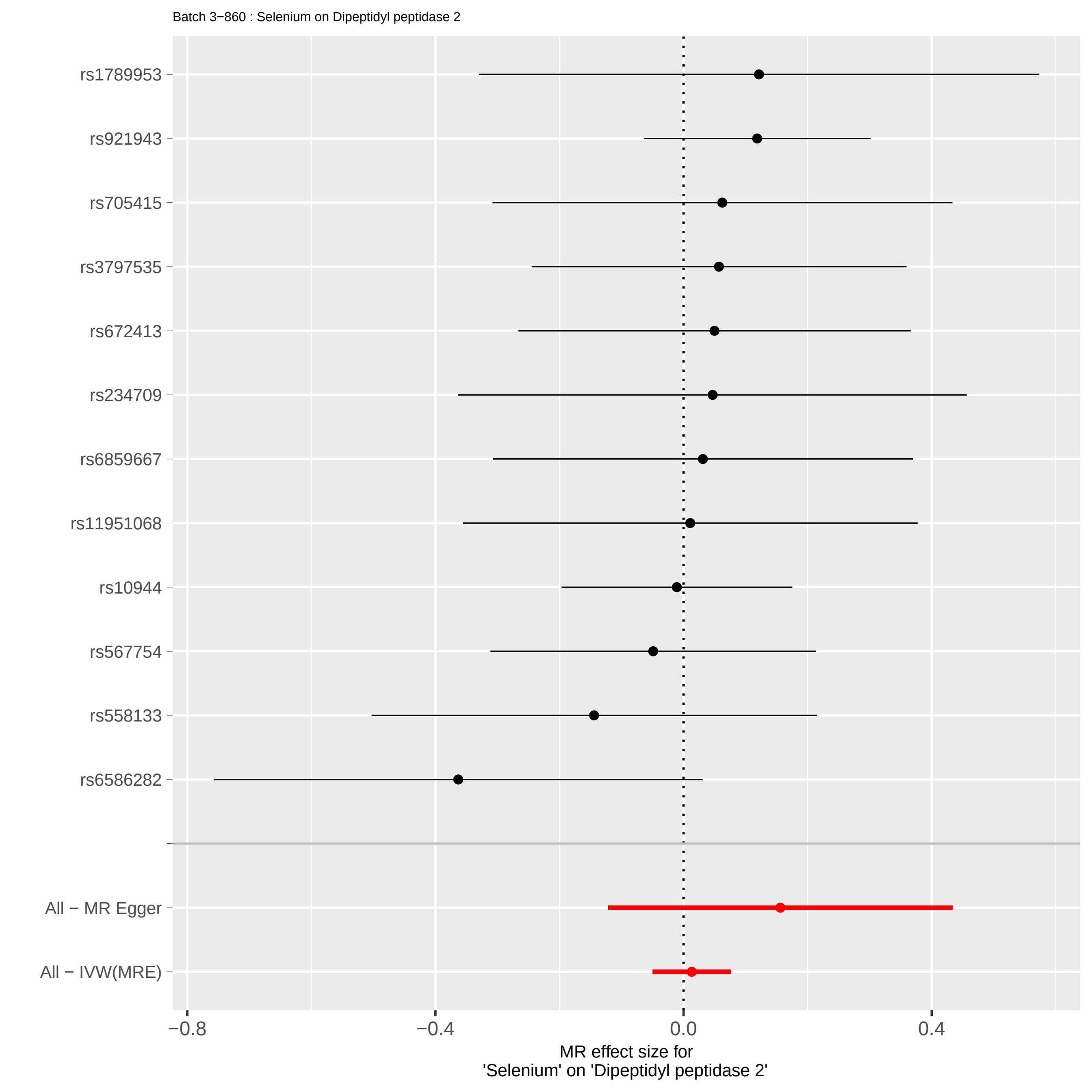

Batch 3−860 : Selenium on Dipeptidyl peptidase 2
rs1789953
rs921943
rs705415
rs3797535
rs672413
rs234709
rs6859667
rs11951068
rs10944
rs567754
rs558133
rs6586282
All − MR Egger
All − IVW(MRE)
−0.8
−0.4
0.0
0.4
MR effect size for
'Selenium' on 'Dipeptidyl peptidase 2'

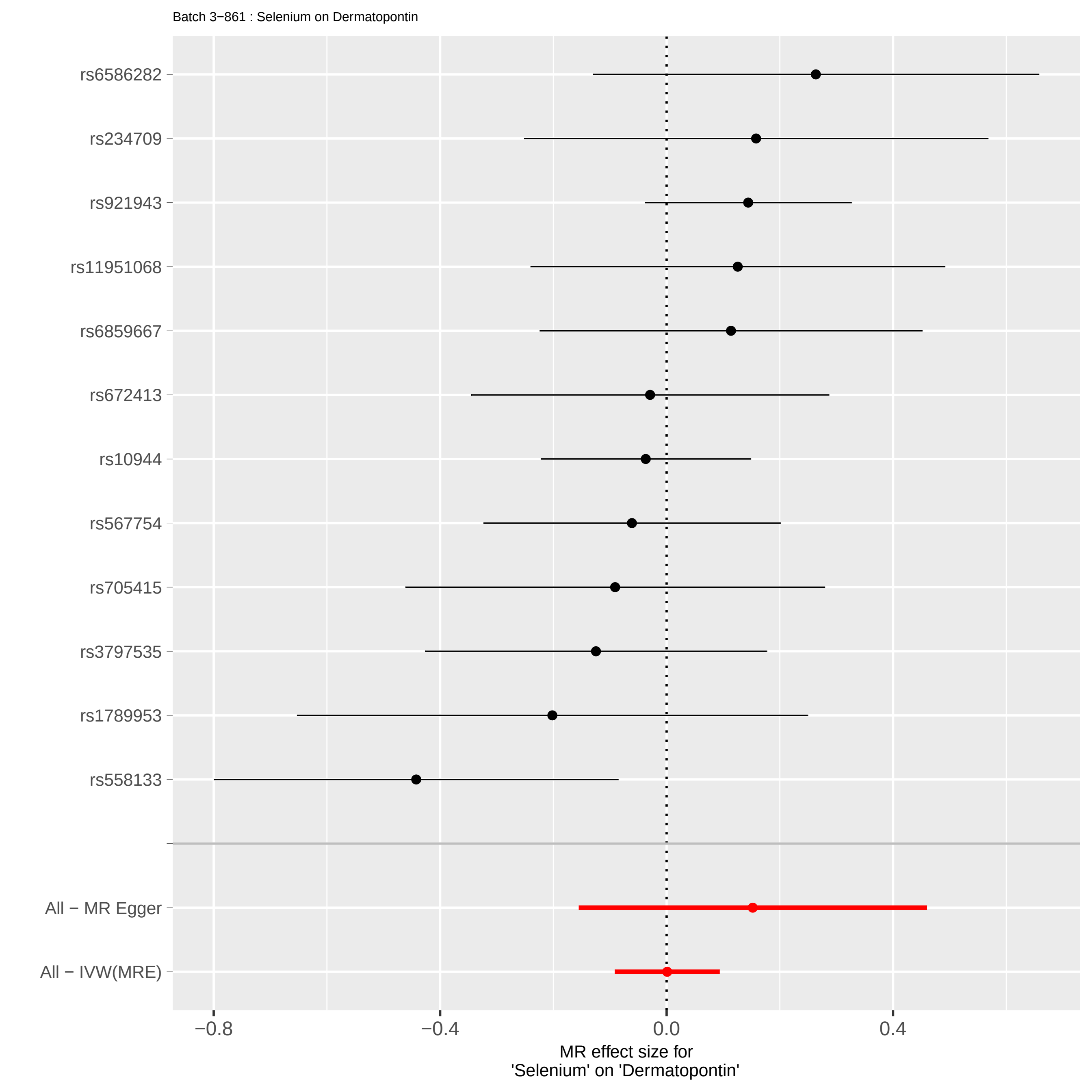
Batch 3−861 : Selenium on Dermatopontin
rs6586282
rs234709
rs921943
rs11951068
rs6859667
rs672413
rs10944
rs567754
rs705415
rs3797535
rs1789953
rs558133
All − MR Egger
All − IVW(MRE)
−0.8
−0.4
0.0
0.4
MR effect size for
'Selenium' on 'Dermatopontin'

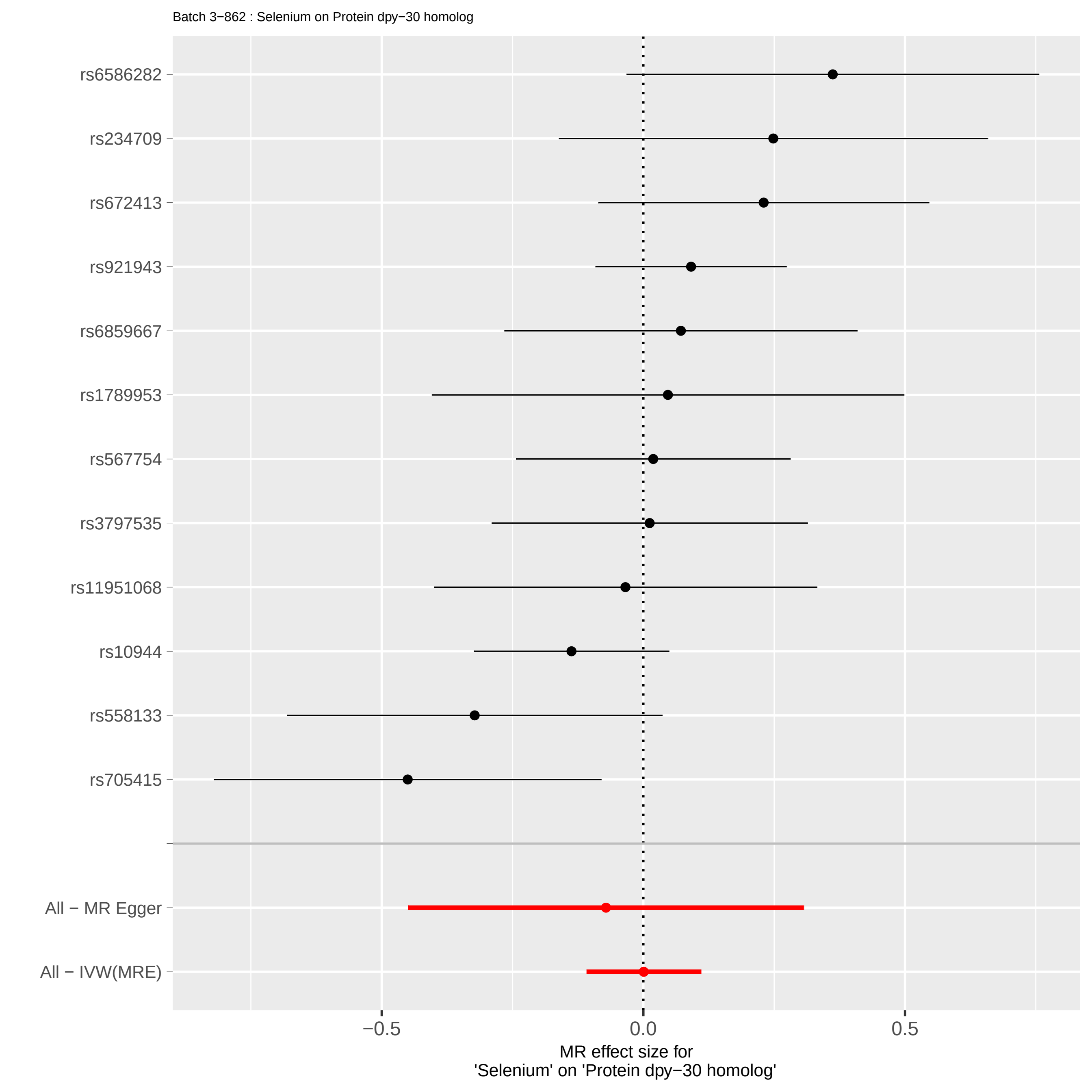

Batch 3−862 : Selenium on Protein dpy−30 homolog
rs6586282
rs234709
rs672413
rs921943
rs6859667
rs1789953
rs567754
rs3797535
rs11951068
rs10944
rs558133
rs705415
All − MR Egger
All − IVW(MRE)
−0.5
0.0
0.5
MR effect size for
'Selenium' on 'Protein dpy−30 homolog'

Batch 3−863 : Selenium on Dihydropyrimidinase−related protein 3

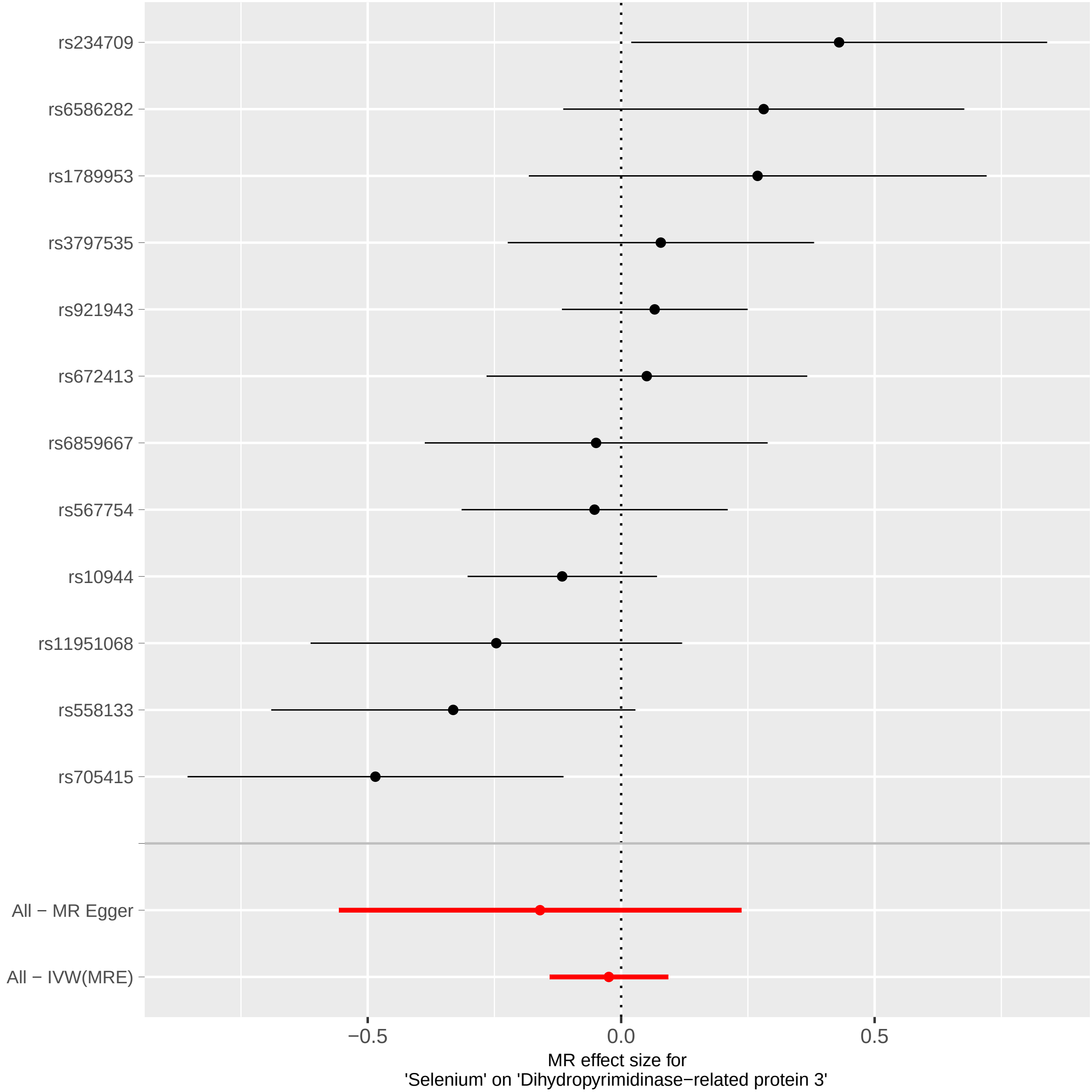

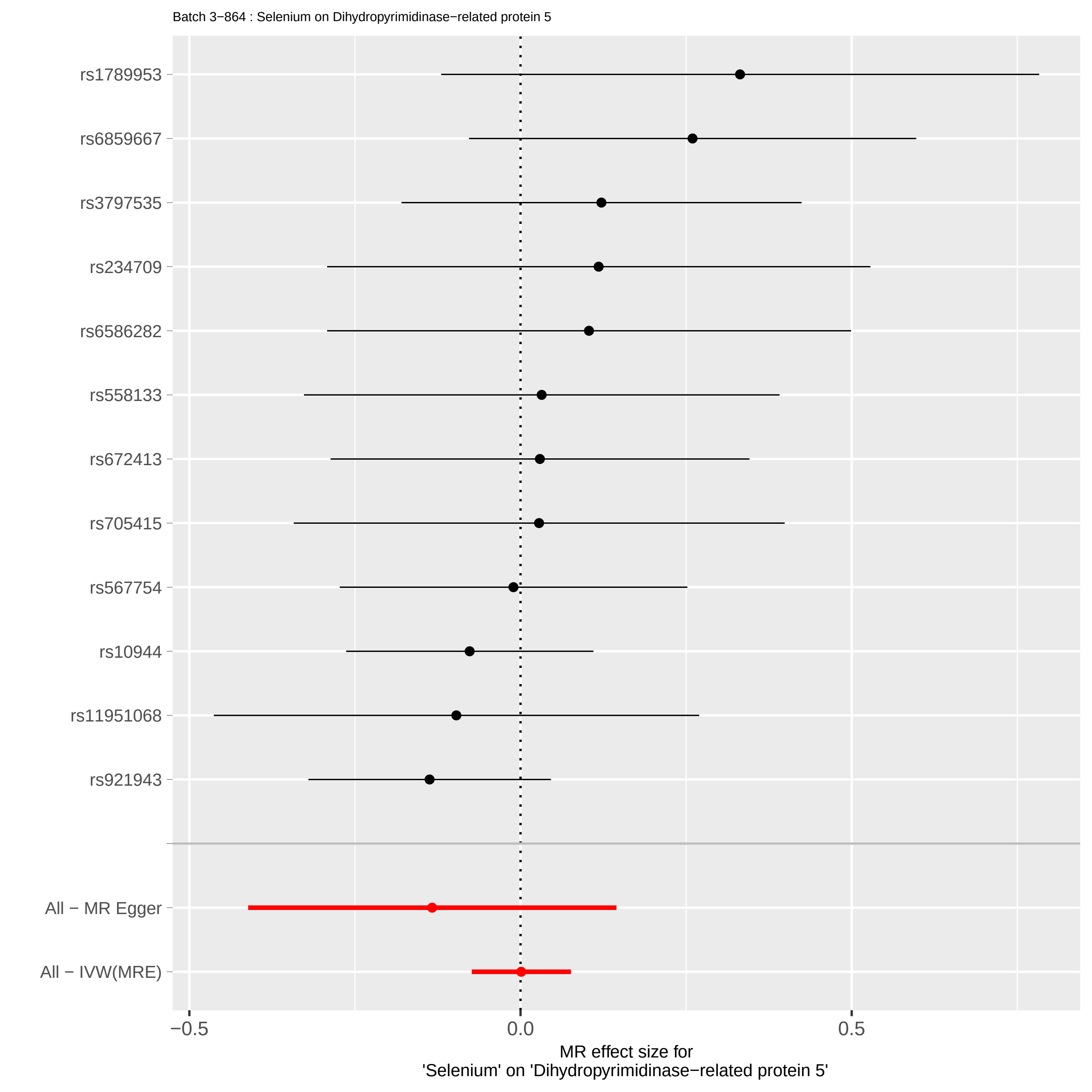
Batch 3−864 : Selenium on Dihydropyrimidinase−related protein 5
rs1789953
rs6859667
rs3797535
rs234709
rs6586282
rs558133
rs672413
rs705415
rs567754
rs10944
rs11951068
rs921943
All − MR Egger
All − IVW(MRE)
−0.5
0.0
0.5
MR effect size for
'Selenium' on 'Dihydropyrimidinase−related protein 5'

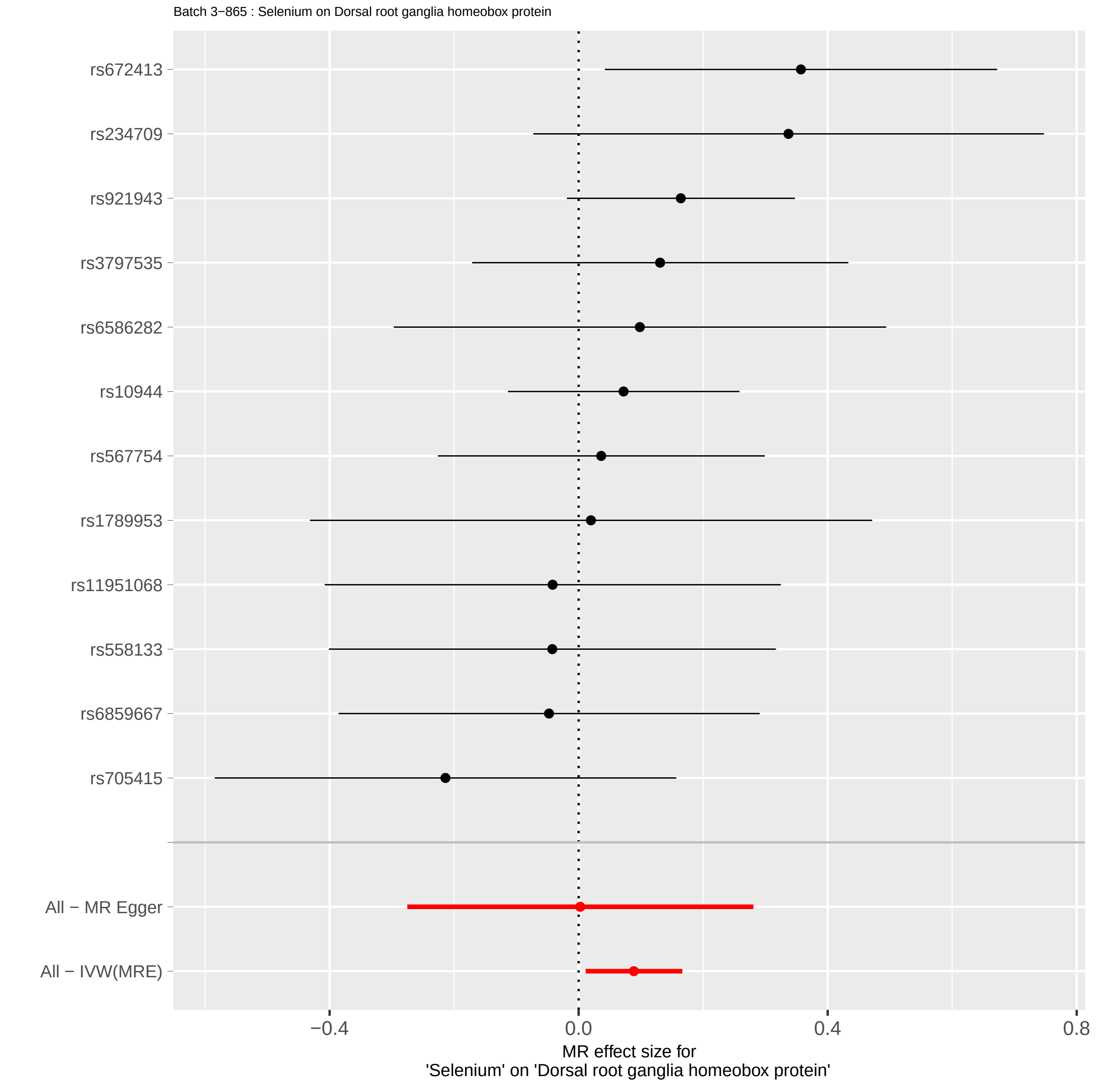

Batch 3−865 : Selenium on Dorsal root ganglia homeobox protein
rs672413
rs234709
rs921943
rs3797535
rs6586282
rs10944
rs567754
rs1789953
rs11951068
rs558133
rs6859667
rs705415
All − MR Egger
All − IVW(MRE)
−0.4
0.0
0.4
0.8
MR effect size for
'Selenium' on 'Dorsal root ganglia homeobox protein'

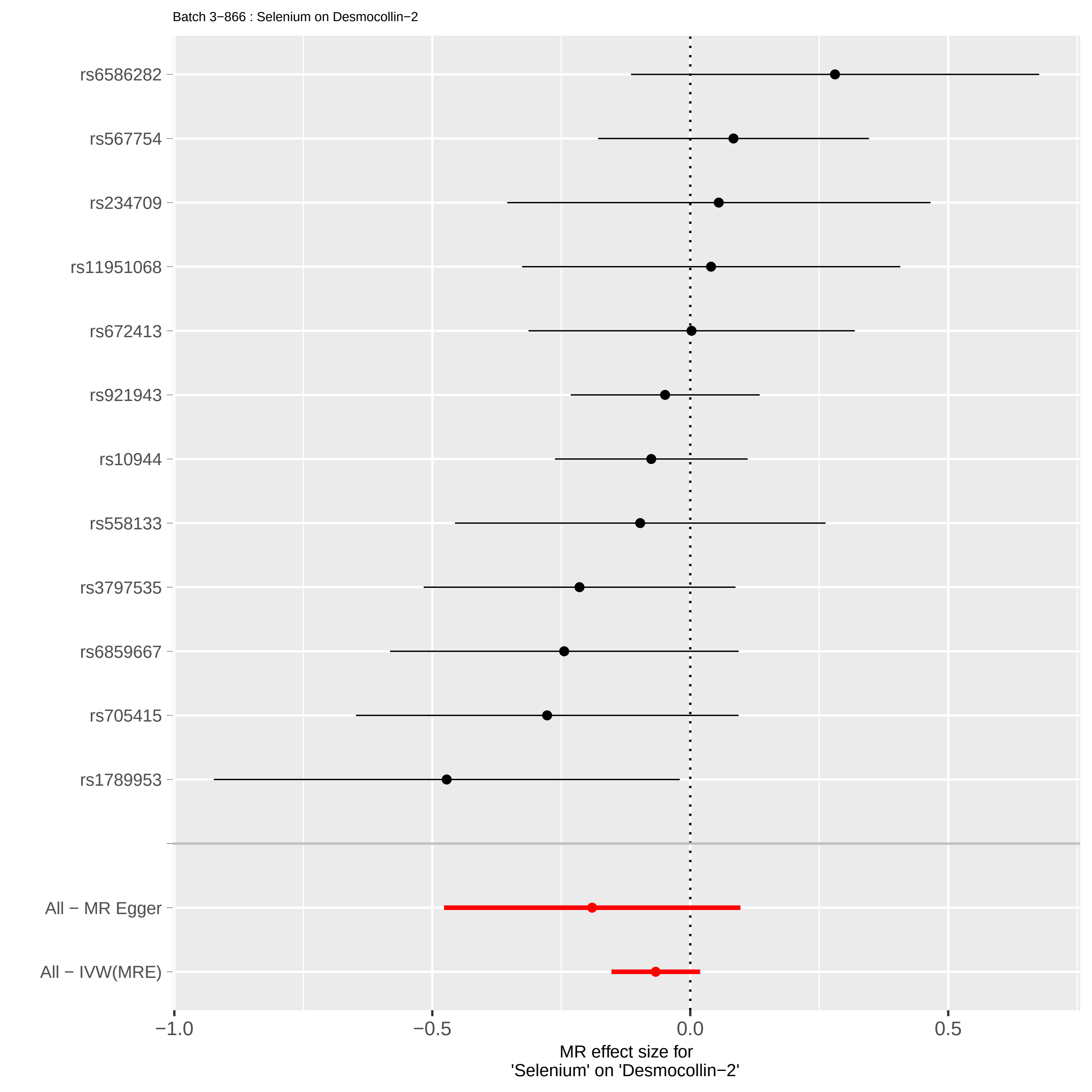

Batch 3−866 : Selenium on Desmocollin−2
rs6586282
rs567754
rs234709
rs11951068
rs672413
rs921943
rs10944
rs558133
rs3797535
rs6859667
rs705415
rs1789953
All − MR Egger
All − IVW(MRE)
−1.0
−0.5
0.0
0.5
MR effect size for
'Selenium' on 'Desmocollin−2'

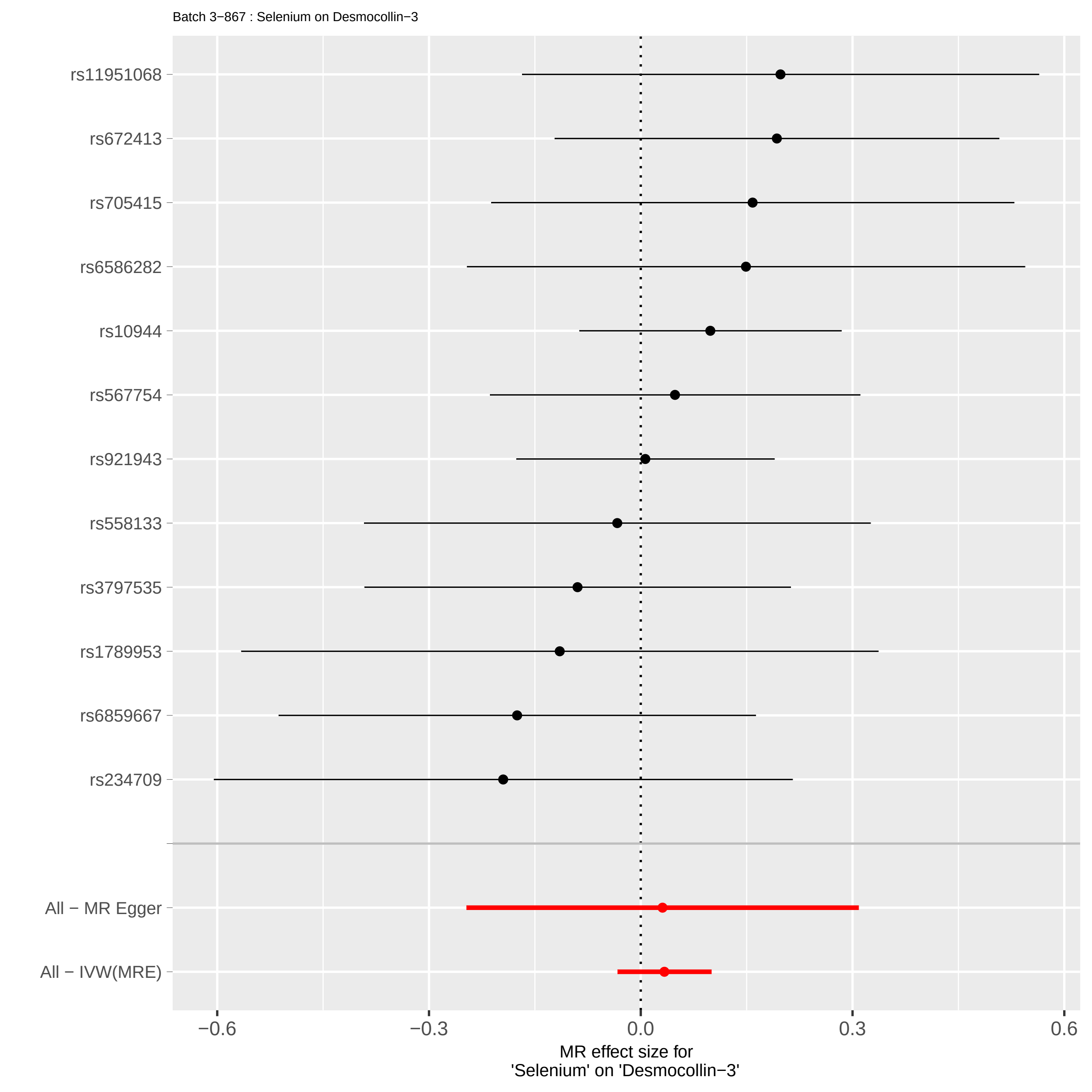

Batch 3−867 : Selenium on Desmocollin−3
rs11951068
rs672413
rs705415
rs6586282
rs10944
rs567754
rs921943
rs558133
rs3797535
rs1789953
rs6859667
rs234709
All − MR Egger
All − IVW(MRE)
−0.6
−0.3
0.0
0.3
0.6
MR effect size for
'Selenium' on 'Desmocollin−3'

Batch 3−868 : Selenium on Down syndrome cell adhesion molecule

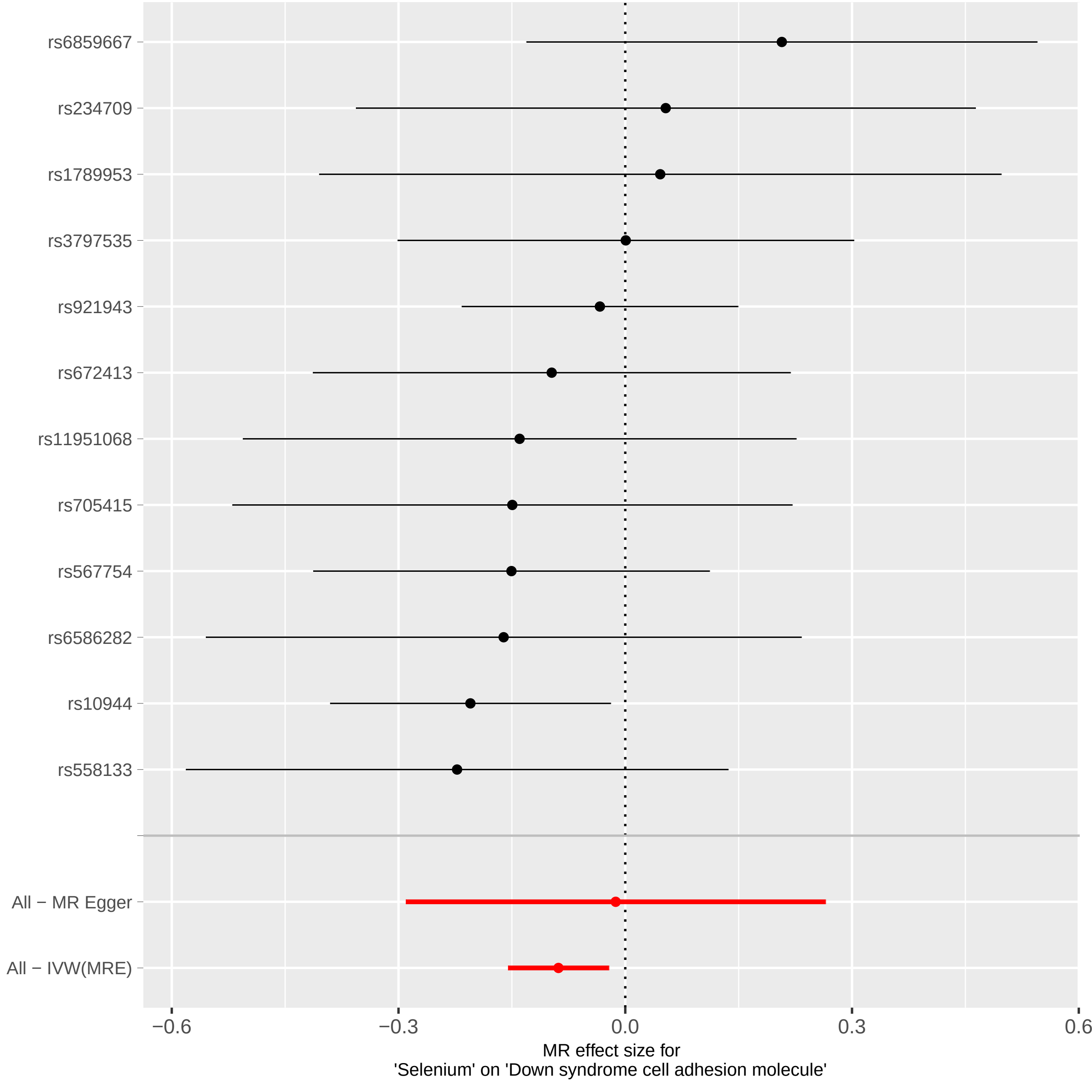

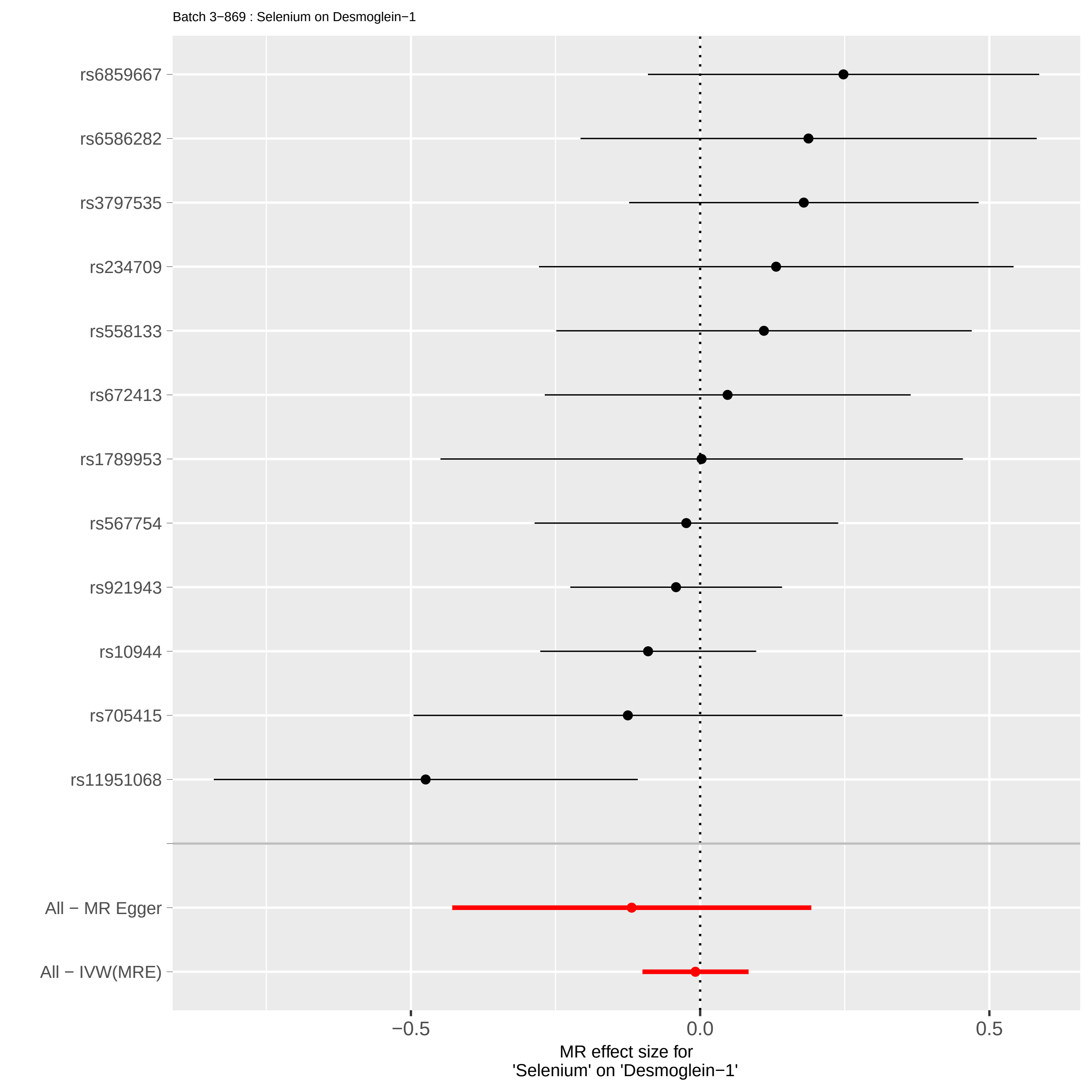
Batch 3−869 : Selenium on Desmoglein−1
rs6859667
rs6586282
rs3797535
rs234709
rs558133
rs672413
rs1789953
rs567754
rs921943
rs10944
rs705415
rs11951068
All − MR Egger
All − IVW(MRE)
−0.5
0.0
0.5
MR effect size for
'Selenium' on 'Desmoglein−1'

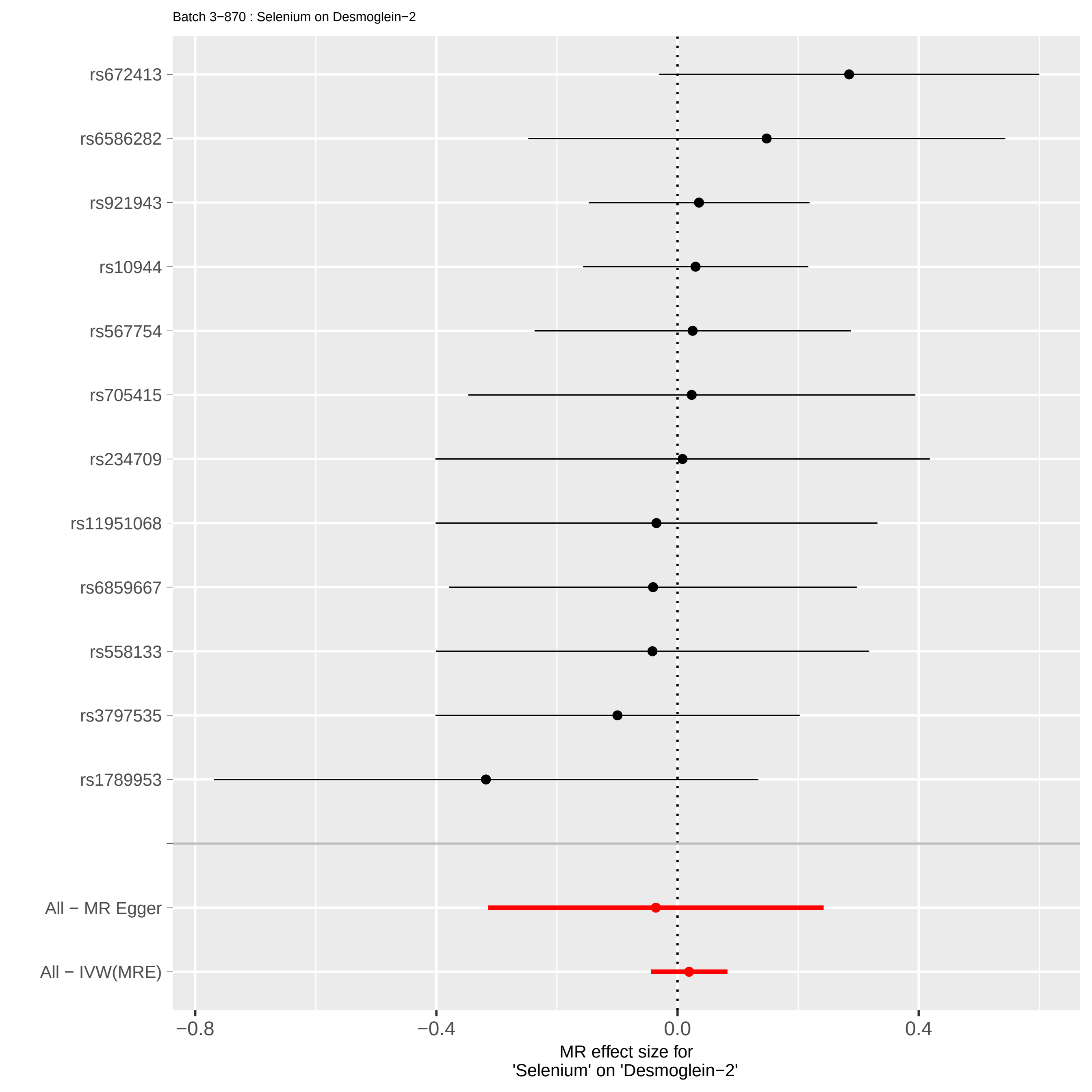

Batch 3−870 : Selenium on Desmoglein−2
rs672413
rs6586282
rs921943
rs10944
rs567754
rs705415
rs234709
rs11951068
rs6859667
rs558133
rs3797535
rs1789953
All − MR Egger
All − IVW(MRE)
−0.8
−0.4
0.0
0.4
MR effect size for
'Selenium' on 'Desmoglein−2'

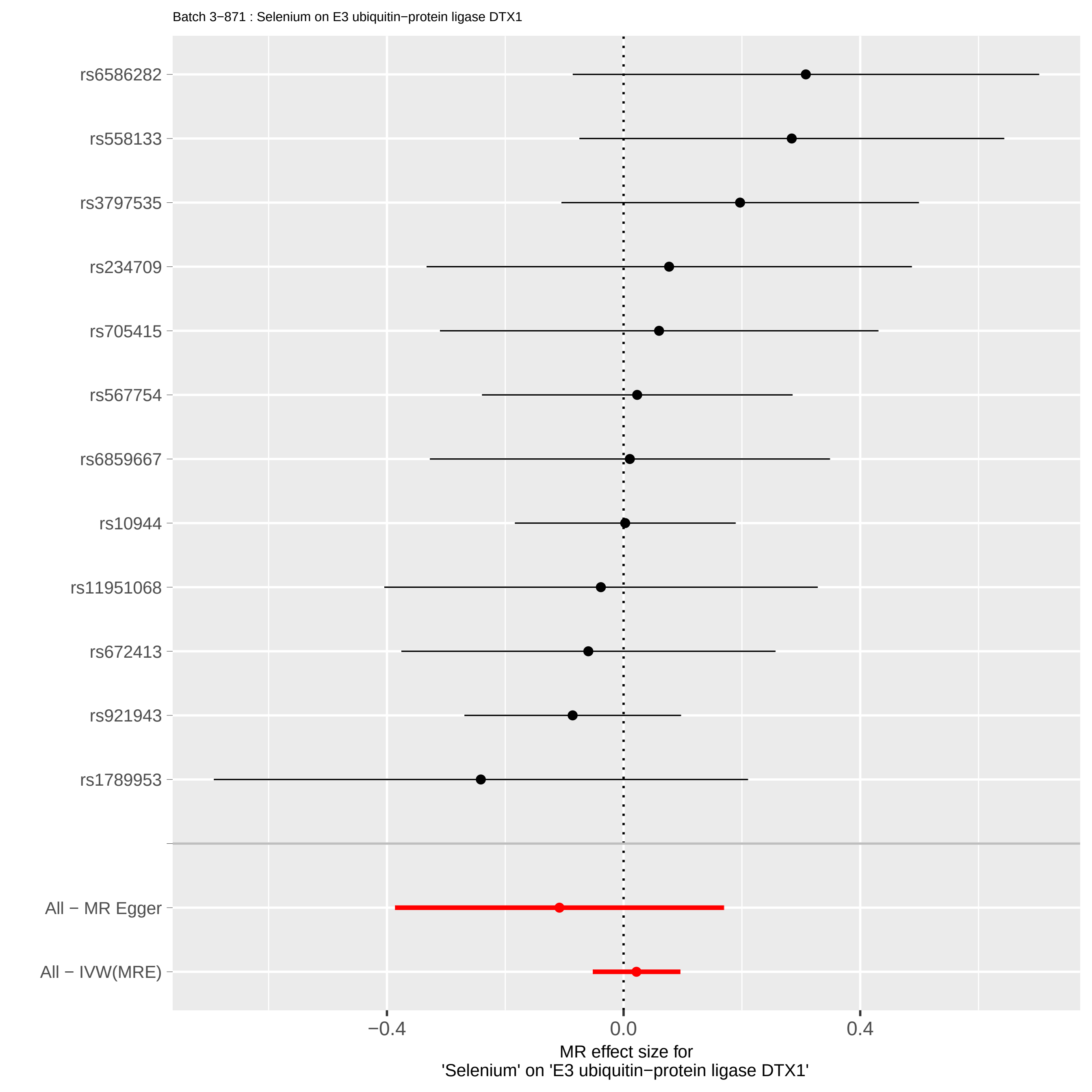

Batch 3−871 : Selenium on E3 ubiquitin−protein ligase DTX1
rs6586282
rs558133
rs3797535
rs234709
rs705415
rs567754
rs6859667
rs10944
rs11951068
rs672413
rs921943
rs1789953
All − MR Egger
All − IVW(MRE)
−0.4
0.0
0.4
MR effect size for
'Selenium' on 'E3 ubiquitin−protein ligase DTX1'

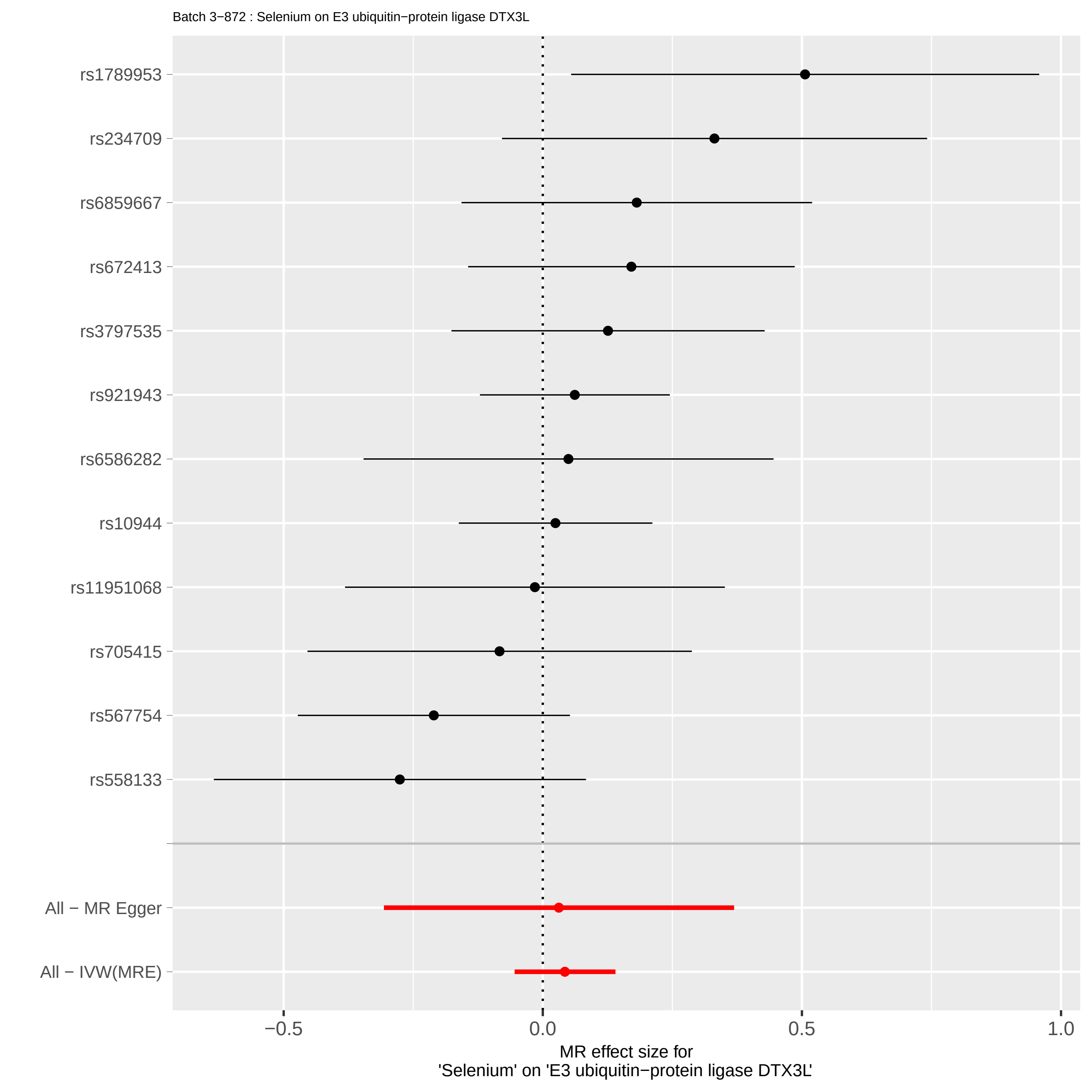

Batch 3−872 : Selenium on E3 ubiquitin−protein ligase DTX3L
rs1789953
rs234709
rs6859667
rs672413
rs3797535
rs921943
rs6586282
rs10944
rs11951068
rs705415
rs567754
rs558133
All − MR Egger
All − IVW(MRE)
−0.5
0.0
0.5
1.0
MR effect size for
'Selenium' on 'E3 ubiquitin−protein ligase DTX3L'

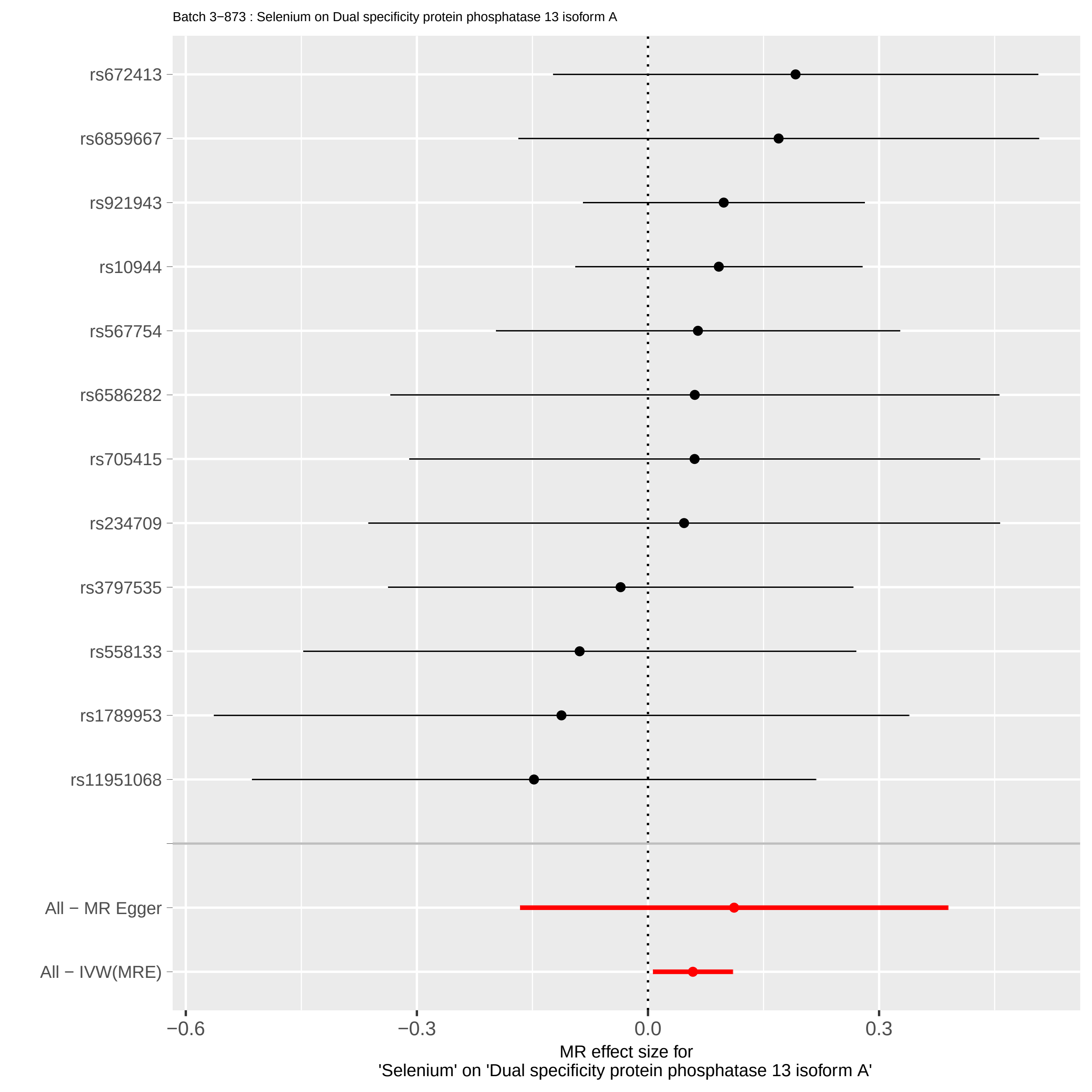

Batch 3−873 : Selenium on Dual specificity protein phosphatase 13 isoform A
rs672413
rs6859667
rs921943
rs10944
rs567754
rs6586282
rs705415
rs234709
rs3797535
rs558133
rs1789953
rs11951068
All − MR Egger
All − IVW(MRE)
−0.6
−0.3
0.0
0.3
MR effect size for
'Selenium' on 'Dual specificity protein phosphatase 13 isoform A'

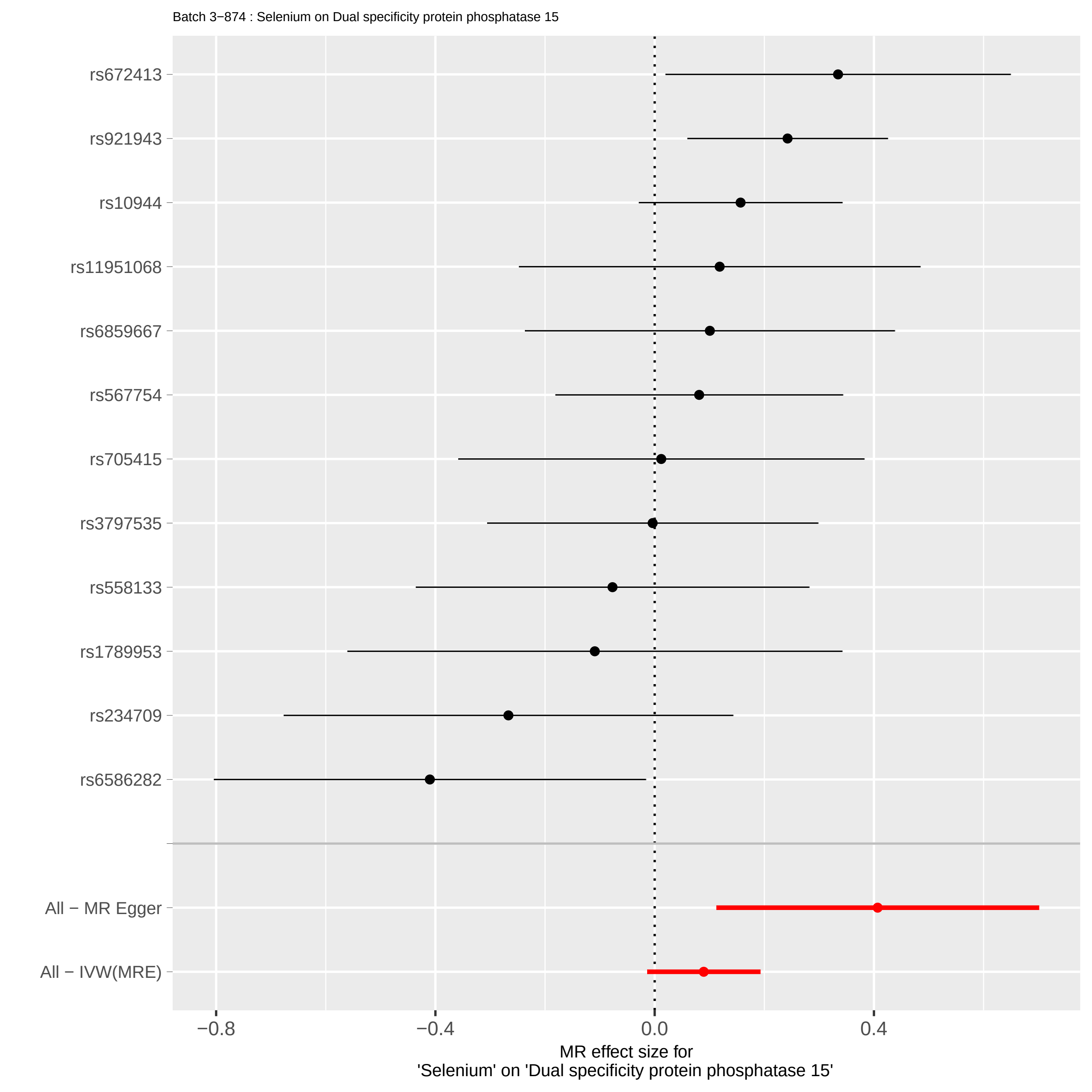
Batch 3−874 : Selenium on Dual specificity protein phosphatase 15
rs672413
rs921943
rs10944
rs11951068
rs6859667
rs567754
rs705415
rs3797535
rs558133
rs1789953
rs234709
rs6586282
All − MR Egger
All − IVW(MRE)
−0.8
−0.4
0.0
0.4
MR effect size for
'Selenium' on 'Dual specificity protein phosphatase 15'

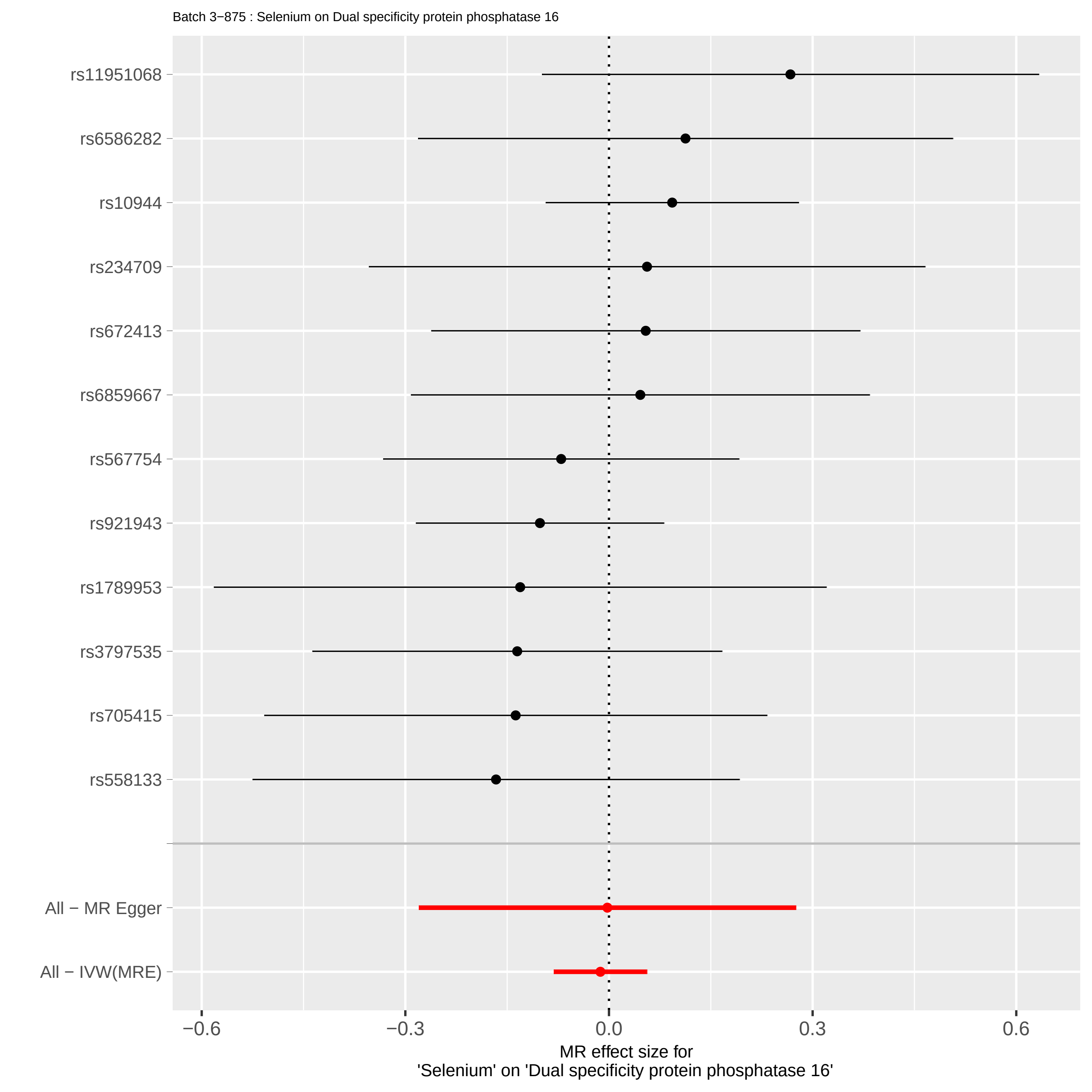

Batch 3−875 : Selenium on Dual specificity protein phosphatase 16
rs11951068
rs6586282
rs10944
rs234709
rs672413
rs6859667
rs567754
rs921943
rs1789953
rs3797535
rs705415
rs558133
All − MR Egger
All − IVW(MRE)
−0.6
−0.3
0.0
0.3
0.6
MR effect size for
'Selenium' on 'Dual specificity protein phosphatase 16'

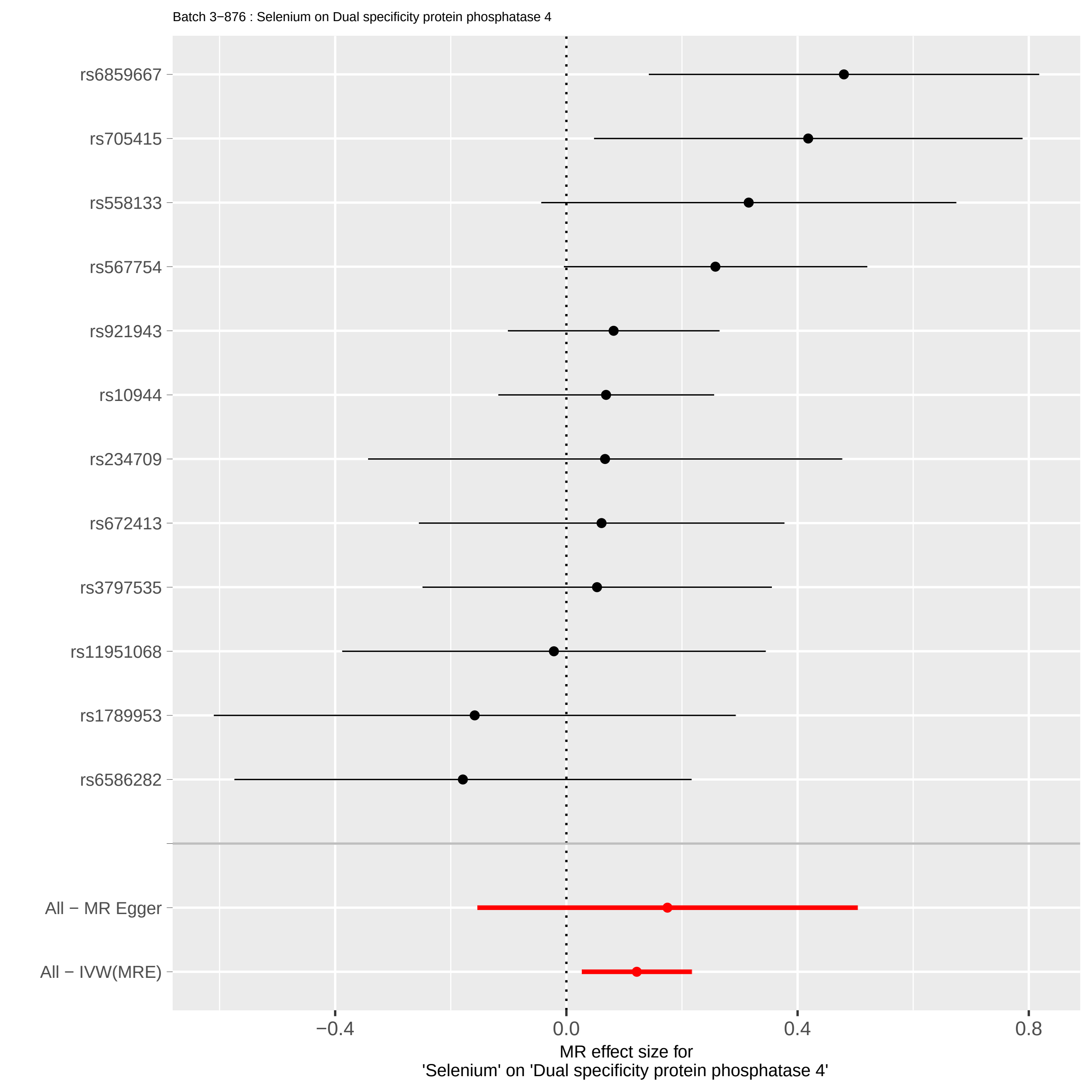

Batch 3−876 : Selenium on Dual specificity protein phosphatase 4
rs6859667
rs705415
rs558133
rs567754
rs921943
rs10944
rs234709
rs672413
rs3797535
rs11951068
rs1789953
rs6586282
All − MR Egger
All − IVW(MRE)
−0.4
0.0
0.4
0.8
MR effect size for
'Selenium' on 'Dual specificity protein phosphatase 4'

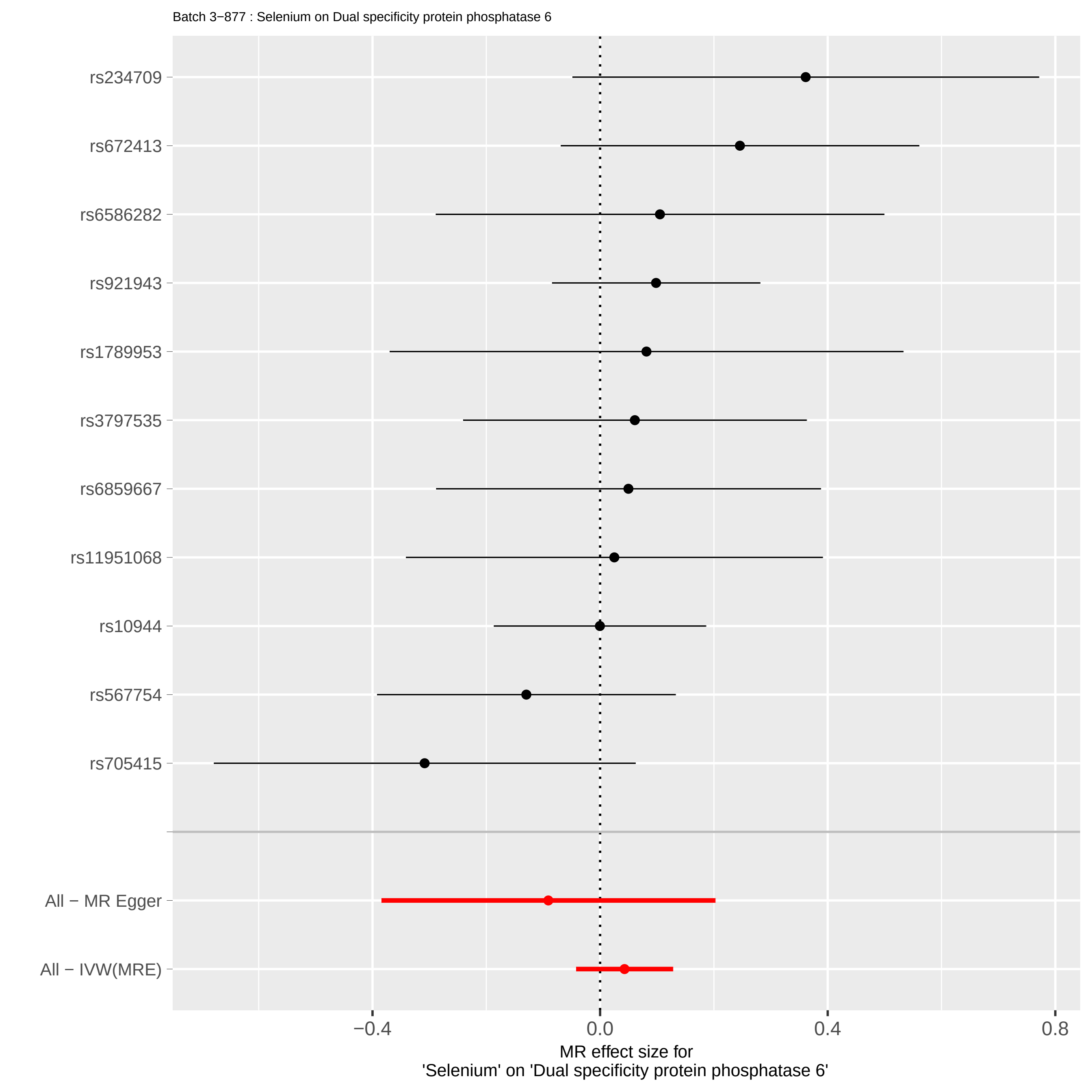
Batch 3−877 : Selenium on Dual specificity protein phosphatase 6
rs234709
rs672413
rs6586282
rs921943
rs1789953
rs3797535
rs6859667
rs11951068
rs10944
rs567754
rs705415
All − MR Egger
All − IVW(MRE)
−0.4
0.0
0.4
0.8
MR effect size for
'Selenium' on 'Dual specificity protein phosphatase 6'

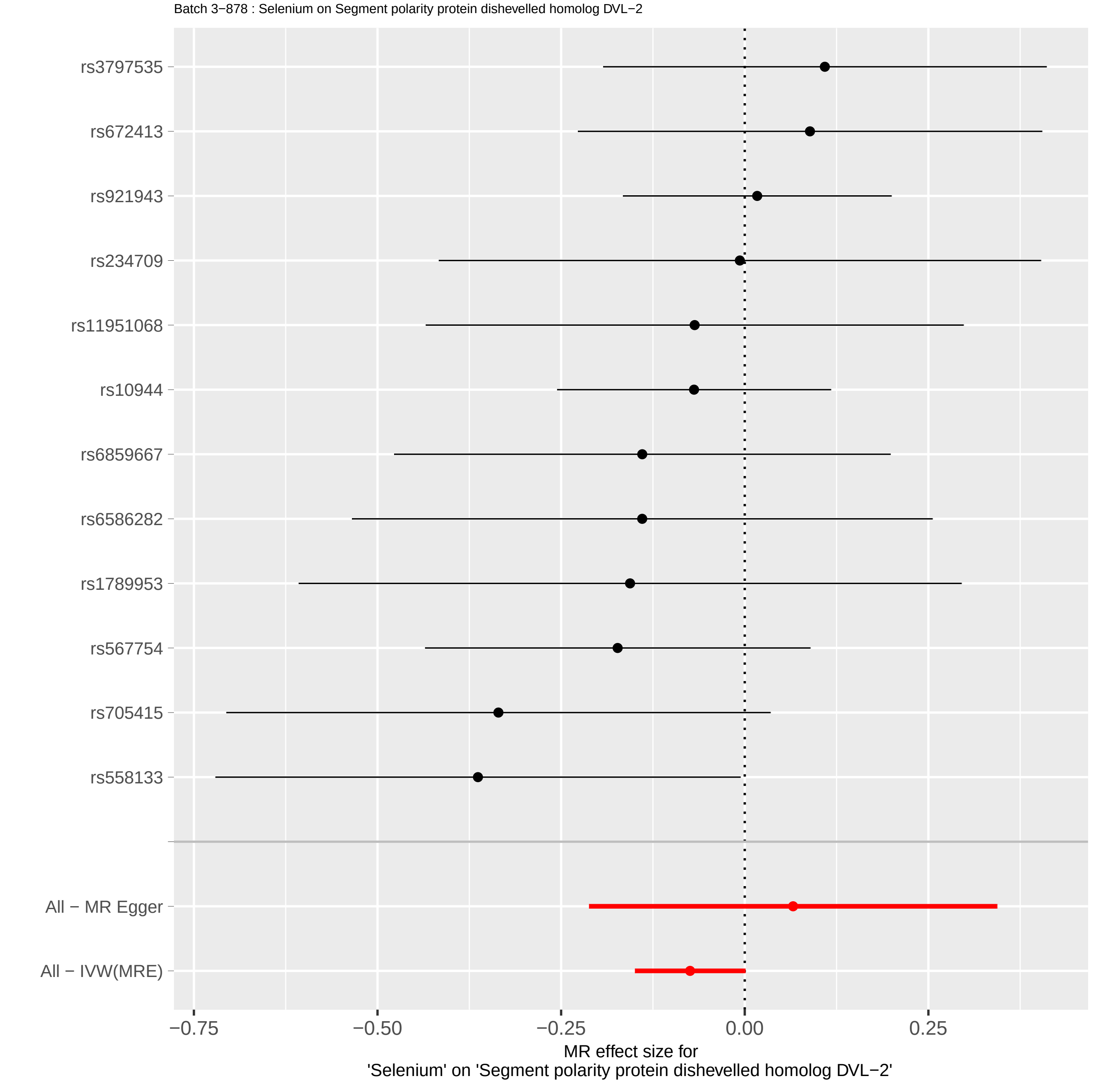

Batch 3−878 : Selenium on Segment polarity protein dishevelled homolog DVL−2
rs3797535
rs672413
rs921943
rs234709
rs11951068
rs10944
rs6859667
rs6586282
rs1789953
rs567754
rs705415
rs558133
All − MR Egger
All − IVW(MRE)
−0.75
−0.50
−0.25
0.00
0.25
MR effect size for
'Selenium' on 'Segment polarity protein dishevelled homolog DVL−2'

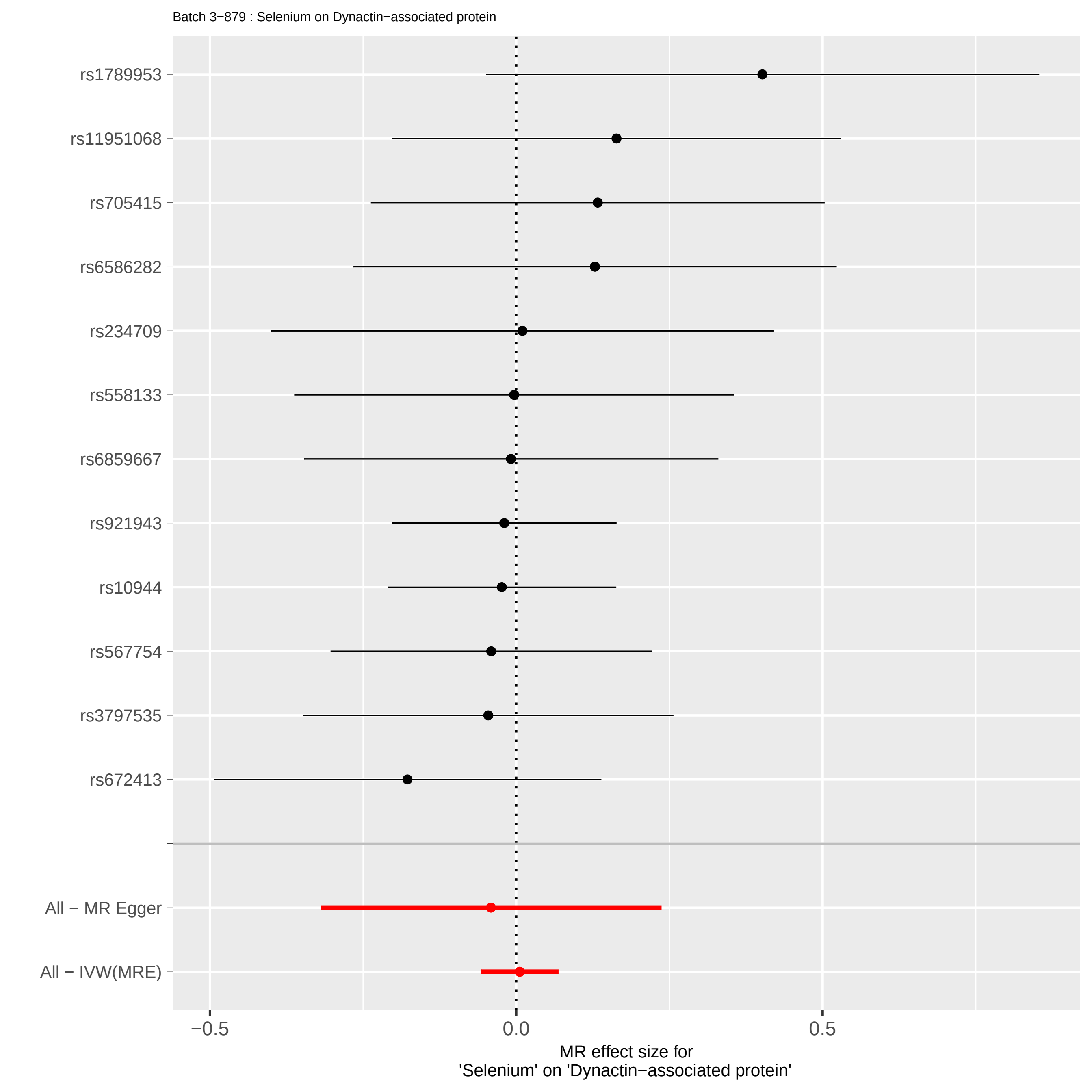
Batch 3−879 : Selenium on Dynactin−associated protein
rs1789953
rs11951068
rs705415
rs6586282
rs234709
rs558133
rs6859667
rs921943
rs10944
rs567754
rs3797535
rs672413
All − MR Egger
All − IVW(MRE)
−0.5
0.0
0.5
MR effect size for
'Selenium' on 'Dynactin−associated protein'

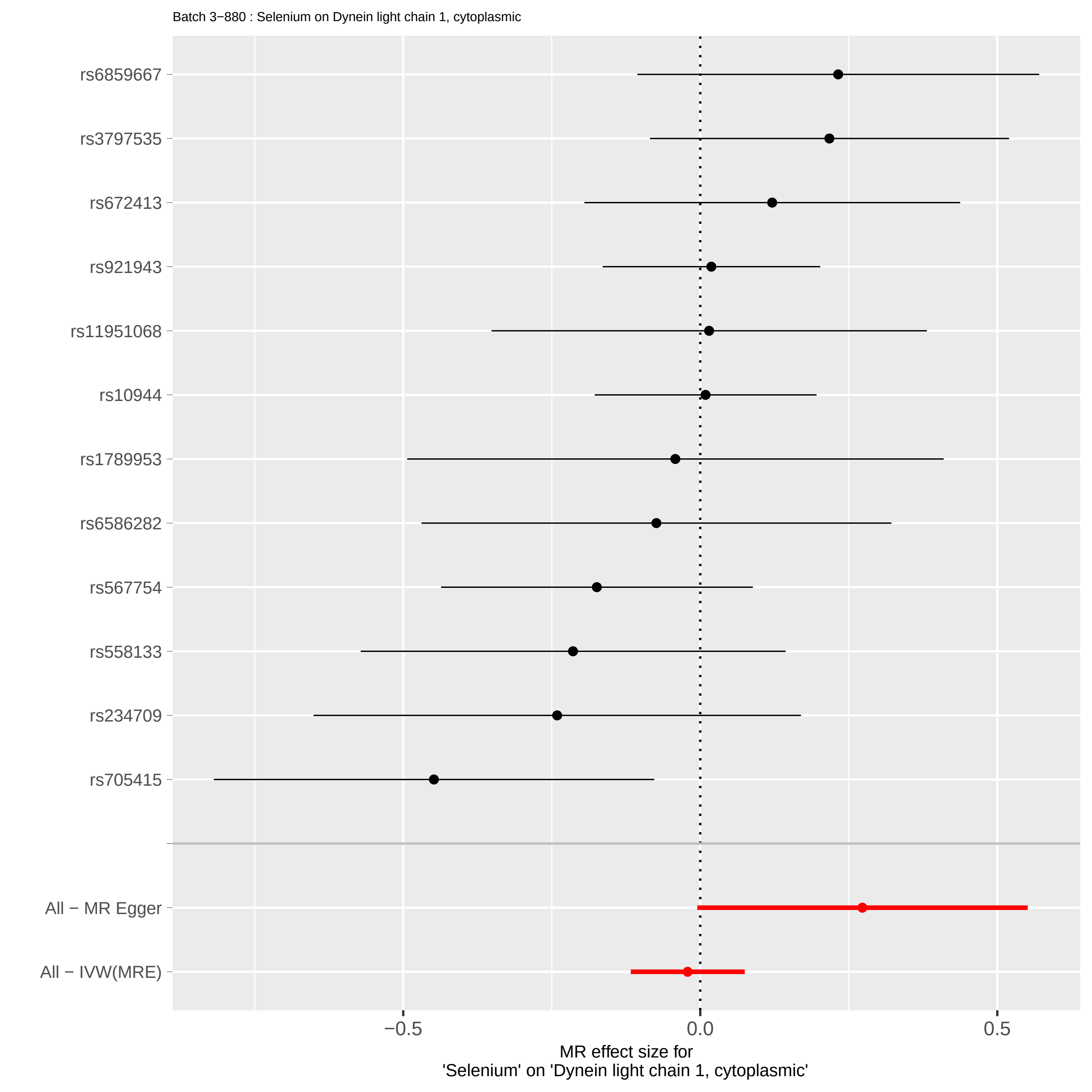

Batch 3−880 : Selenium on Dynein light chain 1, cytoplasmic
rs6859667
rs3797535
rs672413
rs921943
rs11951068
rs10944
rs1789953
rs6586282
rs567754
rs558133
rs234709
rs705415
All − MR Egger
All − IVW(MRE)
−0.5
0.0
0.5
MR effect size for
'Selenium' on 'Dynein light chain 1, cytoplasmic'

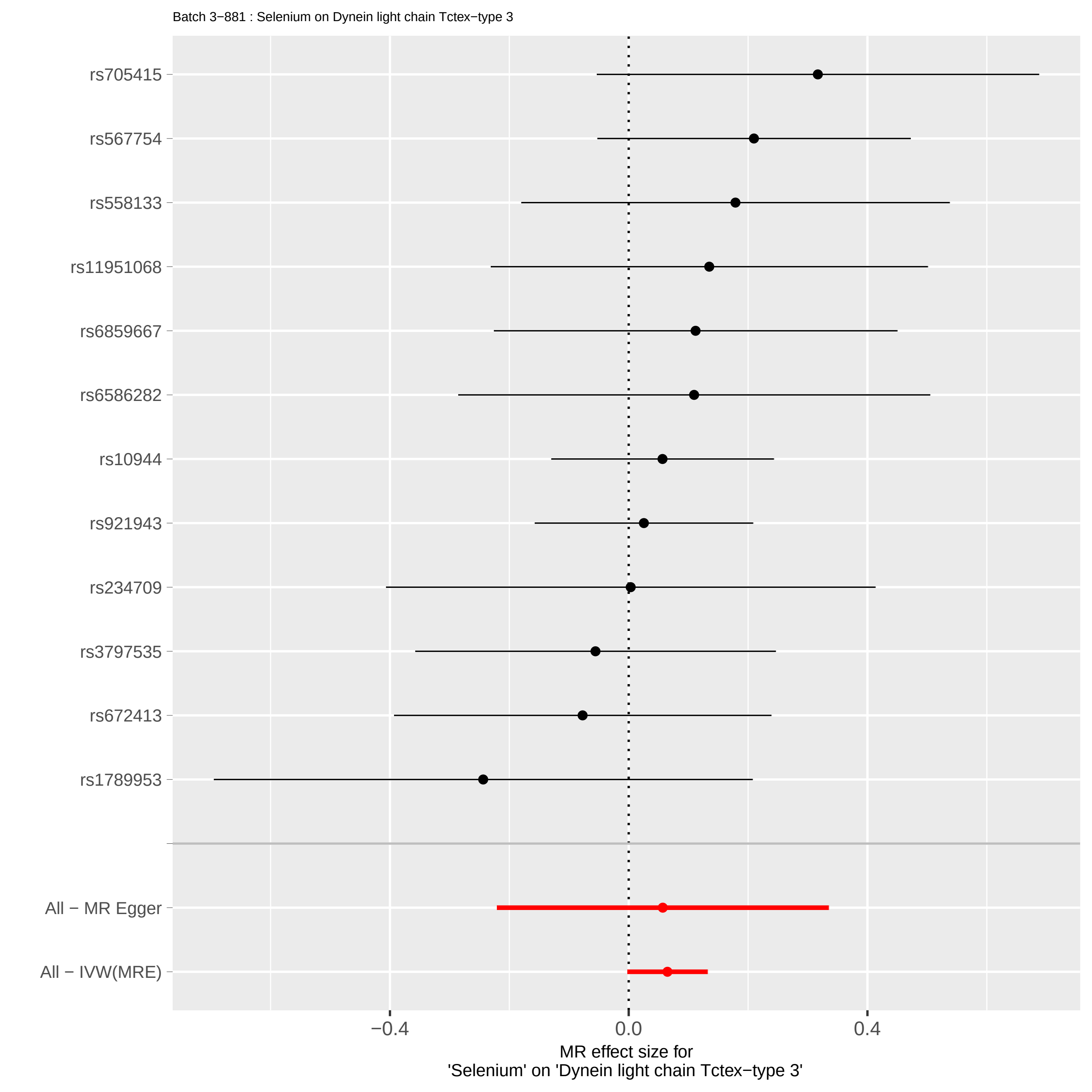

Batch 3−881 : Selenium on Dynein light chain Tctex−type 3
rs705415
rs567754
rs558133
rs11951068
rs6859667
rs6586282
rs10944
rs921943
rs234709
rs3797535
rs672413
rs1789953
All − MR Egger
All − IVW(MRE)
−0.4
0.0
0.4
MR effect size for
'Selenium' on 'Dynein light chain Tctex−type 3'

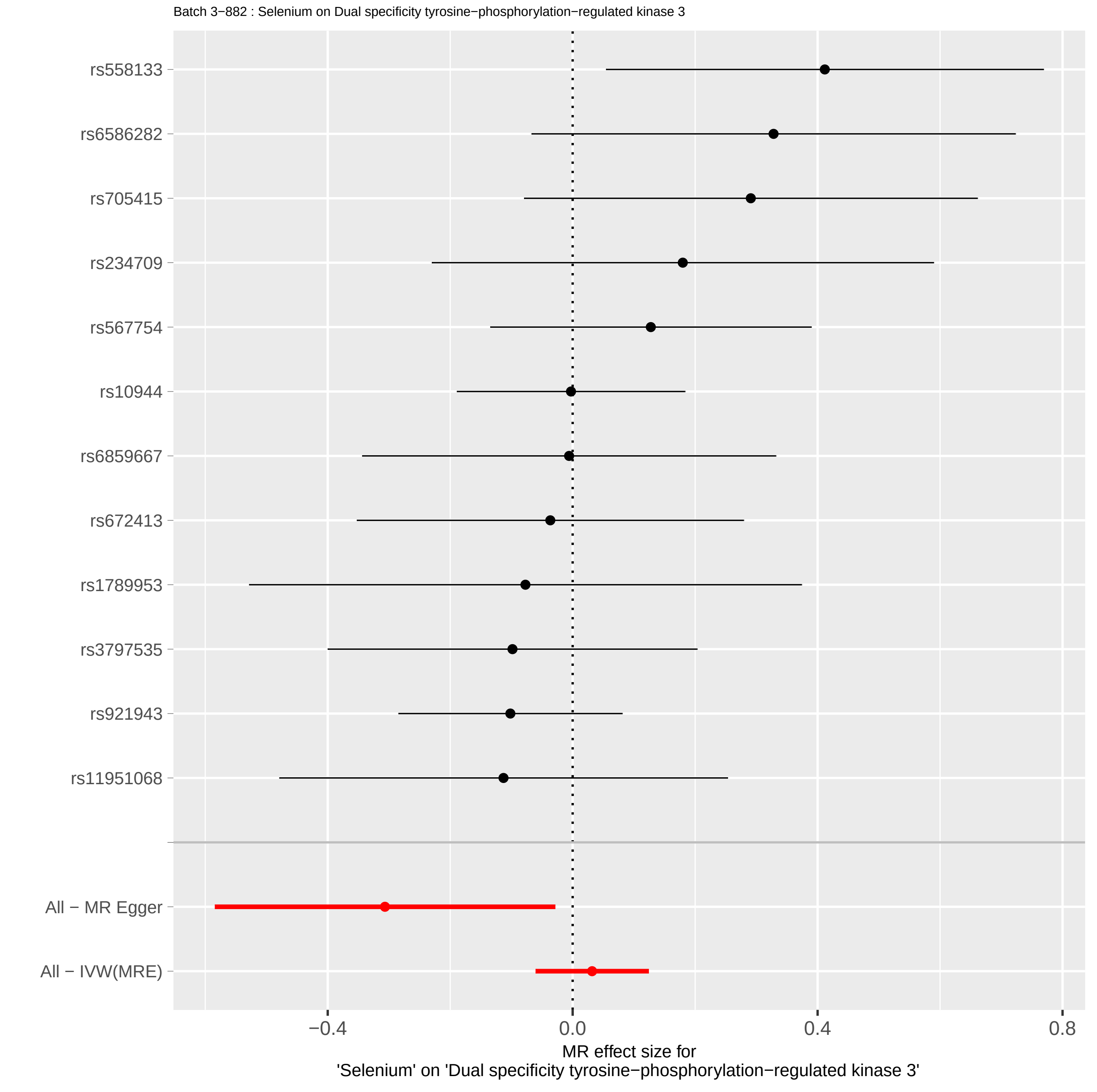

Batch 3−882 : Selenium on Dual specificity tyrosine−phosphorylation−regulated kinase 3
rs558133
rs6586282
rs705415
rs234709
rs567754
rs10944
rs6859667
rs672413
rs1789953
rs3797535
rs921943
rs11951068
All − MR Egger
All − IVW(MRE)
−0.4
0.0
0.4
0.8
MR effect size for
'Selenium' on 'Dual specificity tyrosine−phosphorylation−regulated kinase 3'

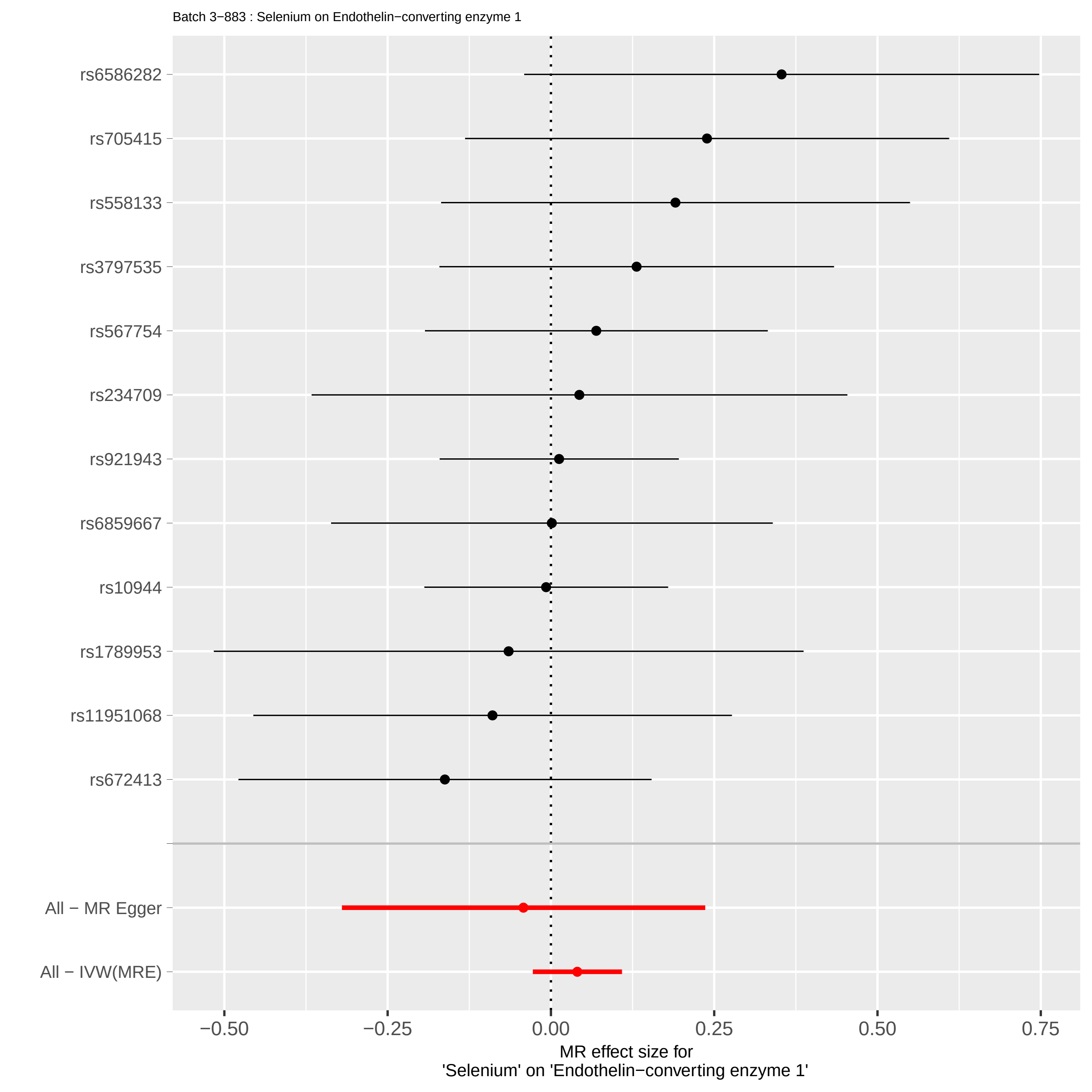

Batch 3−883 : Selenium on Endothelin−converting enzyme 1
rs6586282
rs705415
rs558133
rs3797535
rs567754
rs234709
rs921943
rs6859667
rs10944
rs1789953
rs11951068
rs672413
All − MR Egger
All − IVW(MRE)
−0.50
−0.25
0.00
0.25
0.50
0.75
MR effect size for
'Selenium' on 'Endothelin−converting enzyme 1'

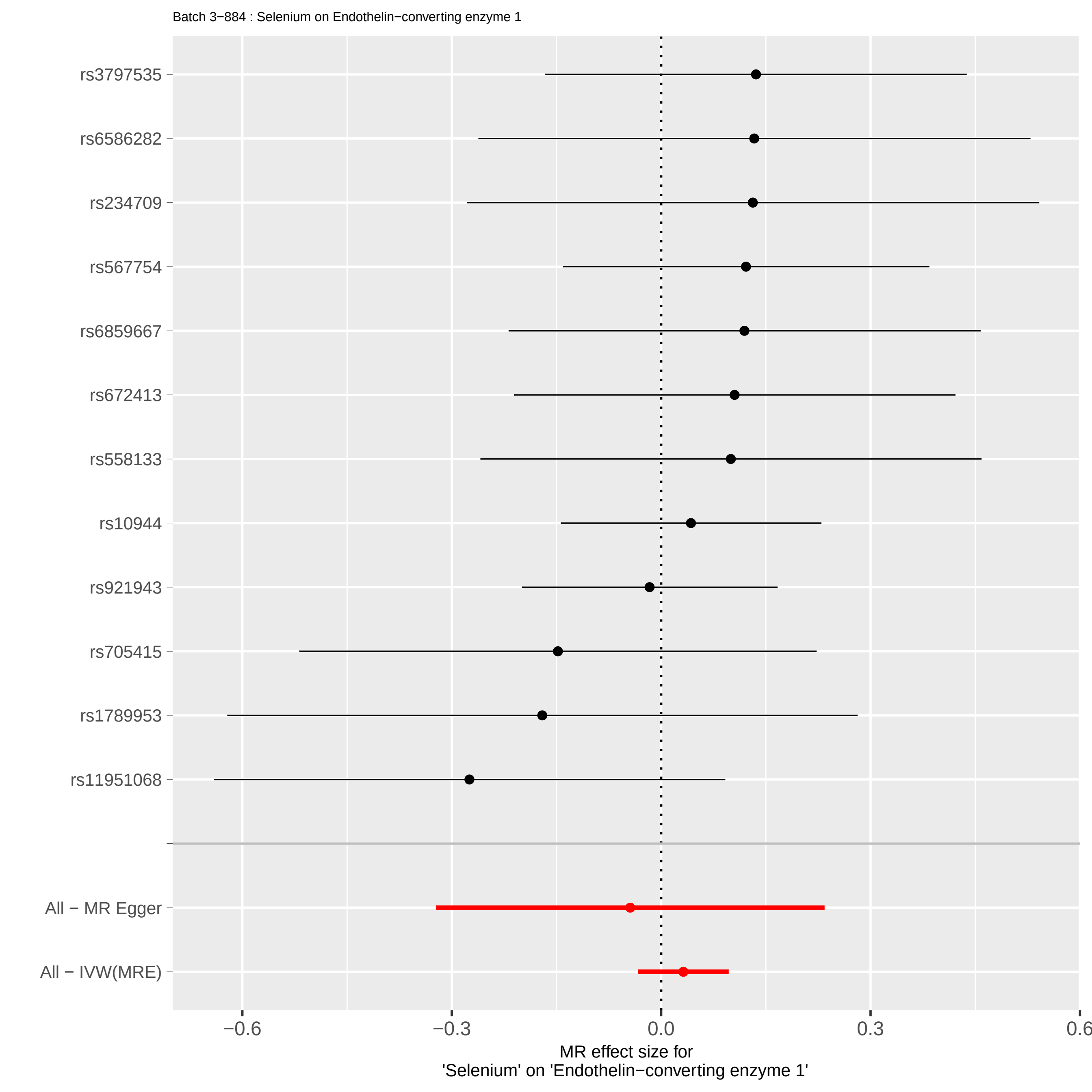
Batch 3−884 : Selenium on Endothelin−converting enzyme 1
rs3797535
rs6586282
rs234709
rs567754
rs6859667
rs672413
rs558133
rs10944
rs921943
rs705415
rs1789953
rs11951068
All − MR Egger
All − IVW(MRE)
−0.6
−0.3
0.0
0.3
0.6
MR effect size for
'Selenium' on 'Endothelin−converting enzyme 1'

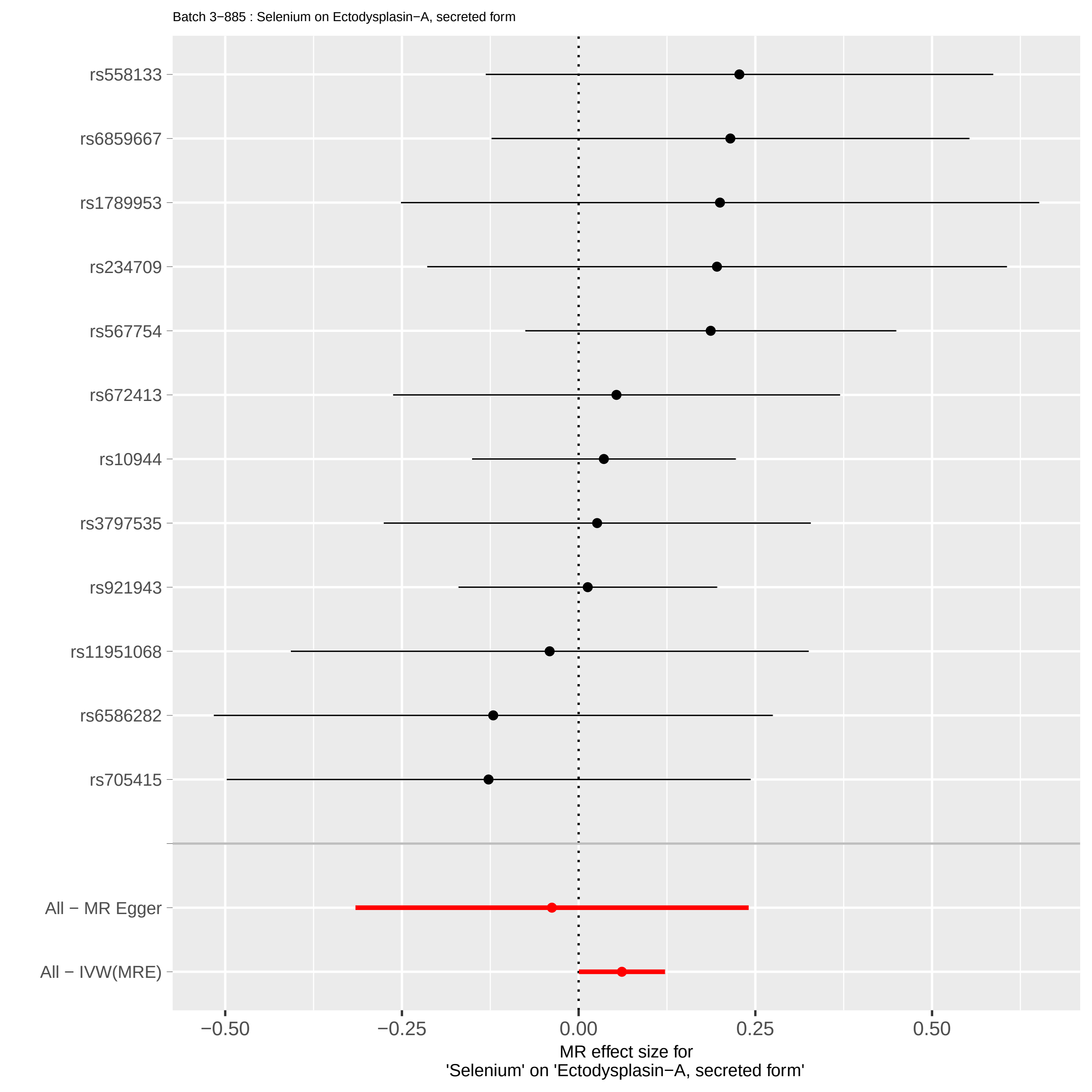

Batch 3−885 : Selenium on Ectodysplasin−A, secreted form
rs558133
rs6859667
rs1789953
rs234709
rs567754
rs672413
rs10944
rs3797535
rs921943
rs11951068
rs6586282
rs705415
All − MR Egger
All − IVW(MRE)
−0.50
−0.25
0.00
0.25
0.50
MR effect size for
'Selenium' on 'Ectodysplasin−A, secreted form'

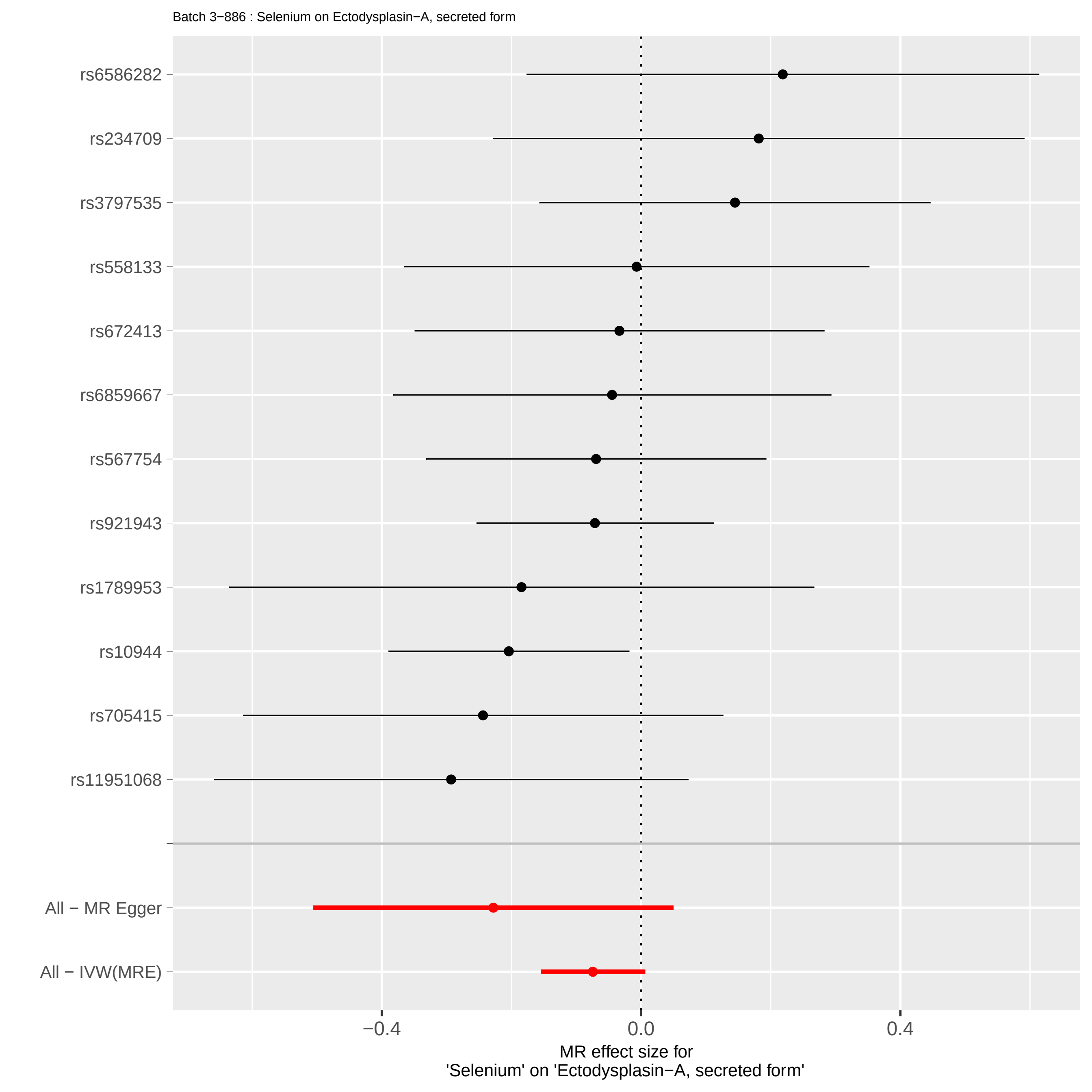
Batch 3−886 : Selenium on Ectodysplasin−A, secreted form
rs6586282
rs234709
rs3797535
rs558133
rs672413
rs6859667
rs567754
rs921943
rs1789953
rs10944
rs705415
rs11951068
All − MR Egger
All − IVW(MRE)
−0.4
0.0
0.4
MR effect size for
'Selenium' on 'Ectodysplasin−A, secreted form'

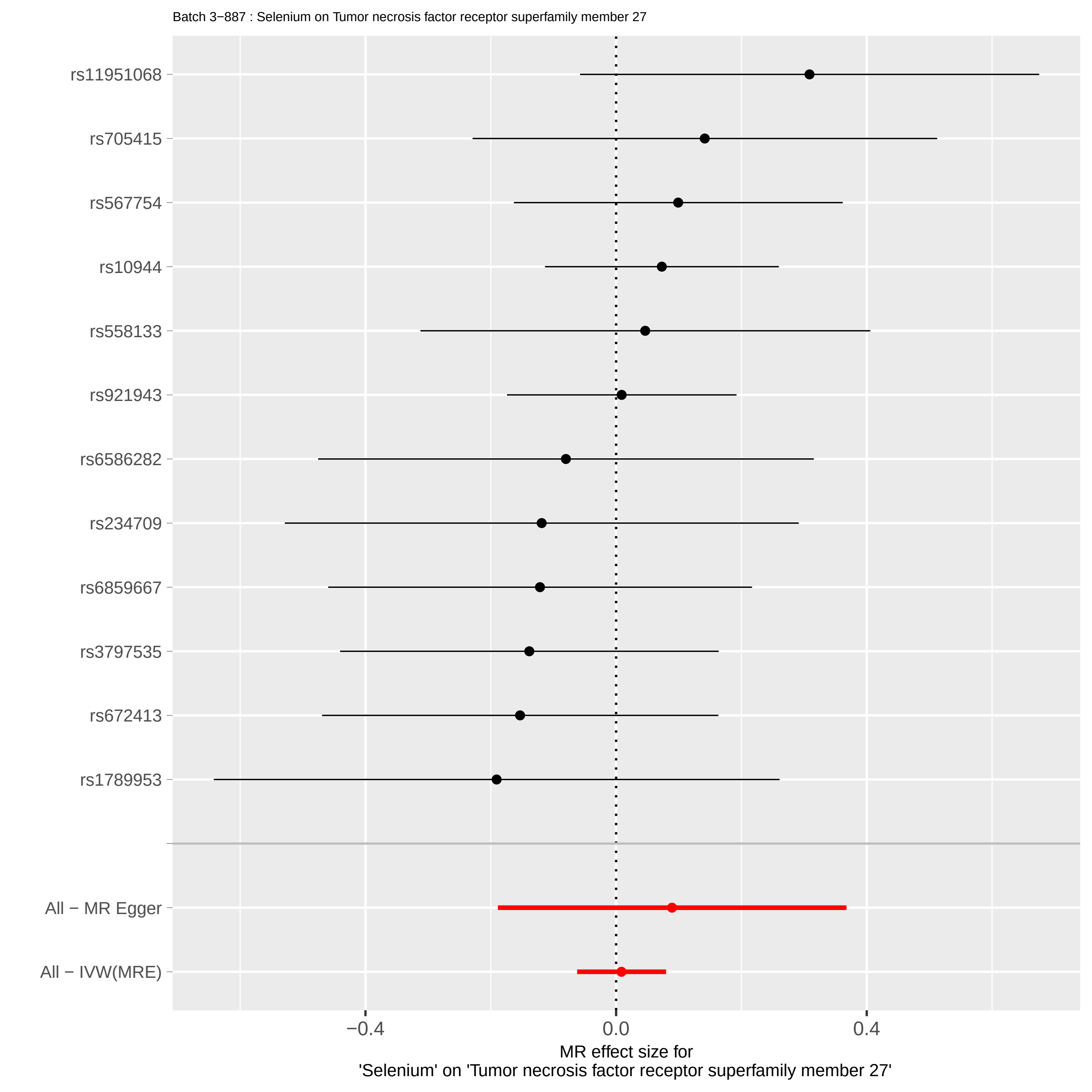
Batch 3−887 : Selenium on Tumor necrosis factor receptor superfamily member 27
rs11951068
rs705415
rs567754
rs10944
rs558133
rs921943
rs6586282
rs234709
rs6859667
rs3797535
rs672413
rs1789953
All − MR Egger
All − IVW(MRE)
−0.4
0.0
0.4
MR effect size for
'Selenium' on 'Tumor necrosis factor receptor superfamily member 27'

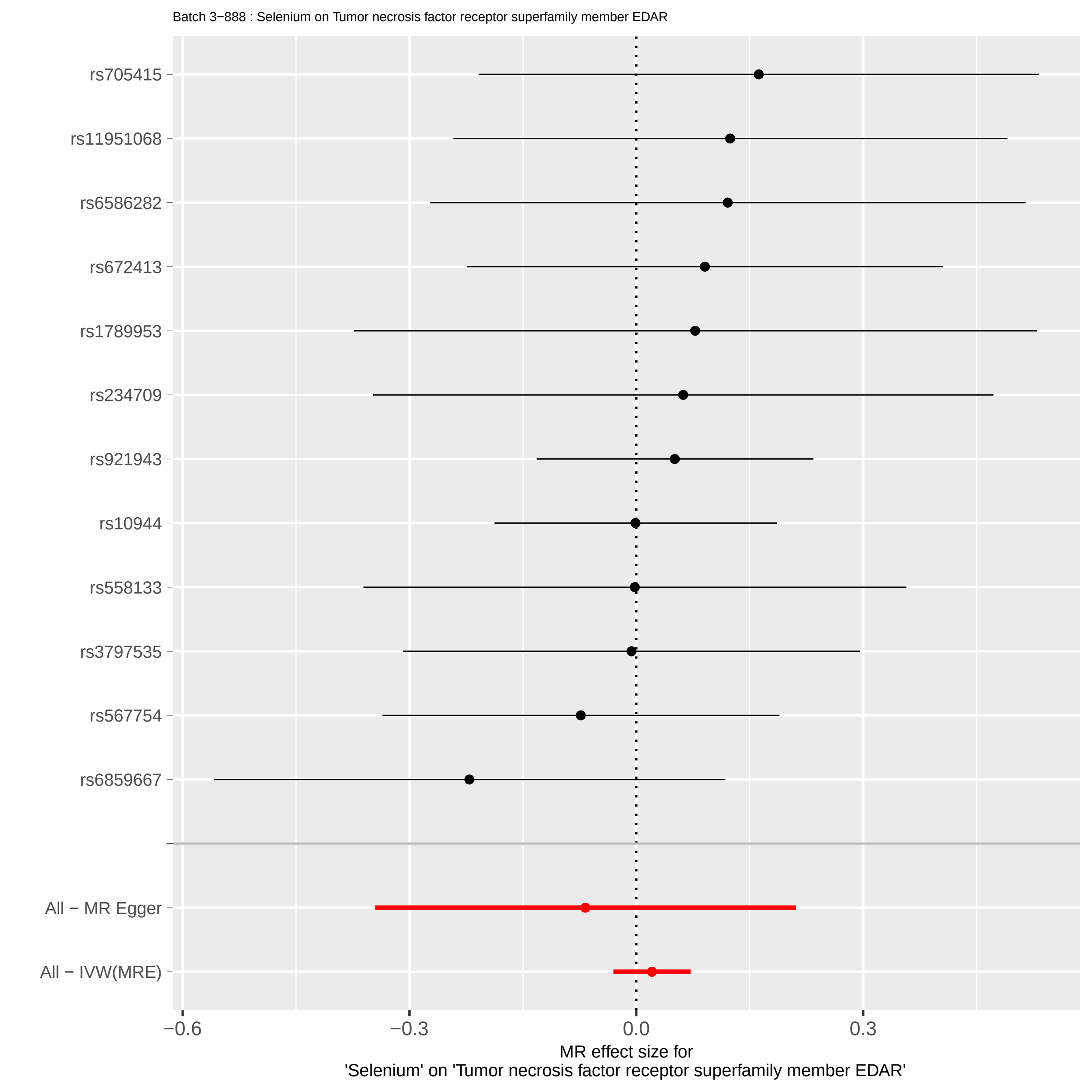

Batch 3−888 : Selenium on Tumor necrosis factor receptor superfamily member EDAR
rs705415
rs11951068
rs6586282
rs672413
rs1789953
rs234709
rs921943
rs10944
rs558133
rs3797535
rs567754
rs6859667
All − MR Egger
All − IVW(MRE)
−0.6
−0.3
0.0
0.3
MR effect size for
'Selenium' on 'Tumor necrosis factor receptor superfamily member EDAR'

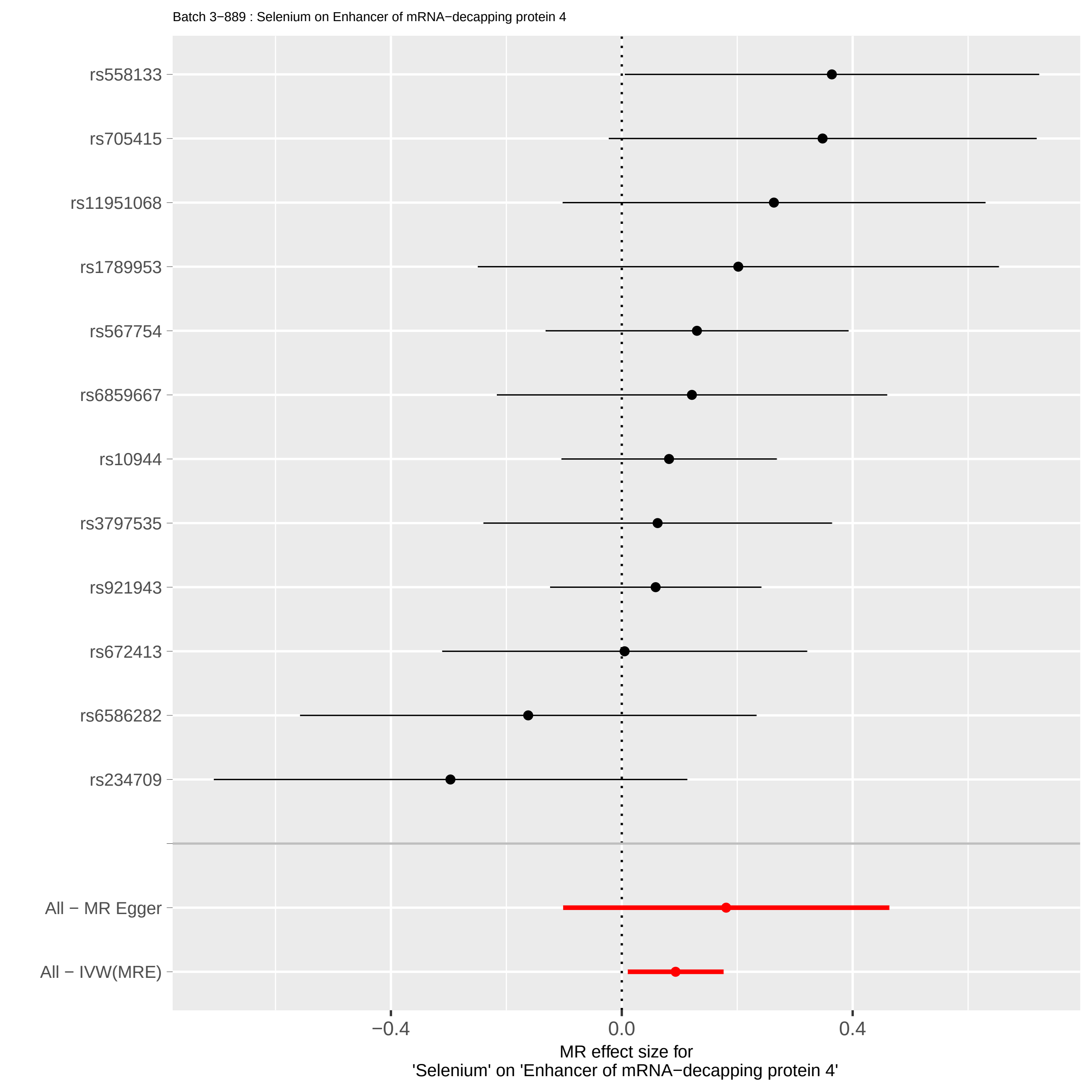

Batch 3−889 : Selenium on Enhancer of mRNA−decapping protein 4
rs558133
rs705415
rs11951068
rs1789953
rs567754
rs6859667
rs10944
rs3797535
rs921943
rs672413
rs6586282
rs234709
All − MR Egger
All − IVW(MRE)
−0.4
0.0
0.4
MR effect size for
'Selenium' on 'Enhancer of mRNA−decapping protein 4'

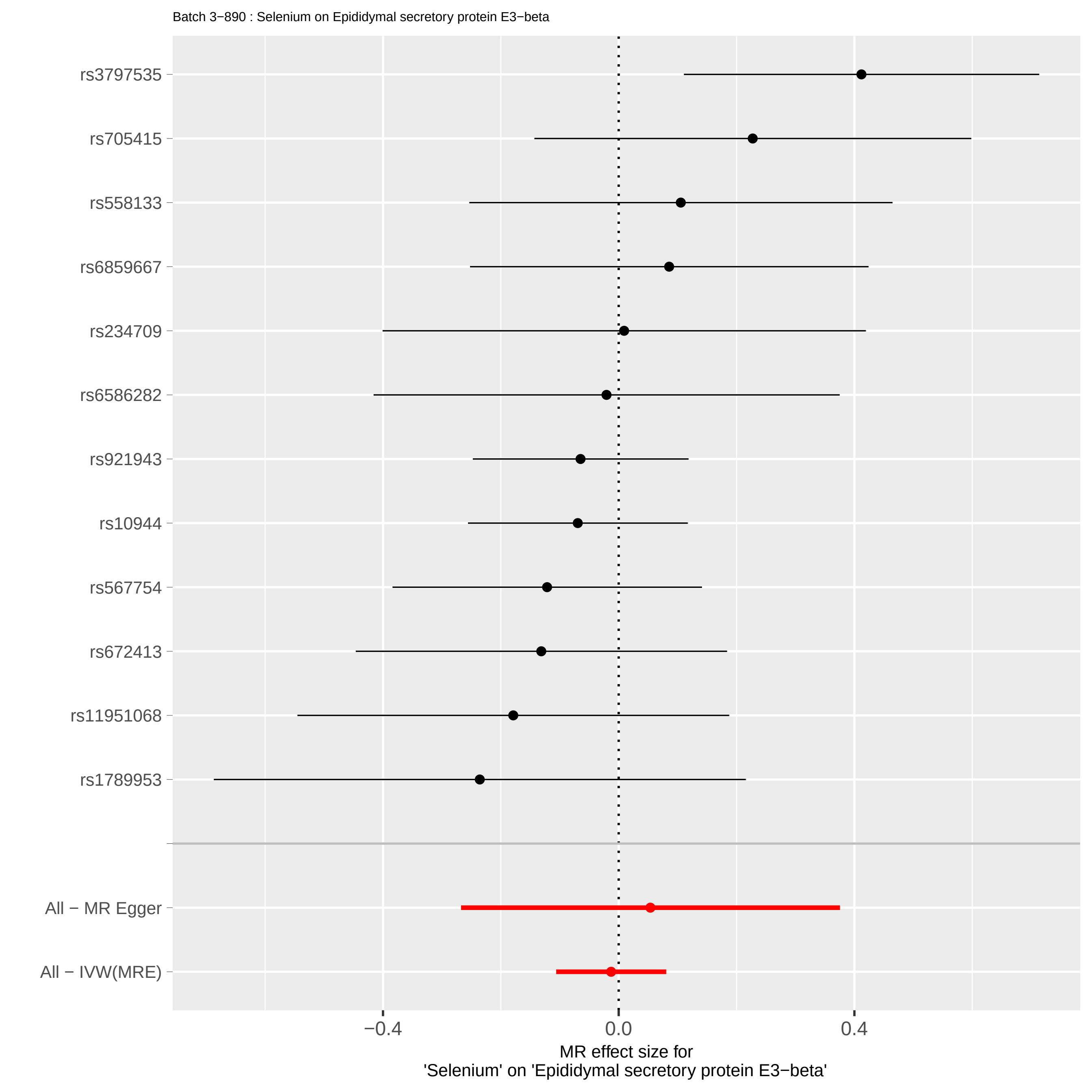
Batch 3−890 : Selenium on Epididymal secretory protein E3−beta
rs3797535
rs705415
rs558133
rs6859667
rs234709
rs6586282
rs921943
rs10944
rs567754
rs672413
rs11951068
rs1789953
All − MR Egger
All − IVW(MRE)
−0.4
0.0
0.4
MR effect size for
'Selenium' on 'Epididymal secretory protein E3−beta'

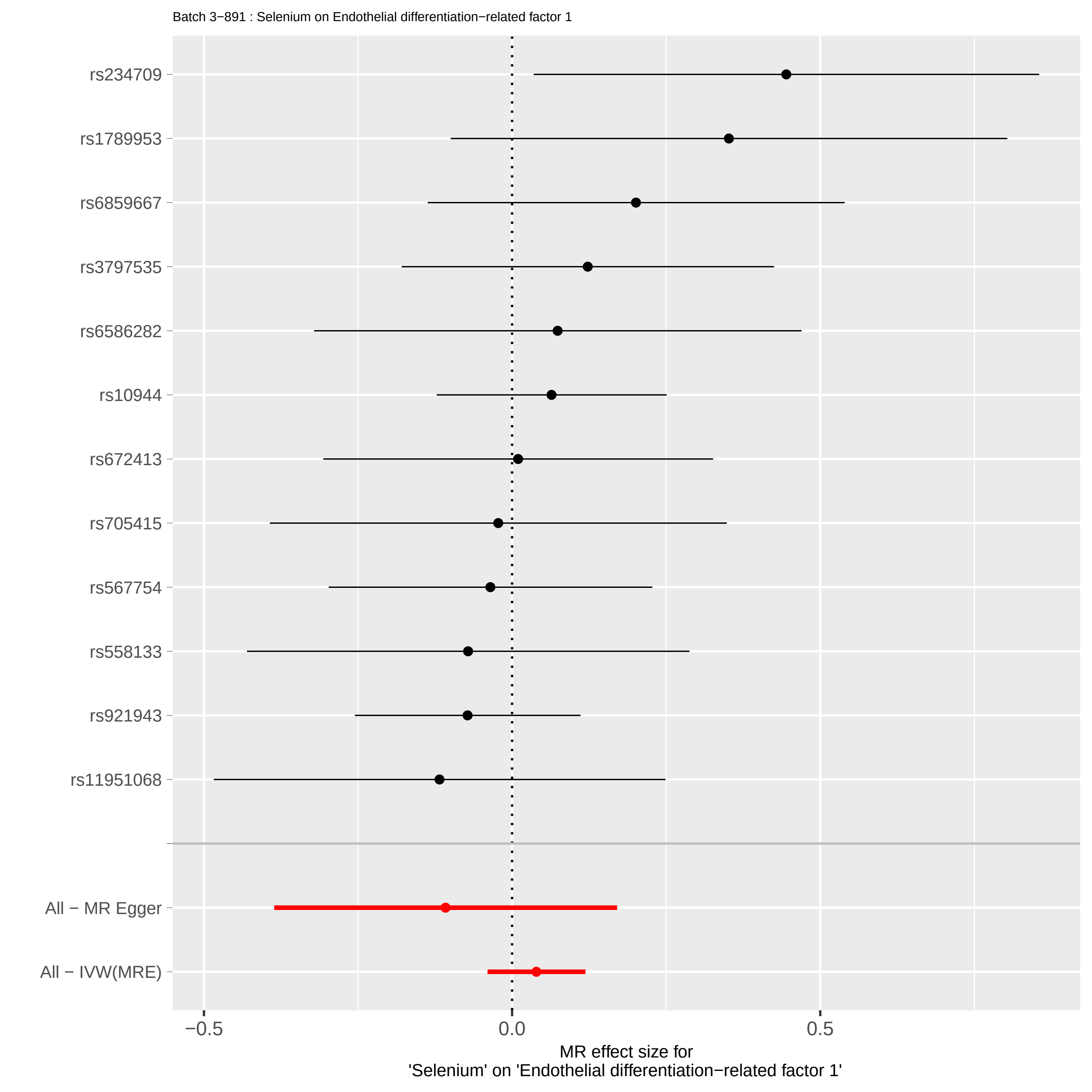

Batch 3−891 : Selenium on Endothelial differentiation−related factor 1
rs234709
rs1789953
rs6859667
rs3797535
rs6586282
rs10944
rs672413
rs705415
rs567754
rs558133
rs921943
rs11951068
All − MR Egger
All − IVW(MRE)
−0.5
0.0
0.5
MR effect size for
'Selenium' on 'Endothelial differentiation−related factor 1'

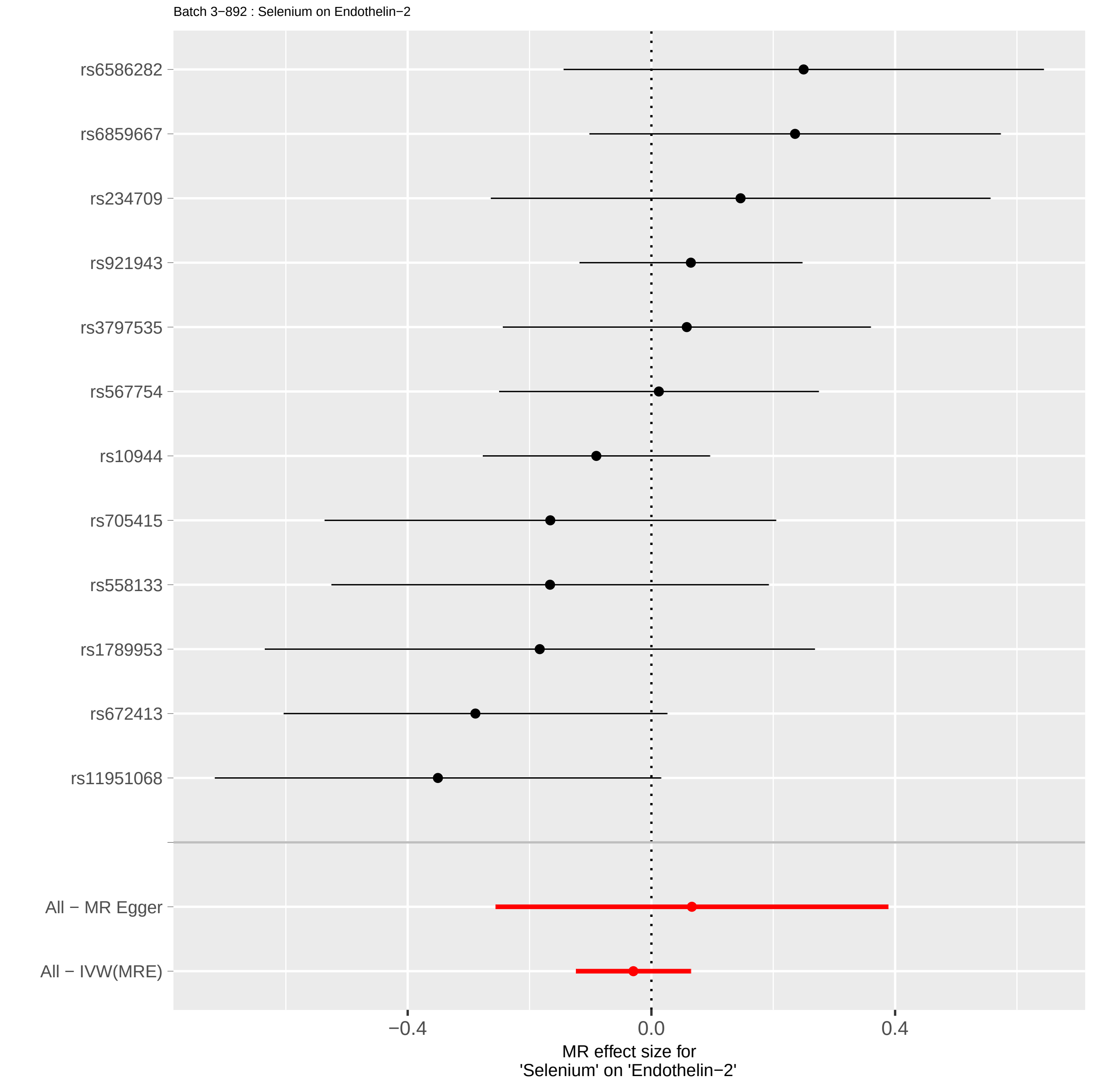

Batch 3−892 : Selenium on Endothelin−2
rs6586282
rs6859667
rs234709
rs921943
rs3797535
rs567754
rs10944
rs705415
rs558133
rs1789953
rs672413
rs11951068
All − MR Egger
All − IVW(MRE)
−0.4
0.0
0.4
MR effect size for
'Selenium' on 'Endothelin−2'

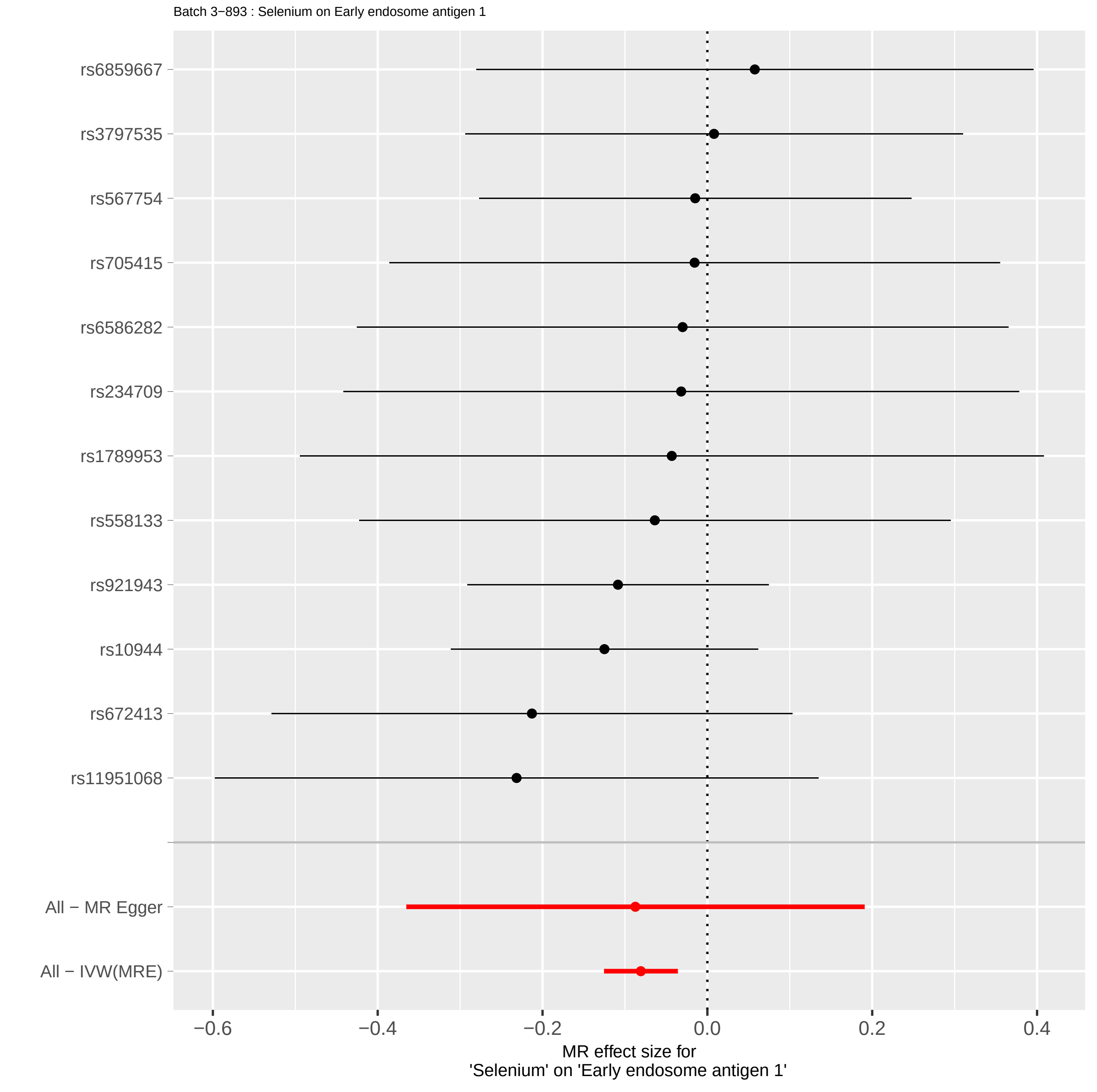
Batch 3−893 : Selenium on Early endosome antigen 1
rs6859667
rs3797535
rs567754
rs705415
rs6586282
rs234709
rs1789953
rs558133
rs921943
rs10944
rs672413
rs11951068
All − MR Egger
All − IVW(MRE)
−0.6
−0.4
−0.2
0.0
0.2
0.4
MR effect size for
'Selenium' on 'Early endosome antigen 1'

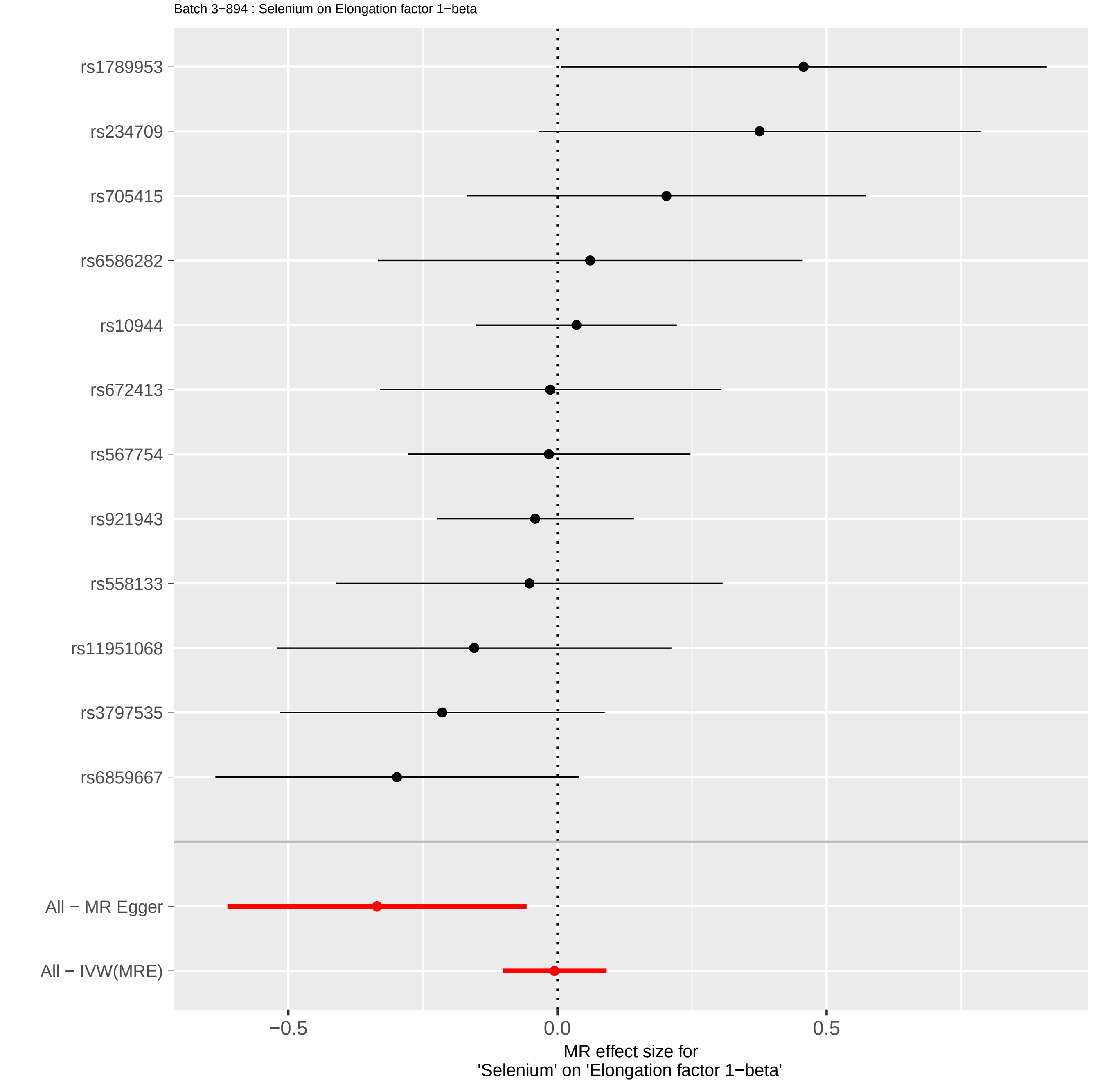
Batch 3−894 : Selenium on Elongation factor 1−beta
rs1789953
rs234709
rs705415
rs6586282
rs10944
rs672413
rs567754
rs921943
rs558133
rs11951068
rs3797535
rs6859667
All − MR Egger
All − IVW(MRE)
−0.5
0.0
0.5
MR effect size for
'Selenium' on 'Elongation factor 1−beta'

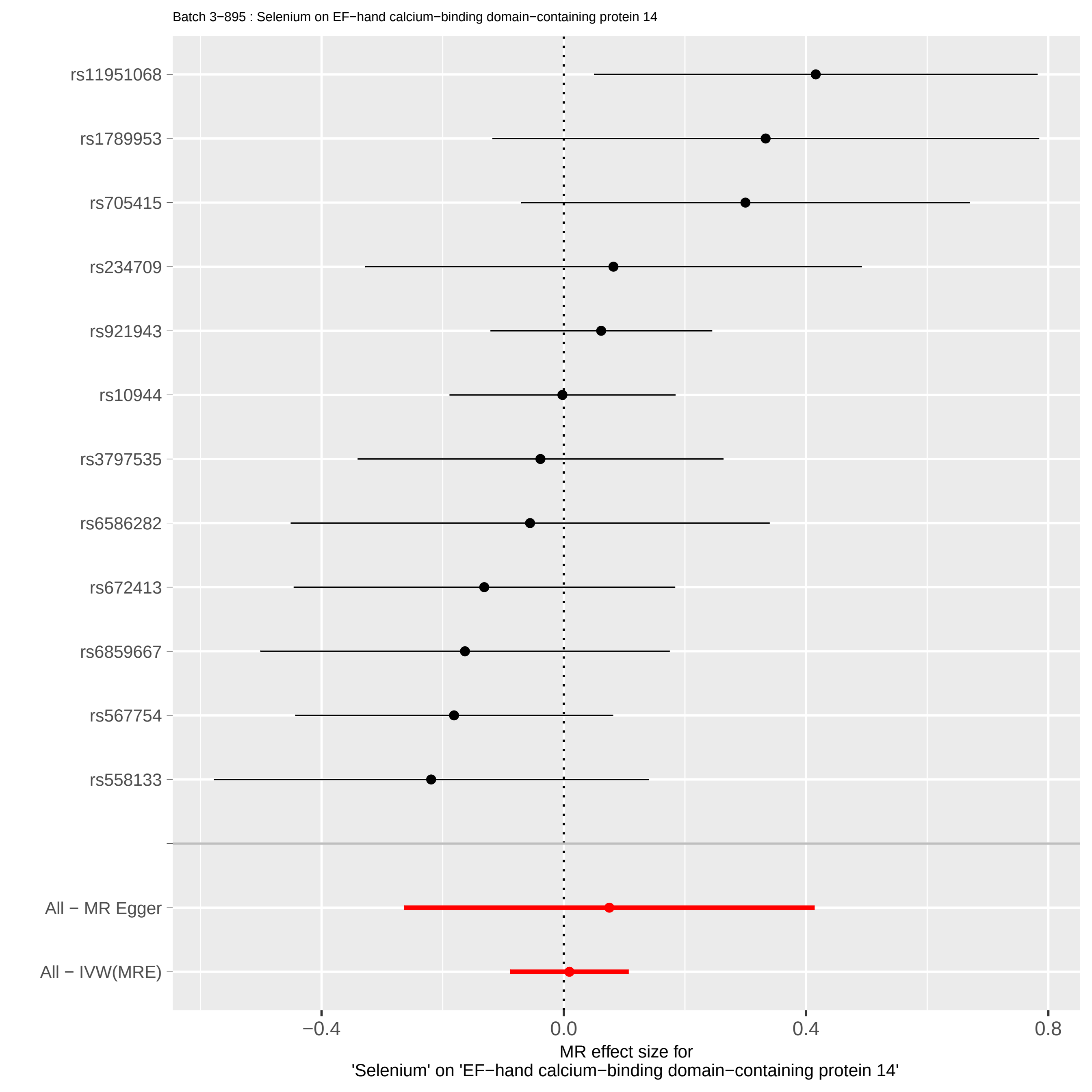

Batch 3−895 : Selenium on EF−hand calcium−binding domain−containing protein 14
rs11951068
rs1789953
rs705415
rs234709
rs921943
rs10944
rs3797535
rs6586282
rs672413
rs6859667
rs567754
rs558133
All − MR Egger
All − IVW(MRE)
−0.4
0.0
0.4
0.8
MR effect size for
'Selenium' on 'EF−hand calcium−binding domain−containing protein 14'

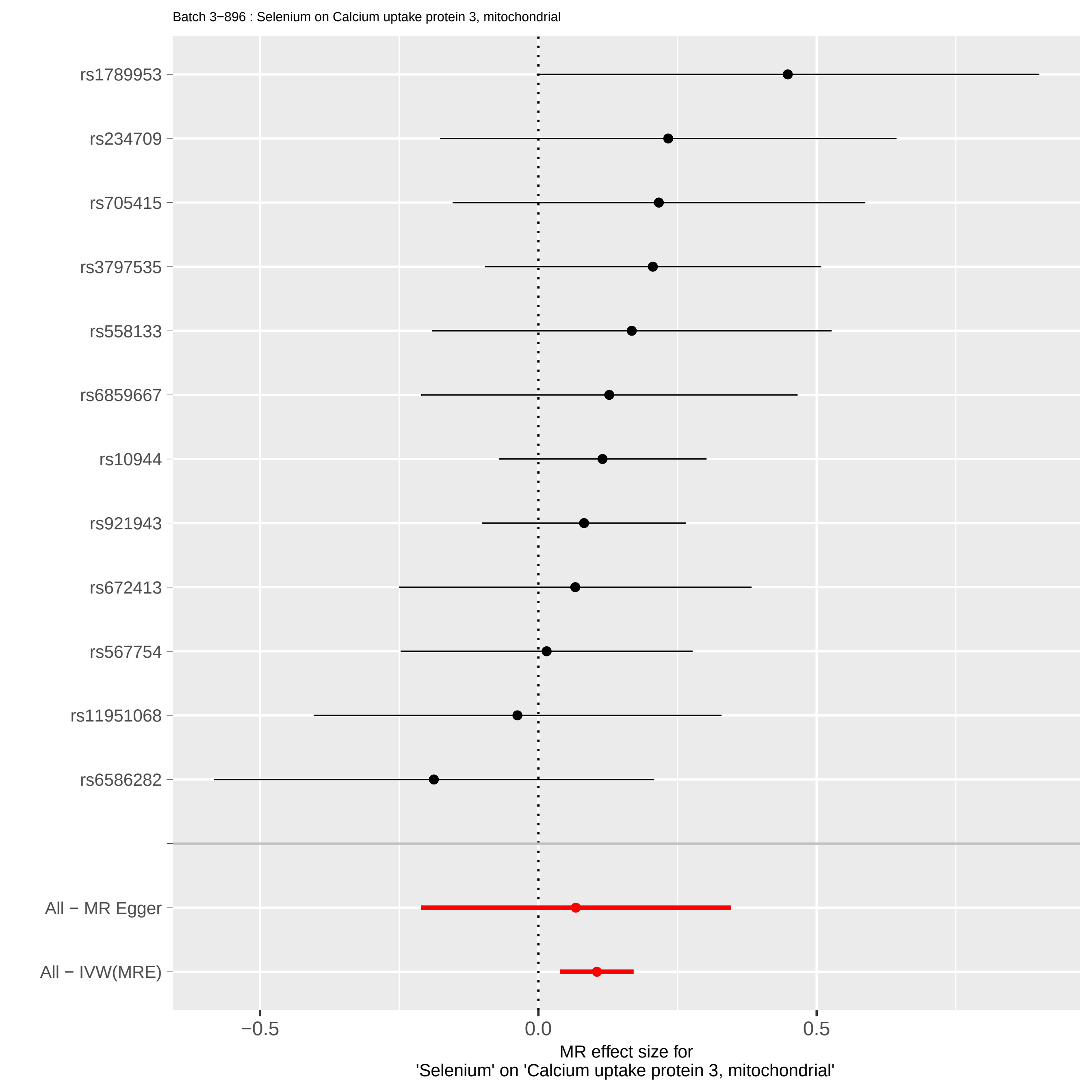
Batch 3−896 : Selenium on Calcium uptake protein 3, mitochondrial
rs1789953
rs234709
rs705415
rs3797535
rs558133
rs6859667
rs10944
rs921943
rs672413
rs567754
rs11951068
rs6586282
All − MR Egger
All − IVW(MRE)
−0.5
0.0
0.5
MR effect size for
'Selenium' on 'Calcium uptake protein 3, mitochondrial'

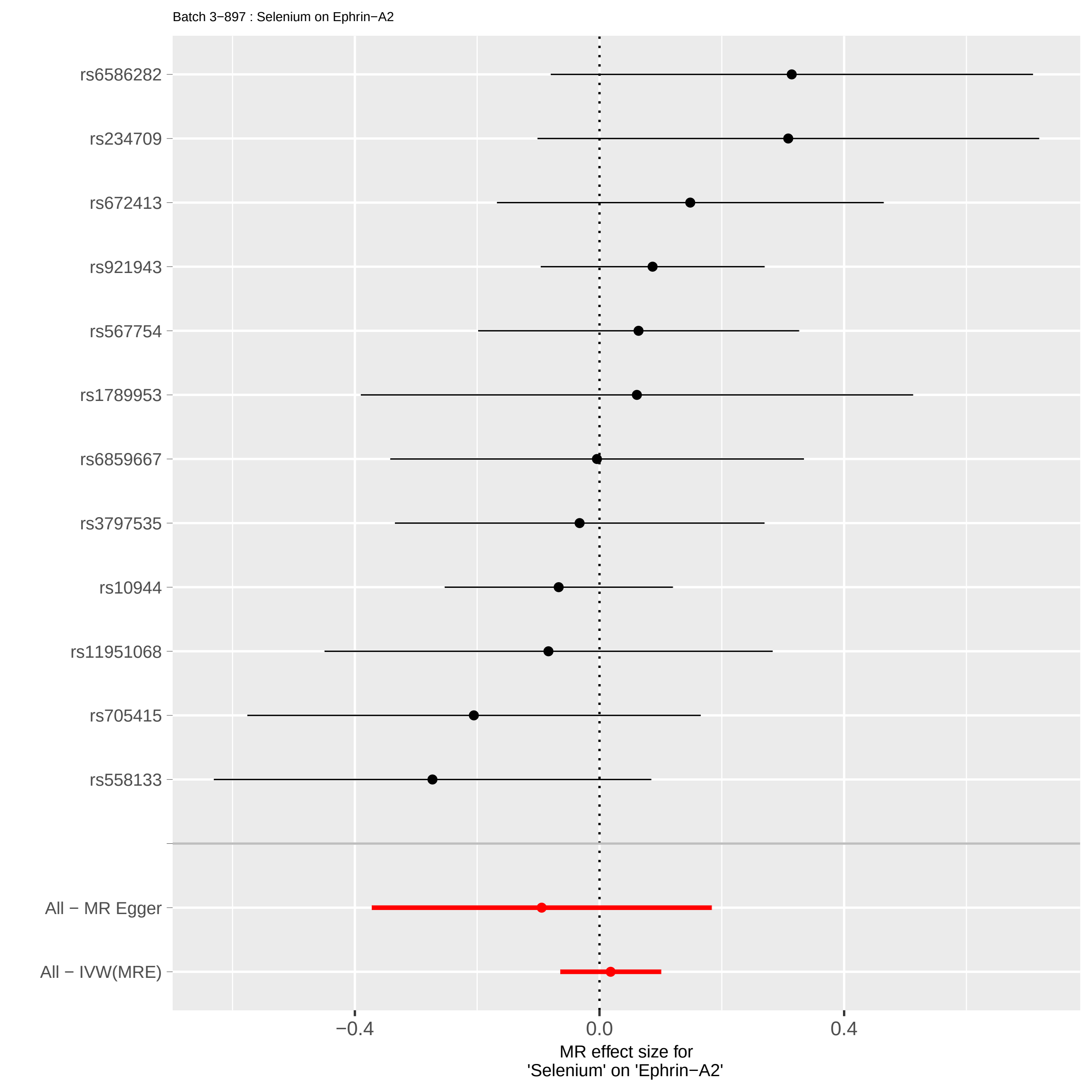
Batch 3−897 : Selenium on Ephrin−A2
rs6586282
rs234709
rs672413
rs921943
rs567754
rs1789953
rs6859667
rs3797535
rs10944
rs11951068
rs705415
rs558133
All − MR Egger
All − IVW(MRE)
−0.4
0.0
0.4
MR effect size for
'Selenium' on 'Ephrin−A2'

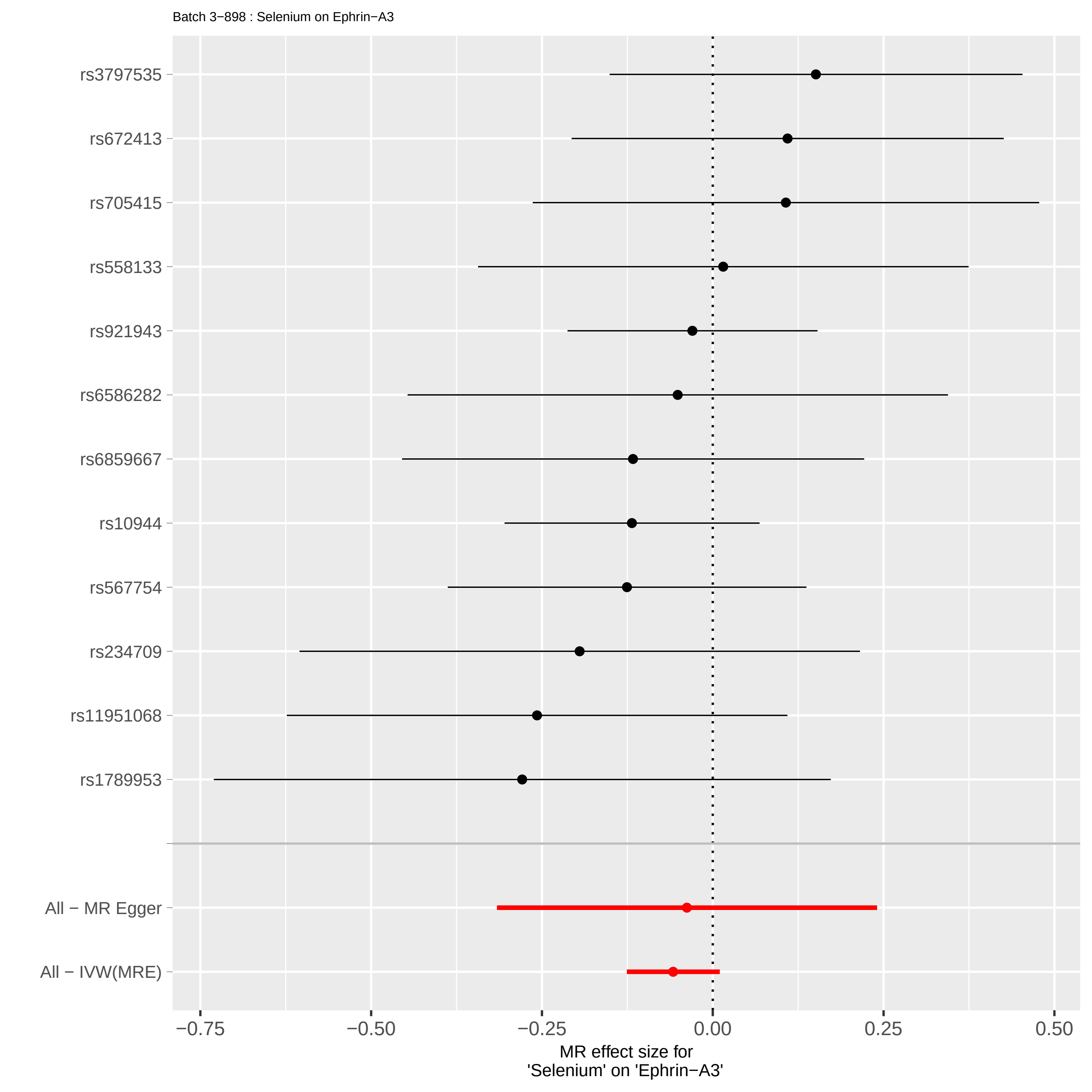

Batch 3−898 : Selenium on Ephrin−A3
rs3797535
rs672413
rs705415
rs558133
rs921943
rs6586282
rs6859667
rs10944
rs567754
rs234709
rs11951068
rs1789953
All − MR Egger
All − IVW(MRE)
−0.75
−0.50
−0.25
0.00
0.25
0.50
MR effect size for
'Selenium' on 'Ephrin−A3'

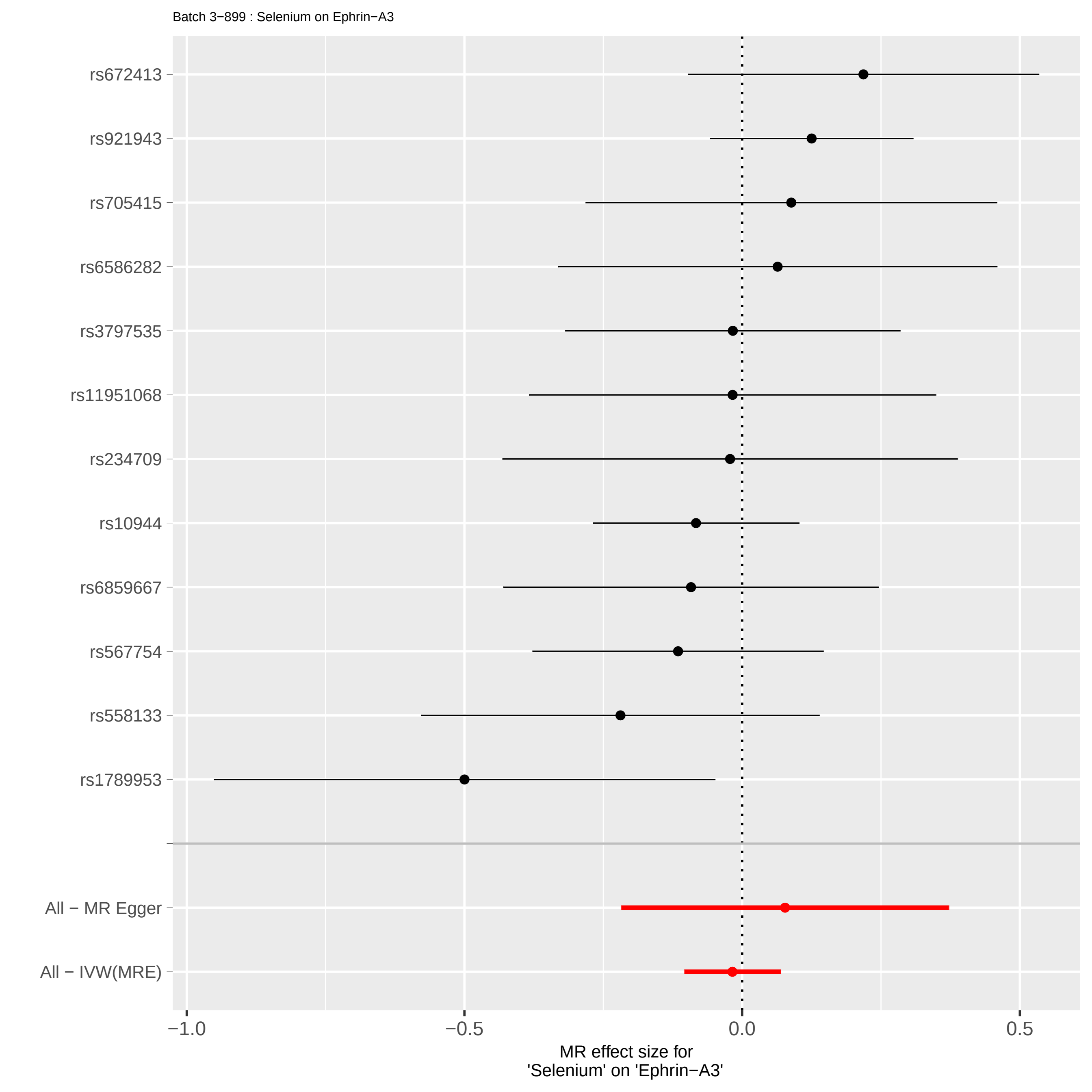

Batch 3−899 : Selenium on Ephrin−A3
rs672413
rs921943
rs705415
rs6586282
rs3797535
rs11951068
rs234709
rs10944
rs6859667
rs567754
rs558133
rs1789953
All − MR Egger
All − IVW(MRE)
−1.0
−0.5
0.0
0.5
MR effect size for
'Selenium' on 'Ephrin−A3'

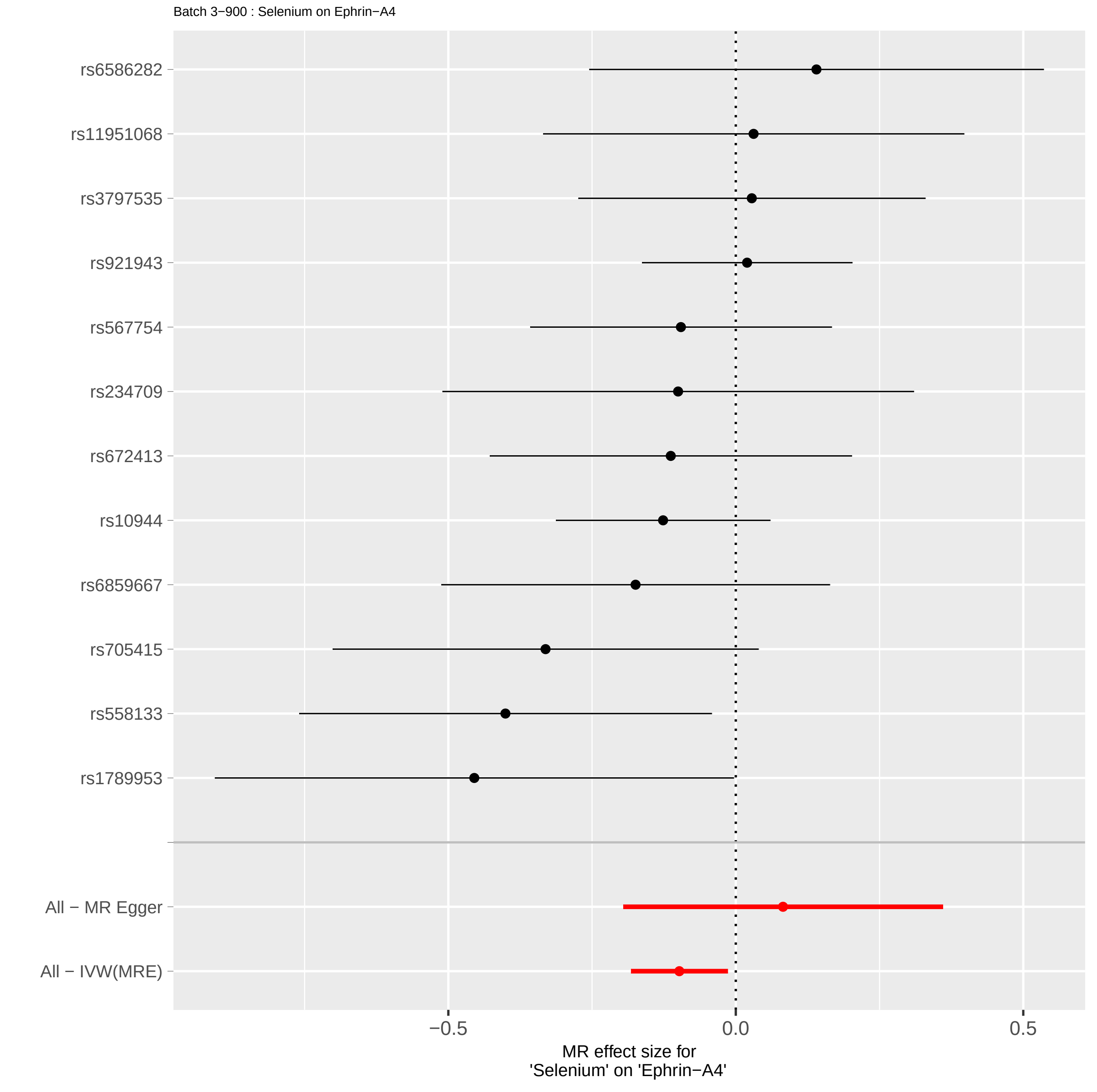

Batch 3−900 : Selenium on Ephrin−A4
rs6586282
rs11951068
rs3797535
rs921943
rs567754
rs234709
rs672413
rs10944
rs6859667
rs705415
rs558133
rs1789953
All − MR Egger
All − IVW(MRE)
−0.5
0.0
0.5
MR effect size for
'Selenium' on 'Ephrin−A4'

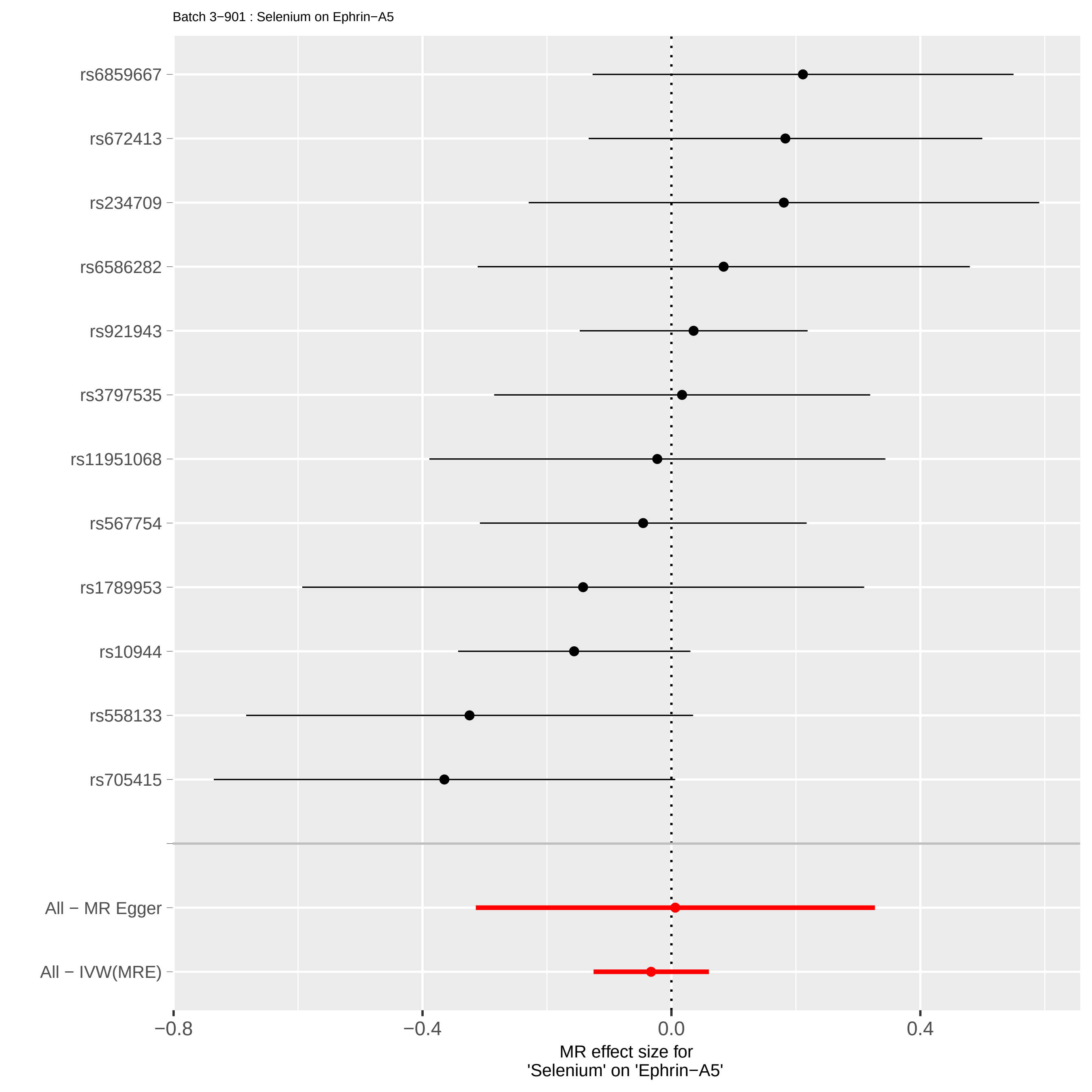

Batch 3−901 : Selenium on Ephrin−A5
rs6859667
rs672413
rs234709
rs6586282
rs921943
rs3797535
rs11951068
rs567754
rs1789953
rs10944
rs558133
rs705415
All − MR Egger
All − IVW(MRE)
−0.8
−0.4
0.0
0.4
MR effect size for
'Selenium' on 'Ephrin−A5'

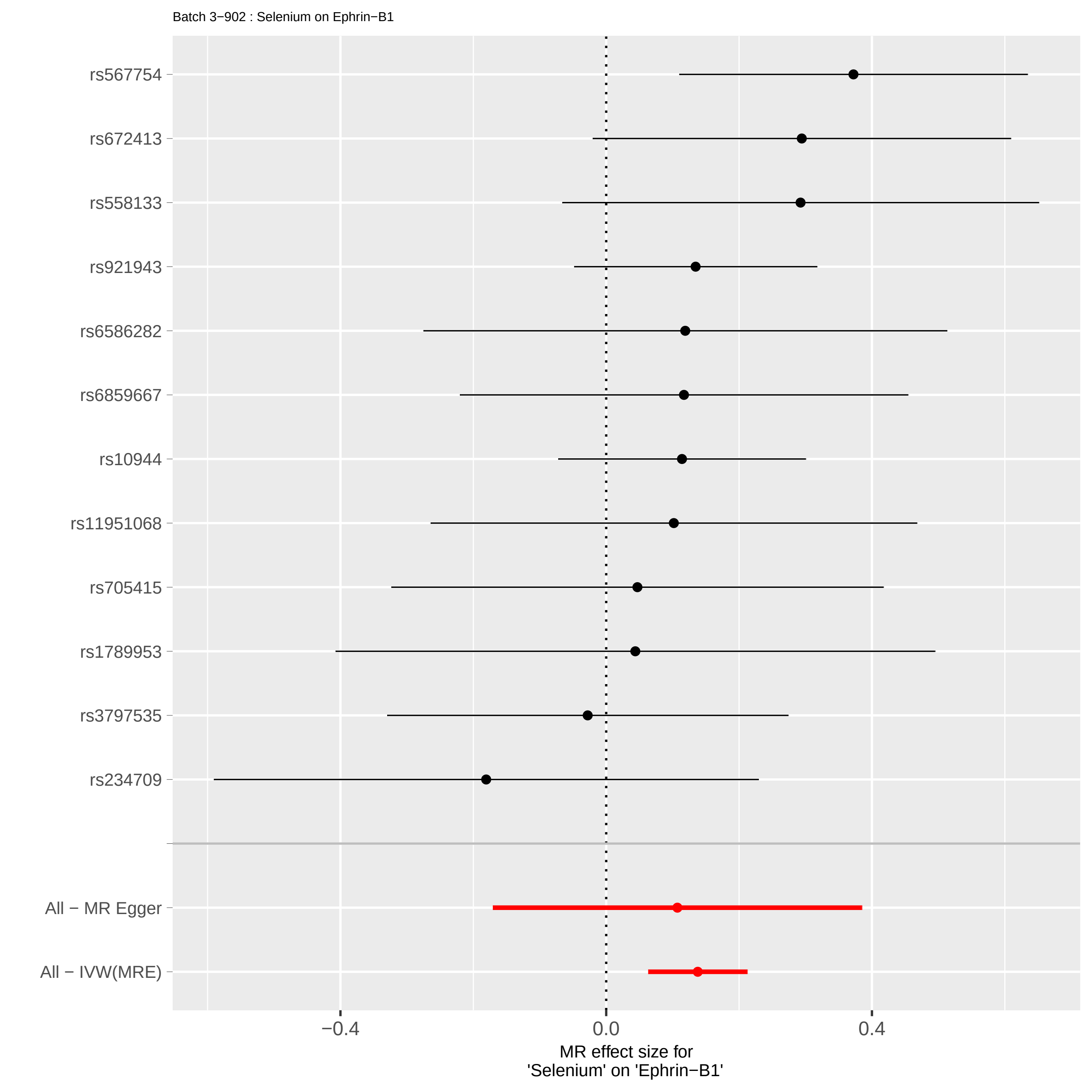

Batch 3−902 : Selenium on Ephrin−B1
rs567754
rs672413
rs558133
rs921943
rs6586282
rs6859667
rs10944
rs11951068
rs705415
rs1789953
rs3797535
rs234709
All − MR Egger
All − IVW(MRE)
−0.4
0.0
0.4
MR effect size for
'Selenium' on 'Ephrin−B1'

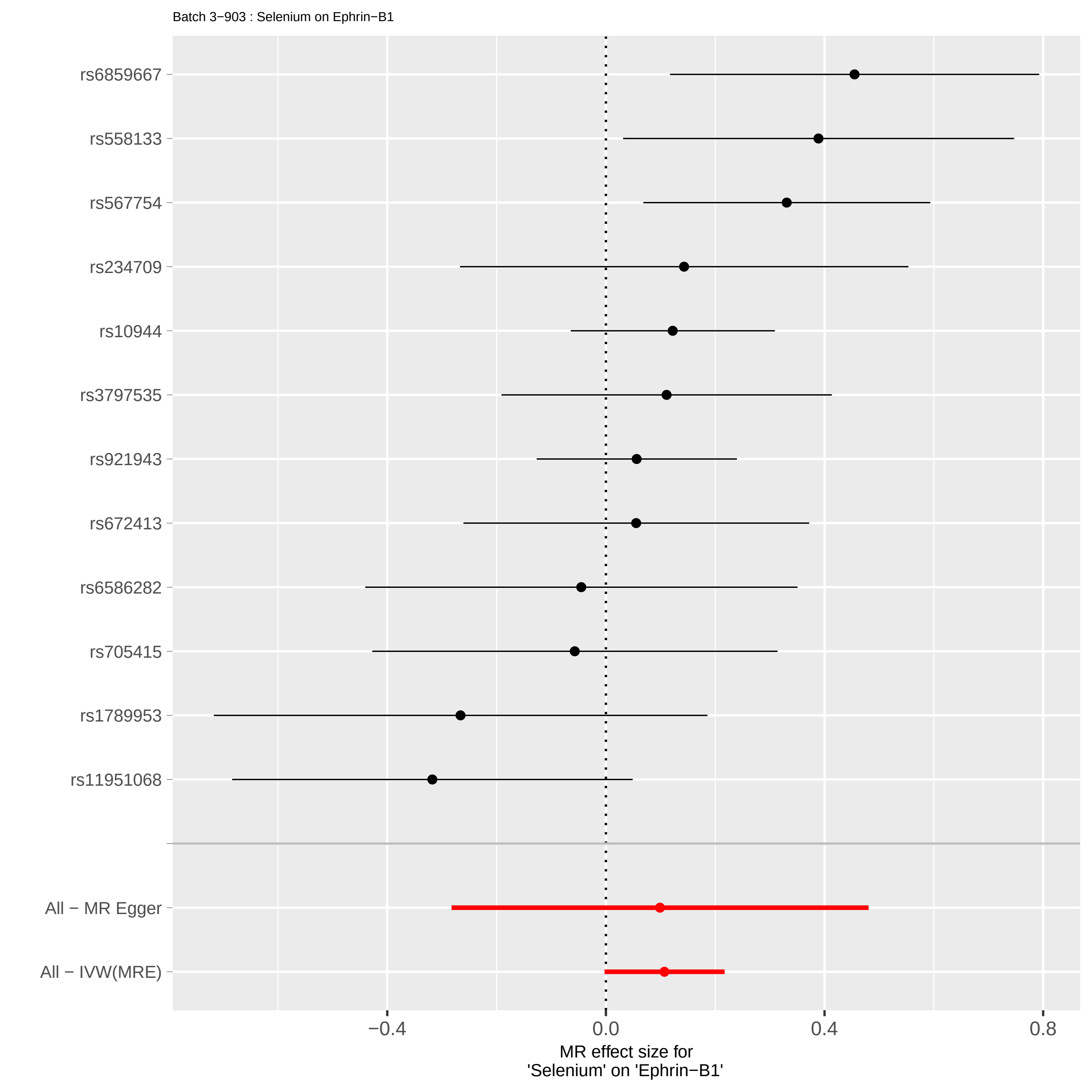

Batch 3−903 : Selenium on Ephrin−B1
rs6859667
rs558133
rs567754
rs234709
rs10944
rs3797535
rs921943
rs672413
rs6586282
rs705415
rs1789953
rs11951068
All − MR Egger
All − IVW(MRE)
−0.4
0.0
0.4
0.8
MR effect size for
'Selenium' on 'Ephrin−B1'

Batch 3−904 : Selenium on Ephrin−B2

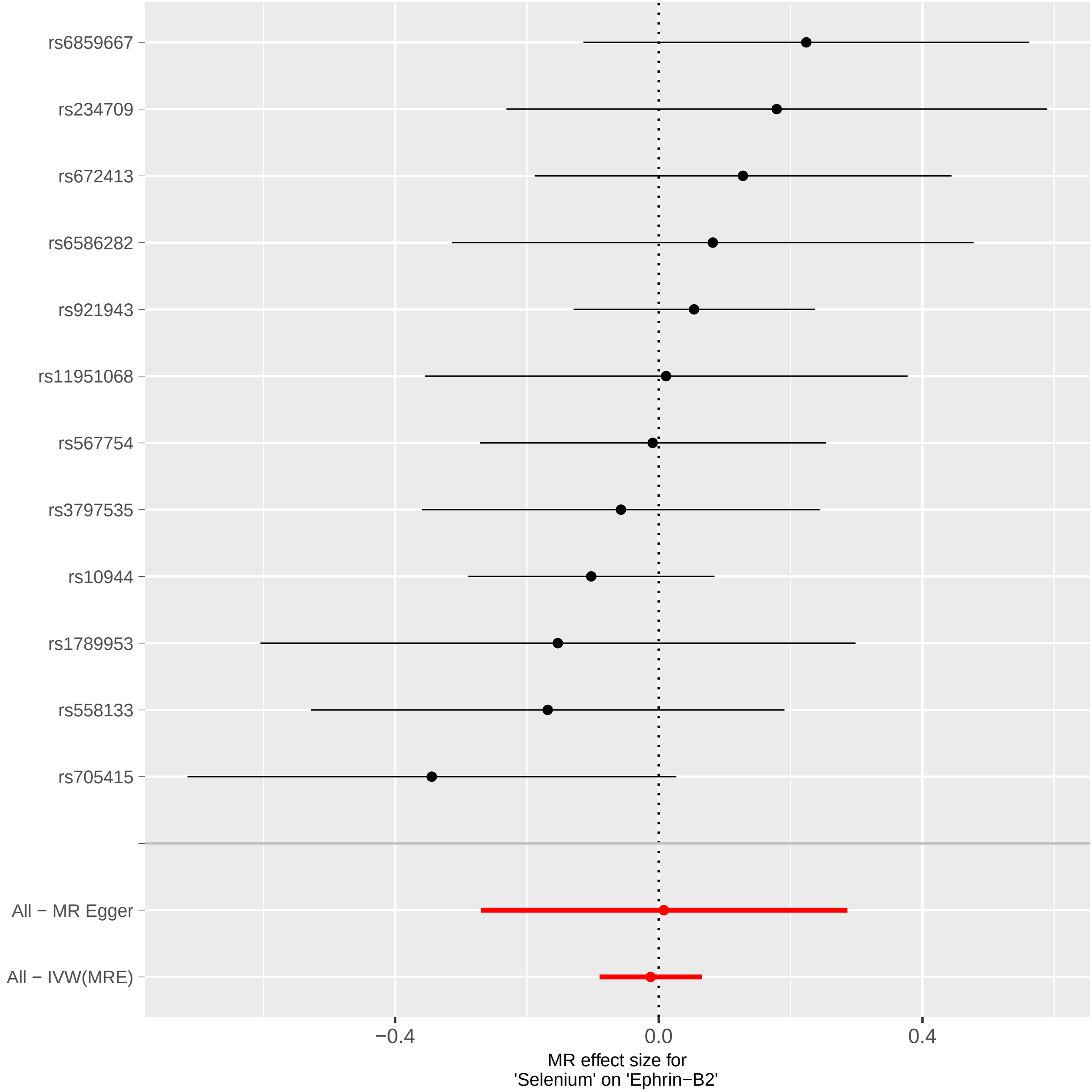

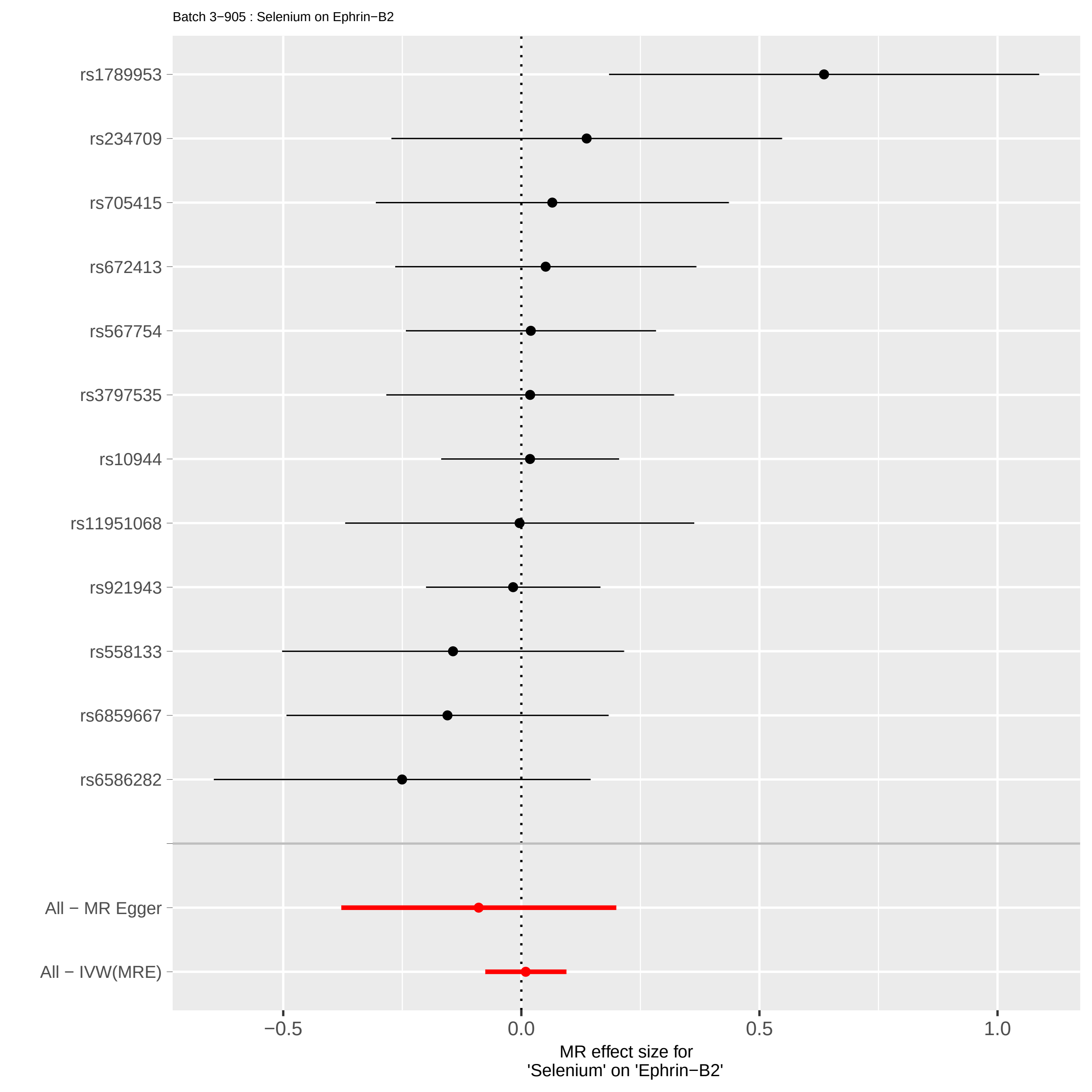
Batch 3−905 : Selenium on Ephrin−B2
rs1789953
rs234709
rs705415
rs672413
rs567754
rs3797535
rs10944
rs11951068
rs921943
rs558133
rs6859667
rs6586282
All − MR Egger
All − IVW(MRE)
−0.5
0.0
0.5
1.0
MR effect size for
'Selenium' on 'Ephrin−B2'

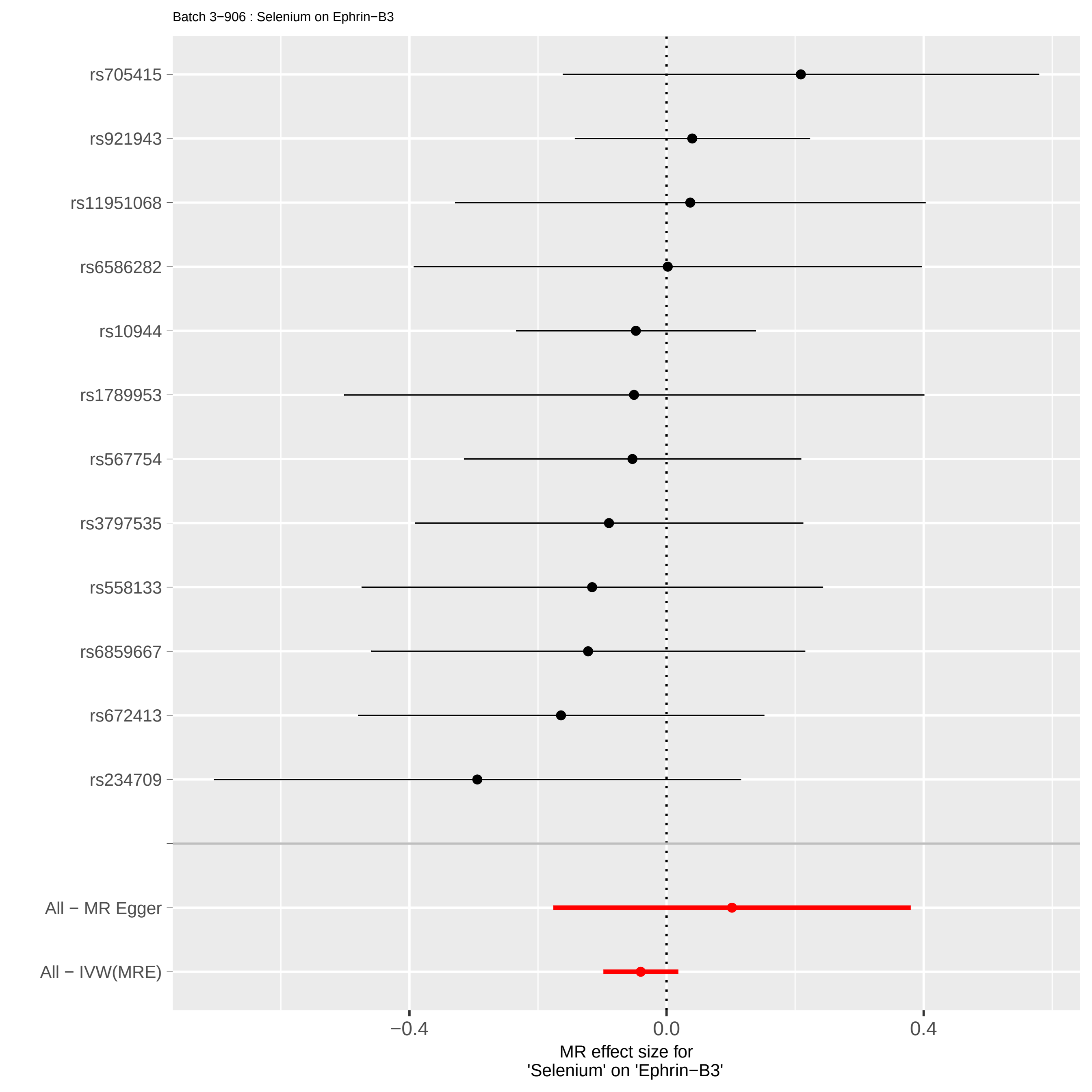

Batch 3−906 : Selenium on Ephrin−B3
rs705415
rs921943
rs11951068
rs6586282
rs10944
rs1789953
rs567754
rs3797535
rs558133
rs6859667
rs672413
rs234709
All − MR Egger
All − IVW(MRE)
−0.4
0.0
0.4
MR effect size for
'Selenium' on 'Ephrin−B3'

Batch 3−907 : Selenium on Ephrin−B3

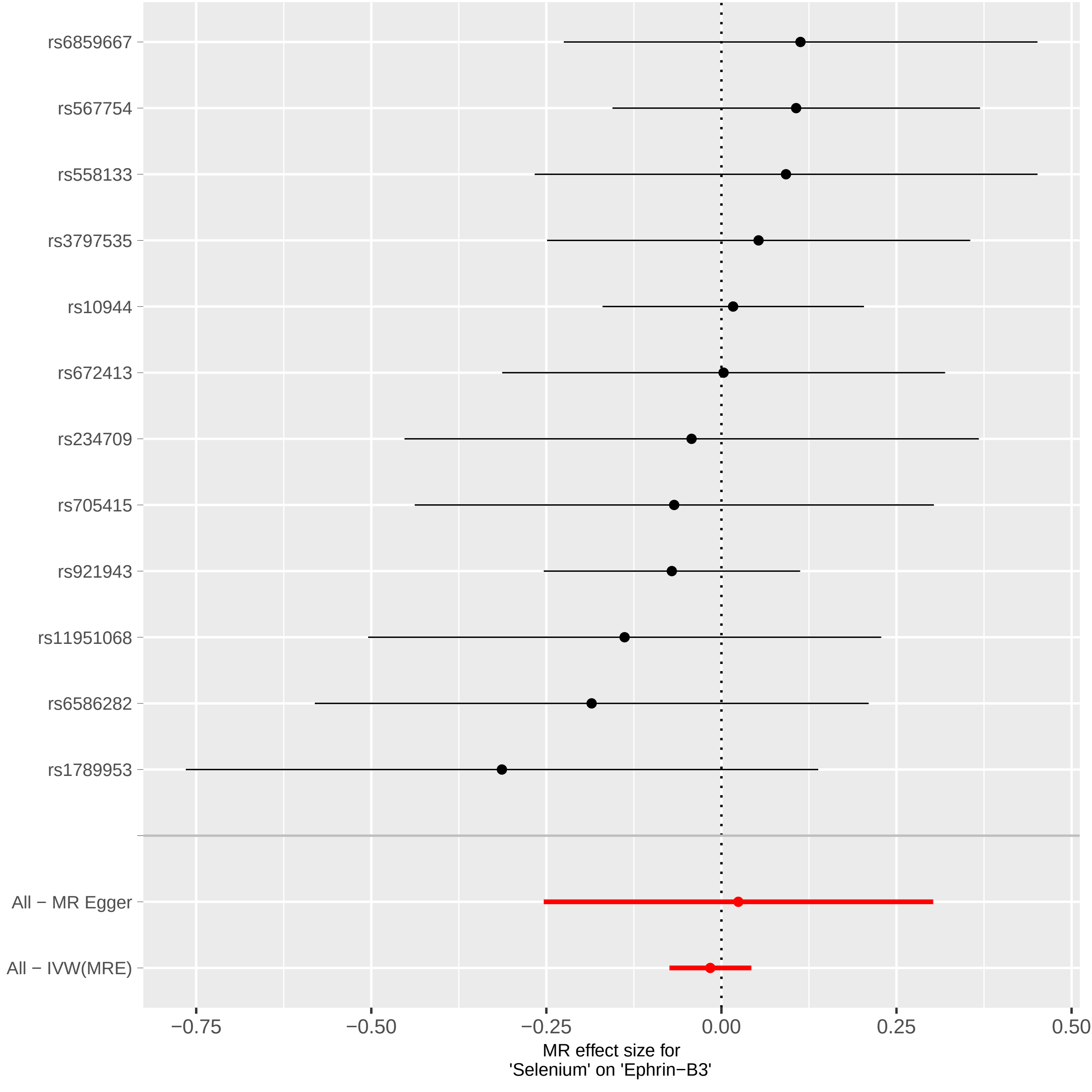

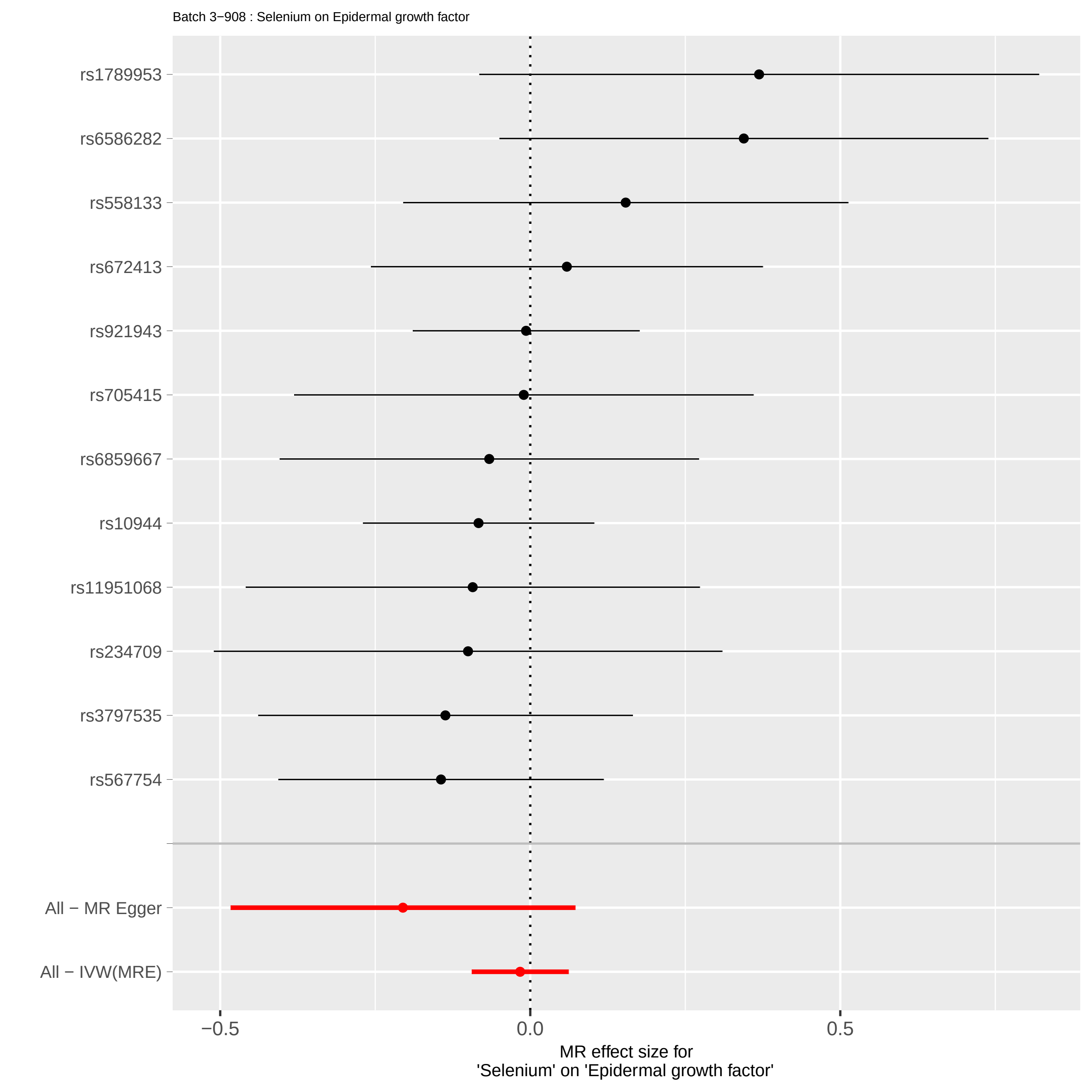

Batch 3−908 : Selenium on Epidermal growth factor
rs1789953
rs6586282
rs558133
rs672413
rs921943
rs705415
rs6859667
rs10944
rs11951068
rs234709
rs3797535
rs567754
All − MR Egger
All − IVW(MRE)
−0.5
0.0
0.5
MR effect size for
'Selenium' on 'Epidermal growth factor'

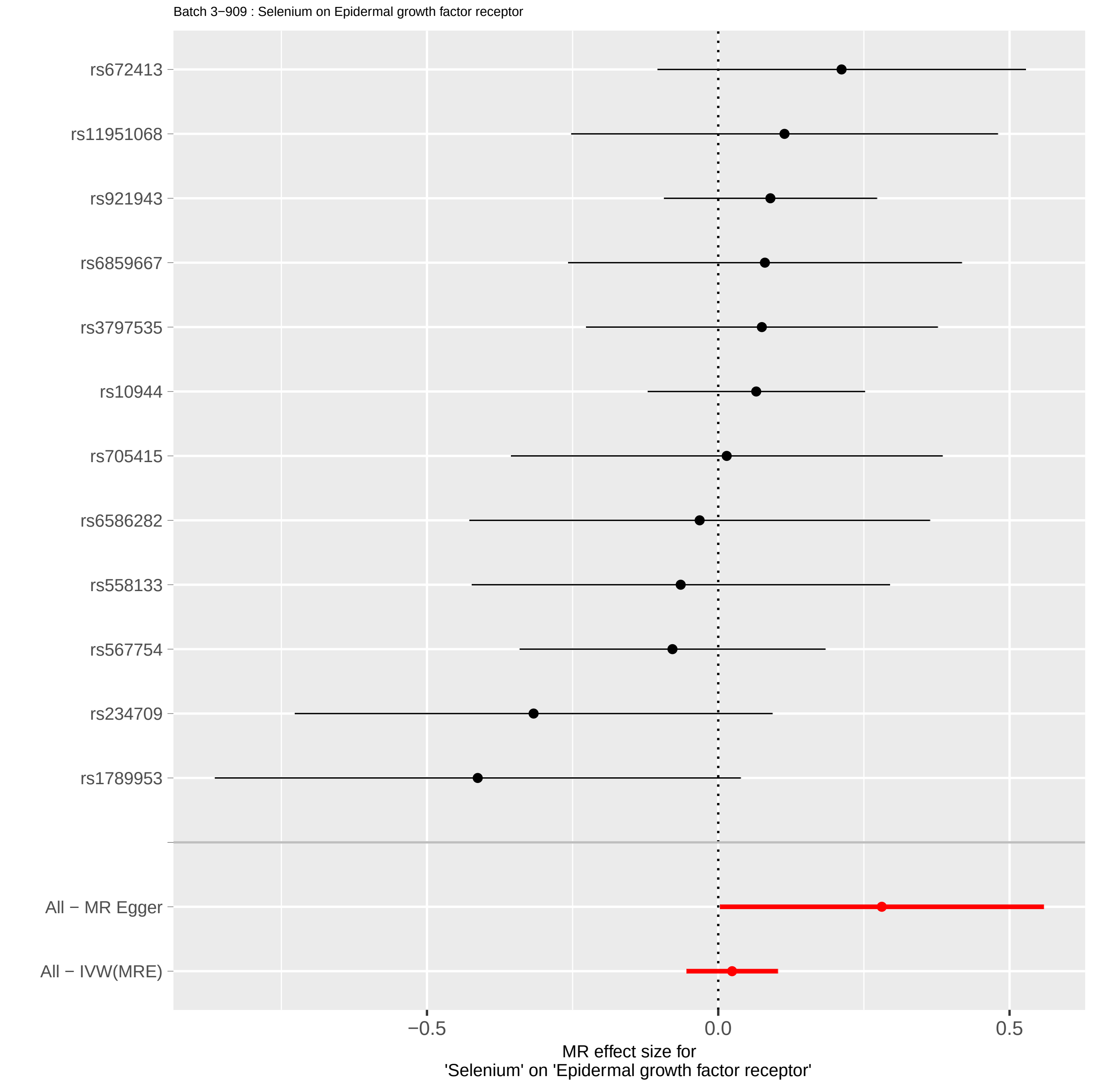

Batch 3−909 : Selenium on Epidermal growth factor receptor
rs672413
rs11951068
rs921943
rs6859667
rs3797535
rs10944
rs705415
rs6586282
rs558133
rs567754
rs234709
rs1789953
All − MR Egger
All − IVW(MRE)
−0.5
0.0
0.5
MR effect size for
'Selenium' on 'Epidermal growth factor receptor'

Batch 3−910 : Selenium on Epidermal growth factor receptor variant III

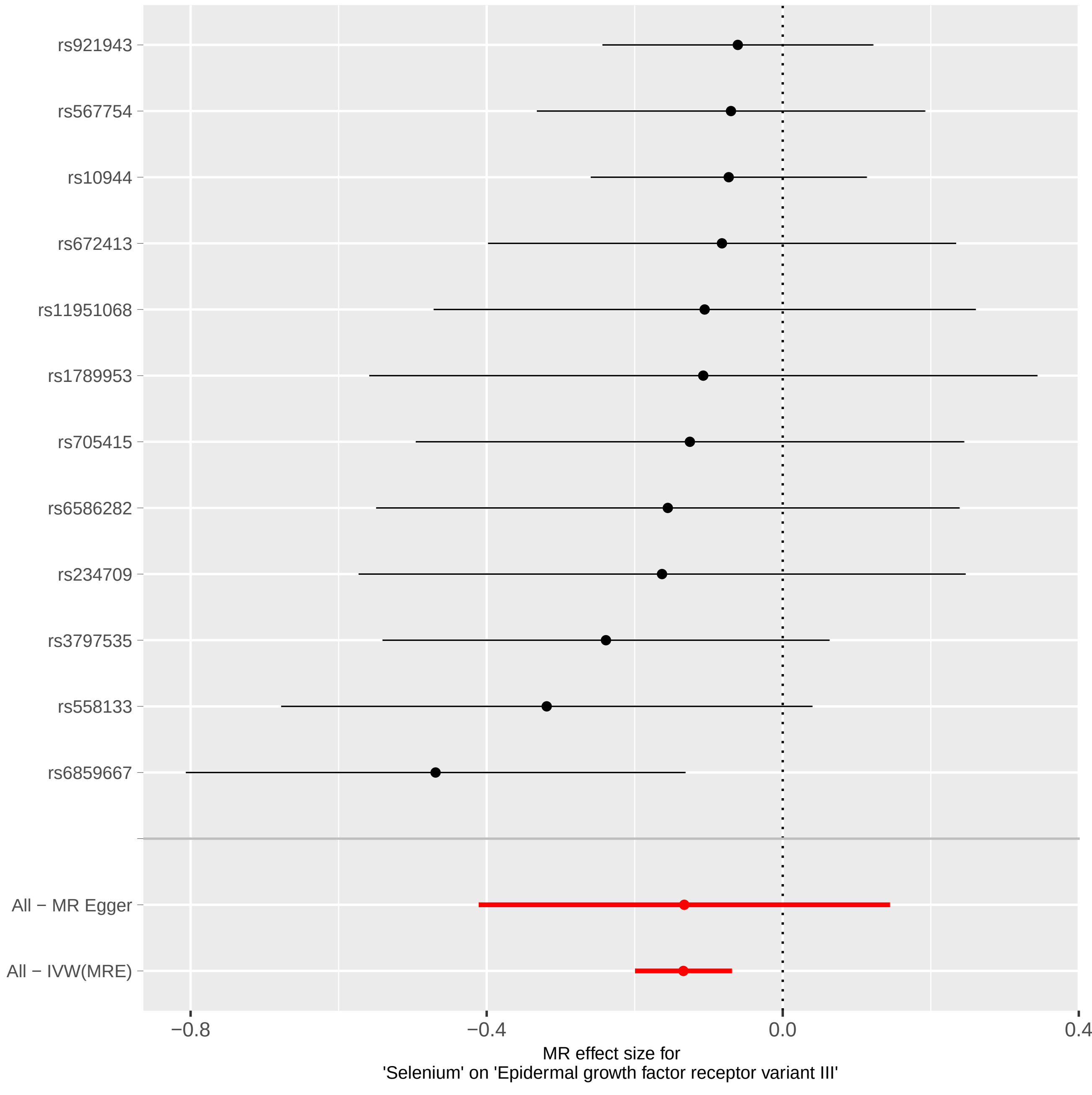

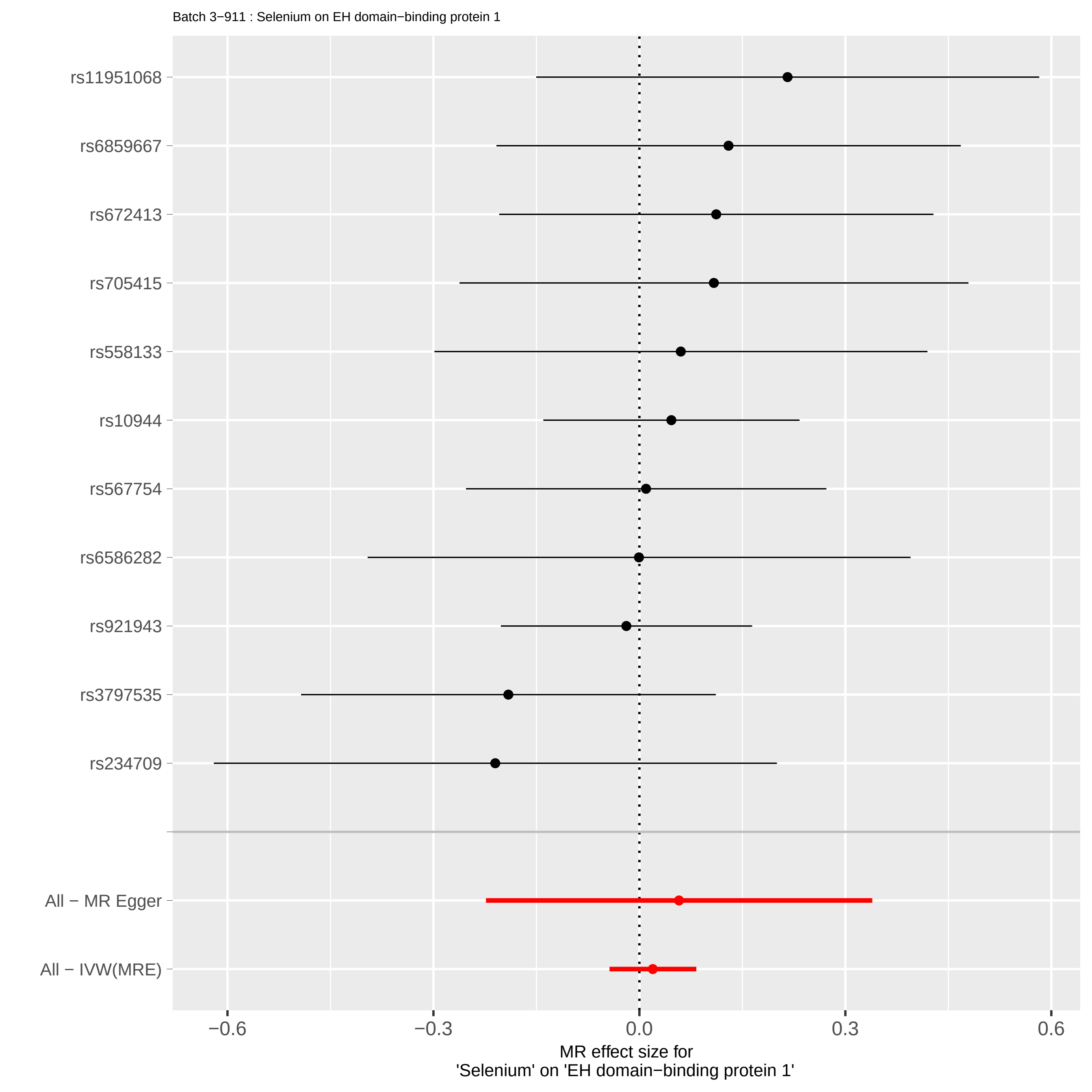
Batch 3−911 : Selenium on EH domain−binding protein 1
rs11951068
rs6859667
rs672413
rs705415
rs558133
rs10944
rs567754
rs6586282
rs921943
rs3797535
rs234709
All − MR Egger
All − IVW(MRE)
−0.6
−0.3
0.0
0.3
0.6
MR effect size for
'Selenium' on 'EH domain−binding protein 1'

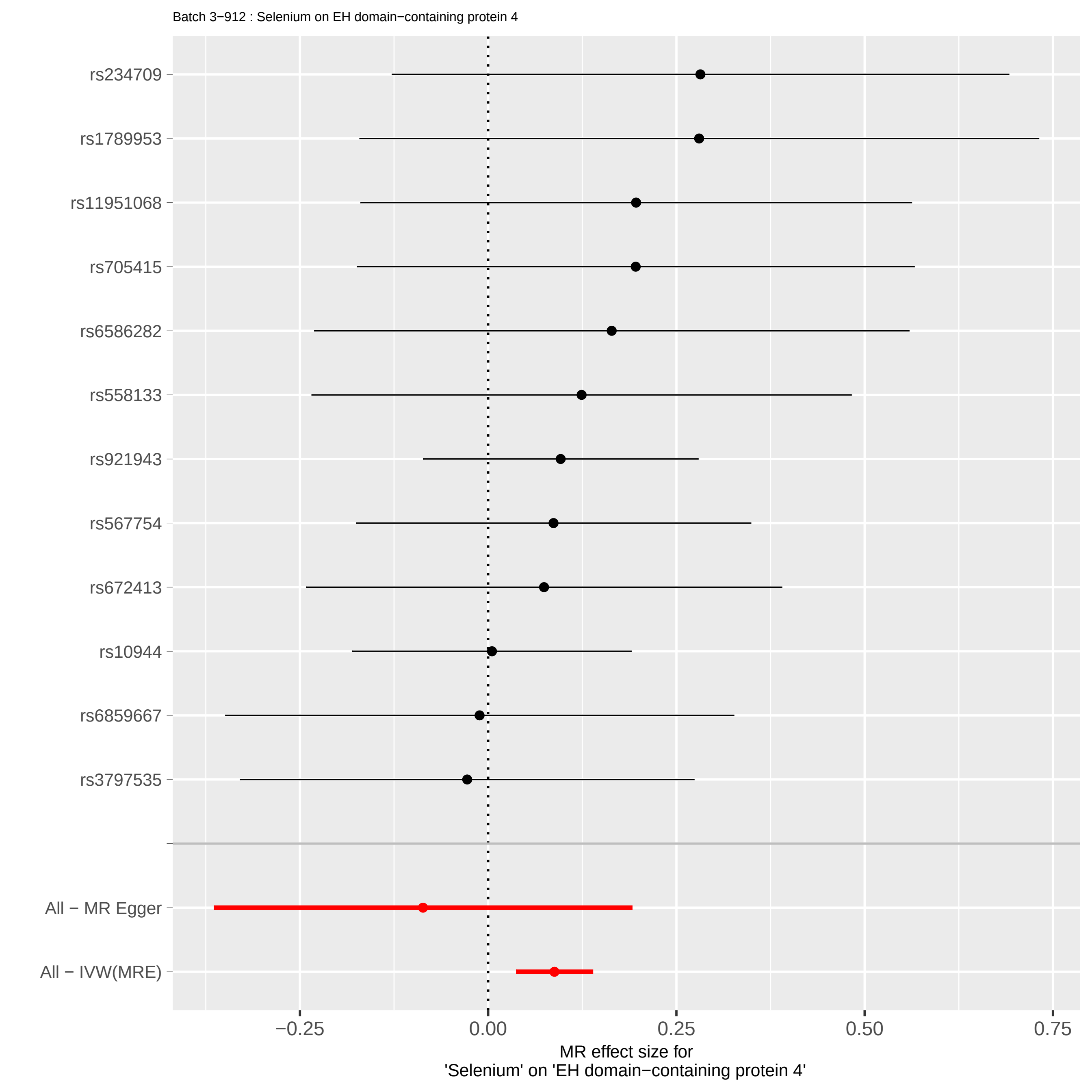

Batch 3−912 : Selenium on EH domain−containing protein 4
rs234709
rs1789953
rs11951068
rs705415
rs6586282
rs558133
rs921943
rs567754
rs672413
rs10944
rs6859667
rs3797535
All − MR Egger
All − IVW(MRE)
−0.25
0.00
0.25
0.50
0.75
MR effect size for
'Selenium' on 'EH domain−containing protein 4'

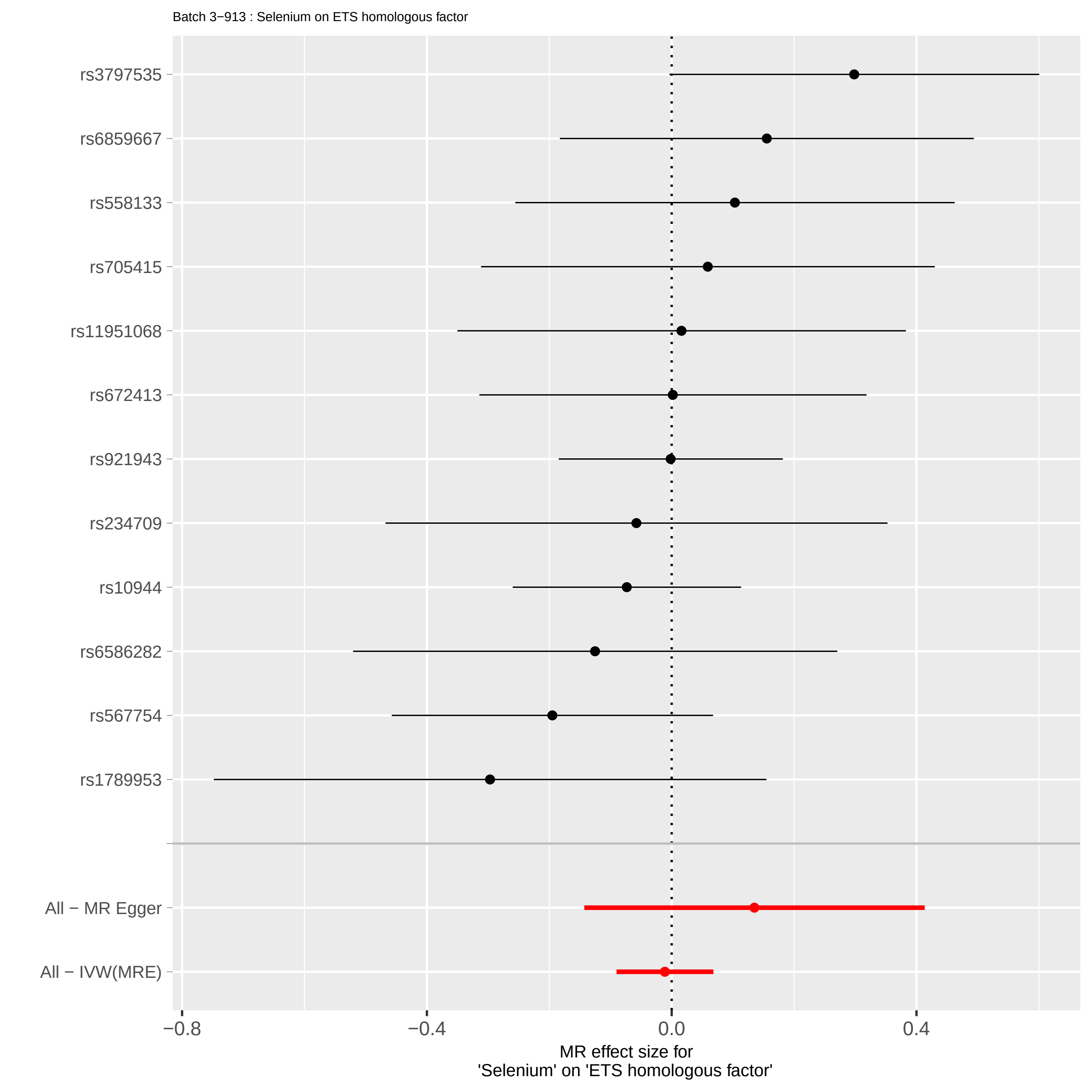

Batch 3−913 : Selenium on ETS homologous factor
rs3797535
rs6859667
rs558133
rs705415
rs11951068
rs672413
rs921943
rs234709
rs10944
rs6586282
rs567754
rs1789953
All − MR Egger
All − IVW(MRE)
−0.8
−0.4
0.0
0.4
MR effect size for
'Selenium' on 'ETS homologous factor'

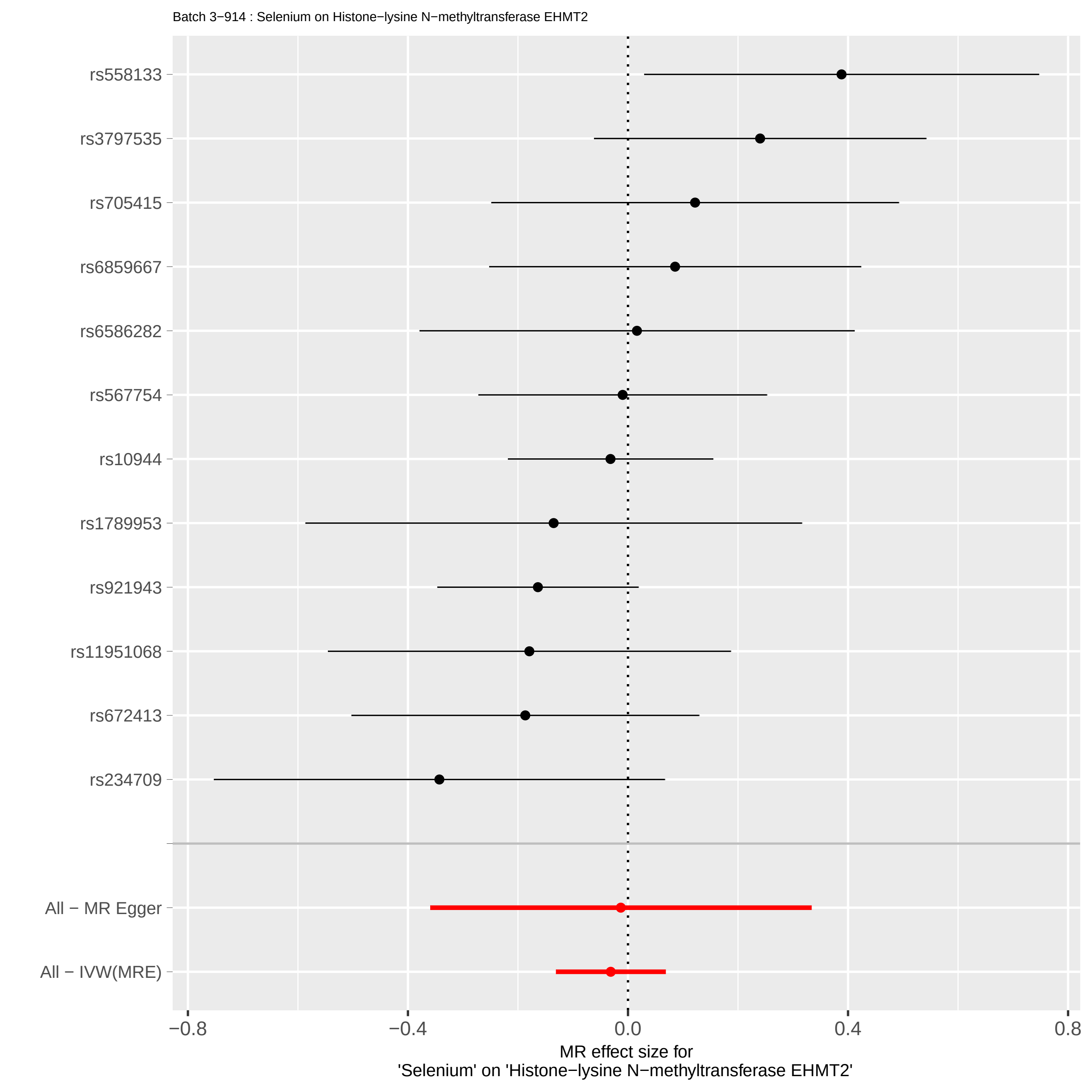
Batch 3−914 : Selenium on Histone−lysine N−methyltransferase EHMT2
rs558133
rs3797535
rs705415
rs6859667
rs6586282
rs567754
rs10944
rs1789953
rs921943
rs11951068
rs672413
rs234709
All − MR Egger
All − IVW(MRE)
−0.8
−0.4
0.0
0.4
0.8
MR effect size for
'Selenium' on 'Histone−lysine N−methyltransferase EHMT2'

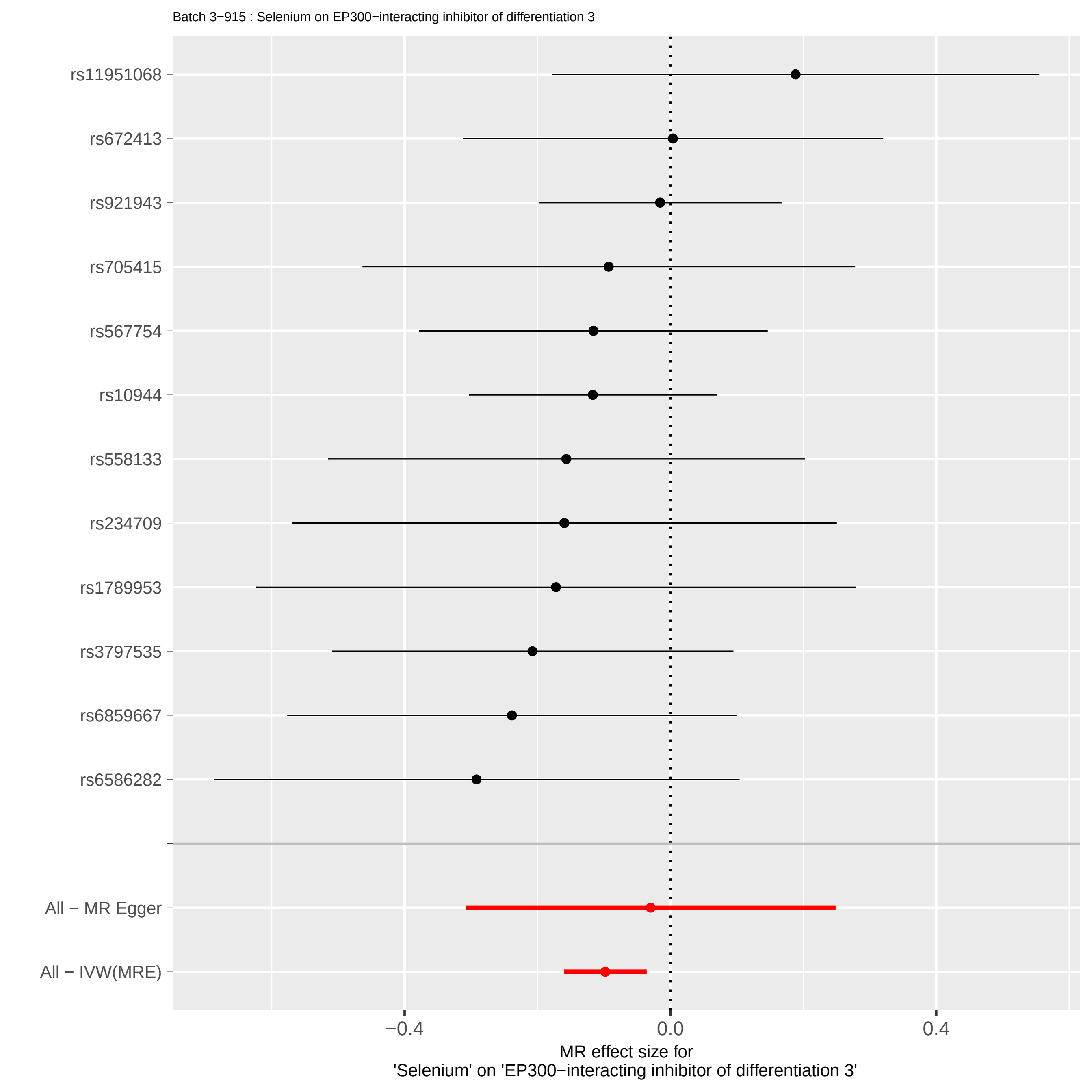

Batch 3−915 : Selenium on EP300−interacting inhibitor of differentiation 3
rs11951068
rs672413
rs921943
rs705415
rs567754
rs10944
rs558133
rs234709
rs1789953
rs3797535
rs6859667
rs6586282
All − MR Egger
All − IVW(MRE)
−0.4
0.0
0.4
MR effect size for
'Selenium' on 'EP300−interacting inhibitor of differentiation 3'

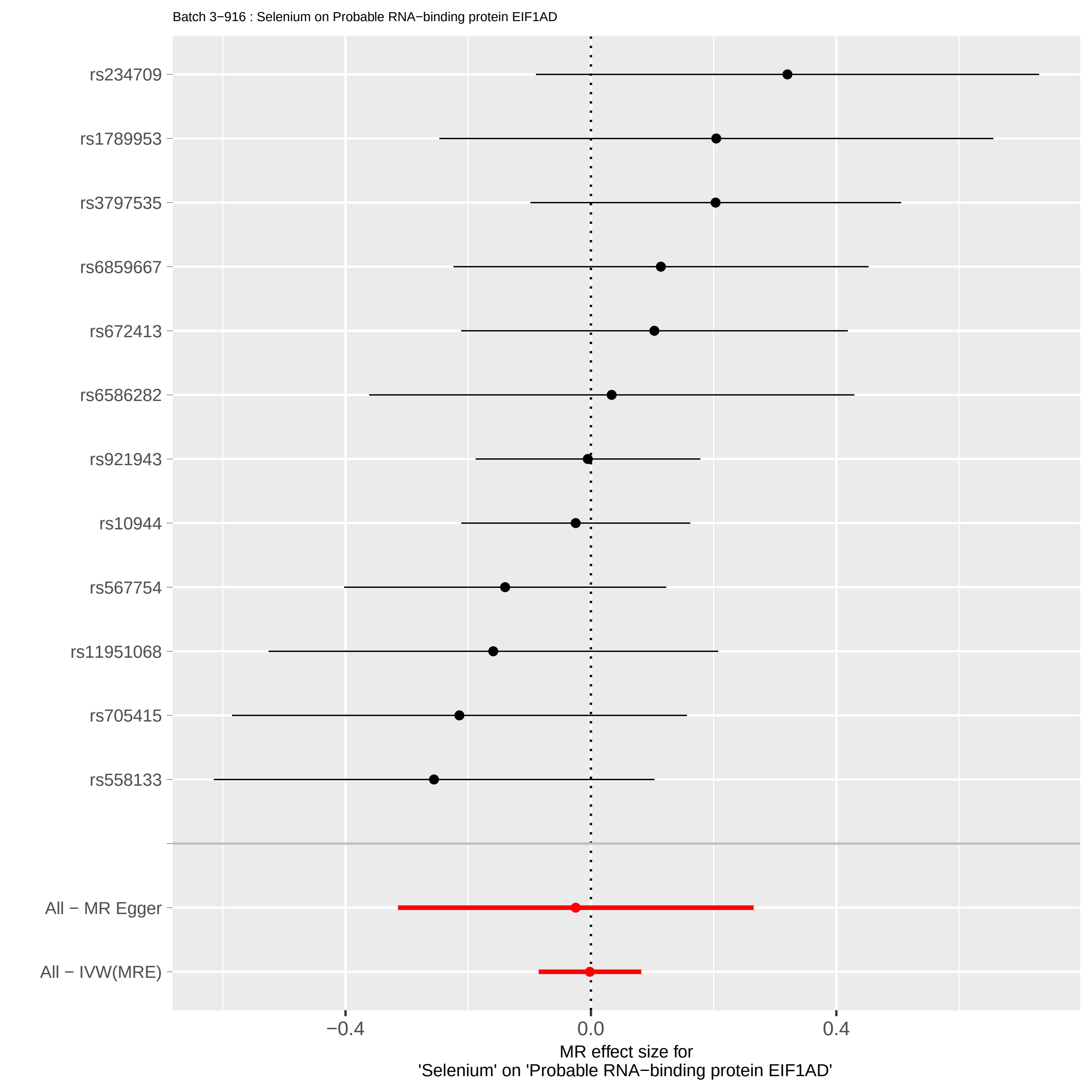

Batch 3−916 : Selenium on Probable RNA−binding protein EIF1AD
rs234709
rs1789953
rs3797535
rs6859667
rs672413
rs6586282
rs921943
rs10944
rs567754
rs11951068
rs705415
rs558133
All − MR Egger
All − IVW(MRE)
−0.4
0.0
0.4
MR effect size for
'Selenium' on 'Probable RNA−binding protein EIF1AD'

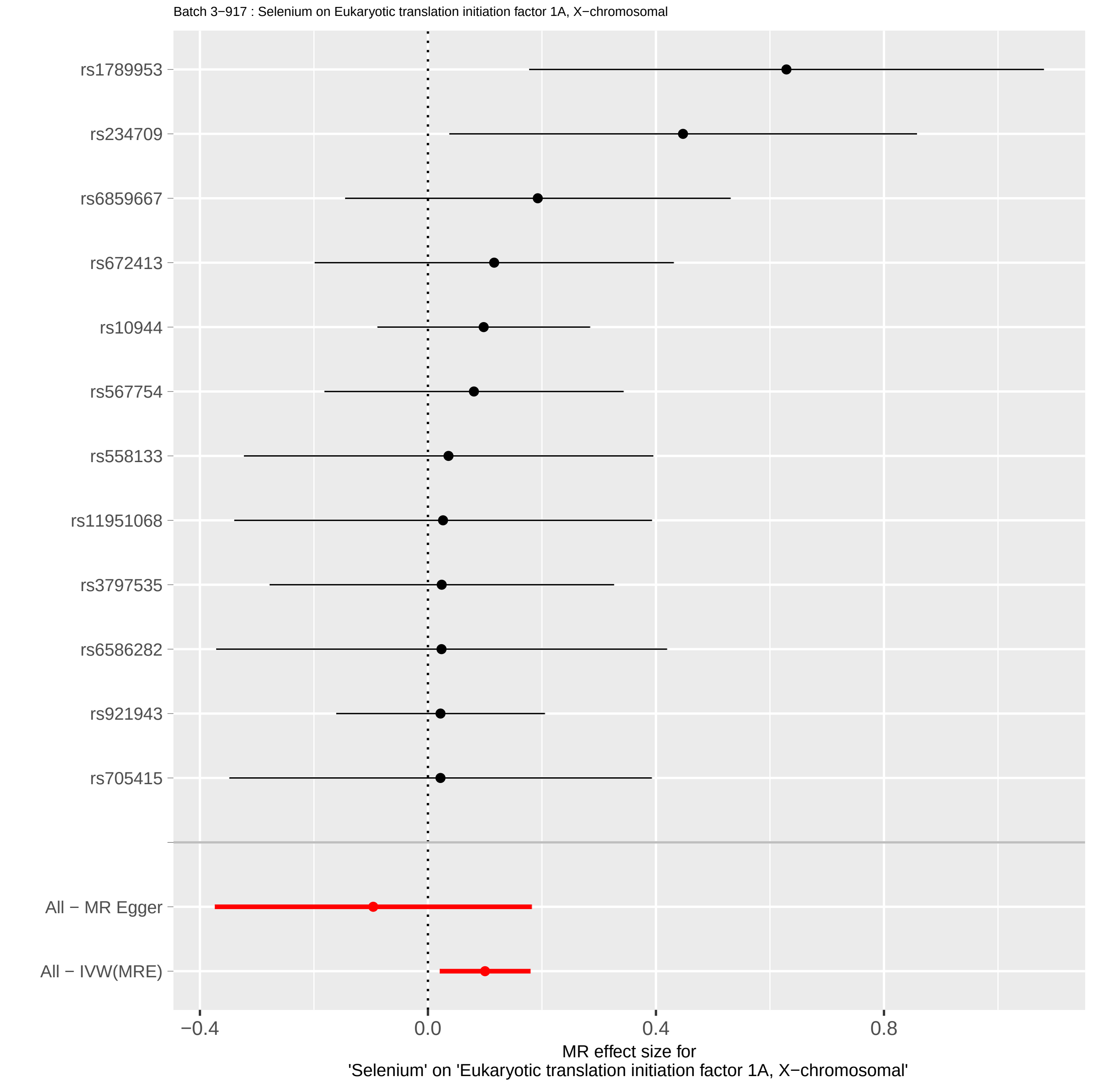

Batch 3−917 : Selenium on Eukaryotic translation initiation factor 1A, X−chromosomal
rs1789953
rs234709
rs6859667
rs672413
rs10944
rs567754
rs558133
rs11951068
rs3797535
rs6586282
rs921943
rs705415
All − MR Egger
All − IVW(MRE)
−0.4
0.0
0.4
0.8
MR effect size for
'Selenium' on 'Eukaryotic translation initiation factor 1A, X−chromosomal'

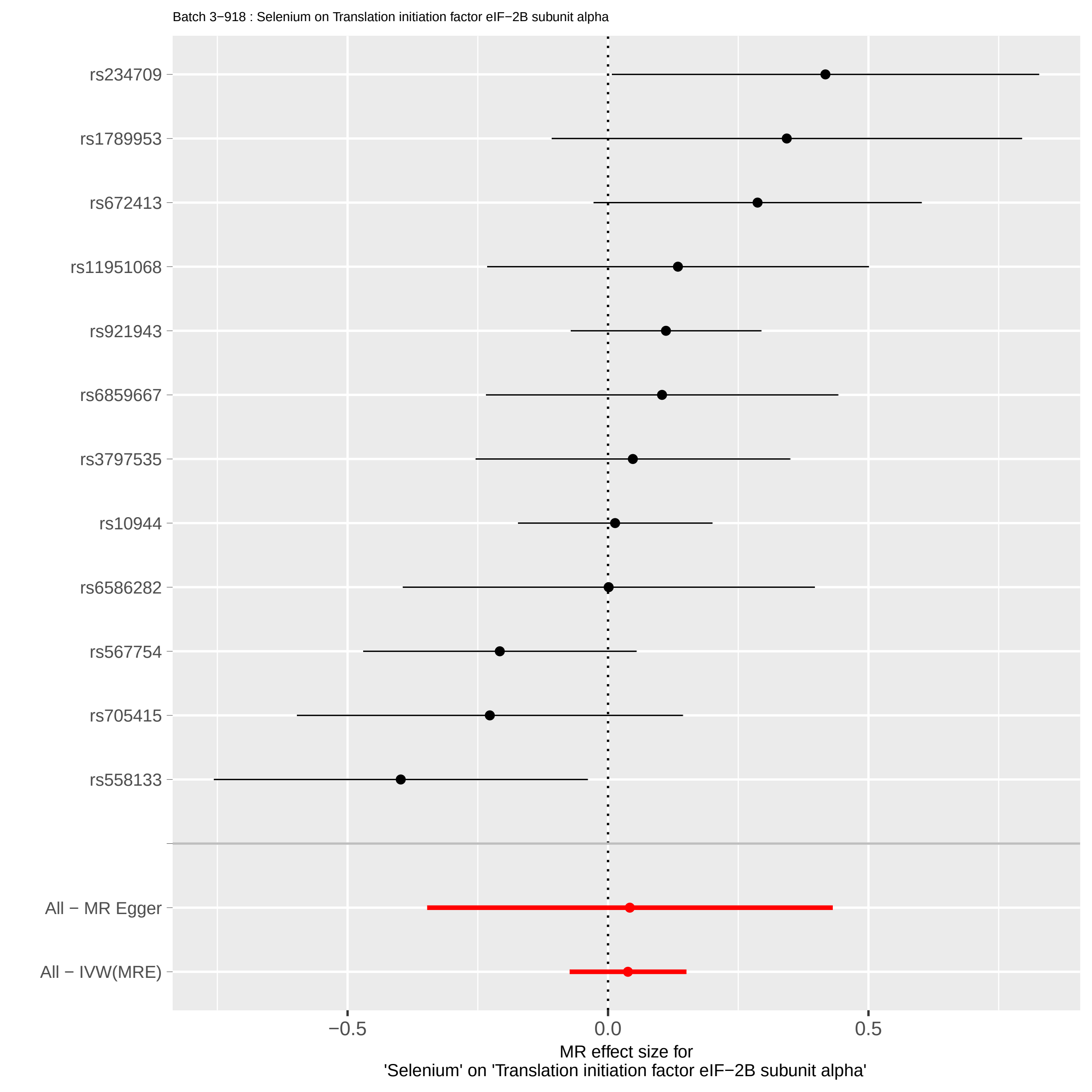
Batch 3−918 : Selenium on Translation initiation factor eIF−2B subunit alpha
rs234709
rs1789953
rs672413
rs11951068
rs921943
rs6859667
rs3797535
rs10944
rs6586282
rs567754
rs705415
rs558133
All − MR Egger
All − IVW(MRE)
−0.5
0.0
0.5
MR effect size for
'Selenium' on 'Translation initiation factor eIF−2B subunit alpha'

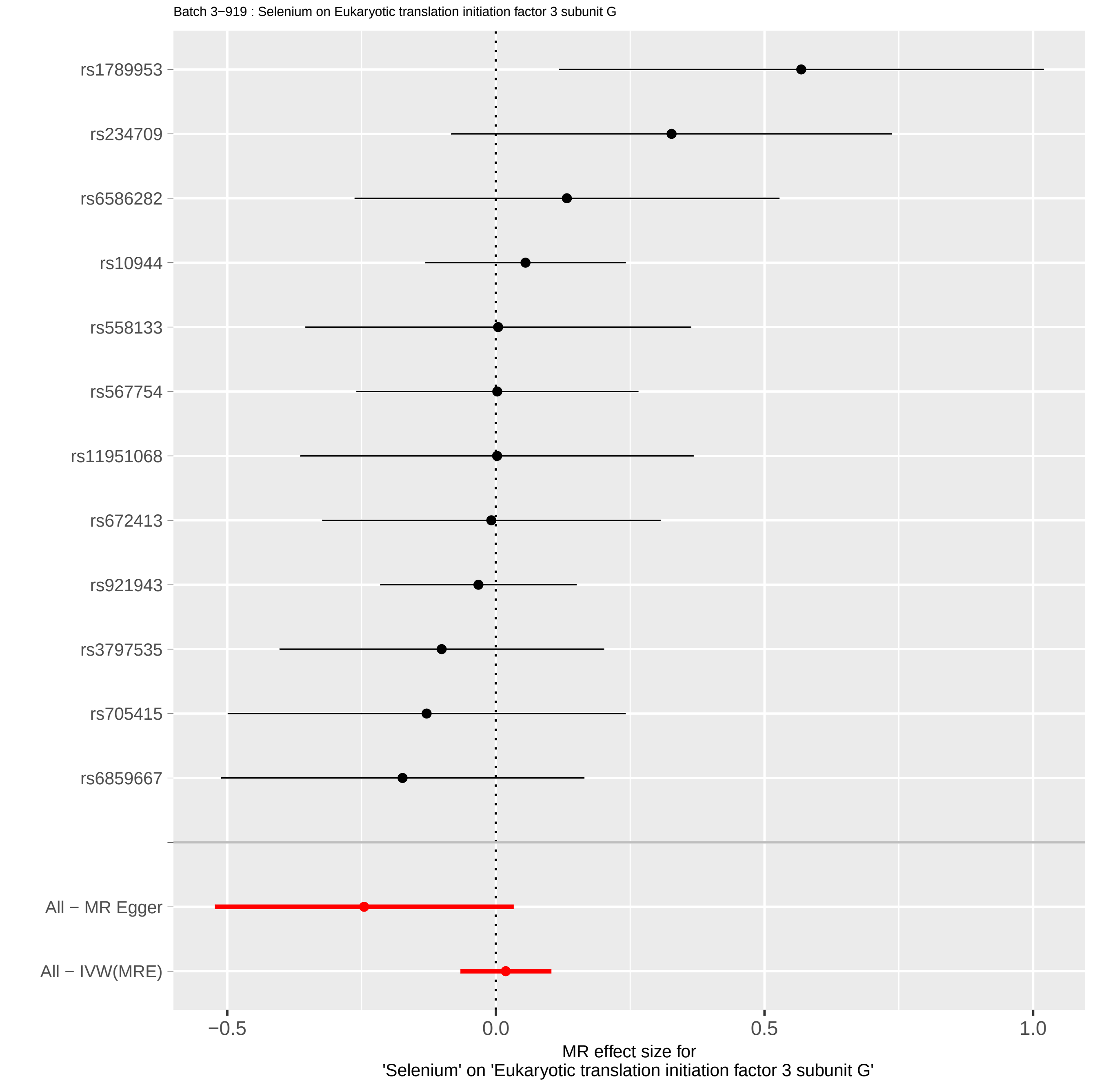
Batch 3−919 : Selenium on Eukaryotic translation initiation factor 3 subunit G
rs1789953
rs234709
rs6586282
rs10944
rs558133
rs567754
rs11951068
rs672413
rs921943
rs3797535
rs705415
rs6859667
All − MR Egger
All − IVW(MRE)
−0.5
0.0
0.5
1.0
MR effect size for
'Selenium' on 'Eukaryotic translation initiation factor 3 subunit G'

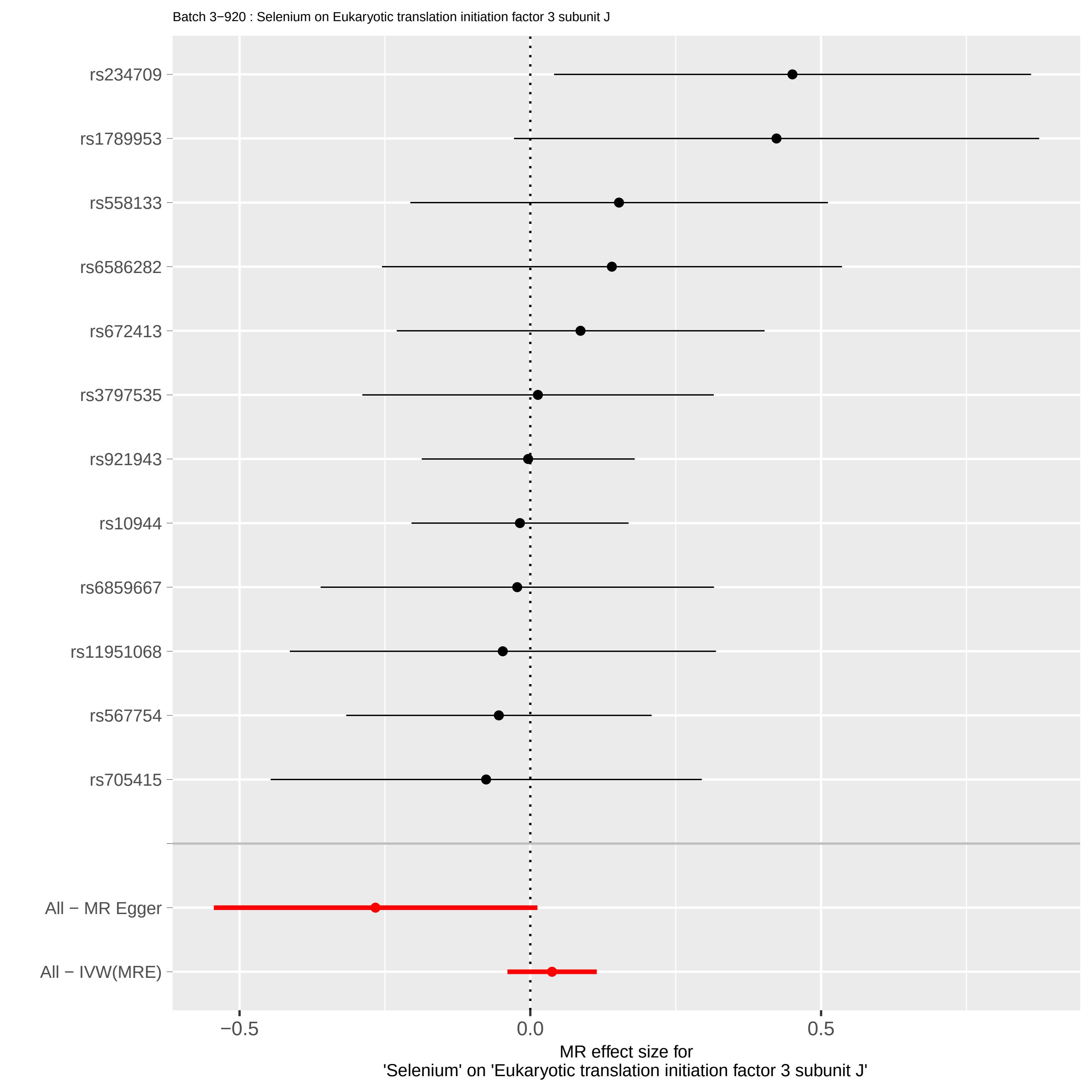

Batch 3−920 : Selenium on Eukaryotic translation initiation factor 3 subunit J
rs234709
rs1789953
rs558133
rs6586282
rs672413
rs3797535
rs921943
rs10944
rs6859667
rs11951068
rs567754
rs705415
All − MR Egger
All − IVW(MRE)
−0.5
0.0
0.5
MR effect size for
'Selenium' on 'Eukaryotic translation initiation factor 3 subunit J'

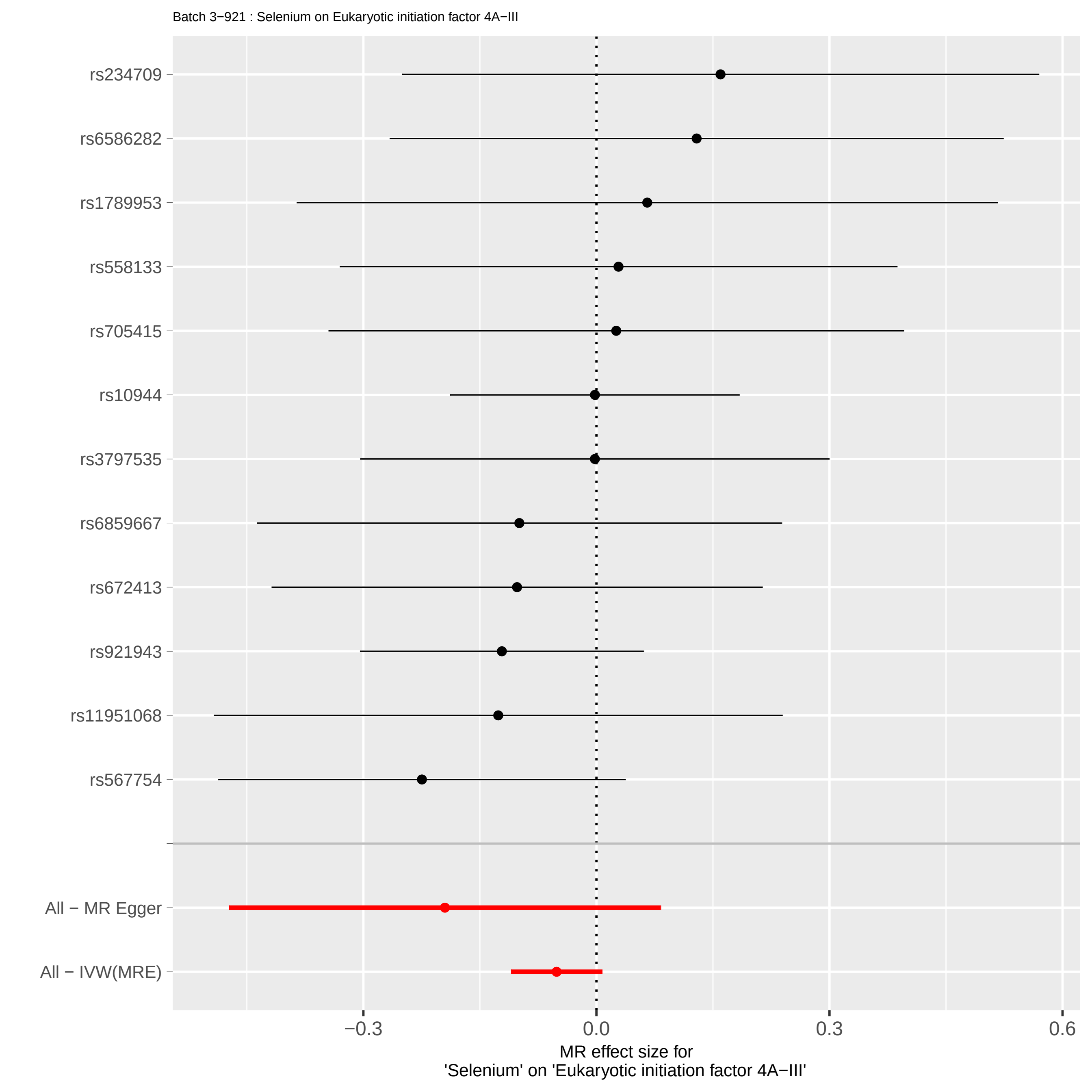
Batch 3−921 : Selenium on Eukaryotic initiation factor 4A−III
rs234709
rs6586282
rs1789953
rs558133
rs705415
rs10944
rs3797535
rs6859667
rs672413
rs921943
rs11951068
rs567754
All − MR Egger
All − IVW(MRE)
−0.3
0.0
0.3
0.6
MR effect size for
'Selenium' on 'Eukaryotic initiation factor 4A−III'

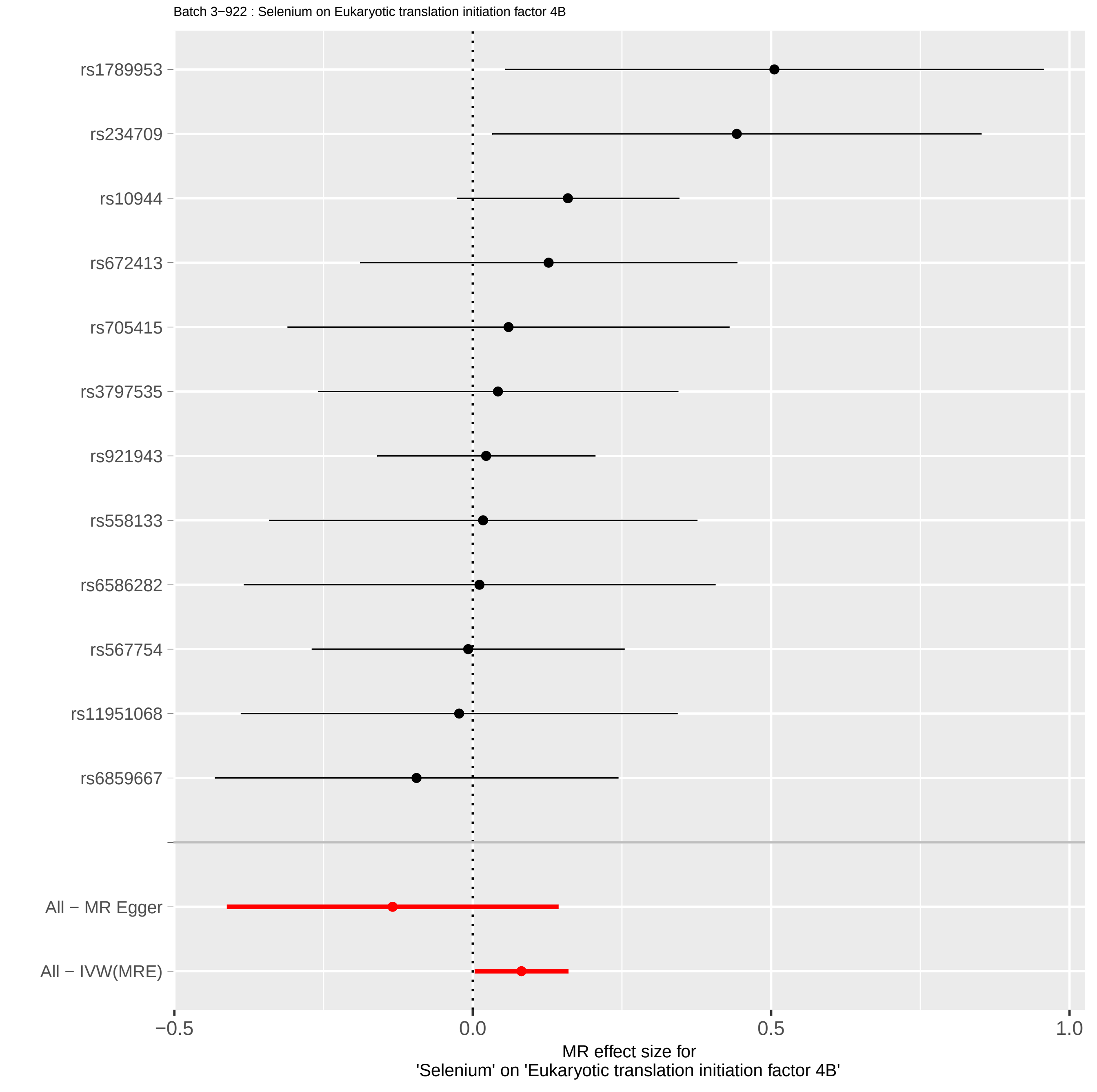

Batch 3−922 : Selenium on Eukaryotic translation initiation factor 4B
rs1789953
rs234709
rs10944
rs672413
rs705415
rs3797535
rs921943
rs558133
rs6586282
rs567754
rs11951068
rs6859667
All − MR Egger
All − IVW(MRE)
−0.5
0.0
0.5
1.0
MR effect size for
'Selenium' on 'Eukaryotic translation initiation factor 4B'

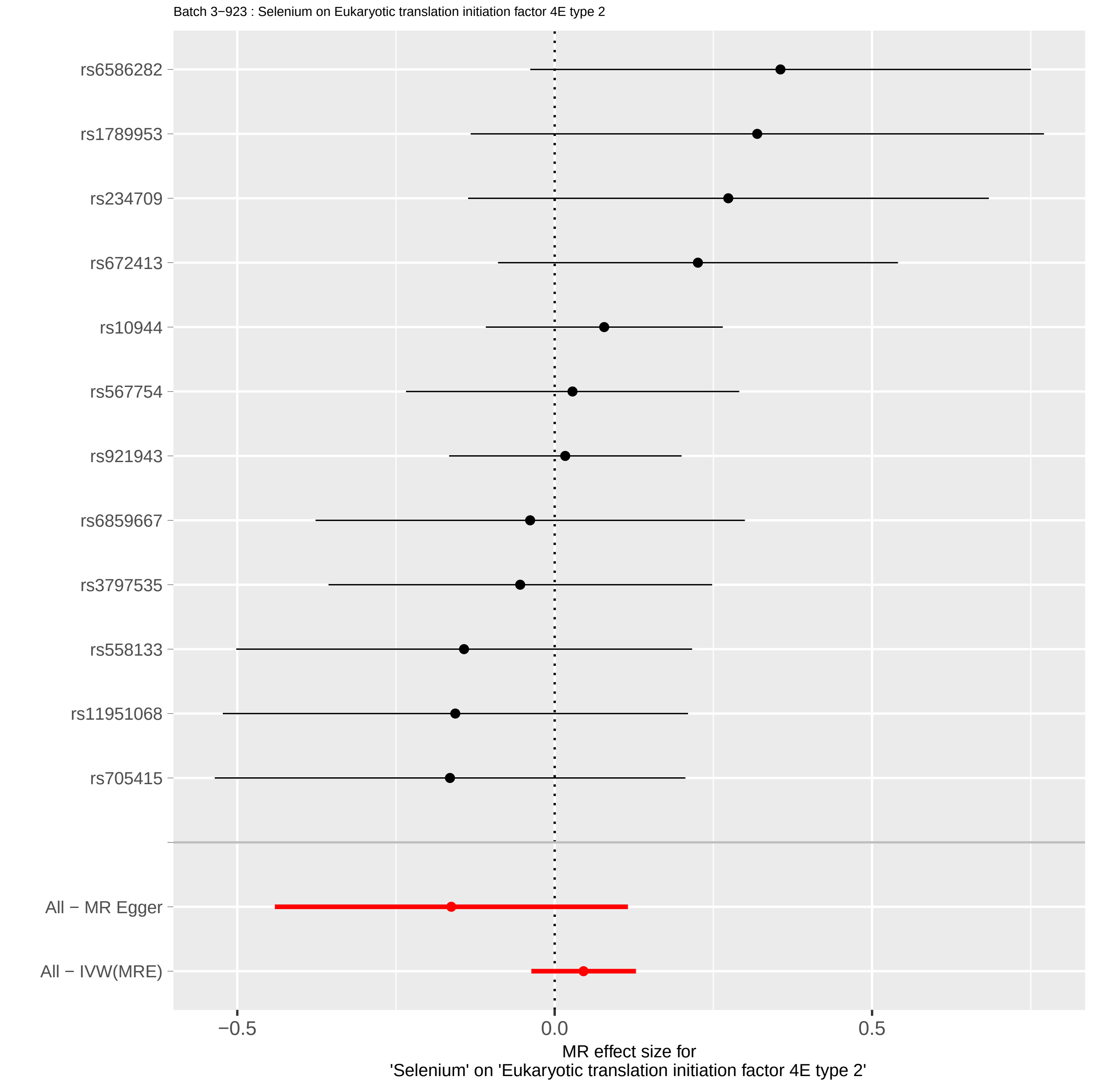
Batch 3−923 : Selenium on Eukaryotic translation initiation factor 4E type 2
rs6586282
rs1789953
rs234709
rs672413
rs10944
rs567754
rs921943
rs6859667
rs3797535
rs558133
rs11951068
rs705415
All − MR Egger
All − IVW(MRE)
−0.5
0.0
0.5
MR effect size for
'Selenium' on 'Eukaryotic translation initiation factor 4E type 2'

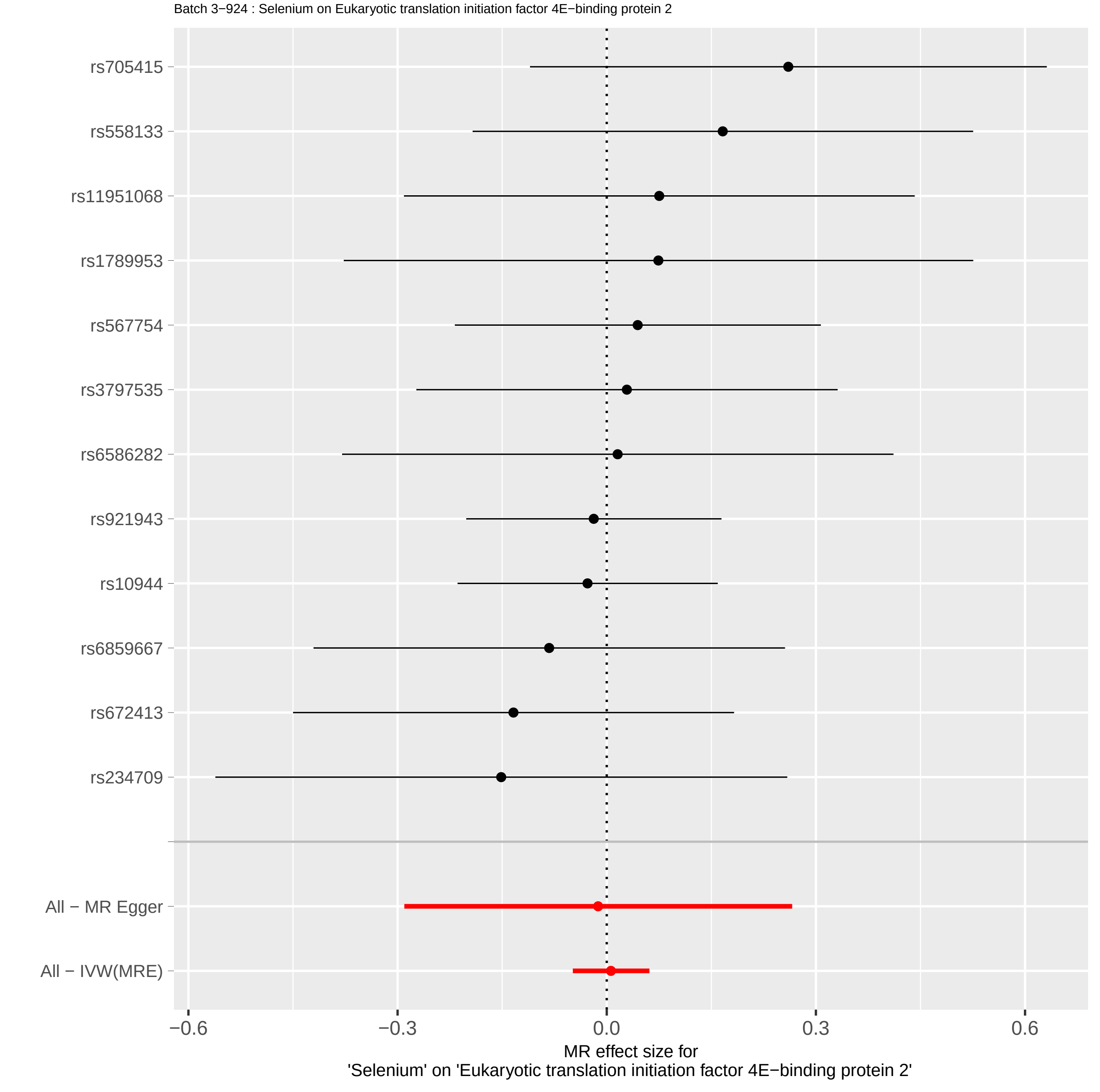
Batch 3−924 : Selenium on Eukaryotic translation initiation factor 4E−binding protein 2
rs705415
rs558133
rs11951068
rs1789953
rs567754
rs3797535
rs6586282
rs921943
rs10944
rs6859667
rs672413
rs234709
All − MR Egger
All − IVW(MRE)
−0.6
−0.3
0.0
0.3
0.6
MR effect size for
'Selenium' on 'Eukaryotic translation initiation factor 4E−binding protein 2'

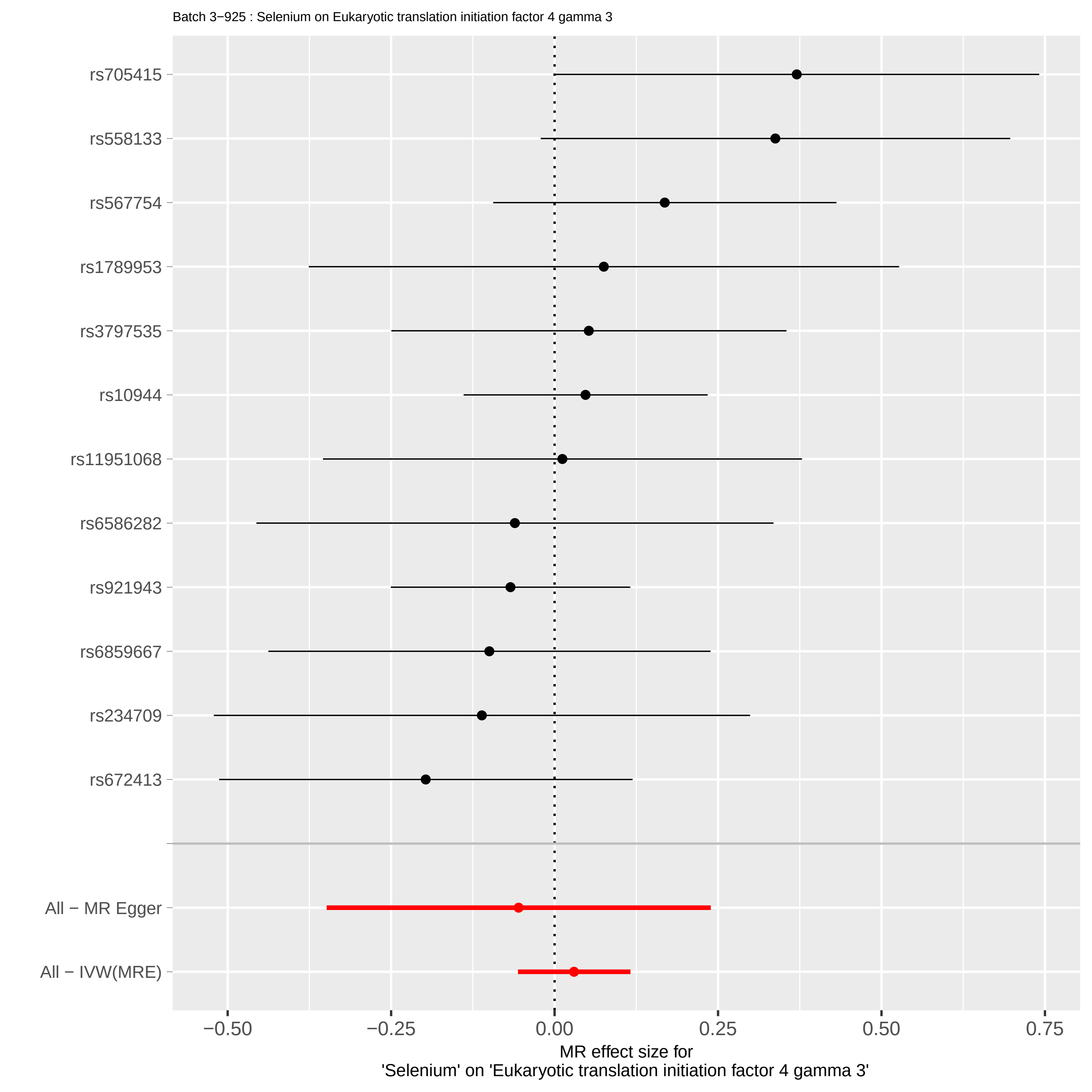
Batch 3−925 : Selenium on Eukaryotic translation initiation factor 4 gamma 3
rs705415
rs558133
rs567754
rs1789953
rs3797535
rs10944
rs11951068
rs6586282
rs921943
rs6859667
rs234709
rs672413
All − MR Egger
All − IVW(MRE)
−0.50
−0.25
0.00
0.25
0.50
0.75
MR effect size for
'Selenium' on 'Eukaryotic translation initiation factor 4 gamma 3'

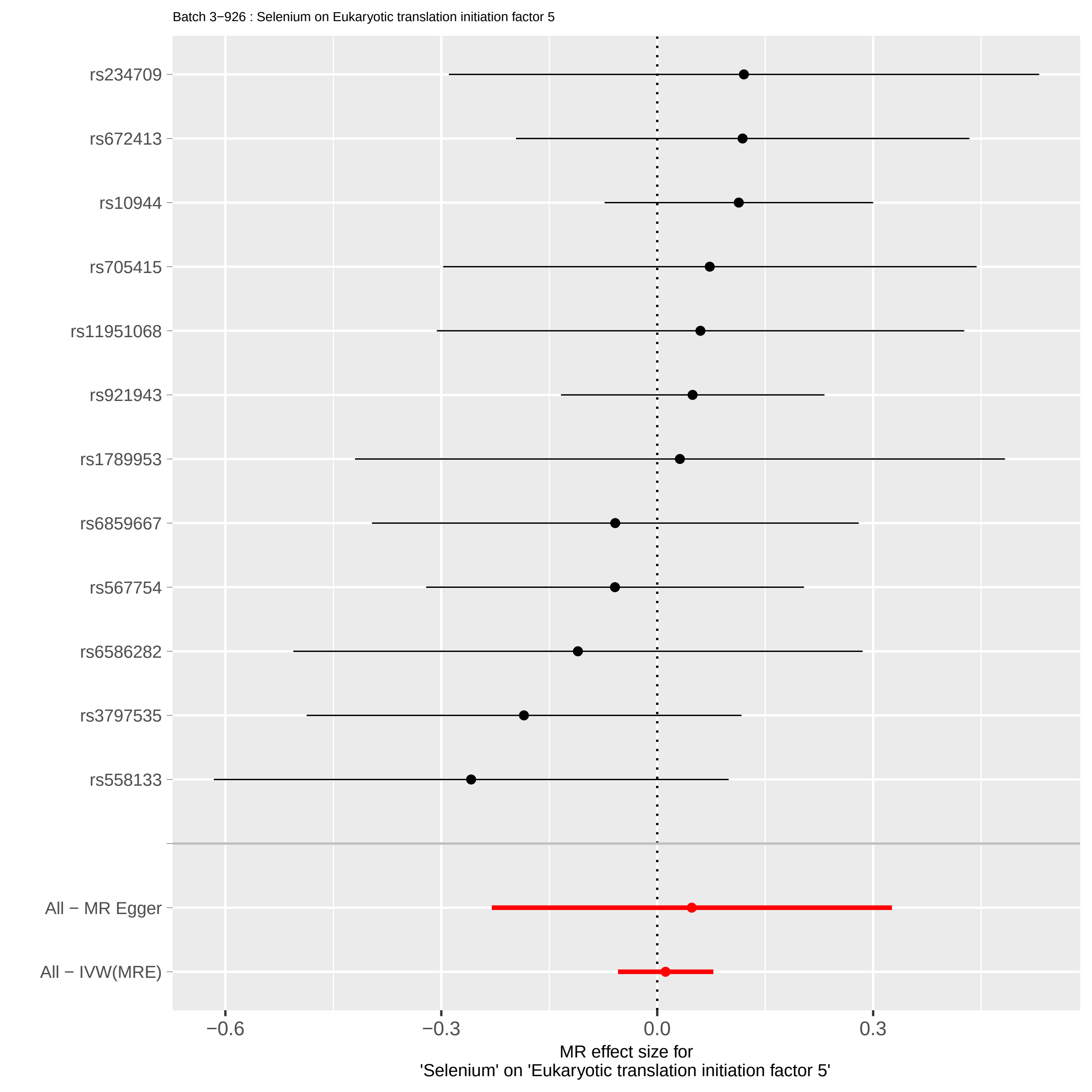

Batch 3−926 : Selenium on Eukaryotic translation initiation factor 5
rs234709
rs672413
rs10944
rs705415
rs11951068
rs921943
rs1789953
rs6859667
rs567754
rs6586282
rs3797535
rs558133
All − MR Egger
All − IVW(MRE)
−0.6
−0.3
0.0
0.3
MR effect size for
'Selenium' on 'Eukaryotic translation initiation factor 5'

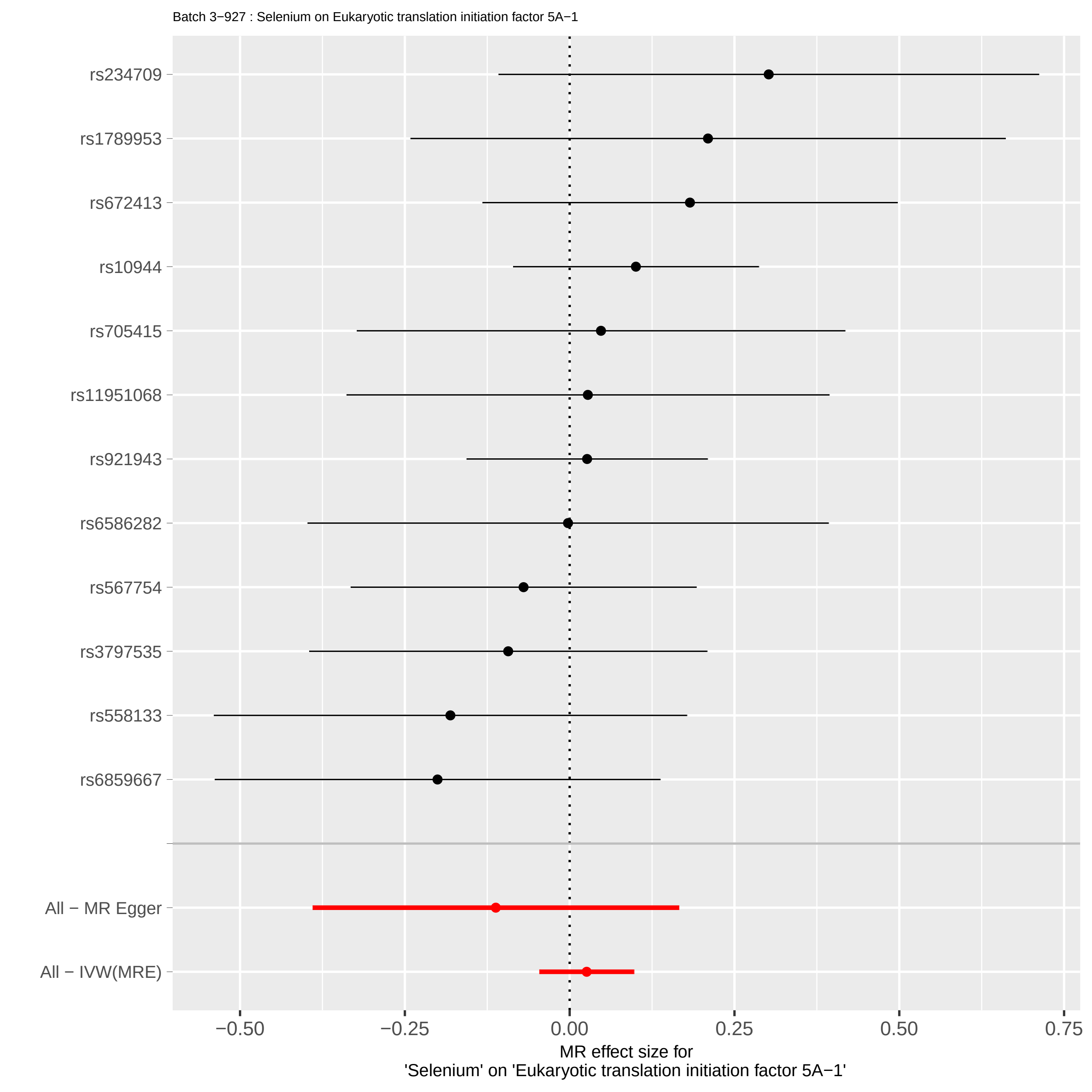
Batch 3−927 : Selenium on Eukaryotic translation initiation factor 5A−1
rs234709
rs1789953
rs672413
rs10944
rs705415
rs11951068
rs921943
rs6586282
rs567754
rs3797535
rs558133
rs6859667
All − MR Egger
All − IVW(MRE)
−0.50
−0.25
0.00
0.25
0.50
0.75
MR effect size for
'Selenium' on 'Eukaryotic translation initiation factor 5A−1'

Batch 3−928 : Selenium on ELAV−like protein 1

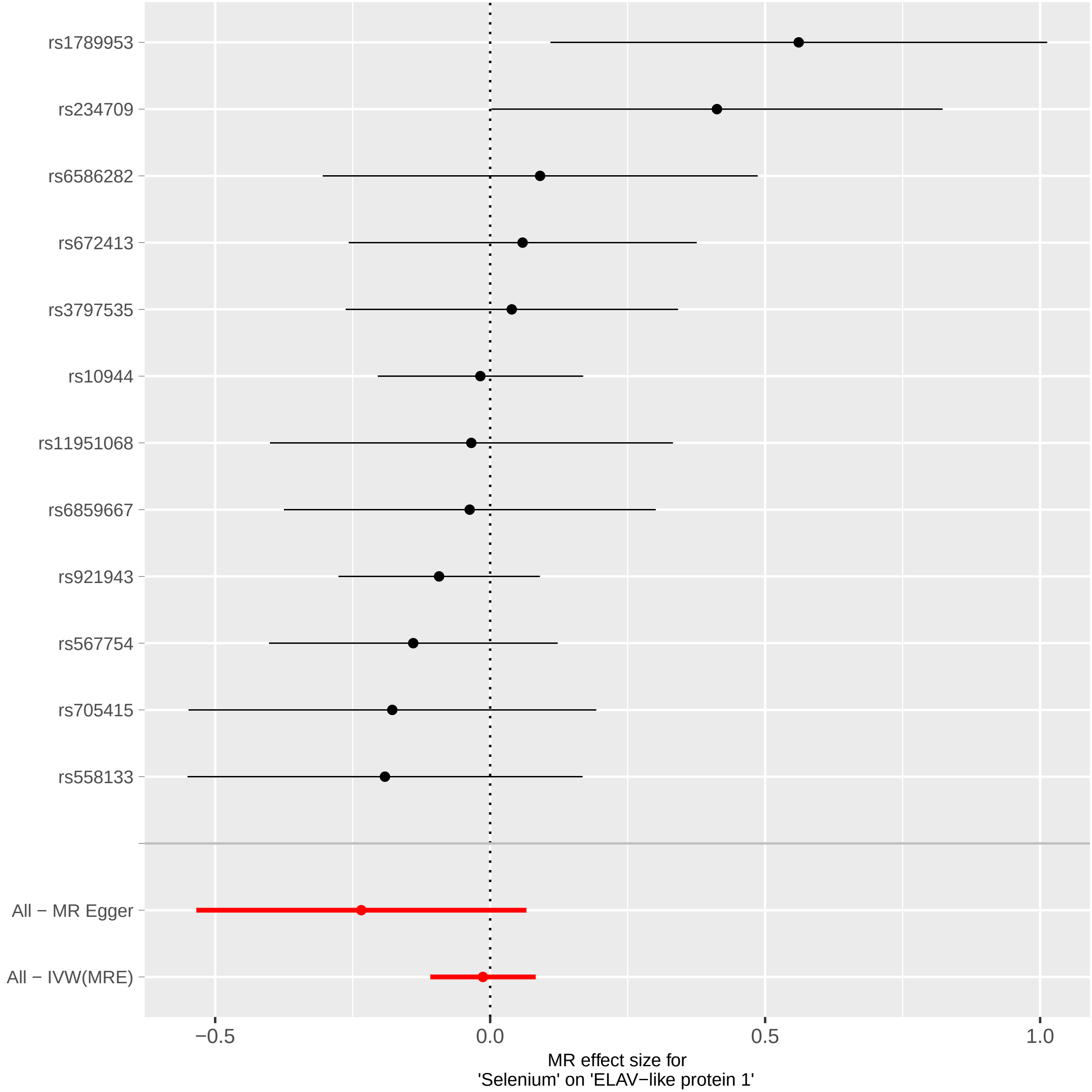

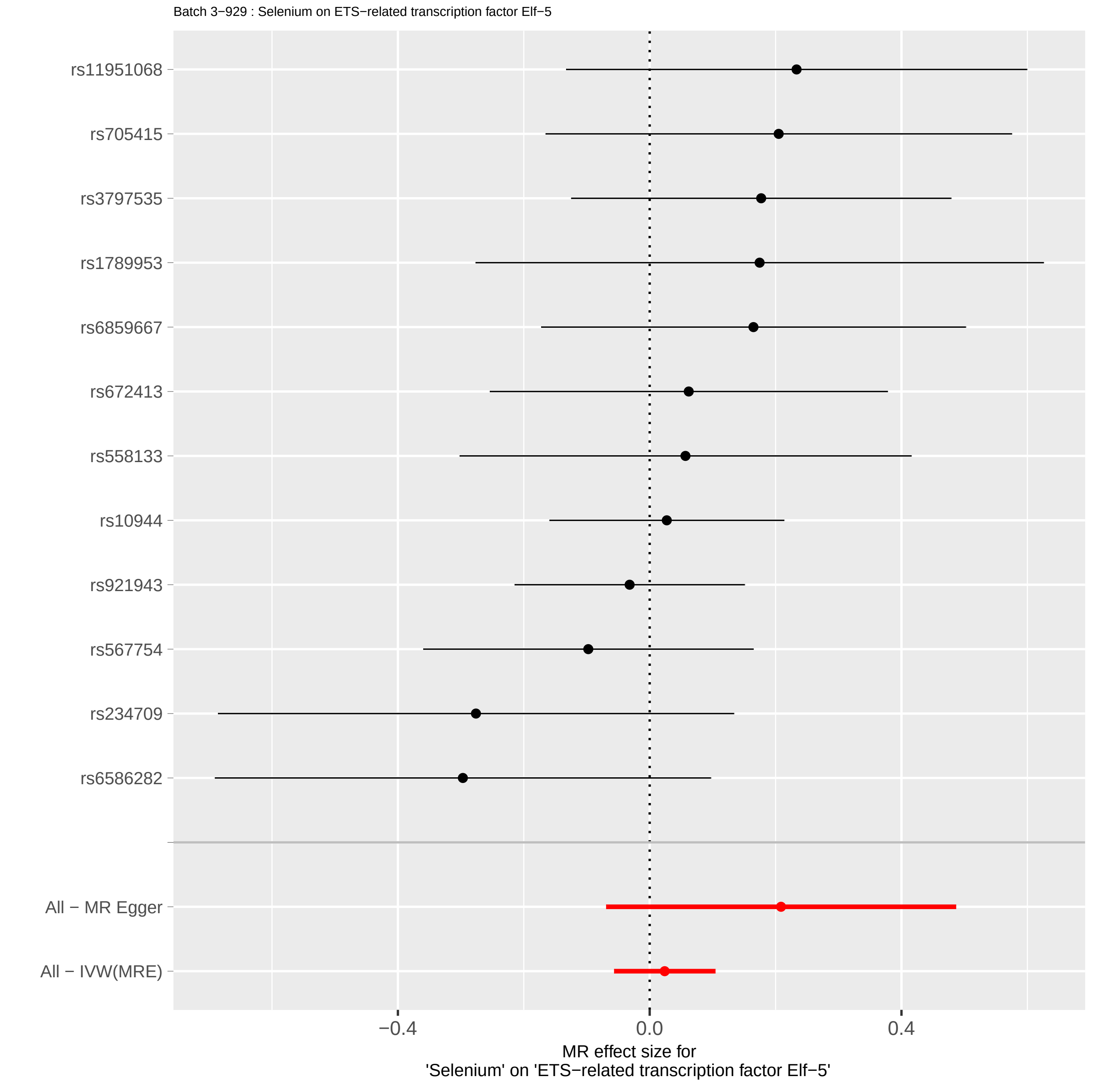

Batch 3−929 : Selenium on ETS−related transcription factor Elf−5
rs11951068
rs705415
rs3797535
rs1789953
rs6859667
rs672413
rs558133
rs10944
rs921943
rs567754
rs234709
rs6586282
All − MR Egger
All − IVW(MRE)
−0.4
0.0
0.4
MR effect size for
'Selenium' on 'ETS−related transcription factor Elf−5'

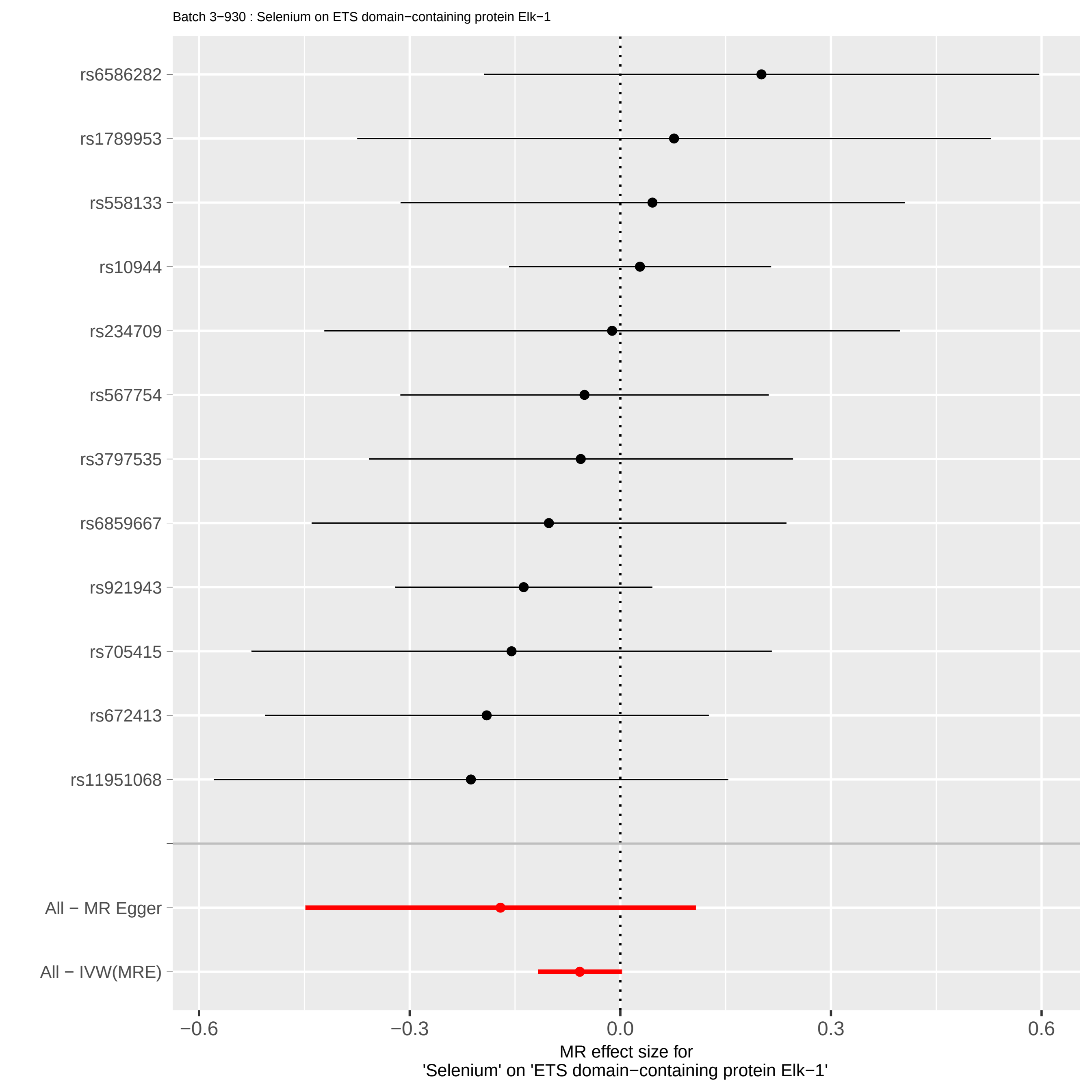
Batch 3−930 : Selenium on ETS domain−containing protein Elk−1
rs6586282
rs1789953
rs558133
rs10944
rs234709
rs567754
rs3797535
rs6859667
rs921943
rs705415
rs672413
rs11951068
All − MR Egger
All − IVW(MRE)
−0.6
−0.3
0.0
0.3
0.6
MR effect size for
'Selenium' on 'ETS domain−containing protein Elk−1'

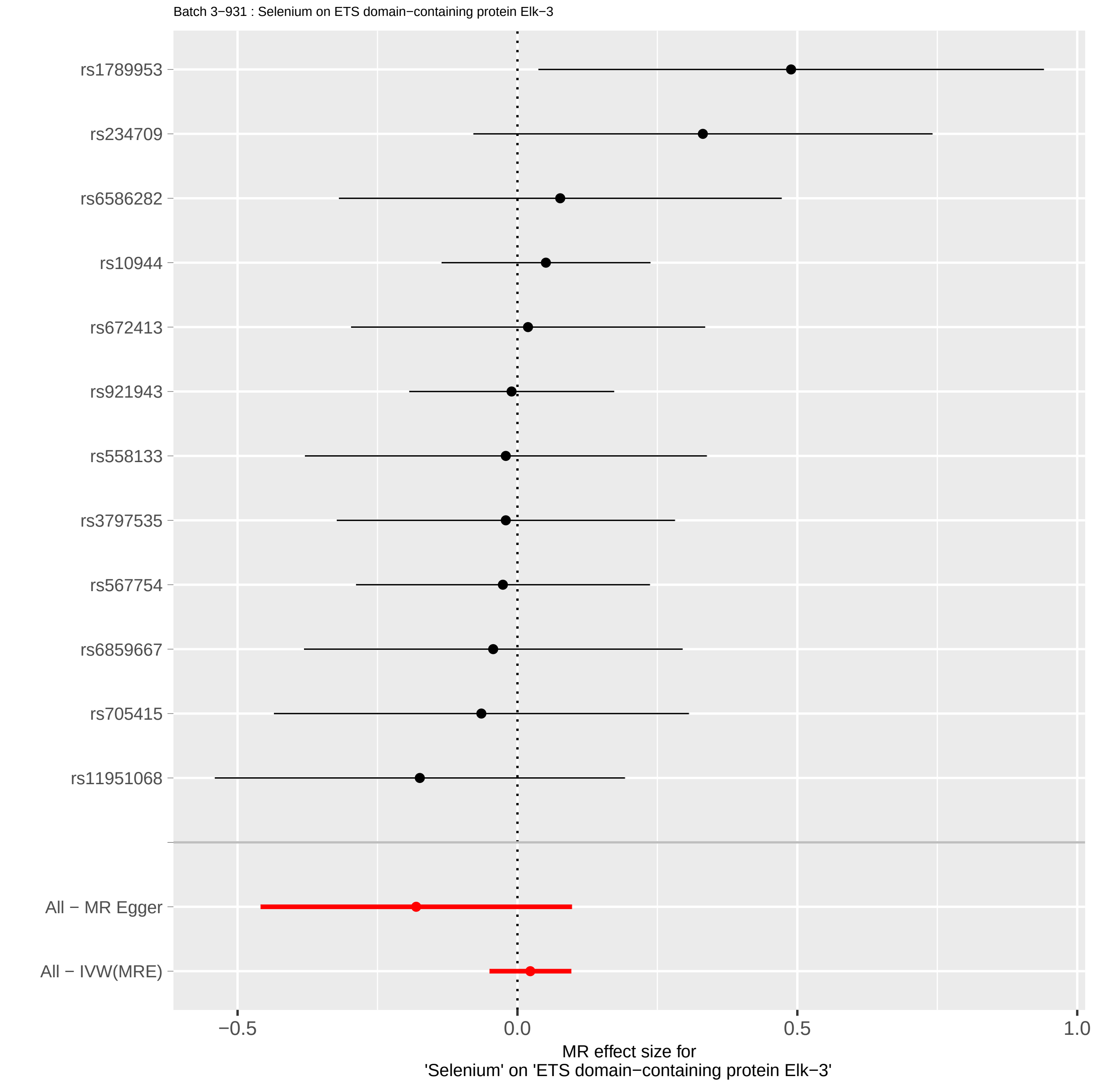

Batch 3−931 : Selenium on ETS domain−containing protein Elk−3
rs1789953
rs234709
rs6586282
rs10944
rs672413
rs921943
rs558133
rs3797535
rs567754
rs6859667
rs705415
rs11951068
All − MR Egger
All − IVW(MRE)
−0.5
0.0
0.5
1.0
MR effect size for
'Selenium' on 'ETS domain−containing protein Elk−3'

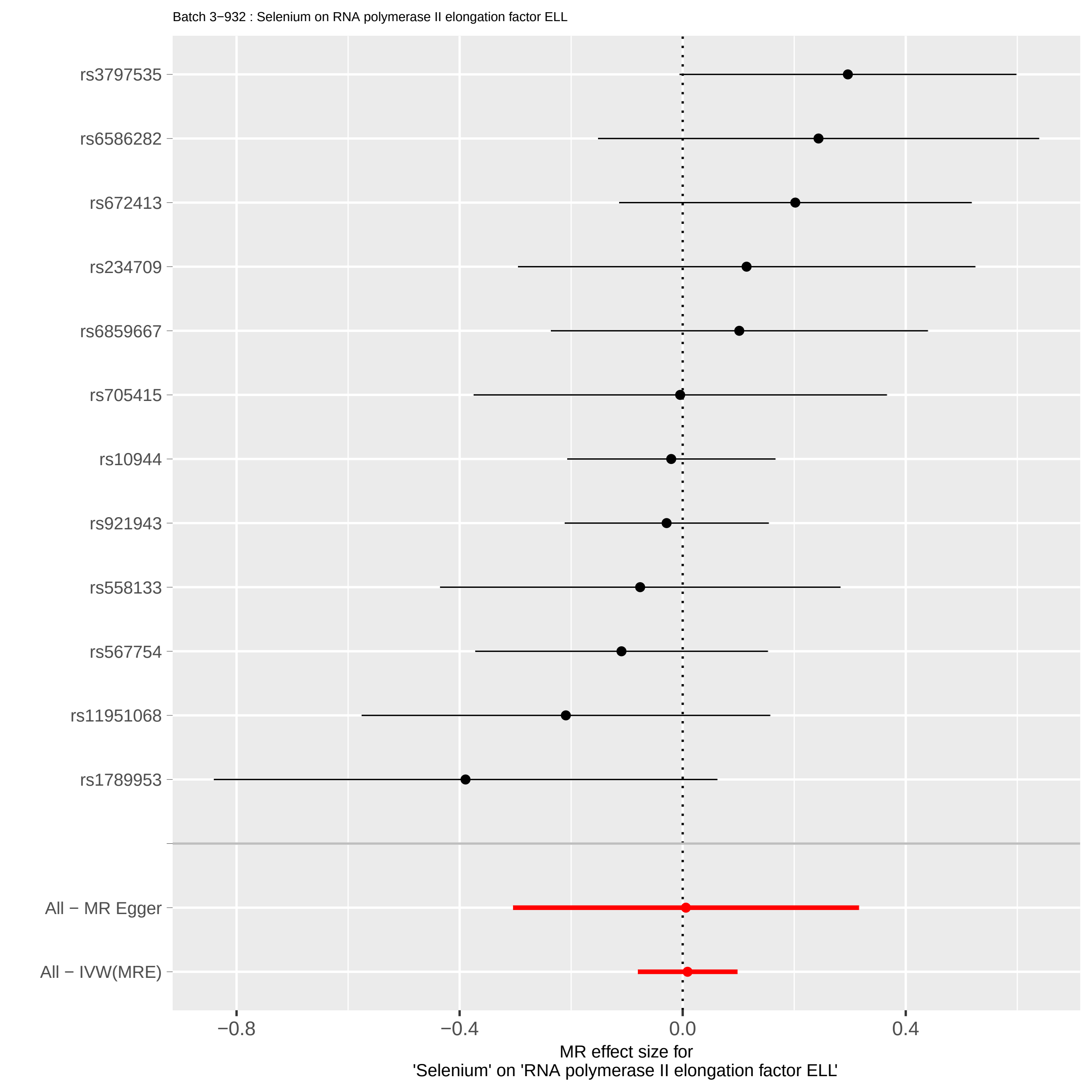
Batch 3−932 : Selenium on RNA polymerase II elongation factor ELL
rs3797535
rs6586282
rs672413
rs234709
rs6859667
rs705415
rs10944
rs921943
rs558133
rs567754
rs11951068
rs1789953
All − MR Egger
All − IVW(MRE)
−0.8
−0.4
0.0
0.4
MR effect size for
'Selenium' on 'RNA polymerase II elongation factor ELL'

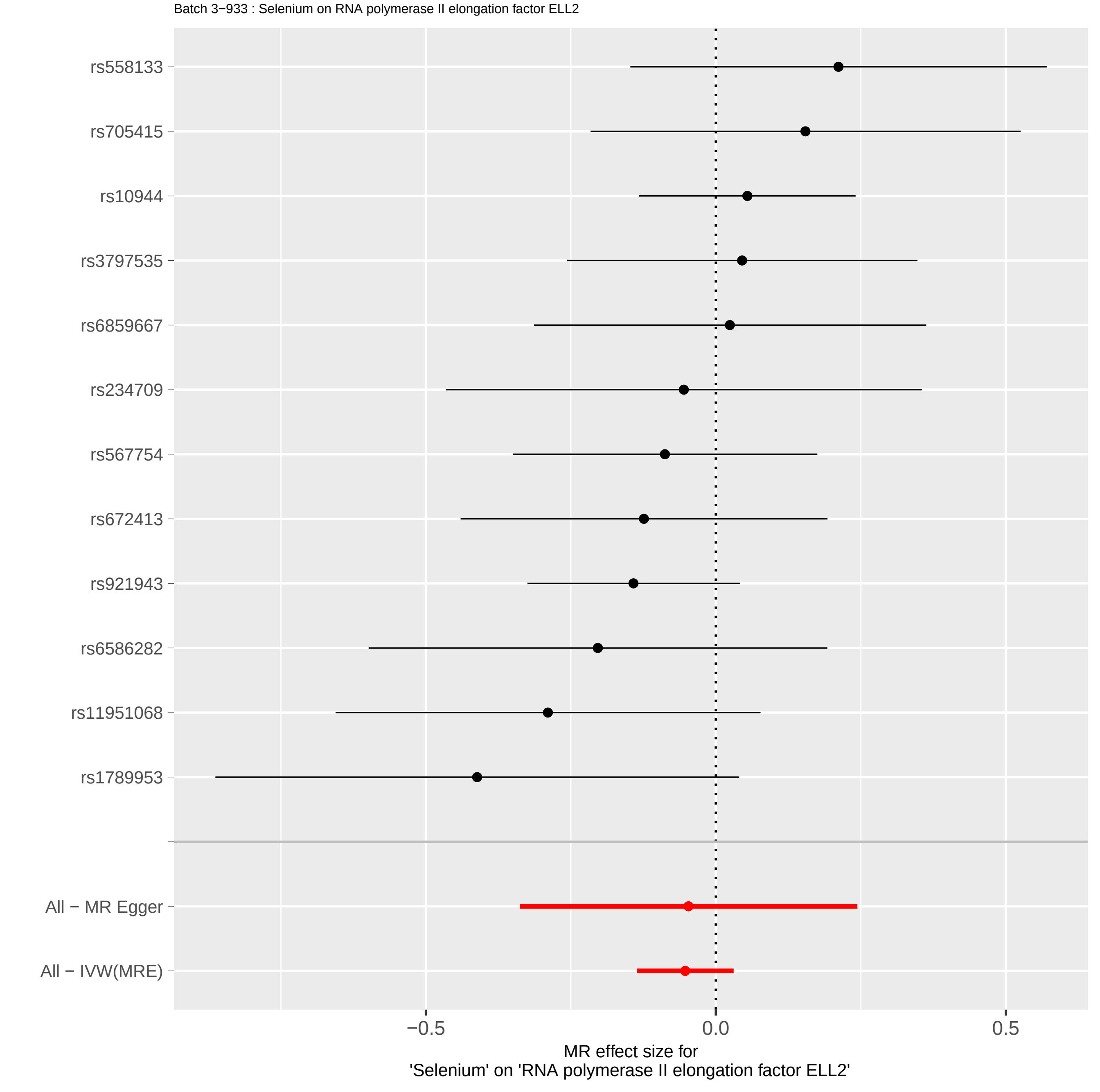
Batch 3−933 : Selenium on RNA polymerase II elongation factor ELL2
rs558133
rs705415
rs10944
rs3797535
rs6859667
rs234709
rs567754
rs672413
rs921943
rs6586282
rs11951068
rs1789953
All − MR Egger
All − IVW(MRE)
−0.5
0.0
0.5
MR effect size for
'Selenium' on 'RNA polymerase II elongation factor ELL2'

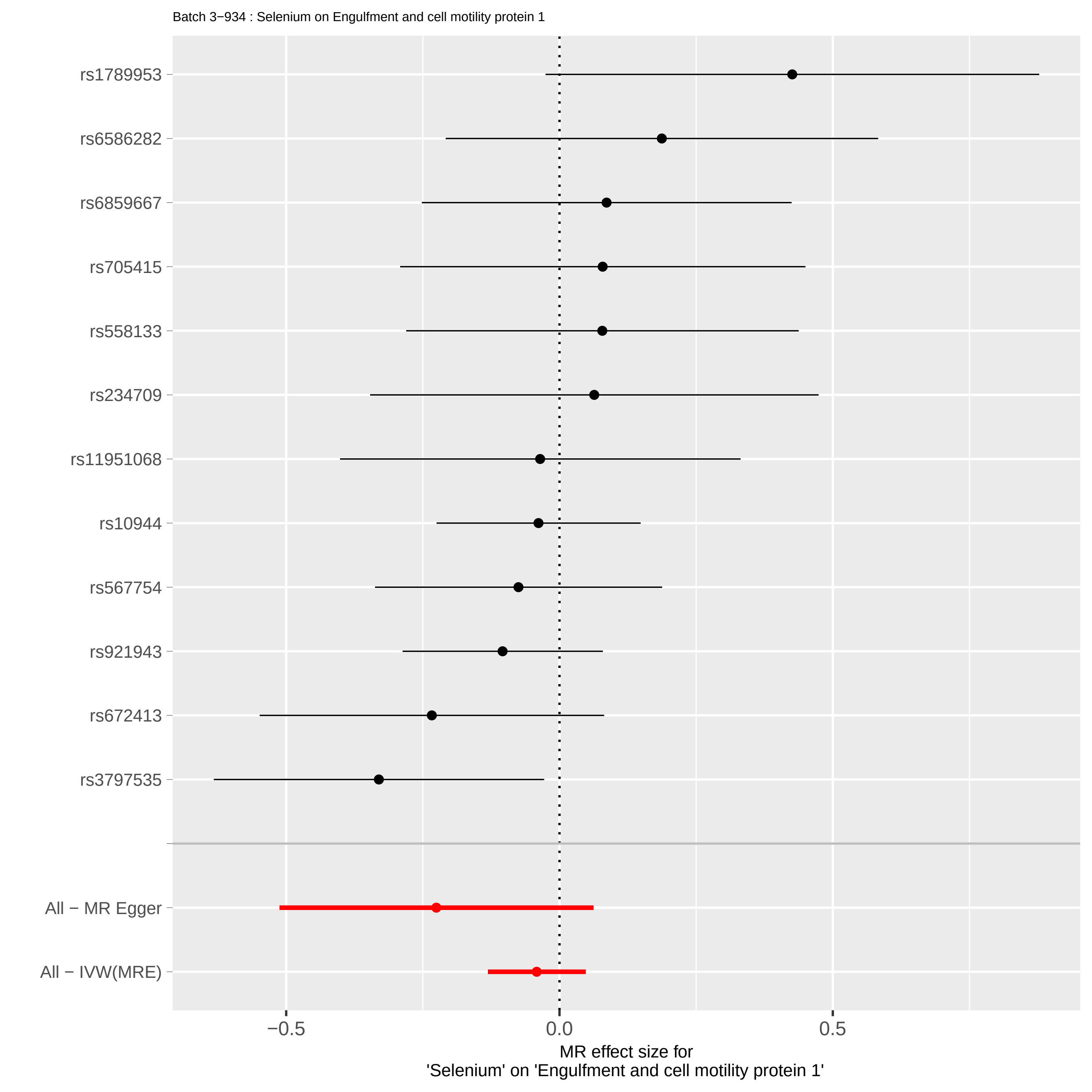

Batch 3−934 : Selenium on Engulfment and cell motility protein 1
rs1789953
rs6586282
rs6859667
rs705415
rs558133
rs234709
rs11951068
rs10944
rs567754
rs921943
rs672413
rs3797535
All − MR Egger
All − IVW(MRE)
−0.5
0.0
0.5
MR effect size for
'Selenium' on 'Engulfment and cell motility protein 1'

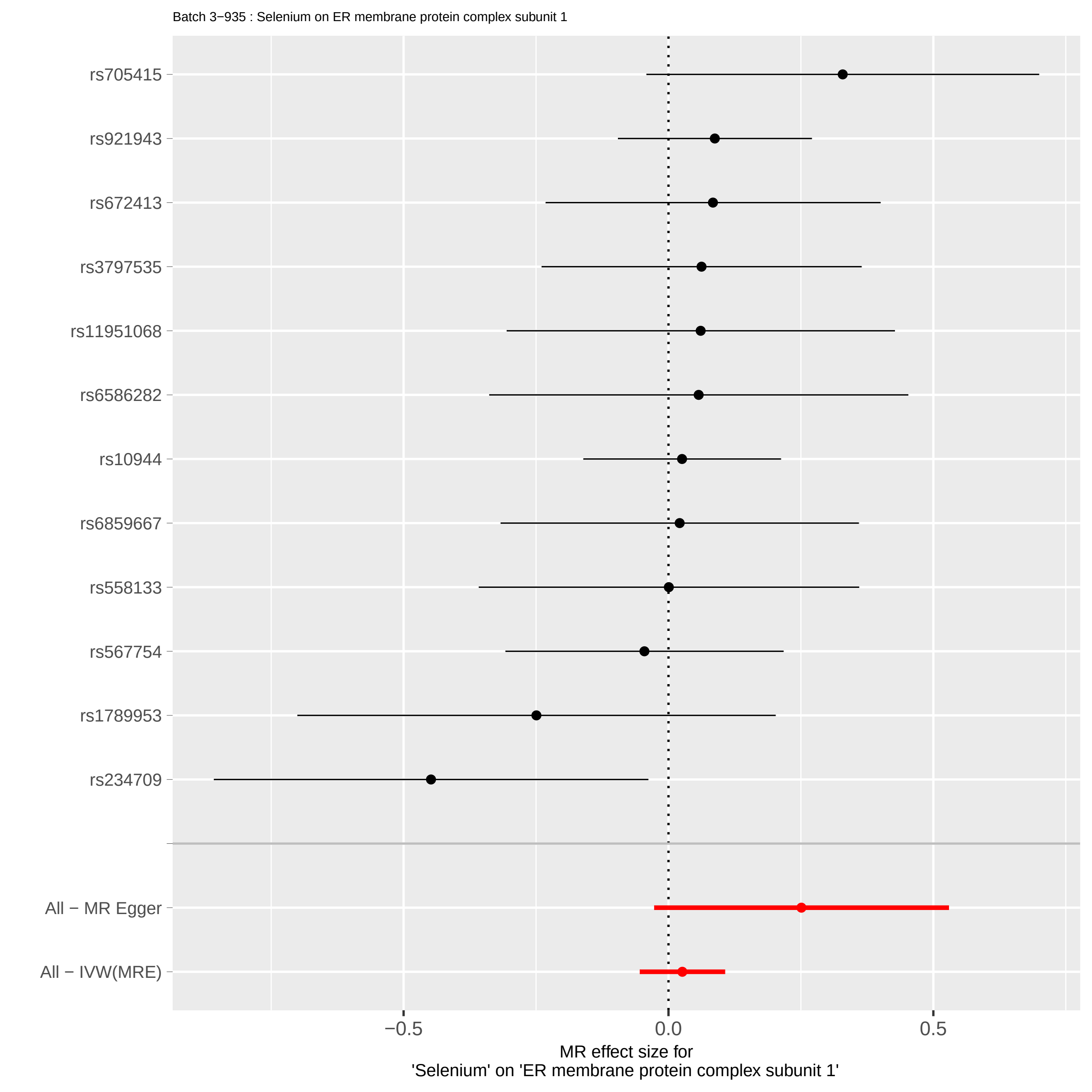

Batch 3−935 : Selenium on ER membrane protein complex subunit 1
rs705415
rs921943
rs672413
rs3797535
rs11951068
rs6586282
rs10944
rs6859667
rs558133
rs567754
rs1789953
rs234709
All − MR Egger
All − IVW(MRE)
−0.5
0.0
0.5
MR effect size for
'Selenium' on 'ER membrane protein complex subunit 1'

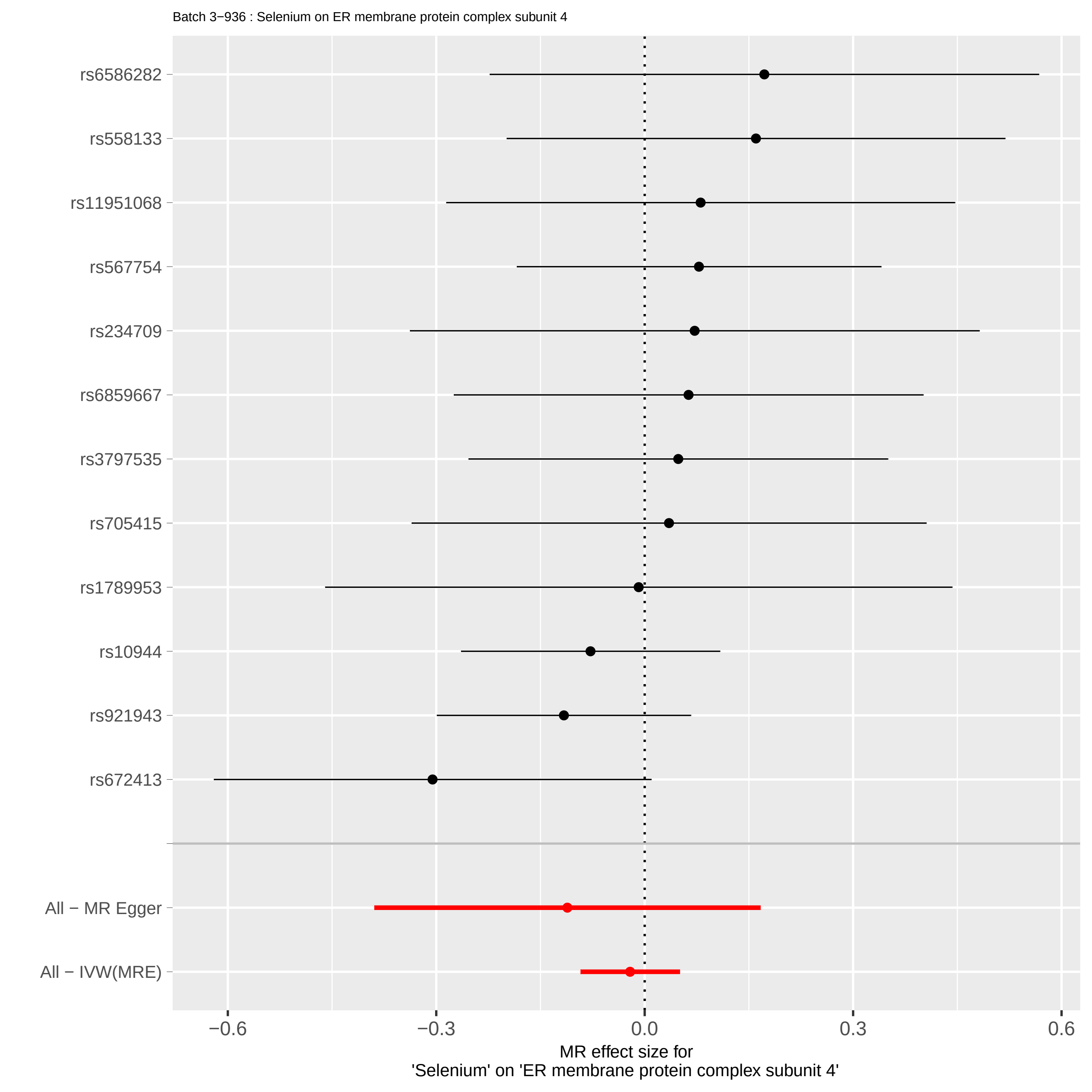
Batch 3−936 : Selenium on ER membrane protein complex subunit 4
rs6586282
rs558133
rs11951068
rs567754
rs234709
rs6859667
rs3797535
rs705415
rs1789953
rs10944
rs921943
rs672413
All − MR Egger
All − IVW(MRE)
−0.6
−0.3
0.0
0.3
0.6
MR effect size for
'Selenium' on 'ER membrane protein complex subunit 4'

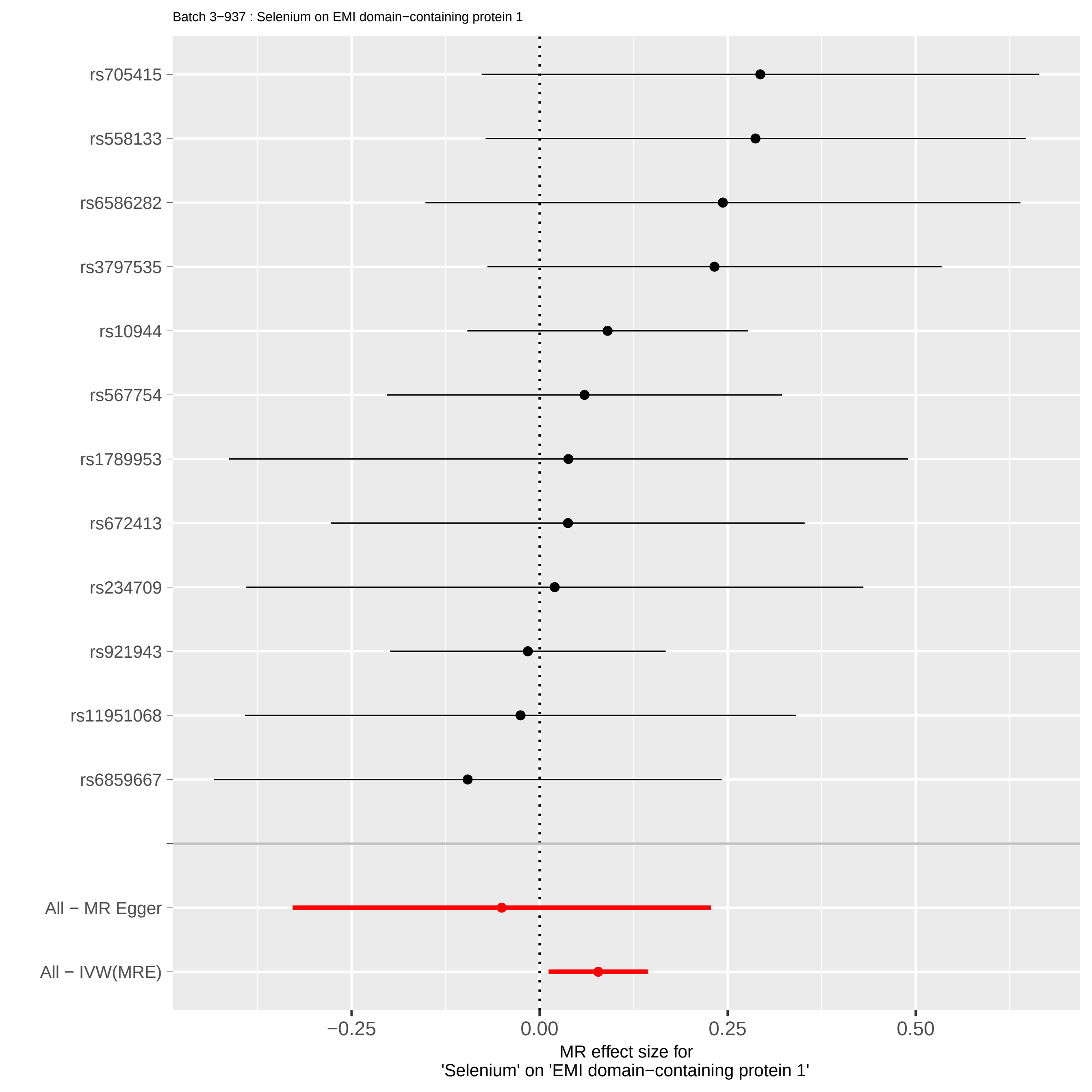
Batch 3−937 : Selenium on EMI domain−containing protein 1
rs705415
rs558133
rs6586282
rs3797535
rs10944
rs567754
rs1789953
rs672413
rs234709
rs921943
rs11951068
rs6859667
All − MR Egger
All − IVW(MRE)
−0.25
0.00
0.25
0.50
MR effect size for
'Selenium' on 'EMI domain−containing protein 1'

Batch 3−938 : Selenium on EMILIN−3

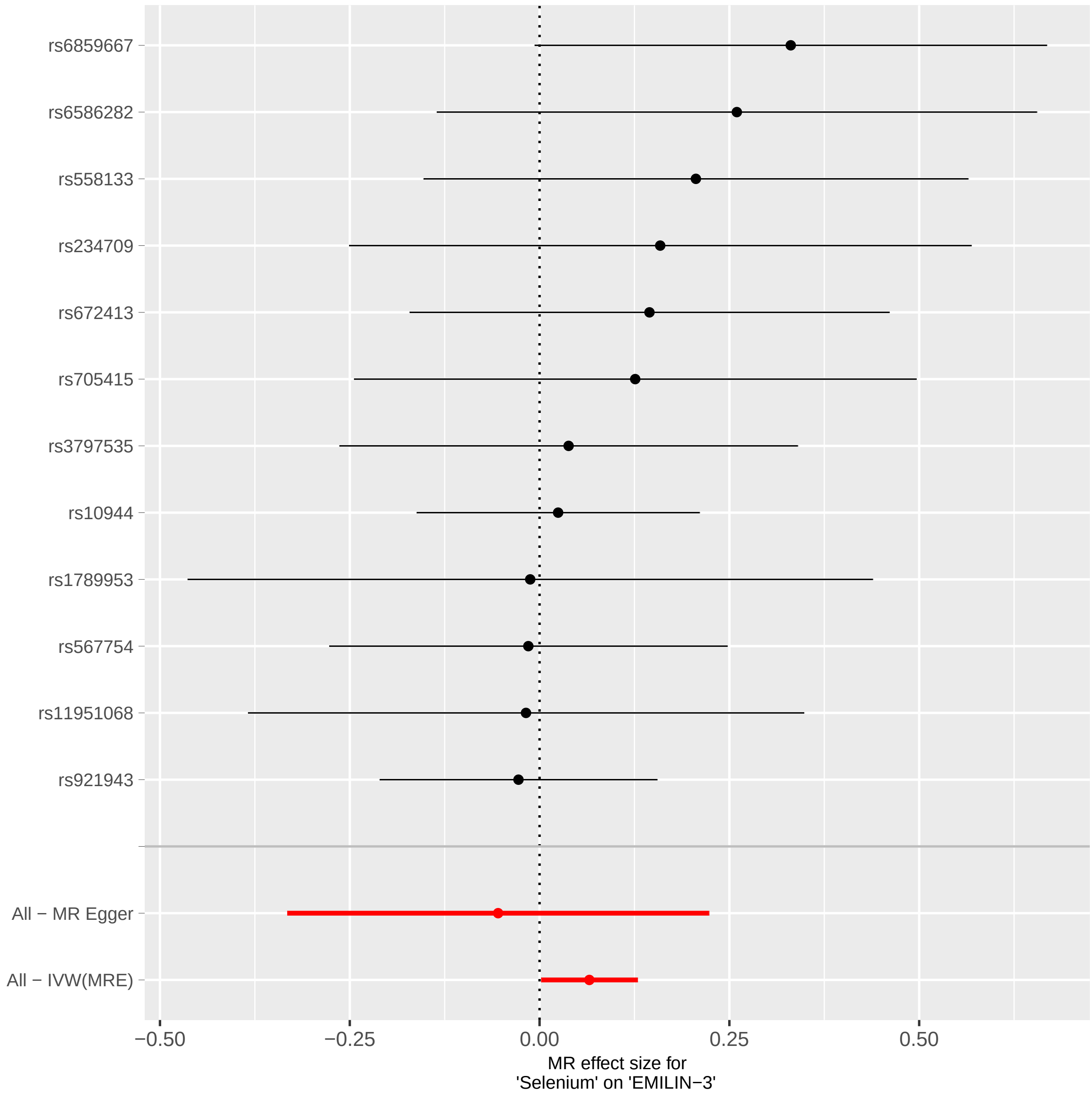

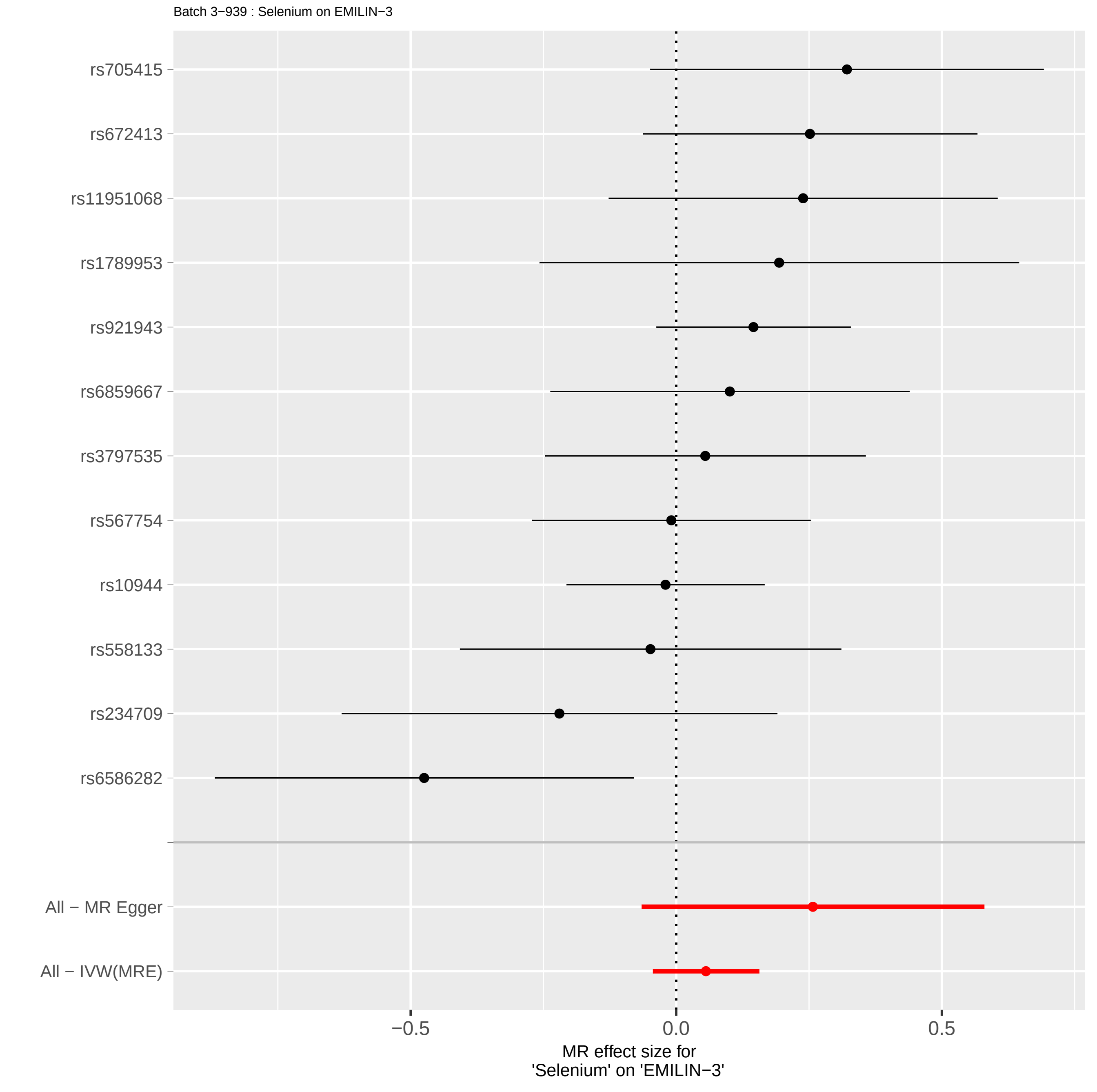

Batch 3−939 : Selenium on EMILIN−3
rs705415
rs672413
rs11951068
rs1789953
rs921943
rs6859667
rs3797535
rs567754
rs10944
rs558133
rs234709
rs6586282
All − MR Egger
All − IVW(MRE)
−0.5
0.0
0.5
MR effect size for
'Selenium' on 'EMILIN−3'

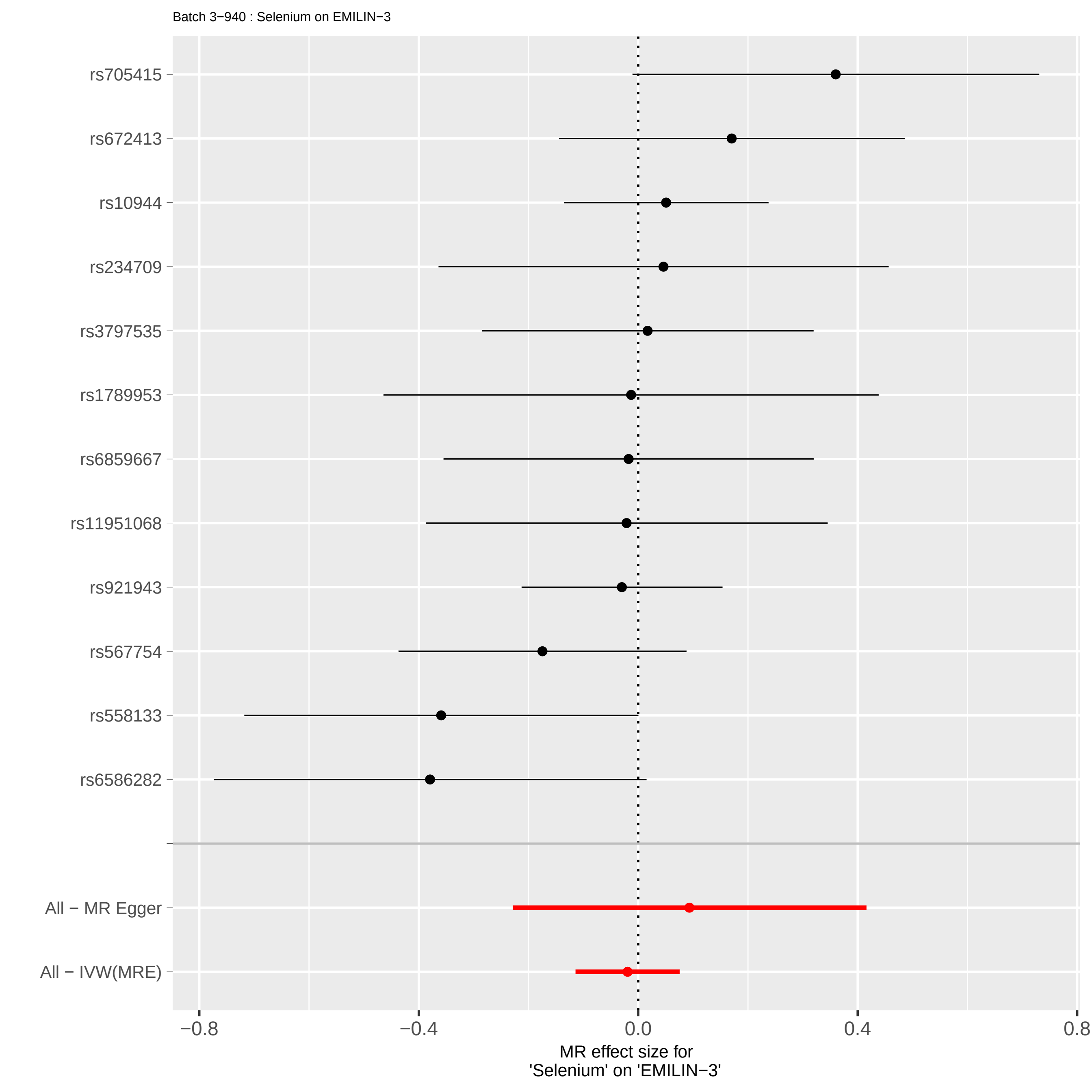

Batch 3−940 : Selenium on EMILIN−3
rs705415
rs672413
rs10944
rs234709
rs3797535
rs1789953
rs6859667
rs11951068
rs921943
rs567754
rs558133
rs6586282
All − MR Egger
All − IVW(MRE)
−0.8
−0.4
0.0
0.4
0.8
MR effect size for
'Selenium' on 'EMILIN−3'

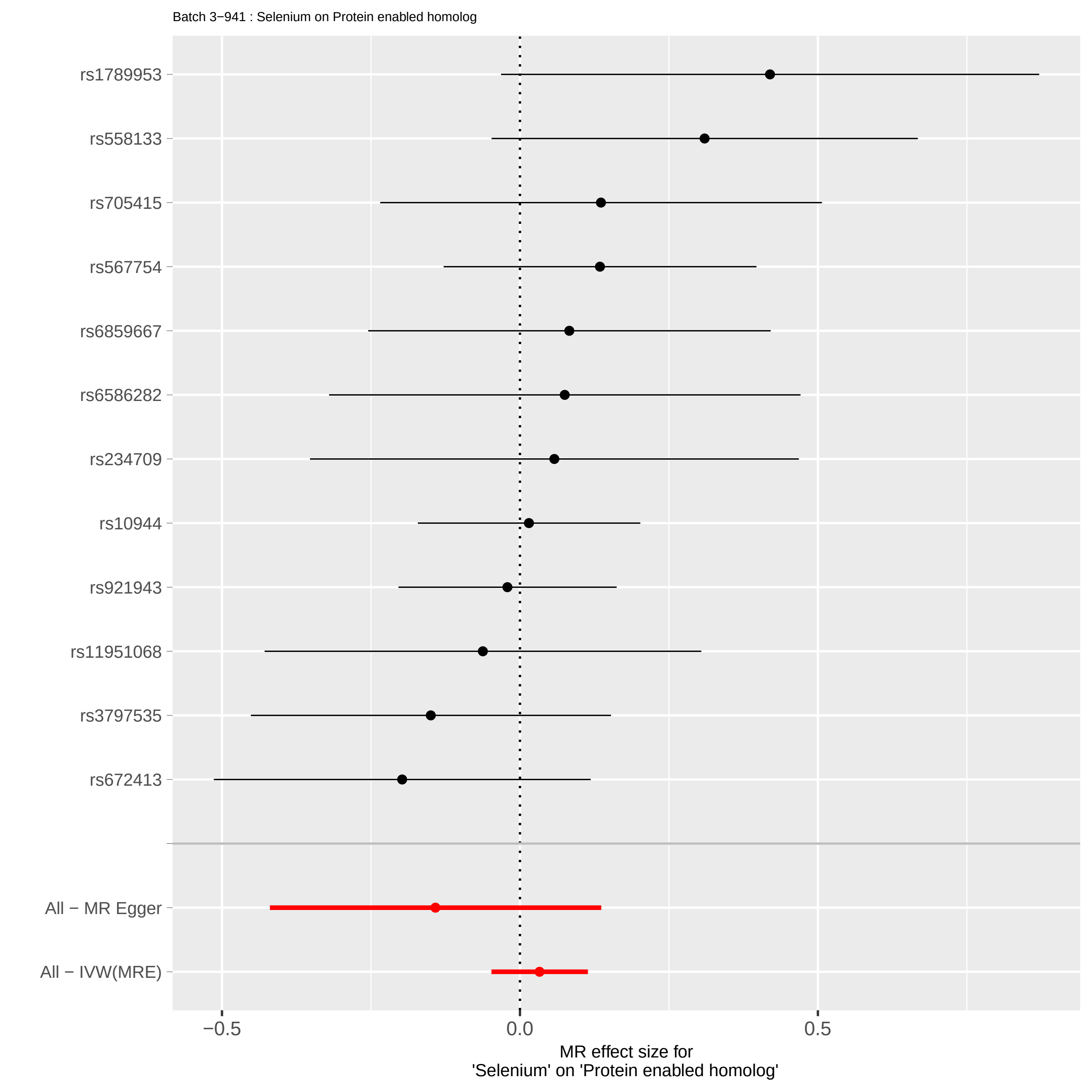
Batch 3−941 : Selenium on Protein enabled homolog
rs1789953
rs558133
rs705415
rs567754
rs6859667
rs6586282
rs234709
rs10944
rs921943
rs11951068
rs3797535
rs672413
All − MR Egger
All − IVW(MRE)
−0.5
0.0
0.5
MR effect size for
'Selenium' on 'Protein enabled homolog'

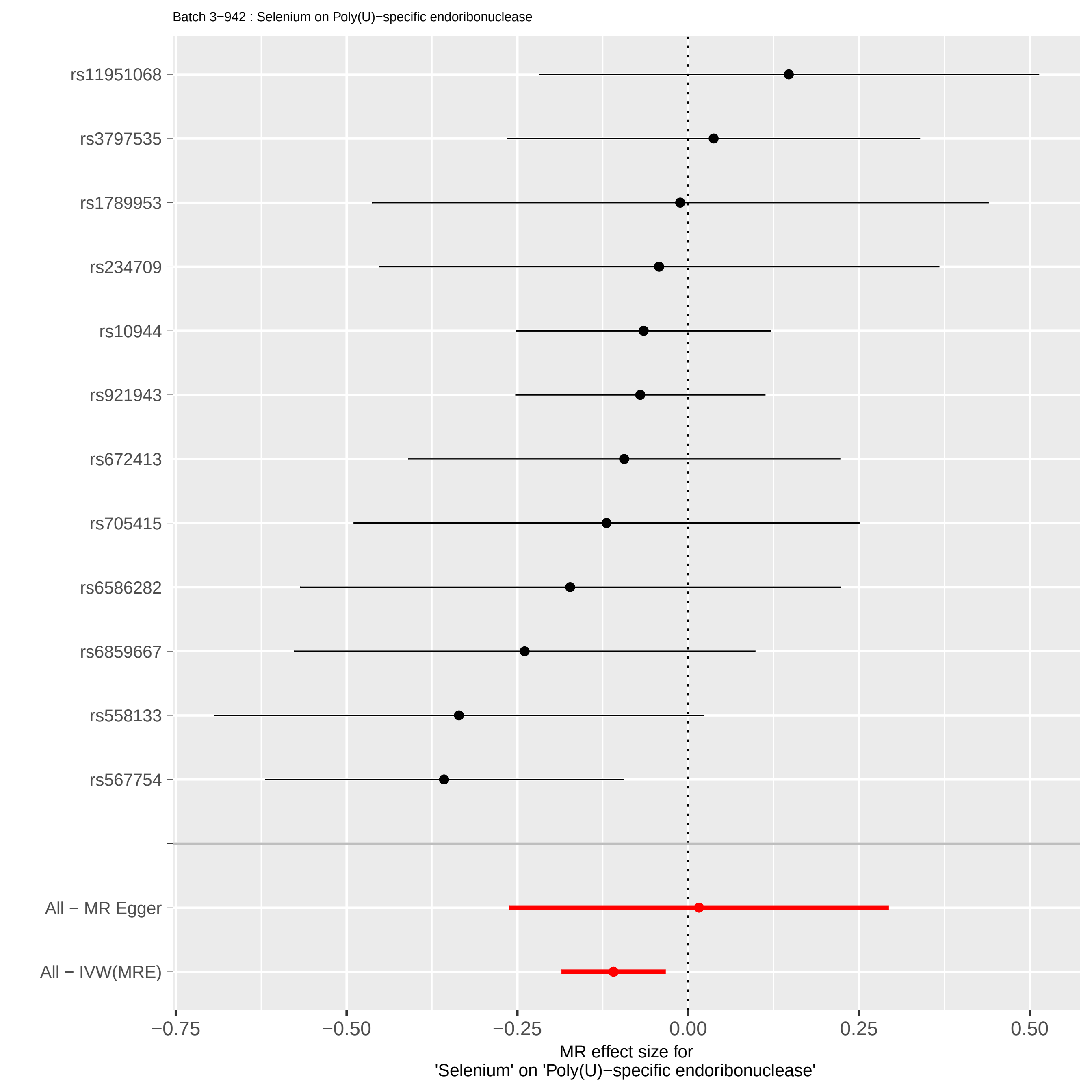
Batch 3−942 : Selenium on Poly(U)−specific endoribonuclease
rs11951068
rs3797535
rs1789953
rs234709
rs10944
rs921943
rs672413
rs705415
rs6586282
rs6859667
rs558133
rs567754
All − MR Egger
All − IVW(MRE)
−0.75
−0.50
−0.25
0.00
0.25
0.50
MR effect size for
'Selenium' on 'Poly(U)−specific endoribonuclease'

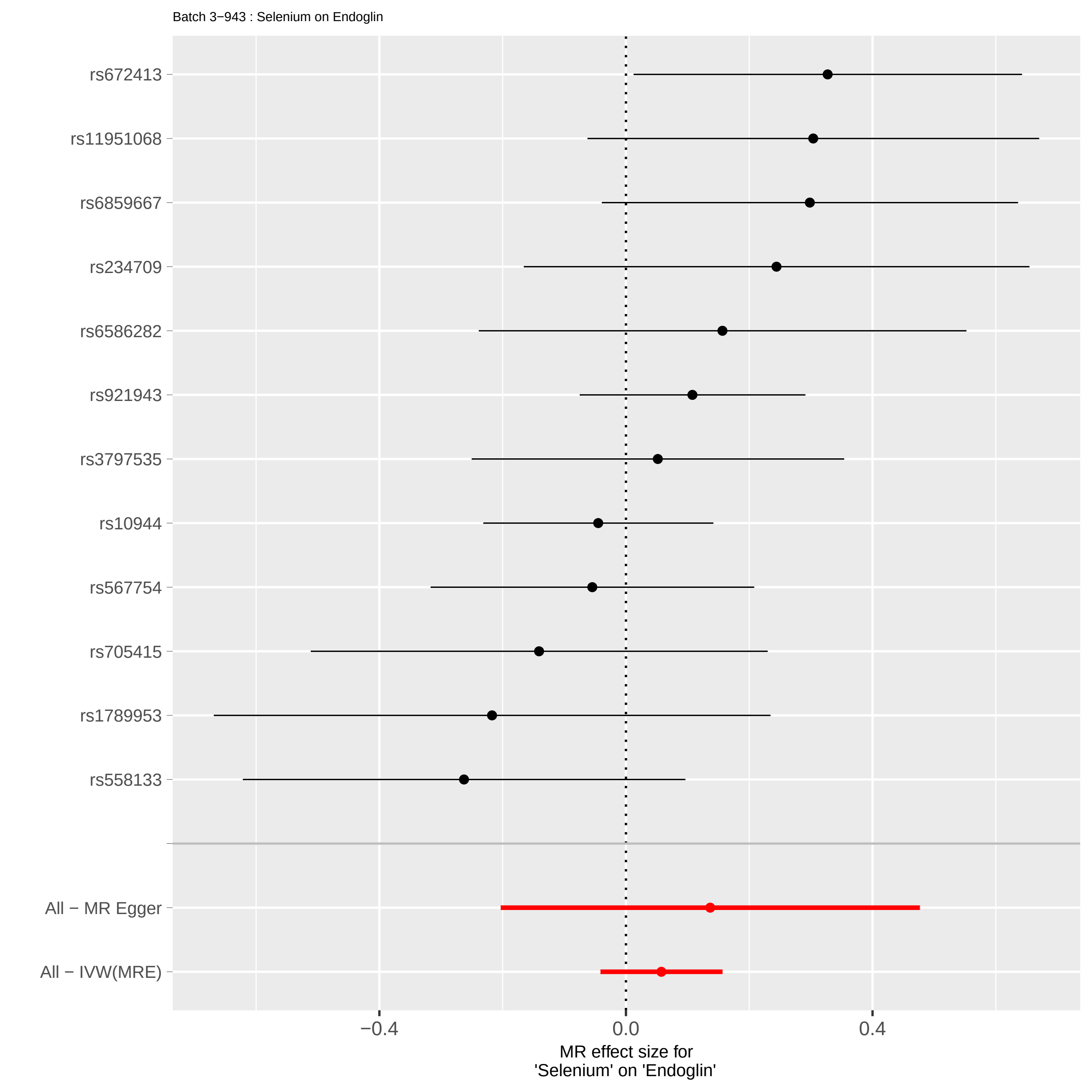
Batch 3−943 : Selenium on Endoglin
rs672413
rs11951068
rs6859667
rs234709
rs6586282
rs921943
rs3797535
rs10944
rs567754
rs705415
rs1789953
rs558133
All − MR Egger
All − IVW(MRE)
−0.4
0.0
0.4
MR effect size for
'Selenium' on 'Endoglin'

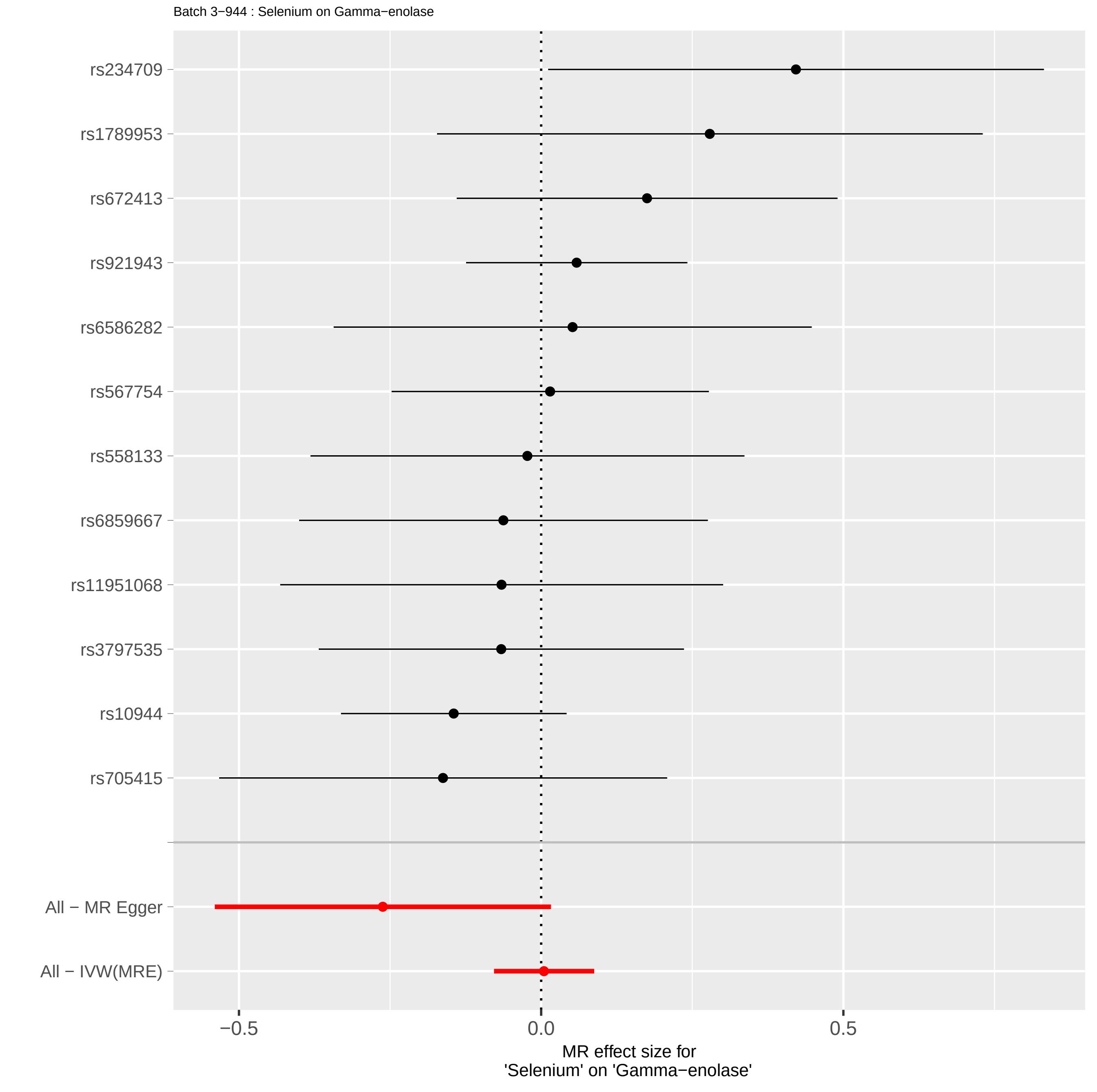

Batch 3−944 : Selenium on Gamma−enolase
rs234709
rs1789953
rs672413
rs921943
rs6586282
rs567754
rs558133
rs6859667
rs11951068
rs3797535
rs10944
rs705415
All − MR Egger
All − IVW(MRE)
−0.5
0.0
0.5
MR effect size for
'Selenium' on 'Gamma−enolase'

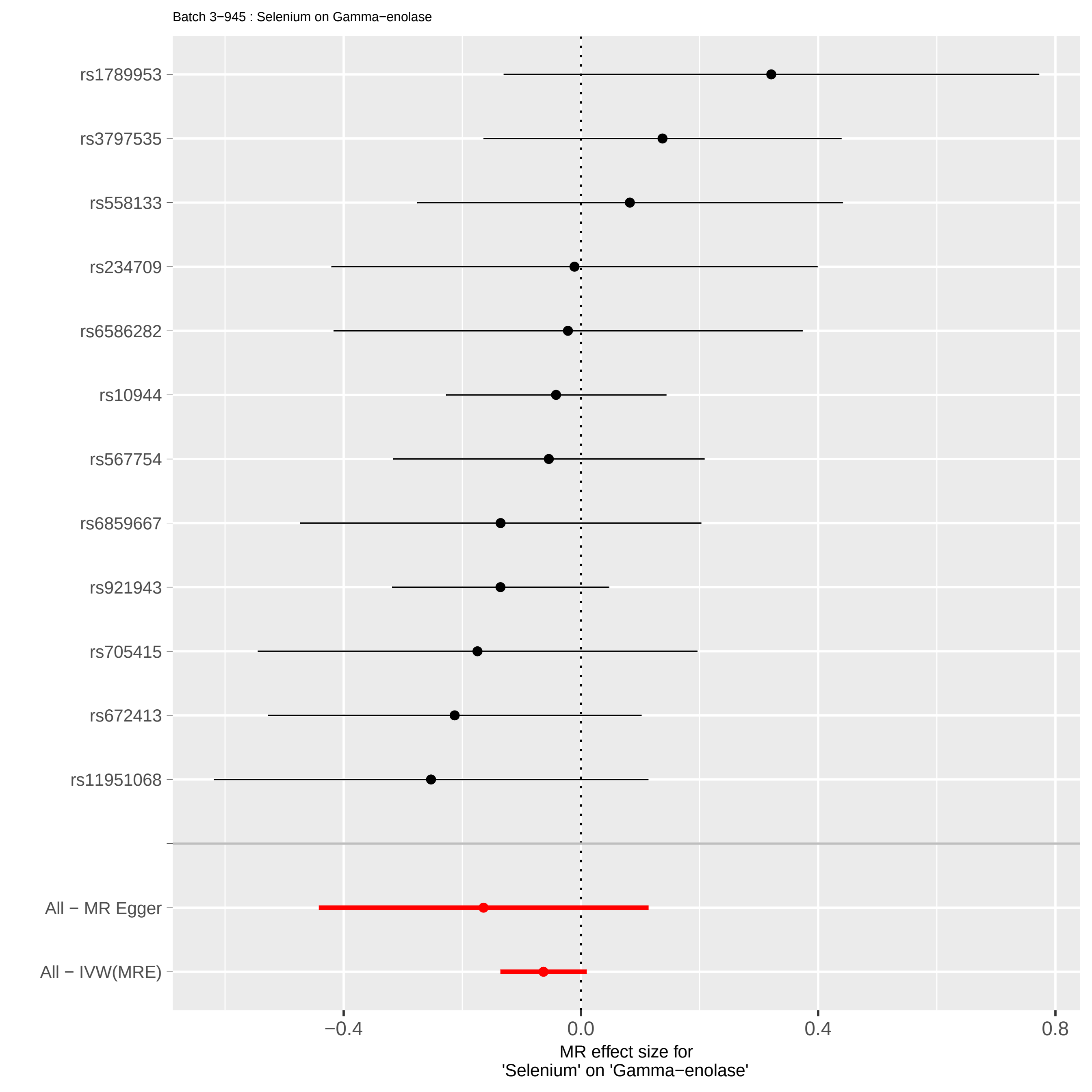
Batch 3−945 : Selenium on Gamma−enolase
rs1789953
rs3797535
rs558133
rs234709
rs6586282
rs10944
rs567754
rs6859667
rs921943
rs705415
rs672413
rs11951068
All − MR Egger
All − IVW(MRE)
−0.4
0.0
0.4
0.8
MR effect size for
'Selenium' on 'Gamma−enolase'

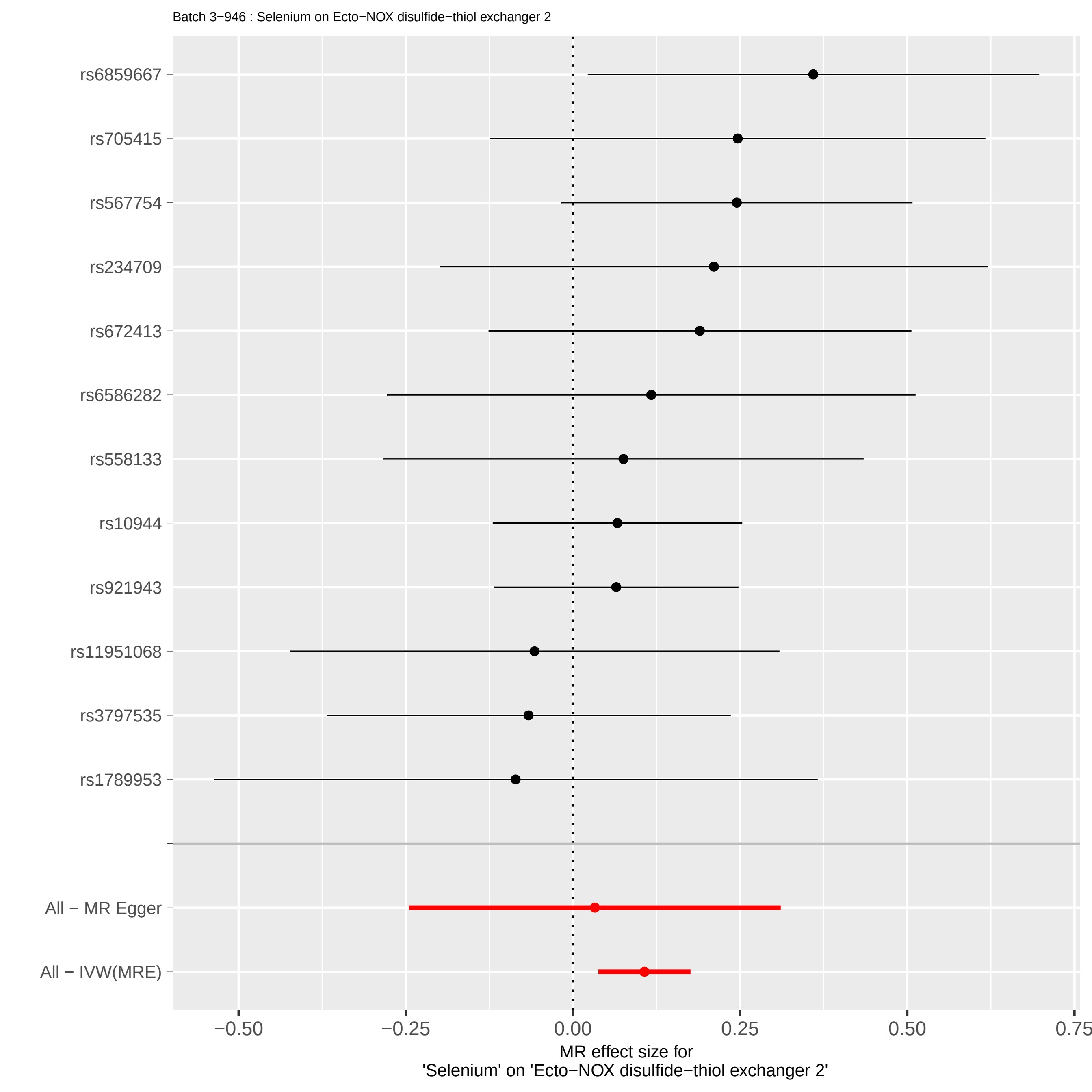

Batch 3−946 : Selenium on Ecto−NOX disulfide−thiol exchanger 2
rs6859667
rs705415
rs567754
rs234709
rs672413
rs6586282
rs558133
rs10944
rs921943
rs11951068
rs3797535
rs1789953
All − MR Egger
All − IVW(MRE)
−0.50
−0.25
0.00
0.25
0.50
0.75
MR effect size for
'Selenium' on 'Ecto−NOX disulfide−thiol exchanger 2'

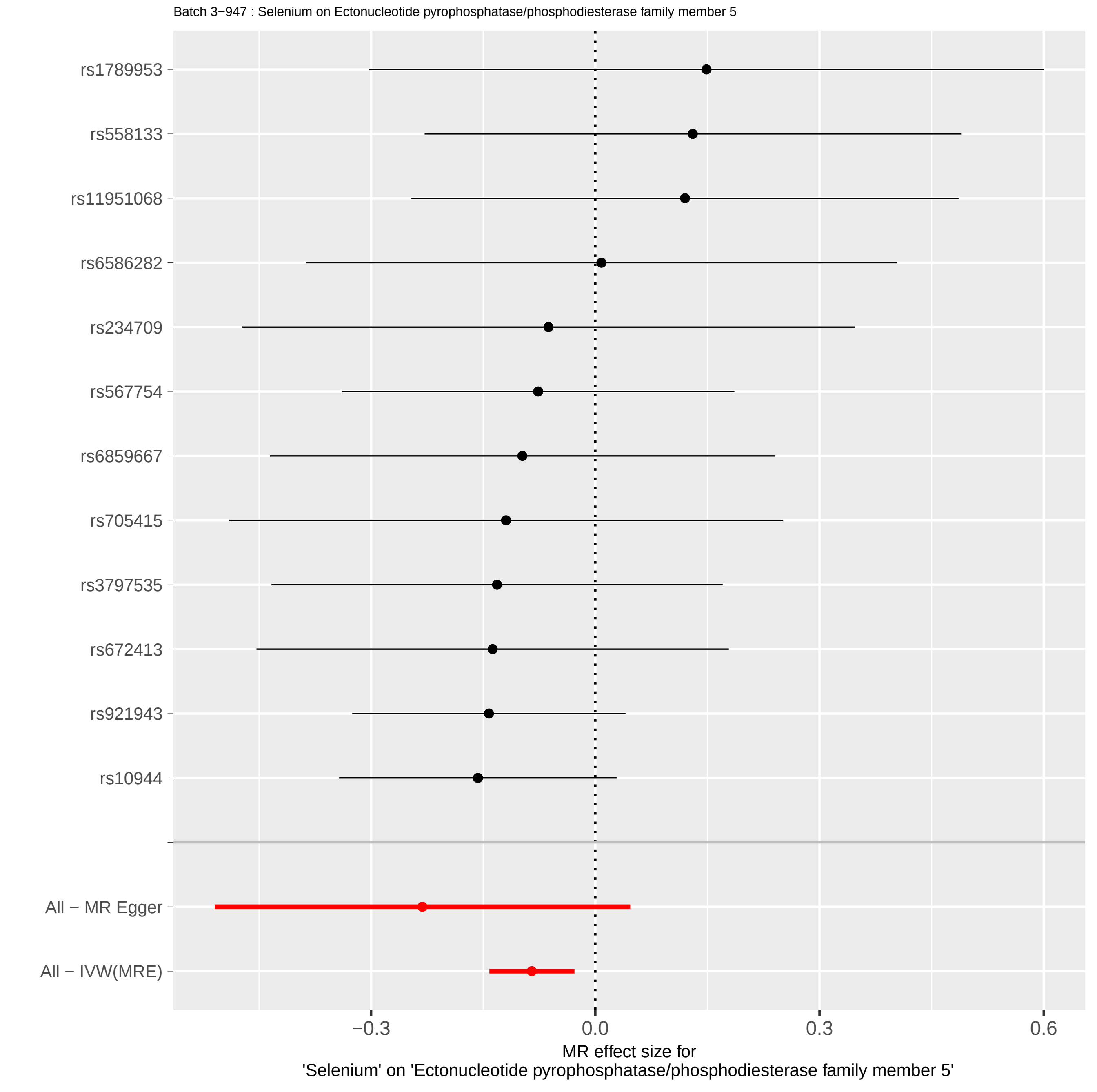

Batch 3−947 : Selenium on Ectonucleotide pyrophosphatase/phosphodiesterase family member 5
rs1789953
rs558133
rs11951068
rs6586282
rs234709
rs567754
rs6859667
rs705415
rs3797535
rs672413
rs921943
rs10944
All − MR Egger
All − IVW(MRE)
−0.3
0.0
0.3
0.6
MR effect size for
'Selenium' on 'Ectonucleotide pyrophosphatase/phosphodiesterase family member 5'

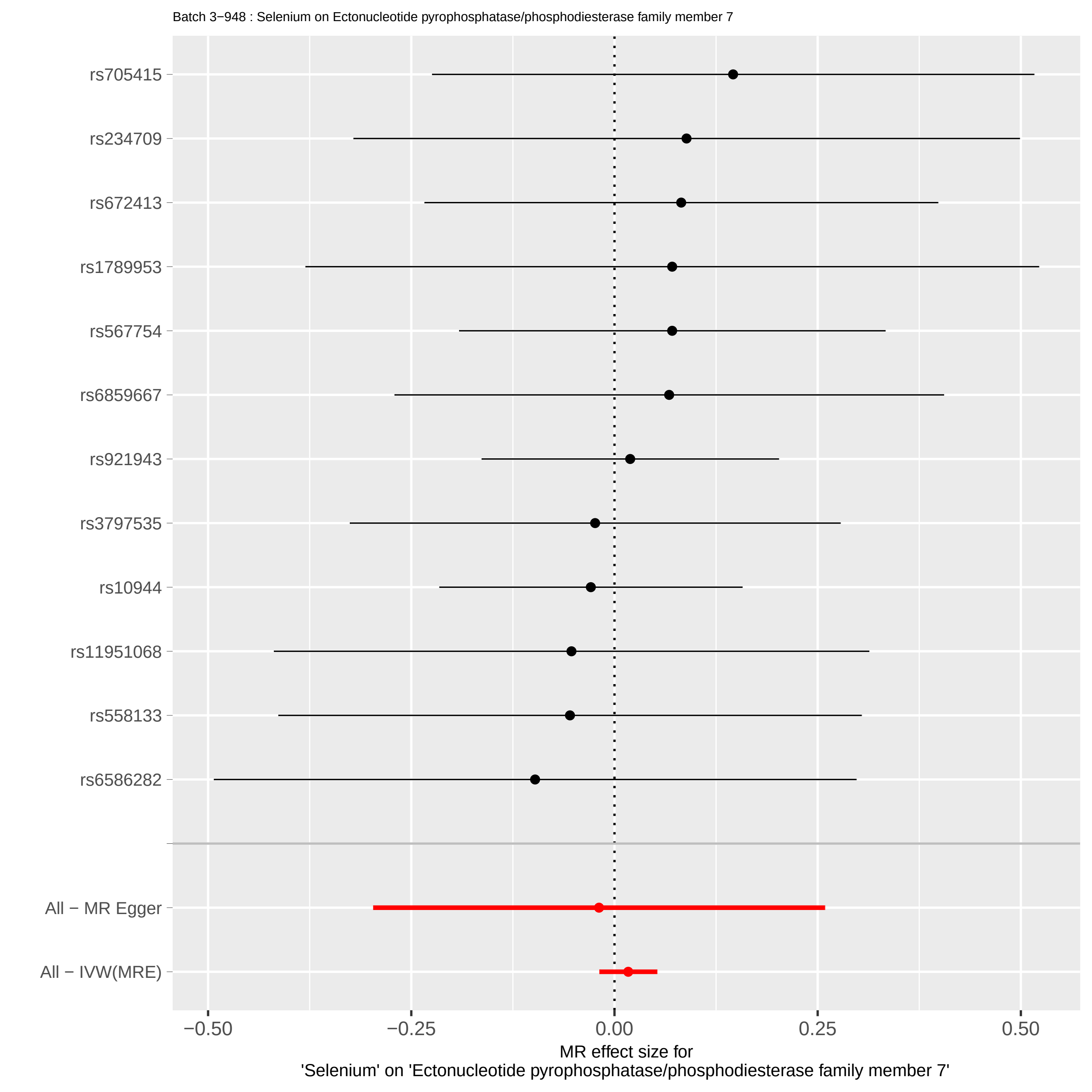

Batch 3−948 : Selenium on Ectonucleotide pyrophosphatase/phosphodiesterase family member 7
rs705415
rs234709
rs672413
rs1789953
rs567754
rs6859667
rs921943
rs3797535
rs10944
rs11951068
rs558133
rs6586282
All − MR Egger
All − IVW(MRE)
−0.50
−0.25
0.00
0.25
0.50
MR effect size for
'Selenium' on 'Ectonucleotide pyrophosphatase/phosphodiesterase family member 7'

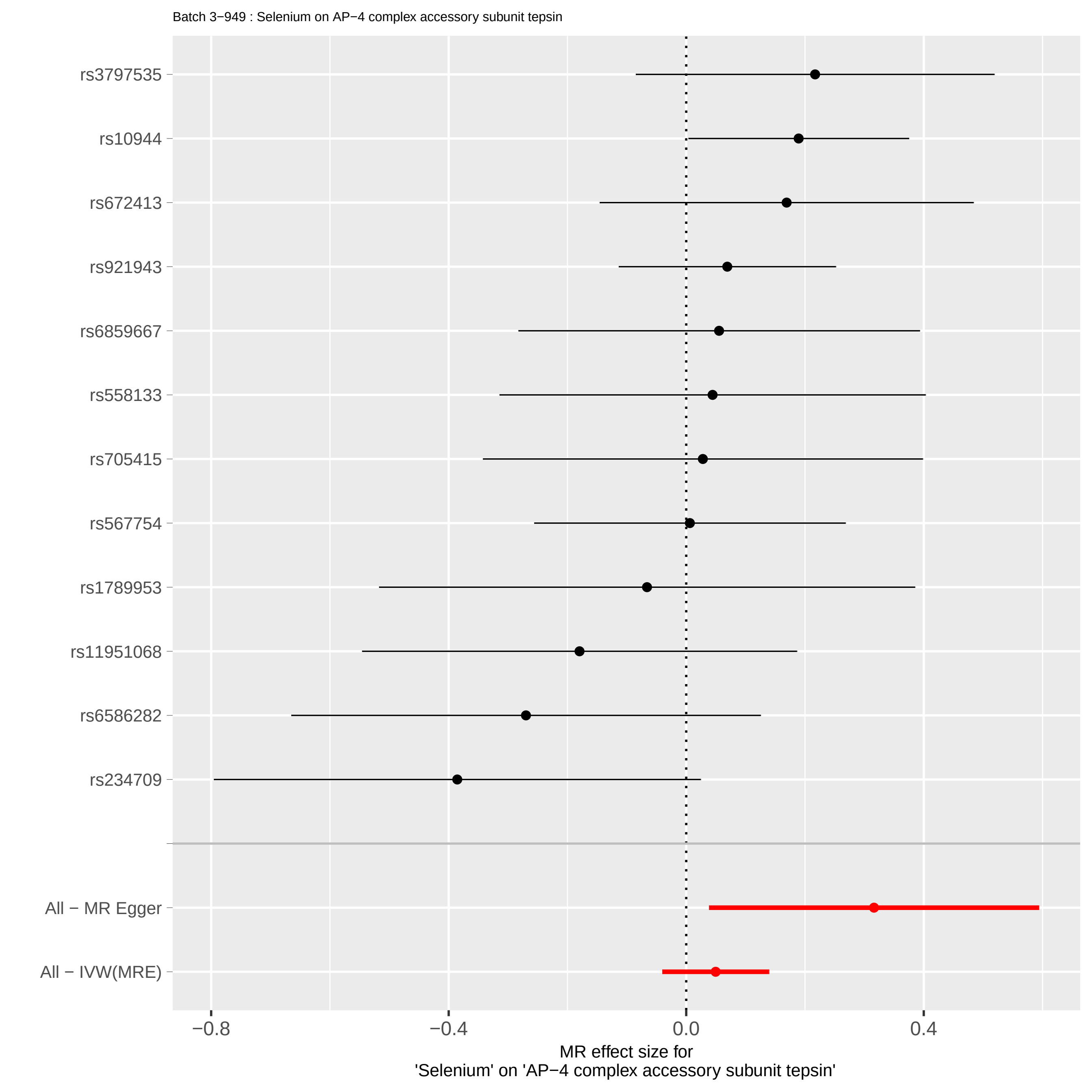
Batch 3−949 : Selenium on AP−4 complex accessory subunit tepsin
rs3797535
rs10944
rs672413
rs921943
rs6859667
rs558133
rs705415
rs567754
rs1789953
rs11951068
rs6586282
rs234709
All − MR Egger
All − IVW(MRE)
−0.8
−0.4
0.0
0.4
MR effect size for
'Selenium' on 'AP−4 complex accessory subunit tepsin'

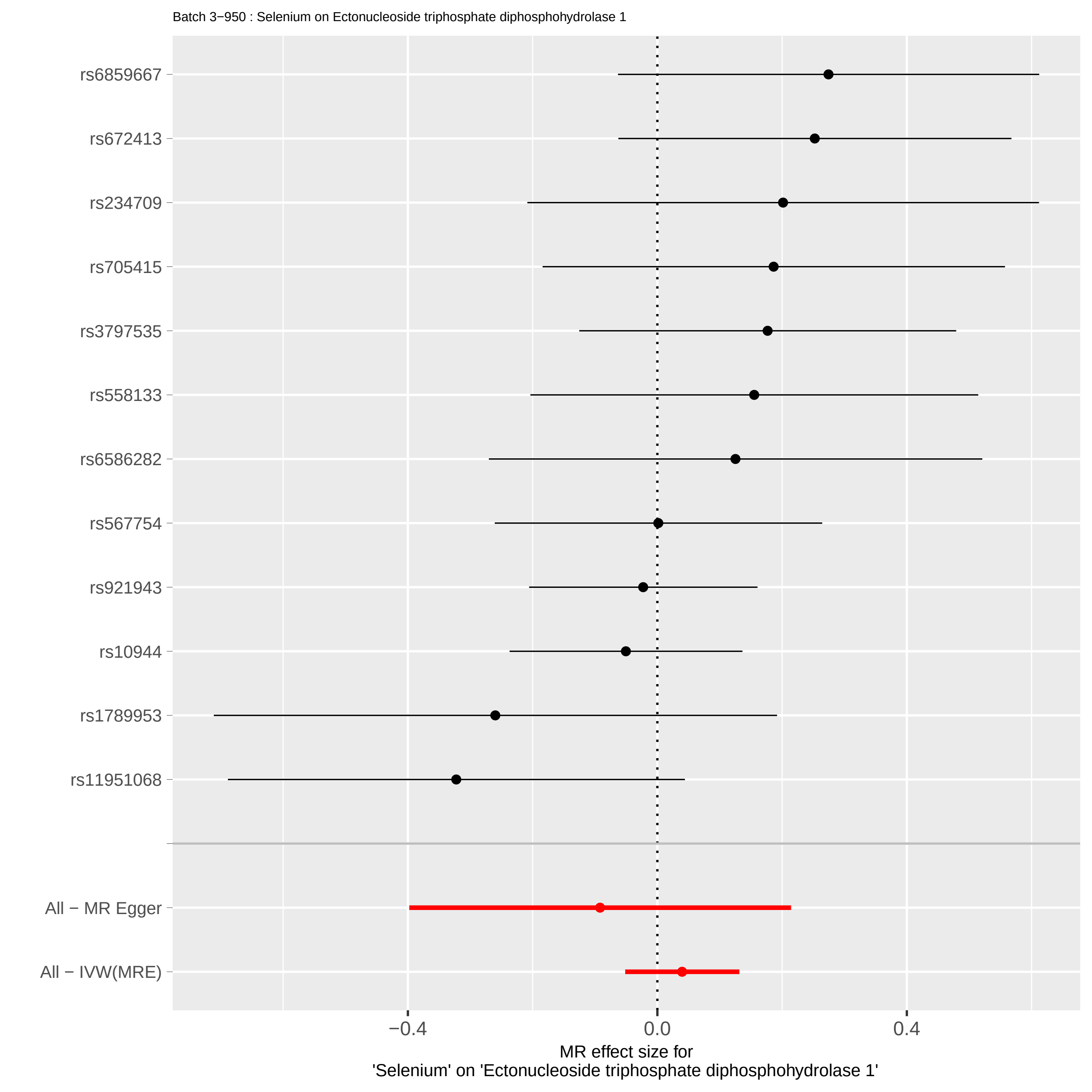
Batch 3−950 : Selenium on Ectonucleoside triphosphate diphosphohydrolase 1
rs6859667
rs672413
rs234709
rs705415
rs3797535
rs558133
rs6586282
rs567754
rs921943
rs10944
rs1789953
rs11951068
All − MR Egger
All − IVW(MRE)
−0.4
0.0
0.4
MR effect size for
'Selenium' on 'Ectonucleoside triphosphate diphosphohydrolase 1'

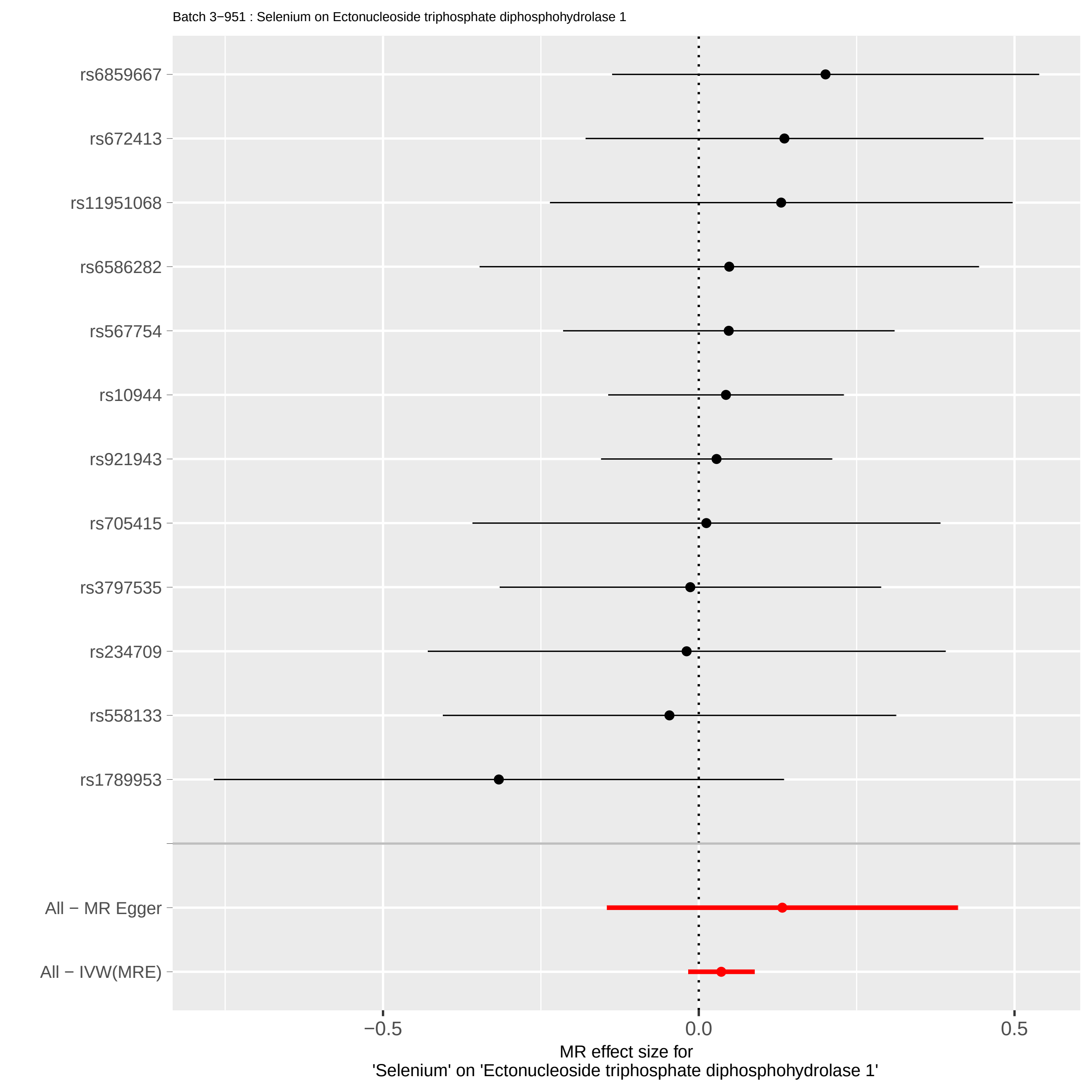
Batch 3−951 : Selenium on Ectonucleoside triphosphate diphosphohydrolase 1
rs6859667
rs672413
rs11951068
rs6586282
rs567754
rs10944
rs921943
rs705415
rs3797535
rs234709
rs558133
rs1789953
All − MR Egger
All − IVW(MRE)
−0.5
0.0
0.5
MR effect size for
'Selenium' on 'Ectonucleoside triphosphate diphosphohydrolase 1'

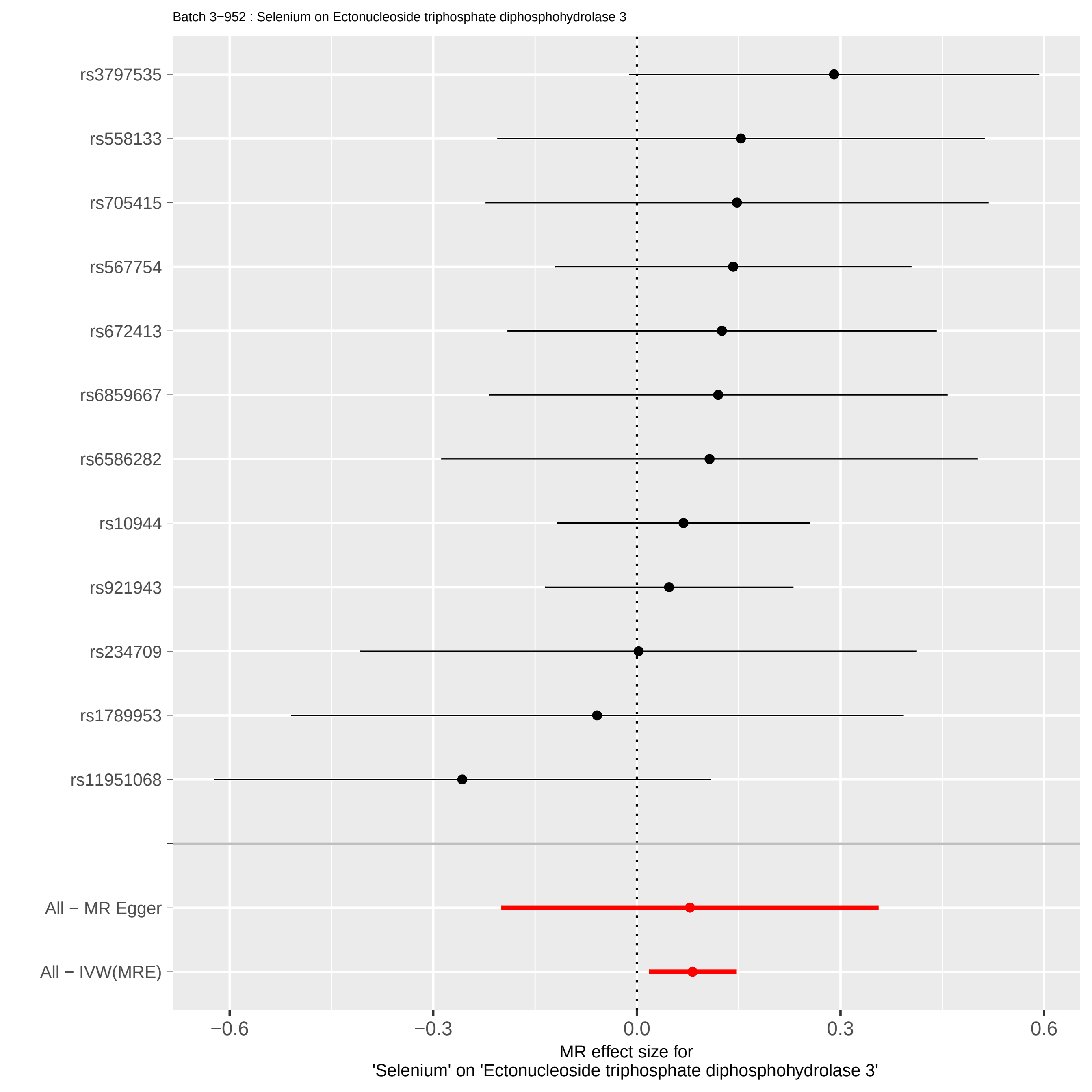

Batch 3−952 : Selenium on Ectonucleoside triphosphate diphosphohydrolase 3
rs3797535
rs558133
rs705415
rs567754
rs672413
rs6859667
rs6586282
rs10944
rs921943
rs234709
rs1789953
rs11951068
All − MR Egger
All − IVW(MRE)
−0.6
−0.3
0.0
0.3
0.6
MR effect size for
'Selenium' on 'Ectonucleoside triphosphate diphosphohydrolase 3'

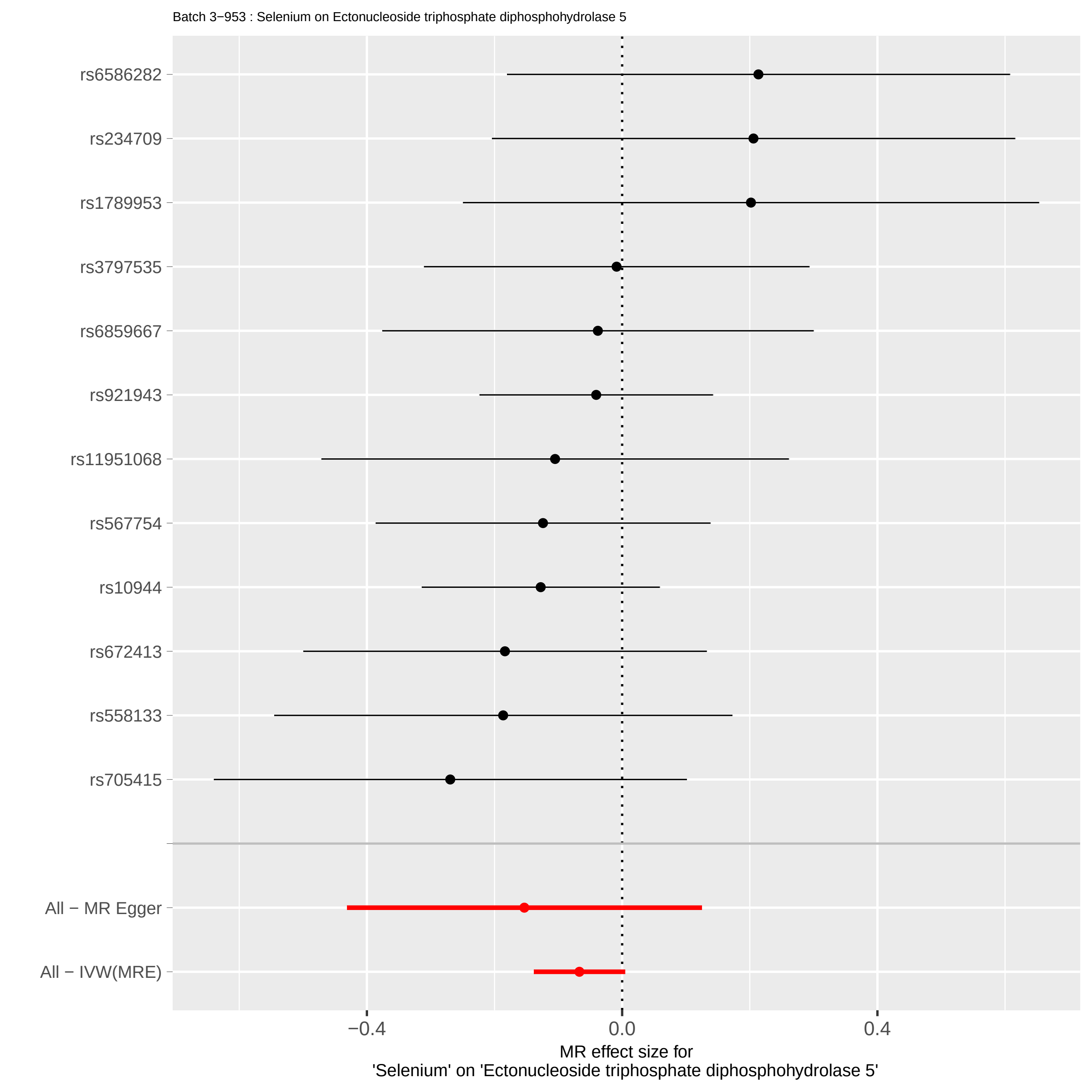

Batch 3−953 : Selenium on Ectonucleoside triphosphate diphosphohydrolase 5
rs6586282
rs234709
rs1789953
rs3797535
rs6859667
rs921943
rs11951068
rs567754
rs10944
rs672413
rs558133
rs705415
All − MR Egger
All − IVW(MRE)
−0.4
0.0
0.4
MR effect size for
'Selenium' on 'Ectonucleoside triphosphate diphosphohydrolase 5'

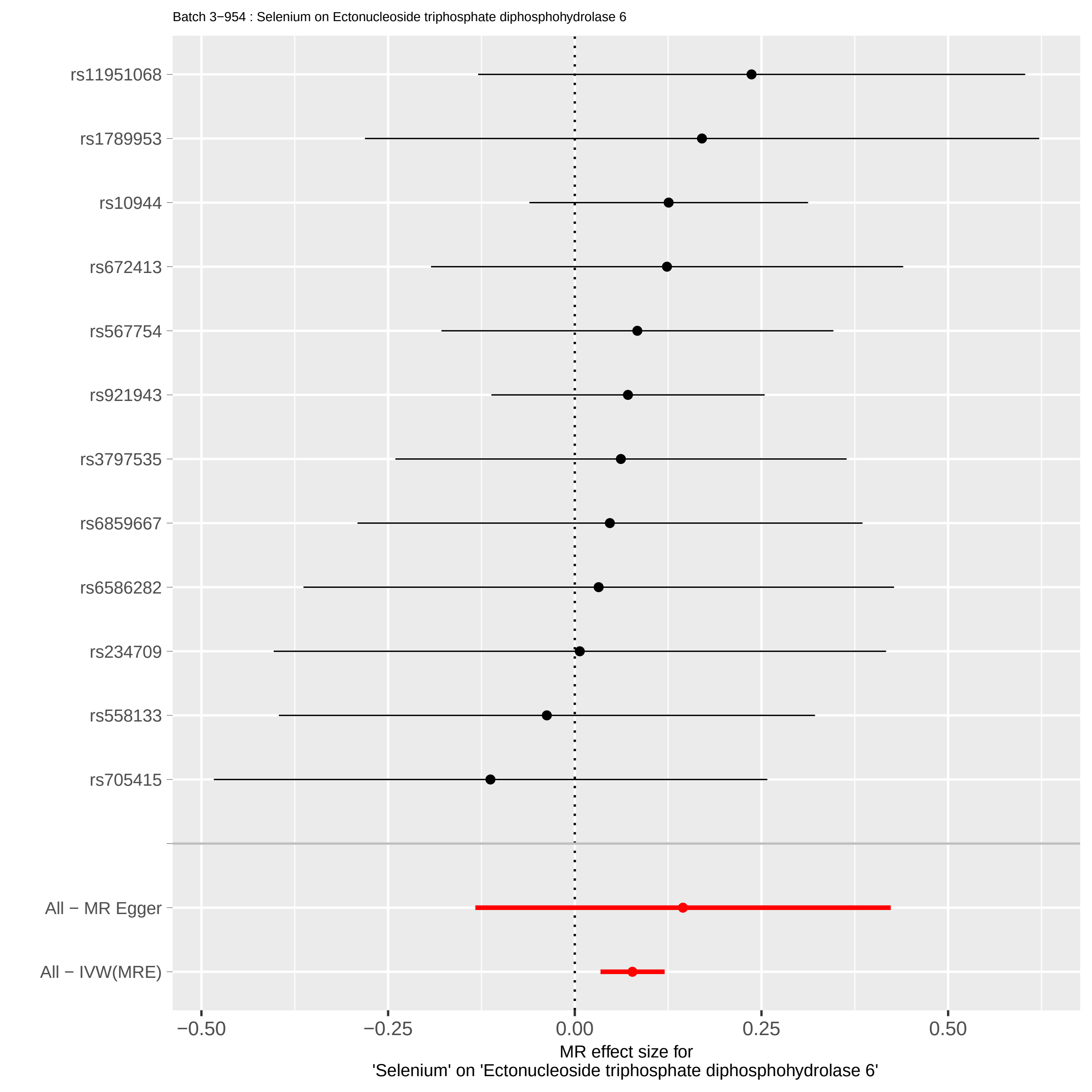
Batch 3−954 : Selenium on Ectonucleoside triphosphate diphosphohydrolase 6
rs11951068
rs1789953
rs10944
rs672413
rs567754
rs921943
rs3797535
rs6859667
rs6586282
rs234709
rs558133
rs705415
All − MR Egger
All − IVW(MRE)
−0.50
−0.25
0.00
0.25
0.50
MR effect size for
'Selenium' on 'Ectonucleoside triphosphate diphosphohydrolase 6'

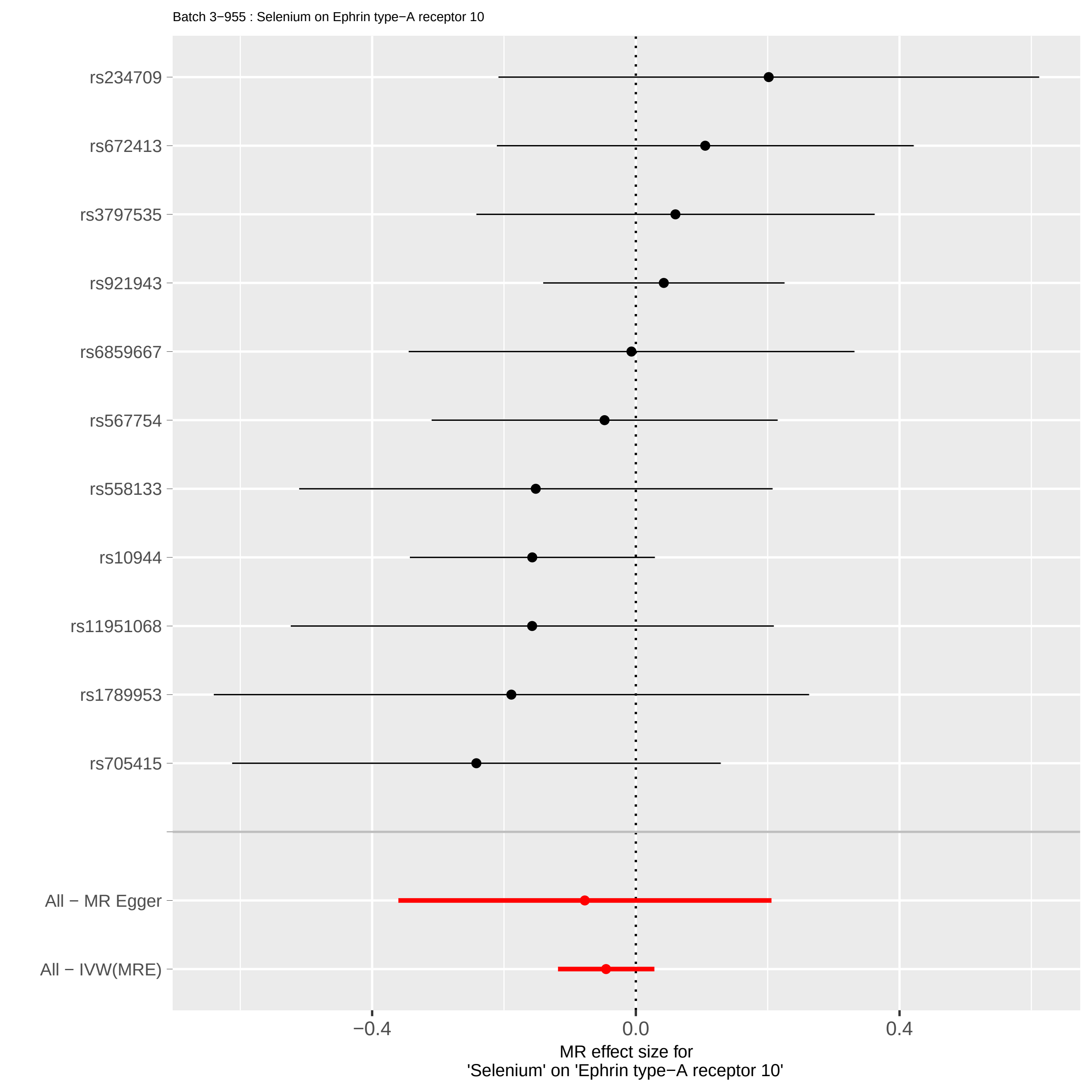
Batch 3−955 : Selenium on Ephrin type−A receptor 10
rs234709
rs672413
rs3797535
rs921943
rs6859667
rs567754
rs558133
rs10944
rs11951068
rs1789953
rs705415
All − MR Egger
All − IVW(MRE)
−0.4
0.0
0.4
MR effect size for
'Selenium' on 'Ephrin type−A receptor 10'

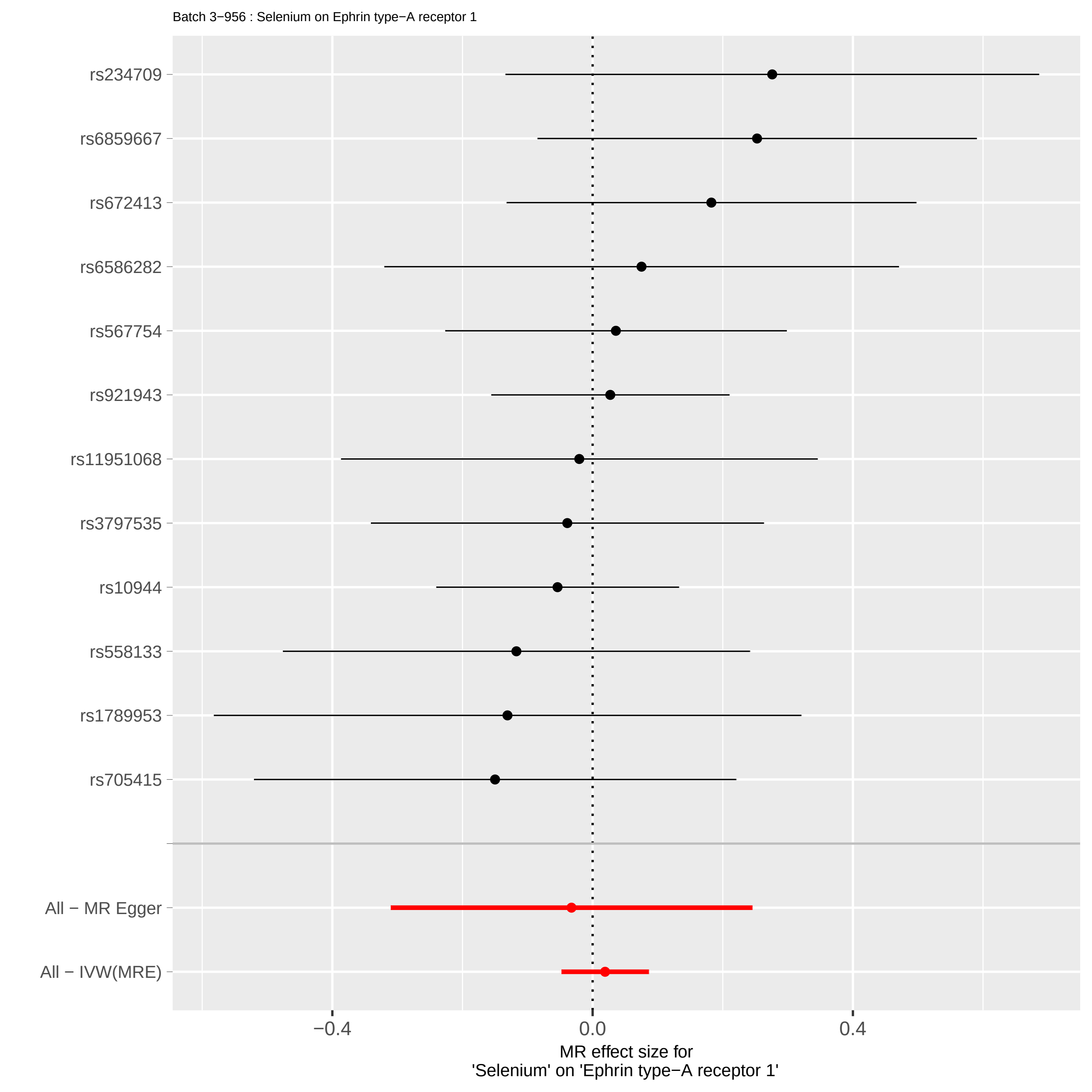

Batch 3−956 : Selenium on Ephrin type−A receptor 1
rs234709
rs6859667
rs672413
rs6586282
rs567754
rs921943
rs11951068
rs3797535
rs10944
rs558133
rs1789953
rs705415
All − MR Egger
All − IVW(MRE)
−0.4
0.0
0.4
MR effect size for
'Selenium' on 'Ephrin type−A receptor 1'

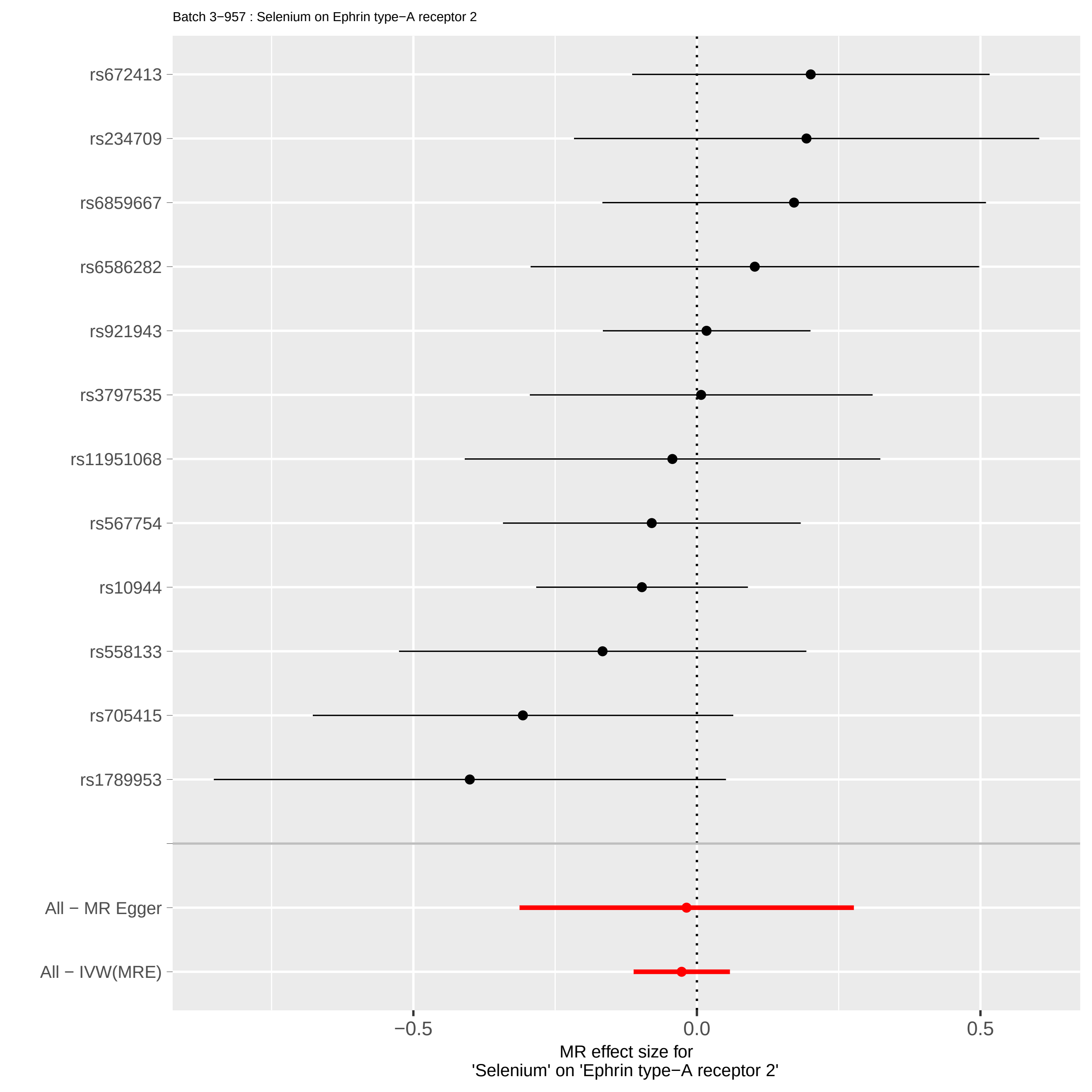

Batch 3−957 : Selenium on Ephrin type−A receptor 2
rs672413
rs234709
rs6859667
rs6586282
rs921943
rs3797535
rs11951068
rs567754
rs10944
rs558133
rs705415
rs1789953
All − MR Egger
All − IVW(MRE)
−0.5
0.0
0.5
MR effect size for
'Selenium' on 'Ephrin type−A receptor 2'

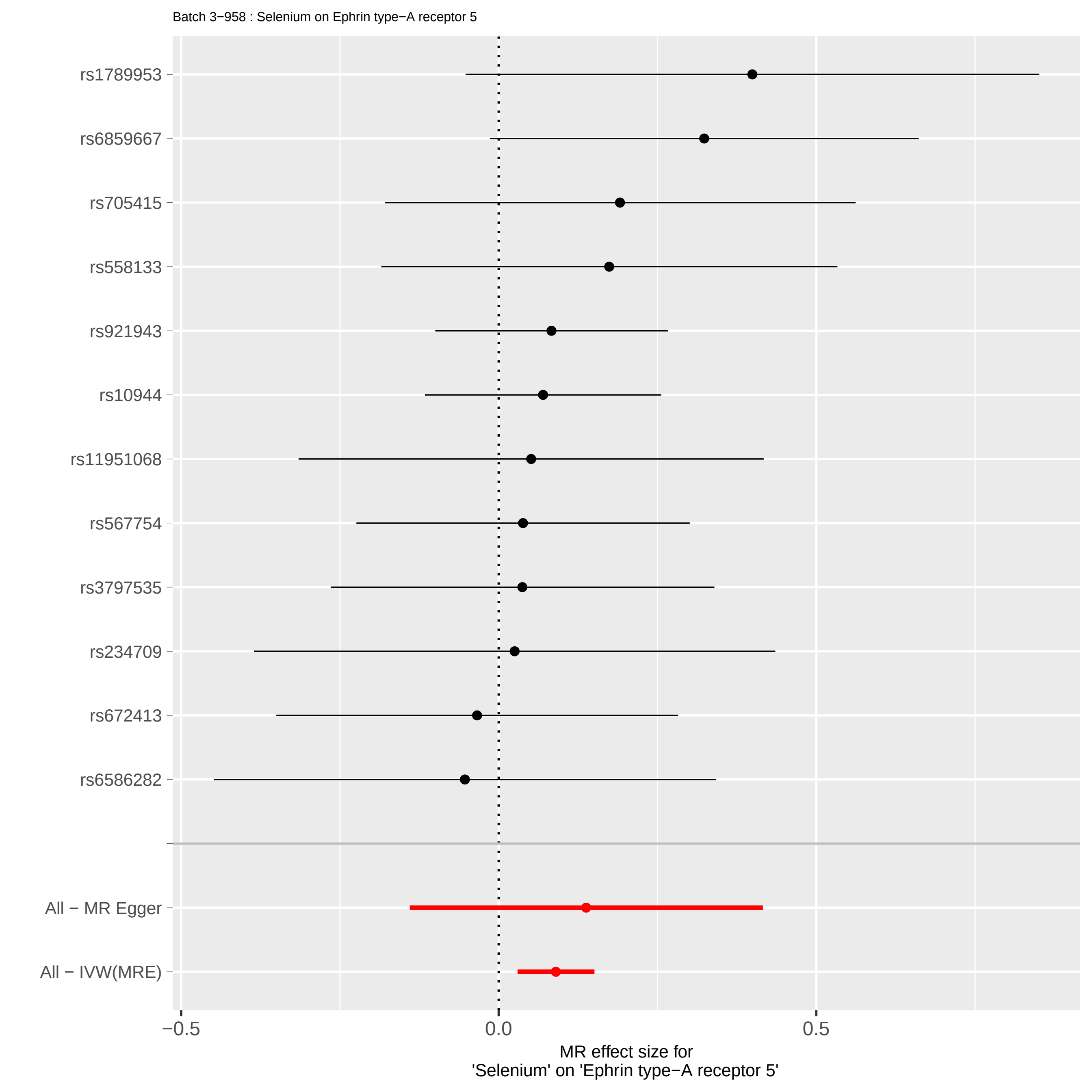

Batch 3−958 : Selenium on Ephrin type−A receptor 5
rs1789953
rs6859667
rs705415
rs558133
rs921943
rs10944
rs11951068
rs567754
rs3797535
rs234709
rs672413
rs6586282
All − MR Egger
All − IVW(MRE)
−0.5
0.0
0.5
MR effect size for
'Selenium' on 'Ephrin type−A receptor 5'

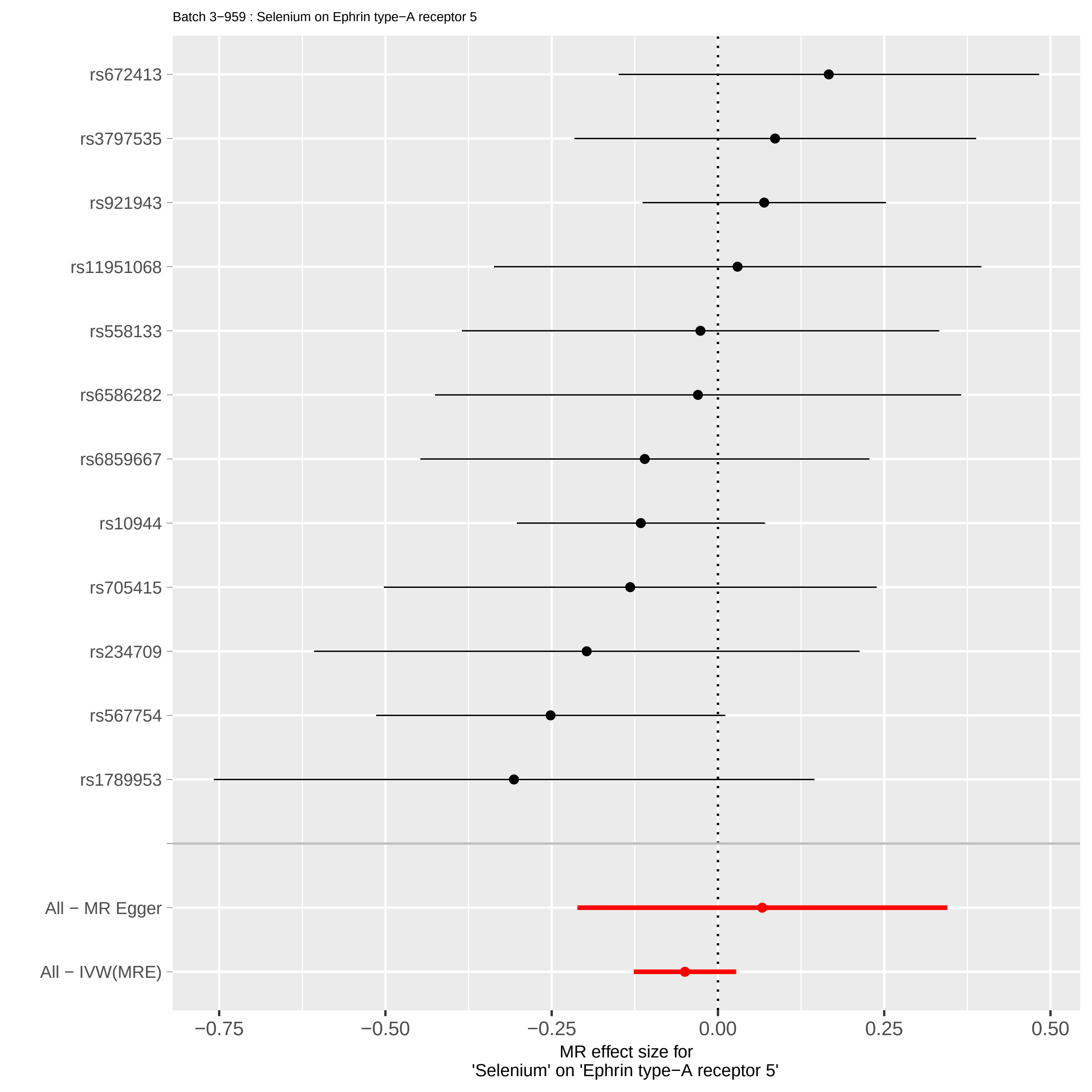
Batch 3−959 : Selenium on Ephrin type−A receptor 5
rs672413
rs3797535
rs921943
rs11951068
rs558133
rs6586282
rs6859667
rs10944
rs705415
rs234709
rs567754
rs1789953
All − MR Egger
All − IVW(MRE)
−0.75
−0.50
−0.25
0.00
0.25
0.50
MR effect size for
'Selenium' on 'Ephrin type−A receptor 5'

Batch 3−960 : Selenium on Ephrin type−B receptor 2

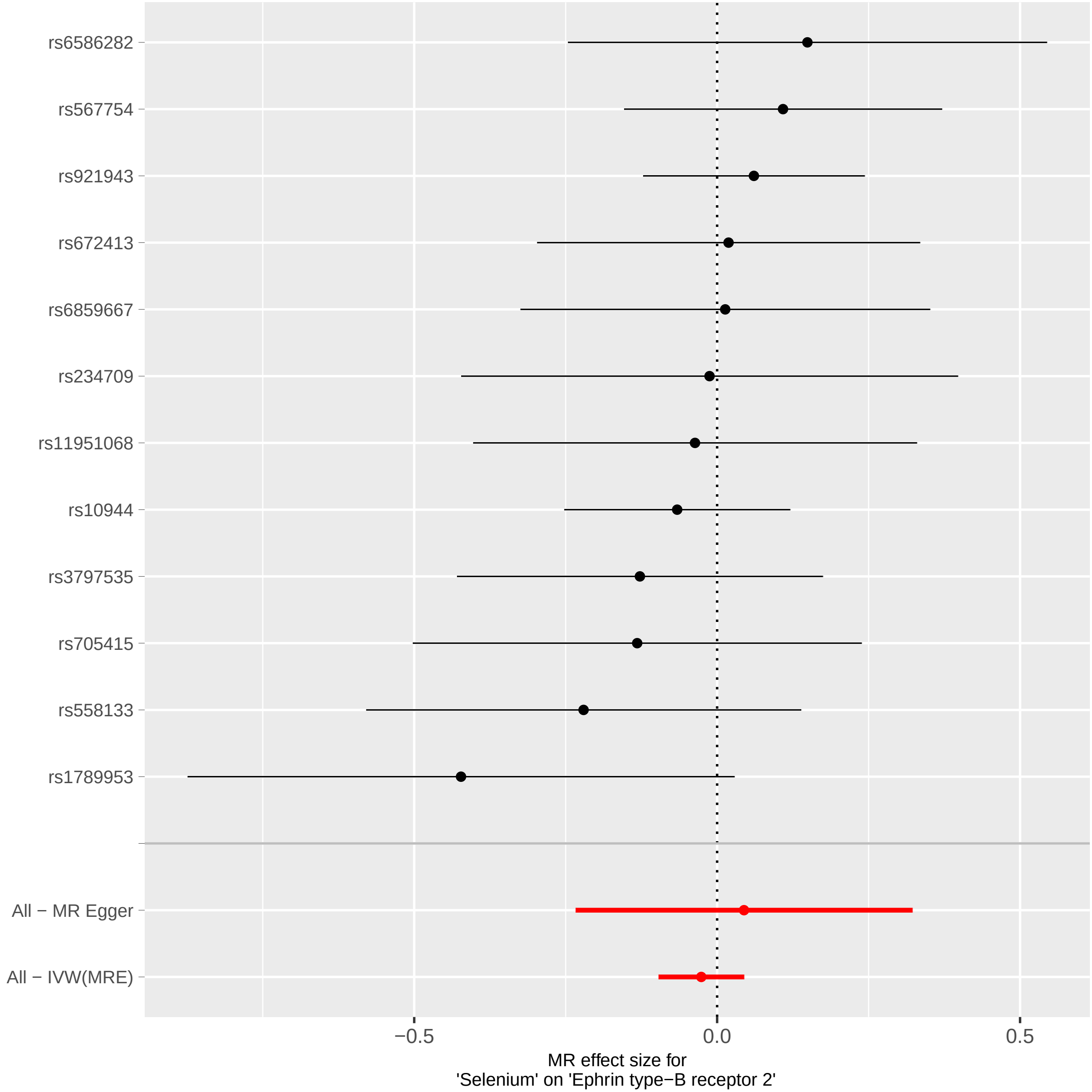

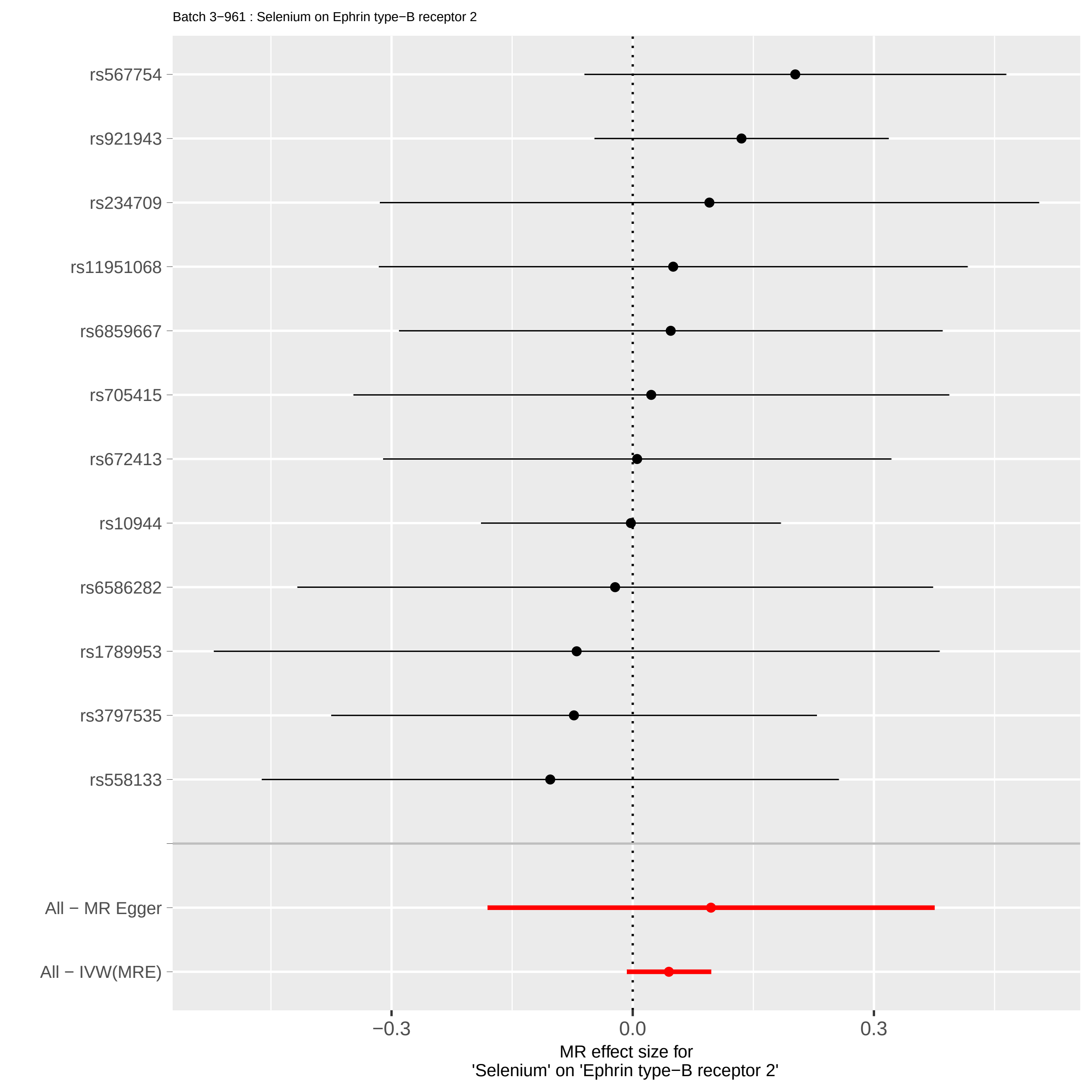

Batch 3−961 : Selenium on Ephrin type−B receptor 2
rs567754
rs921943
rs234709
rs11951068
rs6859667
rs705415
rs672413
rs10944
rs6586282
rs1789953
rs3797535
rs558133
All − MR Egger
All − IVW(MRE)
−0.3
0.0
0.3
MR effect size for
'Selenium' on 'Ephrin type−B receptor 2'

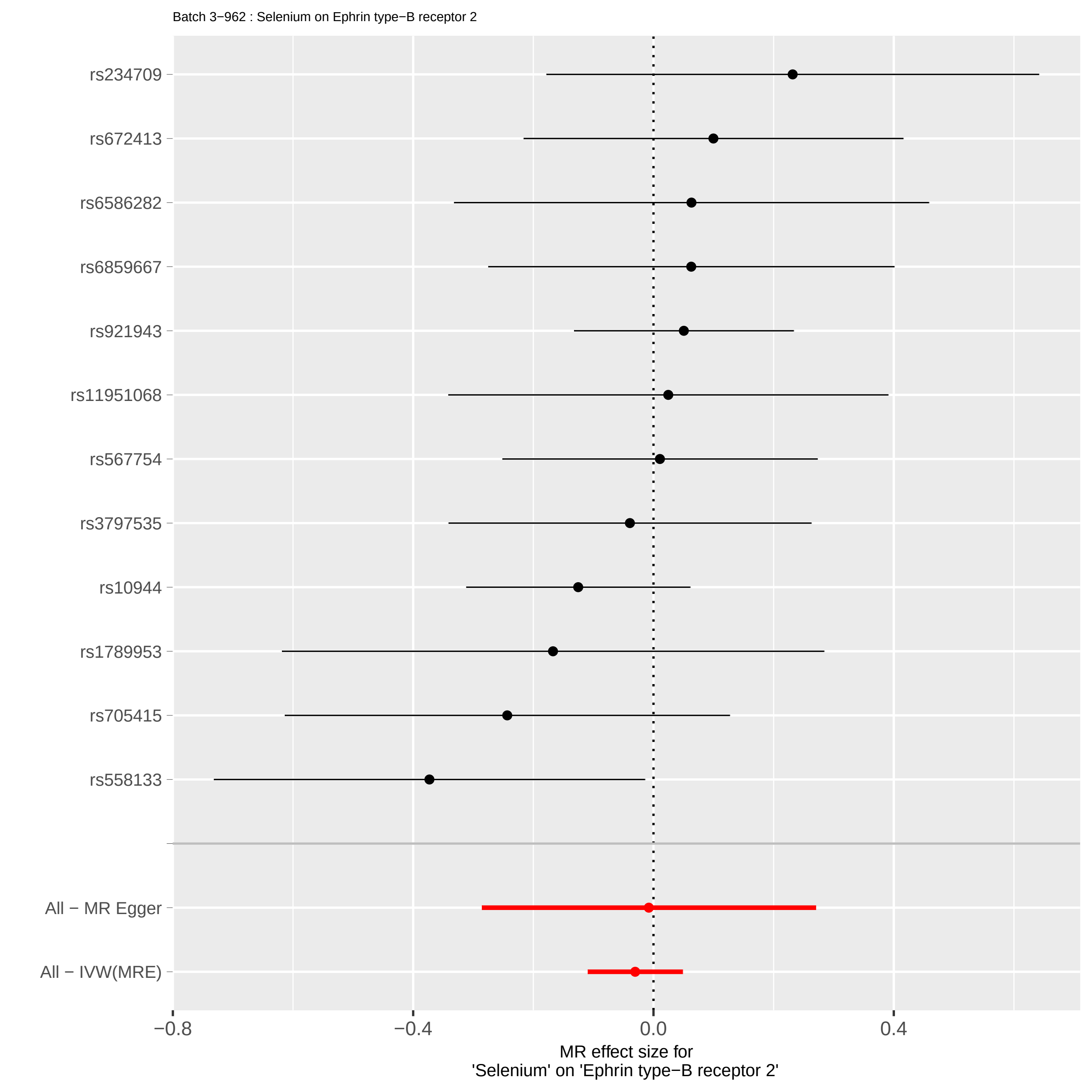

Batch 3−962 : Selenium on Ephrin type−B receptor 2
rs234709
rs672413
rs6586282
rs6859667
rs921943
rs11951068
rs567754
rs3797535
rs10944
rs1789953
rs705415
rs558133
All − MR Egger
All − IVW(MRE)
−0.8
−0.4
0.0
0.4
MR effect size for
'Selenium' on 'Ephrin type−B receptor 2'

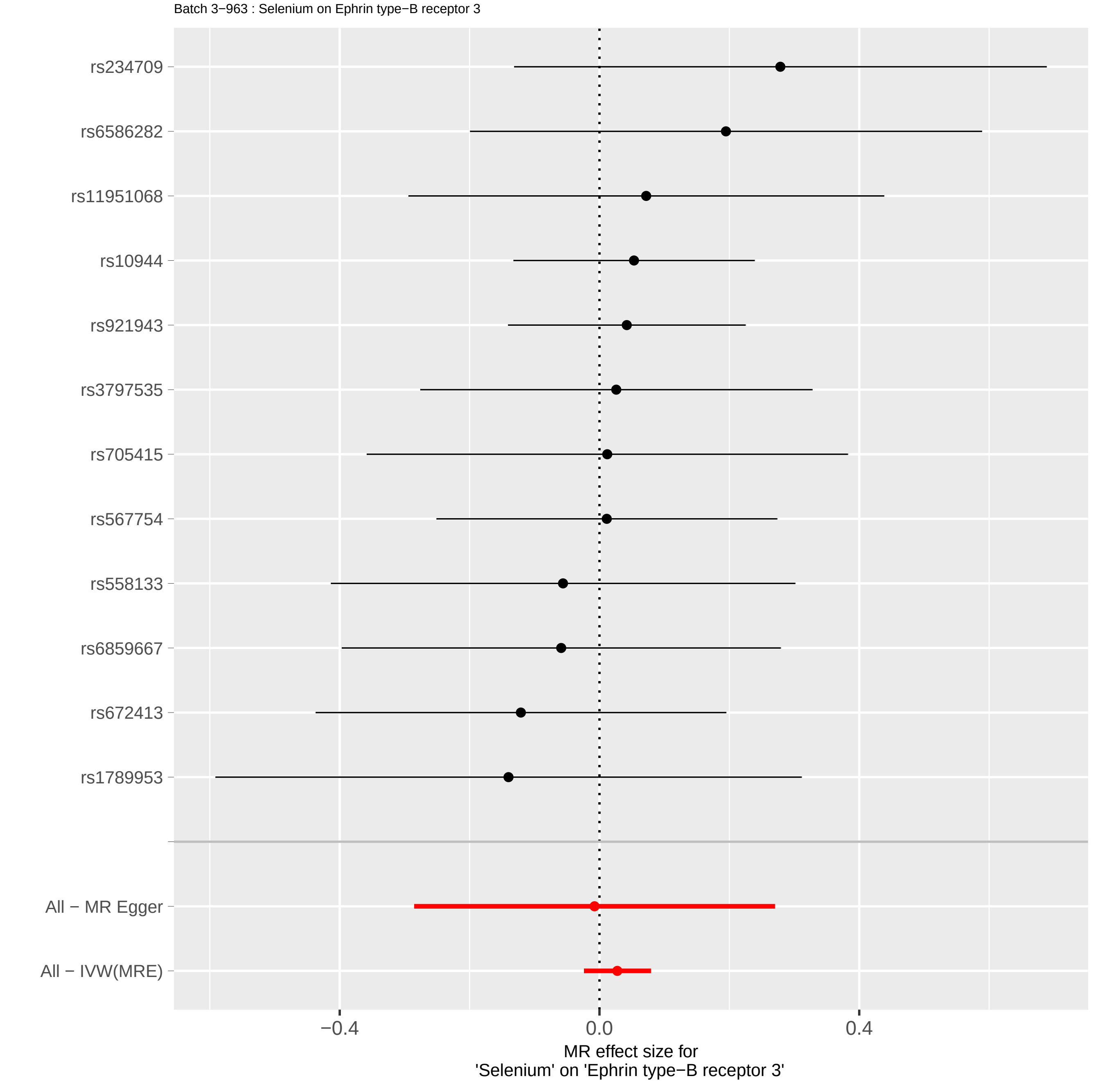

Batch 3−963 : Selenium on Ephrin type−B receptor 3
rs234709
rs6586282
rs11951068
rs10944
rs921943
rs3797535
rs705415
rs567754
rs558133
rs6859667
rs672413
rs1789953
All − MR Egger
All − IVW(MRE)
−0.4
0.0
0.4
MR effect size for
'Selenium' on 'Ephrin type−B receptor 3'

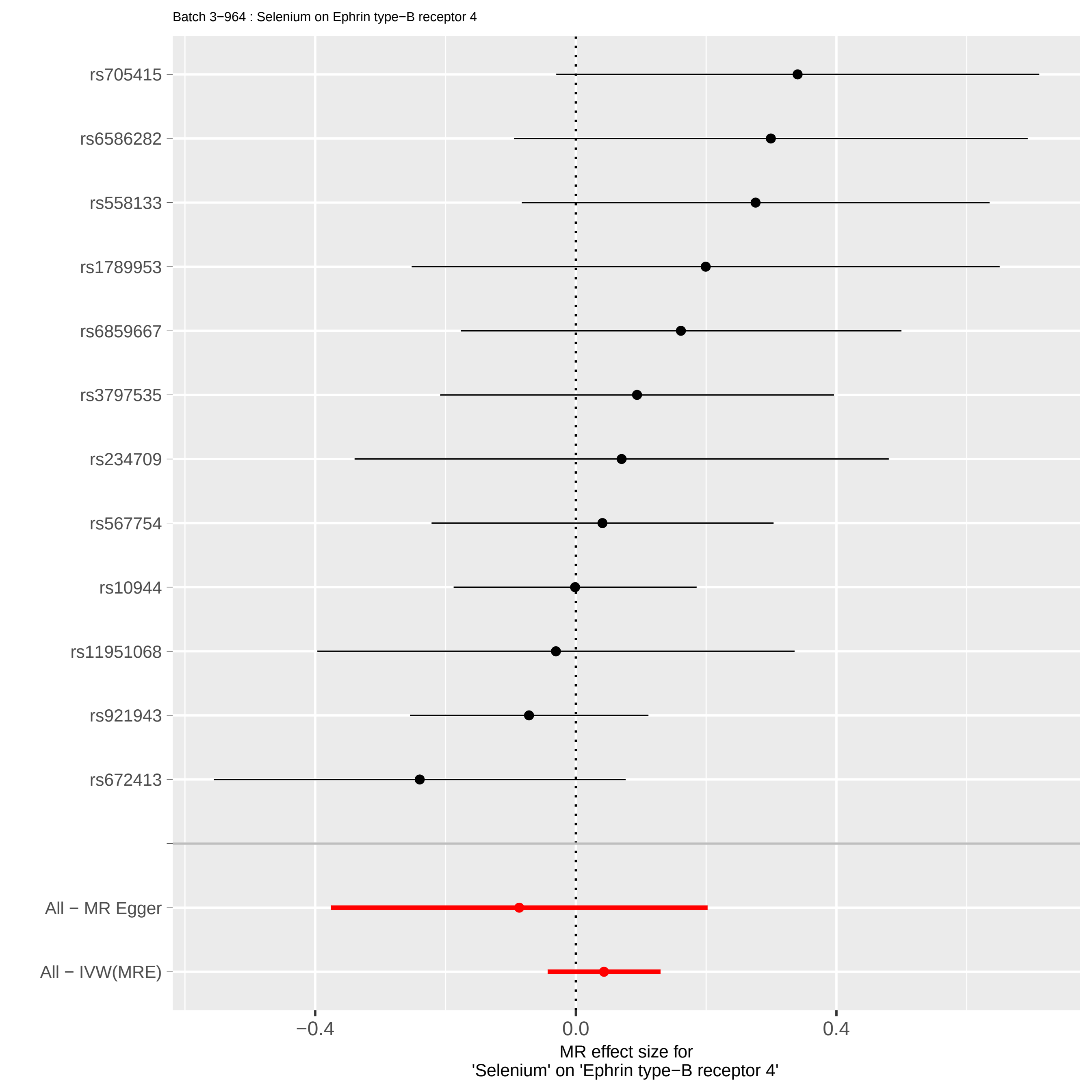

Batch 3−964 : Selenium on Ephrin type−B receptor 4
rs705415
rs6586282
rs558133
rs1789953
rs6859667
rs3797535
rs234709
rs567754
rs10944
rs11951068
rs921943
rs672413
All − MR Egger
All − IVW(MRE)
−0.4
0.0
0.4
MR effect size for
'Selenium' on 'Ephrin type−B receptor 4'

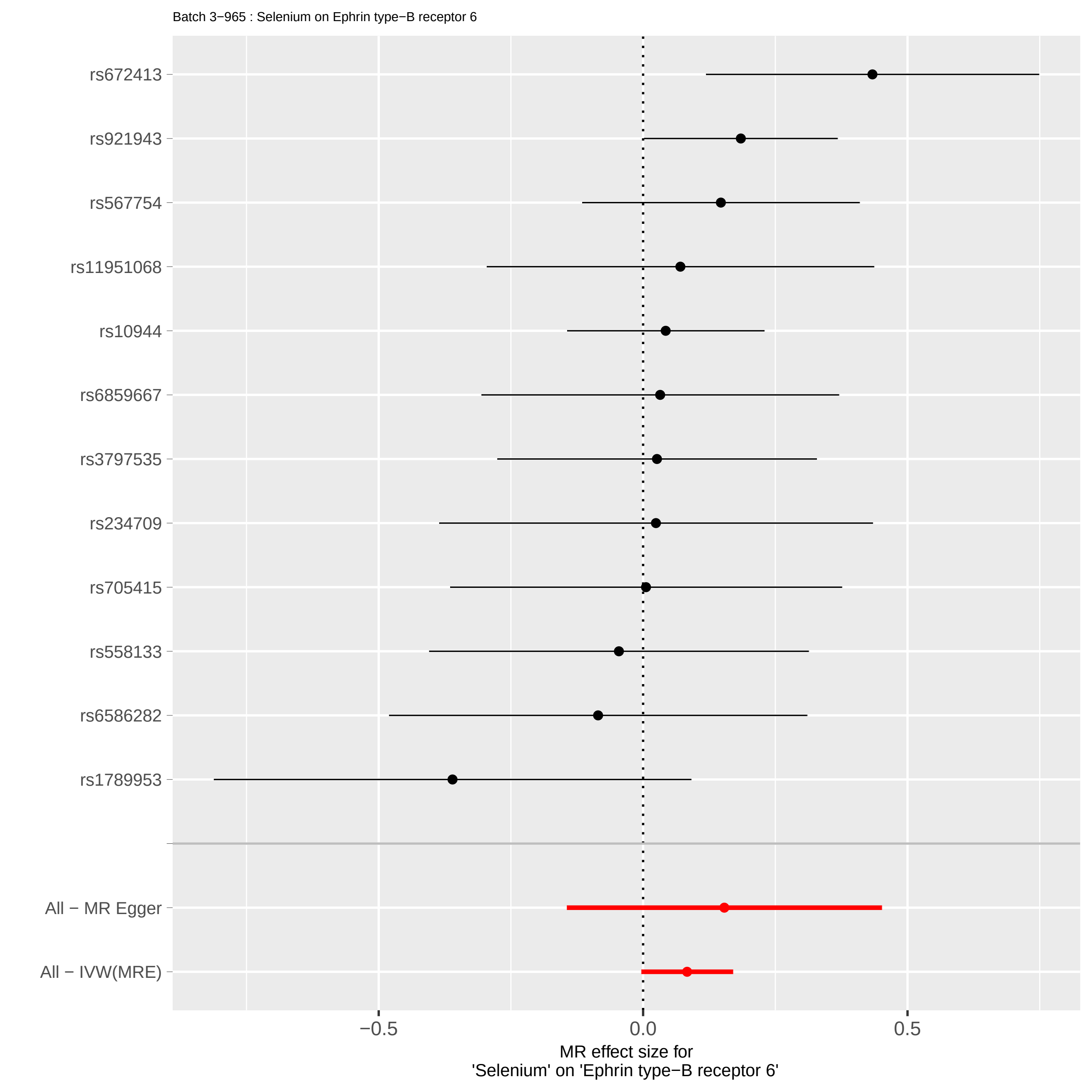

Batch 3−965 : Selenium on Ephrin type−B receptor 6
rs672413
rs921943
rs567754
rs11951068
rs10944
rs6859667
rs3797535
rs234709
rs705415
rs558133
rs6586282
rs1789953
All − MR Egger
All − IVW(MRE)
−0.5
0.0
0.5
MR effect size for
'Selenium' on 'Ephrin type−B receptor 6'

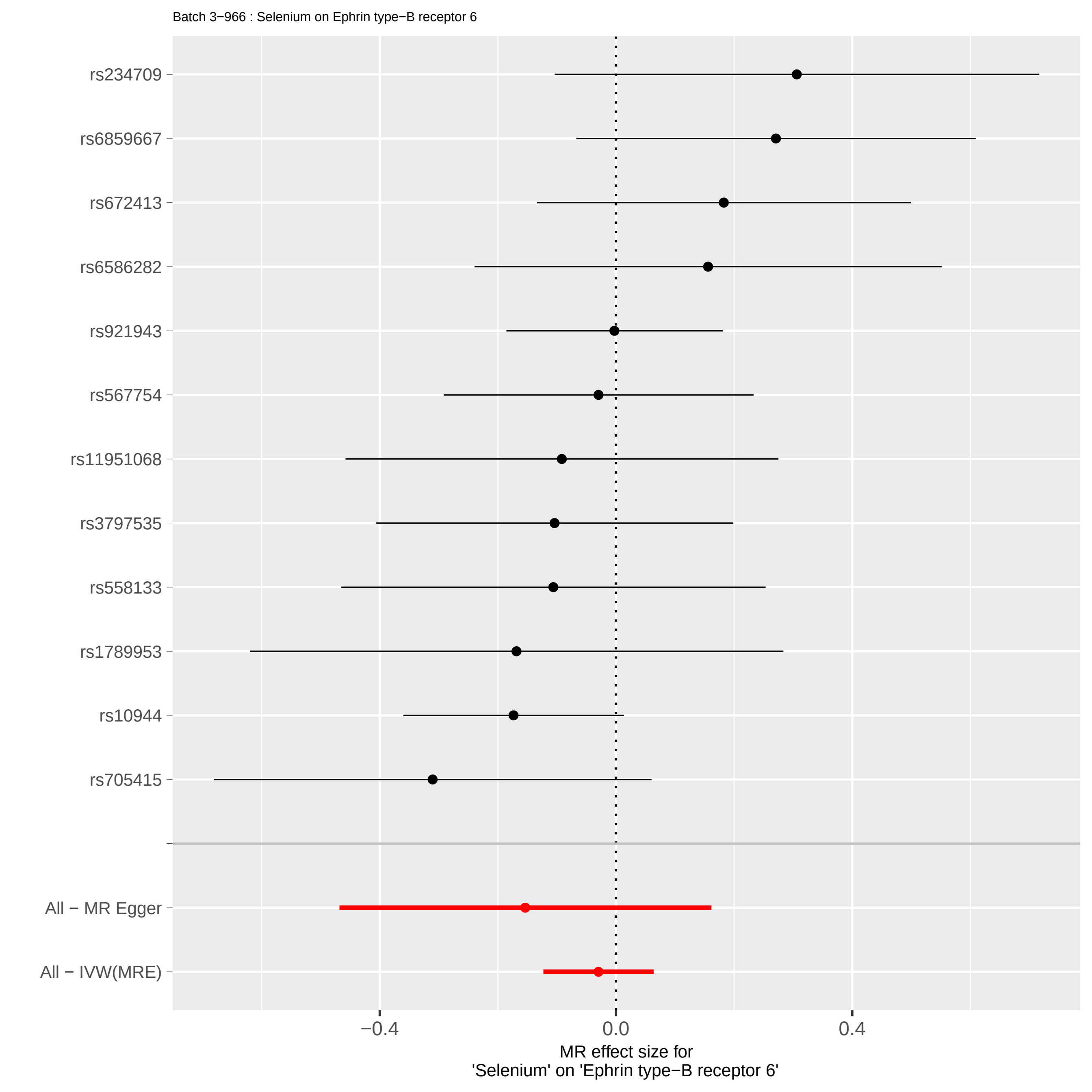
Batch 3−966 : Selenium on Ephrin type−B receptor 6
rs234709
rs6859667
rs672413
rs6586282
rs921943
rs567754
rs11951068
rs3797535
rs558133
rs1789953
rs10944
rs705415
All − MR Egger
All − IVW(MRE)
−0.4
0.0
0.4
MR effect size for
'Selenium' on 'Ephrin type−B receptor 6'

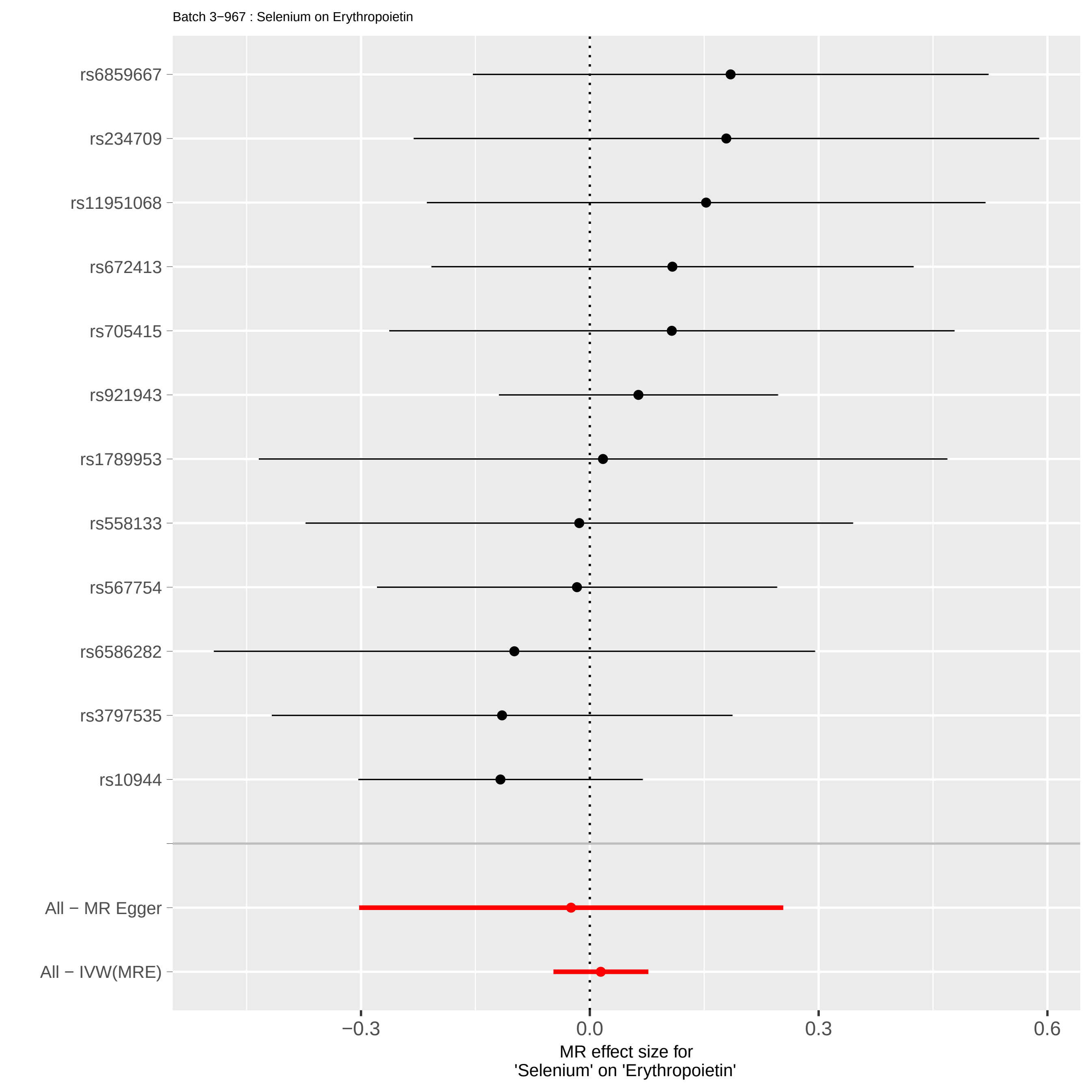

Batch 3−967 : Selenium on Erythropoietin
rs6859667
rs234709
rs11951068
rs672413
rs705415
rs921943
rs1789953
rs558133
rs567754
rs6586282
rs3797535
rs10944
All − MR Egger
All − IVW(MRE)
−0.3
0.0
0.3
0.6
MR effect size for
'Selenium' on 'Erythropoietin'

Batch 3−968 : Selenium on Erythropoietin receptor

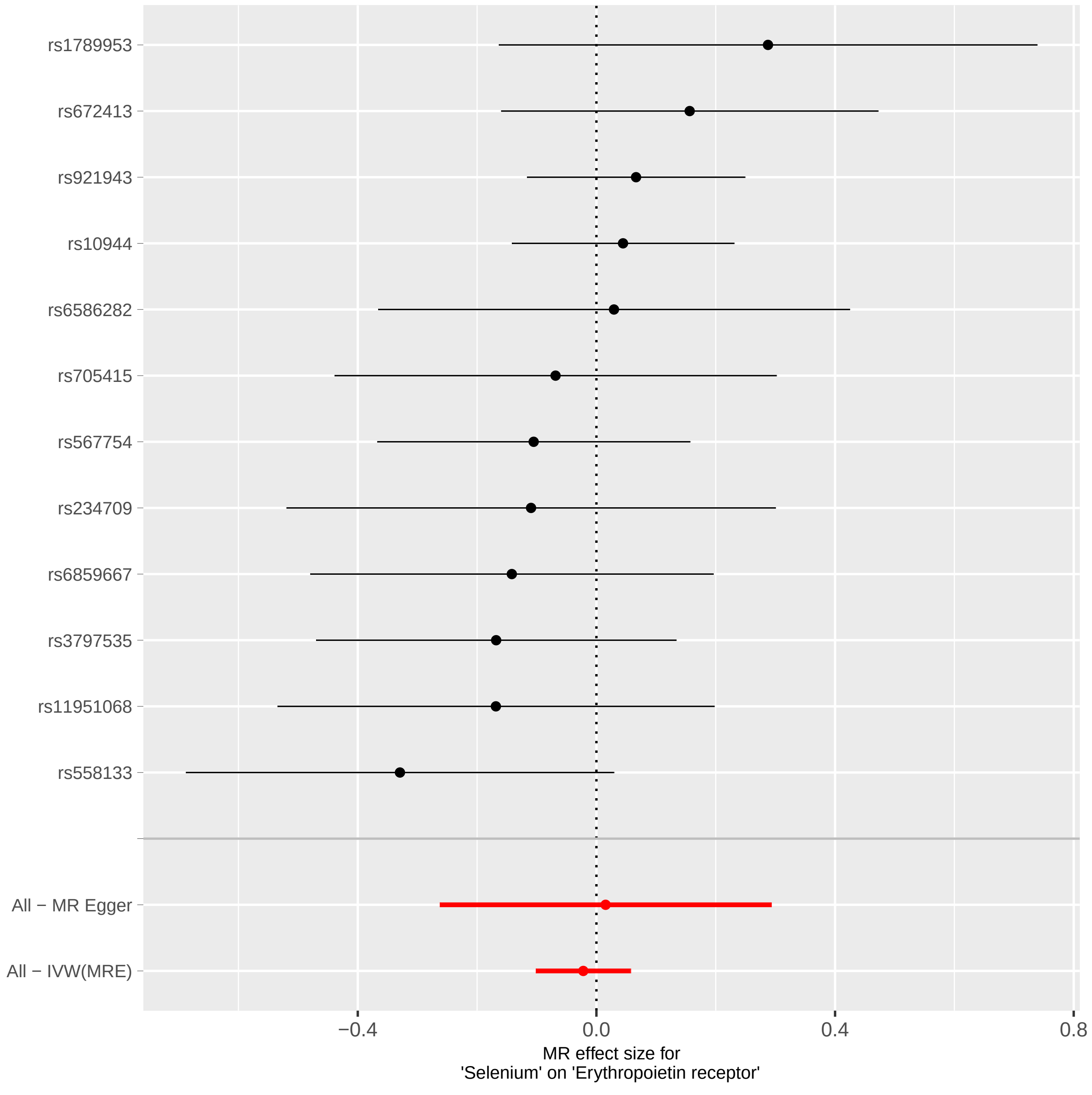

Batch 3−969 : Selenium on Epidermal growth factor receptor substrate 15−like 1

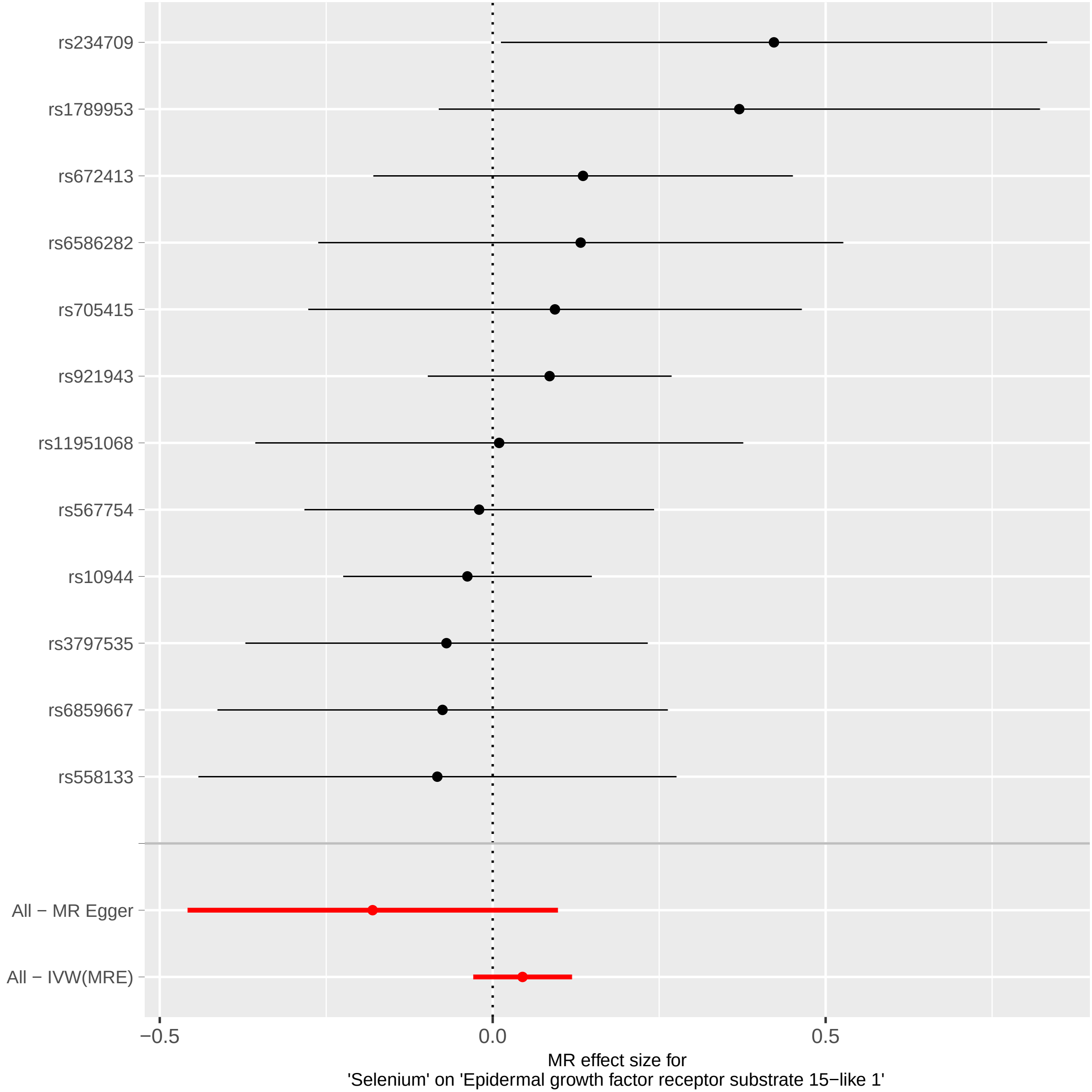

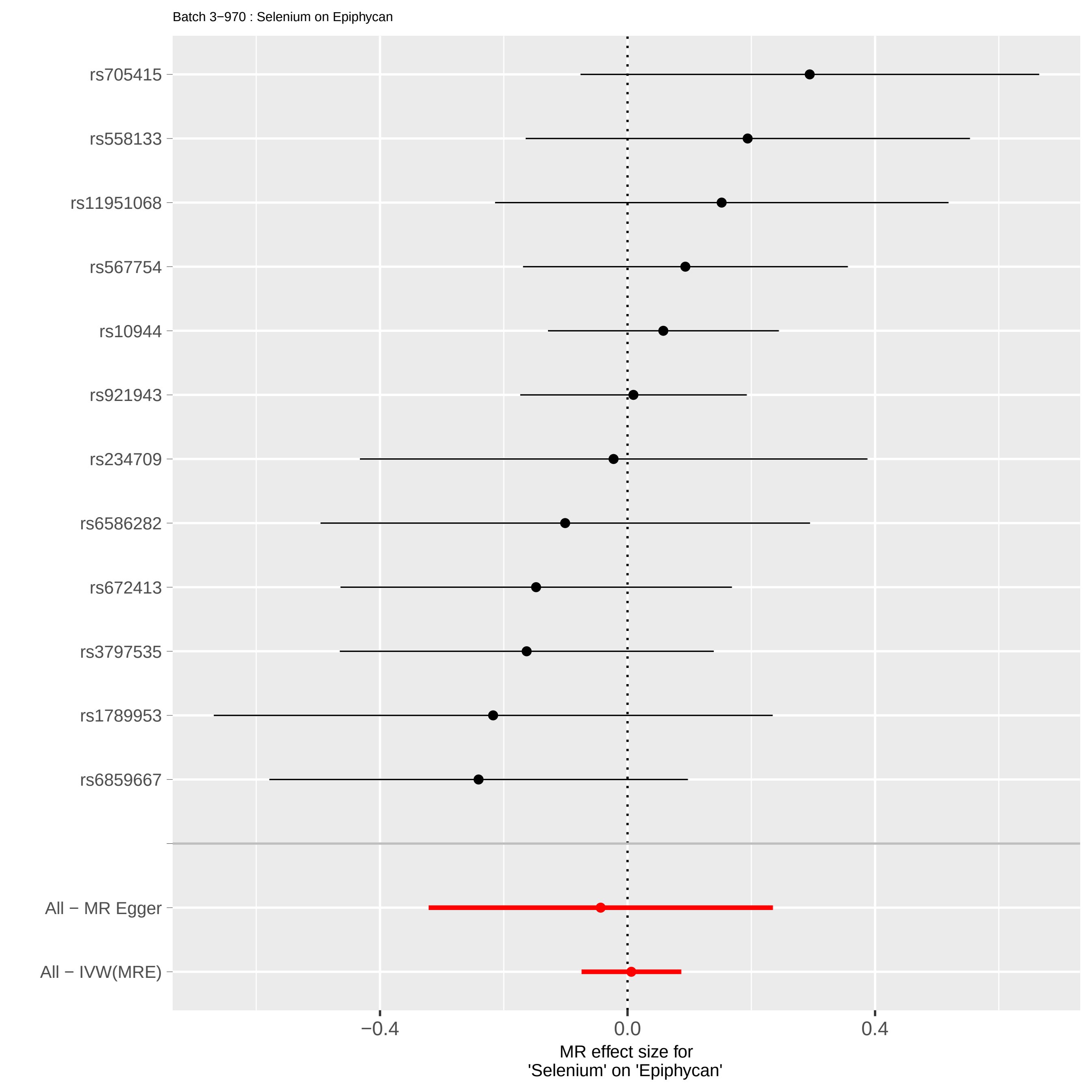
Batch 3−970 : Selenium on Epiphycan
rs705415
rs558133
rs11951068
rs567754
rs10944
rs921943
rs234709
rs6586282
rs672413
rs3797535
rs1789953
rs6859667
All − MR Egger
All − IVW(MRE)
−0.4
0.0
0.4
MR effect size for
'Selenium' on 'Epiphycan'

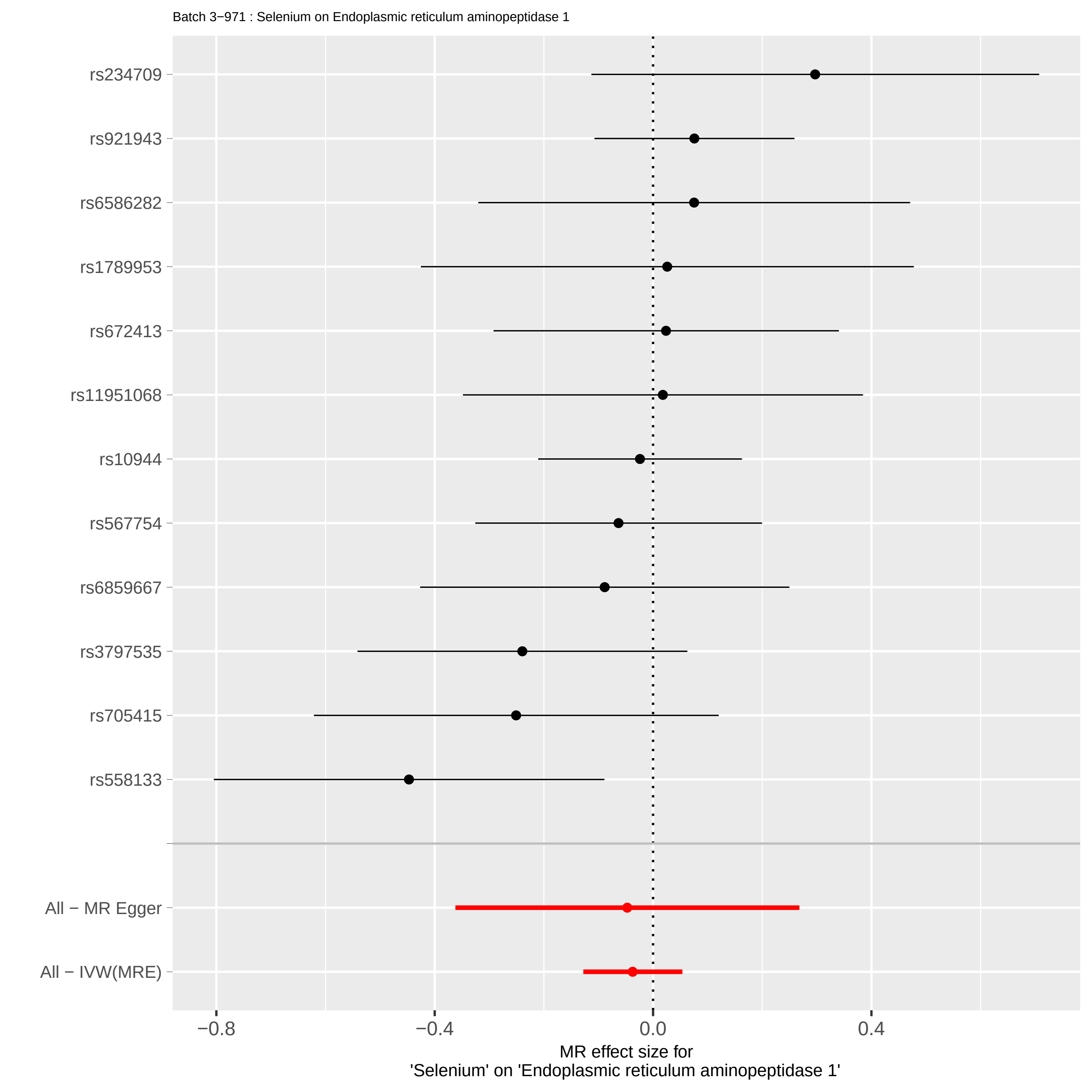
Batch 3−971 : Selenium on Endoplasmic reticulum aminopeptidase 1
rs234709
rs921943
rs6586282
rs1789953
rs672413
rs11951068
rs10944
rs567754
rs6859667
rs3797535
rs705415
rs558133
All − MR Egger
All − IVW(MRE)
−0.8
−0.4
0.0
0.4
MR effect size for
'Selenium' on 'Endoplasmic reticulum aminopeptidase 1'

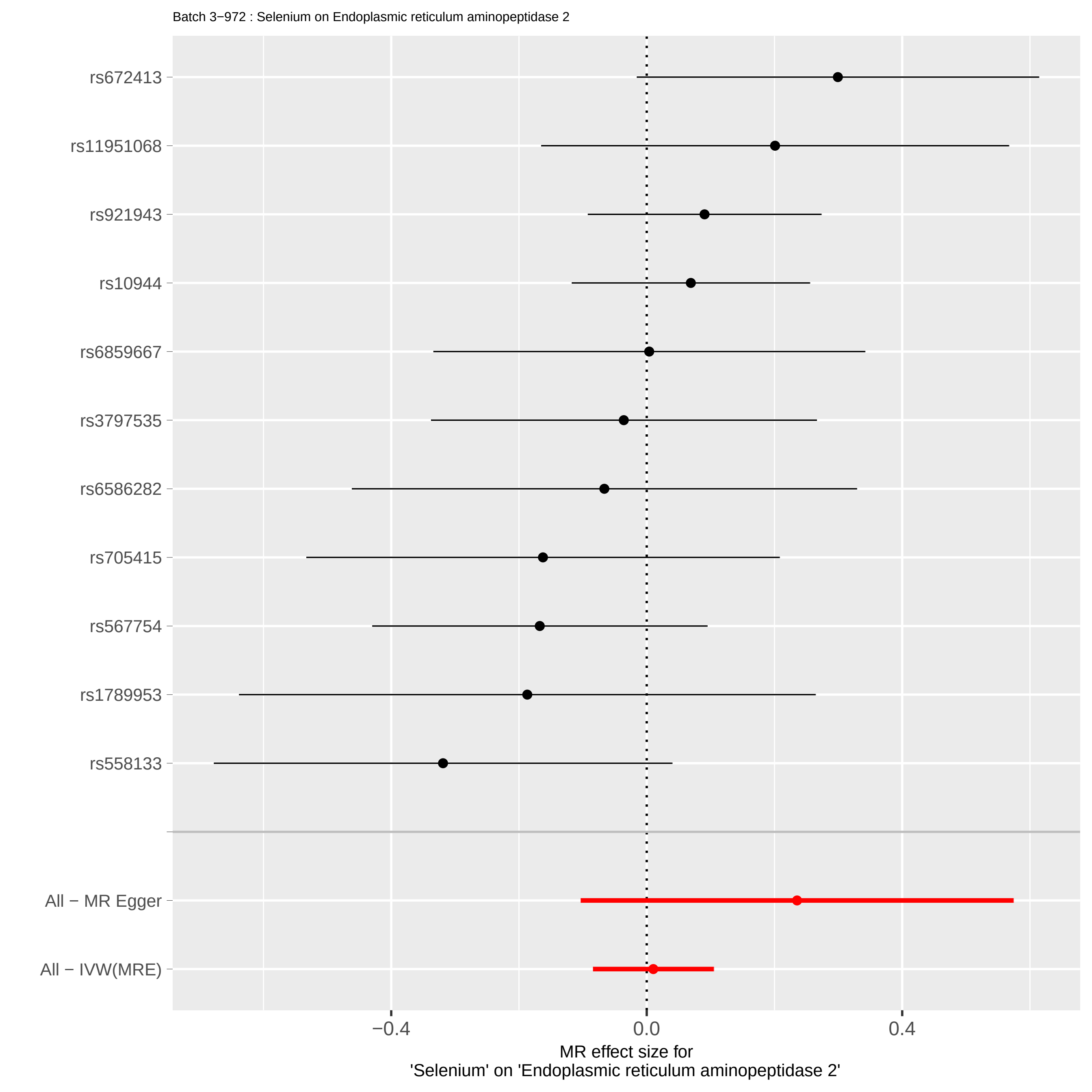
Batch 3−972 : Selenium on Endoplasmic reticulum aminopeptidase 2
rs672413
rs11951068
rs921943
rs10944
rs6859667
rs3797535
rs6586282
rs705415
rs567754
rs1789953
rs558133
All − MR Egger
All − IVW(MRE)
−0.4
0.0
0.4
MR effect size for
'Selenium' on 'Endoplasmic reticulum aminopeptidase 2'

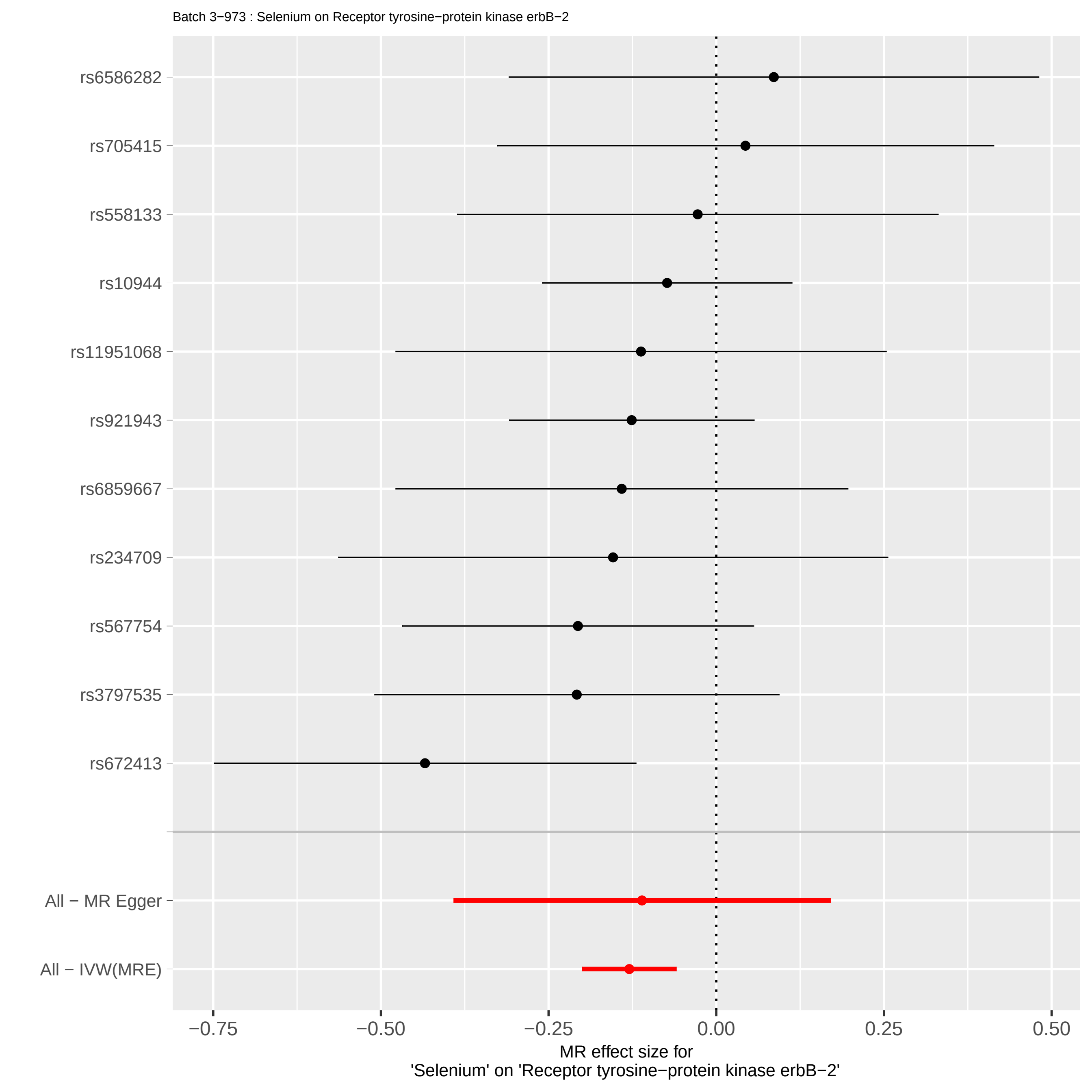

Batch 3−973 : Selenium on Receptor tyrosine−protein kinase erbB−2
rs6586282
rs705415
rs558133
rs10944
rs11951068
rs921943
rs6859667
rs234709
rs567754
rs3797535
rs672413
All − MR Egger
All − IVW(MRE)
−0.75
−0.50
−0.25
0.00
0.25
0.50
MR effect size for
'Selenium' on 'Receptor tyrosine−protein kinase erbB−2'

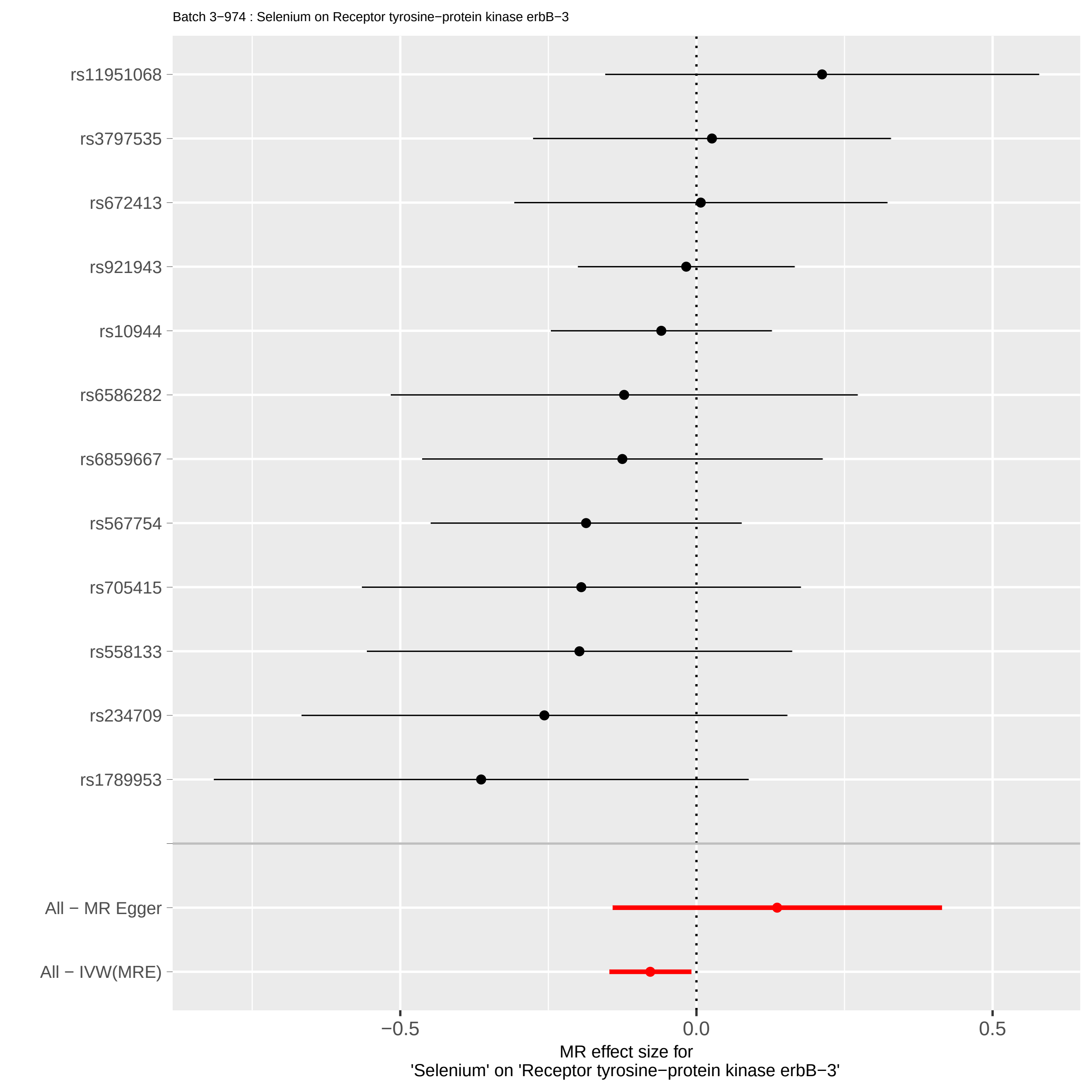

Batch 3−974 : Selenium on Receptor tyrosine−protein kinase erbB−3
rs11951068
rs3797535
rs672413
rs921943
rs10944
rs6586282
rs6859667
rs567754
rs705415
rs558133
rs234709
rs1789953
All − MR Egger
All − IVW(MRE)
−0.5
0.0
0.5
MR effect size for
'Selenium' on 'Receptor tyrosine−protein kinase erbB−3'

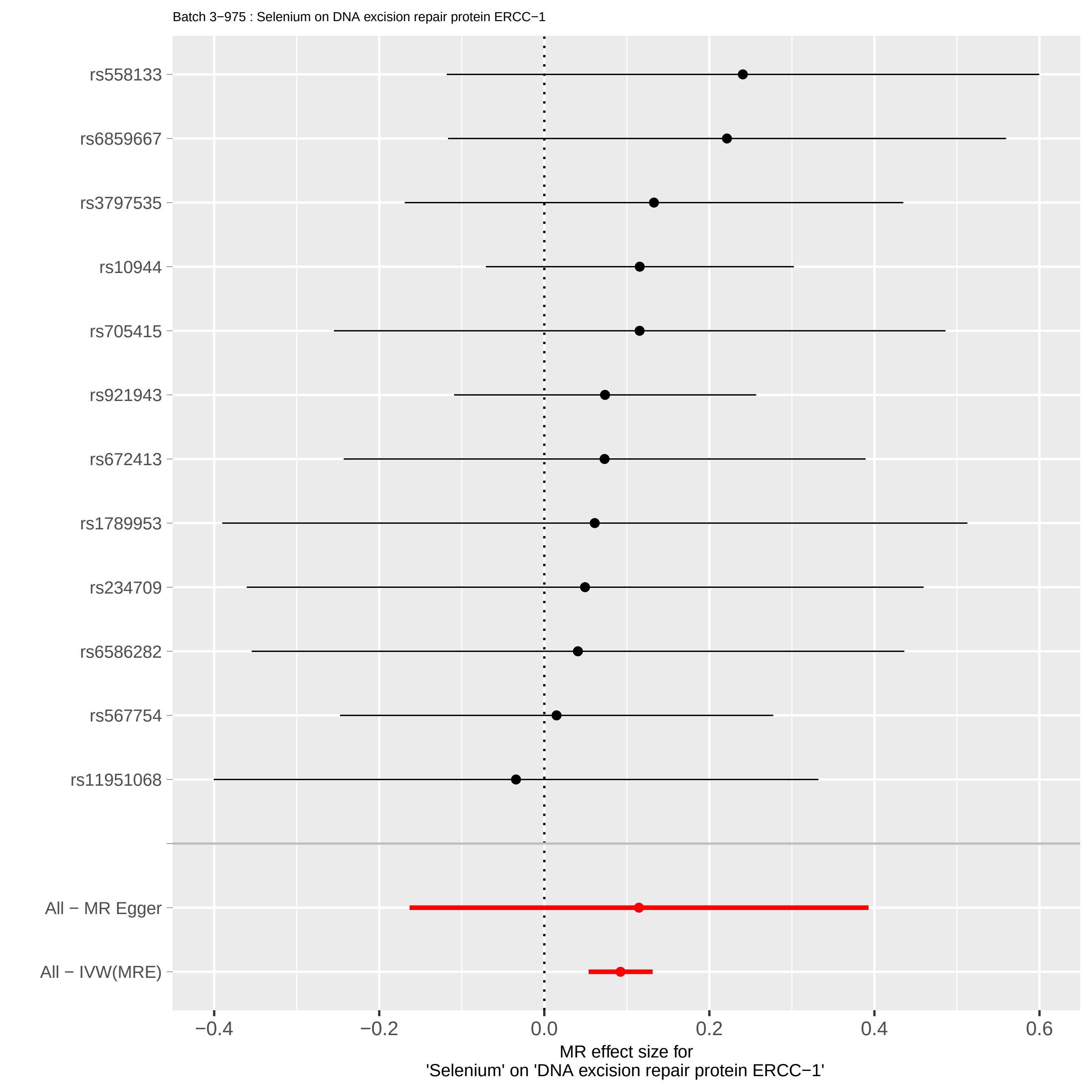

Batch 3−975 : Selenium on DNA excision repair protein ERCC−1
rs558133
rs6859667
rs3797535
rs10944
rs705415
rs921943
rs672413
rs1789953
rs234709
rs6586282
rs567754
rs11951068
All − MR Egger
All − IVW(MRE)
−0.4
−0.2
0.0
0.2
0.4
0.6
MR effect size for
'Selenium' on 'DNA excision repair protein ERCC−1'

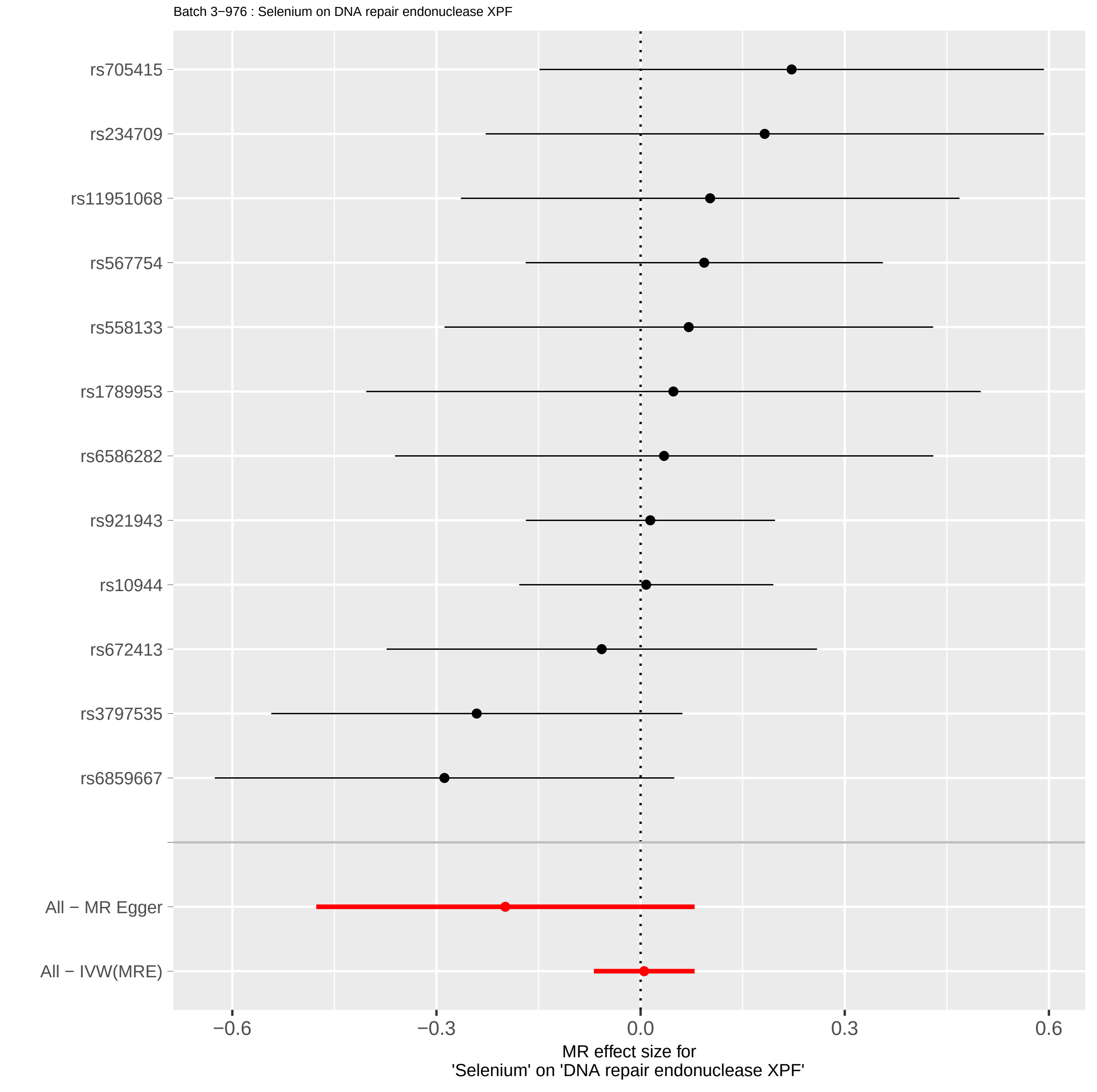
Batch 3−976 : Selenium on DNA repair endonuclease XPF
rs705415
rs234709
rs11951068
rs567754
rs558133
rs1789953
rs6586282
rs921943
rs10944
rs672413
rs3797535
rs6859667
All − MR Egger
All − IVW(MRE)
−0.6
−0.3
0.0
0.3
0.6
MR effect size for
'Selenium' on 'DNA repair endonuclease XPF'

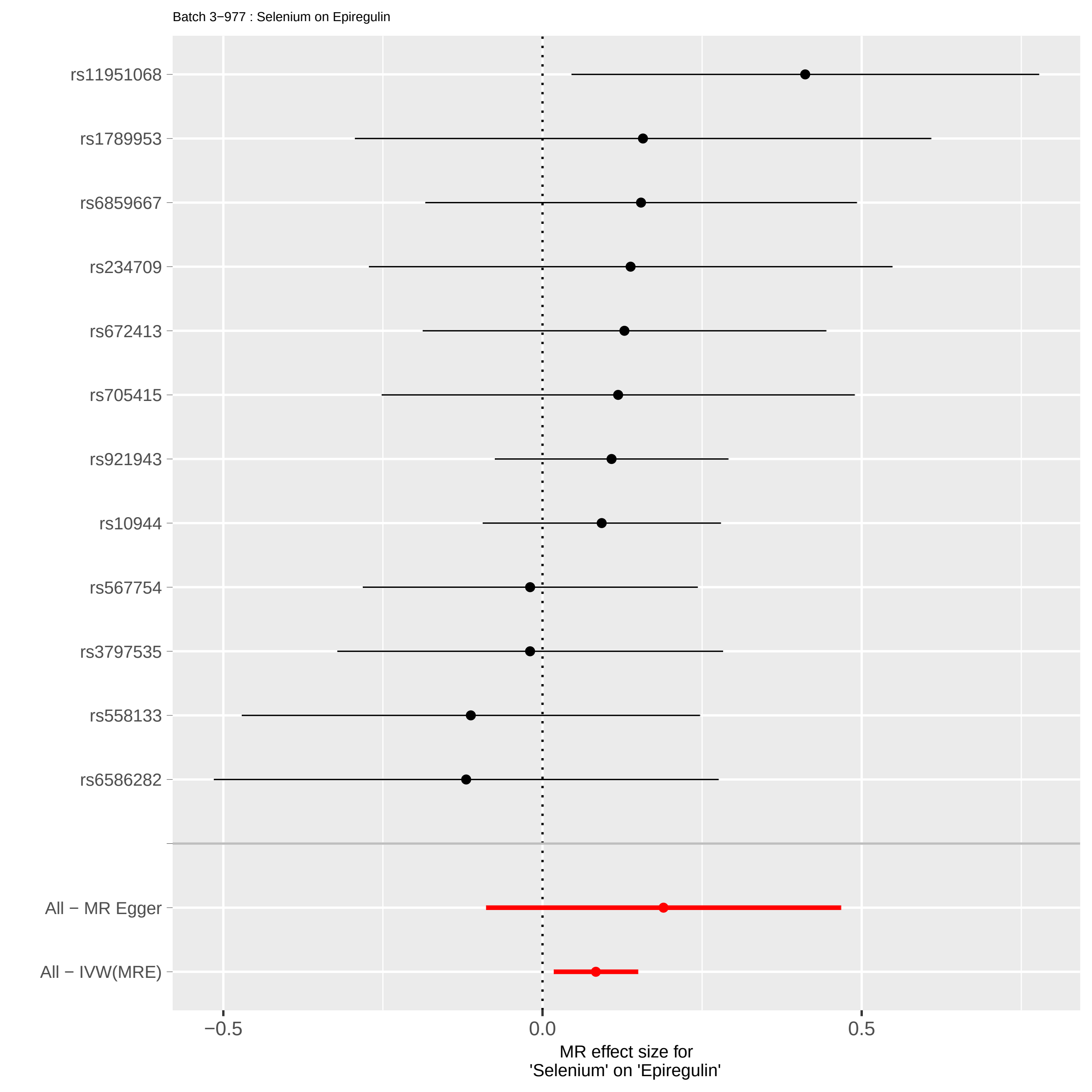
Batch 3−977 : Selenium on Epiregulin
rs11951068
rs1789953
rs6859667
rs234709
rs672413
rs705415
rs921943
rs10944
rs567754
rs3797535
rs558133
rs6586282
All − MR Egger
All − IVW(MRE)
−0.5
0.0
0.5
MR effect size for
'Selenium' on 'Epiregulin'

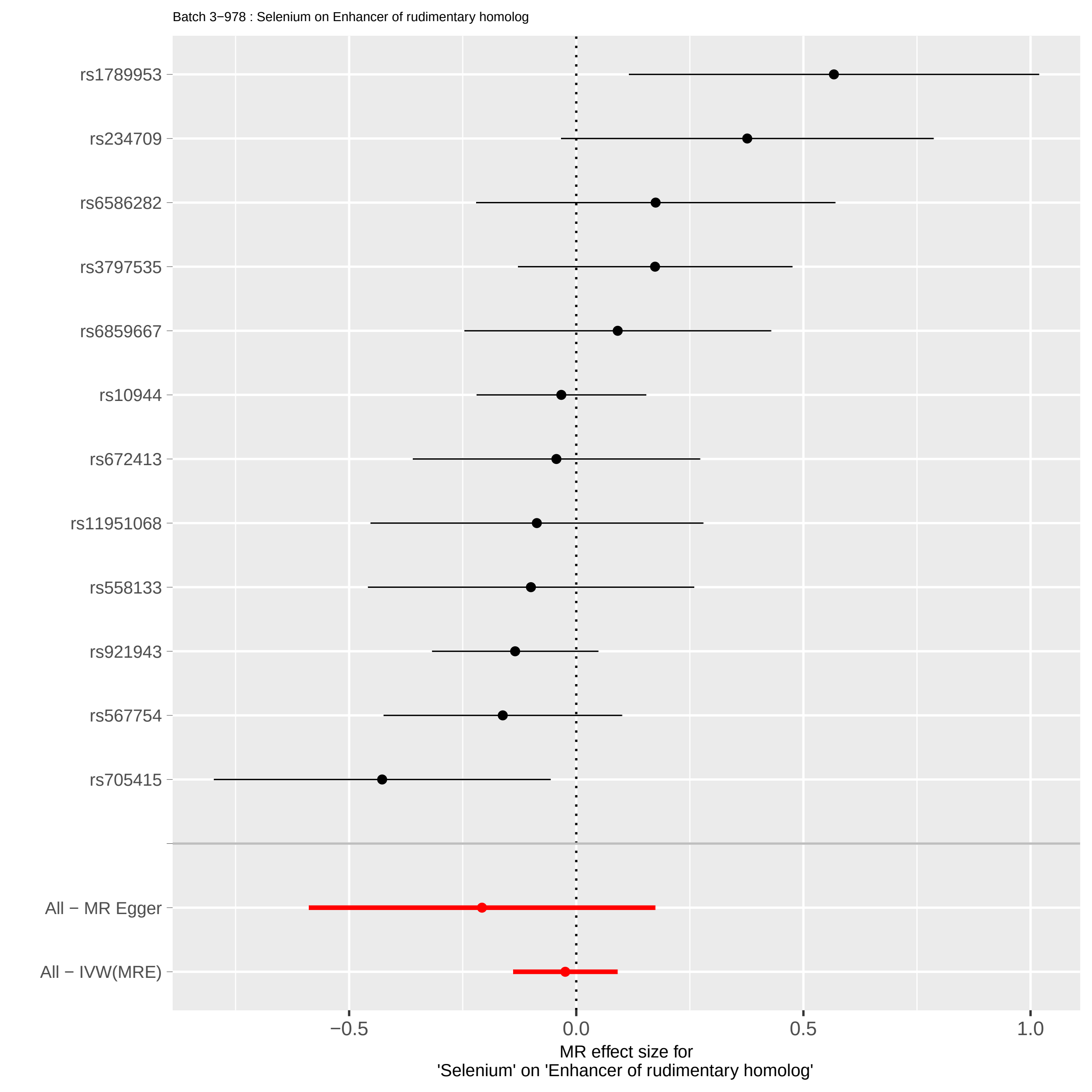

Batch 3−978 : Selenium on Enhancer of rudimentary homolog
rs1789953
rs234709
rs6586282
rs3797535
rs6859667
rs10944
rs672413
rs11951068
rs558133
rs921943
rs567754
rs705415
All − MR Egger
All − IVW(MRE)
−0.5
0.0
0.5
1.0
MR effect size for
'Selenium' on 'Enhancer of rudimentary homolog'

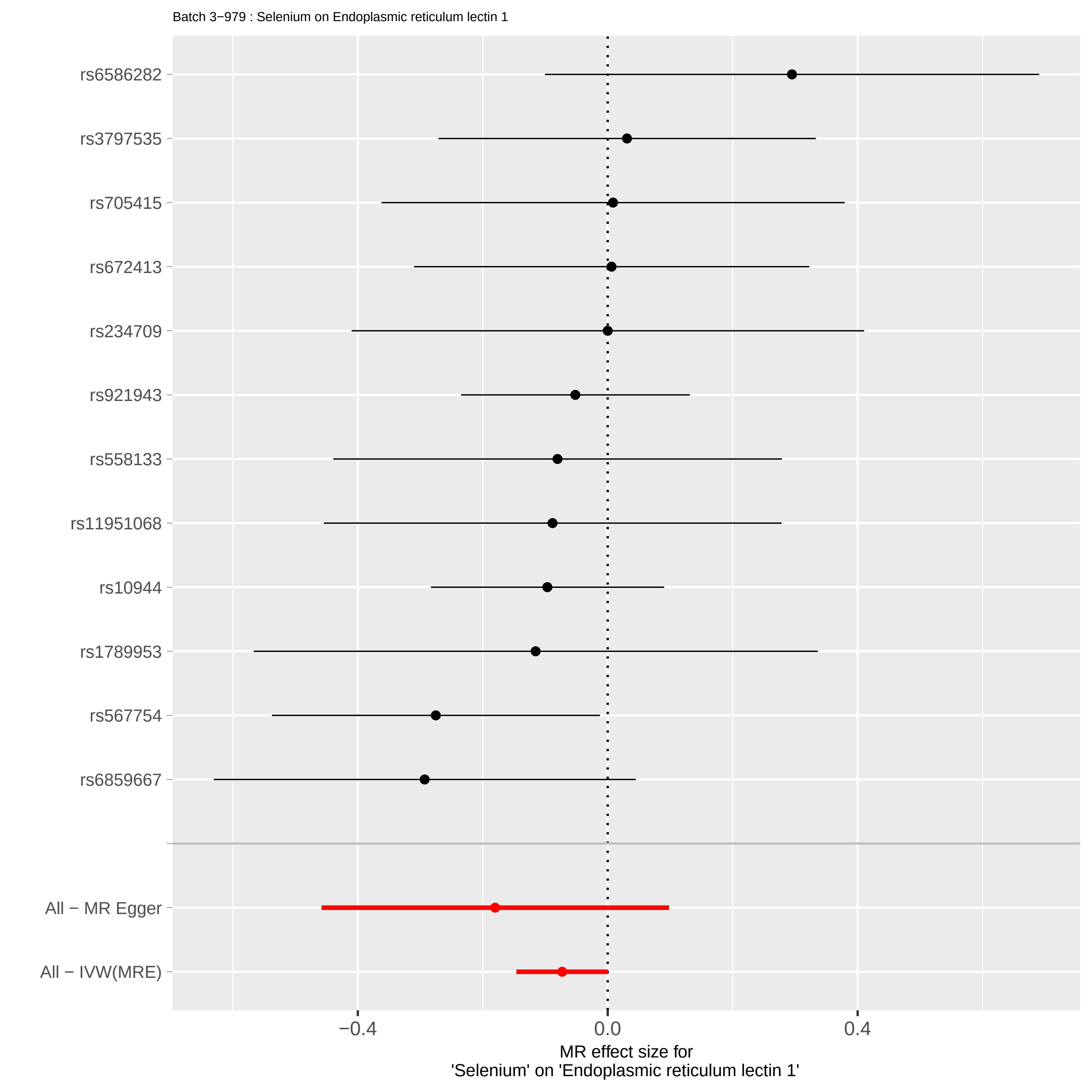
Batch 3−979 : Selenium on Endoplasmic reticulum lectin 1
rs6586282
rs3797535
rs705415
rs672413
rs234709
rs921943
rs558133
rs11951068
rs10944
rs1789953
rs567754
rs6859667
All − MR Egger
All − IVW(MRE)
−0.4
0.0
0.4
MR effect size for
'Selenium' on 'Endoplasmic reticulum lectin 1'

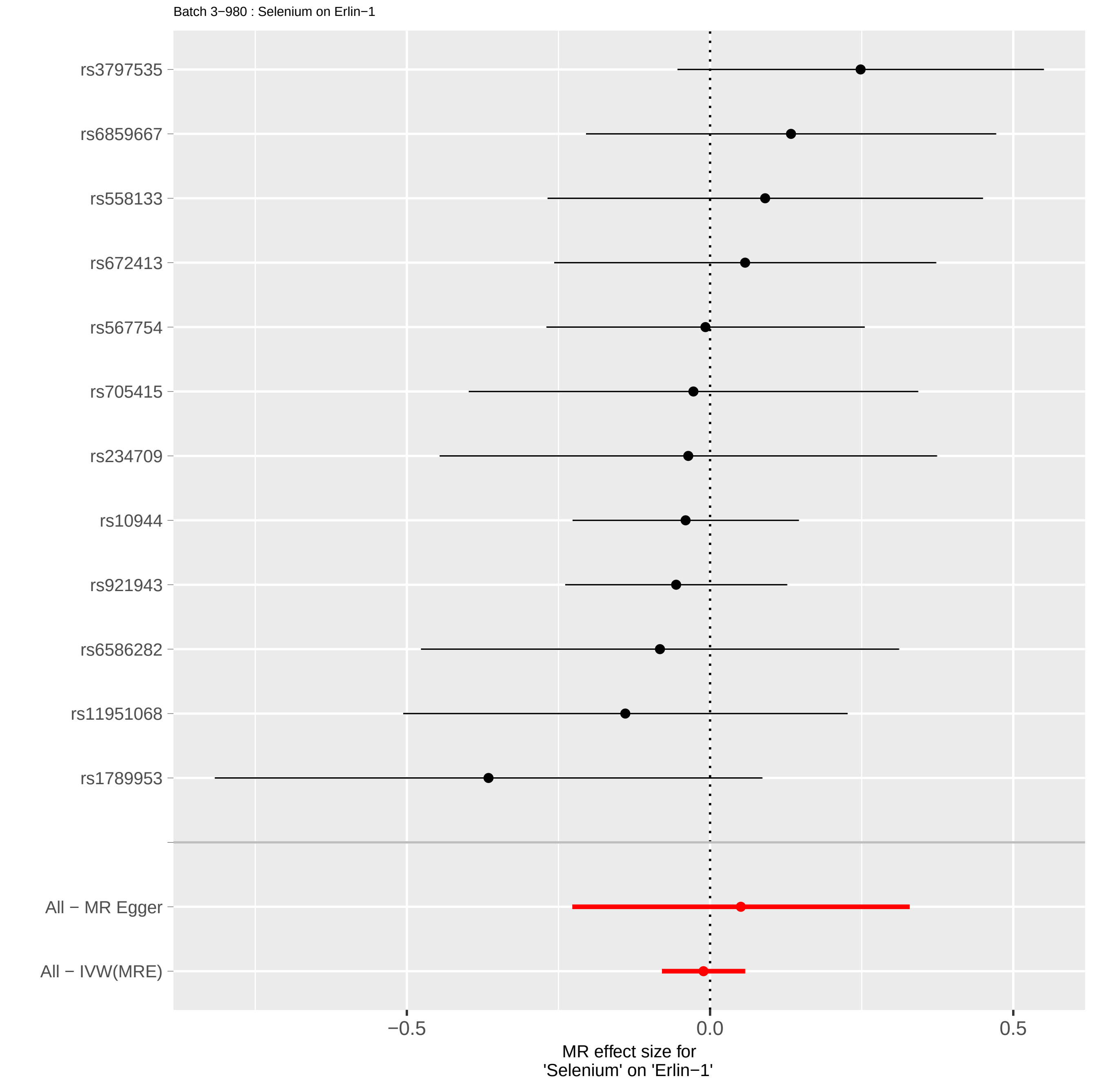

Batch 3−980 : Selenium on Erlin−1
rs3797535
rs6859667
rs558133
rs672413
rs567754
rs705415
rs234709
rs10944
rs921943
rs6586282
rs11951068
rs1789953
All − MR Egger
All − IVW(MRE)
−0.5
0.0
0.5
MR effect size for
'Selenium' on 'Erlin−1'

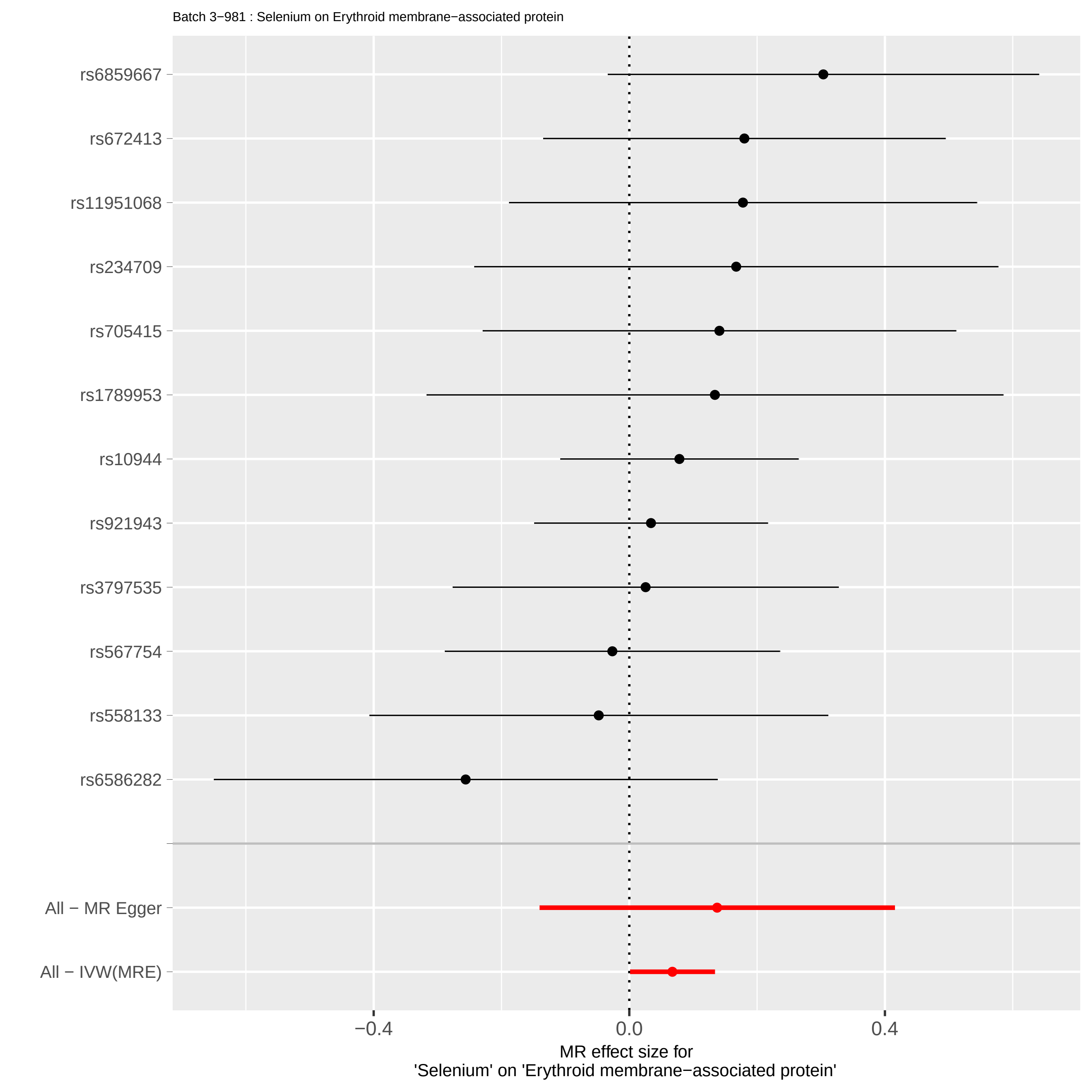
Batch 3−981 : Selenium on Erythroid membrane−associated protein
rs6859667
rs672413
rs11951068
rs234709
rs705415
rs1789953
rs10944
rs921943
rs3797535
rs567754
rs558133
rs6586282
All − MR Egger
All − IVW(MRE)
−0.4
0.0
0.4
MR effect size for
'Selenium' on 'Erythroid membrane−associated protein'

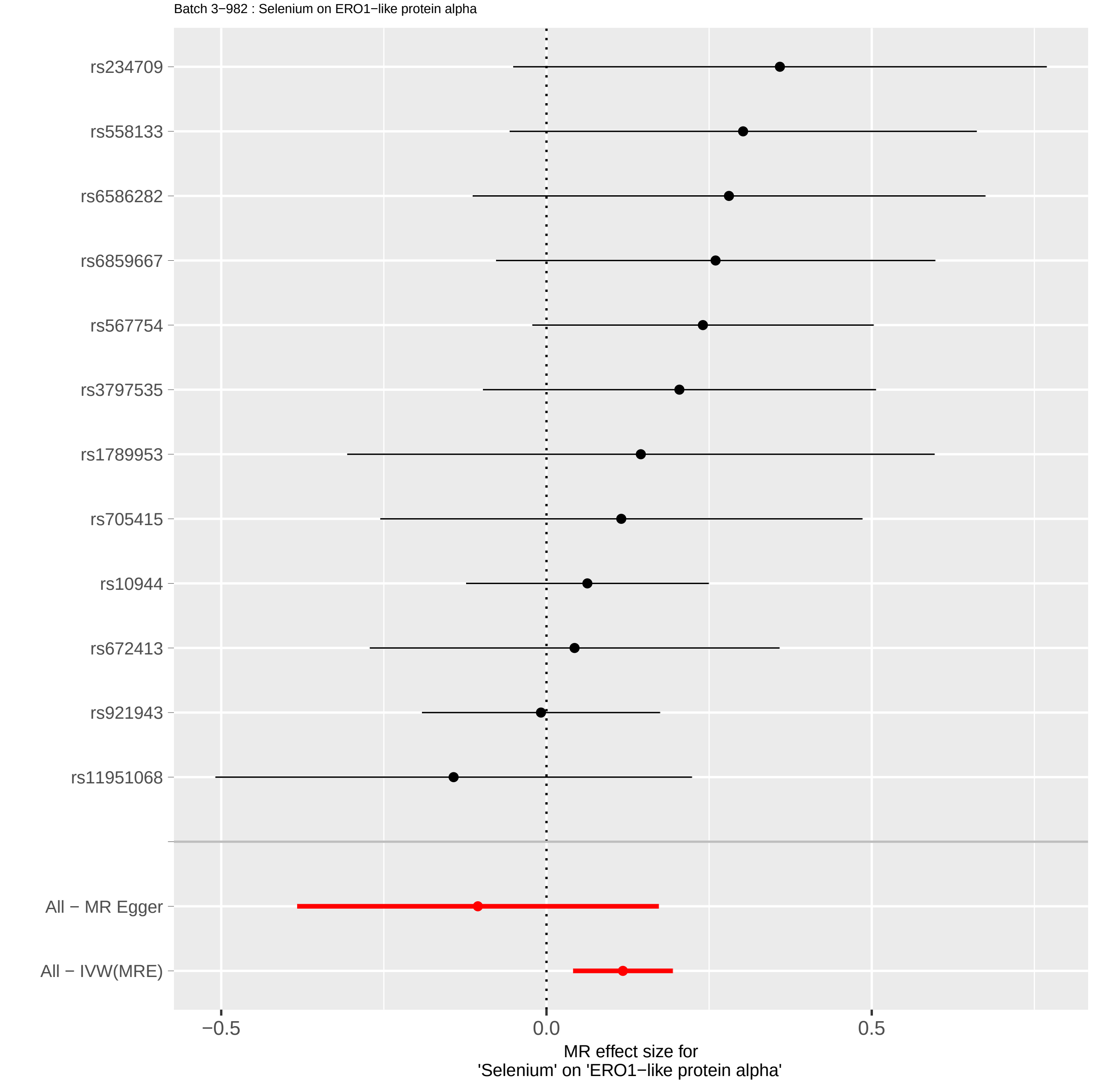

Batch 3−982 : Selenium on ERO1−like protein alpha
rs234709
rs558133
rs6586282
rs6859667
rs567754
rs3797535
rs1789953
rs705415
rs10944
rs672413
rs921943
rs11951068
All − MR Egger
All − IVW(MRE)
−0.5
0.0
0.5
MR effect size for
'Selenium' on 'ERO1−like protein alpha'

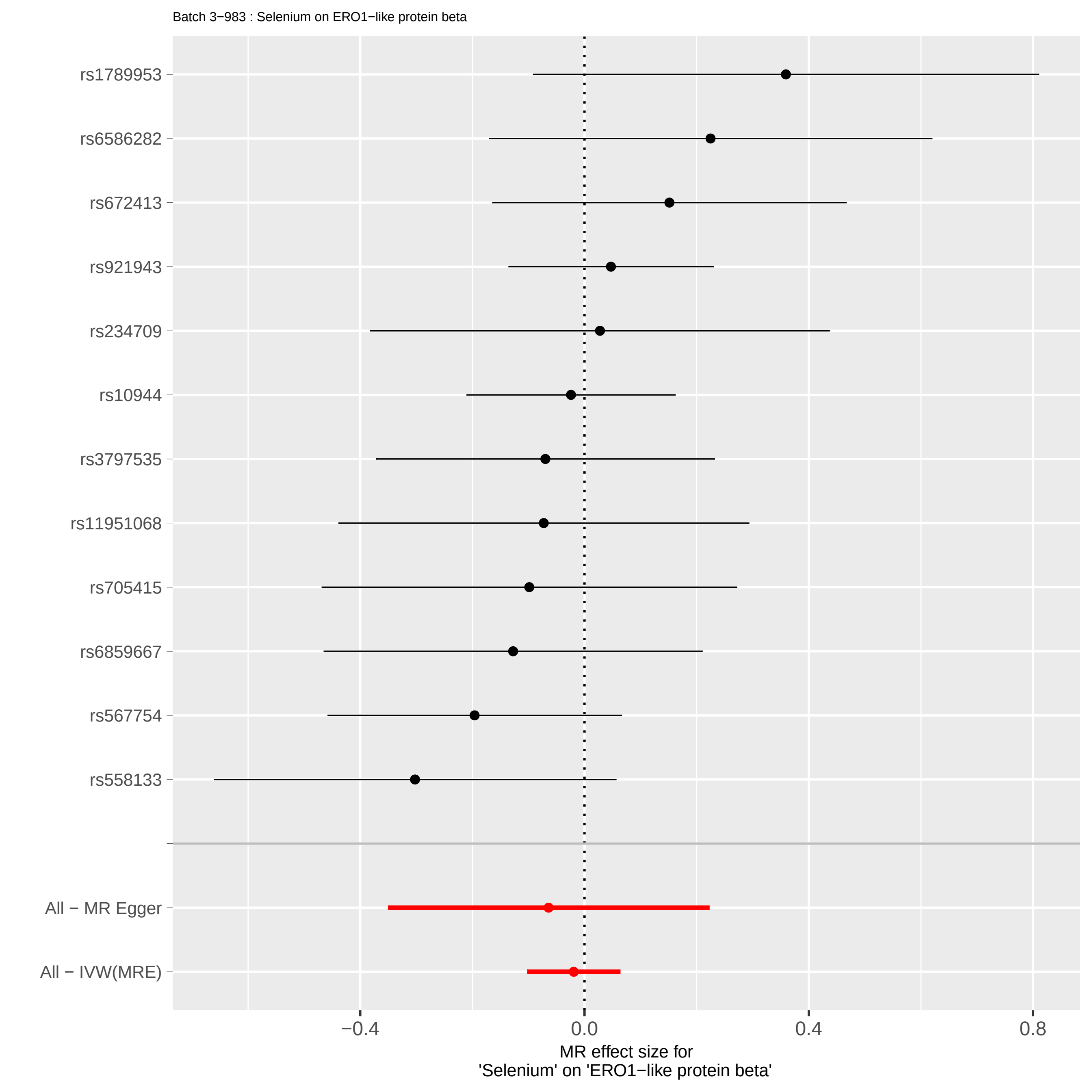
Batch 3−983 : Selenium on ERO1−like protein beta
rs1789953
rs6586282
rs672413
rs921943
rs234709
rs10944
rs3797535
rs11951068
rs705415
rs6859667
rs567754
rs558133
All − MR Egger
All − IVW(MRE)
−0.4
0.0
0.4
0.8
MR effect size for
'Selenium' on 'ERO1−like protein beta'

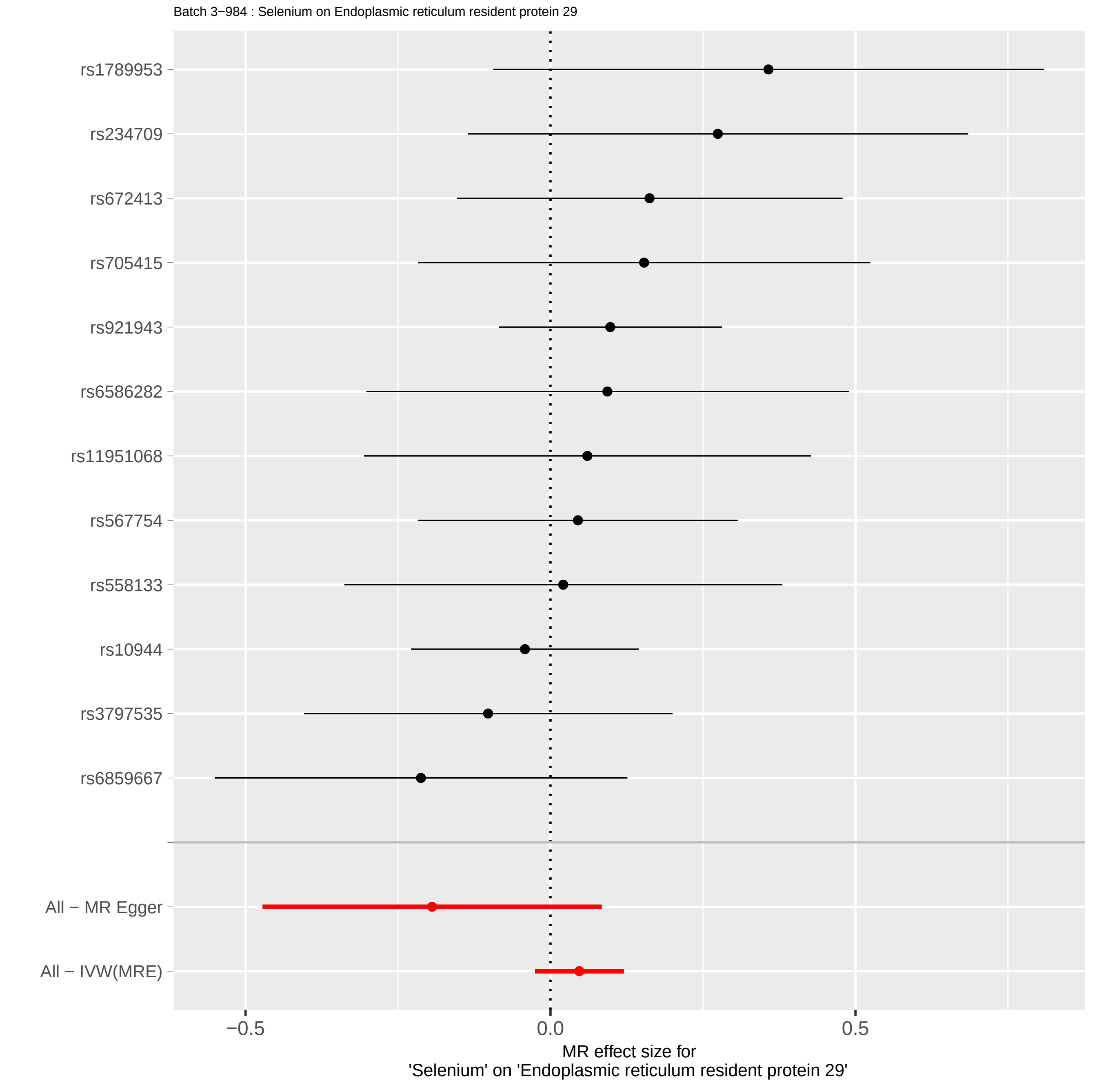

Batch 3−984 : Selenium on Endoplasmic reticulum resident protein 29
rs1789953
rs234709
rs672413
rs705415
rs921943
rs6586282
rs11951068
rs567754
rs558133
rs10944
rs3797535
rs6859667
All − MR Egger
All − IVW(MRE)
−0.5
0.0
0.5
MR effect size for
'Selenium' on 'Endoplasmic reticulum resident protein 29'

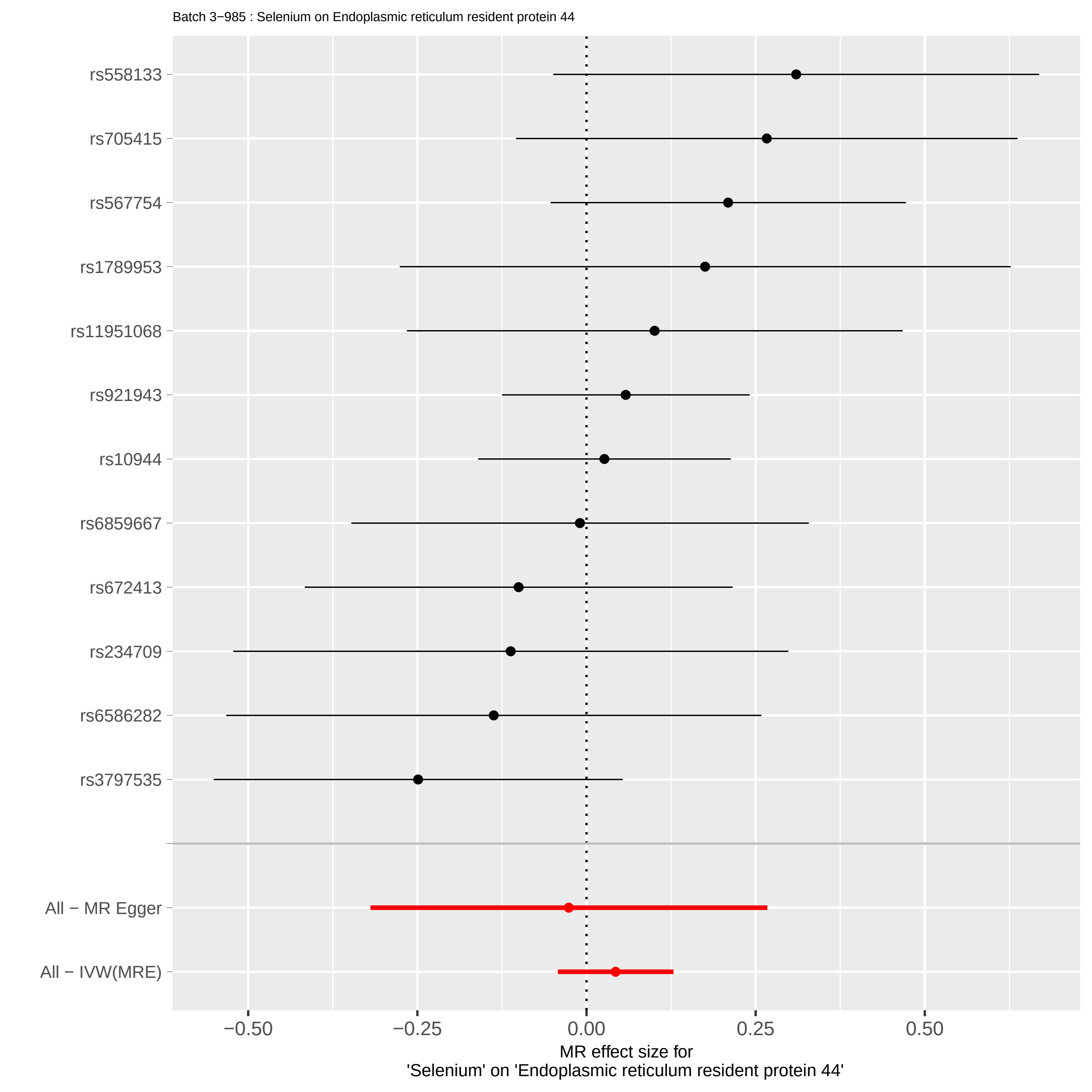
Batch 3−985 : Selenium on Endoplasmic reticulum resident protein 44
rs558133
rs705415
rs567754
rs1789953
rs11951068
rs921943
rs10944
rs6859667
rs672413
rs234709
rs6586282
rs3797535
All − MR Egger
All − IVW(MRE)
−0.50
−0.25
0.00
0.25
0.50
MR effect size for
'Selenium' on 'Endoplasmic reticulum resident protein 44'

Batch 3−986 : Selenium on Endogenous retrovirus group V member 1 Env polyprotein

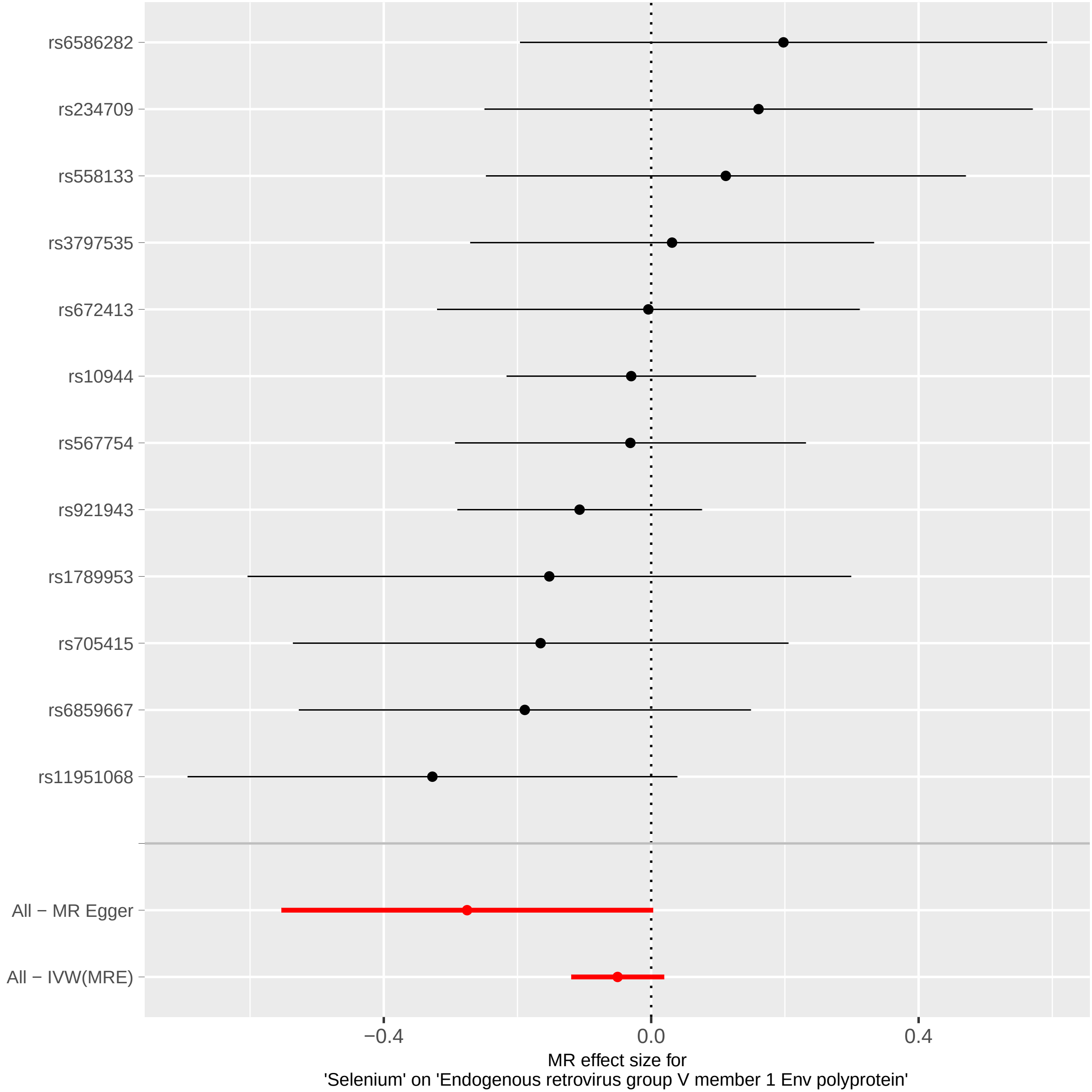

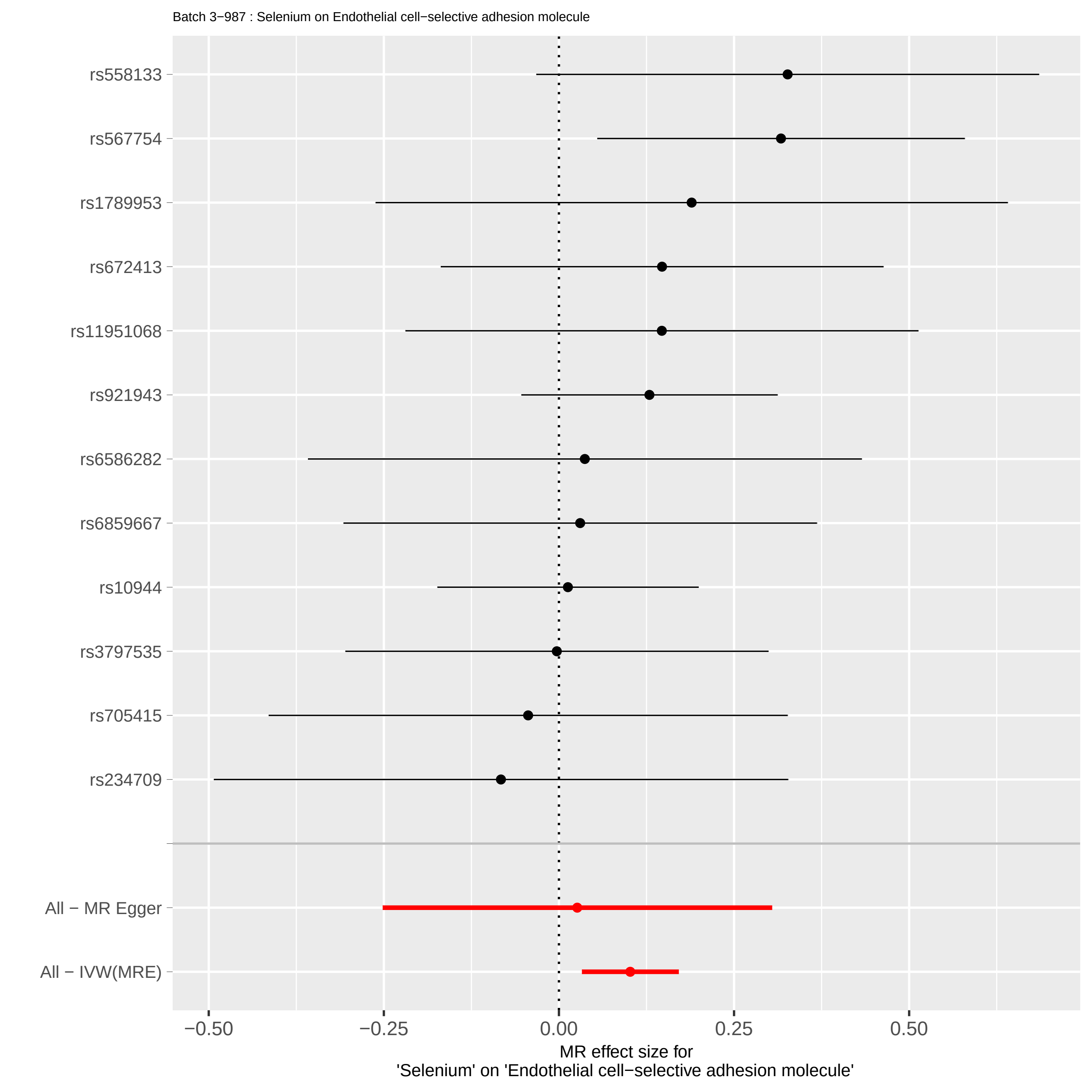

Batch 3−987 : Selenium on Endothelial cell−selective adhesion molecule
rs558133
rs567754
rs1789953
rs672413
rs11951068
rs921943
rs6586282
rs6859667
rs10944
rs3797535
rs705415
rs234709
All − MR Egger
All − IVW(MRE)
−0.50
−0.25
0.00
0.25
0.50
MR effect size for
'Selenium' on 'Endothelial cell−selective adhesion molecule'

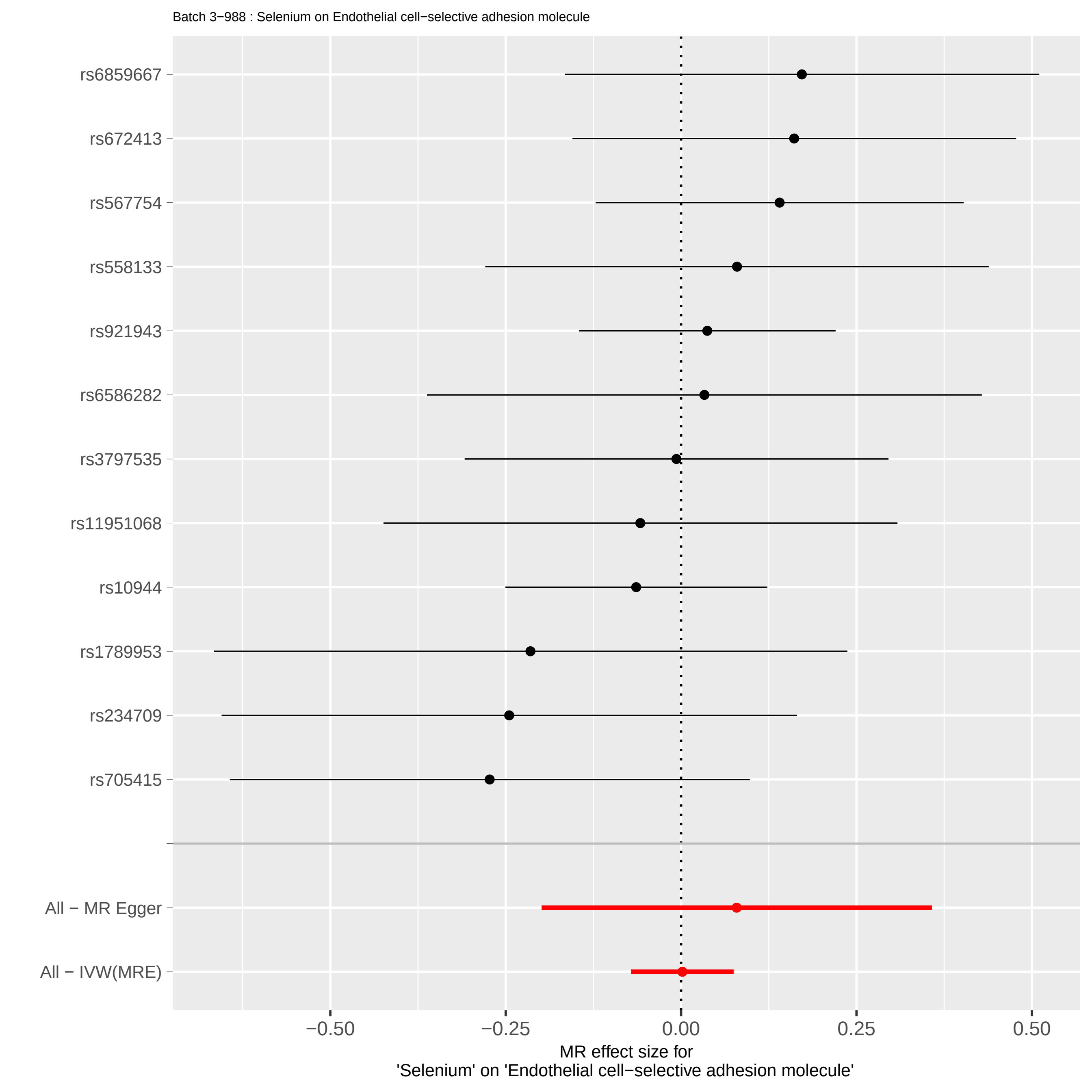

Batch 3−988 : Selenium on Endothelial cell−selective adhesion molecule
rs6859667
rs672413
rs567754
rs558133
rs921943
rs6586282
rs3797535
rs11951068
rs10944
rs1789953
rs234709
rs705415
All − MR Egger
All − IVW(MRE)
−0.50
−0.25
0.00
0.25
0.50
MR effect size for
'Selenium' on 'Endothelial cell−selective adhesion molecule'

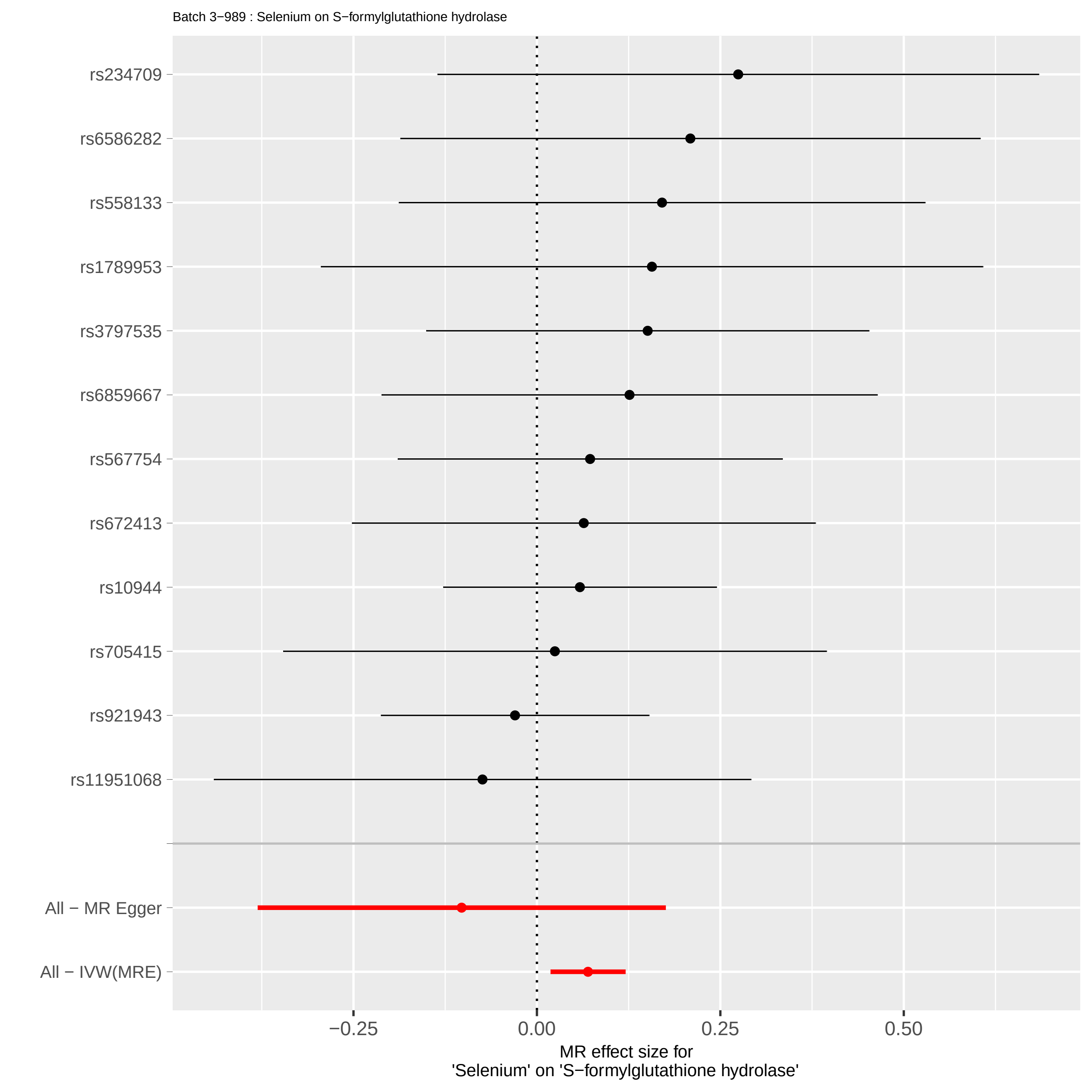

Batch 3−989 : Selenium on S−formylglutathione hydrolase
rs234709
rs6586282
rs558133
rs1789953
rs3797535
rs6859667
rs567754
rs672413
rs10944
rs705415
rs921943
rs11951068
All − MR Egger
All − IVW(MRE)
−0.25
0.00
0.25
0.50
MR effect size for
'Selenium' on 'S−formylglutathione hydrolase'

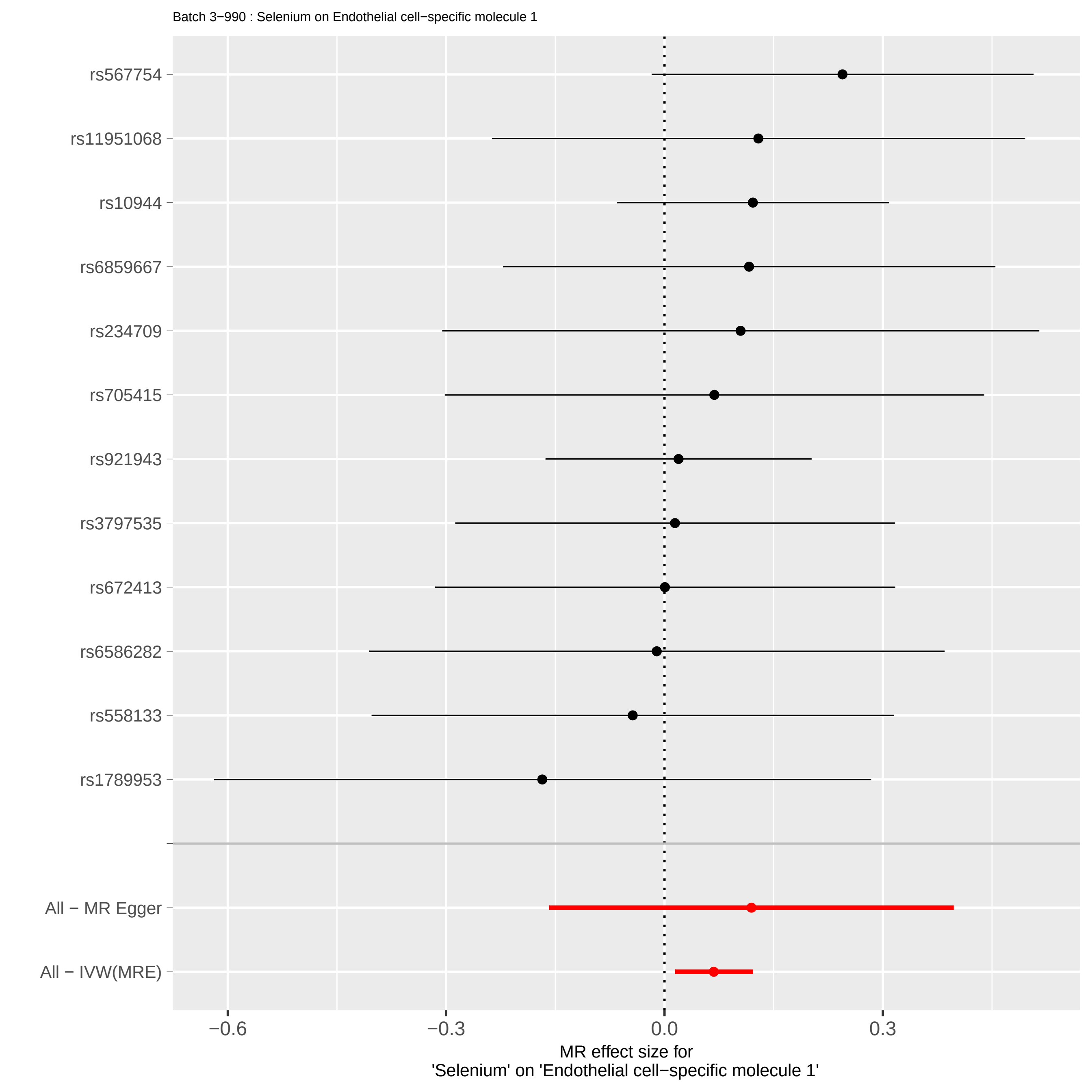

Batch 3−990 : Selenium on Endothelial cell−specific molecule 1
rs567754
rs11951068
rs10944
rs6859667
rs234709
rs705415
rs921943
rs3797535
rs672413
rs6586282
rs558133
rs1789953
All − MR Egger
All − IVW(MRE)
−0.6
−0.3
0.0
0.3
MR effect size for
'Selenium' on 'Endothelial cell−specific molecule 1'

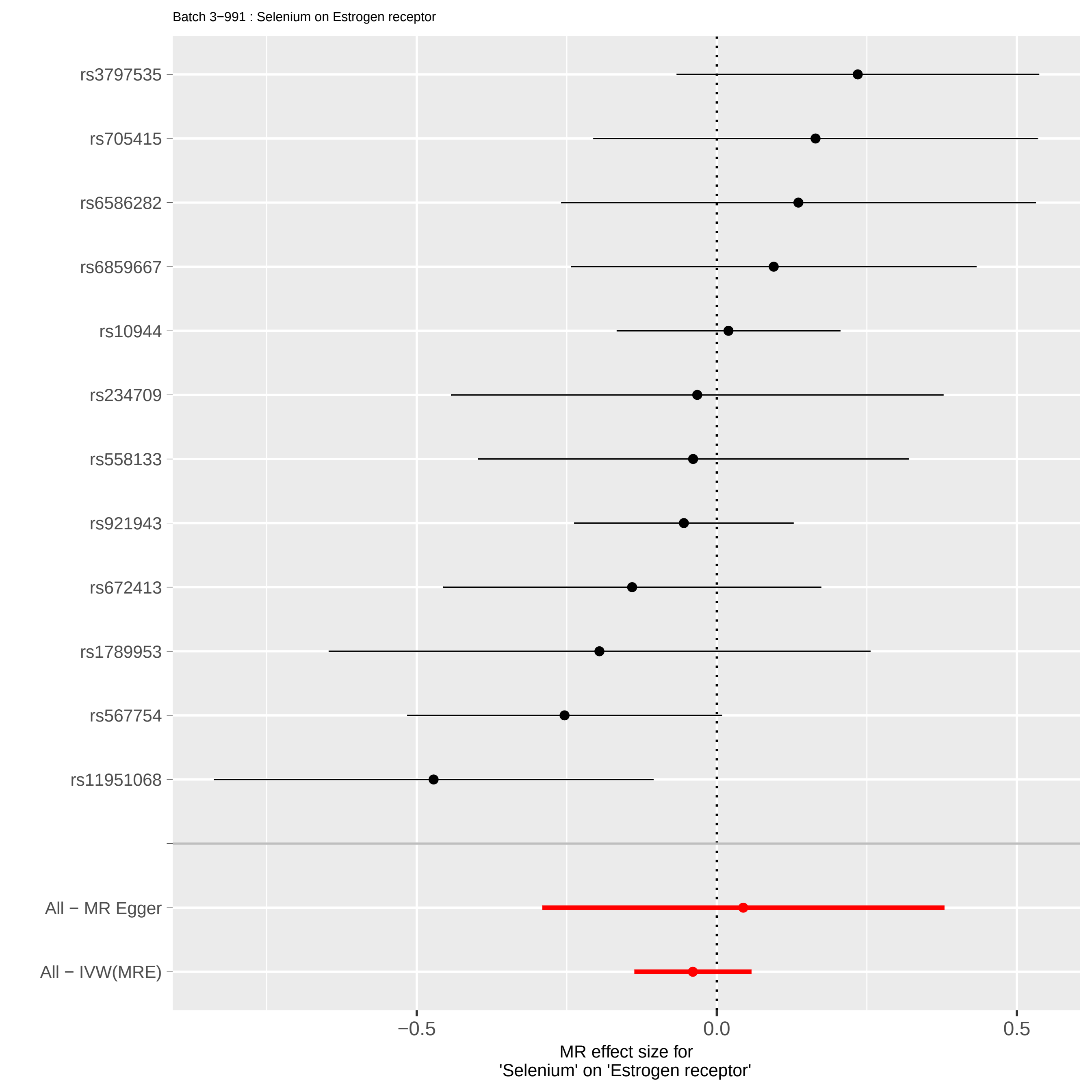
Batch 3−991 : Selenium on Estrogen receptor
rs3797535
rs705415
rs6586282
rs6859667
rs10944
rs234709
rs558133
rs921943
rs672413
rs1789953
rs567754
rs11951068
All − MR Egger
All − IVW(MRE)
−0.5
0.0
0.5
MR effect size for
'Selenium' on 'Estrogen receptor'

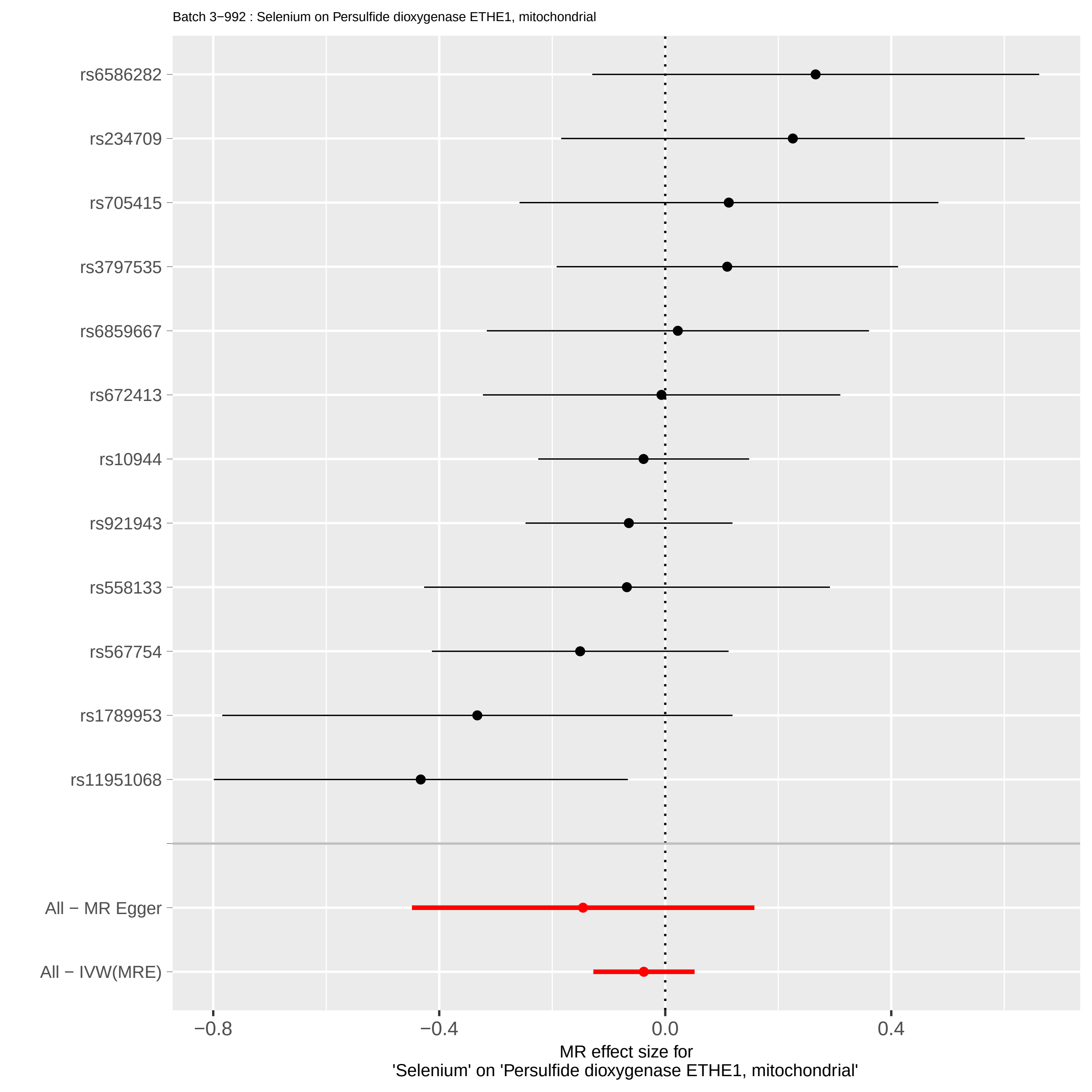

Batch 3−992 : Selenium on Persulfide dioxygenase ETHE1, mitochondrial
rs6586282
rs234709
rs705415
rs3797535
rs6859667
rs672413
rs10944
rs921943
rs558133
rs567754
rs1789953
rs11951068
All − MR Egger
All − IVW(MRE)
−0.8
−0.4
0.0
0.4
MR effect size for
'Selenium' on 'Persulfide dioxygenase ETHE1, mitochondrial'

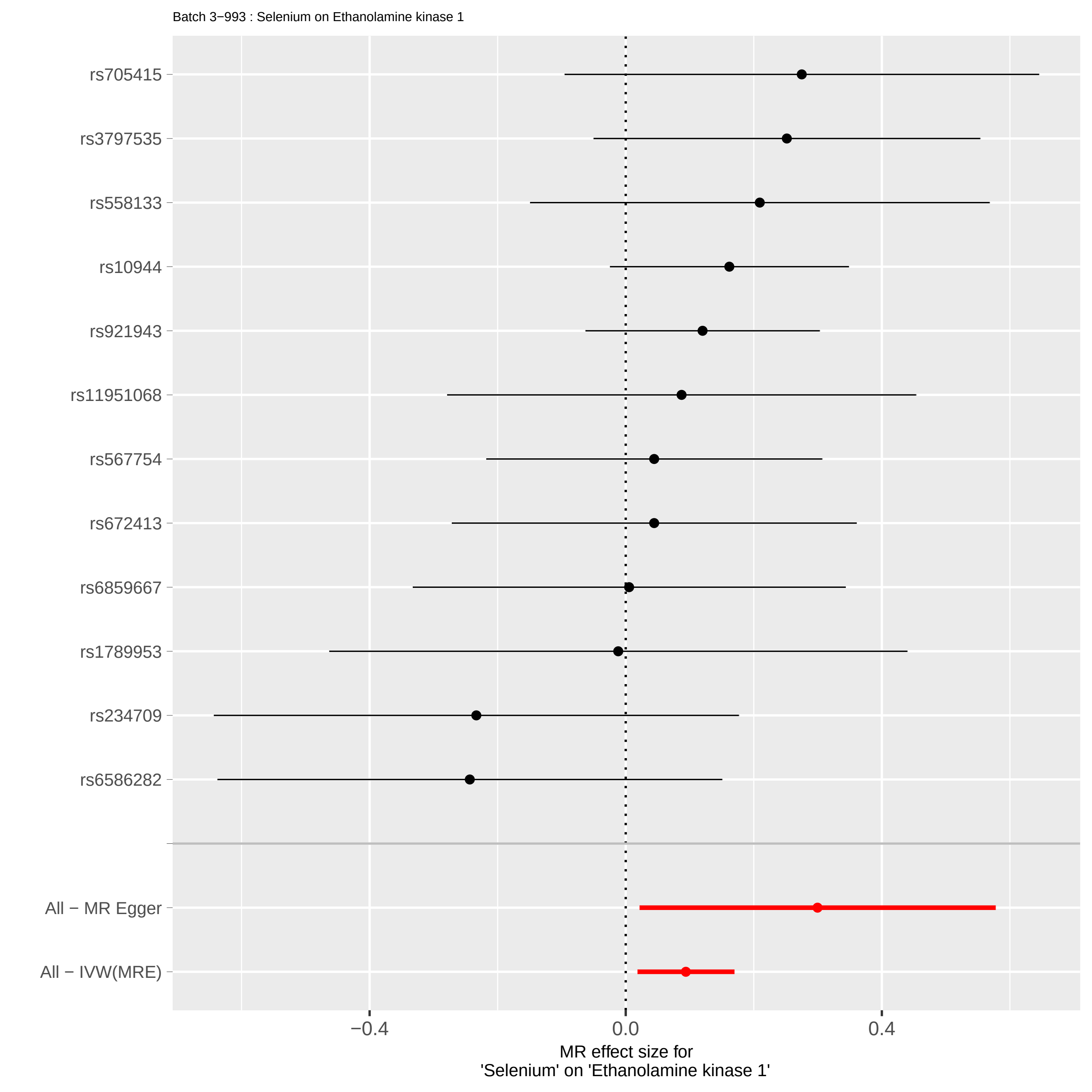
Batch 3−993 : Selenium on Ethanolamine kinase 1
rs705415
rs3797535
rs558133
rs10944
rs921943
rs11951068
rs567754
rs672413
rs6859667
rs1789953
rs234709
rs6586282
All − MR Egger
All − IVW(MRE)
−0.4
0.0
0.4
MR effect size for
'Selenium' on 'Ethanolamine kinase 1'

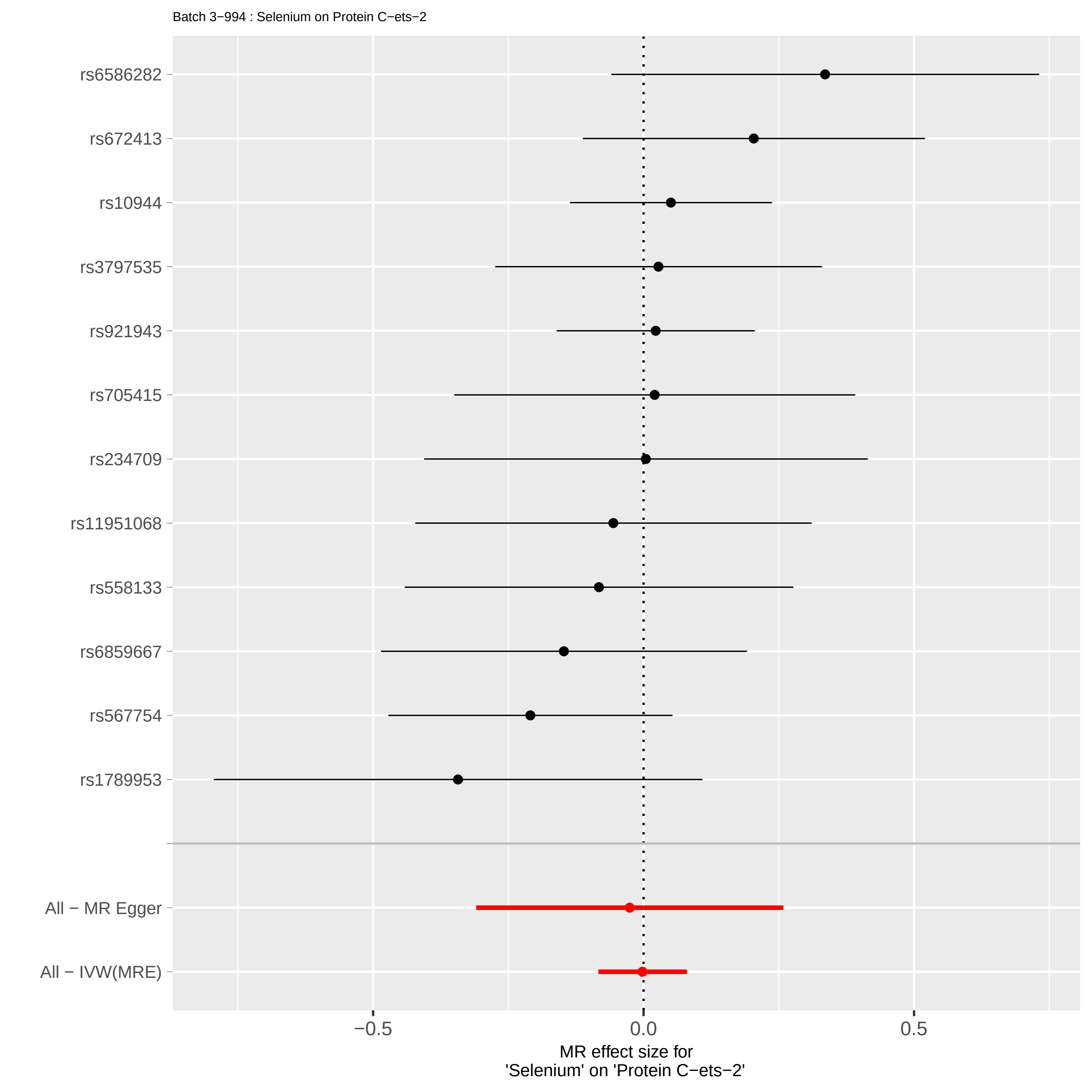
Batch 3−994 : Selenium on Protein C−ets−2
rs6586282
rs672413
rs10944
rs3797535
rs921943
rs705415
rs234709
rs11951068
rs558133
rs6859667
rs567754
rs1789953
All − MR Egger
All − IVW(MRE)
−0.5
0.0
0.5
MR effect size for
'Selenium' on 'Protein C−ets−2'

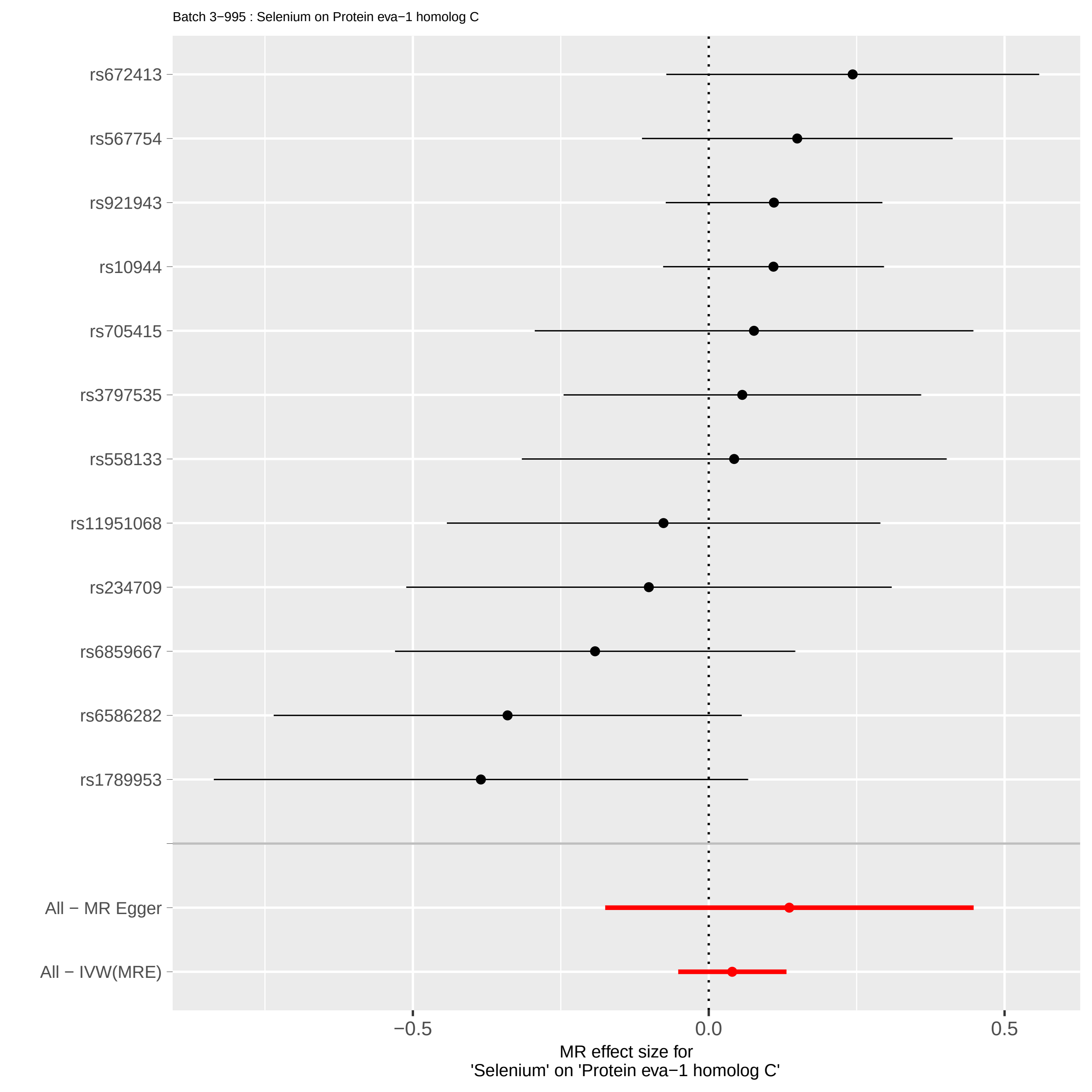
Batch 3−995 : Selenium on Protein eva−1 homolog C
rs672413
rs567754
rs921943
rs10944
rs705415
rs3797535
rs558133
rs11951068
rs234709
rs6859667
rs6586282
rs1789953
All − MR Egger
All − IVW(MRE)
−0.5
0.0
0.5
MR effect size for
'Selenium' on 'Protein eva−1 homolog C'

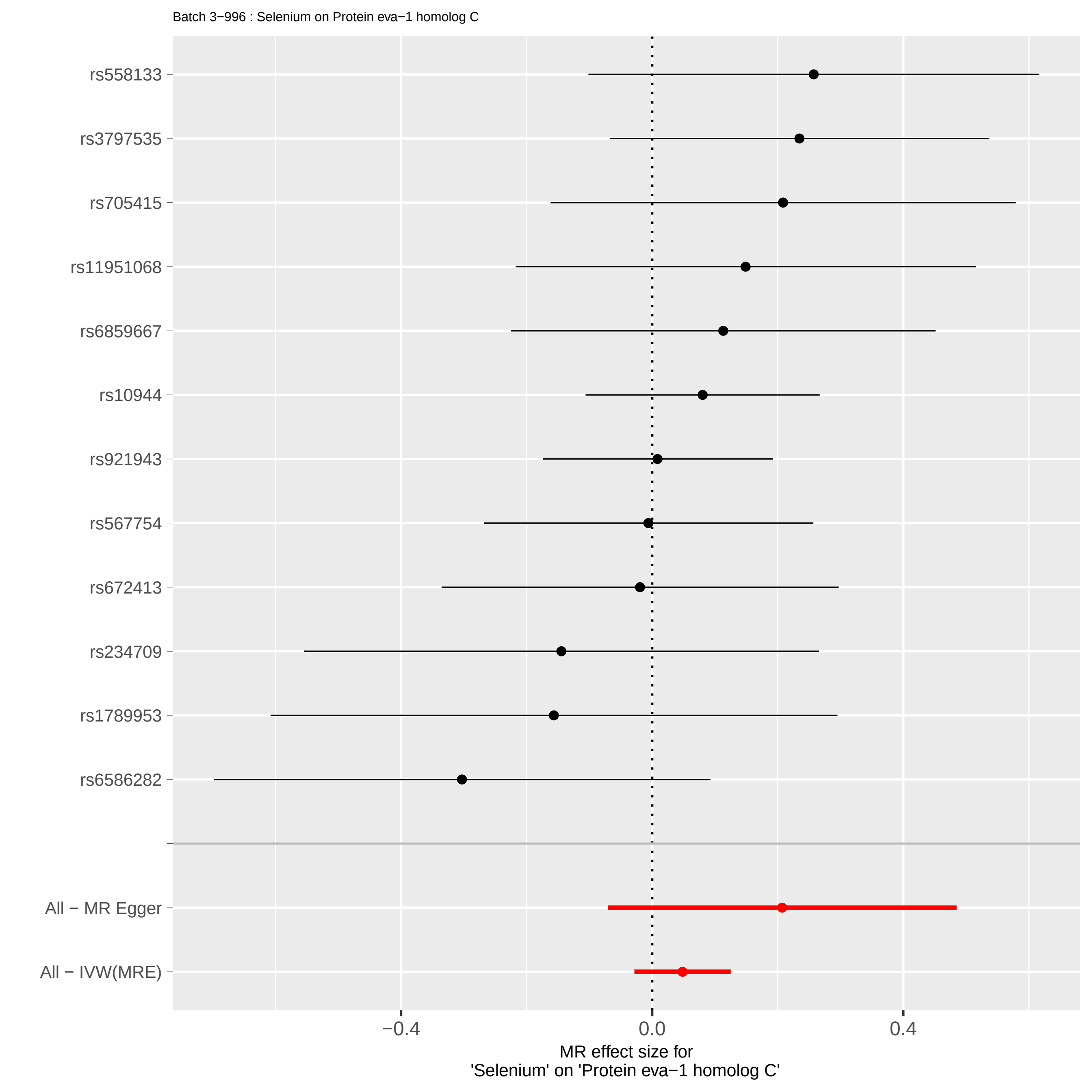

Batch 3−996 : Selenium on Protein eva−1 homolog C
rs558133
rs3797535
rs705415
rs11951068
rs6859667
rs10944
rs921943
rs567754
rs672413
rs234709
rs1789953
rs6586282
All − MR Egger
All − IVW(MRE)
−0.4
0.0
0.4
MR effect size for
'Selenium' on 'Protein eva−1 homolog C'

Batch 3−997 : Selenium on Protein EVI2B

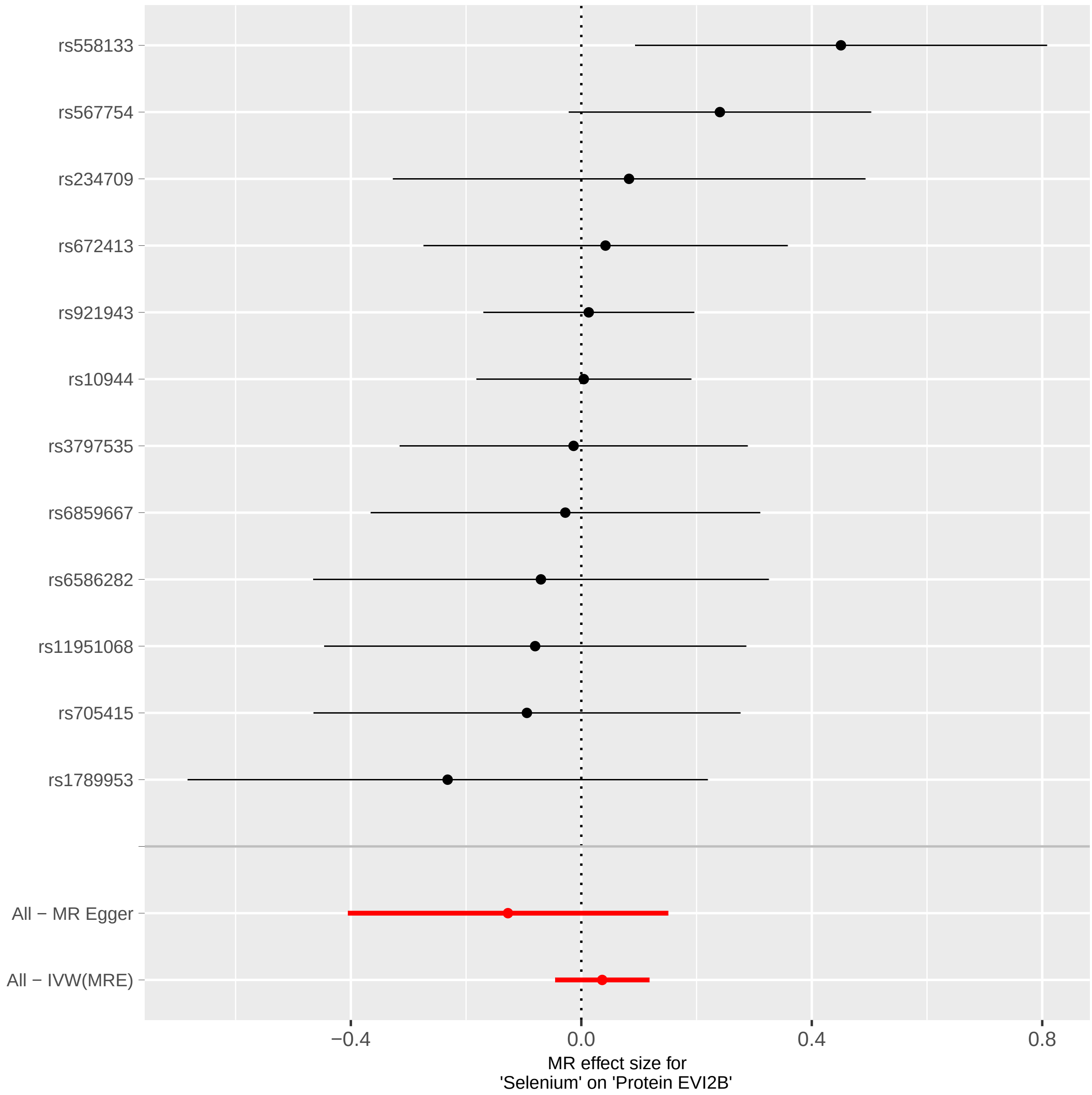

Batch 3−998 : Selenium on Ena/VASP−like protein

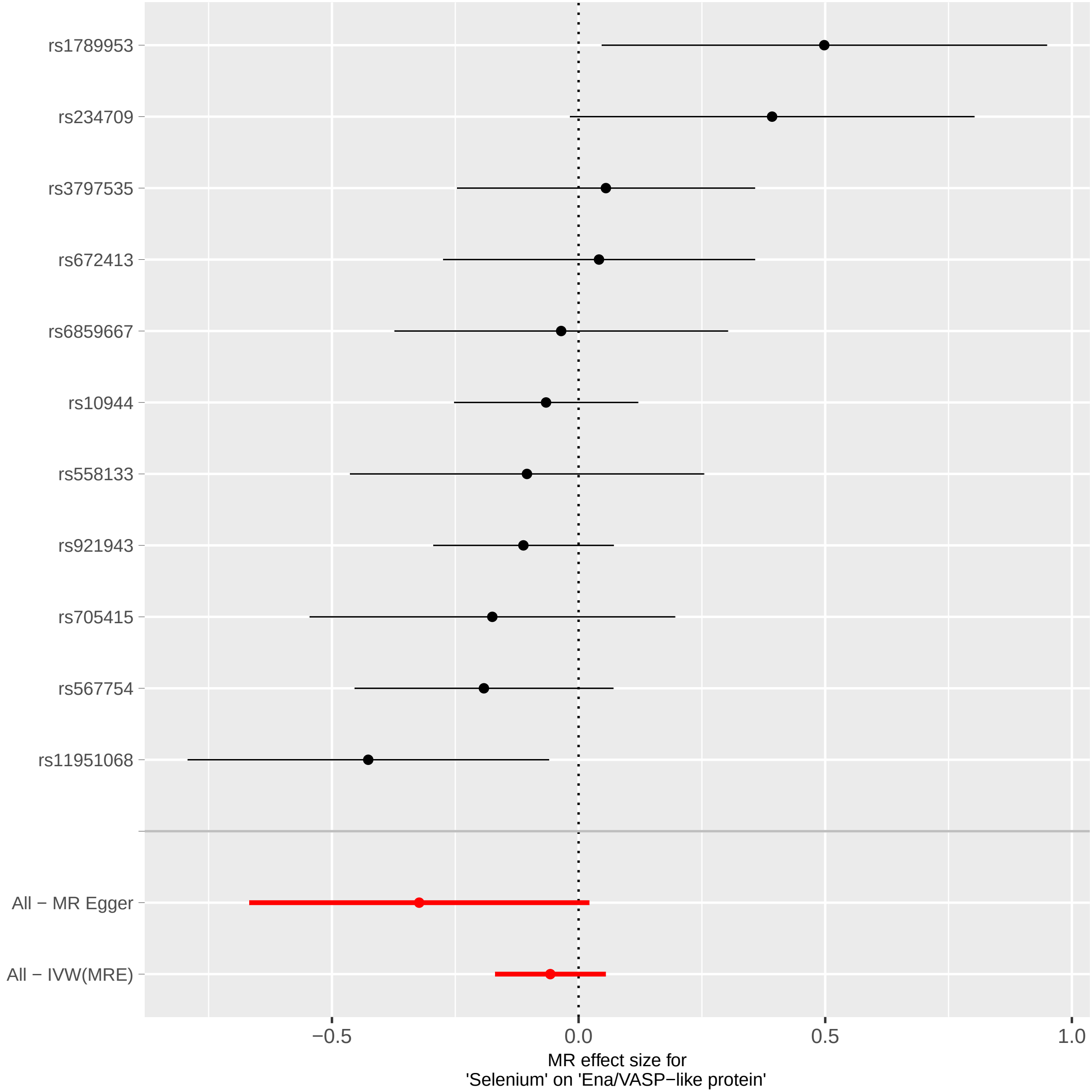

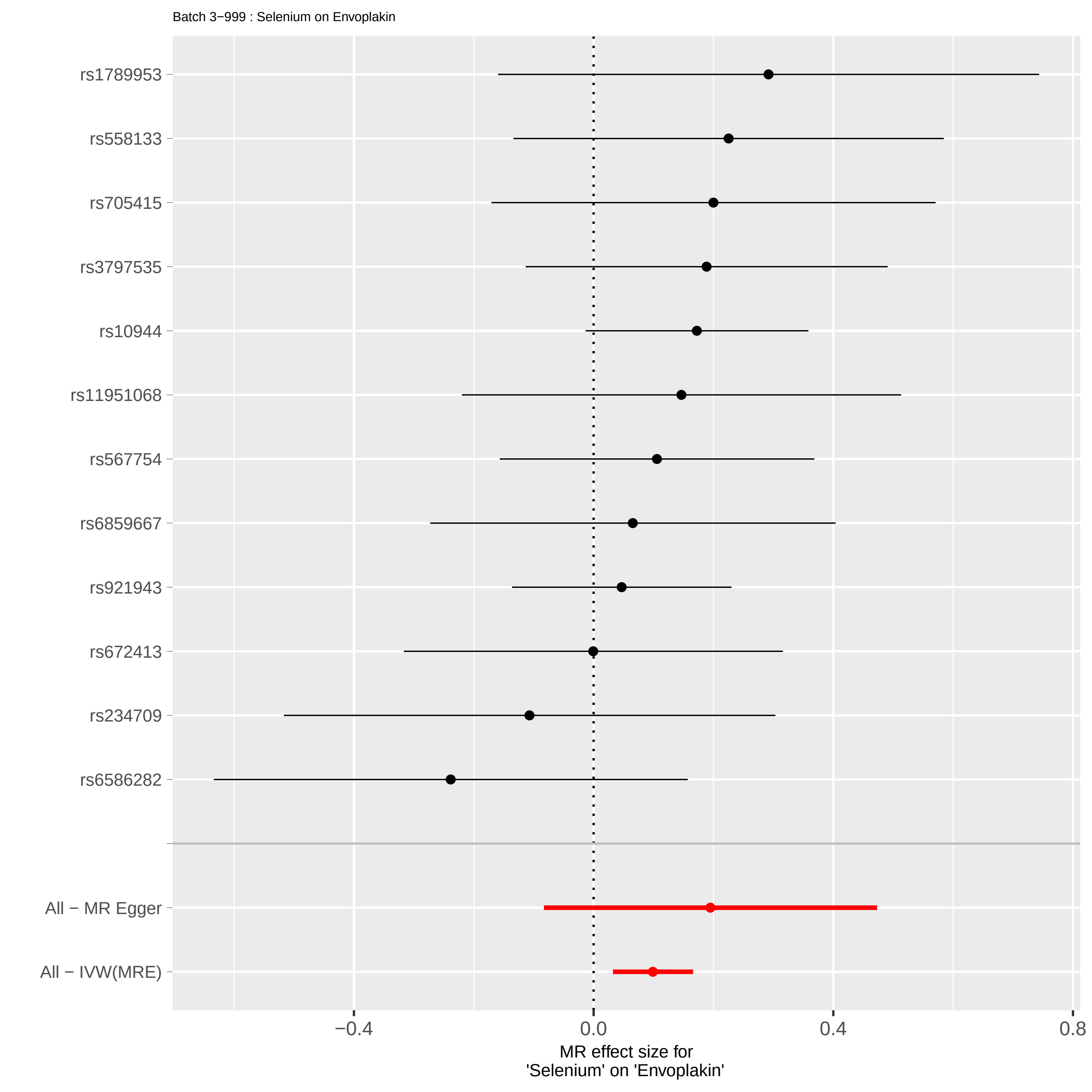

Batch 3−999 : Selenium on Envoplakin
rs1789953
rs558133
rs705415
rs3797535
rs10944
rs11951068
rs567754
rs6859667
rs921943
rs672413
rs234709
rs6586282
All − MR Egger
All − IVW(MRE)
−0.4
0.0
0.4
0.8
MR effect size for
'Selenium' on 'Envoplakin'

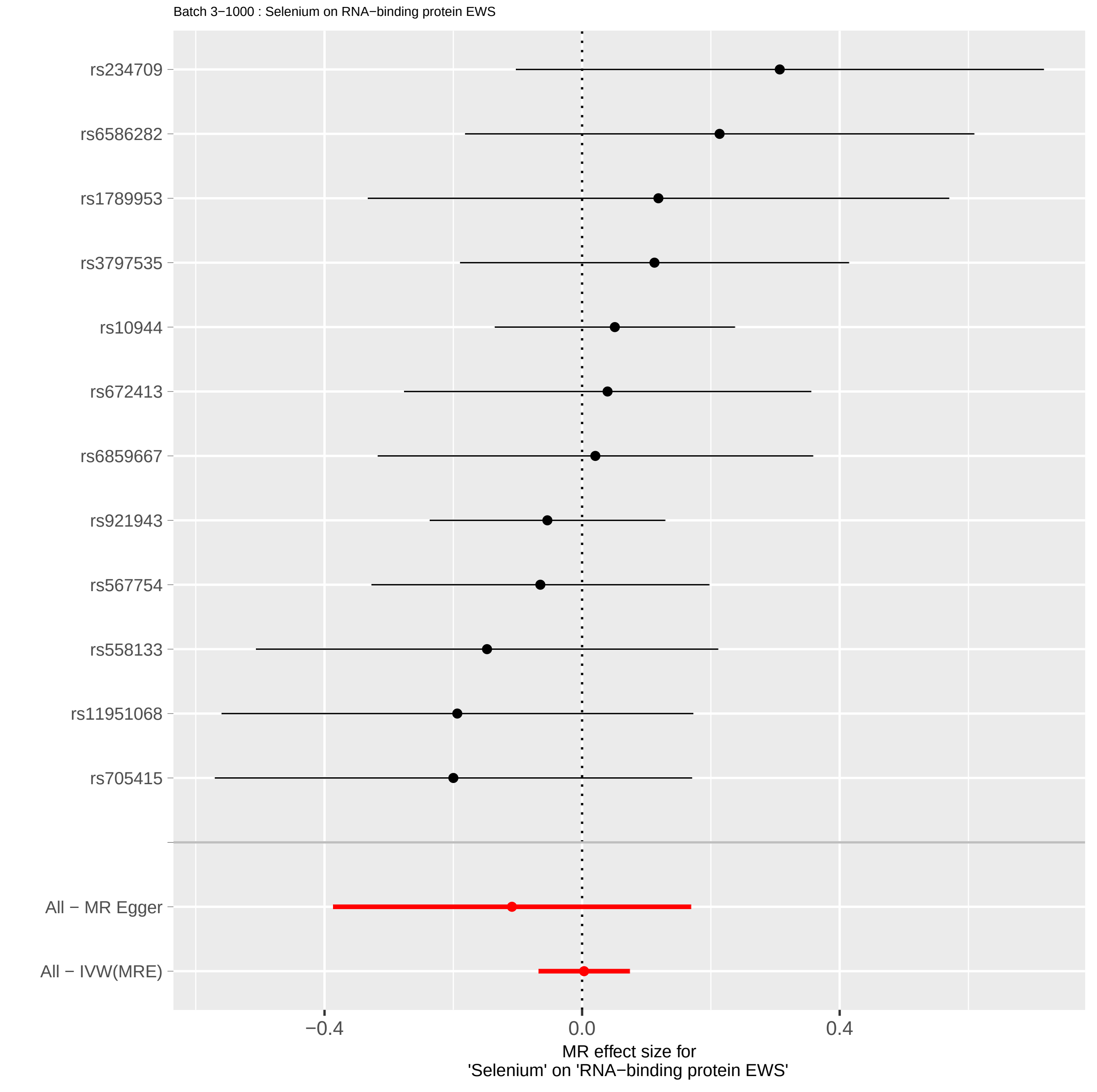

Batch 3−1000 : Selenium on RNA−binding protein EWS
rs234709
rs6586282
rs1789953
rs3797535
rs10944
rs672413
rs6859667
rs921943
rs567754
rs558133
rs11951068
rs705415
All − MR Egger
All − IVW(MRE)
−0.4
0.0
0.4
MR effect size for
'Selenium' on 'RNA−binding protein EWS'

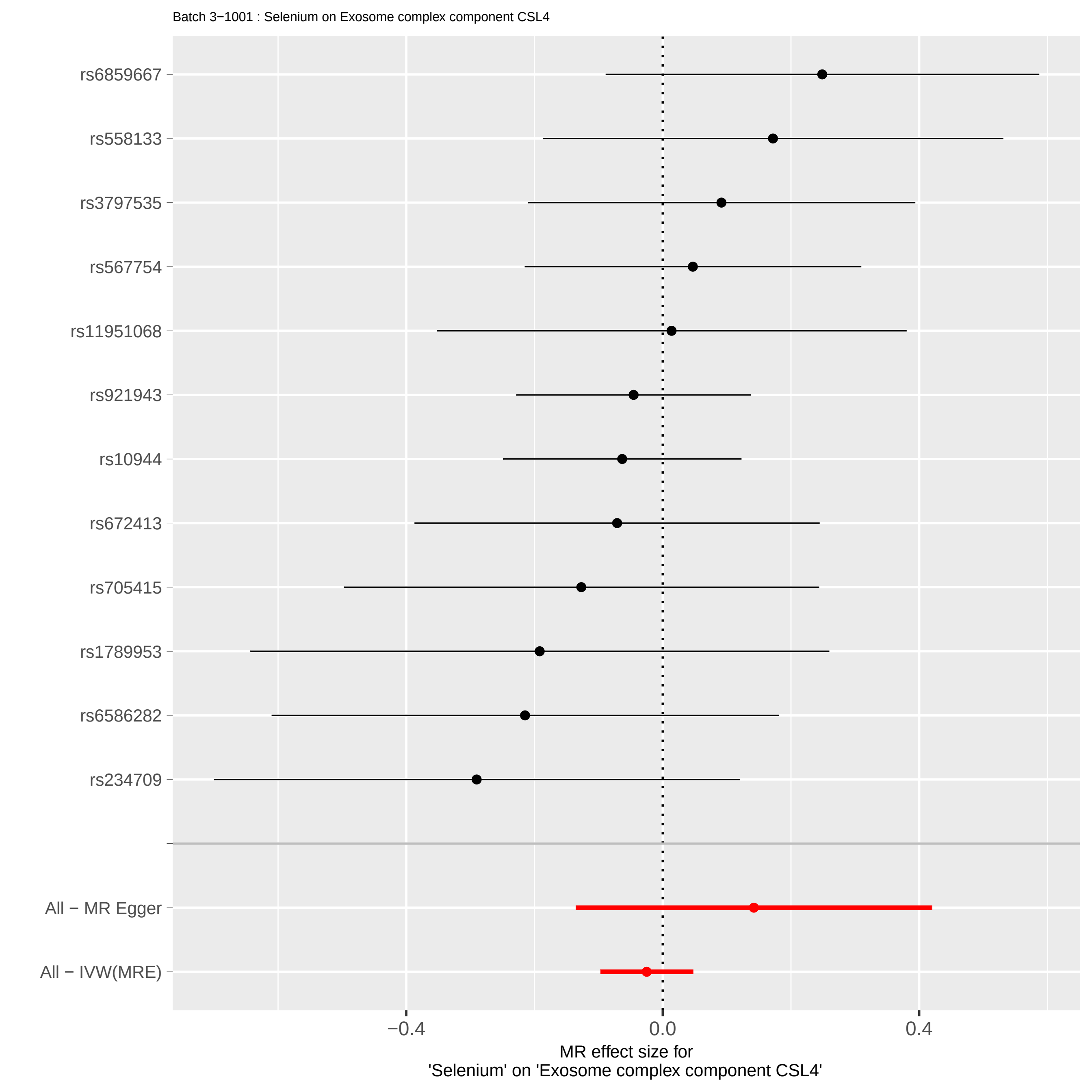
Batch 3−1001 : Selenium on Exosome complex component CSL4
rs6859667
rs558133
rs3797535
rs567754
rs11951068
rs921943
rs10944
rs672413
rs705415
rs1789953
rs6586282
rs234709
All − MR Egger
All − IVW(MRE)
−0.4
0.0
0.4
MR effect size for
'Selenium' on 'Exosome complex component CSL4'

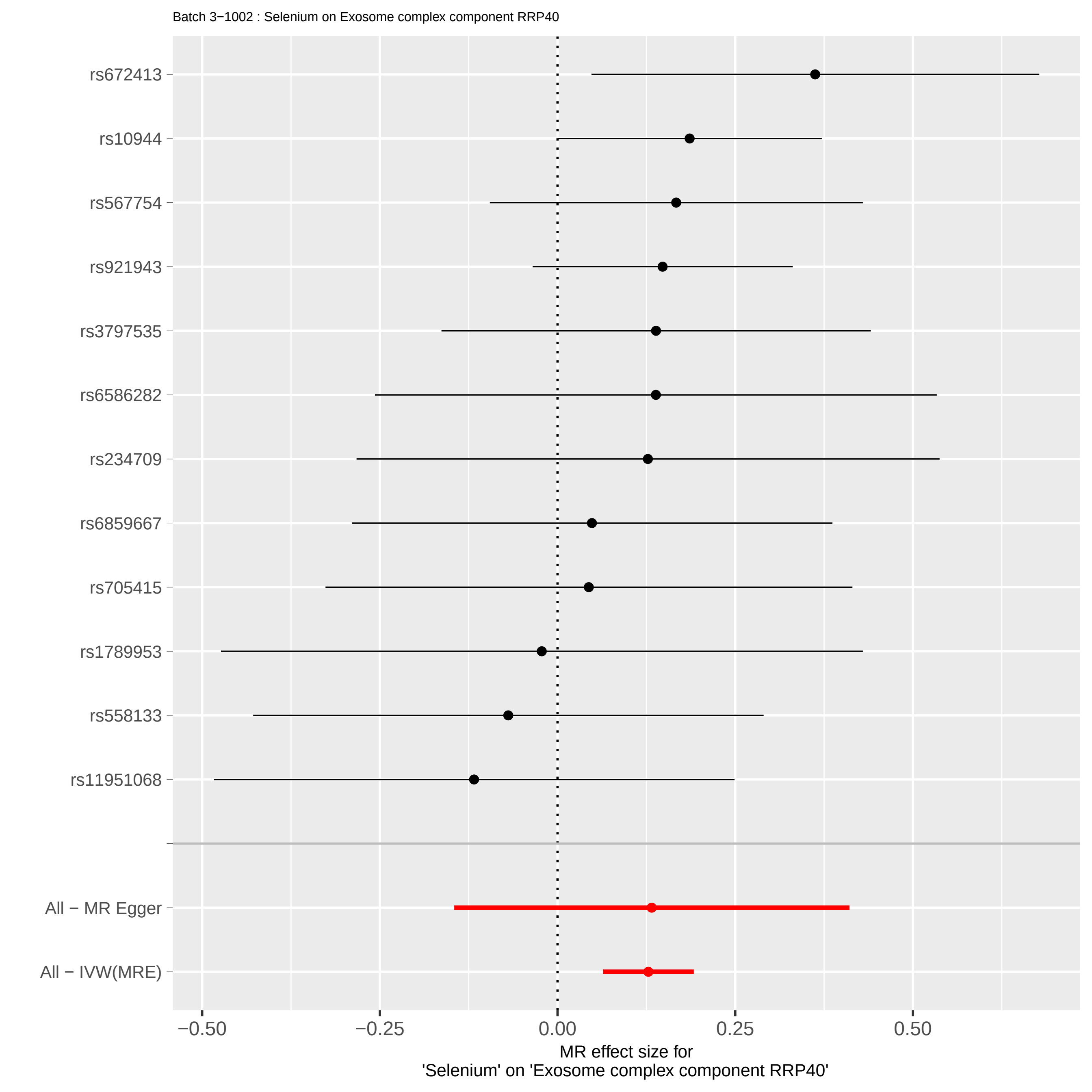

Batch 3−1002 : Selenium on Exosome complex component RRP40
rs672413
rs10944
rs567754
rs921943
rs3797535
rs6586282
rs234709
rs6859667
rs705415
rs1789953
rs558133
rs11951068
All − MR Egger
All − IVW(MRE)
−0.50
−0.25
0.00
0.25
0.50
MR effect size for
'Selenium' on 'Exosome complex component RRP40'

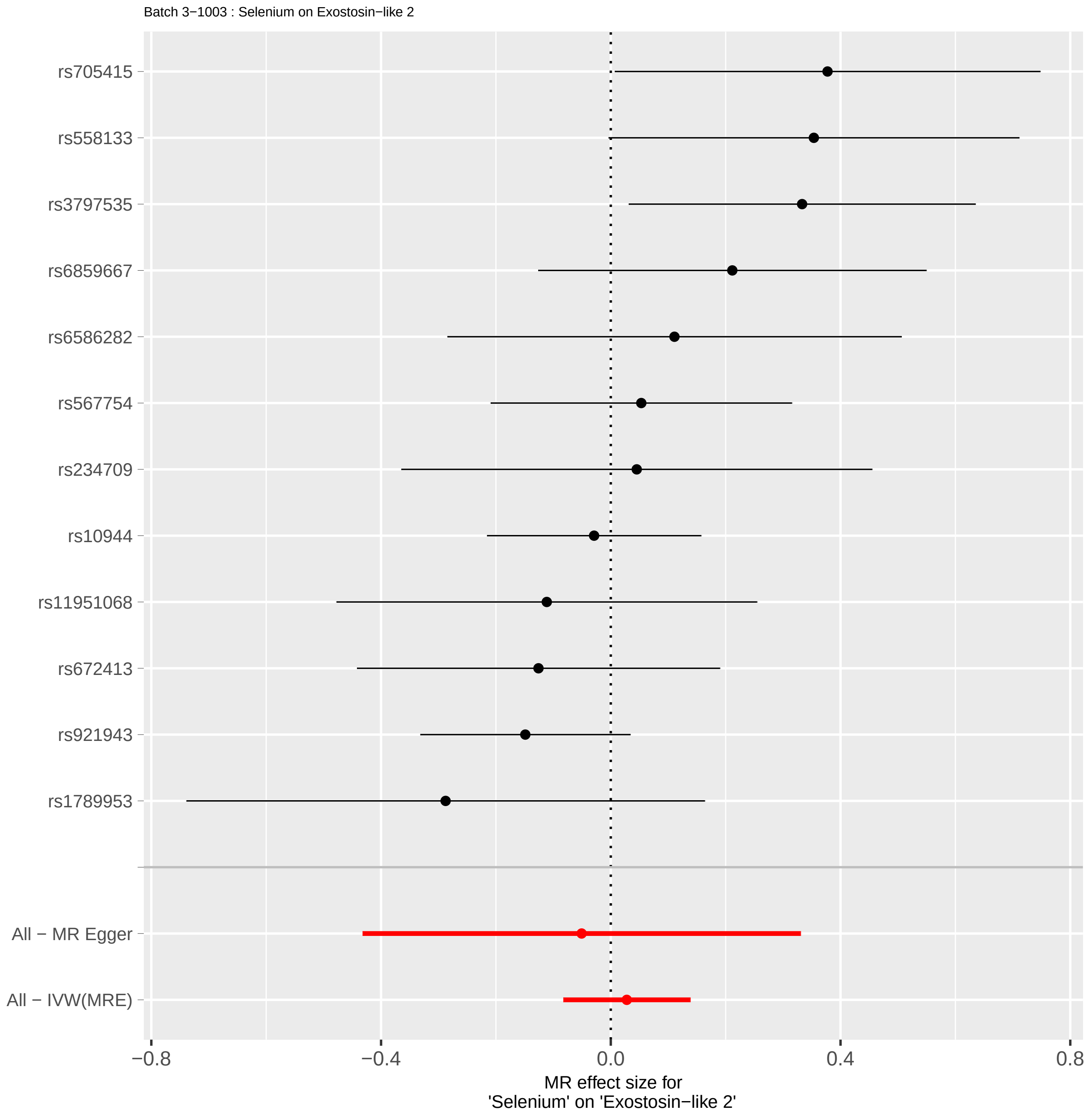

Batch 3−1003 : Selenium on Exostosin−like 2
rs705415
rs558133
rs3797535
rs6859667
rs6586282
rs567754
rs234709
rs10944
rs11951068
rs672413
rs921943
rs1789953
All − MR Egger
All − IVW(MRE)
−0.8
−0.4
0.0
0.4
0.8
MR effect size for
'Selenium' on 'Exostosin−like 2'

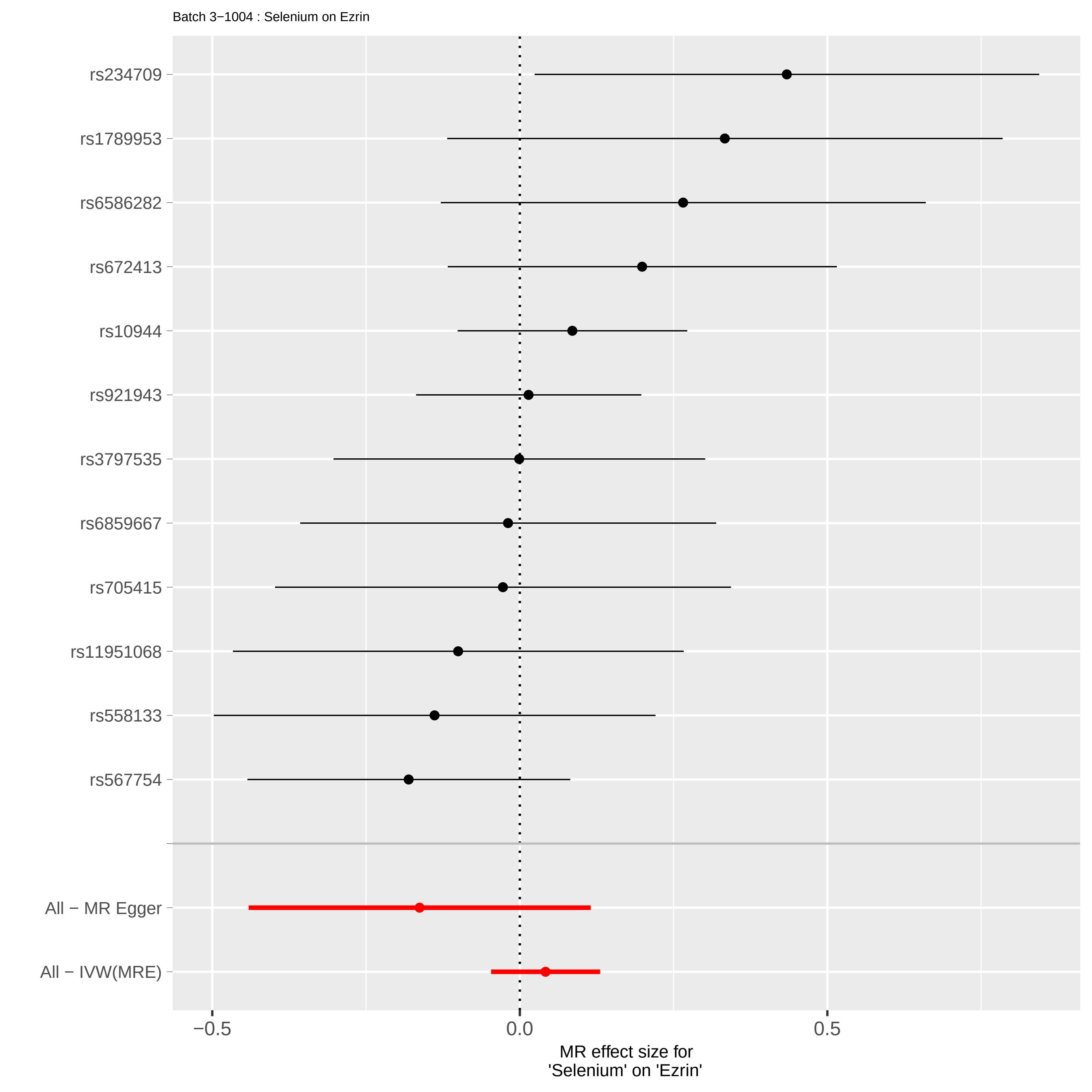

Batch 3−1004 : Selenium on Ezrin
rs234709
rs1789953
rs6586282
rs672413
rs10944
rs921943
rs3797535
rs6859667
rs705415
rs11951068
rs558133
rs567754
All − MR Egger
All − IVW(MRE)
−0.5
0.0
0.5
MR effect size for
'Selenium' on 'Ezrin'

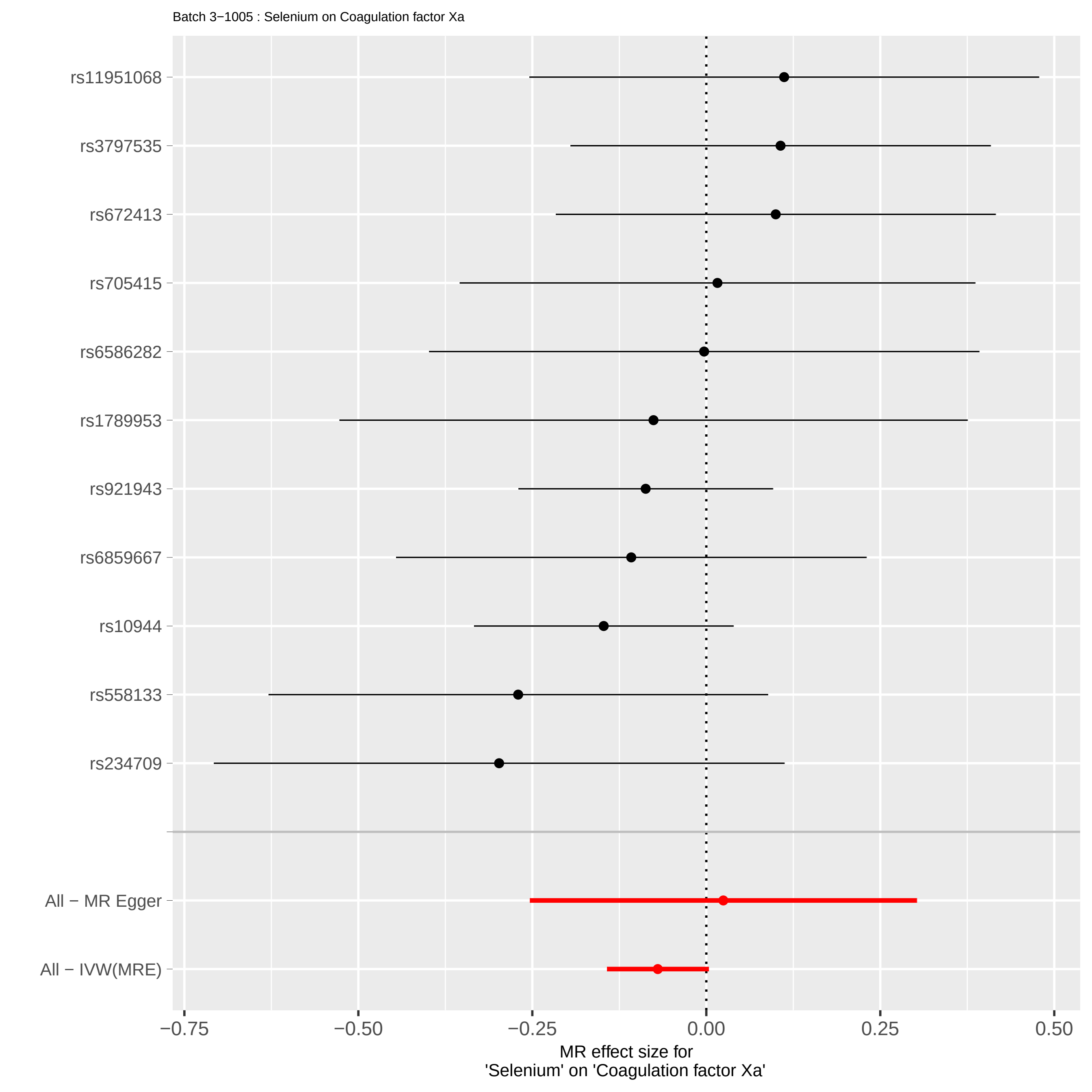

Batch 3−1005 : Selenium on Coagulation factor Xa
rs11951068
rs3797535
rs672413
rs705415
rs6586282
rs1789953
rs921943
rs6859667
rs10944
rs558133
rs234709
All − MR Egger
All − IVW(MRE)
−0.75
−0.50
−0.25
0.00
0.25
0.50
MR effect size for
'Selenium' on 'Coagulation factor Xa'

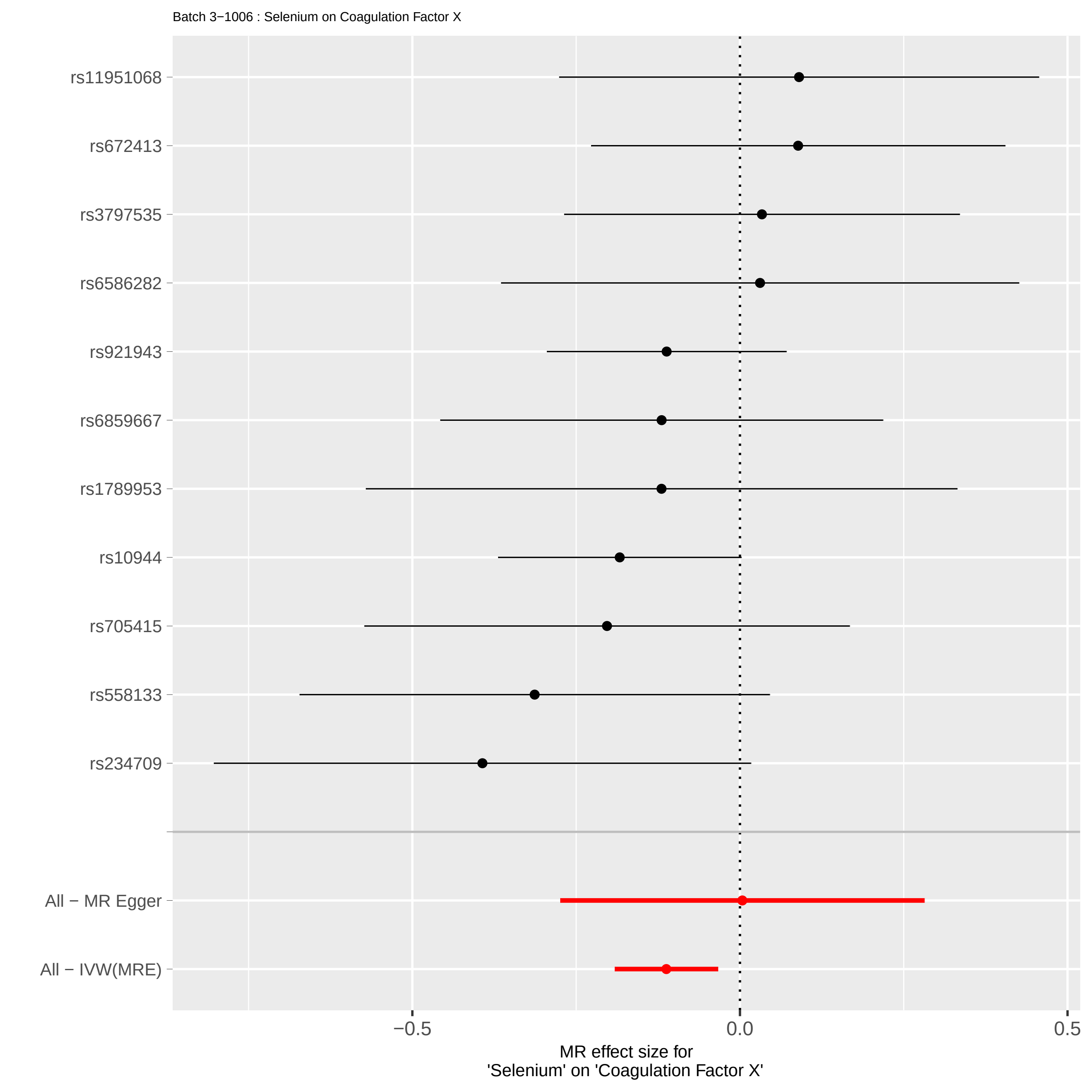

Batch 3−1006 : Selenium on Coagulation Factor X
rs11951068
rs672413
rs3797535
rs6586282
rs921943
rs6859667
rs1789953
rs10944
rs705415
rs558133
rs234709
All − MR Egger
All − IVW(MRE)
−0.5
0.0
0.5
MR effect size for
'Selenium' on 'Coagulation Factor X'

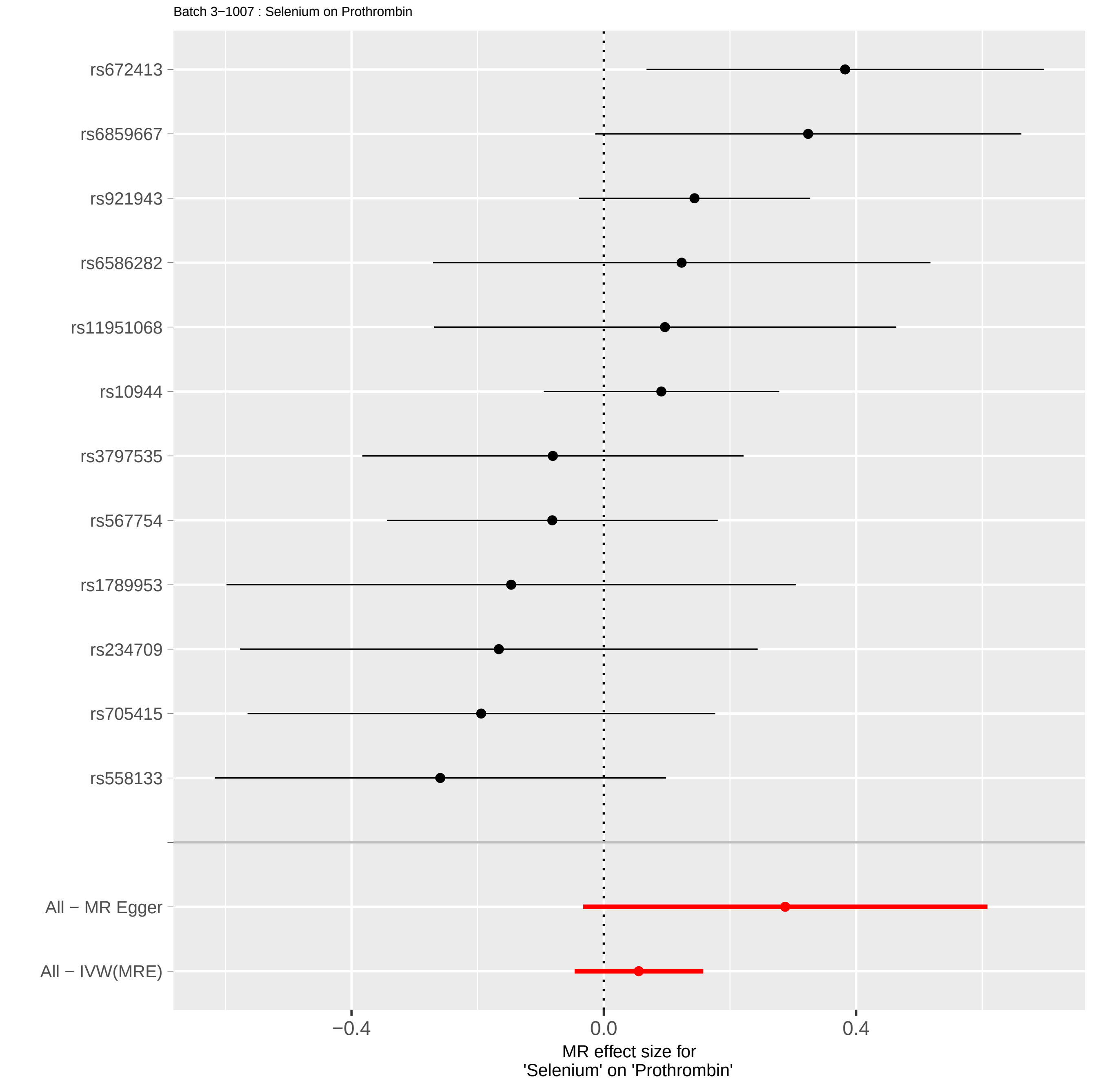

Batch 3−1007 : Selenium on Prothrombin
rs672413
rs6859667
rs921943
rs6586282
rs11951068
rs10944
rs3797535
rs567754
rs1789953
rs234709
rs705415
rs558133
All − MR Egger
All − IVW(MRE)
−0.4
0.0
0.4
MR effect size for
'Selenium' on 'Prothrombin'

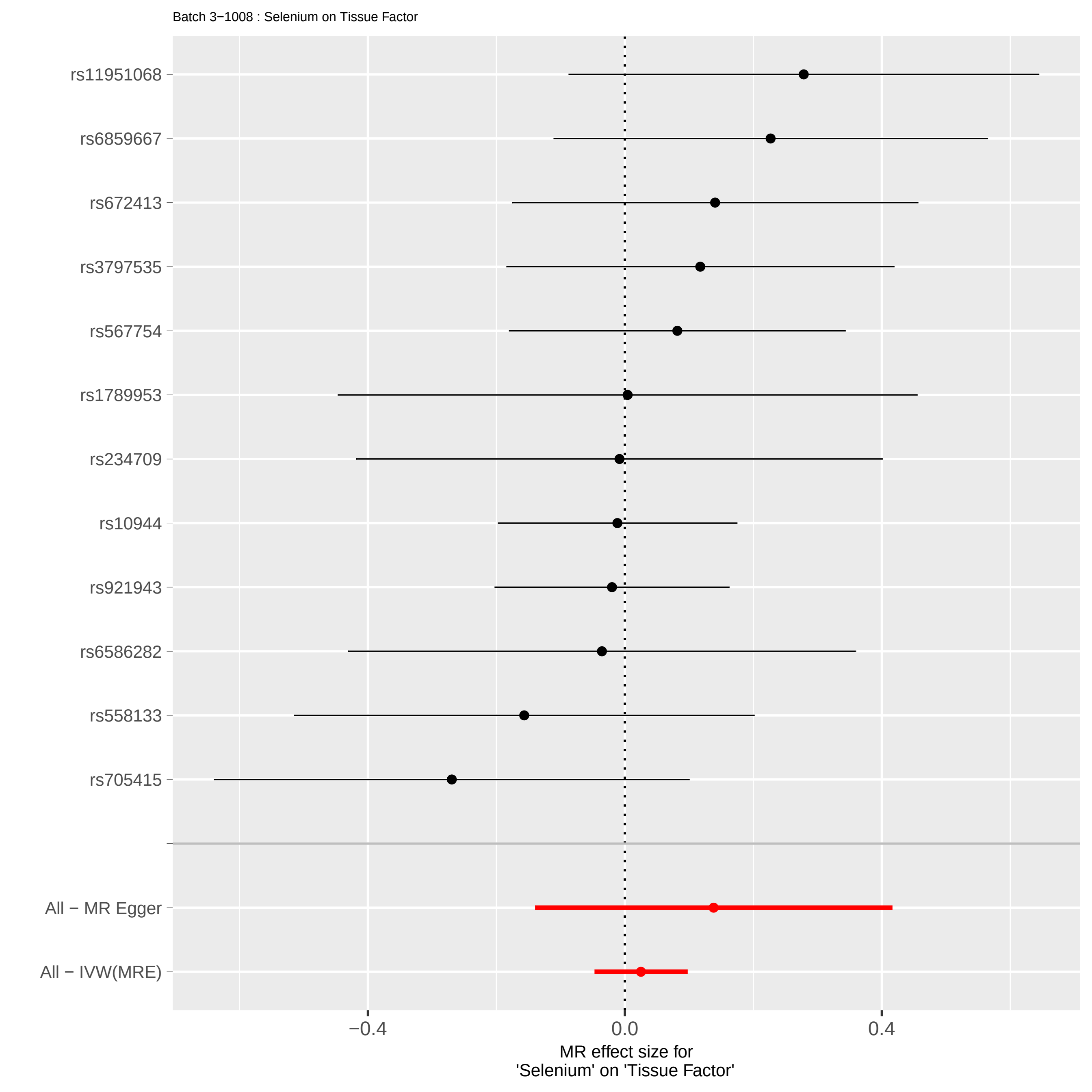
Batch 3−1008 : Selenium on Tissue Factor
rs11951068
rs6859667
rs672413
rs3797535
rs567754
rs1789953
rs234709
rs10944
rs921943
rs6586282
rs558133
rs705415
All − MR Egger
All − IVW(MRE)
−0.4
0.0
0.4
MR effect size for
'Selenium' on 'Tissue Factor'

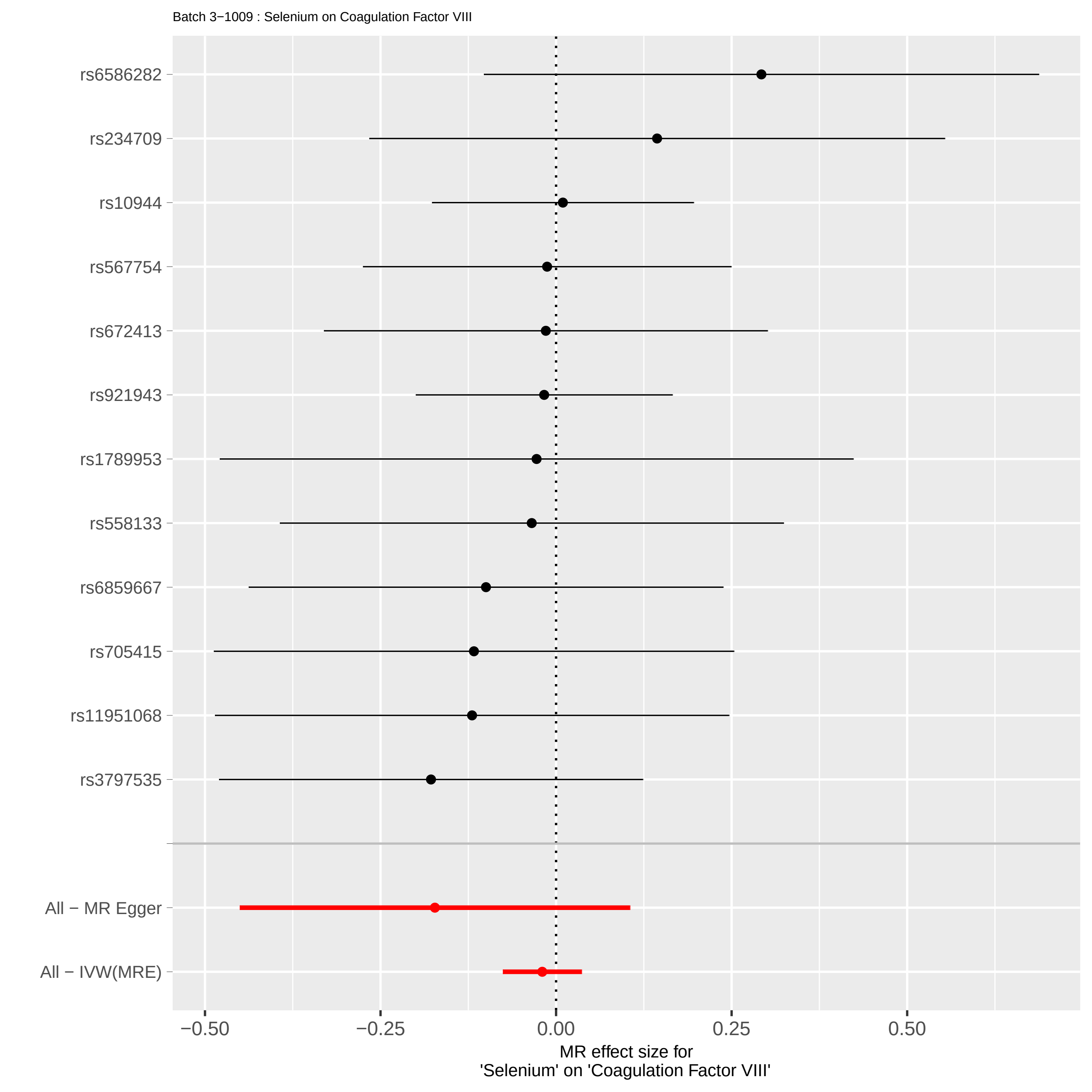
Batch 3−1009 : Selenium on Coagulation Factor VIII
rs6586282
rs234709
rs10944
rs567754
rs672413
rs921943
rs1789953
rs558133
rs6859667
rs705415
rs11951068
rs3797535
All − MR Egger
All − IVW(MRE)
−0.50
−0.25
0.00
0.25
0.50
MR effect size for
'Selenium' on 'Coagulation Factor VIII'

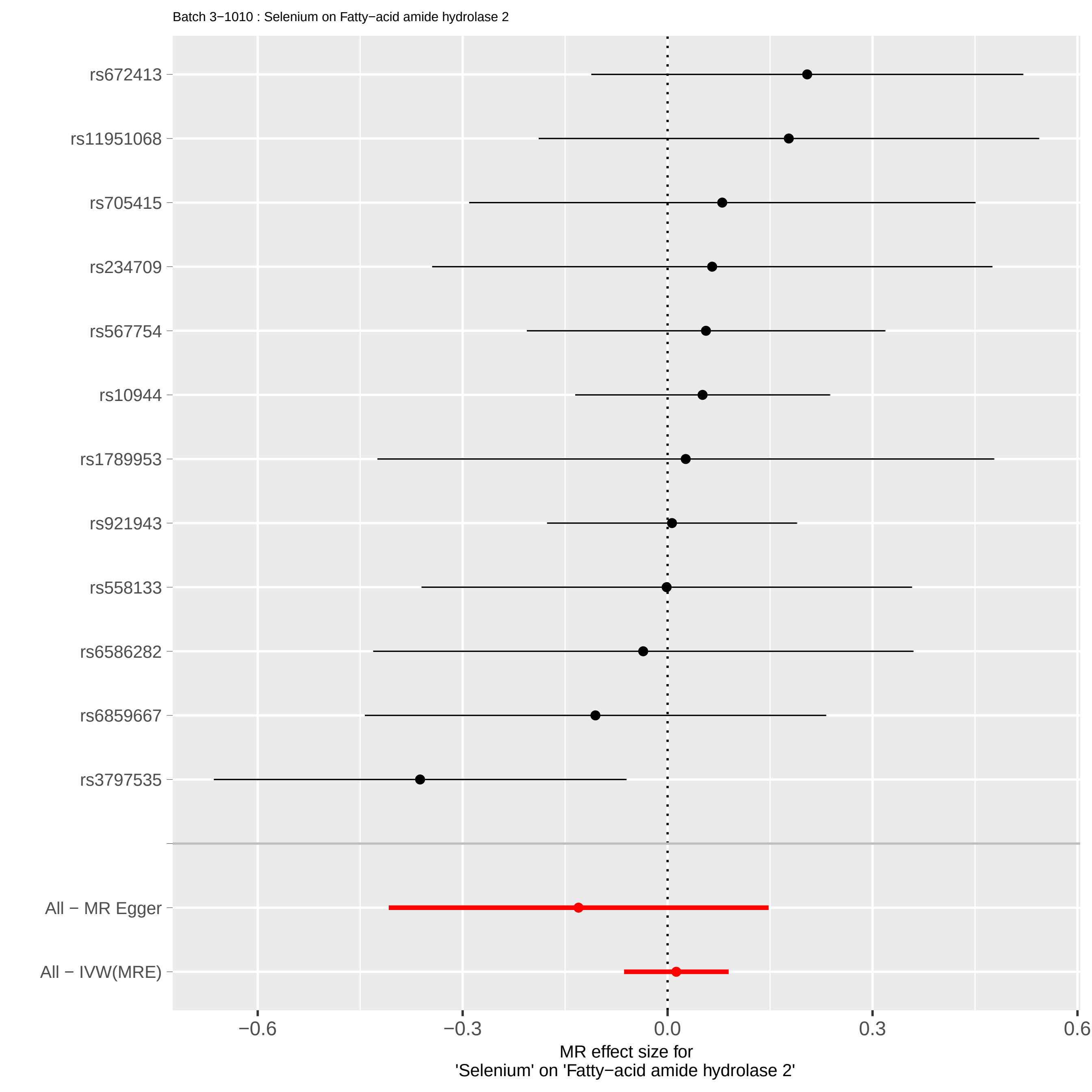

Batch 3−1010 : Selenium on Fatty−acid amide hydrolase 2
rs672413
rs11951068
rs705415
rs234709
rs567754
rs10944
rs1789953
rs921943
rs558133
rs6586282
rs6859667
rs3797535
All − MR Egger
All − IVW(MRE)
−0.6
−0.3
0.0
0.3
0.6
MR effect size for
'Selenium' on 'Fatty−acid amide hydrolase 2'

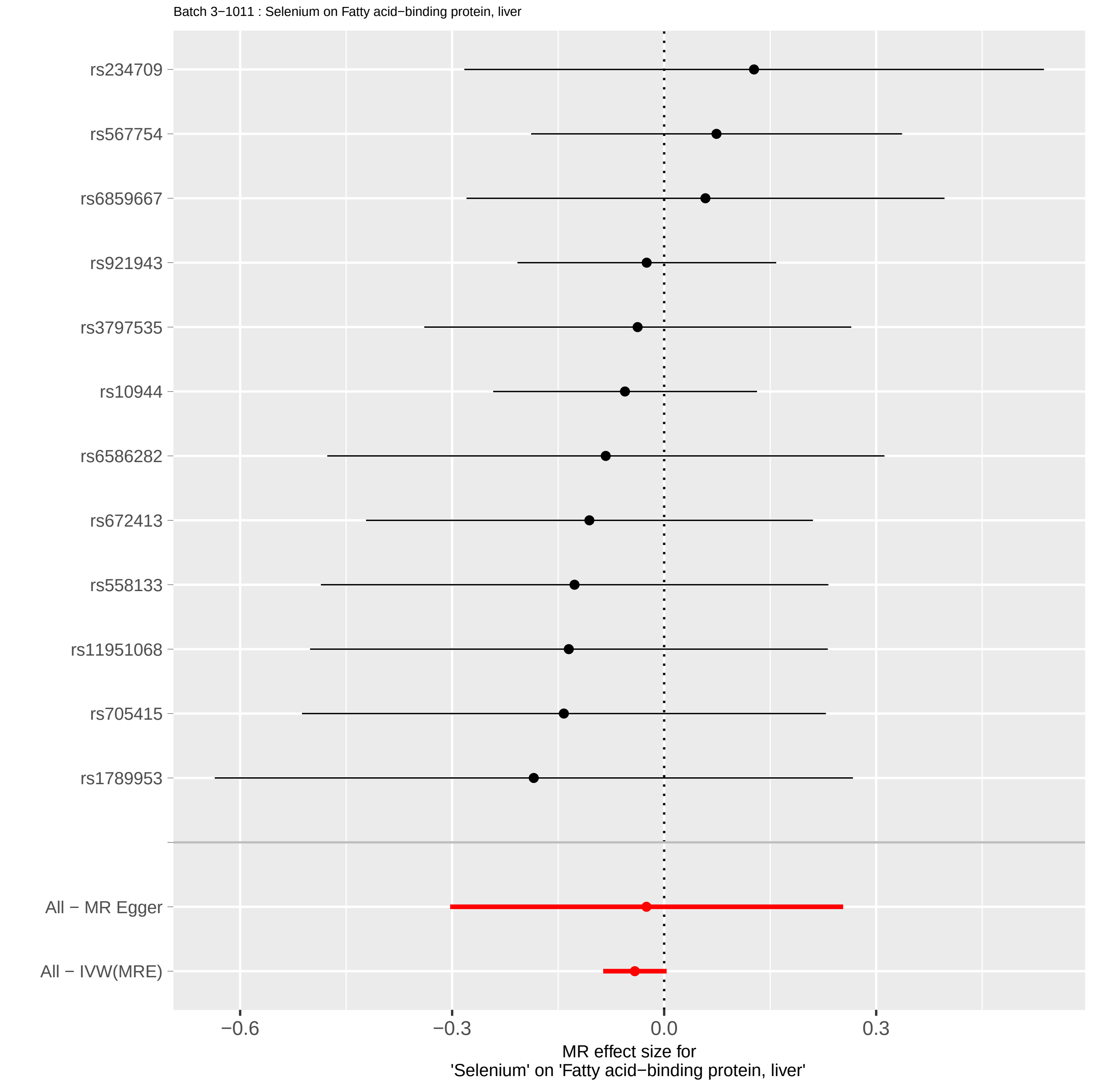
Batch 3−1011 : Selenium on Fatty acid−binding protein, liver
rs234709
rs567754
rs6859667
rs921943
rs3797535
rs10944
rs6586282
rs672413
rs558133
rs11951068
rs705415
rs1789953
All − MR Egger
All − IVW(MRE)
−0.6
−0.3
0.0
0.3
MR effect size for
'Selenium' on 'Fatty acid−binding protein, liver'

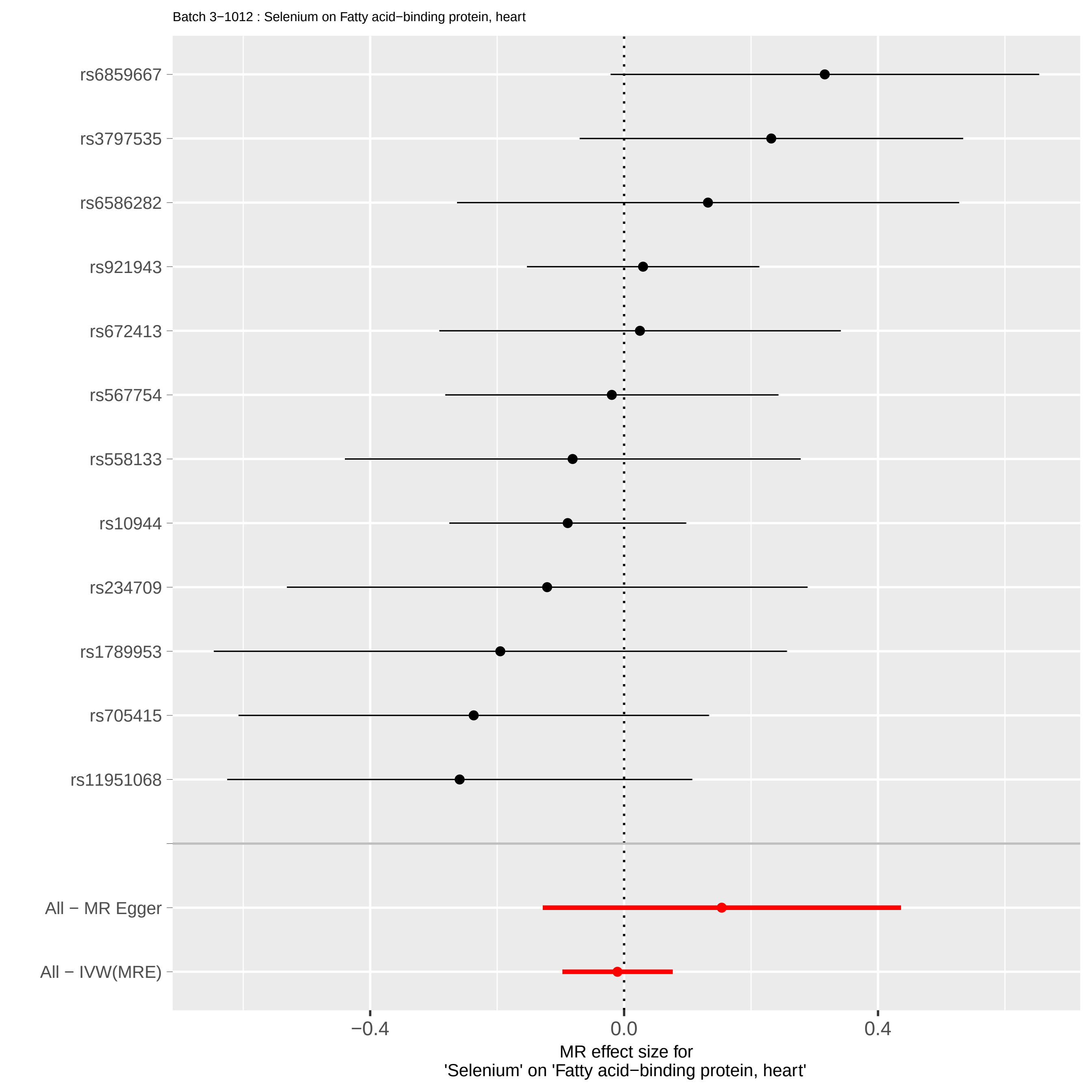
Batch 3−1012 : Selenium on Fatty acid−binding protein, heart
rs6859667
rs3797535
rs6586282
rs921943
rs672413
rs567754
rs558133
rs10944
rs234709
rs1789953
rs705415
rs11951068
All − MR Egger
All − IVW(MRE)
−0.4
0.0
0.4
MR effect size for
'Selenium' on 'Fatty acid−binding protein, heart'

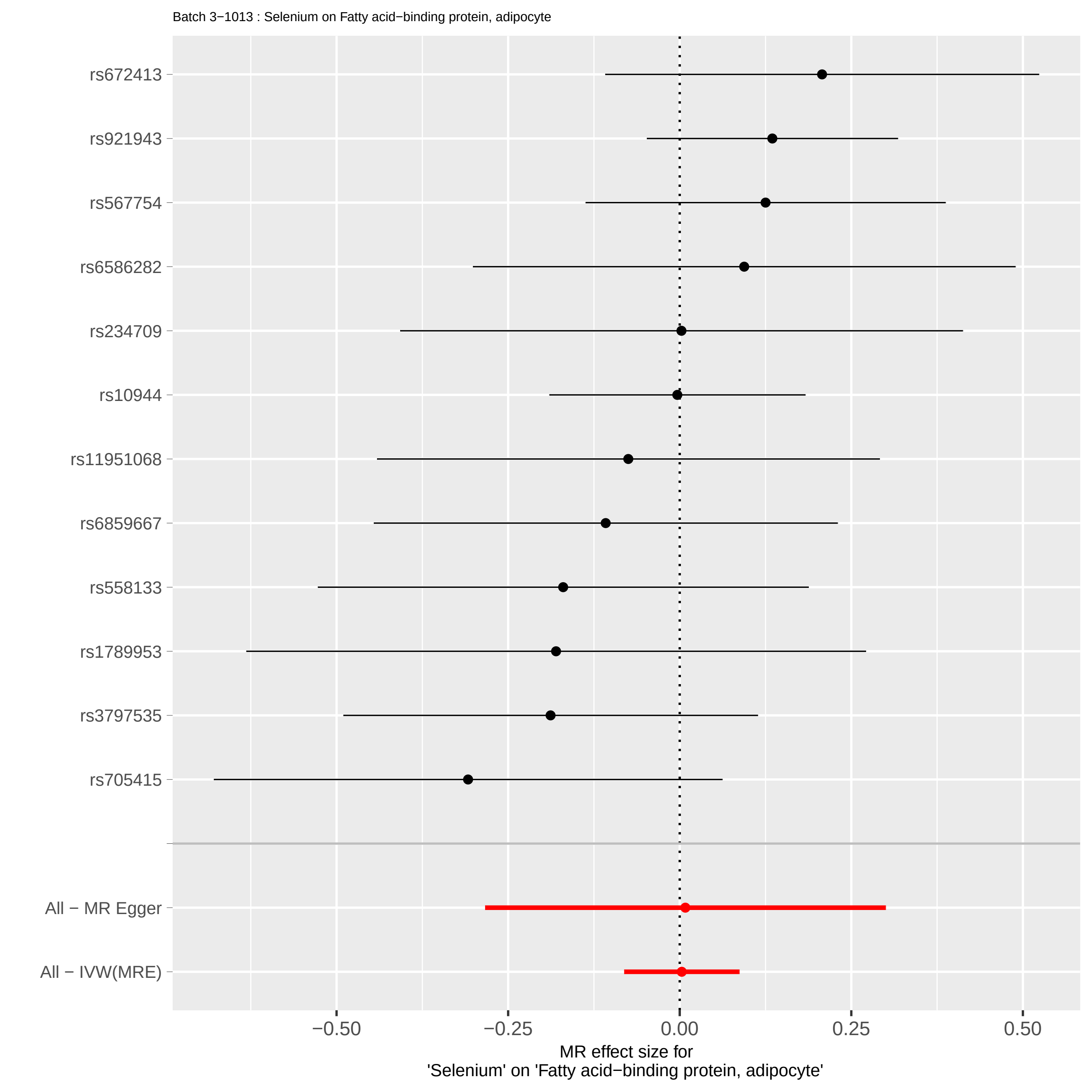
Batch 3−1013 : Selenium on Fatty acid−binding protein, adipocyte
rs672413
rs921943
rs567754
rs6586282
rs234709
rs10944
rs11951068
rs6859667
rs558133
rs1789953
rs3797535
rs705415
All − MR Egger
All − IVW(MRE)
−0.50
−0.25
0.00
0.25
0.50
MR effect size for
'Selenium' on 'Fatty acid−binding protein, adipocyte'

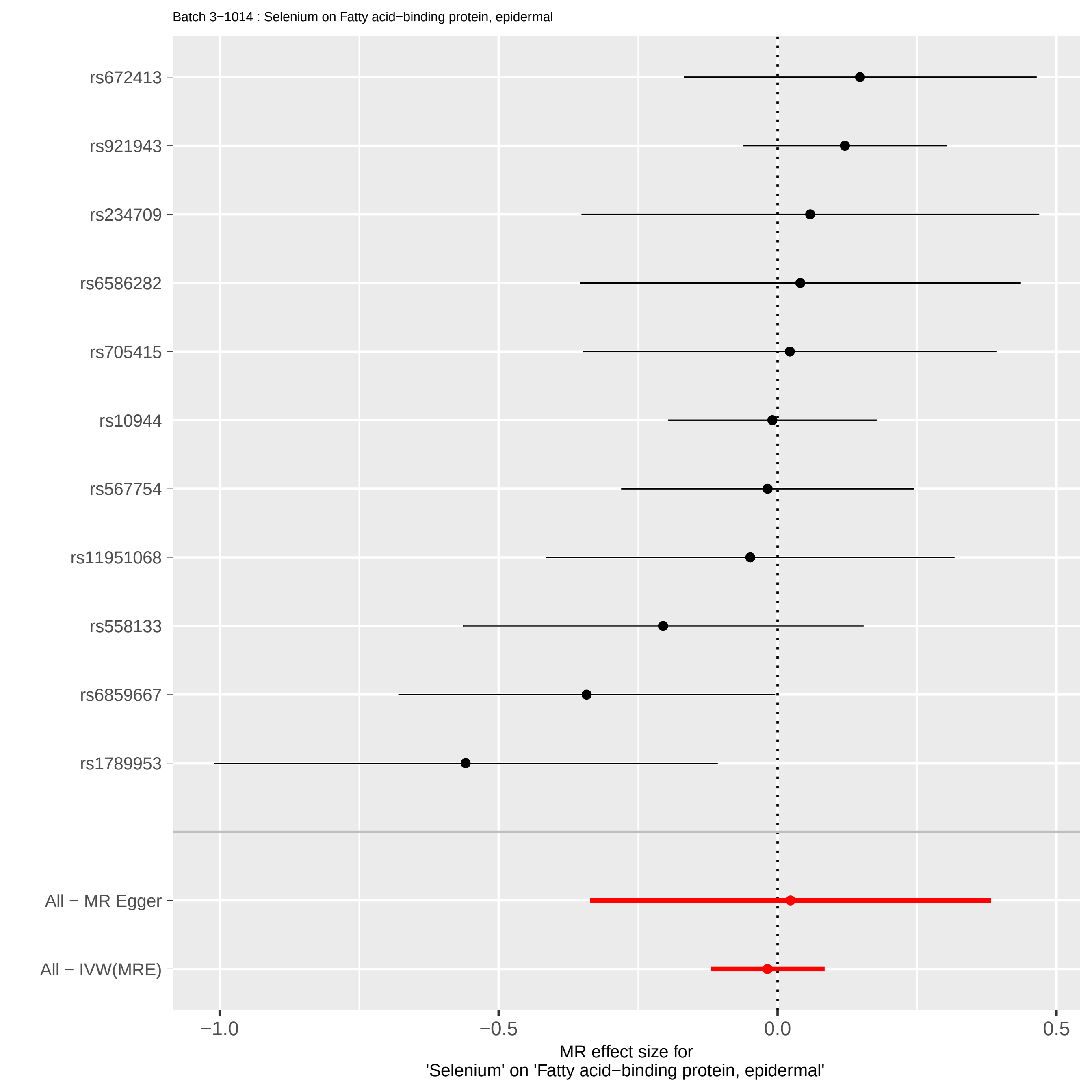

Batch 3−1014 : Selenium on Fatty acid−binding protein, epidermal
rs672413
rs921943
rs234709
rs6586282
rs705415
rs10944
rs567754
rs11951068
rs558133
rs6859667
rs1789953
All − MR Egger
All − IVW(MRE)
−1.0
−0.5
0.0
0.5
MR effect size for
'Selenium' on 'Fatty acid−binding protein, epidermal'

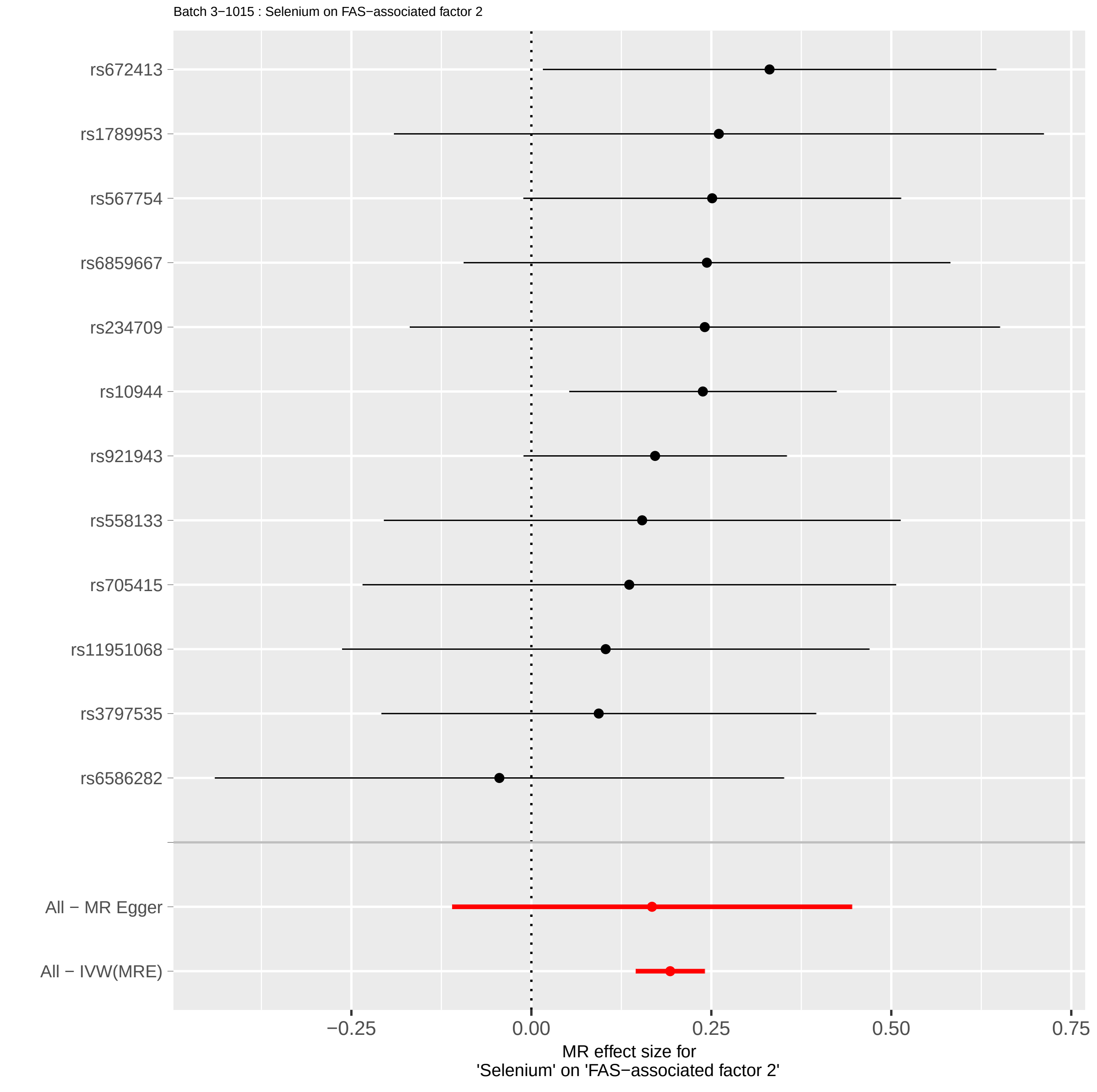

Batch 3−1015 : Selenium on FAS−associated factor 2
rs672413
rs1789953
rs567754
rs6859667
rs234709
rs10944
rs921943
rs558133
rs705415
rs11951068
rs3797535
rs6586282
All − MR Egger
All − IVW(MRE)
−0.25
0.00
0.25
0.50
0.75
MR effect size for
'Selenium' on 'FAS−associated factor 2'

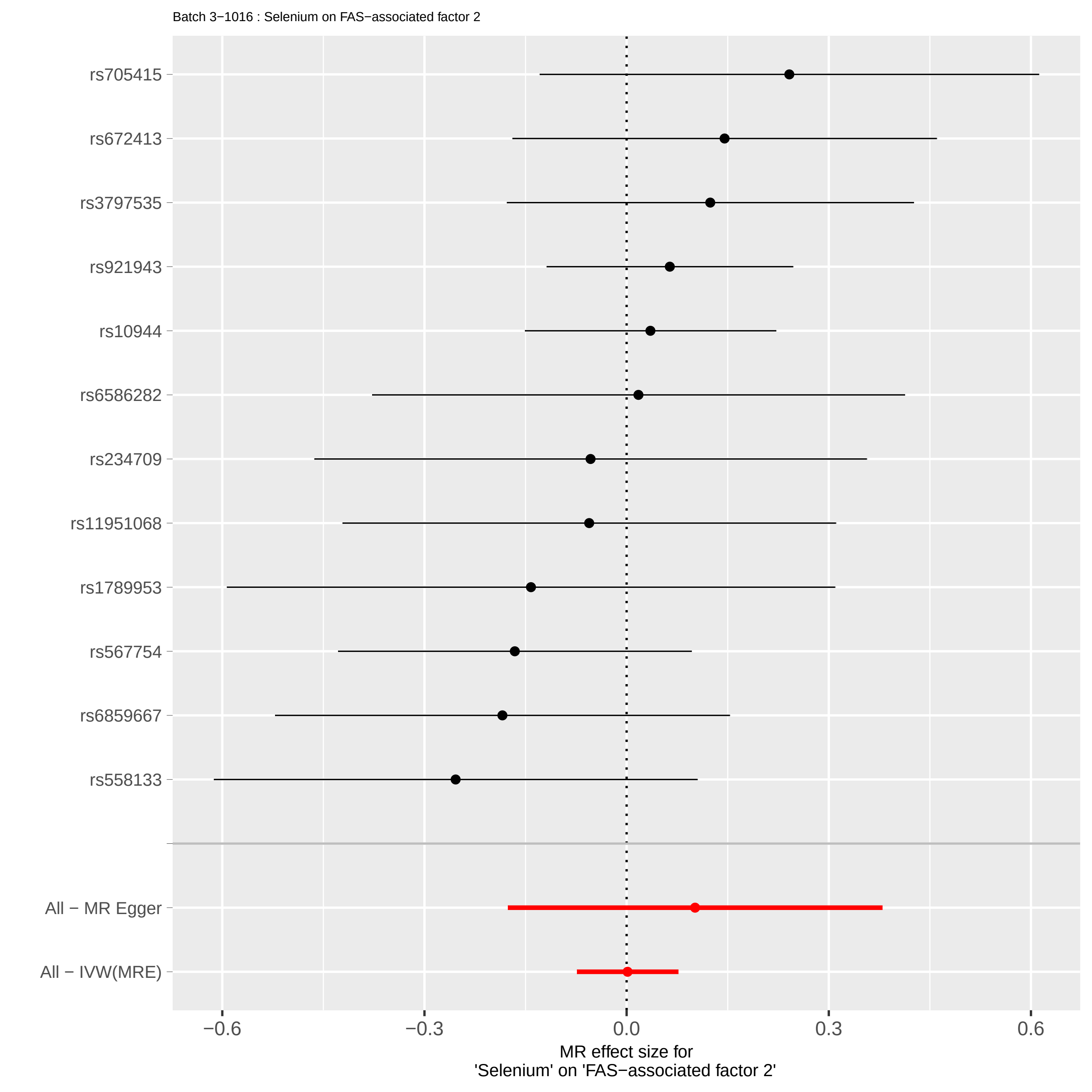

Batch 3−1016 : Selenium on FAS−associated factor 2
rs705415
rs672413
rs3797535
rs921943
rs10944
rs6586282
rs234709
rs11951068
rs1789953
rs567754
rs6859667
rs558133
All − MR Egger
All − IVW(MRE)
−0.6
−0.3
0.0
0.3
0.6
MR effect size for
'Selenium' on 'FAS−associated factor 2'

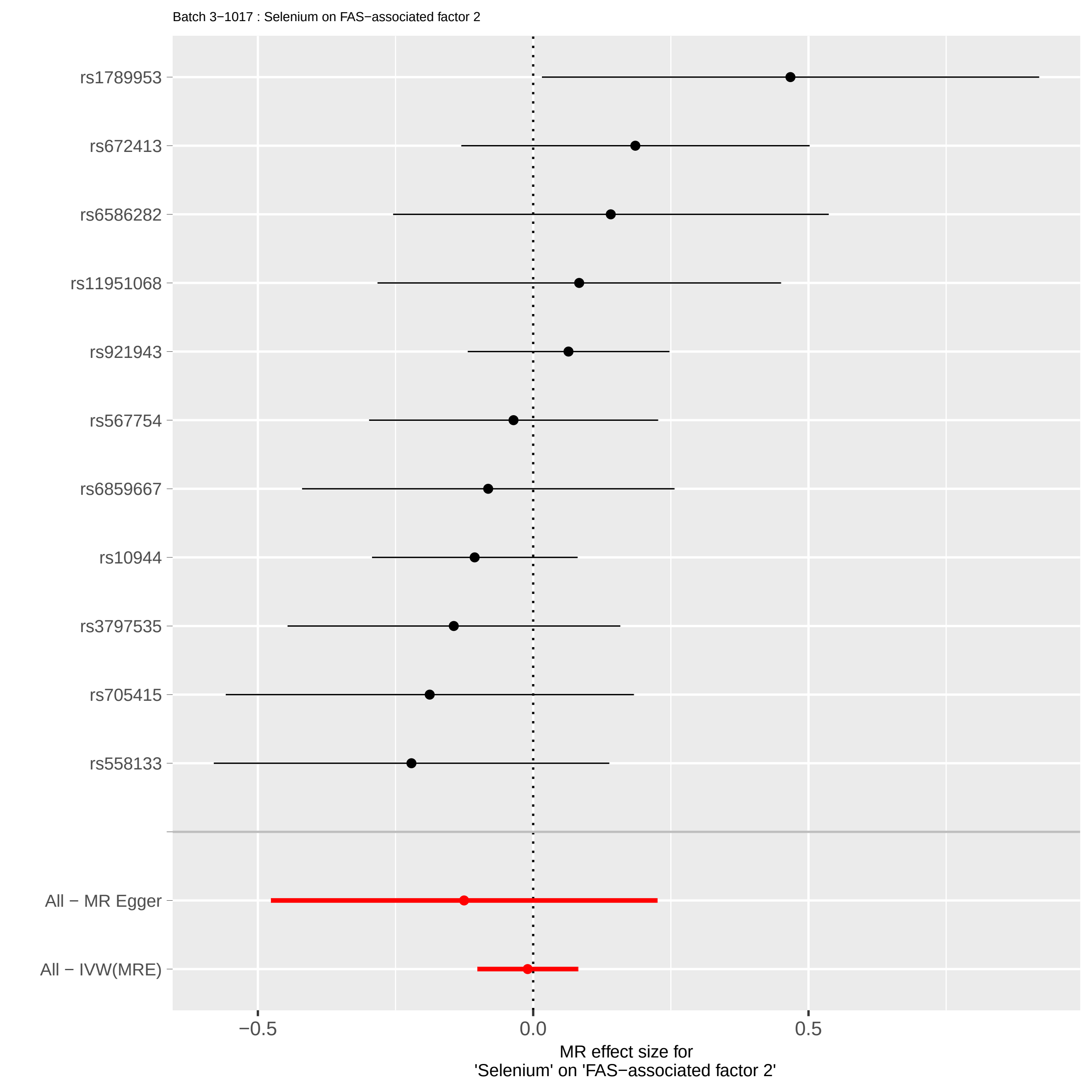

Batch 3−1017 : Selenium on FAS−associated factor 2
rs1789953
rs672413
rs6586282
rs11951068
rs921943
rs567754
rs6859667
rs10944
rs3797535
rs705415
rs558133
All − MR Egger
All − IVW(MRE)
−0.5
0.0
0.5
MR effect size for
'Selenium' on 'FAS−associated factor 2'

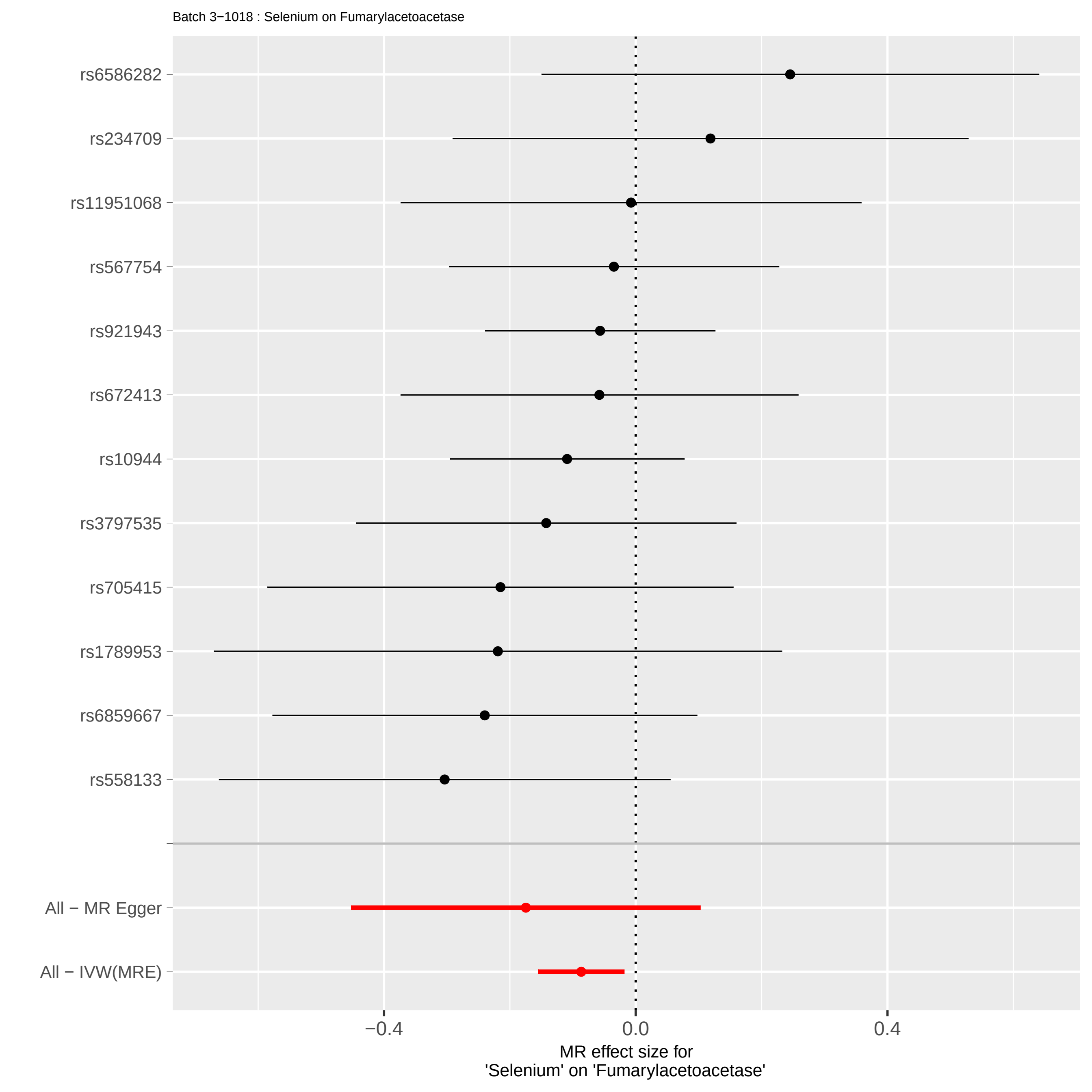

Batch 3−1018 : Selenium on Fumarylacetoacetase
rs6586282
rs234709
rs11951068
rs567754
rs921943
rs672413
rs10944
rs3797535
rs705415
rs1789953
rs6859667
rs558133
All − MR Egger
All − IVW(MRE)
−0.4
0.0
0.4
MR effect size for
'Selenium' on 'Fumarylacetoacetase'

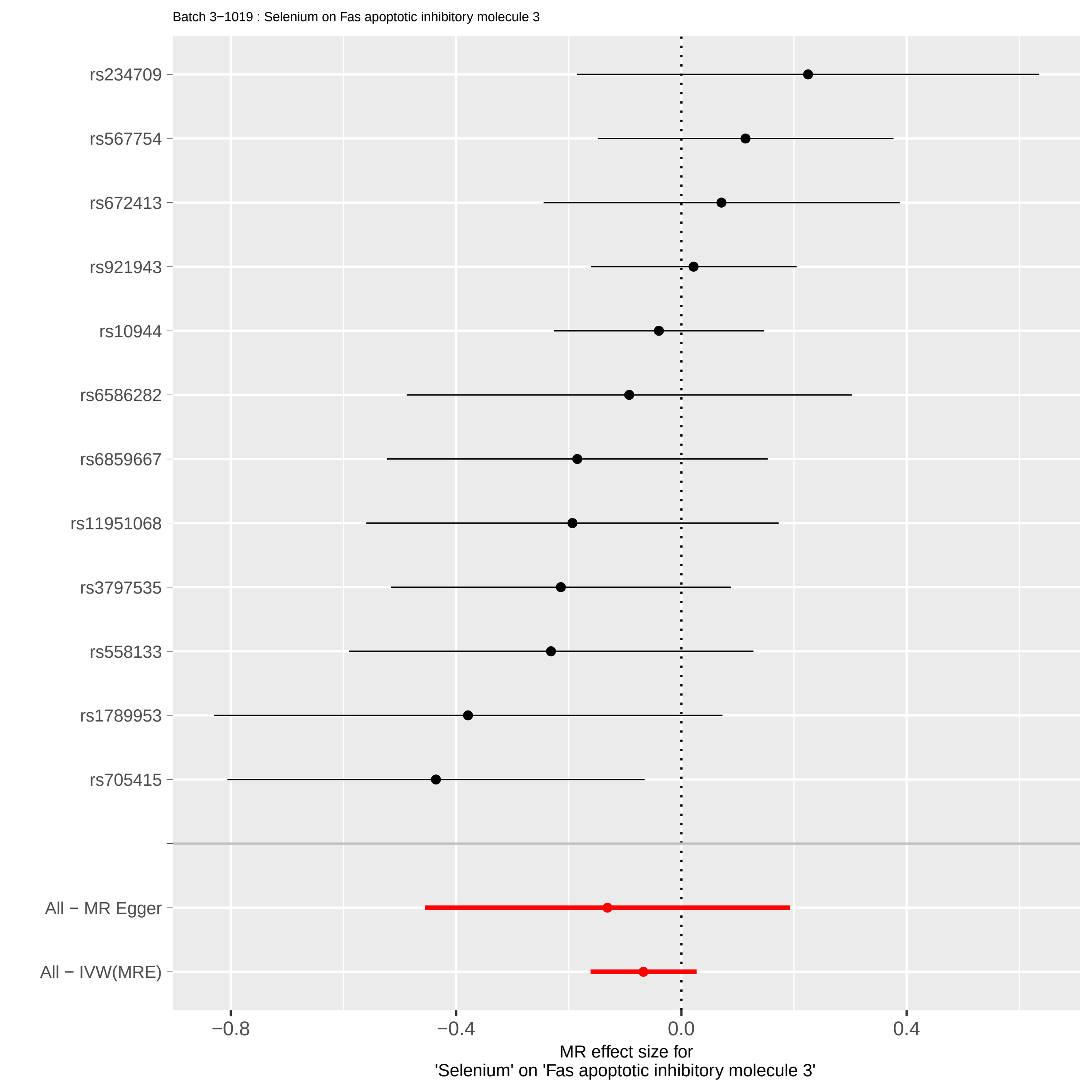

Batch 3−1019 : Selenium on Fas apoptotic inhibitory molecule 3
rs234709
rs567754
rs672413
rs921943
rs10944
rs6586282
rs6859667
rs11951068
rs3797535
rs558133
rs1789953
rs705415
All − MR Egger
All − IVW(MRE)
−0.8
−0.4
0.0
0.4
MR effect size for
'Selenium' on 'Fas apoptotic inhibitory molecule 3'

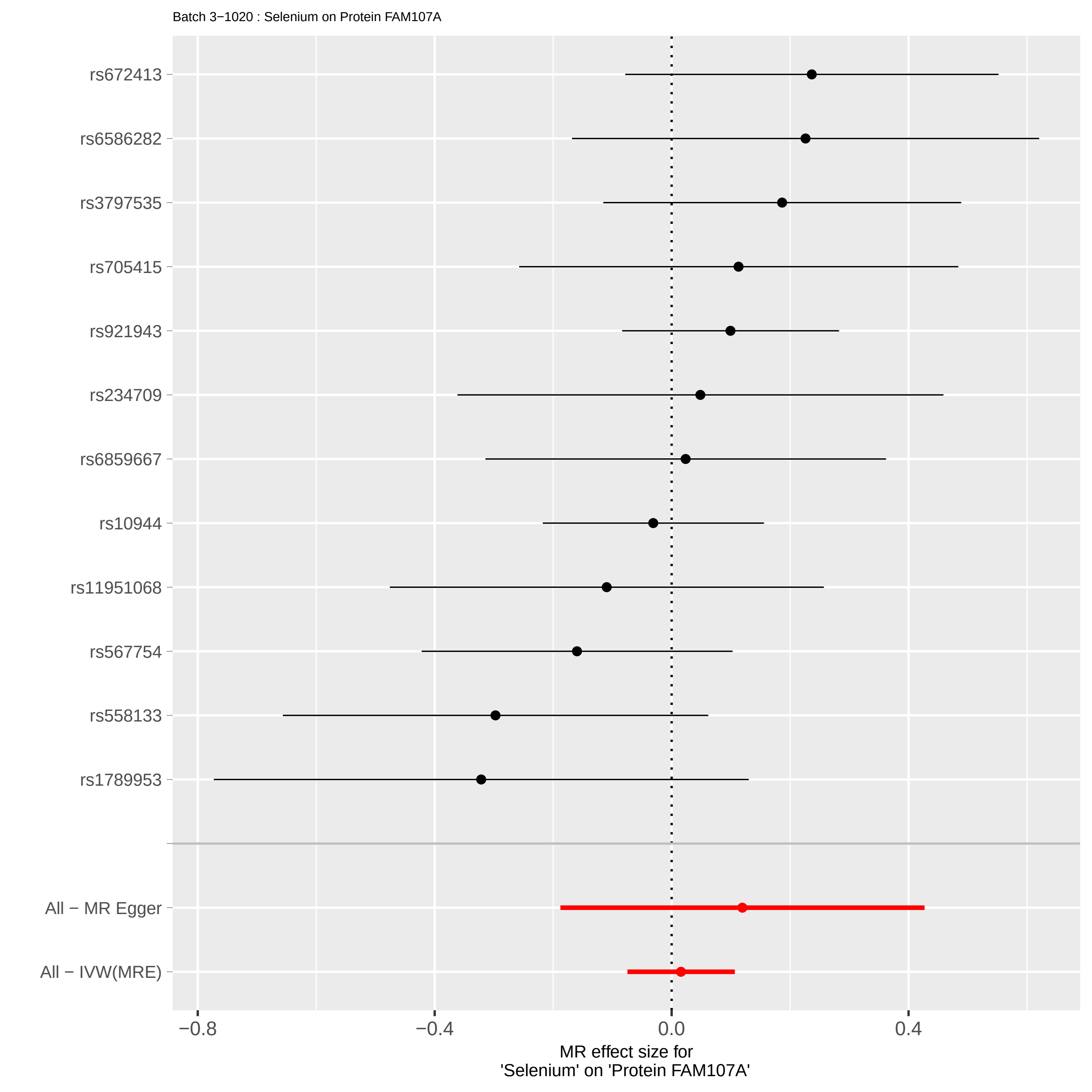

Batch 3−1020 : Selenium on Protein FAM107A
rs672413
rs6586282
rs3797535
rs705415
rs921943
rs234709
rs6859667
rs10944
rs11951068
rs567754
rs558133
rs1789953
All − MR Egger
All − IVW(MRE)
−0.8
−0.4
0.0
0.4
MR effect size for
'Selenium' on 'Protein FAM107A'

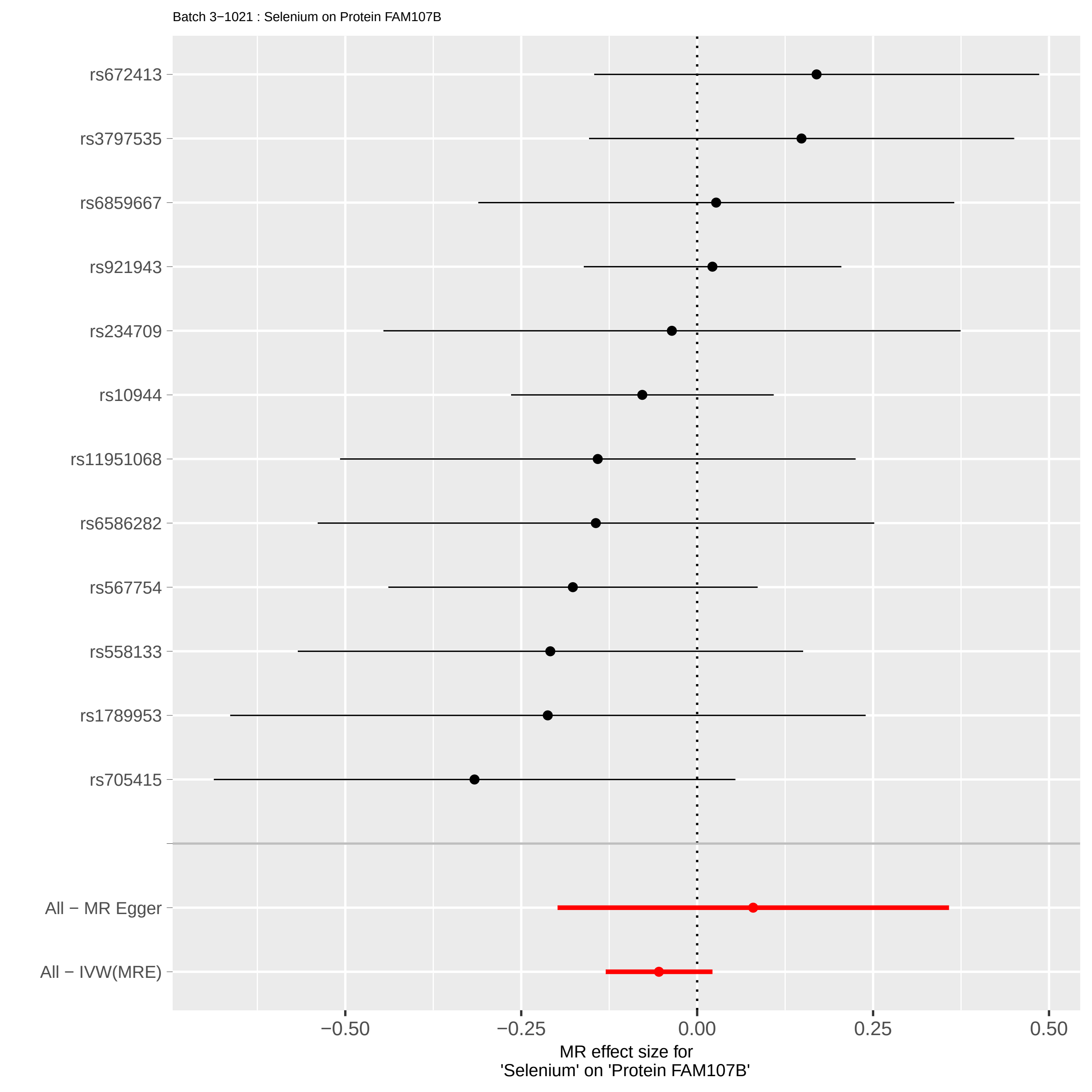

Batch 3−1021 : Selenium on Protein FAM107B
rs672413
rs3797535
rs6859667
rs921943
rs234709
rs10944
rs11951068
rs6586282
rs567754
rs558133
rs1789953
rs705415
All − MR Egger
All − IVW(MRE)
−0.50
−0.25
0.00
0.25
0.50
MR effect size for
'Selenium' on 'Protein FAM107B'

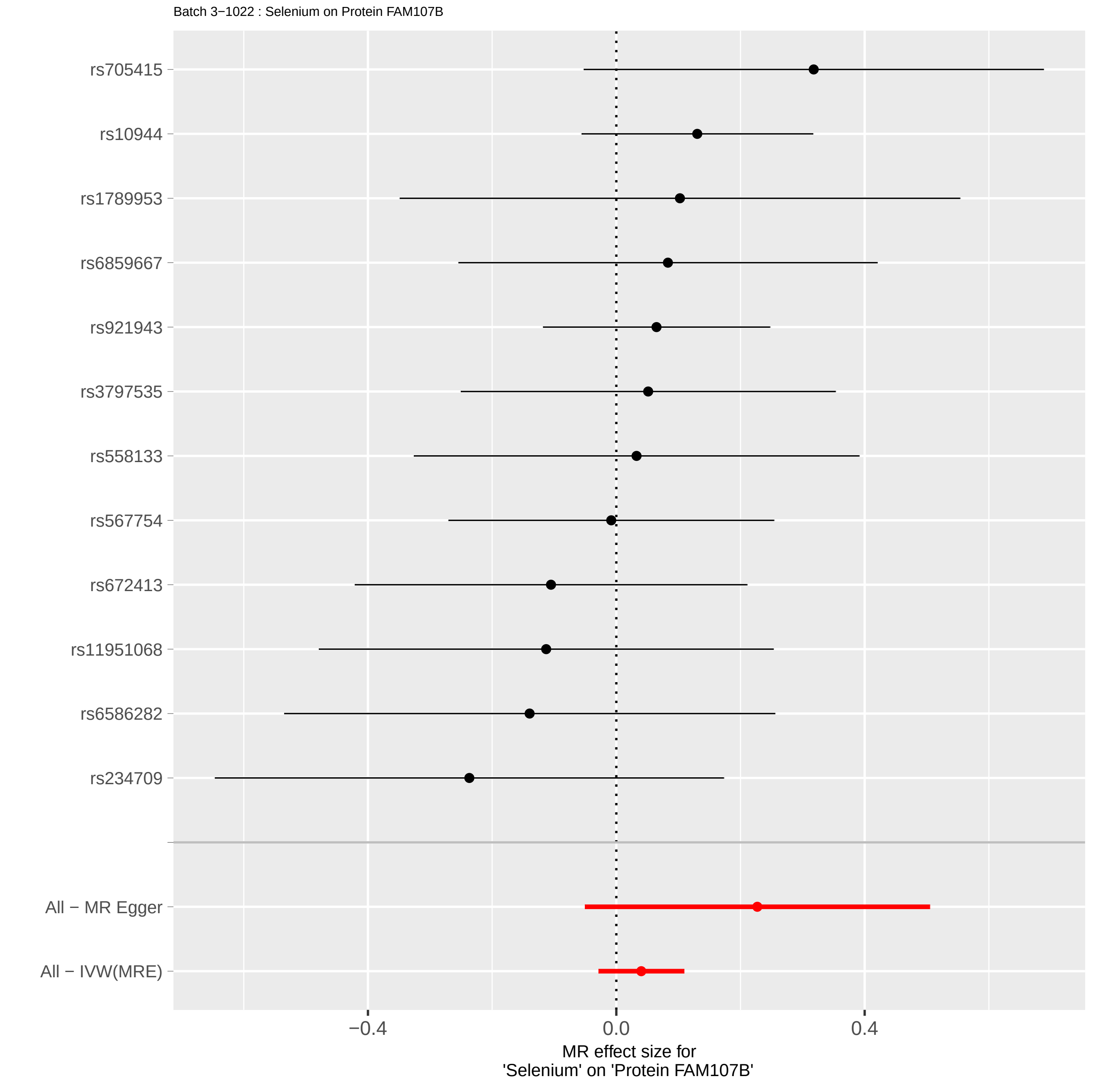

Batch 3−1022 : Selenium on Protein FAM107B
rs705415
rs10944
rs1789953
rs6859667
rs921943
rs3797535
rs558133
rs567754
rs672413
rs11951068
rs6586282
rs234709
All − MR Egger
All − IVW(MRE)
−0.4
0.0
0.4
MR effect size for
'Selenium' on 'Protein FAM107B'

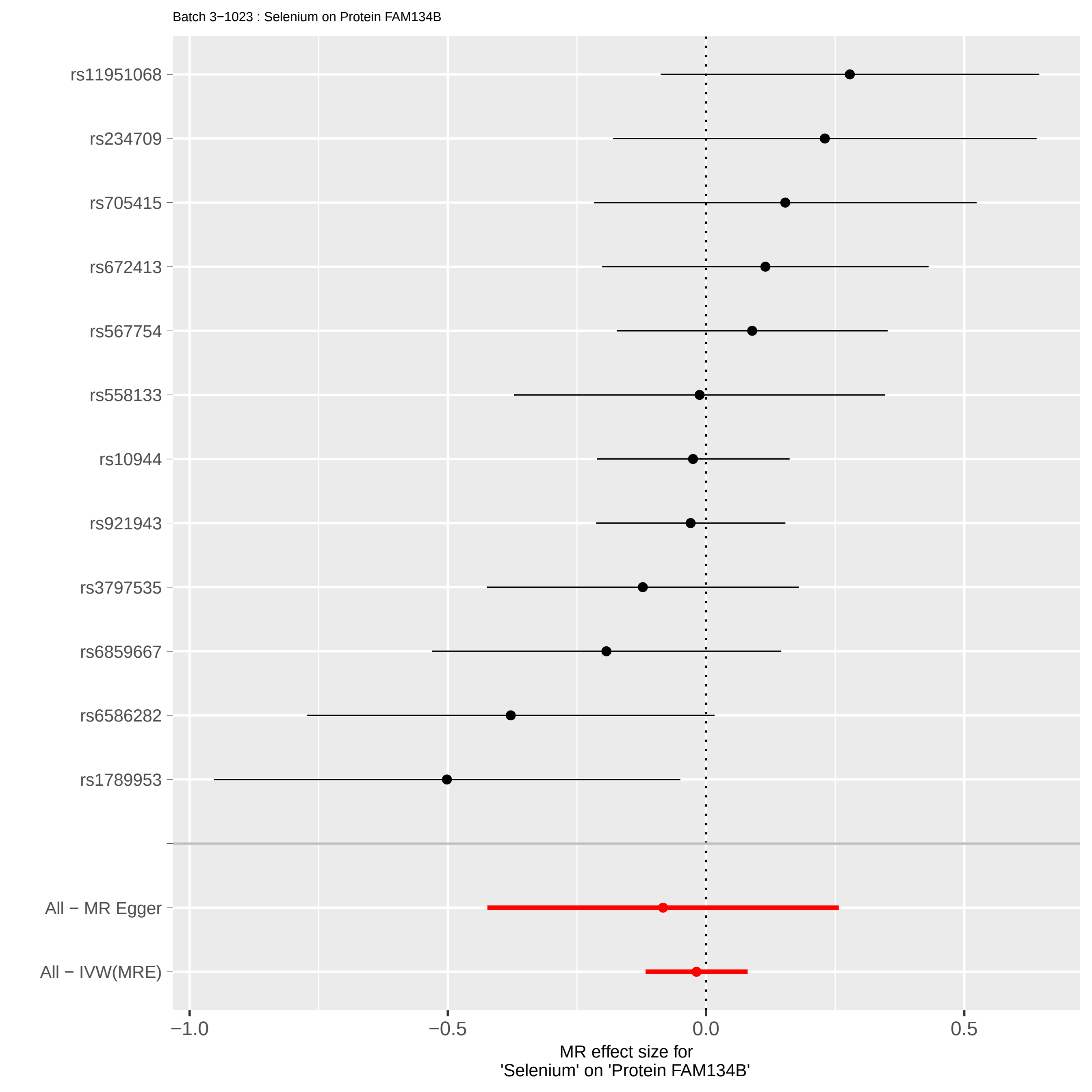
Batch 3−1023 : Selenium on Protein FAM134B
rs11951068
rs234709
rs705415
rs672413
rs567754
rs558133
rs10944
rs921943
rs3797535
rs6859667
rs6586282
rs1789953
All − MR Egger
All − IVW(MRE)
−1.0
−0.5
0.0
0.5
MR effect size for
'Selenium' on 'Protein FAM134B'

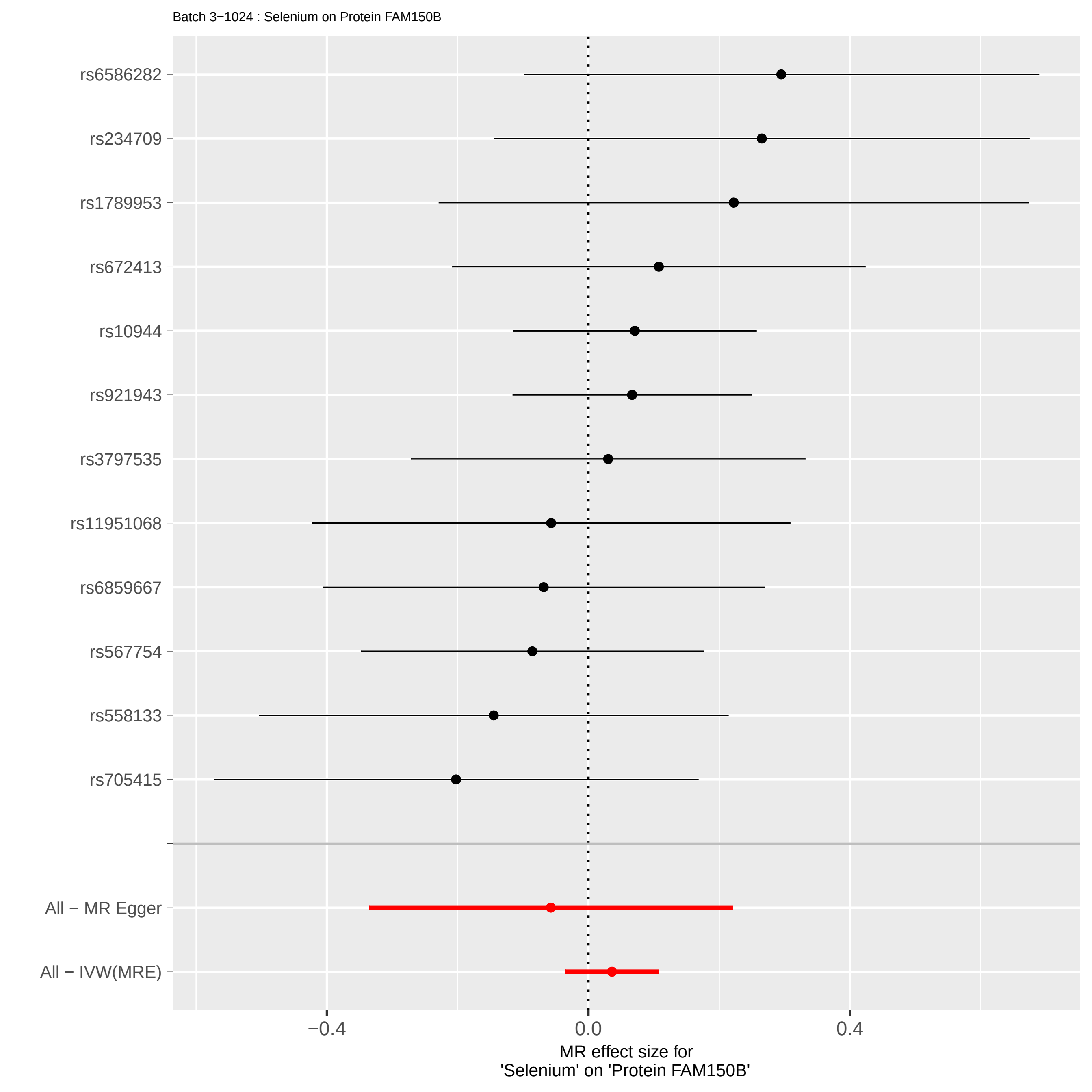

Batch 3−1024 : Selenium on Protein FAM150B
rs6586282
rs234709
rs1789953
rs672413
rs10944
rs921943
rs3797535
rs11951068
rs6859667
rs567754
rs558133
rs705415
All − MR Egger
All − IVW(MRE)
−0.4
0.0
0.4
MR effect size for
'Selenium' on 'Protein FAM150B'

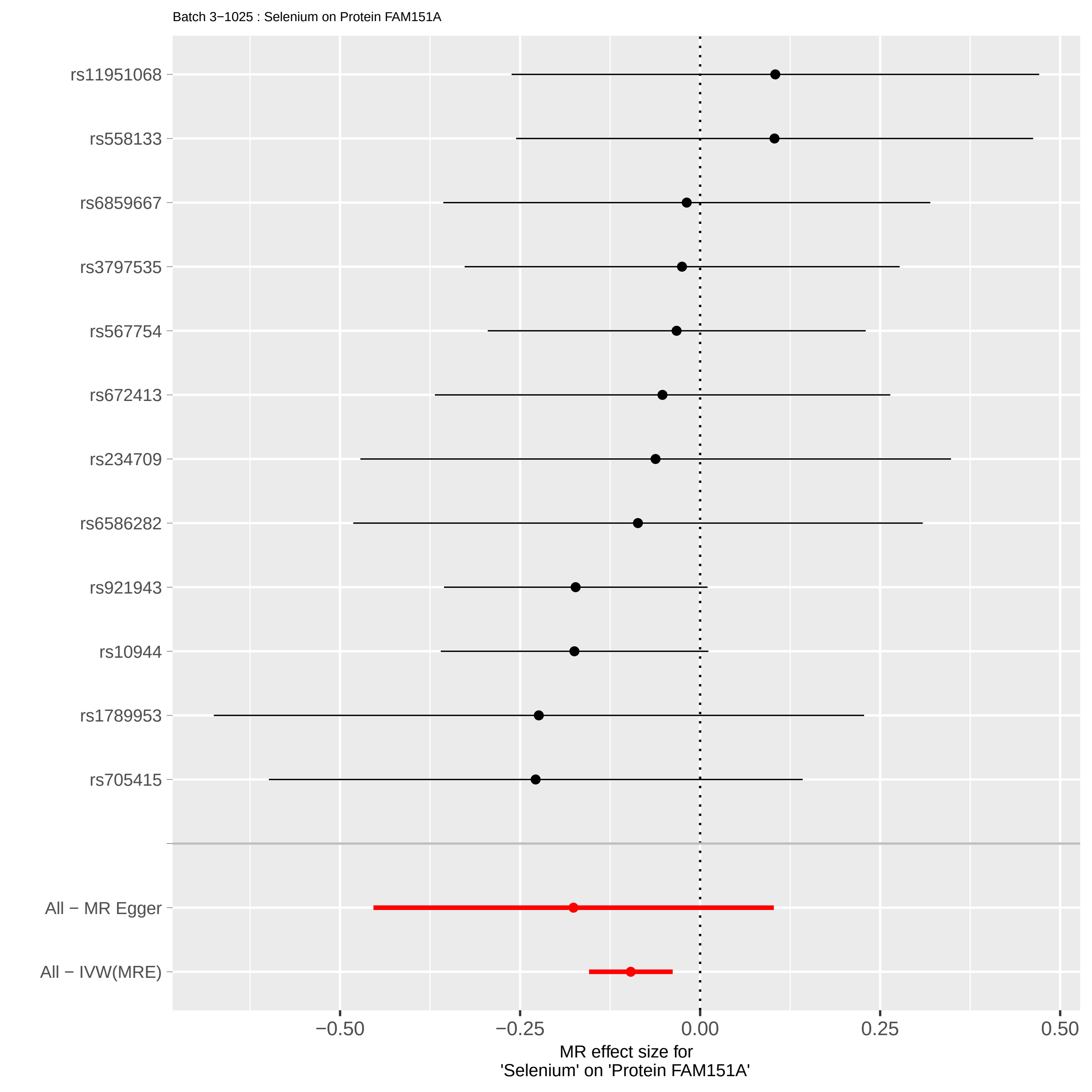
Batch 3−1025 : Selenium on Protein FAM151A
rs11951068
rs558133
rs6859667
rs3797535
rs567754
rs672413
rs234709
rs6586282
rs921943
rs10944
rs1789953
rs705415
All − MR Egger
All − IVW(MRE)
−0.50
−0.25
0.00
0.25
0.50
MR effect size for
'Selenium' on 'Protein FAM151A'

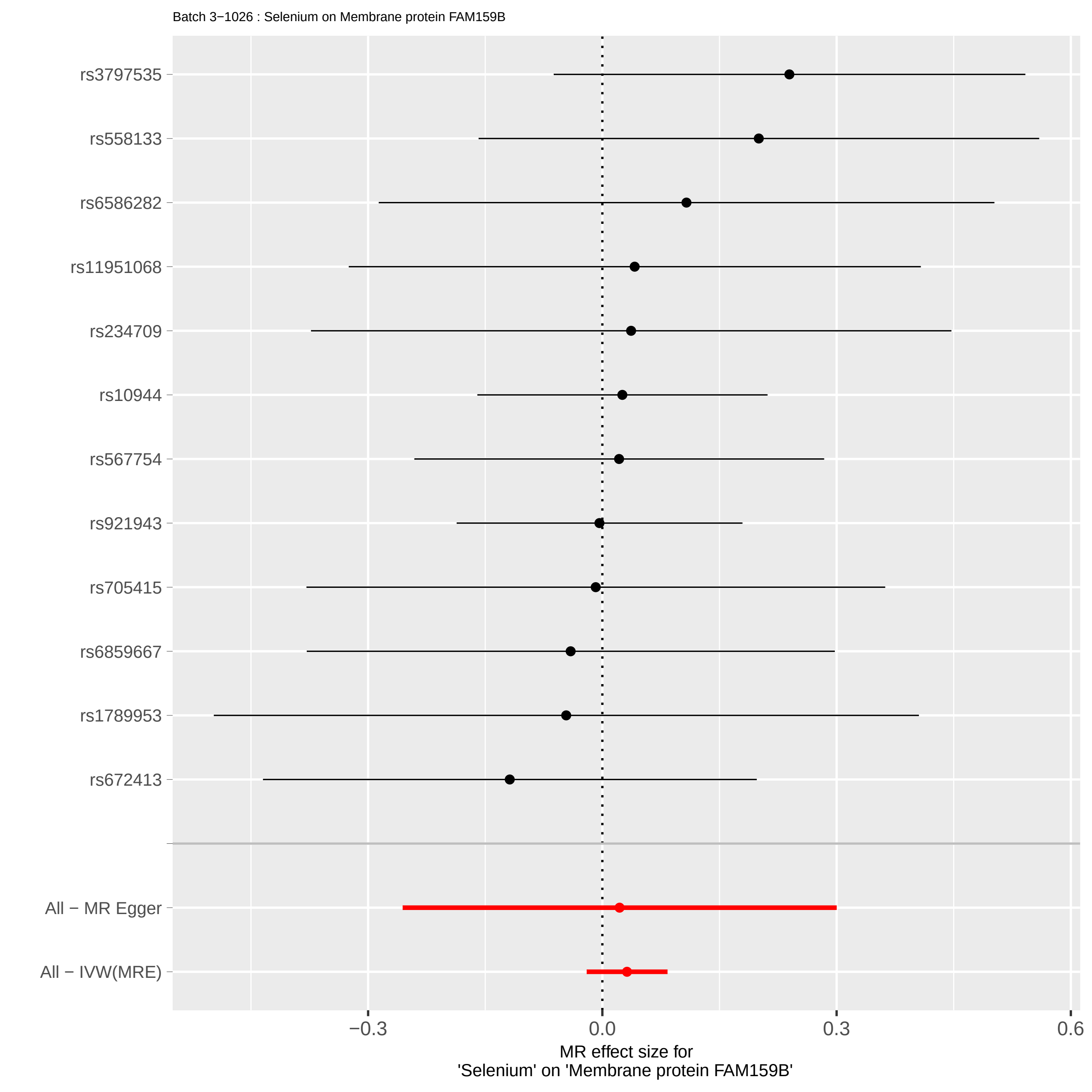

Batch 3−1026 : Selenium on Membrane protein FAM159B
rs3797535
rs558133
rs6586282
rs11951068
rs234709
rs10944
rs567754
rs921943
rs705415
rs6859667
rs1789953
rs672413
All − MR Egger
All − IVW(MRE)
−0.3
0.0
0.3
0.6
MR effect size for
'Selenium' on 'Membrane protein FAM159B'

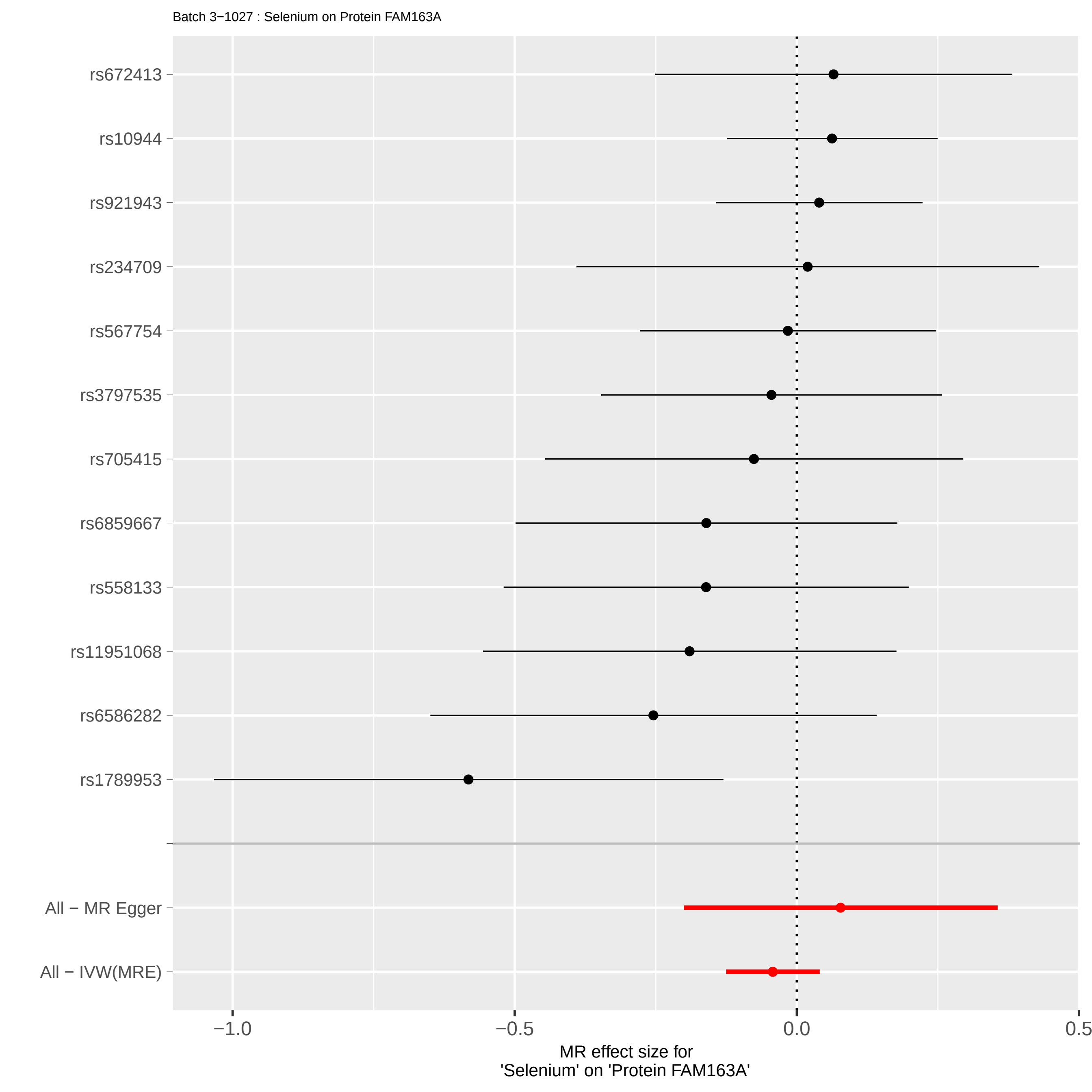

Batch 3−1027 : Selenium on Protein FAM163A
rs672413
rs10944
rs921943
rs234709
rs567754
rs3797535
rs705415
rs6859667
rs558133
rs11951068
rs6586282
rs1789953
All − MR Egger
All − IVW(MRE)
−1.0
−0.5
0.0
0.5
MR effect size for
'Selenium' on 'Protein FAM163A'

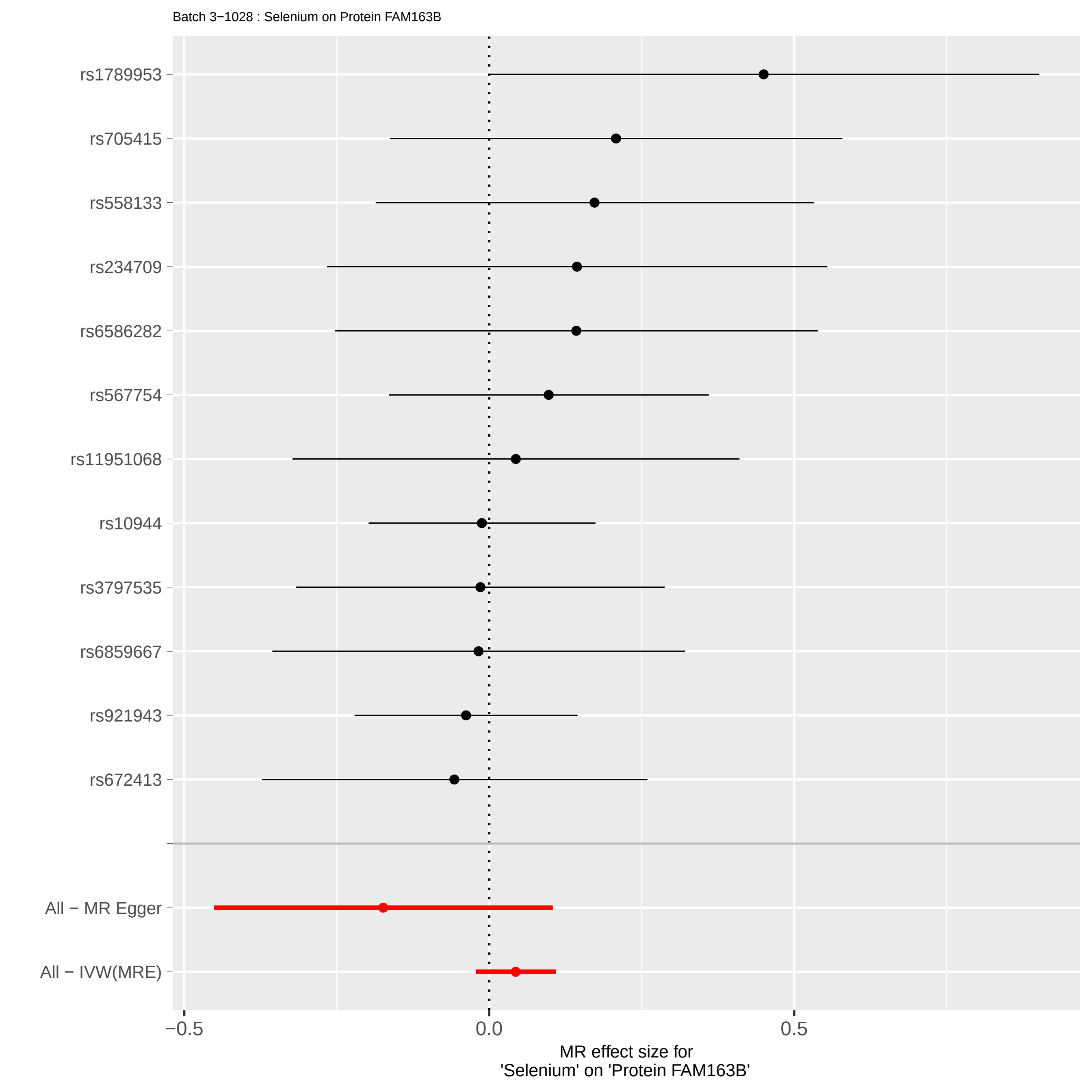

Batch 3−1028 : Selenium on Protein FAM163B
rs1789953
rs705415
rs558133
rs234709
rs6586282
rs567754
rs11951068
rs10944
rs3797535
rs6859667
rs921943
rs672413
All − MR Egger
All − IVW(MRE)
−0.5
0.0
0.5
MR effect size for
'Selenium' on 'Protein FAM163B'

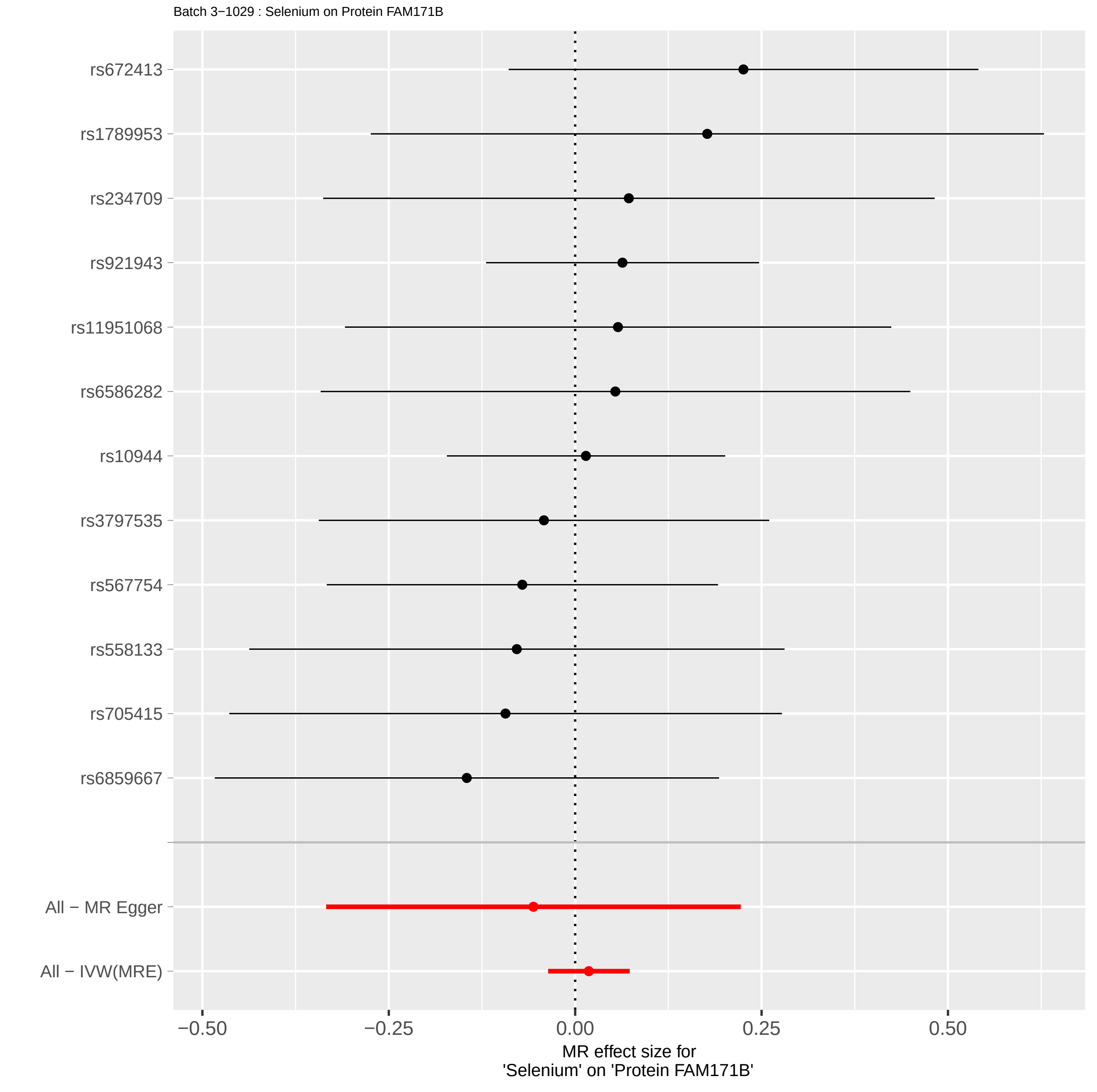

Batch 3−1029 : Selenium on Protein FAM171B
rs672413
rs1789953
rs234709
rs921943
rs11951068
rs6586282
rs10944
rs3797535
rs567754
rs558133
rs705415
rs6859667
All − MR Egger
All − IVW(MRE)
−0.50
−0.25
0.00
0.25
0.50
MR effect size for
'Selenium' on 'Protein FAM171B'

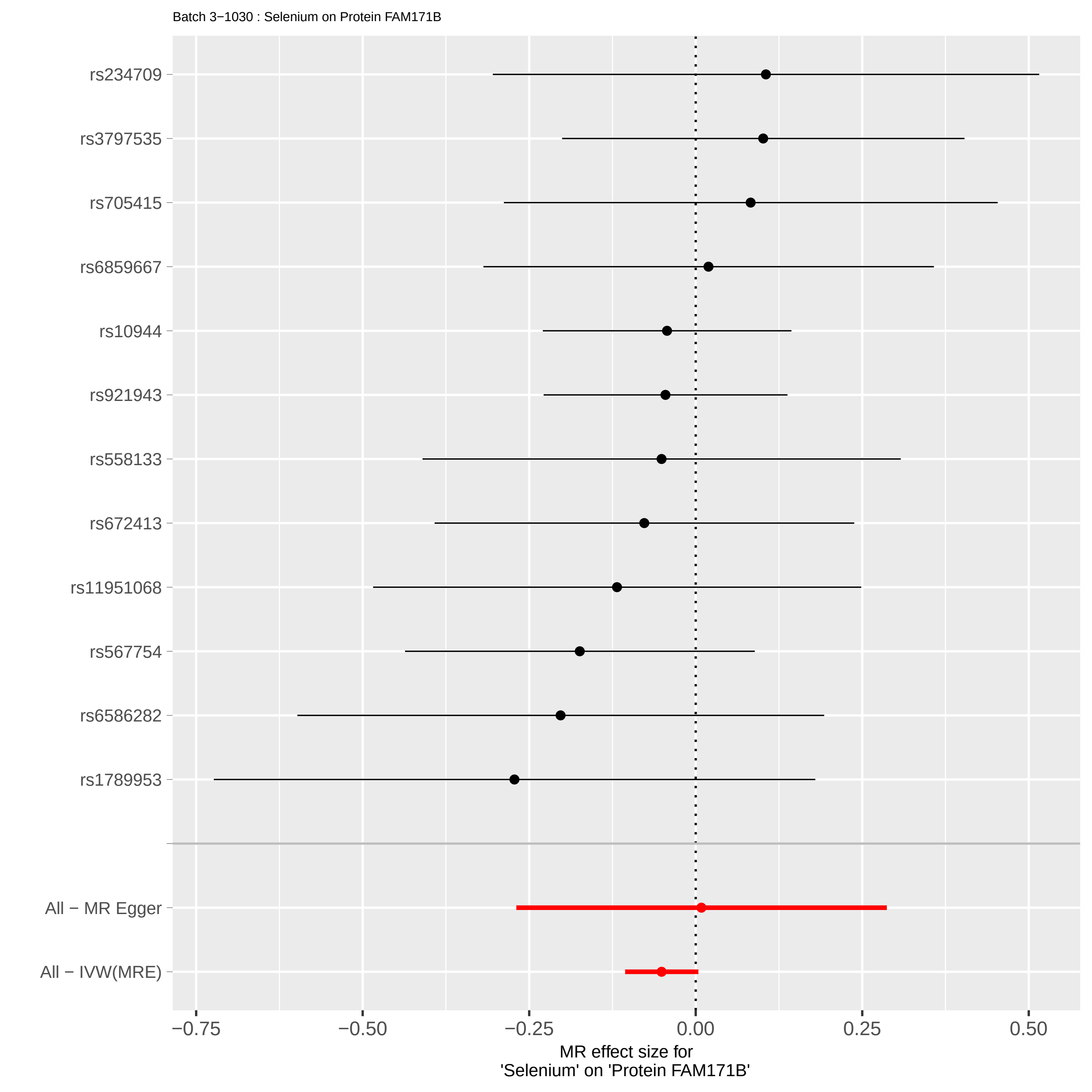

Batch 3−1030 : Selenium on Protein FAM171B
rs234709
rs3797535
rs705415
rs6859667
rs10944
rs921943
rs558133
rs672413
rs11951068
rs567754
rs6586282
rs1789953
All − MR Egger
All − IVW(MRE)
−0.75
−0.50
−0.25
0.00
0.25
0.50
MR effect size for
'Selenium' on 'Protein FAM171B'

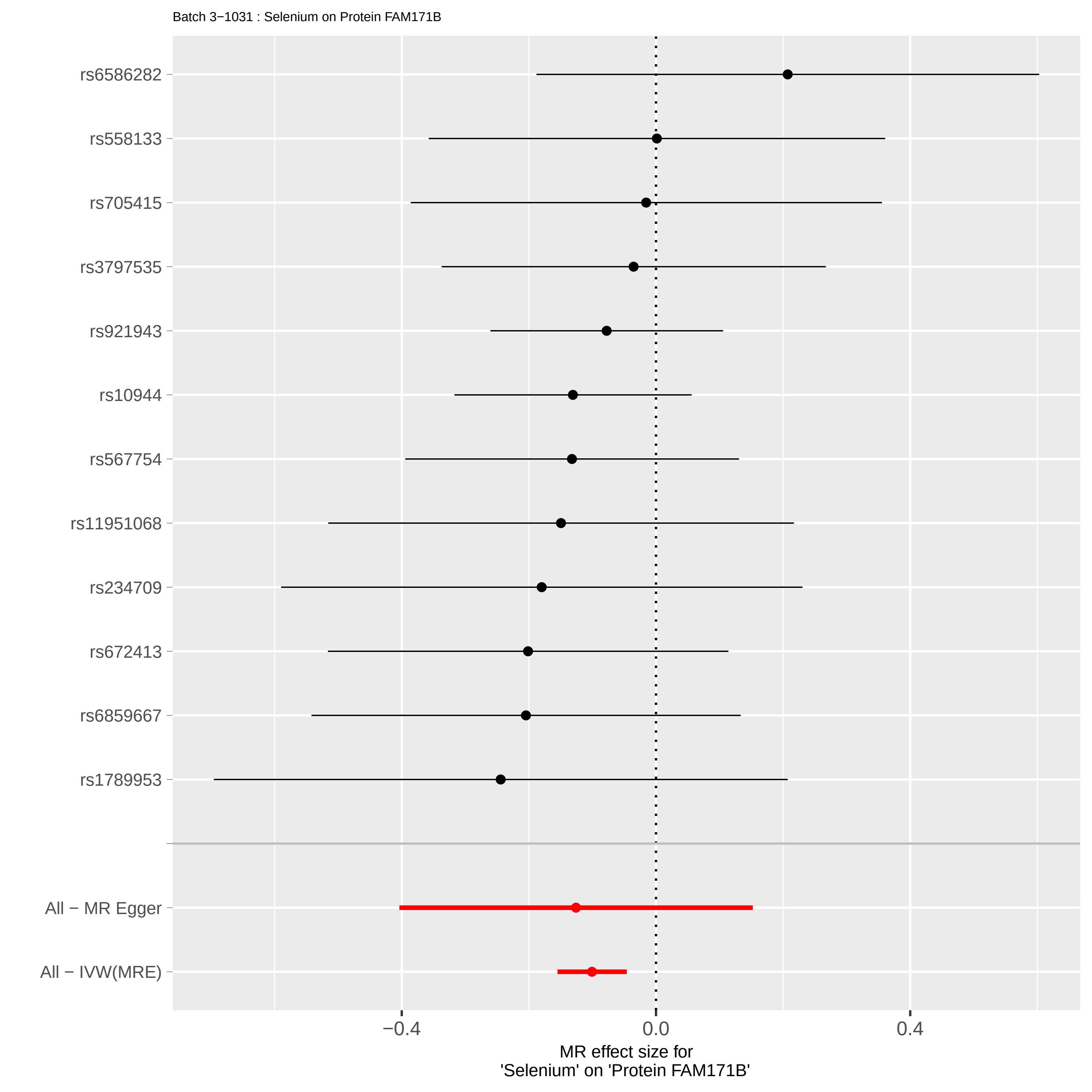

Batch 3−1031 : Selenium on Protein FAM171B
rs6586282
rs558133
rs705415
rs3797535
rs921943
rs10944
rs567754
rs11951068
rs234709
rs672413
rs6859667
rs1789953
All − MR Egger
All − IVW(MRE)
−0.4
0.0
0.4
MR effect size for
'Selenium' on 'Protein FAM171B'

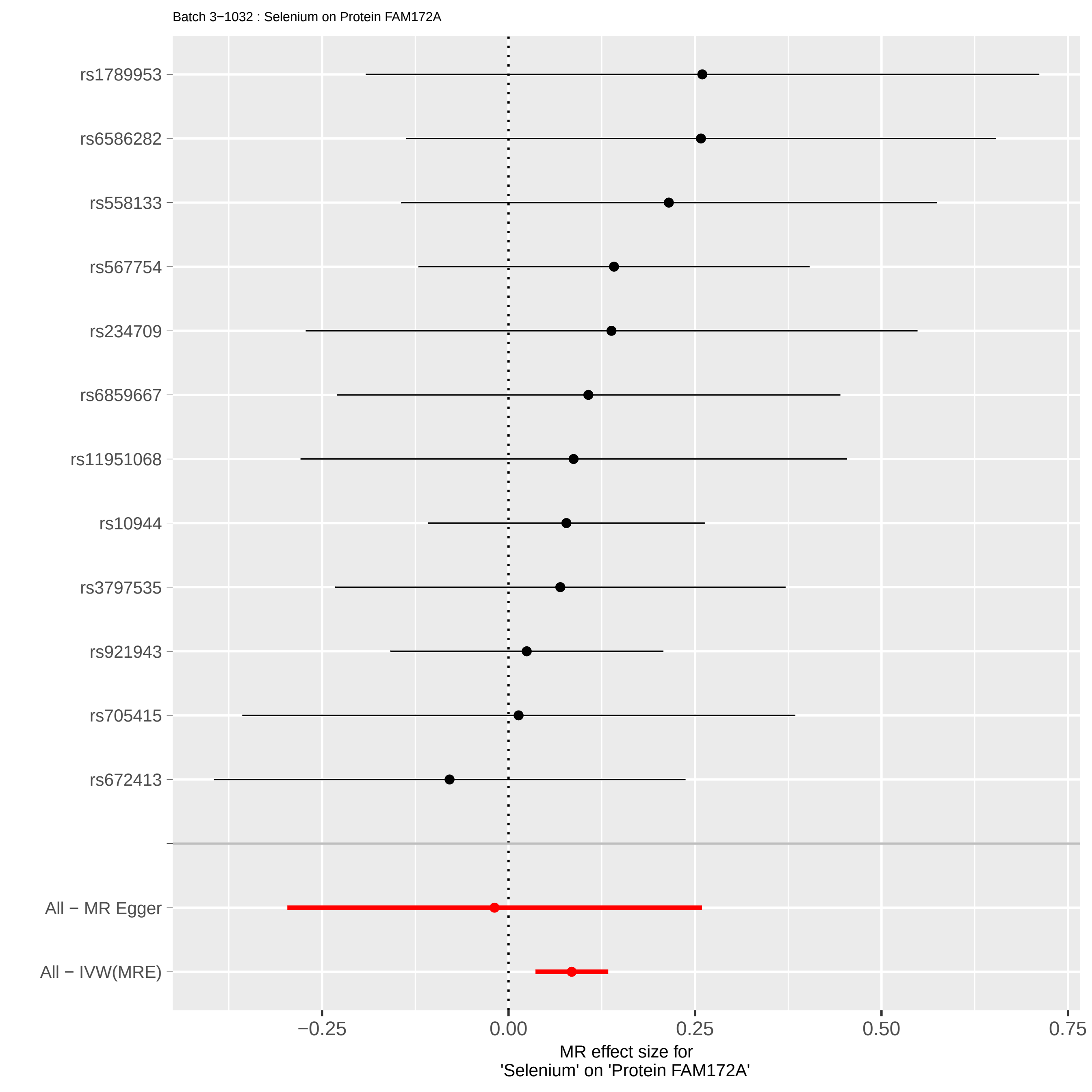

Batch 3−1032 : Selenium on Protein FAM172A
rs1789953
rs6586282
rs558133
rs567754
rs234709
rs6859667
rs11951068
rs10944
rs3797535
rs921943
rs705415
rs672413
All − MR Egger
All − IVW(MRE)
−0.25
0.00
0.25
0.50
0.75
MR effect size for
'Selenium' on 'Protein FAM172A'

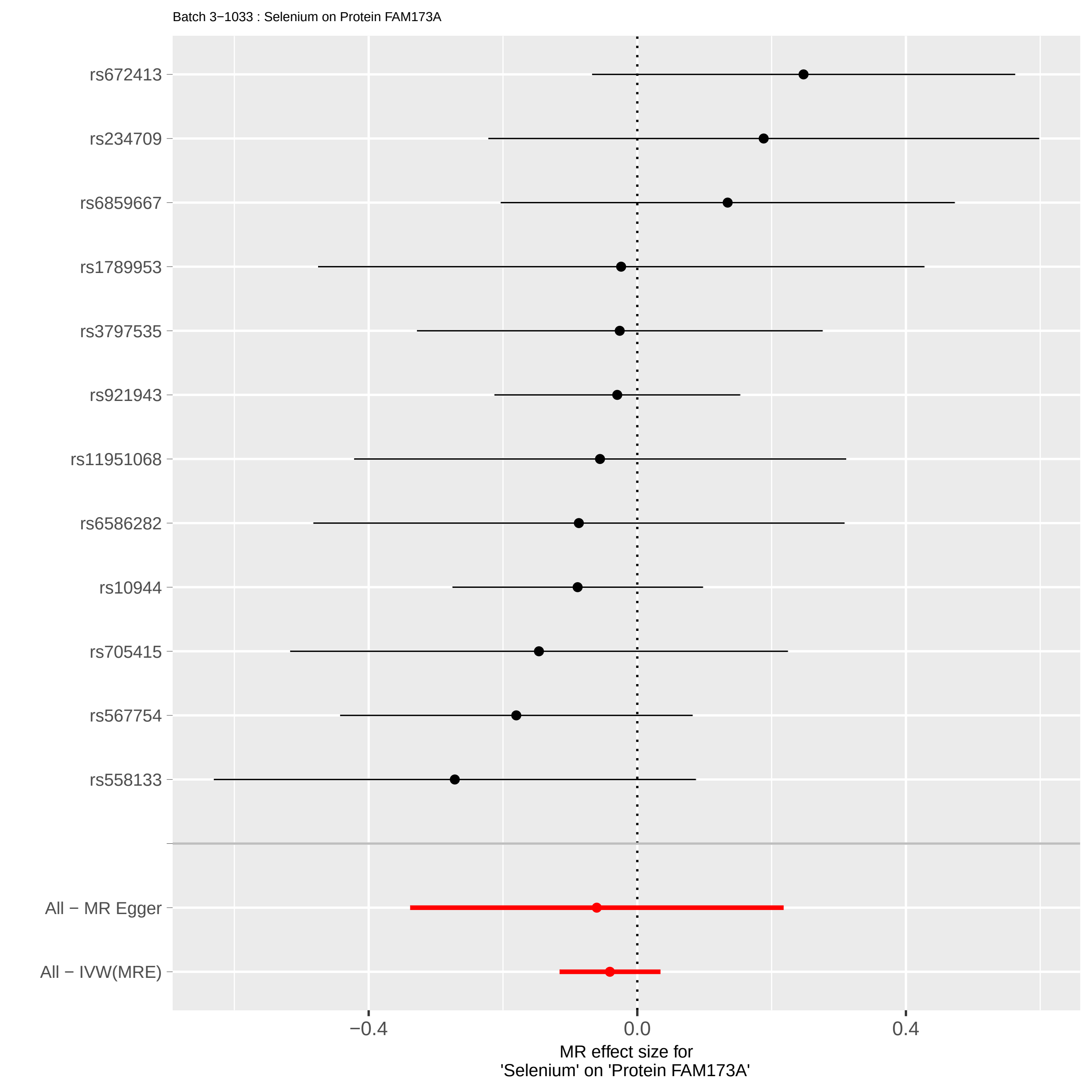

Batch 3−1033 : Selenium on Protein FAM173A
rs672413
rs234709
rs6859667
rs1789953
rs3797535
rs921943
rs11951068
rs6586282
rs10944
rs705415
rs567754
rs558133
All − MR Egger
All − IVW(MRE)
−0.4
0.0
0.4
MR effect size for
'Selenium' on 'Protein FAM173A'

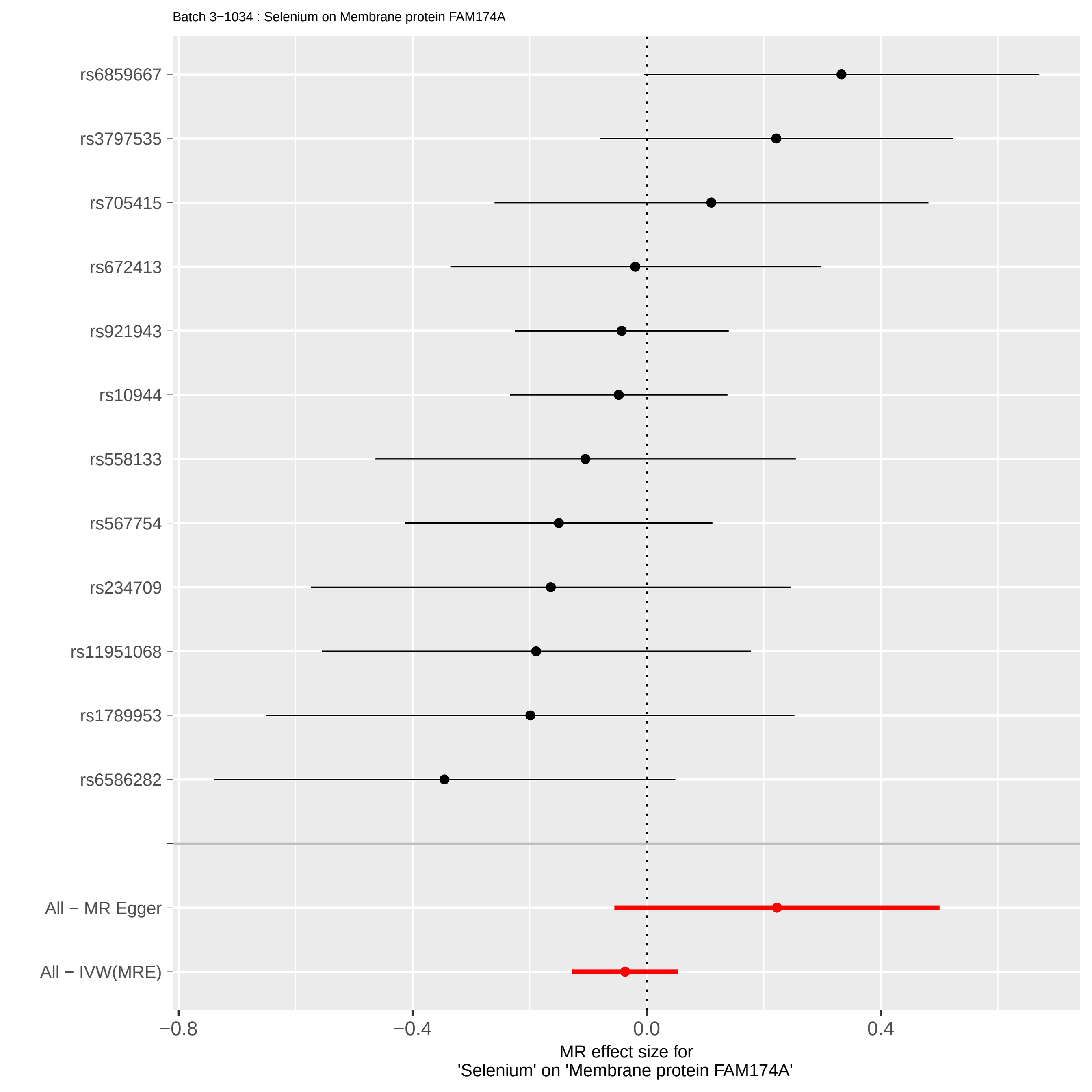

Batch 3−1034 : Selenium on Membrane protein FAM174A
rs6859667
rs3797535
rs705415
rs672413
rs921943
rs10944
rs558133
rs567754
rs234709
rs11951068
rs1789953
rs6586282
All − MR Egger
All − IVW(MRE)
−0.8
−0.4
0.0
0.4
MR effect size for
'Selenium' on 'Membrane protein FAM174A'

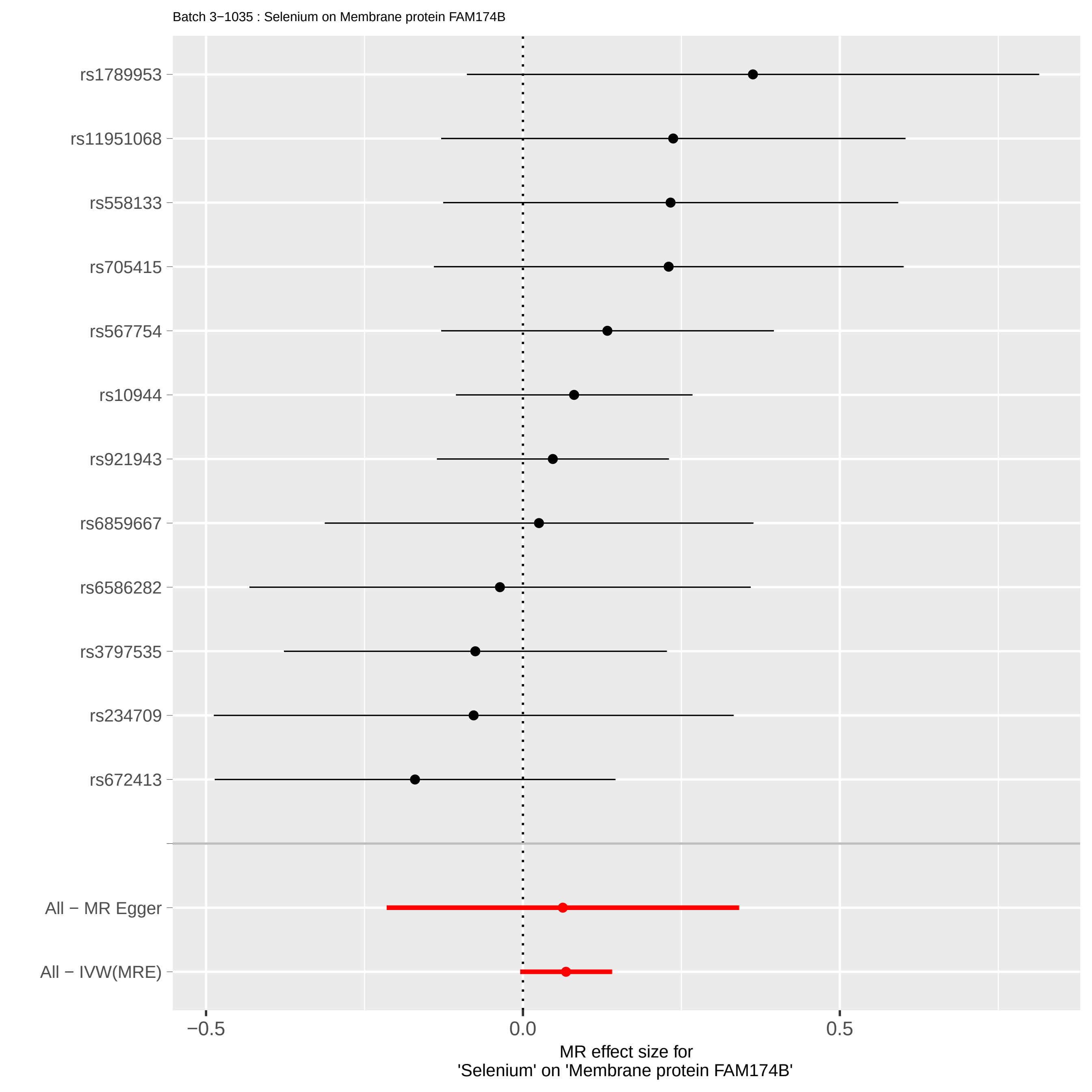

Batch 3−1035 : Selenium on Membrane protein FAM174B
rs1789953
rs11951068
rs558133
rs705415
rs567754
rs10944
rs921943
rs6859667
rs6586282
rs3797535
rs234709
rs672413
All − MR Egger
All − IVW(MRE)
−0.5
0.0
0.5
MR effect size for
'Selenium' on 'Membrane protein FAM174B'

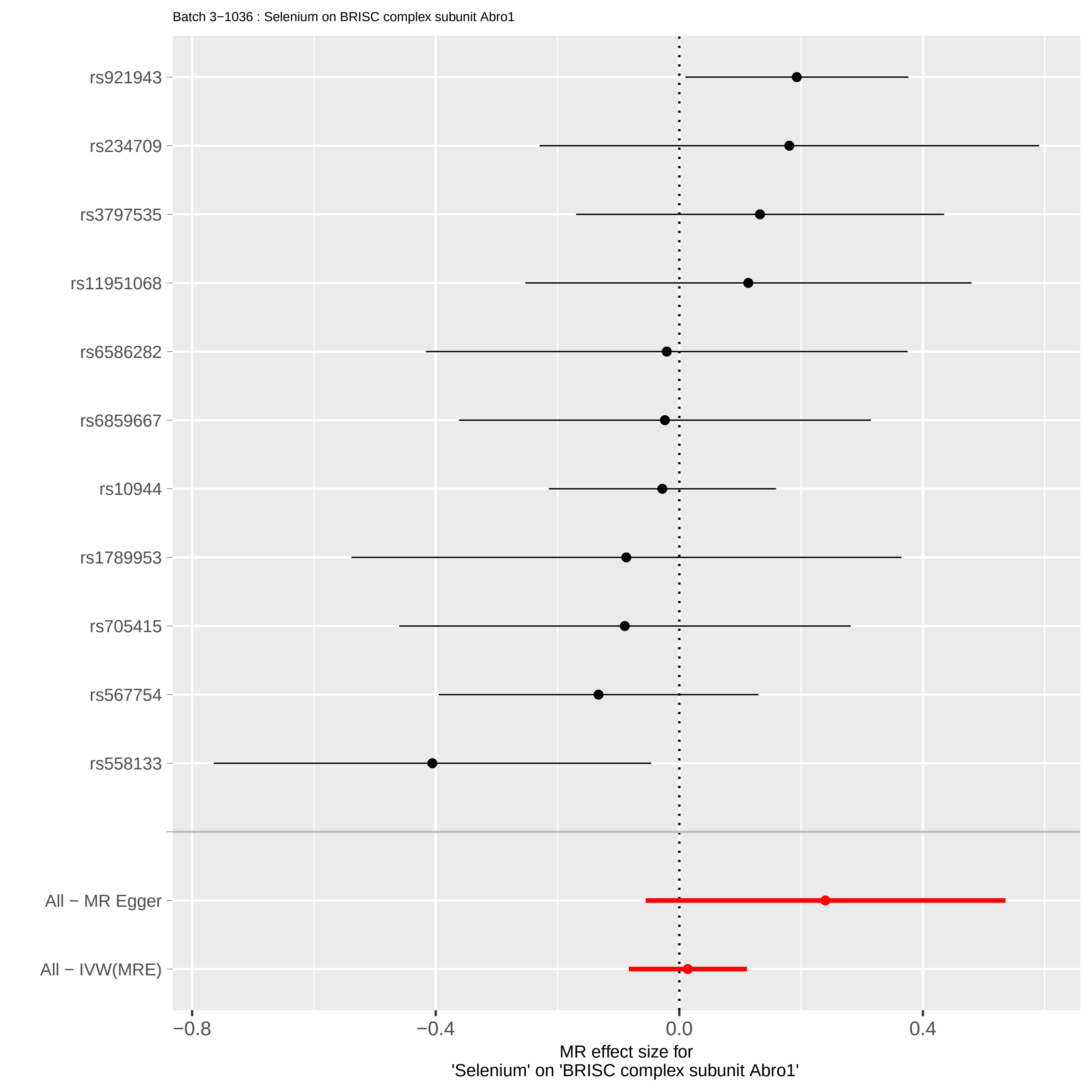

Batch 3−1036 : Selenium on BRISC complex subunit Abro1
rs921943
rs234709
rs3797535
rs11951068
rs6586282
rs6859667
rs10944
rs1789953
rs705415
rs567754
rs558133
All − MR Egger
All − IVW(MRE)
−0.8
−0.4
0.0
0.4
MR effect size for
'Selenium' on 'BRISC complex subunit Abro1'

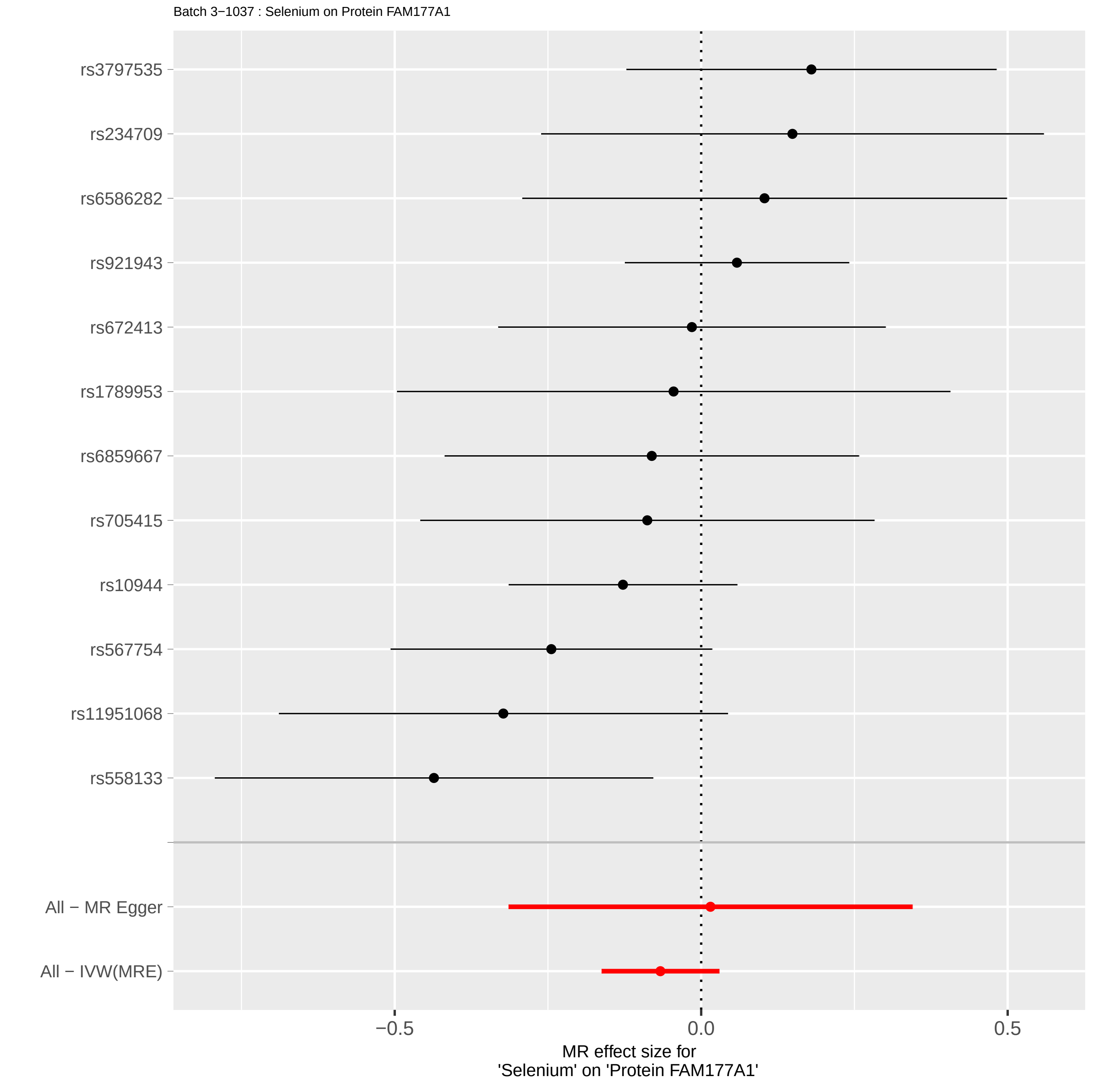

Batch 3−1037 : Selenium on Protein FAM177A1
rs3797535
rs234709
rs6586282
rs921943
rs672413
rs1789953
rs6859667
rs705415
rs10944
rs567754
rs11951068
rs558133
All − MR Egger
All − IVW(MRE)
−0.5
0.0
0.5
MR effect size for
'Selenium' on 'Protein FAM177A1'

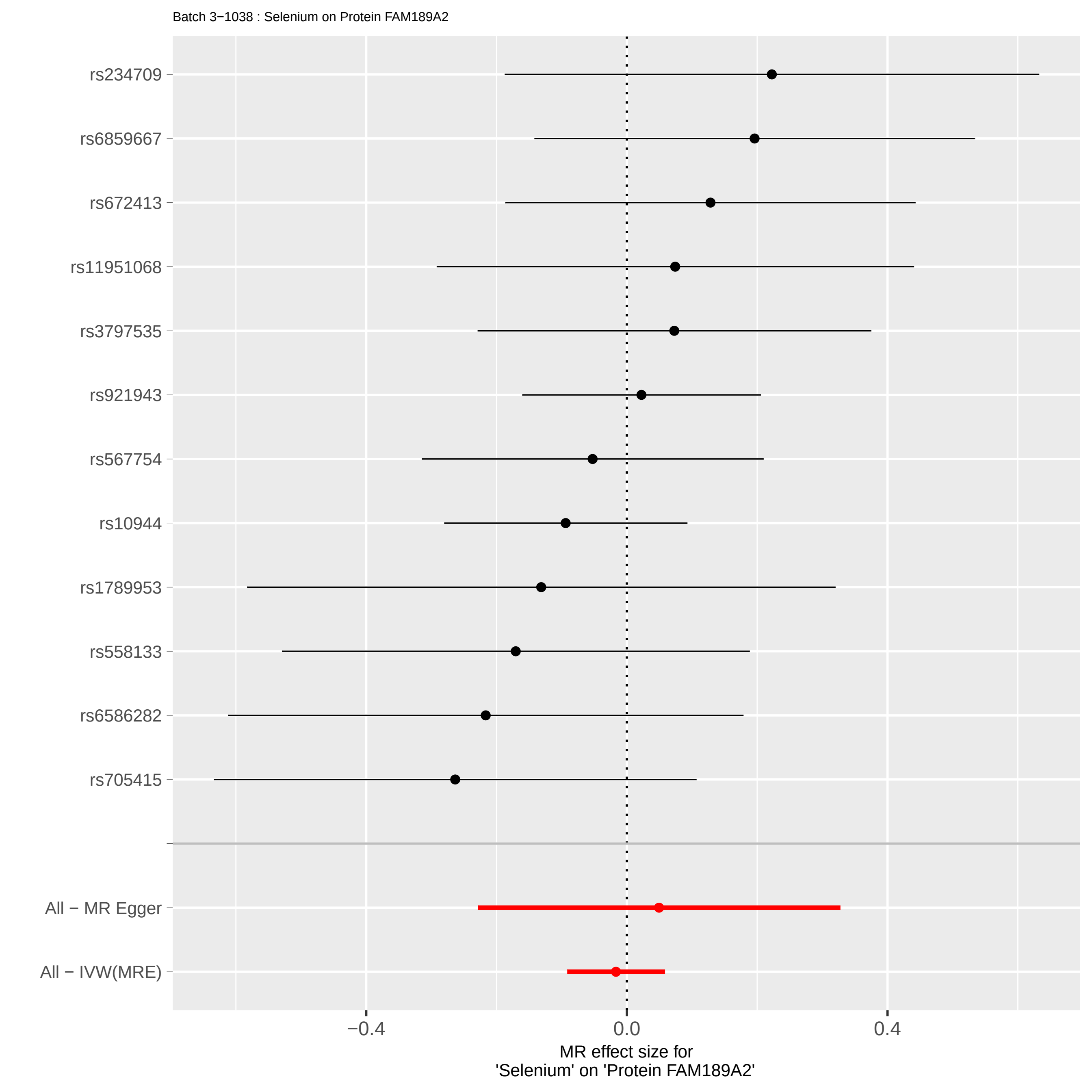

Batch 3−1038 : Selenium on Protein FAM189A2
rs234709
rs6859667
rs672413
rs11951068
rs3797535
rs921943
rs567754
rs10944
rs1789953
rs558133
rs6586282
rs705415
All − MR Egger
All − IVW(MRE)
−0.4
0.0
0.4
MR effect size for
'Selenium' on 'Protein FAM189A2'

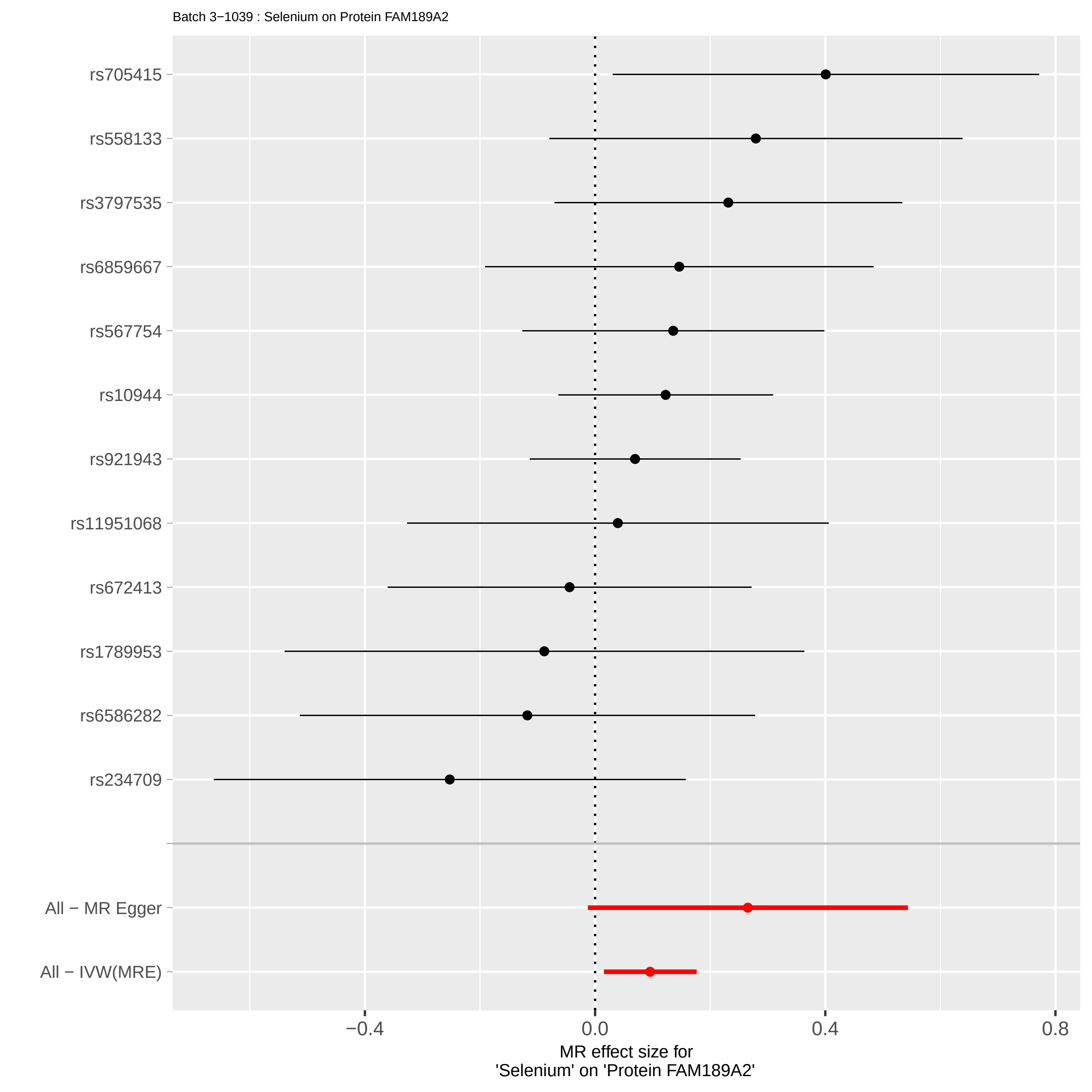

Batch 3−1039 : Selenium on Protein FAM189A2
rs705415
rs558133
rs3797535
rs6859667
rs567754
rs10944
rs921943
rs11951068
rs672413
rs1789953
rs6586282
rs234709
All − MR Egger
All − IVW(MRE)
−0.4
0.0
0.4
0.8
MR effect size for
'Selenium' on 'Protein FAM189A2'

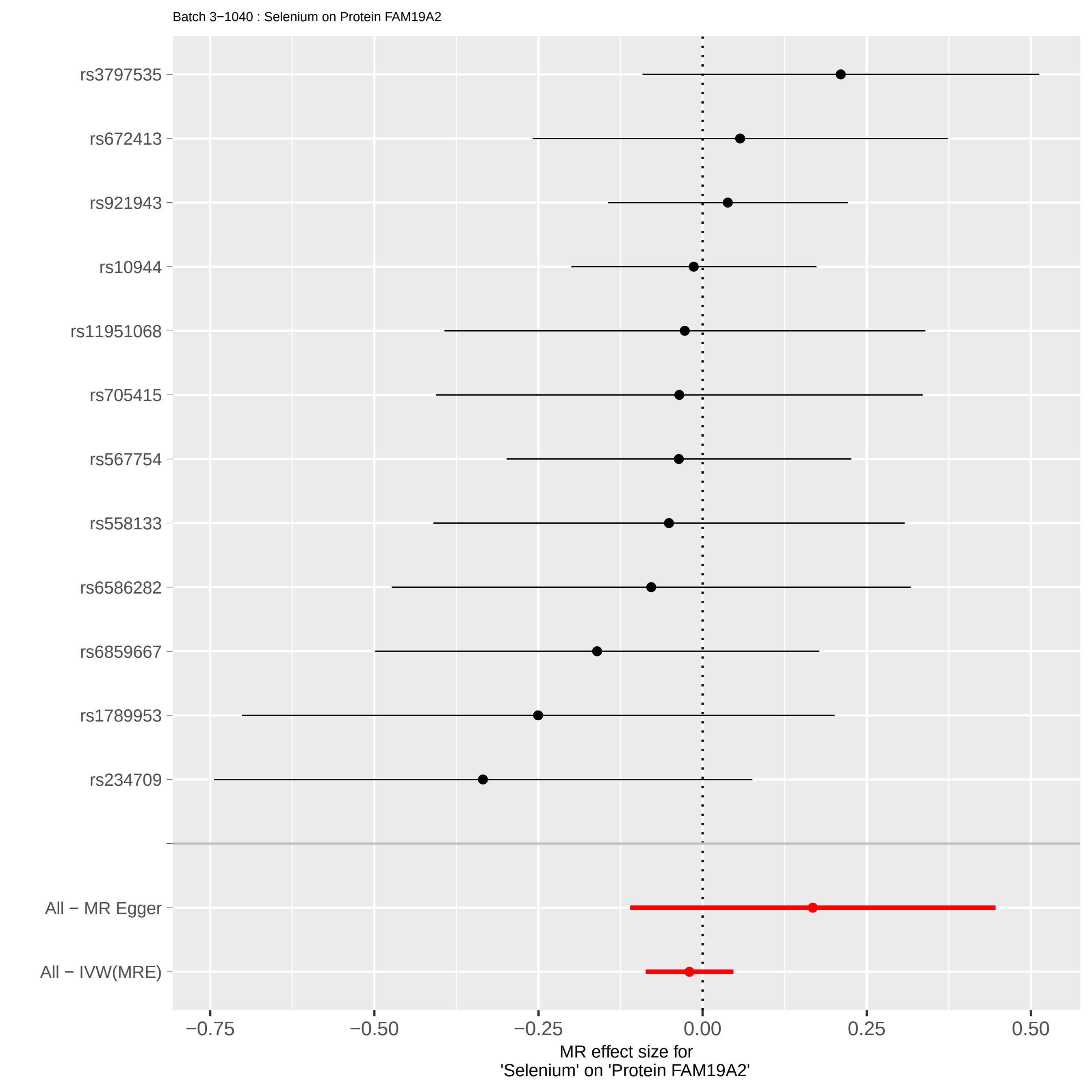

Batch 3−1040 : Selenium on Protein FAM19A2
rs3797535
rs672413
rs921943
rs10944
rs11951068
rs705415
rs567754
rs558133
rs6586282
rs6859667
rs1789953
rs234709
All − MR Egger
All − IVW(MRE)
−0.75
−0.50
−0.25
0.00
0.25
0.50
MR effect size for
'Selenium' on 'Protein FAM19A2'

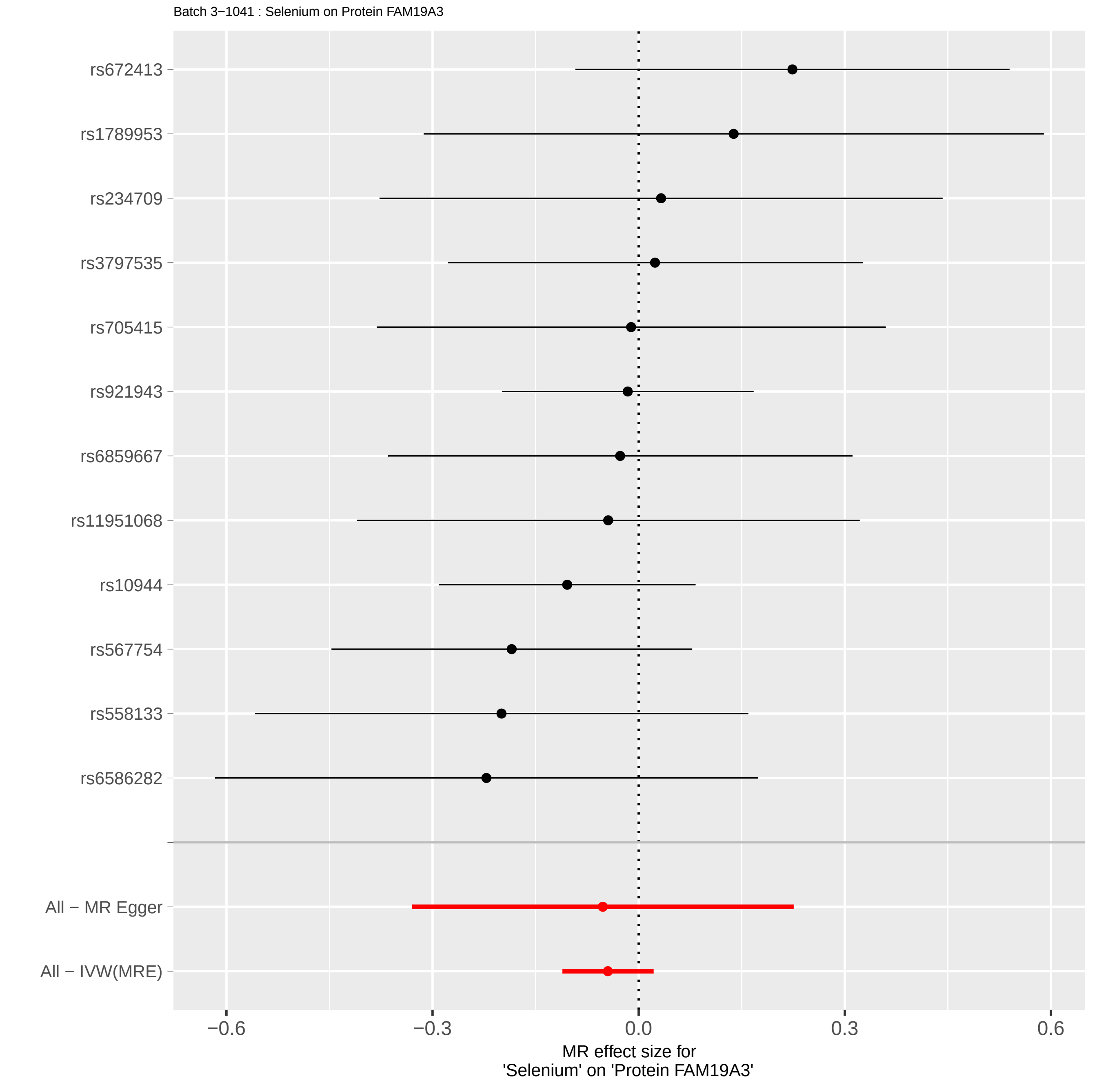

Batch 3−1041 : Selenium on Protein FAM19A3
rs672413
rs1789953
rs234709
rs3797535
rs705415
rs921943
rs6859667
rs11951068
rs10944
rs567754
rs558133
rs6586282
All − MR Egger
All − IVW(MRE)
−0.6
−0.3
0.0
0.3
0.6
MR effect size for
'Selenium' on 'Protein FAM19A3'

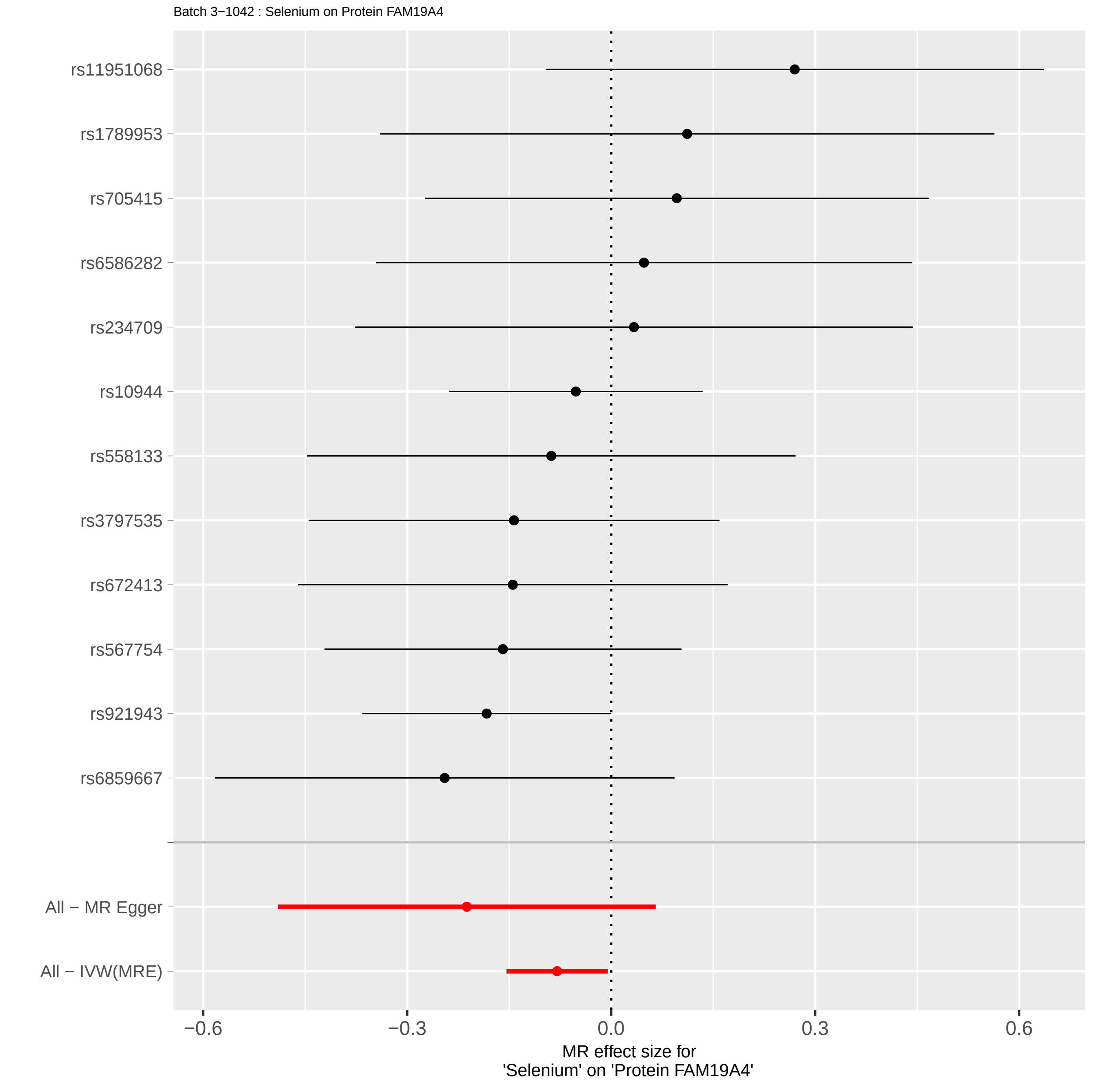

Batch 3−1042 : Selenium on Protein FAM19A4
rs11951068
rs1789953
rs705415
rs6586282
rs234709
rs10944
rs558133
rs3797535
rs672413
rs567754
rs921943
rs6859667
All − MR Egger
All − IVW(MRE)
−0.6
−0.3
0.0
0.3
0.6
MR effect size for
'Selenium' on 'Protein FAM19A4'

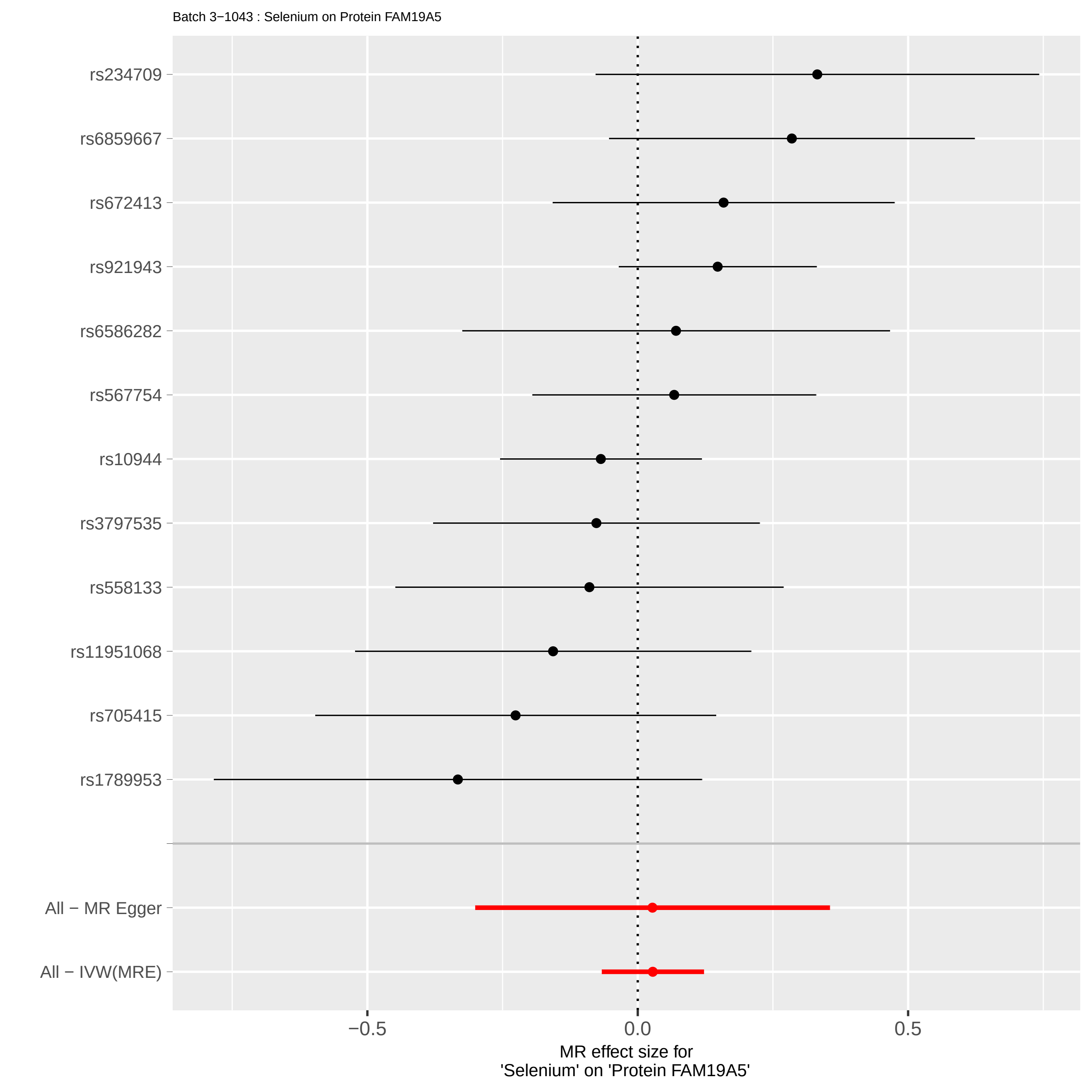

Batch 3−1043 : Selenium on Protein FAM19A5
rs234709
rs6859667
rs672413
rs921943
rs6586282
rs567754
rs10944
rs3797535
rs558133
rs11951068
rs705415
rs1789953
All − MR Egger
All − IVW(MRE)
−0.5
0.0
0.5
MR effect size for
'Selenium' on 'Protein FAM19A5'

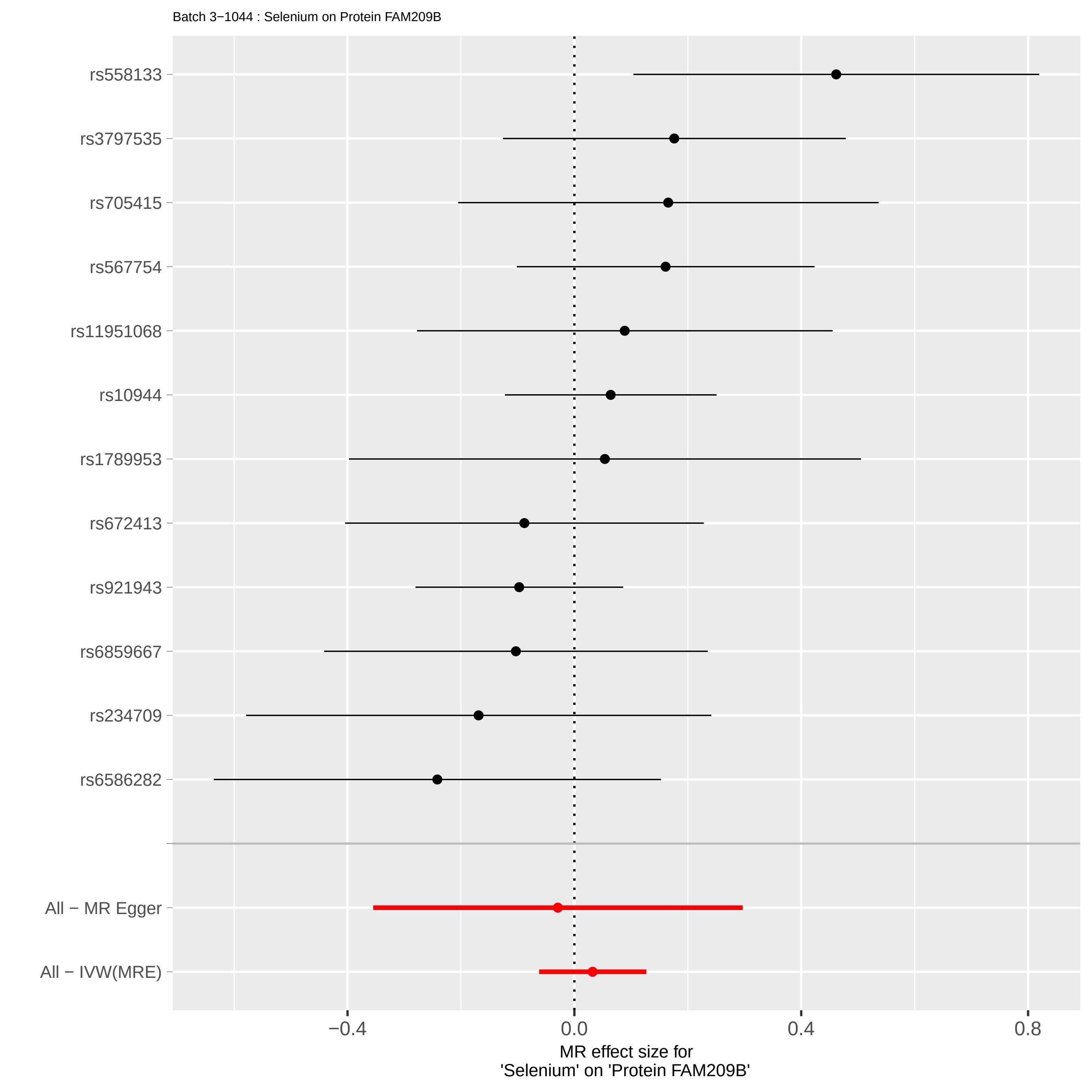

Batch 3−1044 : Selenium on Protein FAM209B
rs558133
rs3797535
rs705415
rs567754
rs11951068
rs10944
rs1789953
rs672413
rs921943
rs6859667
rs234709
rs6586282
All − MR Egger
All − IVW(MRE)
−0.4
0.0
0.4
0.8
MR effect size for
'Selenium' on 'Protein FAM209B'

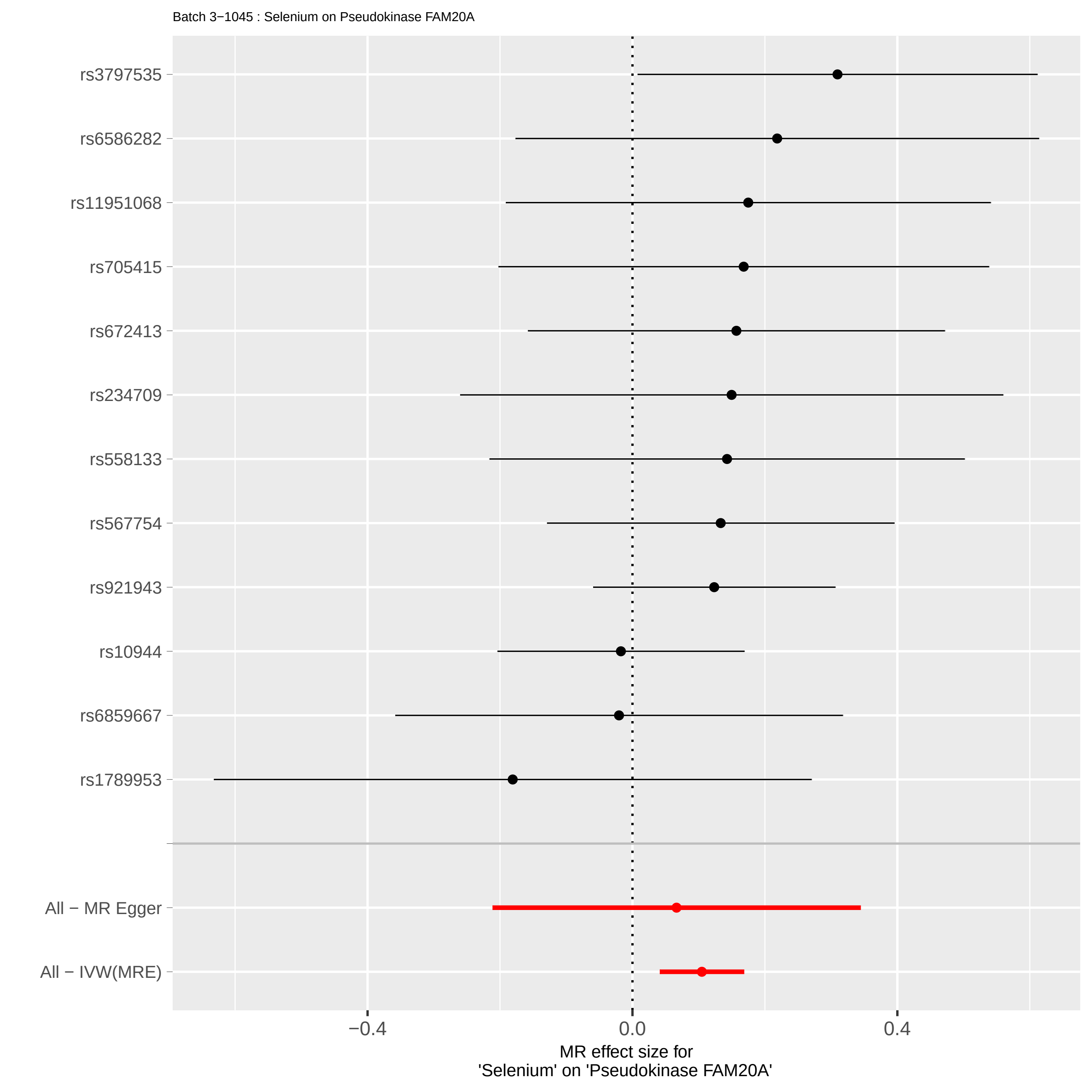
Batch 3−1045 : Selenium on Pseudokinase FAM20A
rs3797535
rs6586282
rs11951068
rs705415
rs672413
rs234709
rs558133
rs567754
rs921943
rs10944
rs6859667
rs1789953
All − MR Egger
All − IVW(MRE)
−0.4
0.0
0.4
MR effect size for
'Selenium' on 'Pseudokinase FAM20A'

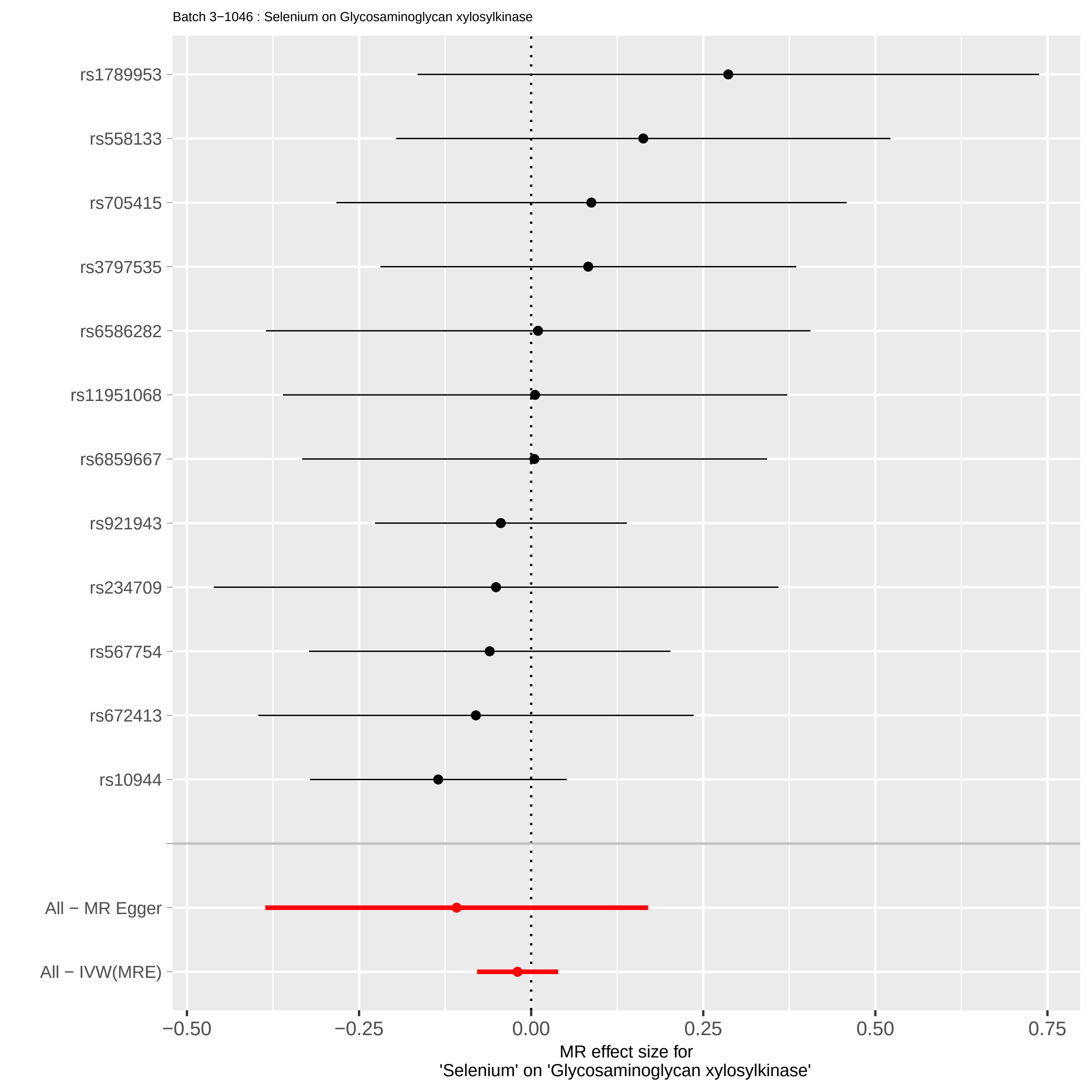

Batch 3−1046 : Selenium on Glycosaminoglycan xylosylkinase
rs1789953
rs558133
rs705415
rs3797535
rs6586282
rs11951068
rs6859667
rs921943
rs234709
rs567754
rs672413
rs10944
All − MR Egger
All − IVW(MRE)
−0.50
−0.25
0.00
0.25
0.50
0.75
MR effect size for
'Selenium' on 'Glycosaminoglycan xylosylkinase'

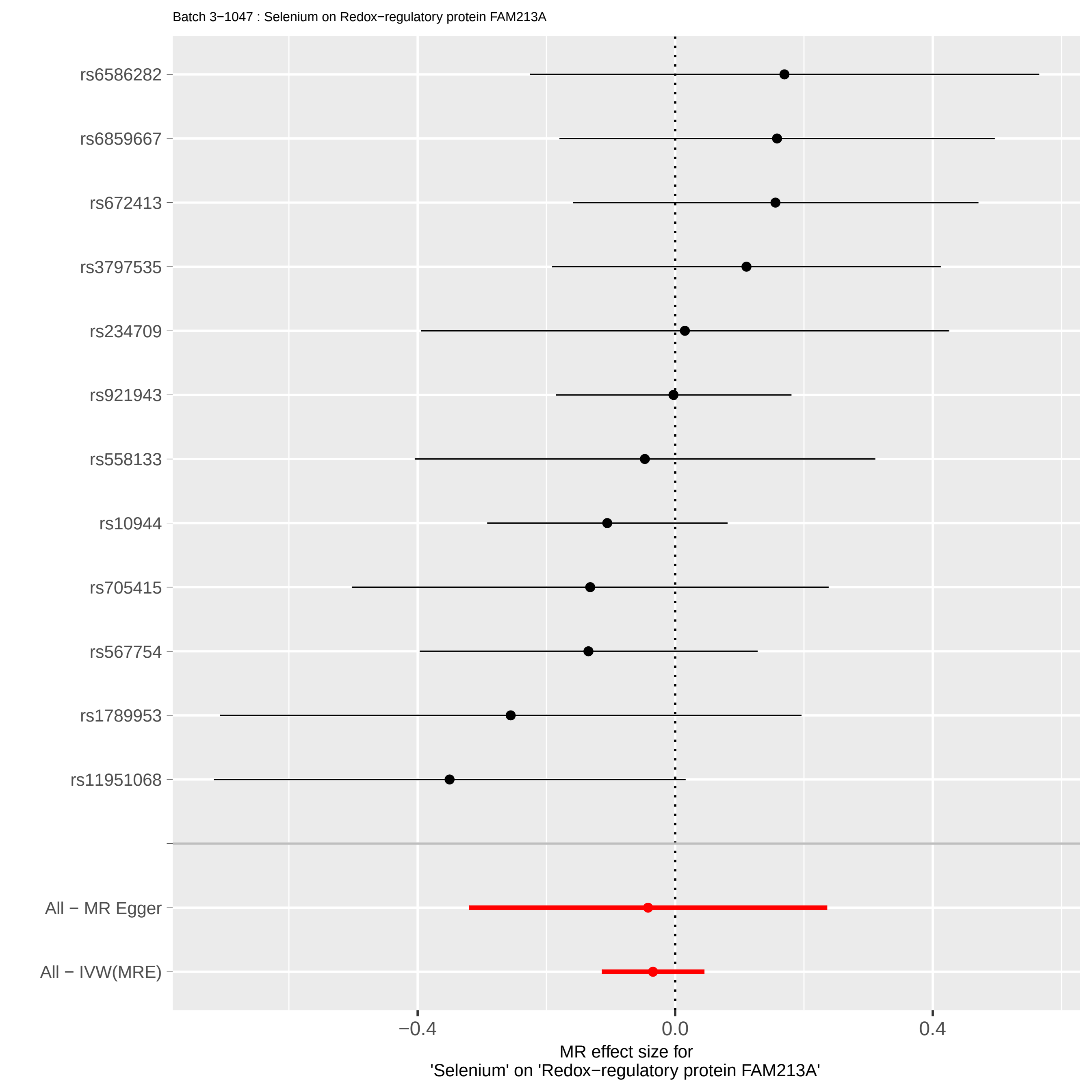
Batch 3−1047 : Selenium on Redox−regulatory protein FAM213A
rs6586282
rs6859667
rs672413
rs3797535
rs234709
rs921943
rs558133
rs10944
rs705415
rs567754
rs1789953
rs11951068
All − MR Egger
All − IVW(MRE)
−0.4
0.0
0.4
MR effect size for
'Selenium' on 'Redox−regulatory protein FAM213A'

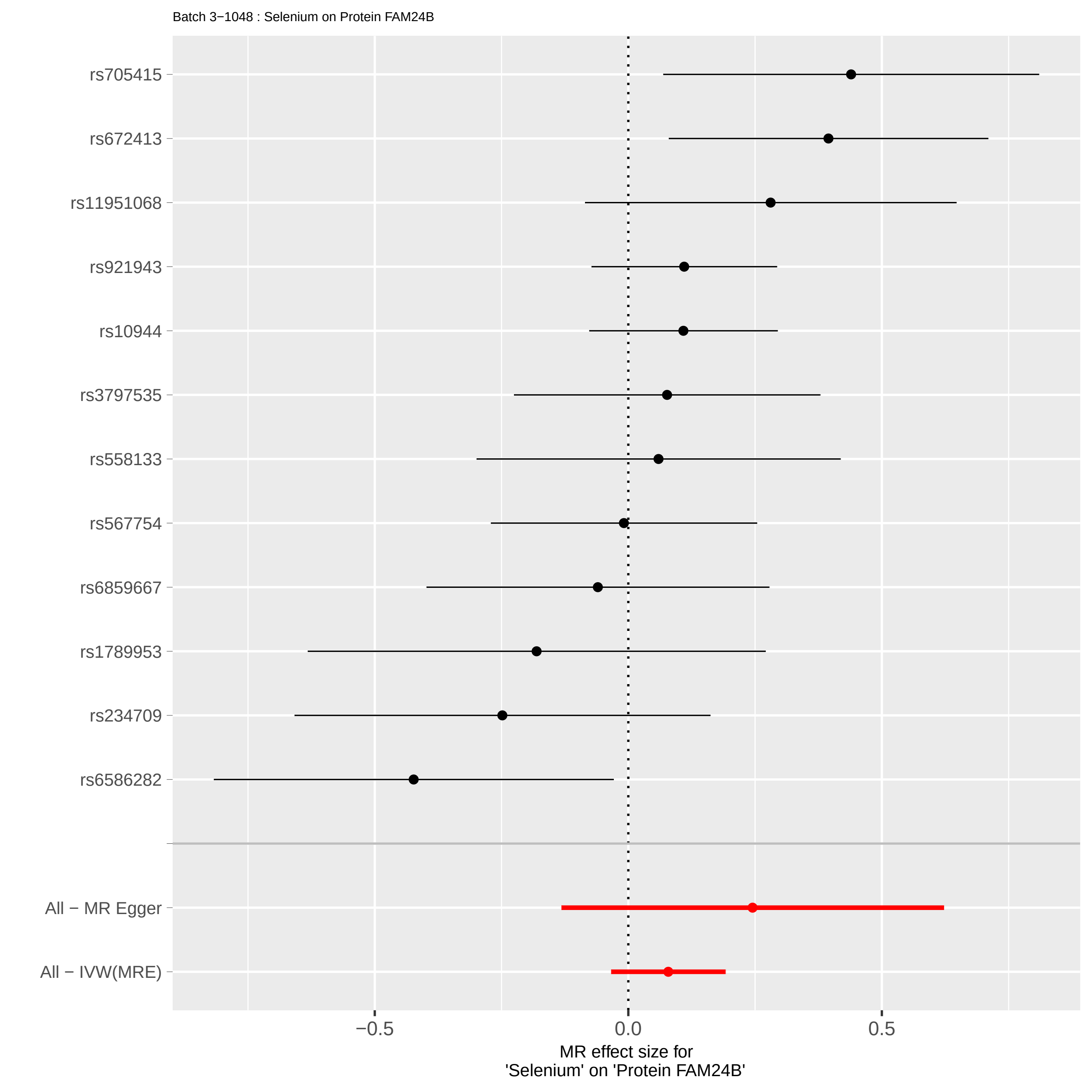

Batch 3−1048 : Selenium on Protein FAM24B
rs705415
rs672413
rs11951068
rs921943
rs10944
rs3797535
rs558133
rs567754
rs6859667
rs1789953
rs234709
rs6586282
All − MR Egger
All − IVW(MRE)
−0.5
0.0
0.5
MR effect size for
'Selenium' on 'Protein FAM24B'

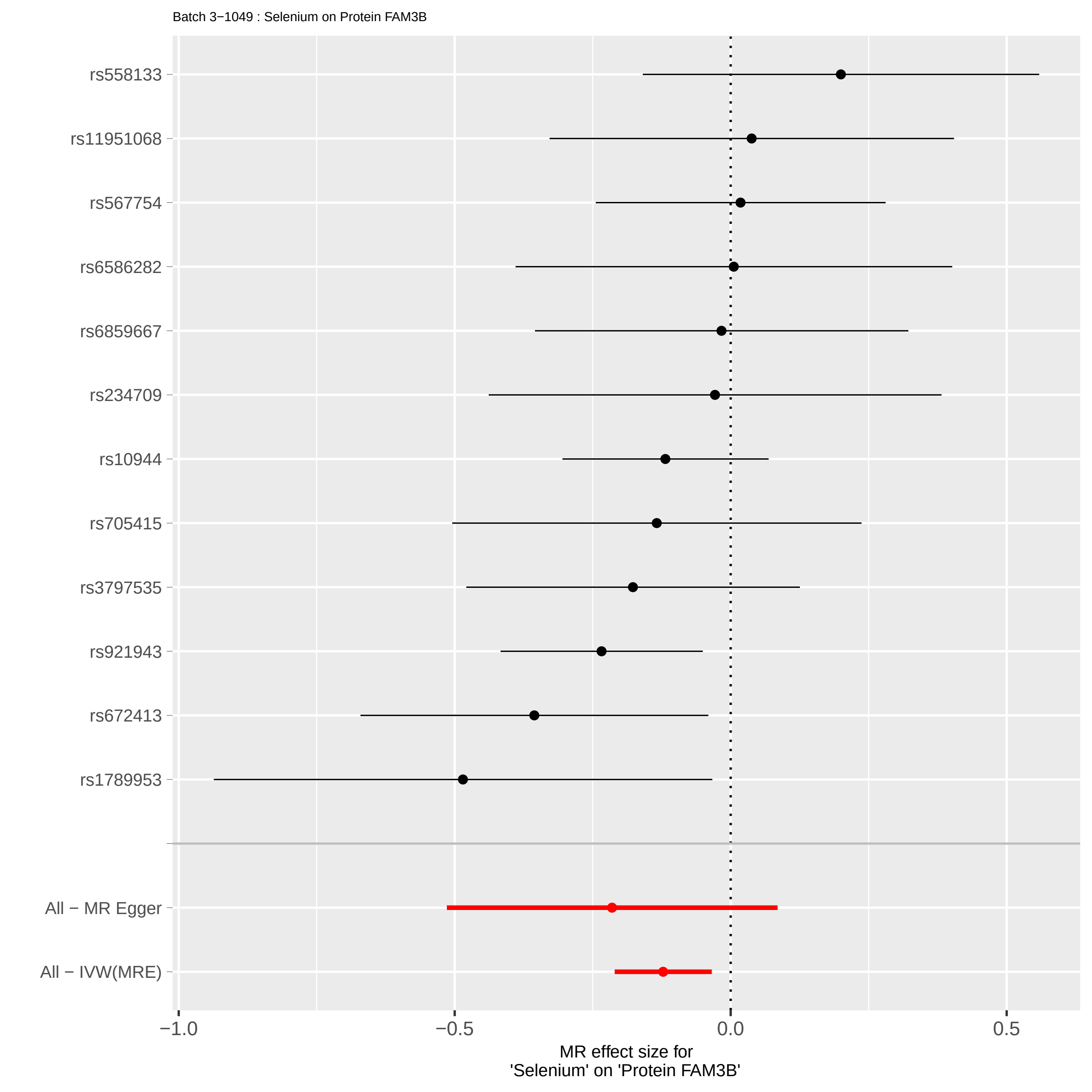

Batch 3−1049 : Selenium on Protein FAM3B
rs558133
rs11951068
rs567754
rs6586282
rs6859667
rs234709
rs10944
rs705415
rs3797535
rs921943
rs672413
rs1789953
All − MR Egger
All − IVW(MRE)
−1.0
−0.5
0.0
0.5
MR effect size for
'Selenium' on 'Protein FAM3B'

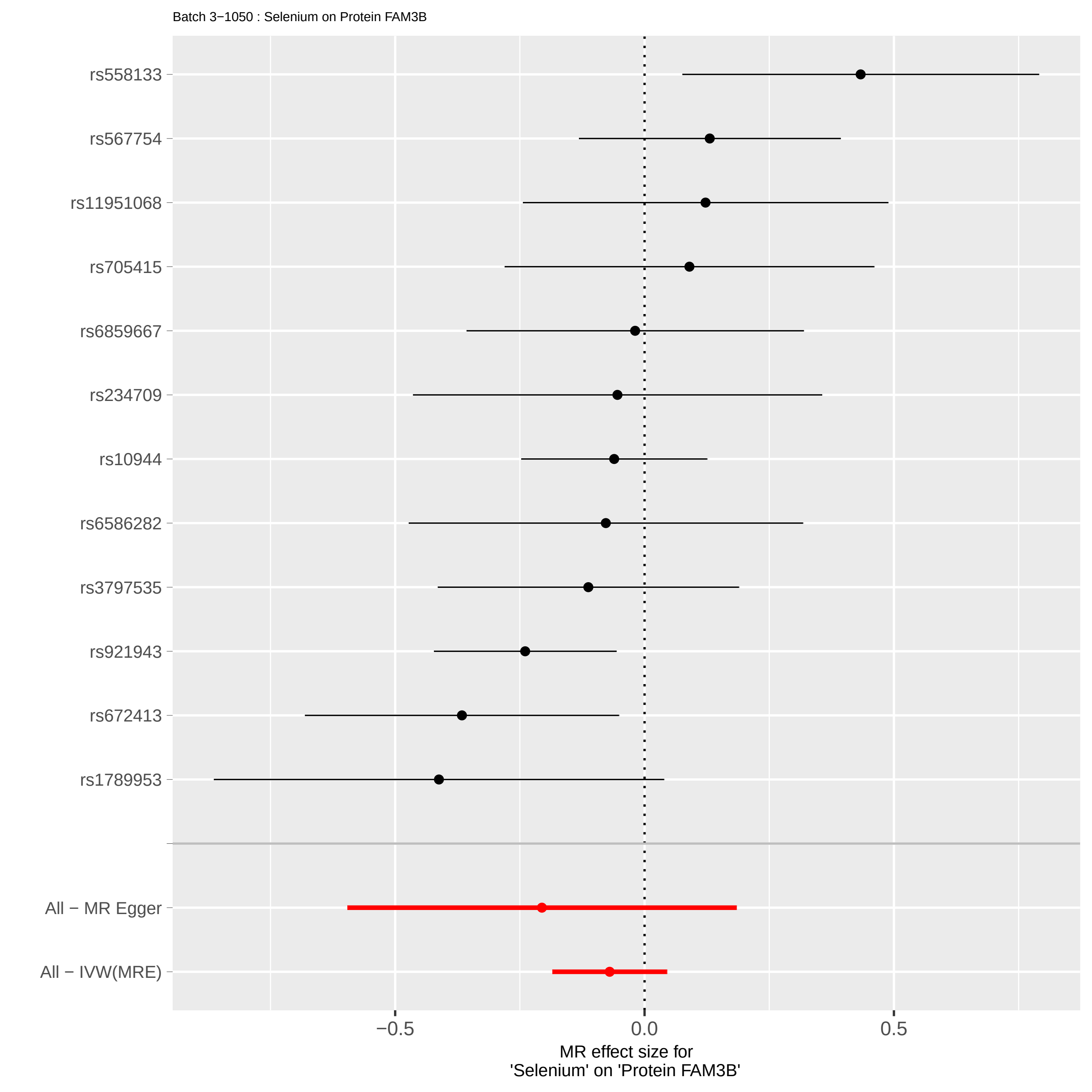
Batch 3−1050 : Selenium on Protein FAM3B
rs558133
rs567754
rs11951068
rs705415
rs6859667
rs234709
rs10944
rs6586282
rs3797535
rs921943
rs672413
rs1789953
All − MR Egger
All − IVW(MRE)
−0.5
0.0
0.5
MR effect size for
'Selenium' on 'Protein FAM3B'

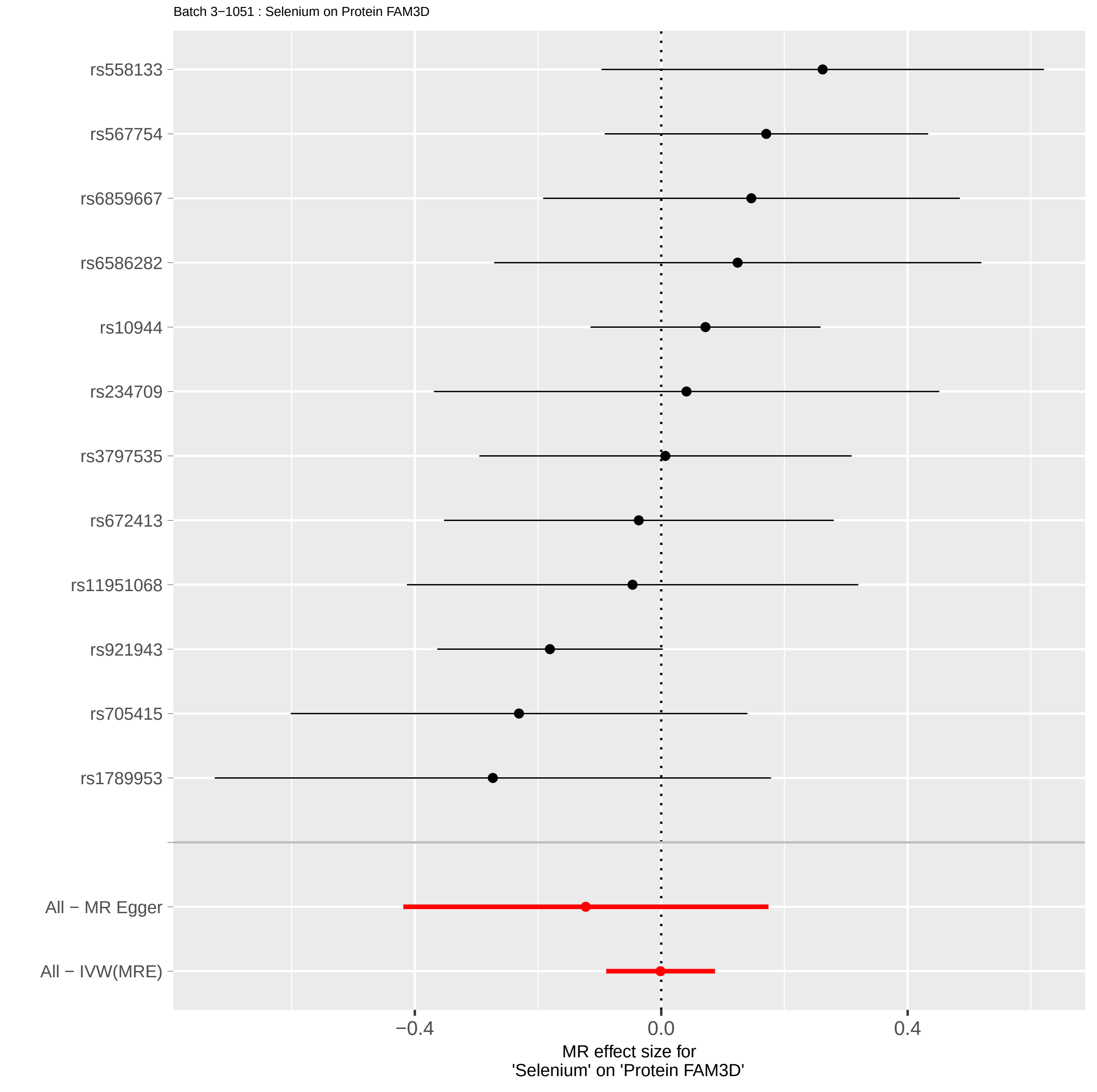

Batch 3−1051 : Selenium on Protein FAM3D
rs558133
rs567754
rs6859667
rs6586282
rs10944
rs234709
rs3797535
rs672413
rs11951068
rs921943
rs705415
rs1789953
All − MR Egger
All − IVW(MRE)
−0.4
0.0
0.4
MR effect size for
'Selenium' on 'Protein FAM3D'

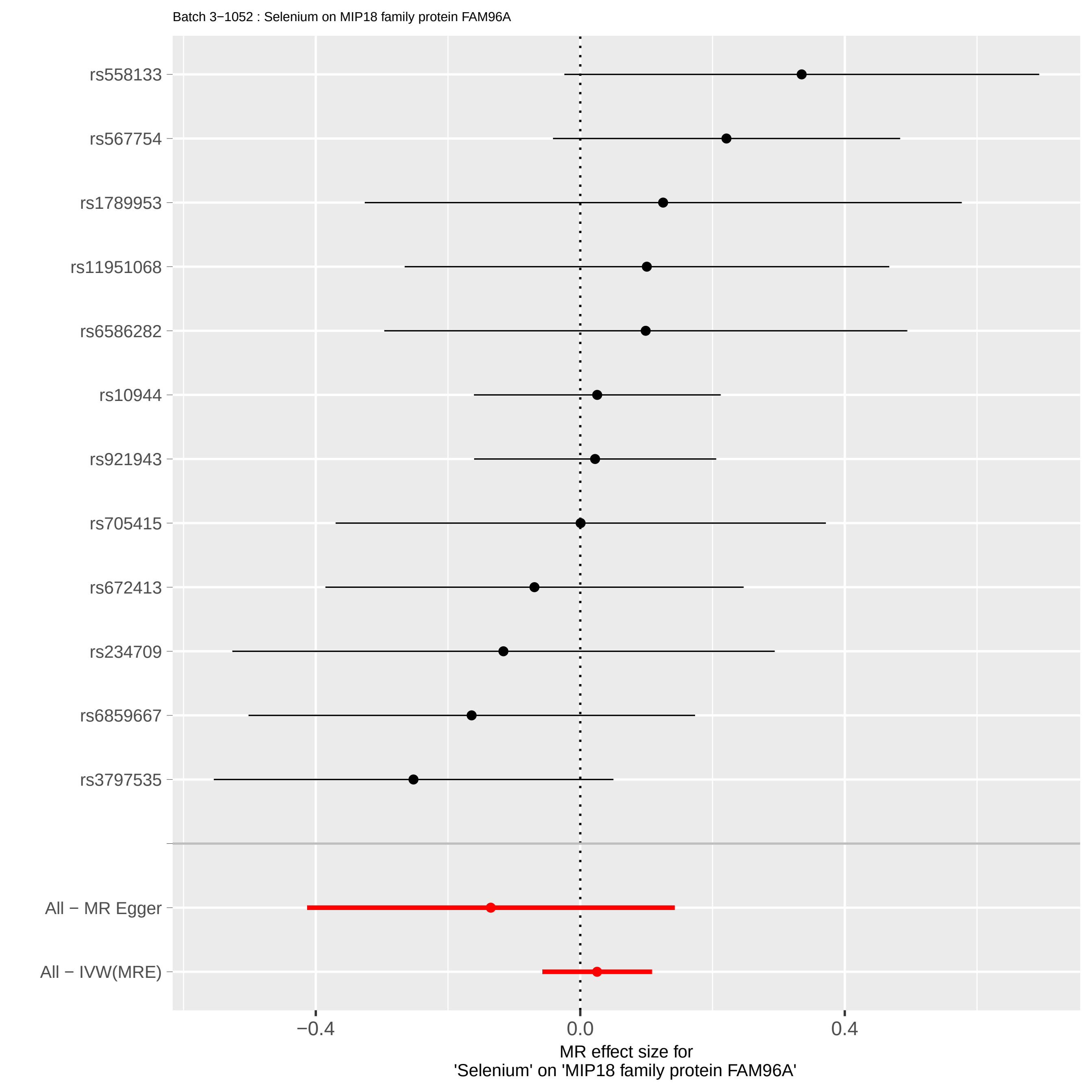

Batch 3−1052 : Selenium on MIP18 family protein FAM96A
rs558133
rs567754
rs1789953
rs11951068
rs6586282
rs10944
rs921943
rs705415
rs672413
rs234709
rs6859667
rs3797535
All − MR Egger
All − IVW(MRE)
−0.4
0.0
0.4
MR effect size for
'Selenium' on 'MIP18 family protein FAM96A'

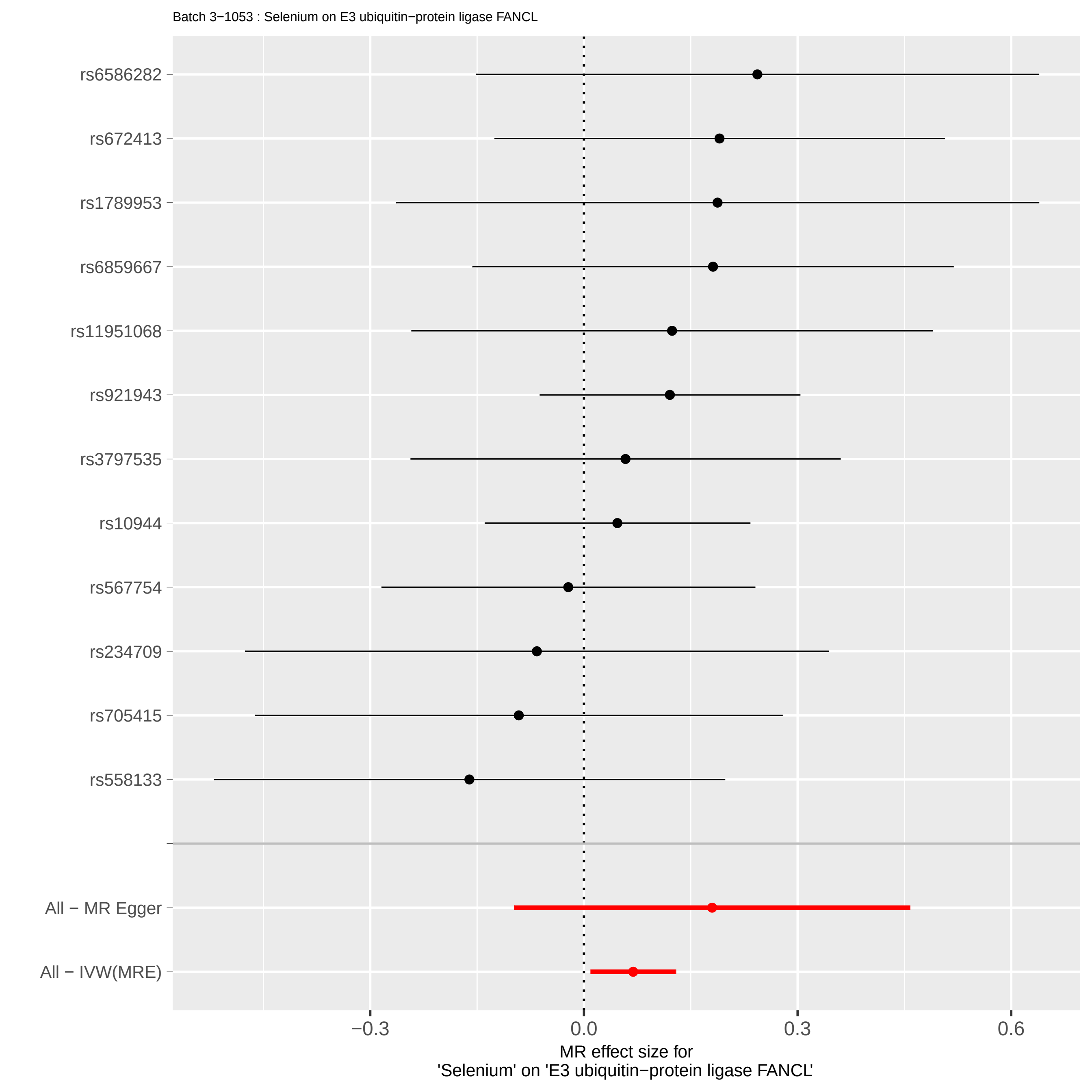

Batch 3−1053 : Selenium on E3 ubiquitin−protein ligase FANCL
rs6586282
rs672413
rs1789953
rs6859667
rs11951068
rs921943
rs3797535
rs10944
rs567754
rs234709
rs705415
rs558133
All − MR Egger
All − IVW(MRE)
−0.3
0.0
0.3
0.6
MR effect size for
'Selenium' on 'E3 ubiquitin−protein ligase FANCL'

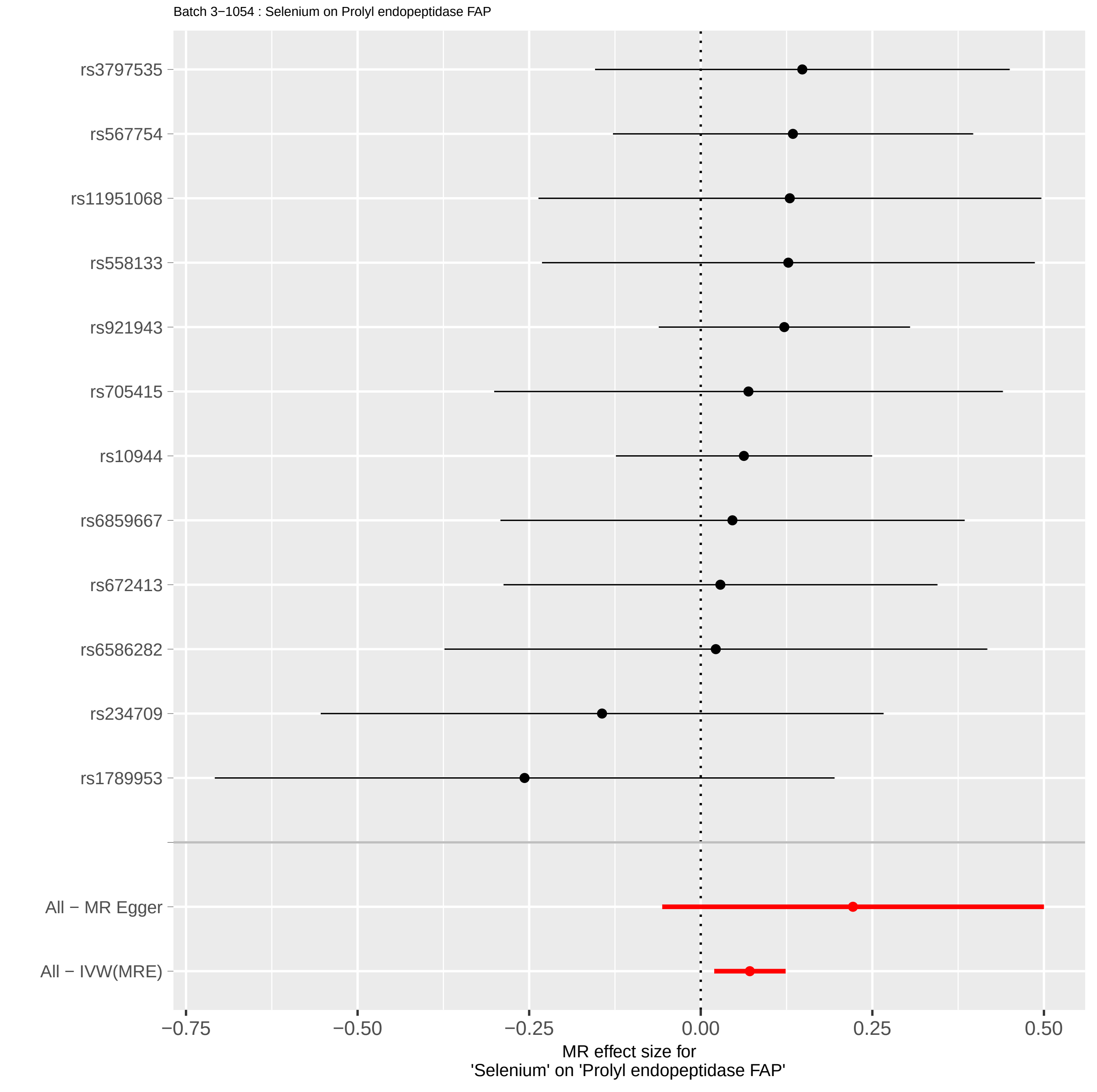

Batch 3−1054 : Selenium on Prolyl endopeptidase FAP
rs3797535
rs567754
rs11951068
rs558133
rs921943
rs705415
rs10944
rs6859667
rs672413
rs6586282
rs234709
rs1789953
All − MR Egger
All − IVW(MRE)
−0.75
−0.50
−0.25
0.00
0.25
0.50
MR effect size for
'Selenium' on 'Prolyl endopeptidase FAP'

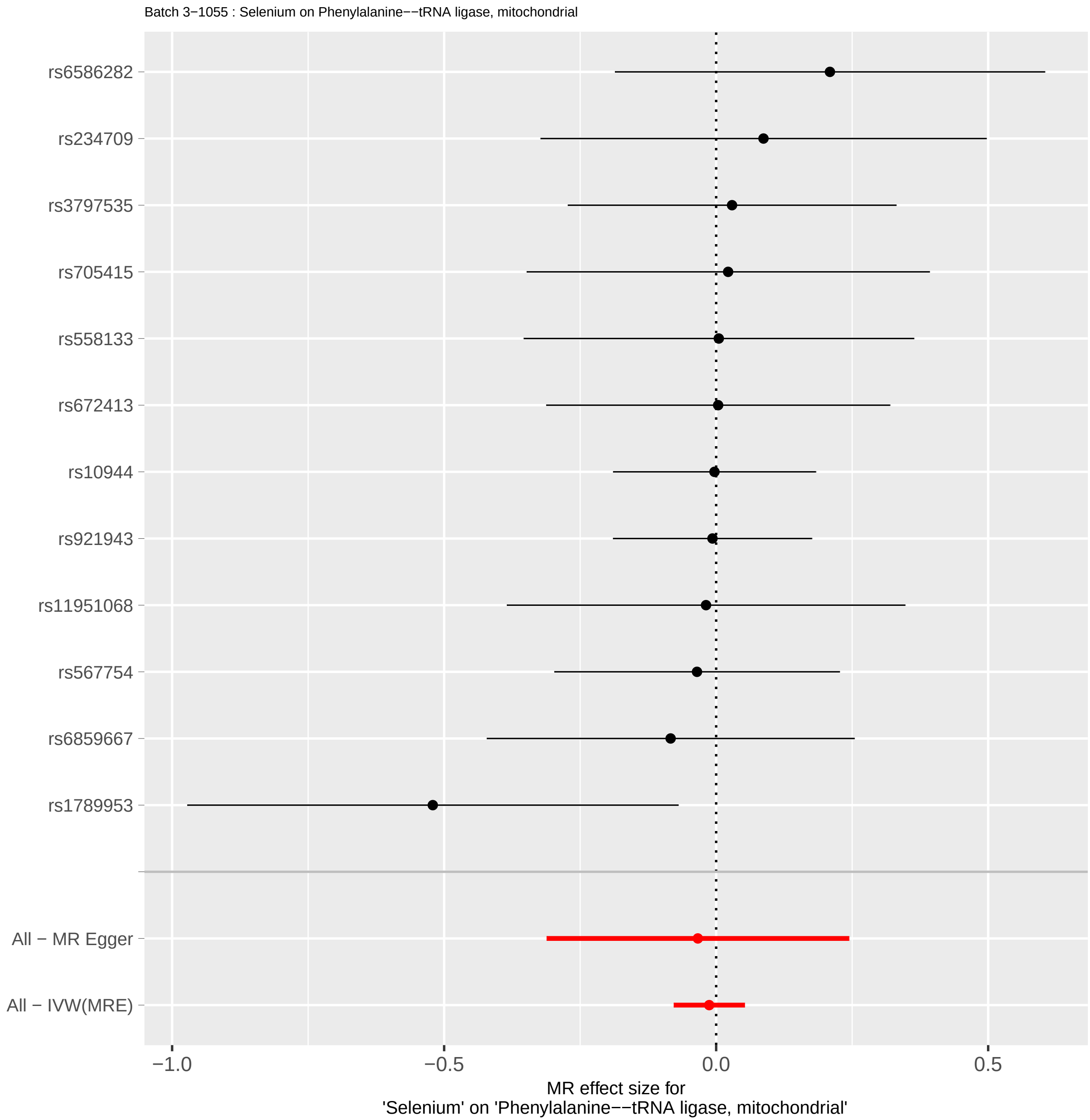

Batch 3−1055 : Selenium on Phenylalanine−−tRNA ligase, mitochondrial
rs6586282
rs234709
rs3797535
rs705415
rs558133
rs672413
rs10944
rs921943
rs11951068
rs567754
rs6859667
rs1789953
All − MR Egger
All − IVW(MRE)
−1.0
−0.5
0.0
0.5
MR effect size for
'Selenium' on 'Phenylalanine−−tRNA ligase, mitochondrial'

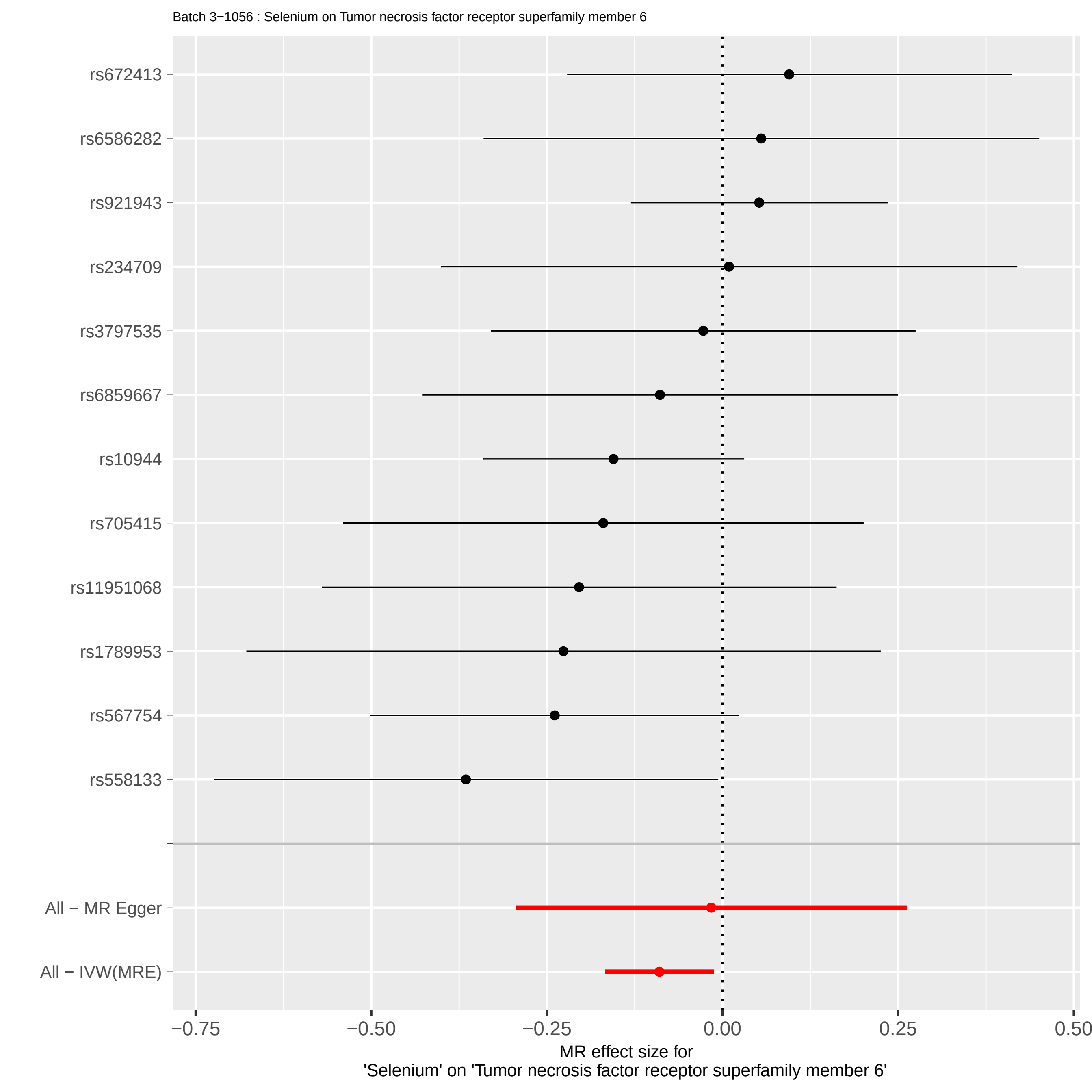

Batch 3−1056 : Selenium on Tumor necrosis factor receptor superfamily member 6
rs672413
rs6586282
rs921943
rs234709
rs3797535
rs6859667
rs10944
rs705415
rs11951068
rs1789953
rs567754
rs558133
All − MR Egger
All − IVW(MRE)
−0.75
−0.50
−0.25
0.00
0.25
0.50
MR effect size for
'Selenium' on 'Tumor necrosis factor receptor superfamily member 6'

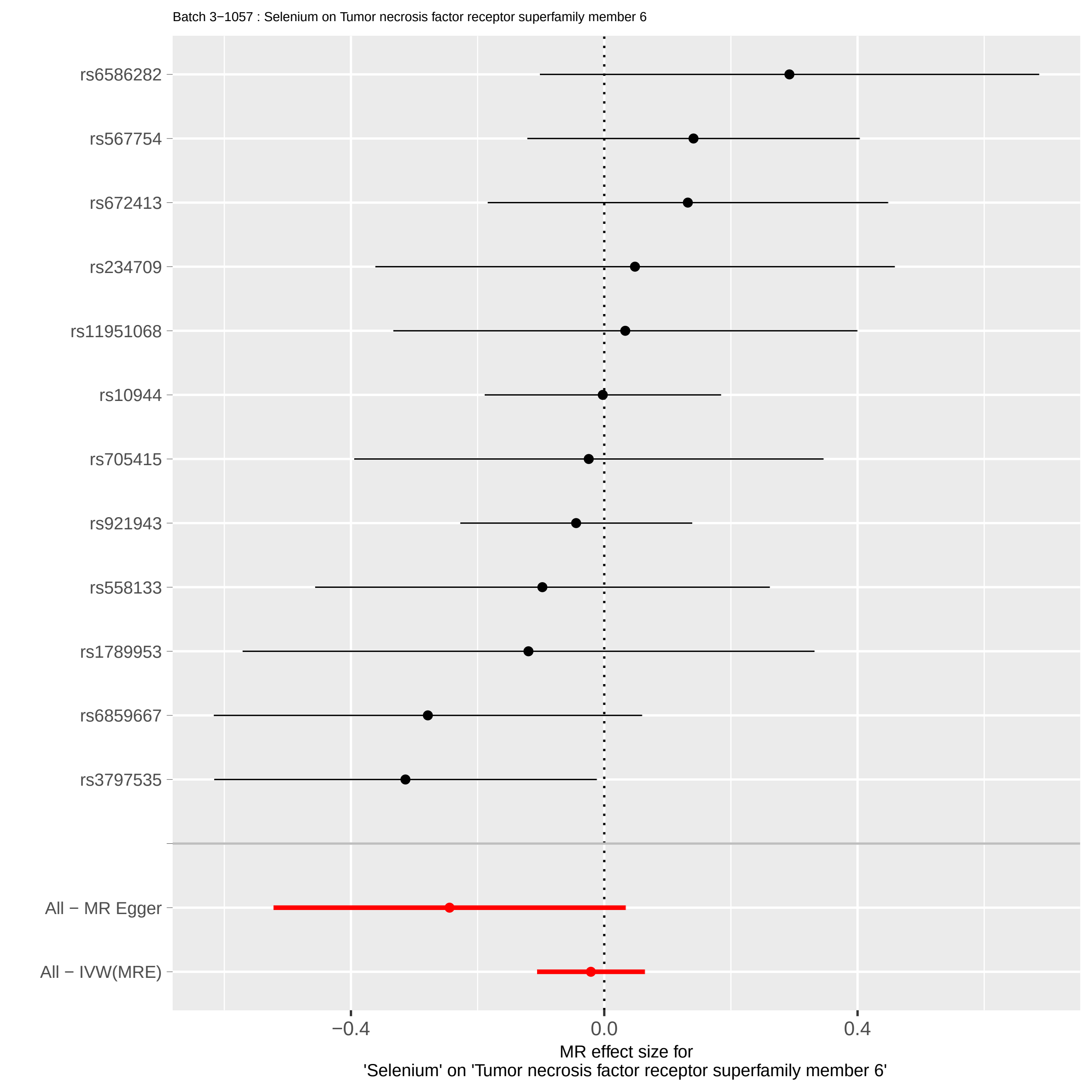

Batch 3−1057 : Selenium on Tumor necrosis factor receptor superfamily member 6
rs6586282
rs567754
rs672413
rs234709
rs11951068
rs10944
rs705415
rs921943
rs558133
rs1789953
rs6859667
rs3797535
All − MR Egger
All − IVW(MRE)
−0.4
0.0
0.4
MR effect size for
'Selenium' on 'Tumor necrosis factor receptor superfamily member 6'

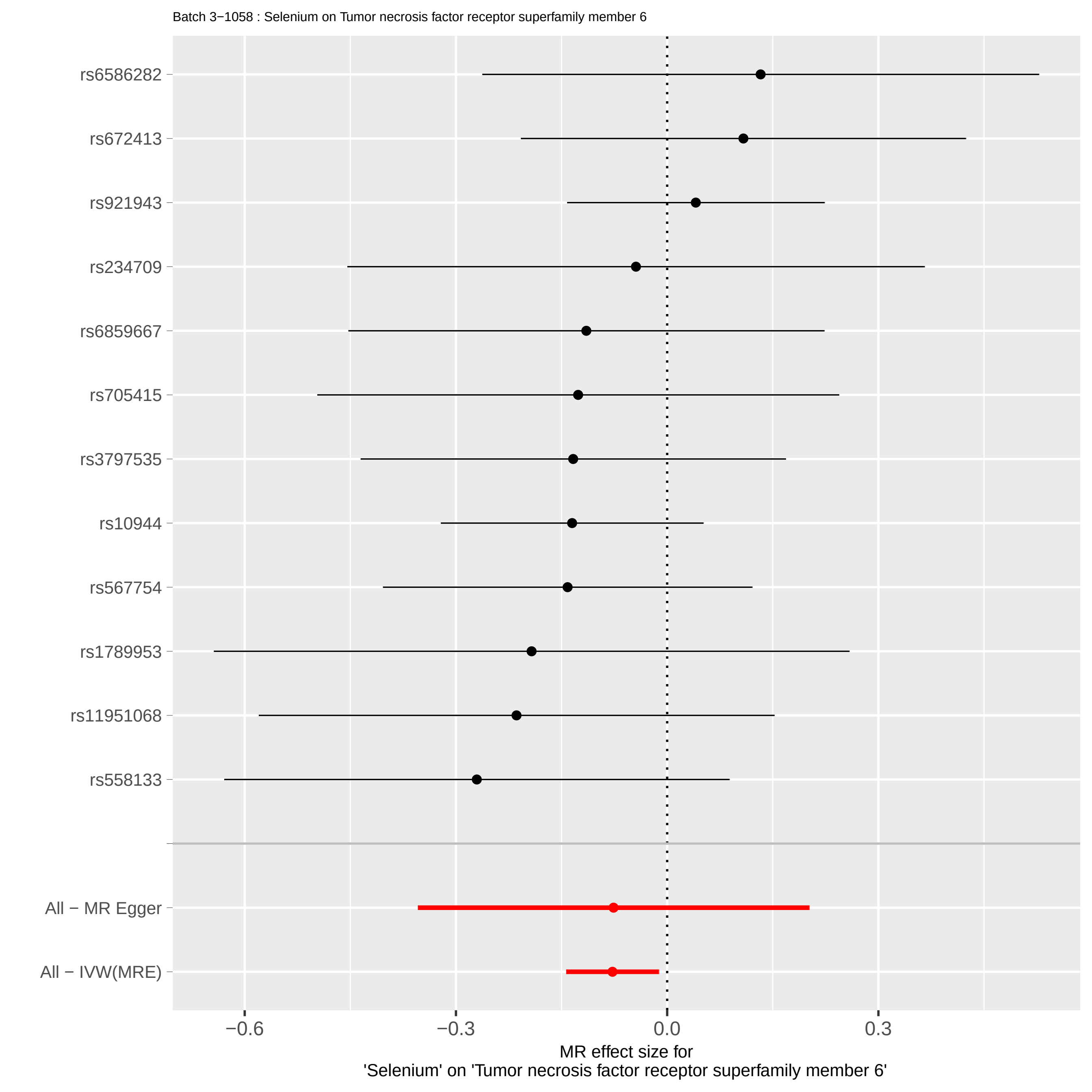
Batch 3−1058 : Selenium on Tumor necrosis factor receptor superfamily member 6
rs6586282
rs672413
rs921943
rs234709
rs6859667
rs705415
rs3797535
rs10944
rs567754
rs1789953
rs11951068
rs558133
All − MR Egger
All − IVW(MRE)
−0.6
−0.3
0.0
0.3
MR effect size for
'Selenium' on 'Tumor necrosis factor receptor superfamily member 6'

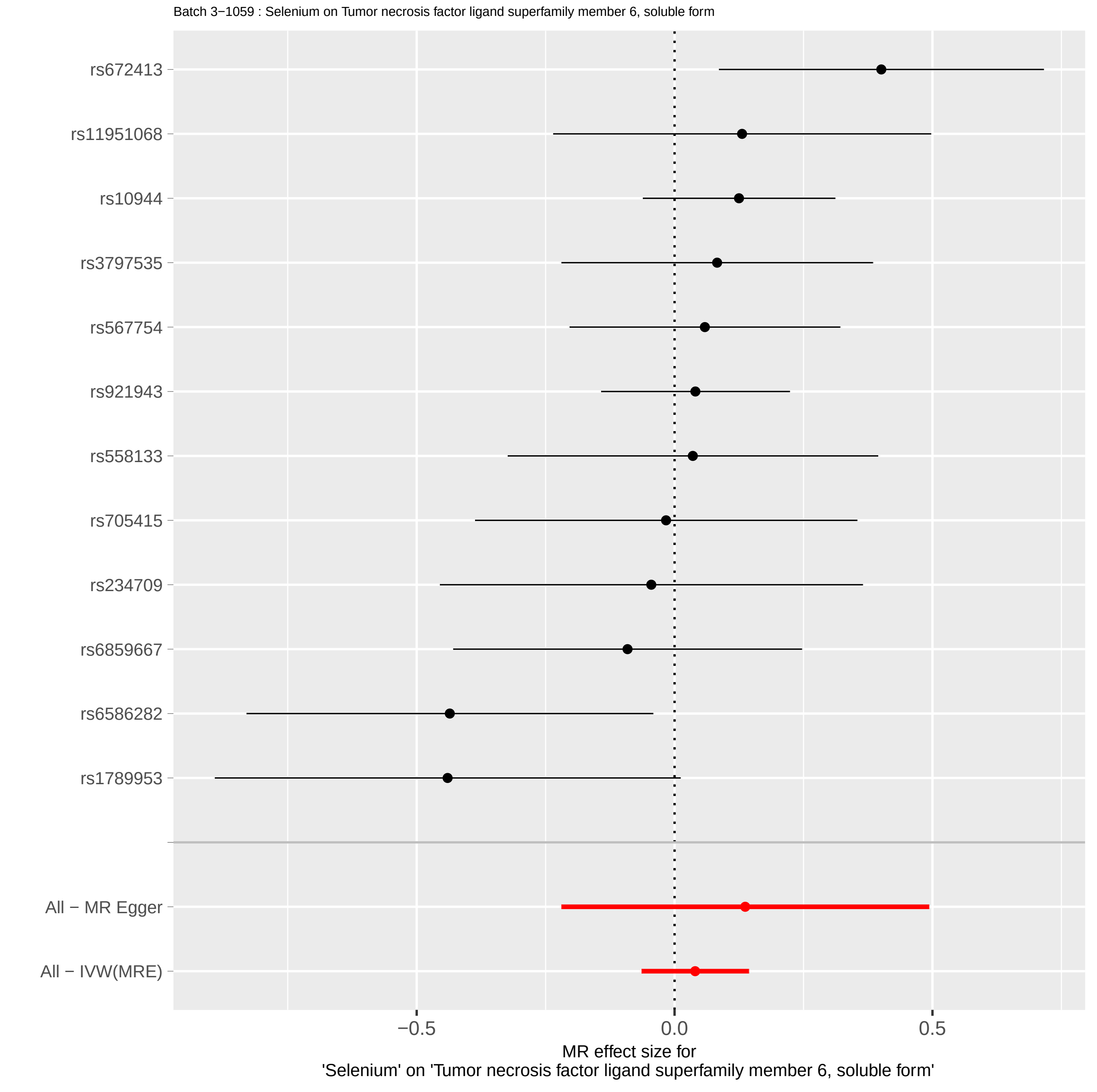
Batch 3−1059 : Selenium on Tumor necrosis factor ligand superfamily member 6, soluble form
rs672413
rs11951068
rs10944
rs3797535
rs567754
rs921943
rs558133
rs705415
rs234709
rs6859667
rs6586282
rs1789953
All − MR Egger
All − IVW(MRE)
−0.5
0.0
0.5
MR effect size for
'Selenium' on 'Tumor necrosis factor ligand superfamily member 6, soluble form'

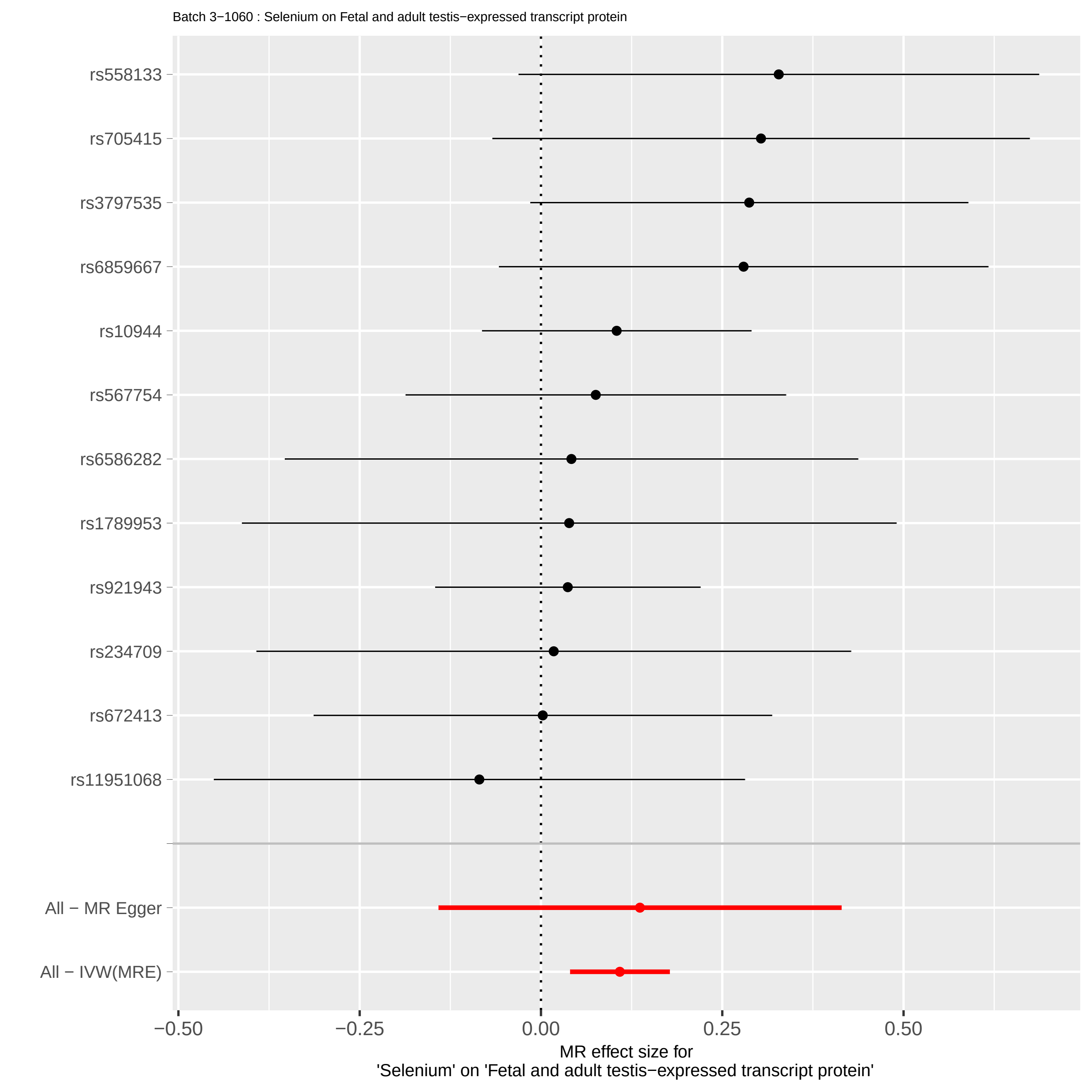

Batch 3−1060 : Selenium on Fetal and adult testis−expressed transcript protein
rs558133
rs705415
rs3797535
rs6859667
rs10944
rs567754
rs6586282
rs1789953
rs921943
rs234709
rs672413
rs11951068
All − MR Egger
All − IVW(MRE)
−0.50
−0.25
0.00
0.25
0.50
MR effect size for
'Selenium' on 'Fetal and adult testis−expressed transcript protein'

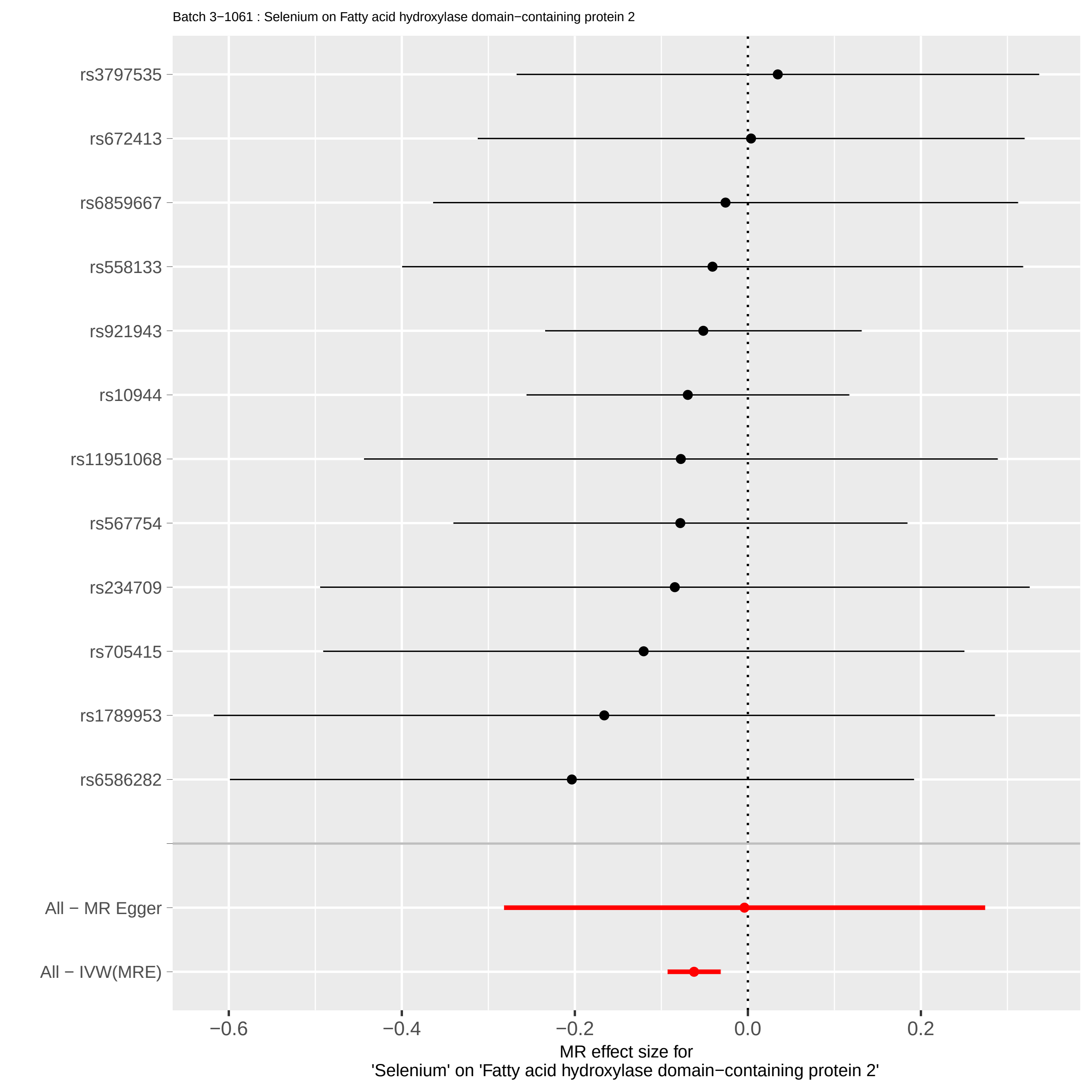

Batch 3−1061 : Selenium on Fatty acid hydroxylase domain−containing protein 2
rs3797535
rs672413
rs6859667
rs558133
rs921943
rs10944
rs11951068
rs567754
rs234709
rs705415
rs1789953
rs6586282
All − MR Egger
All − IVW(MRE)
−0.6
−0.4
−0.2
0.0
0.2
MR effect size for
'Selenium' on 'Fatty acid hydroxylase domain−containing protein 2'

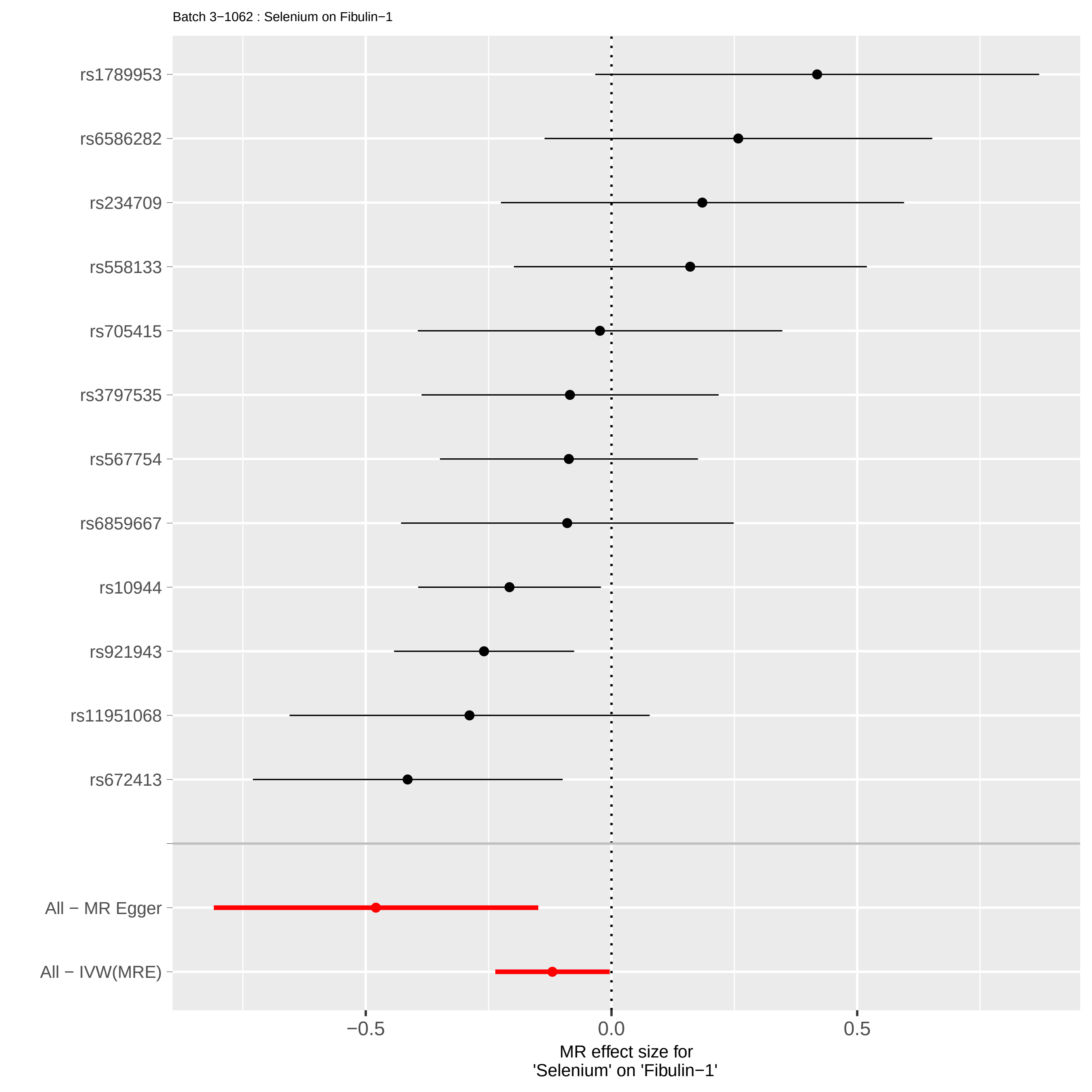

Batch 3−1062 : Selenium on Fibulin−1
rs1789953
rs6586282
rs234709
rs558133
rs705415
rs3797535
rs567754
rs6859667
rs10944
rs921943
rs11951068
rs672413
All − MR Egger
All − IVW(MRE)
−0.5
0.0
0.5
MR effect size for
'Selenium' on 'Fibulin−1'

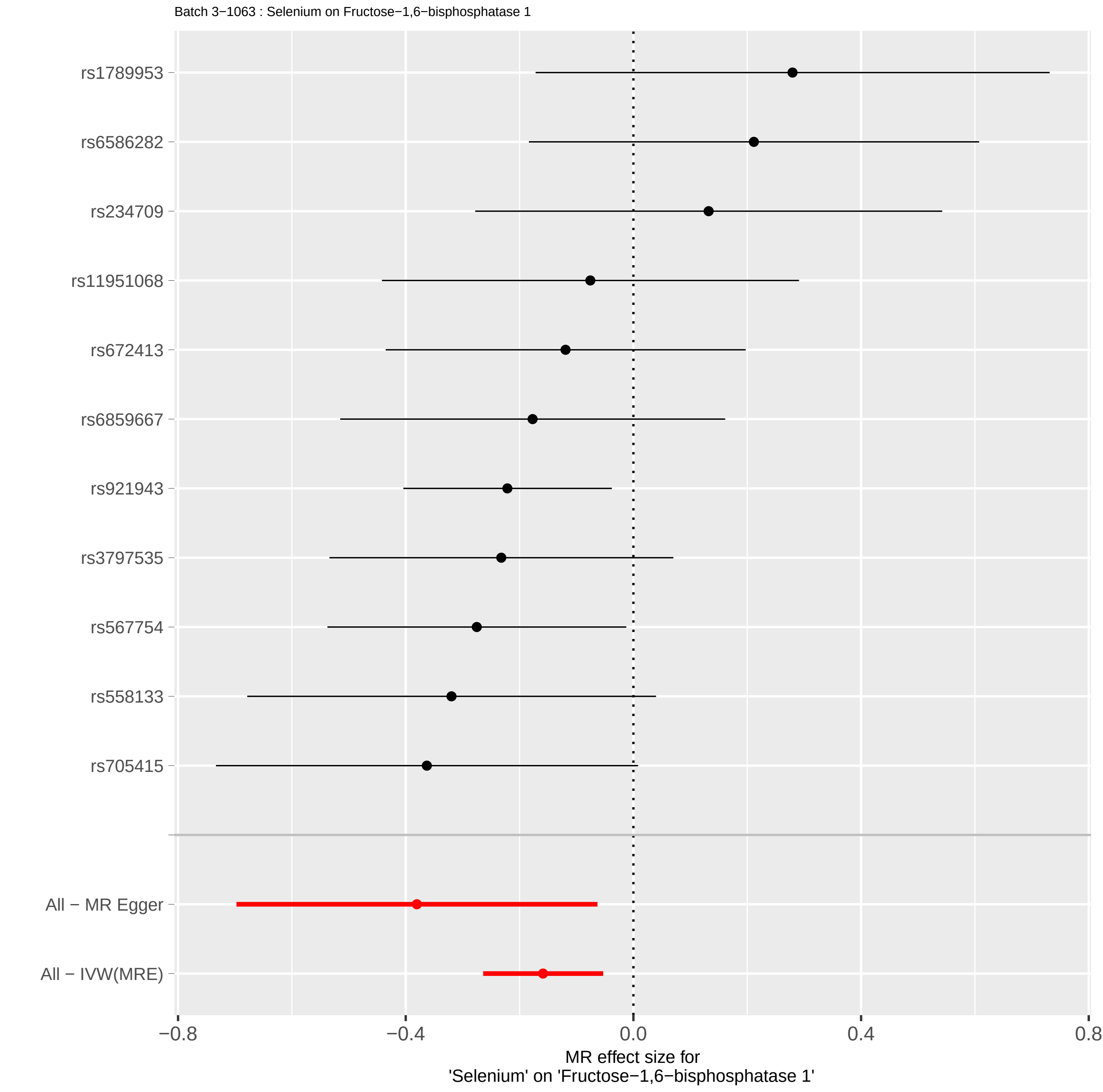

Batch 3−1063 : Selenium on Fructose−1,6−bisphosphatase 1
rs1789953
rs6586282
rs234709
rs11951068
rs672413
rs6859667
rs921943
rs3797535
rs567754
rs558133
rs705415
All − MR Egger
All − IVW(MRE)
−0.8
−0.4
0.0
0.4
0.8
MR effect size for
'Selenium' on 'Fructose−1,6−bisphosphatase 1'

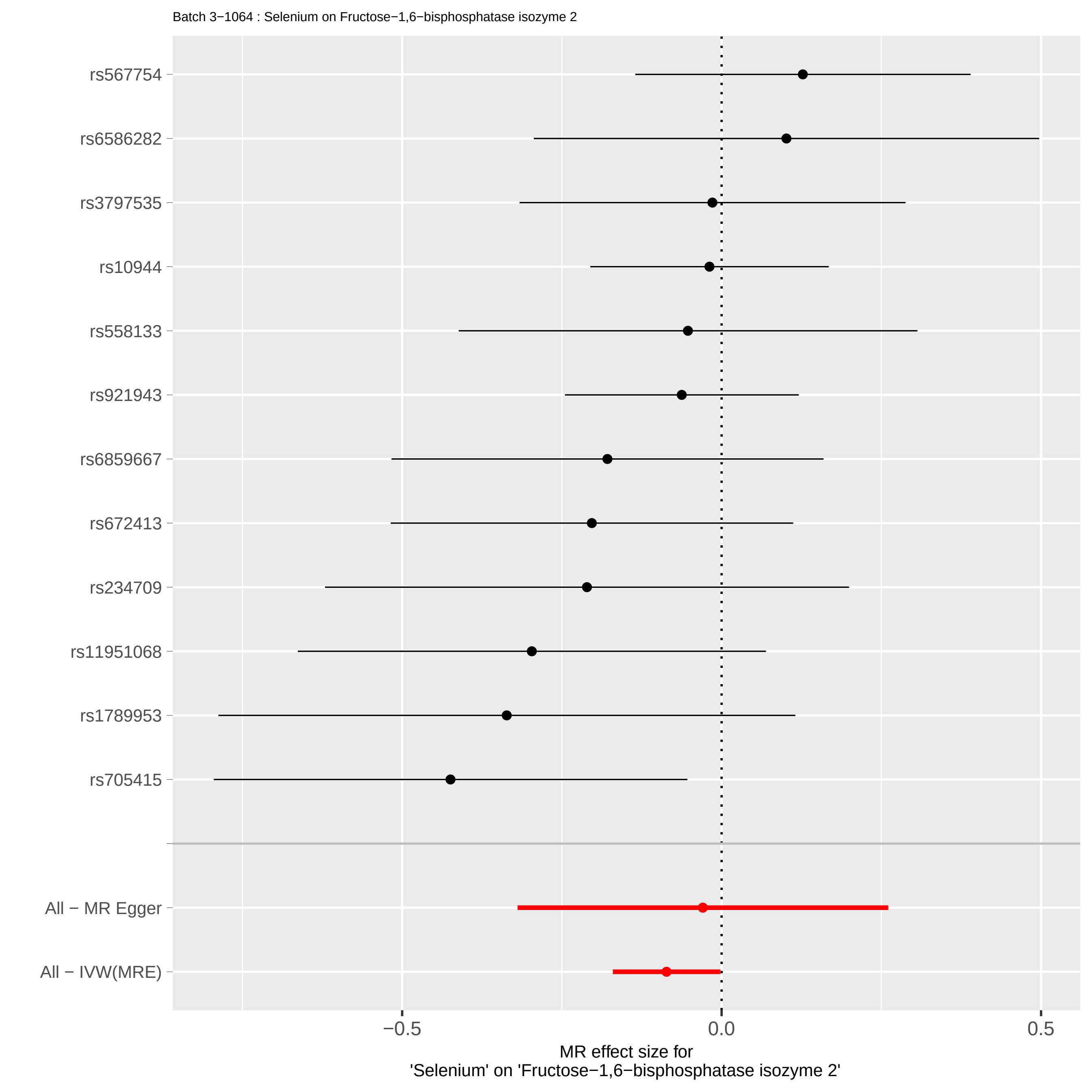
Batch 3−1064 : Selenium on Fructose−1,6−bisphosphatase isozyme 2
rs567754
rs6586282
rs3797535
rs10944
rs558133
rs921943
rs6859667
rs672413
rs234709
rs11951068
rs1789953
rs705415
All − MR Egger
All − IVW(MRE)
−0.5
0.0
0.5
MR effect size for
'Selenium' on 'Fructose−1,6−bisphosphatase isozyme 2'

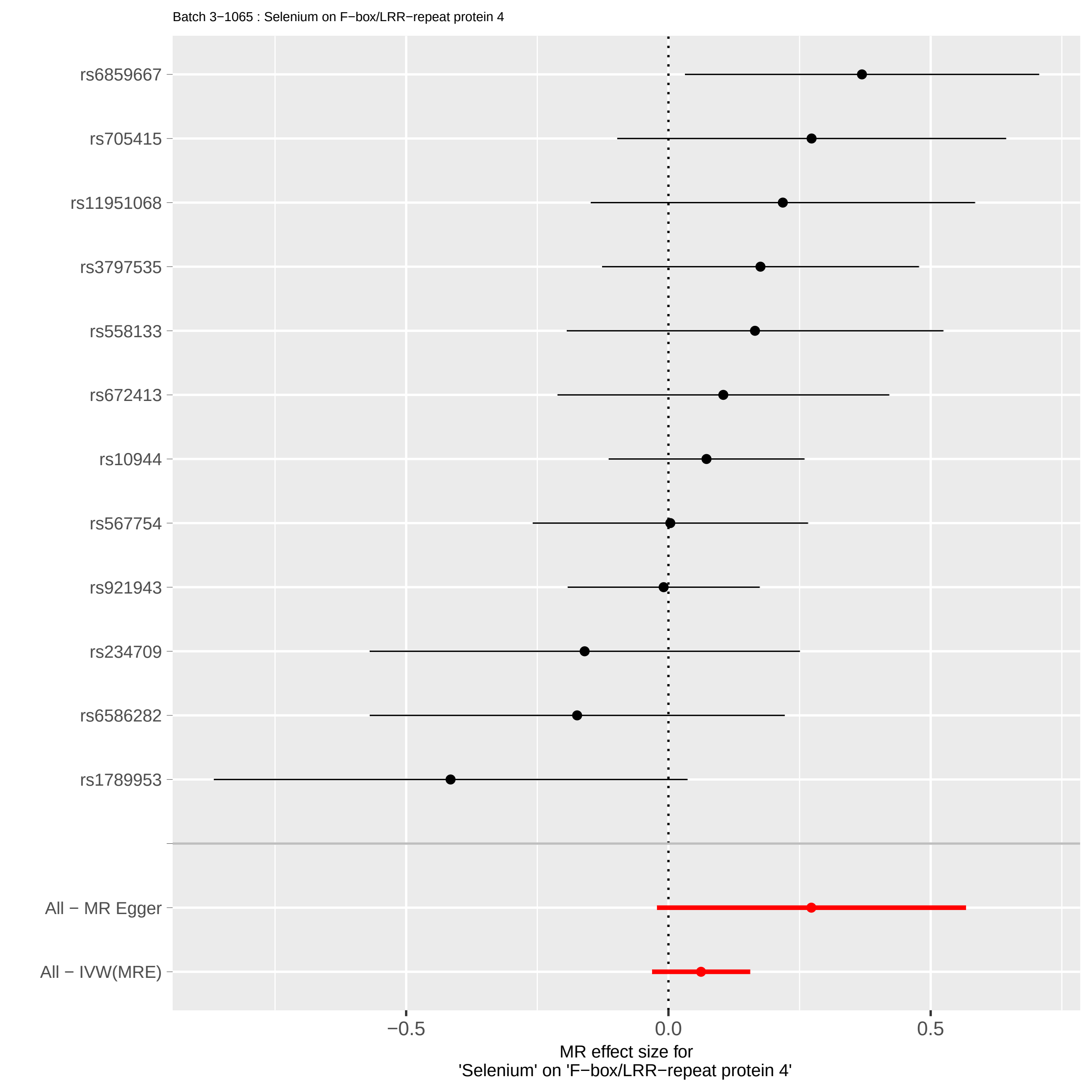

Batch 3−1065 : Selenium on F−box/LRR−repeat protein 4
rs6859667
rs705415
rs11951068
rs3797535
rs558133
rs672413
rs10944
rs567754
rs921943
rs234709
rs6586282
rs1789953
All − MR Egger
All − IVW(MRE)
−0.5
0.0
0.5
MR effect size for
'Selenium' on 'F−box/LRR−repeat protein 4'

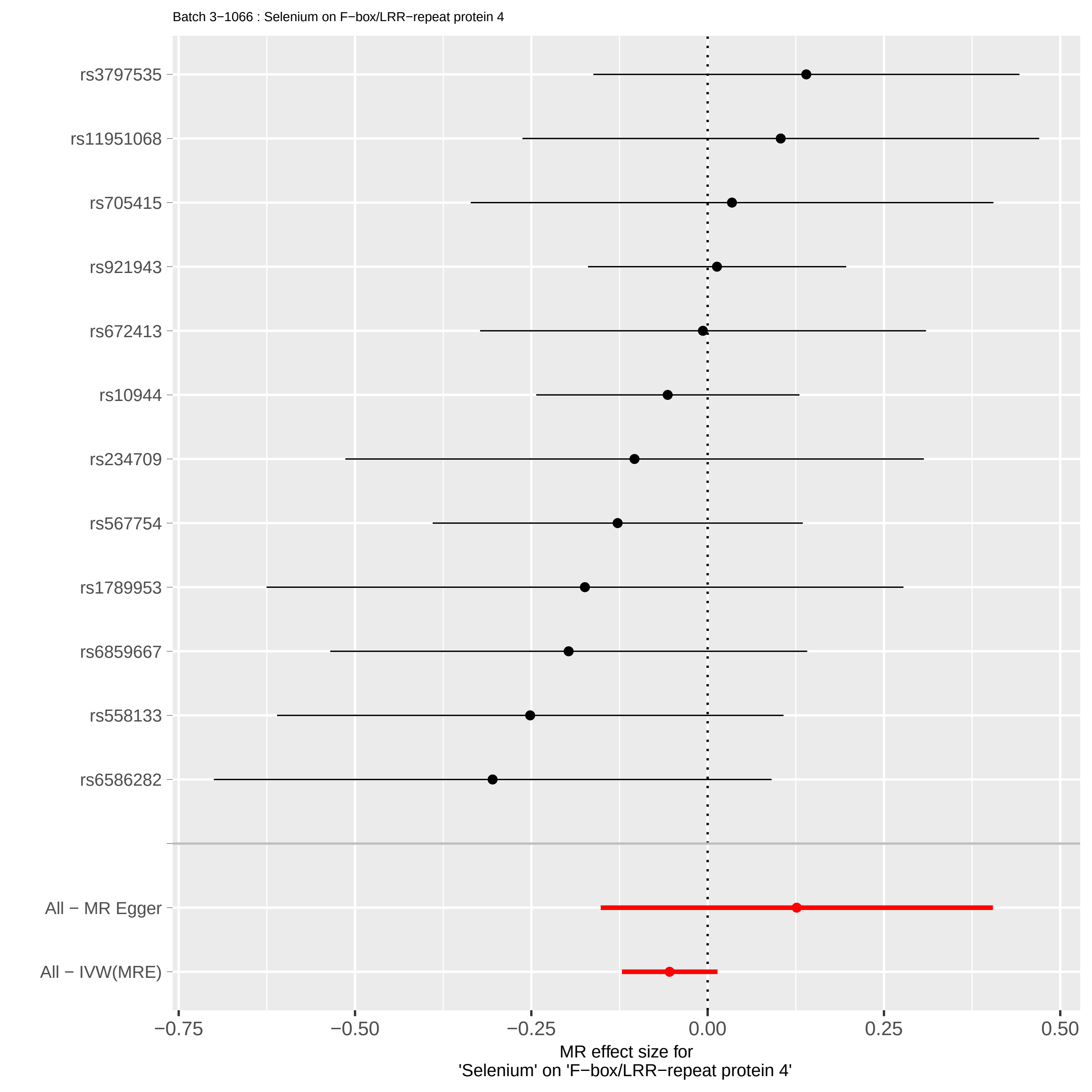

Batch 3−1066 : Selenium on F−box/LRR−repeat protein 4
rs3797535
rs11951068
rs705415
rs921943
rs672413
rs10944
rs234709
rs567754
rs1789953
rs6859667
rs558133
rs6586282
All − MR Egger
All − IVW(MRE)
−0.75
−0.50
−0.25
0.00
0.25
0.50
MR effect size for
'Selenium' on 'F−box/LRR−repeat protein 4'

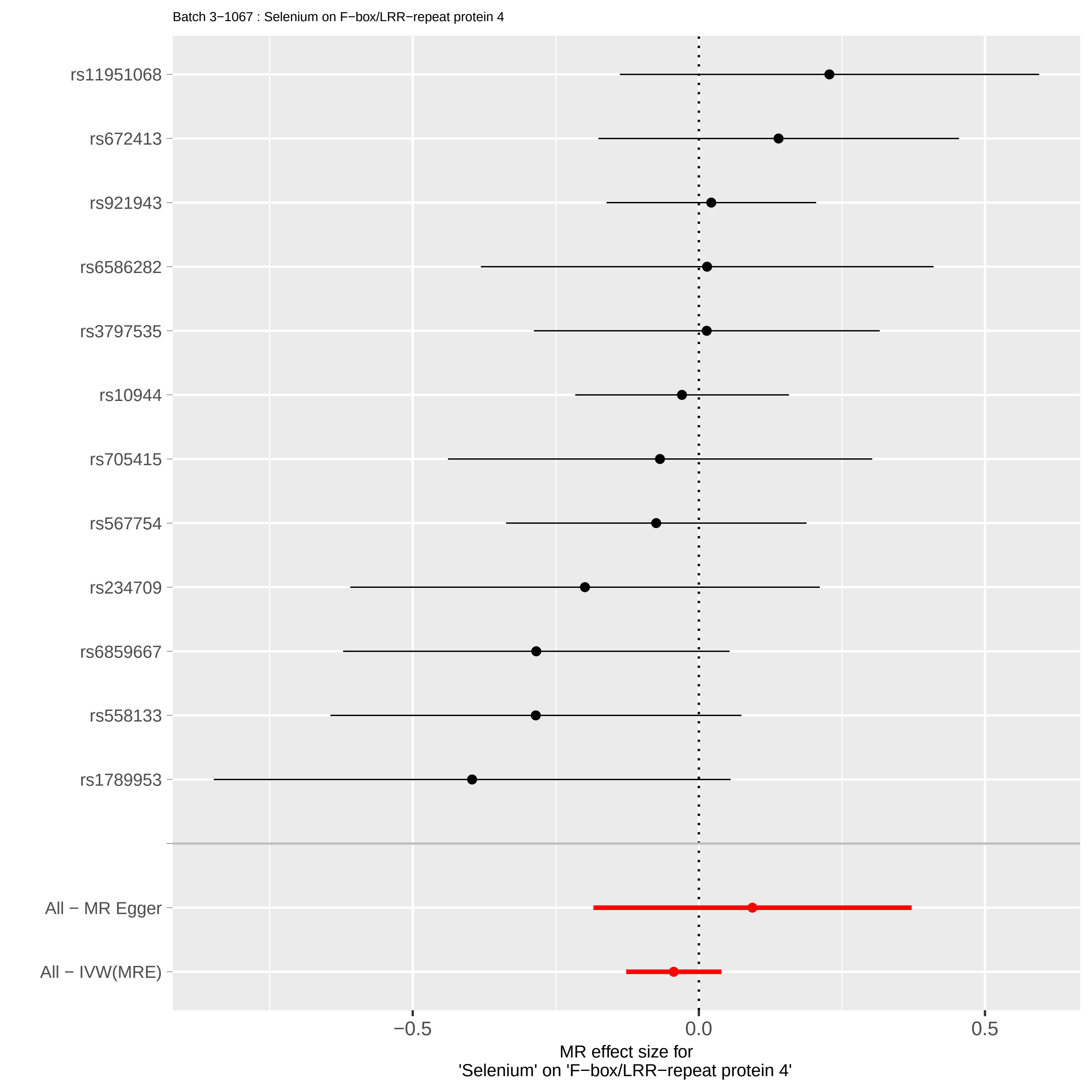

Batch 3−1067 : Selenium on F−box/LRR−repeat protein 4
rs11951068
rs672413
rs921943
rs6586282
rs3797535
rs10944
rs705415
rs567754
rs234709
rs6859667
rs558133
rs1789953
All − MR Egger
All − IVW(MRE)
−0.5
0.0
0.5
MR effect size for
'Selenium' on 'F−box/LRR−repeat protein 4'

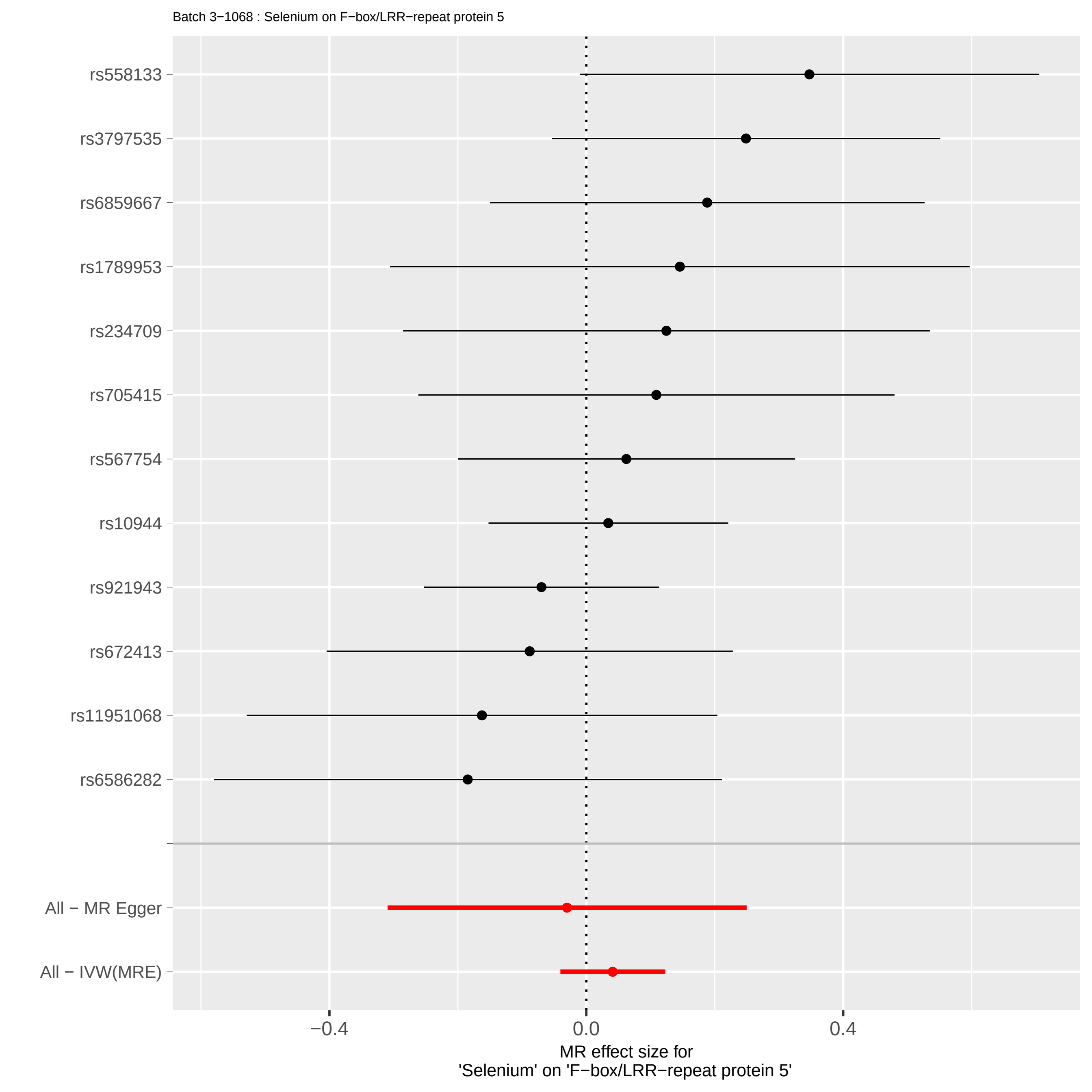

Batch 3−1068 : Selenium on F−box/LRR−repeat protein 5
rs558133
rs3797535
rs6859667
rs1789953
rs234709
rs705415
rs567754
rs10944
rs921943
rs672413
rs11951068
rs6586282
All − MR Egger
All − IVW(MRE)
−0.4
0.0
0.4
MR effect size for
'Selenium' on 'F−box/LRR−repeat protein 5'

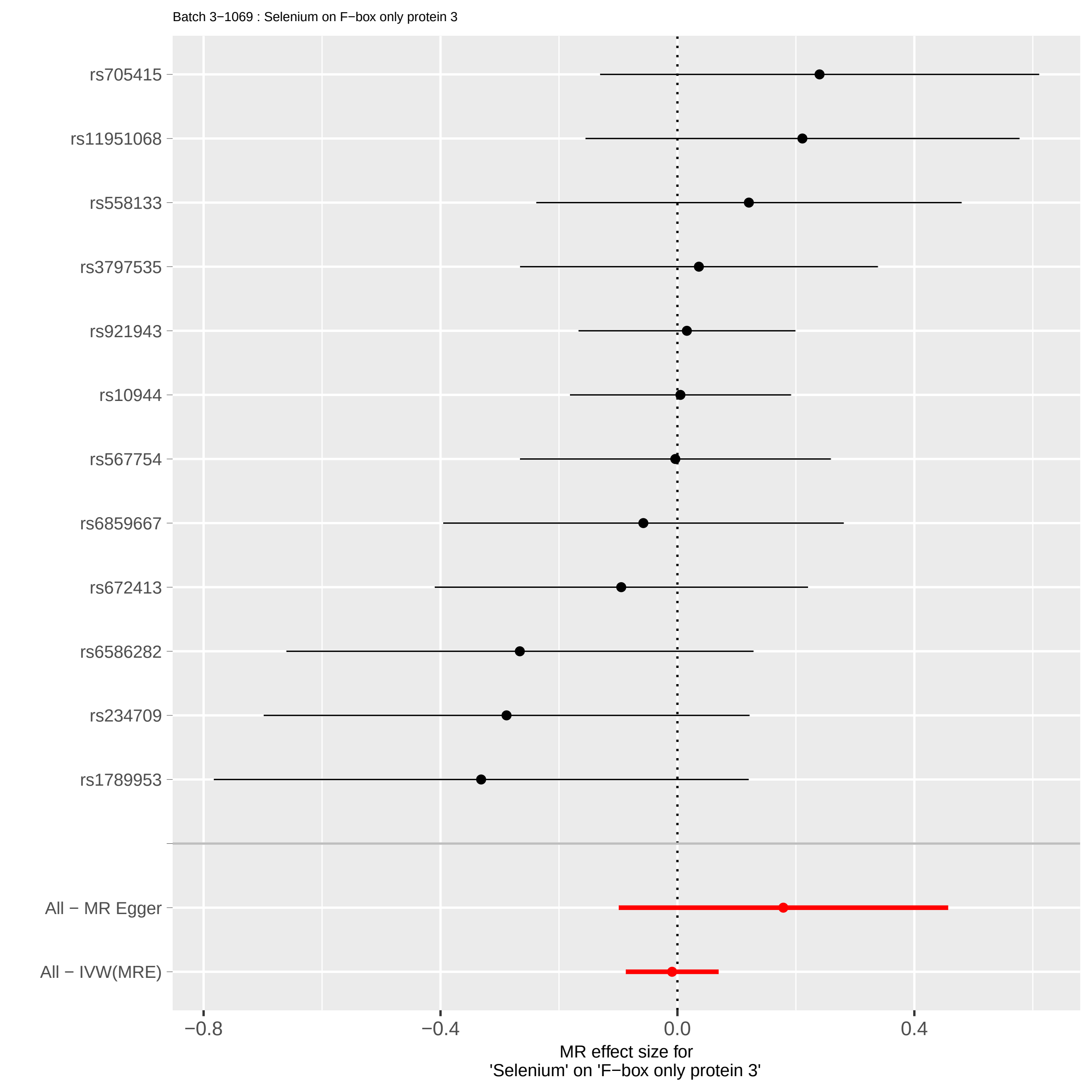

Batch 3−1069 : Selenium on F−box only protein 3
rs705415
rs11951068
rs558133
rs3797535
rs921943
rs10944
rs567754
rs6859667
rs672413
rs6586282
rs234709
rs1789953
All − MR Egger
All − IVW(MRE)
−0.8
−0.4
0.0
0.4
MR effect size for
'Selenium' on 'F−box only protein 3'

Batch 3−1070 : Selenium on High affinity immunoglobulin alpha and immunoglobulin mu Fc receptor

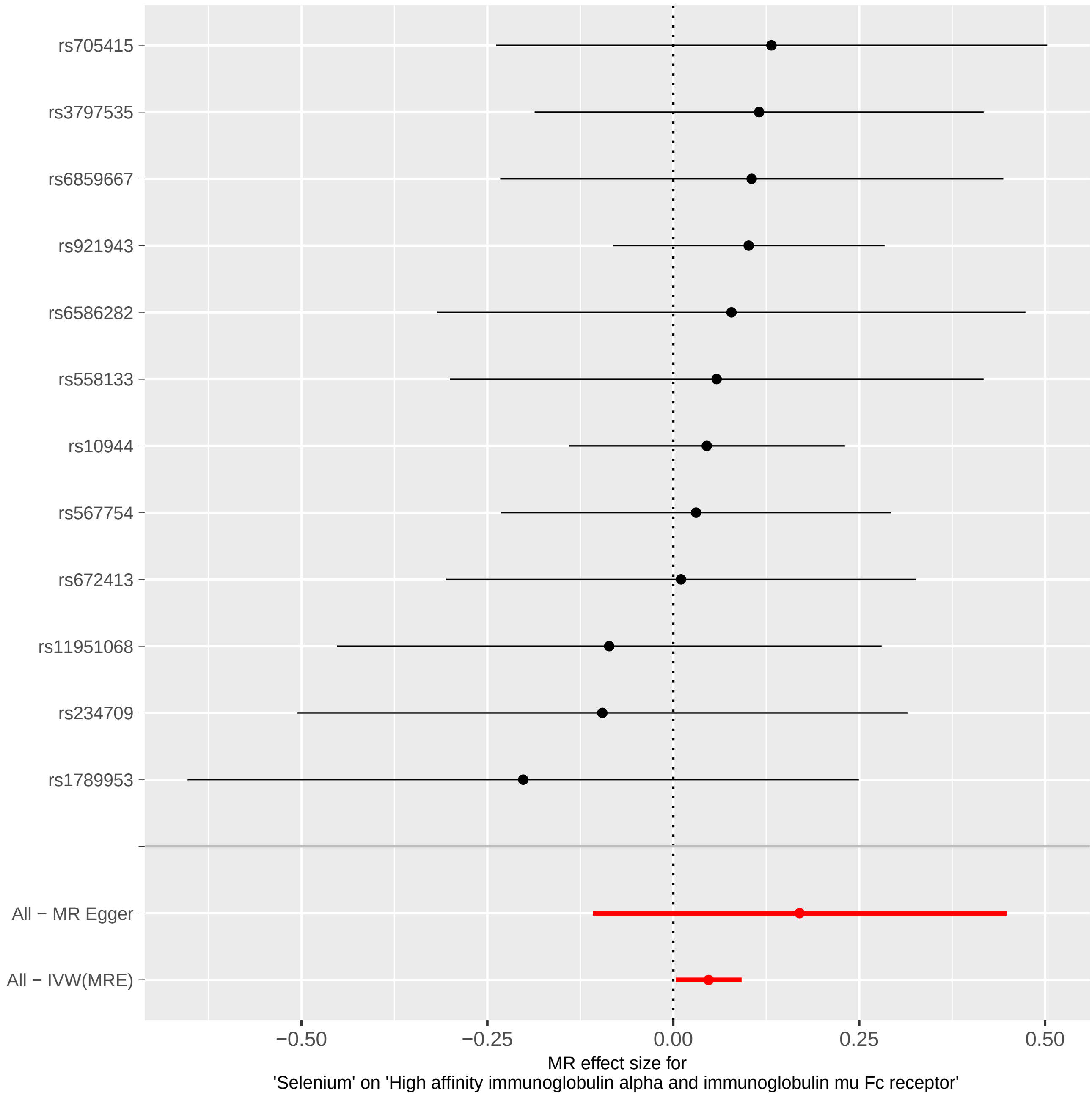

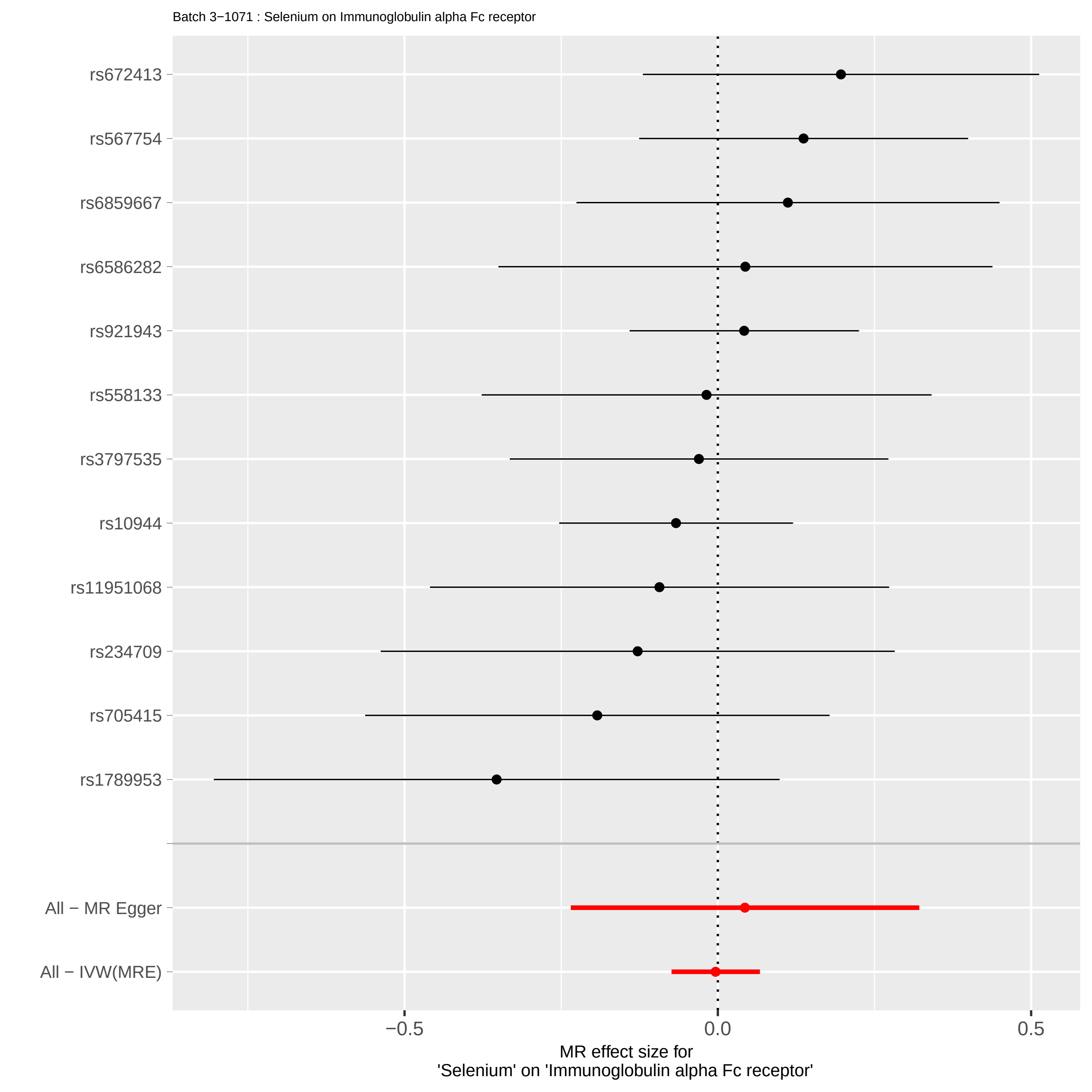

Batch 3−1071 : Selenium on Immunoglobulin alpha Fc receptor
rs672413
rs567754
rs6859667
rs6586282
rs921943
rs558133
rs3797535
rs10944
rs11951068
rs234709
rs705415
rs1789953
All − MR Egger
All − IVW(MRE)
−0.5
0.0
0.5
MR effect size for
'Selenium' on 'Immunoglobulin alpha Fc receptor'

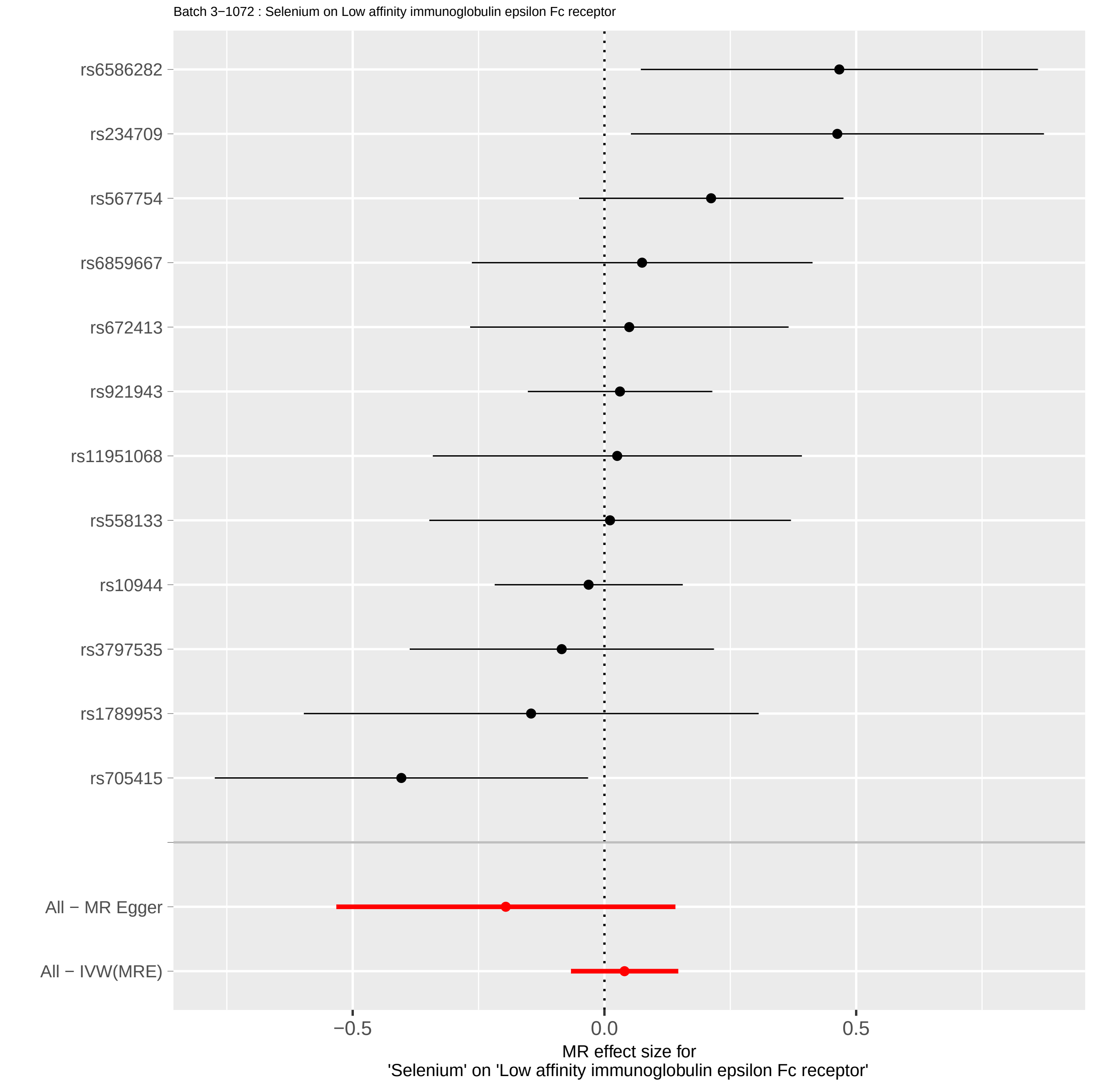

Batch 3−1072 : Selenium on Low affinity immunoglobulin epsilon Fc receptor
rs6586282
rs234709
rs567754
rs6859667
rs672413
rs921943
rs11951068
rs558133
rs10944
rs3797535
rs1789953
rs705415
All − MR Egger
All − IVW(MRE)
−0.5
0.0
0.5
MR effect size for
'Selenium' on 'Low affinity immunoglobulin epsilon Fc receptor'

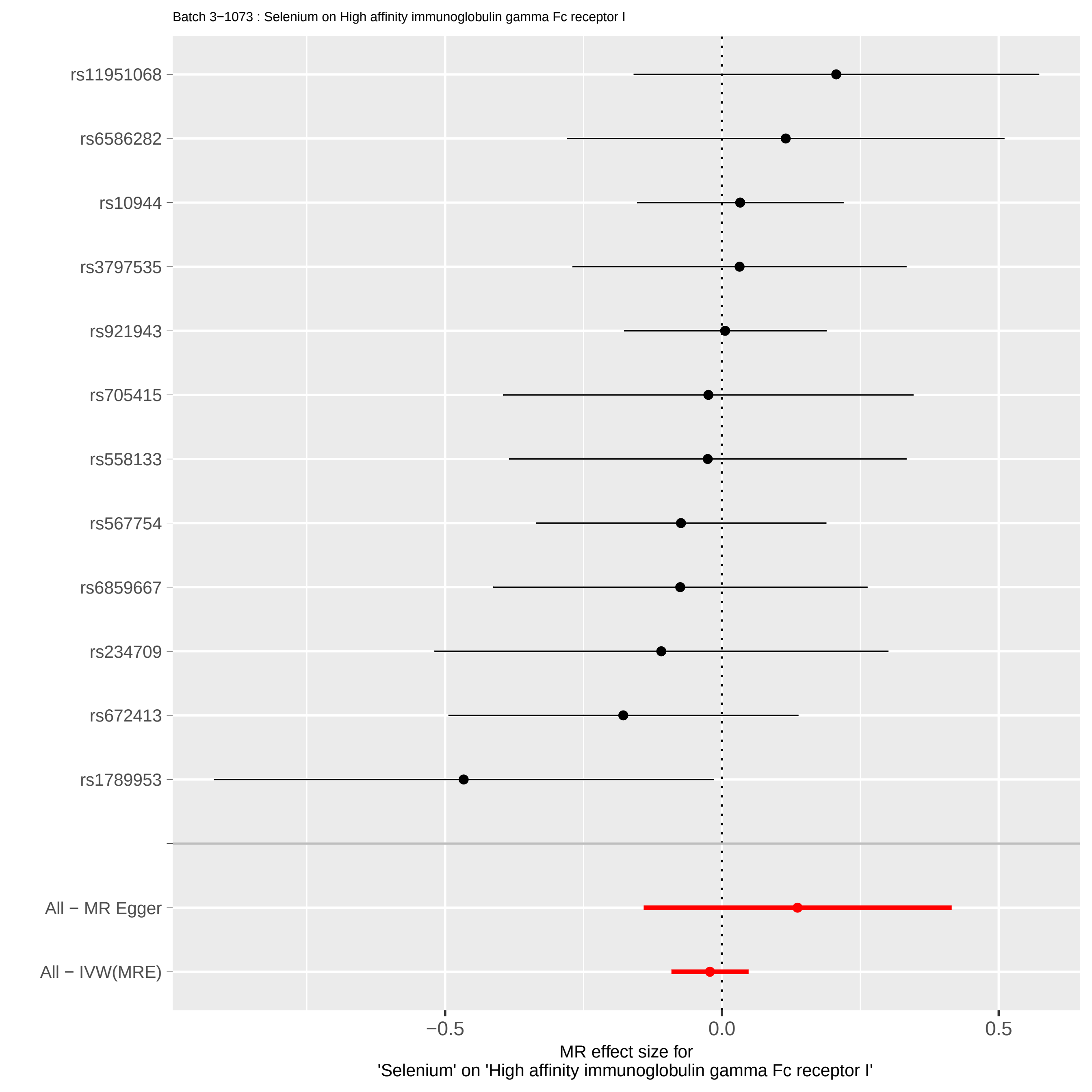
Batch 3−1073 : Selenium on High affinity immunoglobulin gamma Fc receptor I
rs11951068
rs6586282
rs10944
rs3797535
rs921943
rs705415
rs558133
rs567754
rs6859667
rs234709
rs672413
rs1789953
All − MR Egger
All − IVW(MRE)
−0.5
0.0
0.5
MR effect size for
'Selenium' on 'High affinity immunoglobulin gamma Fc receptor I'

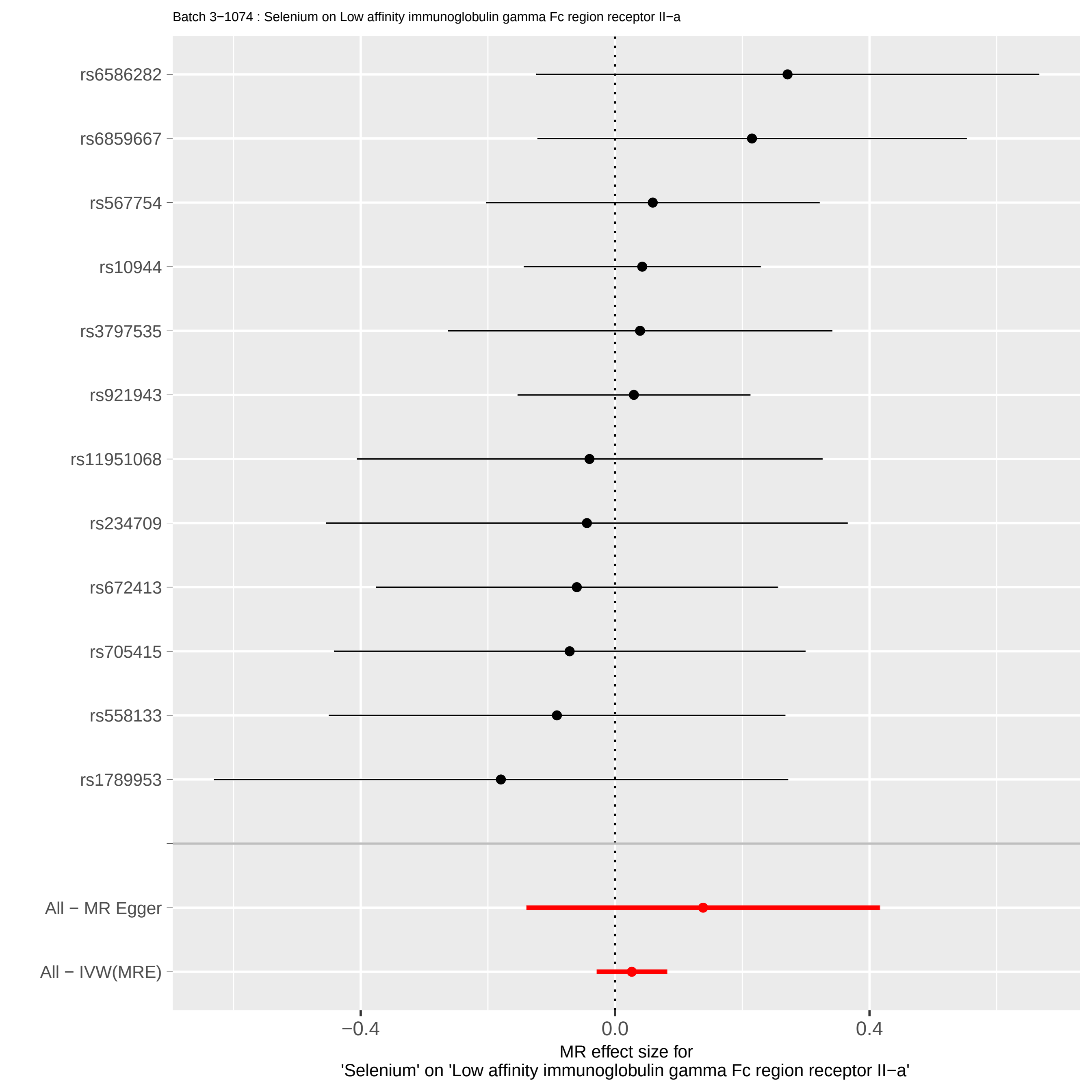
Batch 3−1074 : Selenium on Low affinity immunoglobulin gamma Fc region receptor II−a
rs6586282
rs6859667
rs567754
rs10944
rs3797535
rs921943
rs11951068
rs234709
rs672413
rs705415
rs558133
rs1789953
All − MR Egger
All − IVW(MRE)
−0.4
0.0
0.4
MR effect size for
'Selenium' on 'Low affinity immunoglobulin gamma Fc region receptor II−a'

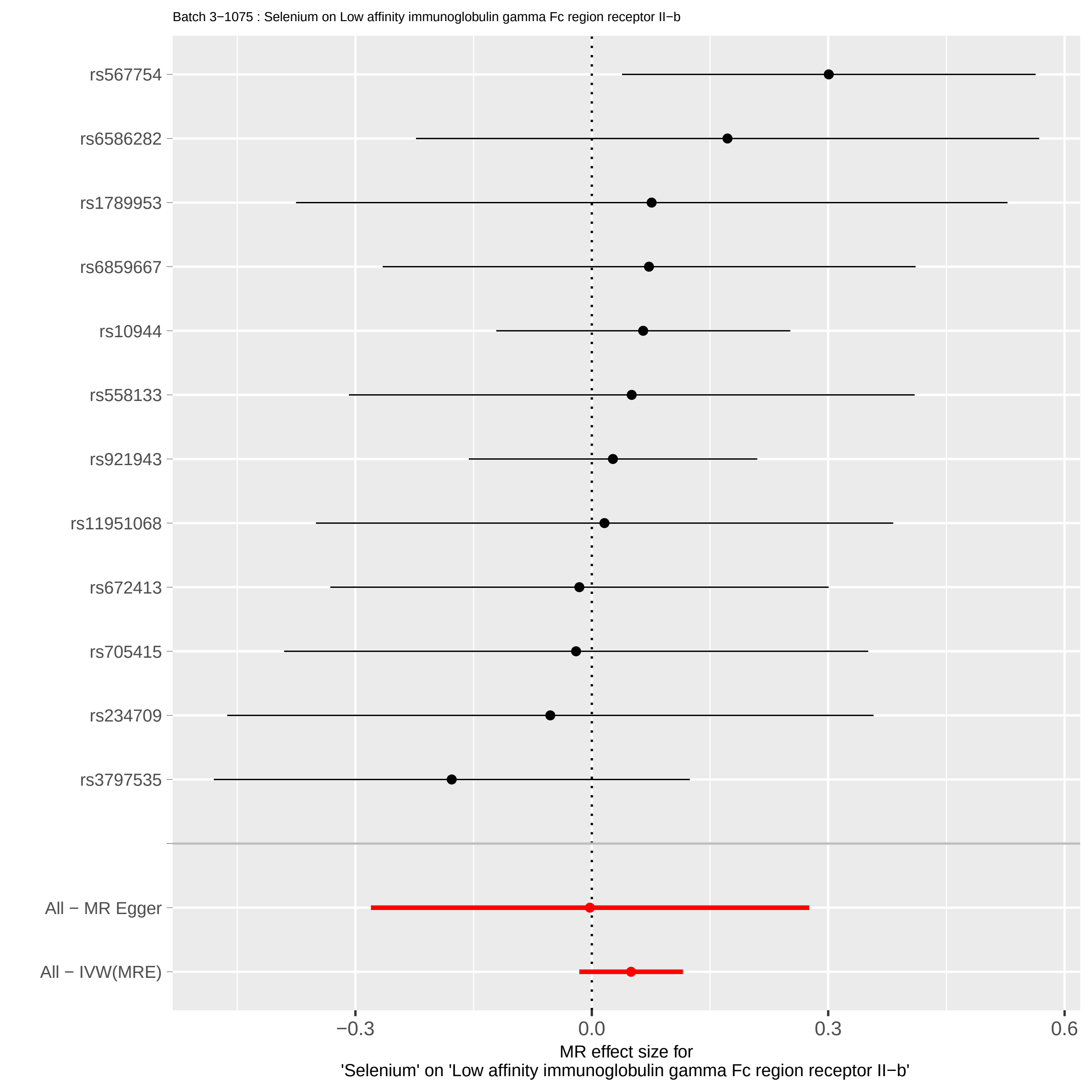
Batch 3−1075 : Selenium on Low affinity immunoglobulin gamma Fc region receptor II−b
rs567754
rs6586282
rs1789953
rs6859667
rs10944
rs558133
rs921943
rs11951068
rs672413
rs705415
rs234709
rs3797535
All − MR Egger
All − IVW(MRE)
−0.3
0.0
0.3
0.6
MR effect size for
'Selenium' on 'Low affinity immunoglobulin gamma Fc region receptor II−b'

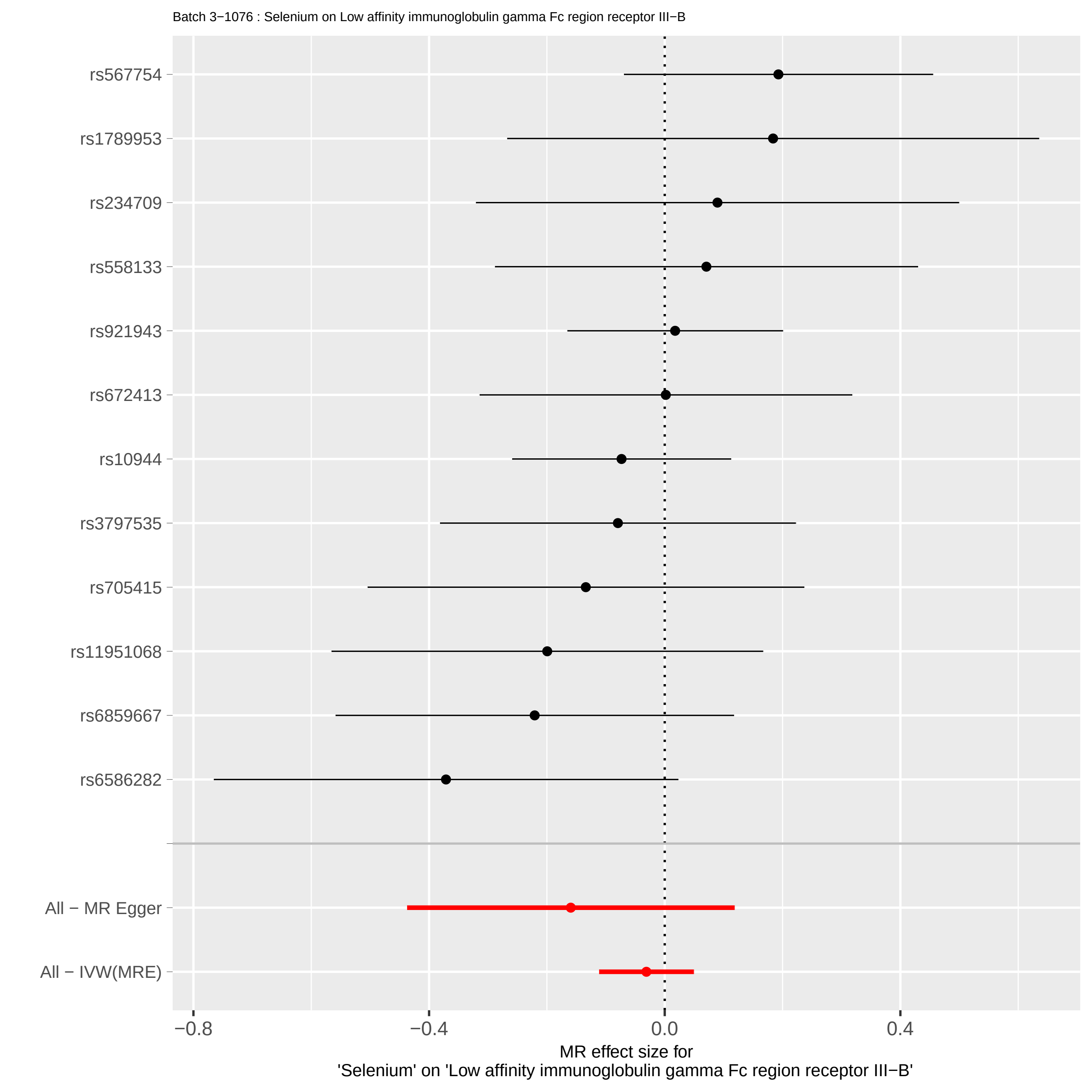

Batch 3−1076 : Selenium on Low affinity immunoglobulin gamma Fc region receptor III−B
rs567754
rs1789953
rs234709
rs558133
rs921943
rs672413
rs10944
rs3797535
rs705415
rs11951068
rs6859667
rs6586282
All − MR Egger
All − IVW(MRE)
−0.8
−0.4
0.0
0.4
MR effect size for
'Selenium' on 'Low affinity immunoglobulin gamma Fc region receptor III−B'

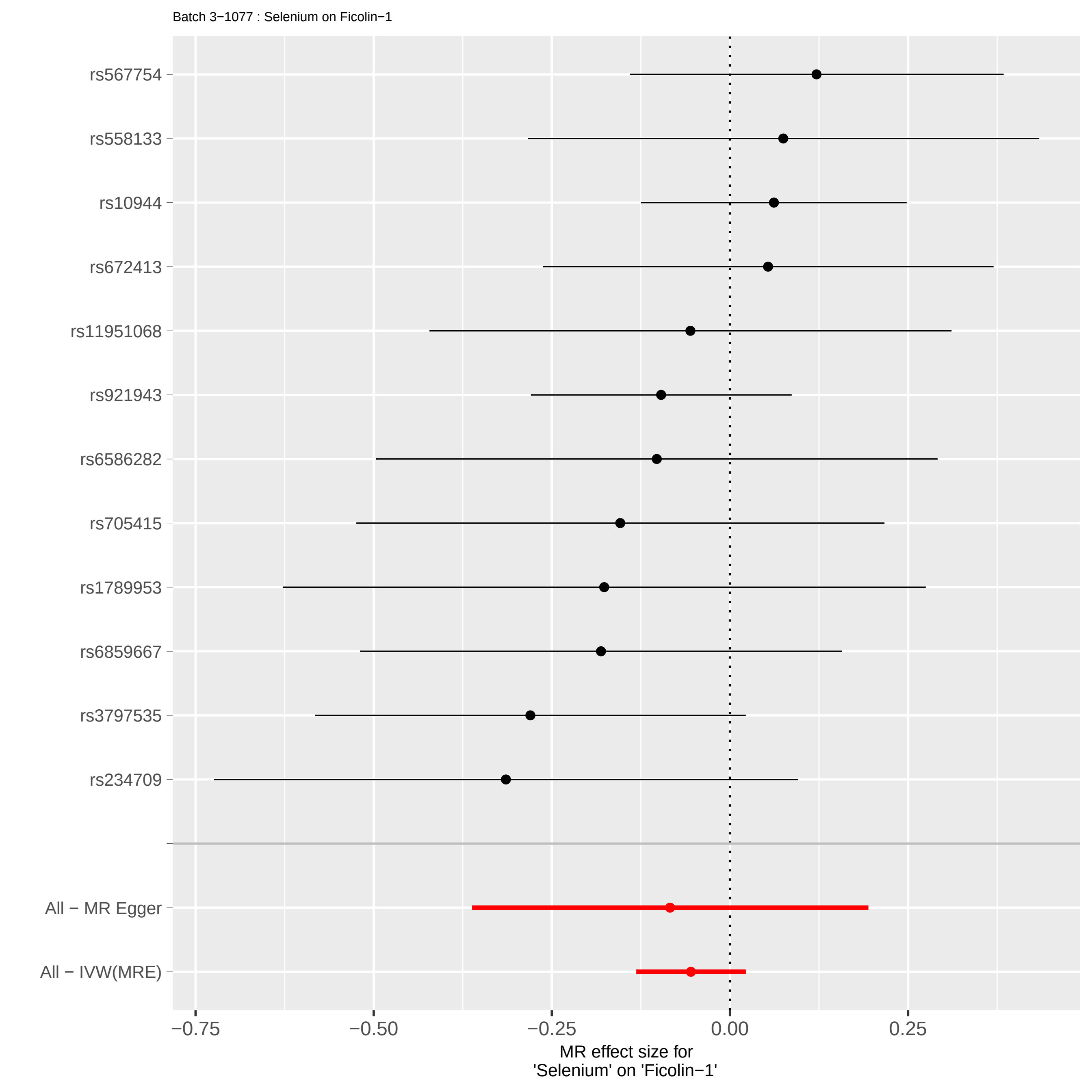
Batch 3−1077 : Selenium on Ficolin−1
rs567754
rs558133
rs10944
rs672413
rs11951068
rs921943
rs6586282
rs705415
rs1789953
rs6859667
rs3797535
rs234709
All − MR Egger
All − IVW(MRE)
−0.75
−0.50
−0.25
0.00
0.25
MR effect size for
'Selenium' on 'Ficolin−1'

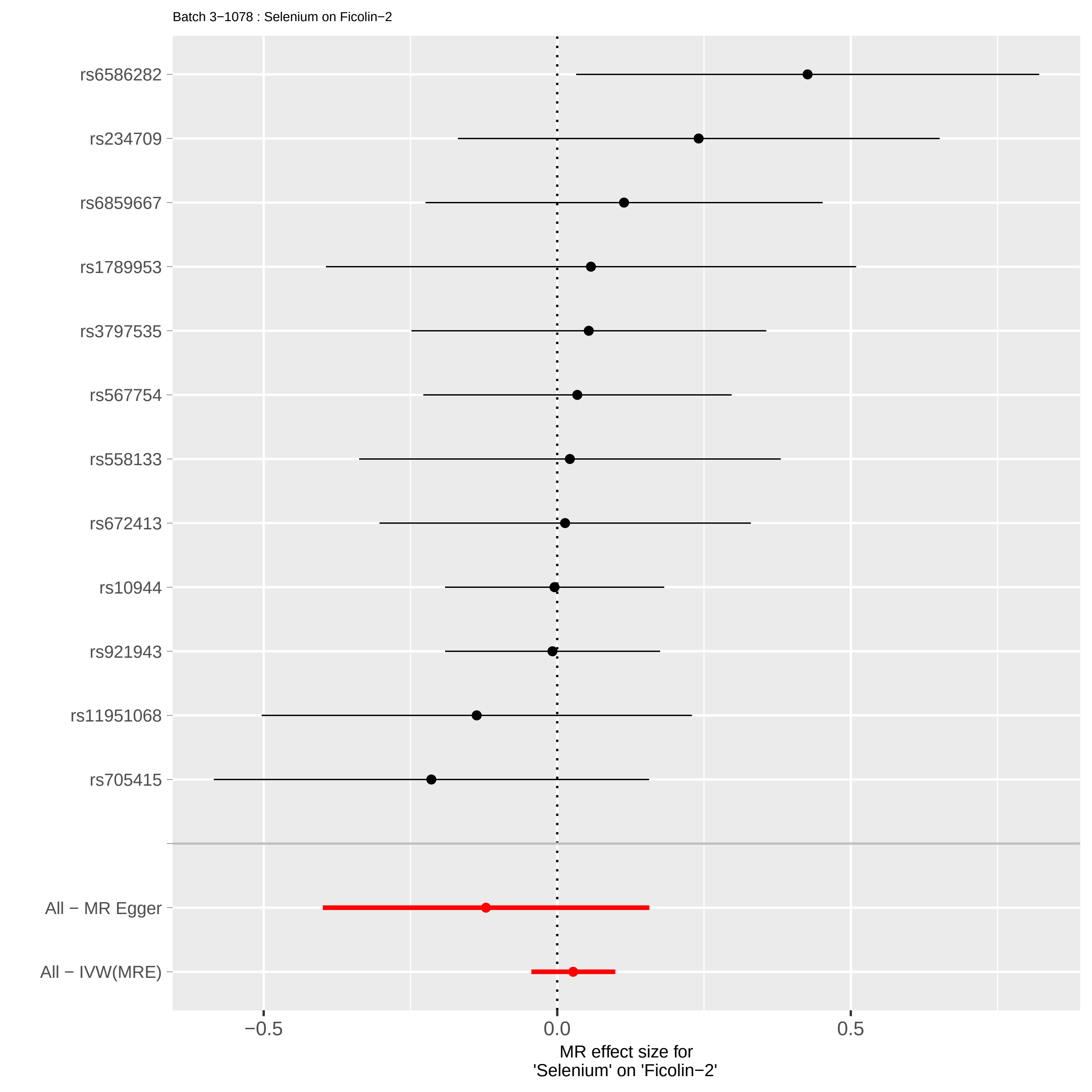
Batch 3−1078 : Selenium on Ficolin−2
rs6586282
rs234709
rs6859667
rs1789953
rs3797535
rs567754
rs558133
rs672413
rs10944
rs921943
rs11951068
rs705415
All − MR Egger
All − IVW(MRE)
−0.5
0.0
0.5
MR effect size for
'Selenium' on 'Ficolin−2'

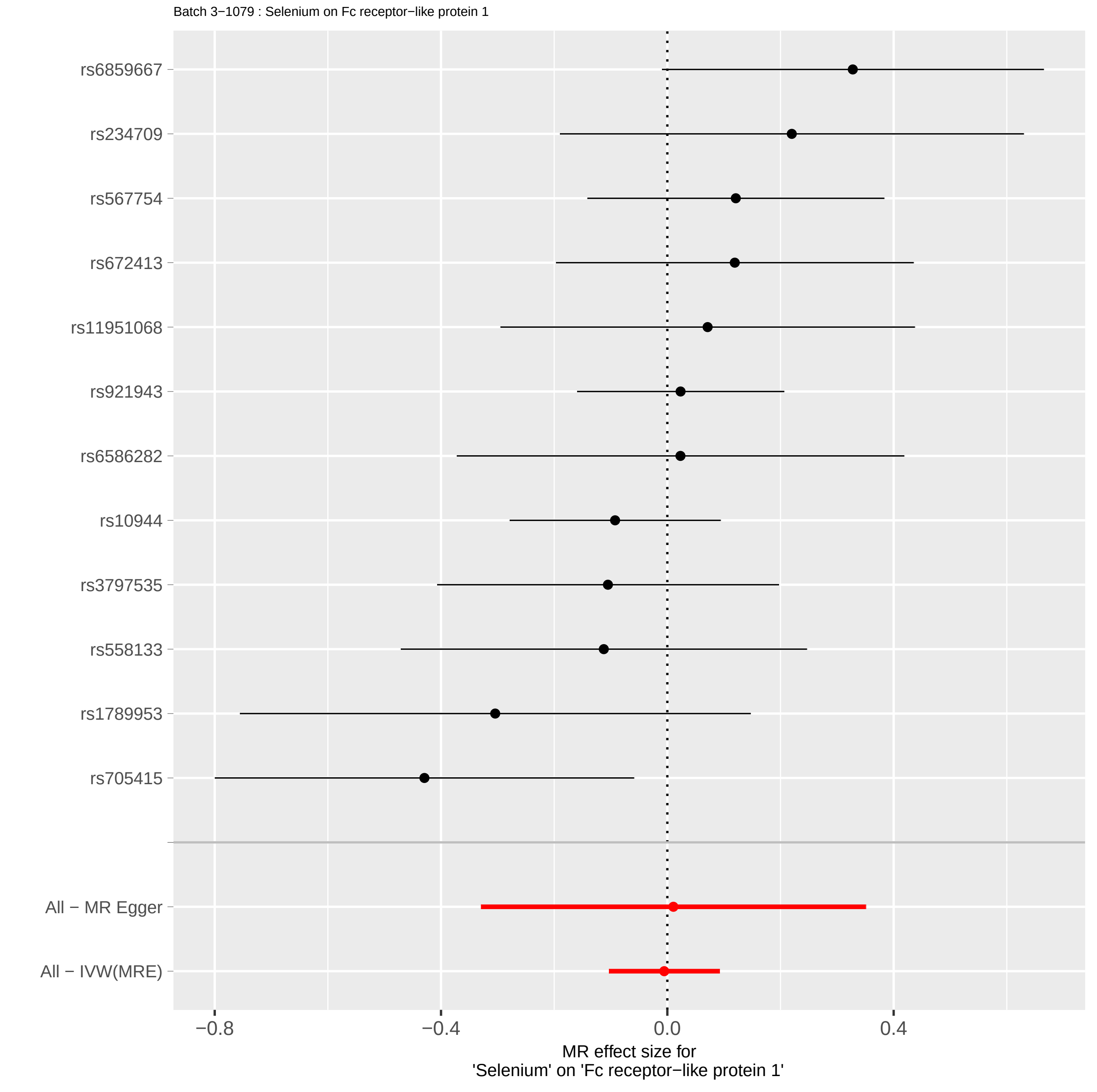

Batch 3−1079 : Selenium on Fc receptor−like protein 1
rs6859667
rs234709
rs567754
rs672413
rs11951068
rs921943
rs6586282
rs10944
rs3797535
rs558133
rs1789953
rs705415
All − MR Egger
All − IVW(MRE)
−0.8
−0.4
0.0
0.4
MR effect size for
'Selenium' on 'Fc receptor−like protein 1'

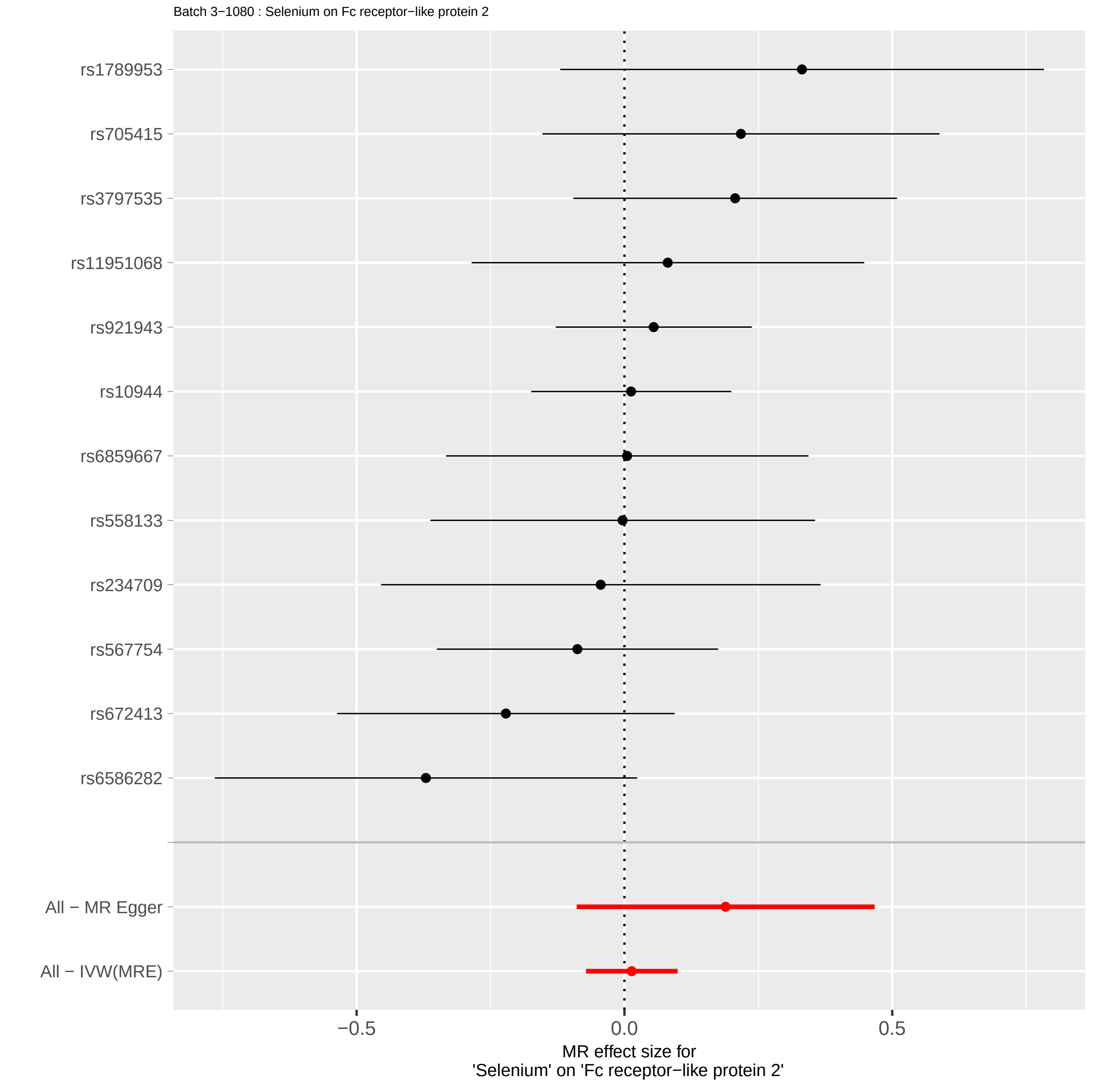

Batch 3−1080 : Selenium on Fc receptor−like protein 2
rs1789953
rs705415
rs3797535
rs11951068
rs921943
rs10944
rs6859667
rs558133
rs234709
rs567754
rs672413
rs6586282
All − MR Egger
All − IVW(MRE)
−0.5
0.0
0.5
MR effect size for
'Selenium' on 'Fc receptor−like protein 2'

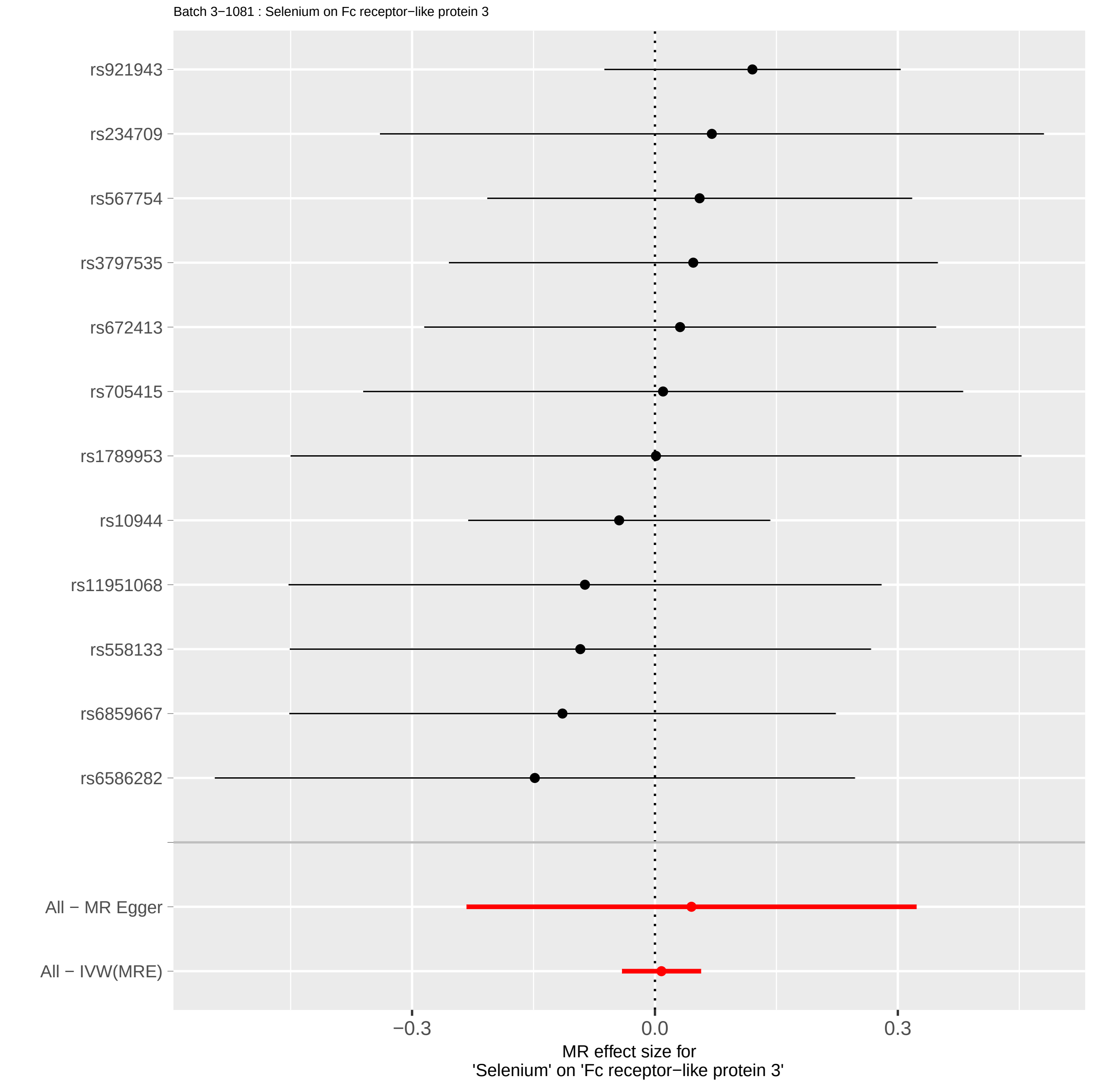

Batch 3−1081 : Selenium on Fc receptor−like protein 3
rs921943
rs234709
rs567754
rs3797535
rs672413
rs705415
rs1789953
rs10944
rs11951068
rs558133
rs6859667
rs6586282
All − MR Egger
All − IVW(MRE)
−0.3
0.0
0.3
MR effect size for
'Selenium' on 'Fc receptor−like protein 3'

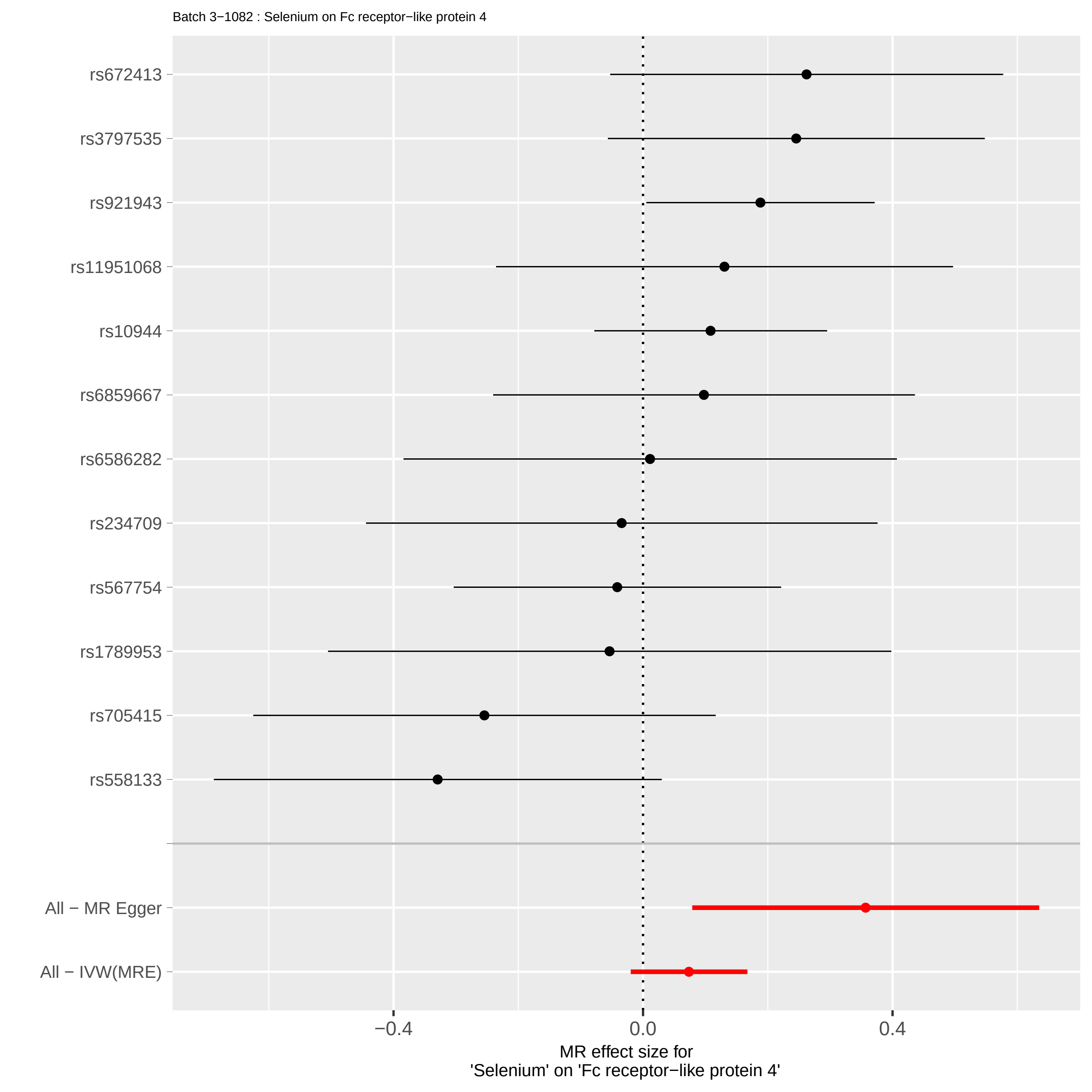

Batch 3−1082 : Selenium on Fc receptor−like protein 4
rs672413
rs3797535
rs921943
rs11951068
rs10944
rs6859667
rs6586282
rs234709
rs567754
rs1789953
rs705415
rs558133
All − MR Egger
All − IVW(MRE)
−0.4
0.0
0.4
MR effect size for
'Selenium' on 'Fc receptor−like protein 4'

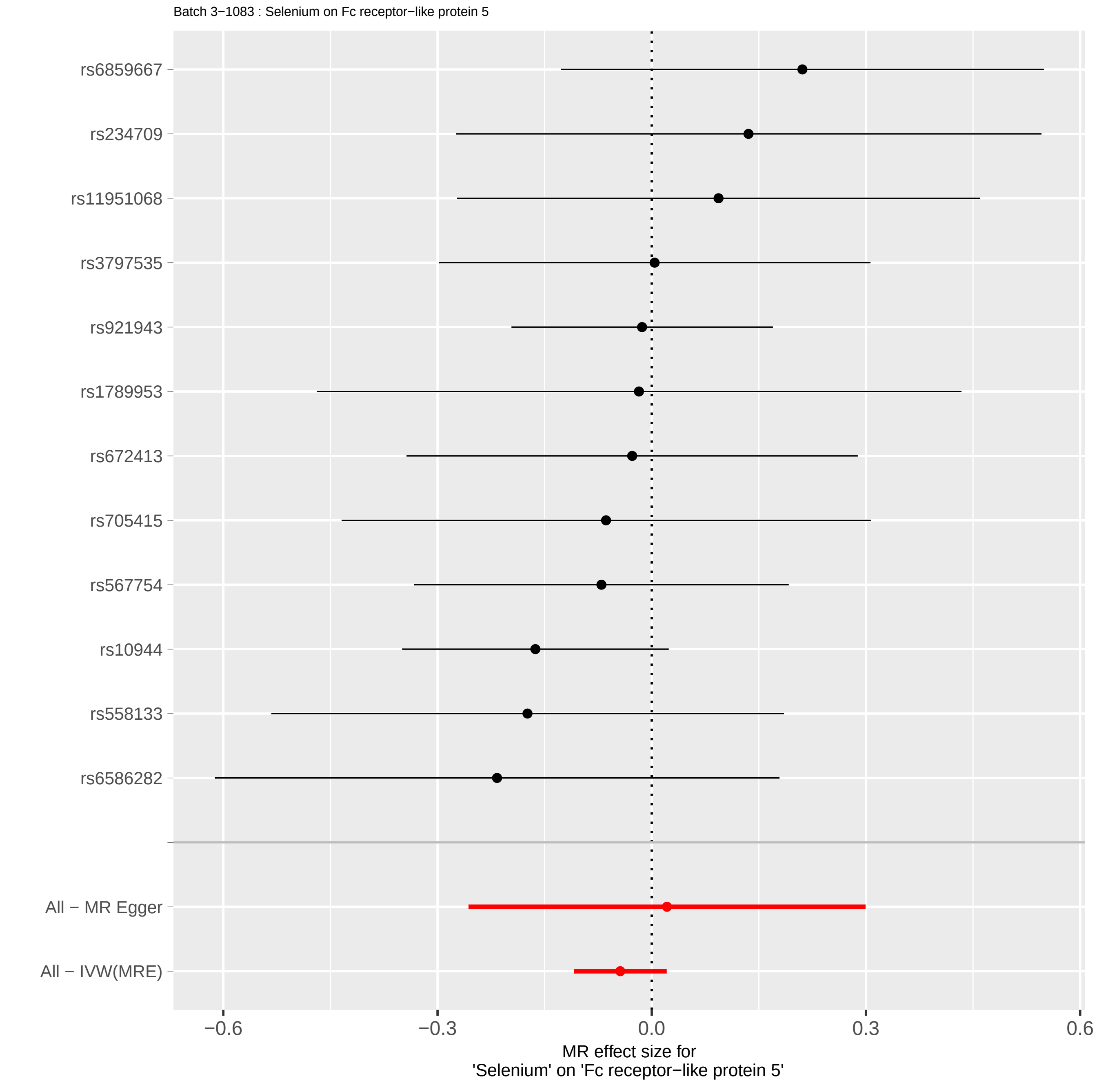

Batch 3−1083 : Selenium on Fc receptor−like protein 5
rs6859667
rs234709
rs11951068
rs3797535
rs921943
rs1789953
rs672413
rs705415
rs567754
rs10944
rs558133
rs6586282
All − MR Egger
All − IVW(MRE)
−0.6
−0.3
0.0
0.3
0.6
MR effect size for
'Selenium' on 'Fc receptor−like protein 5'

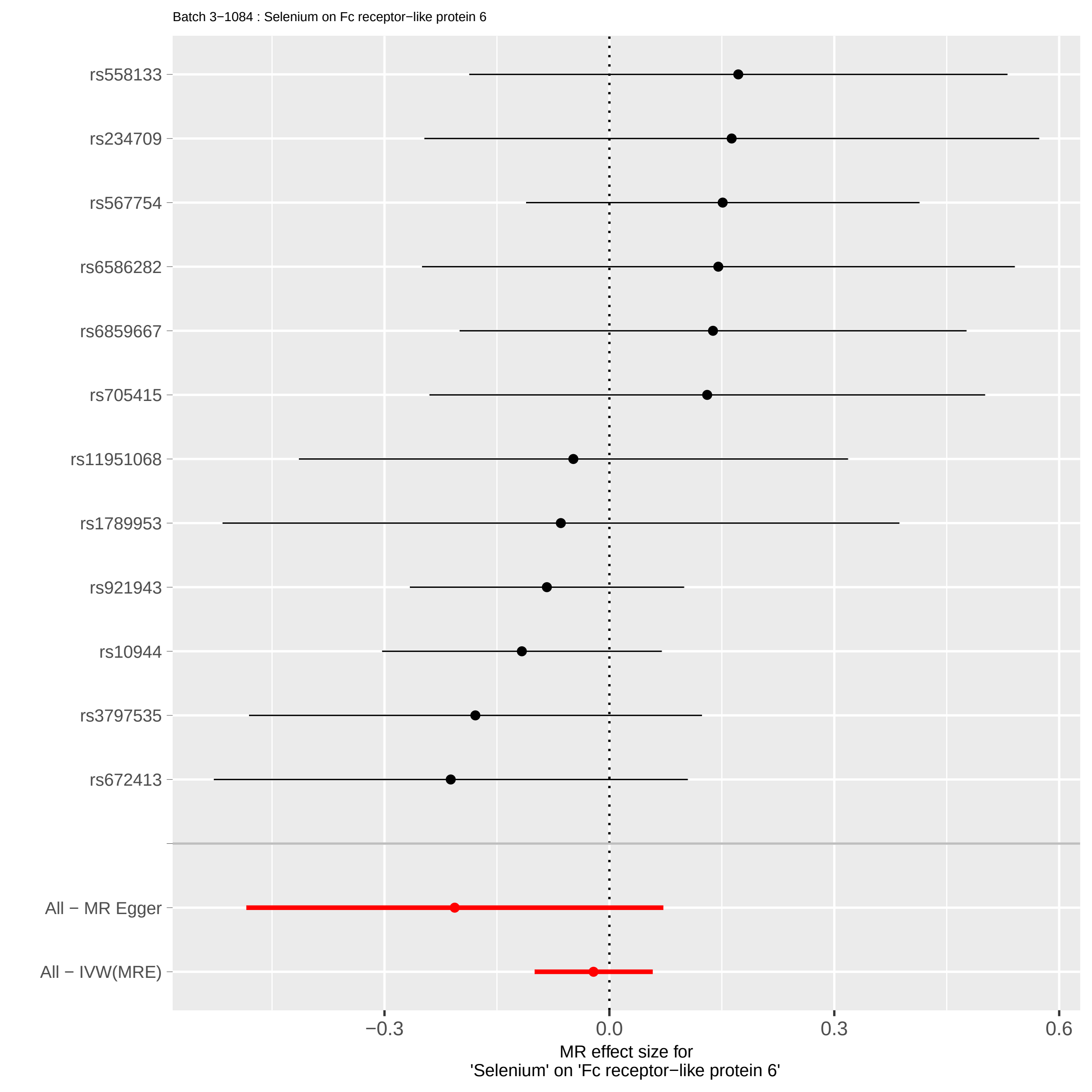

Batch 3−1084 : Selenium on Fc receptor−like protein 6
rs558133
rs234709
rs567754
rs6586282
rs6859667
rs705415
rs11951068
rs1789953
rs921943
rs10944
rs3797535
rs672413
All − MR Egger
All − IVW(MRE)
−0.3
0.0
0.3
0.6
MR effect size for
'Selenium' on 'Fc receptor−like protein 6'

Batch 3−1085 : Selenium on Flap endonuclease 1

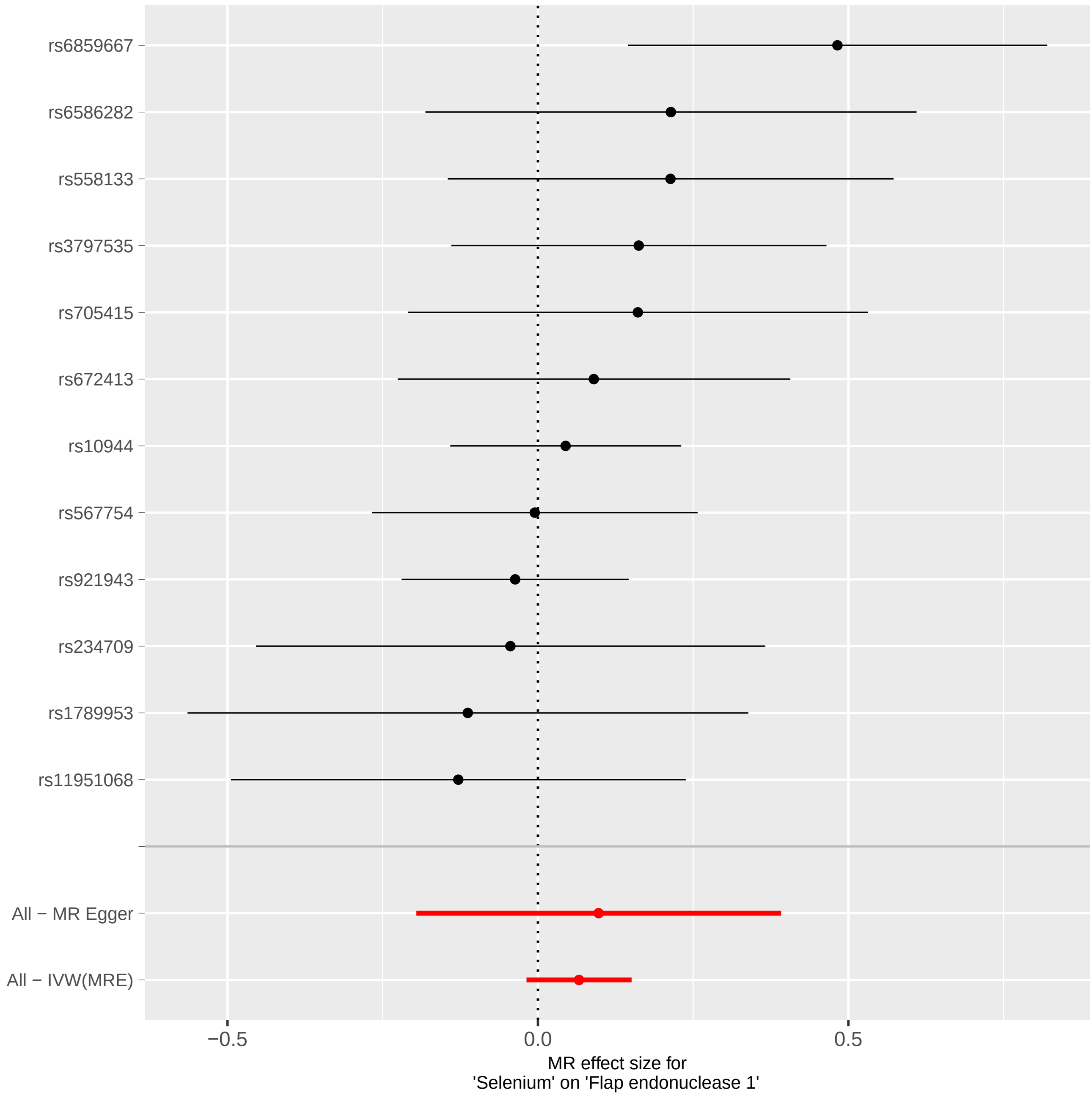

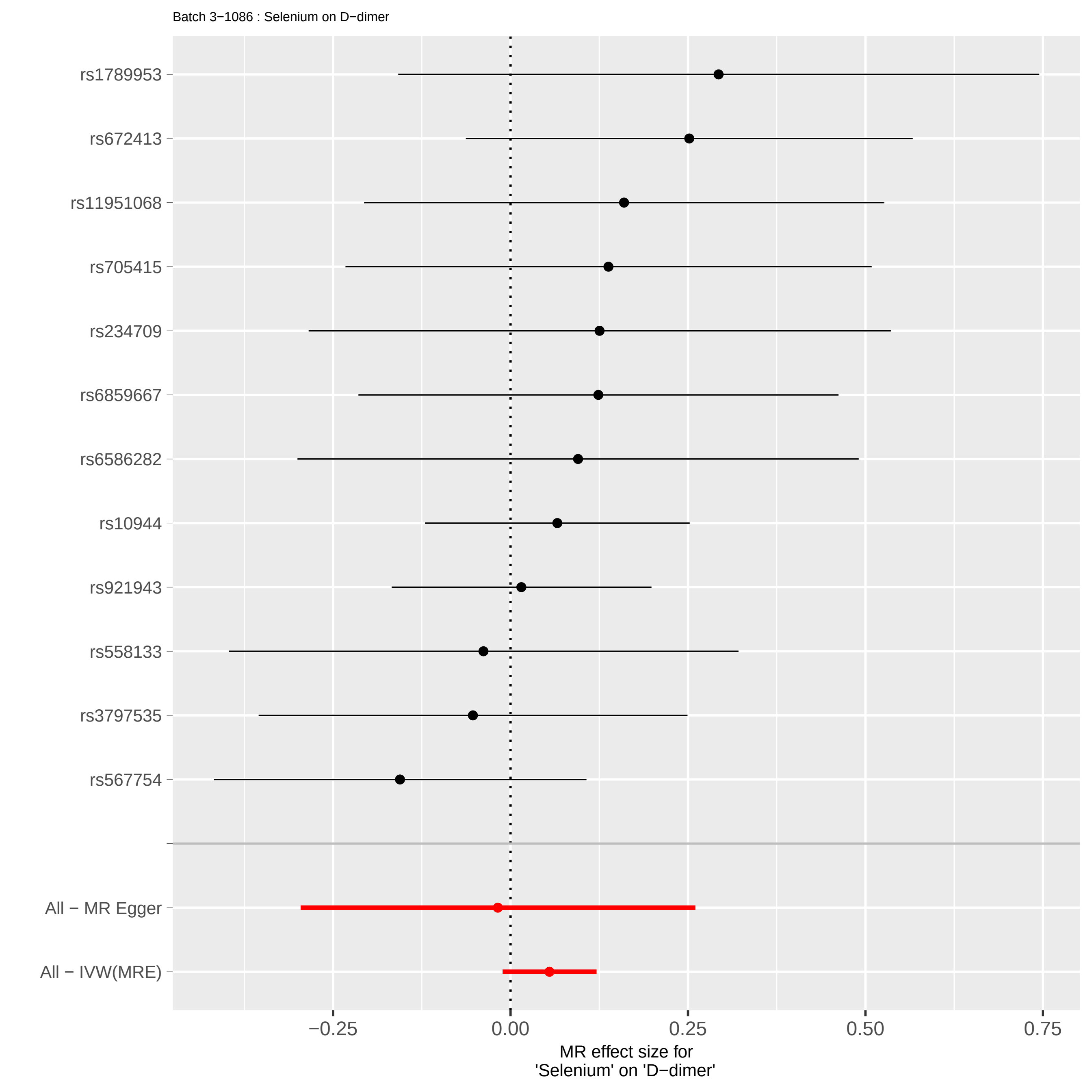

Batch 3−1086 : Selenium on D−dimer
rs1789953
rs672413
rs11951068
rs705415
rs234709
rs6859667
rs6586282
rs10944
rs921943
rs558133
rs3797535
rs567754
All − MR Egger
All − IVW(MRE)
−0.25
0.00
0.25
0.50
0.75
MR effect size for
'Selenium' on 'D−dimer'

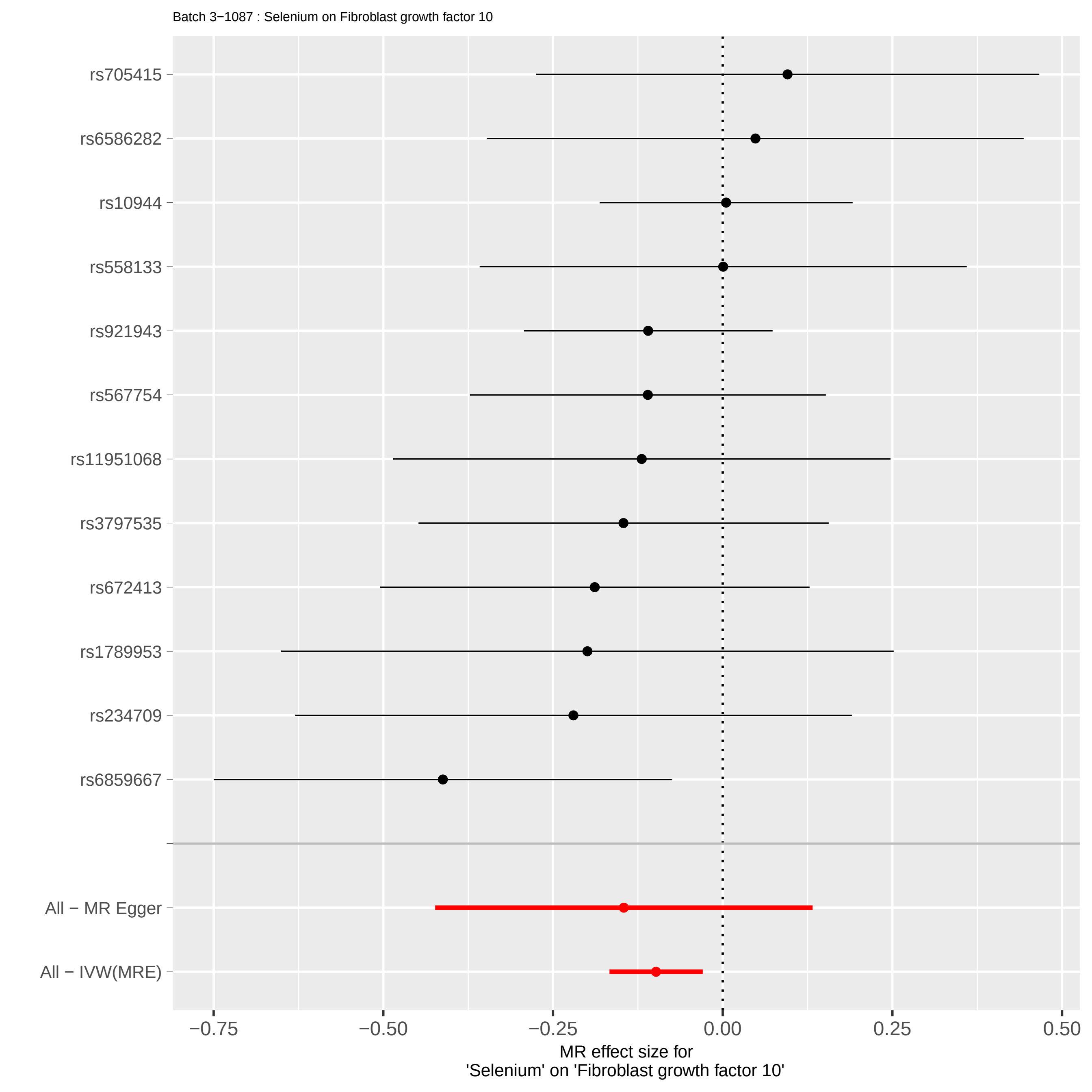

Batch 3−1087 : Selenium on Fibroblast growth factor 10
rs705415
rs6586282
rs10944
rs558133
rs921943
rs567754
rs11951068
rs3797535
rs672413
rs1789953
rs234709
rs6859667
All − MR Egger
All − IVW(MRE)
−0.75
−0.50
−0.25
0.00
0.25
0.50
MR effect size for
'Selenium' on 'Fibroblast growth factor 10'

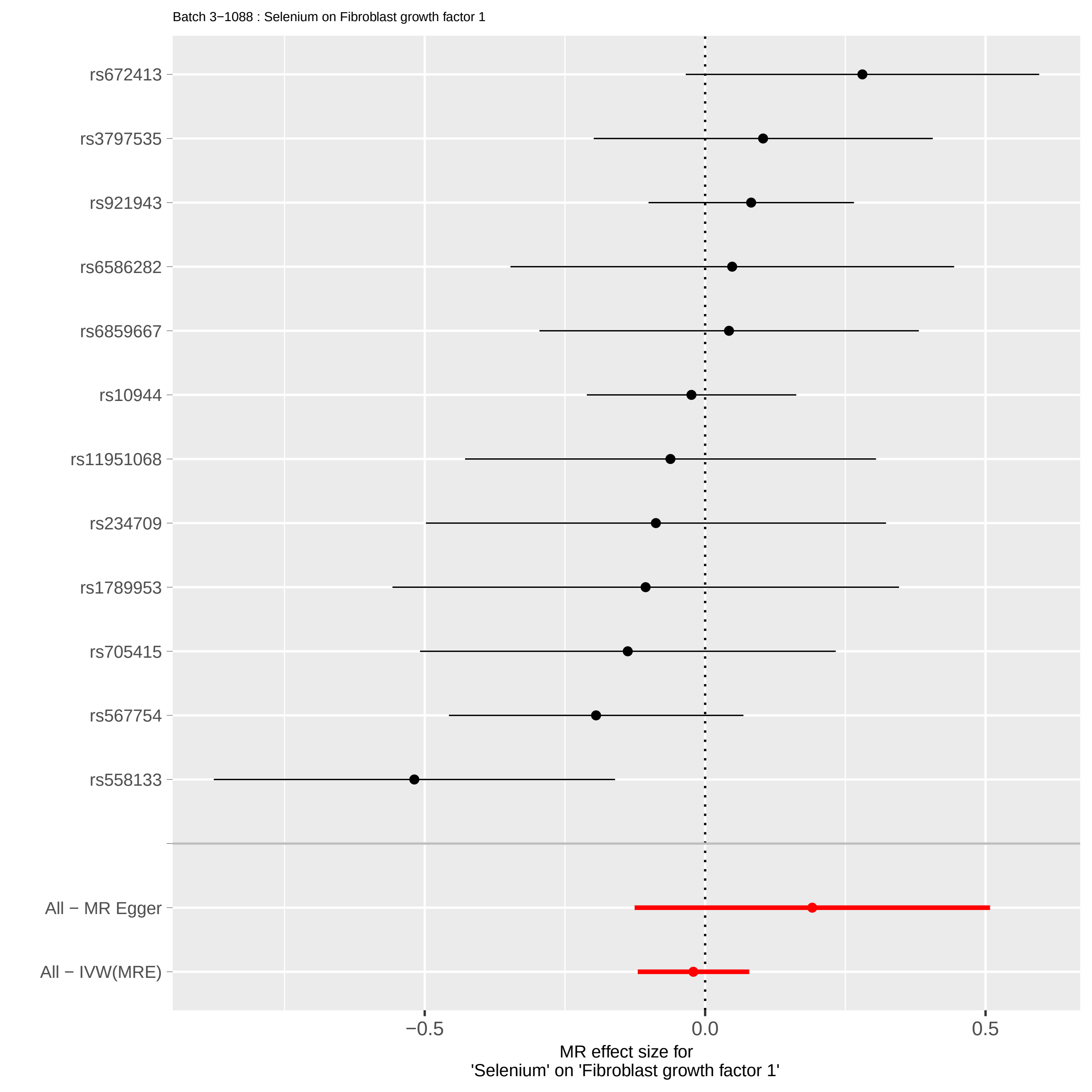
Batch 3−1088 : Selenium on Fibroblast growth factor 1
rs672413
rs3797535
rs921943
rs6586282
rs6859667
rs10944
rs11951068
rs234709
rs1789953
rs705415
rs567754
rs558133
All − MR Egger
All − IVW(MRE)
−0.5
0.0
0.5
MR effect size for
'Selenium' on 'Fibroblast growth factor 1'

Batch 3−1089 : Selenium on Fibroblast growth factor 16

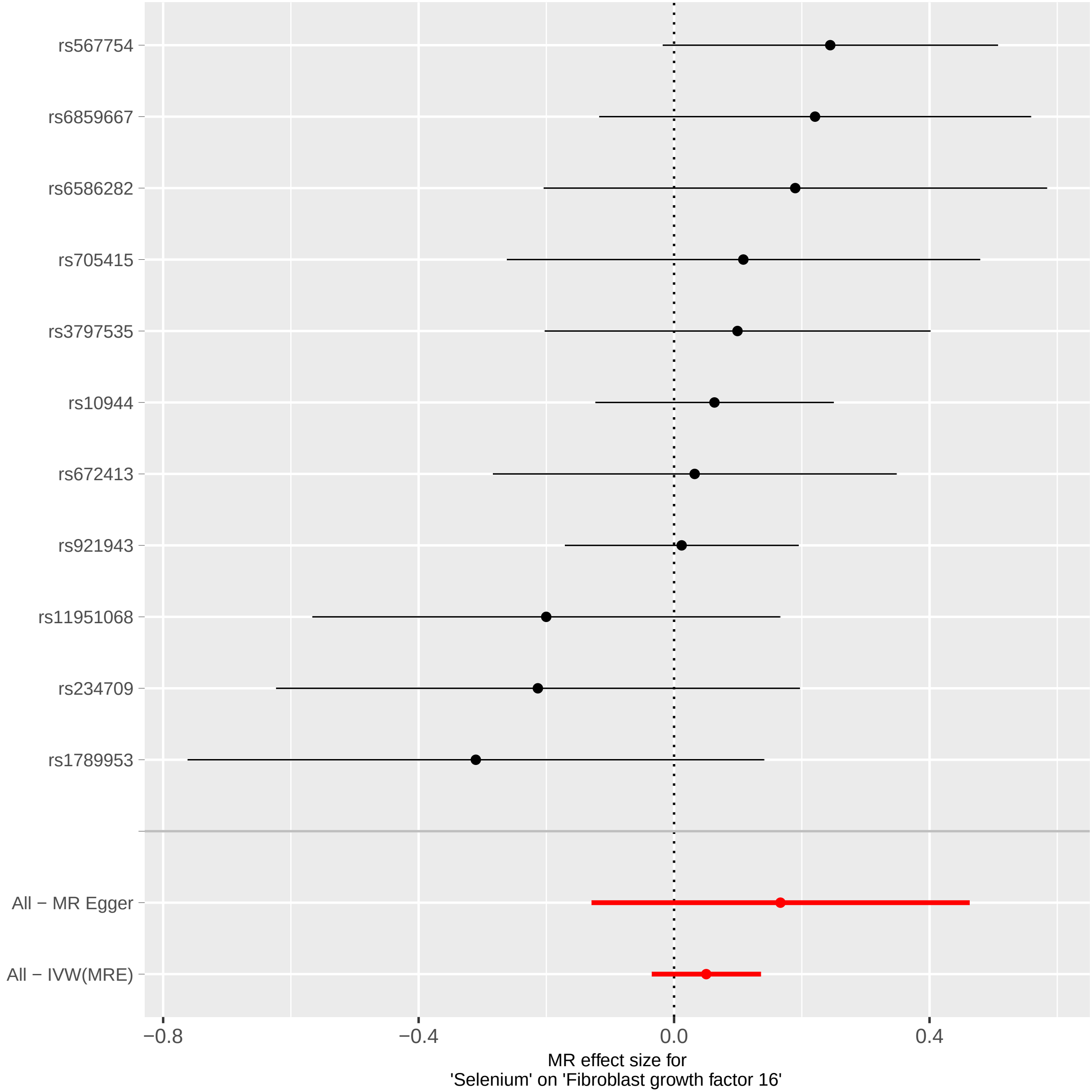

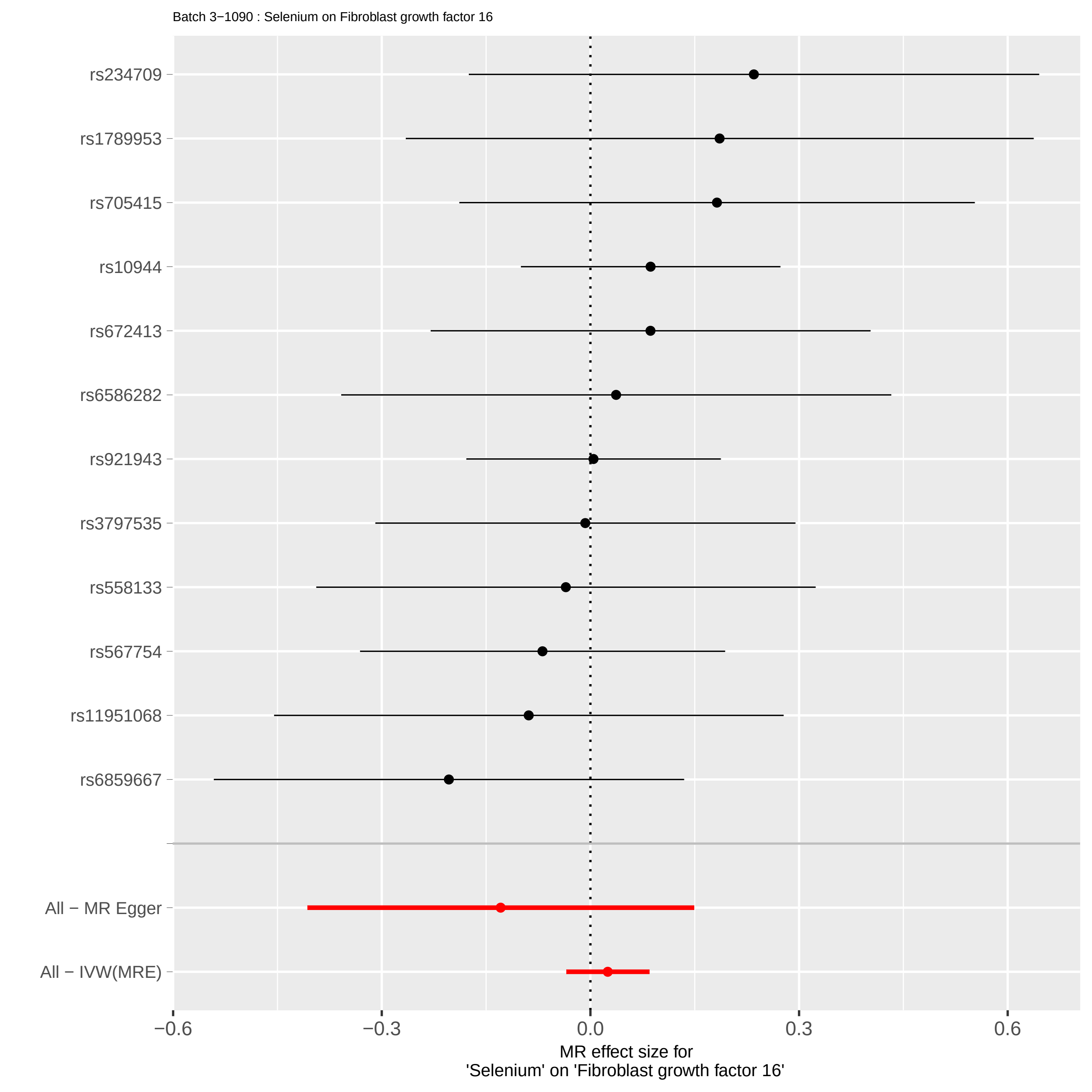
Batch 3−1090 : Selenium on Fibroblast growth factor 16
rs234709
rs1789953
rs705415
rs10944
rs672413
rs6586282
rs921943
rs3797535
rs558133
rs567754
rs11951068
rs6859667
All − MR Egger
All − IVW(MRE)
−0.6
−0.3
0.0
0.3
0.6
MR effect size for
'Selenium' on 'Fibroblast growth factor 16'

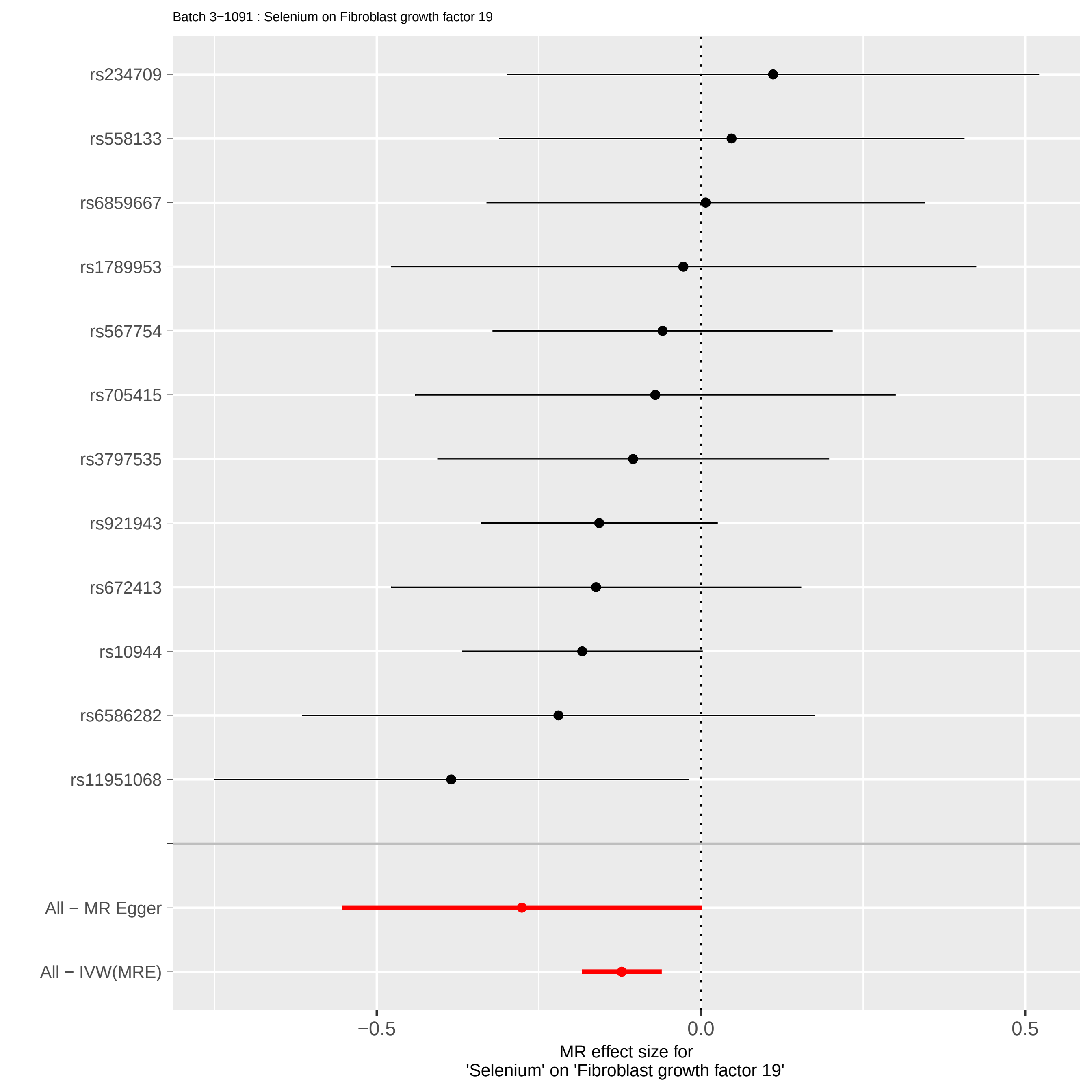

Batch 3−1091 : Selenium on Fibroblast growth factor 19
rs234709
rs558133
rs6859667
rs1789953
rs567754
rs705415
rs3797535
rs921943
rs672413
rs10944
rs6586282
rs11951068
All − MR Egger
All − IVW(MRE)
−0.5
0.0
0.5
MR effect size for
'Selenium' on 'Fibroblast growth factor 19'

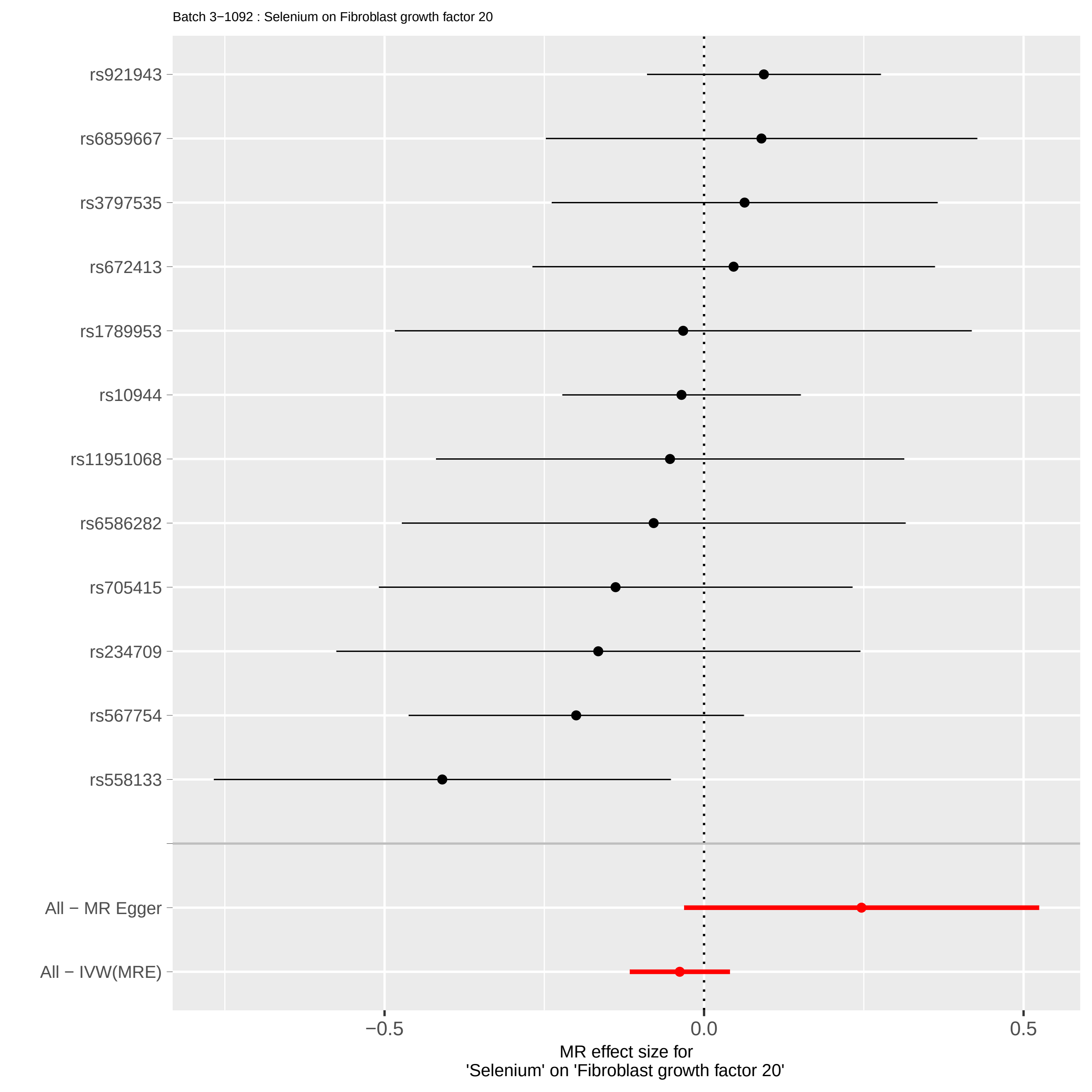
Batch 3−1092 : Selenium on Fibroblast growth factor 20
rs921943
rs6859667
rs3797535
rs672413
rs1789953
rs10944
rs11951068
rs6586282
rs705415
rs234709
rs567754
rs558133
All − MR Egger
All − IVW(MRE)
−0.5
0.0
0.5
MR effect size for
'Selenium' on 'Fibroblast growth factor 20'

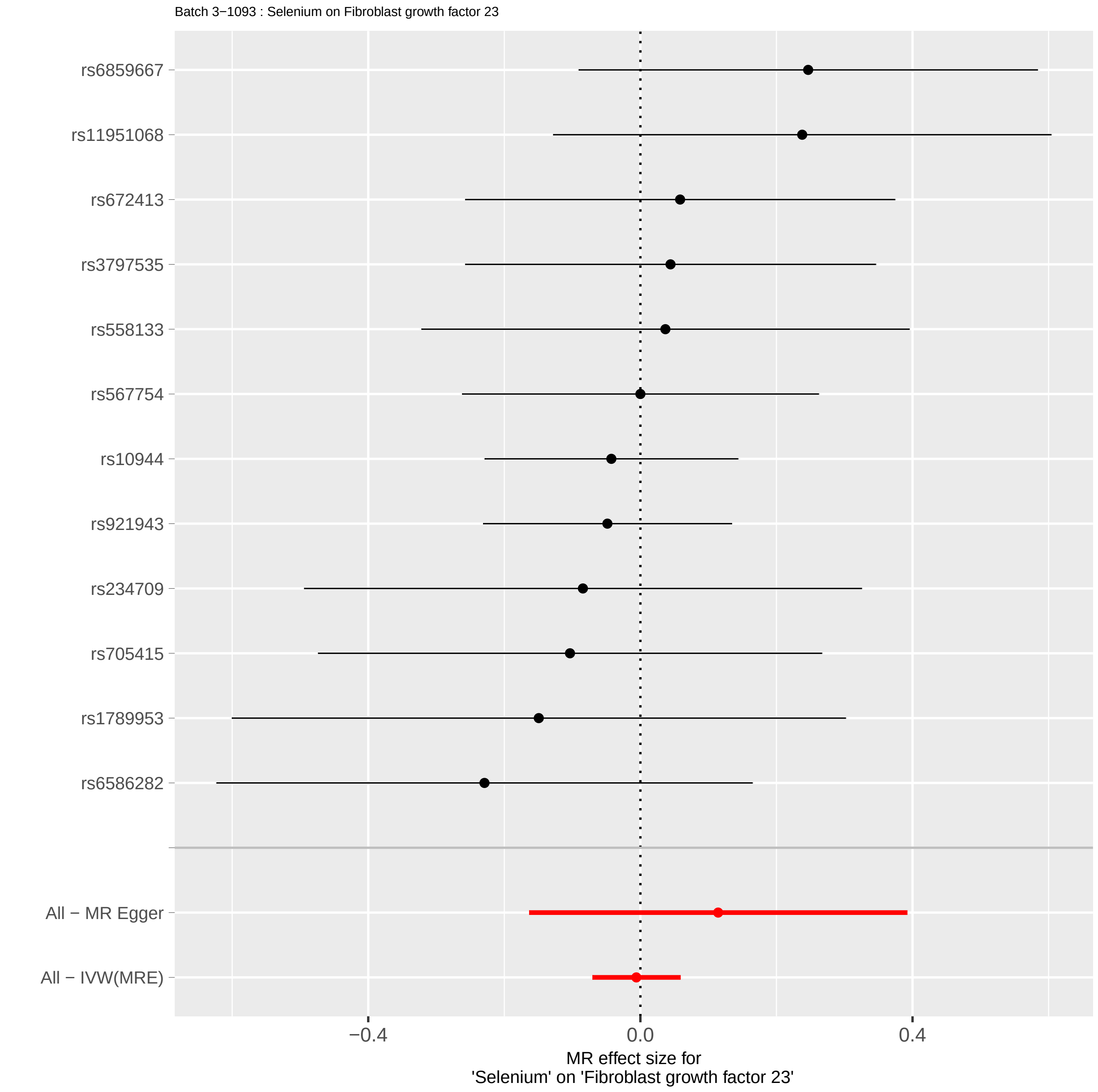

Batch 3−1093 : Selenium on Fibroblast growth factor 23
rs6859667
rs11951068
rs672413
rs3797535
rs558133
rs567754
rs10944
rs921943
rs234709
rs705415
rs1789953
rs6586282
All − MR Egger
All − IVW(MRE)
−0.4
0.0
0.4
MR effect size for
'Selenium' on 'Fibroblast growth factor 23'

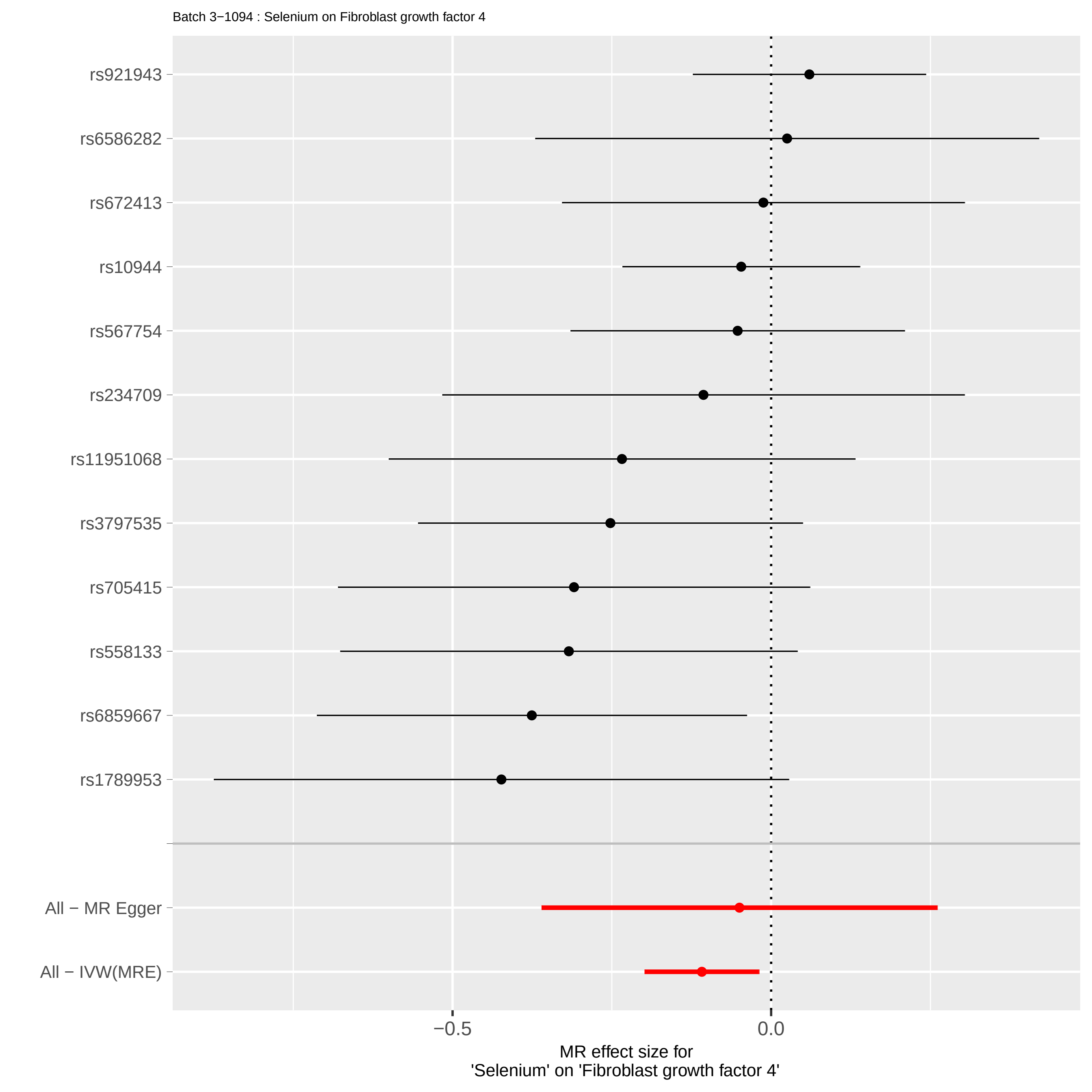

Batch 3−1094 : Selenium on Fibroblast growth factor 4
rs921943
rs6586282
rs672413
rs10944
rs567754
rs234709
rs11951068
rs3797535
rs705415
rs558133
rs6859667
rs1789953
All − MR Egger
All − IVW(MRE)
−0.5
0.0
MR effect size for
'Selenium' on 'Fibroblast growth factor 4'

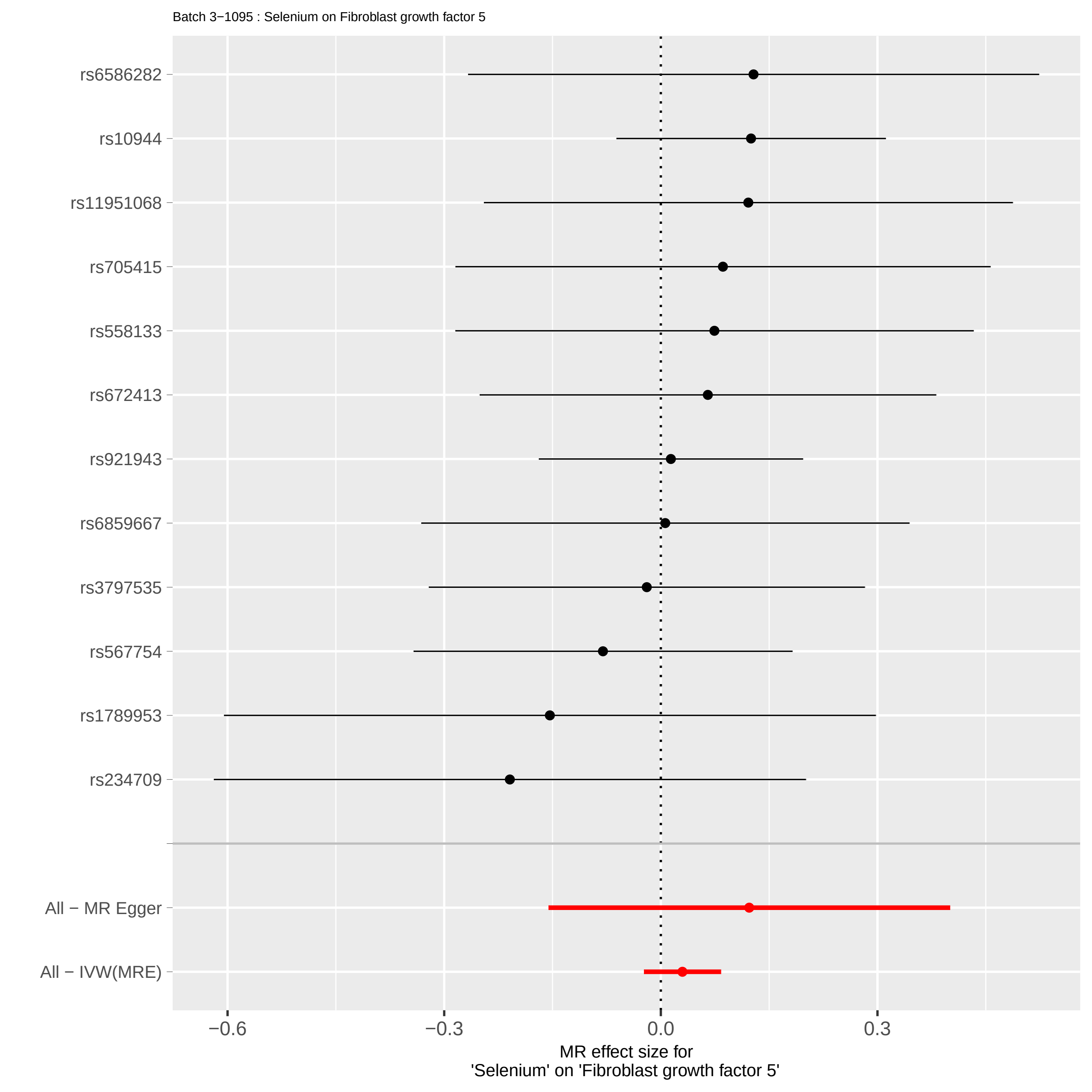

Batch 3−1095 : Selenium on Fibroblast growth factor 5
rs6586282
rs10944
rs11951068
rs705415
rs558133
rs672413
rs921943
rs6859667
rs3797535
rs567754
rs1789953
rs234709
All − MR Egger
All − IVW(MRE)
−0.6
−0.3
0.0
0.3
MR effect size for
'Selenium' on 'Fibroblast growth factor 5'

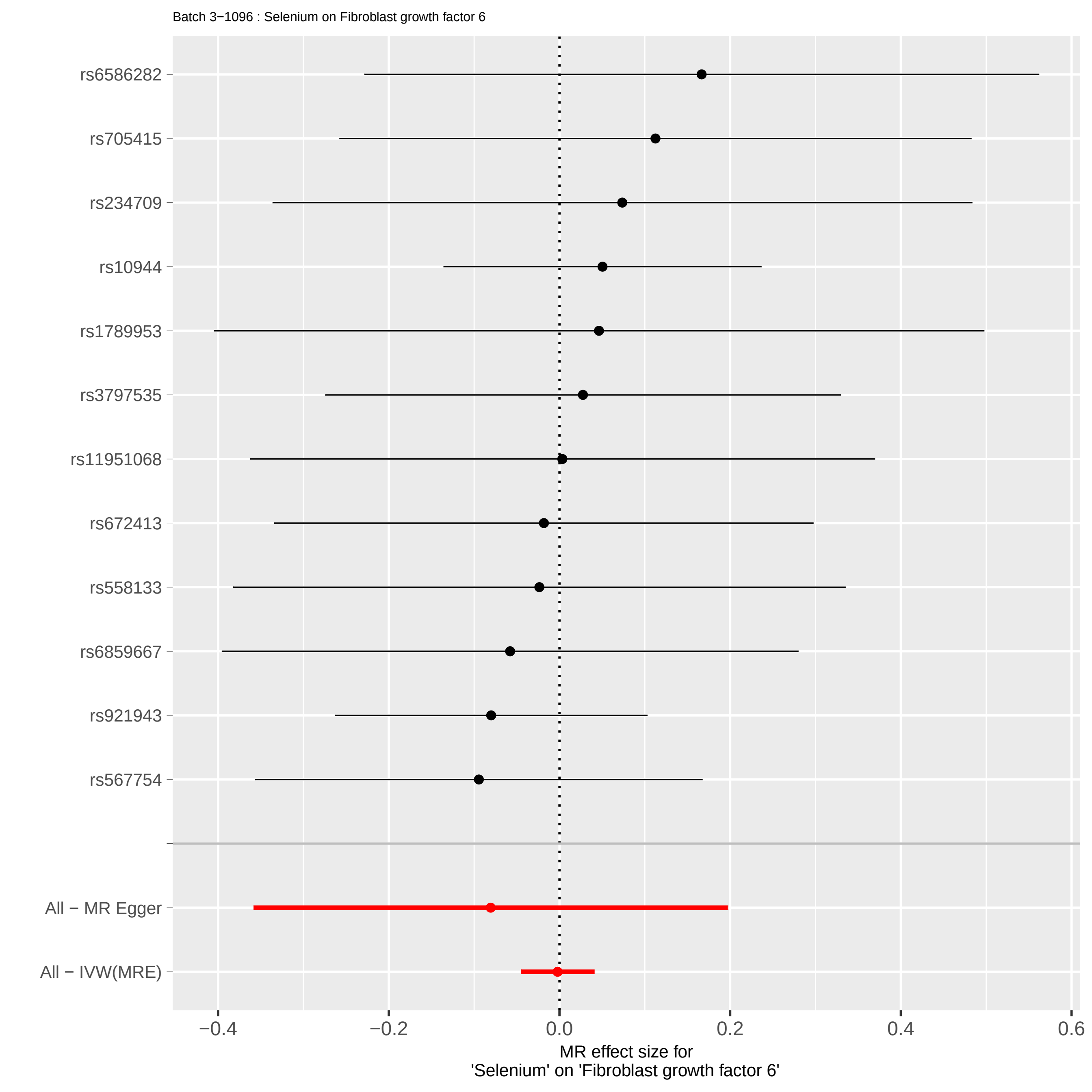
Batch 3−1096 : Selenium on Fibroblast growth factor 6
rs6586282
rs705415
rs234709
rs10944
rs1789953
rs3797535
rs11951068
rs672413
rs558133
rs6859667
rs921943
rs567754
All − MR Egger
All − IVW(MRE)
−0.4
−0.2
0.0
0.2
0.4
0.6
MR effect size for
'Selenium' on 'Fibroblast growth factor 6'

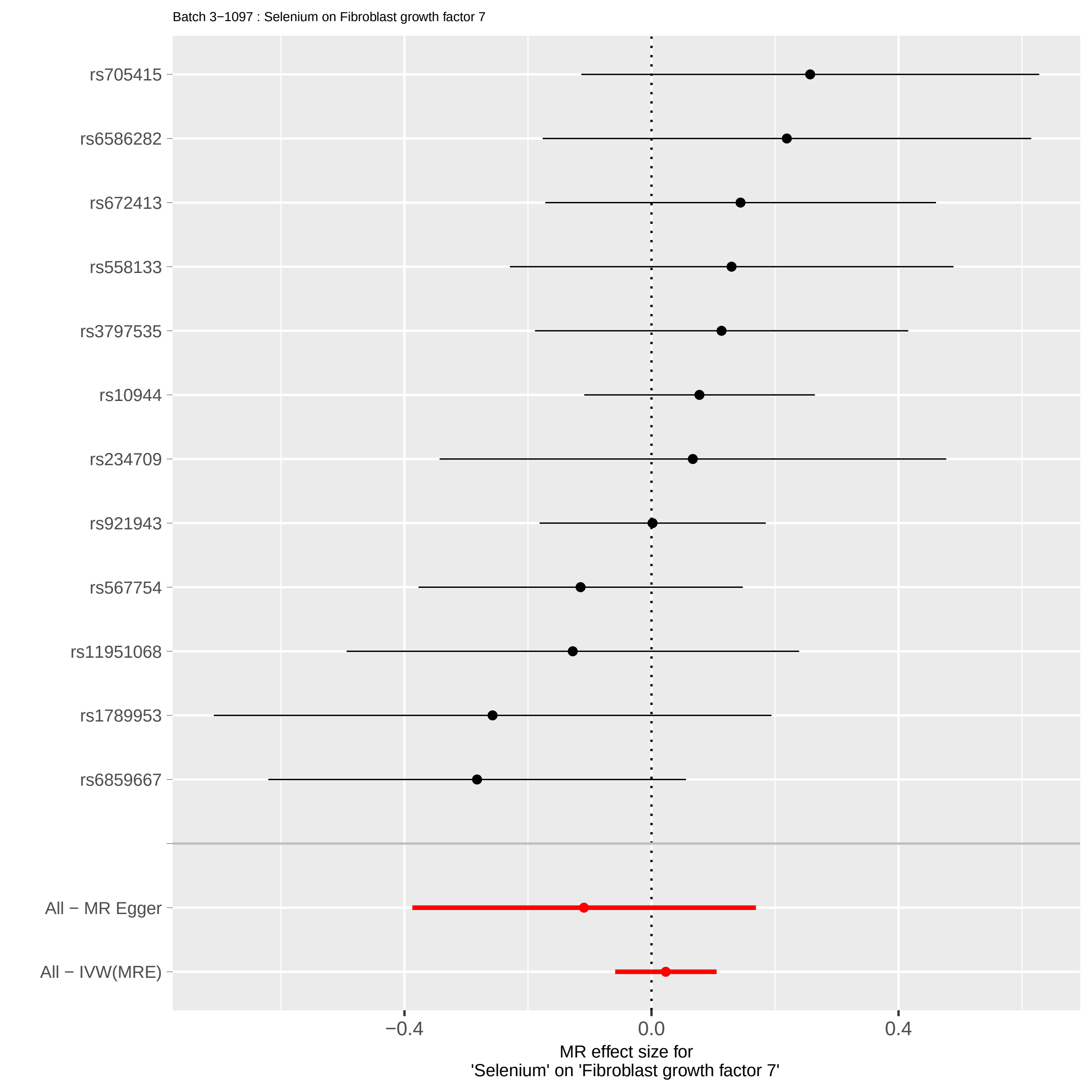
Batch 3−1097 : Selenium on Fibroblast growth factor 7
rs705415
rs6586282
rs672413
rs558133
rs3797535
rs10944
rs234709
rs921943
rs567754
rs11951068
rs1789953
rs6859667
All − MR Egger
All − IVW(MRE)
−0.4
0.0
0.4
MR effect size for
'Selenium' on 'Fibroblast growth factor 7'

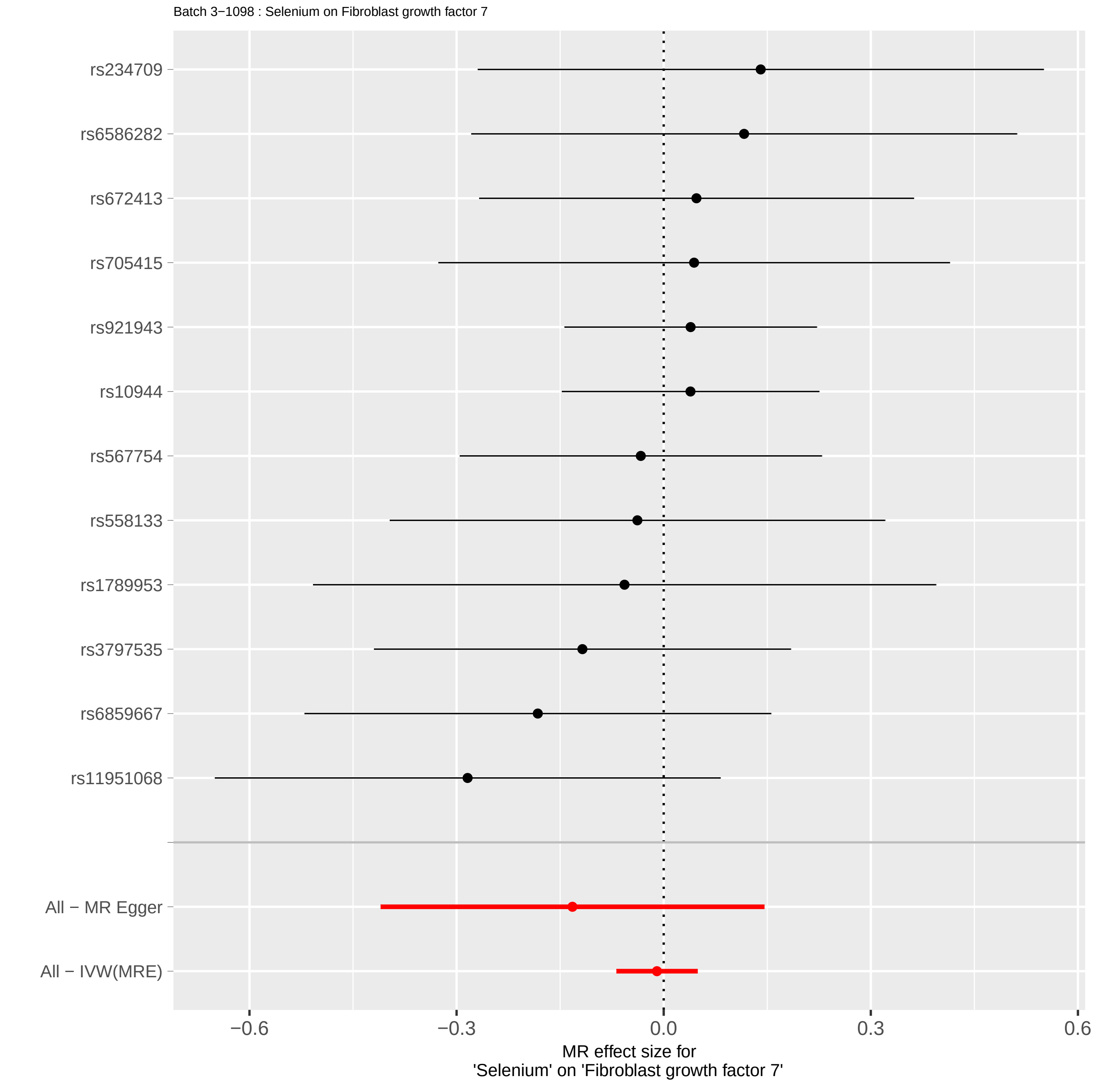

Batch 3−1098 : Selenium on Fibroblast growth factor 7
rs234709
rs6586282
rs672413
rs705415
rs921943
rs10944
rs567754
rs558133
rs1789953
rs3797535
rs6859667
rs11951068
All − MR Egger
All − IVW(MRE)
−0.6
−0.3
0.0
0.3
0.6
MR effect size for
'Selenium' on 'Fibroblast growth factor 7'

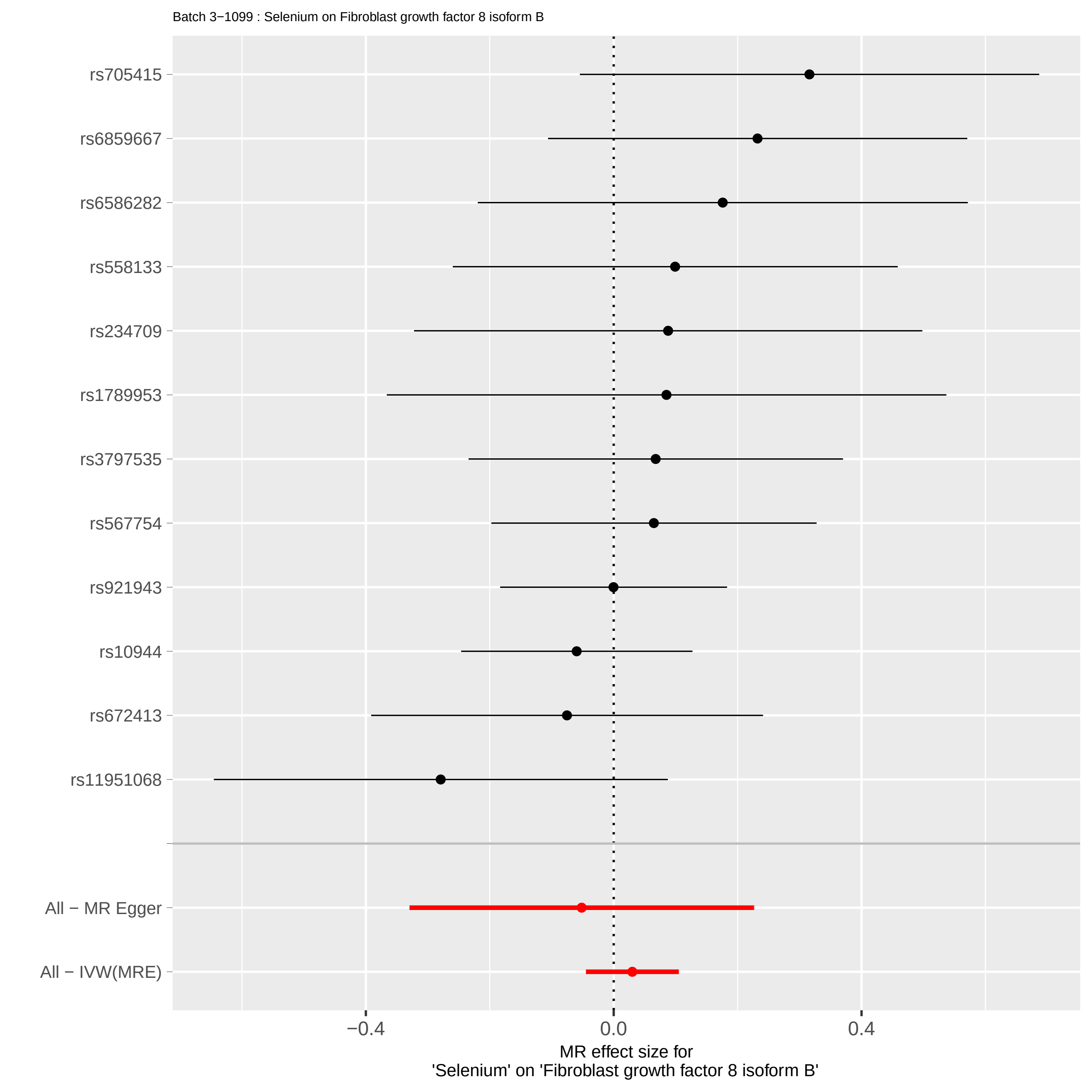

Batch 3−1099 : Selenium on Fibroblast growth factor 8 isoform B
rs705415
rs6859667
rs6586282
rs558133
rs234709
rs1789953
rs3797535
rs567754
rs921943
rs10944
rs672413
rs11951068
All − MR Egger
All − IVW(MRE)
−0.4
0.0
0.4
MR effect size for
'Selenium' on 'Fibroblast growth factor 8 isoform B'

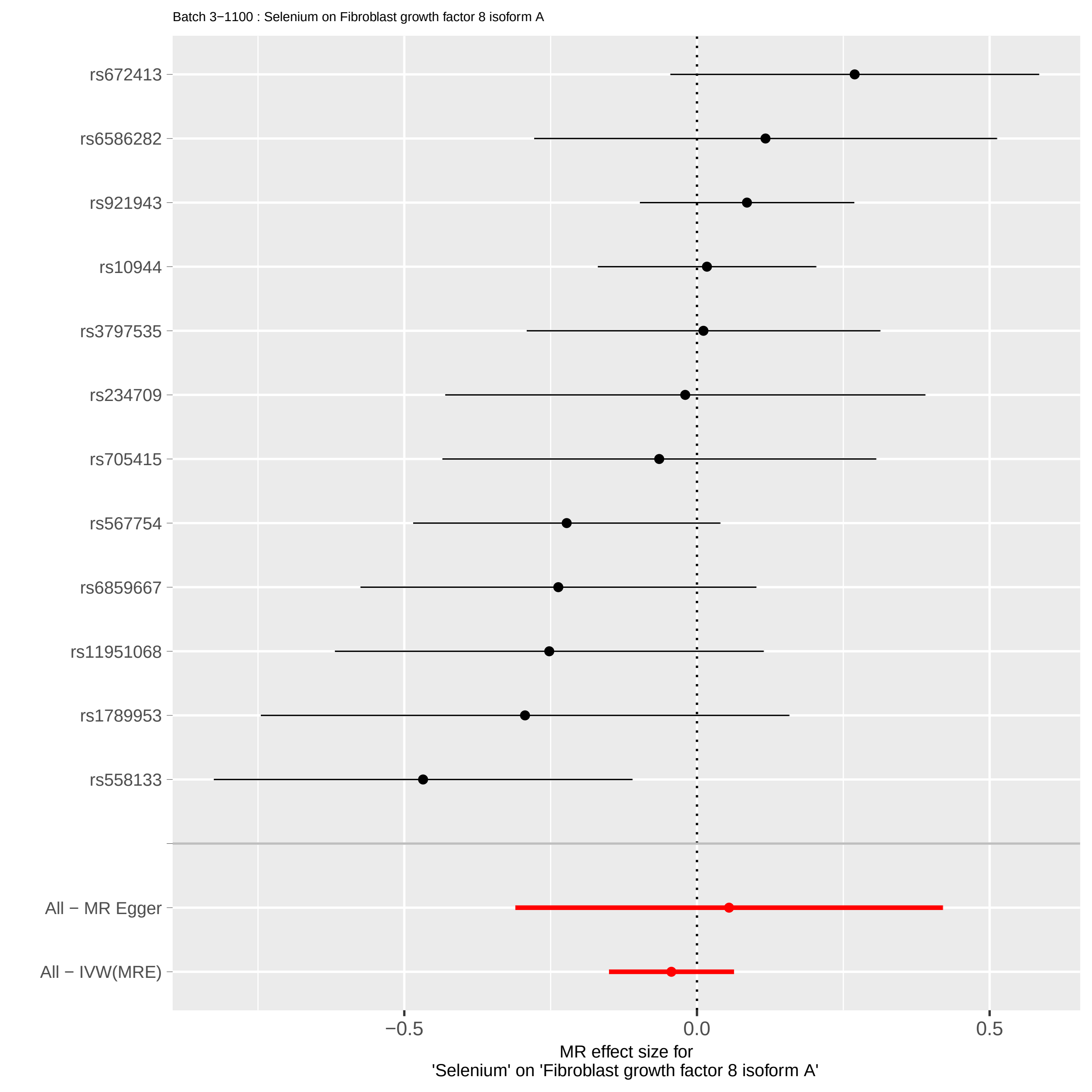
Batch 3−1100 : Selenium on Fibroblast growth factor 8 isoform A
rs672413
rs6586282
rs921943
rs10944
rs3797535
rs234709
rs705415
rs567754
rs6859667
rs11951068
rs1789953
rs558133
All − MR Egger
All − IVW(MRE)
−0.5
0.0
0.5
MR effect size for
'Selenium' on 'Fibroblast growth factor 8 isoform A'

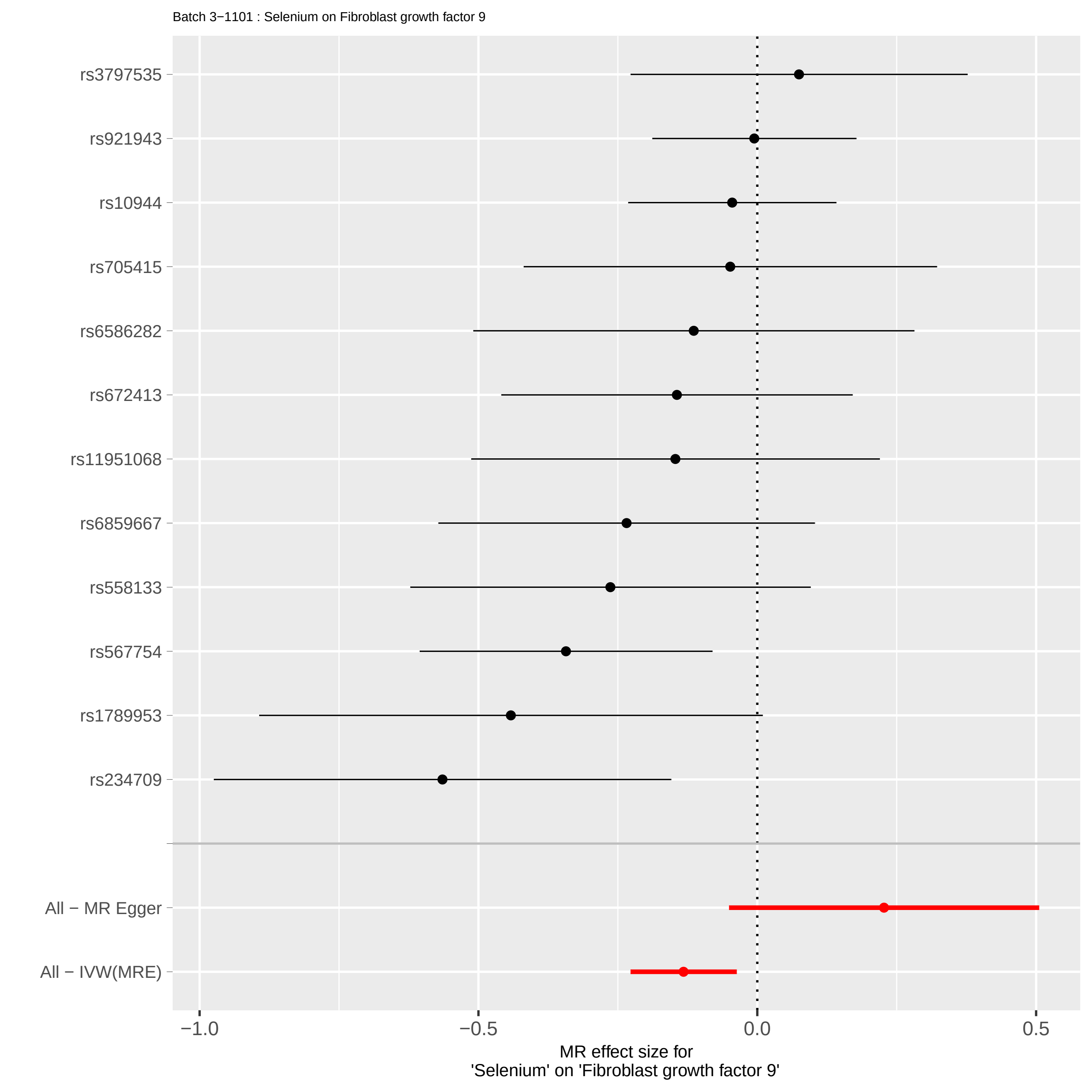

Batch 3−1101 : Selenium on Fibroblast growth factor 9
rs3797535
rs921943
rs10944
rs705415
rs6586282
rs672413
rs11951068
rs6859667
rs558133
rs567754
rs1789953
rs234709
All − MR Egger
All − IVW(MRE)
−1.0
−0.5
0.0
0.5
MR effect size for
'Selenium' on 'Fibroblast growth factor 9'

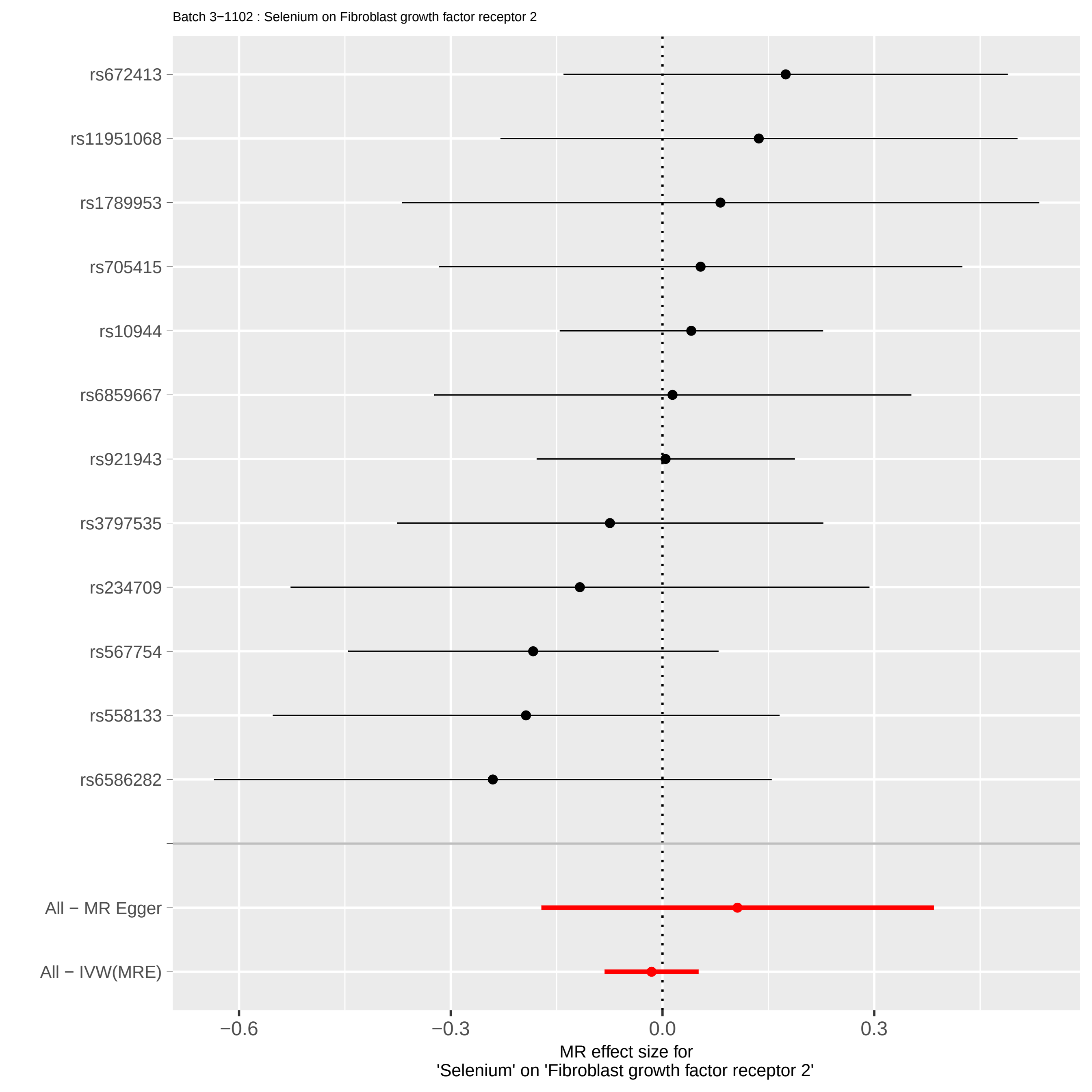

Batch 3−1102 : Selenium on Fibroblast growth factor receptor 2
rs672413
rs11951068
rs1789953
rs705415
rs10944
rs6859667
rs921943
rs3797535
rs234709
rs567754
rs558133
rs6586282
All − MR Egger
All − IVW(MRE)
−0.6
−0.3
0.0
0.3
MR effect size for
'Selenium' on 'Fibroblast growth factor receptor 2'

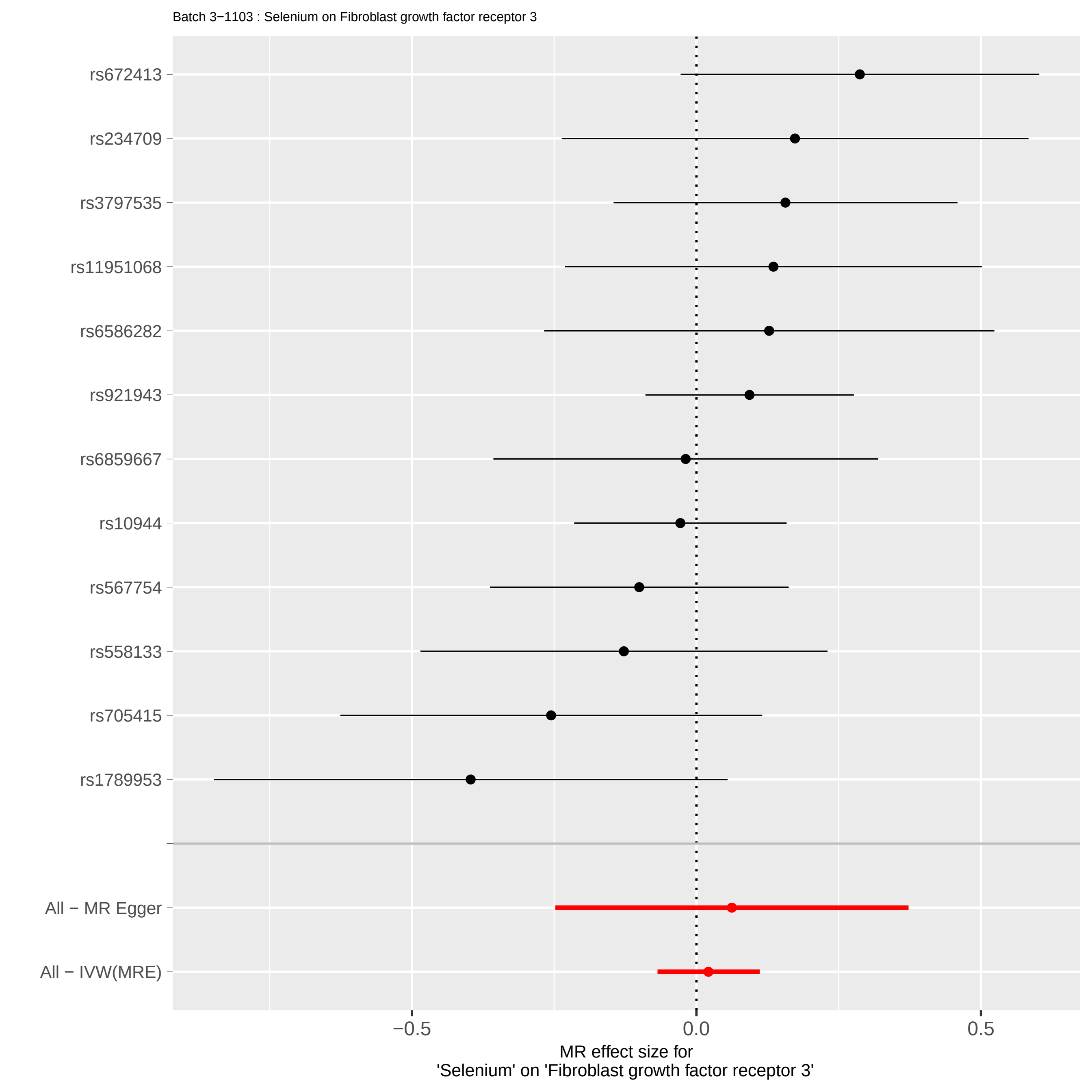
Batch 3−1103 : Selenium on Fibroblast growth factor receptor 3
rs672413
rs234709
rs3797535
rs11951068
rs6586282
rs921943
rs6859667
rs10944
rs567754
rs558133
rs705415
rs1789953
All − MR Egger
All − IVW(MRE)
−0.5
0.0
0.5
MR effect size for
'Selenium' on 'Fibroblast growth factor receptor 3'

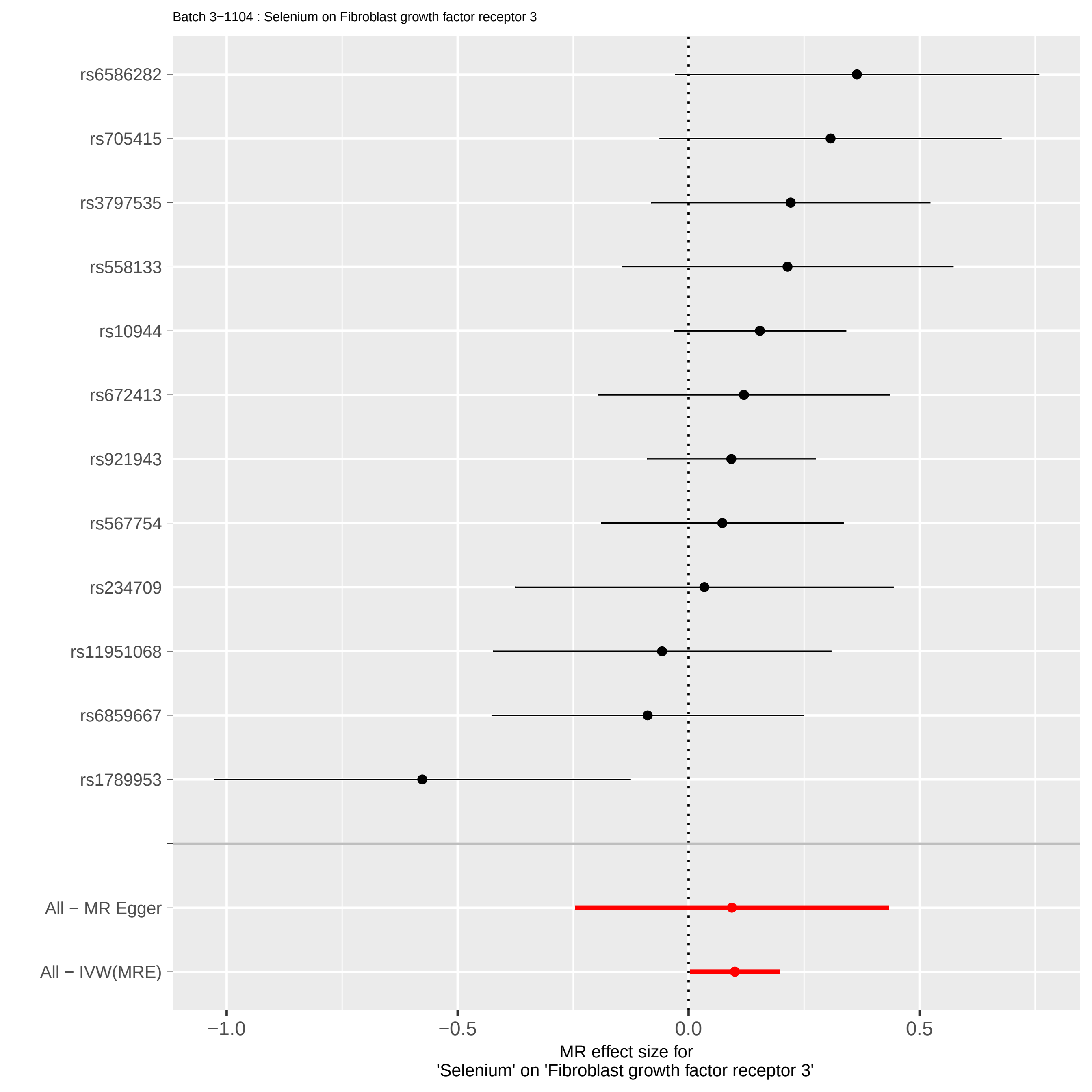

Batch 3−1104 : Selenium on Fibroblast growth factor receptor 3
rs6586282
rs705415
rs3797535
rs558133
rs10944
rs672413
rs921943
rs567754
rs234709
rs11951068
rs6859667
rs1789953
All − MR Egger
All − IVW(MRE)
−1.0
−0.5
0.0
0.5
MR effect size for
'Selenium' on 'Fibroblast growth factor receptor 3'

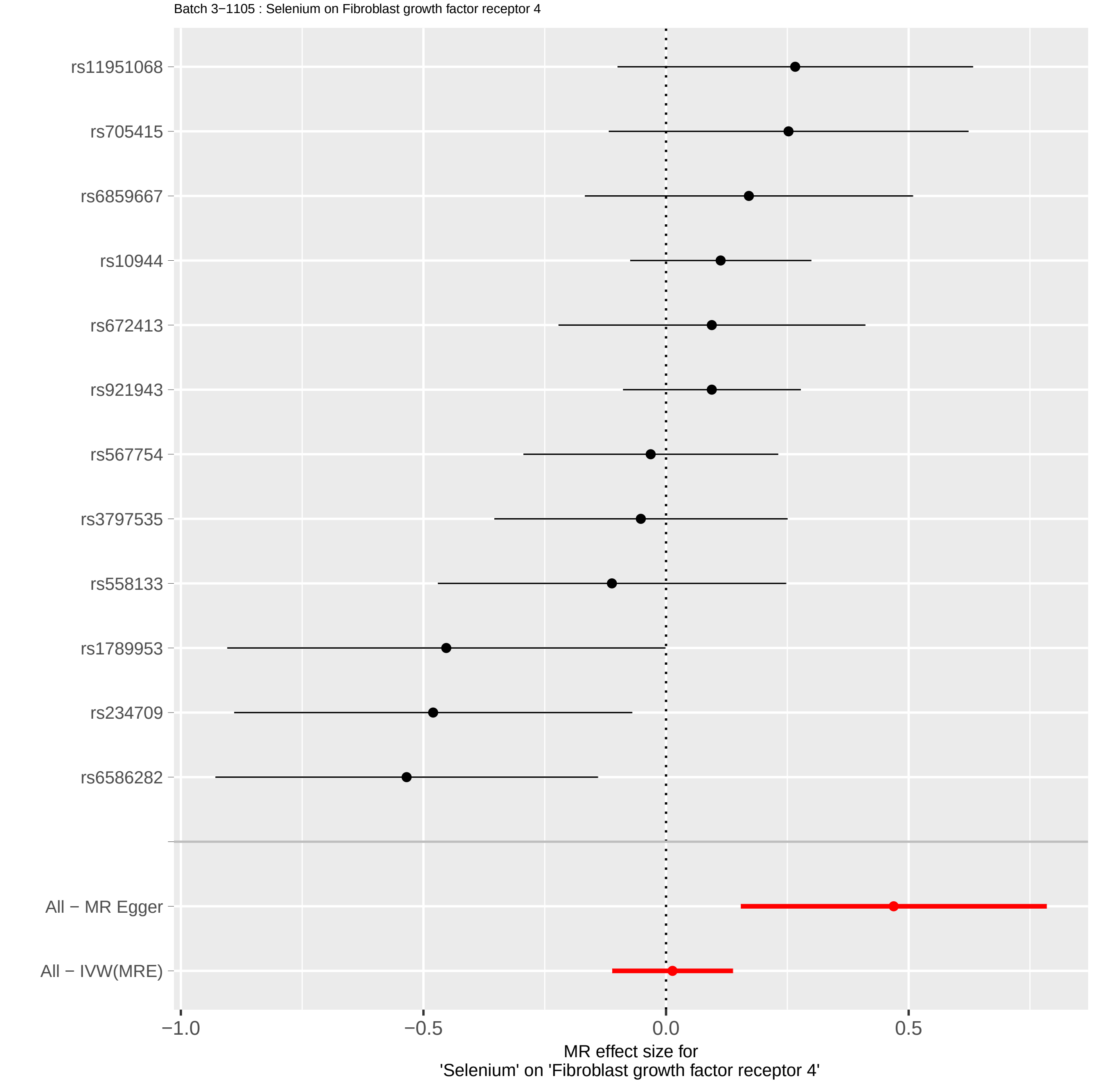

Batch 3−1105 : Selenium on Fibroblast growth factor receptor 4
rs11951068
rs705415
rs6859667
rs10944
rs672413
rs921943
rs567754
rs3797535
rs558133
rs1789953
rs234709
rs6586282
All − MR Egger
All − IVW(MRE)
−1.0
−0.5
0.0
0.5
MR effect size for
'Selenium' on 'Fibroblast growth factor receptor 4'

Batch 3−1106 : Selenium on Tyrosine−protein kinase Fgr

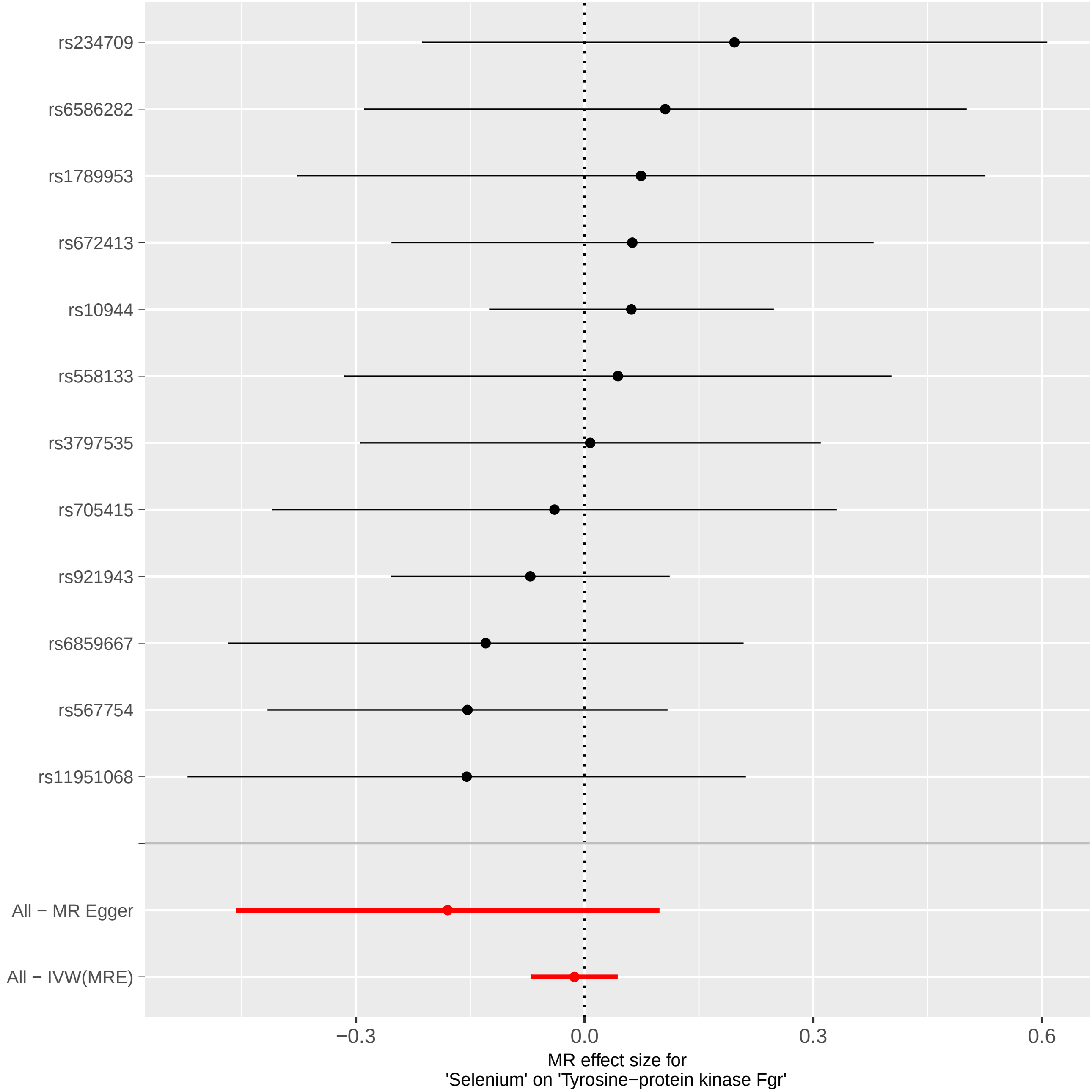

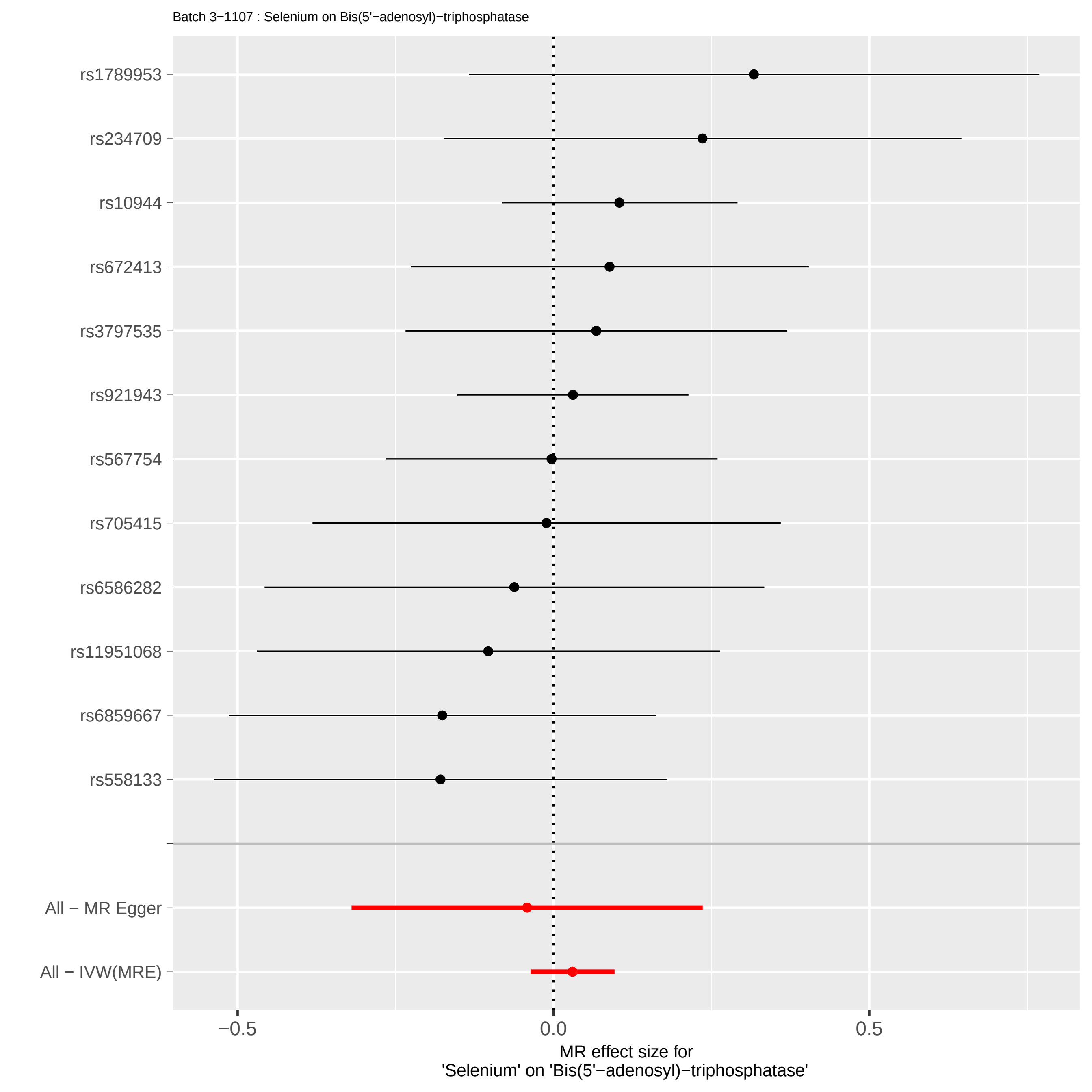

Batch 3−1107 : Selenium on Bis(5'−adenosyl)−triphosphatase
rs1789953
rs234709
rs10944
rs672413
rs3797535
rs921943
rs567754
rs705415
rs6586282
rs11951068
rs6859667
rs558133
All − MR Egger
All − IVW(MRE)
−0.5
0.0
0.5
MR effect size for
'Selenium' on 'Bis(5'−adenosyl)−triphosphatase'

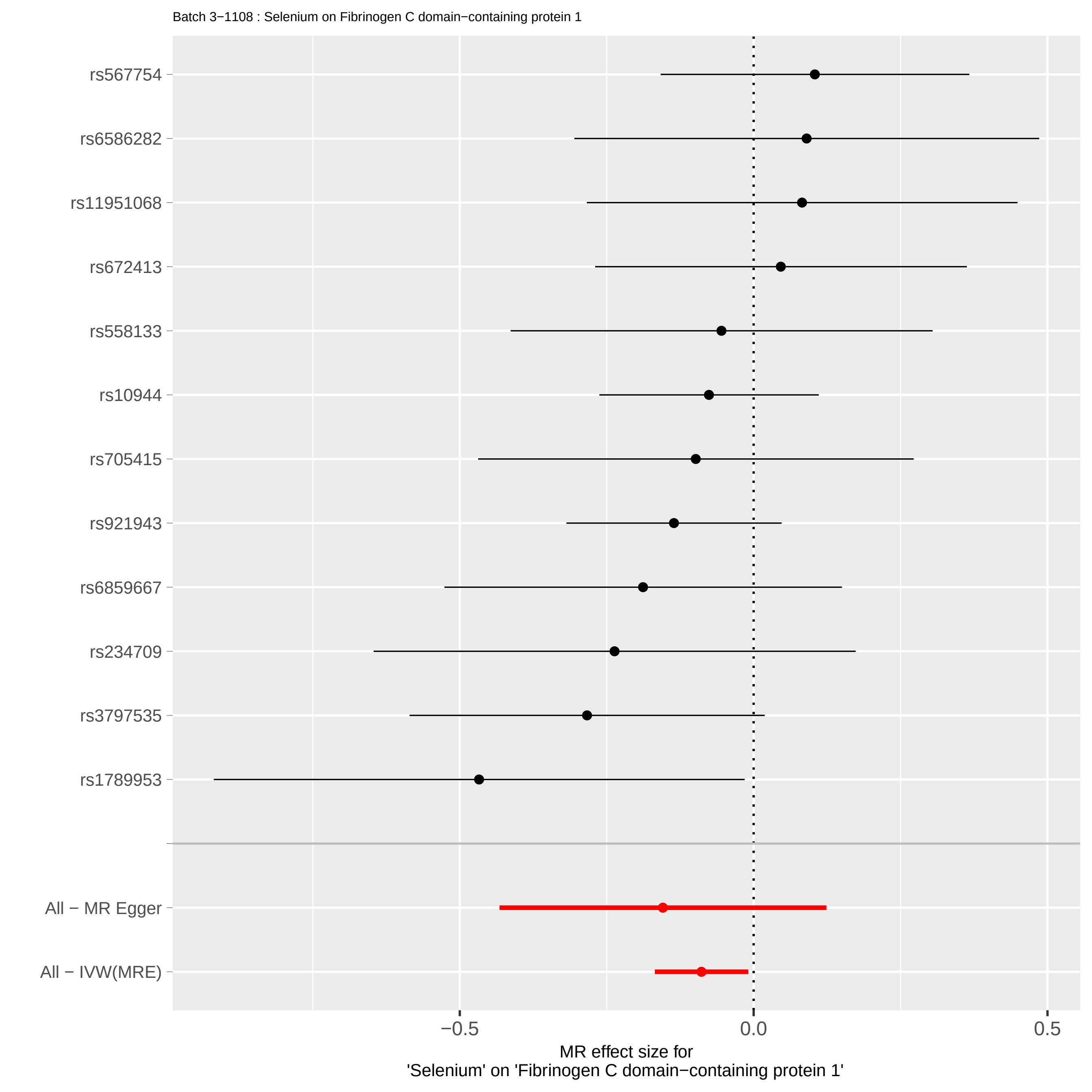

Batch 3−1108 : Selenium on Fibrinogen C domain−containing protein 1
rs567754
rs6586282
rs11951068
rs672413
rs558133
rs10944
rs705415
rs921943
rs6859667
rs234709
rs3797535
rs1789953
All − MR Egger
All − IVW(MRE)
−0.5
0.0
0.5
MR effect size for
'Selenium' on 'Fibrinogen C domain−containing protein 1'

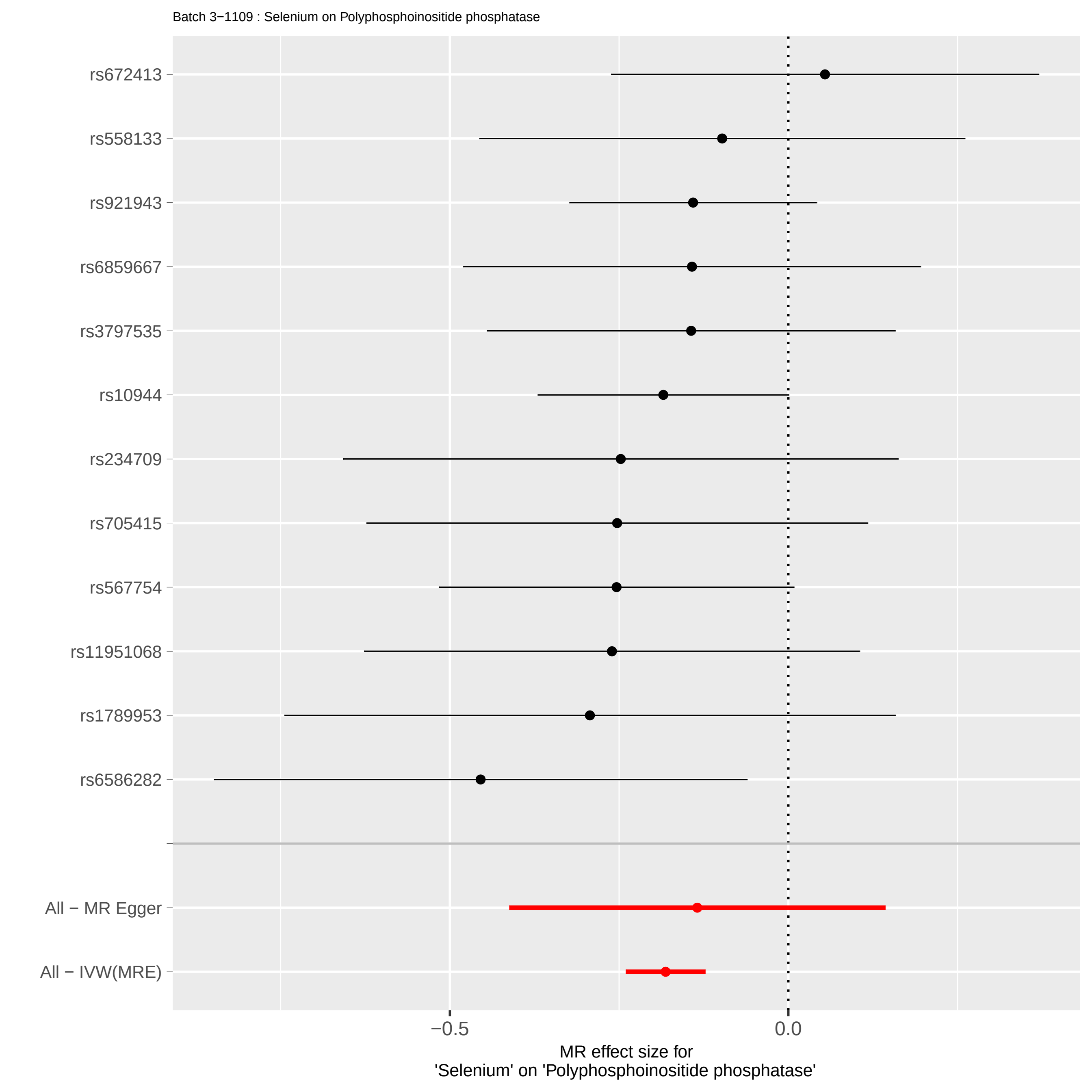

Batch 3−1109 : Selenium on Polyphosphoinositide phosphatase
rs672413
rs558133
rs921943
rs6859667
rs3797535
rs10944
rs234709
rs705415
rs567754
rs11951068
rs1789953
rs6586282
All − MR Egger
All − IVW(MRE)
−0.5
0.0
MR effect size for
'Selenium' on 'Polyphosphoinositide phosphatase'

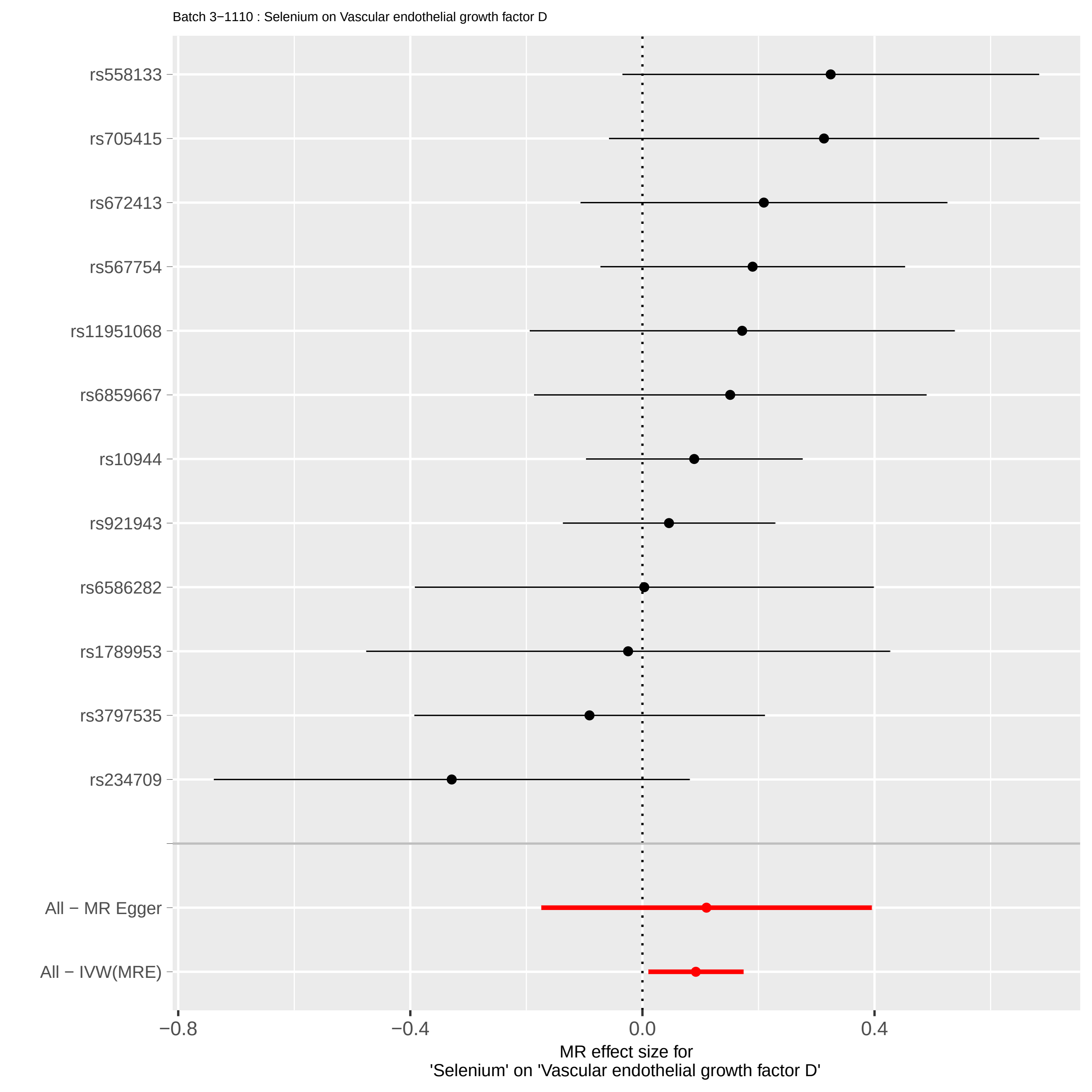
Batch 3−1110 : Selenium on Vascular endothelial growth factor D
rs558133
rs705415
rs672413
rs567754
rs11951068
rs6859667
rs10944
rs921943
rs6586282
rs1789953
rs3797535
rs234709
All − MR Egger
All − IVW(MRE)
−0.8
−0.4
0.0
0.4
MR effect size for
'Selenium' on 'Vascular endothelial growth factor D'

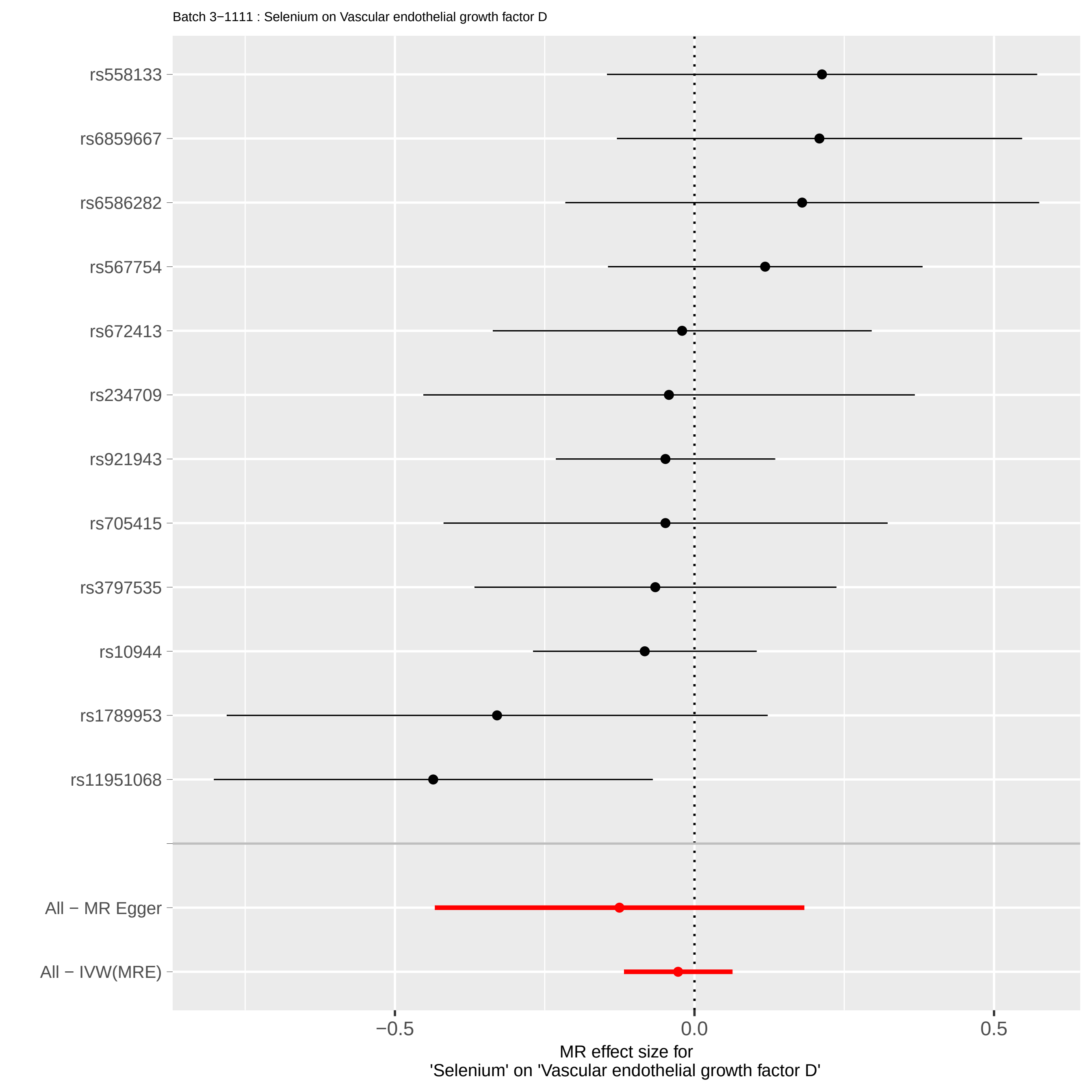

Batch 3−1111 : Selenium on Vascular endothelial growth factor D
rs558133
rs6859667
rs6586282
rs567754
rs672413
rs234709
rs921943
rs705415
rs3797535
rs10944
rs1789953
rs11951068
All − MR Egger
All − IVW(MRE)
−0.5
0.0
0.5
MR effect size for
'Selenium' on 'Vascular endothelial growth factor D'

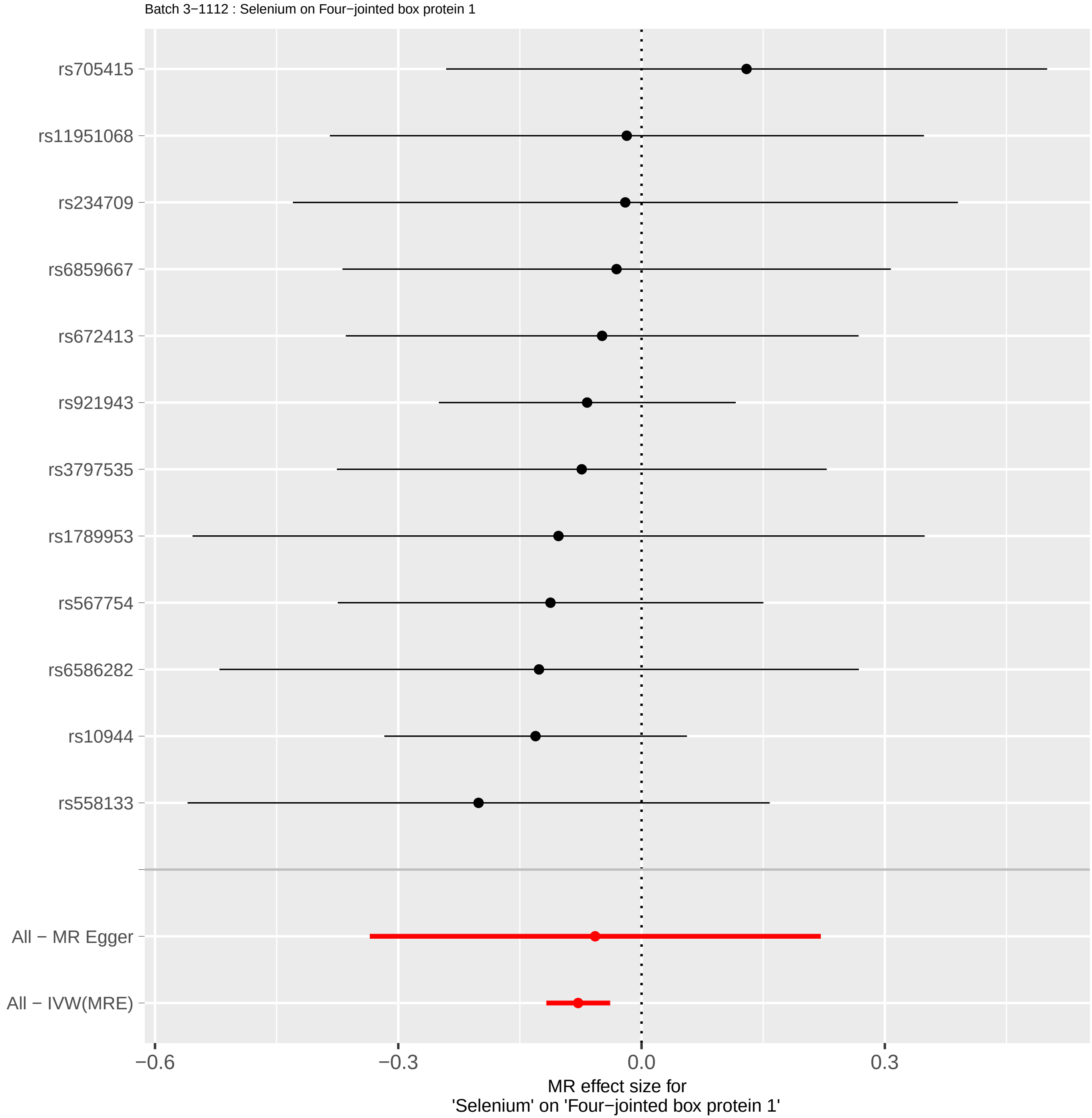

Batch 3−1112 : Selenium on Four−jointed box protein 1
rs705415
rs11951068
rs234709
rs6859667
rs672413
rs921943
rs3797535
rs1789953
rs567754
rs6586282
rs10944
rs558133
All − MR Egger
All − IVW(MRE)
−0.6
−0.3
0.0
0.3
MR effect size for
'Selenium' on 'Four−jointed box protein 1'

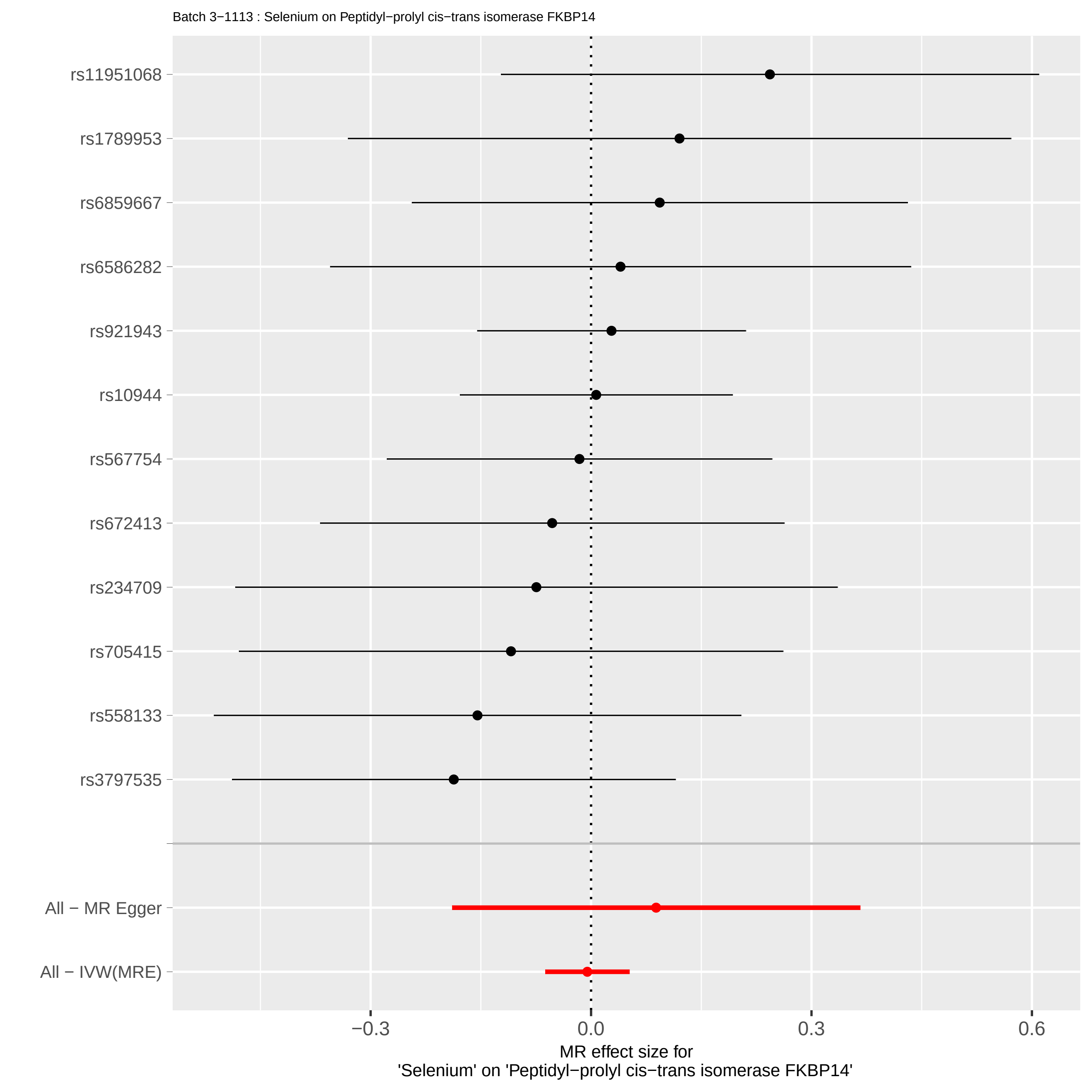
Batch 3−1113 : Selenium on Peptidyl−prolyl cis−trans isomerase FKBP14
rs11951068
rs1789953
rs6859667
rs6586282
rs921943
rs10944
rs567754
rs672413
rs234709
rs705415
rs558133
rs3797535
All − MR Egger
All − IVW(MRE)
−0.3
0.0
0.3
0.6
MR effect size for
'Selenium' on 'Peptidyl−prolyl cis−trans isomerase FKBP14'

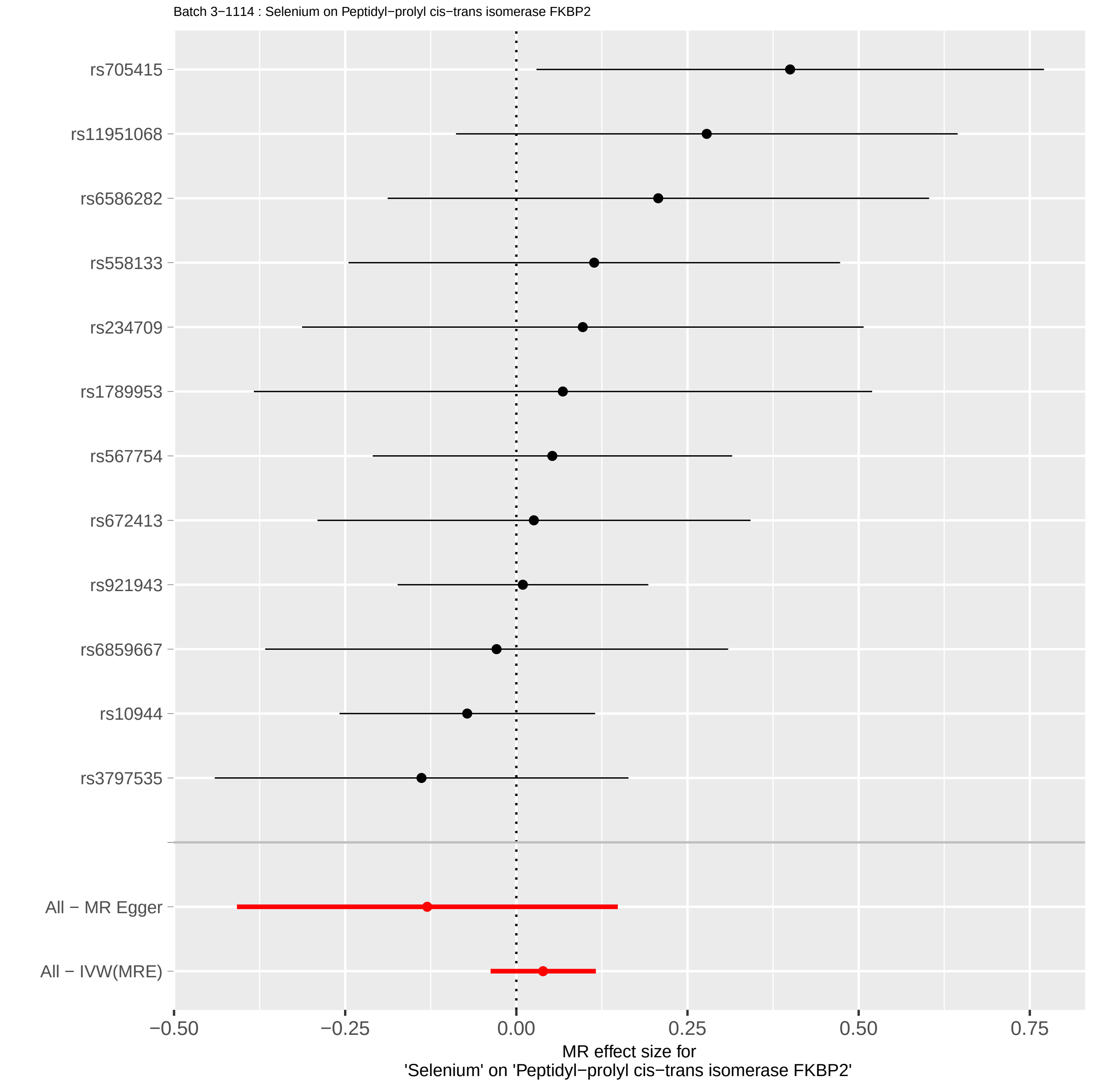

Batch 3−1114 : Selenium on Peptidyl−prolyl cis−trans isomerase FKBP2
rs705415
rs11951068
rs6586282
rs558133
rs234709
rs1789953
rs567754
rs672413
rs921943
rs6859667
rs10944
rs3797535
All − MR Egger
All − IVW(MRE)
−0.50
−0.25
0.00
0.25
0.50
0.75
MR effect size for
'Selenium' on 'Peptidyl−prolyl cis−trans isomerase FKBP2'

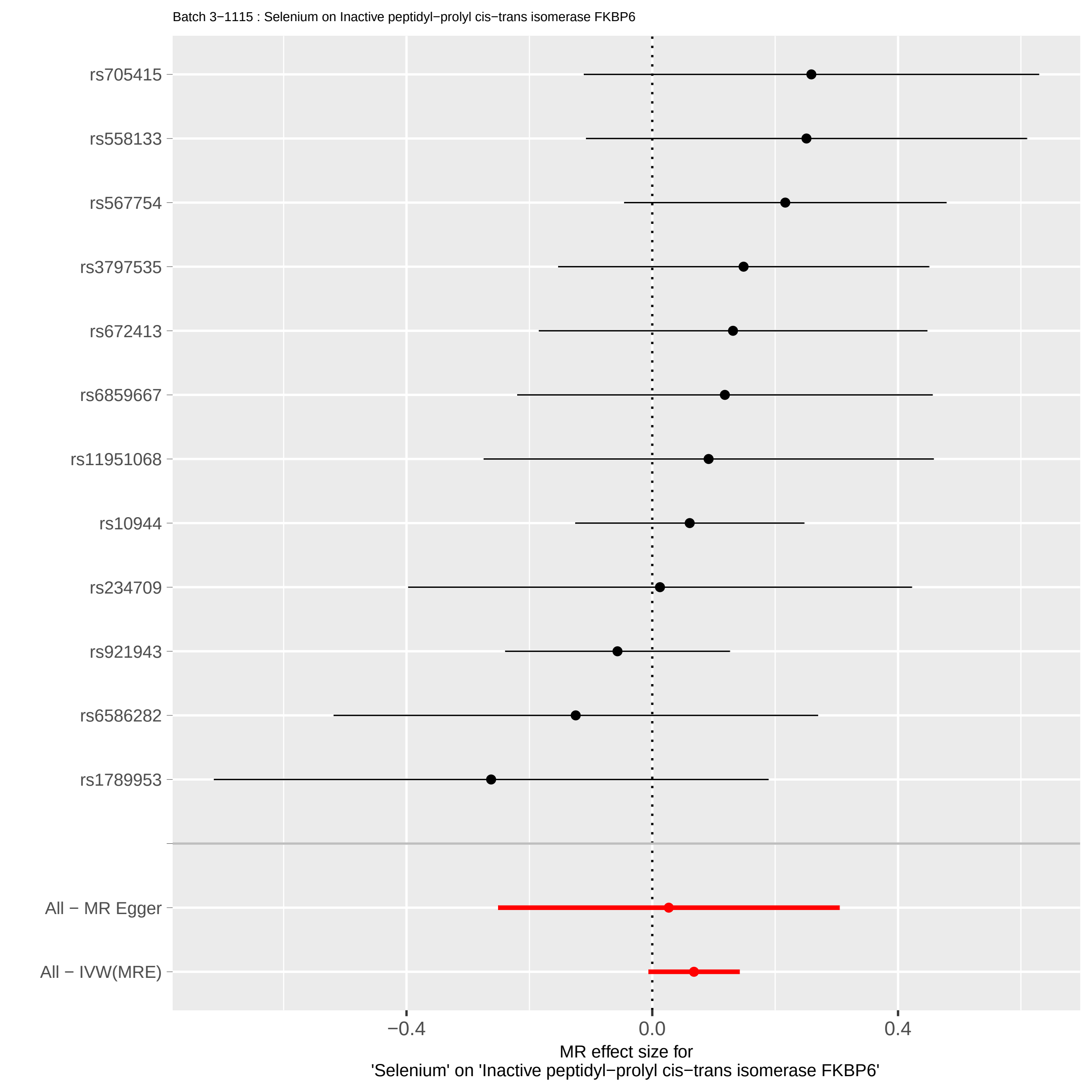
Batch 3−1115 : Selenium on Inactive peptidyl−prolyl cis−trans isomerase FKBP6
rs705415
rs558133
rs567754
rs3797535
rs672413
rs6859667
rs11951068
rs10944
rs234709
rs921943
rs6586282
rs1789953
All − MR Egger
All − IVW(MRE)
−0.4
0.0
0.4
MR effect size for
'Selenium' on 'Inactive peptidyl−prolyl cis−trans isomerase FKBP6'

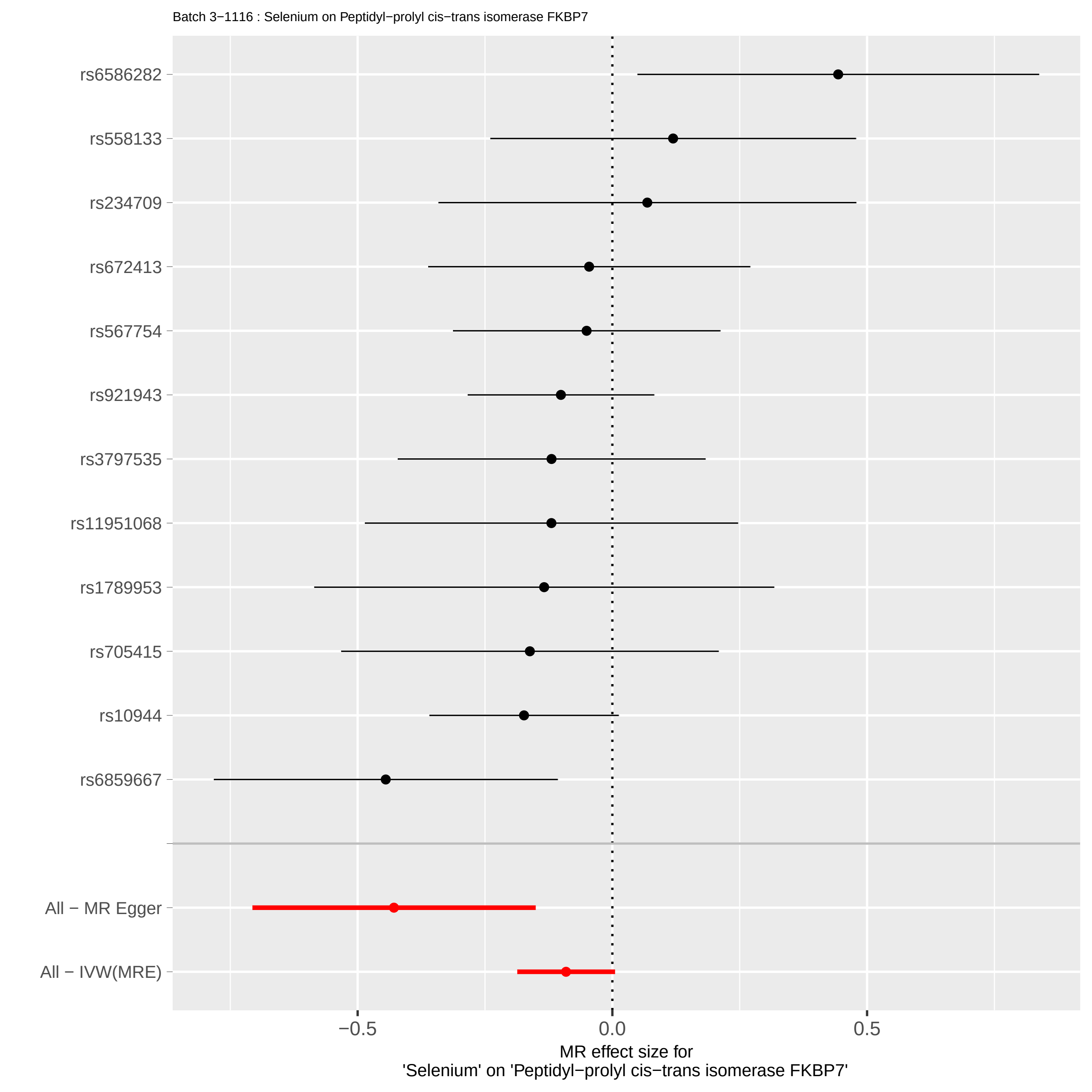

Batch 3−1116 : Selenium on Peptidyl−prolyl cis−trans isomerase FKBP7
rs6586282
rs558133
rs234709
rs672413
rs567754
rs921943
rs3797535
rs11951068
rs1789953
rs705415
rs10944
rs6859667
All − MR Egger
All − IVW(MRE)
−0.5
0.0
0.5
MR effect size for
'Selenium' on 'Peptidyl−prolyl cis−trans isomerase FKBP7'

Batch 3−1117 : Selenium on Filamin−A

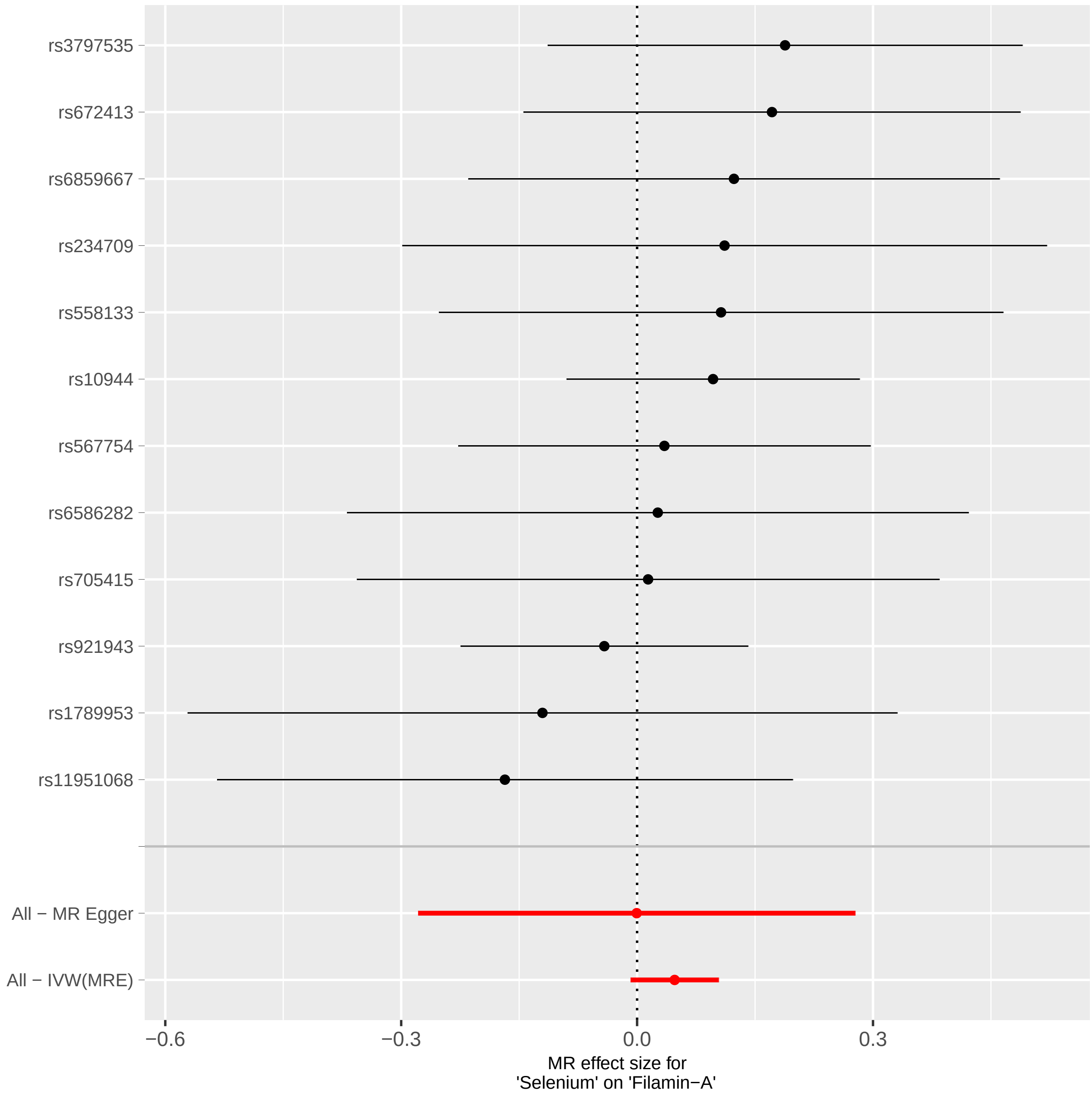

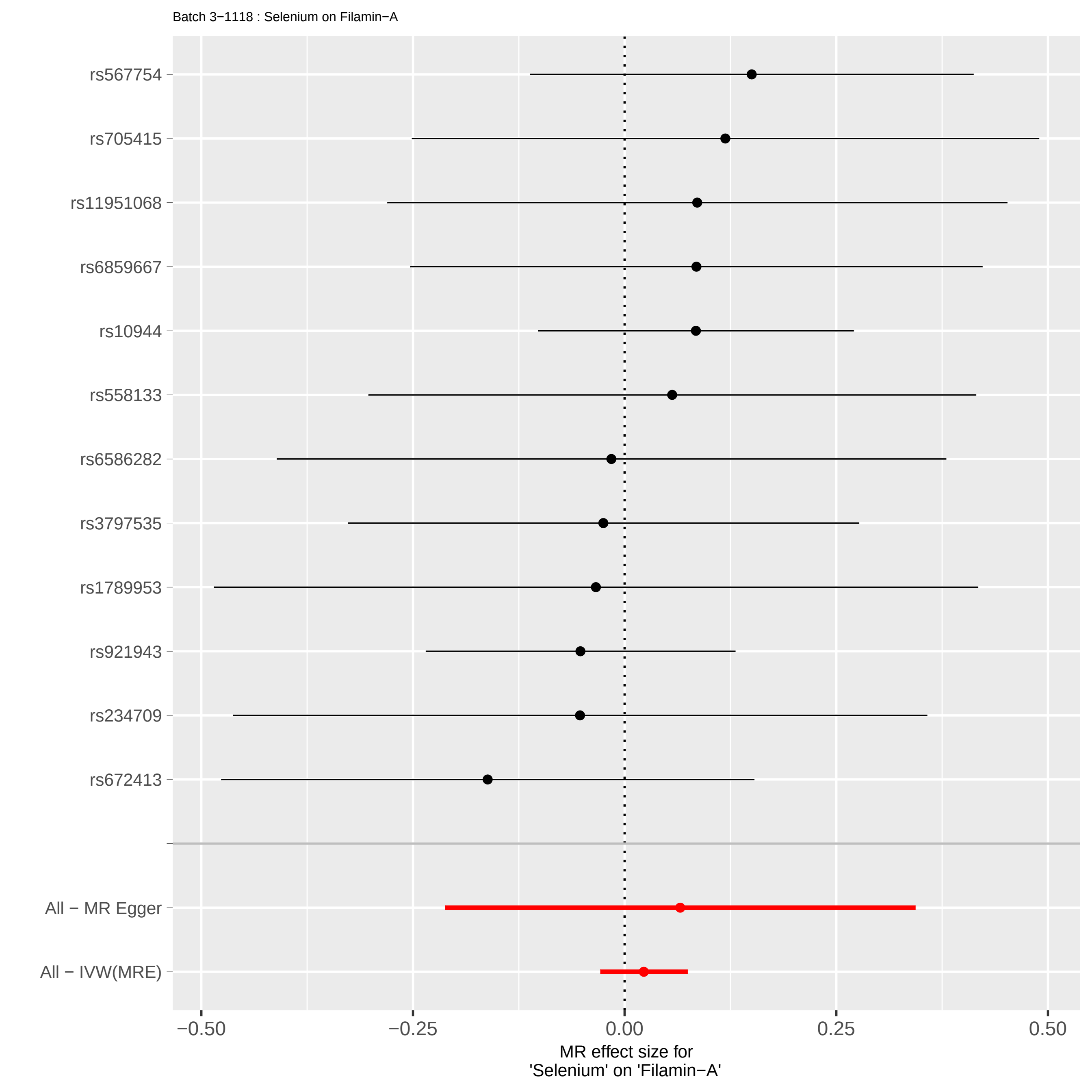

Batch 3−1118 : Selenium on Filamin−A
rs567754
rs705415
rs11951068
rs6859667
rs10944
rs558133
rs6586282
rs3797535
rs1789953
rs921943
rs234709
rs672413
All − MR Egger
All − IVW(MRE)
−0.50
−0.25
0.00
0.25
0.50
MR effect size for
'Selenium' on 'Filamin−A'

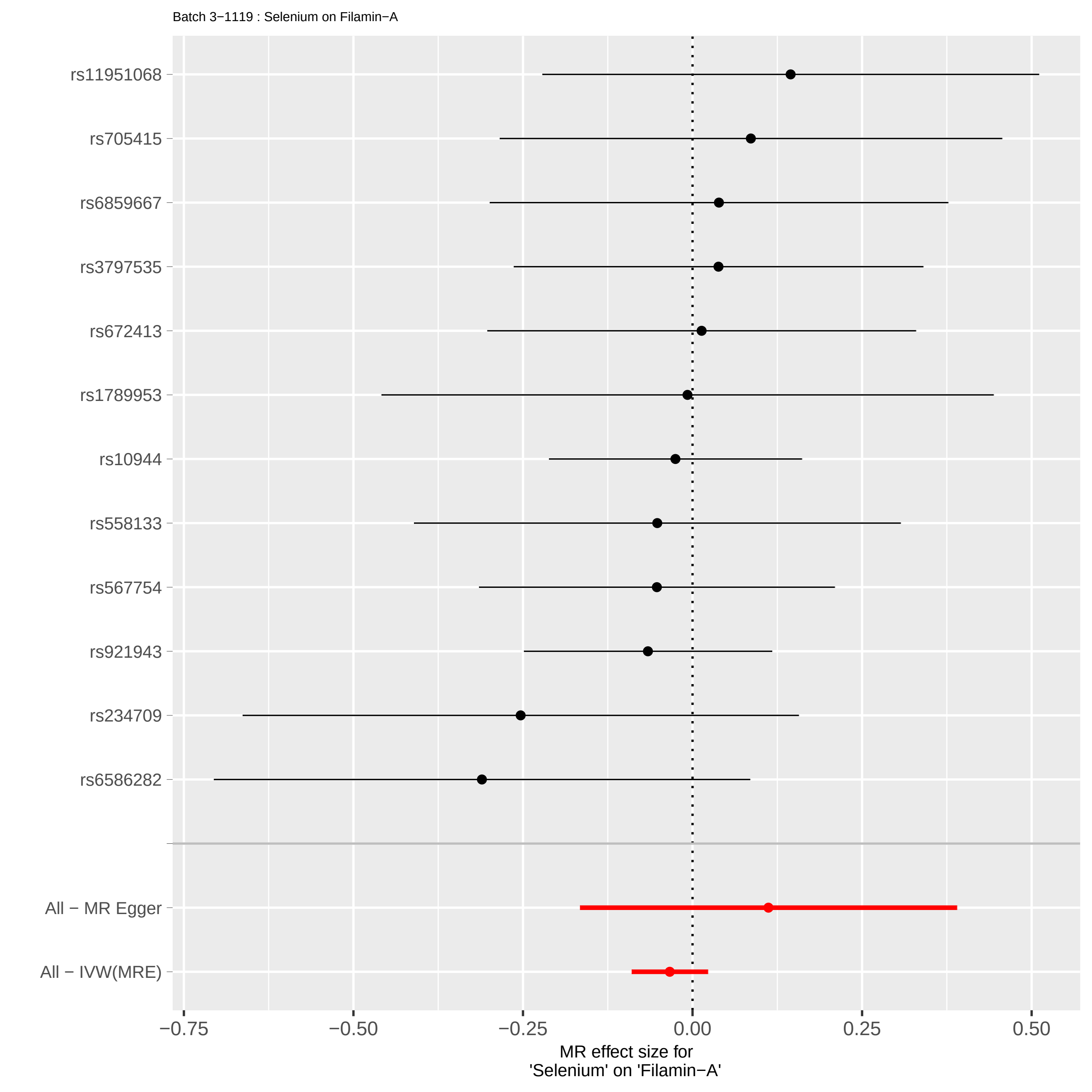
Batch 3−1119 : Selenium on Filamin−A
rs11951068
rs705415
rs6859667
rs3797535
rs672413
rs1789953
rs10944
rs558133
rs567754
rs921943
rs234709
rs6586282
All − MR Egger
All − IVW(MRE)
−0.75
−0.50
−0.25
0.00
0.25
0.50
MR effect size for
'Selenium' on 'Filamin−A'

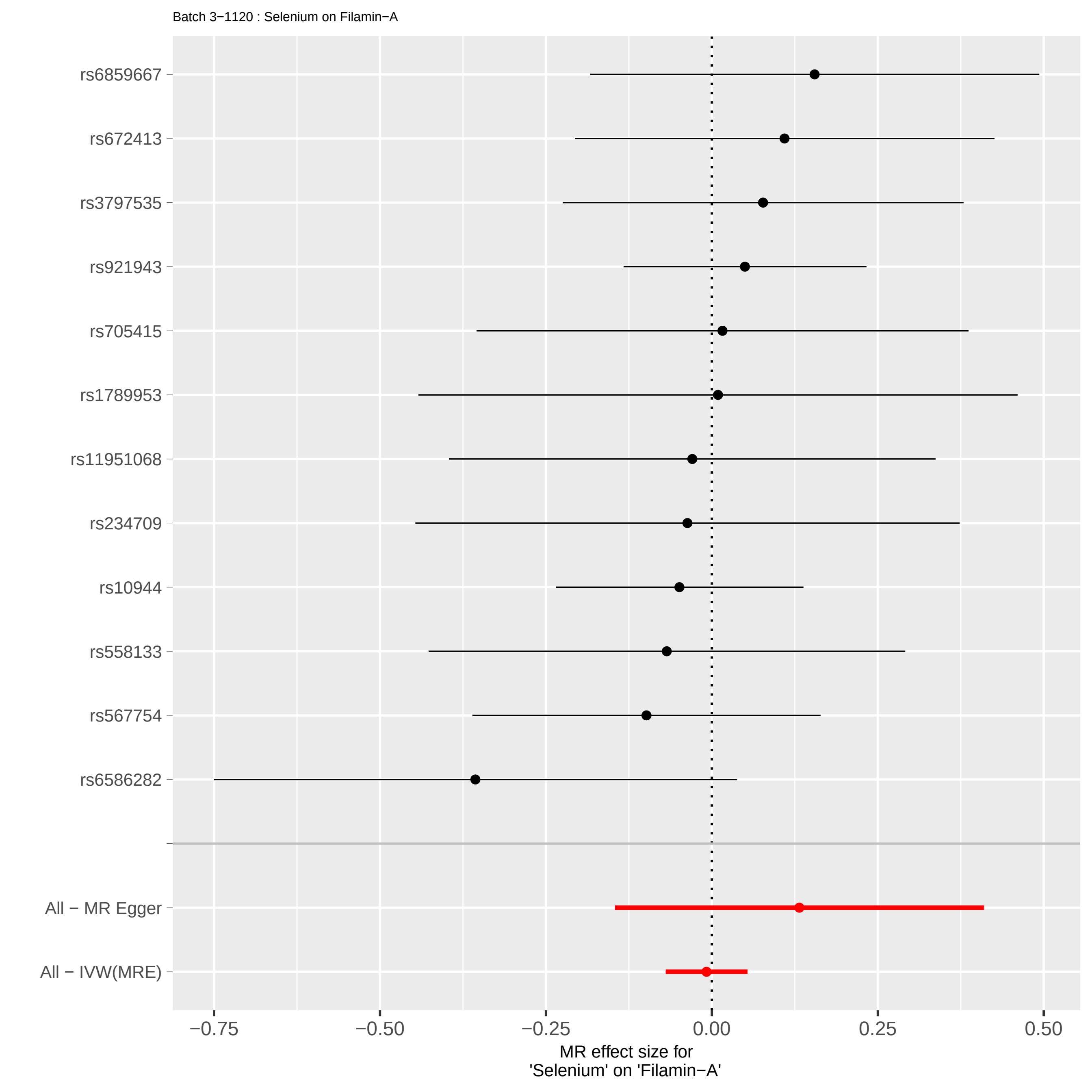

Batch 3−1120 : Selenium on Filamin−A
rs6859667
rs672413
rs3797535
rs921943
rs705415
rs1789953
rs11951068
rs234709
rs10944
rs558133
rs567754
rs6586282
All − MR Egger
All − IVW(MRE)
−0.75
−0.50
−0.25
0.00
0.25
0.50
MR effect size for
'Selenium' on 'Filamin−A'

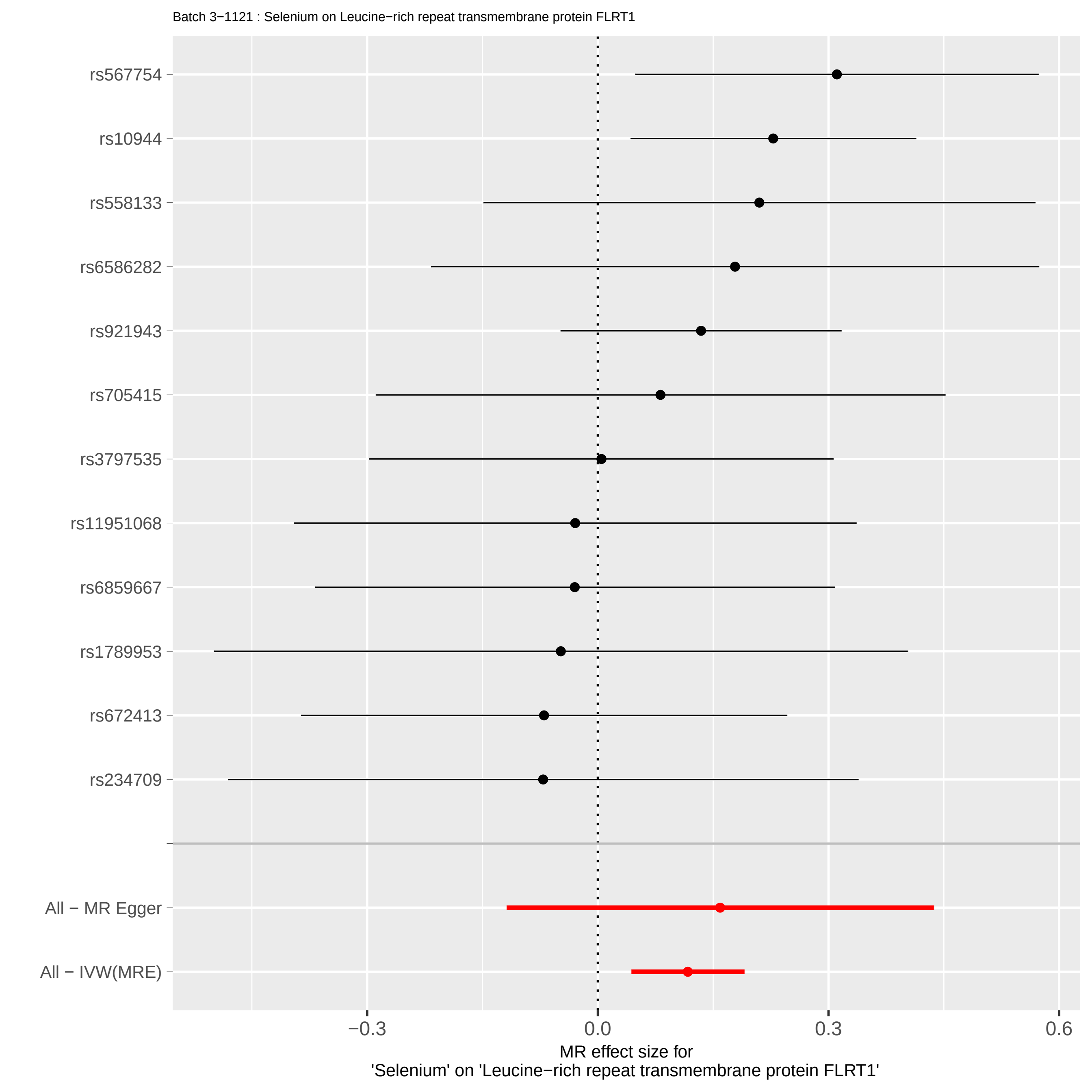

Batch 3−1121 : Selenium on Leucine−rich repeat transmembrane protein FLRT1
rs567754
rs10944
rs558133
rs6586282
rs921943
rs705415
rs3797535
rs11951068
rs6859667
rs1789953
rs672413
rs234709
All − MR Egger
All − IVW(MRE)
−0.3
0.0
0.3
0.6
MR effect size for
'Selenium' on 'Leucine−rich repeat transmembrane protein FLRT1'

Batch 3−1122 : Selenium on Leucine−rich repeat transmembrane protein FLRT1

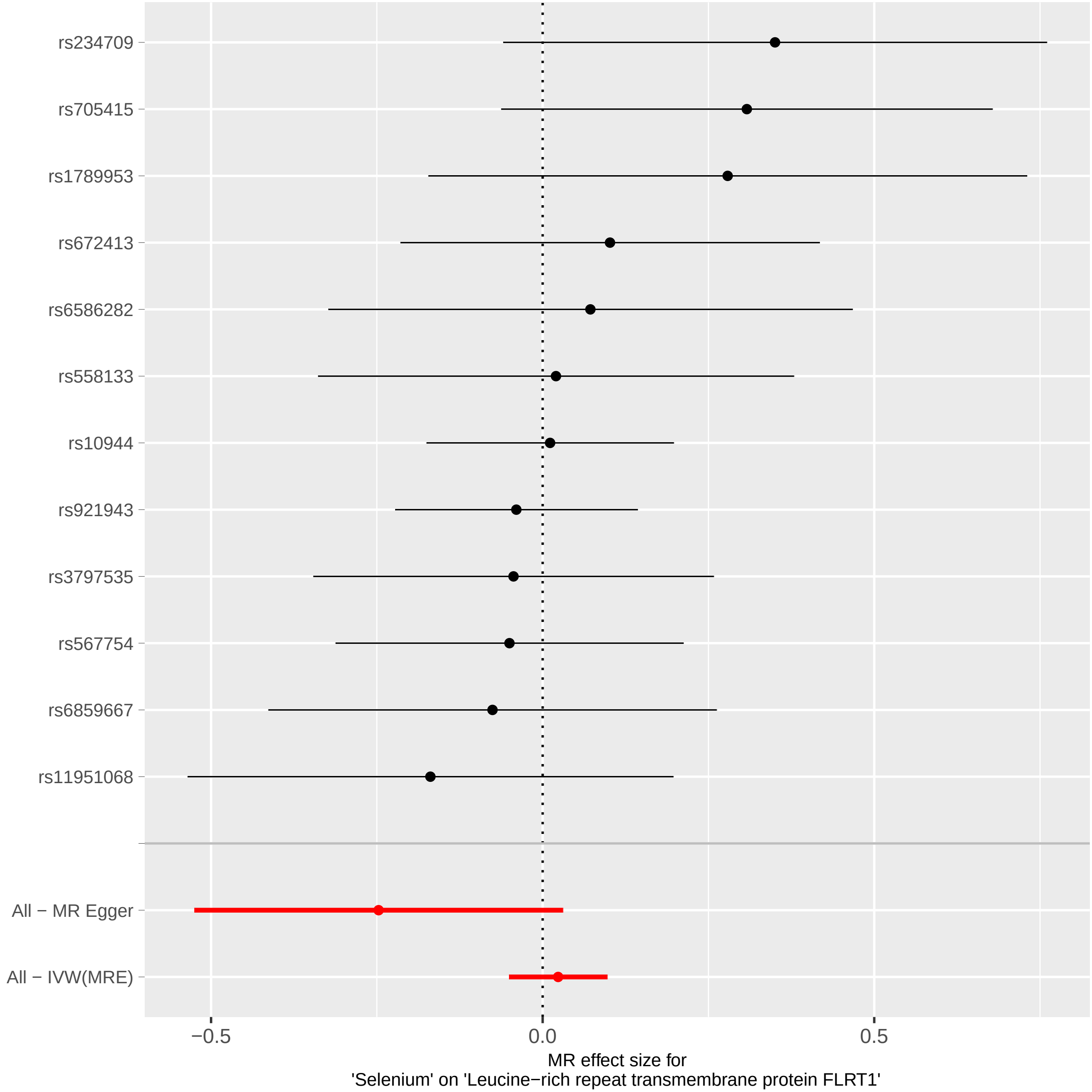

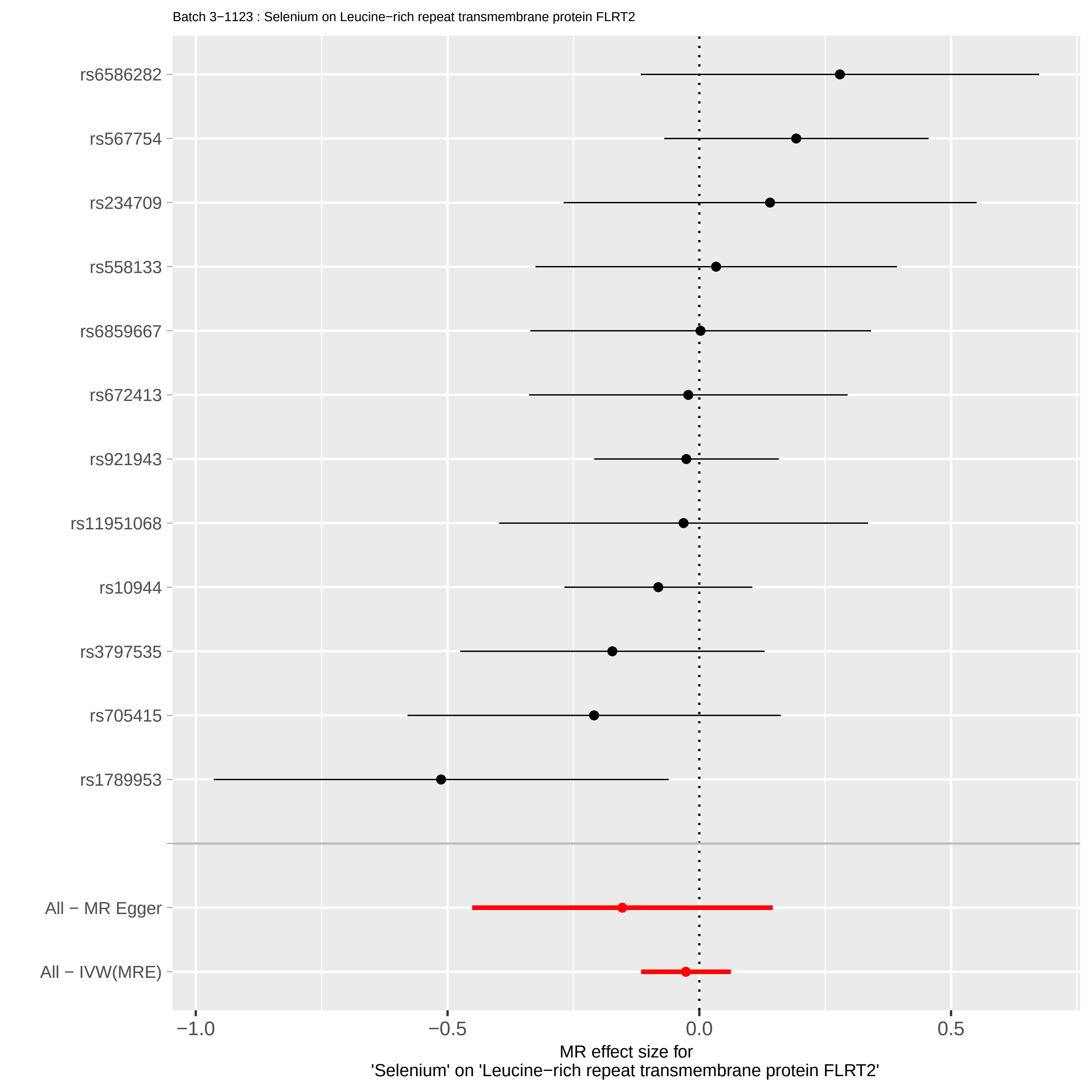

Batch 3−1123 : Selenium on Leucine−rich repeat transmembrane protein FLRT2
rs6586282
rs567754
rs234709
rs558133
rs6859667
rs672413
rs921943
rs11951068
rs10944
rs3797535
rs705415
rs1789953
All − MR Egger
All − IVW(MRE)
−1.0
−0.5
0.0
0.5
MR effect size for
'Selenium' on 'Leucine−rich repeat transmembrane protein FLRT2'

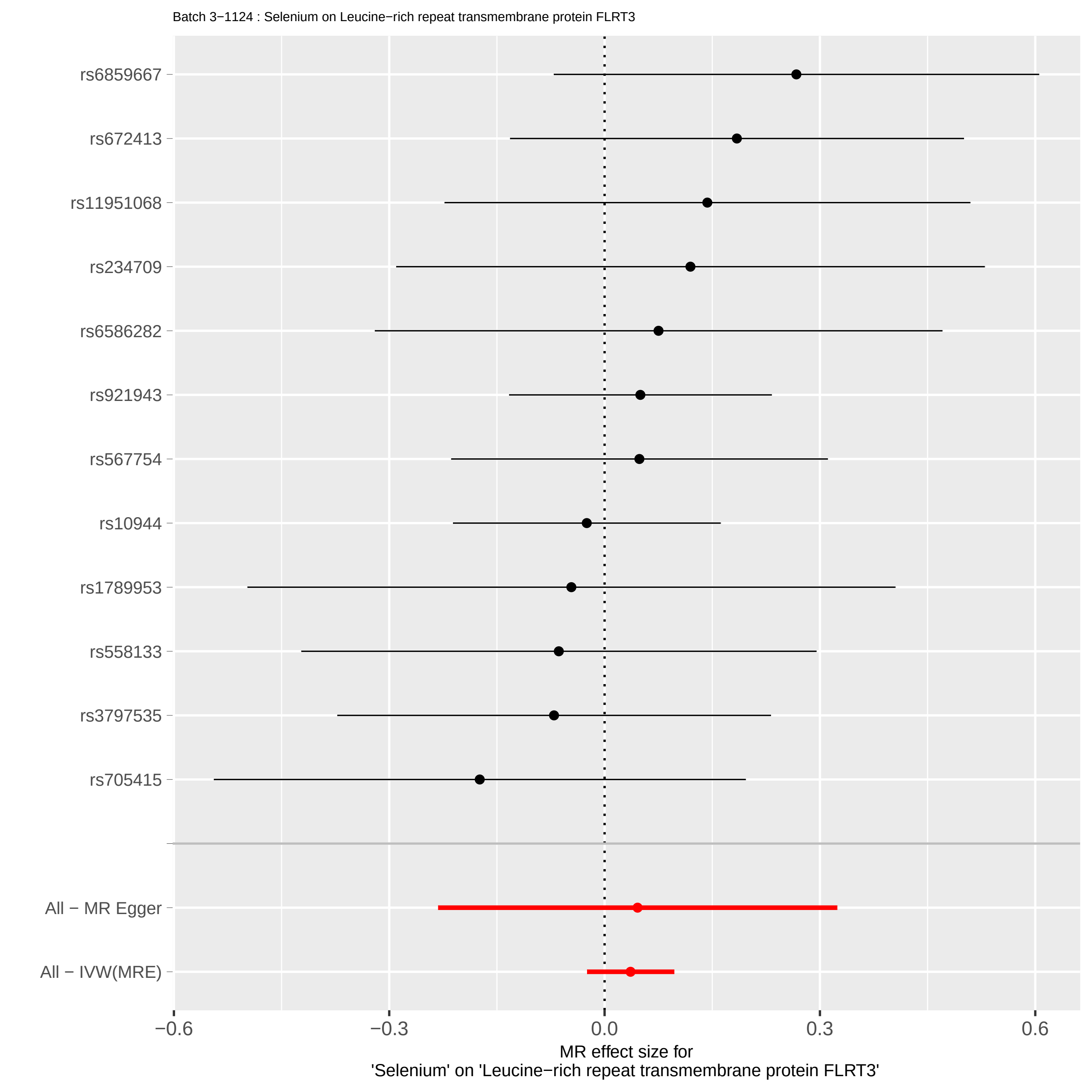

Batch 3−1124 : Selenium on Leucine−rich repeat transmembrane protein FLRT3
rs6859667
rs672413
rs11951068
rs234709
rs6586282
rs921943
rs567754
rs10944
rs1789953
rs558133
rs3797535
rs705415
All − MR Egger
All − IVW(MRE)
−0.6
−0.3
0.0
0.3
0.6
MR effect size for
'Selenium' on 'Leucine−rich repeat transmembrane protein FLRT3'

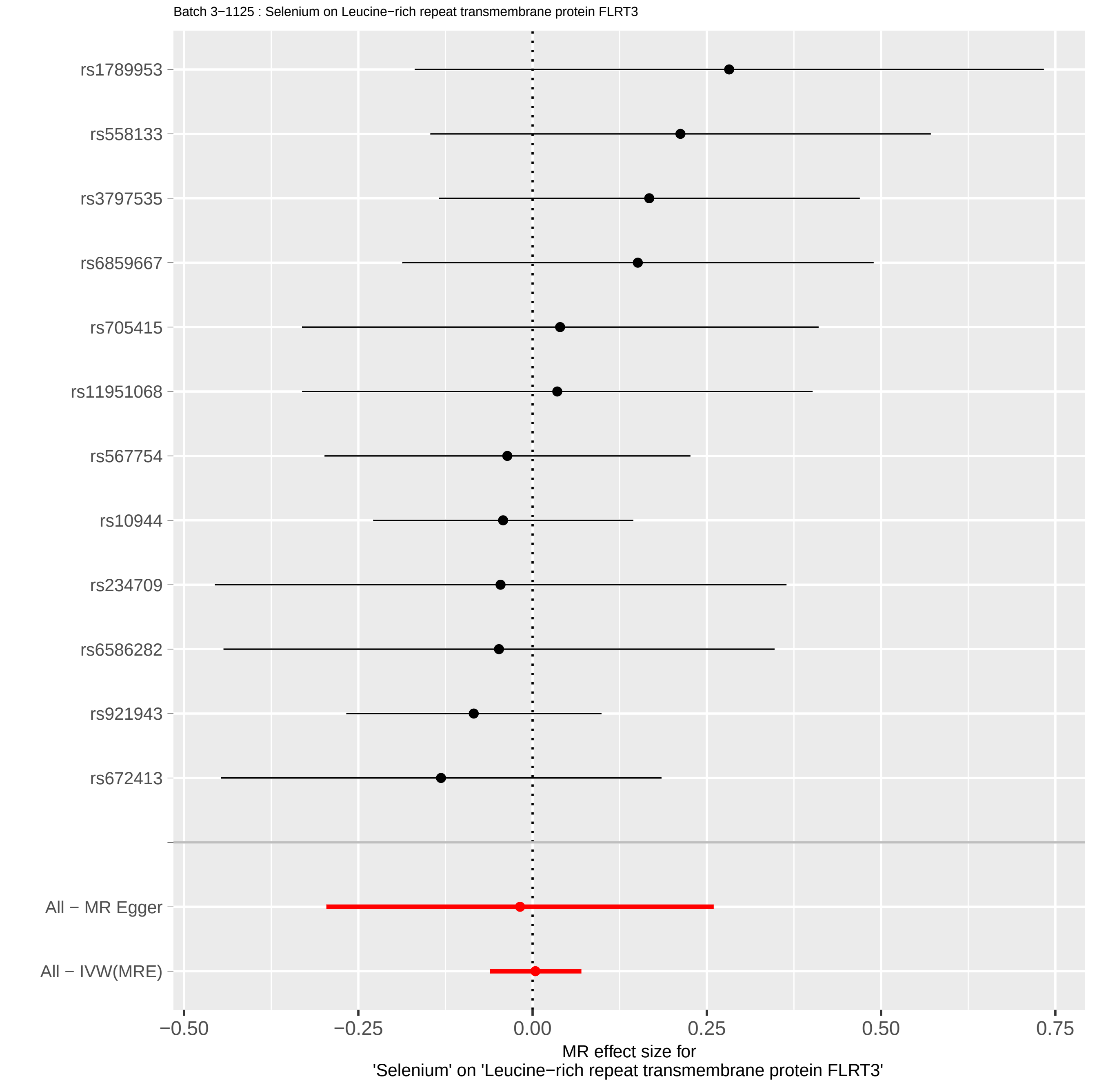
Batch 3−1125 : Selenium on Leucine−rich repeat transmembrane protein FLRT3
rs1789953
rs558133
rs3797535
rs6859667
rs705415
rs11951068
rs567754
rs10944
rs234709
rs6586282
rs921943
rs672413
All − MR Egger
All − IVW(MRE)
−0.50
−0.25
0.00
0.25
0.50
0.75
MR effect size for
'Selenium' on 'Leucine−rich repeat transmembrane protein FLRT3'

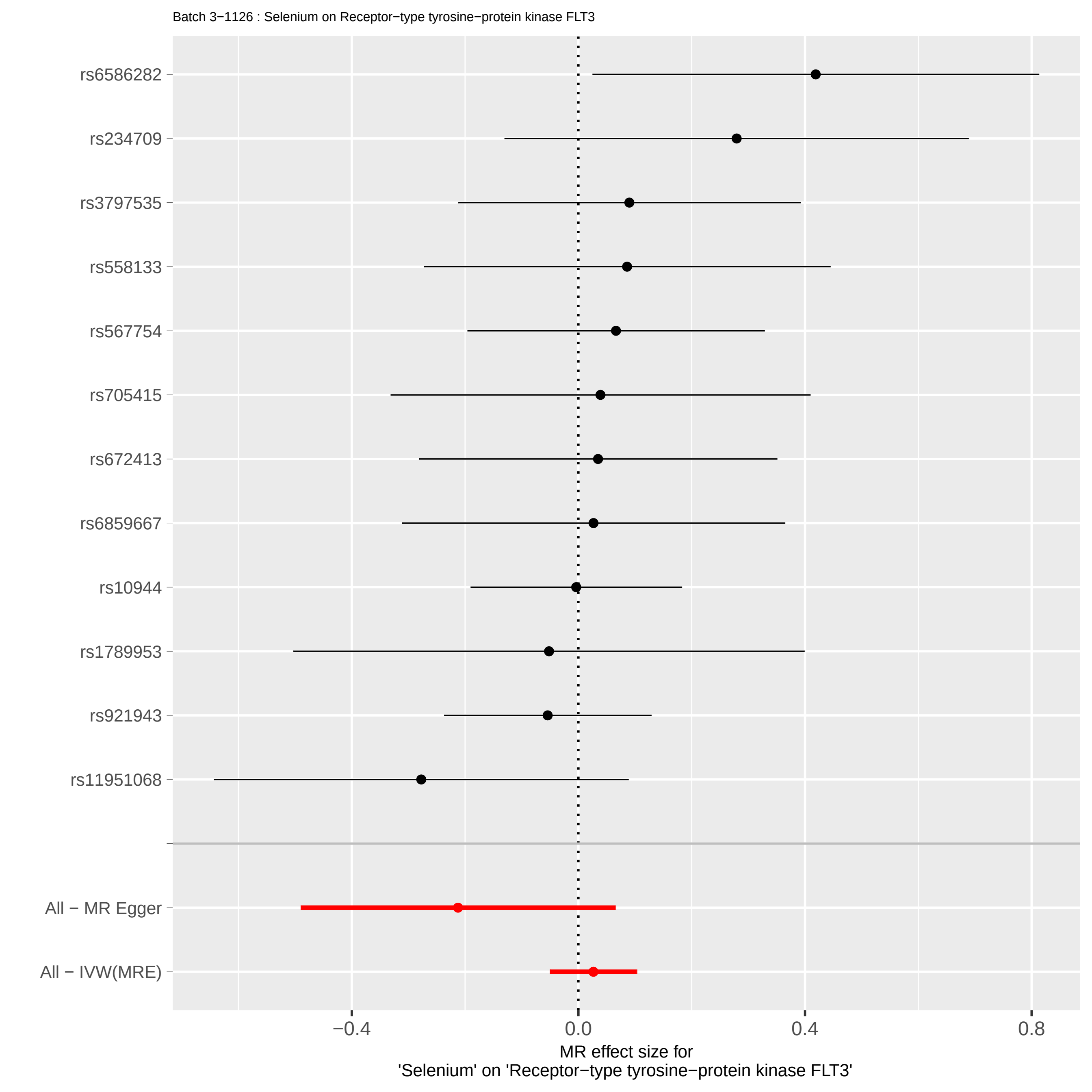

Batch 3−1126 : Selenium on Receptor−type tyrosine−protein kinase FLT3
rs6586282
rs234709
rs3797535
rs558133
rs567754
rs705415
rs672413
rs6859667
rs10944
rs1789953
rs921943
rs11951068
All − MR Egger
All − IVW(MRE)
−0.4
0.0
0.4
0.8
MR effect size for
'Selenium' on 'Receptor−type tyrosine−protein kinase FLT3'

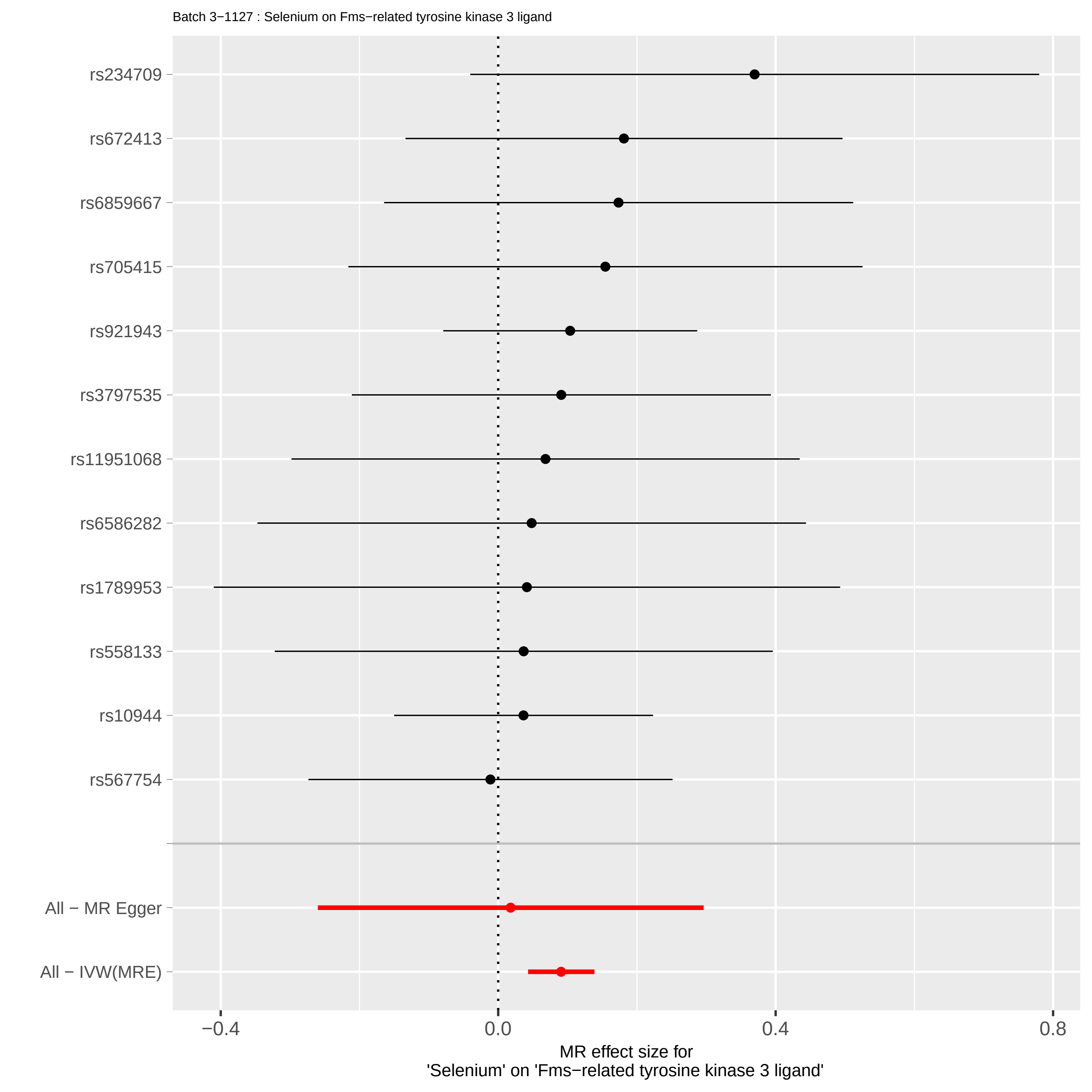

Batch 3−1127 : Selenium on Fms−related tyrosine kinase 3 ligand
rs234709
rs672413
rs6859667
rs705415
rs921943
rs3797535
rs11951068
rs6586282
rs1789953
rs558133
rs10944
rs567754
All − MR Egger
All − IVW(MRE)
−0.4
0.0
0.4
0.8
MR effect size for
'Selenium' on 'Fms−related tyrosine kinase 3 ligand'

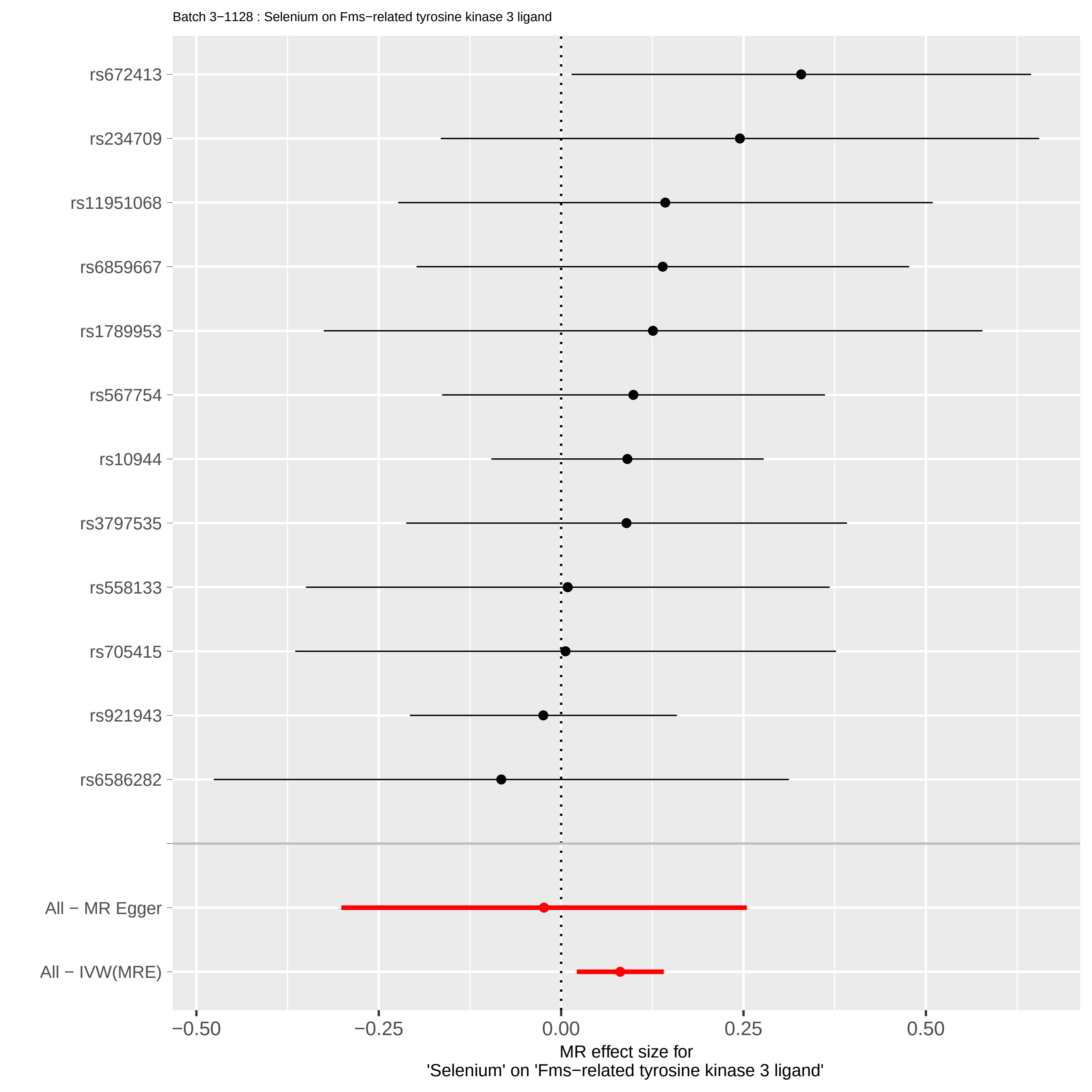

Batch 3−1128 : Selenium on Fms−related tyrosine kinase 3 ligand
rs672413
rs234709
rs11951068
rs6859667
rs1789953
rs567754
rs10944
rs3797535
rs558133
rs705415
rs921943
rs6586282
All − MR Egger
All − IVW(MRE)
−0.50
−0.25
0.00
0.25
0.50
MR effect size for
'Selenium' on 'Fms−related tyrosine kinase 3 ligand'

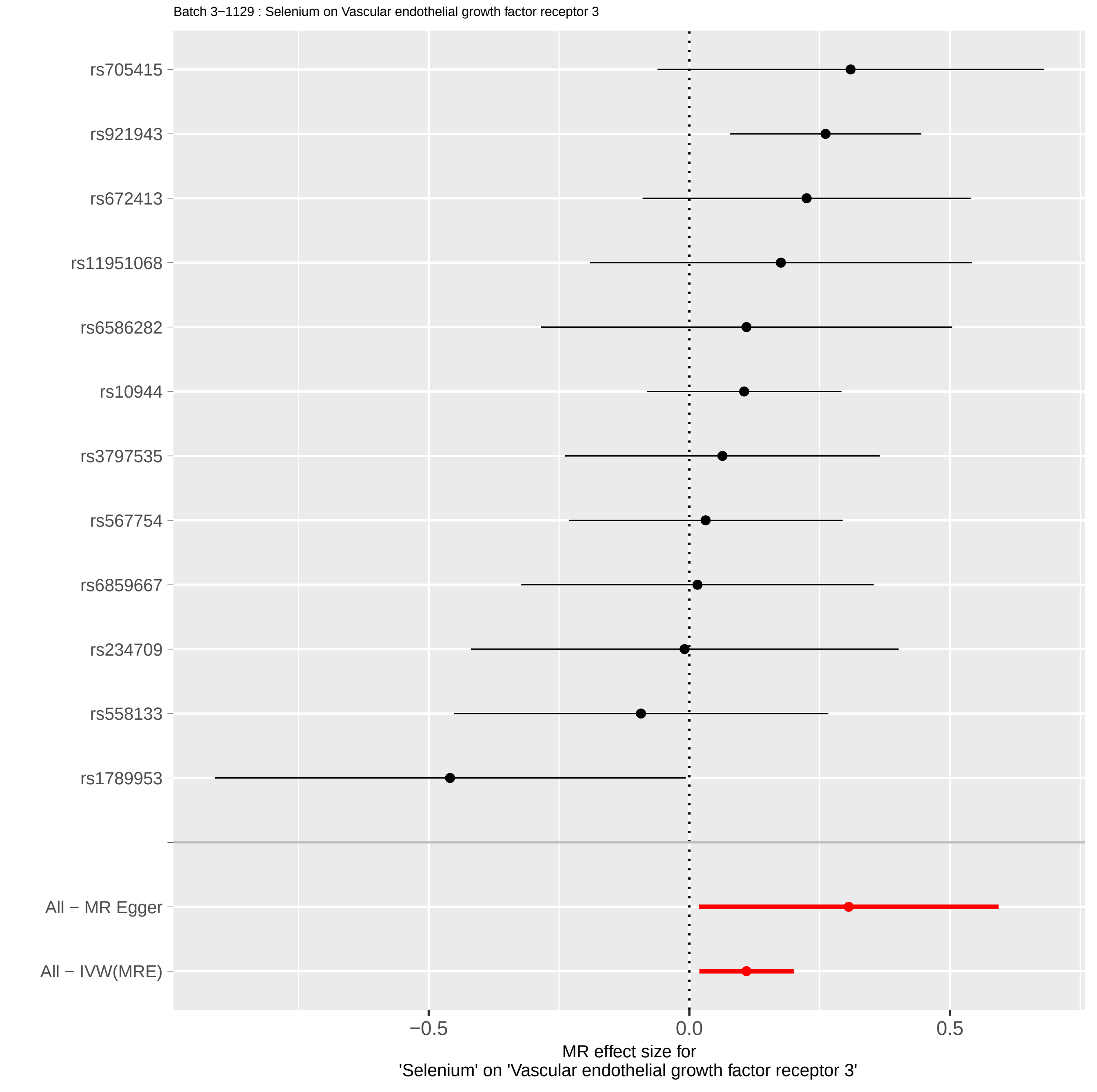
Batch 3−1129 : Selenium on Vascular endothelial growth factor receptor 3
rs705415
rs921943
rs672413
rs11951068
rs6586282
rs10944
rs3797535
rs567754
rs6859667
rs234709
rs558133
rs1789953
All − MR Egger
All − IVW(MRE)
−0.5
0.0
0.5
MR effect size for
'Selenium' on 'Vascular endothelial growth factor receptor 3'

Batch 3−1130 : Selenium on Feline leukemia virus subgroup C receptor−related protein 1

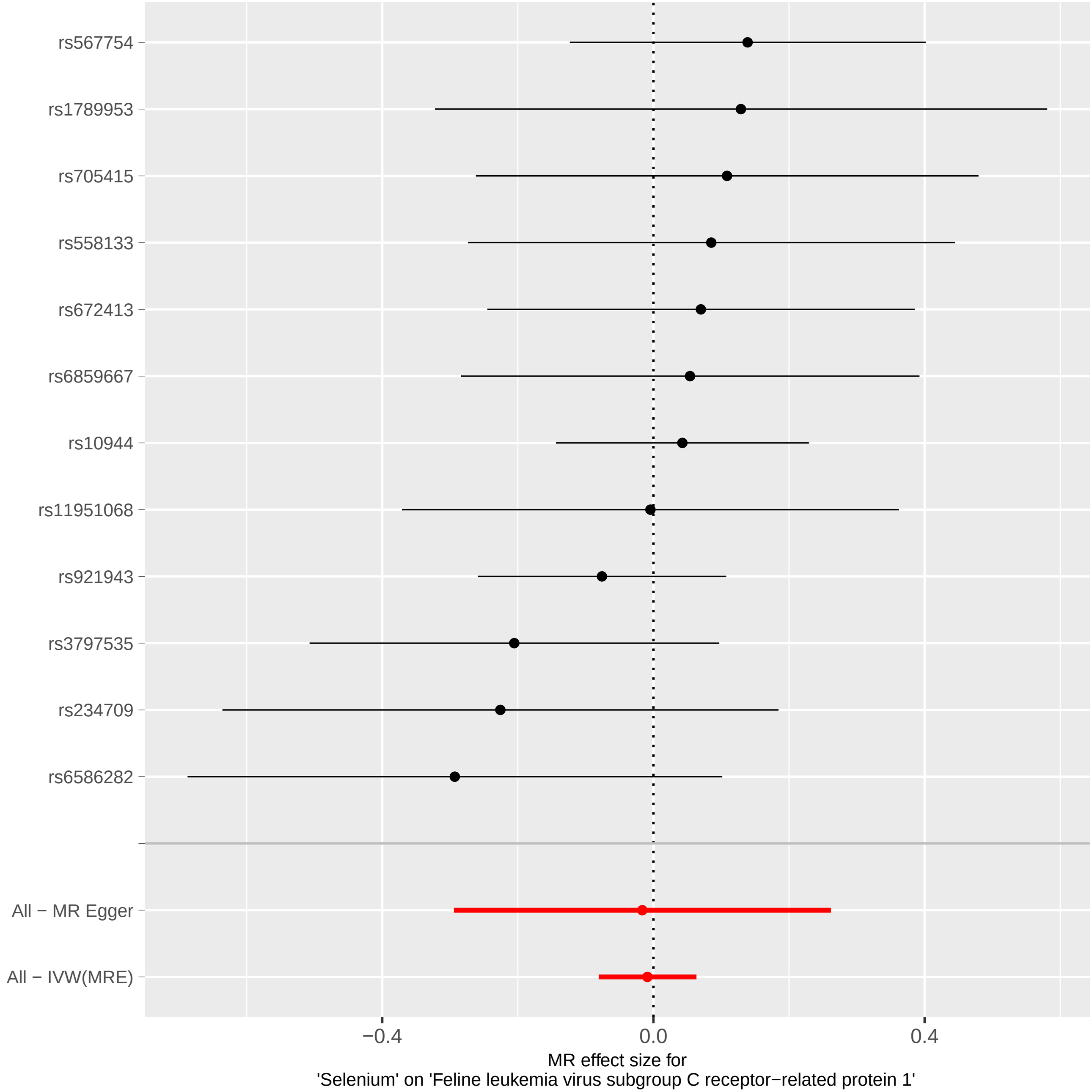

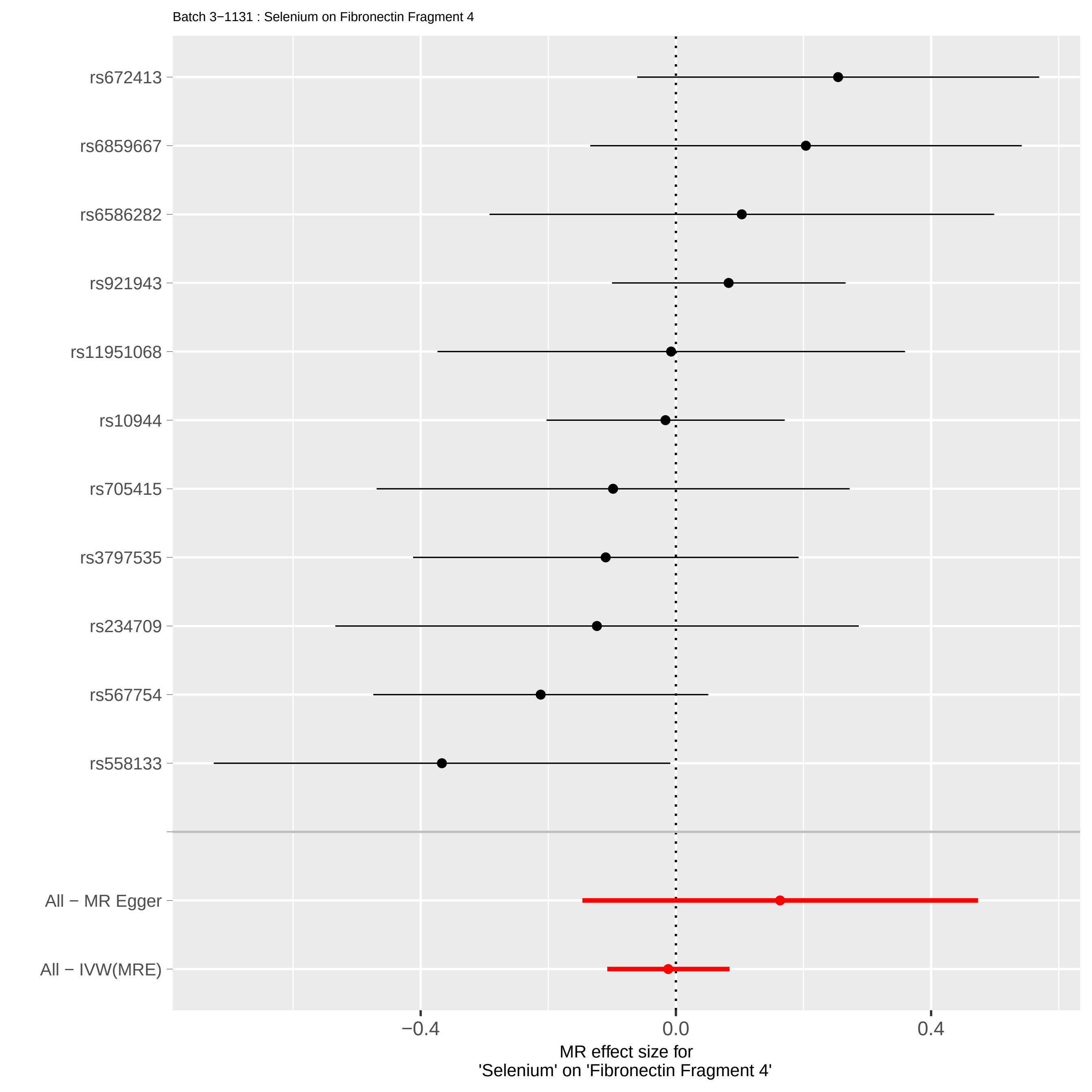

Batch 3−1131 : Selenium on Fibronectin Fragment 4
rs672413
rs6859667
rs6586282
rs921943
rs11951068
rs10944
rs705415
rs3797535
rs234709
rs567754
rs558133
All − MR Egger
All − IVW(MRE)
−0.4
0.0
0.4
MR effect size for
'Selenium' on 'Fibronectin Fragment 4'

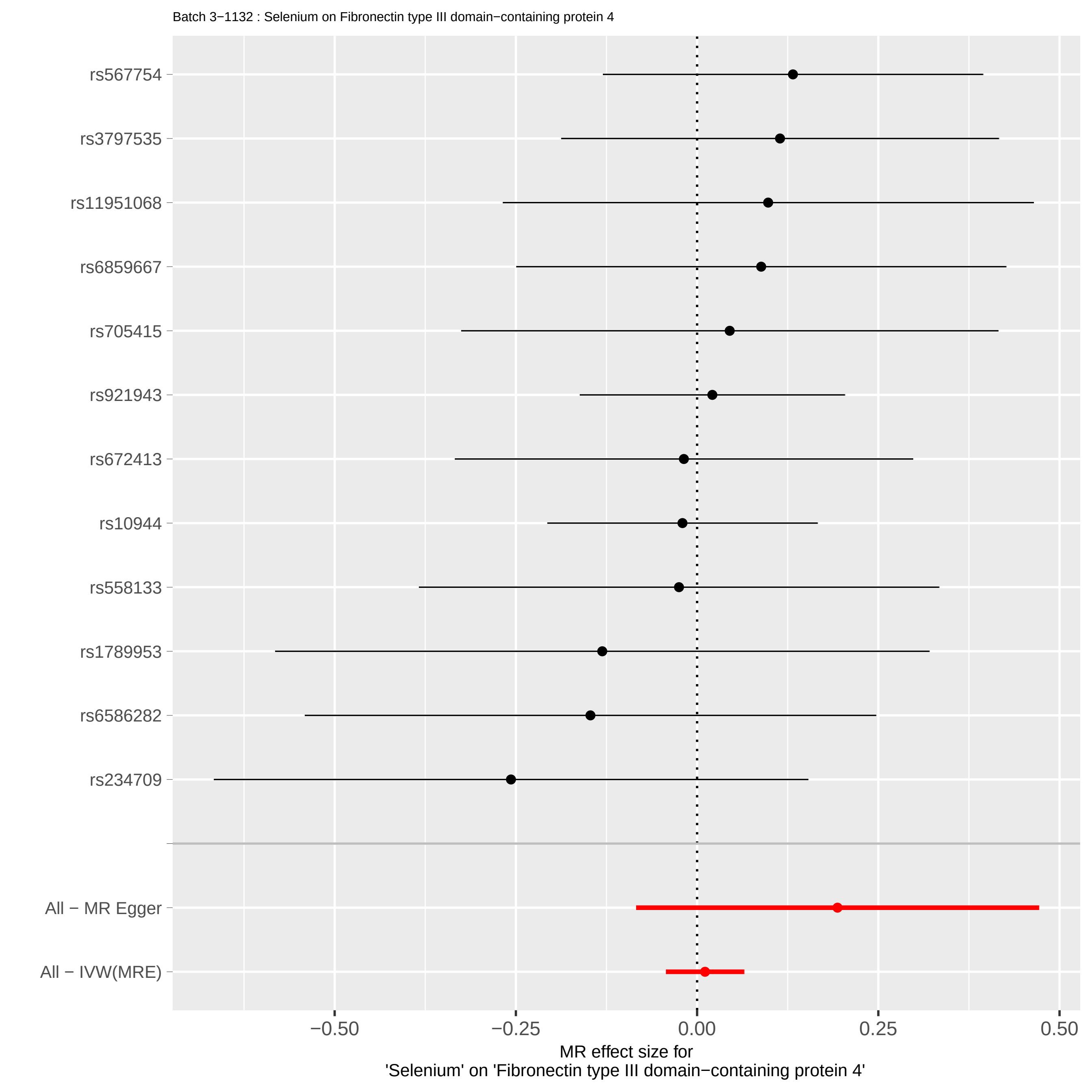
Batch 3−1132 : Selenium on Fibronectin type III domain−containing protein 4
rs567754
rs3797535
rs11951068
rs6859667
rs705415
rs921943
rs672413
rs10944
rs558133
rs1789953
rs6586282
rs234709
All − MR Egger
All − IVW(MRE)
−0.50
−0.25
0.00
0.25
0.50
MR effect size for
'Selenium' on 'Fibronectin type III domain−containing protein 4'

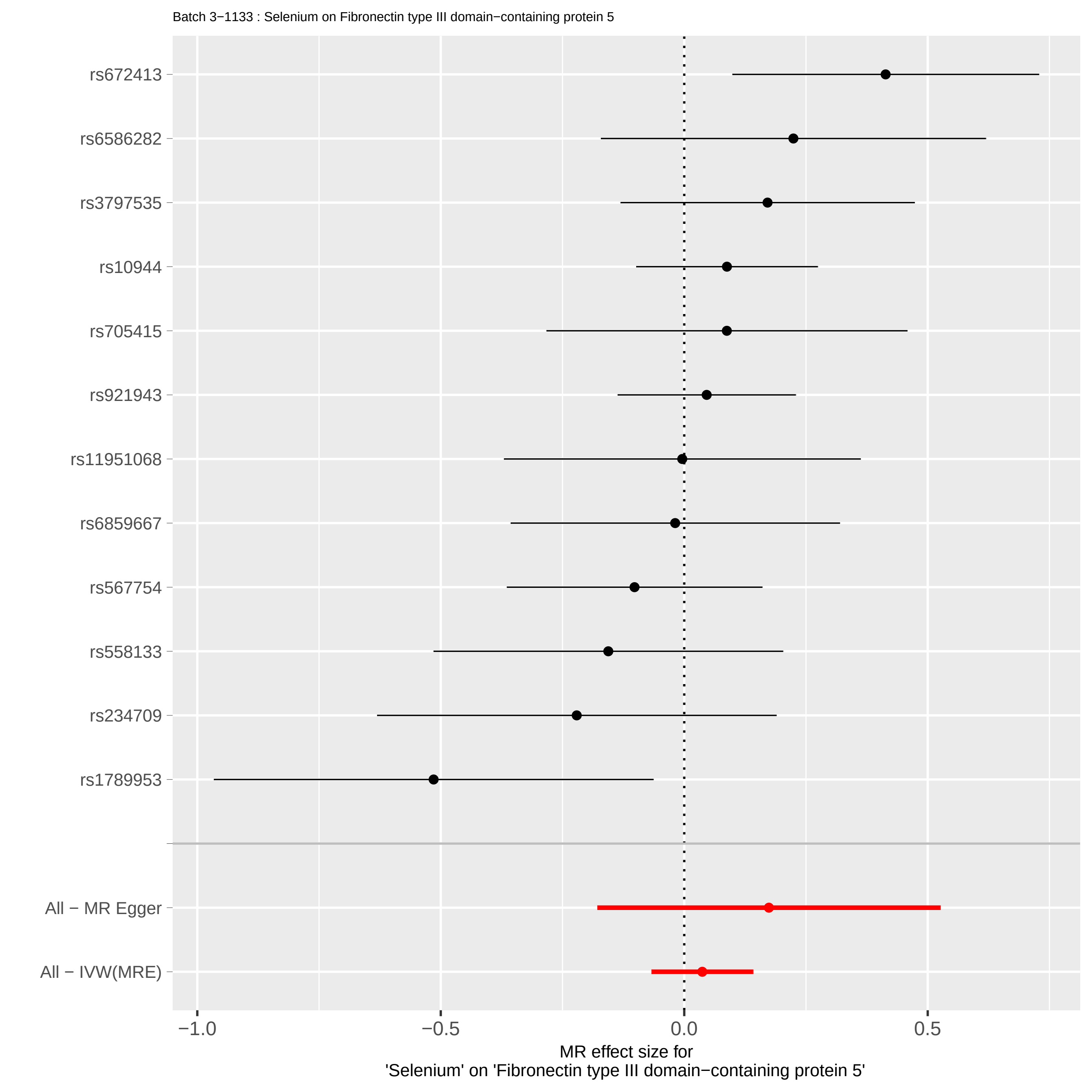

Batch 3−1133 : Selenium on Fibronectin type III domain−containing protein 5
rs672413
rs6586282
rs3797535
rs10944
rs705415
rs921943
rs11951068
rs6859667
rs567754
rs558133
rs234709
rs1789953
All − MR Egger
All − IVW(MRE)
−1.0
−0.5
0.0
0.5
MR effect size for
'Selenium' on 'Fibronectin type III domain−containing protein 5'

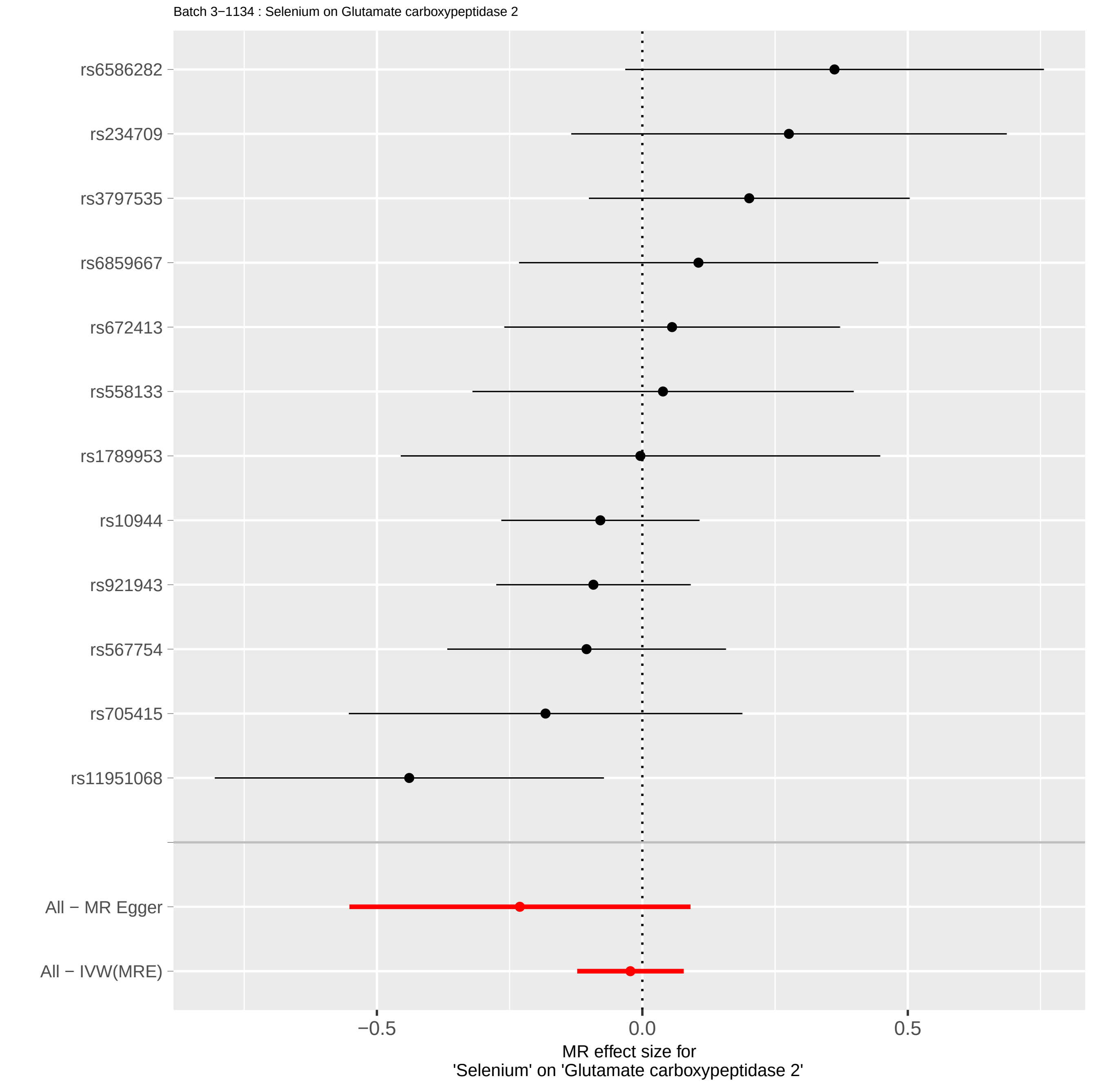

Batch 3−1134 : Selenium on Glutamate carboxypeptidase 2
rs6586282
rs234709
rs3797535
rs6859667
rs672413
rs558133
rs1789953
rs10944
rs921943
rs567754
rs705415
rs11951068
All − MR Egger
All − IVW(MRE)
−0.5
0.0
0.5
MR effect size for
'Selenium' on 'Glutamate carboxypeptidase 2'

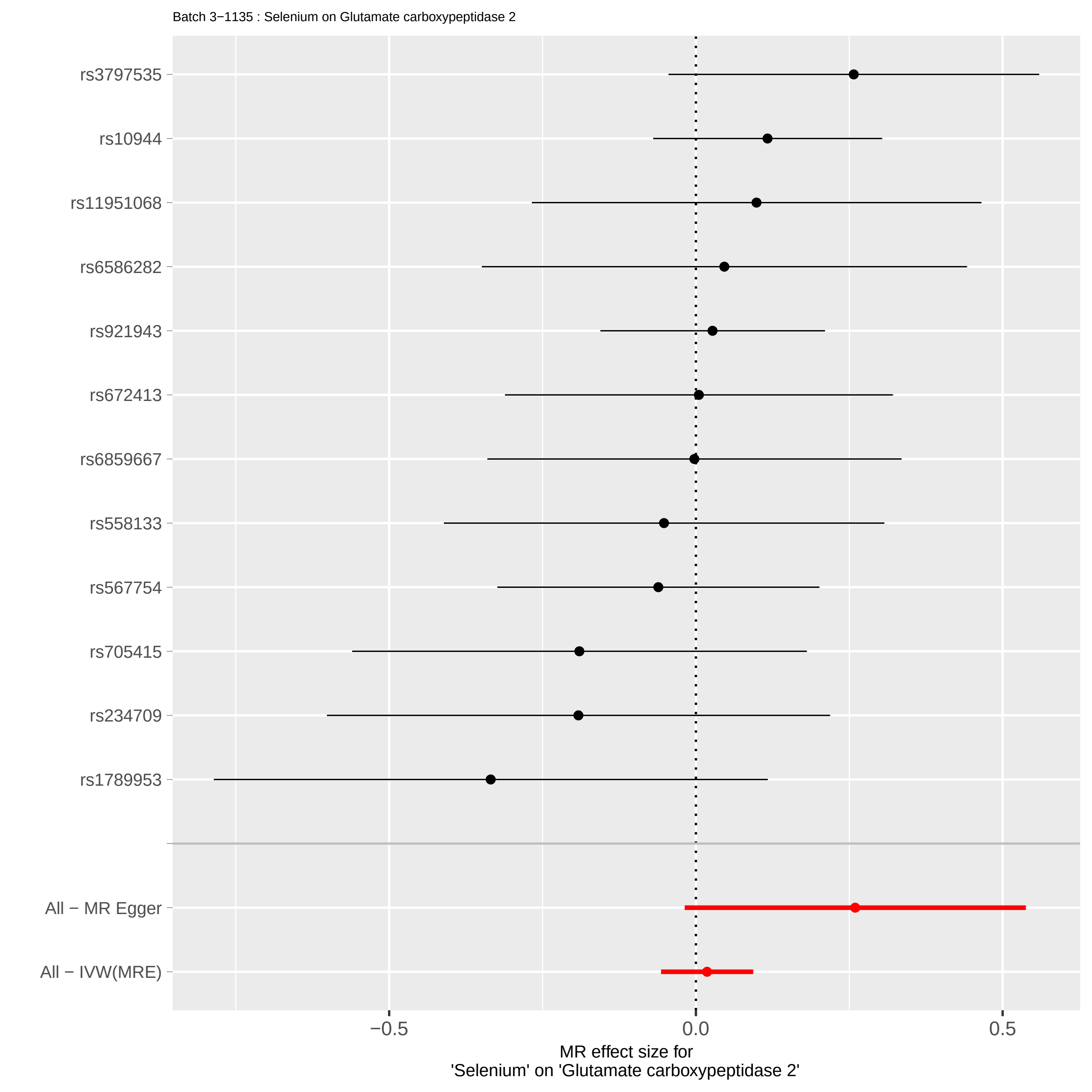
Batch 3−1135 : Selenium on Glutamate carboxypeptidase 2
rs3797535
rs10944
rs11951068
rs6586282
rs921943
rs672413
rs6859667
rs558133
rs567754
rs705415
rs234709
rs1789953
All − MR Egger
All − IVW(MRE)
−0.5
0.0
0.5
MR effect size for
'Selenium' on 'Glutamate carboxypeptidase 2'

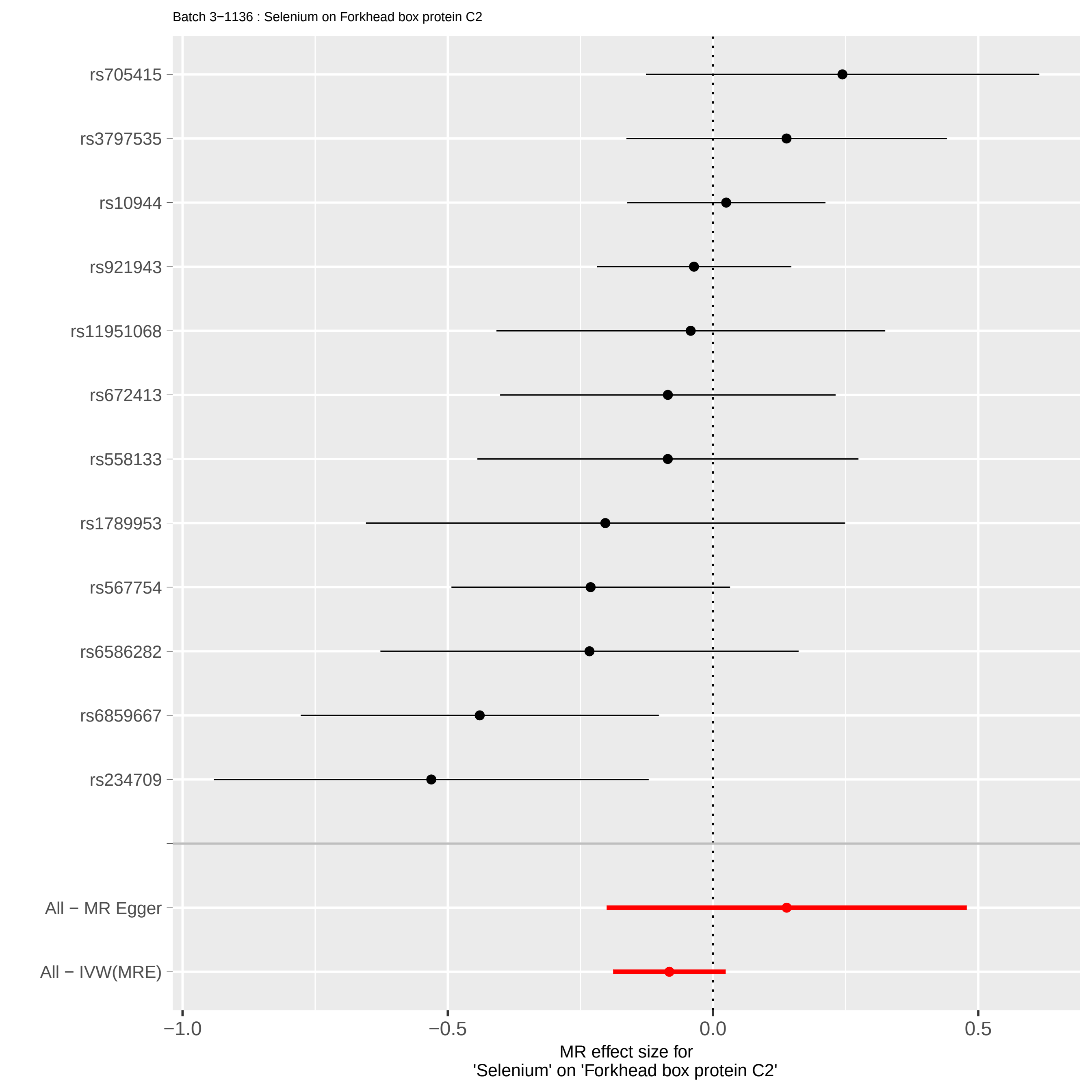

Batch 3−1136 : Selenium on Forkhead box protein C2
rs705415
rs3797535
rs10944
rs921943
rs11951068
rs672413
rs558133
rs1789953
rs567754
rs6586282
rs6859667
rs234709
All − MR Egger
All − IVW(MRE)
−1.0
−0.5
0.0
0.5
MR effect size for
'Selenium' on 'Forkhead box protein C2'

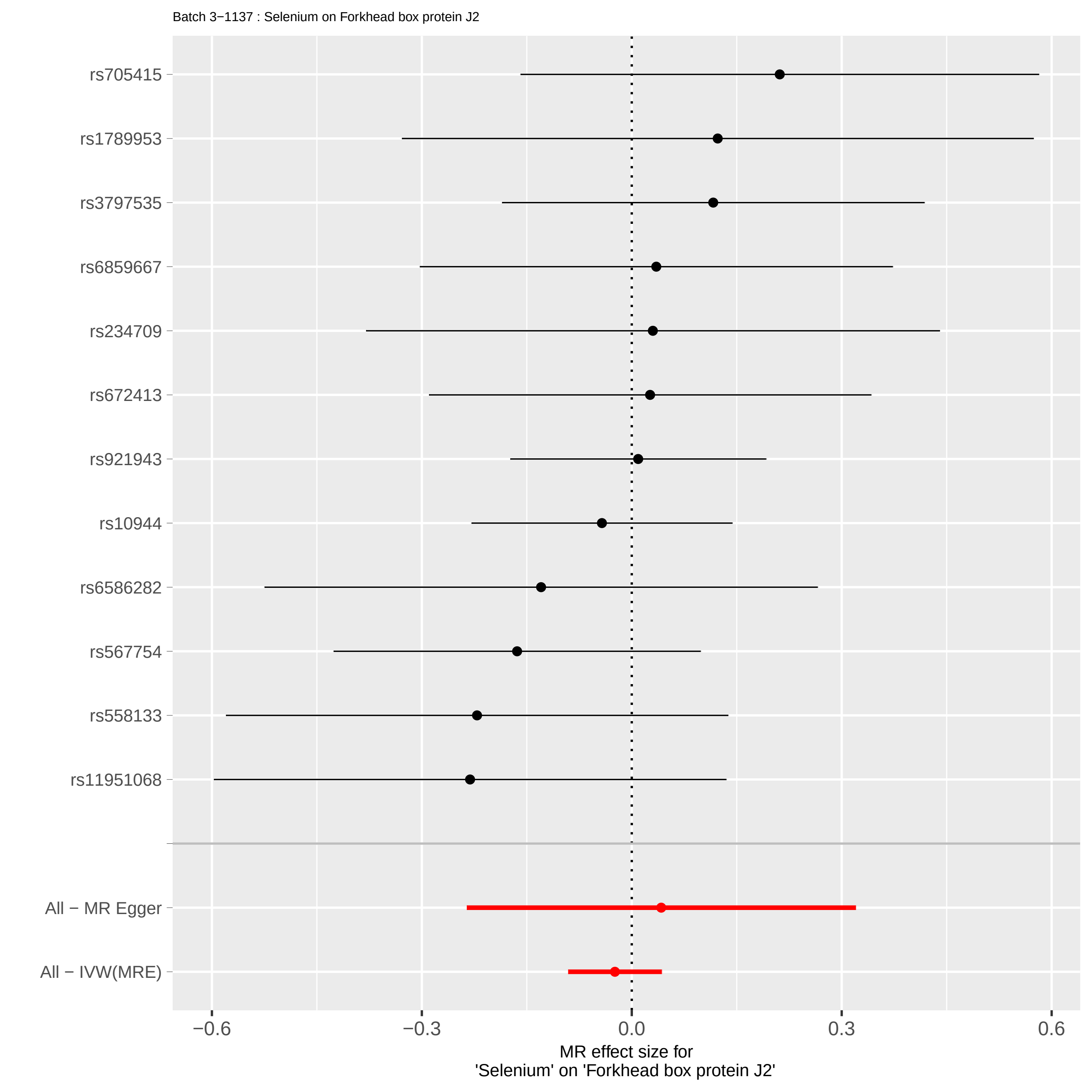

Batch 3−1137 : Selenium on Forkhead box protein J2
rs705415
rs1789953
rs3797535
rs6859667
rs234709
rs672413
rs921943
rs10944
rs6586282
rs567754
rs558133
rs11951068
All − MR Egger
All − IVW(MRE)
−0.6
−0.3
0.0
0.3
0.6
MR effect size for
'Selenium' on 'Forkhead box protein J2'

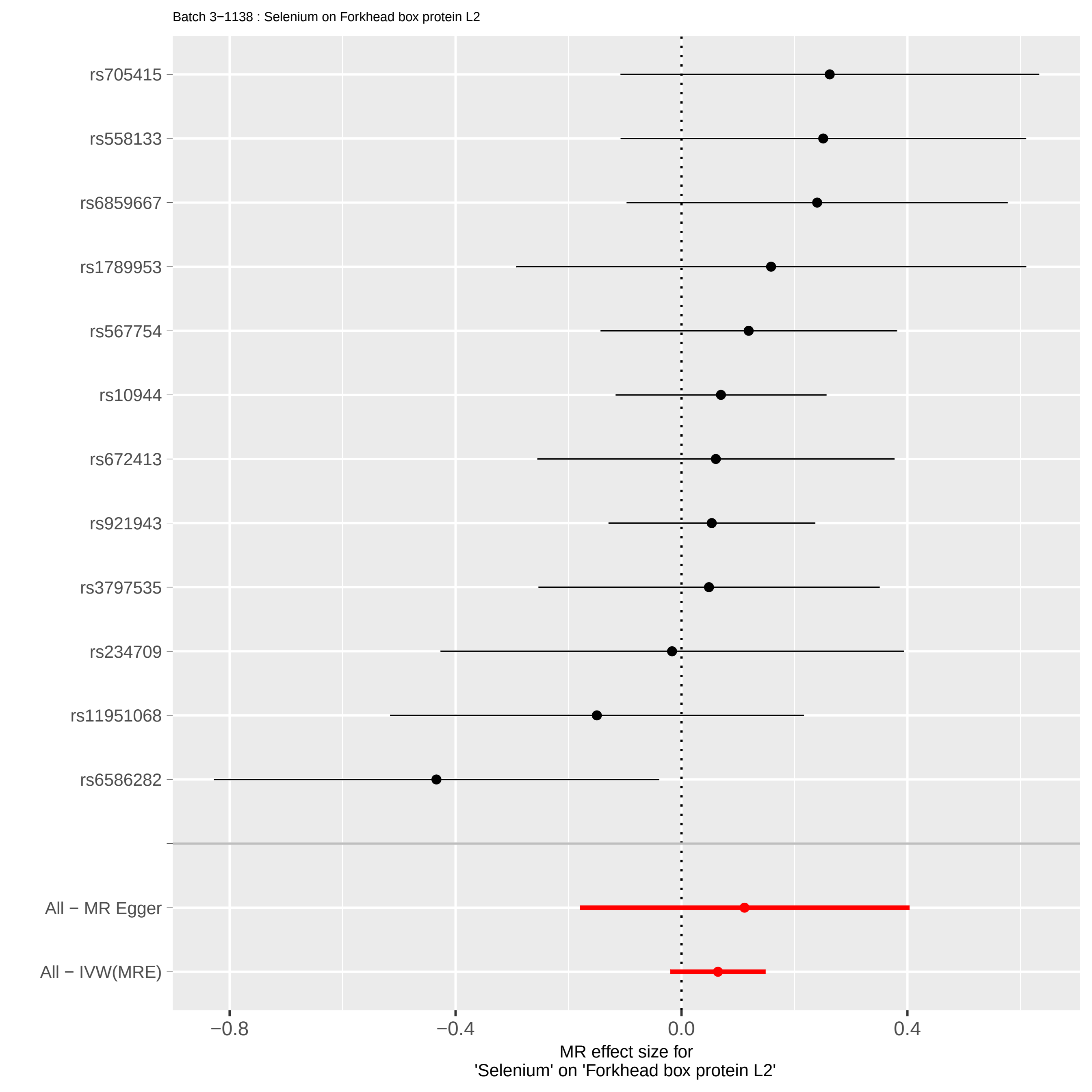
Batch 3−1138 : Selenium on Forkhead box protein L2
rs705415
rs558133
rs6859667
rs1789953
rs567754
rs10944
rs672413
rs921943
rs3797535
rs234709
rs11951068
rs6586282
All − MR Egger
All − IVW(MRE)
−0.8
−0.4
0.0
0.4
MR effect size for
'Selenium' on 'Forkhead box protein L2'

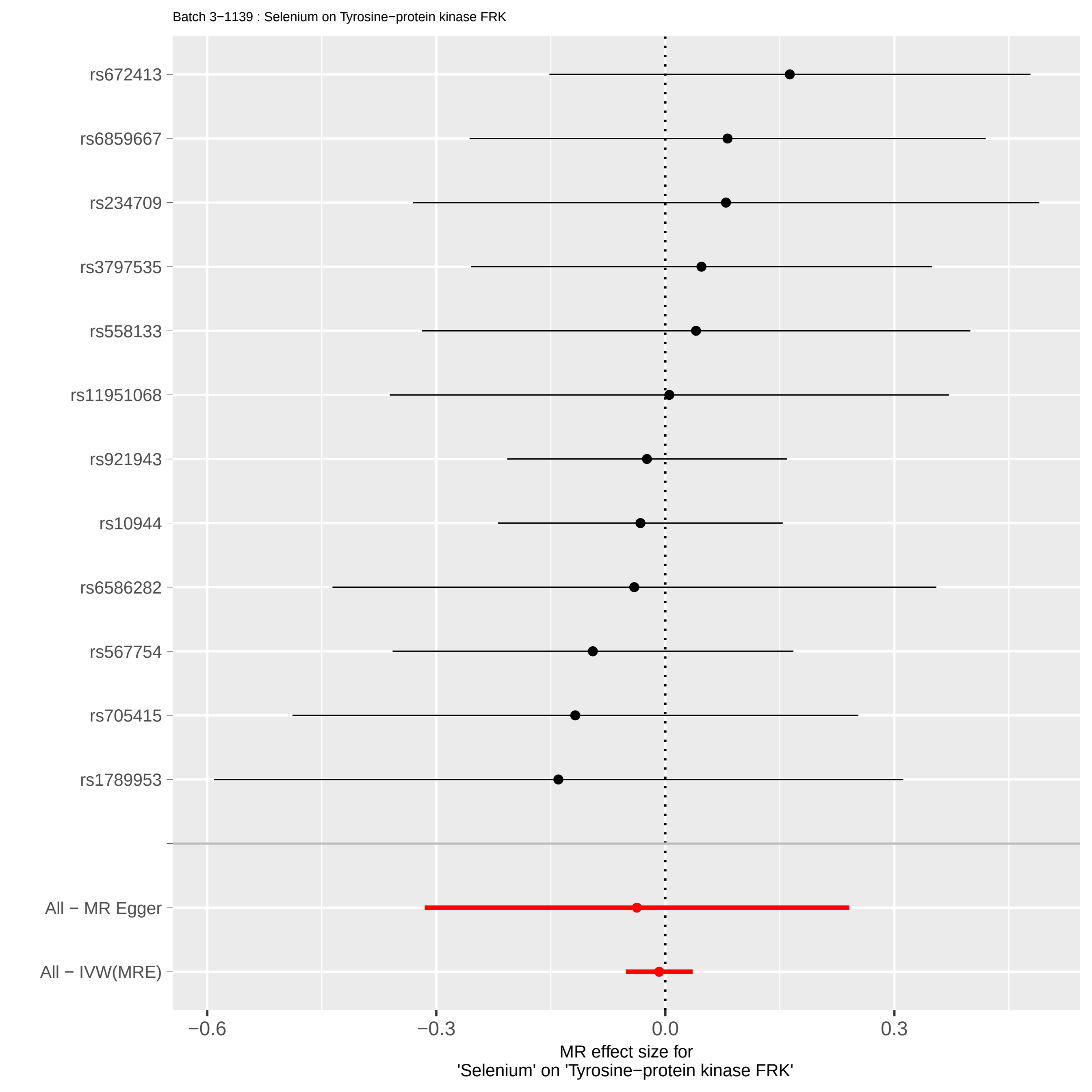
Batch 3−1139 : Selenium on Tyrosine−protein kinase FRK
rs672413
rs6859667
rs234709
rs3797535
rs558133
rs11951068
rs921943
rs10944
rs6586282
rs567754
rs705415
rs1789953
All − MR Egger
All − IVW(MRE)
−0.6
−0.3
0.0
0.3
MR effect size for
'Selenium' on 'Tyrosine−protein kinase FRK'

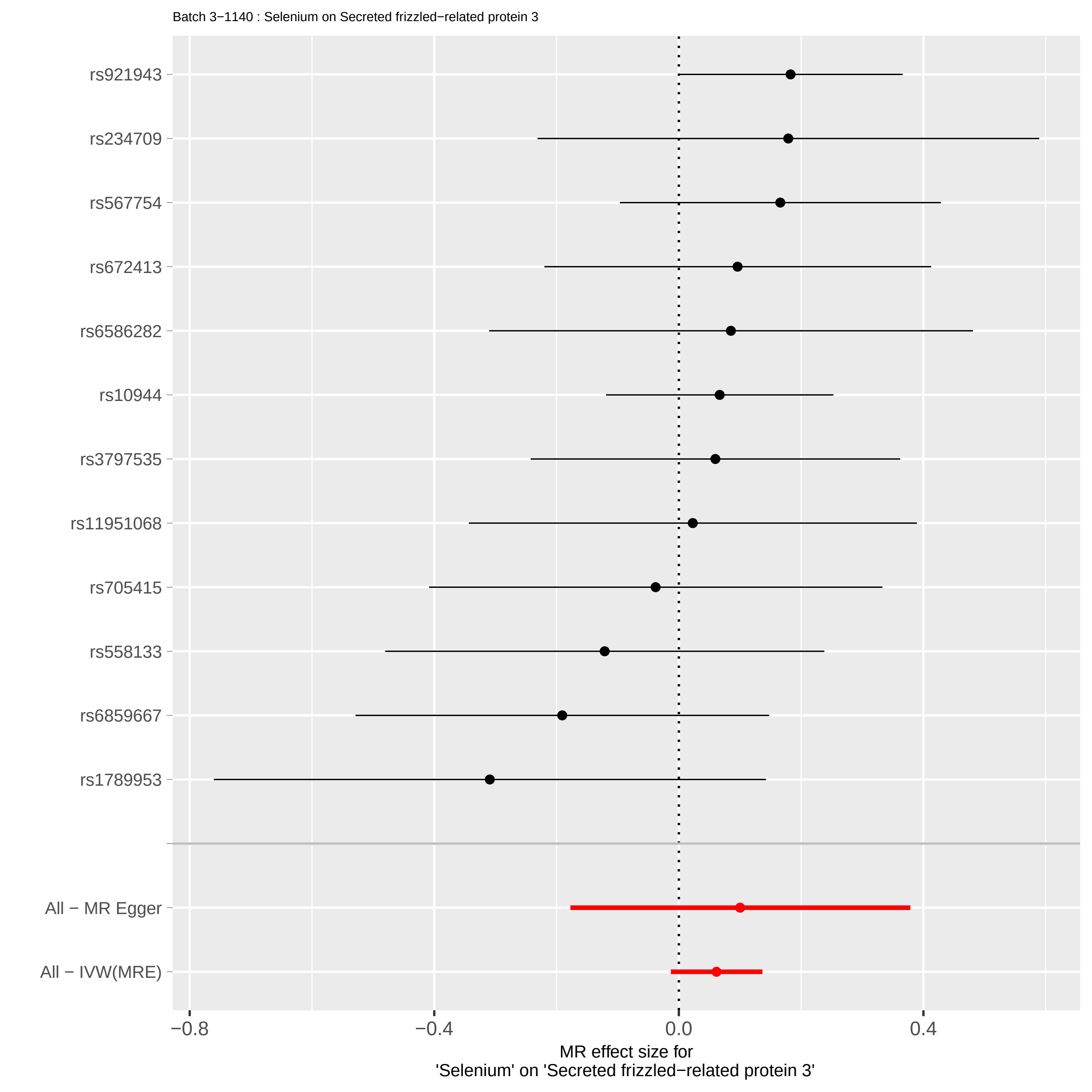

Batch 3−1140 : Selenium on Secreted frizzled−related protein 3
rs921943
rs234709
rs567754
rs672413
rs6586282
rs10944
rs3797535
rs11951068
rs705415
rs558133
rs6859667
rs1789953
All − MR Egger
All − IVW(MRE)
−0.8
−0.4
0.0
0.4
MR effect size for
'Selenium' on 'Secreted frizzled−related protein 3'

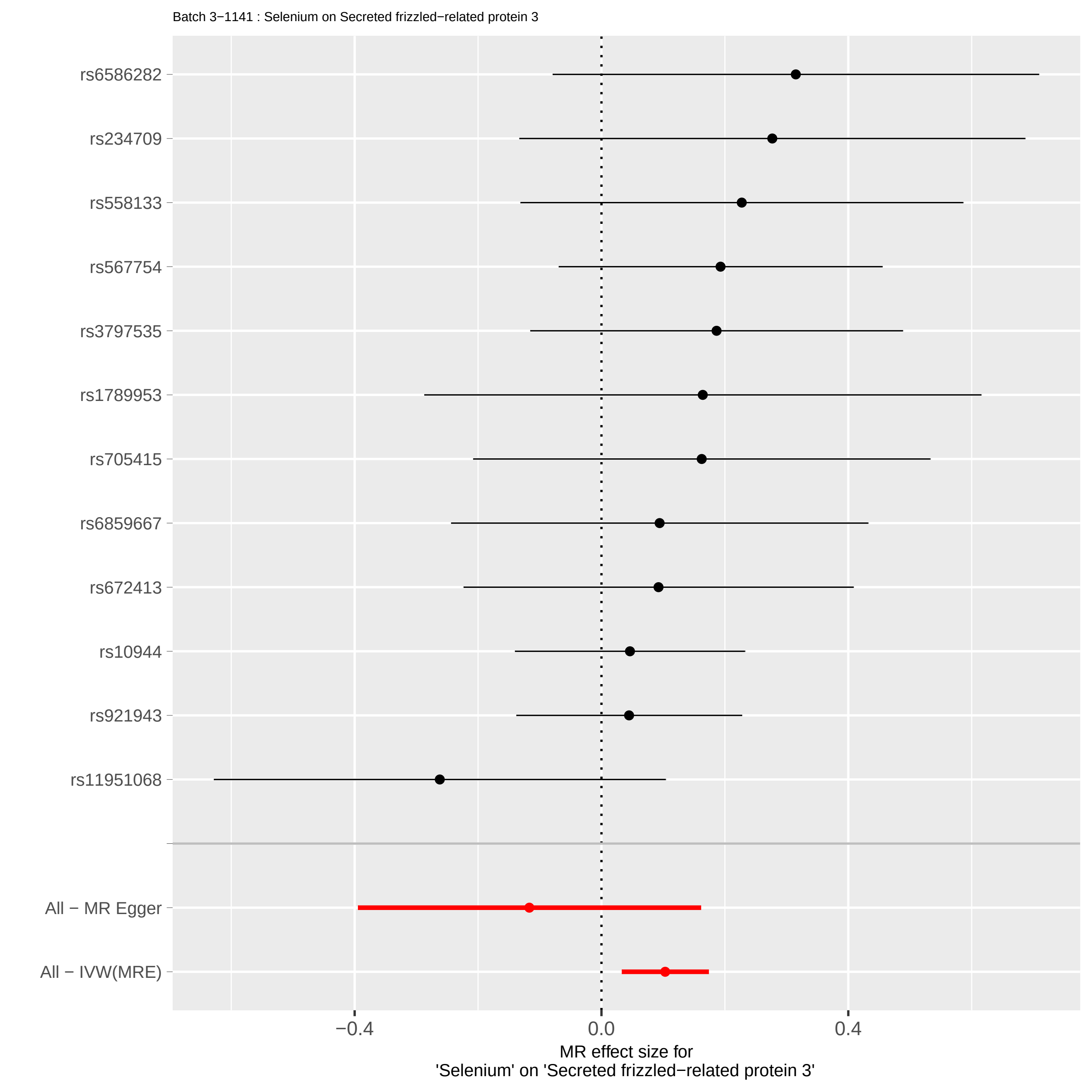
Batch 3−1141 : Selenium on Secreted frizzled−related protein 3
rs6586282
rs234709
rs558133
rs567754
rs3797535
rs1789953
rs705415
rs6859667
rs672413
rs10944
rs921943
rs11951068
All − MR Egger
All − IVW(MRE)
−0.4
0.0
0.4
MR effect size for
'Selenium' on 'Secreted frizzled−related protein 3'

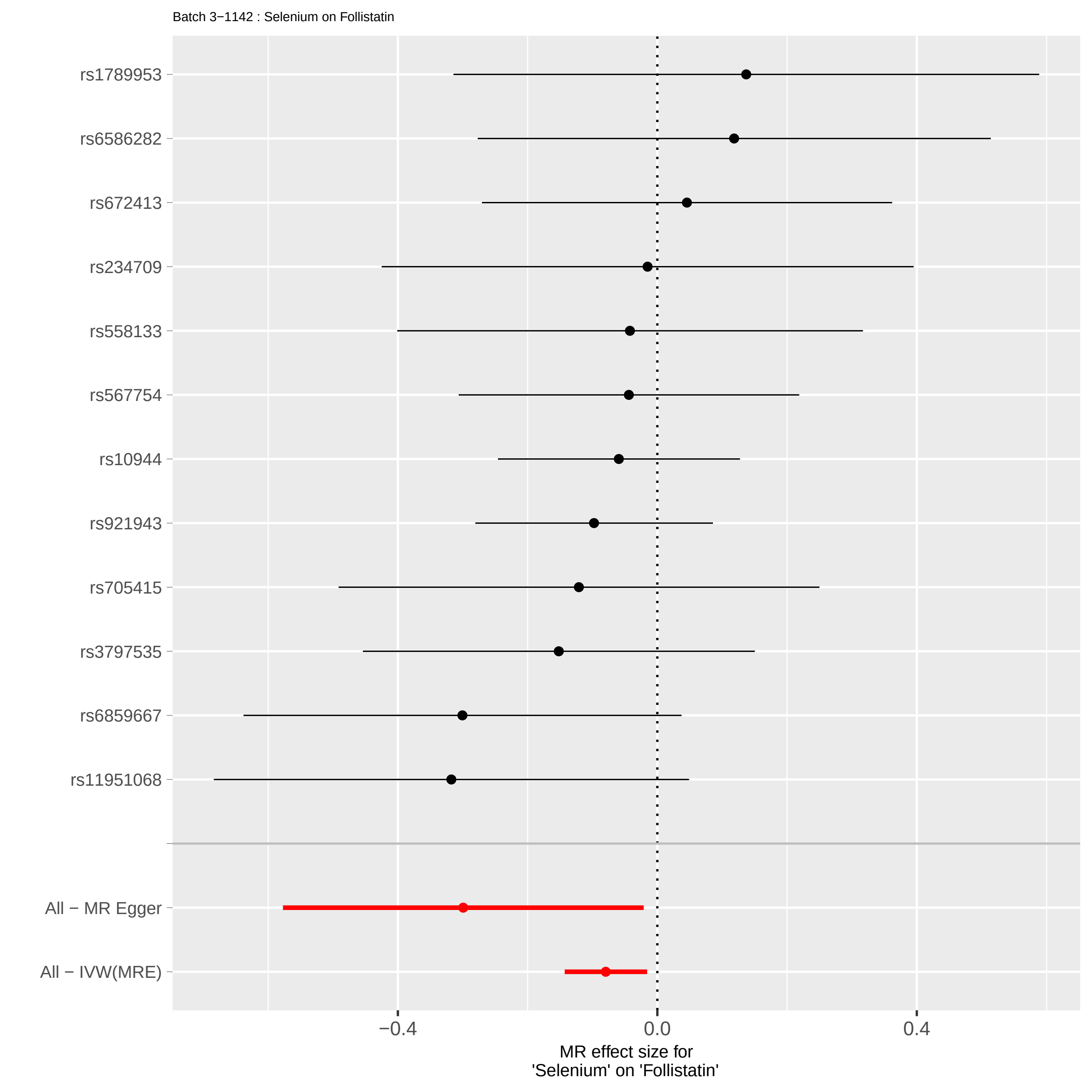
Batch 3−1142 : Selenium on Follistatin
rs1789953
rs6586282
rs672413
rs234709
rs558133
rs567754
rs10944
rs921943
rs705415
rs3797535
rs6859667
rs11951068
All − MR Egger
All − IVW(MRE)
−0.4
0.0
0.4
MR effect size for
'Selenium' on 'Follistatin'

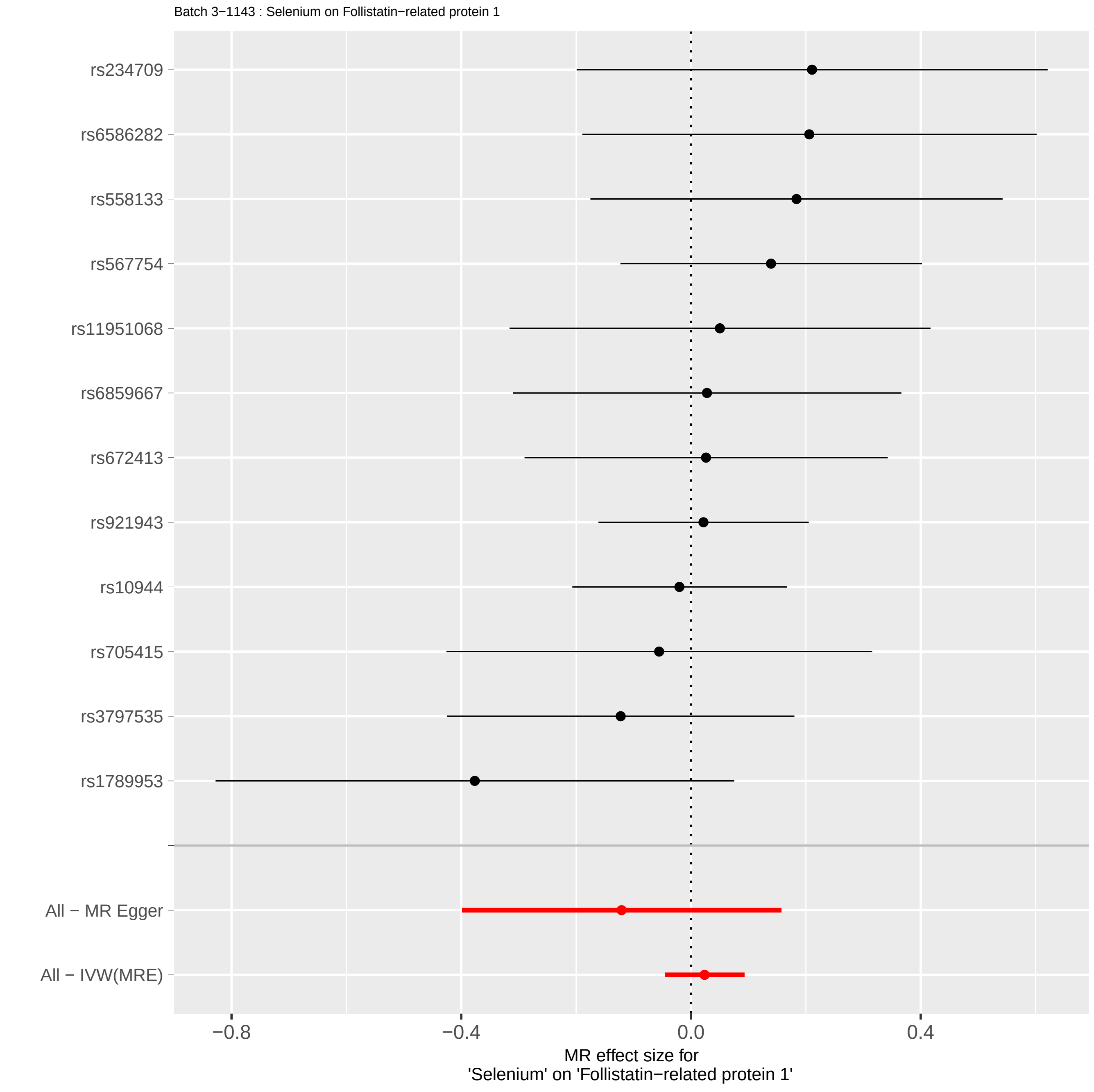

Batch 3−1143 : Selenium on Follistatin−related protein 1
rs234709
rs6586282
rs558133
rs567754
rs11951068
rs6859667
rs672413
rs921943
rs10944
rs705415
rs3797535
rs1789953
All − MR Egger
All − IVW(MRE)
−0.8
−0.4
0.0
0.4
MR effect size for
'Selenium' on 'Follistatin−related protein 1'

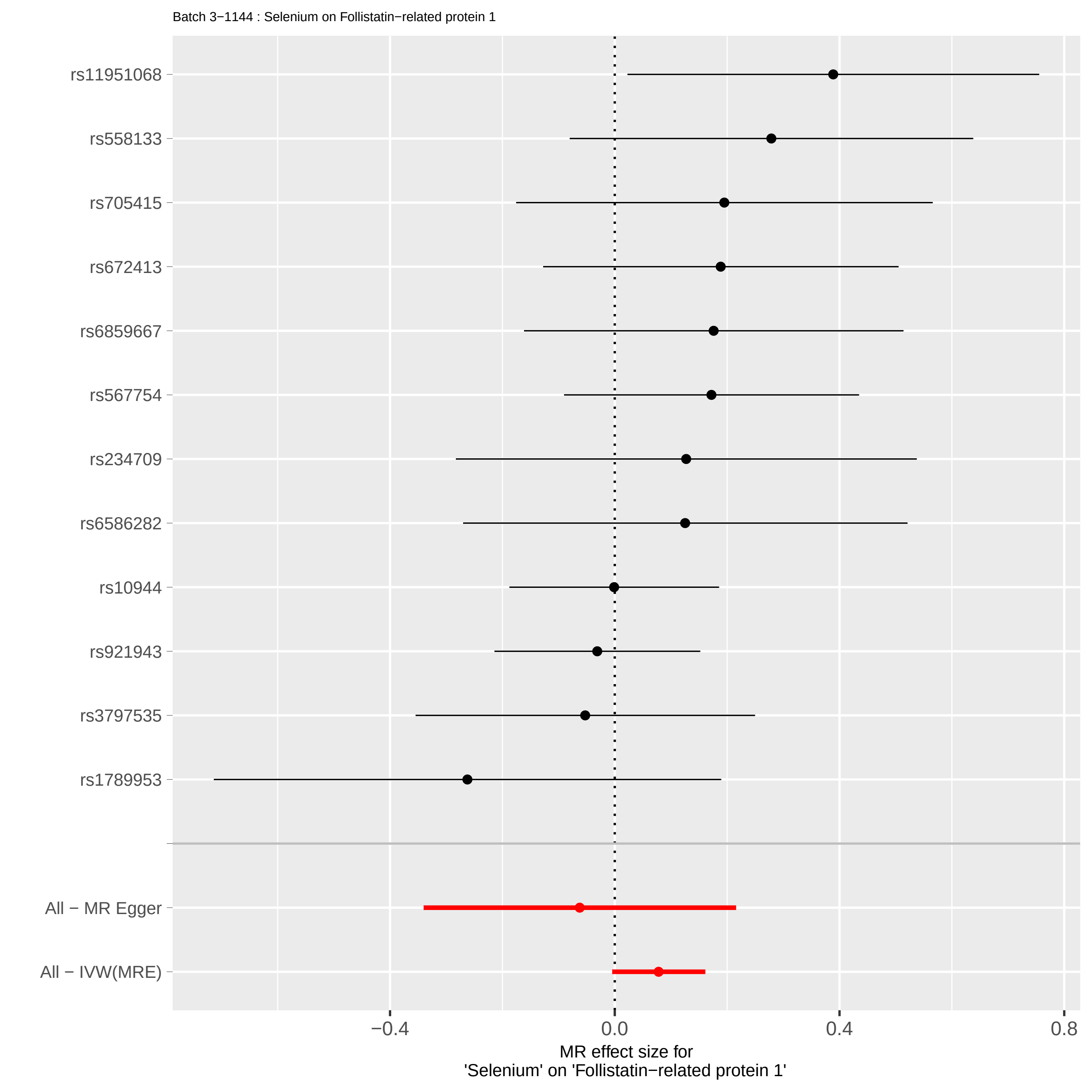
Batch 3−1144 : Selenium on Follistatin−related protein 1
rs11951068
rs558133
rs705415
rs672413
rs6859667
rs567754
rs234709
rs6586282
rs10944
rs921943
rs3797535
rs1789953
All − MR Egger
All − IVW(MRE)
−0.4
0.0
0.4
0.8
MR effect size for
'Selenium' on 'Follistatin−related protein 1'

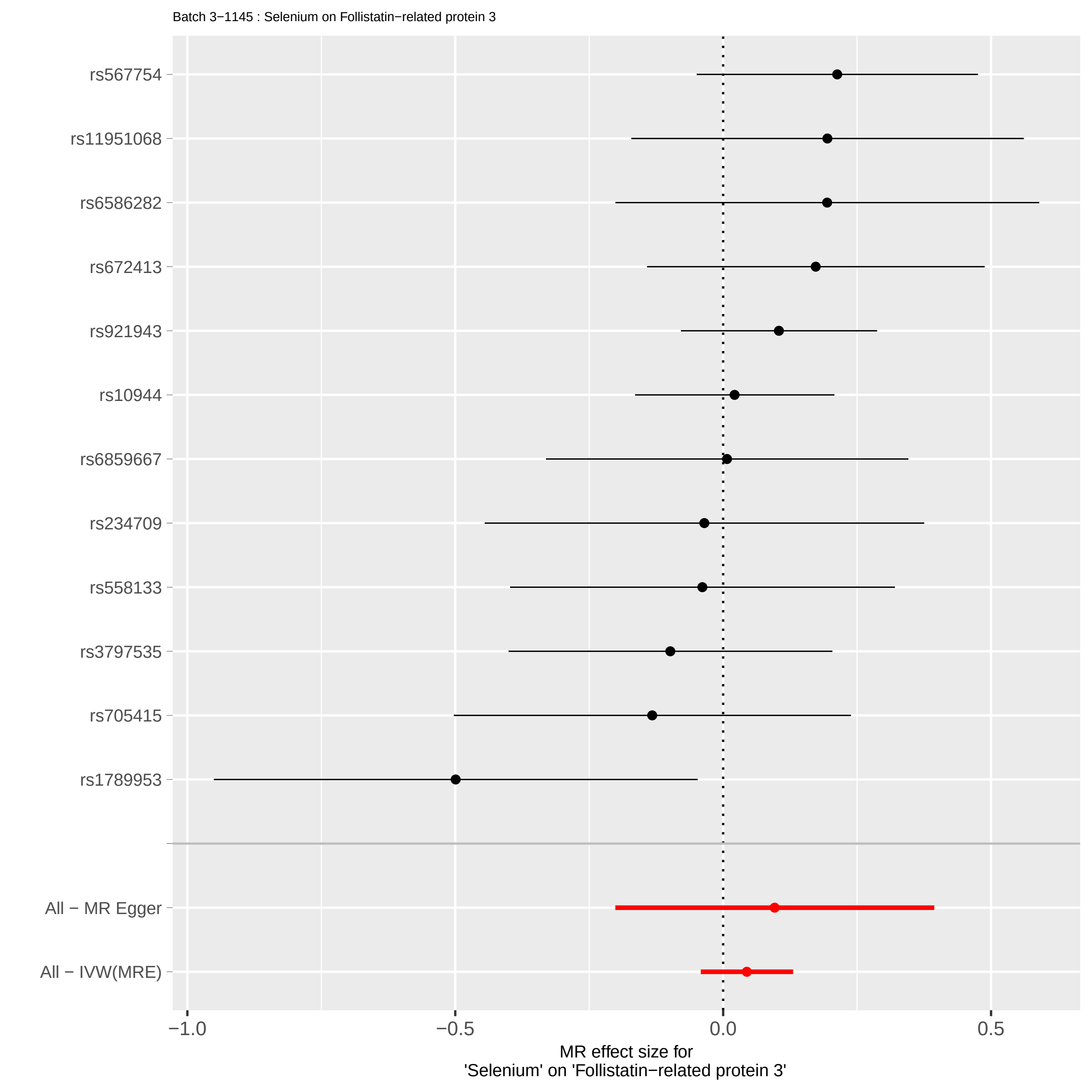

Batch 3−1145 : Selenium on Follistatin−related protein 3
rs567754
rs11951068
rs6586282
rs672413
rs921943
rs10944
rs6859667
rs234709
rs558133
rs3797535
rs705415
rs1789953
All − MR Egger
All − IVW(MRE)
−1.0
−0.5
0.0
0.5
MR effect size for
'Selenium' on 'Follistatin−related protein 3'

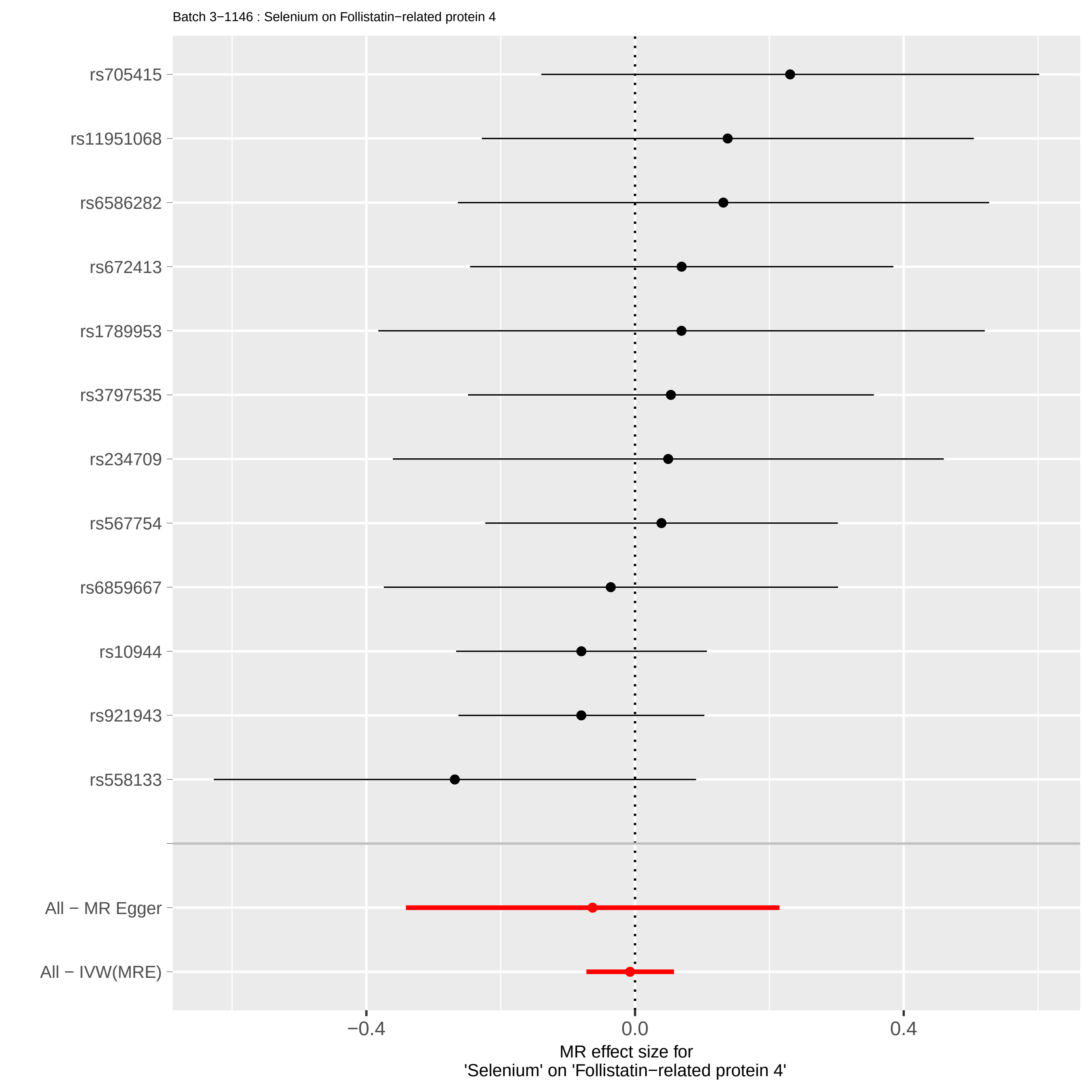
Batch 3−1146 : Selenium on Follistatin−related protein 4
rs705415
rs11951068
rs6586282
rs672413
rs1789953
rs3797535
rs234709
rs567754
rs6859667
rs10944
rs921943
rs558133
All − MR Egger
All − IVW(MRE)
−0.4
0.0
0.4
MR effect size for
'Selenium' on 'Follistatin−related protein 4'

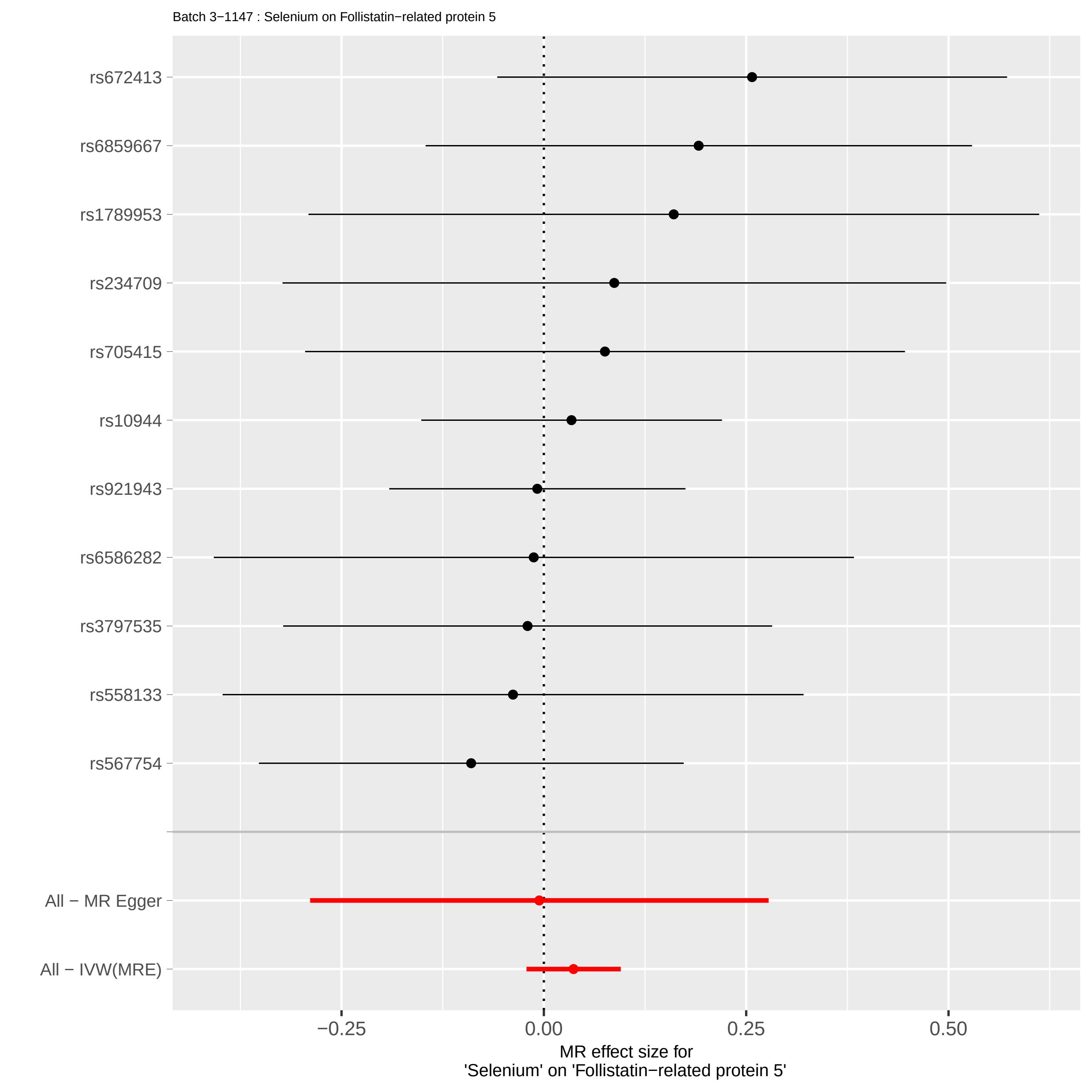
Batch 3−1147 : Selenium on Follistatin−related protein 5
rs672413
rs6859667
rs1789953
rs234709
rs705415
rs10944
rs921943
rs6586282
rs3797535
rs558133
rs567754
All − MR Egger
All − IVW(MRE)
−0.25
0.00
0.25
0.50
MR effect size for
'Selenium' on 'Follistatin−related protein 5'

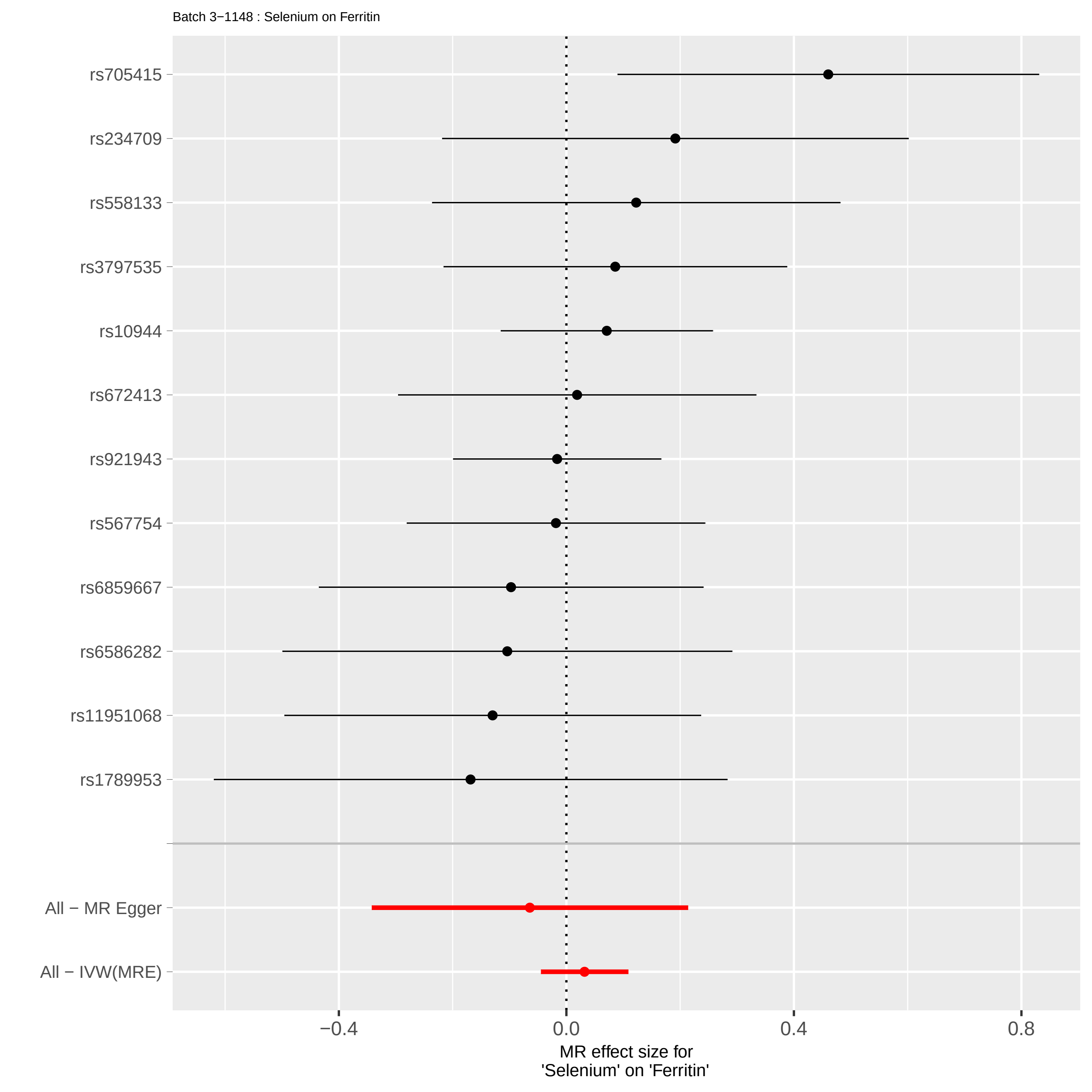

Batch 3−1148 : Selenium on Ferritin
rs705415
rs234709
rs558133
rs3797535
rs10944
rs672413
rs921943
rs567754
rs6859667
rs6586282
rs11951068
rs1789953
All − MR Egger
All − IVW(MRE)
−0.4
0.0
0.4
0.8
MR effect size for
'Selenium' on 'Ferritin'

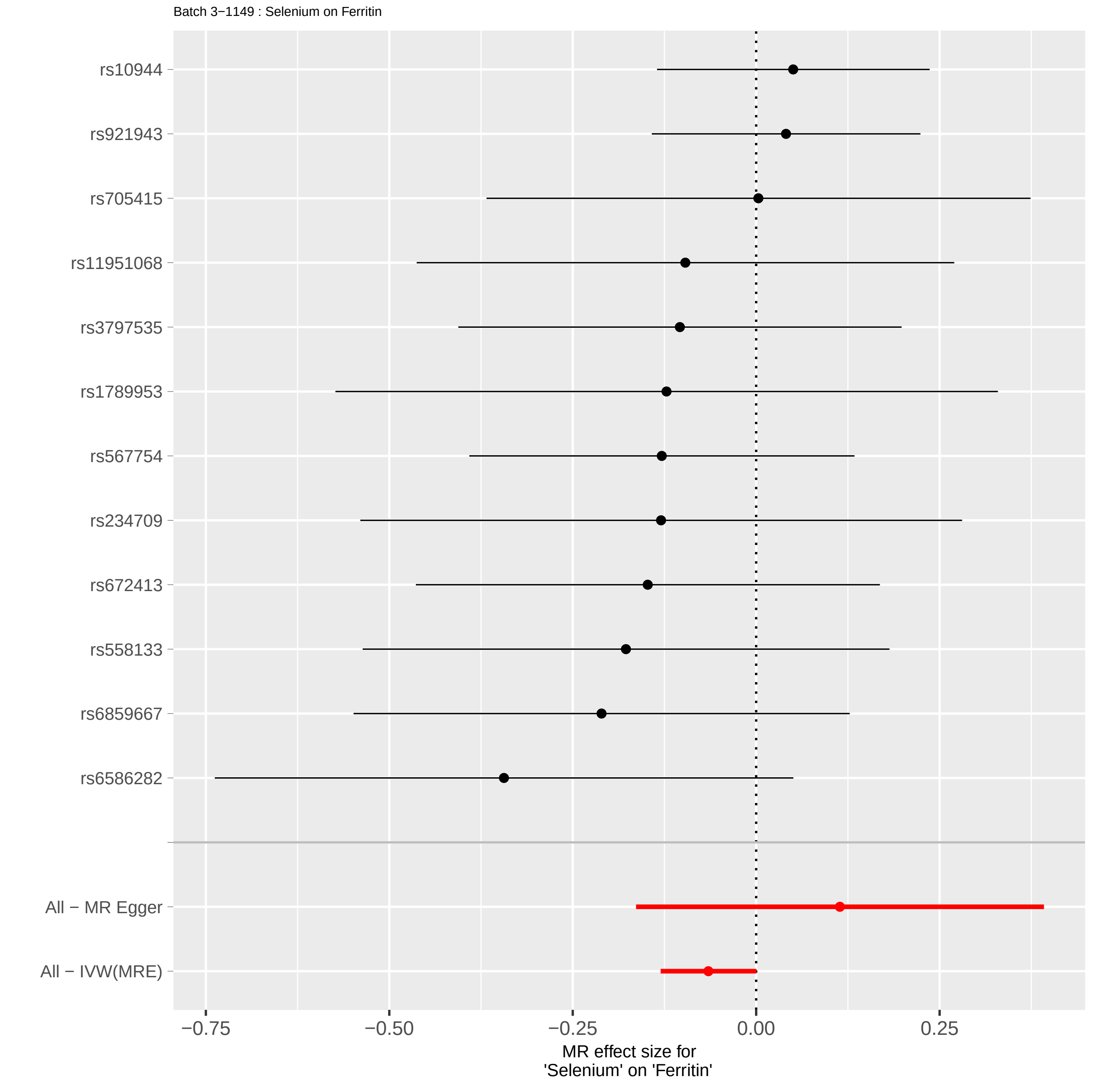

Batch 3−1149 : Selenium on Ferritin
rs10944
rs921943
rs705415
rs11951068
rs3797535
rs1789953
rs567754
rs234709
rs672413
rs558133
rs6859667
rs6586282
All − MR Egger
All − IVW(MRE)
−0.75
−0.50
−0.25
0.00
0.25
MR effect size for
'Selenium' on 'Ferritin'

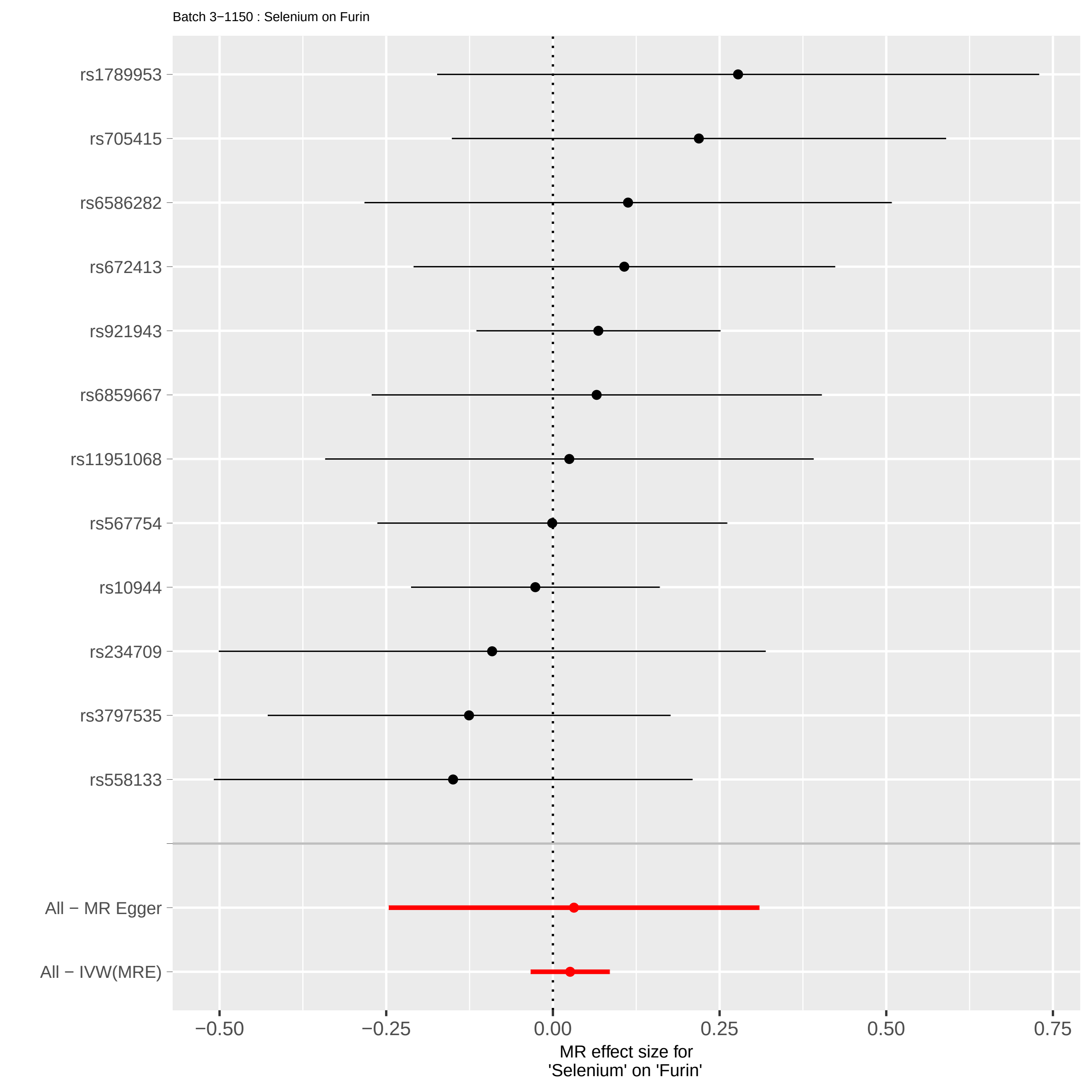
Batch 3−1150 : Selenium on Furin
rs1789953
rs705415
rs6586282
rs672413
rs921943
rs6859667
rs11951068
rs567754
rs10944
rs234709
rs3797535
rs558133
All − MR Egger
All − IVW(MRE)
−0.50
−0.25
0.00
0.25
0.50
0.75
MR effect size for
'Selenium' on 'Furin'

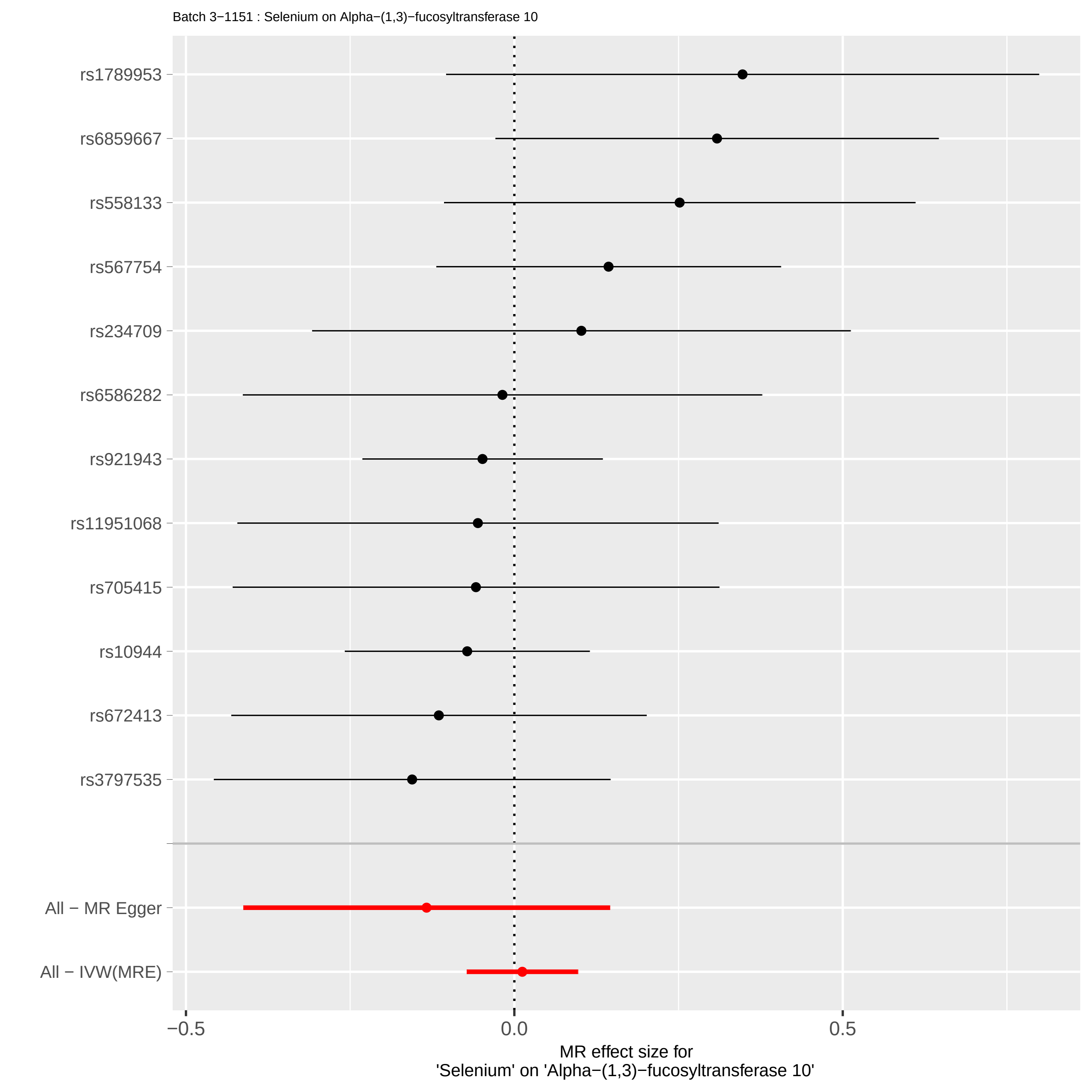
Batch 3−1151 : Selenium on Alpha−(1,3)−fucosyltransferase 10
rs1789953
rs6859667
rs558133
rs567754
rs234709
rs6586282
rs921943
rs11951068
rs705415
rs10944
rs672413
rs3797535
All − MR Egger
All − IVW(MRE)
−0.5
0.0
0.5
MR effect size for
'Selenium' on 'Alpha−(1,3)−fucosyltransferase 10'

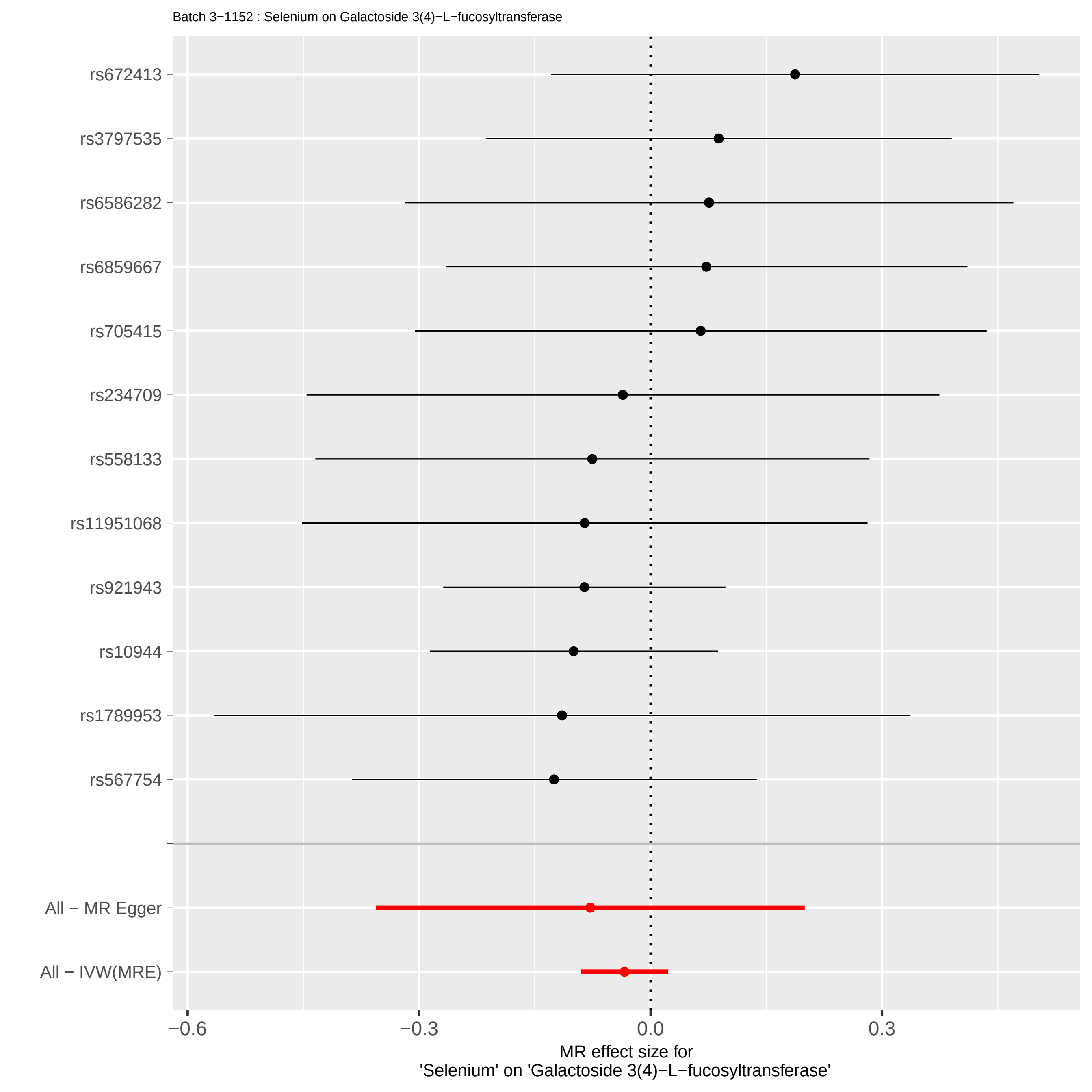

Batch 3−1152 : Selenium on Galactoside 3(4)−L−fucosyltransferase
rs672413
rs3797535
rs6586282
rs6859667
rs705415
rs234709
rs558133
rs11951068
rs921943
rs10944
rs1789953
rs567754
All − MR Egger
All − IVW(MRE)
−0.6
−0.3
0.0
0.3
MR effect size for
'Selenium' on 'Galactoside 3(4)−L−fucosyltransferase'

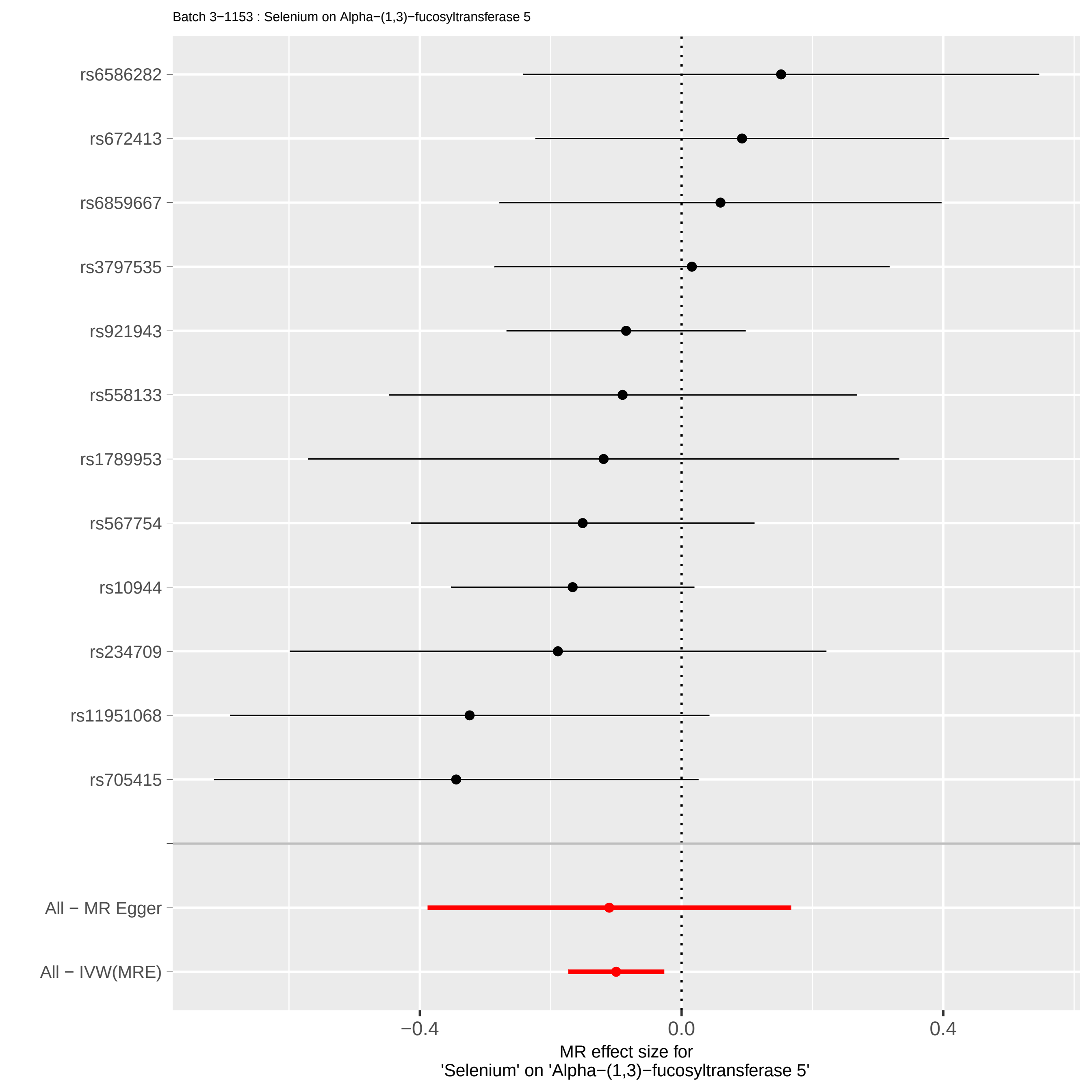

Batch 3−1153 : Selenium on Alpha−(1,3)−fucosyltransferase 5
rs6586282
rs672413
rs6859667
rs3797535
rs921943
rs558133
rs1789953
rs567754
rs10944
rs234709
rs11951068
rs705415
All − MR Egger
All − IVW(MRE)
−0.4
0.0
0.4
MR effect size for
'Selenium' on 'Alpha−(1,3)−fucosyltransferase 5'

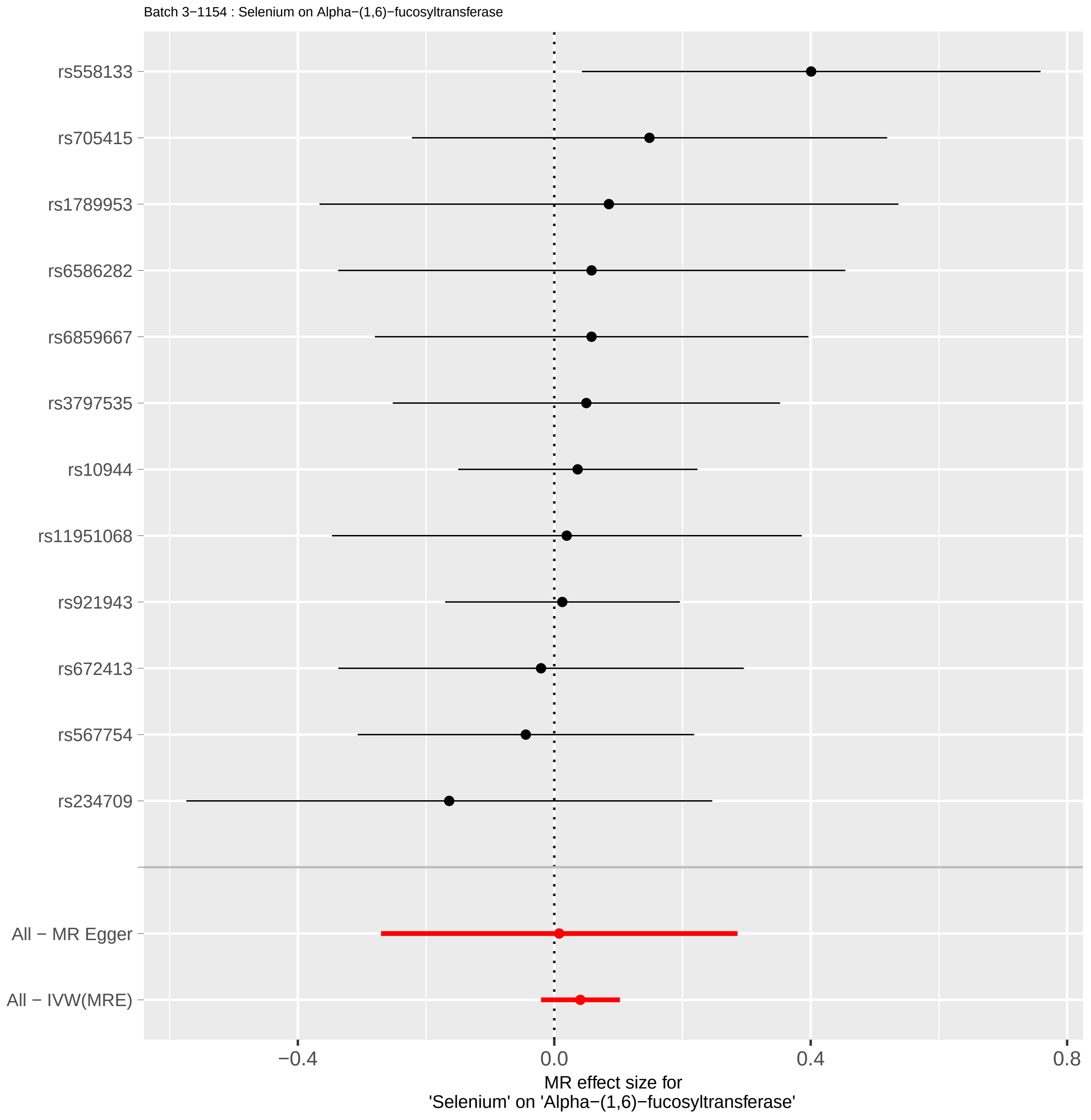

Batch 3−1154 : Selenium on Alpha−(1,6)−fucosyltransferase
rs558133
rs705415
rs1789953
rs6586282
rs6859667
rs3797535
rs10944
rs11951068
rs921943
rs672413
rs567754
rs234709
All − MR Egger
All − IVW(MRE)
−0.4
0.0
0.4
0.8
MR effect size for
'Selenium' on 'Alpha−(1,6)−fucosyltransferase'

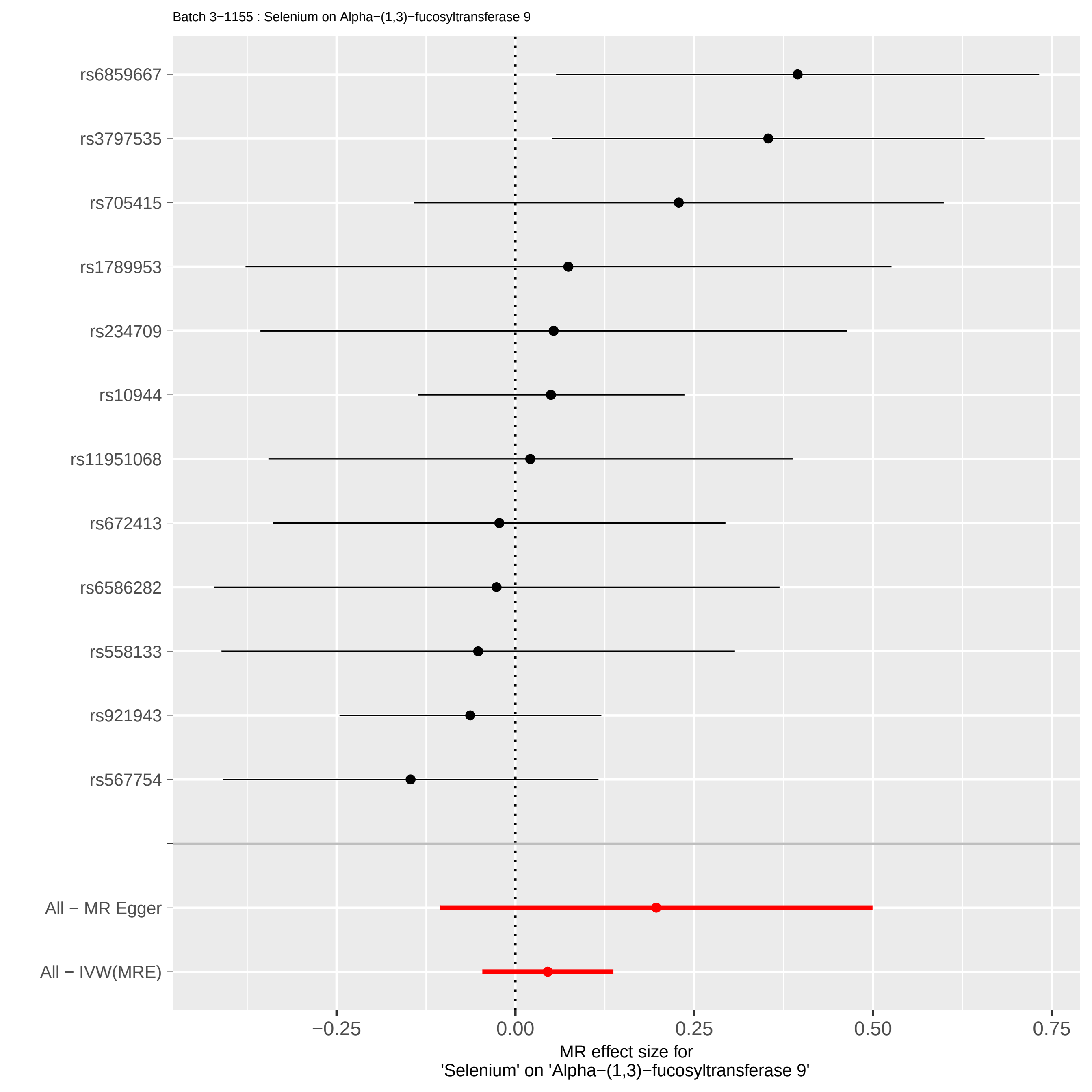

Batch 3−1155 : Selenium on Alpha−(1,3)−fucosyltransferase 9
rs6859667
rs3797535
rs705415
rs1789953
rs234709
rs10944
rs11951068
rs672413
rs6586282
rs558133
rs921943
rs567754
All − MR Egger
All − IVW(MRE)
−0.25
0.00
0.25
0.50
0.75
MR effect size for
'Selenium' on 'Alpha−(1,3)−fucosyltransferase 9'

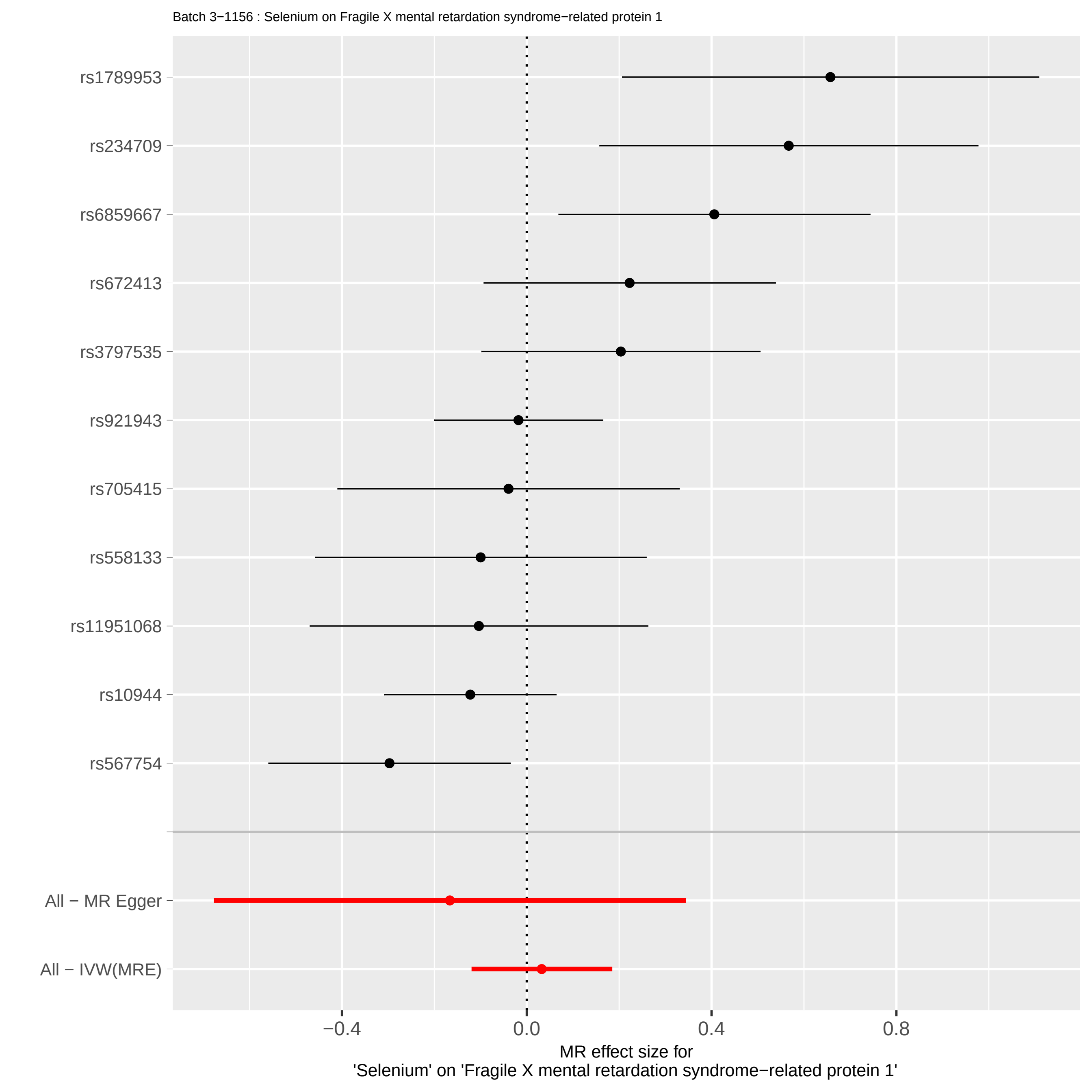

Batch 3−1156 : Selenium on Fragile X mental retardation syndrome−related protein 1
rs1789953
rs234709
rs6859667
rs672413
rs3797535
rs921943
rs705415
rs558133
rs11951068
rs10944
rs567754
All − MR Egger
All − IVW(MRE)
−0.4
0.0
0.4
0.8
MR effect size for
'Selenium' on 'Fragile X mental retardation syndrome−related protein 1'

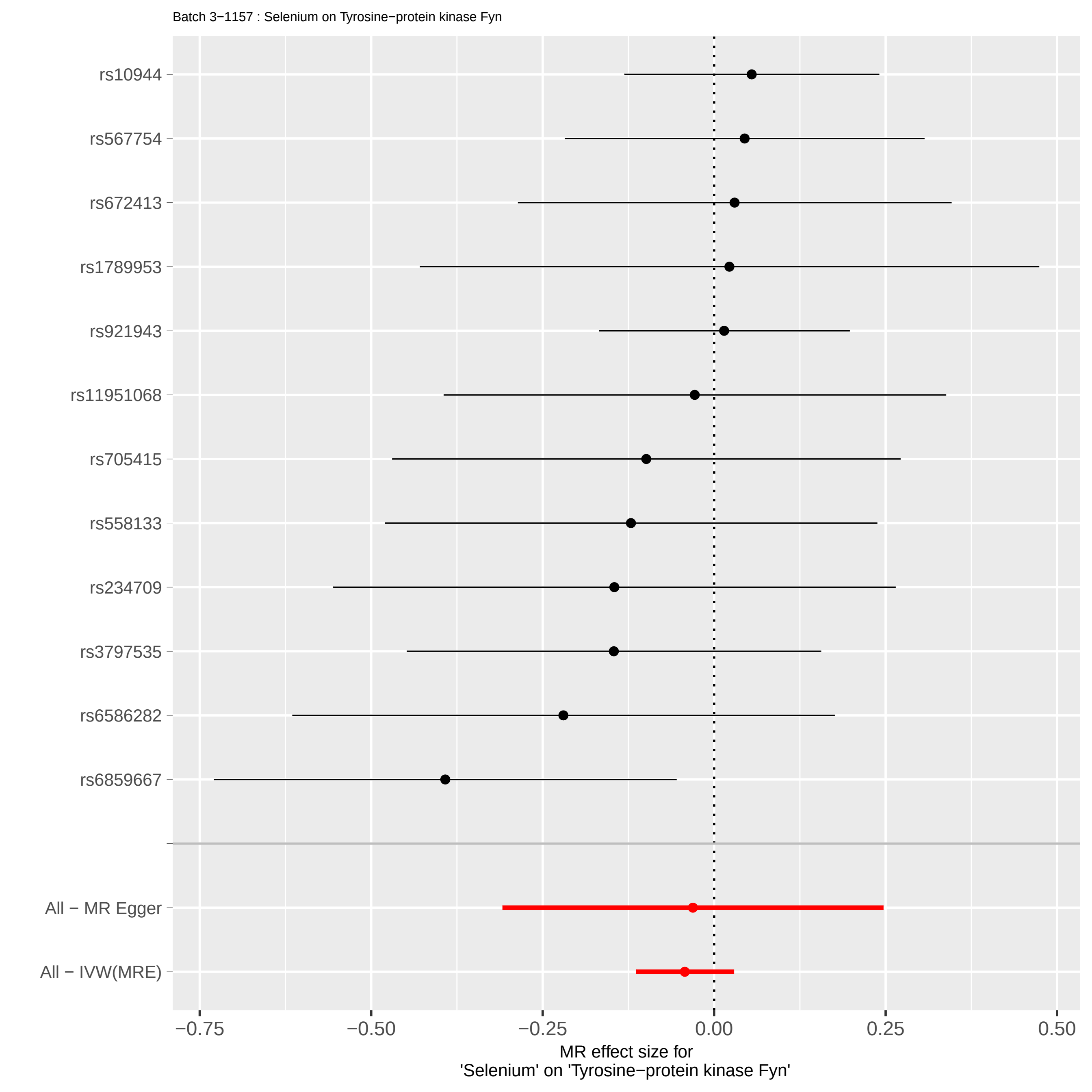
Batch 3−1157 : Selenium on Tyrosine−protein kinase Fyn
rs10944
rs567754
rs672413
rs1789953
rs921943
rs11951068
rs705415
rs558133
rs234709
rs3797535
rs6586282
rs6859667
All − MR Egger
All − IVW(MRE)
−0.75
−0.50
−0.25
0.00
0.25
0.50
MR effect size for
'Selenium' on 'Tyrosine−protein kinase Fyn'

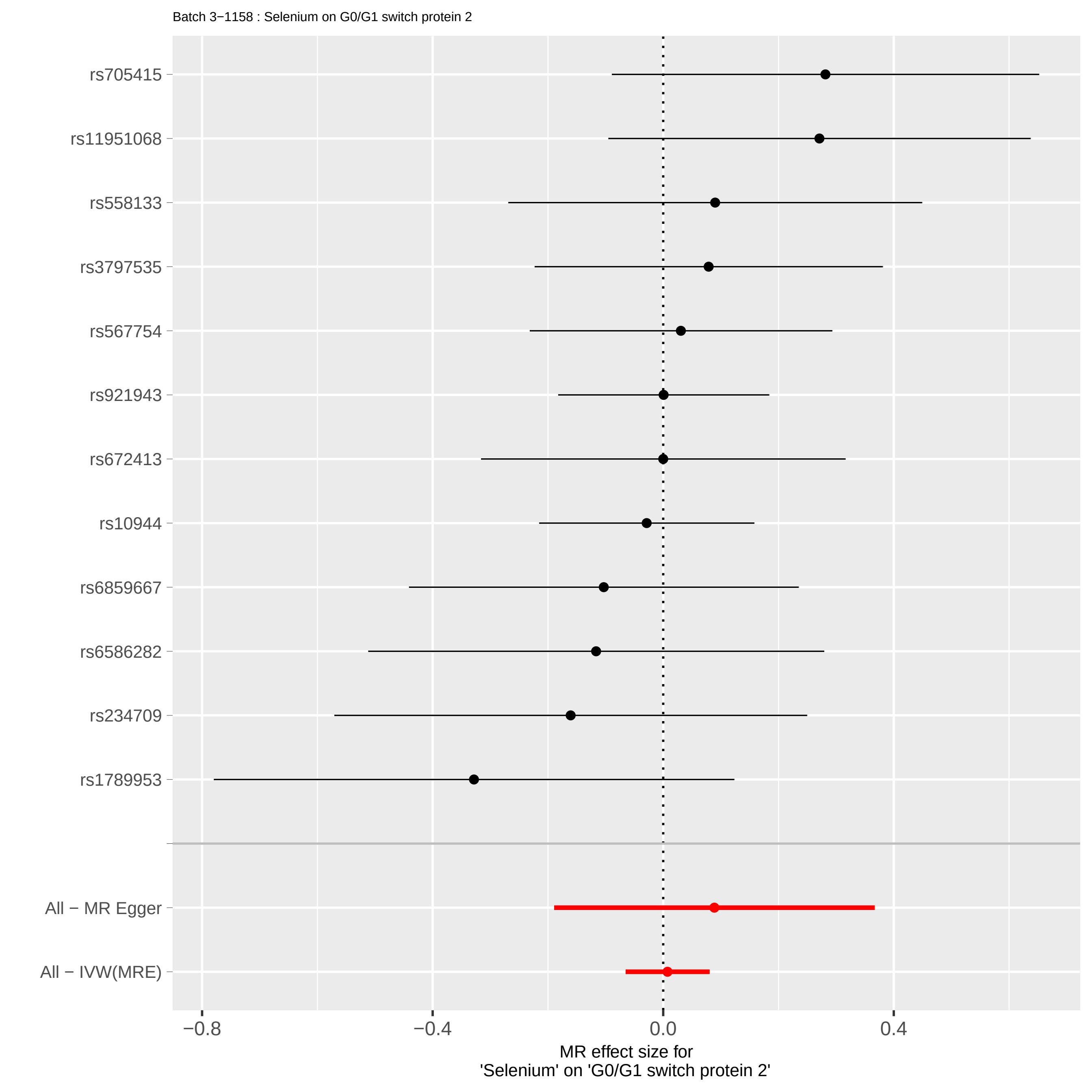
Batch 3−1158 : Selenium on G0/G1 switch protein 2
rs705415
rs11951068
rs558133
rs3797535
rs567754
rs921943
rs672413
rs10944
rs6859667
rs6586282
rs234709
rs1789953
All − MR Egger
All − IVW(MRE)
−0.8
−0.4
0.0
0.4
MR effect size for
'Selenium' on 'G0/G1 switch protein 2'

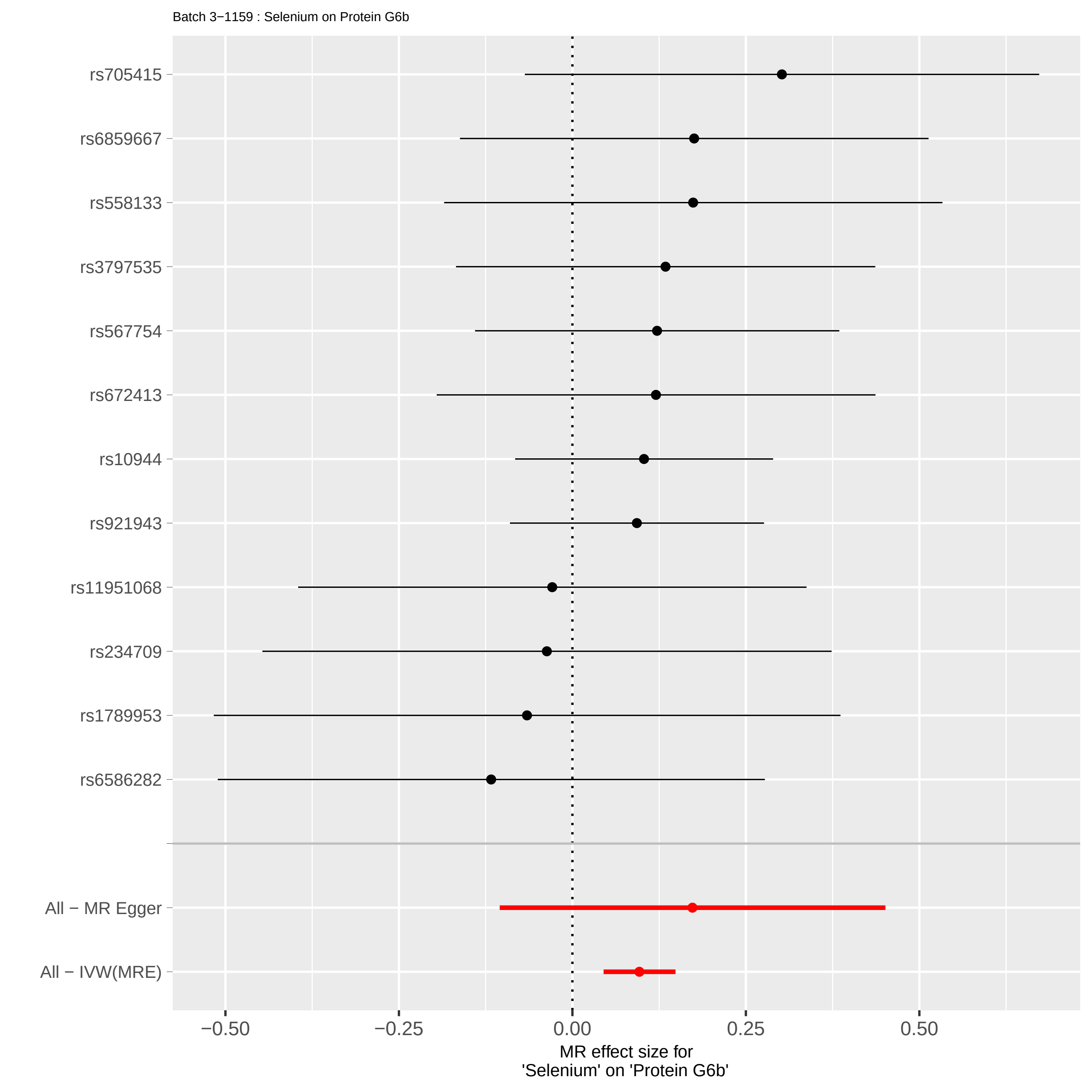
Batch 3−1159 : Selenium on Protein G6b
rs705415
rs6859667
rs558133
rs3797535
rs567754
rs672413
rs10944
rs921943
rs11951068
rs234709
rs1789953
rs6586282
All − MR Egger
All − IVW(MRE)
−0.50
−0.25
0.00
0.25
0.50
MR effect size for
'Selenium' on 'Protein G6b'

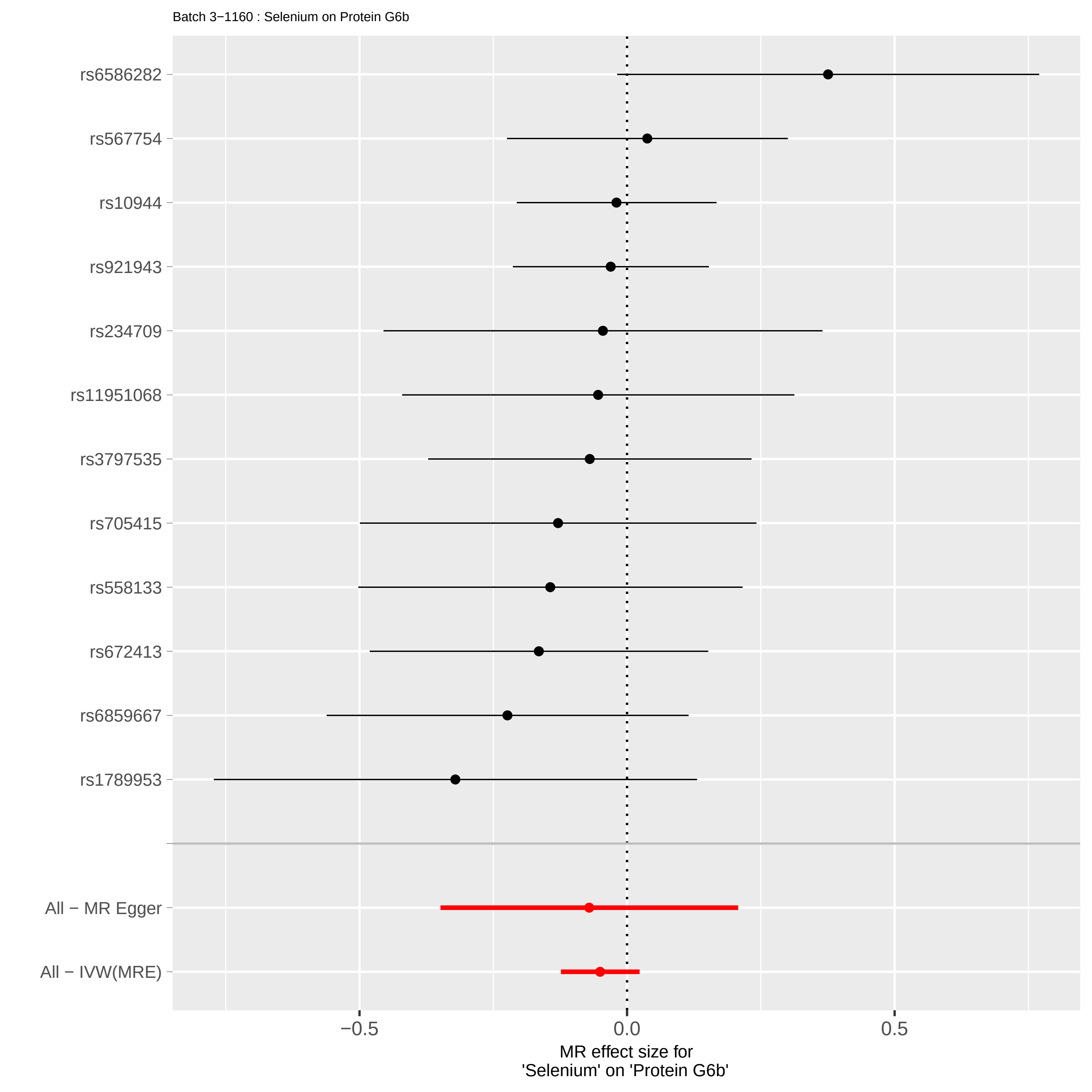
Batch 3−1160 : Selenium on Protein G6b
rs6586282
rs567754
rs10944
rs921943
rs234709
rs11951068
rs3797535
rs705415
rs558133
rs672413
rs6859667
rs1789953
All − MR Egger
All − IVW(MRE)
−0.5
0.0
0.5
MR effect size for
'Selenium' on 'Protein G6b'

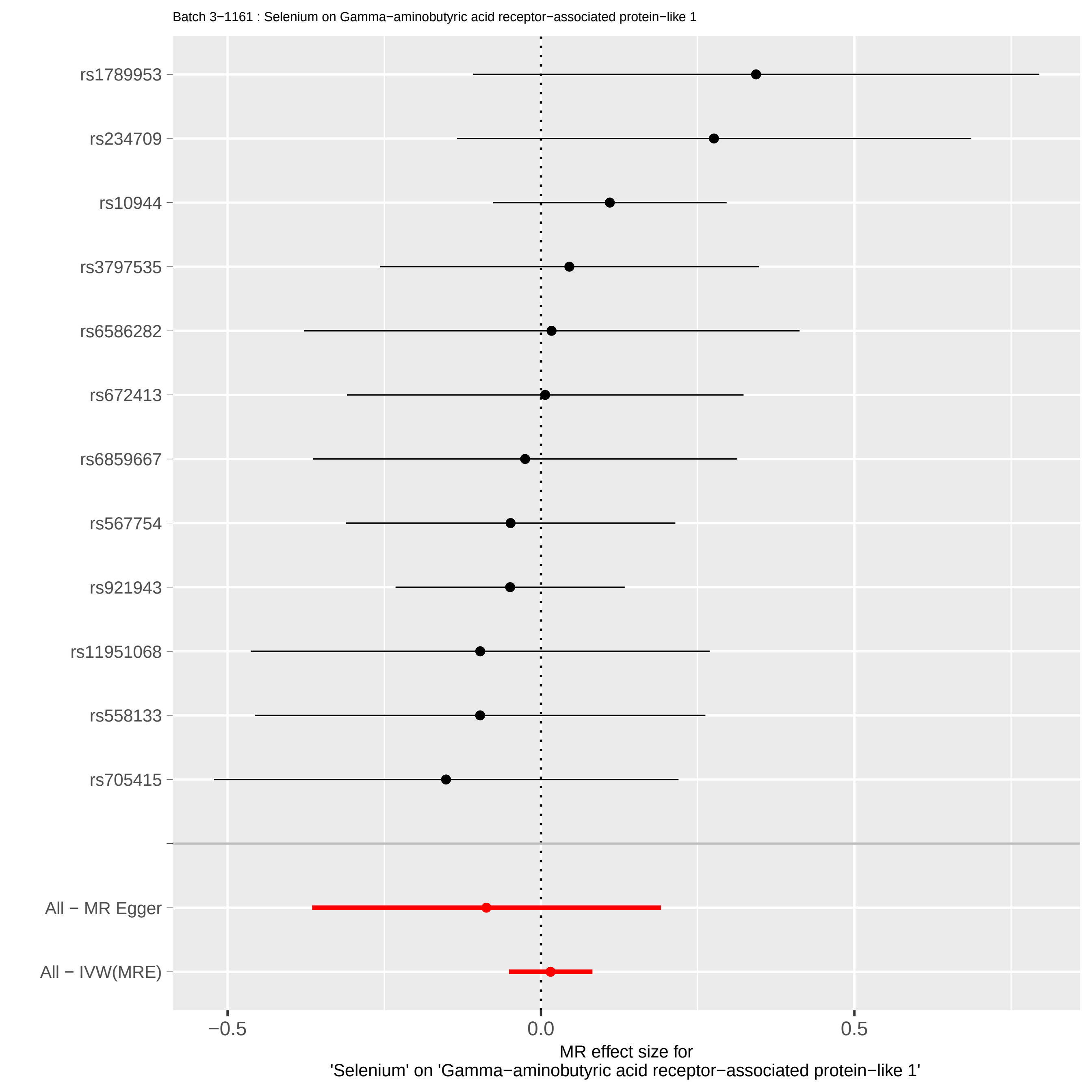
Batch 3−1161 : Selenium on Gamma−aminobutyric acid receptor−associated protein−like 1
rs1789953
rs234709
rs10944
rs3797535
rs6586282
rs672413
rs6859667
rs567754
rs921943
rs11951068
rs558133
rs705415
All − MR Egger
All − IVW(MRE)
−0.5
0.0
0.5
MR effect size for
'Selenium' on 'Gamma−aminobutyric acid receptor−associated protein−like 1'

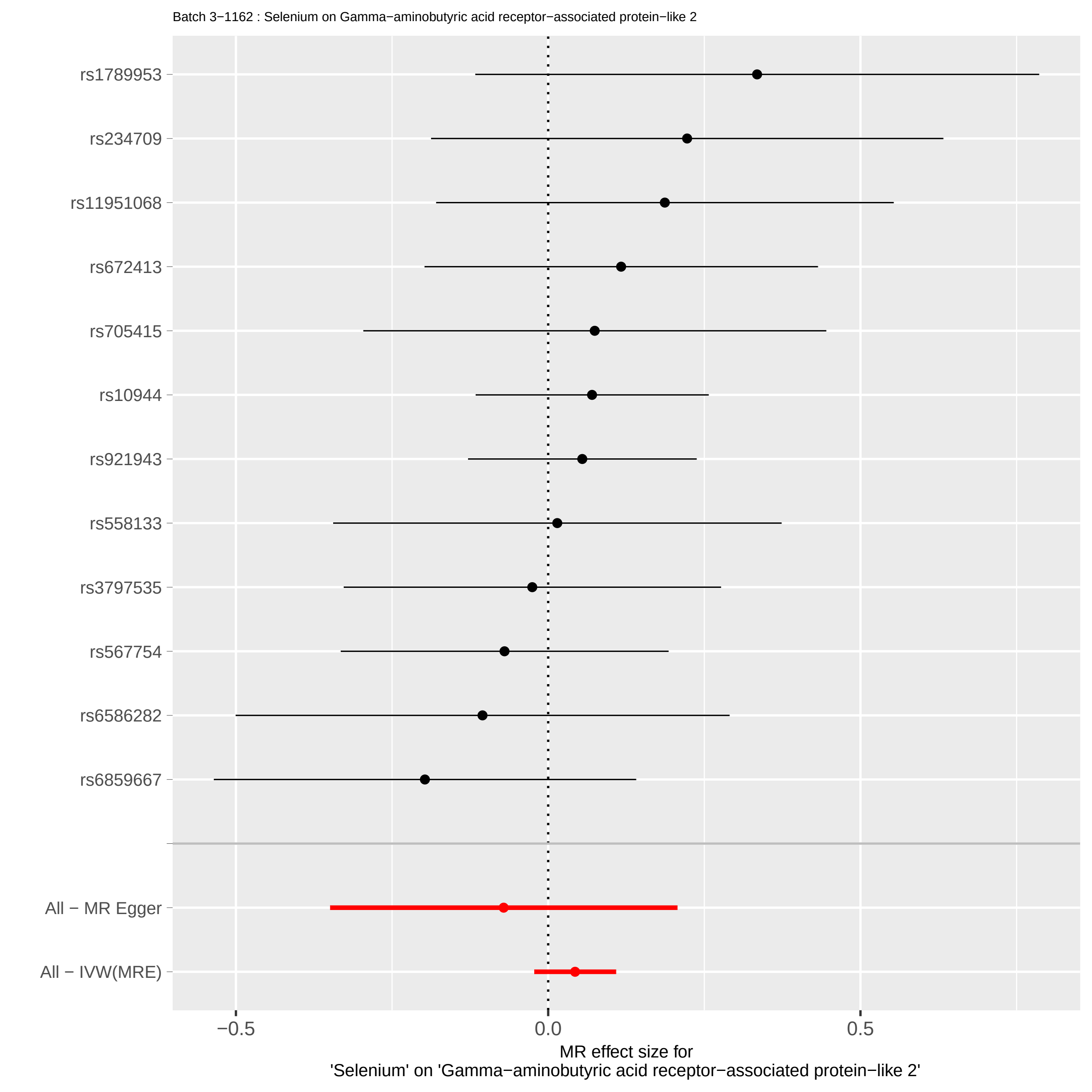

Batch 3−1162 : Selenium on Gamma−aminobutyric acid receptor−associated protein−like 2
rs1789953
rs234709
rs11951068
rs672413
rs705415
rs10944
rs921943
rs558133
rs3797535
rs567754
rs6586282
rs6859667
All − MR Egger
All − IVW(MRE)
−0.5
0.0
0.5
MR effect size for
'Selenium' on 'Gamma−aminobutyric acid receptor−associated protein−like 2'

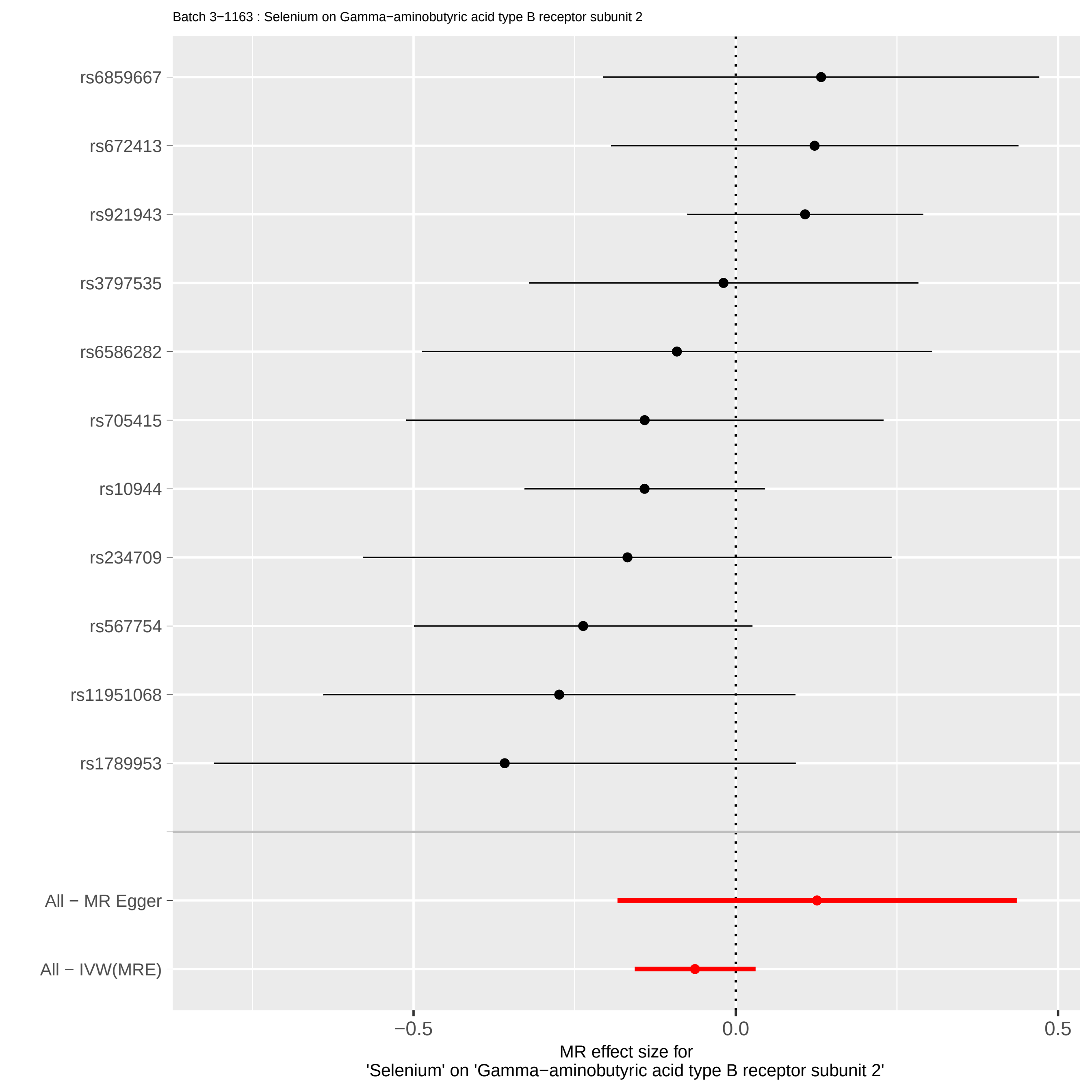
Batch 3−1163 : Selenium on Gamma−aminobutyric acid type B receptor subunit 2
rs6859667
rs672413
rs921943
rs3797535
rs6586282
rs705415
rs10944
rs234709
rs567754
rs11951068
rs1789953
All − MR Egger
All − IVW(MRE)
−0.5
0.0
0.5
MR effect size for
'Selenium' on 'Gamma−aminobutyric acid type B receptor subunit 2'

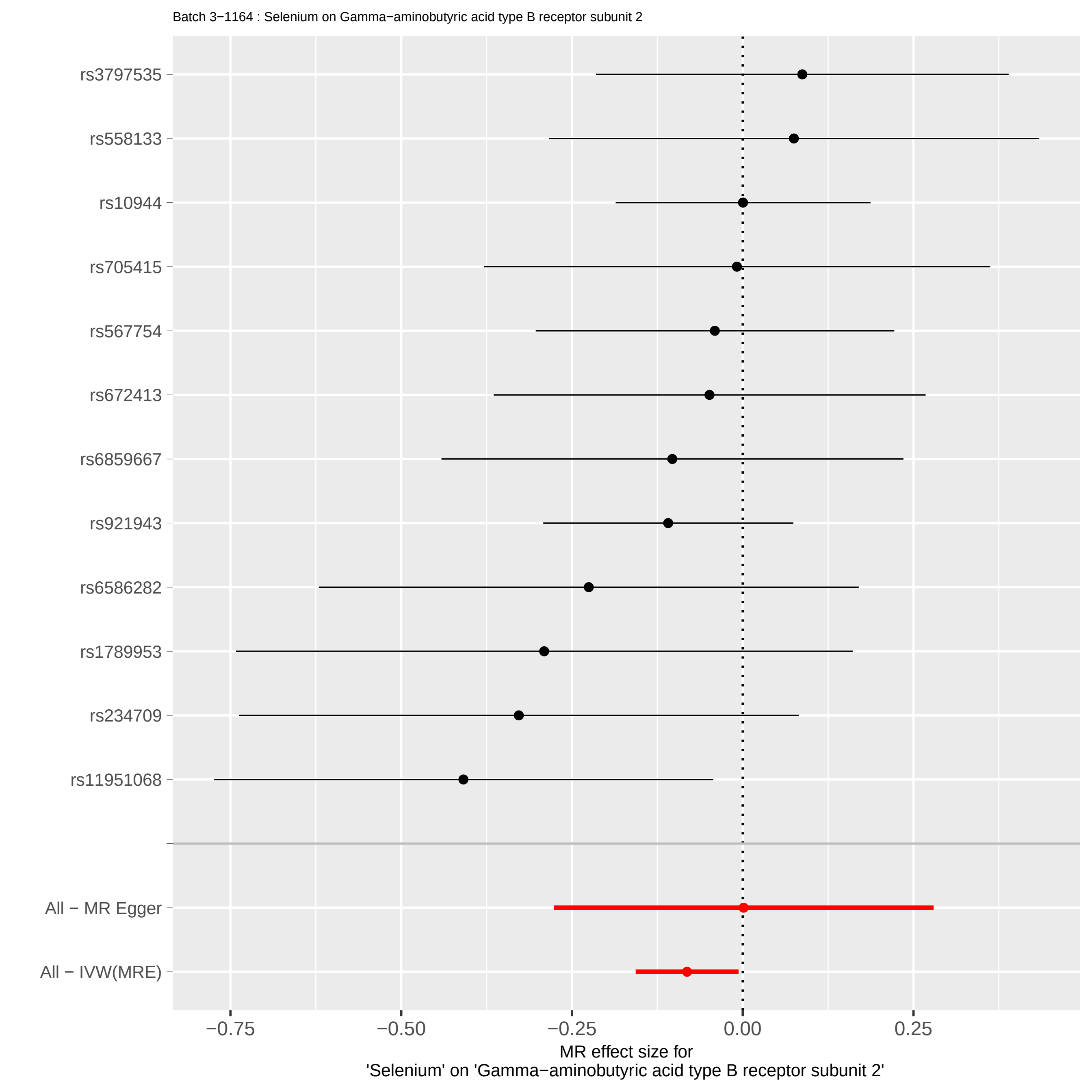
Batch 3−1164 : Selenium on Gamma−aminobutyric acid type B receptor subunit 2
rs3797535
rs558133
rs10944
rs705415
rs567754
rs672413
rs6859667
rs921943
rs6586282
rs1789953
rs234709
rs11951068
All − MR Egger
All − IVW(MRE)
−0.75
−0.50
−0.25
0.00
0.25
MR effect size for
'Selenium' on 'Gamma−aminobutyric acid type B receptor subunit 2'

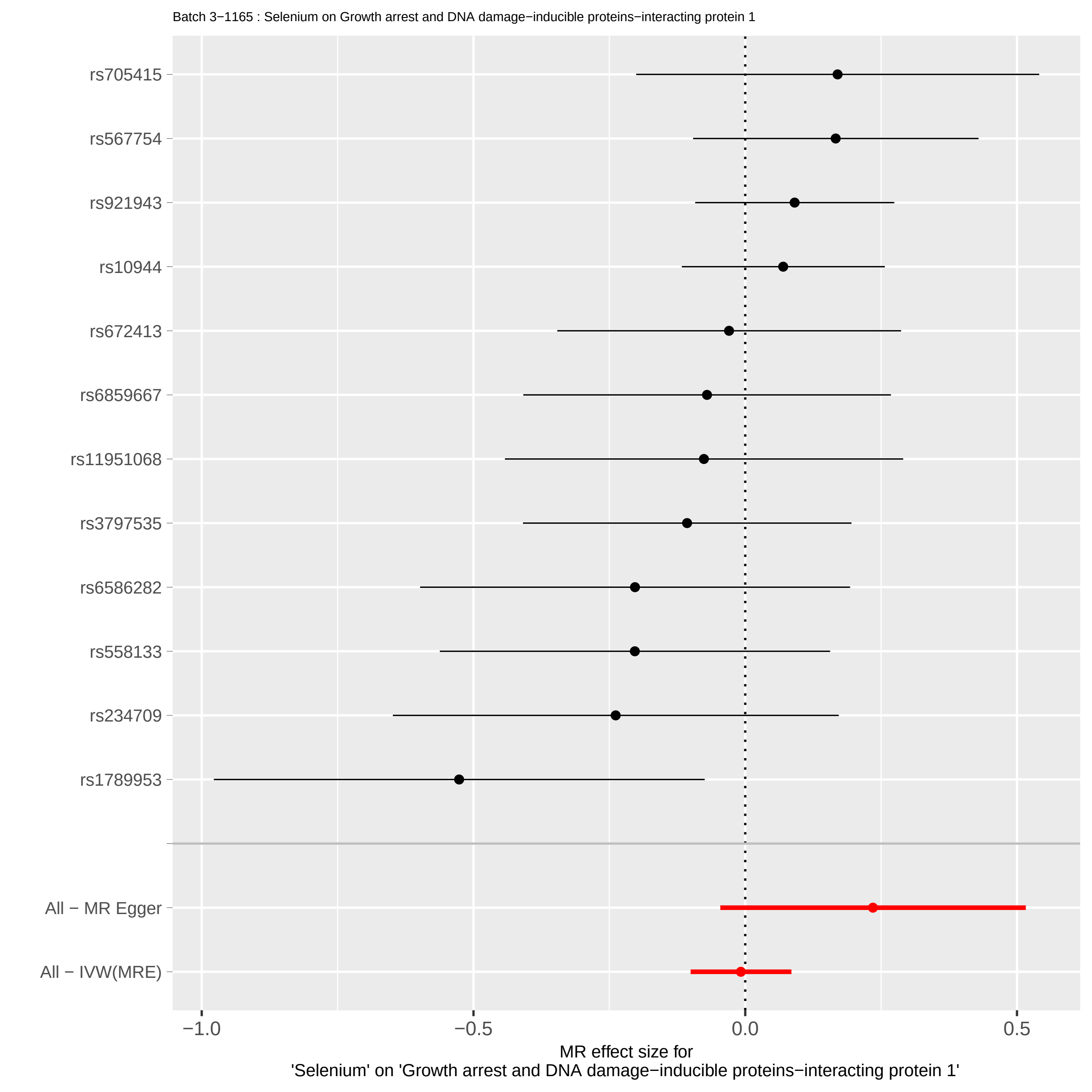
Batch 3−1165 : Selenium on Growth arrest and DNA damage−inducible proteins−interacting protein 1
rs705415
rs567754
rs921943
rs10944
rs672413
rs6859667
rs11951068
rs3797535
rs6586282
rs558133
rs234709
rs1789953
All − MR Egger
All − IVW(MRE)
−1.0
−0.5
0.0
0.5
MR effect size for
'Selenium' on 'Growth arrest and DNA damage−inducible proteins−interacting protein 1'

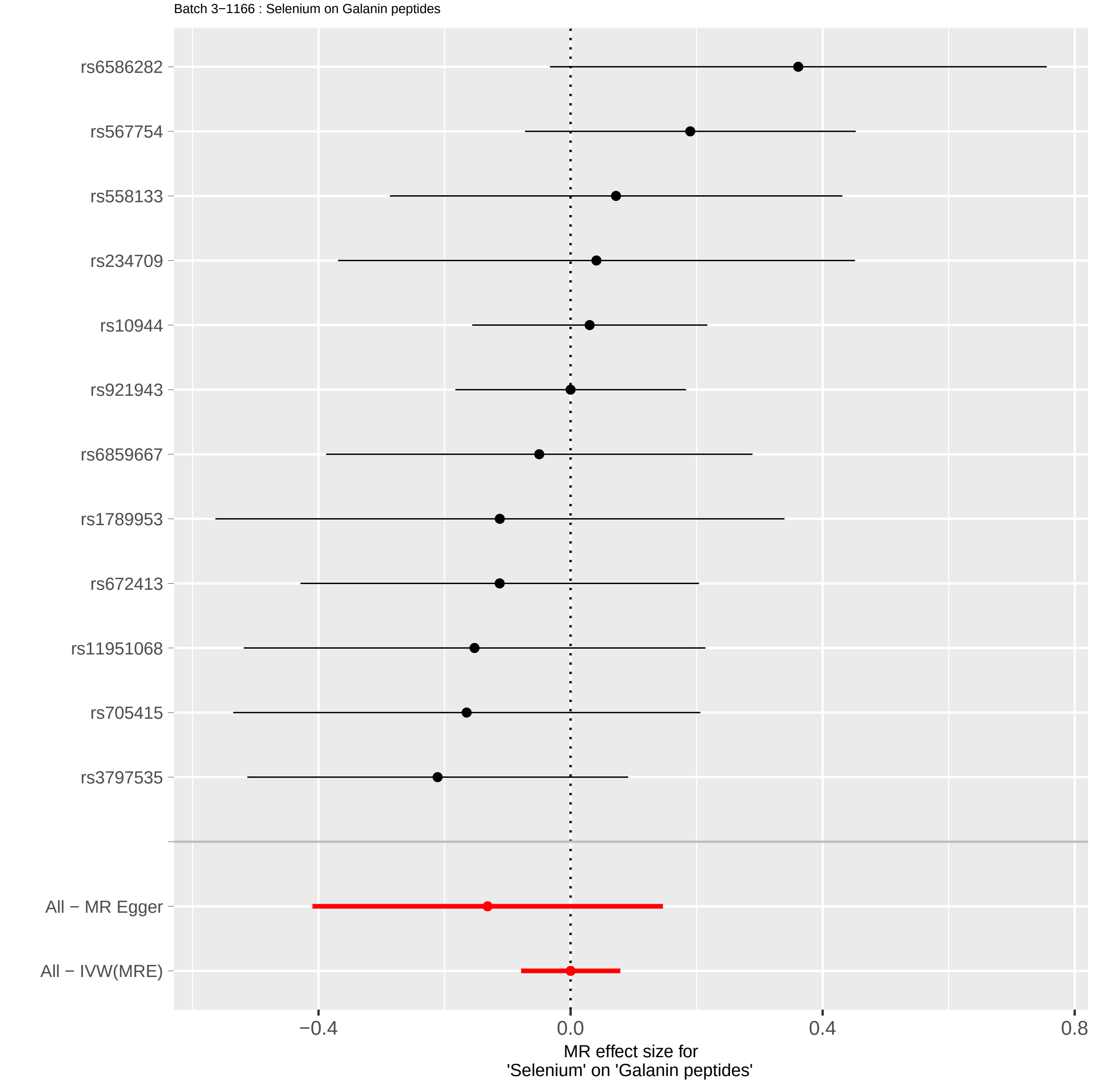
Batch 3−1166 : Selenium on Galanin peptides
rs6586282
rs567754
rs558133
rs234709
rs10944
rs921943
rs6859667
rs1789953
rs672413
rs11951068
rs705415
rs3797535
All − MR Egger
All − IVW(MRE)
−0.4
0.0
0.4
0.8
MR effect size for
'Selenium' on 'Galanin peptides'

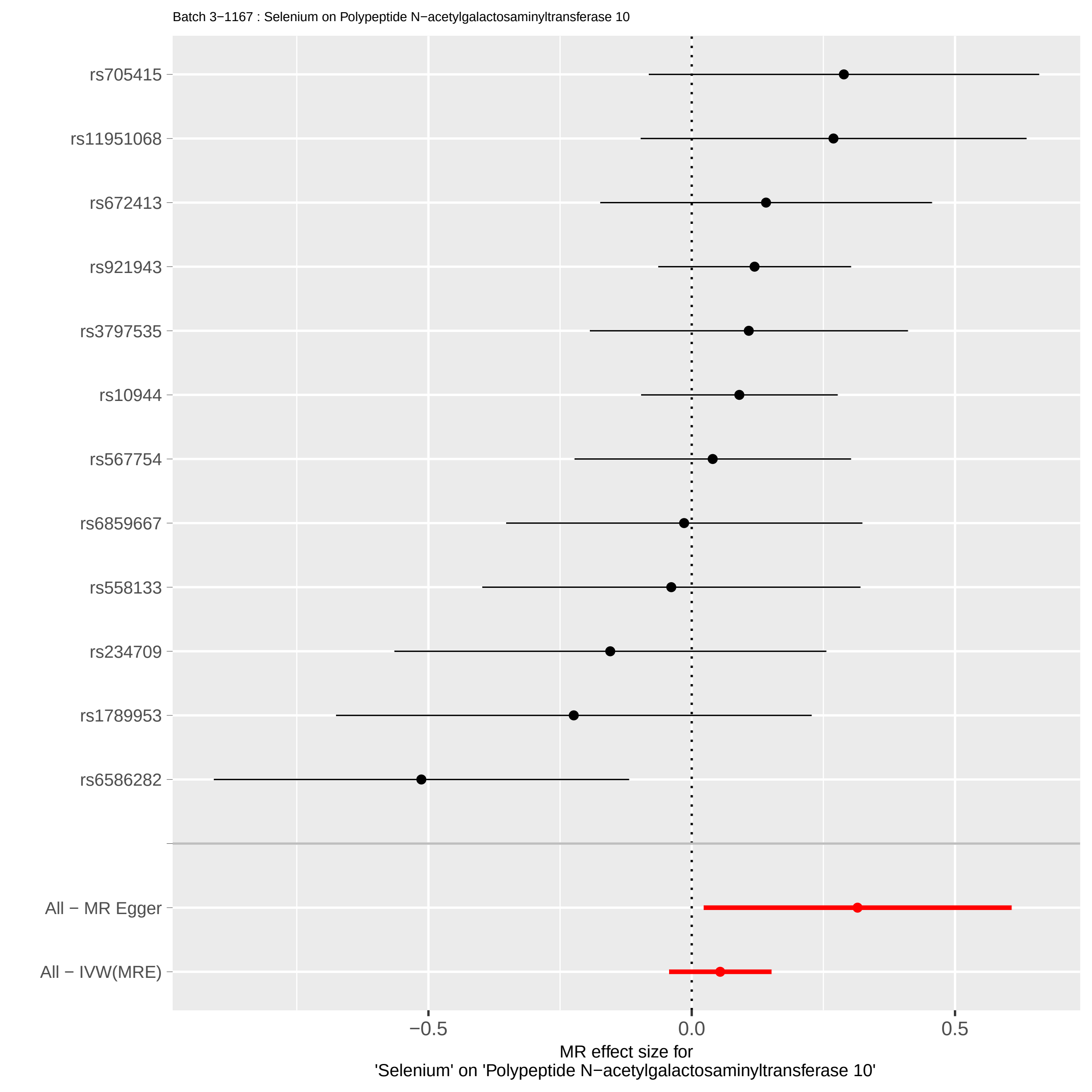

Batch 3−1167 : Selenium on Polypeptide N−acetylgalactosaminyltransferase 10
rs705415
rs11951068
rs672413
rs921943
rs3797535
rs10944
rs567754
rs6859667
rs558133
rs234709
rs1789953
rs6586282
All − MR Egger
All − IVW(MRE)
−0.5
0.0
0.5
MR effect size for
'Selenium' on 'Polypeptide N−acetylgalactosaminyltransferase 10'

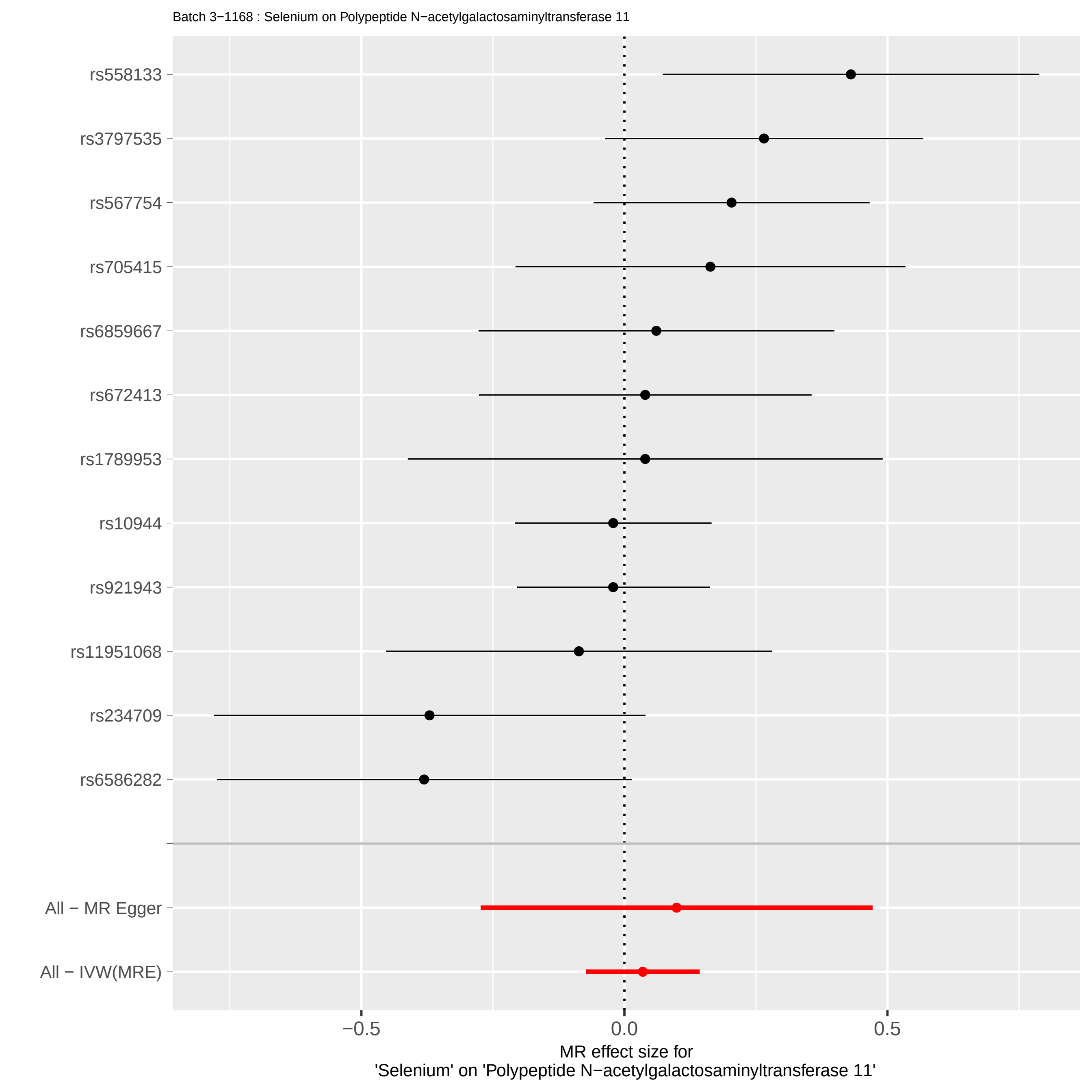
Batch 3−1168 : Selenium on Polypeptide N−acetylgalactosaminyltransferase 11
rs558133
rs3797535
rs567754
rs705415
rs6859667
rs672413
rs1789953
rs10944
rs921943
rs11951068
rs234709
rs6586282
All − MR Egger
All − IVW(MRE)
−0.5
0.0
0.5
MR effect size for
'Selenium' on 'Polypeptide N−acetylgalactosaminyltransferase 11'

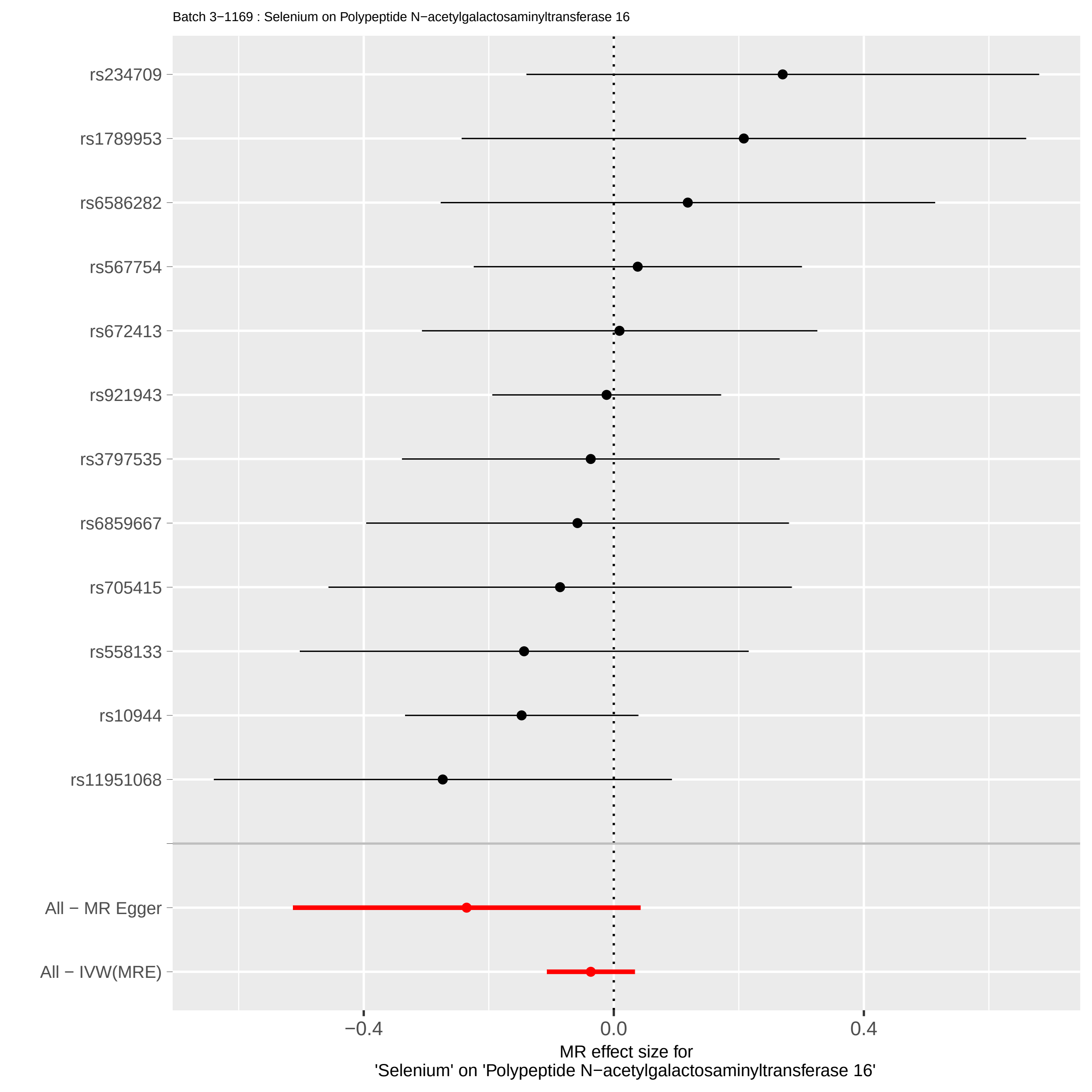

Batch 3−1169 : Selenium on Polypeptide N−acetylgalactosaminyltransferase 16
rs234709
rs1789953
rs6586282
rs567754
rs672413
rs921943
rs3797535
rs6859667
rs705415
rs558133
rs10944
rs11951068
All − MR Egger
All − IVW(MRE)
−0.4
0.0
0.4
MR effect size for
'Selenium' on 'Polypeptide N−acetylgalactosaminyltransferase 16'

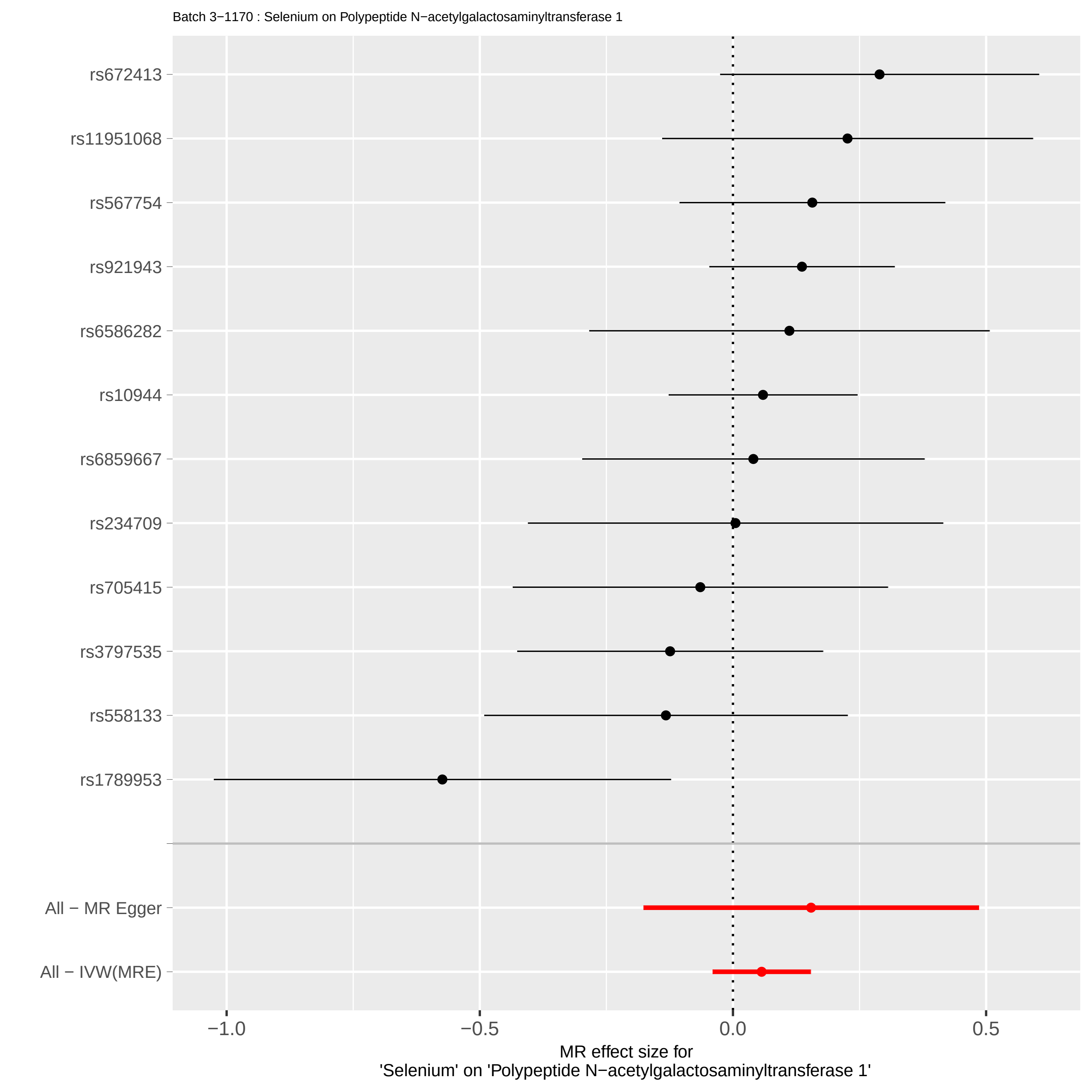

Batch 3−1170 : Selenium on Polypeptide N−acetylgalactosaminyltransferase 1
rs672413
rs11951068
rs567754
rs921943
rs6586282
rs10944
rs6859667
rs234709
rs705415
rs3797535
rs558133
rs1789953
All − MR Egger
All − IVW(MRE)
−1.0
−0.5
0.0
0.5
MR effect size for
'Selenium' on 'Polypeptide N−acetylgalactosaminyltransferase 1'

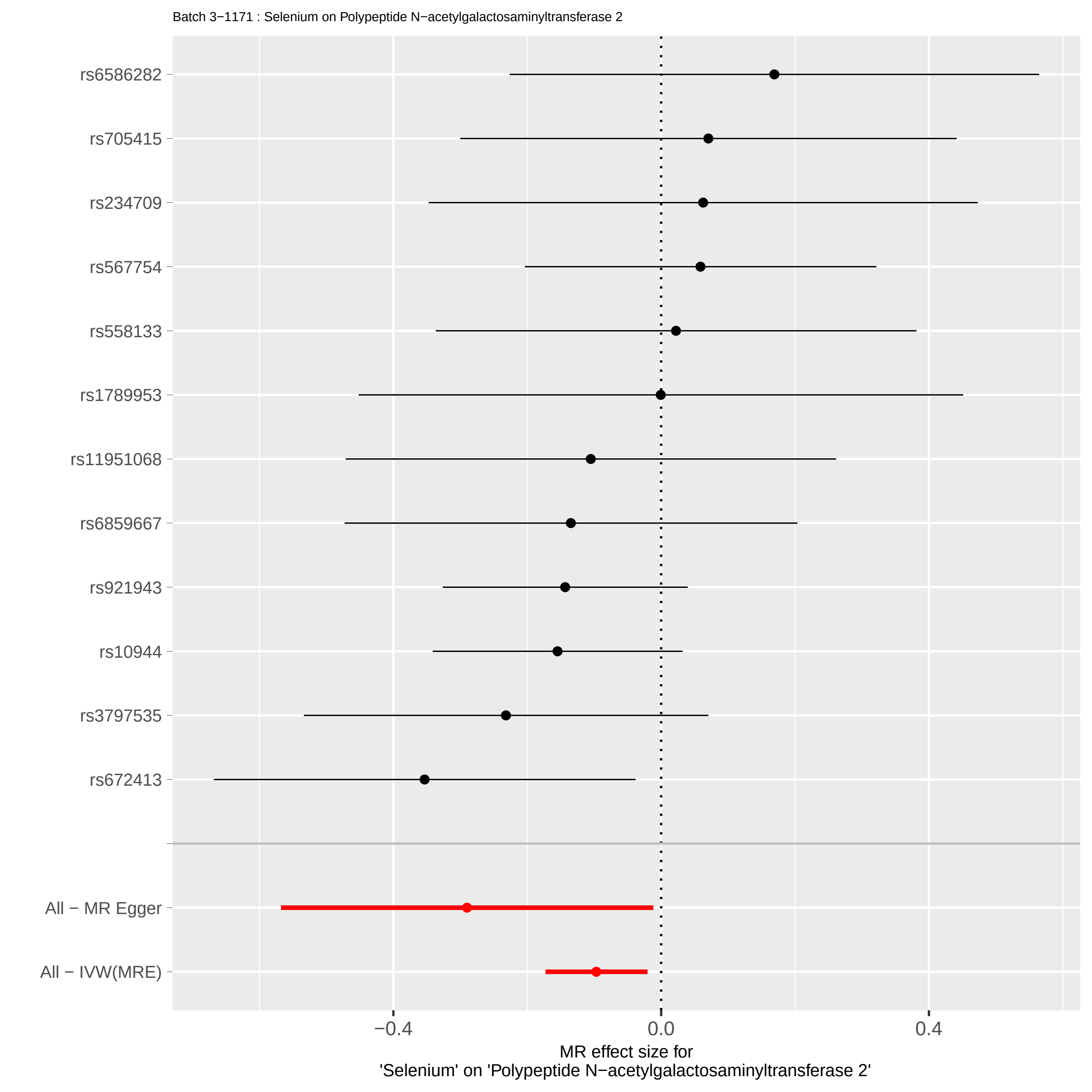

Batch 3−1171 : Selenium on Polypeptide N−acetylgalactosaminyltransferase 2
rs6586282
rs705415
rs234709
rs567754
rs558133
rs1789953
rs11951068
rs6859667
rs921943
rs10944
rs3797535
rs672413
All − MR Egger
All − IVW(MRE)
−0.4
0.0
0.4
MR effect size for
'Selenium' on 'Polypeptide N−acetylgalactosaminyltransferase 2'

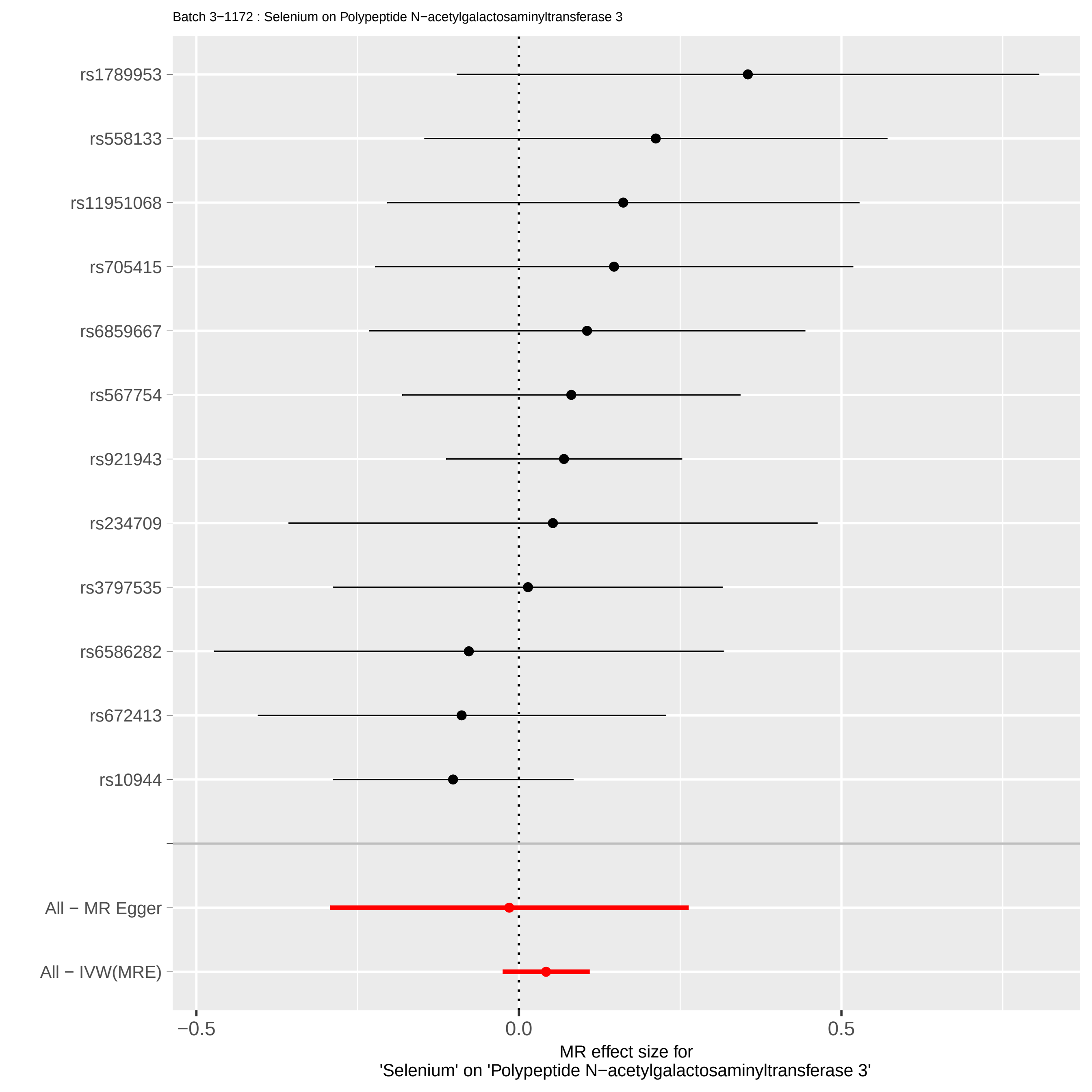

Batch 3−1172 : Selenium on Polypeptide N−acetylgalactosaminyltransferase 3
rs1789953
rs558133
rs11951068
rs705415
rs6859667
rs567754
rs921943
rs234709
rs3797535
rs6586282
rs672413
rs10944
All − MR Egger
All − IVW(MRE)
−0.5
0.0
0.5
MR effect size for
'Selenium' on 'Polypeptide N−acetylgalactosaminyltransferase 3'

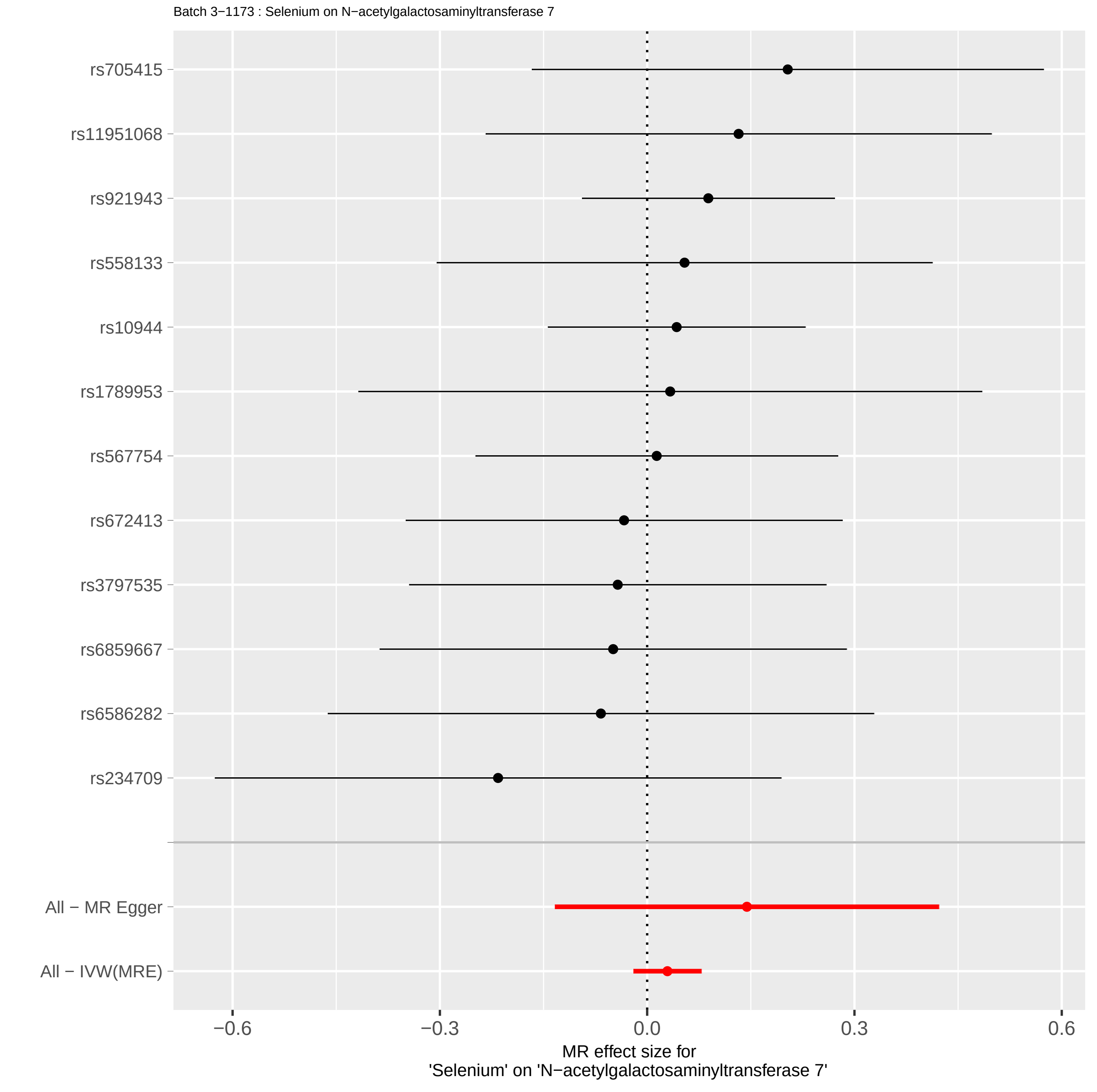
Batch 3−1173 : Selenium on N−acetylgalactosaminyltransferase 7
rs705415
rs11951068
rs921943
rs558133
rs10944
rs1789953
rs567754
rs672413
rs3797535
rs6859667
rs6586282
rs234709
All − MR Egger
All − IVW(MRE)
−0.6
−0.3
0.0
0.3
0.6
MR effect size for
'Selenium' on 'N−acetylgalactosaminyltransferase 7'

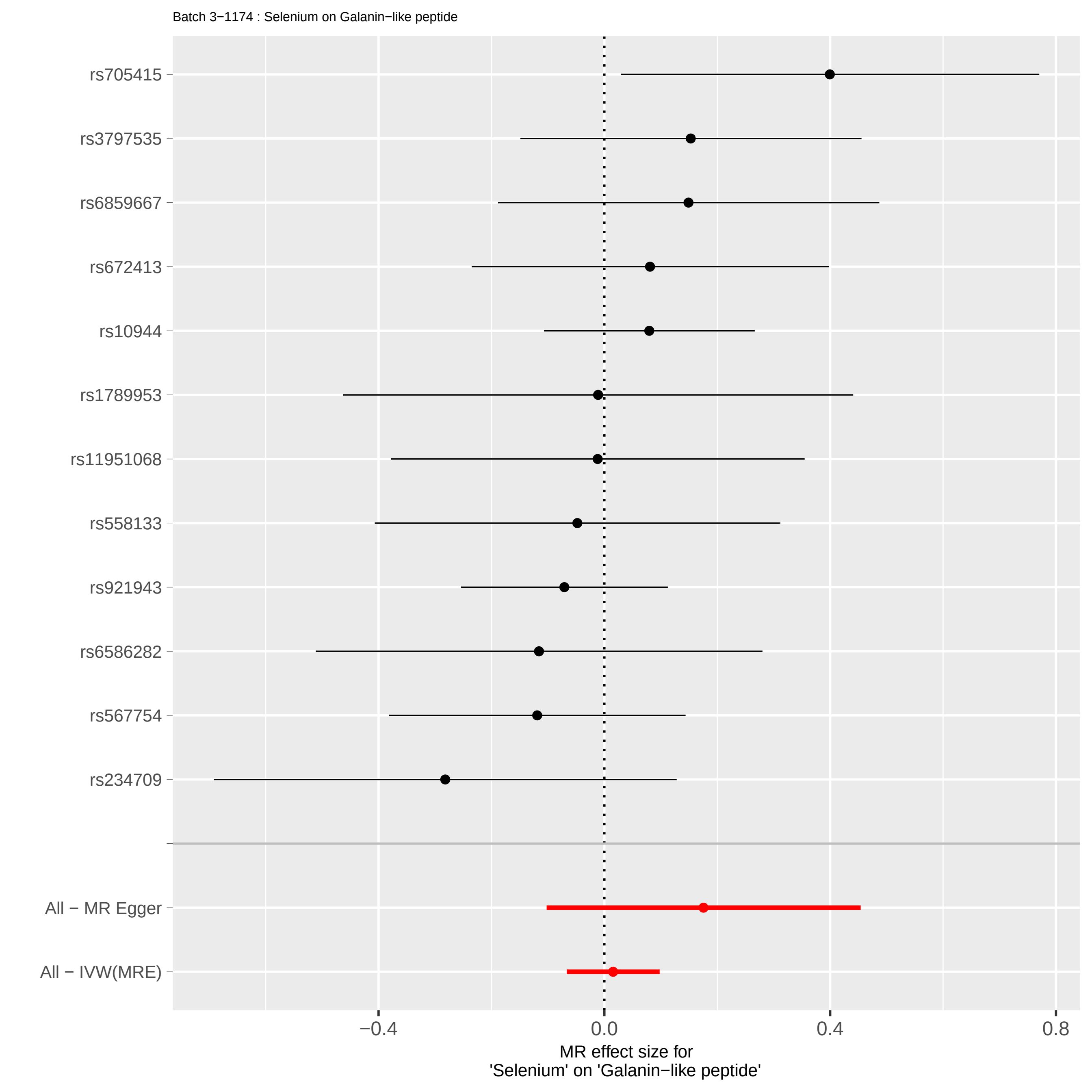

Batch 3−1174 : Selenium on Galanin−like peptide
rs705415
rs3797535
rs6859667
rs672413
rs10944
rs1789953
rs11951068
rs558133
rs921943
rs6586282
rs567754
rs234709
All − MR Egger
All − IVW(MRE)
−0.4
0.0
0.4
0.8
MR effect size for
'Selenium' on 'Galanin−like peptide'

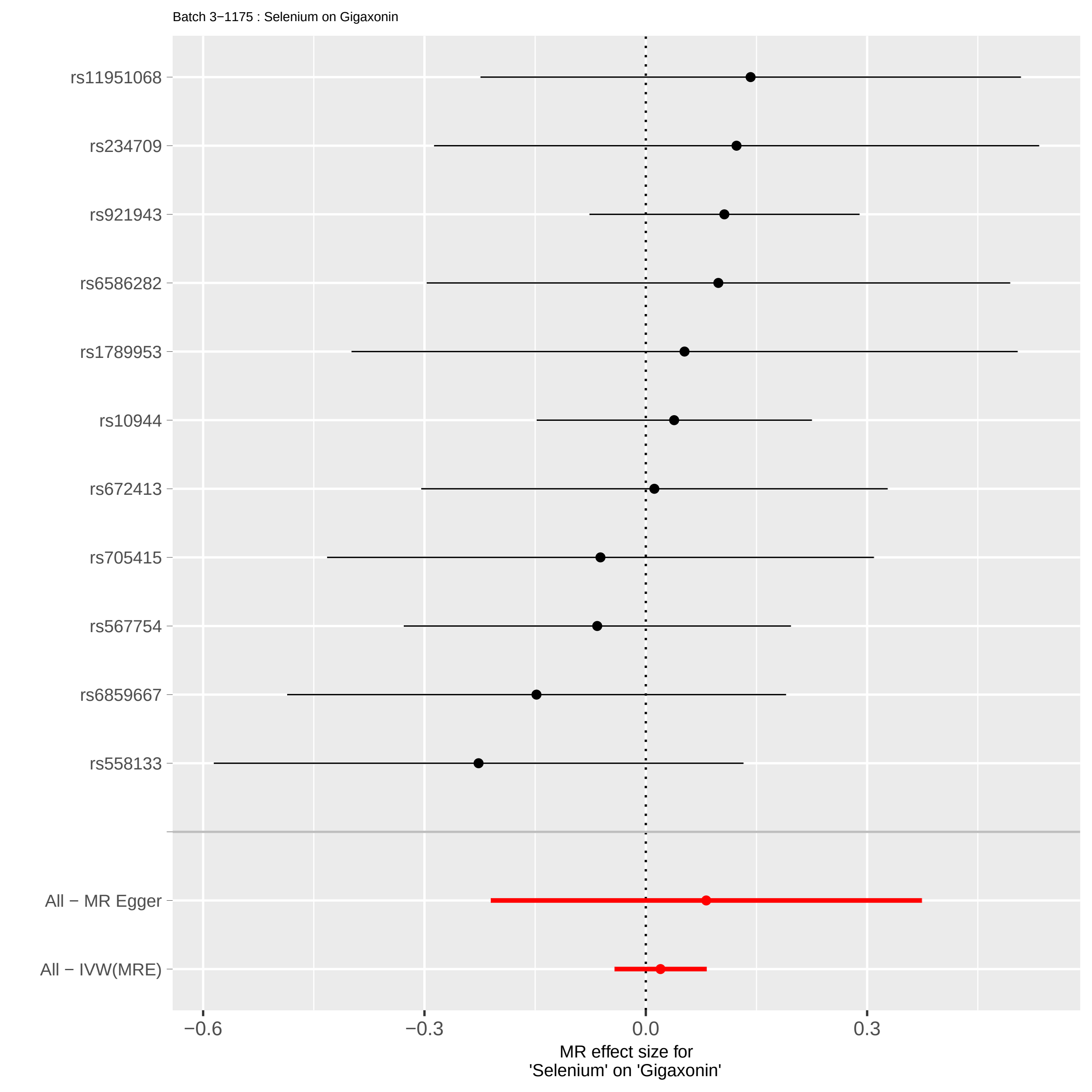
Batch 3−1175 : Selenium on Gigaxonin
rs11951068
rs234709
rs921943
rs6586282
rs1789953
rs10944
rs672413
rs705415
rs567754
rs6859667
rs558133
All − MR Egger
All − IVW(MRE)
−0.6
−0.3
0.0
0.3
MR effect size for
'Selenium' on 'Gigaxonin'

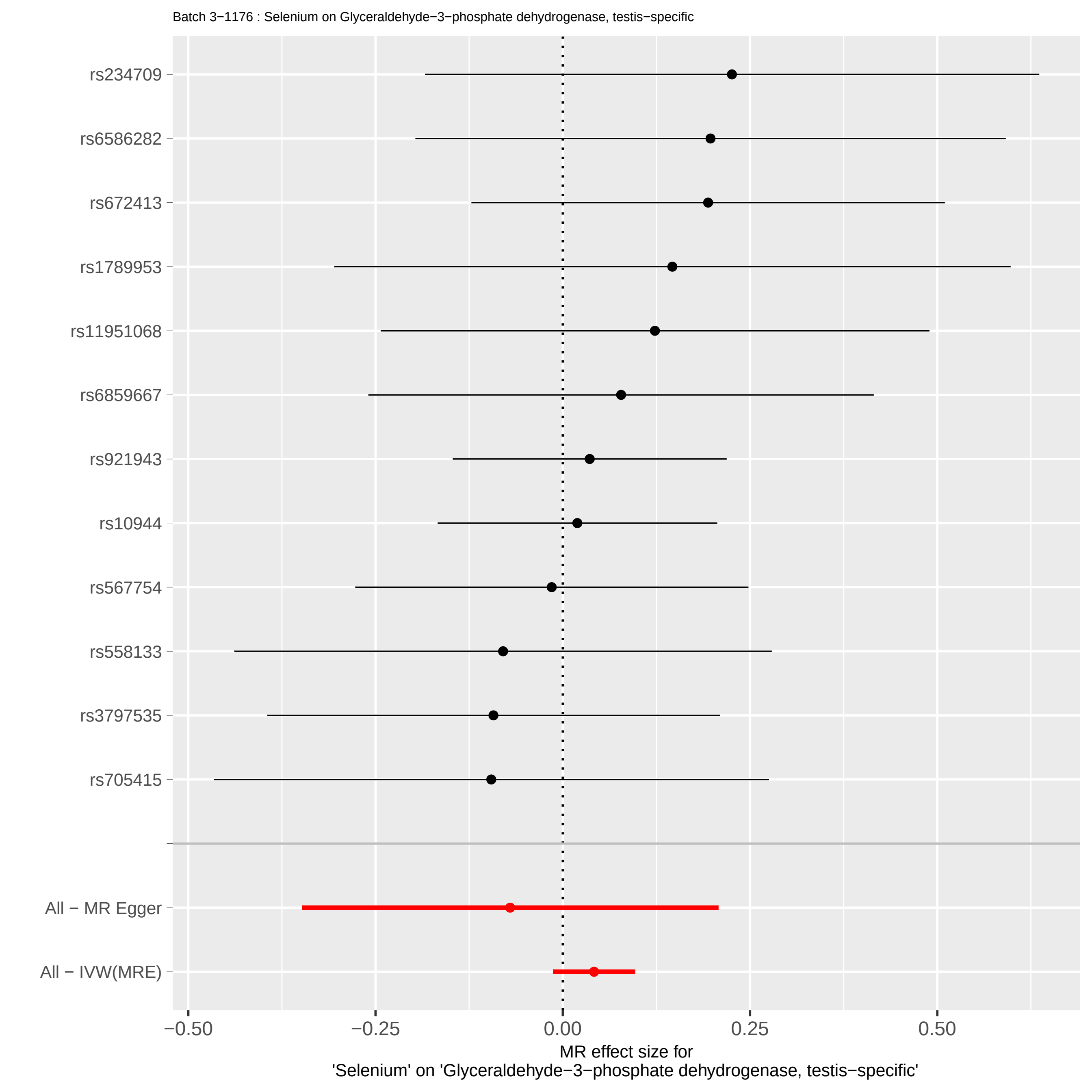

Batch 3−1176 : Selenium on Glyceraldehyde−3−phosphate dehydrogenase, testis−specific
rs234709
rs6586282
rs672413
rs1789953
rs11951068
rs6859667
rs921943
rs10944
rs567754
rs558133
rs3797535
rs705415
All − MR Egger
All − IVW(MRE)
−0.50
−0.25
0.00
0.25
0.50
MR effect size for
'Selenium' on 'Glyceraldehyde−3−phosphate dehydrogenase, testis−specific'

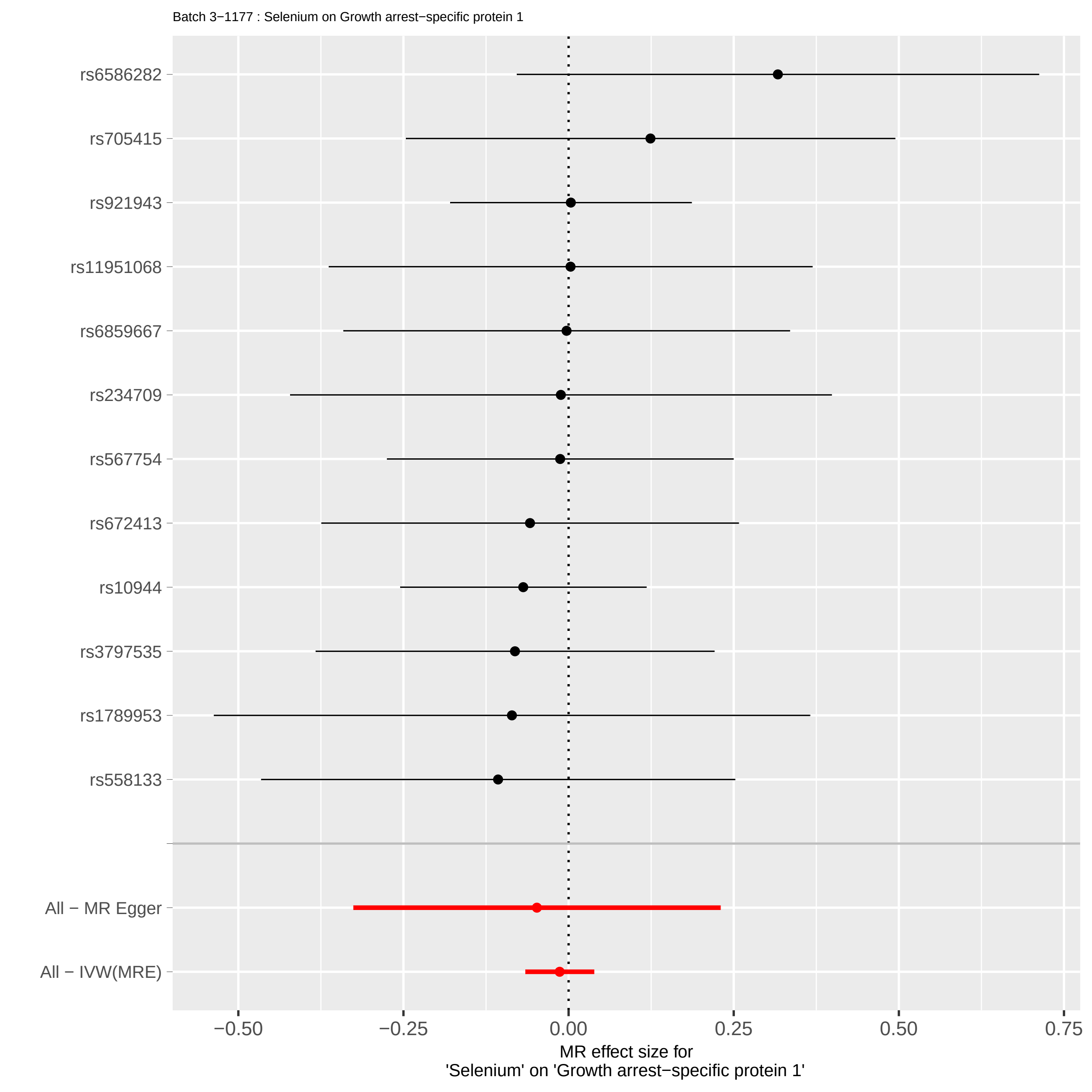

Batch 3−1177 : Selenium on Growth arrest−specific protein 1
rs6586282
rs705415
rs921943
rs11951068
rs6859667
rs234709
rs567754
rs672413
rs10944
rs3797535
rs1789953
rs558133
All − MR Egger
All − IVW(MRE)
−0.50
−0.25
0.00
0.25
0.50
0.75
MR effect size for
'Selenium' on 'Growth arrest−specific protein 1'

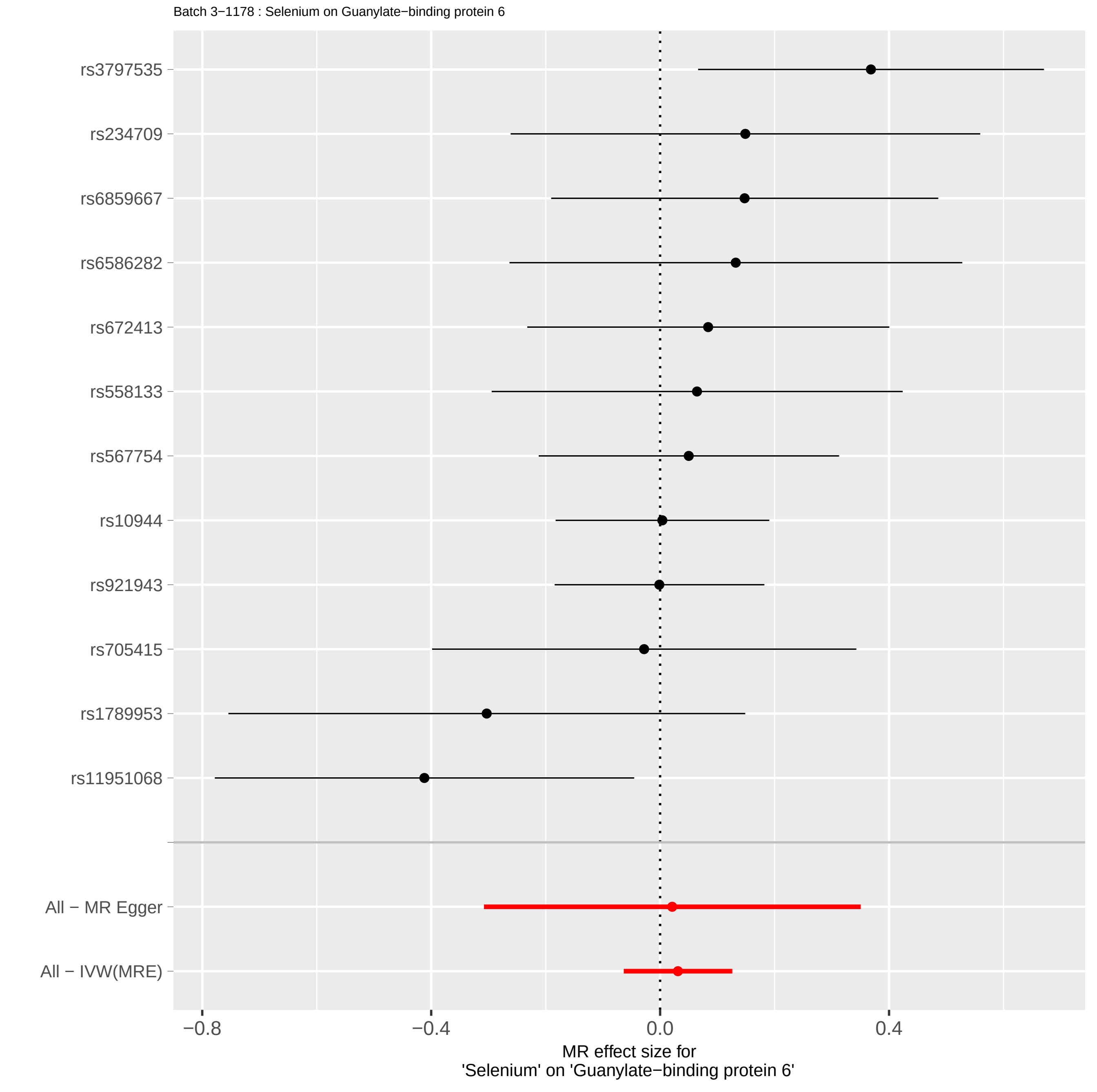
Batch 3−1178 : Selenium on Guanylate−binding protein 6
rs3797535
rs234709
rs6859667
rs6586282
rs672413
rs558133
rs567754
rs10944
rs921943
rs705415
rs1789953
rs11951068
All − MR Egger
All − IVW(MRE)
−0.8
−0.4
0.0
0.4
MR effect size for
'Selenium' on 'Guanylate−binding protein 6'

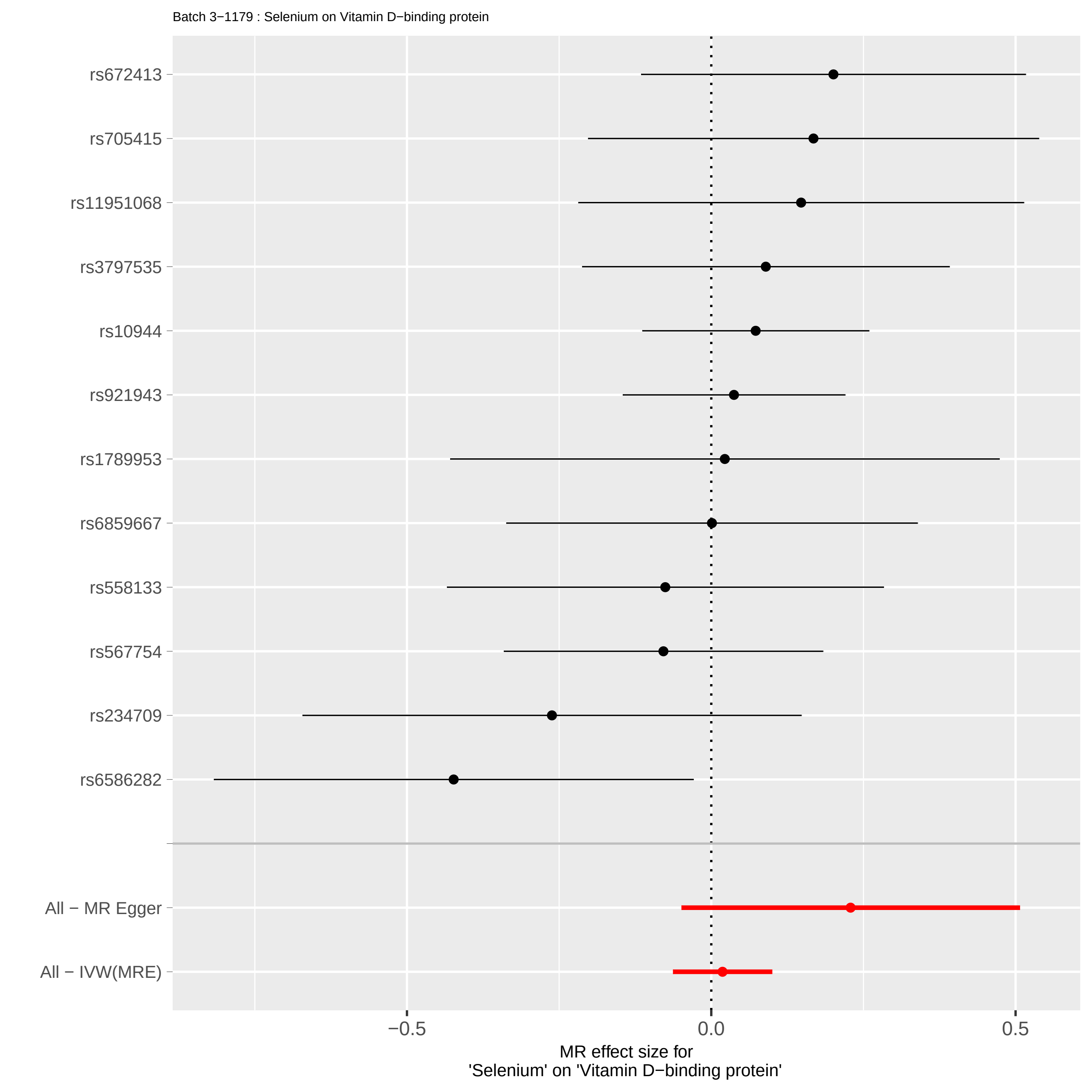

Batch 3−1179 : Selenium on Vitamin D−binding protein
rs672413
rs705415
rs11951068
rs3797535
rs10944
rs921943
rs1789953
rs6859667
rs558133
rs567754
rs234709
rs6586282
All − MR Egger
All − IVW(MRE)
−0.5
0.0
0.5
MR effect size for
'Selenium' on 'Vitamin D−binding protein'

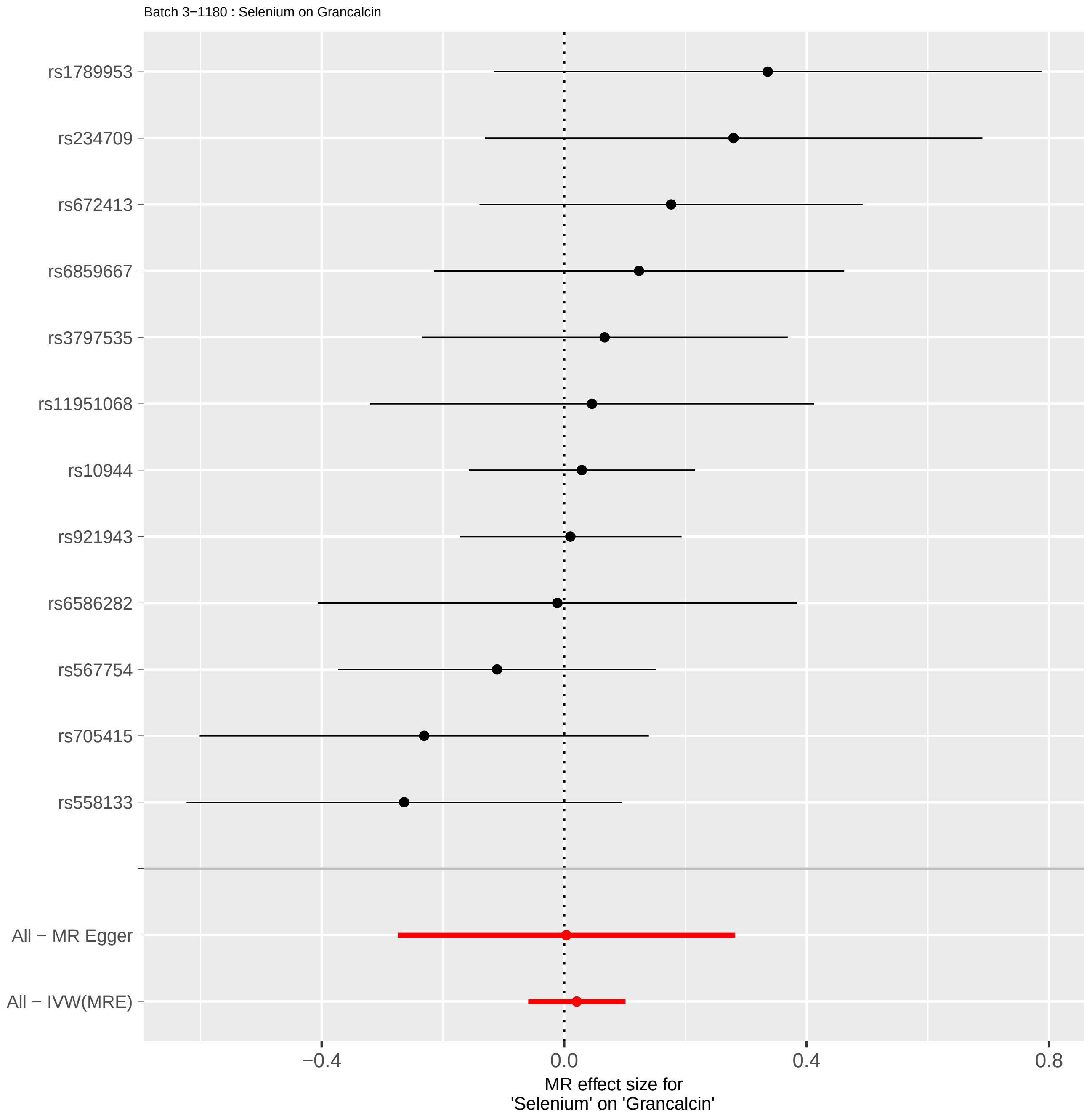

Batch 3−1180 : Selenium on Grancalcin
rs1789953
rs234709
rs672413
rs6859667
rs3797535
rs11951068
rs10944
rs921943
rs6586282
rs567754
rs705415
rs558133
All − MR Egger
All − IVW(MRE)
−0.4
0.0
0.4
0.8
MR effect size for
'Selenium' on 'Grancalcin'

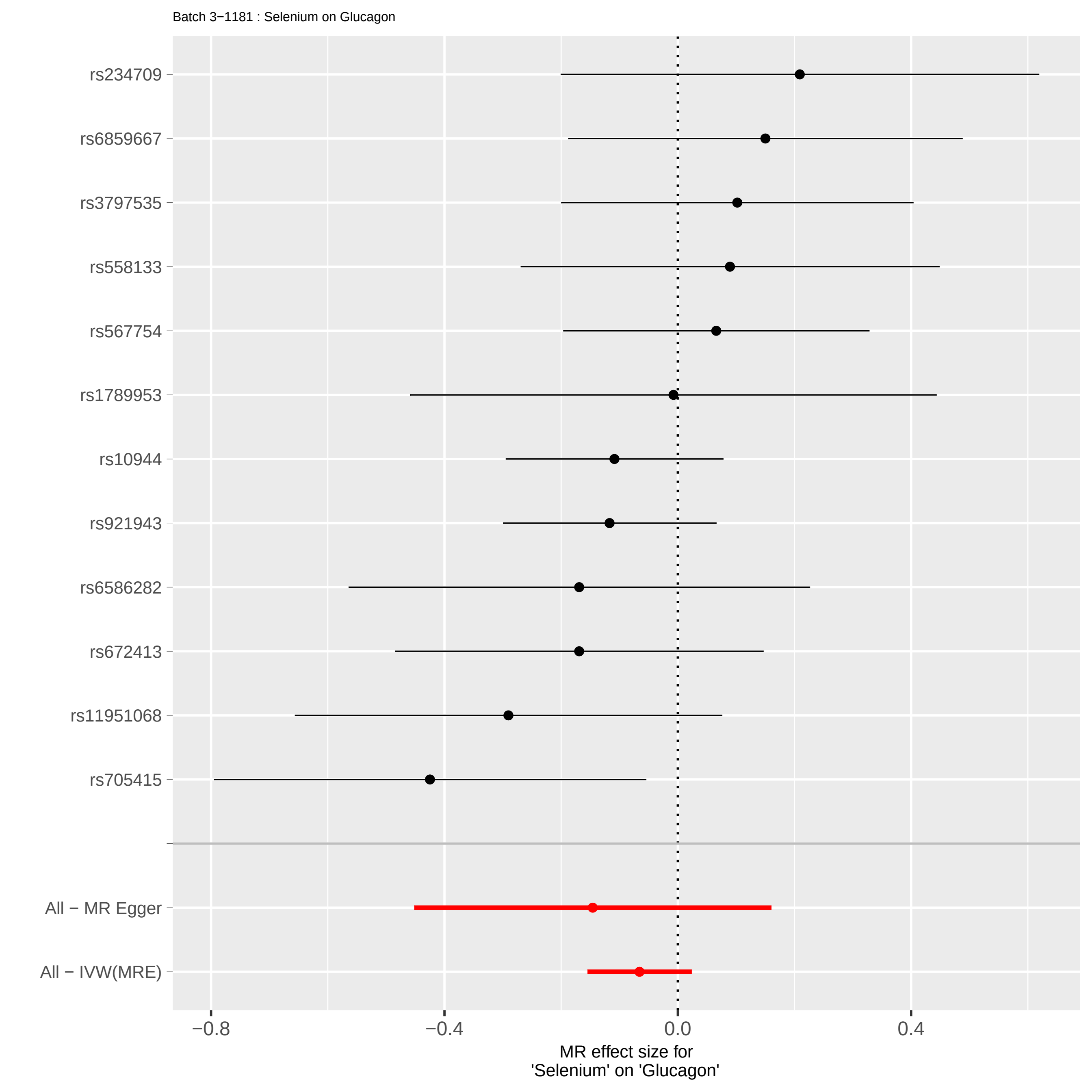

Batch 3−1181 : Selenium on Glucagon
rs234709
rs6859667
rs3797535
rs558133
rs567754
rs1789953
rs10944
rs921943
rs6586282
rs672413
rs11951068
rs705415
All − MR Egger
All − IVW(MRE)
−0.8
−0.4
0.0
0.4
MR effect size for
'Selenium' on 'Glucagon'

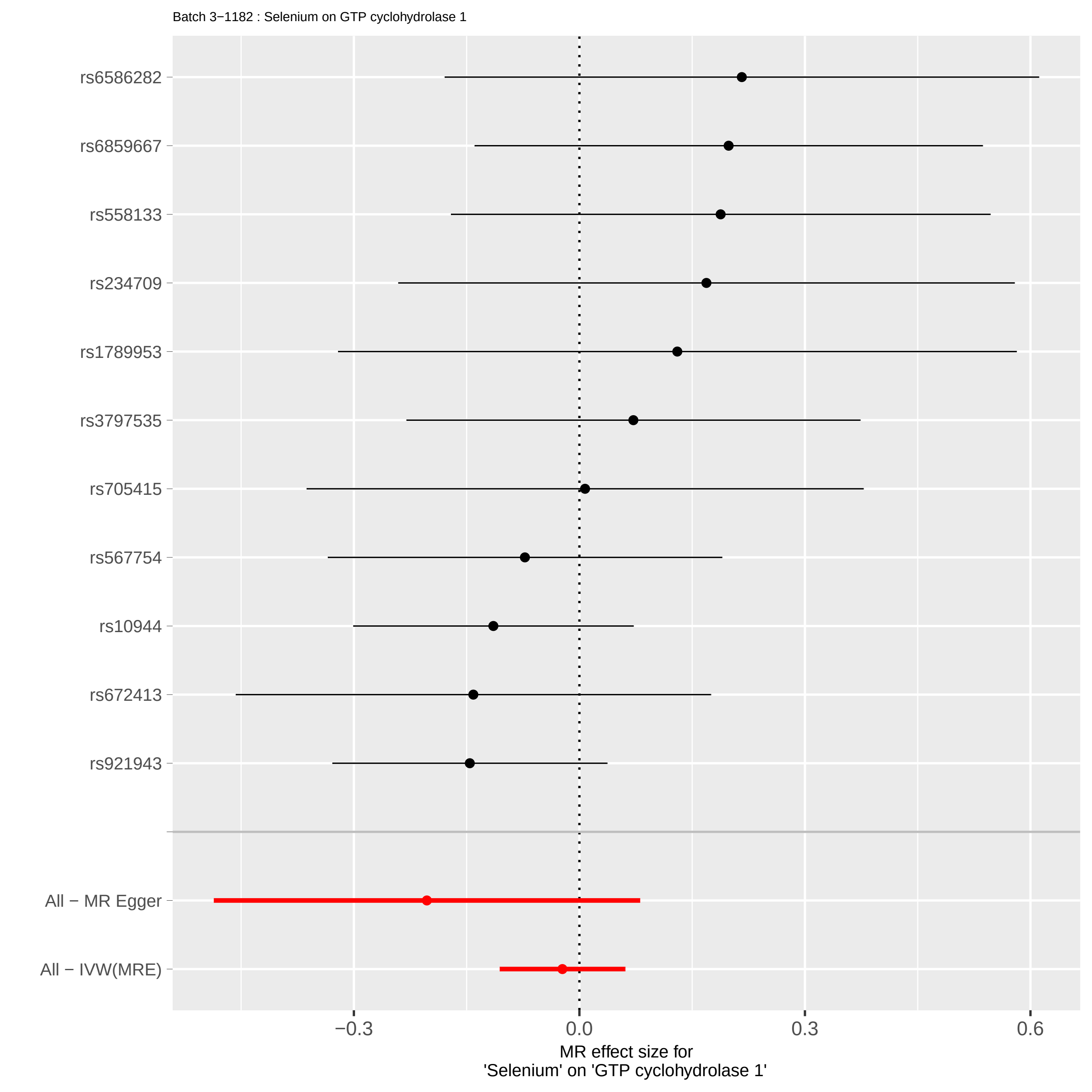

Batch 3−1182 : Selenium on GTP cyclohydrolase 1
rs6586282
rs6859667
rs558133
rs234709
rs1789953
rs3797535
rs705415
rs567754
rs10944
rs672413
rs921943
All − MR Egger
All − IVW(MRE)
−0.3
0.0
0.3
0.6
MR effect size for
'Selenium' on 'GTP cyclohydrolase 1'

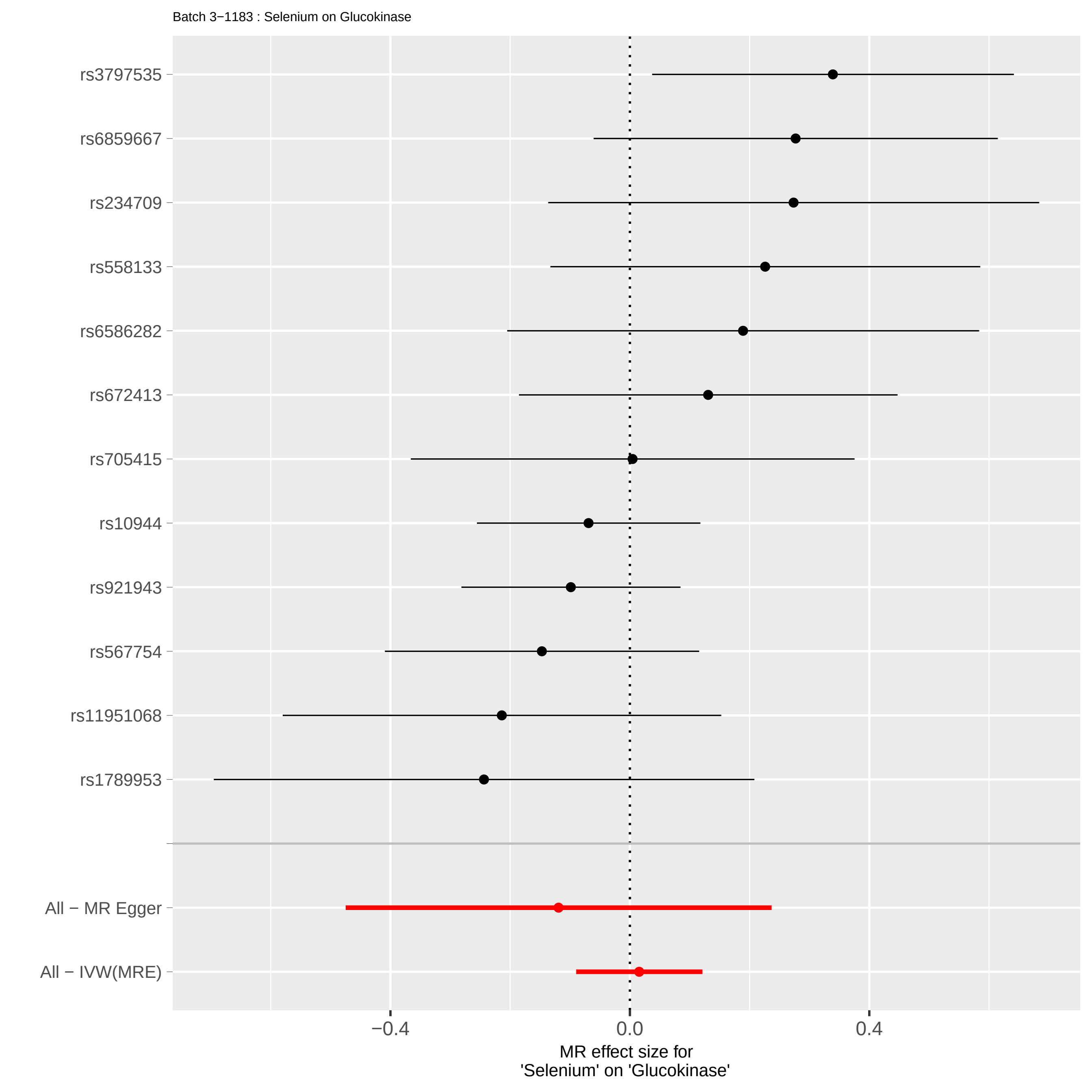
Batch 3−1183 : Selenium on Glucokinase
rs3797535
rs6859667
rs234709
rs558133
rs6586282
rs672413
rs705415
rs10944
rs921943
rs567754
rs11951068
rs1789953
All − MR Egger
All − IVW(MRE)
−0.4
0.0
0.4
MR effect size for
'Selenium' on 'Glucokinase'

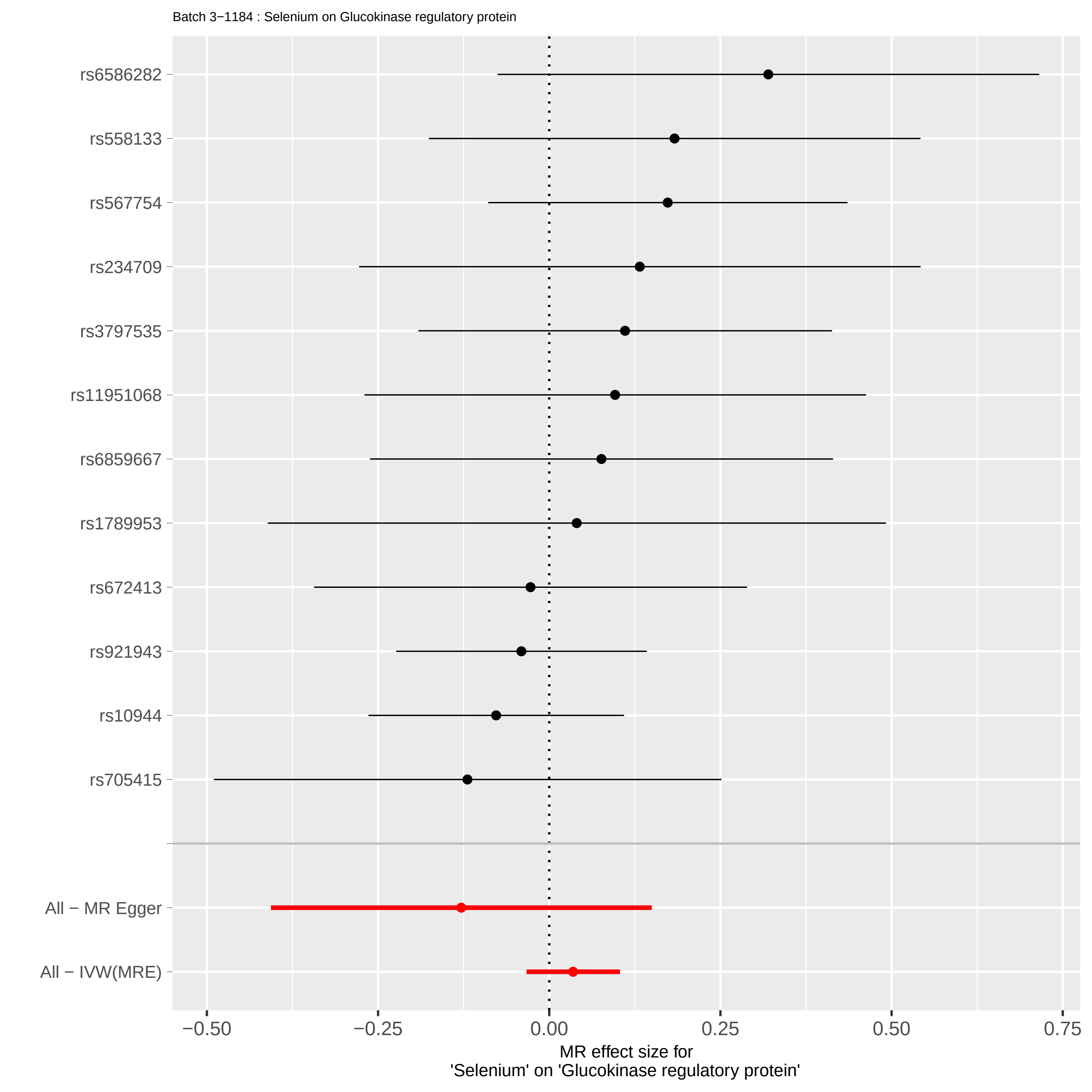

Batch 3−1184 : Selenium on Glucokinase regulatory protein
rs6586282
rs558133
rs567754
rs234709
rs3797535
rs11951068
rs6859667
rs1789953
rs672413
rs921943
rs10944
rs705415
All − MR Egger
All − IVW(MRE)
−0.50
−0.25
0.00
0.25
0.50
0.75
MR effect size for
'Selenium' on 'Glucokinase regulatory protein'

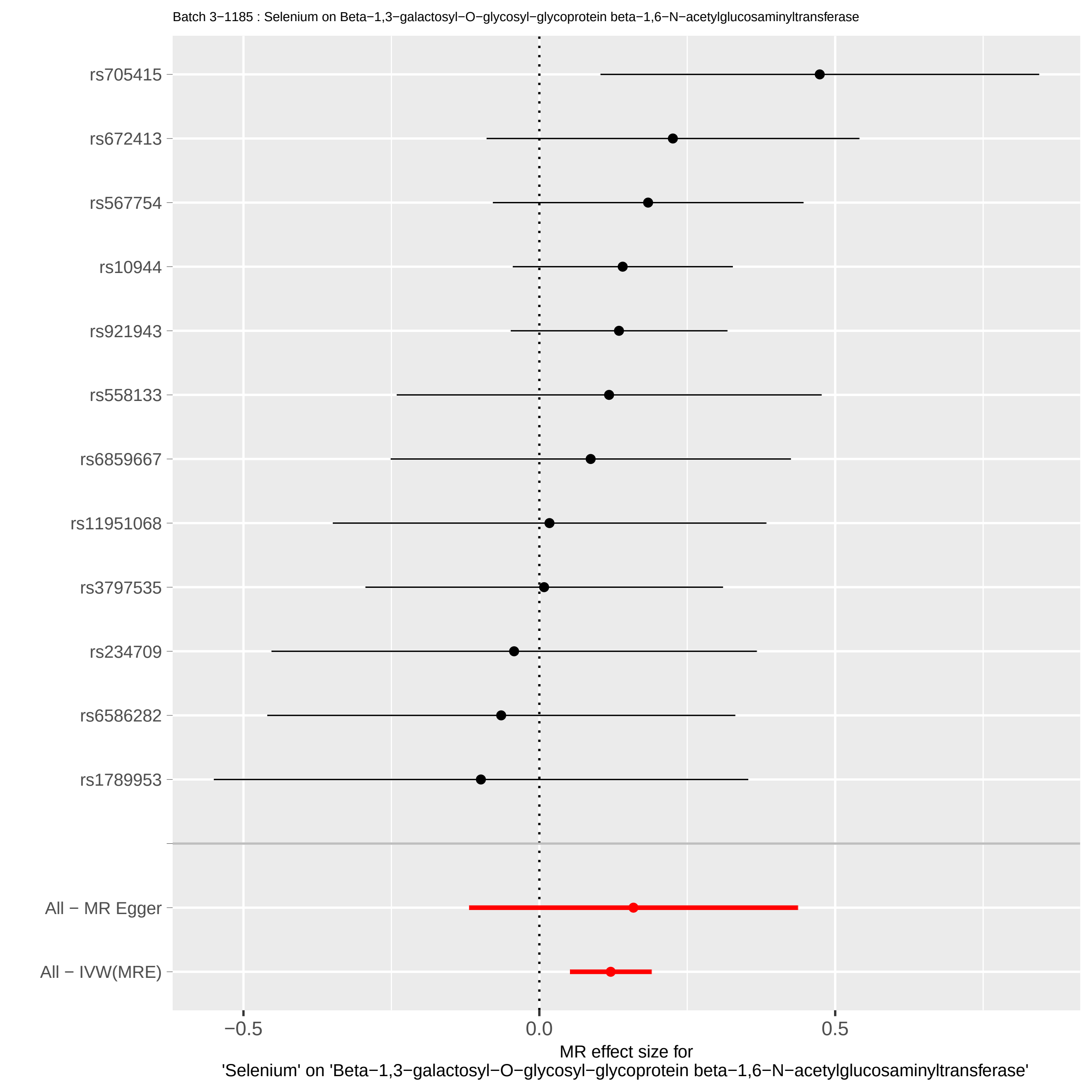

Batch 3−1185 : Selenium on Beta−1,3−galactosyl−O−glycosyl−glycoprotein beta−1,6−N−acetylglucosaminyltransferase
rs705415
rs672413
rs567754
rs10944
rs921943
rs558133
rs6859667
rs11951068
rs3797535
rs234709
rs6586282
rs1789953
All − MR Egger
All − IVW(MRE)
−0.5
0.0
0.5
MR effect size for
'Selenium' on 'Beta−1,3−galactosyl−O−glycosyl−glycoprotein beta−1,6−N−acetylglucosaminyltransferase'

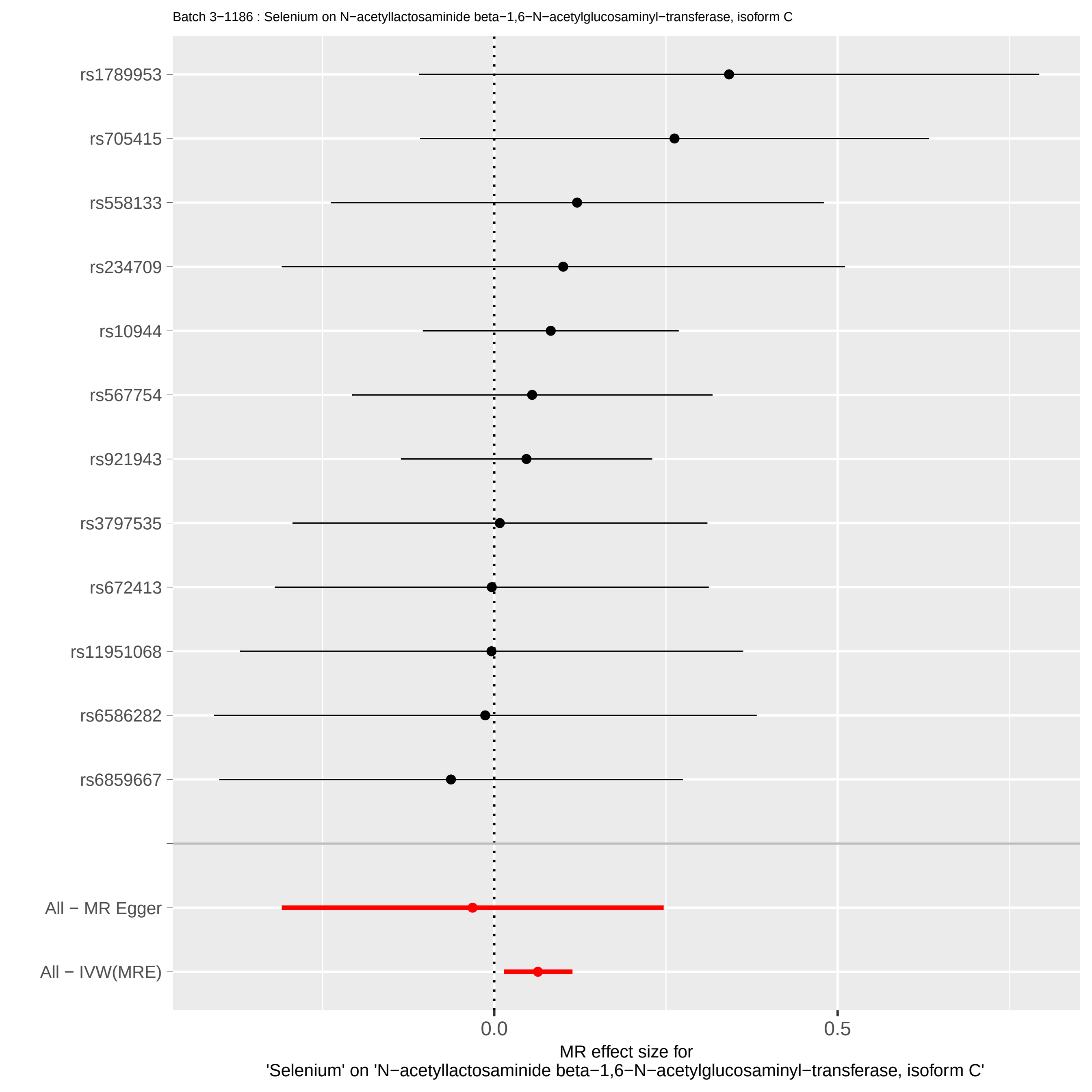
Batch 3−1186 : Selenium on N−acetyllactosaminide beta−1,6−N−acetylglucosaminyl−transferase, isoform C
rs1789953
rs705415
rs558133
rs234709
rs10944
rs567754
rs921943
rs3797535
rs672413
rs11951068
rs6586282
rs6859667
All − MR Egger
All − IVW(MRE)
0.0
0.5
MR effect size for
'Selenium' on 'N−acetyllactosaminide beta−1,6−N−acetylglucosaminyl−transferase, isoform C'

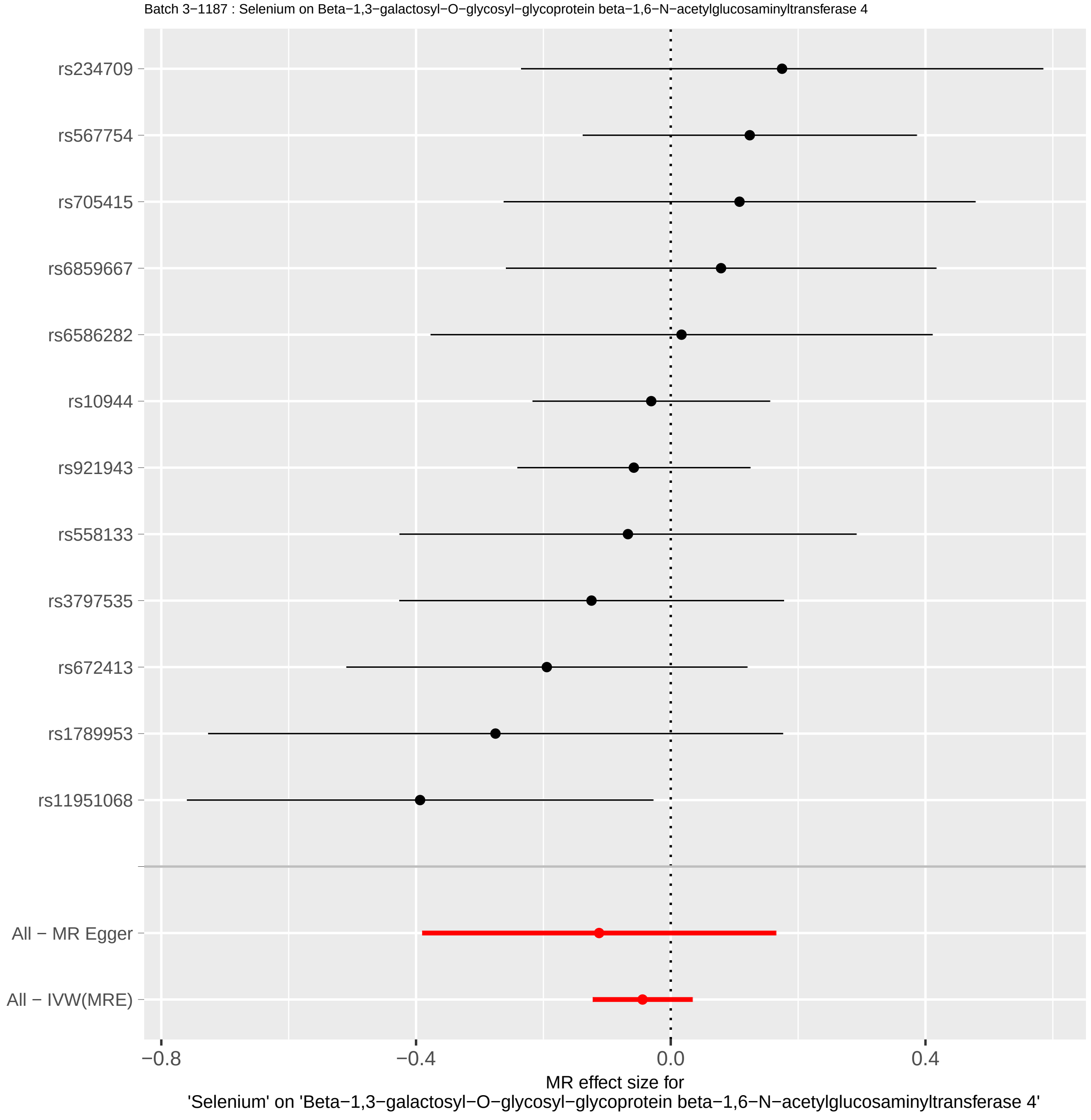
Batch 3−1187 : Selenium on Beta−1,3−galactosyl−O−glycosyl−glycoprotein beta−1,6−N−acetylglucosaminyltransferase 4
rs234709
rs567754
rs705415
rs6859667
rs6586282
rs10944
rs921943
rs558133
rs3797535
rs672413
rs1789953
rs11951068
All − MR Egger
All − IVW(MRE)
−0.8
−0.4
0.0
0.4
MR effect size for
'Selenium' on 'Beta−1,3−galactosyl−O−glycosyl−glycoprotein beta−1,6−N−acetylglucosaminyltransferase 4'

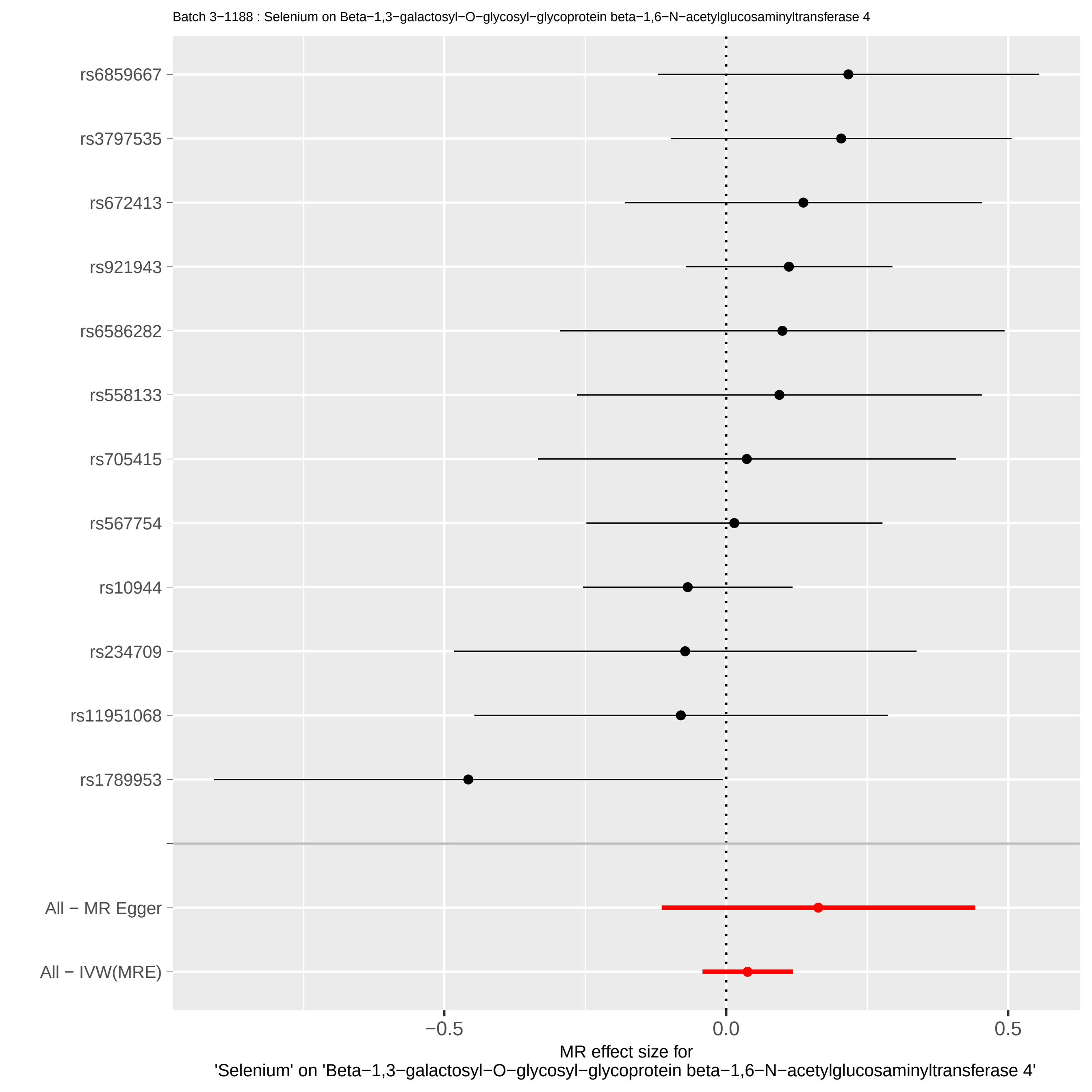

Batch 3−1188 : Selenium on Beta−1,3−galactosyl−O−glycosyl−glycoprotein beta−1,6−N−acetylglucosaminyltransferase 4
rs6859667
rs3797535
rs672413
rs921943
rs6586282
rs558133
rs705415
rs567754
rs10944
rs234709
rs11951068
rs1789953
All − MR Egger
All − IVW(MRE)
−0.5
0.0
0.5
MR effect size for
'Selenium' on 'Beta−1,3−galactosyl−O−glycosyl−glycoprotein beta−1,6−N−acetylglucosaminyltransferase 4'

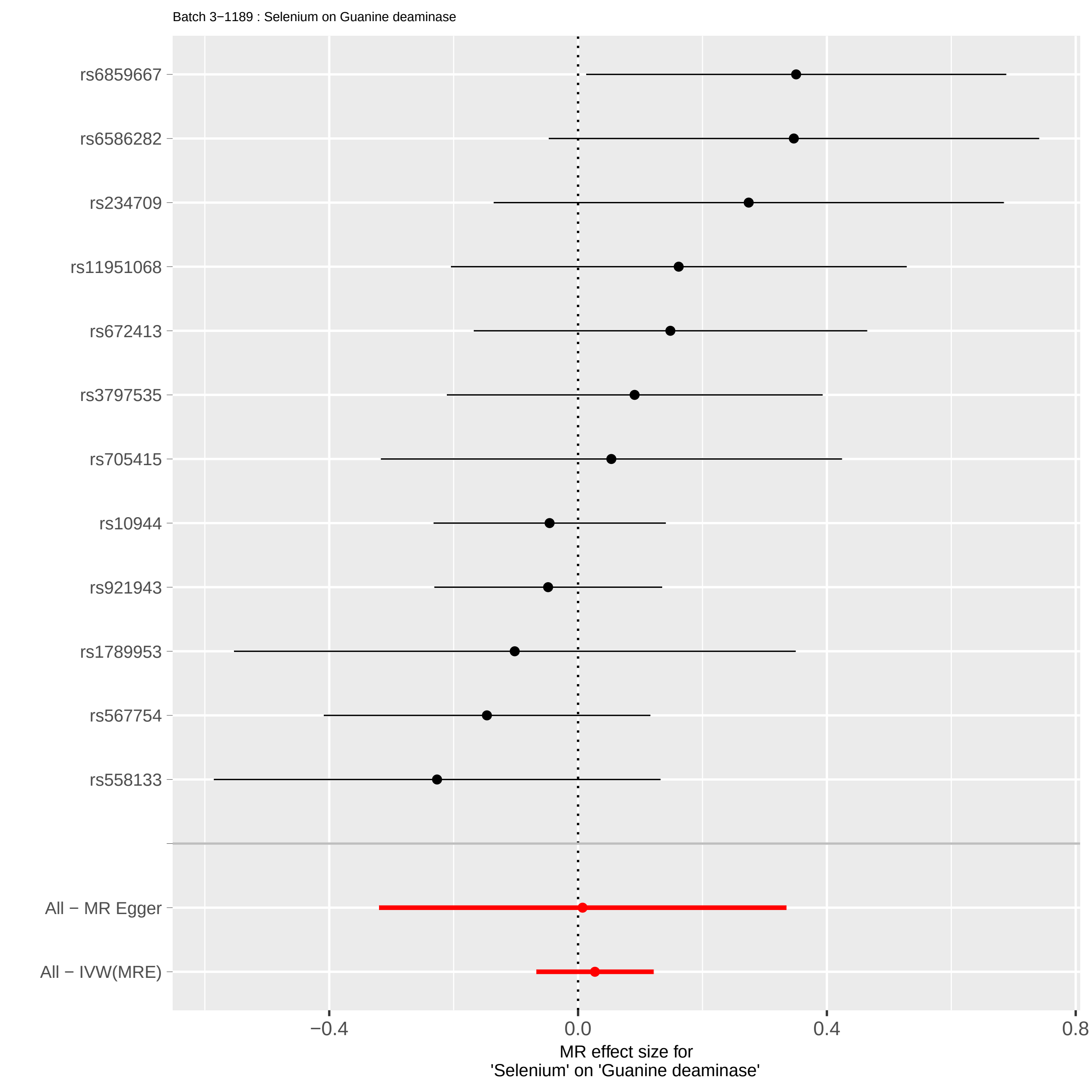
Batch 3−1189 : Selenium on Guanine deaminase
rs6859667
rs6586282
rs234709
rs11951068
rs672413
rs3797535
rs705415
rs10944
rs921943
rs1789953
rs567754
rs558133
All − MR Egger
All − IVW(MRE)
−0.4
0.0
0.4
0.8
MR effect size for
'Selenium' on 'Guanine deaminase'

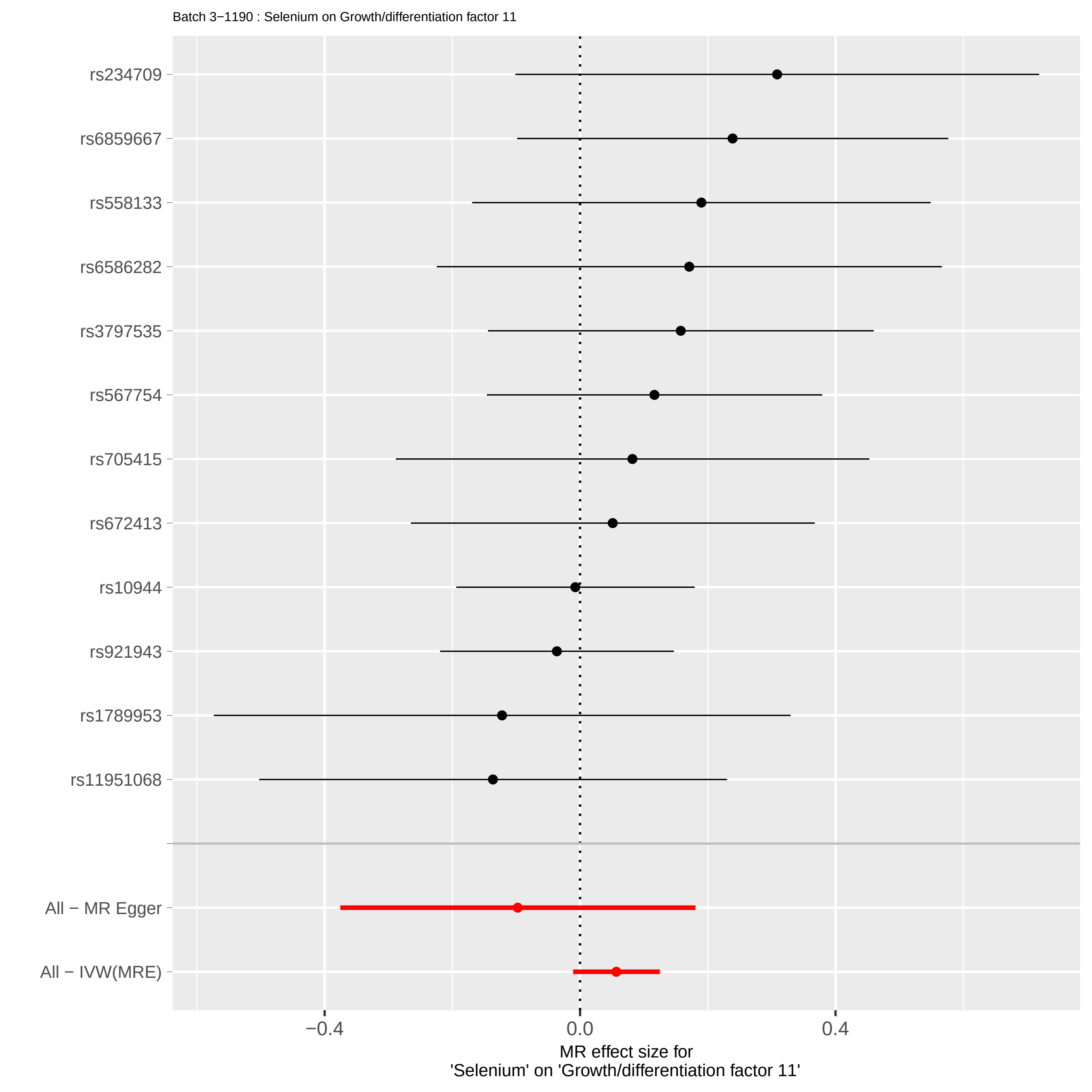

Batch 3−1190 : Selenium on Growth/differentiation factor 11
rs234709
rs6859667
rs558133
rs6586282
rs3797535
rs567754
rs705415
rs672413
rs10944
rs921943
rs1789953
rs11951068
All − MR Egger
All − IVW(MRE)
−0.4
0.0
0.4
MR effect size for
'Selenium' on 'Growth/differentiation factor 11'

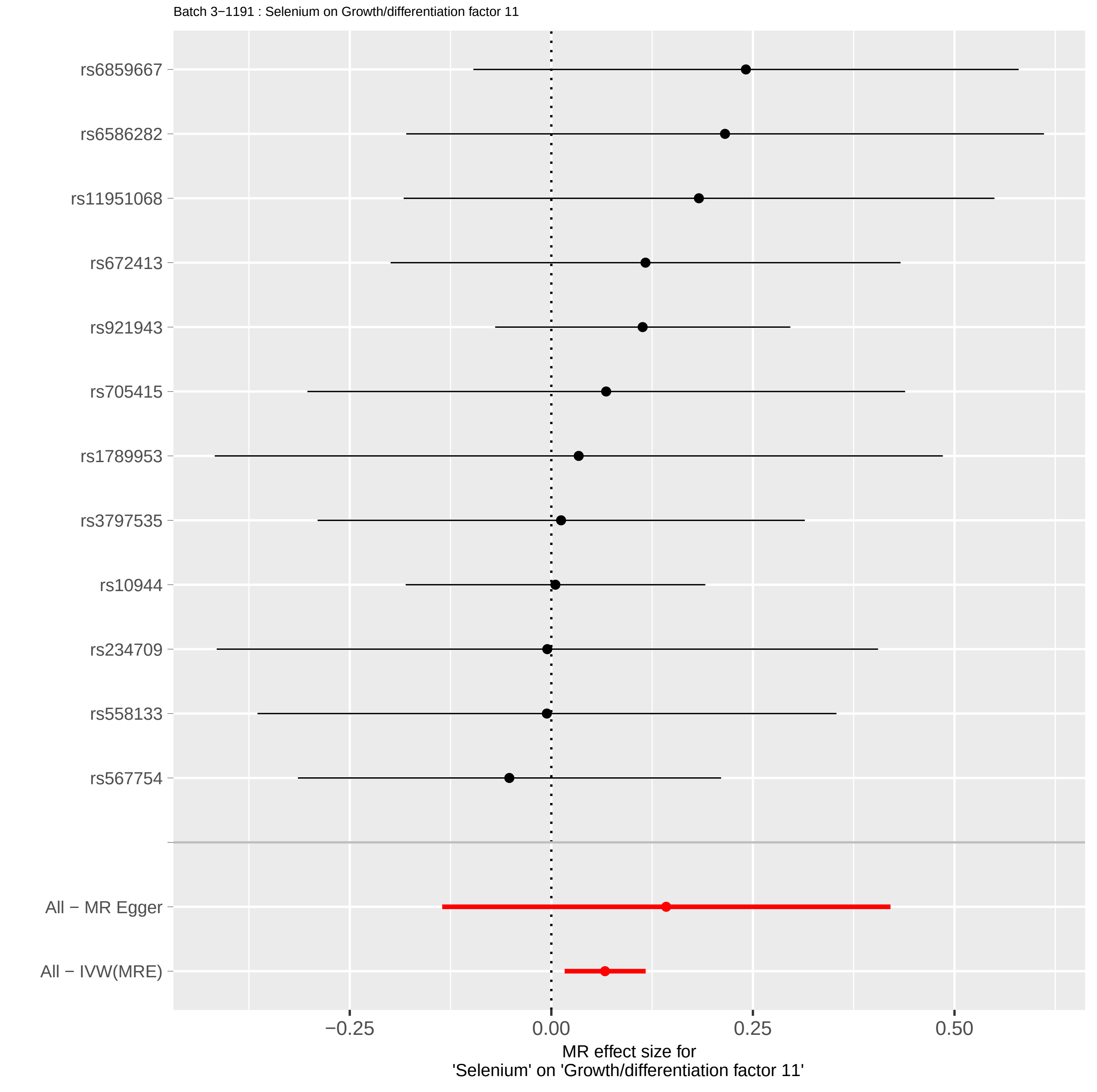
Batch 3−1191 : Selenium on Growth/differentiation factor 11
rs6859667
rs6586282
rs11951068
rs672413
rs921943
rs705415
rs1789953
rs3797535
rs10944
rs234709
rs558133
rs567754
All − MR Egger
All − IVW(MRE)
−0.25
0.00
0.25
0.50
MR effect size for
'Selenium' on 'Growth/differentiation factor 11'

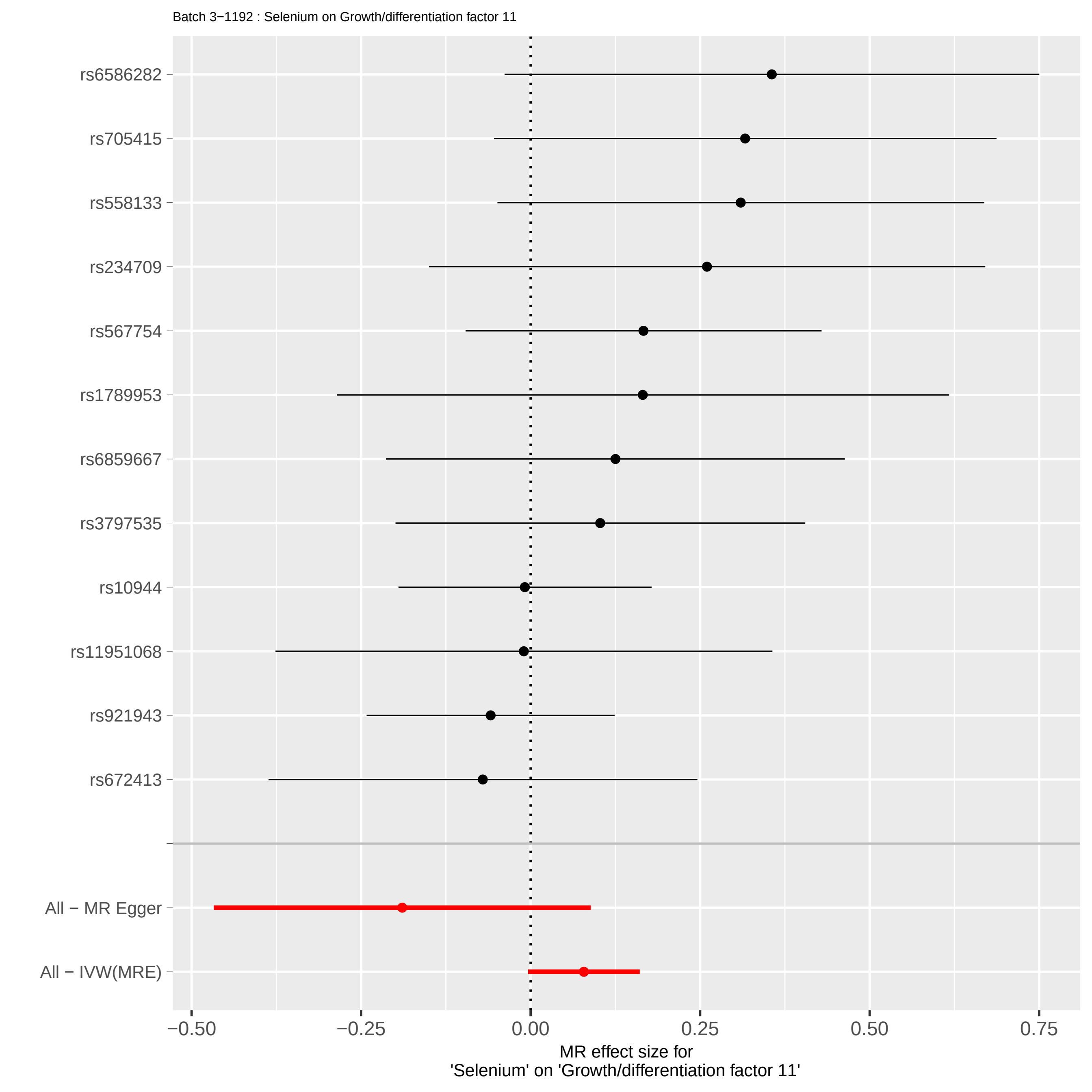
Batch 3−1192 : Selenium on Growth/differentiation factor 11
rs6586282
rs705415
rs558133
rs234709
rs567754
rs1789953
rs6859667
rs3797535
rs10944
rs11951068
rs921943
rs672413
All − MR Egger
All − IVW(MRE)
−0.50
−0.25
0.00
0.25
0.50
0.75
MR effect size for
'Selenium' on 'Growth/differentiation factor 11'

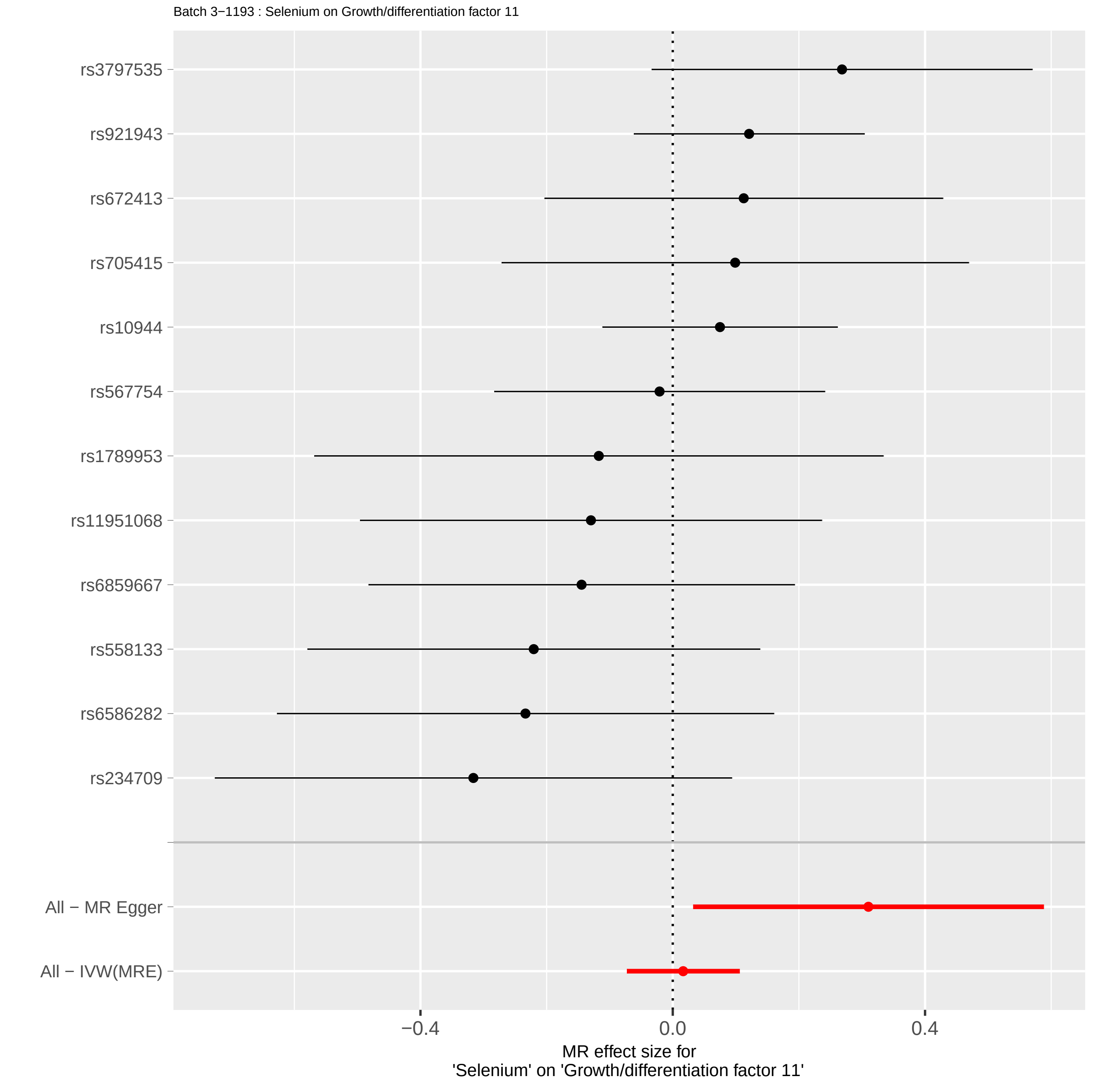

Batch 3−1193 : Selenium on Growth/differentiation factor 11
rs3797535
rs921943
rs672413
rs705415
rs10944
rs567754
rs1789953
rs11951068
rs6859667
rs558133
rs6586282
rs234709
All − MR Egger
All − IVW(MRE)
−0.4
0.0
0.4
MR effect size for
'Selenium' on 'Growth/differentiation factor 11'

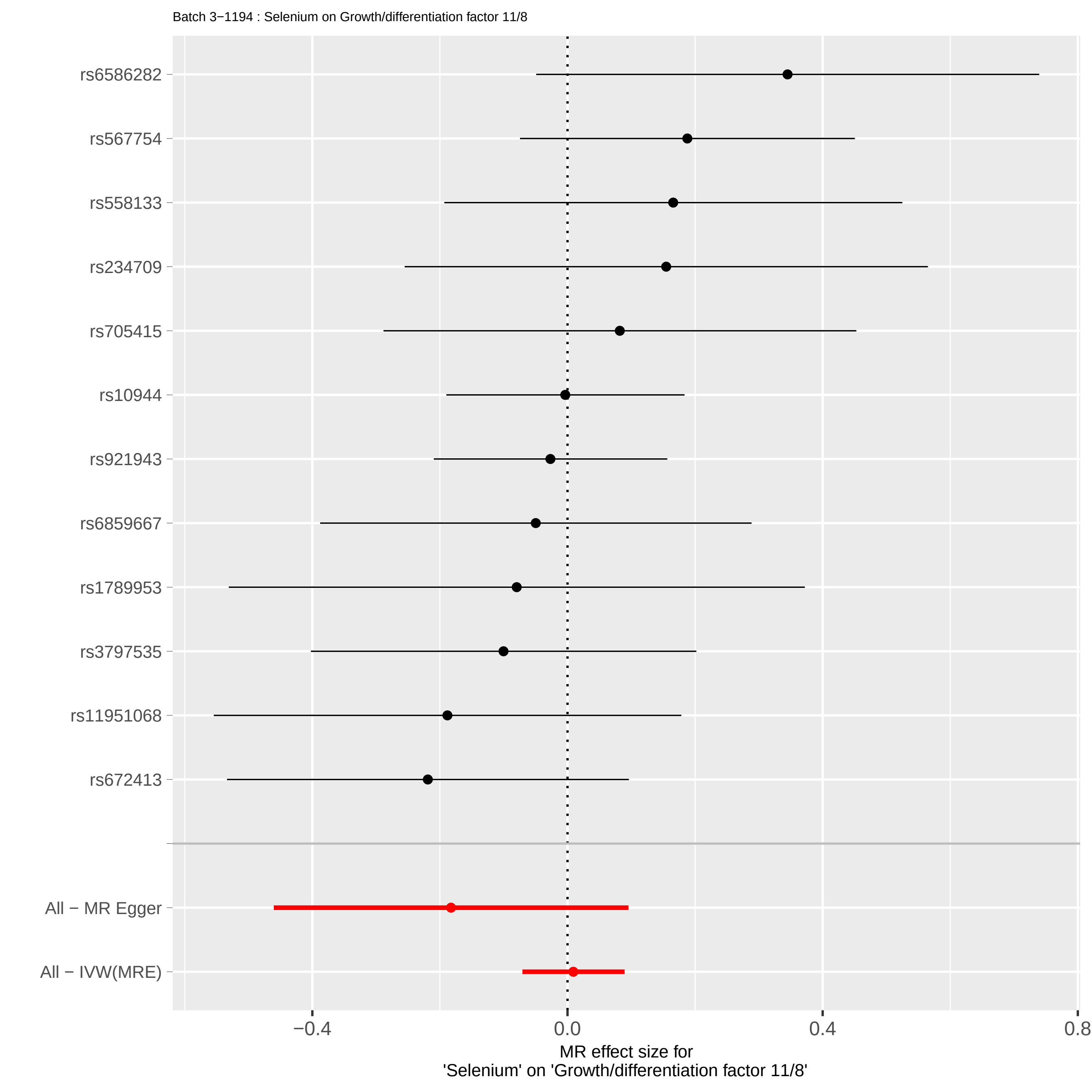
Batch 3−1194 : Selenium on Growth/differentiation factor 11/8
rs6586282
rs567754
rs558133
rs234709
rs705415
rs10944
rs921943
rs6859667
rs1789953
rs3797535
rs11951068
rs672413
All − MR Egger
All − IVW(MRE)
−0.4
0.0
0.4
0.8
MR effect size for
'Selenium' on 'Growth/differentiation factor 11/8'

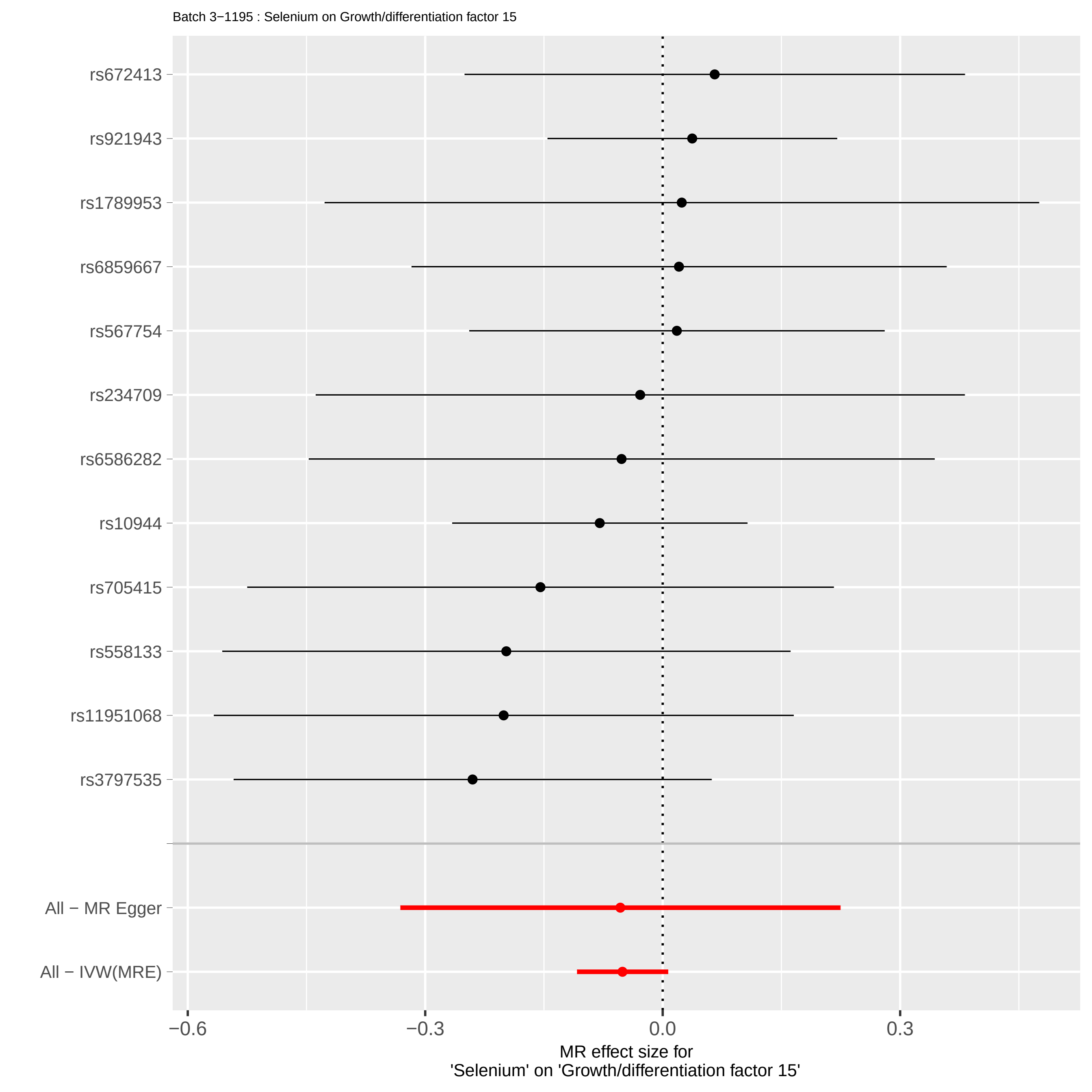

Batch 3−1195 : Selenium on Growth/differentiation factor 15
rs672413
rs921943
rs1789953
rs6859667
rs567754
rs234709
rs6586282
rs10944
rs705415
rs558133
rs11951068
rs3797535
All − MR Egger
All − IVW(MRE)
−0.6
−0.3
0.0
0.3
MR effect size for
'Selenium' on 'Growth/differentiation factor 15'

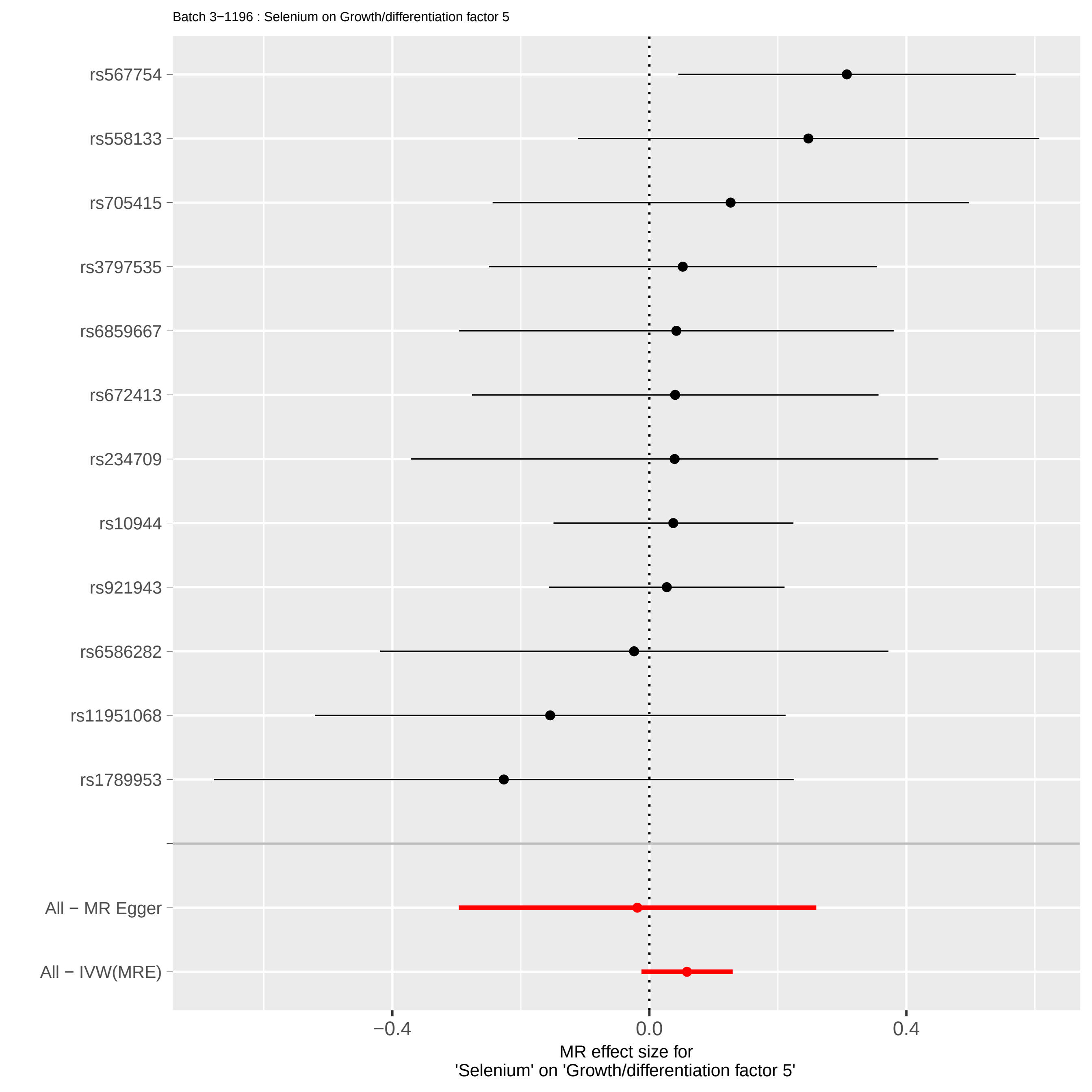

Batch 3−1196 : Selenium on Growth/differentiation factor 5
rs567754
rs558133
rs705415
rs3797535
rs6859667
rs672413
rs234709
rs10944
rs921943
rs6586282
rs11951068
rs1789953
All − MR Egger
All − IVW(MRE)
−0.4
0.0
0.4
MR effect size for
'Selenium' on 'Growth/differentiation factor 5'

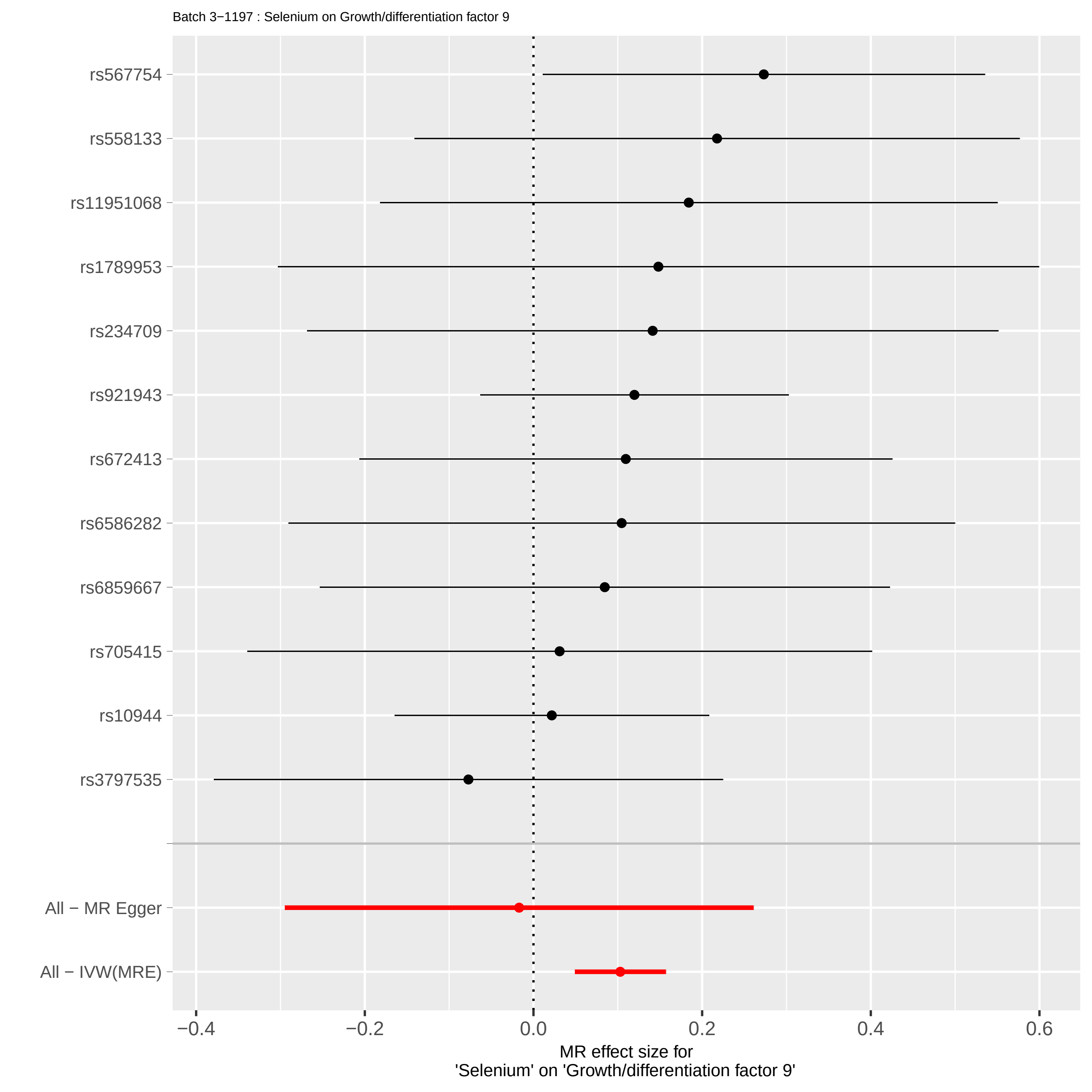
Batch 3−1197 : Selenium on Growth/differentiation factor 9
rs567754
rs558133
rs11951068
rs1789953
rs234709
rs921943
rs672413
rs6586282
rs6859667
rs705415
rs10944
rs3797535
All − MR Egger
All − IVW(MRE)
−0.4
−0.2
0.0
0.2
0.4
0.6
MR effect size for
'Selenium' on 'Growth/differentiation factor 9'

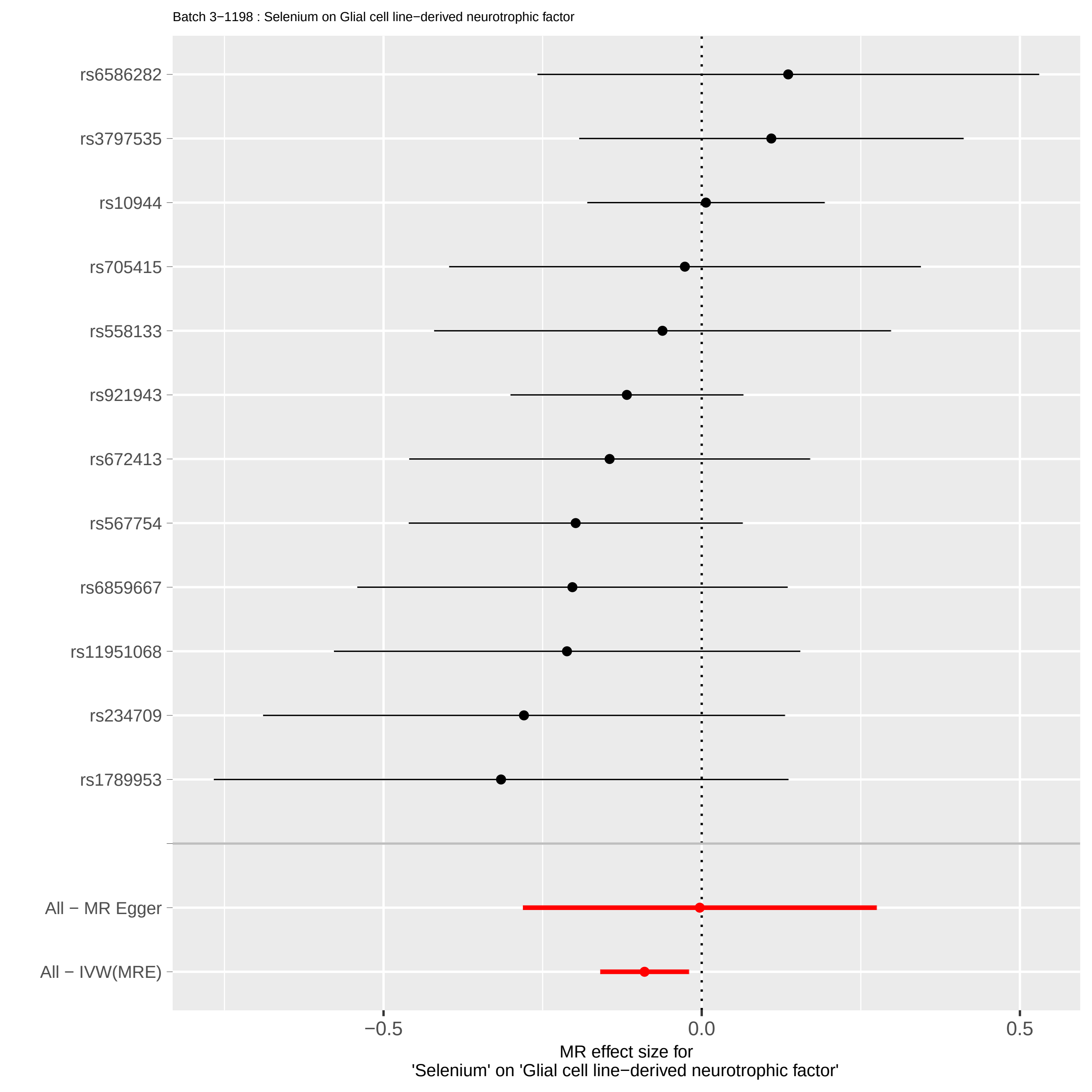

Batch 3−1198 : Selenium on Glial cell line−derived neurotrophic factor
rs6586282
rs3797535
rs10944
rs705415
rs558133
rs921943
rs672413
rs567754
rs6859667
rs11951068
rs234709
rs1789953
All − MR Egger
All − IVW(MRE)
−0.5
0.0
0.5
MR effect size for
'Selenium' on 'Glial cell line−derived neurotrophic factor'

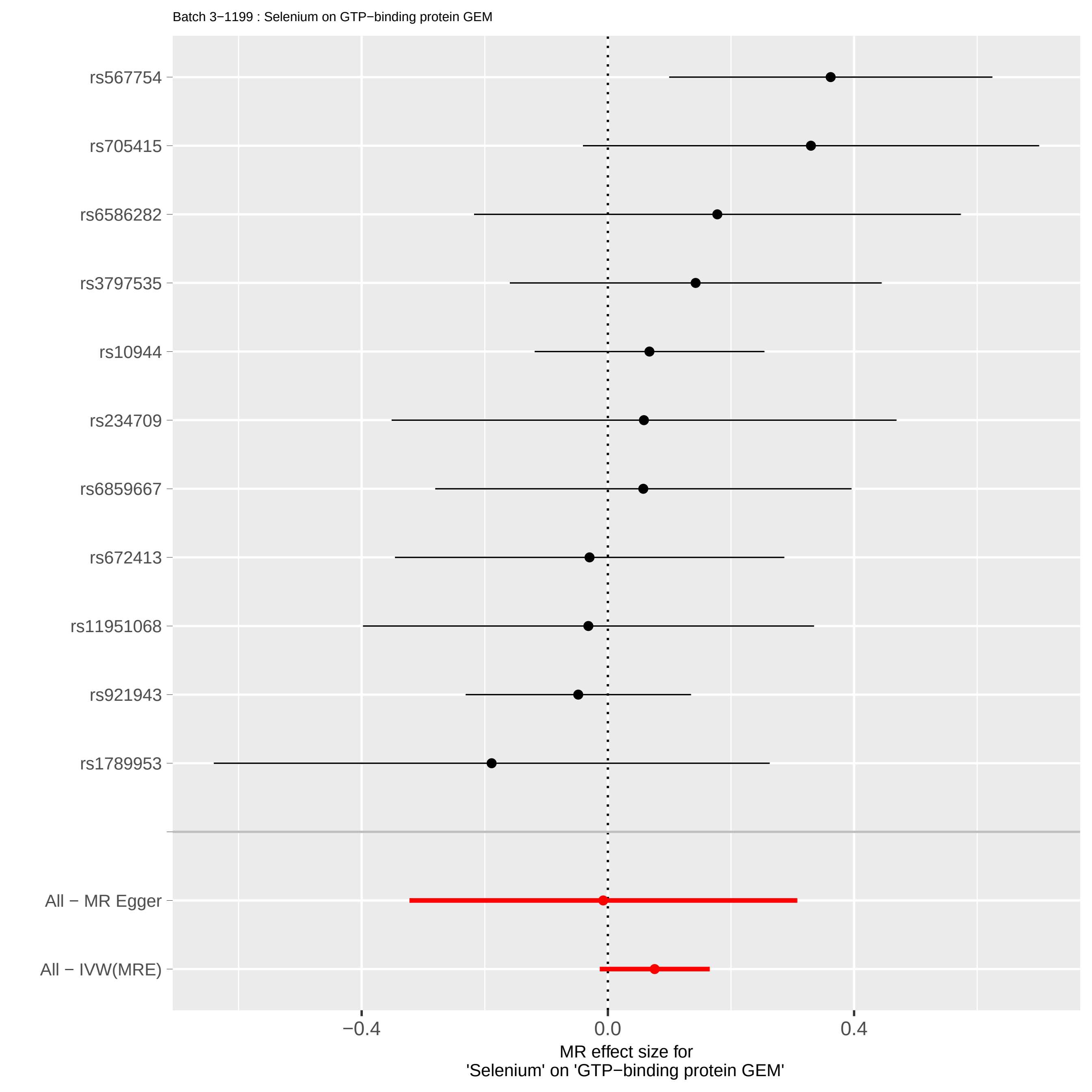
Batch 3−1199 : Selenium on GTP−binding protein GEM
rs567754
rs705415
rs6586282
rs3797535
rs10944
rs234709
rs6859667
rs672413
rs11951068
rs921943
rs1789953
All − MR Egger
All − IVW(MRE)
−0.4
0.0
0.4
MR effect size for
'Selenium' on 'GTP−binding protein GEM'

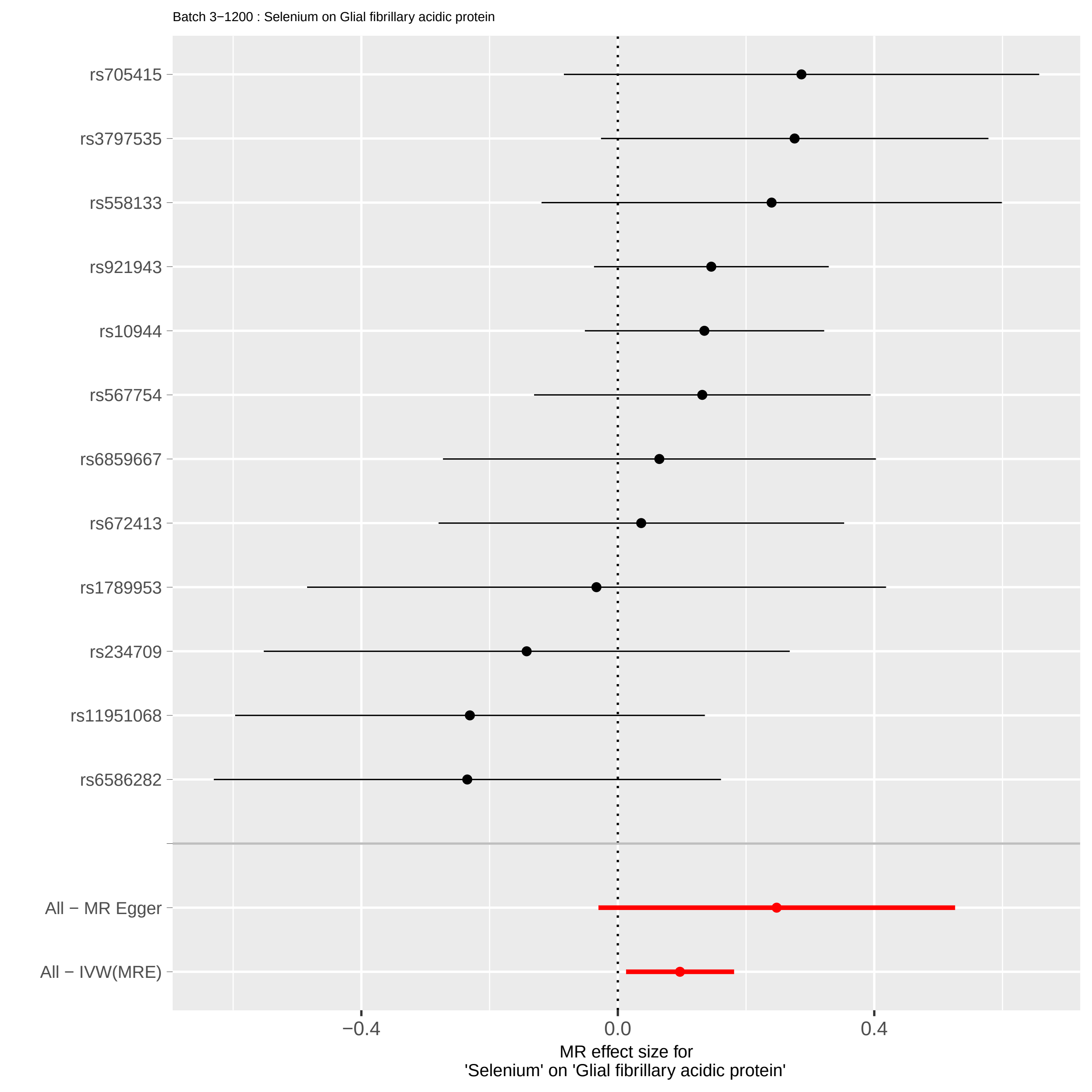

Batch 3−1200 : Selenium on Glial fibrillary acidic protein
rs705415
rs3797535
rs558133
rs921943
rs10944
rs567754
rs6859667
rs672413
rs1789953
rs234709
rs11951068
rs6586282
All − MR Egger
All − IVW(MRE)
−0.4
0.0
0.4
MR effect size for
'Selenium' on 'Glial fibrillary acidic protein'

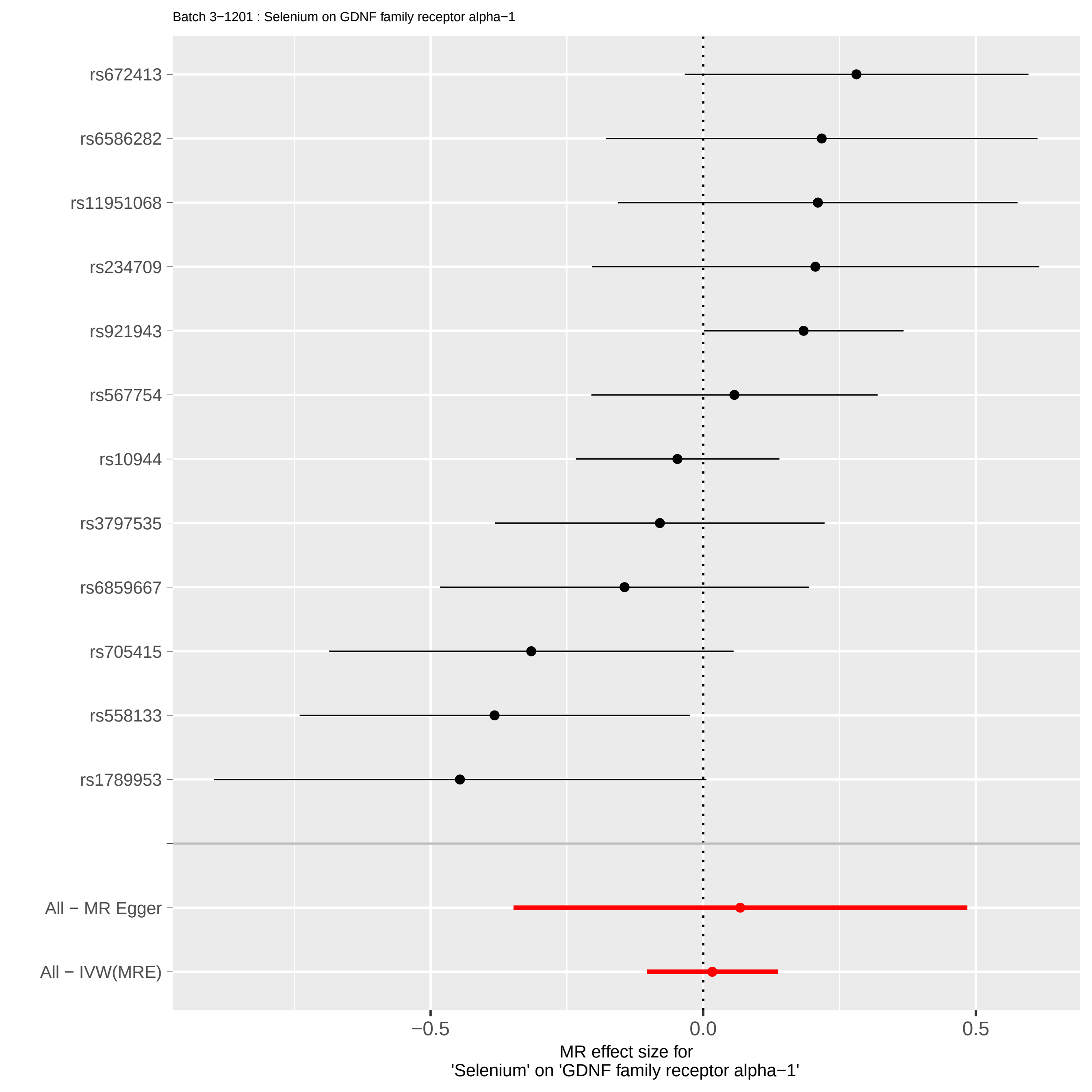

Batch 3−1201 : Selenium on GDNF family receptor alpha−1
rs672413
rs6586282
rs11951068
rs234709
rs921943
rs567754
rs10944
rs3797535
rs6859667
rs705415
rs558133
rs1789953
All − MR Egger
All − IVW(MRE)
−0.5
0.0
0.5
MR effect size for
'Selenium' on 'GDNF family receptor alpha−1'

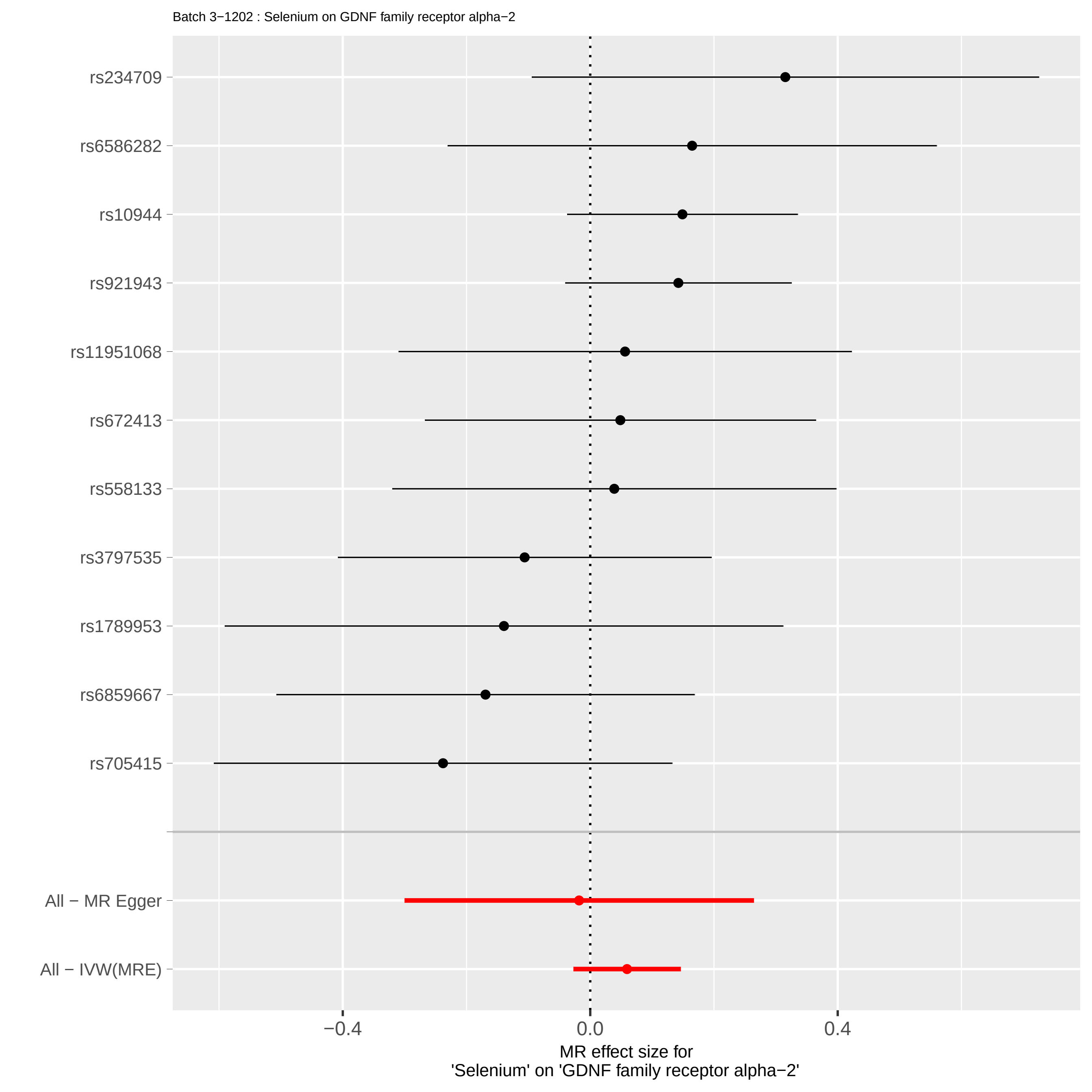
Batch 3−1202 : Selenium on GDNF family receptor alpha−2
rs234709
rs6586282
rs10944
rs921943
rs11951068
rs672413
rs558133
rs3797535
rs1789953
rs6859667
rs705415
All − MR Egger
All − IVW(MRE)
−0.4
0.0
0.4
MR effect size for
'Selenium' on 'GDNF family receptor alpha−2'

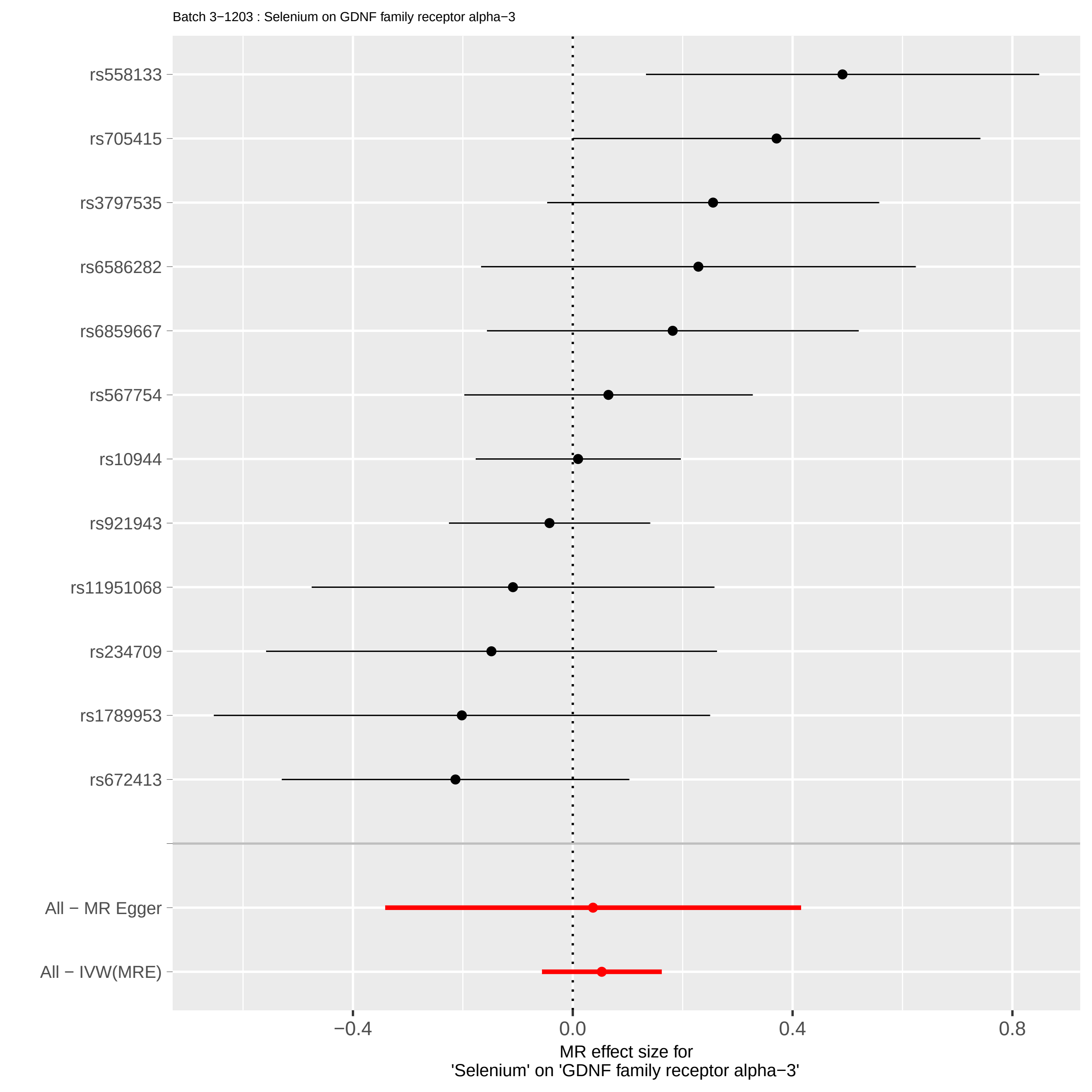
Batch 3−1203 : Selenium on GDNF family receptor alpha−3
rs558133
rs705415
rs3797535
rs6586282
rs6859667
rs567754
rs10944
rs921943
rs11951068
rs234709
rs1789953
rs672413
All − MR Egger
All − IVW(MRE)
−0.4
0.0
0.4
0.8
MR effect size for
'Selenium' on 'GDNF family receptor alpha−3'

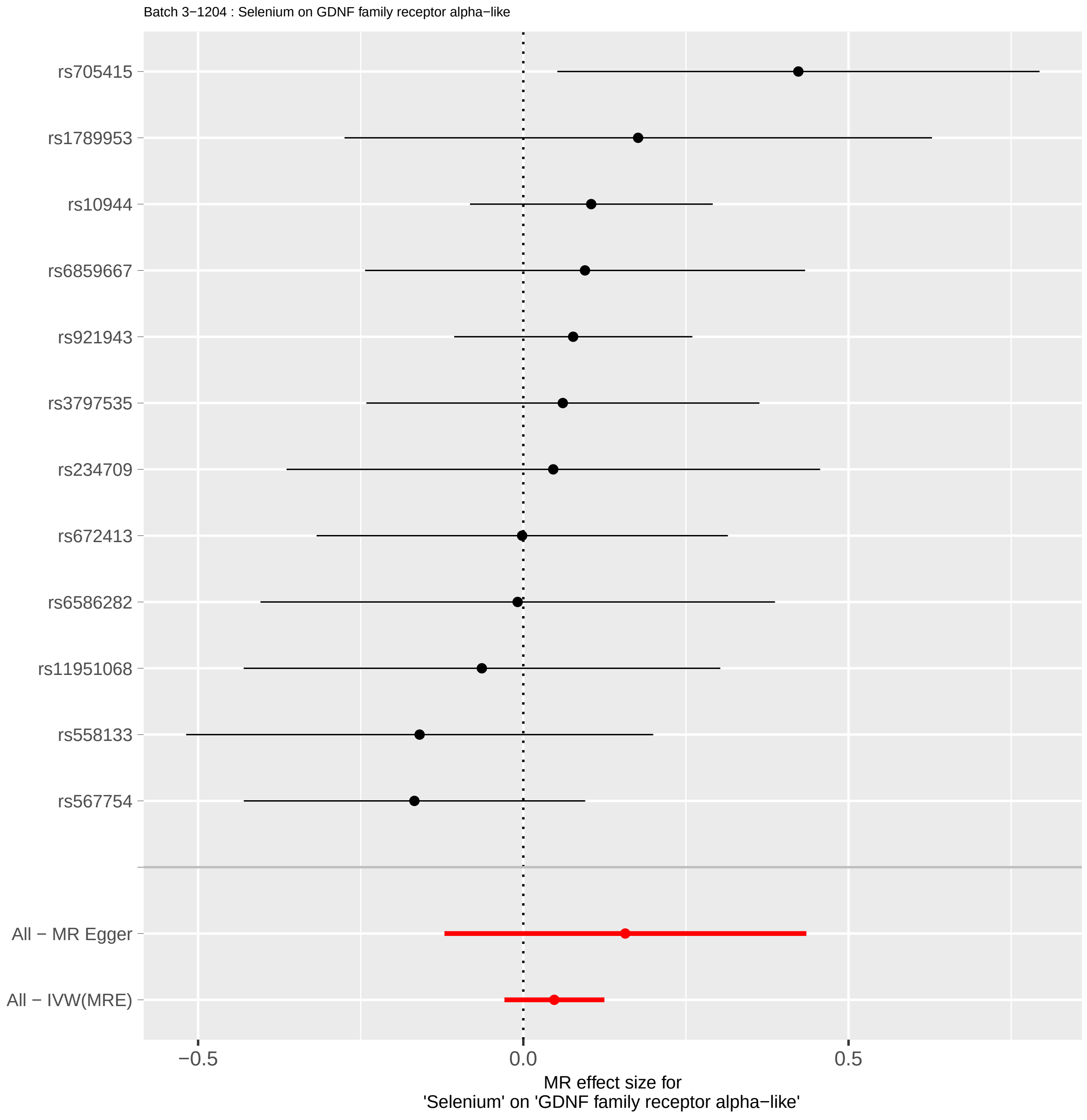

Batch 3−1204 : Selenium on GDNF family receptor alpha−like
rs705415
rs1789953
rs10944
rs6859667
rs921943
rs3797535
rs234709
rs672413
rs6586282
rs11951068
rs558133
rs567754
All − MR Egger
All − IVW(MRE)
−0.5
0.0
0.5
MR effect size for
'Selenium' on 'GDNF family receptor alpha−like'

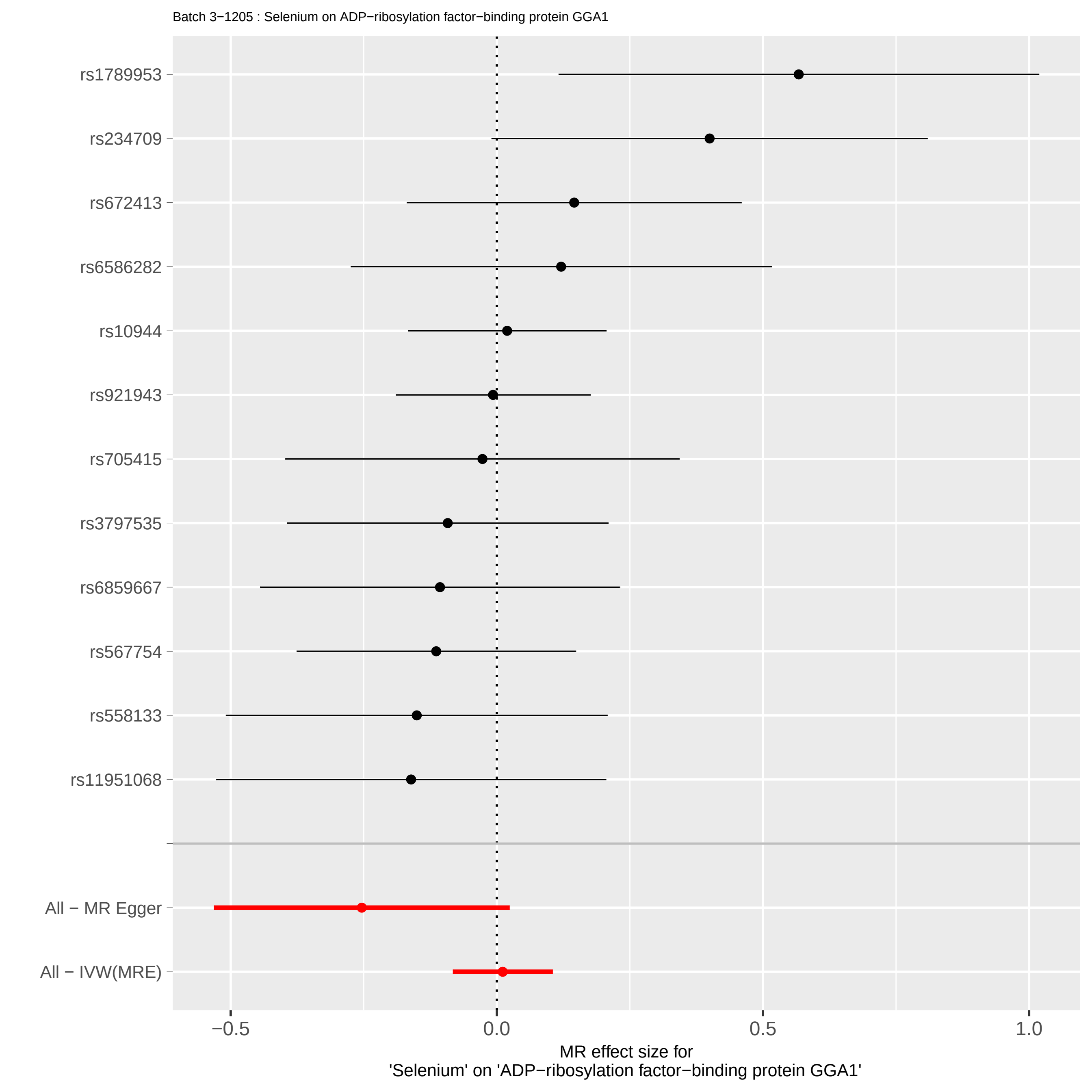

Batch 3−1205 : Selenium on ADP−ribosylation factor−binding protein GGA1
rs1789953
rs234709
rs672413
rs6586282
rs10944
rs921943
rs705415
rs3797535
rs6859667
rs567754
rs558133
rs11951068
All − MR Egger
All − IVW(MRE)
−0.5
0.0
0.5
1.0
MR effect size for
'Selenium' on 'ADP−ribosylation factor−binding protein GGA1'

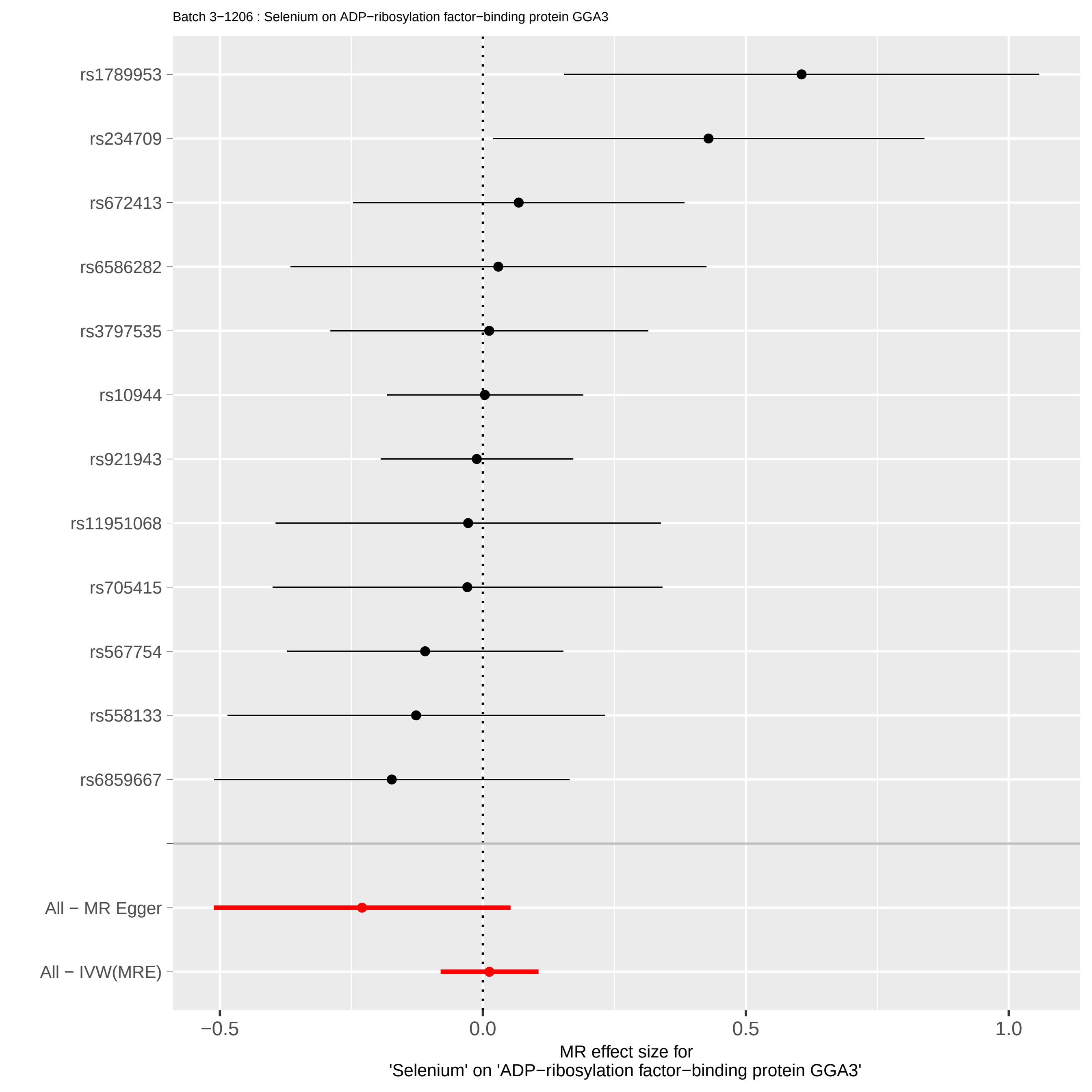

Batch 3−1206 : Selenium on ADP−ribosylation factor−binding protein GGA3
rs1789953
rs234709
rs672413
rs6586282
rs3797535
rs10944
rs921943
rs11951068
rs705415
rs567754
rs558133
rs6859667
All − MR Egger
All − IVW(MRE)
−0.5
0.0
0.5
1.0
MR effect size for
'Selenium' on 'ADP−ribosylation factor−binding protein GGA3'

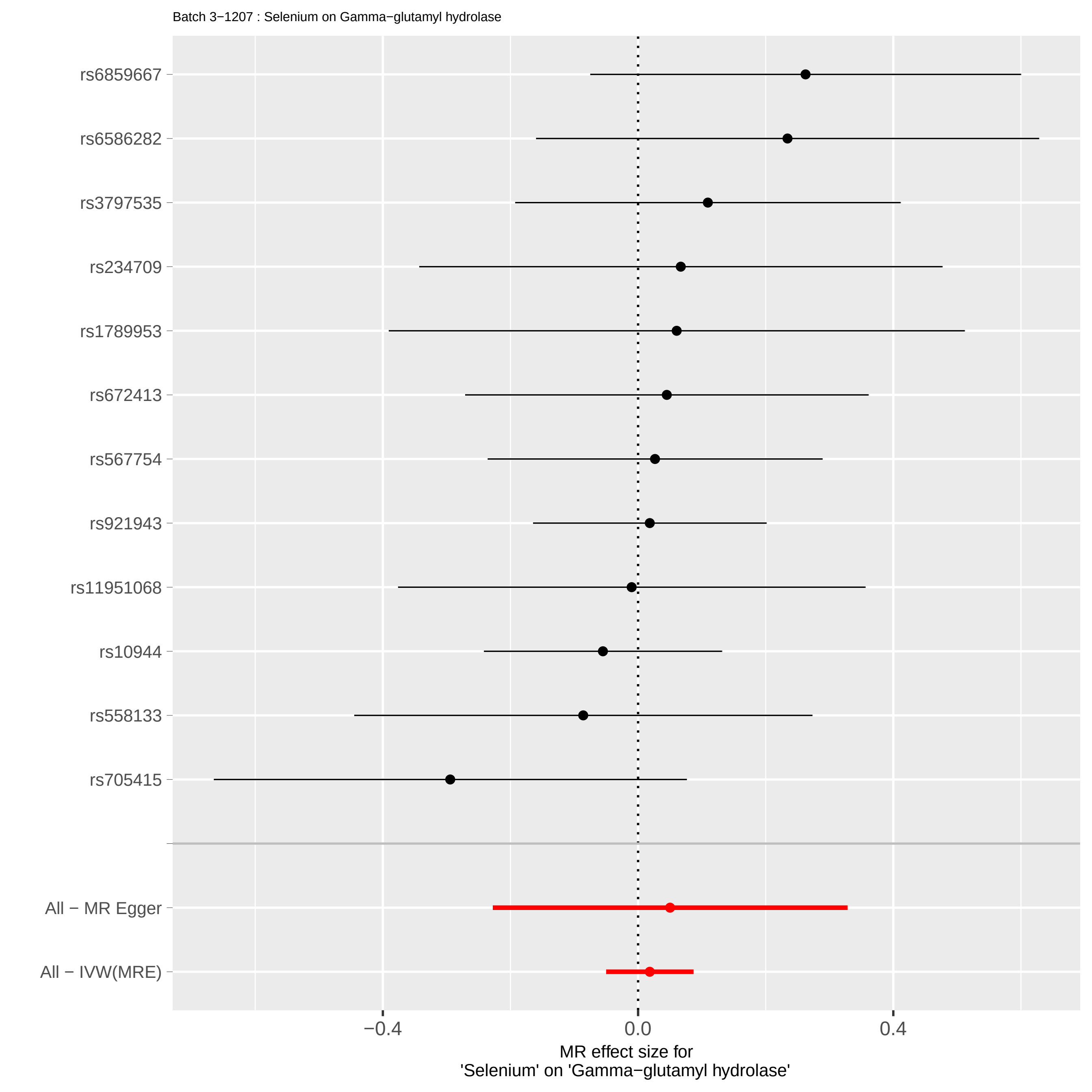

Batch 3−1207 : Selenium on Gamma−glutamyl hydrolase
rs6859667
rs6586282
rs3797535
rs234709
rs1789953
rs672413
rs567754
rs921943
rs11951068
rs10944
rs558133
rs705415
All − MR Egger
All − IVW(MRE)
−0.4
0.0
0.4
MR effect size for
'Selenium' on 'Gamma−glutamyl hydrolase'

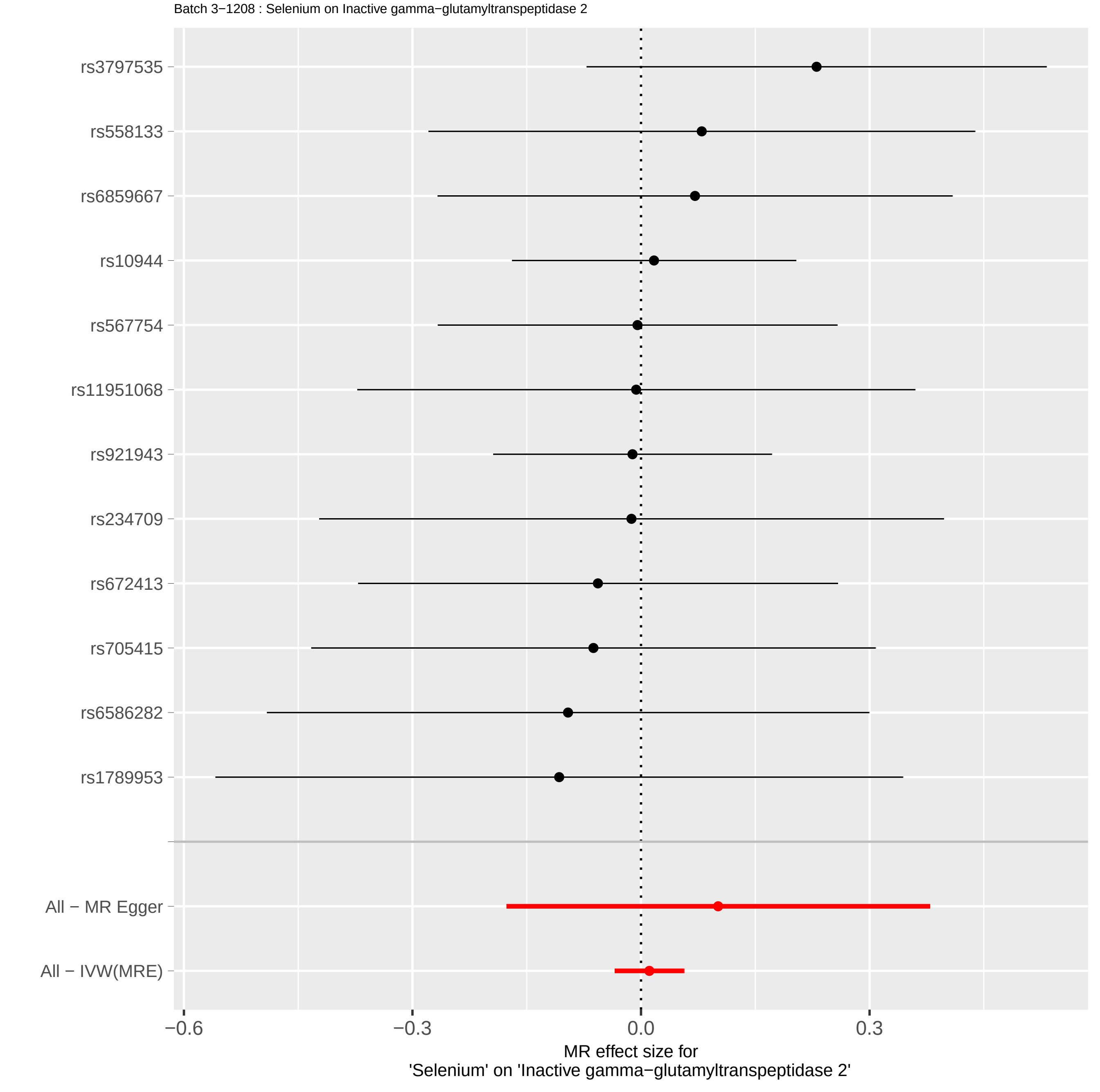

Batch 3−1208 : Selenium on Inactive gamma−glutamyltranspeptidase 2
rs3797535
rs558133
rs6859667
rs10944
rs567754
rs11951068
rs921943
rs234709
rs672413
rs705415
rs6586282
rs1789953
All − MR Egger
All − IVW(MRE)
−0.6
−0.3
0.0
0.3
MR effect size for
'Selenium' on 'Inactive gamma−glutamyltranspeptidase 2'

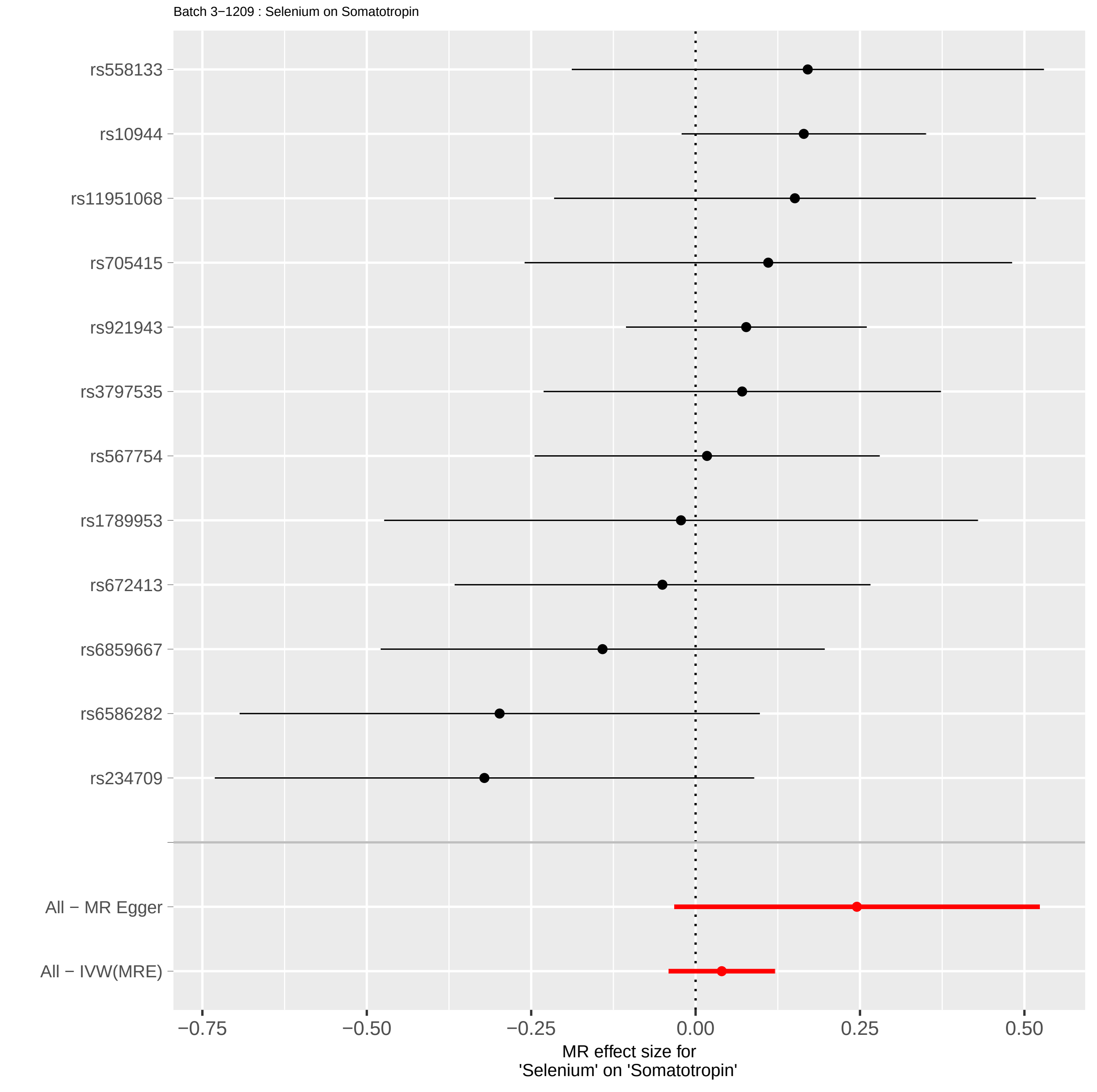

Batch 3−1209 : Selenium on Somatotropin
rs558133
rs10944
rs11951068
rs705415
rs921943
rs3797535
rs567754
rs1789953
rs672413
rs6859667
rs6586282
rs234709
All − MR Egger
All − IVW(MRE)
−0.75
−0.50
−0.25
0.00
0.25
0.50
MR effect size for
'Selenium' on 'Somatotropin'

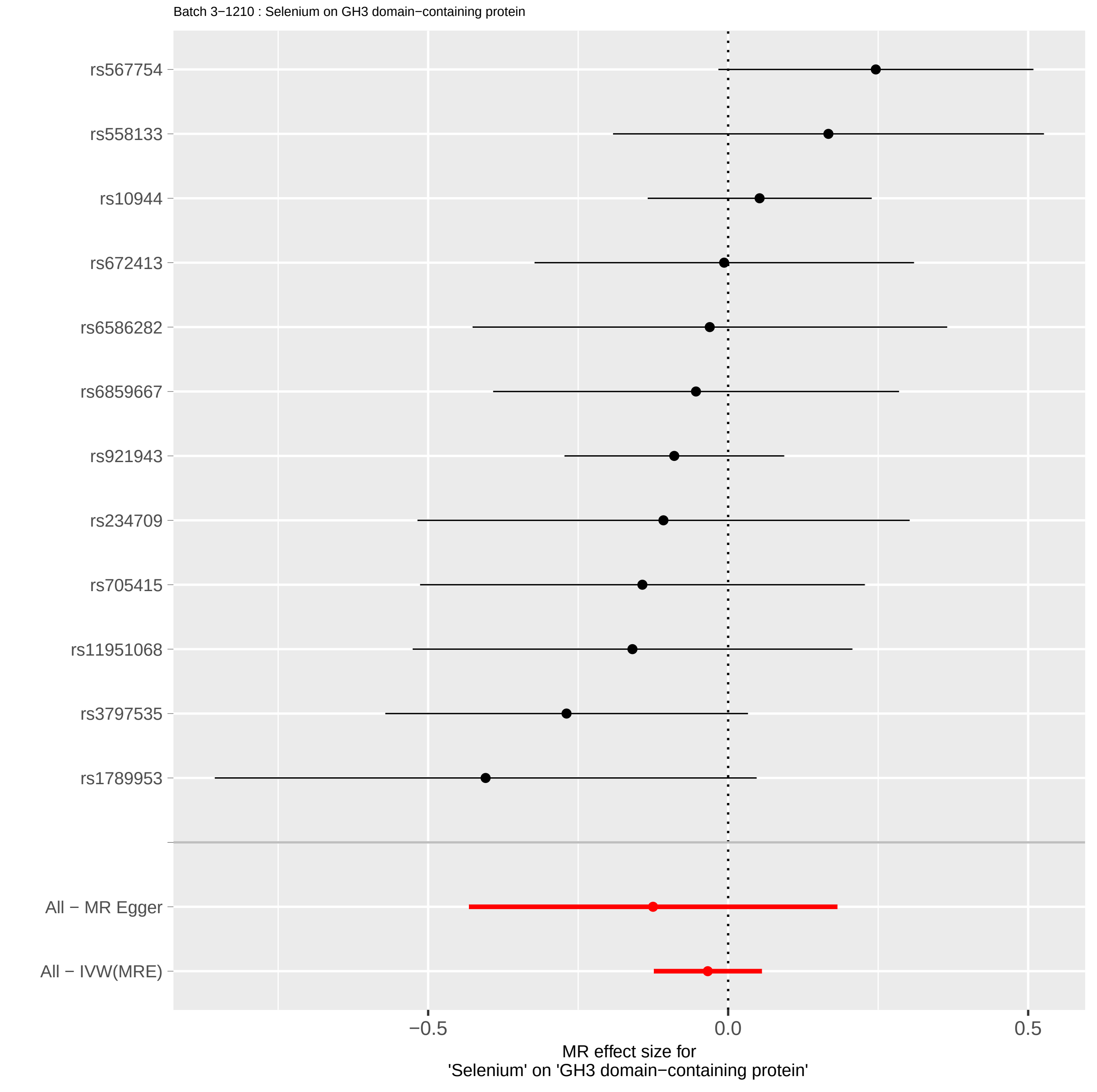

Batch 3−1210 : Selenium on GH3 domain−containing protein
rs567754
rs558133
rs10944
rs672413
rs6586282
rs6859667
rs921943
rs234709
rs705415
rs11951068
rs3797535
rs1789953
All − MR Egger
All − IVW(MRE)
−0.5
0.0
0.5
MR effect size for
'Selenium' on 'GH3 domain−containing protein'

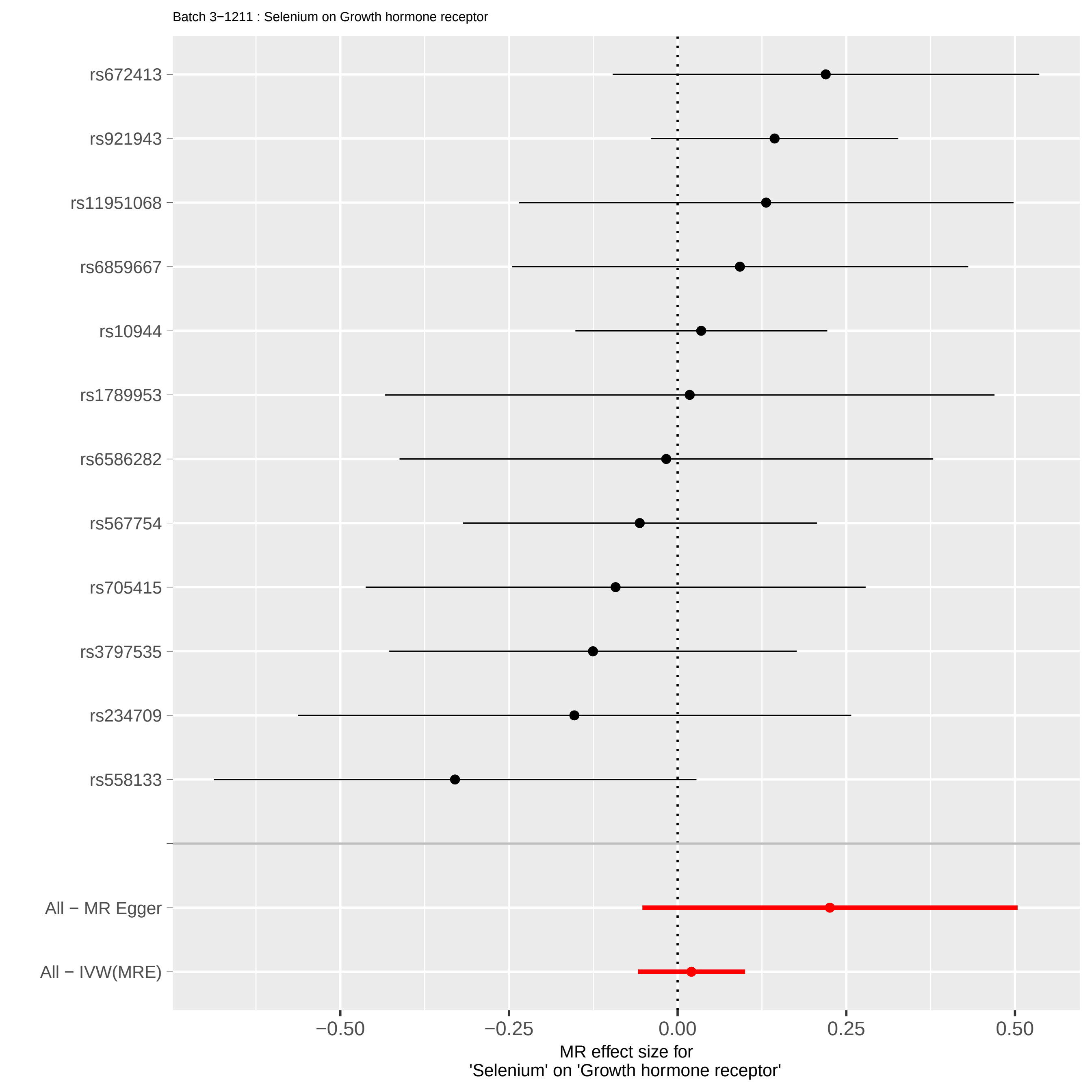

Batch 3−1211 : Selenium on Growth hormone receptor
rs672413
rs921943
rs11951068
rs6859667
rs10944
rs1789953
rs6586282
rs567754
rs705415
rs3797535
rs234709
rs558133
All − MR Egger
All − IVW(MRE)
−0.50
−0.25
0.00
0.25
0.50
MR effect size for
'Selenium' on 'Growth hormone receptor'

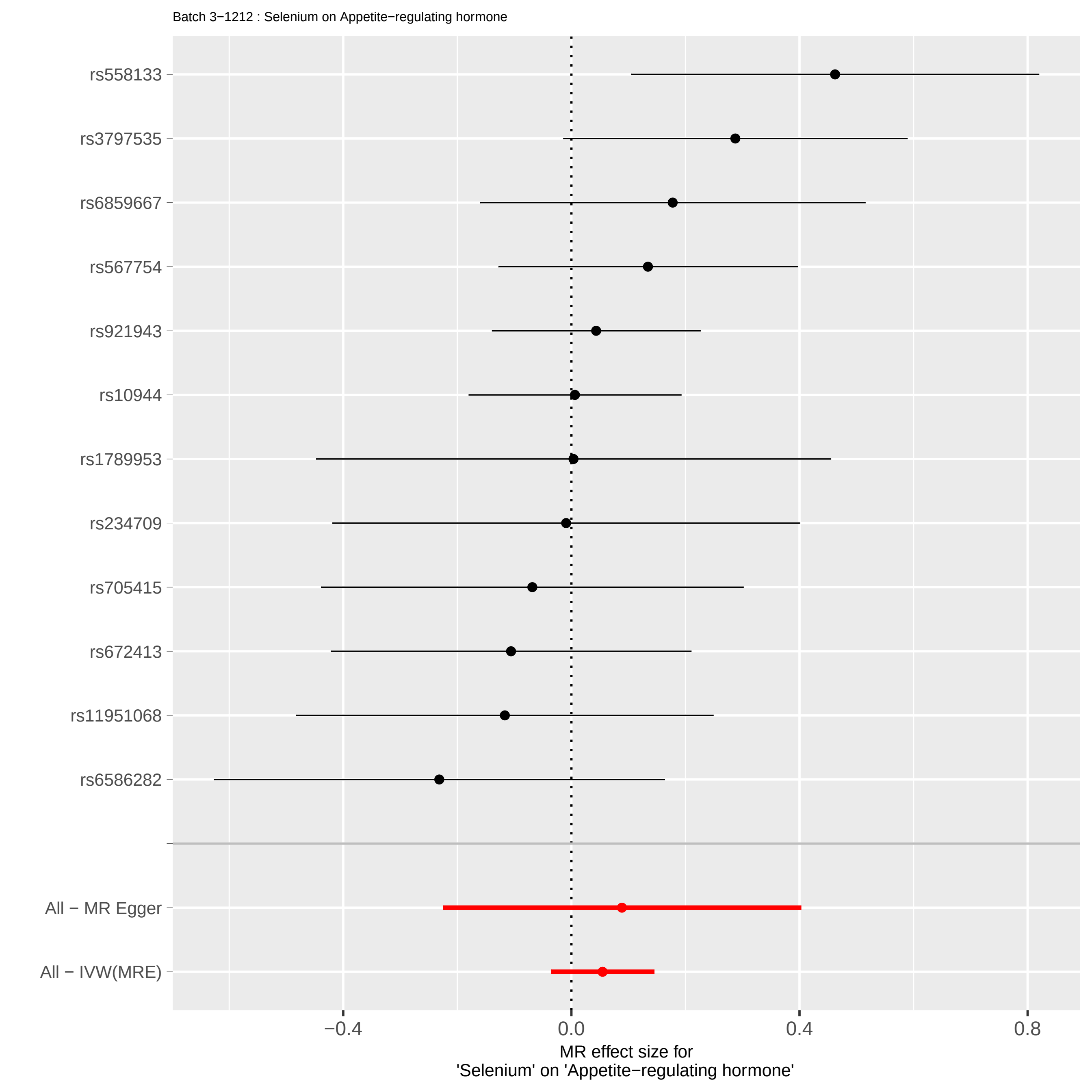

Batch 3−1212 : Selenium on Appetite−regulating hormone
rs558133
rs3797535
rs6859667
rs567754
rs921943
rs10944
rs1789953
rs234709
rs705415
rs672413
rs11951068
rs6586282
All − MR Egger
All − IVW(MRE)
−0.4
0.0
0.4
0.8
MR effect size for
'Selenium' on 'Appetite−regulating hormone'

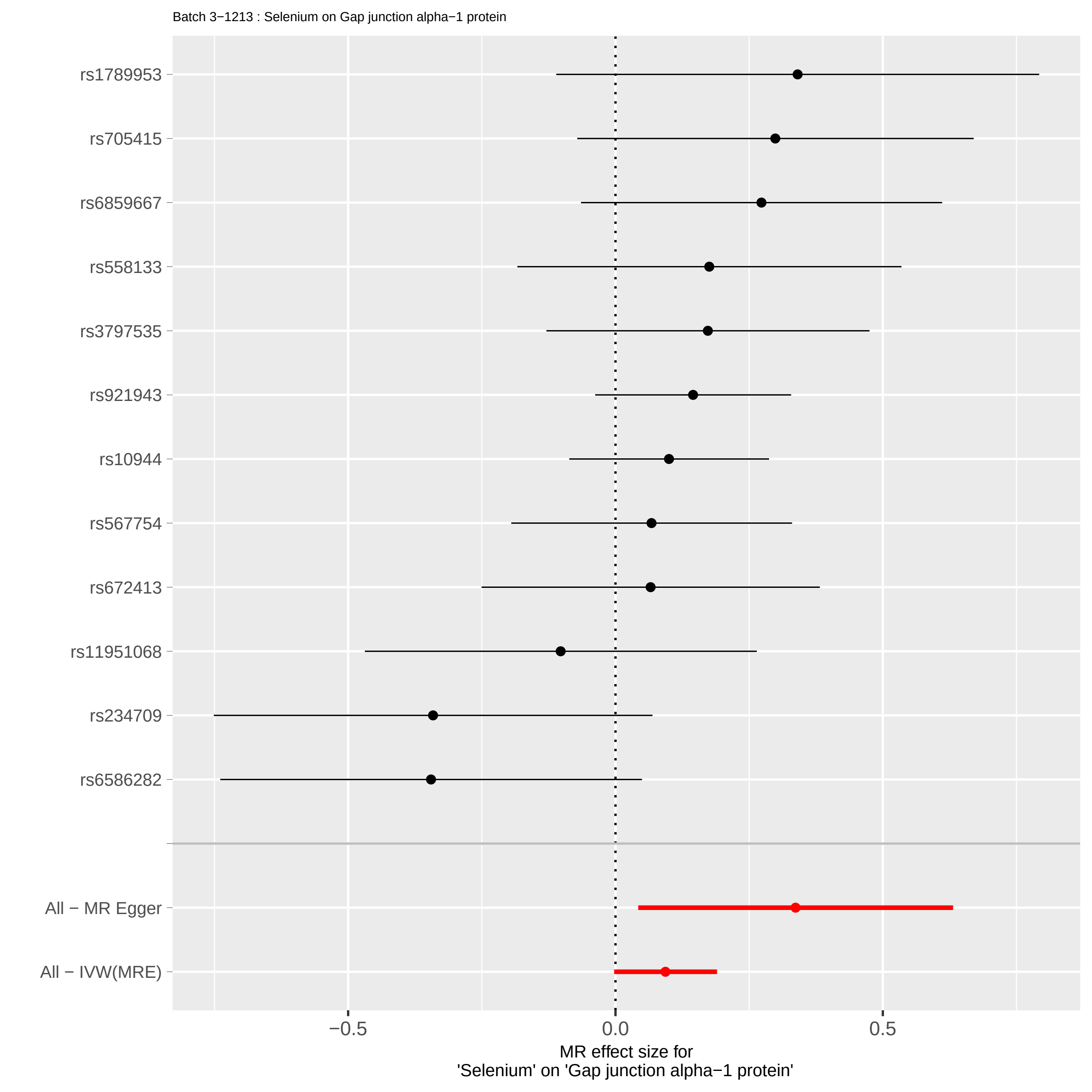

Batch 3−1213 : Selenium on Gap junction alpha−1 protein
rs1789953
rs705415
rs6859667
rs558133
rs3797535
rs921943
rs10944
rs567754
rs672413
rs11951068
rs234709
rs6586282
All − MR Egger
All − IVW(MRE)
−0.5
0.0
0.5
MR effect size for
'Selenium' on 'Gap junction alpha−1 protein'

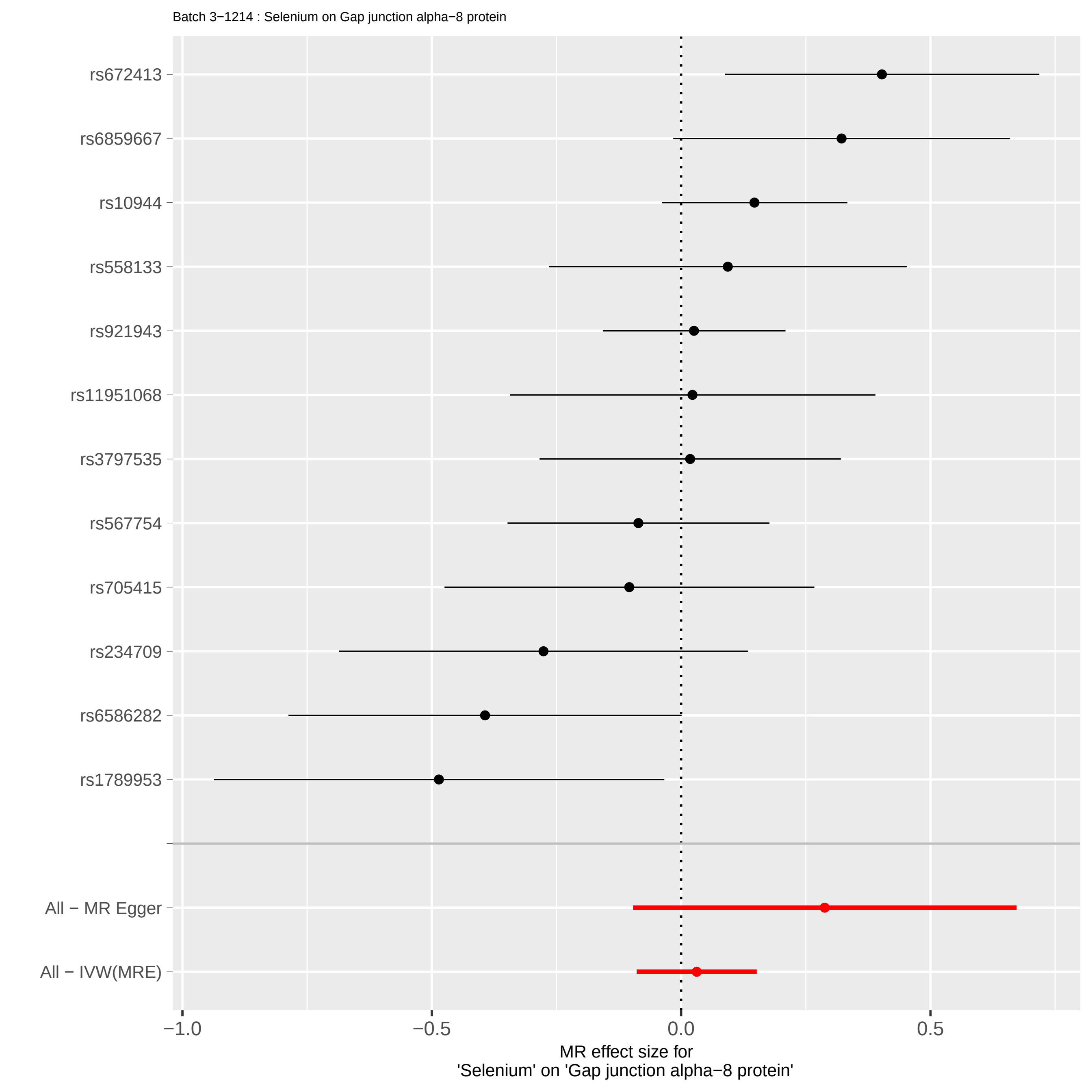

Batch 3−1214 : Selenium on Gap junction alpha−8 protein
rs672413
rs6859667
rs10944
rs558133
rs921943
rs11951068
rs3797535
rs567754
rs705415
rs234709
rs6586282
rs1789953
All − MR Egger
All − IVW(MRE)
−1.0
−0.5
0.0
0.5
MR effect size for
'Selenium' on 'Gap junction alpha−8 protein'

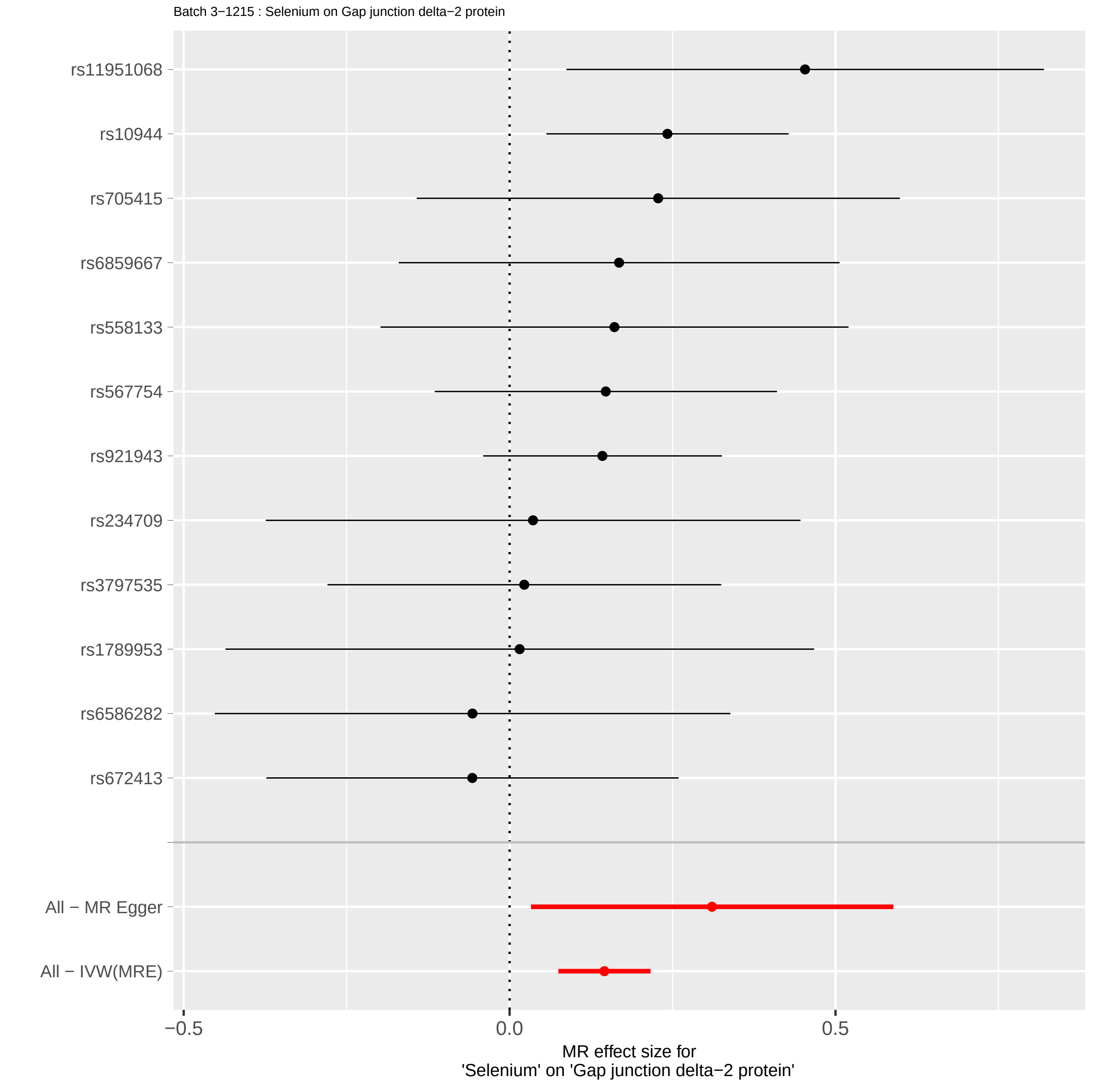

Batch 3−1215 : Selenium on Gap junction delta−2 protein
rs11951068
rs10944
rs705415
rs6859667
rs558133
rs567754
rs921943
rs234709
rs3797535
rs1789953
rs6586282
rs672413
All − MR Egger
All − IVW(MRE)
−0.5
0.0
0.5
MR effect size for
'Selenium' on 'Gap junction delta−2 protein'

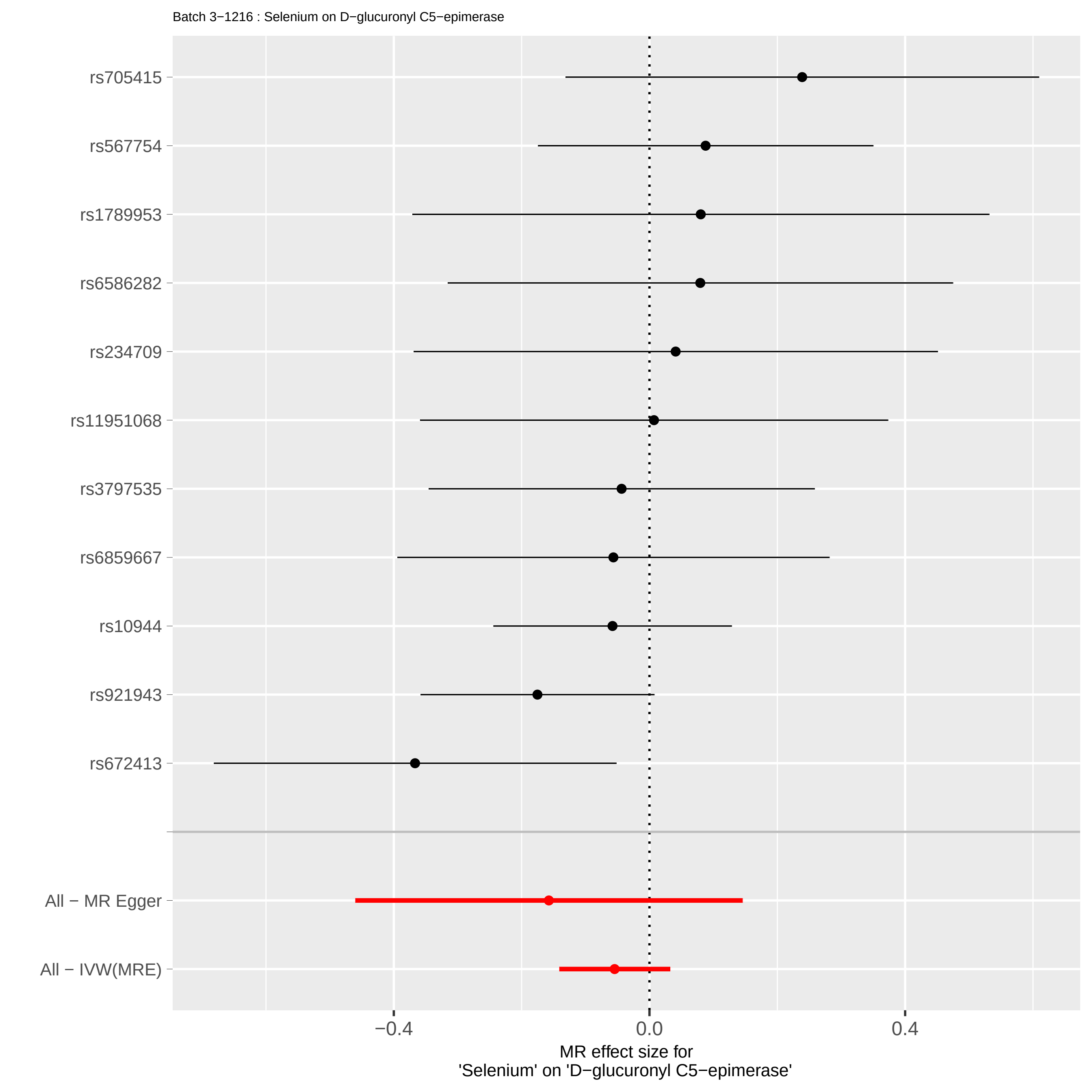
Batch 3−1216 : Selenium on D−glucuronyl C5−epimerase
rs705415
rs567754
rs1789953
rs6586282
rs234709
rs11951068
rs3797535
rs6859667
rs10944
rs921943
rs672413
All − MR Egger
All − IVW(MRE)
−0.4
0.0
0.4
MR effect size for
'Selenium' on 'D−glucuronyl C5−epimerase'

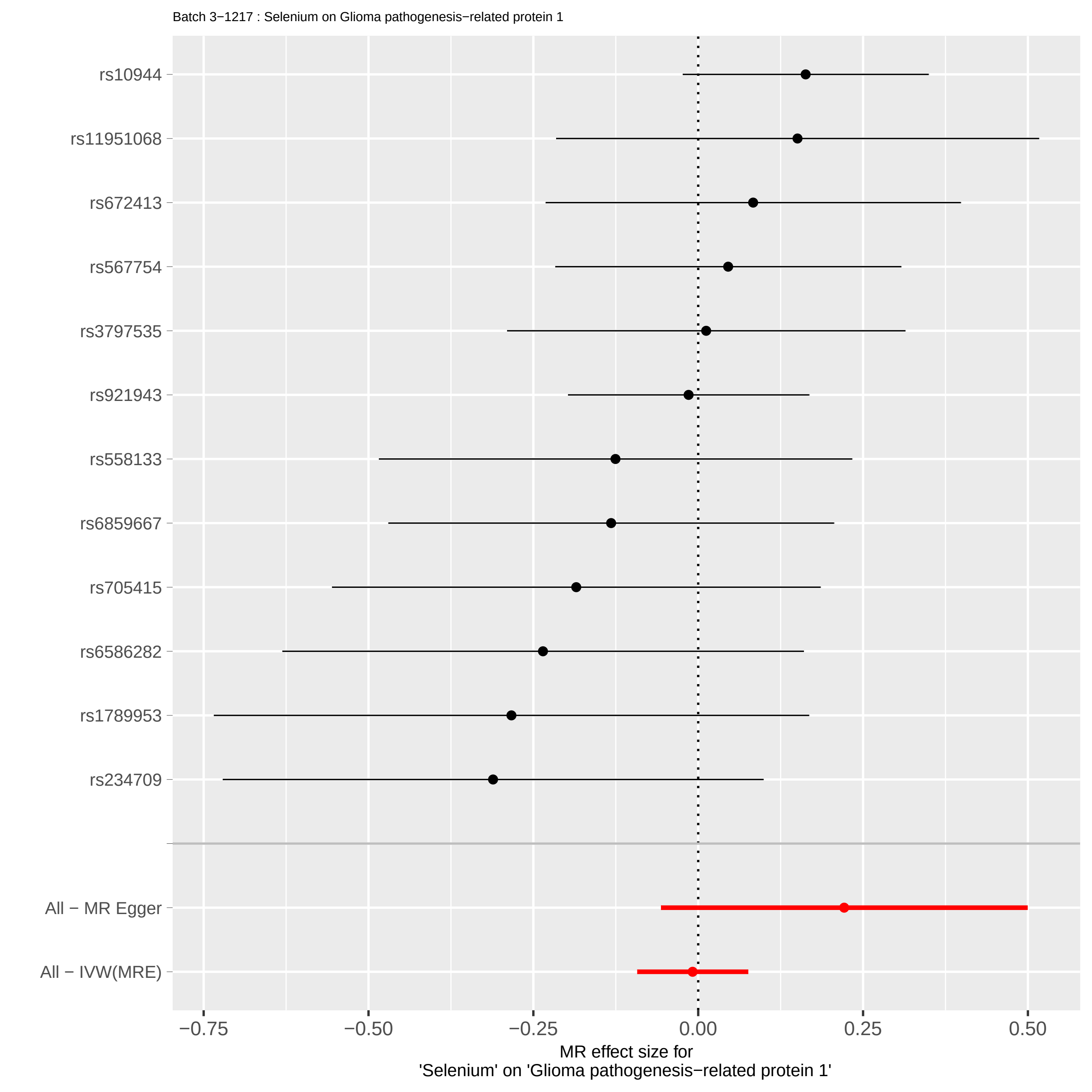

Batch 3−1217 : Selenium on Glioma pathogenesis−related protein 1
rs10944
rs11951068
rs672413
rs567754
rs3797535
rs921943
rs558133
rs6859667
rs705415
rs6586282
rs1789953
rs234709
All − MR Egger
All − IVW(MRE)
−0.75
−0.50
−0.25
0.00
0.25
0.50
MR effect size for
'Selenium' on 'Glioma pathogenesis−related protein 1'

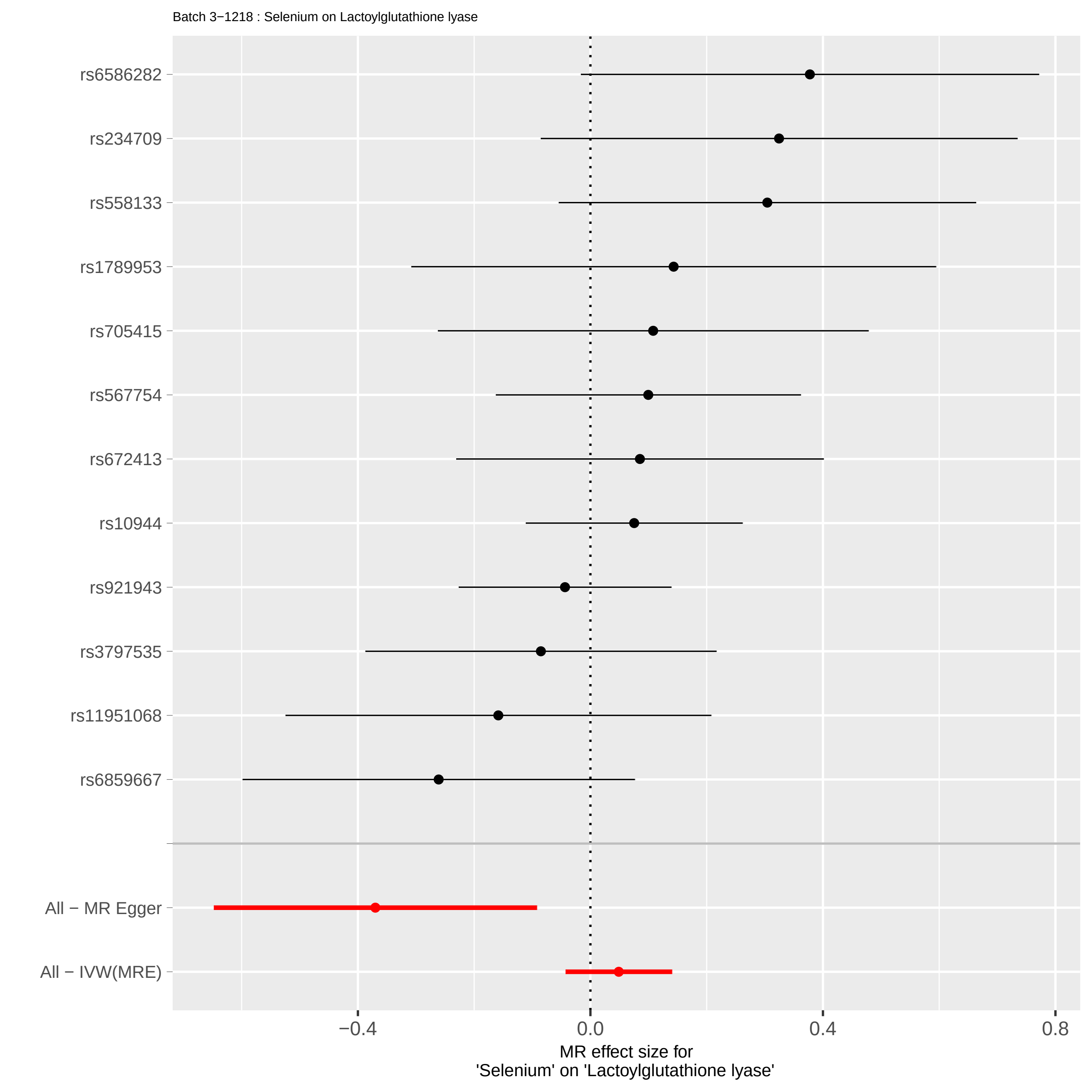

Batch 3−1218 : Selenium on Lactoylglutathione lyase
rs6586282
rs234709
rs558133
rs1789953
rs705415
rs567754
rs672413
rs10944
rs921943
rs3797535
rs11951068
rs6859667
All − MR Egger
All − IVW(MRE)
−0.4
0.0
0.4
0.8
MR effect size for
'Selenium' on 'Lactoylglutathione lyase'

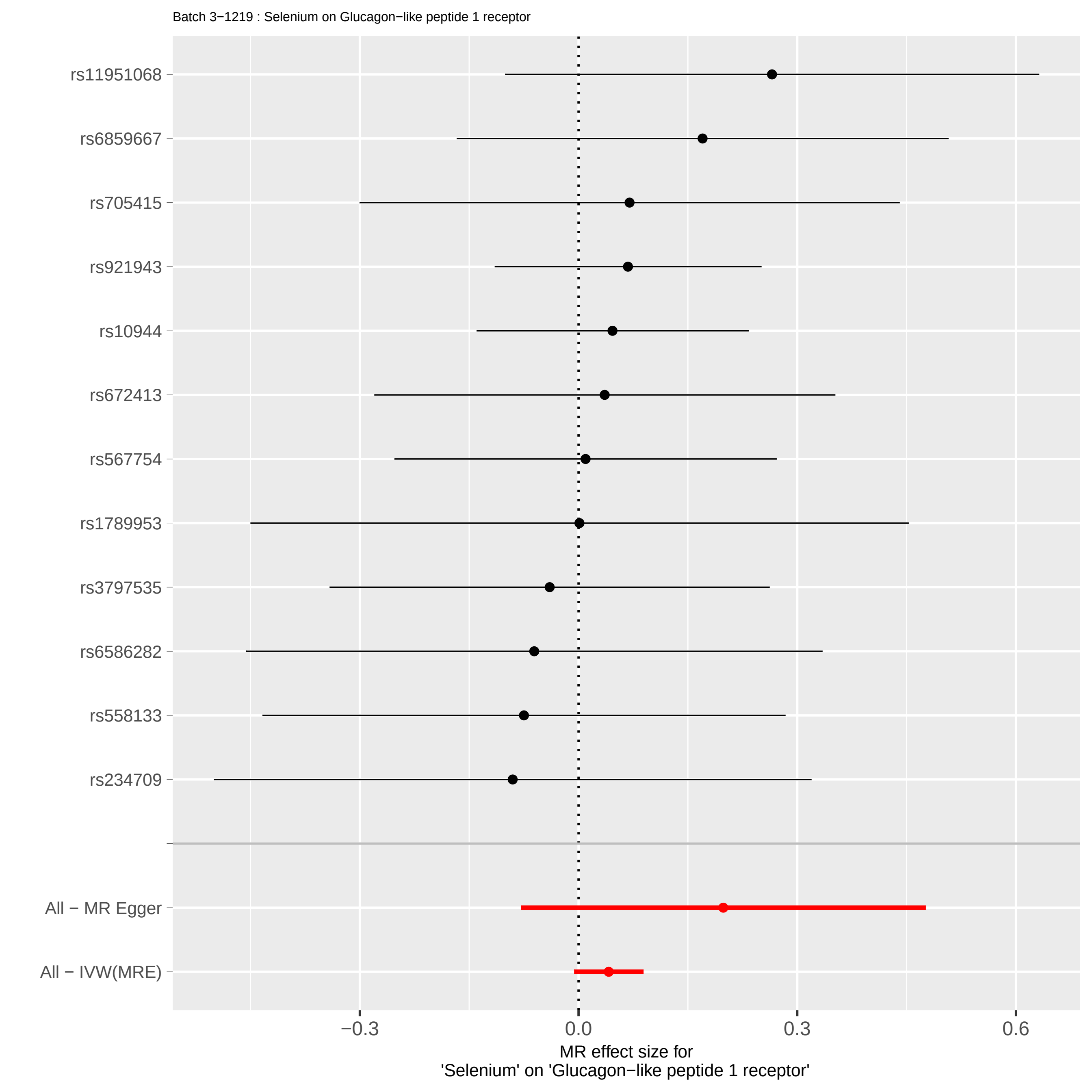
Batch 3−1219 : Selenium on Glucagon−like peptide 1 receptor
rs11951068
rs6859667
rs705415
rs921943
rs10944
rs672413
rs567754
rs1789953
rs3797535
rs6586282
rs558133
rs234709
All − MR Egger
All − IVW(MRE)
−0.3
0.0
0.3
0.6
MR effect size for
'Selenium' on 'Glucagon−like peptide 1 receptor'

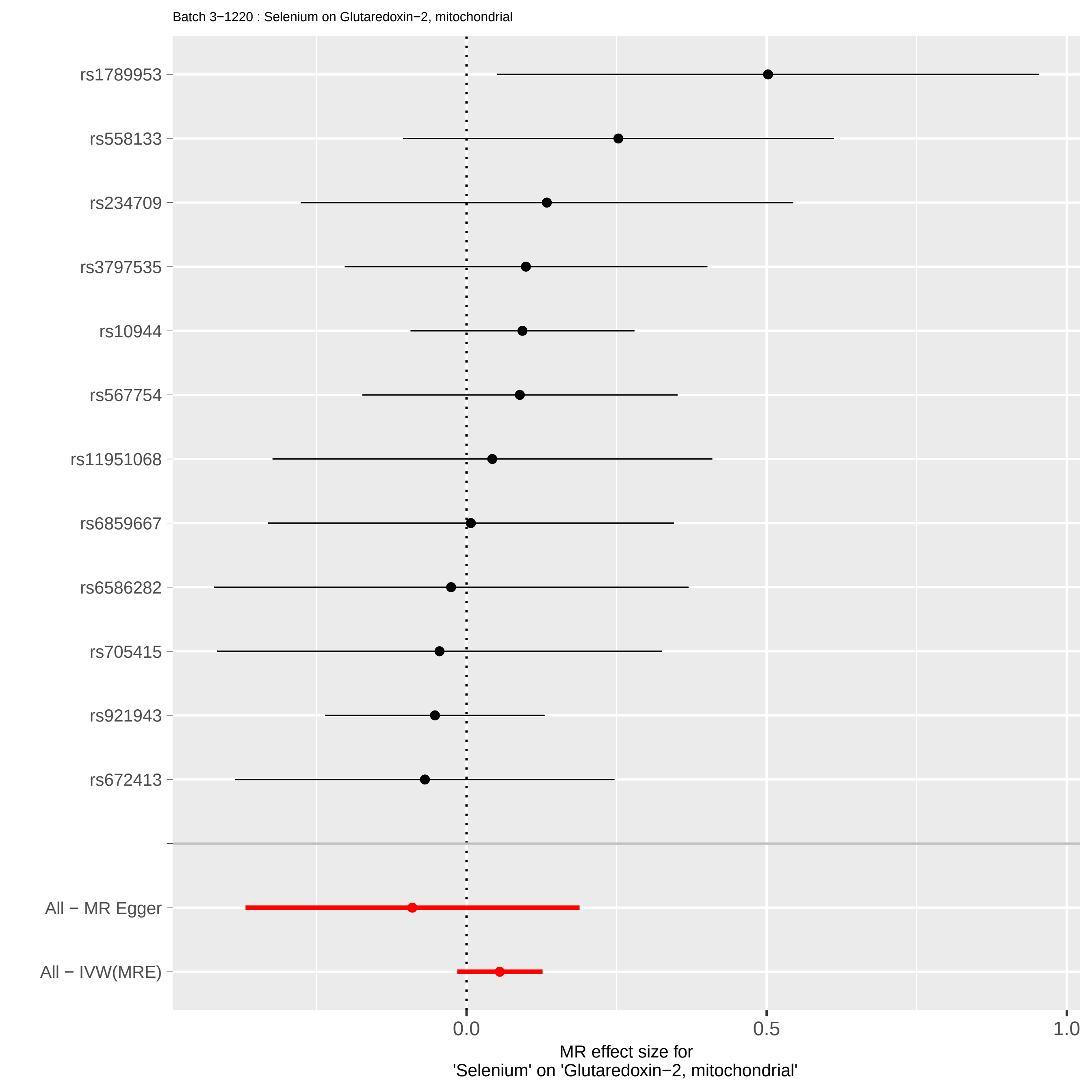

Batch 3−1220 : Selenium on Glutaredoxin−2, mitochondrial
rs1789953
rs558133
rs234709
rs3797535
rs10944
rs567754
rs11951068
rs6859667
rs6586282
rs705415
rs921943
rs672413
All − MR Egger
All − IVW(MRE)
0.0
0.5
1.0
MR effect size for
'Selenium' on 'Glutaredoxin−2, mitochondrial'

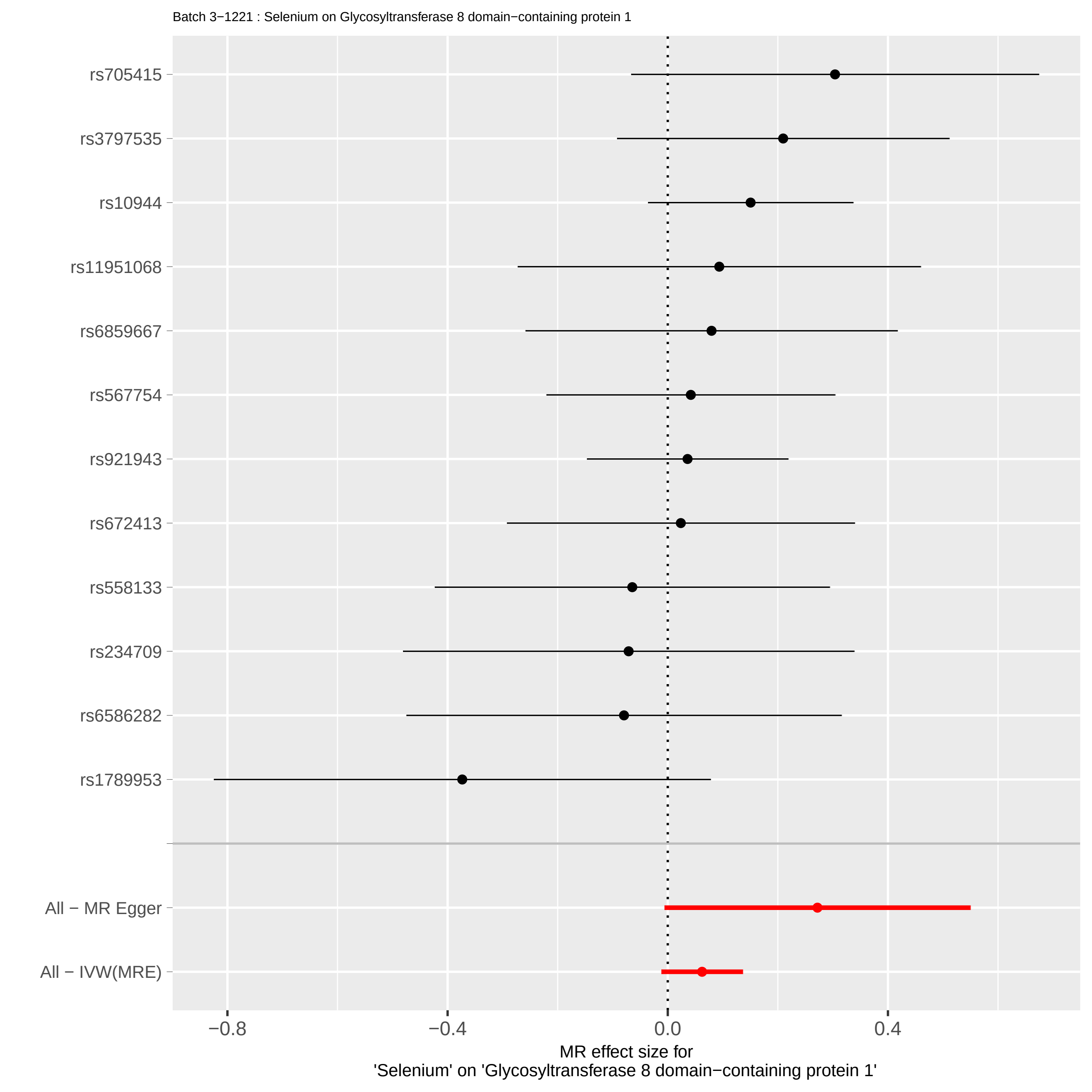

Batch 3−1221 : Selenium on Glycosyltransferase 8 domain−containing protein 1
rs705415
rs3797535
rs10944
rs11951068
rs6859667
rs567754
rs921943
rs672413
rs558133
rs234709
rs6586282
rs1789953
All − MR Egger
All − IVW(MRE)
−0.8
−0.4
0.0
0.4
MR effect size for
'Selenium' on 'Glycosyltransferase 8 domain−containing protein 1'

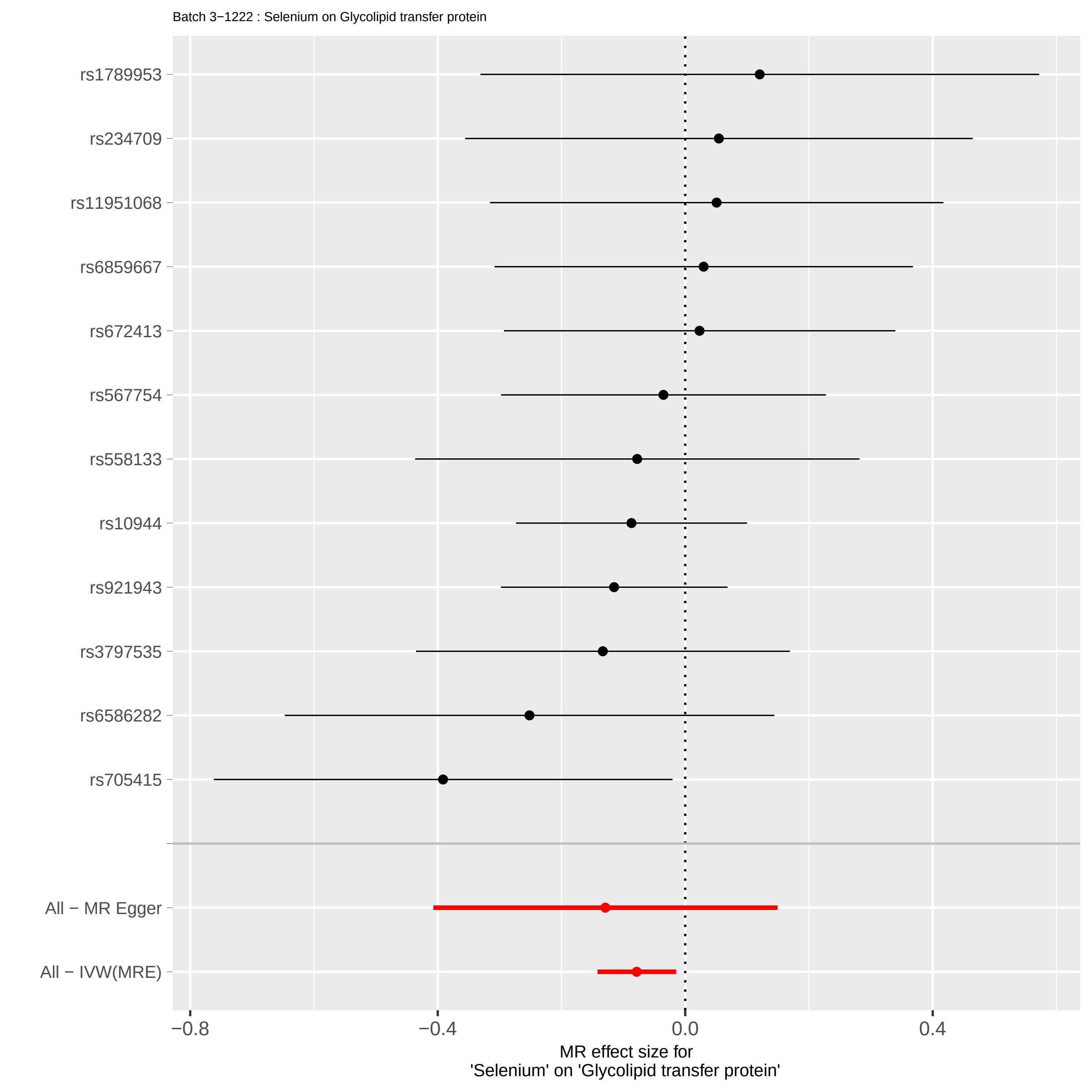

Batch 3−1222 : Selenium on Glycolipid transfer protein
rs1789953
rs234709
rs11951068
rs6859667
rs672413
rs567754
rs558133
rs10944
rs921943
rs3797535
rs6586282
rs705415
All − MR Egger
All − IVW(MRE)
−0.8
−0.4
0.0
0.4
MR effect size for
'Selenium' on 'Glycolipid transfer protein'

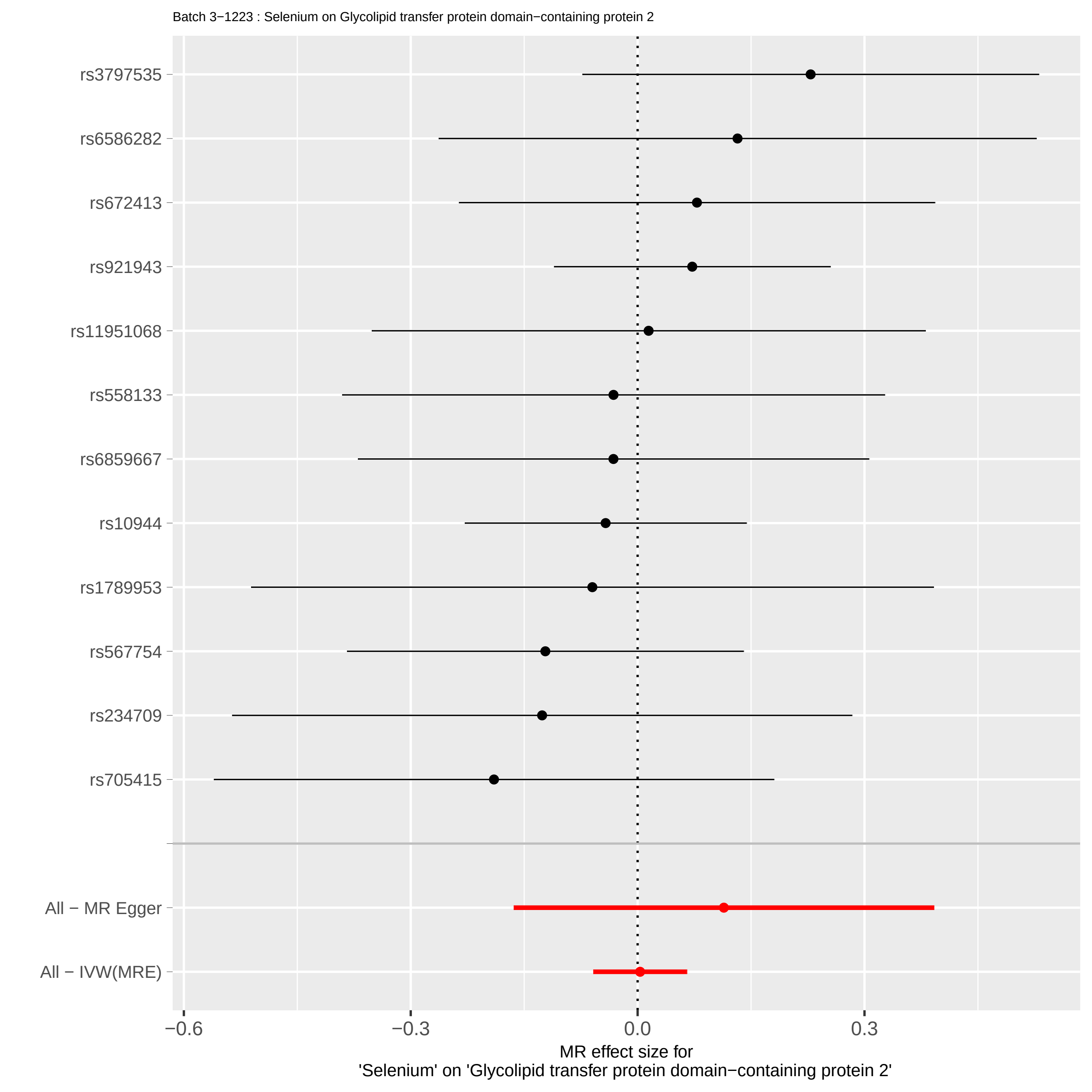
Batch 3−1223 : Selenium on Glycolipid transfer protein domain−containing protein 2
rs3797535
rs6586282
rs672413
rs921943
rs11951068
rs558133
rs6859667
rs10944
rs1789953
rs567754
rs234709
rs705415
All − MR Egger
All − IVW(MRE)
−0.6
−0.3
0.0
0.3
MR effect size for
'Selenium' on 'Glycolipid transfer protein domain−containing protein 2'

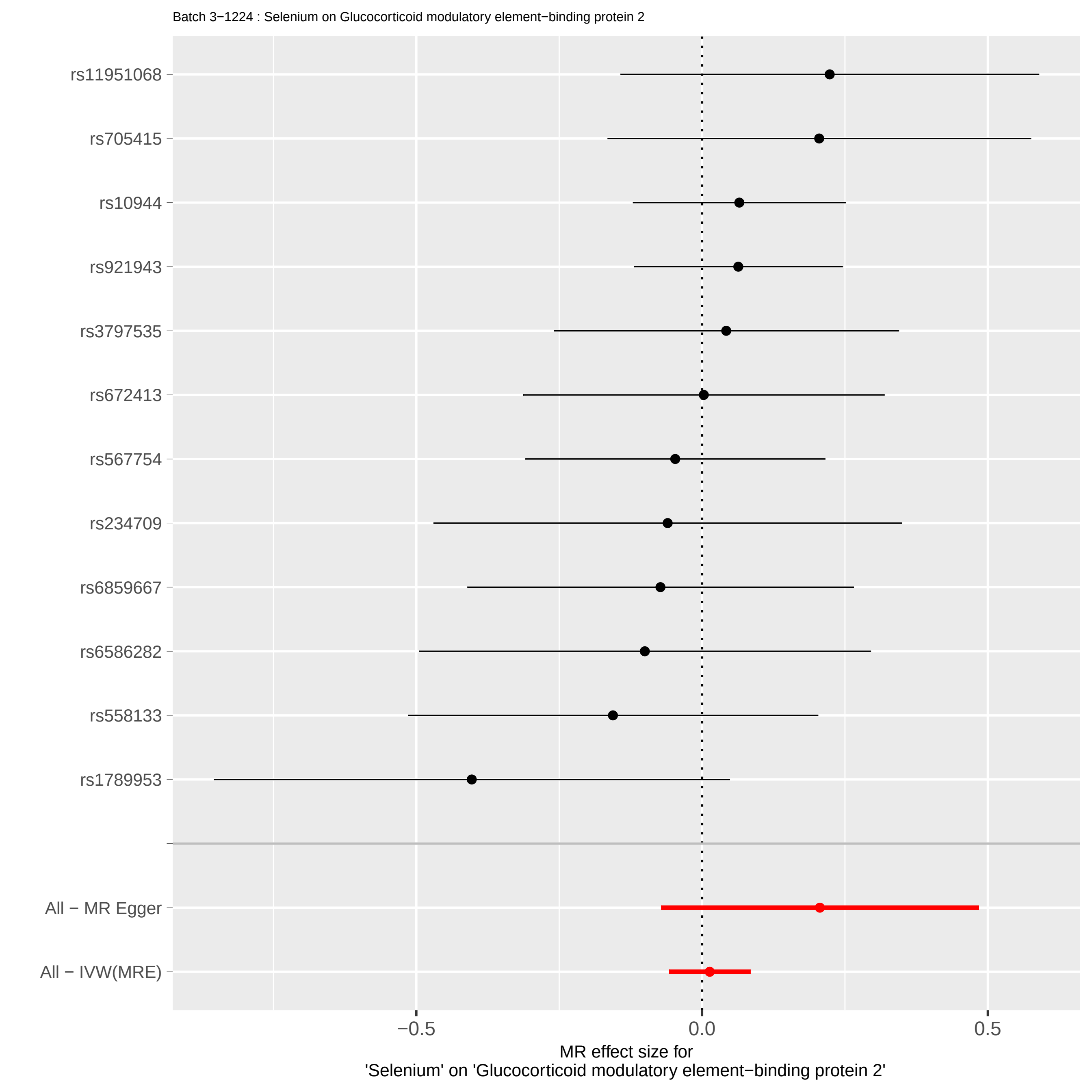

Batch 3−1224 : Selenium on Glucocorticoid modulatory element−binding protein 2
rs11951068
rs705415
rs10944
rs921943
rs3797535
rs672413
rs567754
rs234709
rs6859667
rs6586282
rs558133
rs1789953
All − MR Egger
All − IVW(MRE)
−0.5
0.0
0.5
MR effect size for
'Selenium' on 'Glucocorticoid modulatory element−binding protein 2'

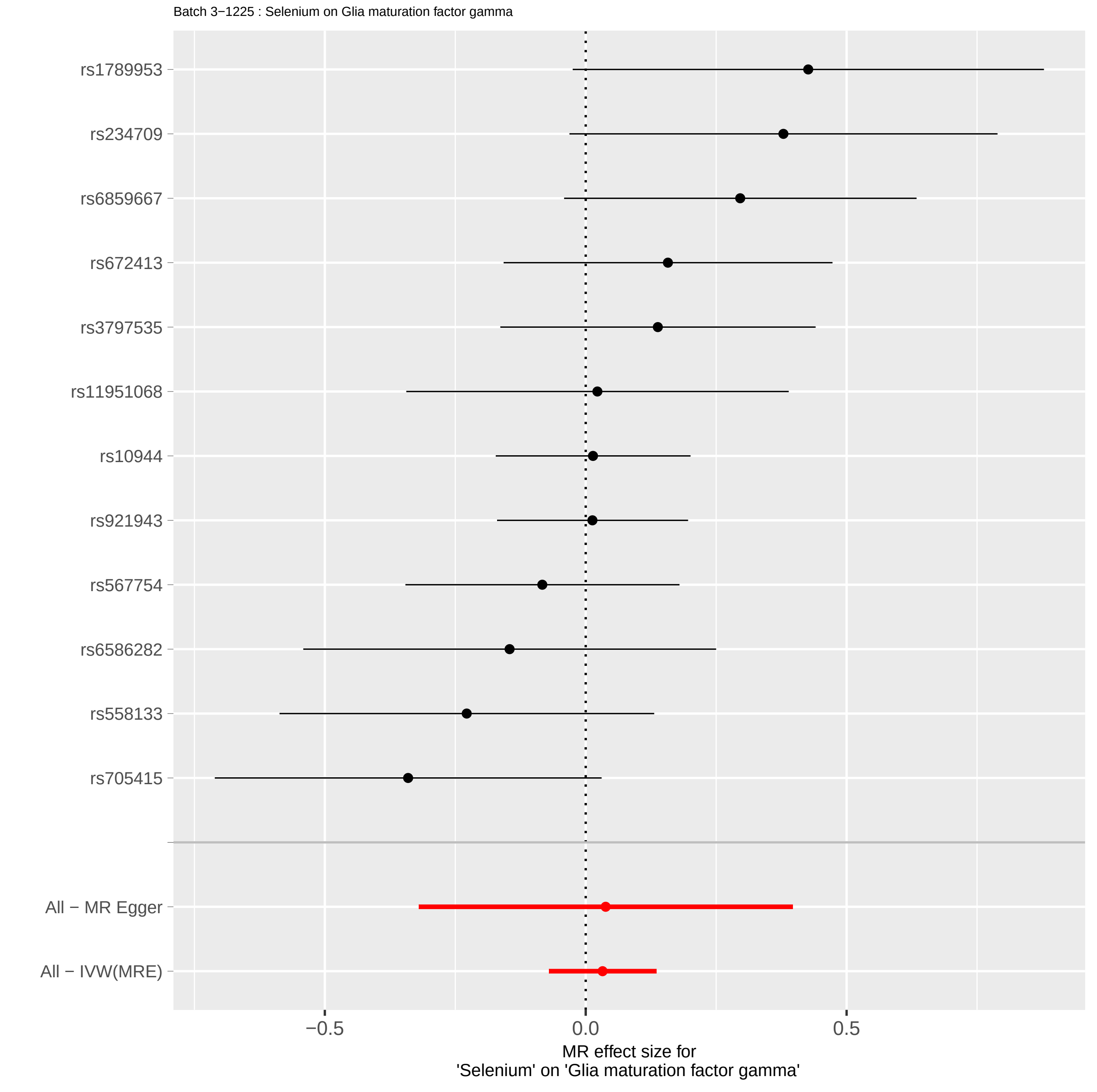

Batch 3−1225 : Selenium on Glia maturation factor gamma
rs1789953
rs234709
rs6859667
rs672413
rs3797535
rs11951068
rs10944
rs921943
rs567754
rs6586282
rs558133
rs705415
All − MR Egger
All − IVW(MRE)
−0.5
0.0
0.5
MR effect size for
'Selenium' on 'Glia maturation factor gamma'

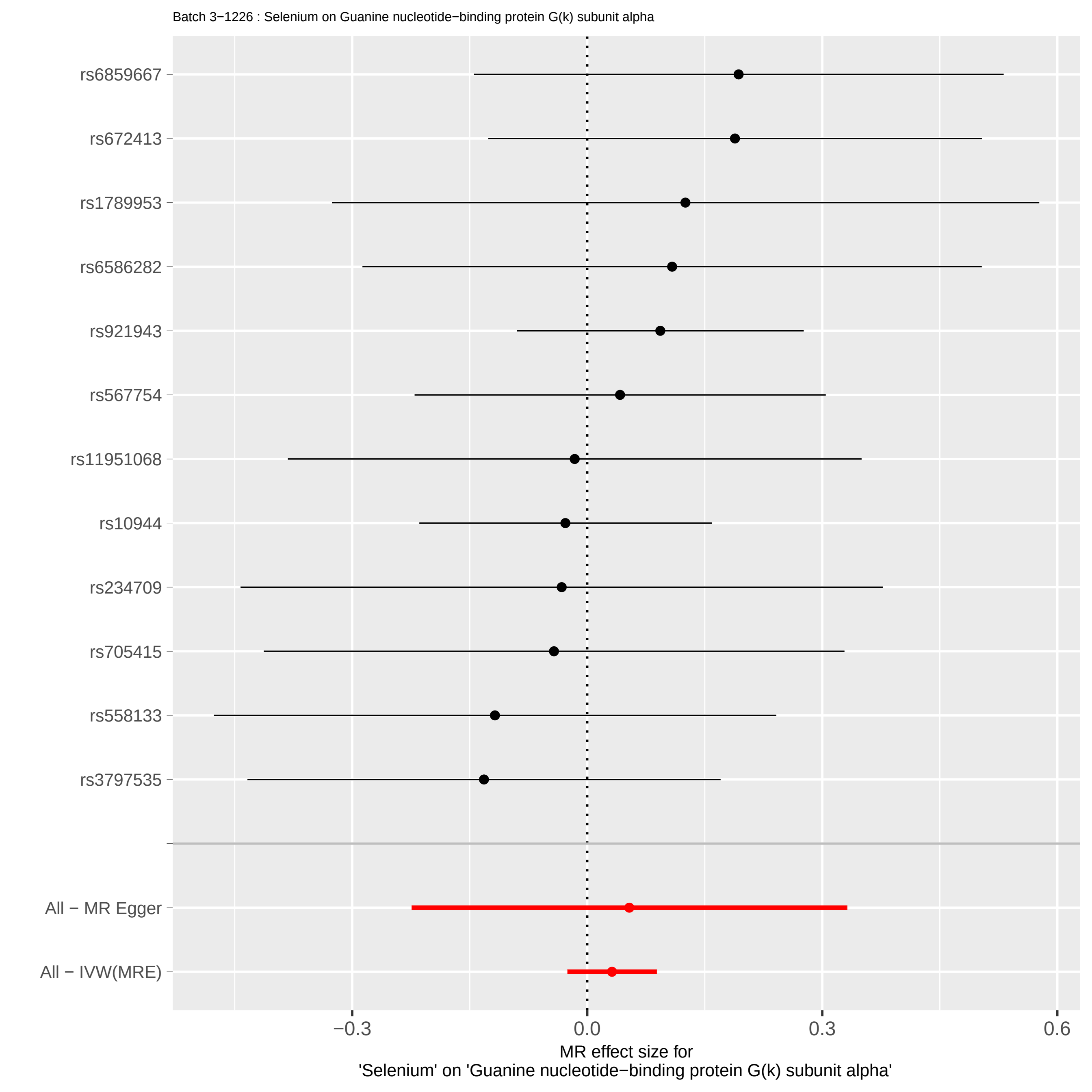
Batch 3−1226 : Selenium on Guanine nucleotide−binding protein G(k) subunit alpha
rs6859667
rs672413
rs1789953
rs6586282
rs921943
rs567754
rs11951068
rs10944
rs234709
rs705415
rs558133
rs3797535
All − MR Egger
All − IVW(MRE)
−0.3
0.0
0.3
0.6
MR effect size for
'Selenium' on 'Guanine nucleotide−binding protein G(k) subunit alpha'

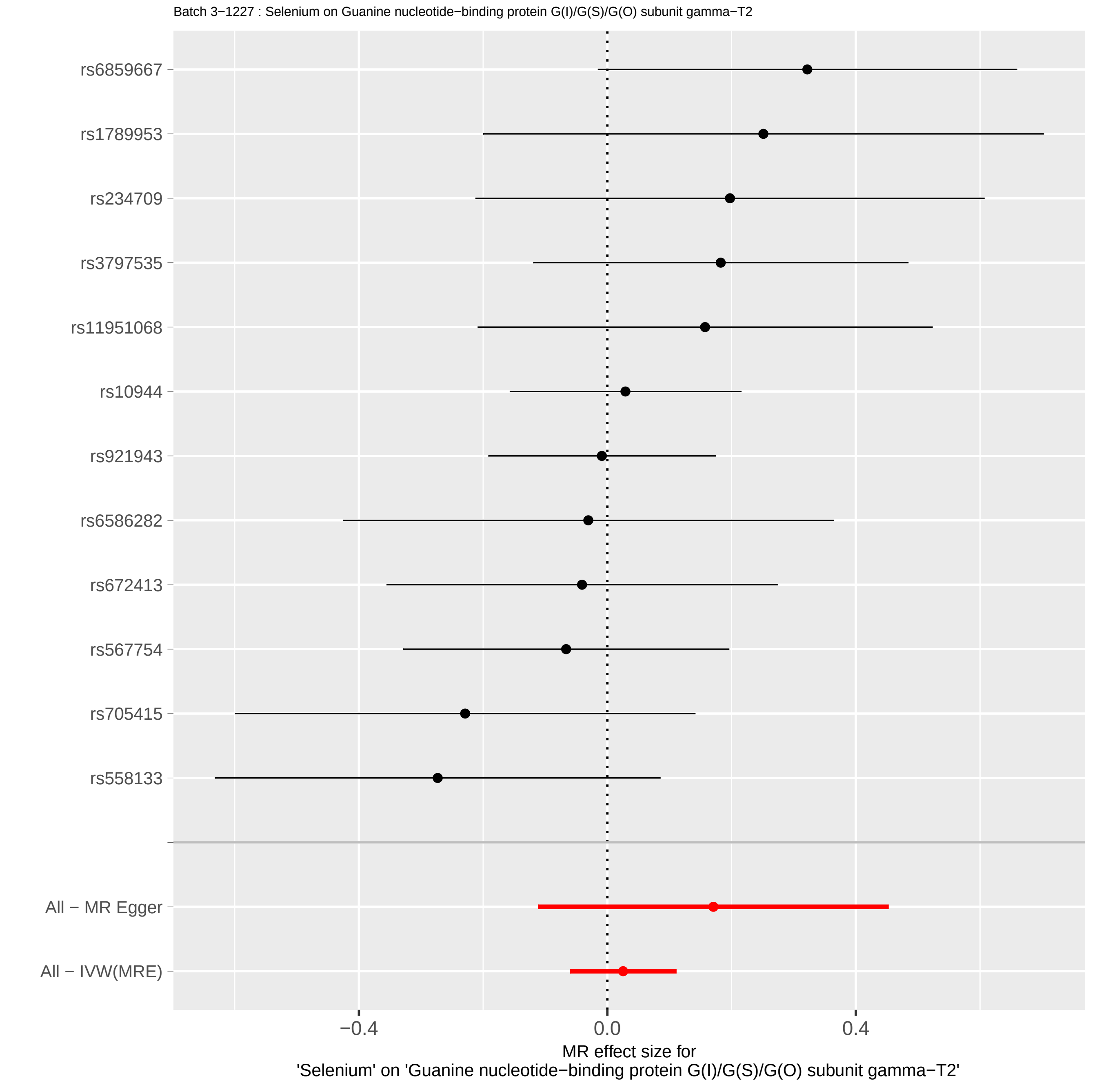

Batch 3−1227 : Selenium on Guanine nucleotide−binding protein G(I)/G(S)/G(O) subunit gamma−T2
rs6859667
rs1789953
rs234709
rs3797535
rs11951068
rs10944
rs921943
rs6586282
rs672413
rs567754
rs705415
rs558133
All − MR Egger
All − IVW(MRE)
−0.4
0.0
0.4
MR effect size for
'Selenium' on 'Guanine nucleotide−binding protein G(I)/G(S)/G(O) subunit gamma−T2'

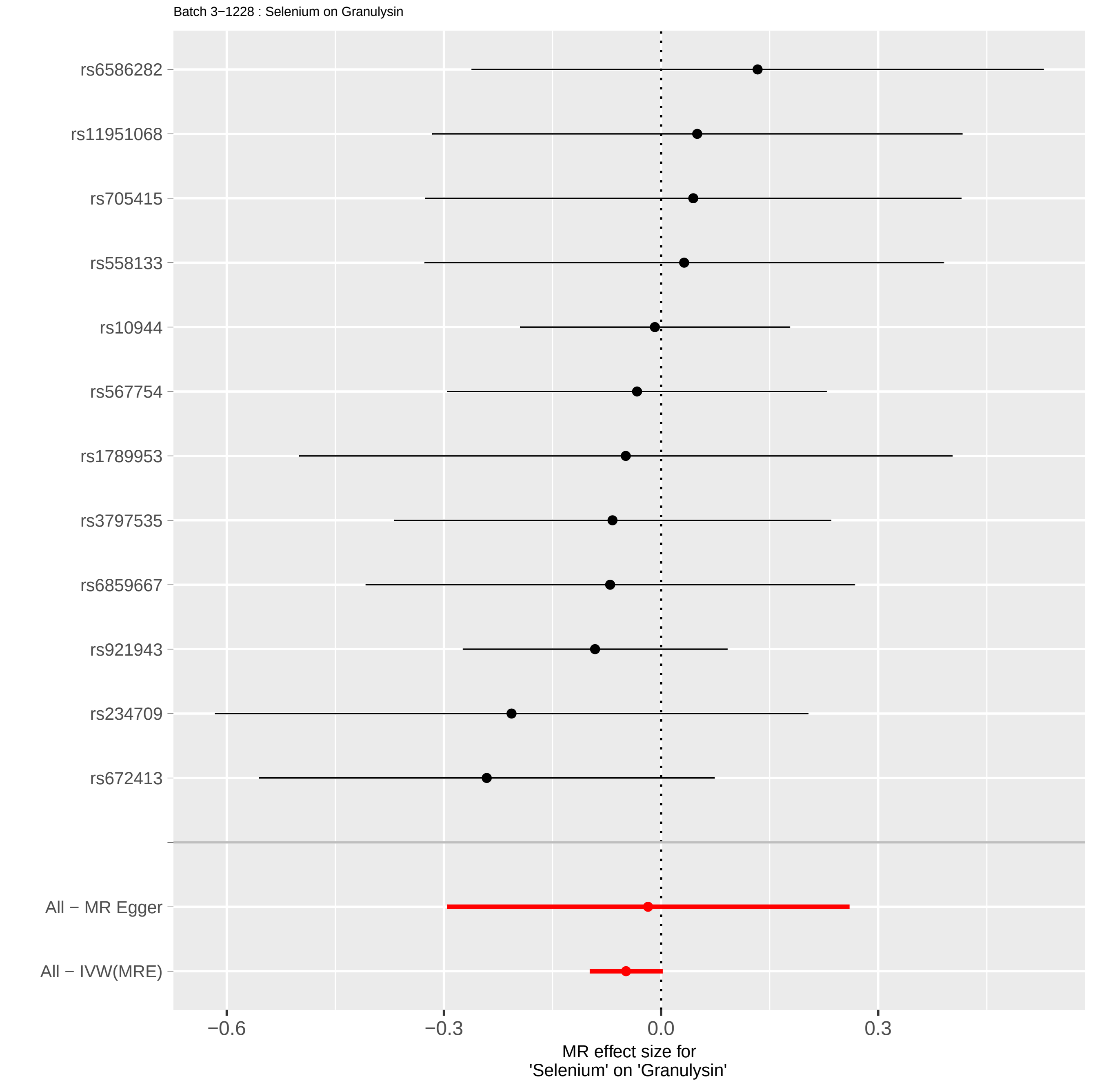

Batch 3−1228 : Selenium on Granulysin
rs6586282
rs11951068
rs705415
rs558133
rs10944
rs567754
rs1789953
rs3797535
rs6859667
rs921943
rs234709
rs672413
All − MR Egger
All − IVW(MRE)
−0.6
−0.3
0.0
0.3
MR effect size for
'Selenium' on 'Granulysin'

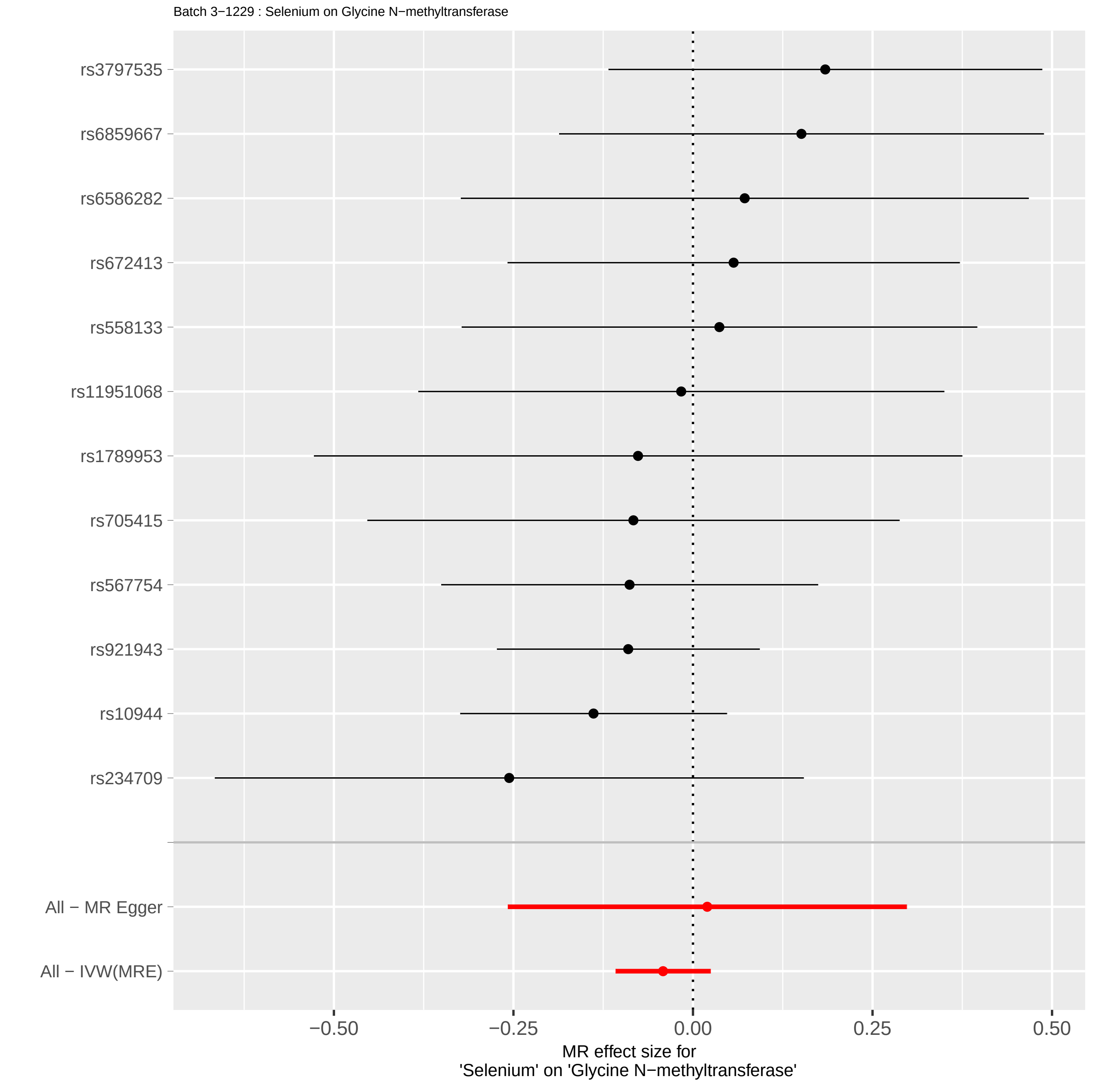

Batch 3−1229 : Selenium on Glycine N−methyltransferase
rs3797535
rs6859667
rs6586282
rs672413
rs558133
rs11951068
rs1789953
rs705415
rs567754
rs921943
rs10944
rs234709
All − MR Egger
All − IVW(MRE)
−0.50
−0.25
0.00
0.25
0.50
MR effect size for
'Selenium' on 'Glycine N−methyltransferase'

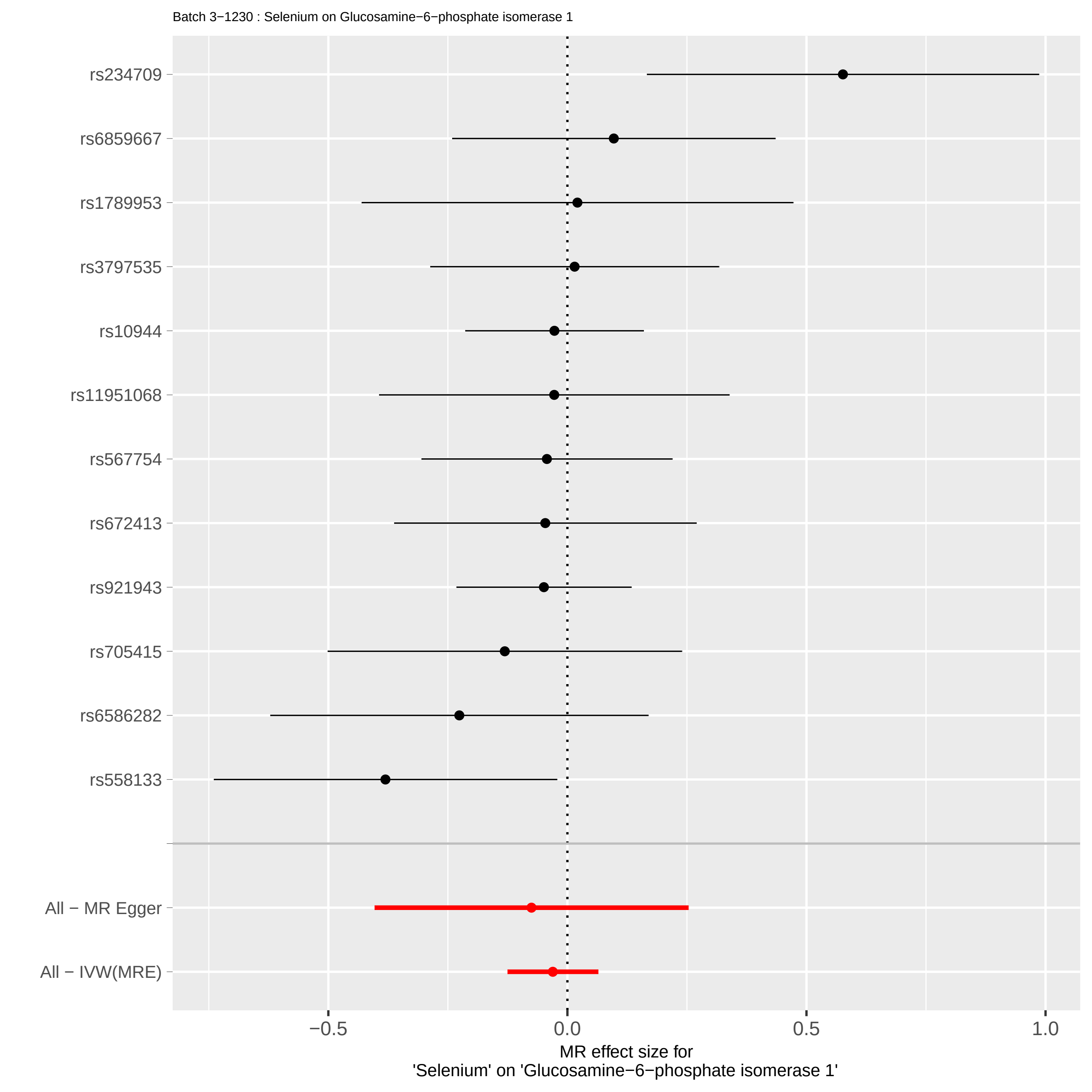
Batch 3−1230 : Selenium on Glucosamine−6−phosphate isomerase 1
rs234709
rs6859667
rs1789953
rs3797535
rs10944
rs11951068
rs567754
rs672413
rs921943
rs705415
rs6586282
rs558133
All − MR Egger
All − IVW(MRE)
−0.5
0.0
0.5
1.0
MR effect size for
'Selenium' on 'Glucosamine−6−phosphate isomerase 1'

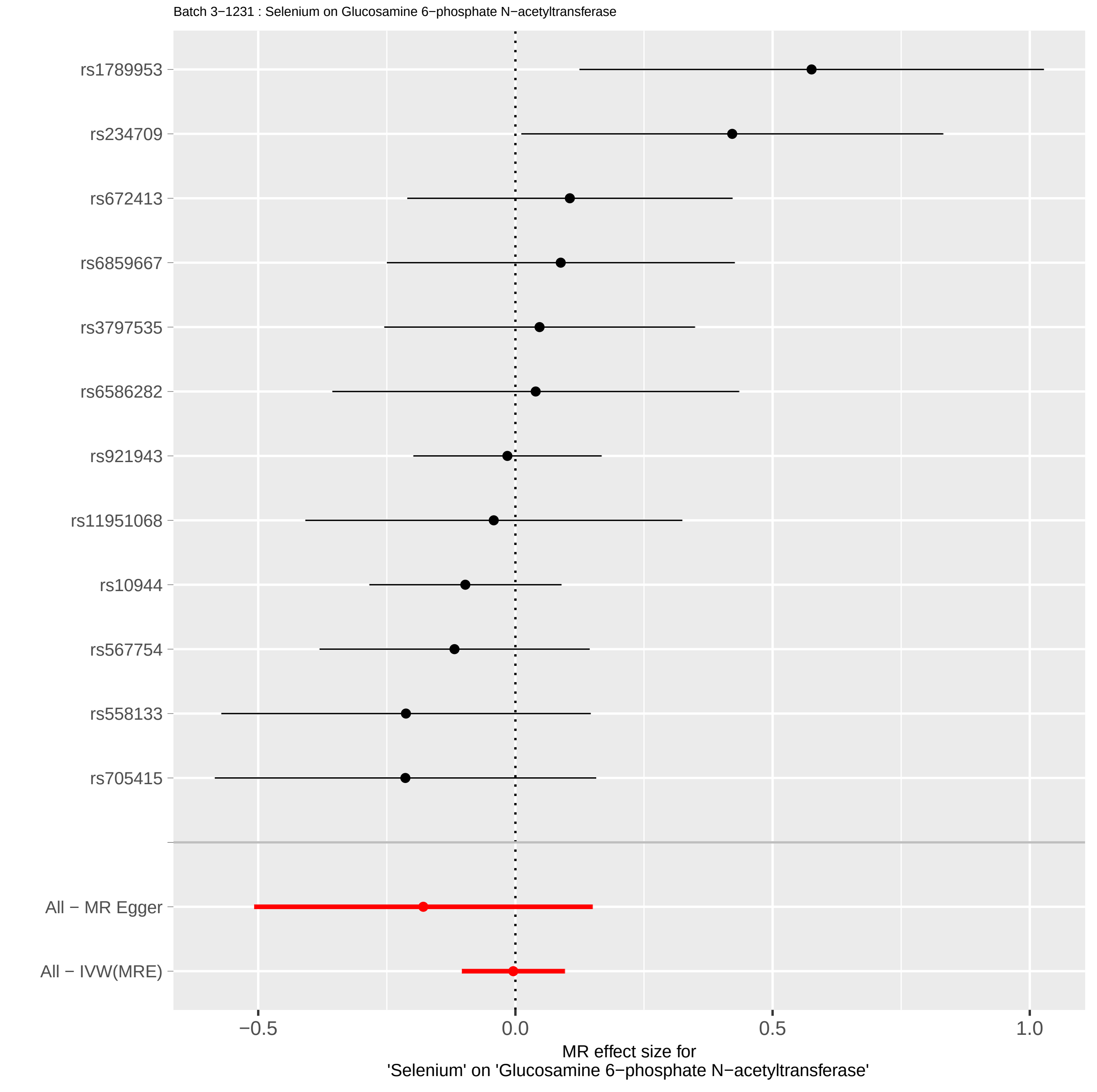

Batch 3−1231 : Selenium on Glucosamine 6−phosphate N−acetyltransferase
rs1789953
rs234709
rs672413
rs6859667
rs3797535
rs6586282
rs921943
rs11951068
rs10944
rs567754
rs558133
rs705415
All − MR Egger
All − IVW(MRE)
−0.5
0.0
0.5
1.0
MR effect size for
'Selenium' on 'Glucosamine 6−phosphate N−acetyltransferase'

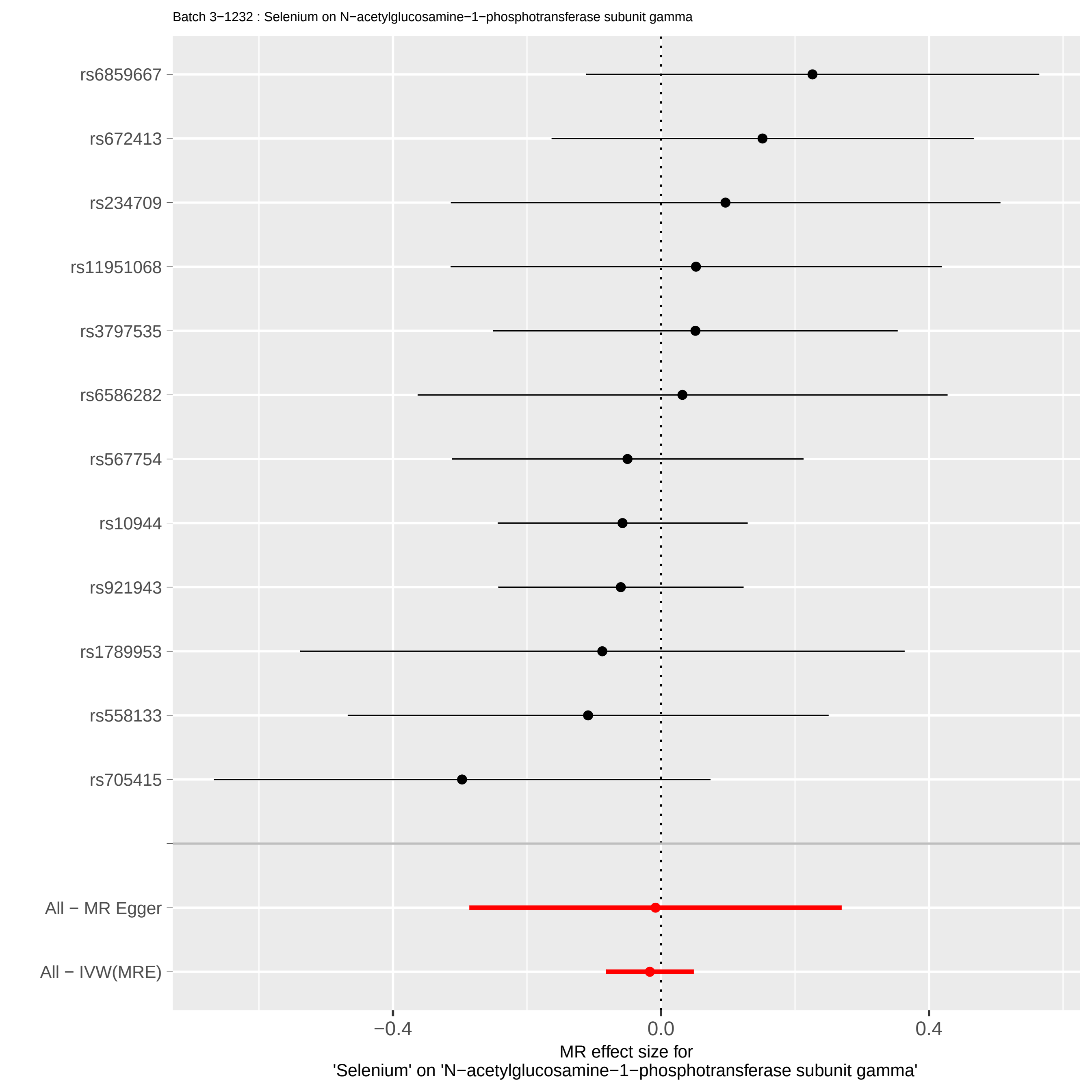

Batch 3−1232 : Selenium on N−acetylglucosamine−1−phosphotransferase subunit gamma
rs6859667
rs672413
rs234709
rs11951068
rs3797535
rs6586282
rs567754
rs10944
rs921943
rs1789953
rs558133
rs705415
All − MR Egger
All − IVW(MRE)
−0.4
0.0
0.4
MR effect size for
'Selenium' on 'N−acetylglucosamine−1−phosphotransferase subunit gamma'

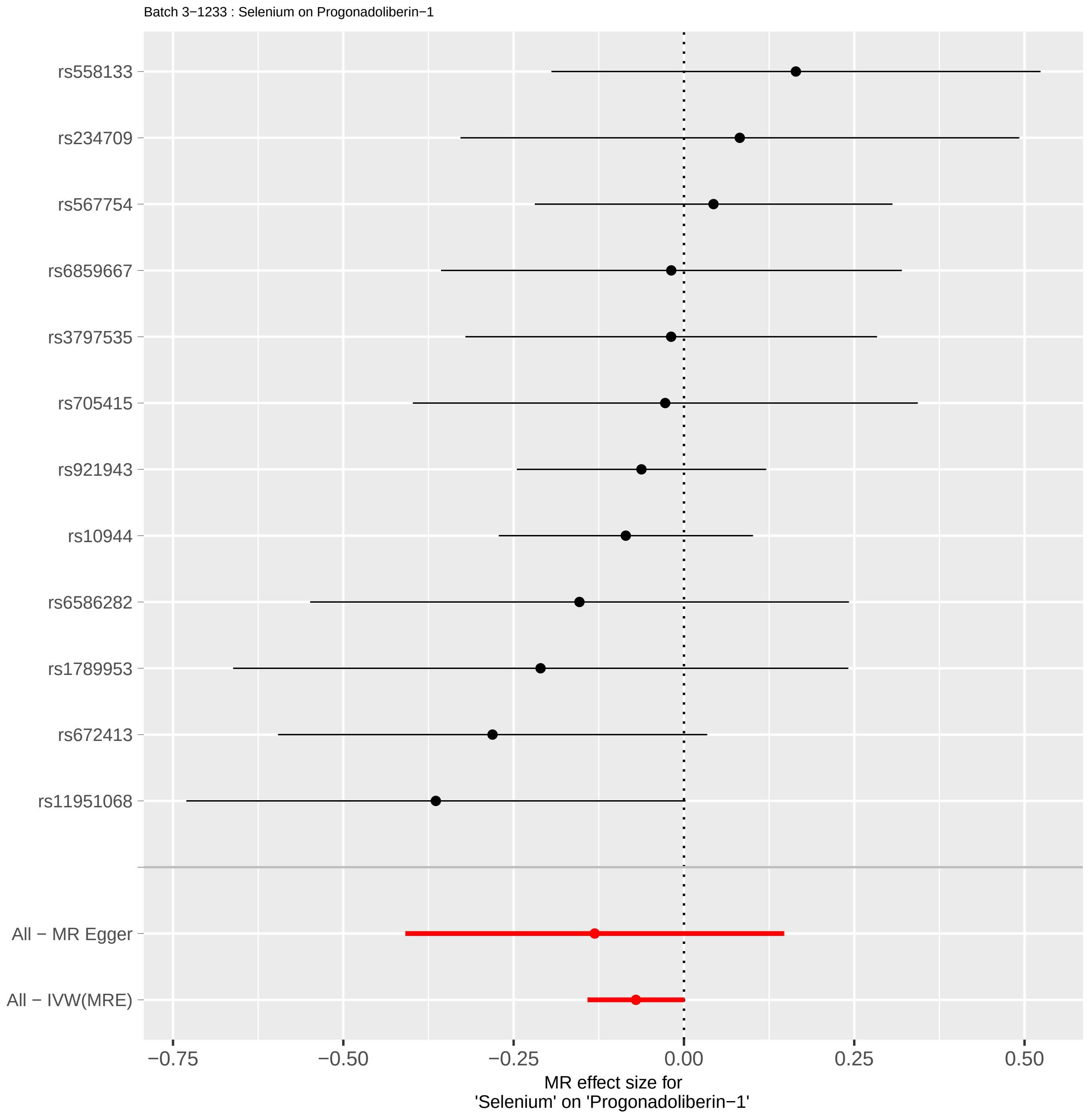

Batch 3−1233 : Selenium on Progonadoliberin−1
rs558133
rs234709
rs567754
rs6859667
rs3797535
rs705415
rs921943
rs10944
rs6586282
rs1789953
rs672413
rs11951068
All − MR Egger
All − IVW(MRE)
−0.75
−0.50
−0.25
0.00
0.25
0.50
MR effect size for
'Selenium' on 'Progonadoliberin−1'

Batch 3−1234 : Selenium on Progonadoliberin−2

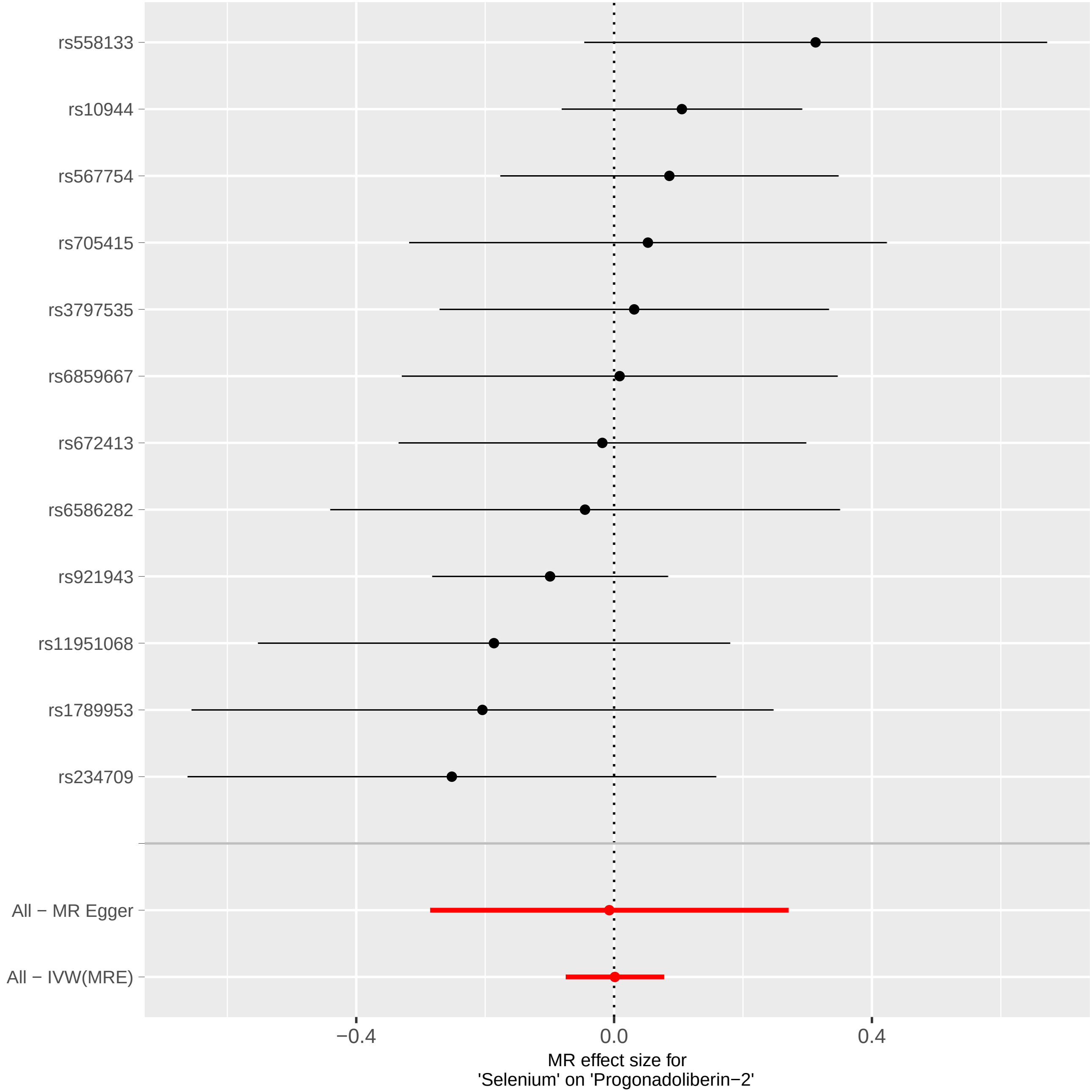

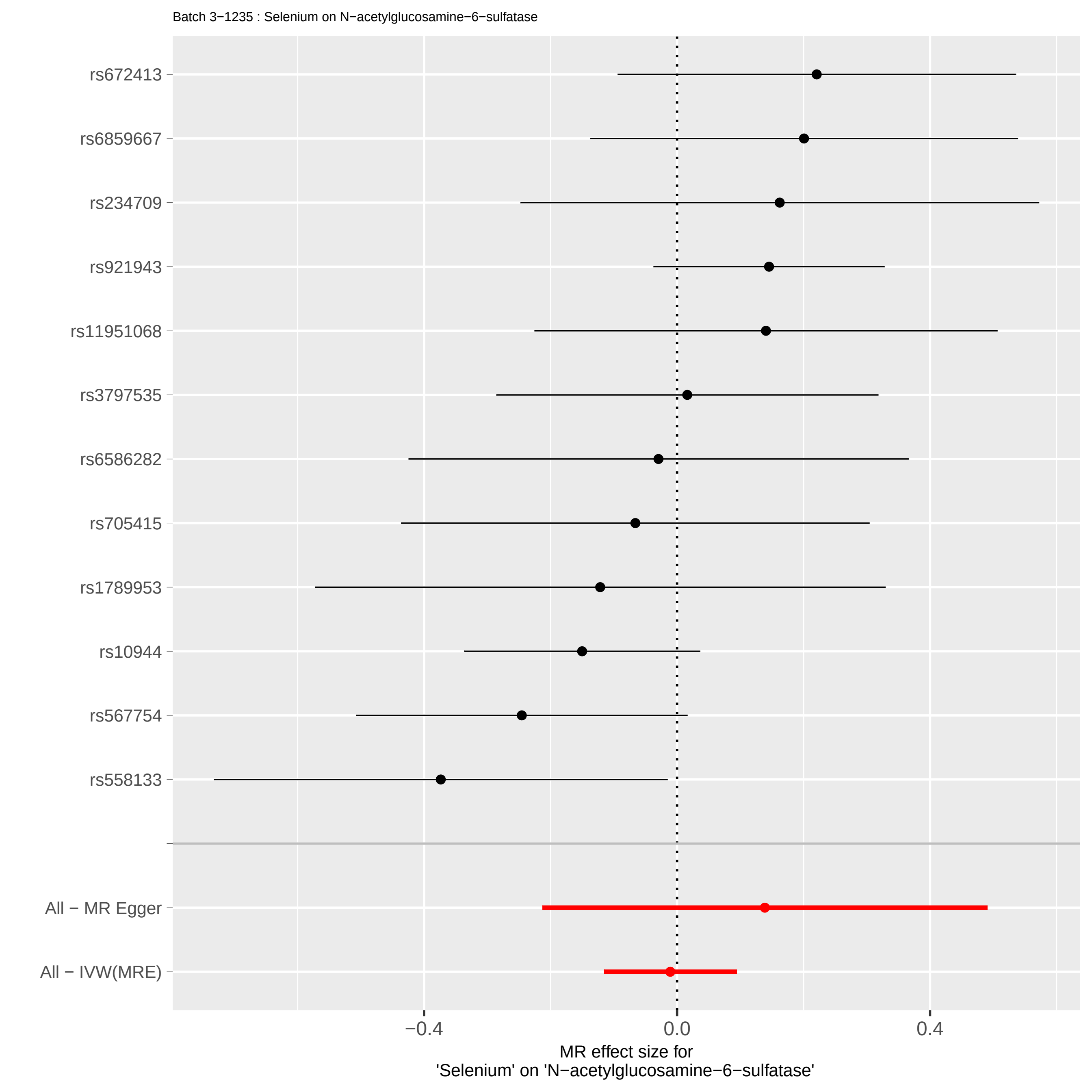

Batch 3−1235 : Selenium on N−acetylglucosamine−6−sulfatase
rs672413
rs6859667
rs234709
rs921943
rs11951068
rs3797535
rs6586282
rs705415
rs1789953
rs10944
rs567754
rs558133
All − MR Egger
All − IVW(MRE)
−0.4
0.0
0.4
MR effect size for
'Selenium' on 'N−acetylglucosamine−6−sulfatase'

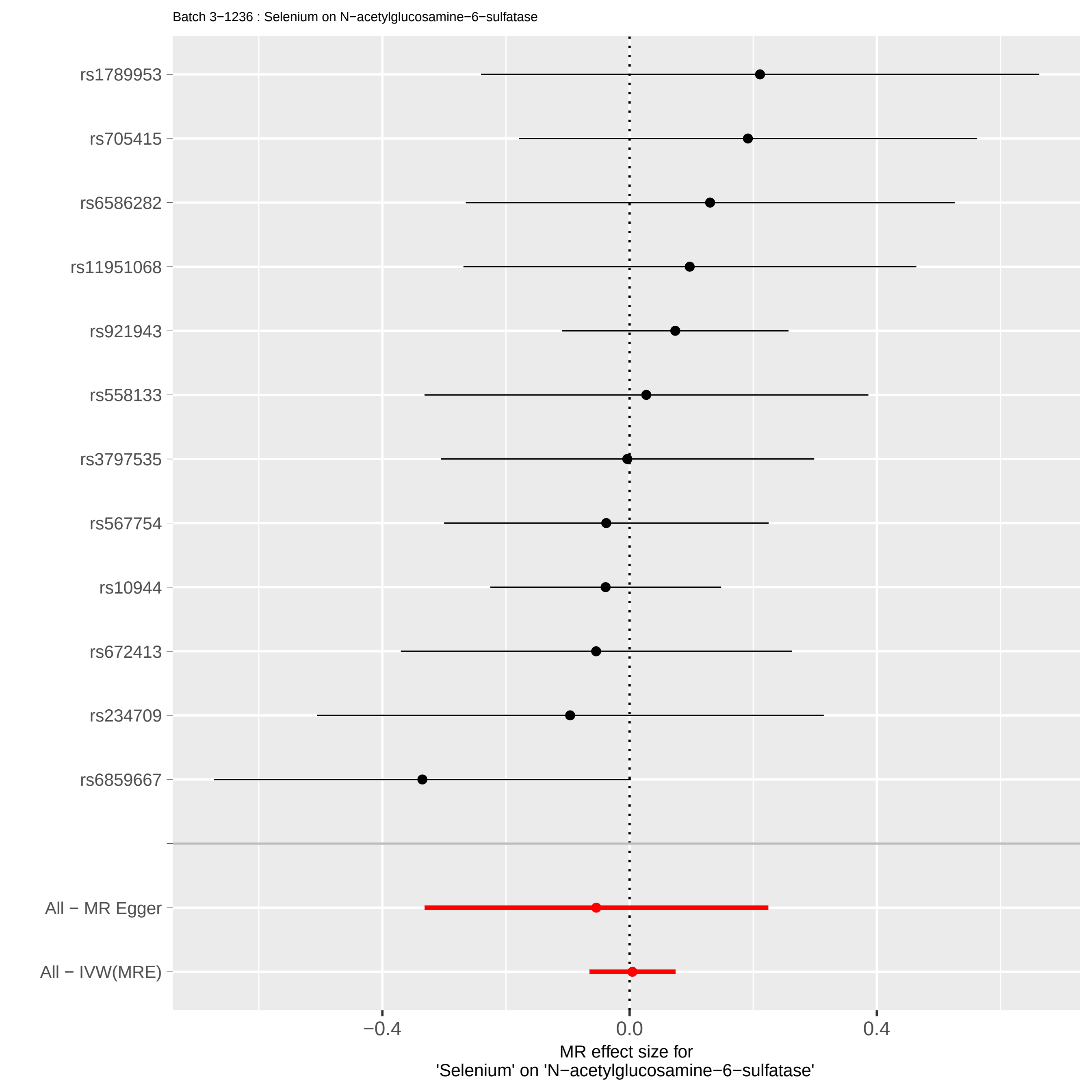

Batch 3−1236 : Selenium on N−acetylglucosamine−6−sulfatase
rs1789953
rs705415
rs6586282
rs11951068
rs921943
rs558133
rs3797535
rs567754
rs10944
rs672413
rs234709
rs6859667
All − MR Egger
All − IVW(MRE)
−0.4
0.0
0.4
MR effect size for
'Selenium' on 'N−acetylglucosamine−6−sulfatase'

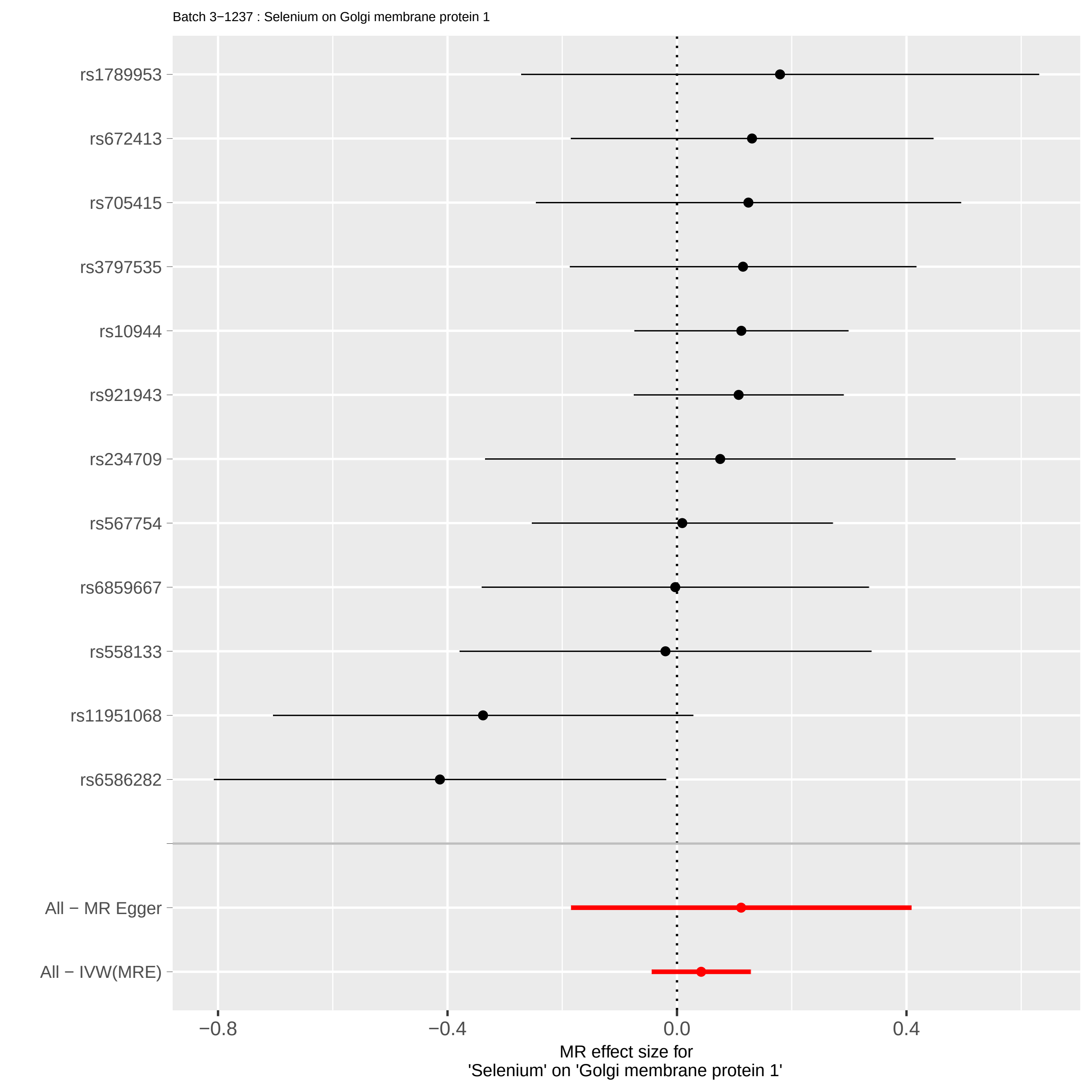
Batch 3−1237 : Selenium on Golgi membrane protein 1
rs1789953
rs672413
rs705415
rs3797535
rs10944
rs921943
rs234709
rs567754
rs6859667
rs558133
rs11951068
rs6586282
All − MR Egger
All − IVW(MRE)
−0.8
−0.4
0.0
0.4
MR effect size for
'Selenium' on 'Golgi membrane protein 1'

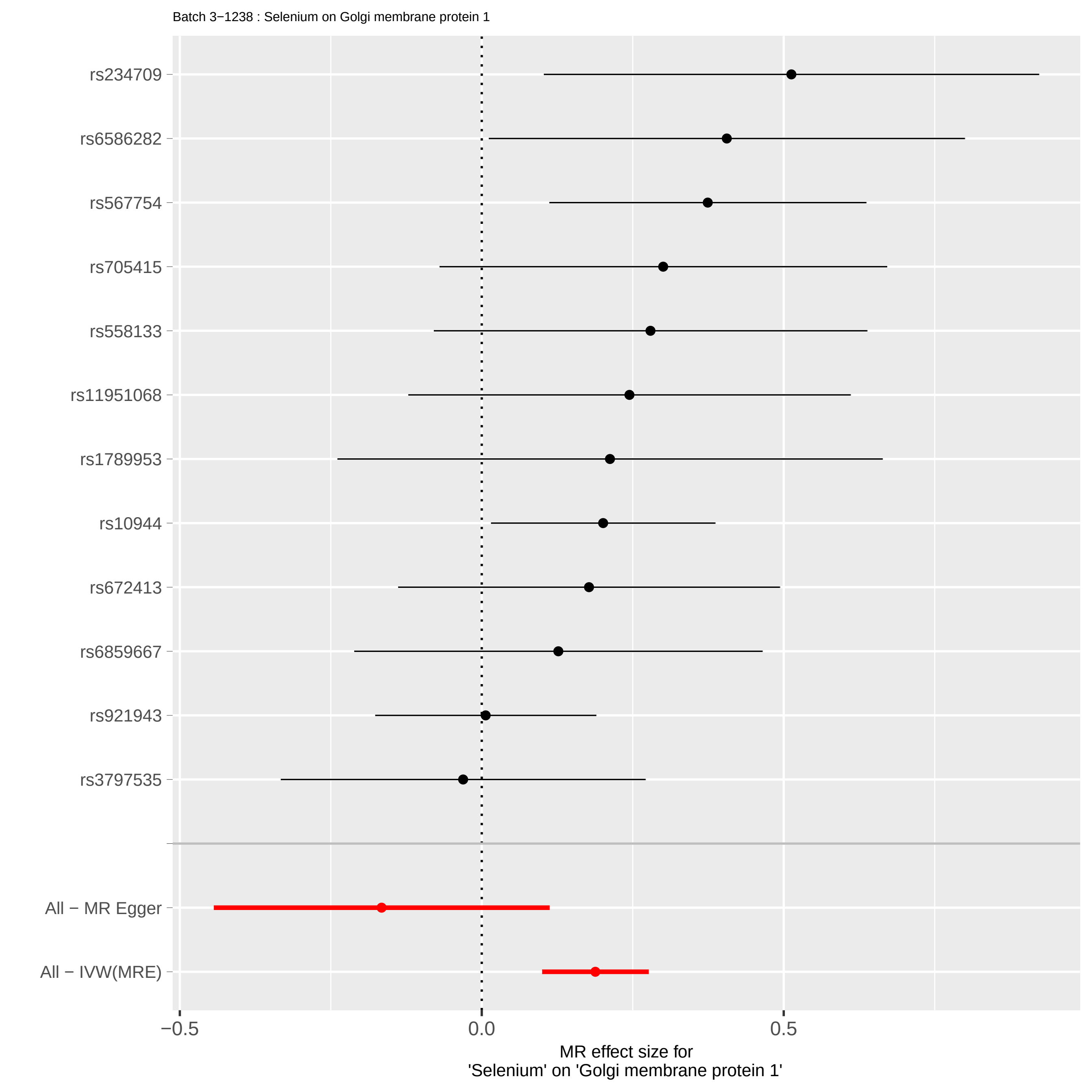

Batch 3−1238 : Selenium on Golgi membrane protein 1
rs234709
rs6586282
rs567754
rs705415
rs558133
rs11951068
rs1789953
rs10944
rs672413
rs6859667
rs921943
rs3797535
All − MR Egger
All − IVW(MRE)
−0.5
0.0
0.5
MR effect size for
'Selenium' on 'Golgi membrane protein 1'

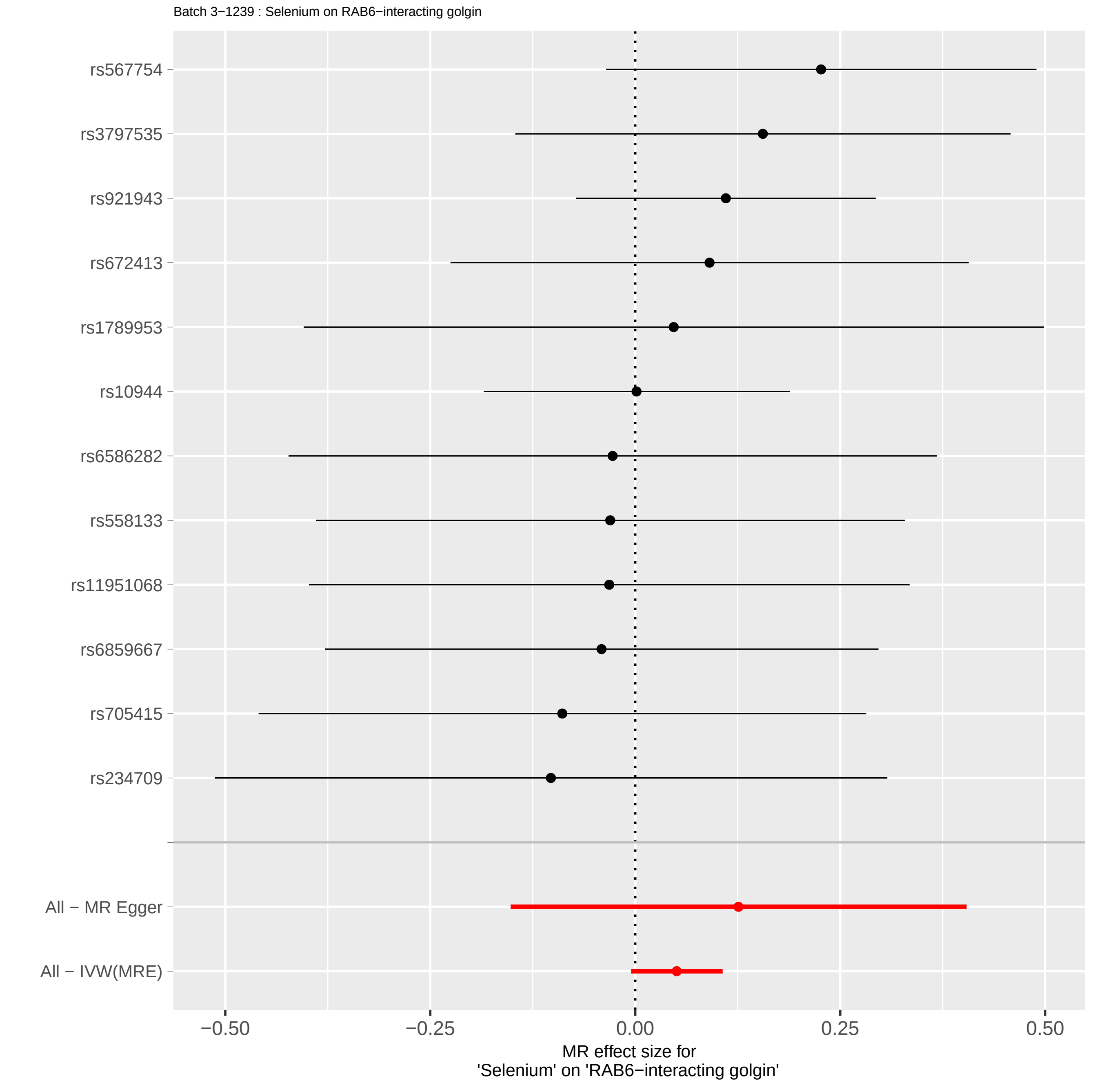
Batch 3−1239 : Selenium on RAB6−interacting golgin
rs567754
rs3797535
rs921943
rs672413
rs1789953
rs10944
rs6586282
rs558133
rs11951068
rs6859667
rs705415
rs234709
All − MR Egger
All − IVW(MRE)
−0.50
−0.25
0.00
0.25
0.50
MR effect size for
'Selenium' on 'RAB6−interacting golgin'

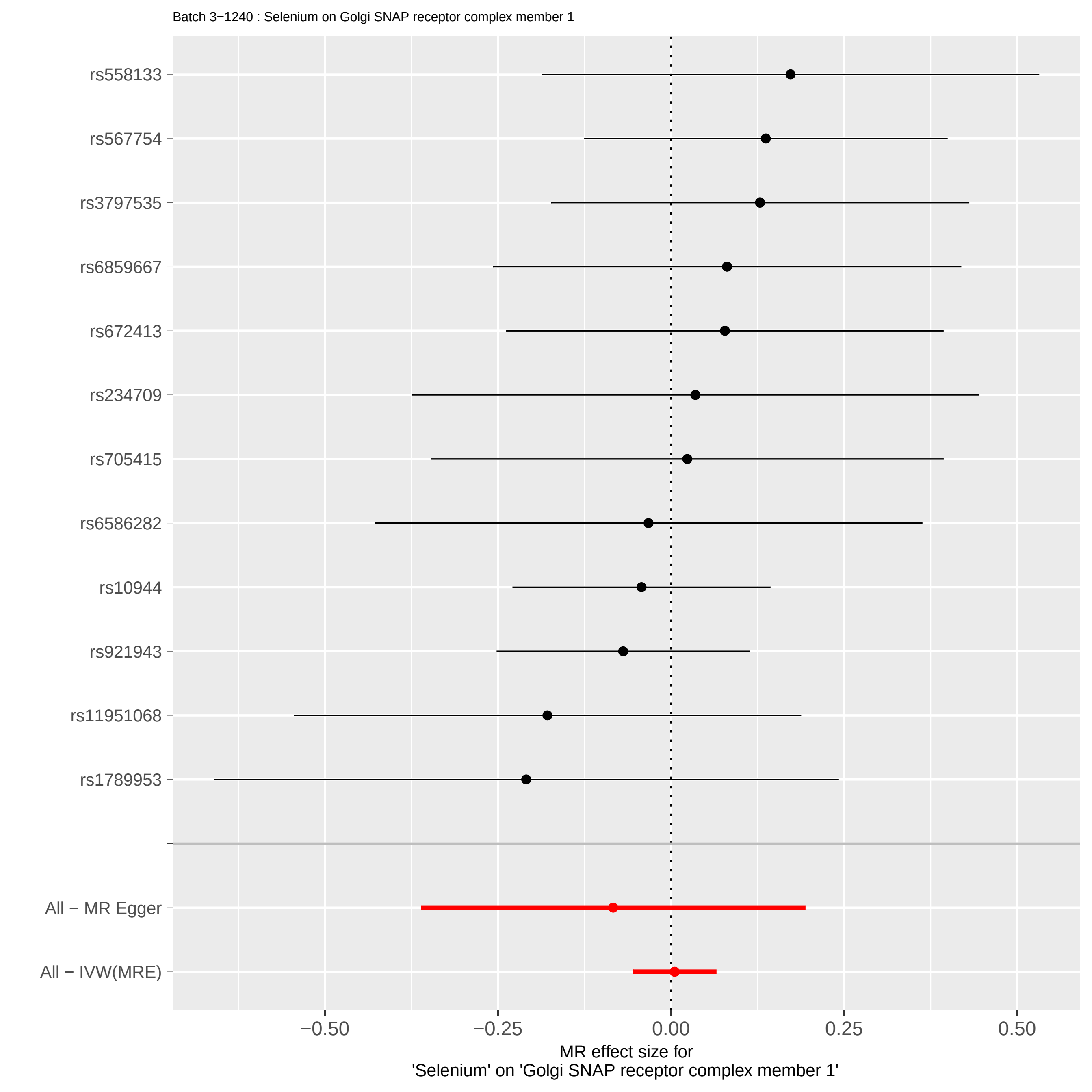

Batch 3−1240 : Selenium on Golgi SNAP receptor complex member 1
rs558133
rs567754
rs3797535
rs6859667
rs672413
rs234709
rs705415
rs6586282
rs10944
rs921943
rs11951068
rs1789953
All − MR Egger
All − IVW(MRE)
−0.50
−0.25
0.00
0.25
0.50
MR effect size for
'Selenium' on 'Golgi SNAP receptor complex member 1'

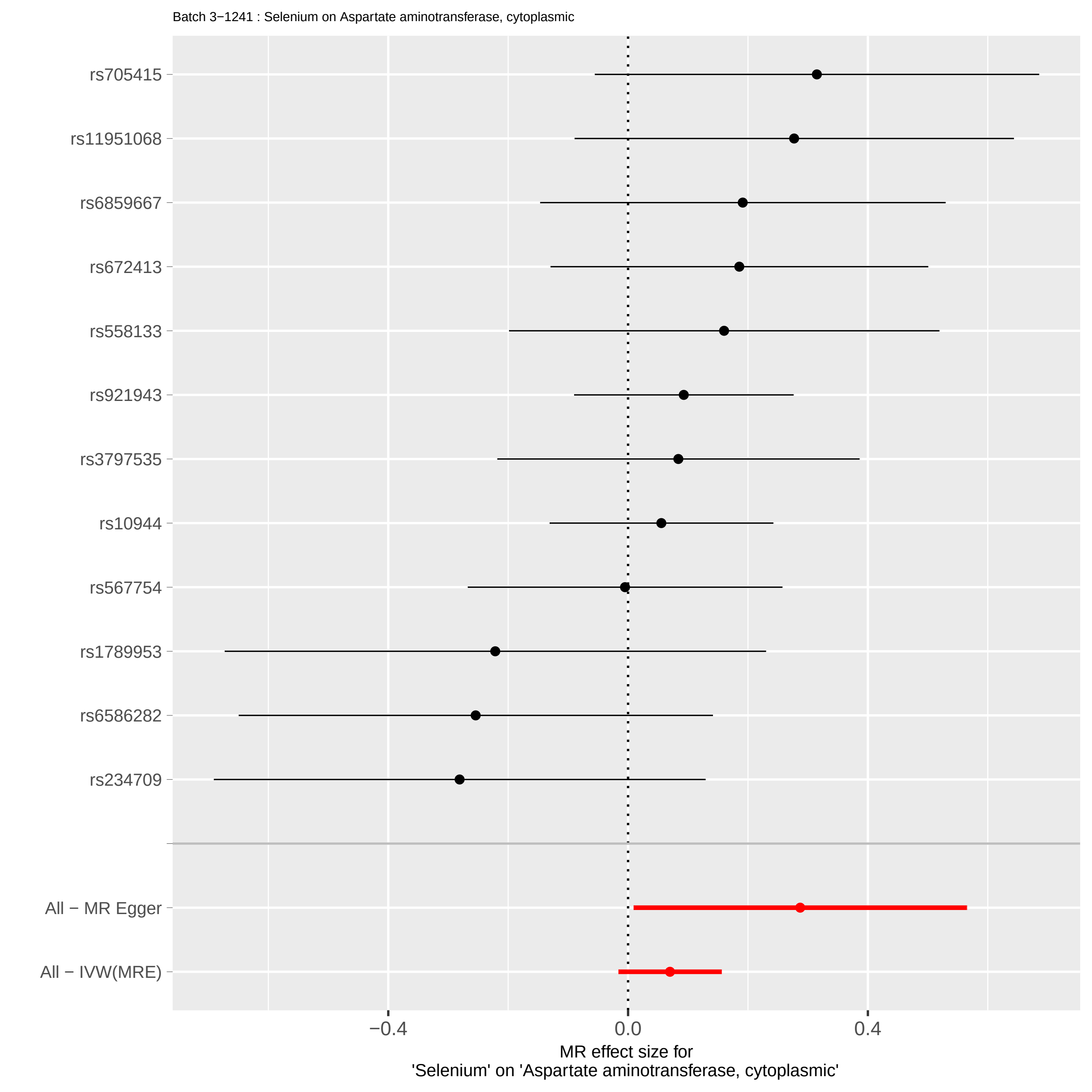

Batch 3−1241 : Selenium on Aspartate aminotransferase, cytoplasmic
rs705415
rs11951068
rs6859667
rs672413
rs558133
rs921943
rs3797535
rs10944
rs567754
rs1789953
rs6586282
rs234709
All − MR Egger
All − IVW(MRE)
−0.4
0.0
0.4
MR effect size for
'Selenium' on 'Aspartate aminotransferase, cytoplasmic'

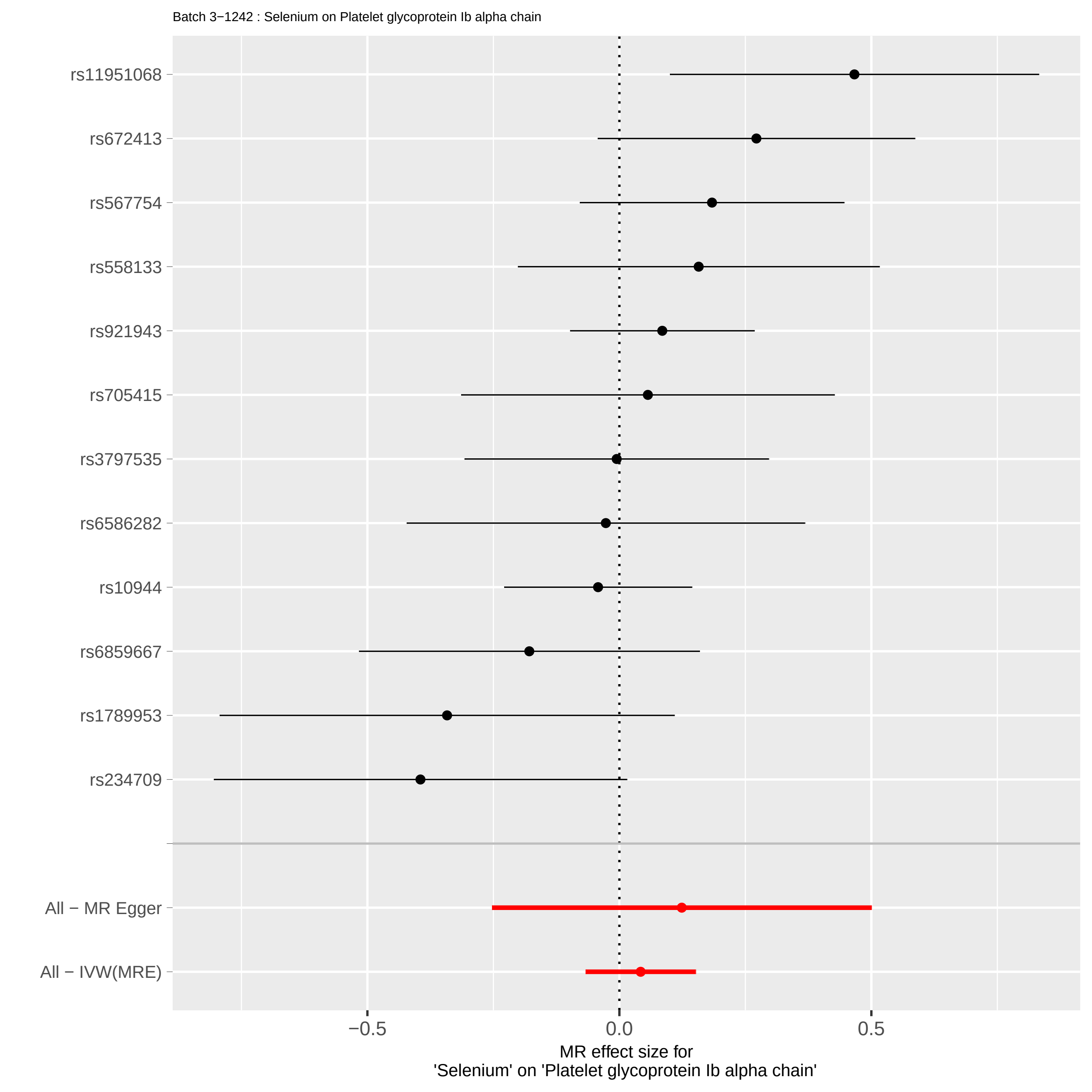

Batch 3−1242 : Selenium on Platelet glycoprotein Ib alpha chain
rs11951068
rs672413
rs567754
rs558133
rs921943
rs705415
rs3797535
rs6586282
rs10944
rs6859667
rs1789953
rs234709
All − MR Egger
All − IVW(MRE)
−0.5
0.0
0.5
MR effect size for
'Selenium' on 'Platelet glycoprotein Ib alpha chain'

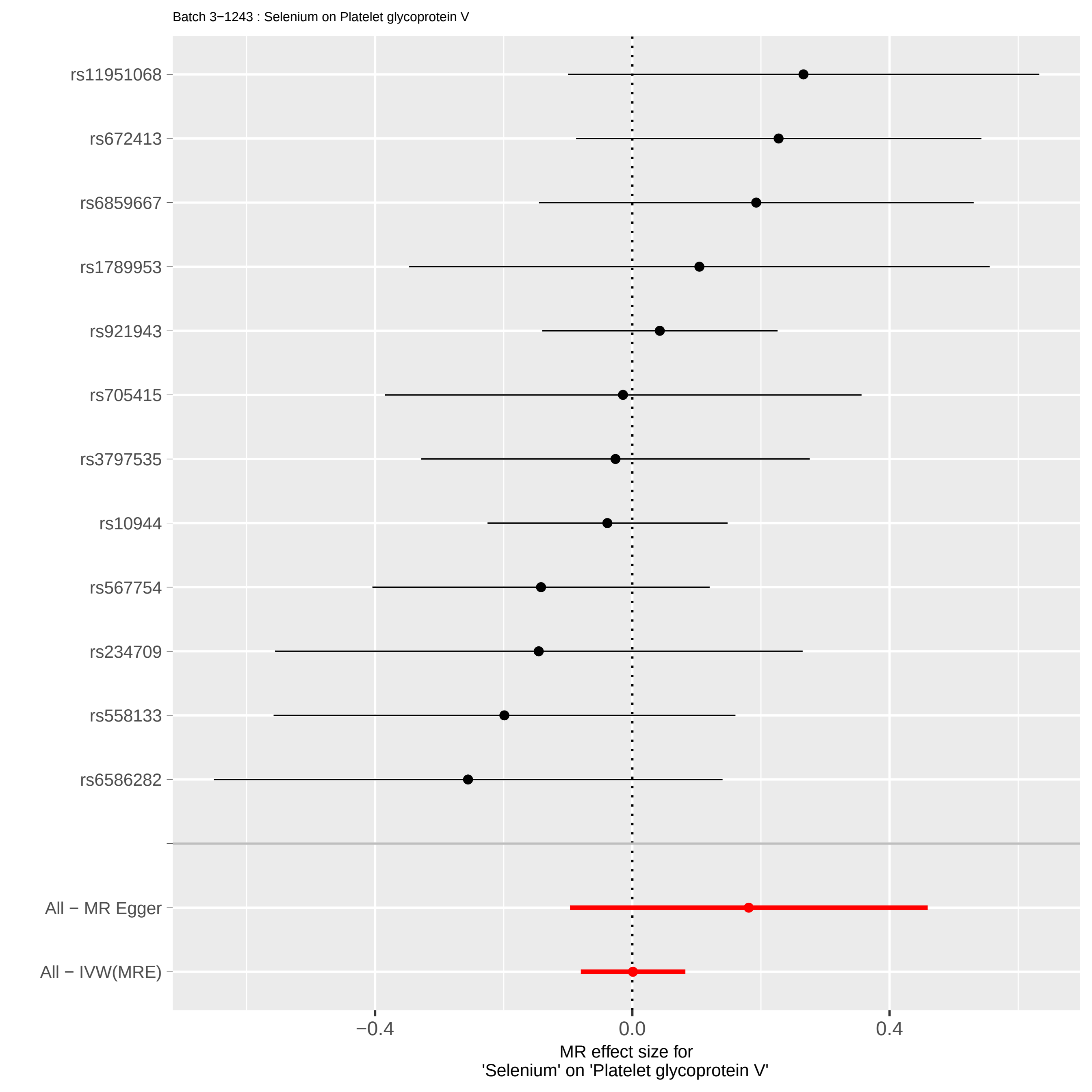
Batch 3−1243 : Selenium on Platelet glycoprotein V
rs11951068
rs672413
rs6859667
rs1789953
rs921943
rs705415
rs3797535
rs10944
rs567754
rs234709
rs558133
rs6586282
All − MR Egger
All − IVW(MRE)
−0.4
0.0
0.4
MR effect size for
'Selenium' on 'Platelet glycoprotein V'

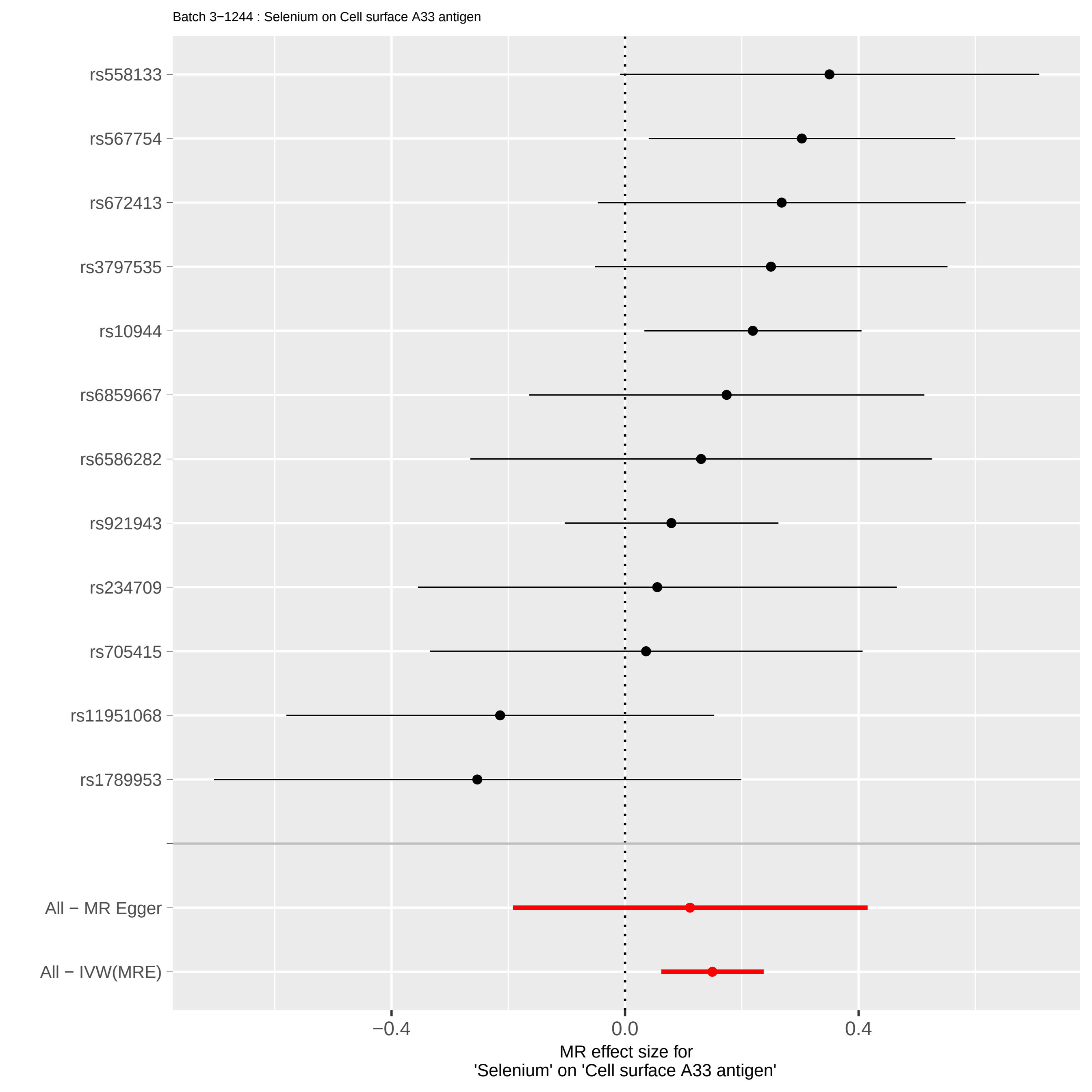

Batch 3−1244 : Selenium on Cell surface A33 antigen
rs558133
rs567754
rs672413
rs3797535
rs10944
rs6859667
rs6586282
rs921943
rs234709
rs705415
rs11951068
rs1789953
All − MR Egger
All − IVW(MRE)
−0.4
0.0
0.4
MR effect size for
'Selenium' on 'Cell surface A33 antigen'

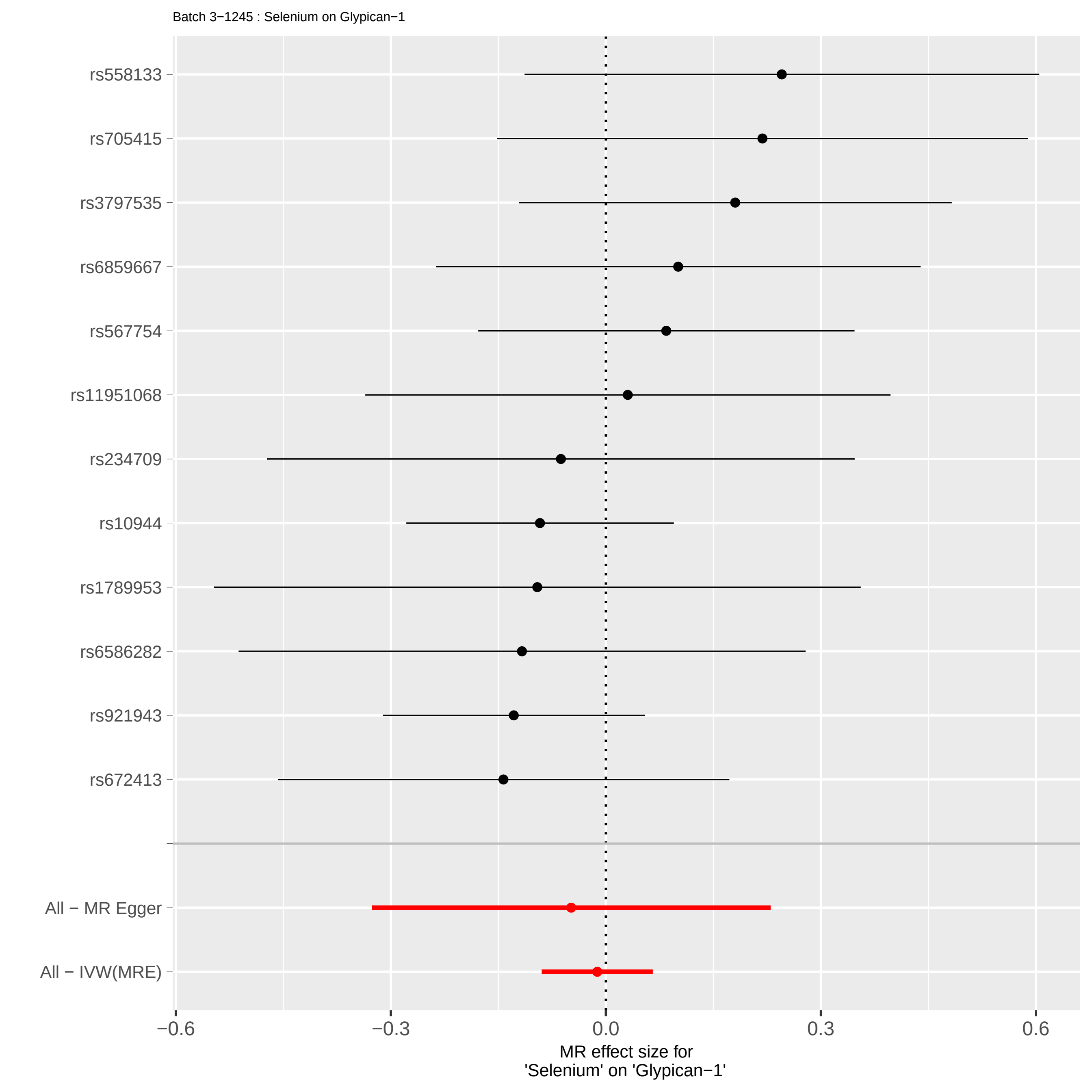

Batch 3−1245 : Selenium on Glypican−1
rs558133
rs705415
rs3797535
rs6859667
rs567754
rs11951068
rs234709
rs10944
rs1789953
rs6586282
rs921943
rs672413
All − MR Egger
All − IVW(MRE)
−0.6
−0.3
0.0
0.3
0.6
MR effect size for
'Selenium' on 'Glypican−1'

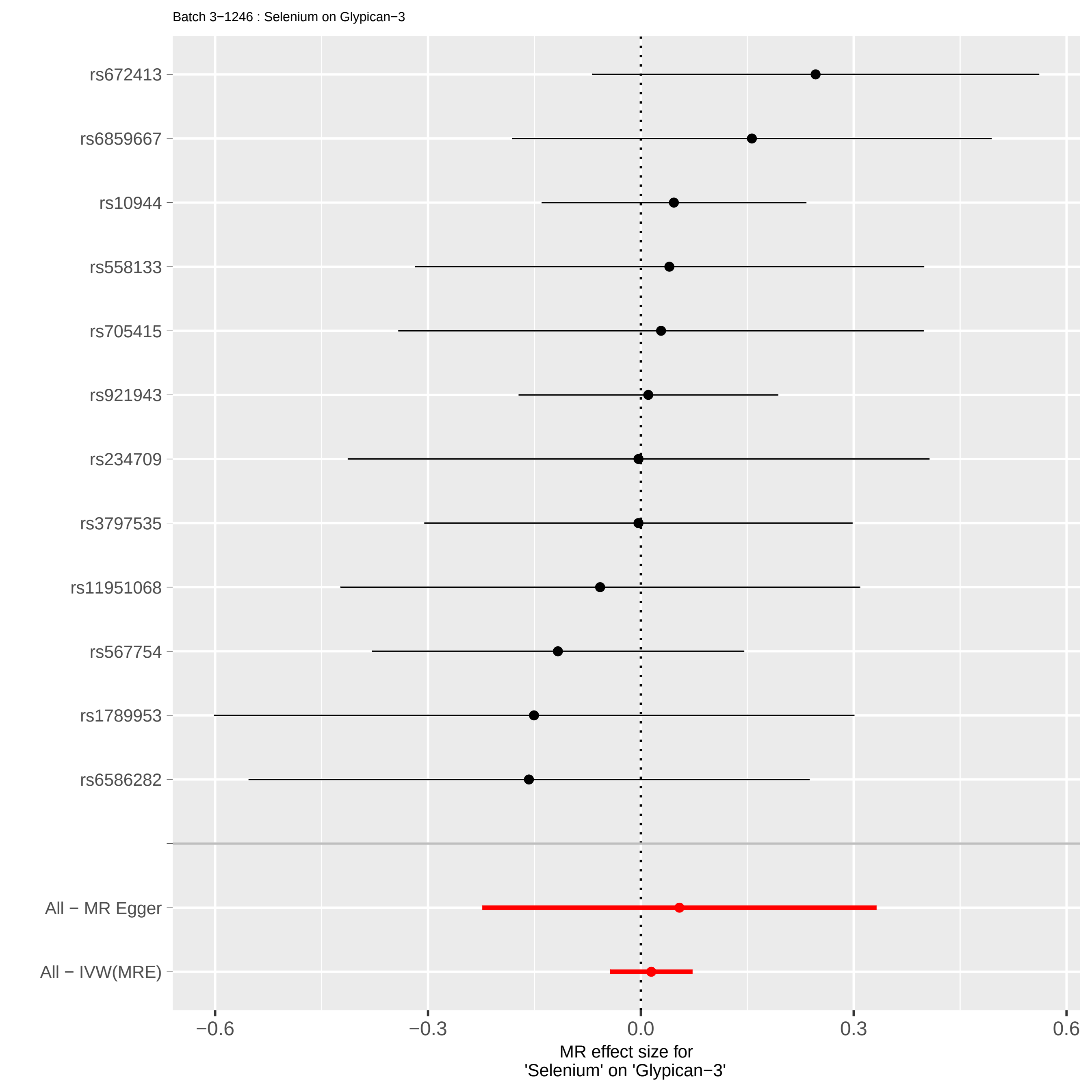

Batch 3−1246 : Selenium on Glypican−3
rs672413
rs6859667
rs10944
rs558133
rs705415
rs921943
rs234709
rs3797535
rs11951068
rs567754
rs1789953
rs6586282
All − MR Egger
All − IVW(MRE)
−0.6
−0.3
0.0
0.3
0.6
MR effect size for
'Selenium' on 'Glypican−3'

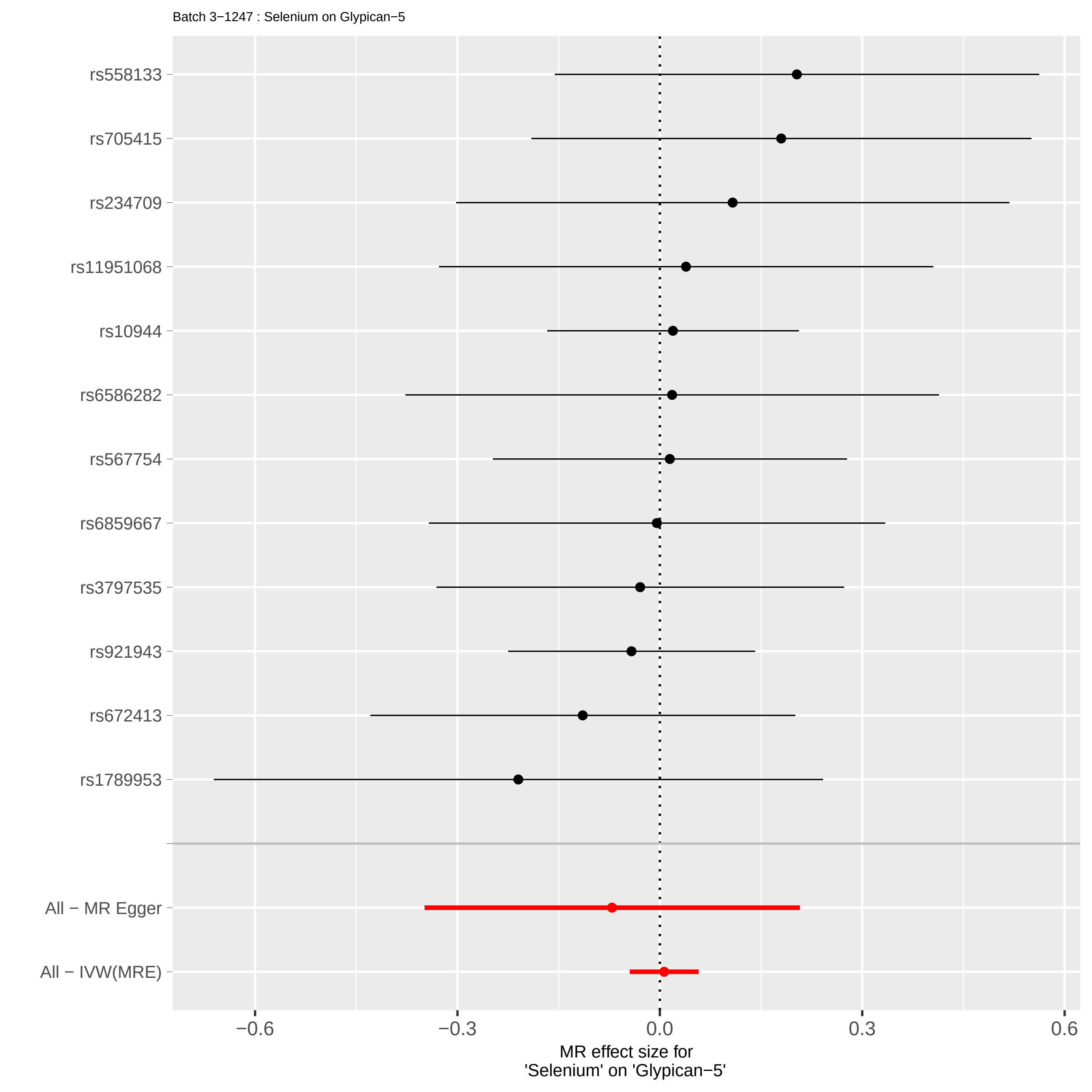
Batch 3−1247 : Selenium on Glypican−5
rs558133
rs705415
rs234709
rs11951068
rs10944
rs6586282
rs567754
rs6859667
rs3797535
rs921943
rs672413
rs1789953
All − MR Egger
All − IVW(MRE)
−0.6
−0.3
0.0
0.3
0.6
MR effect size for
'Selenium' on 'Glypican−5'

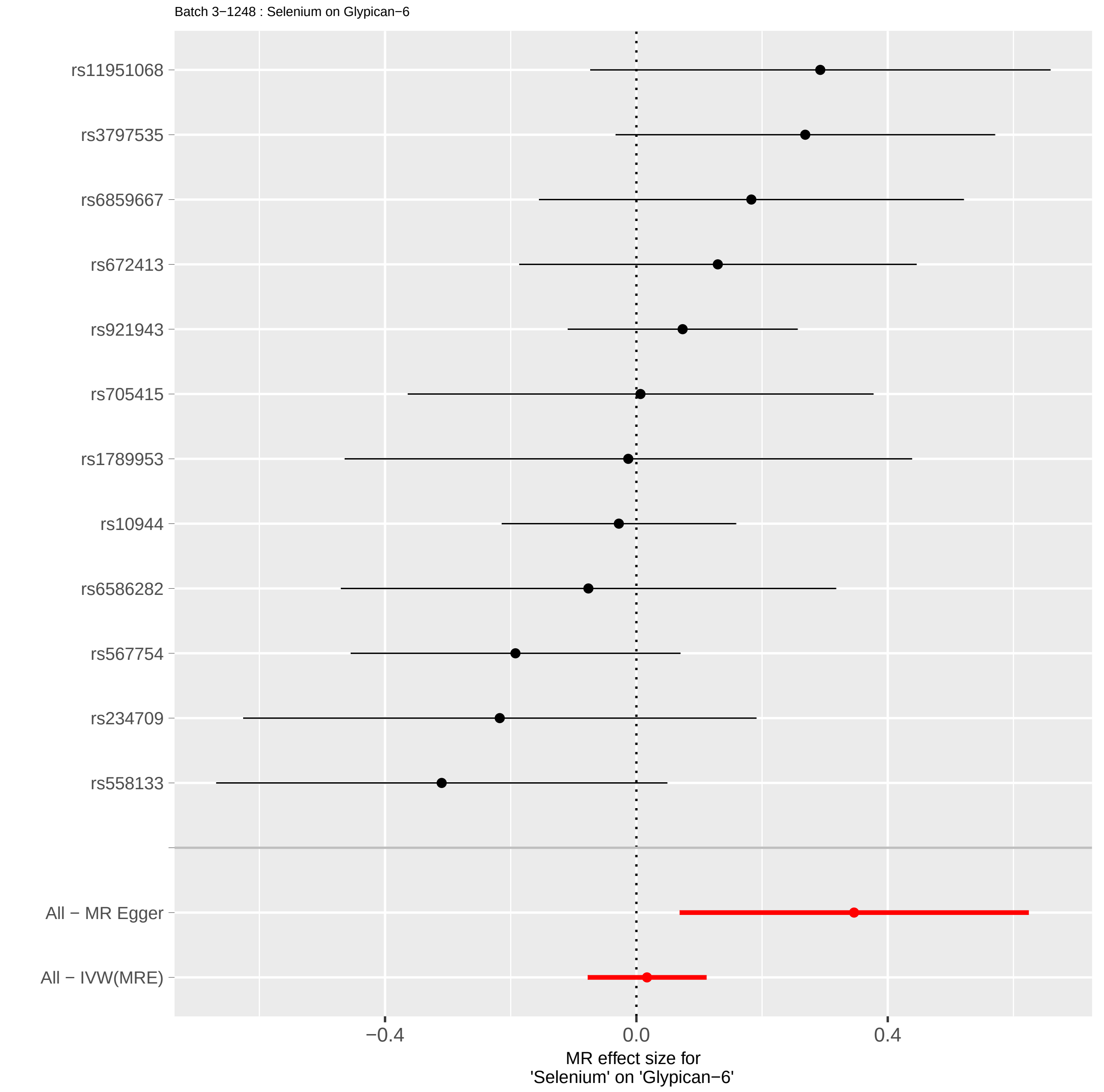
Batch 3−1248 : Selenium on Glypican−6
rs11951068
rs3797535
rs6859667
rs672413
rs921943
rs705415
rs1789953
rs10944
rs6586282
rs567754
rs234709
rs558133
All − MR Egger
All − IVW(MRE)
−0.4
0.0
0.4
MR effect size for
'Selenium' on 'Glypican−6'

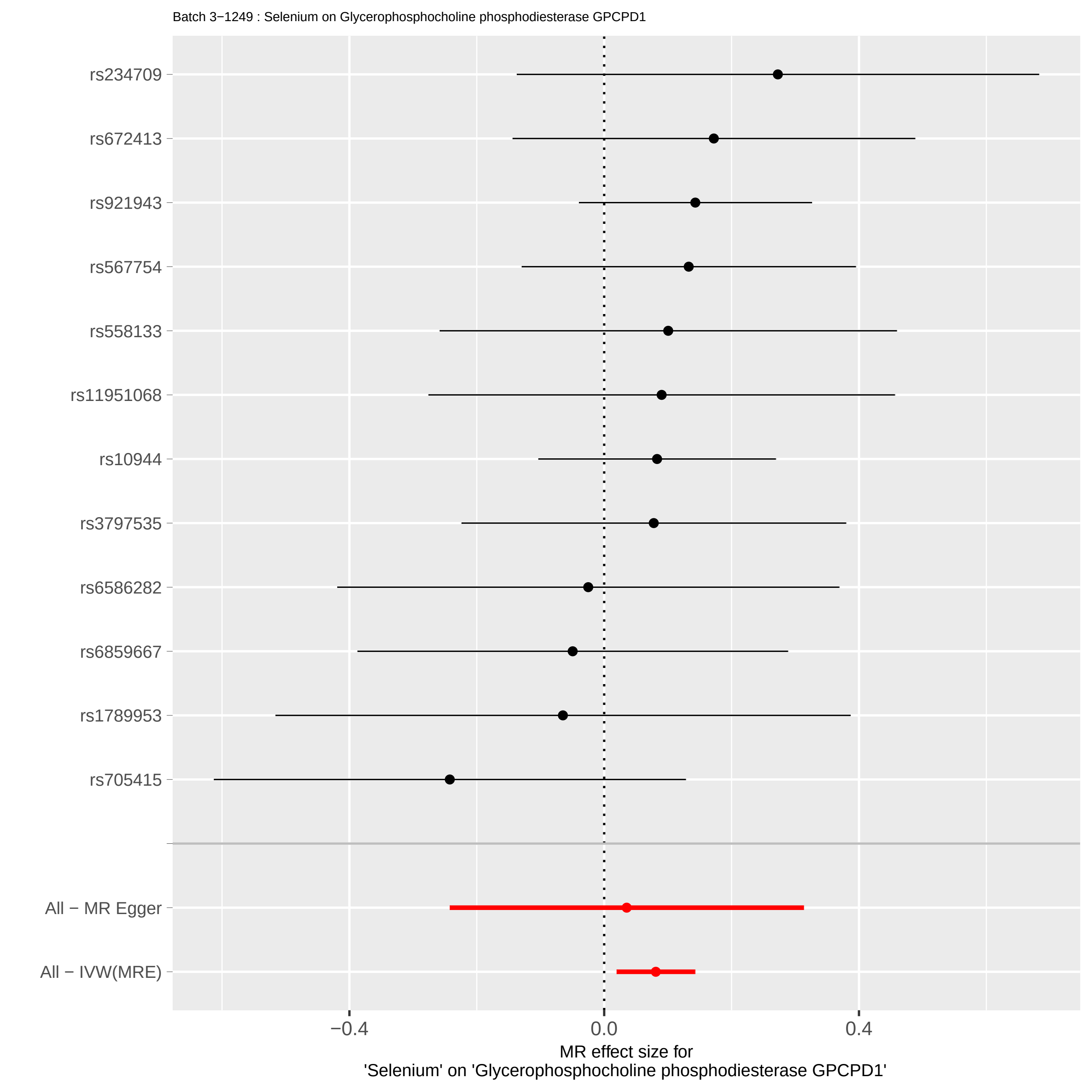

Batch 3−1249 : Selenium on Glycerophosphocholine phosphodiesterase GPCPD1
rs234709
rs672413
rs921943
rs567754
rs558133
rs11951068
rs10944
rs3797535
rs6586282
rs6859667
rs1789953
rs705415
All − MR Egger
All − IVW(MRE)
−0.4
0.0
0.4
MR effect size for
'Selenium' on 'Glycerophosphocholine phosphodiesterase GPCPD1'

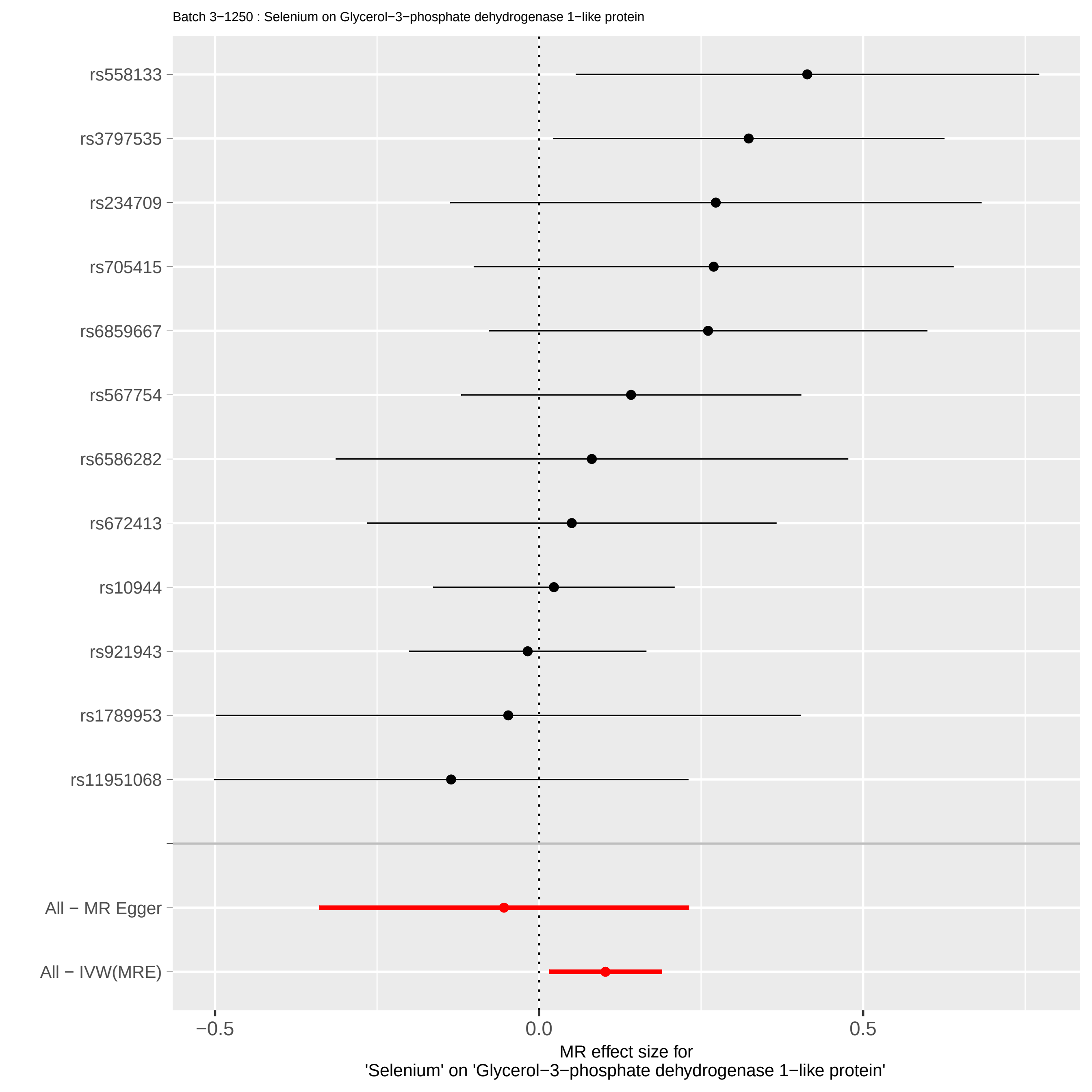
Batch 3−1250 : Selenium on Glycerol−3−phosphate dehydrogenase 1−like protein
rs558133
rs3797535
rs234709
rs705415
rs6859667
rs567754
rs6586282
rs672413
rs10944
rs921943
rs1789953
rs11951068
All − MR Egger
All − IVW(MRE)
−0.5
0.0
0.5
MR effect size for
'Selenium' on 'Glycerol−3−phosphate dehydrogenase 1−like protein'

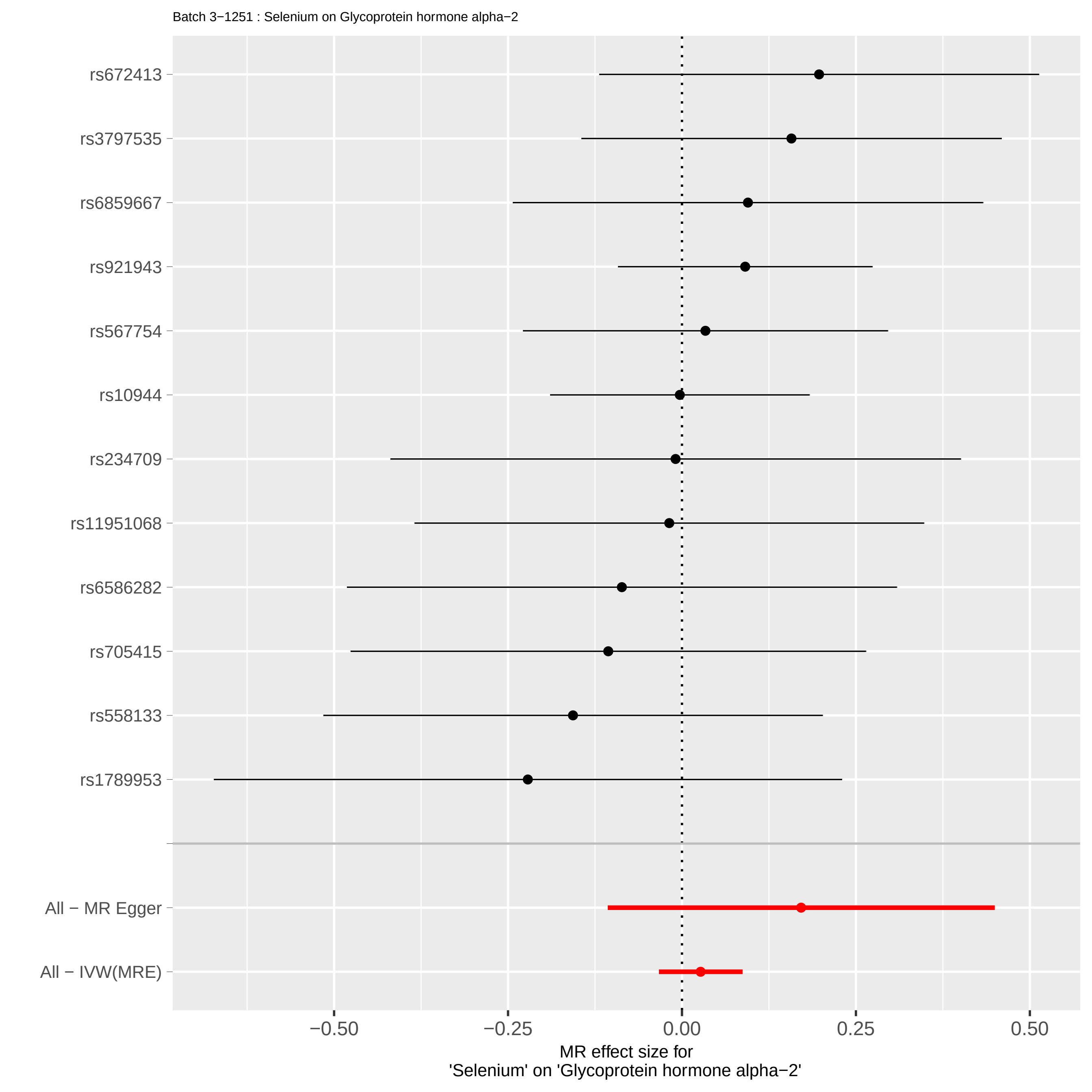

Batch 3−1251 : Selenium on Glycoprotein hormone alpha−2
rs672413
rs3797535
rs6859667
rs921943
rs567754
rs10944
rs234709
rs11951068
rs6586282
rs705415
rs558133
rs1789953
All − MR Egger
All − IVW(MRE)
−0.50
−0.25
0.00
0.25
0.50
MR effect size for
'Selenium' on 'Glycoprotein hormone alpha−2'

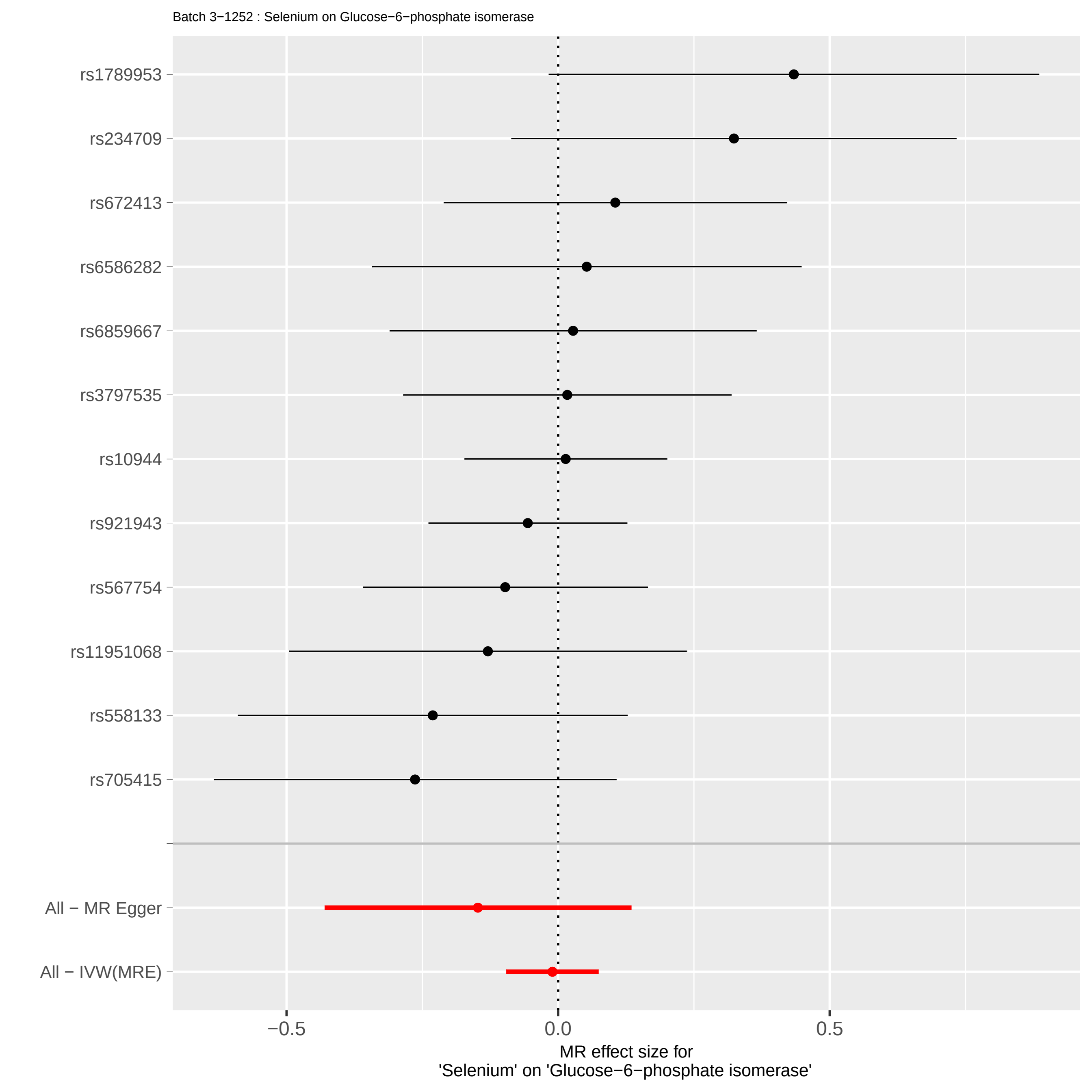
Batch 3−1252 : Selenium on Glucose−6−phosphate isomerase
rs1789953
rs234709
rs672413
rs6586282
rs6859667
rs3797535
rs10944
rs921943
rs567754
rs11951068
rs558133
rs705415
All − MR Egger
All − IVW(MRE)
−0.5
0.0
0.5
MR effect size for
'Selenium' on 'Glucose−6−phosphate isomerase'

Batch 3−1253 : Selenium on Phosphatidylinositol−glycan−specific phospholipase D

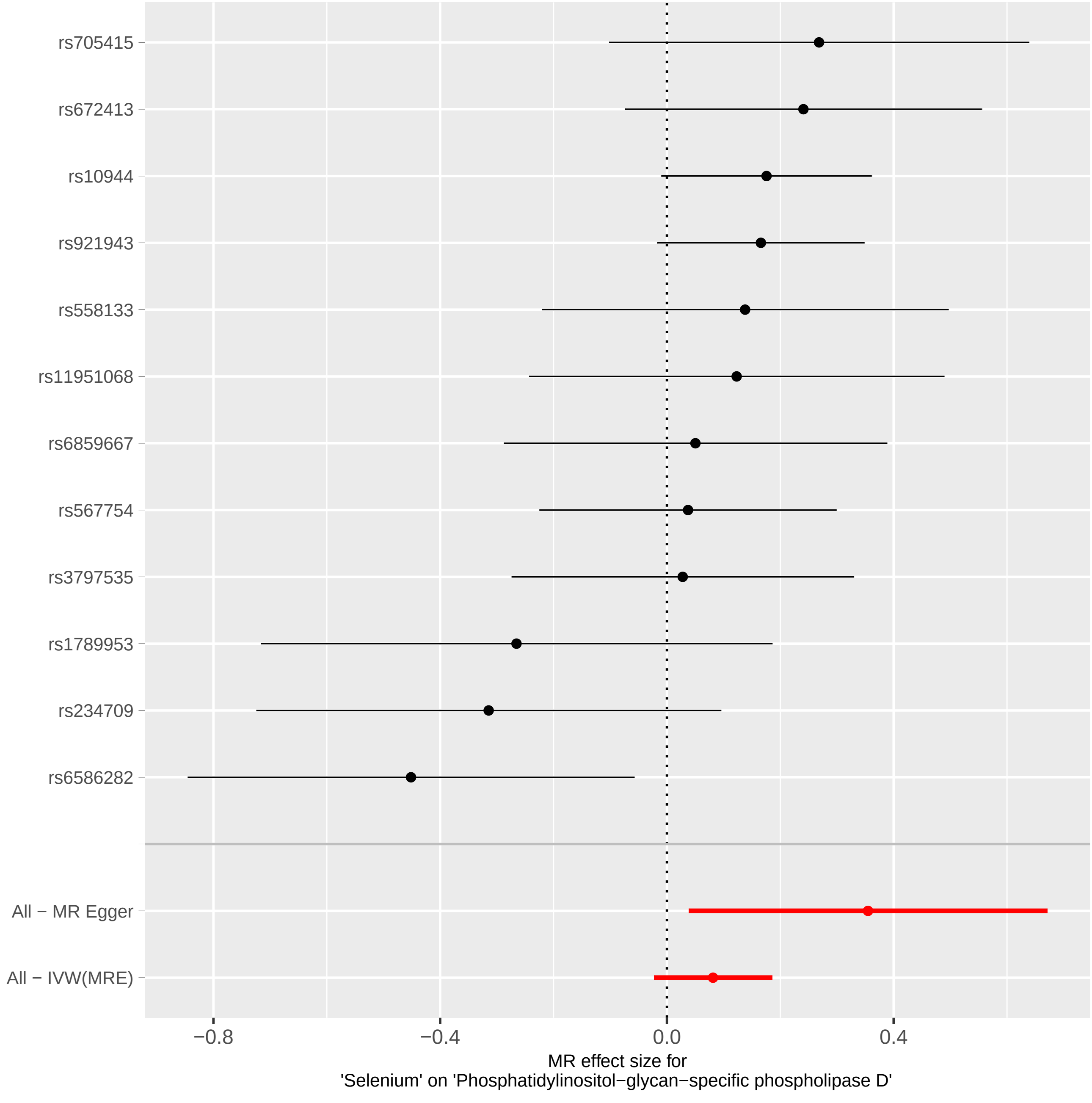

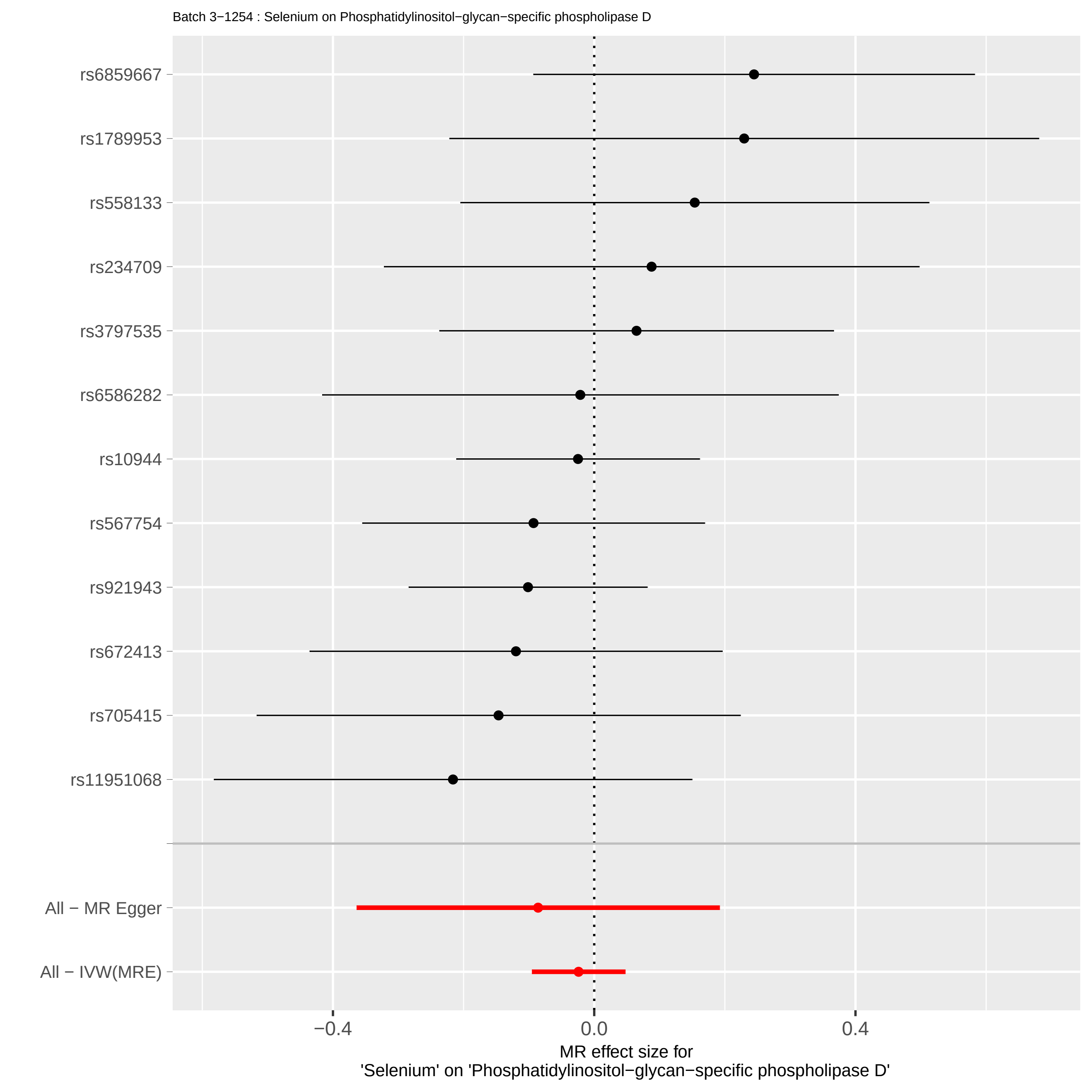
Batch 3−1254 : Selenium on Phosphatidylinositol−glycan−specific phospholipase D
rs6859667
rs1789953
rs558133
rs234709
rs3797535
rs6586282
rs10944
rs567754
rs921943
rs672413
rs705415
rs11951068
All − MR Egger
All − IVW(MRE)
−0.4
0.0
0.4
MR effect size for
'Selenium' on 'Phosphatidylinositol−glycan−specific phospholipase D'

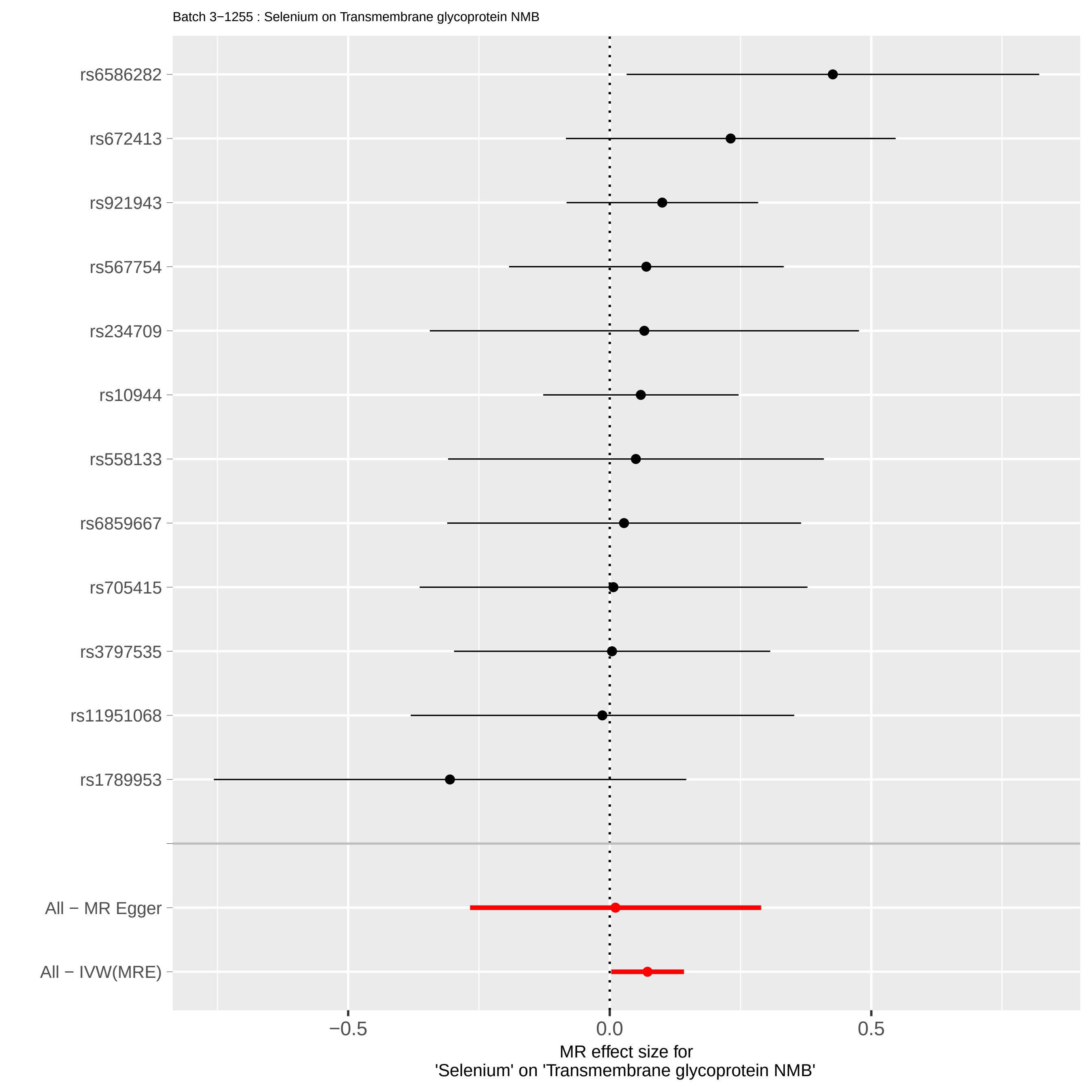

Batch 3−1255 : Selenium on Transmembrane glycoprotein NMB
rs6586282
rs672413
rs921943
rs567754
rs234709
rs10944
rs558133
rs6859667
rs705415
rs3797535
rs11951068
rs1789953
All − MR Egger
All − IVW(MRE)
−0.5
0.0
0.5
MR effect size for
'Selenium' on 'Transmembrane glycoprotein NMB'

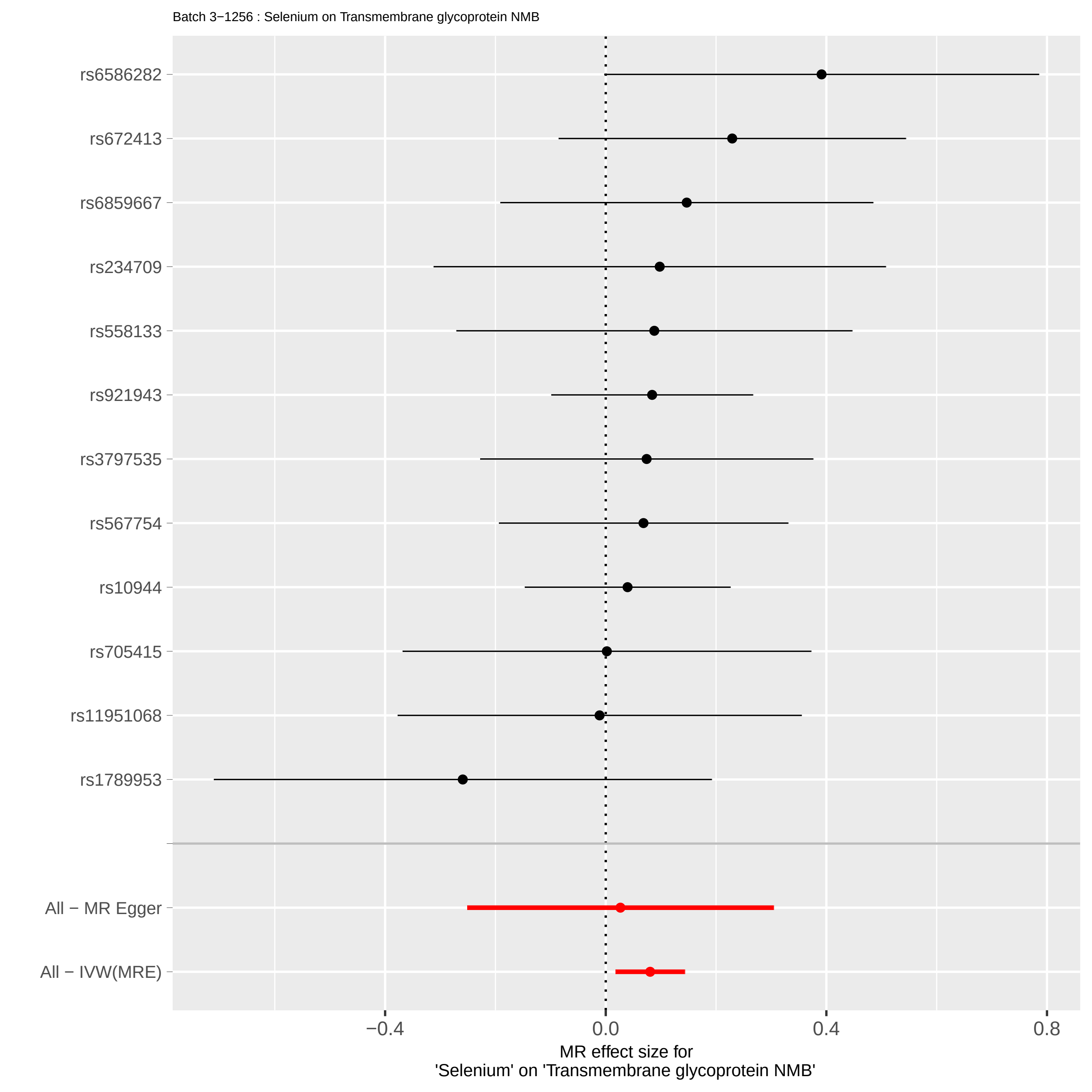
Batch 3−1256 : Selenium on Transmembrane glycoprotein NMB
rs6586282
rs672413
rs6859667
rs234709
rs558133
rs921943
rs3797535
rs567754
rs10944
rs705415
rs11951068
rs1789953
All − MR Egger
All − IVW(MRE)
−0.4
0.0
0.4
0.8
MR effect size for
'Selenium' on 'Transmembrane glycoprotein NMB'

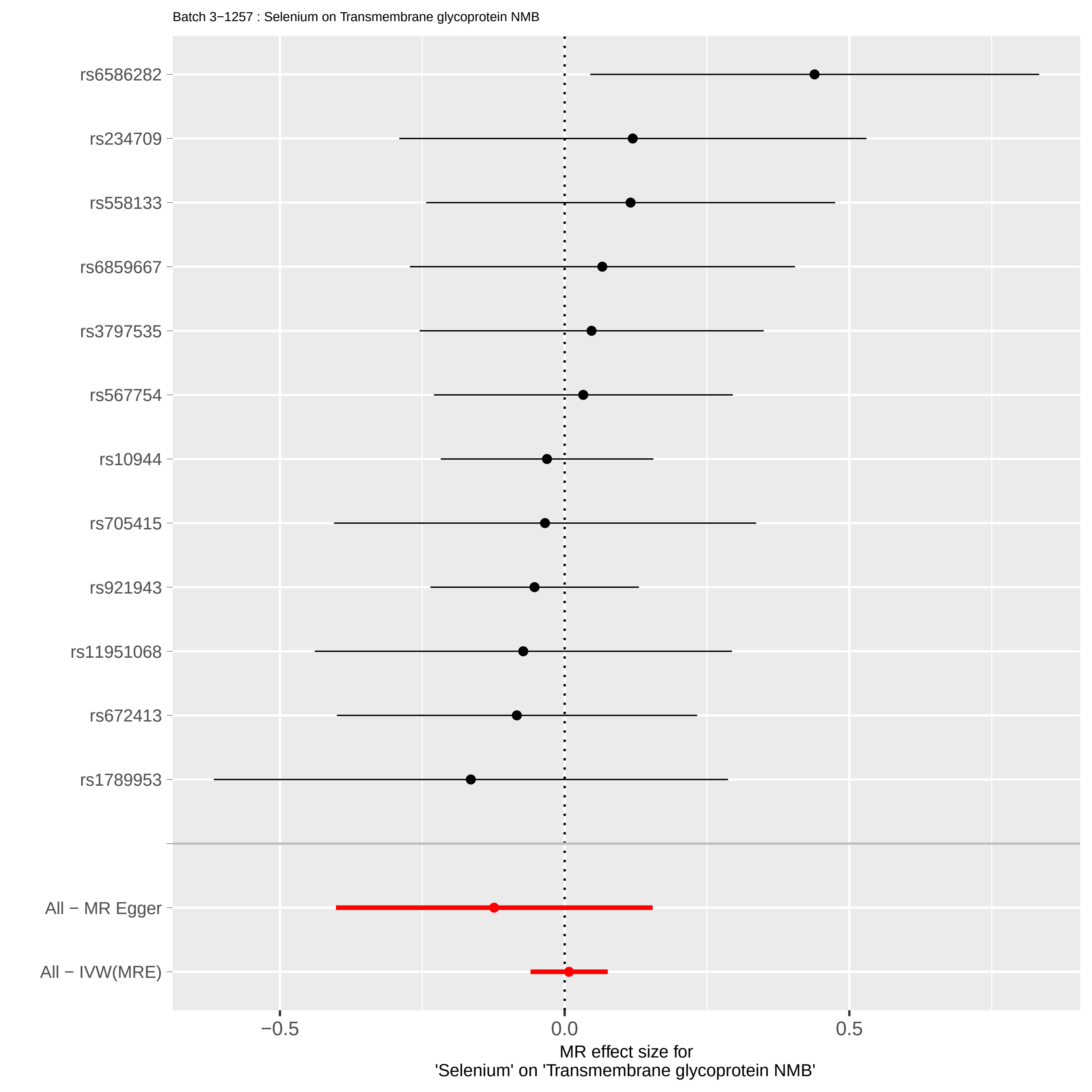
Batch 3−1257 : Selenium on Transmembrane glycoprotein NMB
rs6586282
rs234709
rs558133
rs6859667
rs3797535
rs567754
rs10944
rs705415
rs921943
rs11951068
rs672413
rs1789953
All − MR Egger
All − IVW(MRE)
−0.5
0.0
0.5
MR effect size for
'Selenium' on 'Transmembrane glycoprotein NMB'

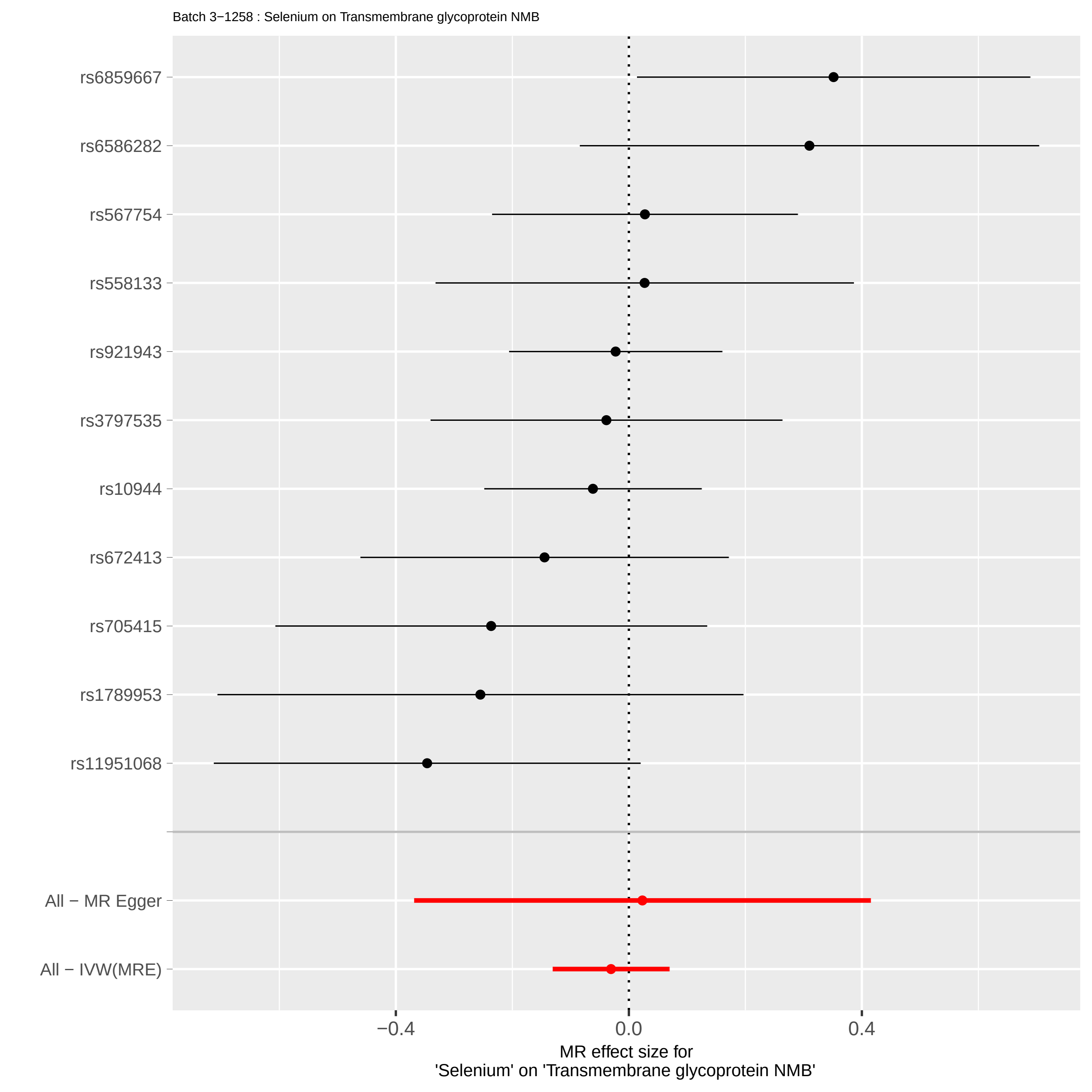
Batch 3−1258 : Selenium on Transmembrane glycoprotein NMB
rs6859667
rs6586282
rs567754
rs558133
rs921943
rs3797535
rs10944
rs672413
rs705415
rs1789953
rs11951068
All − MR Egger
All − IVW(MRE)
−0.4
0.0
0.4
MR effect size for
'Selenium' on 'Transmembrane glycoprotein NMB'

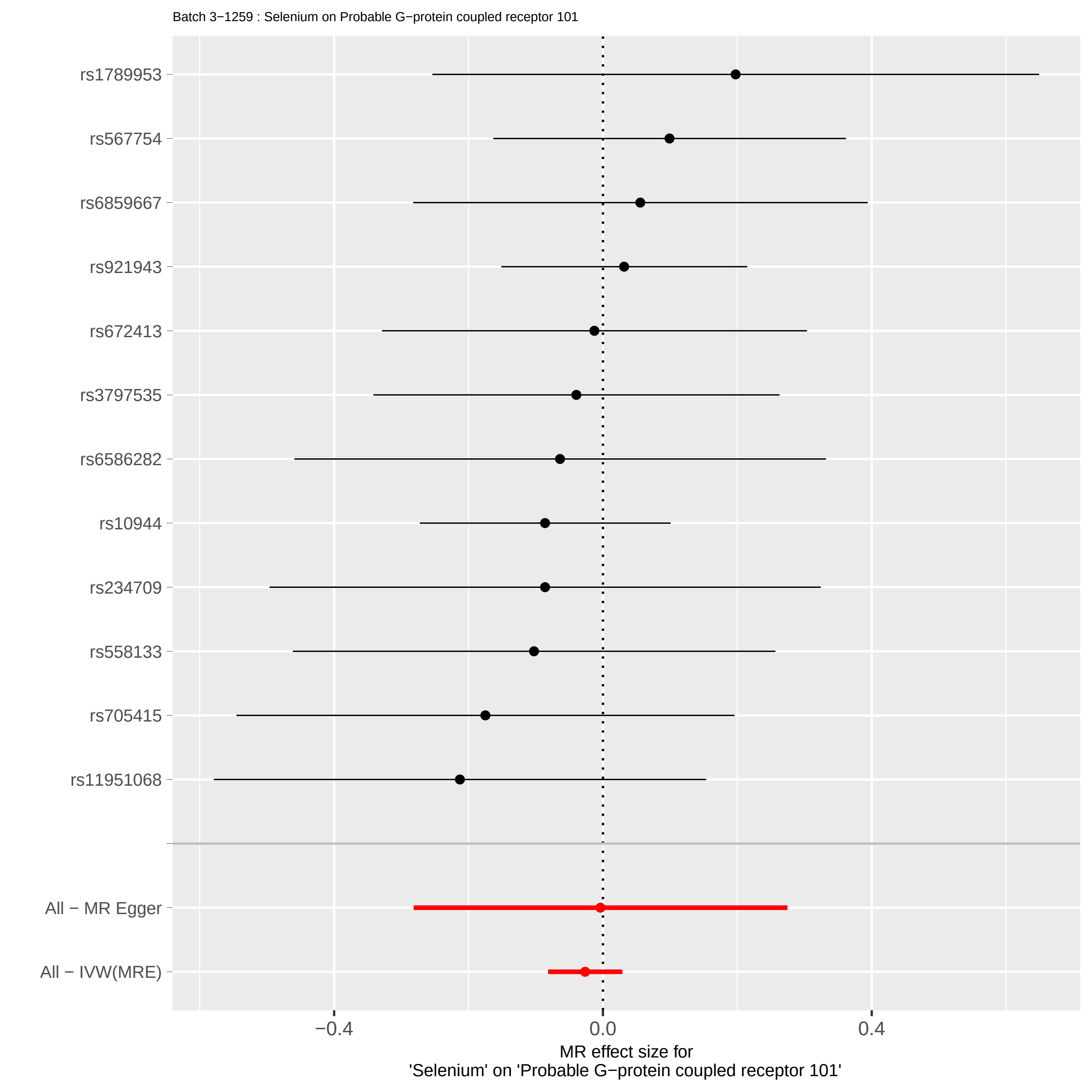
Batch 3−1259 : Selenium on Probable G−protein coupled receptor 101
rs1789953
rs567754
rs6859667
rs921943
rs672413
rs3797535
rs6586282
rs10944
rs234709
rs558133
rs705415
rs11951068
All − MR Egger
All − IVW(MRE)
−0.4
0.0
0.4
MR effect size for
'Selenium' on 'Probable G−protein coupled receptor 101'

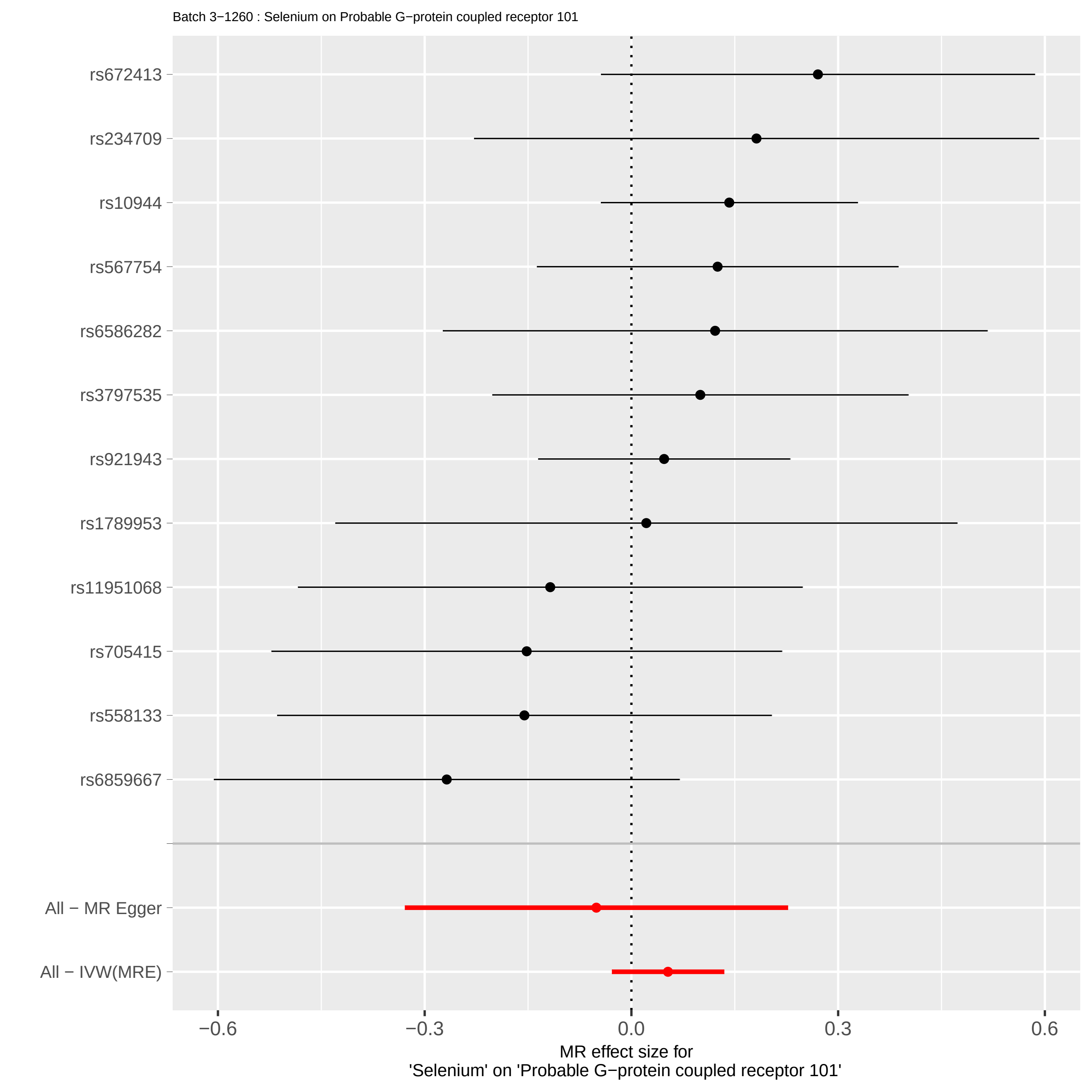

Batch 3−1260 : Selenium on Probable G−protein coupled receptor 101
rs672413
rs234709
rs10944
rs567754
rs6586282
rs3797535
rs921943
rs1789953
rs11951068
rs705415
rs558133
rs6859667
All − MR Egger
All − IVW(MRE)
−0.6
−0.3
0.0
0.3
0.6
MR effect size for
'Selenium' on 'Probable G−protein coupled receptor 101'

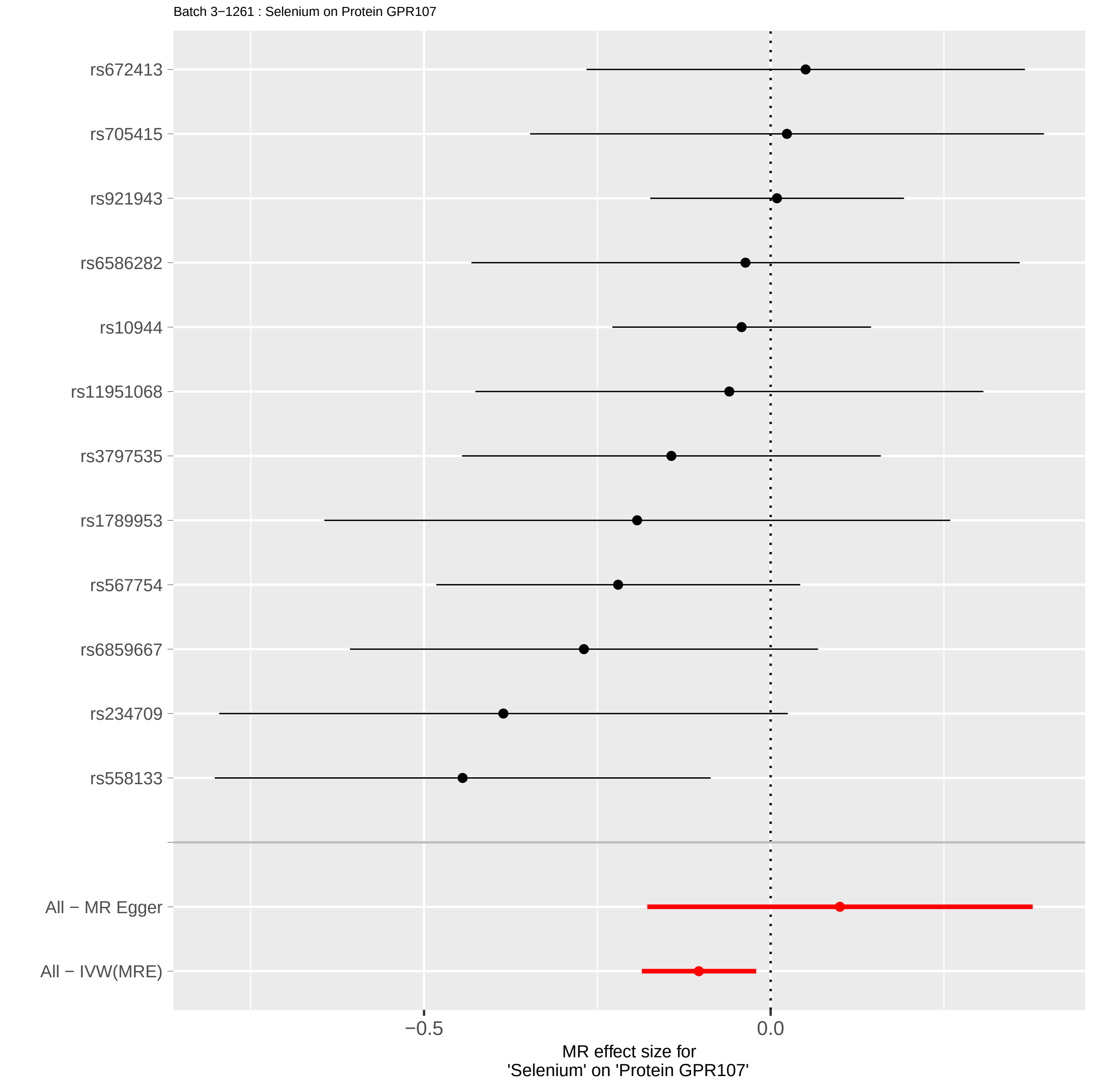

Batch 3−1261 : Selenium on Protein GPR107
rs672413
rs705415
rs921943
rs6586282
rs10944
rs11951068
rs3797535
rs1789953
rs567754
rs6859667
rs234709
rs558133
All − MR Egger
All − IVW(MRE)
−0.5
0.0
MR effect size for
'Selenium' on 'Protein GPR107'

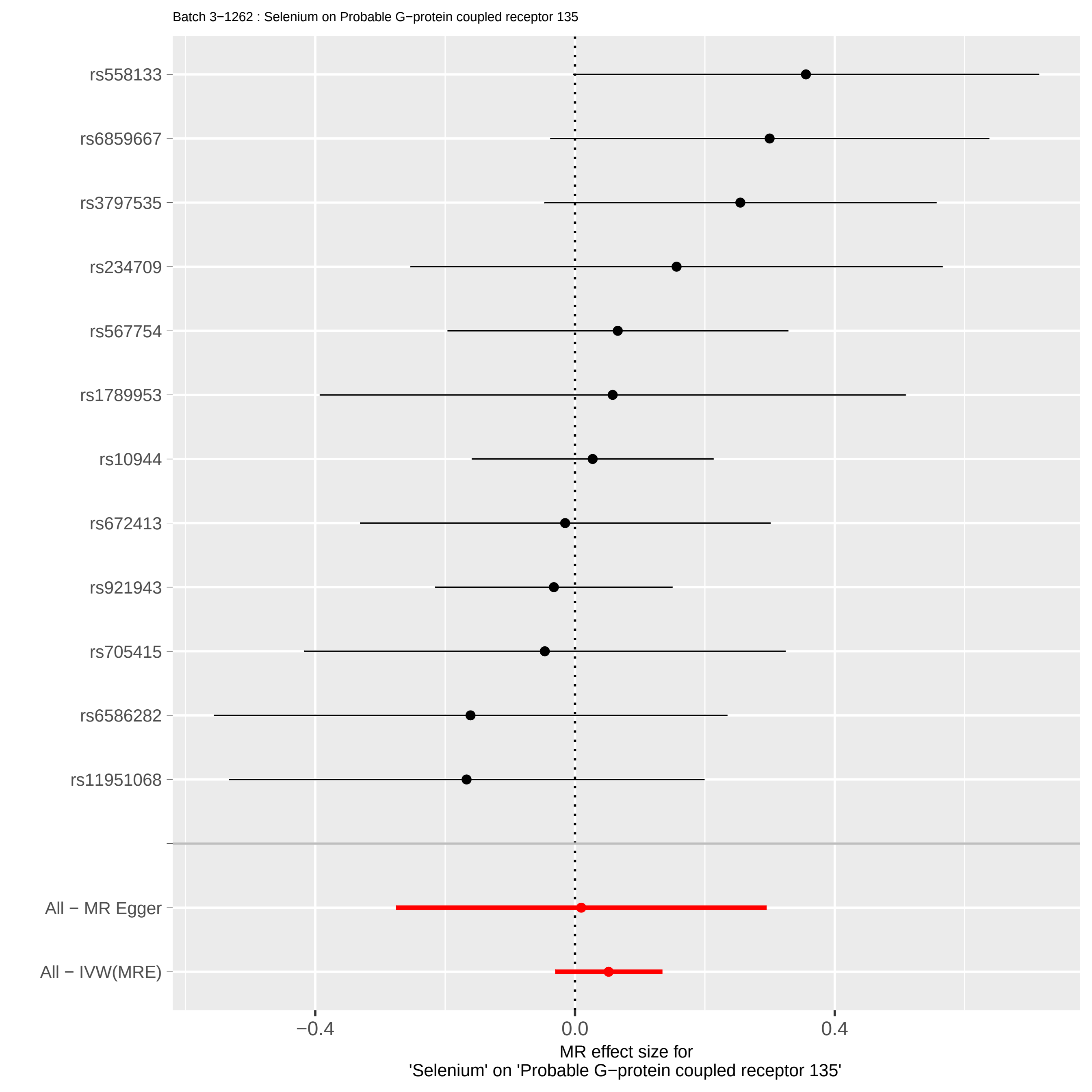

Batch 3−1262 : Selenium on Probable G−protein coupled receptor 135
rs558133
rs6859667
rs3797535
rs234709
rs567754
rs1789953
rs10944
rs672413
rs921943
rs705415
rs6586282
rs11951068
All − MR Egger
All − IVW(MRE)
−0.4
0.0
0.4
MR effect size for
'Selenium' on 'Probable G−protein coupled receptor 135'

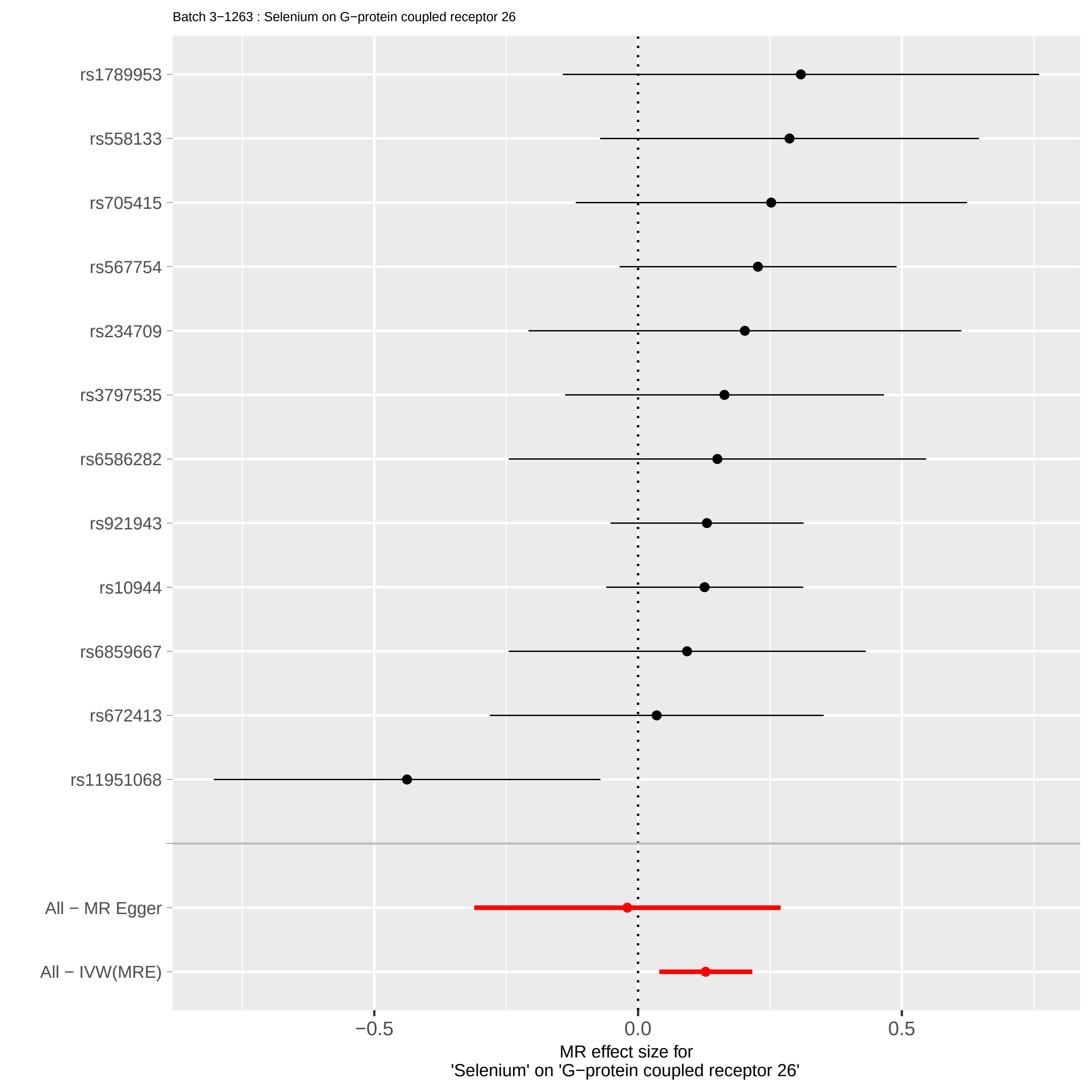

Batch 3−1263 : Selenium on G−protein coupled receptor 26
rs1789953
rs558133
rs705415
rs567754
rs234709
rs3797535
rs6586282
rs921943
rs10944
rs6859667
rs672413
rs11951068
All − MR Egger
All − IVW(MRE)
−0.5
0.0
0.5
MR effect size for
'Selenium' on 'G−protein coupled receptor 26'

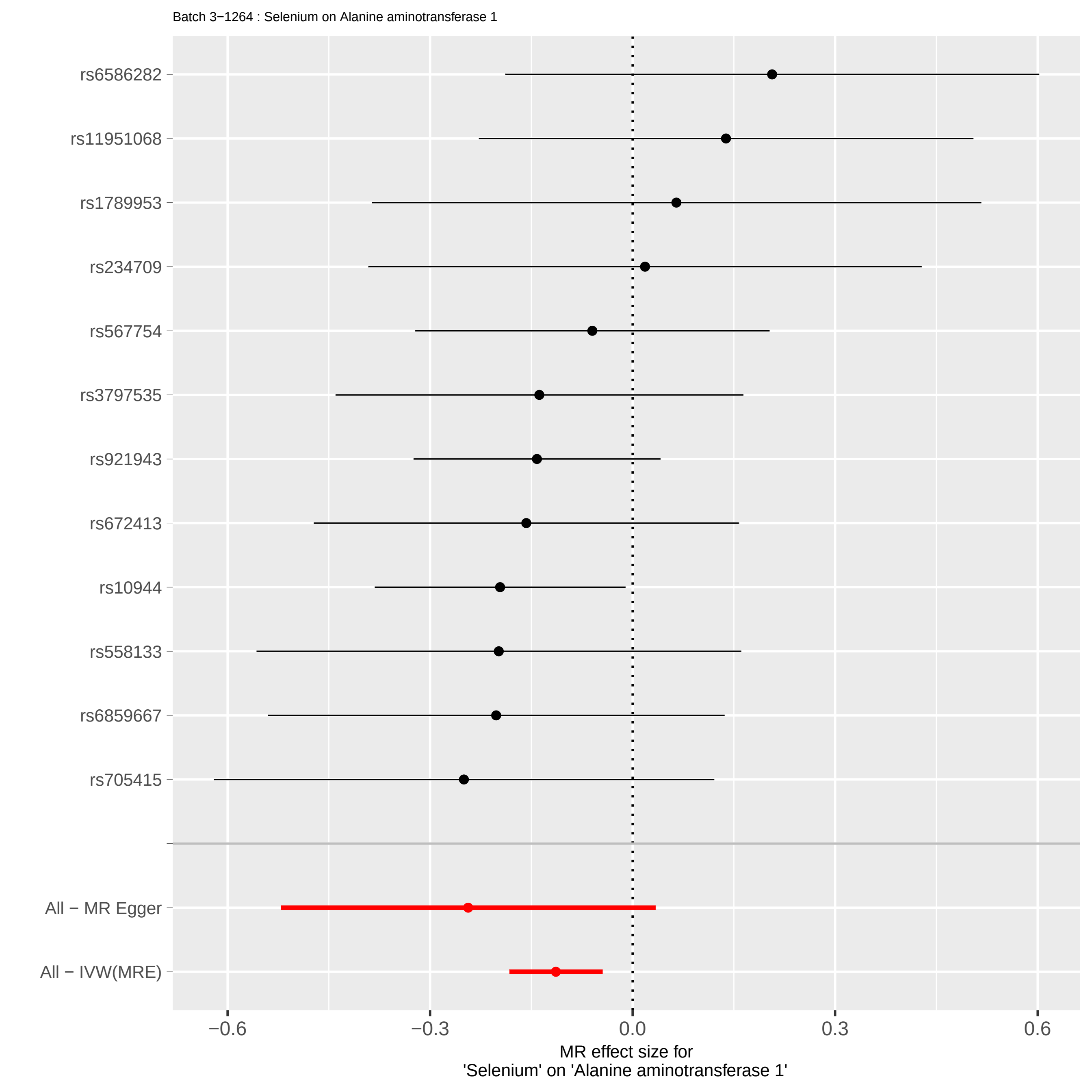

Batch 3−1264 : Selenium on Alanine aminotransferase 1
rs6586282
rs11951068
rs1789953
rs234709
rs567754
rs3797535
rs921943
rs672413
rs10944
rs558133
rs6859667
rs705415
All − MR Egger
All − IVW(MRE)
−0.6
−0.3
0.0
0.3
0.6
MR effect size for
'Selenium' on 'Alanine aminotransferase 1'

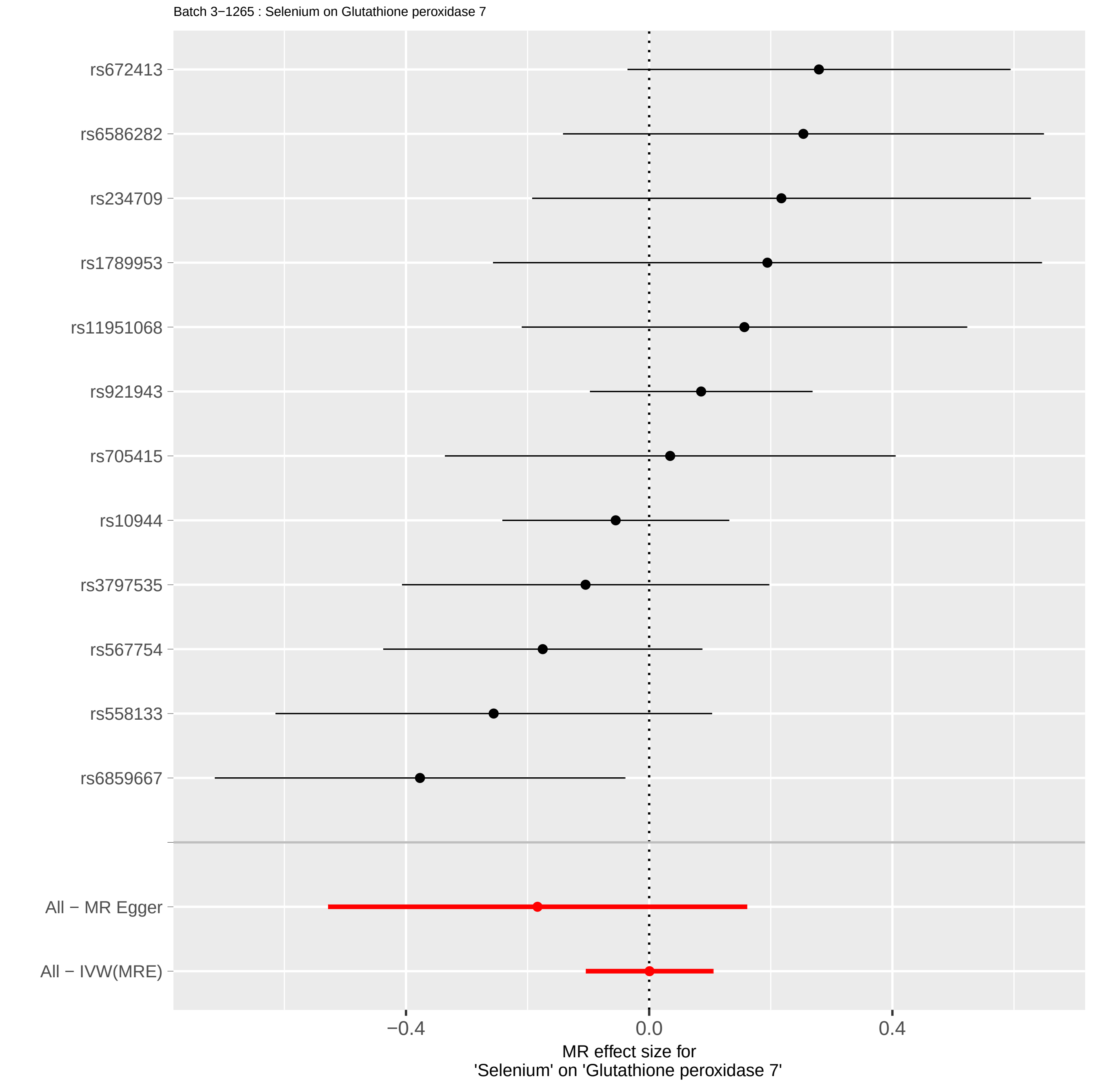
Batch 3−1265 : Selenium on Glutathione peroxidase 7
rs672413
rs6586282
rs234709
rs1789953
rs11951068
rs921943
rs705415
rs10944
rs3797535
rs567754
rs558133
rs6859667
All − MR Egger
All − IVW(MRE)
−0.4
0.0
0.4
MR effect size for
'Selenium' on 'Glutathione peroxidase 7'

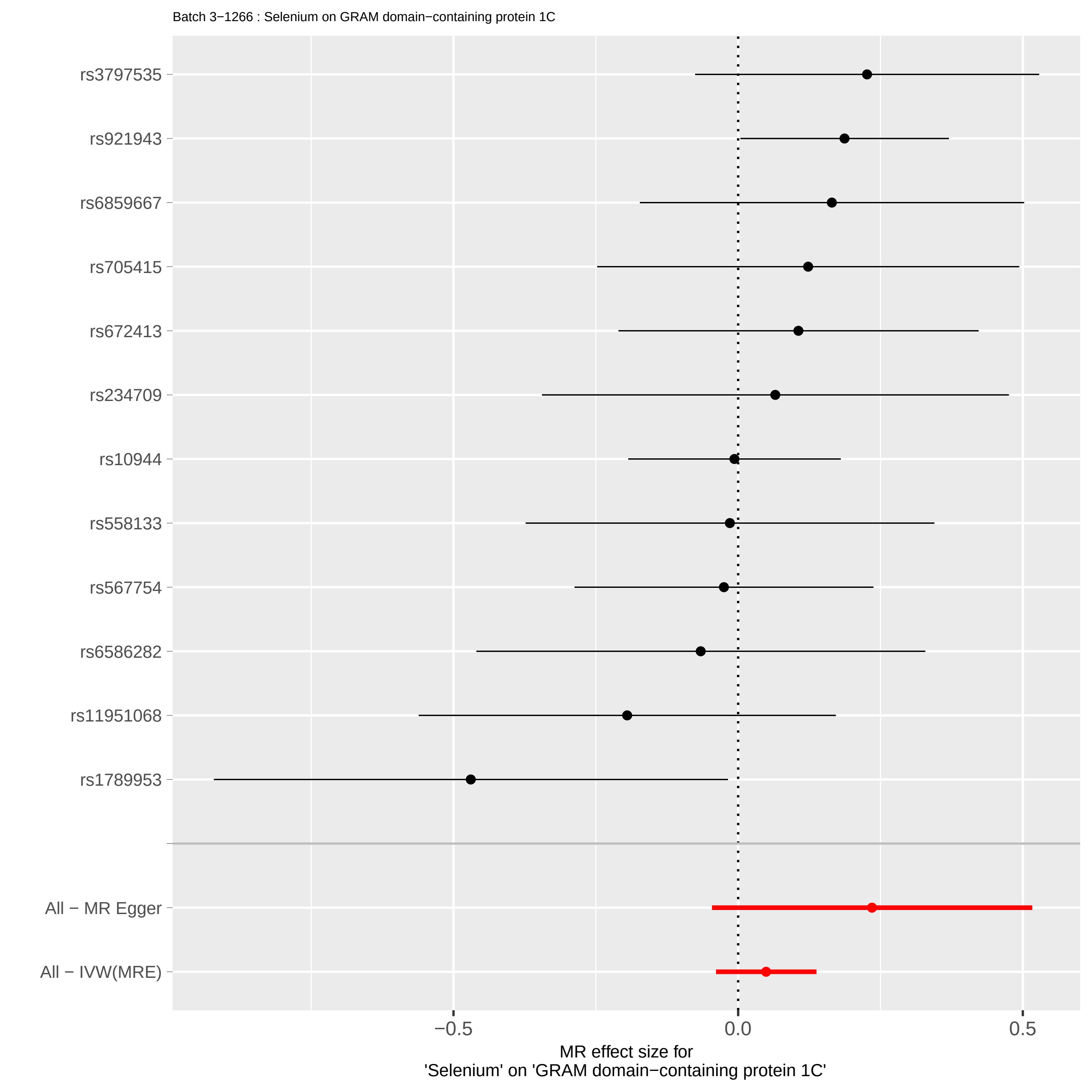

Batch 3−1266 : Selenium on GRAM domain−containing protein 1C
rs3797535
rs921943
rs6859667
rs705415
rs672413
rs234709
rs10944
rs558133
rs567754
rs6586282
rs11951068
rs1789953
All − MR Egger
All − IVW(MRE)
−0.5
0.0
0.5
MR effect size for
'Selenium' on 'GRAM domain−containing protein 1C'

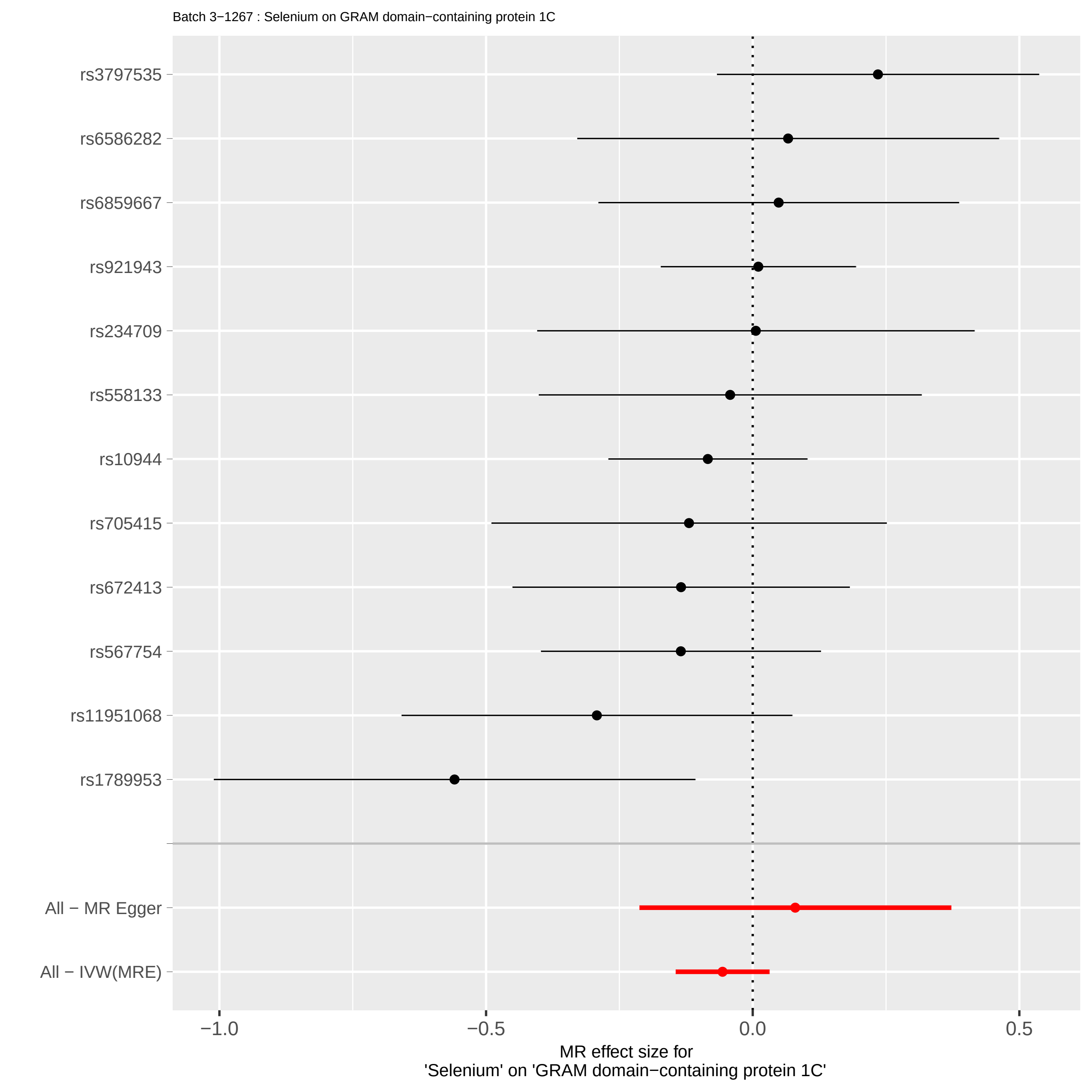
Batch 3−1267 : Selenium on GRAM domain−containing protein 1C
rs3797535
rs6586282
rs6859667
rs921943
rs234709
rs558133
rs10944
rs705415
rs672413
rs567754
rs11951068
rs1789953
All − MR Egger
All − IVW(MRE)
−1.0
−0.5
0.0
0.5
MR effect size for
'Selenium' on 'GRAM domain−containing protein 1C'

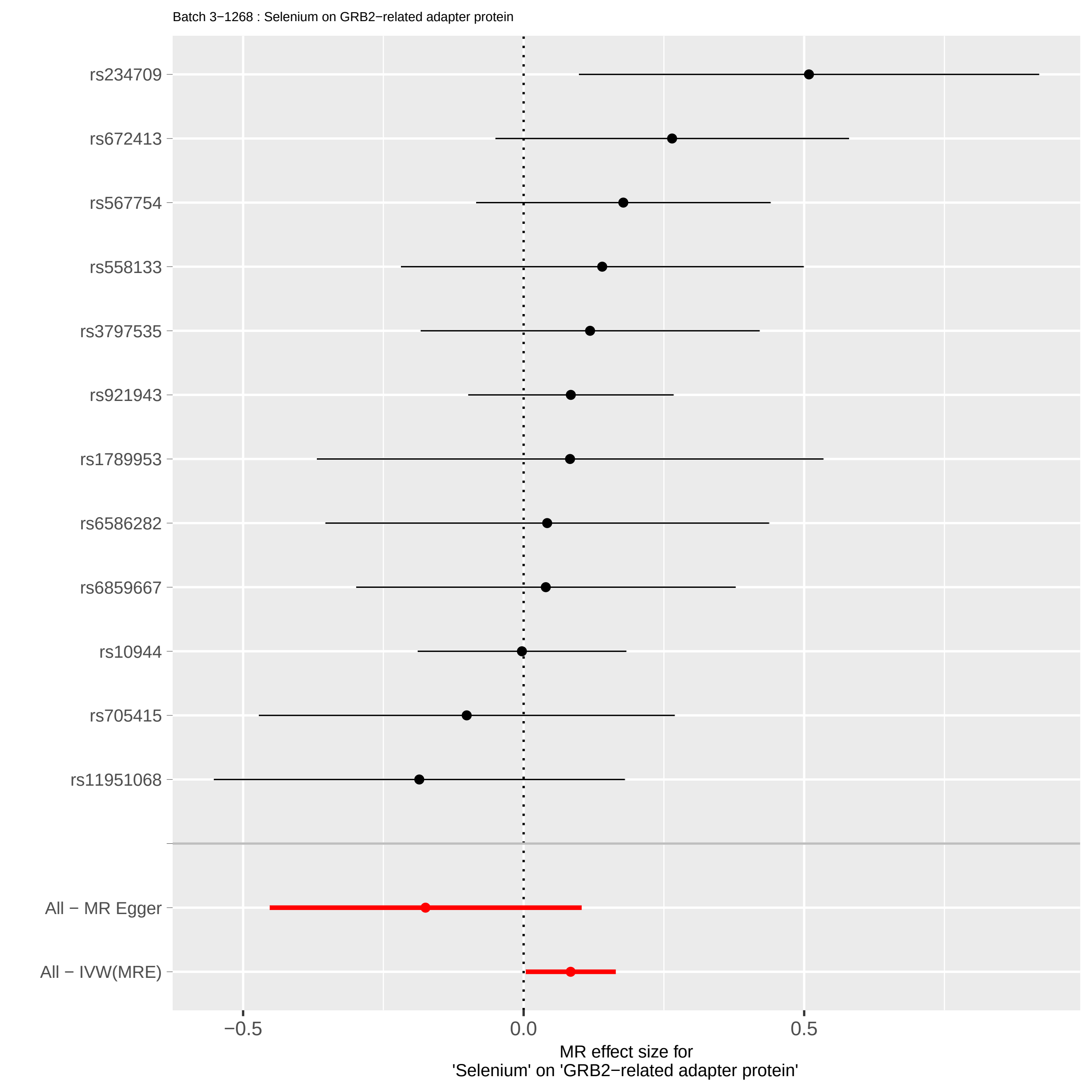

Batch 3−1268 : Selenium on GRB2−related adapter protein
rs234709
rs672413
rs567754
rs558133
rs3797535
rs921943
rs1789953
rs6586282
rs6859667
rs10944
rs705415
rs11951068
All − MR Egger
All − IVW(MRE)
−0.5
0.0
0.5
MR effect size for
'Selenium' on 'GRB2−related adapter protein'

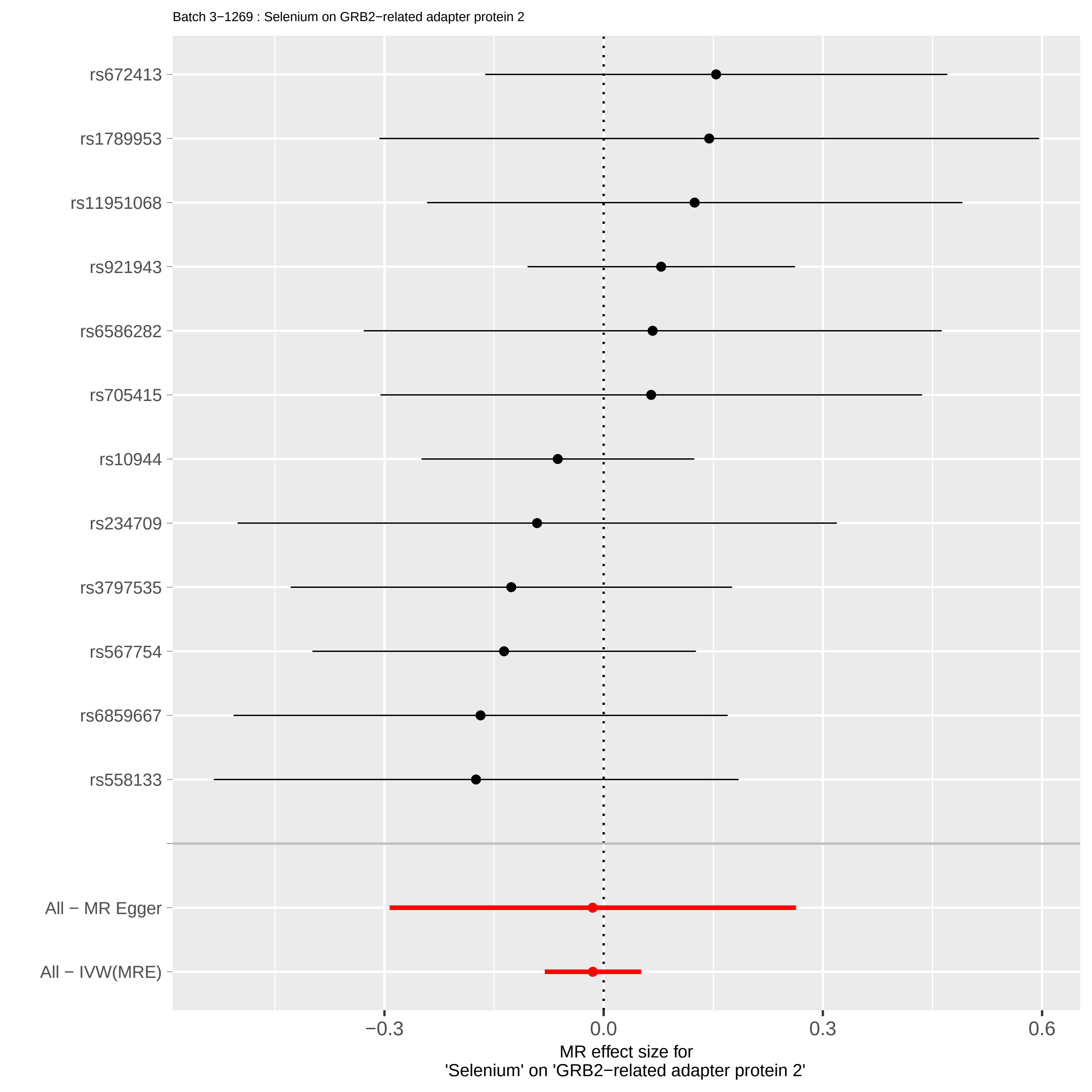

Batch 3−1269 : Selenium on GRB2−related adapter protein 2
rs672413
rs1789953
rs11951068
rs921943
rs6586282
rs705415
rs10944
rs234709
rs3797535
rs567754
rs6859667
rs558133
All − MR Egger
All − IVW(MRE)
−0.3
0.0
0.3
0.6
MR effect size for
'Selenium' on 'GRB2−related adapter protein 2'

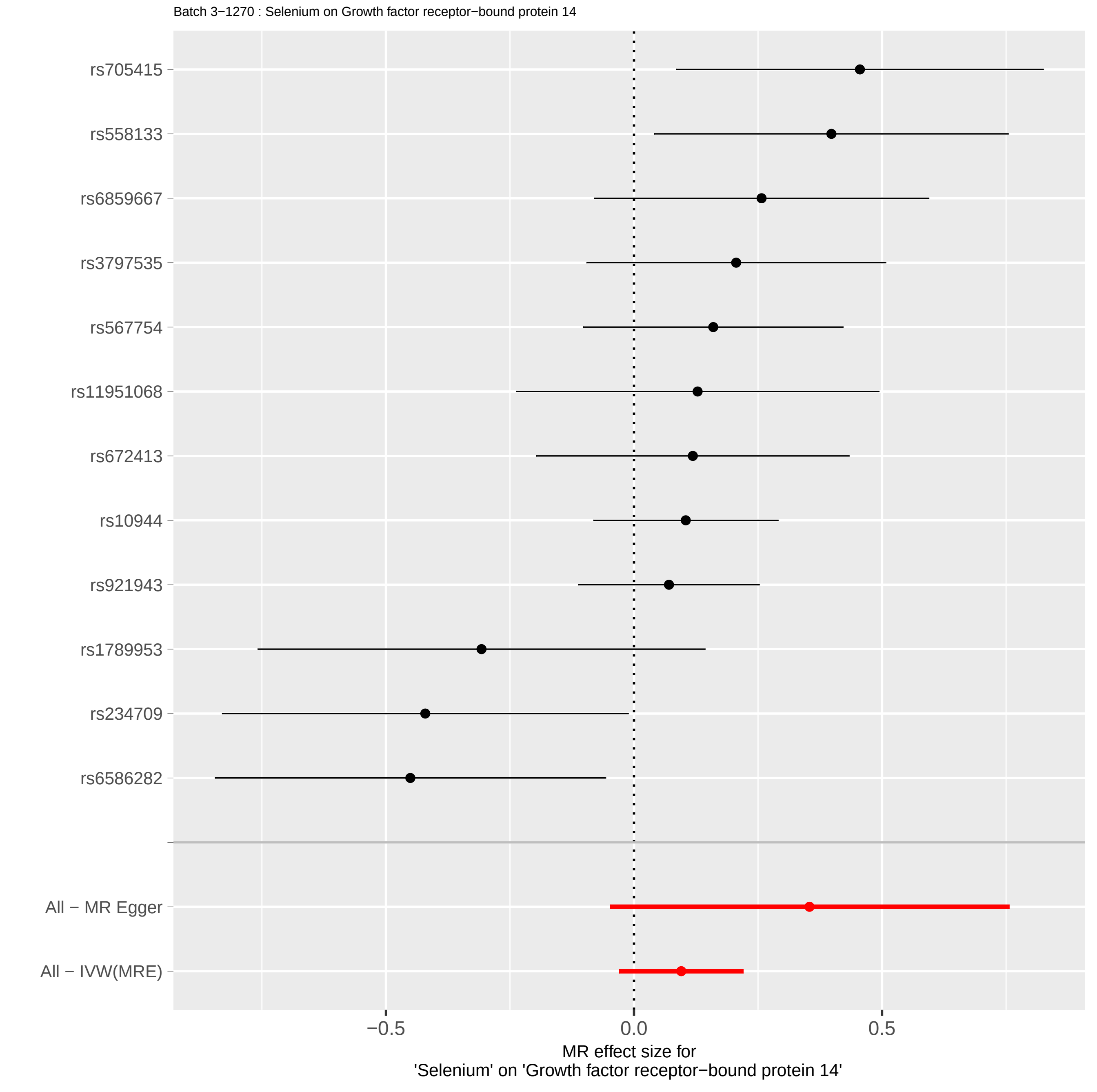

Batch 3−1270 : Selenium on Growth factor receptor−bound protein 14
rs705415
rs558133
rs6859667
rs3797535
rs567754
rs11951068
rs672413
rs10944
rs921943
rs1789953
rs234709
rs6586282
All − MR Egger
All − IVW(MRE)
−0.5
0.0
0.5
MR effect size for
'Selenium' on 'Growth factor receptor−bound protein 14'

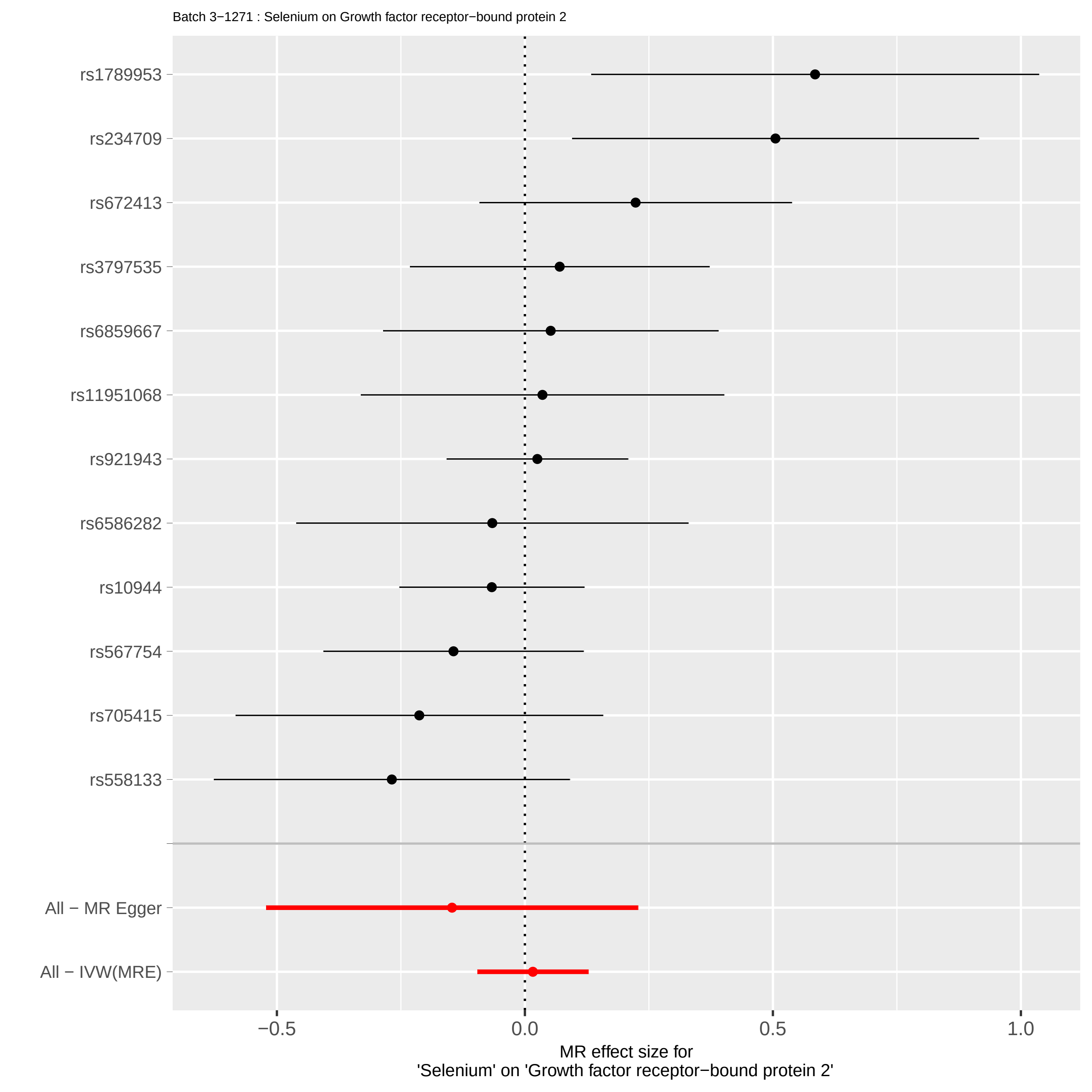

Batch 3−1271 : Selenium on Growth factor receptor−bound protein 2
rs1789953
rs234709
rs672413
rs3797535
rs6859667
rs11951068
rs921943
rs6586282
rs10944
rs567754
rs705415
rs558133
All − MR Egger
All − IVW(MRE)
−0.5
0.0
0.5
1.0
MR effect size for
'Selenium' on 'Growth factor receptor−bound protein 2'

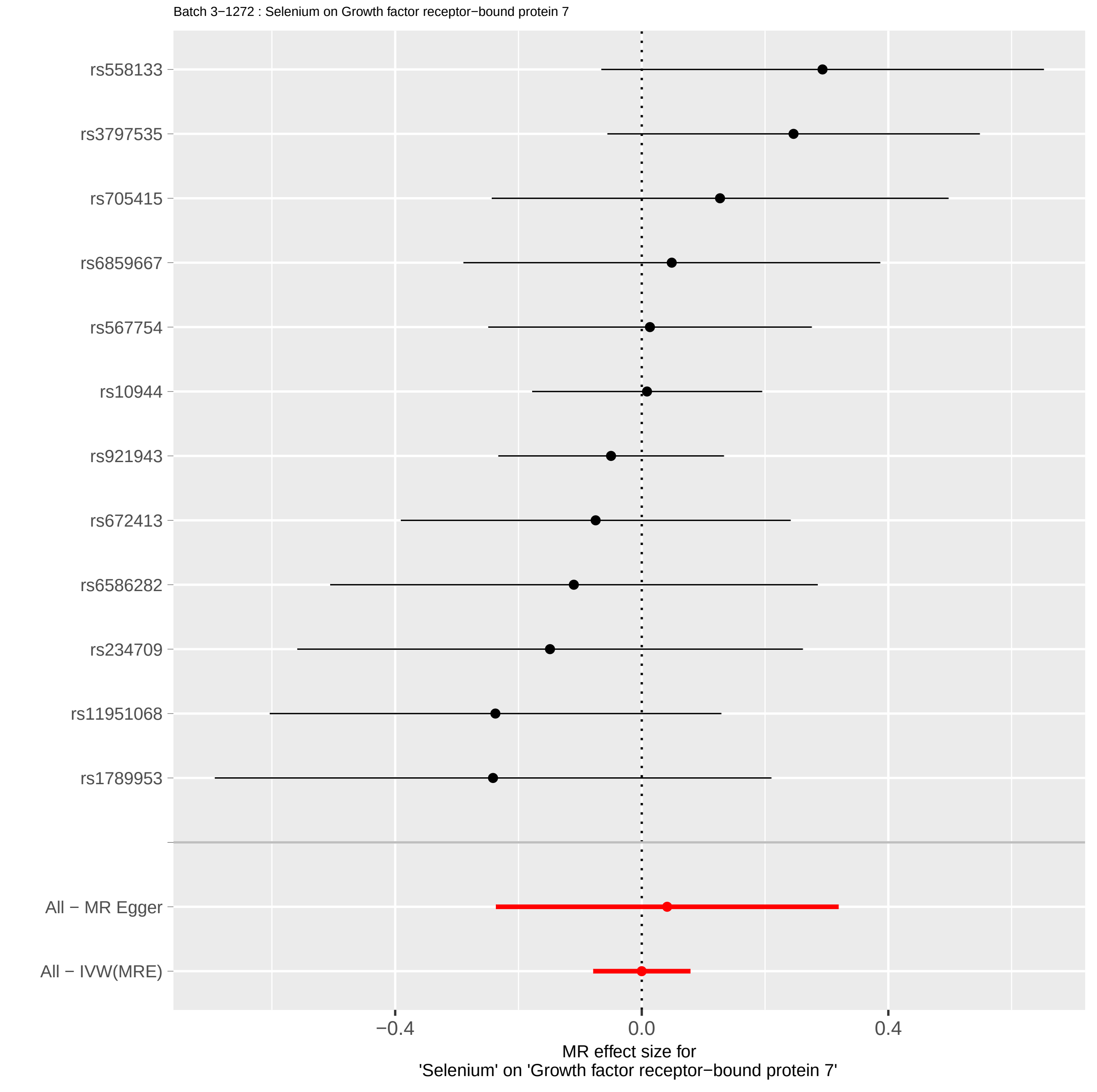

Batch 3−1272 : Selenium on Growth factor receptor−bound protein 7
rs558133
rs3797535
rs705415
rs6859667
rs567754
rs10944
rs921943
rs672413
rs6586282
rs234709
rs11951068
rs1789953
All − MR Egger
All − IVW(MRE)
−0.4
0.0
0.4
MR effect size for
'Selenium' on 'Growth factor receptor−bound protein 7'

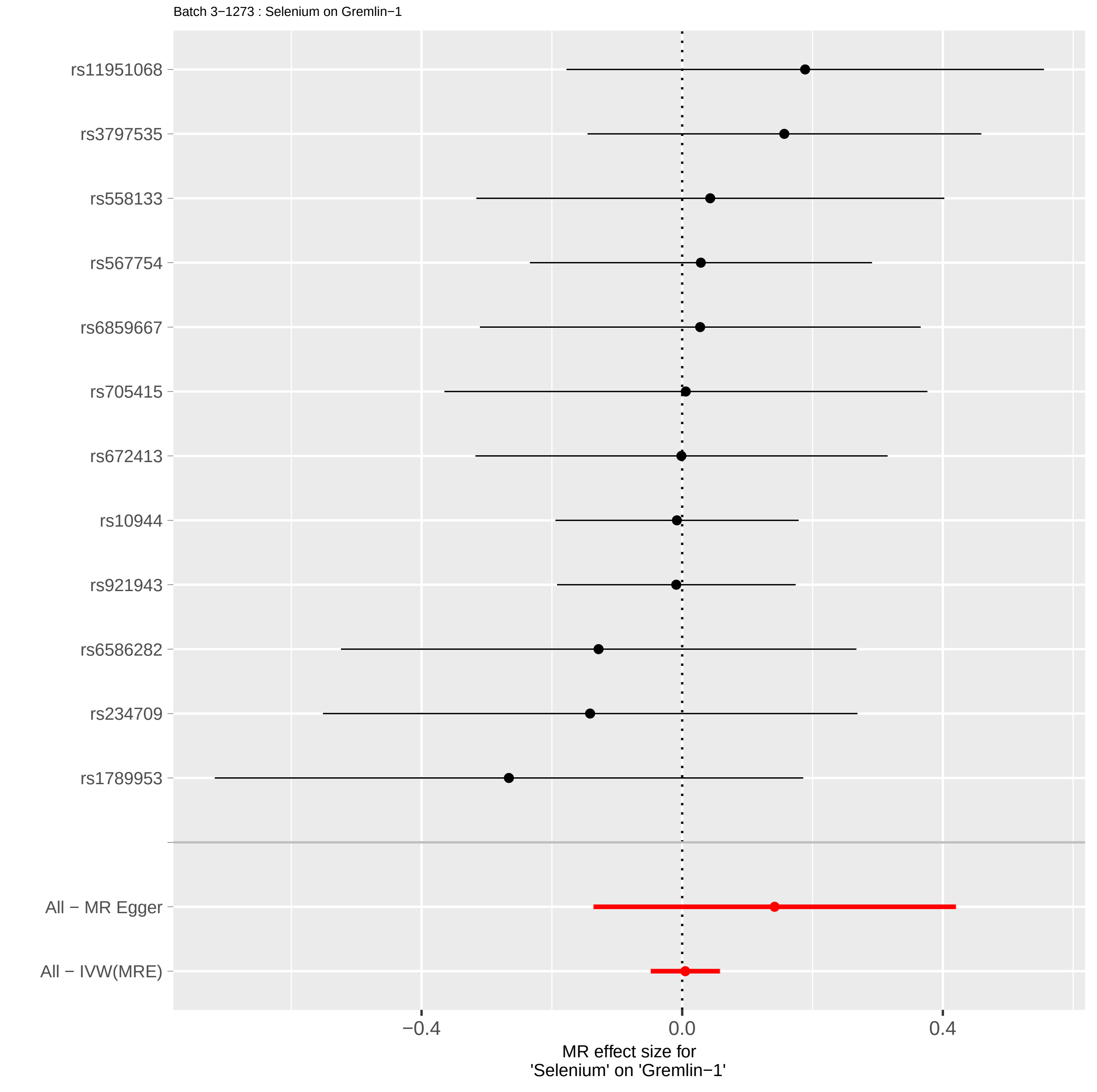

Batch 3−1273 : Selenium on Gremlin−1
rs11951068
rs3797535
rs558133
rs567754
rs6859667
rs705415
rs672413
rs10944
rs921943
rs6586282
rs234709
rs1789953
All − MR Egger
All − IVW(MRE)
−0.4
0.0
0.4
MR effect size for
'Selenium' on 'Gremlin−1'

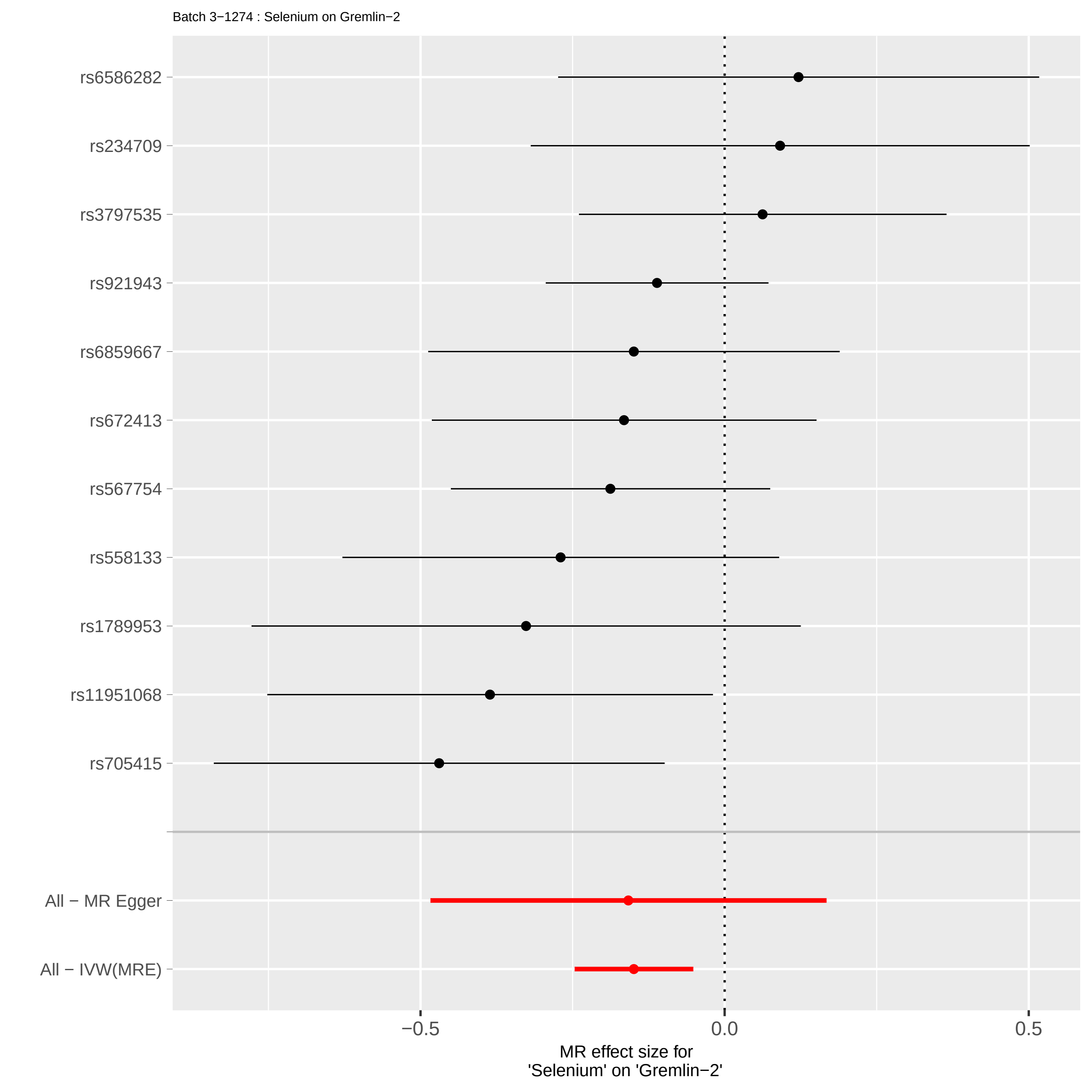
Batch 3−1274 : Selenium on Gremlin−2
rs6586282
rs234709
rs3797535
rs921943
rs6859667
rs672413
rs567754
rs558133
rs1789953
rs11951068
rs705415
All − MR Egger
All − IVW(MRE)
−0.5
0.0
0.5
MR effect size for
'Selenium' on 'Gremlin−2'

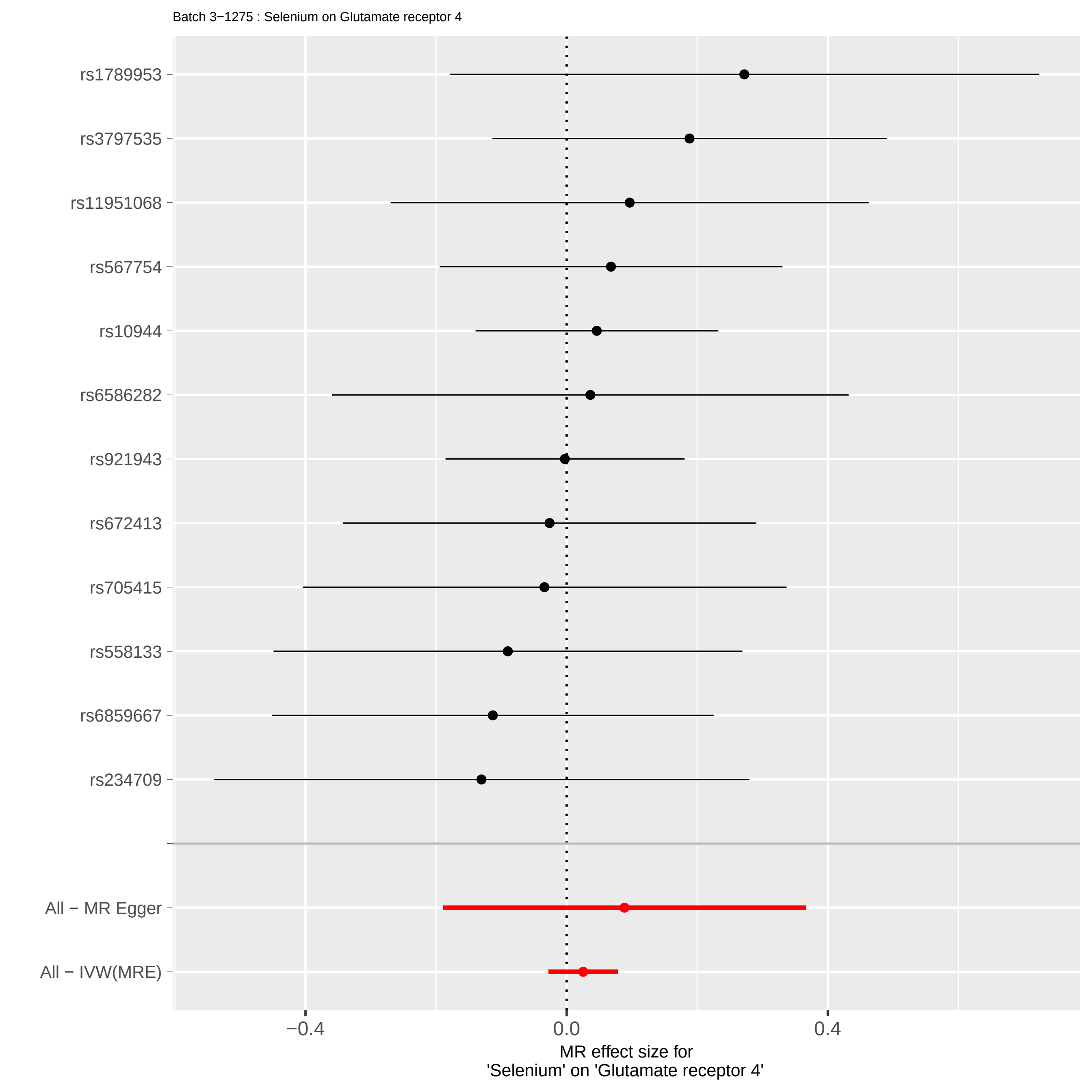

Batch 3−1275 : Selenium on Glutamate receptor 4
rs1789953
rs3797535
rs11951068
rs567754
rs10944
rs6586282
rs921943
rs672413
rs705415
rs558133
rs6859667
rs234709
All − MR Egger
All − IVW(MRE)
−0.4
0.0
0.4
MR effect size for
'Selenium' on 'Glutamate receptor 4'

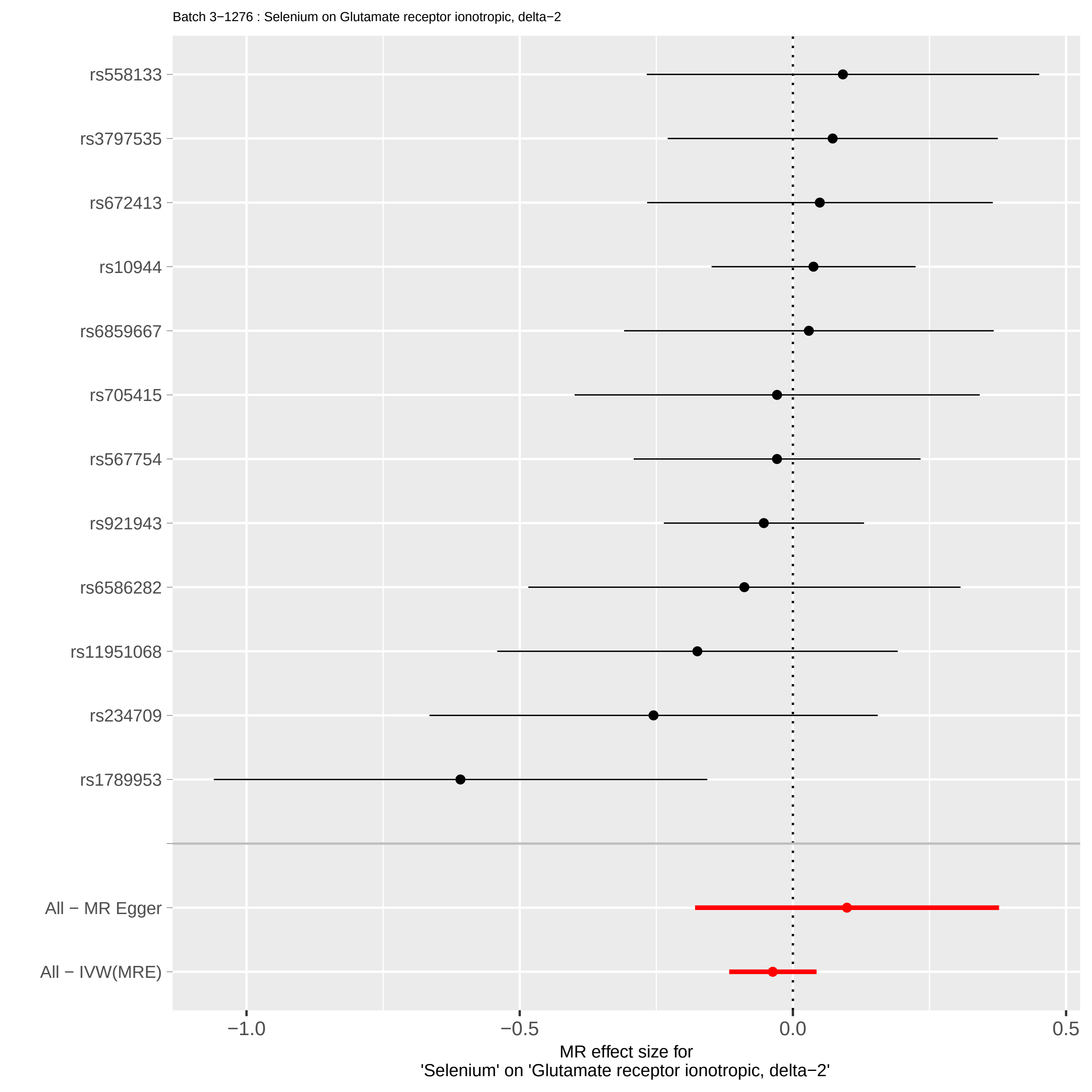

Batch 3−1276 : Selenium on Glutamate receptor ionotropic, delta−2
rs558133
rs3797535
rs672413
rs10944
rs6859667
rs705415
rs567754
rs921943
rs6586282
rs11951068
rs234709
rs1789953
All − MR Egger
All − IVW(MRE)
−1.0
−0.5
0.0
0.5
MR effect size for
'Selenium' on 'Glutamate receptor ionotropic, delta−2'

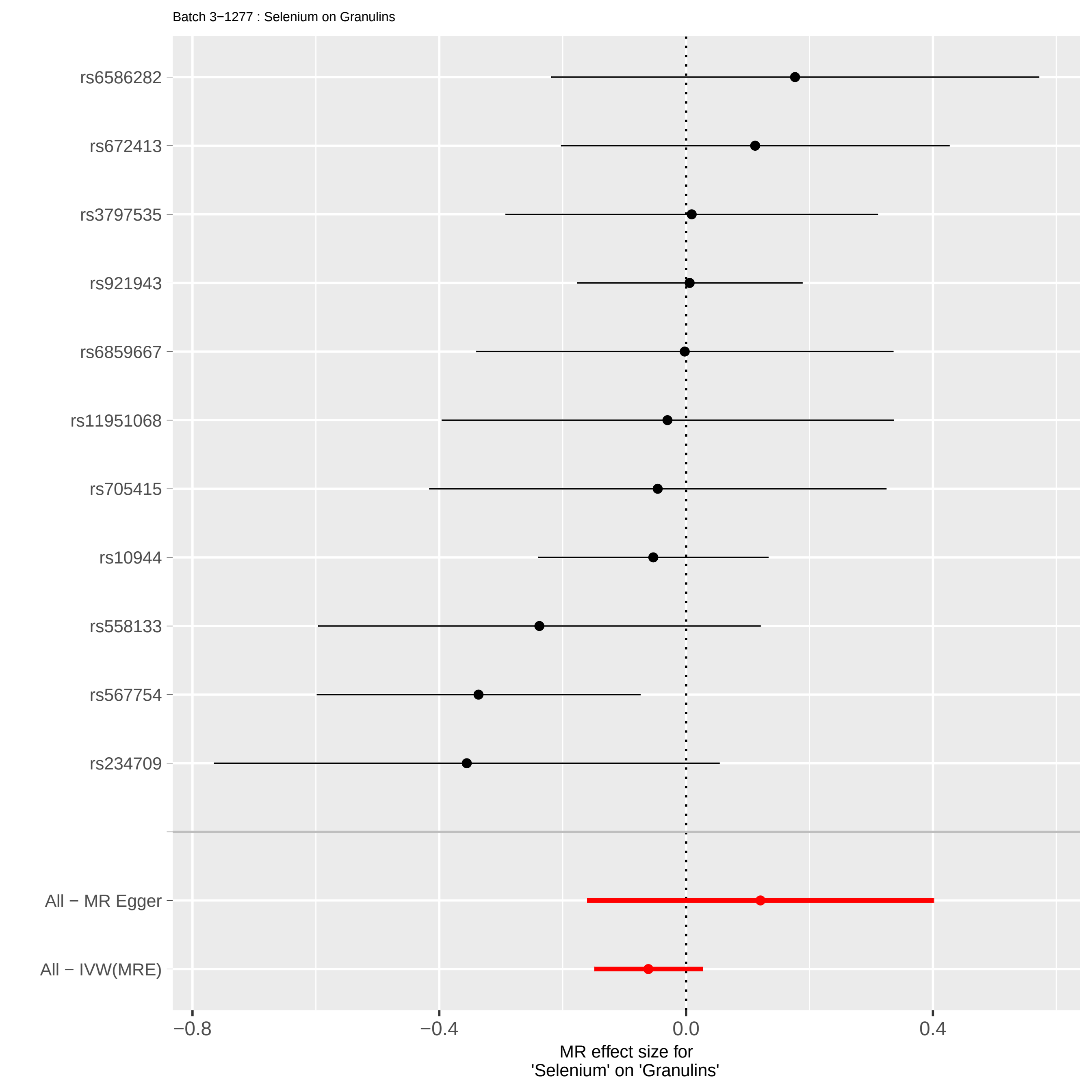

Batch 3−1277 : Selenium on Granulins
rs6586282
rs672413
rs3797535
rs921943
rs6859667
rs11951068
rs705415
rs10944
rs558133
rs567754
rs234709
All − MR Egger
All − IVW(MRE)
−0.8
−0.4
0.0
0.4
MR effect size for
'Selenium' on 'Granulins'

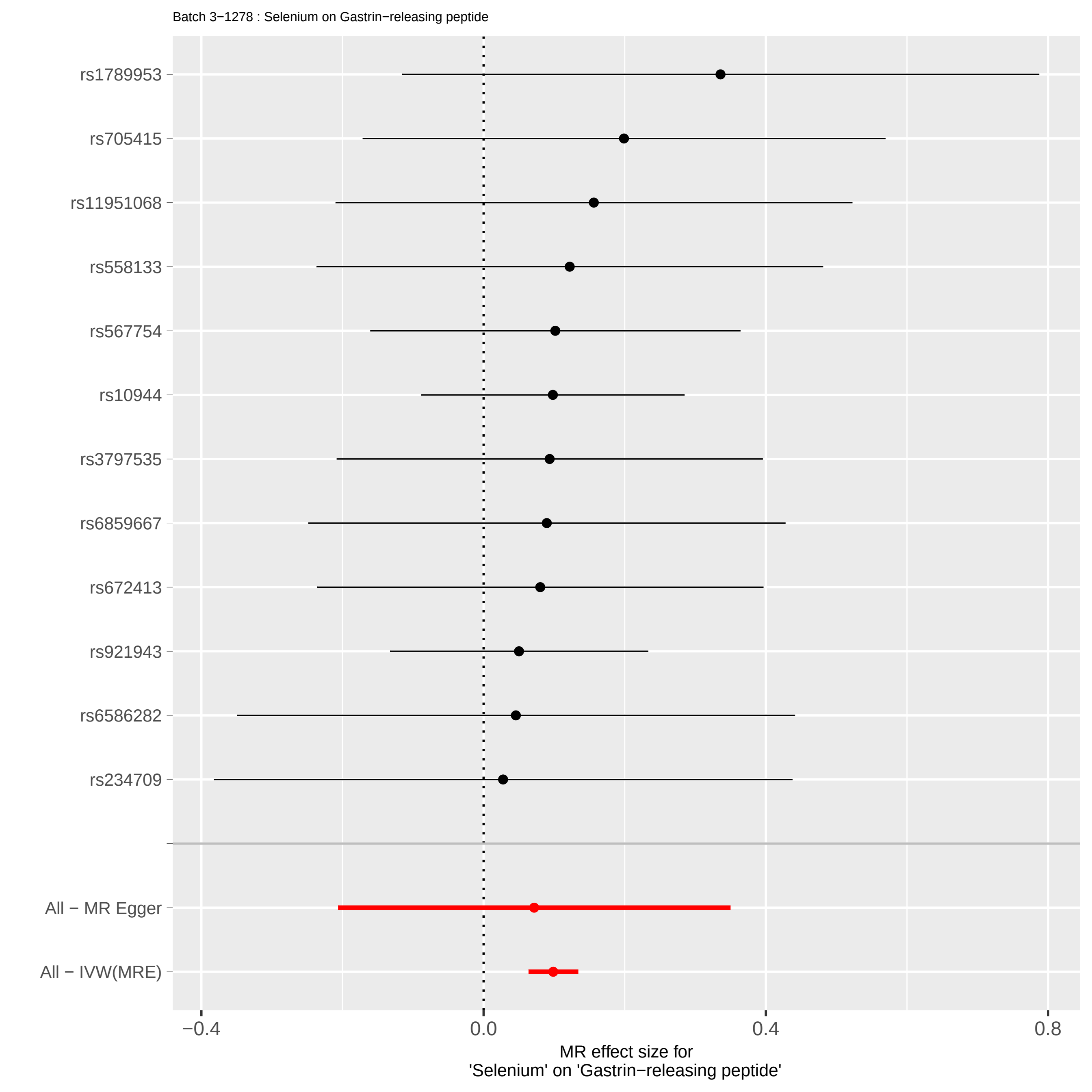

Batch 3−1278 : Selenium on Gastrin−releasing peptide
rs1789953
rs705415
rs11951068
rs558133
rs567754
rs10944
rs3797535
rs6859667
rs672413
rs921943
rs6586282
rs234709
All − MR Egger
All − IVW(MRE)
−0.4
0.0
0.4
0.8
MR effect size for
'Selenium' on 'Gastrin−releasing peptide'

Batch 3−1279 : Selenium on Gastrin−releasing peptide

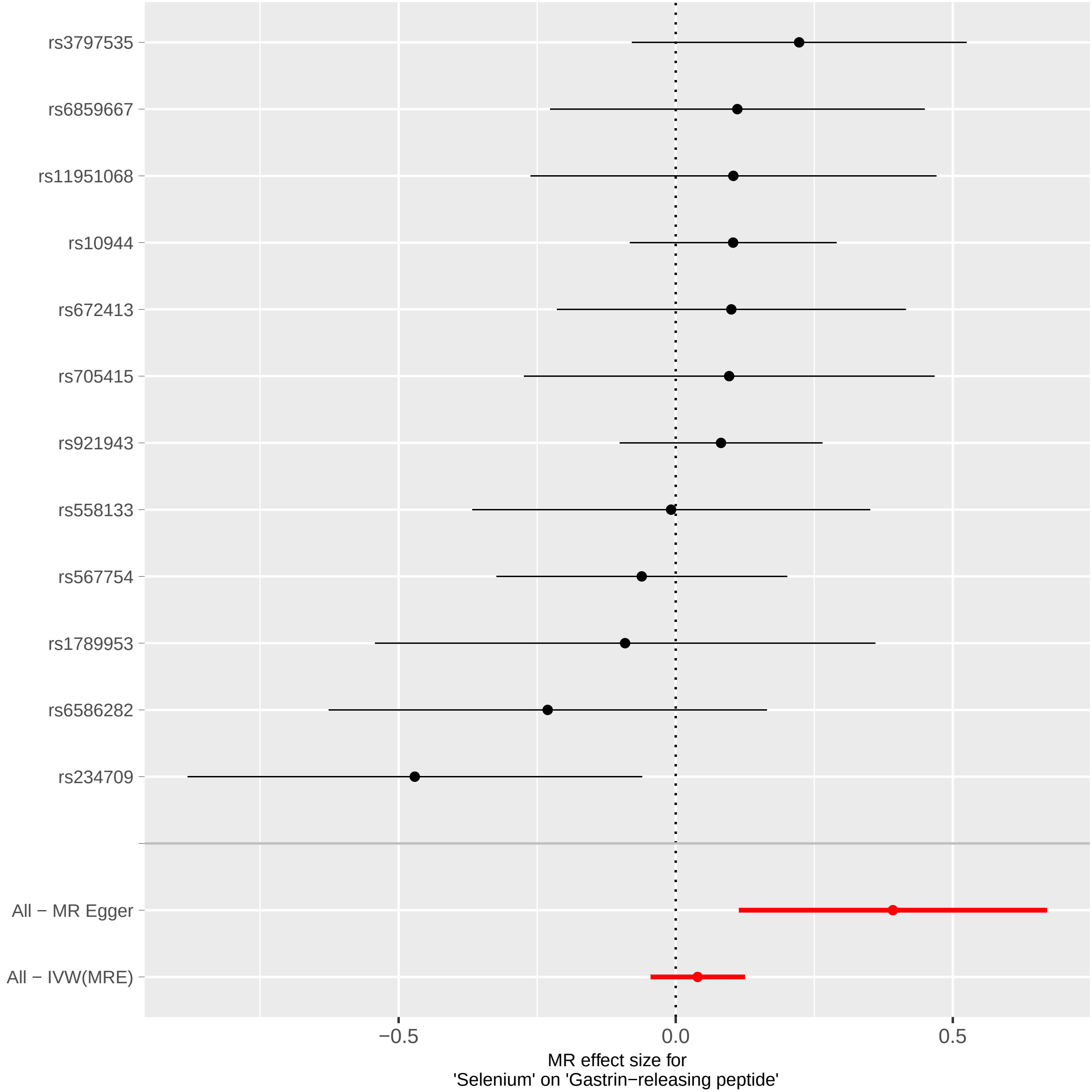

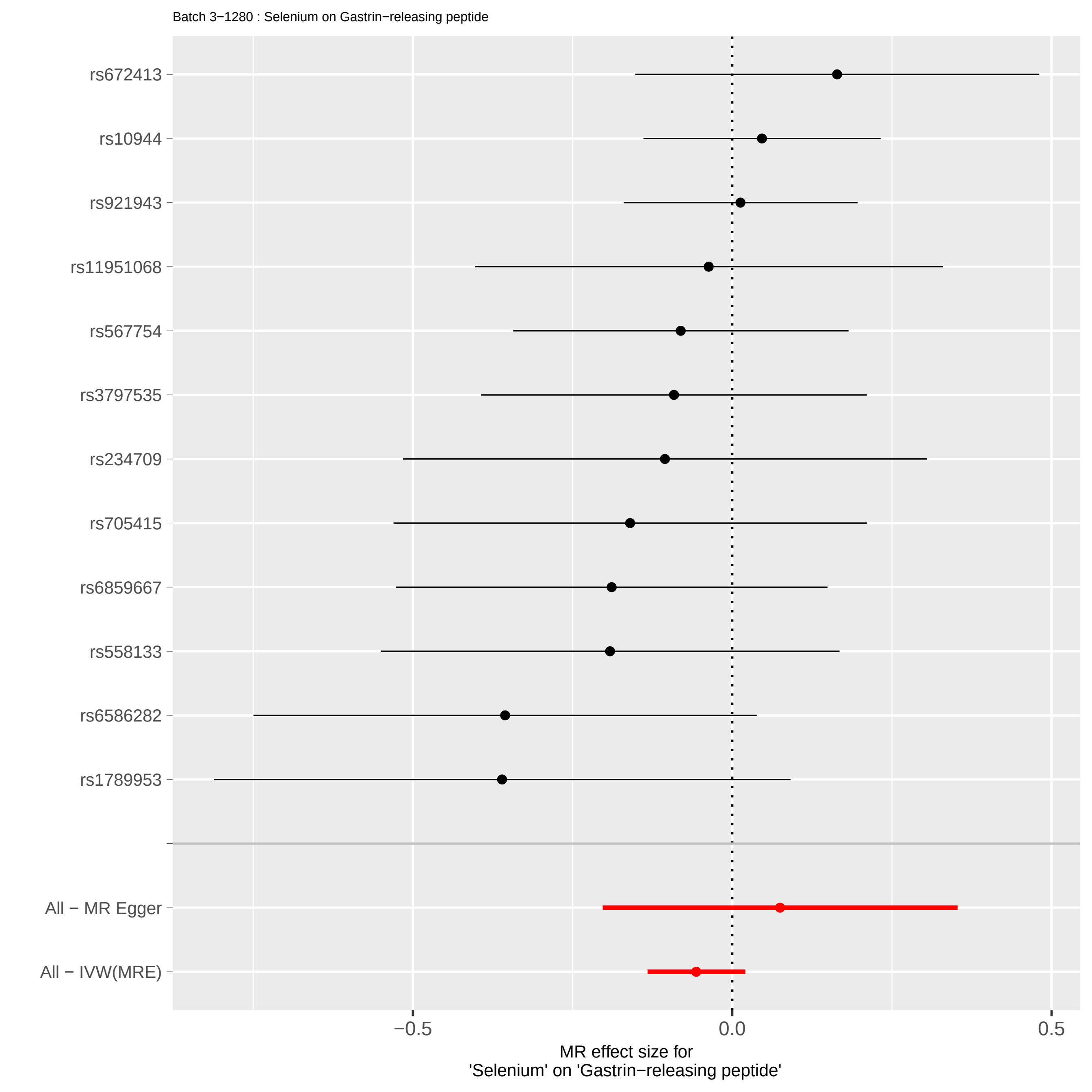
Batch 3−1280 : Selenium on Gastrin−releasing peptide
rs672413
rs10944
rs921943
rs11951068
rs567754
rs3797535
rs234709
rs705415
rs6859667
rs558133
rs6586282
rs1789953
All − MR Egger
All − IVW(MRE)
−0.5
0.0
0.5
MR effect size for
'Selenium' on 'Gastrin−releasing peptide'

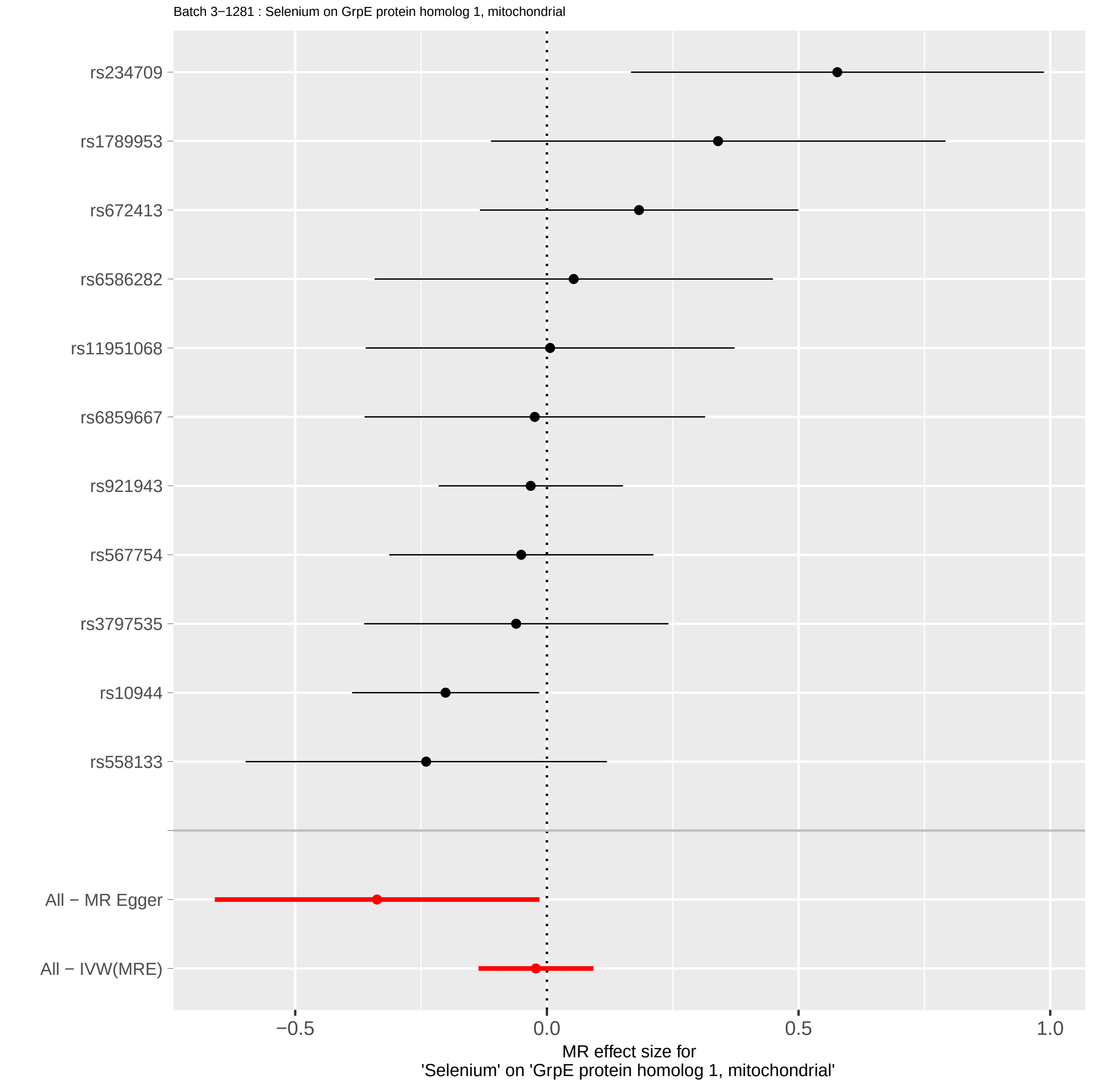

Batch 3−1281 : Selenium on GrpE protein homolog 1, mitochondrial
rs234709
rs1789953
rs672413
rs6586282
rs11951068
rs6859667
rs921943
rs567754
rs3797535
rs10944
rs558133
All − MR Egger
All − IVW(MRE)
−0.5
0.0
0.5
1.0
MR effect size for
'Selenium' on 'GrpE protein homolog 1, mitochondrial'

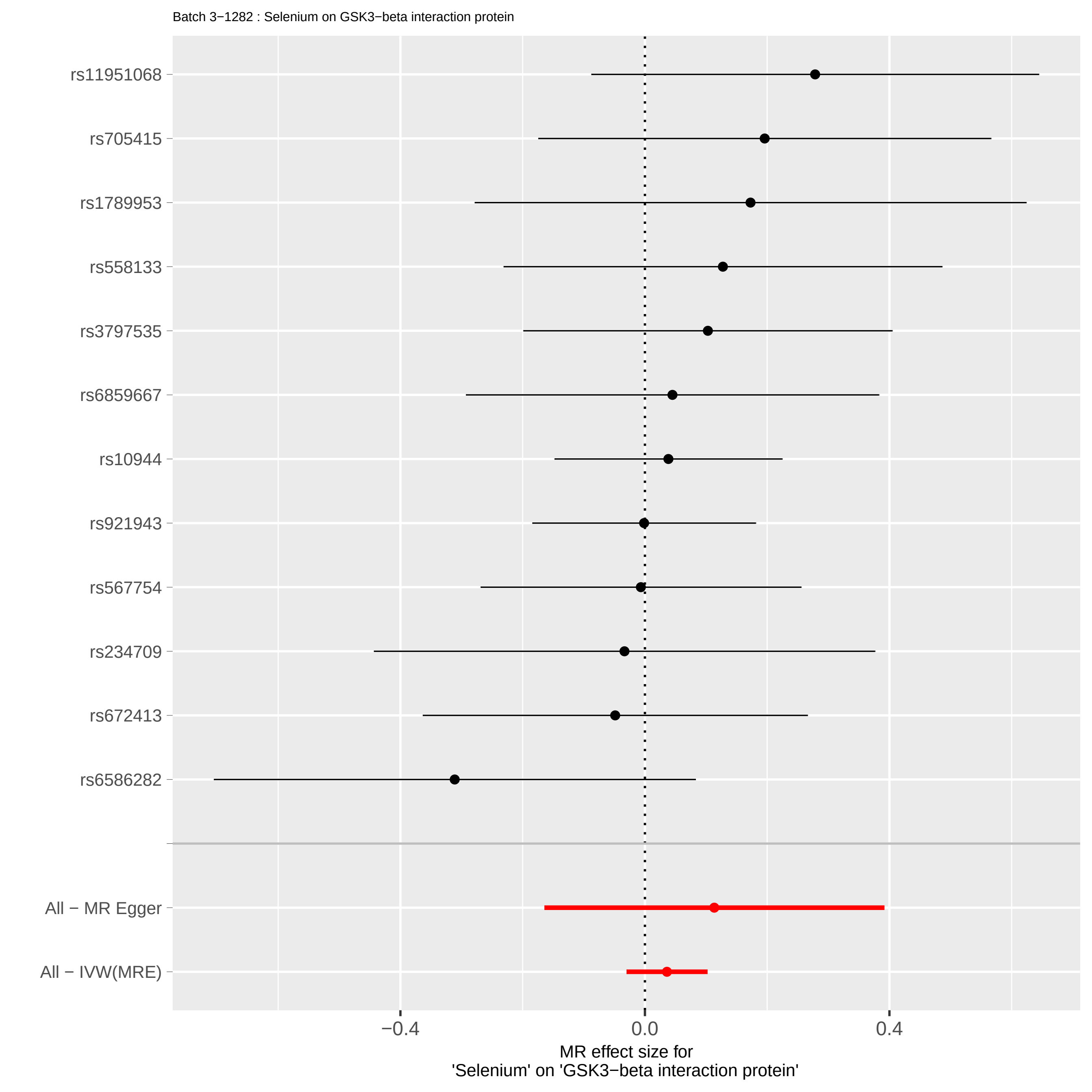

Batch 3−1282 : Selenium on GSK3−beta interaction protein
rs11951068
rs705415
rs1789953
rs558133
rs3797535
rs6859667
rs10944
rs921943
rs567754
rs234709
rs672413
rs6586282
All − MR Egger
All − IVW(MRE)
−0.4
0.0
0.4
MR effect size for
'Selenium' on 'GSK3−beta interaction protein'

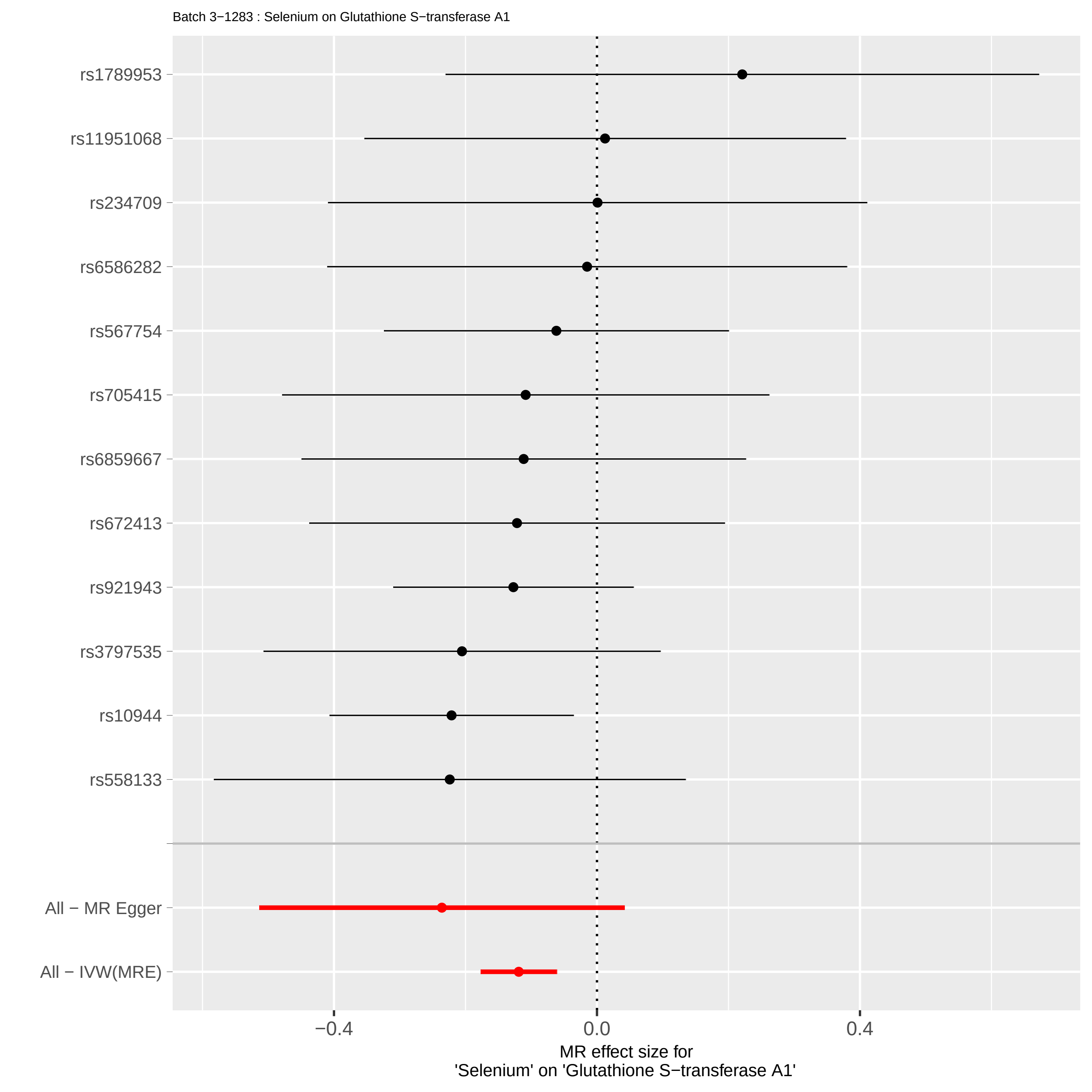

Batch 3−1283 : Selenium on Glutathione S−transferase A1
rs1789953
rs11951068
rs234709
rs6586282
rs567754
rs705415
rs6859667
rs672413
rs921943
rs3797535
rs10944
rs558133
All − MR Egger
All − IVW(MRE)
−0.4
0.0
0.4
MR effect size for
'Selenium' on 'Glutathione S−transferase A1'

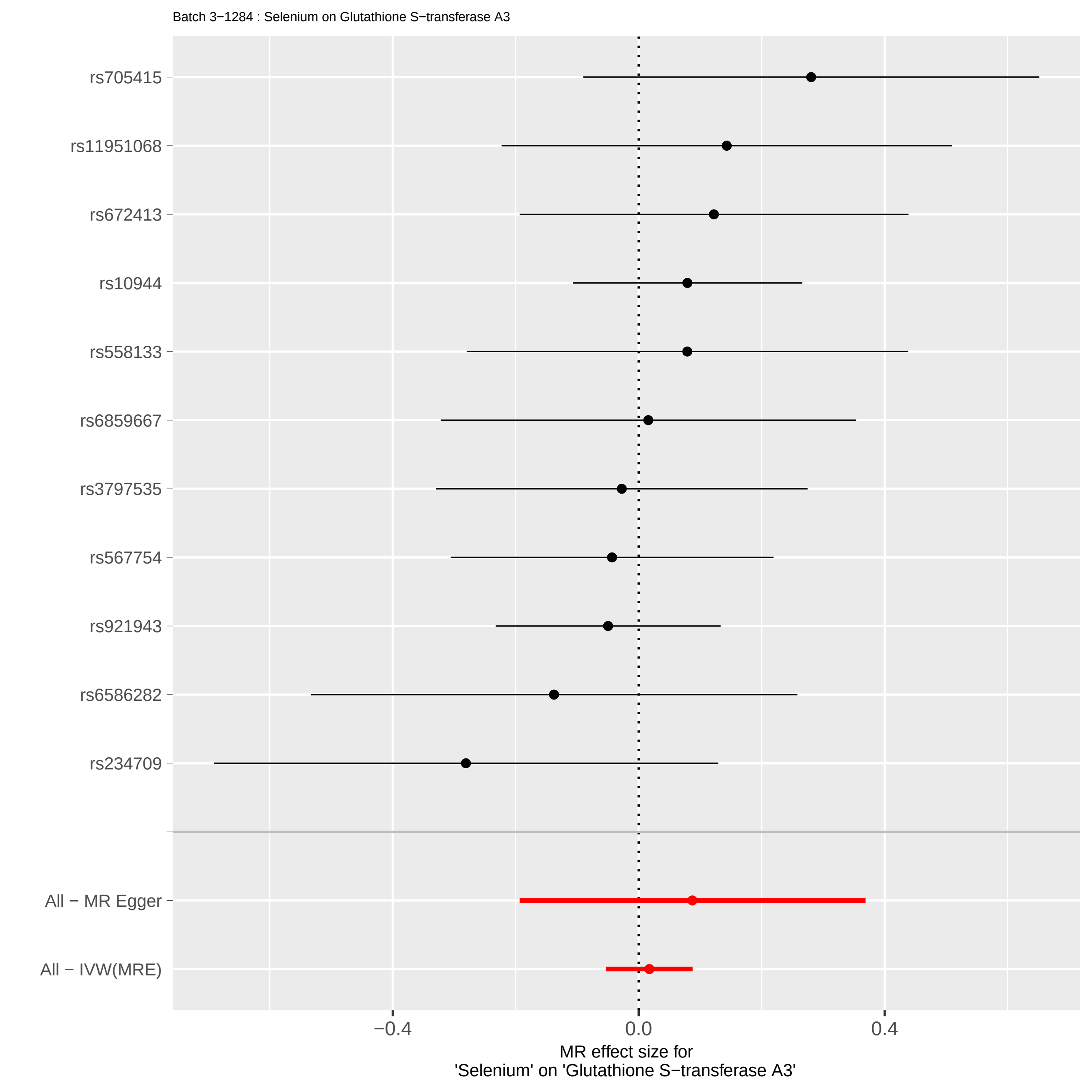

Batch 3−1284 : Selenium on Glutathione S−transferase A3
rs705415
rs11951068
rs672413
rs10944
rs558133
rs6859667
rs3797535
rs567754
rs921943
rs6586282
rs234709
All − MR Egger
All − IVW(MRE)
−0.4
0.0
0.4
MR effect size for
'Selenium' on 'Glutathione S−transferase A3'

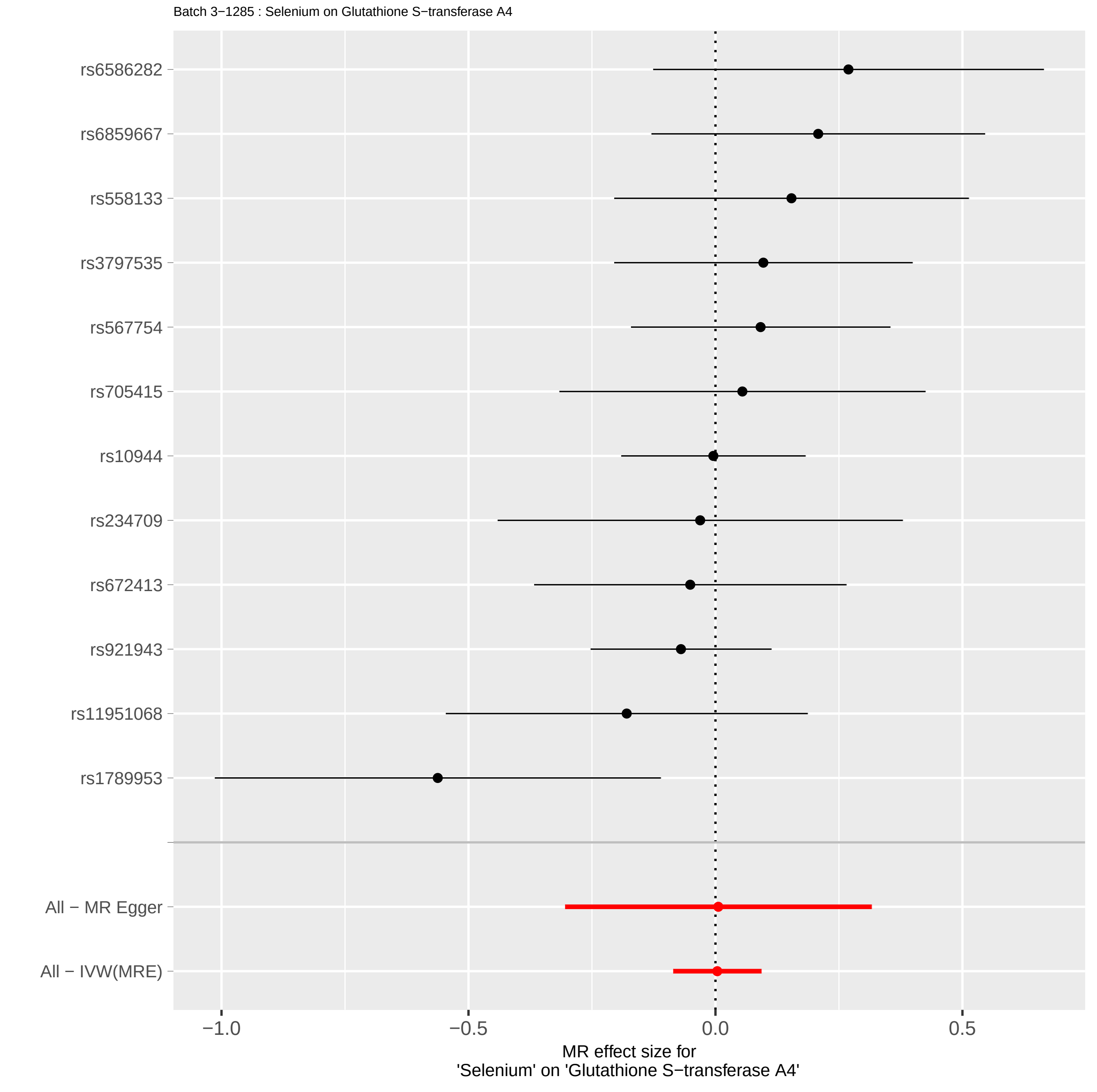

Batch 3−1285 : Selenium on Glutathione S−transferase A4
rs6586282
rs6859667
rs558133
rs3797535
rs567754
rs705415
rs10944
rs234709
rs672413
rs921943
rs11951068
rs1789953
All − MR Egger
All − IVW(MRE)
−1.0
−0.5
0.0
0.5
MR effect size for
'Selenium' on 'Glutathione S−transferase A4'

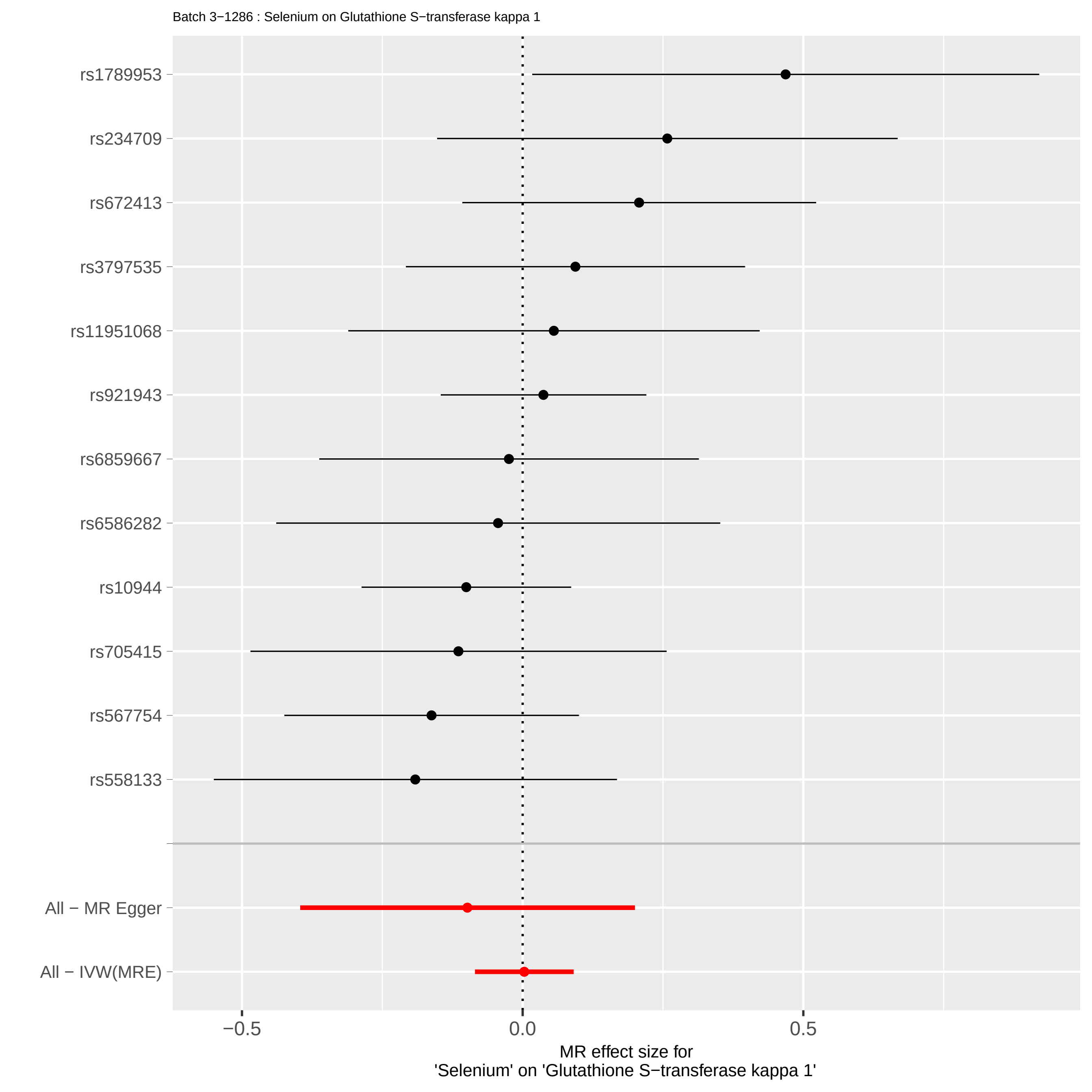
Batch 3−1286 : Selenium on Glutathione S−transferase kappa 1
rs1789953
rs234709
rs672413
rs3797535
rs11951068
rs921943
rs6859667
rs6586282
rs10944
rs705415
rs567754
rs558133
All − MR Egger
All − IVW(MRE)
−0.5
0.0
0.5
MR effect size for
'Selenium' on 'Glutathione S−transferase kappa 1'

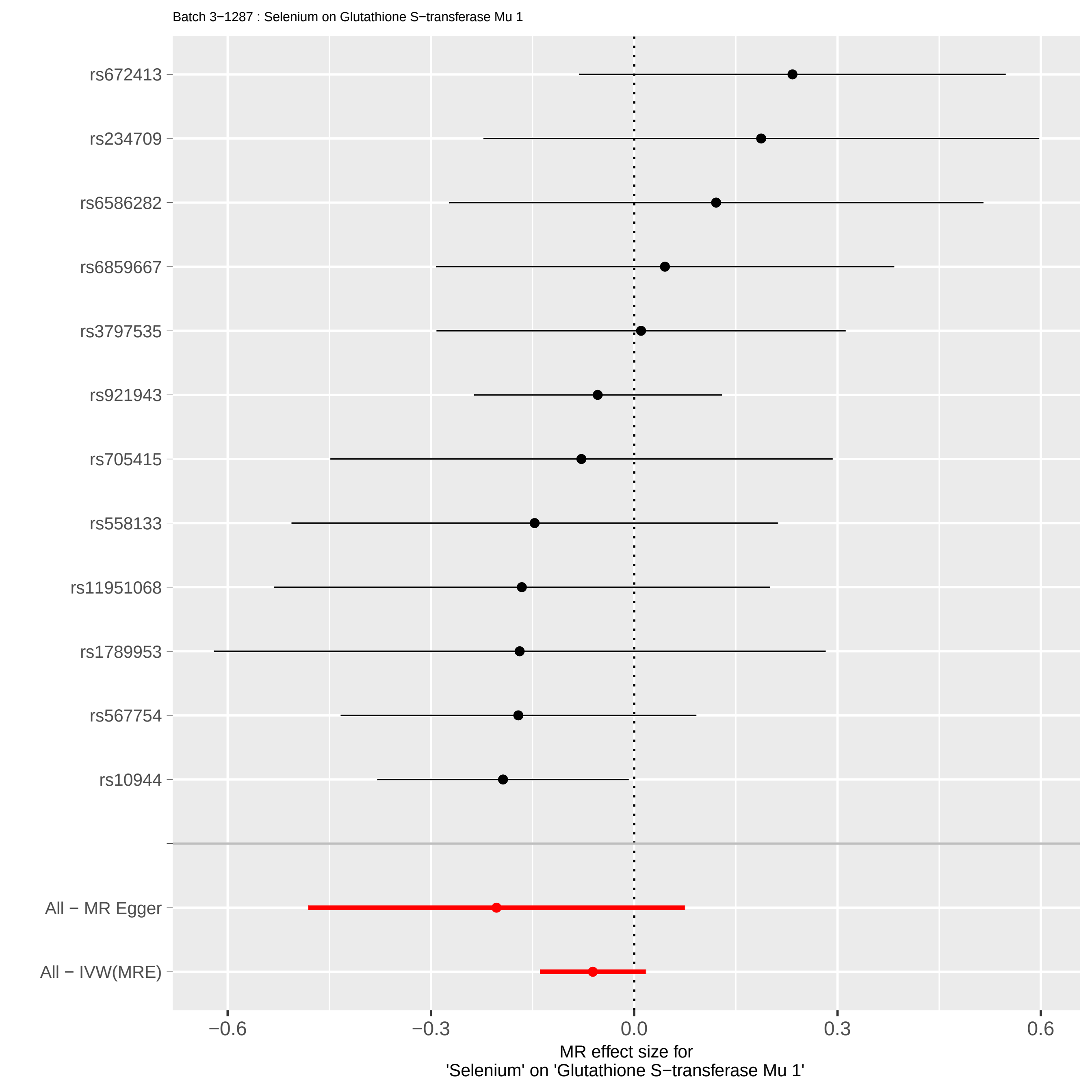

Batch 3−1287 : Selenium on Glutathione S−transferase Mu 1
rs672413
rs234709
rs6586282
rs6859667
rs3797535
rs921943
rs705415
rs558133
rs11951068
rs1789953
rs567754
rs10944
All − MR Egger
All − IVW(MRE)
−0.6
−0.3
0.0
0.3
0.6
MR effect size for
'Selenium' on 'Glutathione S−transferase Mu 1'

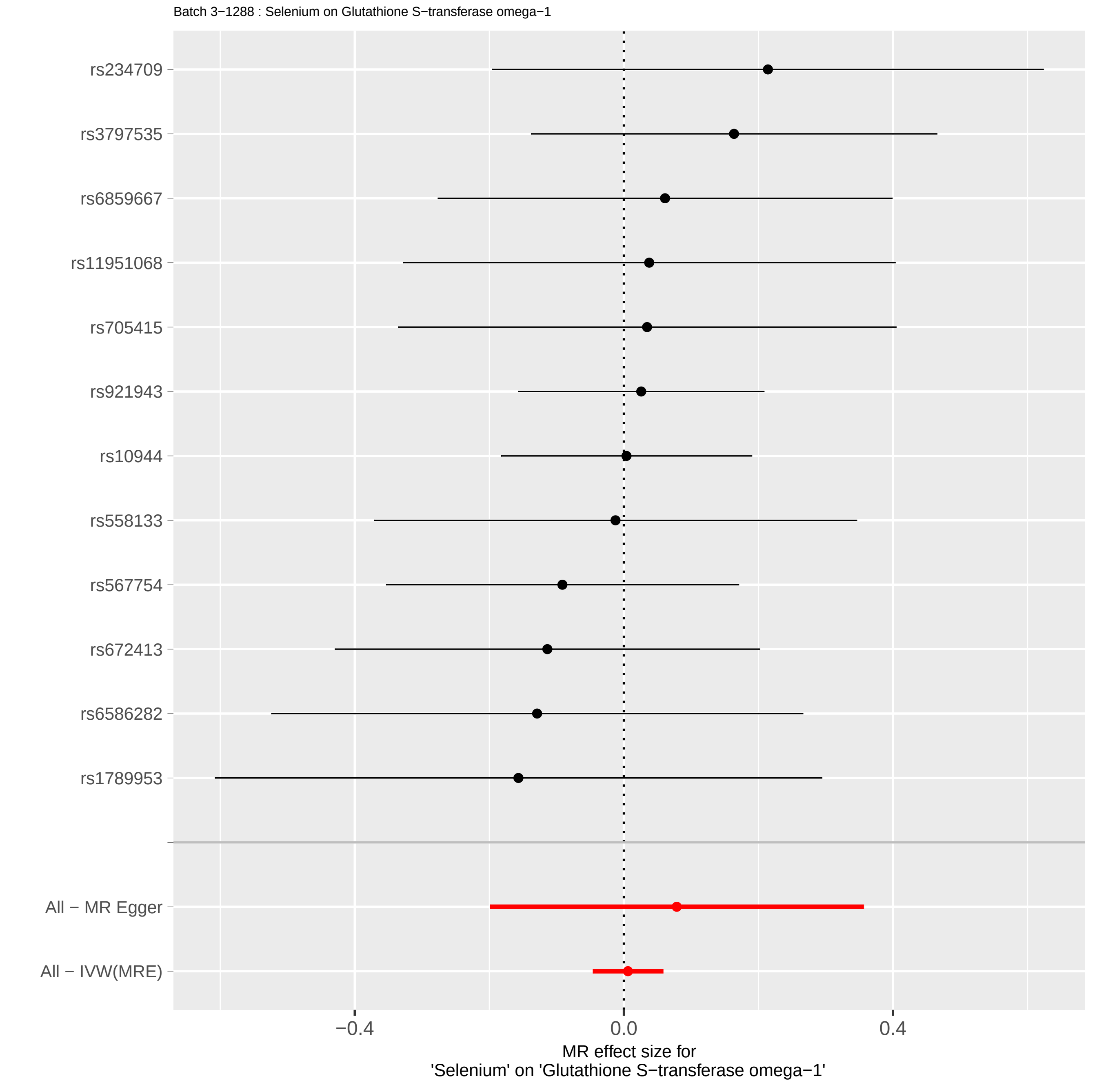

Batch 3−1288 : Selenium on Glutathione S−transferase omega−1
rs234709
rs3797535
rs6859667
rs11951068
rs705415
rs921943
rs10944
rs558133
rs567754
rs672413
rs6586282
rs1789953
All − MR Egger
All − IVW(MRE)
−0.4
0.0
0.4
MR effect size for
'Selenium' on 'Glutathione S−transferase omega−1'

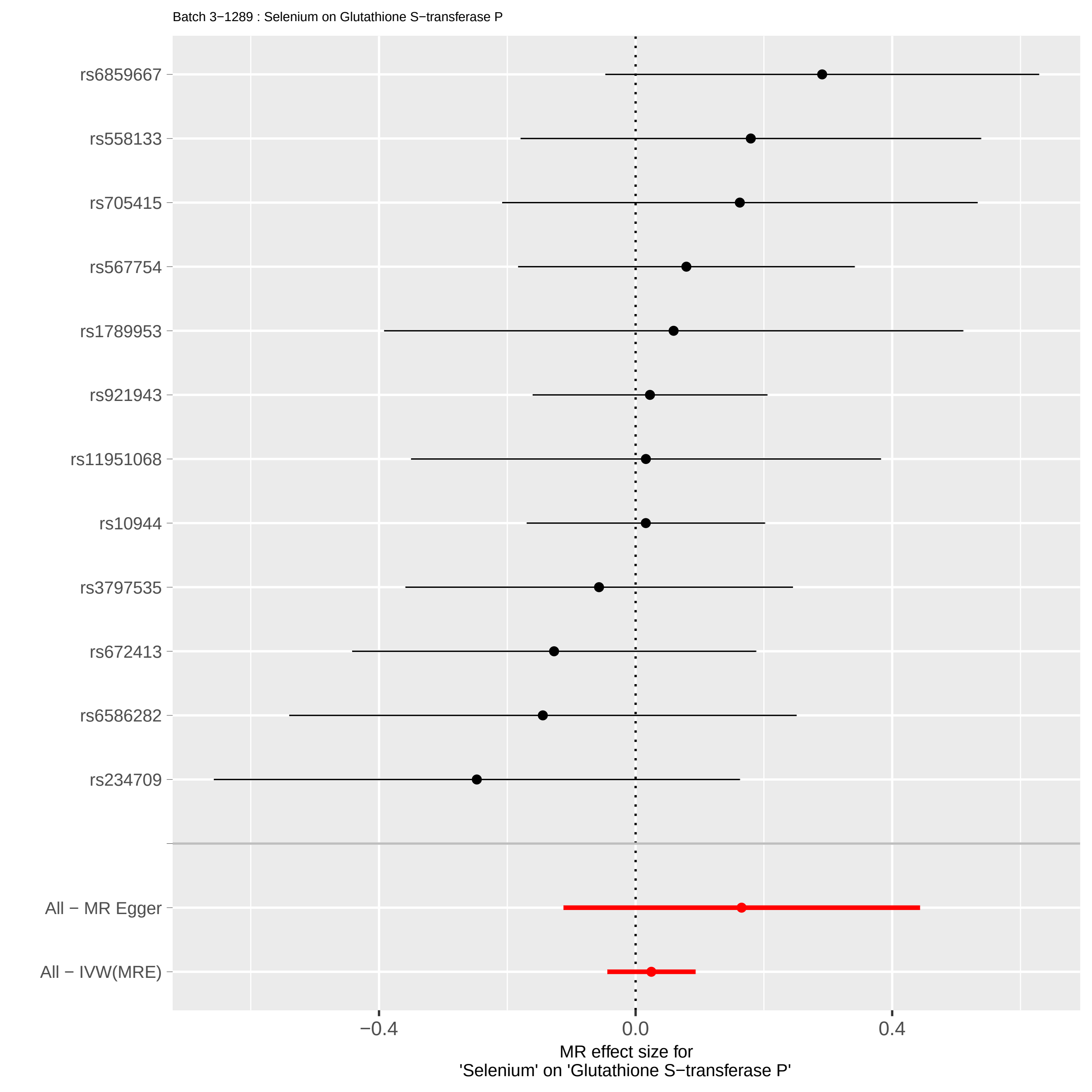
Batch 3−1289 : Selenium on Glutathione S−transferase P
rs6859667
rs558133
rs705415
rs567754
rs1789953
rs921943
rs11951068
rs10944
rs3797535
rs672413
rs6586282
rs234709
All − MR Egger
All − IVW(MRE)
−0.4
0.0
0.4
MR effect size for
'Selenium' on 'Glutathione S−transferase P'

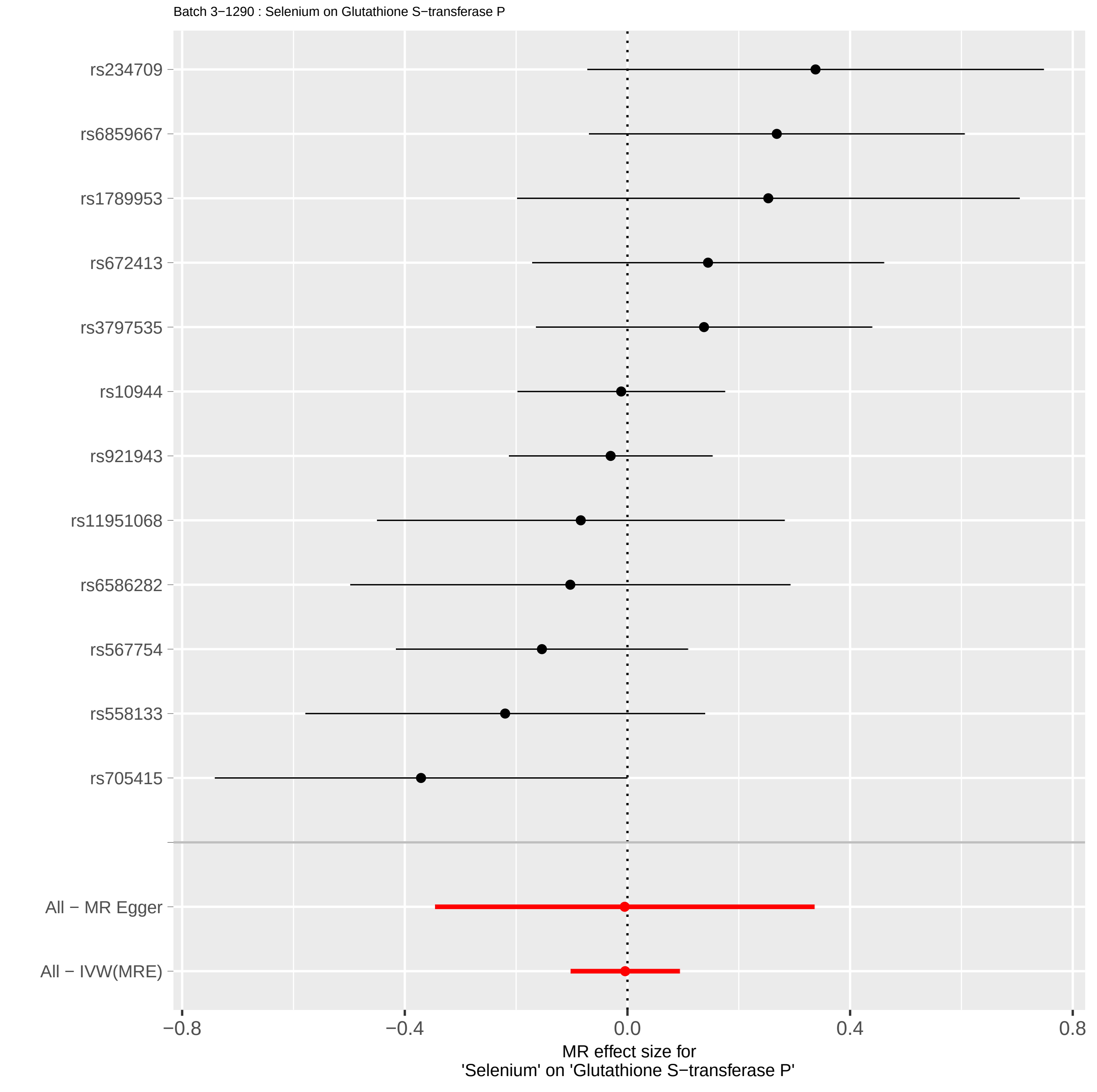

Batch 3−1290 : Selenium on Glutathione S−transferase P
rs234709
rs6859667
rs1789953
rs672413
rs3797535
rs10944
rs921943
rs11951068
rs6586282
rs567754
rs558133
rs705415
All − MR Egger
All − IVW(MRE)
−0.8
−0.4
0.0
0.4
0.8
MR effect size for
'Selenium' on 'Glutathione S−transferase P'

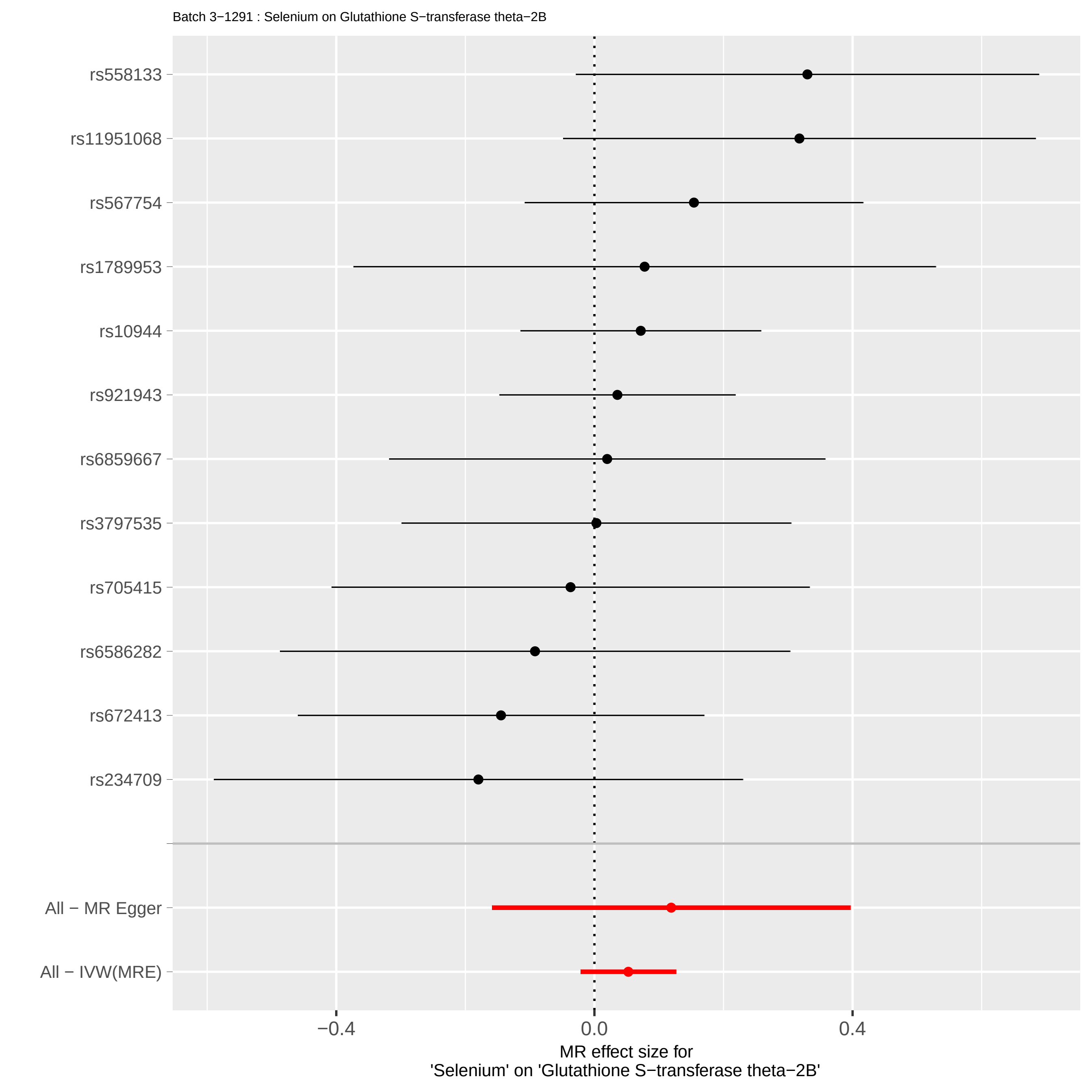

Batch 3−1291 : Selenium on Glutathione S−transferase theta−2B
rs558133
rs11951068
rs567754
rs1789953
rs10944
rs921943
rs6859667
rs3797535
rs705415
rs6586282
rs672413
rs234709
All − MR Egger
All − IVW(MRE)
−0.4
0.0
0.4
MR effect size for
'Selenium' on 'Glutathione S−transferase theta−2B'

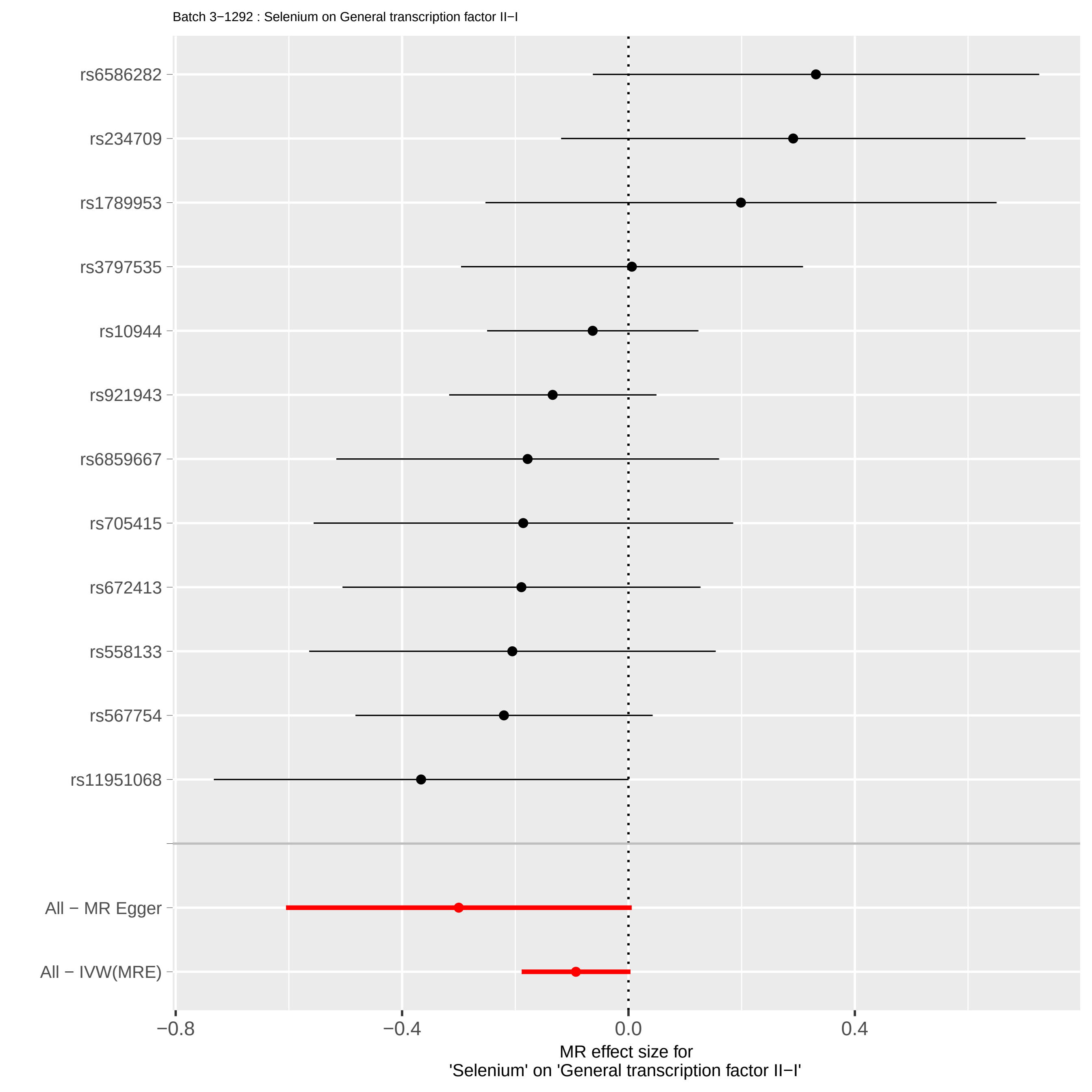

Batch 3−1292 : Selenium on General transcription factor II−I
rs6586282
rs234709
rs1789953
rs3797535
rs10944
rs921943
rs6859667
rs705415
rs672413
rs558133
rs567754
rs11951068
All − MR Egger
All − IVW(MRE)
−0.8
−0.4
0.0
0.4
MR effect size for
'Selenium' on 'General transcription factor II−I'

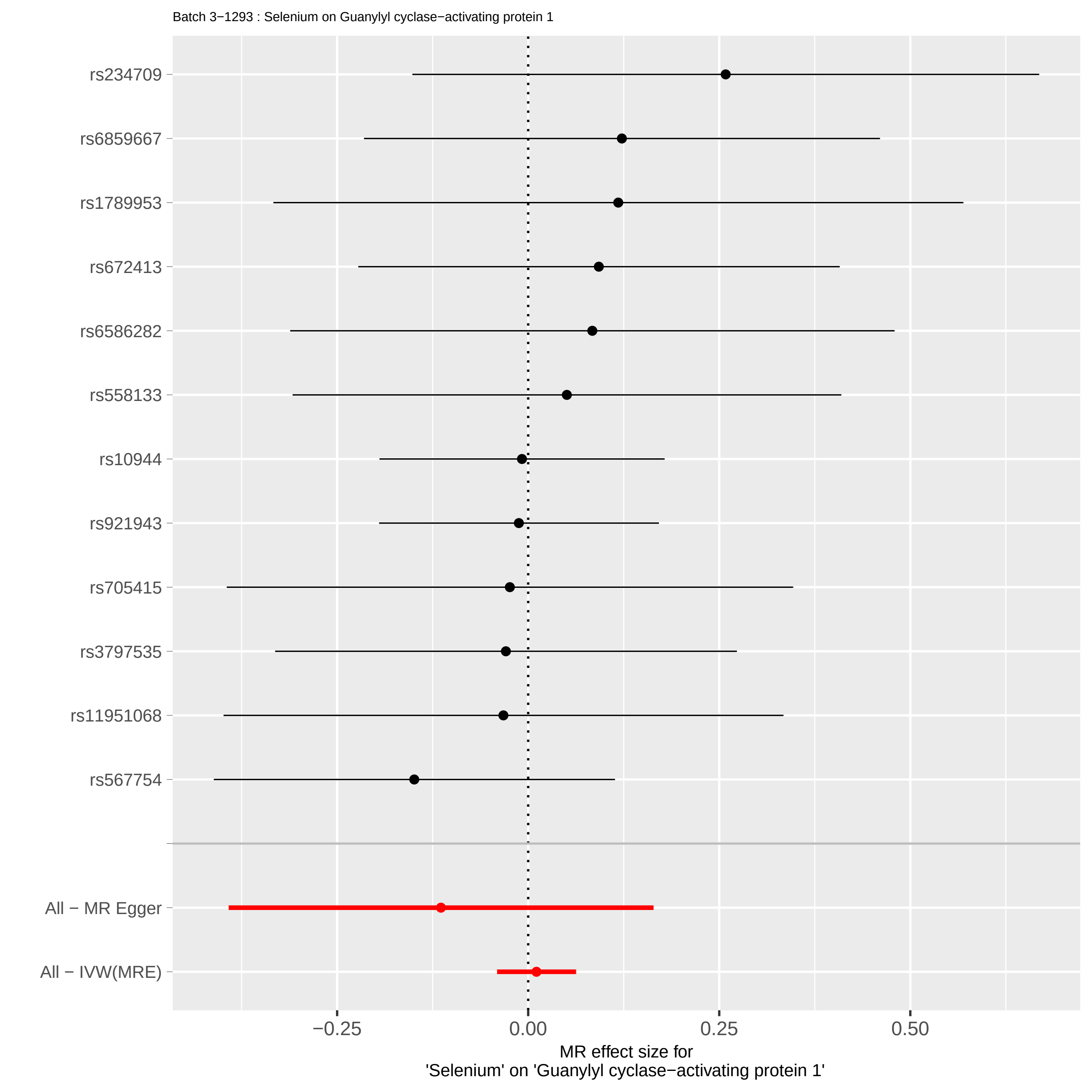

Batch 3−1293 : Selenium on Guanylyl cyclase−activating protein 1
rs234709
rs6859667
rs1789953
rs672413
rs6586282
rs558133
rs10944
rs921943
rs705415
rs3797535
rs11951068
rs567754
All − MR Egger
All − IVW(MRE)
−0.25
0.00
0.25
0.50
MR effect size for
'Selenium' on 'Guanylyl cyclase−activating protein 1'

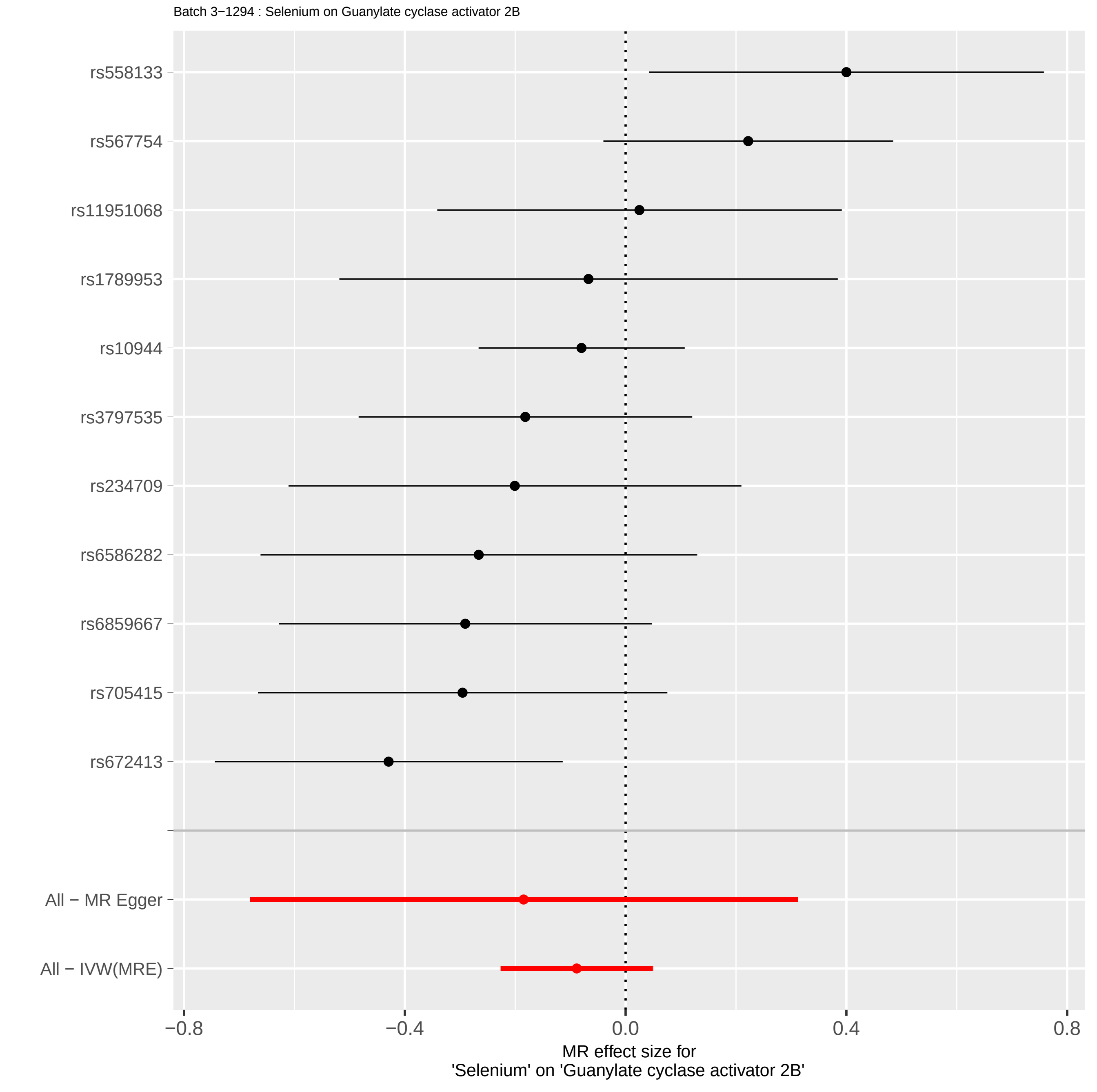
Batch 3−1294 : Selenium on Guanylate cyclase activator 2B
rs558133
rs567754
rs11951068
rs1789953
rs10944
rs3797535
rs234709
rs6586282
rs6859667
rs705415
rs672413
All − MR Egger
All − IVW(MRE)
−0.8
−0.4
0.0
0.4
0.8
MR effect size for
'Selenium' on 'Guanylate cyclase activator 2B'

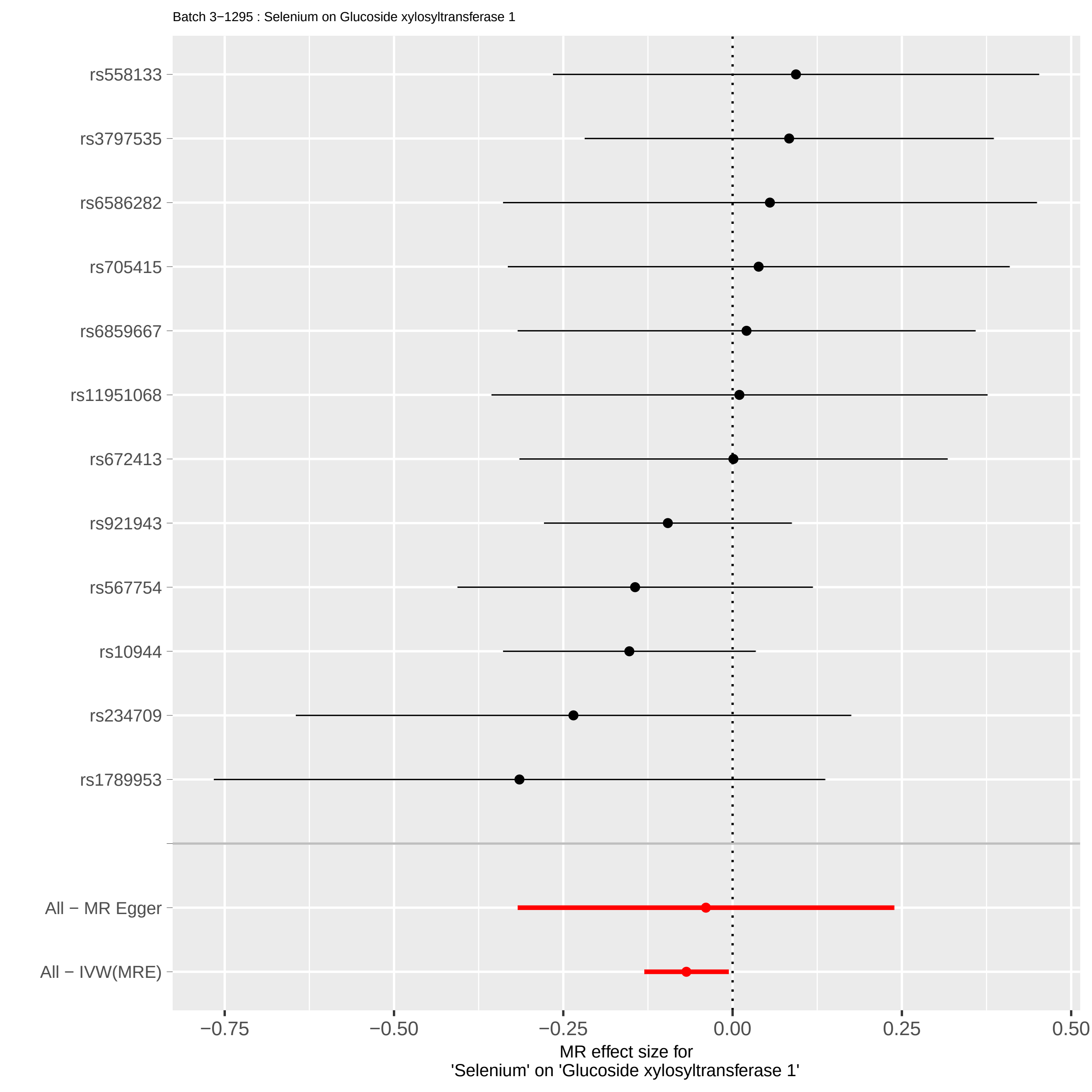

Batch 3−1295 : Selenium on Glucoside xylosyltransferase 1
rs558133
rs3797535
rs6586282
rs705415
rs6859667
rs11951068
rs672413
rs921943
rs567754
rs10944
rs234709
rs1789953
All − MR Egger
All − IVW(MRE)
−0.75
−0.50
−0.25
0.00
0.25
0.50
MR effect size for
'Selenium' on 'Glucoside xylosyltransferase 1'

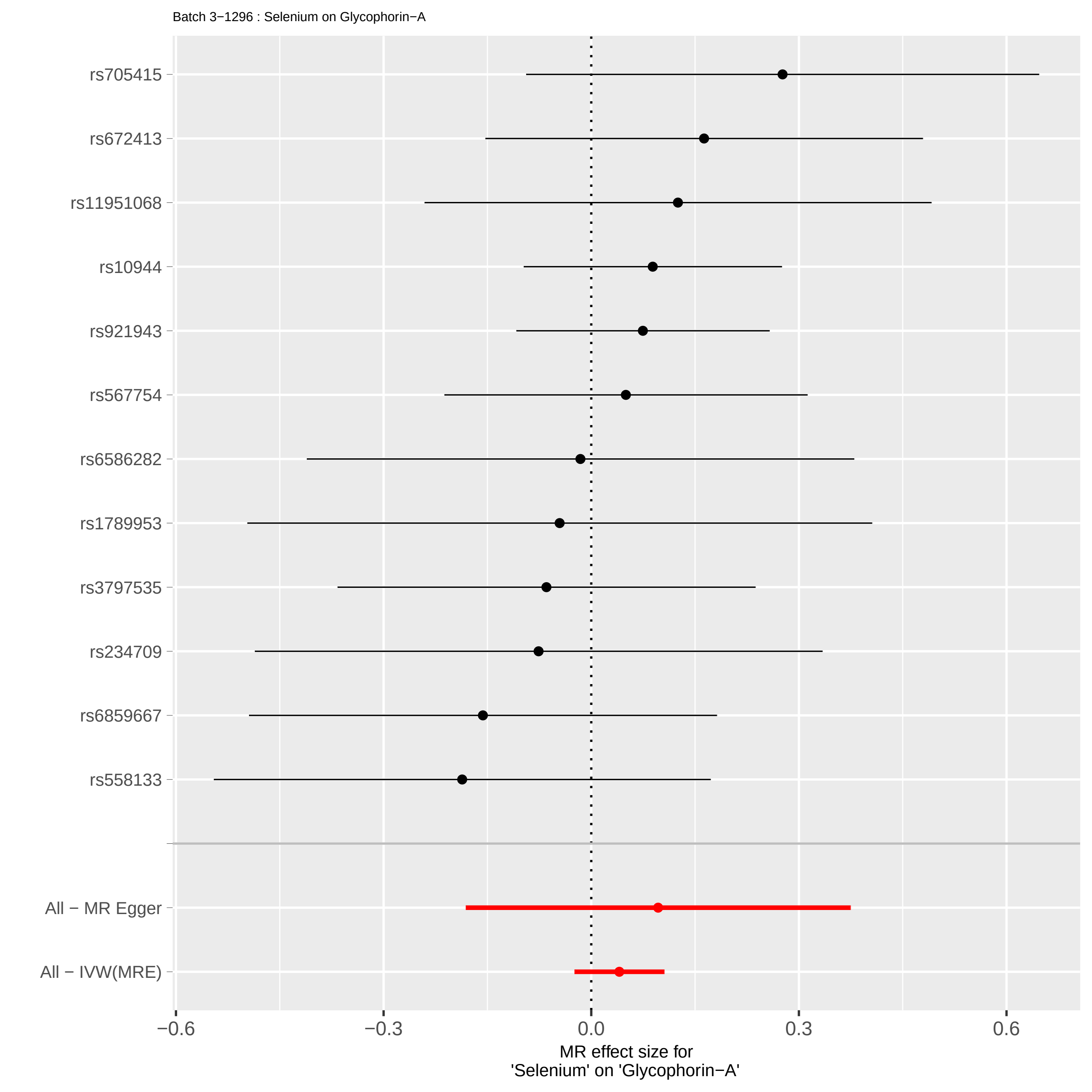

Batch 3−1296 : Selenium on Glycophorin−A
rs705415
rs672413
rs11951068
rs10944
rs921943
rs567754
rs6586282
rs1789953
rs3797535
rs234709
rs6859667
rs558133
All − MR Egger
All − IVW(MRE)
−0.6
−0.3
0.0
0.3
0.6
MR effect size for
'Selenium' on 'Glycophorin−A'

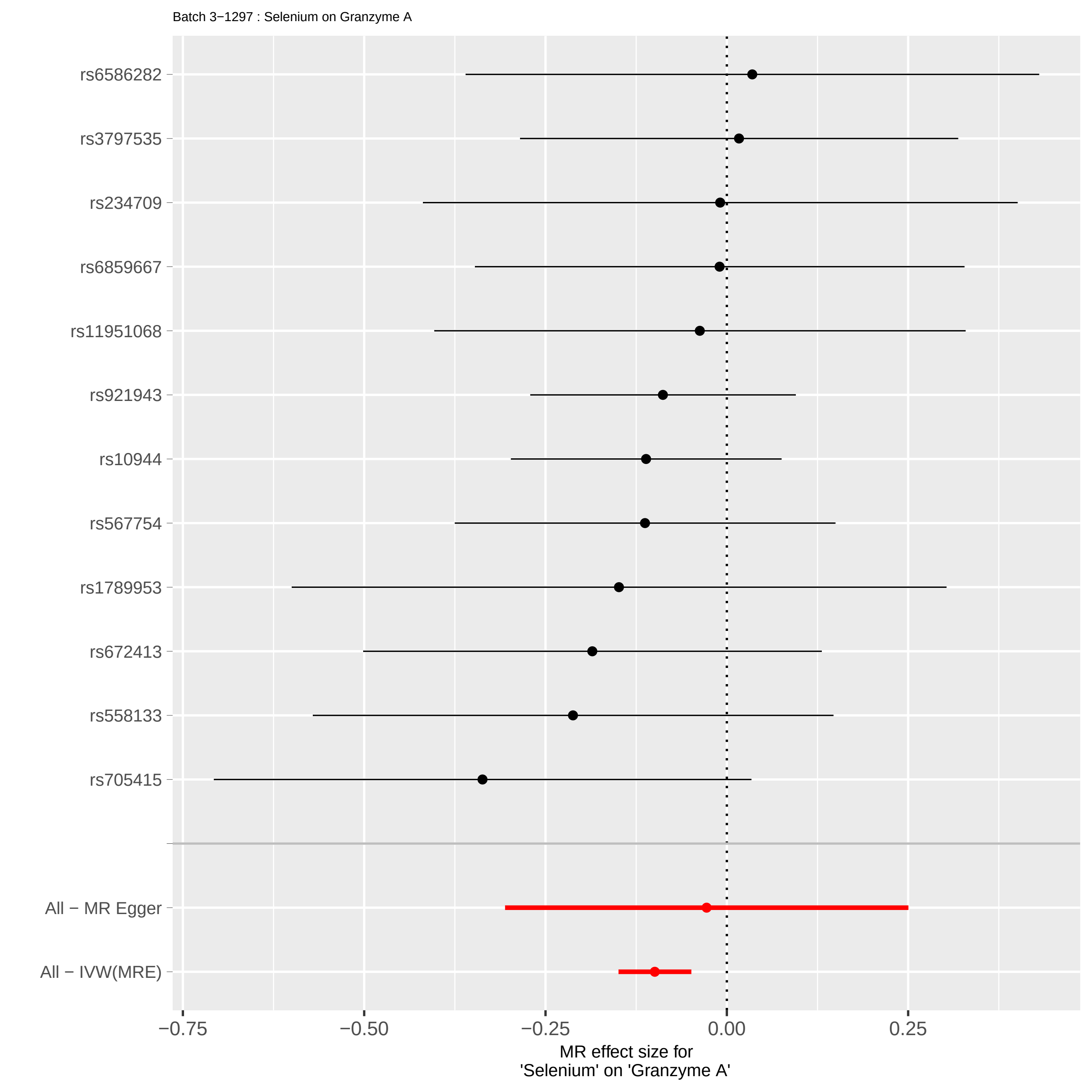

Batch 3−1297 : Selenium on Granzyme A
rs6586282
rs3797535
rs234709
rs6859667
rs11951068
rs921943
rs10944
rs567754
rs1789953
rs672413
rs558133
rs705415
All − MR Egger
All − IVW(MRE)
−0.75
−0.50
−0.25
0.00
0.25
MR effect size for
'Selenium' on 'Granzyme A'

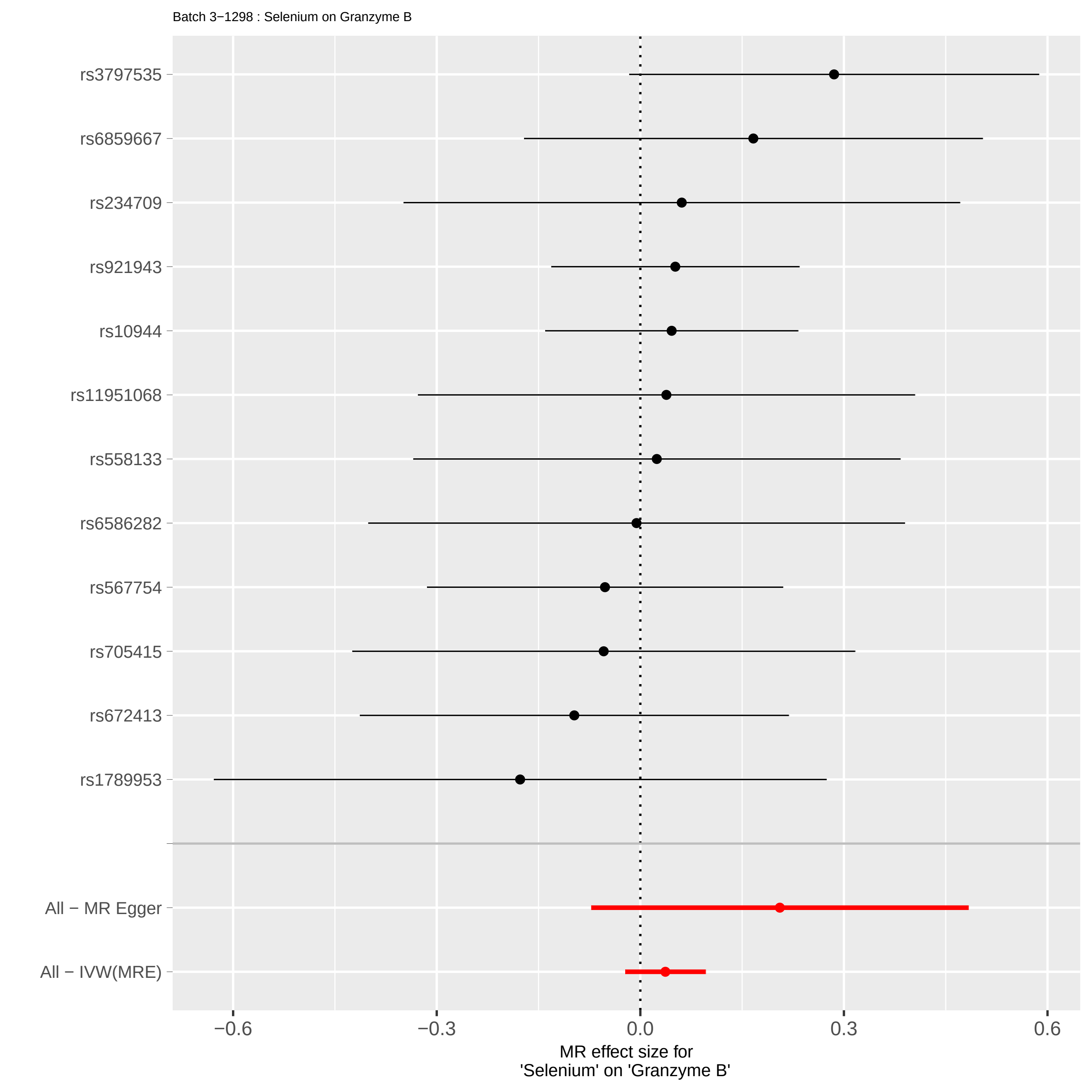

Batch 3−1298 : Selenium on Granzyme B
rs3797535
rs6859667
rs234709
rs921943
rs10944
rs11951068
rs558133
rs6586282
rs567754
rs705415
rs672413
rs1789953
All − MR Egger
All − IVW(MRE)
−0.6
−0.3
0.0
0.3
0.6
MR effect size for
'Selenium' on 'Granzyme B'

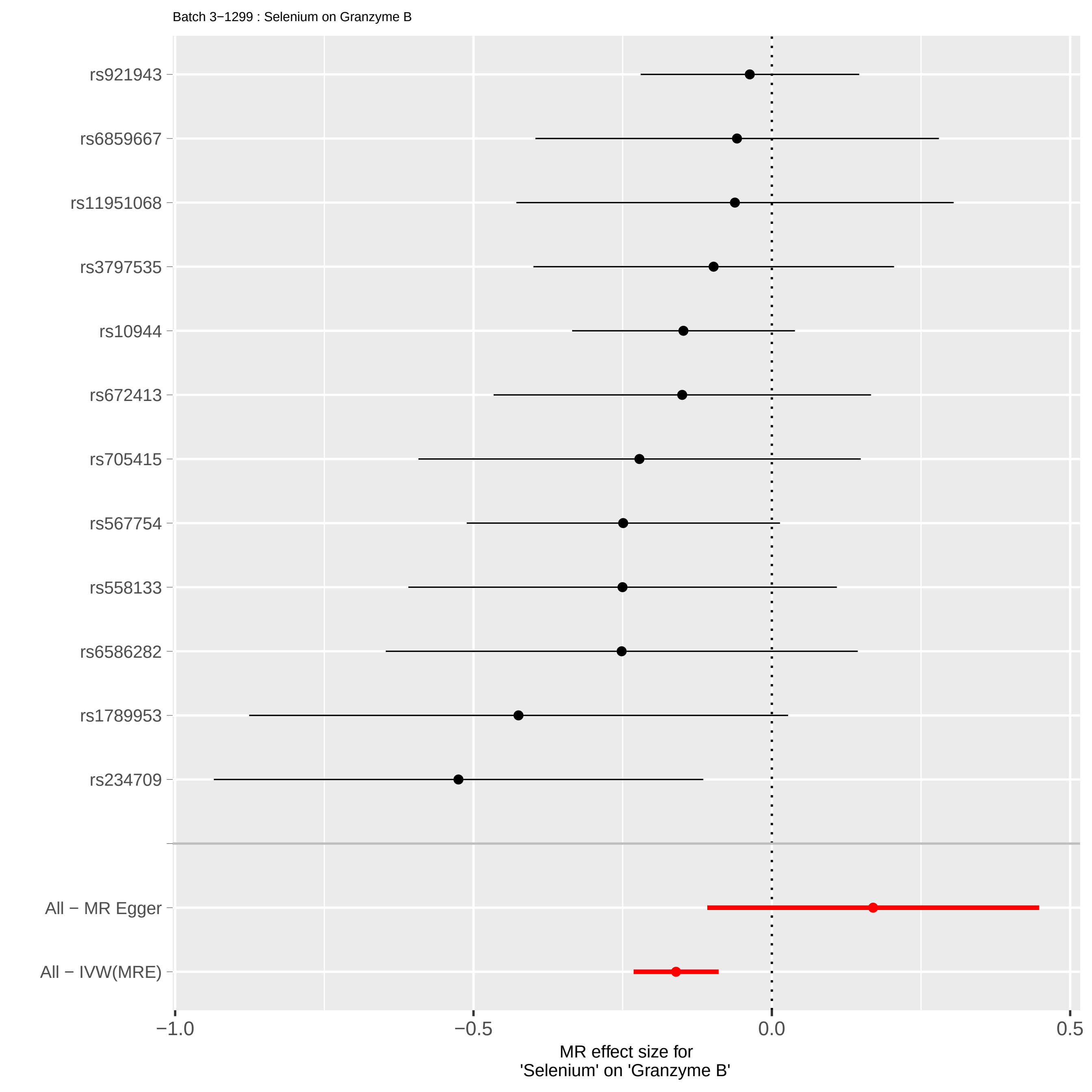

Batch 3−1299 : Selenium on Granzyme B
rs921943
rs6859667
rs11951068
rs3797535
rs10944
rs672413
rs705415
rs567754
rs558133
rs6586282
rs1789953
rs234709
All − MR Egger
All − IVW(MRE)
−1.0
−0.5
0.0
0.5
MR effect size for
'Selenium' on 'Granzyme B'

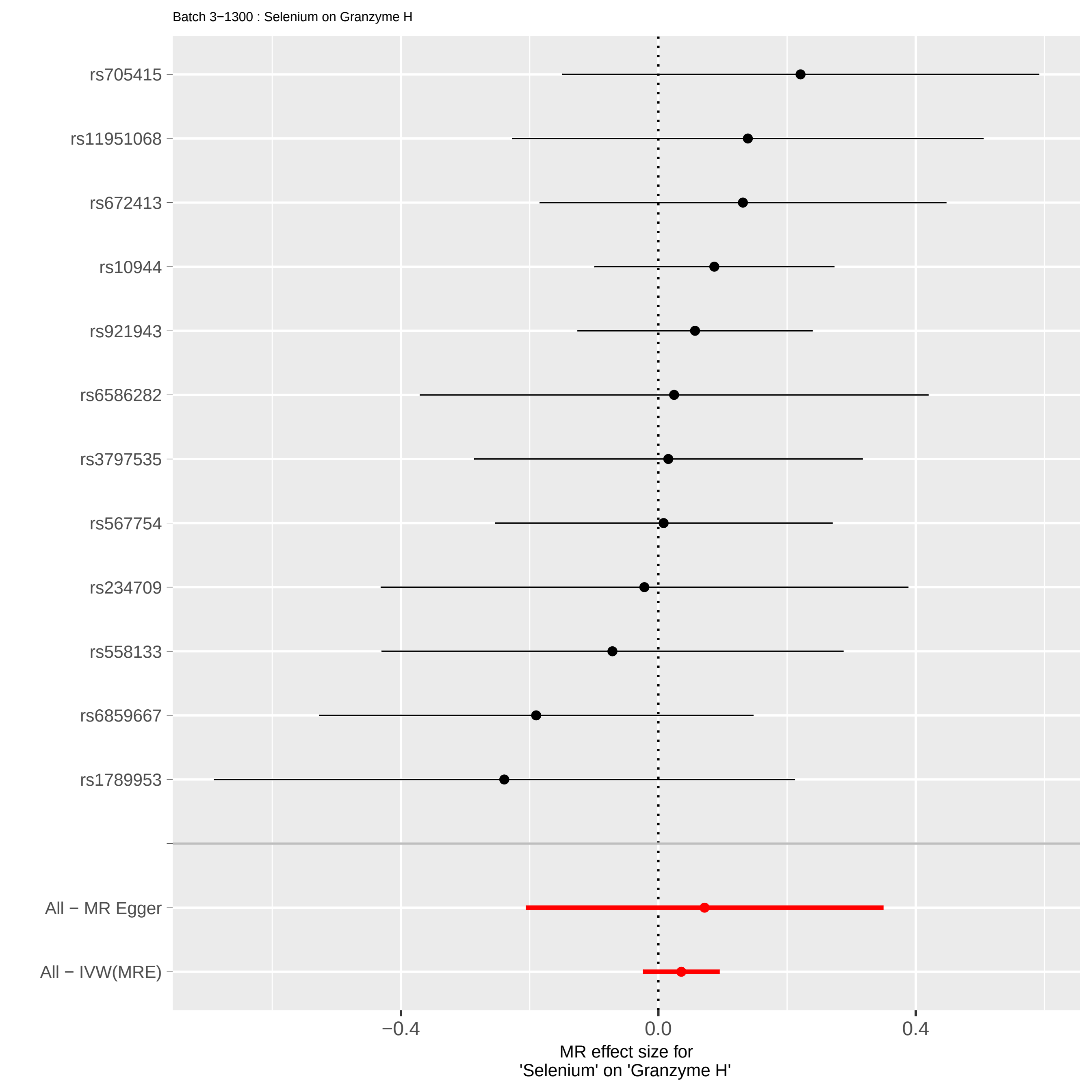
Batch 3−1300 : Selenium on Granzyme H
rs705415
rs11951068
rs672413
rs10944
rs921943
rs6586282
rs3797535
rs567754
rs234709
rs558133
rs6859667
rs1789953
All − MR Egger
All − IVW(MRE)
−0.4
0.0
0.4
MR effect size for
'Selenium' on 'Granzyme H'

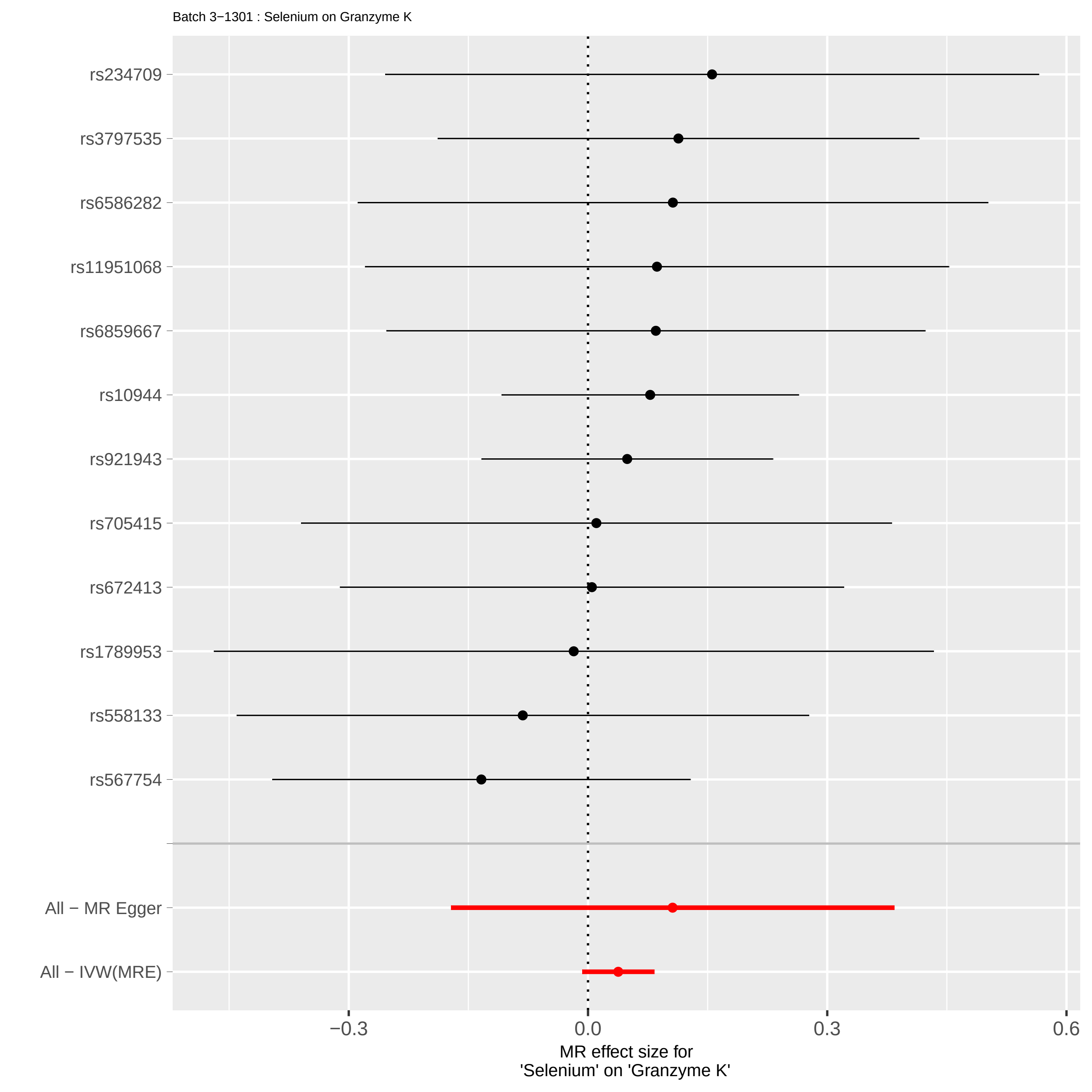

Batch 3−1301 : Selenium on Granzyme K
rs234709
rs3797535
rs6586282
rs11951068
rs6859667
rs10944
rs921943
rs705415
rs672413
rs1789953
rs558133
rs567754
All − MR Egger
All − IVW(MRE)
−0.3
0.0
0.3
0.6
MR effect size for
'Selenium' on 'Granzyme K'

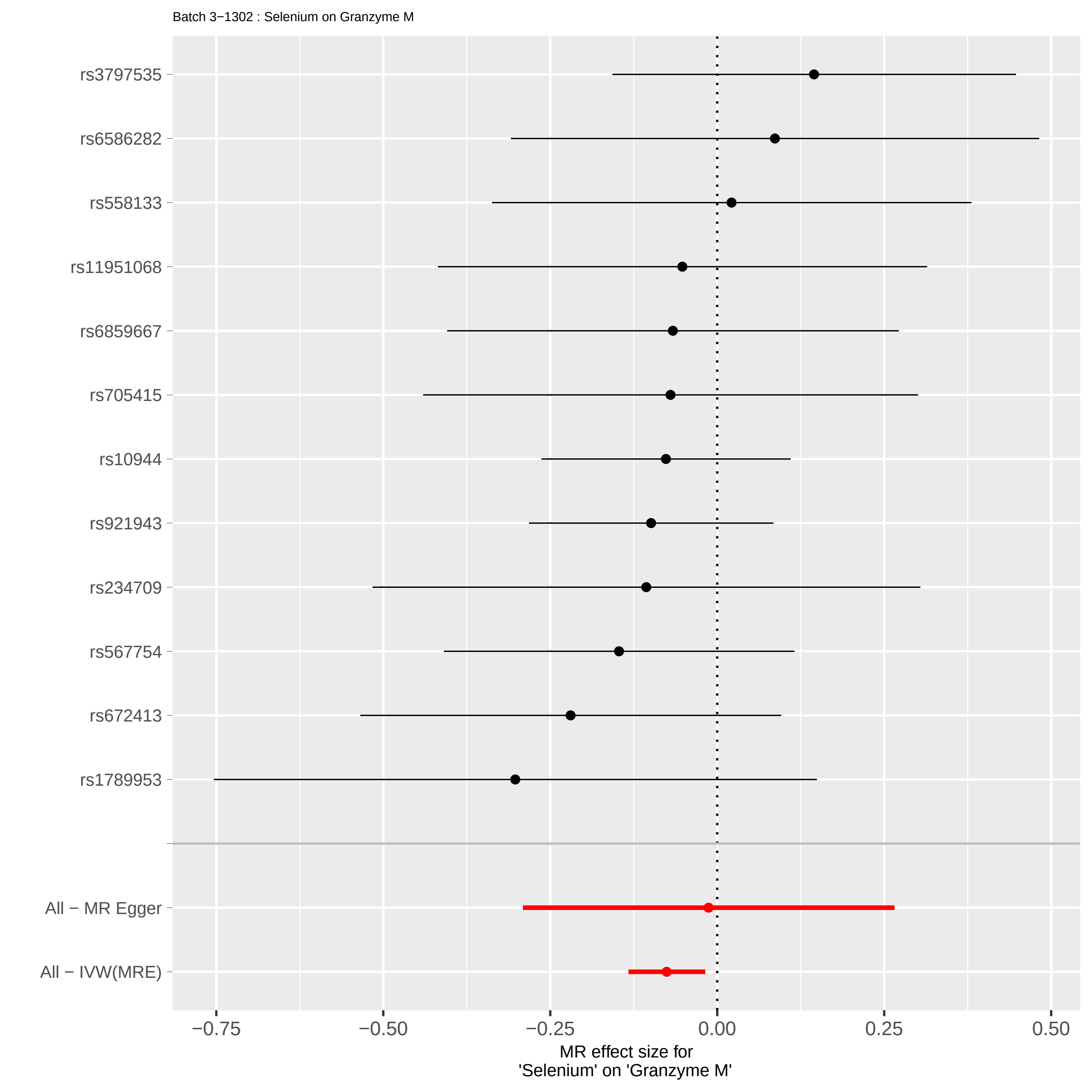

Batch 3−1302 : Selenium on Granzyme M
rs3797535
rs6586282
rs558133
rs11951068
rs6859667
rs705415
rs10944
rs921943
rs234709
rs567754
rs672413
rs1789953
All − MR Egger
All − IVW(MRE)
−0.75
−0.50
−0.25
0.00
0.25
0.50
MR effect size for
'Selenium' on 'Granzyme M'

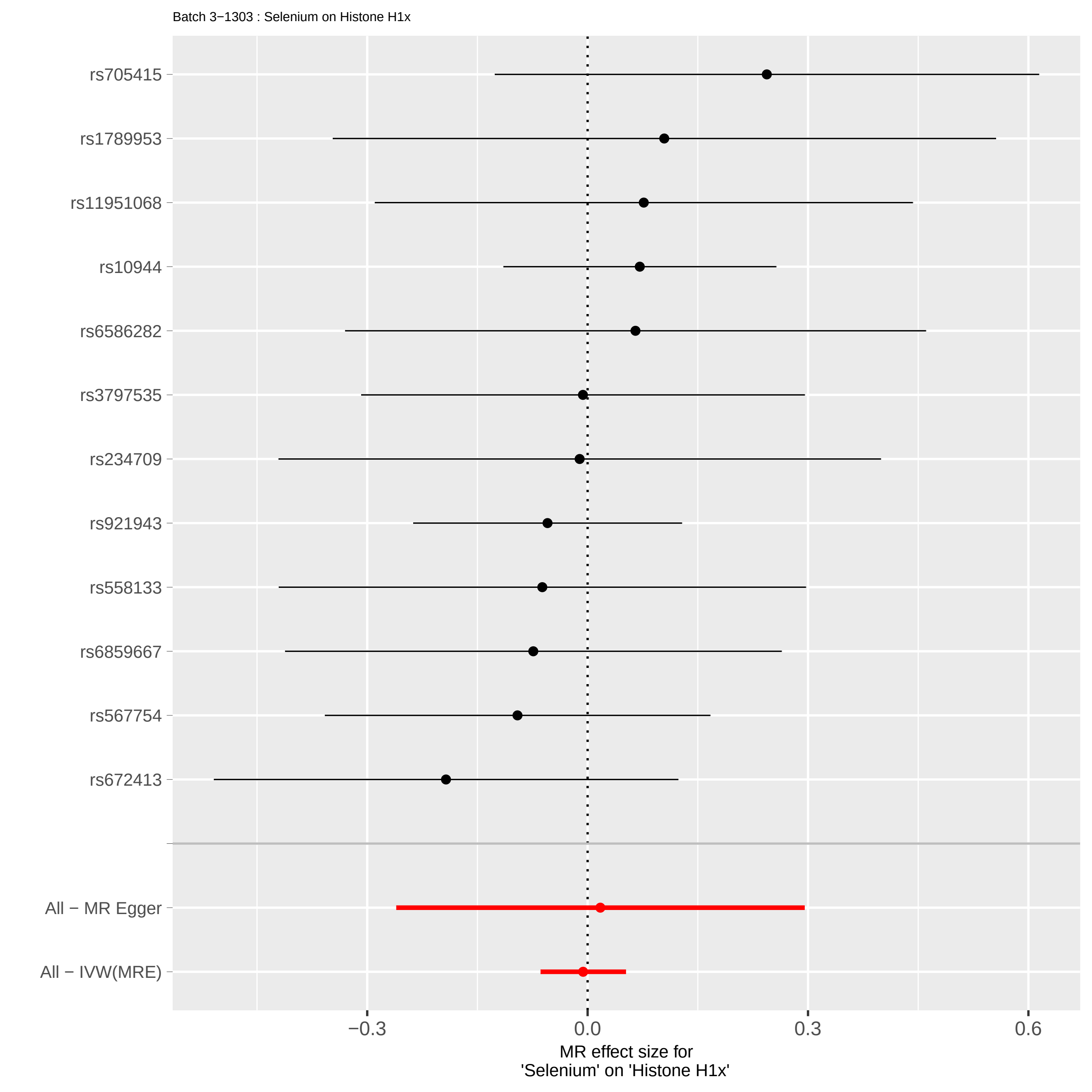

Batch 3−1303 : Selenium on Histone H1x
rs705415
rs1789953
rs11951068
rs10944
rs6586282
rs3797535
rs234709
rs921943
rs558133
rs6859667
rs567754
rs672413
All − MR Egger
All − IVW(MRE)
−0.3
0.0
0.3
0.6
MR effect size for
'Selenium' on 'Histone H1x'

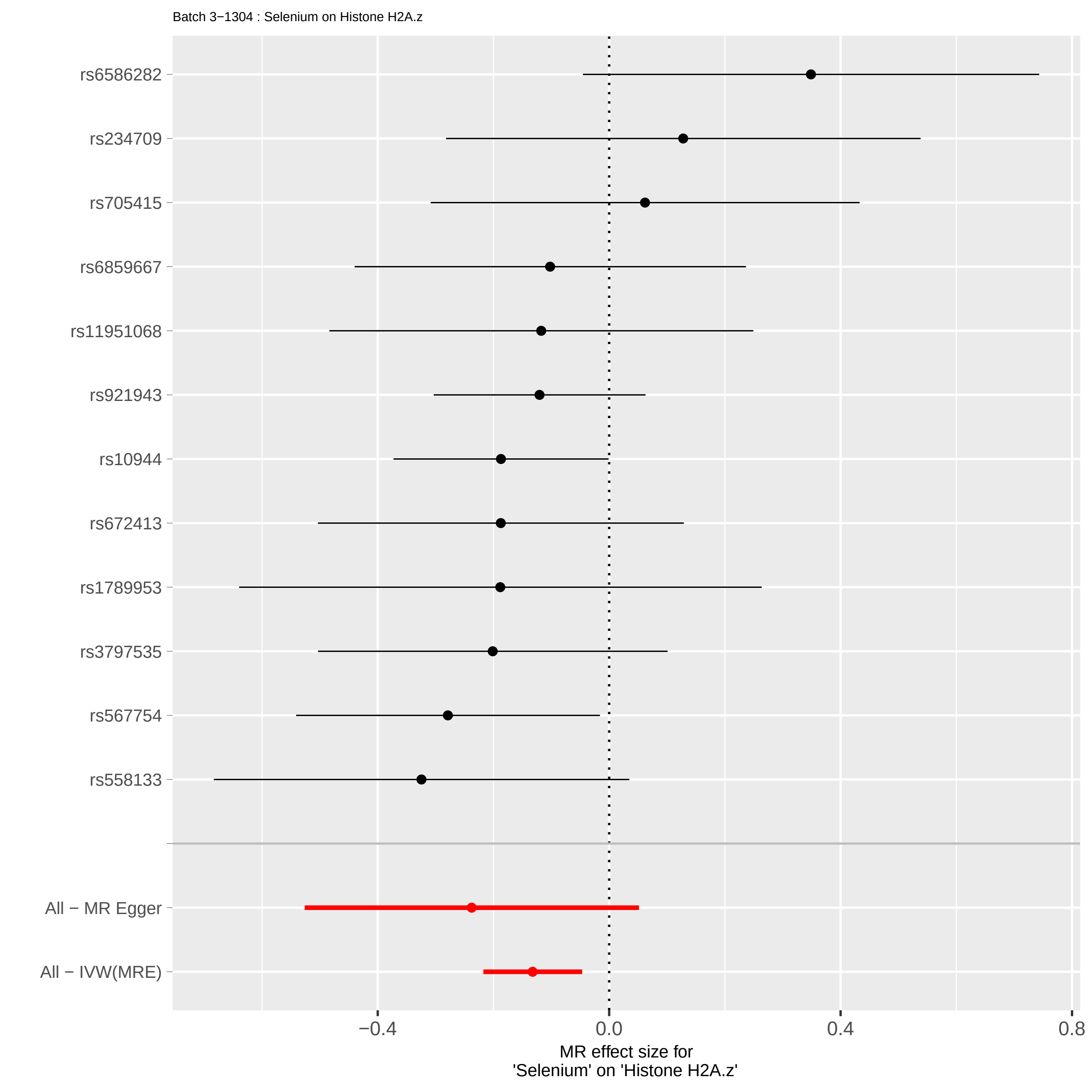

Batch 3−1304 : Selenium on Histone H2A.z
rs6586282
rs234709
rs705415
rs6859667
rs11951068
rs921943
rs10944
rs672413
rs1789953
rs3797535
rs567754
rs558133
All − MR Egger
All − IVW(MRE)
−0.4
0.0
0.4
0.8
MR effect size for
'Selenium' on 'Histone H2A.z'

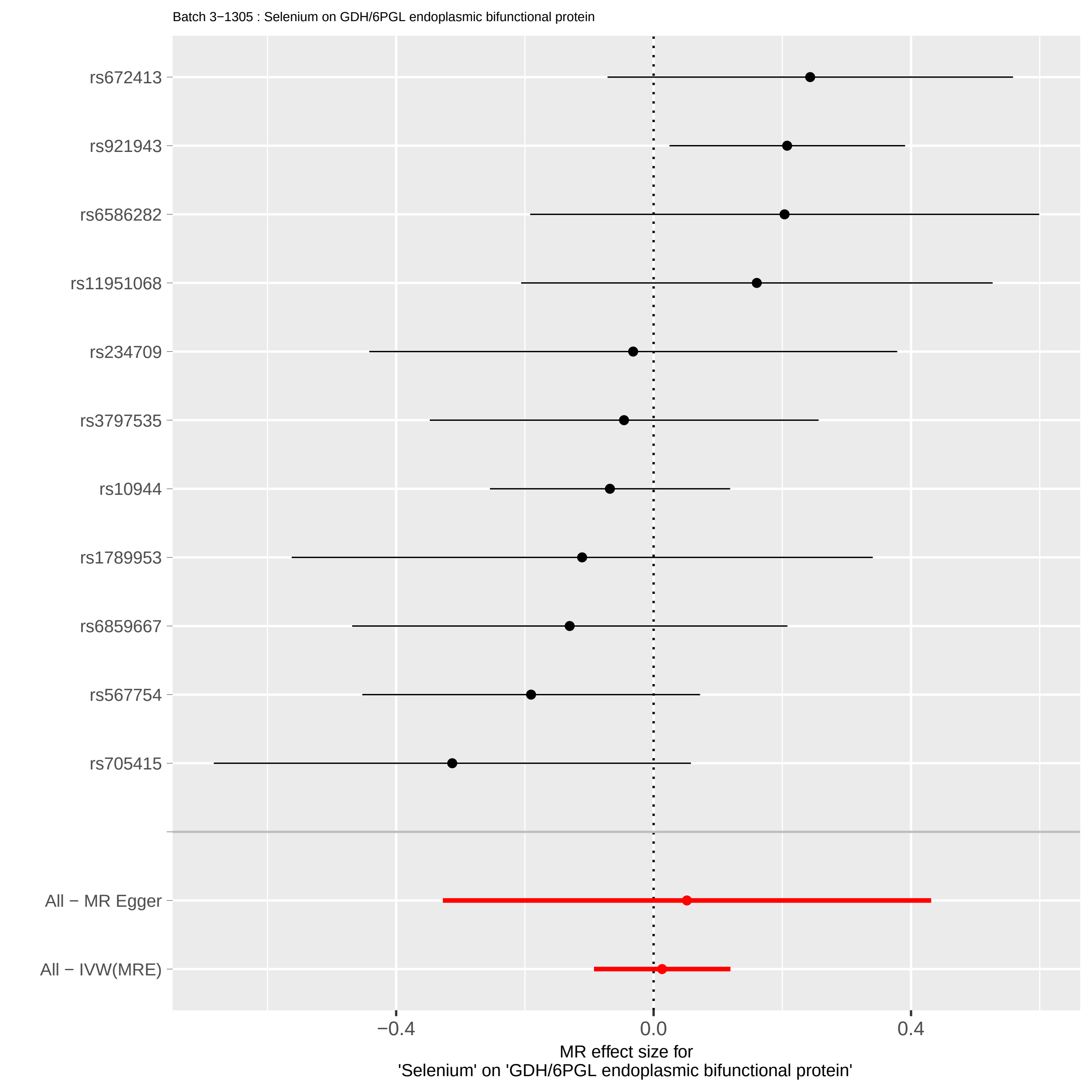

Batch 3−1305 : Selenium on GDH/6PGL endoplasmic bifunctional protein
rs672413
rs921943
rs6586282
rs11951068
rs234709
rs3797535
rs10944
rs1789953
rs6859667
rs567754
rs705415
All − MR Egger
All − IVW(MRE)
−0.4
0.0
0.4
MR effect size for
'Selenium' on 'GDH/6PGL endoplasmic bifunctional protein'

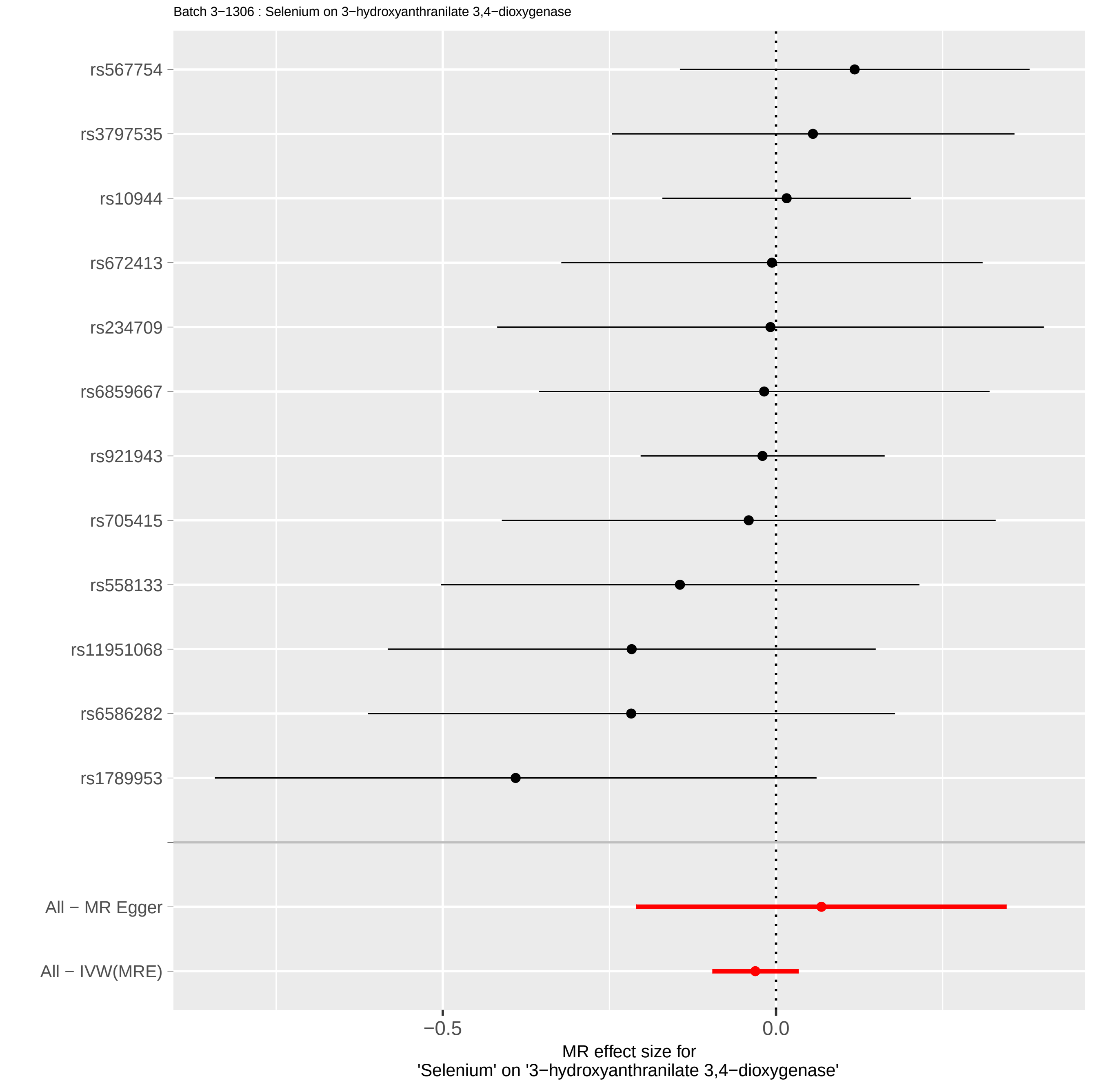

Batch 3−1306 : Selenium on 3−hydroxyanthranilate 3,4−dioxygenase
rs567754
rs3797535
rs10944
rs672413
rs234709
rs6859667
rs921943
rs705415
rs558133
rs11951068
rs6586282
rs1789953
All − MR Egger
All − IVW(MRE)
−0.5
0.0
MR effect size for
'Selenium' on '3−hydroxyanthranilate 3,4−dioxygenase'

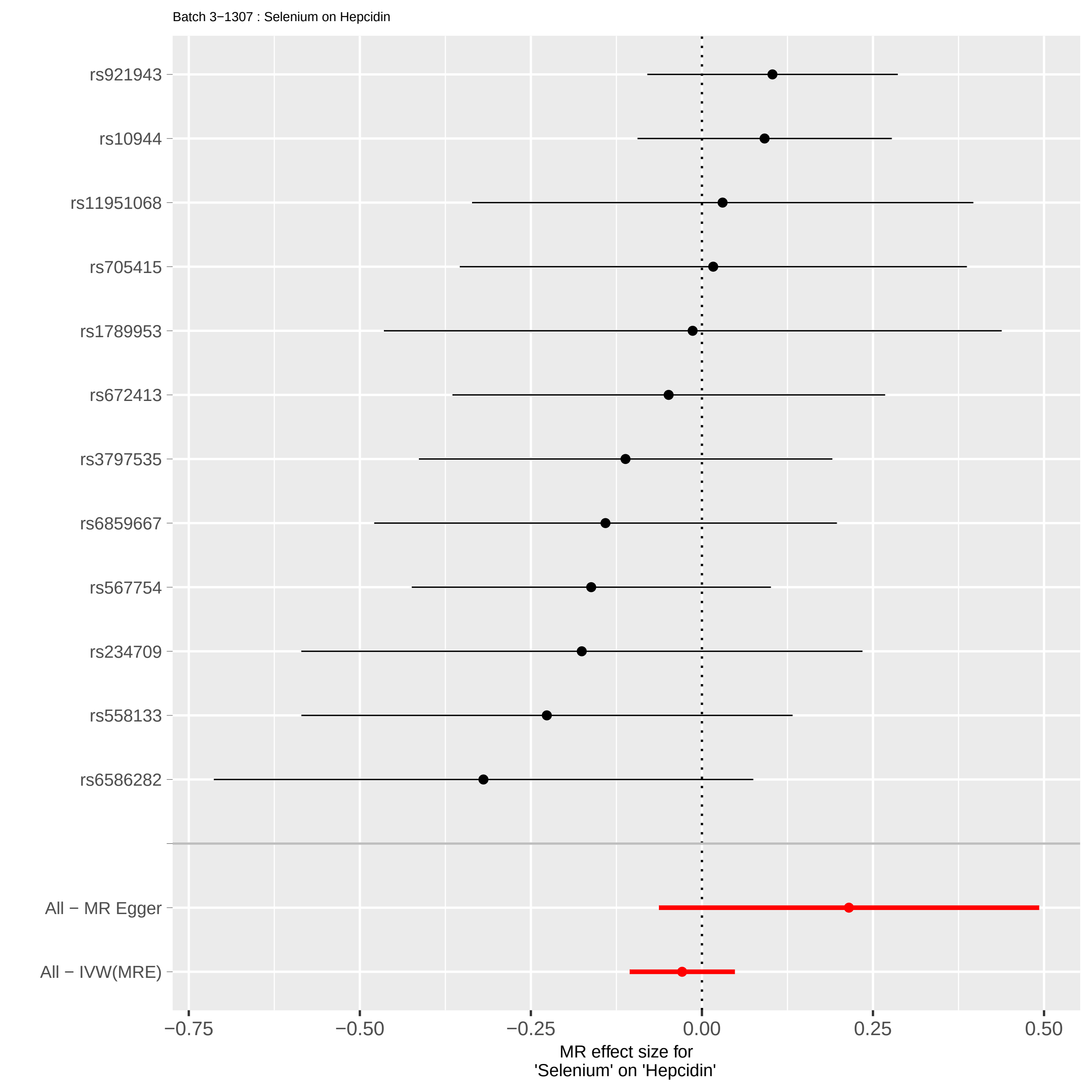

Batch 3−1307 : Selenium on Hepcidin
rs921943
rs10944
rs11951068
rs705415
rs1789953
rs672413
rs3797535
rs6859667
rs567754
rs234709
rs558133
rs6586282
All − MR Egger
All − IVW(MRE)
−0.75
−0.50
−0.25
0.00
0.25
0.50
MR effect size for
'Selenium' on 'Hepcidin'

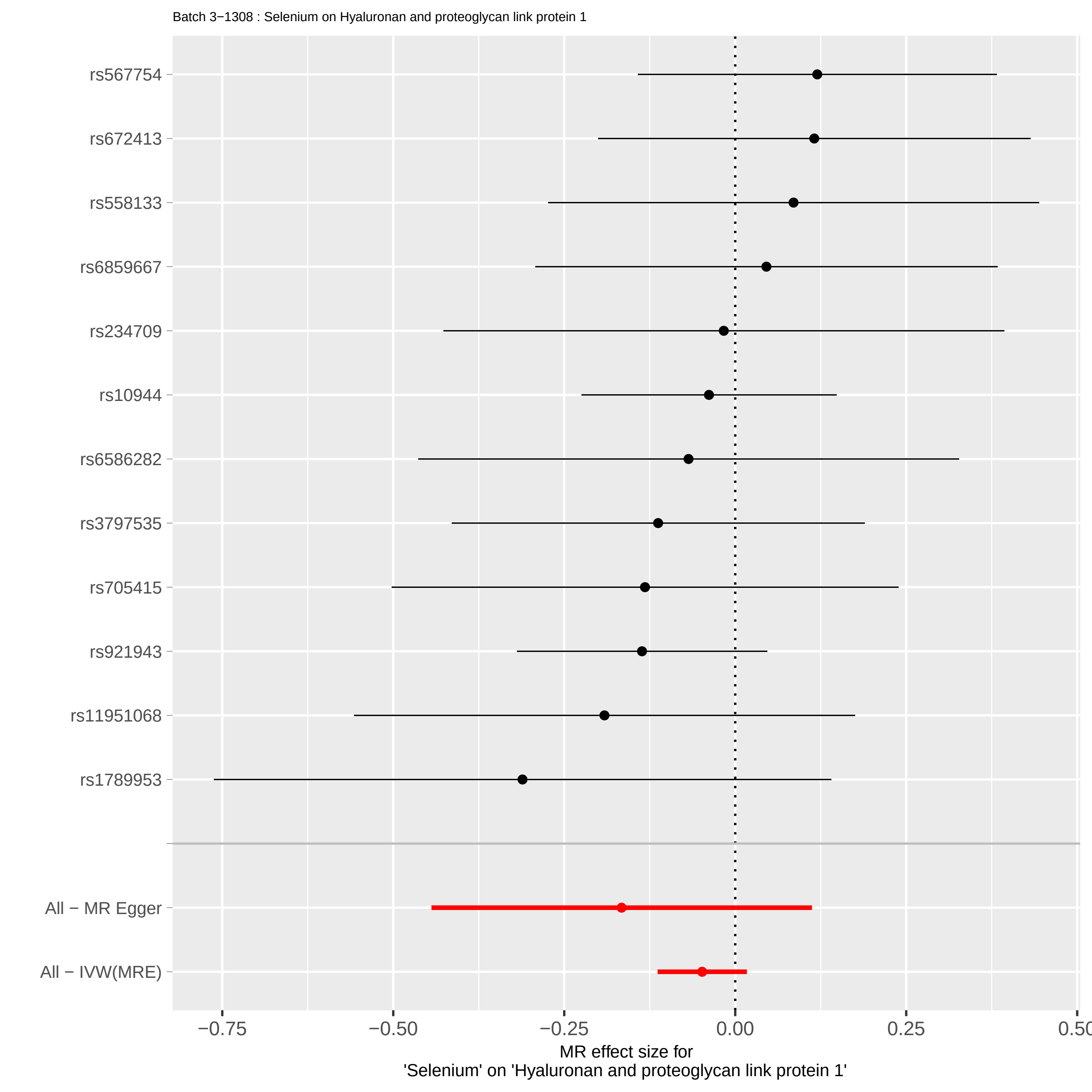

Batch 3−1308 : Selenium on Hyaluronan and proteoglycan link protein 1
rs567754
rs672413
rs558133
rs6859667
rs234709
rs10944
rs6586282
rs3797535
rs705415
rs921943
rs11951068
rs1789953
All − MR Egger
All − IVW(MRE)
−0.75
−0.50
−0.25
0.00
0.25
0.50
MR effect size for
'Selenium' on 'Hyaluronan and proteoglycan link protein 1'

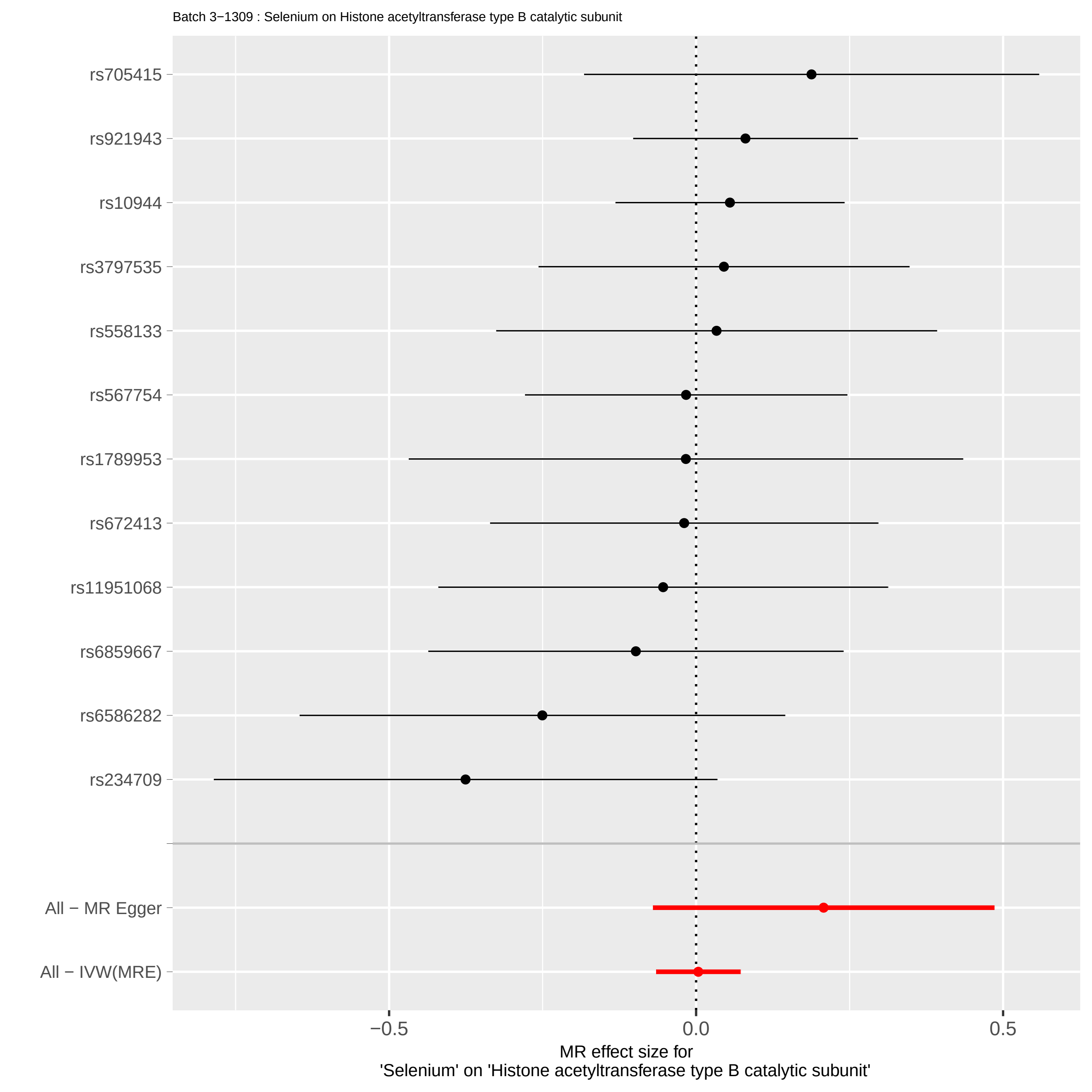
Batch 3−1309 : Selenium on Histone acetyltransferase type B catalytic subunit
rs705415
rs921943
rs10944
rs3797535
rs558133
rs567754
rs1789953
rs672413
rs11951068
rs6859667
rs6586282
rs234709
All − MR Egger
All − IVW(MRE)
−0.5
0.0
0.5
MR effect size for
'Selenium' on 'Histone acetyltransferase type B catalytic subunit'

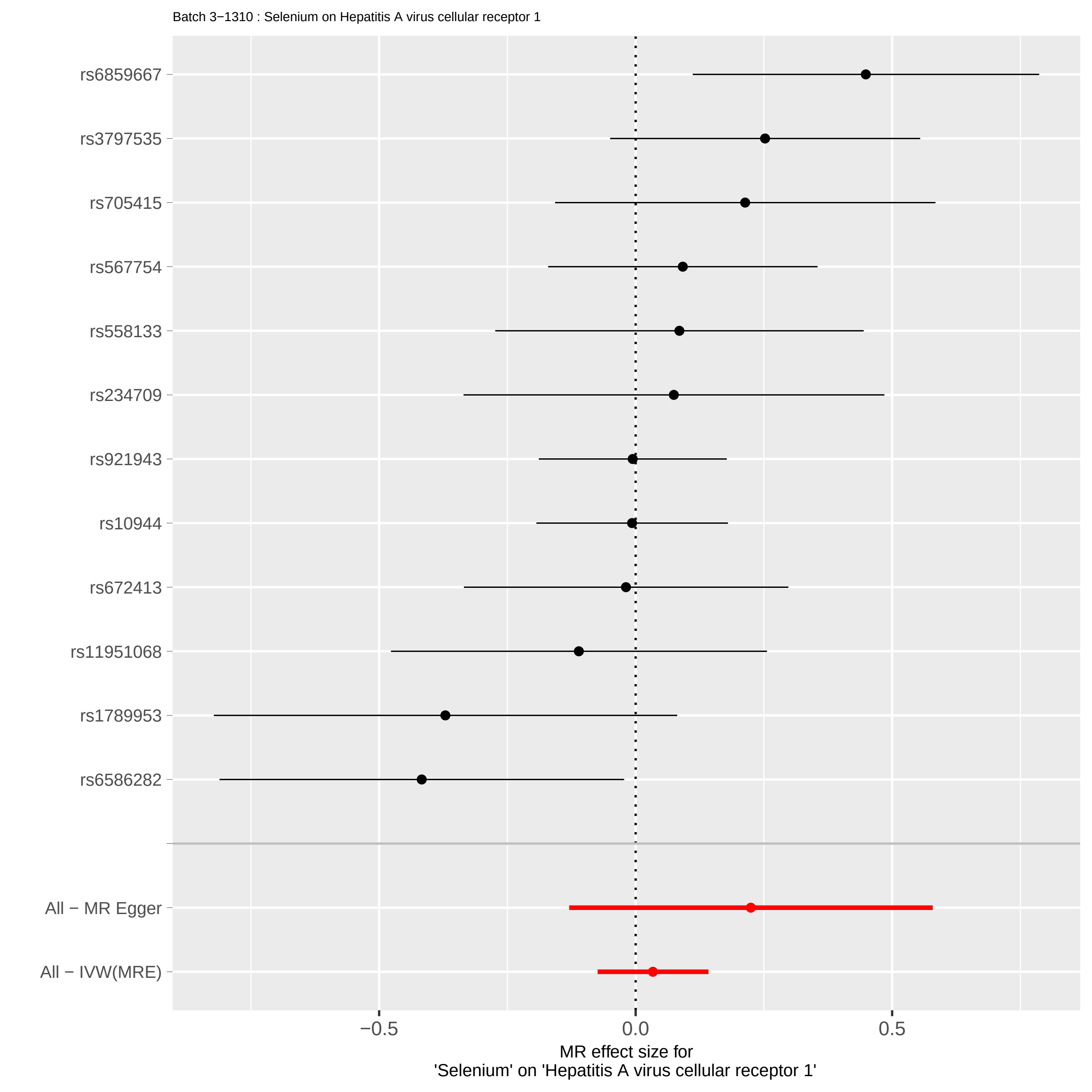
Batch 3−1310 : Selenium on Hepatitis A virus cellular receptor 1
rs6859667
rs3797535
rs705415
rs567754
rs558133
rs234709
rs921943
rs10944
rs672413
rs11951068
rs1789953
rs6586282
All − MR Egger
All − IVW(MRE)
−0.5
0.0
0.5
MR effect size for
'Selenium' on 'Hepatitis A virus cellular receptor 1'

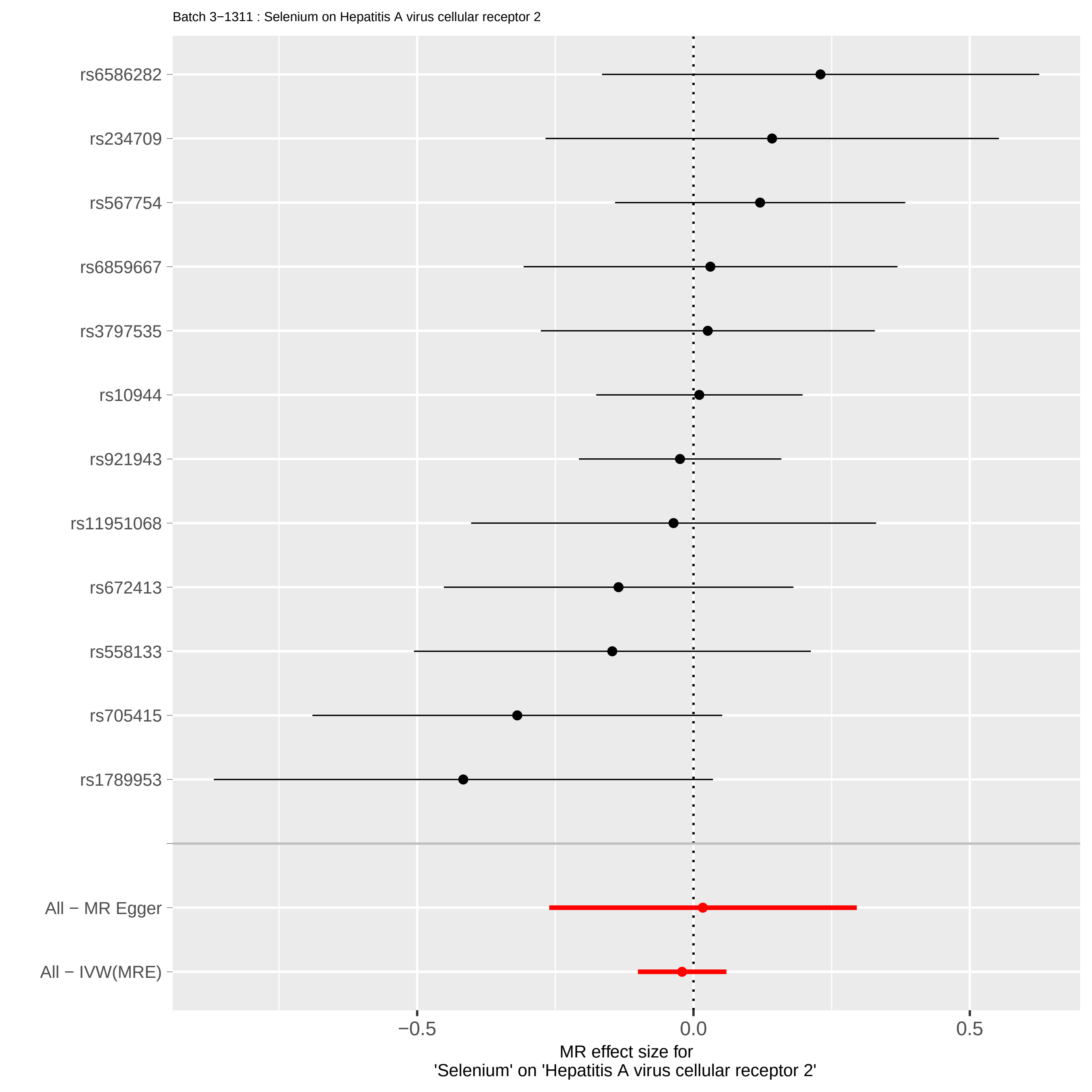
Batch 3−1311 : Selenium on Hepatitis A virus cellular receptor 2
rs6586282
rs234709
rs567754
rs6859667
rs3797535
rs10944
rs921943
rs11951068
rs672413
rs558133
rs705415
rs1789953
All − MR Egger
All − IVW(MRE)
−0.5
0.0
0.5
MR effect size for
'Selenium' on 'Hepatitis A virus cellular receptor 2'

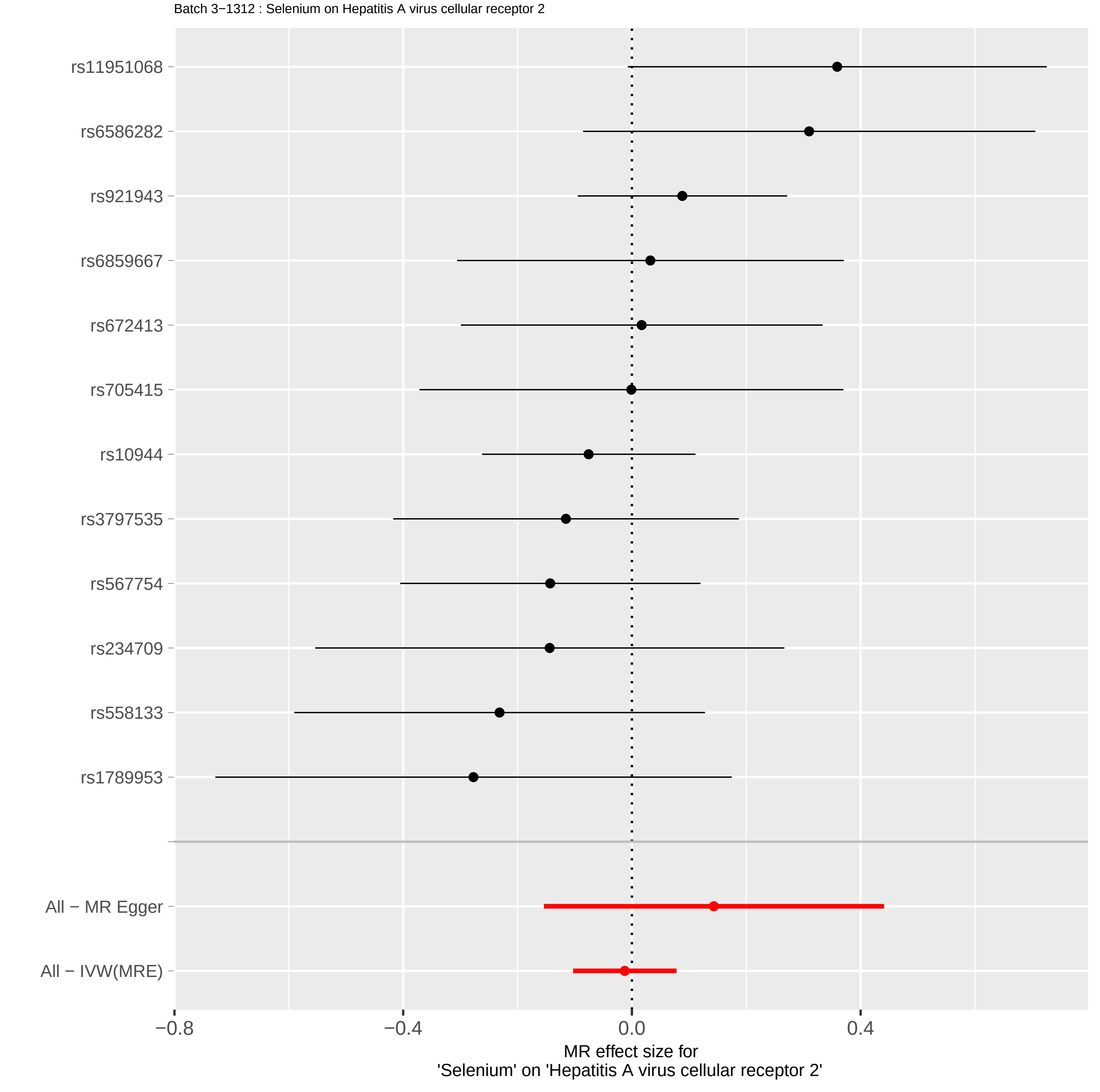

Batch 3−1312 : Selenium on Hepatitis A virus cellular receptor 2
rs11951068
rs6586282
rs921943
rs6859667
rs672413
rs705415
rs10944
rs3797535
rs567754
rs234709
rs558133
rs1789953
All − MR Egger
All − IVW(MRE)
−0.8
−0.4
0.0
0.4
MR effect size for
'Selenium' on 'Hepatitis A virus cellular receptor 2'

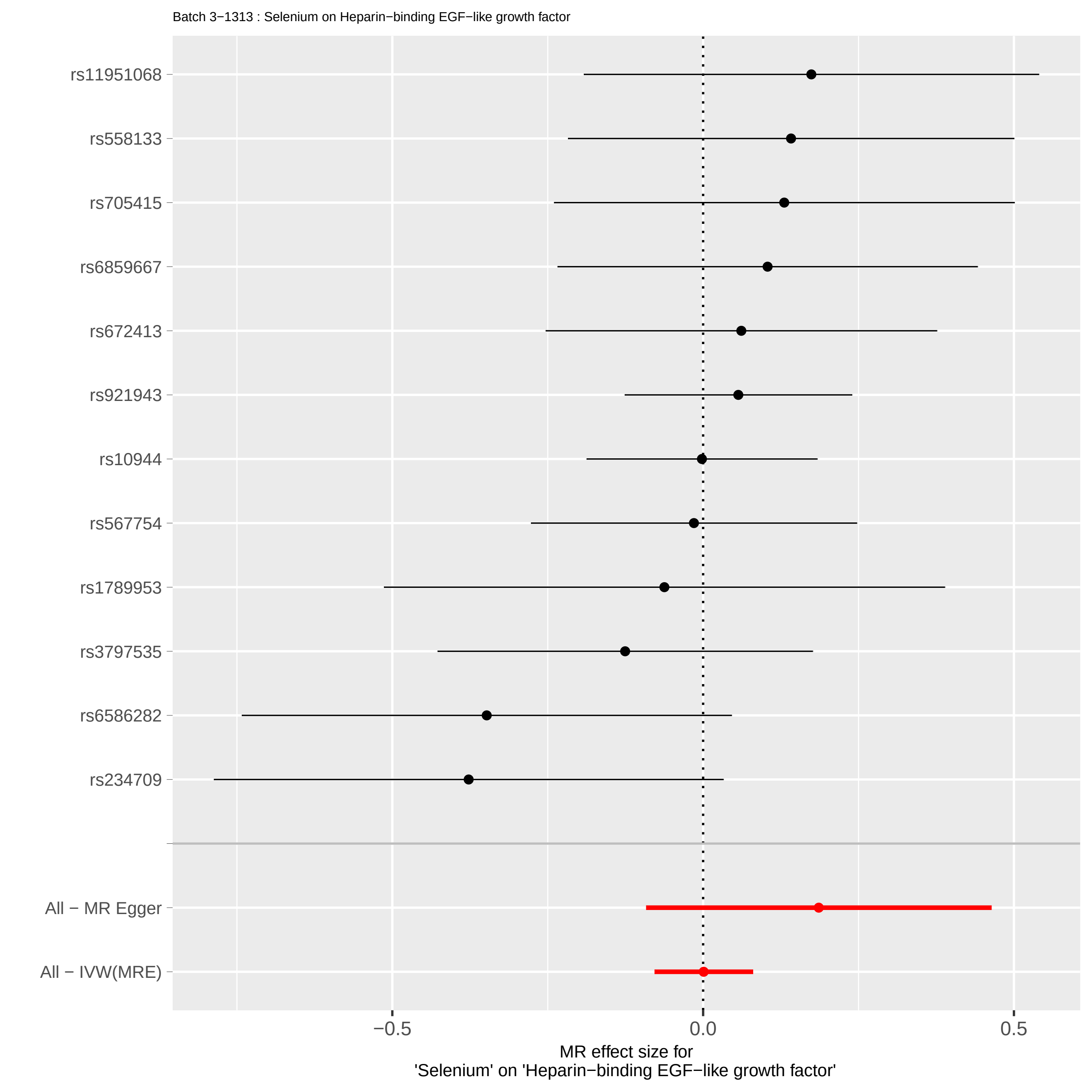

Batch 3−1313 : Selenium on Heparin−binding EGF−like growth factor
rs11951068
rs558133
rs705415
rs6859667
rs672413
rs921943
rs10944
rs567754
rs1789953
rs3797535
rs6586282
rs234709
All − MR Egger
All − IVW(MRE)
−0.5
0.0
0.5
MR effect size for
'Selenium' on 'Heparin−binding EGF−like growth factor'

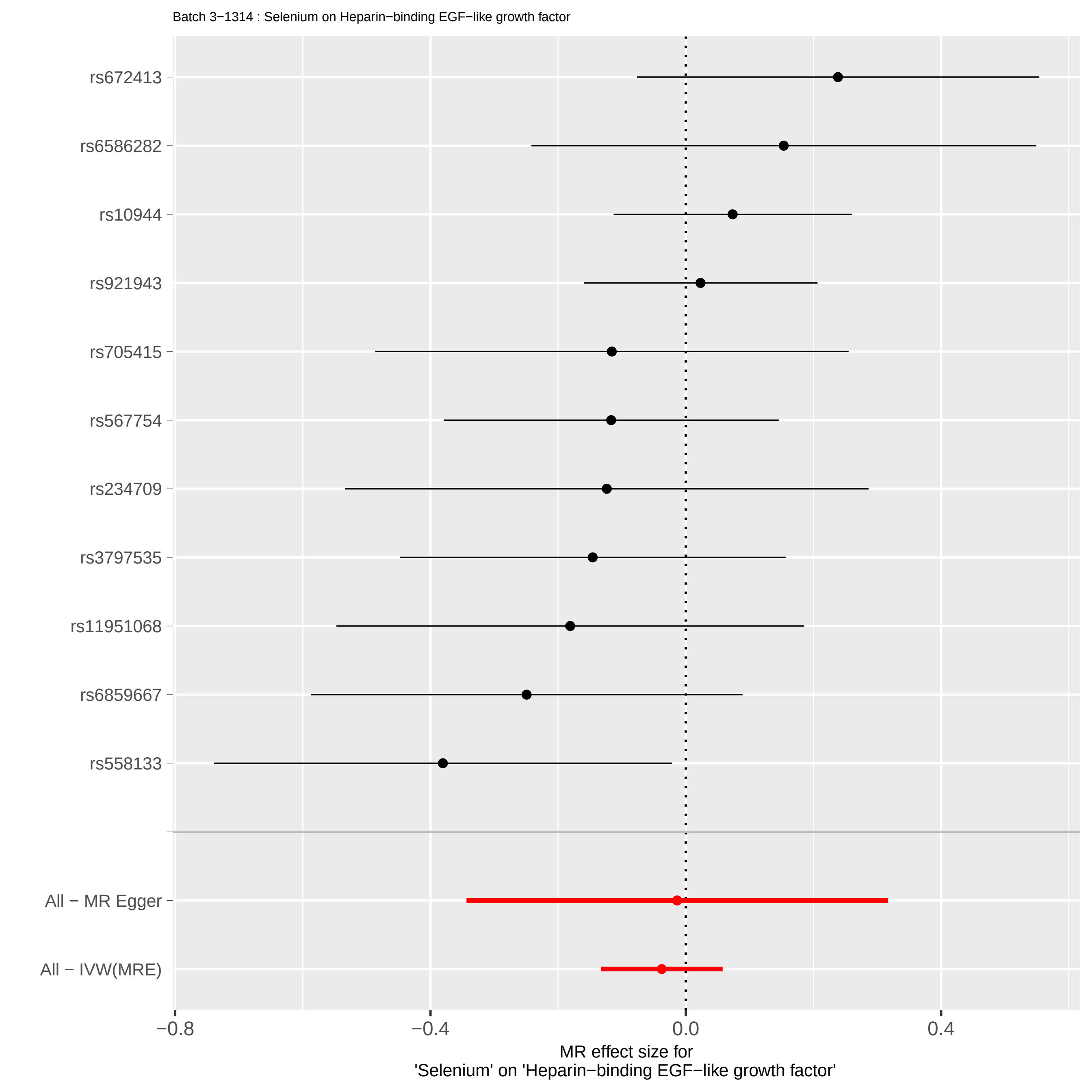

Batch 3−1314 : Selenium on Heparin−binding EGF−like growth factor
rs672413
rs6586282
rs10944
rs921943
rs705415
rs567754
rs234709
rs3797535
rs11951068
rs6859667
rs558133
All − MR Egger
All − IVW(MRE)
−0.8
−0.4
0.0
0.4
MR effect size for
'Selenium' on 'Heparin−binding EGF−like growth factor'

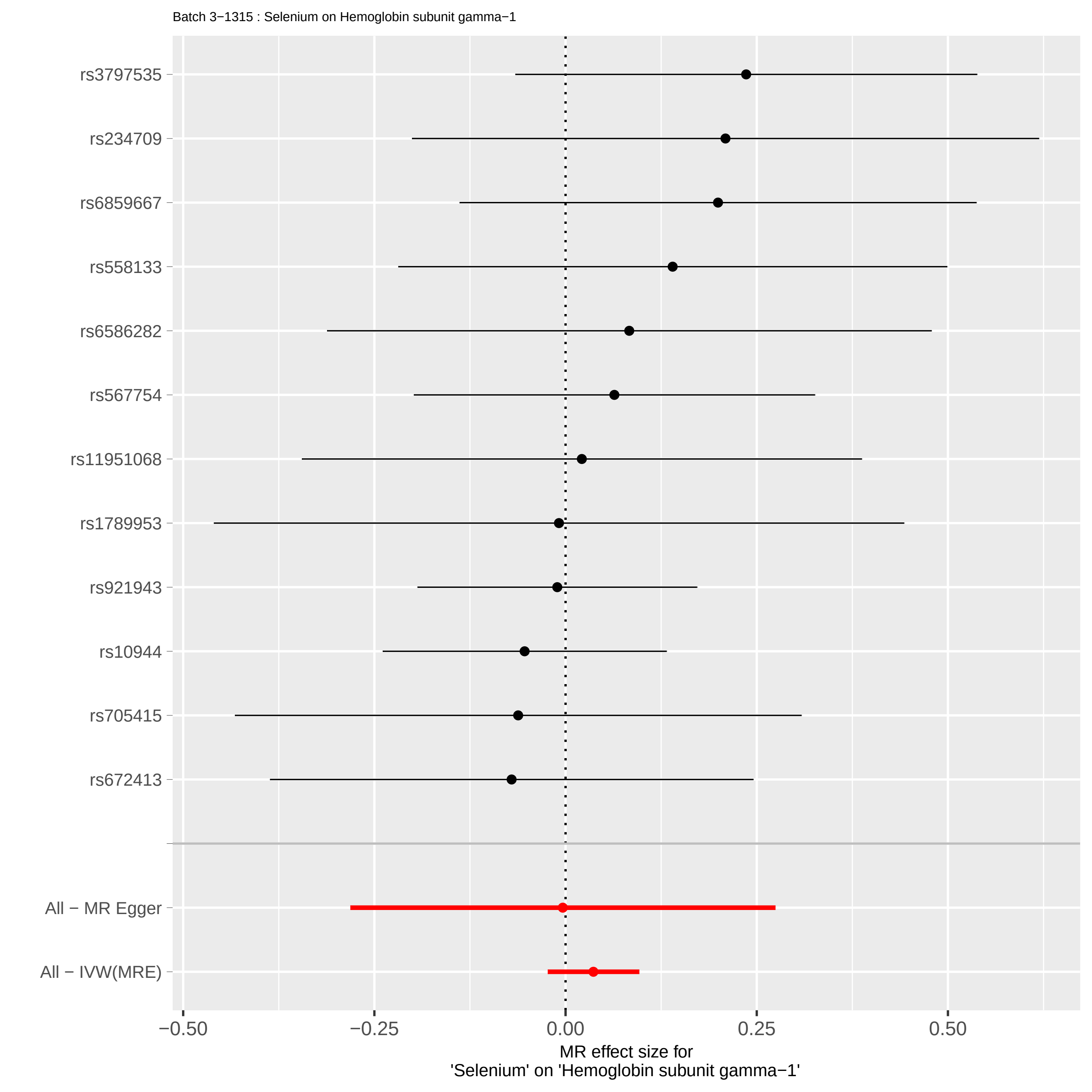

Batch 3−1315 : Selenium on Hemoglobin subunit gamma−1
rs3797535
rs234709
rs6859667
rs558133
rs6586282
rs567754
rs11951068
rs1789953
rs921943
rs10944
rs705415
rs672413
All − MR Egger
All − IVW(MRE)
−0.50
−0.25
0.00
0.25
0.50
MR effect size for
'Selenium' on 'Hemoglobin subunit gamma−1'

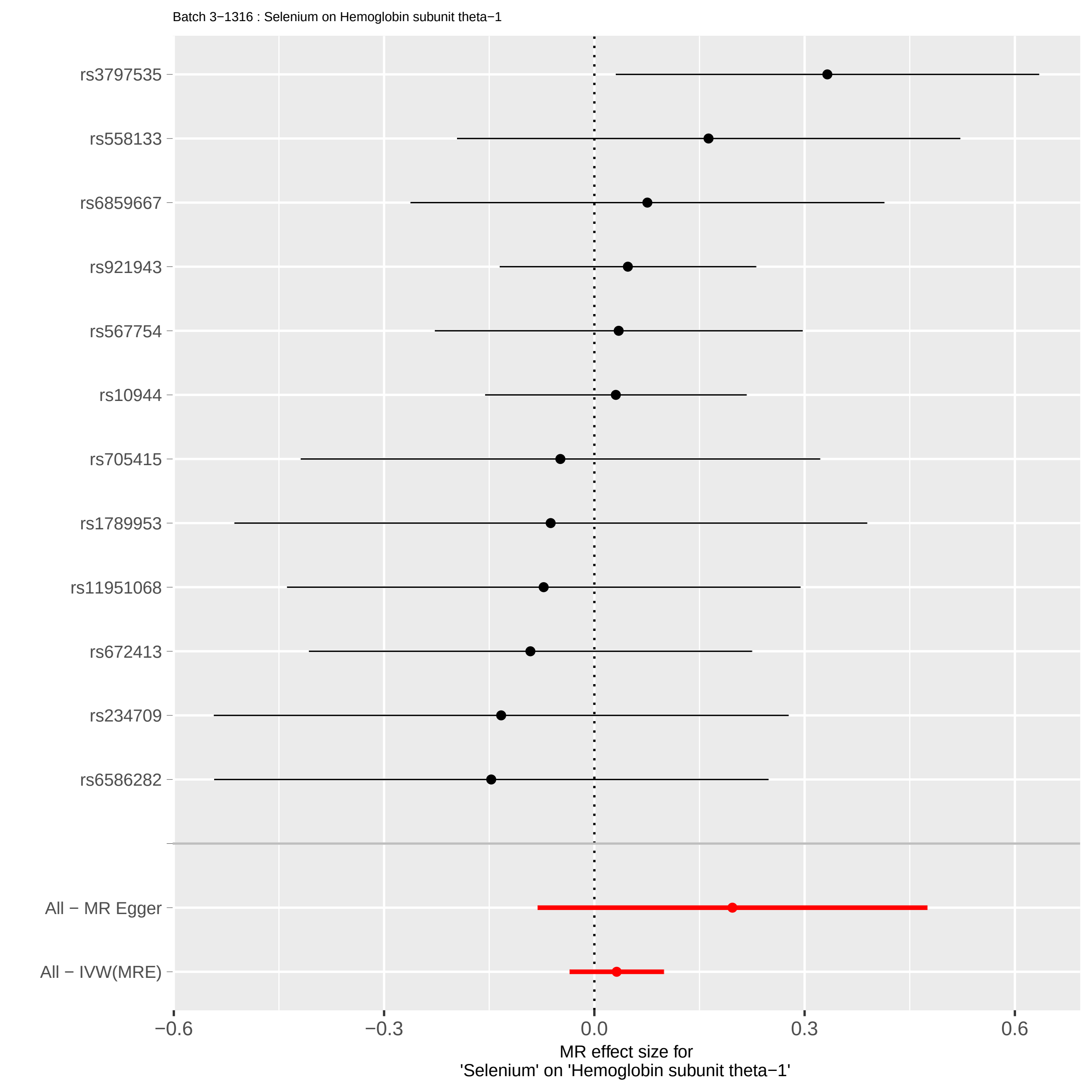

Batch 3−1316 : Selenium on Hemoglobin subunit theta−1
rs3797535
rs558133
rs6859667
rs921943
rs567754
rs10944
rs705415
rs1789953
rs11951068
rs672413
rs234709
rs6586282
All − MR Egger
All − IVW(MRE)
−0.6
−0.3
0.0
0.3
0.6
MR effect size for
'Selenium' on 'Hemoglobin subunit theta−1'

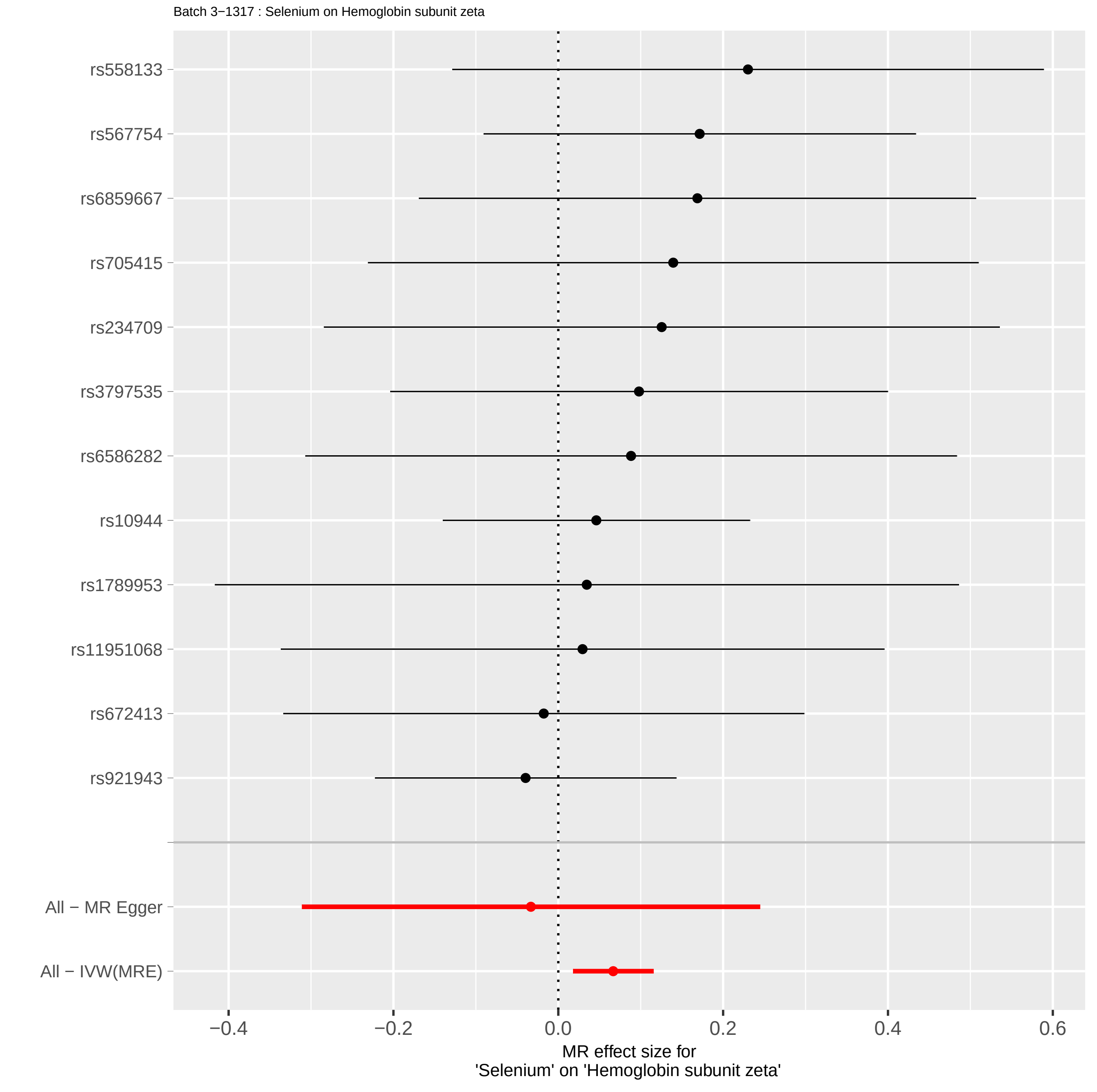
Batch 3−1317 : Selenium on Hemoglobin subunit zeta
rs558133
rs567754
rs6859667
rs705415
rs234709
rs3797535
rs6586282
rs10944
rs1789953
rs11951068
rs672413
rs921943
All − MR Egger
All − IVW(MRE)
−0.4
−0.2
0.0
0.2
0.4
0.6
MR effect size for
'Selenium' on 'Hemoglobin subunit zeta'

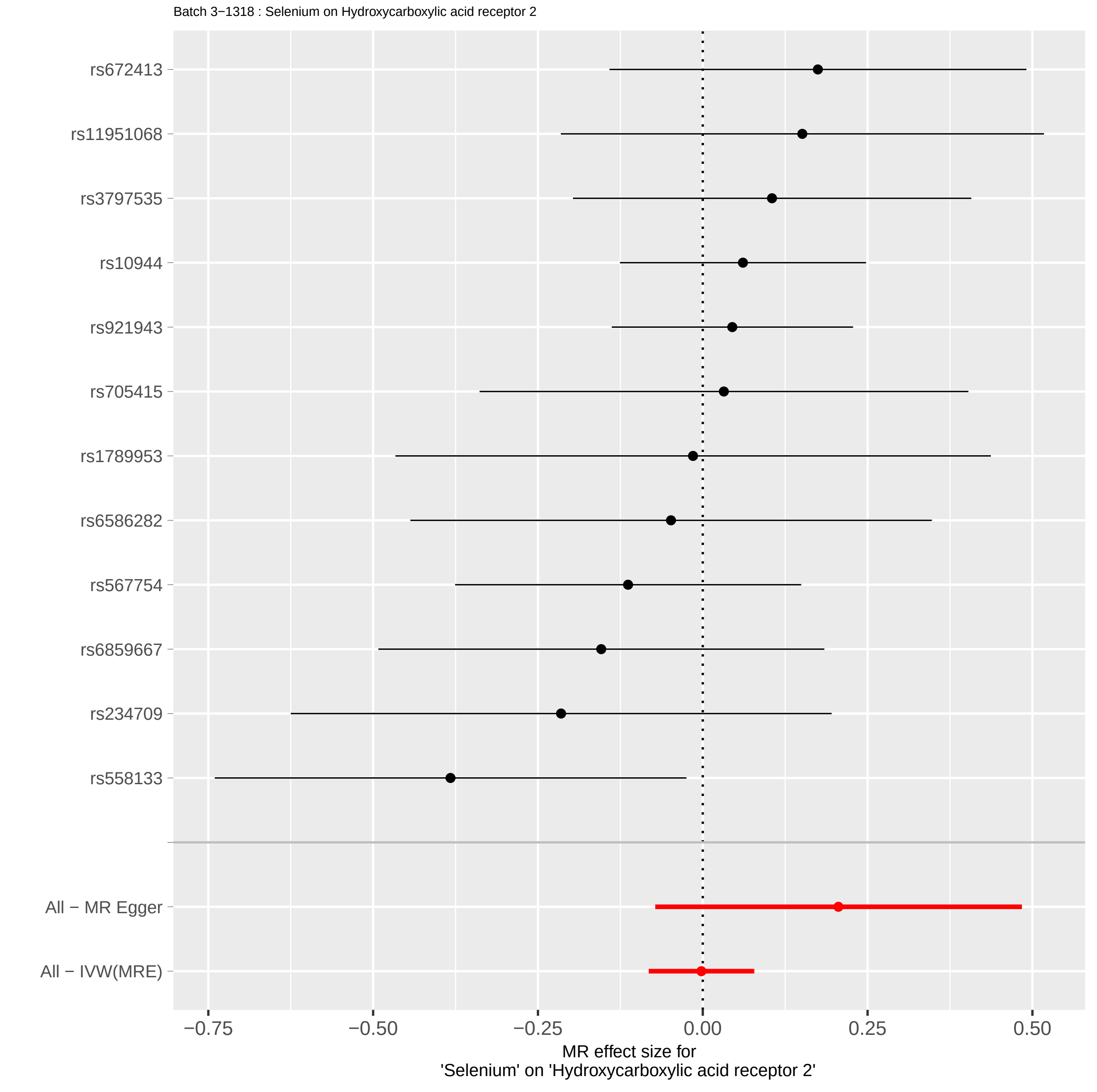

Batch 3−1318 : Selenium on Hydroxycarboxylic acid receptor 2
rs672413
rs11951068
rs3797535
rs10944
rs921943
rs705415
rs1789953
rs6586282
rs567754
rs6859667
rs234709
rs558133
All − MR Egger
All − IVW(MRE)
−0.75
−0.50
−0.25
0.00
0.25
0.50
MR effect size for
'Selenium' on 'Hydroxycarboxylic acid receptor 2'

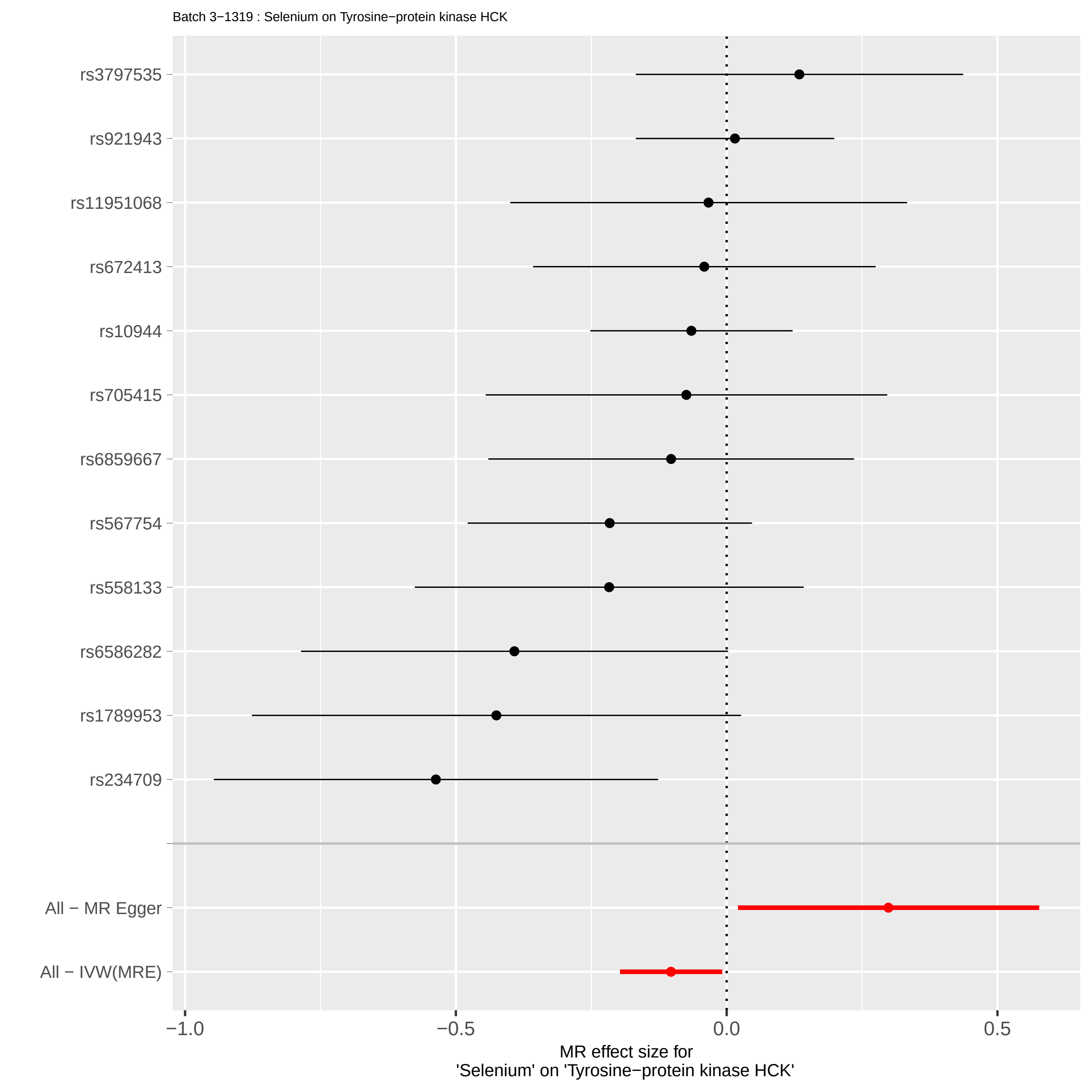

Batch 3−1319 : Selenium on Tyrosine−protein kinase HCK
rs3797535
rs921943
rs11951068
rs672413
rs10944
rs705415
rs6859667
rs567754
rs558133
rs6586282
rs1789953
rs234709
All − MR Egger
All − IVW(MRE)
−1.0
−0.5
0.0
0.5
MR effect size for
'Selenium' on 'Tyrosine−protein kinase HCK'

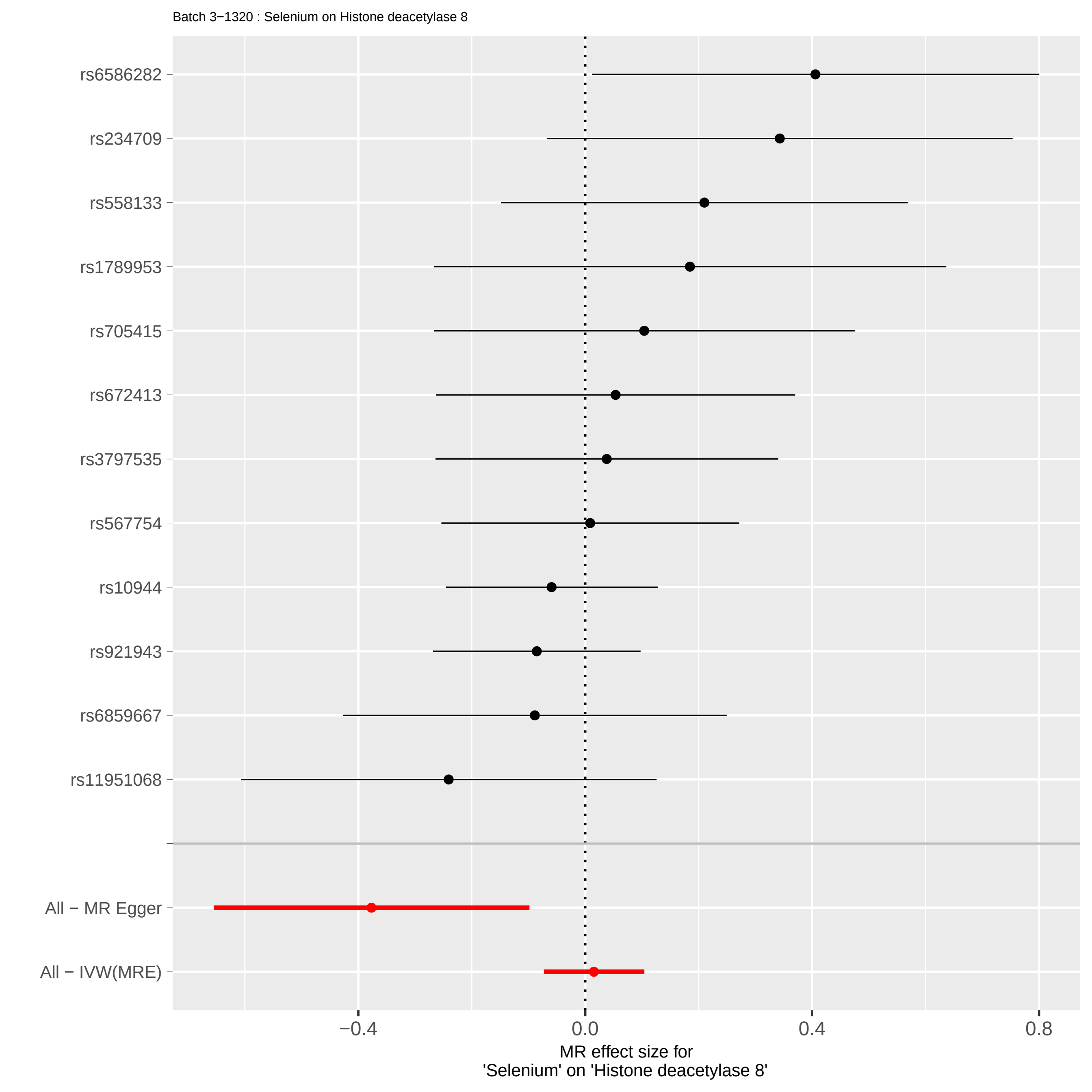
Batch 3−1320 : Selenium on Histone deacetylase 8
rs6586282
rs234709
rs558133
rs1789953
rs705415
rs672413
rs3797535
rs567754
rs10944
rs921943
rs6859667
rs11951068
All − MR Egger
All − IVW(MRE)
−0.4
0.0
0.4
0.8
MR effect size for
'Selenium' on 'Histone deacetylase 8'

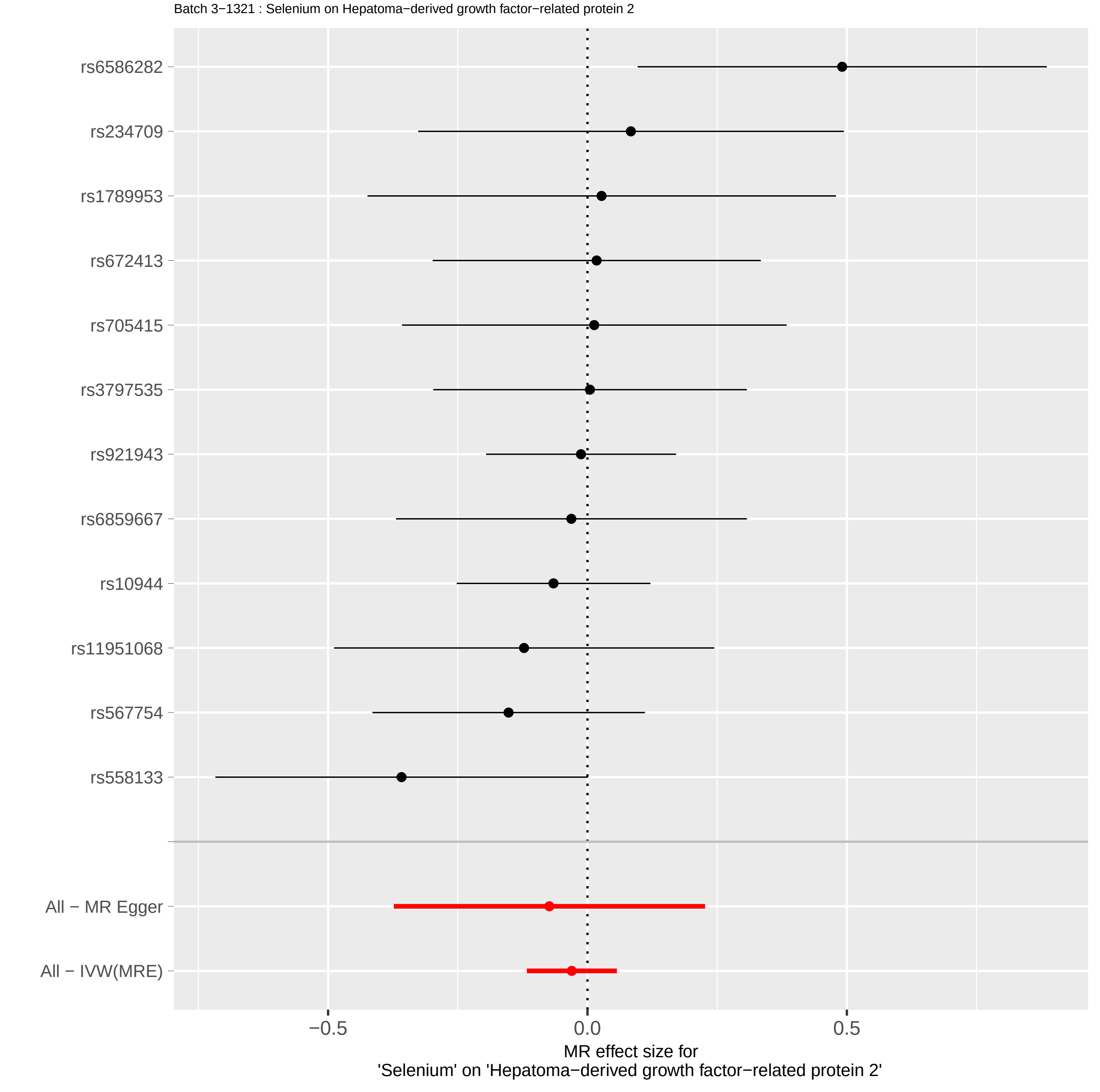

Batch 3−1321 : Selenium on Hepatoma−derived growth factor−related protein 2
rs6586282
rs234709
rs1789953
rs672413
rs705415
rs3797535
rs921943
rs6859667
rs10944
rs11951068
rs567754
rs558133
All − MR Egger
All − IVW(MRE)
−0.5
0.0
0.5
MR effect size for
'Selenium' on 'Hepatoma−derived growth factor−related protein 2'

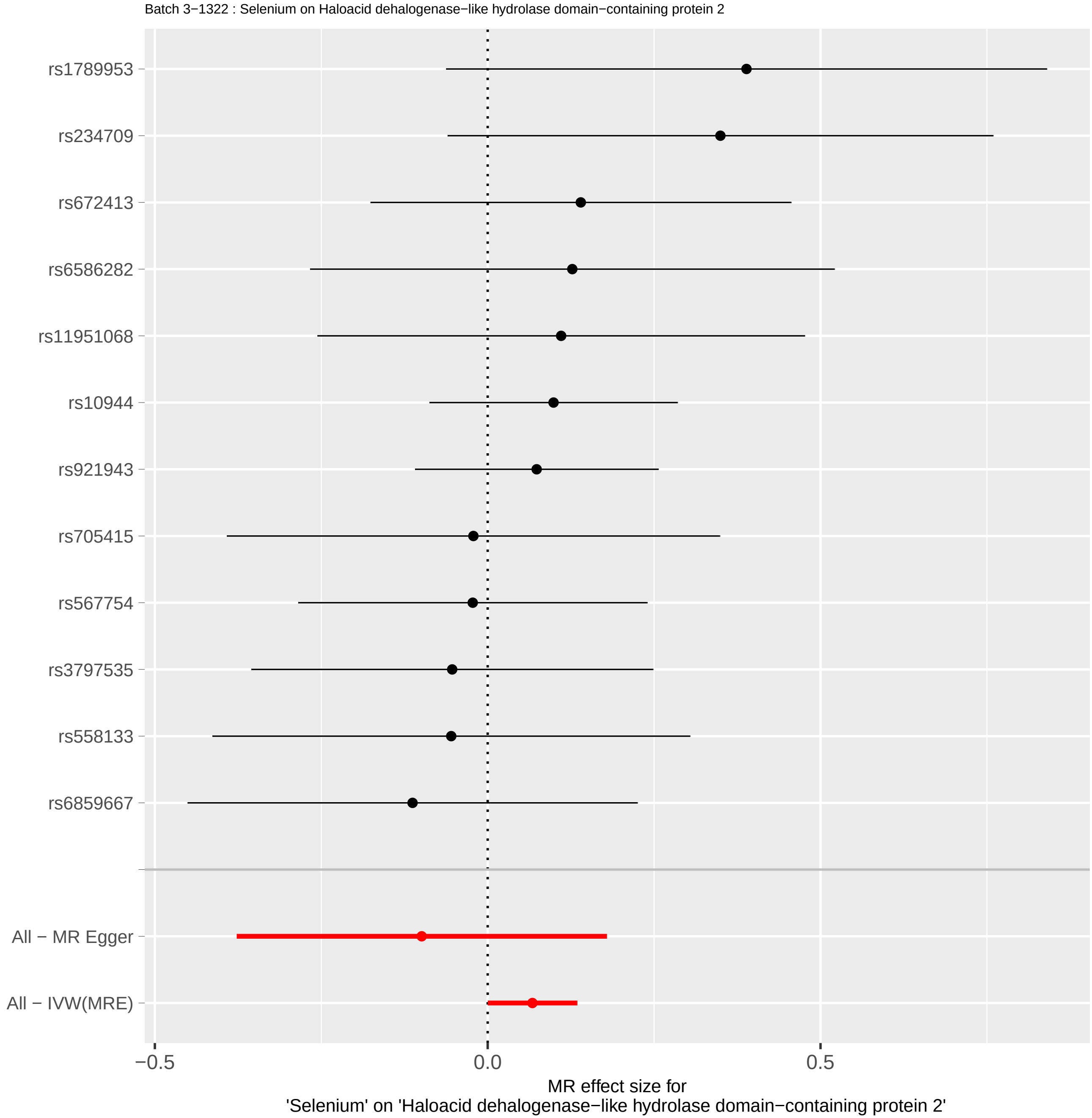

Batch 3−1322 : Selenium on Haloacid dehalogenase−like hydrolase domain−containing protein 2
rs1789953
rs234709
rs672413
rs6586282
rs11951068
rs10944
rs921943
rs705415
rs567754
rs3797535
rs558133
rs6859667
All − MR Egger
All − IVW(MRE)
−0.5
0.0
0.5
MR effect size for
'Selenium' on 'Haloacid dehalogenase−like hydrolase domain−containing protein 2'

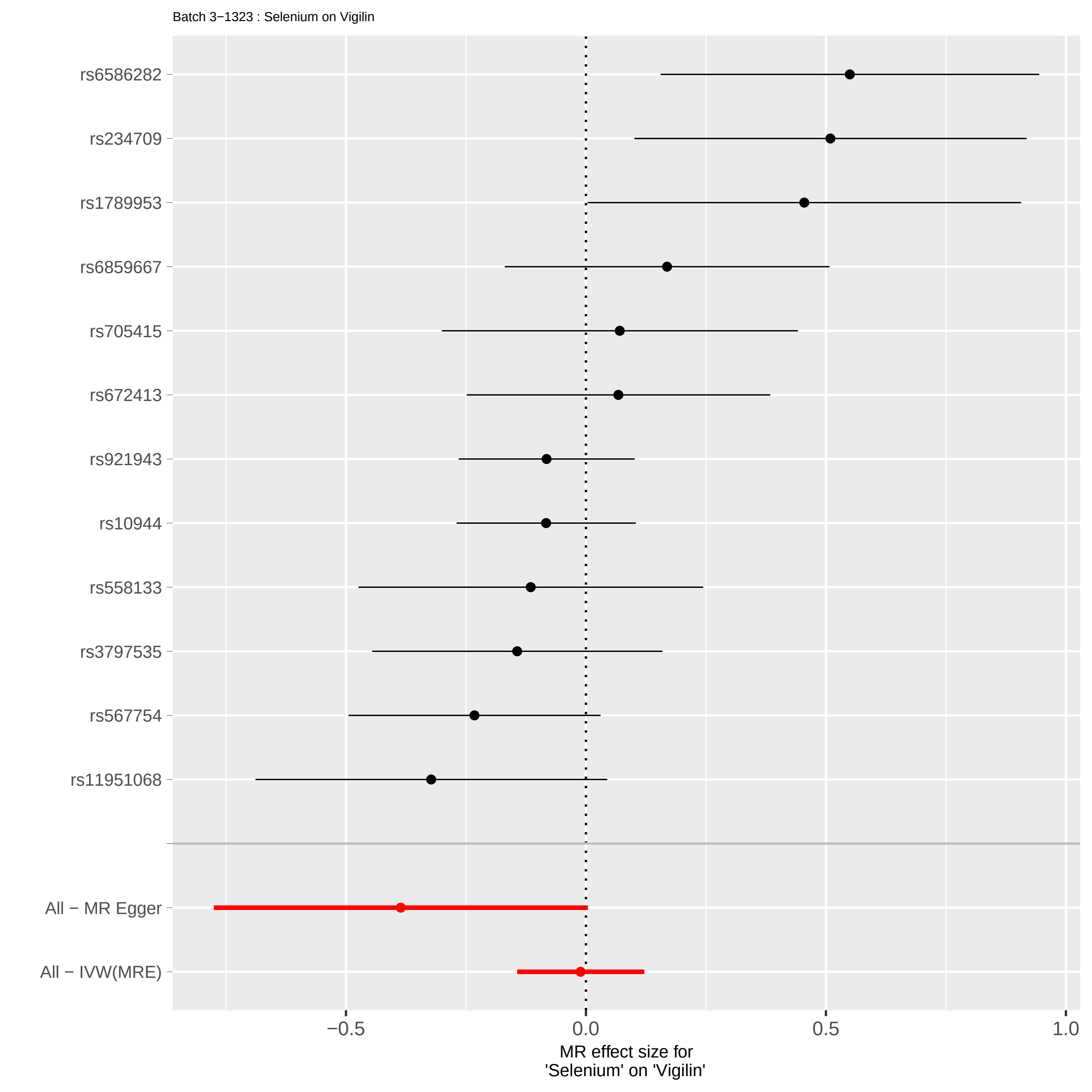
Batch 3−1323 : Selenium on Vigilin
rs6586282
rs234709
rs1789953
rs6859667
rs705415
rs672413
rs921943
rs10944
rs558133
rs3797535
rs567754
rs11951068
All − MR Egger
All − IVW(MRE)
−0.5
0.0
0.5
1.0
MR effect size for
'Selenium' on 'Vigilin'

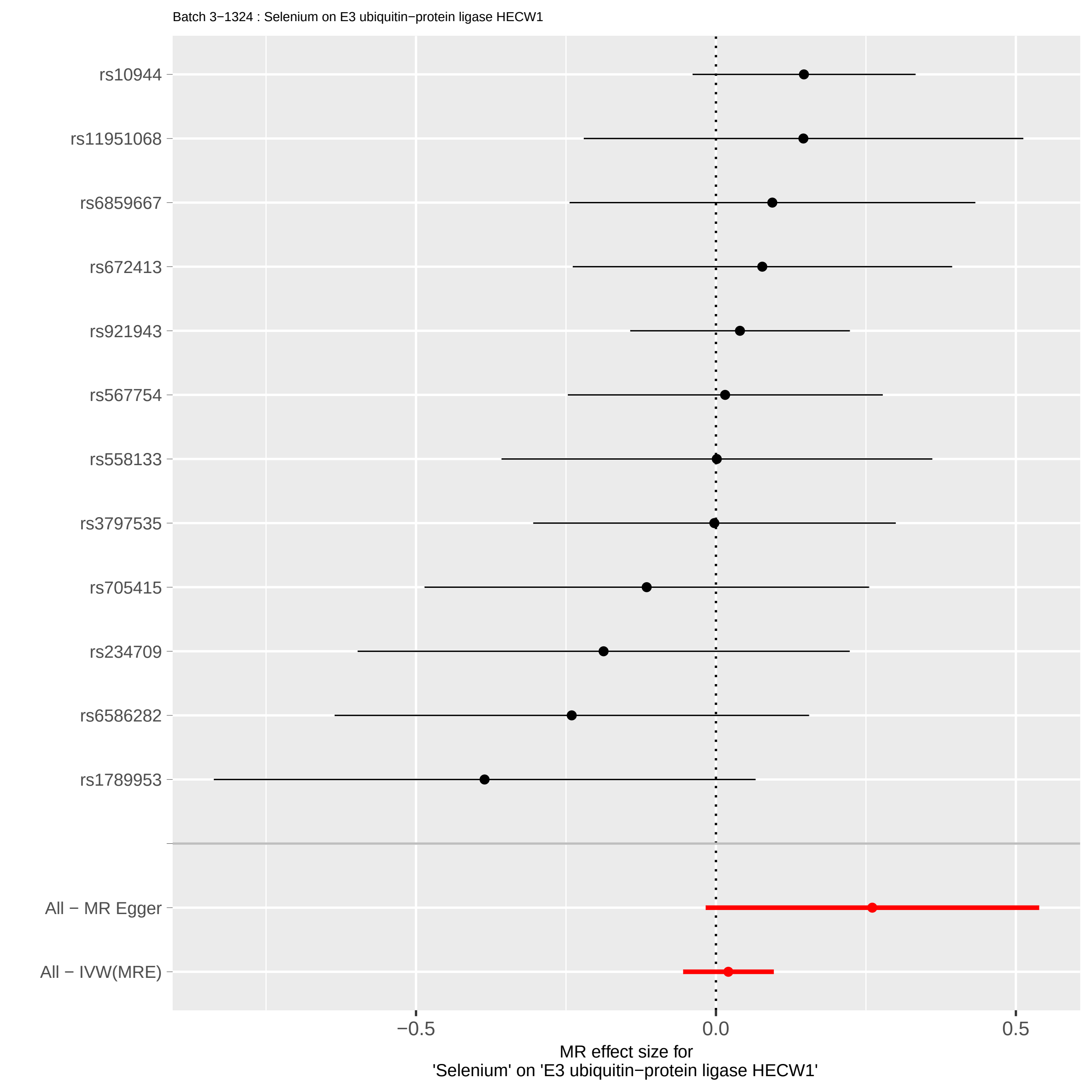
Batch 3−1324 : Selenium on E3 ubiquitin−protein ligase HECW1
rs10944
rs11951068
rs6859667
rs672413
rs921943
rs567754
rs558133
rs3797535
rs705415
rs234709
rs6586282
rs1789953
All − MR Egger
All − IVW(MRE)
−0.5
0.0
0.5
MR effect size for
'Selenium' on 'E3 ubiquitin−protein ligase HECW1'

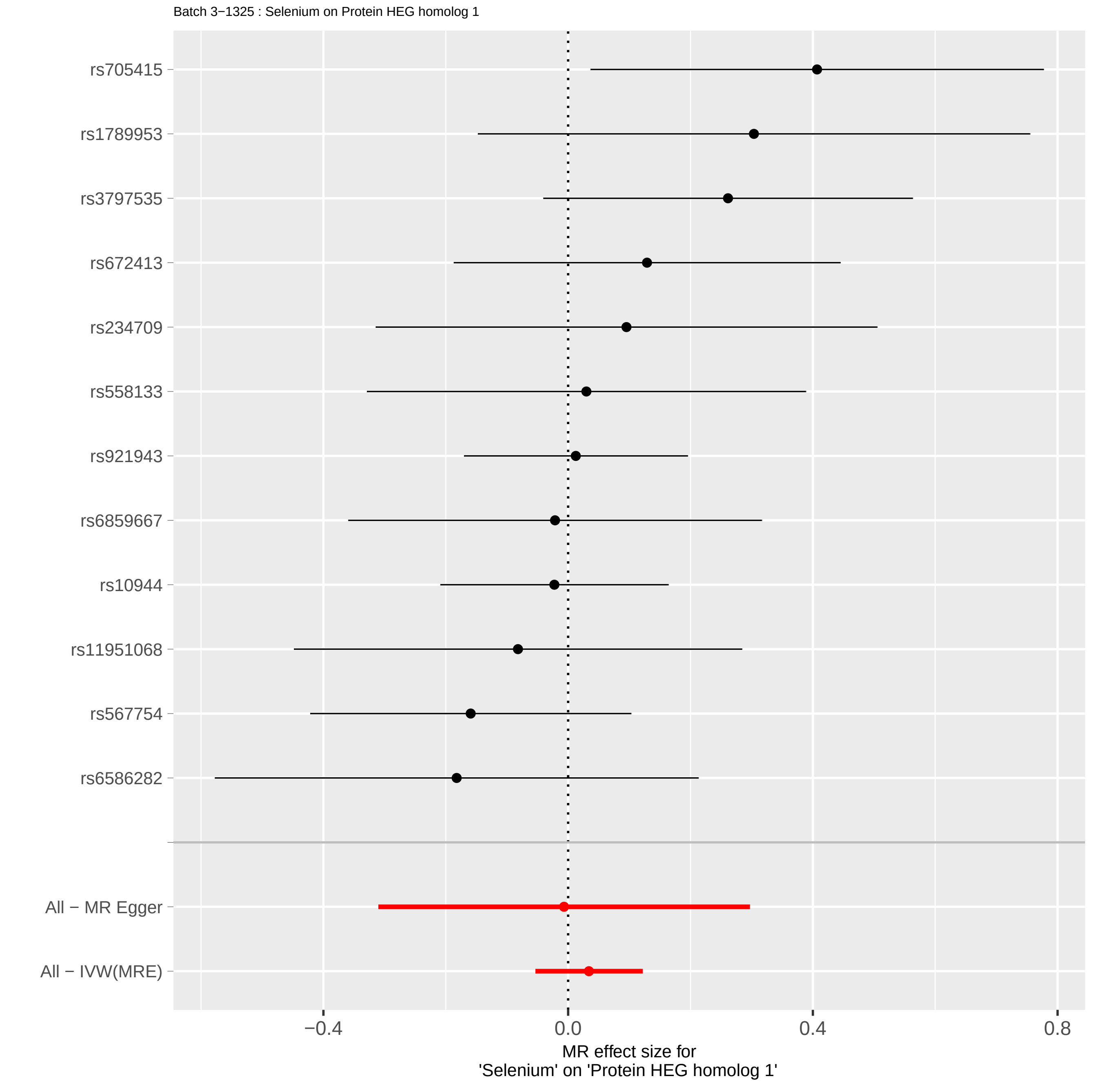

Batch 3−1325 : Selenium on Protein HEG homolog 1
rs705415
rs1789953
rs3797535
rs672413
rs234709
rs558133
rs921943
rs6859667
rs10944
rs11951068
rs567754
rs6586282
All − MR Egger
All − IVW(MRE)
−0.4
0.0
0.4
0.8
MR effect size for
'Selenium' on 'Protein HEG homolog 1'

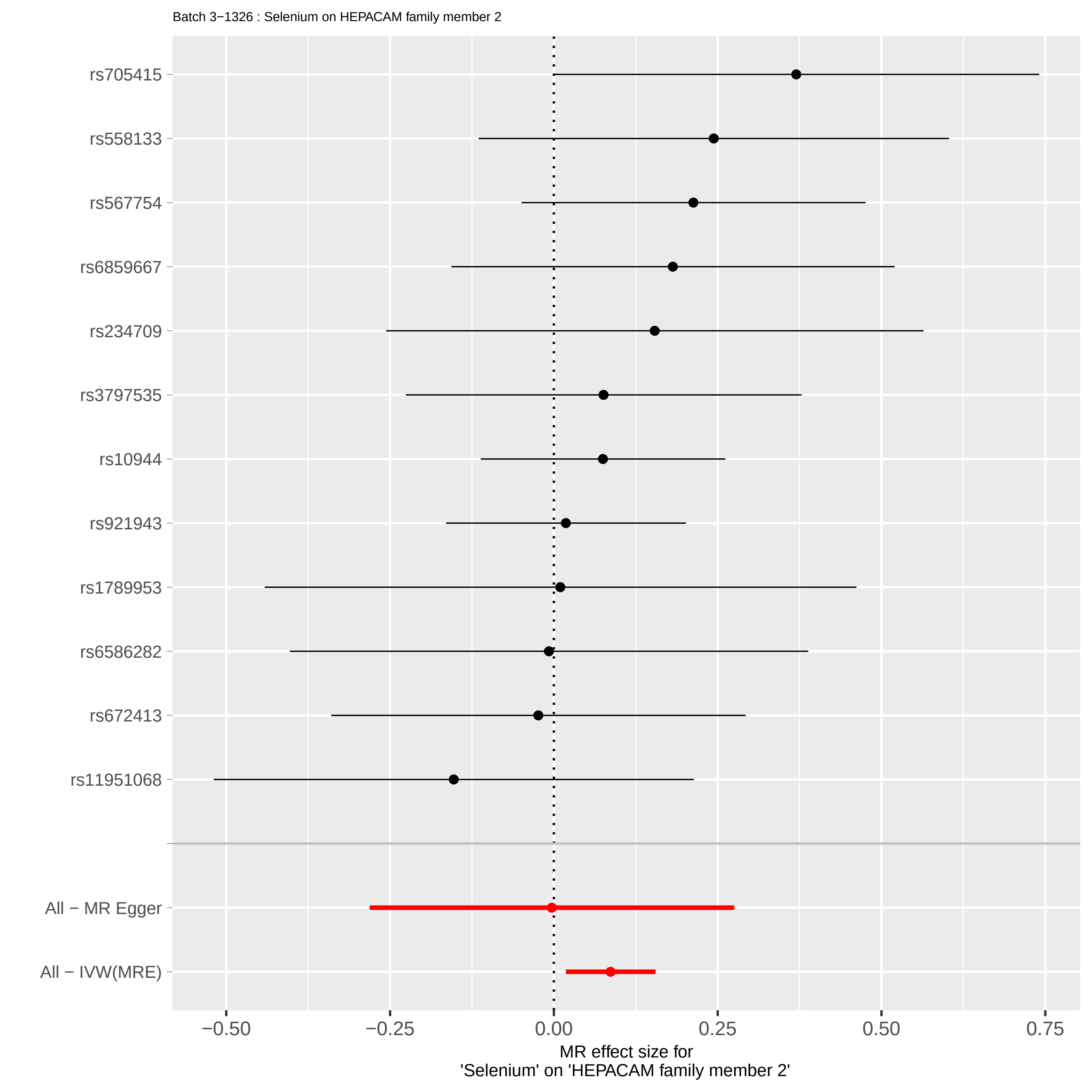

Batch 3−1326 : Selenium on HEPACAM family member 2
rs705415
rs558133
rs567754
rs6859667
rs234709
rs3797535
rs10944
rs921943
rs1789953
rs6586282
rs672413
rs11951068
All − MR Egger
All − IVW(MRE)
−0.50
−0.25
0.00
0.25
0.50
0.75
MR effect size for
'Selenium' on 'HEPACAM family member 2'

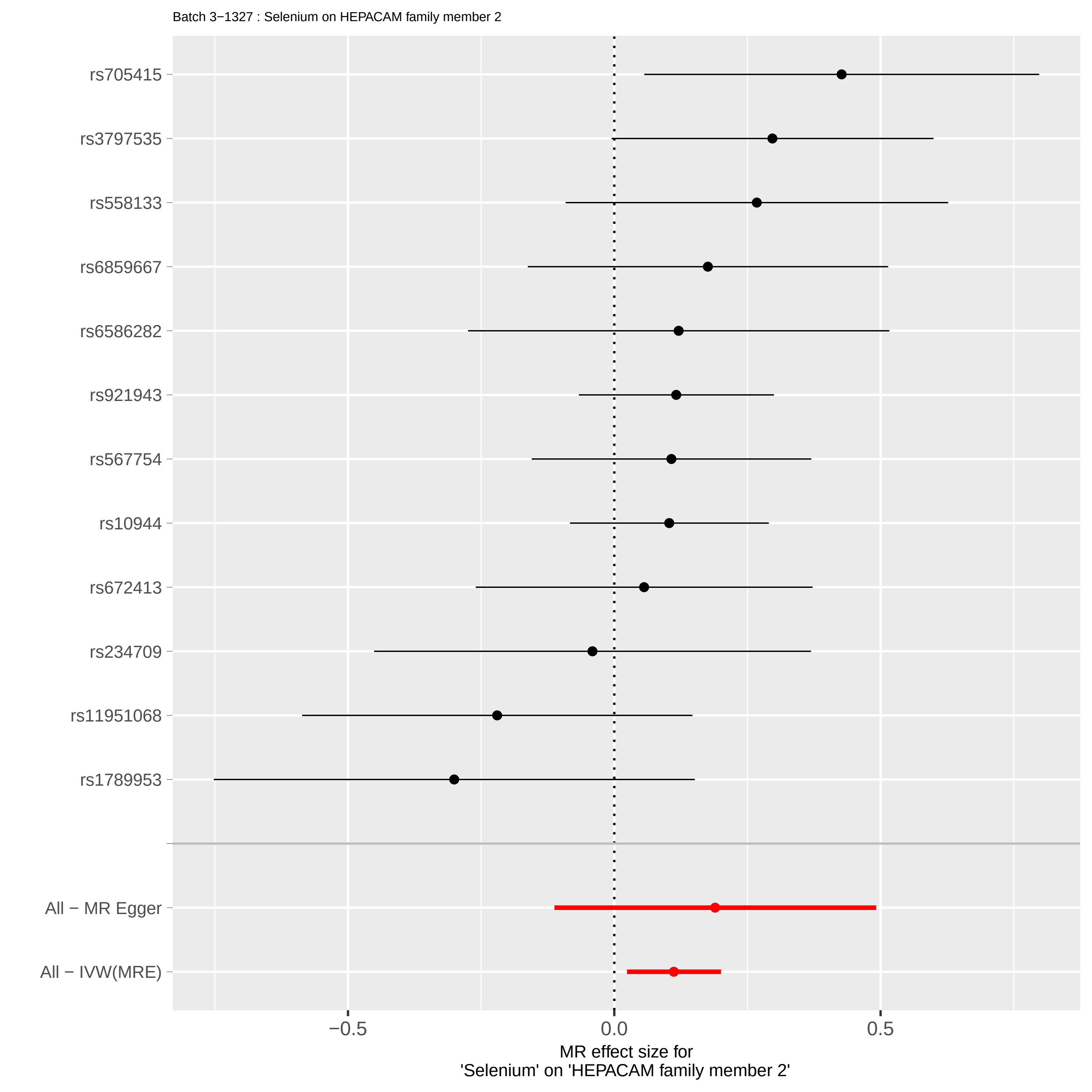

Batch 3−1327 : Selenium on HEPACAM family member 2
rs705415
rs3797535
rs558133
rs6859667
rs6586282
rs921943
rs567754
rs10944
rs672413
rs234709
rs11951068
rs1789953
All − MR Egger
All − IVW(MRE)
−0.5
0.0
0.5
MR effect size for
'Selenium' on 'HEPACAM family member 2'

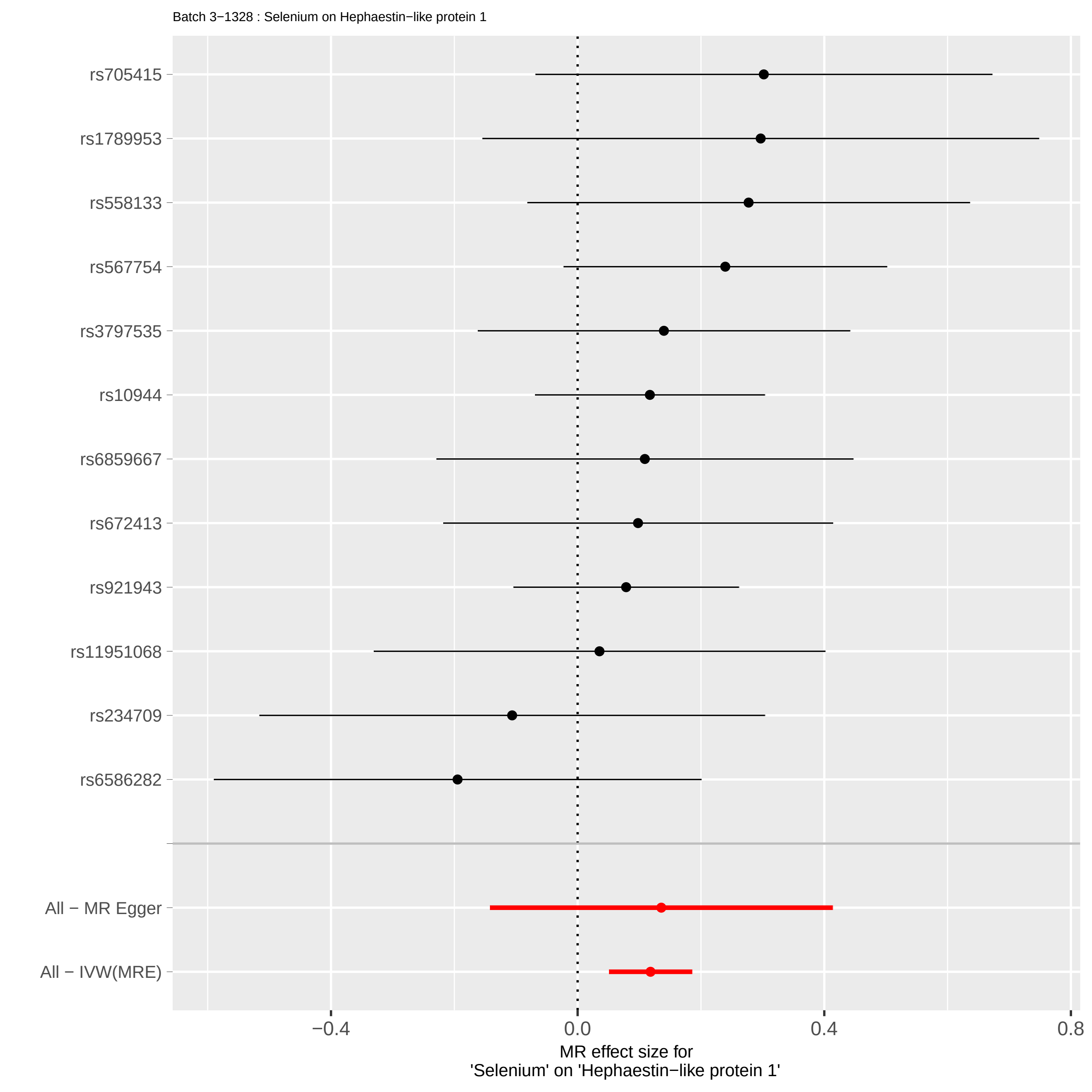

Batch 3−1328 : Selenium on Hephaestin−like protein 1
rs705415
rs1789953
rs558133
rs567754
rs3797535
rs10944
rs6859667
rs672413
rs921943
rs11951068
rs234709
rs6586282
All − MR Egger
All − IVW(MRE)
−0.4
0.0
0.4
0.8
MR effect size for
'Selenium' on 'Hephaestin−like protein 1'

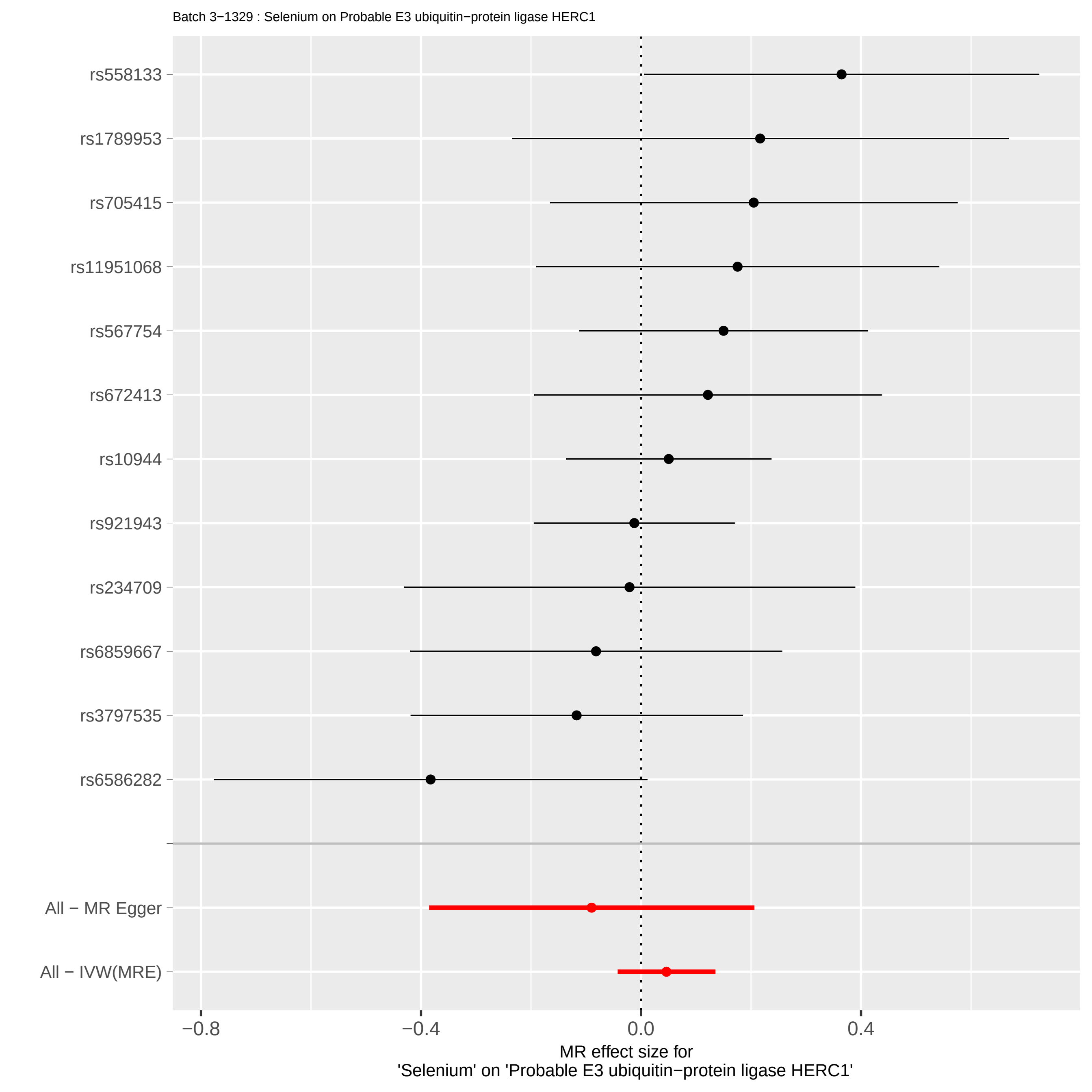

Batch 3−1329 : Selenium on Probable E3 ubiquitin−protein ligase HERC1
rs558133
rs1789953
rs705415
rs11951068
rs567754
rs672413
rs10944
rs921943
rs234709
rs6859667
rs3797535
rs6586282
All − MR Egger
All − IVW(MRE)
−0.8
−0.4
0.0
0.4
MR effect size for
'Selenium' on 'Probable E3 ubiquitin−protein ligase HERC1'

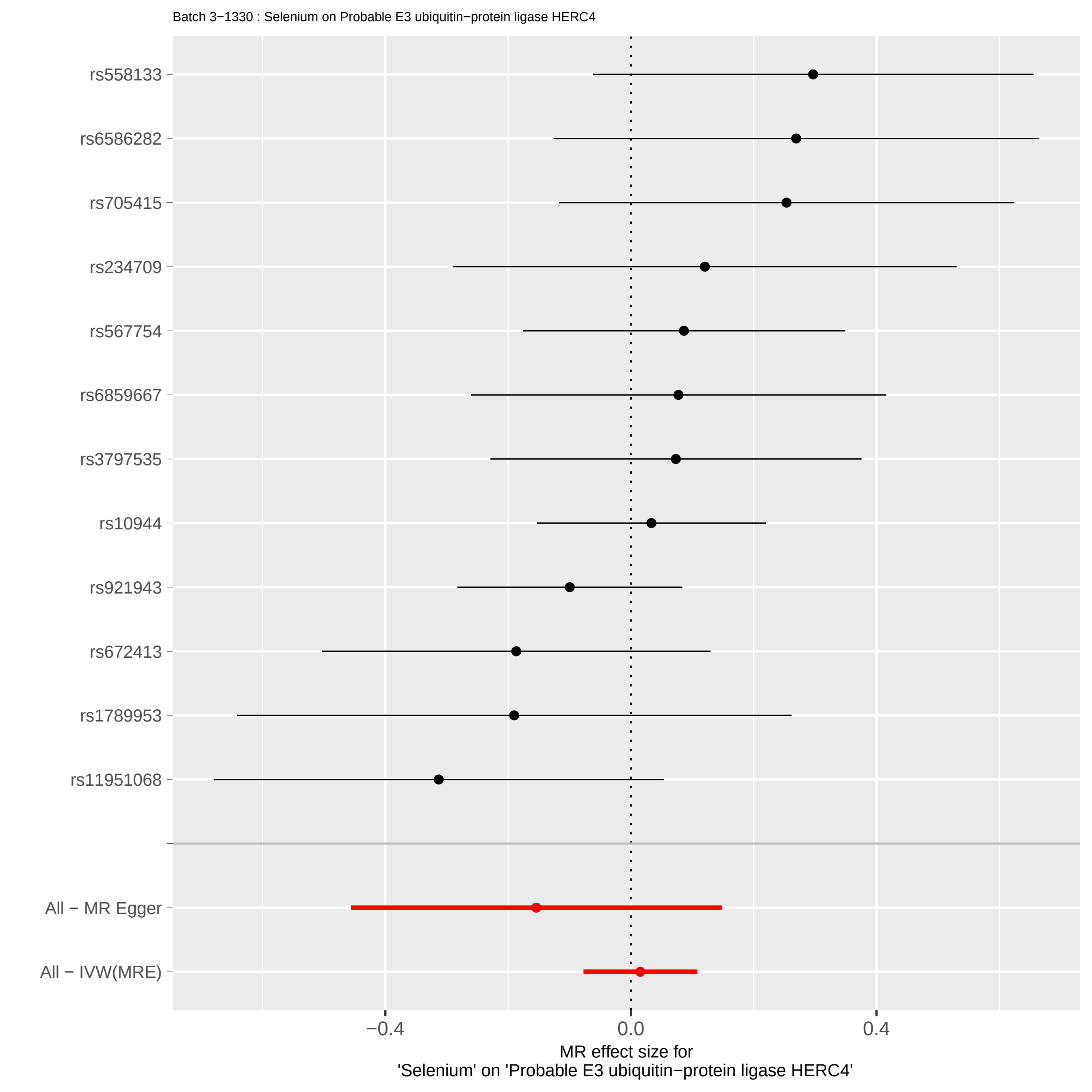

Batch 3−1330 : Selenium on Probable E3 ubiquitin−protein ligase HERC4
rs558133
rs6586282
rs705415
rs234709
rs567754
rs6859667
rs3797535
rs10944
rs921943
rs672413
rs1789953
rs11951068
All − MR Egger
All − IVW(MRE)
−0.4
0.0
0.4
MR effect size for
'Selenium' on 'Probable E3 ubiquitin−protein ligase HERC4'

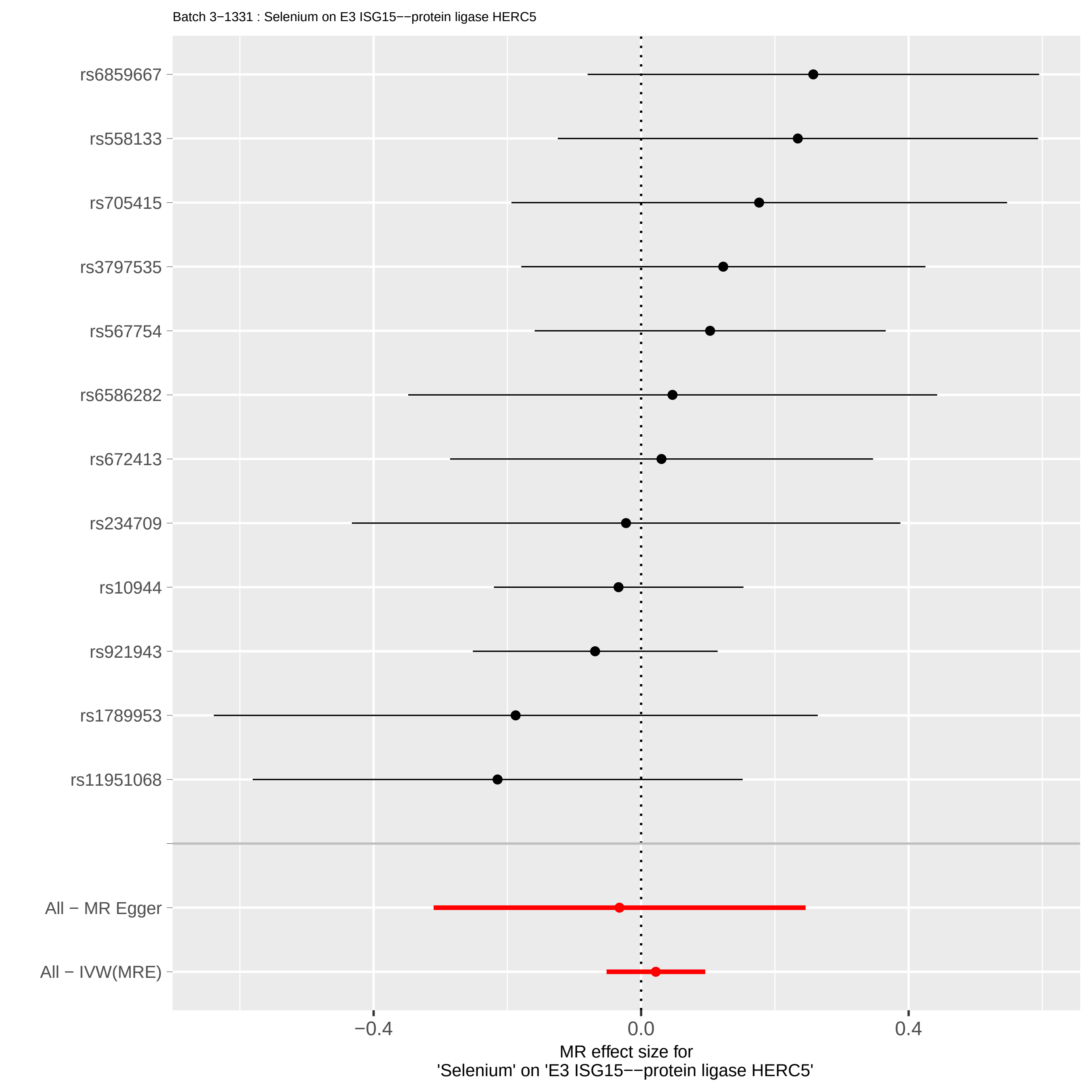

Batch 3−1331 : Selenium on E3 ISG15−−protein ligase HERC5
rs6859667
rs558133
rs705415
rs3797535
rs567754
rs6586282
rs672413
rs234709
rs10944
rs921943
rs1789953
rs11951068
All − MR Egger
All − IVW(MRE)
−0.4
0.0
0.4
MR effect size for
'Selenium' on 'E3 ISG15−−protein ligase HERC5'

Batch 3−1332 : Selenium on Hemojuvelin

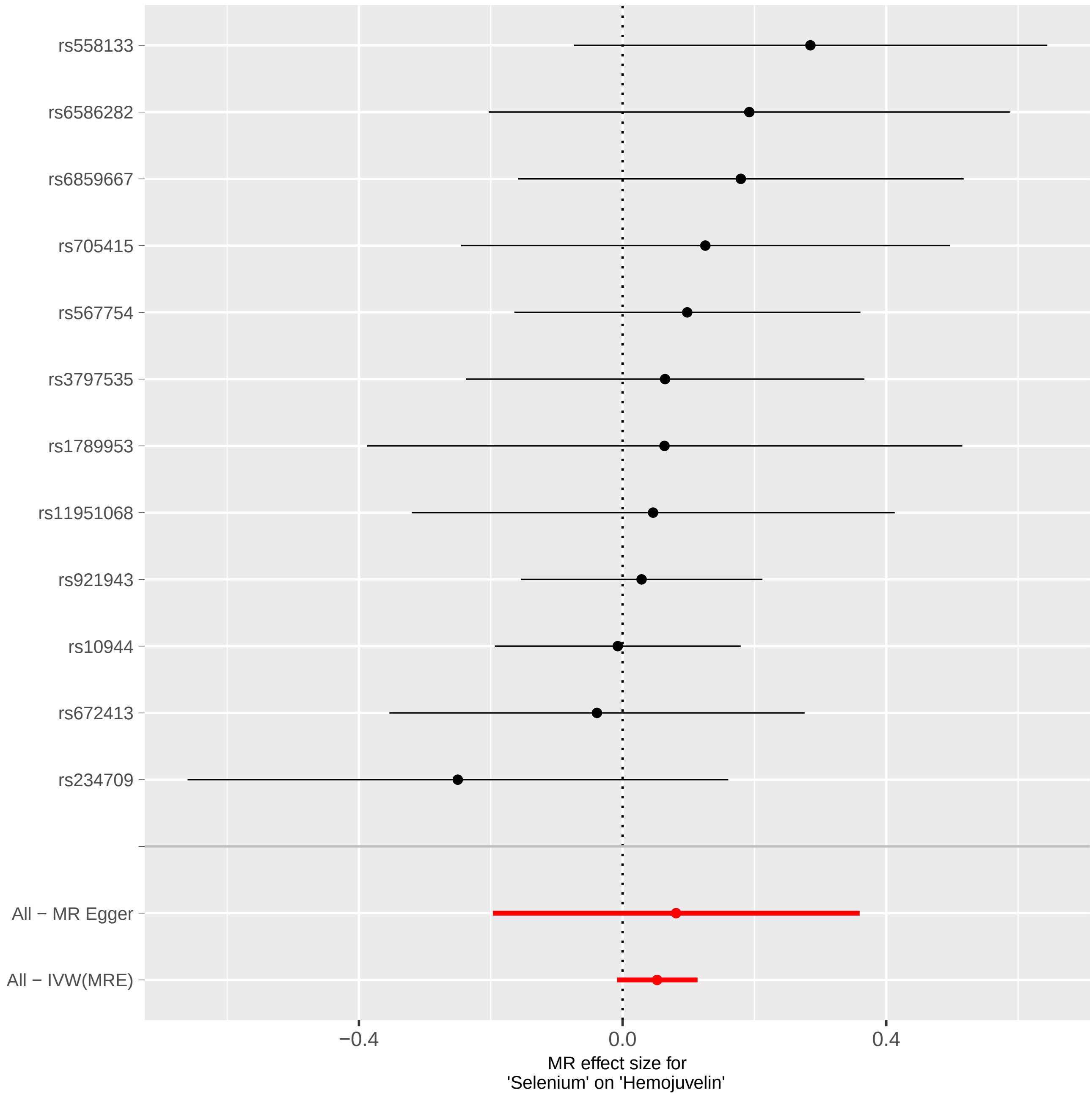

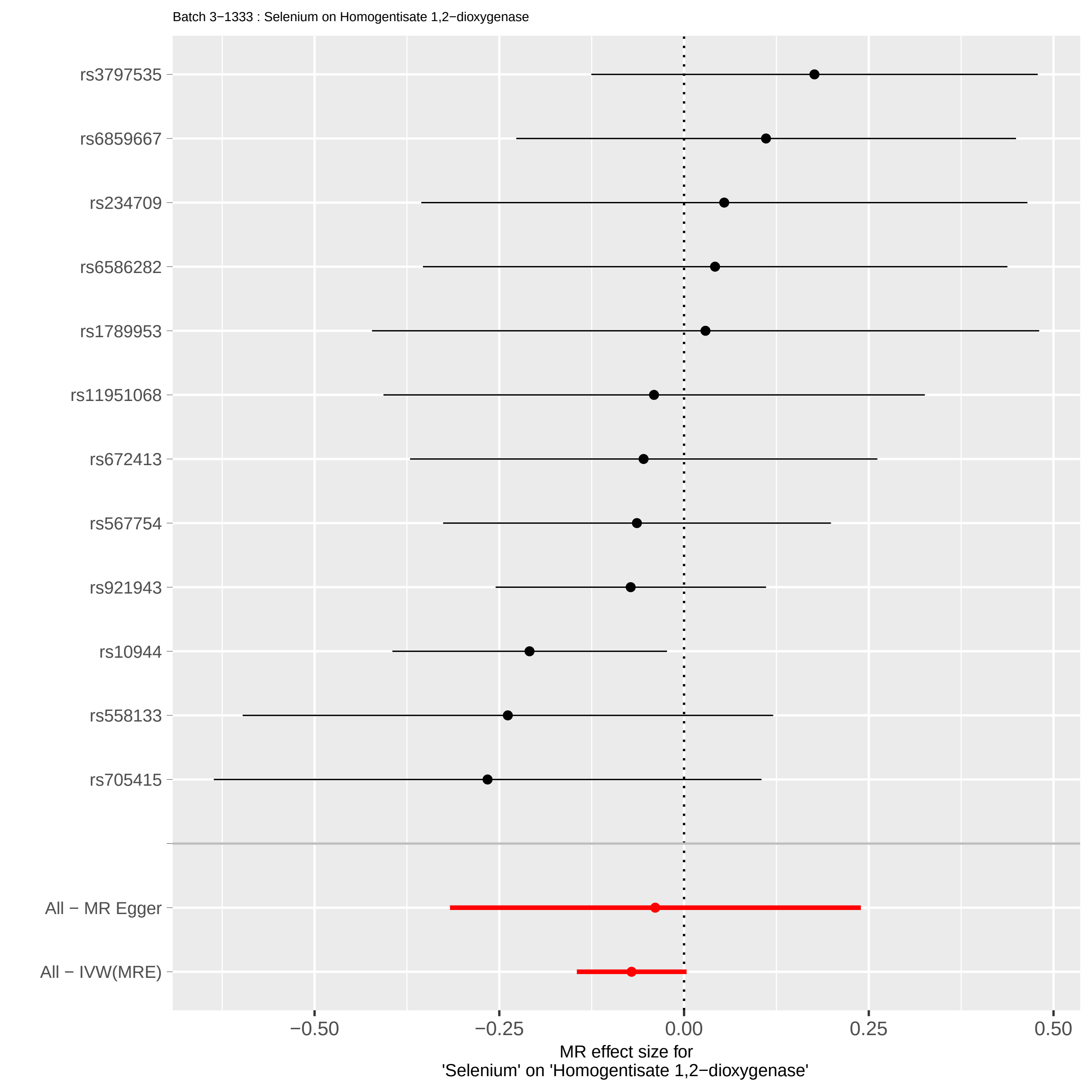
Batch 3−1333 : Selenium on Homogentisate 1,2−dioxygenase
rs3797535
rs6859667
rs234709
rs6586282
rs1789953
rs11951068
rs672413
rs567754
rs921943
rs10944
rs558133
rs705415
All − MR Egger
All − IVW(MRE)
−0.50
−0.25
0.00
0.25
0.50
MR effect size for
'Selenium' on 'Homogentisate 1,2−dioxygenase'

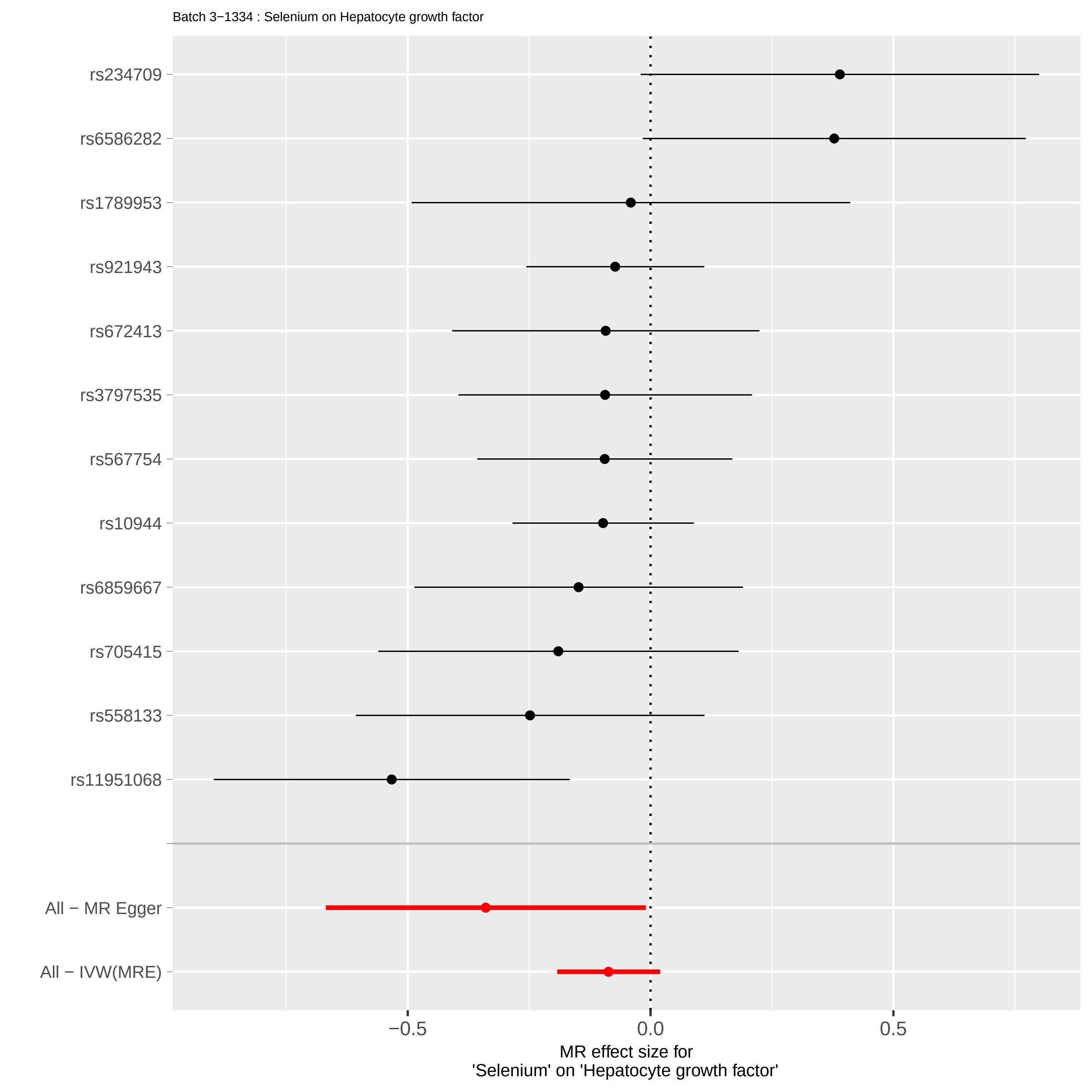

Batch 3−1334 : Selenium on Hepatocyte growth factor
rs234709
rs6586282
rs1789953
rs921943
rs672413
rs3797535
rs567754
rs10944
rs6859667
rs705415
rs558133
rs11951068
All − MR Egger
All − IVW(MRE)
−0.5
0.0
0.5
MR effect size for
'Selenium' on 'Hepatocyte growth factor'

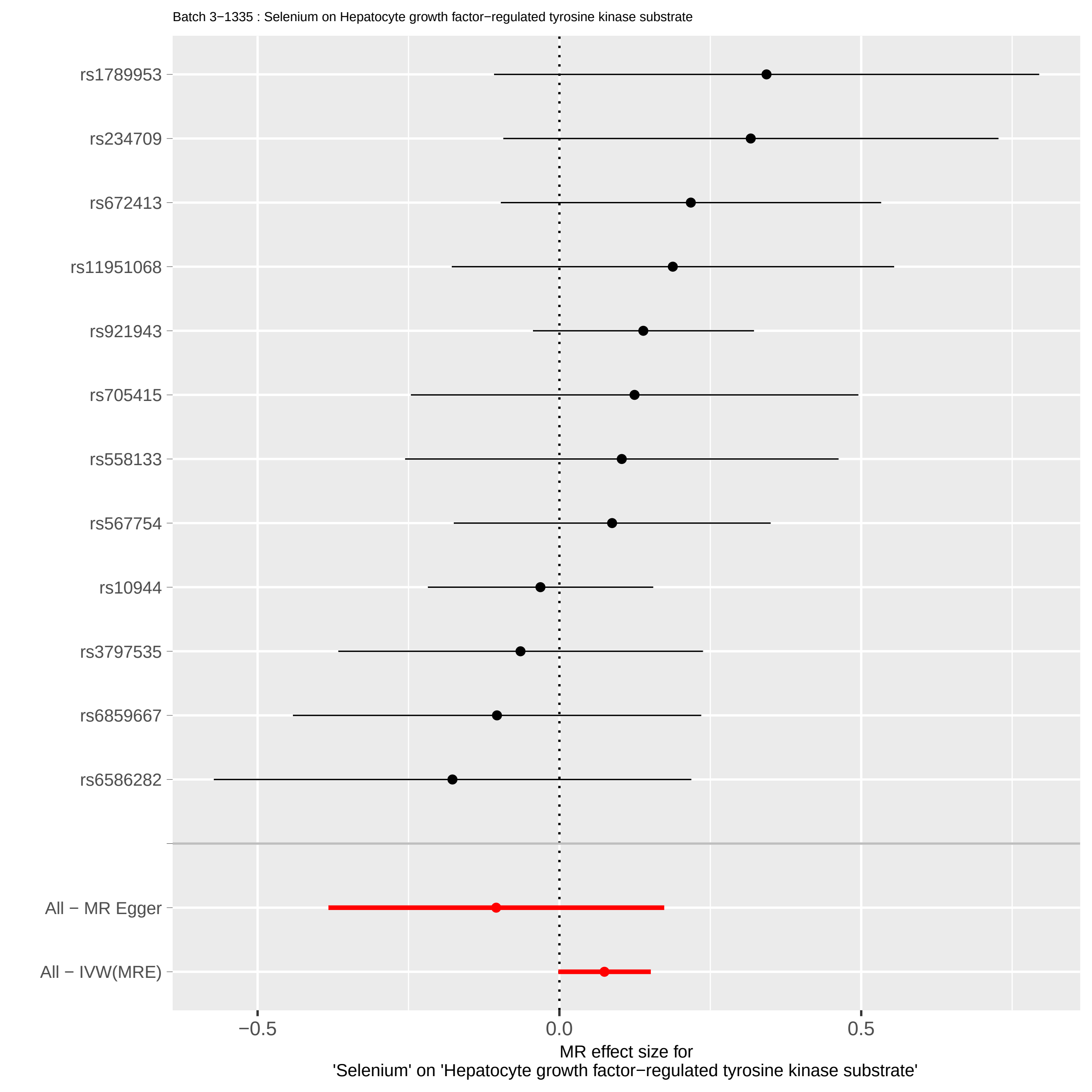
Batch 3−1335 : Selenium on Hepatocyte growth factor−regulated tyrosine kinase substrate
rs1789953
rs234709
rs672413
rs11951068
rs921943
rs705415
rs558133
rs567754
rs10944
rs3797535
rs6859667
rs6586282
All − MR Egger
All − IVW(MRE)
−0.5
0.0
0.5
MR effect size for
'Selenium' on 'Hepatocyte growth factor−regulated tyrosine kinase substrate'

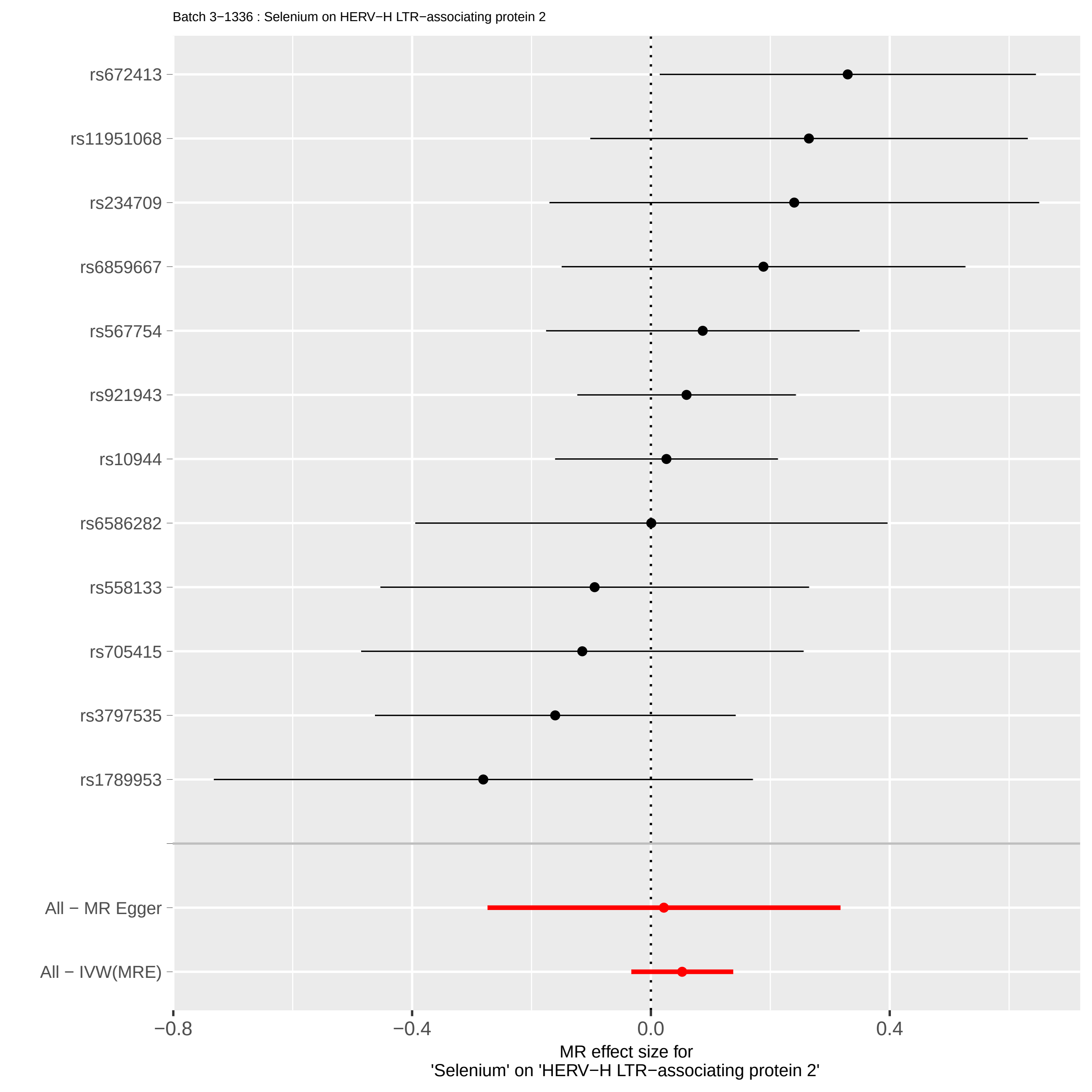

Batch 3−1336 : Selenium on HERV−H LTR−associating protein 2
rs672413
rs11951068
rs234709
rs6859667
rs567754
rs921943
rs10944
rs6586282
rs558133
rs705415
rs3797535
rs1789953
All − MR Egger
All − IVW(MRE)
−0.8
−0.4
0.0
0.4
MR effect size for
'Selenium' on 'HERV−H LTR−associating protein 2'

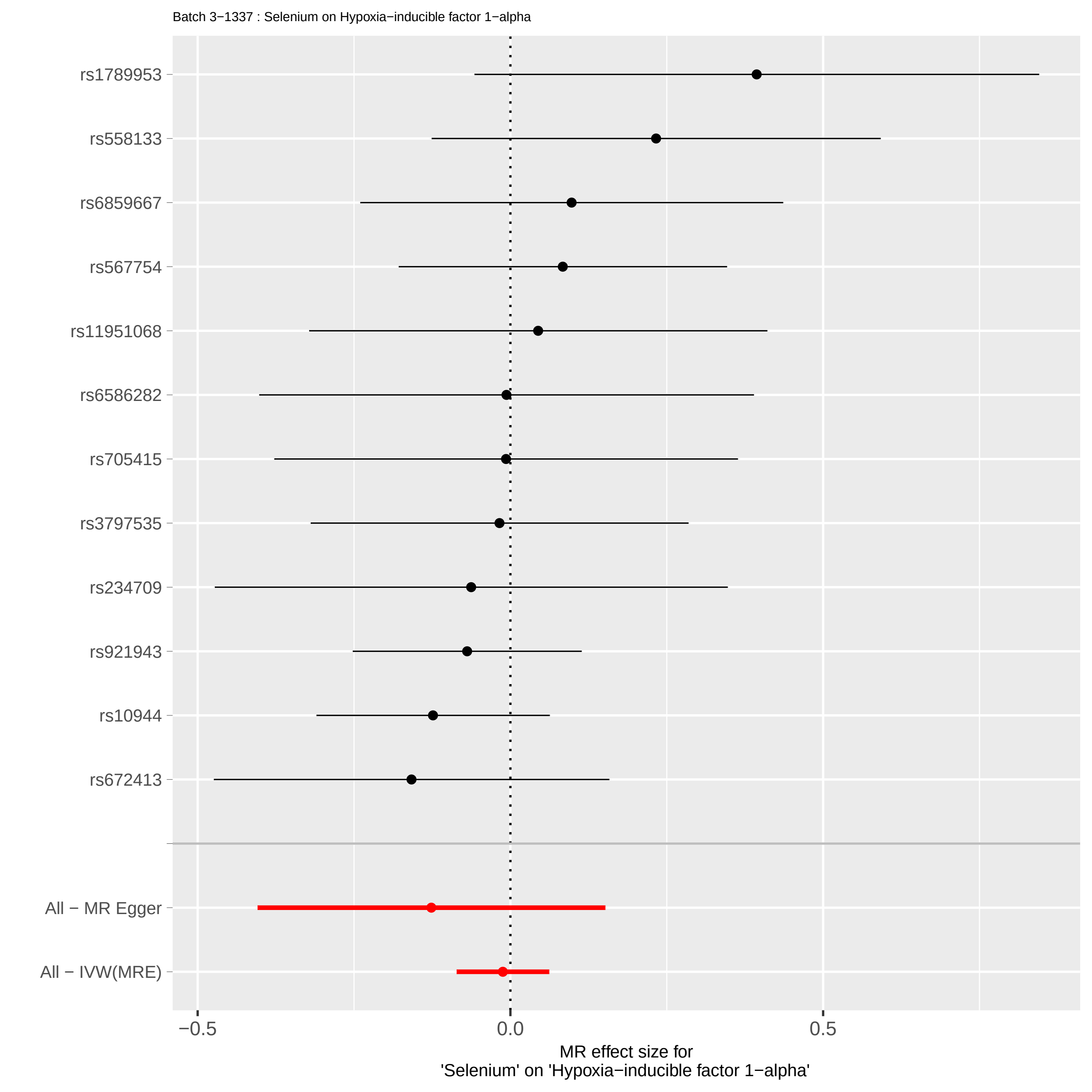
Batch 3−1337 : Selenium on Hypoxia−inducible factor 1−alpha
rs1789953
rs558133
rs6859667
rs567754
rs11951068
rs6586282
rs705415
rs3797535
rs234709
rs921943
rs10944
rs672413
All − MR Egger
All − IVW(MRE)
−0.5
0.0
0.5
MR effect size for
'Selenium' on 'Hypoxia−inducible factor 1−alpha'

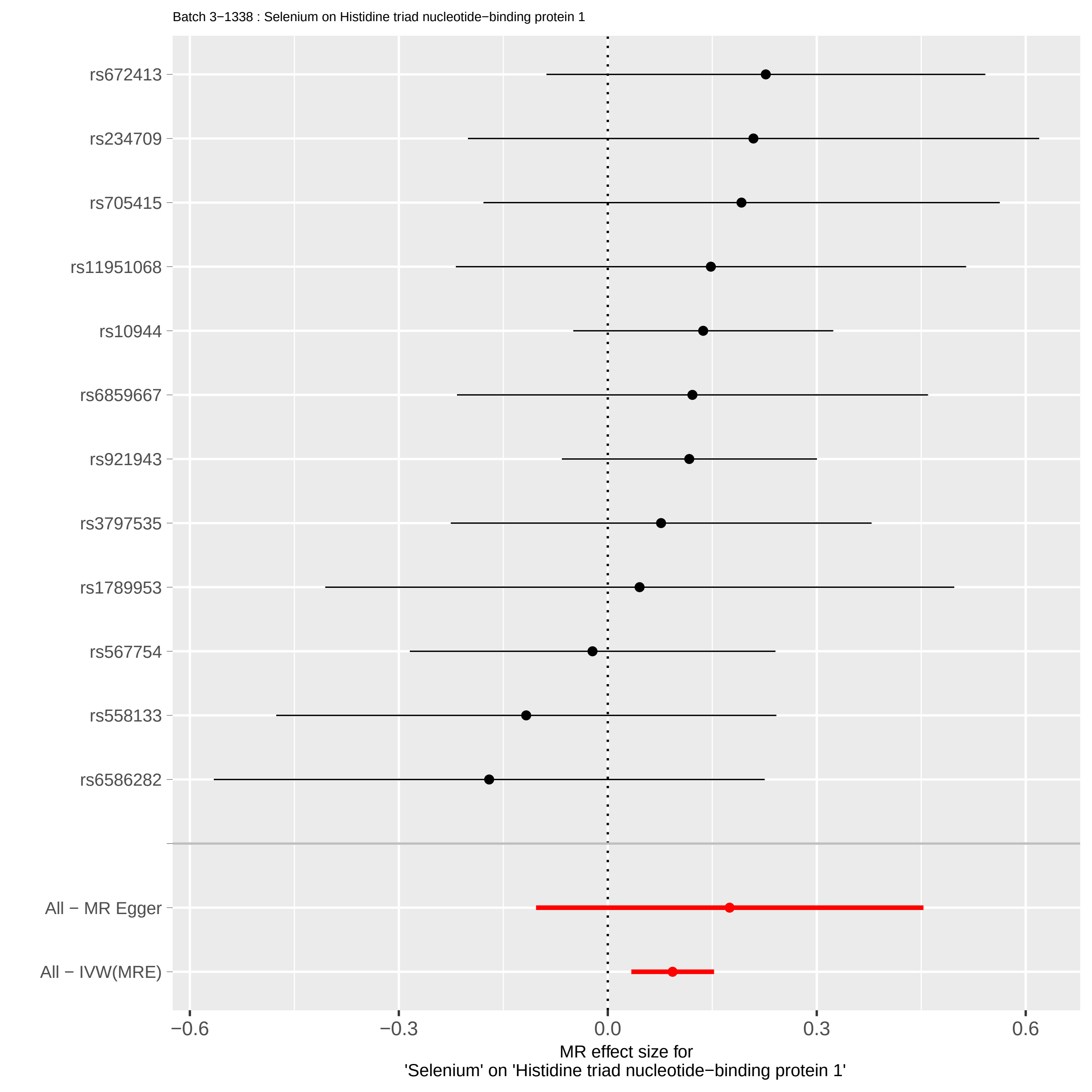

Batch 3−1338 : Selenium on Histidine triad nucleotide−binding protein 1
rs672413
rs234709
rs705415
rs11951068
rs10944
rs6859667
rs921943
rs3797535
rs1789953
rs567754
rs558133
rs6586282
All − MR Egger
All − IVW(MRE)
−0.6
−0.3
0.0
0.3
0.6
MR effect size for
'Selenium' on 'Histidine triad nucleotide−binding protein 1'

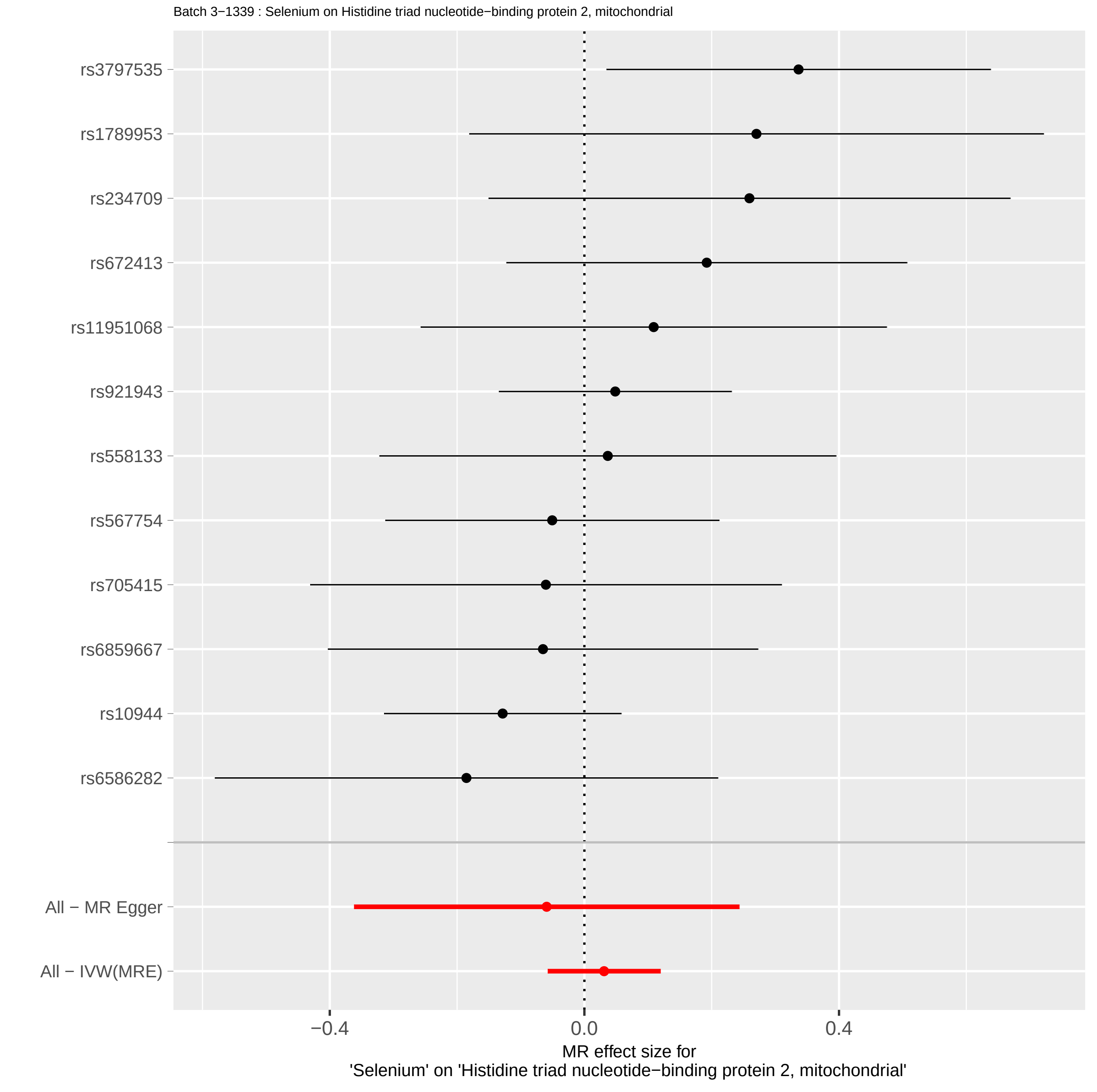
Batch 3−1339 : Selenium on Histidine triad nucleotide−binding protein 2, mitochondrial
rs3797535
rs1789953
rs234709
rs672413
rs11951068
rs921943
rs558133
rs567754
rs705415
rs6859667
rs10944
rs6586282
All − MR Egger
All − IVW(MRE)
−0.4
0.0
0.4
MR effect size for
'Selenium' on 'Histidine triad nucleotide−binding protein 2, mitochondrial'

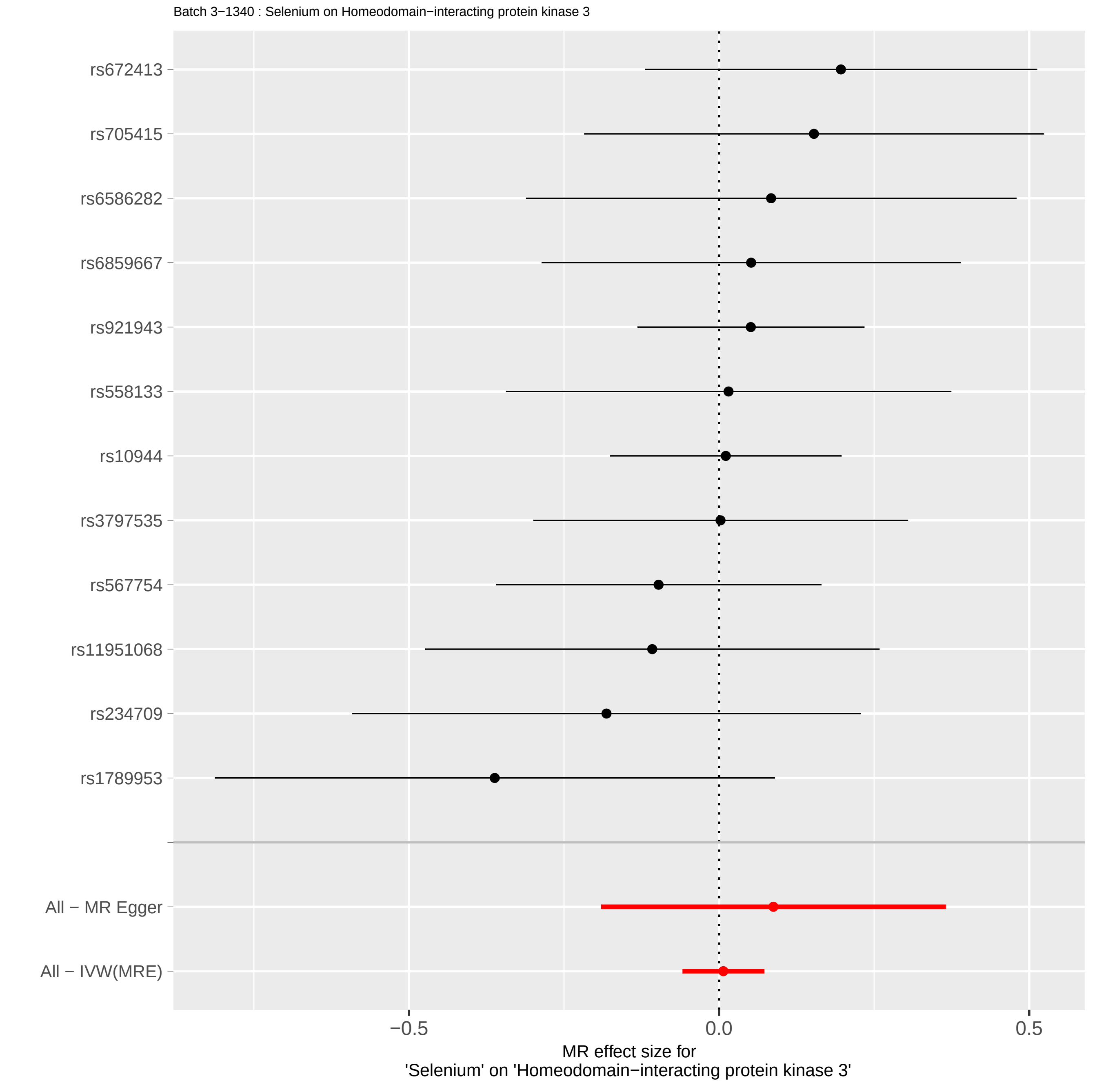

Batch 3−1340 : Selenium on Homeodomain−interacting protein kinase 3
rs672413
rs705415
rs6586282
rs6859667
rs921943
rs558133
rs10944
rs3797535
rs567754
rs11951068
rs234709
rs1789953
All − MR Egger
All − IVW(MRE)
−0.5
0.0
0.5
MR effect size for
'Selenium' on 'Homeodomain−interacting protein kinase 3'

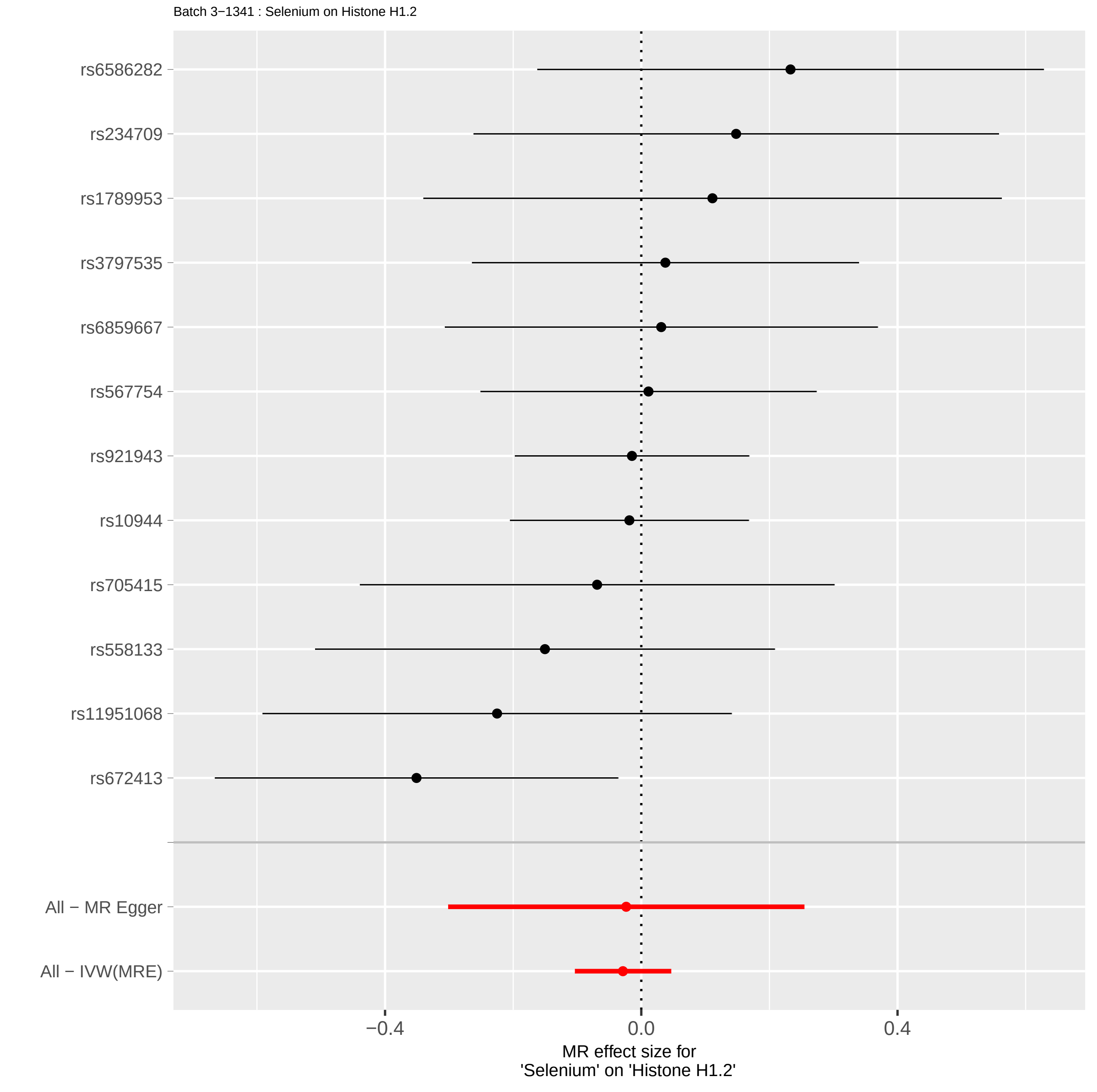
Batch 3−1341 : Selenium on Histone H1.2
rs6586282
rs234709
rs1789953
rs3797535
rs6859667
rs567754
rs921943
rs10944
rs705415
rs558133
rs11951068
rs672413
All − MR Egger
All − IVW(MRE)
−0.4
0.0
0.4
MR effect size for
'Selenium' on 'Histone H1.2'

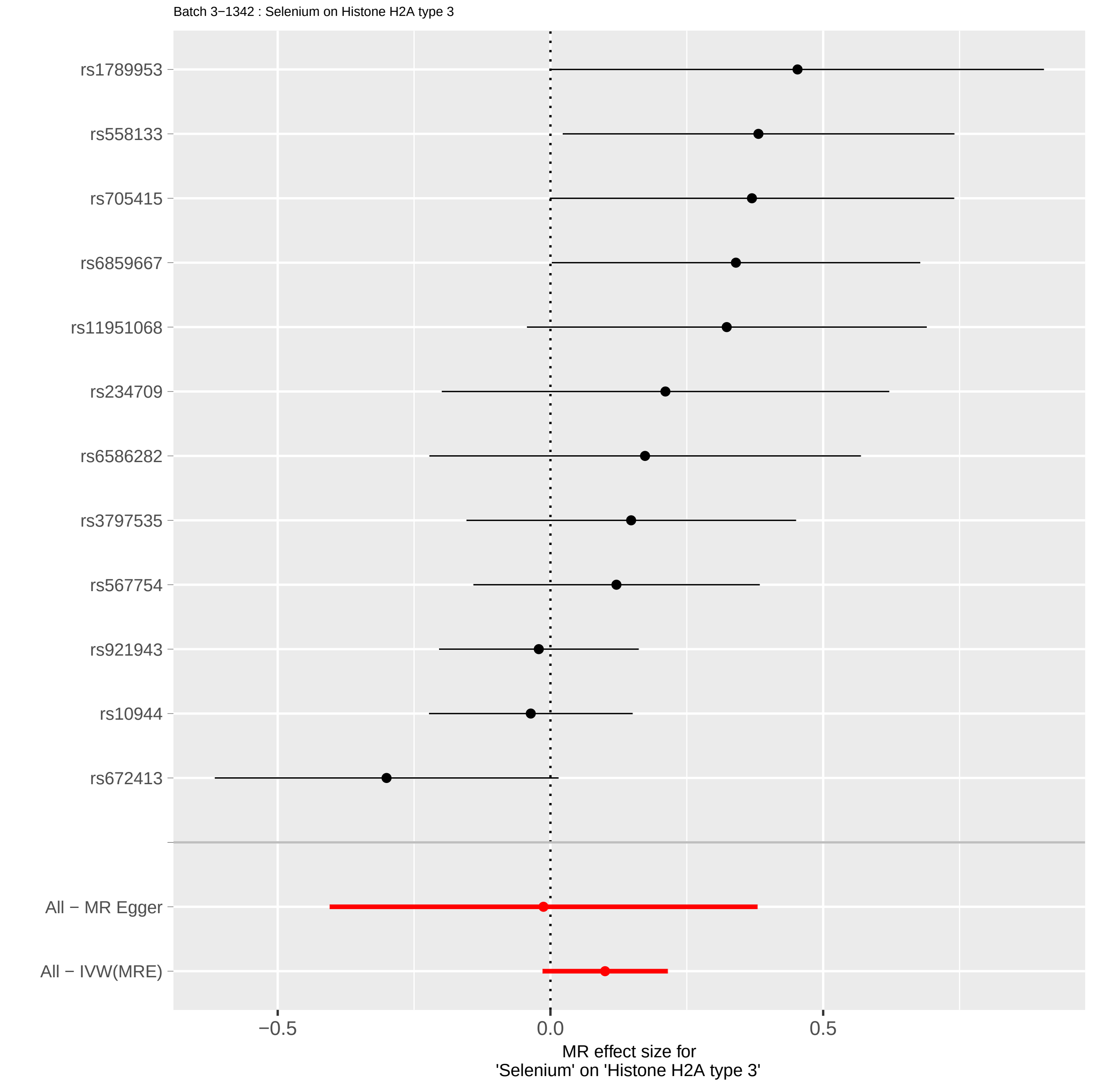

Batch 3−1342 : Selenium on Histone H2A type 3
rs1789953
rs558133
rs705415
rs6859667
rs11951068
rs234709
rs6586282
rs3797535
rs567754
rs921943
rs10944
rs672413
All − MR Egger
All − IVW(MRE)
−0.5
0.0
0.5
MR effect size for
'Selenium' on 'Histone H2A type 3'

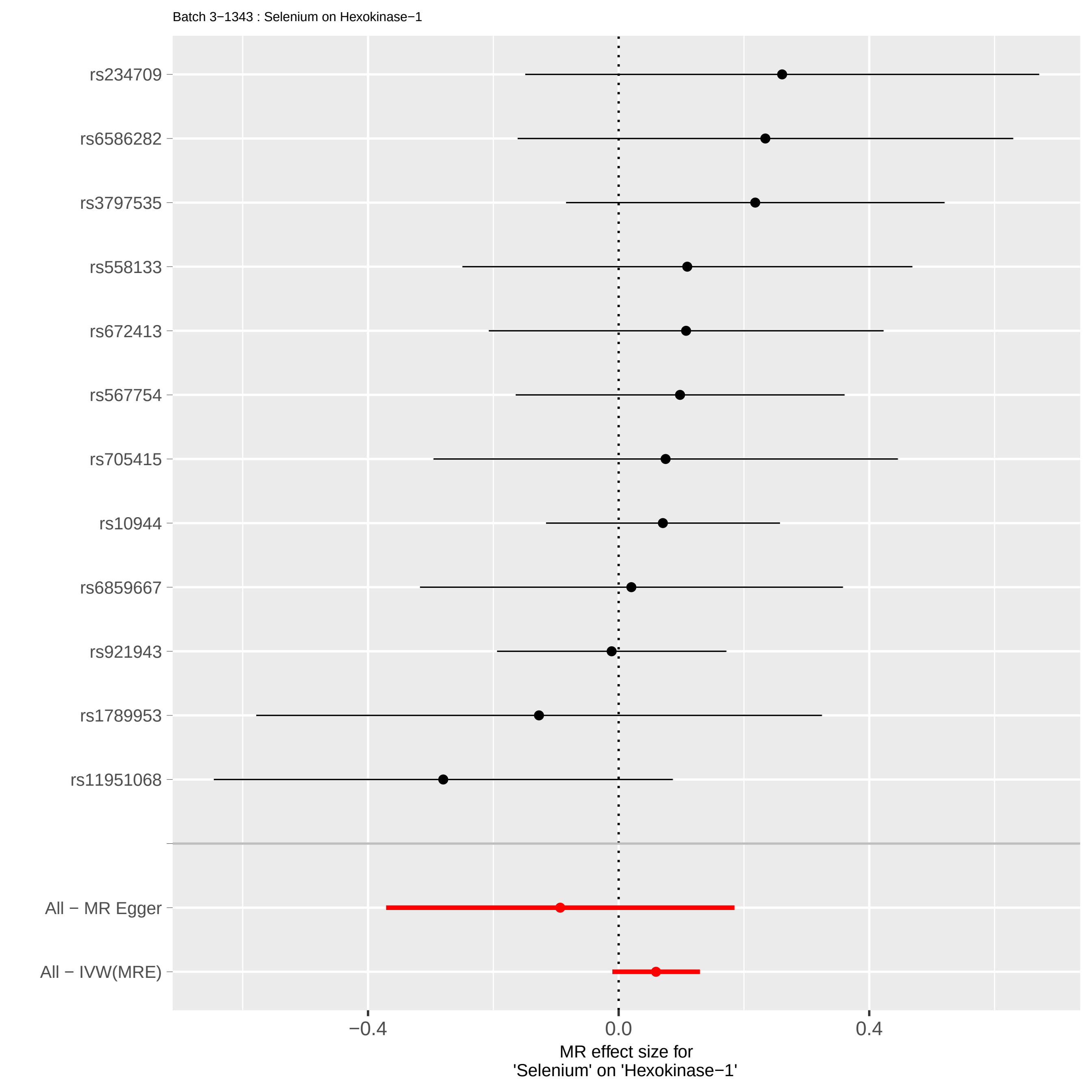

Batch 3−1343 : Selenium on Hexokinase−1
rs234709
rs6586282
rs3797535
rs558133
rs672413
rs567754
rs705415
rs10944
rs6859667
rs921943
rs1789953
rs11951068
All − MR Egger
All − IVW(MRE)
−0.4
0.0
0.4
MR effect size for
'Selenium' on 'Hexokinase−1'

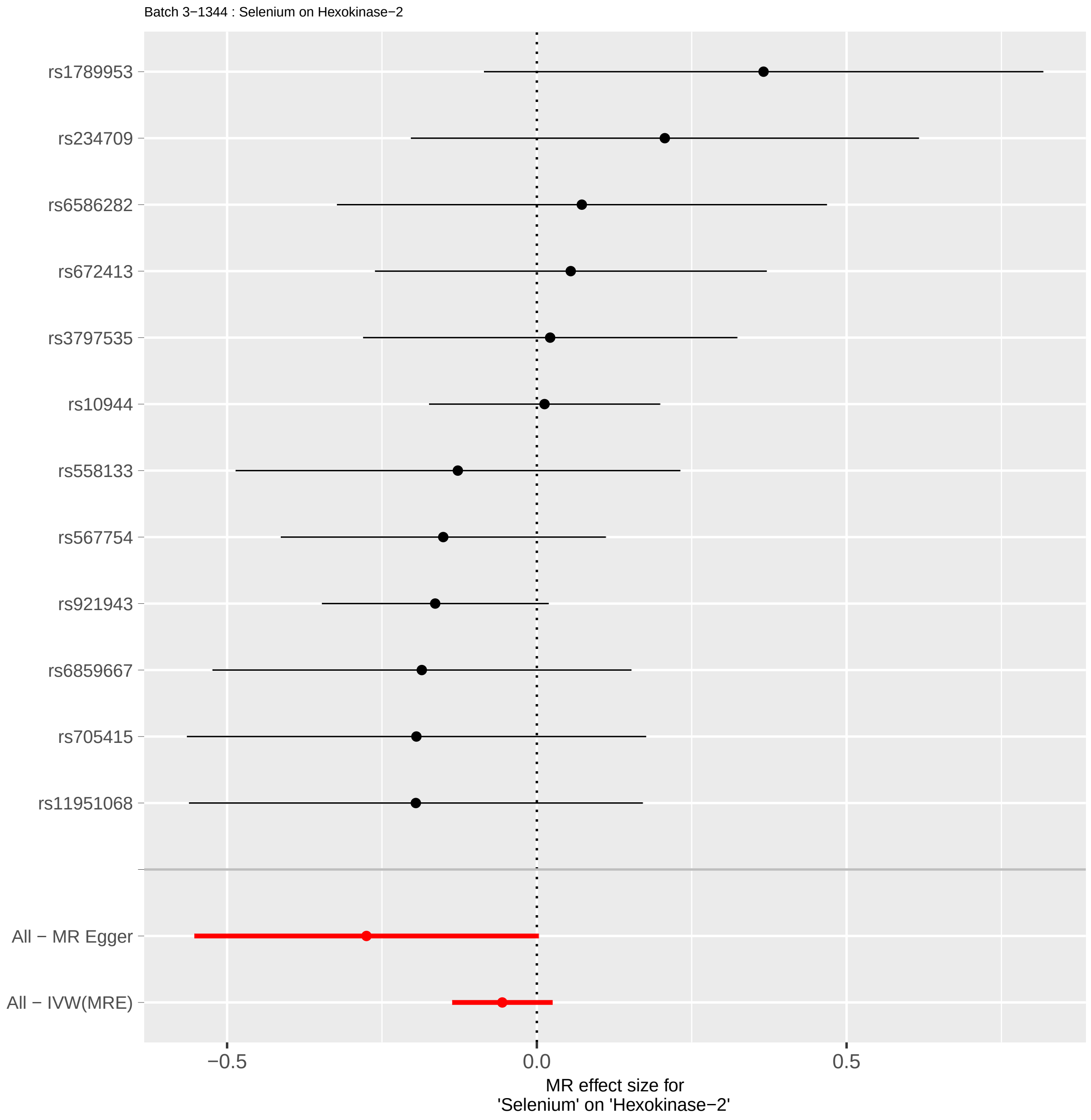
Batch 3−1344 : Selenium on Hexokinase−2
rs1789953
rs234709
rs6586282
rs672413
rs3797535
rs10944
rs558133
rs567754
rs921943
rs6859667
rs705415
rs11951068
All − MR Egger
All − IVW(MRE)
−0.5
0.0
0.5
MR effect size for
'Selenium' on 'Hexokinase−2'

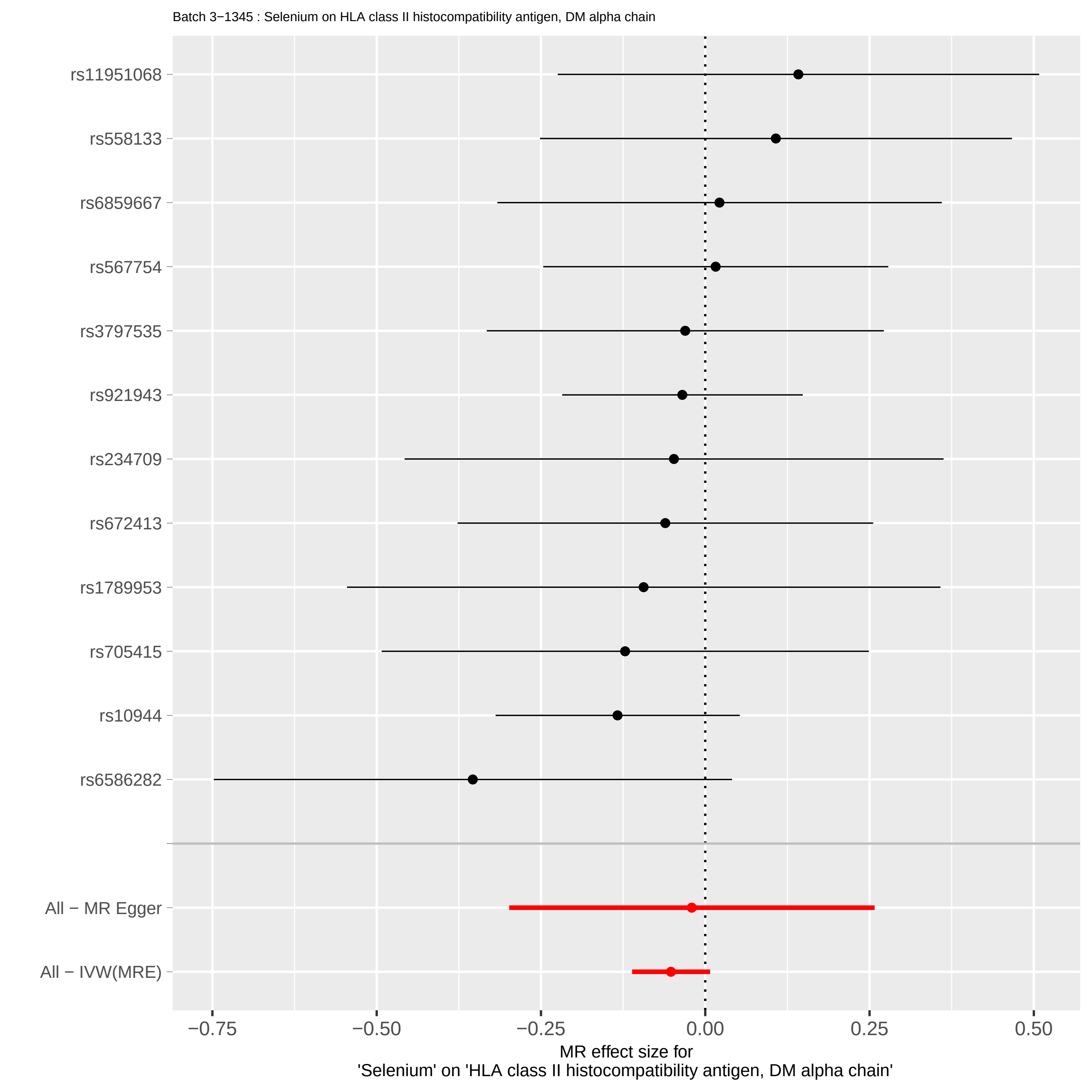

Batch 3−1345 : Selenium on HLA class II histocompatibility antigen, DM alpha chain
rs11951068
rs558133
rs6859667
rs567754
rs3797535
rs921943
rs234709
rs672413
rs1789953
rs705415
rs10944
rs6586282
All − MR Egger
All − IVW(MRE)
−0.75
−0.50
−0.25
0.00
0.25
0.50
MR effect size for
'Selenium' on 'HLA class II histocompatibility antigen, DM alpha chain'

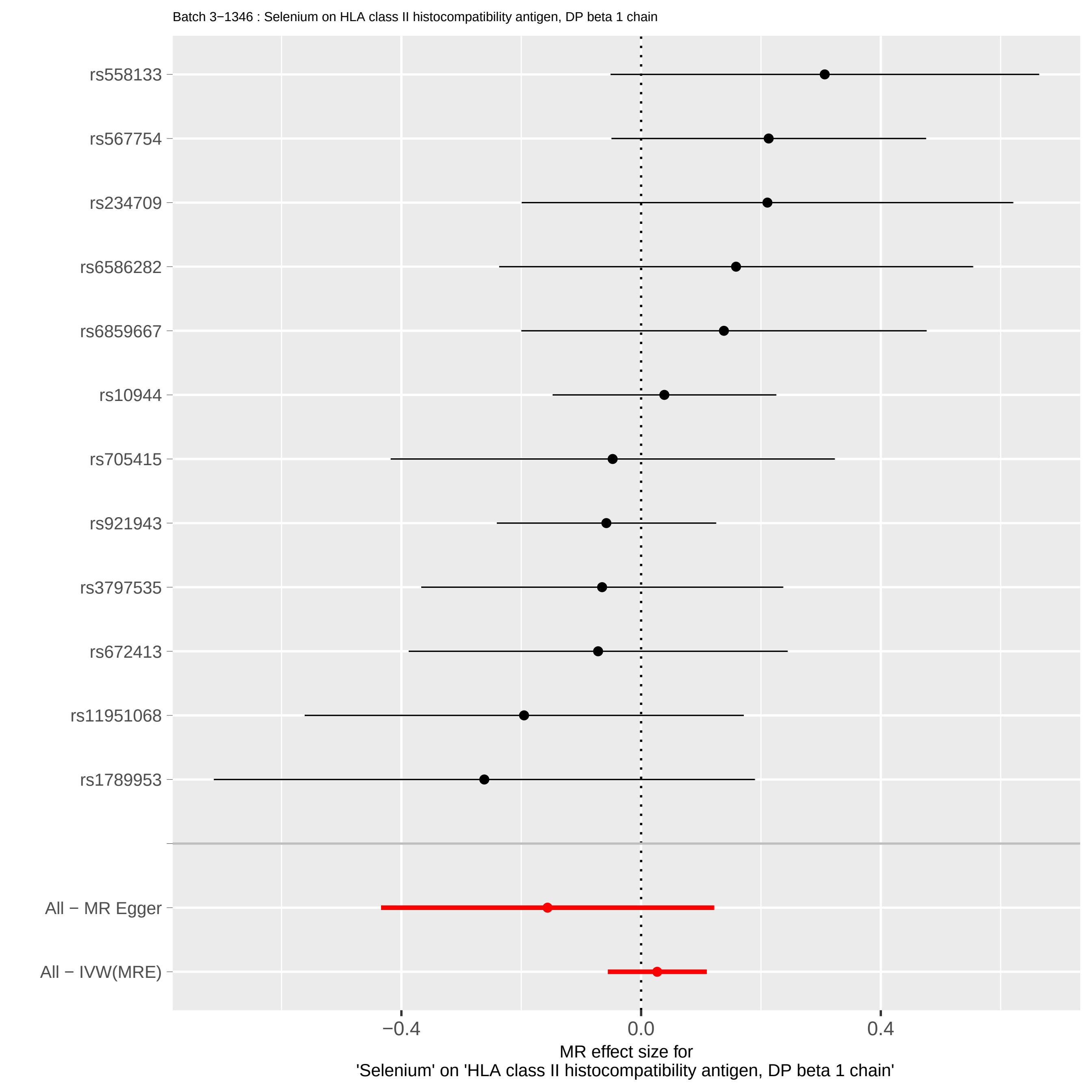

Batch 3−1346 : Selenium on HLA class II histocompatibility antigen, DP beta 1 chain
rs558133
rs567754
rs234709
rs6586282
rs6859667
rs10944
rs705415
rs921943
rs3797535
rs672413
rs11951068
rs1789953
All − MR Egger
All − IVW(MRE)
−0.4
0.0
0.4
MR effect size for
'Selenium' on 'HLA class II histocompatibility antigen, DP beta 1 chain'

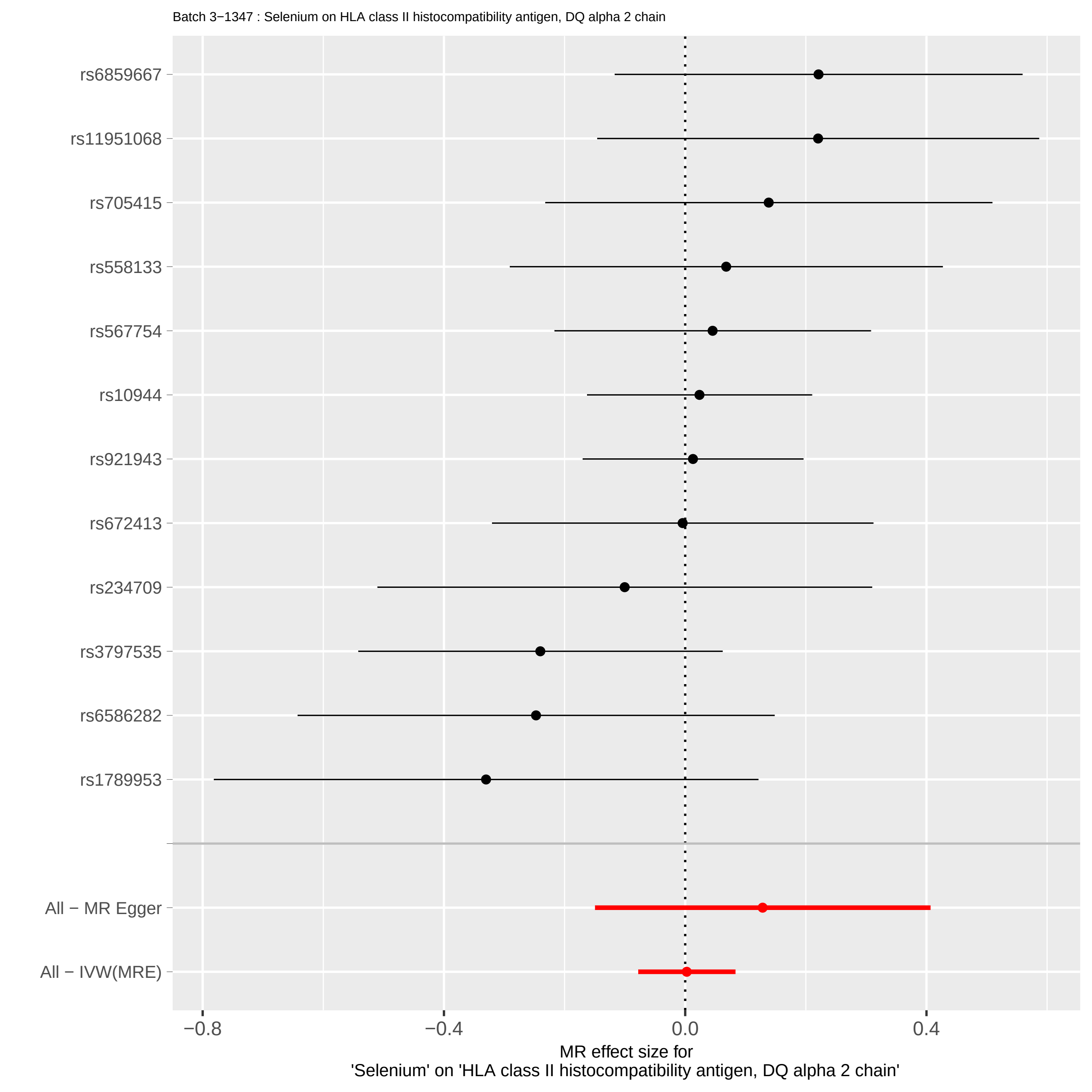

Batch 3−1347 : Selenium on HLA class II histocompatibility antigen, DQ alpha 2 chain
rs6859667
rs11951068
rs705415
rs558133
rs567754
rs10944
rs921943
rs672413
rs234709
rs3797535
rs6586282
rs1789953
All − MR Egger
All − IVW(MRE)
−0.8
−0.4
0.0
0.4
MR effect size for
'Selenium' on 'HLA class II histocompatibility antigen, DQ alpha 2 chain'

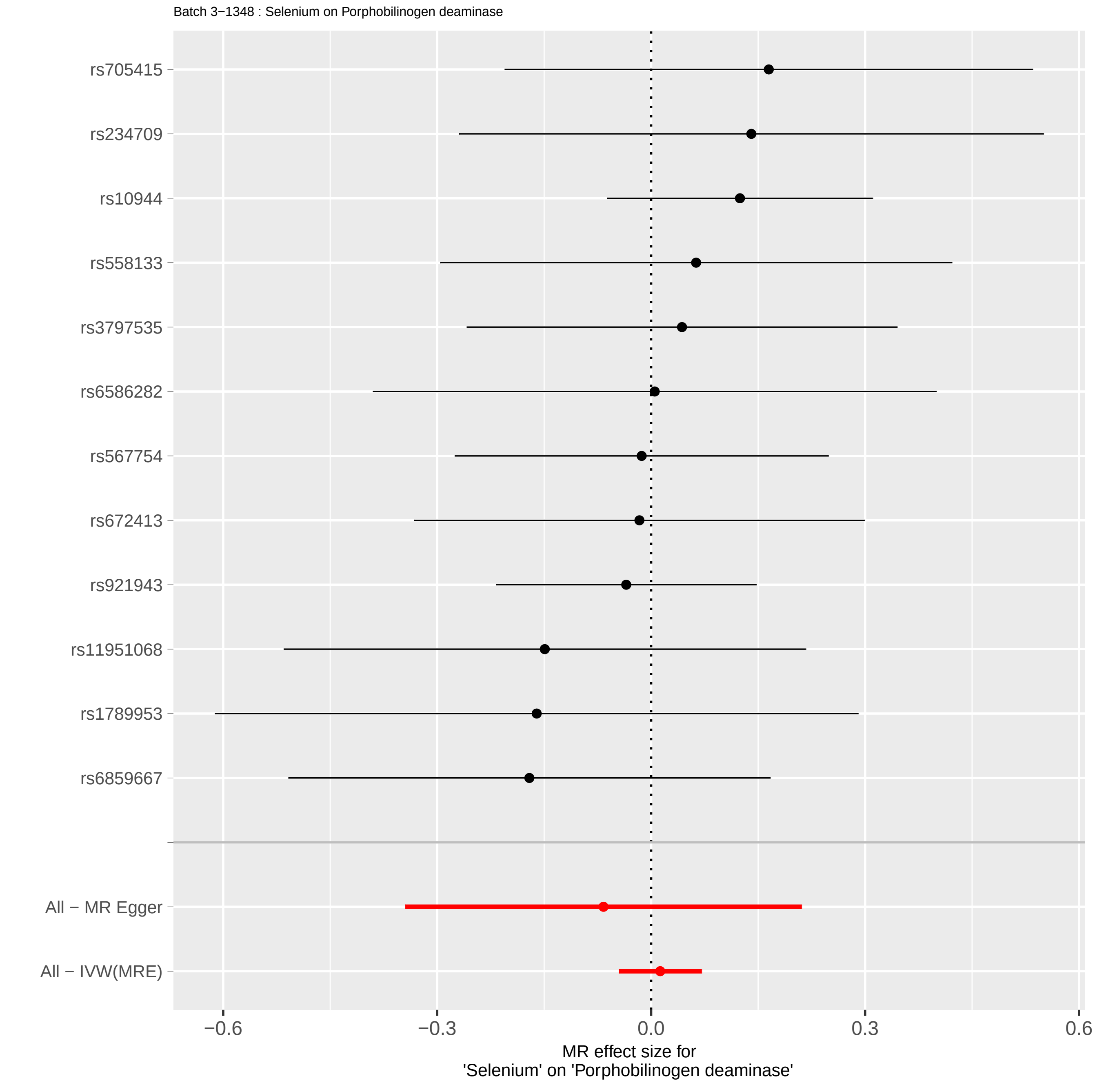

Batch 3−1348 : Selenium on Porphobilinogen deaminase
rs705415
rs234709
rs10944
rs558133
rs3797535
rs6586282
rs567754
rs672413
rs921943
rs11951068
rs1789953
rs6859667
All − MR Egger
All − IVW(MRE)
−0.6
−0.3
0.0
0.3
0.6
MR effect size for
'Selenium' on 'Porphobilinogen deaminase'

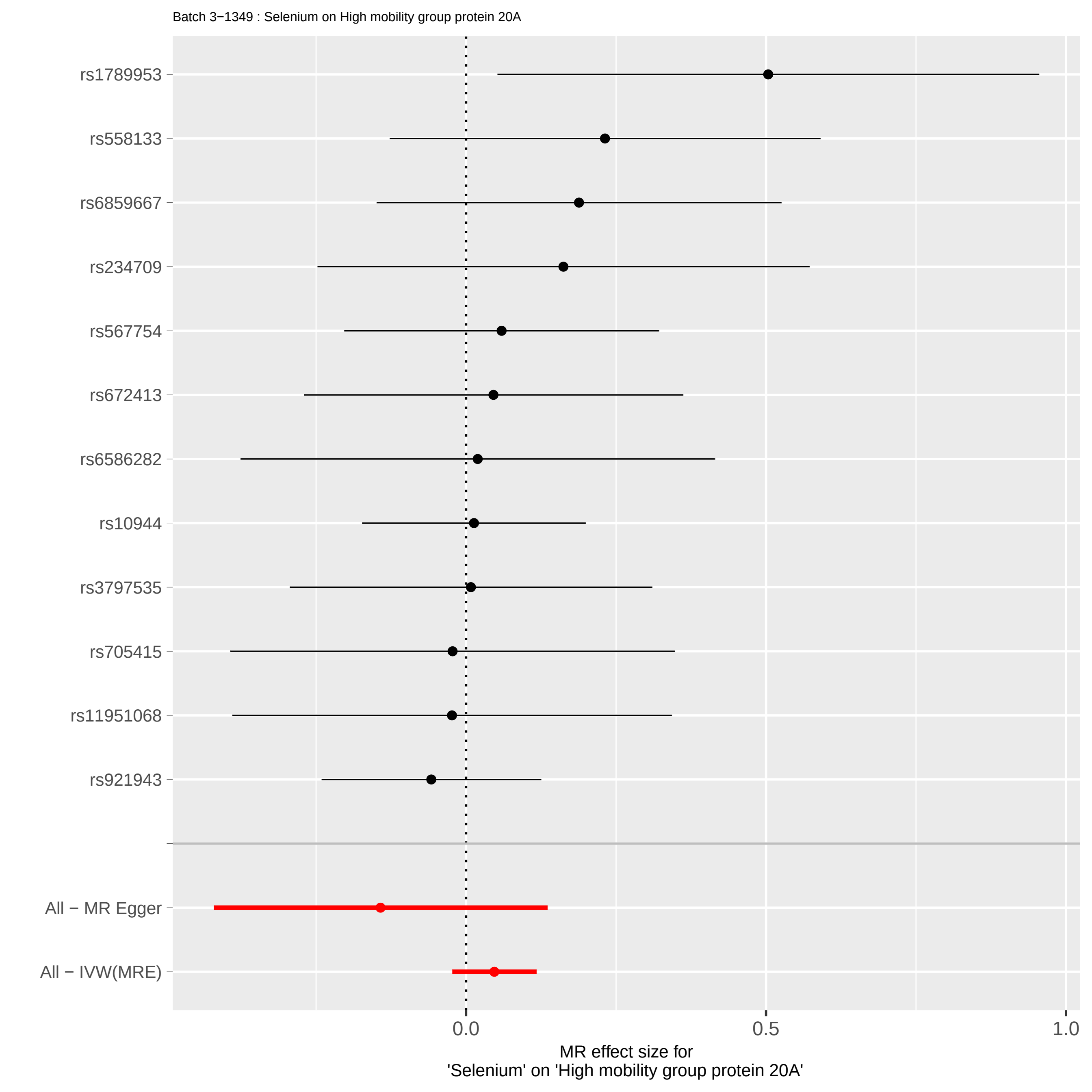

Batch 3−1349 : Selenium on High mobility group protein 20A
rs1789953
rs558133
rs6859667
rs234709
rs567754
rs672413
rs6586282
rs10944
rs3797535
rs705415
rs11951068
rs921943
All − MR Egger
All − IVW(MRE)
0.0
0.5
1.0
MR effect size for
'Selenium' on 'High mobility group protein 20A'

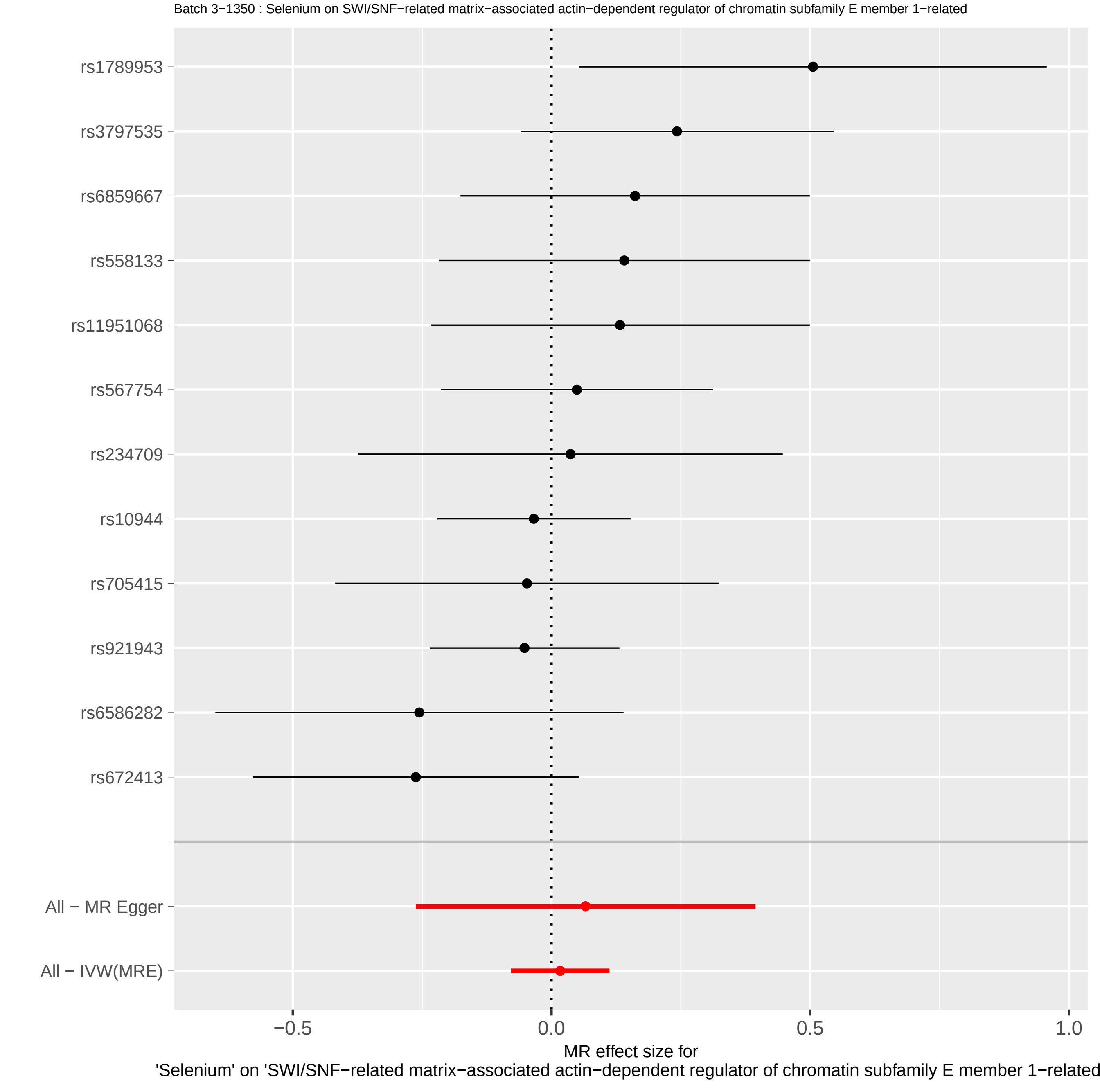

Batch 3−1350 : Selenium on SWI/SNF−related matrix−associated actin−dependent regulator of chromatin subfamily E member 1−related
rs1789953
rs3797535
rs6859667
rs558133
rs11951068
rs567754
rs234709
rs10944
rs705415
rs921943
rs6586282
rs672413
All − MR Egger
All − IVW(MRE)
−0.5
0.0
0.5
1.0
MR effect size for
'Selenium' on 'SWI/SNF−related matrix−associated actin−dependent regulator of chromatin subfamily E member 1−related

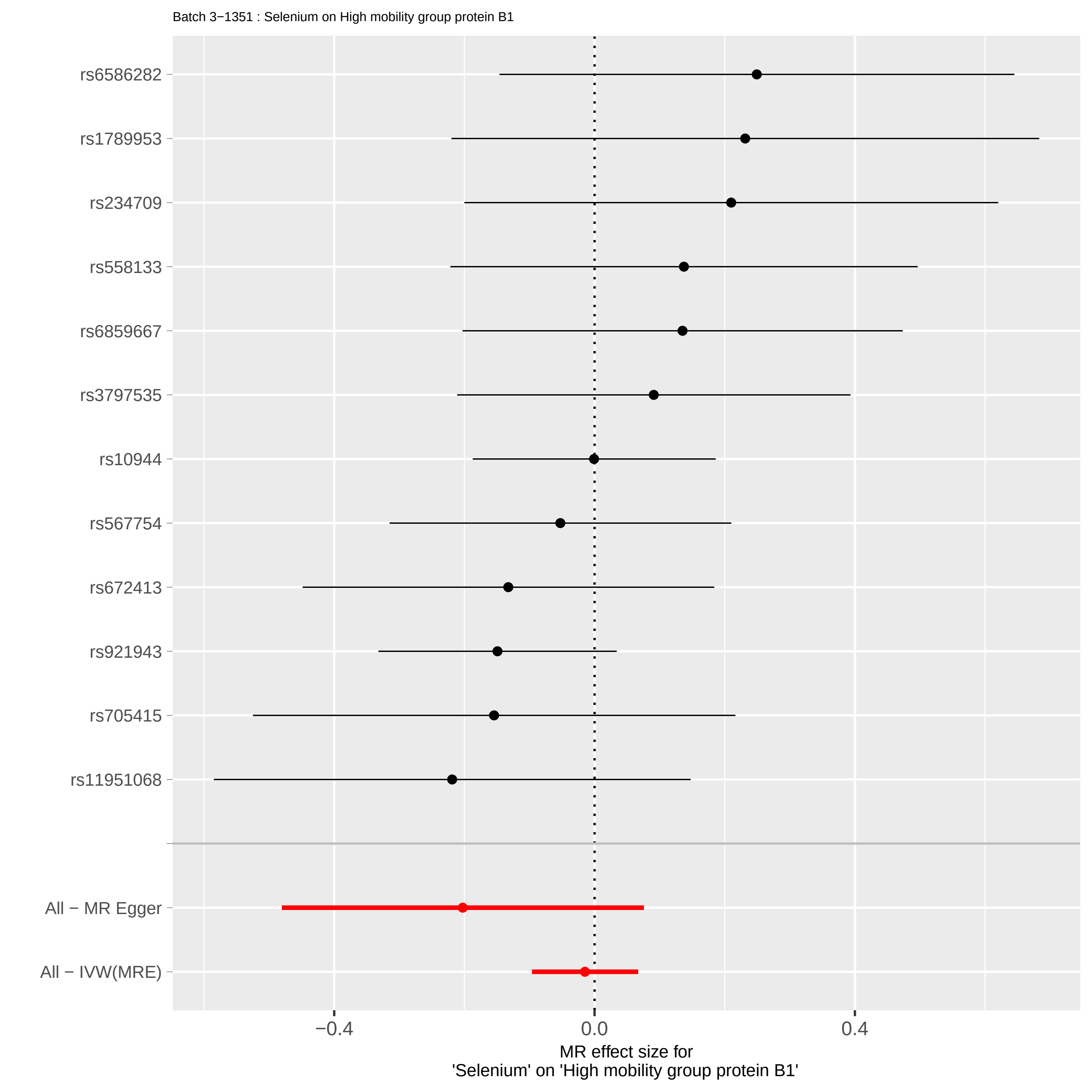

Batch 3−1351 : Selenium on High mobility group protein B1
rs6586282
rs1789953
rs234709
rs558133
rs6859667
rs3797535
rs10944
rs567754
rs672413
rs921943
rs705415
rs11951068
All − MR Egger
All − IVW(MRE)
−0.4
0.0
0.4
MR effect size for
'Selenium' on 'High mobility group protein B1'

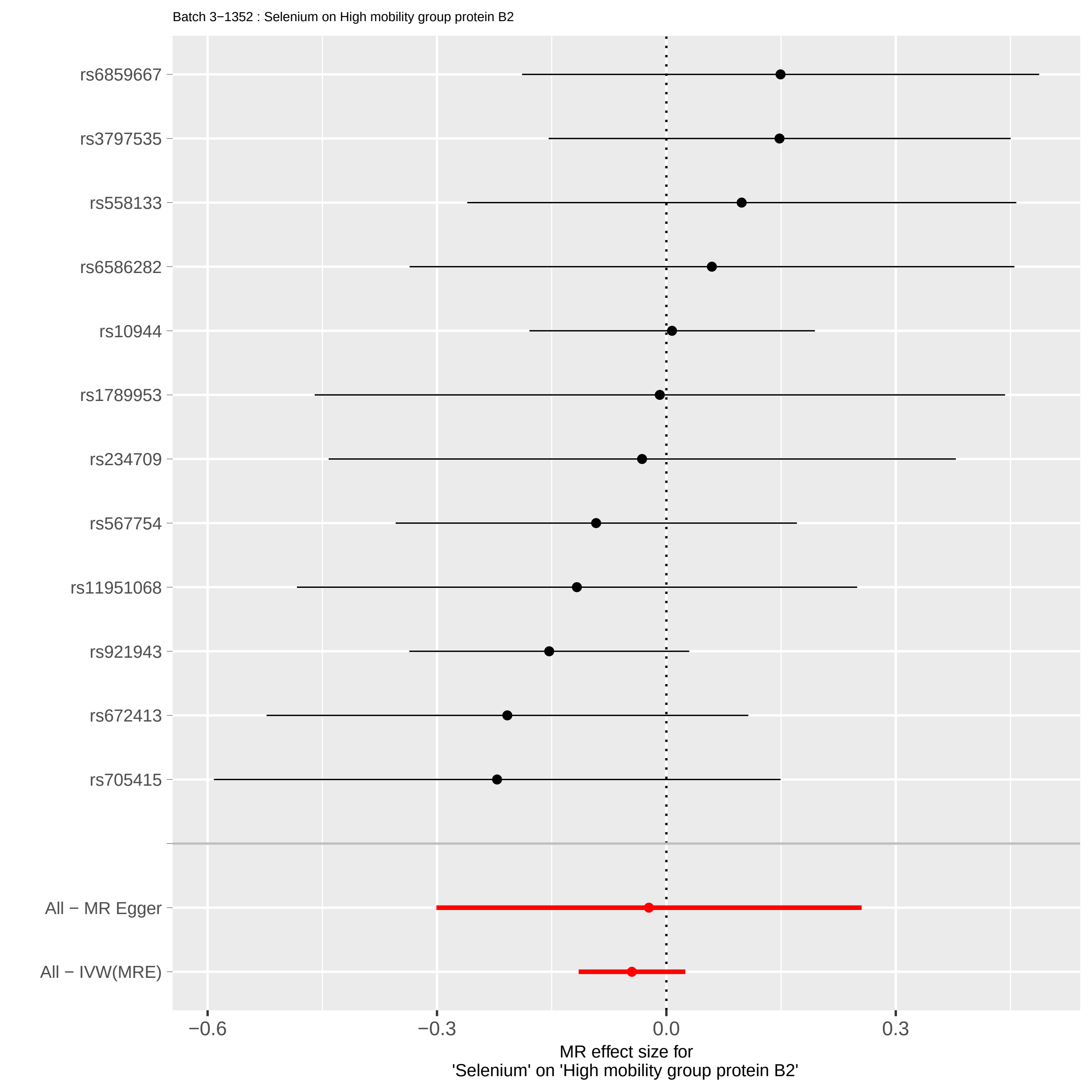

Batch 3−1352 : Selenium on High mobility group protein B2
rs6859667
rs3797535
rs558133
rs6586282
rs10944
rs1789953
rs234709
rs567754
rs11951068
rs921943
rs672413
rs705415
All − MR Egger
All − IVW(MRE)
−0.6
−0.3
0.0
0.3
MR effect size for
'Selenium' on 'High mobility group protein B2'

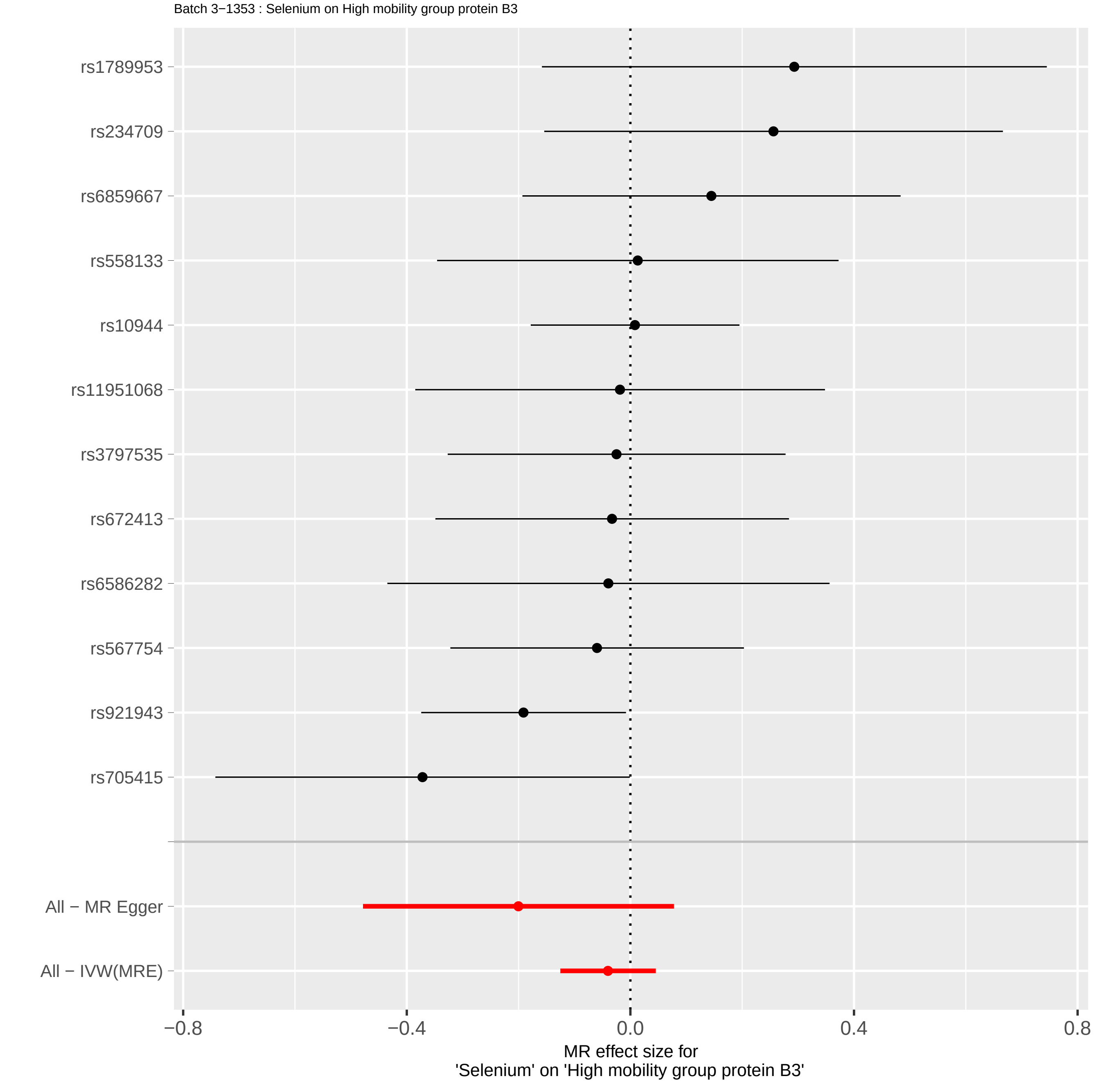

Batch 3−1353 : Selenium on High mobility group protein B3
rs1789953
rs234709
rs6859667
rs558133
rs10944
rs11951068
rs3797535
rs672413
rs6586282
rs567754
rs921943
rs705415
All − MR Egger
All − IVW(MRE)
−0.8
−0.4
0.0
0.4
0.8
MR effect size for
'Selenium' on 'High mobility group protein B3'

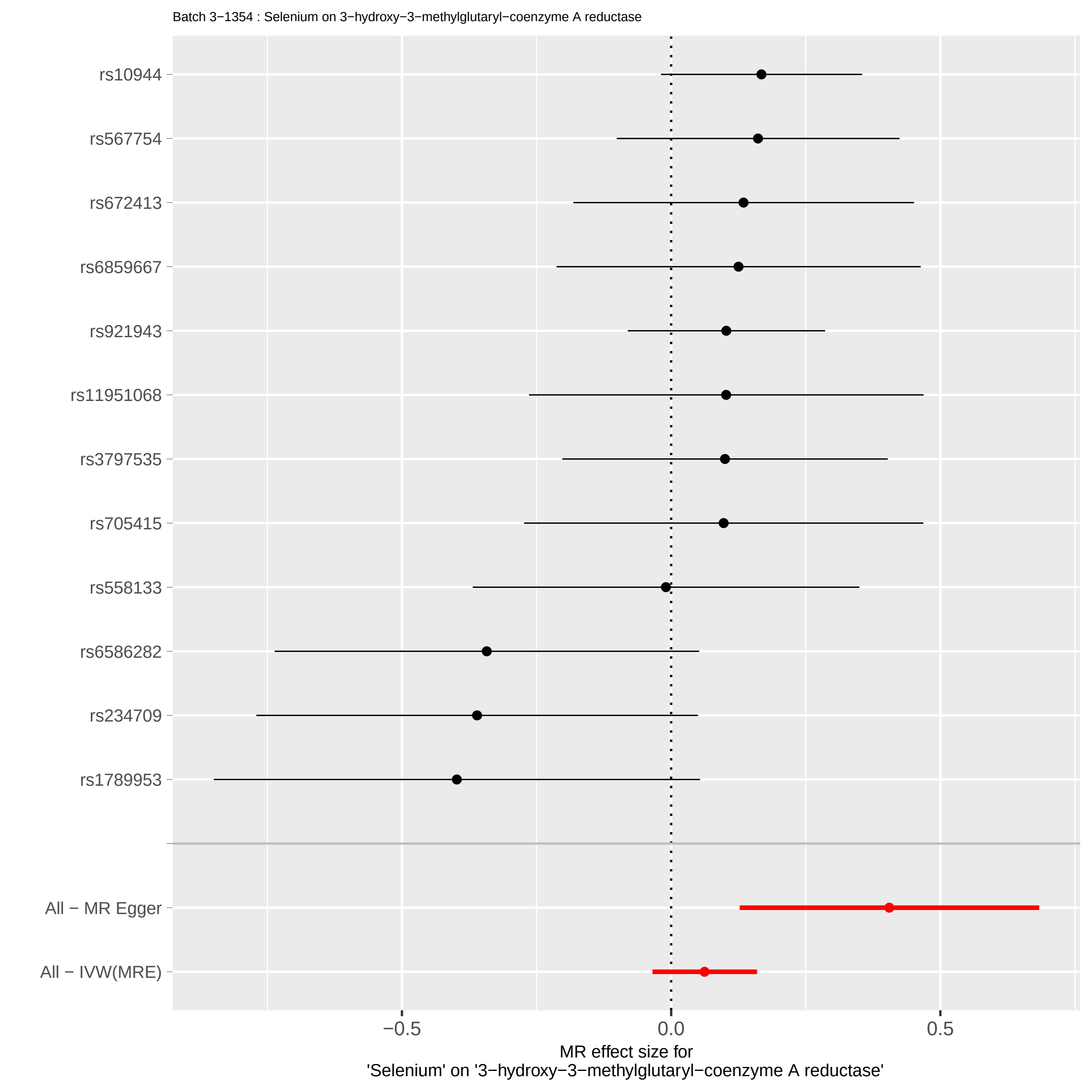
Batch 3−1354 : Selenium on 3−hydroxy−3−methylglutaryl−coenzyme A reductase
rs10944
rs567754
rs672413
rs6859667
rs921943
rs11951068
rs3797535
rs705415
rs558133
rs6586282
rs234709
rs1789953
All − MR Egger
All − IVW(MRE)
−0.5
0.0
0.5
MR effect size for
'Selenium' on '3−hydroxy−3−methylglutaryl−coenzyme A reductase'

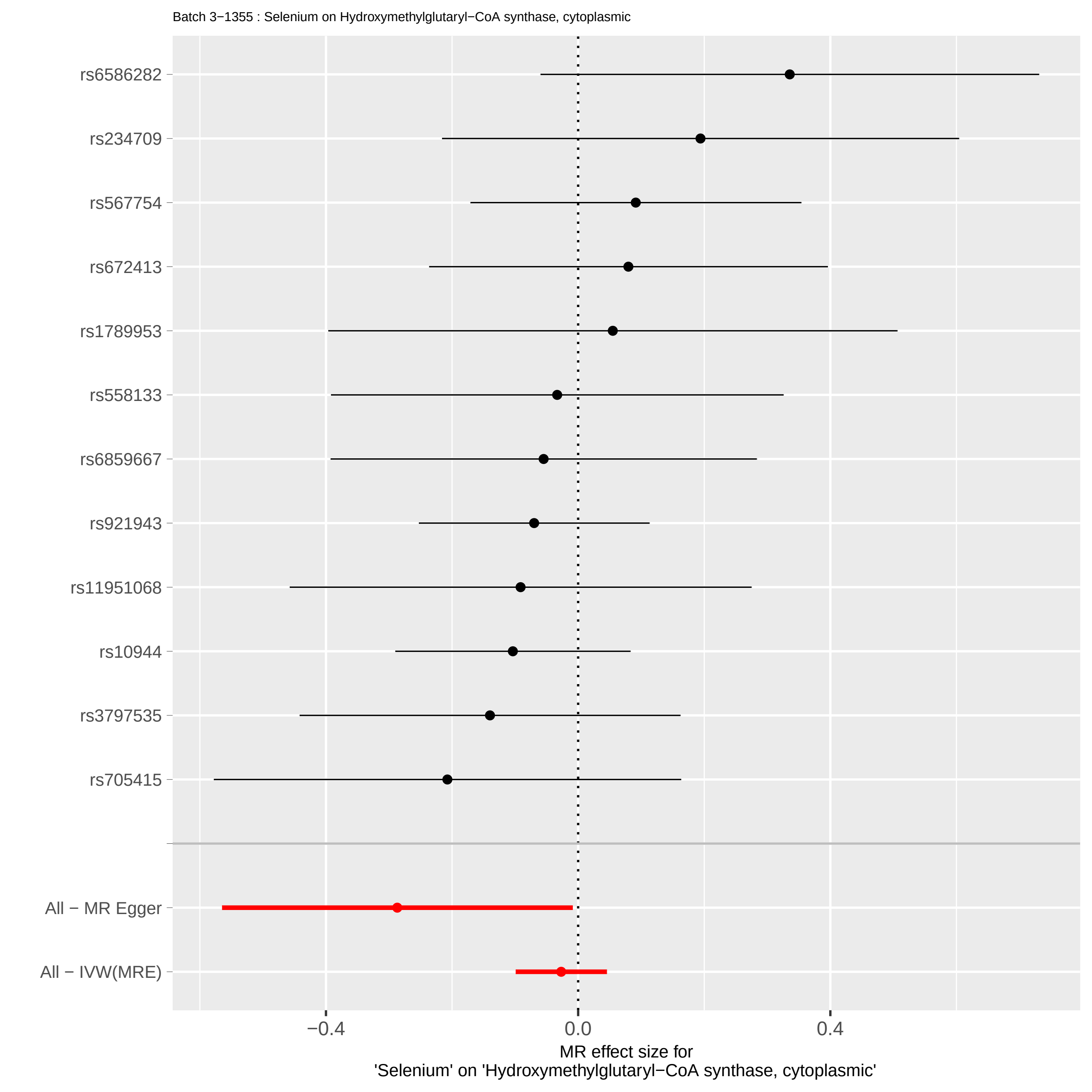
Batch 3−1355 : Selenium on Hydroxymethylglutaryl−CoA synthase, cytoplasmic
rs6586282
rs234709
rs567754
rs672413
rs1789953
rs558133
rs6859667
rs921943
rs11951068
rs10944
rs3797535
rs705415
All − MR Egger
All − IVW(MRE)
−0.4
0.0
0.4
MR effect size for
'Selenium' on 'Hydroxymethylglutaryl−CoA synthase, cytoplasmic'

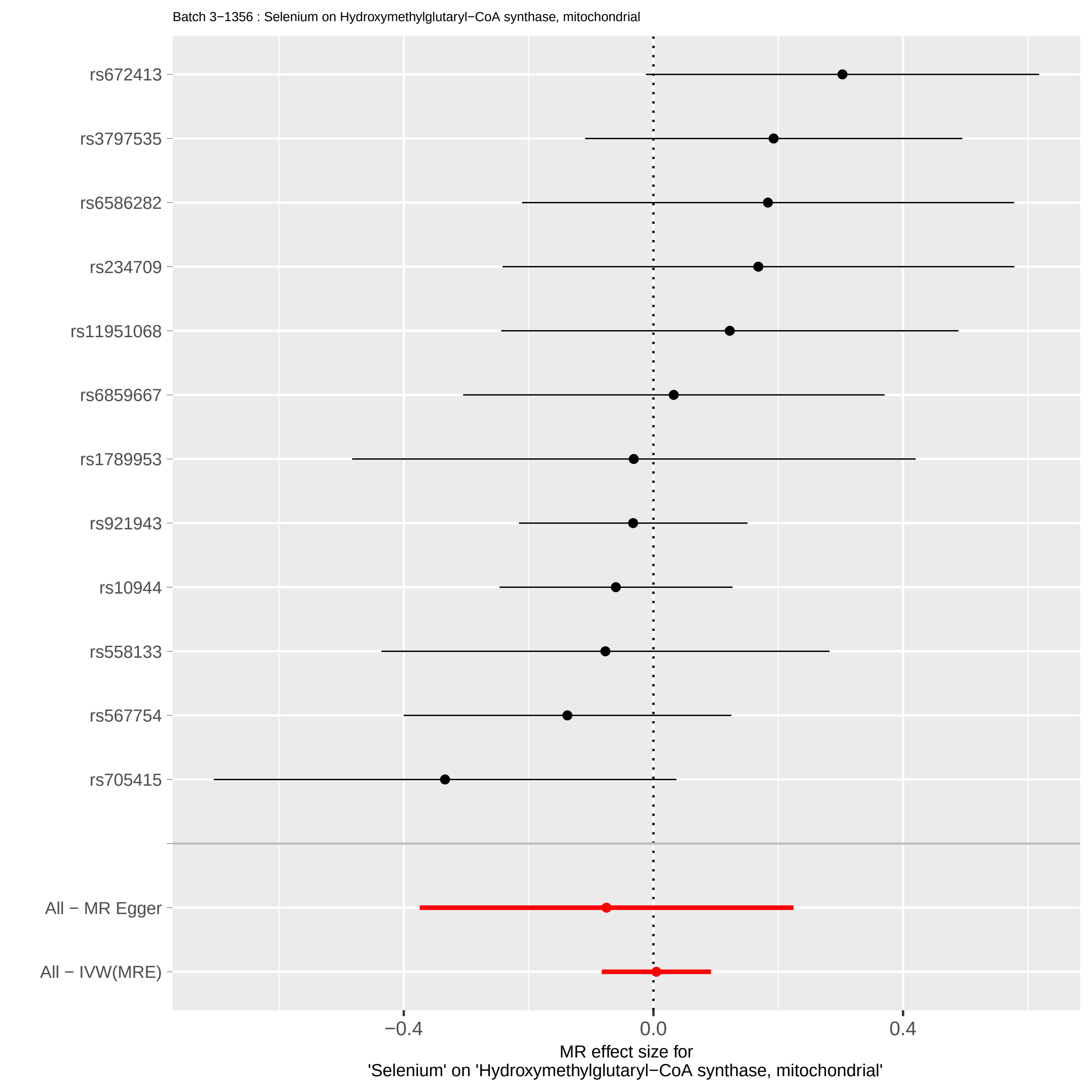

Batch 3−1356 : Selenium on Hydroxymethylglutaryl−CoA synthase, mitochondrial
rs672413
rs3797535
rs6586282
rs234709
rs11951068
rs6859667
rs1789953
rs921943
rs10944
rs558133
rs567754
rs705415
All − MR Egger
All − IVW(MRE)
−0.4
0.0
0.4
MR effect size for
'Selenium' on 'Hydroxymethylglutaryl−CoA synthase, mitochondrial'

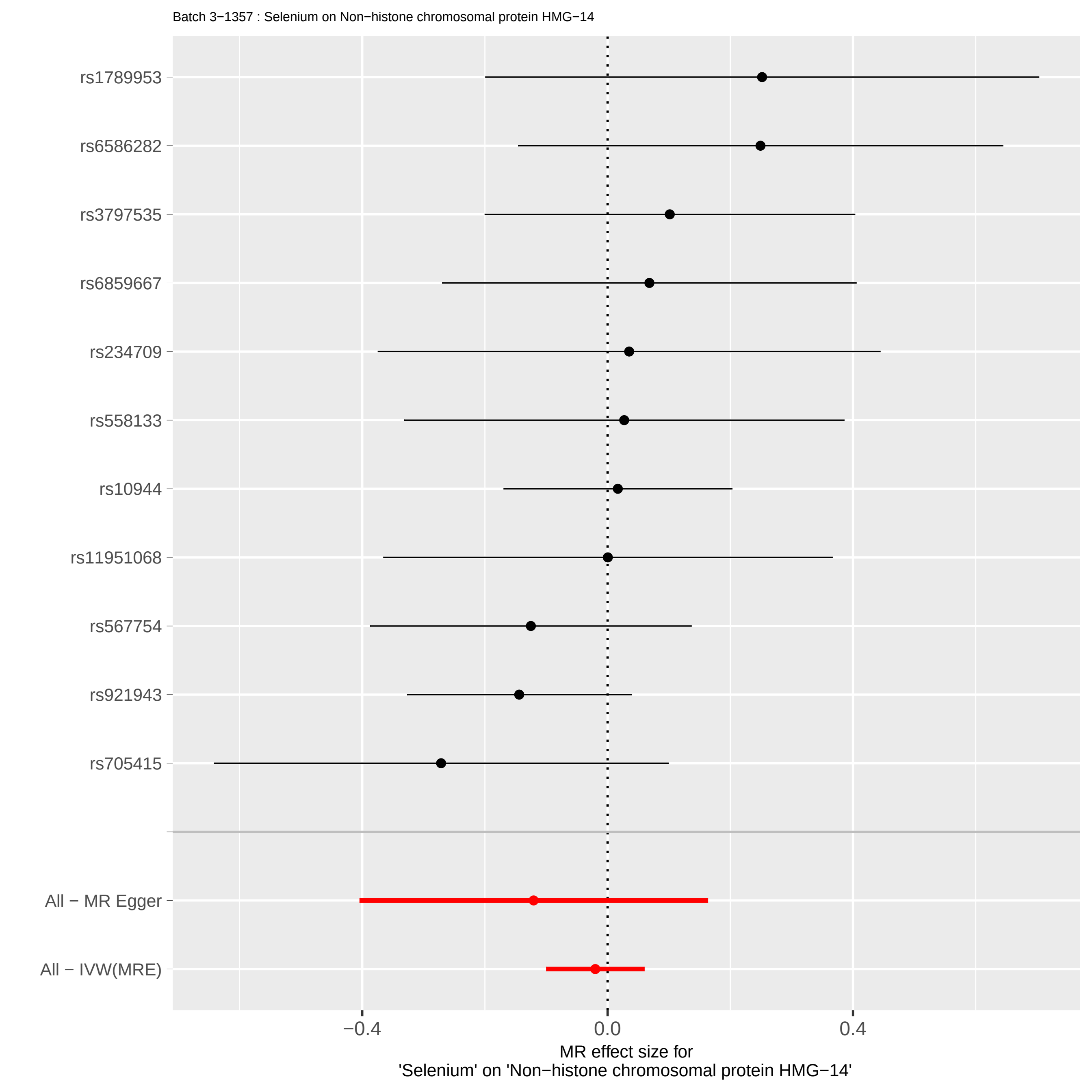
Batch 3−1357 : Selenium on Non−histone chromosomal protein HMG−14
rs1789953
rs6586282
rs3797535
rs6859667
rs234709
rs558133
rs10944
rs11951068
rs567754
rs921943
rs705415
All − MR Egger
All − IVW(MRE)
−0.4
0.0
0.4
MR effect size for
'Selenium' on 'Non−histone chromosomal protein HMG−14'

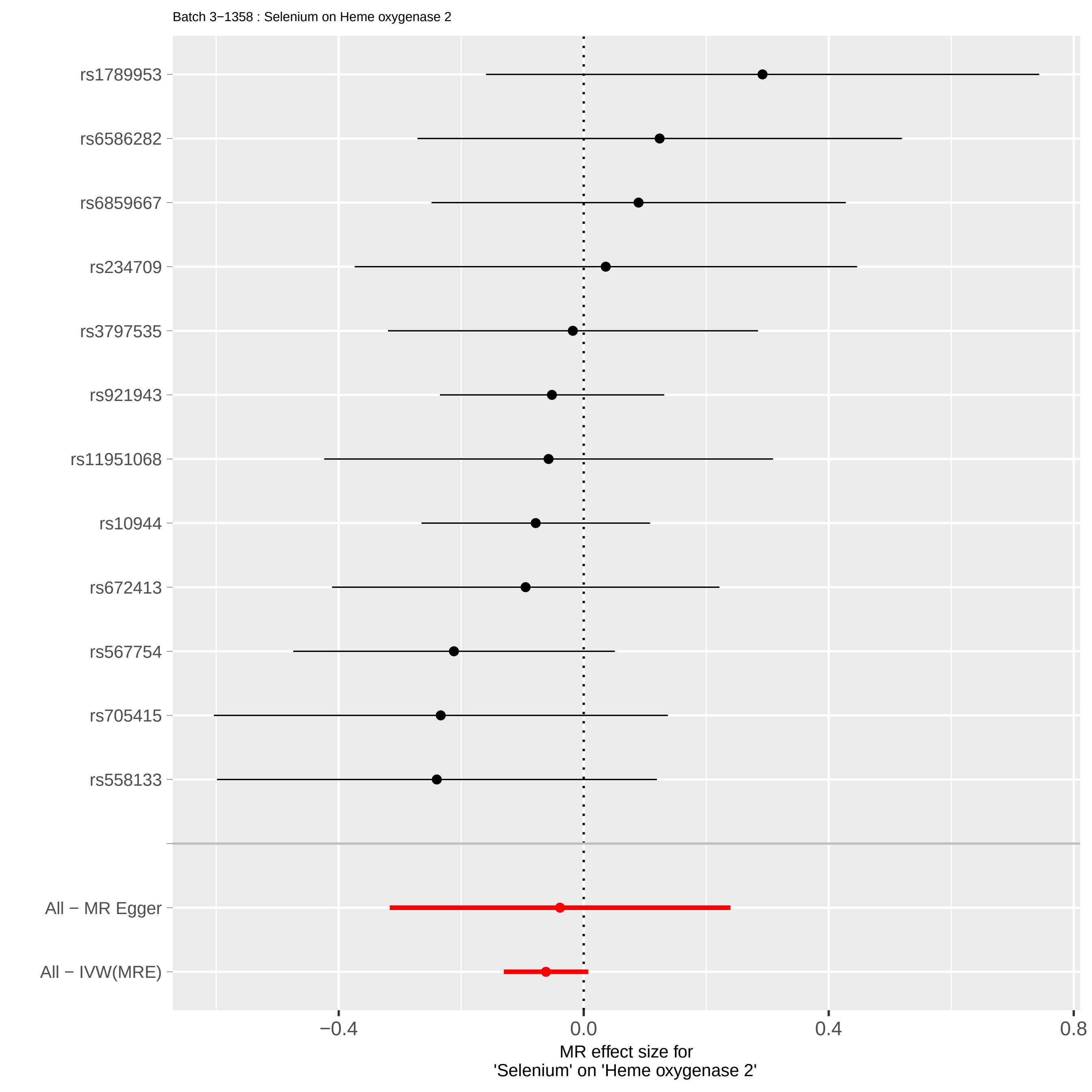

Batch 3−1358 : Selenium on Heme oxygenase 2
rs1789953
rs6586282
rs6859667
rs234709
rs3797535
rs921943
rs11951068
rs10944
rs672413
rs567754
rs705415
rs558133
All − MR Egger
All − IVW(MRE)
−0.4
0.0
0.4
0.8
MR effect size for
'Selenium' on 'Heme oxygenase 2'

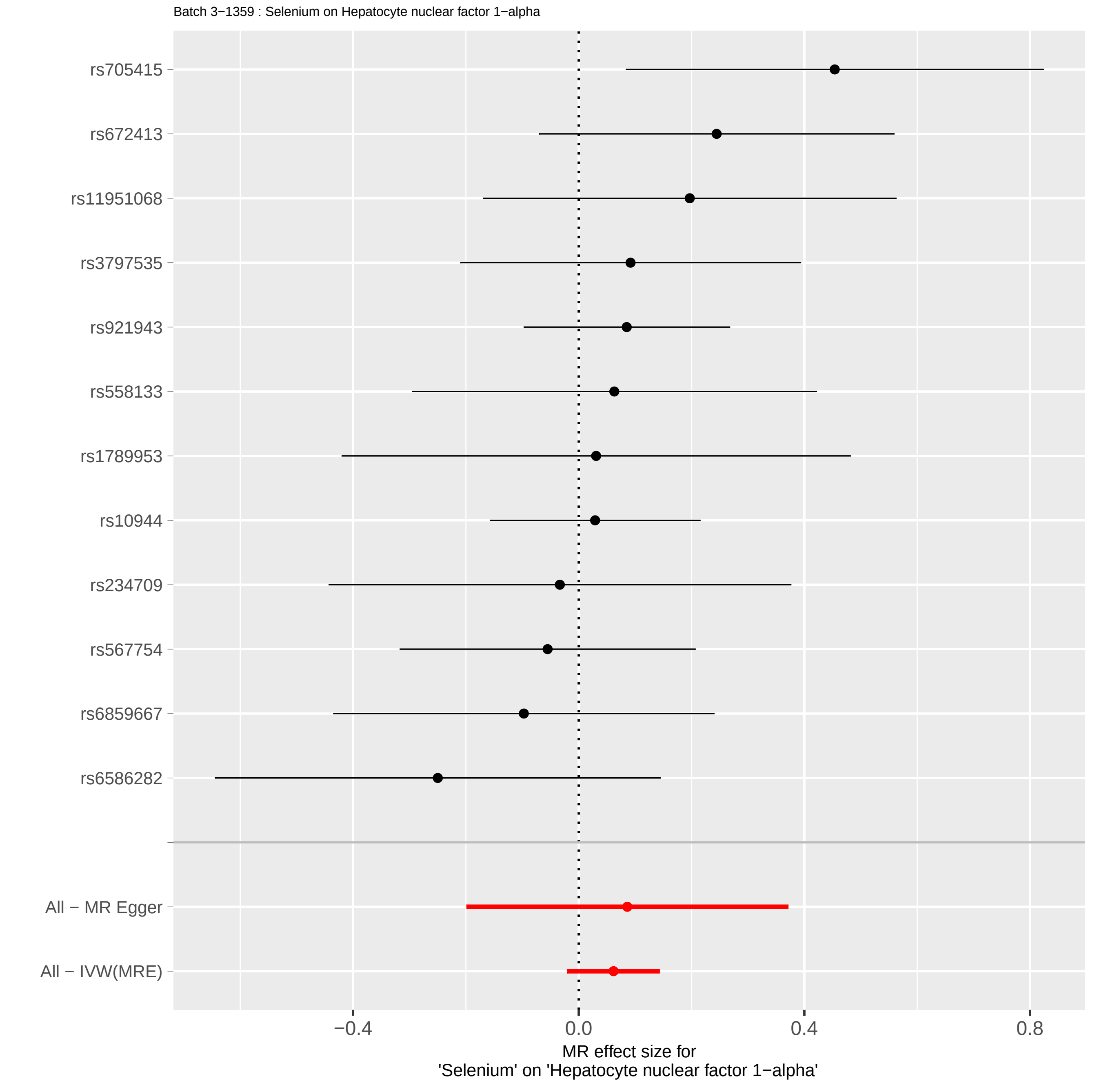

Batch 3−1359 : Selenium on Hepatocyte nuclear factor 1−alpha
rs705415
rs672413
rs11951068
rs3797535
rs921943
rs558133
rs1789953
rs10944
rs234709
rs567754
rs6859667
rs6586282
All − MR Egger
All − IVW(MRE)
−0.4
0.0
0.4
0.8
MR effect size for
'Selenium' on 'Hepatocyte nuclear factor 1−alpha'

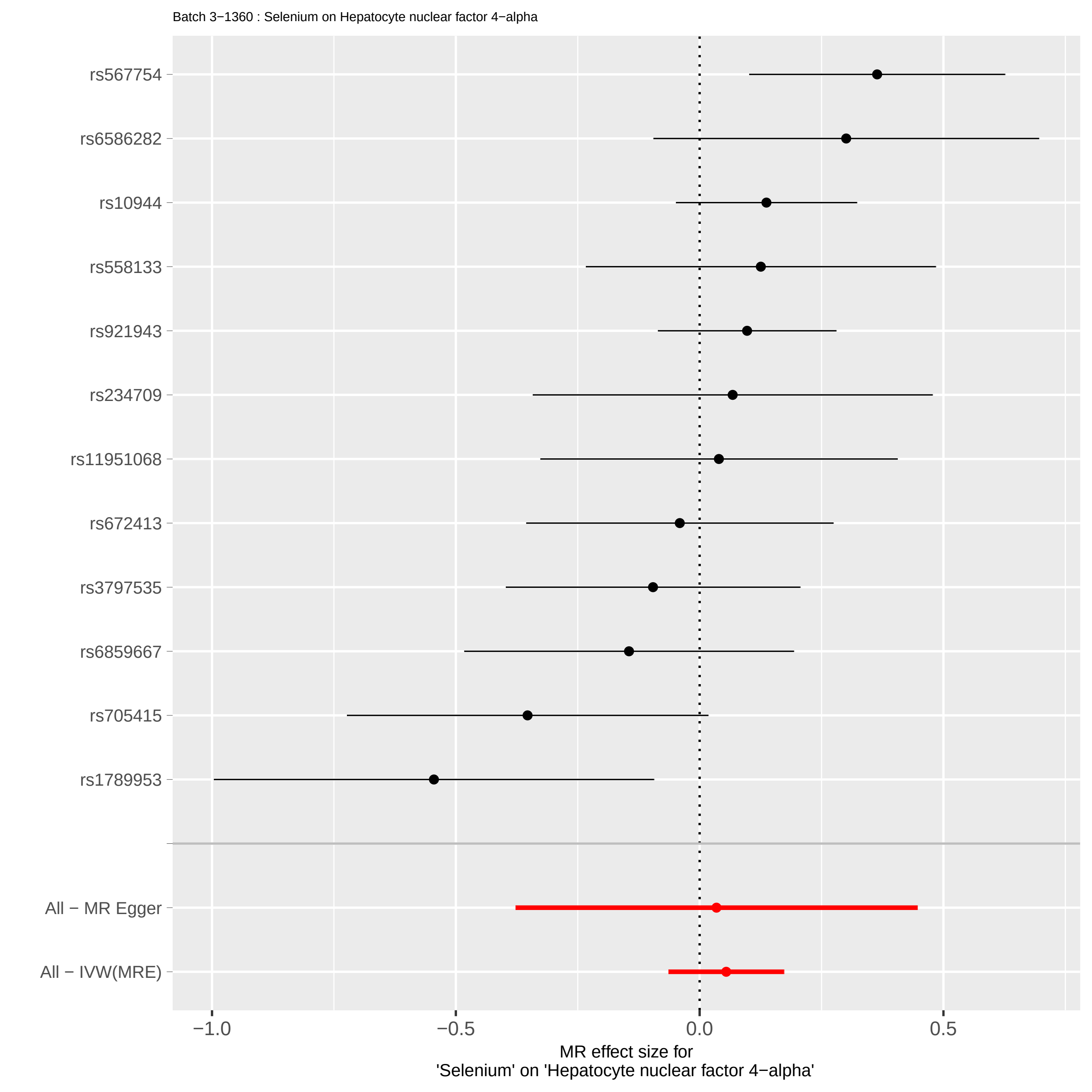
Batch 3−1360 : Selenium on Hepatocyte nuclear factor 4−alpha
rs567754
rs6586282
rs10944
rs558133
rs921943
rs234709
rs11951068
rs672413
rs3797535
rs6859667
rs705415
rs1789953
All − MR Egger
All − IVW(MRE)
−1.0
−0.5
0.0
0.5
MR effect size for
'Selenium' on 'Hepatocyte nuclear factor 4−alpha'

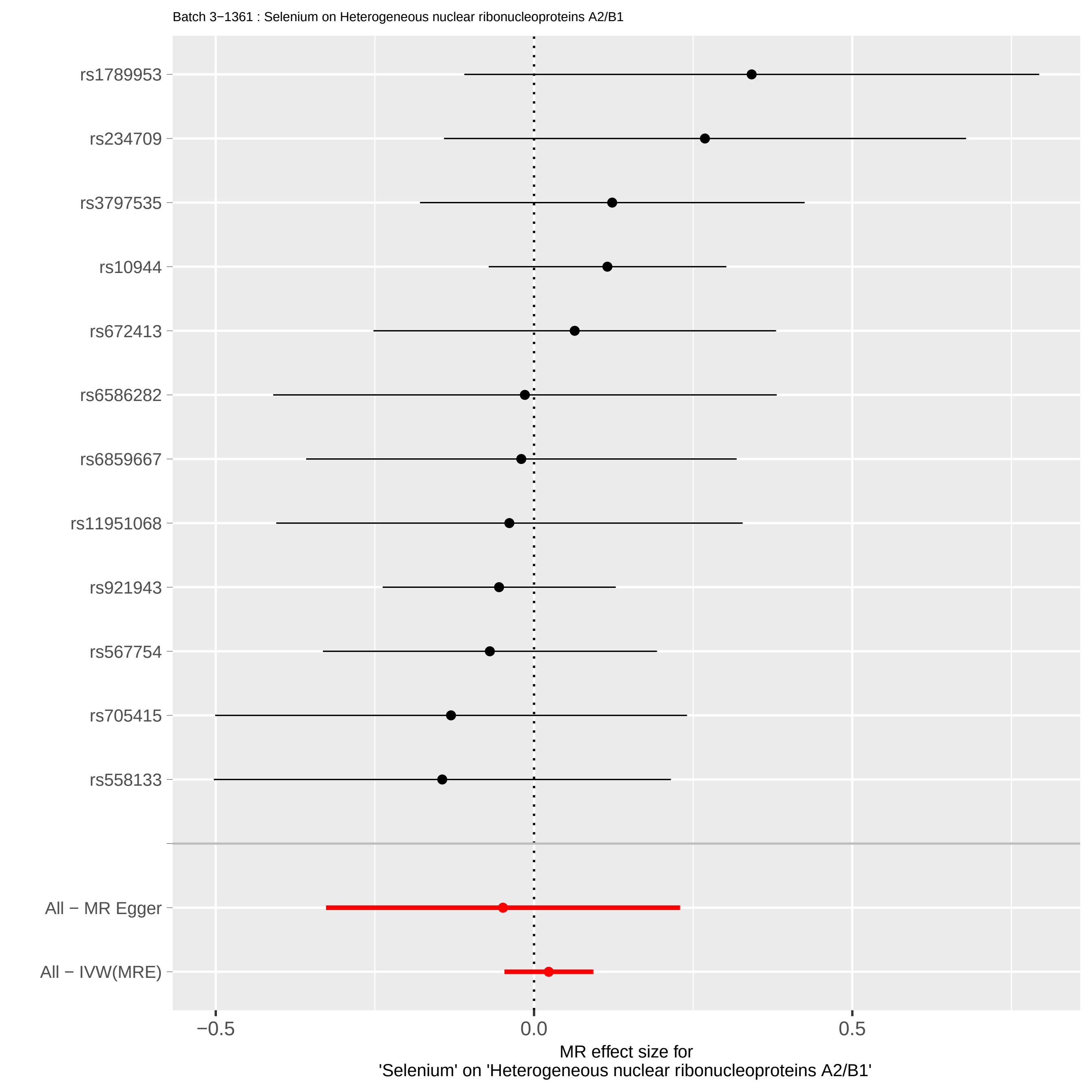
Batch 3−1361 : Selenium on Heterogeneous nuclear ribonucleoproteins A2/B1
rs1789953
rs234709
rs3797535
rs10944
rs672413
rs6586282
rs6859667
rs11951068
rs921943
rs567754
rs705415
rs558133
All − MR Egger
All − IVW(MRE)
−0.5
0.0
0.5
MR effect size for
'Selenium' on 'Heterogeneous nuclear ribonucleoproteins A2/B1'

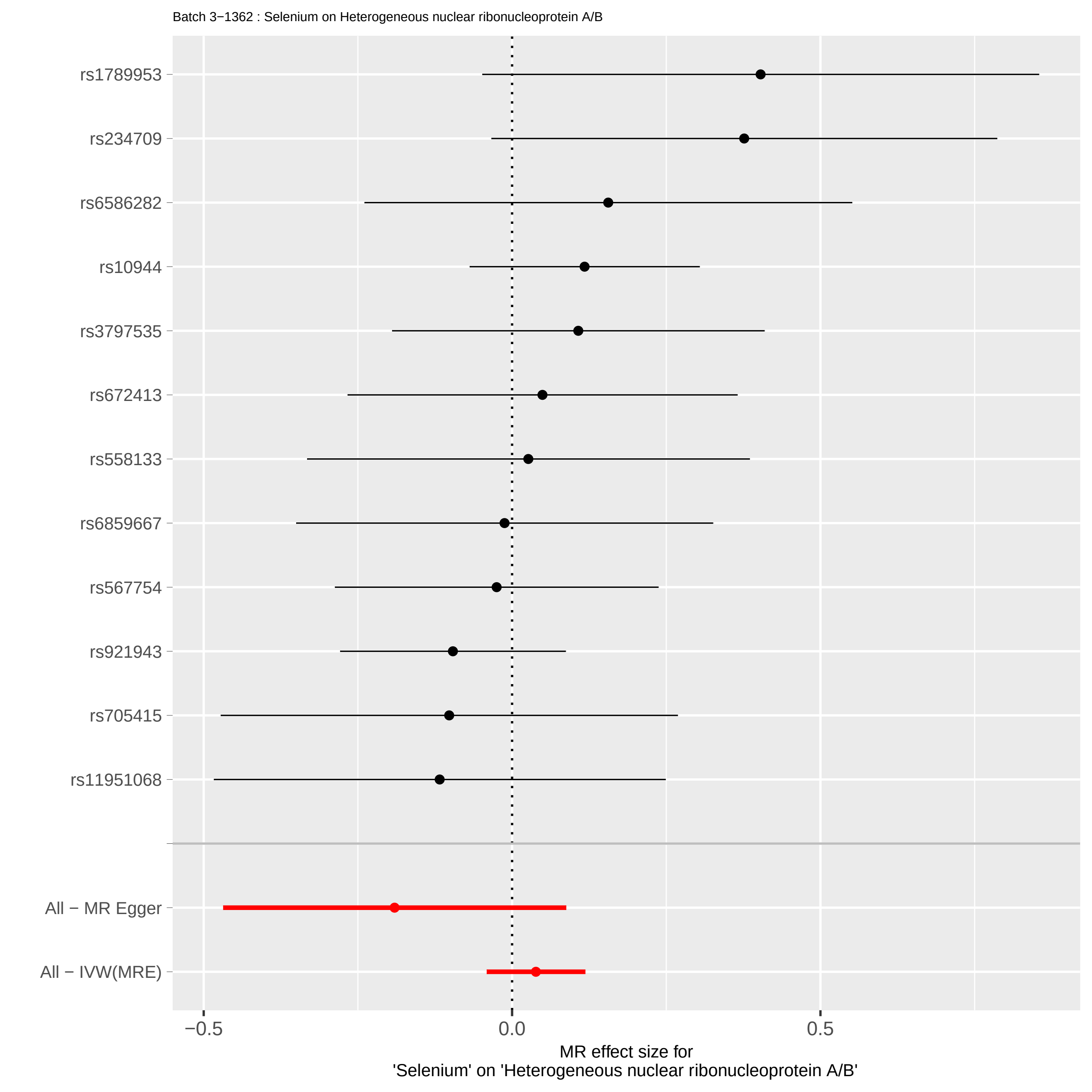

Batch 3−1362 : Selenium on Heterogeneous nuclear ribonucleoprotein A/B
rs1789953
rs234709
rs6586282
rs10944
rs3797535
rs672413
rs558133
rs6859667
rs567754
rs921943
rs705415
rs11951068
All − MR Egger
All − IVW(MRE)
−0.5
0.0
0.5
MR effect size for
'Selenium' on 'Heterogeneous nuclear ribonucleoprotein A/B'

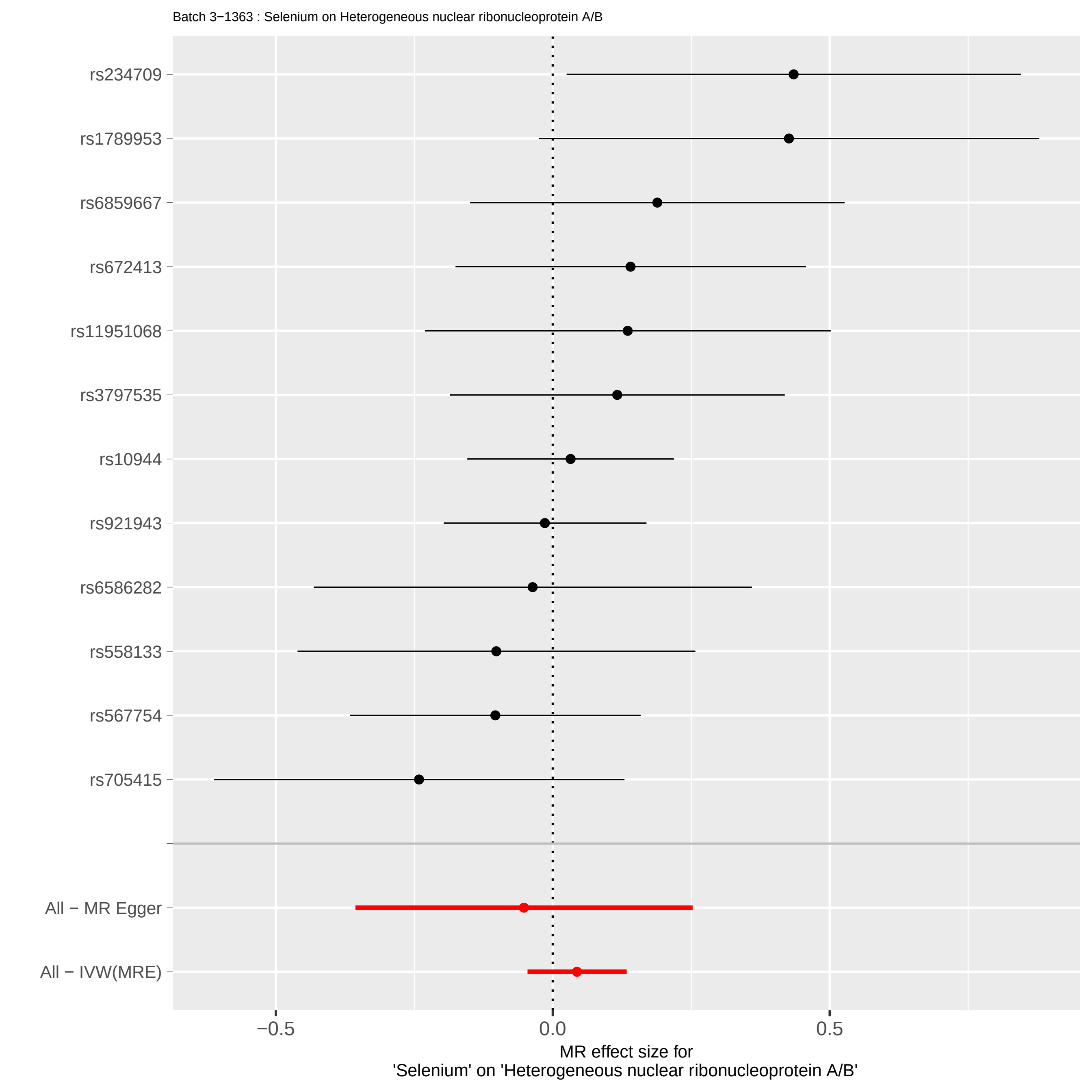
Batch 3−1363 : Selenium on Heterogeneous nuclear ribonucleoprotein A/B
rs234709
rs1789953
rs6859667
rs672413
rs11951068
rs3797535
rs10944
rs921943
rs6586282
rs558133
rs567754
rs705415
All − MR Egger
All − IVW(MRE)
−0.5
0.0
0.5
MR effect size for
'Selenium' on 'Heterogeneous nuclear ribonucleoprotein A/B'

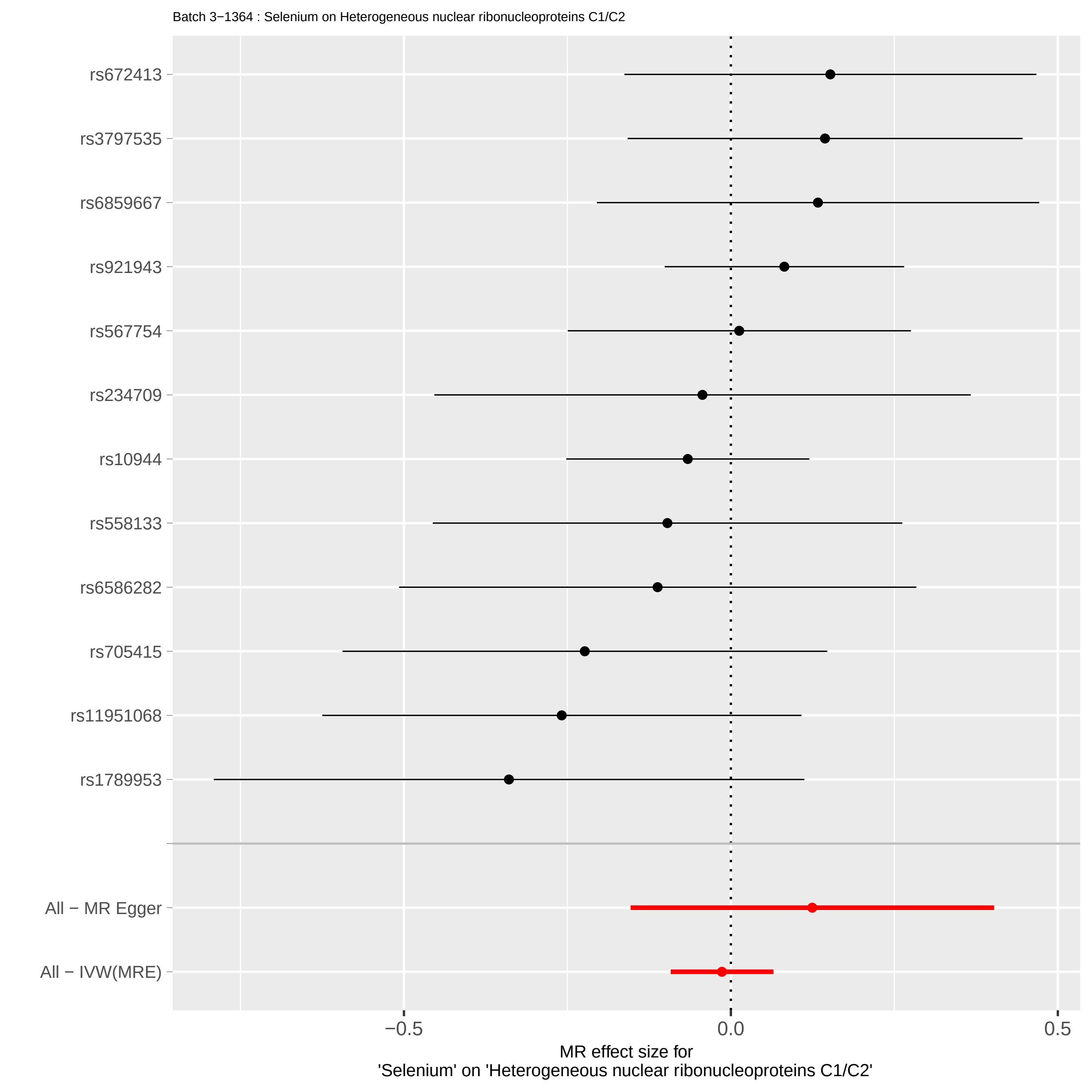

Batch 3−1364 : Selenium on Heterogeneous nuclear ribonucleoproteins C1/C2
rs672413
rs3797535
rs6859667
rs921943
rs567754
rs234709
rs10944
rs558133
rs6586282
rs705415
rs11951068
rs1789953
All − MR Egger
All − IVW(MRE)
−0.5
0.0
0.5
MR effect size for
'Selenium' on 'Heterogeneous nuclear ribonucleoproteins C1/C2'

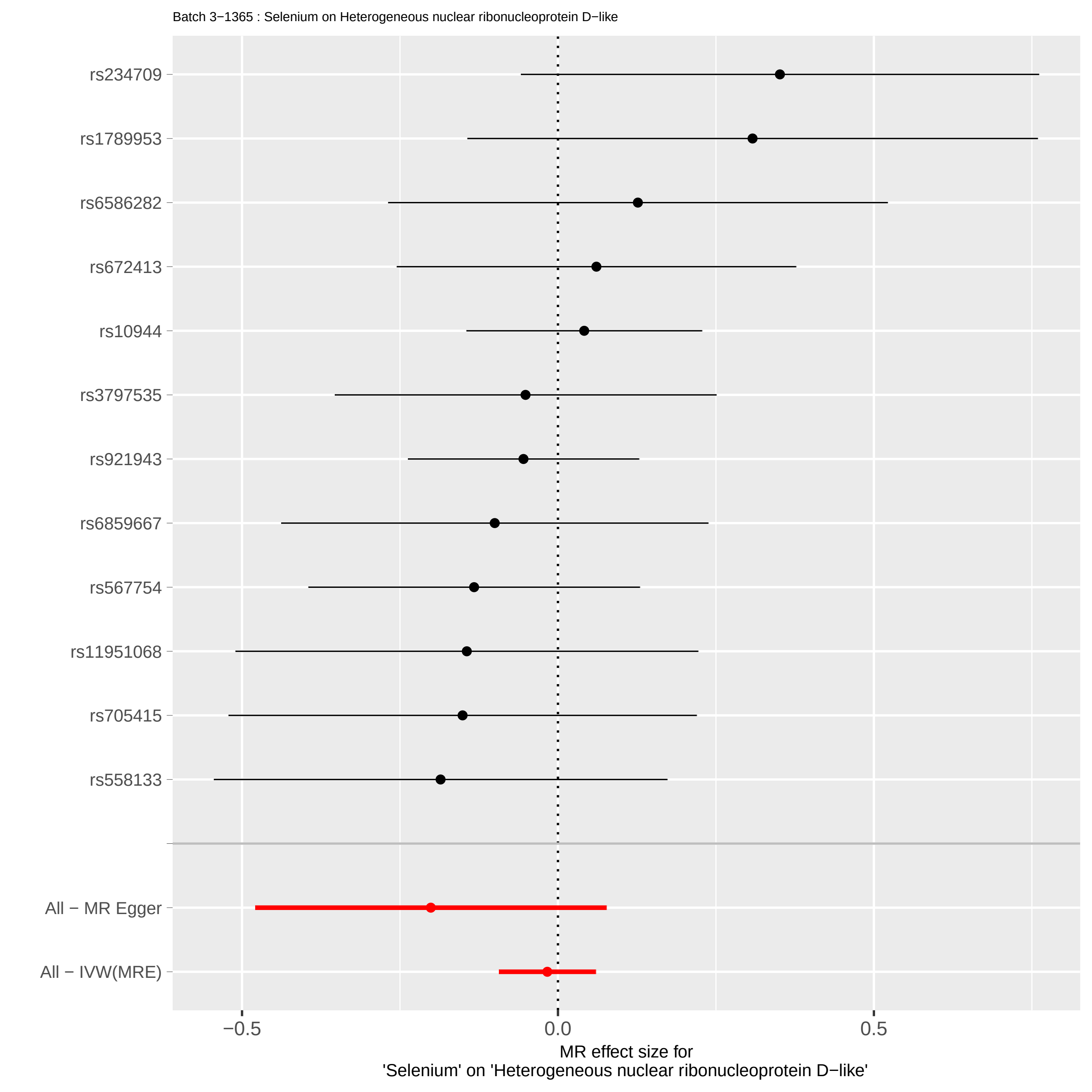

Batch 3−1365 : Selenium on Heterogeneous nuclear ribonucleoprotein D−like
rs234709
rs1789953
rs6586282
rs672413
rs10944
rs3797535
rs921943
rs6859667
rs567754
rs11951068
rs705415
rs558133
All − MR Egger
All − IVW(MRE)
−0.5
0.0
0.5
MR effect size for
'Selenium' on 'Heterogeneous nuclear ribonucleoprotein D−like'

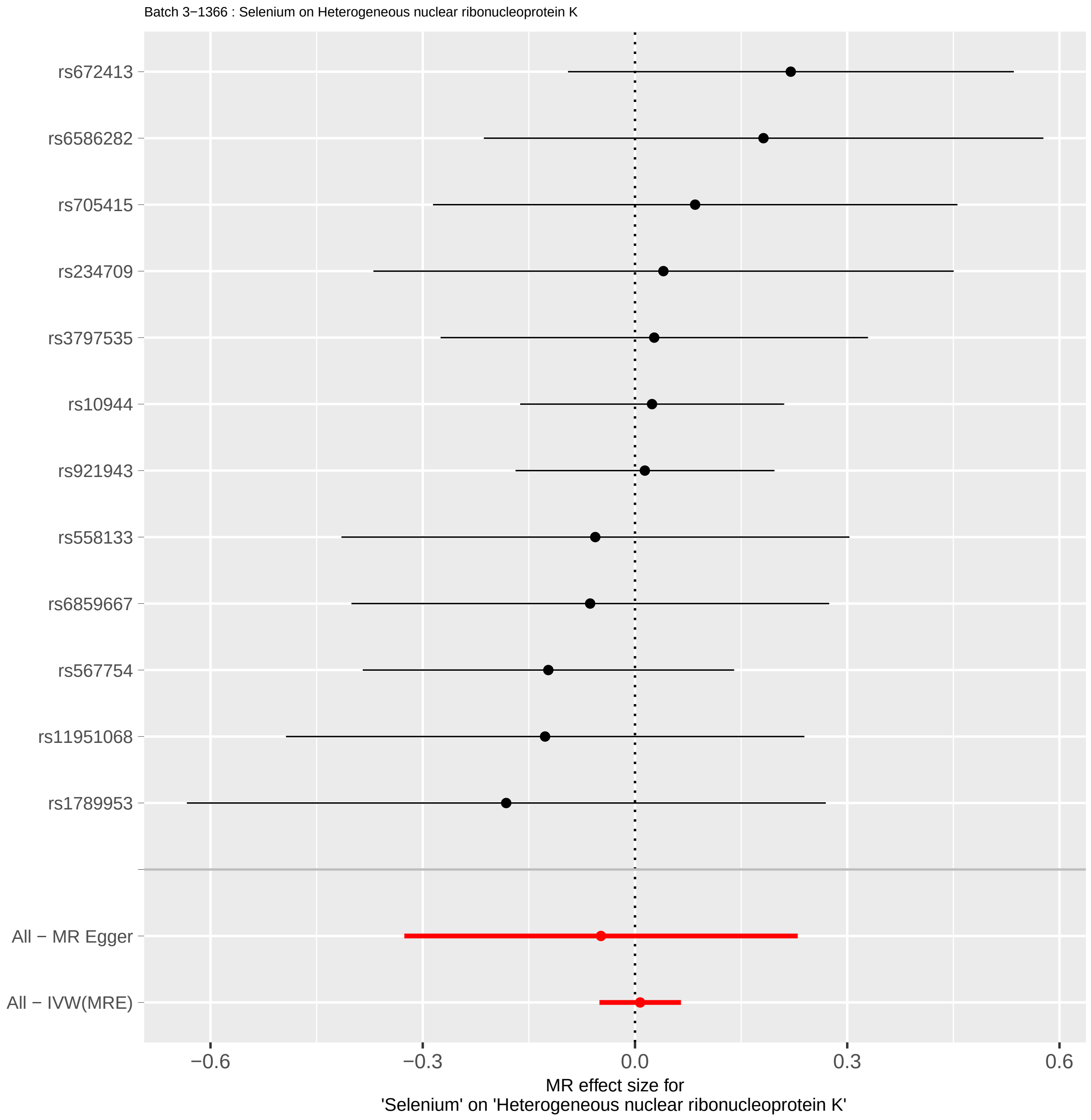

Batch 3−1366 : Selenium on Heterogeneous nuclear ribonucleoprotein K
rs672413
rs6586282
rs705415
rs234709
rs3797535
rs10944
rs921943
rs558133
rs6859667
rs567754
rs11951068
rs1789953
All − MR Egger
All − IVW(MRE)
−0.6
−0.3
0.0
0.3
0.6
MR effect size for
'Selenium' on 'Heterogeneous nuclear ribonucleoprotein K'

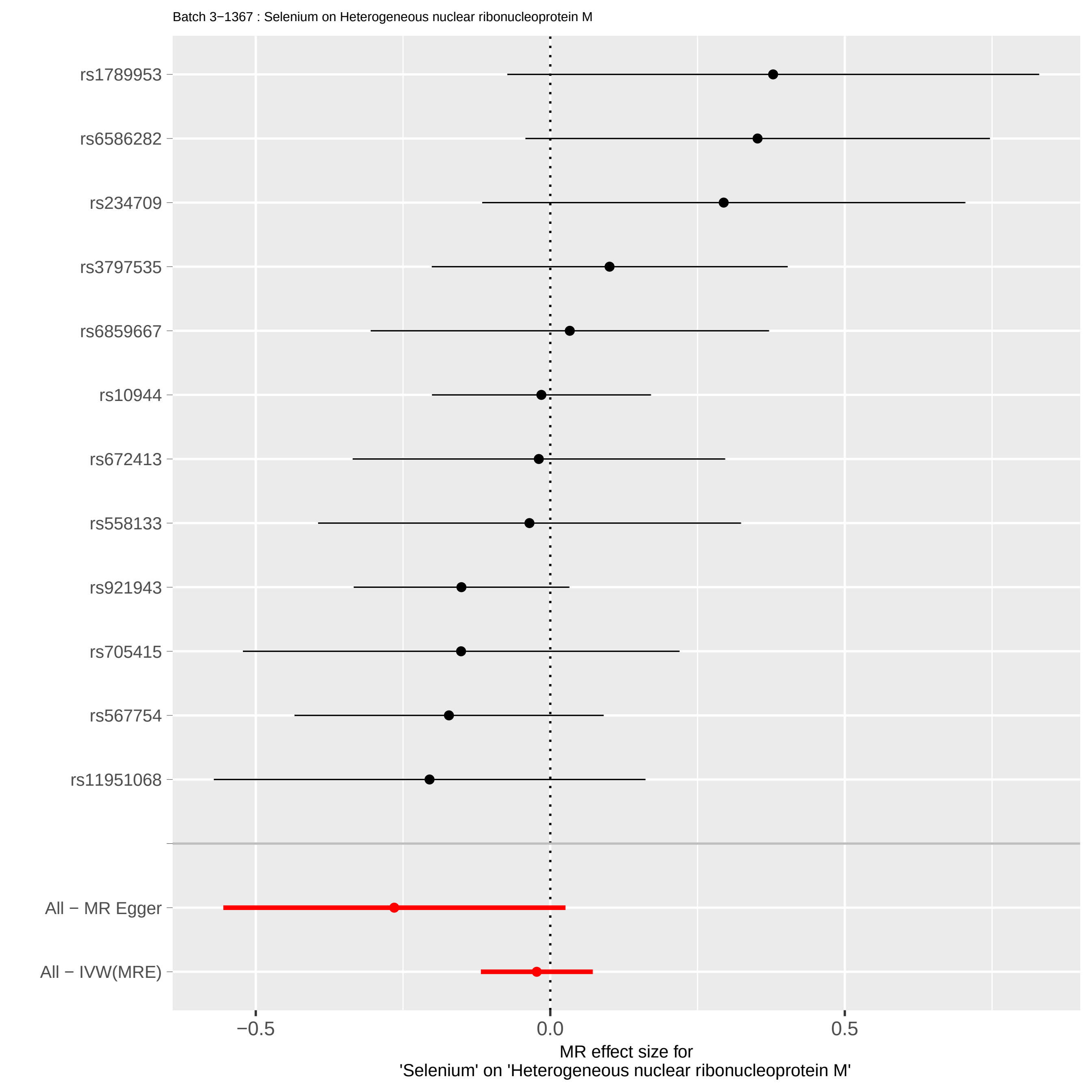

Batch 3−1367 : Selenium on Heterogeneous nuclear ribonucleoprotein M
rs1789953
rs6586282
rs234709
rs3797535
rs6859667
rs10944
rs672413
rs558133
rs921943
rs705415
rs567754
rs11951068
All − MR Egger
All − IVW(MRE)
−0.5
0.0
0.5
MR effect size for
'Selenium' on 'Heterogeneous nuclear ribonucleoprotein M'

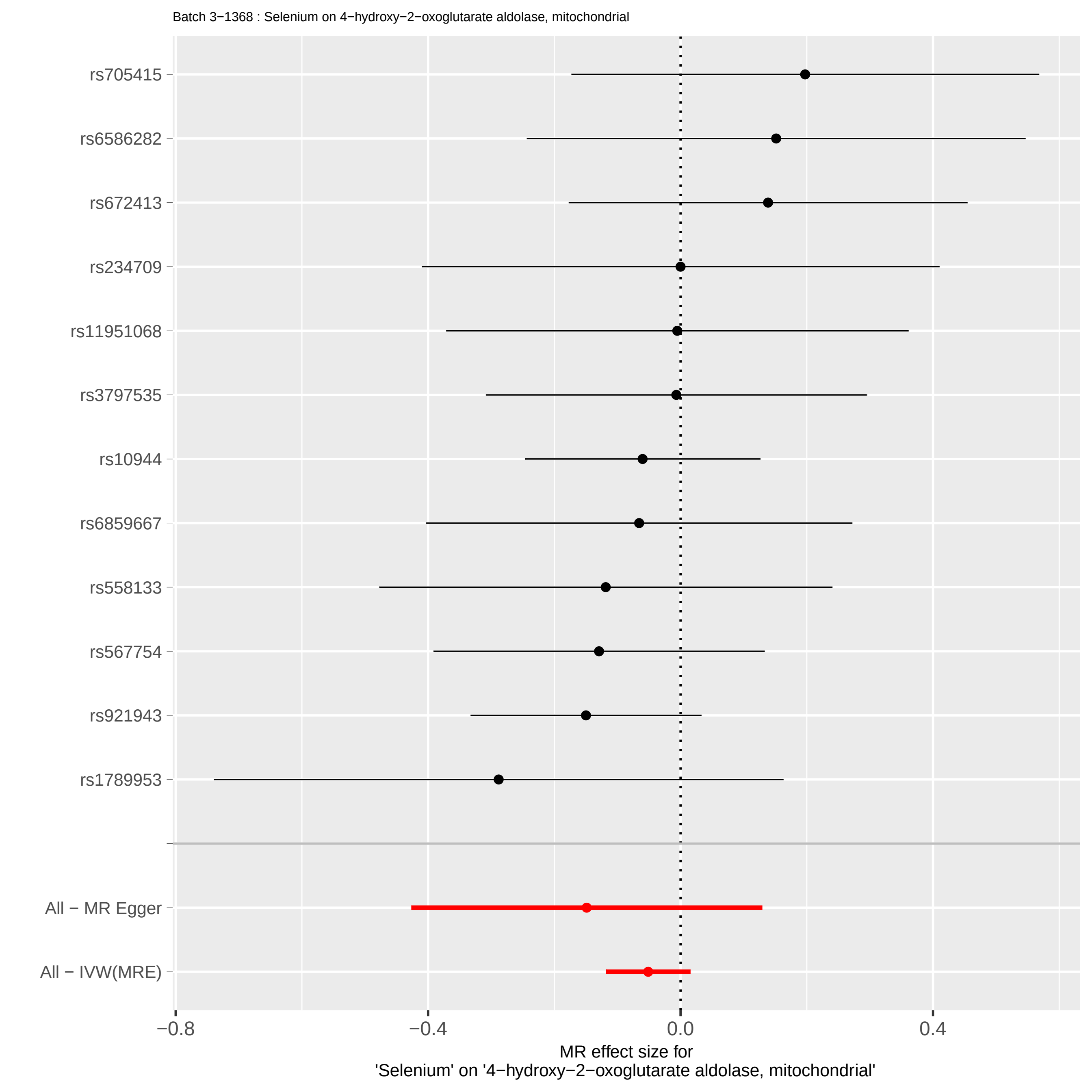

Batch 3−1368 : Selenium on 4−hydroxy−2−oxoglutarate aldolase, mitochondrial
rs705415
rs6586282
rs672413
rs234709
rs11951068
rs3797535
rs10944
rs6859667
rs558133
rs567754
rs921943
rs1789953
All − MR Egger
All − IVW(MRE)
−0.8
−0.4
0.0
0.4
MR effect size for
'Selenium' on '4−hydroxy−2−oxoglutarate aldolase, mitochondrial'

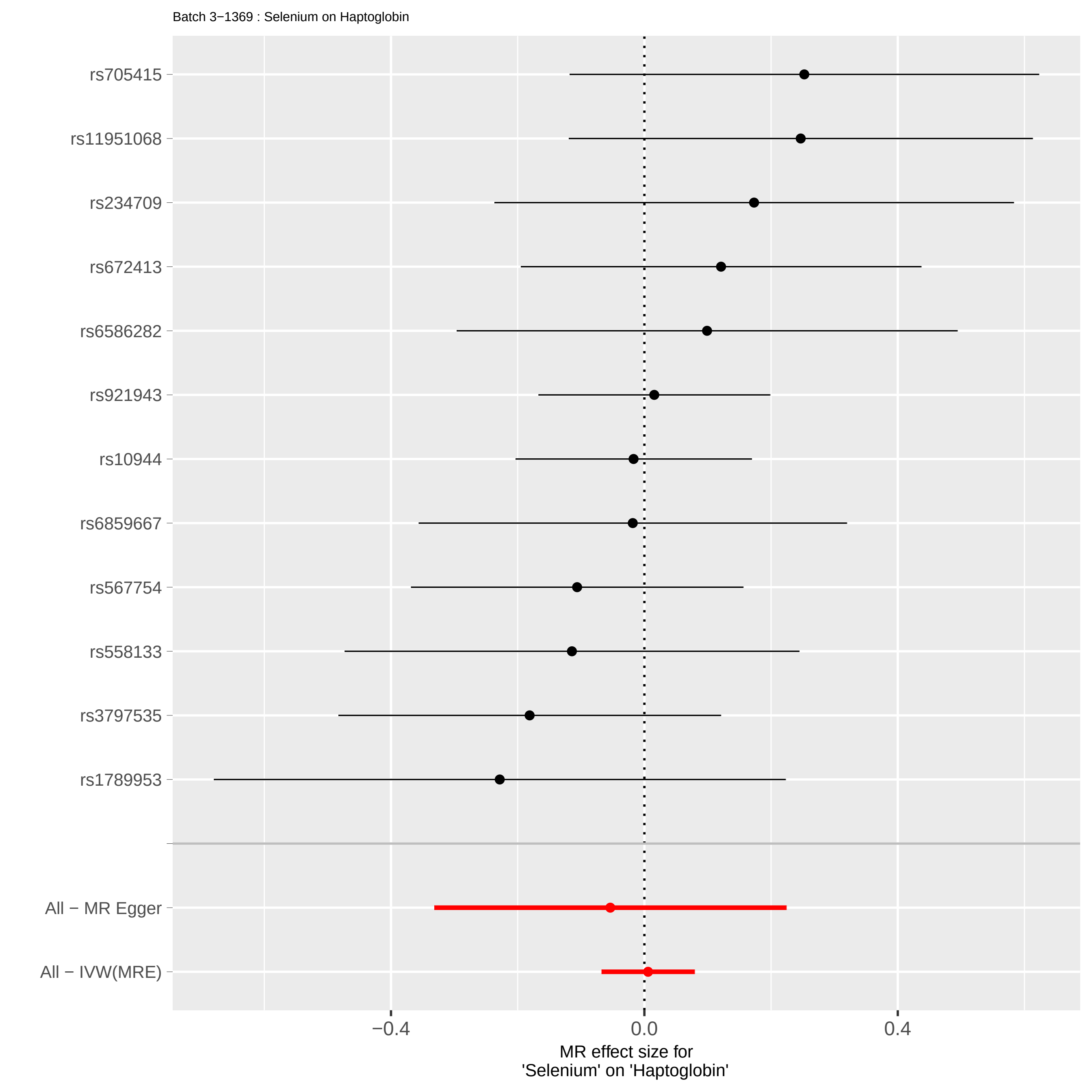

Batch 3−1369 : Selenium on Haptoglobin
rs705415
rs11951068
rs234709
rs672413
rs6586282
rs921943
rs10944
rs6859667
rs567754
rs558133
rs3797535
rs1789953
All − MR Egger
All − IVW(MRE)
−0.4
0.0
0.4
MR effect size for
'Selenium' on 'Haptoglobin'

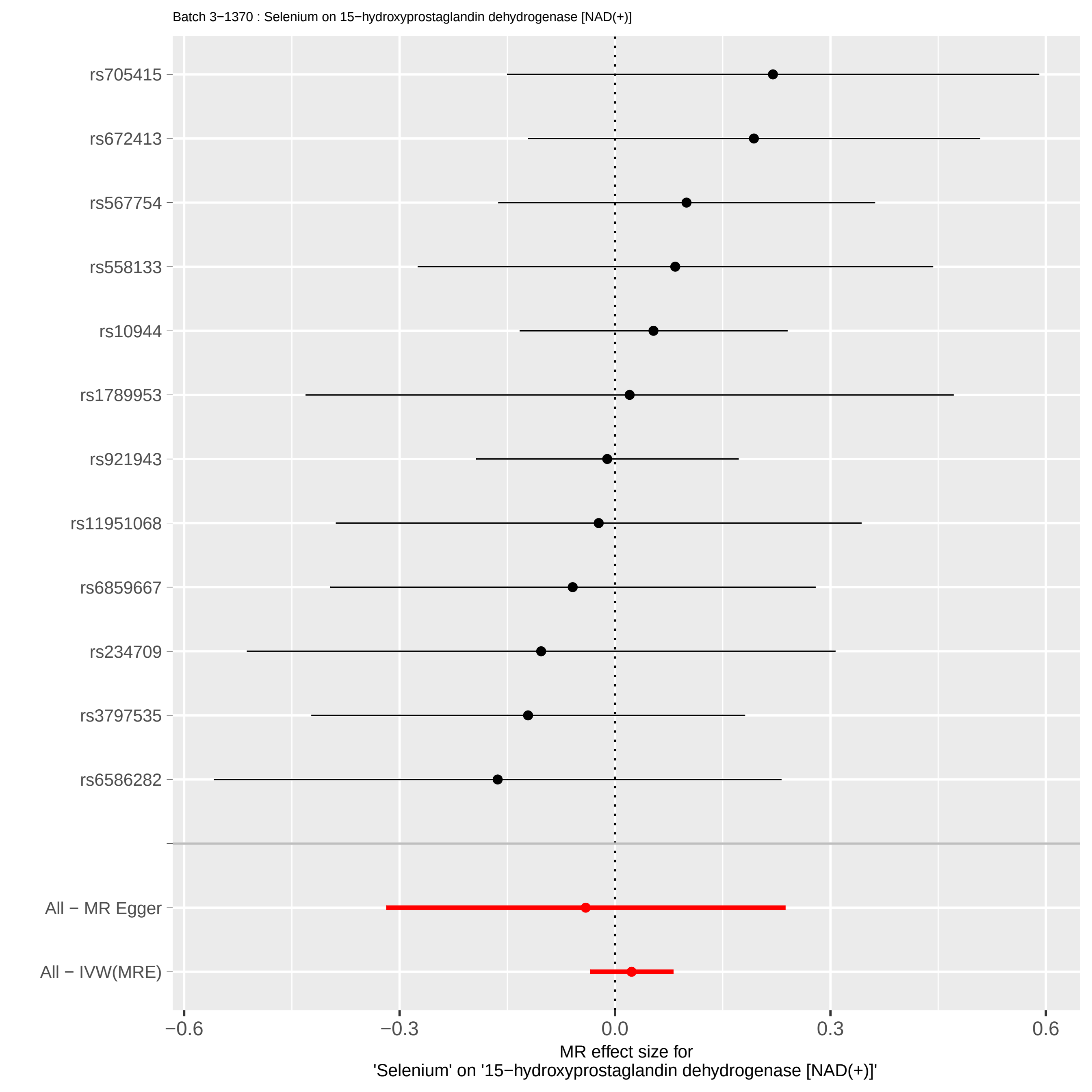

Batch 3−1370 : Selenium on 15−hydroxyprostaglandin dehydrogenase [NAD(+)]
rs705415
rs672413
rs567754
rs558133
rs10944
rs1789953
rs921943
rs11951068
rs6859667
rs234709
rs3797535
rs6586282
All − MR Egger
All − IVW(MRE)
−0.6
−0.3
0.0
0.3
0.6
MR effect size for
'Selenium' on '15−hydroxyprostaglandin dehydrogenase [NAD(+)]'

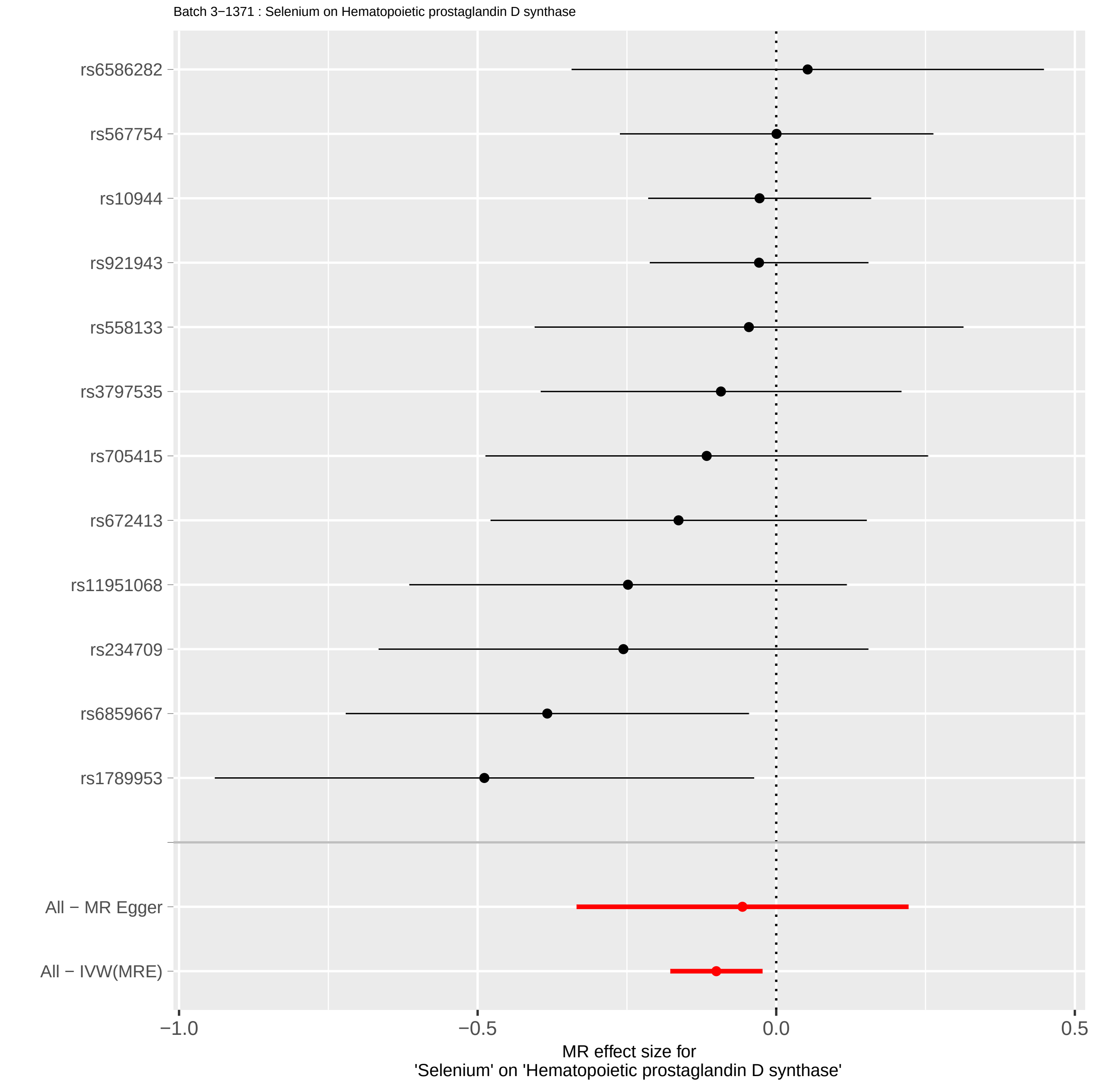

Batch 3−1371 : Selenium on Hematopoietic prostaglandin D synthase
rs6586282
rs567754
rs10944
rs921943
rs558133
rs3797535
rs705415
rs672413
rs11951068
rs234709
rs6859667
rs1789953
All − MR Egger
All − IVW(MRE)
−1.0
−0.5
0.0
0.5
MR effect size for
'Selenium' on 'Hematopoietic prostaglandin D synthase'

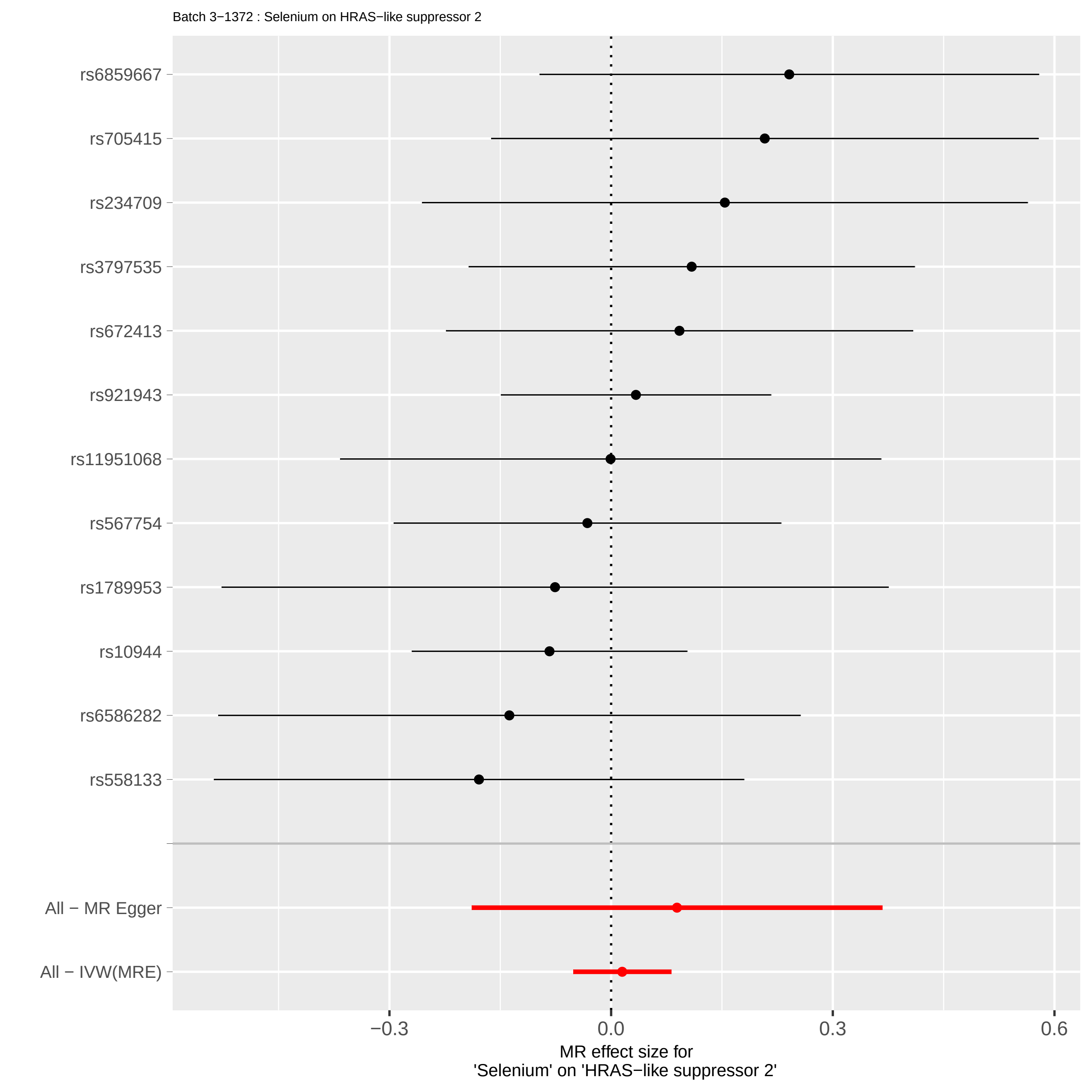

Batch 3−1372 : Selenium on HRAS−like suppressor 2
rs6859667
rs705415
rs234709
rs3797535
rs672413
rs921943
rs11951068
rs567754
rs1789953
rs10944
rs6586282
rs558133
All − MR Egger
All − IVW(MRE)
−0.3
0.0
0.3
0.6
MR effect size for
'Selenium' on 'HRAS−like suppressor 2'

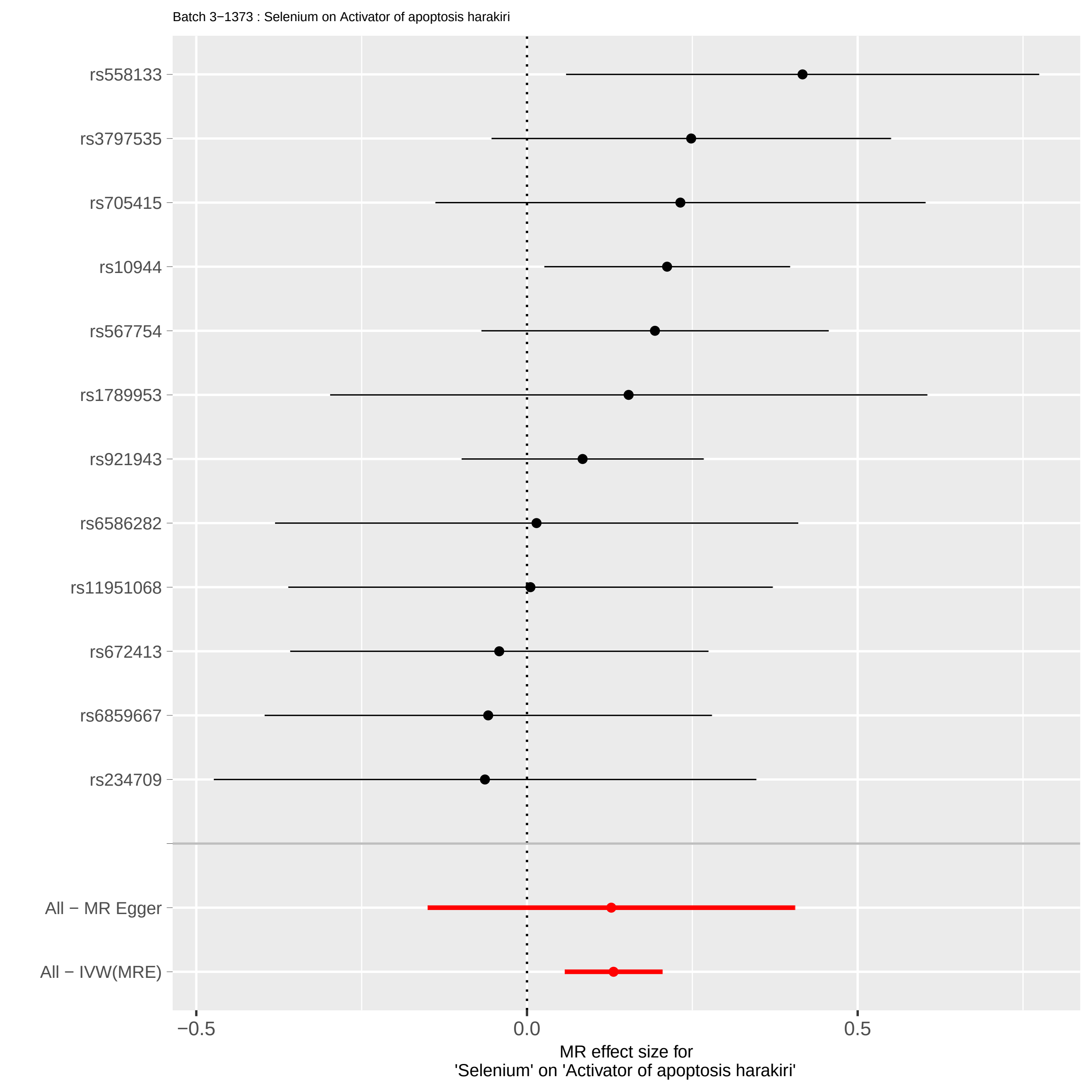
Batch 3−1373 : Selenium on Activator of apoptosis harakiri
rs558133
rs3797535
rs705415
rs10944
rs567754
rs1789953
rs921943
rs6586282
rs11951068
rs672413
rs6859667
rs234709
All − MR Egger
All − IVW(MRE)
−0.5
0.0
0.5
MR effect size for
'Selenium' on 'Activator of apoptosis harakiri'

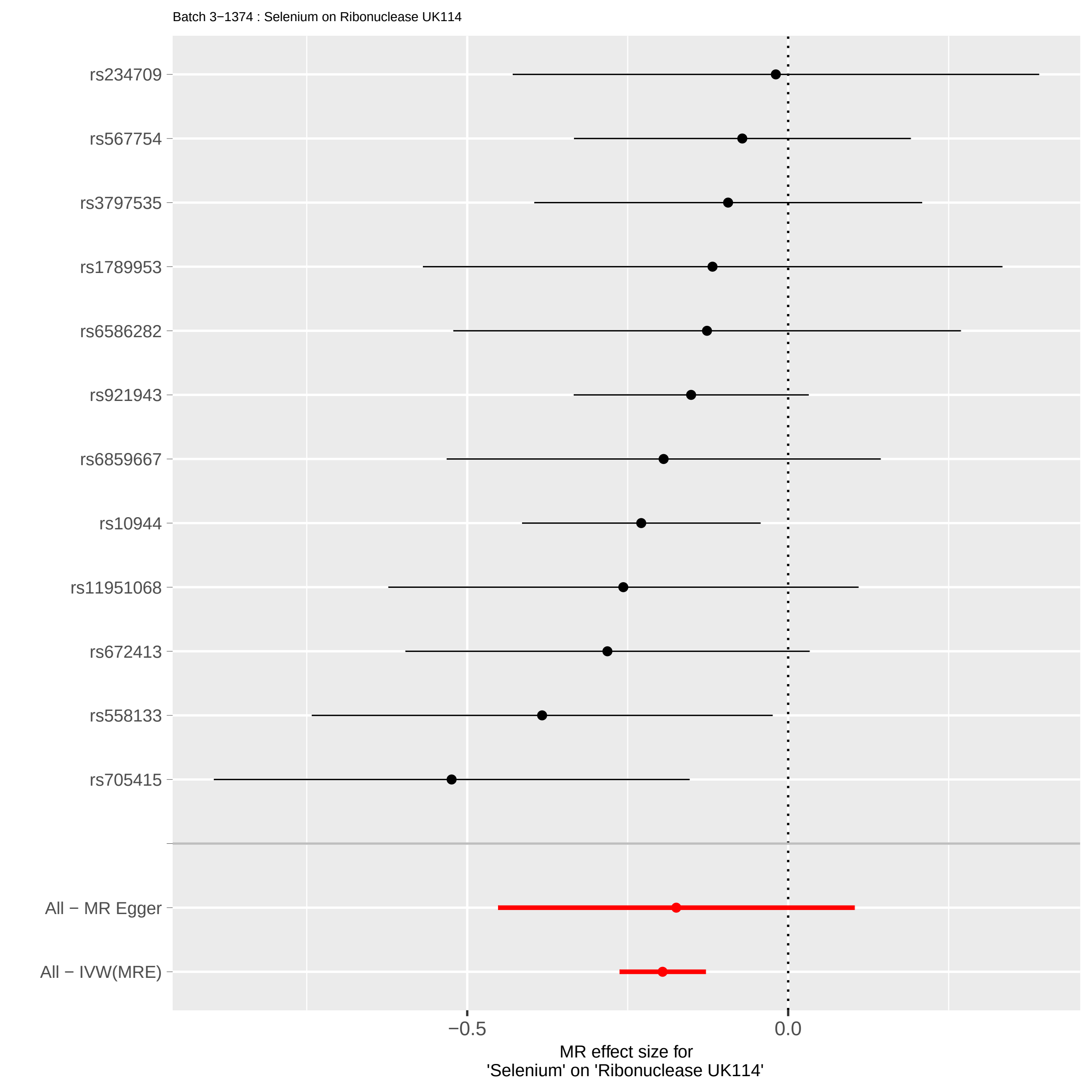

Batch 3−1374 : Selenium on Ribonuclease UK114
rs234709
rs567754
rs3797535
rs1789953
rs6586282
rs921943
rs6859667
rs10944
rs11951068
rs672413
rs558133
rs705415
All − MR Egger
All − IVW(MRE)
−0.5
0.0
MR effect size for
'Selenium' on 'Ribonuclease UK114'

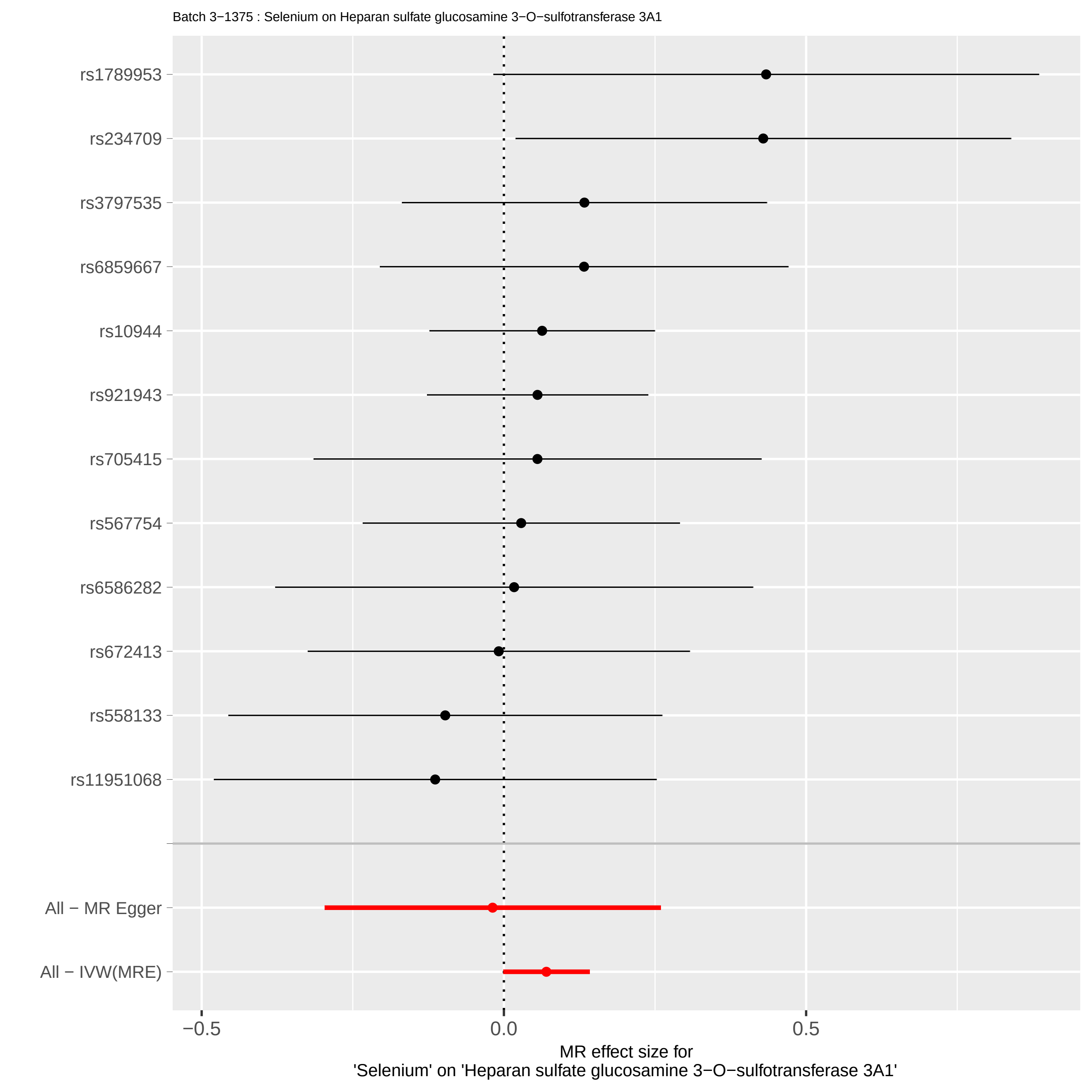

Batch 3−1375 : Selenium on Heparan sulfate glucosamine 3−O−sulfotransferase 3A1
rs1789953
rs234709
rs3797535
rs6859667
rs10944
rs921943
rs705415
rs567754
rs6586282
rs672413
rs558133
rs11951068
All − MR Egger
All − IVW(MRE)
−0.5
0.0
0.5
MR effect size for
'Selenium' on 'Heparan sulfate glucosamine 3−O−sulfotransferase 3A1'

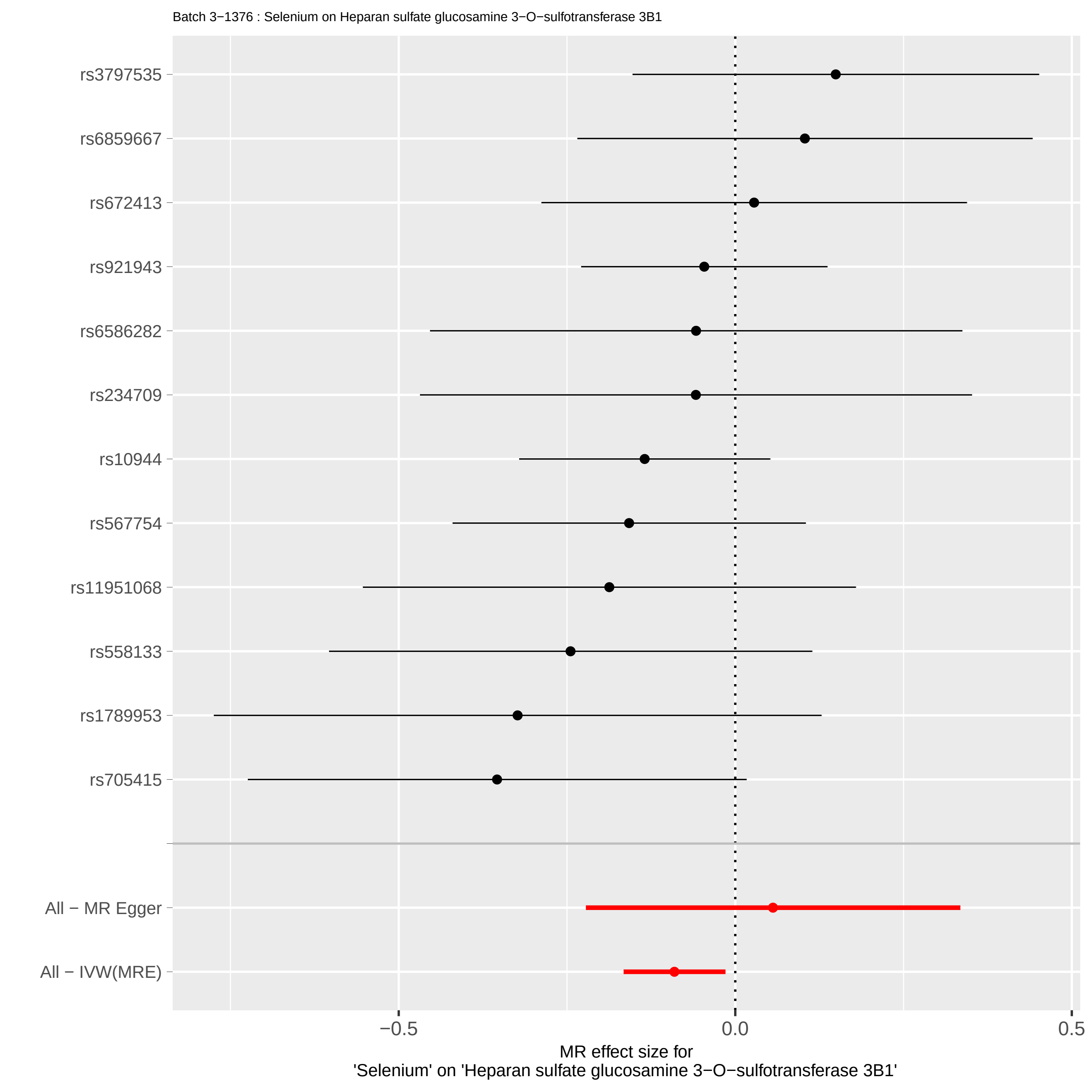

Batch 3−1376 : Selenium on Heparan sulfate glucosamine 3−O−sulfotransferase 3B1
rs3797535
rs6859667
rs672413
rs921943
rs6586282
rs234709
rs10944
rs567754
rs11951068
rs558133
rs1789953
rs705415
All − MR Egger
All − IVW(MRE)
−0.5
0.0
0.5
MR effect size for
'Selenium' on 'Heparan sulfate glucosamine 3−O−sulfotransferase 3B1'

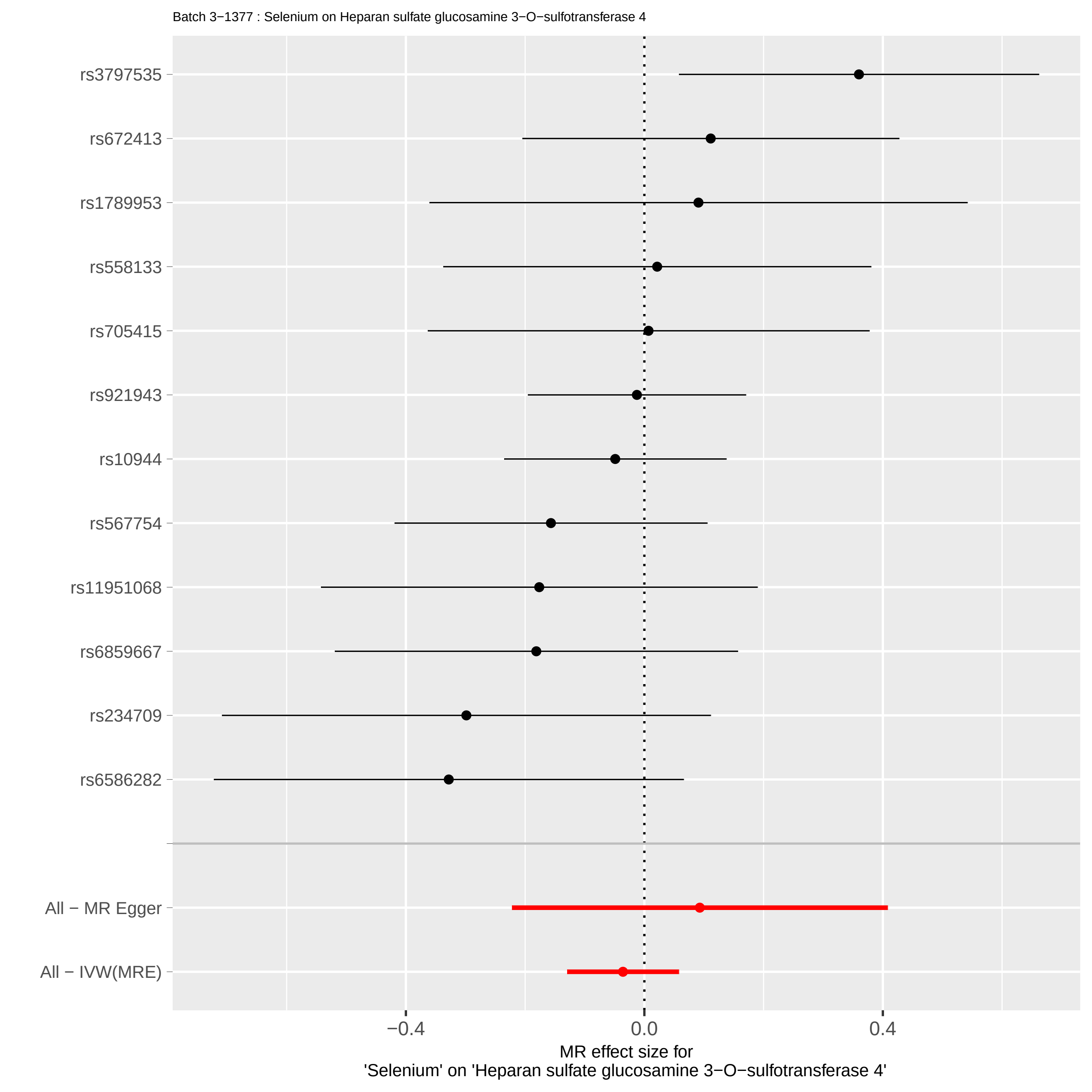

Batch 3−1377 : Selenium on Heparan sulfate glucosamine 3−O−sulfotransferase 4
rs3797535
rs672413
rs1789953
rs558133
rs705415
rs921943
rs10944
rs567754
rs11951068
rs6859667
rs234709
rs6586282
All − MR Egger
All − IVW(MRE)
−0.4
0.0
0.4
MR effect size for
'Selenium' on 'Heparan sulfate glucosamine 3−O−sulfotransferase 4'

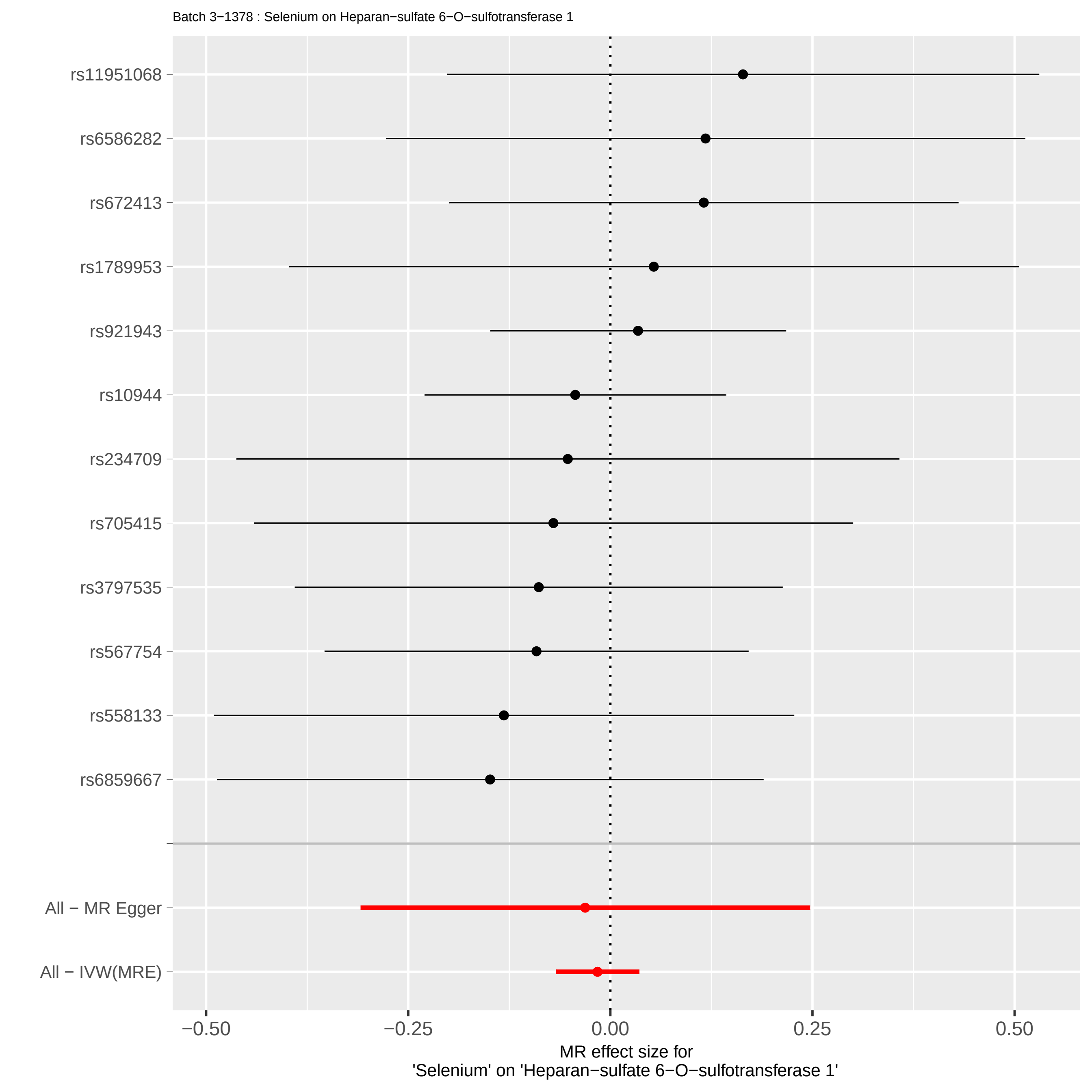

Batch 3−1378 : Selenium on Heparan−sulfate 6−O−sulfotransferase 1
rs11951068
rs6586282
rs672413
rs1789953
rs921943
rs10944
rs234709
rs705415
rs3797535
rs567754
rs558133
rs6859667
All − MR Egger
All − IVW(MRE)
−0.50
−0.25
0.00
0.25
0.50
MR effect size for
'Selenium' on 'Heparan−sulfate 6−O−sulfotransferase 1'

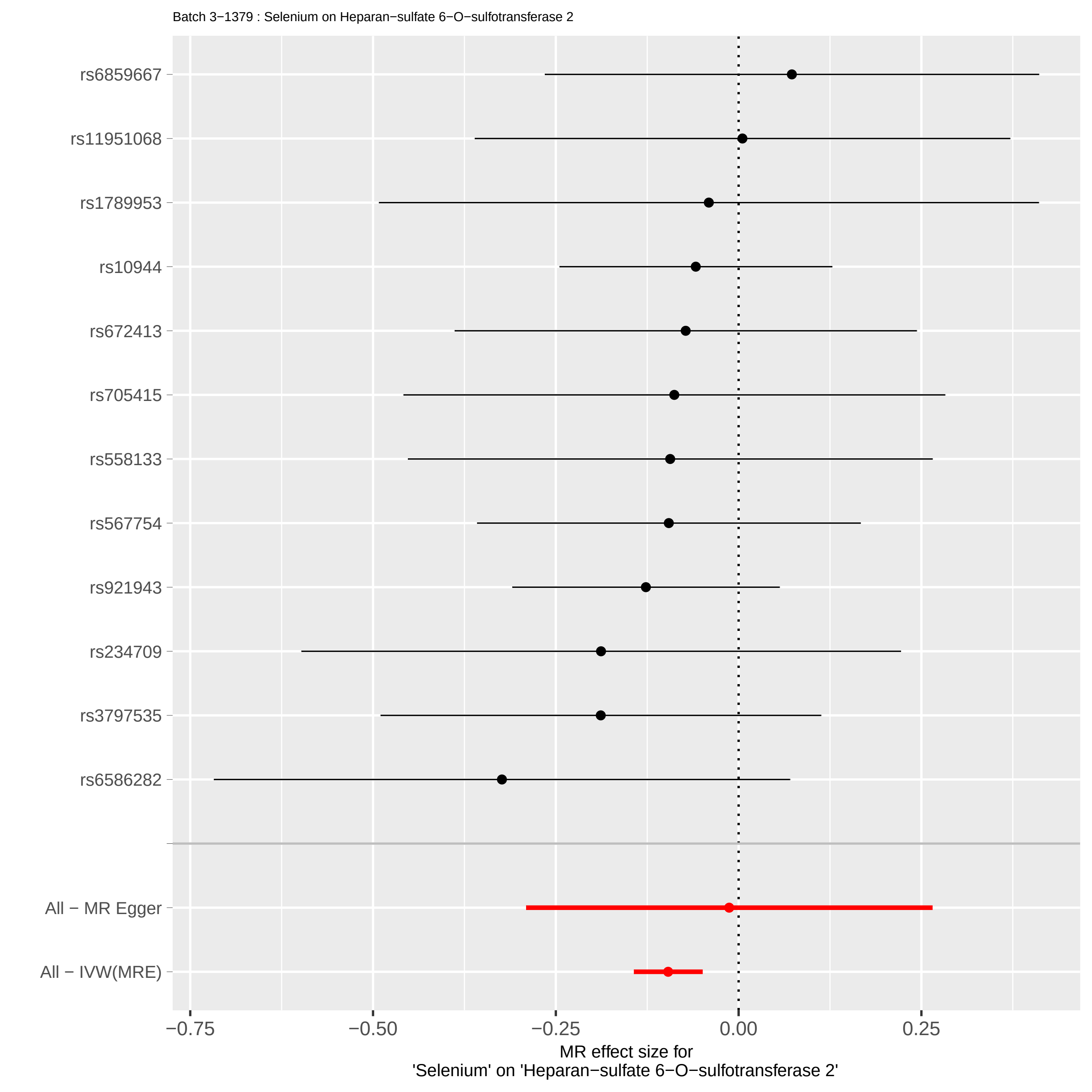

Batch 3−1379 : Selenium on Heparan−sulfate 6−O−sulfotransferase 2
rs6859667
rs11951068
rs1789953
rs10944
rs672413
rs705415
rs558133
rs567754
rs921943
rs234709
rs3797535
rs6586282
All − MR Egger
All − IVW(MRE)
−0.75
−0.50
−0.25
0.00
0.25
MR effect size for
'Selenium' on 'Heparan−sulfate 6−O−sulfotransferase 2'

Batch 3−1380 : Selenium on 3−hydroxyacyl−CoA dehydrogenase type−2

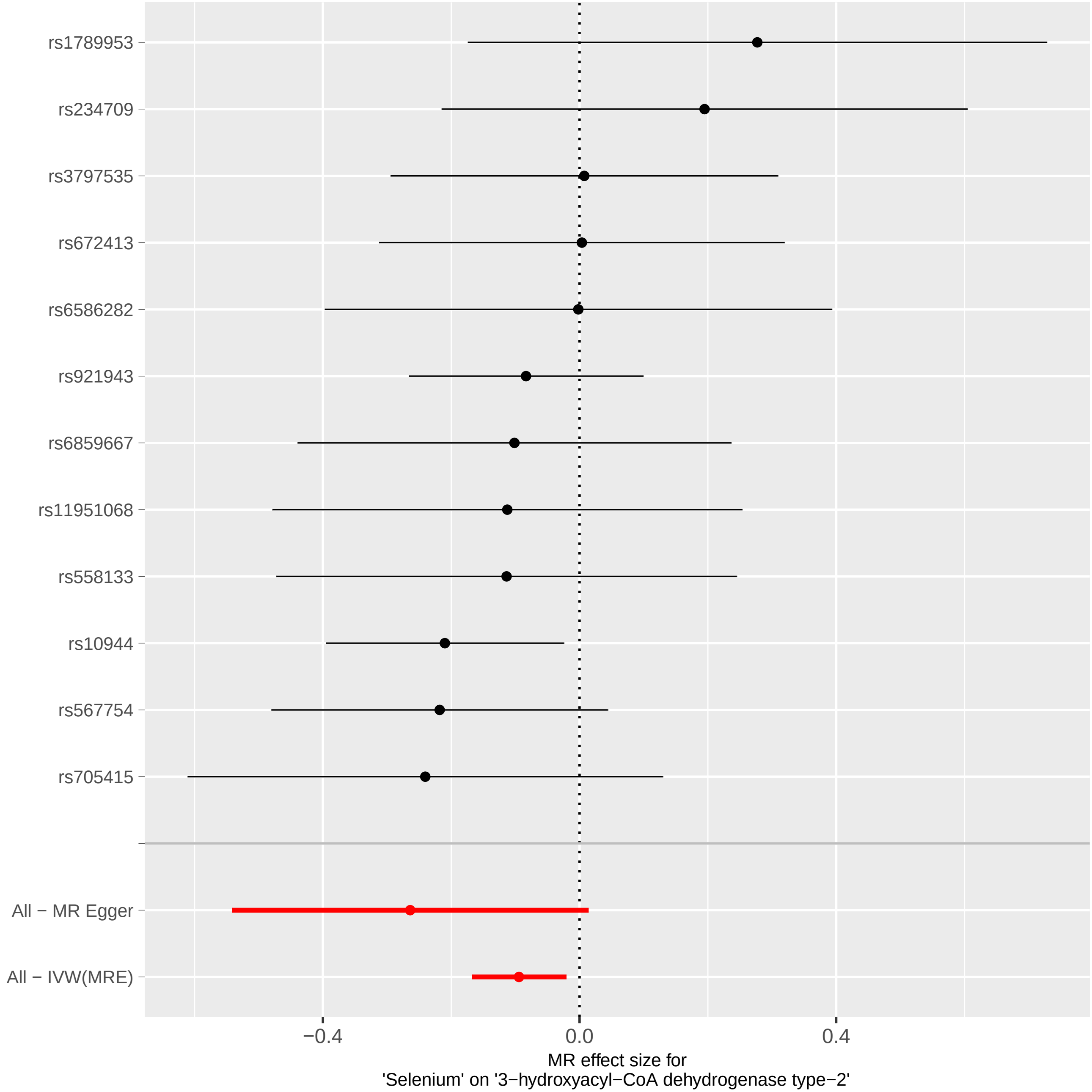

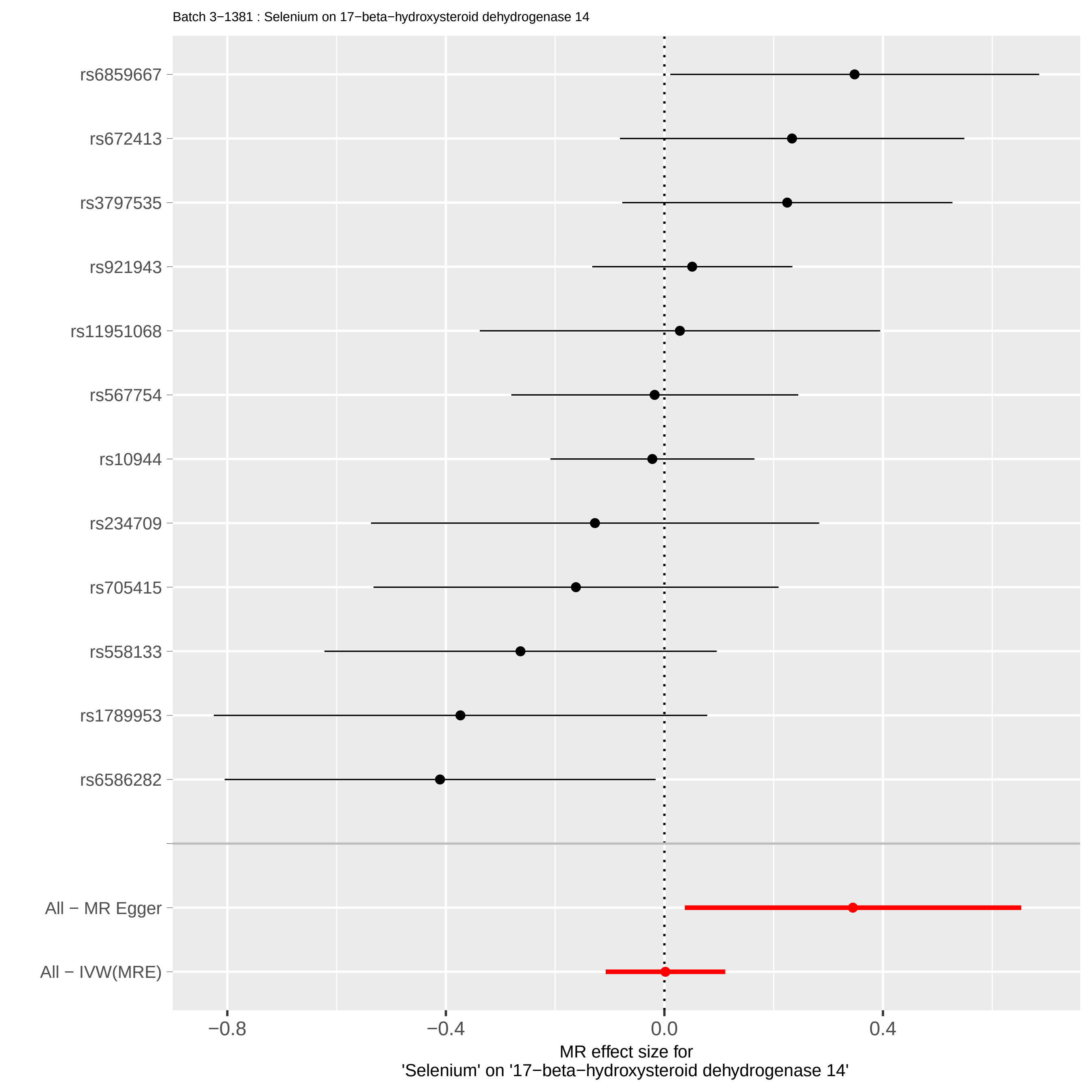

Batch 3−1381 : Selenium on 17−beta−hydroxysteroid dehydrogenase 14
rs6859667
rs672413
rs3797535
rs921943
rs11951068
rs567754
rs10944
rs234709
rs705415
rs558133
rs1789953
rs6586282
All − MR Egger
All − IVW(MRE)
−0.8
−0.4
0.0
0.4
MR effect size for
'Selenium' on '17−beta−hydroxysteroid dehydrogenase 14'

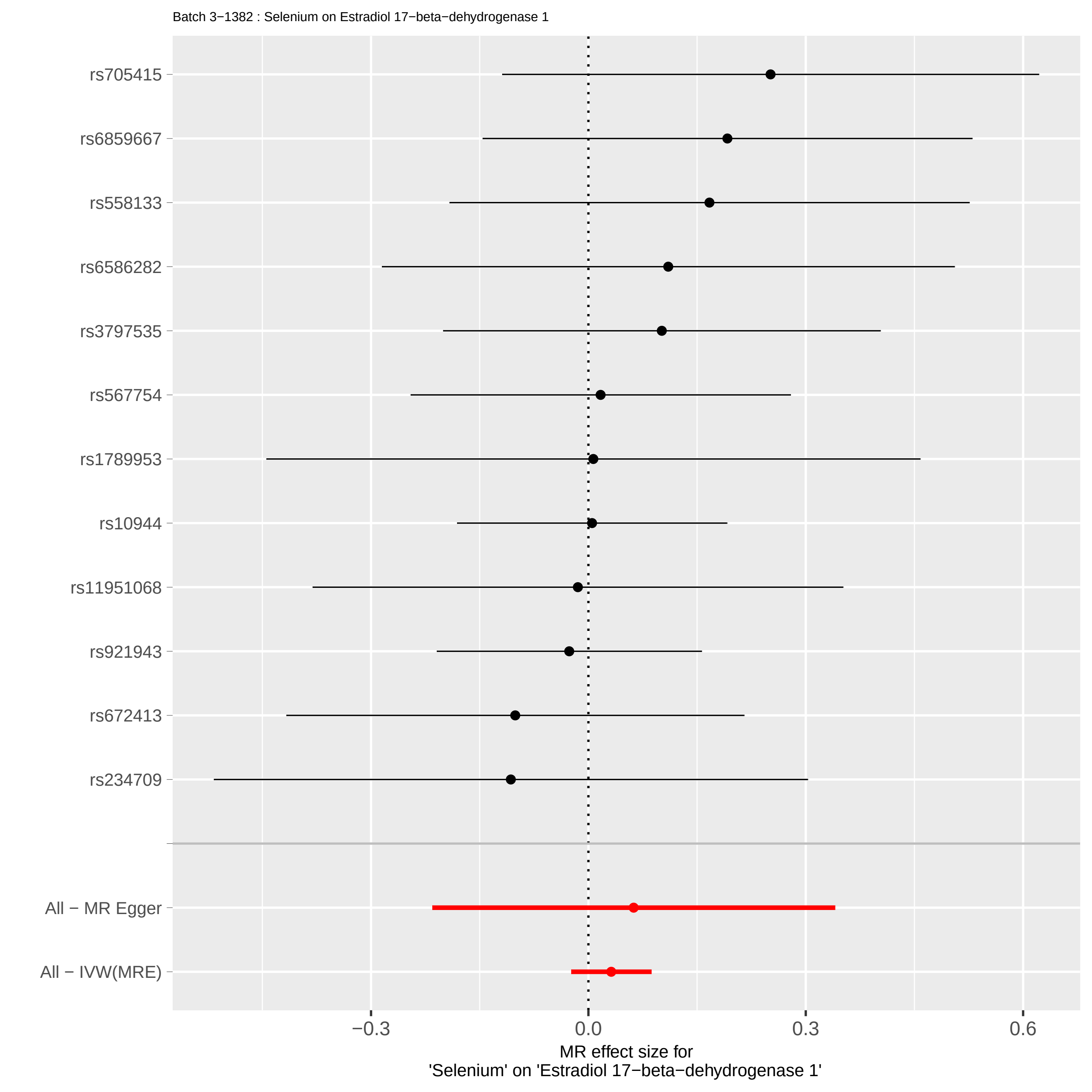

Batch 3−1382 : Selenium on Estradiol 17−beta−dehydrogenase 1
rs705415
rs6859667
rs558133
rs6586282
rs3797535
rs567754
rs1789953
rs10944
rs11951068
rs921943
rs672413
rs234709
All − MR Egger
All − IVW(MRE)
−0.3
0.0
0.3
0.6
MR effect size for
'Selenium' on 'Estradiol 17−beta−dehydrogenase 1'

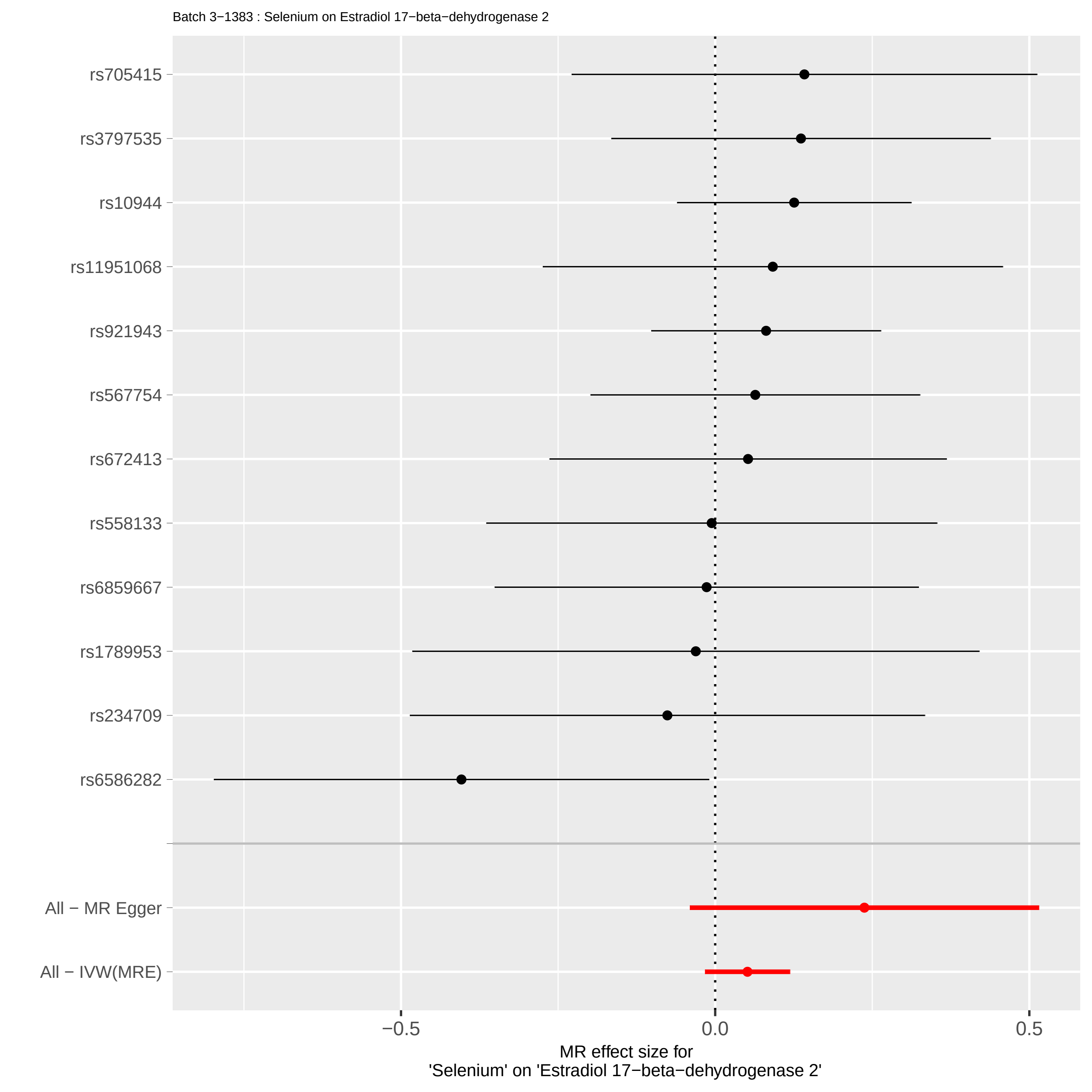

Batch 3−1383 : Selenium on Estradiol 17−beta−dehydrogenase 2
rs705415
rs3797535
rs10944
rs11951068
rs921943
rs567754
rs672413
rs558133
rs6859667
rs1789953
rs234709
rs6586282
All − MR Egger
All − IVW(MRE)
−0.5
0.0
0.5
MR effect size for
'Selenium' on 'Estradiol 17−beta−dehydrogenase 2'

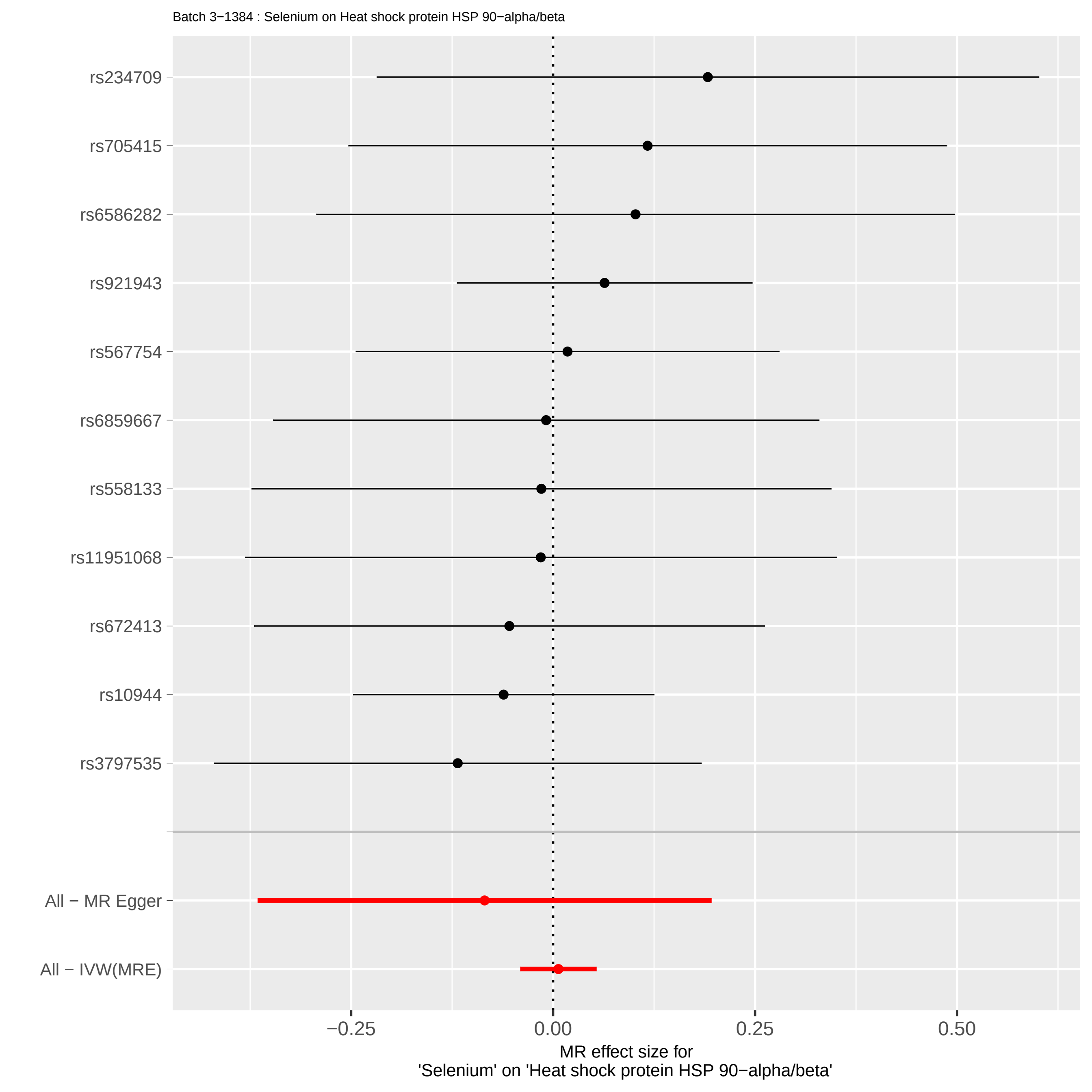

Batch 3−1384 : Selenium on Heat shock protein HSP 90−alpha/beta
rs234709
rs705415
rs6586282
rs921943
rs567754
rs6859667
rs558133
rs11951068
rs672413
rs10944
rs3797535
All − MR Egger
All − IVW(MRE)
−0.25
0.00
0.25
0.50
MR effect size for
'Selenium' on 'Heat shock protein HSP 90−alpha/beta'

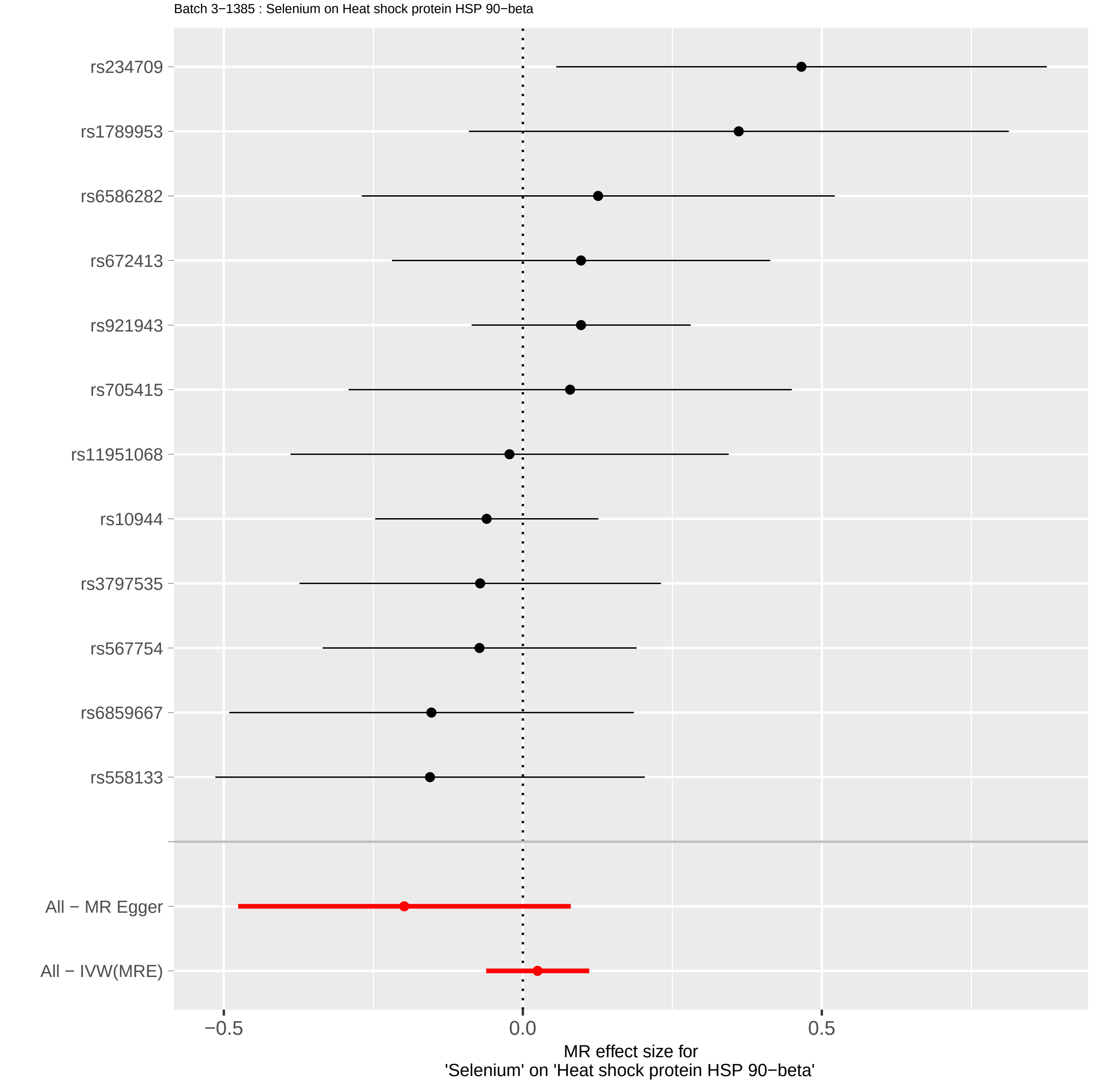
Batch 3−1385 : Selenium on Heat shock protein HSP 90−beta
rs234709
rs1789953
rs6586282
rs672413
rs921943
rs705415
rs11951068
rs10944
rs3797535
rs567754
rs6859667
rs558133
All − MR Egger
All − IVW(MRE)
−0.5
0.0
0.5
MR effect size for
'Selenium' on 'Heat shock protein HSP 90−beta'

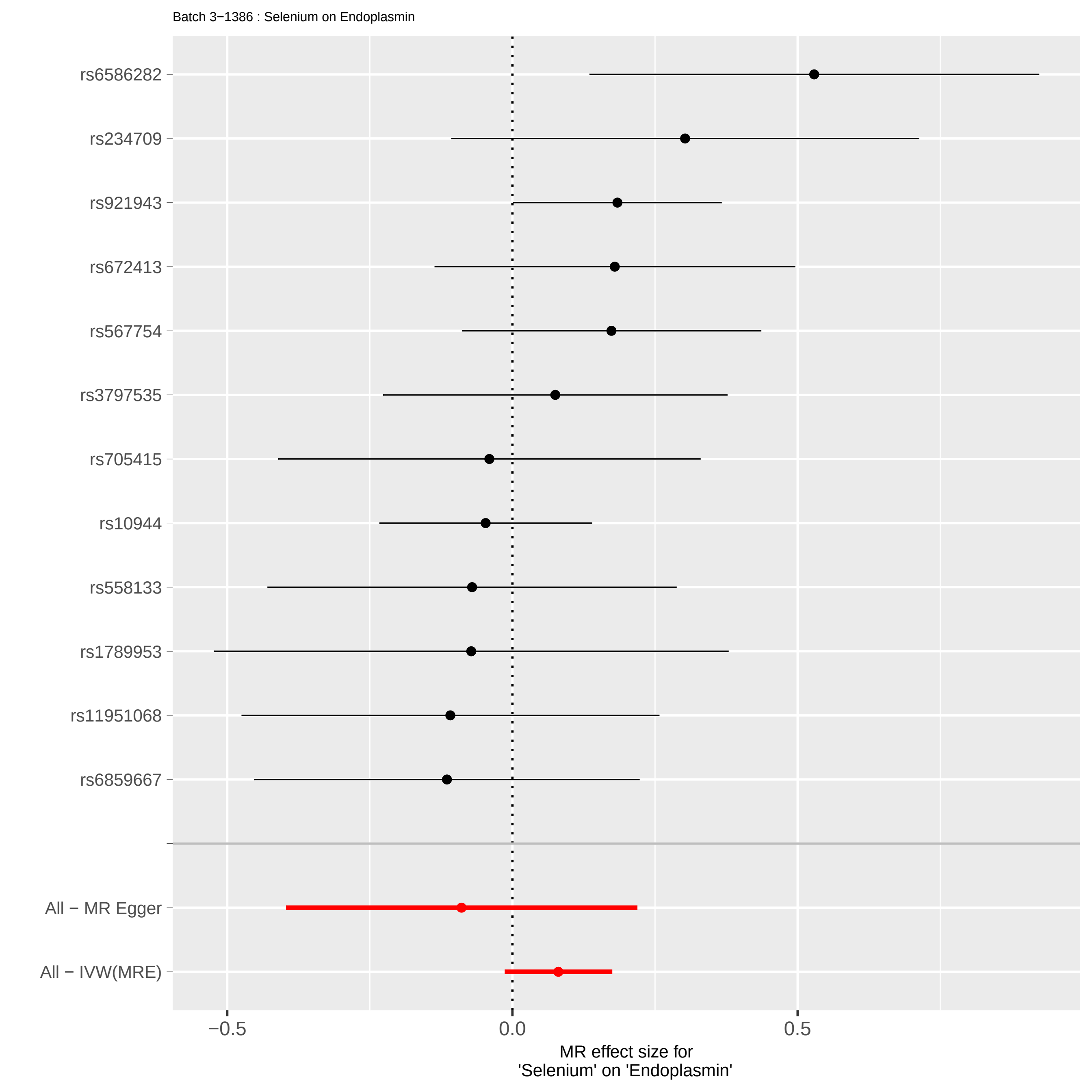

Batch 3−1386 : Selenium on Endoplasmin
rs6586282
rs234709
rs921943
rs672413
rs567754
rs3797535
rs705415
rs10944
rs558133
rs1789953
rs11951068
rs6859667
All − MR Egger
All − IVW(MRE)
−0.5
0.0
0.5
MR effect size for
'Selenium' on 'Endoplasmin'

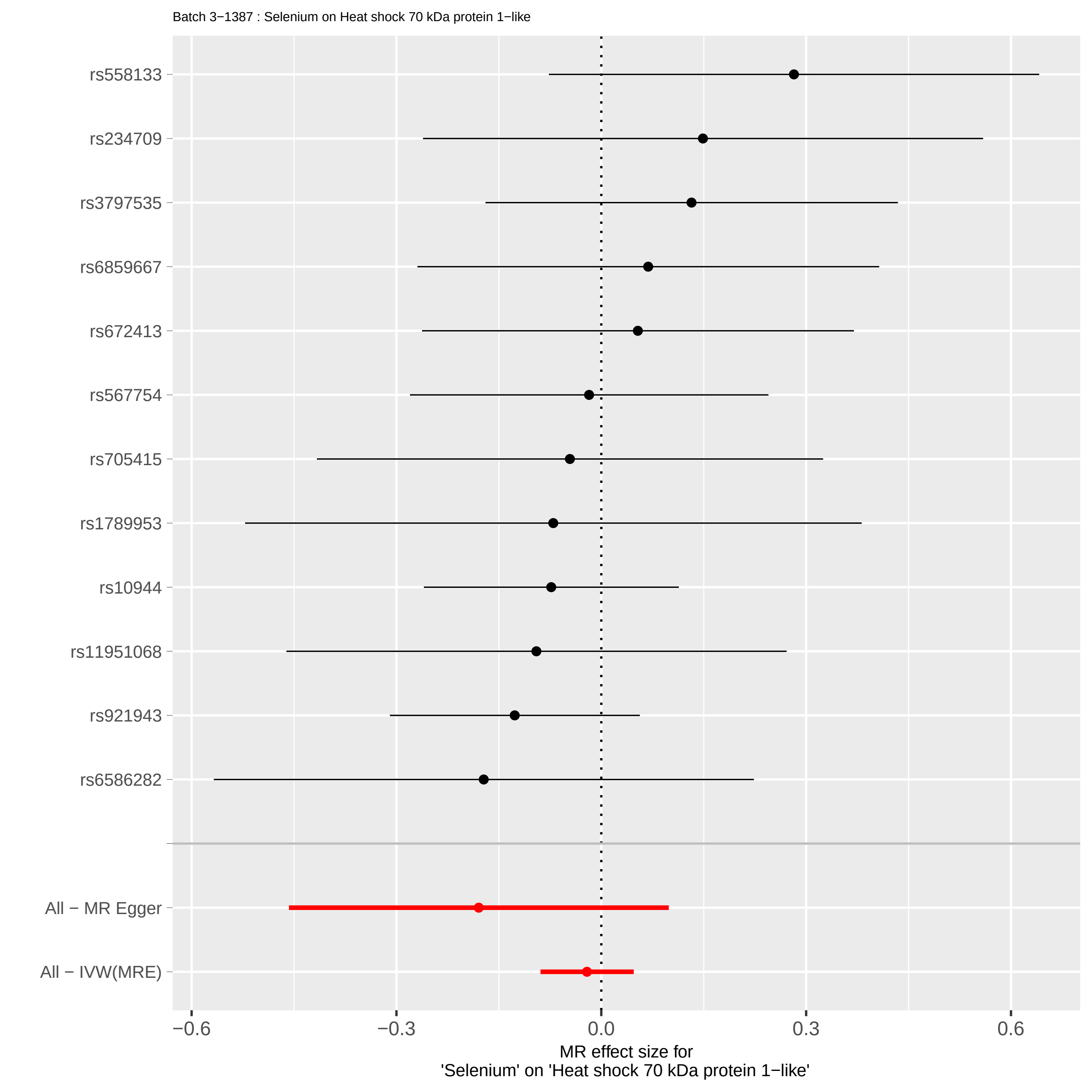
Batch 3−1387 : Selenium on Heat shock 70 kDa protein 1−like
rs558133
rs234709
rs3797535
rs6859667
rs672413
rs567754
rs705415
rs1789953
rs10944
rs11951068
rs921943
rs6586282
All − MR Egger
All − IVW(MRE)
−0.6
−0.3
0.0
0.3
0.6
MR effect size for
'Selenium' on 'Heat shock 70 kDa protein 1−like'

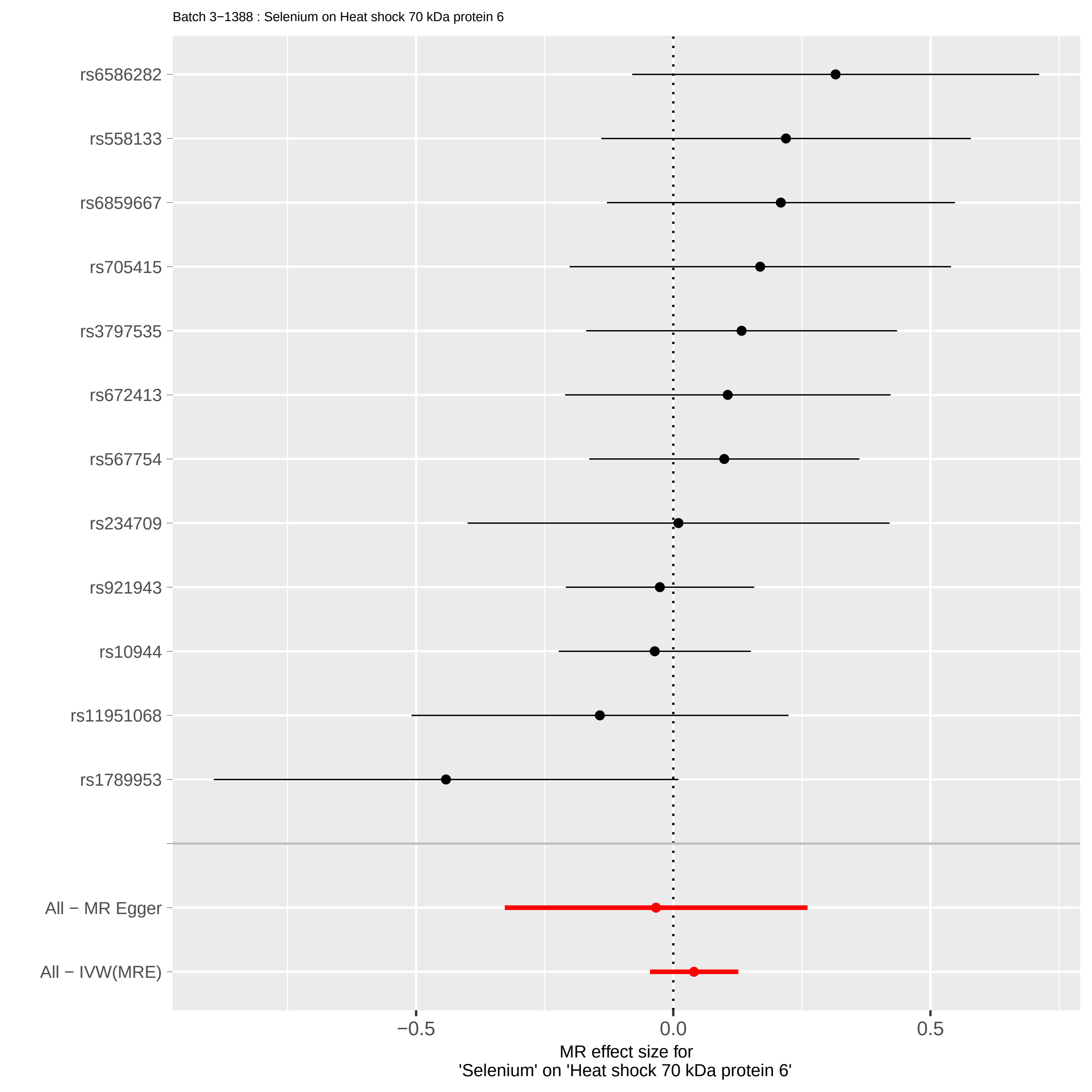
Batch 3−1388 : Selenium on Heat shock 70 kDa protein 6
rs6586282
rs558133
rs6859667
rs705415
rs3797535
rs672413
rs567754
rs234709
rs921943
rs10944
rs11951068
rs1789953
All − MR Egger
All − IVW(MRE)
−0.5
0.0
0.5
MR effect size for
'Selenium' on 'Heat shock 70 kDa protein 6'

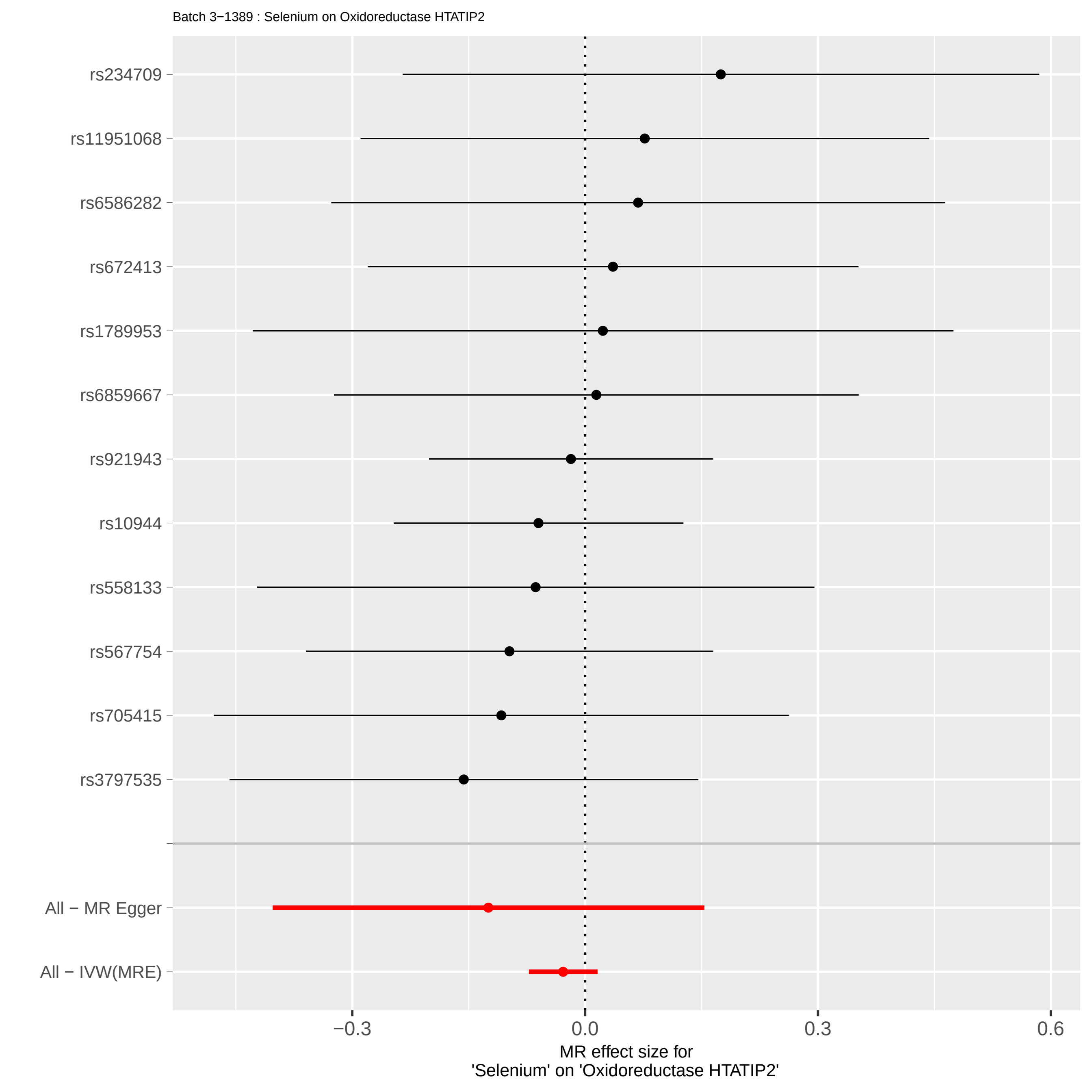

Batch 3−1389 : Selenium on Oxidoreductase HTATIP2
rs234709
rs11951068
rs6586282
rs672413
rs1789953
rs6859667
rs921943
rs10944
rs558133
rs567754
rs705415
rs3797535
All − MR Egger
All − IVW(MRE)
−0.3
0.0
0.3
0.6
MR effect size for
'Selenium' on 'Oxidoreductase HTATIP2'

Batch 3−1390 : Selenium on Oxidoreductase HTATIP2

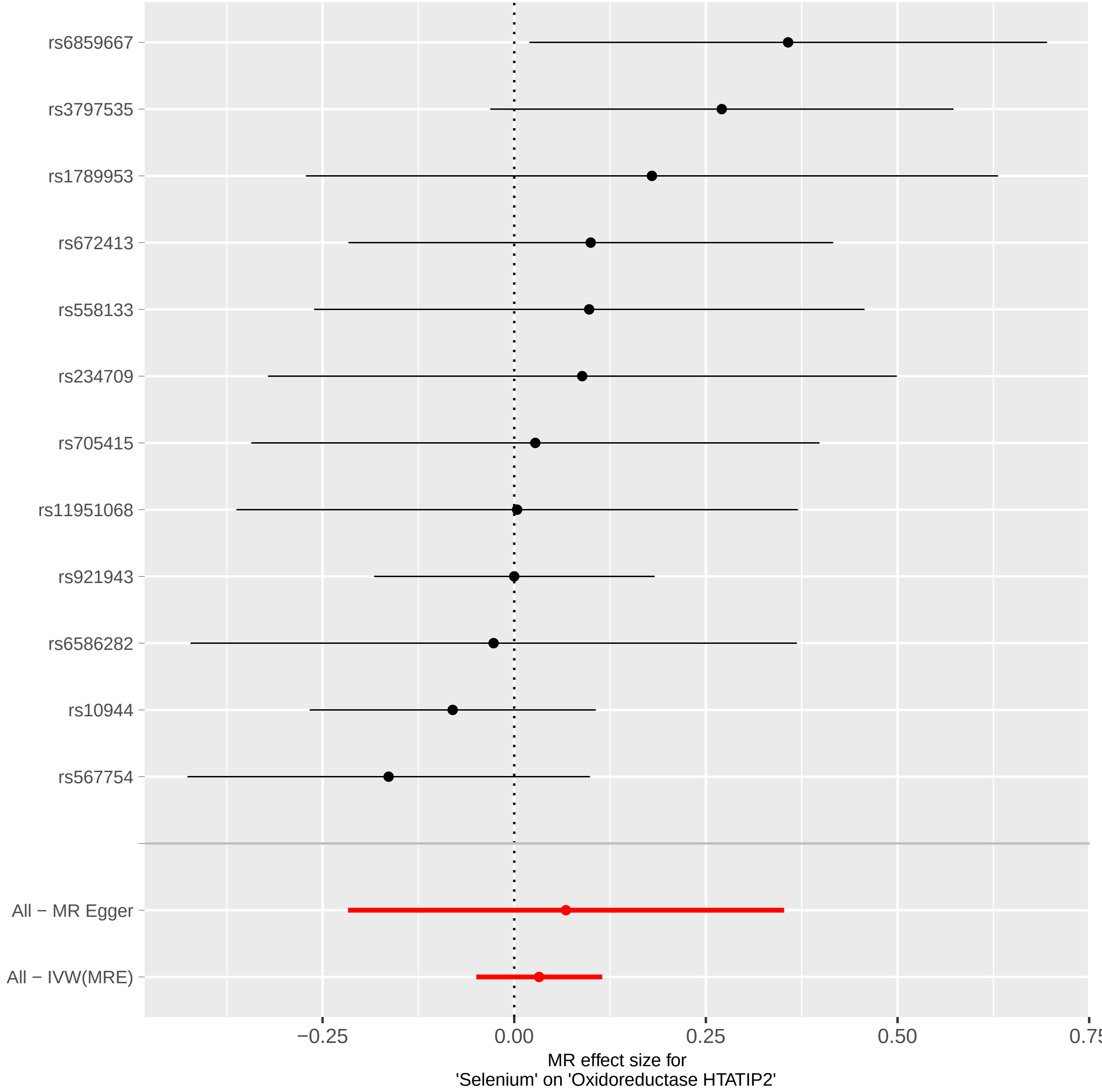

Batch 3−1391 : Selenium on Histatin−1

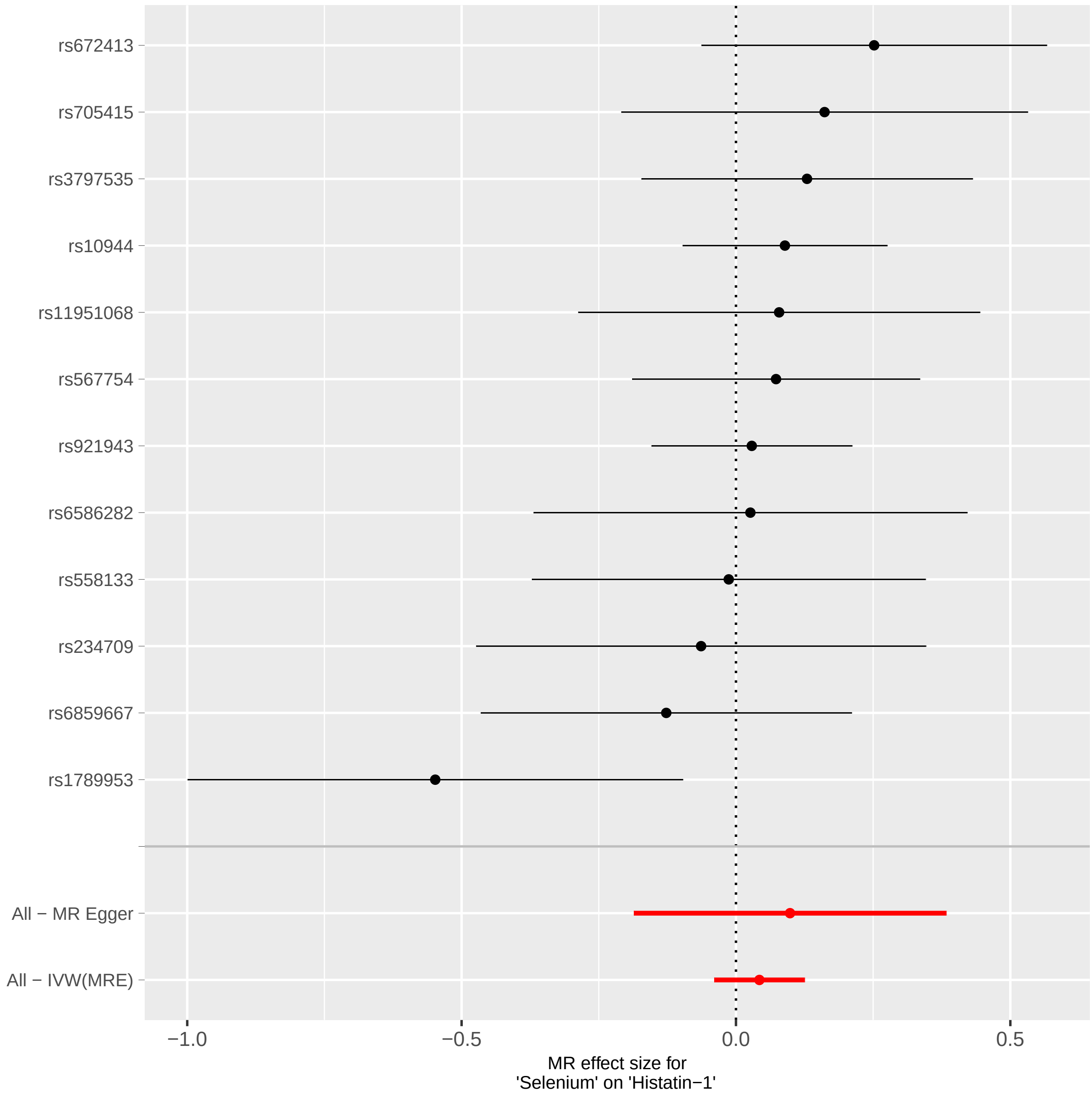

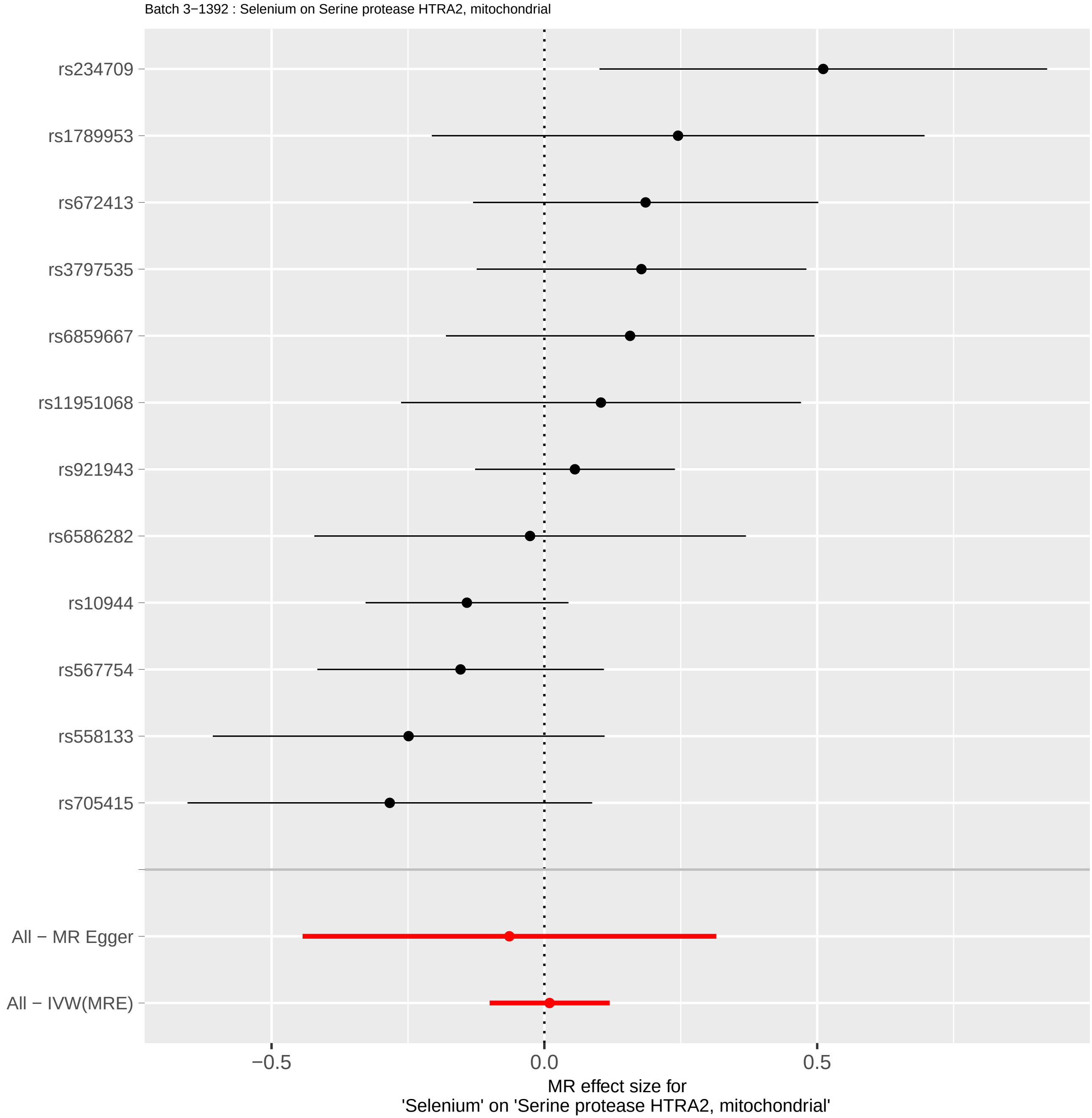

Batch 3−1392 : Selenium on Serine protease HTRA2, mitochondrial
rs234709
rs1789953
rs672413
rs3797535
rs6859667
rs11951068
rs921943
rs6586282
rs10944
rs567754
rs558133
rs705415
All − MR Egger
All − IVW(MRE)
−0.5
0.0
0.5
MR effect size for
'Selenium' on 'Serine protease HTRA2, mitochondrial'

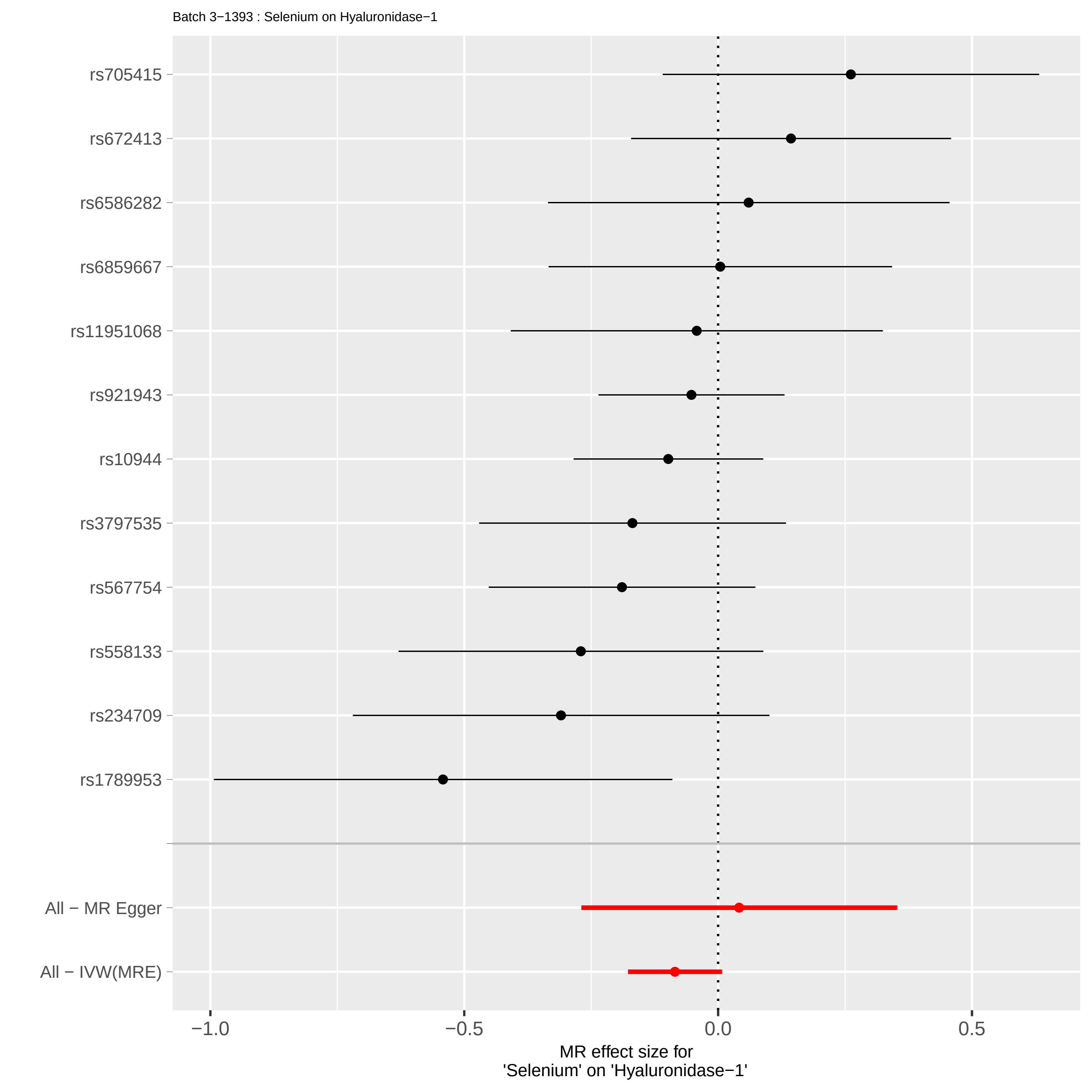
Batch 3−1393 : Selenium on Hyaluronidase−1
rs705415
rs672413
rs6586282
rs6859667
rs11951068
rs921943
rs10944
rs3797535
rs567754
rs558133
rs234709
rs1789953
All − MR Egger
All − IVW(MRE)
−1.0
−0.5
0.0
0.5
MR effect size for
'Selenium' on 'Hyaluronidase−1'

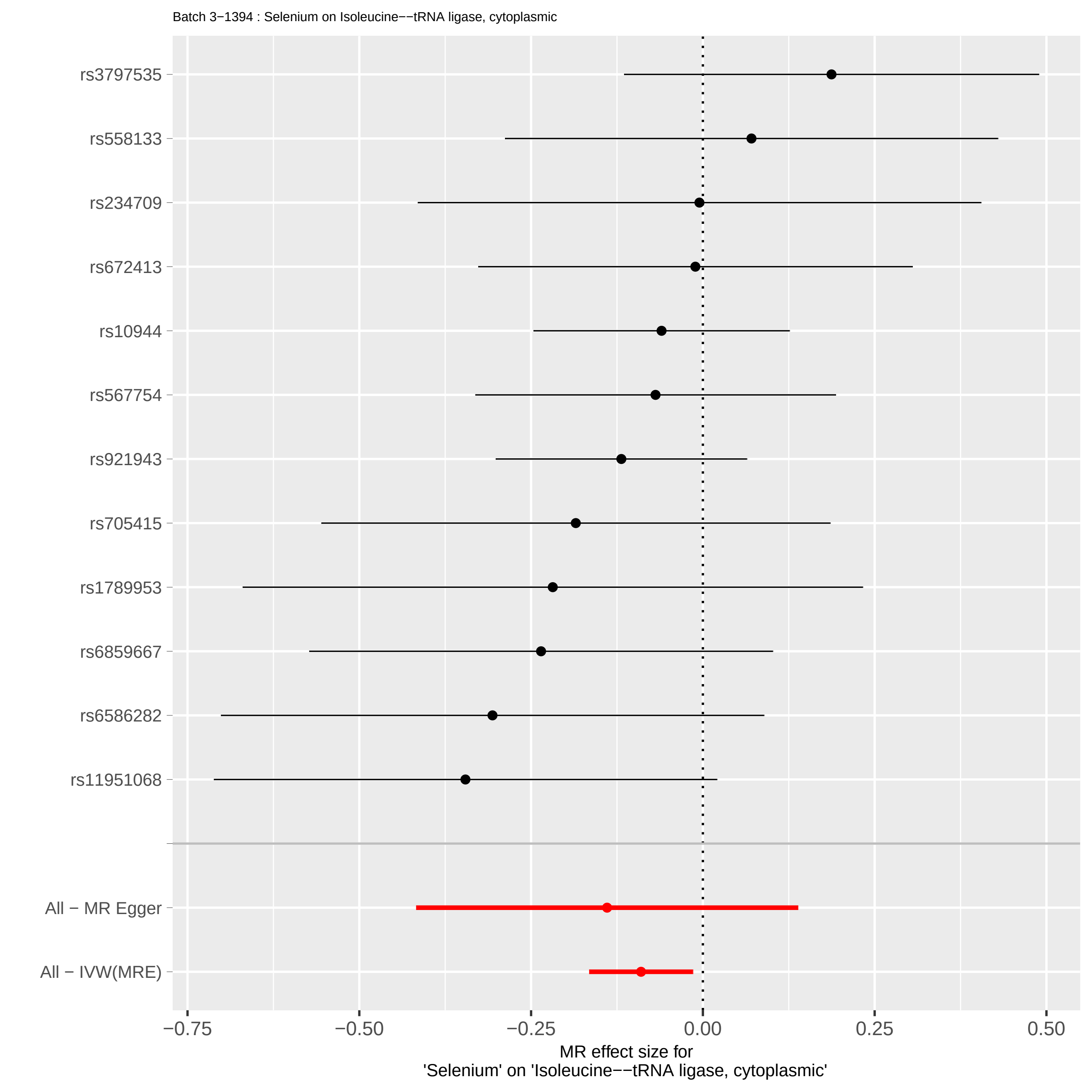

Batch 3−1394 : Selenium on Isoleucine−−tRNA ligase, cytoplasmic
rs3797535
rs558133
rs234709
rs672413
rs10944
rs567754
rs921943
rs705415
rs1789953
rs6859667
rs6586282
rs11951068
All − MR Egger
All − IVW(MRE)
−0.75
−0.50
−0.25
0.00
0.25
0.50
MR effect size for
'Selenium' on 'Isoleucine−−tRNA ligase, cytoplasmic'

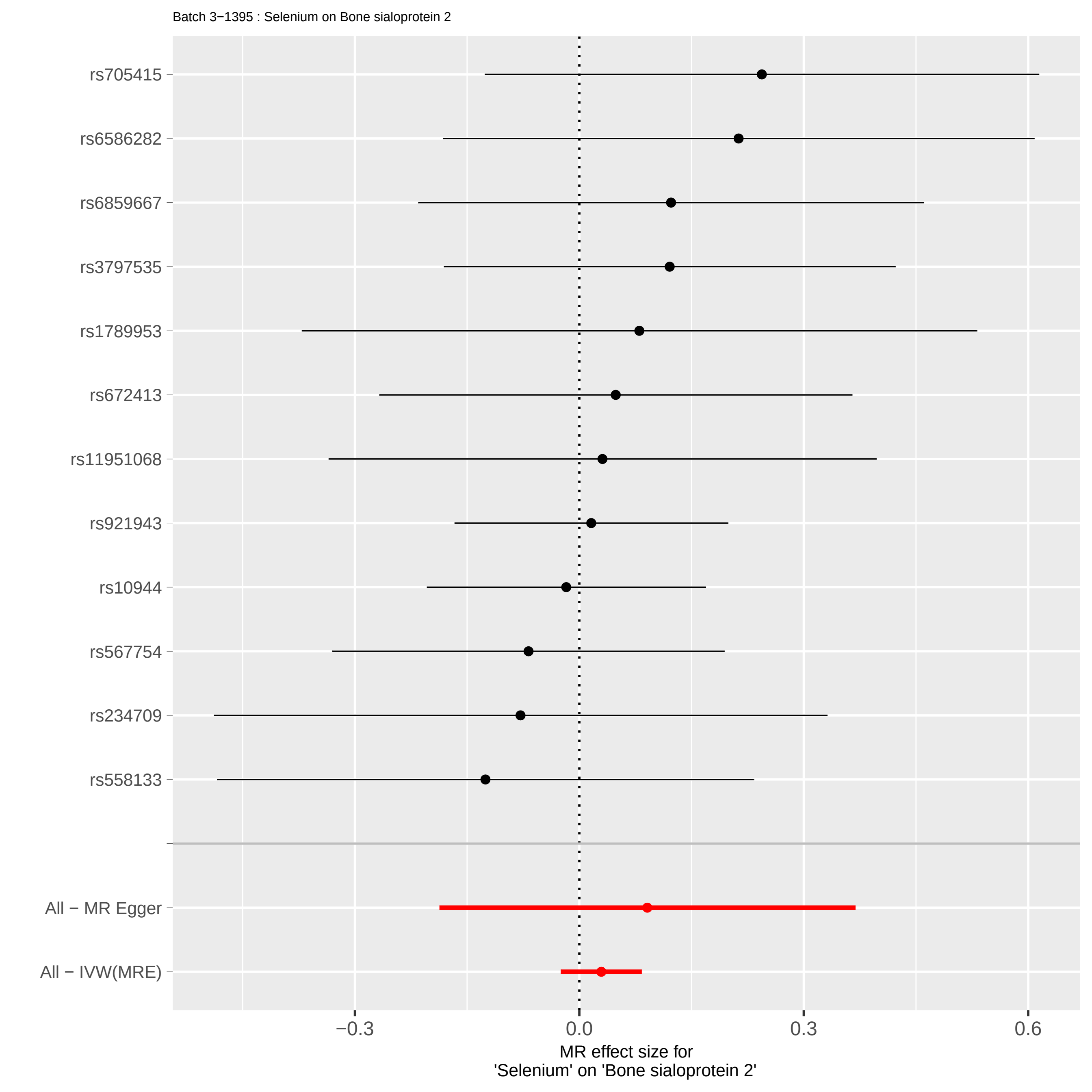

Batch 3−1395 : Selenium on Bone sialoprotein 2
rs705415
rs6586282
rs6859667
rs3797535
rs1789953
rs672413
rs11951068
rs921943
rs10944
rs567754
rs234709
rs558133
All − MR Egger
All − IVW(MRE)
−0.3
0.0
0.3
0.6
MR effect size for
'Selenium' on 'Bone sialoprotein 2'

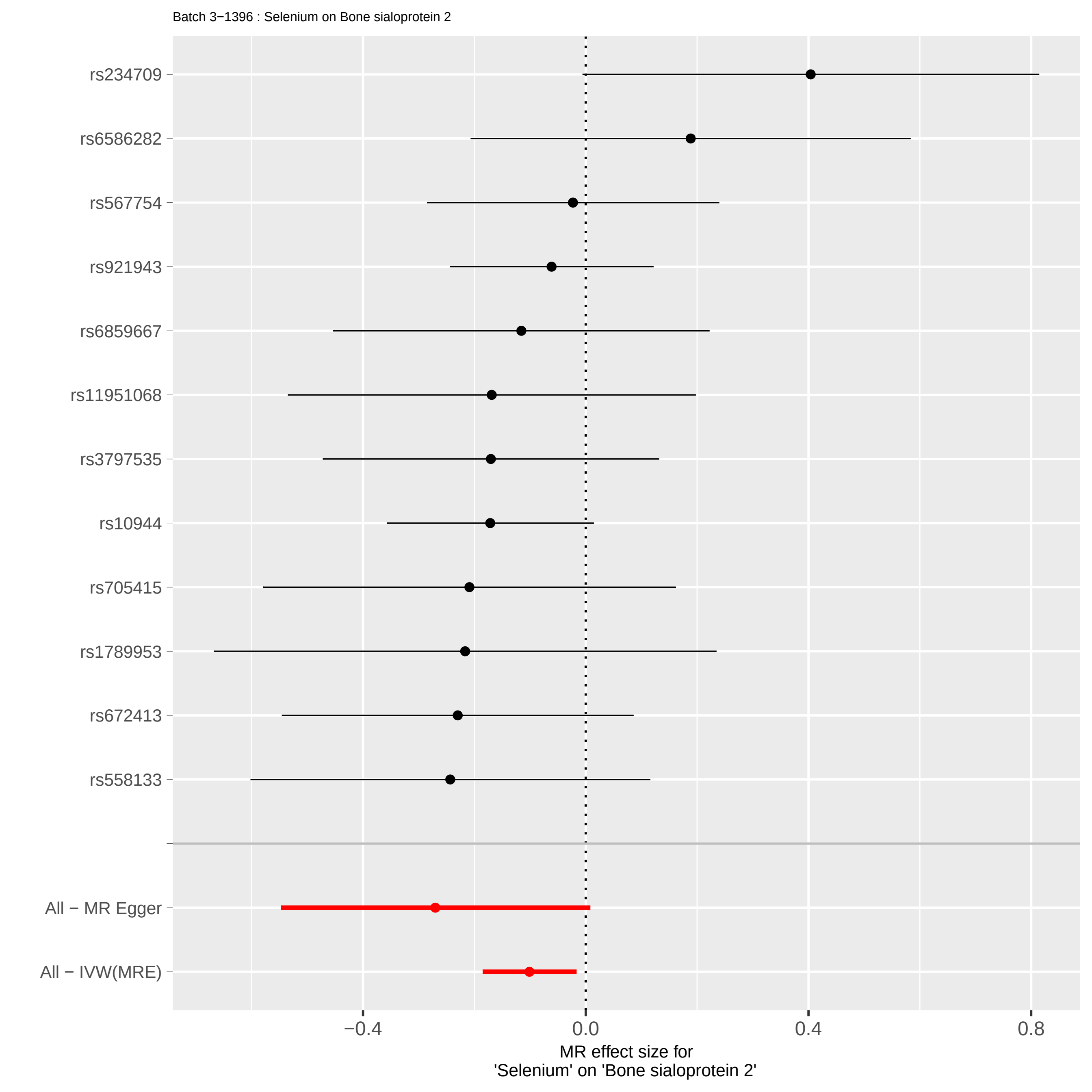

Batch 3−1396 : Selenium on Bone sialoprotein 2
rs234709
rs6586282
rs567754
rs921943
rs6859667
rs11951068
rs3797535
rs10944
rs705415
rs1789953
rs672413
rs558133
All − MR Egger
All − IVW(MRE)
−0.4
0.0
0.4
0.8
MR effect size for
'Selenium' on 'Bone sialoprotein 2'

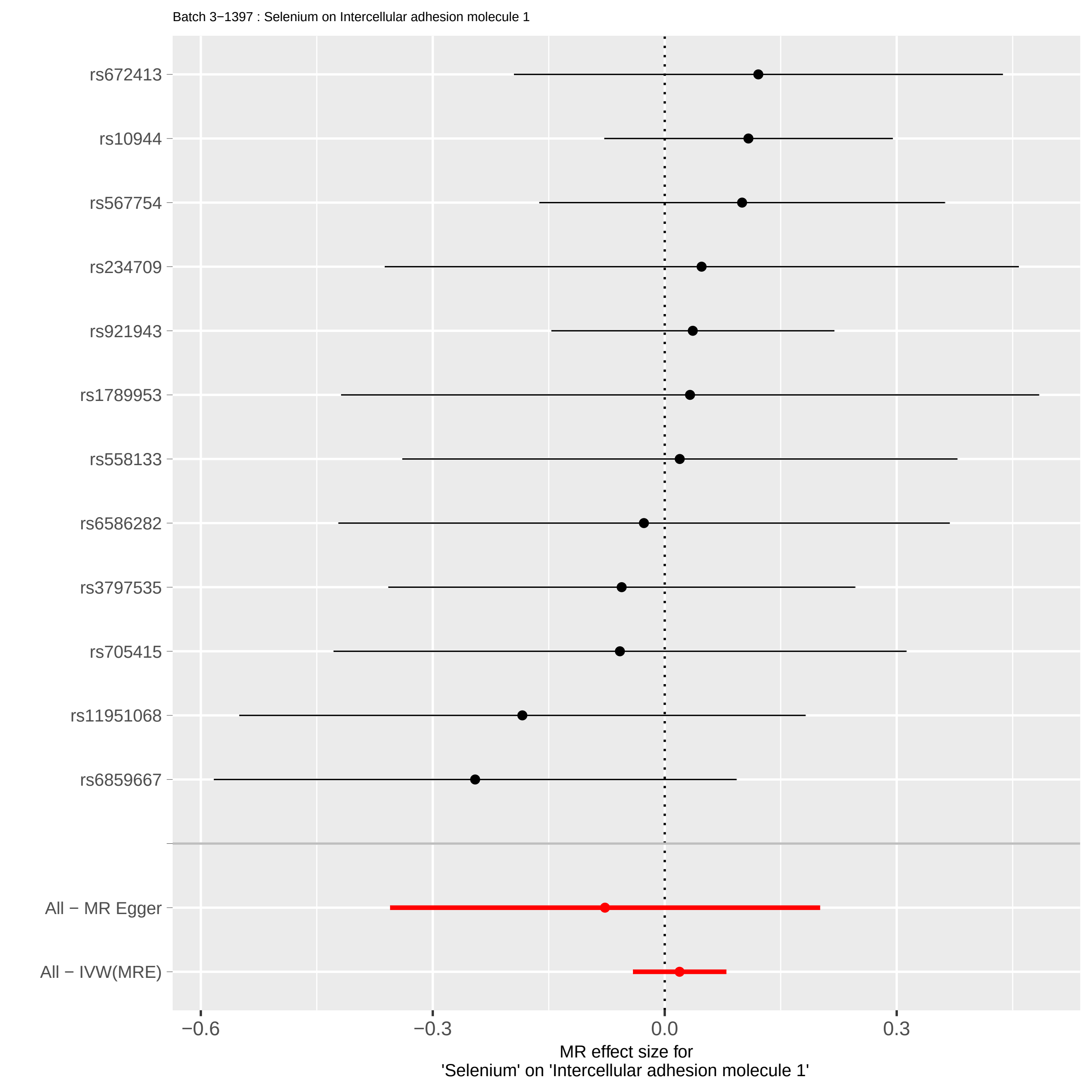

Batch 3−1397 : Selenium on Intercellular adhesion molecule 1
rs672413
rs10944
rs567754
rs234709
rs921943
rs1789953
rs558133
rs6586282
rs3797535
rs705415
rs11951068
rs6859667
All − MR Egger
All − IVW(MRE)
−0.6
−0.3
0.0
0.3
MR effect size for
'Selenium' on 'Intercellular adhesion molecule 1'

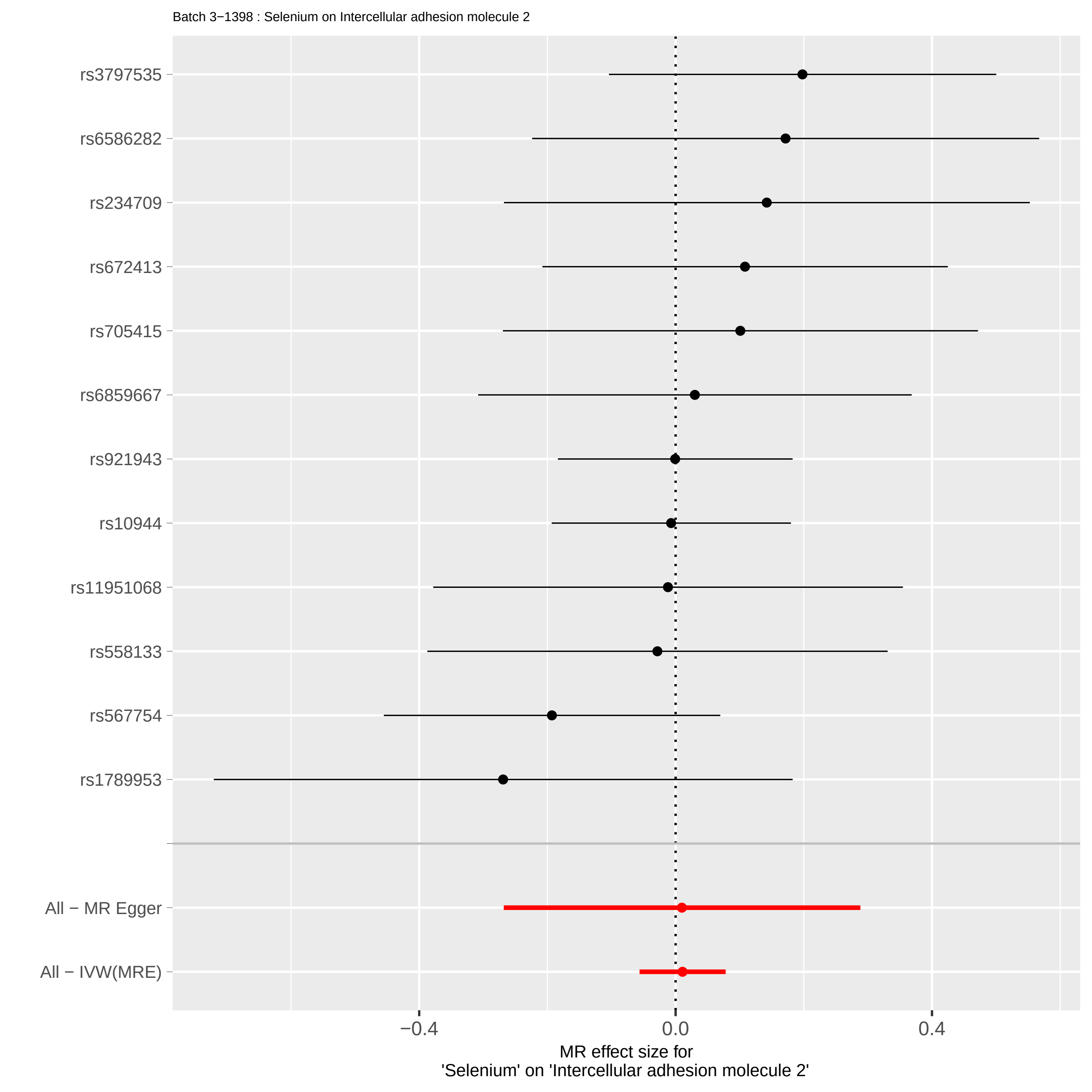
Batch 3−1398 : Selenium on Intercellular adhesion molecule 2
rs3797535
rs6586282
rs234709
rs672413
rs705415
rs6859667
rs921943
rs10944
rs11951068
rs558133
rs567754
rs1789953
All − MR Egger
All − IVW(MRE)
−0.4
0.0
0.4
MR effect size for
'Selenium' on 'Intercellular adhesion molecule 2'

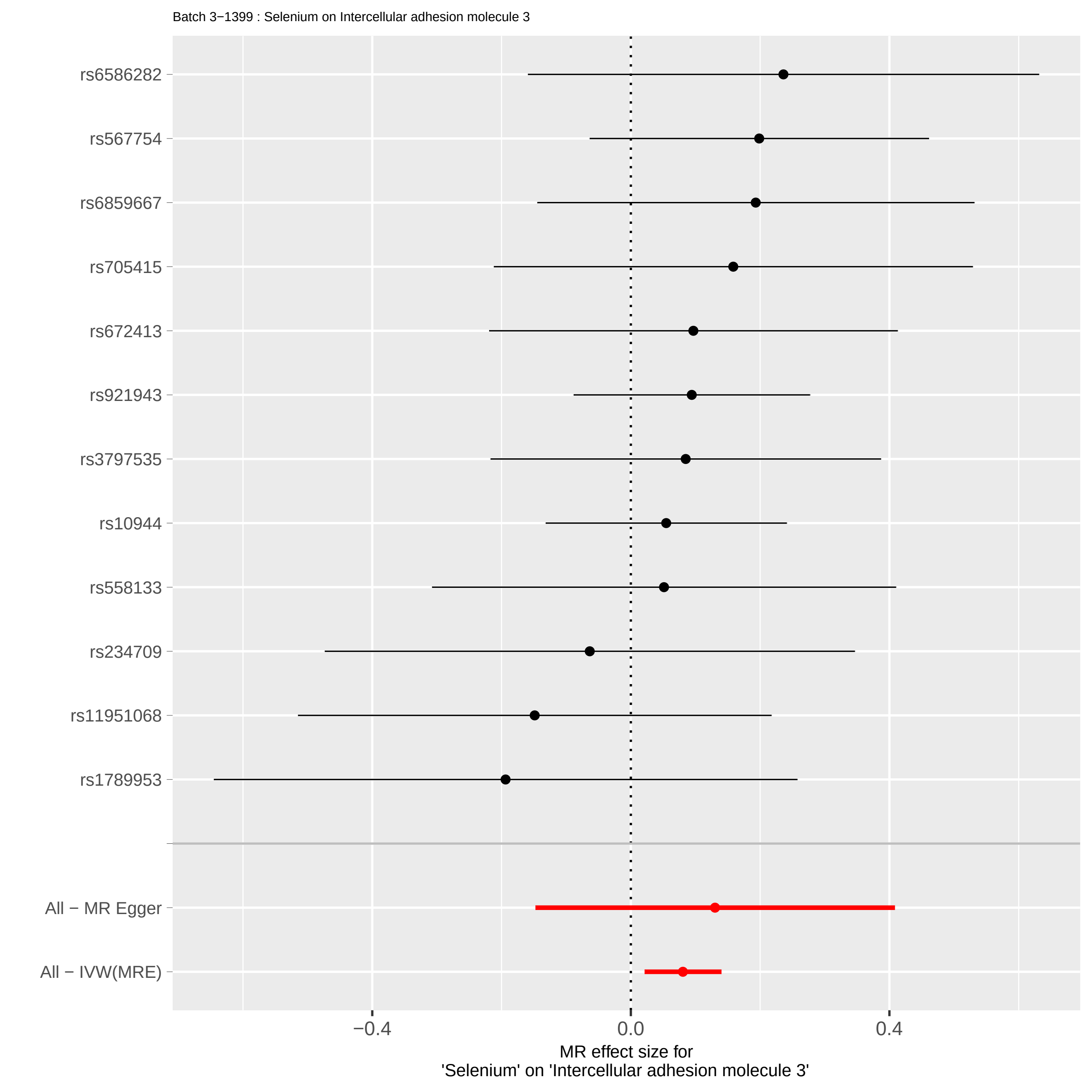
Batch 3−1399 : Selenium on Intercellular adhesion molecule 3
rs6586282
rs567754
rs6859667
rs705415
rs672413
rs921943
rs3797535
rs10944
rs558133
rs234709
rs11951068
rs1789953
All − MR Egger
All − IVW(MRE)
−0.4
0.0
0.4
MR effect size for
'Selenium' on 'Intercellular adhesion molecule 3'

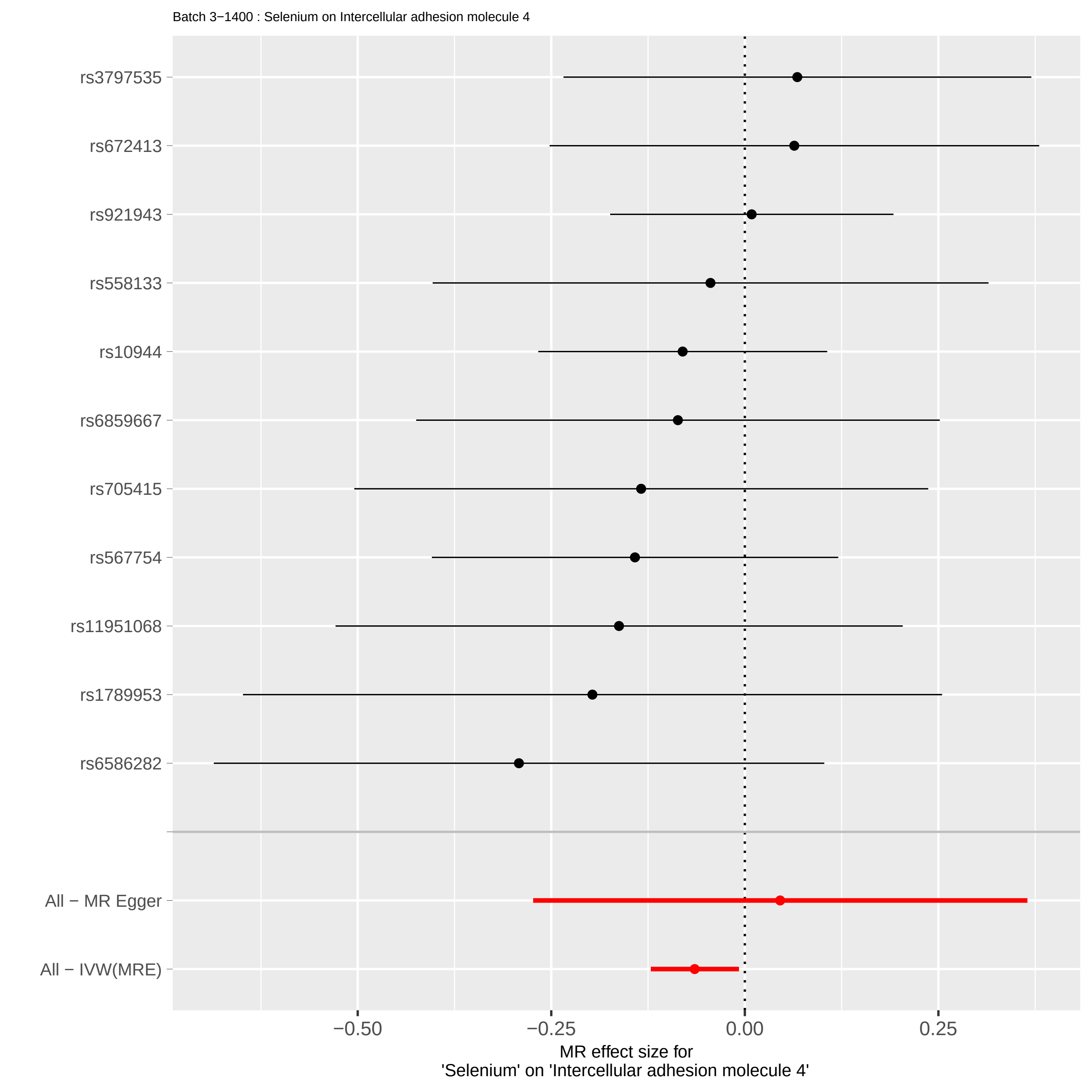

Batch 3−1400 : Selenium on Intercellular adhesion molecule 4
rs3797535
rs672413
rs921943
rs558133
rs10944
rs6859667
rs705415
rs567754
rs11951068
rs1789953
rs6586282
All − MR Egger
All − IVW(MRE)
−0.50
−0.25
0.00
0.25
MR effect size for
'Selenium' on 'Intercellular adhesion molecule 4'

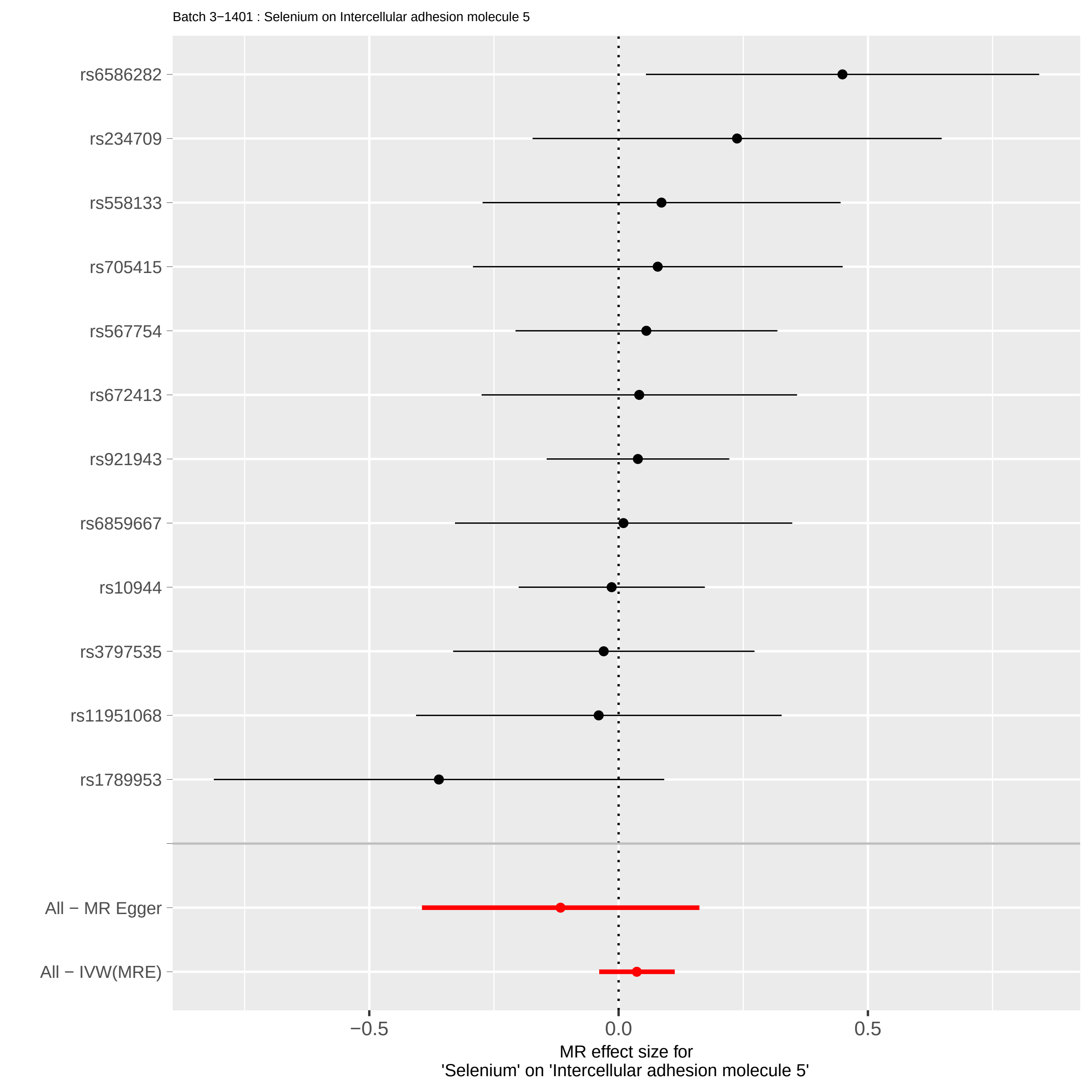
Batch 3−1401 : Selenium on Intercellular adhesion molecule 5
rs6586282
rs234709
rs558133
rs705415
rs567754
rs672413
rs921943
rs6859667
rs10944
rs3797535
rs11951068
rs1789953
All − MR Egger
All − IVW(MRE)
−0.5
0.0
0.5
MR effect size for
'Selenium' on 'Intercellular adhesion molecule 5'

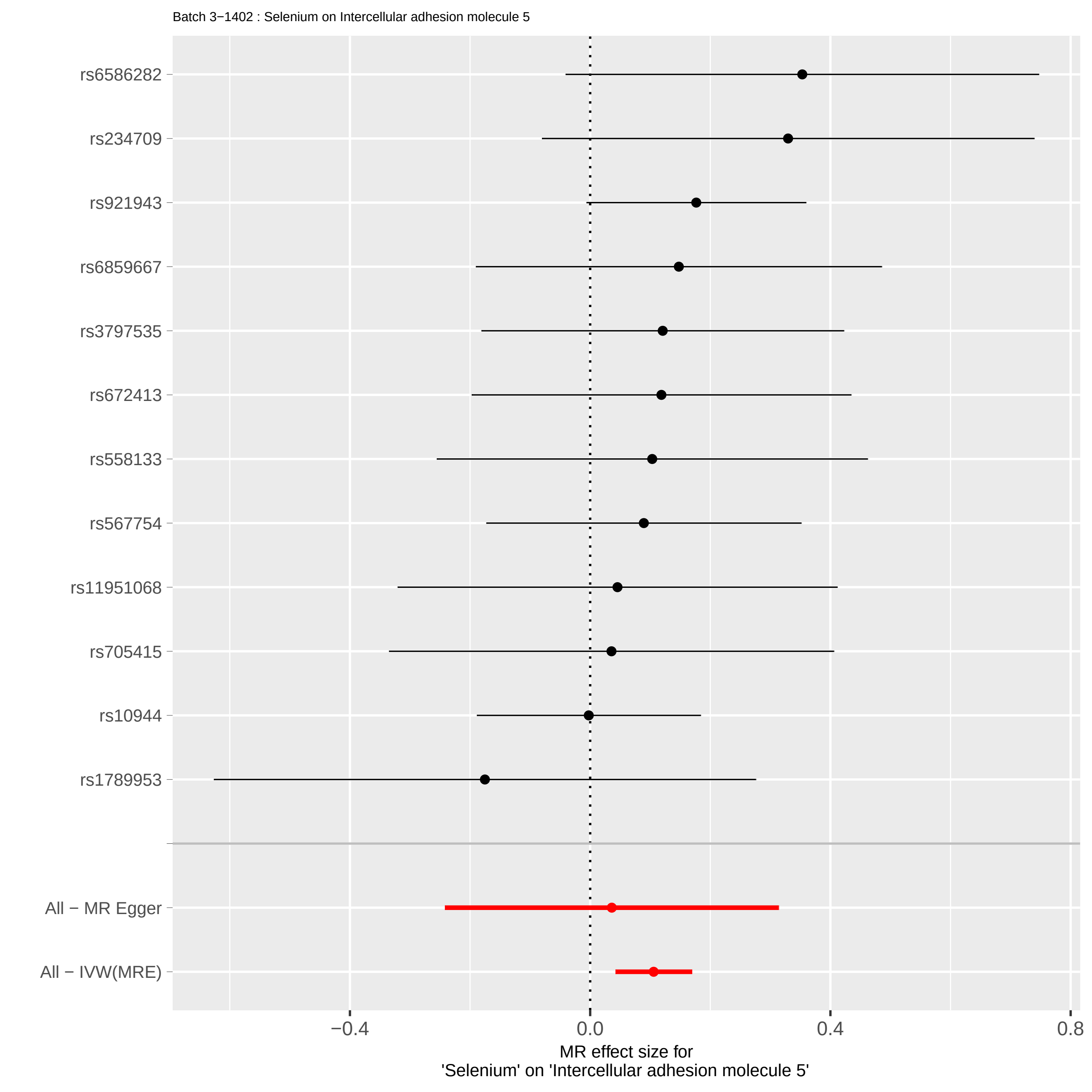
Batch 3−1402 : Selenium on Intercellular adhesion molecule 5
rs6586282
rs234709
rs921943
rs6859667
rs3797535
rs672413
rs558133
rs567754
rs11951068
rs705415
rs10944
rs1789953
All − MR Egger
All − IVW(MRE)
−0.4
0.0
0.4
0.8
MR effect size for
'Selenium' on 'Intercellular adhesion molecule 5'

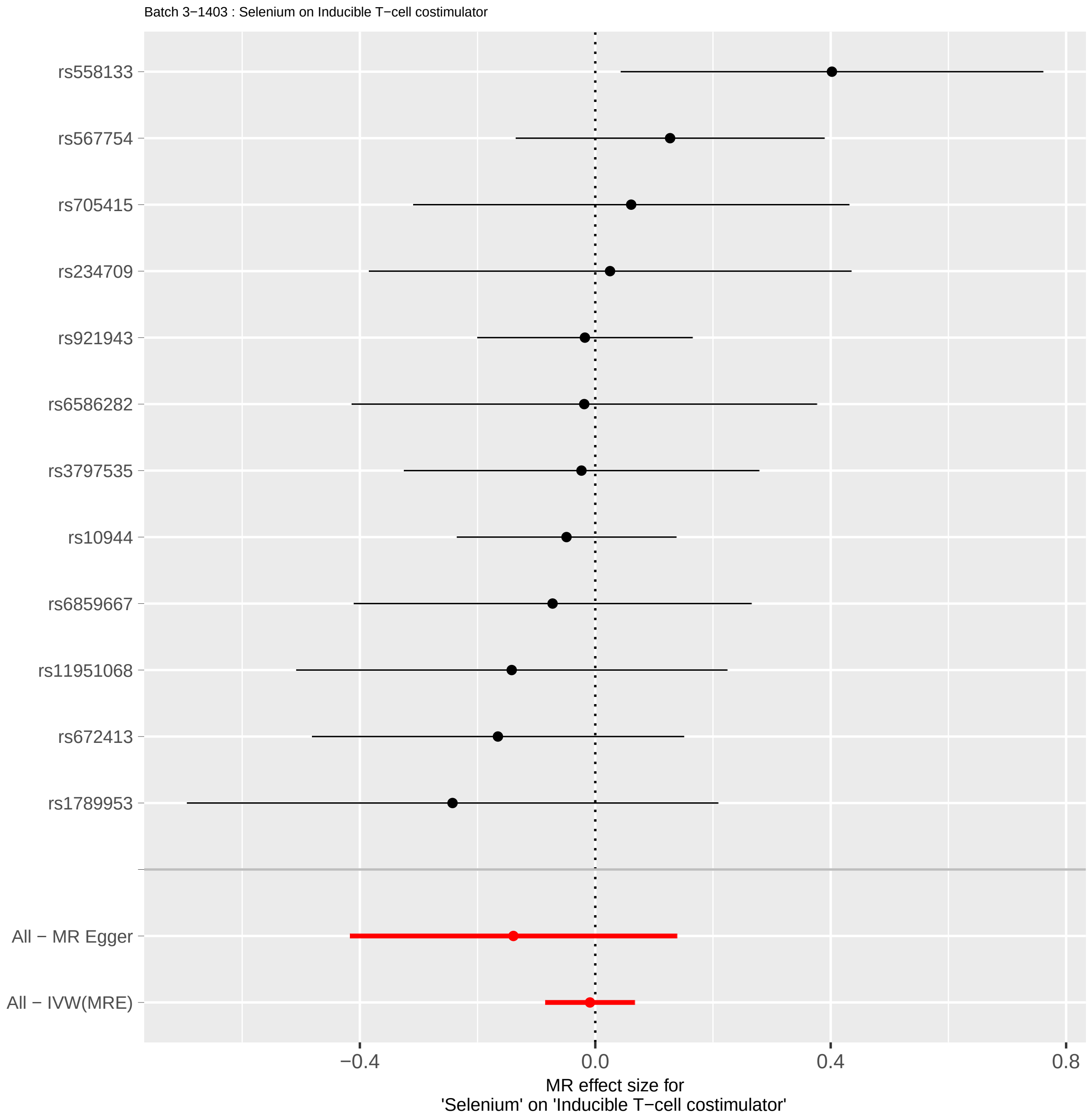

Batch 3−1403 : Selenium on Inducible T−cell costimulator
rs558133
rs567754
rs705415
rs234709
rs921943
rs6586282
rs3797535
rs10944
rs6859667
rs11951068
rs672413
rs1789953
All − MR Egger
All − IVW(MRE)
−0.4
0.0
0.4
0.8
MR effect size for
'Selenium' on 'Inducible T−cell costimulator'

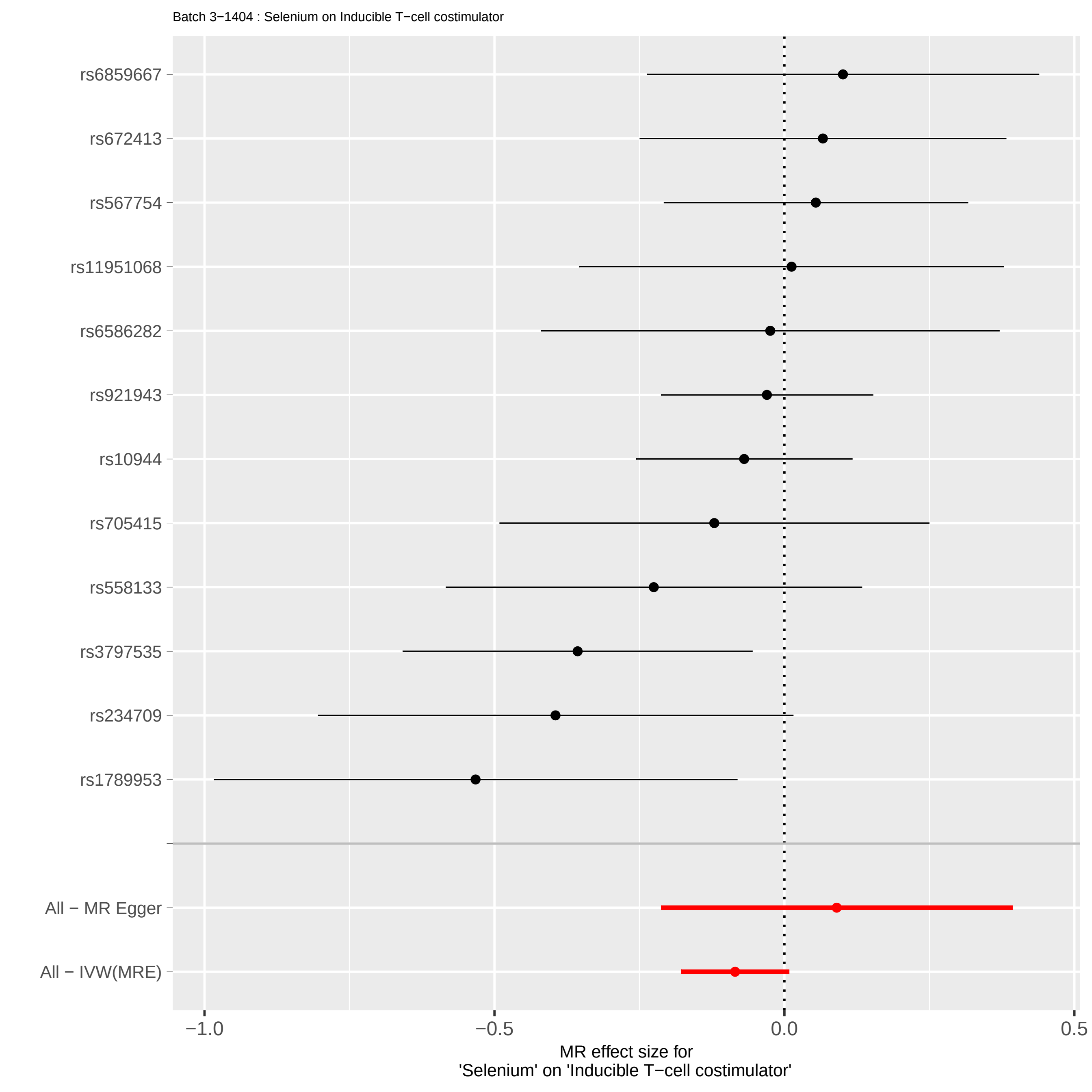

Batch 3−1404 : Selenium on Inducible T−cell costimulator
rs6859667
rs672413
rs567754
rs11951068
rs6586282
rs921943
rs10944
rs705415
rs558133
rs3797535
rs234709
rs1789953
All − MR Egger
All − IVW(MRE)
−1.0
−0.5
0.0
0.5
MR effect size for
'Selenium' on 'Inducible T−cell costimulator'

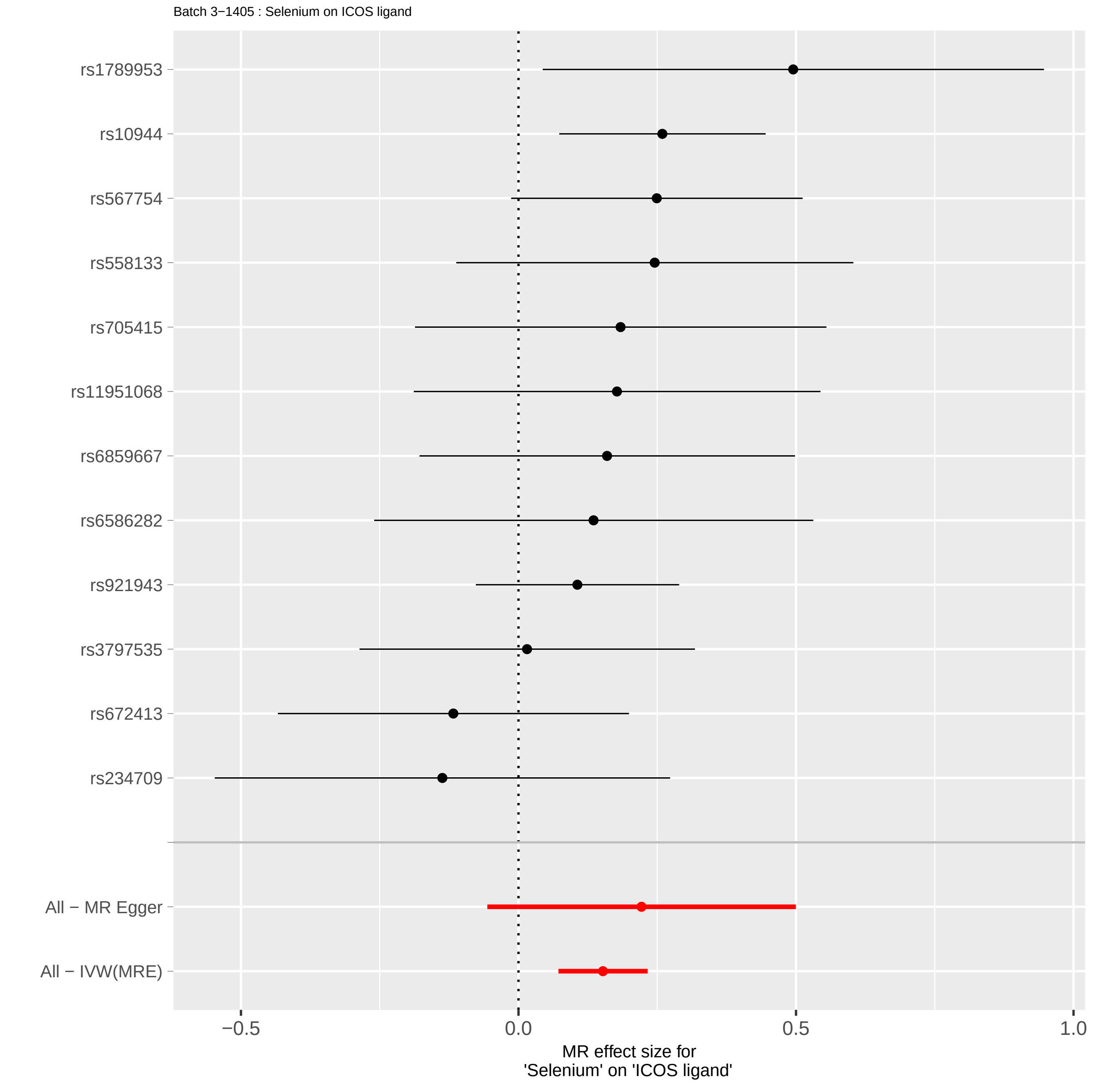
Batch 3−1405 : Selenium on ICOS ligand
rs1789953
rs10944
rs567754
rs558133
rs705415
rs11951068
rs6859667
rs6586282
rs921943
rs3797535
rs672413
rs234709
All − MR Egger
All − IVW(MRE)
−0.5
0.0
0.5
1.0
MR effect size for
'Selenium' on 'ICOS ligand'

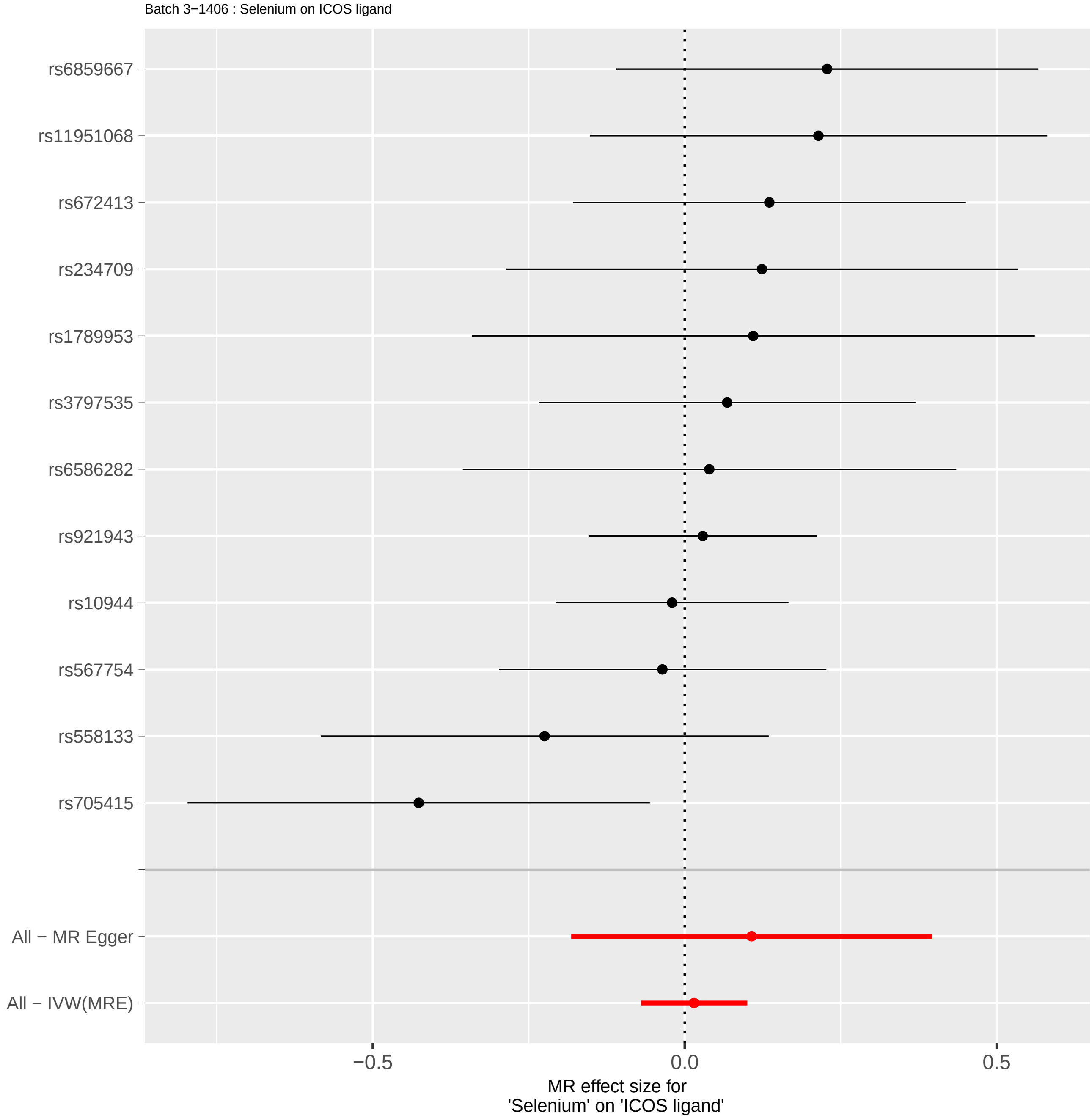
Batch 3−1406 : Selenium on ICOS ligand
rs6859667
rs11951068
rs672413
rs234709
rs1789953
rs3797535
rs6586282
rs921943
rs10944
rs567754
rs558133
rs705415
All − MR Egger
All − IVW(MRE)
−0.5
0.0
0.5
MR effect size for
'Selenium' on 'ICOS ligand'

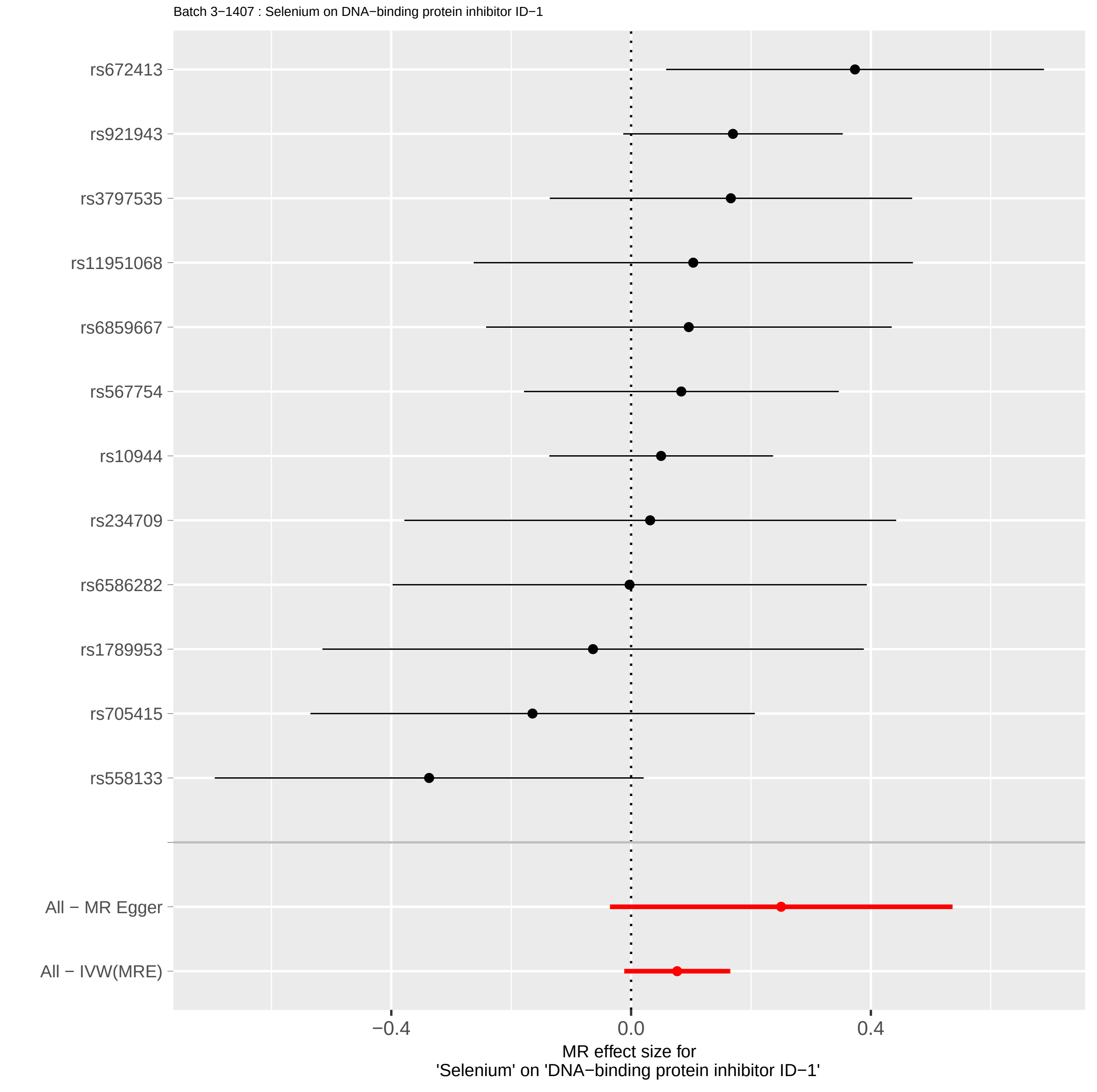

Batch 3−1407 : Selenium on DNA−binding protein inhibitor ID−1
rs672413
rs921943
rs3797535
rs11951068
rs6859667
rs567754
rs10944
rs234709
rs6586282
rs1789953
rs705415
rs558133
All − MR Egger
All − IVW(MRE)
−0.4
0.0
0.4
MR effect size for
'Selenium' on 'DNA−binding protein inhibitor ID−1'

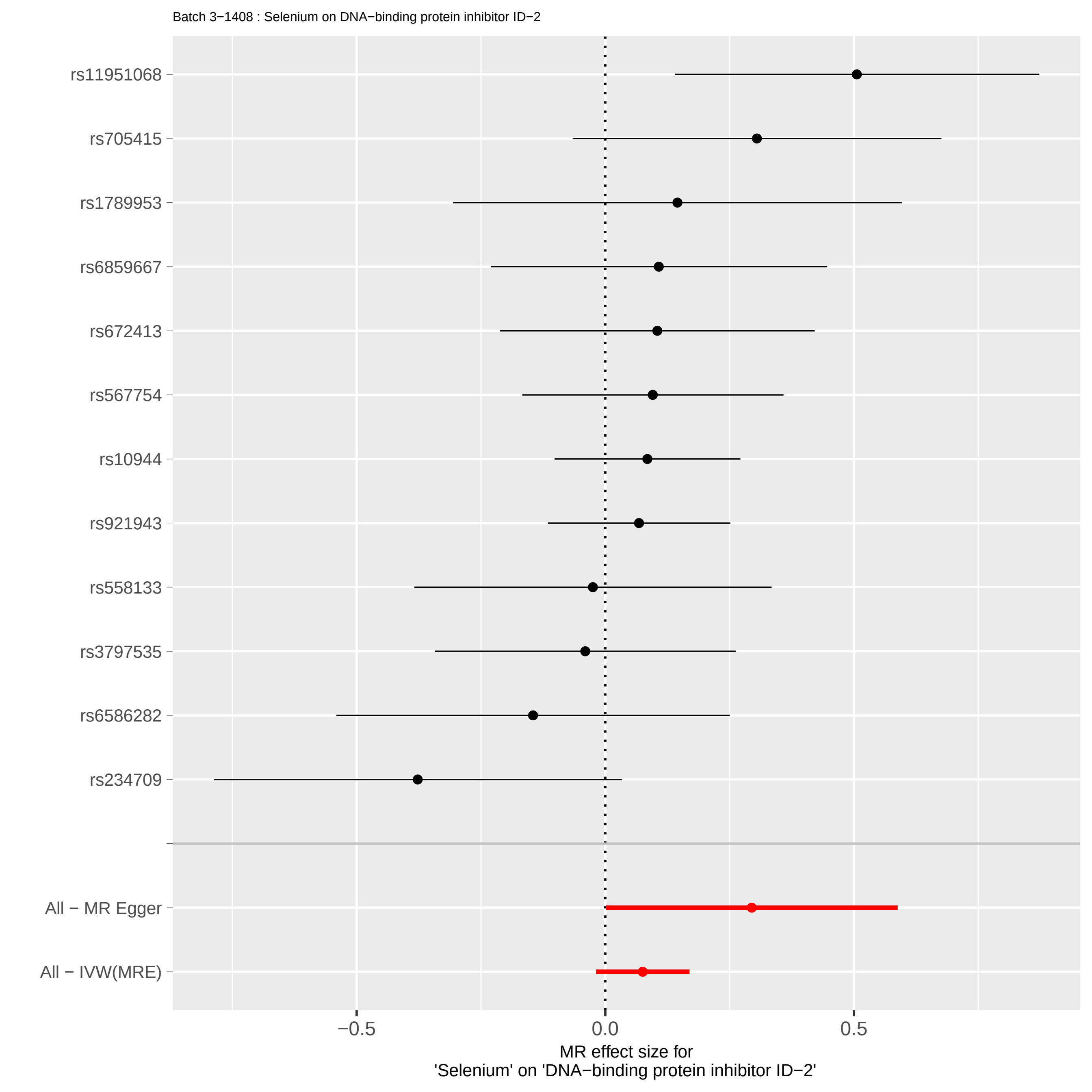

Batch 3−1408 : Selenium on DNA−binding protein inhibitor ID−2
rs11951068
rs705415
rs1789953
rs6859667
rs672413
rs567754
rs10944
rs921943
rs558133
rs3797535
rs6586282
rs234709
All − MR Egger
All − IVW(MRE)
−0.5
0.0
0.5
MR effect size for
'Selenium' on 'DNA−binding protein inhibitor ID−2'

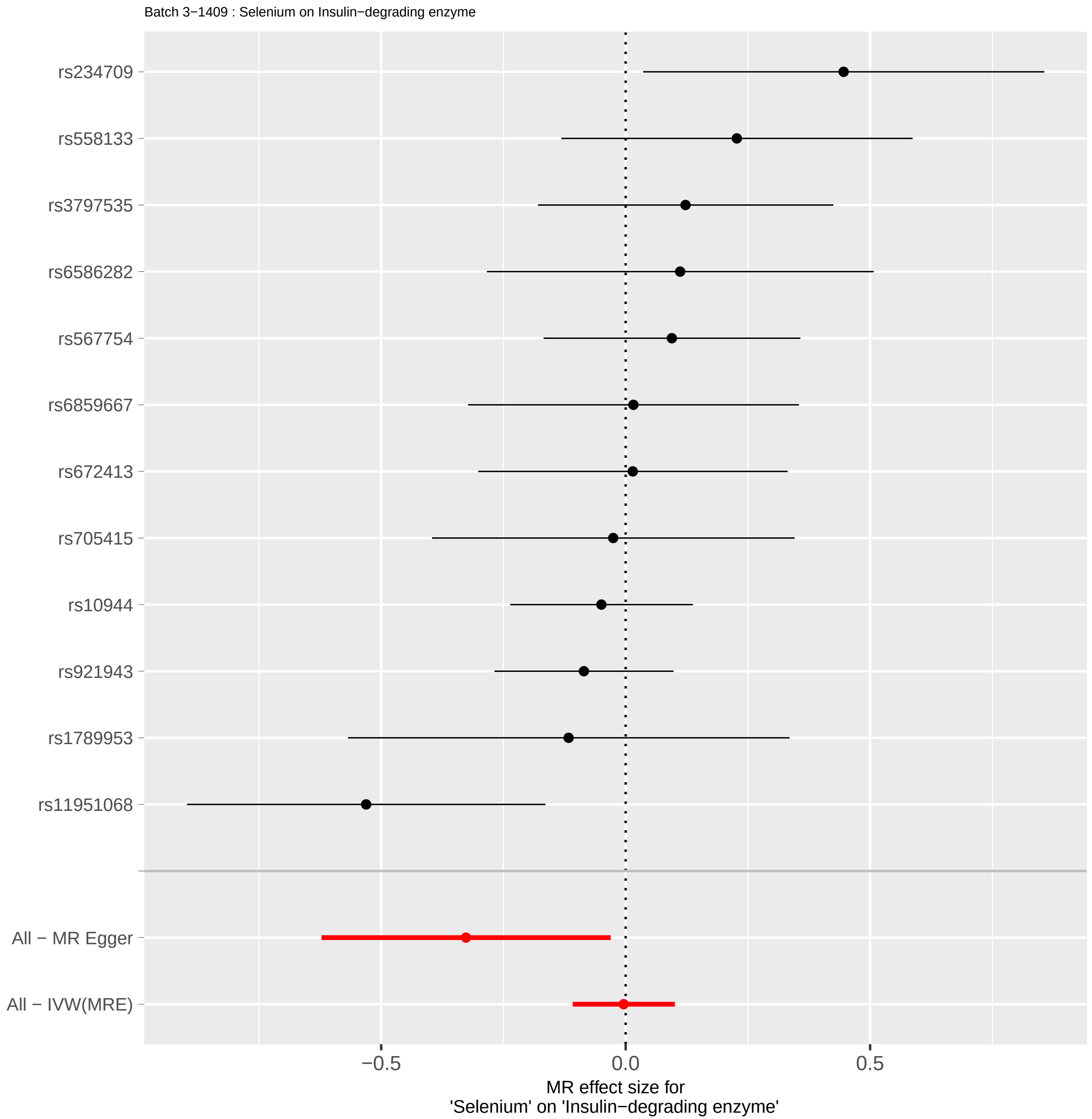
Batch 3−1409 : Selenium on Insulin−degrading enzyme
rs234709
rs558133
rs3797535
rs6586282
rs567754
rs6859667
rs672413
rs705415
rs10944
rs921943
rs1789953
rs11951068
All − MR Egger
All − IVW(MRE)
−0.5
0.0
0.5
MR effect size for
'Selenium' on 'Insulin−degrading enzyme'

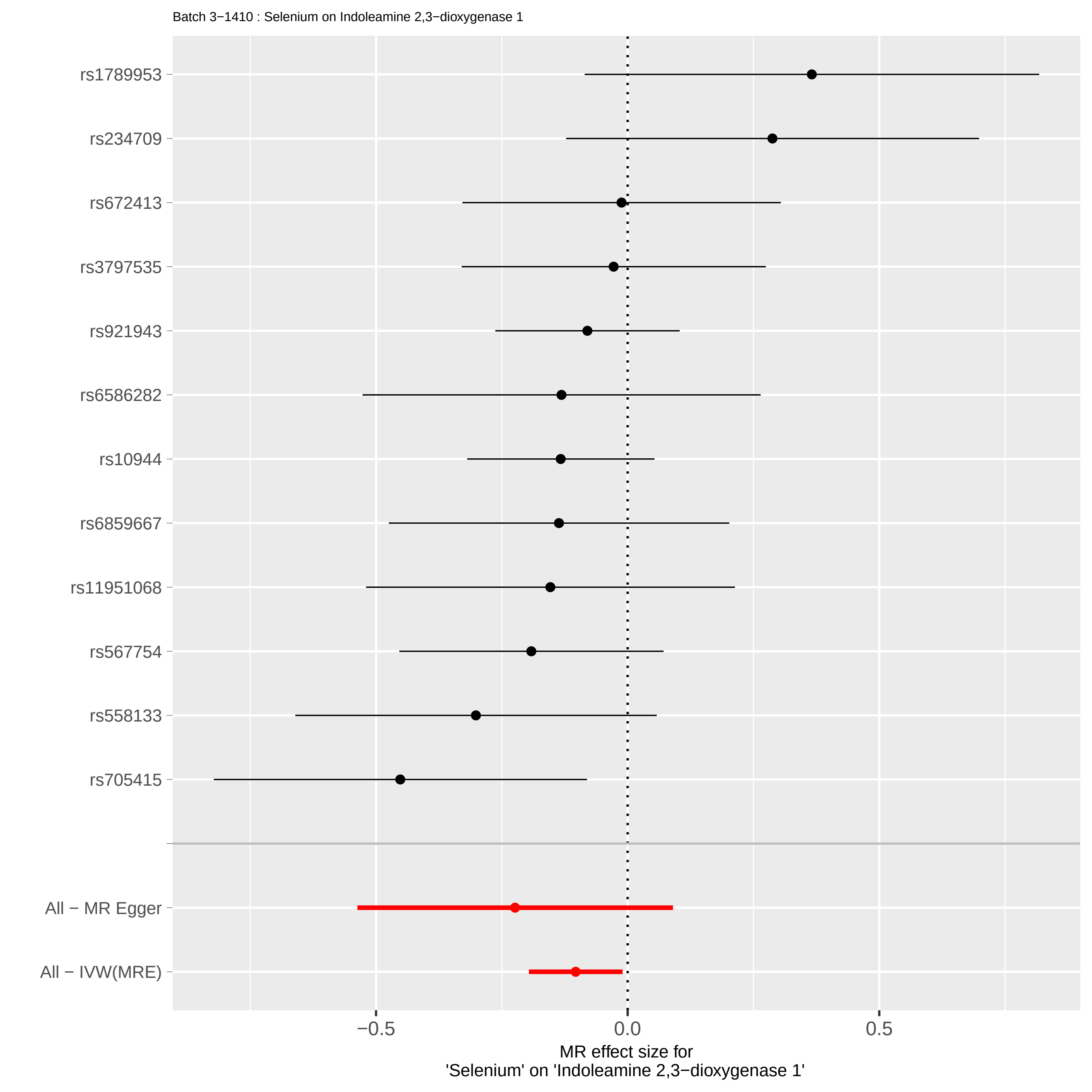
Batch 3−1410 : Selenium on Indoleamine 2,3−dioxygenase 1
rs1789953
rs234709
rs672413
rs3797535
rs921943
rs6586282
rs10944
rs6859667
rs11951068
rs567754
rs558133
rs705415
All − MR Egger
All − IVW(MRE)
−0.5
0.0
0.5
MR effect size for
'Selenium' on 'Indoleamine 2,3−dioxygenase 1'

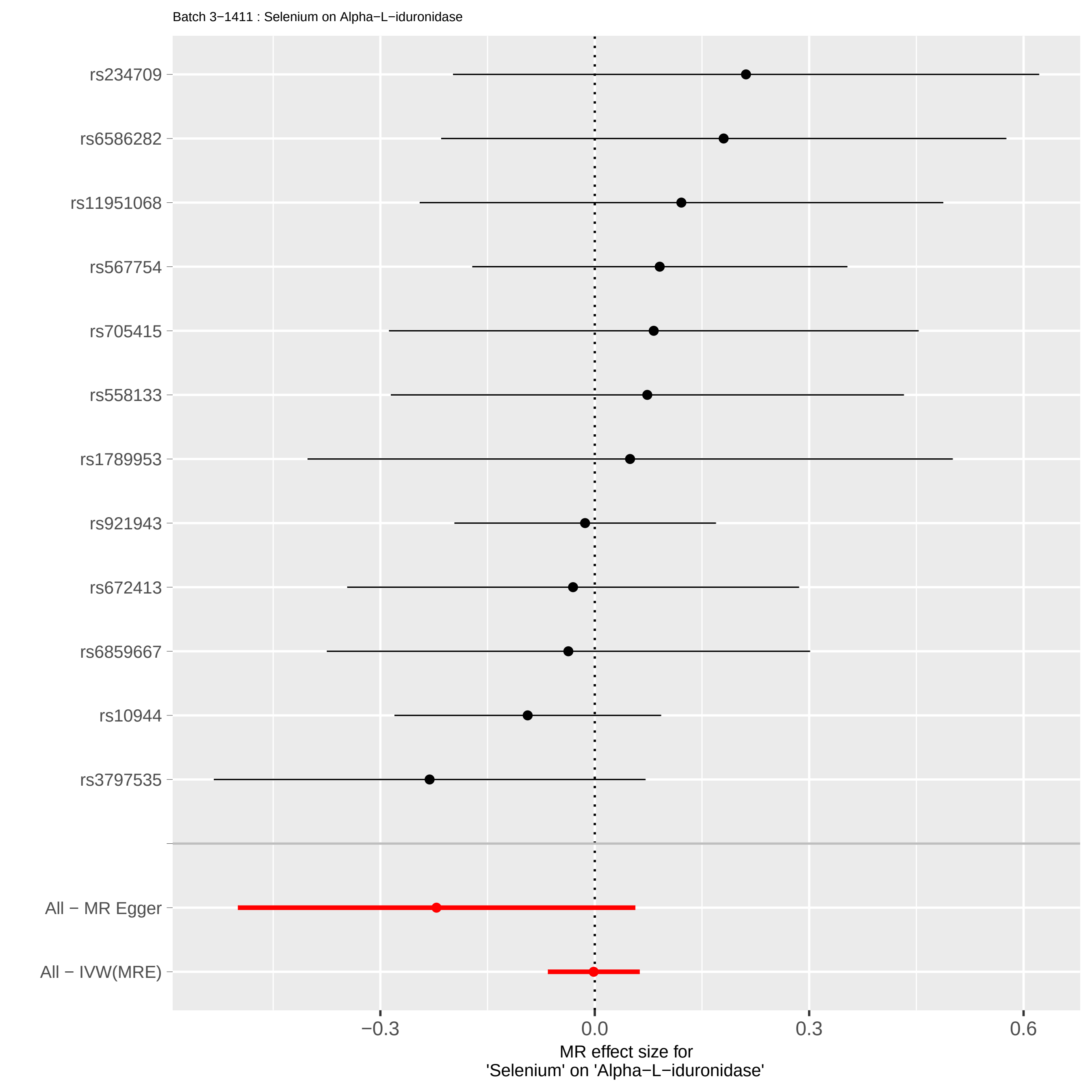

Batch 3−1411 : Selenium on Alpha−L−iduronidase
rs234709
rs6586282
rs11951068
rs567754
rs705415
rs558133
rs1789953
rs921943
rs672413
rs6859667
rs10944
rs3797535
All − MR Egger
All − IVW(MRE)
−0.3
0.0
0.3
0.6
MR effect size for
'Selenium' on 'Alpha−L−iduronidase'

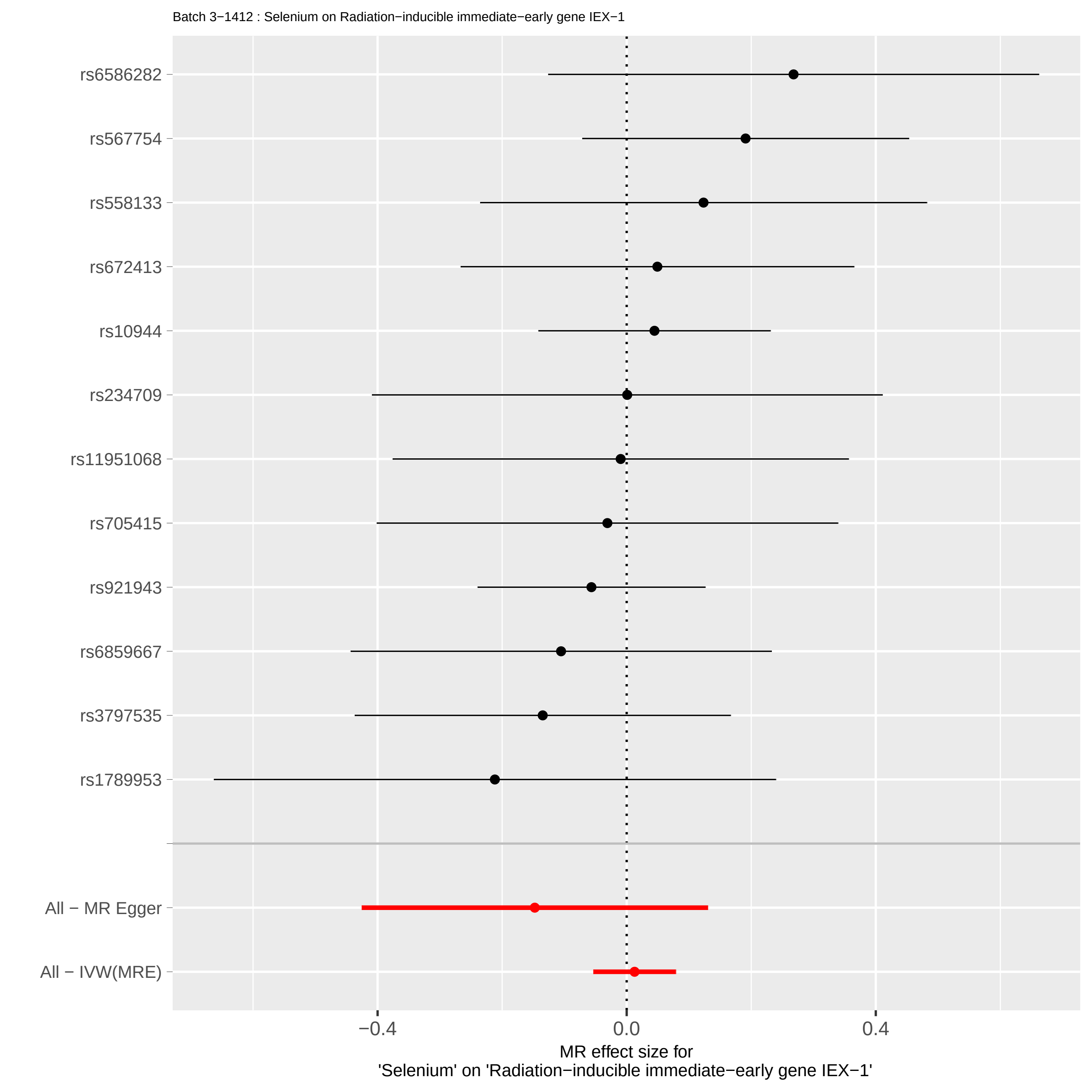

Batch 3−1412 : Selenium on Radiation−inducible immediate−early gene IEX−1
rs6586282
rs567754
rs558133
rs672413
rs10944
rs234709
rs11951068
rs705415
rs921943
rs6859667
rs3797535
rs1789953
All − MR Egger
All − IVW(MRE)
−0.4
0.0
0.4
MR effect size for
'Selenium' on 'Radiation−inducible immediate−early gene IEX−1'

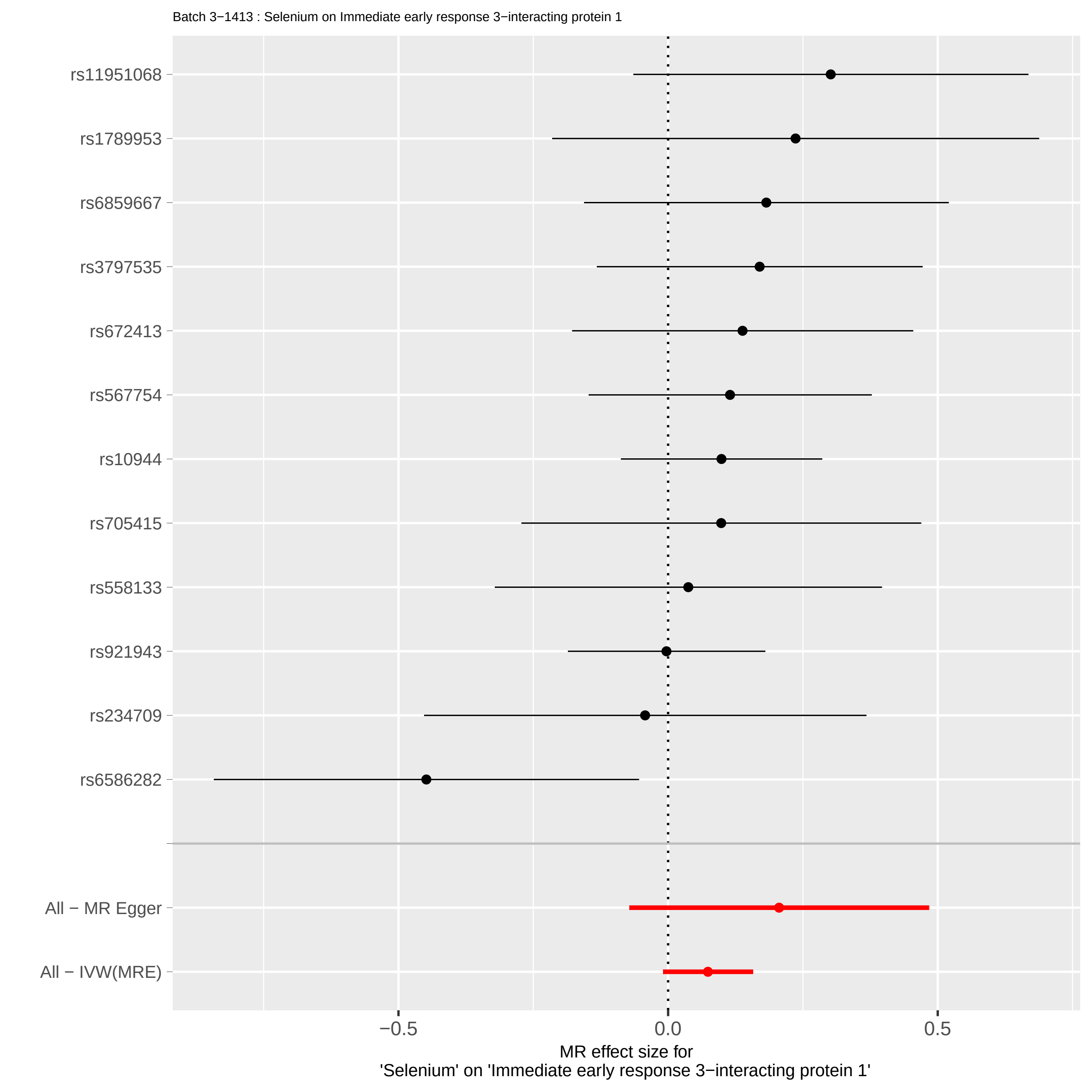

Batch 3−1413 : Selenium on Immediate early response 3−interacting protein 1
rs11951068
rs1789953
rs6859667
rs3797535
rs672413
rs567754
rs10944
rs705415
rs558133
rs921943
rs234709
rs6586282
All − MR Egger
All − IVW(MRE)
−0.5
0.0
0.5
MR effect size for
'Selenium' on 'Immediate early response 3−interacting protein 1'

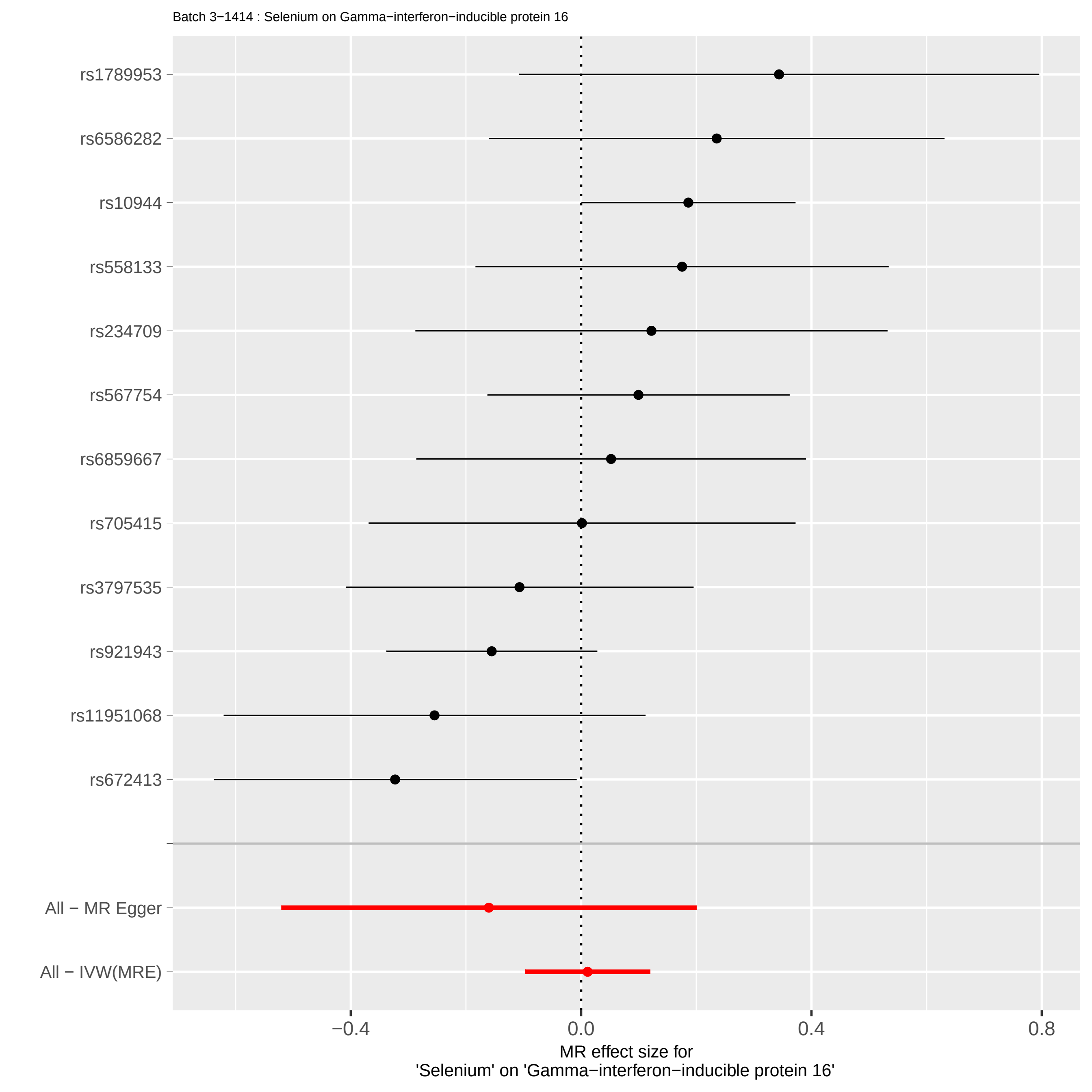
Batch 3−1414 : Selenium on Gamma−interferon−inducible protein 16
rs1789953
rs6586282
rs10944
rs558133
rs234709
rs567754
rs6859667
rs705415
rs3797535
rs921943
rs11951068
rs672413
All − MR Egger
All − IVW(MRE)
−0.4
0.0
0.4
0.8
MR effect size for
'Selenium' on 'Gamma−interferon−inducible protein 16'

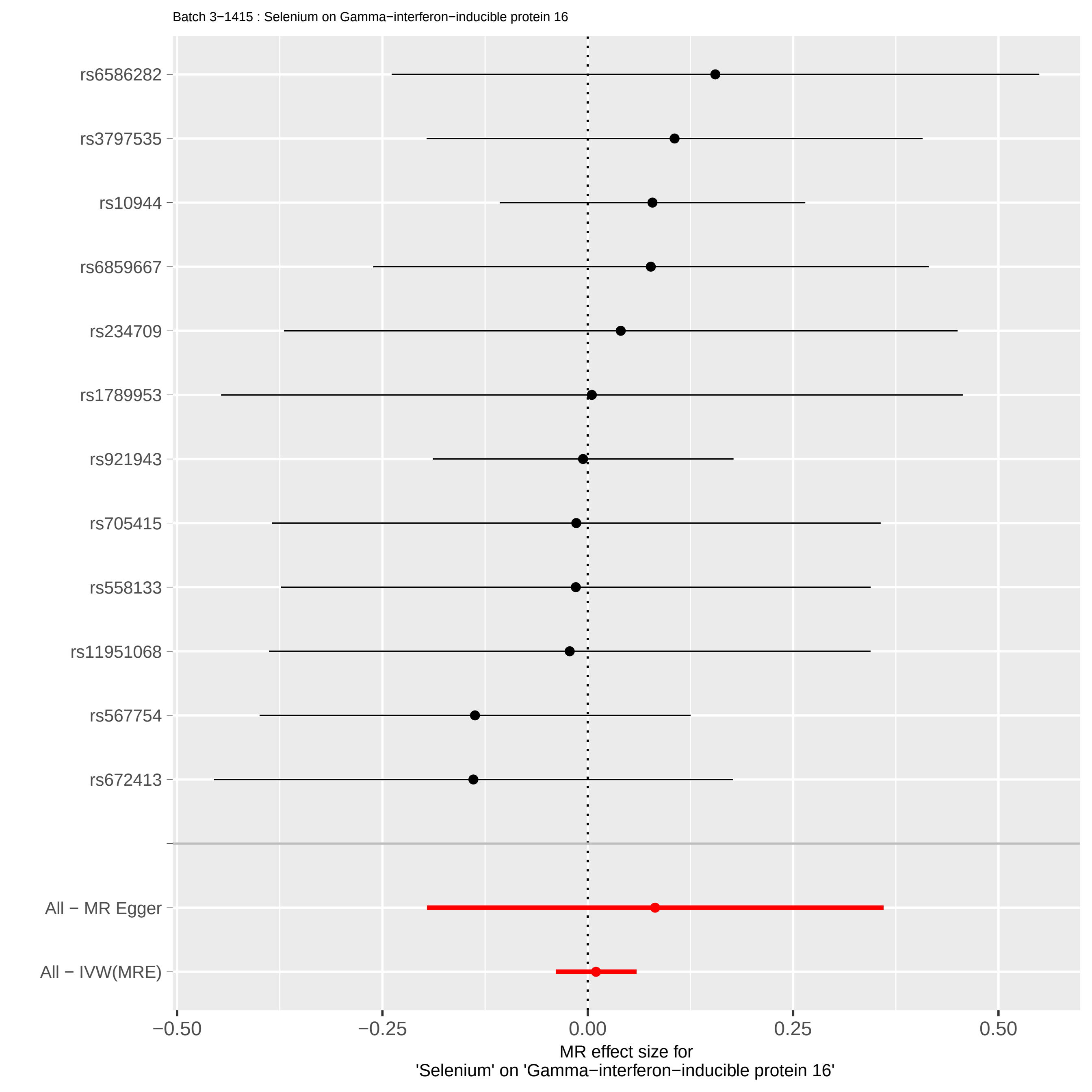
Batch 3−1415 : Selenium on Gamma−interferon−inducible protein 16
rs6586282
rs3797535
rs10944
rs6859667
rs234709
rs1789953
rs921943
rs705415
rs558133
rs11951068
rs567754
rs672413
All − MR Egger
All − IVW(MRE)
−0.50
−0.25
0.00
0.25
0.50
MR effect size for
'Selenium' on 'Gamma−interferon−inducible protein 16'

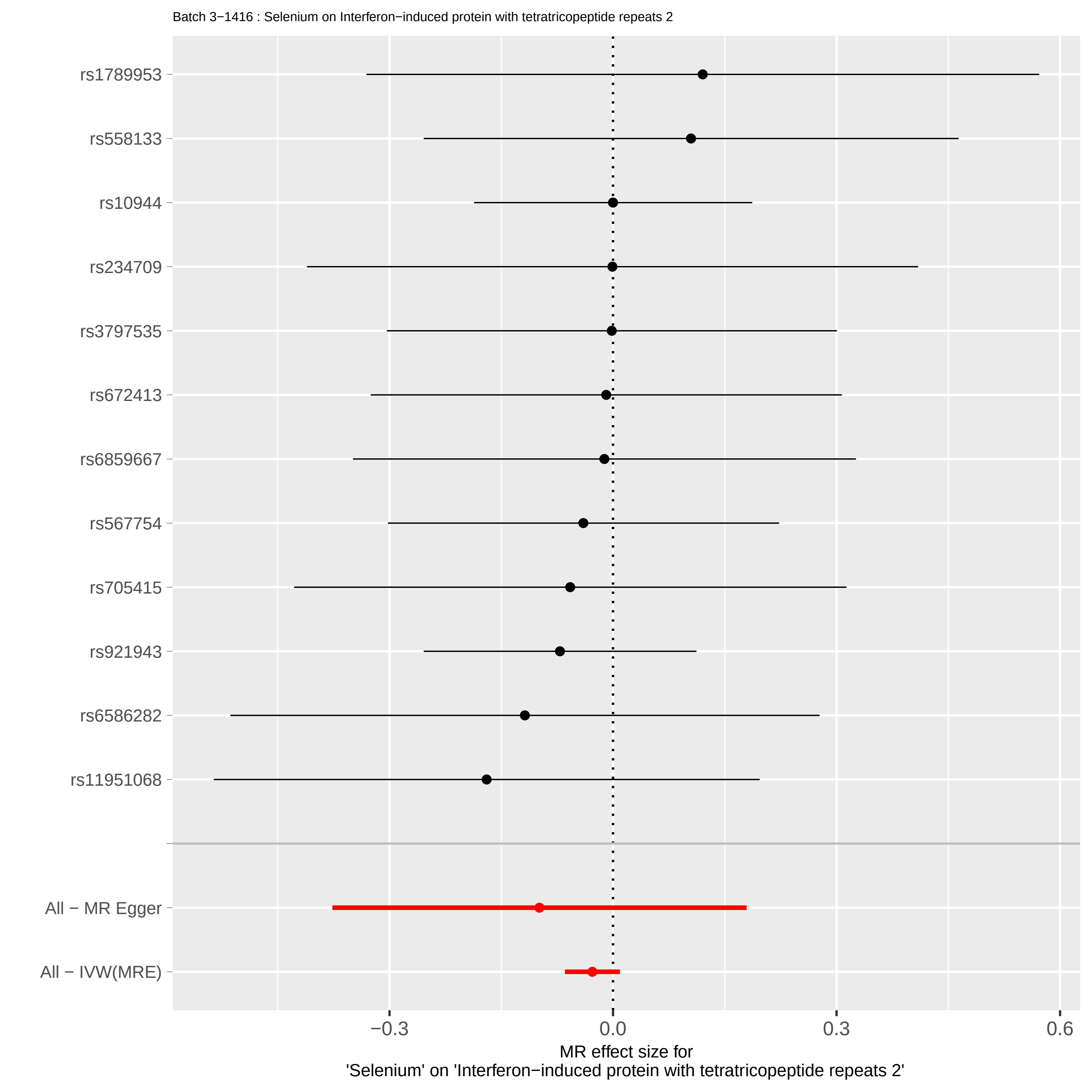
Batch 3−1416 : Selenium on Interferon−induced protein with tetratricopeptide repeats 2
rs1789953
rs558133
rs10944
rs234709
rs3797535
rs672413
rs6859667
rs567754
rs705415
rs921943
rs6586282
rs11951068
All − MR Egger
All − IVW(MRE)
−0.3
0.0
0.3
0.6
MR effect size for
'Selenium' on 'Interferon−induced protein with tetratricopeptide repeats 2'

Batch 3−1417 : Selenium on Interferon−induced protein with tetratricopeptide repeats 3

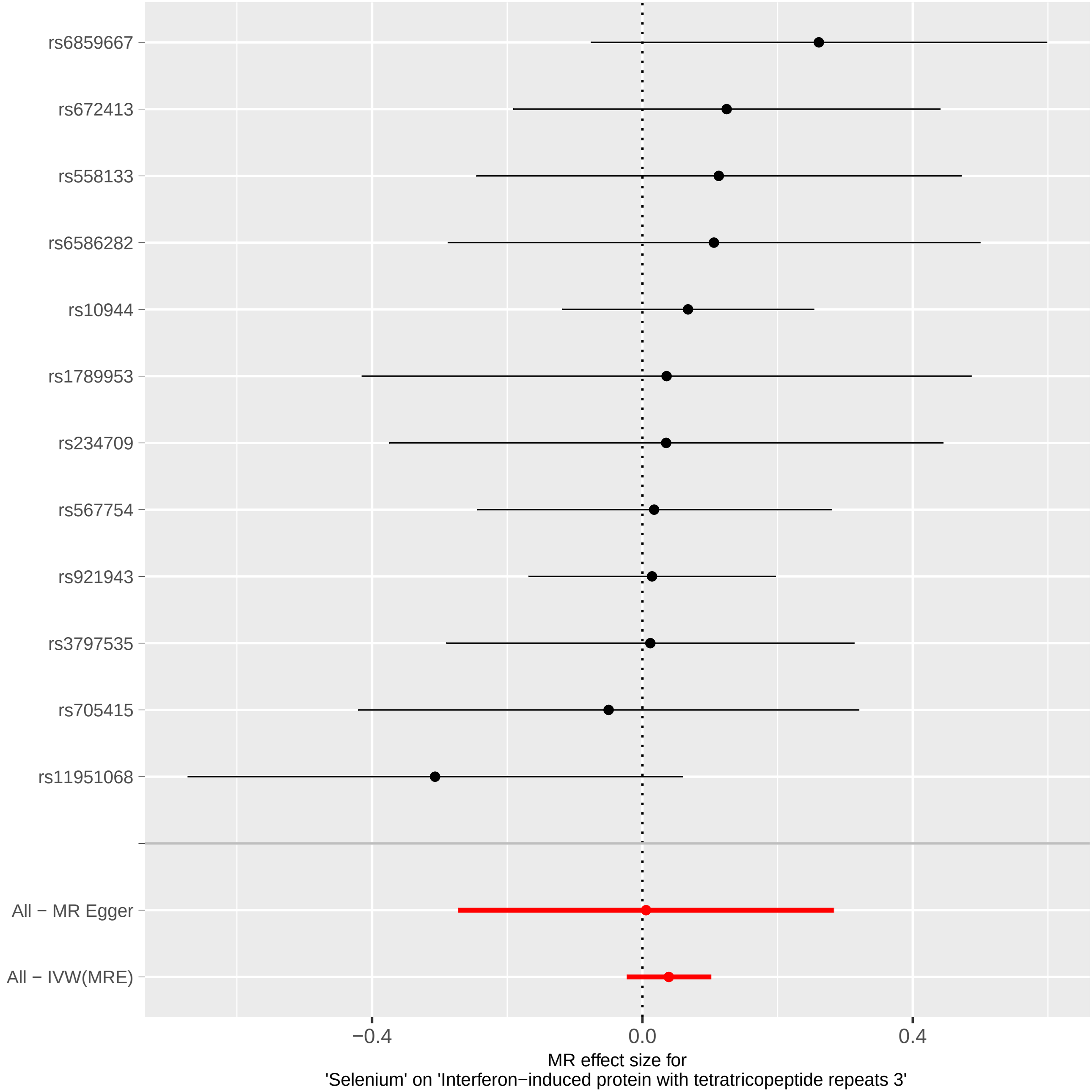

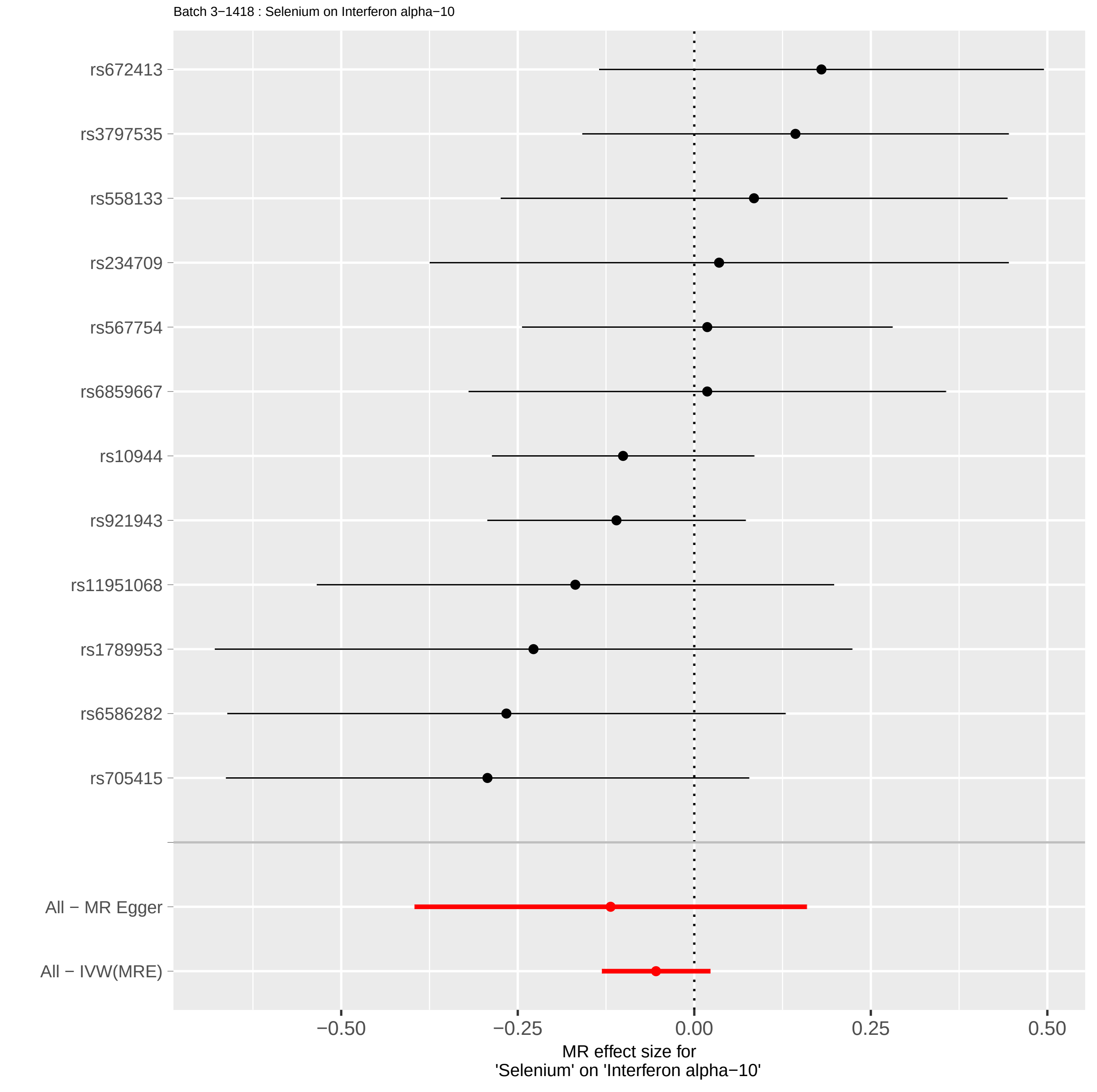

Batch 3−1418 : Selenium on Interferon alpha−10
rs672413
rs3797535
rs558133
rs234709
rs567754
rs6859667
rs10944
rs921943
rs11951068
rs1789953
rs6586282
rs705415
All − MR Egger
All − IVW(MRE)
−0.50
−0.25
0.00
0.25
0.50
MR effect size for
'Selenium' on 'Interferon alpha−10'

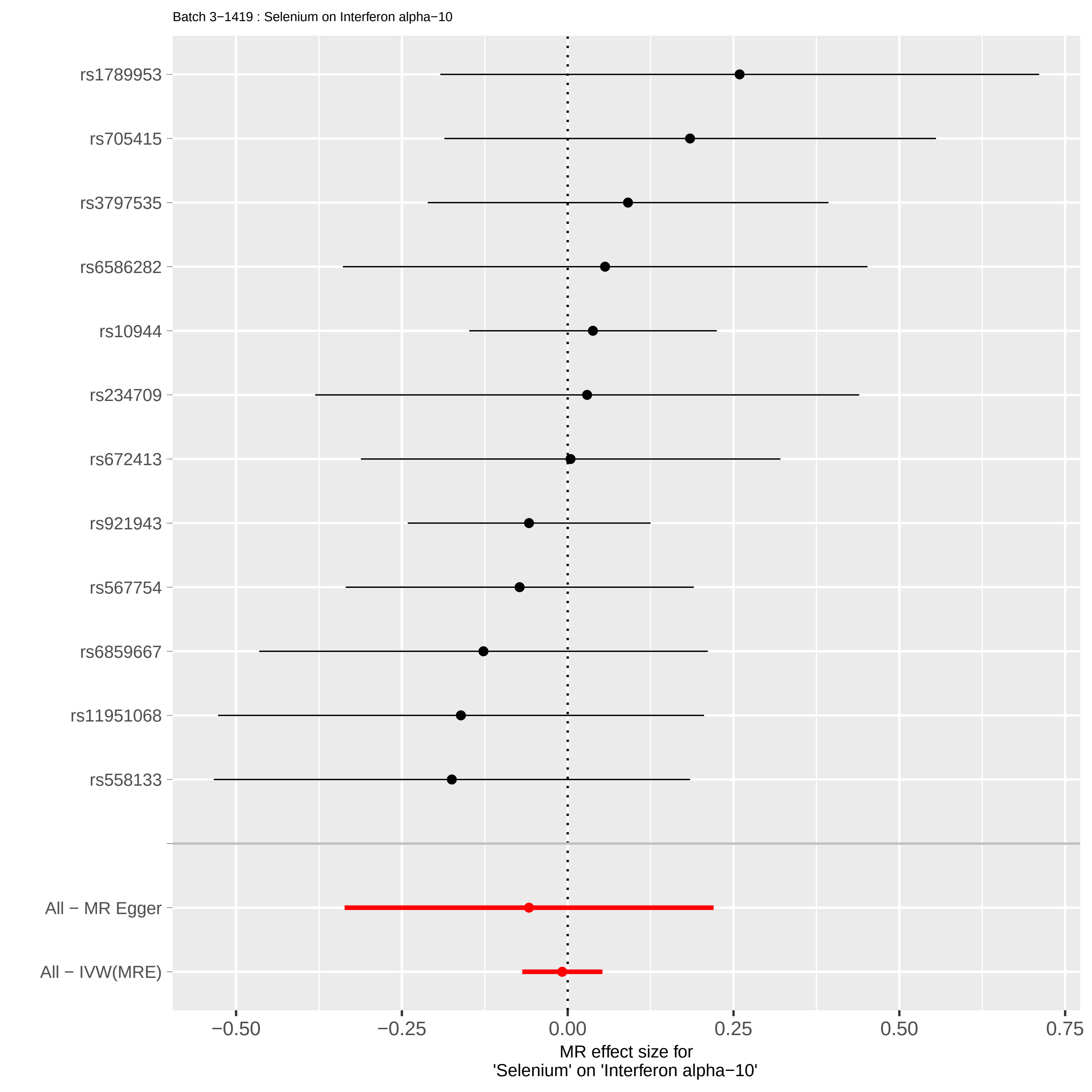

Batch 3−1419 : Selenium on Interferon alpha−10
rs1789953
rs705415
rs3797535
rs6586282
rs10944
rs234709
rs672413
rs921943
rs567754
rs6859667
rs11951068
rs558133
All − MR Egger
All − IVW(MRE)
−0.50
−0.25
0.00
0.25
0.50
0.75
MR effect size for
'Selenium' on 'Interferon alpha−10'

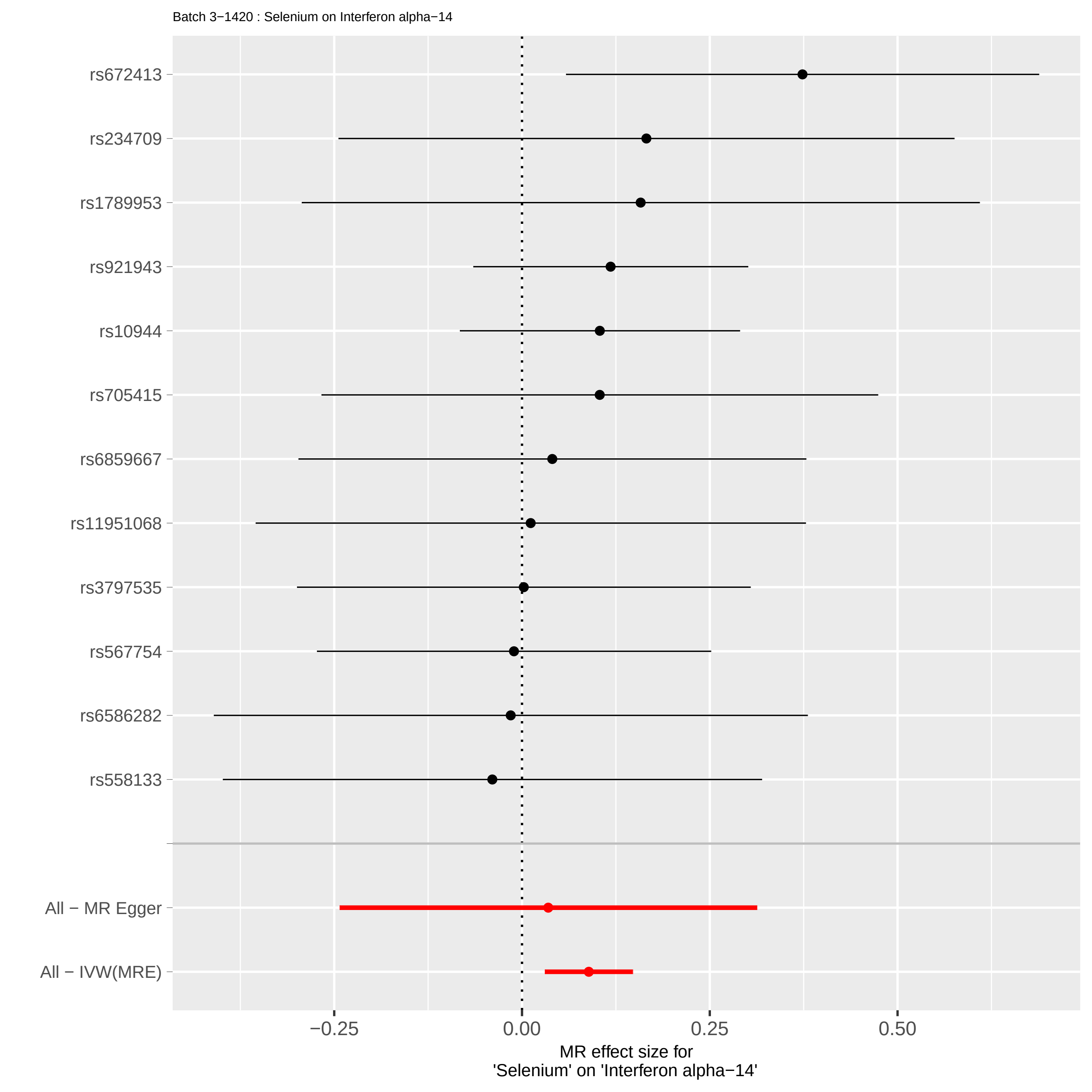

Batch 3−1420 : Selenium on Interferon alpha−14
rs672413
rs234709
rs1789953
rs921943
rs10944
rs705415
rs6859667
rs11951068
rs3797535
rs567754
rs6586282
rs558133
All − MR Egger
All − IVW(MRE)
−0.25
0.00
0.25
0.50
MR effect size for
'Selenium' on 'Interferon alpha−14'

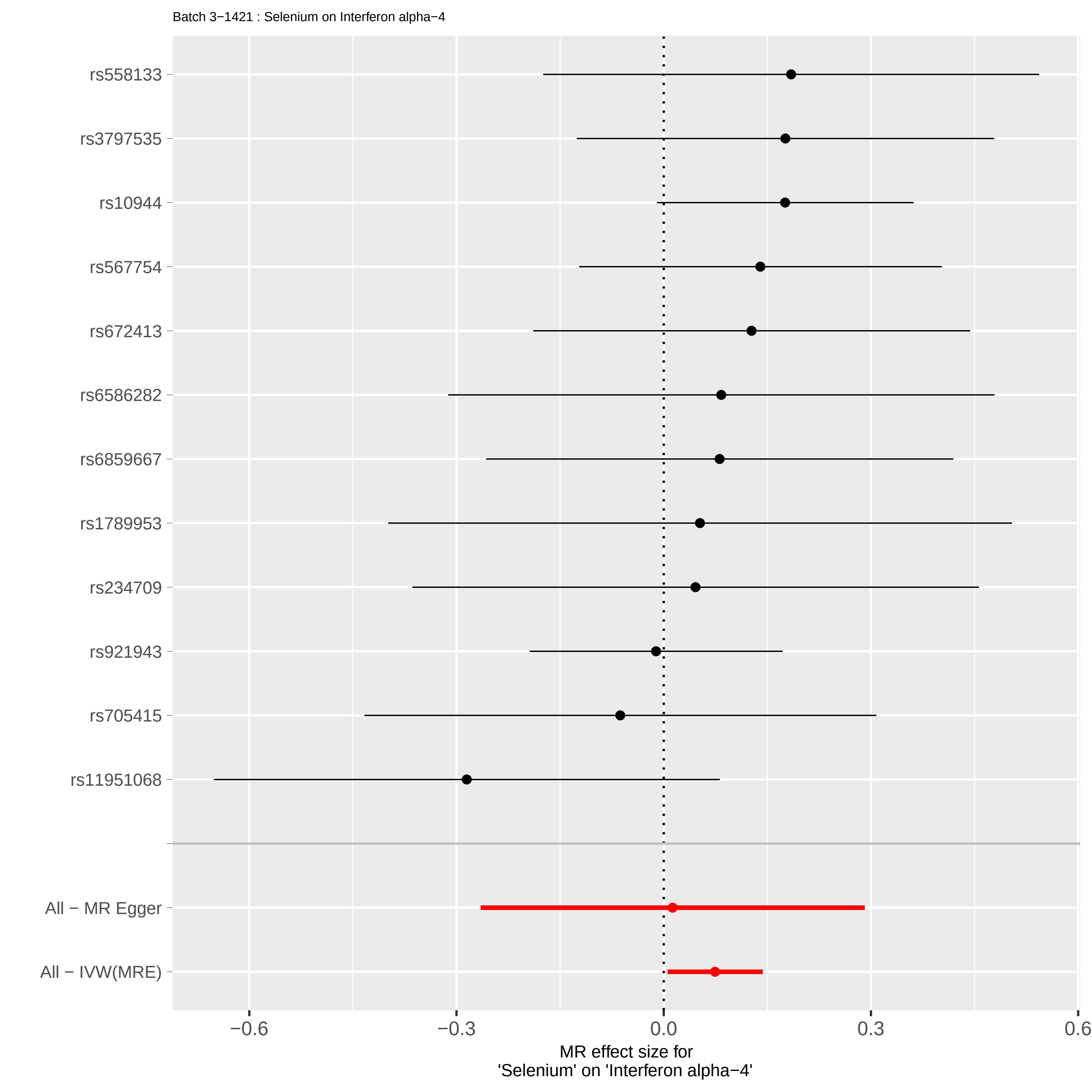

Batch 3−1421 : Selenium on Interferon alpha−4
rs558133
rs3797535
rs10944
rs567754
rs672413
rs6586282
rs6859667
rs1789953
rs234709
rs921943
rs705415
rs11951068
All − MR Egger
All − IVW(MRE)
−0.6
−0.3
0.0
0.3
0.6
MR effect size for
'Selenium' on 'Interferon alpha−4'

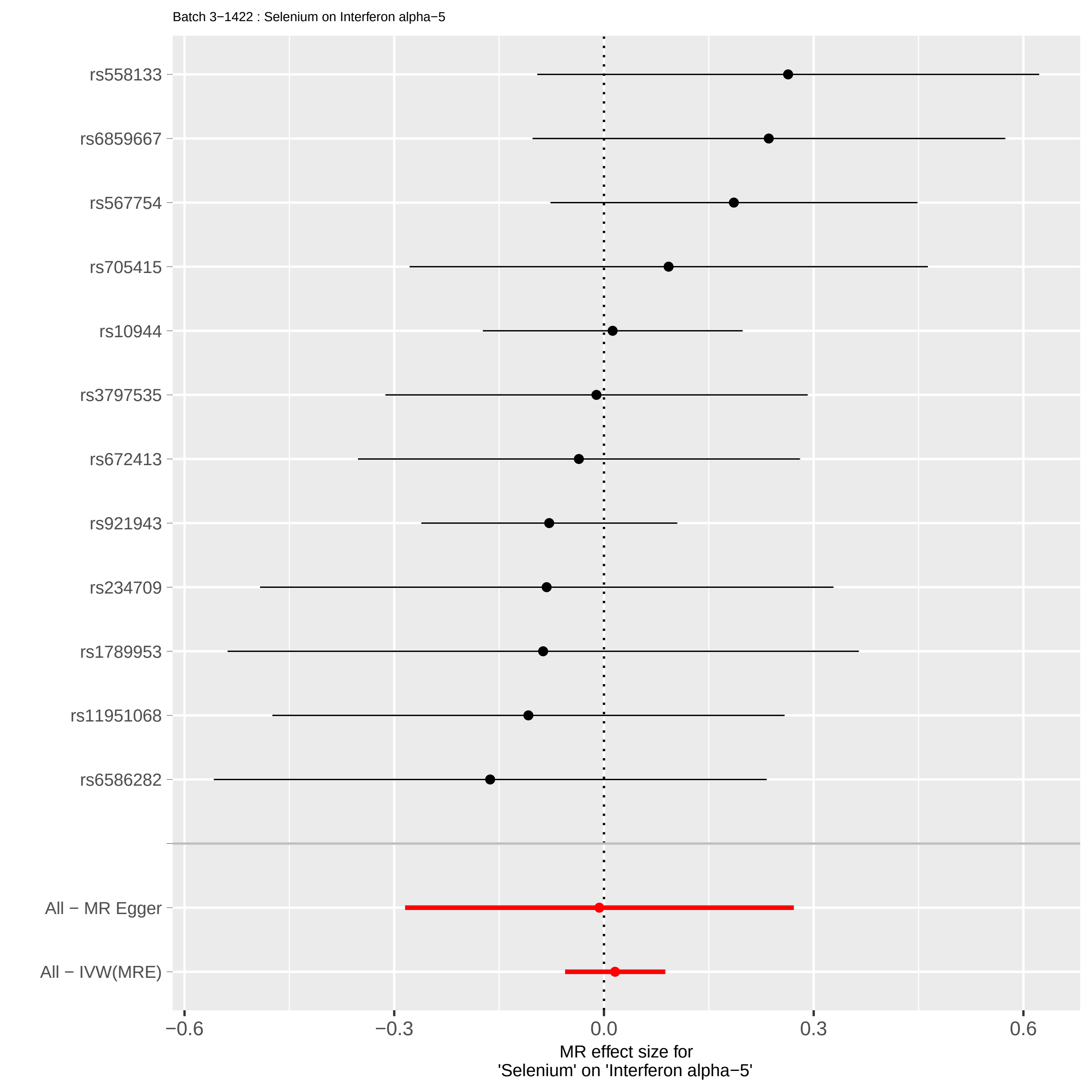

Batch 3−1422 : Selenium on Interferon alpha−5
rs558133
rs6859667
rs567754
rs705415
rs10944
rs3797535
rs672413
rs921943
rs234709
rs1789953
rs11951068
rs6586282
All − MR Egger
All − IVW(MRE)
−0.6
−0.3
0.0
0.3
0.6
MR effect size for
'Selenium' on 'Interferon alpha−5'

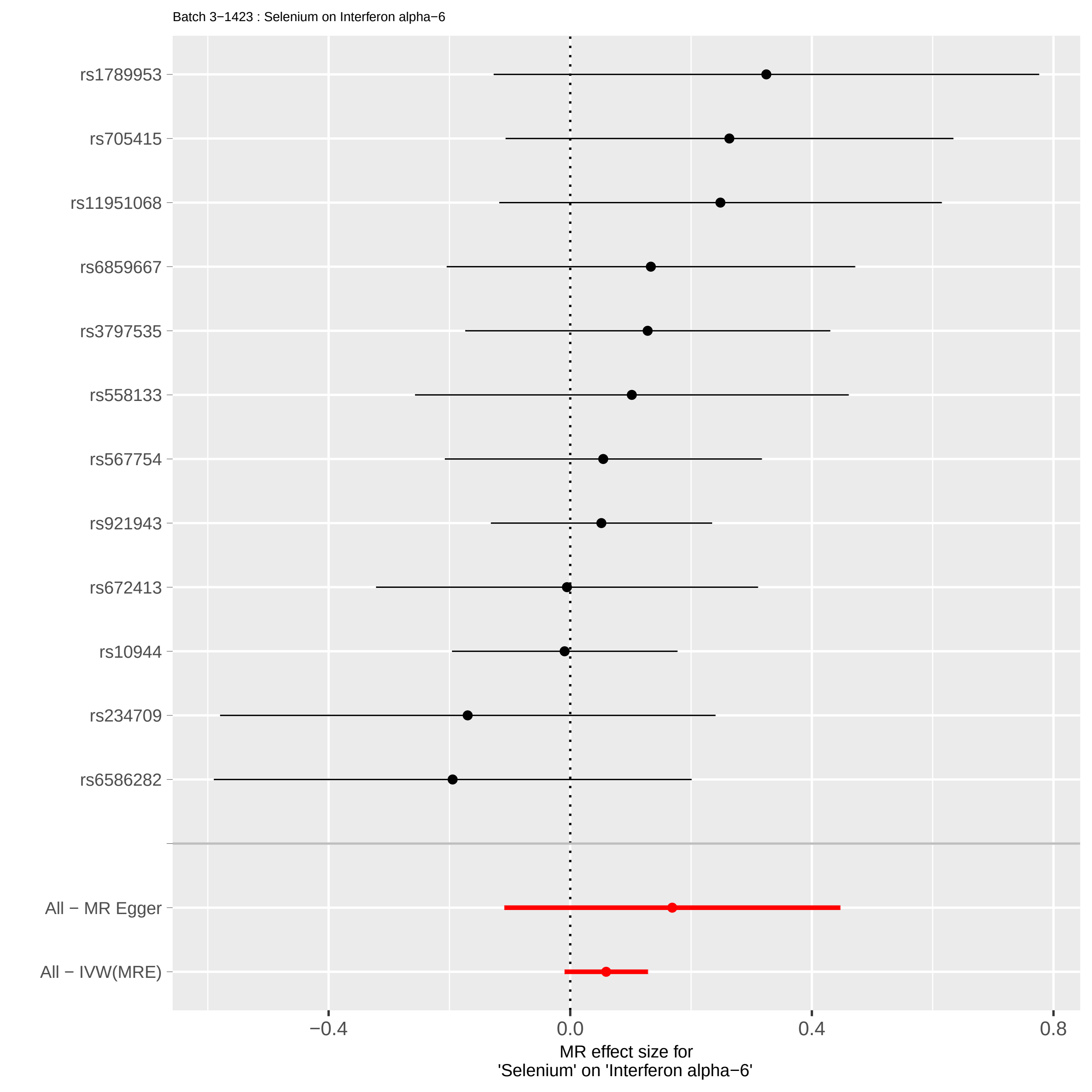
Batch 3−1423 : Selenium on Interferon alpha−6
rs1789953
rs705415
rs11951068
rs6859667
rs3797535
rs558133
rs567754
rs921943
rs672413
rs10944
rs234709
rs6586282
All − MR Egger
All − IVW(MRE)
−0.4
0.0
0.4
0.8
MR effect size for
'Selenium' on 'Interferon alpha−6'

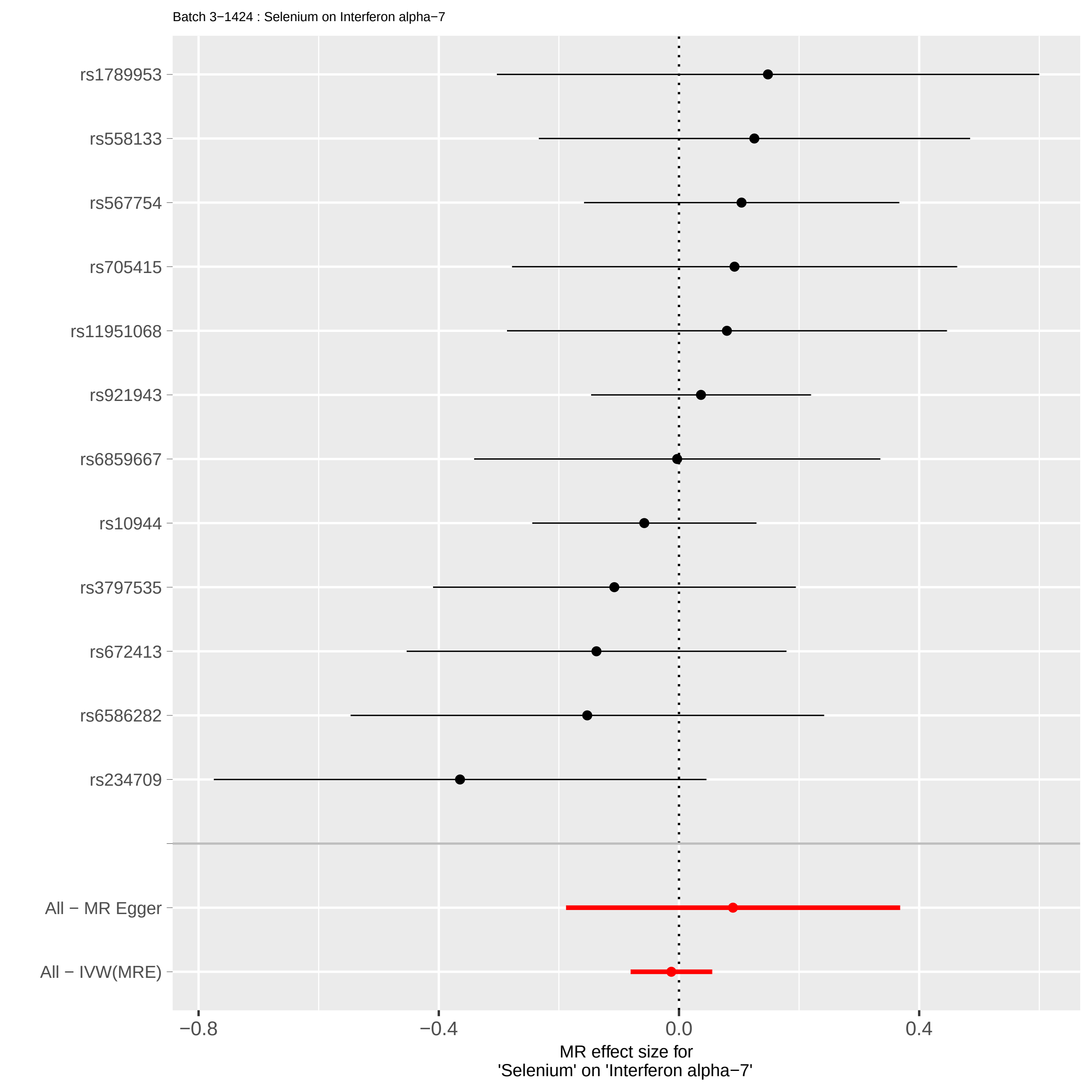

Batch 3−1424 : Selenium on Interferon alpha−7
rs1789953
rs558133
rs567754
rs705415
rs11951068
rs921943
rs6859667
rs10944
rs3797535
rs672413
rs6586282
rs234709
All − MR Egger
All − IVW(MRE)
−0.8
−0.4
0.0
0.4
MR effect size for
'Selenium' on 'Interferon alpha−7'

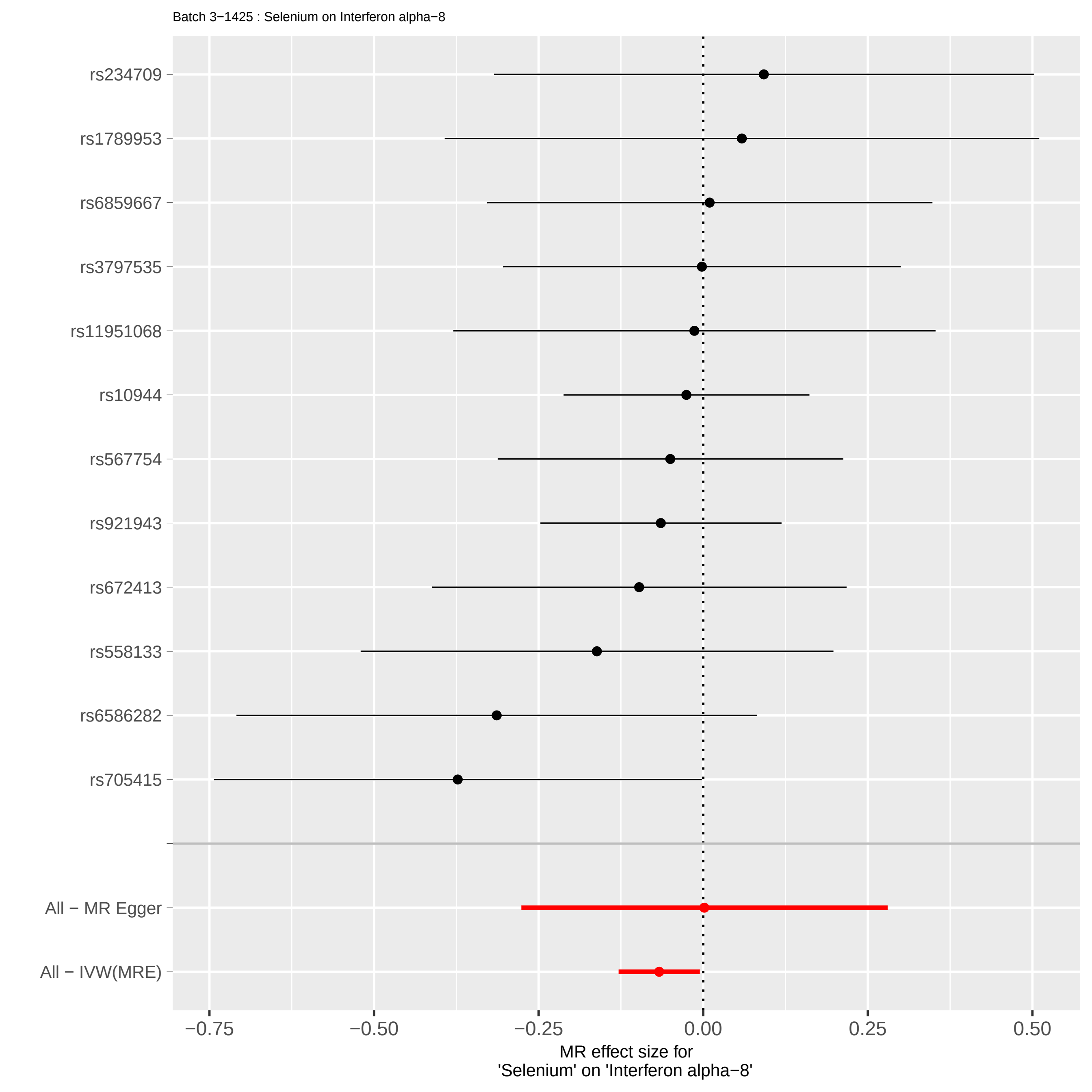

Batch 3−1425 : Selenium on Interferon alpha−8
rs234709
rs1789953
rs6859667
rs3797535
rs11951068
rs10944
rs567754
rs921943
rs672413
rs558133
rs6586282
rs705415
All − MR Egger
All − IVW(MRE)
−0.75
−0.50
−0.25
0.00
0.25
0.50
MR effect size for
'Selenium' on 'Interferon alpha−8'

Batch 3−1426 : Selenium on Interferon alpha/beta receptor 1

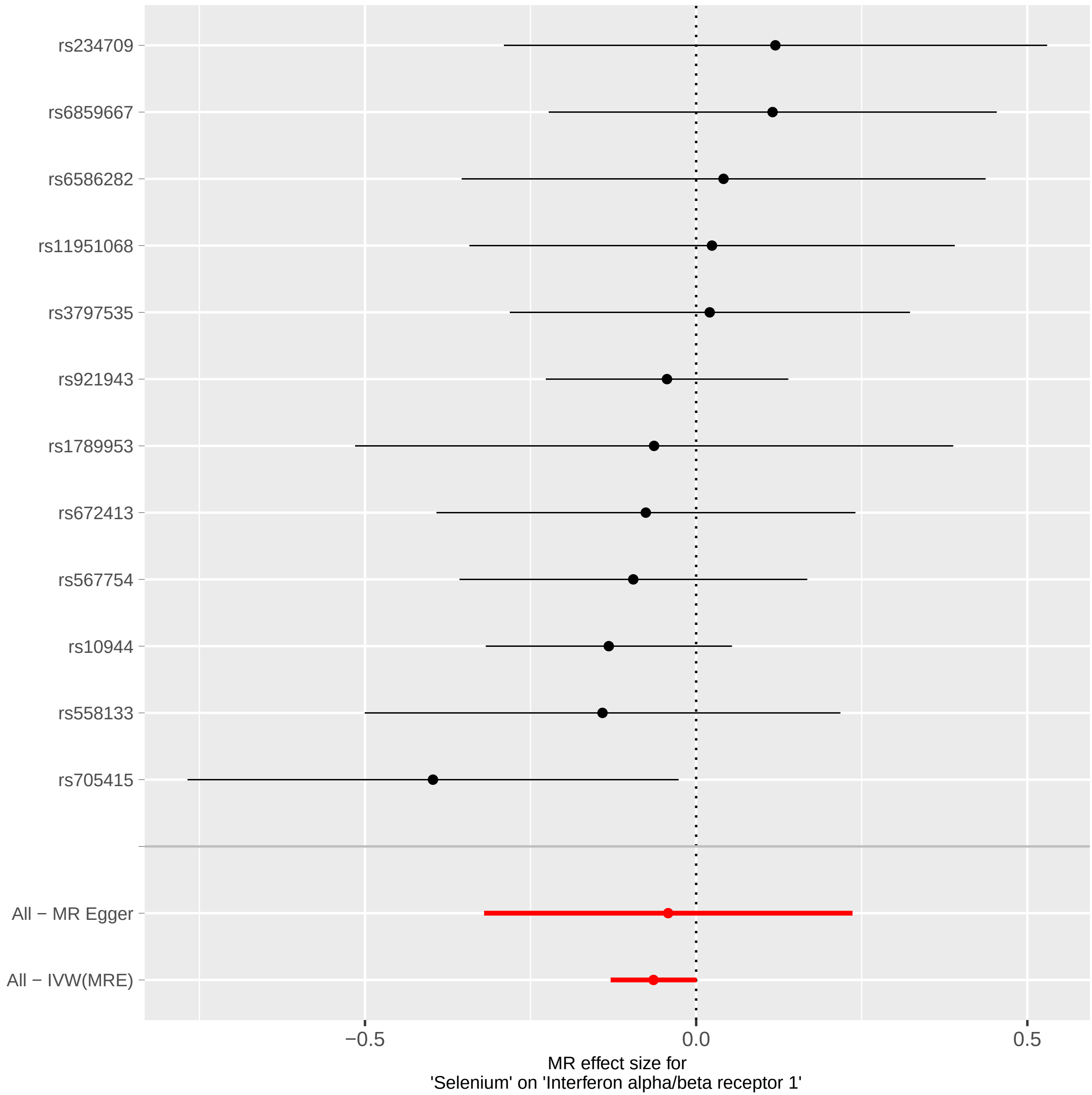

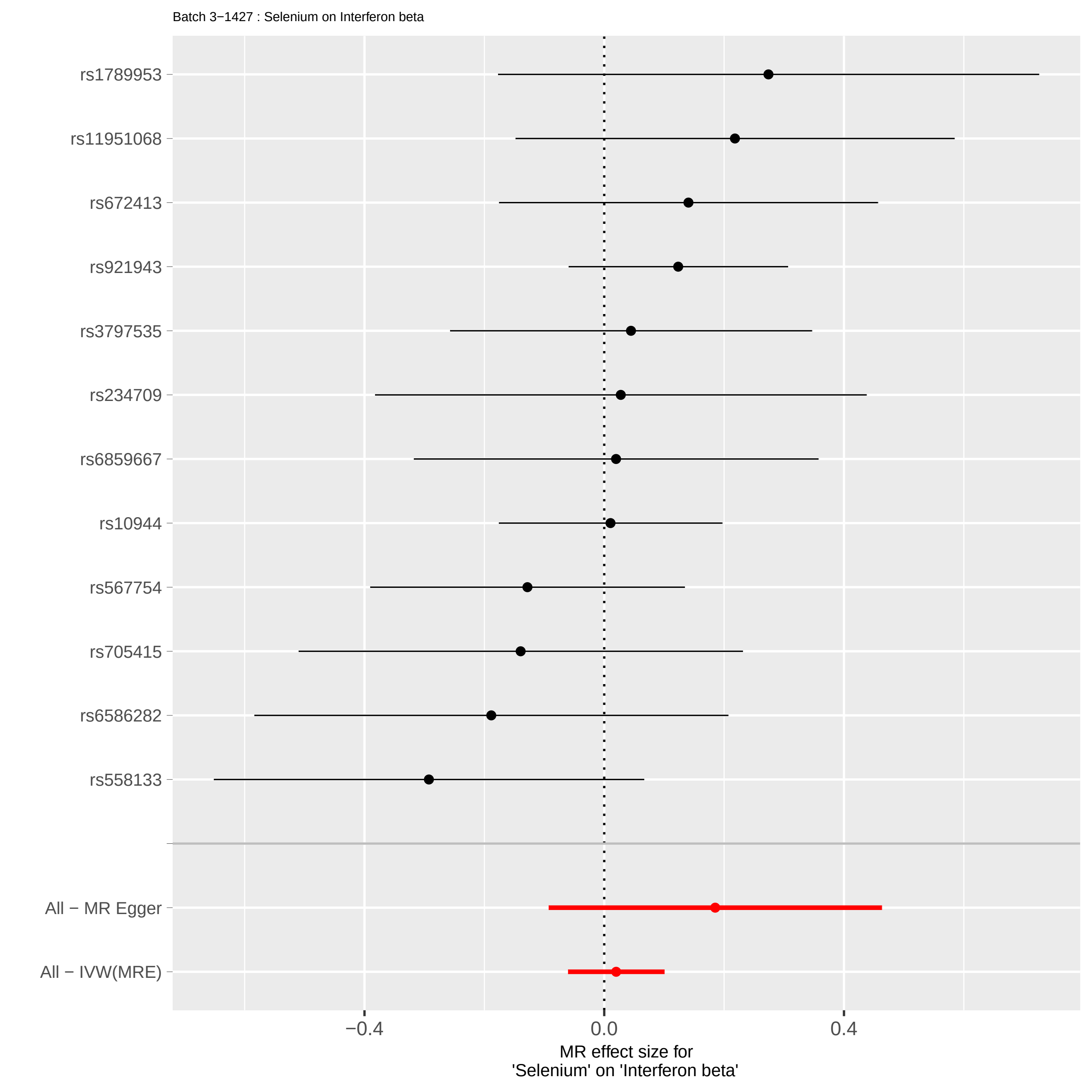

Batch 3−1427 : Selenium on Interferon beta
rs1789953
rs11951068
rs672413
rs921943
rs3797535
rs234709
rs6859667
rs10944
rs567754
rs705415
rs6586282
rs558133
All − MR Egger
All − IVW(MRE)
−0.4
0.0
0.4
MR effect size for
'Selenium' on 'Interferon beta'

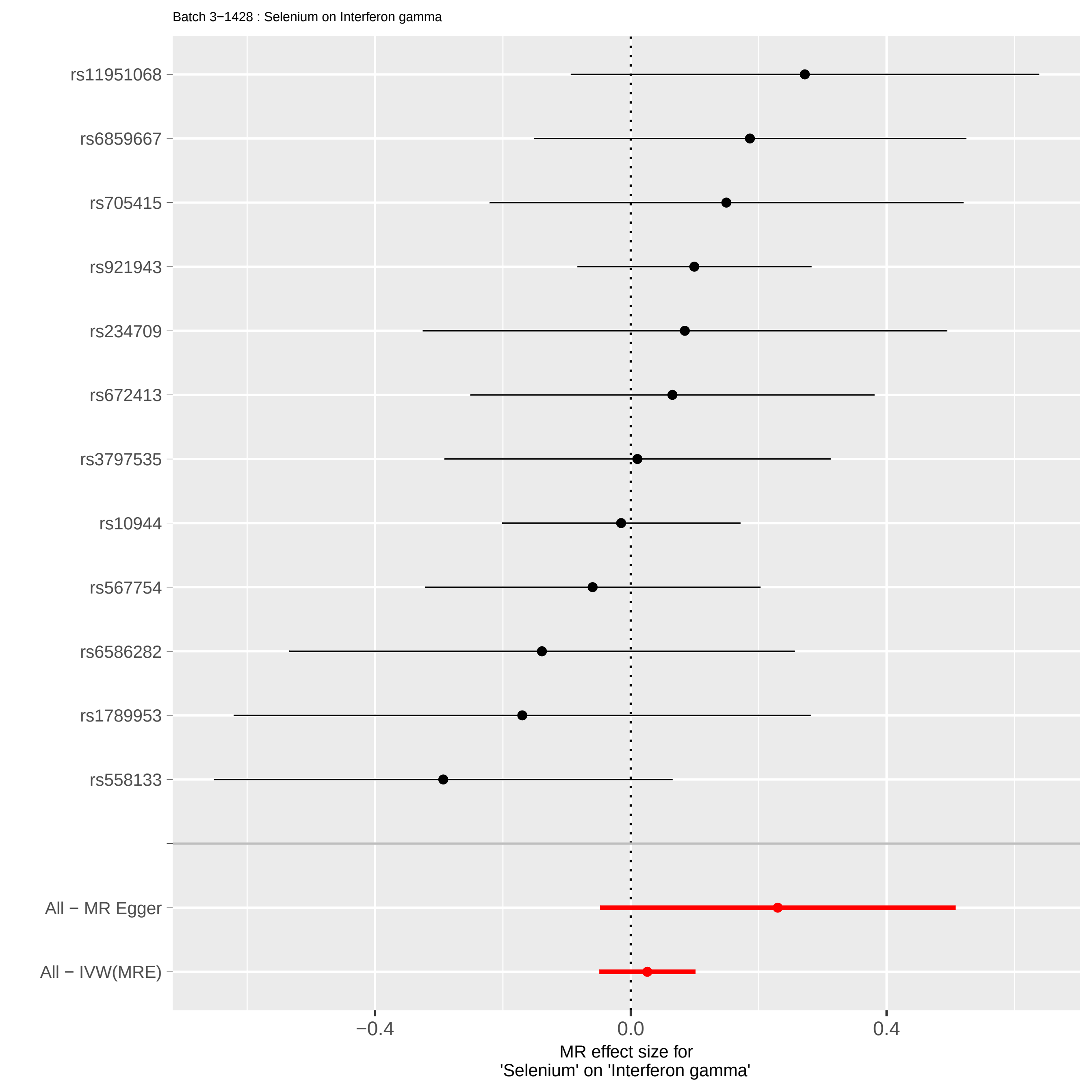
Batch 3−1428 : Selenium on Interferon gamma
rs11951068
rs6859667
rs705415
rs921943
rs234709
rs672413
rs3797535
rs10944
rs567754
rs6586282
rs1789953
rs558133
All − MR Egger
All − IVW(MRE)
−0.4
0.0
0.4
MR effect size for
'Selenium' on 'Interferon gamma'

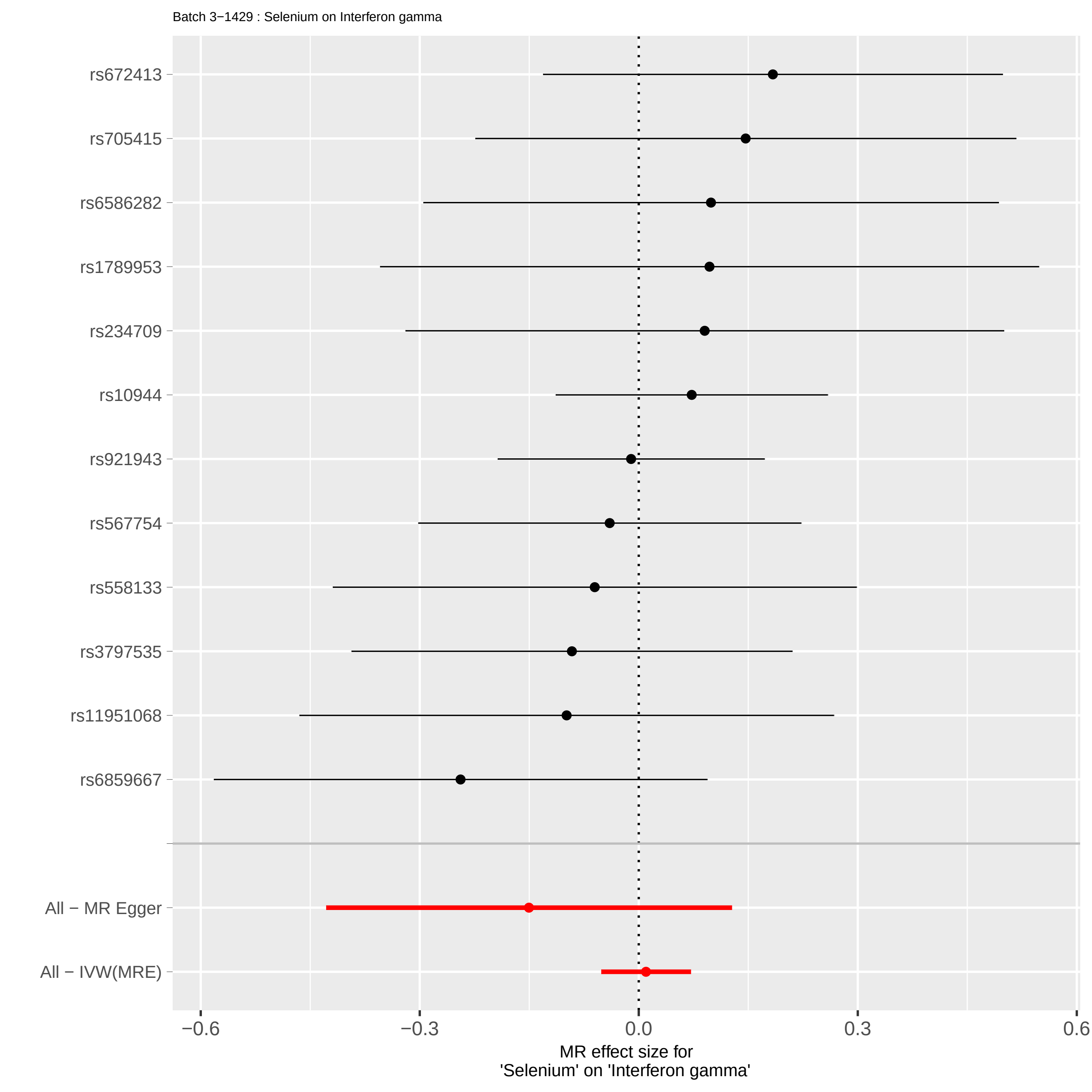

Batch 3−1429 : Selenium on Interferon gamma
rs672413
rs705415
rs6586282
rs1789953
rs234709
rs10944
rs921943
rs567754
rs558133
rs3797535
rs11951068
rs6859667
All − MR Egger
All − IVW(MRE)
−0.6
−0.3
0.0
0.3
0.6
MR effect size for
'Selenium' on 'Interferon gamma'

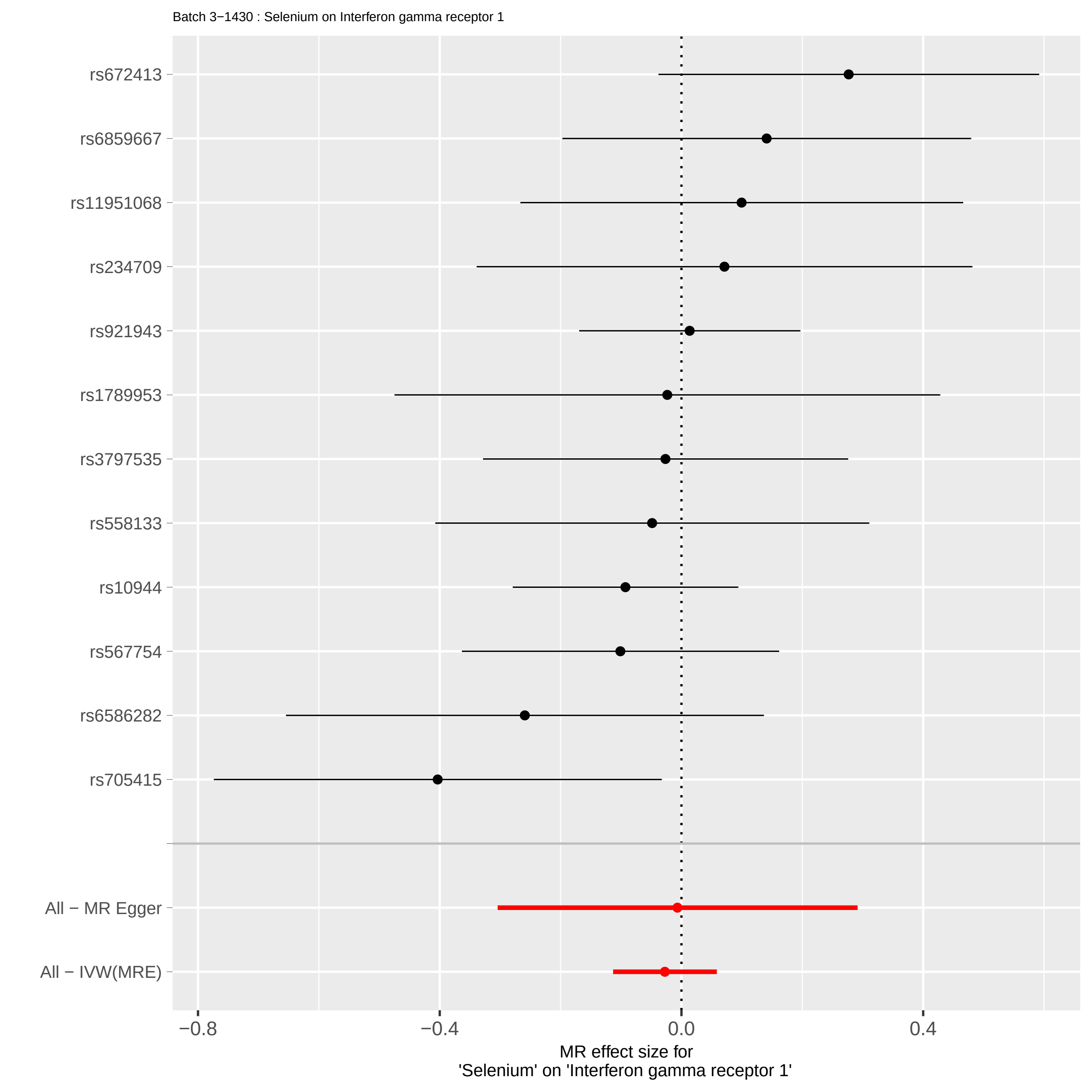

Batch 3−1430 : Selenium on Interferon gamma receptor 1
rs672413
rs6859667
rs11951068
rs234709
rs921943
rs1789953
rs3797535
rs558133
rs10944
rs567754
rs6586282
rs705415
All − MR Egger
All − IVW(MRE)
−0.8
−0.4
0.0
0.4
MR effect size for
'Selenium' on 'Interferon gamma receptor 1'

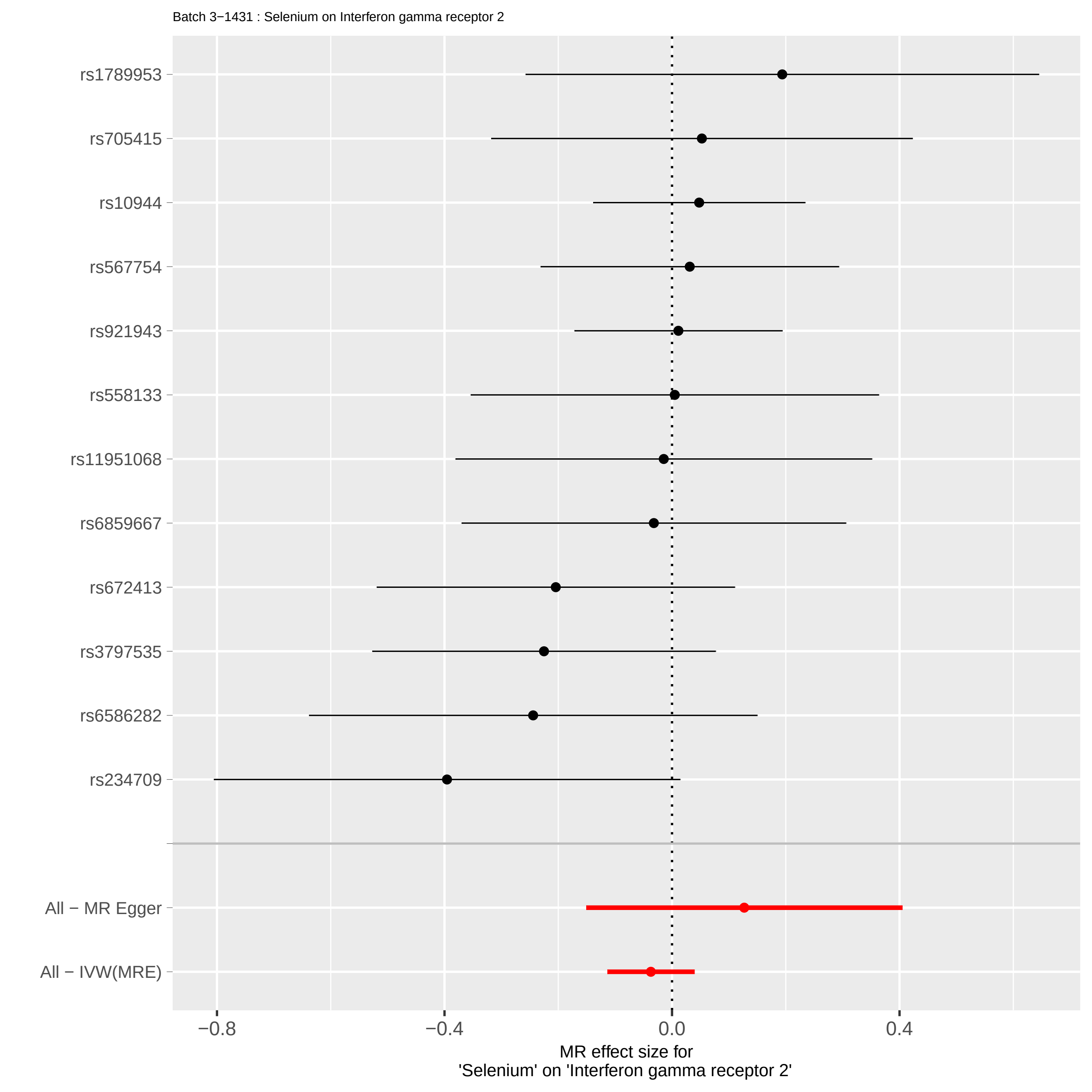

Batch 3−1431 : Selenium on Interferon gamma receptor 2
rs1789953
rs705415
rs10944
rs567754
rs921943
rs558133
rs11951068
rs6859667
rs672413
rs3797535
rs6586282
rs234709
All − MR Egger
All − IVW(MRE)
−0.8
−0.4
0.0
0.4
MR effect size for
'Selenium' on 'Interferon gamma receptor 2'

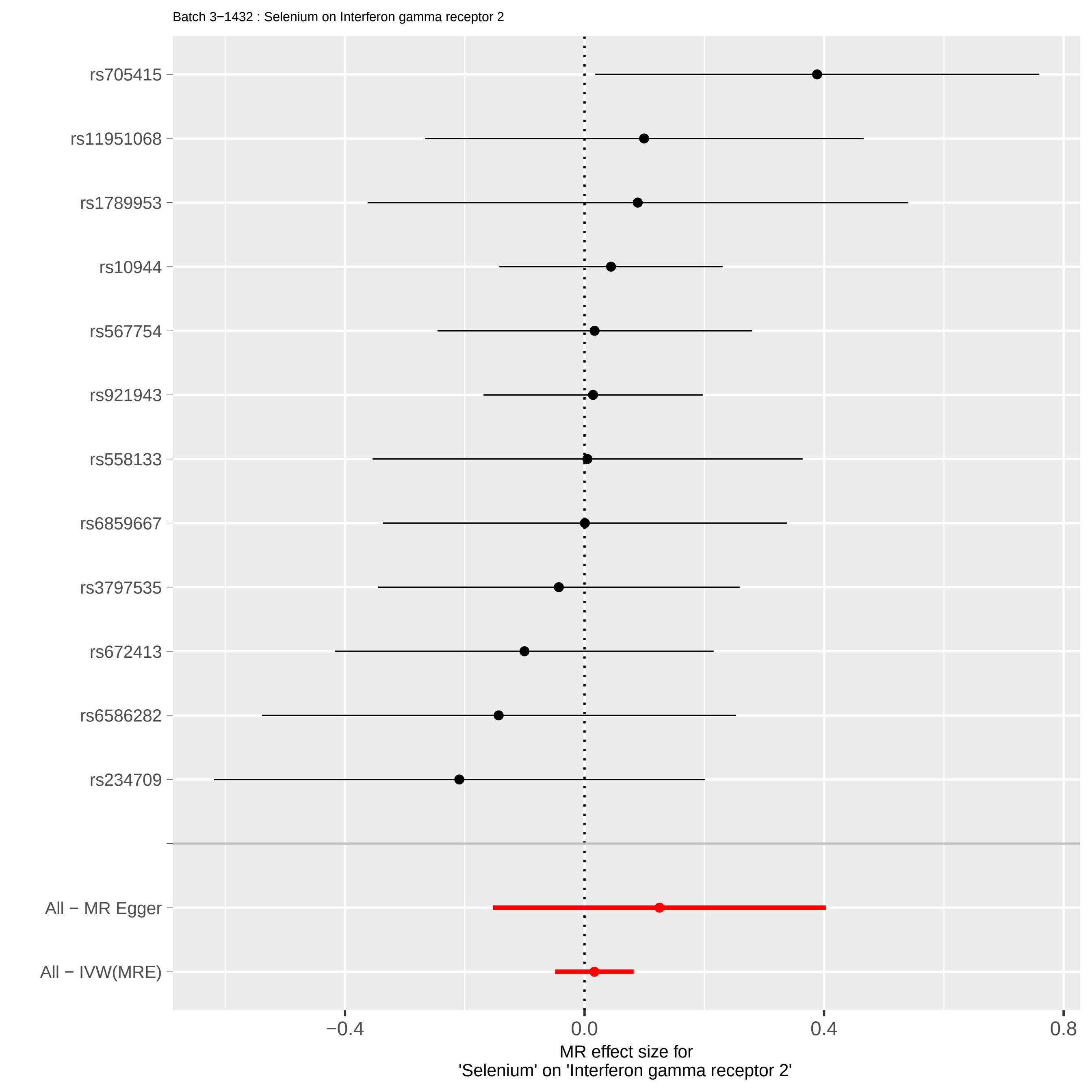

Batch 3−1432 : Selenium on Interferon gamma receptor 2
rs705415
rs11951068
rs1789953
rs10944
rs567754
rs921943
rs558133
rs6859667
rs3797535
rs672413
rs6586282
rs234709
All − MR Egger
All − IVW(MRE)
−0.4
0.0
0.4
0.8
MR effect size for
'Selenium' on 'Interferon gamma receptor 2'

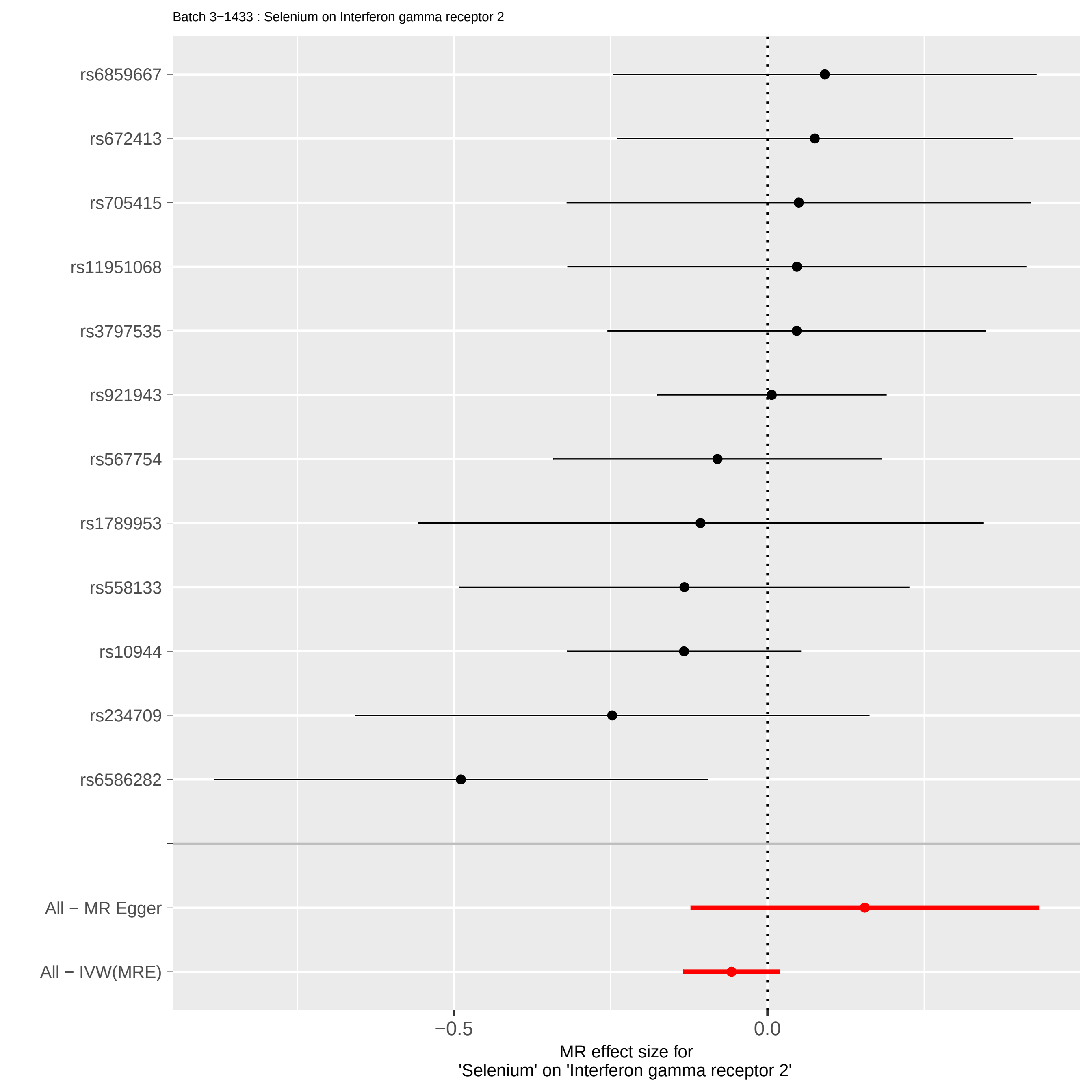
Batch 3−1433 : Selenium on Interferon gamma receptor 2
rs6859667
rs672413
rs705415
rs11951068
rs3797535
rs921943
rs567754
rs1789953
rs558133
rs10944
rs234709
rs6586282
All − MR Egger
All − IVW(MRE)
−0.5
0.0
MR effect size for
'Selenium' on 'Interferon gamma receptor 2'

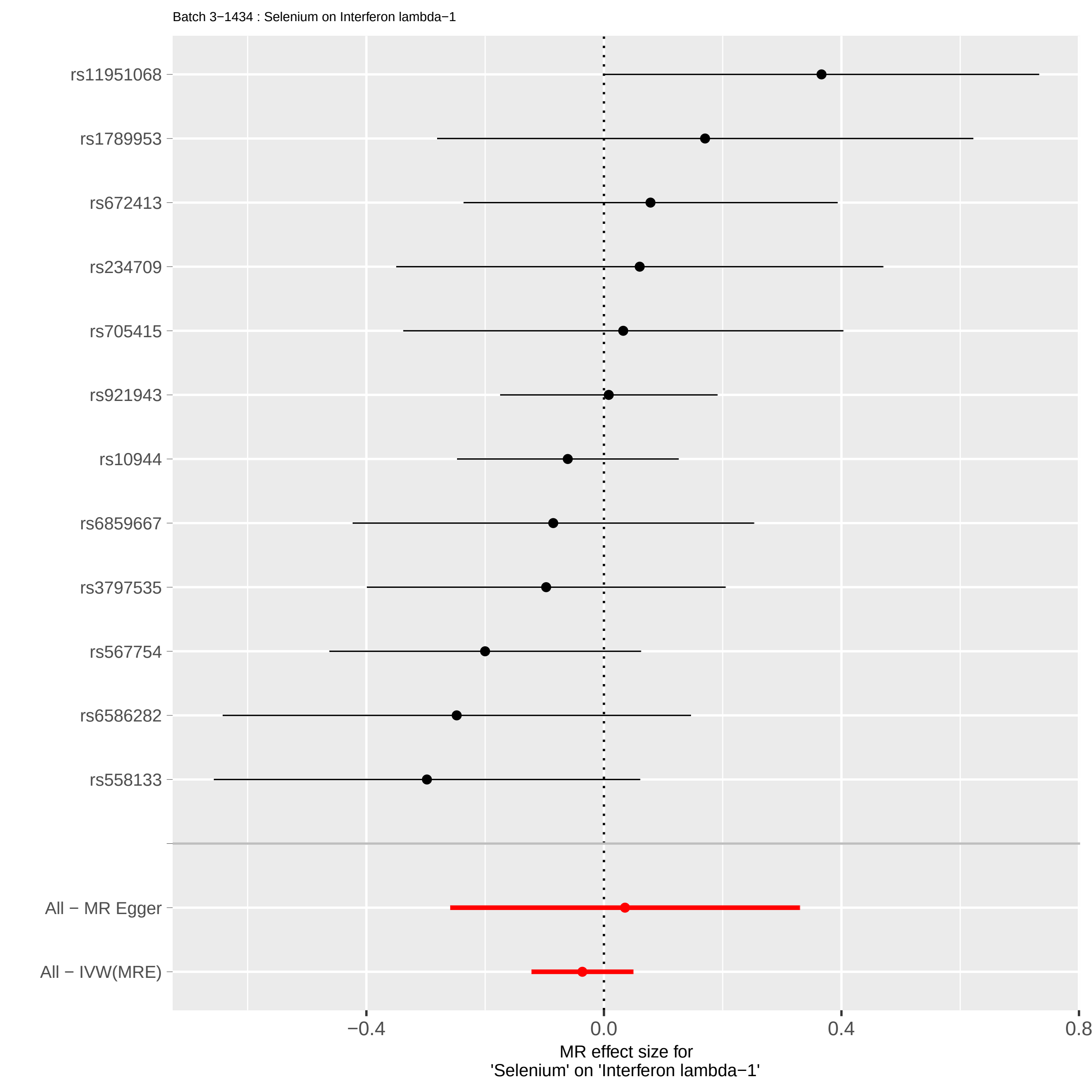
Batch 3−1434 : Selenium on Interferon lambda−1
rs11951068
rs1789953
rs672413
rs234709
rs705415
rs921943
rs10944
rs6859667
rs3797535
rs567754
rs6586282
rs558133
All − MR Egger
All − IVW(MRE)
−0.4
0.0
0.4
0.8
MR effect size for
'Selenium' on 'Interferon lambda−1'

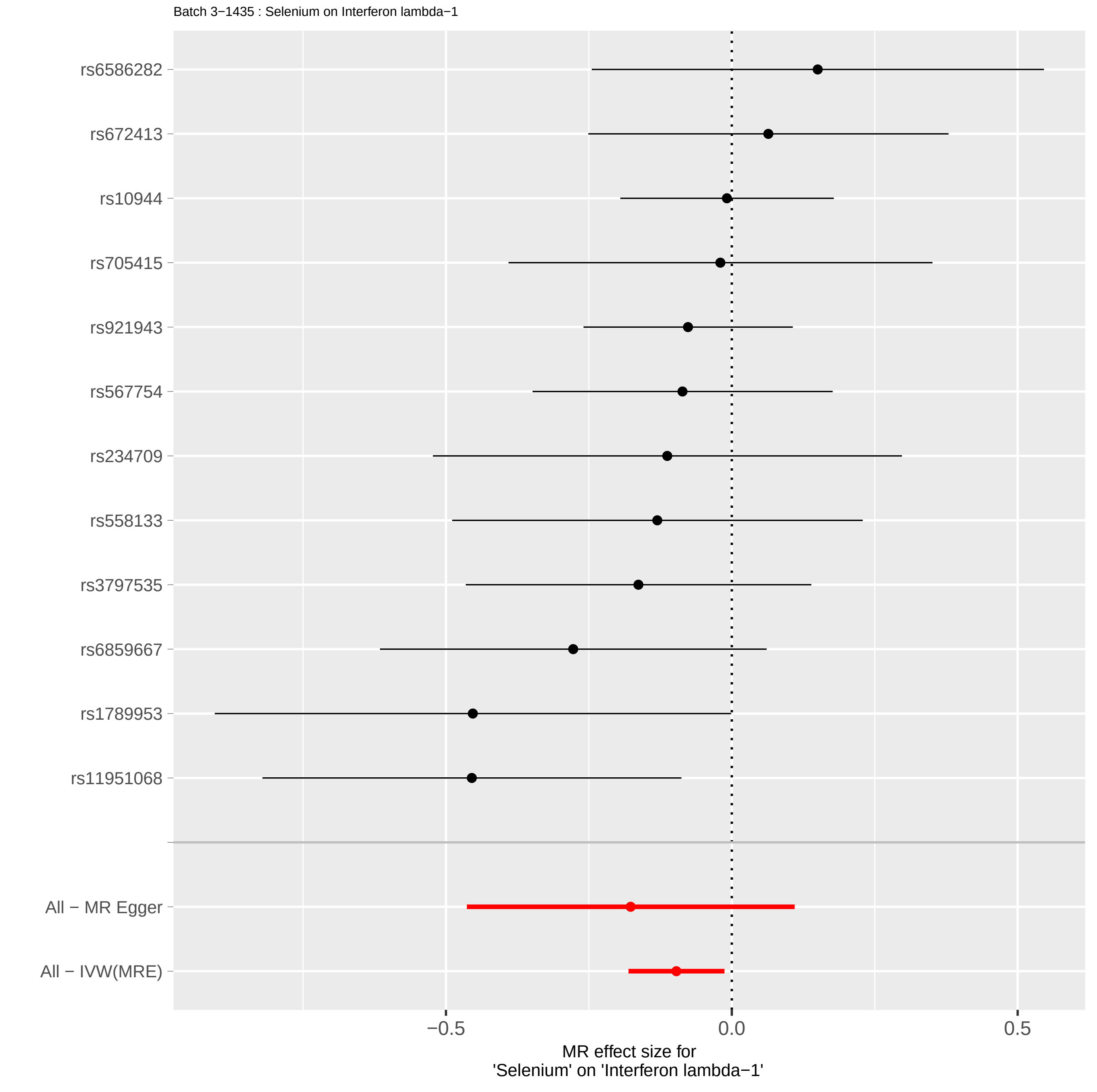

Batch 3−1435 : Selenium on Interferon lambda−1
rs6586282
rs672413
rs10944
rs705415
rs921943
rs567754
rs234709
rs558133
rs3797535
rs6859667
rs1789953
rs11951068
All − MR Egger
All − IVW(MRE)
−0.5
0.0
0.5
MR effect size for
'Selenium' on 'Interferon lambda−1'

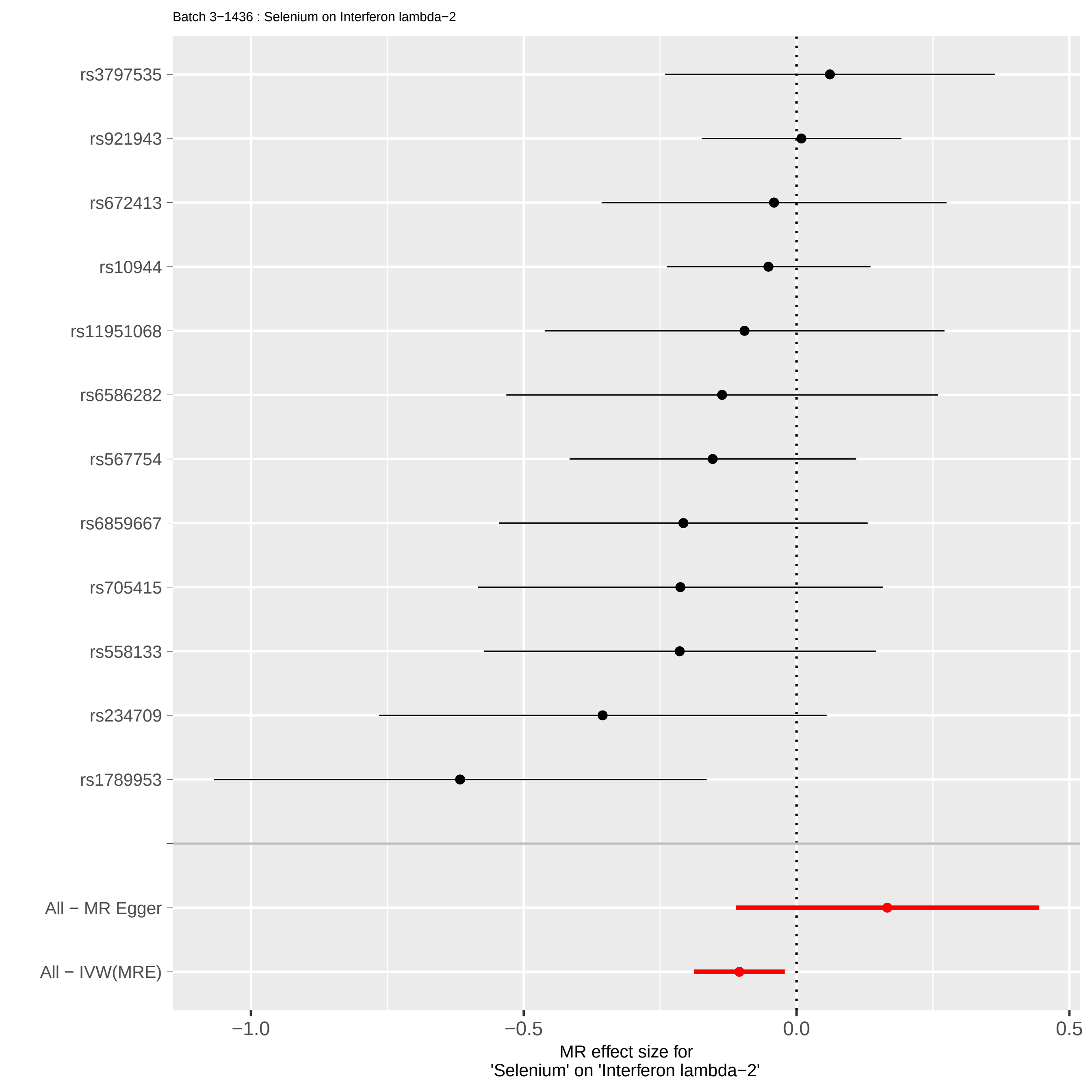

Batch 3−1436 : Selenium on Interferon lambda−2
rs3797535
rs921943
rs672413
rs10944
rs11951068
rs6586282
rs567754
rs6859667
rs705415
rs558133
rs234709
rs1789953
All − MR Egger
All − IVW(MRE)
−1.0
−0.5
0.0
0.5
MR effect size for
'Selenium' on 'Interferon lambda−2'

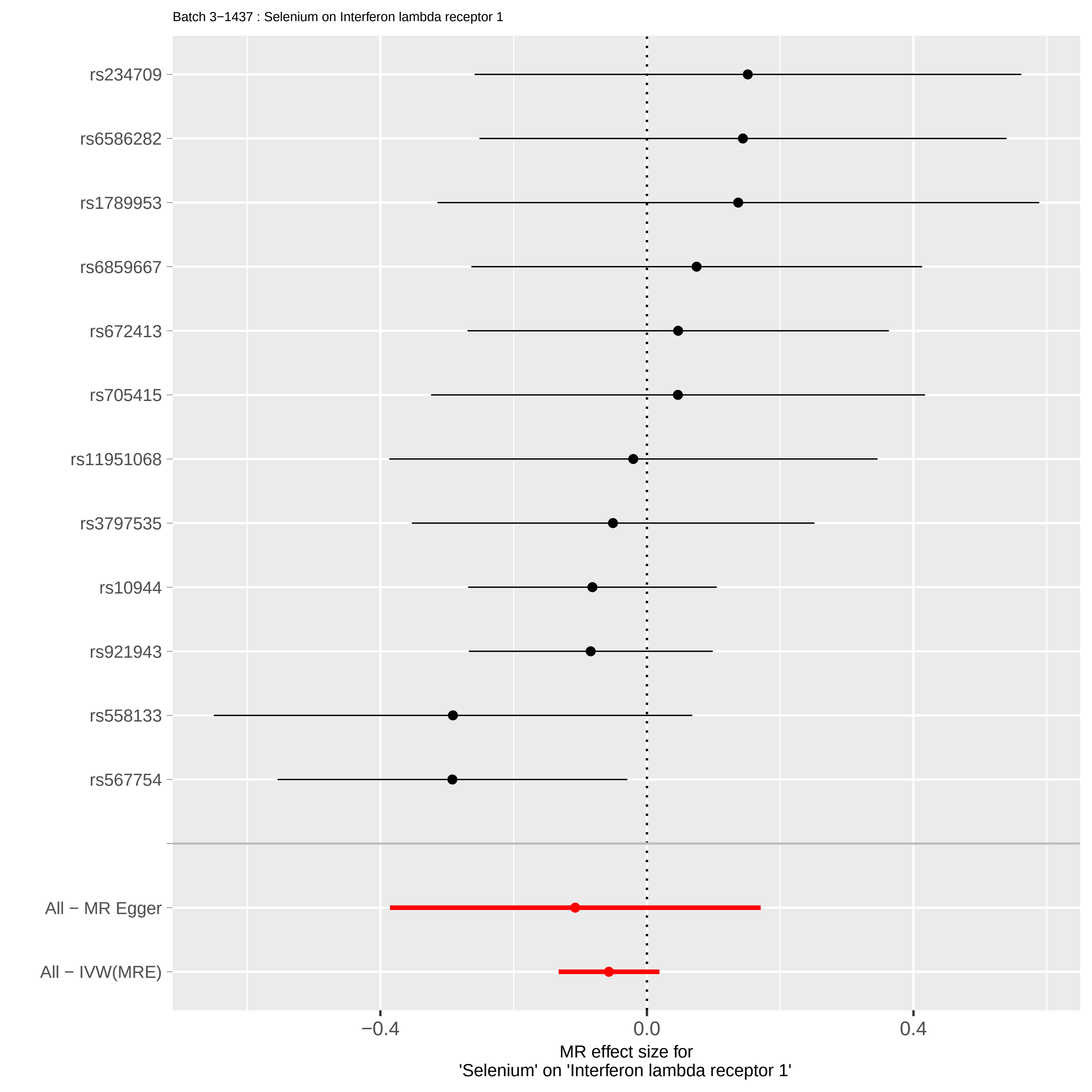

Batch 3−1437 : Selenium on Interferon lambda receptor 1
rs234709
rs6586282
rs1789953
rs6859667
rs672413
rs705415
rs11951068
rs3797535
rs10944
rs921943
rs558133
rs567754
All − MR Egger
All − IVW(MRE)
−0.4
0.0
0.4
MR effect size for
'Selenium' on 'Interferon lambda receptor 1'

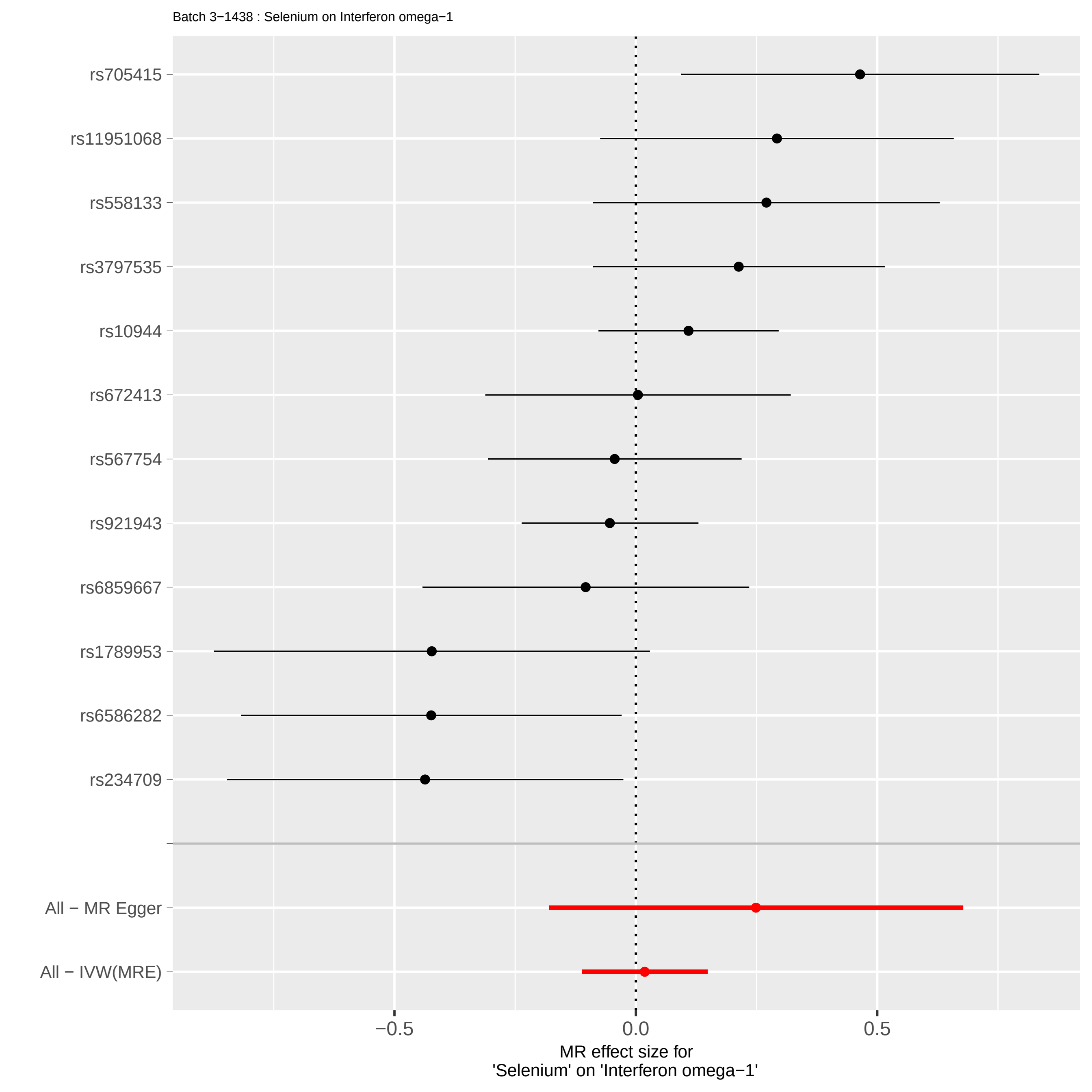
Batch 3−1438 : Selenium on Interferon omega−1
rs705415
rs11951068
rs558133
rs3797535
rs10944
rs672413
rs567754
rs921943
rs6859667
rs1789953
rs6586282
rs234709
All − MR Egger
All − IVW(MRE)
−0.5
0.0
0.5
MR effect size for
'Selenium' on 'Interferon omega−1'

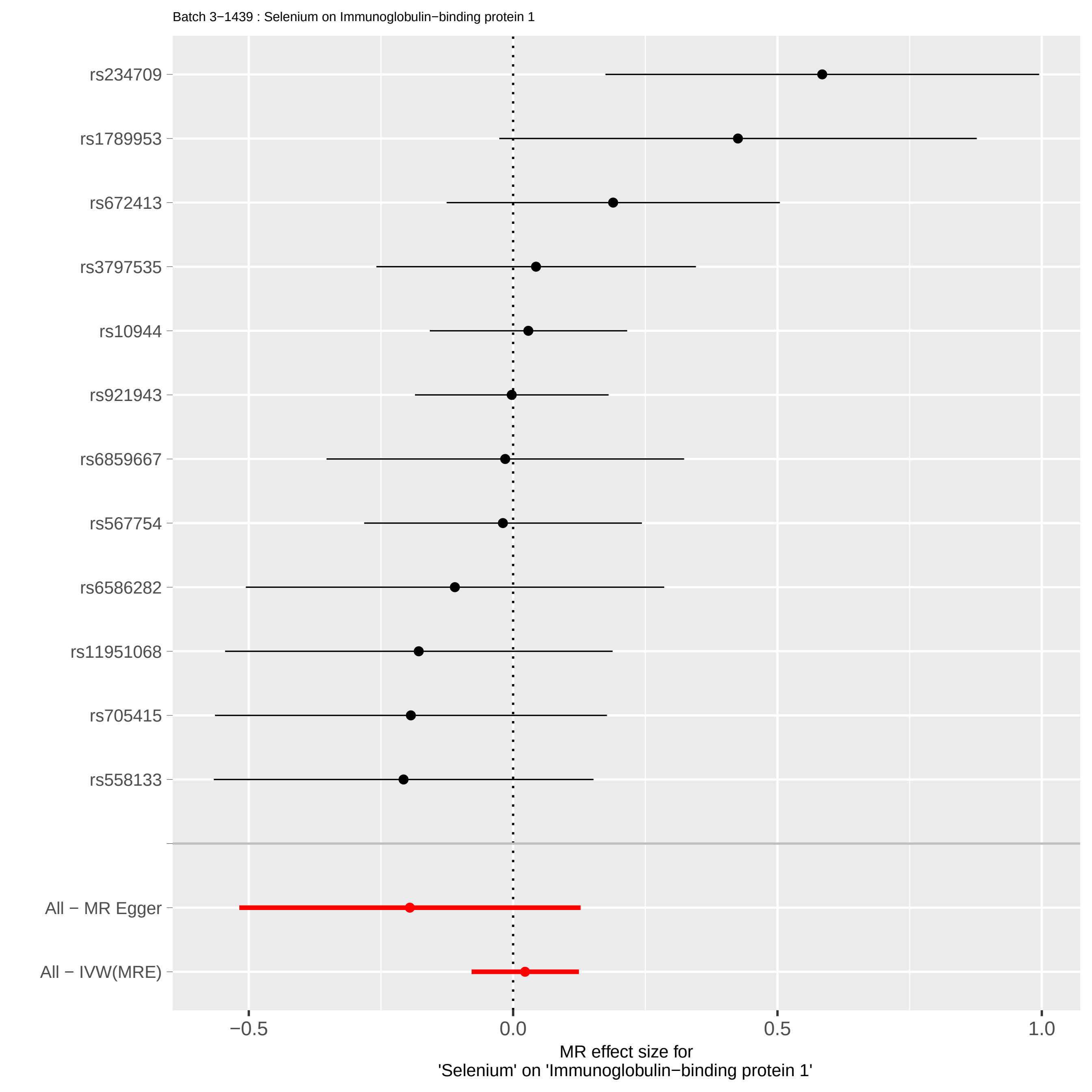

Batch 3−1439 : Selenium on Immunoglobulin−binding protein 1
rs234709
rs1789953
rs672413
rs3797535
rs10944
rs921943
rs6859667
rs567754
rs6586282
rs11951068
rs705415
rs558133
All − MR Egger
All − IVW(MRE)
−0.5
0.0
0.5
1.0
MR effect size for
'Selenium' on 'Immunoglobulin−binding protein 1'

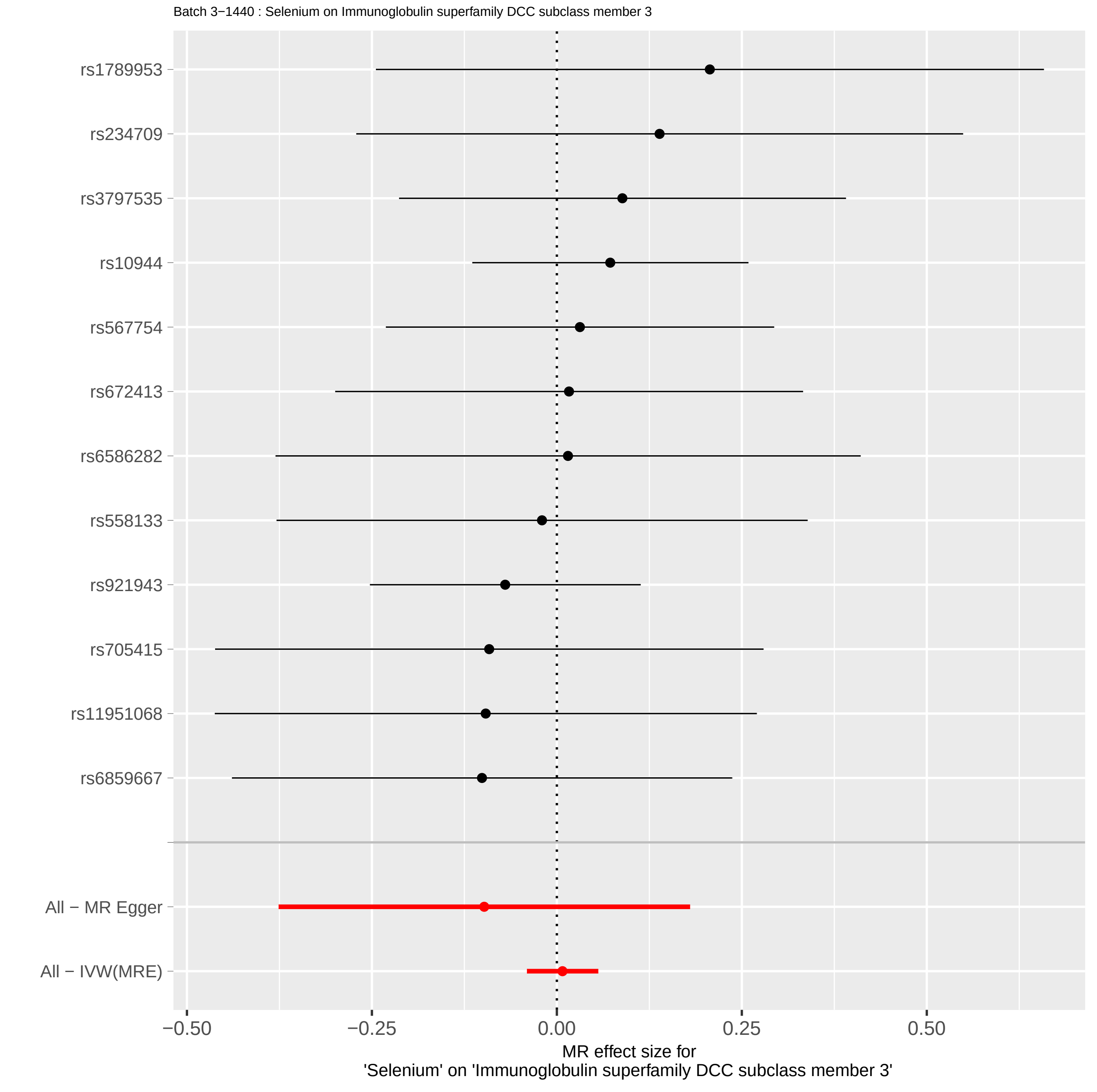
Batch 3−1440 : Selenium on Immunoglobulin superfamily DCC subclass member 3
rs1789953
rs234709
rs3797535
rs10944
rs567754
rs672413
rs6586282
rs558133
rs921943
rs705415
rs11951068
rs6859667
All − MR Egger
All − IVW(MRE)
−0.50
−0.25
0.00
0.25
0.50
MR effect size for
'Selenium' on 'Immunoglobulin superfamily DCC subclass member 3'

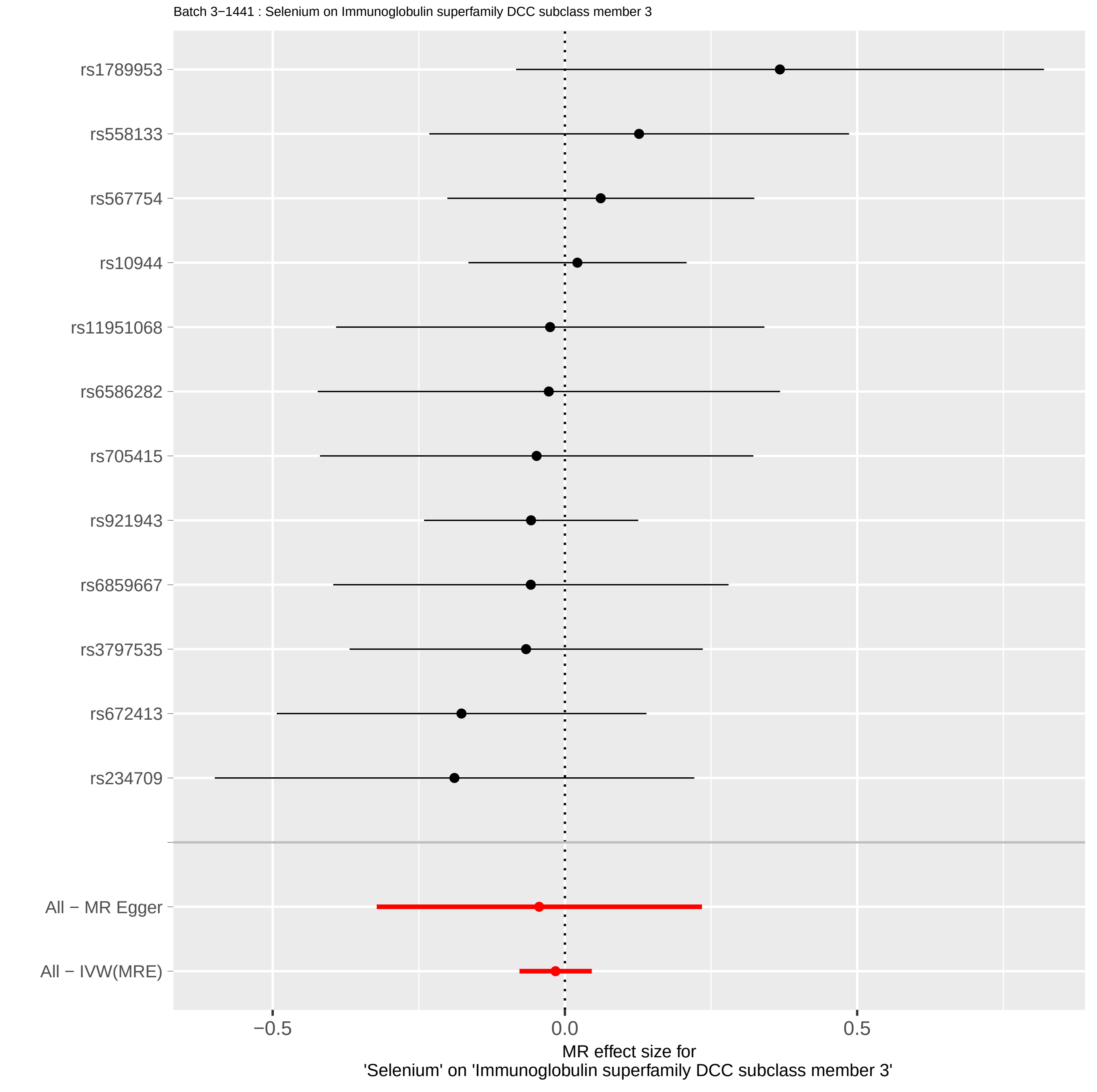

Batch 3−1441 : Selenium on Immunoglobulin superfamily DCC subclass member 3
rs1789953
rs558133
rs567754
rs10944
rs11951068
rs6586282
rs705415
rs921943
rs6859667
rs3797535
rs672413
rs234709
All − MR Egger
All − IVW(MRE)
−0.5
0.0
0.5
MR effect size for
'Selenium' on 'Immunoglobulin superfamily DCC subclass member 3'

Batch 3−1442 : Selenium on Immunoglobulin superfamily DCC subclass member 4

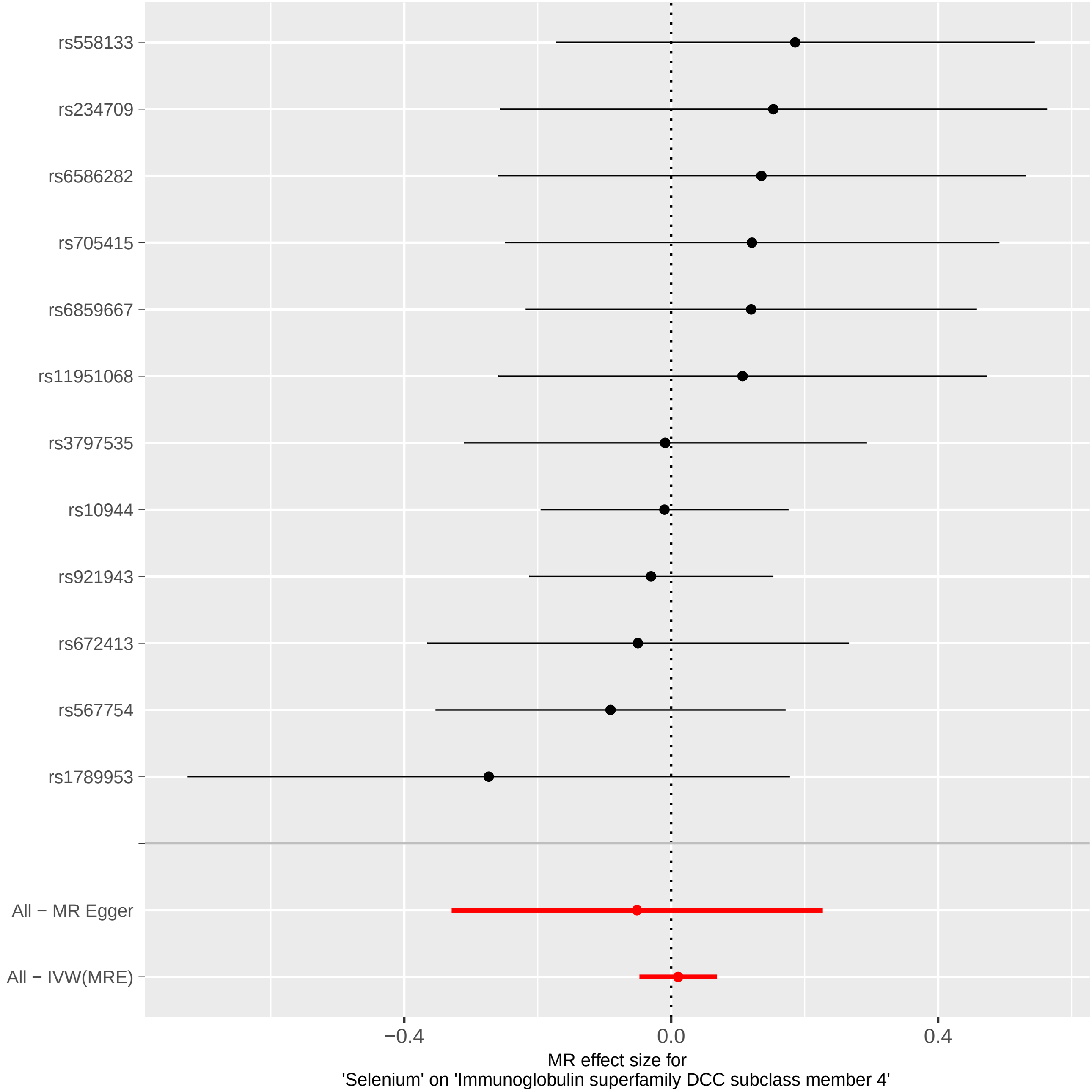

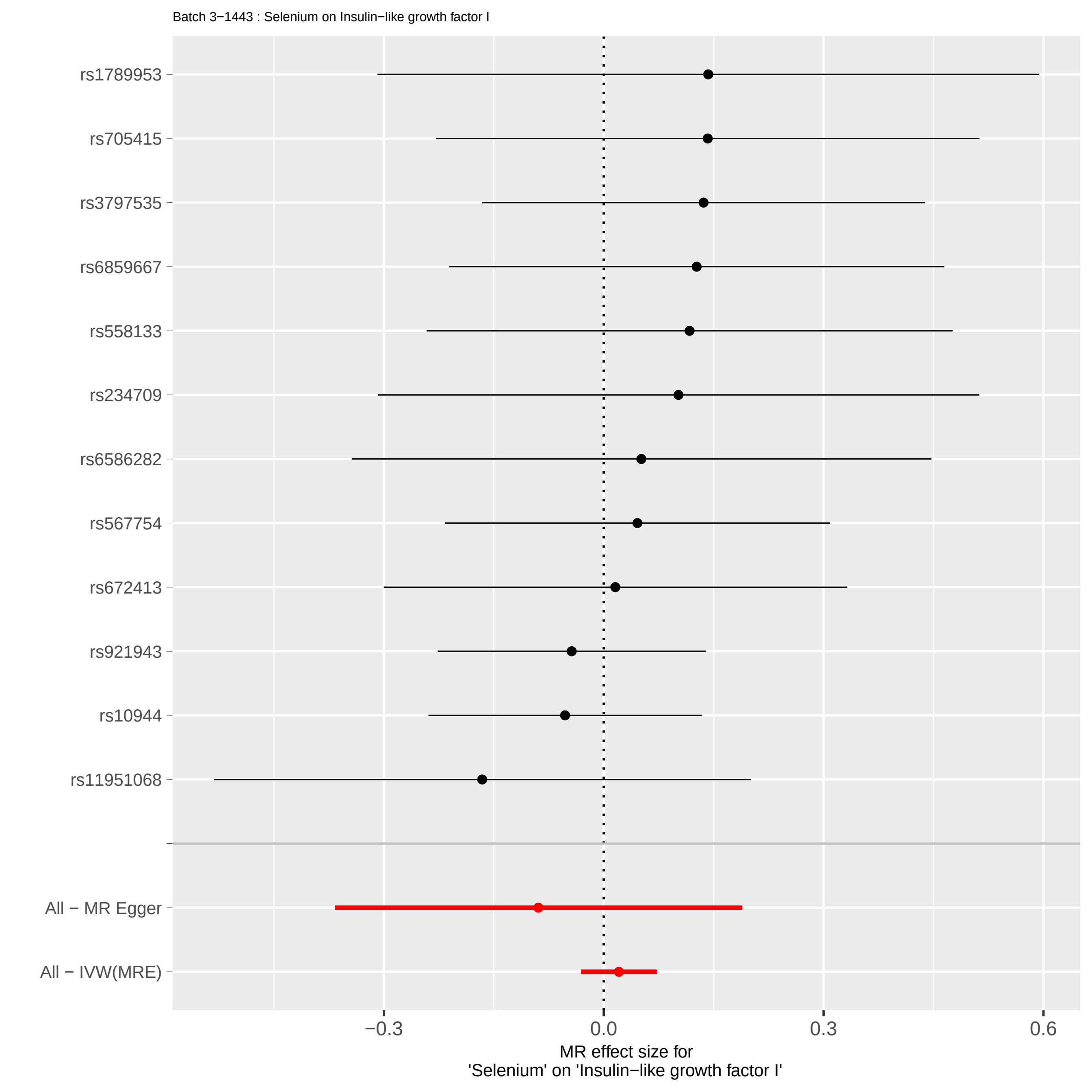

Batch 3−1443 : Selenium on Insulin−like growth factor I
rs1789953
rs705415
rs3797535
rs6859667
rs558133
rs234709
rs6586282
rs567754
rs672413
rs921943
rs10944
rs11951068
All − MR Egger
All − IVW(MRE)
−0.3
0.0
0.3
0.6
MR effect size for
'Selenium' on 'Insulin−like growth factor I'

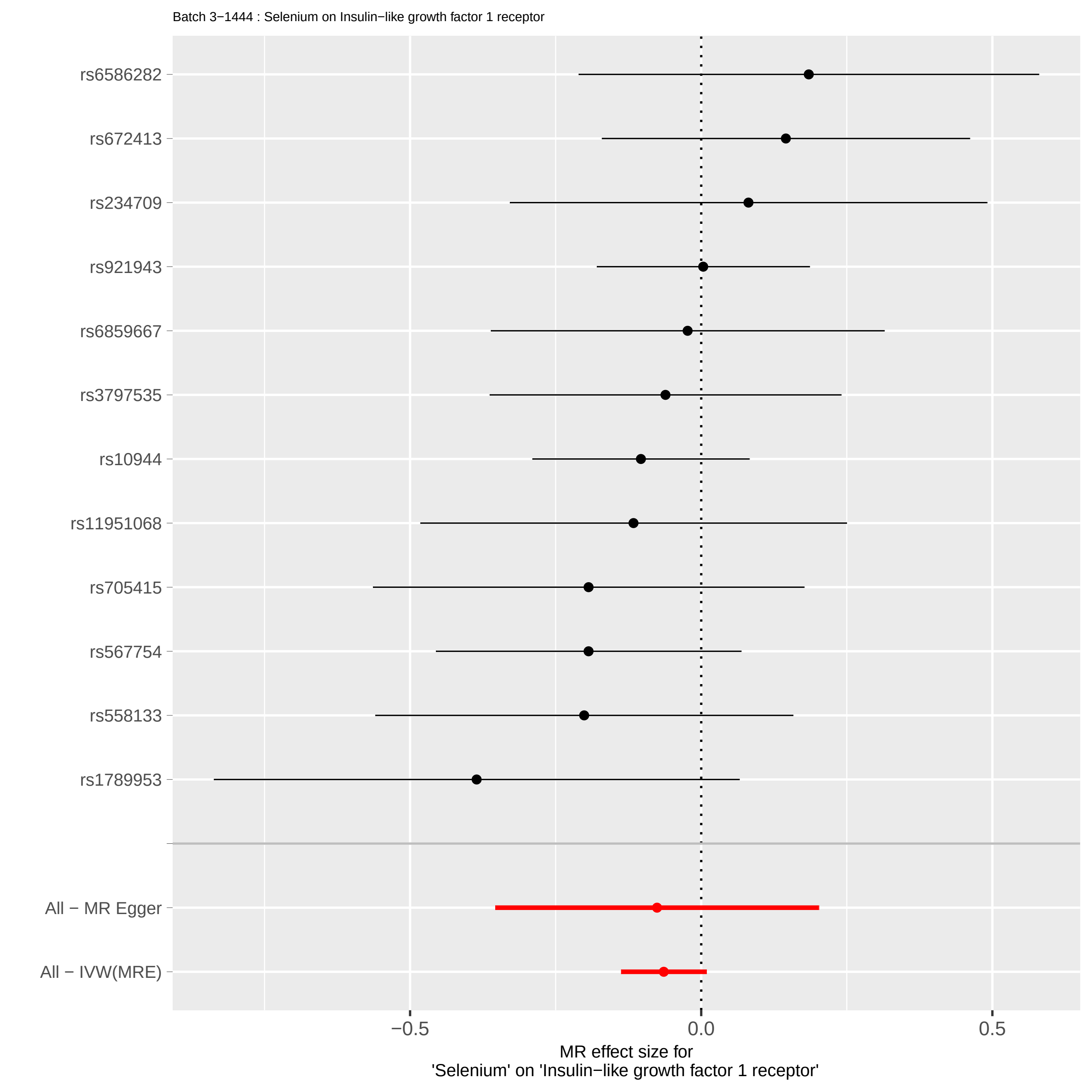

Batch 3−1444 : Selenium on Insulin−like growth factor 1 receptor
rs6586282
rs672413
rs234709
rs921943
rs6859667
rs3797535
rs10944
rs11951068
rs705415
rs567754
rs558133
rs1789953
All − MR Egger
All − IVW(MRE)
−0.5
0.0
0.5
MR effect size for
'Selenium' on 'Insulin−like growth factor 1 receptor'

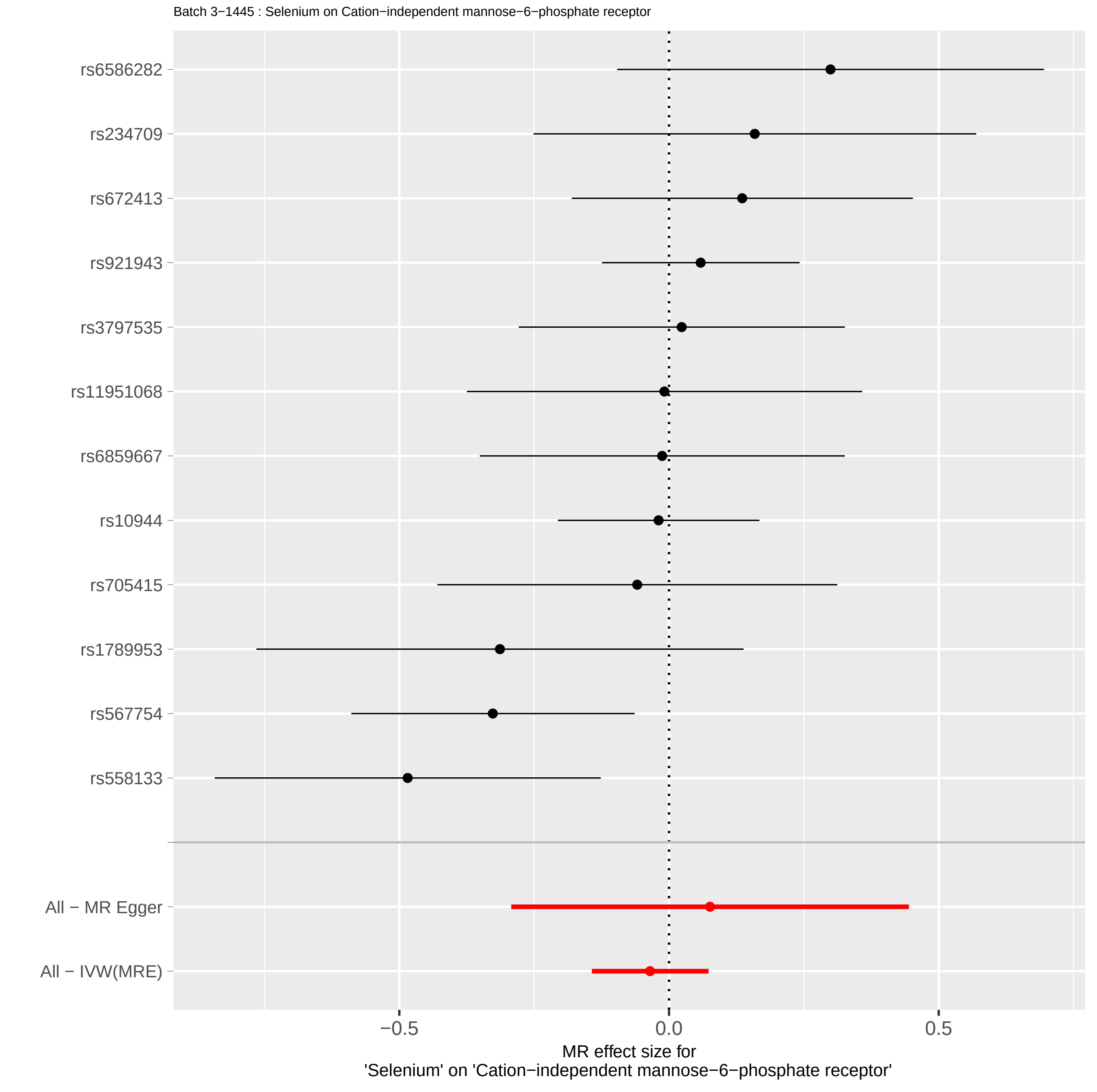

Batch 3−1445 : Selenium on Cation−independent mannose−6−phosphate receptor
rs6586282
rs234709
rs672413
rs921943
rs3797535
rs11951068
rs6859667
rs10944
rs705415
rs1789953
rs567754
rs558133
All − MR Egger
All − IVW(MRE)
−0.5
0.0
0.5
MR effect size for
'Selenium' on 'Cation−independent mannose−6−phosphate receptor'

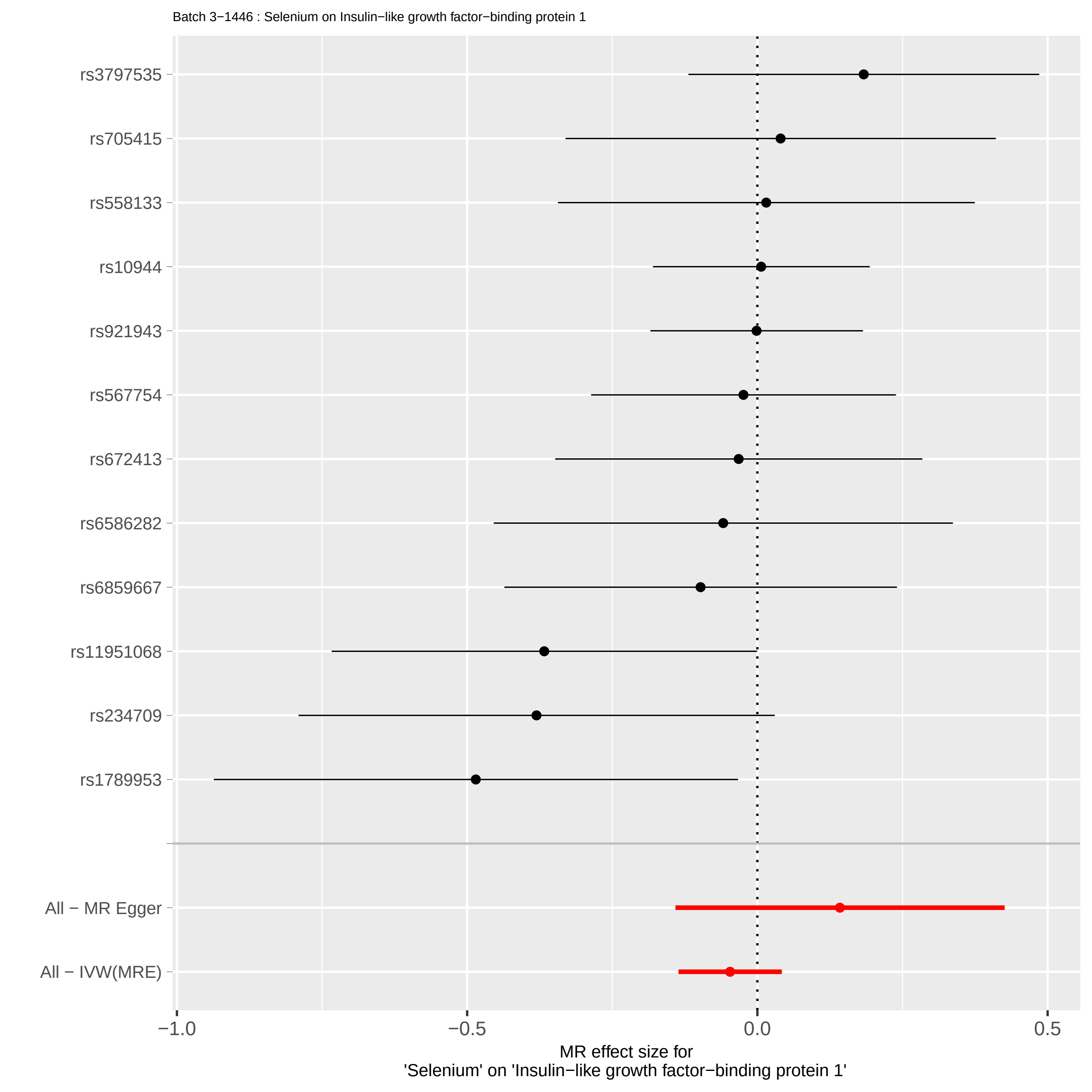

Batch 3−1446 : Selenium on Insulin−like growth factor−binding protein 1
rs3797535
rs705415
rs558133
rs10944
rs921943
rs567754
rs672413
rs6586282
rs6859667
rs11951068
rs234709
rs1789953
All − MR Egger
All − IVW(MRE)
−1.0
−0.5
0.0
0.5
MR effect size for
'Selenium' on 'Insulin−like growth factor−binding protein 1'

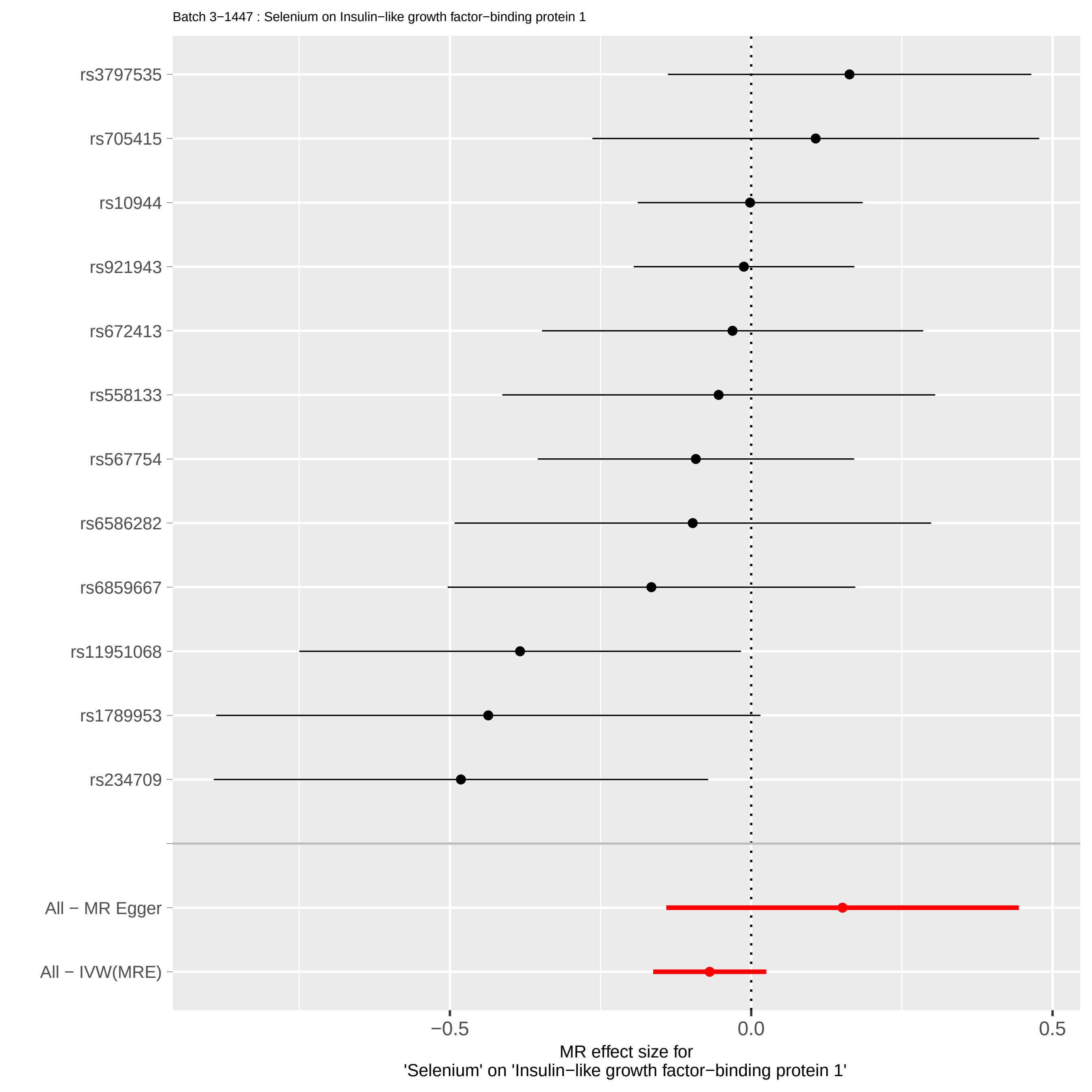
Batch 3−1447 : Selenium on Insulin−like growth factor−binding protein 1
rs3797535
rs705415
rs10944
rs921943
rs672413
rs558133
rs567754
rs6586282
rs6859667
rs11951068
rs1789953
rs234709
All − MR Egger
All − IVW(MRE)
−0.5
0.0
0.5
MR effect size for
'Selenium' on 'Insulin−like growth factor−binding protein 1'

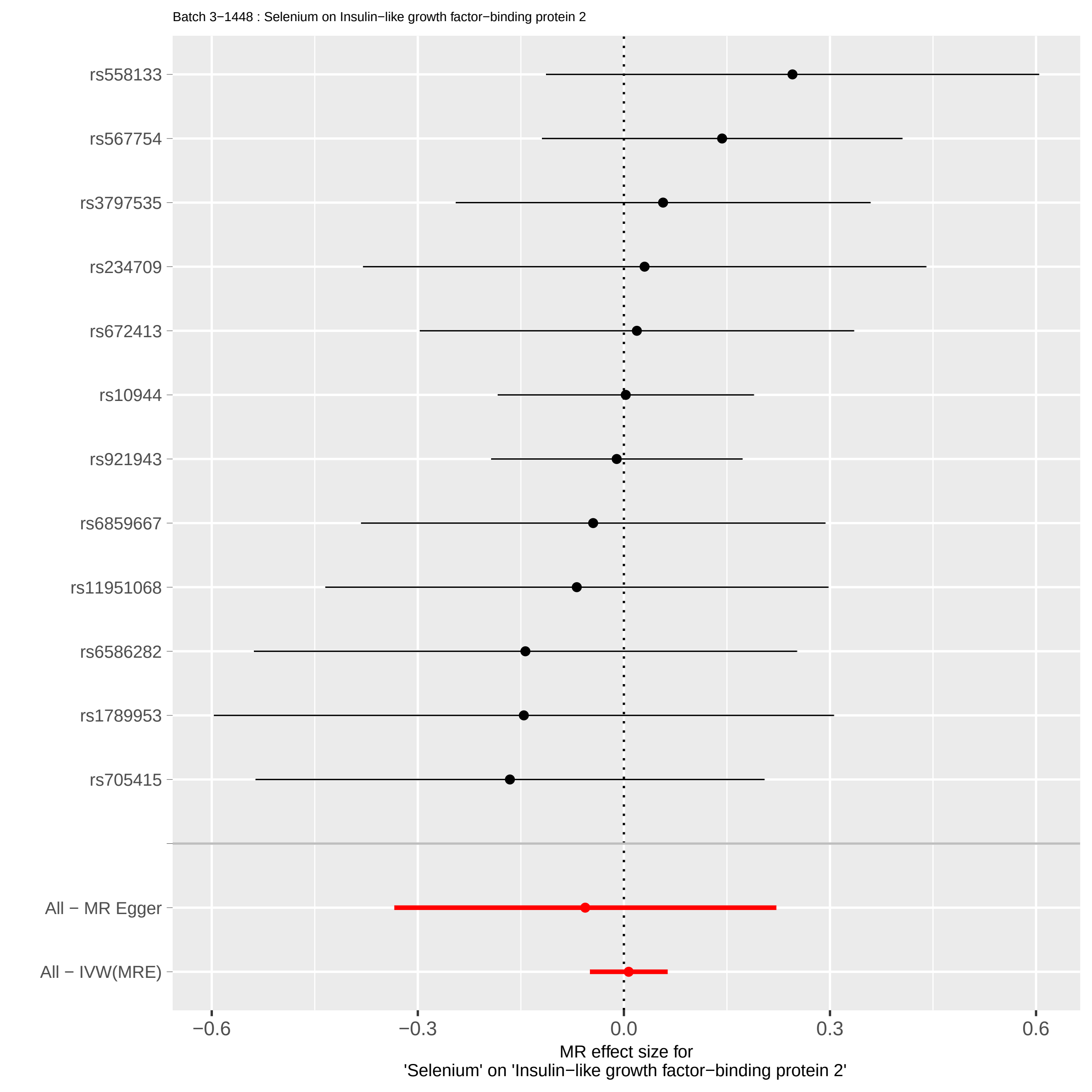
Batch 3−1448 : Selenium on Insulin−like growth factor−binding protein 2
rs558133
rs567754
rs3797535
rs234709
rs672413
rs10944
rs921943
rs6859667
rs11951068
rs6586282
rs1789953
rs705415
All − MR Egger
All − IVW(MRE)
−0.6
−0.3
0.0
0.3
0.6
MR effect size for
'Selenium' on 'Insulin−like growth factor−binding protein 2'

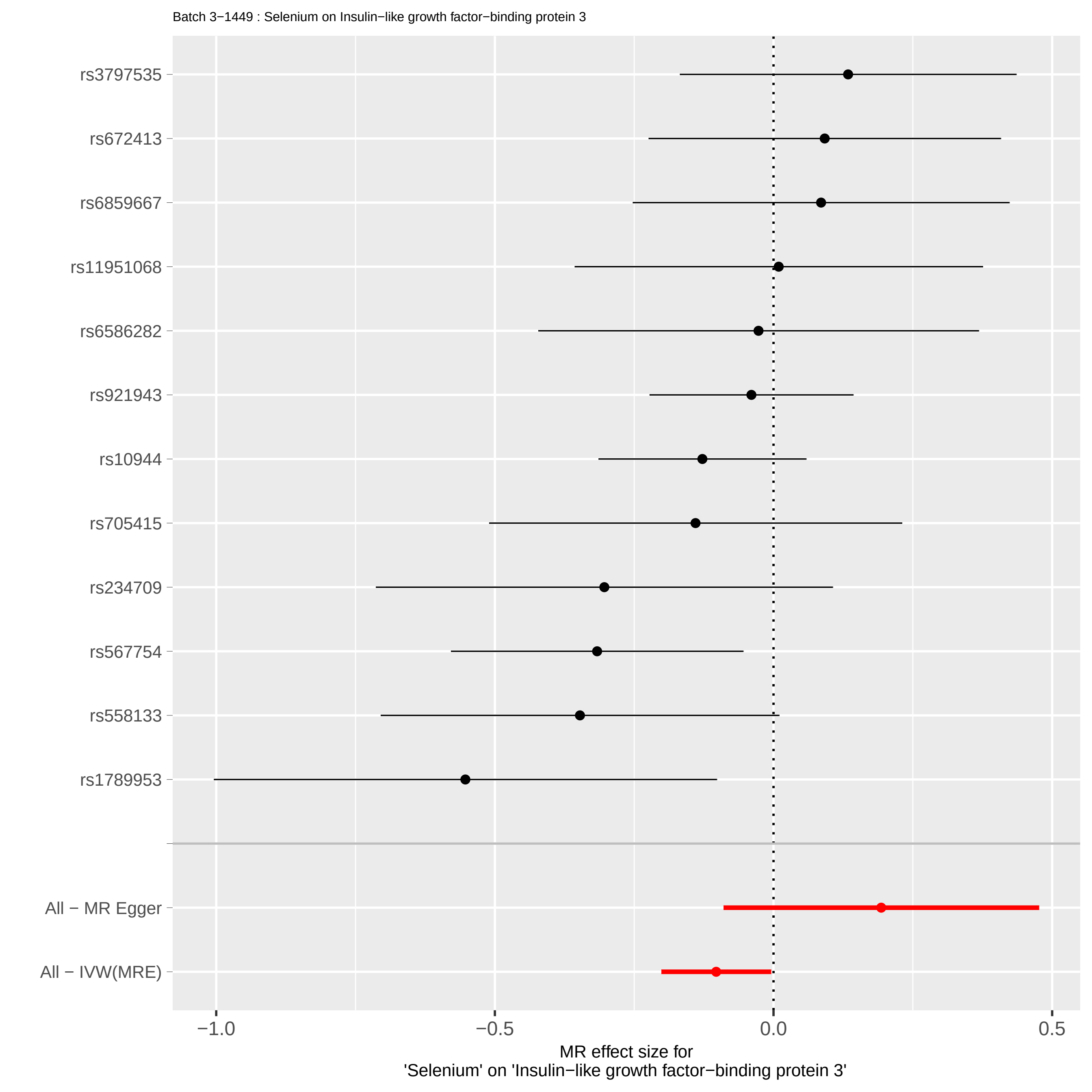
Batch 3−1449 : Selenium on Insulin−like growth factor−binding protein 3
rs3797535
rs672413
rs6859667
rs11951068
rs6586282
rs921943
rs10944
rs705415
rs234709
rs567754
rs558133
rs1789953
All − MR Egger
All − IVW(MRE)
−1.0
−0.5
0.0
0.5
MR effect size for
'Selenium' on 'Insulin−like growth factor−binding protein 3'

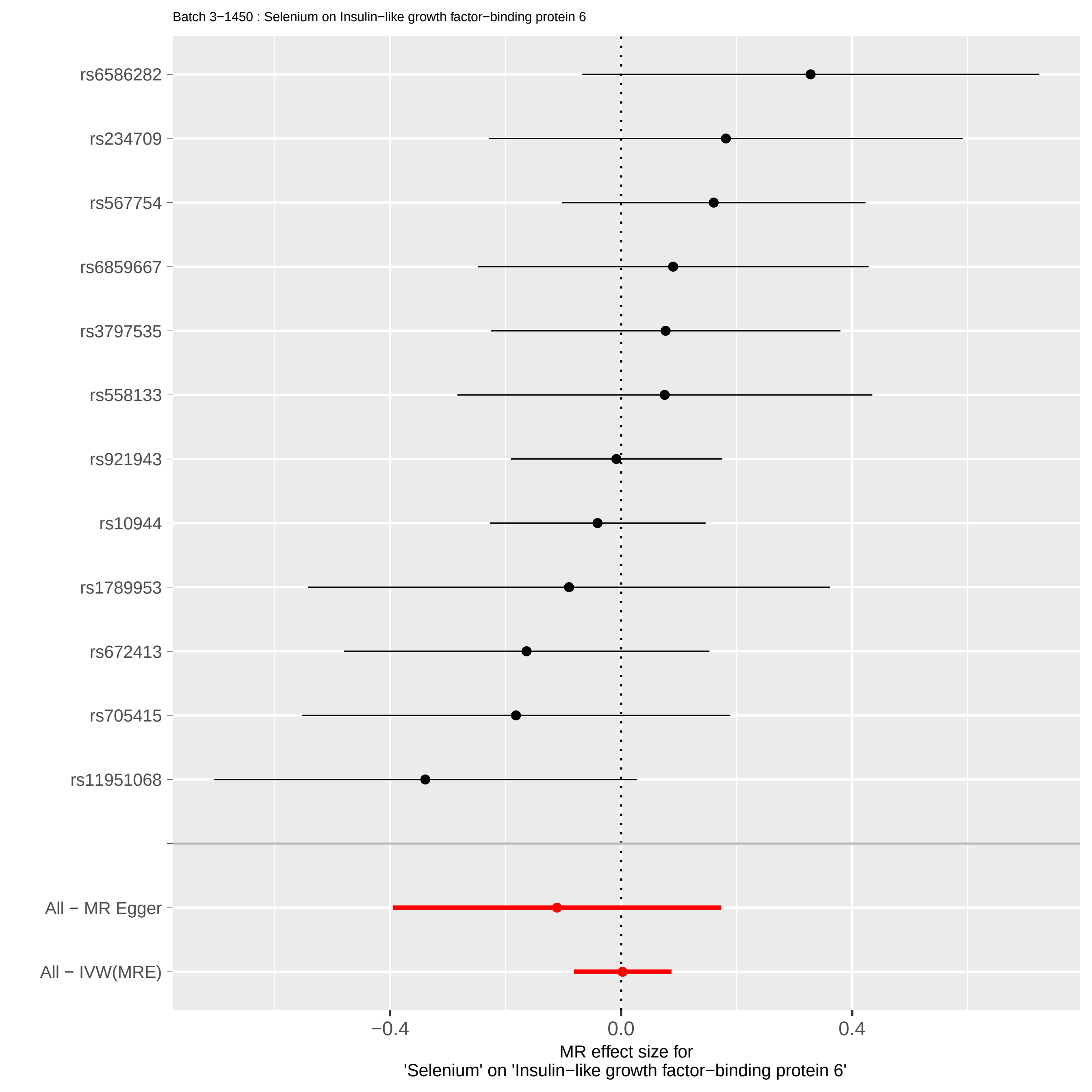

Batch 3−1450 : Selenium on Insulin−like growth factor−binding protein 6
rs6586282
rs234709
rs567754
rs6859667
rs3797535
rs558133
rs921943
rs10944
rs1789953
rs672413
rs705415
rs11951068
All − MR Egger
All − IVW(MRE)
−0.4
0.0
0.4
MR effect size for
'Selenium' on 'Insulin−like growth factor−binding protein 6'

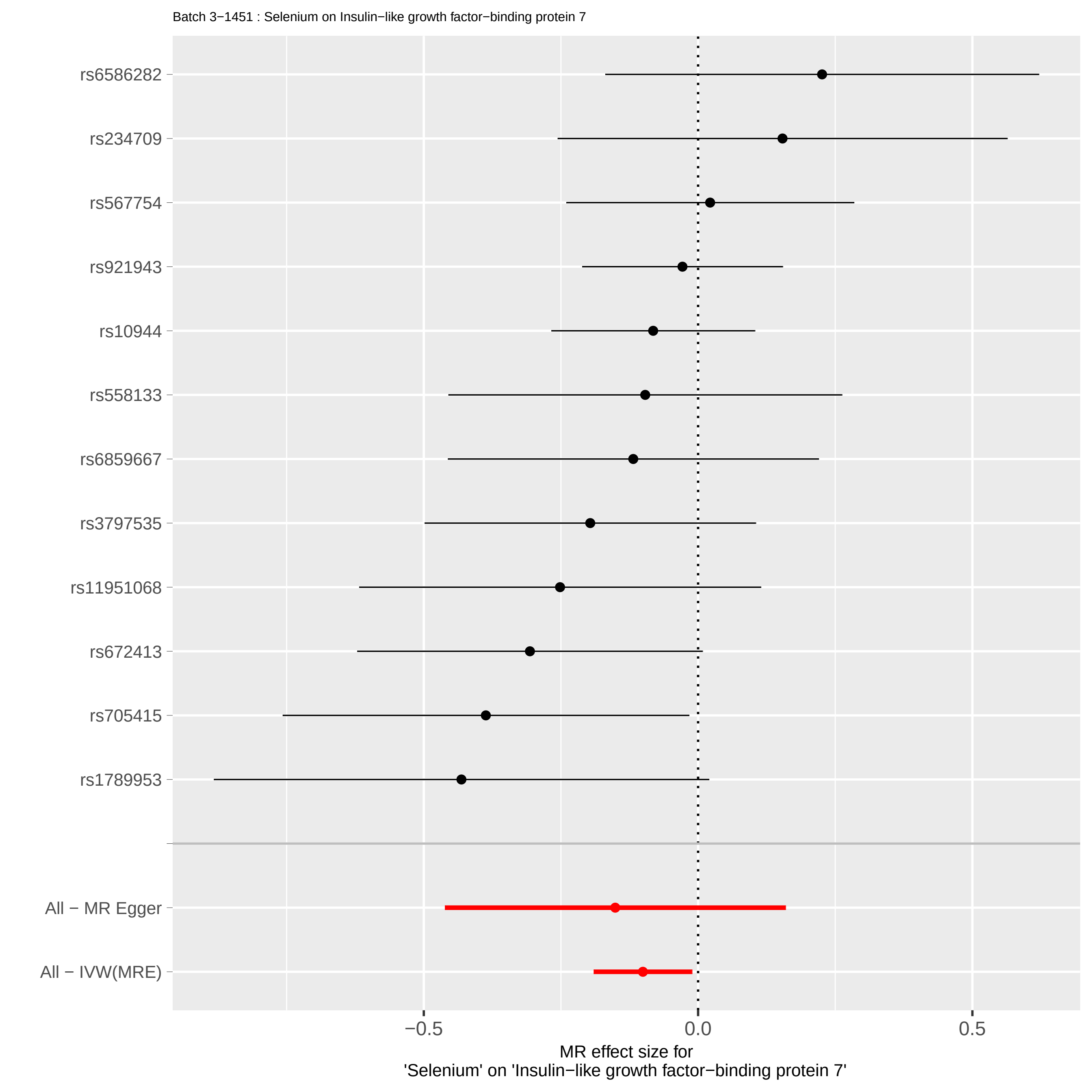
Batch 3−1451 : Selenium on Insulin−like growth factor−binding protein 7
rs6586282
rs234709
rs567754
rs921943
rs10944
rs558133
rs6859667
rs3797535
rs11951068
rs672413
rs705415
rs1789953
All − MR Egger
All − IVW(MRE)
−0.5
0.0
0.5
MR effect size for
'Selenium' on 'Insulin−like growth factor−binding protein 7'

Batch 3−1452 : Selenium on Insulin−like growth factor−binding protein−like 1

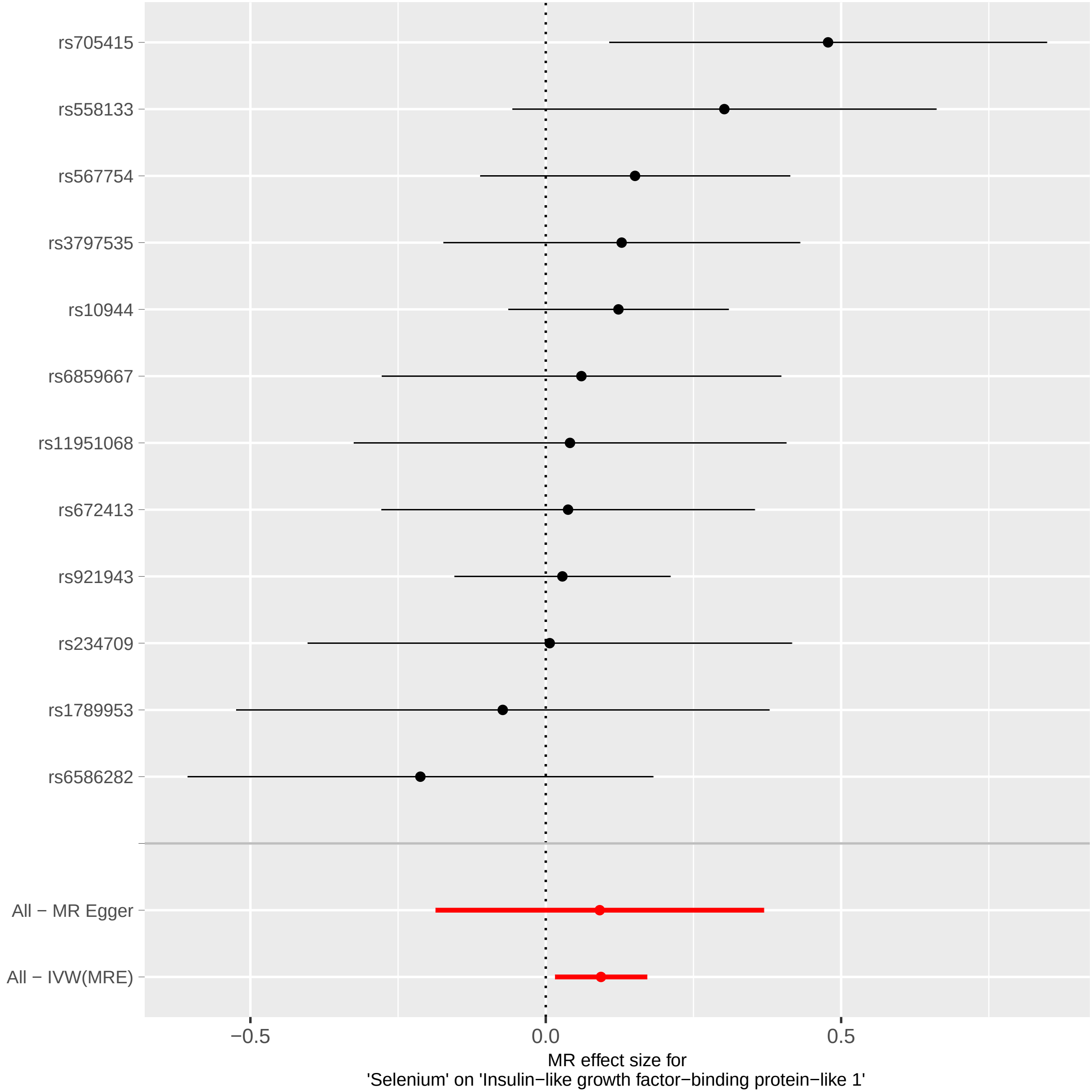

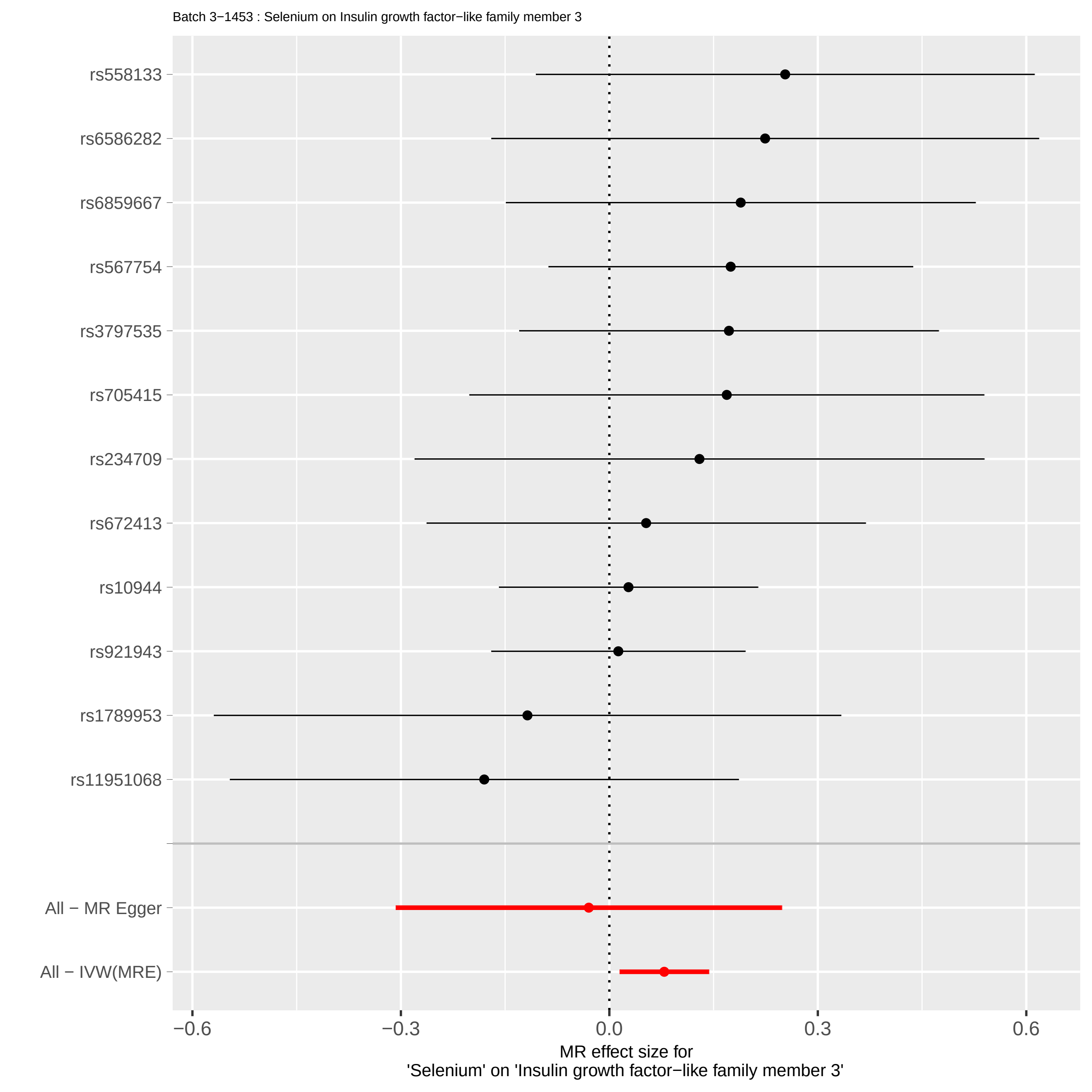

Batch 3−1453 : Selenium on Insulin growth factor−like family member 3
rs558133
rs6586282
rs6859667
rs567754
rs3797535
rs705415
rs234709
rs672413
rs10944
rs921943
rs1789953
rs11951068
All − MR Egger
All − IVW(MRE)
−0.6
−0.3
0.0
0.3
0.6
MR effect size for
'Selenium' on 'Insulin growth factor−like family member 3'

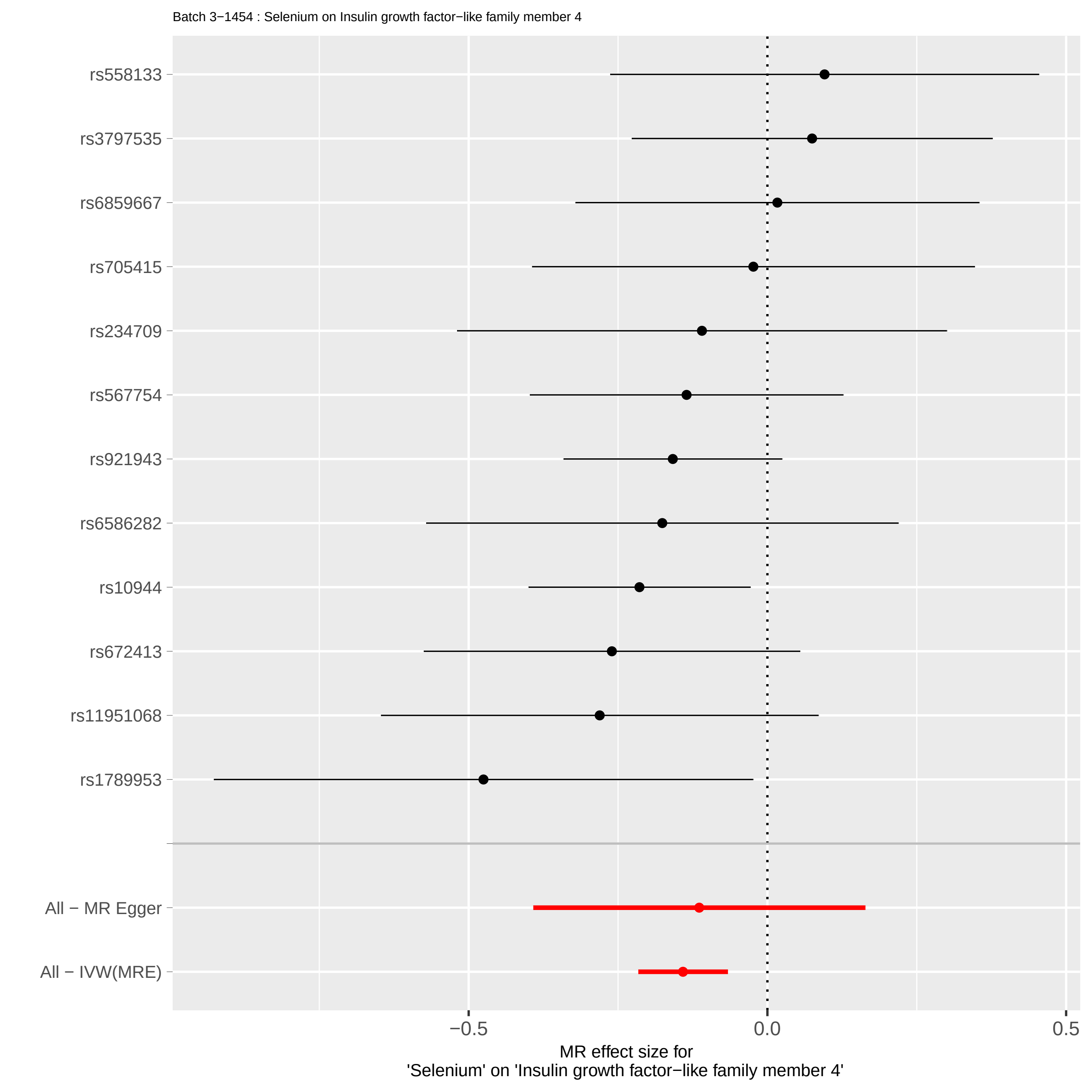

Batch 3−1454 : Selenium on Insulin growth factor−like family member 4
rs558133
rs3797535
rs6859667
rs705415
rs234709
rs567754
rs921943
rs6586282
rs10944
rs672413
rs11951068
rs1789953
All − MR Egger
All − IVW(MRE)
−0.5
0.0
0.5
MR effect size for
'Selenium' on 'Insulin growth factor−like family member 4'

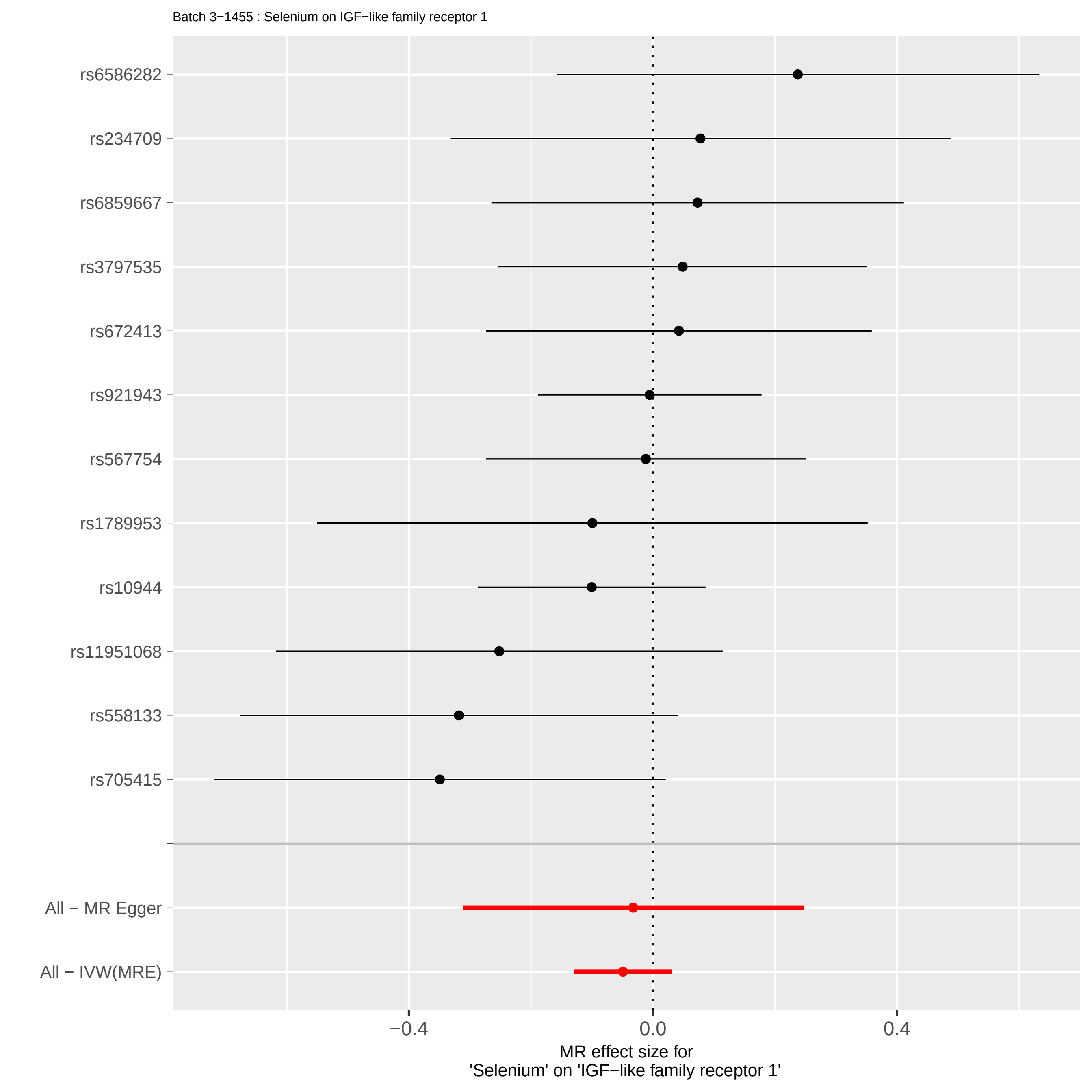

Batch 3−1455 : Selenium on IGF−like family receptor 1
rs6586282
rs234709
rs6859667
rs3797535
rs672413
rs921943
rs567754
rs1789953
rs10944
rs11951068
rs558133
rs705415
All − MR Egger
All − IVW(MRE)
−0.4
0.0
0.4
MR effect size for
'Selenium' on 'IGF−like family receptor 1'

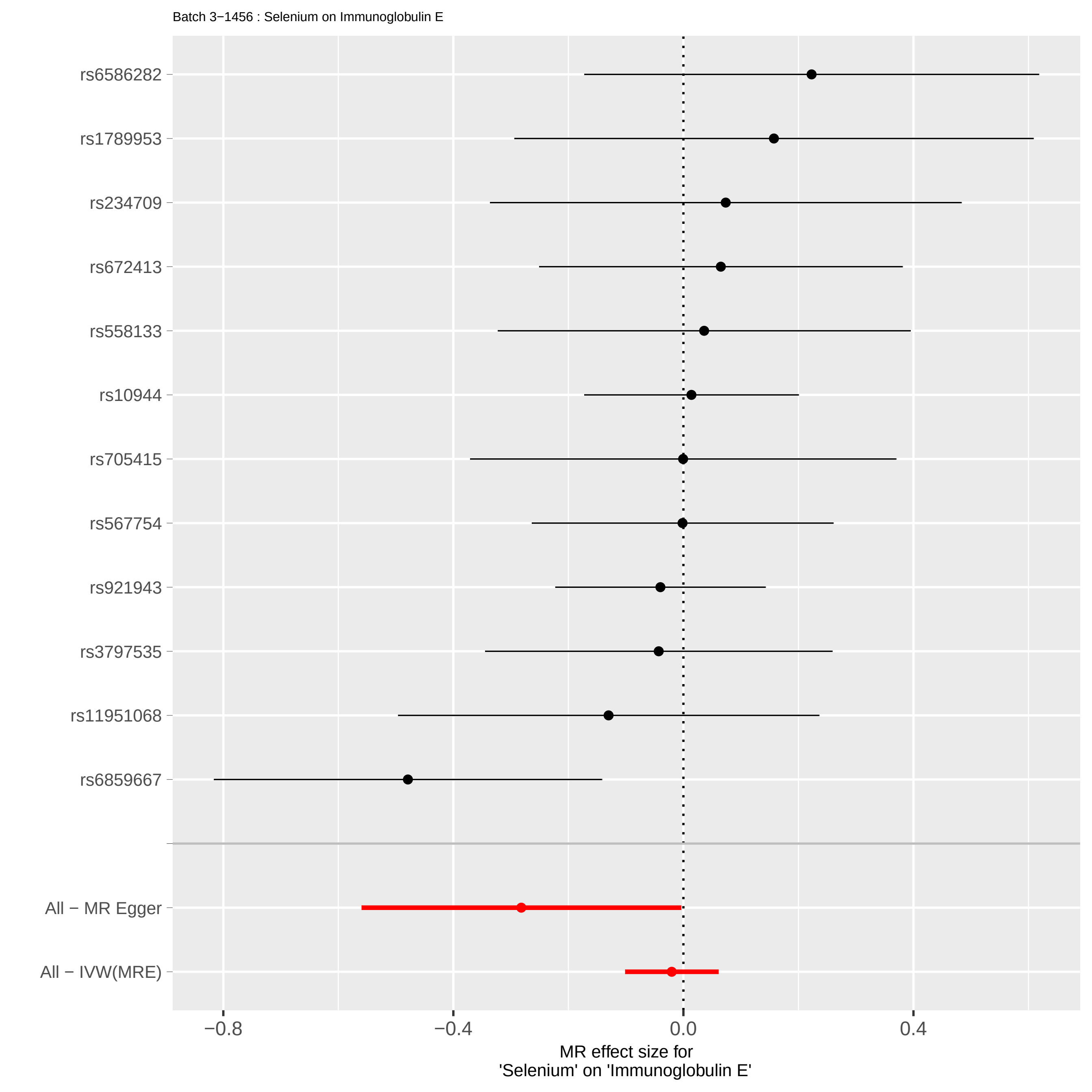
Batch 3−1456 : Selenium on Immunoglobulin E
rs6586282
rs1789953
rs234709
rs672413
rs558133
rs10944
rs705415
rs567754
rs921943
rs3797535
rs11951068
rs6859667
All − MR Egger
All − IVW(MRE)
−0.8
−0.4
0.0
0.4
MR effect size for
'Selenium' on 'Immunoglobulin E'

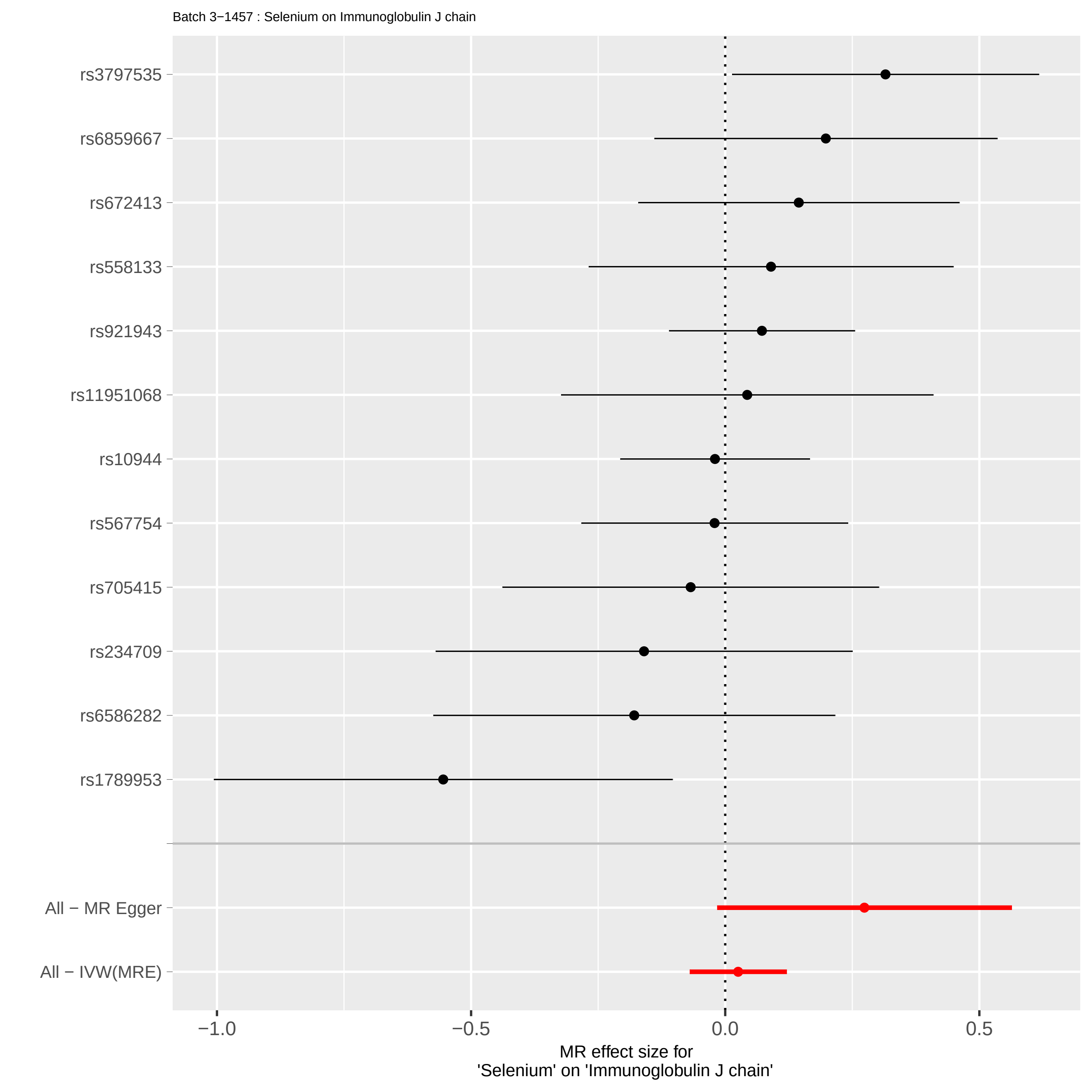
Batch 3−1457 : Selenium on Immunoglobulin J chain
rs3797535
rs6859667
rs672413
rs558133
rs921943
rs11951068
rs10944
rs567754
rs705415
rs234709
rs6586282
rs1789953
All − MR Egger
All − IVW(MRE)
−1.0
−0.5
0.0
0.5
MR effect size for
'Selenium' on 'Immunoglobulin J chain'

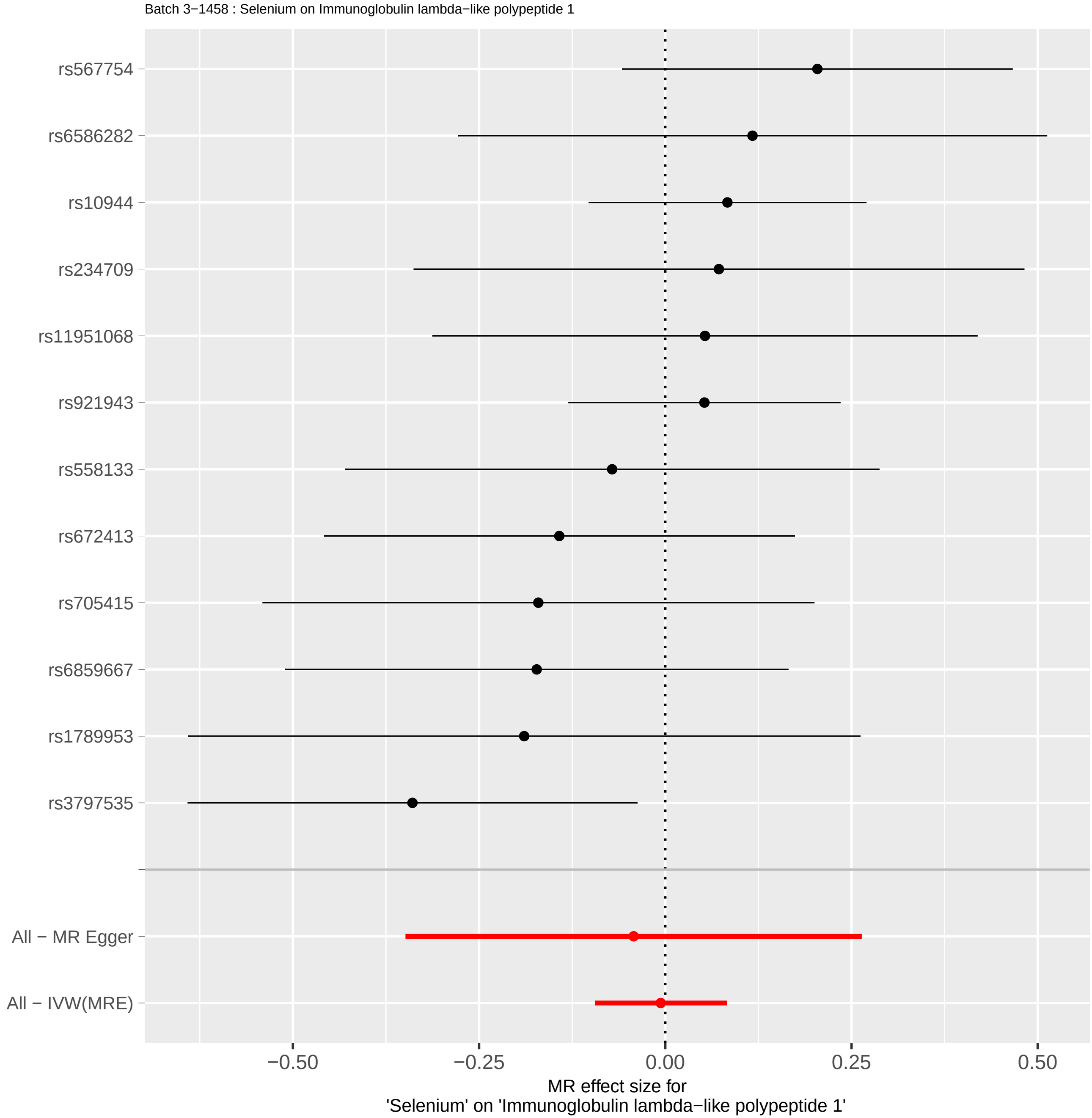

Batch 3−1458 : Selenium on Immunoglobulin lambda−like polypeptide 1
rs567754
rs6586282
rs10944
rs234709
rs11951068
rs921943
rs558133
rs672413
rs705415
rs6859667
rs1789953
rs3797535
All − MR Egger
All − IVW(MRE)
−0.50
−0.25
0.00
0.25
0.50
MR effect size for
'Selenium' on 'Immunoglobulin lambda−like polypeptide 1'

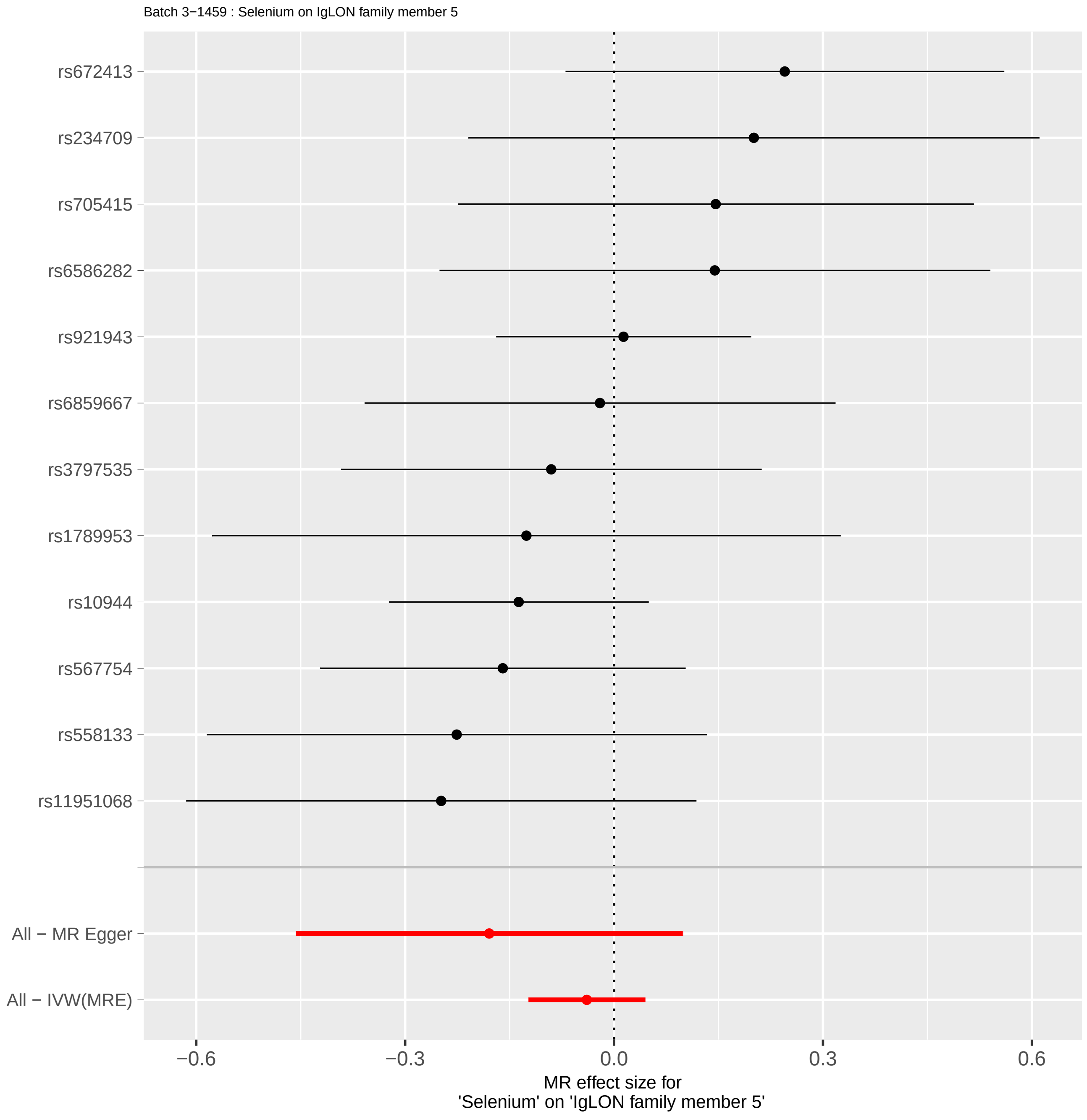

Batch 3−1459 : Selenium on IgLON family member 5
rs672413
rs234709
rs705415
rs6586282
rs921943
rs6859667
rs3797535
rs1789953
rs10944
rs567754
rs558133
rs11951068
All − MR Egger
All − IVW(MRE)
−0.6
−0.3
0.0
0.3
0.6
MR effect size for
'Selenium' on 'IgLON family member 5'

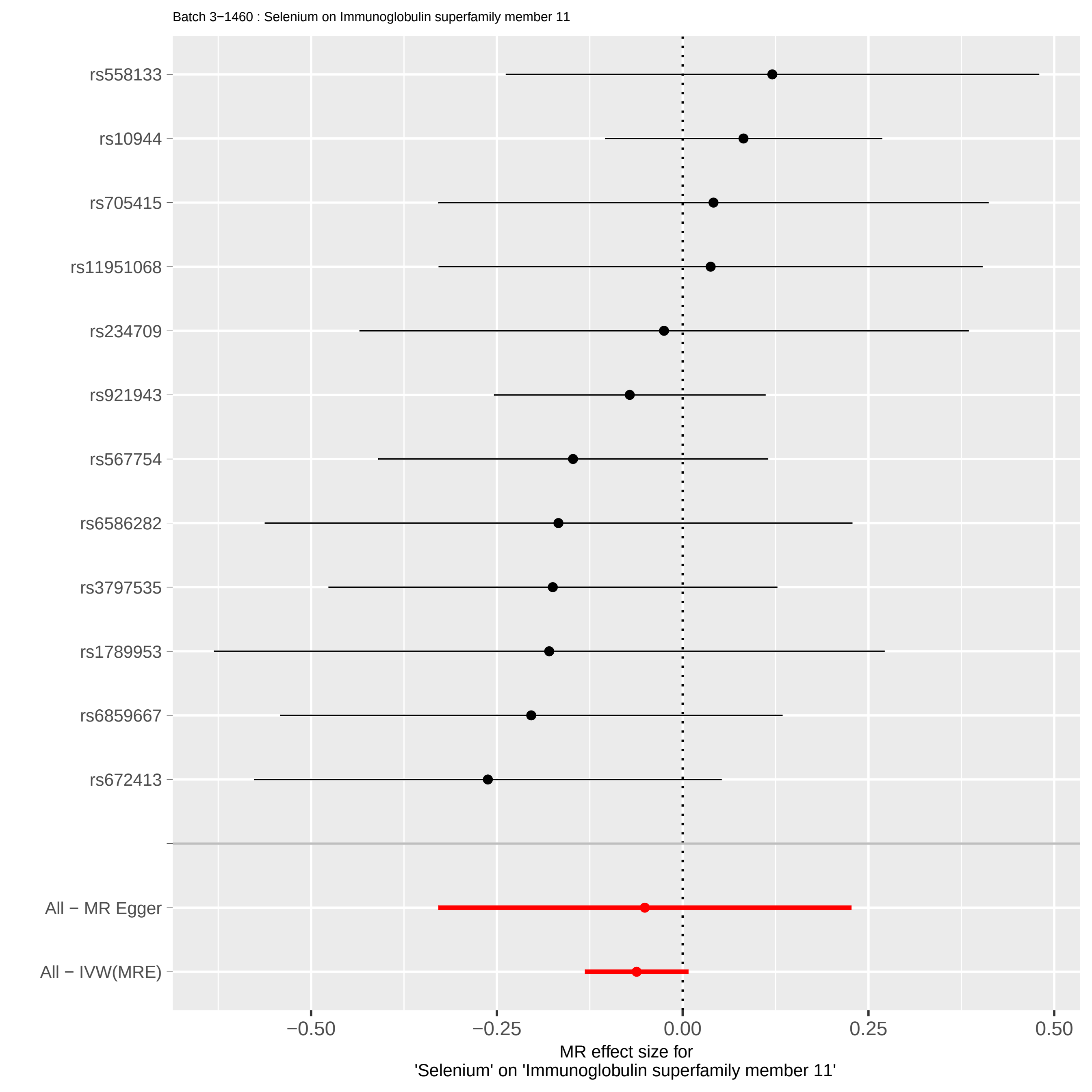
Batch 3−1460 : Selenium on Immunoglobulin superfamily member 11
rs558133
rs10944
rs705415
rs11951068
rs234709
rs921943
rs567754
rs6586282
rs3797535
rs1789953
rs6859667
rs672413
All − MR Egger
All − IVW(MRE)
−0.50
−0.25
0.00
0.25
0.50
MR effect size for
'Selenium' on 'Immunoglobulin superfamily member 11'

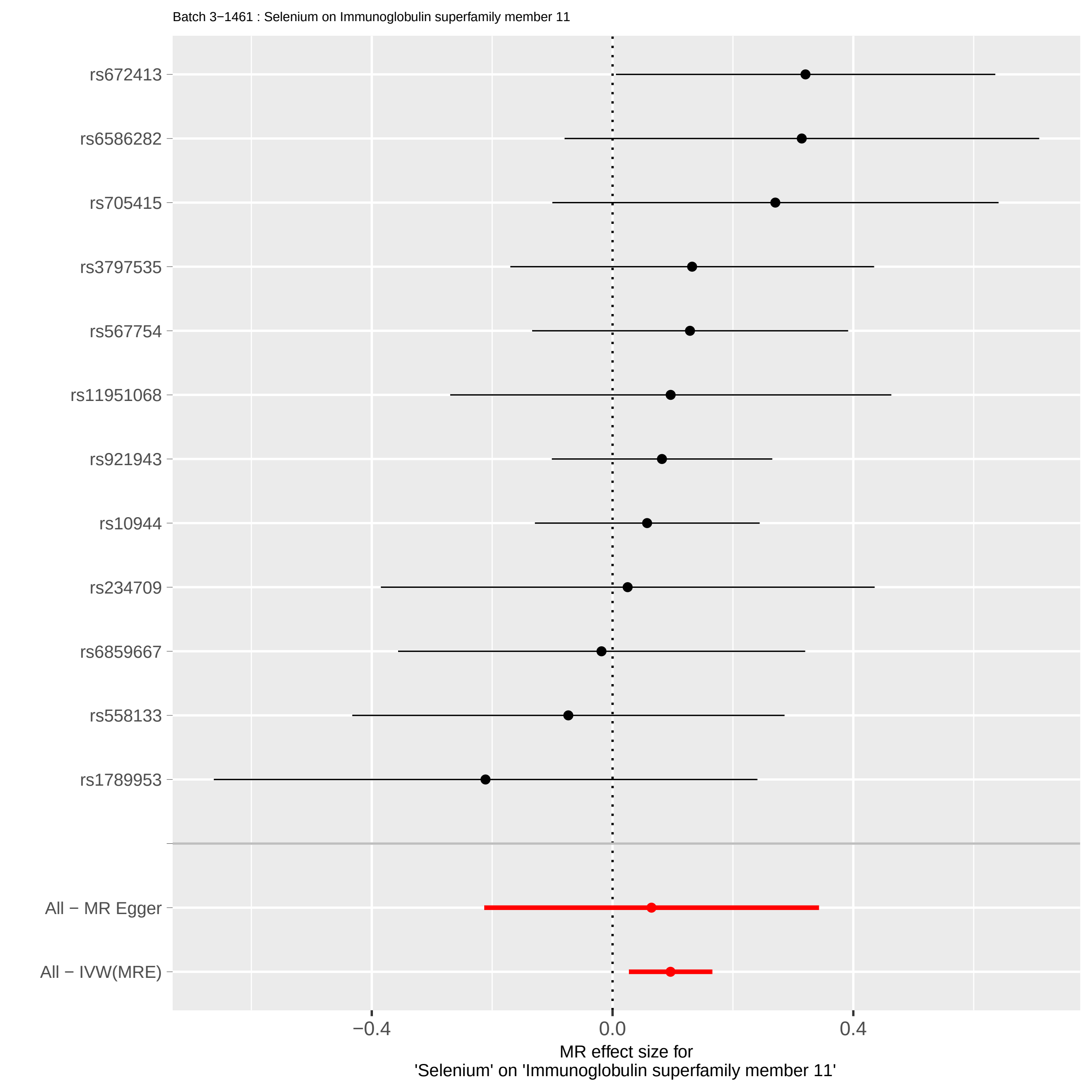
Batch 3−1461 : Selenium on Immunoglobulin superfamily member 11
rs672413
rs6586282
rs705415
rs3797535
rs567754
rs11951068
rs921943
rs10944
rs234709
rs6859667
rs558133
rs1789953
All − MR Egger
All − IVW(MRE)
−0.4
0.0
0.4
MR effect size for
'Selenium' on 'Immunoglobulin superfamily member 11'

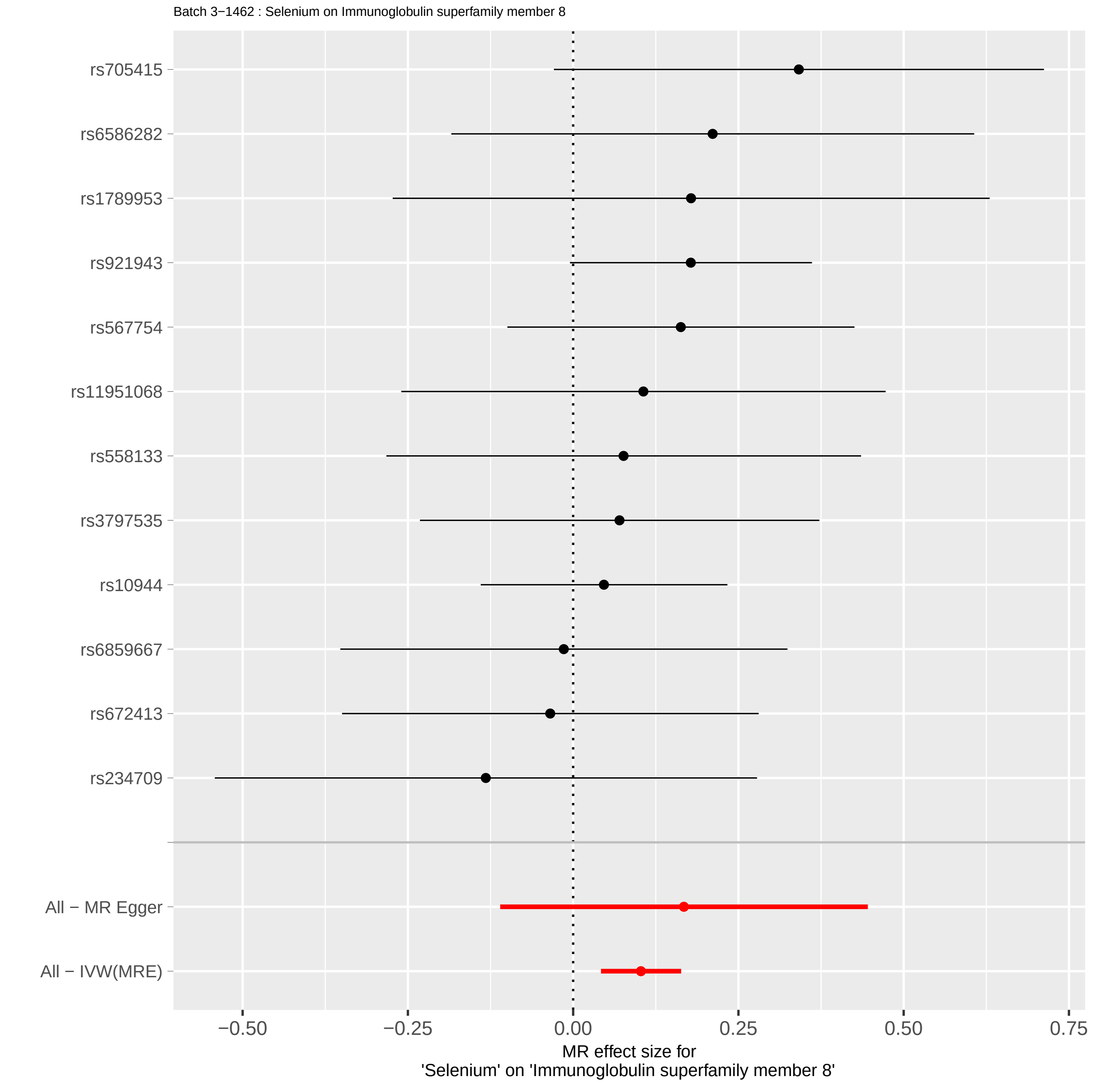

Batch 3−1462 : Selenium on Immunoglobulin superfamily member 8
rs705415
rs6586282
rs1789953
rs921943
rs567754
rs11951068
rs558133
rs3797535
rs10944
rs6859667
rs672413
rs234709
All − MR Egger
All − IVW(MRE)
−0.50
−0.25
0.00
0.25
0.50
0.75
MR effect size for
'Selenium' on 'Immunoglobulin superfamily member 8'

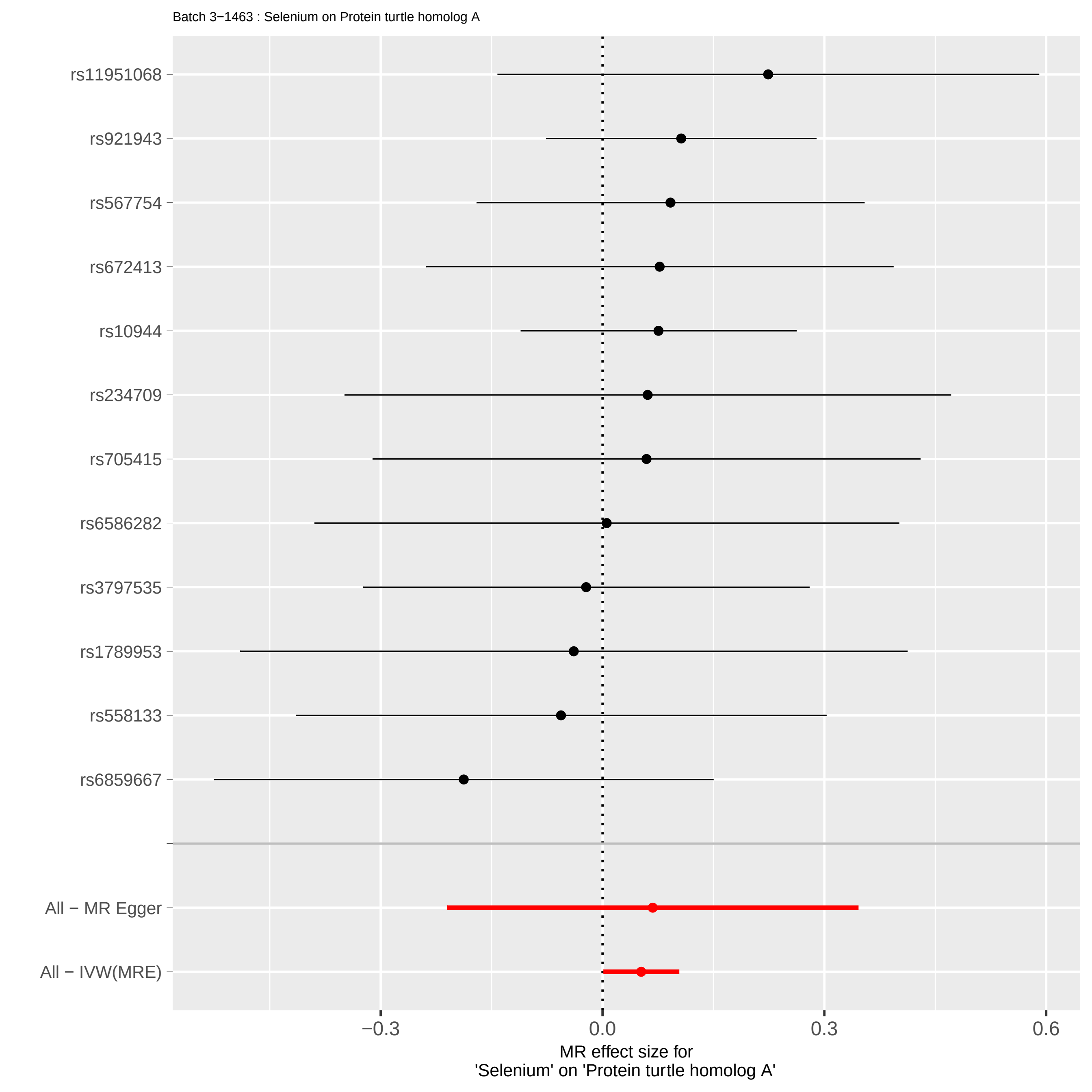
Batch 3−1463 : Selenium on Protein turtle homolog A
rs11951068
rs921943
rs567754
rs672413
rs10944
rs234709
rs705415
rs6586282
rs3797535
rs1789953
rs558133
rs6859667
All − MR Egger
All − IVW(MRE)
−0.3
0.0
0.3
0.6
MR effect size for
'Selenium' on 'Protein turtle homolog A'

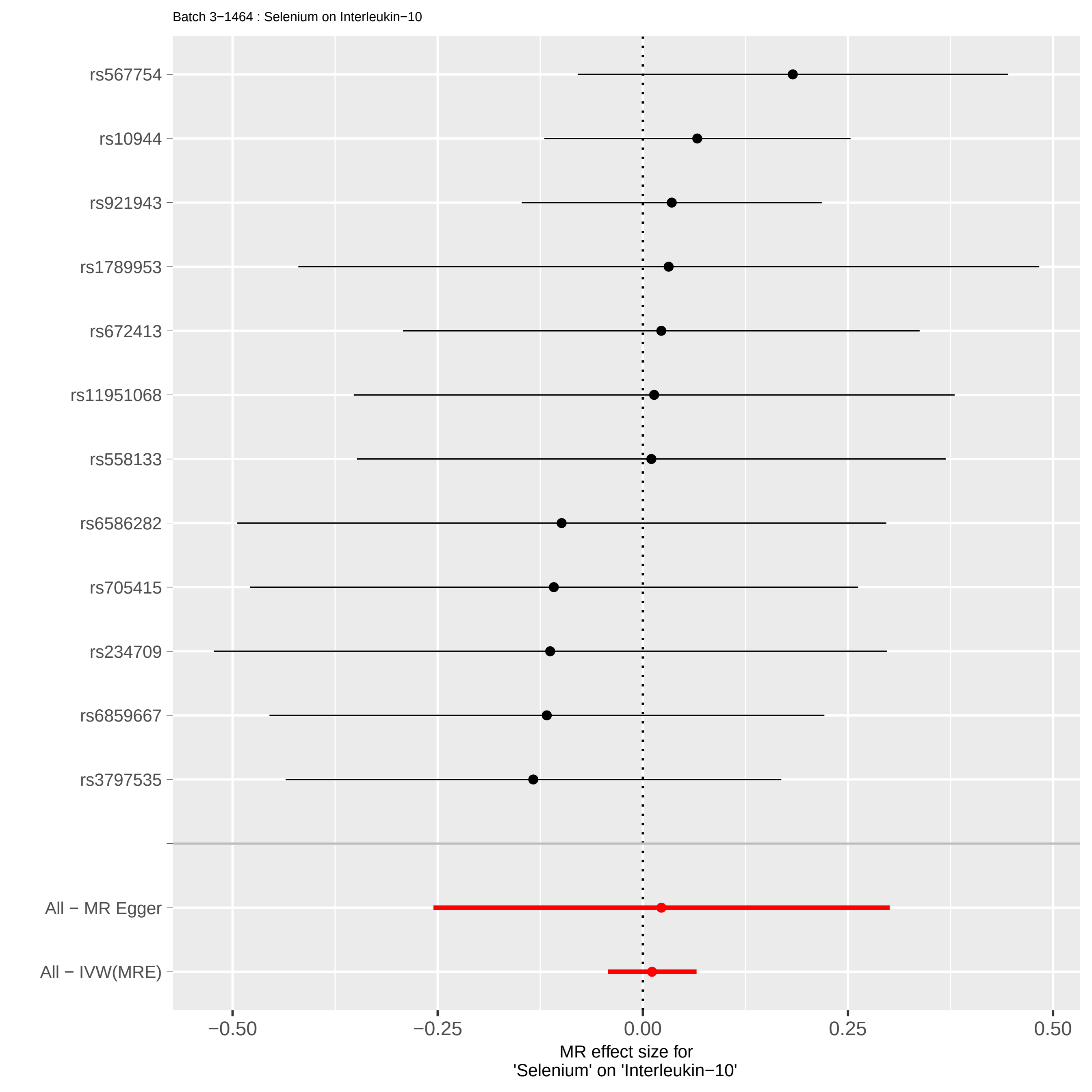

Batch 3−1464 : Selenium on Interleukin−10
rs567754
rs10944
rs921943
rs1789953
rs672413
rs11951068
rs558133
rs6586282
rs705415
rs234709
rs6859667
rs3797535
All − MR Egger
All − IVW(MRE)
−0.50
−0.25
0.00
0.25
0.50
MR effect size for
'Selenium' on 'Interleukin−10'

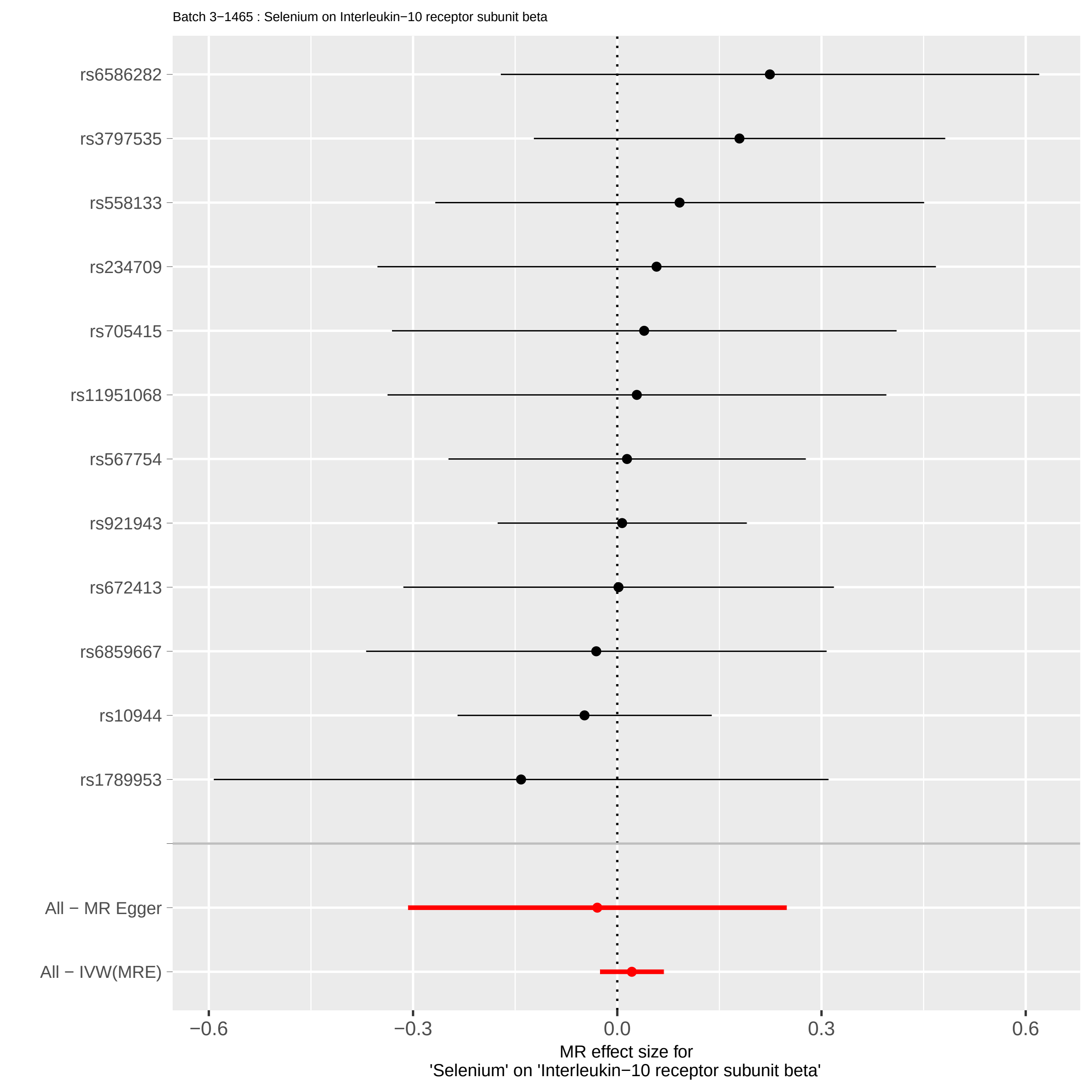

Batch 3−1465 : Selenium on Interleukin−10 receptor subunit beta
rs6586282
rs3797535
rs558133
rs234709
rs705415
rs11951068
rs567754
rs921943
rs672413
rs6859667
rs10944
rs1789953
All − MR Egger
All − IVW(MRE)
−0.6
−0.3
0.0
0.3
0.6
MR effect size for
'Selenium' on 'Interleukin−10 receptor subunit beta'

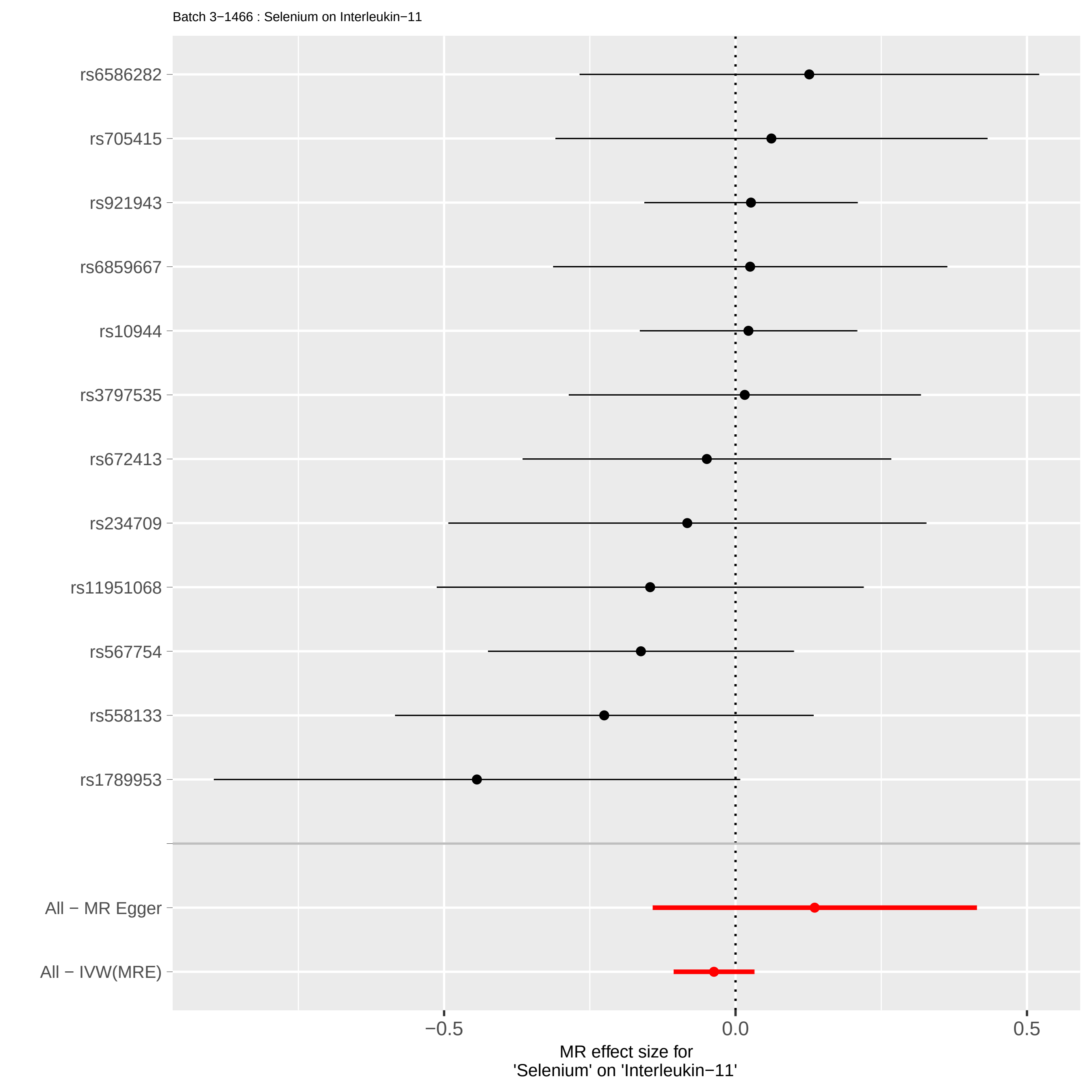
Batch 3−1466 : Selenium on Interleukin−11
rs6586282
rs705415
rs921943
rs6859667
rs10944
rs3797535
rs672413
rs234709
rs11951068
rs567754
rs558133
rs1789953
All − MR Egger
All − IVW(MRE)
−0.5
0.0
0.5
MR effect size for
'Selenium' on 'Interleukin−11'

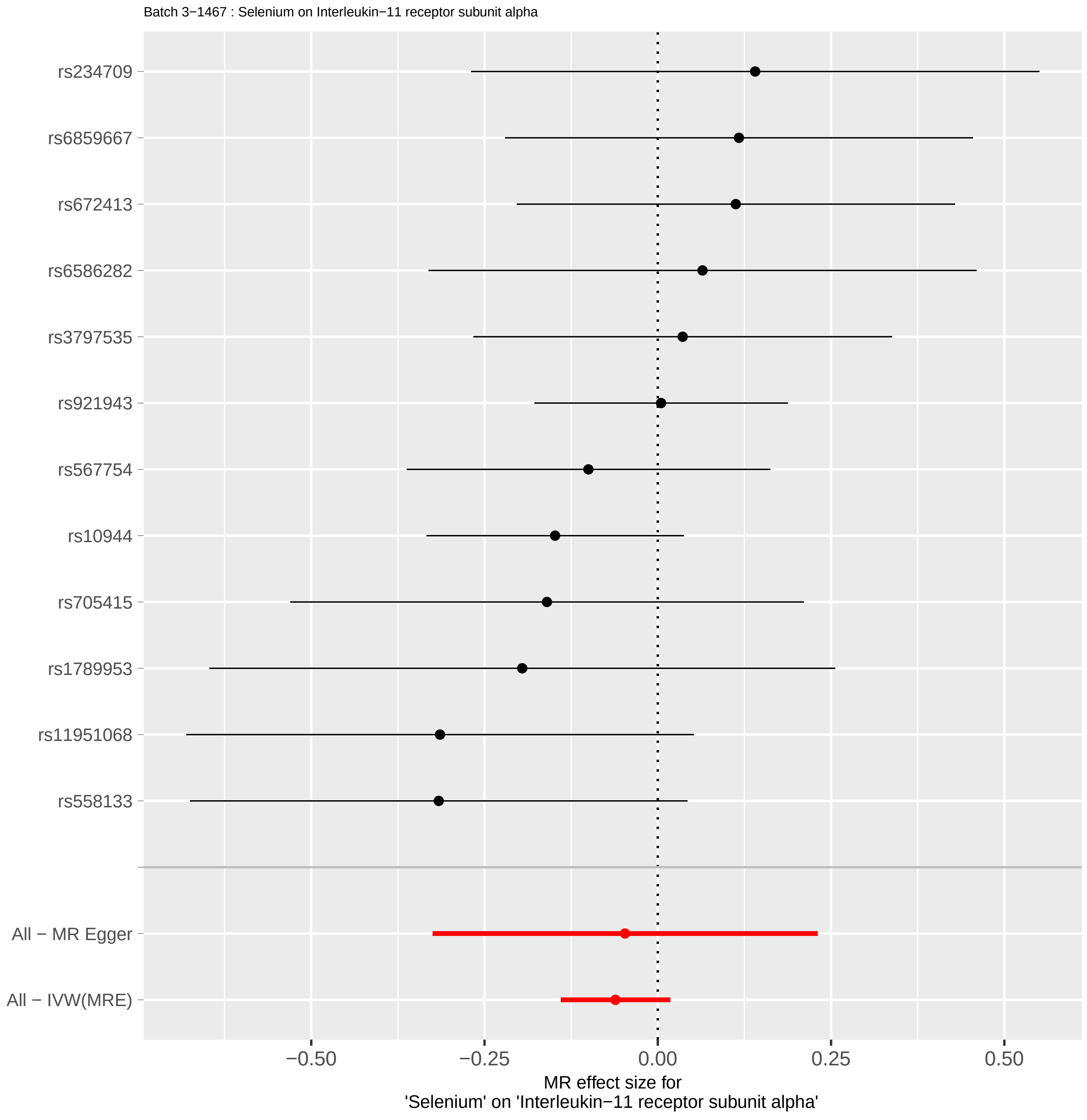

Batch 3−1467 : Selenium on Interleukin−11 receptor subunit alpha
rs234709
rs6859667
rs672413
rs6586282
rs3797535
rs921943
rs567754
rs10944
rs705415
rs1789953
rs11951068
rs558133
All − MR Egger
All − IVW(MRE)
−0.50
−0.25
0.00
0.25
0.50
MR effect size for
'Selenium' on 'Interleukin−11 receptor subunit alpha'

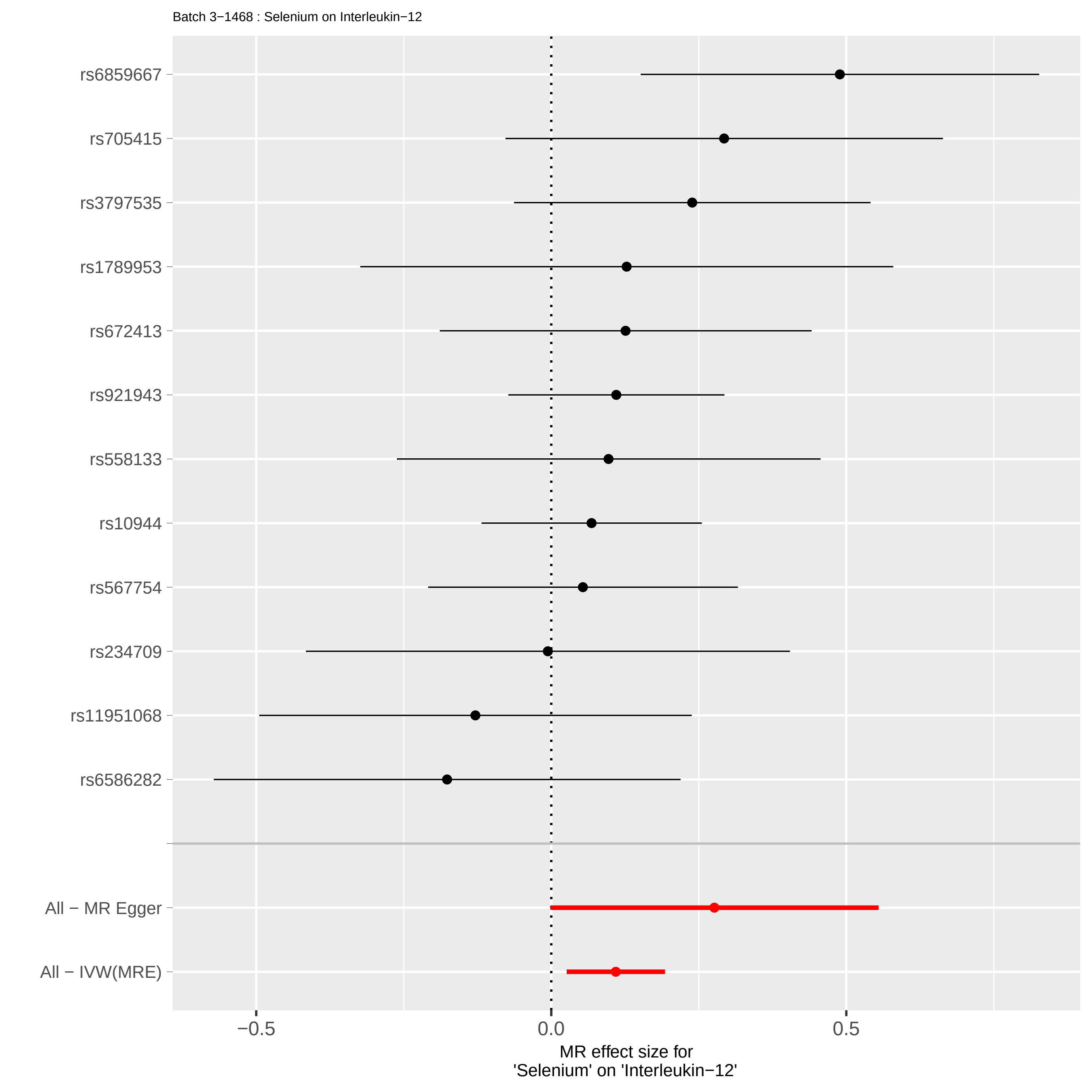

Batch 3−1468 : Selenium on Interleukin−12
rs6859667
rs705415
rs3797535
rs1789953
rs672413
rs921943
rs558133
rs10944
rs567754
rs234709
rs11951068
rs6586282
All − MR Egger
All − IVW(MRE)
−0.5
0.0
0.5
MR effect size for
'Selenium' on 'Interleukin−12'

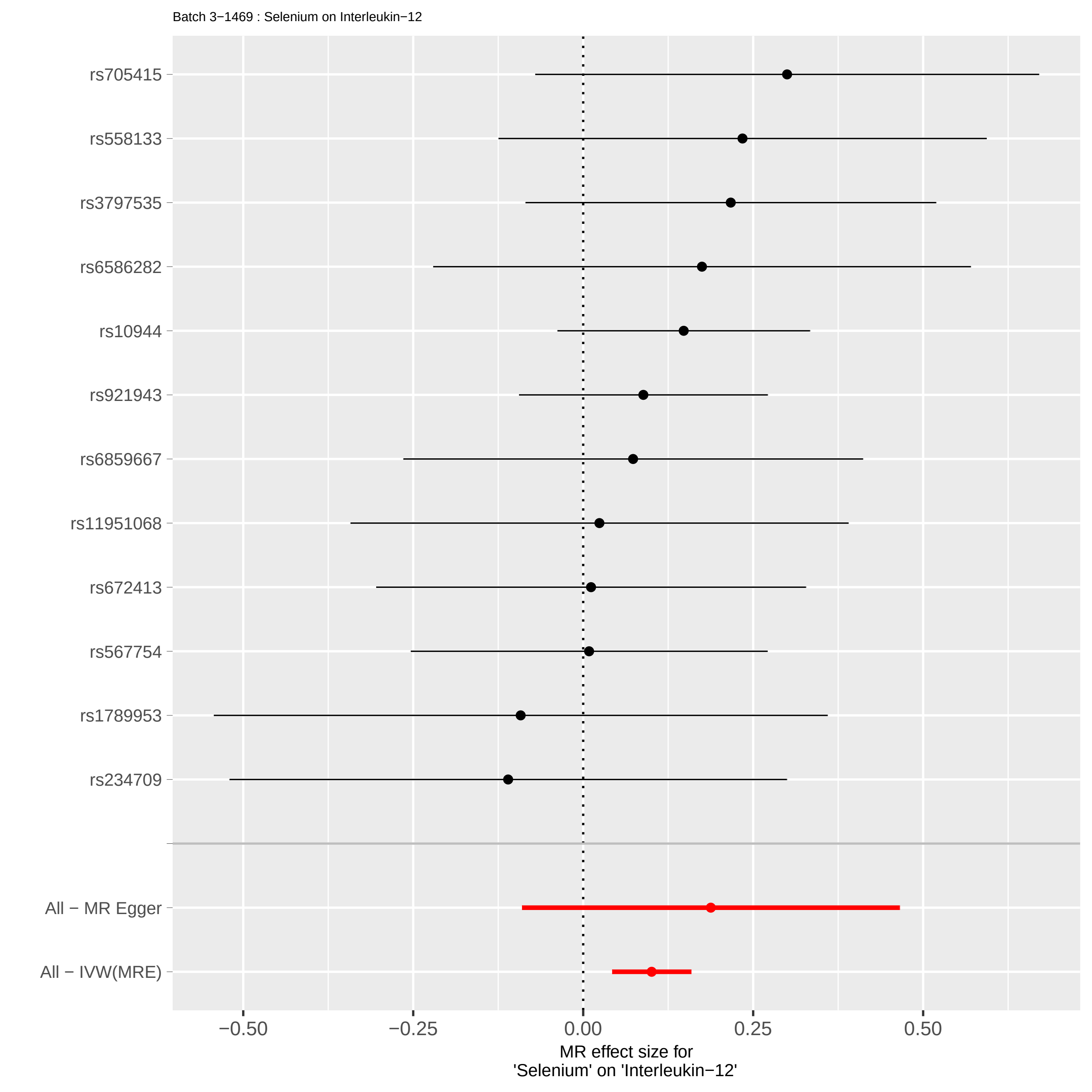

Batch 3−1469 : Selenium on Interleukin−12
rs705415
rs558133
rs3797535
rs6586282
rs10944
rs921943
rs6859667
rs11951068
rs672413
rs567754
rs1789953
rs234709
All − MR Egger
All − IVW(MRE)
−0.50
−0.25
0.00
0.25
0.50
MR effect size for
'Selenium' on 'Interleukin−12'

Batch 3−1470 : Selenium on Interleukin−12

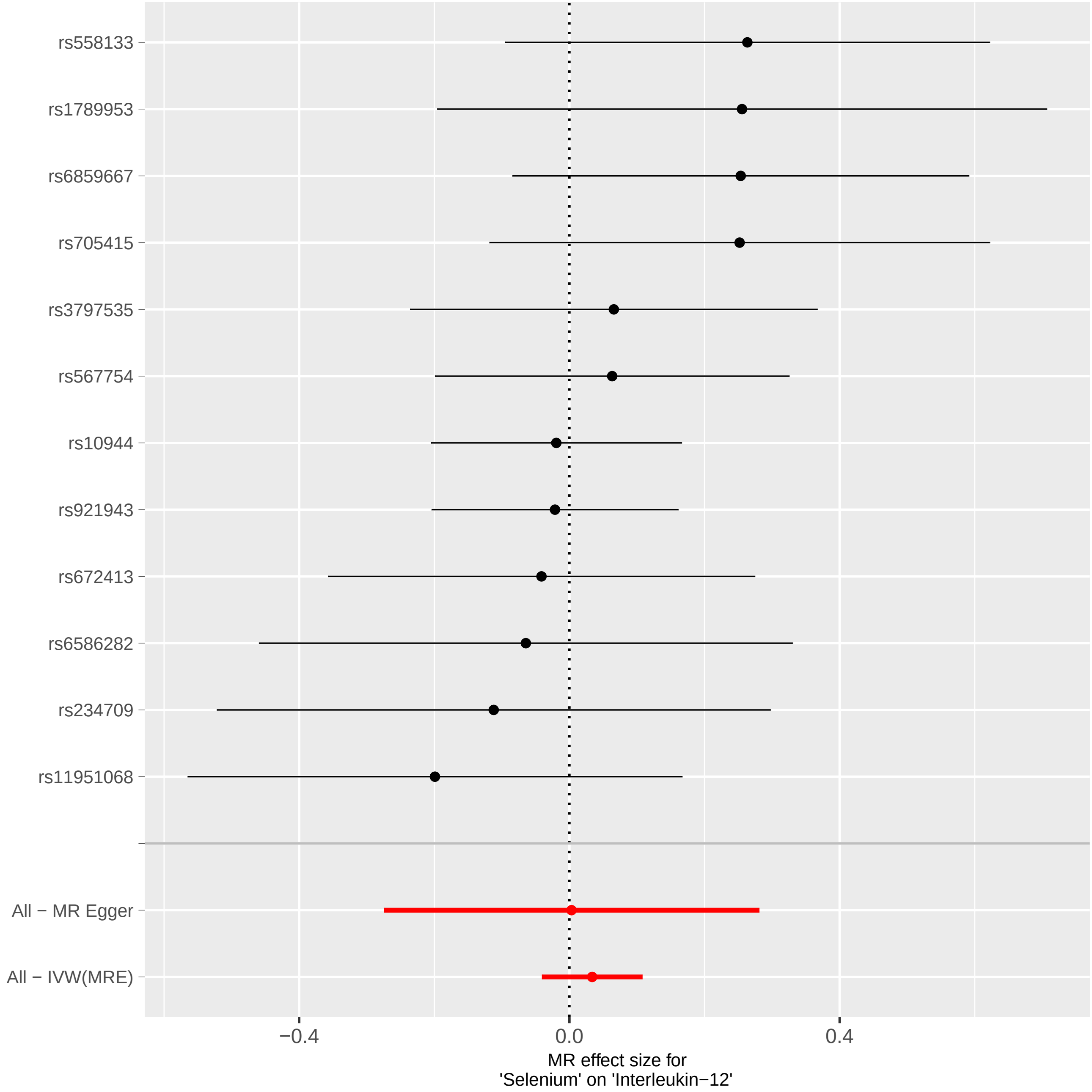

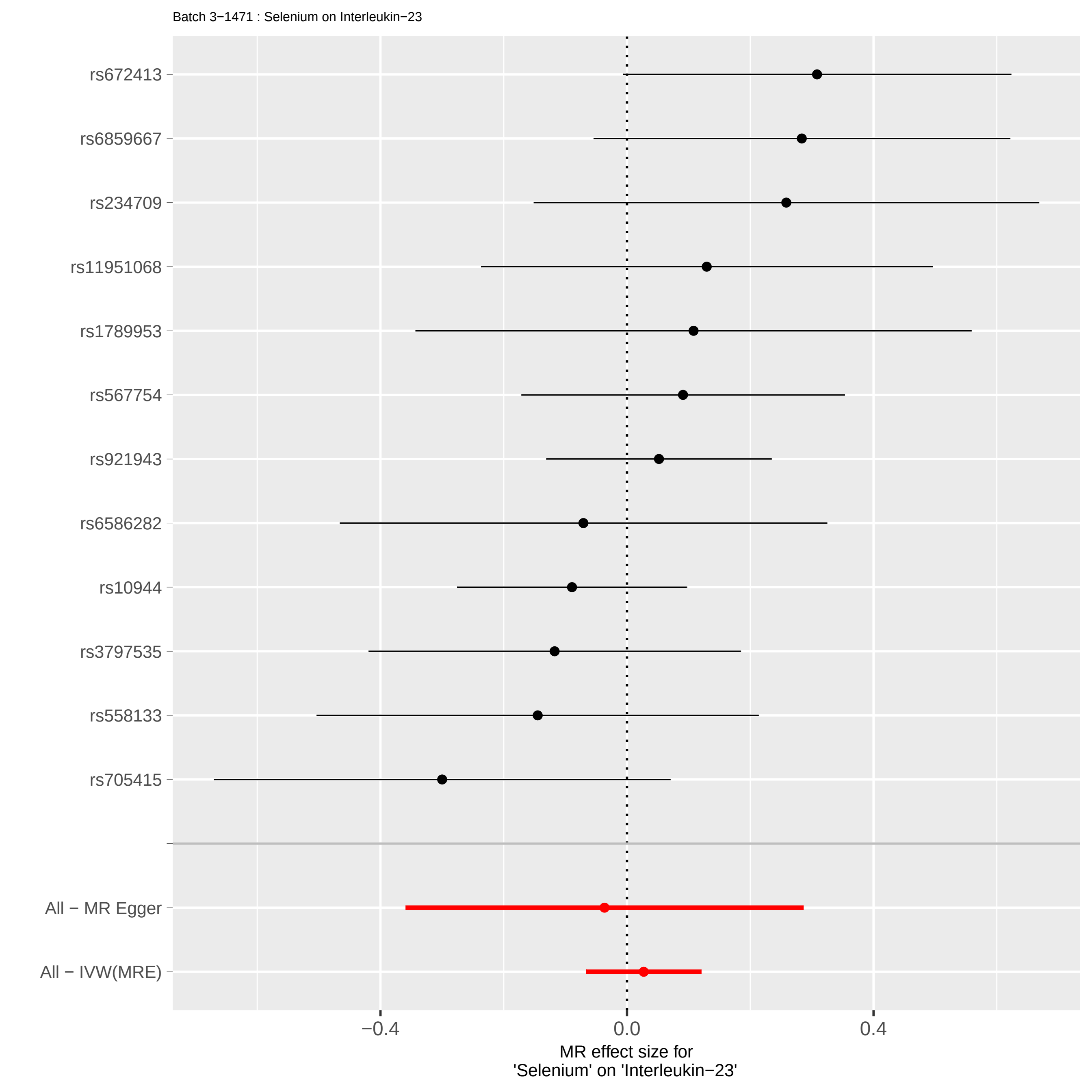
Batch 3−1471 : Selenium on Interleukin−23
rs672413
rs6859667
rs234709
rs11951068
rs1789953
rs567754
rs921943
rs6586282
rs10944
rs3797535
rs558133
rs705415
All − MR Egger
All − IVW(MRE)
−0.4
0.0
0.4
MR effect size for
'Selenium' on 'Interleukin−23'

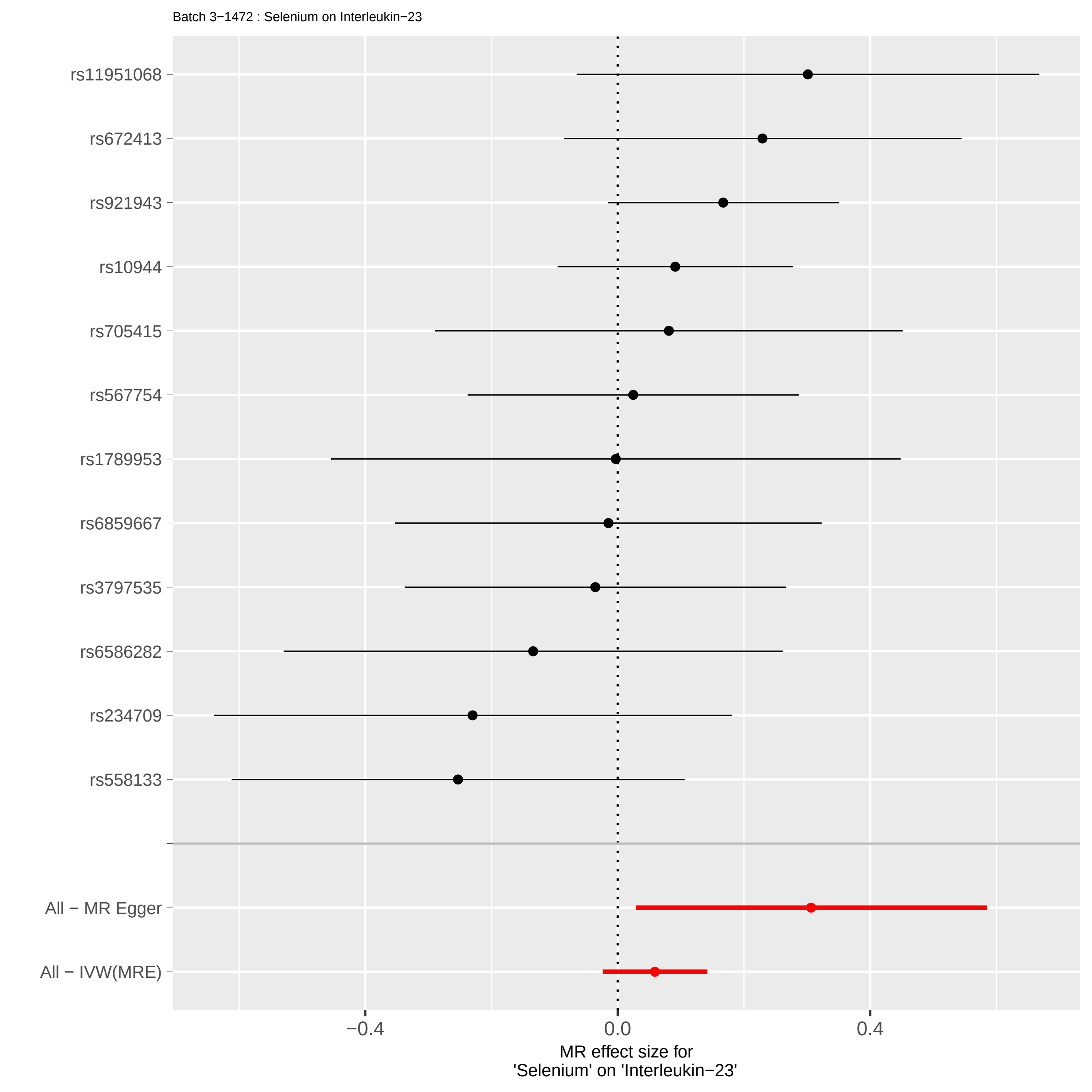
Batch 3−1472 : Selenium on Interleukin−23
rs11951068
rs672413
rs921943
rs10944
rs705415
rs567754
rs1789953
rs6859667
rs3797535
rs6586282
rs234709
rs558133
All − MR Egger
All − IVW(MRE)
−0.4
0.0
0.4
MR effect size for
'Selenium' on 'Interleukin−23'

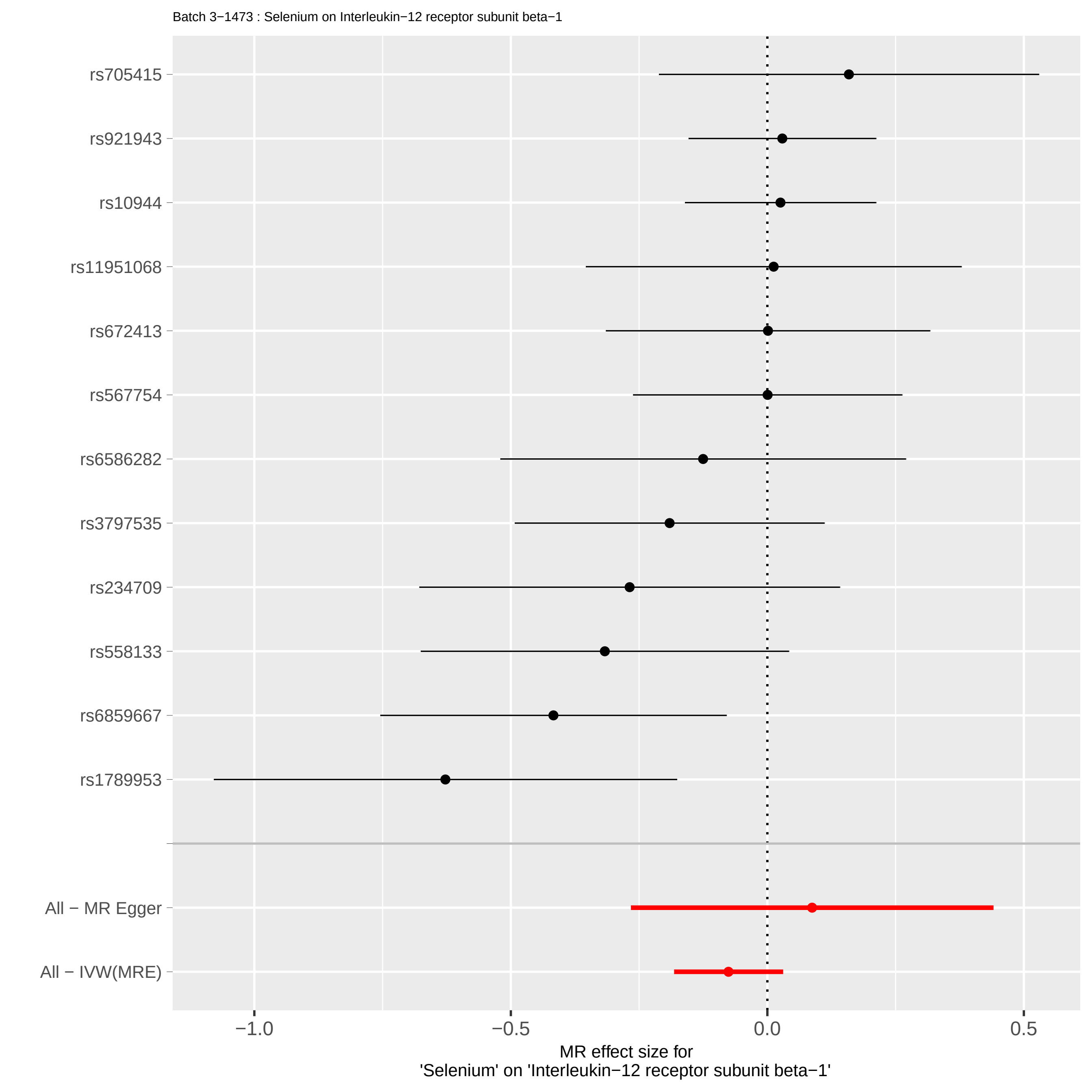
Batch 3−1473 : Selenium on Interleukin−12 receptor subunit beta−1
rs705415
rs921943
rs10944
rs11951068
rs672413
rs567754
rs6586282
rs3797535
rs234709
rs558133
rs6859667
rs1789953
All − MR Egger
All − IVW(MRE)
−1.0
−0.5
0.0
0.5
MR effect size for
'Selenium' on 'Interleukin−12 receptor subunit beta−1'

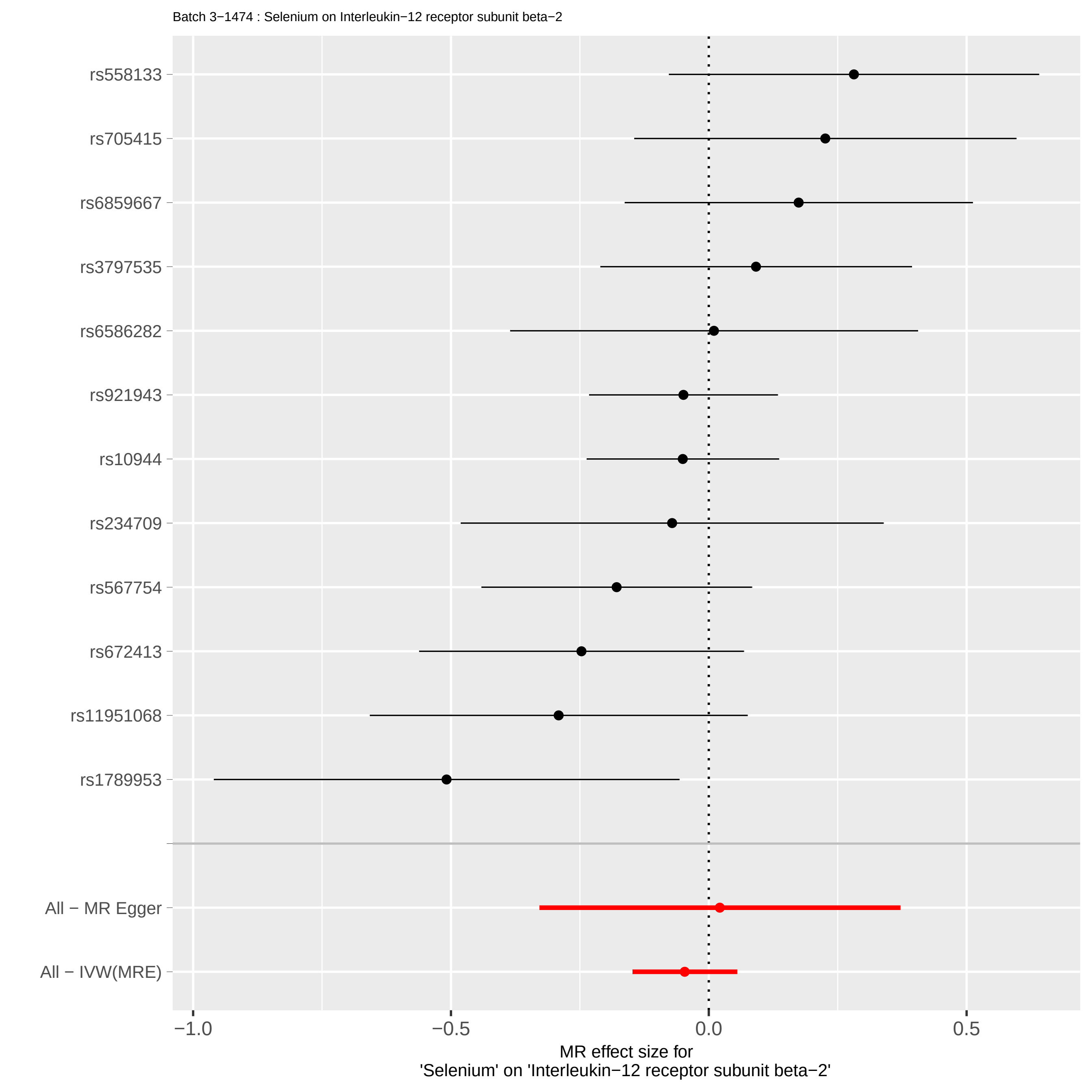

Batch 3−1474 : Selenium on Interleukin−12 receptor subunit beta−2
rs558133
rs705415
rs6859667
rs3797535
rs6586282
rs921943
rs10944
rs234709
rs567754
rs672413
rs11951068
rs1789953
All − MR Egger
All − IVW(MRE)
−1.0
−0.5
0.0
0.5
MR effect size for
'Selenium' on 'Interleukin−12 receptor subunit beta−2'

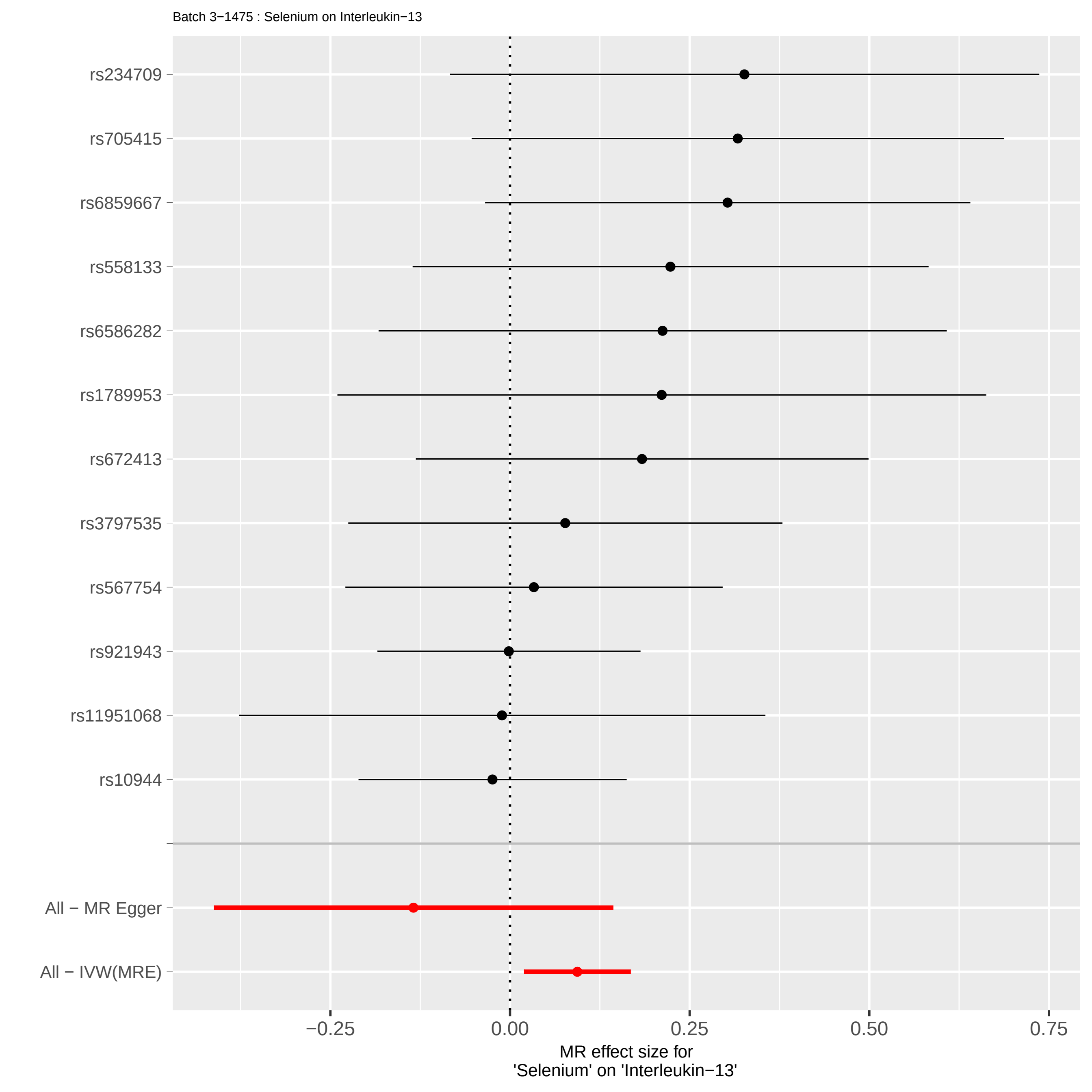

Batch 3−1475 : Selenium on Interleukin−13
rs234709
rs705415
rs6859667
rs558133
rs6586282
rs1789953
rs672413
rs3797535
rs567754
rs921943
rs11951068
rs10944
All − MR Egger
All − IVW(MRE)
−0.25
0.00
0.25
0.50
0.75
MR effect size for
'Selenium' on 'Interleukin−13'

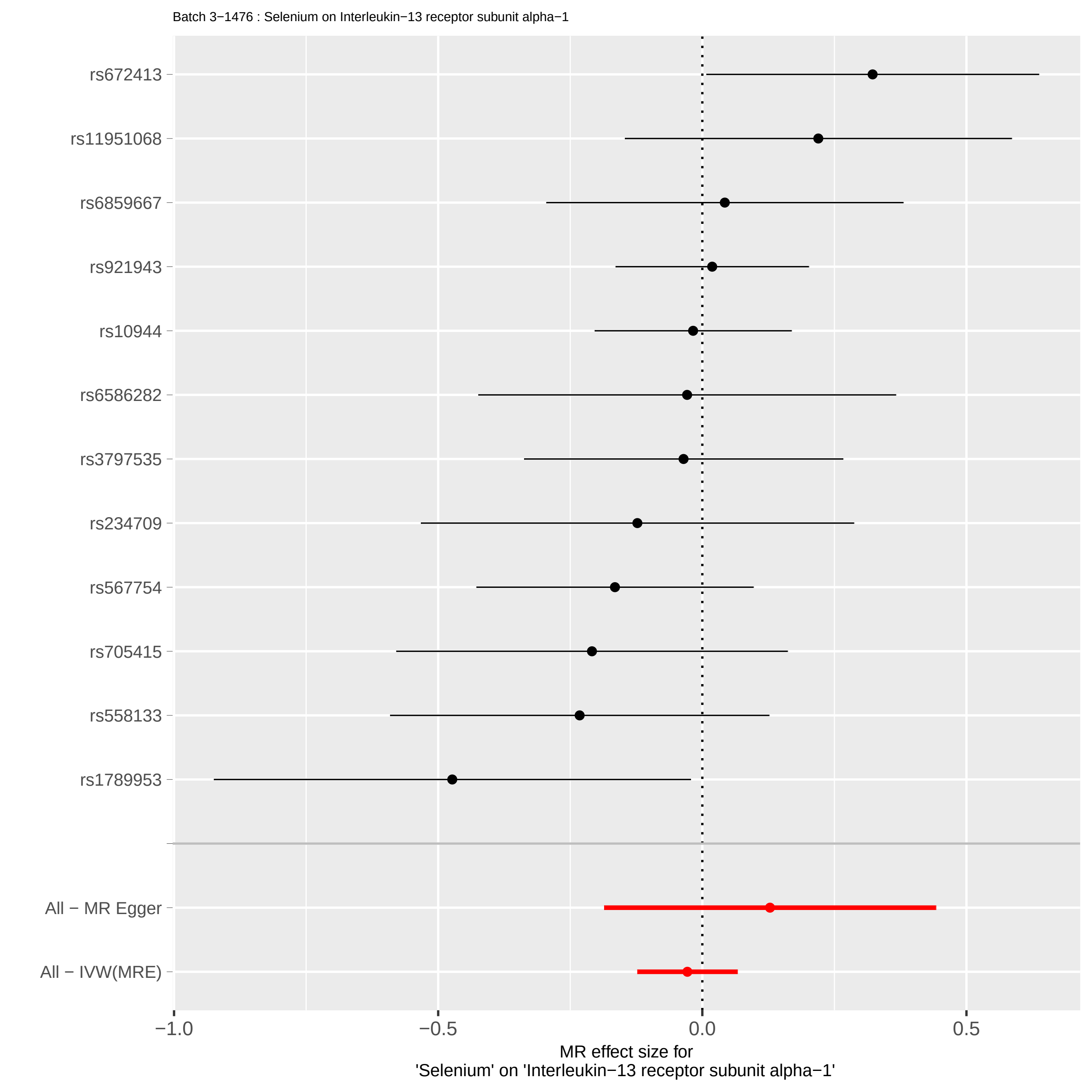

Batch 3−1476 : Selenium on Interleukin−13 receptor subunit alpha−1
rs672413
rs11951068
rs6859667
rs921943
rs10944
rs6586282
rs3797535
rs234709
rs567754
rs705415
rs558133
rs1789953
All − MR Egger
All − IVW(MRE)
−1.0
−0.5
0.0
0.5
MR effect size for
'Selenium' on 'Interleukin−13 receptor subunit alpha−1'

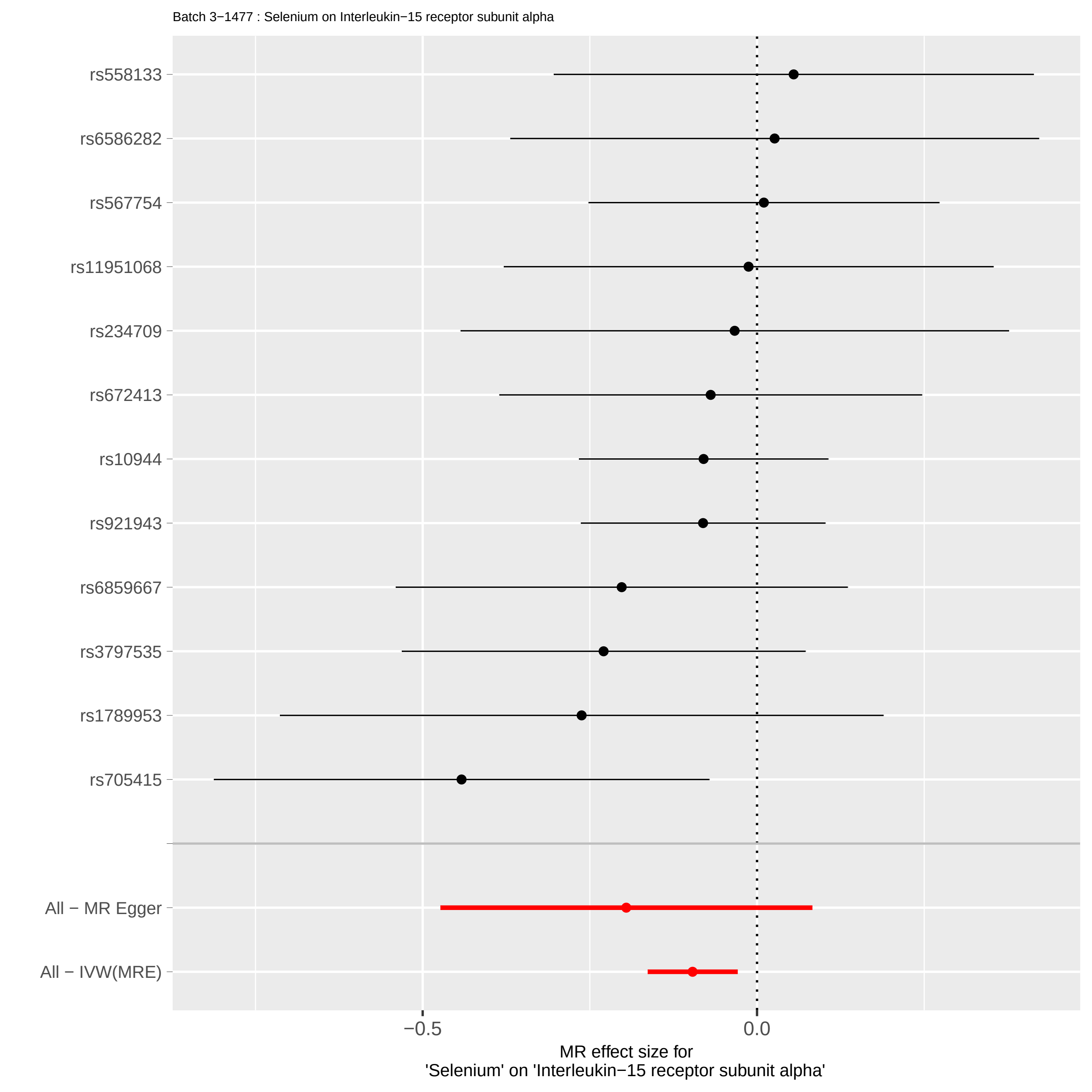
Batch 3−1477 : Selenium on Interleukin−15 receptor subunit alpha
rs558133
rs6586282
rs567754
rs11951068
rs234709
rs672413
rs10944
rs921943
rs6859667
rs3797535
rs1789953
rs705415
All − MR Egger
All − IVW(MRE)
−0.5
0.0
MR effect size for
'Selenium' on 'Interleukin−15 receptor subunit alpha'

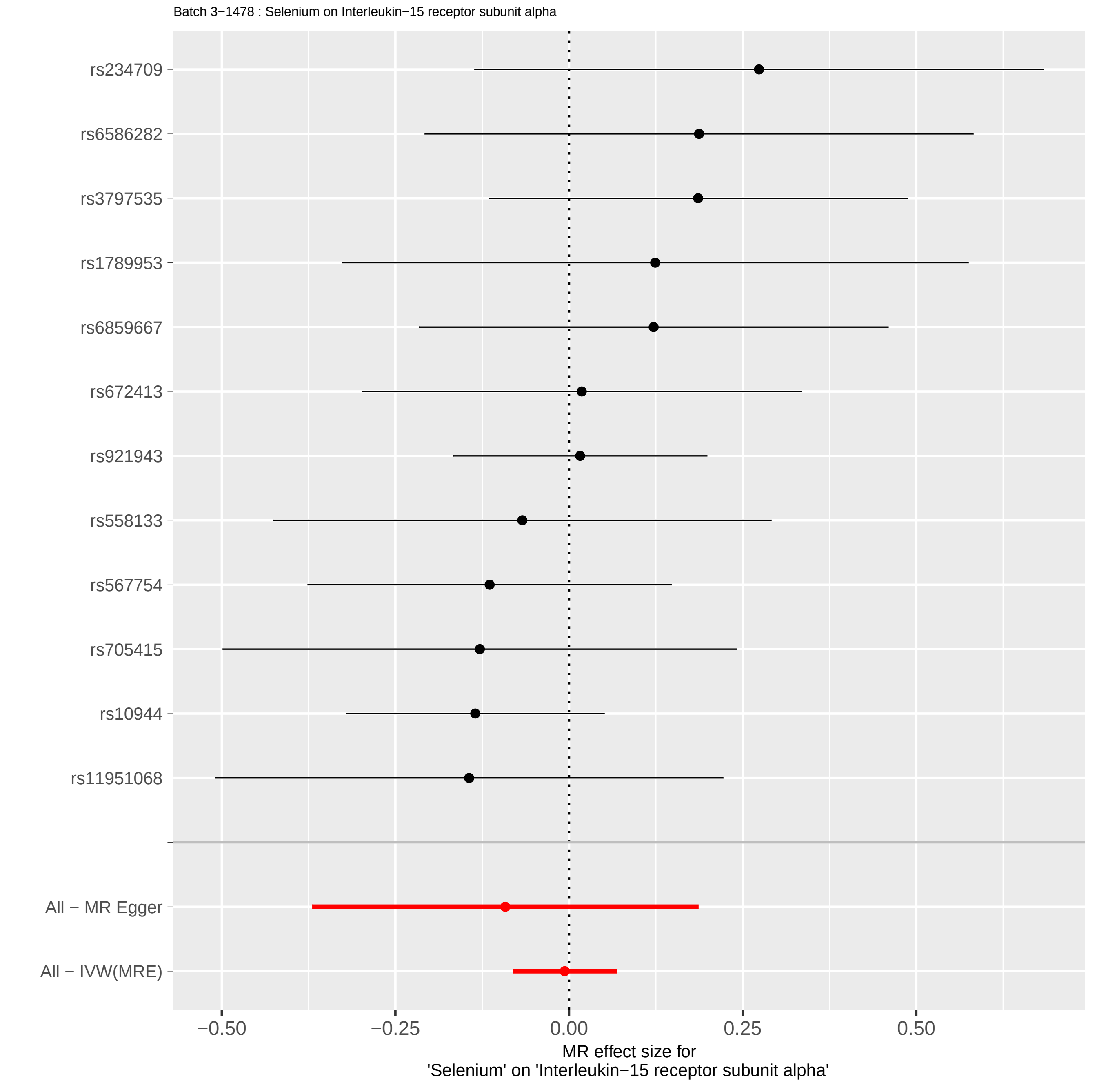

Batch 3−1478 : Selenium on Interleukin−15 receptor subunit alpha
rs234709
rs6586282
rs3797535
rs1789953
rs6859667
rs672413
rs921943
rs558133
rs567754
rs705415
rs10944
rs11951068
All − MR Egger
All − IVW(MRE)
−0.50
−0.25
0.00
0.25
0.50
MR effect size for
'Selenium' on 'Interleukin−15 receptor subunit alpha'

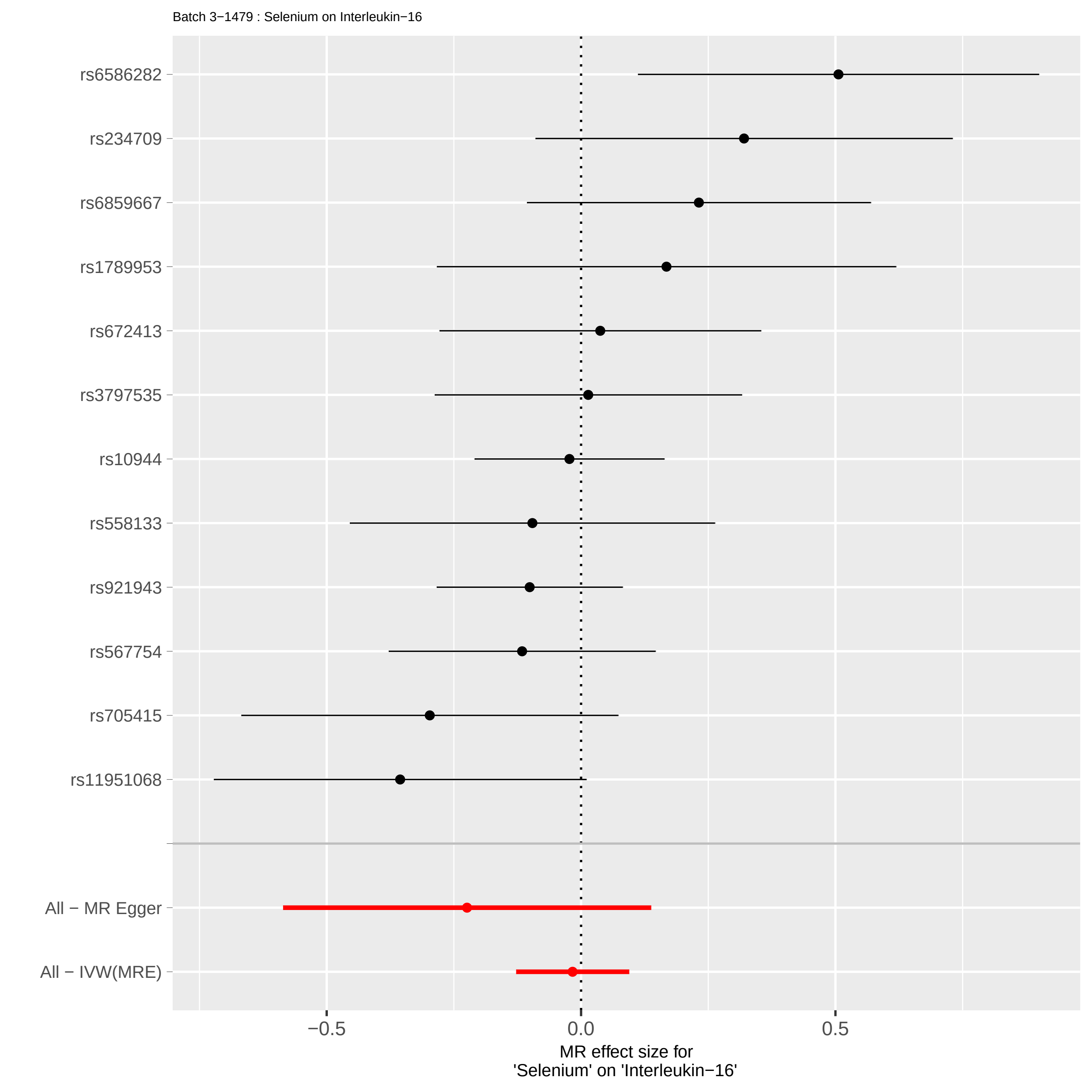

Batch 3−1479 : Selenium on Interleukin−16
rs6586282
rs234709
rs6859667
rs1789953
rs672413
rs3797535
rs10944
rs558133
rs921943
rs567754
rs705415
rs11951068
All − MR Egger
All − IVW(MRE)
−0.5
0.0
0.5
MR effect size for
'Selenium' on 'Interleukin−16'

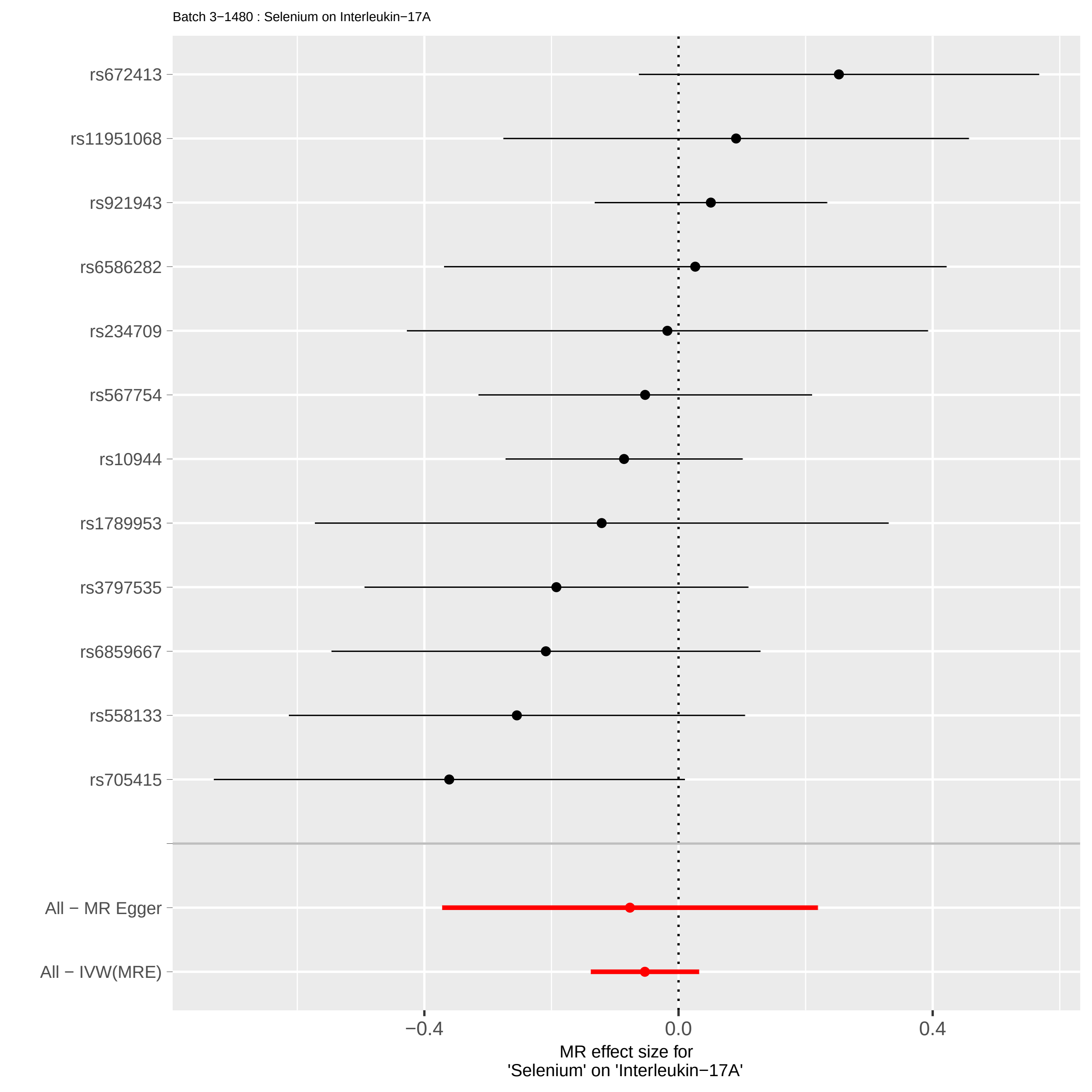

Batch 3−1480 : Selenium on Interleukin−17A
rs672413
rs11951068
rs921943
rs6586282
rs234709
rs567754
rs10944
rs1789953
rs3797535
rs6859667
rs558133
rs705415
All − MR Egger
All − IVW(MRE)
−0.4
0.0
0.4
MR effect size for
'Selenium' on 'Interleukin−17A'

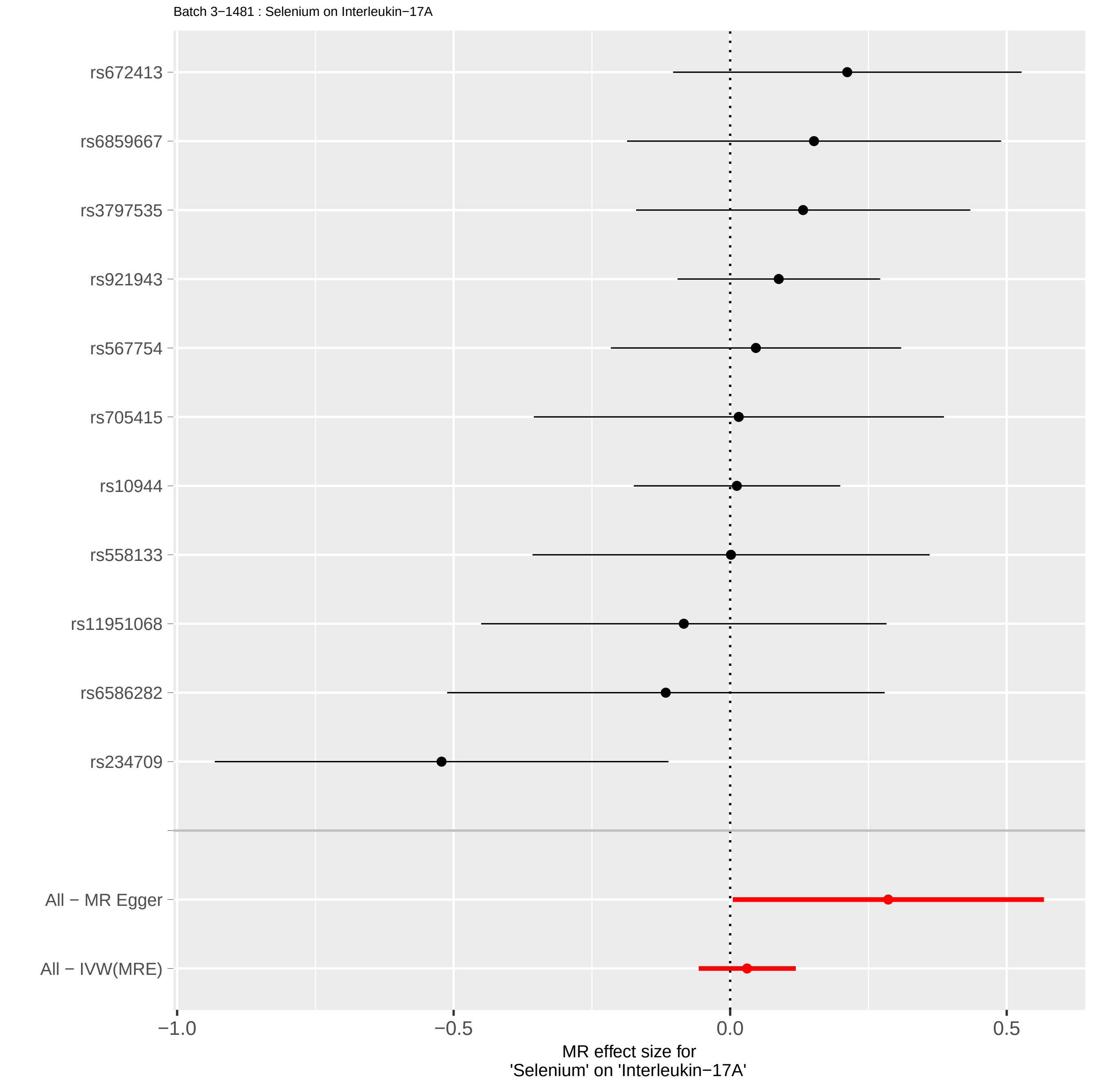
Batch 3−1481 : Selenium on Interleukin−17A
rs672413
rs6859667
rs3797535
rs921943
rs567754
rs705415
rs10944
rs558133
rs11951068
rs6586282
rs234709
All − MR Egger
All − IVW(MRE)
−1.0
−0.5
0.0
0.5
MR effect size for
'Selenium' on 'Interleukin−17A'

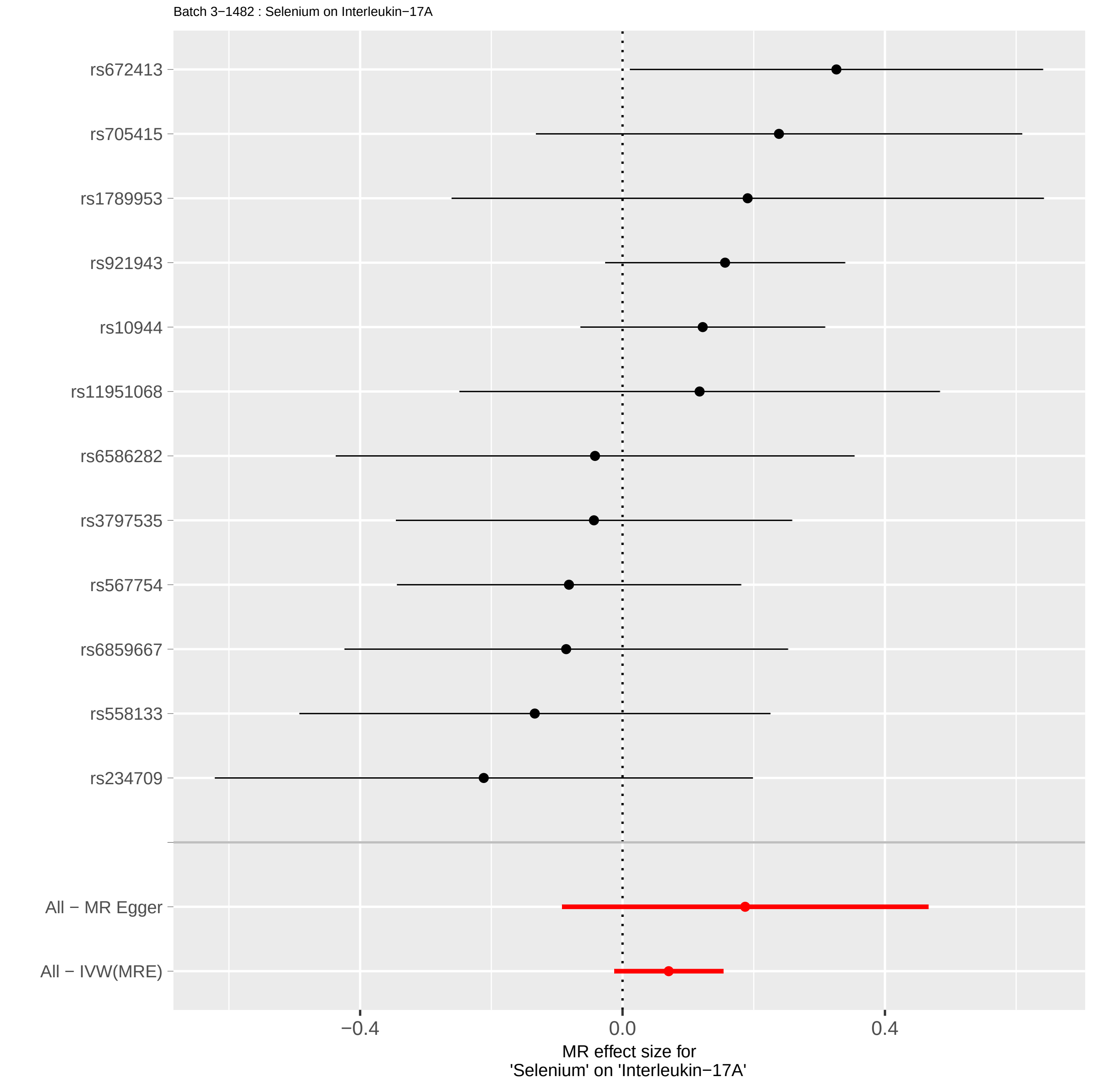

Batch 3−1482 : Selenium on Interleukin−17A
rs672413
rs705415
rs1789953
rs921943
rs10944
rs11951068
rs6586282
rs3797535
rs567754
rs6859667
rs558133
rs234709
All − MR Egger
All − IVW(MRE)
−0.4
0.0
0.4
MR effect size for
'Selenium' on 'Interleukin−17A'

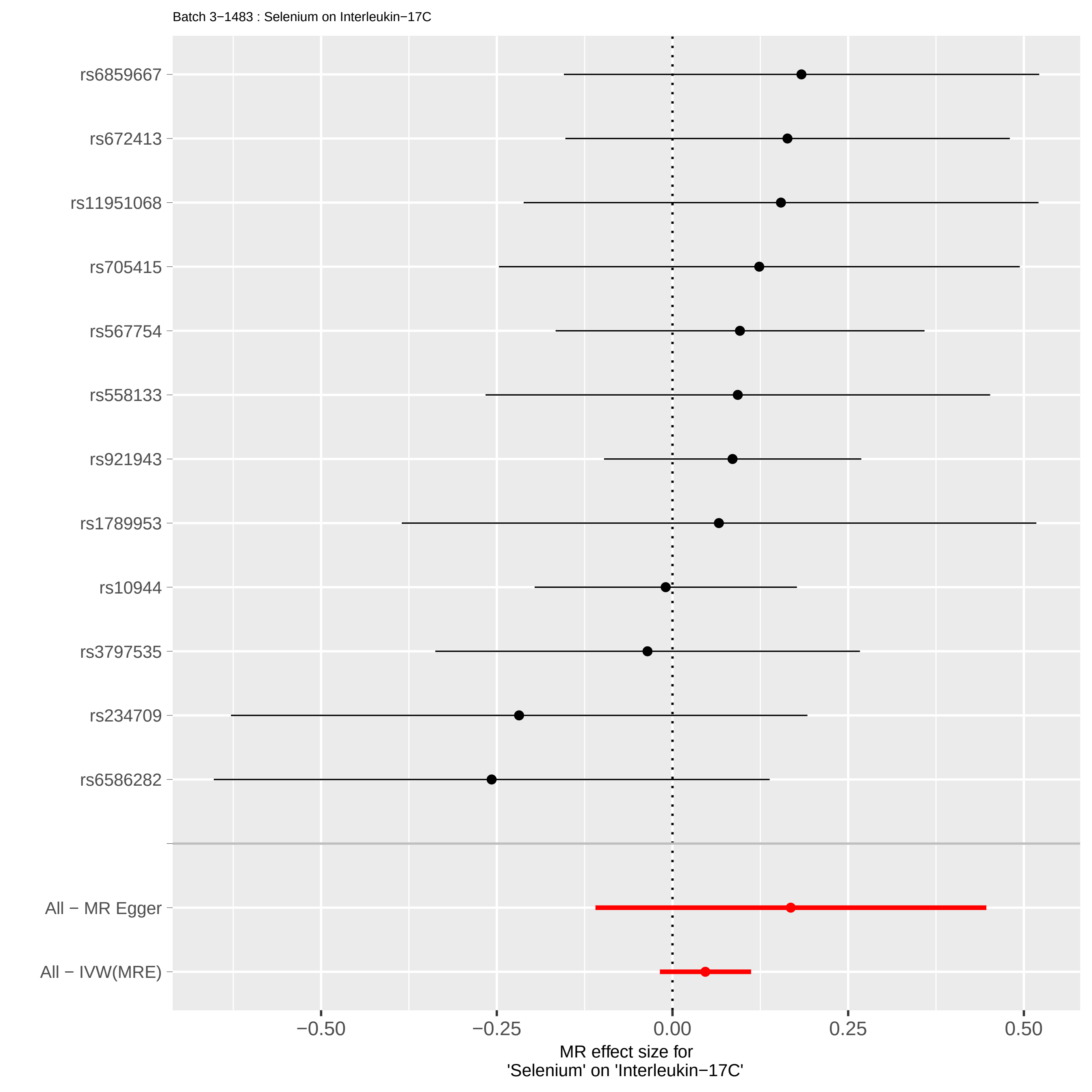

Batch 3−1483 : Selenium on Interleukin−17C
rs6859667
rs672413
rs11951068
rs705415
rs567754
rs558133
rs921943
rs1789953
rs10944
rs3797535
rs234709
rs6586282
All − MR Egger
All − IVW(MRE)
−0.50
−0.25
0.00
0.25
0.50
MR effect size for
'Selenium' on 'Interleukin−17C'

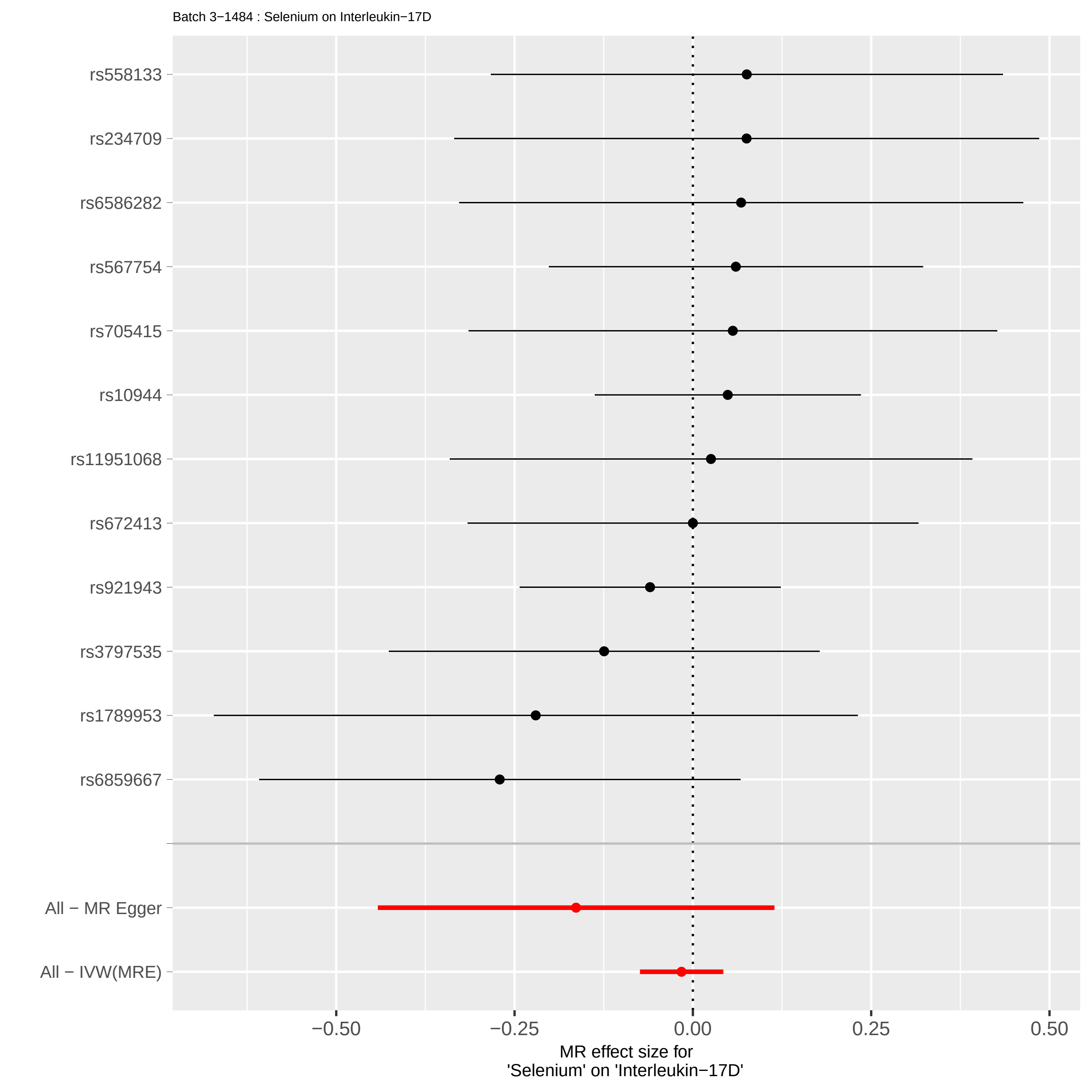

Batch 3−1484 : Selenium on Interleukin−17D
rs558133
rs234709
rs6586282
rs567754
rs705415
rs10944
rs11951068
rs672413
rs921943
rs3797535
rs1789953
rs6859667
All − MR Egger
All − IVW(MRE)
−0.50
−0.25
0.00
0.25
0.50
MR effect size for
'Selenium' on 'Interleukin−17D'

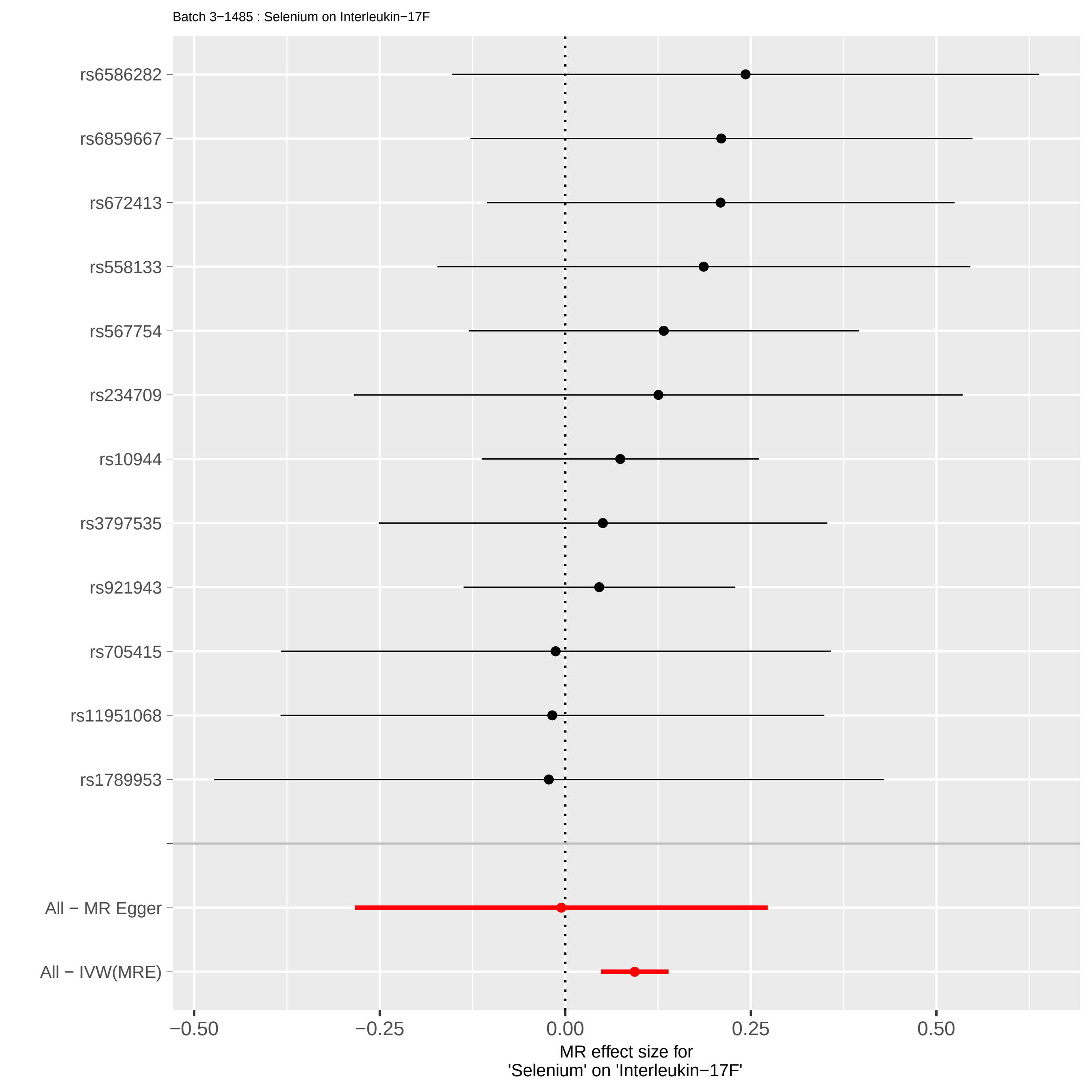
Batch 3−1485 : Selenium on Interleukin−17F
rs6586282
rs6859667
rs672413
rs558133
rs567754
rs234709
rs10944
rs3797535
rs921943
rs705415
rs11951068
rs1789953
All − MR Egger
All − IVW(MRE)
−0.50
−0.25
0.00
0.25
0.50
MR effect size for
'Selenium' on 'Interleukin−17F'

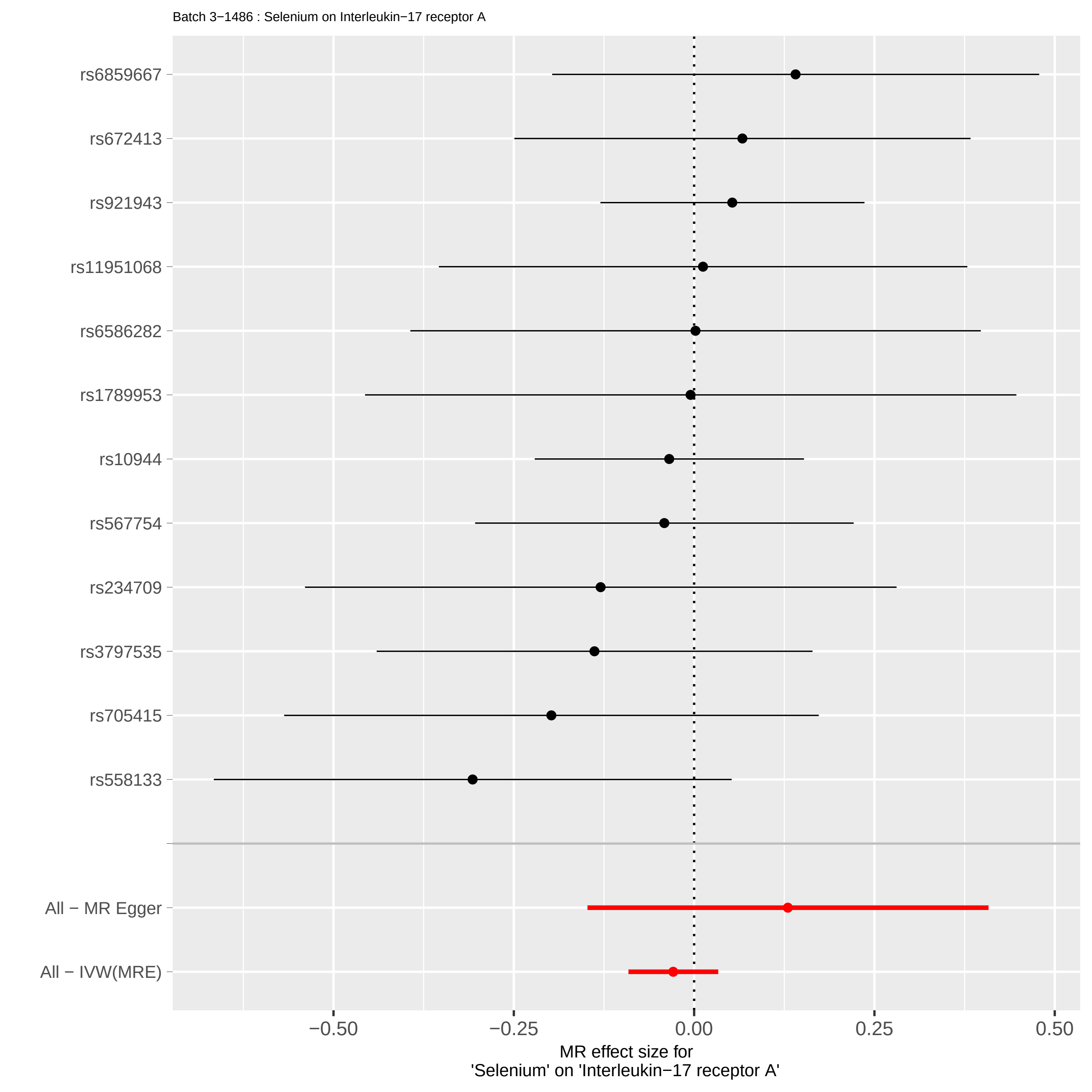
Batch 3−1486 : Selenium on Interleukin−17 receptor A
rs6859667
rs672413
rs921943
rs11951068
rs6586282
rs1789953
rs10944
rs567754
rs234709
rs3797535
rs705415
rs558133
All − MR Egger
All − IVW(MRE)
−0.50
−0.25
0.00
0.25
0.50
MR effect size for
'Selenium' on 'Interleukin−17 receptor A'

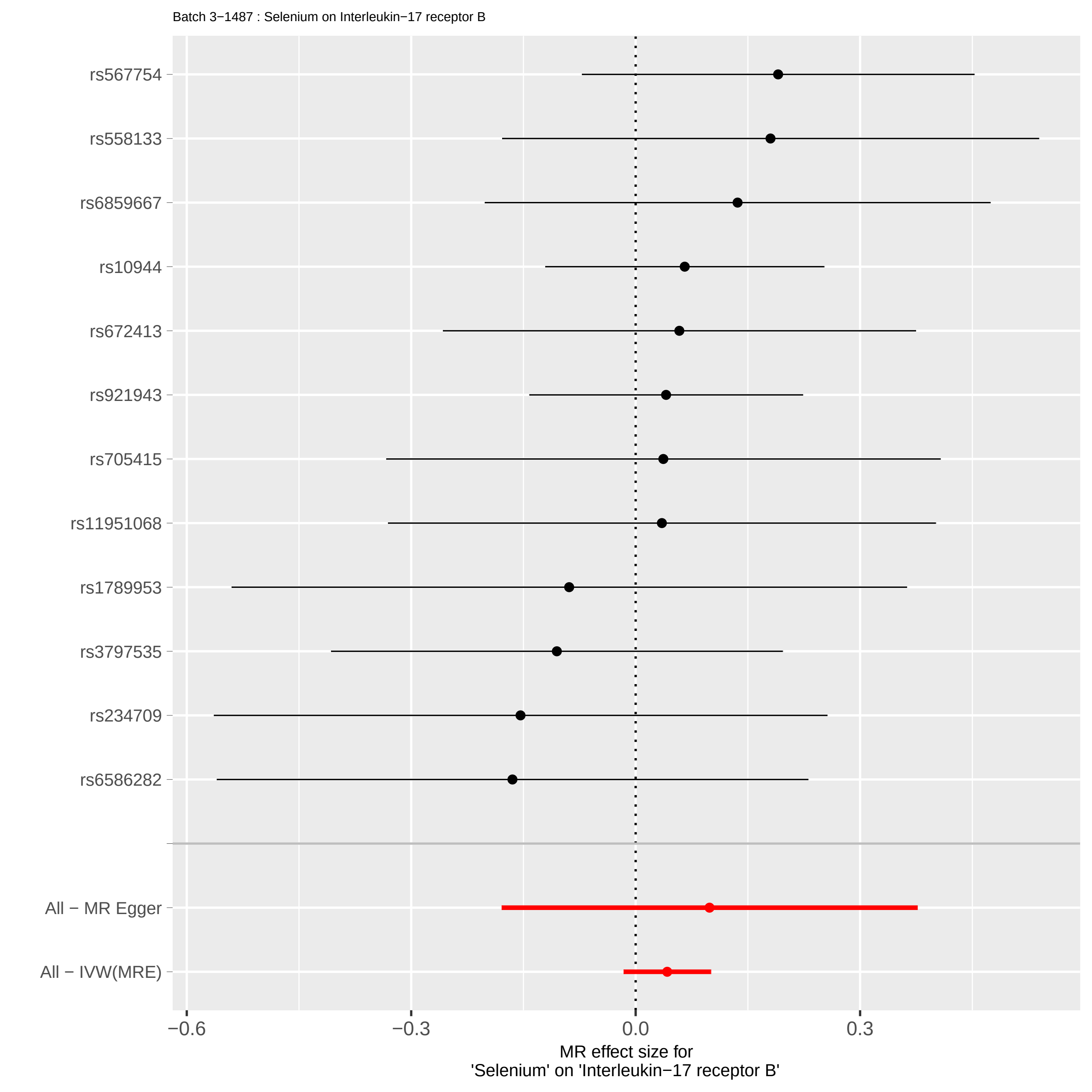

Batch 3−1487 : Selenium on Interleukin−17 receptor B
rs567754
rs558133
rs6859667
rs10944
rs672413
rs921943
rs705415
rs11951068
rs1789953
rs3797535
rs234709
rs6586282
All − MR Egger
All − IVW(MRE)
−0.6
−0.3
0.0
0.3
MR effect size for
'Selenium' on 'Interleukin−17 receptor B'

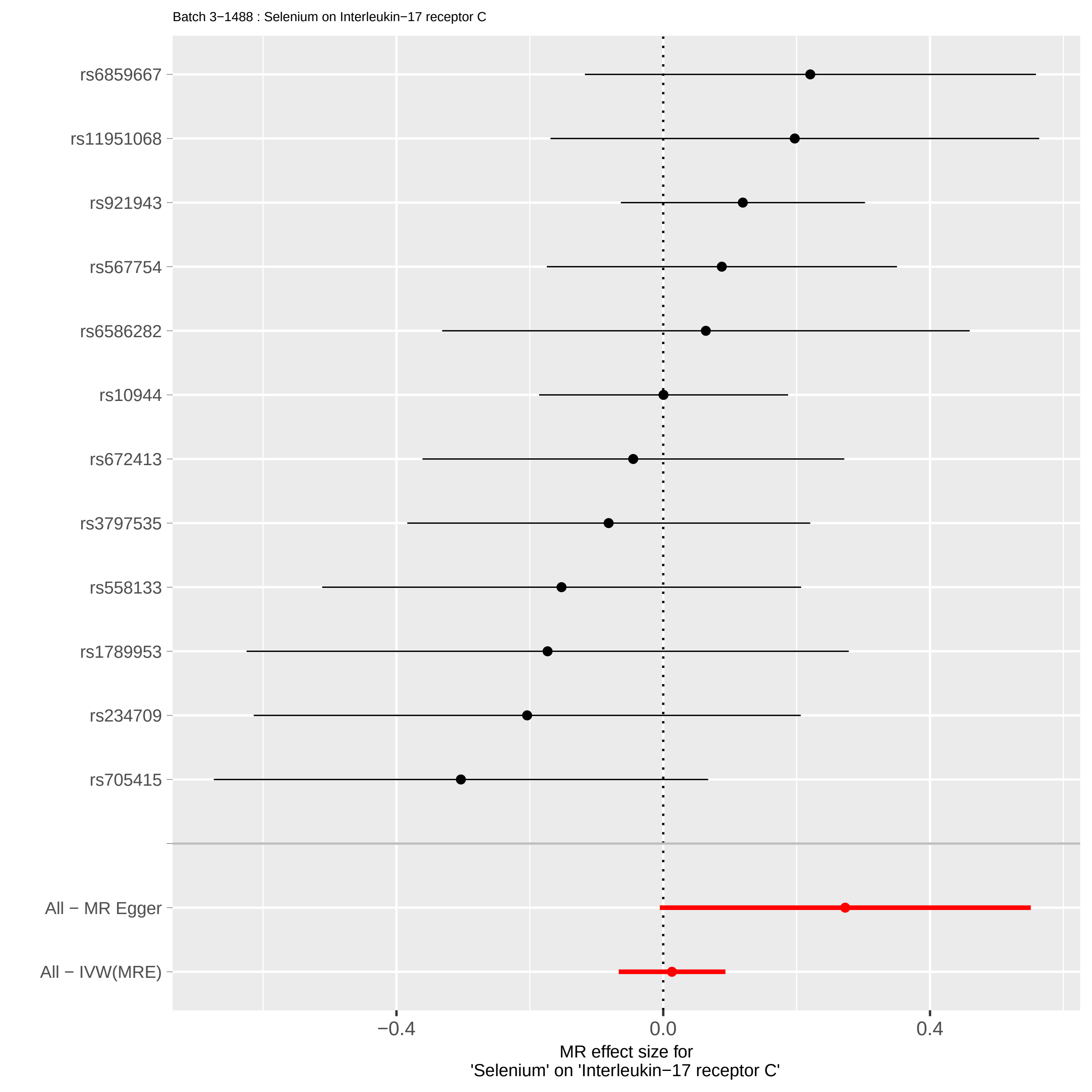

Batch 3−1488 : Selenium on Interleukin−17 receptor C
rs6859667
rs11951068
rs921943
rs567754
rs6586282
rs10944
rs672413
rs3797535
rs558133
rs1789953
rs234709
rs705415
All − MR Egger
All − IVW(MRE)
−0.4
0.0
0.4
MR effect size for
'Selenium' on 'Interleukin−17 receptor C'

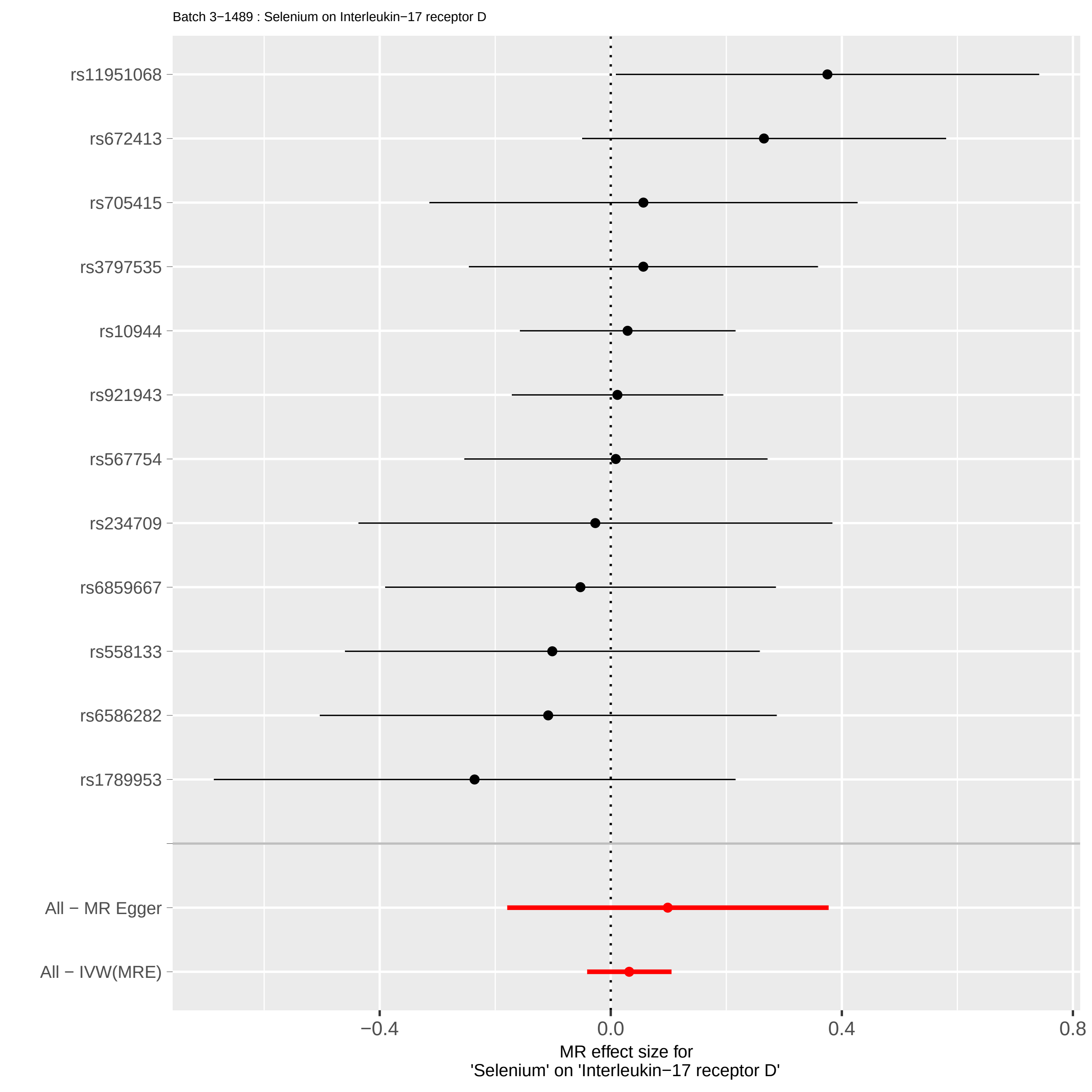
Batch 3−1489 : Selenium on Interleukin−17 receptor D
rs11951068
rs672413
rs705415
rs3797535
rs10944
rs921943
rs567754
rs234709
rs6859667
rs558133
rs6586282
rs1789953
All − MR Egger
All − IVW(MRE)
−0.4
0.0
0.4
0.8
MR effect size for
'Selenium' on 'Interleukin−17 receptor D'

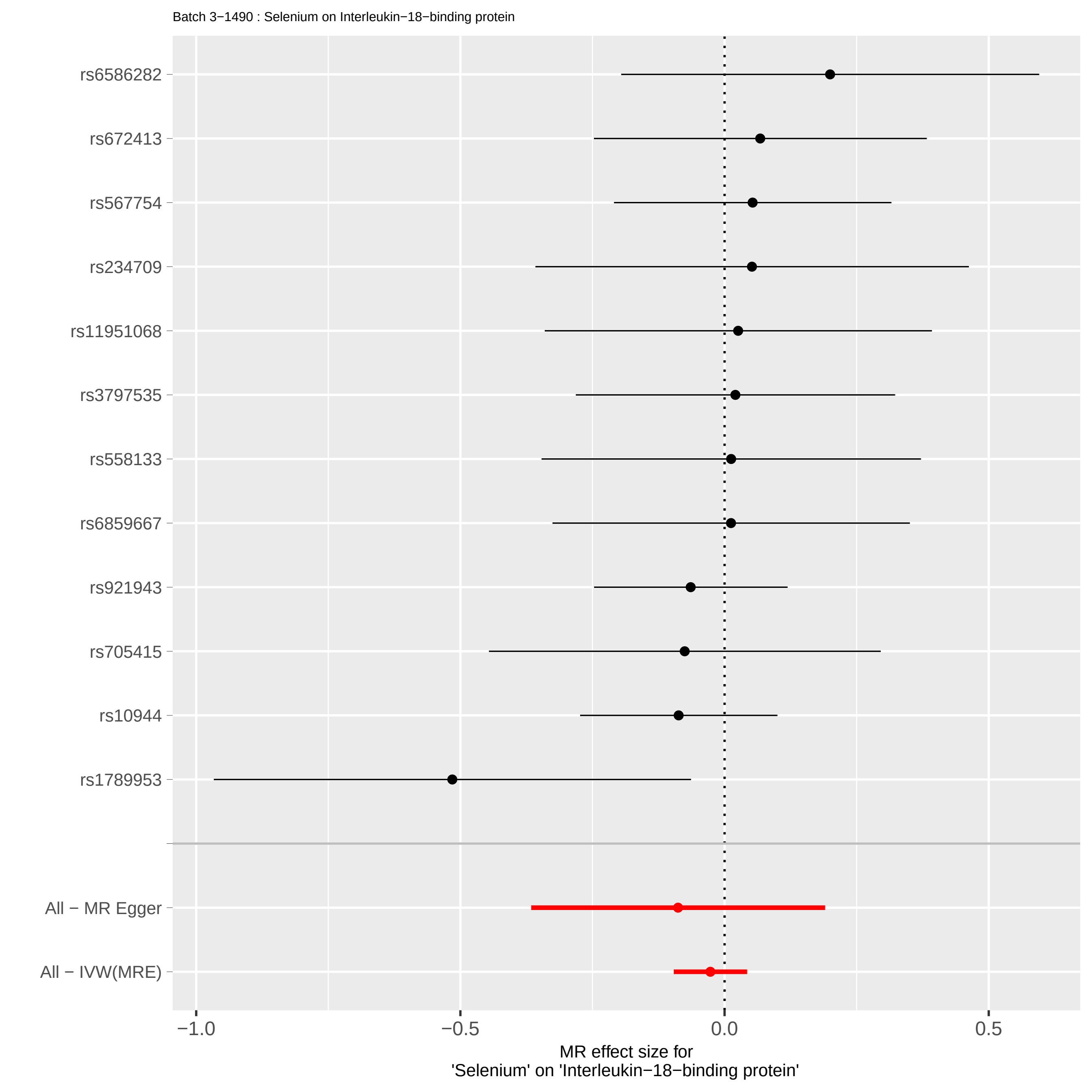

Batch 3−1490 : Selenium on Interleukin−18−binding protein
rs6586282
rs672413
rs567754
rs234709
rs11951068
rs3797535
rs558133
rs6859667
rs921943
rs705415
rs10944
rs1789953
All − MR Egger
All − IVW(MRE)
−1.0
−0.5
0.0
0.5
MR effect size for
'Selenium' on 'Interleukin−18−binding protein'

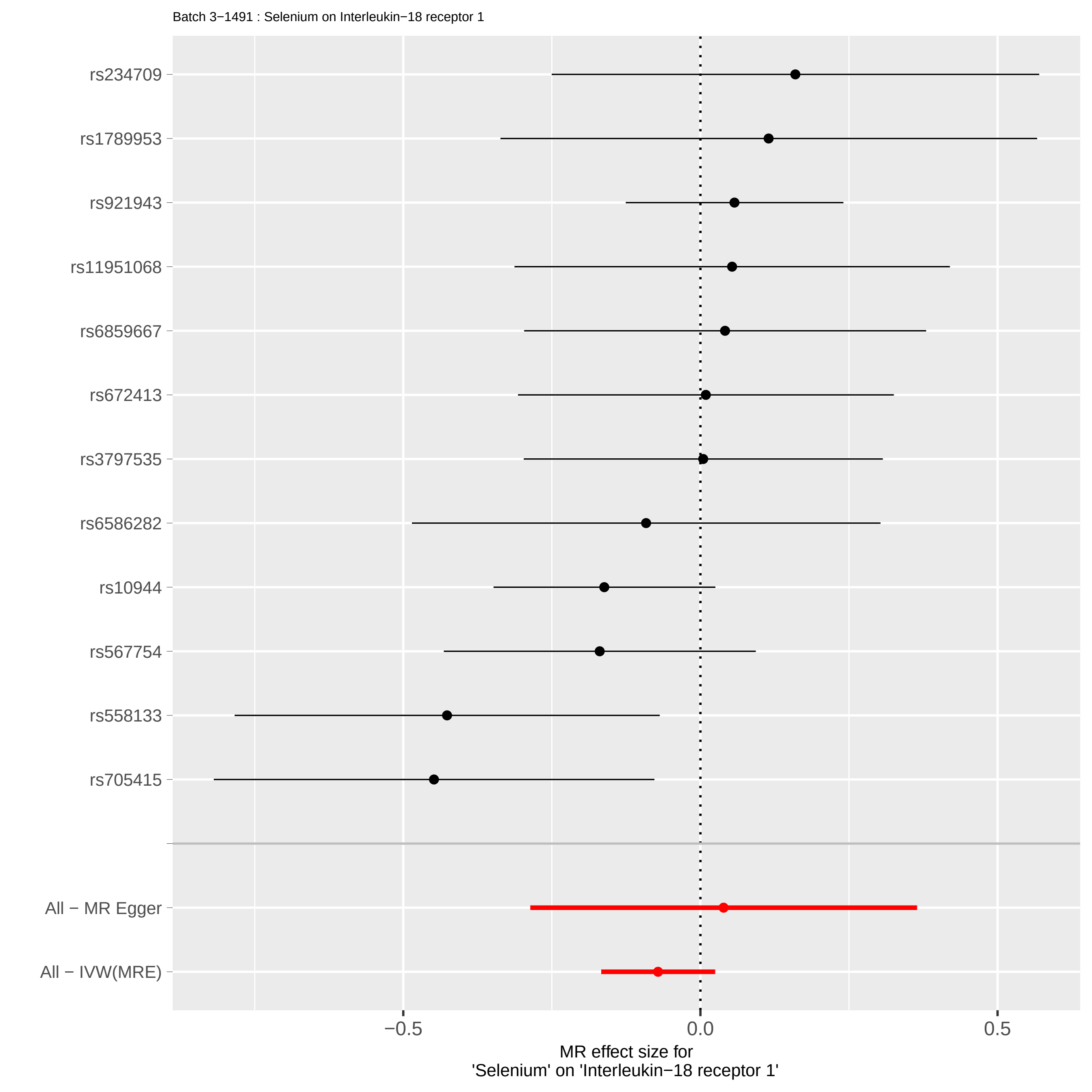
Batch 3−1491 : Selenium on Interleukin−18 receptor 1
rs234709
rs1789953
rs921943
rs11951068
rs6859667
rs672413
rs3797535
rs6586282
rs10944
rs567754
rs558133
rs705415
All − MR Egger
All − IVW(MRE)
−0.5
0.0
0.5
MR effect size for
'Selenium' on 'Interleukin−18 receptor 1'

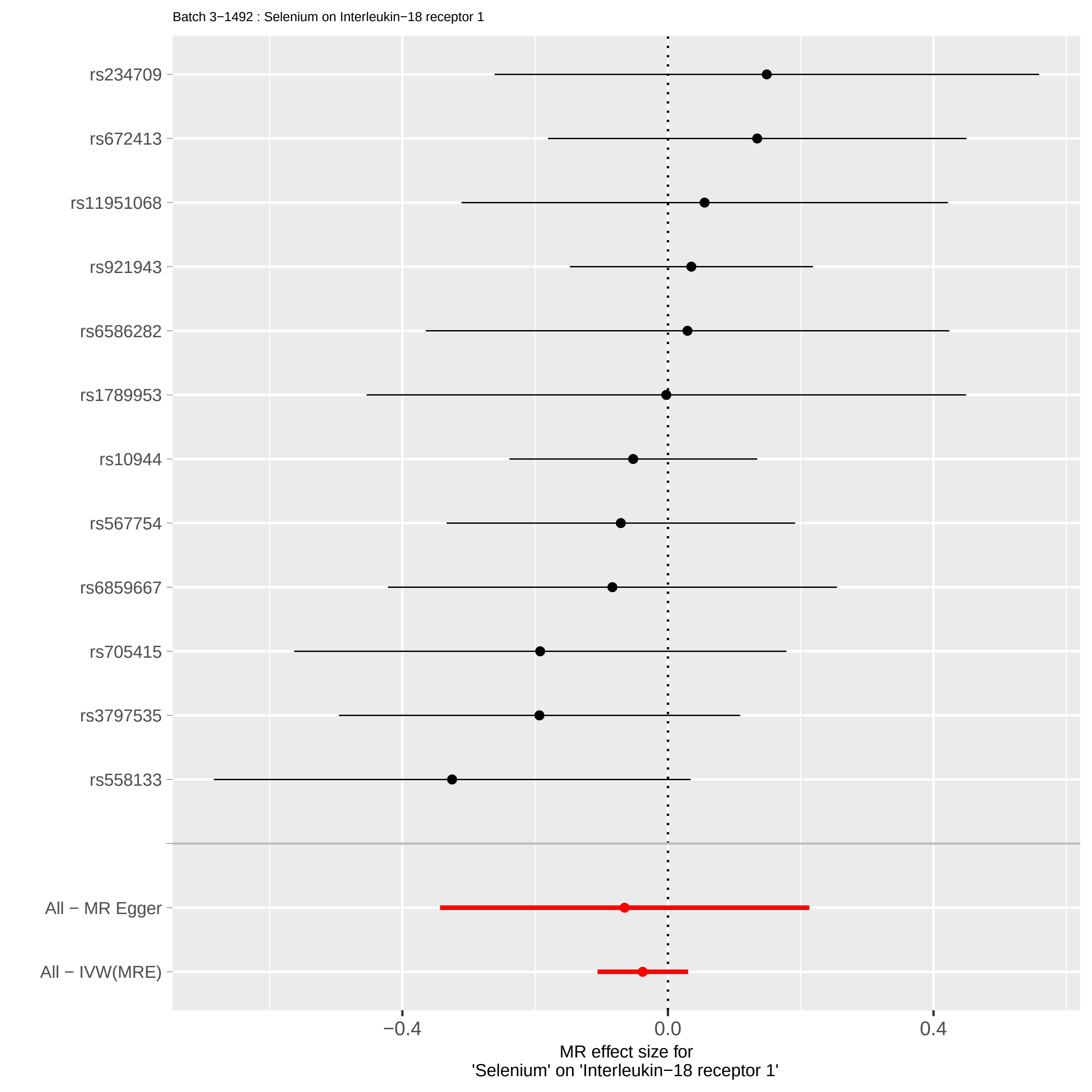

Batch 3−1492 : Selenium on Interleukin−18 receptor 1
rs234709
rs672413
rs11951068
rs921943
rs6586282
rs1789953
rs10944
rs567754
rs6859667
rs705415
rs3797535
rs558133
All − MR Egger
All − IVW(MRE)
−0.4
0.0
0.4
MR effect size for
'Selenium' on 'Interleukin−18 receptor 1'

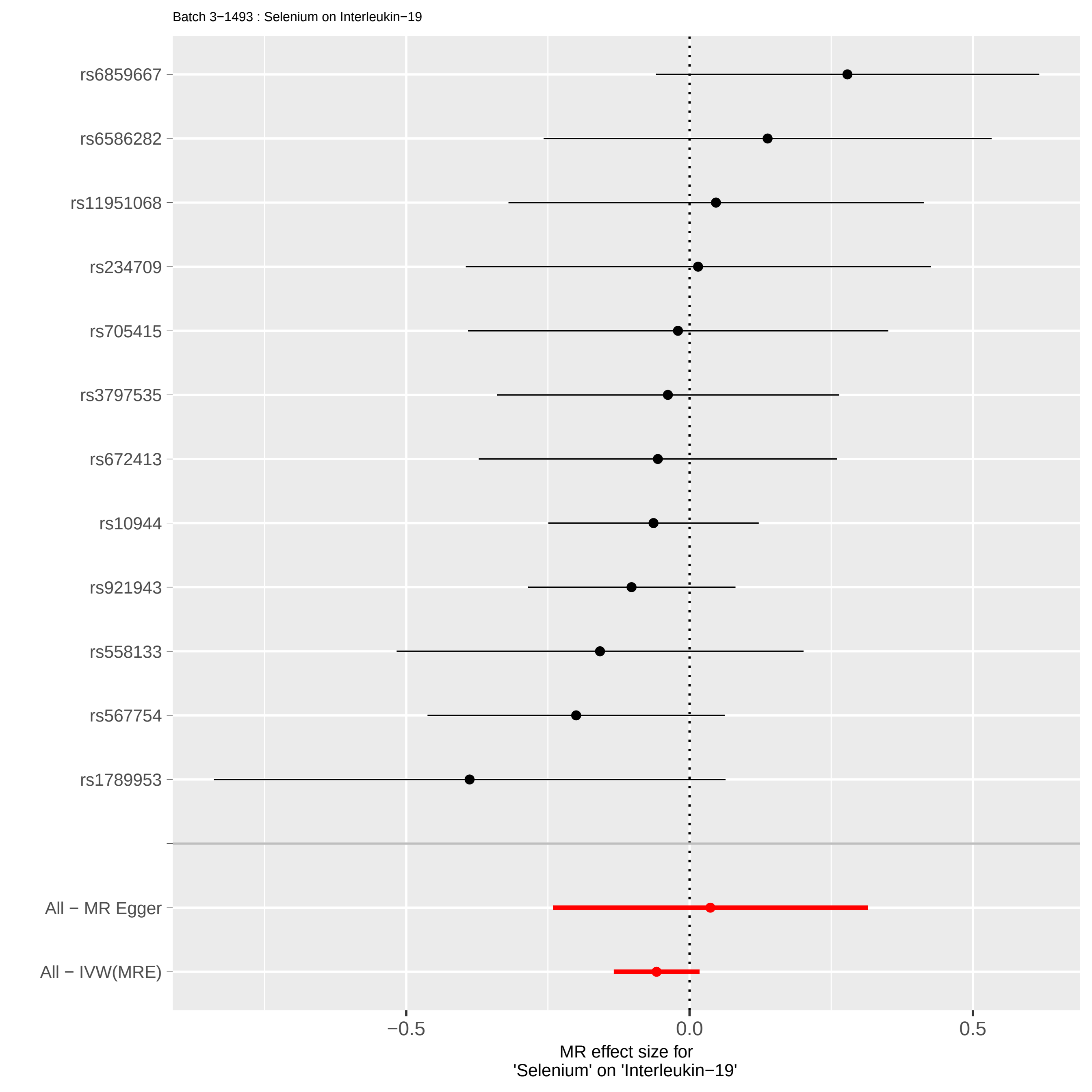
Batch 3−1493 : Selenium on Interleukin−19
rs6859667
rs6586282
rs11951068
rs234709
rs705415
rs3797535
rs672413
rs10944
rs921943
rs558133
rs567754
rs1789953
All − MR Egger
All − IVW(MRE)
−0.5
0.0
0.5
MR effect size for
'Selenium' on 'Interleukin−19'

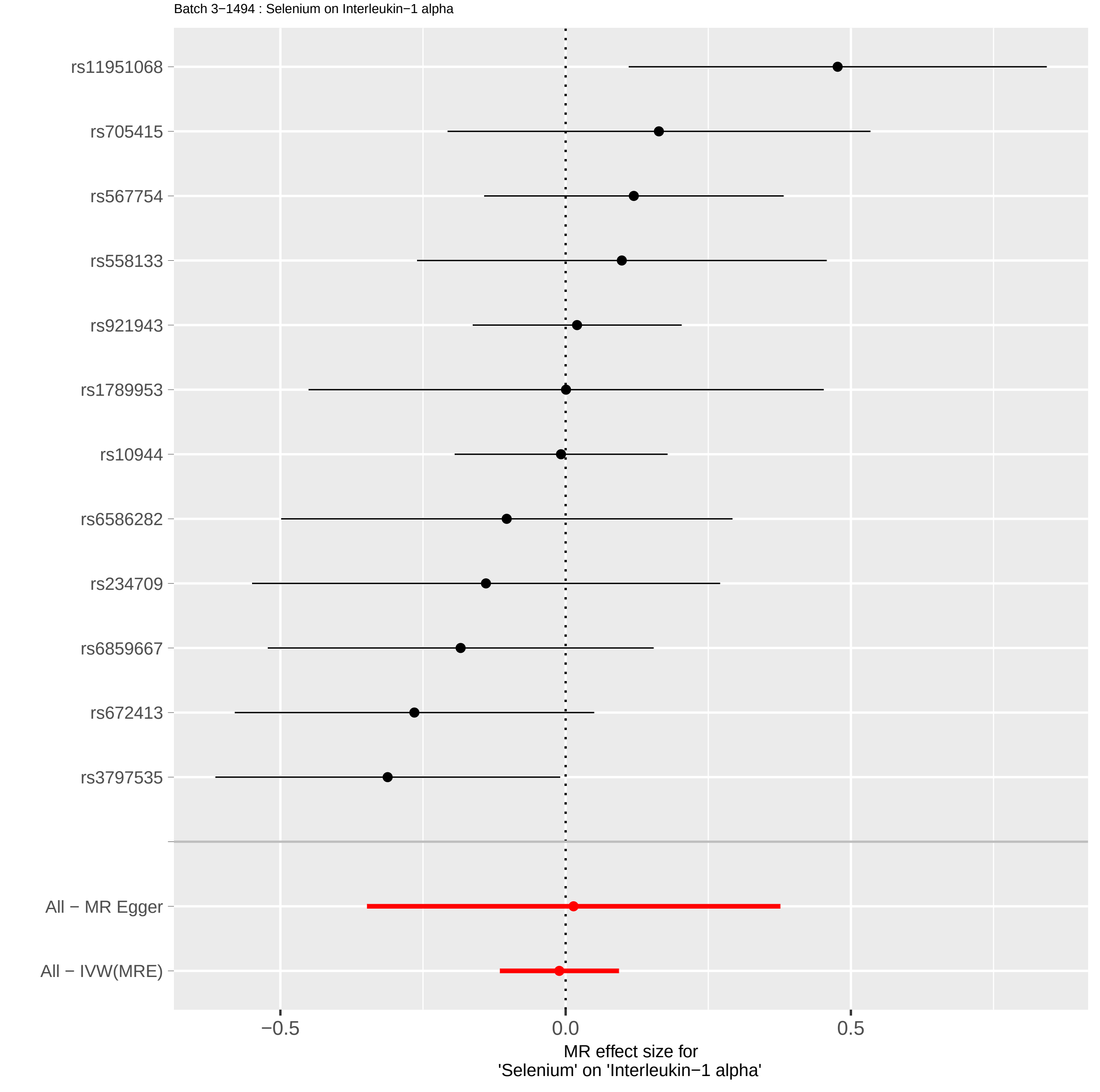

Batch 3−1494 : Selenium on Interleukin−1 alpha
rs11951068
rs705415
rs567754
rs558133
rs921943
rs1789953
rs10944
rs6586282
rs234709
rs6859667
rs672413
rs3797535
All − MR Egger
All − IVW(MRE)
−0.5
0.0
0.5
MR effect size for
'Selenium' on 'Interleukin−1 alpha'

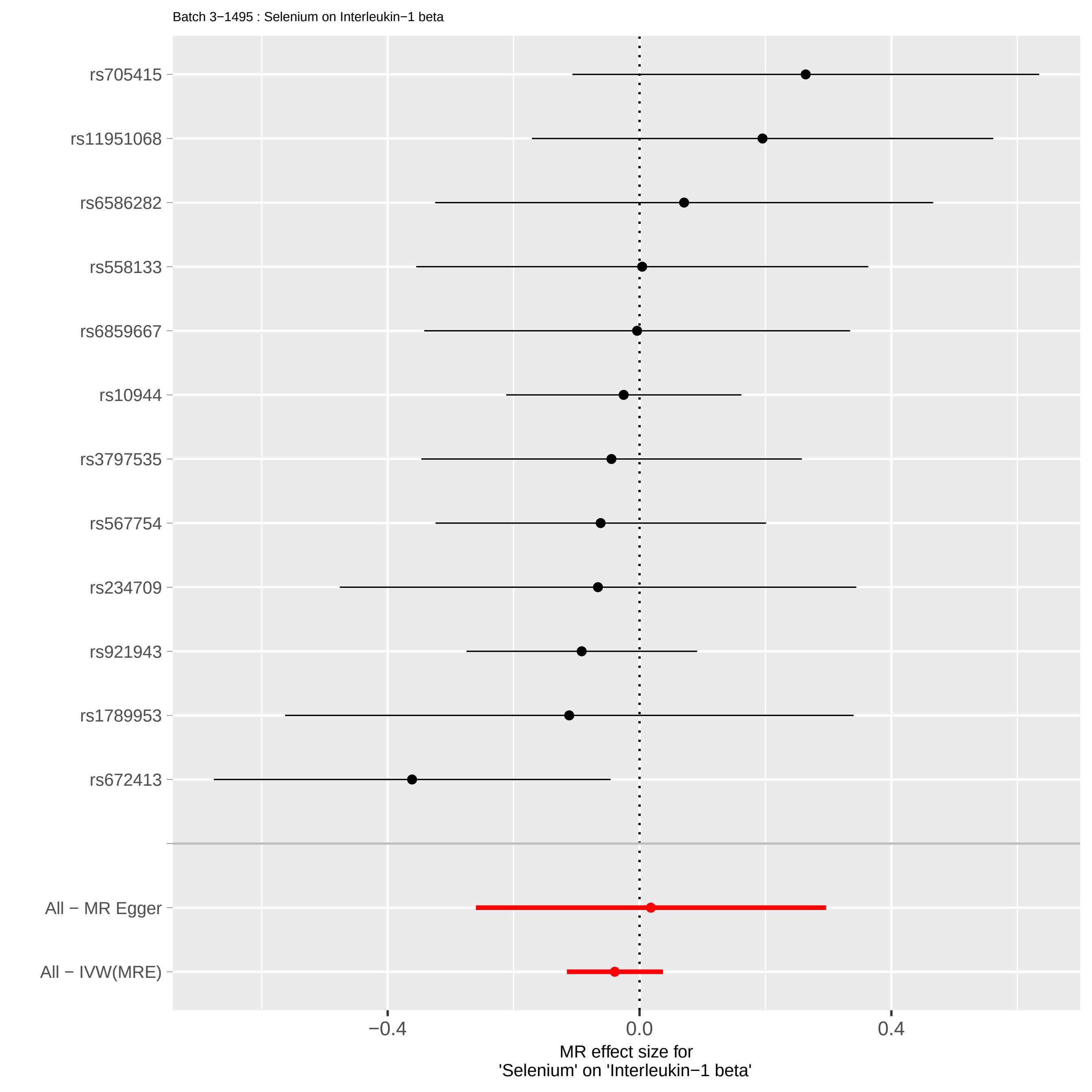

Batch 3−1495 : Selenium on Interleukin−1 beta
rs705415
rs11951068
rs6586282
rs558133
rs6859667
rs10944
rs3797535
rs567754
rs234709
rs921943
rs1789953
rs672413
All − MR Egger
All − IVW(MRE)
−0.4
0.0
0.4
MR effect size for
'Selenium' on 'Interleukin−1 beta'

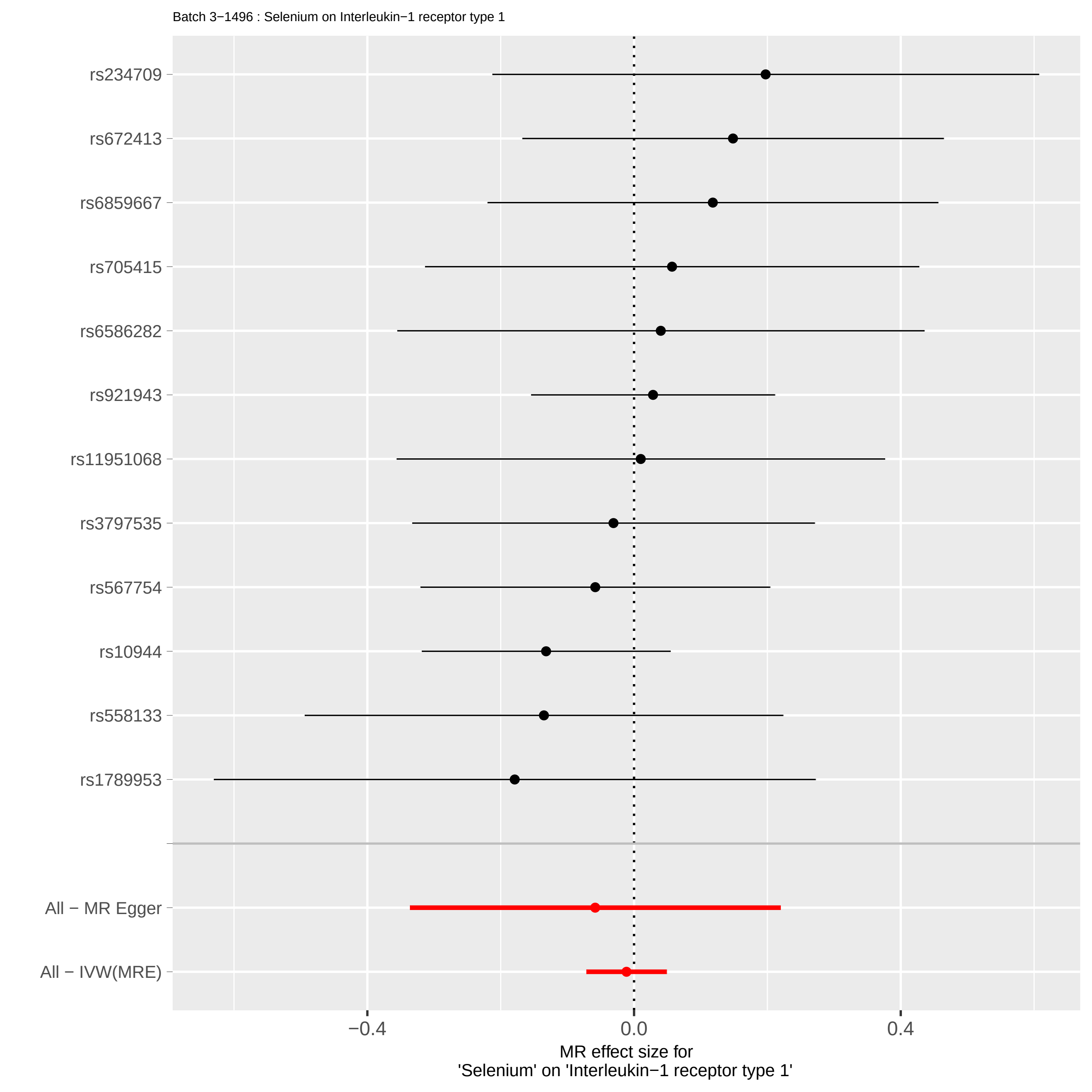

Batch 3−1496 : Selenium on Interleukin−1 receptor type 1
rs234709
rs672413
rs6859667
rs705415
rs6586282
rs921943
rs11951068
rs3797535
rs567754
rs10944
rs558133
rs1789953
All − MR Egger
All − IVW(MRE)
−0.4
0.0
0.4
MR effect size for
'Selenium' on 'Interleukin−1 receptor type 1'

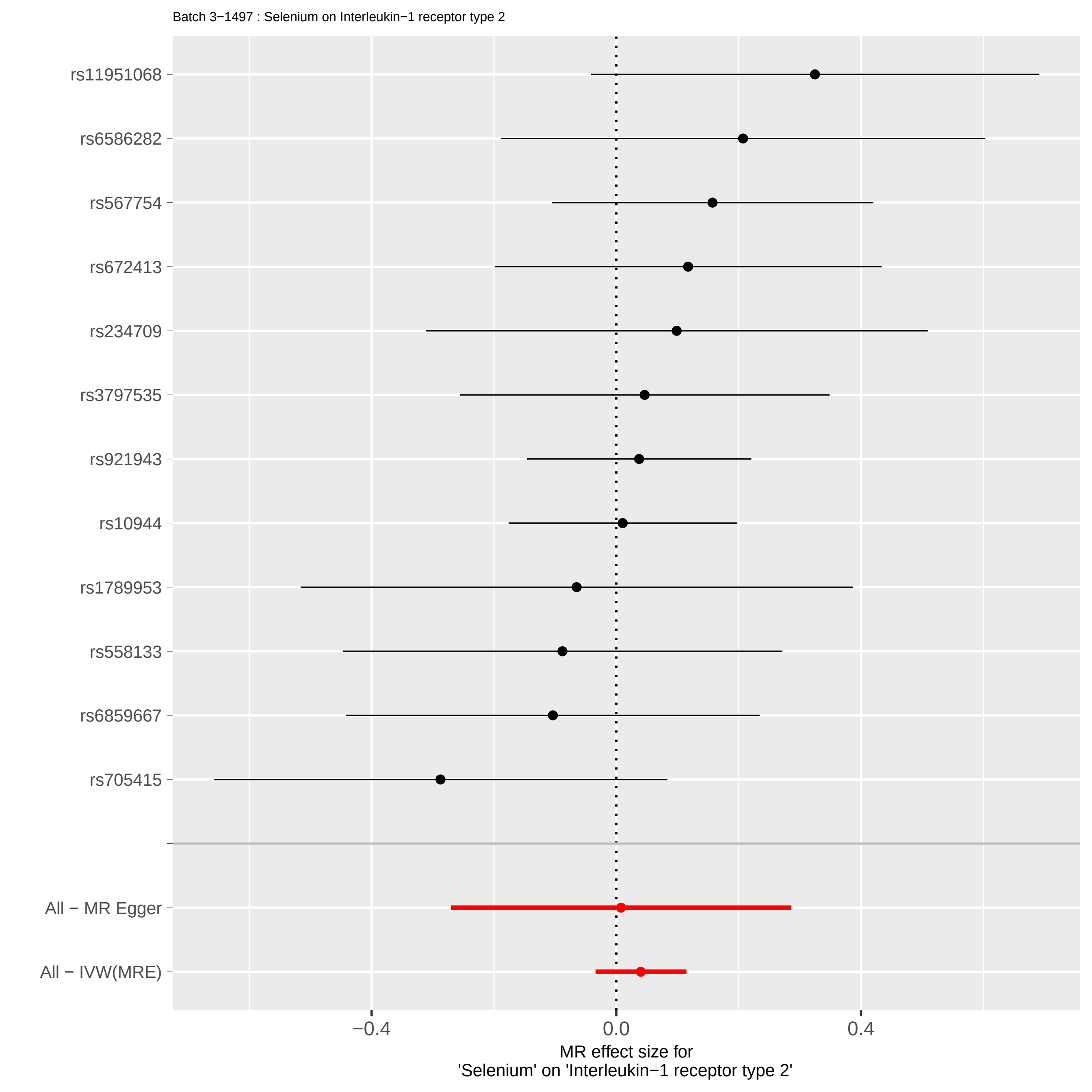

Batch 3−1497 : Selenium on Interleukin−1 receptor type 2
rs11951068
rs6586282
rs567754
rs672413
rs234709
rs3797535
rs921943
rs10944
rs1789953
rs558133
rs6859667
rs705415
All − MR Egger
All − IVW(MRE)
−0.4
0.0
0.4
MR effect size for
'Selenium' on 'Interleukin−1 receptor type 2'

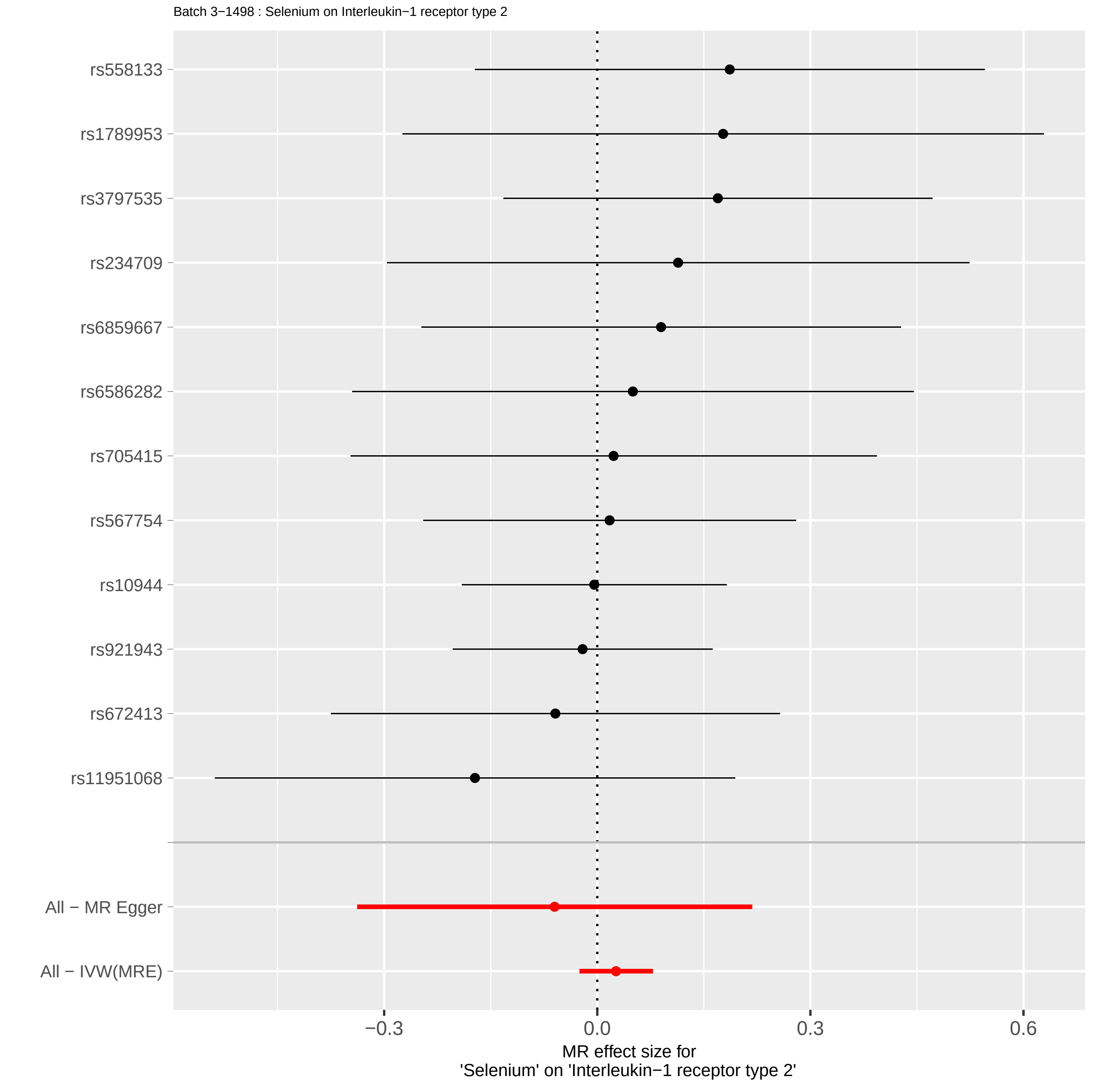

Batch 3−1498 : Selenium on Interleukin−1 receptor type 2
rs558133
rs1789953
rs3797535
rs234709
rs6859667
rs6586282
rs705415
rs567754
rs10944
rs921943
rs672413
rs11951068
All − MR Egger
All − IVW(MRE)
−0.3
0.0
0.3
0.6
MR effect size for
'Selenium' on 'Interleukin−1 receptor type 2'

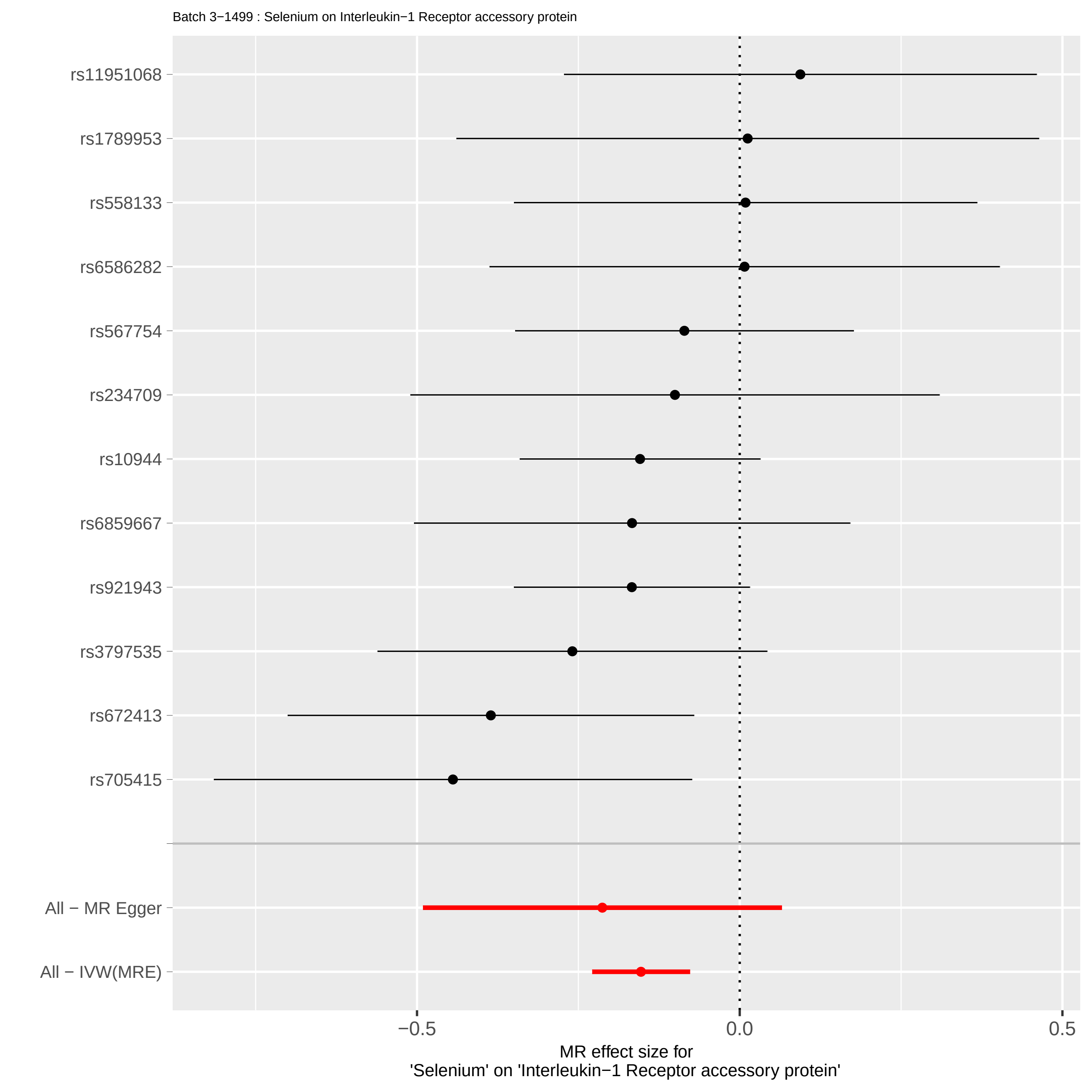

Batch 3−1499 : Selenium on Interleukin−1 Receptor accessory protein
rs11951068
rs1789953
rs558133
rs6586282
rs567754
rs234709
rs10944
rs6859667
rs921943
rs3797535
rs672413
rs705415
All − MR Egger
All − IVW(MRE)
−0.5
0.0
0.5
MR effect size for
'Selenium' on 'Interleukin−1 Receptor accessory protein'

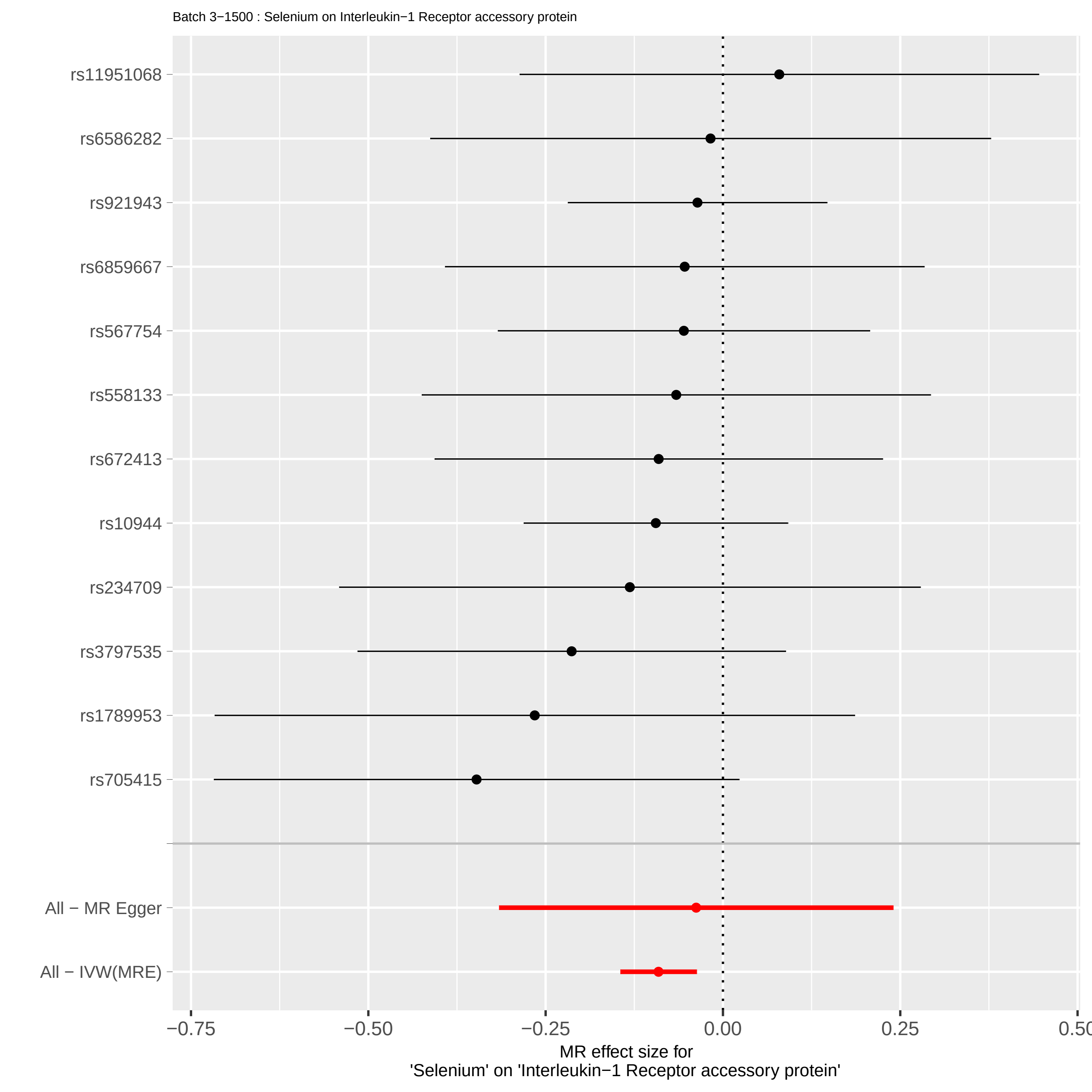

Batch 3−1500 : Selenium on Interleukin−1 Receptor accessory protein
rs11951068
rs6586282
rs921943
rs6859667
rs567754
rs558133
rs672413
rs10944
rs234709
rs3797535
rs1789953
rs705415
All − MR Egger
All − IVW(MRE)
−0.75
−0.50
−0.25
0.00
0.25
0.50
MR effect size for
'Selenium' on 'Interleukin−1 Receptor accessory protein'

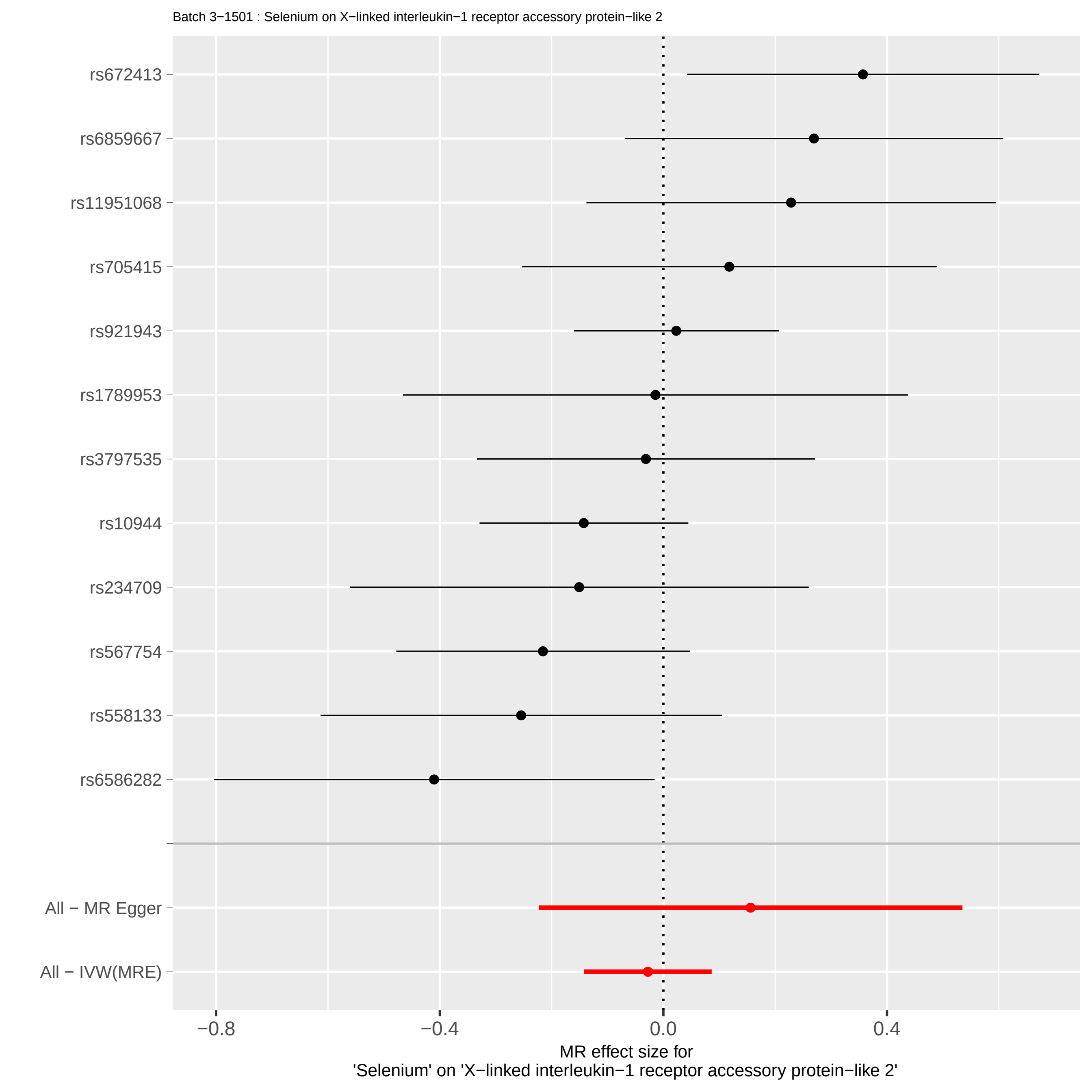

Batch 3−1501 : Selenium on X−linked interleukin−1 receptor accessory protein−like 2
rs672413
rs6859667
rs11951068
rs705415
rs921943
rs1789953
rs3797535
rs10944
rs234709
rs567754
rs558133
rs6586282
All − MR Egger
All − IVW(MRE)
−0.8
−0.4
0.0
0.4
MR effect size for
'Selenium' on 'X−linked interleukin−1 receptor accessory protein−like 2'

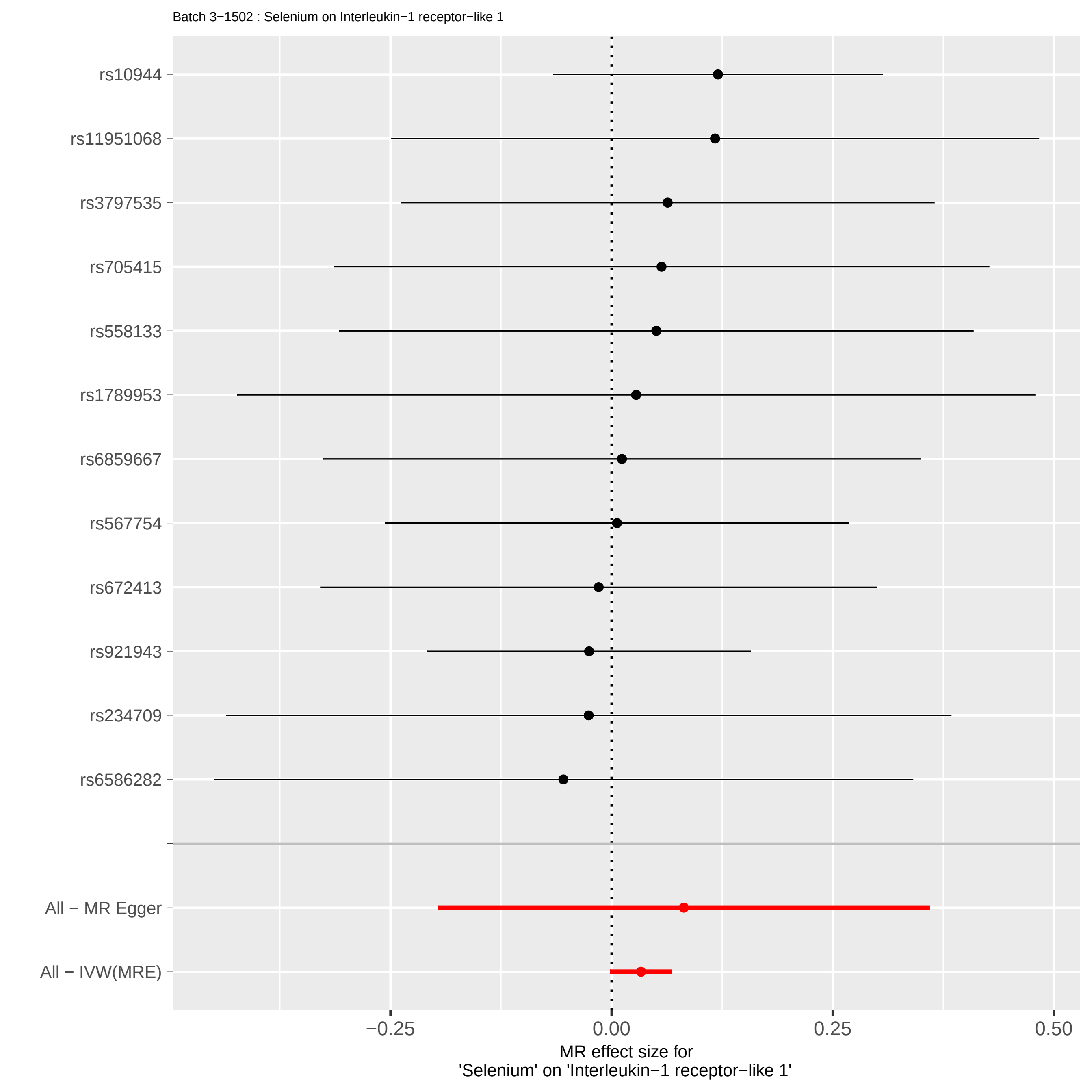

Batch 3−1502 : Selenium on Interleukin−1 receptor−like 1
rs10944
rs11951068
rs3797535
rs705415
rs558133
rs1789953
rs6859667
rs567754
rs672413
rs921943
rs234709
rs6586282
All − MR Egger
All − IVW(MRE)
−0.25
0.00
0.25
0.50
MR effect size for
'Selenium' on 'Interleukin−1 receptor−like 1'

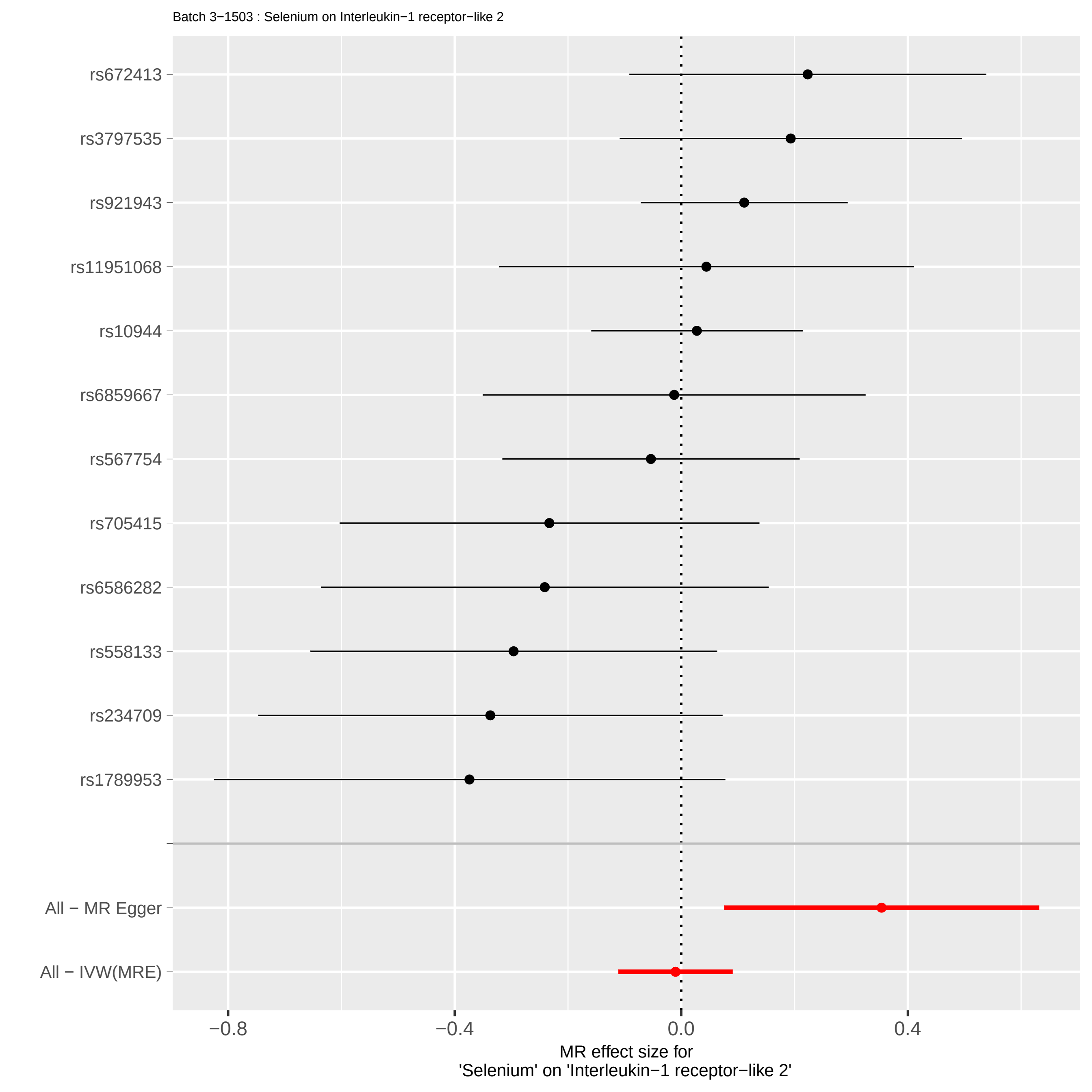

Batch 3−1503 : Selenium on Interleukin−1 receptor−like 2
rs672413
rs3797535
rs921943
rs11951068
rs10944
rs6859667
rs567754
rs705415
rs6586282
rs558133
rs234709
rs1789953
All − MR Egger
All − IVW(MRE)
−0.8
−0.4
0.0
0.4
MR effect size for
'Selenium' on 'Interleukin−1 receptor−like 2'

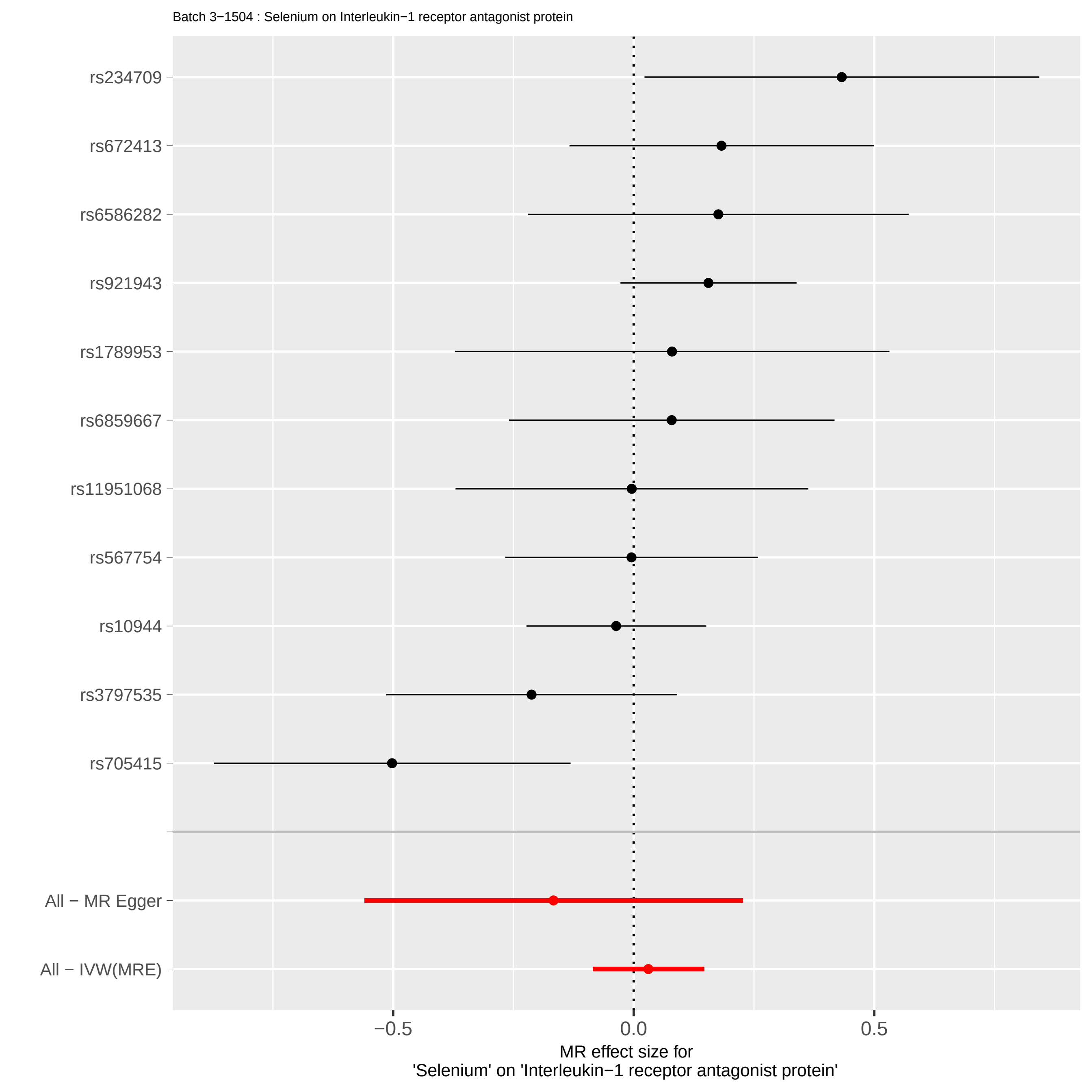

Batch 3−1504 : Selenium on Interleukin−1 receptor antagonist protein
rs234709
rs672413
rs6586282
rs921943
rs1789953
rs6859667
rs11951068
rs567754
rs10944
rs3797535
rs705415
All − MR Egger
All − IVW(MRE)
−0.5
0.0
0.5
MR effect size for
'Selenium' on 'Interleukin−1 receptor antagonist protein'

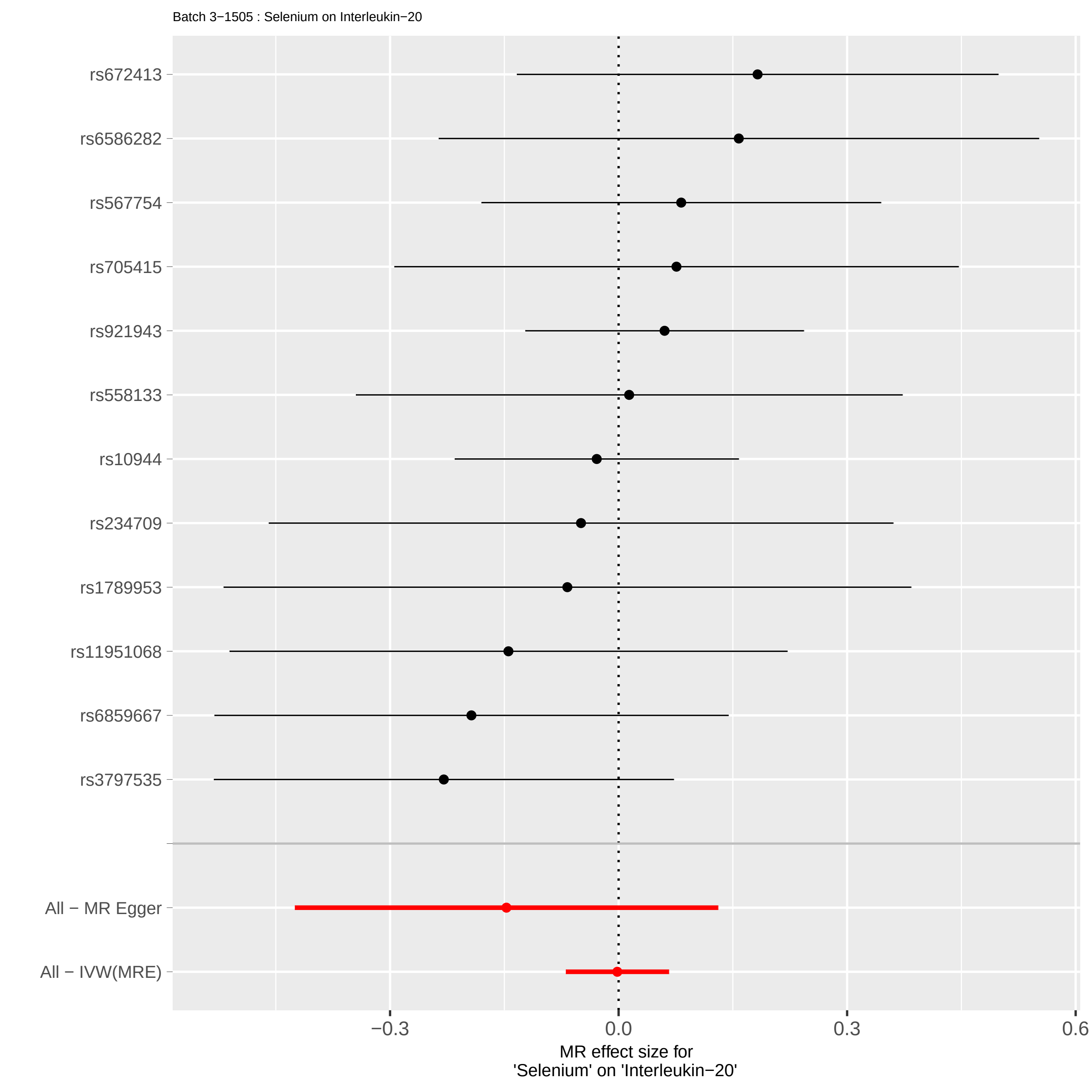

Batch 3−1505 : Selenium on Interleukin−20
rs672413
rs6586282
rs567754
rs705415
rs921943
rs558133
rs10944
rs234709
rs1789953
rs11951068
rs6859667
rs3797535
All − MR Egger
All − IVW(MRE)
−0.3
0.0
0.3
0.6
MR effect size for
'Selenium' on 'Interleukin−20'

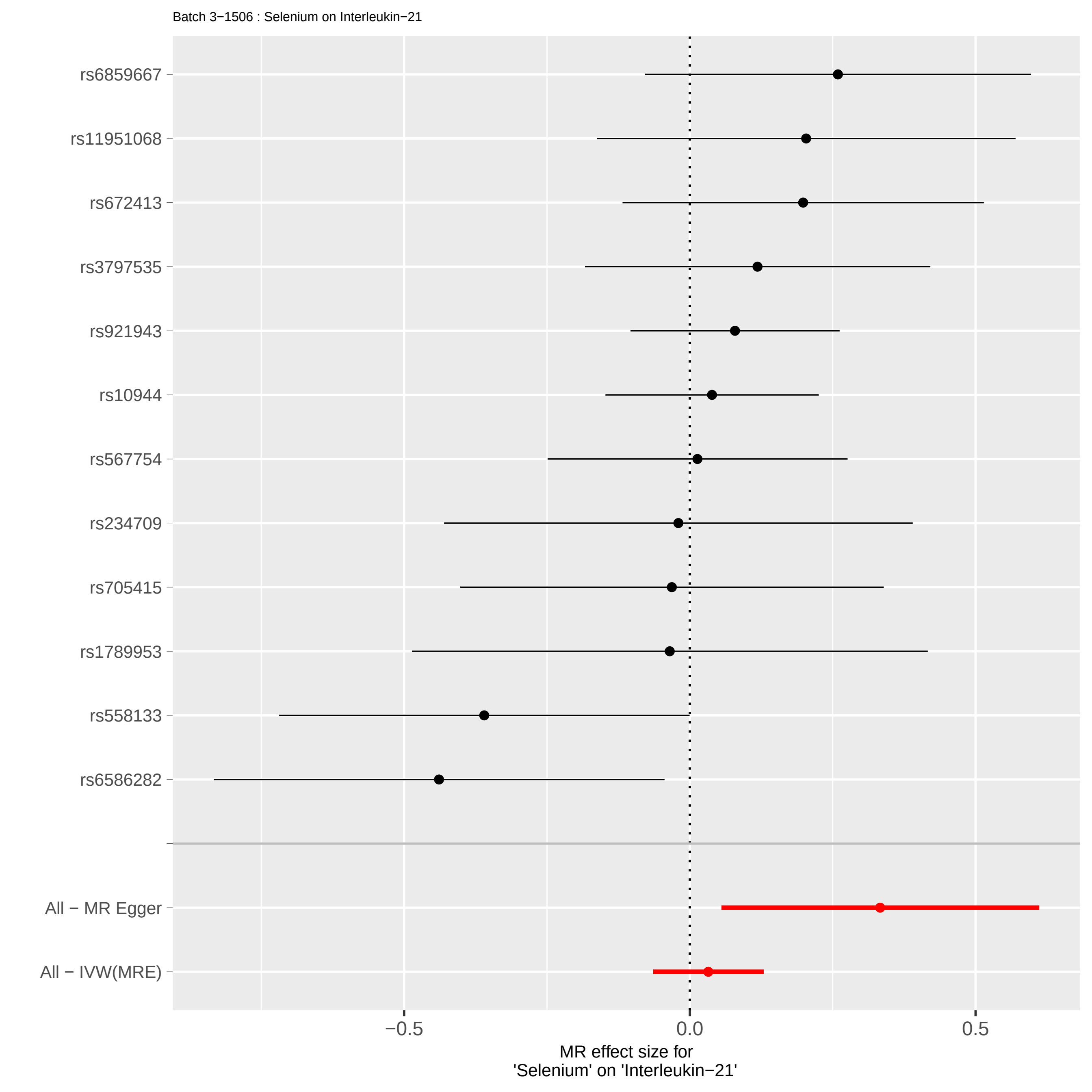
Batch 3−1506 : Selenium on Interleukin−21
rs6859667
rs11951068
rs672413
rs3797535
rs921943
rs10944
rs567754
rs234709
rs705415
rs1789953
rs558133
rs6586282
All − MR Egger
All − IVW(MRE)
−0.5
0.0
0.5
MR effect size for
'Selenium' on 'Interleukin−21'

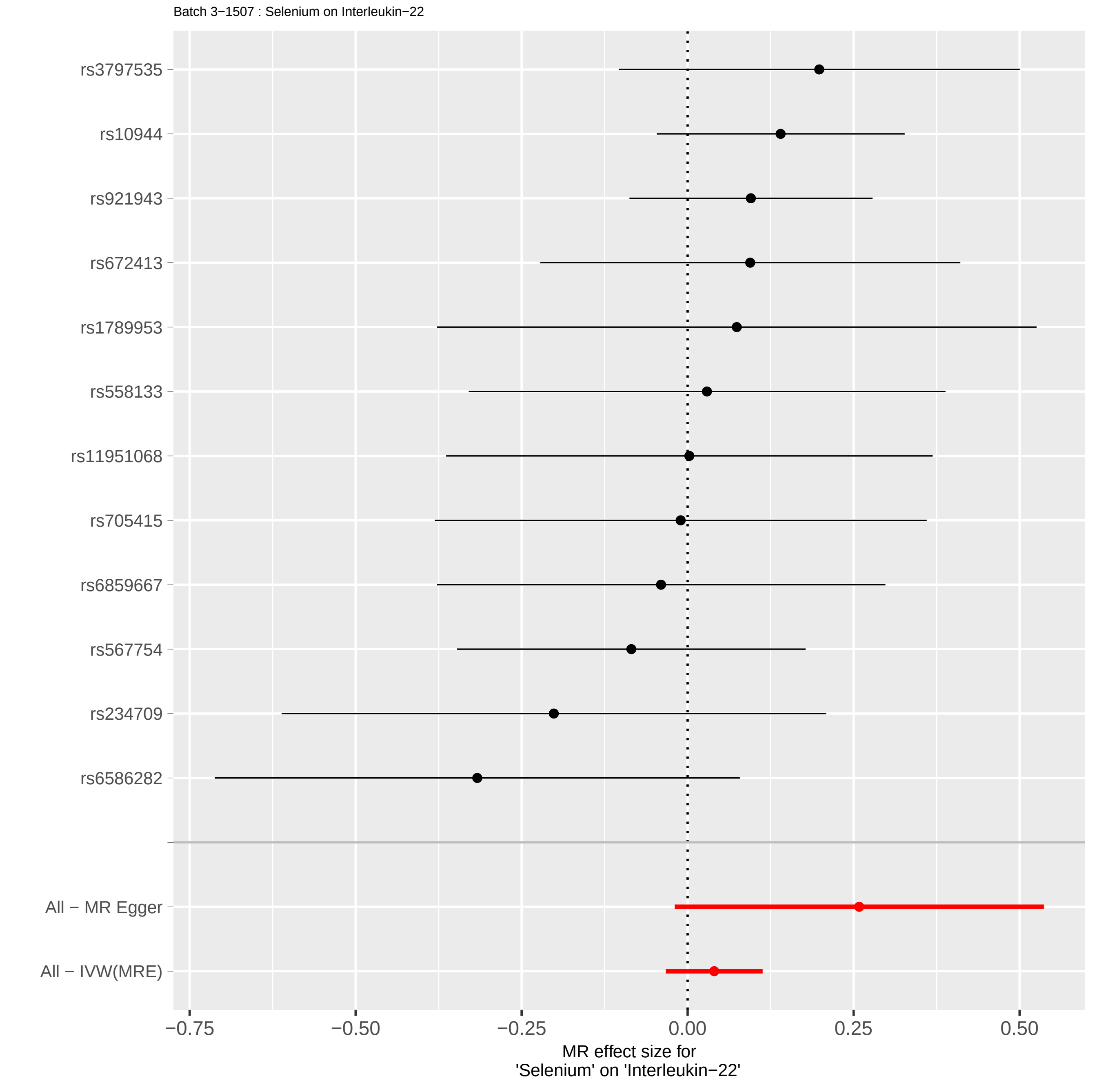
Batch 3−1507 : Selenium on Interleukin−22
rs3797535
rs10944
rs921943
rs672413
rs1789953
rs558133
rs11951068
rs705415
rs6859667
rs567754
rs234709
rs6586282
All − MR Egger
All − IVW(MRE)
−0.75
−0.50
−0.25
0.00
0.25
0.50
MR effect size for
'Selenium' on 'Interleukin−22'

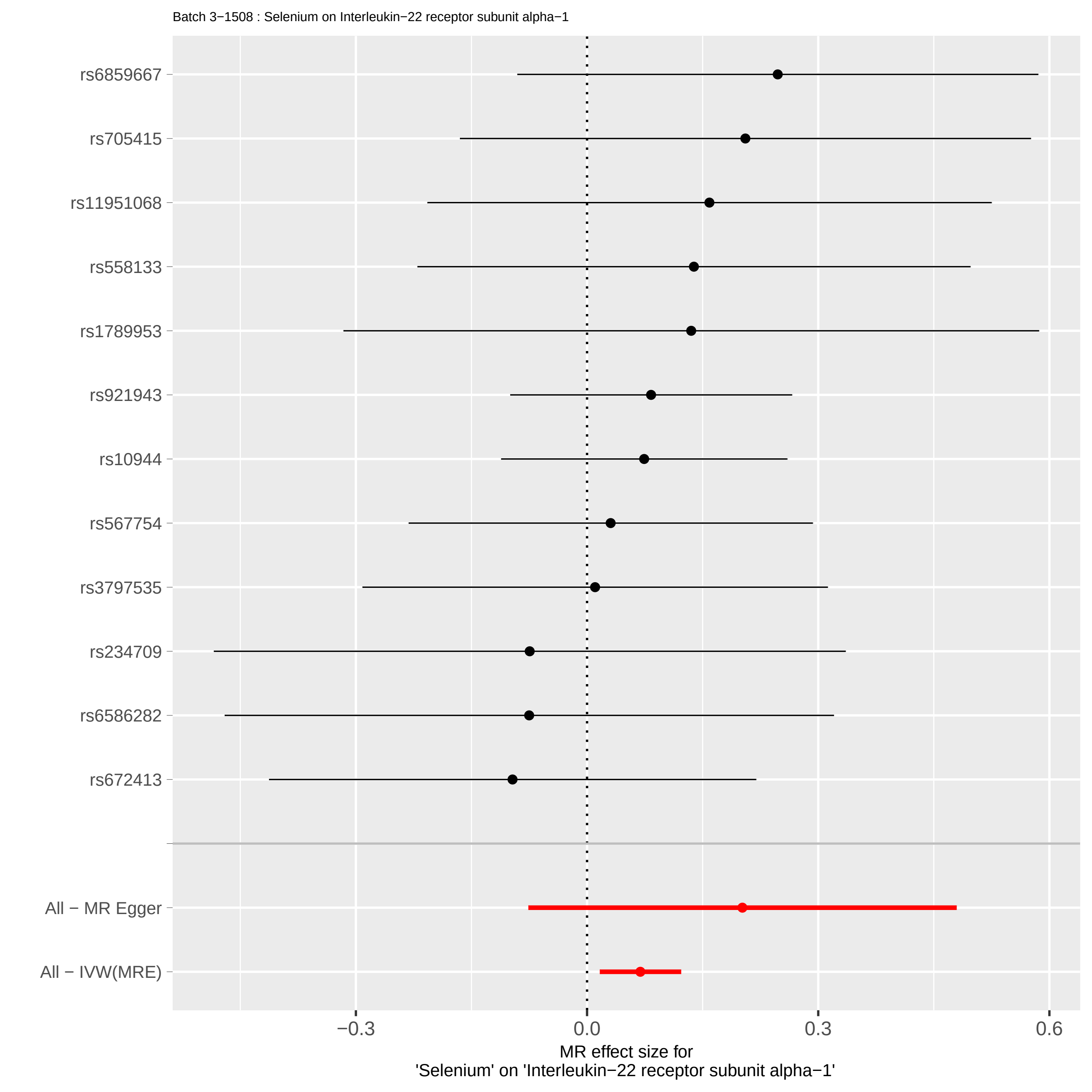

Batch 3−1508 : Selenium on Interleukin−22 receptor subunit alpha−1
rs6859667
rs705415
rs11951068
rs558133
rs1789953
rs921943
rs10944
rs567754
rs3797535
rs234709
rs6586282
rs672413
All − MR Egger
All − IVW(MRE)
−0.3
0.0
0.3
0.6
MR effect size for
'Selenium' on 'Interleukin−22 receptor subunit alpha−1'

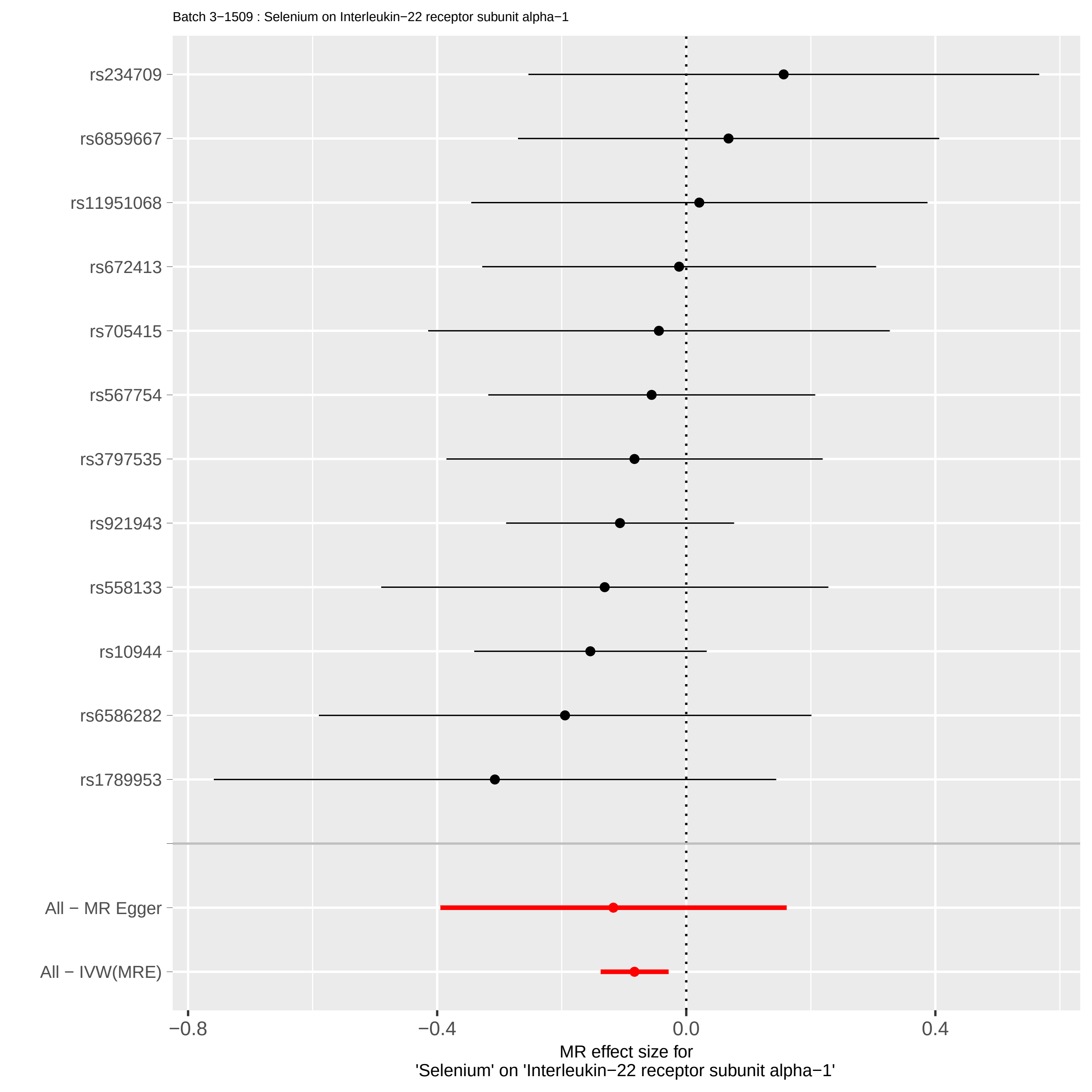

Batch 3−1509 : Selenium on Interleukin−22 receptor subunit alpha−1
rs234709
rs6859667
rs11951068
rs672413
rs705415
rs567754
rs3797535
rs921943
rs558133
rs10944
rs6586282
rs1789953
All − MR Egger
All − IVW(MRE)
−0.8
−0.4
0.0
0.4
MR effect size for
'Selenium' on 'Interleukin−22 receptor subunit alpha−1'

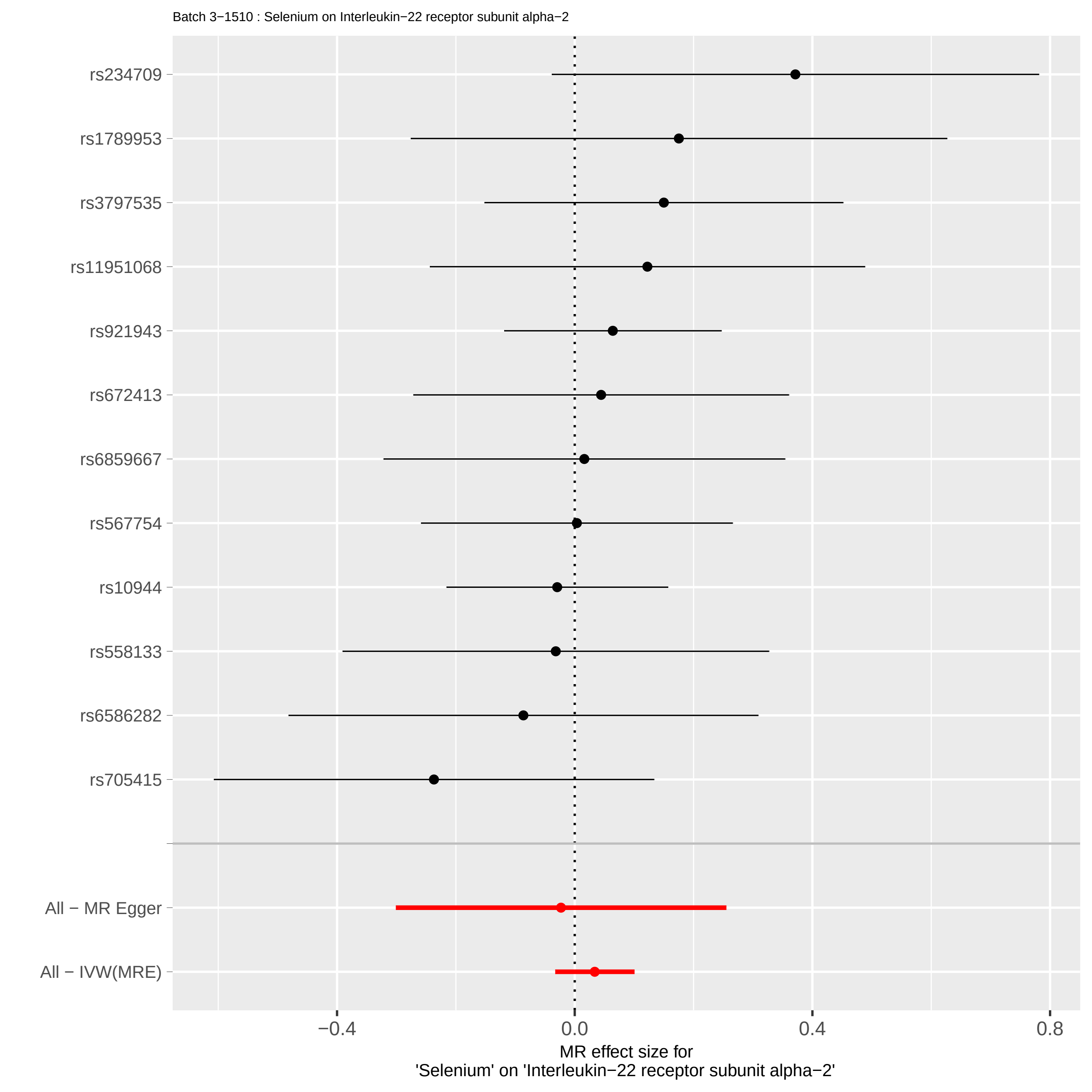

Batch 3−1510 : Selenium on Interleukin−22 receptor subunit alpha−2
rs234709
rs1789953
rs3797535
rs11951068
rs921943
rs672413
rs6859667
rs567754
rs10944
rs558133
rs6586282
rs705415
All − MR Egger
All − IVW(MRE)
−0.4
0.0
0.4
0.8
MR effect size for
'Selenium' on 'Interleukin−22 receptor subunit alpha−2'

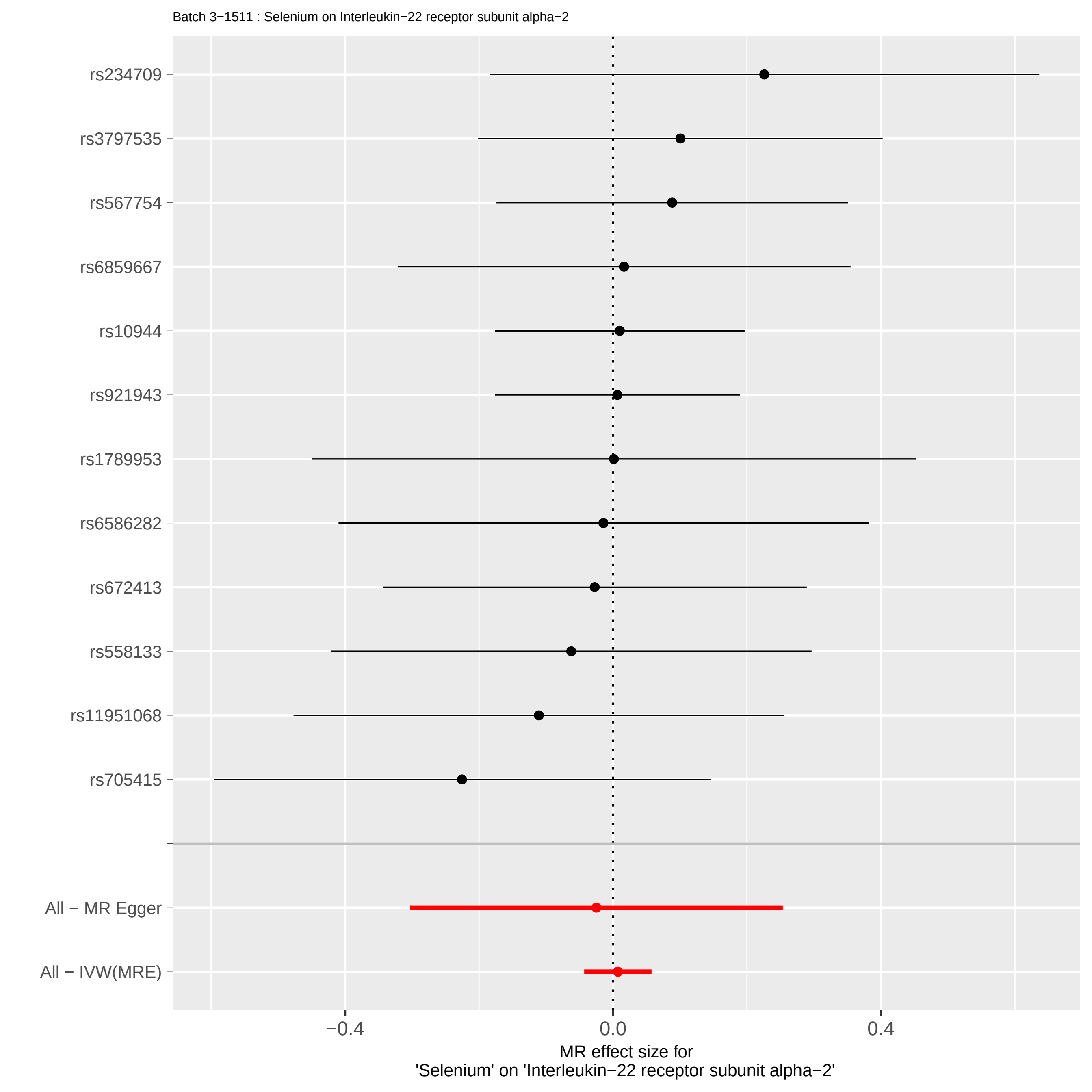

Batch 3−1511 : Selenium on Interleukin−22 receptor subunit alpha−2
rs234709
rs3797535
rs567754
rs6859667
rs10944
rs921943
rs1789953
rs6586282
rs672413
rs558133
rs11951068
rs705415
All − MR Egger
All − IVW(MRE)
−0.4
0.0
0.4
MR effect size for
'Selenium' on 'Interleukin−22 receptor subunit alpha−2'

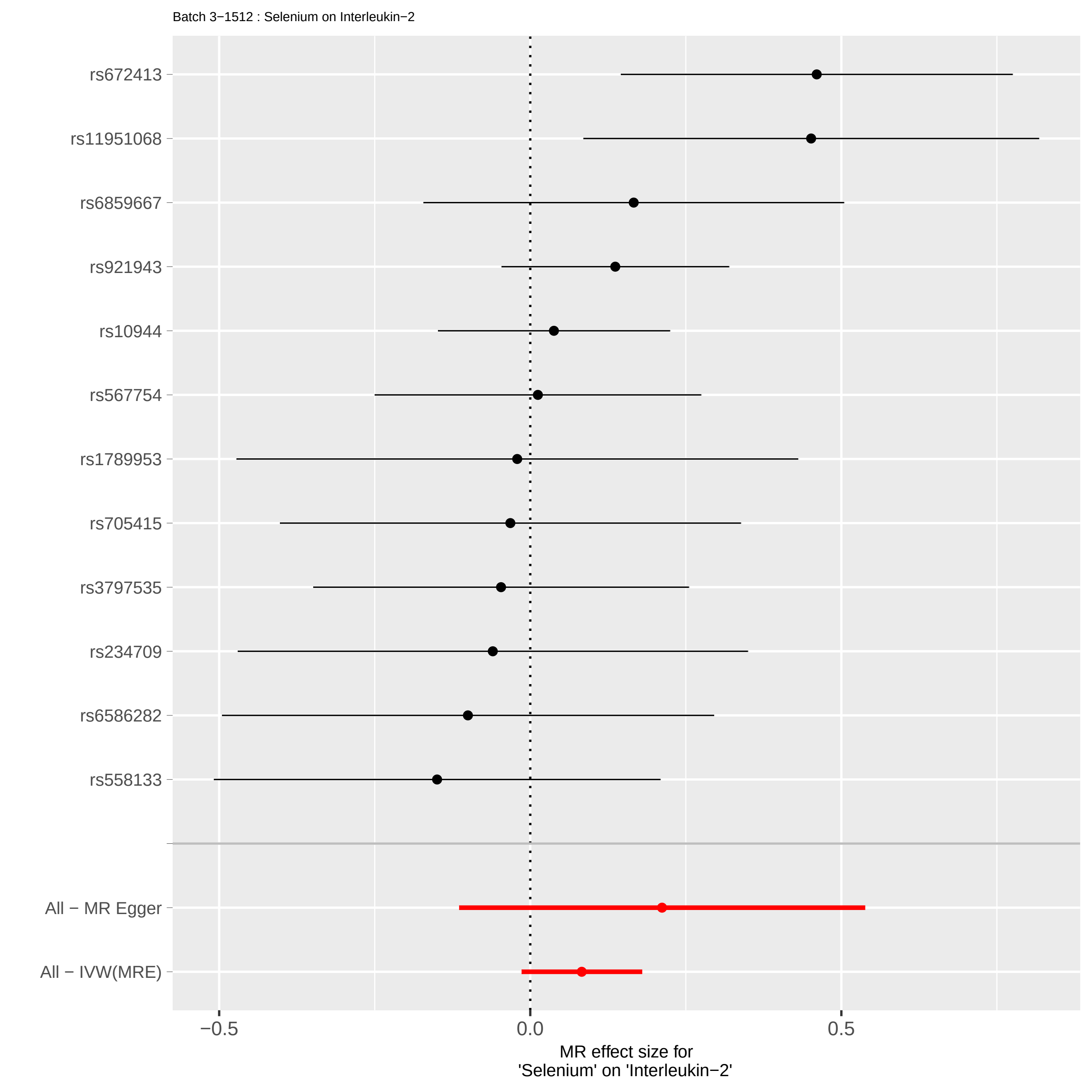
Batch 3−1512 : Selenium on Interleukin−2
rs672413
rs11951068
rs6859667
rs921943
rs10944
rs567754
rs1789953
rs705415
rs3797535
rs234709
rs6586282
rs558133
All − MR Egger
All − IVW(MRE)
−0.5
0.0
0.5
MR effect size for
'Selenium' on 'Interleukin−2'

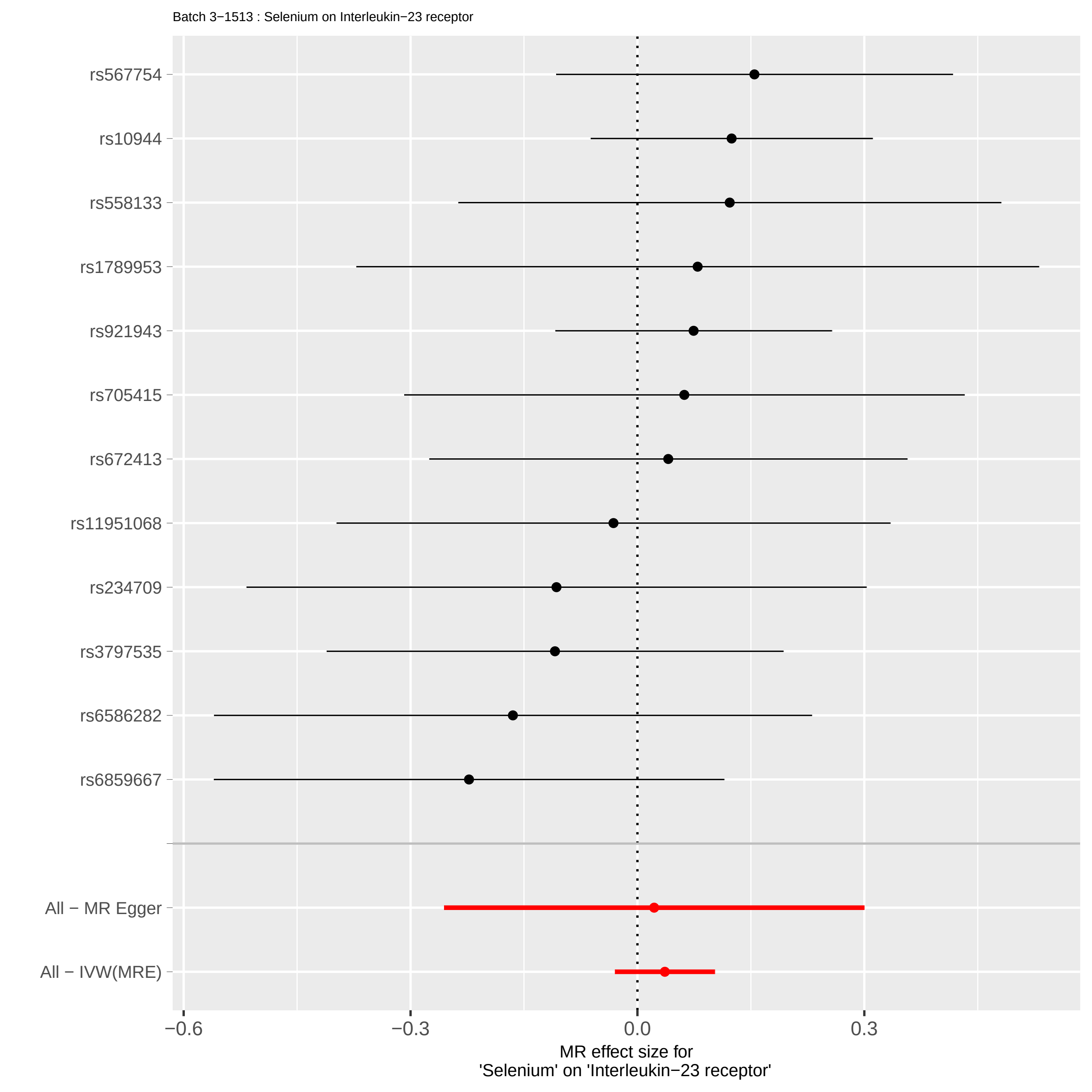

Batch 3−1513 : Selenium on Interleukin−23 receptor
rs567754
rs10944
rs558133
rs1789953
rs921943
rs705415
rs672413
rs11951068
rs234709
rs3797535
rs6586282
rs6859667
All − MR Egger
All − IVW(MRE)
−0.6
−0.3
0.0
0.3
MR effect size for
'Selenium' on 'Interleukin−23 receptor'

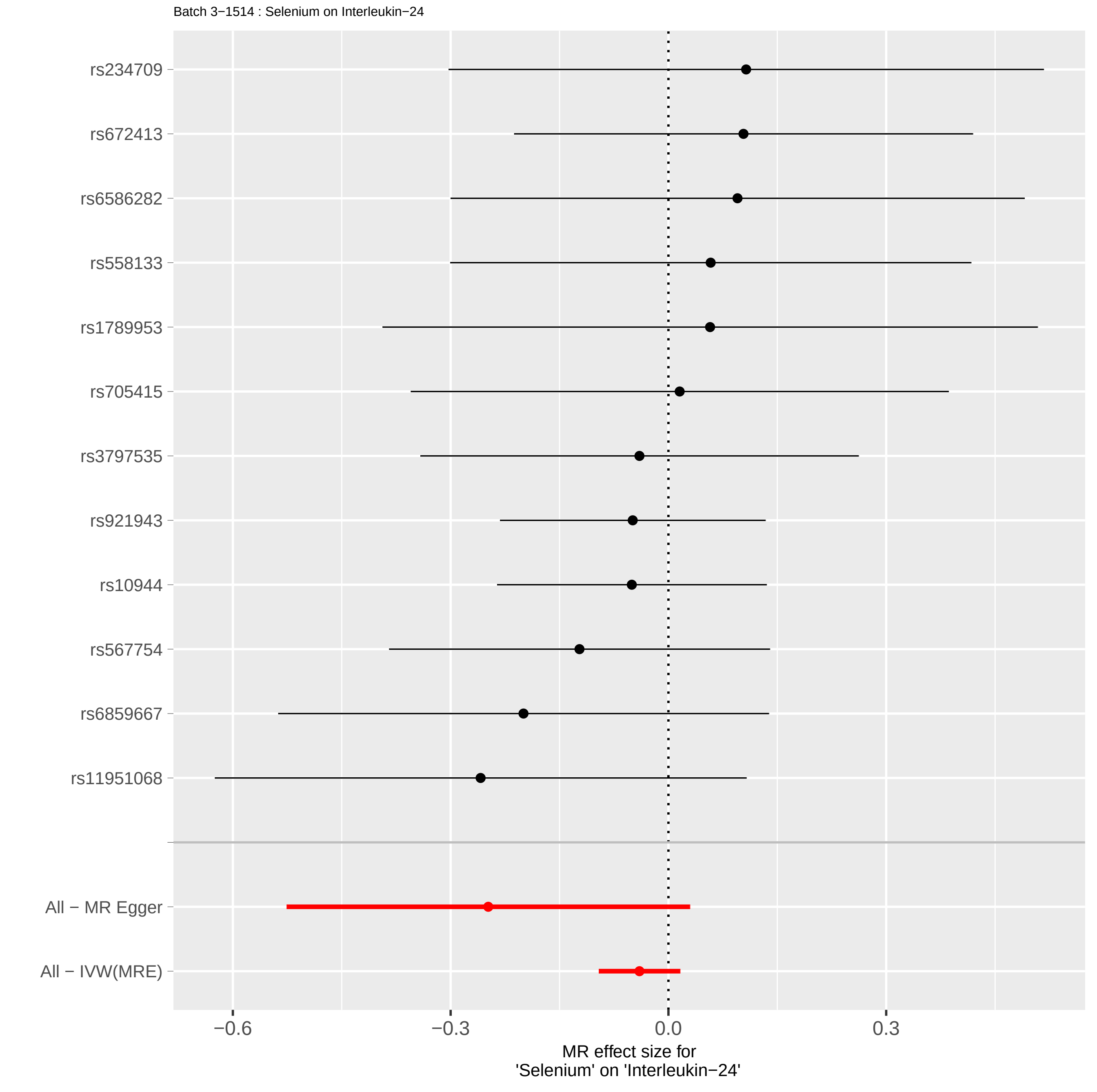

Batch 3−1514 : Selenium on Interleukin−24
rs234709
rs672413
rs6586282
rs558133
rs1789953
rs705415
rs3797535
rs921943
rs10944
rs567754
rs6859667
rs11951068
All − MR Egger
All − IVW(MRE)
−0.6
−0.3
0.0
0.3
MR effect size for
'Selenium' on 'Interleukin−24'

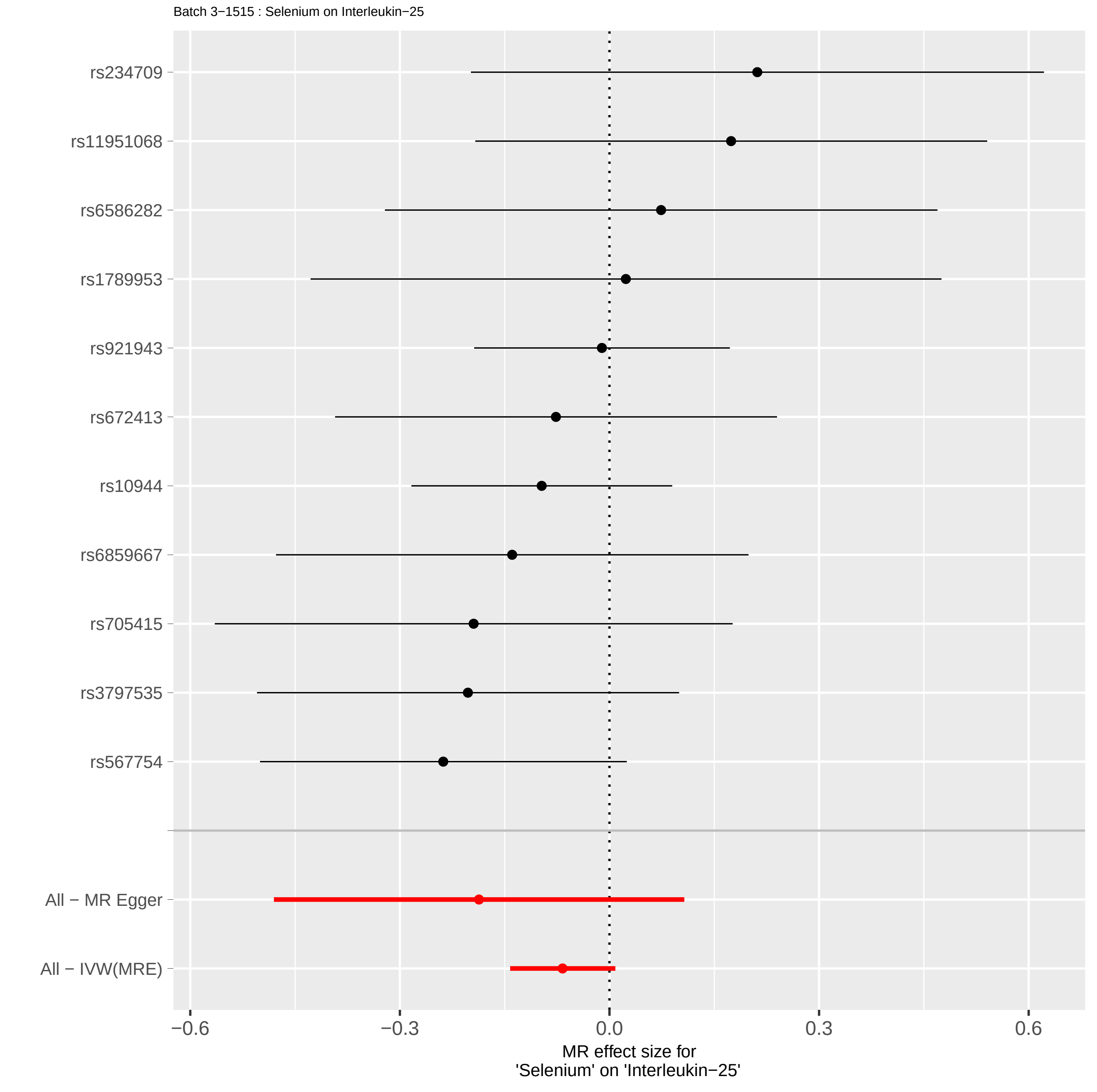

Batch 3−1515 : Selenium on Interleukin−25
rs234709
rs11951068
rs6586282
rs1789953
rs921943
rs672413
rs10944
rs6859667
rs705415
rs3797535
rs567754
All − MR Egger
All − IVW(MRE)
−0.6
−0.3
0.0
0.3
0.6
MR effect size for
'Selenium' on 'Interleukin−25'

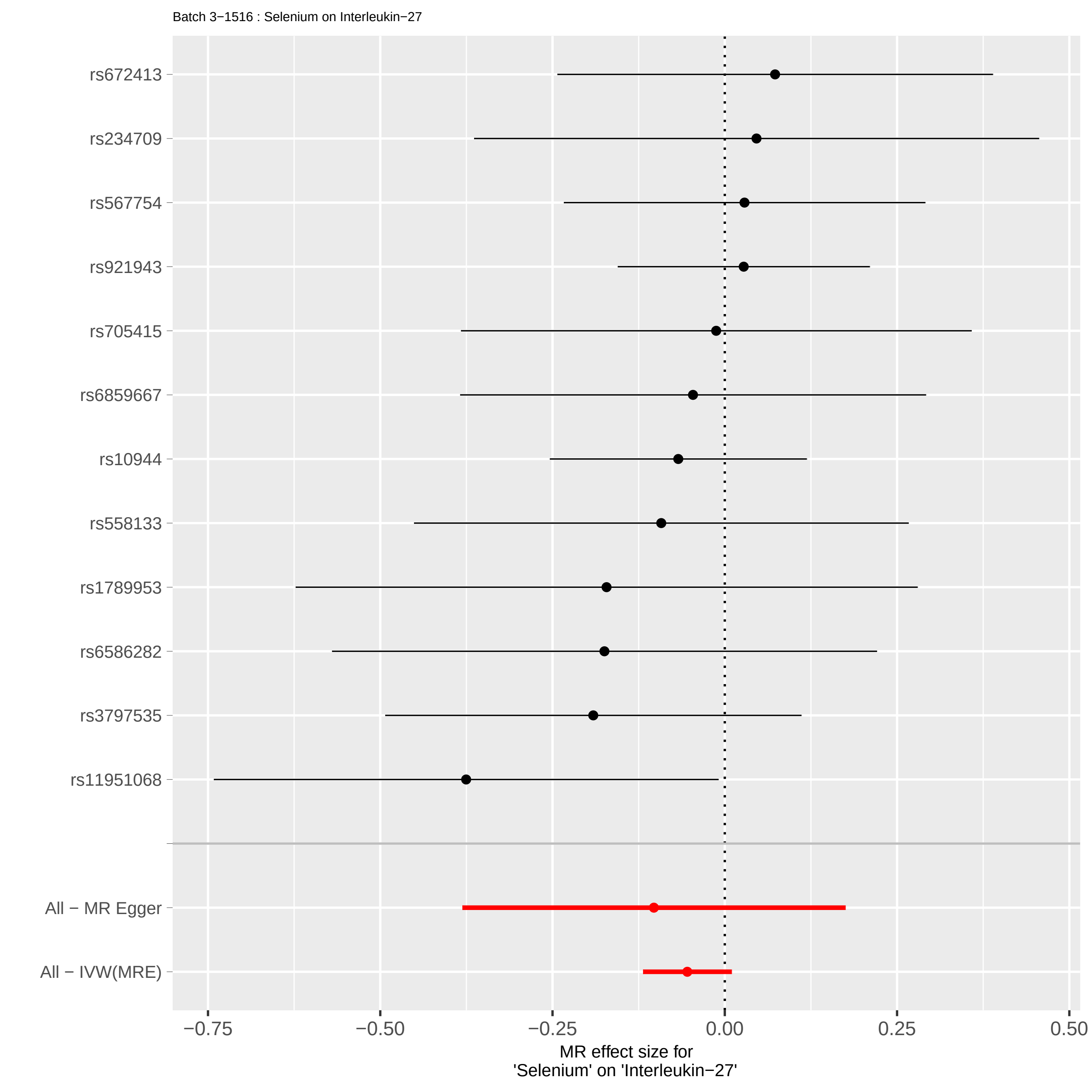

Batch 3−1516 : Selenium on Interleukin−27
rs672413
rs234709
rs567754
rs921943
rs705415
rs6859667
rs10944
rs558133
rs1789953
rs6586282
rs3797535
rs11951068
All − MR Egger
All − IVW(MRE)
−0.75
−0.50
−0.25
0.00
0.25
0.50
MR effect size for
'Selenium' on 'Interleukin−27'

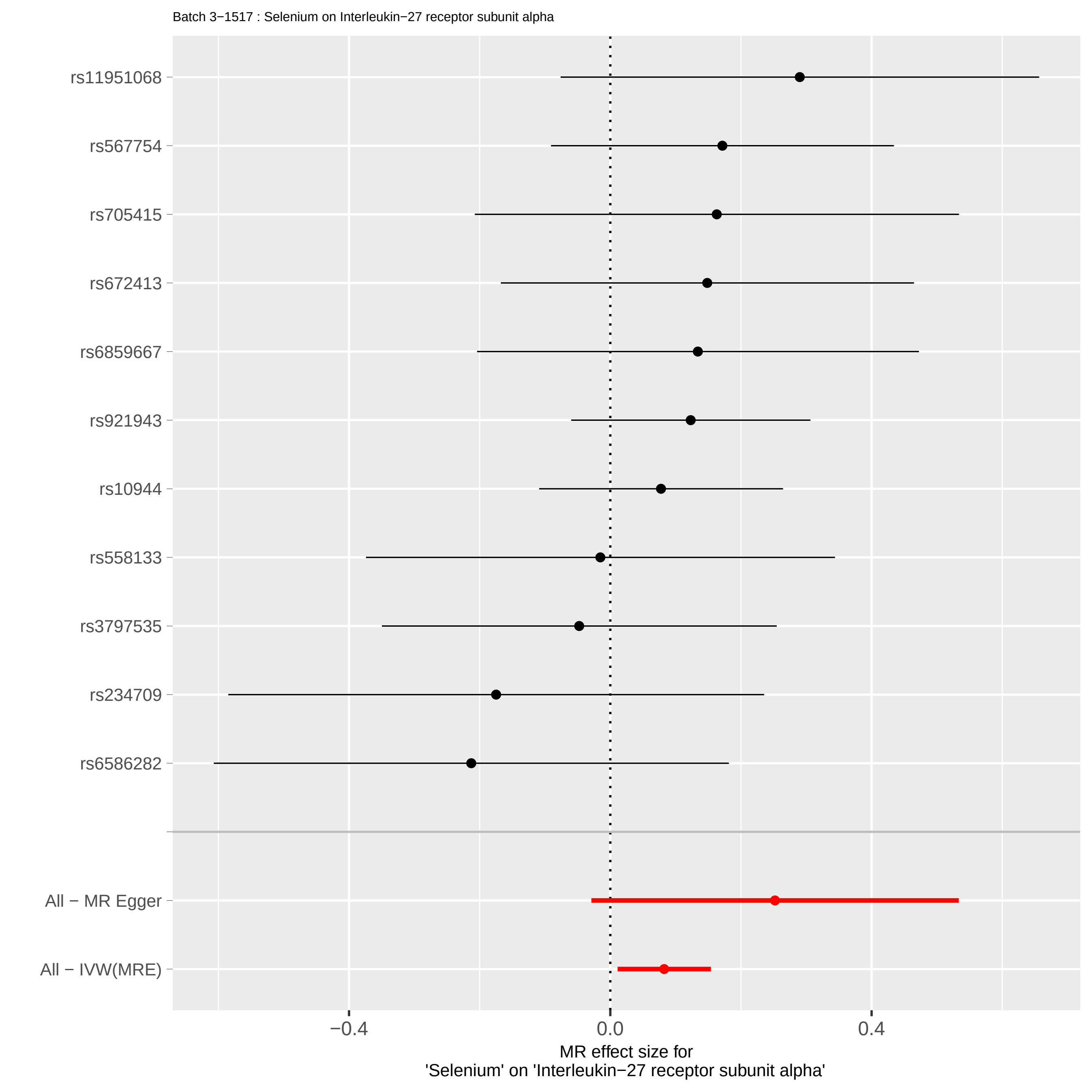
Batch 3−1517 : Selenium on Interleukin−27 receptor subunit alpha
rs11951068
rs567754
rs705415
rs672413
rs6859667
rs921943
rs10944
rs558133
rs3797535
rs234709
rs6586282
All − MR Egger
All − IVW(MRE)
−0.4
0.0
0.4
MR effect size for
'Selenium' on 'Interleukin−27 receptor subunit alpha'

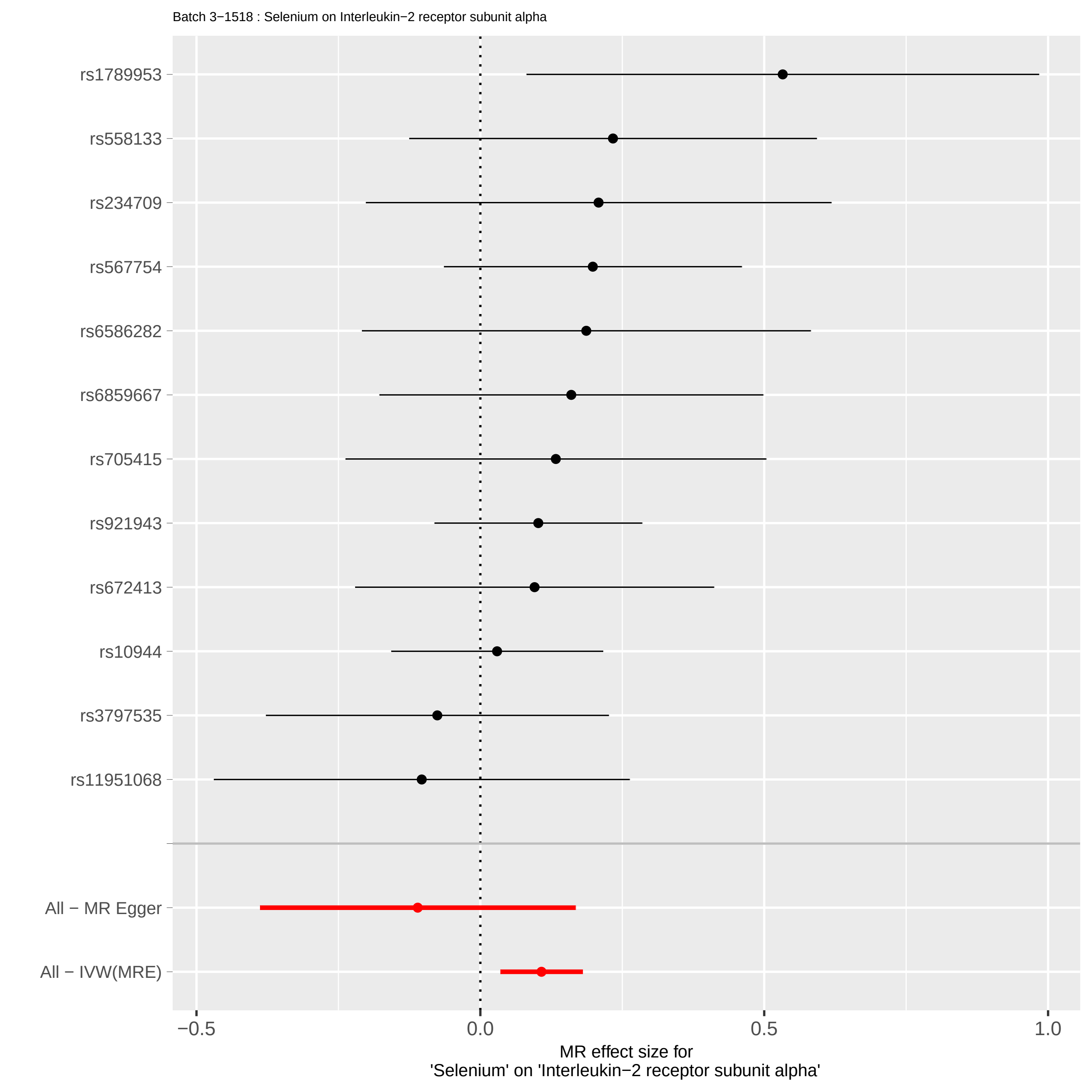

Batch 3−1518 : Selenium on Interleukin−2 receptor subunit alpha
rs1789953
rs558133
rs234709
rs567754
rs6586282
rs6859667
rs705415
rs921943
rs672413
rs10944
rs3797535
rs11951068
All − MR Egger
All − IVW(MRE)
−0.5
0.0
0.5
1.0
MR effect size for
'Selenium' on 'Interleukin−2 receptor subunit alpha'

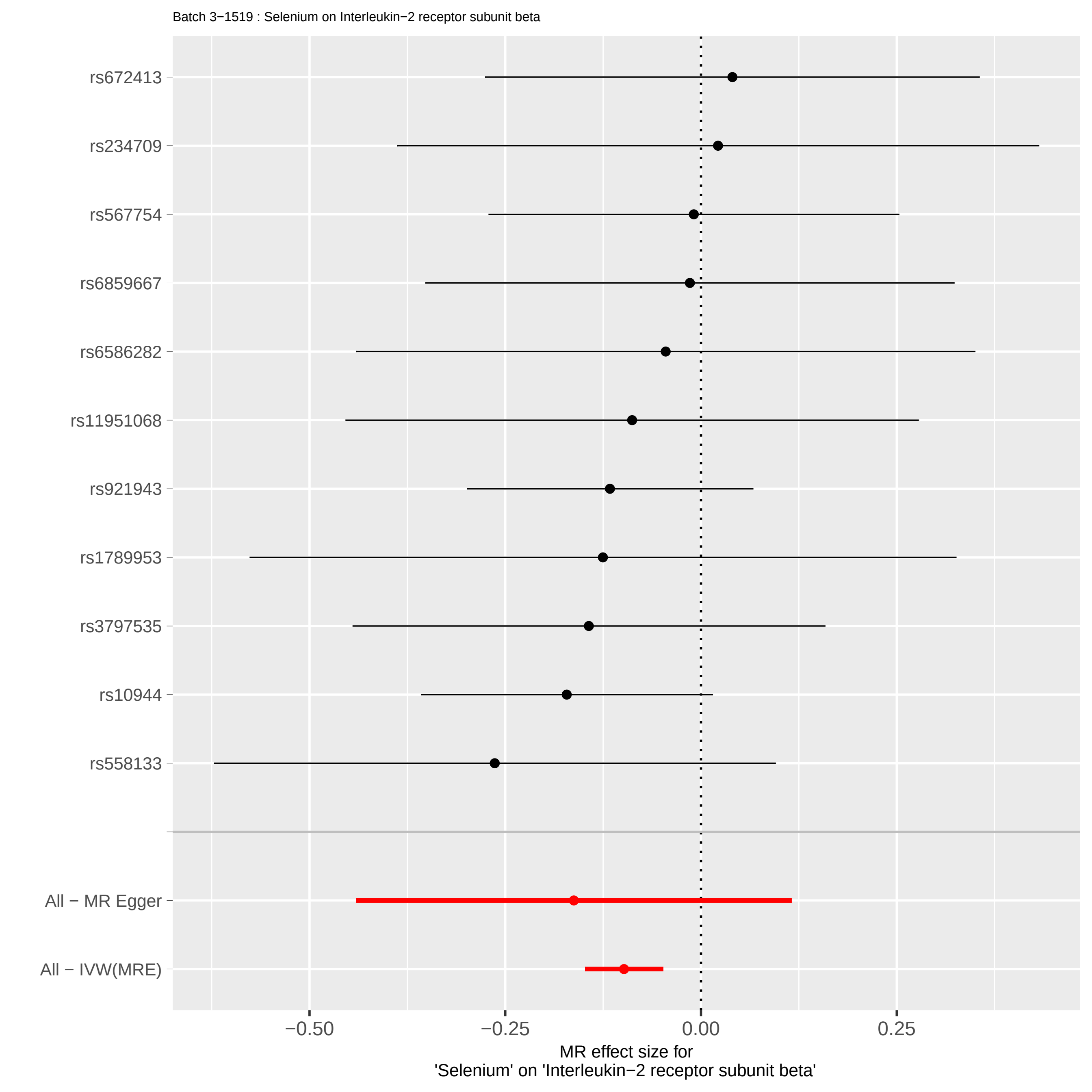

Batch 3−1519 : Selenium on Interleukin−2 receptor subunit beta
rs672413
rs234709
rs567754
rs6859667
rs6586282
rs11951068
rs921943
rs1789953
rs3797535
rs10944
rs558133
All − MR Egger
All − IVW(MRE)
−0.50
−0.25
0.00
0.25
MR effect size for
'Selenium' on 'Interleukin−2 receptor subunit beta'

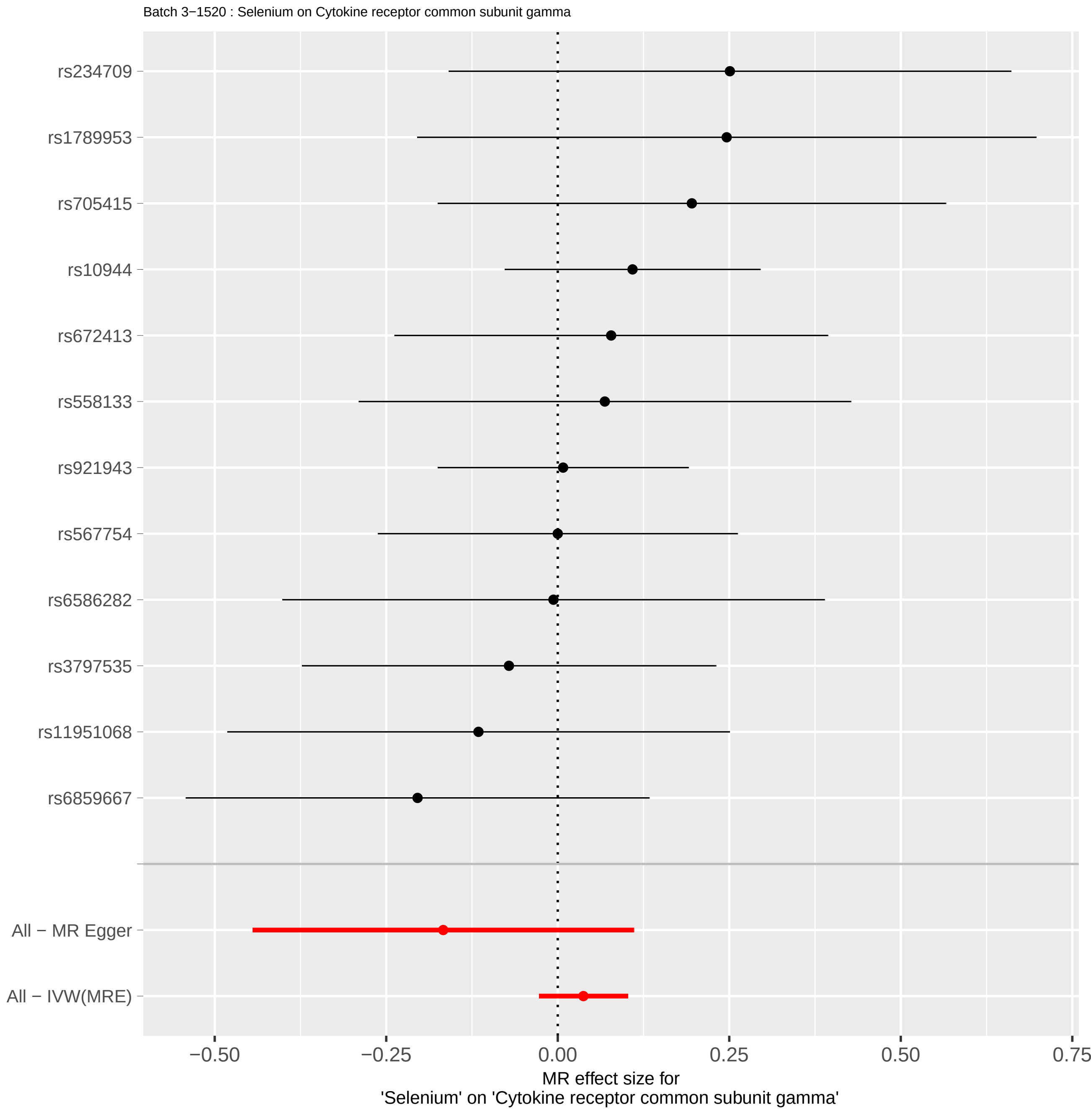
Batch 3−1520 : Selenium on Cytokine receptor common subunit gamma
rs234709
rs1789953
rs705415
rs10944
rs672413
rs558133
rs921943
rs567754
rs6586282
rs3797535
rs11951068
rs6859667
All − MR Egger
All − IVW(MRE)
−0.50
−0.25
0.00
0.25
0.50
0.75
MR effect size for
'Selenium' on 'Cytokine receptor common subunit gamma'

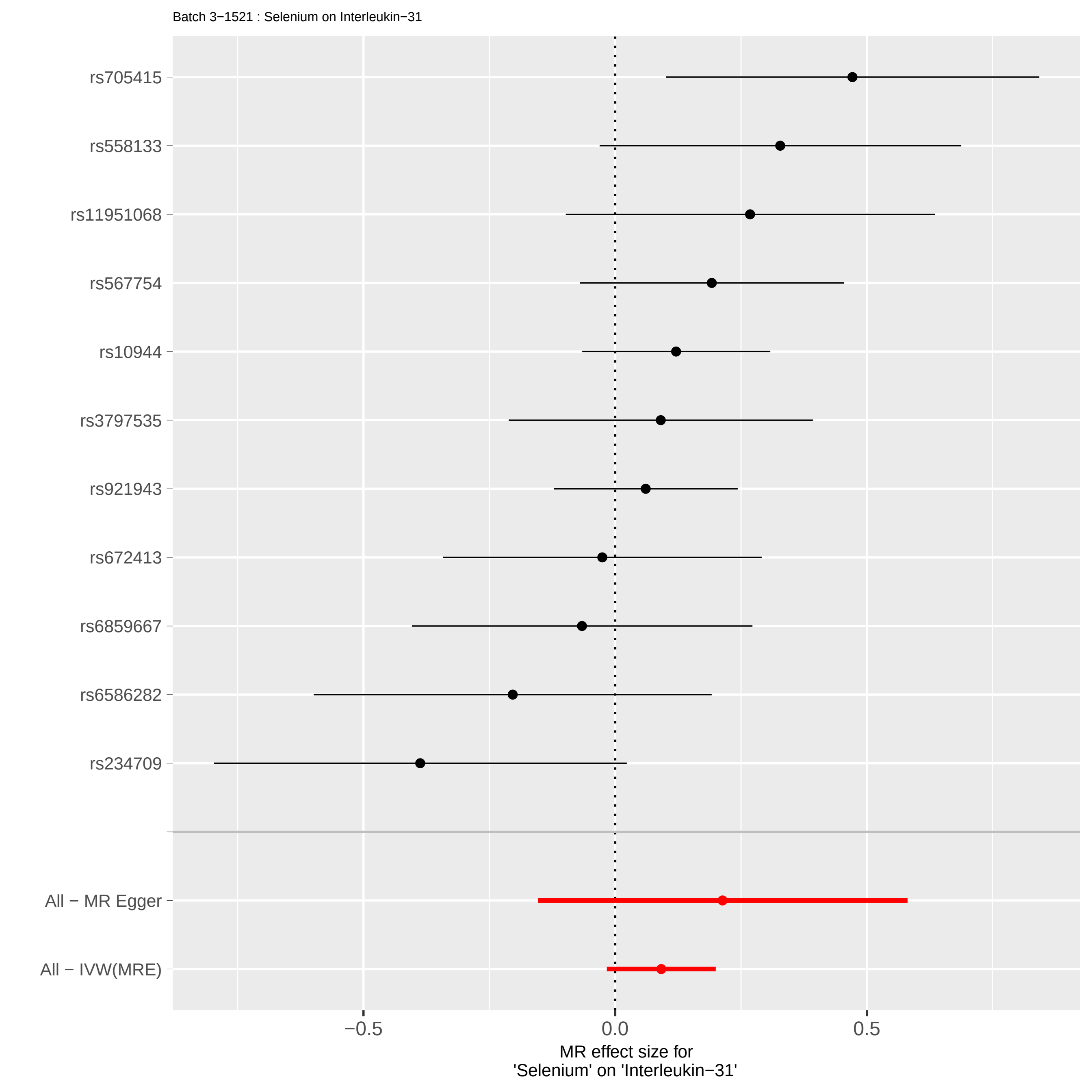

Batch 3−1521 : Selenium on Interleukin−31
rs705415
rs558133
rs11951068
rs567754
rs10944
rs3797535
rs921943
rs672413
rs6859667
rs6586282
rs234709
All − MR Egger
All − IVW(MRE)
−0.5
0.0
0.5
MR effect size for
'Selenium' on 'Interleukin−31'

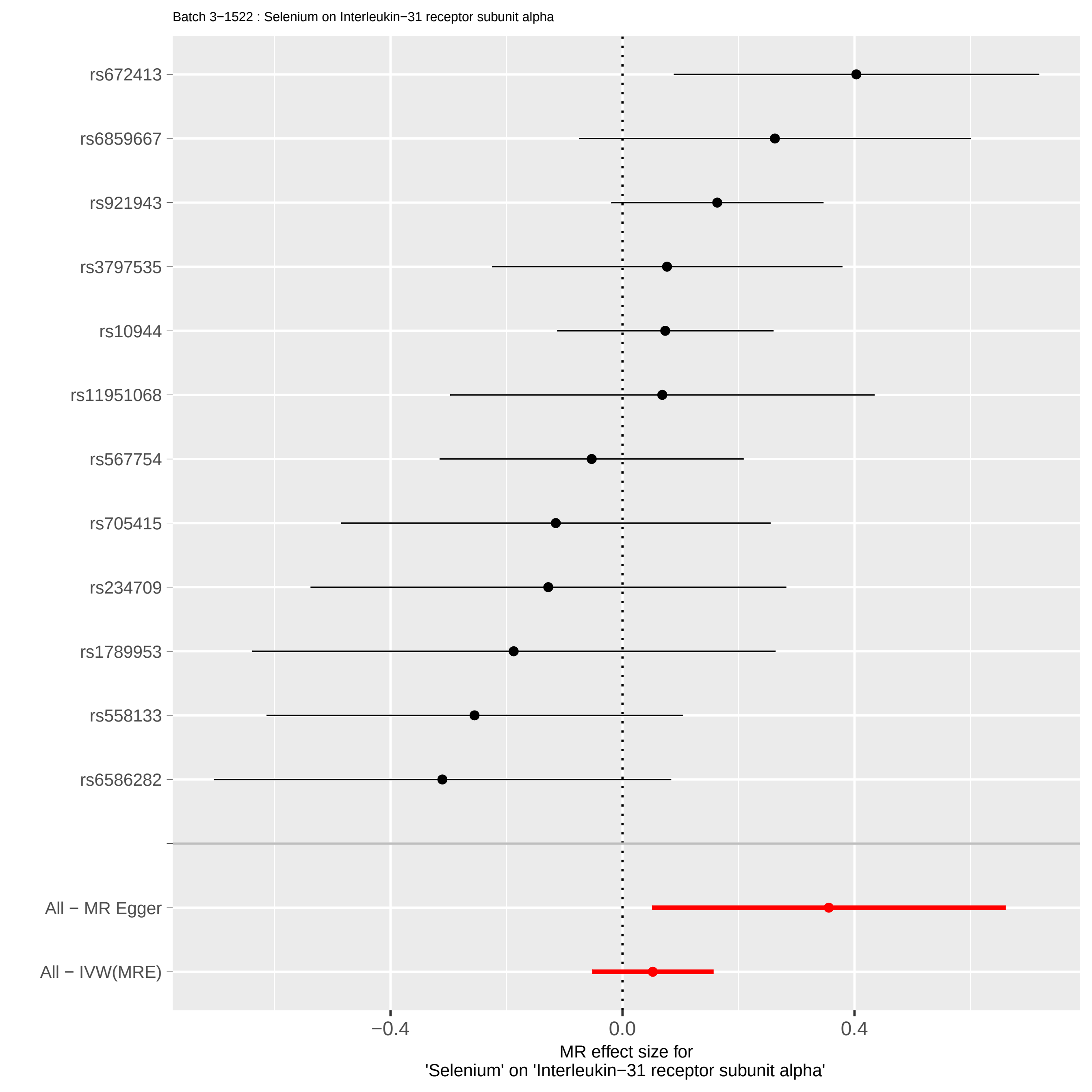
Batch 3−1522 : Selenium on Interleukin−31 receptor subunit alpha
rs672413
rs6859667
rs921943
rs3797535
rs10944
rs11951068
rs567754
rs705415
rs234709
rs1789953
rs558133
rs6586282
All − MR Egger
All − IVW(MRE)
−0.4
0.0
0.4
MR effect size for
'Selenium' on 'Interleukin−31 receptor subunit alpha'

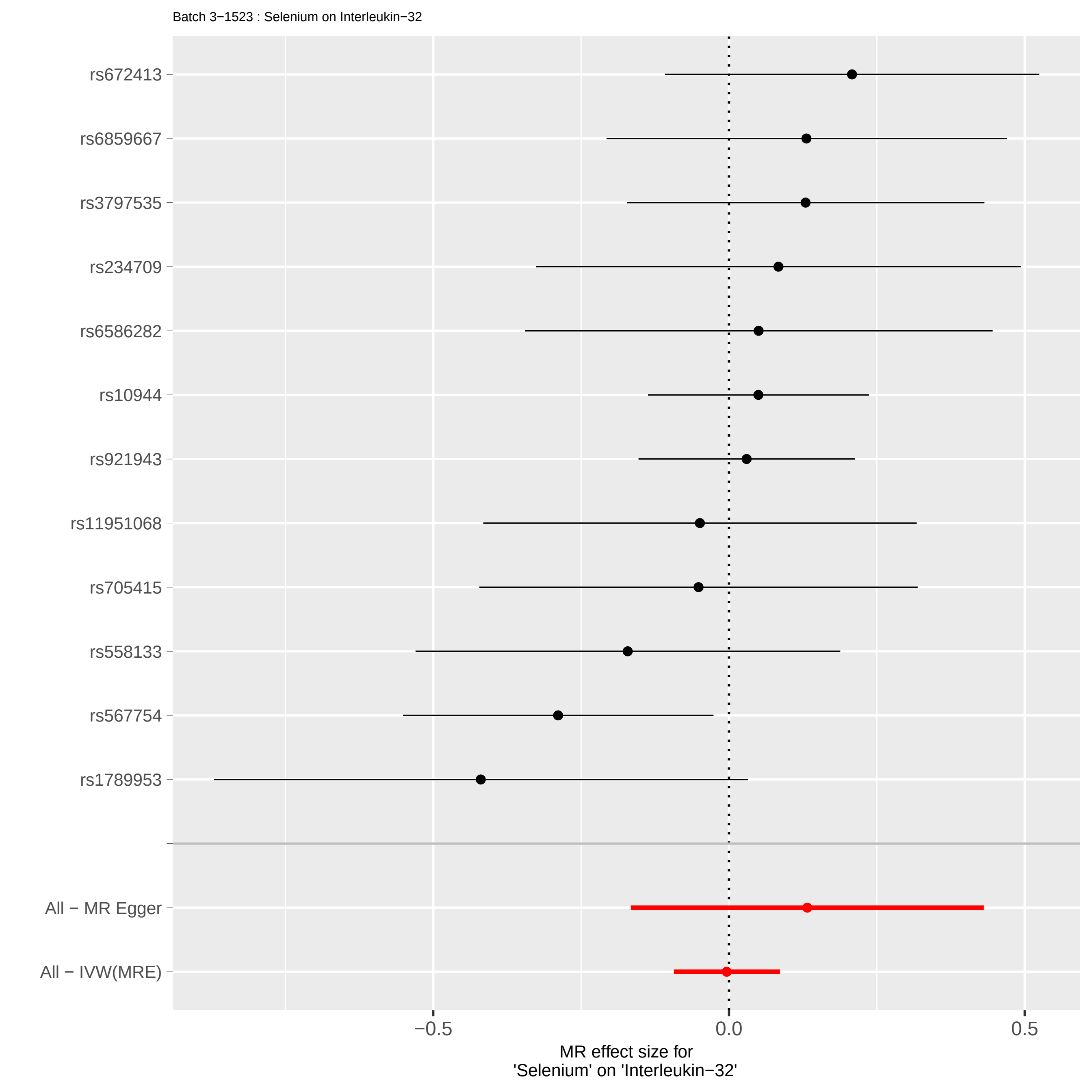

Batch 3−1523 : Selenium on Interleukin−32
rs672413
rs6859667
rs3797535
rs234709
rs6586282
rs10944
rs921943
rs11951068
rs705415
rs558133
rs567754
rs1789953
All − MR Egger
All − IVW(MRE)
−0.5
0.0
0.5
MR effect size for
'Selenium' on 'Interleukin−32'

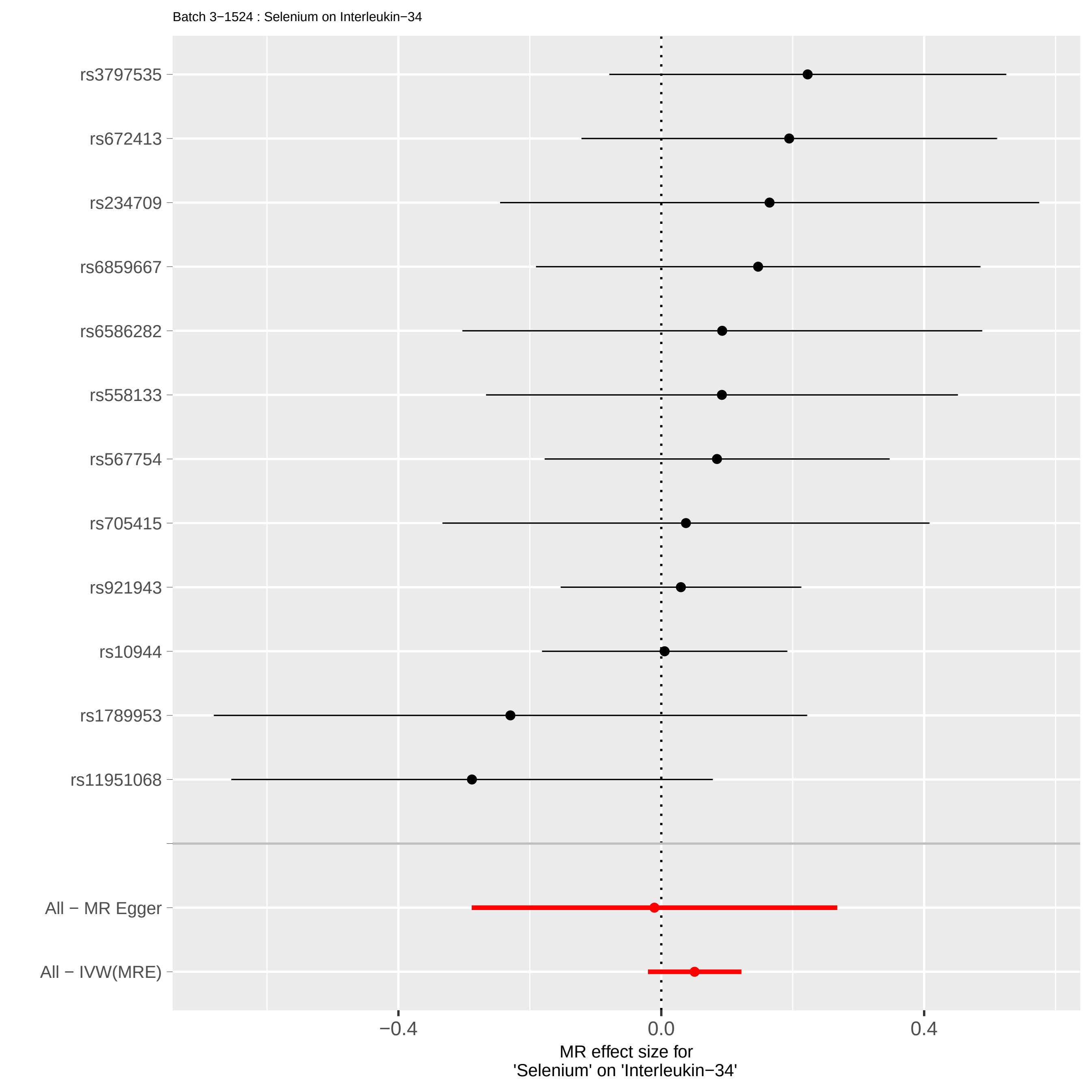

Batch 3−1524 : Selenium on Interleukin−34
rs3797535
rs672413
rs234709
rs6859667
rs6586282
rs558133
rs567754
rs705415
rs921943
rs10944
rs1789953
rs11951068
All − MR Egger
All − IVW(MRE)
−0.4
0.0
0.4
MR effect size for
'Selenium' on 'Interleukin−34'

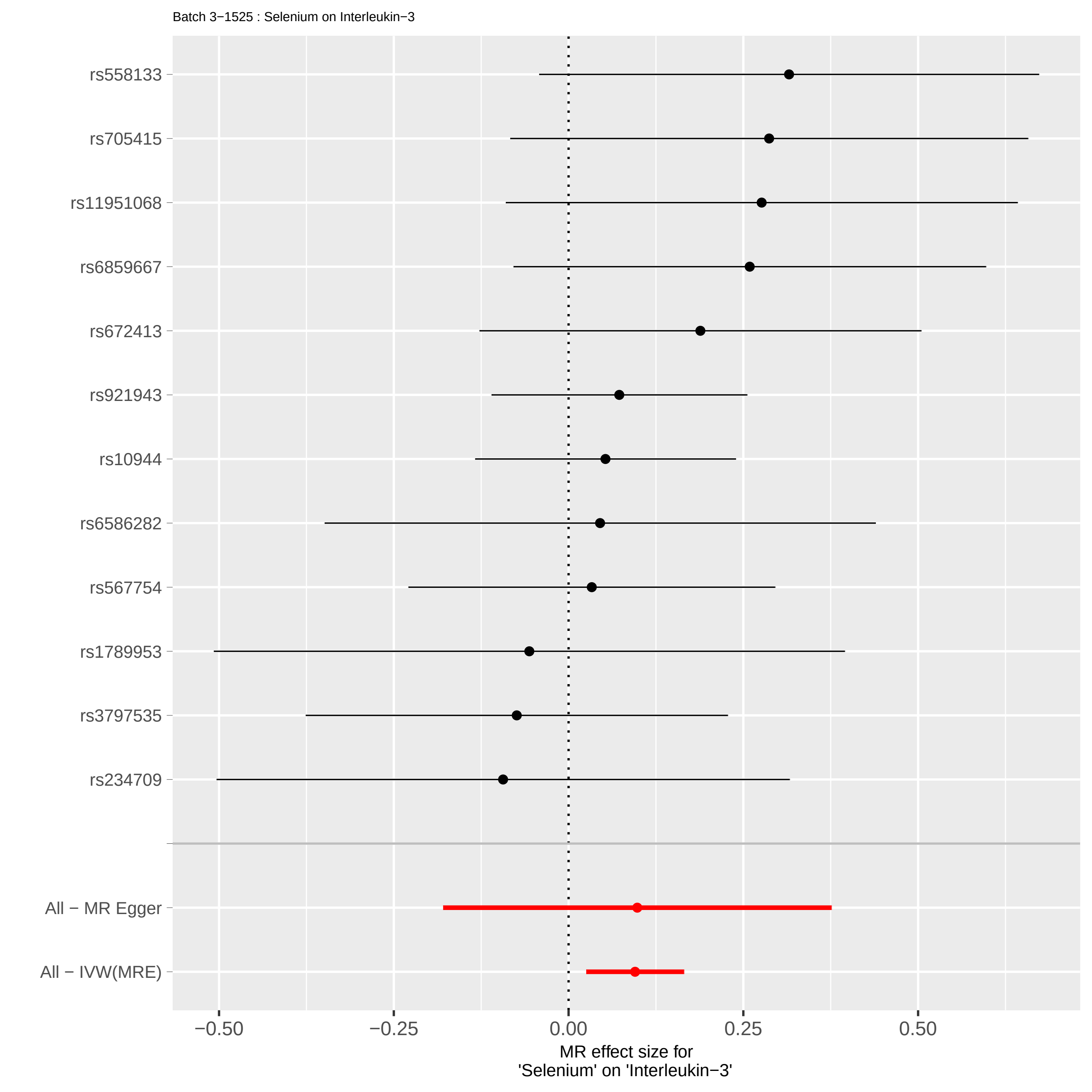

Batch 3−1525 : Selenium on Interleukin−3
rs558133
rs705415
rs11951068
rs6859667
rs672413
rs921943
rs10944
rs6586282
rs567754
rs1789953
rs3797535
rs234709
All − MR Egger
All − IVW(MRE)
−0.50
−0.25
0.00
0.25
0.50
MR effect size for
'Selenium' on 'Interleukin−3'

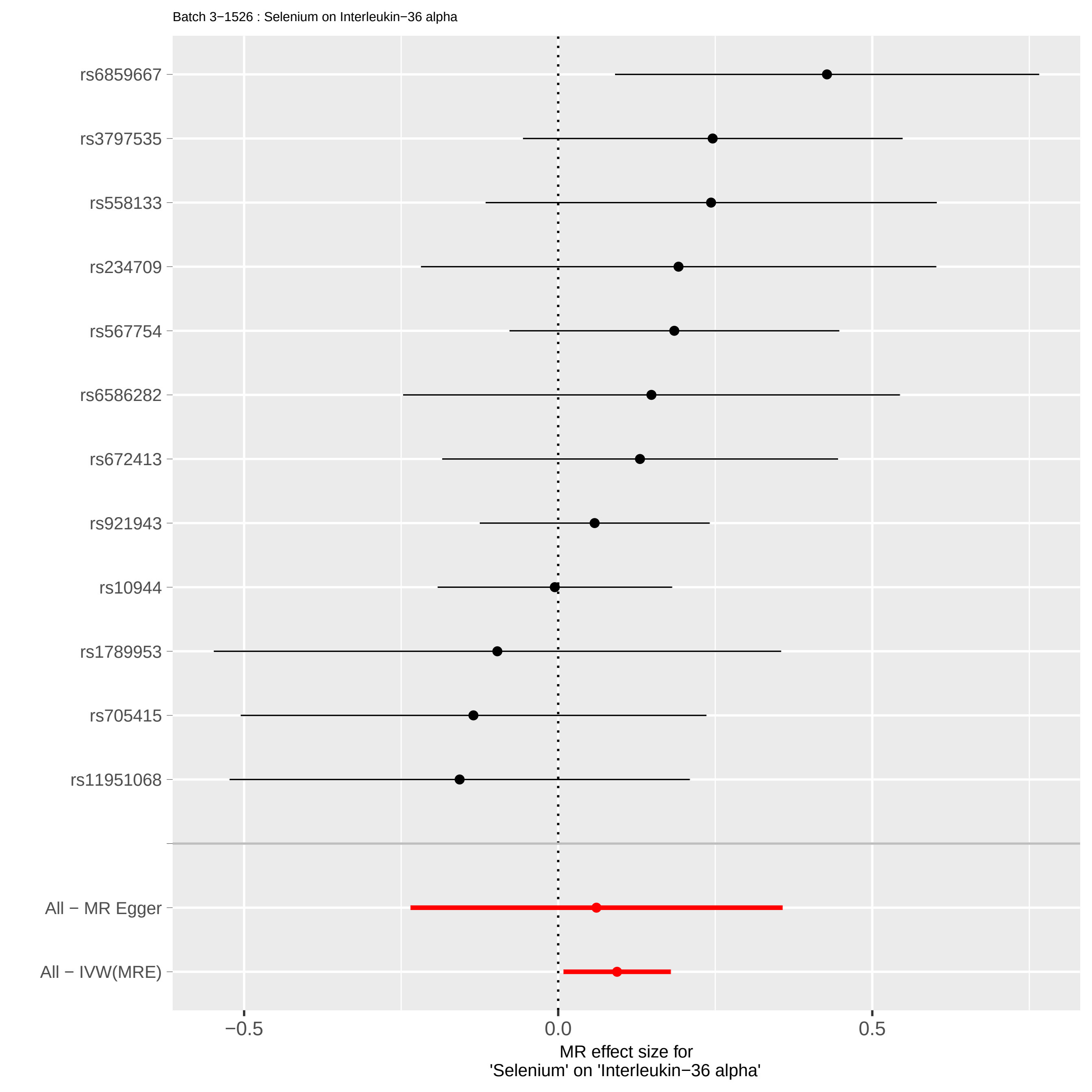
Batch 3−1526 : Selenium on Interleukin−36 alpha
rs6859667
rs3797535
rs558133
rs234709
rs567754
rs6586282
rs672413
rs921943
rs10944
rs1789953
rs705415
rs11951068
All − MR Egger
All − IVW(MRE)
−0.5
0.0
0.5
MR effect size for
'Selenium' on 'Interleukin−36 alpha'

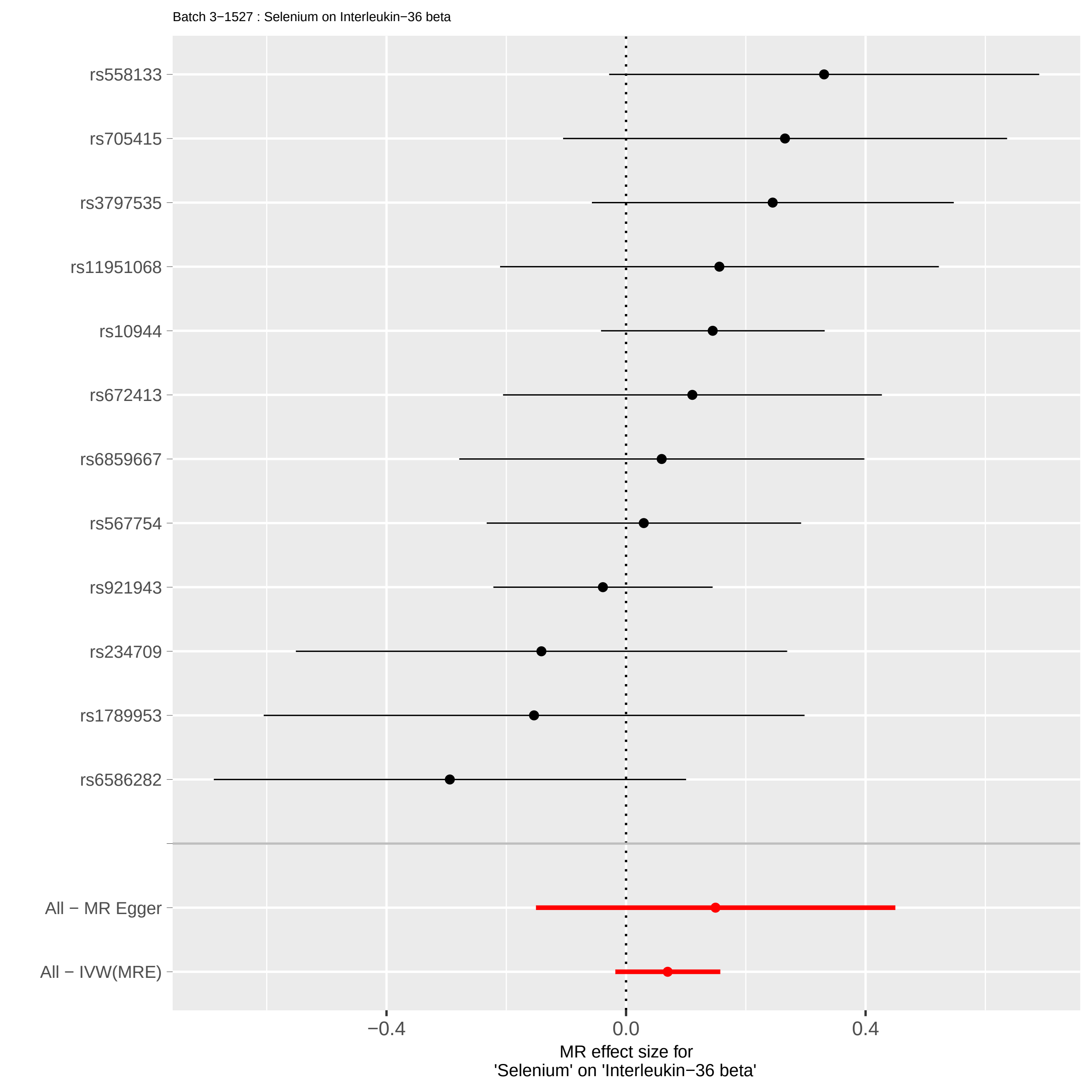

Batch 3−1527 : Selenium on Interleukin−36 beta
rs558133
rs705415
rs3797535
rs11951068
rs10944
rs672413
rs6859667
rs567754
rs921943
rs234709
rs1789953
rs6586282
All − MR Egger
All − IVW(MRE)
−0.4
0.0
0.4
MR effect size for
'Selenium' on 'Interleukin−36 beta'

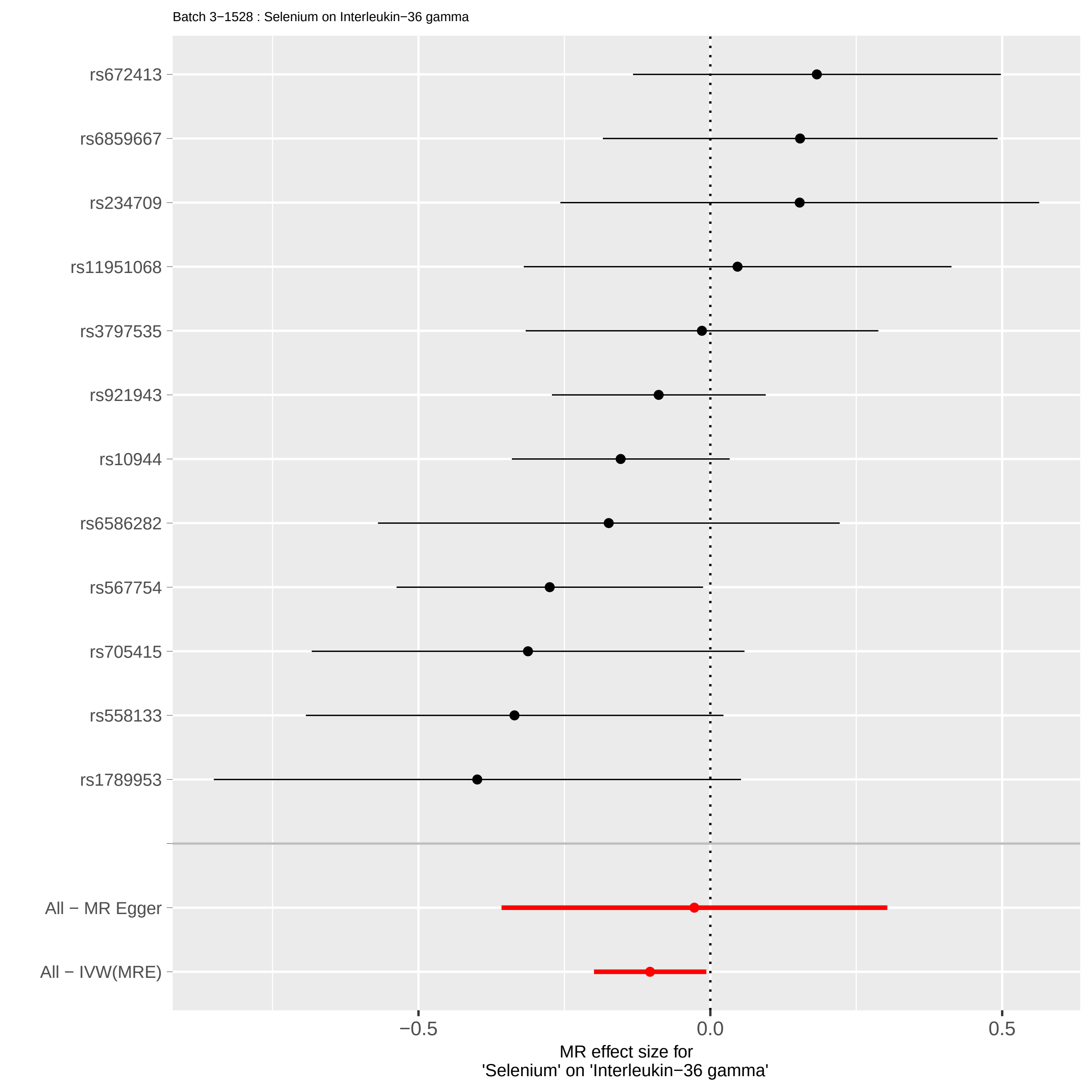

Batch 3−1528 : Selenium on Interleukin−36 gamma
rs672413
rs6859667
rs234709
rs11951068
rs3797535
rs921943
rs10944
rs6586282
rs567754
rs705415
rs558133
rs1789953
All − MR Egger
All − IVW(MRE)
−0.5
0.0
0.5
MR effect size for
'Selenium' on 'Interleukin−36 gamma'

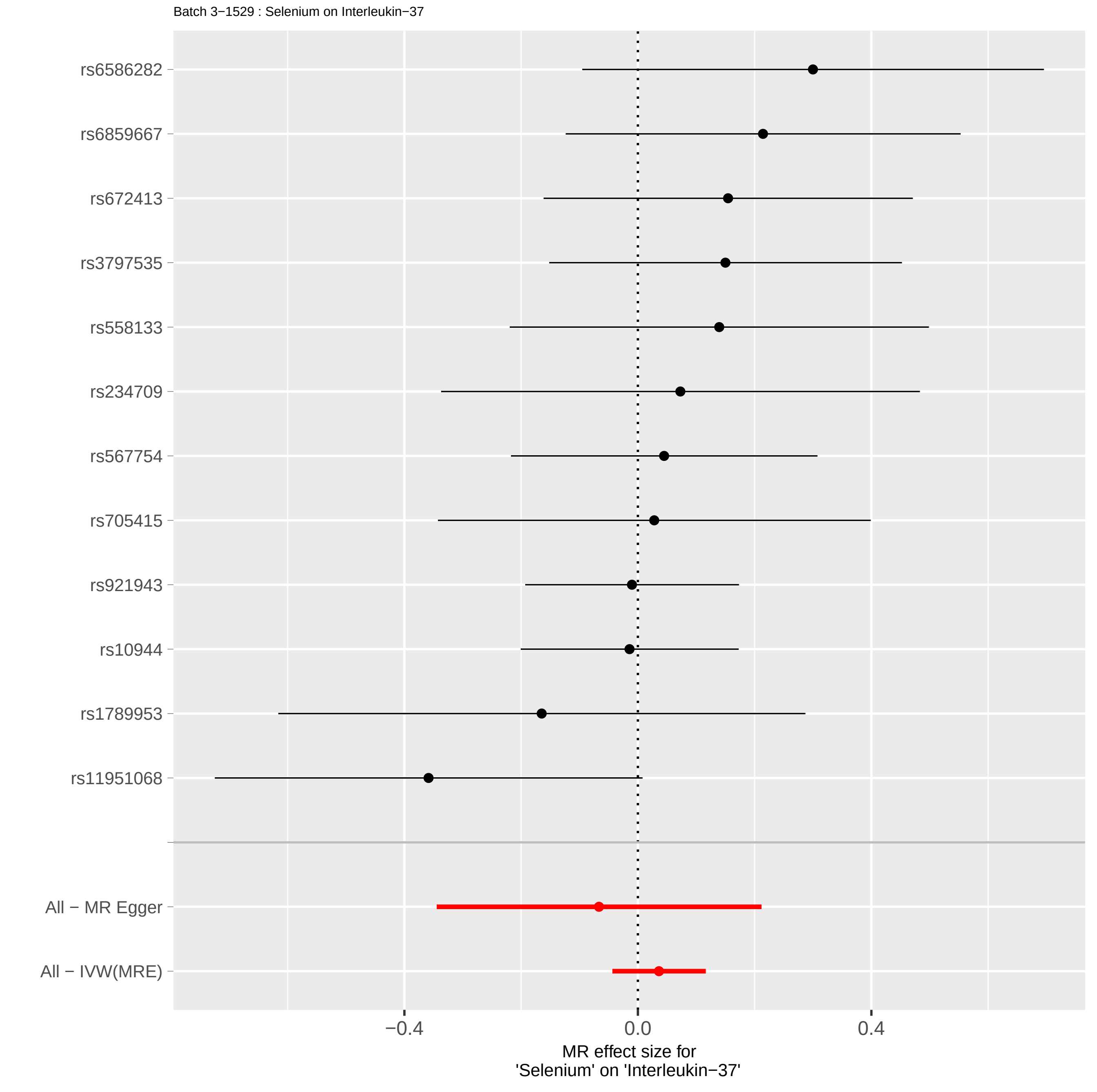
Batch 3−1529 : Selenium on Interleukin−37
rs6586282
rs6859667
rs672413
rs3797535
rs558133
rs234709
rs567754
rs705415
rs921943
rs10944
rs1789953
rs11951068
All − MR Egger
All − IVW(MRE)
−0.4
0.0
0.4
MR effect size for
'Selenium' on 'Interleukin−37'

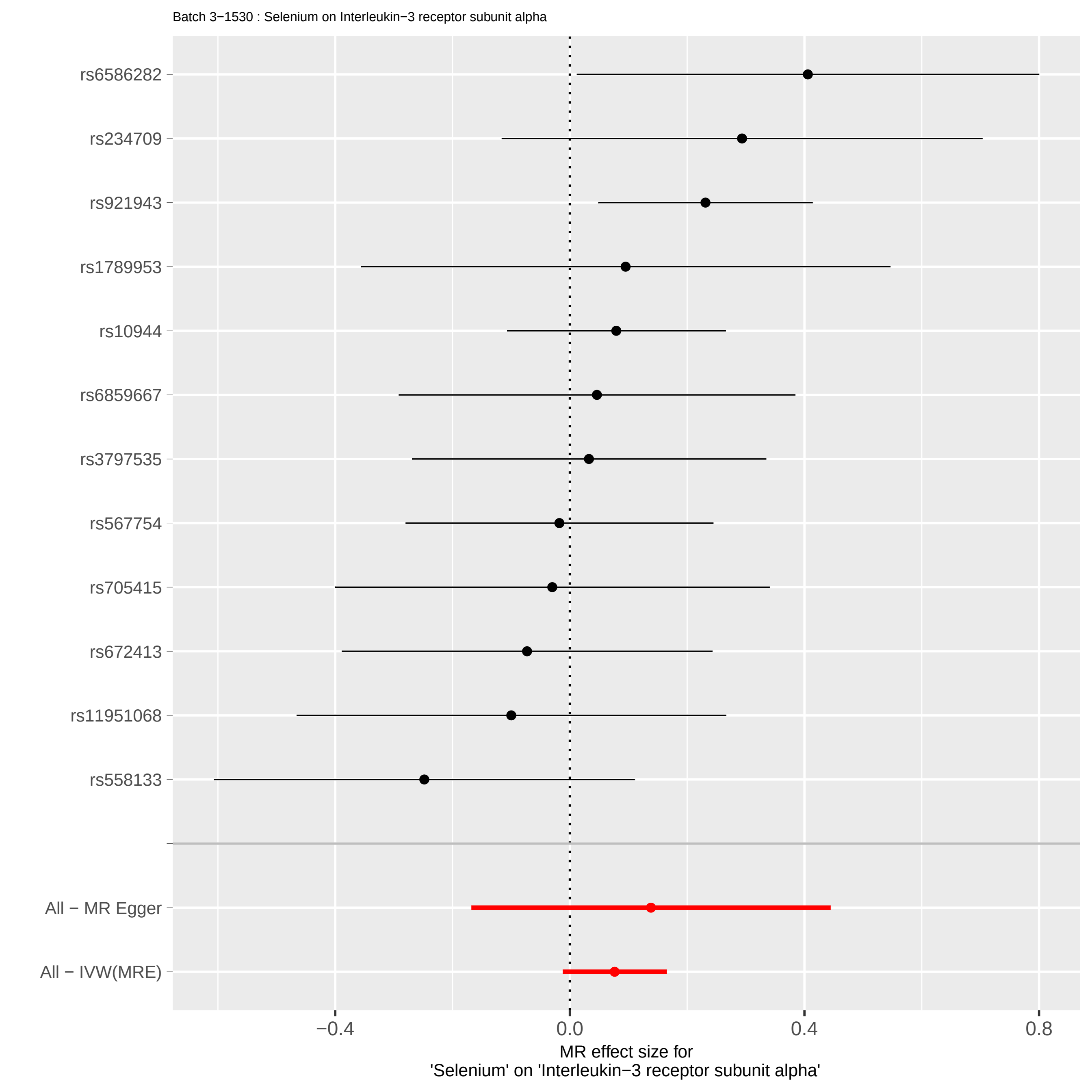

Batch 3−1530 : Selenium on Interleukin−3 receptor subunit alpha
rs6586282
rs234709
rs921943
rs1789953
rs10944
rs6859667
rs3797535
rs567754
rs705415
rs672413
rs11951068
rs558133
All − MR Egger
All − IVW(MRE)
−0.4
0.0
0.4
0.8
MR effect size for
'Selenium' on 'Interleukin−3 receptor subunit alpha'

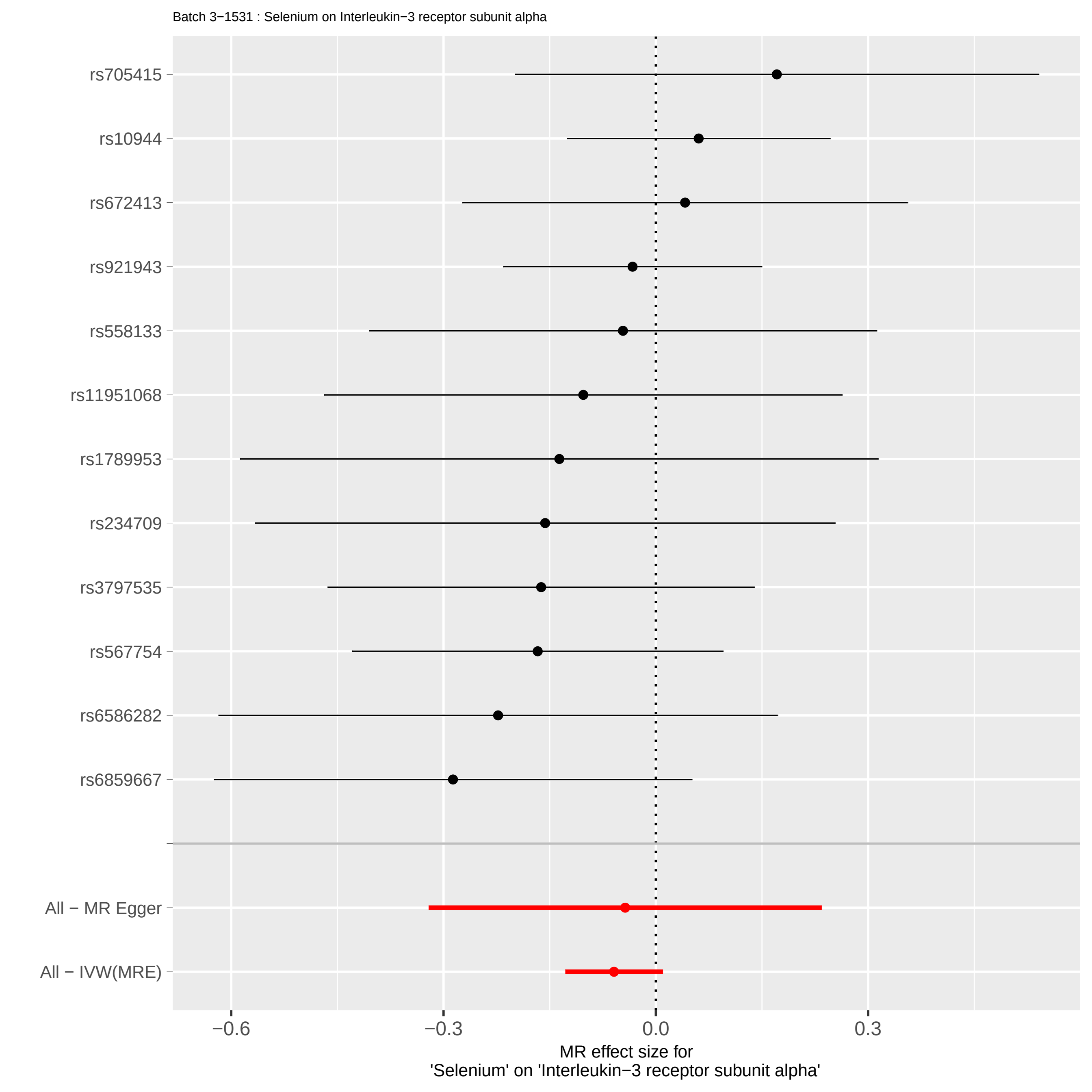

Batch 3−1531 : Selenium on Interleukin−3 receptor subunit alpha
rs705415
rs10944
rs672413
rs921943
rs558133
rs11951068
rs1789953
rs234709
rs3797535
rs567754
rs6586282
rs6859667
All − MR Egger
All − IVW(MRE)
−0.6
−0.3
0.0
0.3
MR effect size for
'Selenium' on 'Interleukin−3 receptor subunit alpha'

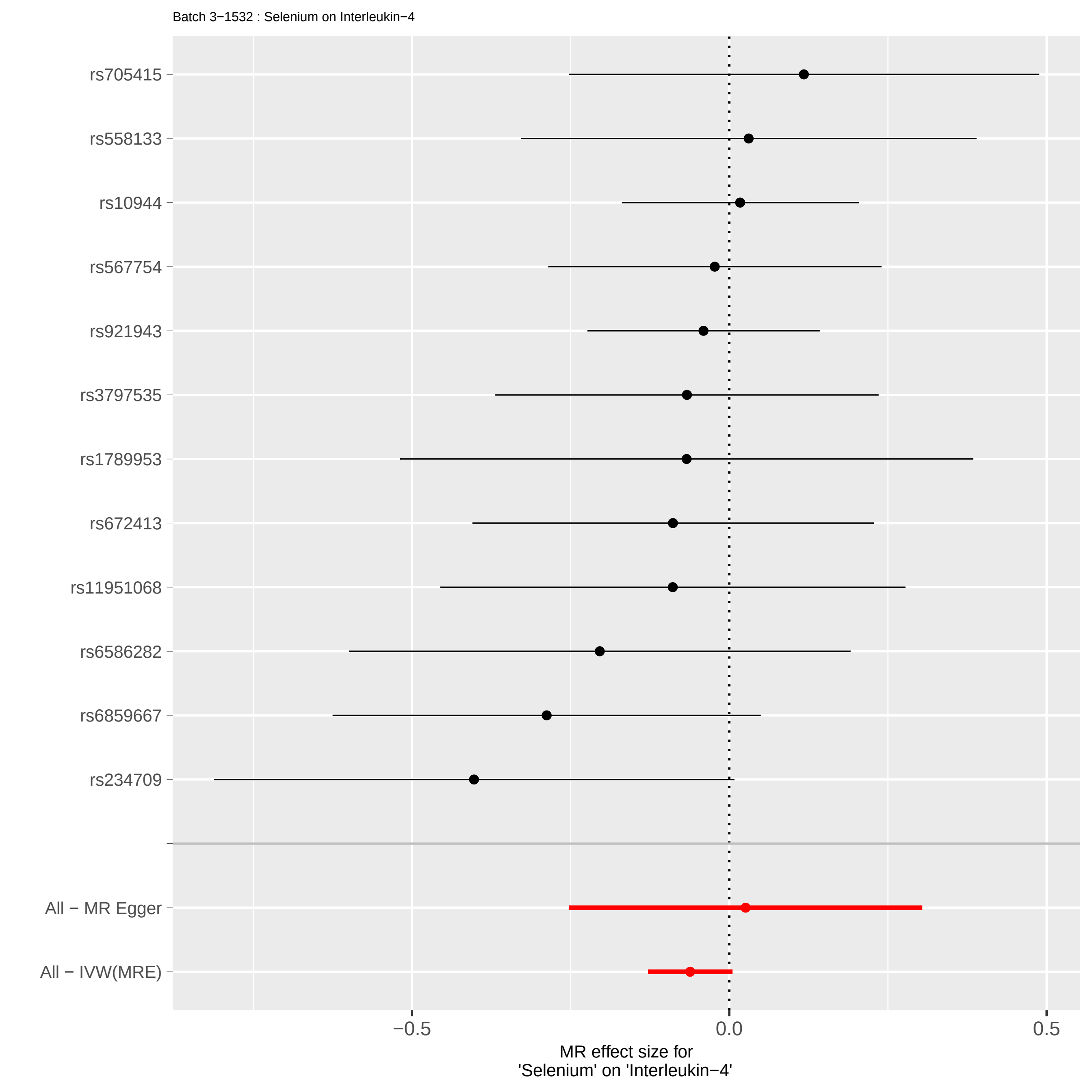

Batch 3−1532 : Selenium on Interleukin−4
rs705415
rs558133
rs10944
rs567754
rs921943
rs3797535
rs1789953
rs672413
rs11951068
rs6586282
rs6859667
rs234709
All − MR Egger
All − IVW(MRE)
−0.5
0.0
0.5
MR effect size for
'Selenium' on 'Interleukin−4'

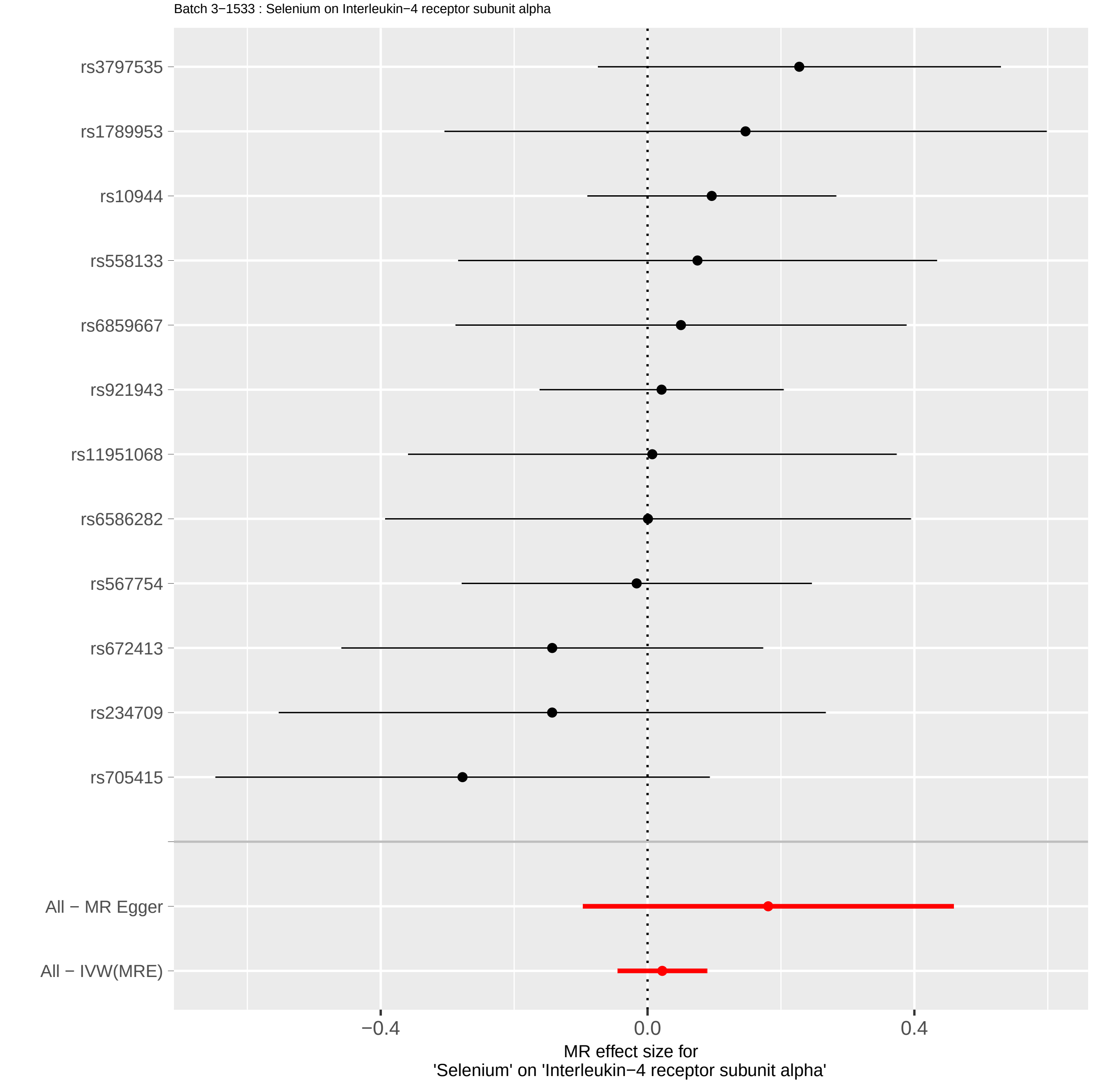

Batch 3−1533 : Selenium on Interleukin−4 receptor subunit alpha
rs3797535
rs1789953
rs10944
rs558133
rs6859667
rs921943
rs11951068
rs6586282
rs567754
rs672413
rs234709
rs705415
All − MR Egger
All − IVW(MRE)
−0.4
0.0
0.4
MR effect size for
'Selenium' on 'Interleukin−4 receptor subunit alpha'

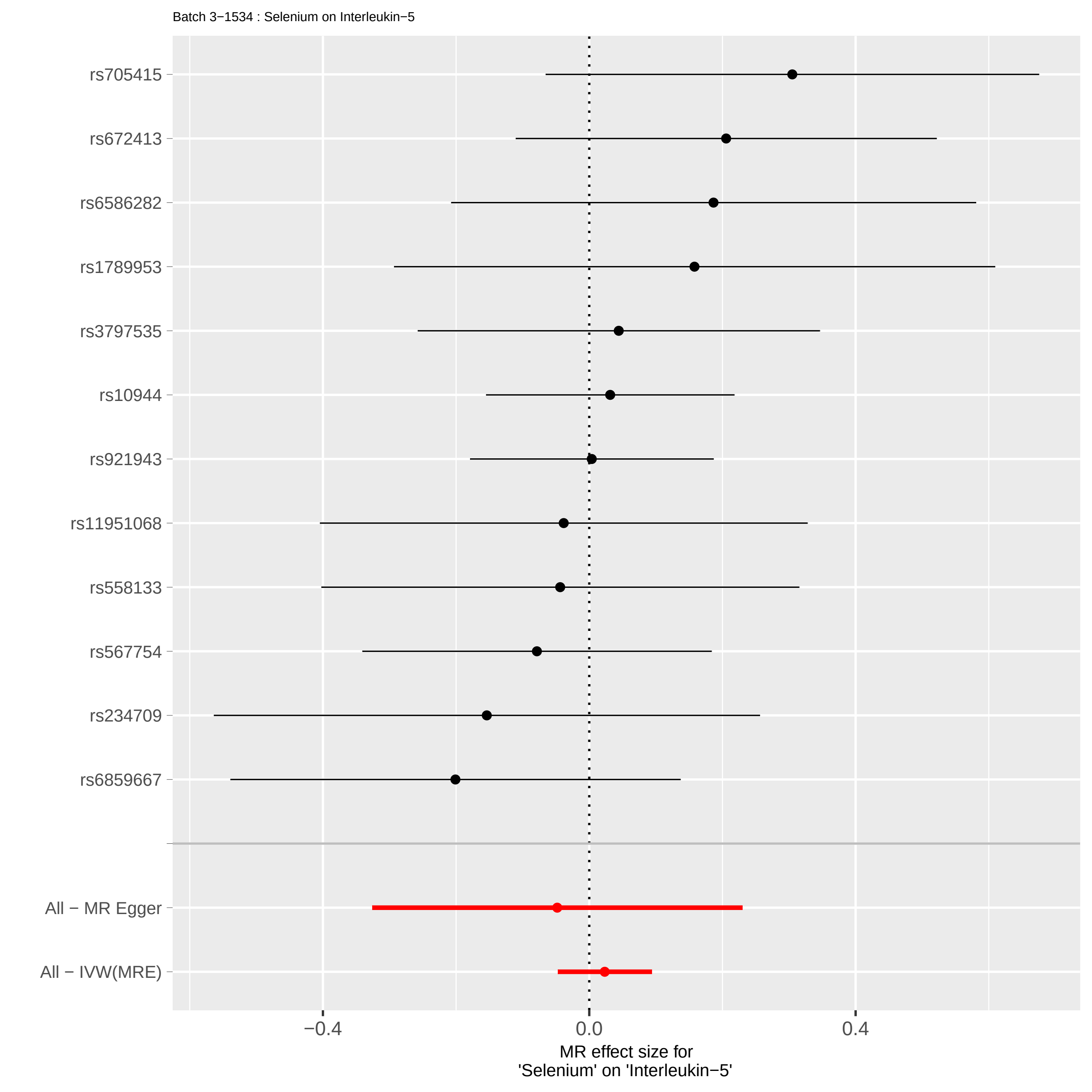

Batch 3−1534 : Selenium on Interleukin−5
rs705415
rs672413
rs6586282
rs1789953
rs3797535
rs10944
rs921943
rs11951068
rs558133
rs567754
rs234709
rs6859667
All − MR Egger
All − IVW(MRE)
−0.4
0.0
0.4
MR effect size for
'Selenium' on 'Interleukin−5'

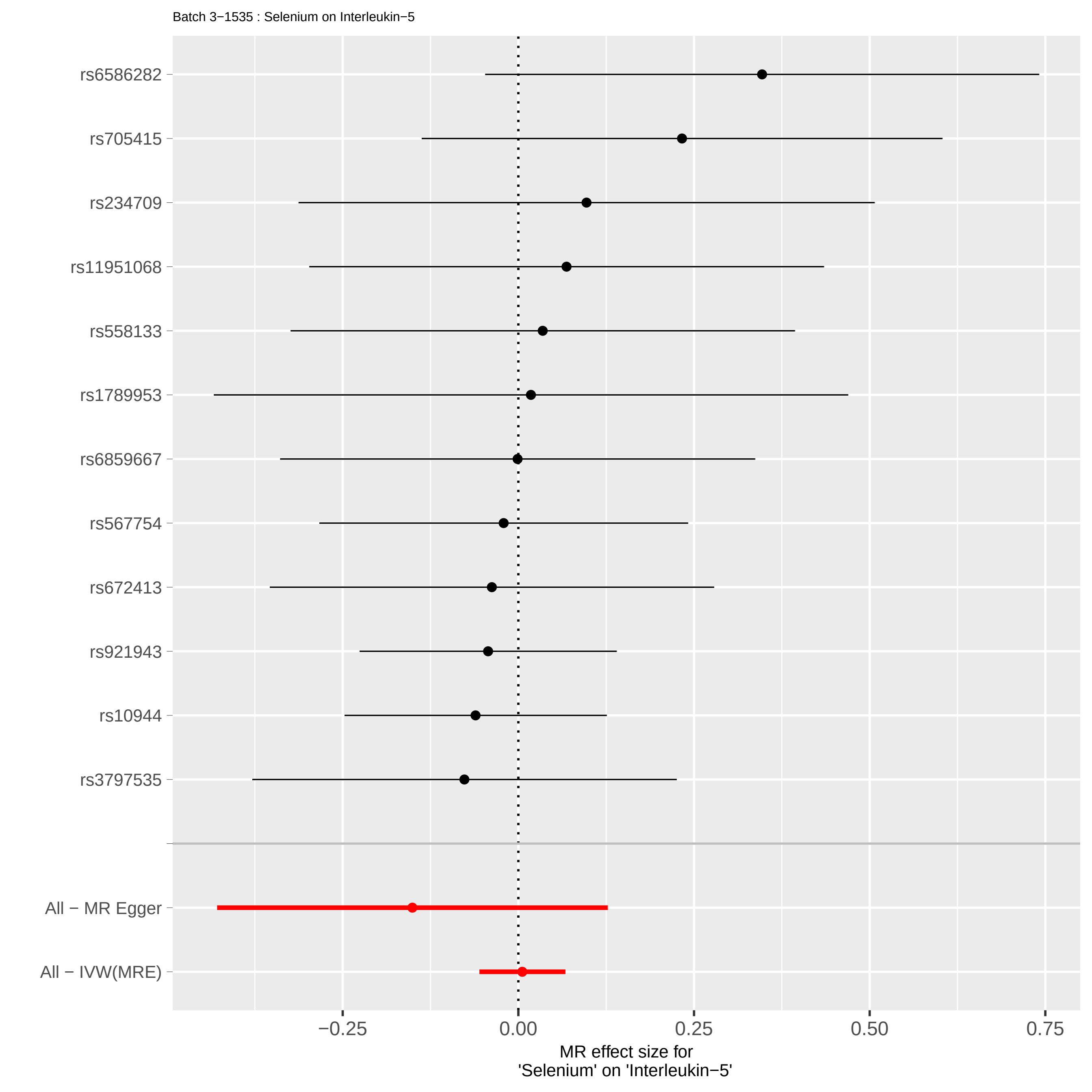

Batch 3−1535 : Selenium on Interleukin−5
rs6586282
rs705415
rs234709
rs11951068
rs558133
rs1789953
rs6859667
rs567754
rs672413
rs921943
rs10944
rs3797535
All − MR Egger
All − IVW(MRE)
−0.25
0.00
0.25
0.50
0.75
MR effect size for
'Selenium' on 'Interleukin−5'

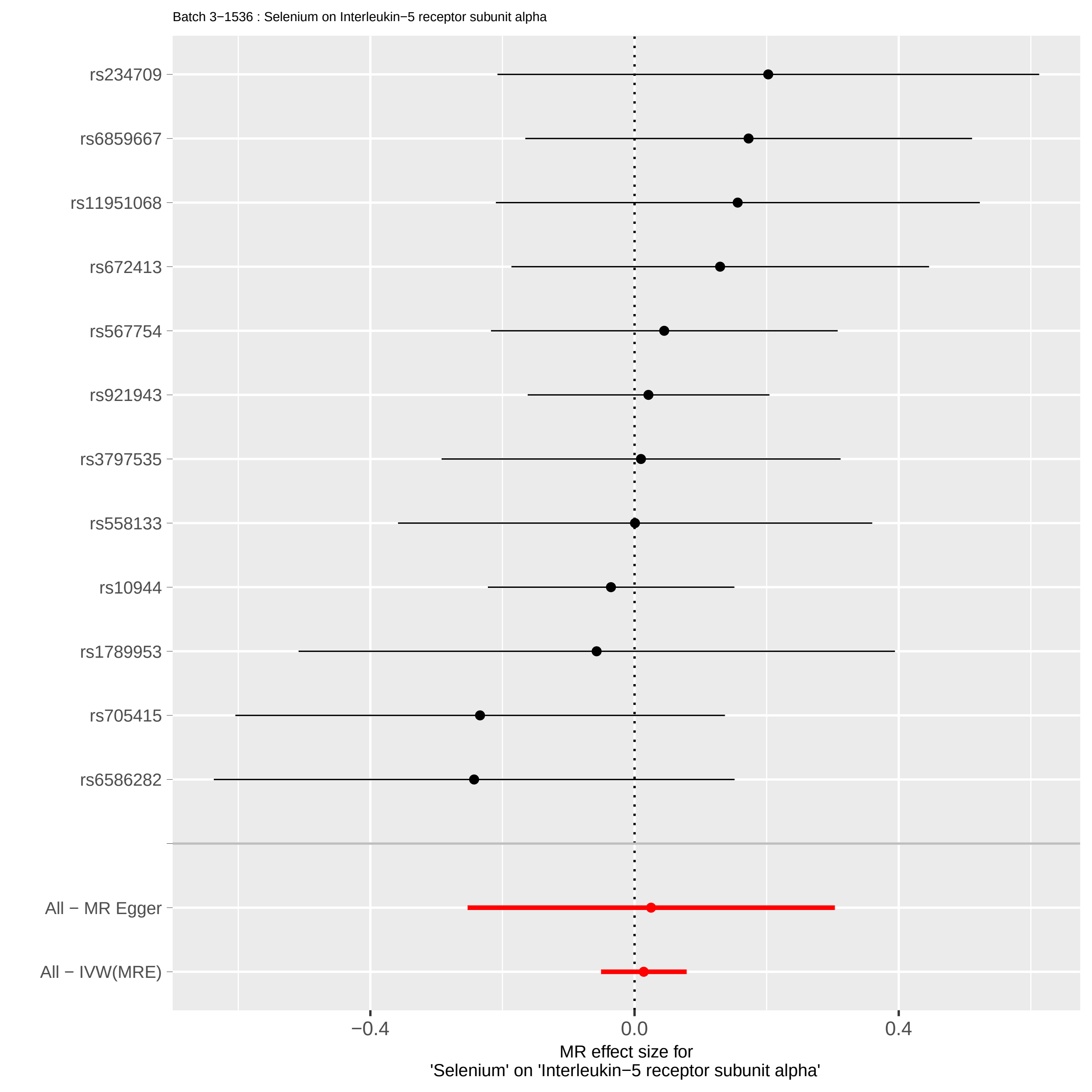
Batch 3−1536 : Selenium on Interleukin−5 receptor subunit alpha
rs234709
rs6859667
rs11951068
rs672413
rs567754
rs921943
rs3797535
rs558133
rs10944
rs1789953
rs705415
rs6586282
All − MR Egger
All − IVW(MRE)
−0.4
0.0
0.4
MR effect size for
'Selenium' on 'Interleukin−5 receptor subunit alpha'

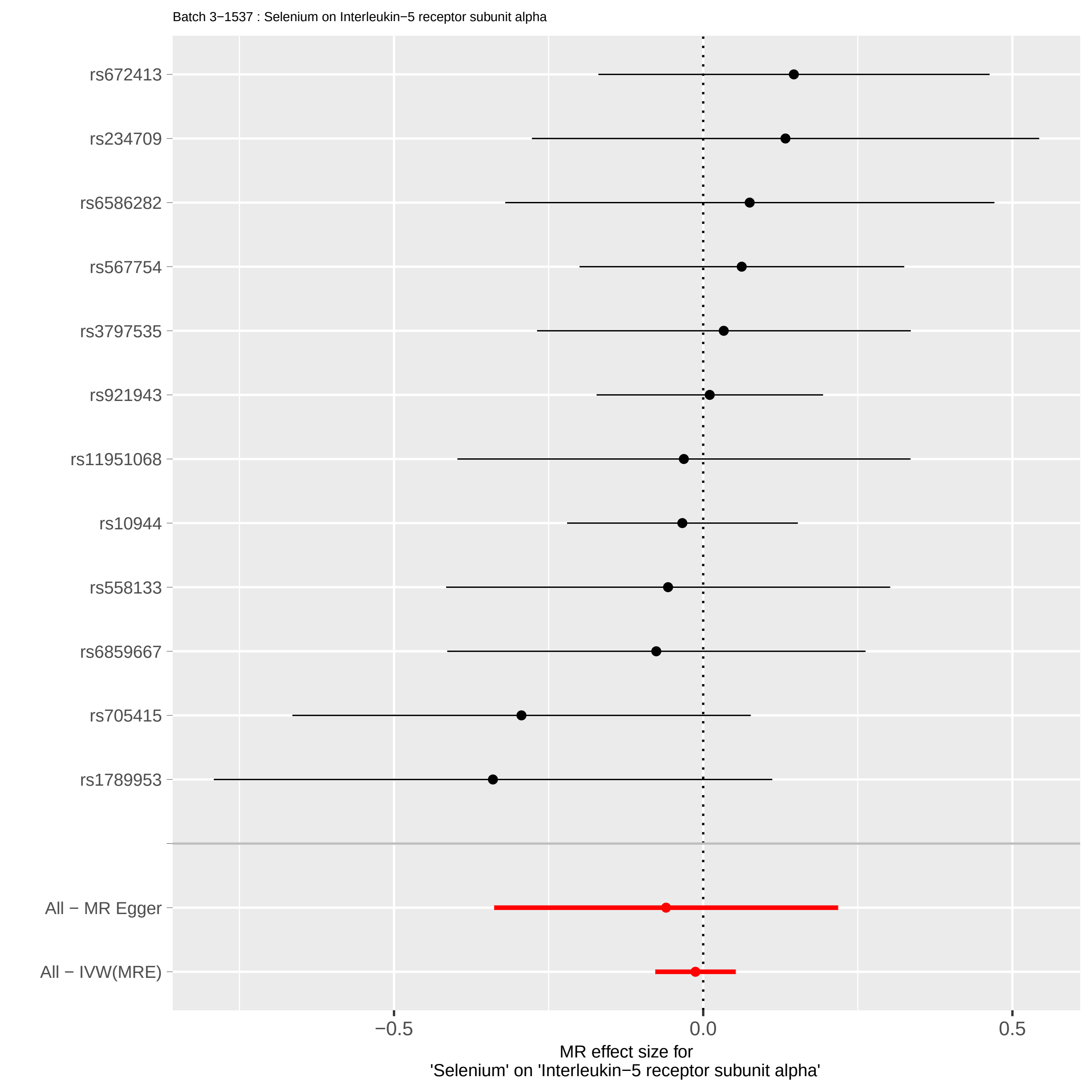

Batch 3−1537 : Selenium on Interleukin−5 receptor subunit alpha
rs672413
rs234709
rs6586282
rs567754
rs3797535
rs921943
rs11951068
rs10944
rs558133
rs6859667
rs705415
rs1789953
All − MR Egger
All − IVW(MRE)
−0.5
0.0
0.5
MR effect size for
'Selenium' on 'Interleukin−5 receptor subunit alpha'

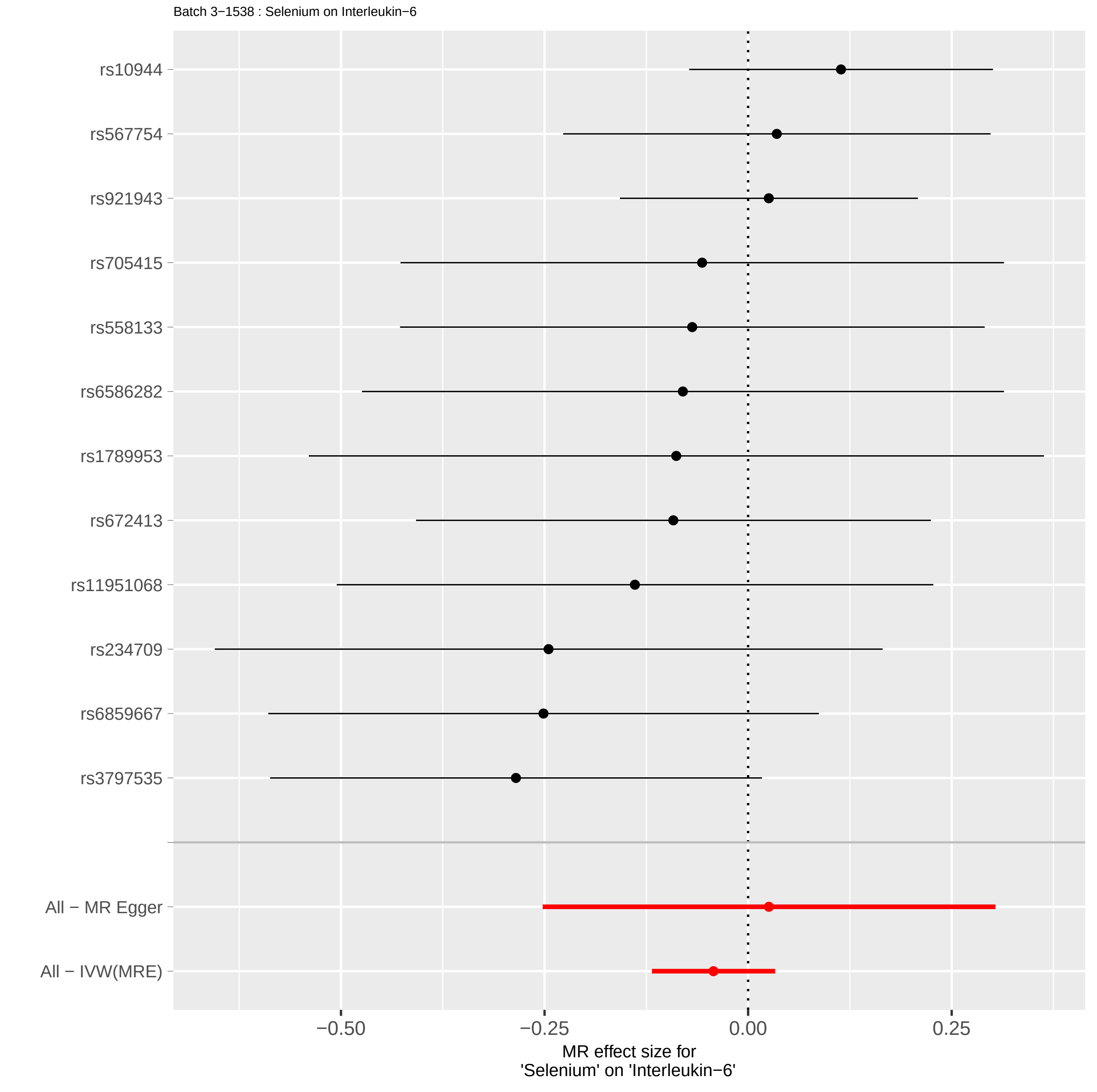

Batch 3−1538 : Selenium on Interleukin−6
rs10944
rs567754
rs921943
rs705415
rs558133
rs6586282
rs1789953
rs672413
rs11951068
rs234709
rs6859667
rs3797535
All − MR Egger
All − IVW(MRE)
−0.50
−0.25
0.00
0.25
MR effect size for
'Selenium' on 'Interleukin−6'

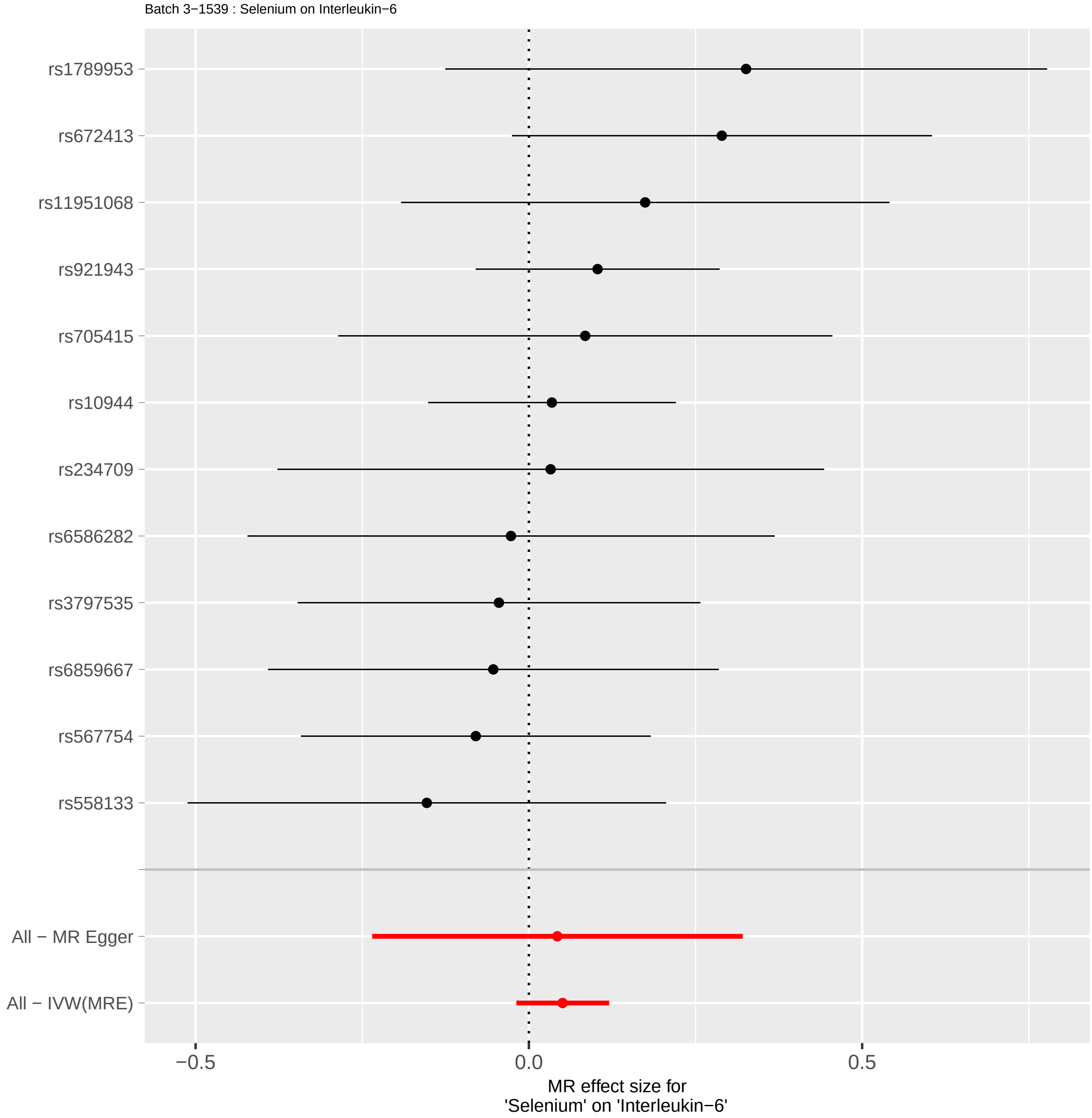

Batch 3−1539 : Selenium on Interleukin−6
rs1789953
rs672413
rs11951068
rs921943
rs705415
rs10944
rs234709
rs6586282
rs3797535
rs6859667
rs567754
rs558133
All − MR Egger
All − IVW(MRE)
−0.5
0.0
0.5
MR effect size for
'Selenium' on 'Interleukin−6'

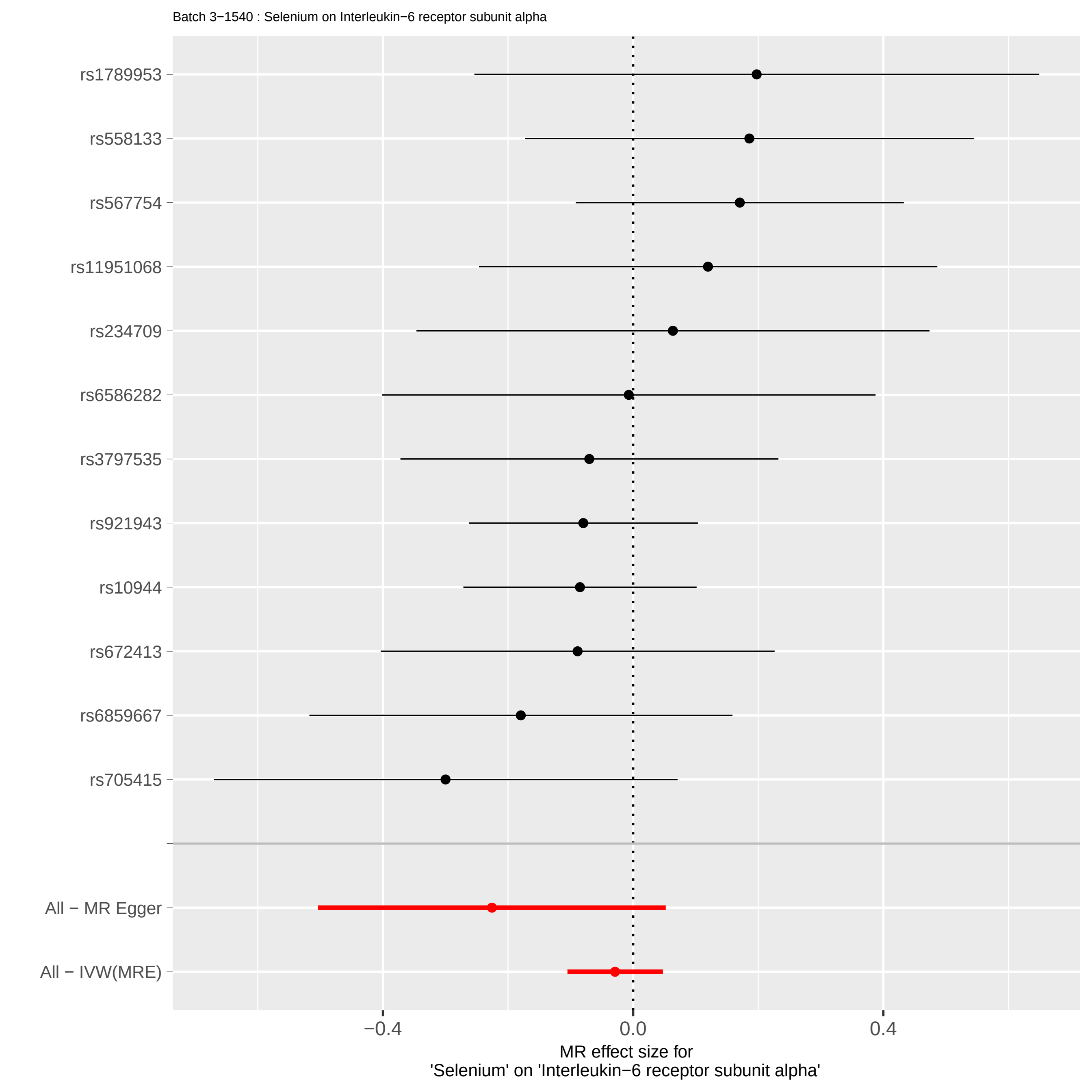
Batch 3−1540 : Selenium on Interleukin−6 receptor subunit alpha
rs1789953
rs558133
rs567754
rs11951068
rs234709
rs6586282
rs3797535
rs921943
rs10944
rs672413
rs6859667
rs705415
All − MR Egger
All − IVW(MRE)
−0.4
0.0
0.4
MR effect size for
'Selenium' on 'Interleukin−6 receptor subunit alpha'

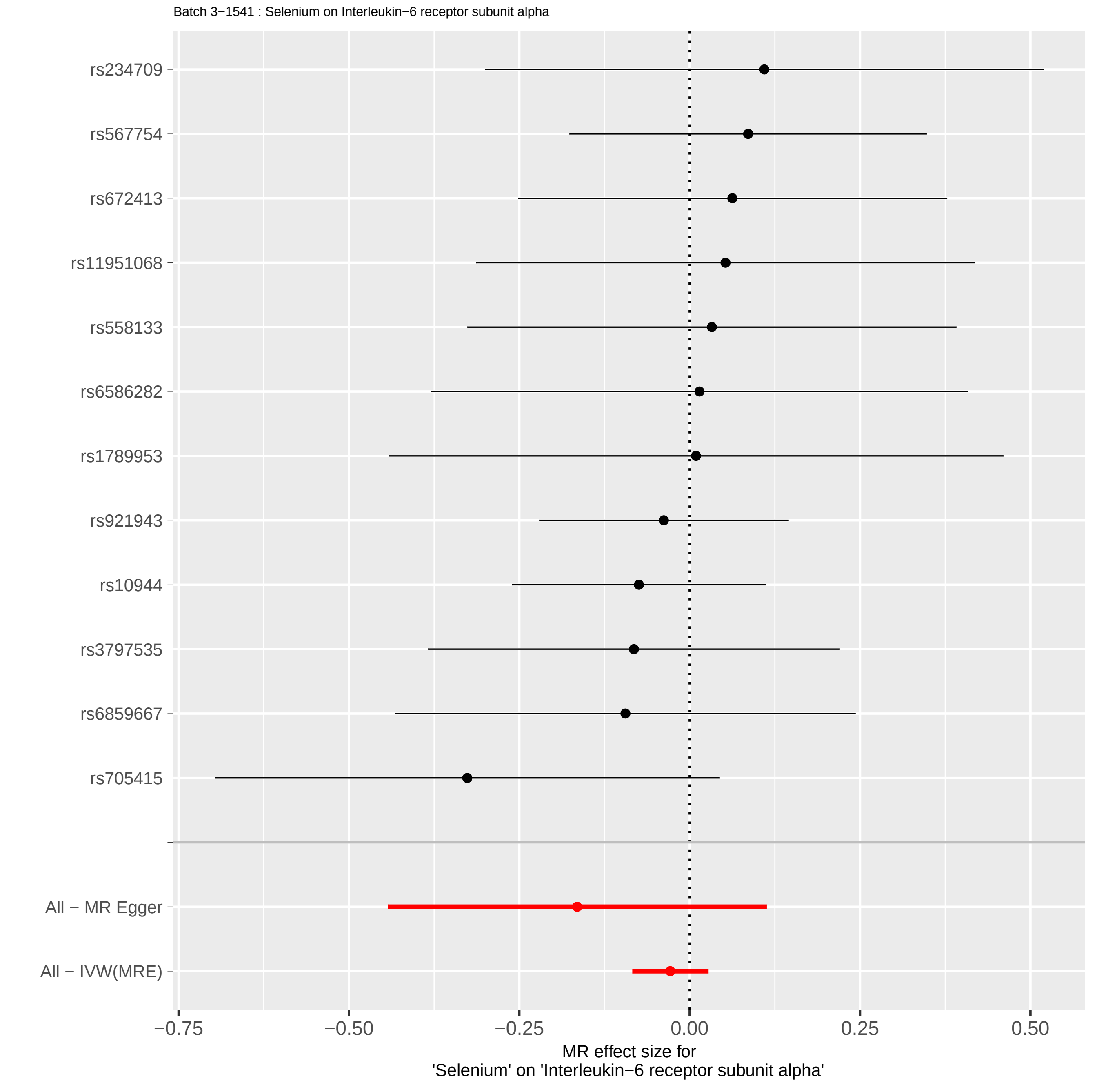

Batch 3−1541 : Selenium on Interleukin−6 receptor subunit alpha
rs234709
rs567754
rs672413
rs11951068
rs558133
rs6586282
rs1789953
rs921943
rs10944
rs3797535
rs6859667
rs705415
All − MR Egger
All − IVW(MRE)
−0.75
−0.50
−0.25
0.00
0.25
0.50
MR effect size for
'Selenium' on 'Interleukin−6 receptor subunit alpha'

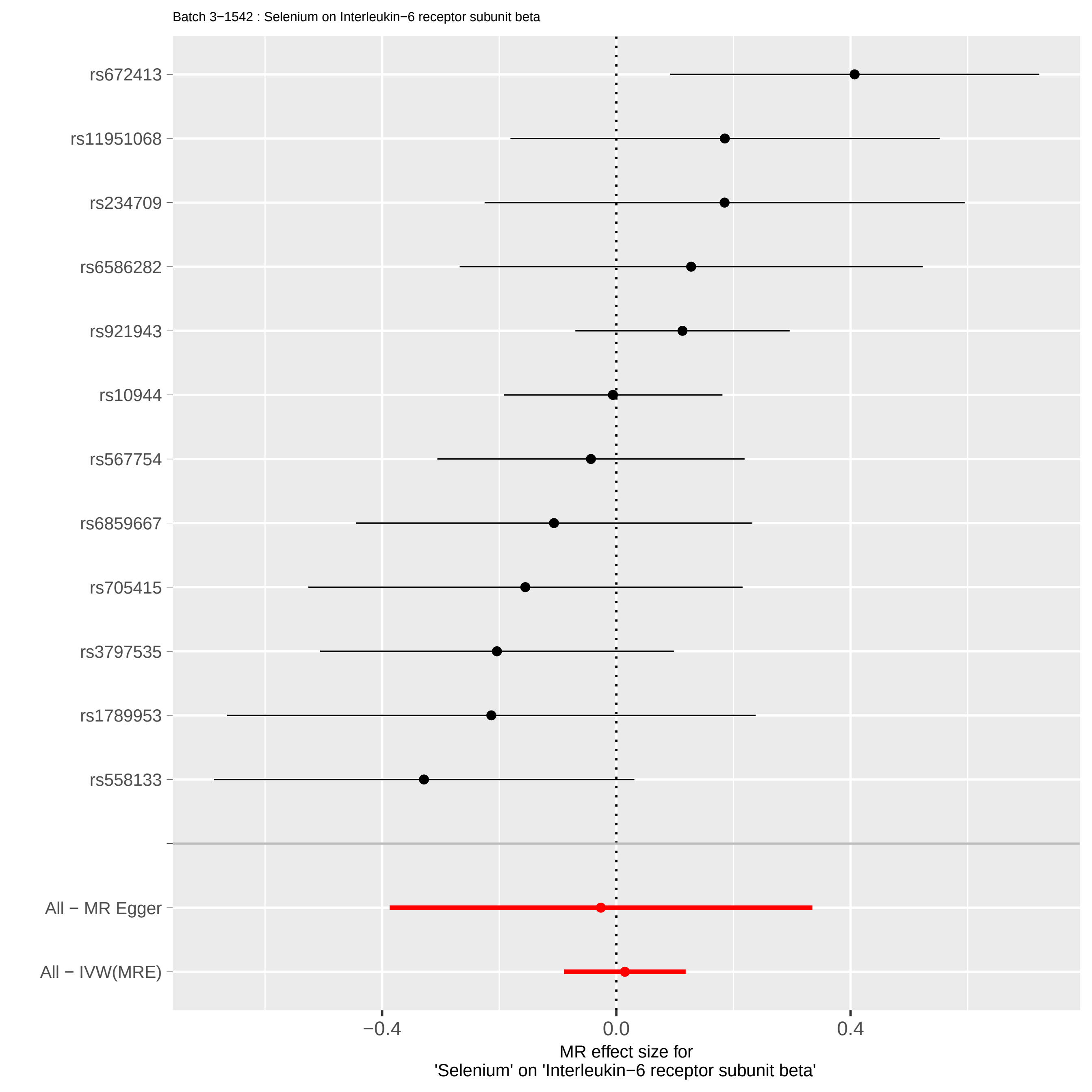
Batch 3−1542 : Selenium on Interleukin−6 receptor subunit beta
rs672413
rs11951068
rs234709
rs6586282
rs921943
rs10944
rs567754
rs6859667
rs705415
rs3797535
rs1789953
rs558133
All − MR Egger
All − IVW(MRE)
−0.4
0.0
0.4
MR effect size for
'Selenium' on 'Interleukin−6 receptor subunit beta'

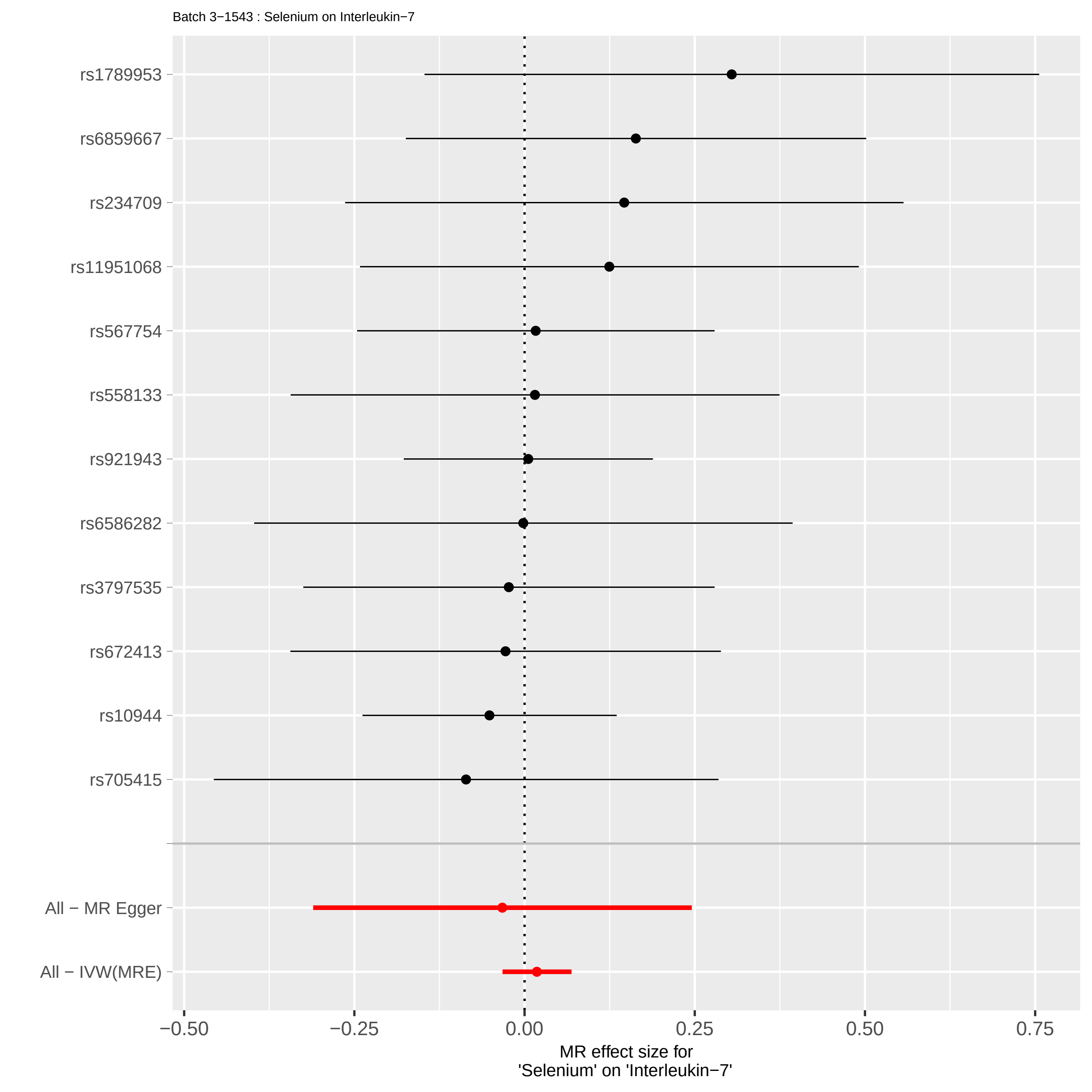

Batch 3−1543 : Selenium on Interleukin−7
rs1789953
rs6859667
rs234709
rs11951068
rs567754
rs558133
rs921943
rs6586282
rs3797535
rs672413
rs10944
rs705415
All − MR Egger
All − IVW(MRE)
−0.50
−0.25
0.00
0.25
0.50
0.75
MR effect size for
'Selenium' on 'Interleukin−7'

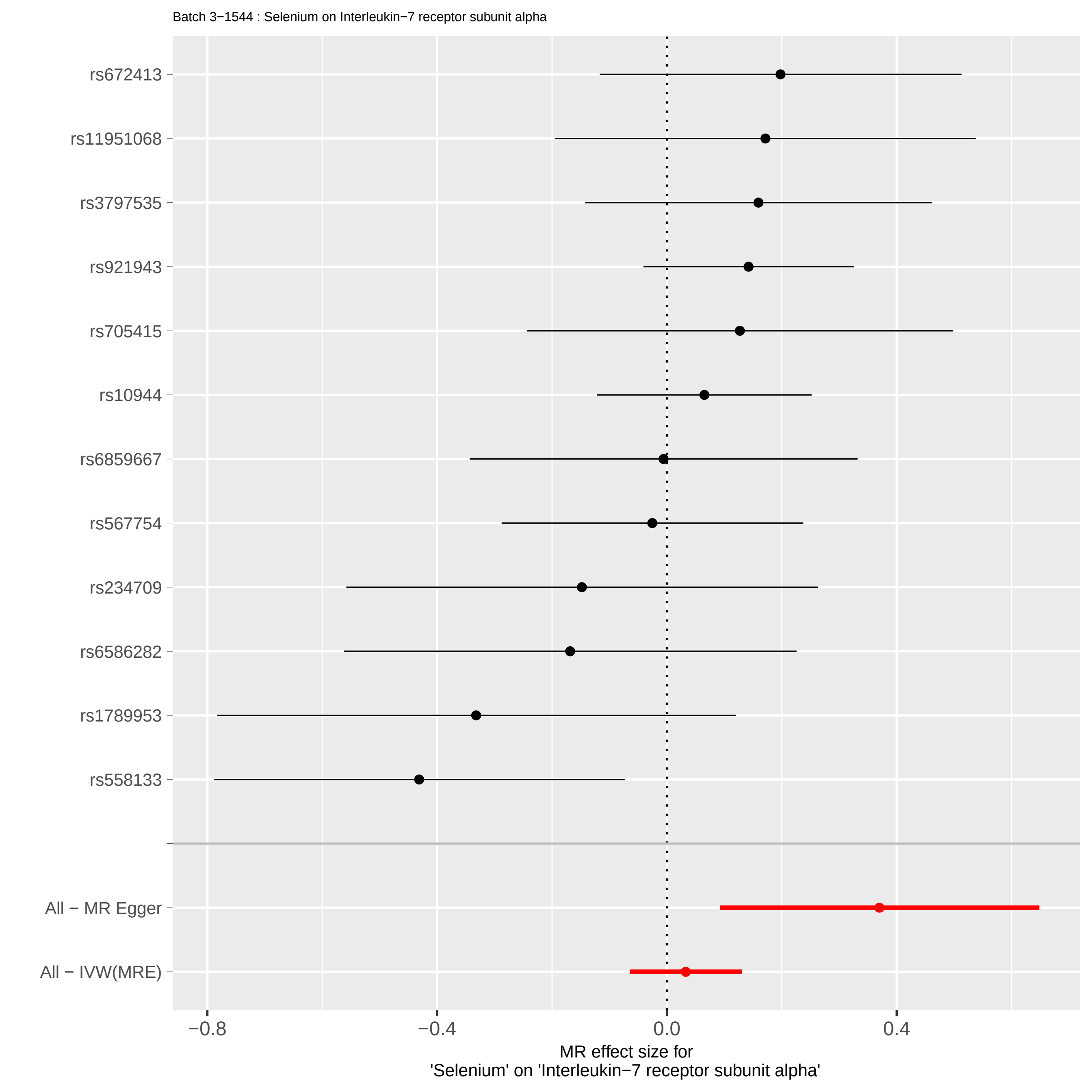

Batch 3−1544 : Selenium on Interleukin−7 receptor subunit alpha
rs672413
rs11951068
rs3797535
rs921943
rs705415
rs10944
rs6859667
rs567754
rs234709
rs6586282
rs1789953
rs558133
All − MR Egger
All − IVW(MRE)
−0.8
−0.4
0.0
0.4
MR effect size for
'Selenium' on 'Interleukin−7 receptor subunit alpha'

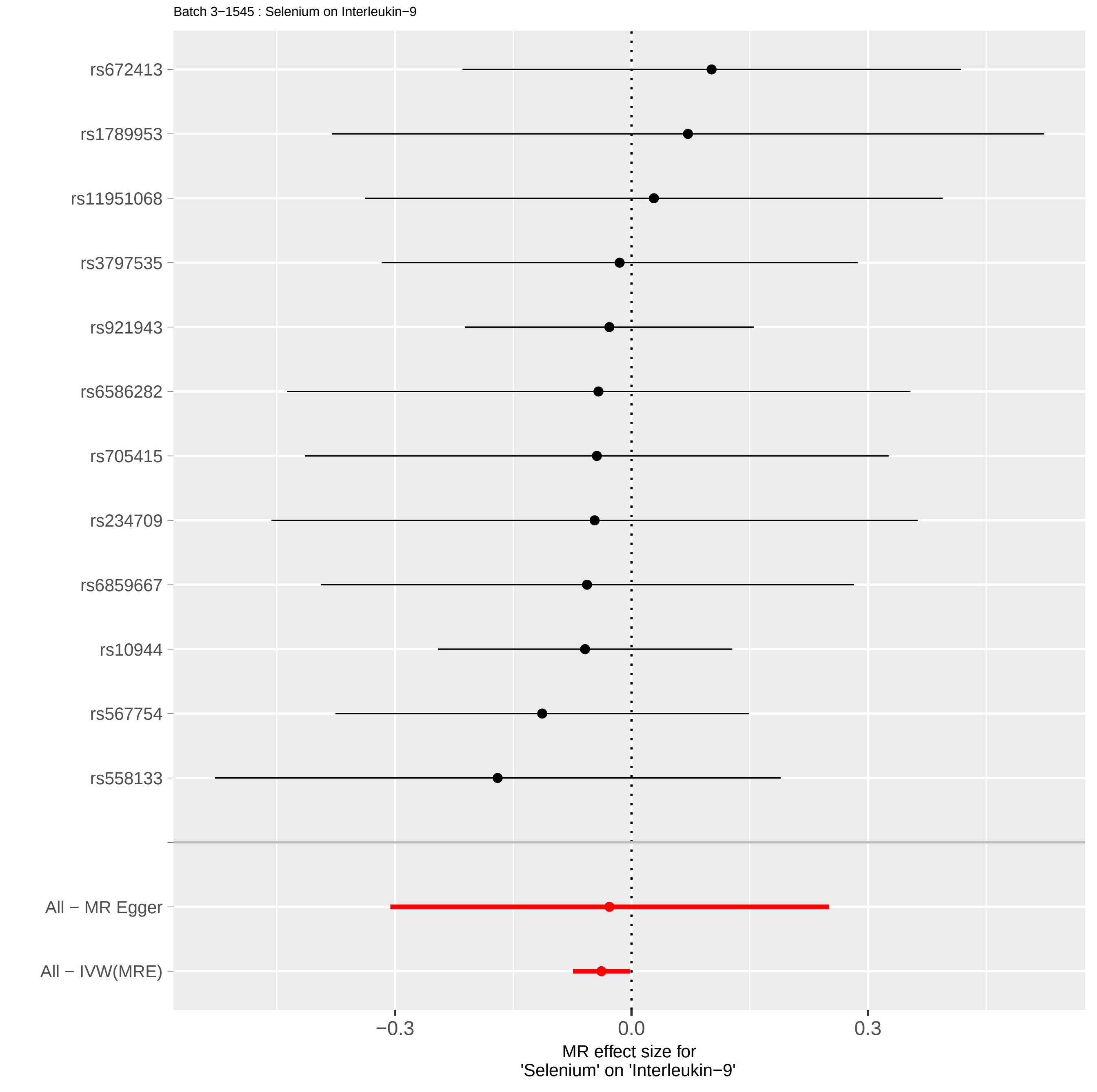

Batch 3−1545 : Selenium on Interleukin−9
rs672413
rs1789953
rs11951068
rs3797535
rs921943
rs6586282
rs705415
rs234709
rs6859667
rs10944
rs567754
rs558133
All − MR Egger
All − IVW(MRE)
−0.3
0.0
0.3
MR effect size for
'Selenium' on 'Interleukin−9'

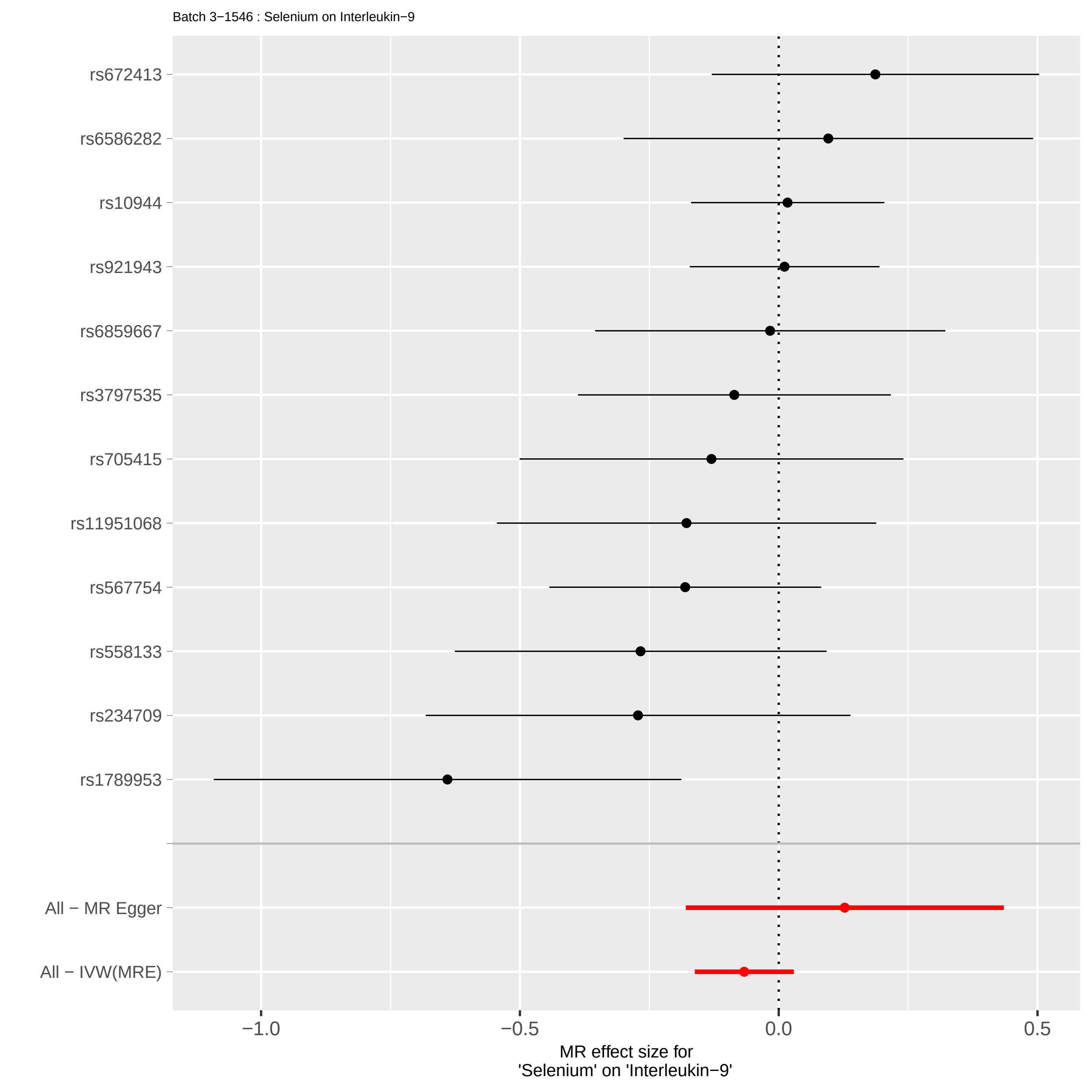

Batch 3−1546 : Selenium on Interleukin−9
rs672413
rs6586282
rs10944
rs921943
rs6859667
rs3797535
rs705415
rs11951068
rs567754
rs558133
rs234709
rs1789953
All − MR Egger
All − IVW(MRE)
−1.0
−0.5
0.0
0.5
MR effect size for
'Selenium' on 'Interleukin−9'

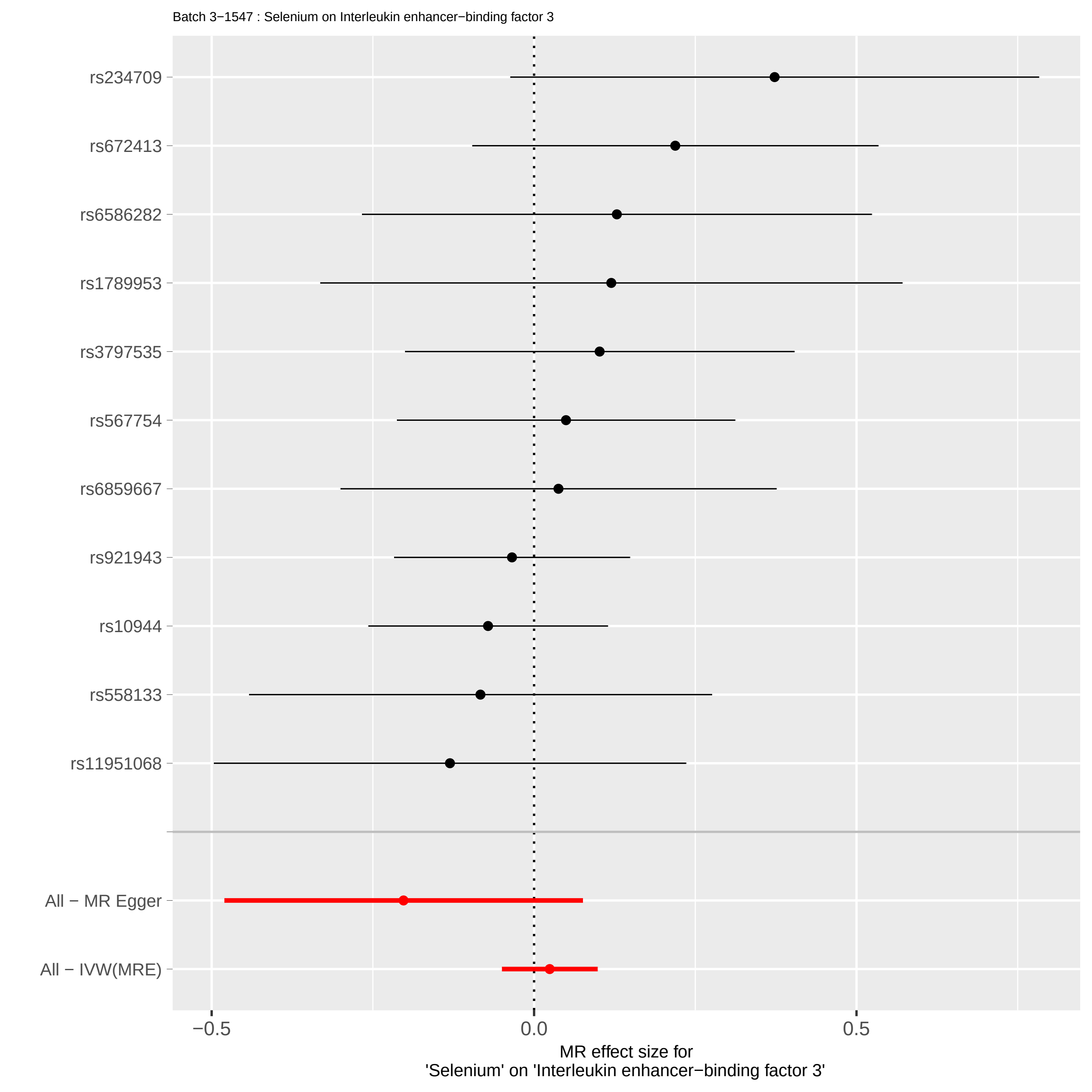
Batch 3−1547 : Selenium on Interleukin enhancer−binding factor 3
rs234709
rs672413
rs6586282
rs1789953
rs3797535
rs567754
rs6859667
rs921943
rs10944
rs558133
rs11951068
All − MR Egger
All − IVW(MRE)
−0.5
0.0
0.5
MR effect size for
'Selenium' on 'Interleukin enhancer−binding factor 3'

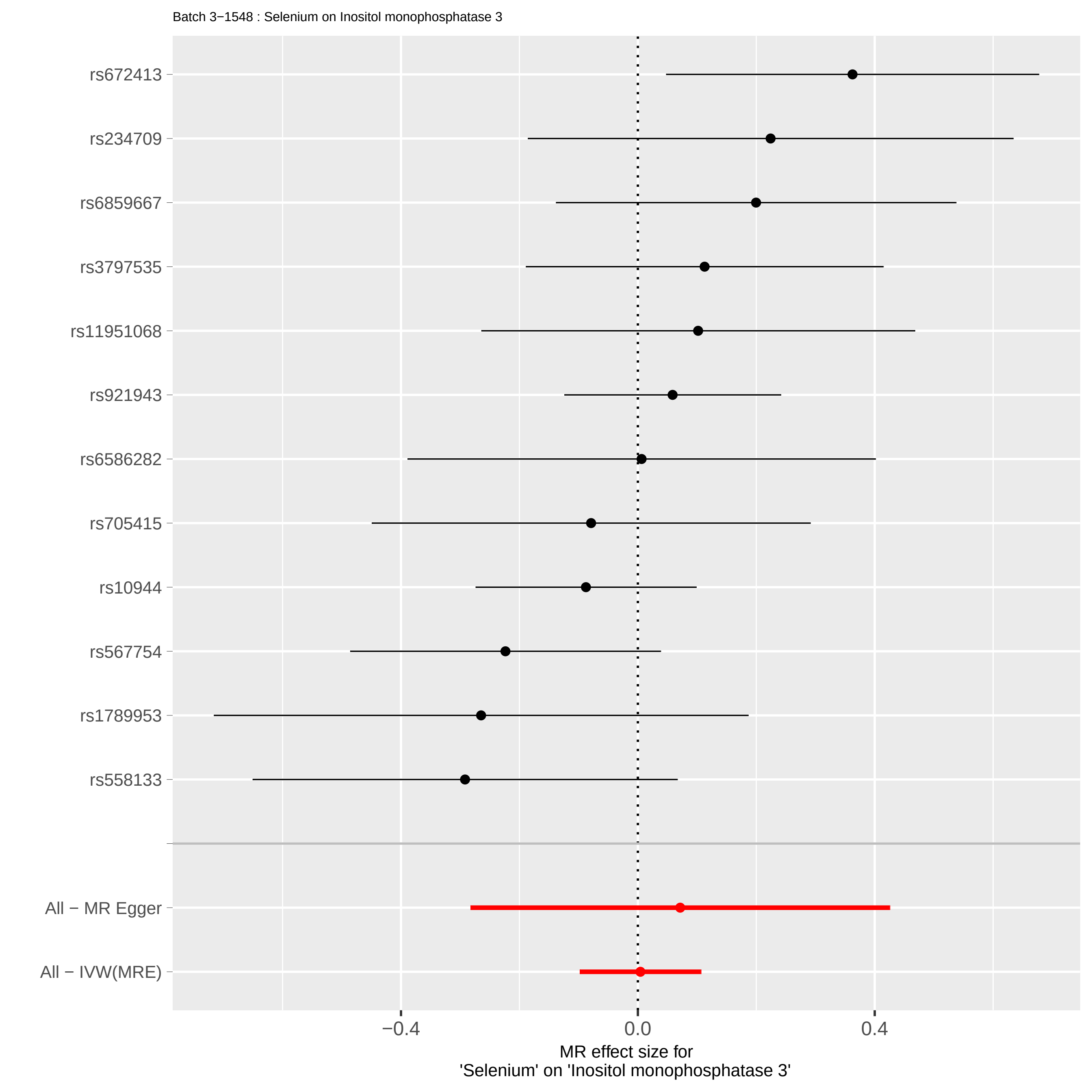

Batch 3−1548 : Selenium on Inositol monophosphatase 3
rs672413
rs234709
rs6859667
rs3797535
rs11951068
rs921943
rs6586282
rs705415
rs10944
rs567754
rs1789953
rs558133
All − MR Egger
All − IVW(MRE)
−0.4
0.0
0.4
MR effect size for
'Selenium' on 'Inositol monophosphatase 3'

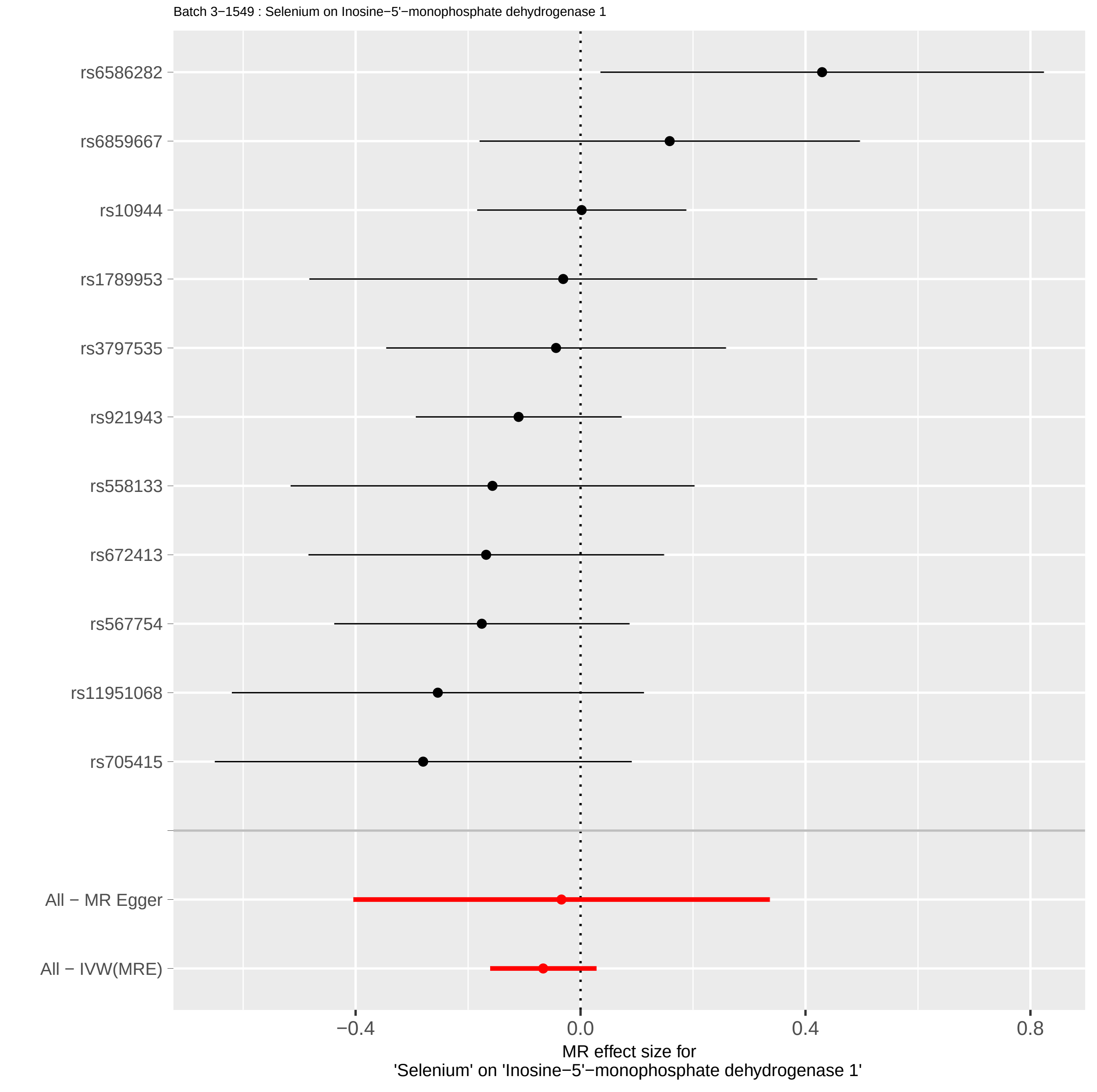

Batch 3−1549 : Selenium on Inosine−5'−monophosphate dehydrogenase 1
rs6586282
rs6859667
rs10944
rs1789953
rs3797535
rs921943
rs558133
rs672413
rs567754
rs11951068
rs705415
All − MR Egger
All − IVW(MRE)
−0.4
0.0
0.4
0.8
MR effect size for
'Selenium' on 'Inosine−5'−monophosphate dehydrogenase 1'

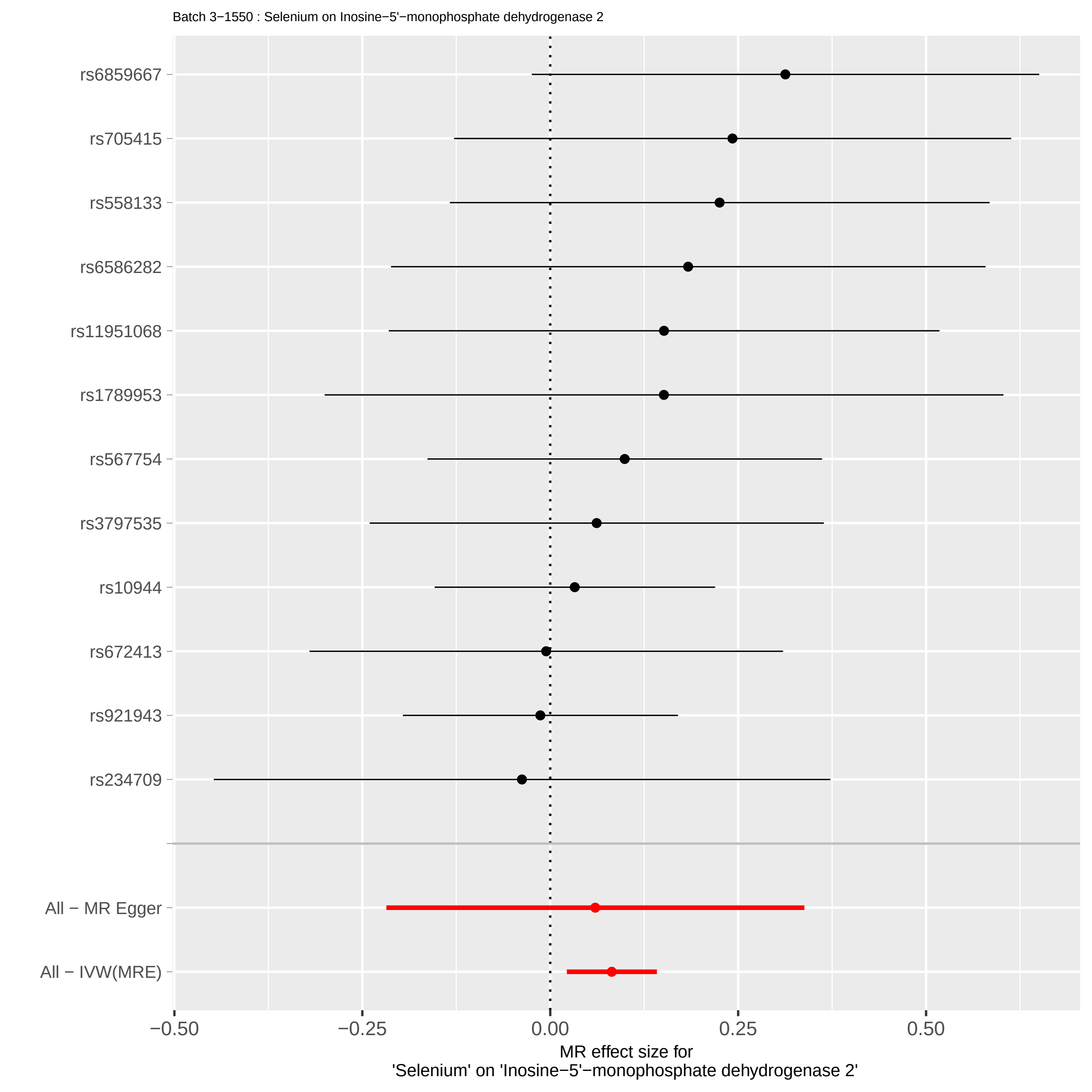

Batch 3−1550 : Selenium on Inosine−5'−monophosphate dehydrogenase 2
rs6859667
rs705415
rs558133
rs6586282
rs11951068
rs1789953
rs567754
rs3797535
rs10944
rs672413
rs921943
rs234709
All − MR Egger
All − IVW(MRE)
−0.50
−0.25
0.00
0.25
0.50
MR effect size for
'Selenium' on 'Inosine−5'−monophosphate dehydrogenase 2'

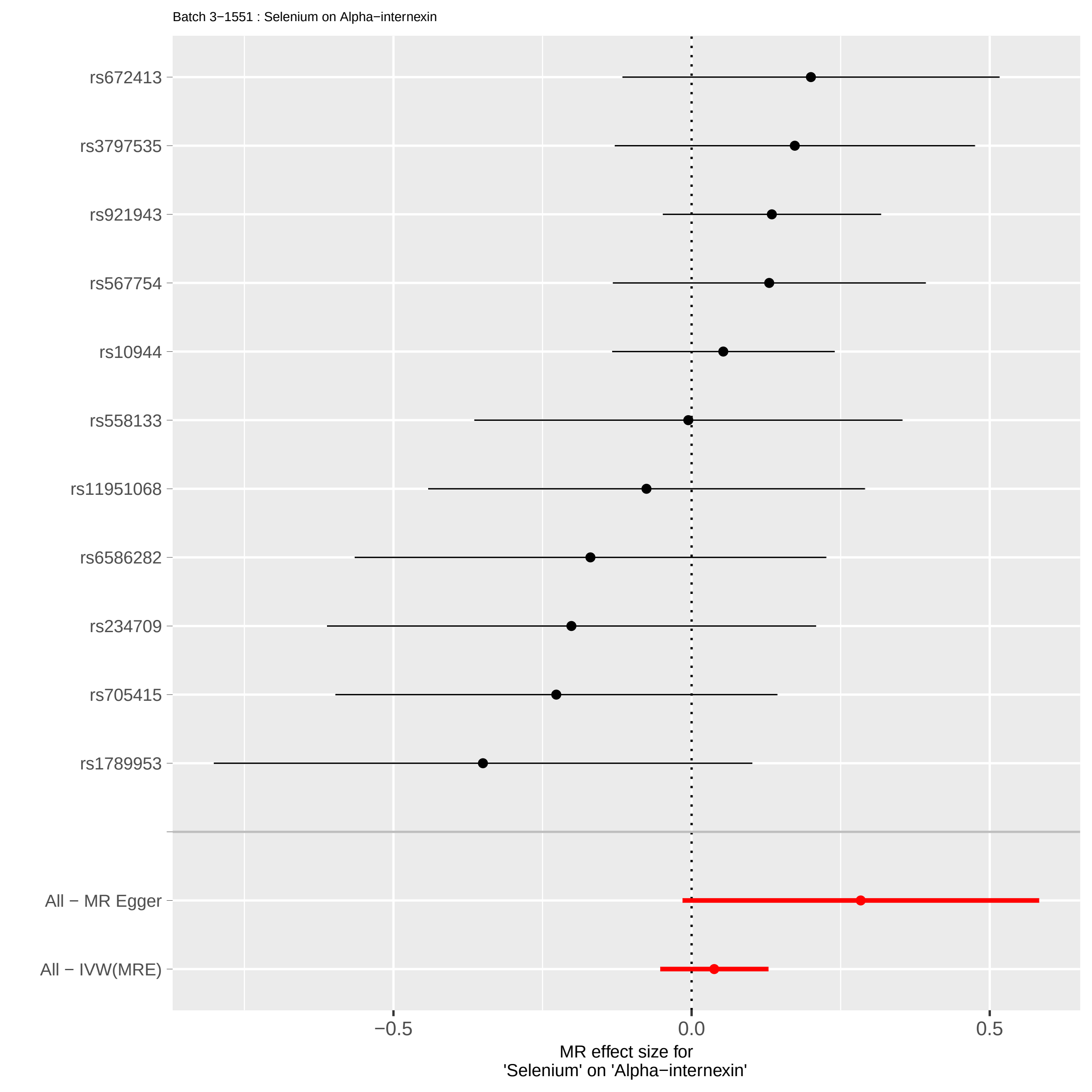

Batch 3−1551 : Selenium on Alpha−internexin
rs672413
rs3797535
rs921943
rs567754
rs10944
rs558133
rs11951068
rs6586282
rs234709
rs705415
rs1789953
All − MR Egger
All − IVW(MRE)
−0.5
0.0
0.5
MR effect size for
'Selenium' on 'Alpha−internexin'

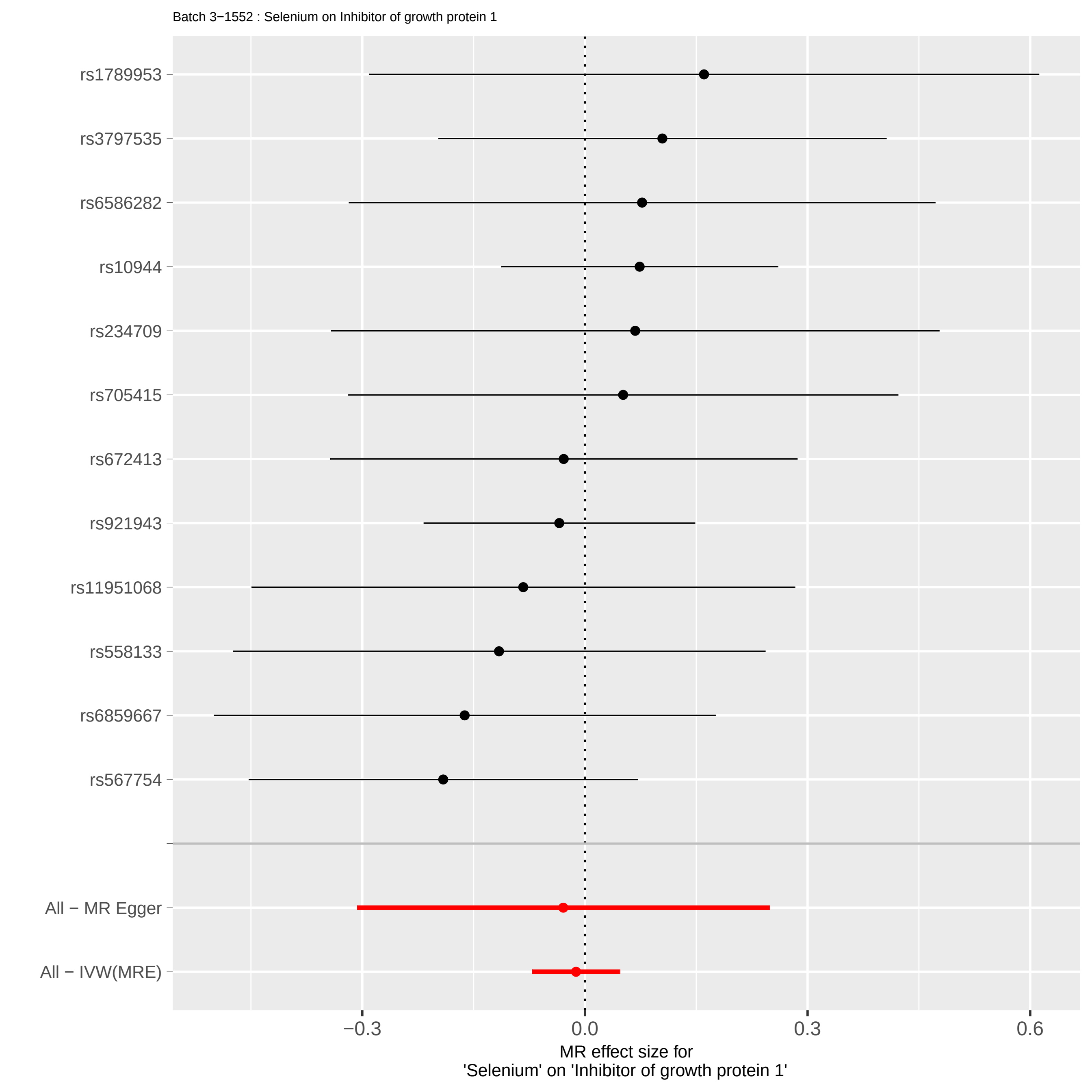

Batch 3−1552 : Selenium on Inhibitor of growth protein 1
rs1789953
rs3797535
rs6586282
rs10944
rs234709
rs705415
rs672413
rs921943
rs11951068
rs558133
rs6859667
rs567754
All − MR Egger
All − IVW(MRE)
−0.3
0.0
0.3
0.6
MR effect size for
'Selenium' on 'Inhibitor of growth protein 1'

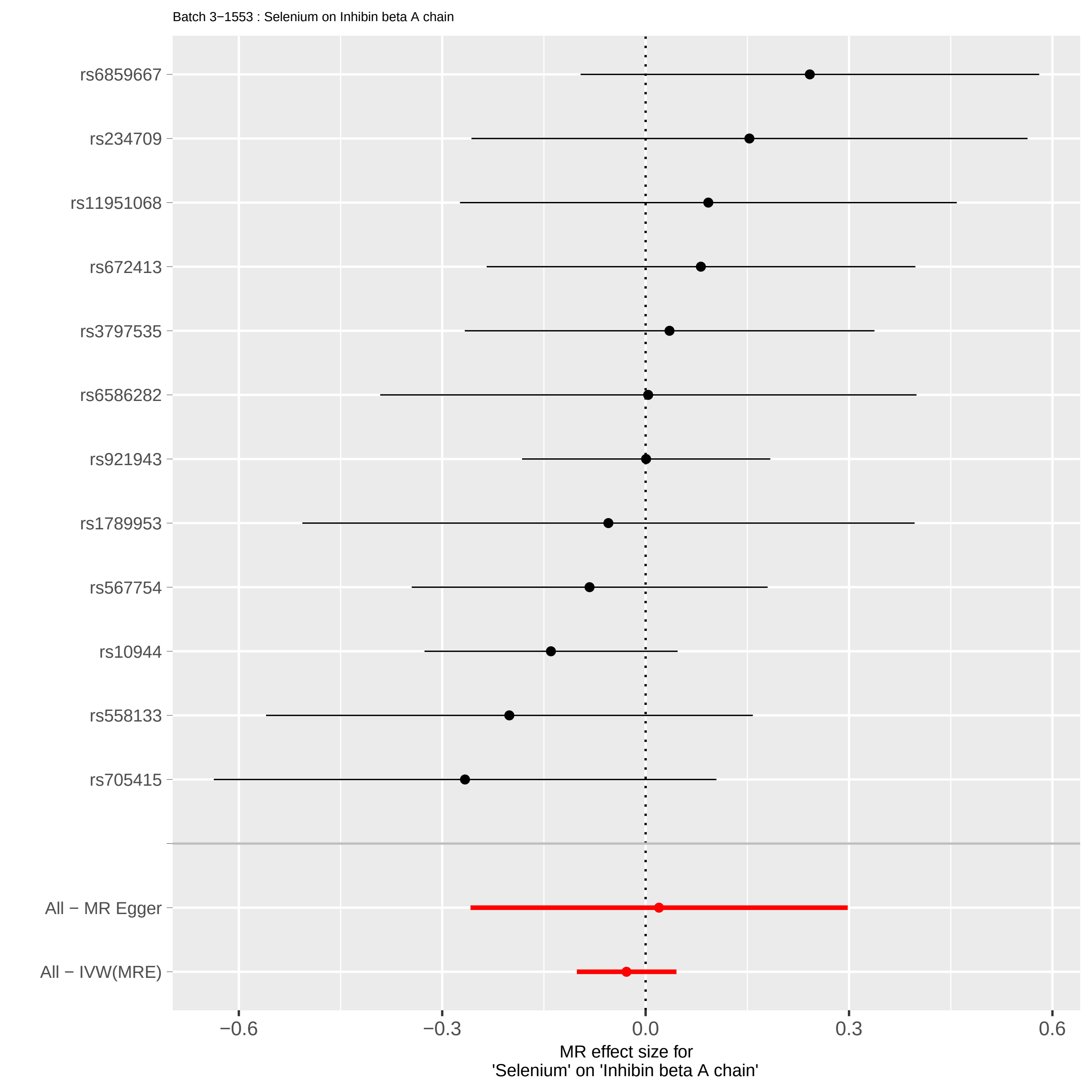

Batch 3−1553 : Selenium on Inhibin beta A chain
rs6859667
rs234709
rs11951068
rs672413
rs3797535
rs6586282
rs921943
rs1789953
rs567754
rs10944
rs558133
rs705415
All − MR Egger
All − IVW(MRE)
−0.6
−0.3
0.0
0.3
0.6
MR effect size for
'Selenium' on 'Inhibin beta A chain'

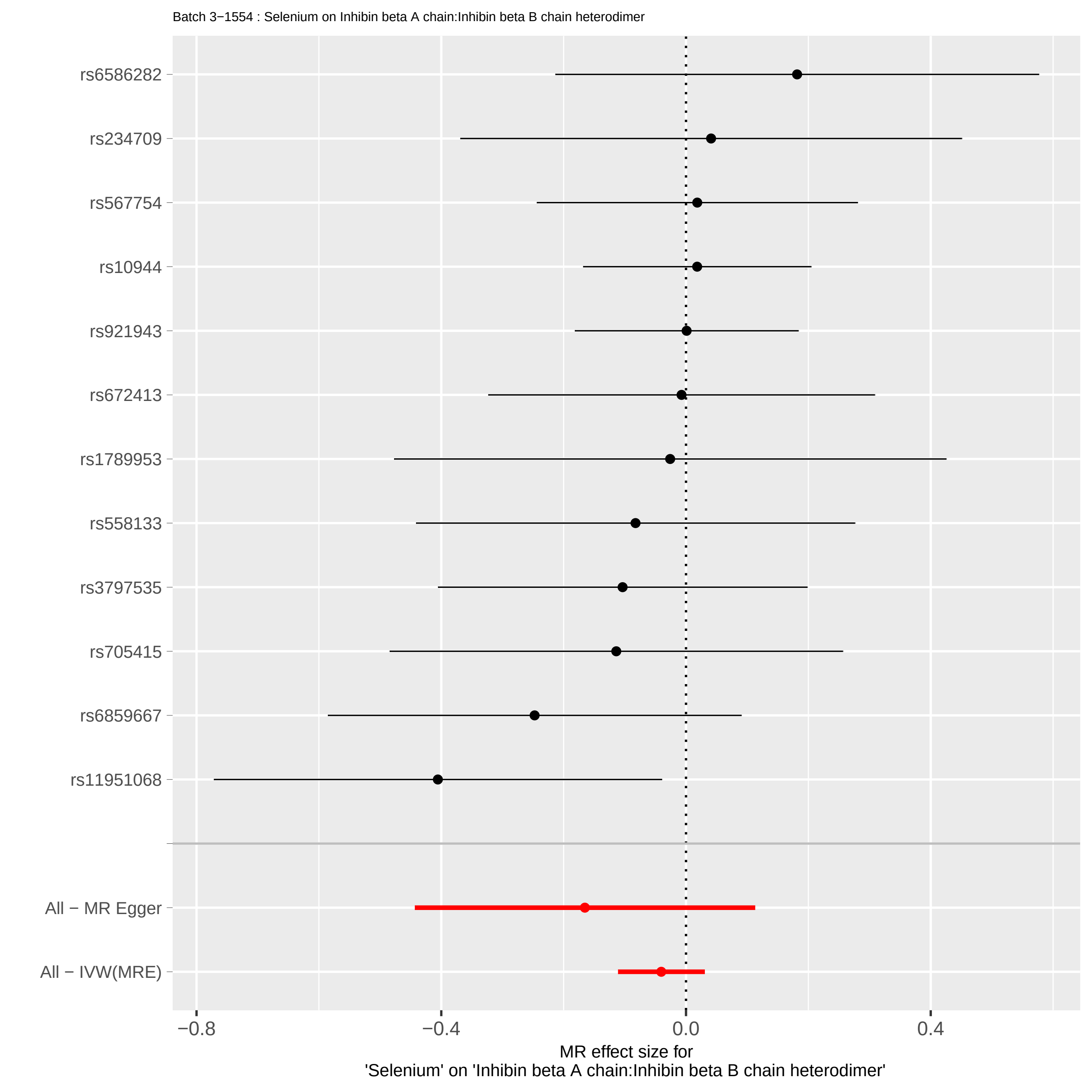
Batch 3−1554 : Selenium on Inhibin beta A chain:Inhibin beta B chain heterodimer
rs6586282
rs234709
rs567754
rs10944
rs921943
rs672413
rs1789953
rs558133
rs3797535
rs705415
rs6859667
rs11951068
All − MR Egger
All − IVW(MRE)
−0.8
−0.4
0.0
0.4
MR effect size for
'Selenium' on 'Inhibin beta A chain:Inhibin beta B chain heterodimer'

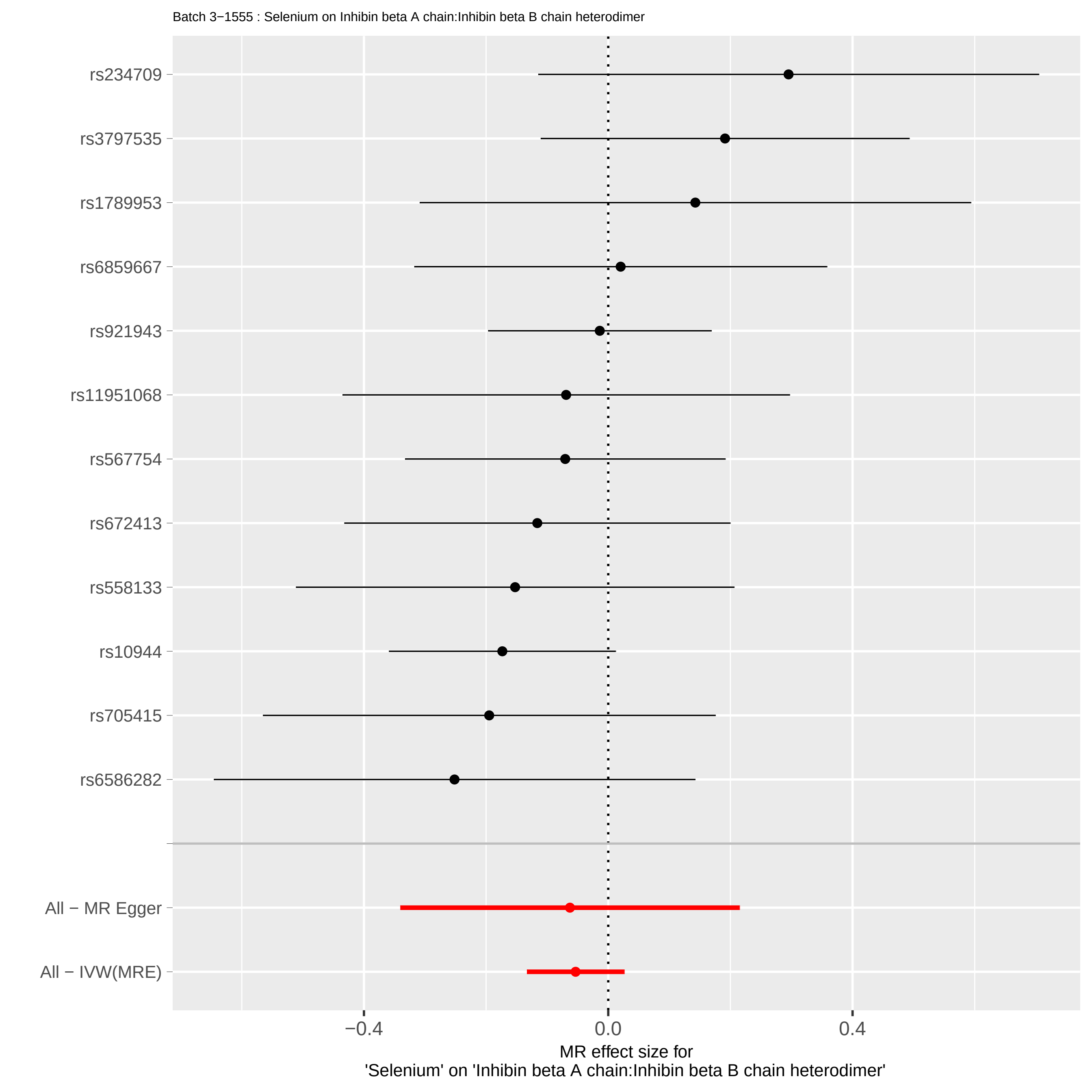
Batch 3−1555 : Selenium on Inhibin beta A chain:Inhibin beta B chain heterodimer
rs234709
rs3797535
rs1789953
rs6859667
rs921943
rs11951068
rs567754
rs672413
rs558133
rs10944
rs705415
rs6586282
All − MR Egger
All − IVW(MRE)
−0.4
0.0
0.4
MR effect size for
'Selenium' on 'Inhibin beta A chain:Inhibin beta B chain heterodimer'

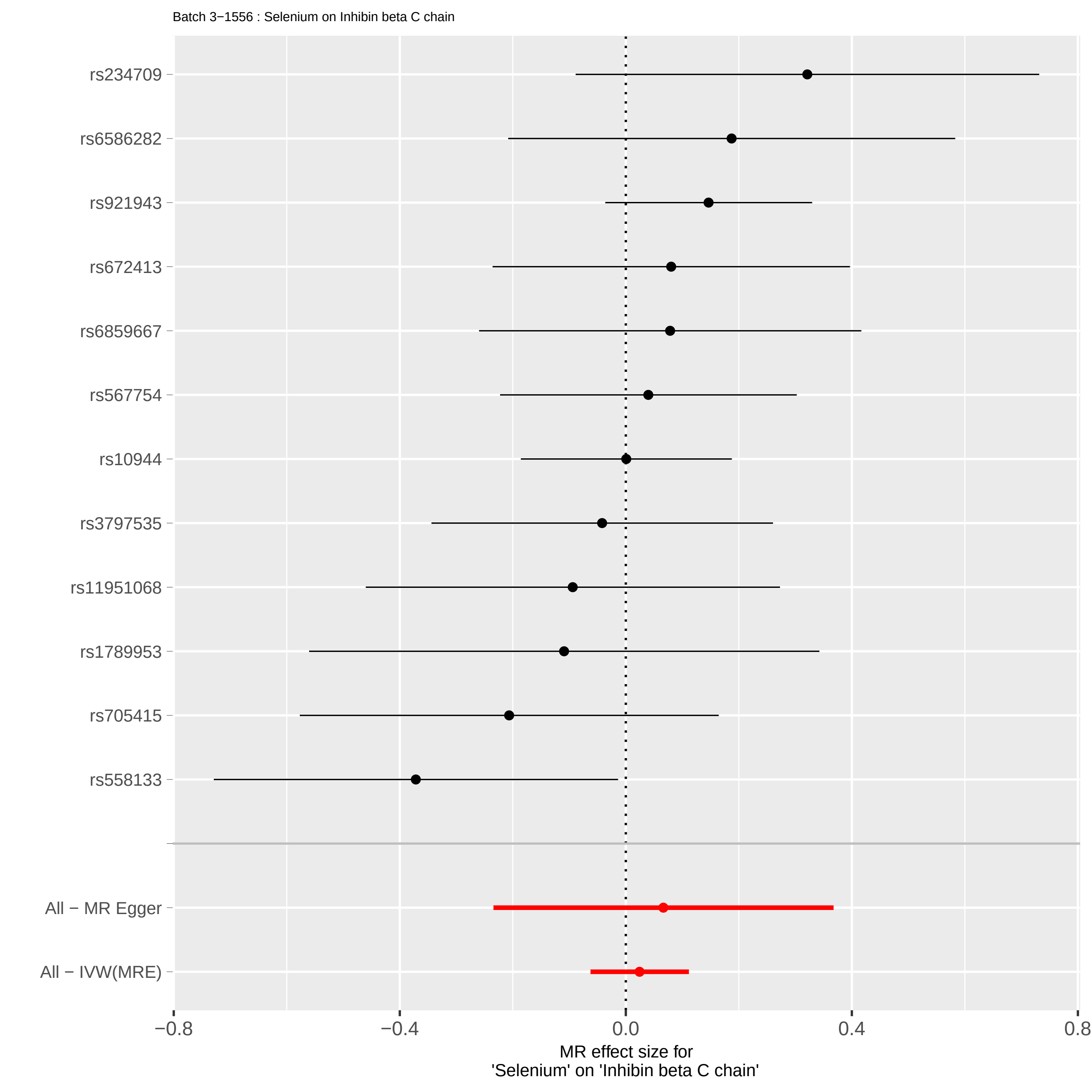
Batch 3−1556 : Selenium on Inhibin beta C chain
rs234709
rs6586282
rs921943
rs672413
rs6859667
rs567754
rs10944
rs3797535
rs11951068
rs1789953
rs705415
rs558133
All − MR Egger
All − IVW(MRE)
−0.8
−0.4
0.0
0.4
0.8
MR effect size for
'Selenium' on 'Inhibin beta C chain'

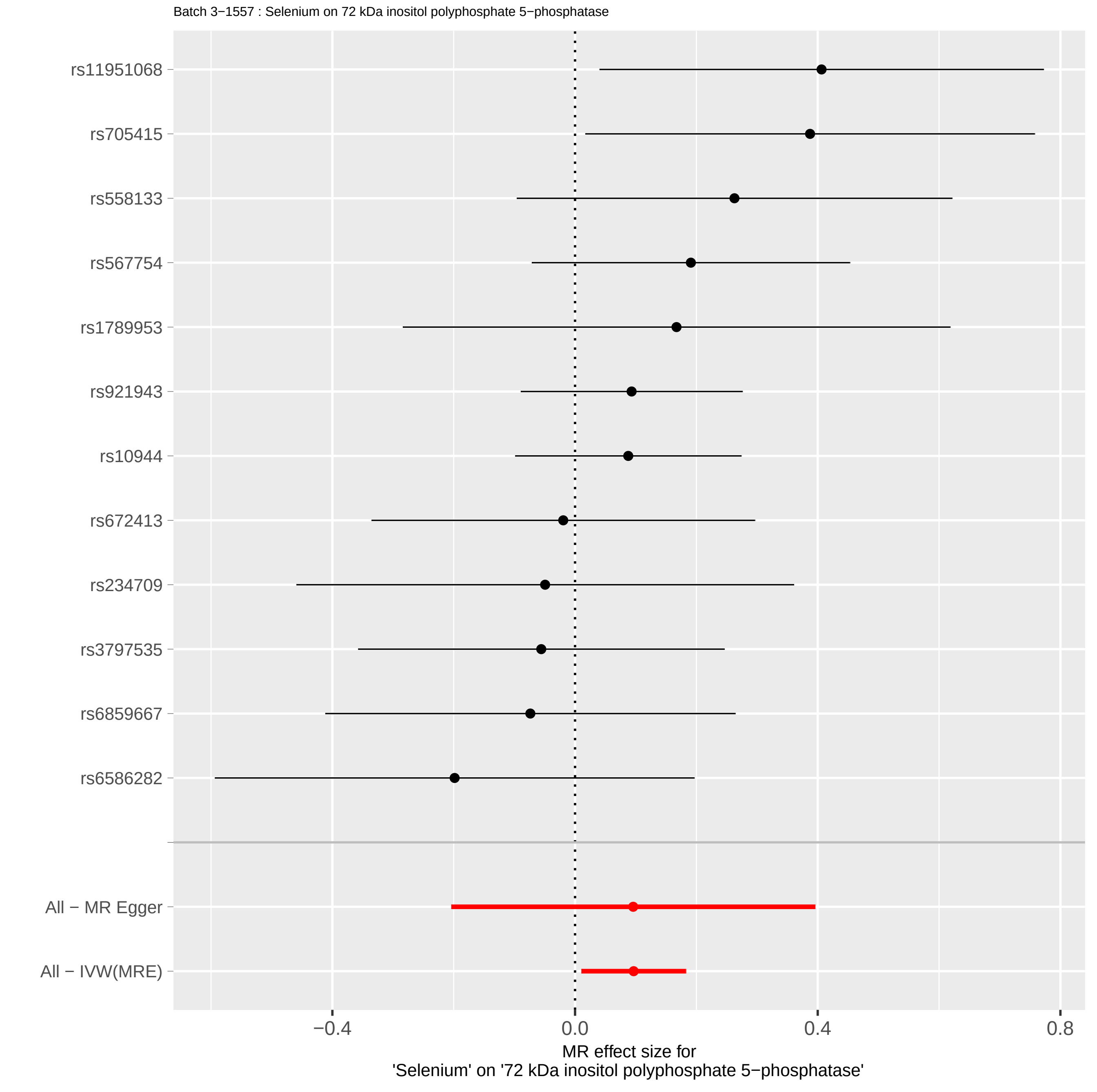

Batch 3−1557 : Selenium on 72 kDa inositol polyphosphate 5−phosphatase
rs11951068
rs705415
rs558133
rs567754
rs1789953
rs921943
rs10944
rs672413
rs234709
rs3797535
rs6859667
rs6586282
All − MR Egger
All − IVW(MRE)
−0.4
0.0
0.4
0.8
MR effect size for
'Selenium' on '72 kDa inositol polyphosphate 5−phosphatase'

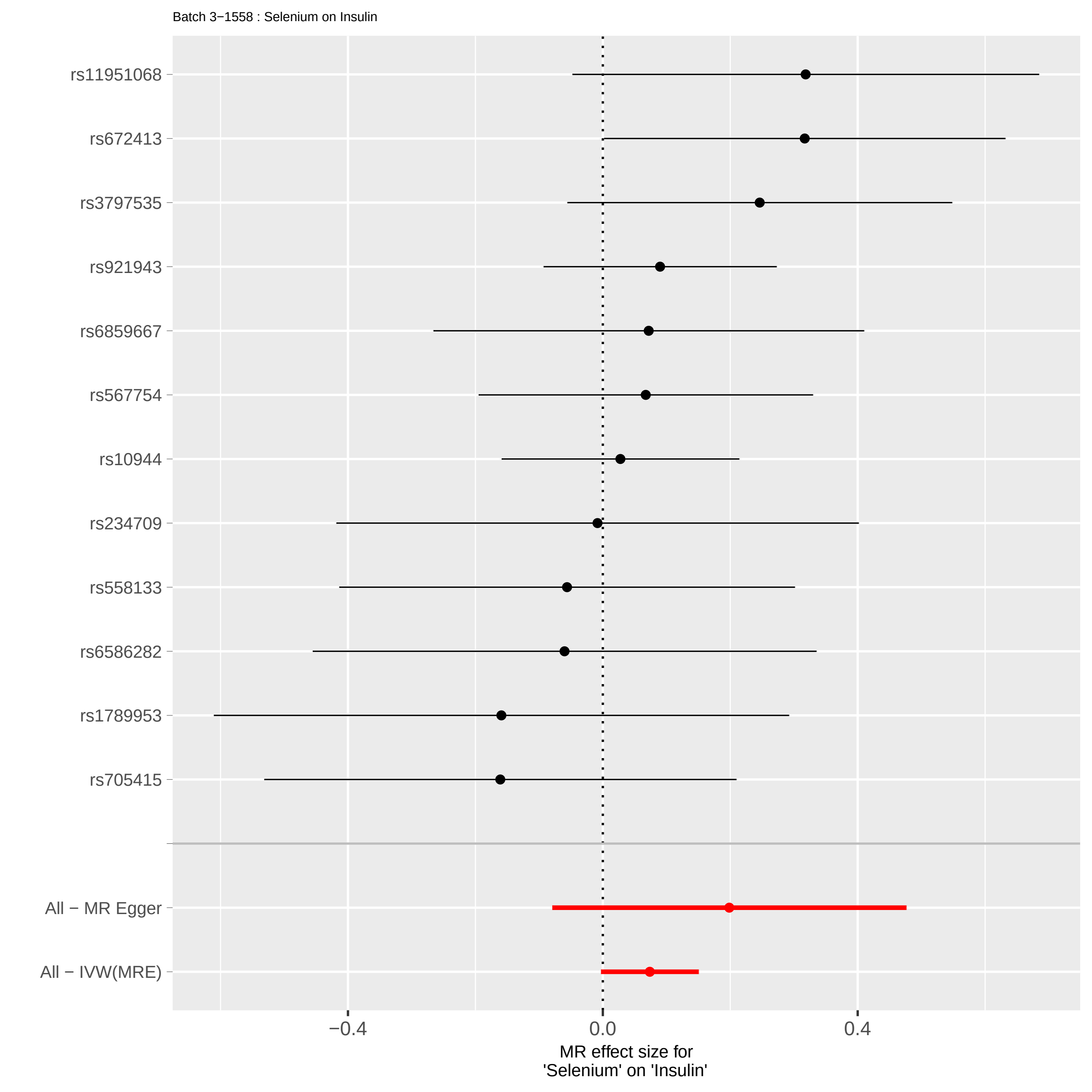
Batch 3−1558 : Selenium on Insulin
rs11951068
rs672413
rs3797535
rs921943
rs6859667
rs567754
rs10944
rs234709
rs558133
rs6586282
rs1789953
rs705415
All − MR Egger
All − IVW(MRE)
−0.4
0.0
0.4
MR effect size for
'Selenium' on 'Insulin'

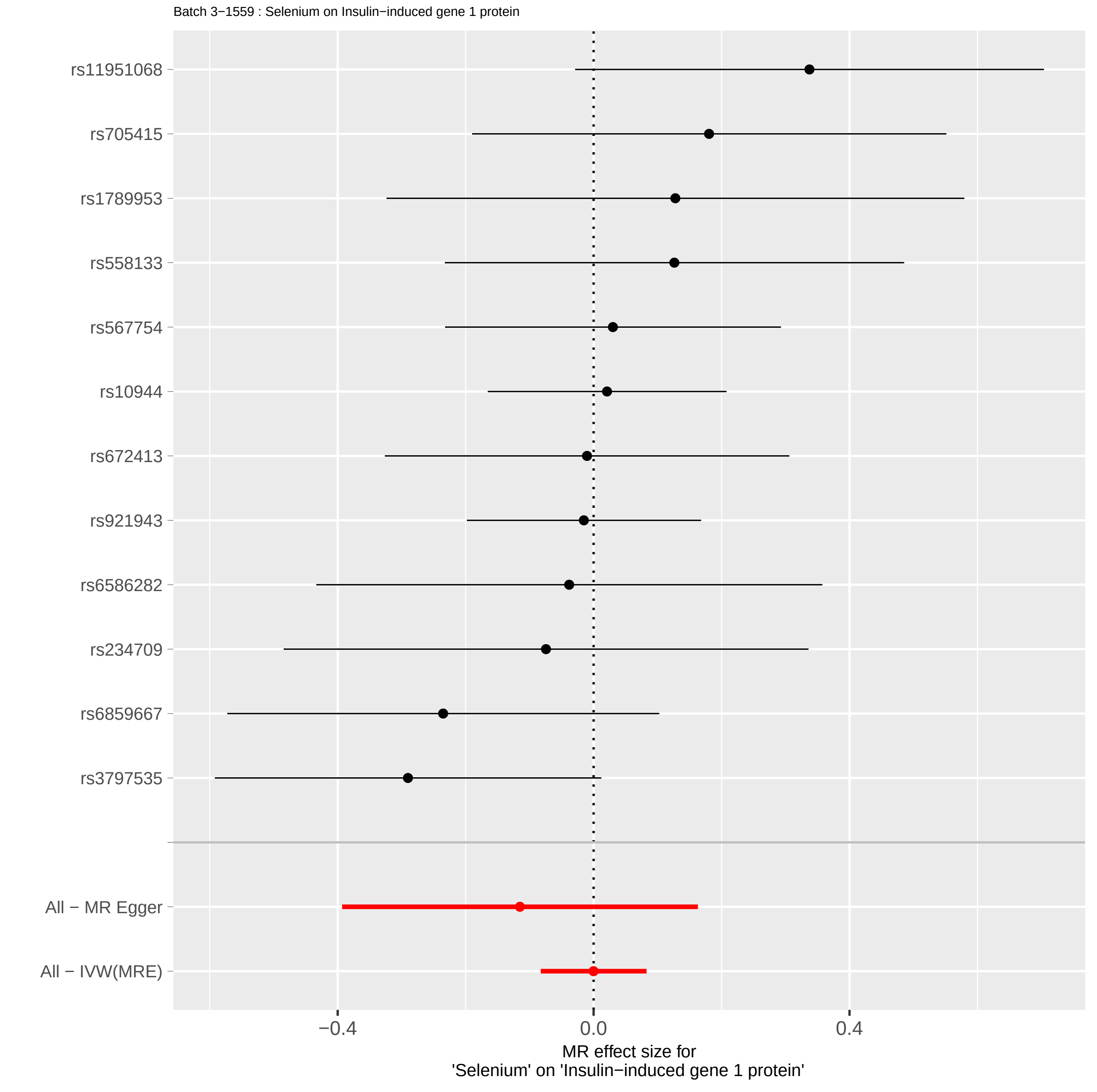

Batch 3−1559 : Selenium on Insulin−induced gene 1 protein
rs11951068
rs705415
rs1789953
rs558133
rs567754
rs10944
rs672413
rs921943
rs6586282
rs234709
rs6859667
rs3797535
All − MR Egger
All − IVW(MRE)
−0.4
0.0
0.4
MR effect size for
'Selenium' on 'Insulin−induced gene 1 protein'

Batch 3−1560 : Selenium on Insulin−like 3

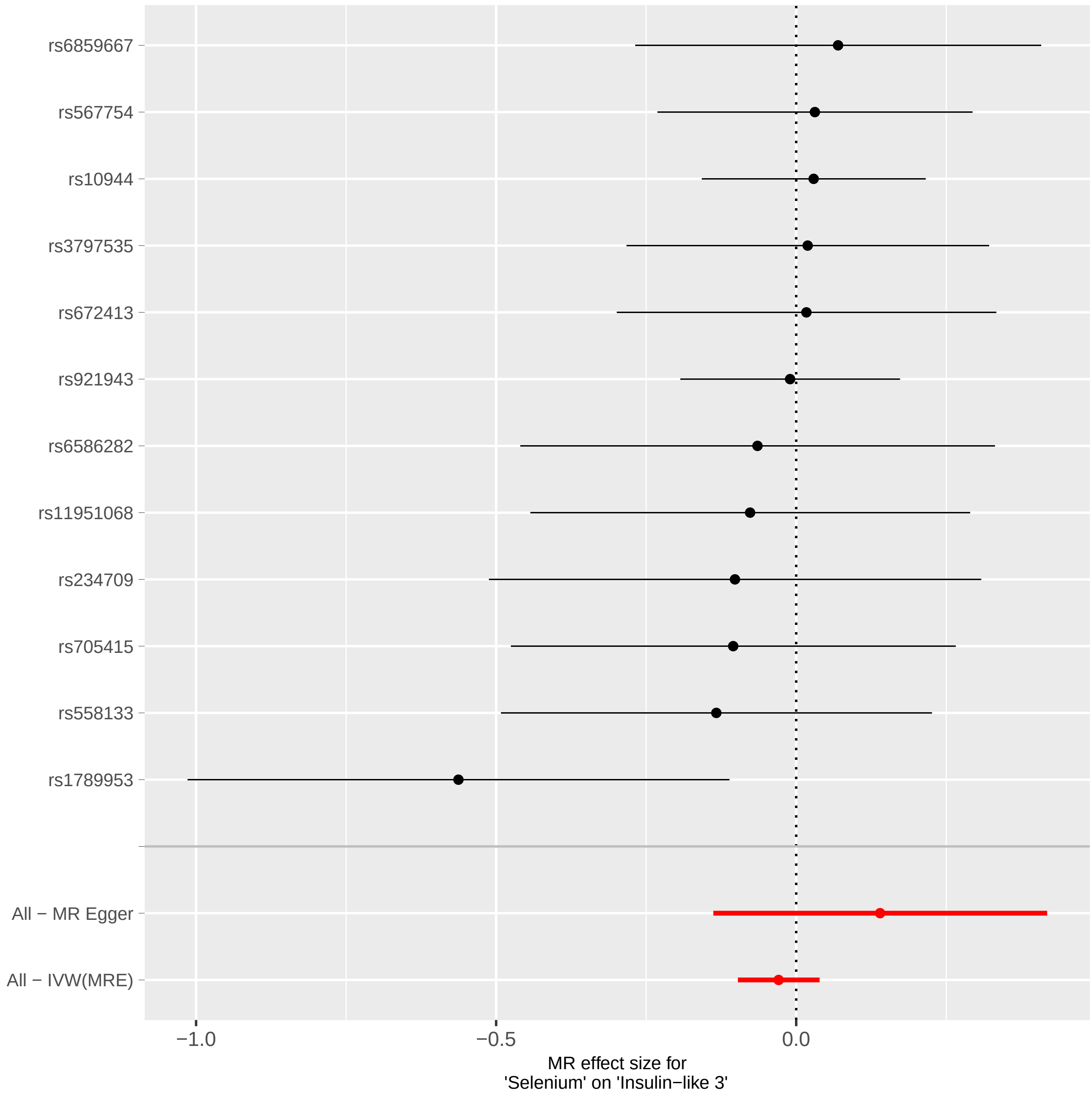

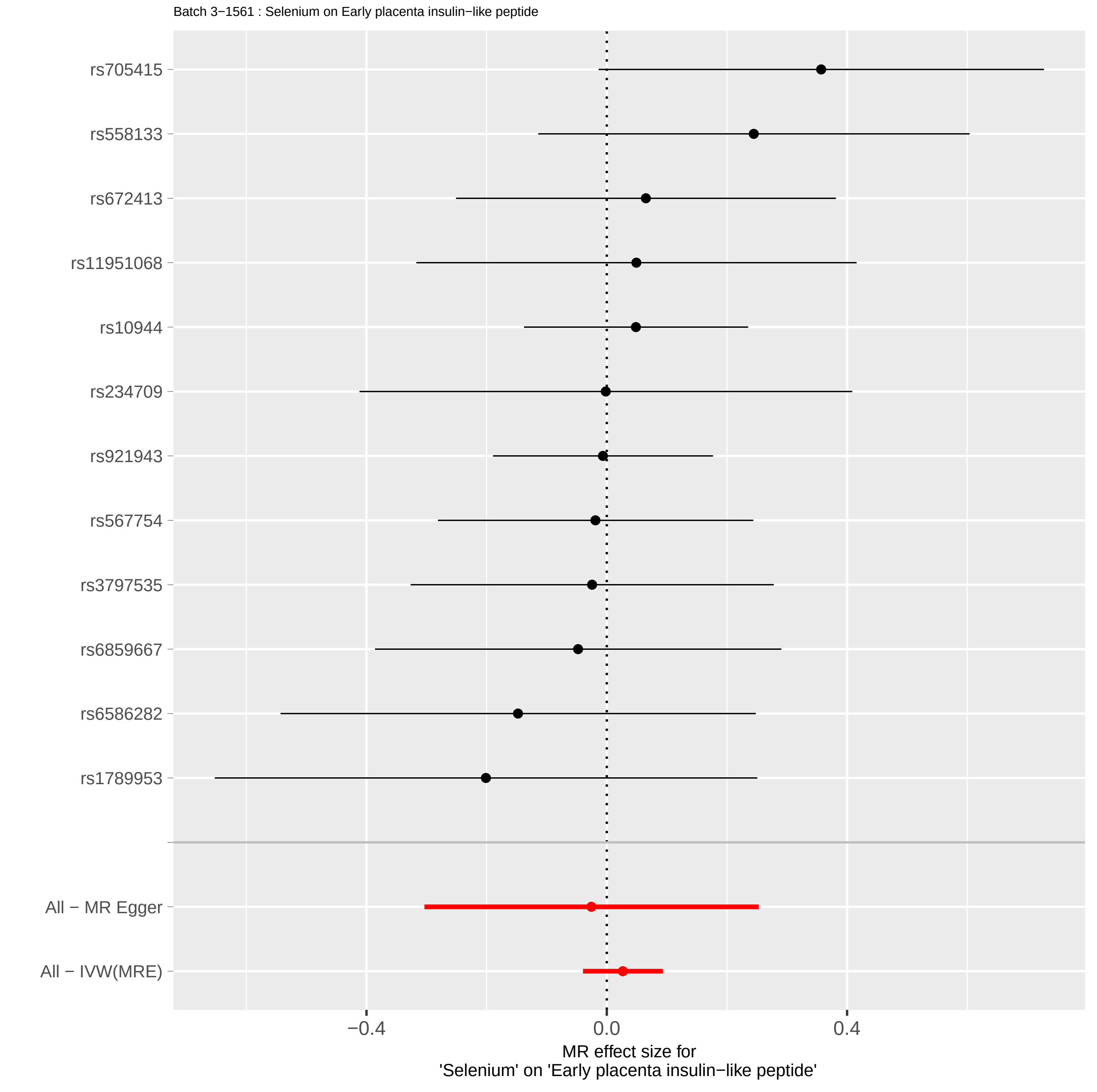
Batch 3−1561 : Selenium on Early placenta insulin−like peptide
rs705415
rs558133
rs672413
rs11951068
rs10944
rs234709
rs921943
rs567754
rs3797535
rs6859667
rs6586282
rs1789953
All − MR Egger
All − IVW(MRE)
−0.4
0.0
0.4
MR effect size for
'Selenium' on 'Early placenta insulin−like peptide'

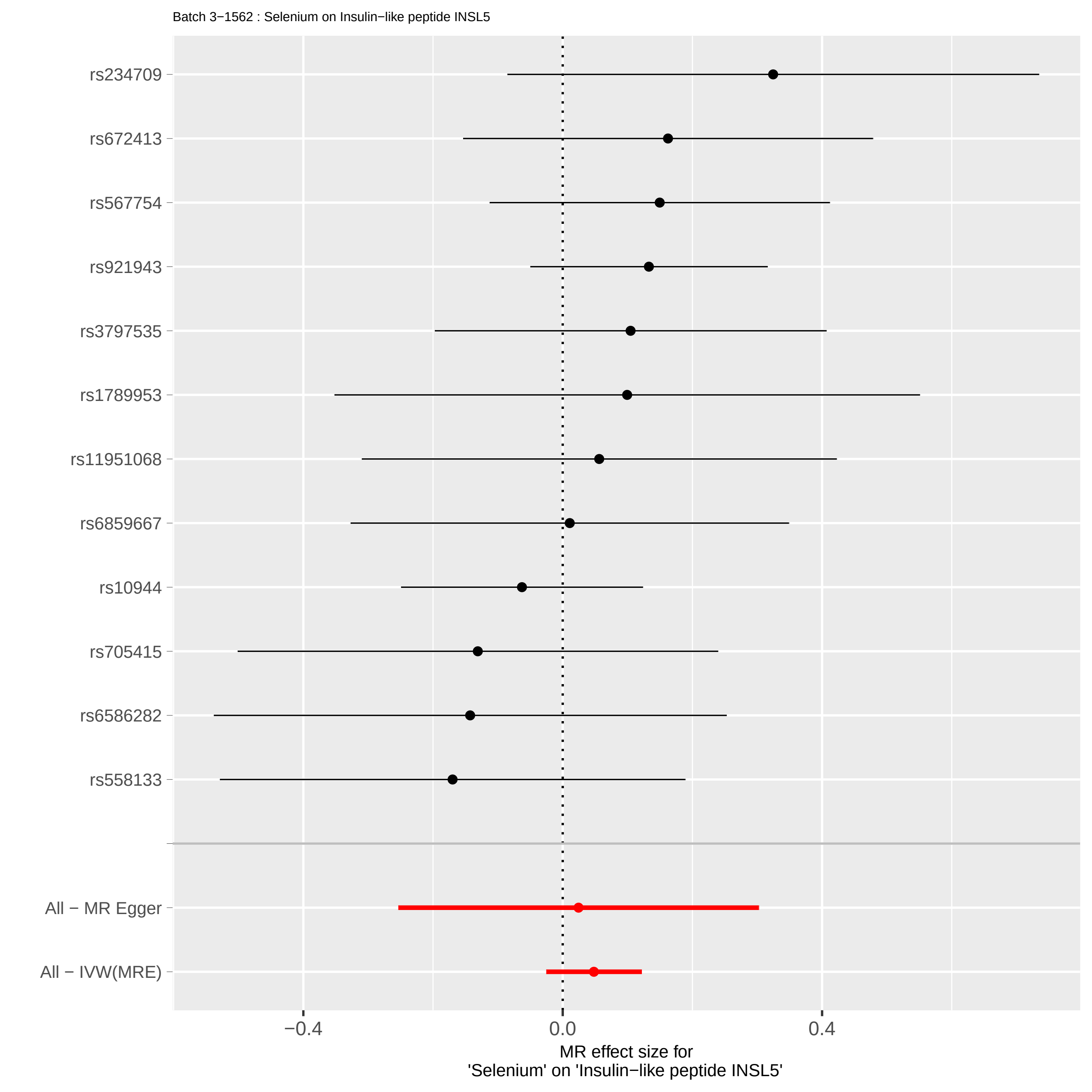

Batch 3−1562 : Selenium on Insulin−like peptide INSL5
rs234709
rs672413
rs567754
rs921943
rs3797535
rs1789953
rs11951068
rs6859667
rs10944
rs705415
rs6586282
rs558133
All − MR Egger
All − IVW(MRE)
−0.4
0.0
0.4
MR effect size for
'Selenium' on 'Insulin−like peptide INSL5'

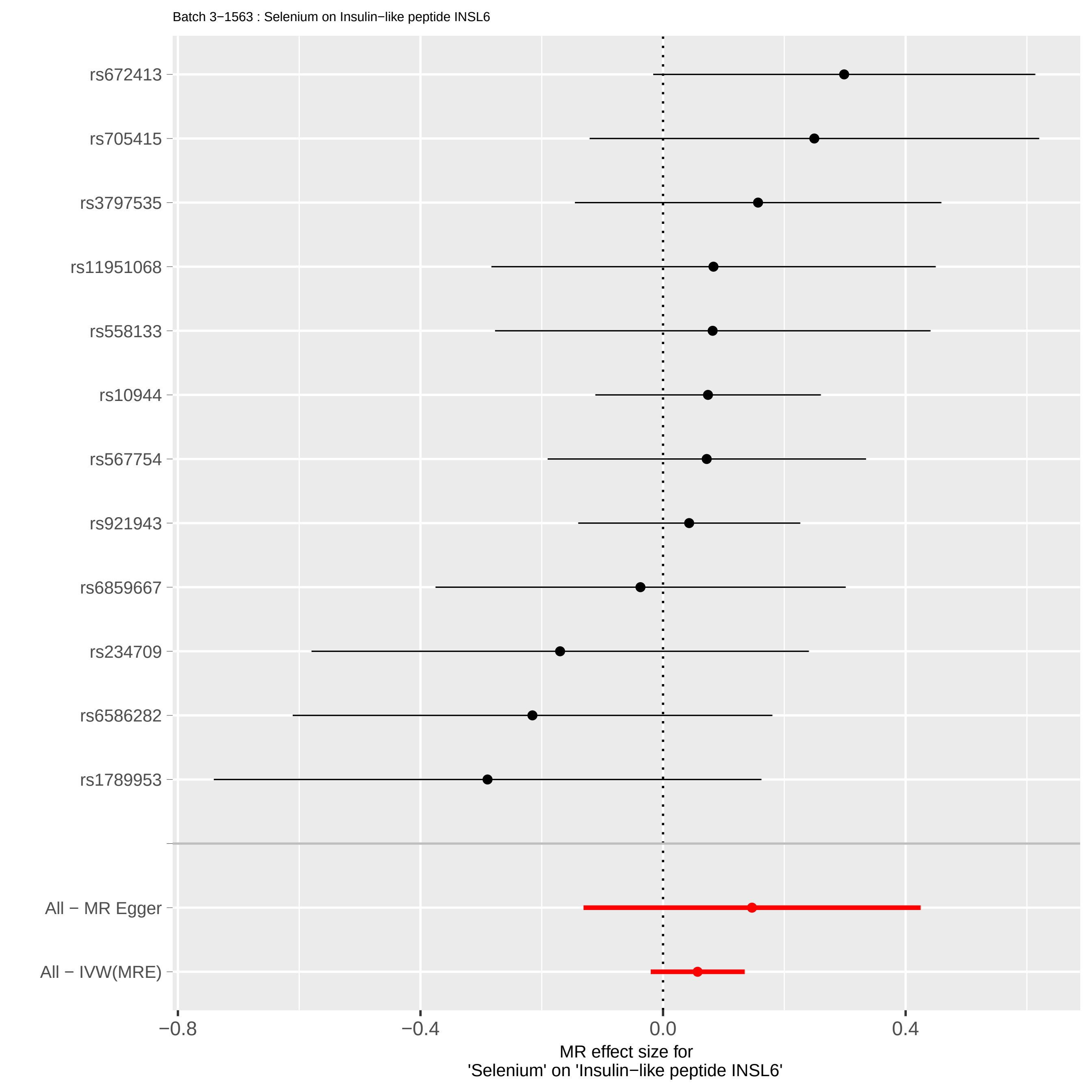

Batch 3−1563 : Selenium on Insulin−like peptide INSL6
rs672413
rs705415
rs3797535
rs11951068
rs558133
rs10944
rs567754
rs921943
rs6859667
rs234709
rs6586282
rs1789953
All − MR Egger
All − IVW(MRE)
−0.8
−0.4
0.0
0.4
MR effect size for
'Selenium' on 'Insulin−like peptide INSL6'

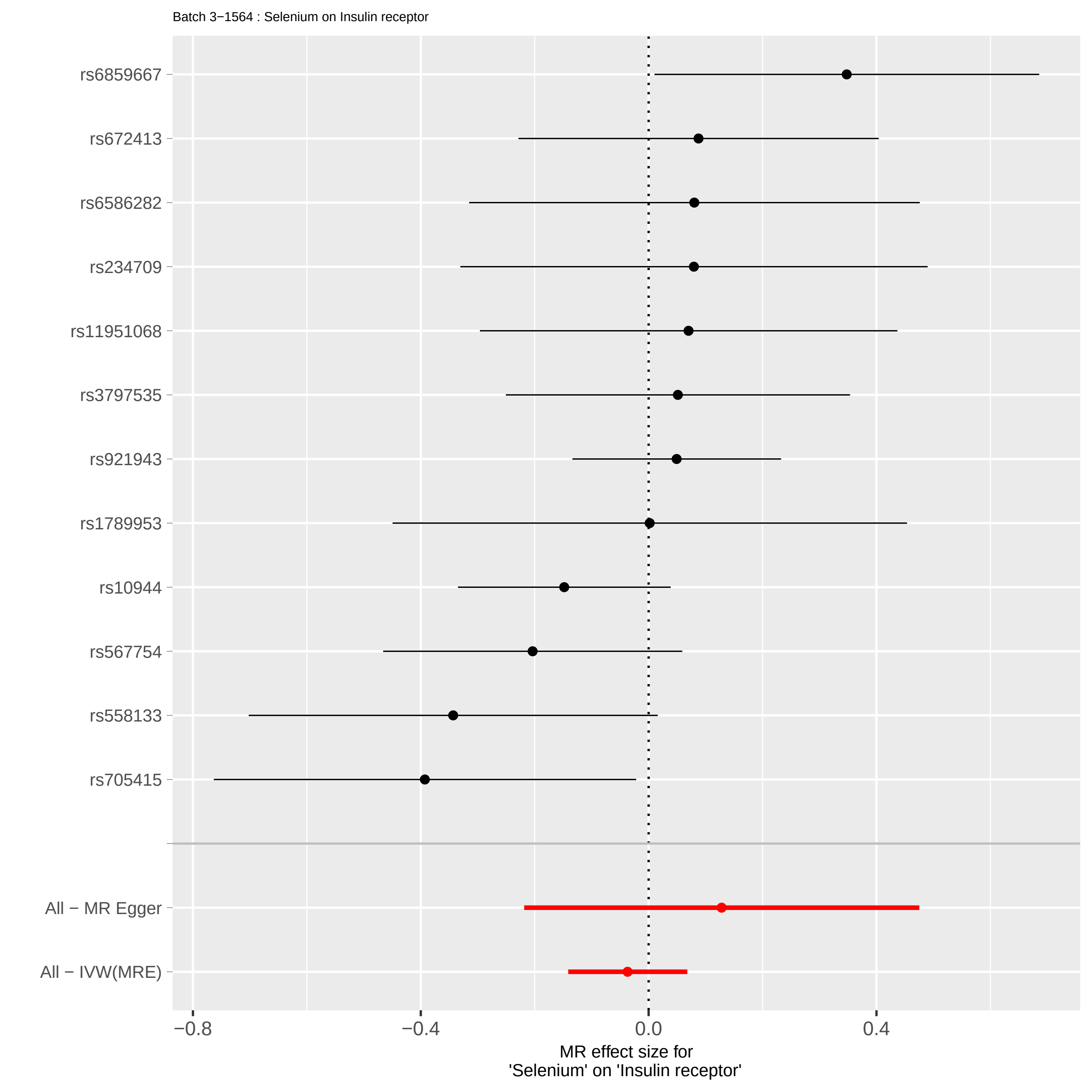

Batch 3−1564 : Selenium on Insulin receptor
rs6859667
rs672413
rs6586282
rs234709
rs11951068
rs3797535
rs921943
rs1789953
rs10944
rs567754
rs558133
rs705415
All − MR Egger
All − IVW(MRE)
−0.8
−0.4
0.0
0.4
MR effect size for
'Selenium' on 'Insulin receptor'

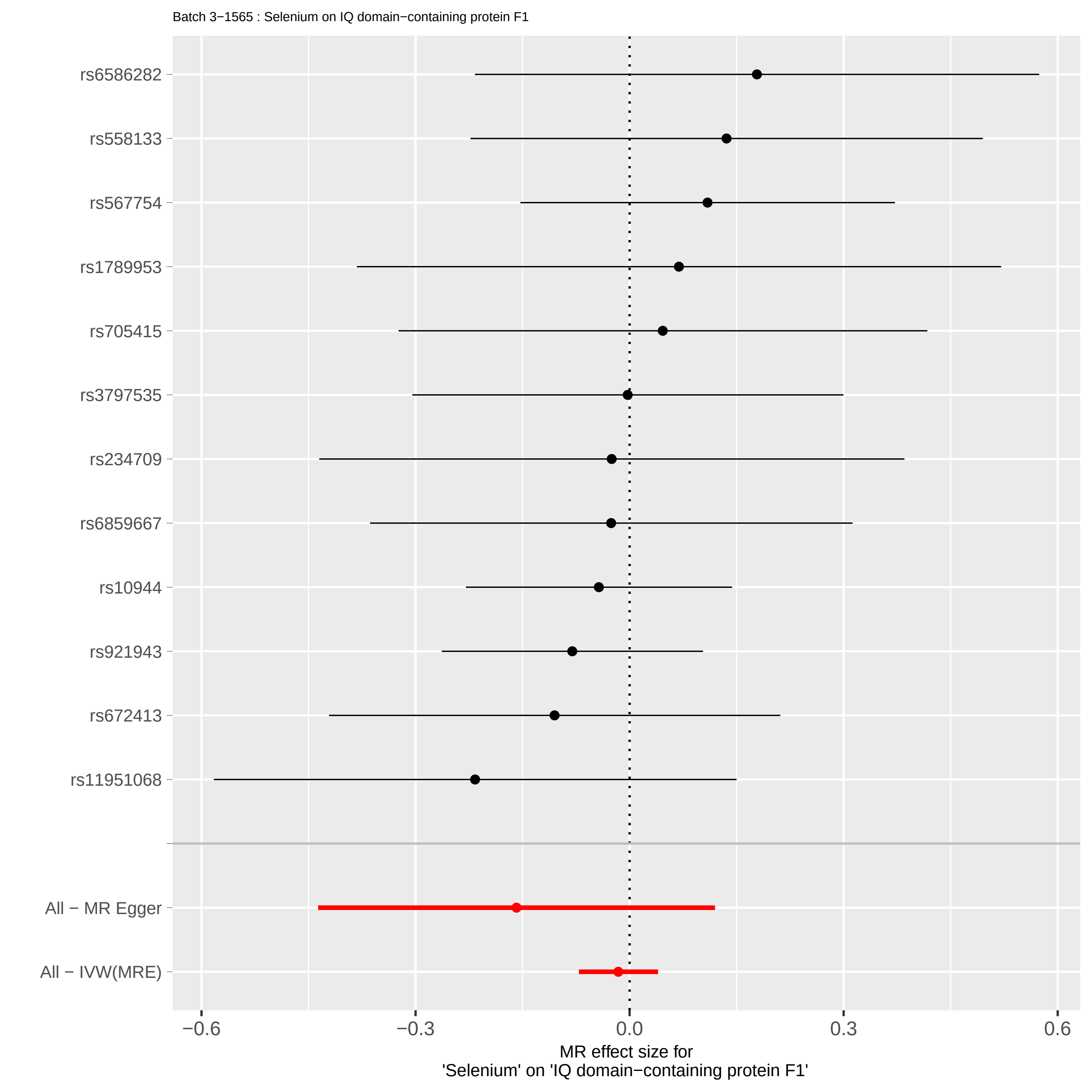

Batch 3−1565 : Selenium on IQ domain−containing protein F1
rs6586282
rs558133
rs567754
rs1789953
rs705415
rs3797535
rs234709
rs6859667
rs10944
rs921943
rs672413
rs11951068
All − MR Egger
All − IVW(MRE)
−0.6
−0.3
0.0
0.3
0.6
MR effect size for
'Selenium' on 'IQ domain−containing protein F1'

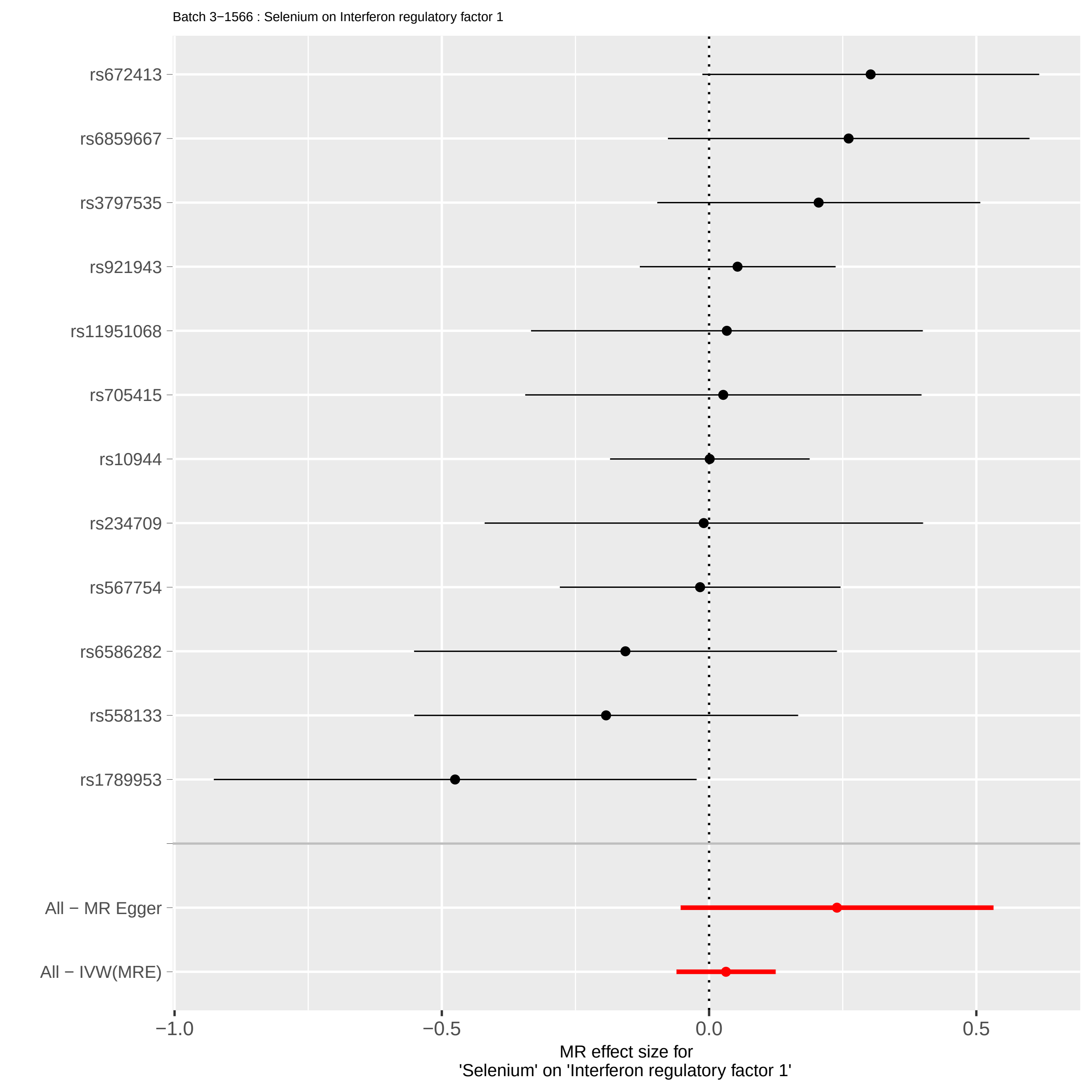

Batch 3−1566 : Selenium on Interferon regulatory factor 1
rs672413
rs6859667
rs3797535
rs921943
rs11951068
rs705415
rs10944
rs234709
rs567754
rs6586282
rs558133
rs1789953
All − MR Egger
All − IVW(MRE)
−1.0
−0.5
0.0
0.5
MR effect size for
'Selenium' on 'Interferon regulatory factor 1'

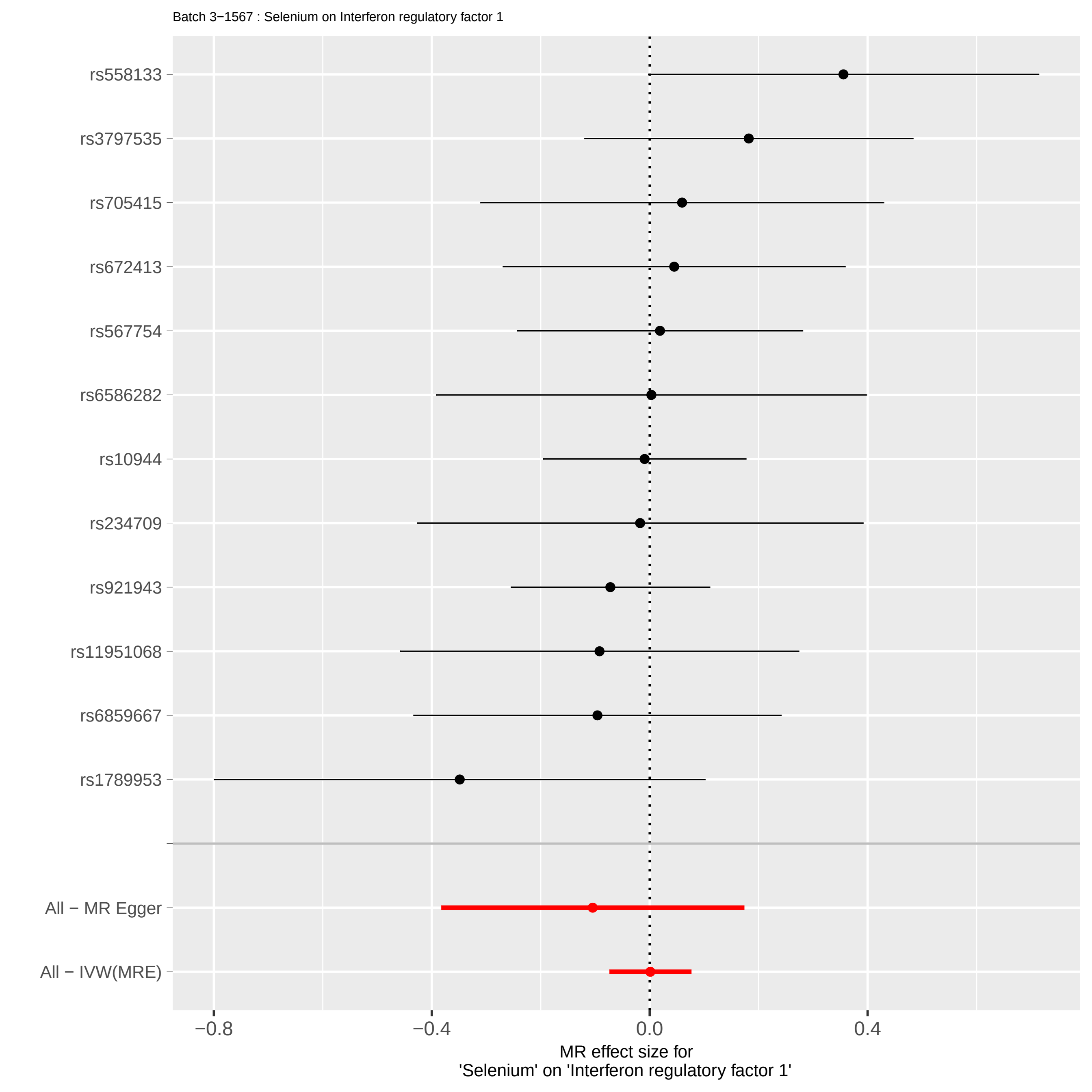

Batch 3−1567 : Selenium on Interferon regulatory factor 1
rs558133
rs3797535
rs705415
rs672413
rs567754
rs6586282
rs10944
rs234709
rs921943
rs11951068
rs6859667
rs1789953
All − MR Egger
All − IVW(MRE)
−0.8
−0.4
0.0
0.4
MR effect size for
'Selenium' on 'Interferon regulatory factor 1'

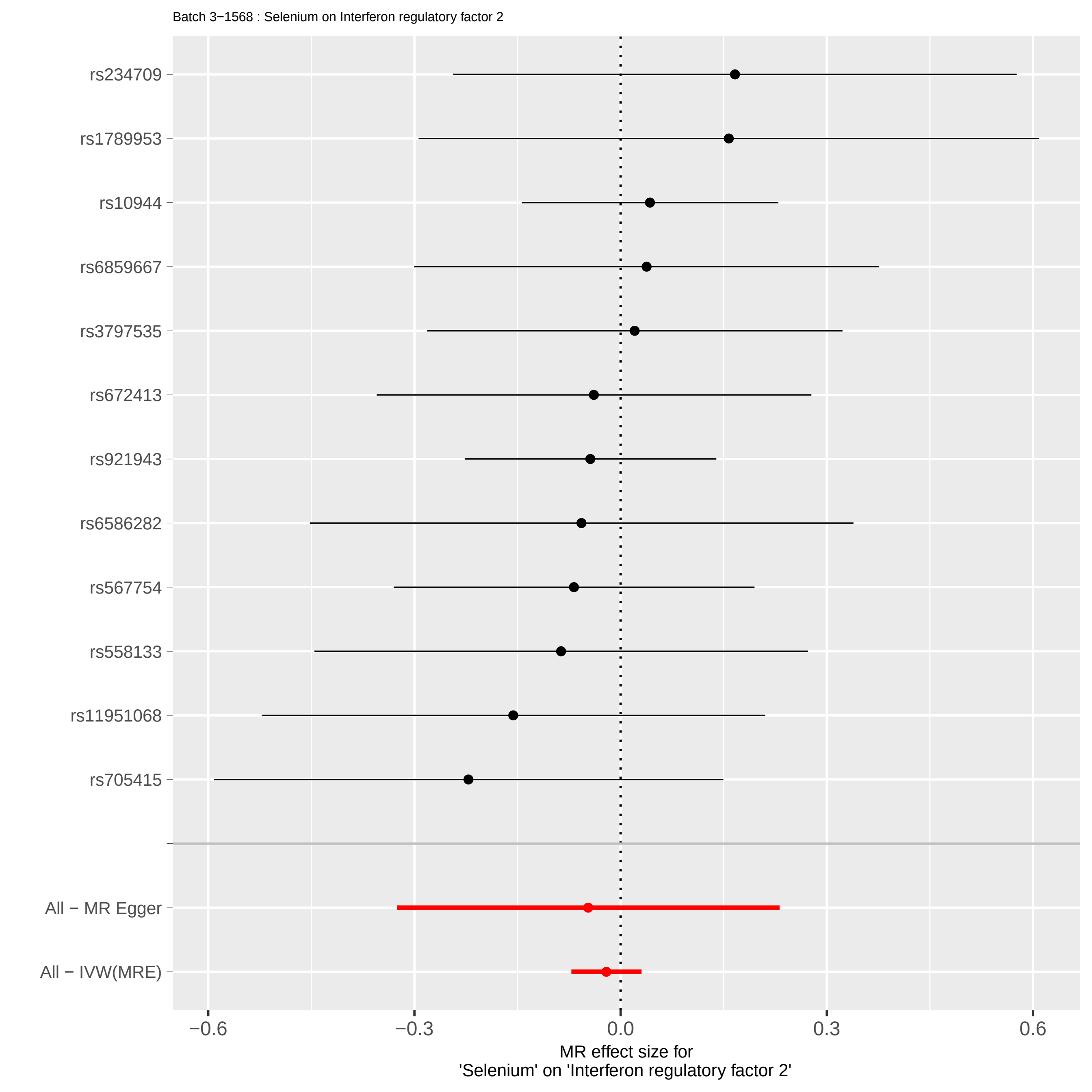

Batch 3−1568 : Selenium on Interferon regulatory factor 2
rs234709
rs1789953
rs10944
rs6859667
rs3797535
rs672413
rs921943
rs6586282
rs567754
rs558133
rs11951068
rs705415
All − MR Egger
All − IVW(MRE)
−0.6
−0.3
0.0
0.3
0.6
MR effect size for
'Selenium' on 'Interferon regulatory factor 2'

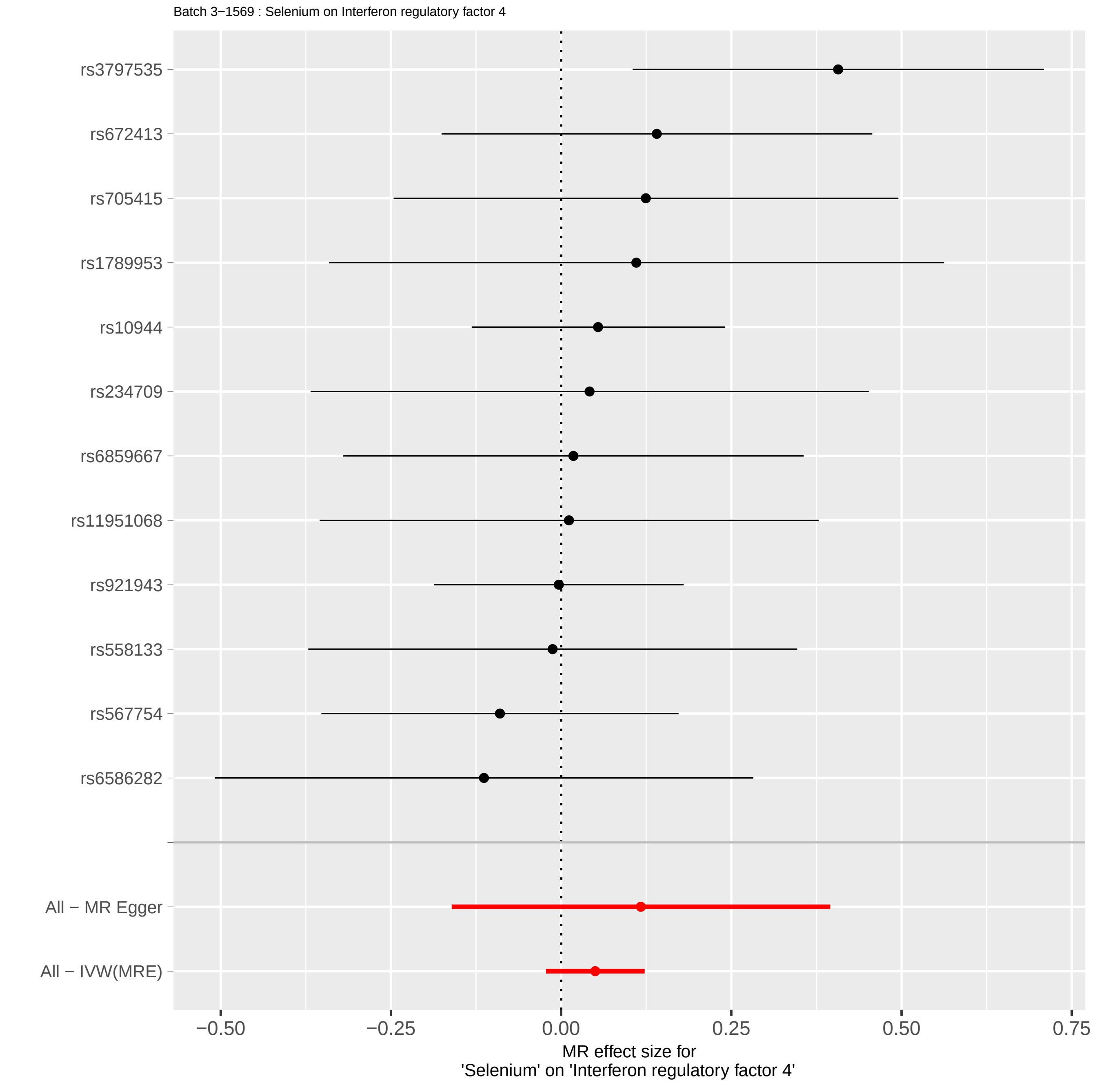

Batch 3−1569 : Selenium on Interferon regulatory factor 4
rs3797535
rs672413
rs705415
rs1789953
rs10944
rs234709
rs6859667
rs11951068
rs921943
rs558133
rs567754
rs6586282
All − MR Egger
All − IVW(MRE)
−0.50
−0.25
0.00
0.25
0.50
0.75
MR effect size for
'Selenium' on 'Interferon regulatory factor 4'

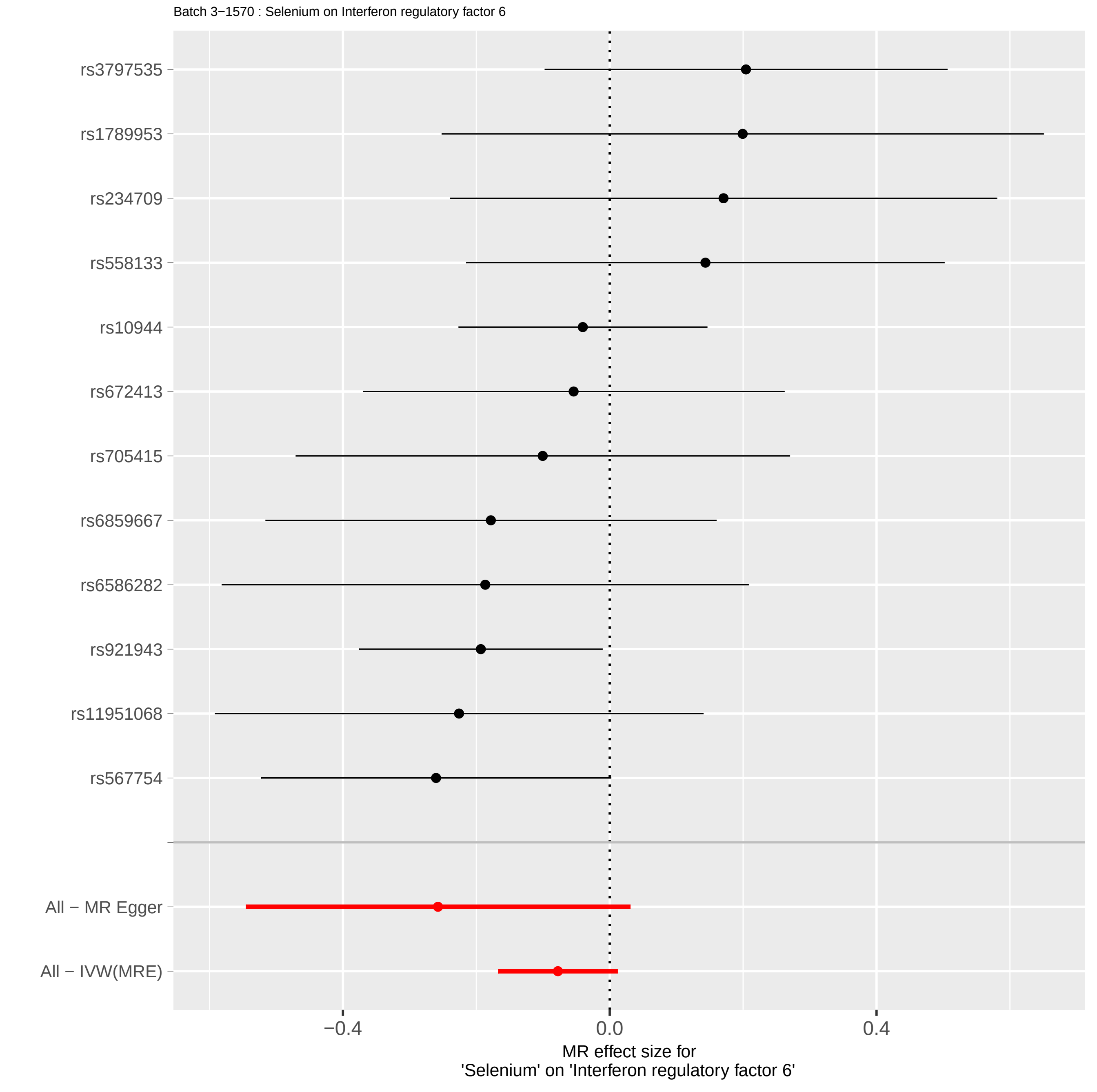

Batch 3−1570 : Selenium on Interferon regulatory factor 6
rs3797535
rs1789953
rs234709
rs558133
rs10944
rs672413
rs705415
rs6859667
rs6586282
rs921943
rs11951068
rs567754
All − MR Egger
All − IVW(MRE)
−0.4
0.0
0.4
MR effect size for
'Selenium' on 'Interferon regulatory factor 6'

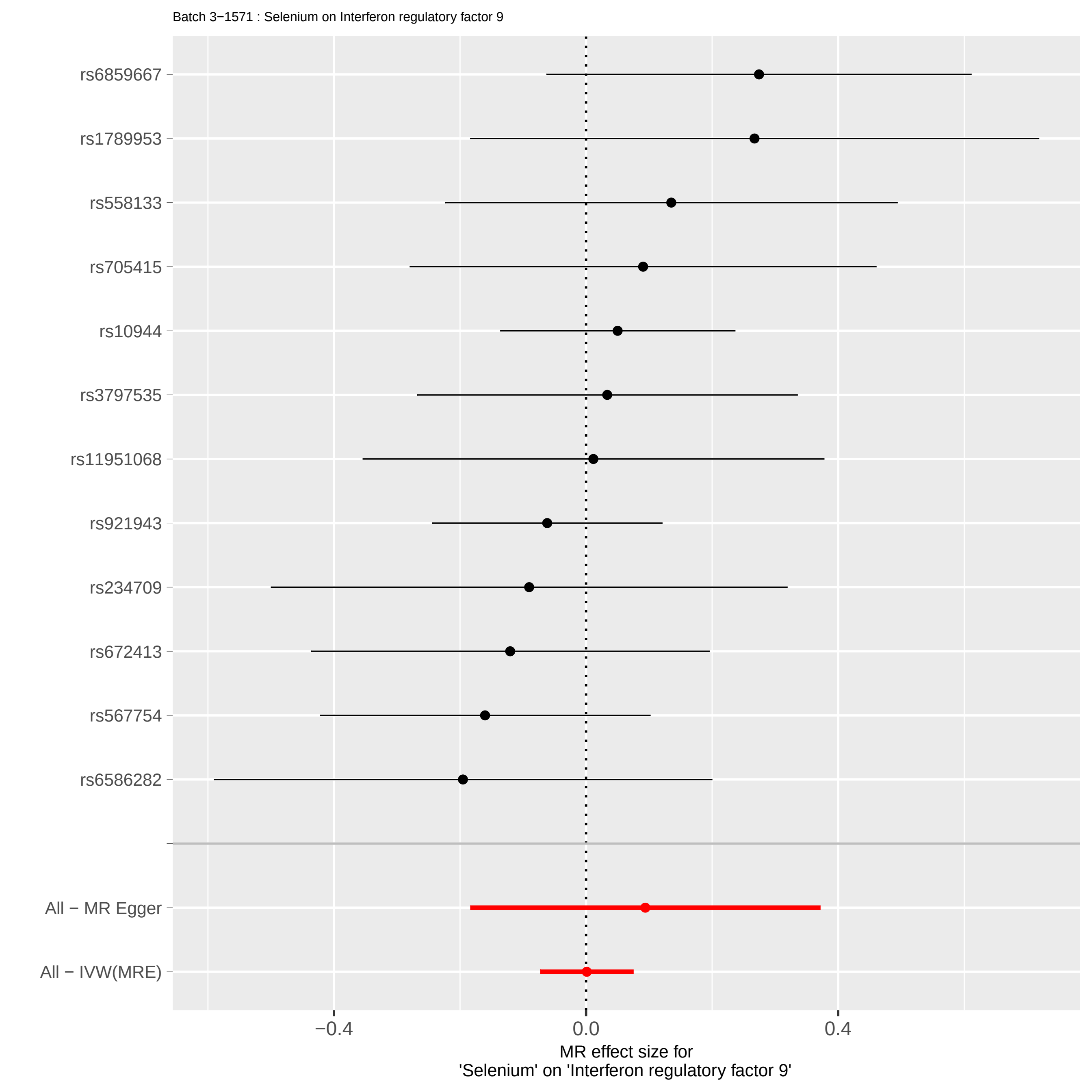

Batch 3−1571 : Selenium on Interferon regulatory factor 9
rs6859667
rs1789953
rs558133
rs705415
rs10944
rs3797535
rs11951068
rs921943
rs234709
rs672413
rs567754
rs6586282
All − MR Egger
All − IVW(MRE)
−0.4
0.0
0.4
MR effect size for
'Selenium' on 'Interferon regulatory factor 9'

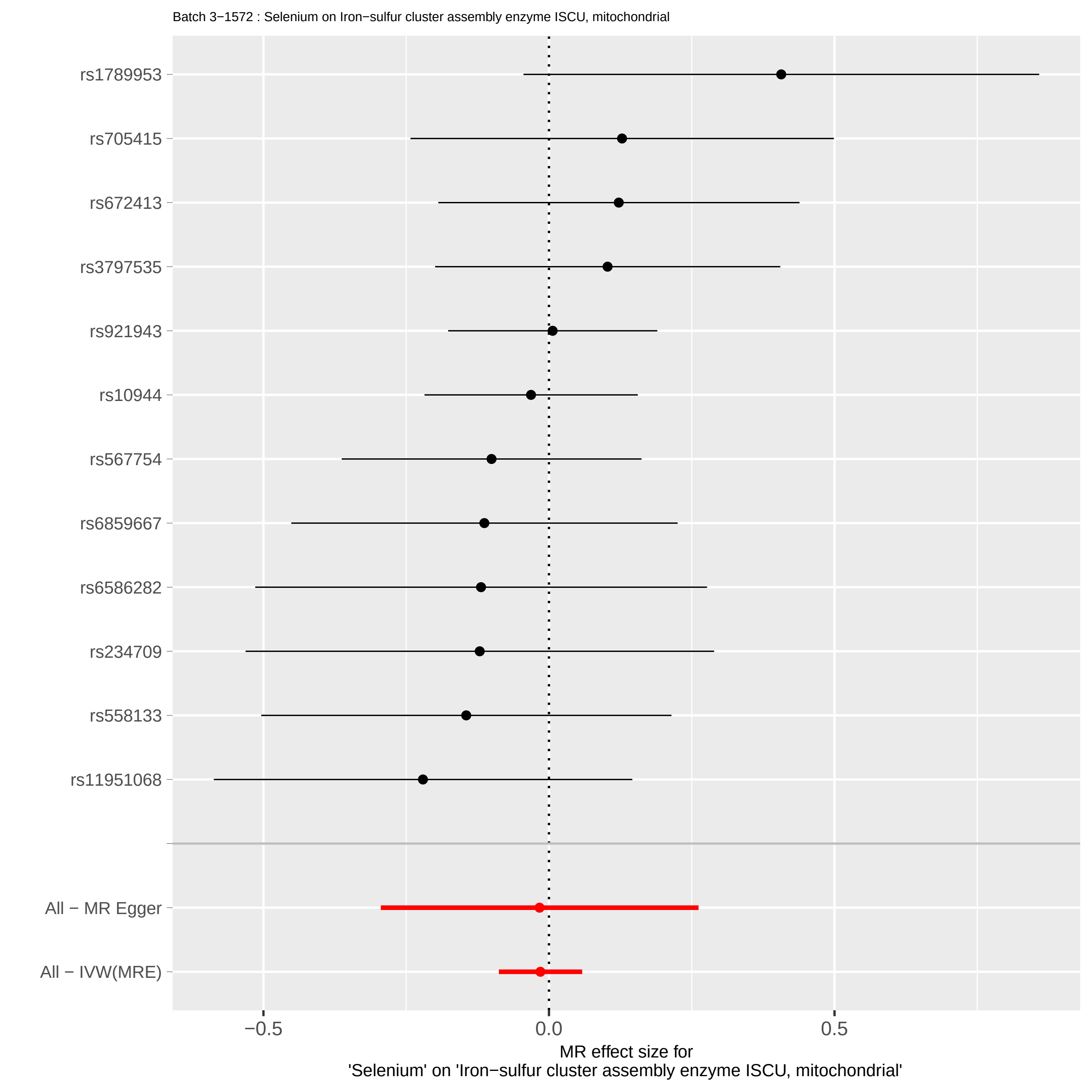

Batch 3−1572 : Selenium on Iron−sulfur cluster assembly enzyme ISCU, mitochondrial
rs1789953
rs705415
rs672413
rs3797535
rs921943
rs10944
rs567754
rs6859667
rs6586282
rs234709
rs558133
rs11951068
All − MR Egger
All − IVW(MRE)
−0.5
0.0
0.5
MR effect size for
'Selenium' on 'Iron−sulfur cluster assembly enzyme ISCU, mitochondrial'

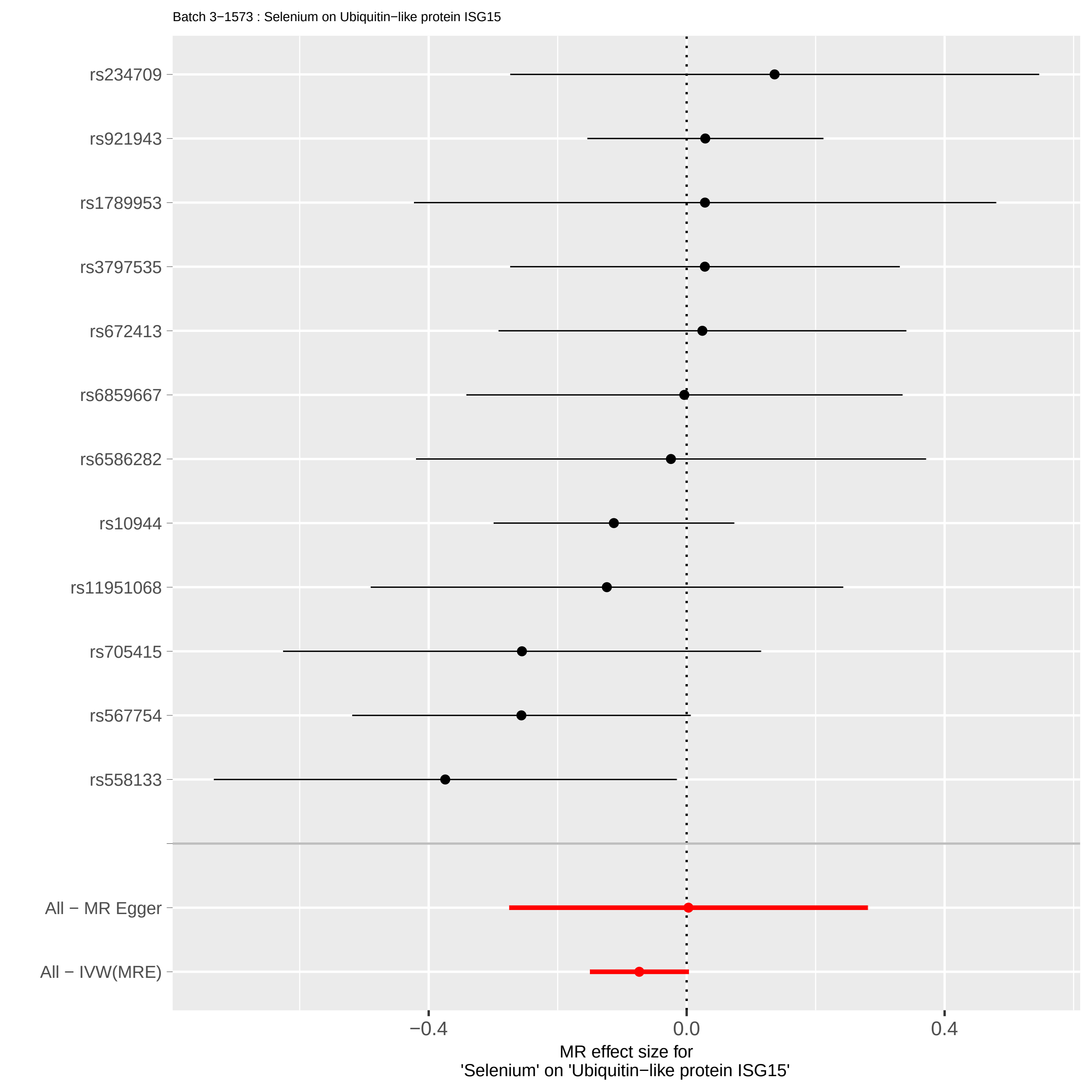

Batch 3−1573 : Selenium on Ubiquitin−like protein ISG15
rs234709
rs921943
rs1789953
rs3797535
rs672413
rs6859667
rs6586282
rs10944
rs11951068
rs705415
rs567754
rs558133
All − MR Egger
All − IVW(MRE)
−0.4
0.0
0.4
MR effect size for
'Selenium' on 'Ubiquitin−like protein ISG15'

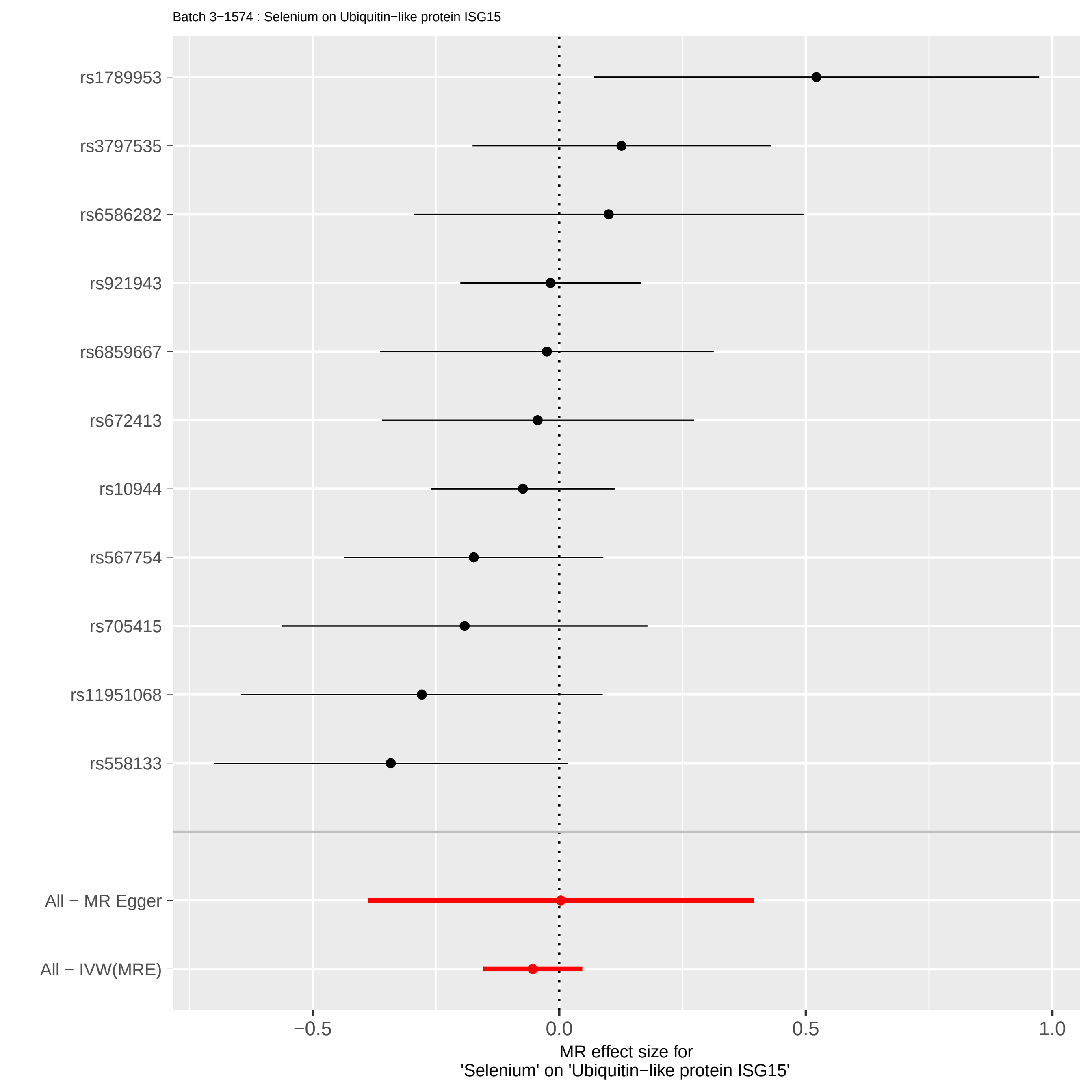
Batch 3−1574 : Selenium on Ubiquitin−like protein ISG15
rs1789953
rs3797535
rs6586282
rs921943
rs6859667
rs672413
rs10944
rs567754
rs705415
rs11951068
rs558133
All − MR Egger
All − IVW(MRE)
−0.5
0.0
0.5
1.0
MR effect size for
'Selenium' on 'Ubiquitin−like protein ISG15'

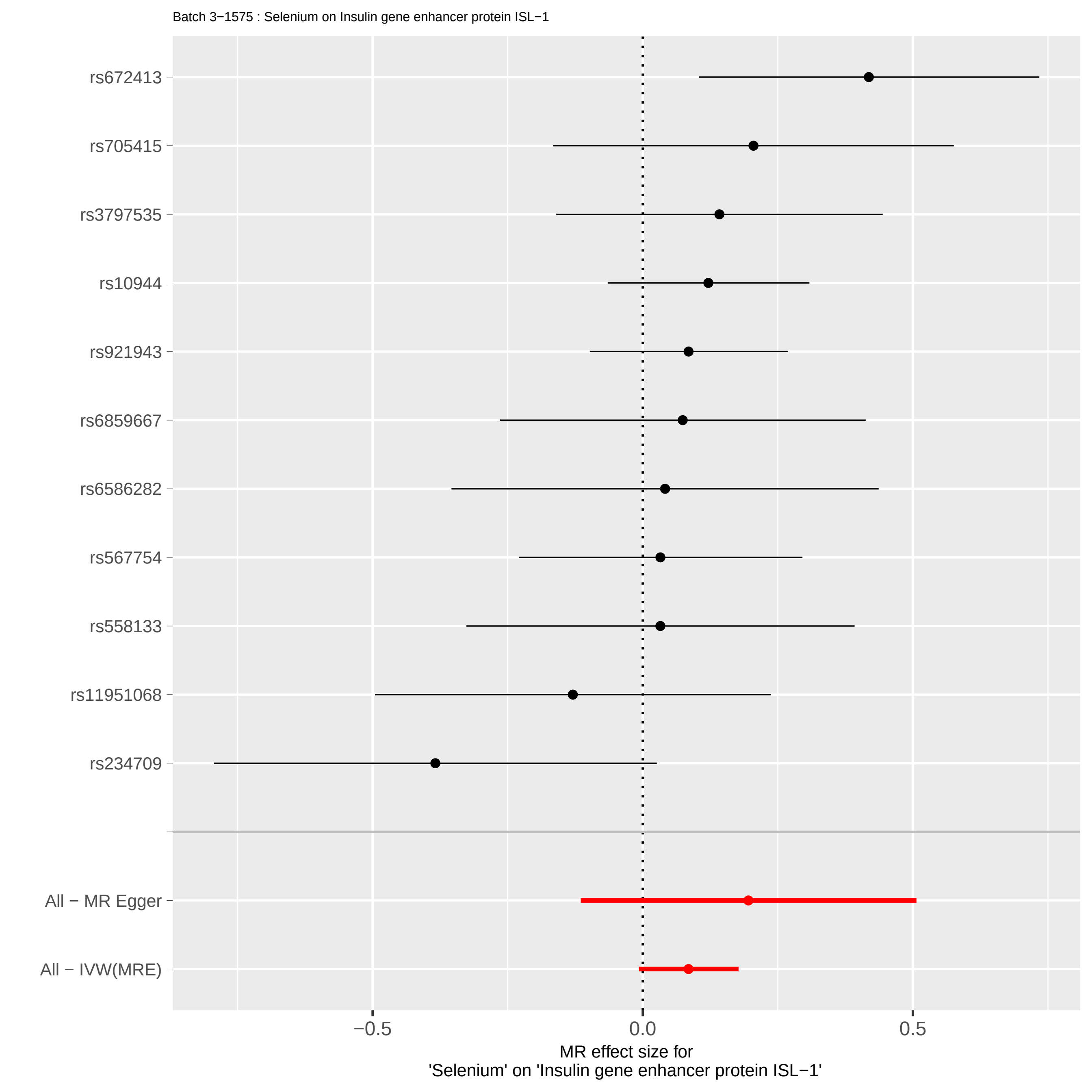
Batch 3−1575 : Selenium on Insulin gene enhancer protein ISL−1
rs672413
rs705415
rs3797535
rs10944
rs921943
rs6859667
rs6586282
rs567754
rs558133
rs11951068
rs234709
All − MR Egger
All − IVW(MRE)
−0.5
0.0
0.5
MR effect size for
'Selenium' on 'Insulin gene enhancer protein ISL−1'

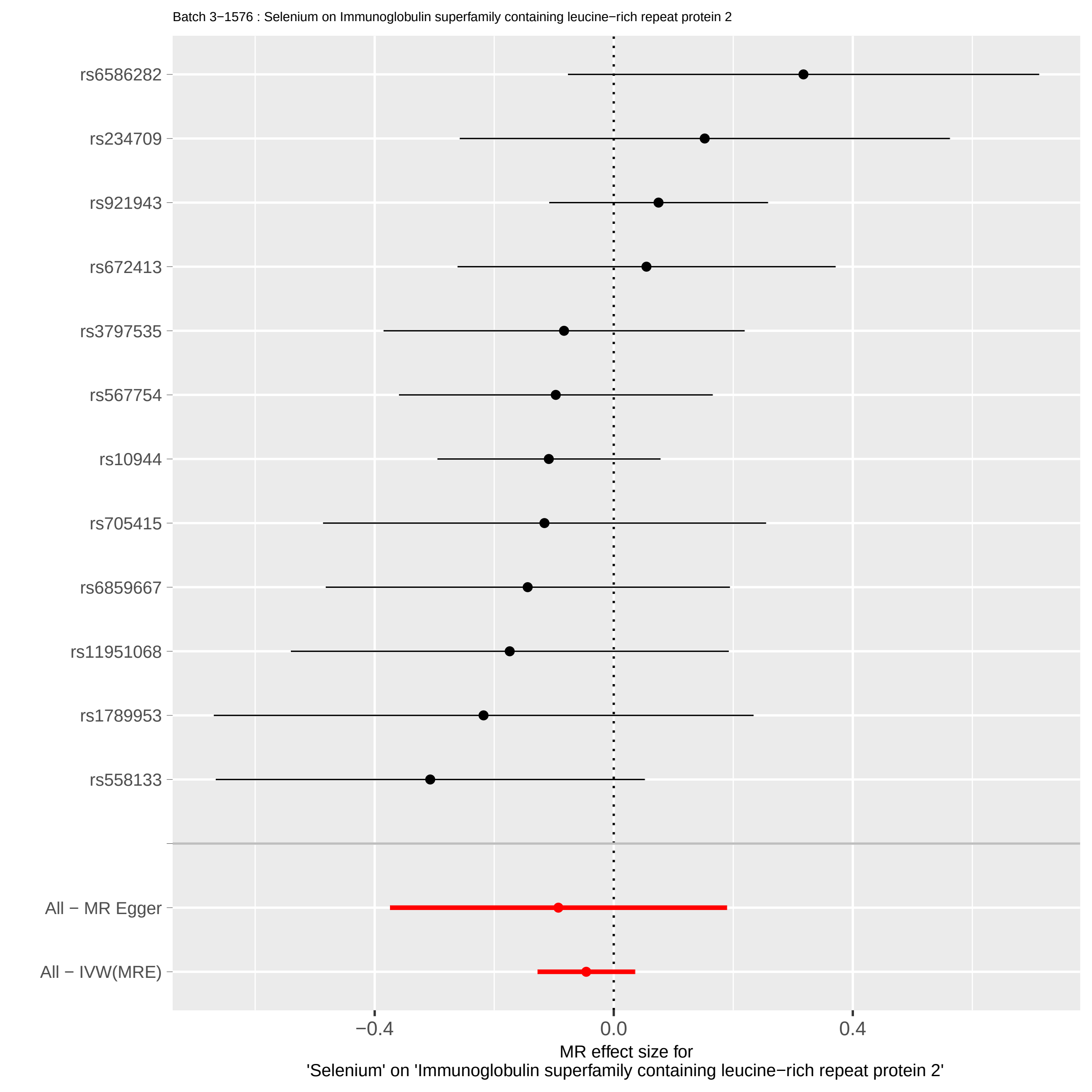
Batch 3−1576 : Selenium on Immunoglobulin superfamily containing leucine−rich repeat protein 2
rs6586282
rs234709
rs921943
rs672413
rs3797535
rs567754
rs10944
rs705415
rs6859667
rs11951068
rs1789953
rs558133
All − MR Egger
All − IVW(MRE)
−0.4
0.0
0.4
MR effect size for
'Selenium' on 'Immunoglobulin superfamily containing leucine−rich repeat protein 2'

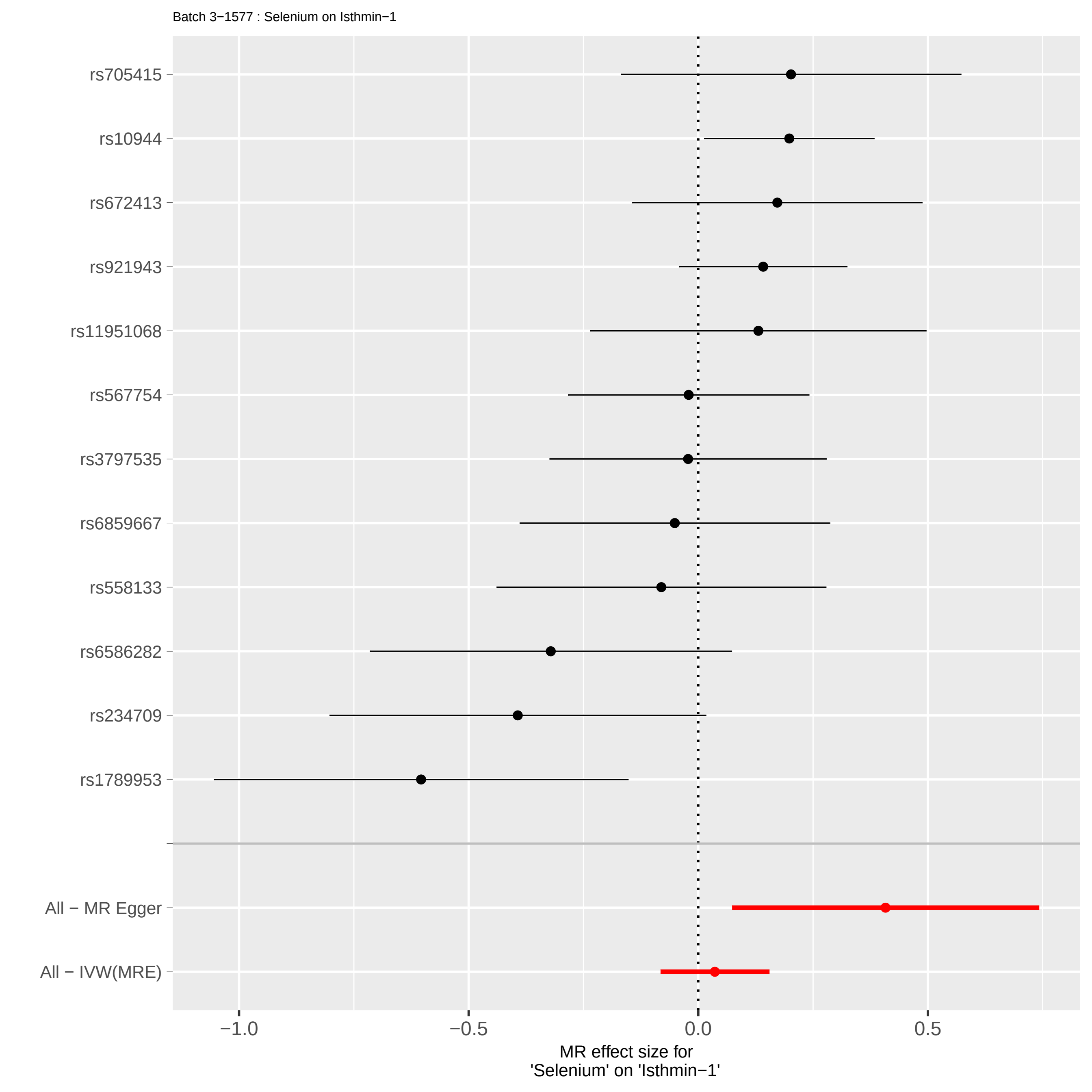
Batch 3−1577 : Selenium on Isthmin−1
rs705415
rs10944
rs672413
rs921943
rs11951068
rs567754
rs3797535
rs6859667
rs558133
rs6586282
rs234709
rs1789953
All − MR Egger
All − IVW(MRE)
−1.0
−0.5
0.0
0.5
MR effect size for
'Selenium' on 'Isthmin−1'

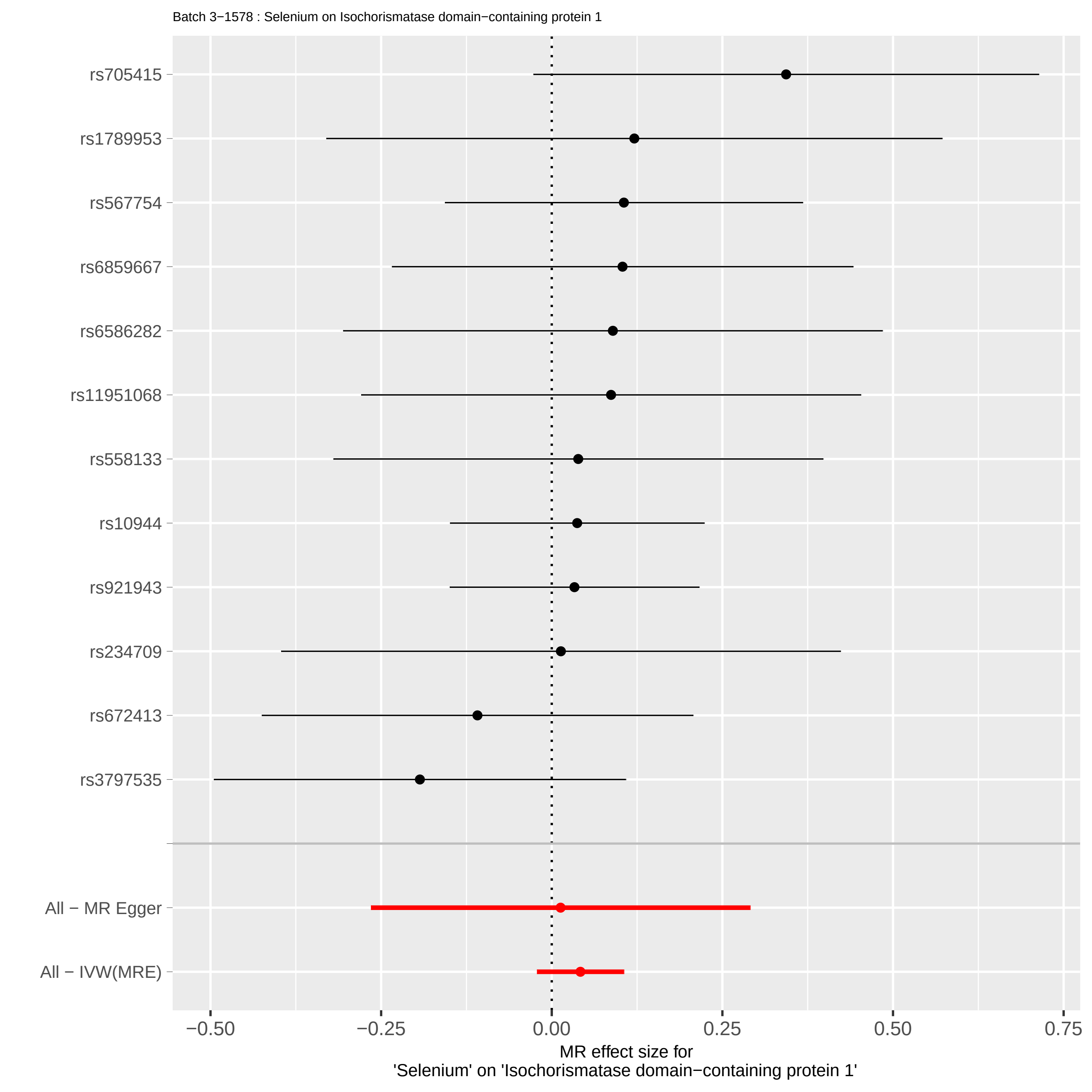

Batch 3−1578 : Selenium on Isochorismatase domain−containing protein 1
rs705415
rs1789953
rs567754
rs6859667
rs6586282
rs11951068
rs558133
rs10944
rs921943
rs234709
rs672413
rs3797535
All − MR Egger
All − IVW(MRE)
−0.50
−0.25
0.00
0.25
0.50
0.75
MR effect size for
'Selenium' on 'Isochorismatase domain−containing protein 1'

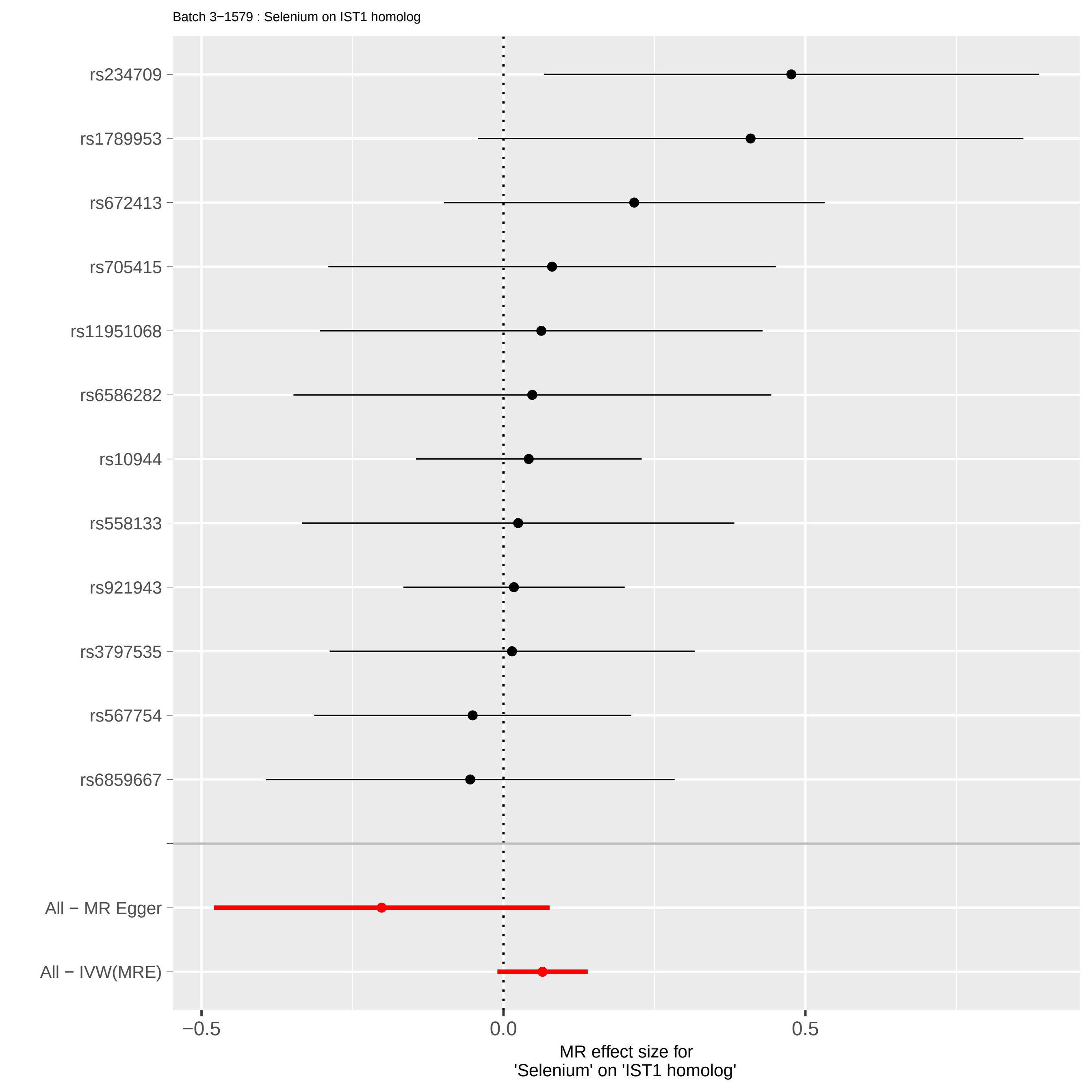

Batch 3−1579 : Selenium on IST1 homolog
rs234709
rs1789953
rs672413
rs705415
rs11951068
rs6586282
rs10944
rs558133
rs921943
rs3797535
rs567754
rs6859667
All − MR Egger
All − IVW(MRE)
−0.5
0.0
0.5
MR effect size for
'Selenium' on 'IST1 homolog'

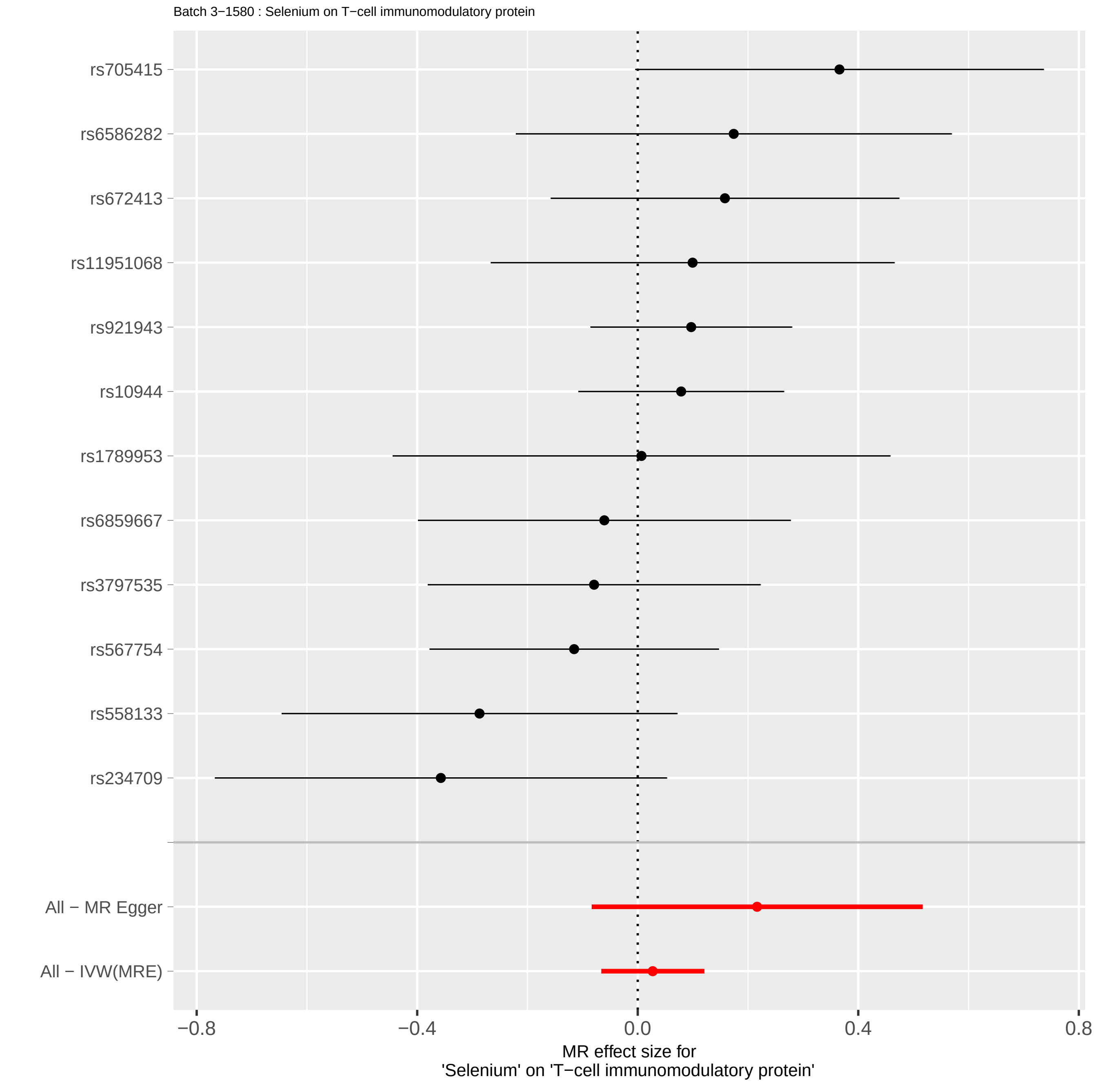
Batch 3−1580 : Selenium on T−cell immunomodulatory protein
rs705415
rs6586282
rs672413
rs11951068
rs921943
rs10944
rs1789953
rs6859667
rs3797535
rs567754
rs558133
rs234709
All − MR Egger
All − IVW(MRE)
−0.8
−0.4
0.0
0.4
0.8
MR effect size for
'Selenium' on 'T−cell immunomodulatory protein'

Batch 3−1581 : Selenium on Integrin alpha−I: beta−1 complex

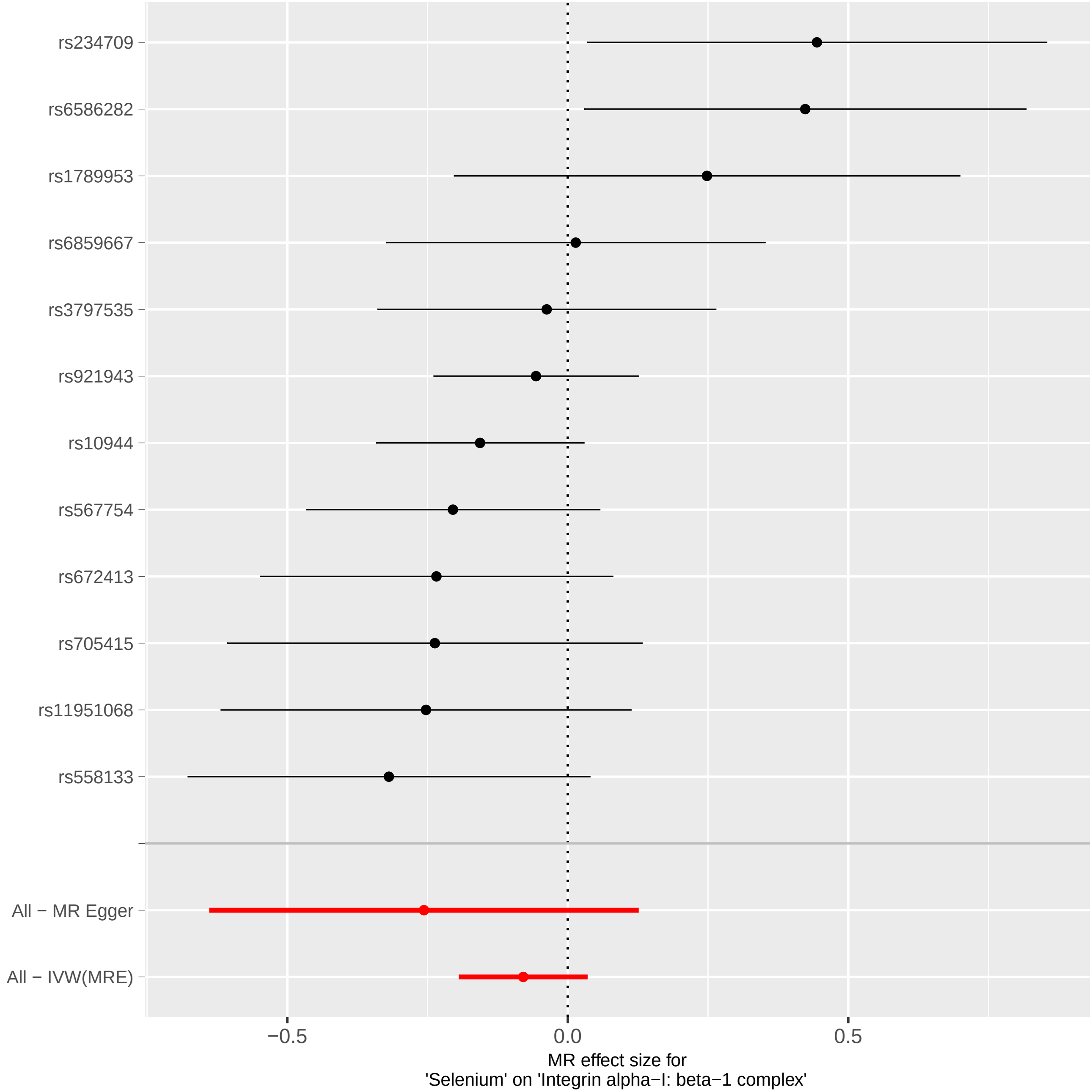

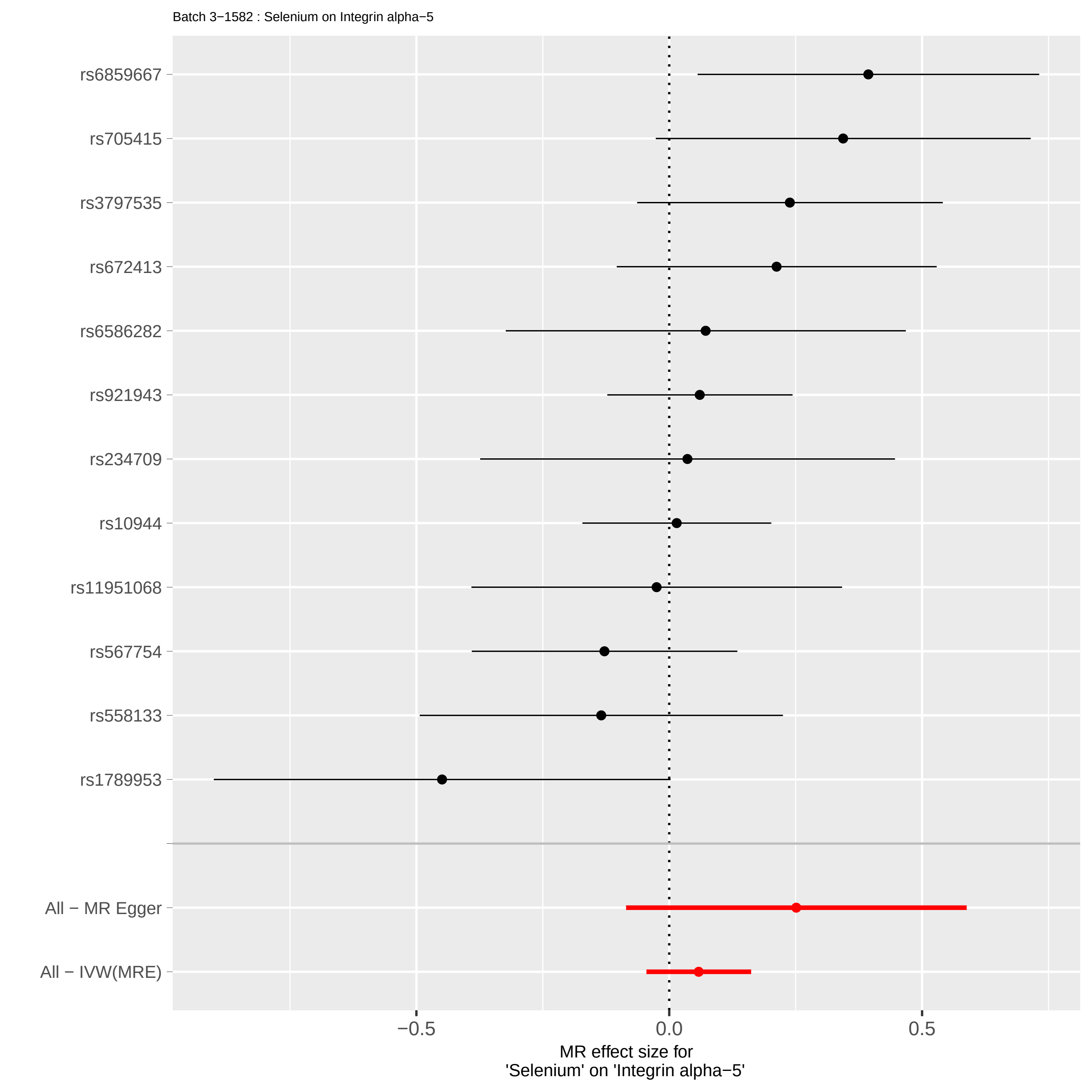

Batch 3−1582 : Selenium on Integrin alpha−5
rs6859667
rs705415
rs3797535
rs672413
rs6586282
rs921943
rs234709
rs10944
rs11951068
rs567754
rs558133
rs1789953
All − MR Egger
All − IVW(MRE)
−0.5
0.0
0.5
MR effect size for
'Selenium' on 'Integrin alpha−5'

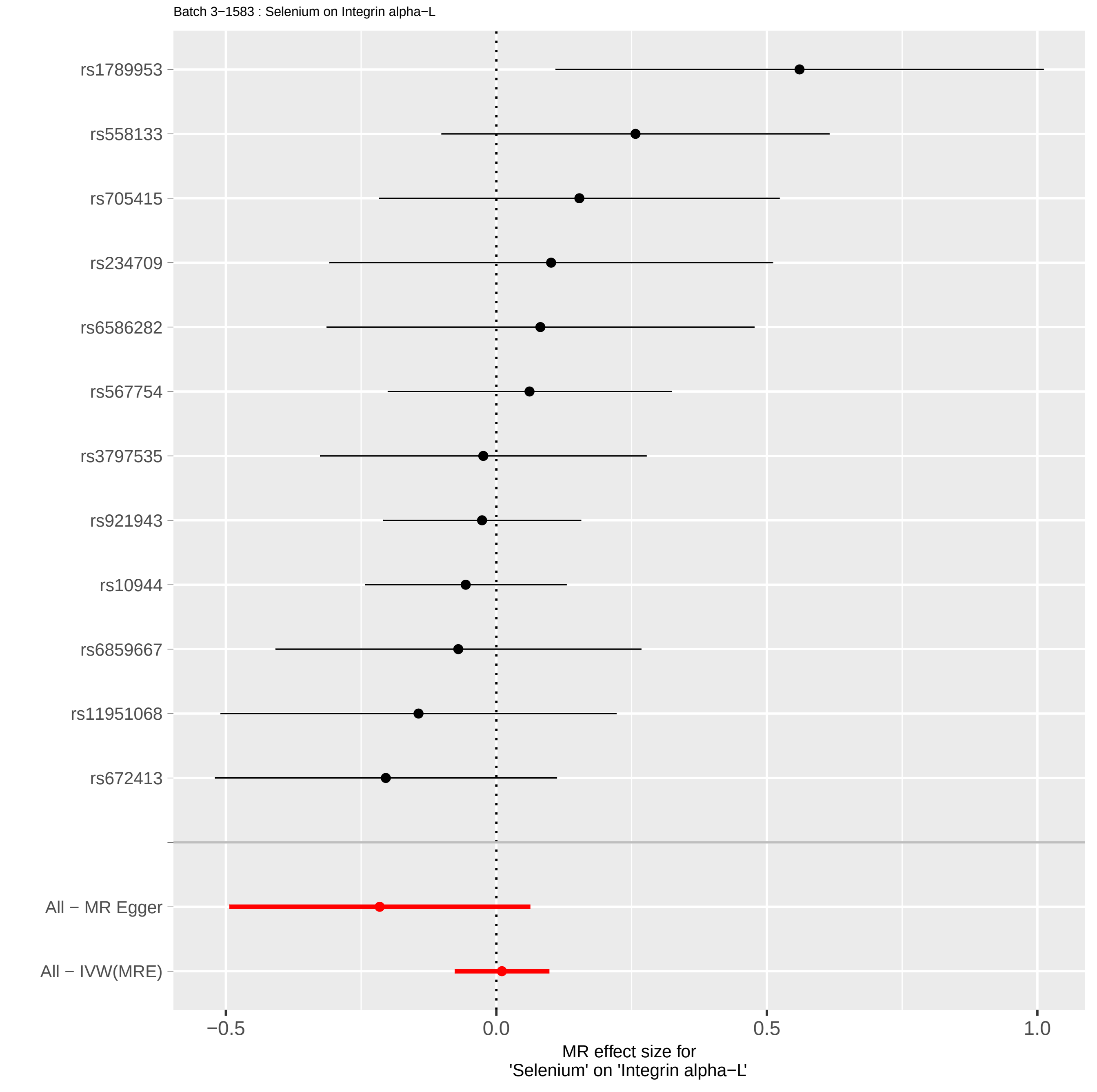
Batch 3−1583 : Selenium on Integrin alpha−L
rs1789953
rs558133
rs705415
rs234709
rs6586282
rs567754
rs3797535
rs921943
rs10944
rs6859667
rs11951068
rs672413
All − MR Egger
All − IVW(MRE)
−0.5
0.0
0.5
1.0
MR effect size for
'Selenium' on 'Integrin alpha−L'

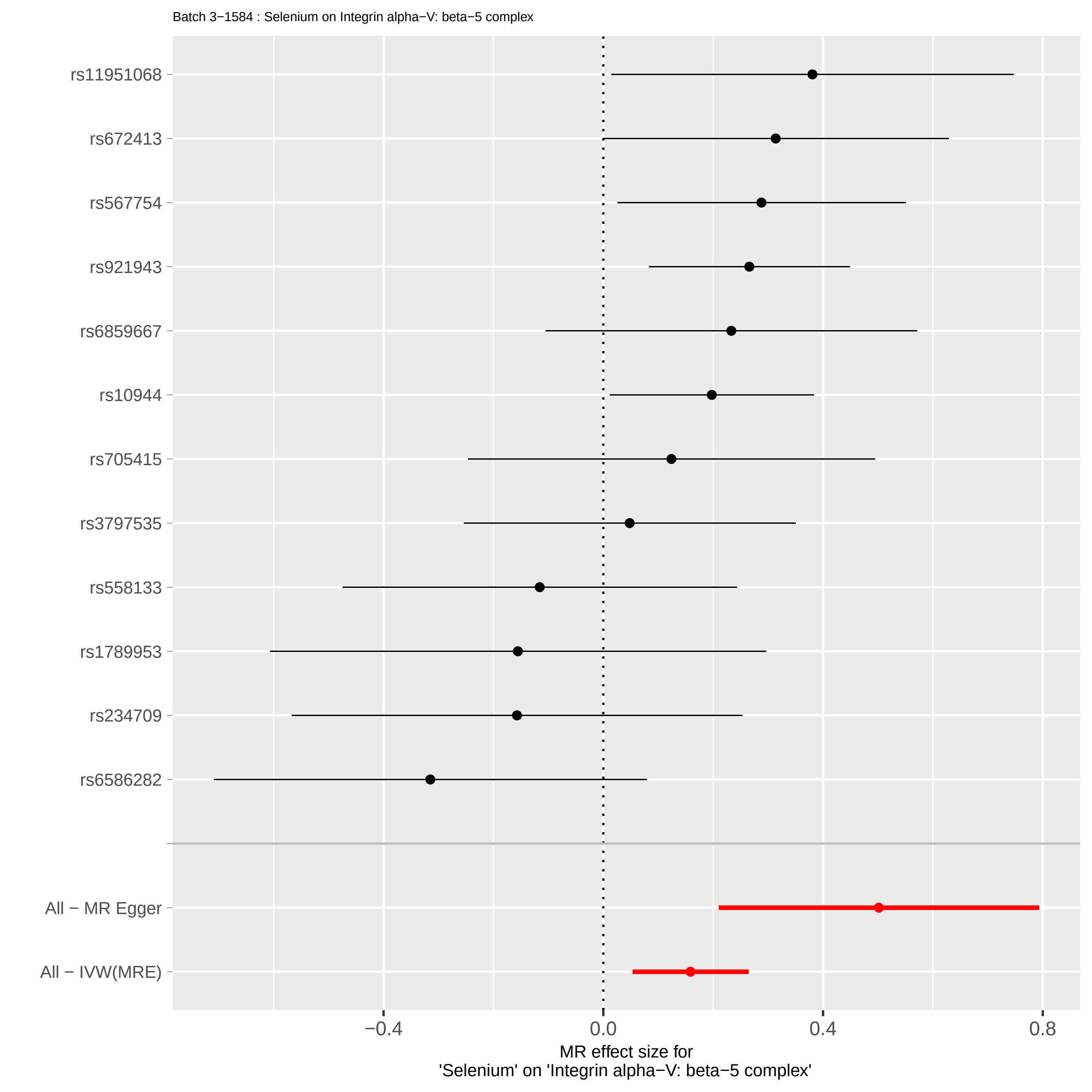

Batch 3−1584 : Selenium on Integrin alpha−V: beta−5 complex
rs11951068
rs672413
rs567754
rs921943
rs6859667
rs10944
rs705415
rs3797535
rs558133
rs1789953
rs234709
rs6586282
All − MR Egger
All − IVW(MRE)
−0.4
0.0
0.4
0.8
MR effect size for
'Selenium' on 'Integrin alpha−V: beta−5 complex'

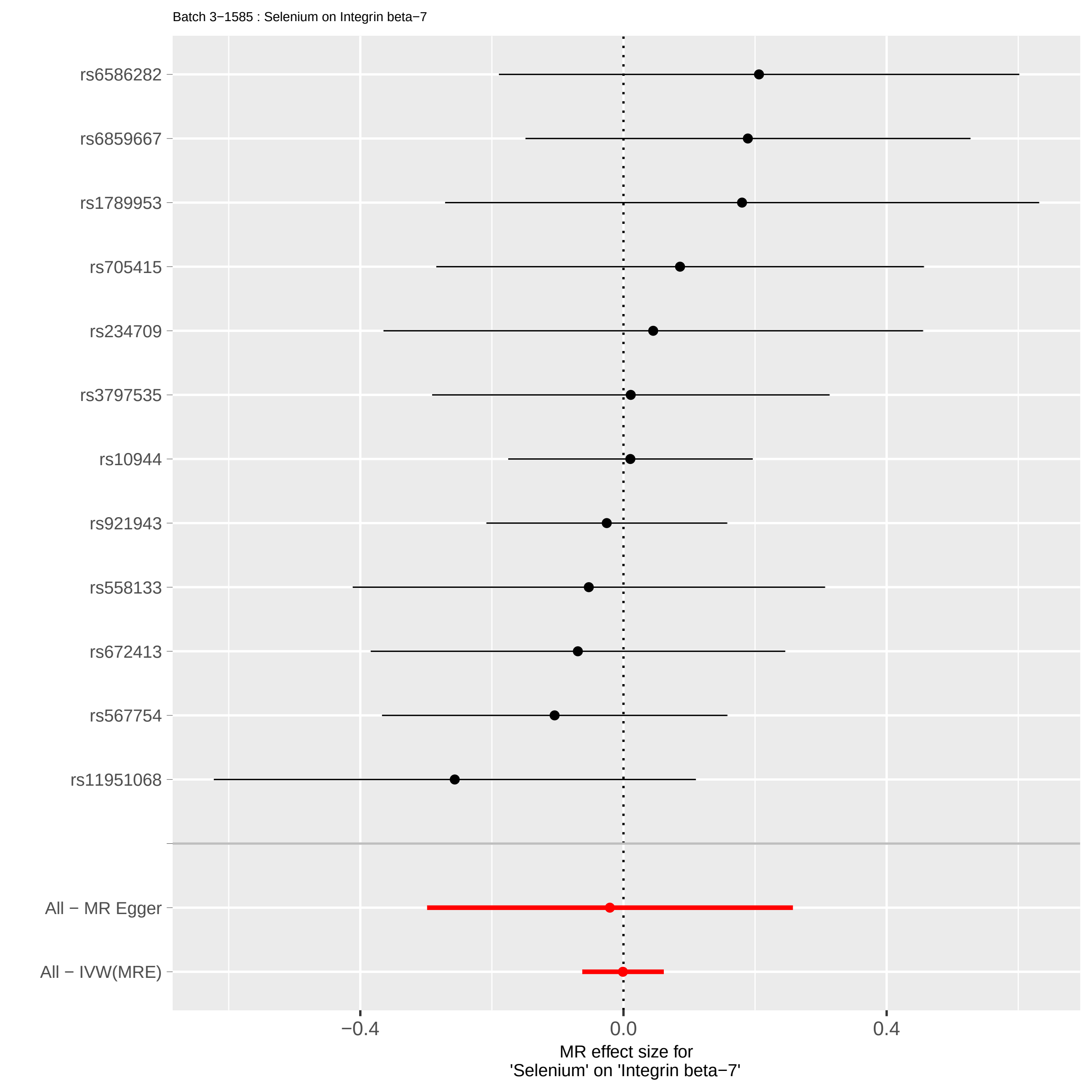

Batch 3−1585 : Selenium on Integrin beta−7
rs6586282
rs6859667
rs1789953
rs705415
rs234709
rs3797535
rs10944
rs921943
rs558133
rs672413
rs567754
rs11951068
All − MR Egger
All − IVW(MRE)
−0.4
0.0
0.4
MR effect size for
'Selenium' on 'Integrin beta−7'

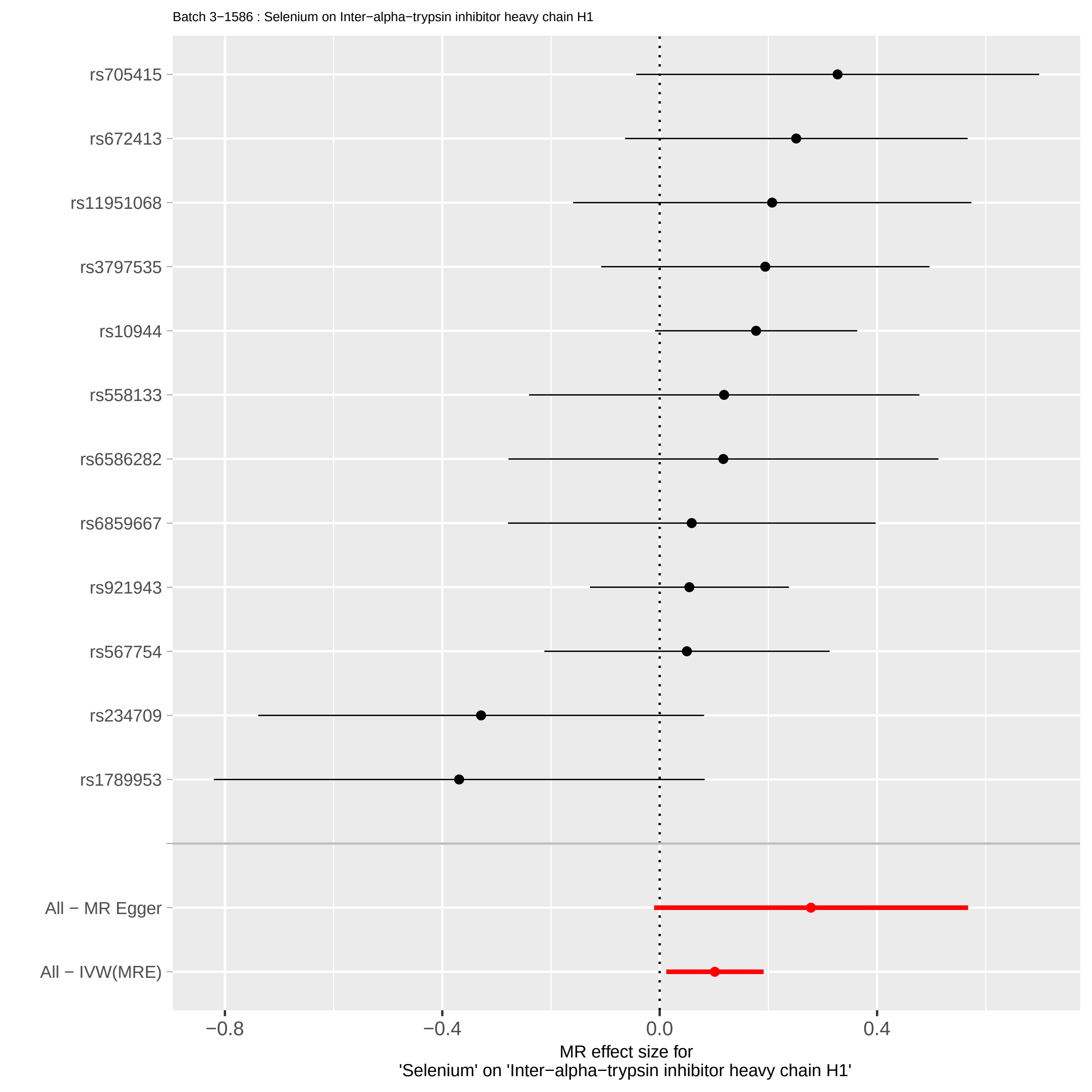

Batch 3−1586 : Selenium on Inter−alpha−trypsin inhibitor heavy chain H1
rs705415
rs672413
rs11951068
rs3797535
rs10944
rs558133
rs6586282
rs6859667
rs921943
rs567754
rs234709
rs1789953
All − MR Egger
All − IVW(MRE)
−0.8
−0.4
0.0
0.4
MR effect size for
'Selenium' on 'Inter−alpha−trypsin inhibitor heavy chain H1'

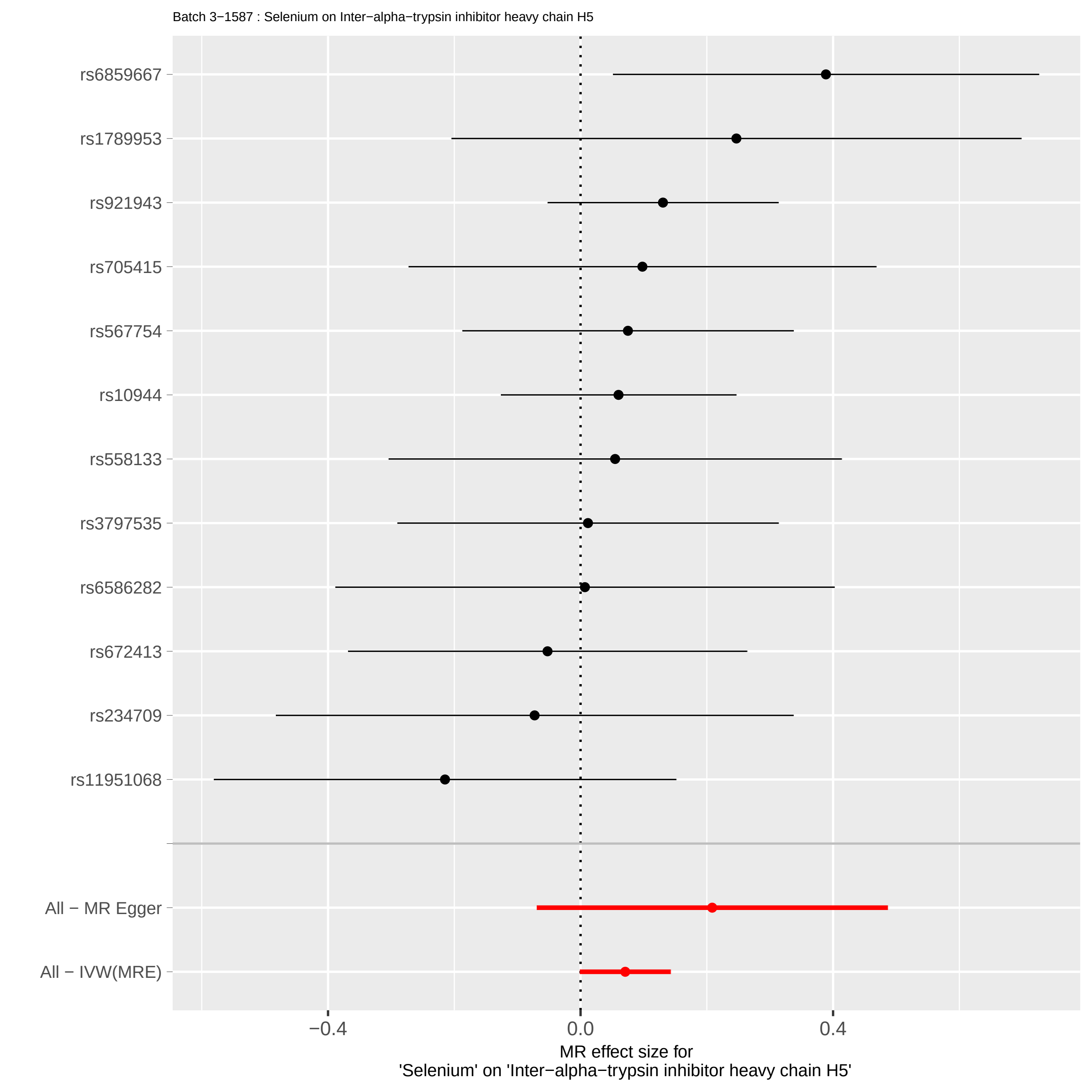

Batch 3−1587 : Selenium on Inter−alpha−trypsin inhibitor heavy chain H5
rs6859667
rs1789953
rs921943
rs705415
rs567754
rs10944
rs558133
rs3797535
rs6586282
rs672413
rs234709
rs11951068
All − MR Egger
All − IVW(MRE)
−0.4
0.0
0.4
MR effect size for
'Selenium' on 'Inter−alpha−trypsin inhibitor heavy chain H5'

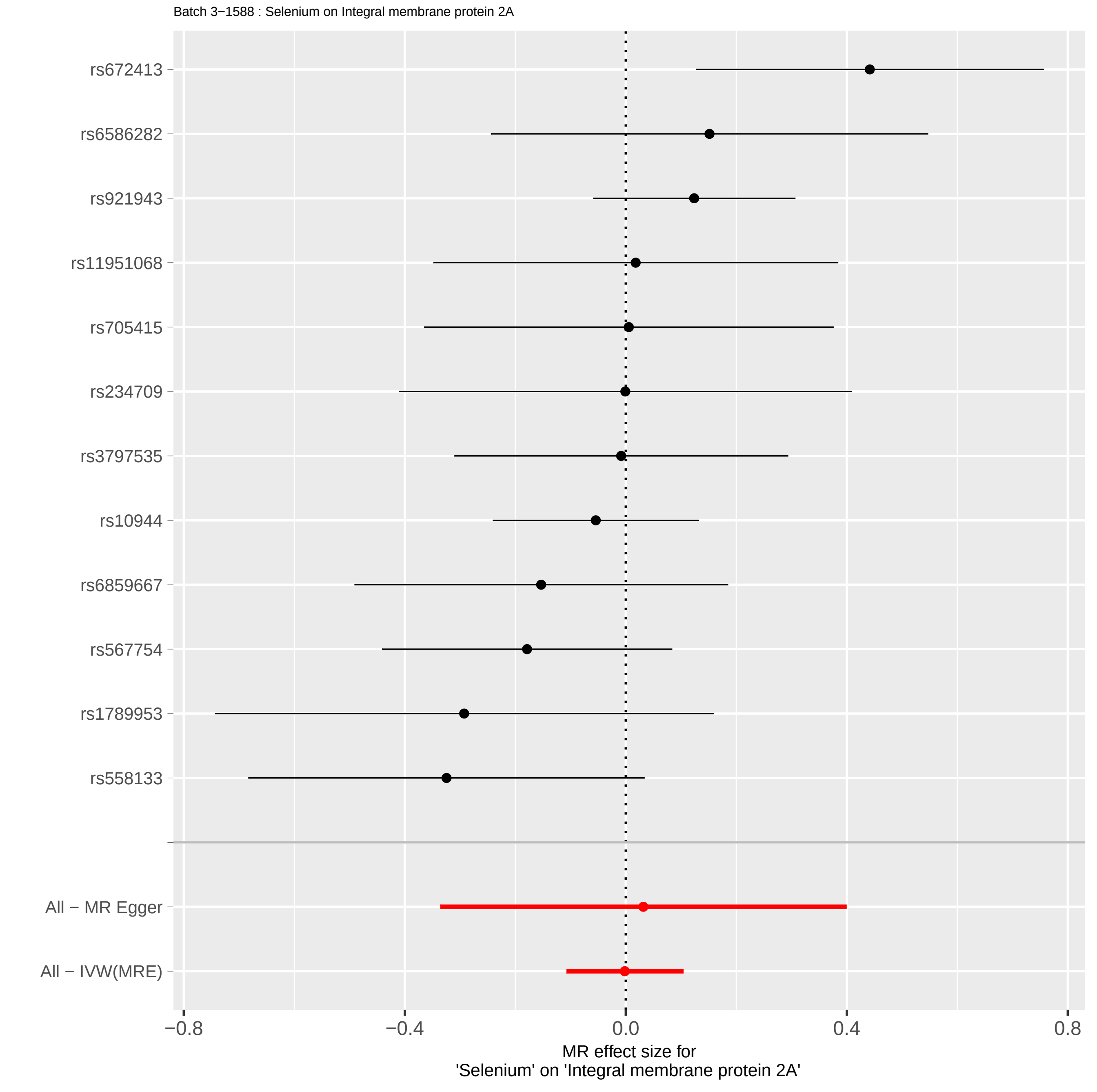

Batch 3−1588 : Selenium on Integral membrane protein 2A
rs672413
rs6586282
rs921943
rs11951068
rs705415
rs234709
rs3797535
rs10944
rs6859667
rs567754
rs1789953
rs558133
All − MR Egger
All − IVW(MRE)
−0.8
−0.4
0.0
0.4
0.8
MR effect size for
'Selenium' on 'Integral membrane protein 2A'

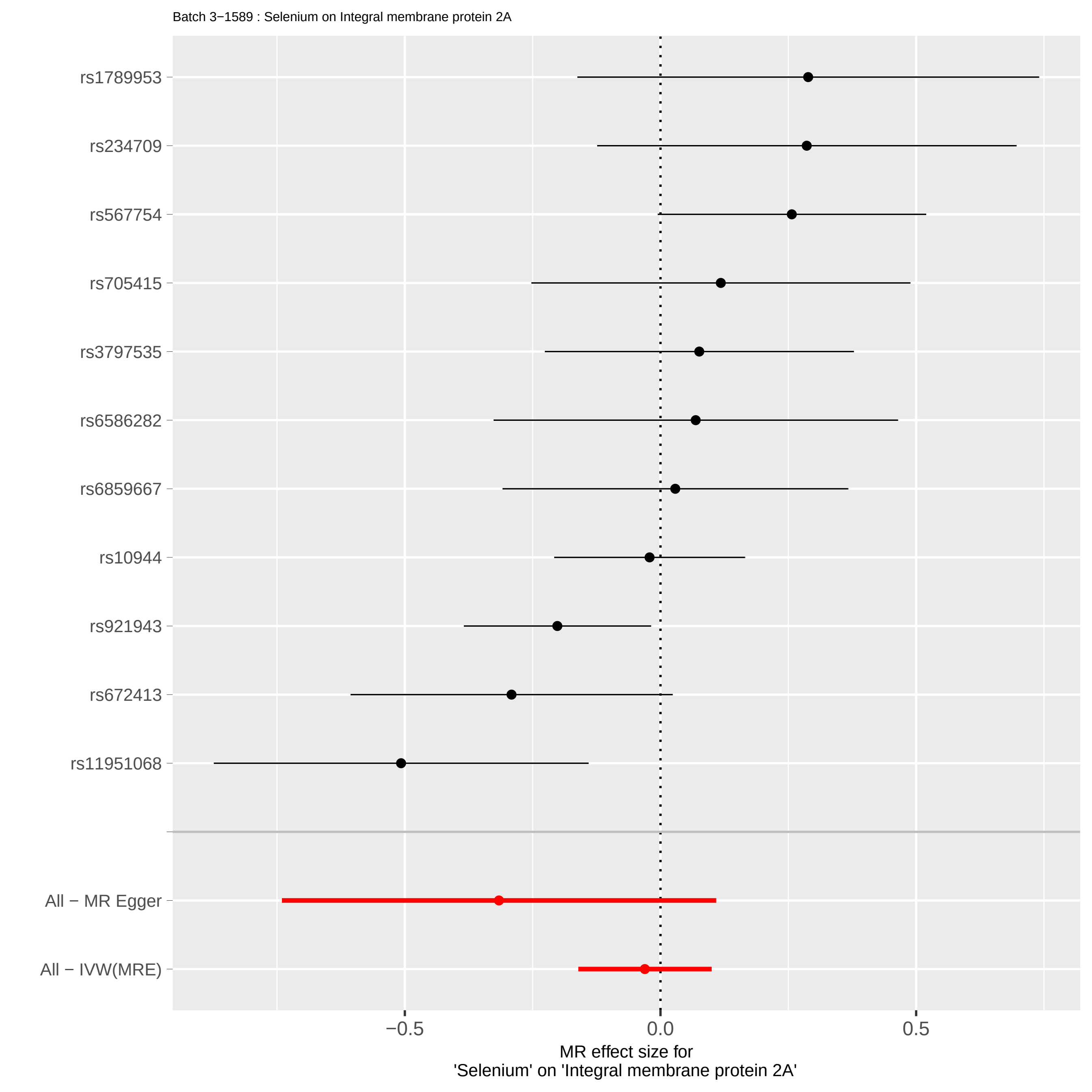

Batch 3−1589 : Selenium on Integral membrane protein 2A
rs1789953
rs234709
rs567754
rs705415
rs3797535
rs6586282
rs6859667
rs10944
rs921943
rs672413
rs11951068
All − MR Egger
All − IVW(MRE)
−0.5
0.0
0.5
MR effect size for
'Selenium' on 'Integral membrane protein 2A'

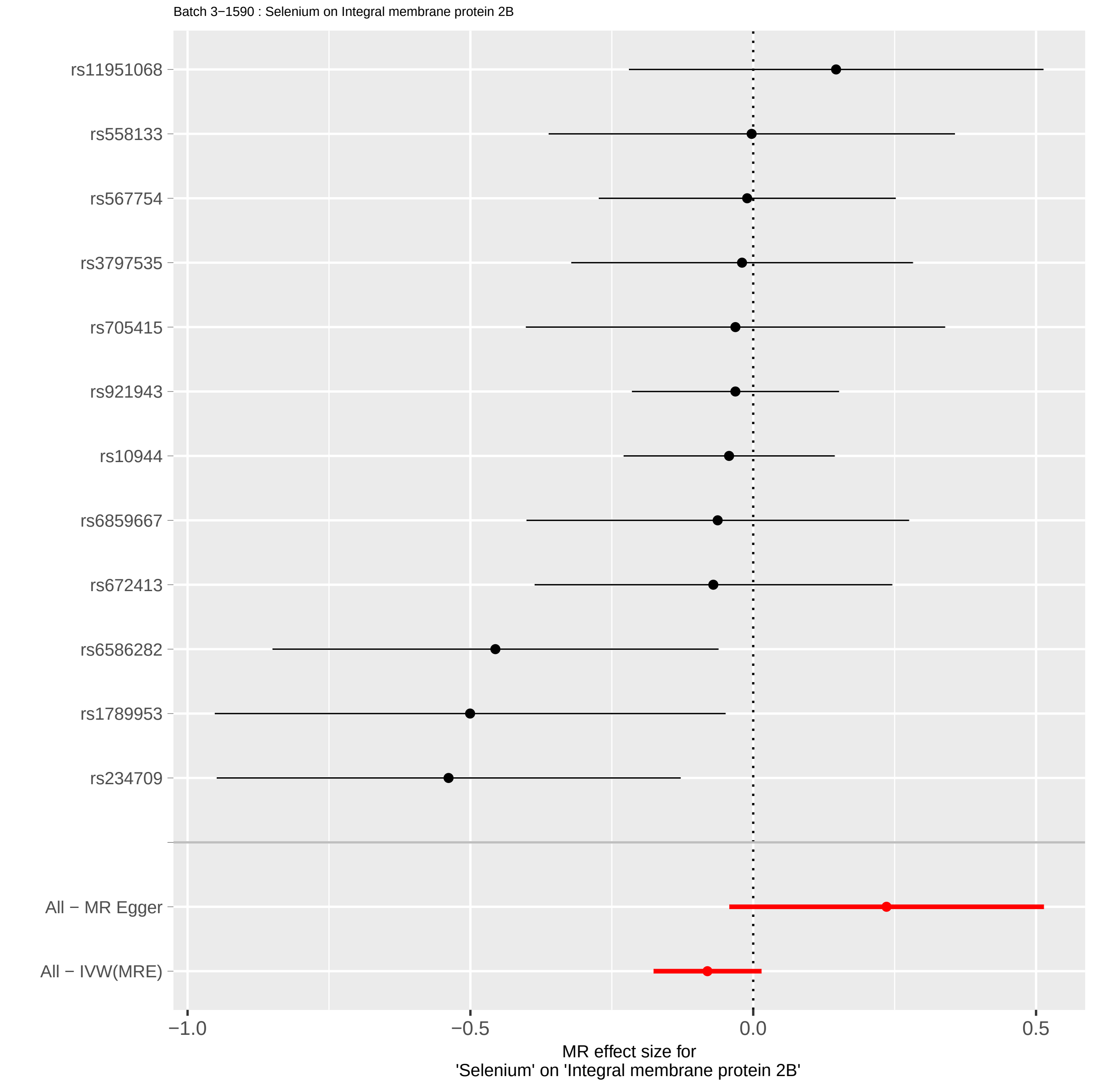
Batch 3−1590 : Selenium on Integral membrane protein 2B
rs11951068
rs558133
rs567754
rs3797535
rs705415
rs921943
rs10944
rs6859667
rs672413
rs6586282
rs1789953
rs234709
All − MR Egger
All − IVW(MRE)
−1.0
−0.5
0.0
0.5
MR effect size for
'Selenium' on 'Integral membrane protein 2B'

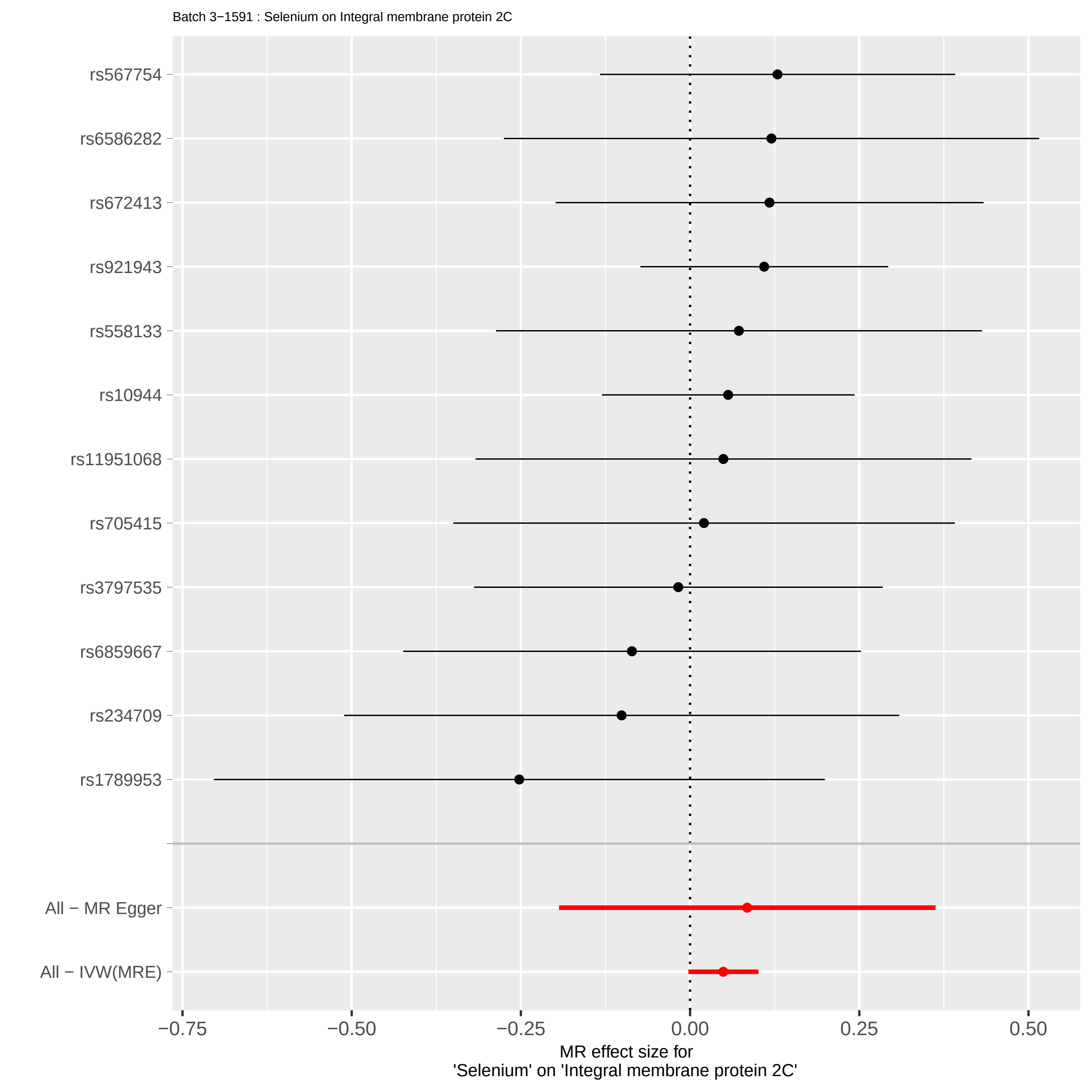
Batch 3−1591 : Selenium on Integral membrane protein 2C
rs567754
rs6586282
rs672413
rs921943
rs558133
rs10944
rs11951068
rs705415
rs3797535
rs6859667
rs234709
rs1789953
All − MR Egger
All − IVW(MRE)
−0.75
−0.50
−0.25
0.00
0.25
0.50
MR effect size for
'Selenium' on 'Integral membrane protein 2C'

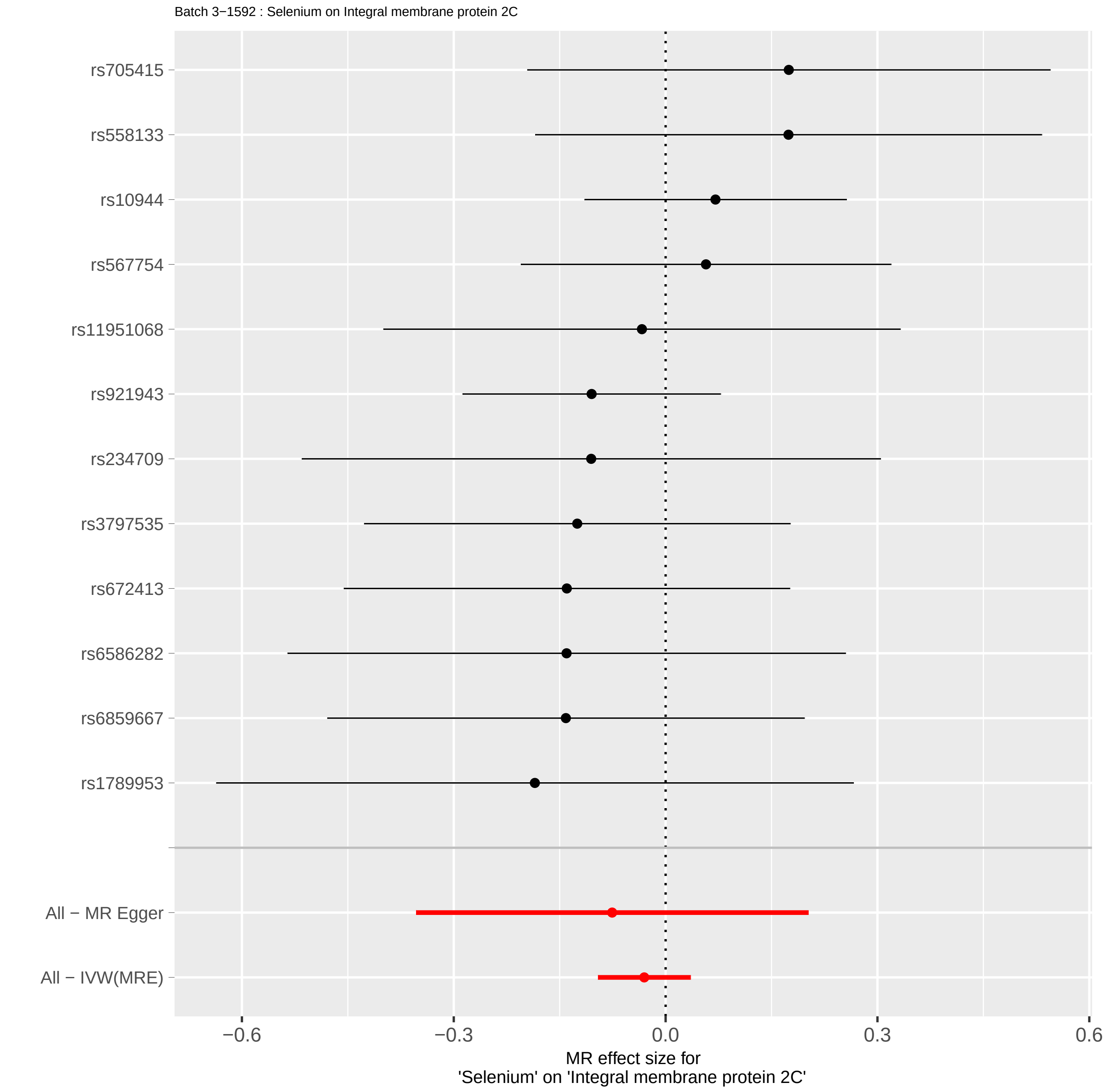

Batch 3−1592 : Selenium on Integral membrane protein 2C
rs705415
rs558133
rs10944
rs567754
rs11951068
rs921943
rs234709
rs3797535
rs672413
rs6586282
rs6859667
rs1789953
All − MR Egger
All − IVW(MRE)
−0.6
−0.3
0.0
0.3
0.6
MR effect size for
'Selenium' on 'Integral membrane protein 2C'

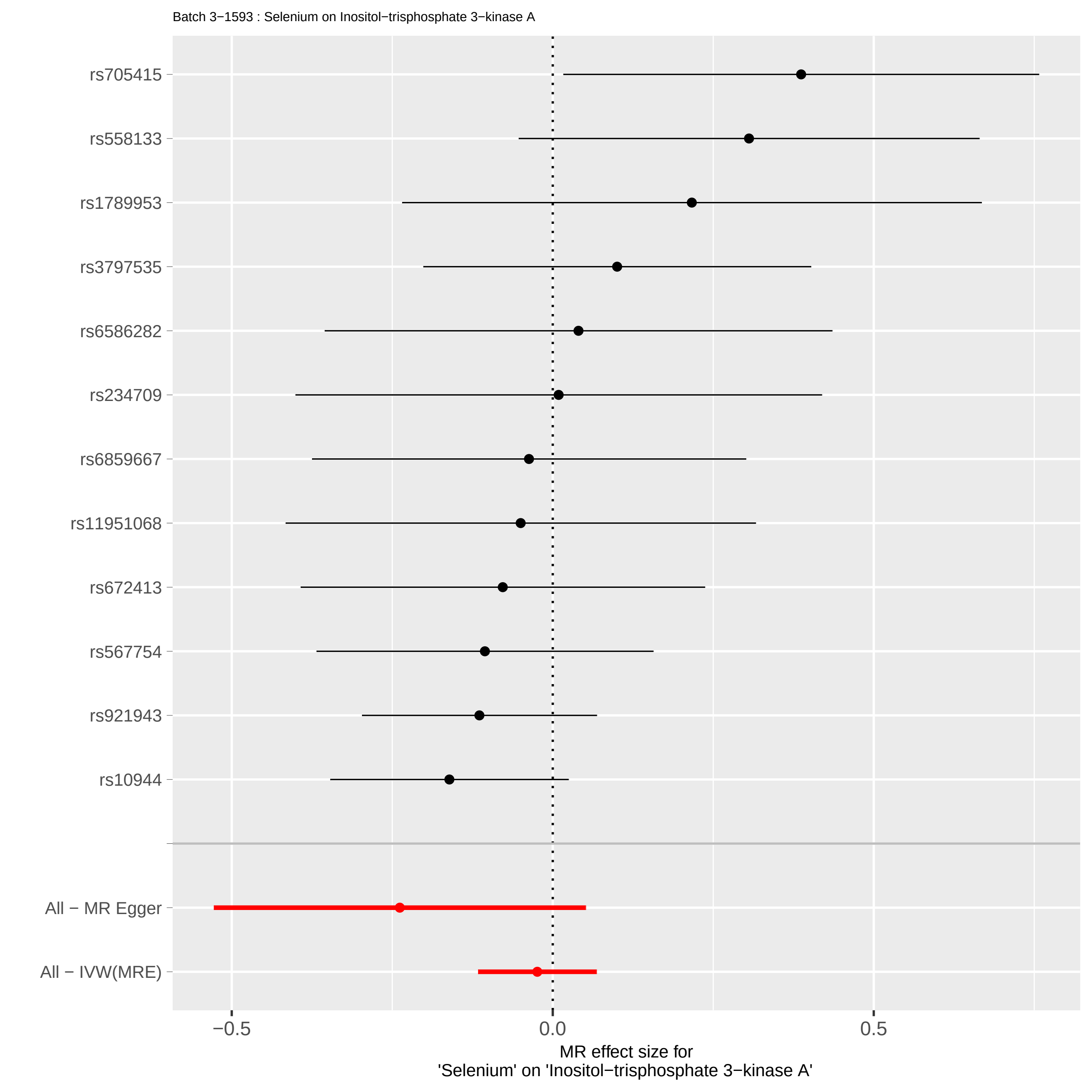
Batch 3−1593 : Selenium on Inositol−trisphosphate 3−kinase A
rs705415
rs558133
rs1789953
rs3797535
rs6586282
rs234709
rs6859667
rs11951068
rs672413
rs567754
rs921943
rs10944
All − MR Egger
All − IVW(MRE)
−0.5
0.0
0.5
MR effect size for
'Selenium' on 'Inositol−trisphosphate 3−kinase A'

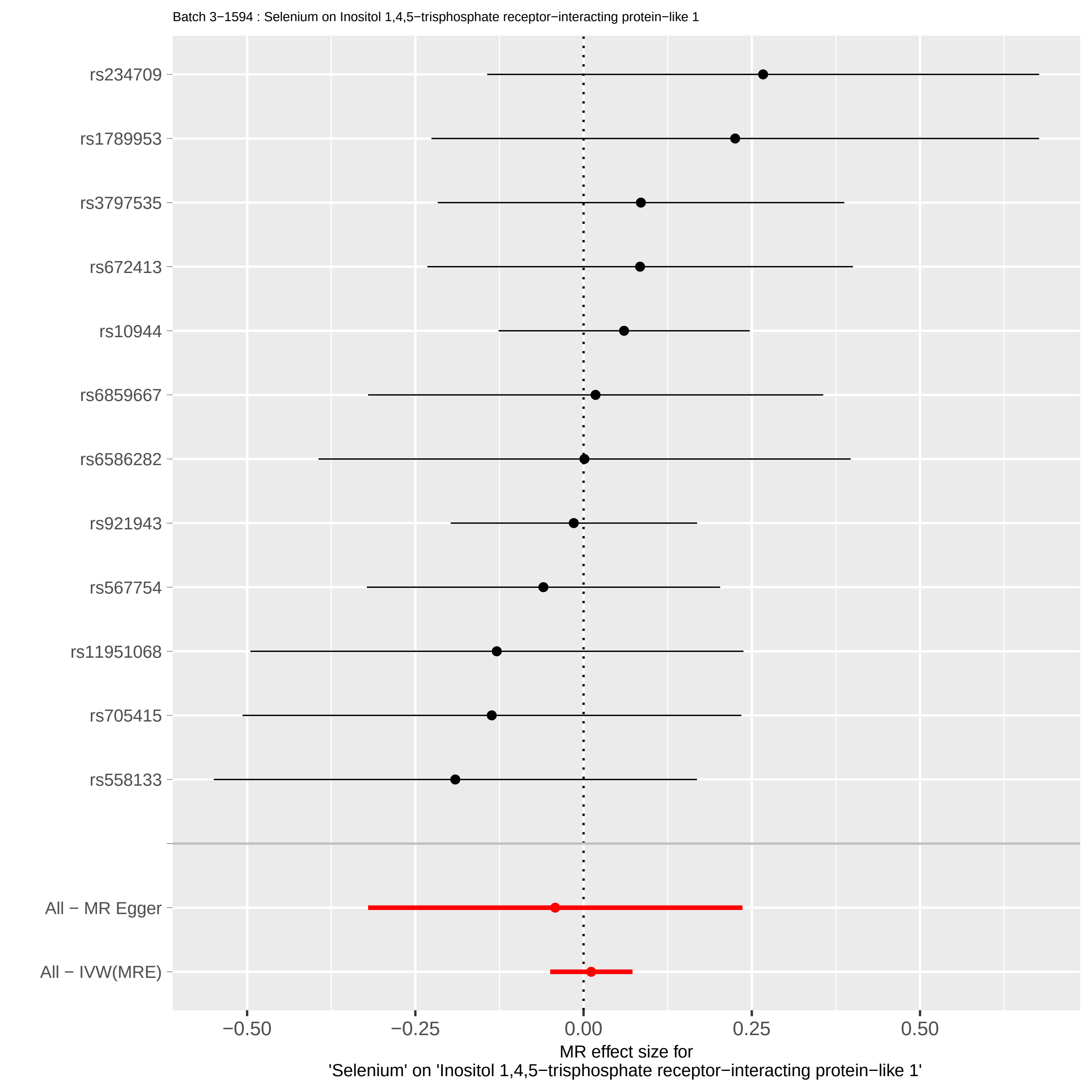

Batch 3−1594 : Selenium on Inositol 1,4,5−trisphosphate receptor−interacting protein−like 1
rs234709
rs1789953
rs3797535
rs672413
rs10944
rs6859667
rs6586282
rs921943
rs567754
rs11951068
rs705415
rs558133
All − MR Egger
All − IVW(MRE)
−0.50
−0.25
0.00
0.25
0.50
MR effect size for
'Selenium' on 'Inositol 1,4,5−trisphosphate receptor−interacting protein−like 1'

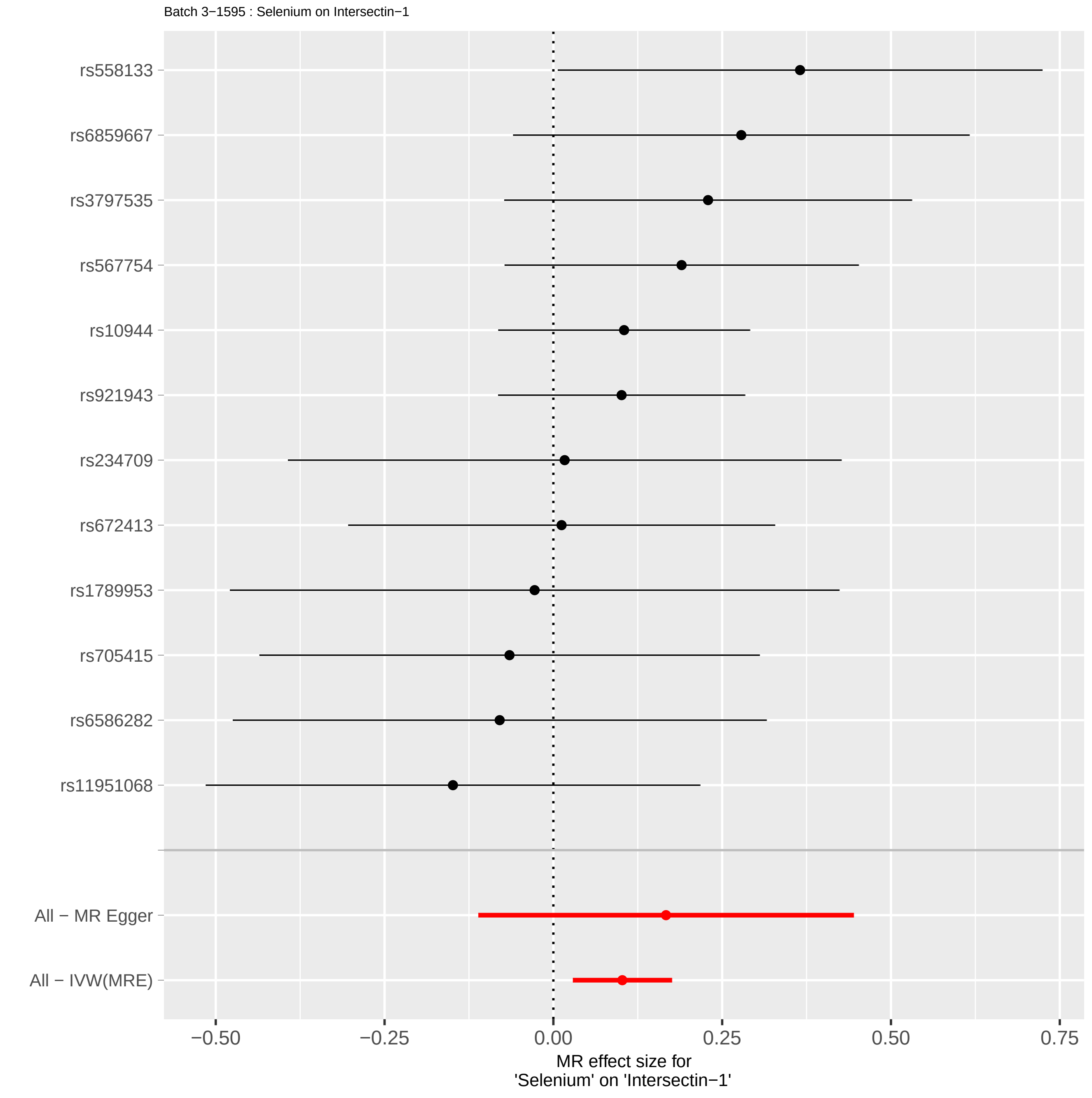

Batch 3−1595 : Selenium on Intersectin−1
rs558133
rs6859667
rs3797535
rs567754
rs10944
rs921943
rs234709
rs672413
rs1789953
rs705415
rs6586282
rs11951068
All − MR Egger
All − IVW(MRE)
−0.50
−0.25
0.00
0.25
0.50
0.75
MR effect size for
'Selenium' on 'Intersectin−1'

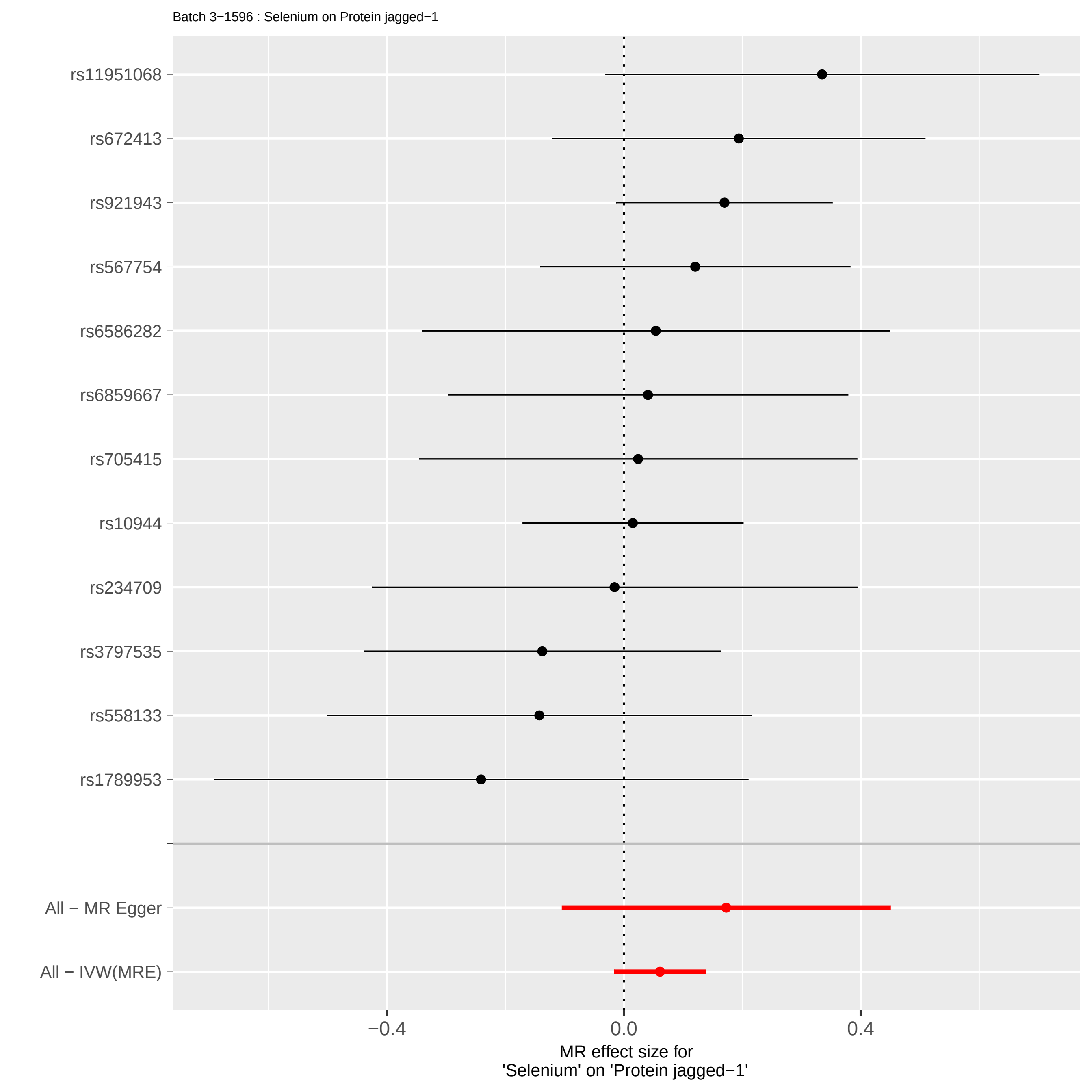

Batch 3−1596 : Selenium on Protein jagged−1
rs11951068
rs672413
rs921943
rs567754
rs6586282
rs6859667
rs705415
rs10944
rs234709
rs3797535
rs558133
rs1789953
All − MR Egger
All − IVW(MRE)
−0.4
0.0
0.4
MR effect size for
'Selenium' on 'Protein jagged−1'

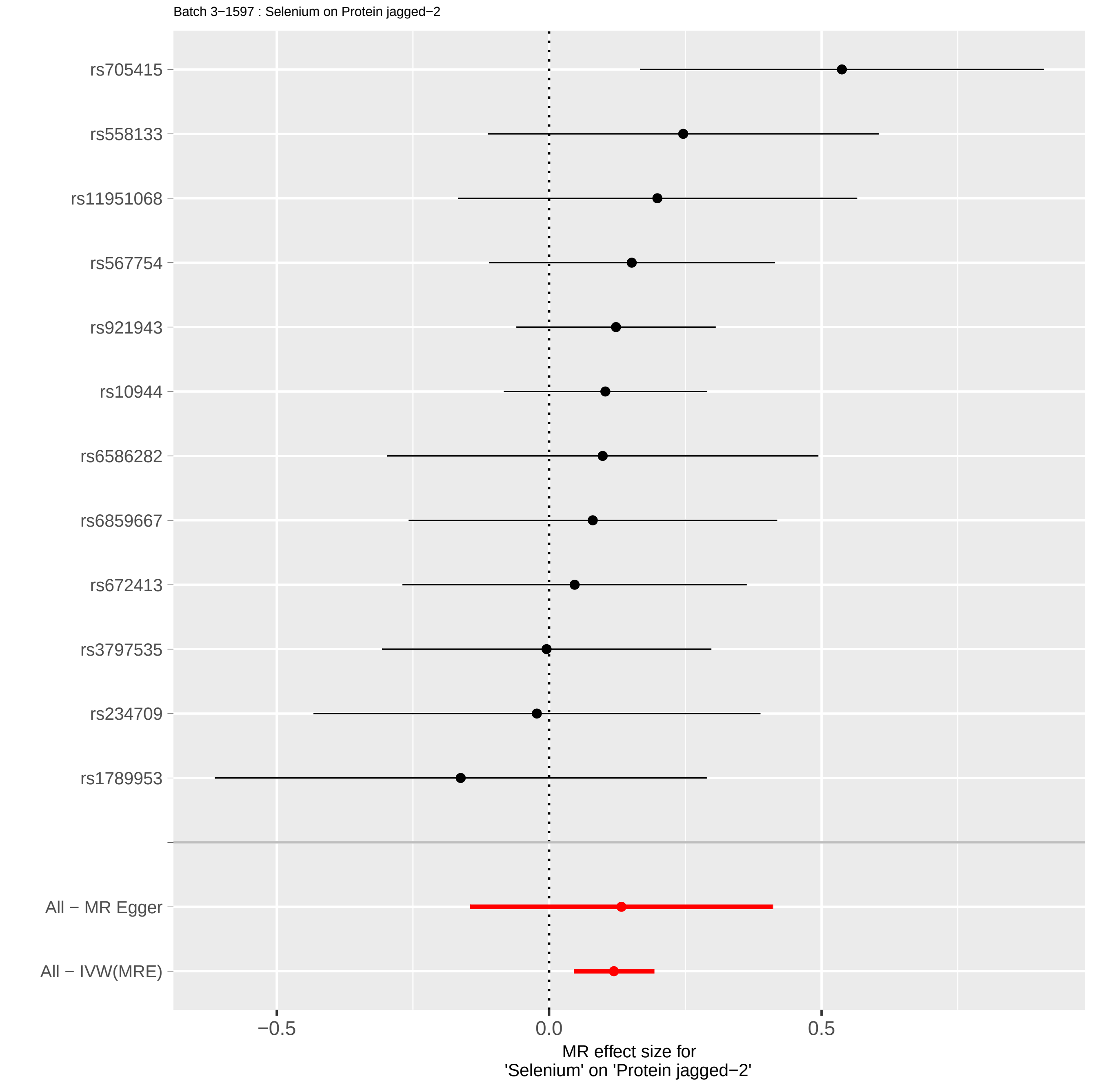

Batch 3−1597 : Selenium on Protein jagged−2
rs705415
rs558133
rs11951068
rs567754
rs921943
rs10944
rs6586282
rs6859667
rs672413
rs3797535
rs234709
rs1789953
All − MR Egger
All − IVW(MRE)
−0.5
0.0
0.5
MR effect size for
'Selenium' on 'Protein jagged−2'

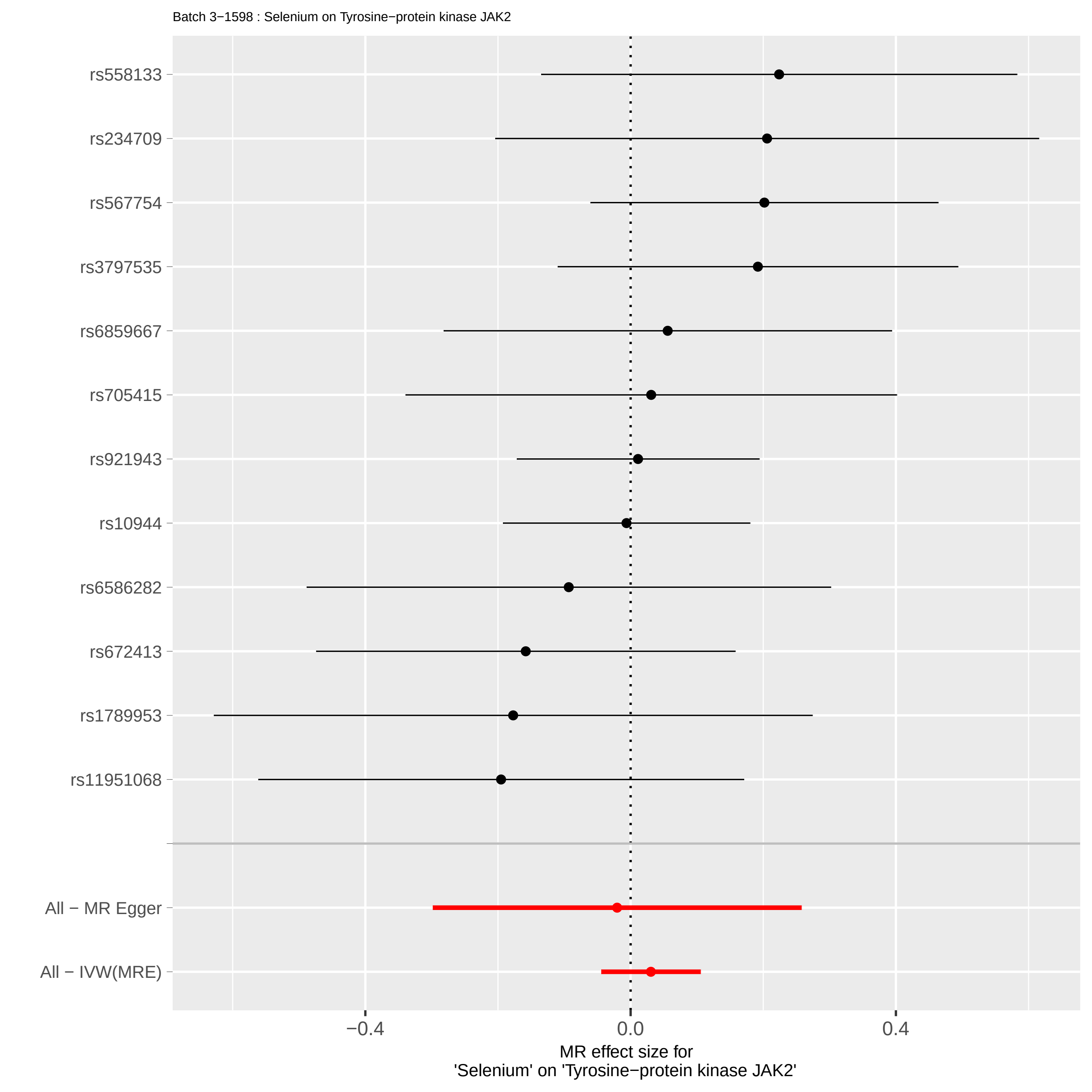

Batch 3−1598 : Selenium on Tyrosine−protein kinase JAK2
rs558133
rs234709
rs567754
rs3797535
rs6859667
rs705415
rs921943
rs10944
rs6586282
rs672413
rs1789953
rs11951068
All − MR Egger
All − IVW(MRE)
−0.4
0.0
0.4
MR effect size for
'Selenium' on 'Tyrosine−protein kinase JAK2'

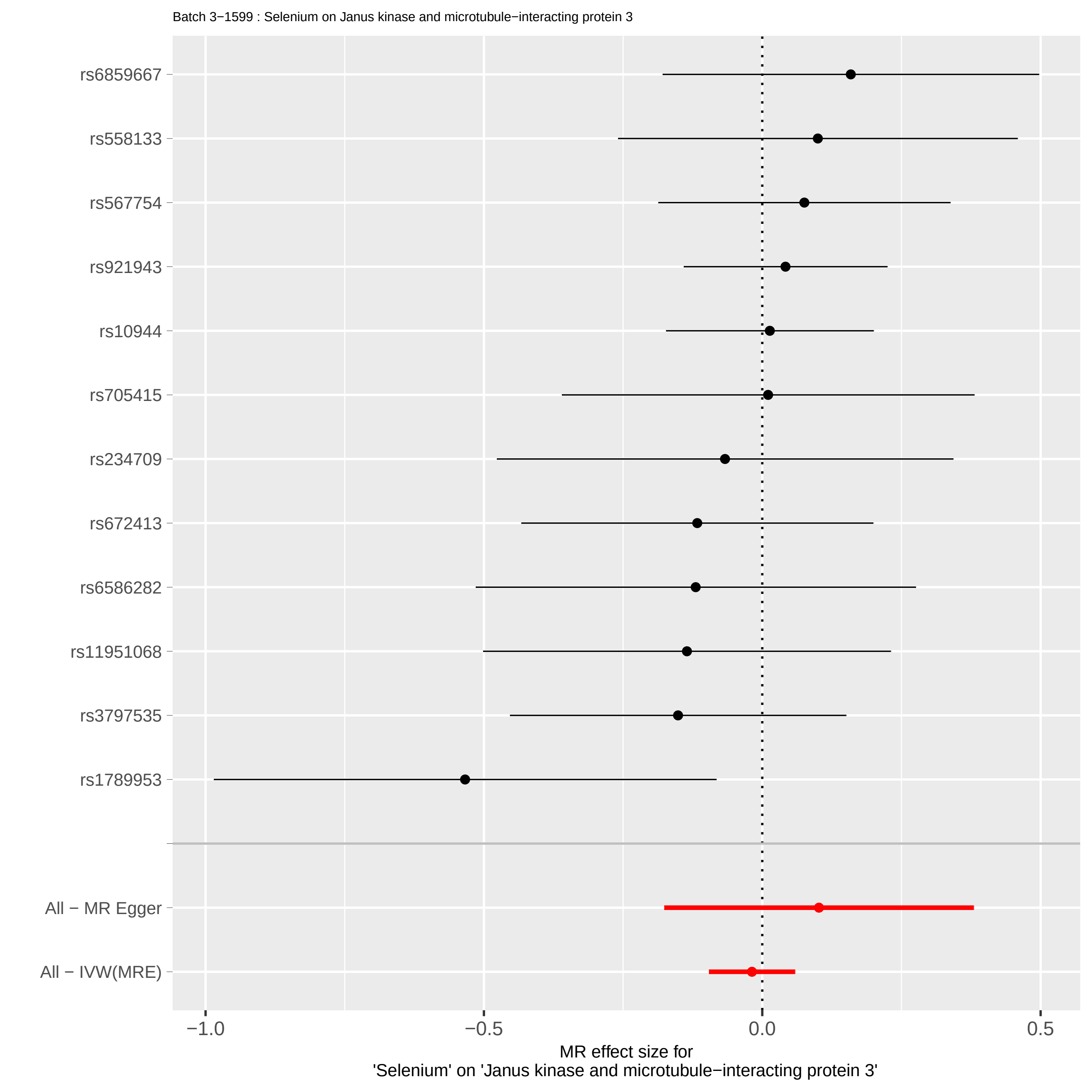

Batch 3−1599 : Selenium on Janus kinase and microtubule−interacting protein 3
rs6859667
rs558133
rs567754
rs921943
rs10944
rs705415
rs234709
rs672413
rs6586282
rs11951068
rs3797535
rs1789953
All − MR Egger
All − IVW(MRE)
−1.0
−0.5
0.0
0.5
MR effect size for
'Selenium' on 'Janus kinase and microtubule−interacting protein 3'

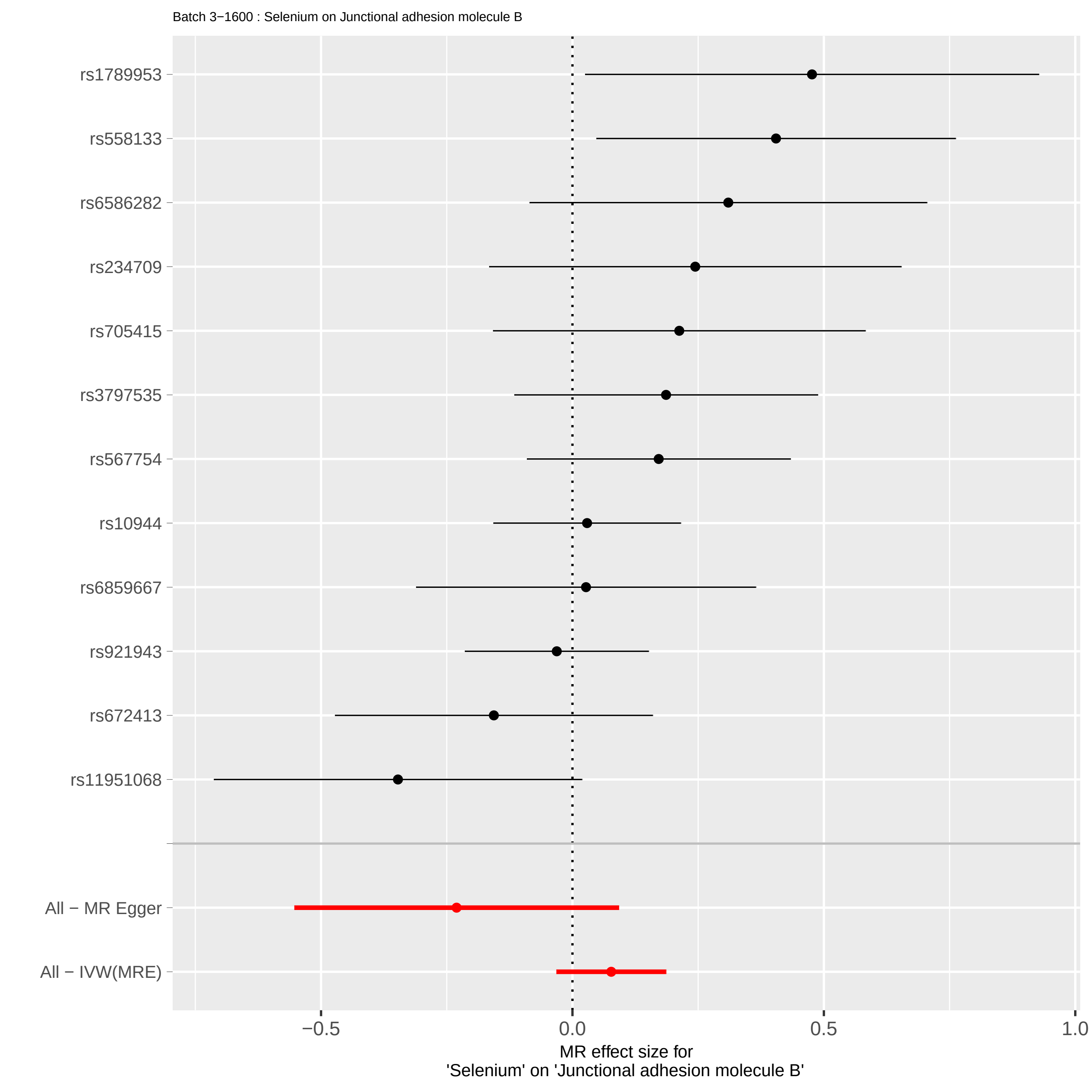
Batch 3−1600 : Selenium on Junctional adhesion molecule B
rs1789953
rs558133
rs6586282
rs234709
rs705415
rs3797535
rs567754
rs10944
rs6859667
rs921943
rs672413
rs11951068
All − MR Egger
All − IVW(MRE)
−0.5
0.0
0.5
1.0
MR effect size for
'Selenium' on 'Junctional adhesion molecule B'

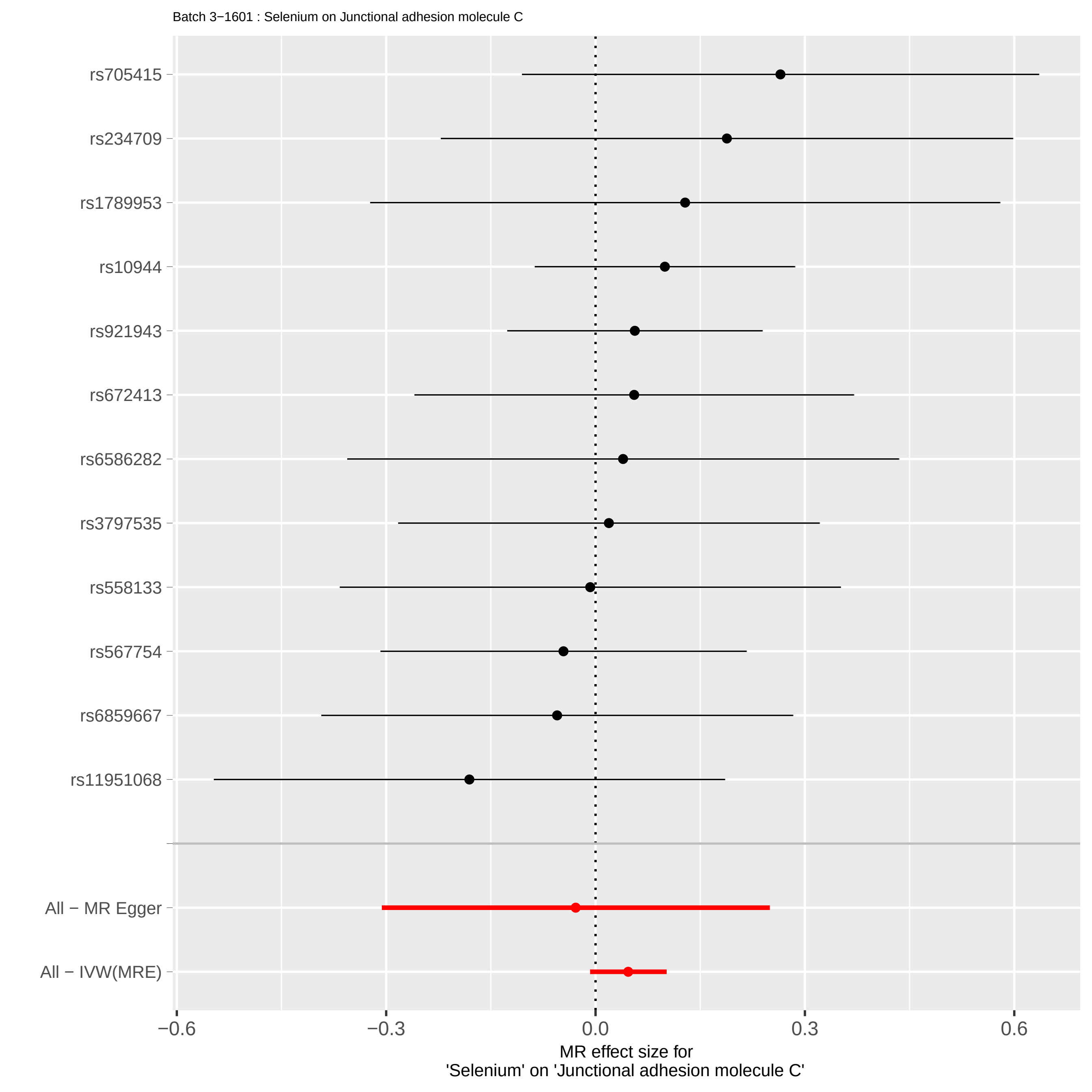
Batch 3−1601 : Selenium on Junctional adhesion molecule C
rs705415
rs234709
rs1789953
rs10944
rs921943
rs672413
rs6586282
rs3797535
rs558133
rs567754
rs6859667
rs11951068
All − MR Egger
All − IVW(MRE)
−0.6
−0.3
0.0
0.3
0.6
MR effect size for
'Selenium' on 'Junctional adhesion molecule C'

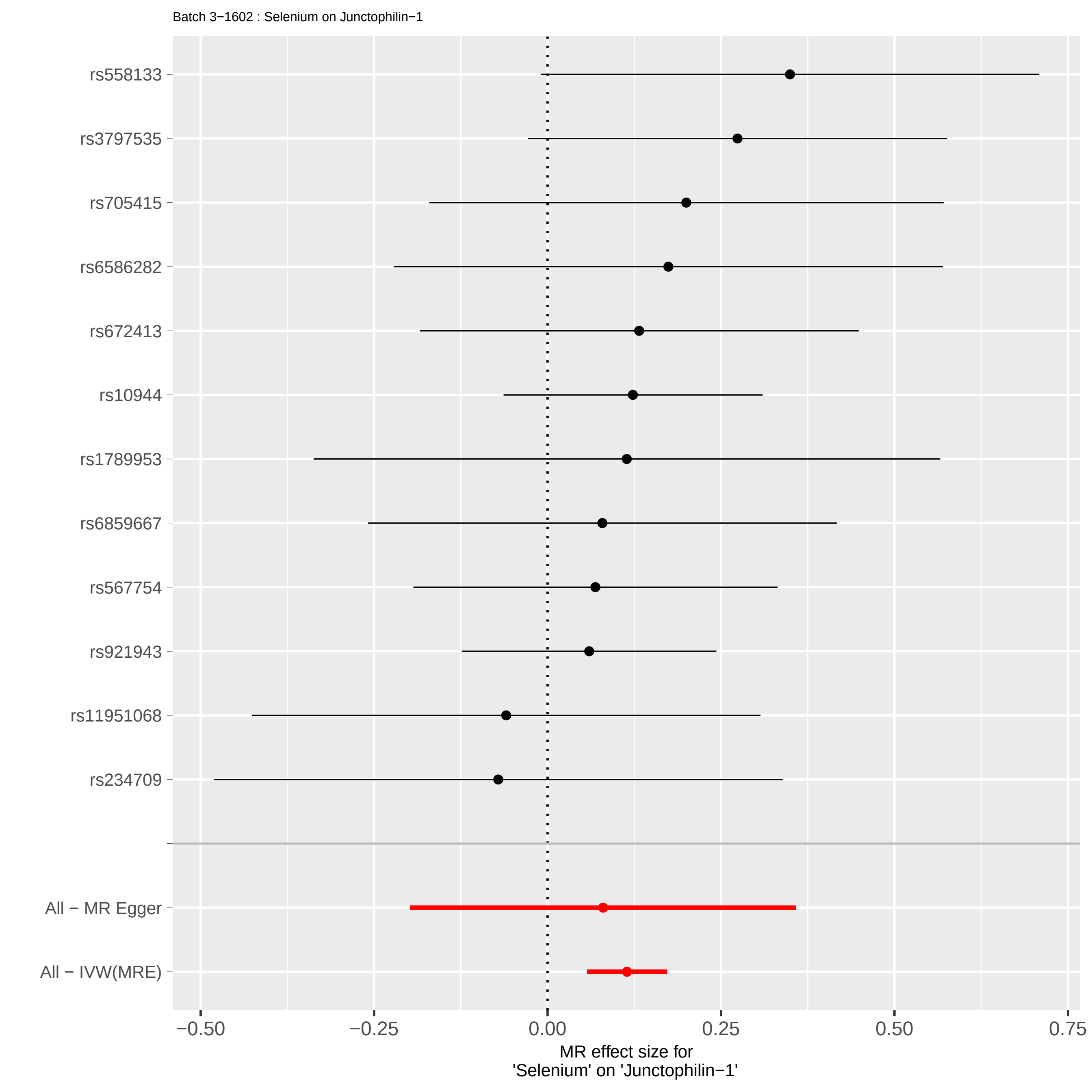

Batch 3−1602 : Selenium on Junctophilin−1
rs558133
rs3797535
rs705415
rs6586282
rs672413
rs10944
rs1789953
rs6859667
rs567754
rs921943
rs11951068
rs234709
All − MR Egger
All − IVW(MRE)
−0.50
−0.25
0.00
0.25
0.50
0.75
MR effect size for
'Selenium' on 'Junctophilin−1'

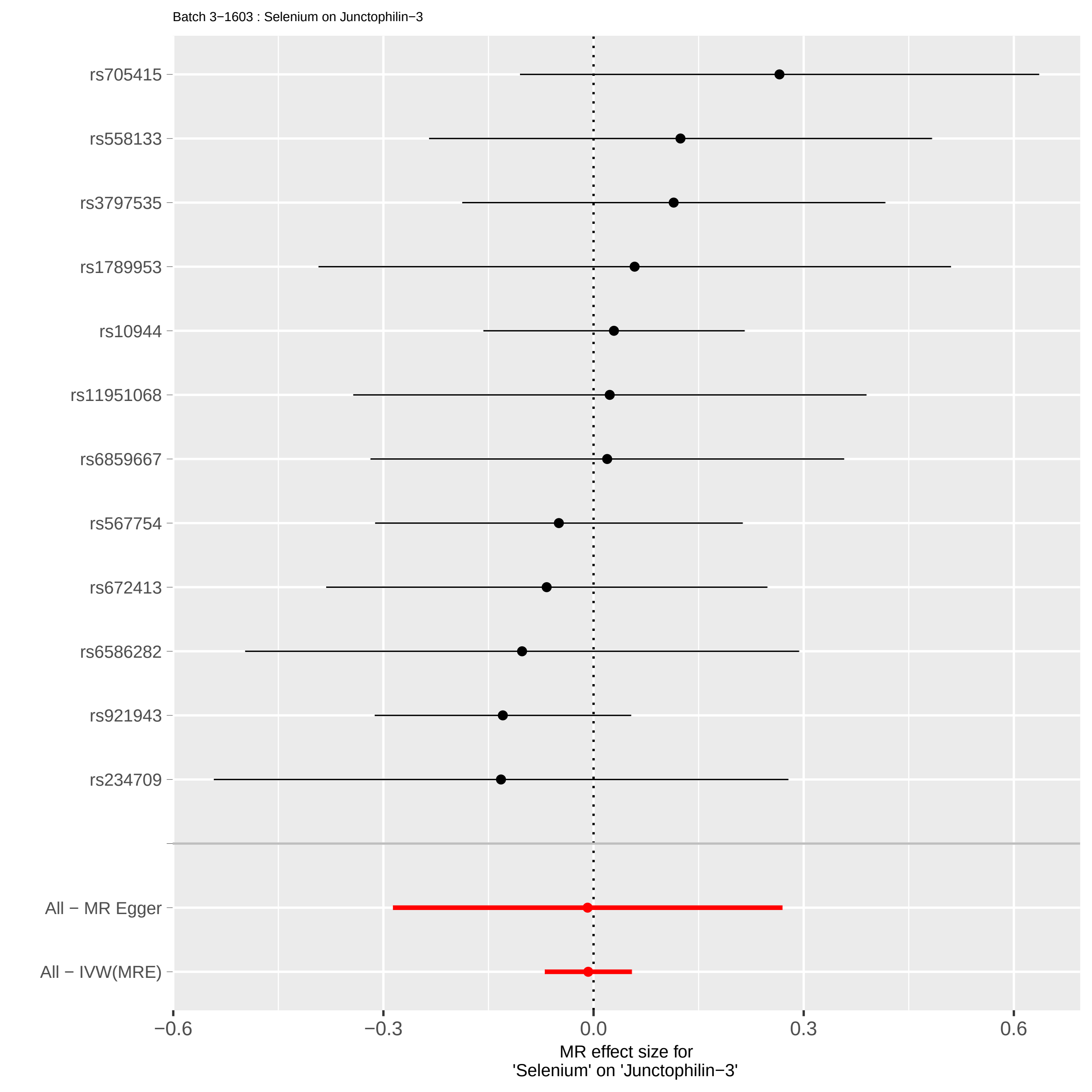

Batch 3−1603 : Selenium on Junctophilin−3
rs705415
rs558133
rs3797535
rs1789953
rs10944
rs11951068
rs6859667
rs567754
rs672413
rs6586282
rs921943
rs234709
All − MR Egger
All − IVW(MRE)
−0.6
−0.3
0.0
0.3
0.6
MR effect size for
'Selenium' on 'Junctophilin−3'

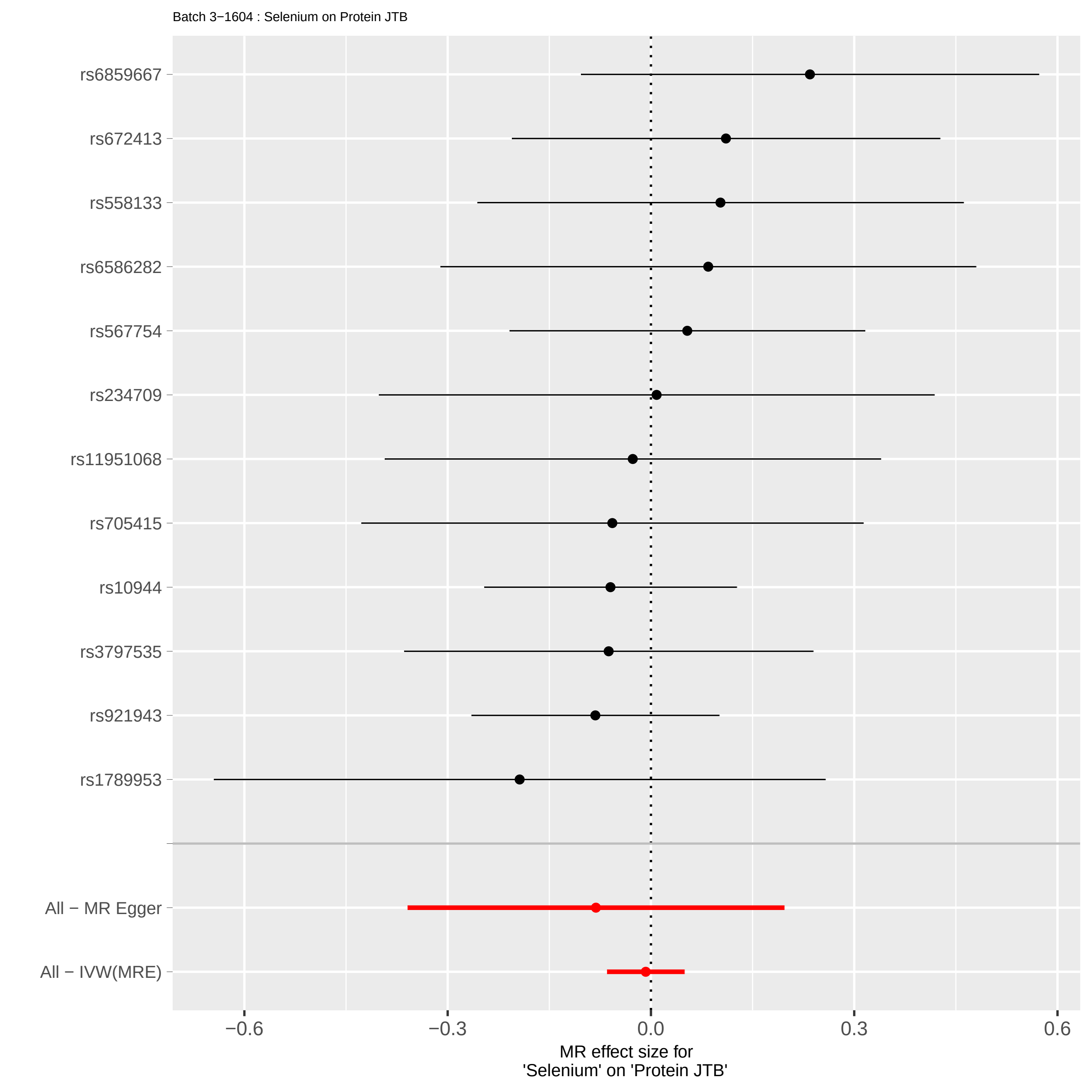

Batch 3−1604 : Selenium on Protein JTB
rs6859667
rs672413
rs558133
rs6586282
rs567754
rs234709
rs11951068
rs705415
rs10944
rs3797535
rs921943
rs1789953
All − MR Egger
All − IVW(MRE)
−0.6
−0.3
0.0
0.3
0.6
MR effect size for
'Selenium' on 'Protein JTB'

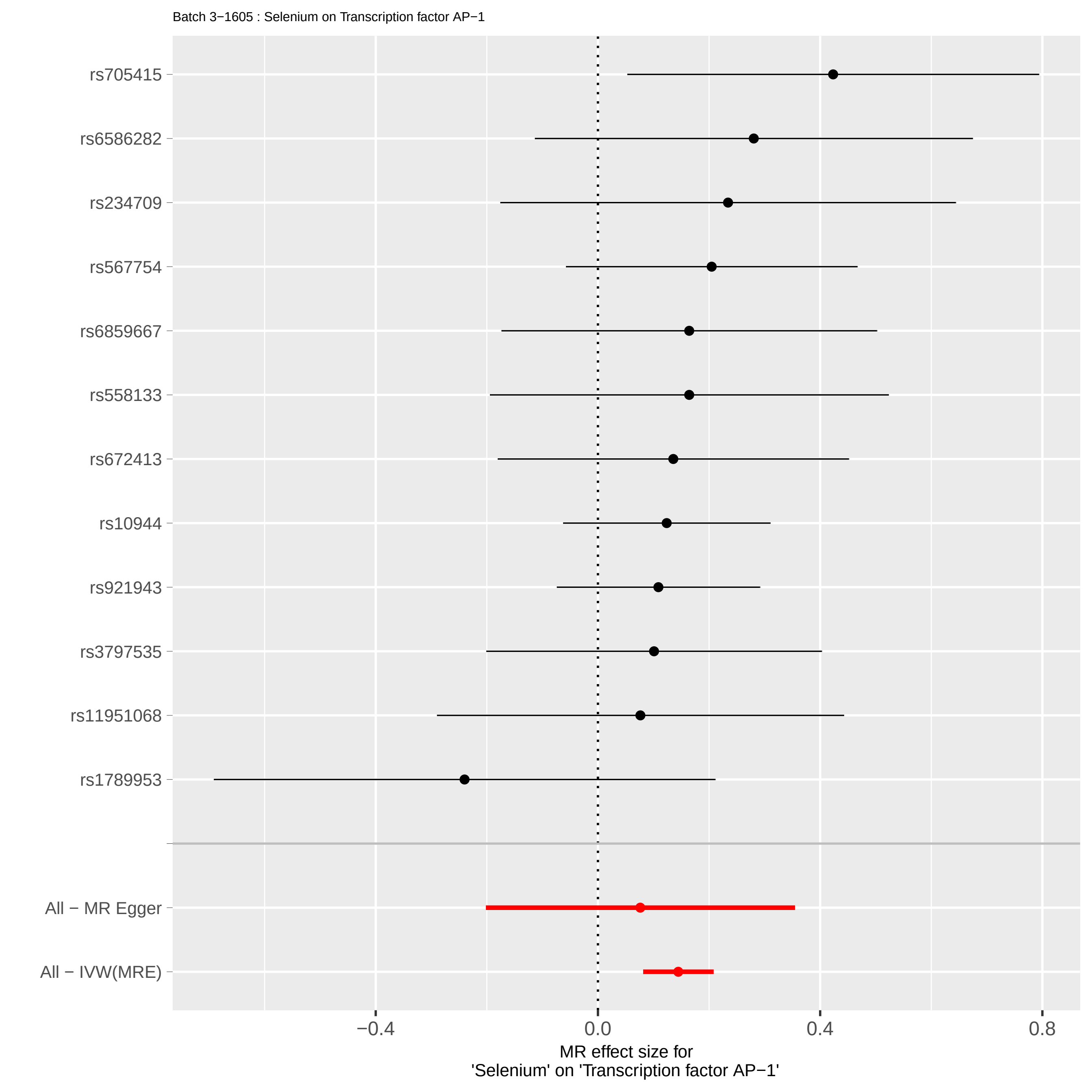
Batch 3−1605 : Selenium on Transcription factor AP−1
rs705415
rs6586282
rs234709
rs567754
rs6859667
rs558133
rs672413
rs10944
rs921943
rs3797535
rs11951068
rs1789953
All − MR Egger
All − IVW(MRE)
−0.4
0.0
0.4
0.8
MR effect size for
'Selenium' on 'Transcription factor AP−1'

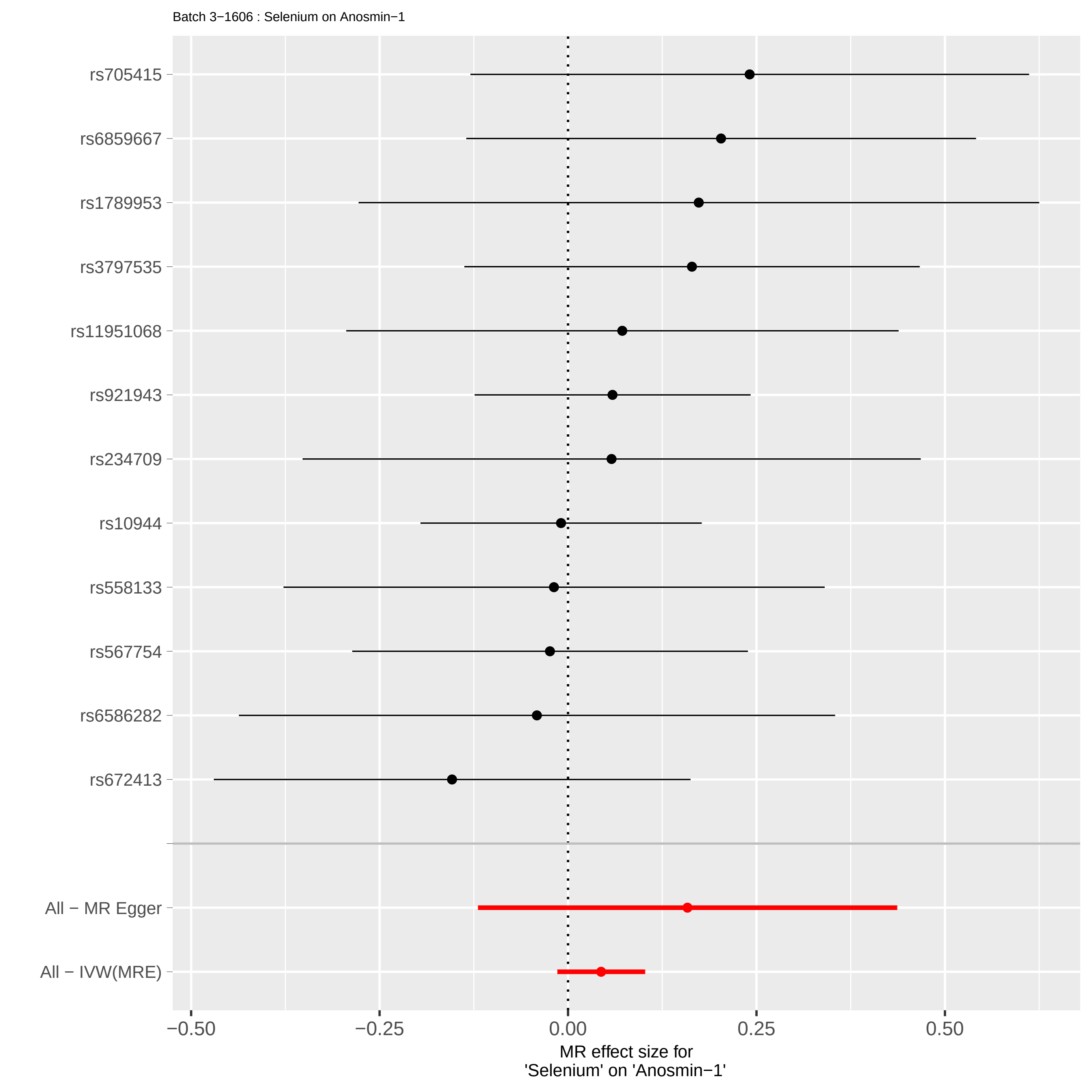

Batch 3−1606 : Selenium on Anosmin−1
rs705415
rs6859667
rs1789953
rs3797535
rs11951068
rs921943
rs234709
rs10944
rs558133
rs567754
rs6586282
rs672413
All − MR Egger
All − IVW(MRE)
−0.50
−0.25
0.00
0.25
0.50
MR effect size for
'Selenium' on 'Anosmin−1'

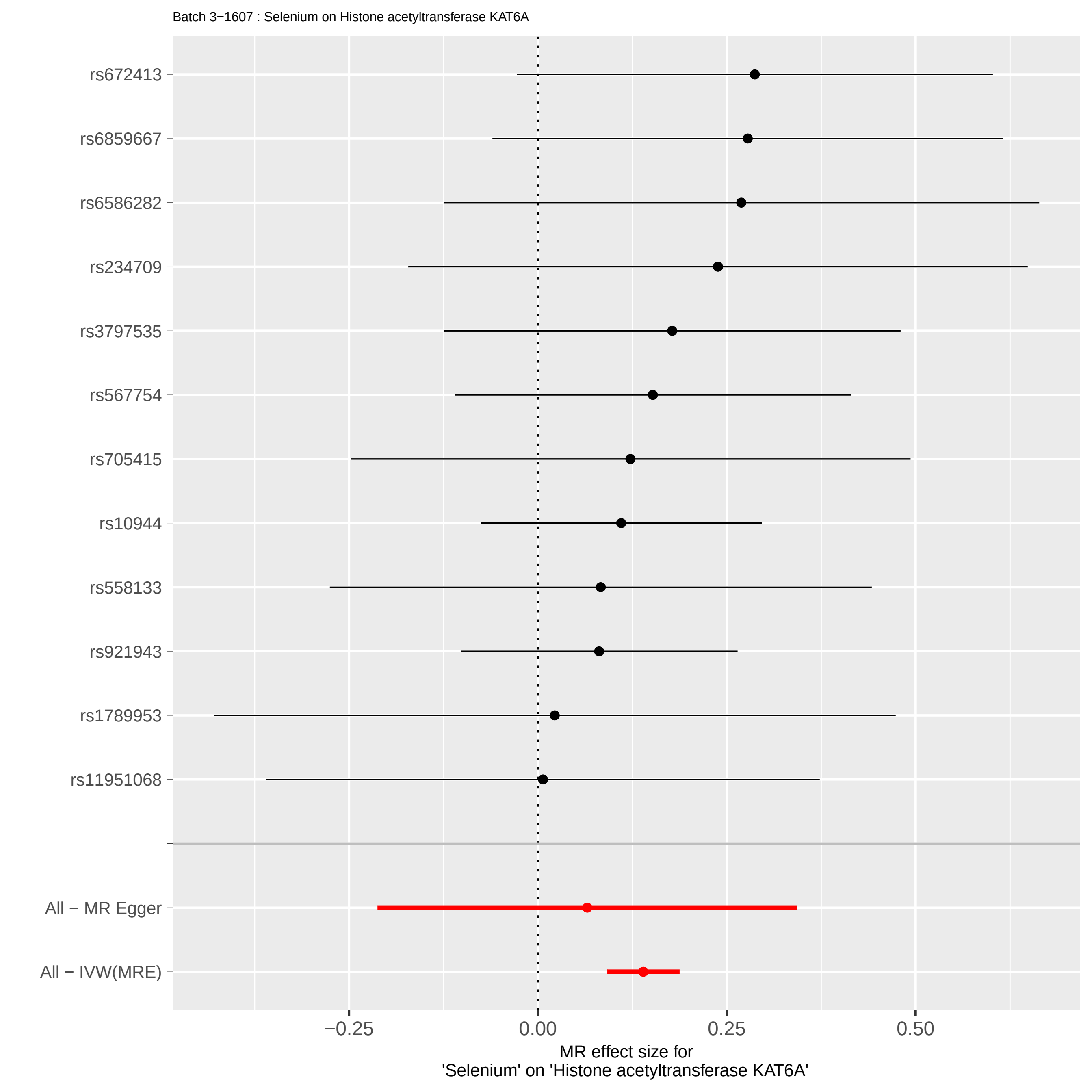

Batch 3−1607 : Selenium on Histone acetyltransferase KAT6A
rs672413
rs6859667
rs6586282
rs234709
rs3797535
rs567754
rs705415
rs10944
rs558133
rs921943
rs1789953
rs11951068
All − MR Egger
All − IVW(MRE)
−0.25
0.00
0.25
0.50
MR effect size for
'Selenium' on 'Histone acetyltransferase KAT6A'

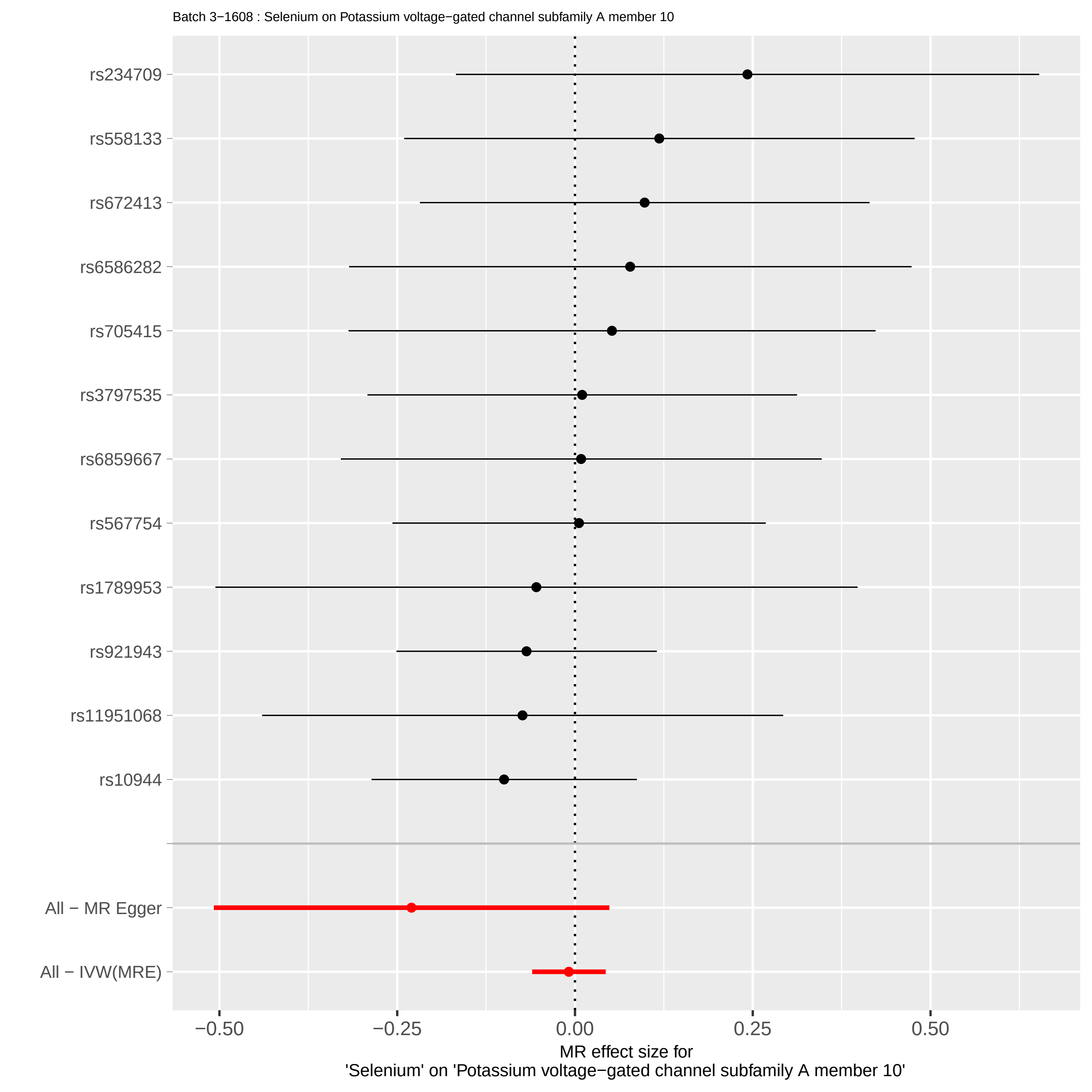

Batch 3−1608 : Selenium on Potassium voltage−gated channel subfamily A member 10
rs234709
rs558133
rs672413
rs6586282
rs705415
rs3797535
rs6859667
rs567754
rs1789953
rs921943
rs11951068
rs10944
All − MR Egger
All − IVW(MRE)
−0.50
−0.25
0.00
0.25
0.50
MR effect size for
'Selenium' on 'Potassium voltage−gated channel subfamily A member 10'

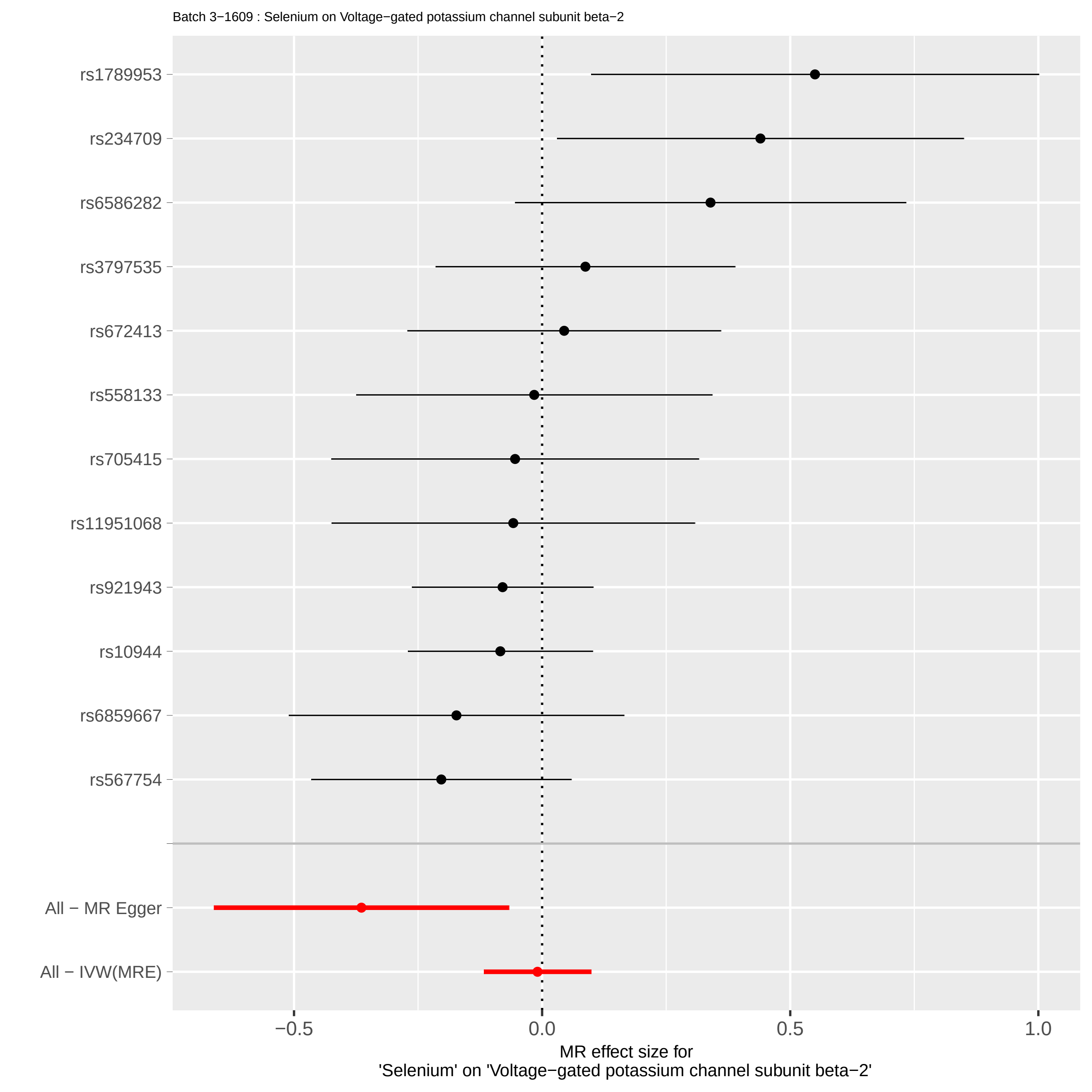

Batch 3−1609 : Selenium on Voltage−gated potassium channel subunit beta−2
rs1789953
rs234709
rs6586282
rs3797535
rs672413
rs558133
rs705415
rs11951068
rs921943
rs10944
rs6859667
rs567754
All − MR Egger
All − IVW(MRE)
−0.5
0.0
0.5
1.0
MR effect size for
'Selenium' on 'Voltage−gated potassium channel subunit beta−2'

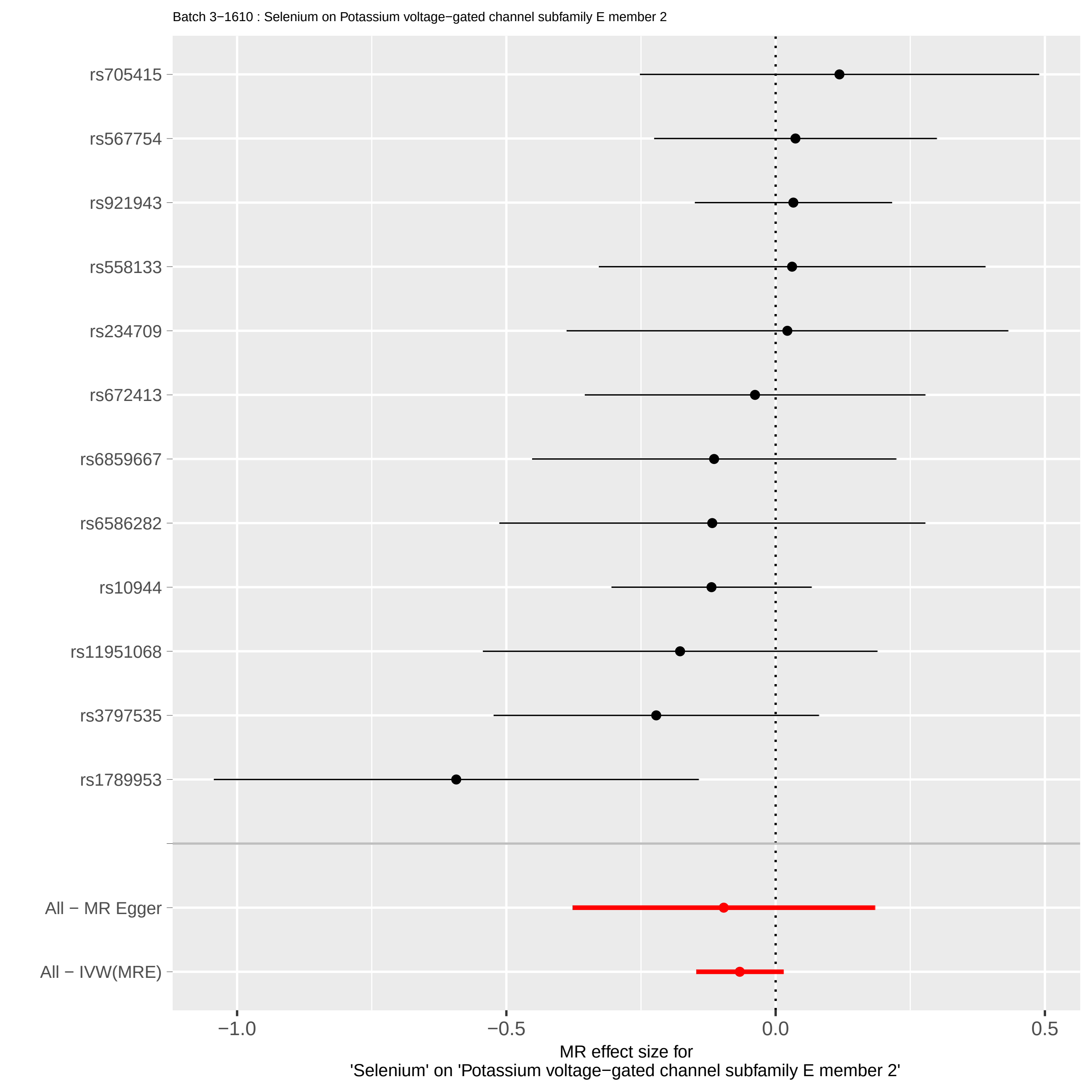

Batch 3−1610 : Selenium on Potassium voltage−gated channel subfamily E member 2
rs705415
rs567754
rs921943
rs558133
rs234709
rs672413
rs6859667
rs6586282
rs10944
rs11951068
rs3797535
rs1789953
All − MR Egger
All − IVW(MRE)
−1.0
−0.5
0.0
0.5
MR effect size for
'Selenium' on 'Potassium voltage−gated channel subfamily E member 2'

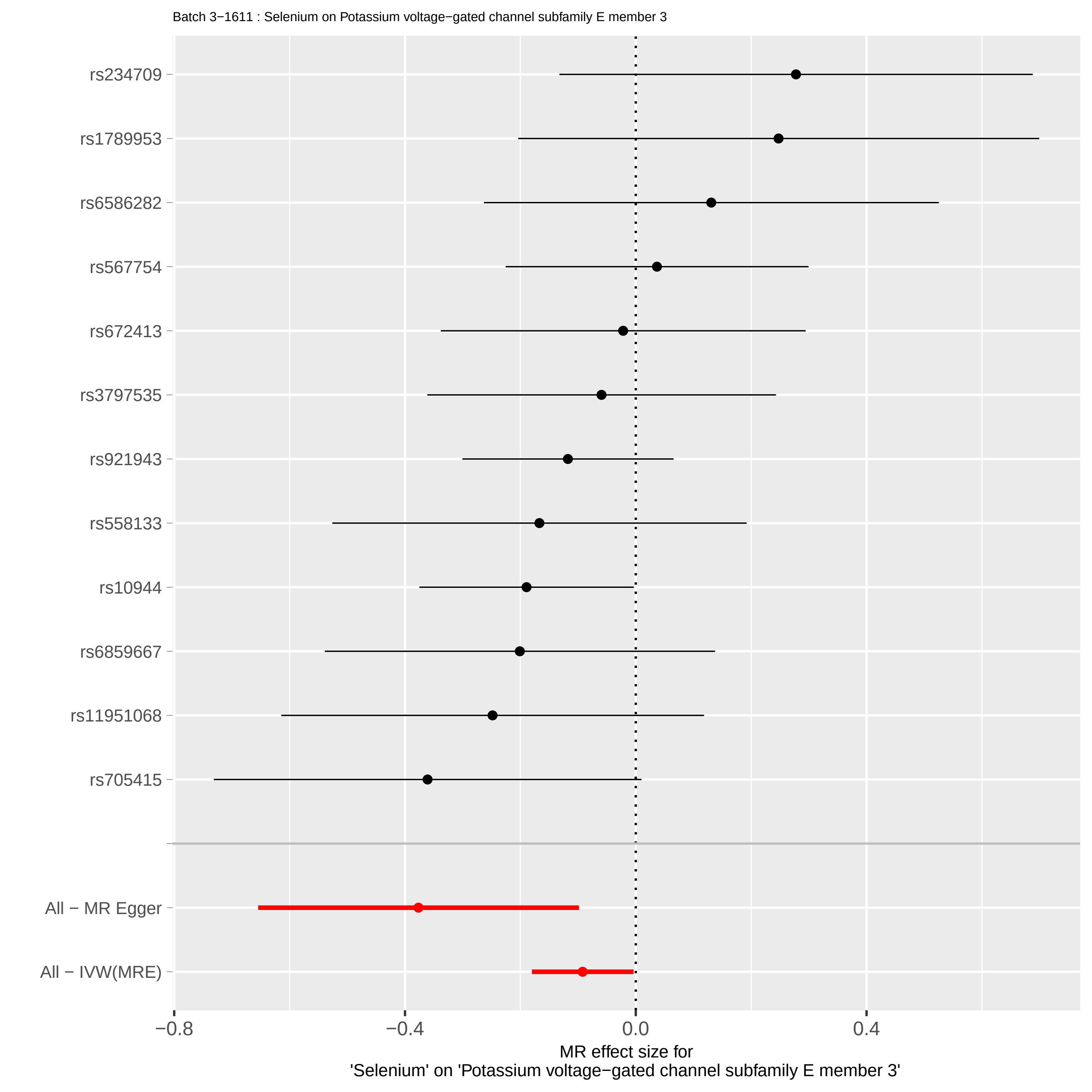

Batch 3−1611 : Selenium on Potassium voltage−gated channel subfamily E member 3
rs234709
rs1789953
rs6586282
rs567754
rs672413
rs3797535
rs921943
rs558133
rs10944
rs6859667
rs11951068
rs705415
All − MR Egger
All − IVW(MRE)
−0.8
−0.4
0.0
0.4
MR effect size for
'Selenium' on 'Potassium voltage−gated channel subfamily E member 3'

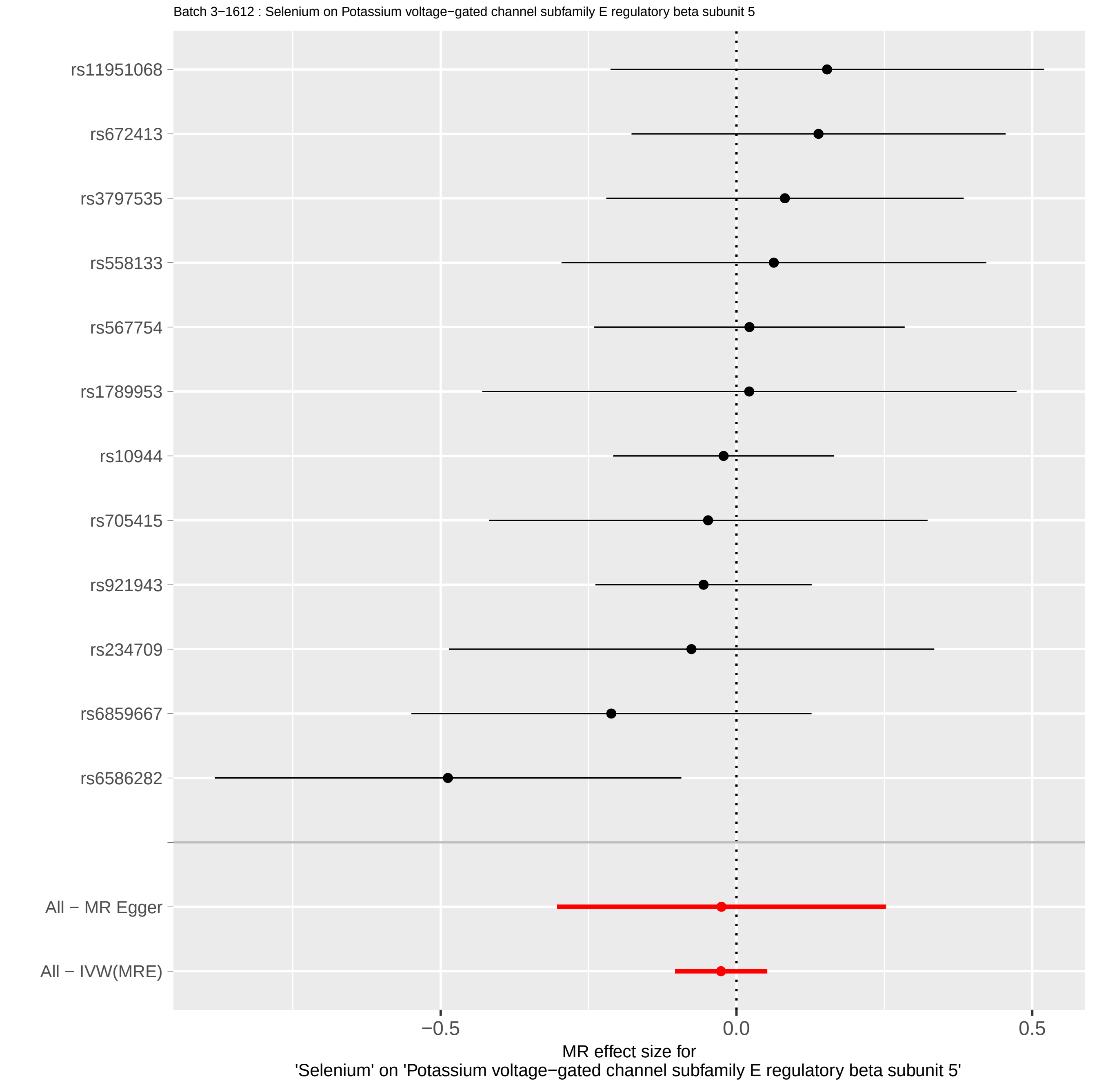

Batch 3−1612 : Selenium on Potassium voltage−gated channel subfamily E regulatory beta subunit 5
rs11951068
rs672413
rs3797535
rs558133
rs567754
rs1789953
rs10944
rs705415
rs921943
rs234709
rs6859667
rs6586282
All − MR Egger
All − IVW(MRE)
−0.5
0.0
0.5
MR effect size for
'Selenium' on 'Potassium voltage−gated channel subfamily E regulatory beta subunit 5'

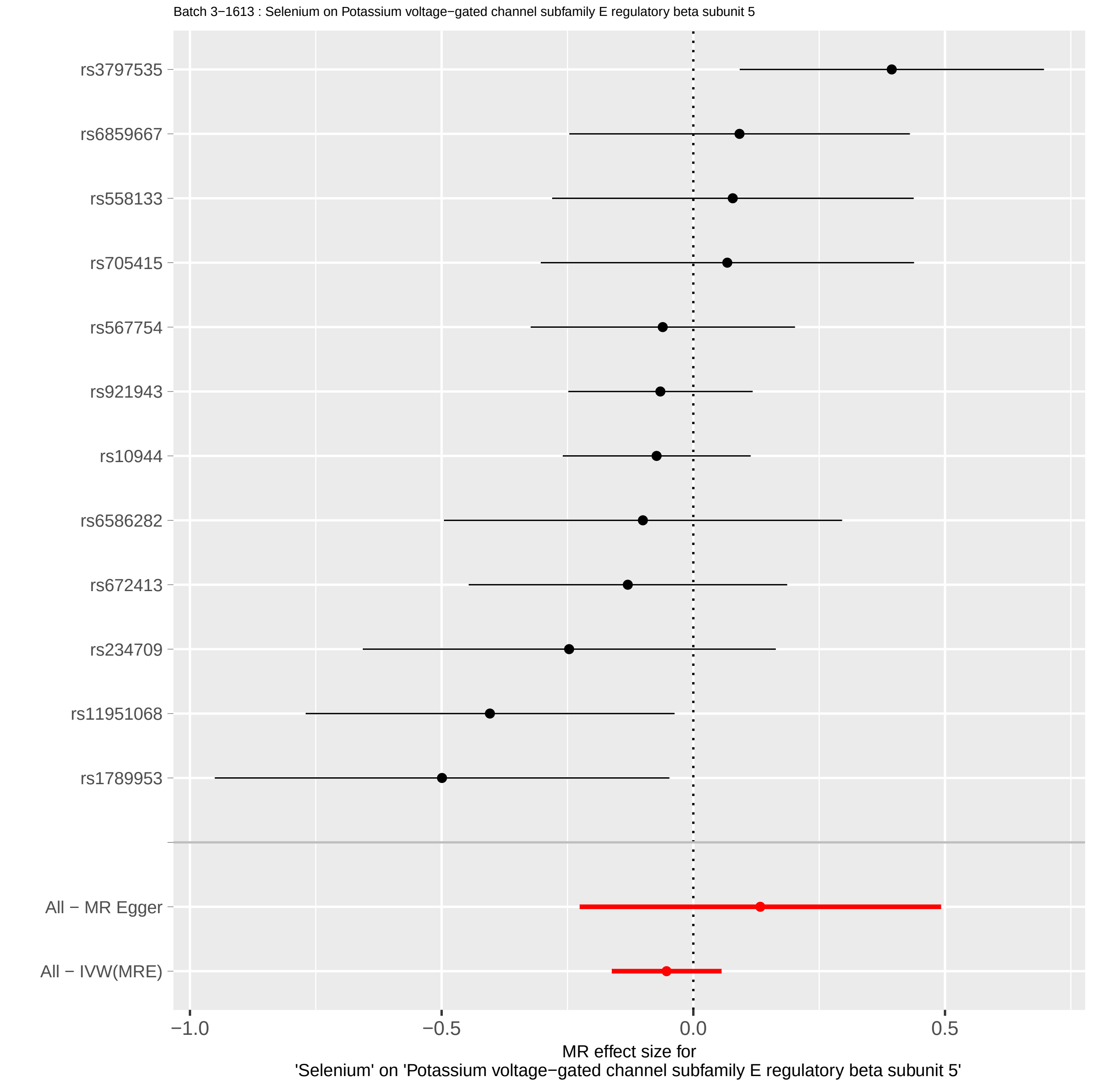

Batch 3−1613 : Selenium on Potassium voltage−gated channel subfamily E regulatory beta subunit 5
rs3797535
rs6859667
rs558133
rs705415
rs567754
rs921943
rs10944
rs6586282
rs672413
rs234709
rs11951068
rs1789953
All − MR Egger
All − IVW(MRE)
−1.0
−0.5
0.0
0.5
MR effect size for
'Selenium' on 'Potassium voltage−gated channel subfamily E regulatory beta subunit 5'

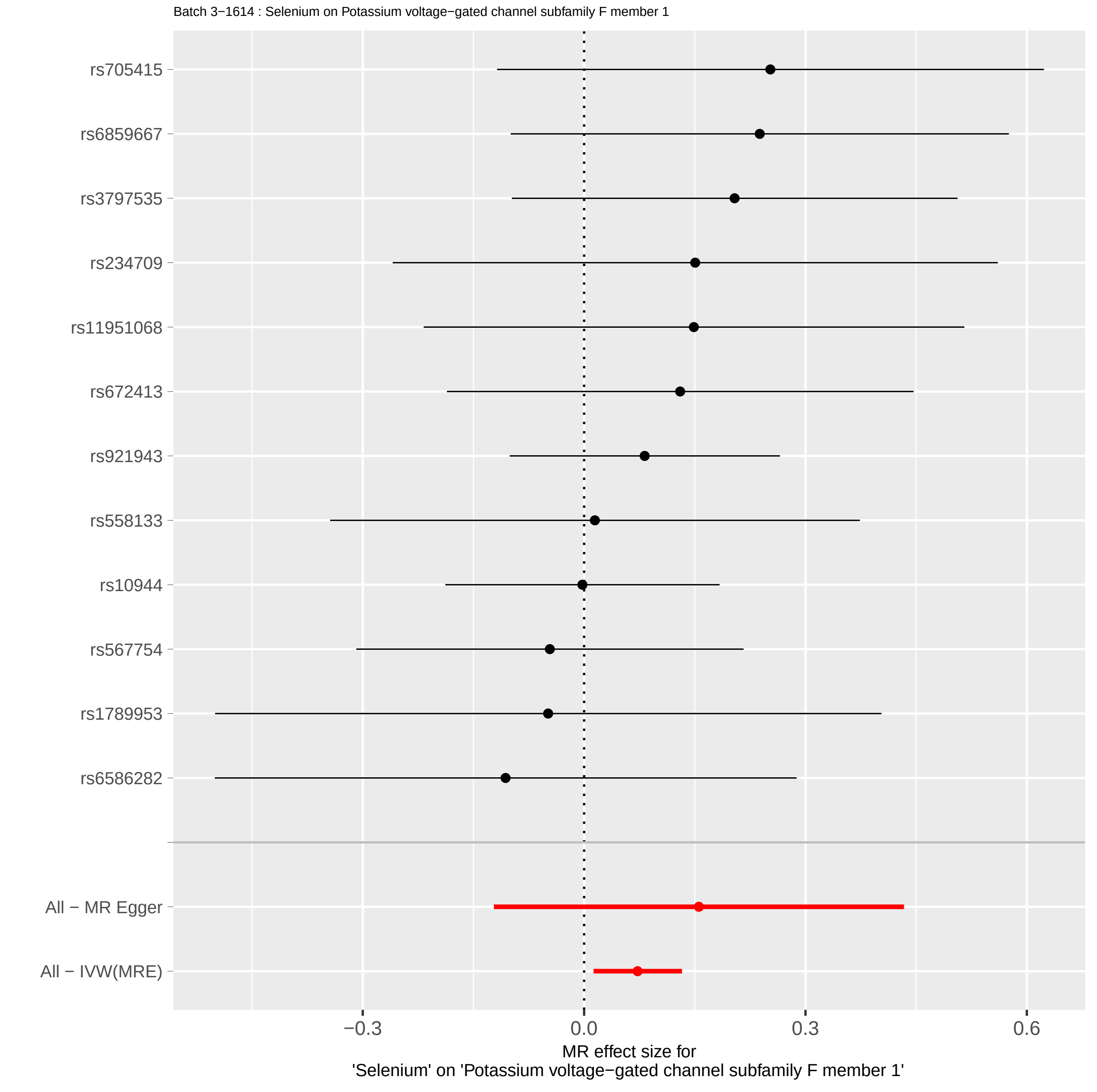

Batch 3−1614 : Selenium on Potassium voltage−gated channel subfamily F member 1
rs705415
rs6859667
rs3797535
rs234709
rs11951068
rs672413
rs921943
rs558133
rs10944
rs567754
rs1789953
rs6586282
All − MR Egger
All − IVW(MRE)
−0.3
0.0
0.3
0.6
MR effect size for
'Selenium' on 'Potassium voltage−gated channel subfamily F member 1'

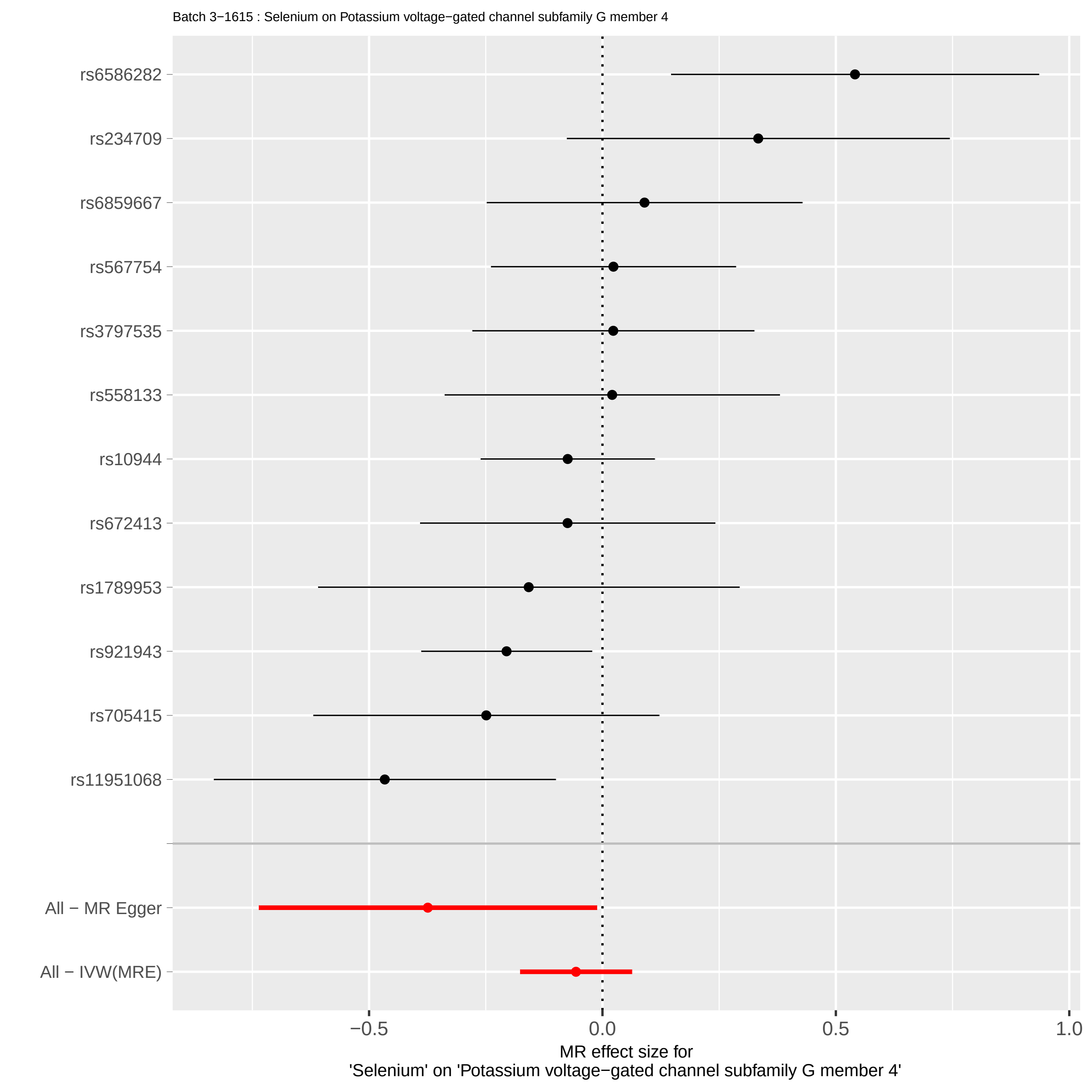
Batch 3−1615 : Selenium on Potassium voltage−gated channel subfamily G member 4
rs6586282
rs234709
rs6859667
rs567754
rs3797535
rs558133
rs10944
rs672413
rs1789953
rs921943
rs705415
rs11951068
All − MR Egger
All − IVW(MRE)
−0.5
0.0
0.5
1.0
MR effect size for
'Selenium' on 'Potassium voltage−gated channel subfamily G member 4'

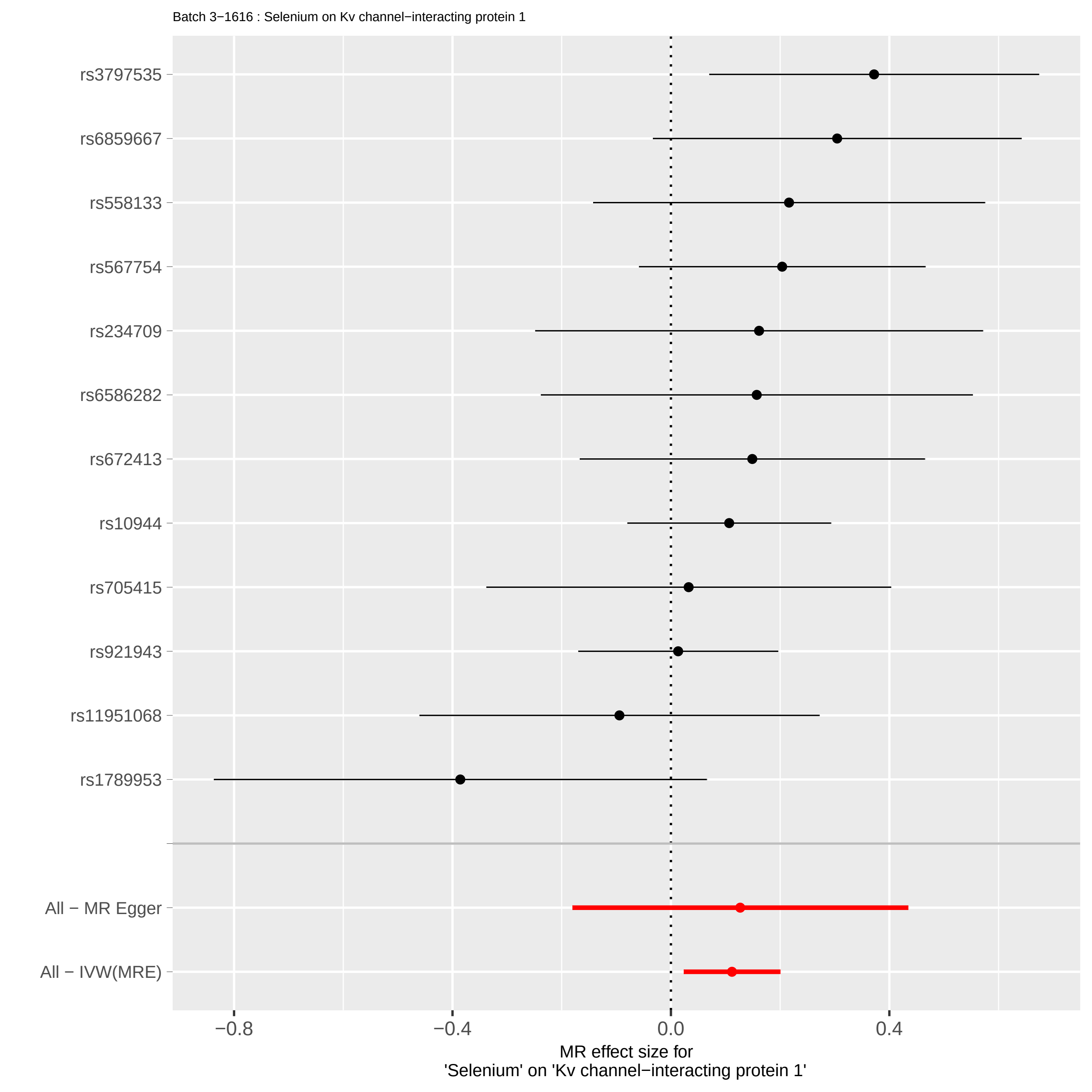

Batch 3−1616 : Selenium on Kv channel−interacting protein 1
rs3797535
rs6859667
rs558133
rs567754
rs234709
rs6586282
rs672413
rs10944
rs705415
rs921943
rs11951068
rs1789953
All − MR Egger
All − IVW(MRE)
−0.8
−0.4
0.0
0.4
MR effect size for
'Selenium' on 'Kv channel−interacting protein 1'

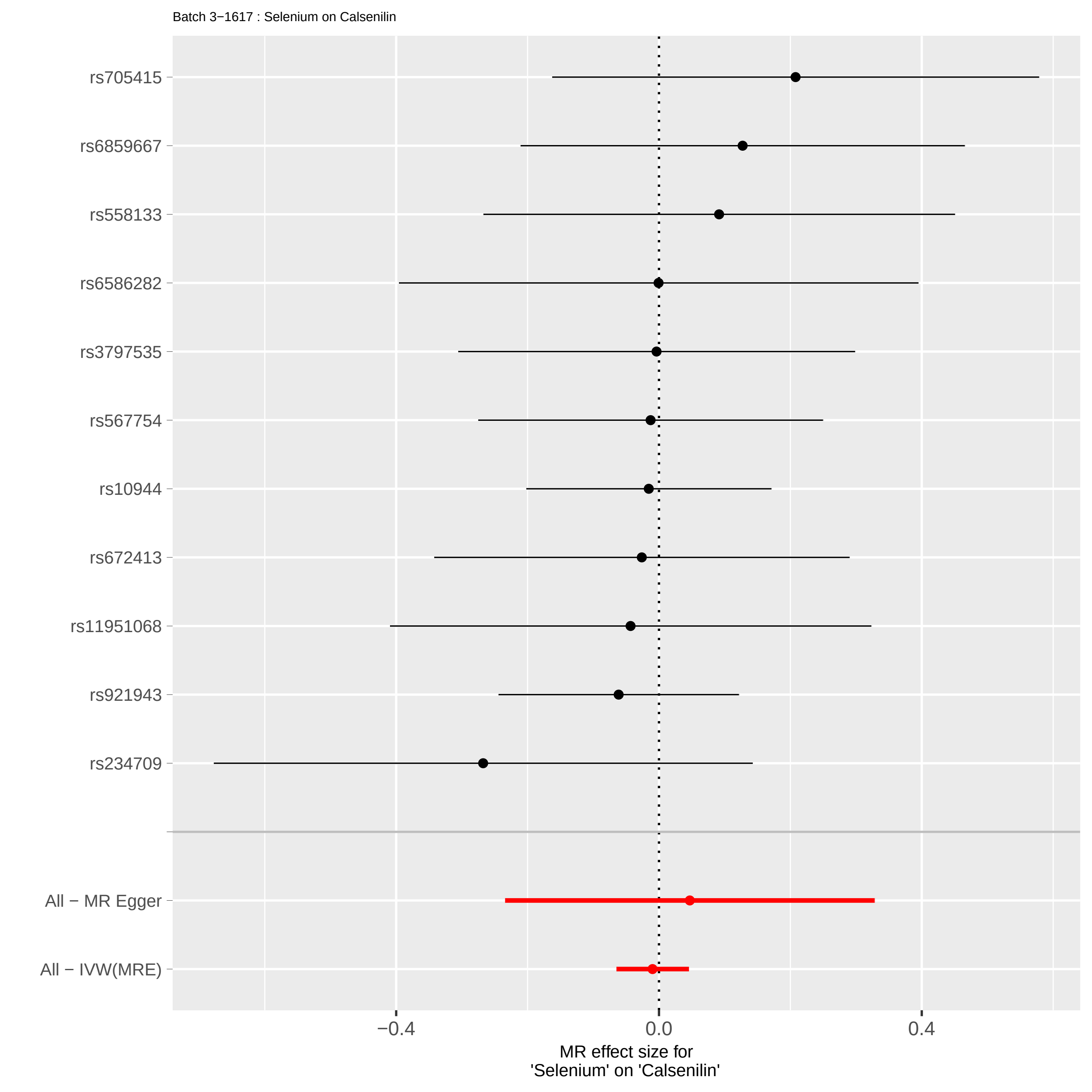

Batch 3−1617 : Selenium on Calsenilin
rs705415
rs6859667
rs558133
rs6586282
rs3797535
rs567754
rs10944
rs672413
rs11951068
rs921943
rs234709
All − MR Egger
All − IVW(MRE)
−0.4
0.0
0.4
MR effect size for
'Selenium' on 'Calsenilin'

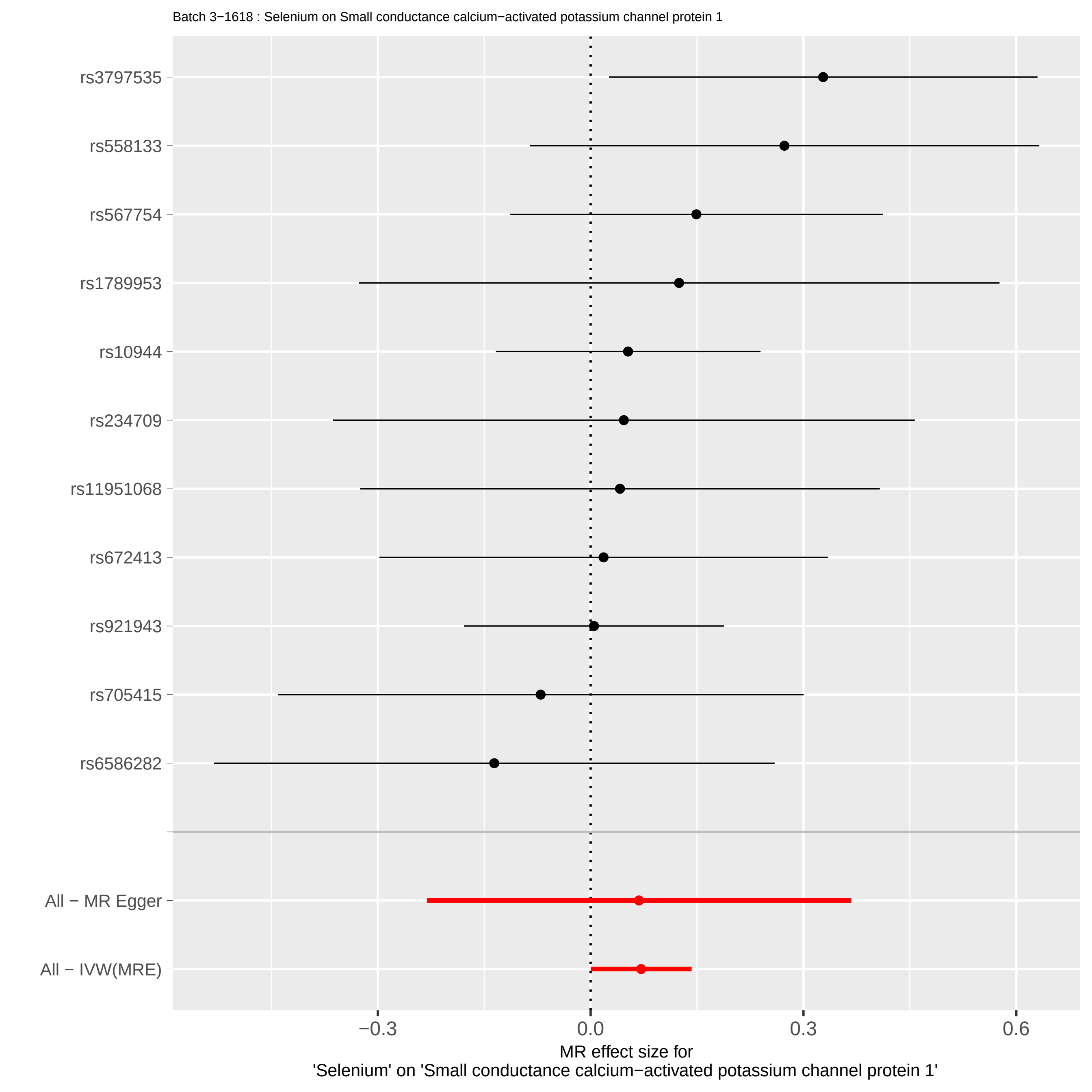

Batch 3−1618 : Selenium on Small conductance calcium−activated potassium channel protein 1
rs3797535
rs558133
rs567754
rs1789953
rs10944
rs234709
rs11951068
rs672413
rs921943
rs705415
rs6586282
All − MR Egger
All − IVW(MRE)
−0.3
0.0
0.3
0.6
MR effect size for
'Selenium' on 'Small conductance calcium−activated potassium channel protein 1'

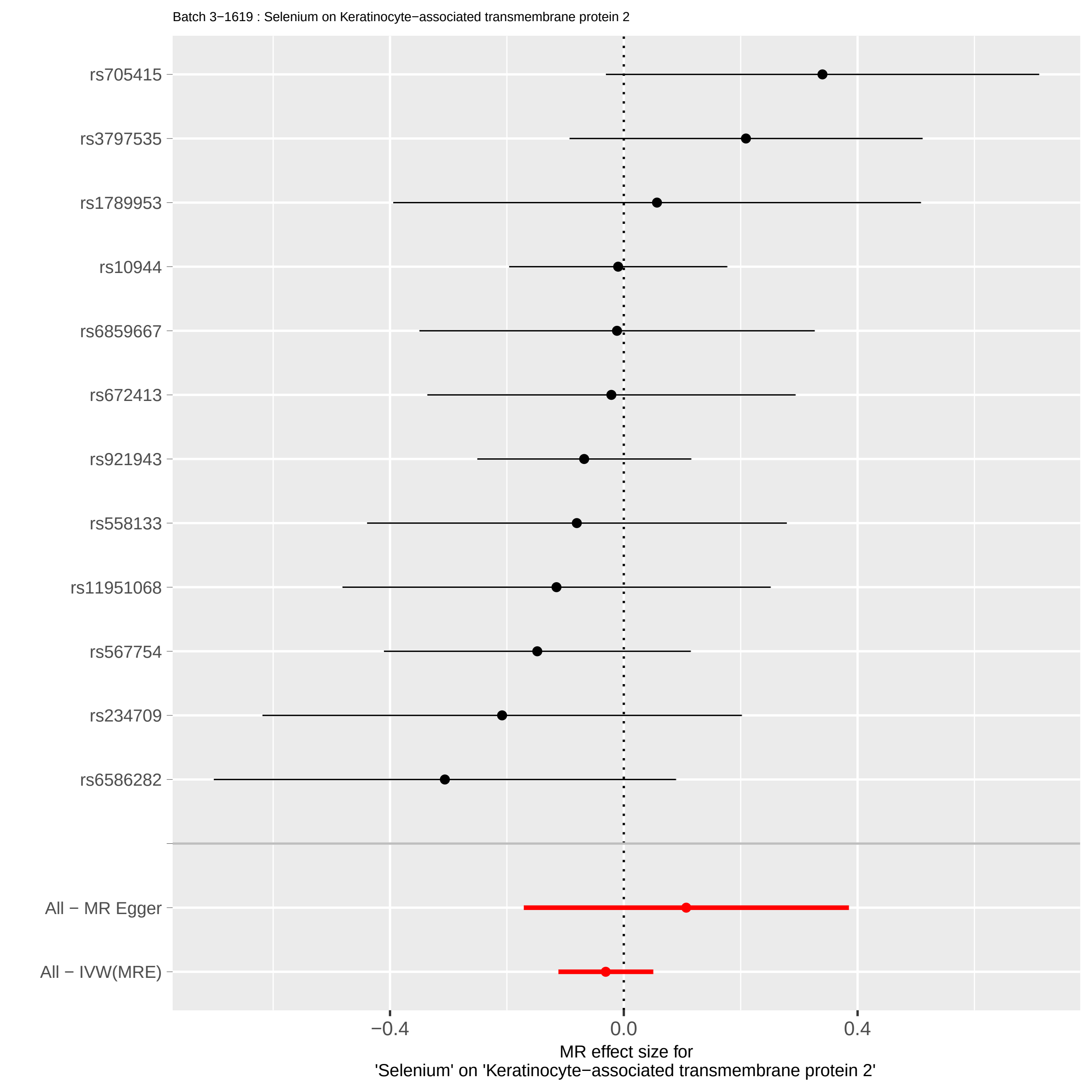

Batch 3−1619 : Selenium on Keratinocyte−associated transmembrane protein 2
rs705415
rs3797535
rs1789953
rs10944
rs6859667
rs672413
rs921943
rs558133
rs11951068
rs567754
rs234709
rs6586282
All − MR Egger
All − IVW(MRE)
−0.4
0.0
0.4
MR effect size for
'Selenium' on 'Keratinocyte−associated transmembrane protein 2'

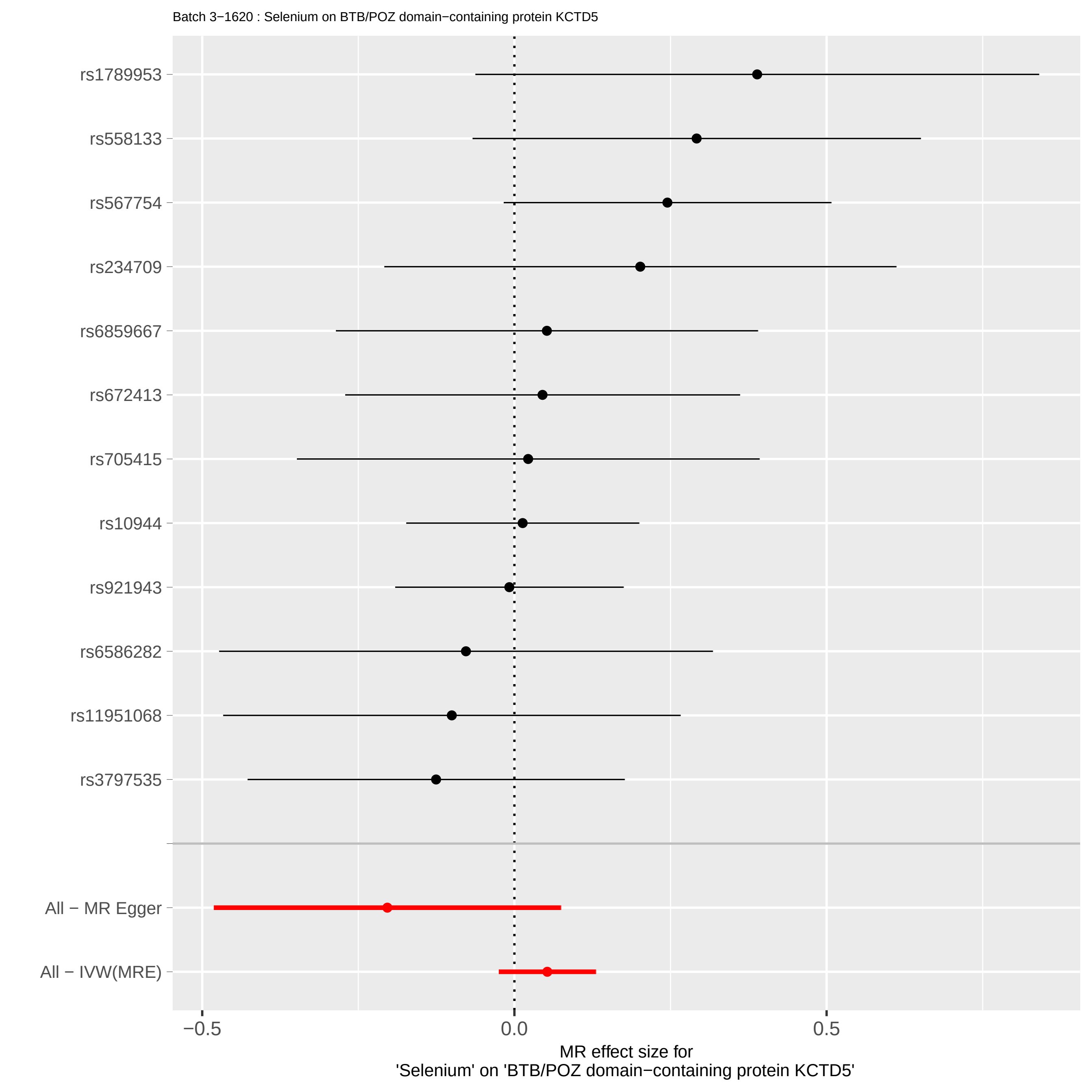

Batch 3−1620 : Selenium on BTB/POZ domain−containing protein KCTD5
rs1789953
rs558133
rs567754
rs234709
rs6859667
rs672413
rs705415
rs10944
rs921943
rs6586282
rs11951068
rs3797535
All − MR Egger
All − IVW(MRE)
−0.5
0.0
0.5
MR effect size for
'Selenium' on 'BTB/POZ domain−containing protein KCTD5'

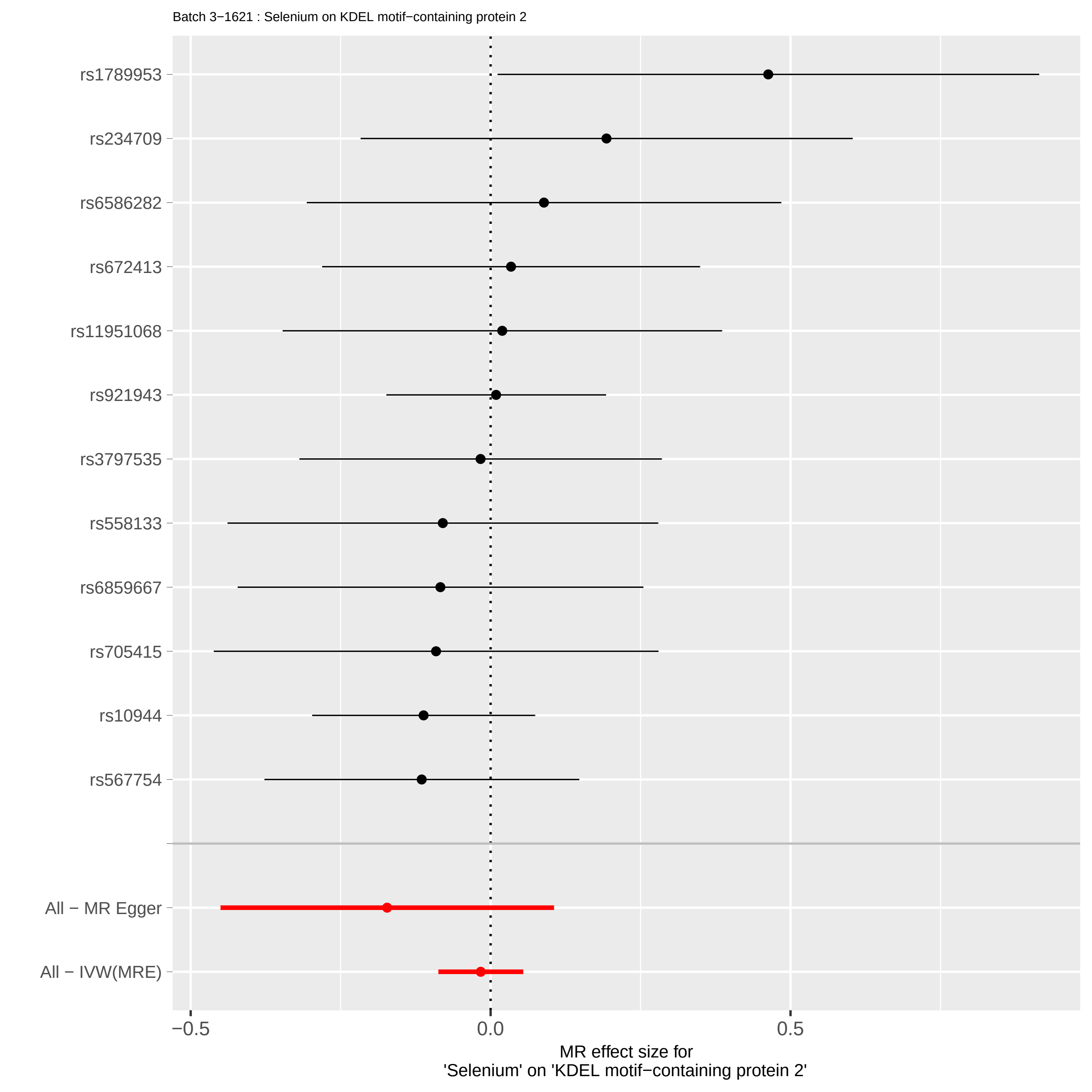

Batch 3−1621 : Selenium on KDEL motif−containing protein 2
rs1789953
rs234709
rs6586282
rs672413
rs11951068
rs921943
rs3797535
rs558133
rs6859667
rs705415
rs10944
rs567754
All − MR Egger
All − IVW(MRE)
−0.5
0.0
0.5
MR effect size for
'Selenium' on 'KDEL motif−containing protein 2'

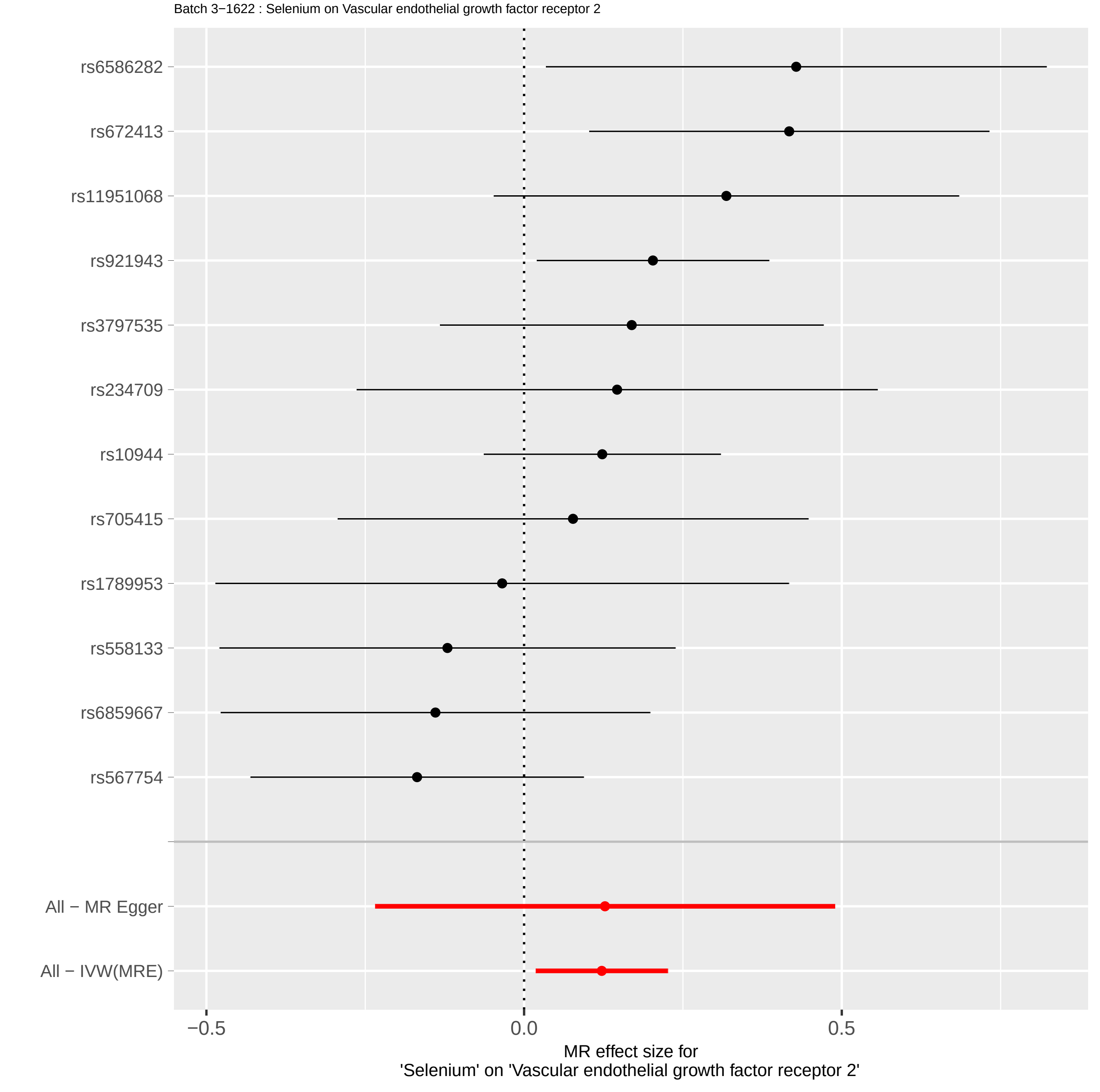

Batch 3−1622 : Selenium on Vascular endothelial growth factor receptor 2
rs6586282
rs672413
rs11951068
rs921943
rs3797535
rs234709
rs10944
rs705415
rs1789953
rs558133
rs6859667
rs567754
All − MR Egger
All − IVW(MRE)
−0.5
0.0
0.5
MR effect size for
'Selenium' on 'Vascular endothelial growth factor receptor 2'

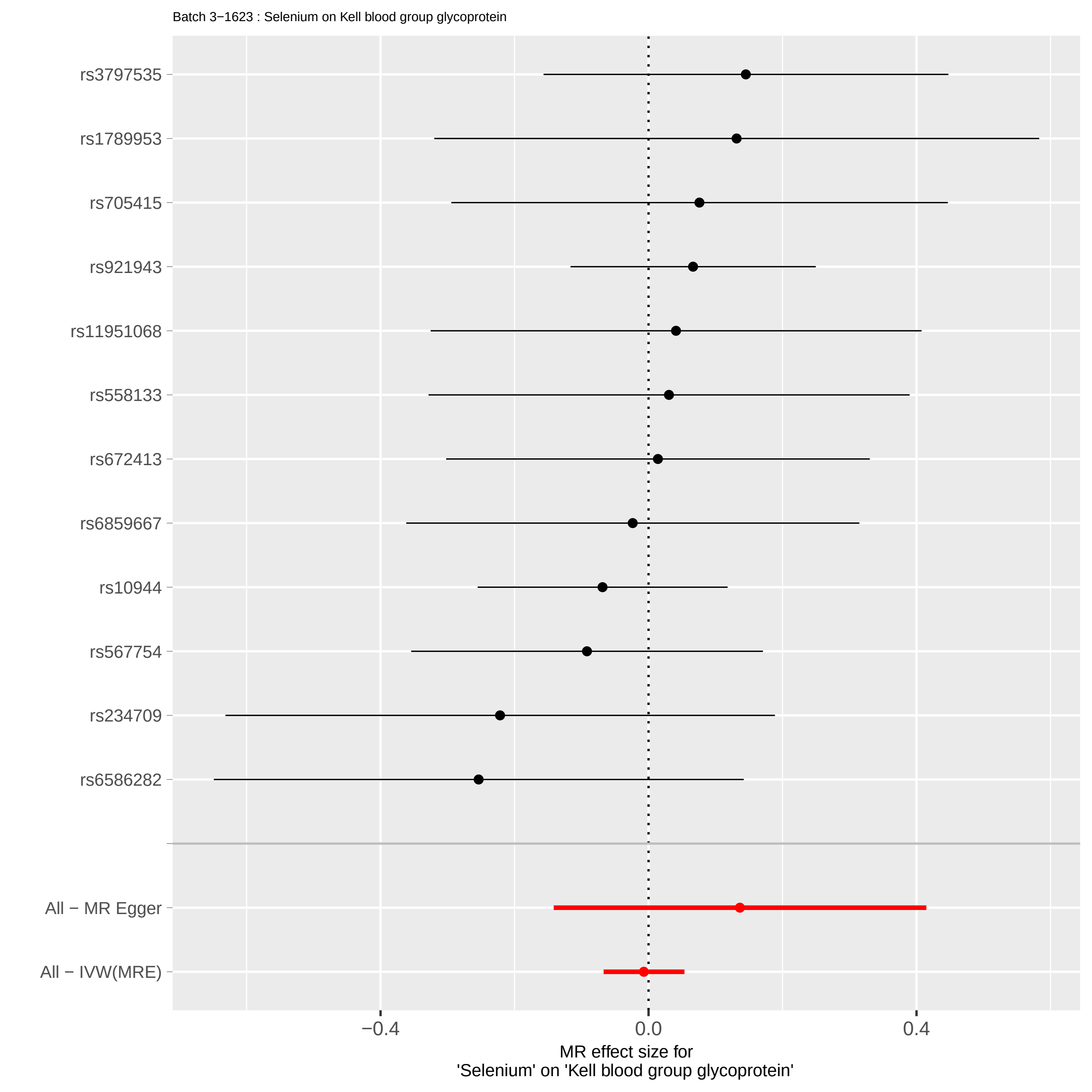

Batch 3−1623 : Selenium on Kell blood group glycoprotein
rs3797535
rs1789953
rs705415
rs921943
rs11951068
rs558133
rs672413
rs6859667
rs10944
rs567754
rs234709
rs6586282
All − MR Egger
All − IVW(MRE)
−0.4
0.0
0.4
MR effect size for
'Selenium' on 'Kell blood group glycoprotein'

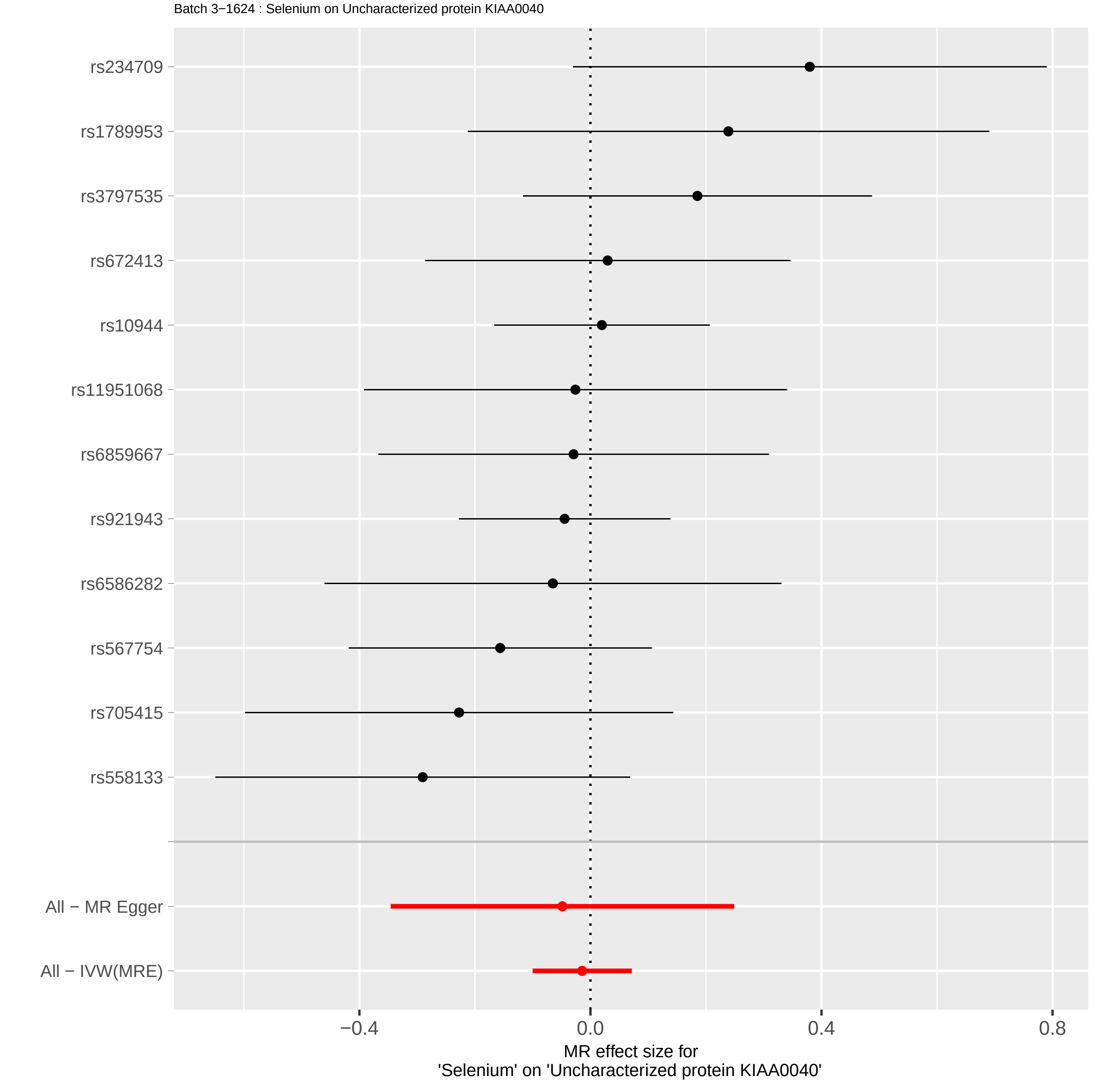

Batch 3−1624 : Selenium on Uncharacterized protein KIAA0040
rs234709
rs1789953
rs3797535
rs672413
rs10944
rs11951068
rs6859667
rs921943
rs6586282
rs567754
rs705415
rs558133
All − MR Egger
All − IVW(MRE)
−0.4
0.0
0.4
0.8
MR effect size for
'Selenium' on 'Uncharacterized protein KIAA0040'

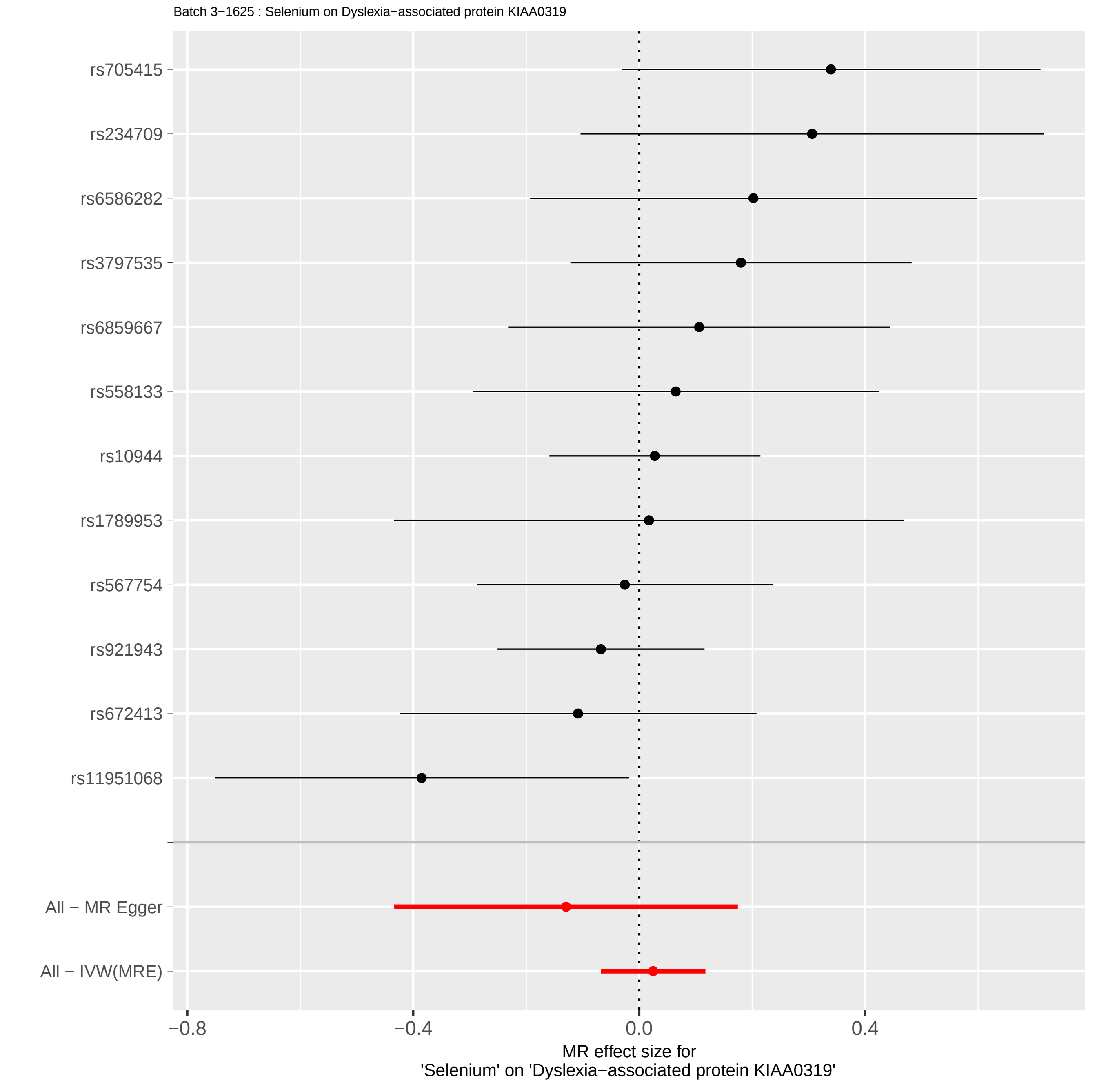
Batch 3−1625 : Selenium on Dyslexia−associated protein KIAA0319
rs705415
rs234709
rs6586282
rs3797535
rs6859667
rs558133
rs10944
rs1789953
rs567754
rs921943
rs672413
rs11951068
All − MR Egger
All − IVW(MRE)
−0.8
−0.4
0.0
0.4
MR effect size for
'Selenium' on 'Dyslexia−associated protein KIAA0319'

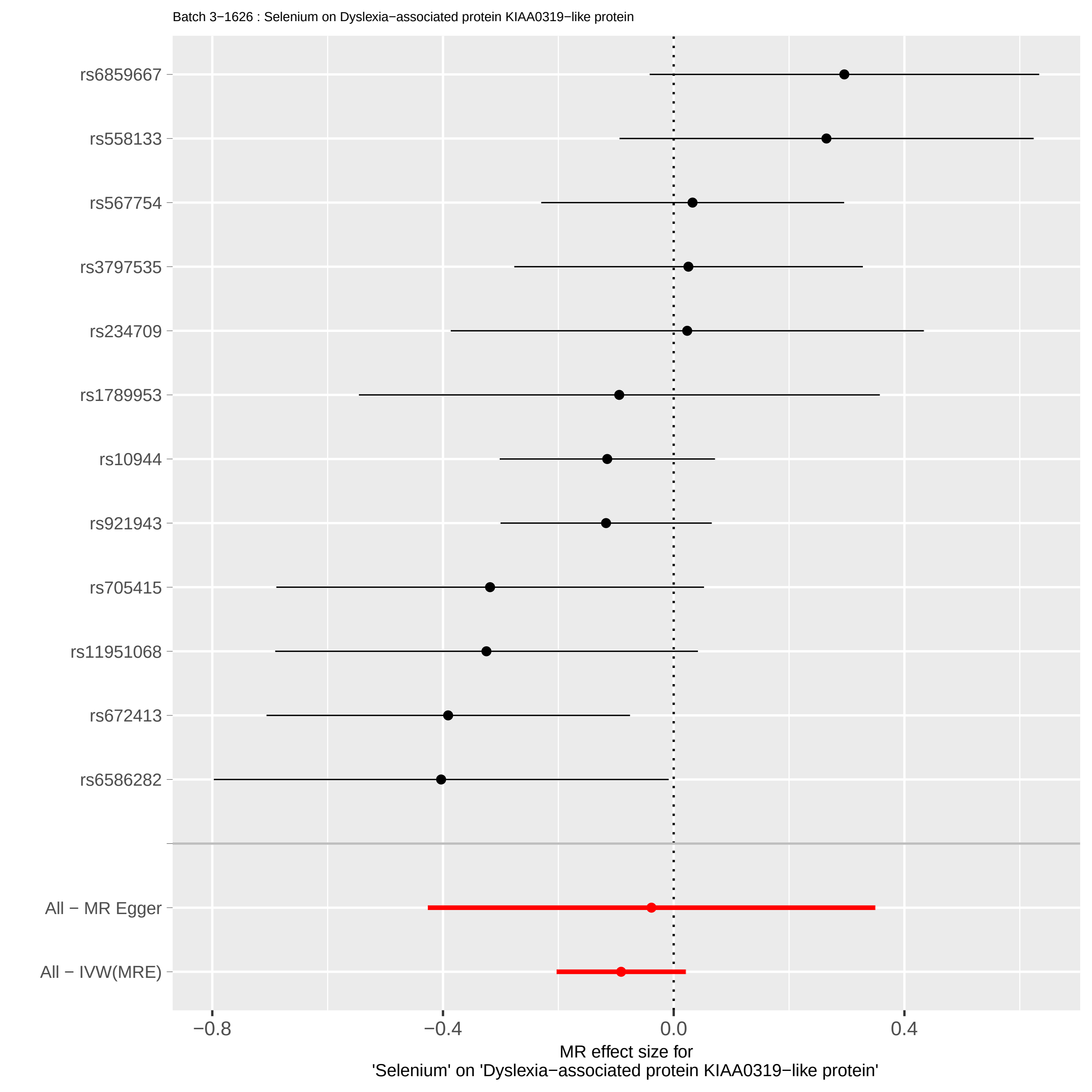
Batch 3−1626 : Selenium on Dyslexia−associated protein KIAA0319−like protein
rs6859667
rs558133
rs567754
rs3797535
rs234709
rs1789953
rs10944
rs921943
rs705415
rs11951068
rs672413
rs6586282
All − MR Egger
All − IVW(MRE)
−0.8
−0.4
0.0
0.4
MR effect size for
'Selenium' on 'Dyslexia−associated protein KIAA0319−like protein'

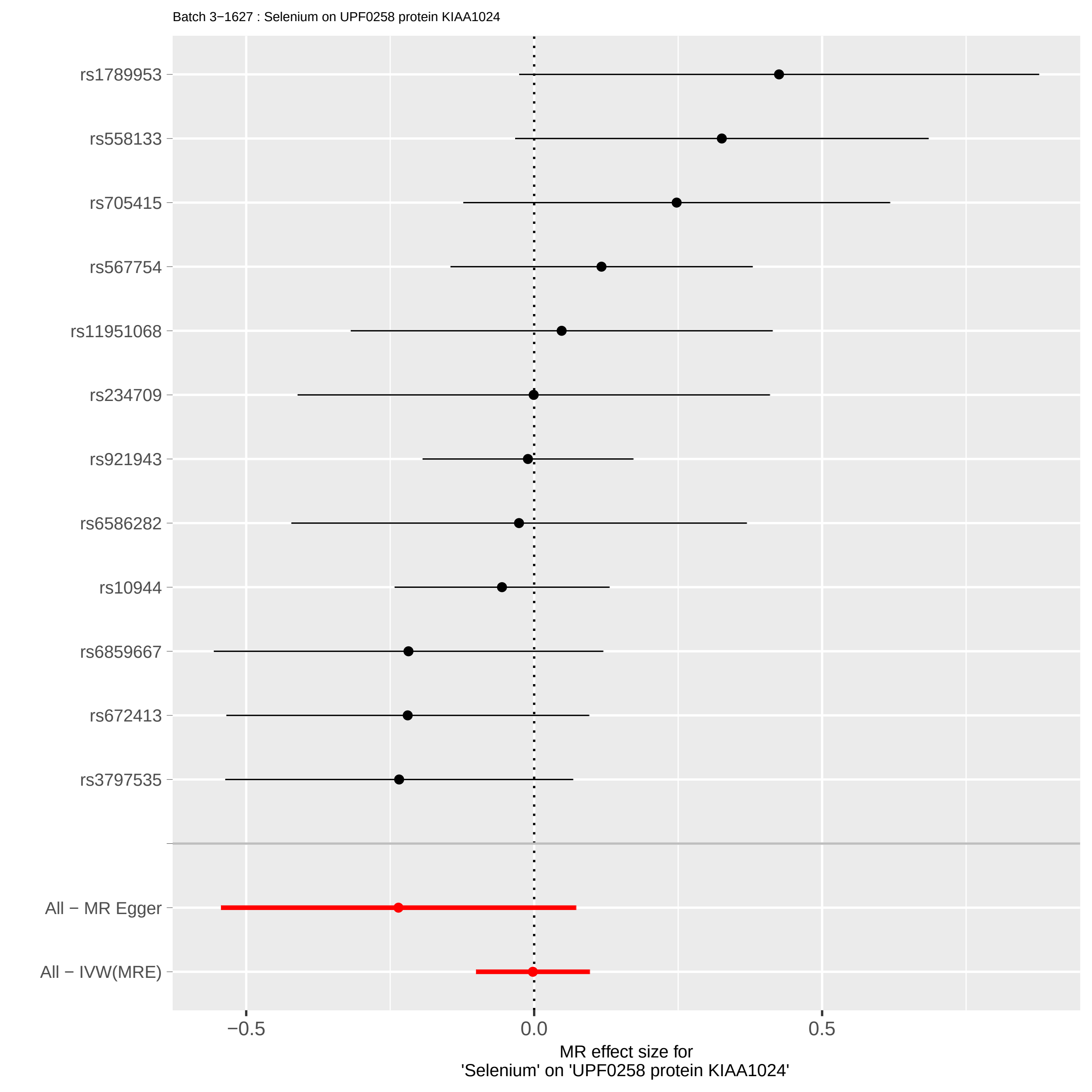

Batch 3−1627 : Selenium on UPF0258 protein KIAA1024
rs1789953
rs558133
rs705415
rs567754
rs11951068
rs234709
rs921943
rs6586282
rs10944
rs6859667
rs672413
rs3797535
All − MR Egger
All − IVW(MRE)
−0.5
0.0
0.5
MR effect size for
'Selenium' on 'UPF0258 protein KIAA1024'

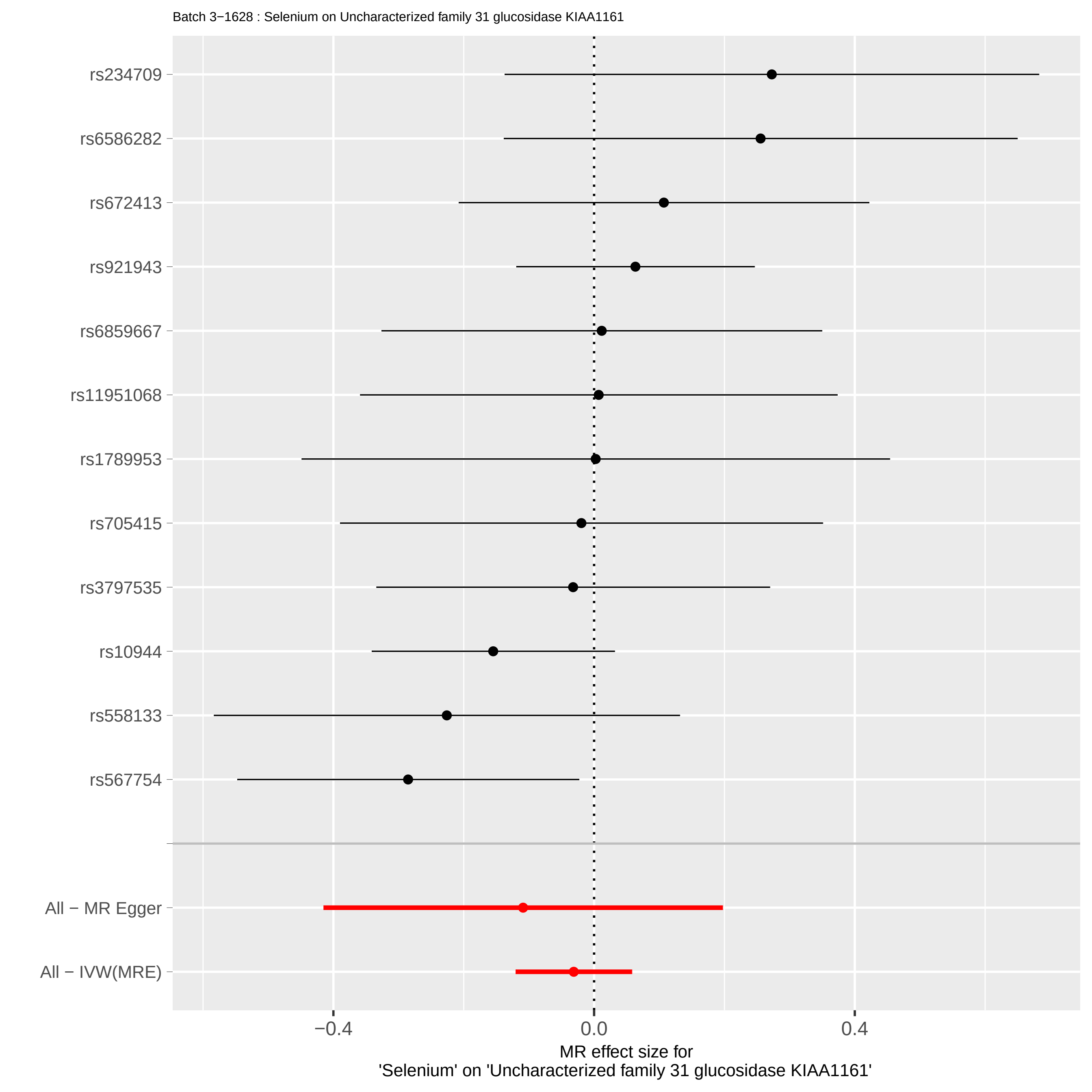

Batch 3−1628 : Selenium on Uncharacterized family 31 glucosidase KIAA1161
rs234709
rs6586282
rs672413
rs921943
rs6859667
rs11951068
rs1789953
rs705415
rs3797535
rs10944
rs558133
rs567754
All − MR Egger
All − IVW(MRE)
−0.4
0.0
0.4
MR effect size for
'Selenium' on 'Uncharacterized family 31 glucosidase KIAA1161'

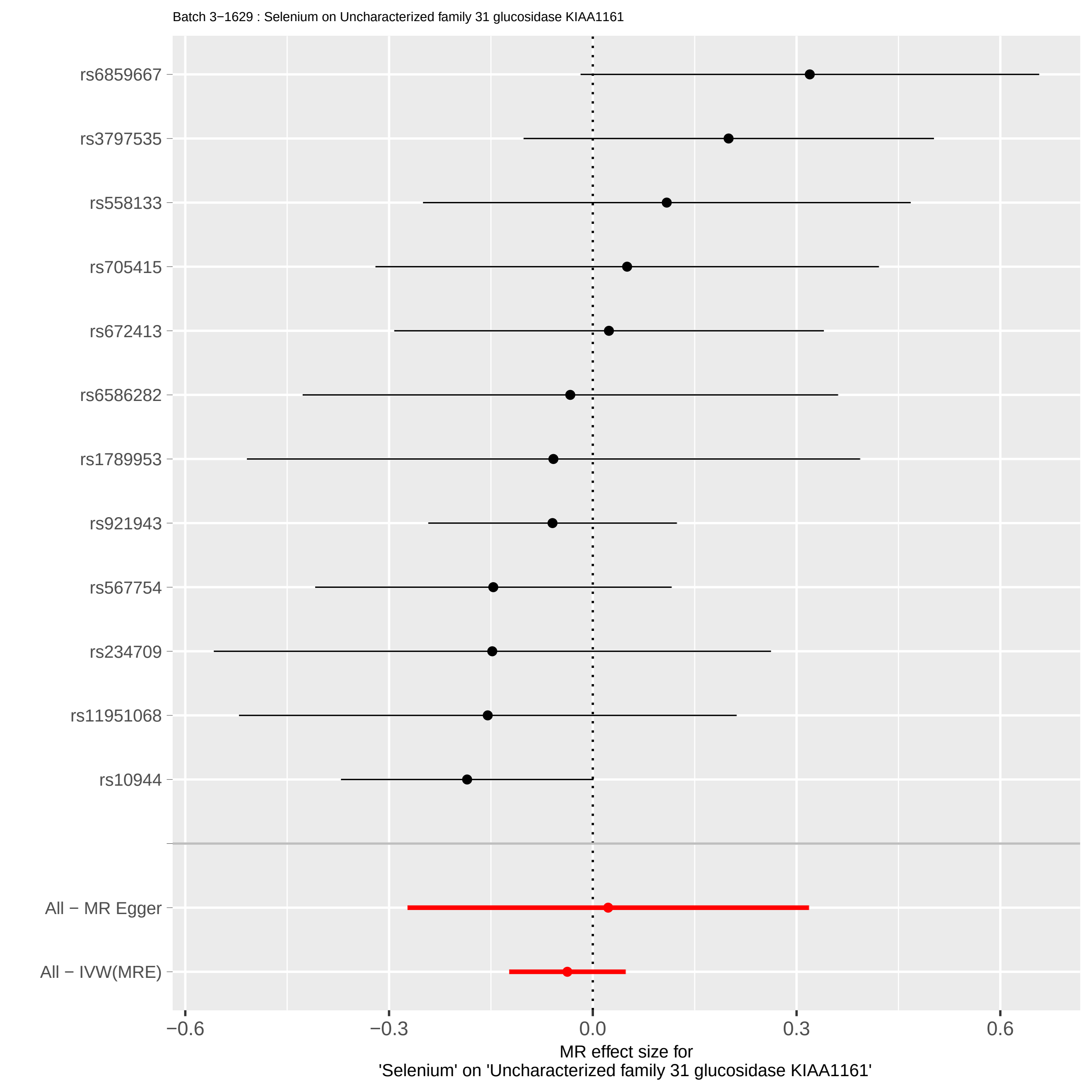

Batch 3−1629 : Selenium on Uncharacterized family 31 glucosidase KIAA1161
rs6859667
rs3797535
rs558133
rs705415
rs672413
rs6586282
rs1789953
rs921943
rs567754
rs234709
rs11951068
rs10944
All − MR Egger
All − IVW(MRE)
−0.6
−0.3
0.0
0.3
0.6
MR effect size for
'Selenium' on 'Uncharacterized family 31 glucosidase KIAA1161'

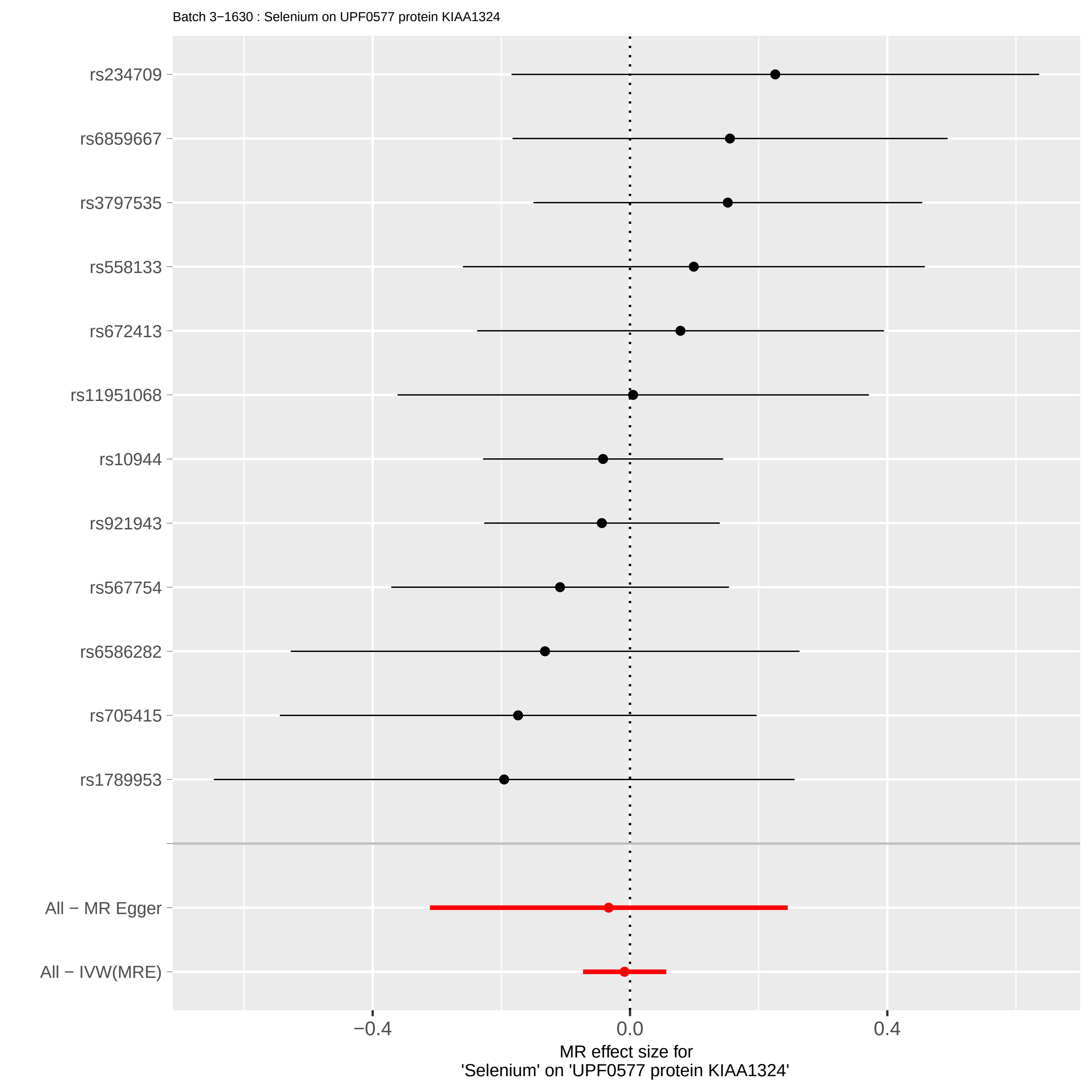

Batch 3−1630 : Selenium on UPF0577 protein KIAA1324
rs234709
rs6859667
rs3797535
rs558133
rs672413
rs11951068
rs10944
rs921943
rs567754
rs6586282
rs705415
rs1789953
All − MR Egger
All − IVW(MRE)
−0.4
0.0
0.4
MR effect size for
'Selenium' on 'UPF0577 protein KIAA1324'

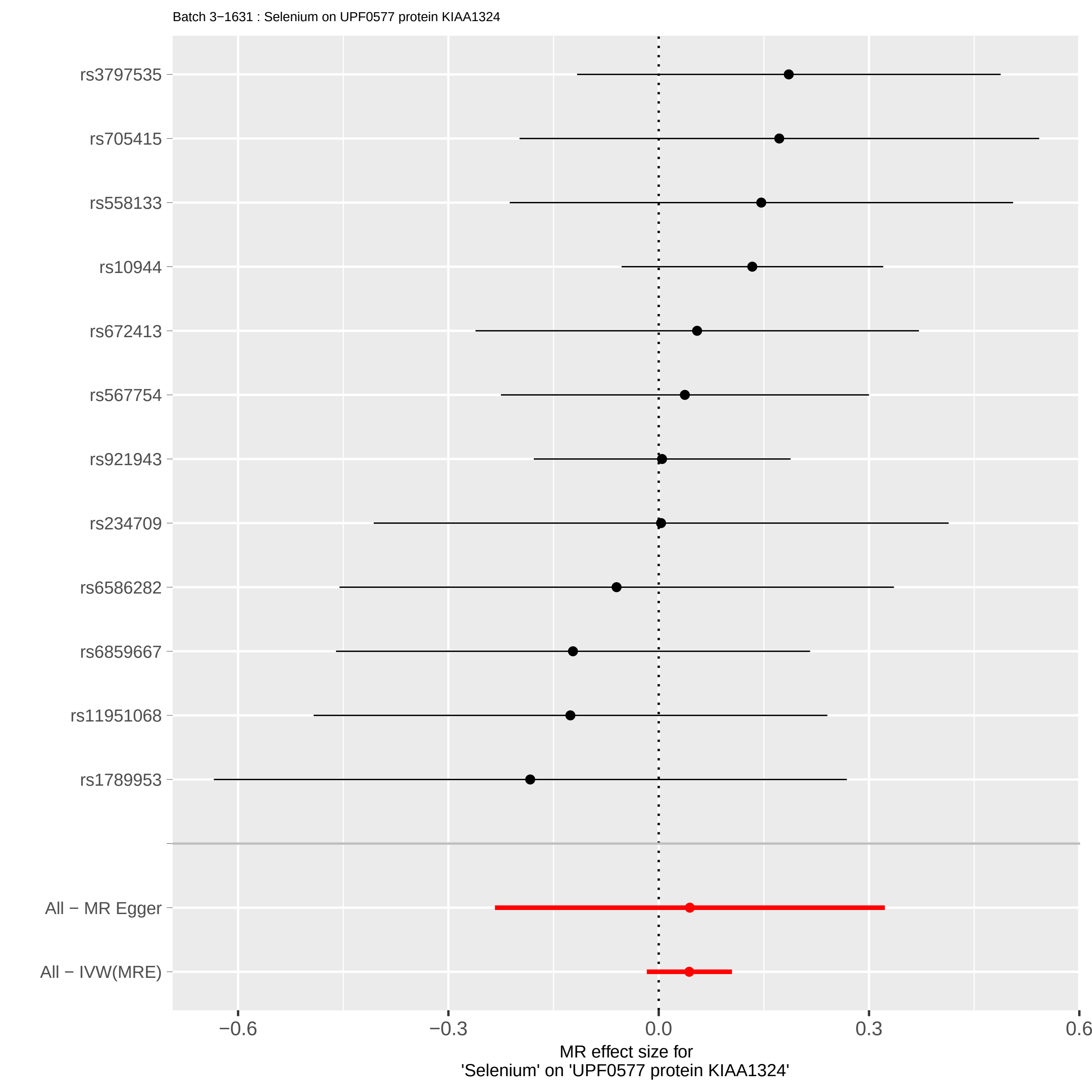
Batch 3−1631 : Selenium on UPF0577 protein KIAA1324
rs3797535
rs705415
rs558133
rs10944
rs672413
rs567754
rs921943
rs234709
rs6586282
rs6859667
rs11951068
rs1789953
All − MR Egger
All − IVW(MRE)
−0.6
−0.3
0.0
0.3
0.6
MR effect size for
'Selenium' on 'UPF0577 protein KIAA1324'

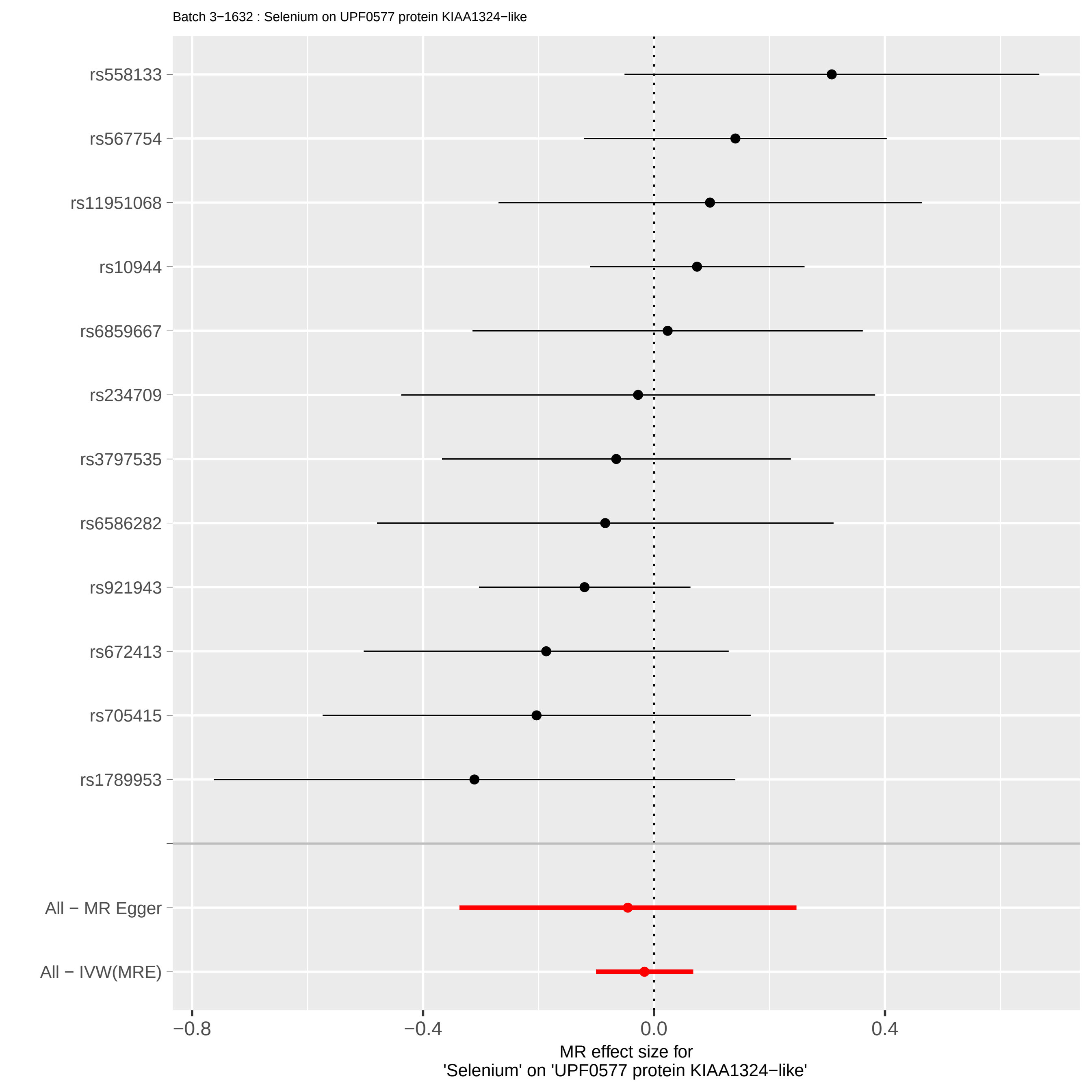
Batch 3−1632 : Selenium on UPF0577 protein KIAA1324−like
rs558133
rs567754
rs11951068
rs10944
rs6859667
rs234709
rs3797535
rs6586282
rs921943
rs672413
rs705415
rs1789953
All − MR Egger
All − IVW(MRE)
−0.8
−0.4
0.0
0.4
MR effect size for
'Selenium' on 'UPF0577 protein KIAA1324−like'

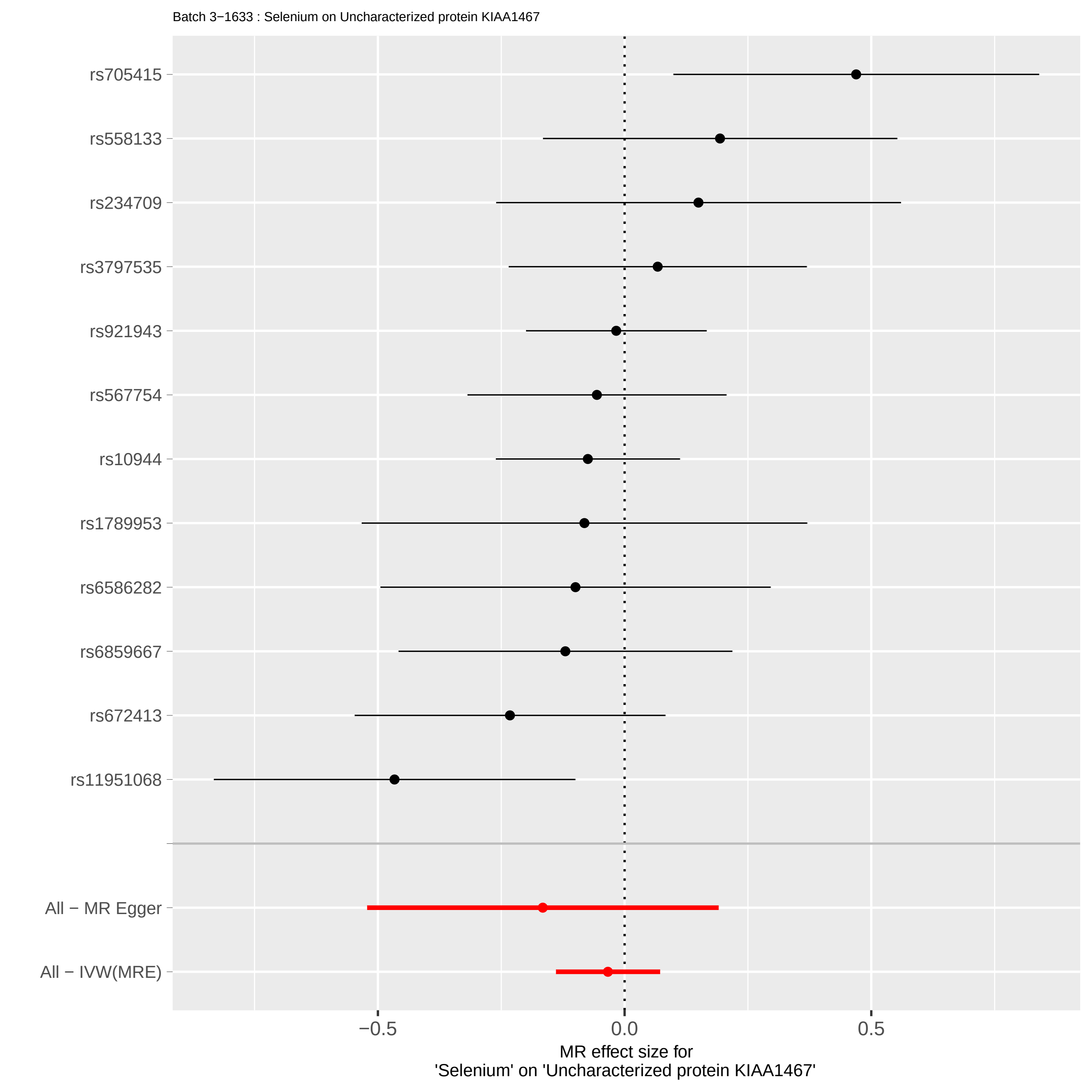

Batch 3−1633 : Selenium on Uncharacterized protein KIAA1467
rs705415
rs558133
rs234709
rs3797535
rs921943
rs567754
rs10944
rs1789953
rs6586282
rs6859667
rs672413
rs11951068
All − MR Egger
All − IVW(MRE)
−0.5
0.0
0.5
MR effect size for
'Selenium' on 'Uncharacterized protein KIAA1467'

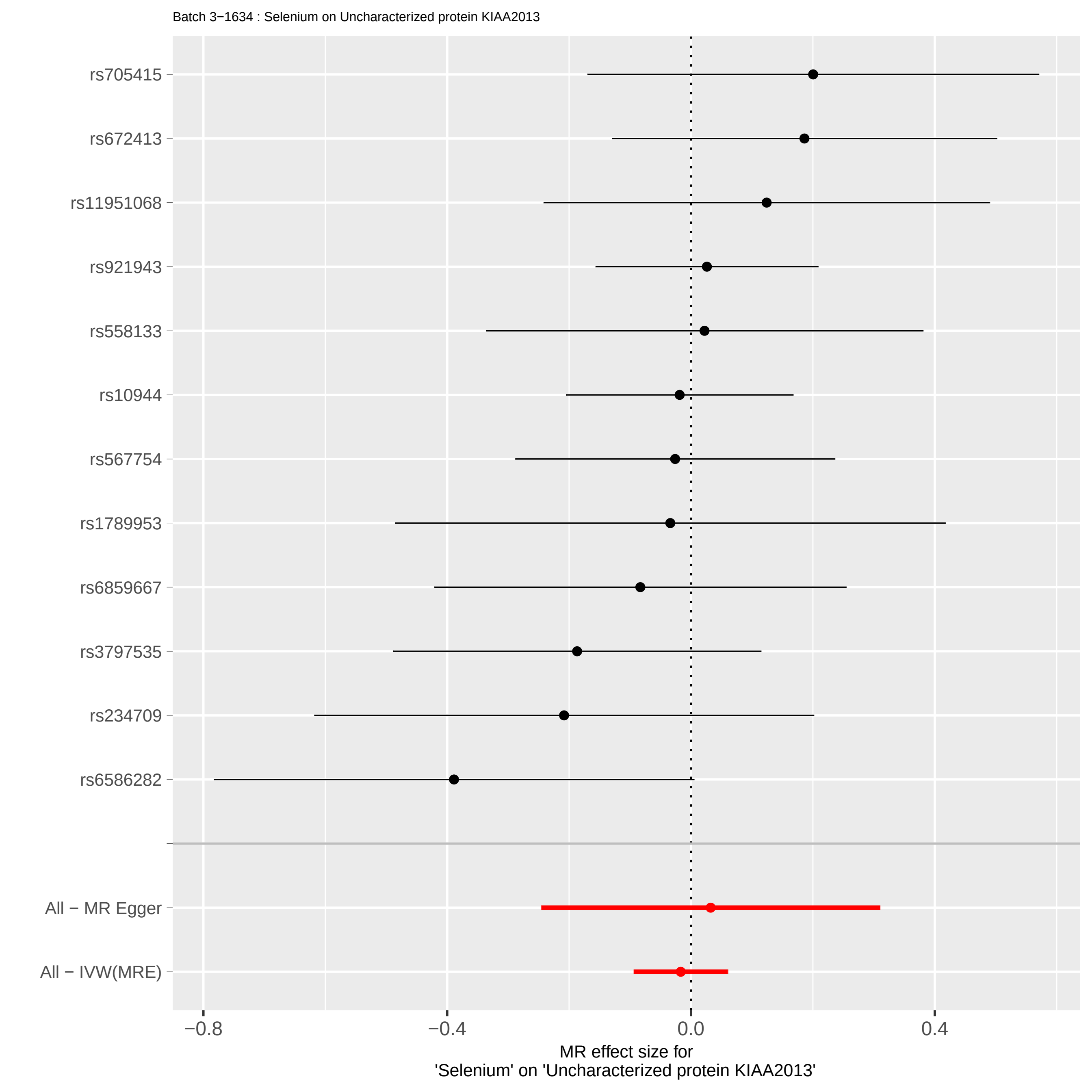
Batch 3−1634 : Selenium on Uncharacterized protein KIAA2013
rs705415
rs672413
rs11951068
rs921943
rs558133
rs10944
rs567754
rs1789953
rs6859667
rs3797535
rs234709
rs6586282
All − MR Egger
All − IVW(MRE)
−0.8
−0.4
0.0
0.4
MR effect size for
'Selenium' on 'Uncharacterized protein KIAA2013'

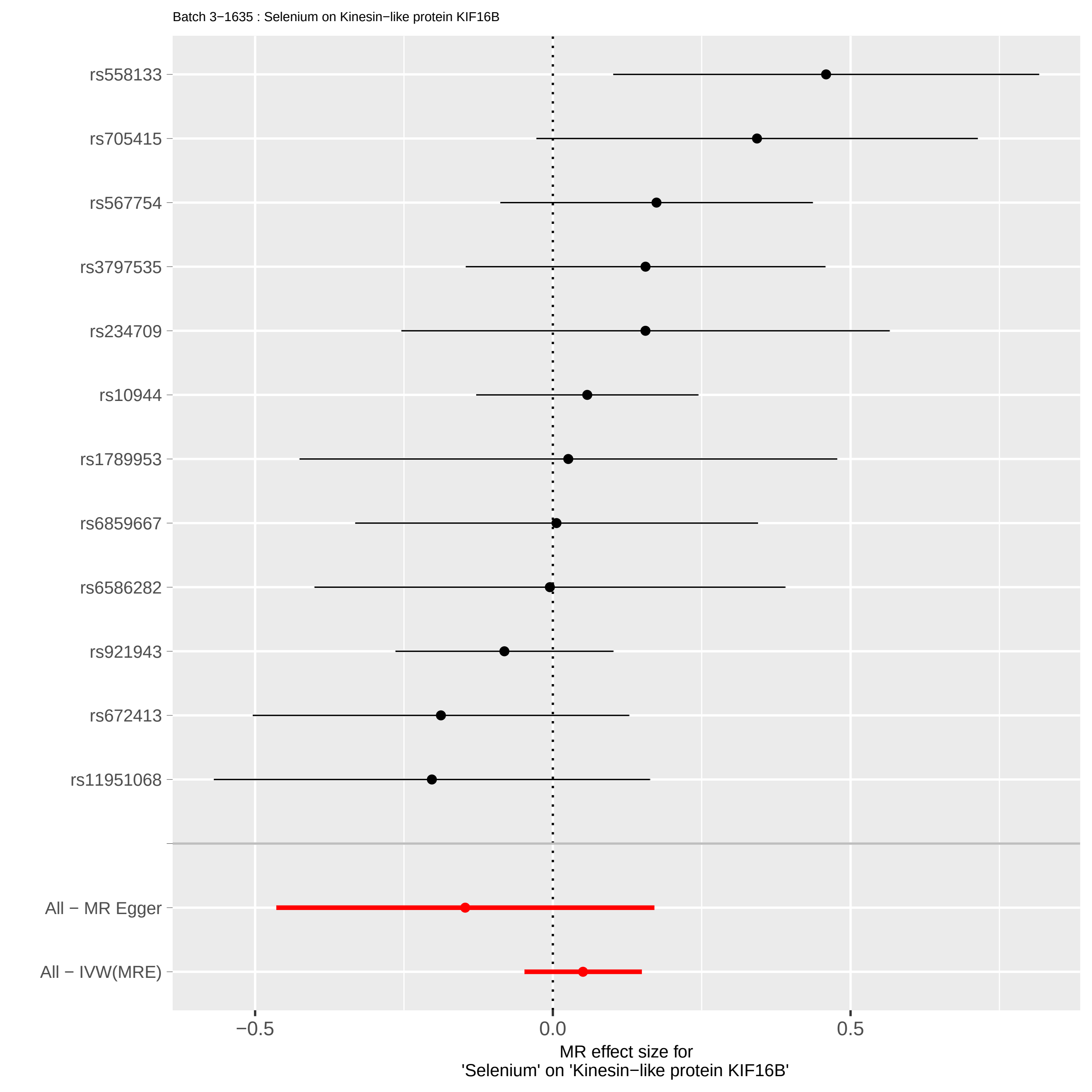

Batch 3−1635 : Selenium on Kinesin−like protein KIF16B
rs558133
rs705415
rs567754
rs3797535
rs234709
rs10944
rs1789953
rs6859667
rs6586282
rs921943
rs672413
rs11951068
All − MR Egger
All − IVW(MRE)
−0.5
0.0
0.5
MR effect size for
'Selenium' on 'Kinesin−like protein KIF16B'

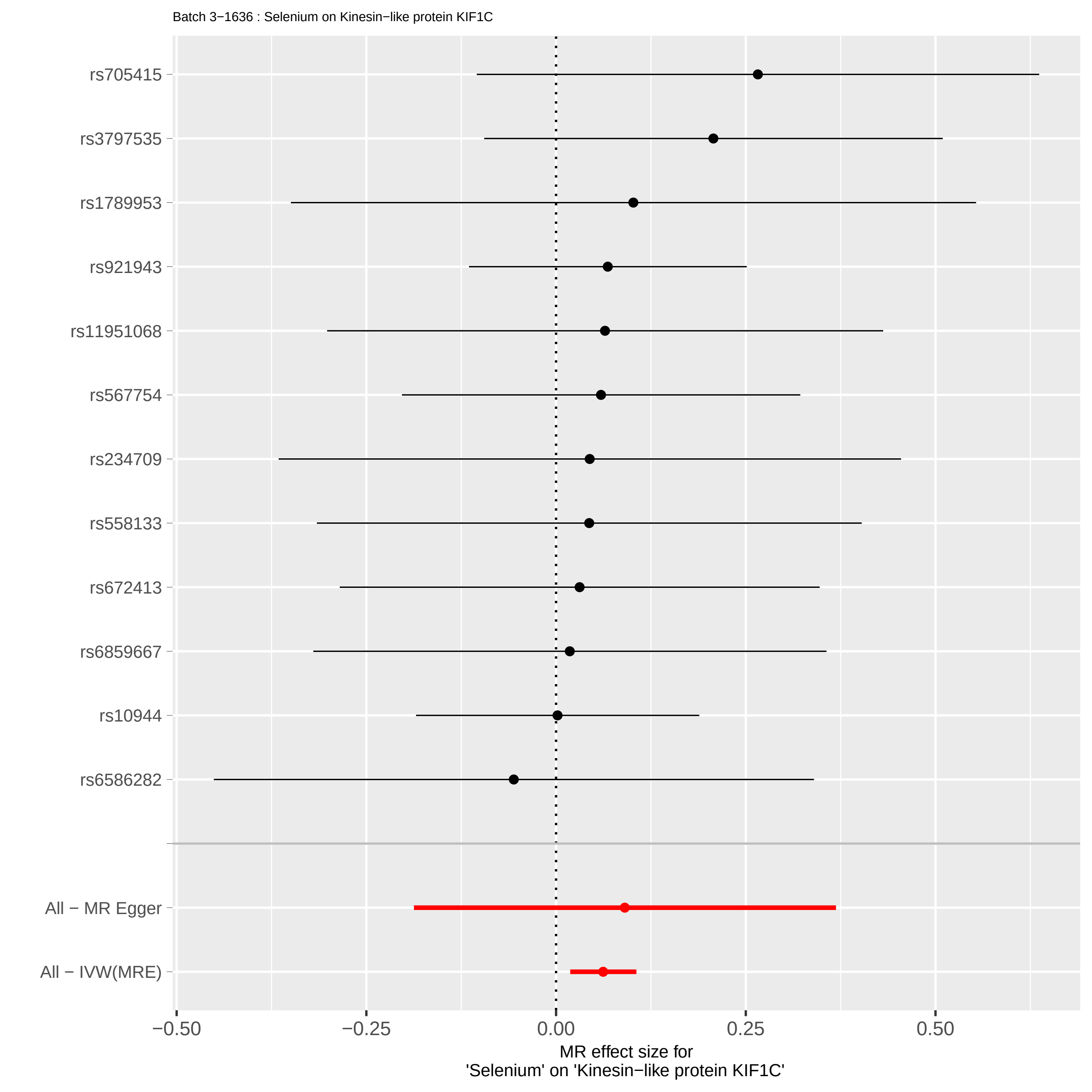
Batch 3−1636 : Selenium on Kinesin−like protein KIF1C
rs705415
rs3797535
rs1789953
rs921943
rs11951068
rs567754
rs234709
rs558133
rs672413
rs6859667
rs10944
rs6586282
All − MR Egger
All − IVW(MRE)
−0.50
−0.25
0.00
0.25
0.50
MR effect size for
'Selenium' on 'Kinesin−like protein KIF1C'

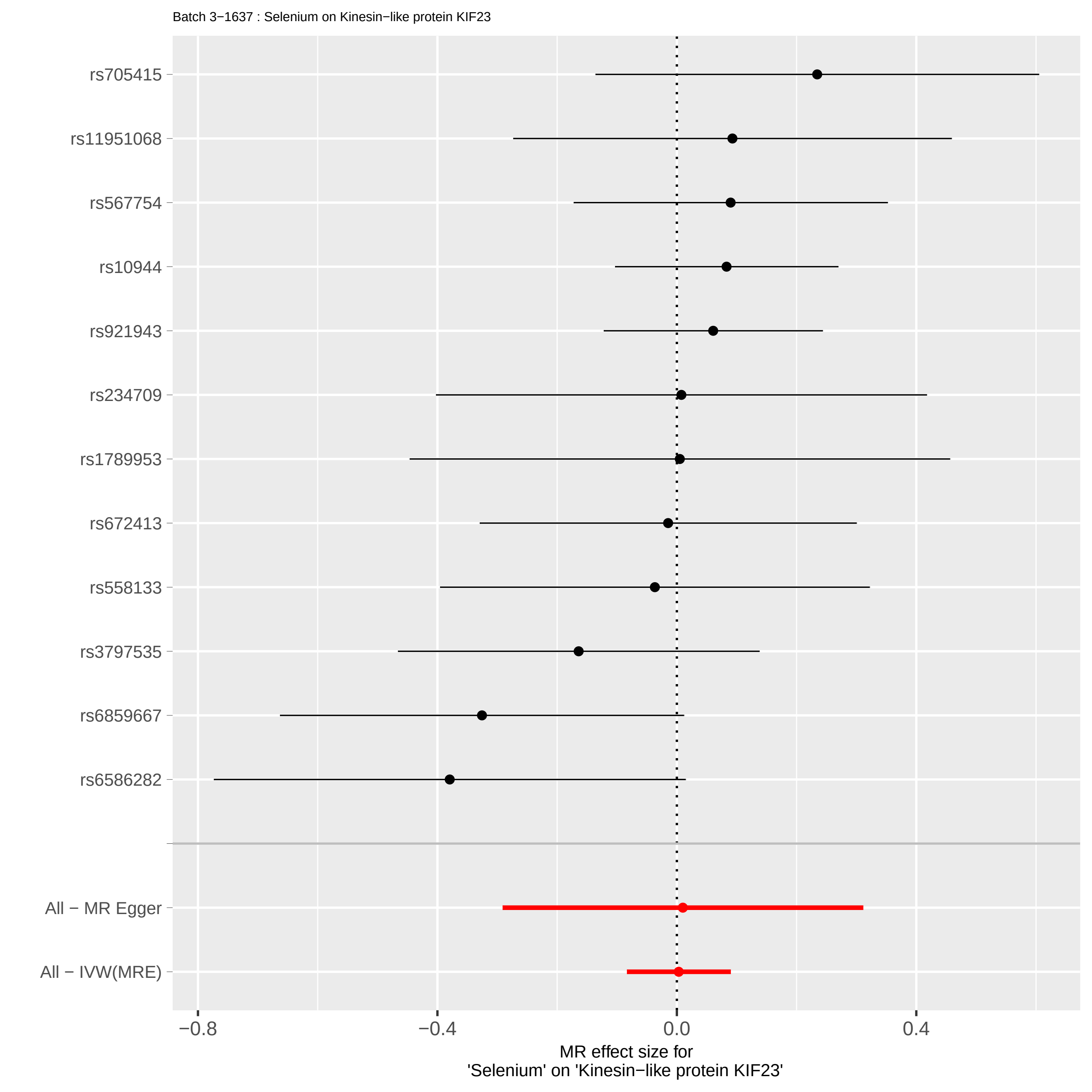
Batch 3−1637 : Selenium on Kinesin−like protein KIF23
rs705415
rs11951068
rs567754
rs10944
rs921943
rs234709
rs1789953
rs672413
rs558133
rs3797535
rs6859667
rs6586282
All − MR Egger
All − IVW(MRE)
−0.8
−0.4
0.0
0.4
MR effect size for
'Selenium' on 'Kinesin−like protein KIF23'

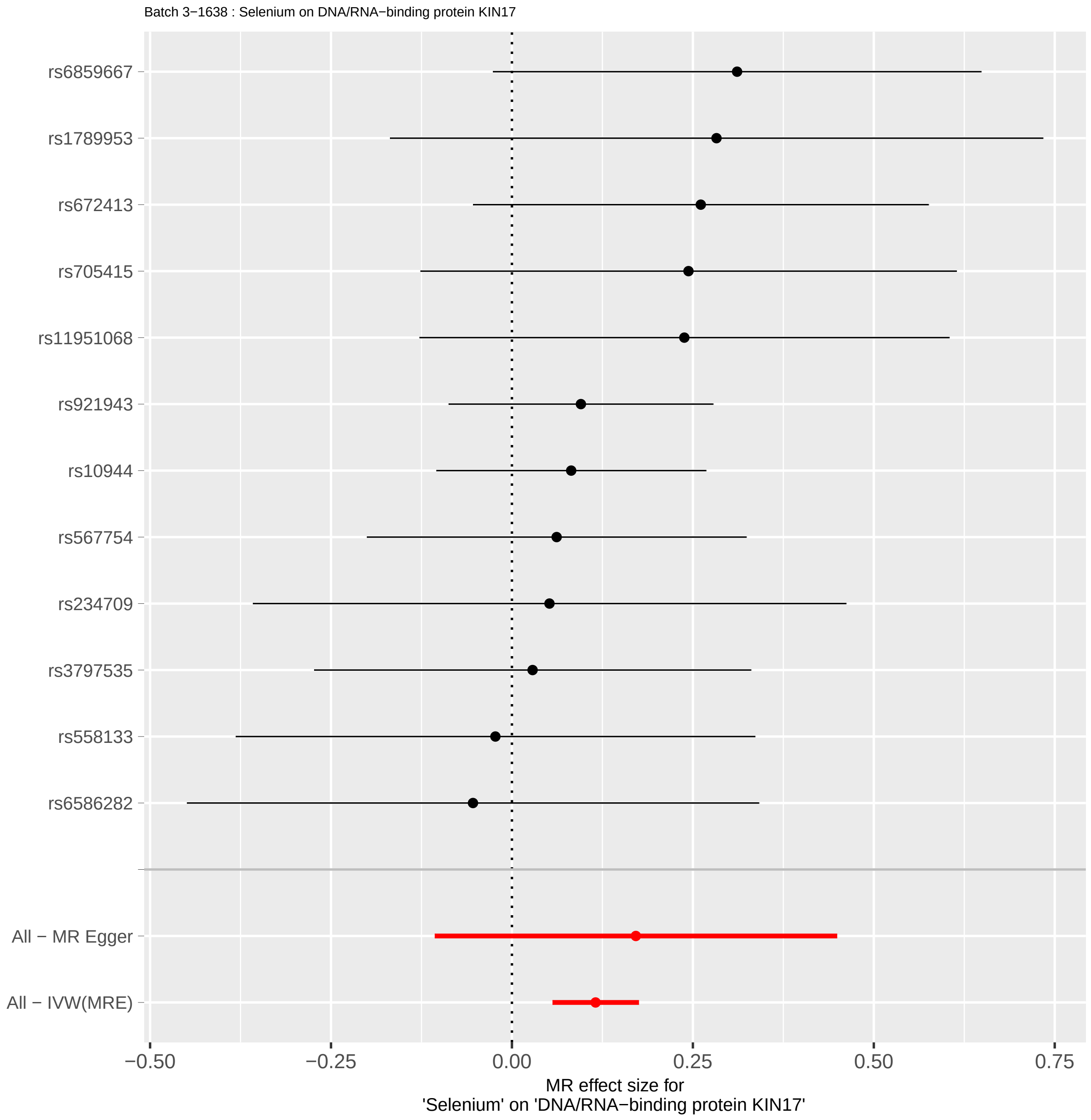

Batch 3−1638 : Selenium on DNA/RNA−binding protein KIN17
rs6859667
rs1789953
rs672413
rs705415
rs11951068
rs921943
rs10944
rs567754
rs234709
rs3797535
rs558133
rs6586282
All − MR Egger
All − IVW(MRE)
−0.50
−0.25
0.00
0.25
0.50
0.75
MR effect size for
'Selenium' on 'DNA/RNA−binding protein KIN17'

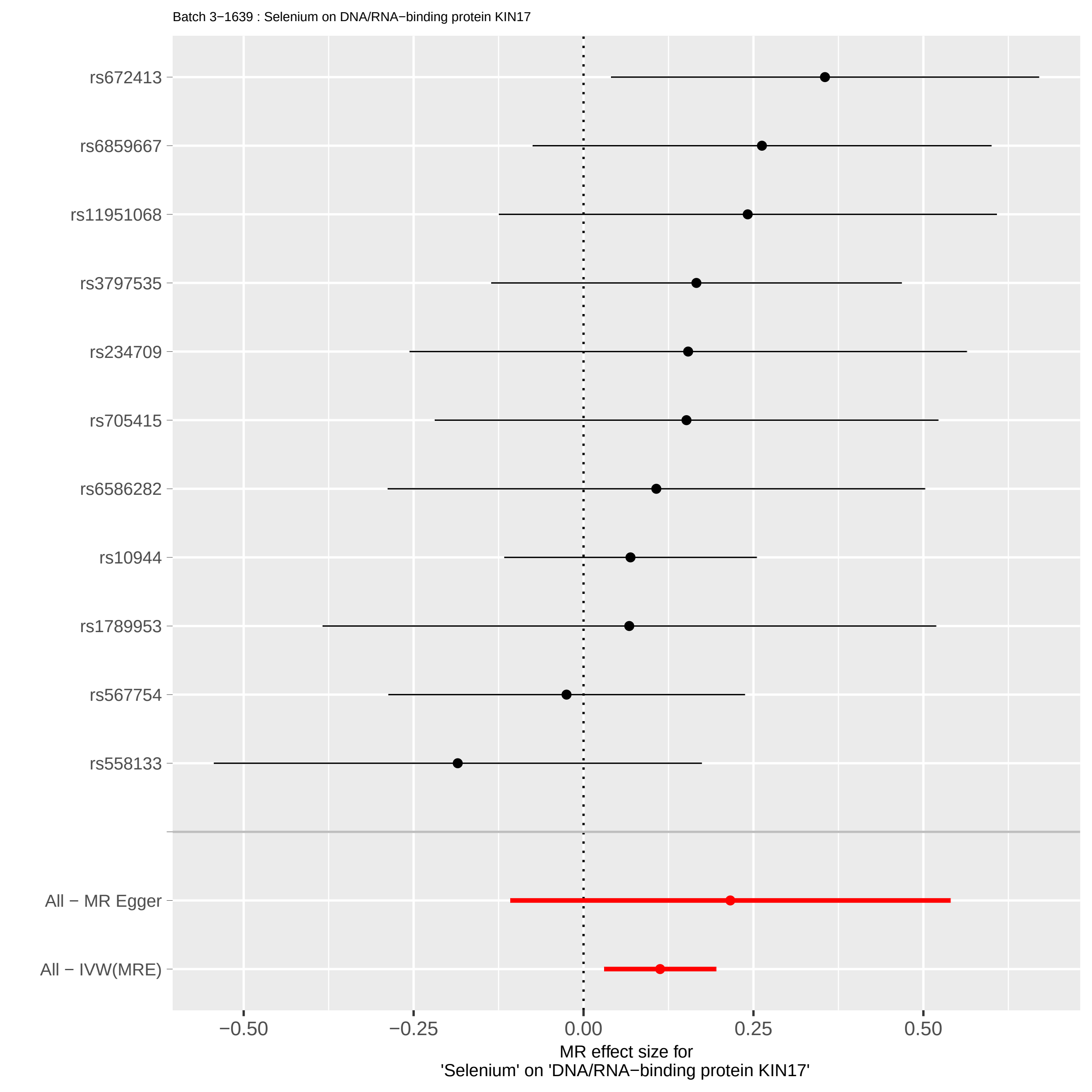
Batch 3−1639 : Selenium on DNA/RNA−binding protein KIN17
rs672413
rs6859667
rs11951068
rs3797535
rs234709
rs705415
rs6586282
rs10944
rs1789953
rs567754
rs558133
All − MR Egger
All − IVW(MRE)
−0.50
−0.25
0.00
0.25
0.50
MR effect size for
'Selenium' on 'DNA/RNA−binding protein KIN17'

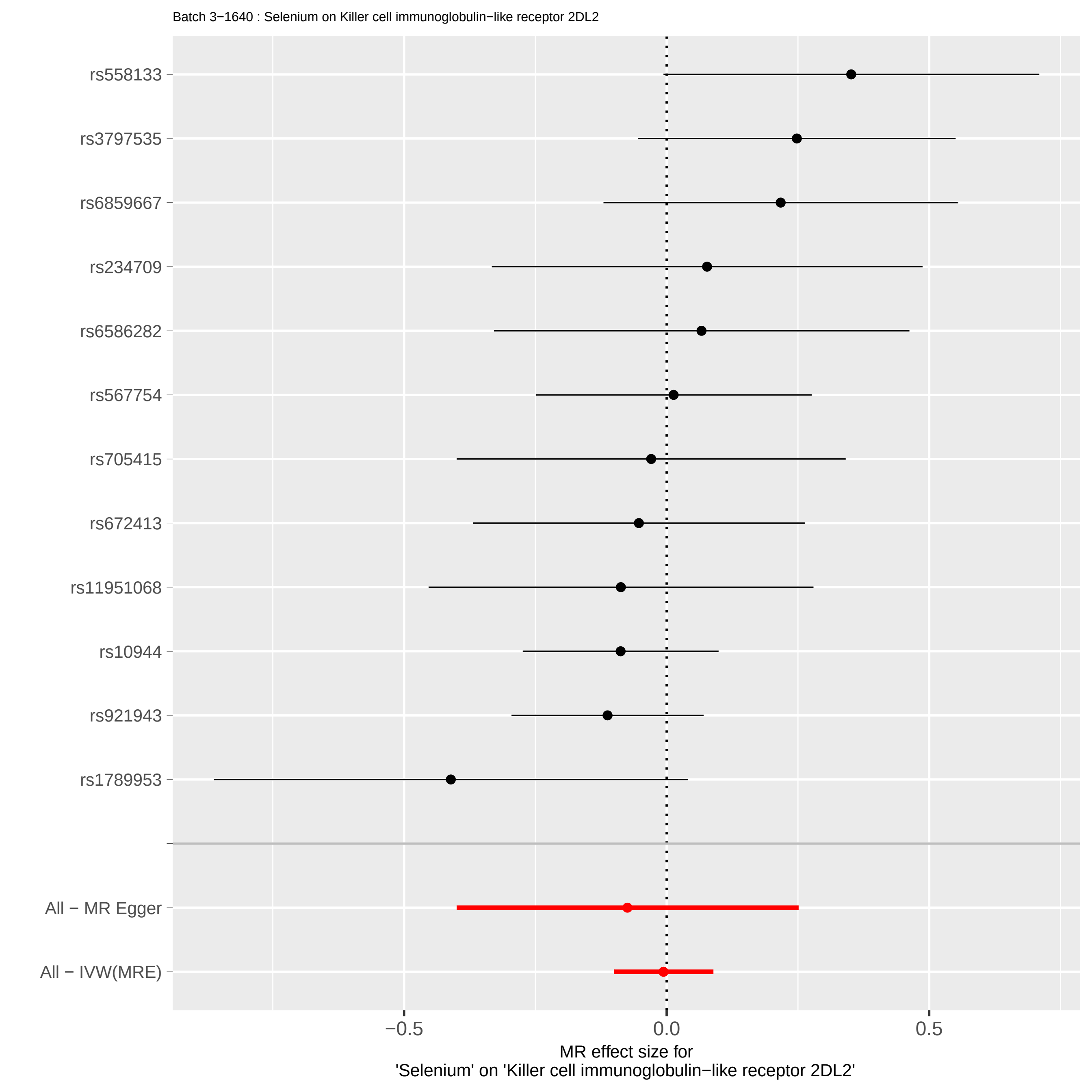
Batch 3−1640 : Selenium on Killer cell immunoglobulin−like receptor 2DL2
rs558133
rs3797535
rs6859667
rs234709
rs6586282
rs567754
rs705415
rs672413
rs11951068
rs10944
rs921943
rs1789953
All − MR Egger
All − IVW(MRE)
−0.5
0.0
0.5
MR effect size for
'Selenium' on 'Killer cell immunoglobulin−like receptor 2DL2'

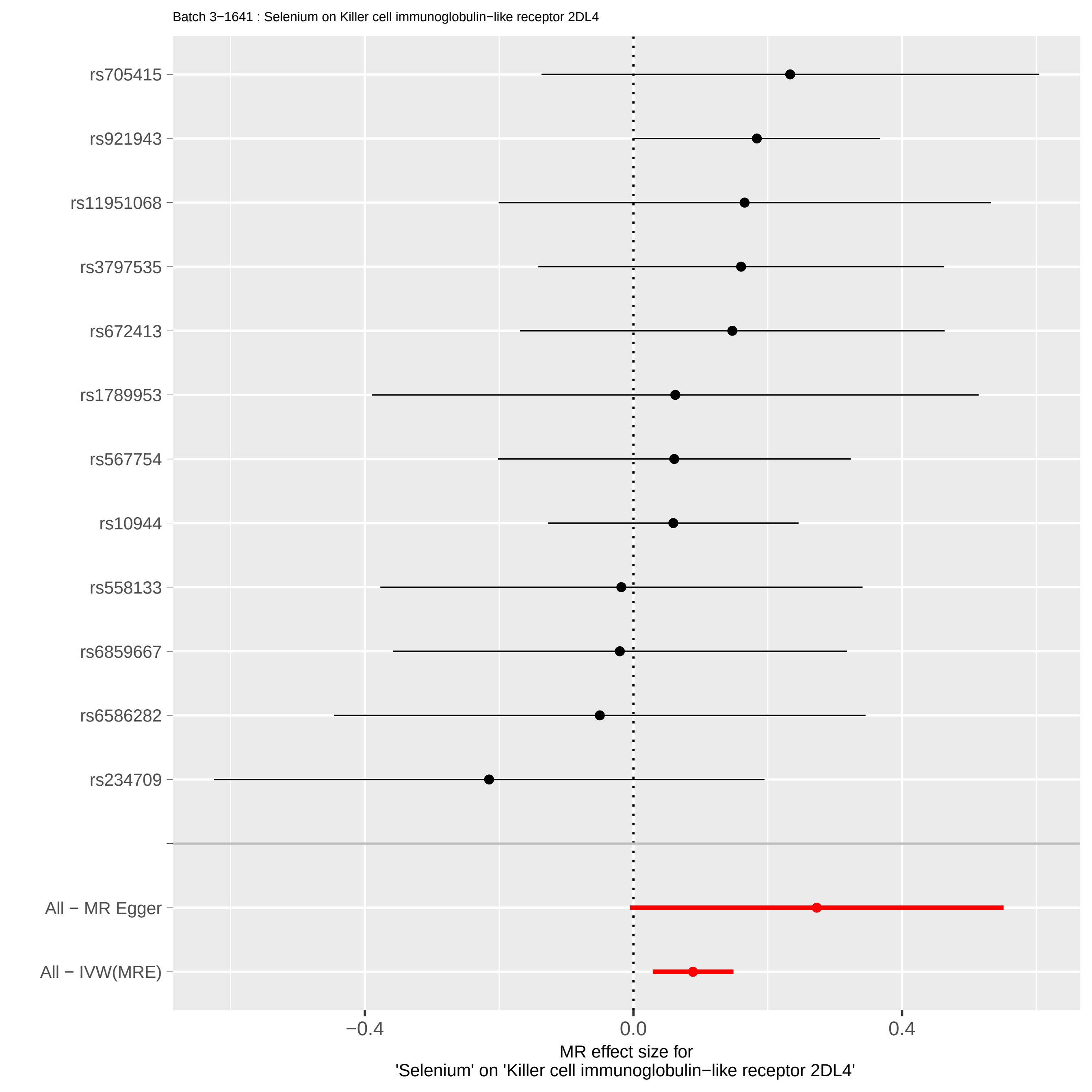

Batch 3−1641 : Selenium on Killer cell immunoglobulin−like receptor 2DL4
rs705415
rs921943
rs11951068
rs3797535
rs672413
rs1789953
rs567754
rs10944
rs558133
rs6859667
rs6586282
rs234709
All − MR Egger
All − IVW(MRE)
−0.4
0.0
0.4
MR effect size for
'Selenium' on 'Killer cell immunoglobulin−like receptor 2DL4'

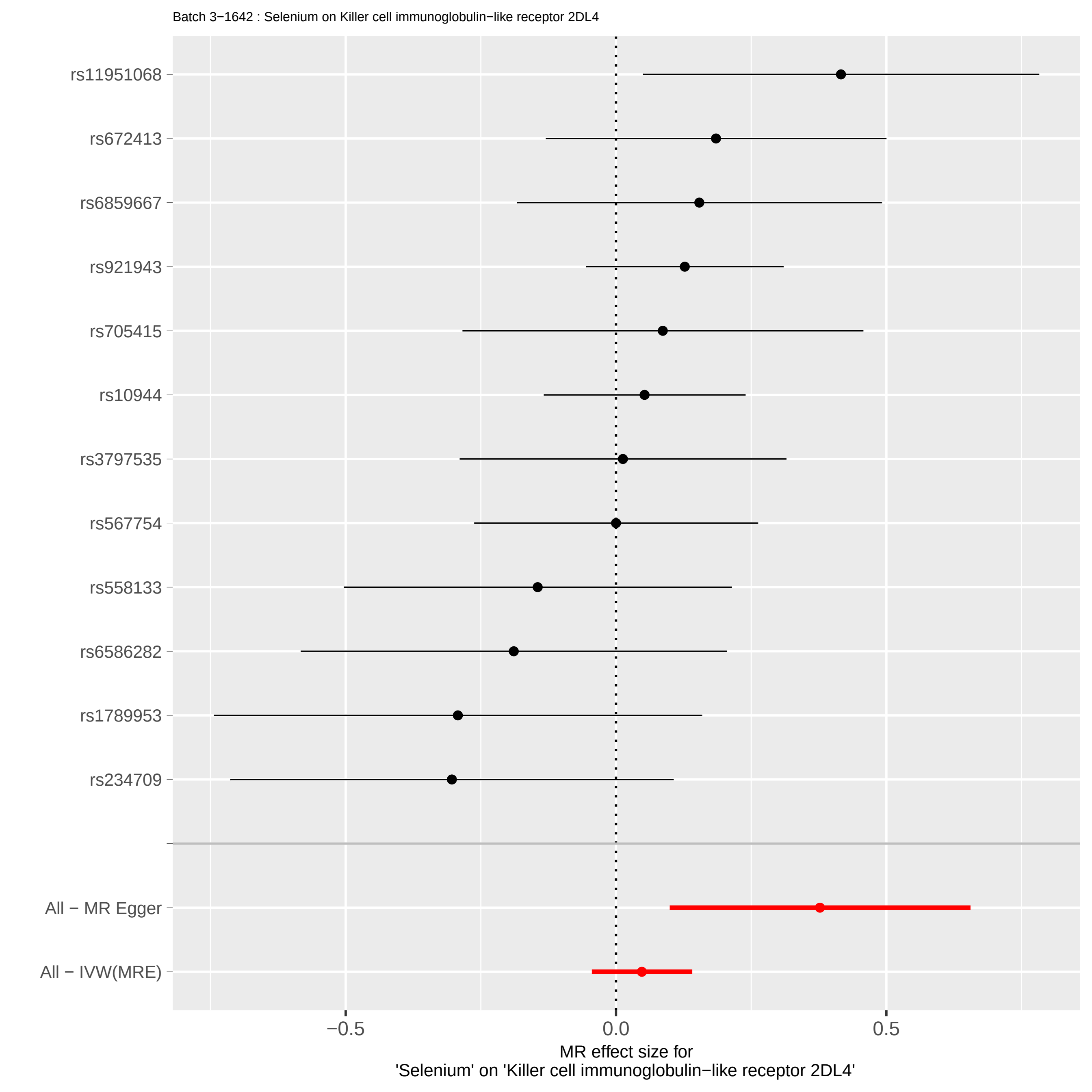

Batch 3−1642 : Selenium on Killer cell immunoglobulin−like receptor 2DL4
rs11951068
rs672413
rs6859667
rs921943
rs705415
rs10944
rs3797535
rs567754
rs558133
rs6586282
rs1789953
rs234709
All − MR Egger
All − IVW(MRE)
−0.5
0.0
0.5
MR effect size for
'Selenium' on 'Killer cell immunoglobulin−like receptor 2DL4'

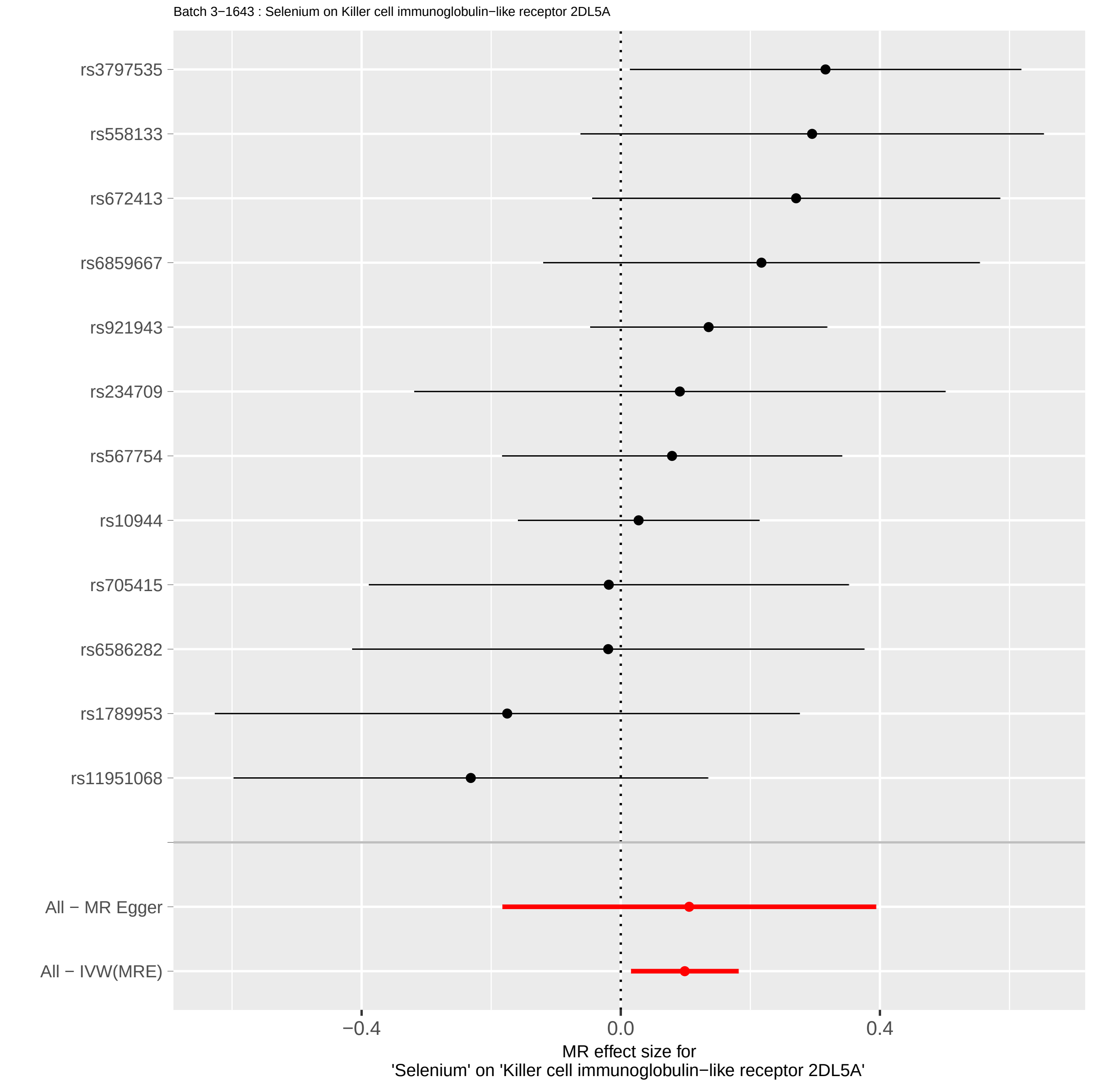

Batch 3−1643 : Selenium on Killer cell immunoglobulin−like receptor 2DL5A
rs3797535
rs558133
rs672413
rs6859667
rs921943
rs234709
rs567754
rs10944
rs705415
rs6586282
rs1789953
rs11951068
All − MR Egger
All − IVW(MRE)
−0.4
0.0
0.4
MR effect size for
'Selenium' on 'Killer cell immunoglobulin−like receptor 2DL5A'

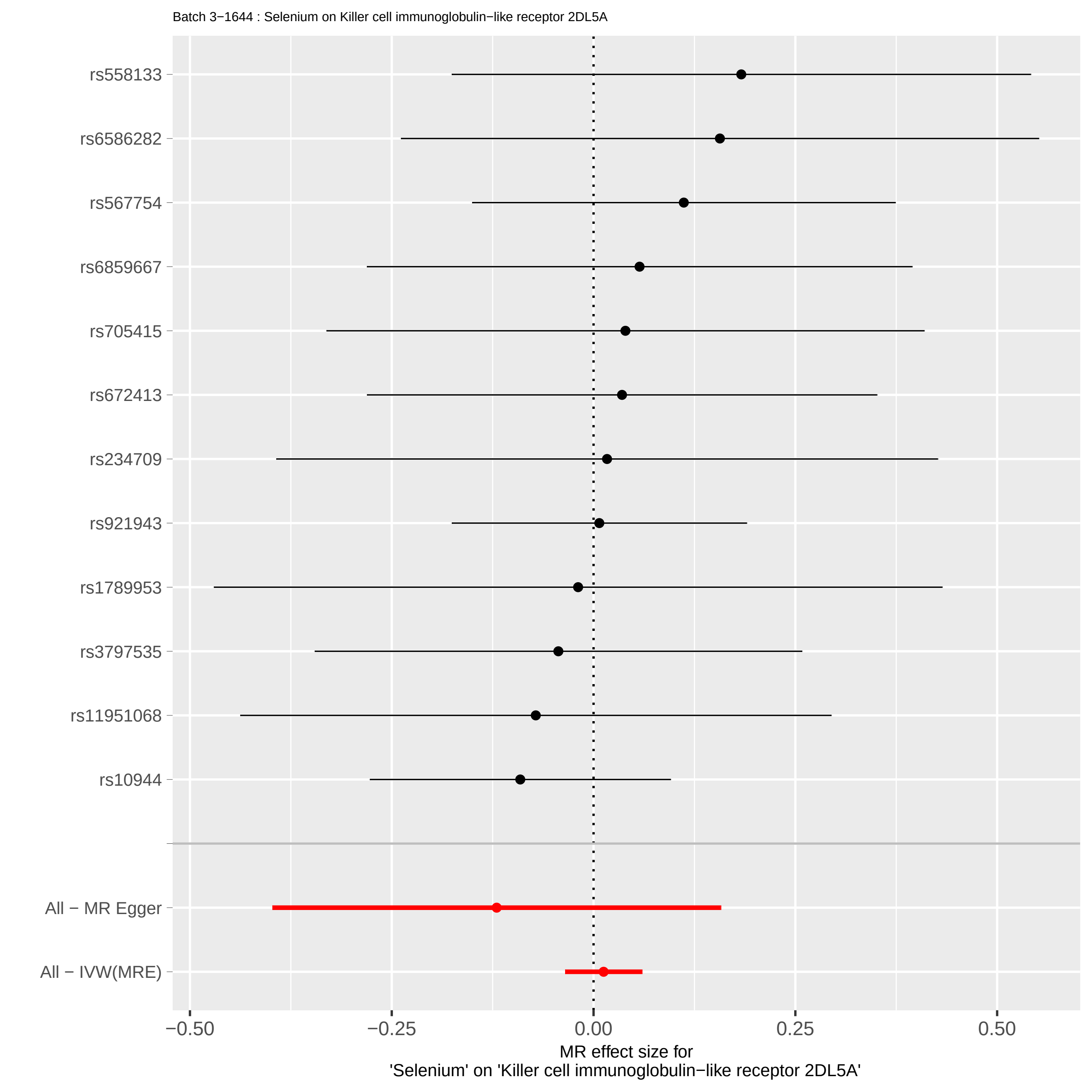

Batch 3−1644 : Selenium on Killer cell immunoglobulin−like receptor 2DL5A
rs558133
rs6586282
rs567754
rs6859667
rs705415
rs672413
rs234709
rs921943
rs1789953
rs3797535
rs11951068
rs10944
All − MR Egger
All − IVW(MRE)
−0.50
−0.25
0.00
0.25
0.50
MR effect size for
'Selenium' on 'Killer cell immunoglobulin−like receptor 2DL5A'

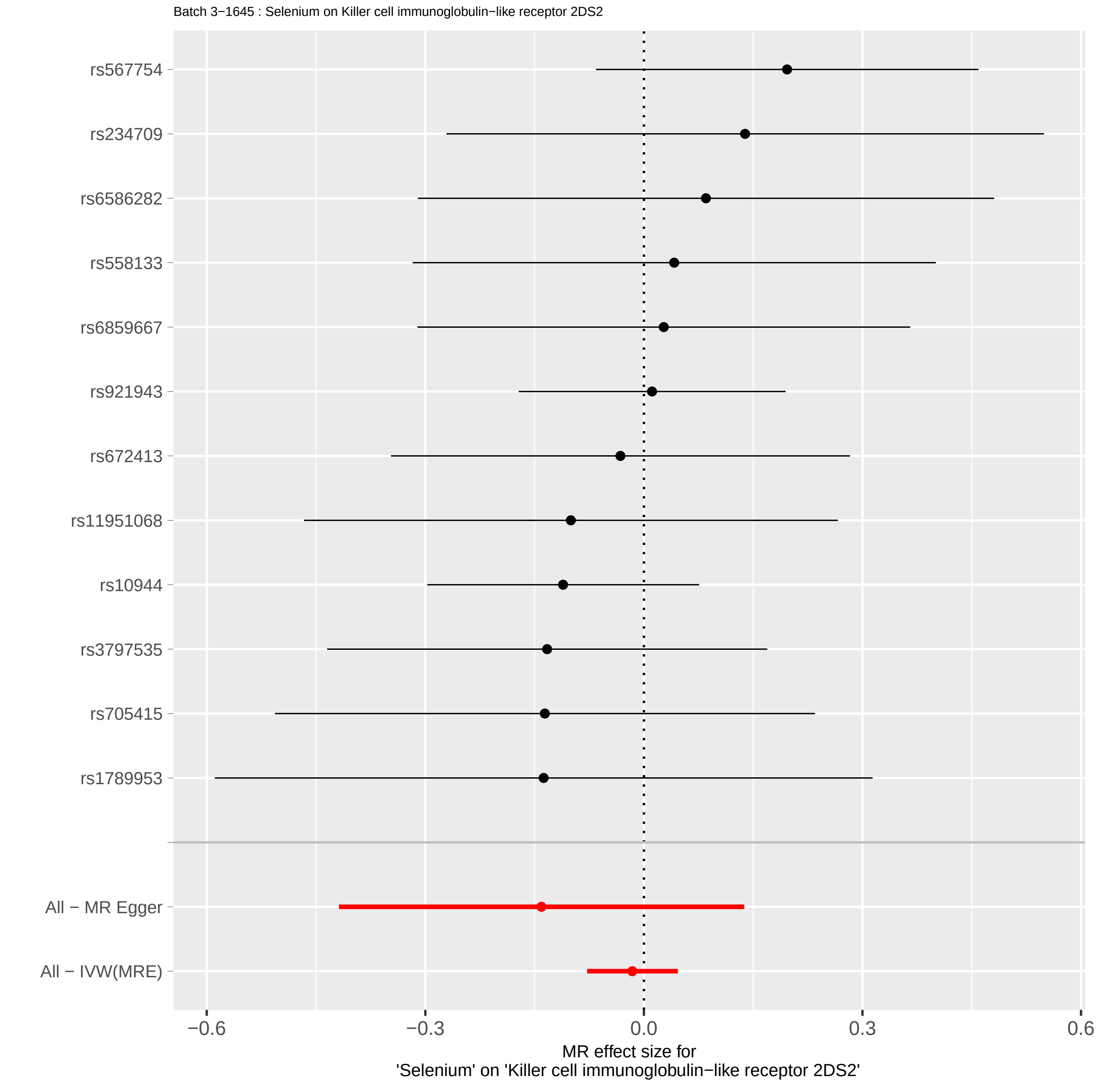

Batch 3−1645 : Selenium on Killer cell immunoglobulin−like receptor 2DS2
rs567754
rs234709
rs6586282
rs558133
rs6859667
rs921943
rs672413
rs11951068
rs10944
rs3797535
rs705415
rs1789953
All − MR Egger
All − IVW(MRE)
−0.6
−0.3
0.0
0.3
0.6
MR effect size for
'Selenium' on 'Killer cell immunoglobulin−like receptor 2DS2'

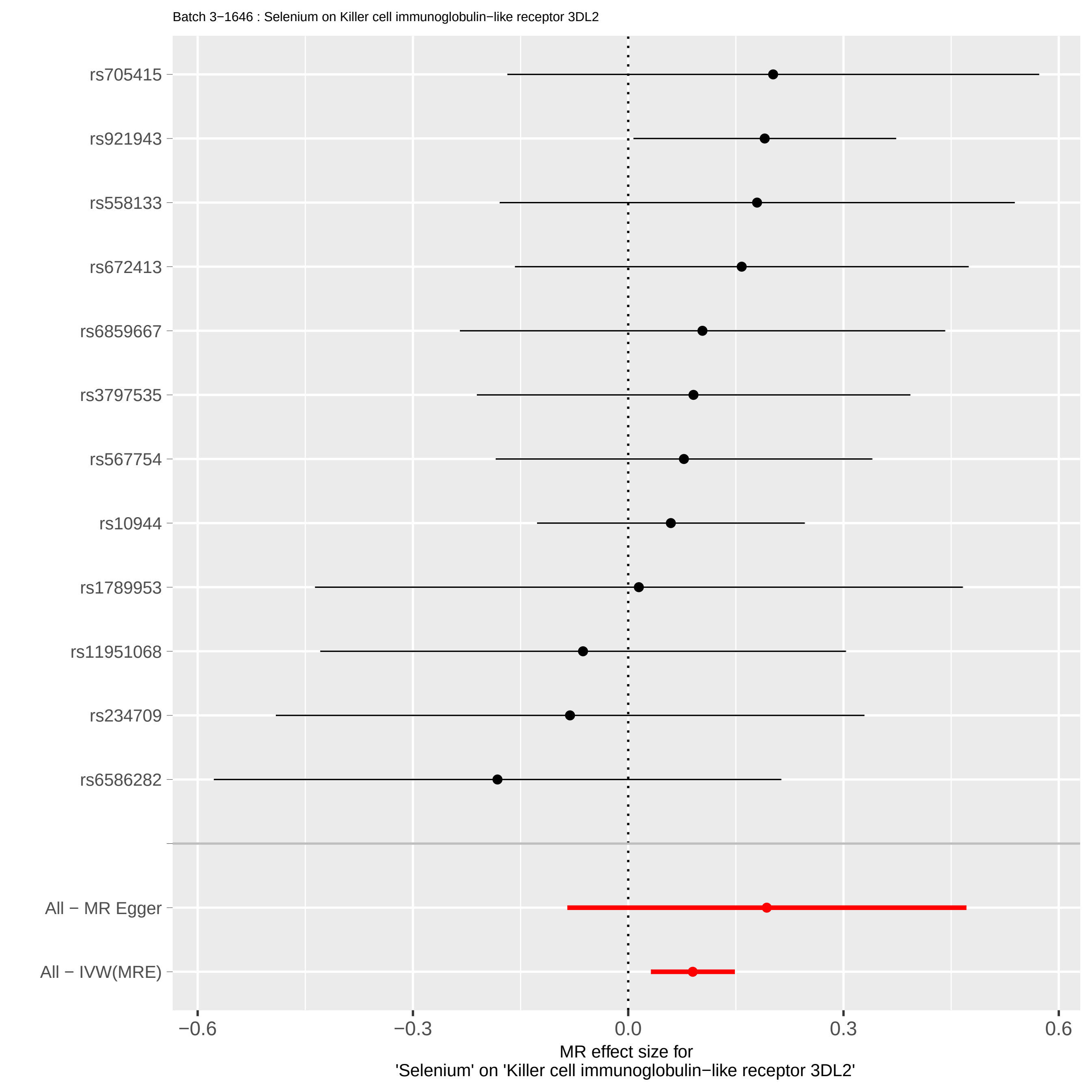

Batch 3−1646 : Selenium on Killer cell immunoglobulin−like receptor 3DL2
rs705415
rs921943
rs558133
rs672413
rs6859667
rs3797535
rs567754
rs10944
rs1789953
rs11951068
rs234709
rs6586282
All − MR Egger
All − IVW(MRE)
−0.6
−0.3
0.0
0.3
0.6
MR effect size for
'Selenium' on 'Killer cell immunoglobulin−like receptor 3DL2'

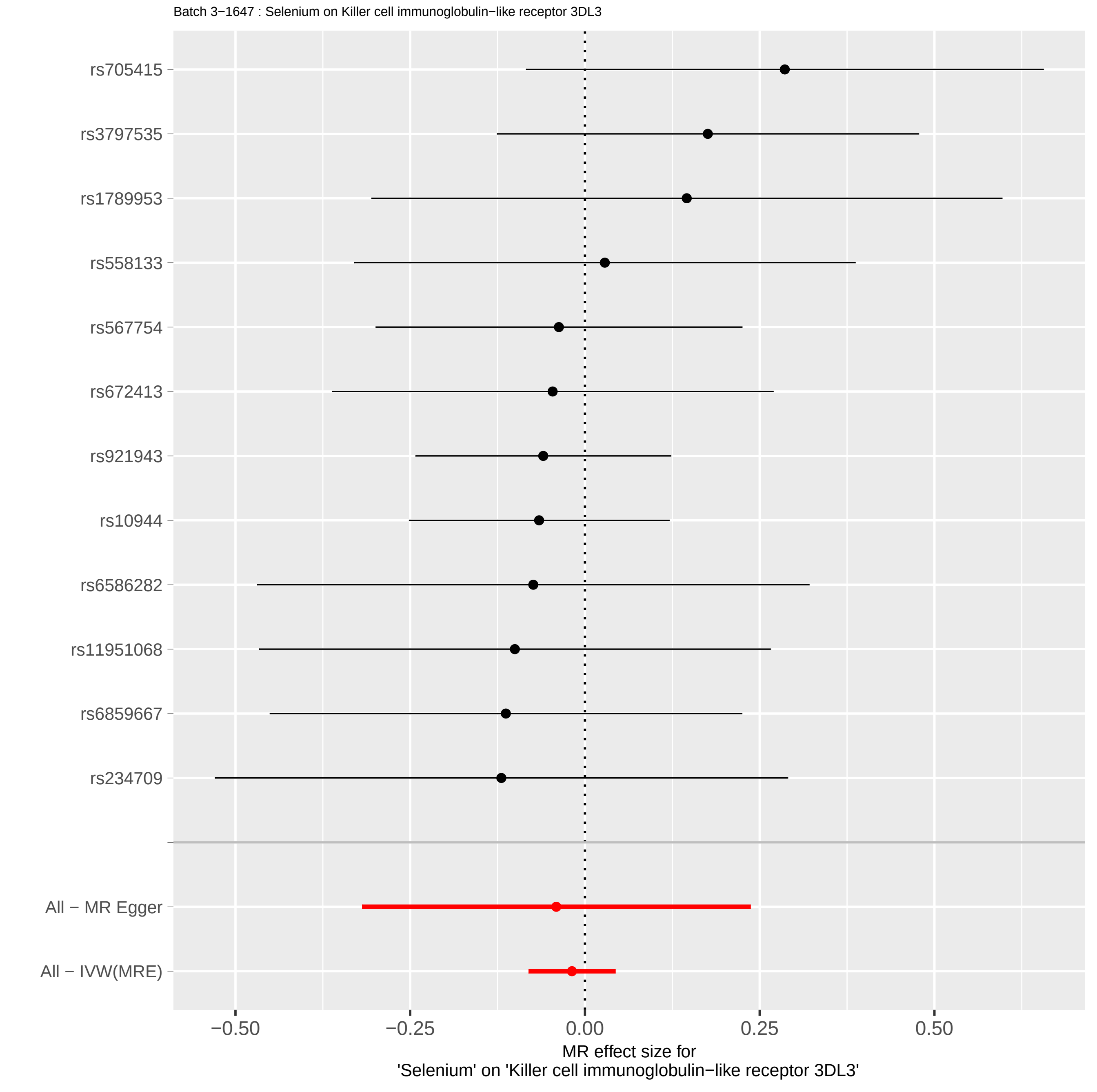
Batch 3−1647 : Selenium on Killer cell immunoglobulin−like receptor 3DL3
rs705415
rs3797535
rs1789953
rs558133
rs567754
rs672413
rs921943
rs10944
rs6586282
rs11951068
rs6859667
rs234709
All − MR Egger
All − IVW(MRE)
−0.50
−0.25
0.00
0.25
0.50
MR effect size for
'Selenium' on 'Killer cell immunoglobulin−like receptor 3DL3'

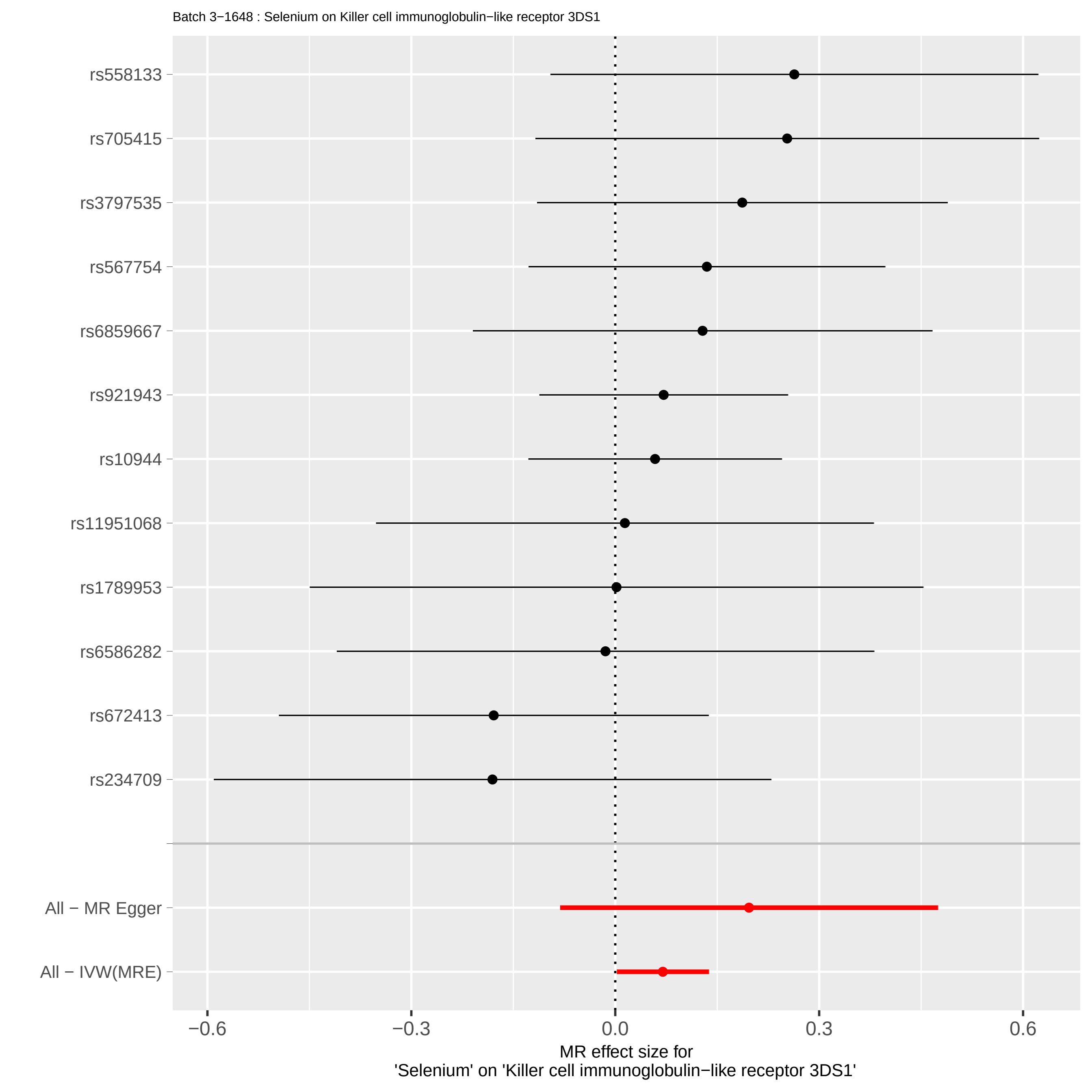

Batch 3−1648 : Selenium on Killer cell immunoglobulin−like receptor 3DS1
rs558133
rs705415
rs3797535
rs567754
rs6859667
rs921943
rs10944
rs11951068
rs1789953
rs6586282
rs672413
rs234709
All − MR Egger
All − IVW(MRE)
−0.6
−0.3
0.0
0.3
0.6
MR effect size for
'Selenium' on 'Killer cell immunoglobulin−like receptor 3DS1'

Batch 3−1649 : Selenium on Kin of IRRE−like protein 2

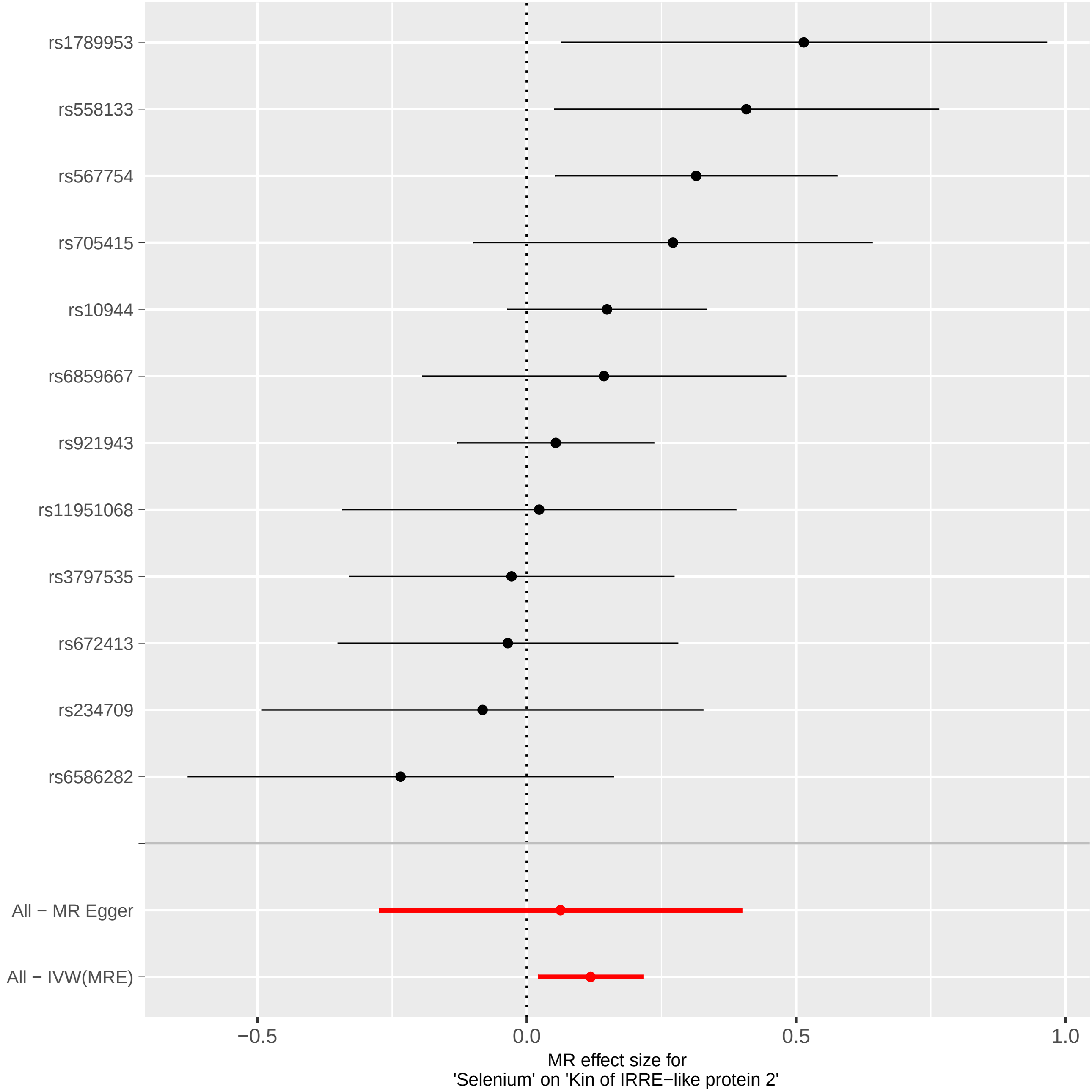

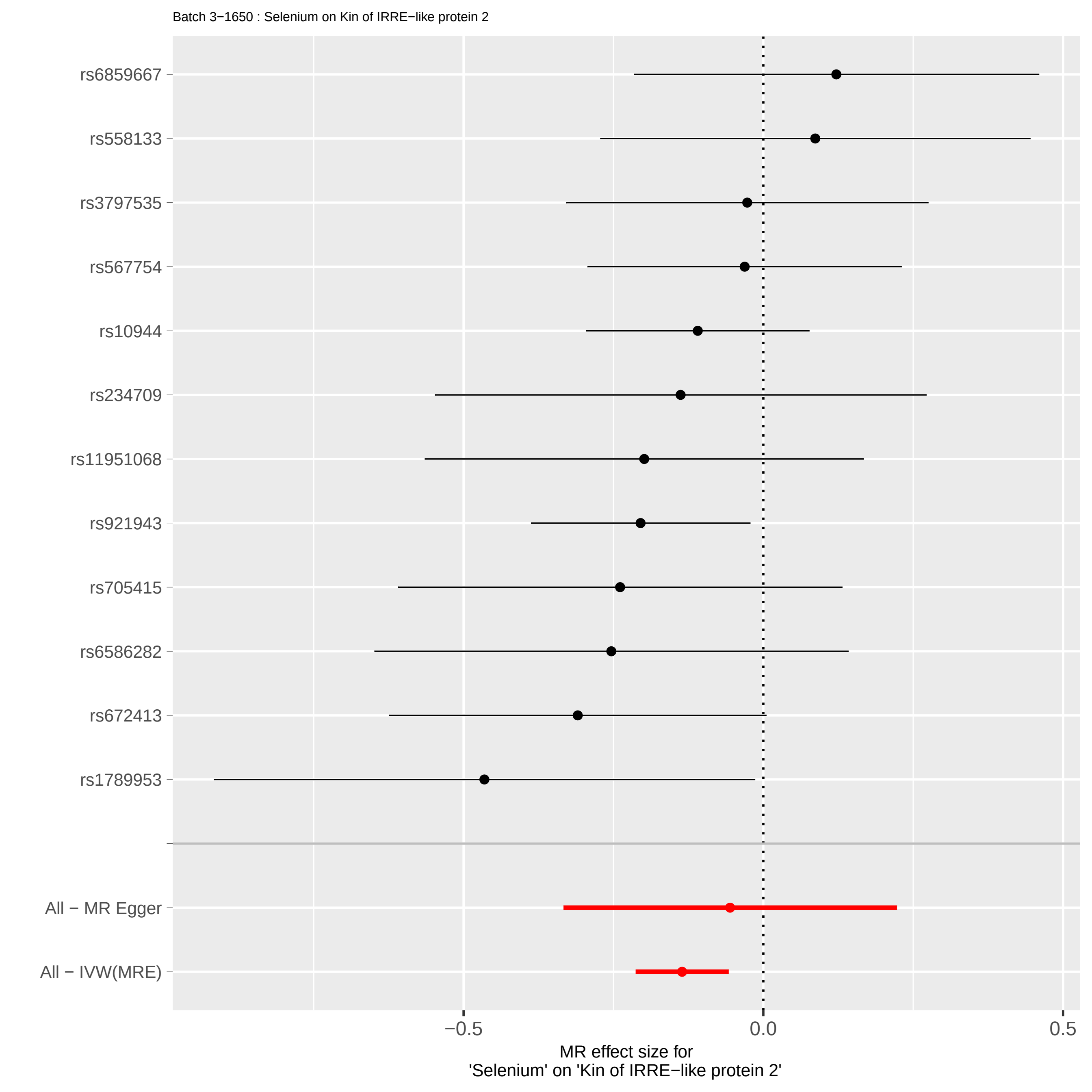

Batch 3−1650 : Selenium on Kin of IRRE−like protein 2
rs6859667
rs558133
rs3797535
rs567754
rs10944
rs234709
rs11951068
rs921943
rs705415
rs6586282
rs672413
rs1789953
All − MR Egger
All − IVW(MRE)
−0.5
0.0
0.5
MR effect size for
'Selenium' on 'Kin of IRRE−like protein 2'

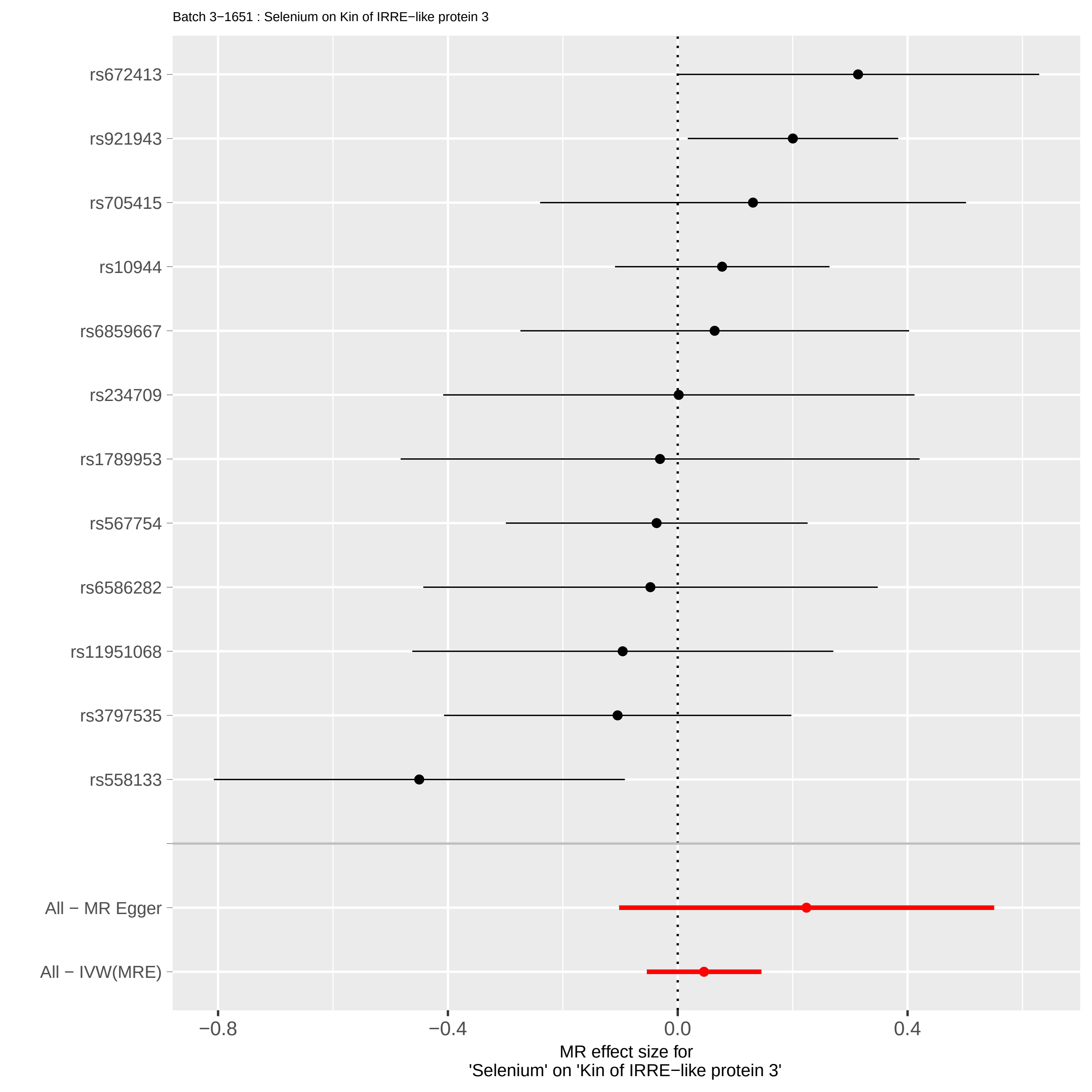

Batch 3−1651 : Selenium on Kin of IRRE−like protein 3
rs672413
rs921943
rs705415
rs10944
rs6859667
rs234709
rs1789953
rs567754
rs6586282
rs11951068
rs3797535
rs558133
All − MR Egger
All − IVW(MRE)
−0.8
−0.4
0.0
0.4
MR effect size for
'Selenium' on 'Kin of IRRE−like protein 3'

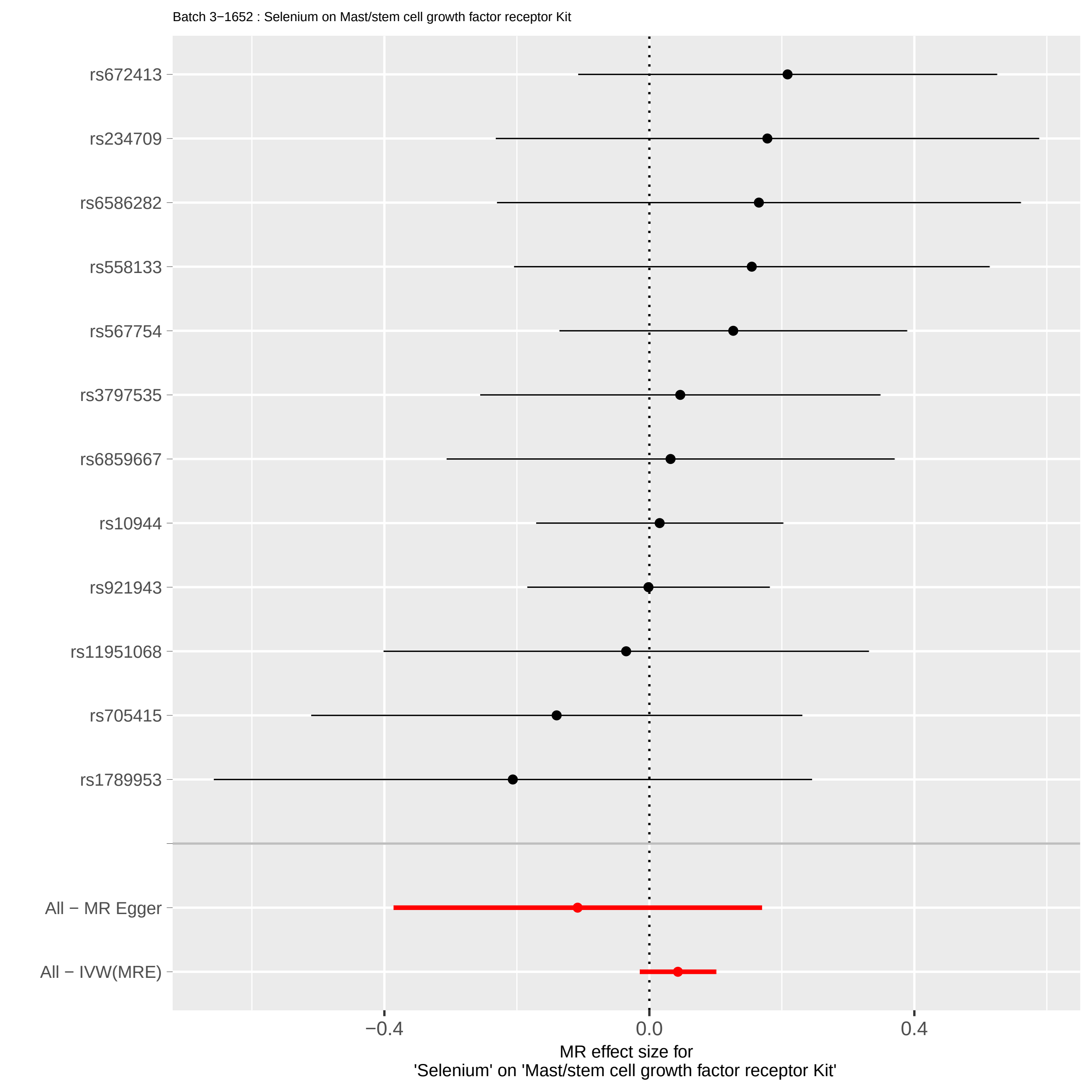

Batch 3−1652 : Selenium on Mast/stem cell growth factor receptor Kit
rs672413
rs234709
rs6586282
rs558133
rs567754
rs3797535
rs6859667
rs10944
rs921943
rs11951068
rs705415
rs1789953
All − MR Egger
All − IVW(MRE)
−0.4
0.0
0.4
MR effect size for
'Selenium' on 'Mast/stem cell growth factor receptor Kit'

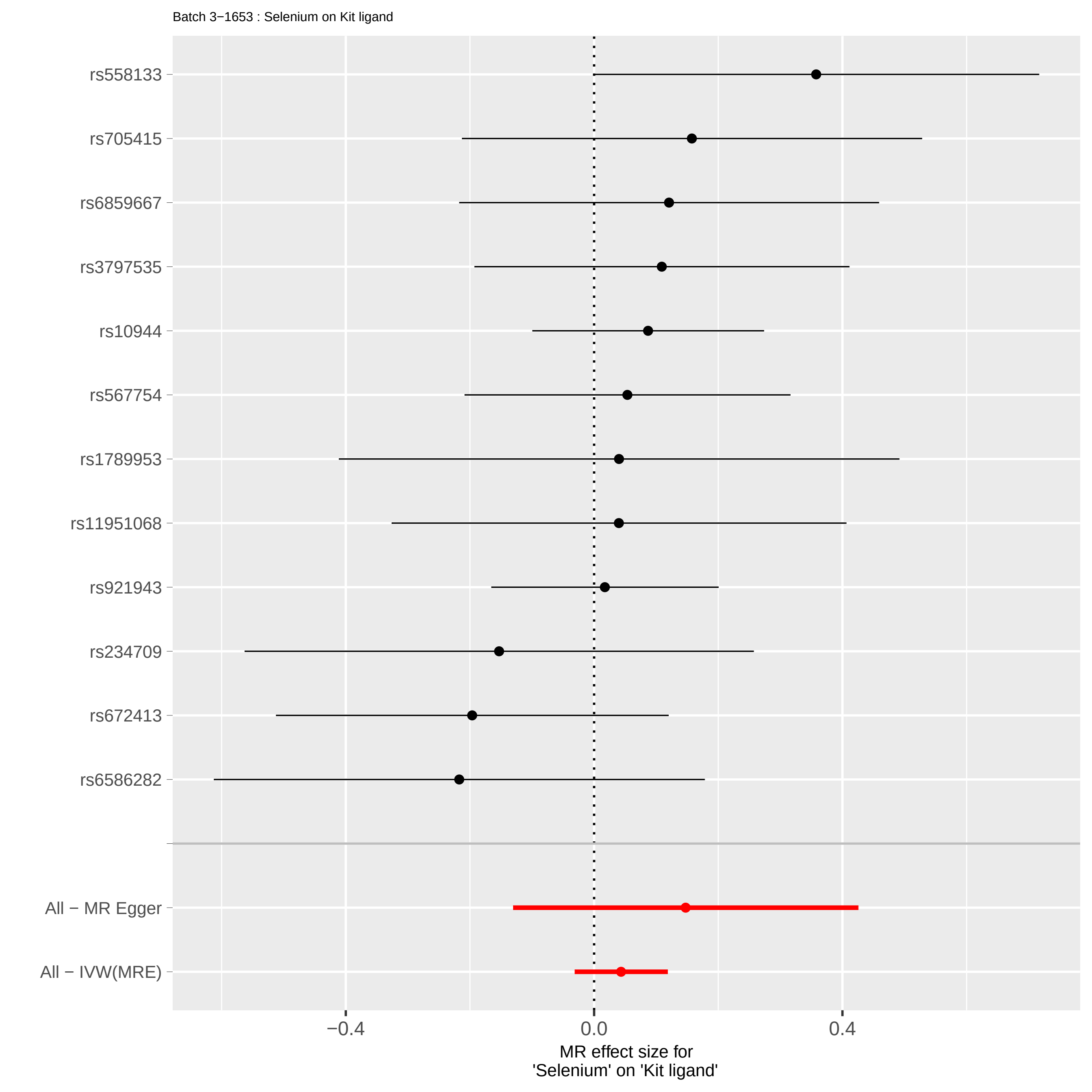

Batch 3−1653 : Selenium on Kit ligand
rs558133
rs705415
rs6859667
rs3797535
rs10944
rs567754
rs1789953
rs11951068
rs921943
rs234709
rs672413
rs6586282
All − MR Egger
All − IVW(MRE)
−0.4
0.0
0.4
MR effect size for
'Selenium' on 'Kit ligand'

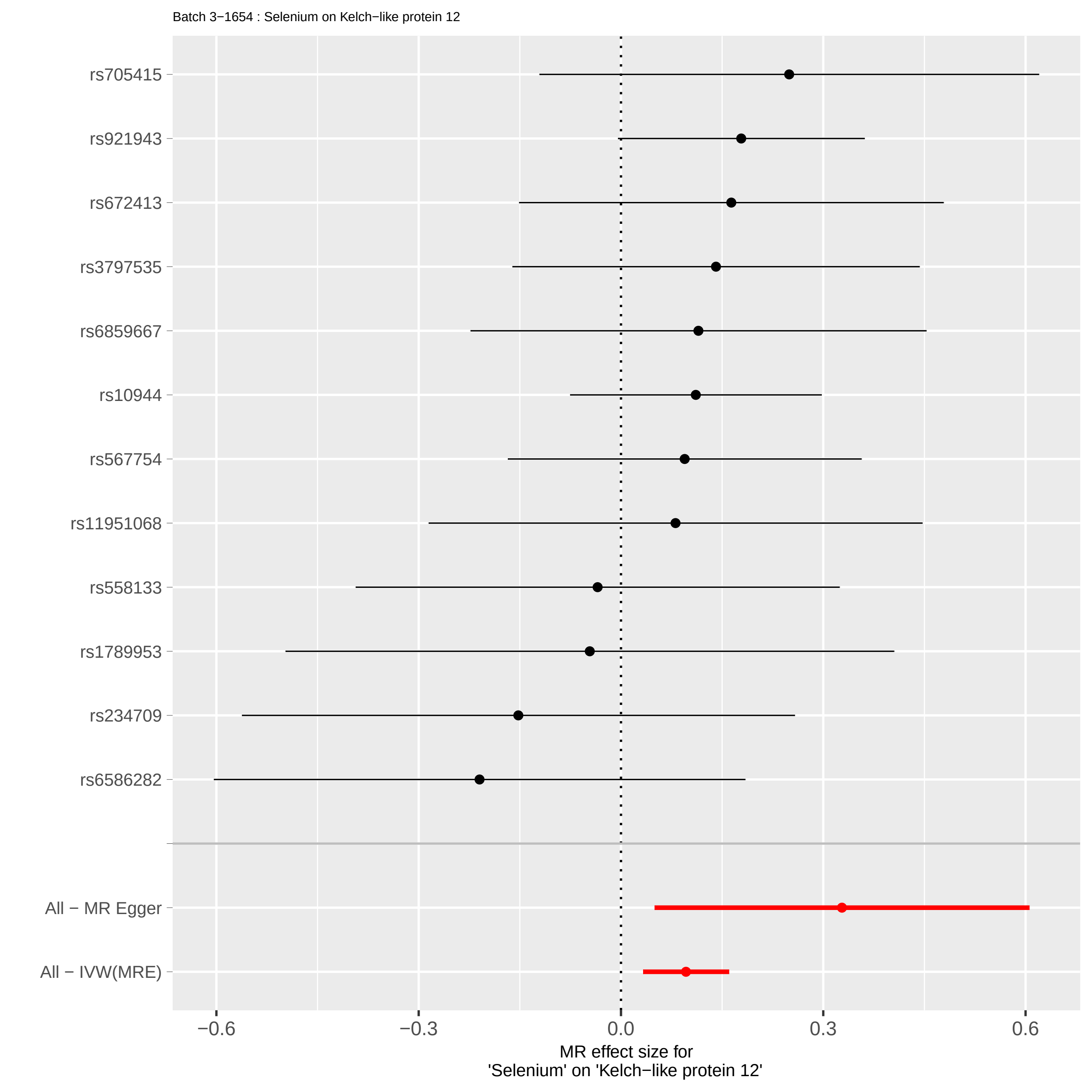

Batch 3−1654 : Selenium on Kelch−like protein 12
rs705415
rs921943
rs672413
rs3797535
rs6859667
rs10944
rs567754
rs11951068
rs558133
rs1789953
rs234709
rs6586282
All − MR Egger
All − IVW(MRE)
−0.6
−0.3
0.0
0.3
0.6
MR effect size for
'Selenium' on 'Kelch−like protein 12'

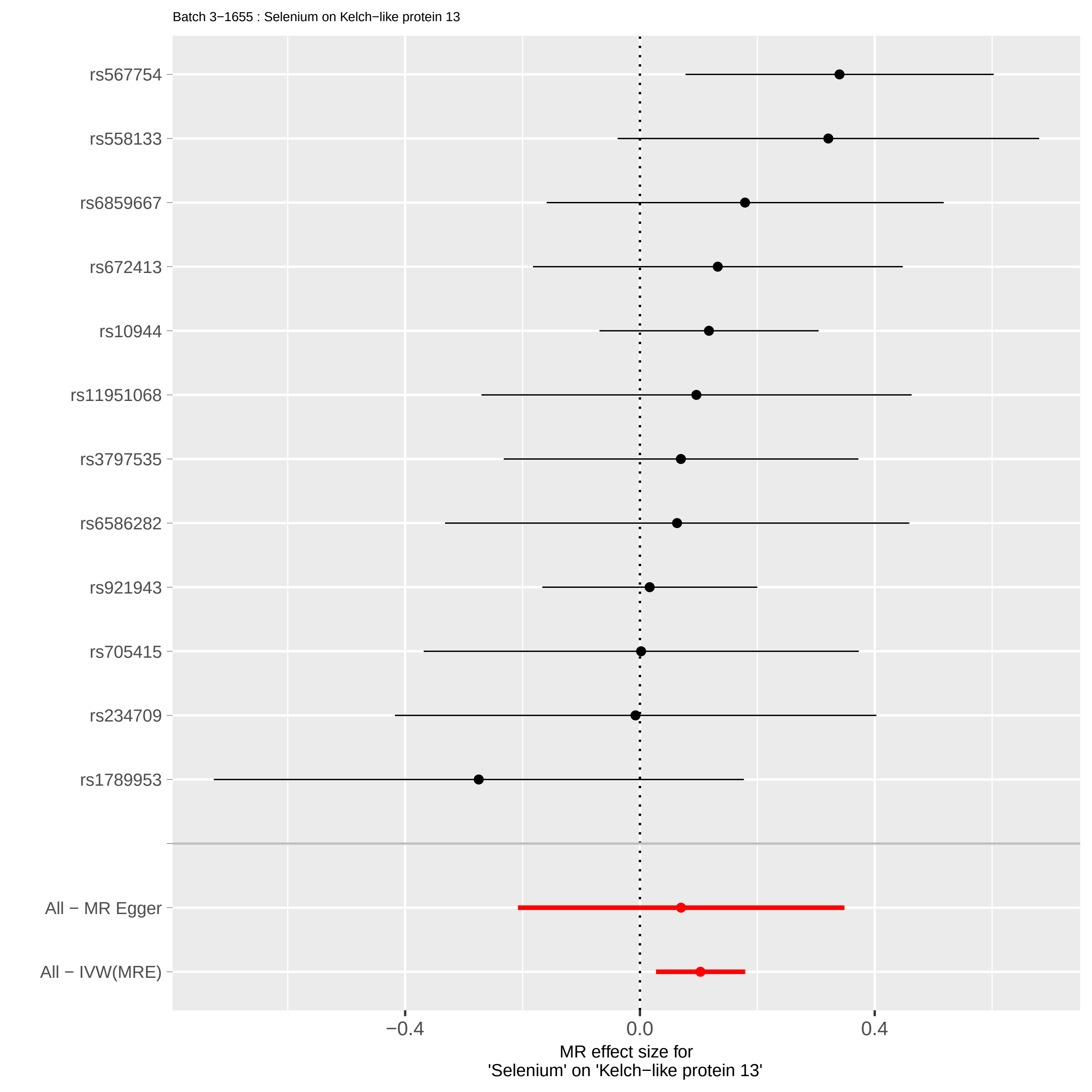

Batch 3−1655 : Selenium on Kelch−like protein 13
rs567754
rs558133
rs6859667
rs672413
rs10944
rs11951068
rs3797535
rs6586282
rs921943
rs705415
rs234709
rs1789953
All − MR Egger
All − IVW(MRE)
−0.4
0.0
0.4
MR effect size for
'Selenium' on 'Kelch−like protein 13'

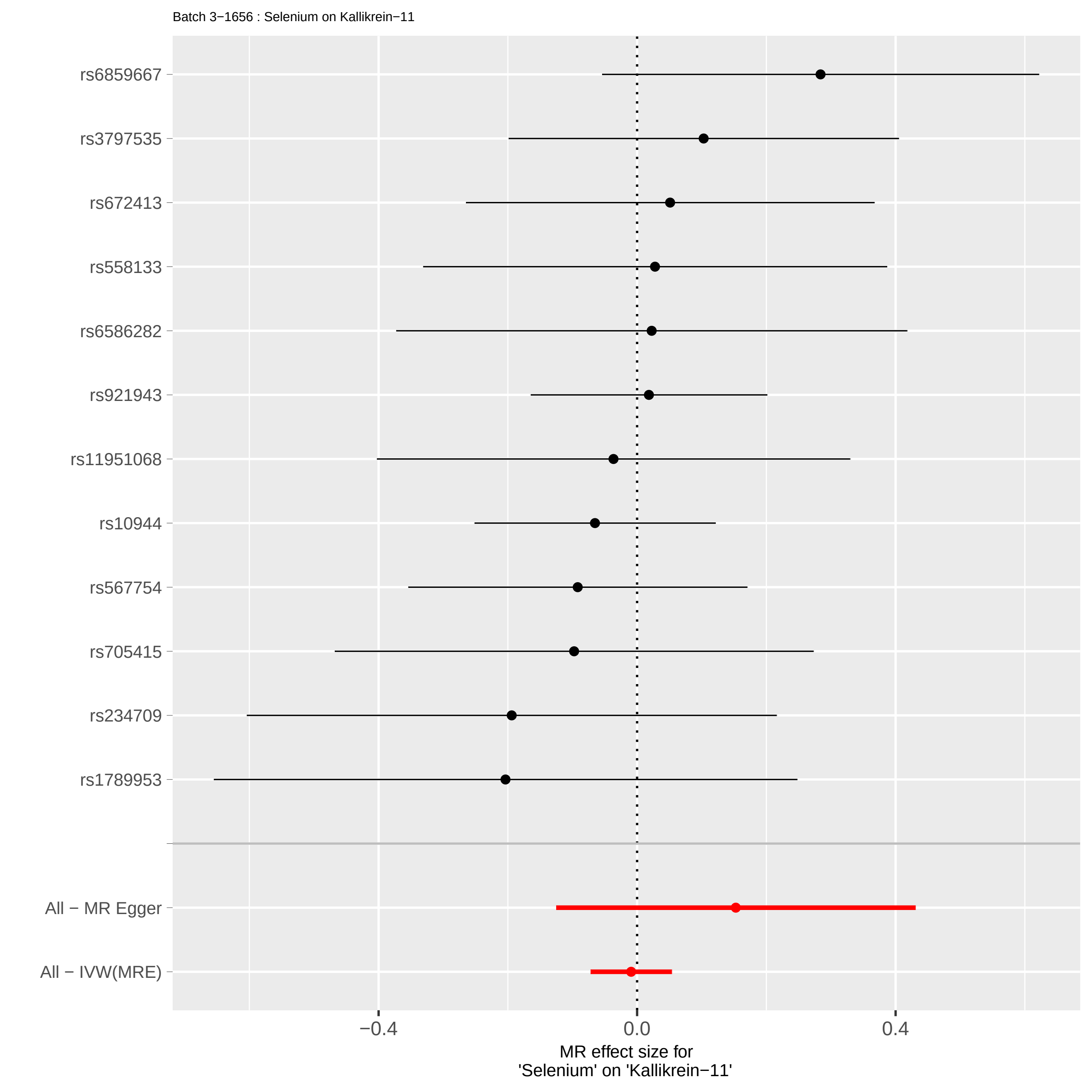

Batch 3−1656 : Selenium on Kallikrein−11
rs6859667
rs3797535
rs672413
rs558133
rs6586282
rs921943
rs11951068
rs10944
rs567754
rs705415
rs234709
rs1789953
All − MR Egger
All − IVW(MRE)
−0.4
0.0
0.4
MR effect size for
'Selenium' on 'Kallikrein−11'

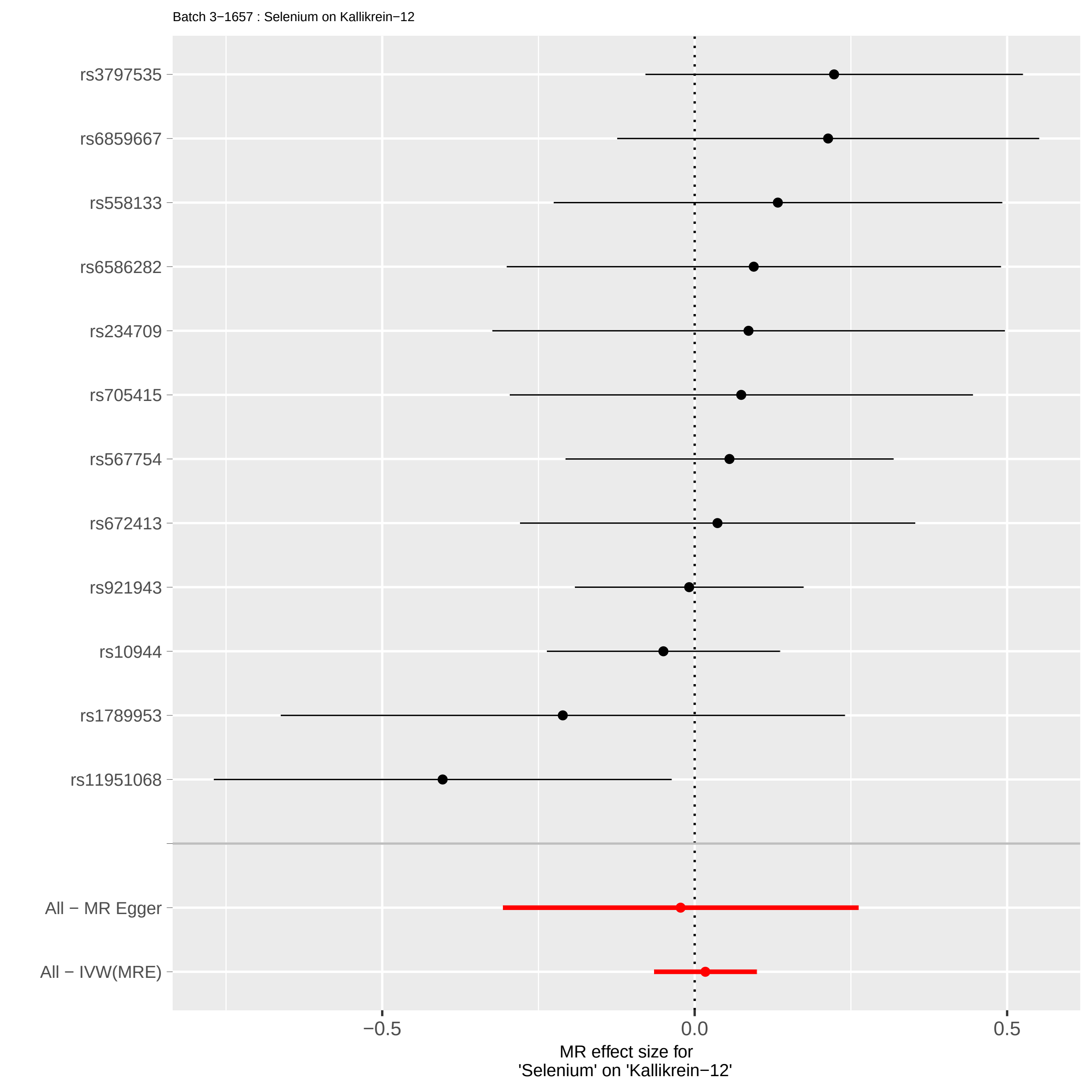
Batch 3−1657 : Selenium on Kallikrein−12
rs3797535
rs6859667
rs558133
rs6586282
rs234709
rs705415
rs567754
rs672413
rs921943
rs10944
rs1789953
rs11951068
All − MR Egger
All − IVW(MRE)
−0.5
0.0
0.5
MR effect size for
'Selenium' on 'Kallikrein−12'

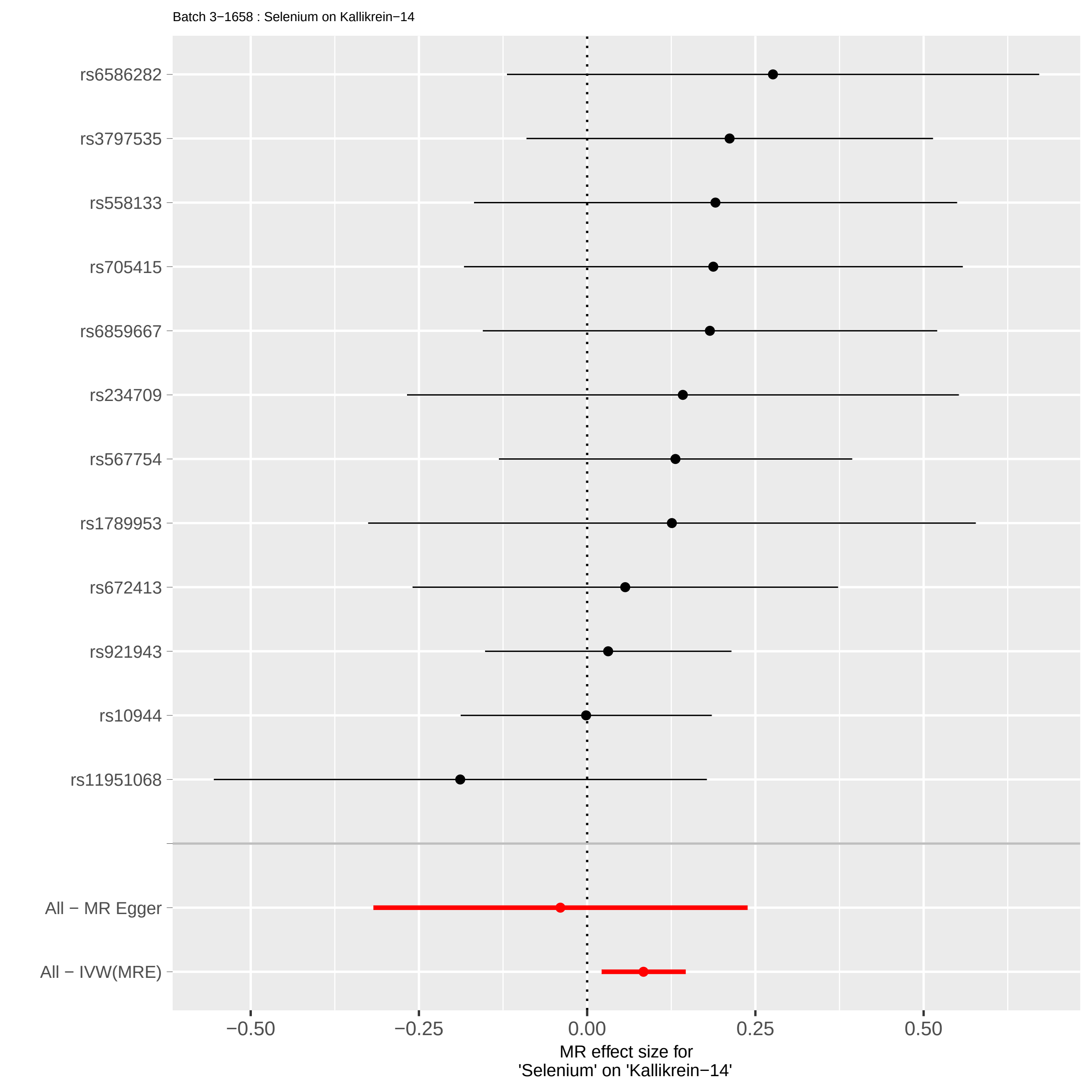

Batch 3−1658 : Selenium on Kallikrein−14
rs6586282
rs3797535
rs558133
rs705415
rs6859667
rs234709
rs567754
rs1789953
rs672413
rs921943
rs10944
rs11951068
All − MR Egger
All − IVW(MRE)
−0.50
−0.25
0.00
0.25
0.50
MR effect size for
'Selenium' on 'Kallikrein−14'

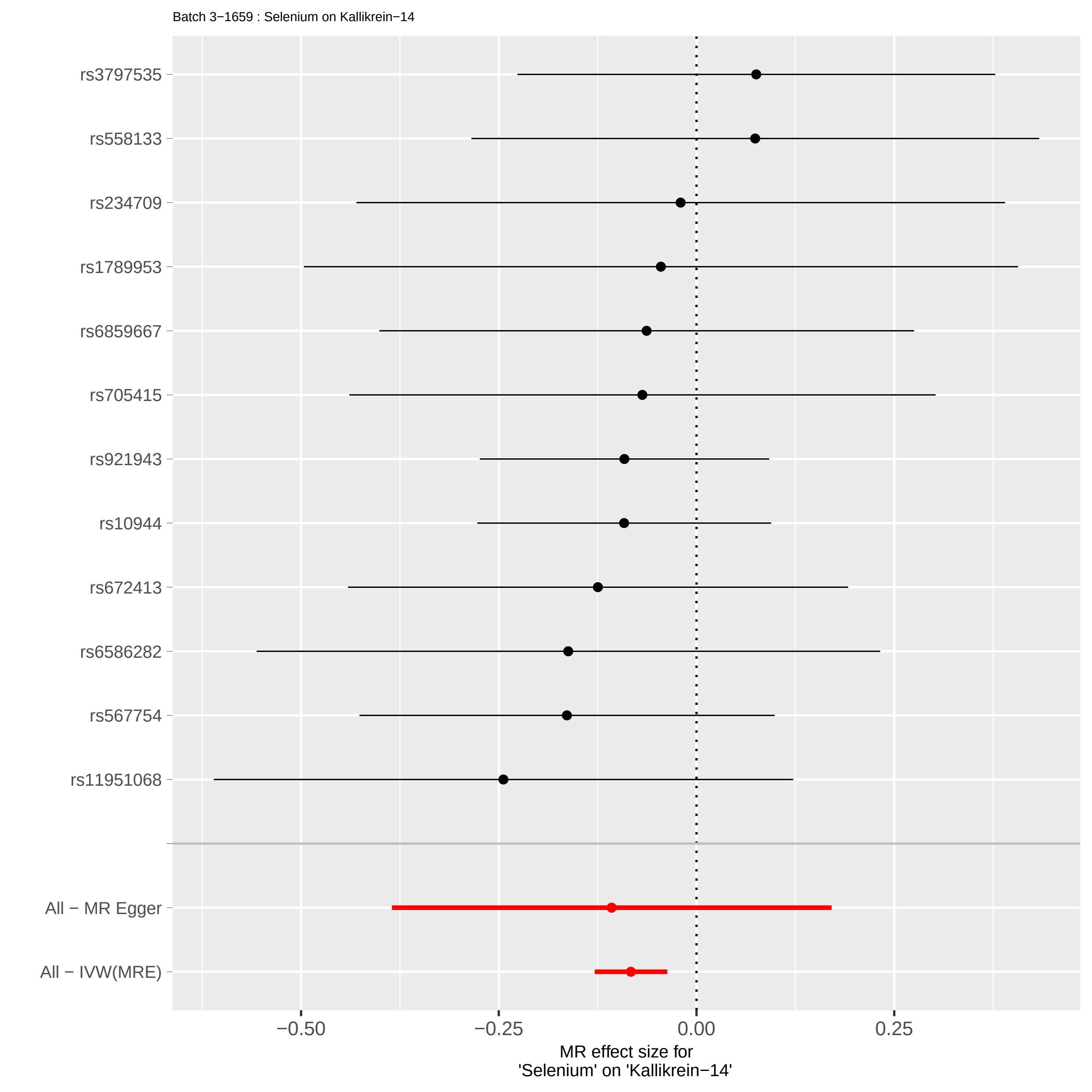

Batch 3−1659 : Selenium on Kallikrein−14
rs3797535
rs558133
rs234709
rs1789953
rs6859667
rs705415
rs921943
rs10944
rs672413
rs6586282
rs567754
rs11951068
All − MR Egger
All − IVW(MRE)
−0.50
−0.25
0.00
0.25
MR effect size for
'Selenium' on 'Kallikrein−14'

Batch 3−1660 : Selenium on Kallikrein−15

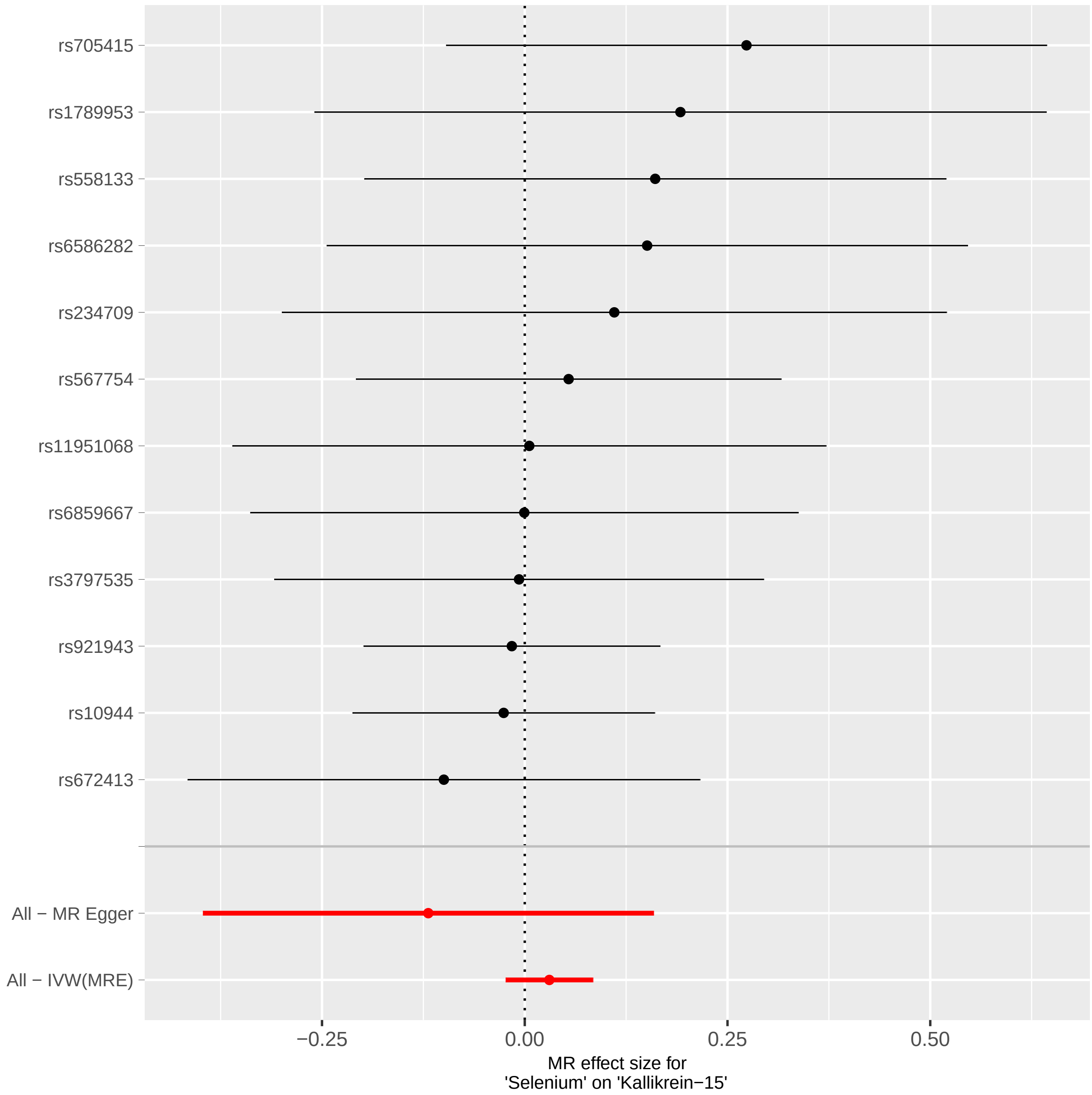

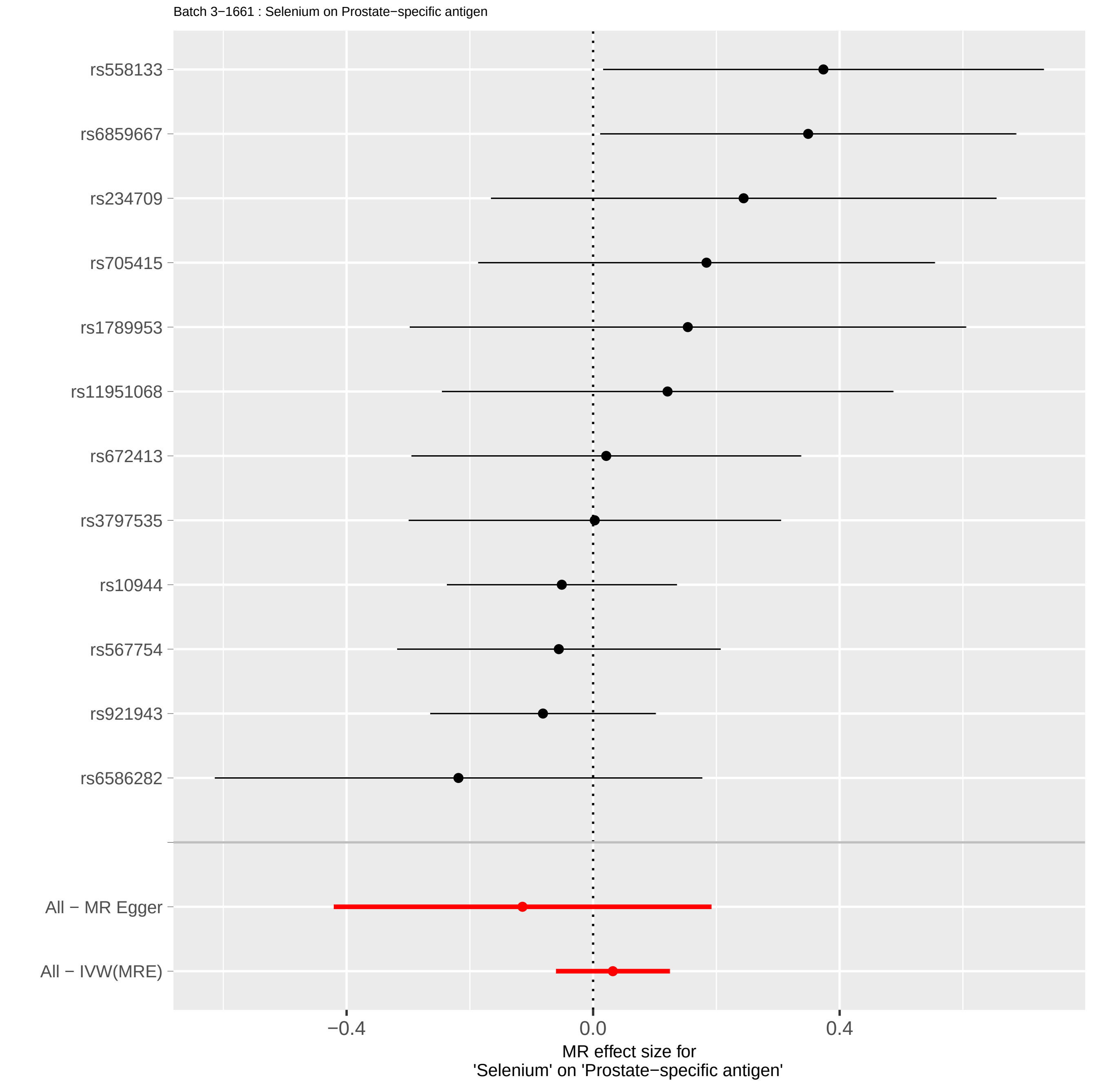

Batch 3−1661 : Selenium on Prostate−specific antigen
rs558133
rs6859667
rs234709
rs705415
rs1789953
rs11951068
rs672413
rs3797535
rs10944
rs567754
rs921943
rs6586282
All − MR Egger
All − IVW(MRE)
−0.4
0.0
0.4
MR effect size for
'Selenium' on 'Prostate−specific antigen'

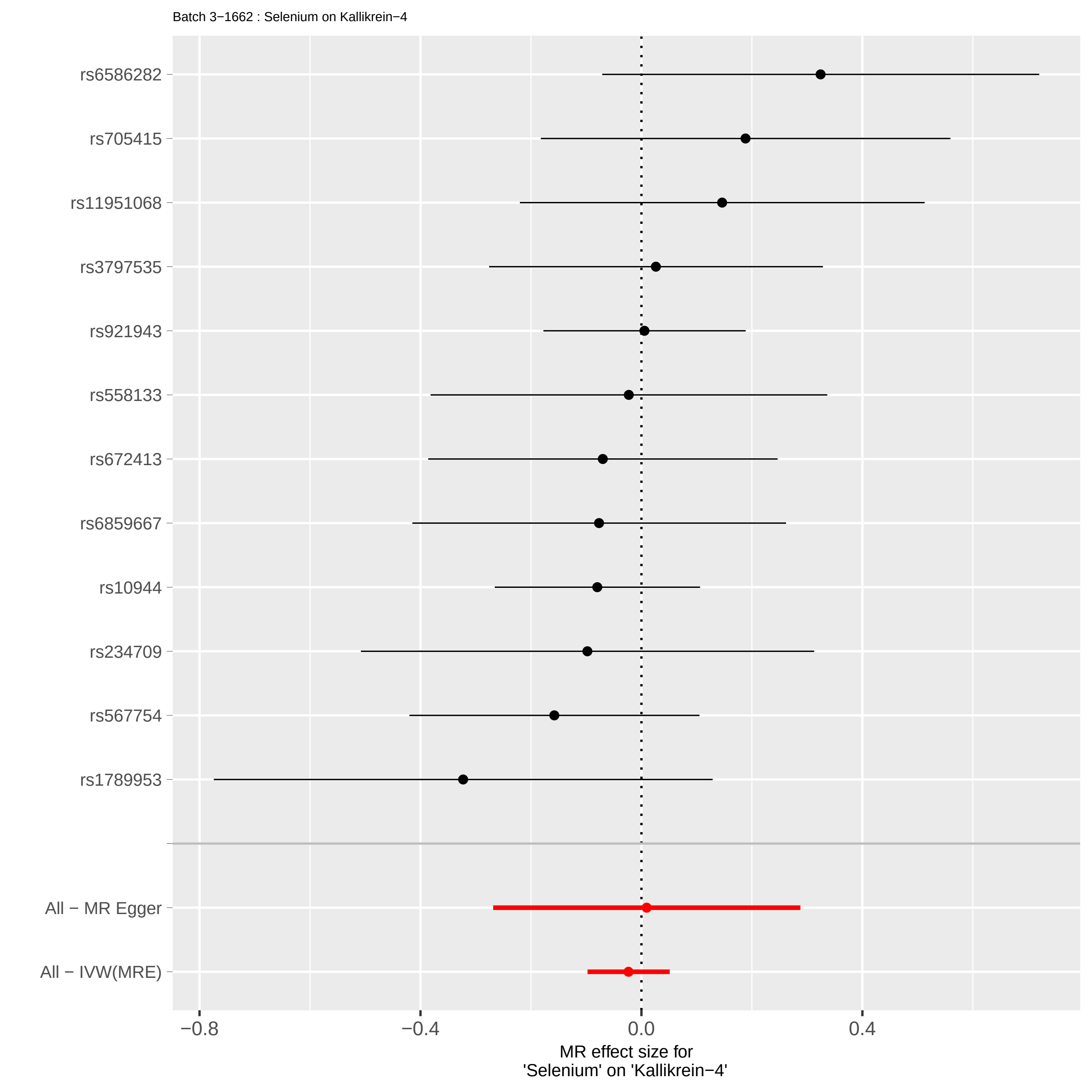
Batch 3−1662 : Selenium on Kallikrein−4
rs6586282
rs705415
rs11951068
rs3797535
rs921943
rs558133
rs672413
rs6859667
rs10944
rs234709
rs567754
rs1789953
All − MR Egger
All − IVW(MRE)
−0.8
−0.4
0.0
0.4
MR effect size for
'Selenium' on 'Kallikrein−4'

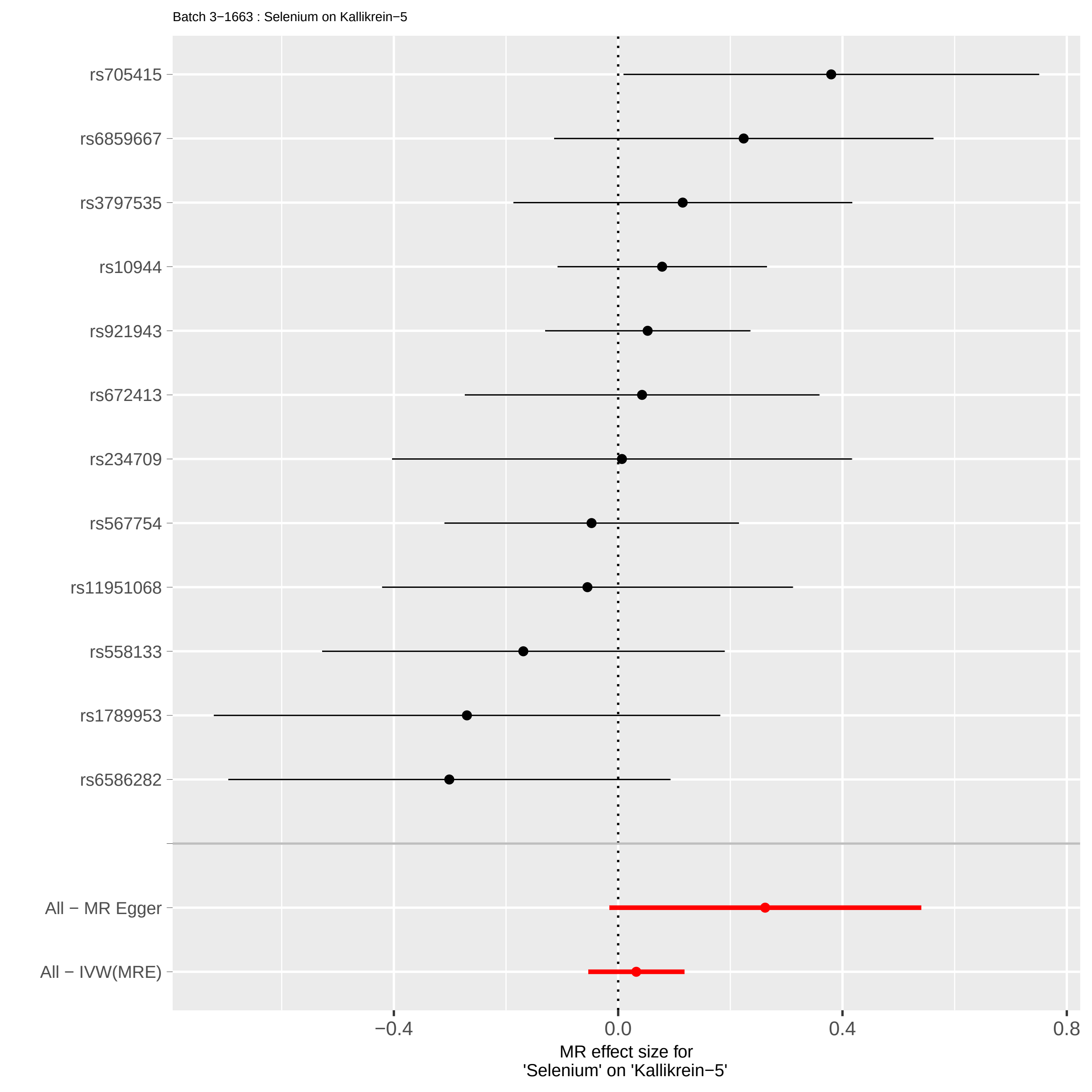

Batch 3−1663 : Selenium on Kallikrein−5
rs705415
rs6859667
rs3797535
rs10944
rs921943
rs672413
rs234709
rs567754
rs11951068
rs558133
rs1789953
rs6586282
All − MR Egger
All − IVW(MRE)
−0.4
0.0
0.4
0.8
MR effect size for
'Selenium' on 'Kallikrein−5'

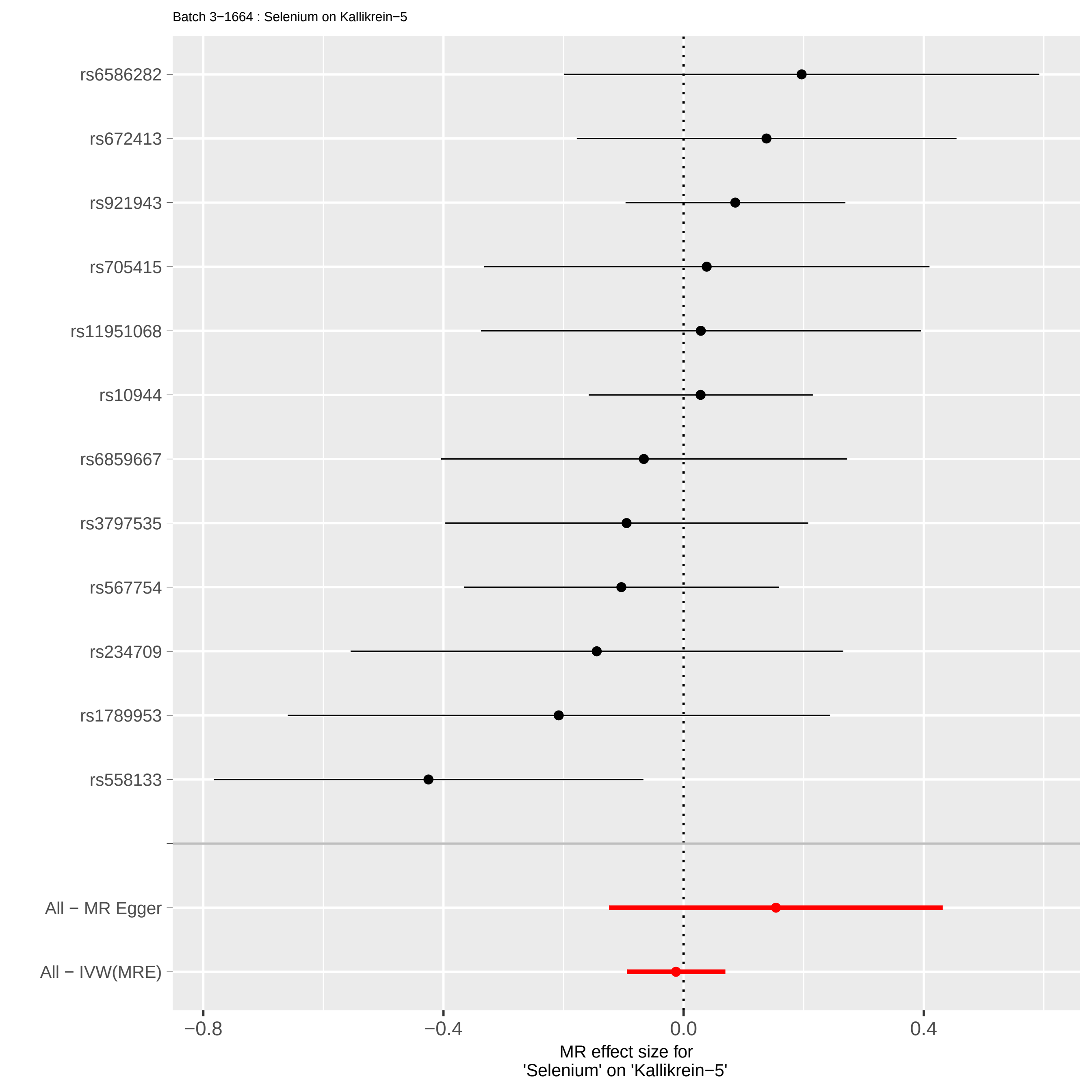

Batch 3−1664 : Selenium on Kallikrein−5
rs6586282
rs672413
rs921943
rs705415
rs11951068
rs10944
rs6859667
rs3797535
rs567754
rs234709
rs1789953
rs558133
All − MR Egger
All − IVW(MRE)
−0.8
−0.4
0.0
0.4
MR effect size for
'Selenium' on 'Kallikrein−5'

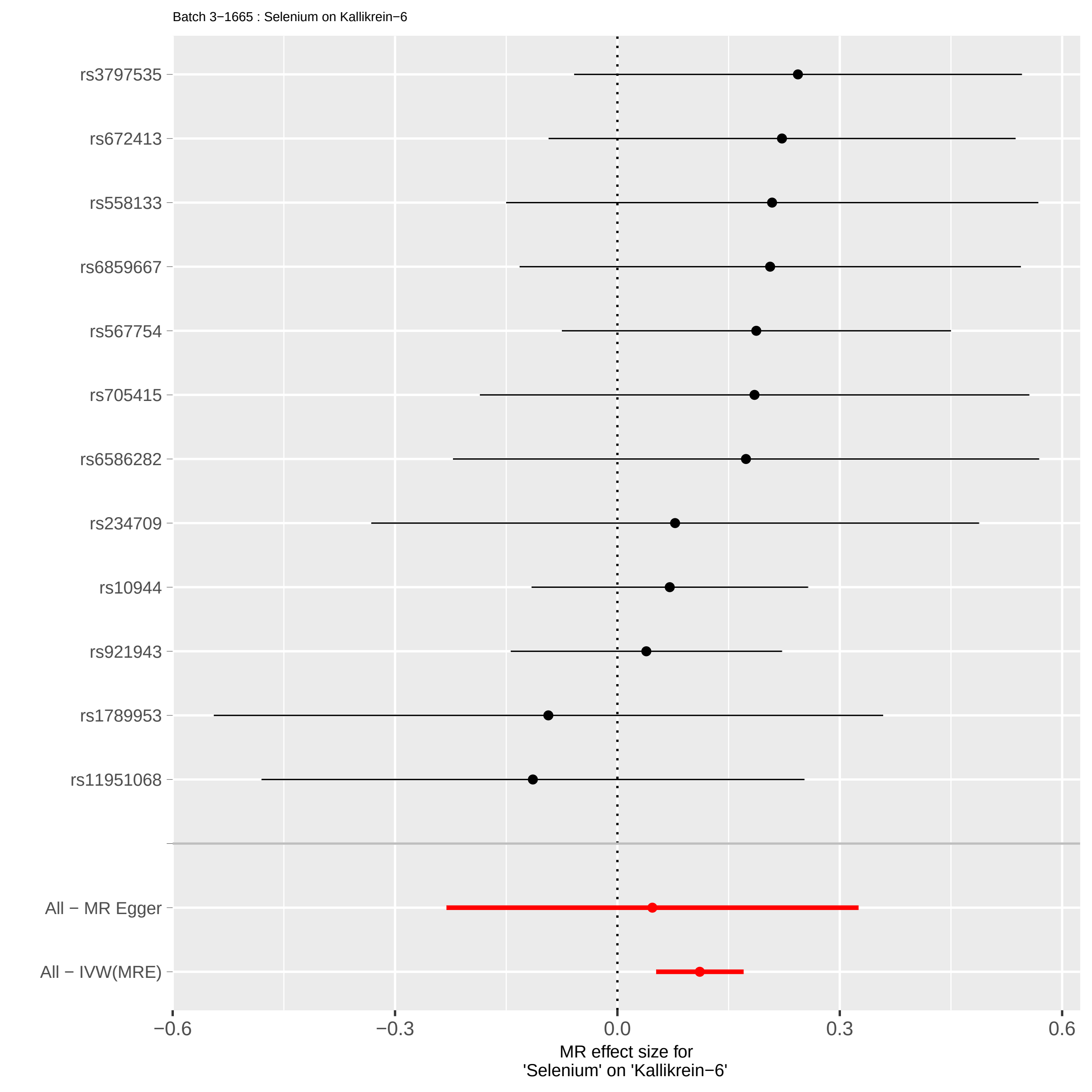
Batch 3−1665 : Selenium on Kallikrein−6
rs3797535
rs672413
rs558133
rs6859667
rs567754
rs705415
rs6586282
rs234709
rs10944
rs921943
rs1789953
rs11951068
All − MR Egger
All − IVW(MRE)
−0.6
−0.3
0.0
0.3
0.6
MR effect size for
'Selenium' on 'Kallikrein−6'

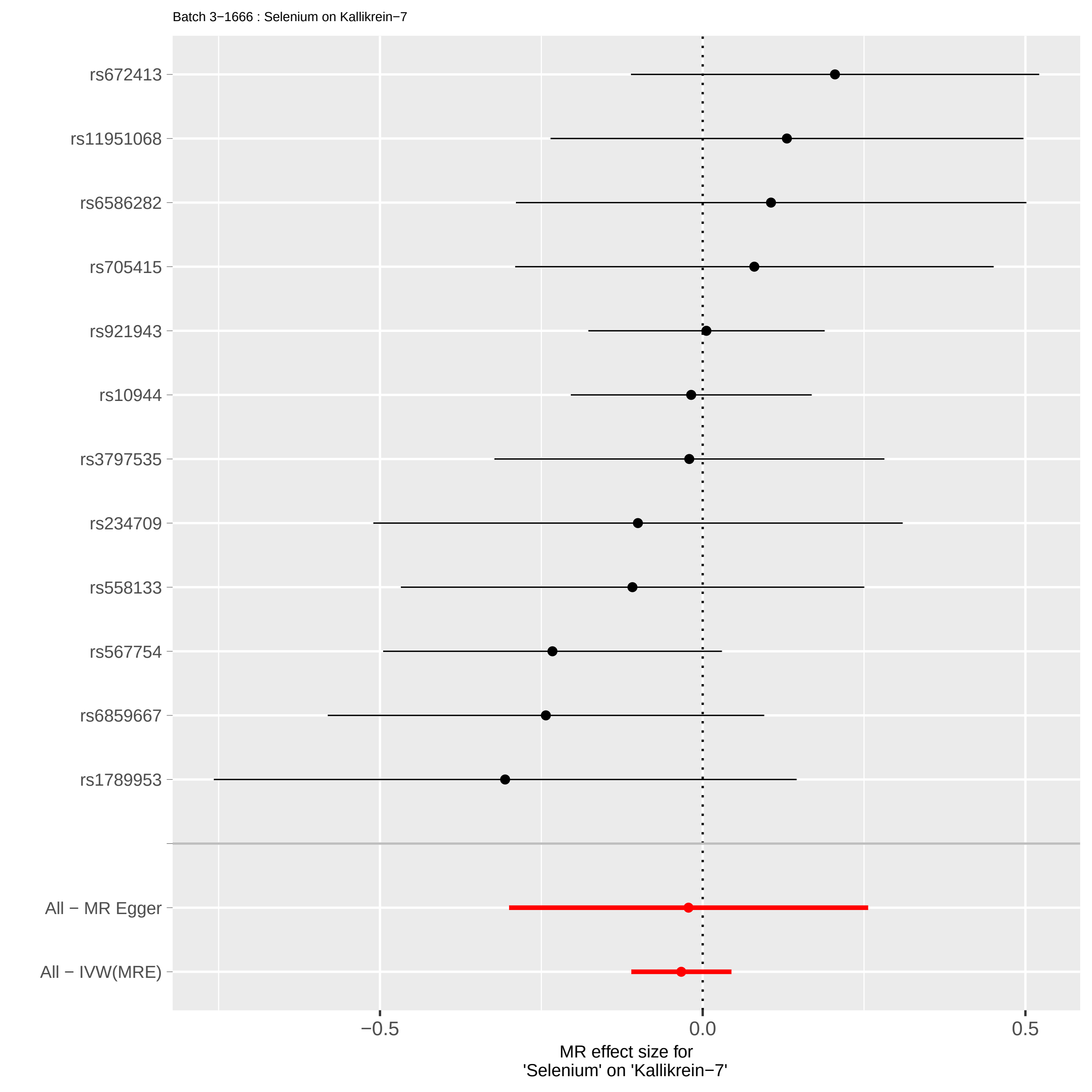
Batch 3−1666 : Selenium on Kallikrein−7
rs672413
rs11951068
rs6586282
rs705415
rs921943
rs10944
rs3797535
rs234709
rs558133
rs567754
rs6859667
rs1789953
All − MR Egger
All − IVW(MRE)
−0.5
0.0
0.5
MR effect size for
'Selenium' on 'Kallikrein−7'

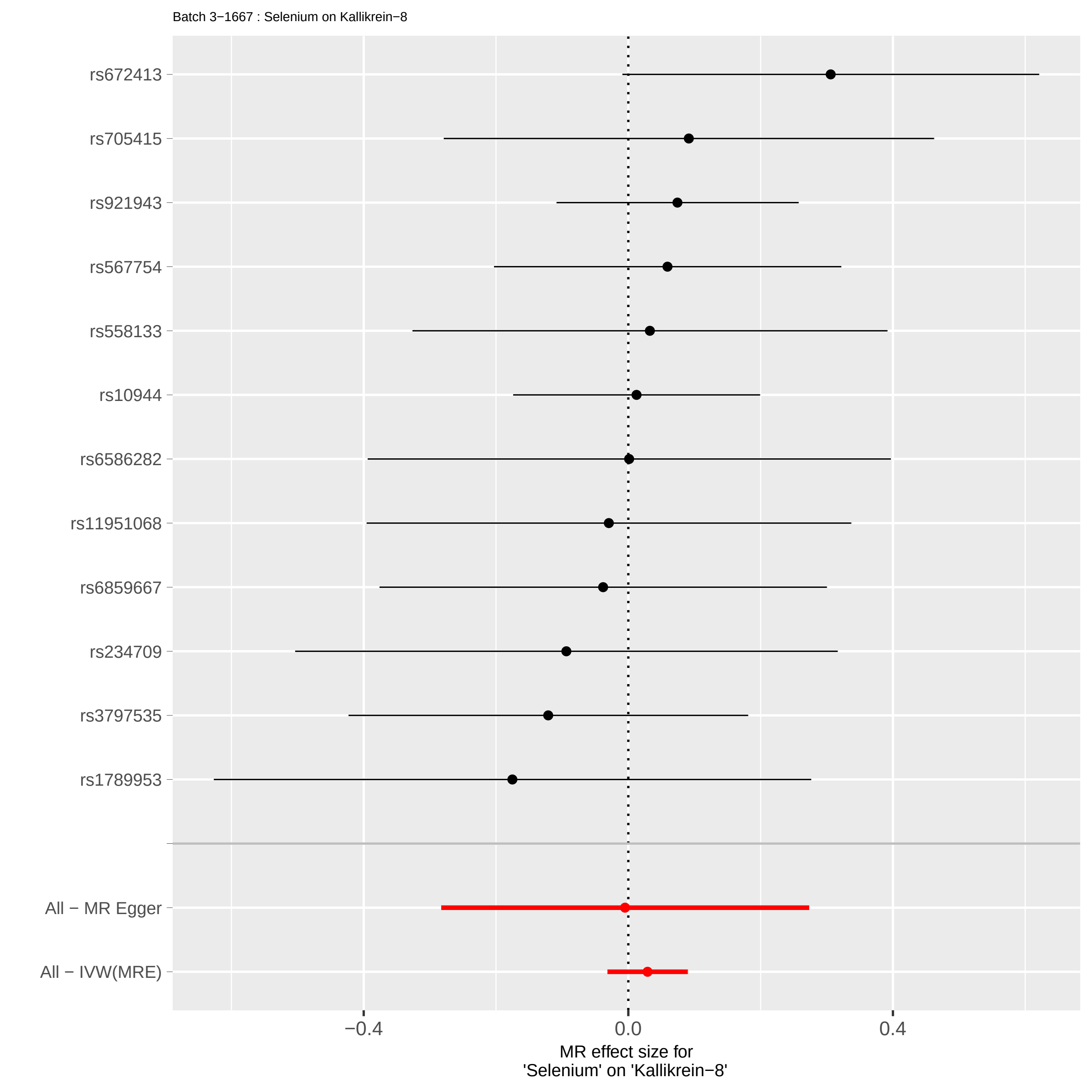

Batch 3−1667 : Selenium on Kallikrein−8
rs672413
rs705415
rs921943
rs567754
rs558133
rs10944
rs6586282
rs11951068
rs6859667
rs234709
rs3797535
rs1789953
All − MR Egger
All − IVW(MRE)
−0.4
0.0
0.4
MR effect size for
'Selenium' on 'Kallikrein−8'

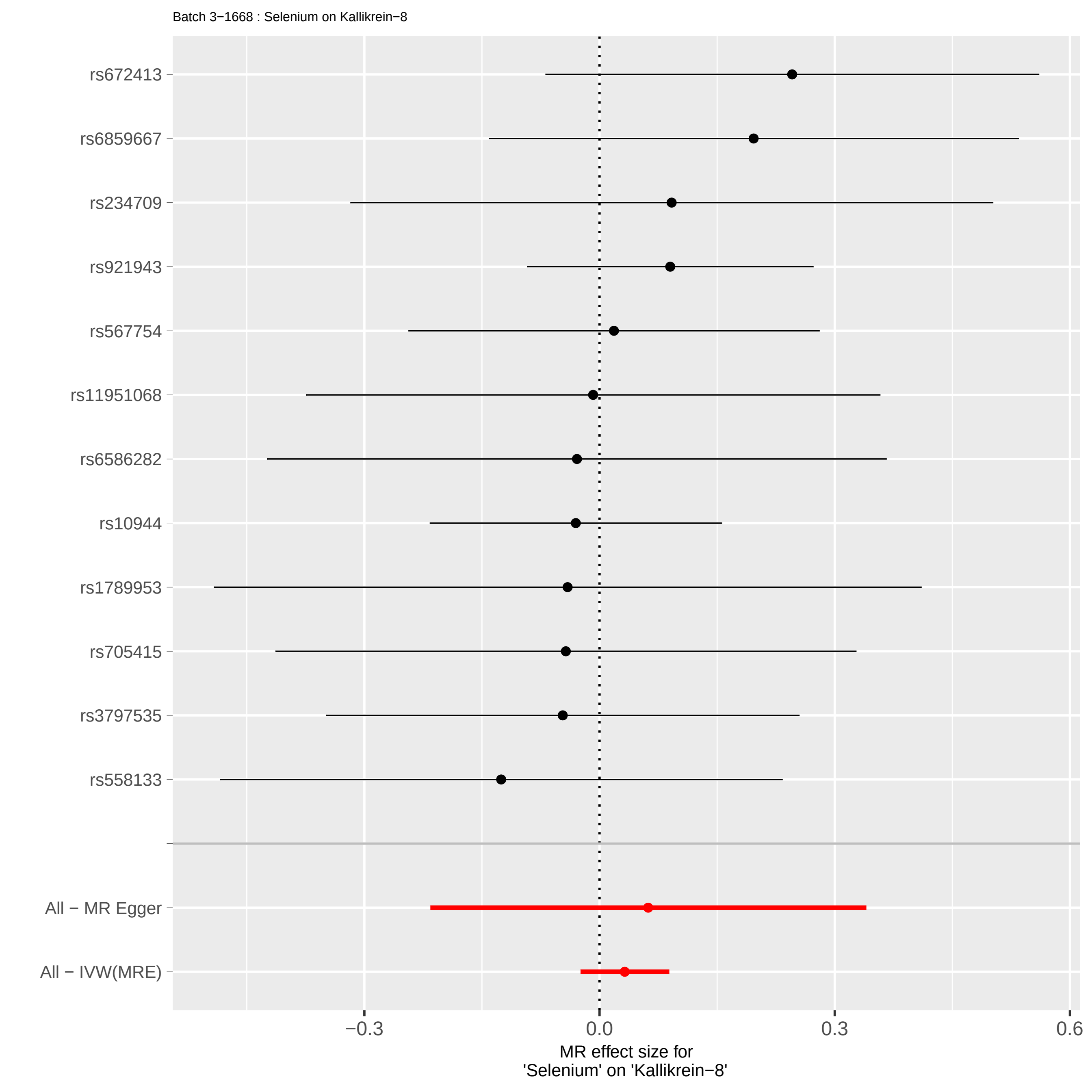

Batch 3−1668 : Selenium on Kallikrein−8
rs672413
rs6859667
rs234709
rs921943
rs567754
rs11951068
rs6586282
rs10944
rs1789953
rs705415
rs3797535
rs558133
All − MR Egger
All − IVW(MRE)
−0.3
0.0
0.3
0.6
MR effect size for
'Selenium' on 'Kallikrein−8'

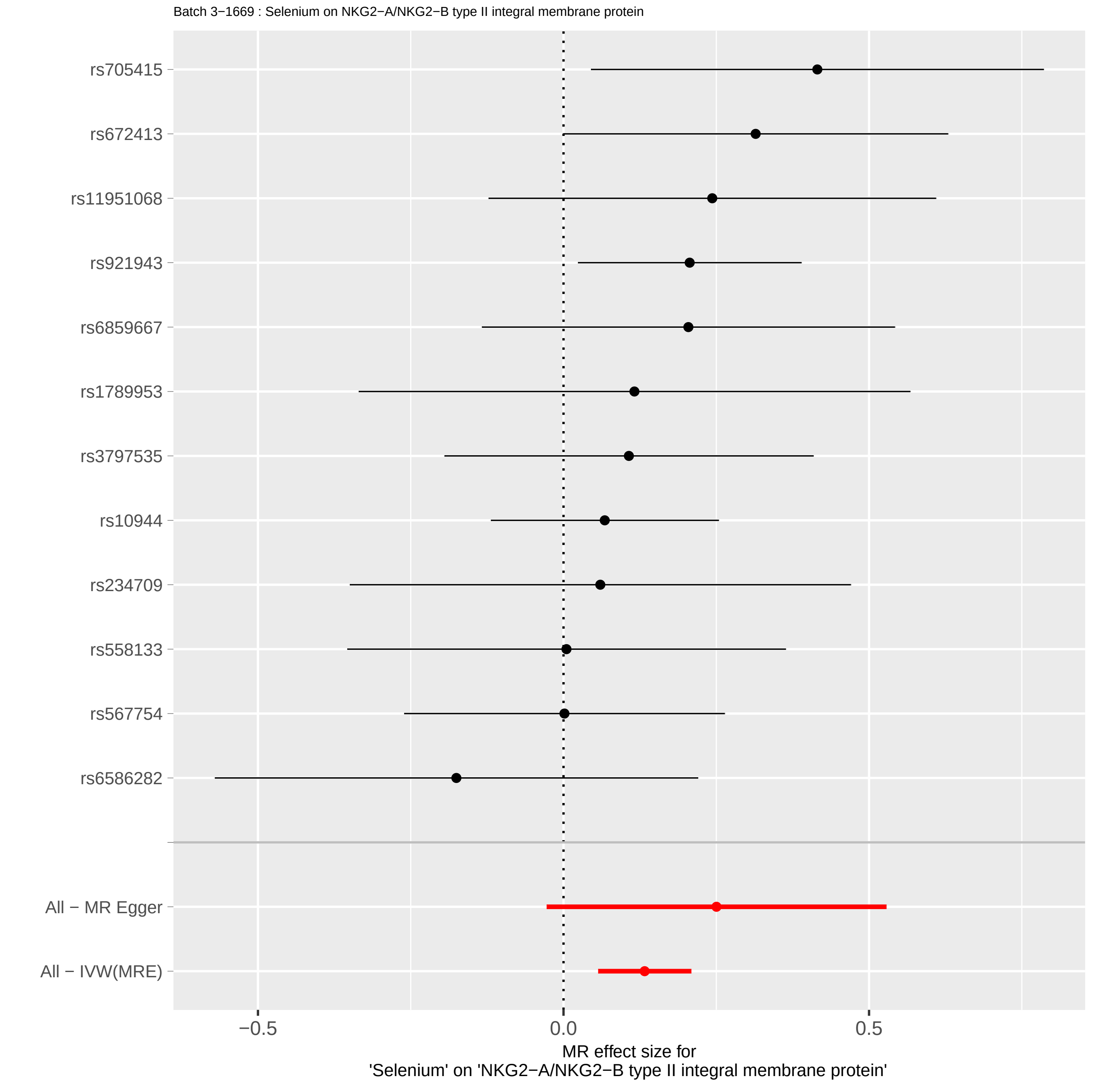
Batch 3−1669 : Selenium on NKG2−A/NKG2−B type II integral membrane protein
rs705415
rs672413
rs11951068
rs921943
rs6859667
rs1789953
rs3797535
rs10944
rs234709
rs558133
rs567754
rs6586282
All − MR Egger
All − IVW(MRE)
−0.5
0.0
0.5
MR effect size for
'Selenium' on 'NKG2−A/NKG2−B type II integral membrane protein'

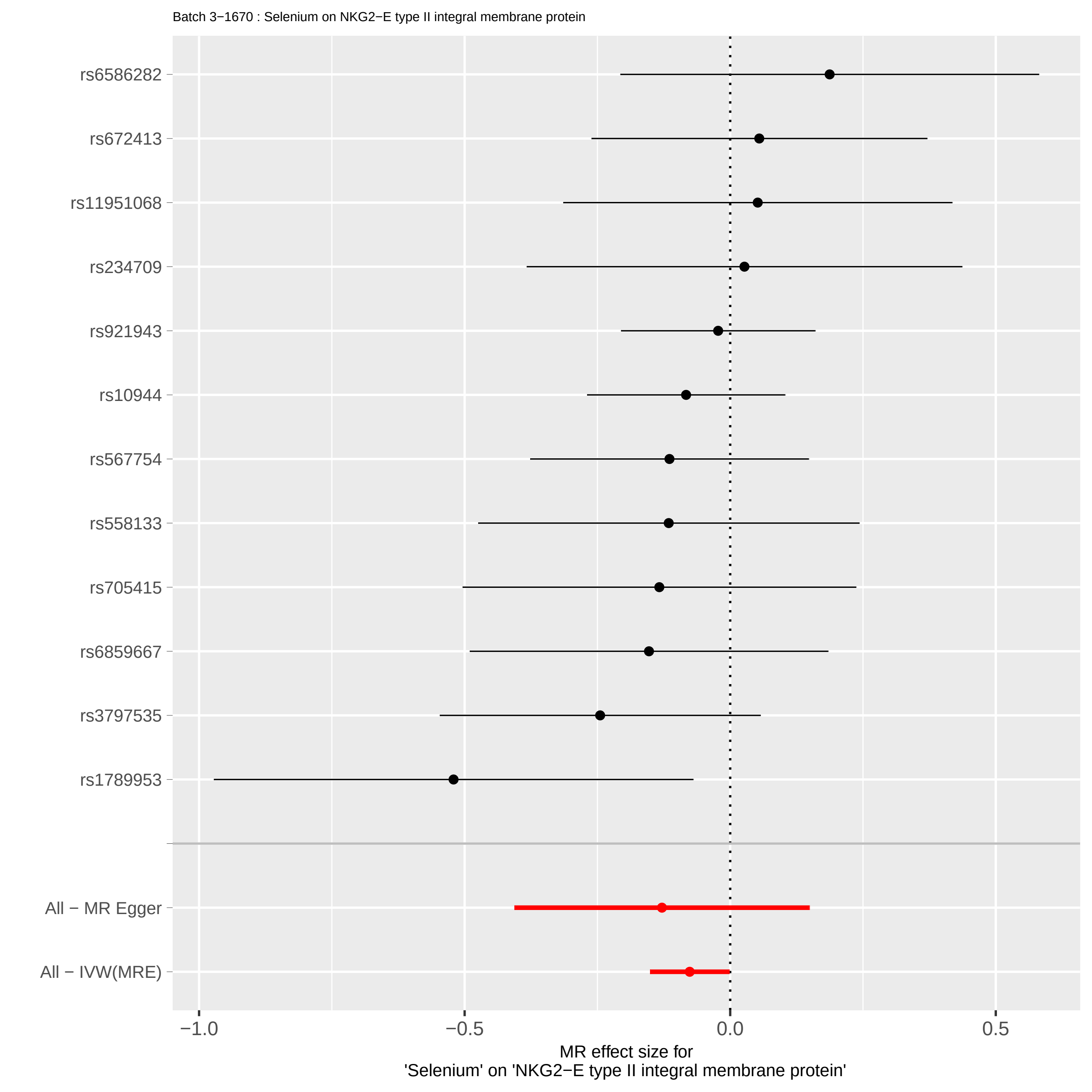

Batch 3−1670 : Selenium on NKG2−E type II integral membrane protein
rs6586282
rs672413
rs11951068
rs234709
rs921943
rs10944
rs567754
rs558133
rs705415
rs6859667
rs3797535
rs1789953
All − MR Egger
All − IVW(MRE)
−1.0
−0.5
0.0
0.5
MR effect size for
'Selenium' on 'NKG2−E type II integral membrane protein'

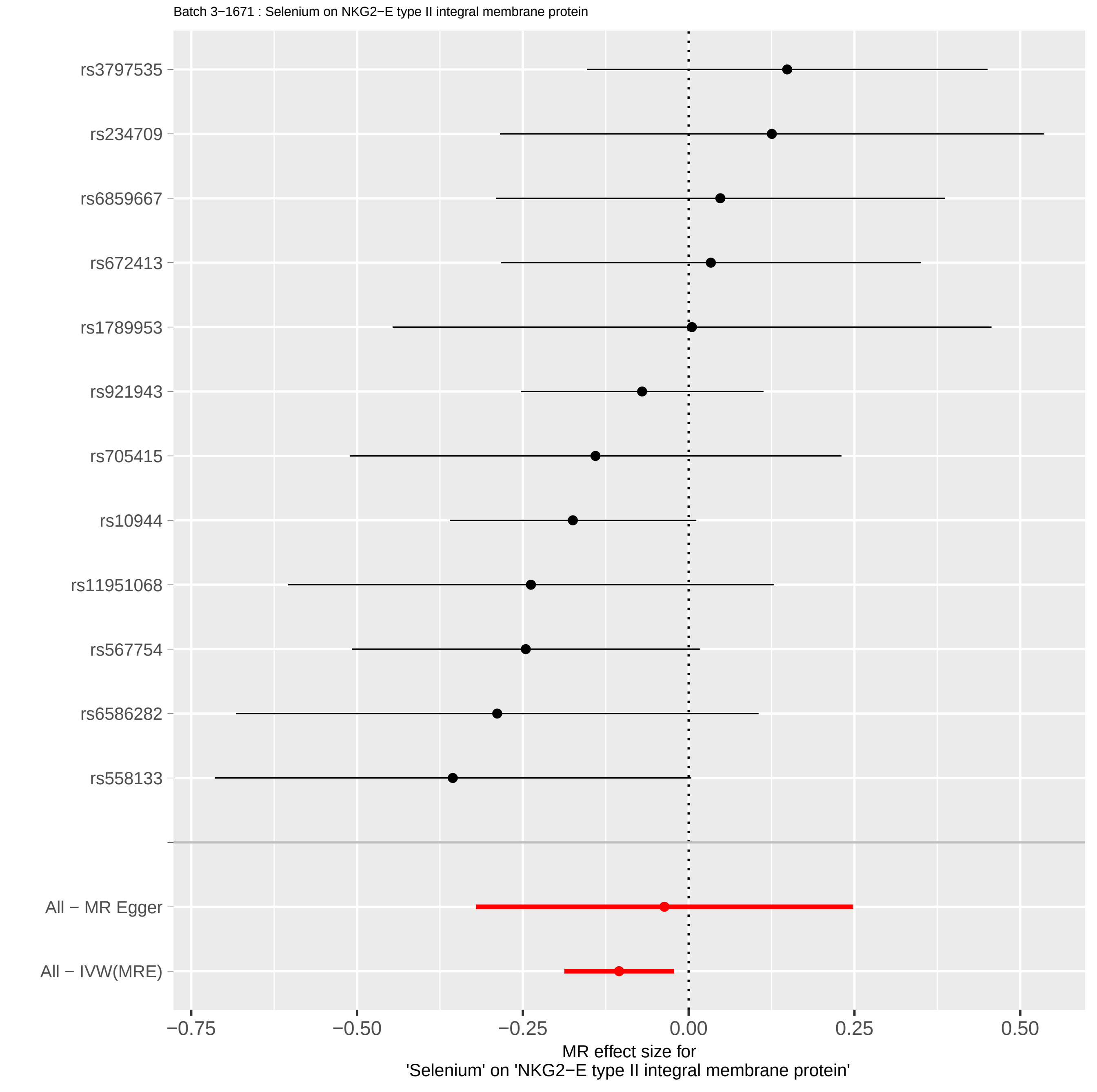

Batch 3−1671 : Selenium on NKG2−E type II integral membrane protein
rs3797535
rs234709
rs6859667
rs672413
rs1789953
rs921943
rs705415
rs10944
rs11951068
rs567754
rs6586282
rs558133
All − MR Egger
All − IVW(MRE)
−0.75
−0.50
−0.25
0.00
0.25
0.50
MR effect size for
'Selenium' on 'NKG2−E type II integral membrane protein'

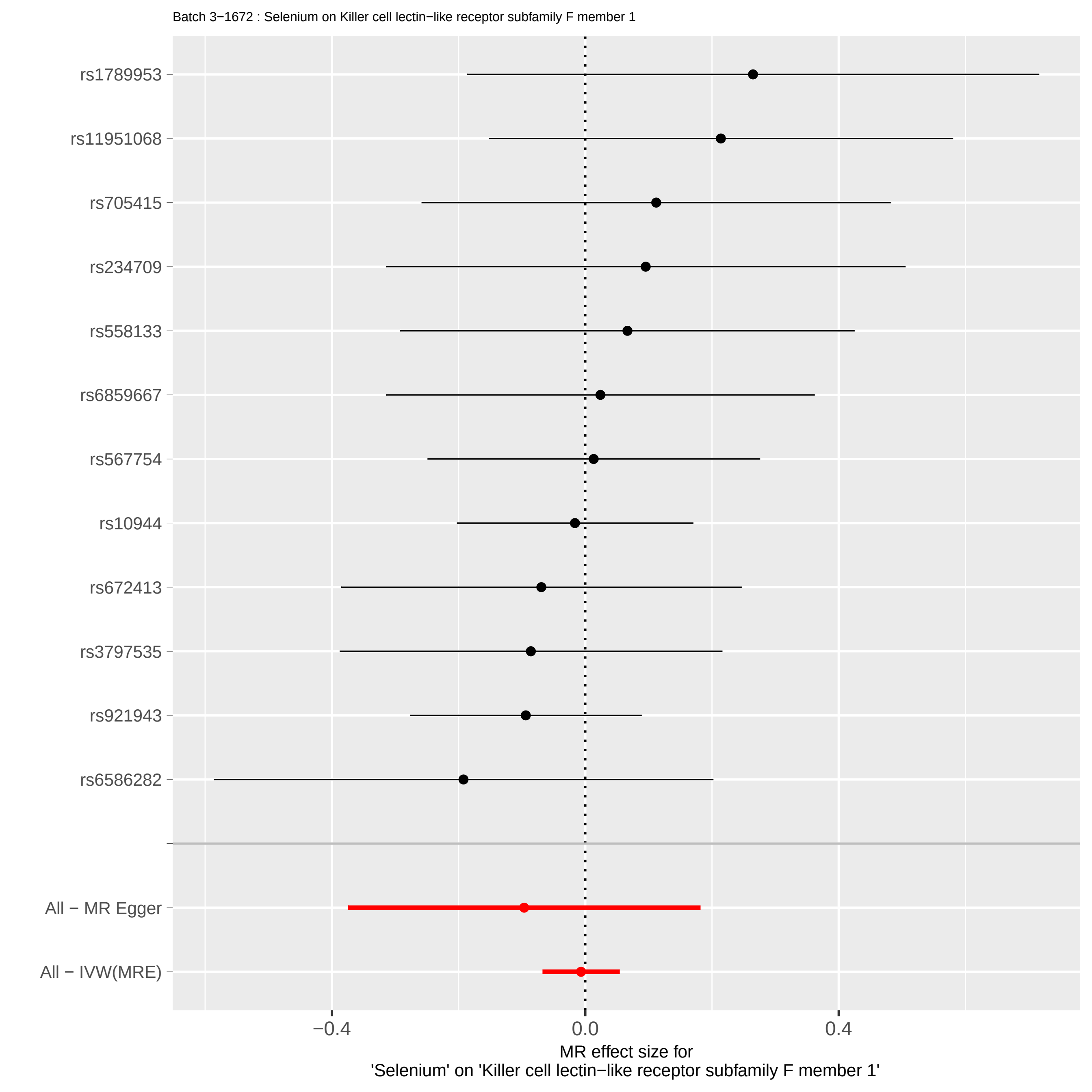

Batch 3−1672 : Selenium on Killer cell lectin−like receptor subfamily F member 1
rs1789953
rs11951068
rs705415
rs234709
rs558133
rs6859667
rs567754
rs10944
rs672413
rs3797535
rs921943
rs6586282
All − MR Egger
All − IVW(MRE)
−0.4
0.0
0.4
MR effect size for
'Selenium' on 'Killer cell lectin−like receptor subfamily F member 1'

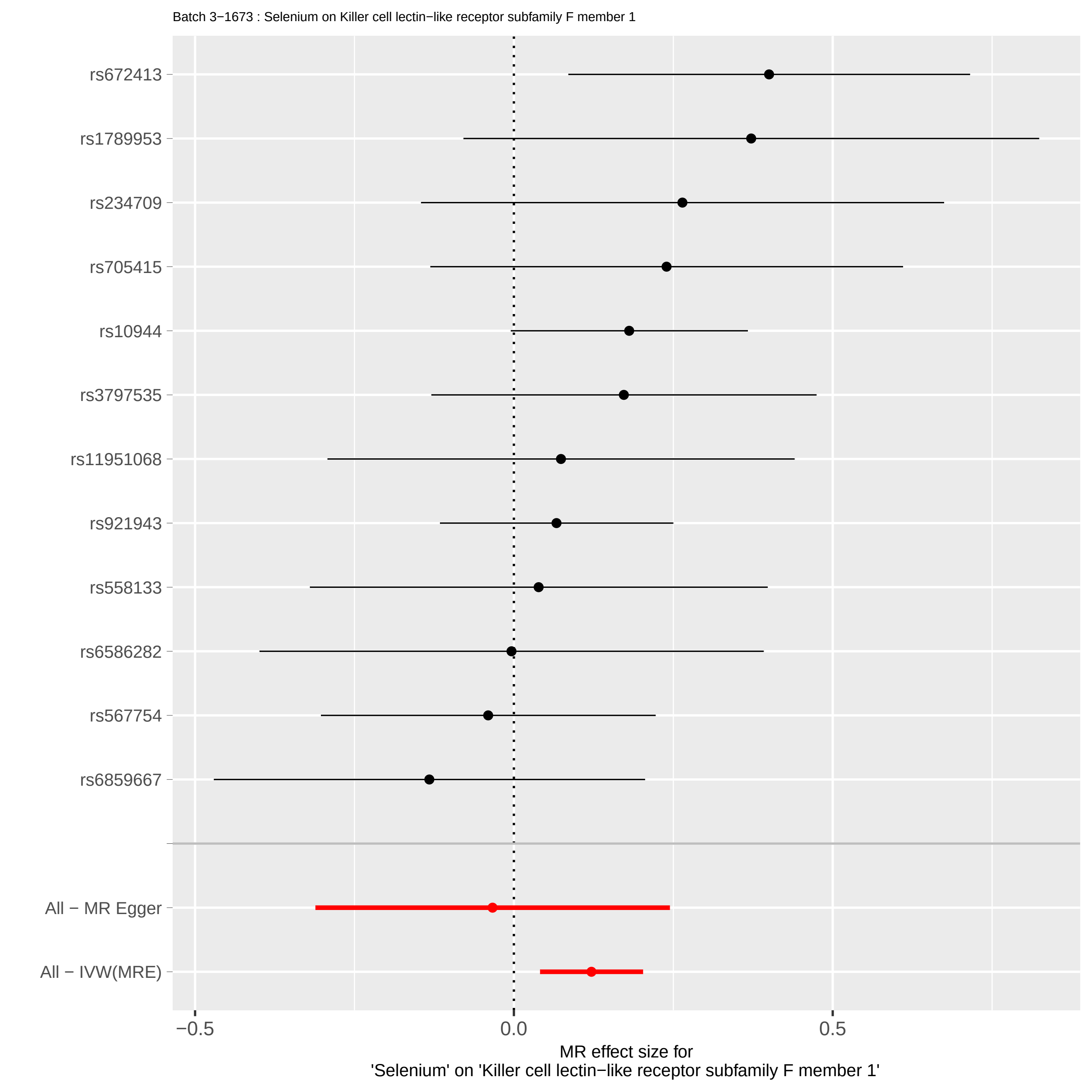
Batch 3−1673 : Selenium on Killer cell lectin−like receptor subfamily F member 1
rs672413
rs1789953
rs234709
rs705415
rs10944
rs3797535
rs11951068
rs921943
rs558133
rs6586282
rs567754
rs6859667
All − MR Egger
All − IVW(MRE)
−0.5
0.0
0.5
MR effect size for
'Selenium' on 'Killer cell lectin−like receptor subfamily F member 1'

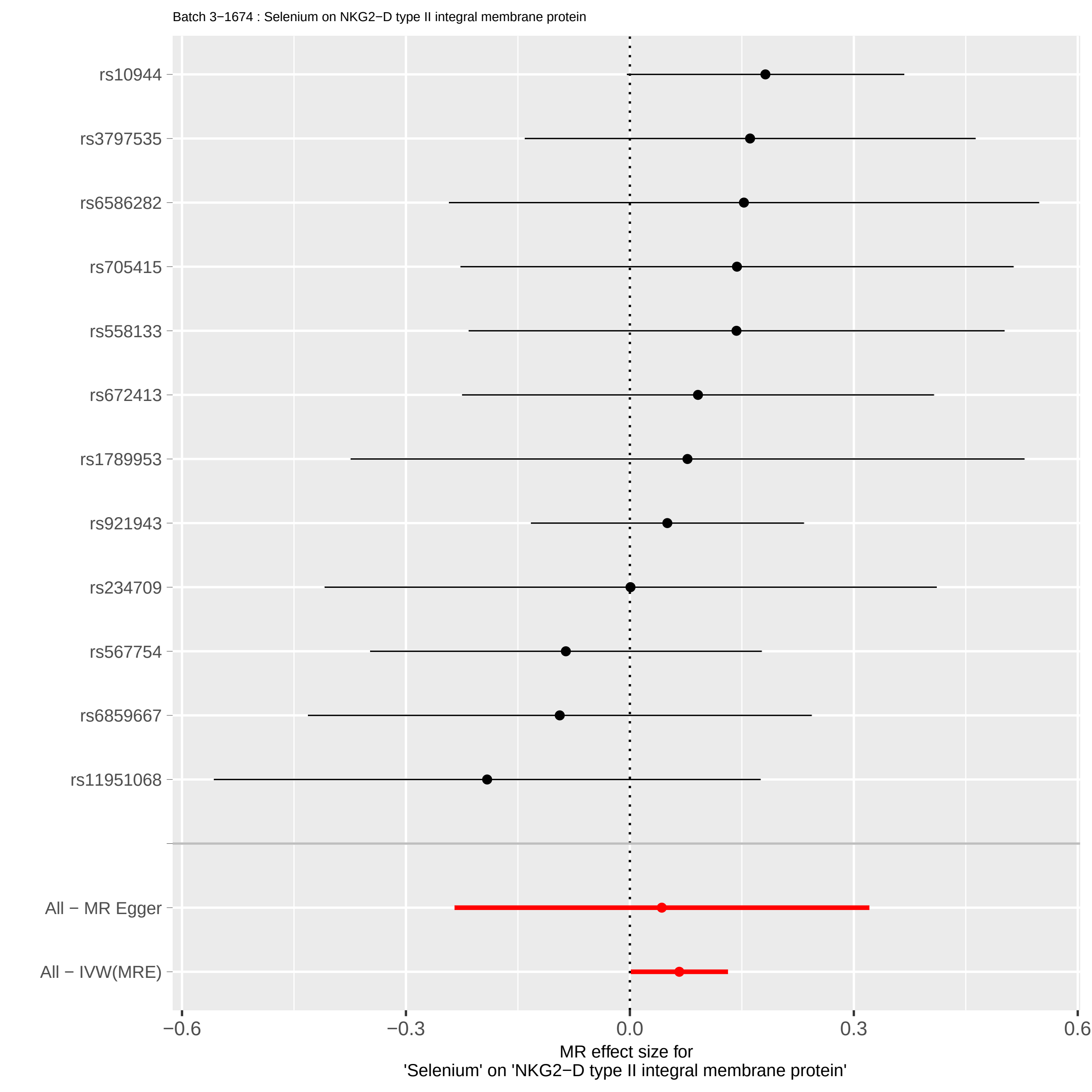
Batch 3−1674 : Selenium on NKG2−D type II integral membrane protein
rs10944
rs3797535
rs6586282
rs705415
rs558133
rs672413
rs1789953
rs921943
rs234709
rs567754
rs6859667
rs11951068
All − MR Egger
All − IVW(MRE)
−0.6
−0.3
0.0
0.3
0.6
MR effect size for
'Selenium' on 'NKG2−D type II integral membrane protein'

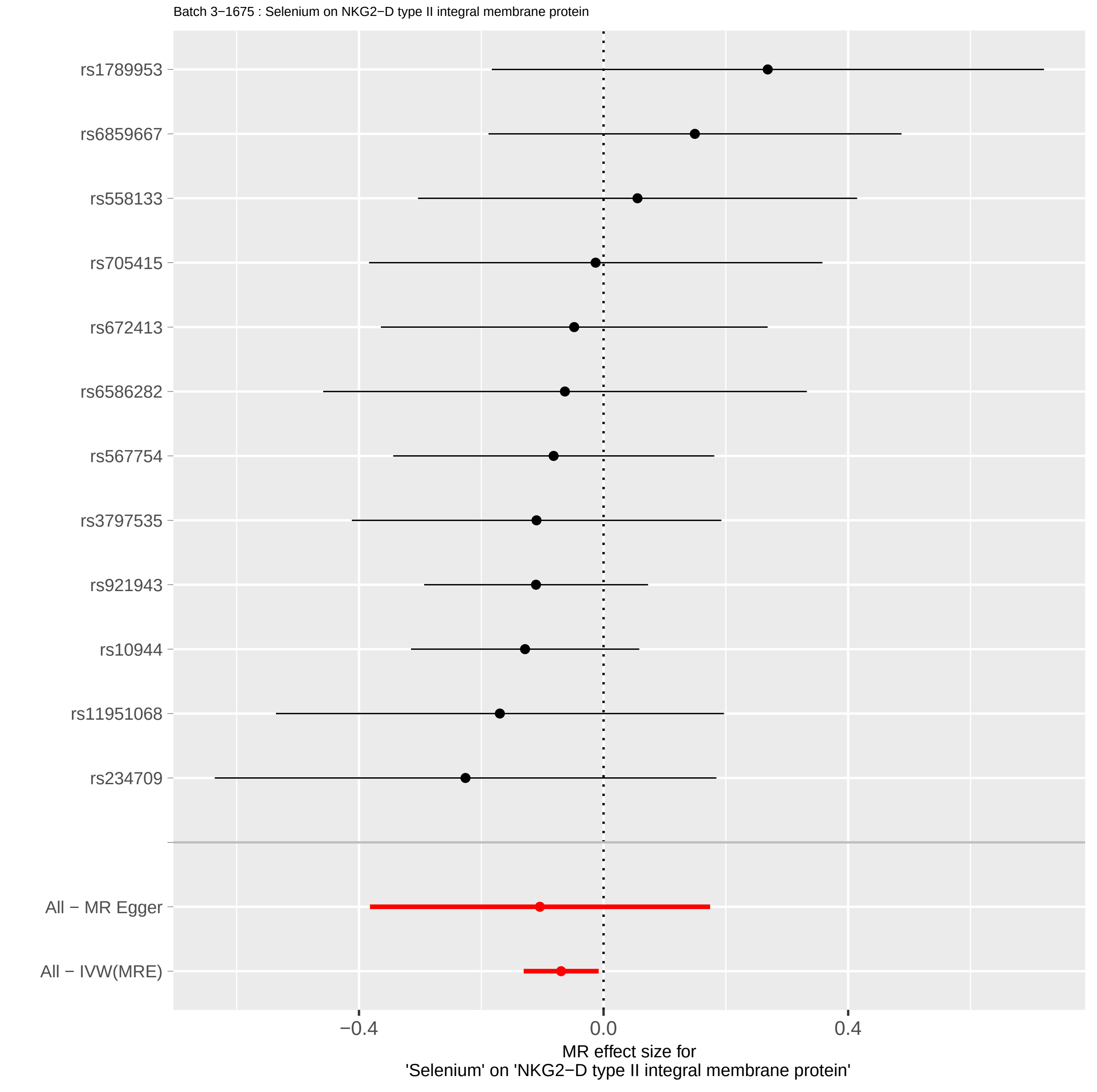

Batch 3−1675 : Selenium on NKG2−D type II integral membrane protein
rs1789953
rs6859667
rs558133
rs705415
rs672413
rs6586282
rs567754
rs3797535
rs921943
rs10944
rs11951068
rs234709
All − MR Egger
All − IVW(MRE)
−0.4
0.0
0.4
MR effect size for
'Selenium' on 'NKG2−D type II integral membrane protein'

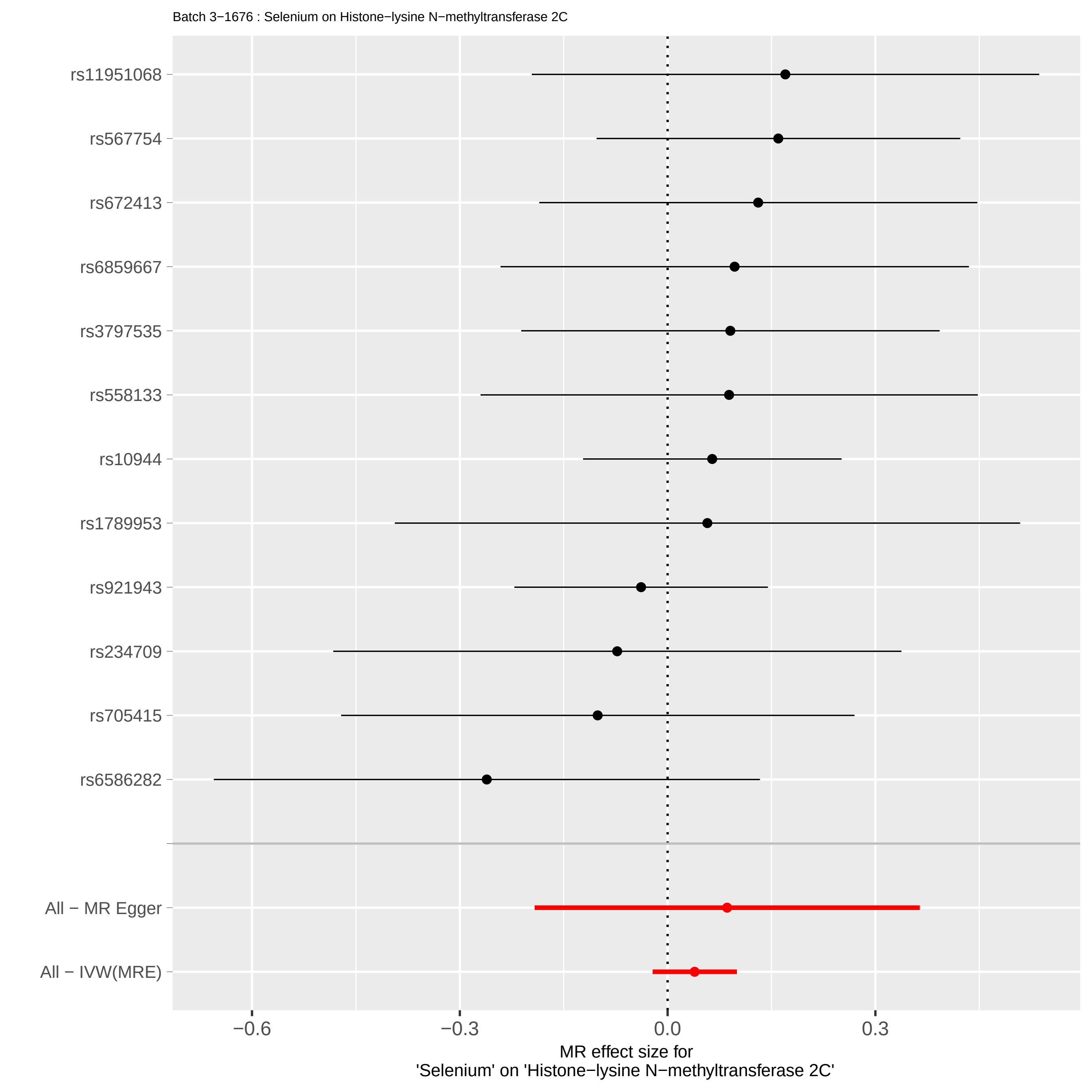
Batch 3−1676 : Selenium on Histone−lysine N−methyltransferase 2C
rs11951068
rs567754
rs672413
rs6859667
rs3797535
rs558133
rs10944
rs1789953
rs921943
rs234709
rs705415
rs6586282
All − MR Egger
All − IVW(MRE)
−0.6
−0.3
0.0
0.3
MR effect size for
'Selenium' on 'Histone−lysine N−methyltransferase 2C'

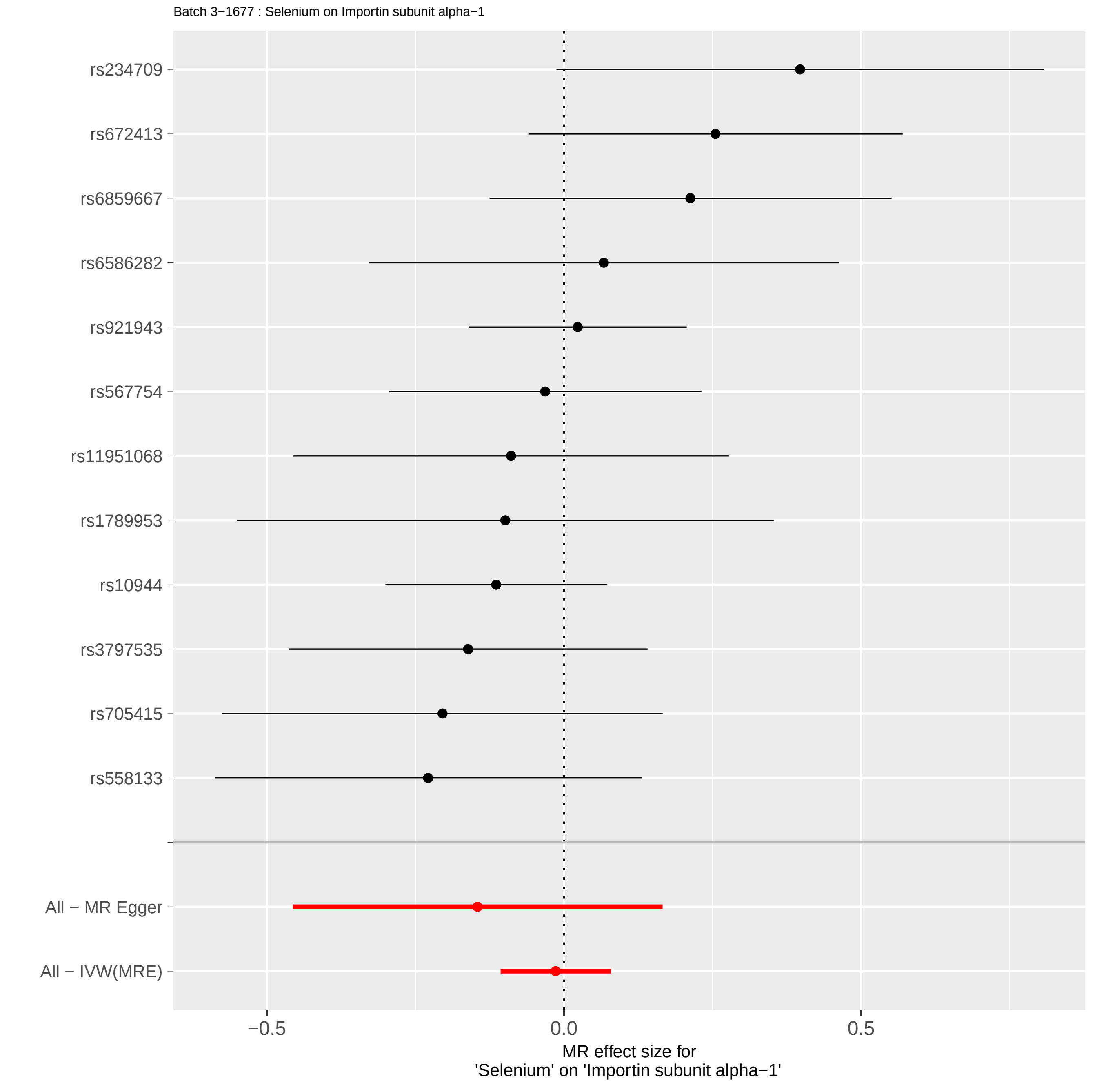

Batch 3−1677 : Selenium on Importin subunit alpha−1
rs234709
rs672413
rs6859667
rs6586282
rs921943
rs567754
rs11951068
rs1789953
rs10944
rs3797535
rs705415
rs558133
All − MR Egger
All − IVW(MRE)
−0.5
0.0
0.5
MR effect size for
'Selenium' on 'Importin subunit alpha−1'

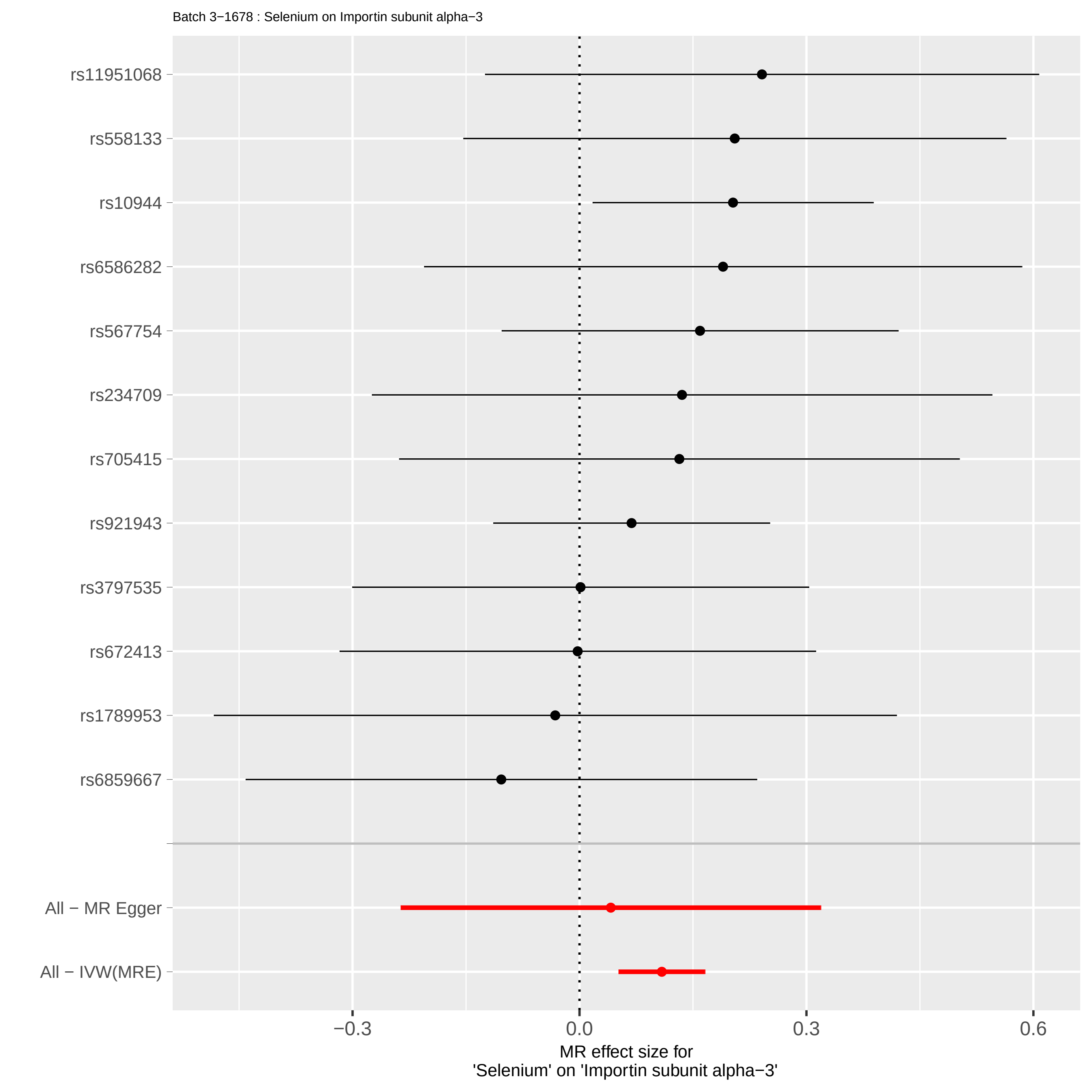

Batch 3−1678 : Selenium on Importin subunit alpha−3
rs11951068
rs558133
rs10944
rs6586282
rs567754
rs234709
rs705415
rs921943
rs3797535
rs672413
rs1789953
rs6859667
All − MR Egger
All − IVW(MRE)
−0.3
0.0
0.3
0.6
MR effect size for
'Selenium' on 'Importin subunit alpha−3'

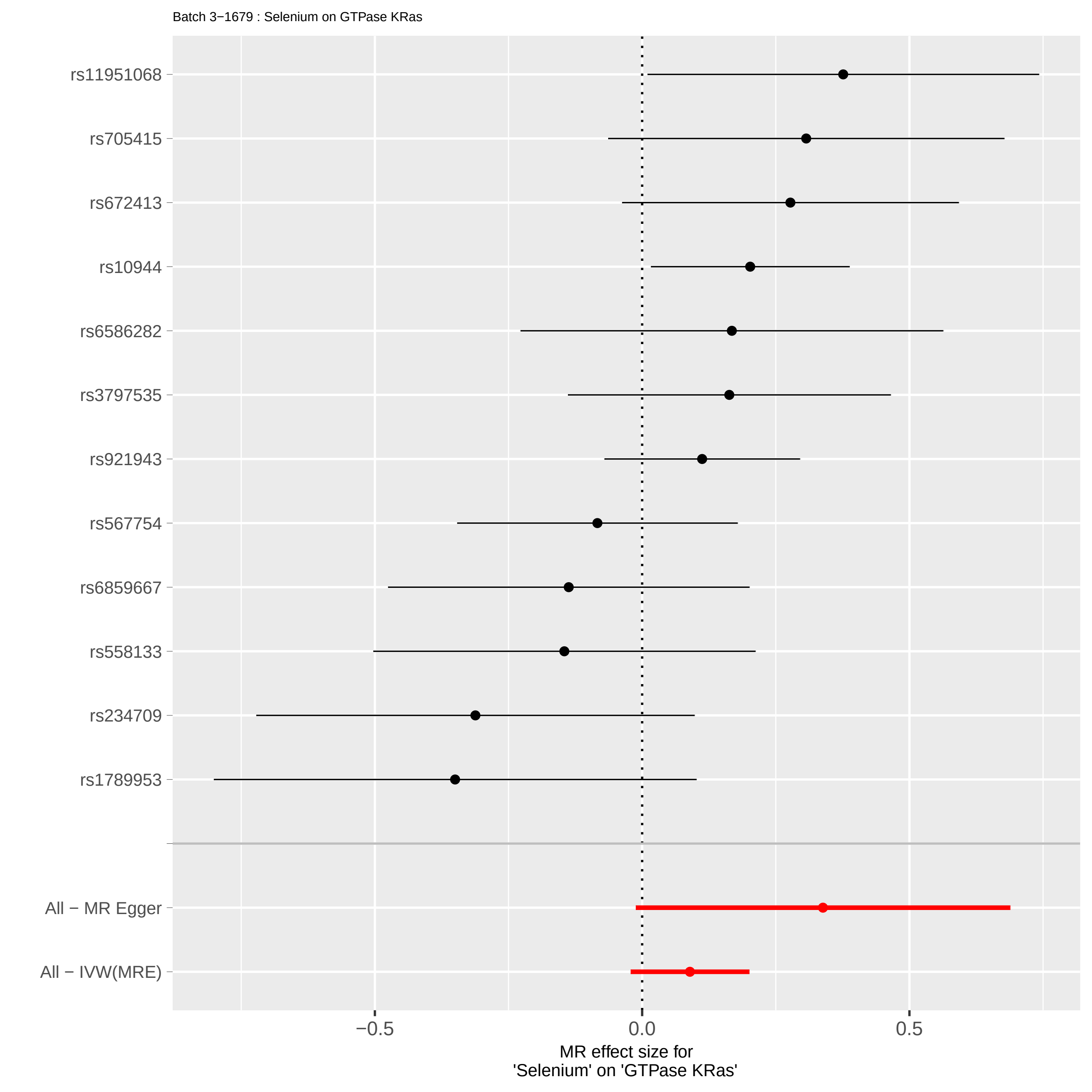

Batch 3−1679 : Selenium on GTPase KRas
rs11951068
rs705415
rs672413
rs10944
rs6586282
rs3797535
rs921943
rs567754
rs6859667
rs558133
rs234709
rs1789953
All − MR Egger
All − IVW(MRE)
−0.5
0.0
0.5
MR effect size for
'Selenium' on 'GTPase KRas'

Batch 3−1680 : Selenium on Kremen protein 1

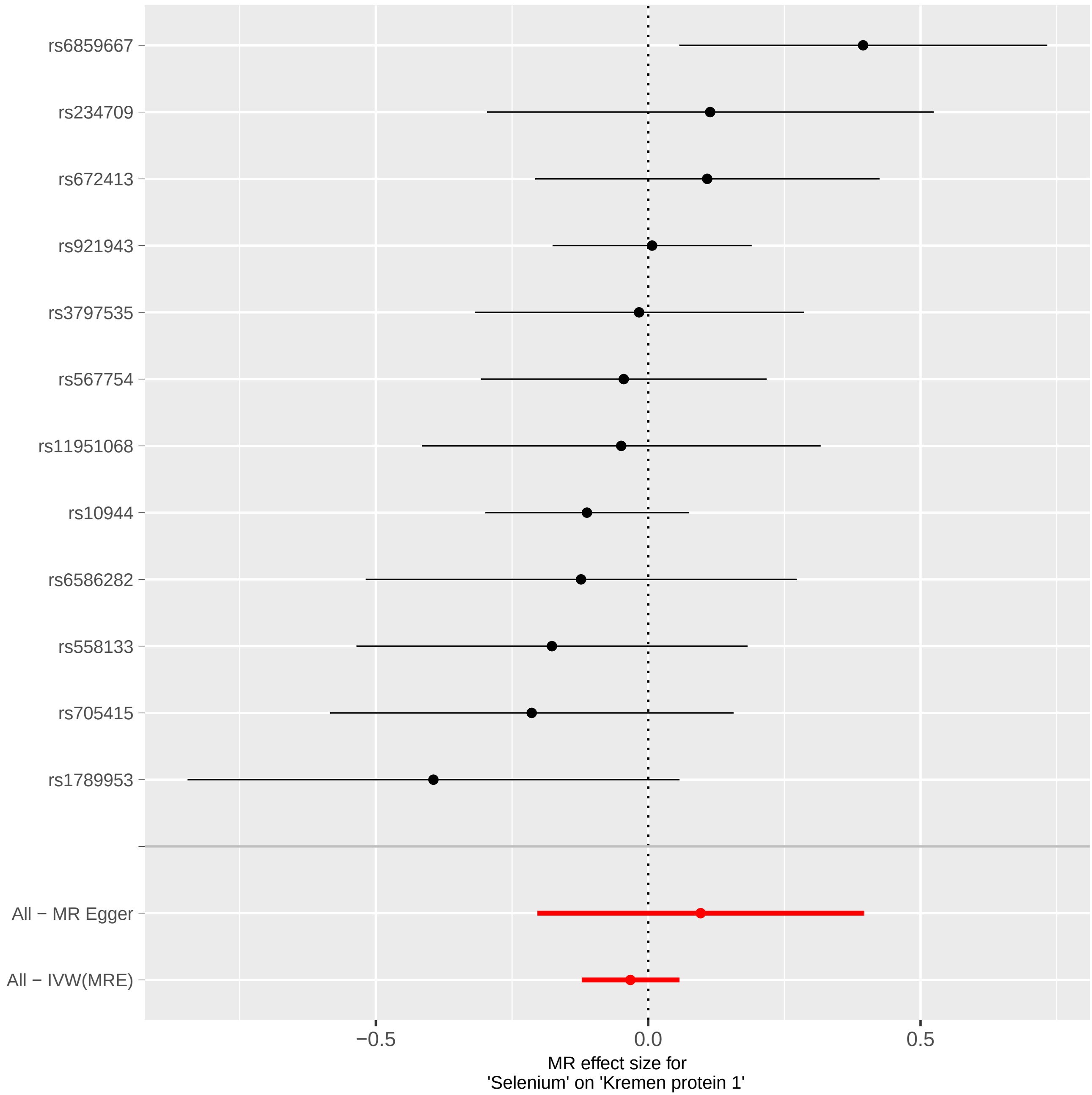

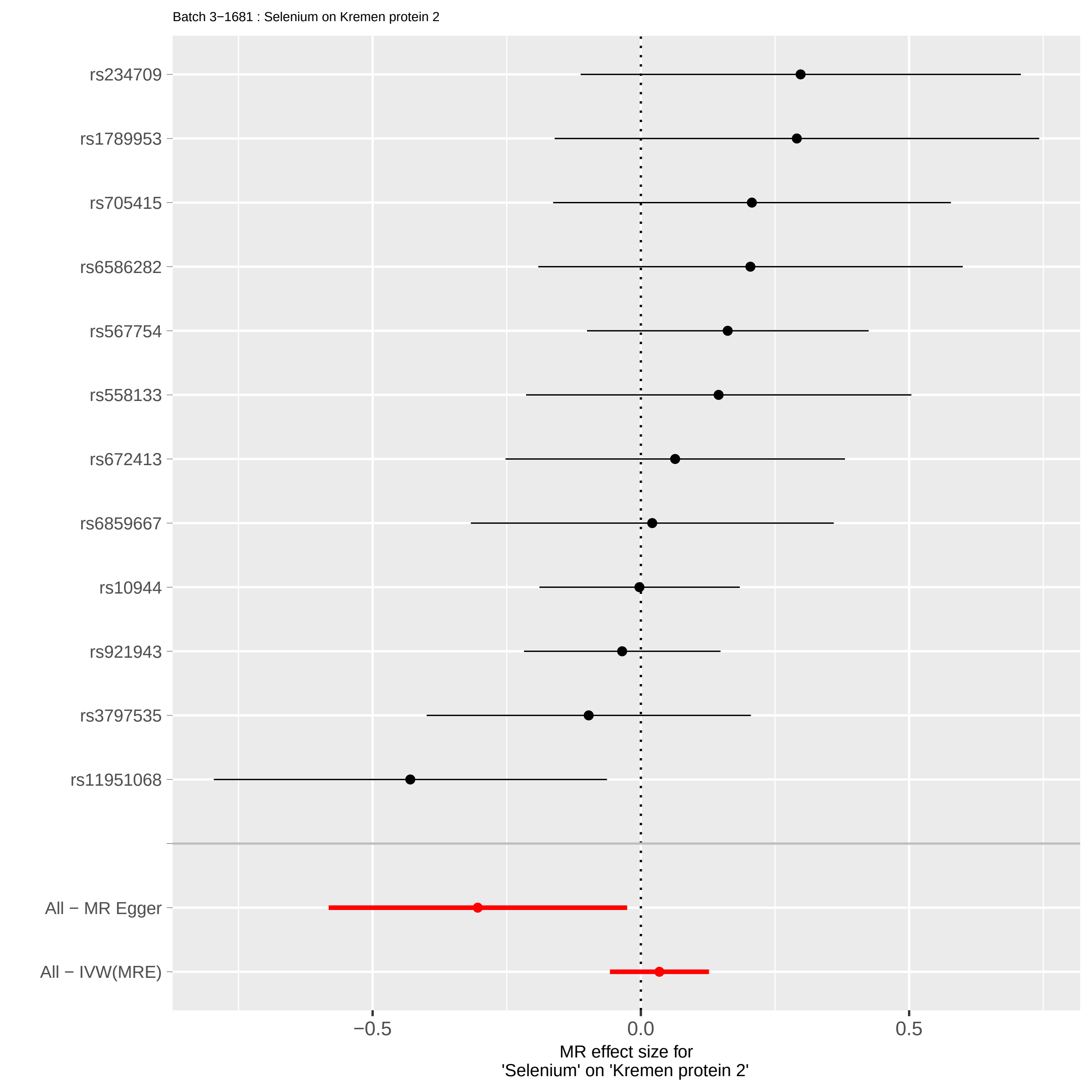
Batch 3−1681 : Selenium on Kremen protein 2
rs234709
rs1789953
rs705415
rs6586282
rs567754
rs558133
rs672413
rs6859667
rs10944
rs921943
rs3797535
rs11951068
All − MR Egger
All − IVW(MRE)
−0.5
0.0
0.5
MR effect size for
'Selenium' on 'Kremen protein 2'

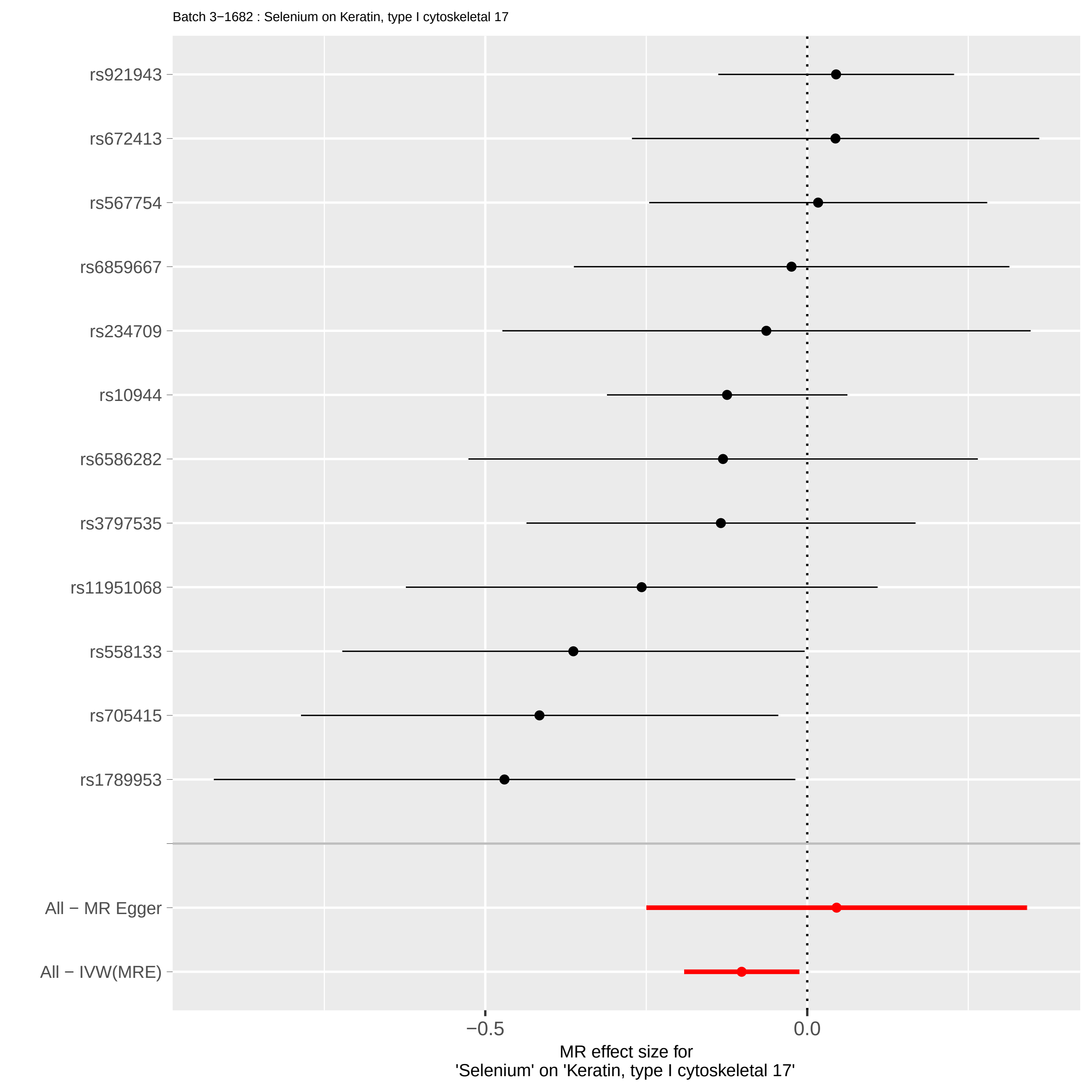

Batch 3−1682 : Selenium on Keratin, type I cytoskeletal 17
rs921943
rs672413
rs567754
rs6859667
rs234709
rs10944
rs6586282
rs3797535
rs11951068
rs558133
rs705415
rs1789953
All − MR Egger
All − IVW(MRE)
−0.5
0.0
MR effect size for
'Selenium' on 'Keratin, type I cytoskeletal 17'

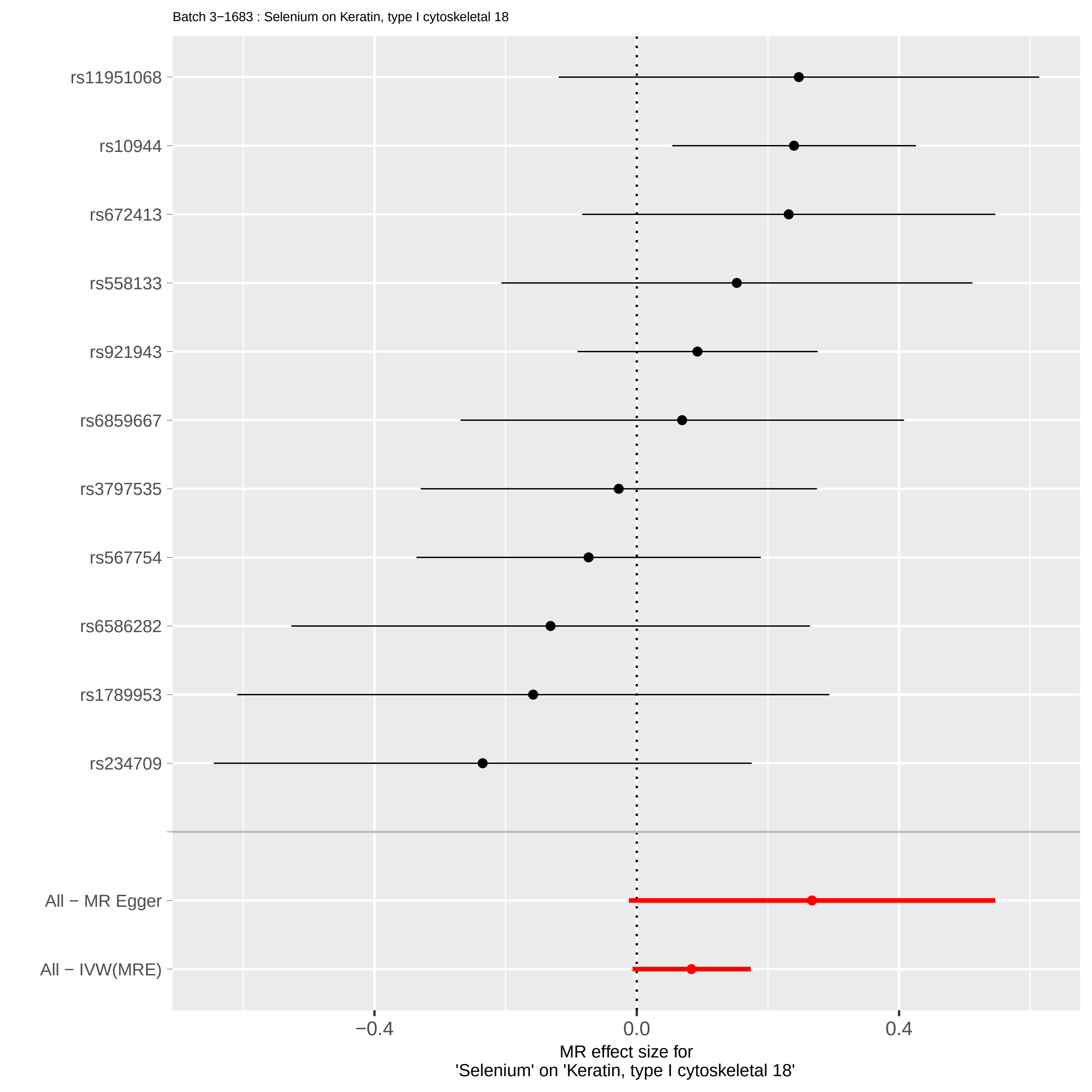

Batch 3−1683 : Selenium on Keratin, type I cytoskeletal 18
rs11951068
rs10944
rs672413
rs558133
rs921943
rs6859667
rs3797535
rs567754
rs6586282
rs1789953
rs234709
All − MR Egger
All − IVW(MRE)
−0.4
0.0
0.4
MR effect size for
'Selenium' on 'Keratin, type I cytoskeletal 18'

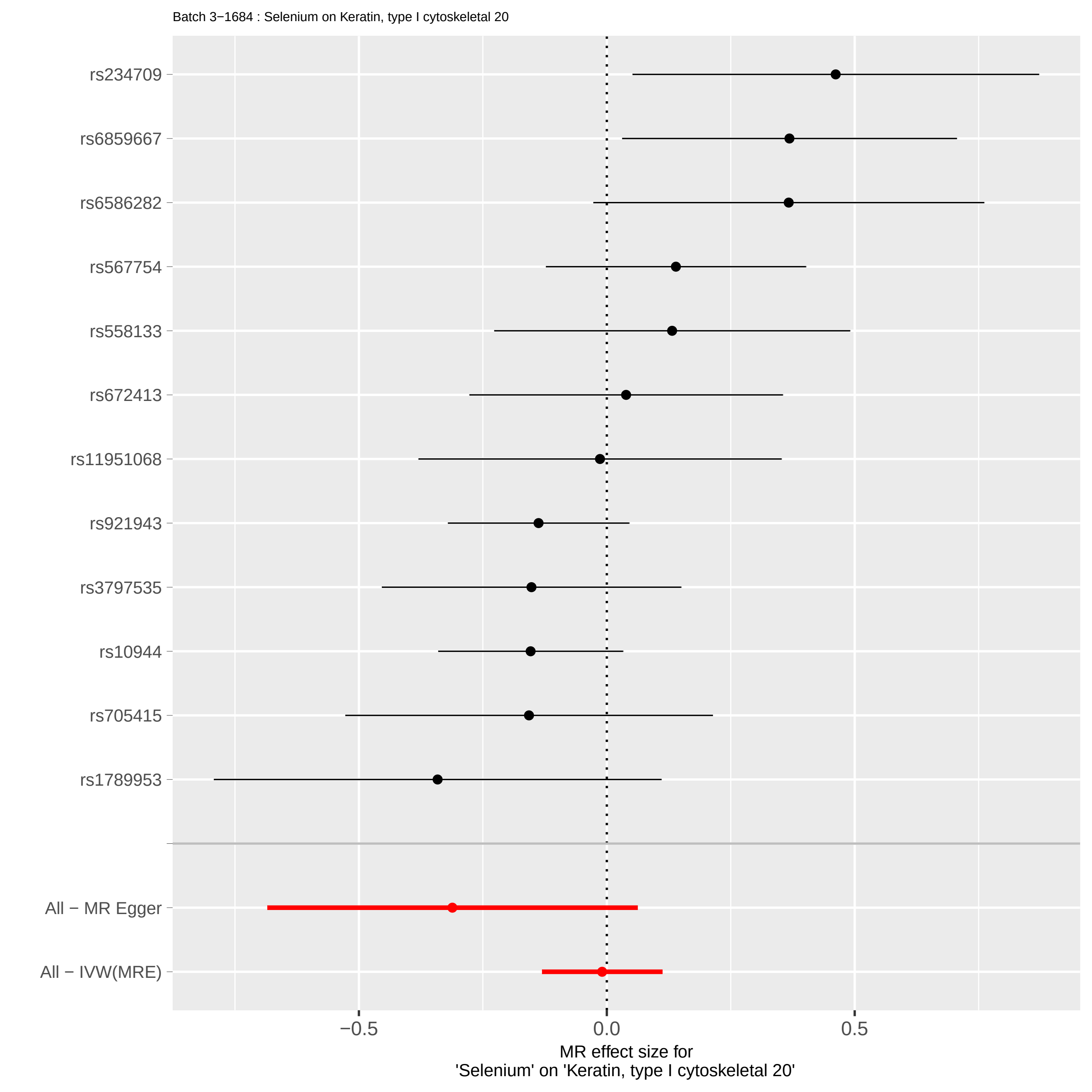

Batch 3−1684 : Selenium on Keratin, type I cytoskeletal 20
rs234709
rs6859667
rs6586282
rs567754
rs558133
rs672413
rs11951068
rs921943
rs3797535
rs10944
rs705415
rs1789953
All − MR Egger
All − IVW(MRE)
−0.5
0.0
0.5
MR effect size for
'Selenium' on 'Keratin, type I cytoskeletal 20'

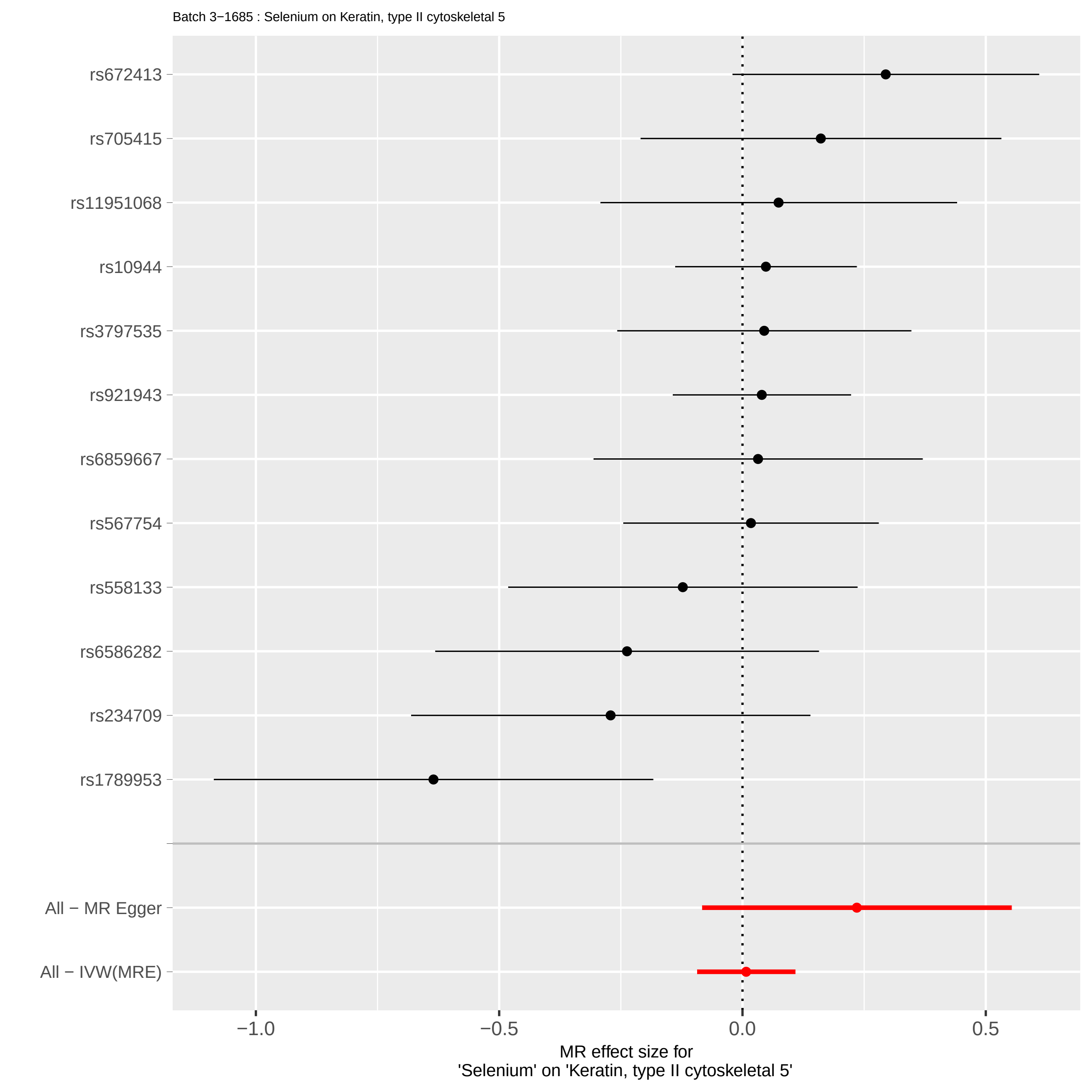

Batch 3−1685 : Selenium on Keratin, type II cytoskeletal 5
rs672413
rs705415
rs11951068
rs10944
rs3797535
rs921943
rs6859667
rs567754
rs558133
rs6586282
rs234709
rs1789953
All − MR Egger
All − IVW(MRE)
−1.0
−0.5
0.0
0.5
MR effect size for
'Selenium' on 'Keratin, type II cytoskeletal 5'

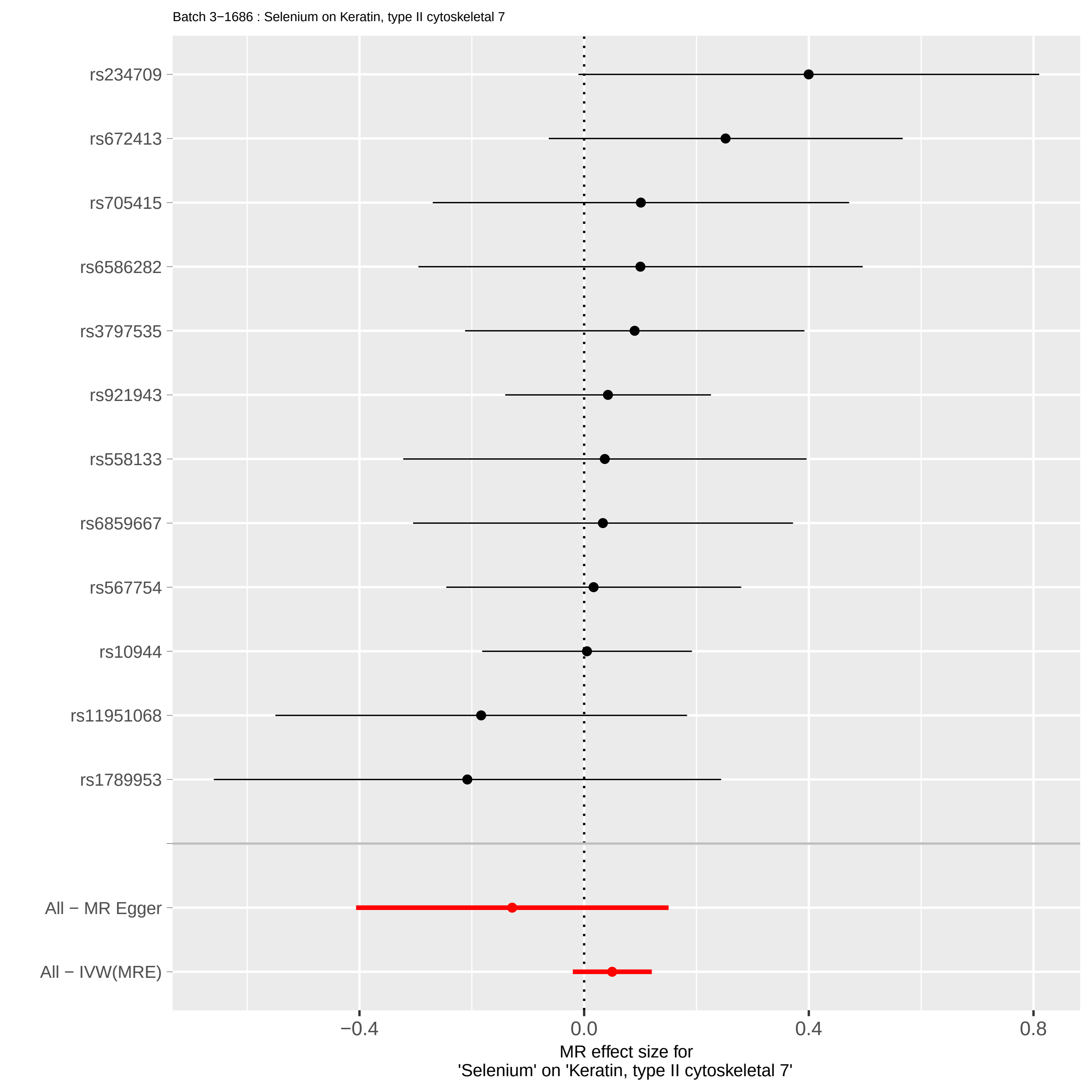

Batch 3−1686 : Selenium on Keratin, type II cytoskeletal 7
rs234709
rs672413
rs705415
rs6586282
rs3797535
rs921943
rs558133
rs6859667
rs567754
rs10944
rs11951068
rs1789953
All − MR Egger
All − IVW(MRE)
−0.4
0.0
0.4
0.8
MR effect size for
'Selenium' on 'Keratin, type II cytoskeletal 7'

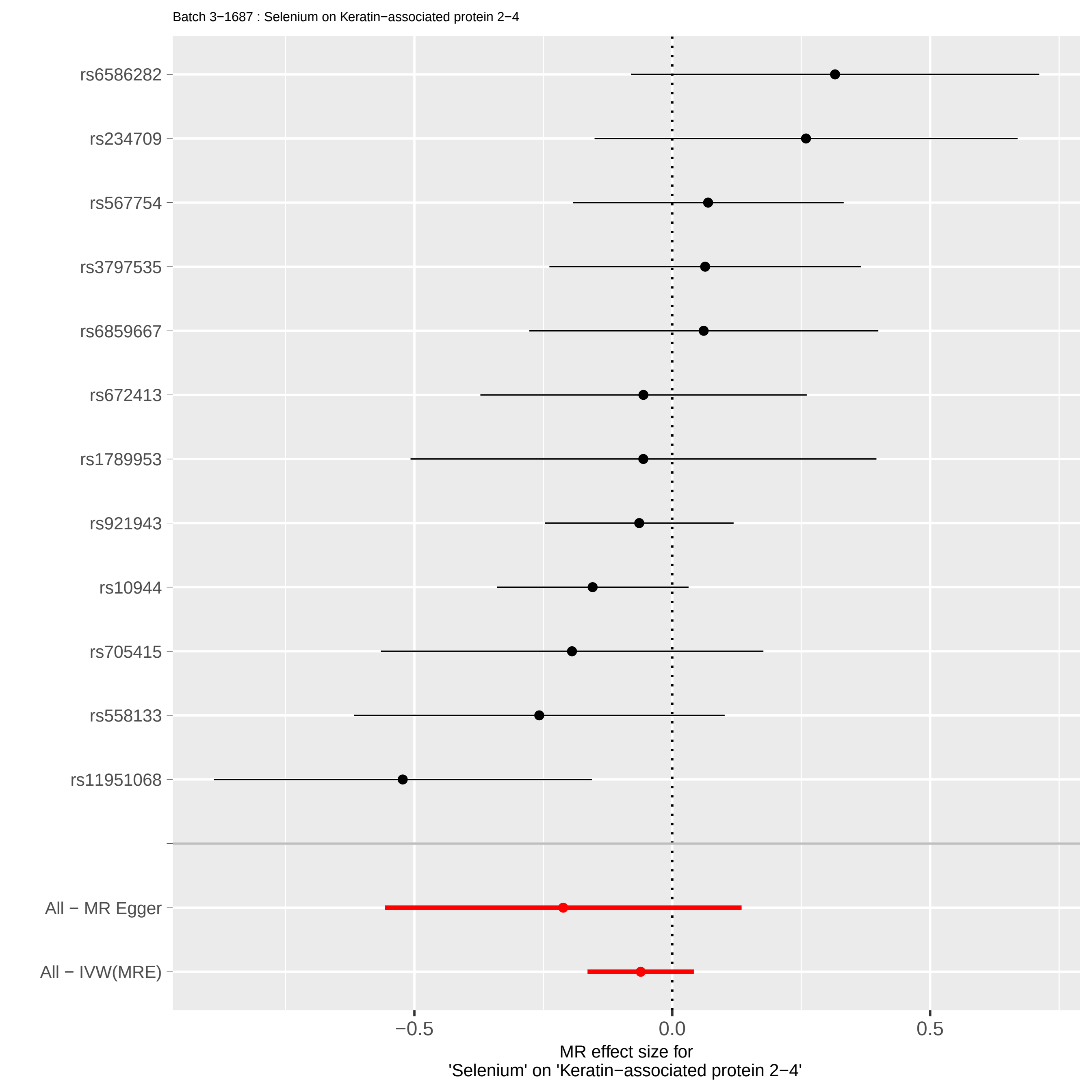
Batch 3−1687 : Selenium on Keratin−associated protein 2−4
rs6586282
rs234709
rs567754
rs3797535
rs6859667
rs672413
rs1789953
rs921943
rs10944
rs705415
rs558133
rs11951068
All − MR Egger
All − IVW(MRE)
−0.5
0.0
0.5
MR effect size for
'Selenium' on 'Keratin−associated protein 2−4'

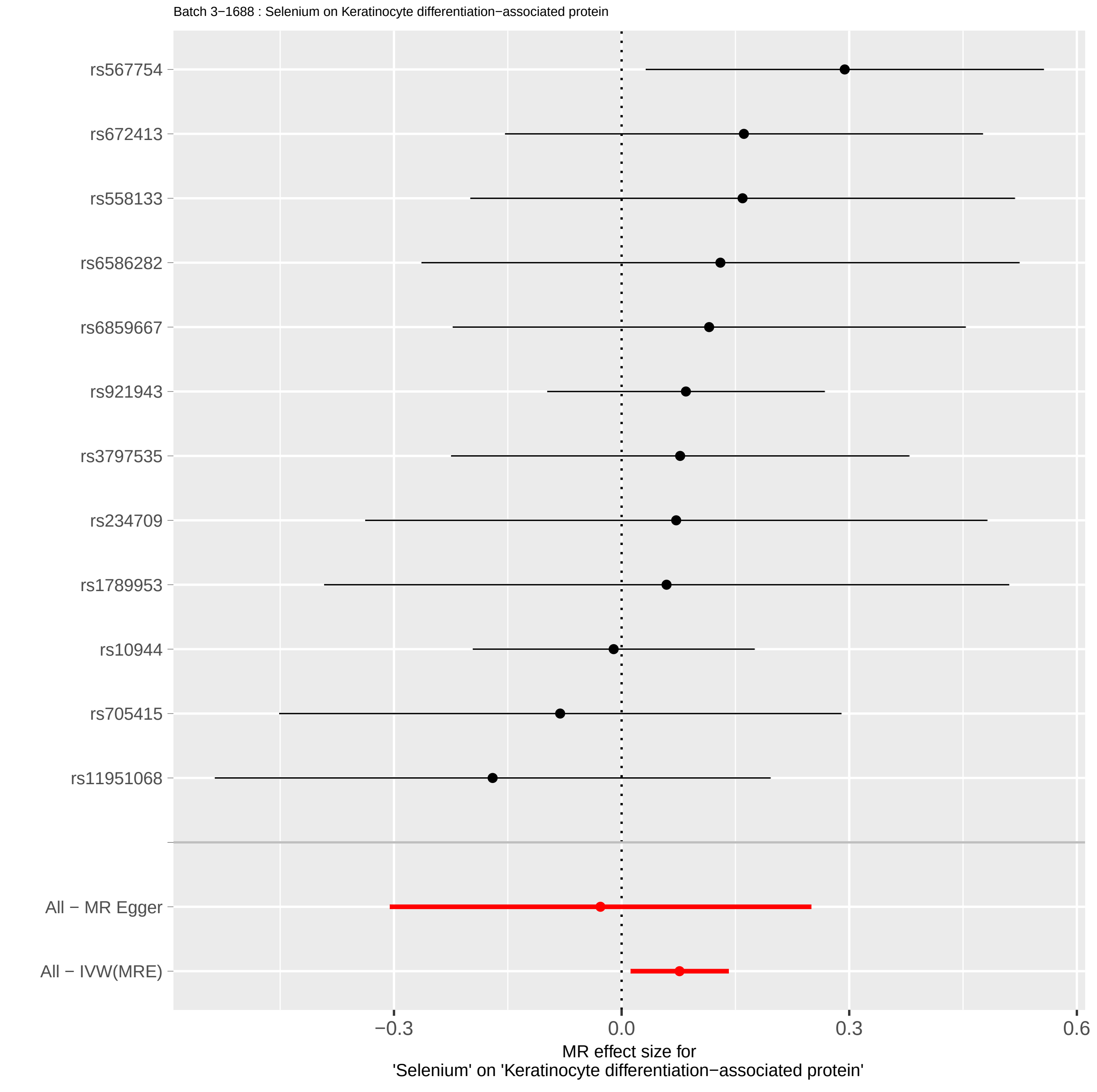

Batch 3−1688 : Selenium on Keratinocyte differentiation−associated protein
rs567754
rs672413
rs558133
rs6586282
rs6859667
rs921943
rs3797535
rs234709
rs1789953
rs10944
rs705415
rs11951068
All − MR Egger
All − IVW(MRE)
−0.3
0.0
0.3
0.6
MR effect size for
'Selenium' on 'Keratinocyte differentiation−associated protein'

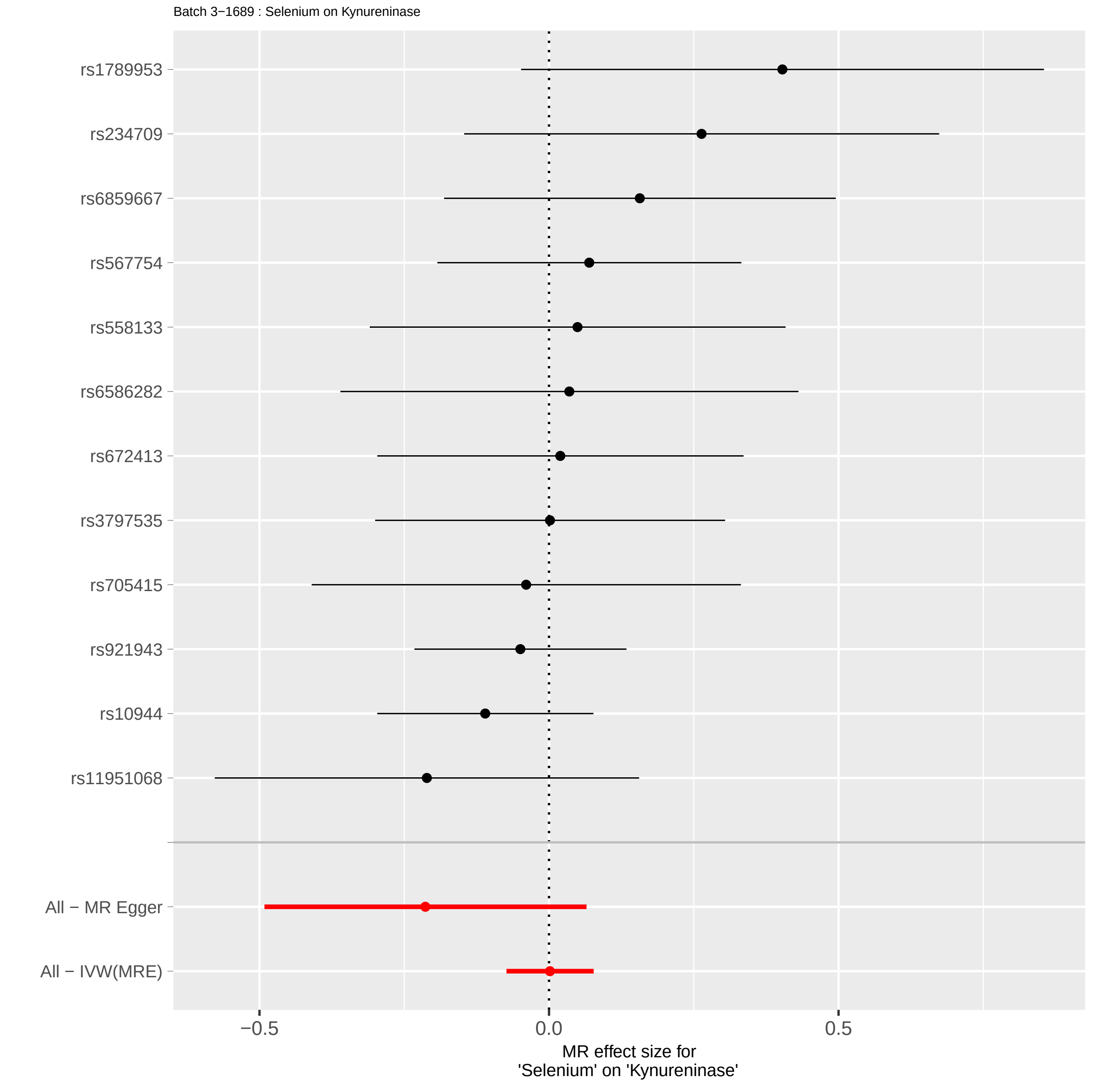

Batch 3−1689 : Selenium on Kynureninase
rs1789953
rs234709
rs6859667
rs567754
rs558133
rs6586282
rs672413
rs3797535
rs705415
rs921943
rs10944
rs11951068
All − MR Egger
All − IVW(MRE)
−0.5
0.0
0.5
MR effect size for
'Selenium' on 'Kynureninase'

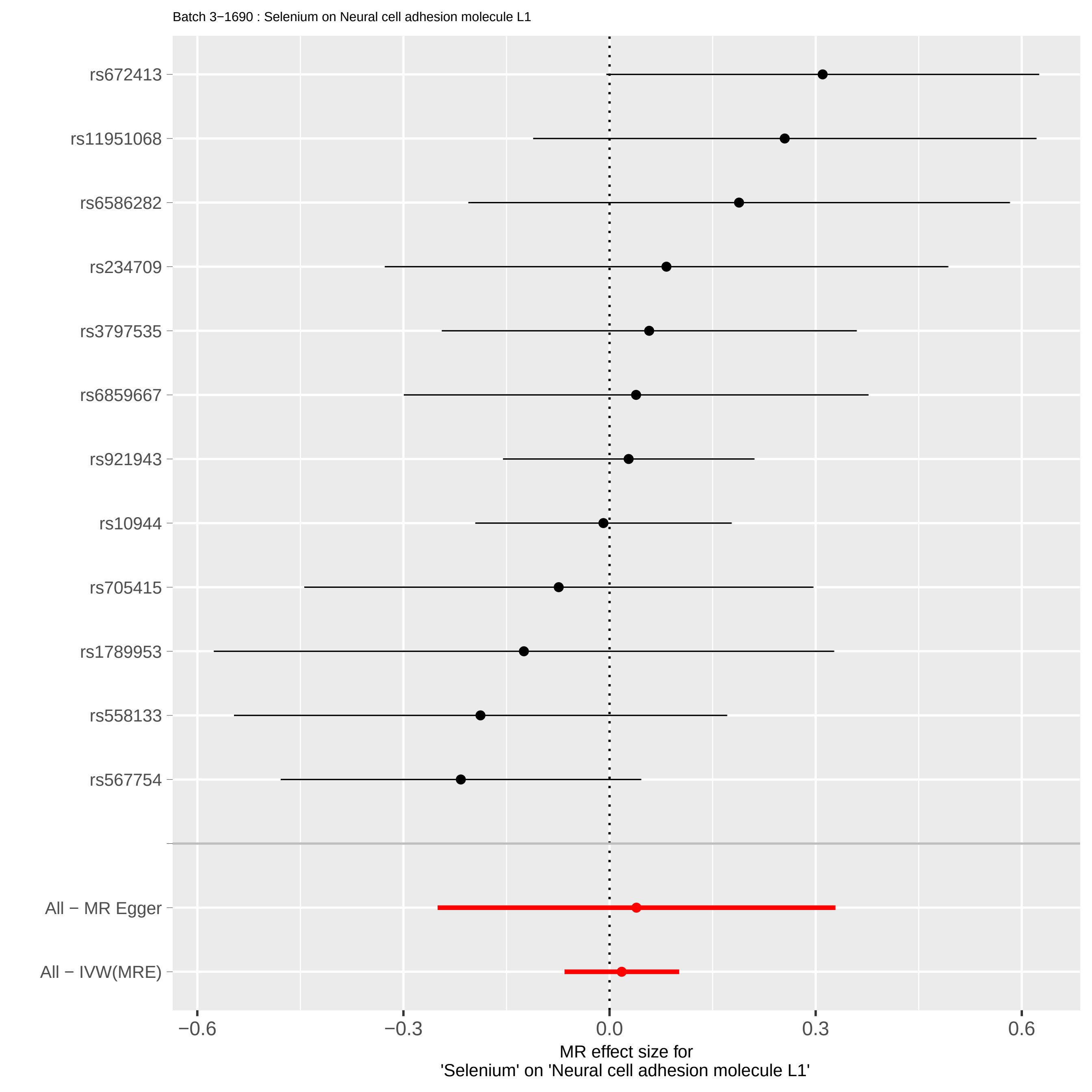

Batch 3−1690 : Selenium on Neural cell adhesion molecule L1
rs672413
rs11951068
rs6586282
rs234709
rs3797535
rs6859667
rs921943
rs10944
rs705415
rs1789953
rs558133
rs567754
All − MR Egger
All − IVW(MRE)
−0.6
−0.3
0.0
0.3
0.6
MR effect size for
'Selenium' on 'Neural cell adhesion molecule L1'

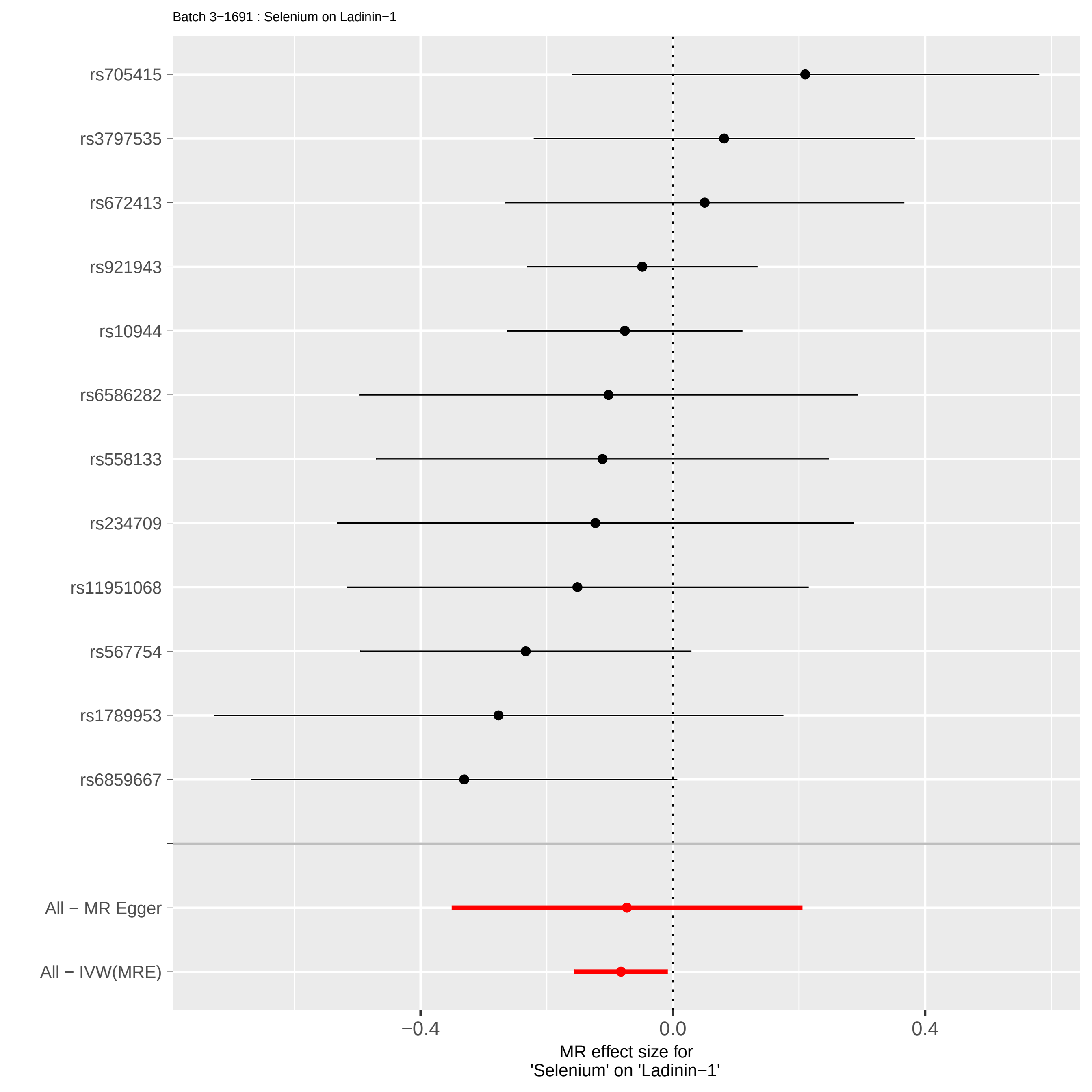
Batch 3−1691 : Selenium on Ladinin−1
rs705415
rs3797535
rs672413
rs921943
rs10944
rs6586282
rs558133
rs234709
rs11951068
rs567754
rs1789953
rs6859667
All − MR Egger
All − IVW(MRE)
−0.4
0.0
0.4
MR effect size for
'Selenium' on 'Ladinin−1'

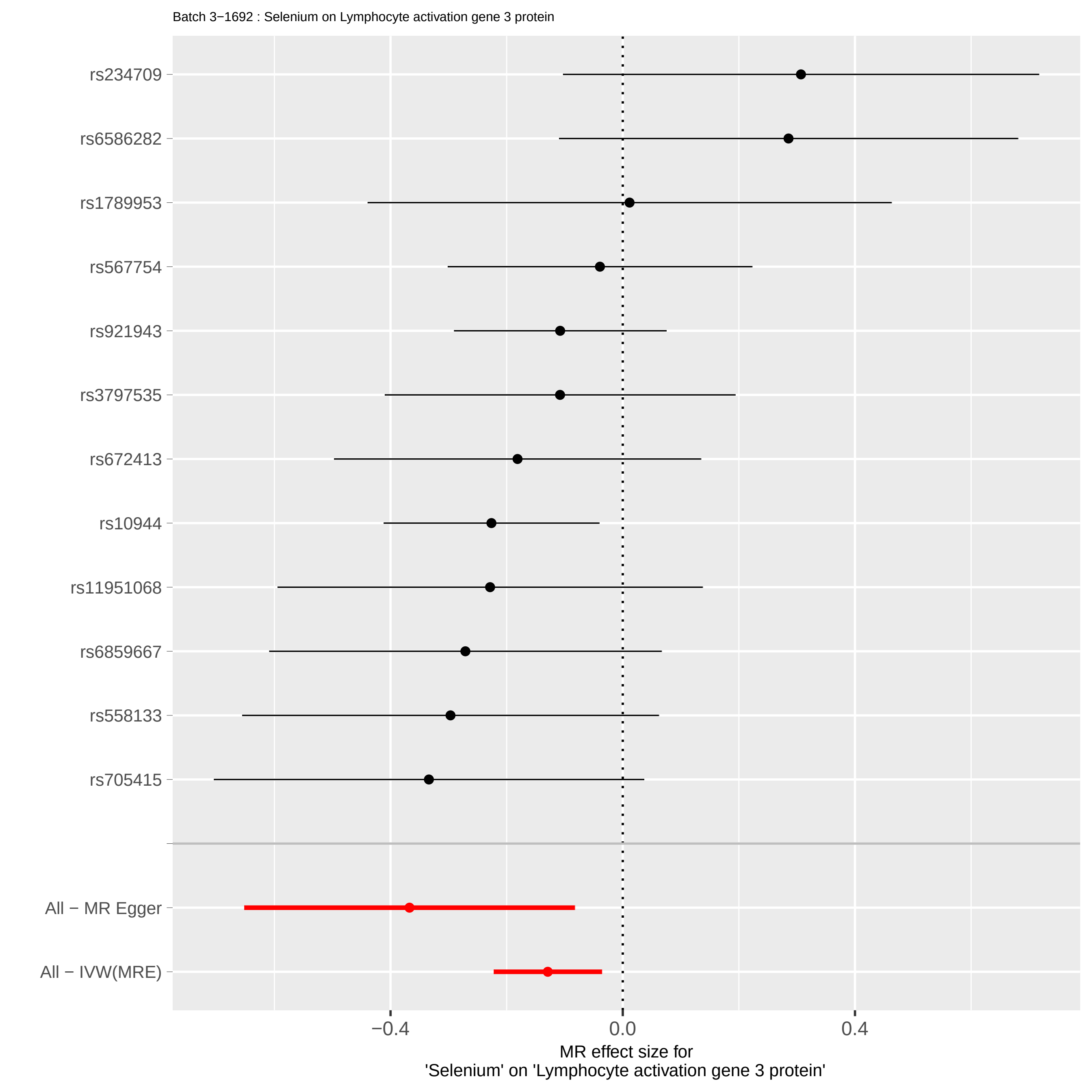

Batch 3−1692 : Selenium on Lymphocyte activation gene 3 protein
rs234709
rs6586282
rs1789953
rs567754
rs921943
rs3797535
rs672413
rs10944
rs11951068
rs6859667
rs558133
rs705415
All − MR Egger
All − IVW(MRE)
−0.4
0.0
0.4
MR effect size for
'Selenium' on 'Lymphocyte activation gene 3 protein'

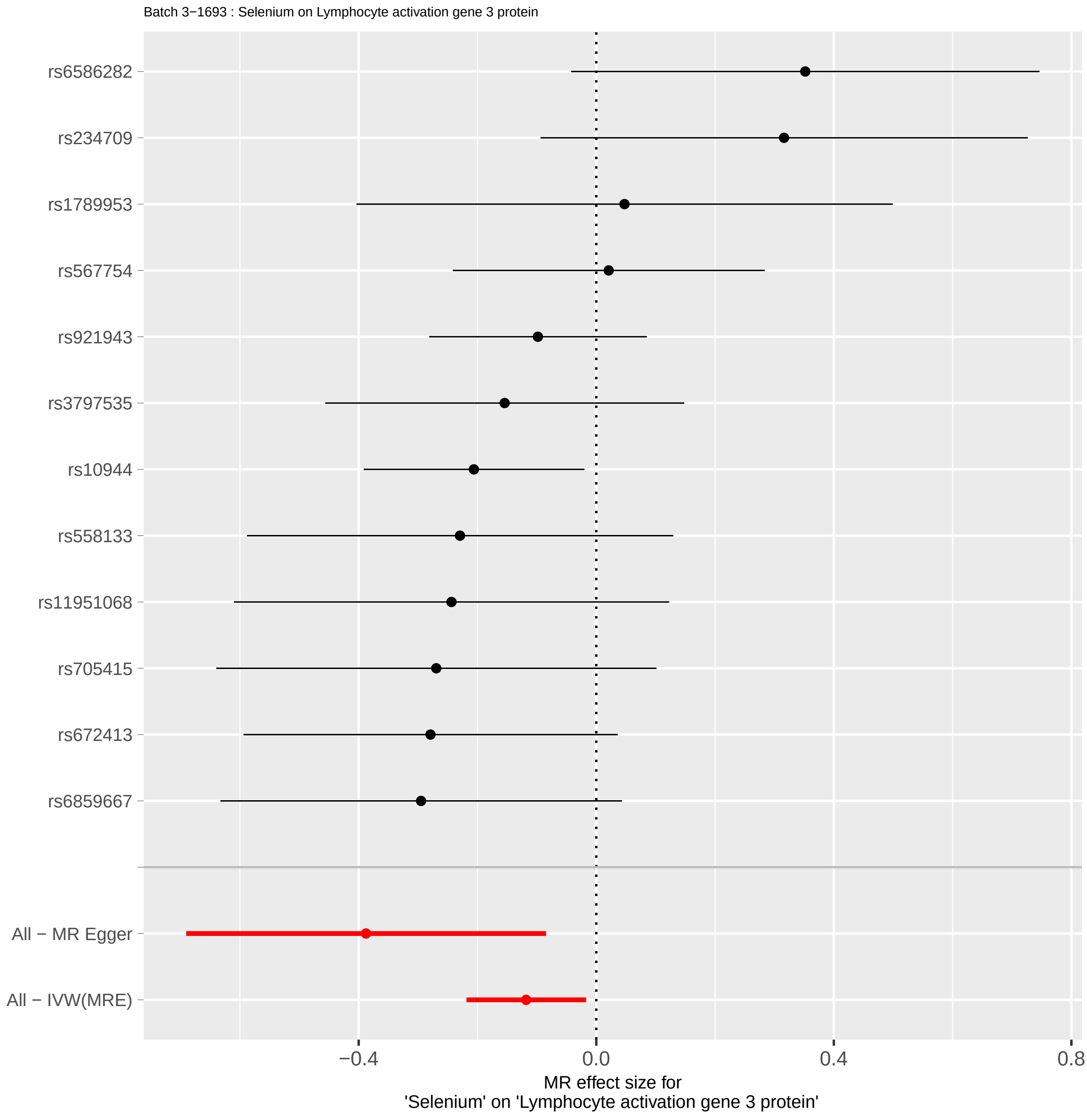
Batch 3−1693 : Selenium on Lymphocyte activation gene 3 protein
rs6586282
rs234709
rs1789953
rs567754
rs921943
rs3797535
rs10944
rs558133
rs11951068
rs705415
rs672413
rs6859667
All − MR Egger
All − IVW(MRE)
−0.4
0.0
0.4
0.8
MR effect size for
'Selenium' on 'Lymphocyte activation gene 3 protein'

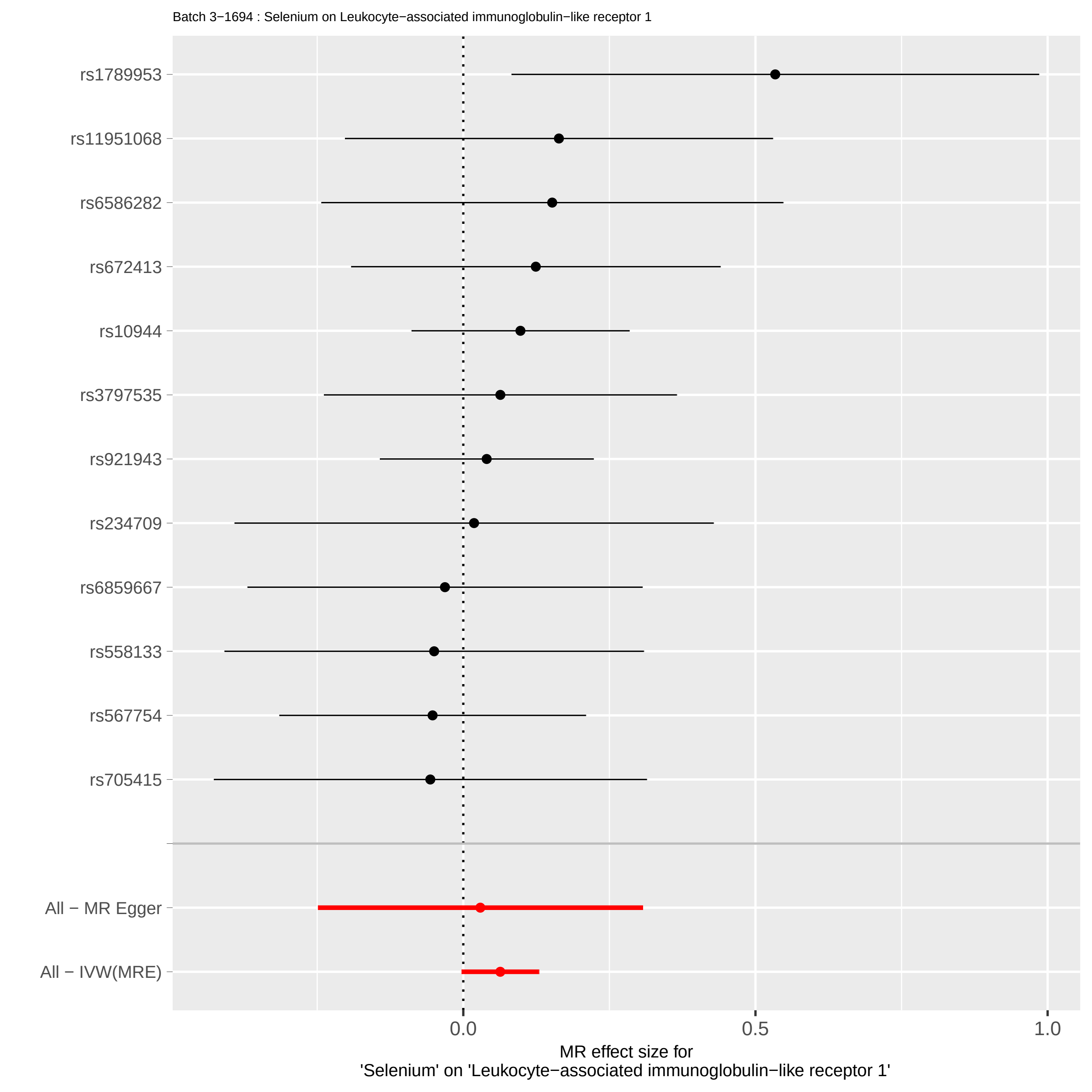
Batch 3−1694 : Selenium on Leukocyte−associated immunoglobulin−like receptor 1
rs1789953
rs11951068
rs6586282
rs672413
rs10944
rs3797535
rs921943
rs234709
rs6859667
rs558133
rs567754
rs705415
All − MR Egger
All − IVW(MRE)
0.0
0.5
1.0
MR effect size for
'Selenium' on 'Leukocyte−associated immunoglobulin−like receptor 1'

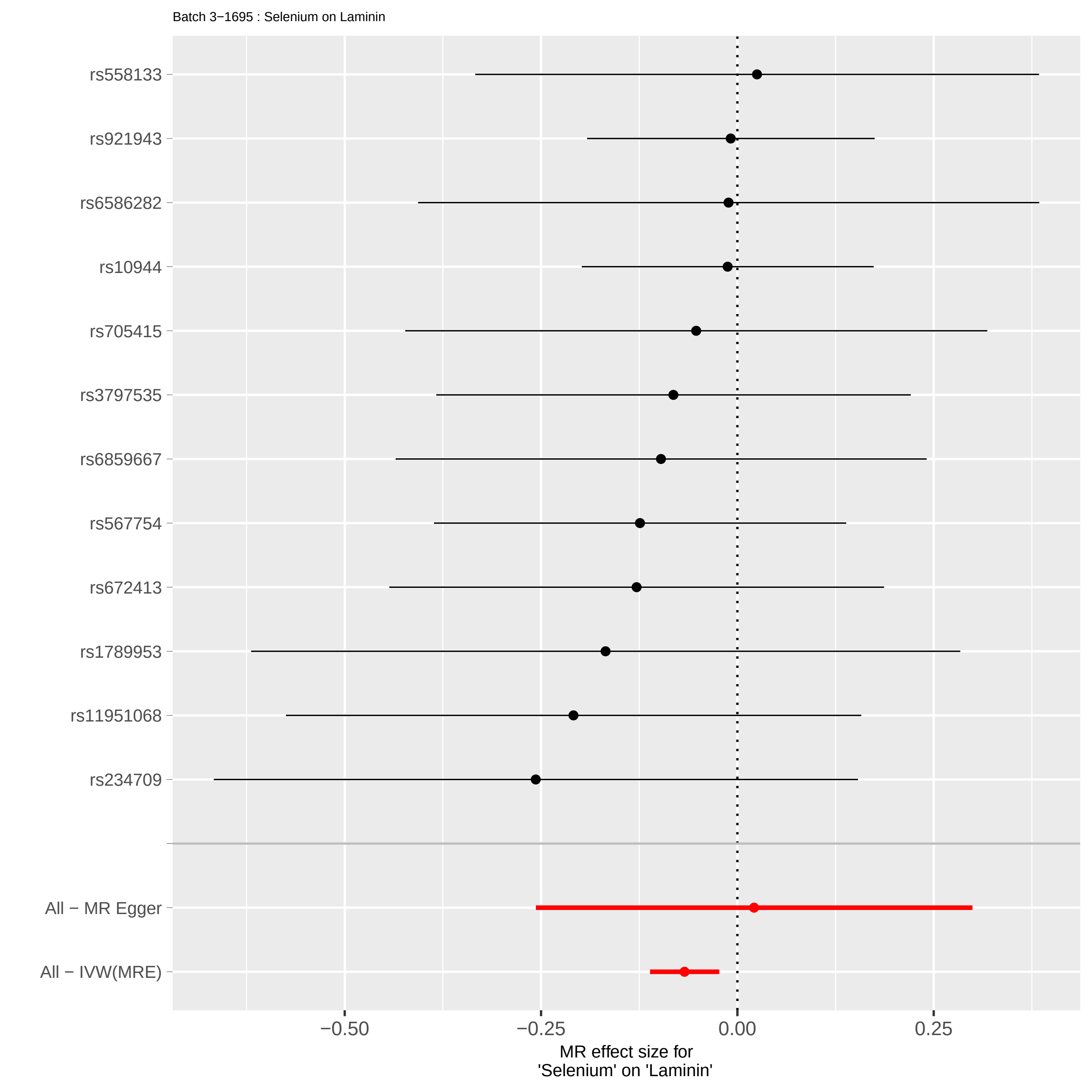

Batch 3−1695 : Selenium on Laminin
rs558133
rs921943
rs6586282
rs10944
rs705415
rs3797535
rs6859667
rs567754
rs672413
rs1789953
rs11951068
rs234709
All − MR Egger
All − IVW(MRE)
−0.50
−0.25
0.00
0.25
MR effect size for
'Selenium' on 'Laminin'

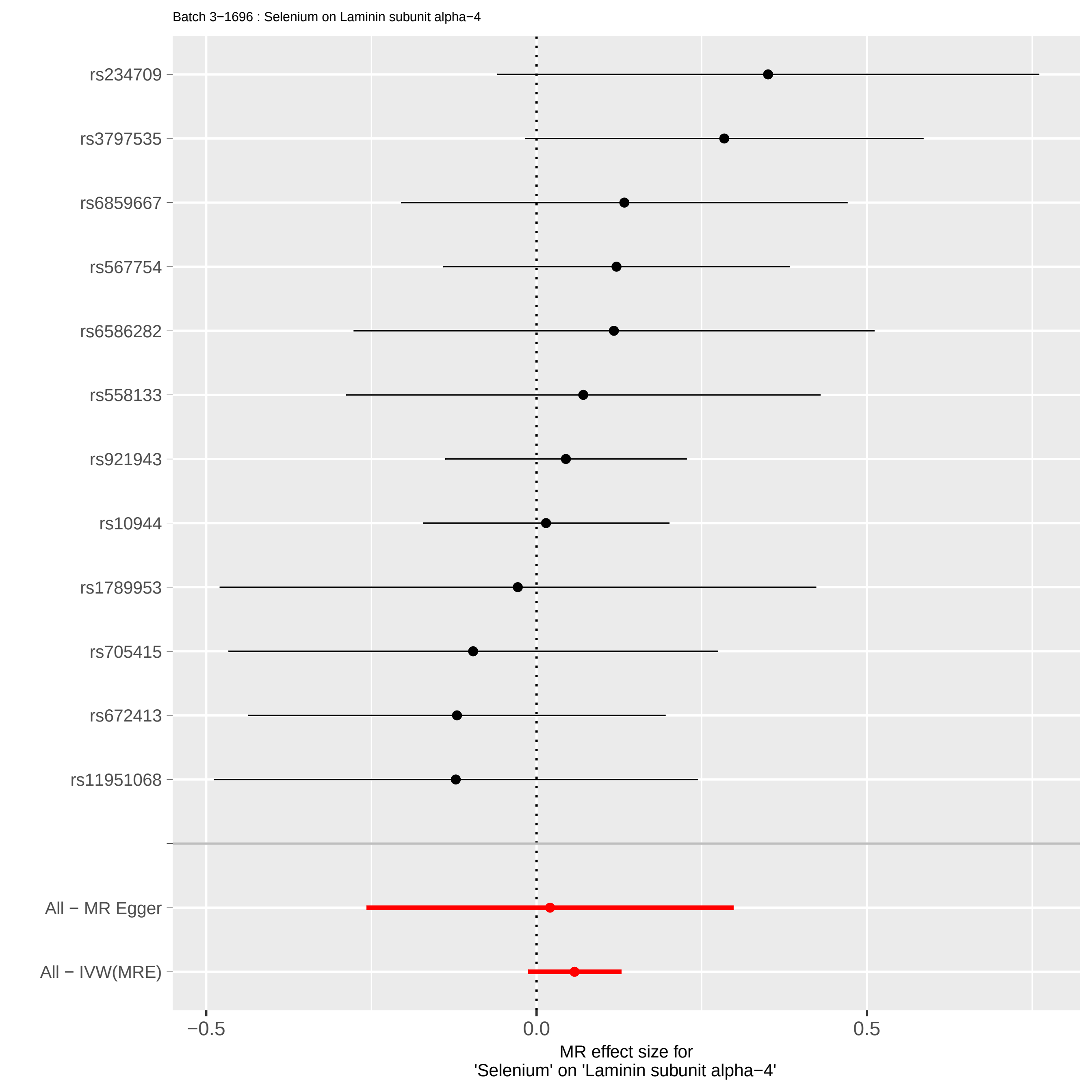

Batch 3−1696 : Selenium on Laminin subunit alpha−4
rs234709
rs3797535
rs6859667
rs567754
rs6586282
rs558133
rs921943
rs10944
rs1789953
rs705415
rs672413
rs11951068
All − MR Egger
All − IVW(MRE)
−0.5
0.0
0.5
MR effect size for
'Selenium' on 'Laminin subunit alpha−4'

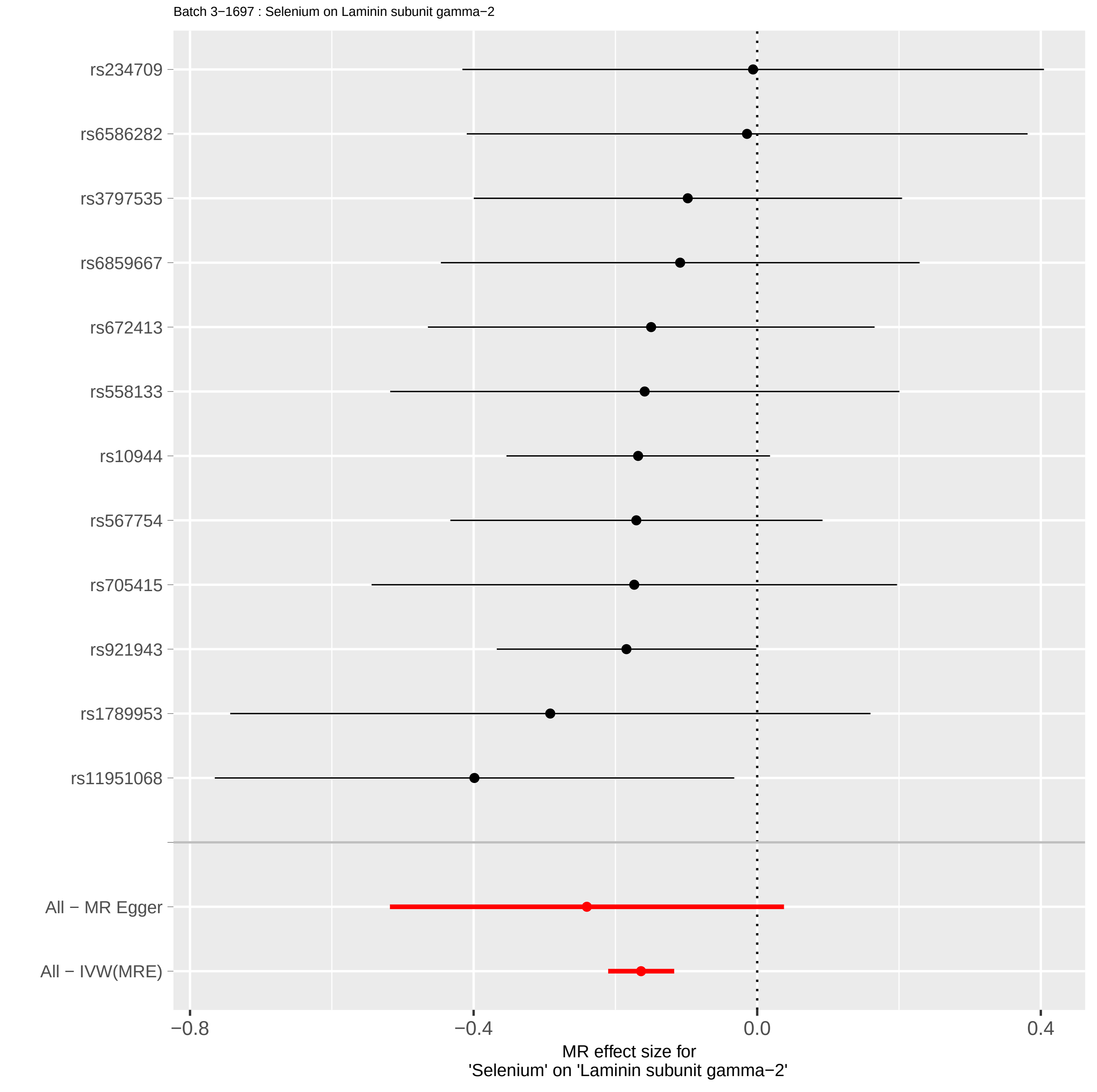

Batch 3−1697 : Selenium on Laminin subunit gamma−2
rs234709
rs6586282
rs3797535
rs6859667
rs672413
rs558133
rs10944
rs567754
rs705415
rs921943
rs1789953
rs11951068
All − MR Egger
All − IVW(MRE)
−0.8
−0.4
0.0
0.4
MR effect size for
'Selenium' on 'Laminin subunit gamma−2'

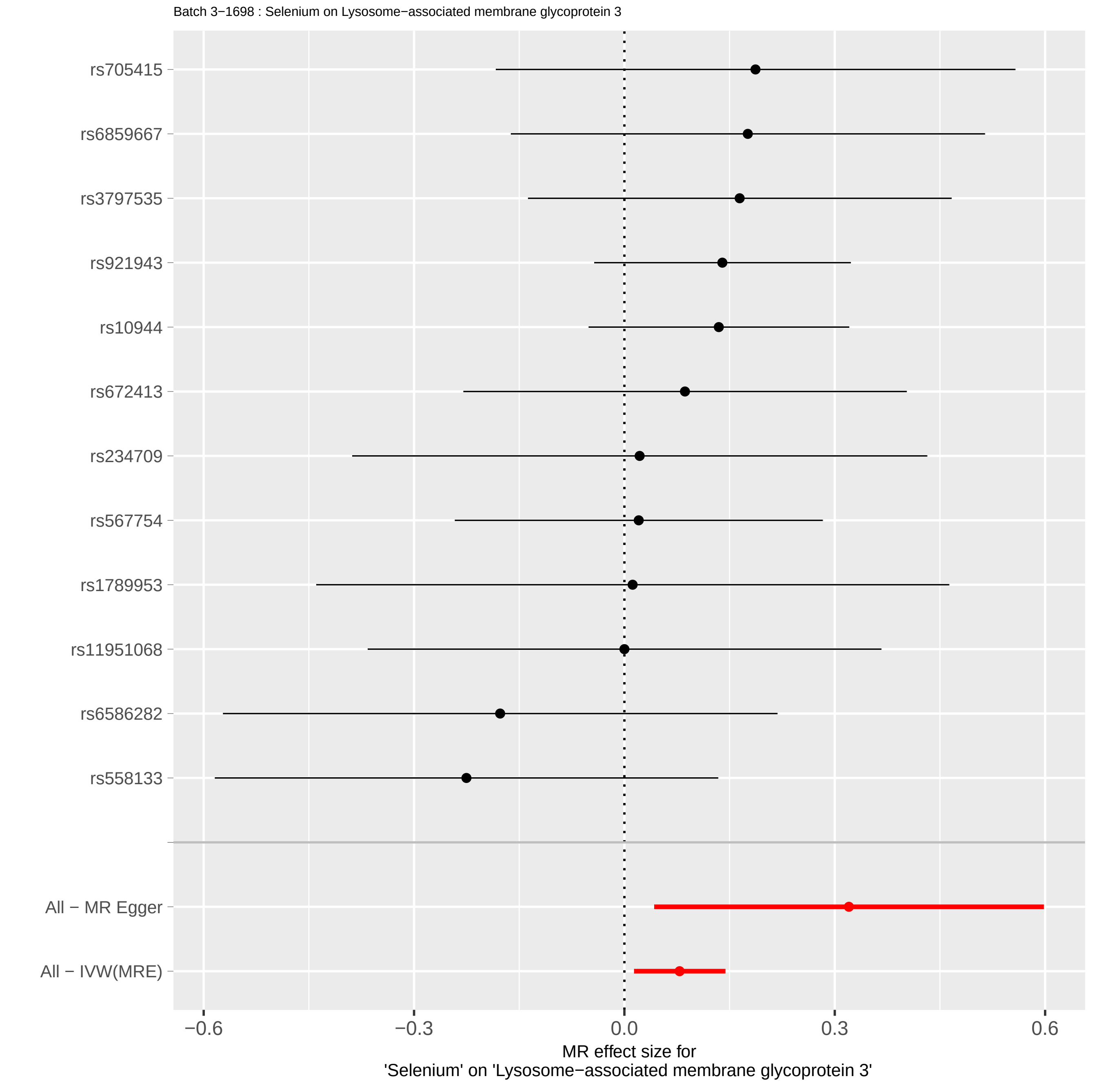

Batch 3−1698 : Selenium on Lysosome−associated membrane glycoprotein 3
rs705415
rs6859667
rs3797535
rs921943
rs10944
rs672413
rs234709
rs567754
rs1789953
rs11951068
rs6586282
rs558133
All − MR Egger
All − IVW(MRE)
−0.6
−0.3
0.0
0.3
0.6
MR effect size for
'Selenium' on 'Lysosome−associated membrane glycoprotein 3'

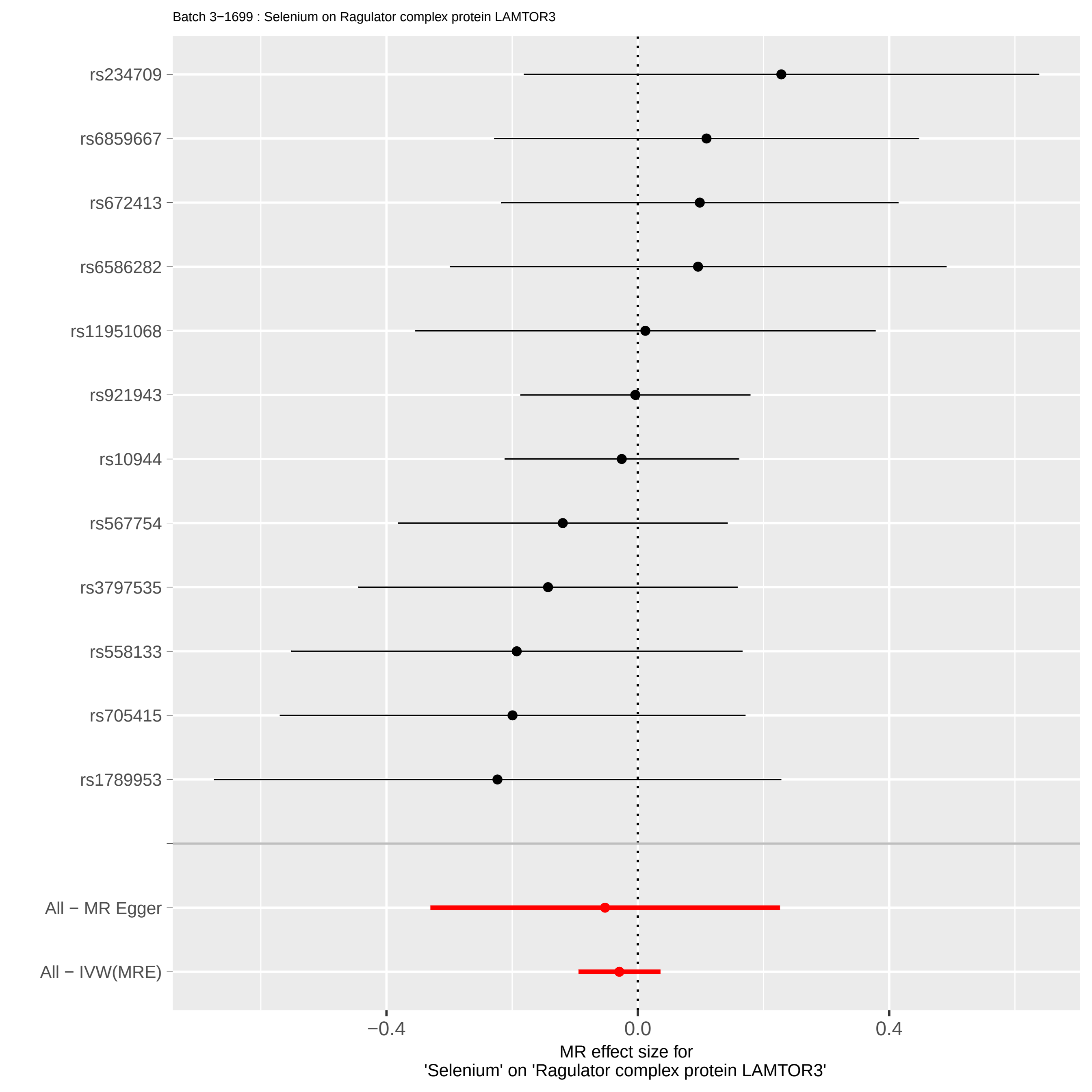
Batch 3−1699 : Selenium on Ragulator complex protein LAMTOR3
rs234709
rs6859667
rs672413
rs6586282
rs11951068
rs921943
rs10944
rs567754
rs3797535
rs558133
rs705415
rs1789953
All − MR Egger
All − IVW(MRE)
−0.4
0.0
0.4
MR effect size for
'Selenium' on 'Ragulator complex protein LAMTOR3'

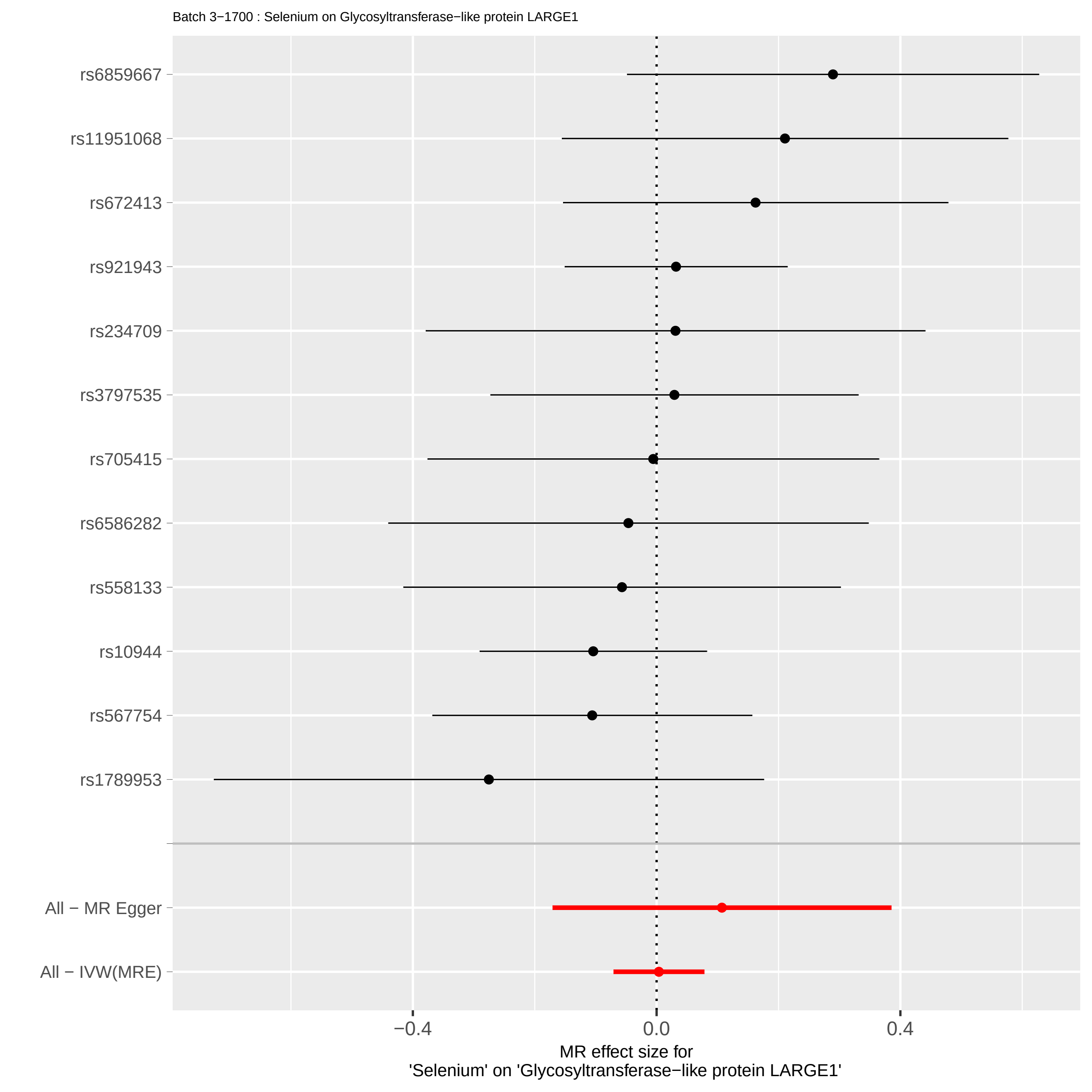

Batch 3−1700 : Selenium on Glycosyltransferase−like protein LARGE1
rs6859667
rs11951068
rs672413
rs921943
rs234709
rs3797535
rs705415
rs6586282
rs558133
rs10944
rs567754
rs1789953
All − MR Egger
All − IVW(MRE)
−0.4
0.0
0.4
MR effect size for
'Selenium' on 'Glycosyltransferase−like protein LARGE1'

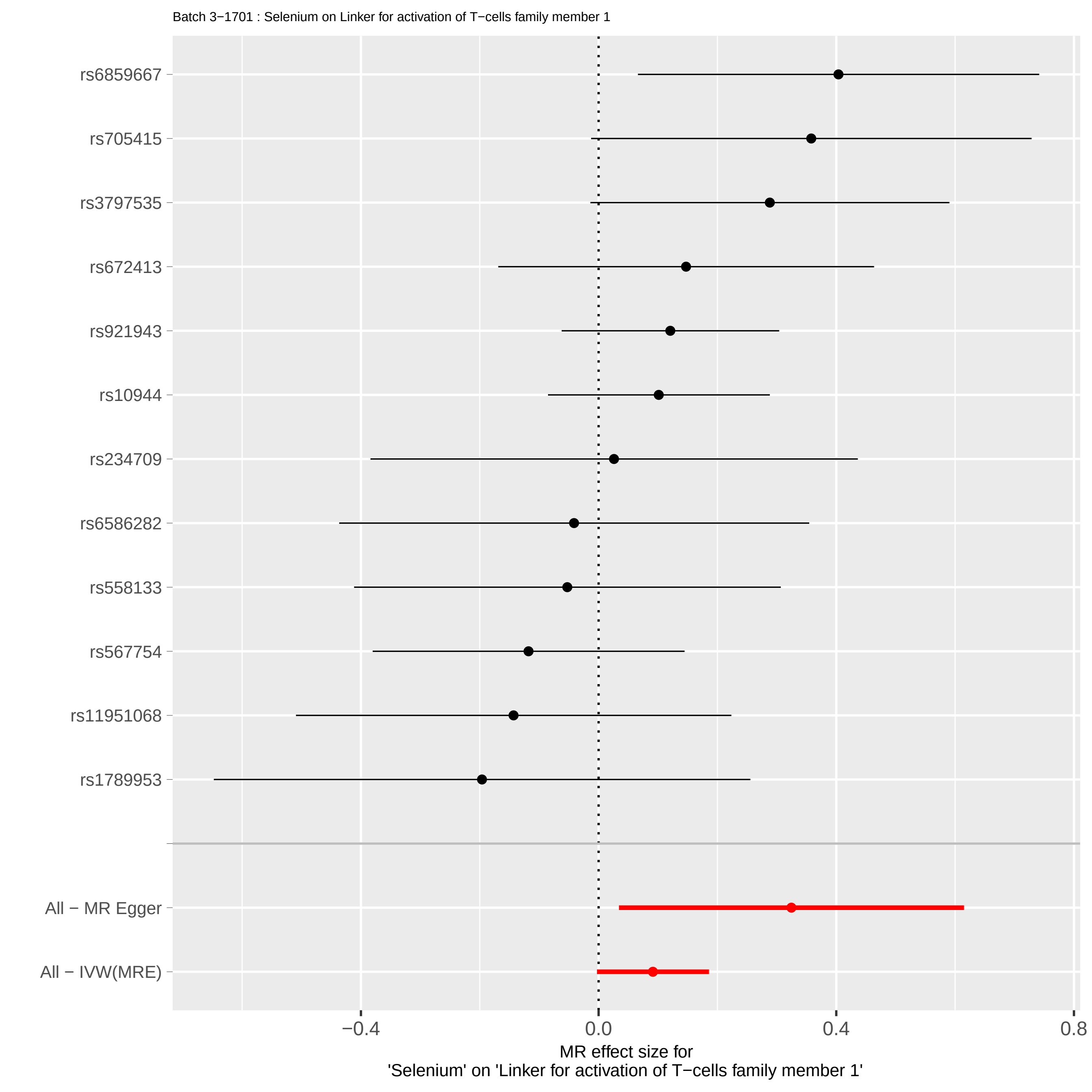
Batch 3−1701 : Selenium on Linker for activation of T−cells family member 1
rs6859667
rs705415
rs3797535
rs672413
rs921943
rs10944
rs234709
rs6586282
rs558133
rs567754
rs11951068
rs1789953
All − MR Egger
All − IVW(MRE)
−0.4
0.0
0.4
0.8
MR effect size for
'Selenium' on 'Linker for activation of T−cells family member 1'

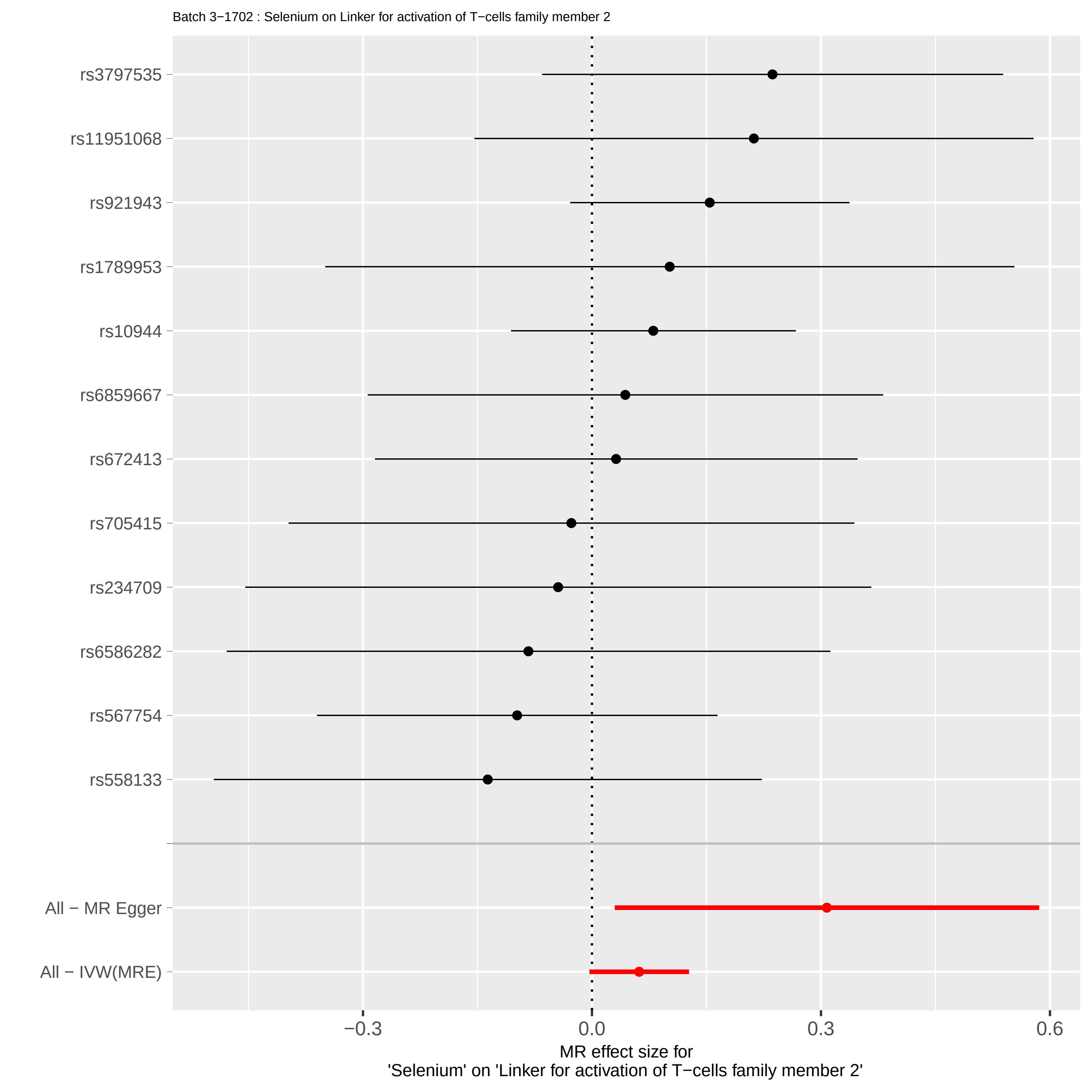

Batch 3−1702 : Selenium on Linker for activation of T−cells family member 2
rs3797535
rs11951068
rs921943
rs1789953
rs10944
rs6859667
rs672413
rs705415
rs234709
rs6586282
rs567754
rs558133
All − MR Egger
All − IVW(MRE)
−0.3
0.0
0.3
0.6
MR effect size for
'Selenium' on 'Linker for activation of T−cells family member 2'

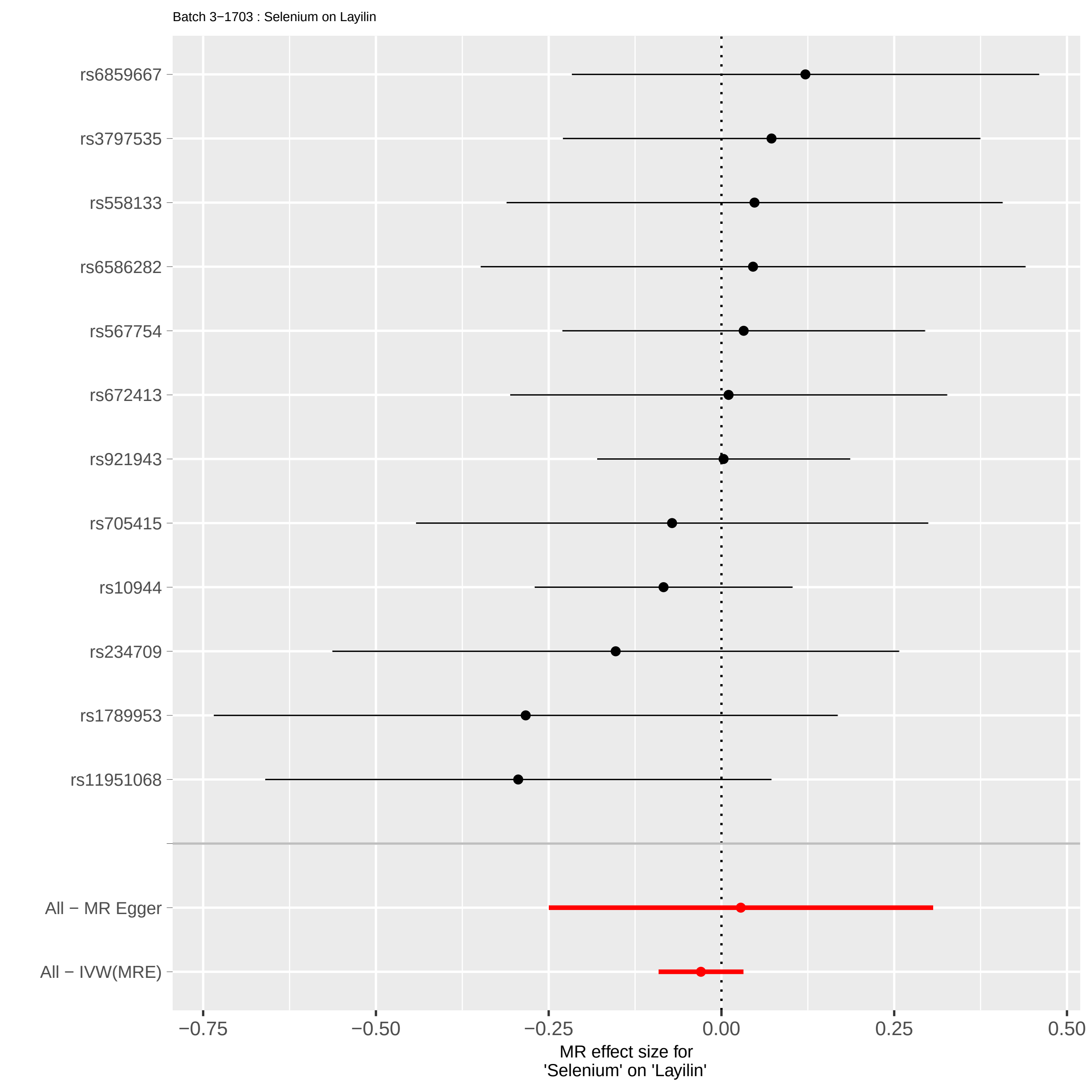
Batch 3−1703 : Selenium on Layilin
rs6859667
rs3797535
rs558133
rs6586282
rs567754
rs672413
rs921943
rs705415
rs10944
rs234709
rs1789953
rs11951068
All − MR Egger
All − IVW(MRE)
−0.75
−0.50
−0.25
0.00
0.25
0.50
MR effect size for
'Selenium' on 'Layilin'

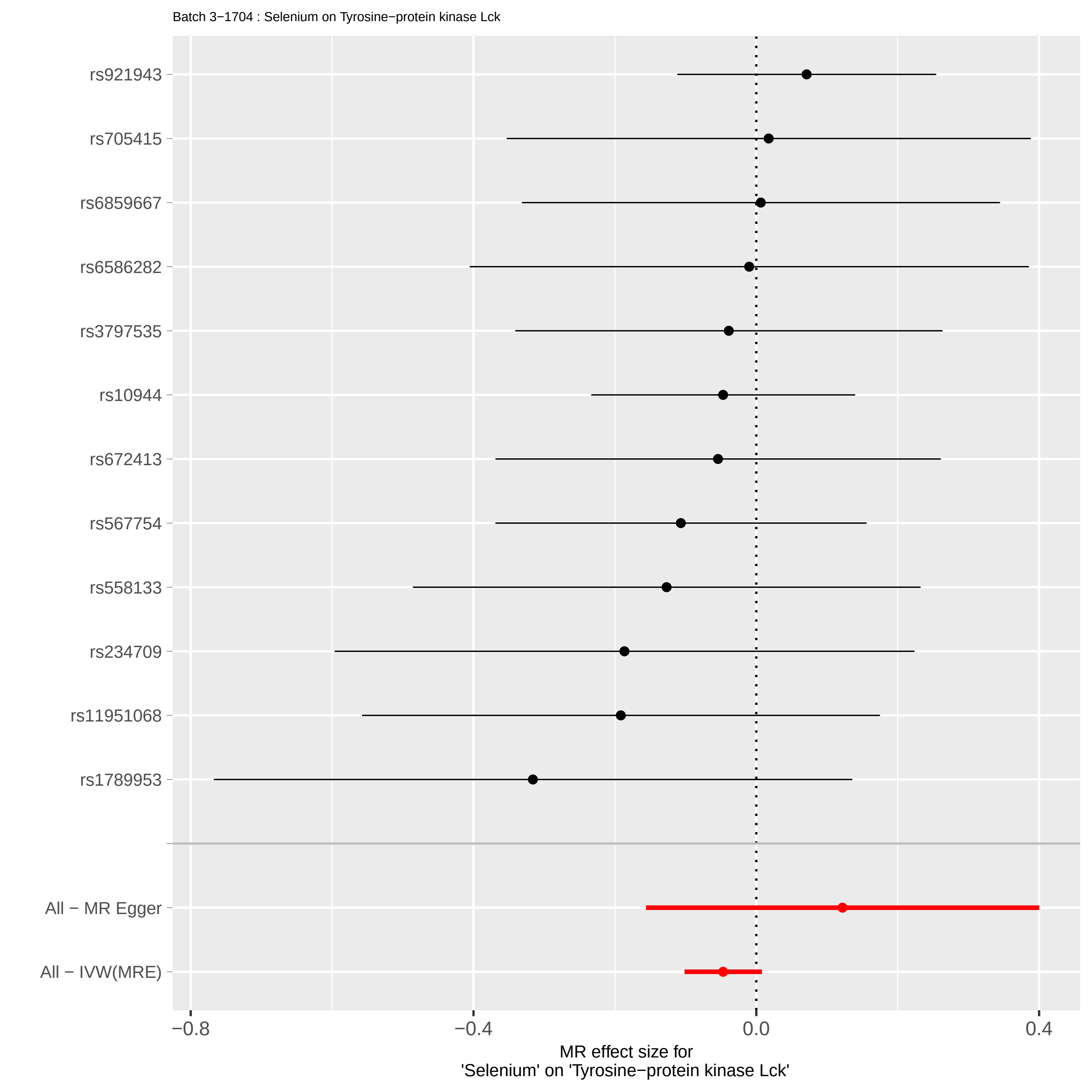

Batch 3−1704 : Selenium on Tyrosine−protein kinase Lck
rs921943
rs705415
rs6859667
rs6586282
rs3797535
rs10944
rs672413
rs567754
rs558133
rs234709
rs11951068
rs1789953
All − MR Egger
All − IVW(MRE)
−0.8
−0.4
0.0
0.4
MR effect size for
'Selenium' on 'Tyrosine−protein kinase Lck'

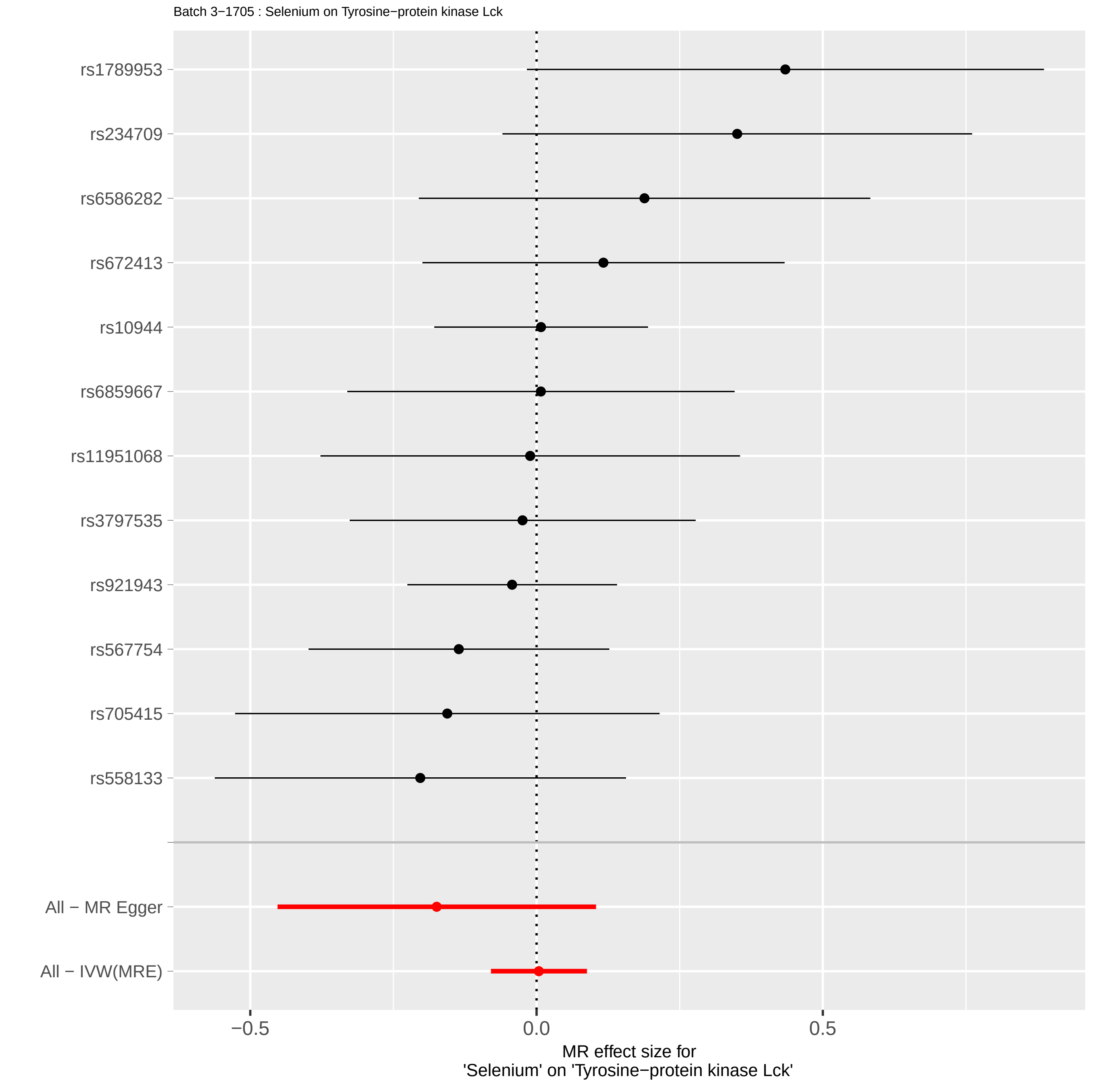
Batch 3−1705 : Selenium on Tyrosine−protein kinase Lck
rs1789953
rs234709
rs6586282
rs672413
rs10944
rs6859667
rs11951068
rs3797535
rs921943
rs567754
rs705415
rs558133
All − MR Egger
All − IVW(MRE)
−0.5
0.0
0.5
MR effect size for
'Selenium' on 'Tyrosine−protein kinase Lck'

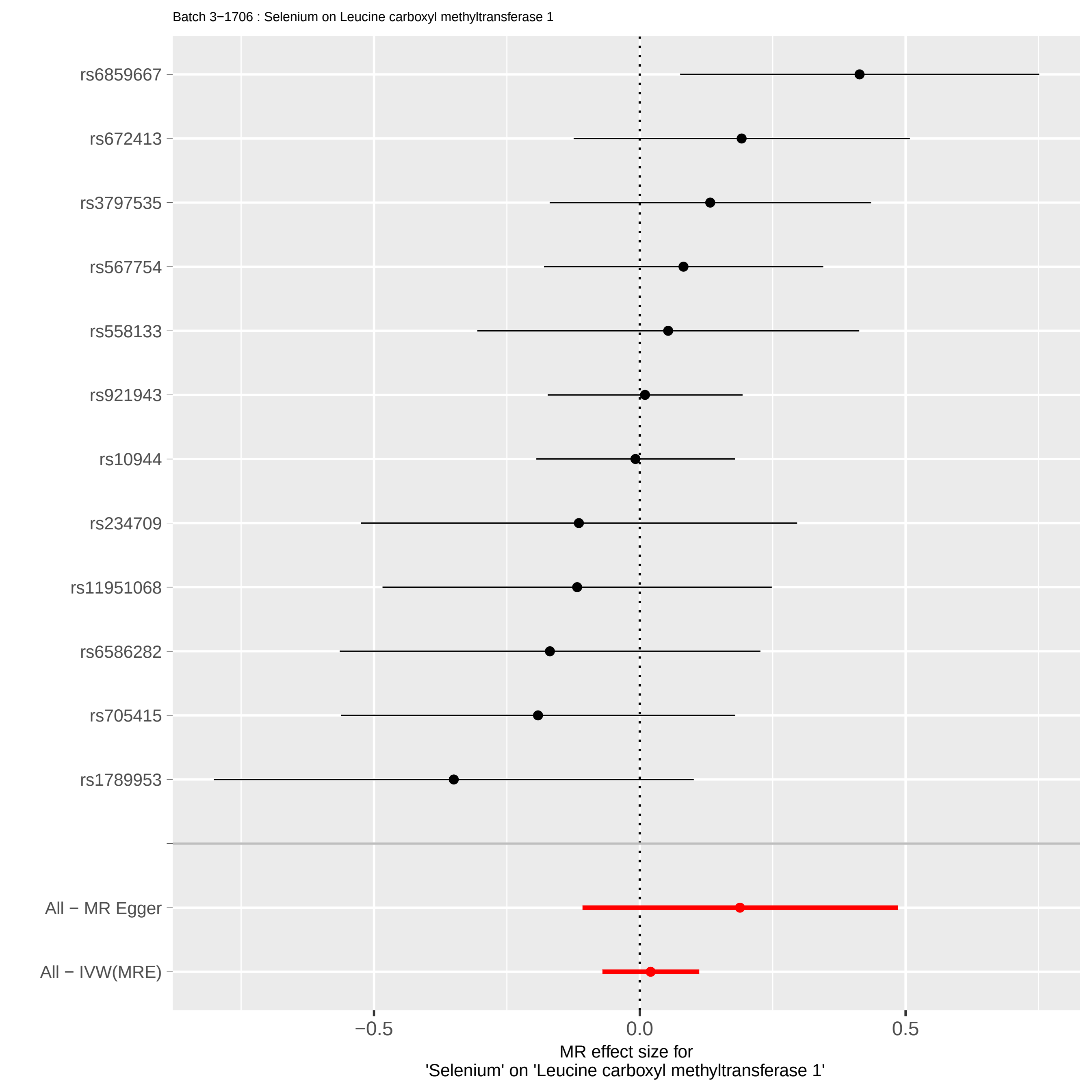

Batch 3−1706 : Selenium on Leucine carboxyl methyltransferase 1
rs6859667
rs672413
rs3797535
rs567754
rs558133
rs921943
rs10944
rs234709
rs11951068
rs6586282
rs705415
rs1789953
All − MR Egger
All − IVW(MRE)
−0.5
0.0
0.5
MR effect size for
'Selenium' on 'Leucine carboxyl methyltransferase 1'

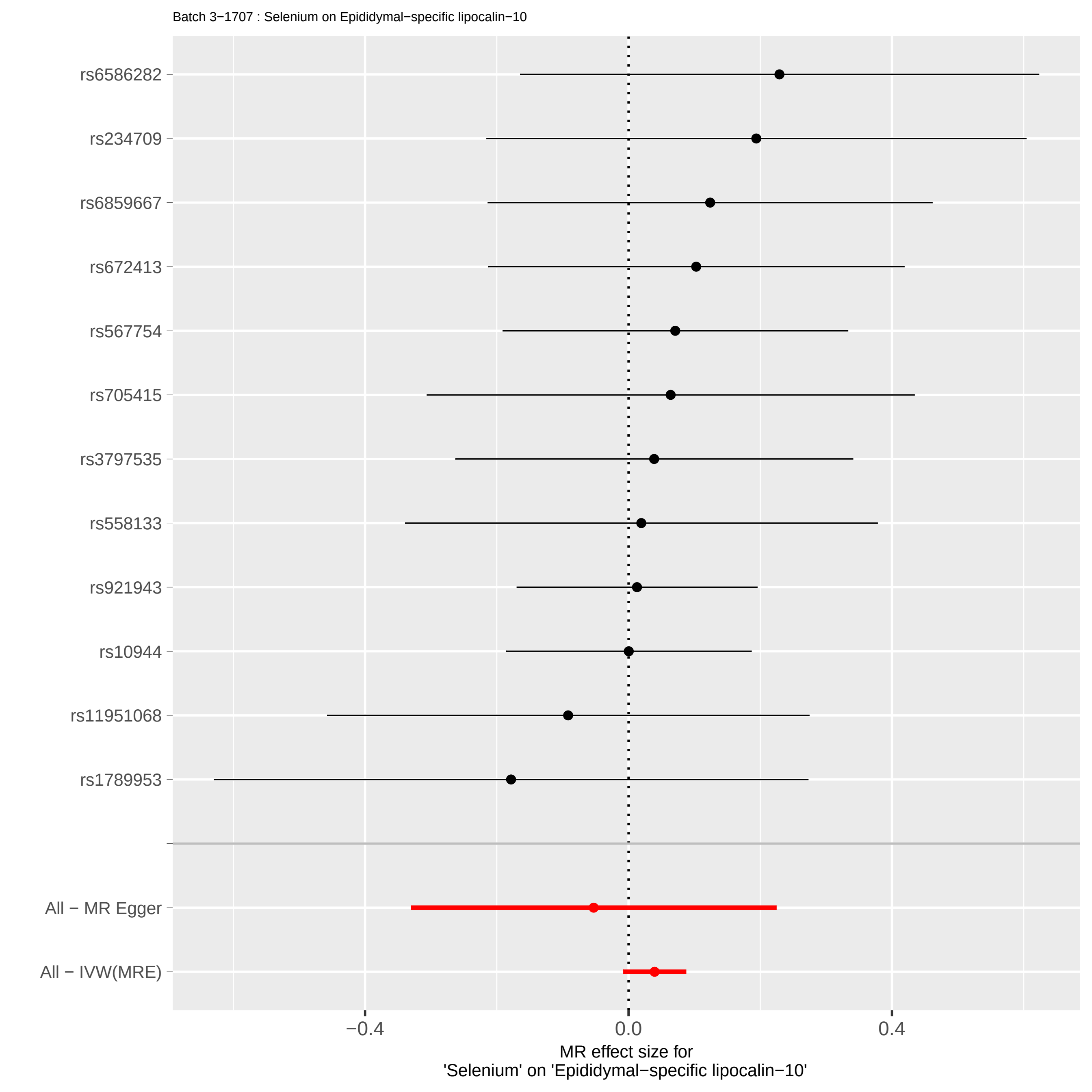

Batch 3−1707 : Selenium on Epididymal−specific lipocalin−10
rs6586282
rs234709
rs6859667
rs672413
rs567754
rs705415
rs3797535
rs558133
rs921943
rs10944
rs11951068
rs1789953
All − MR Egger
All − IVW(MRE)
−0.4
0.0
0.4
MR effect size for
'Selenium' on 'Epididymal−specific lipocalin−10'

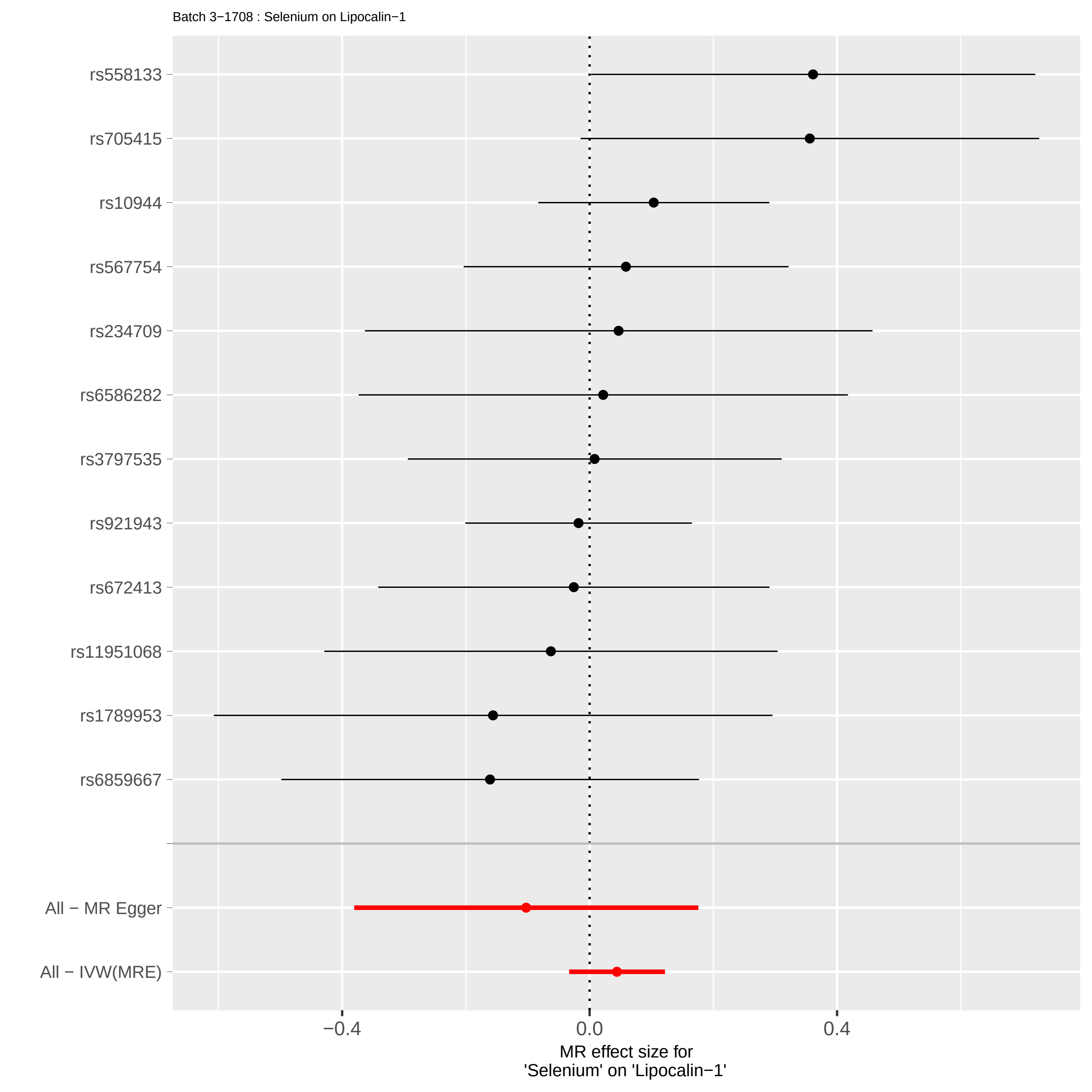
Batch 3−1708 : Selenium on Lipocalin−1
rs558133
rs705415
rs10944
rs567754
rs234709
rs6586282
rs3797535
rs921943
rs672413
rs11951068
rs1789953
rs6859667
All − MR Egger
All − IVW(MRE)
−0.4
0.0
0.4
MR effect size for
'Selenium' on 'Lipocalin−1'

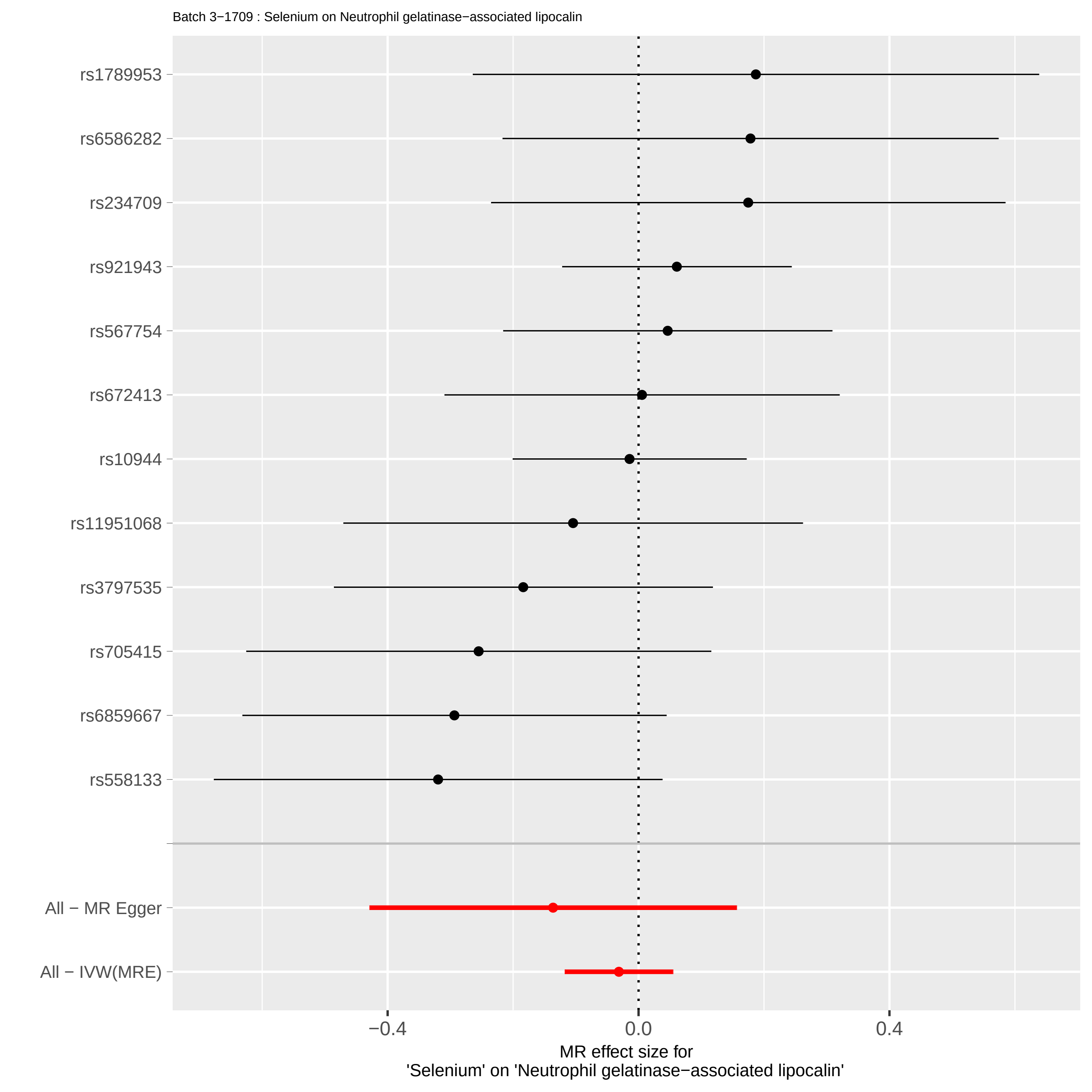
Batch 3−1709 : Selenium on Neutrophil gelatinase−associated lipocalin
rs1789953
rs6586282
rs234709
rs921943
rs567754
rs672413
rs10944
rs11951068
rs3797535
rs705415
rs6859667
rs558133
All − MR Egger
All − IVW(MRE)
−0.4
0.0
0.4
MR effect size for
'Selenium' on 'Neutrophil gelatinase−associated lipocalin'

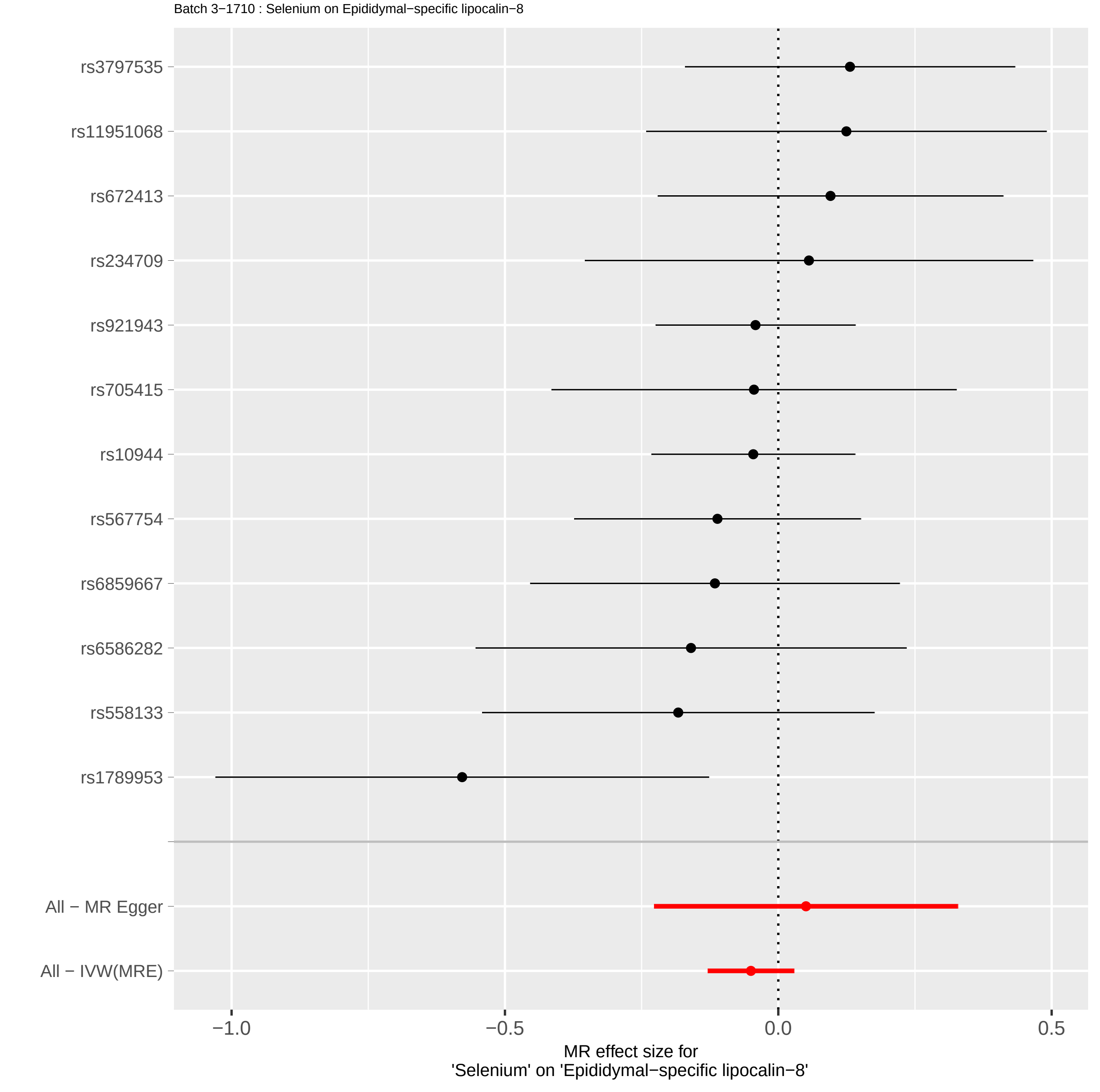

Batch 3−1710 : Selenium on Epididymal−specific lipocalin−8
rs3797535
rs11951068
rs672413
rs234709
rs921943
rs705415
rs10944
rs567754
rs6859667
rs6586282
rs558133
rs1789953
All − MR Egger
All − IVW(MRE)
−1.0
−0.5
0.0
0.5
MR effect size for
'Selenium' on 'Epididymal−specific lipocalin−8'

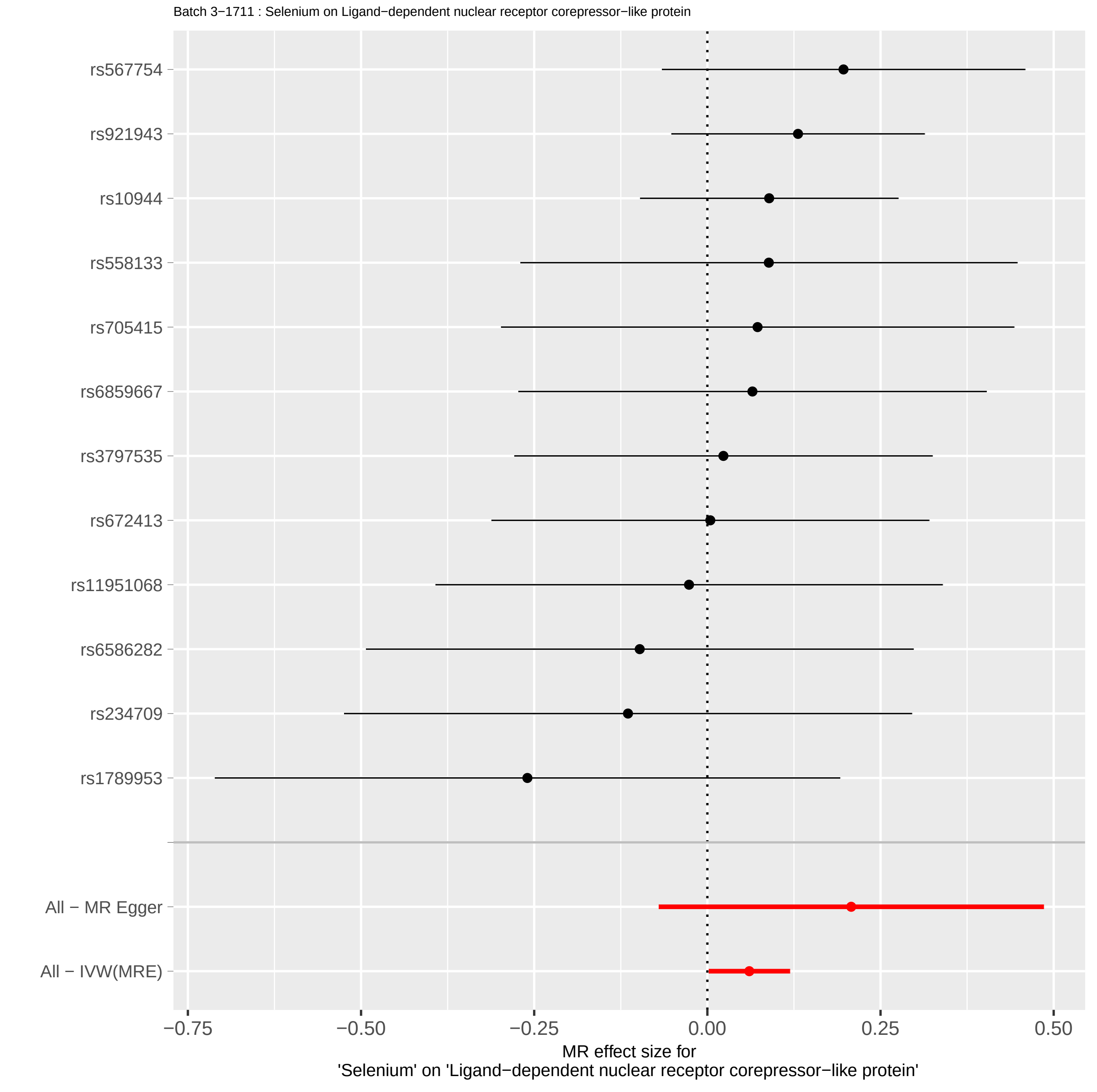
Batch 3−1711 : Selenium on Ligand−dependent nuclear receptor corepressor−like protein
rs567754
rs921943
rs10944
rs558133
rs705415
rs6859667
rs3797535
rs672413
rs11951068
rs6586282
rs234709
rs1789953
All − MR Egger
All − IVW(MRE)
−0.75
−0.50
−0.25
0.00
0.25
0.50
MR effect size for
'Selenium' on 'Ligand−dependent nuclear receptor corepressor−like protein'

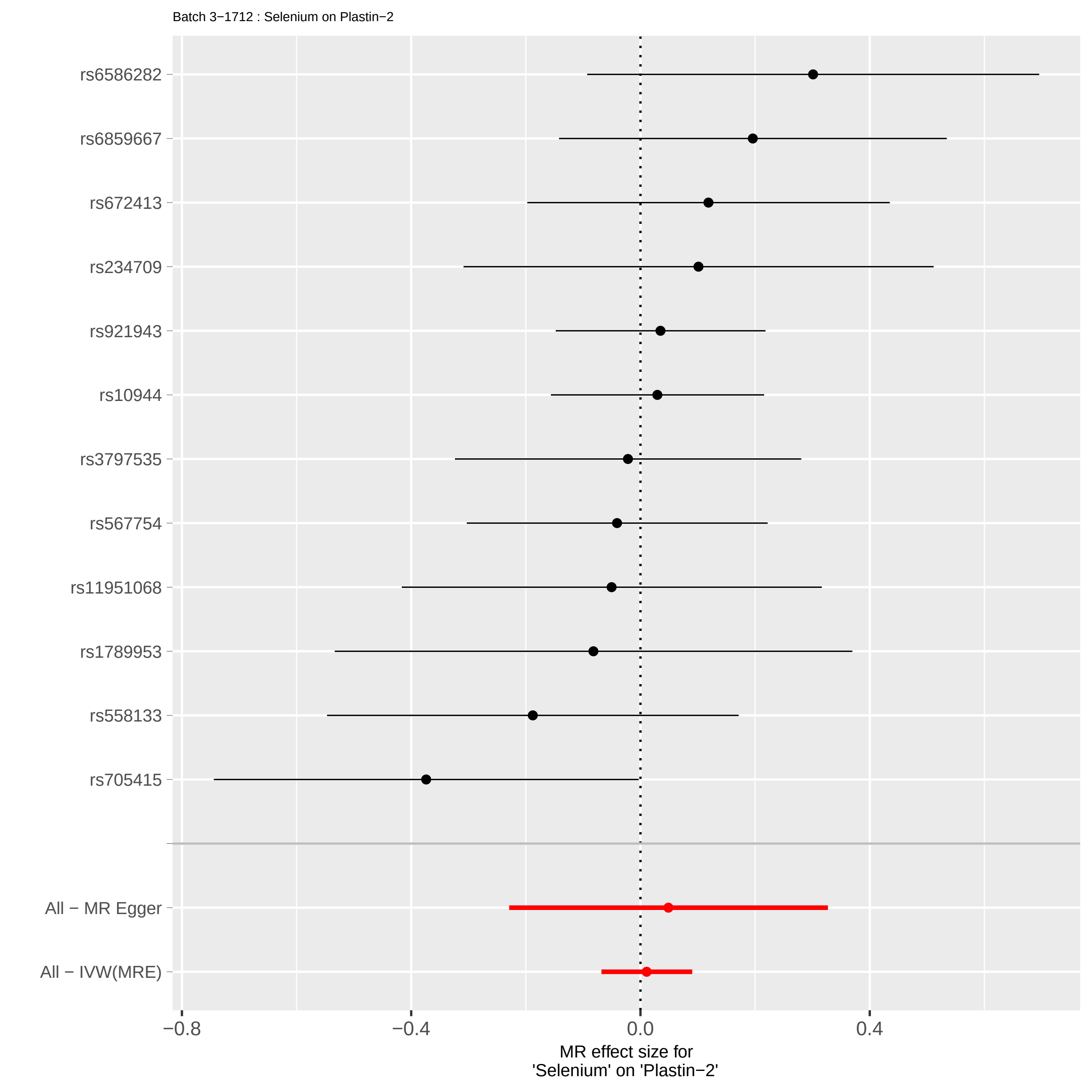
Batch 3−1712 : Selenium on Plastin−2
rs6586282
rs6859667
rs672413
rs234709
rs921943
rs10944
rs3797535
rs567754
rs11951068
rs1789953
rs558133
rs705415
All − MR Egger
All − IVW(MRE)
−0.8
−0.4
0.0
0.4
MR effect size for
'Selenium' on 'Plastin−2'

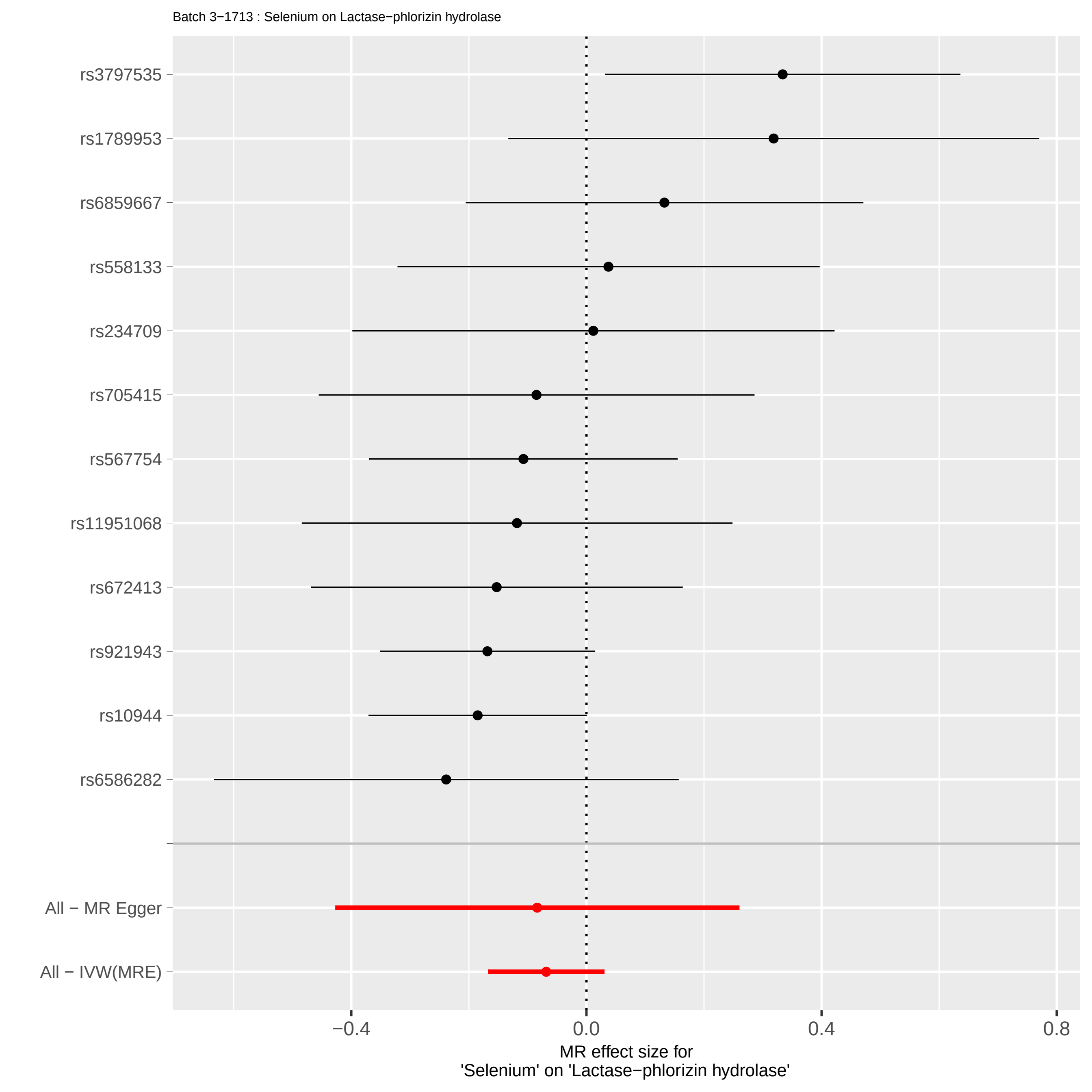

Batch 3−1713 : Selenium on Lactase−phlorizin hydrolase
rs3797535
rs1789953
rs6859667
rs558133
rs234709
rs705415
rs567754
rs11951068
rs672413
rs921943
rs10944
rs6586282
All − MR Egger
All − IVW(MRE)
−0.4
0.0
0.4
0.8
MR effect size for
'Selenium' on 'Lactase−phlorizin hydrolase'

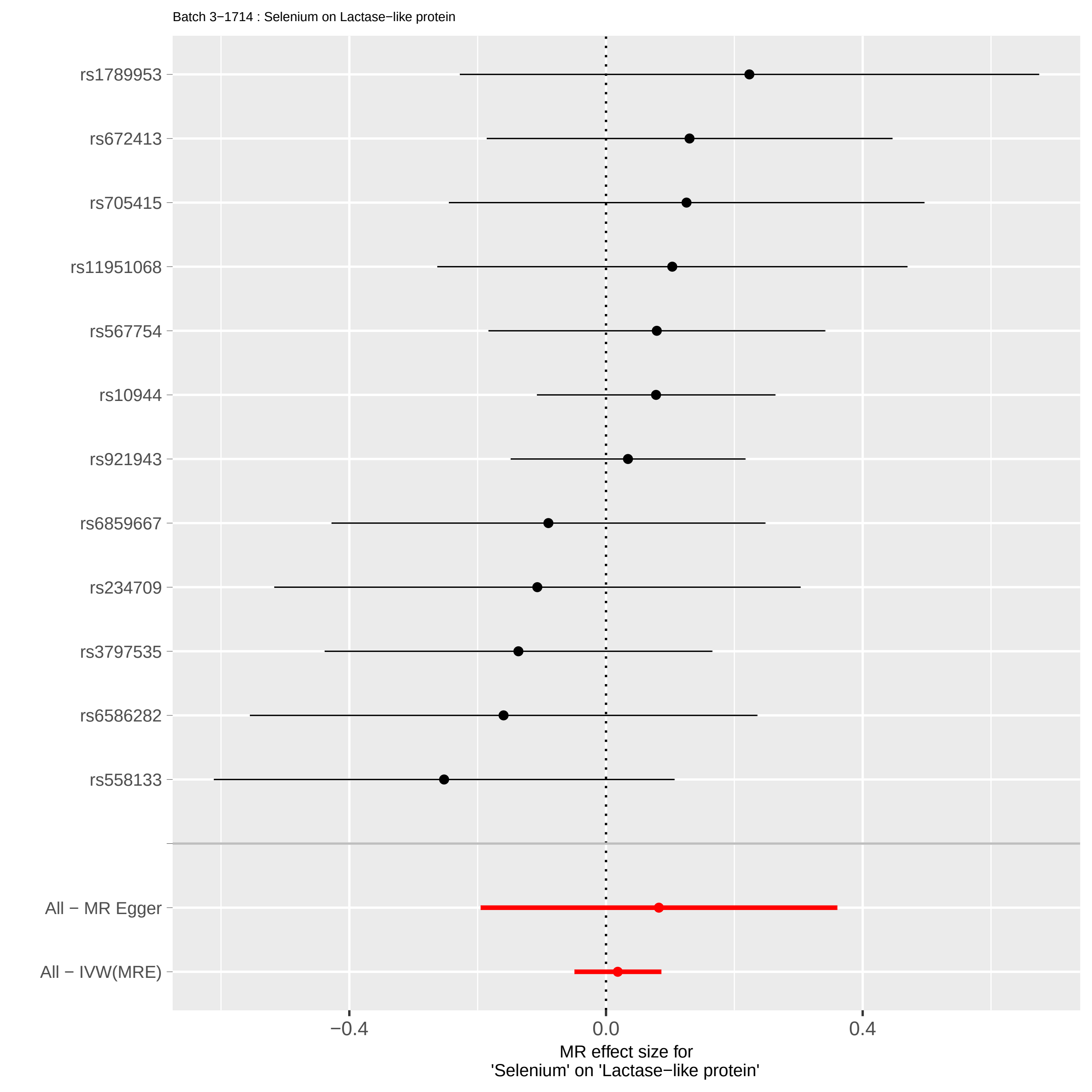

Batch 3−1714 : Selenium on Lactase−like protein
rs1789953
rs672413
rs705415
rs11951068
rs567754
rs10944
rs921943
rs6859667
rs234709
rs3797535
rs6586282
rs558133
All − MR Egger
All − IVW(MRE)
−0.4
0.0
0.4
MR effect size for
'Selenium' on 'Lactase−like protein'

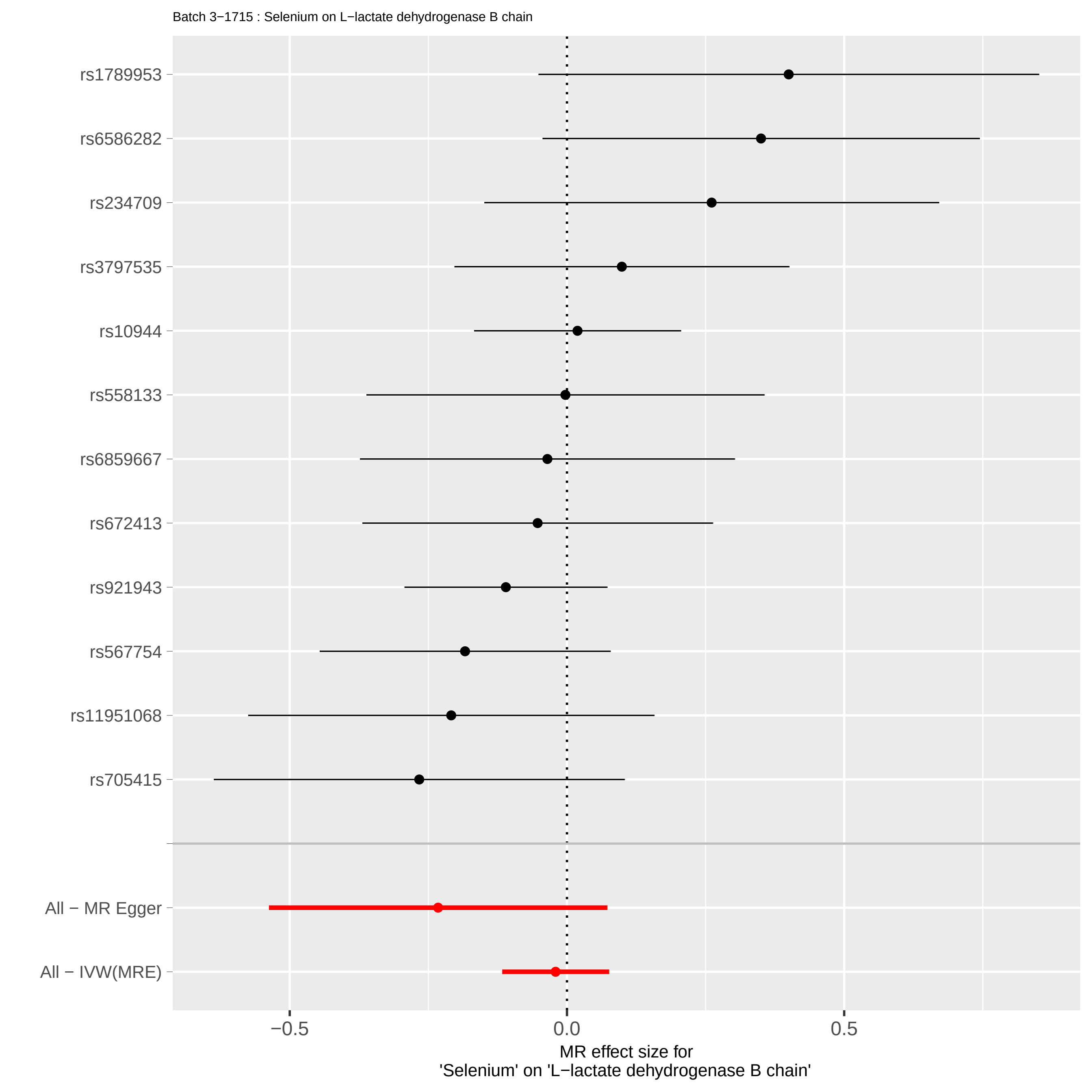

Batch 3−1715 : Selenium on L−lactate dehydrogenase B chain
rs1789953
rs6586282
rs234709
rs3797535
rs10944
rs558133
rs6859667
rs672413
rs921943
rs567754
rs11951068
rs705415
All − MR Egger
All − IVW(MRE)
−0.5
0.0
0.5
MR effect size for
'Selenium' on 'L−lactate dehydrogenase B chain'

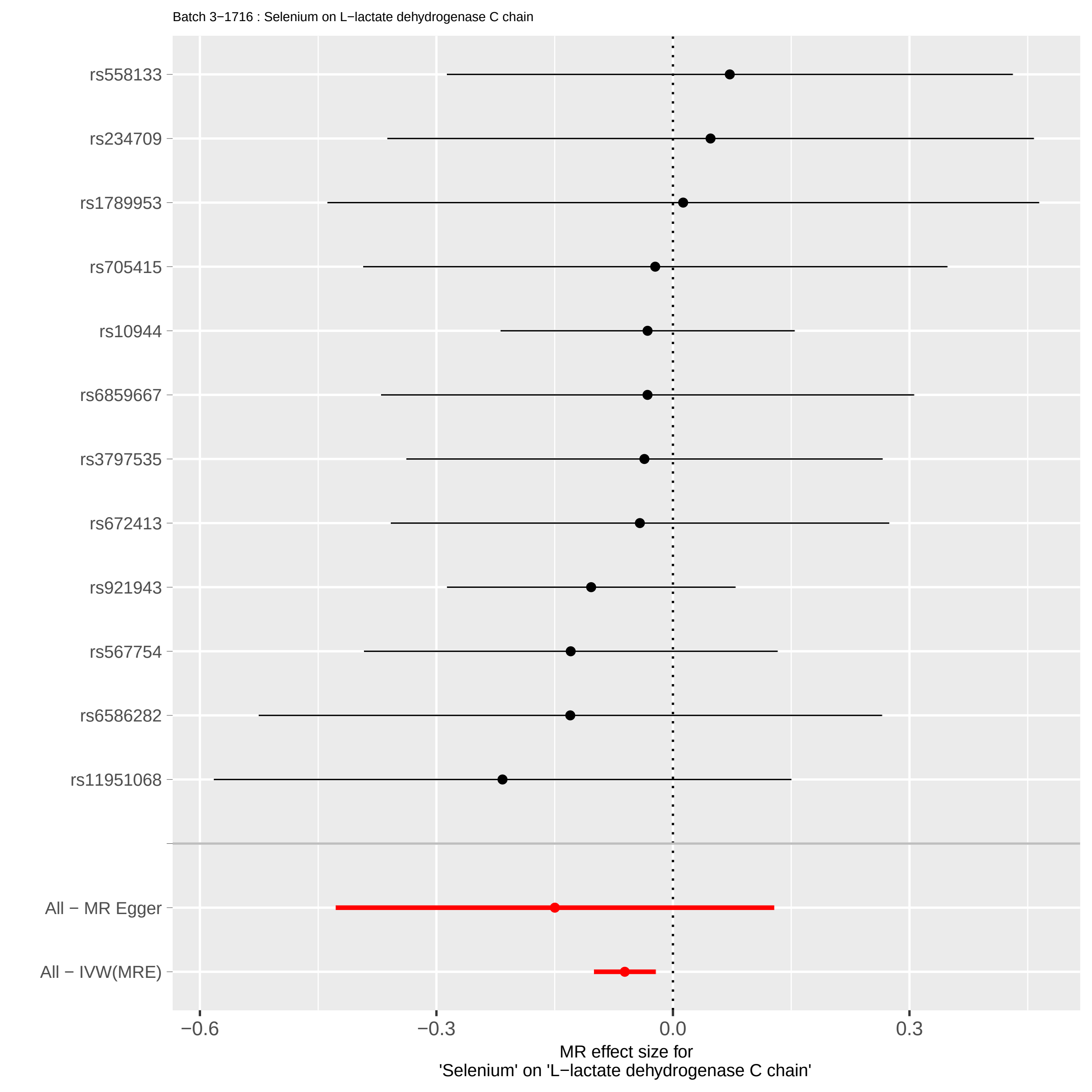

Batch 3−1716 : Selenium on L−lactate dehydrogenase C chain
rs558133
rs234709
rs1789953
rs705415
rs10944
rs6859667
rs3797535
rs672413
rs921943
rs567754
rs6586282
rs11951068
All − MR Egger
All − IVW(MRE)
−0.6
−0.3
0.0
0.3
MR effect size for
'Selenium' on 'L−lactate dehydrogenase C chain'

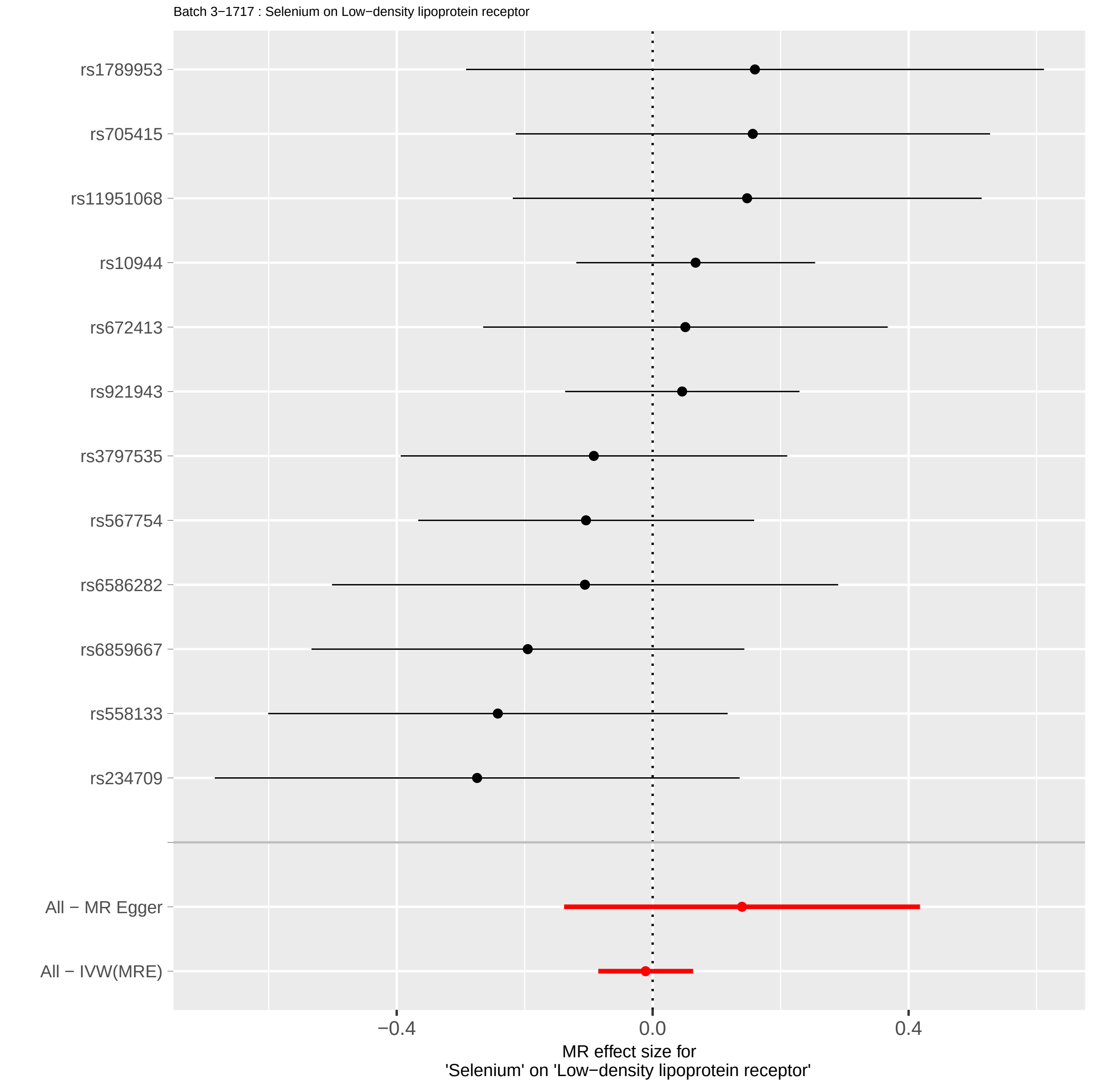

Batch 3−1717 : Selenium on Low−density lipoprotein receptor
rs1789953
rs705415
rs11951068
rs10944
rs672413
rs921943
rs3797535
rs567754
rs6586282
rs6859667
rs558133
rs234709
All − MR Egger
All − IVW(MRE)
−0.4
0.0
0.4
MR effect size for
'Selenium' on 'Low−density lipoprotein receptor'

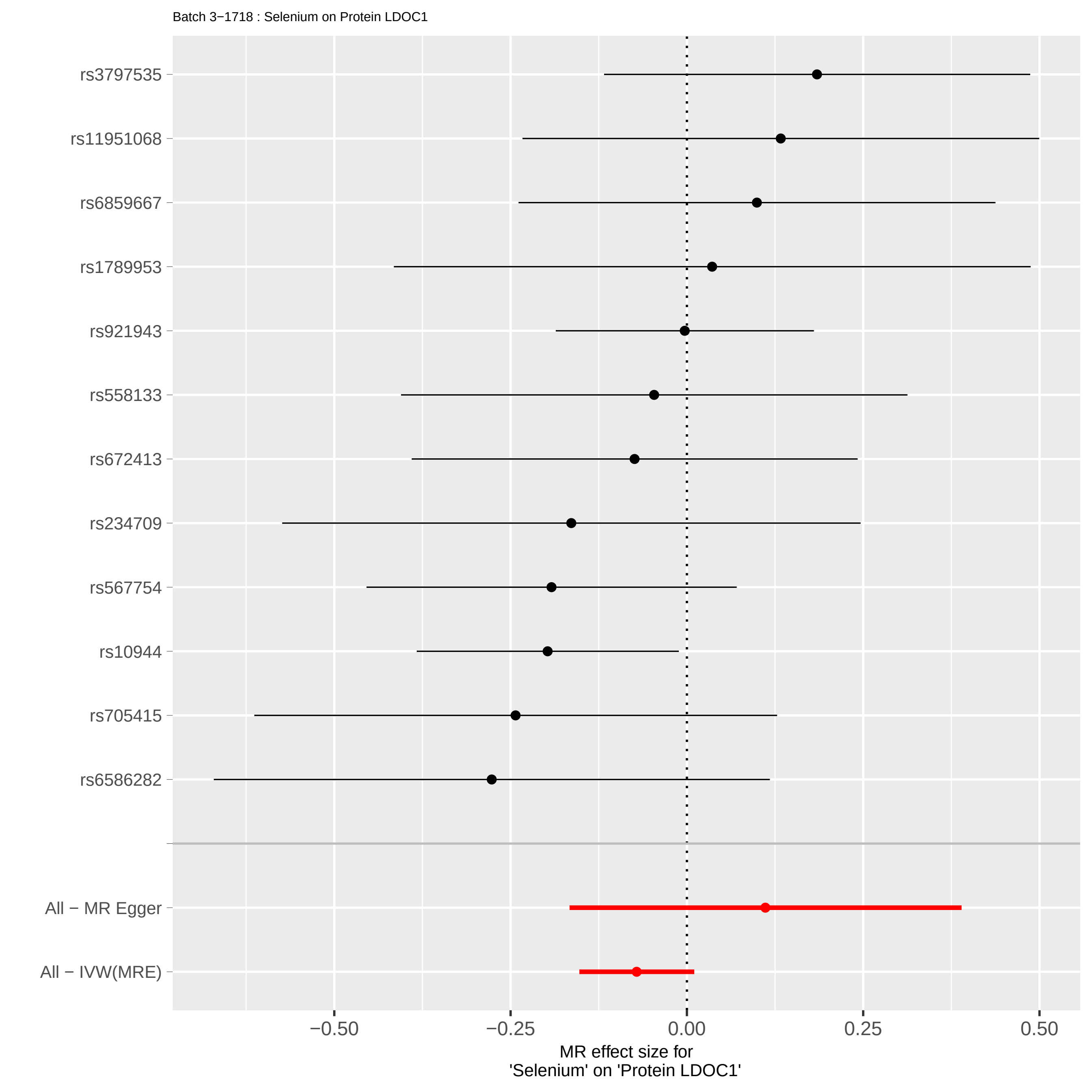
Batch 3−1718 : Selenium on Protein LDOC1
rs3797535
rs11951068
rs6859667
rs1789953
rs921943
rs558133
rs672413
rs234709
rs567754
rs10944
rs705415
rs6586282
All − MR Egger
All − IVW(MRE)
−0.50
−0.25
0.00
0.25
0.50
MR effect size for
'Selenium' on 'Protein LDOC1'

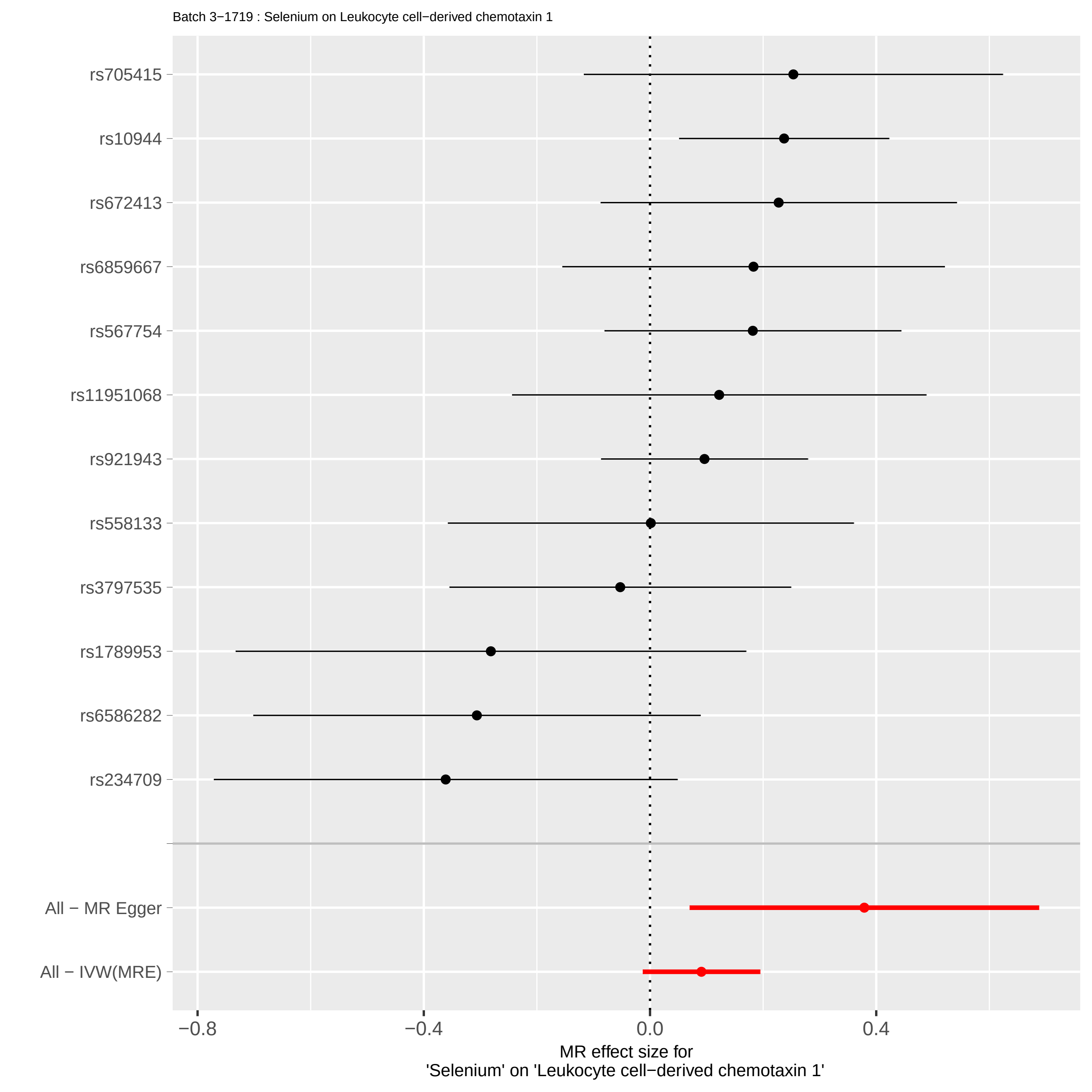

Batch 3−1719 : Selenium on Leukocyte cell−derived chemotaxin 1
rs705415
rs10944
rs672413
rs6859667
rs567754
rs11951068
rs921943
rs558133
rs3797535
rs1789953
rs6586282
rs234709
All − MR Egger
All − IVW(MRE)
−0.8
−0.4
0.0
0.4
MR effect size for
'Selenium' on 'Leukocyte cell−derived chemotaxin 1'

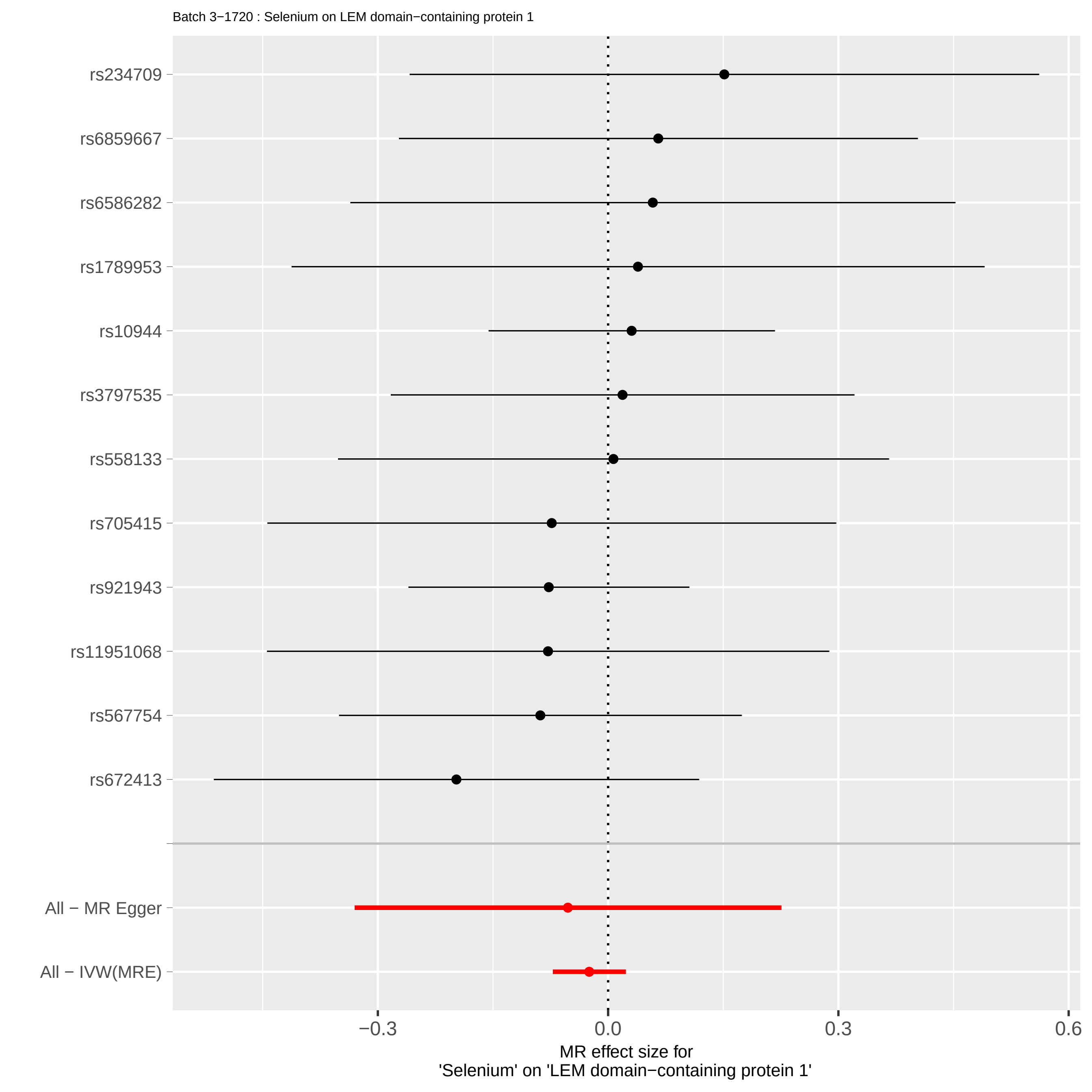
Batch 3−1720 : Selenium on LEM domain−containing protein 1
rs234709
rs6859667
rs6586282
rs1789953
rs10944
rs3797535
rs558133
rs705415
rs921943
rs11951068
rs567754
rs672413
All − MR Egger
All − IVW(MRE)
−0.3
0.0
0.3
0.6
MR effect size for
'Selenium' on 'LEM domain−containing protein 1'

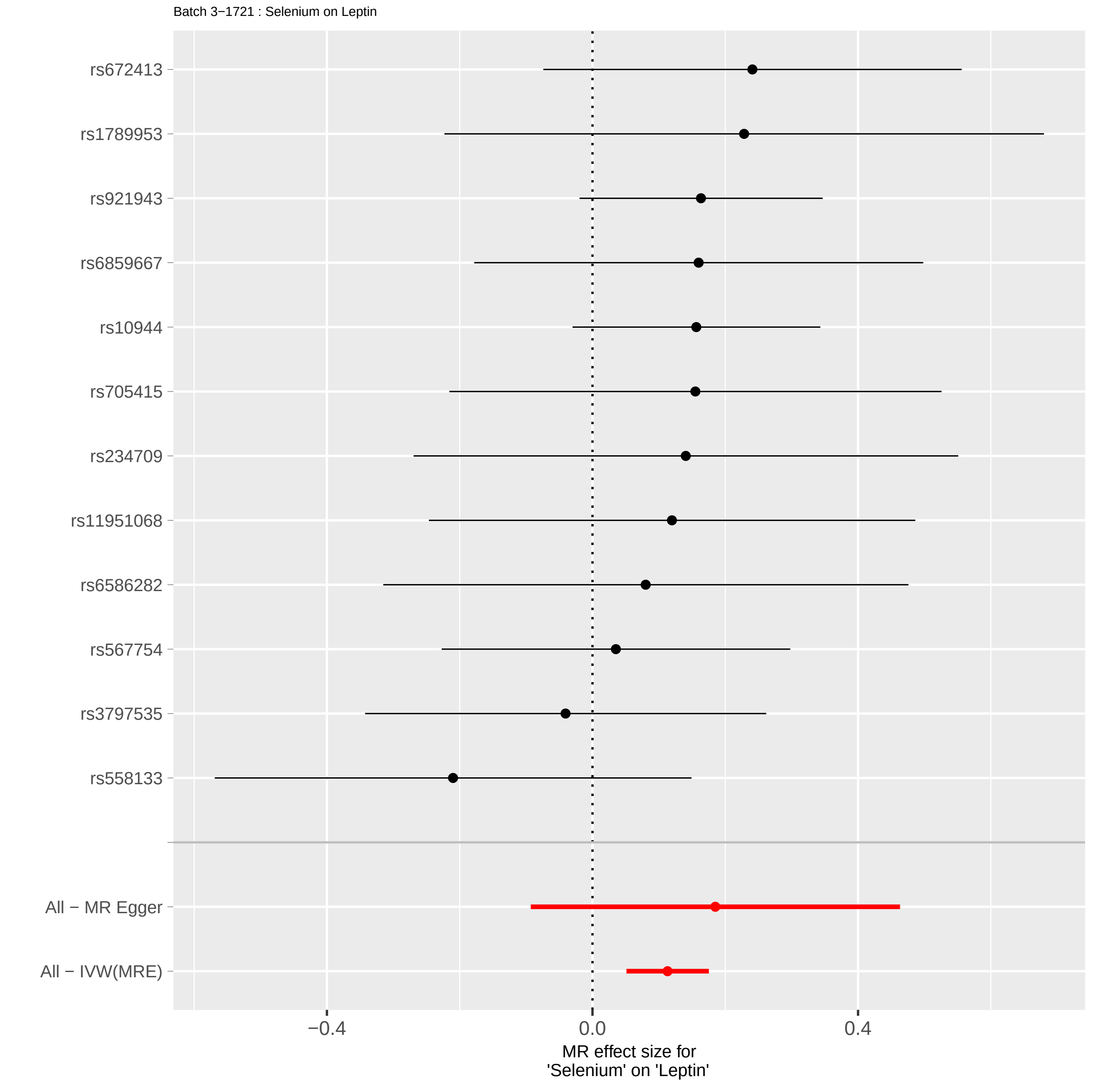
Batch 3−1721 : Selenium on Leptin
rs672413
rs1789953
rs921943
rs6859667
rs10944
rs705415
rs234709
rs11951068
rs6586282
rs567754
rs3797535
rs558133
All − MR Egger
All − IVW(MRE)
−0.4
0.0
0.4
MR effect size for
'Selenium' on 'Leptin'

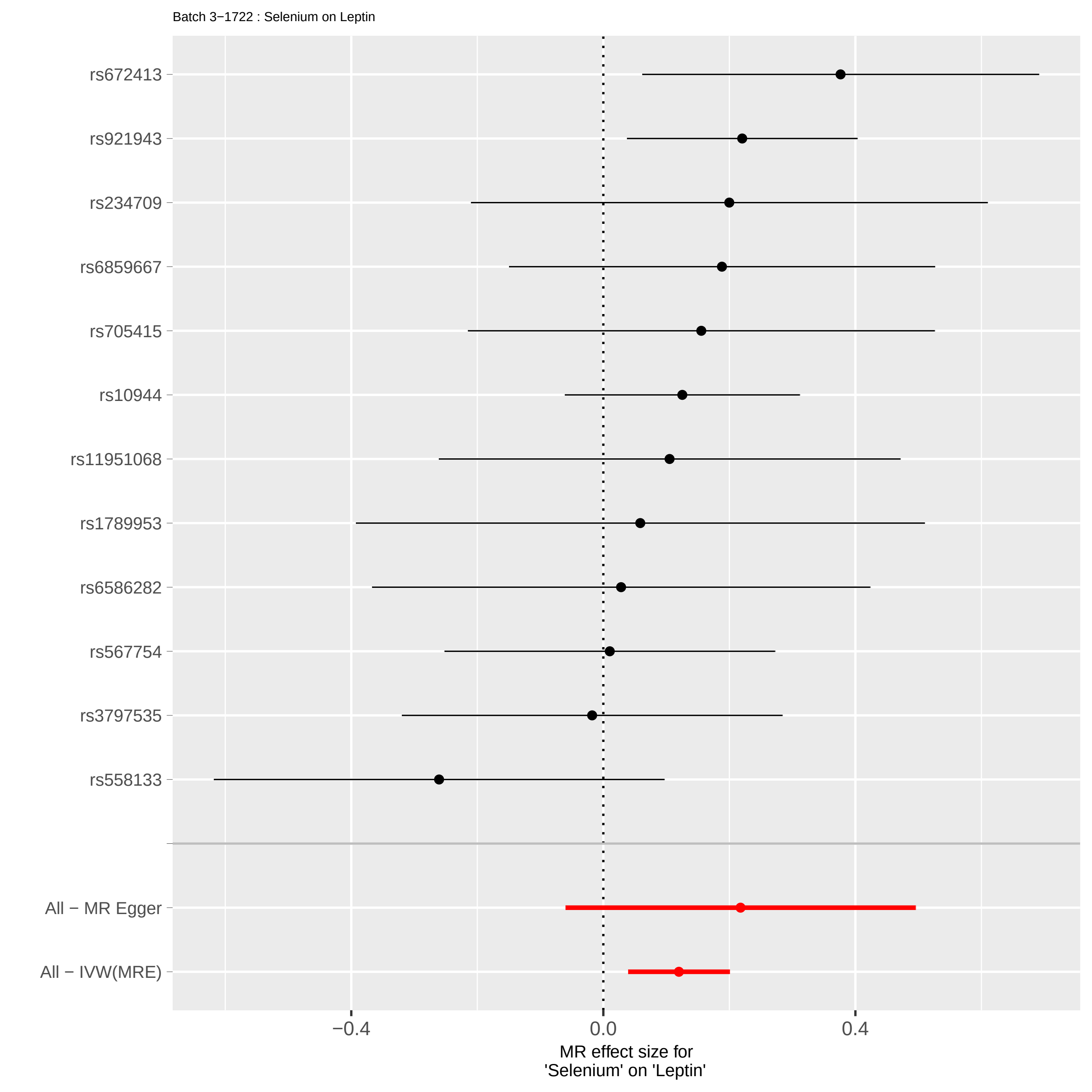

Batch 3−1722 : Selenium on Leptin
rs672413
rs921943
rs234709
rs6859667
rs705415
rs10944
rs11951068
rs1789953
rs6586282
rs567754
rs3797535
rs558133
All − MR Egger
All − IVW(MRE)
−0.4
0.0
0.4
MR effect size for
'Selenium' on 'Leptin'

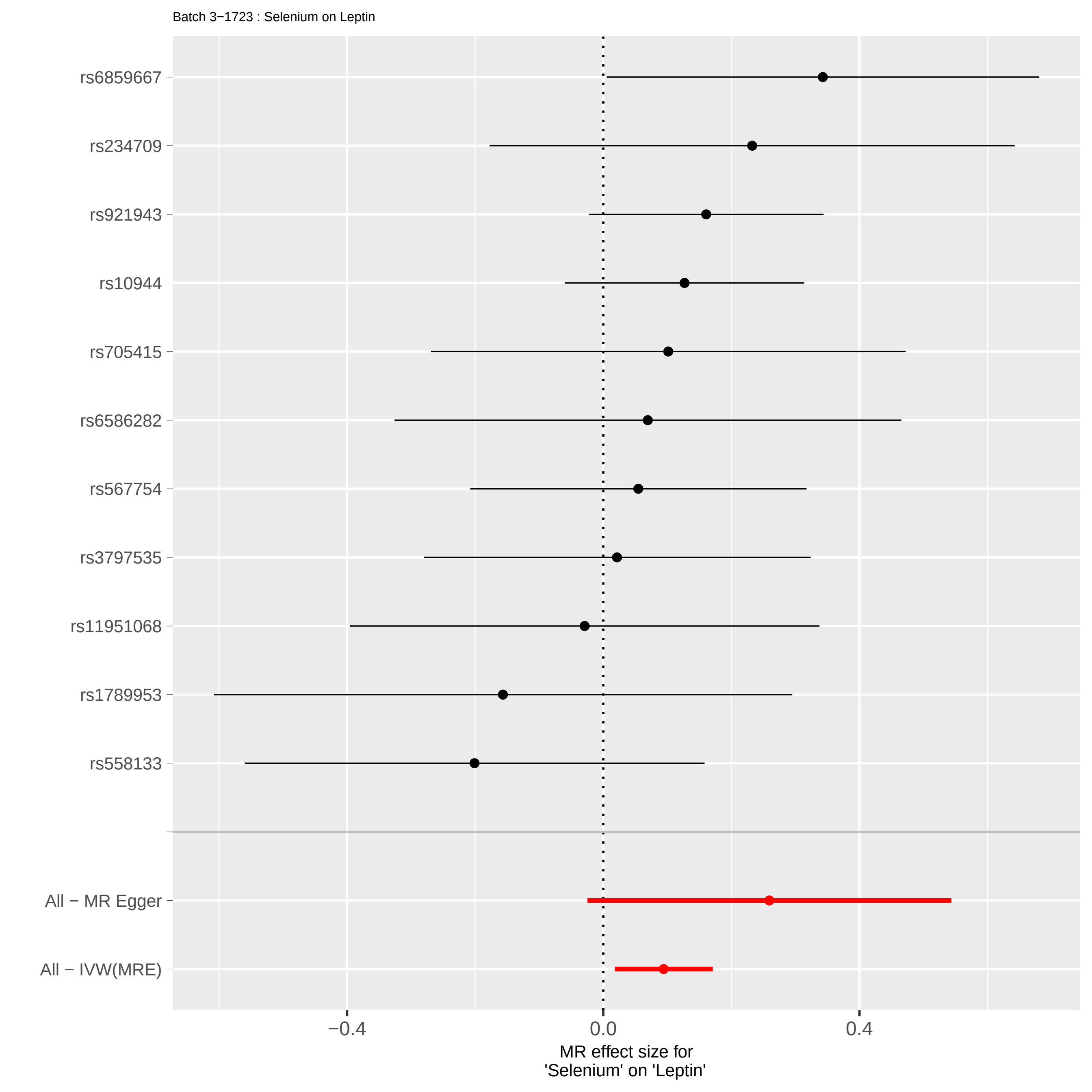
Batch 3−1723 : Selenium on Leptin
rs6859667
rs234709
rs921943
rs10944
rs705415
rs6586282
rs567754
rs3797535
rs11951068
rs1789953
rs558133
All − MR Egger
All − IVW(MRE)
−0.4
0.0
0.4
MR effect size for
'Selenium' on 'Leptin'

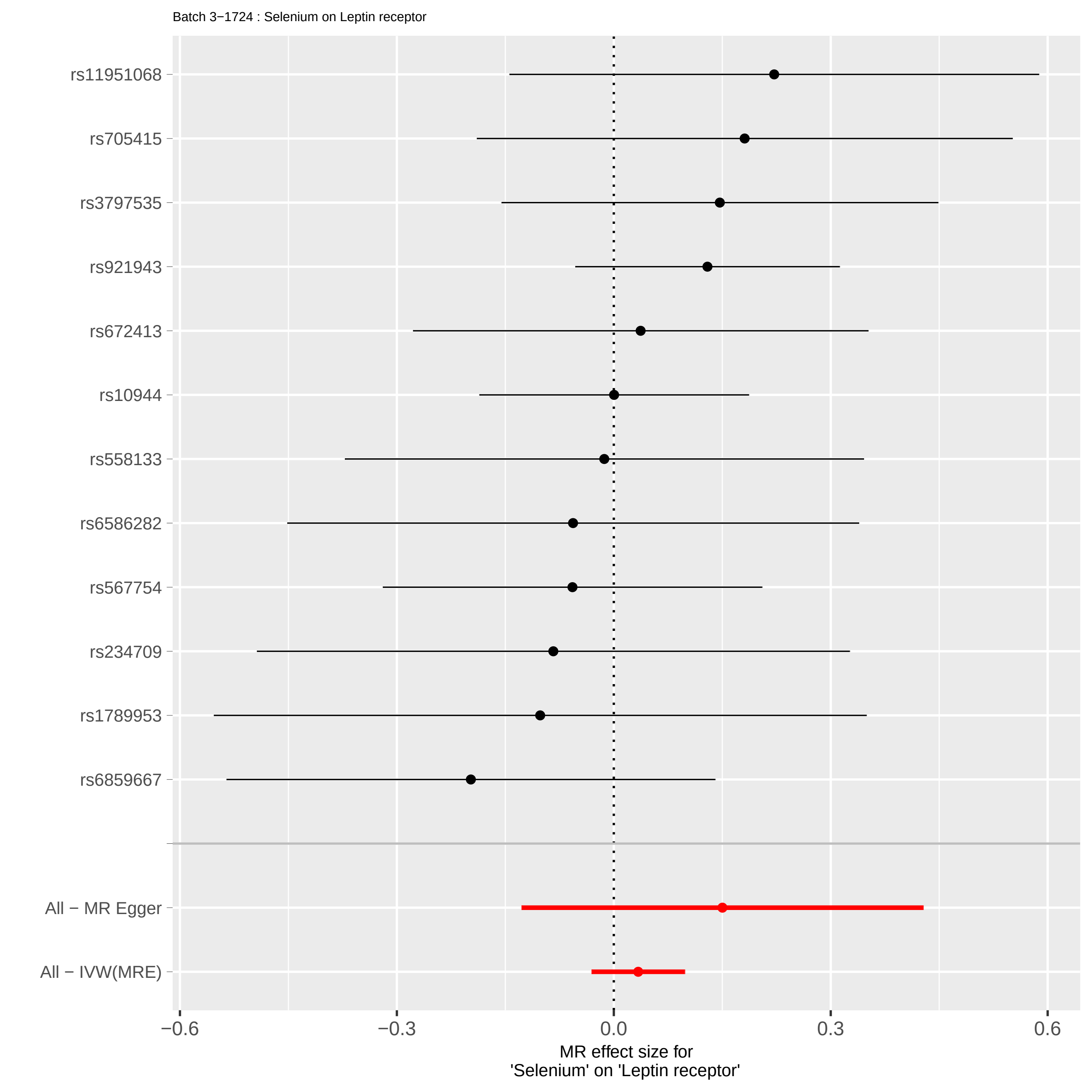

Batch 3−1724 : Selenium on Leptin receptor
rs11951068
rs705415
rs3797535
rs921943
rs672413
rs10944
rs558133
rs6586282
rs567754
rs234709
rs1789953
rs6859667
All − MR Egger
All − IVW(MRE)
−0.6
−0.3
0.0
0.3
0.6
MR effect size for
'Selenium' on 'Leptin receptor'

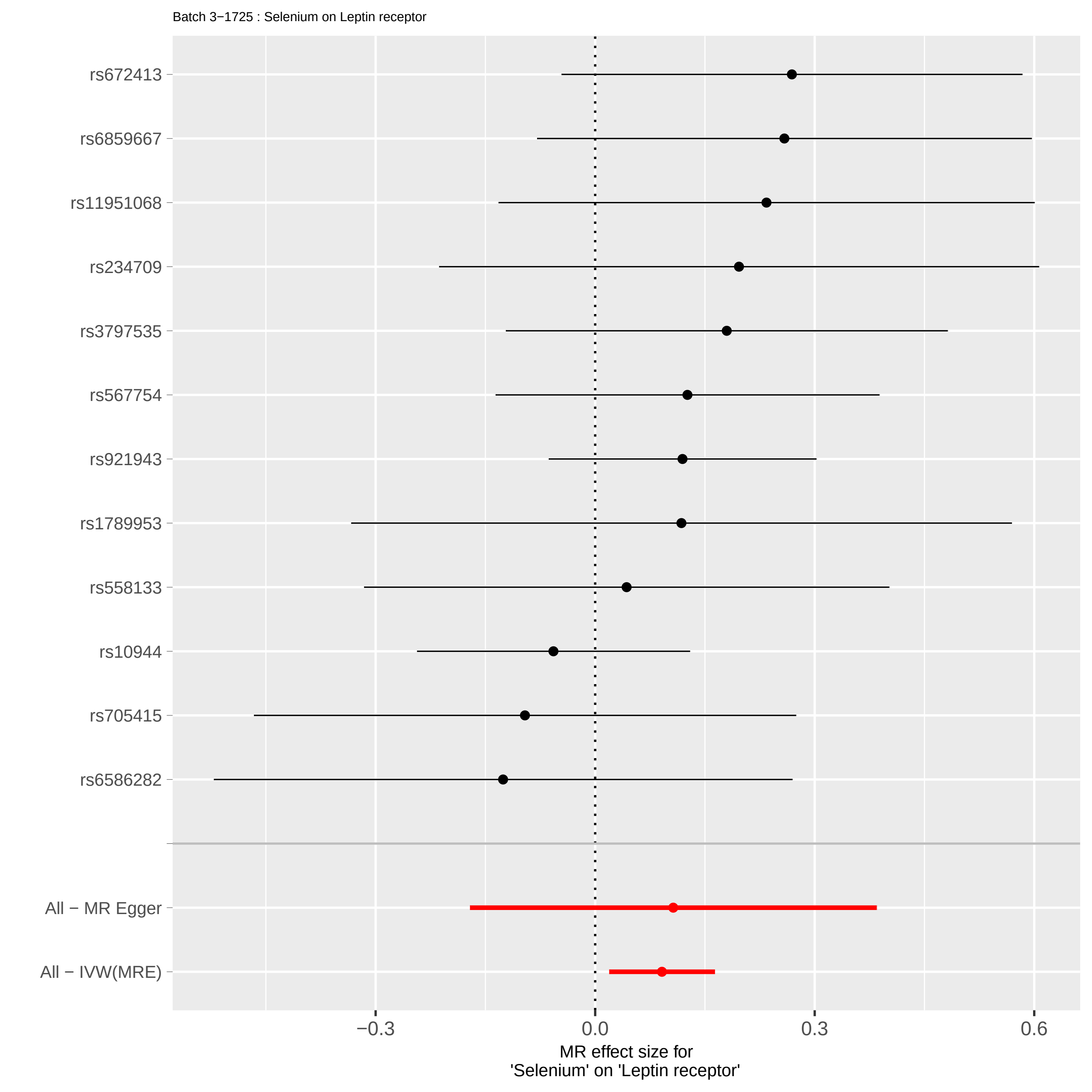

Batch 3−1725 : Selenium on Leptin receptor
rs672413
rs6859667
rs11951068
rs234709
rs3797535
rs567754
rs921943
rs1789953
rs558133
rs10944
rs705415
rs6586282
All − MR Egger
All − IVW(MRE)
−0.3
0.0
0.3
0.6
MR effect size for
'Selenium' on 'Leptin receptor'

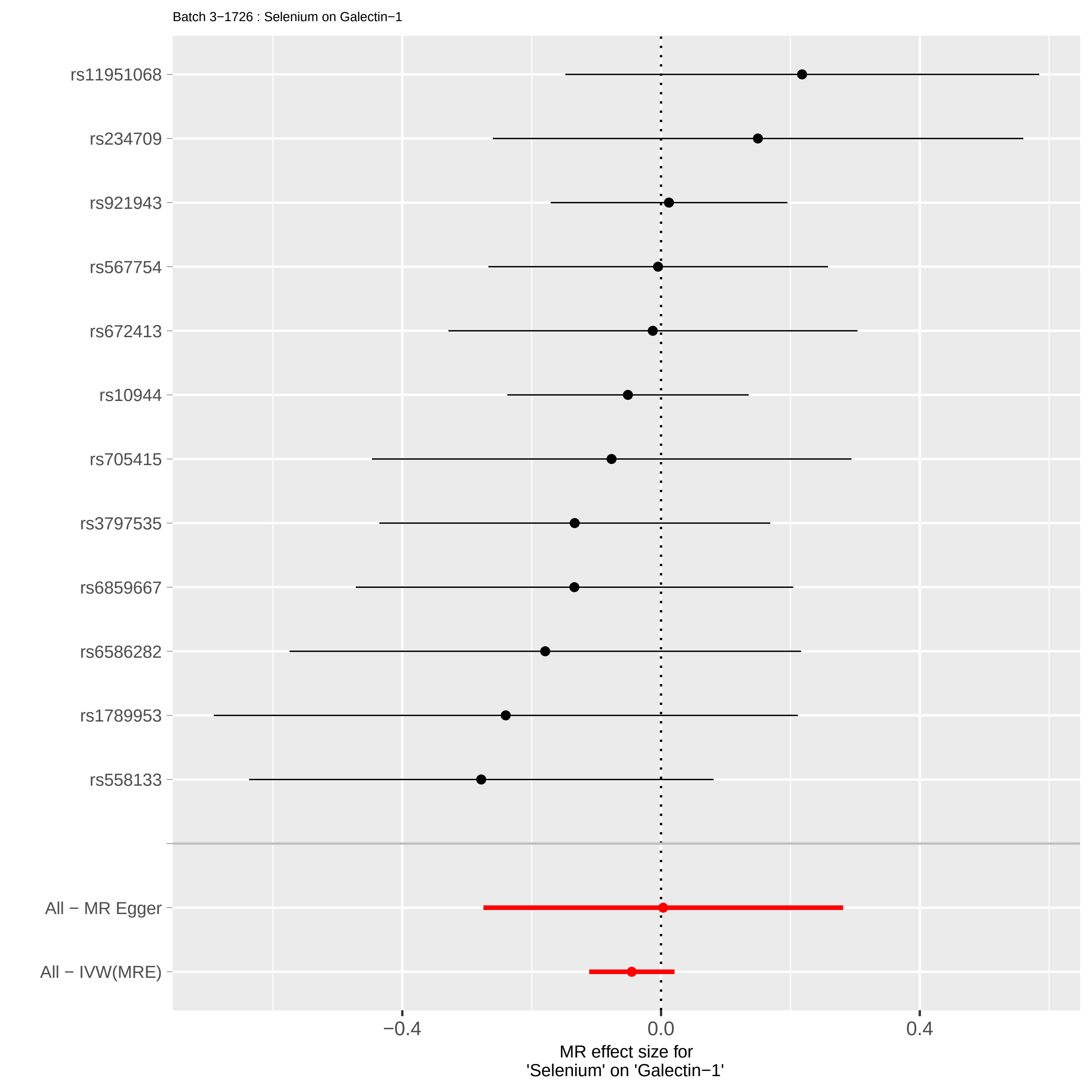

Batch 3−1726 : Selenium on Galectin−1
rs11951068
rs234709
rs921943
rs567754
rs672413
rs10944
rs705415
rs3797535
rs6859667
rs6586282
rs1789953
rs558133
All − MR Egger
All − IVW(MRE)
−0.4
0.0
0.4
MR effect size for
'Selenium' on 'Galectin−1'

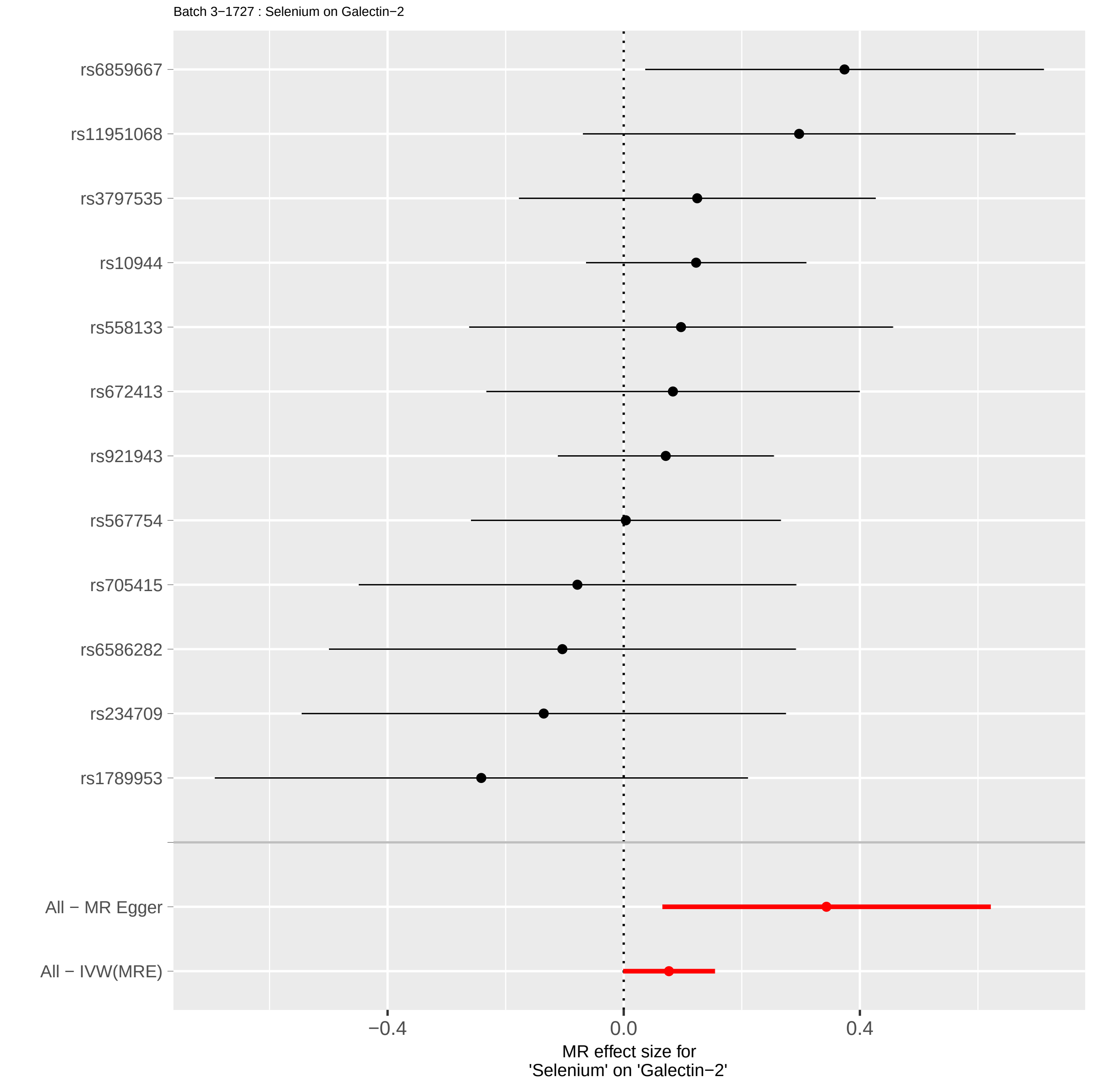
Batch 3−1727 : Selenium on Galectin−2
rs6859667
rs11951068
rs3797535
rs10944
rs558133
rs672413
rs921943
rs567754
rs705415
rs6586282
rs234709
rs1789953
All − MR Egger
All − IVW(MRE)
−0.4
0.0
0.4
MR effect size for
'Selenium' on 'Galectin−2'

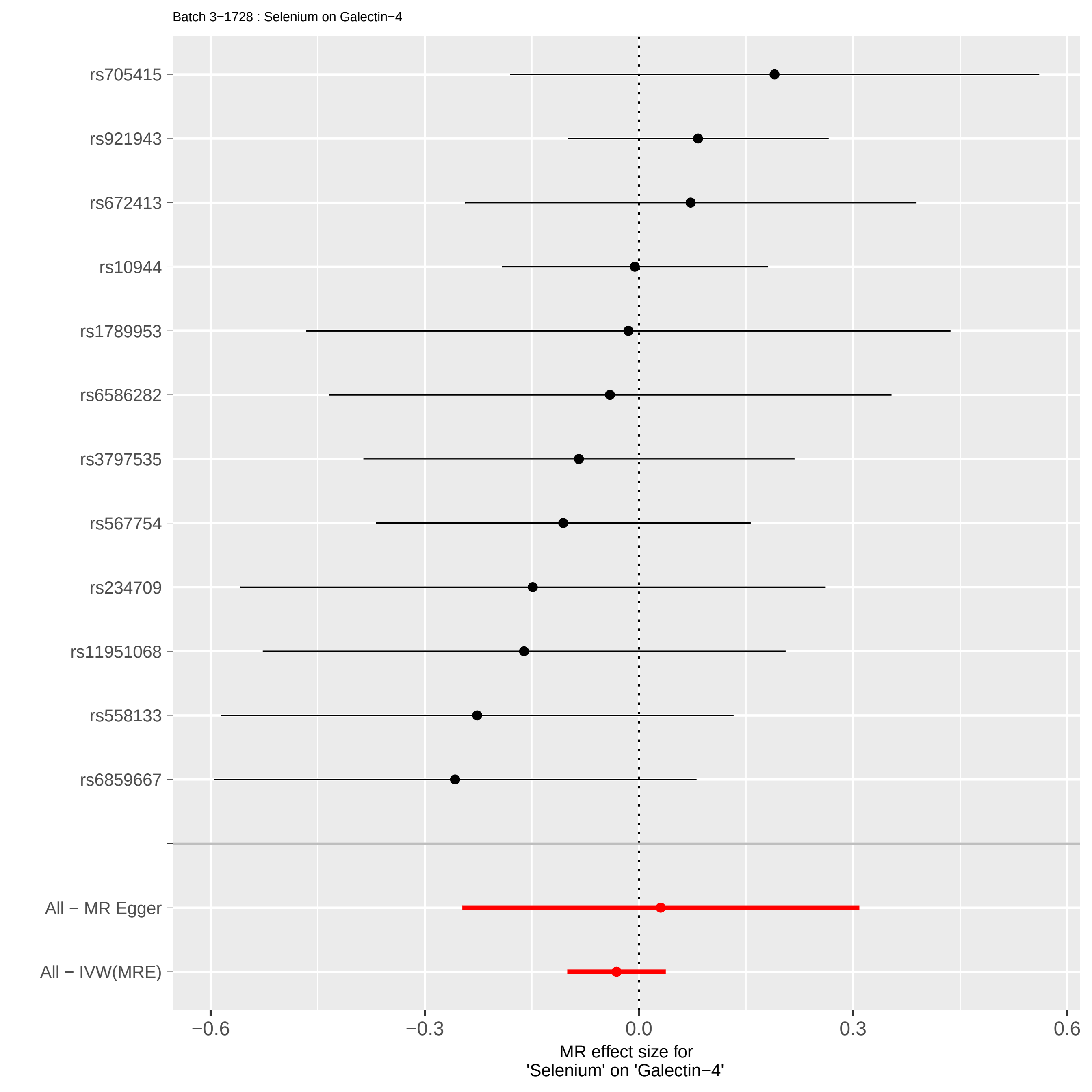
Batch 3−1728 : Selenium on Galectin−4
rs705415
rs921943
rs672413
rs10944
rs1789953
rs6586282
rs3797535
rs567754
rs234709
rs11951068
rs558133
rs6859667
All − MR Egger
All − IVW(MRE)
−0.6
−0.3
0.0
0.3
0.6
MR effect size for
'Selenium' on 'Galectin−4'

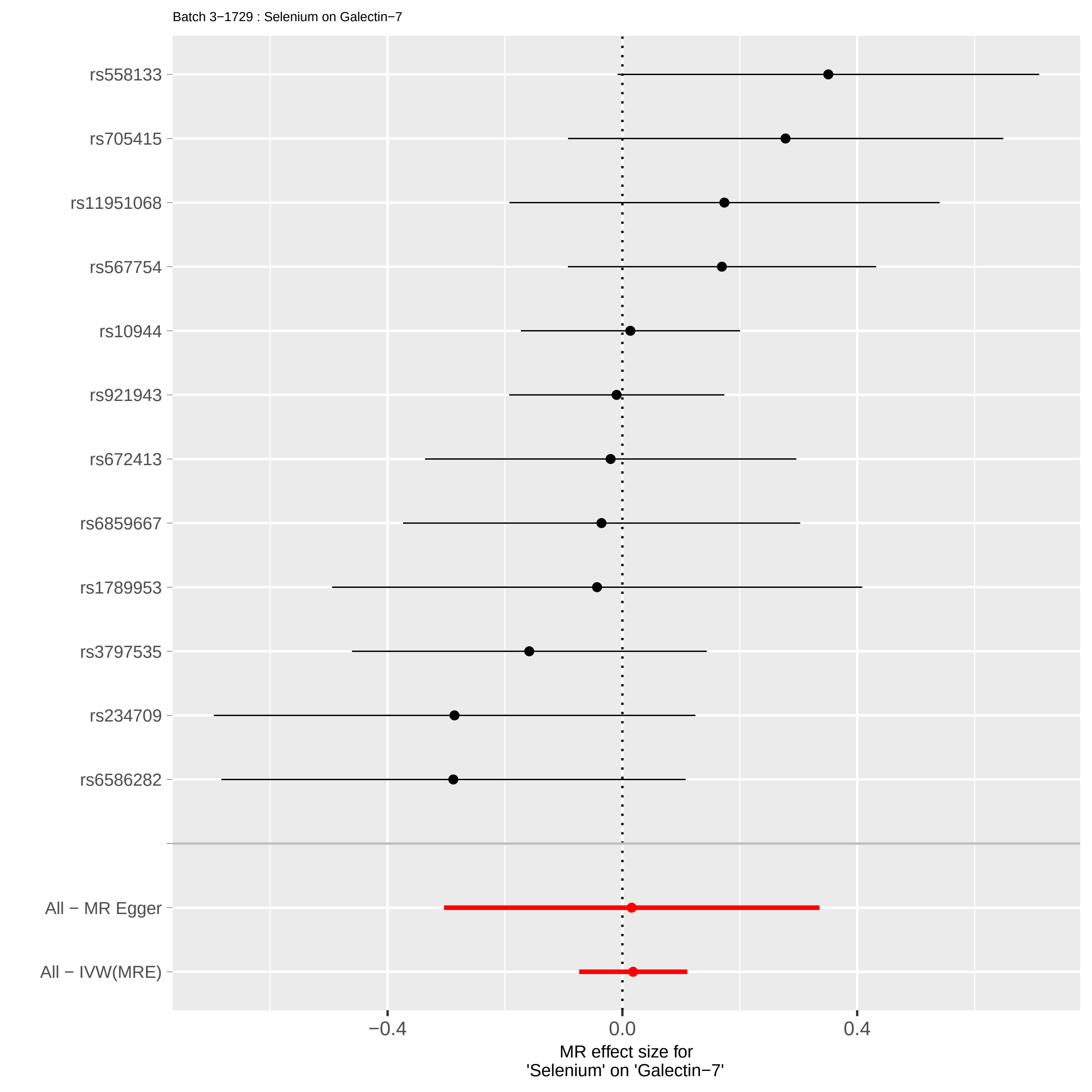

Batch 3−1729 : Selenium on Galectin−7
rs558133
rs705415
rs11951068
rs567754
rs10944
rs921943
rs672413
rs6859667
rs1789953
rs3797535
rs234709
rs6586282
All − MR Egger
All − IVW(MRE)
−0.4
0.0
0.4
MR effect size for
'Selenium' on 'Galectin−7'

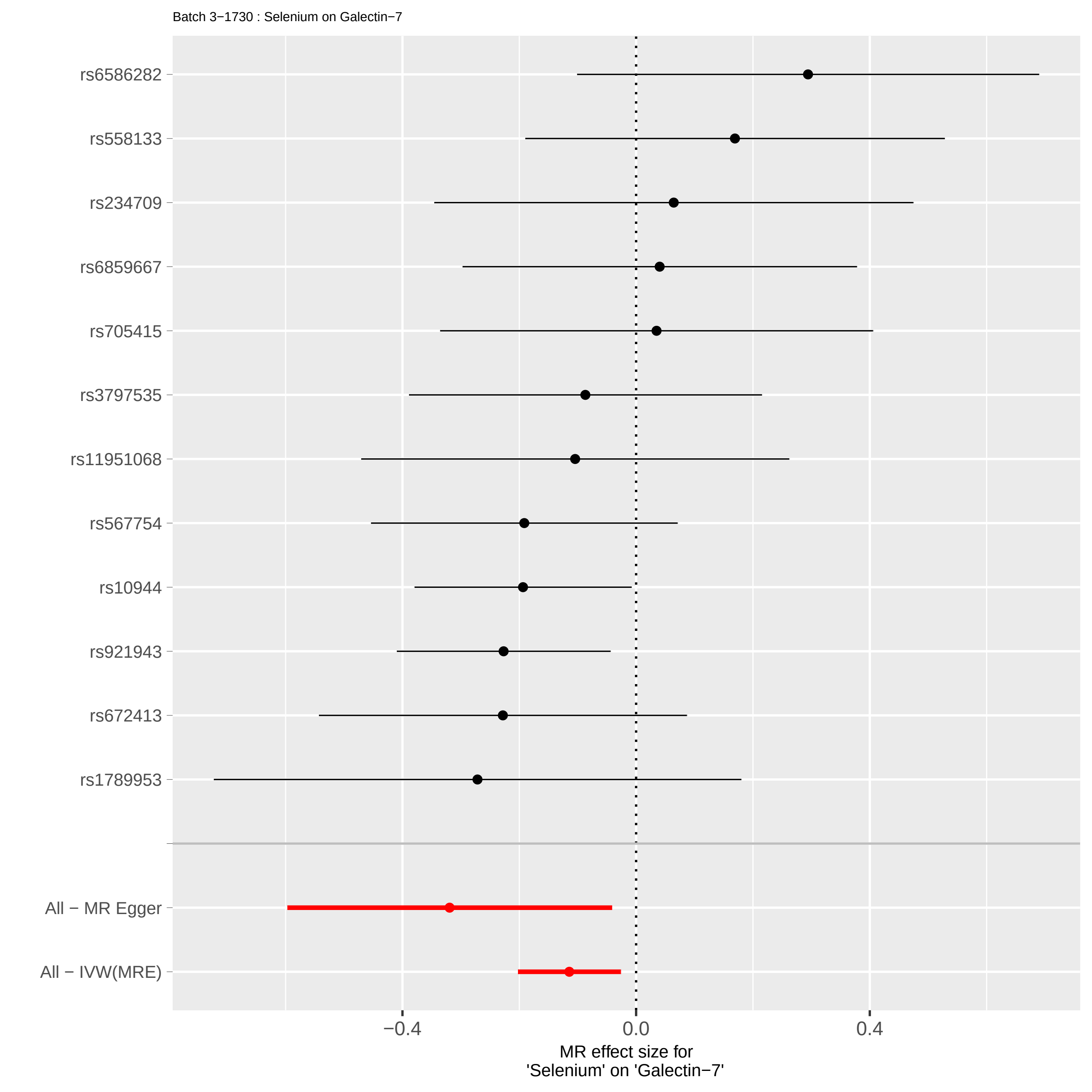
Batch 3−1730 : Selenium on Galectin−7
rs6586282
rs558133
rs234709
rs6859667
rs705415
rs3797535
rs11951068
rs567754
rs10944
rs921943
rs672413
rs1789953
All − MR Egger
All − IVW(MRE)
−0.4
0.0
0.4
MR effect size for
'Selenium' on 'Galectin−7'

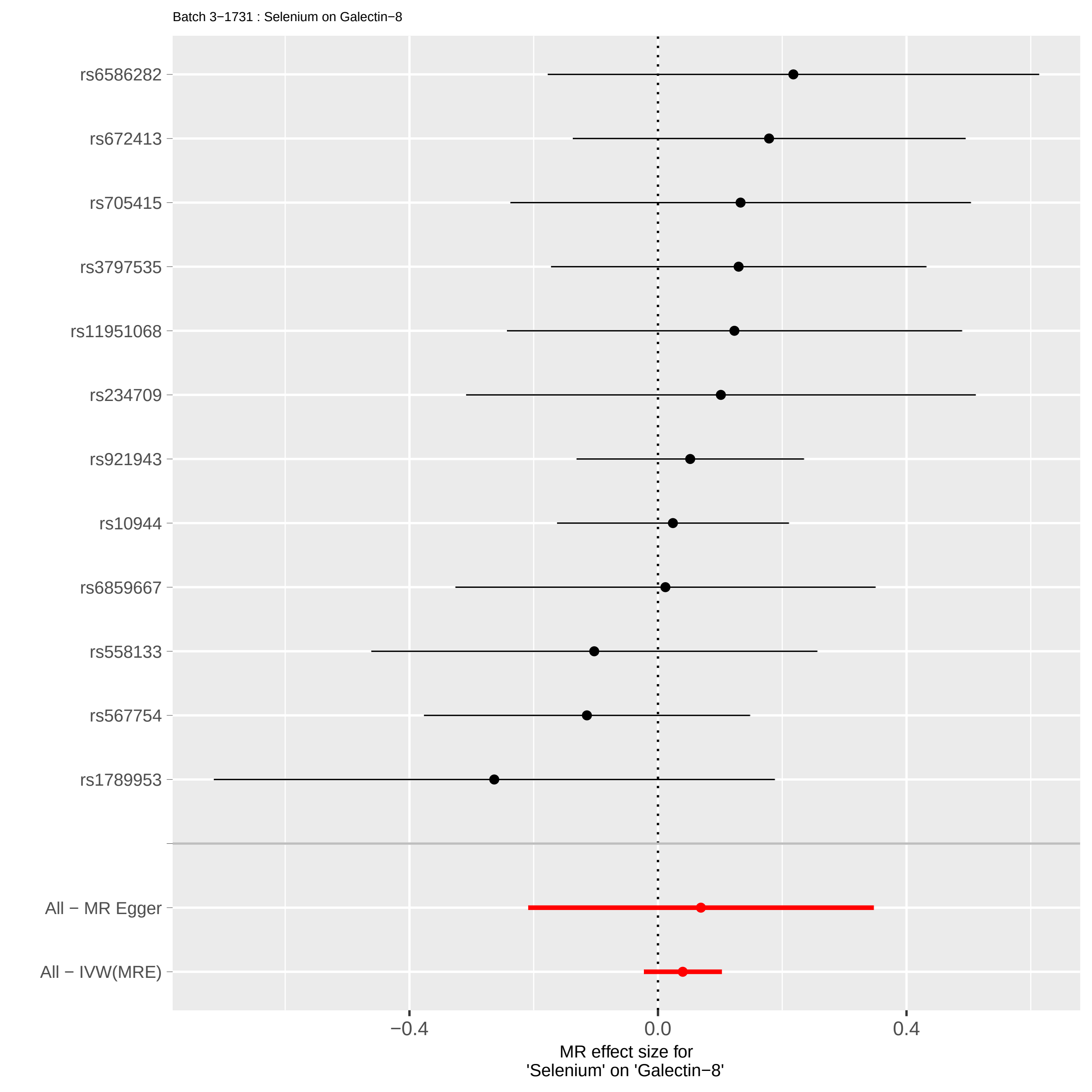

Batch 3−1731 : Selenium on Galectin−8
rs6586282
rs672413
rs705415
rs3797535
rs11951068
rs234709
rs921943
rs10944
rs6859667
rs558133
rs567754
rs1789953
All − MR Egger
All − IVW(MRE)
−0.4
0.0
0.4
MR effect size for
'Selenium' on 'Galectin−8'

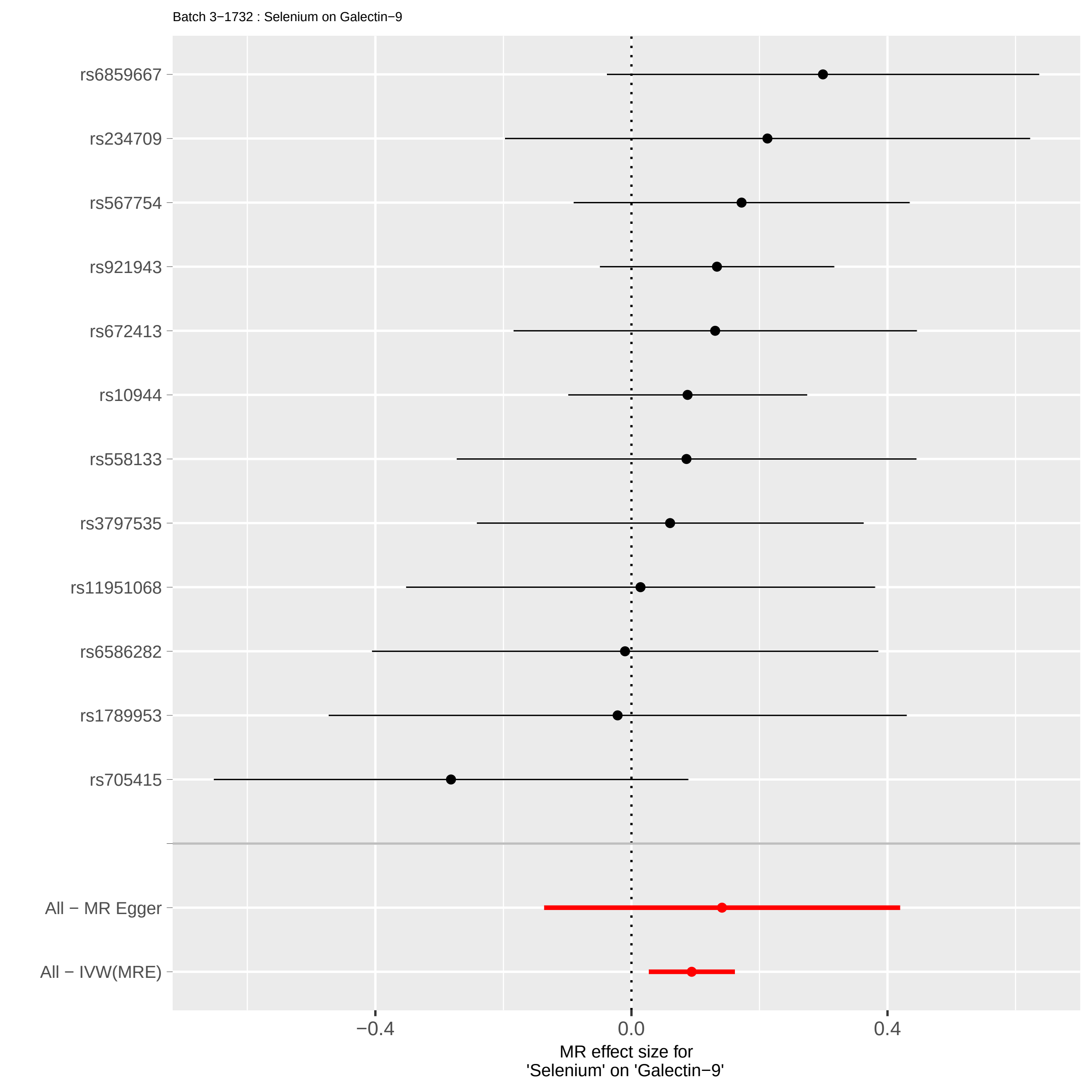

Batch 3−1732 : Selenium on Galectin−9
rs6859667
rs234709
rs567754
rs921943
rs672413
rs10944
rs558133
rs3797535
rs11951068
rs6586282
rs1789953
rs705415
All − MR Egger
All − IVW(MRE)
−0.4
0.0
0.4
MR effect size for
'Selenium' on 'Galectin−9'

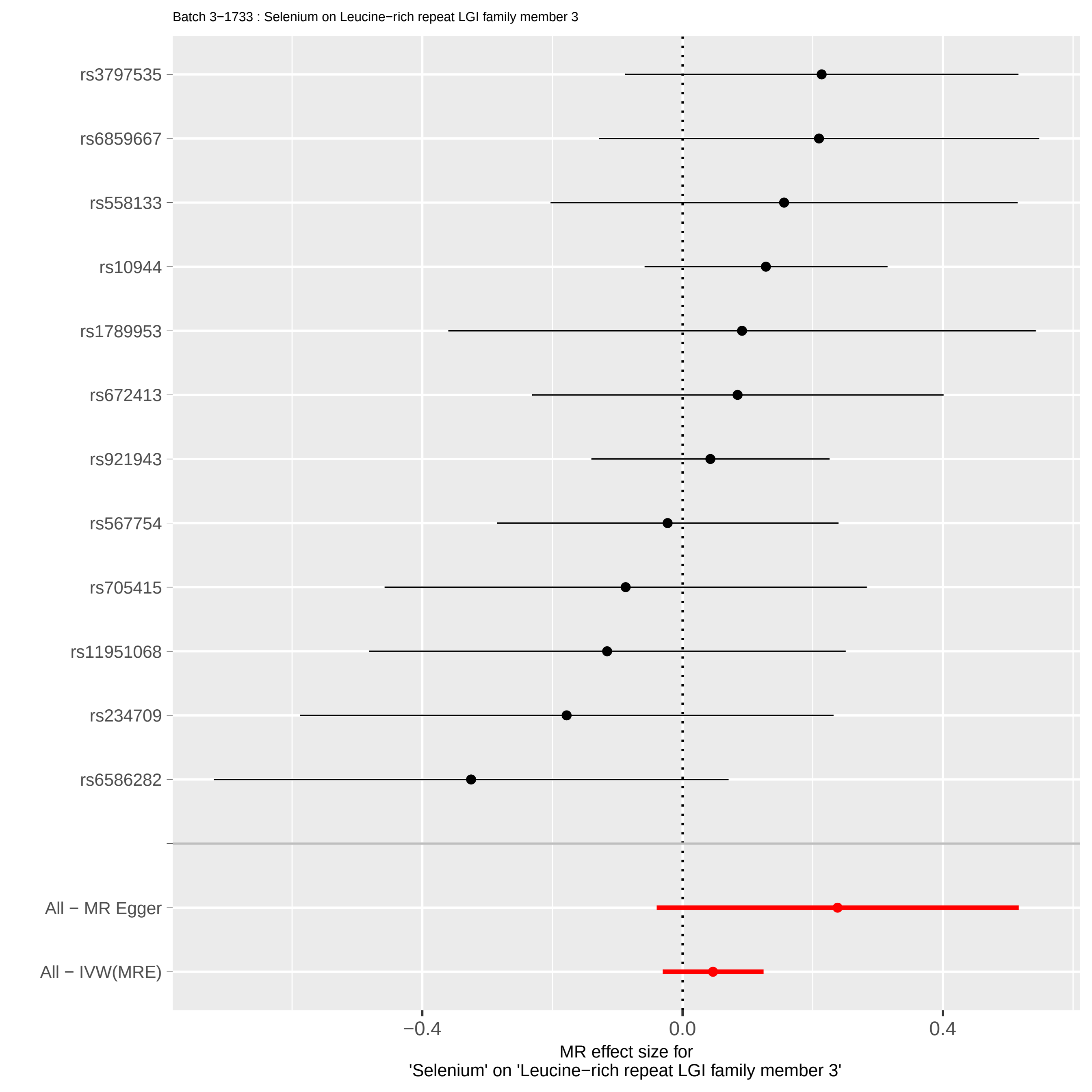

Batch 3−1733 : Selenium on Leucine−rich repeat LGI family member 3
rs3797535
rs6859667
rs558133
rs10944
rs1789953
rs672413
rs921943
rs567754
rs705415
rs11951068
rs234709
rs6586282
All − MR Egger
All − IVW(MRE)
−0.4
0.0
0.4
MR effect size for
'Selenium' on 'Leucine−rich repeat LGI family member 3'

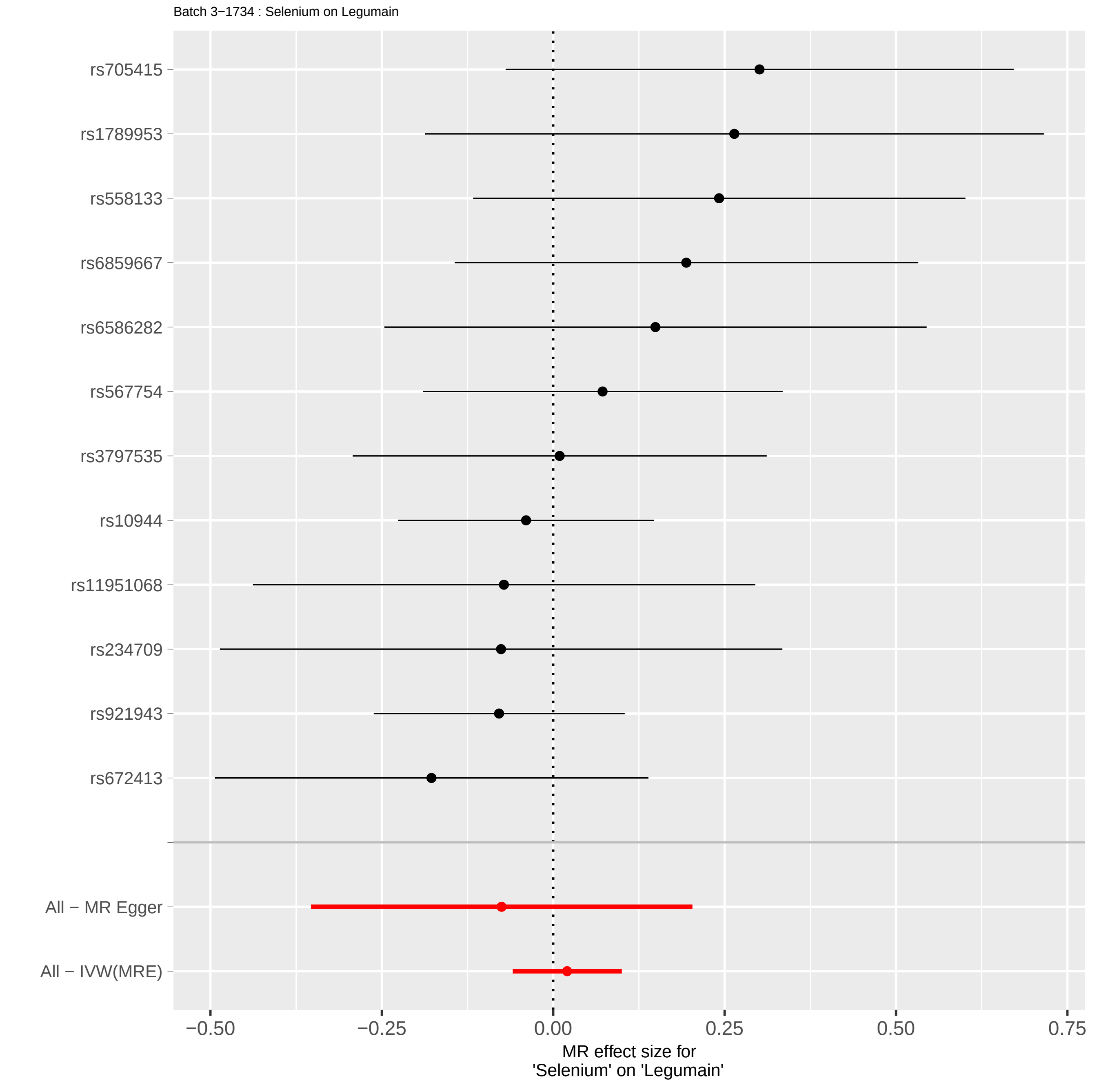

Batch 3−1734 : Selenium on Legumain
rs705415
rs1789953
rs558133
rs6859667
rs6586282
rs567754
rs3797535
rs10944
rs11951068
rs234709
rs921943
rs672413
All − MR Egger
All − IVW(MRE)
−0.50
−0.25
0.00
0.25
0.50
0.75
MR effect size for
'Selenium' on 'Legumain'

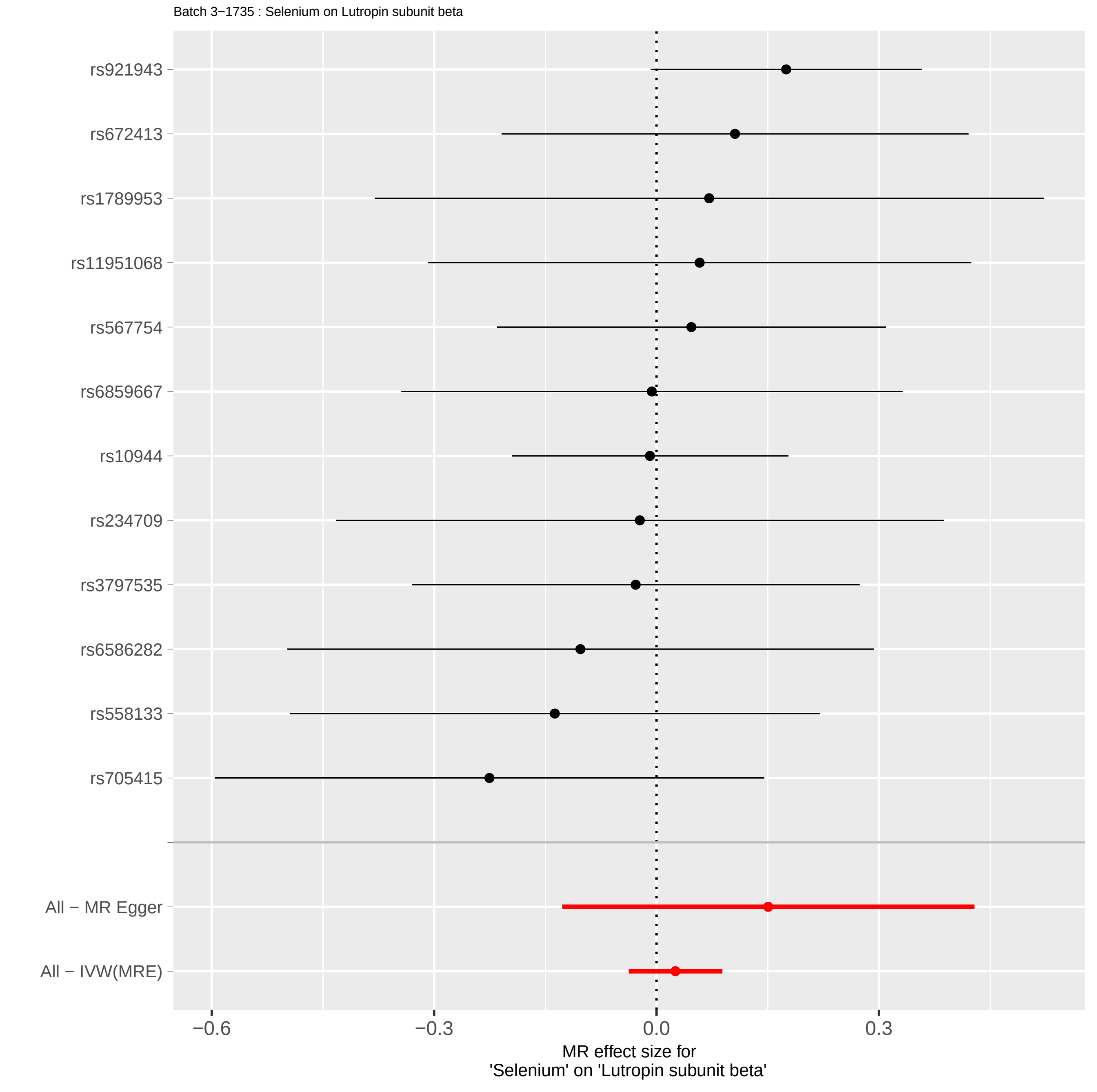

Batch 3−1735 : Selenium on Lutropin subunit beta
rs921943
rs672413
rs1789953
rs11951068
rs567754
rs6859667
rs10944
rs234709
rs3797535
rs6586282
rs558133
rs705415
All − MR Egger
All − IVW(MRE)
−0.6
−0.3
0.0
0.3
MR effect size for
'Selenium' on 'Lutropin subunit beta'

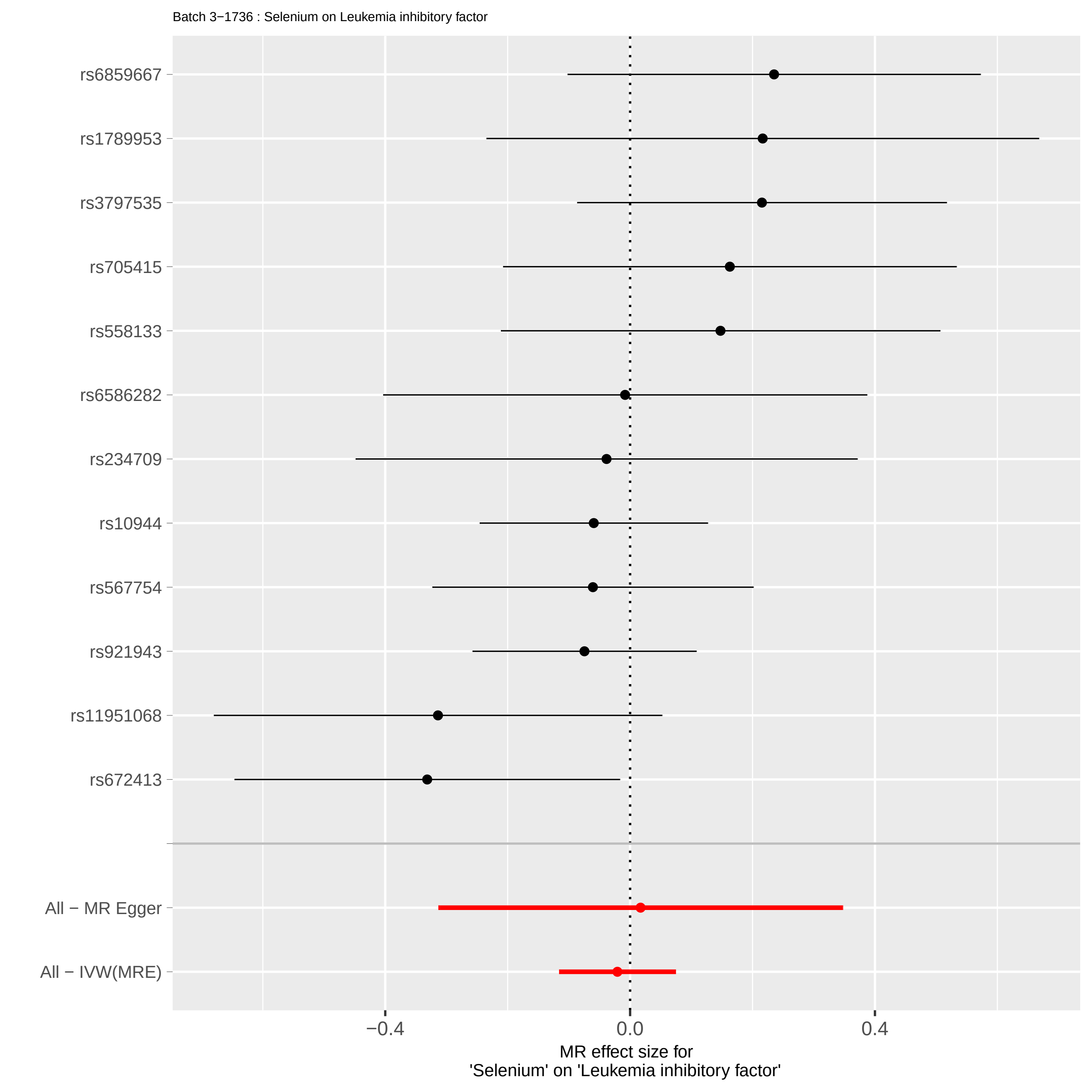

Batch 3−1736 : Selenium on Leukemia inhibitory factor
rs6859667
rs1789953
rs3797535
rs705415
rs558133
rs6586282
rs234709
rs10944
rs567754
rs921943
rs11951068
rs672413
All − MR Egger
All − IVW(MRE)
−0.4
0.0
0.4
MR effect size for
'Selenium' on 'Leukemia inhibitory factor'

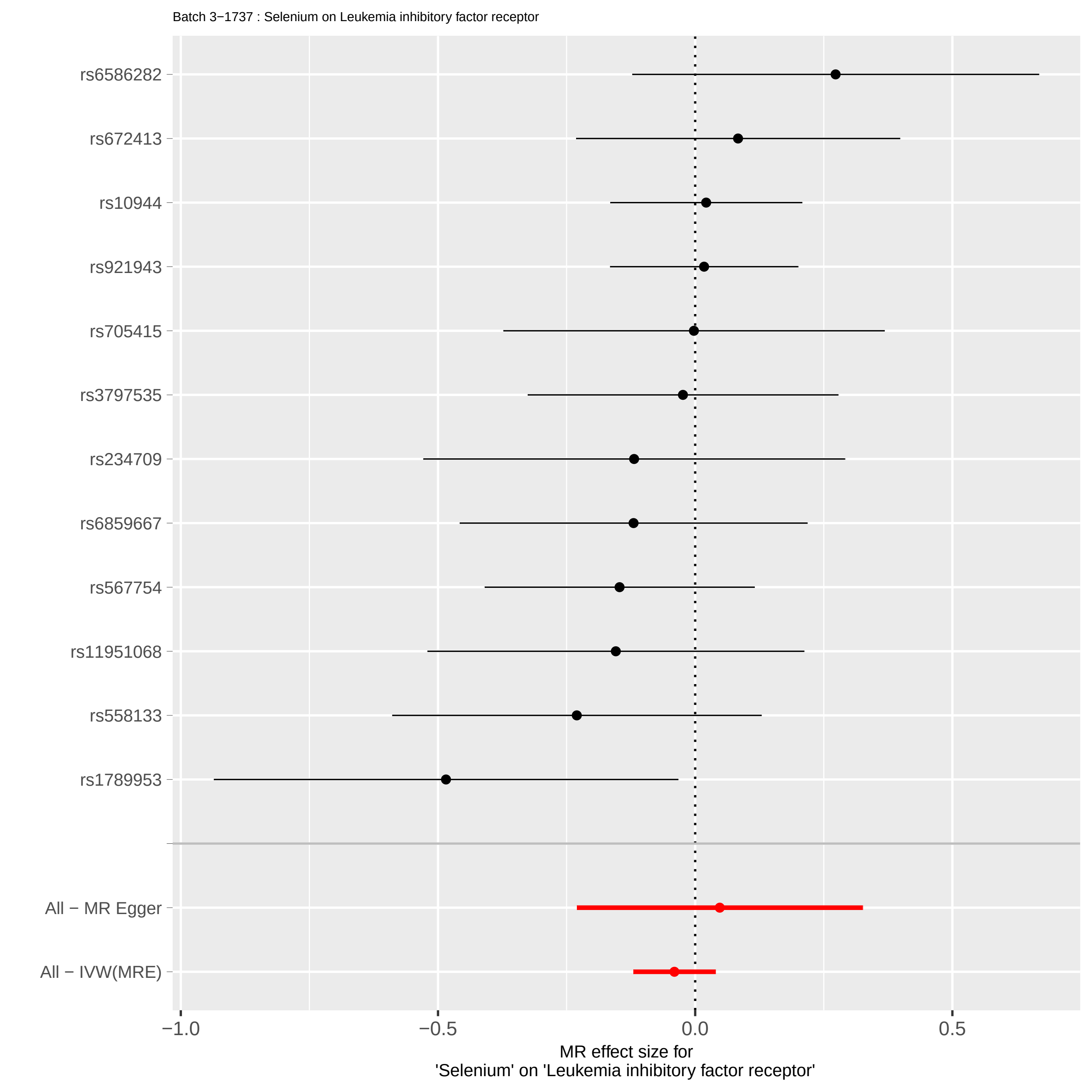
Batch 3−1737 : Selenium on Leukemia inhibitory factor receptor
rs6586282
rs672413
rs10944
rs921943
rs705415
rs3797535
rs234709
rs6859667
rs567754
rs11951068
rs558133
rs1789953
All − MR Egger
All − IVW(MRE)
−1.0
−0.5
0.0
0.5
MR effect size for
'Selenium' on 'Leukemia inhibitory factor receptor'

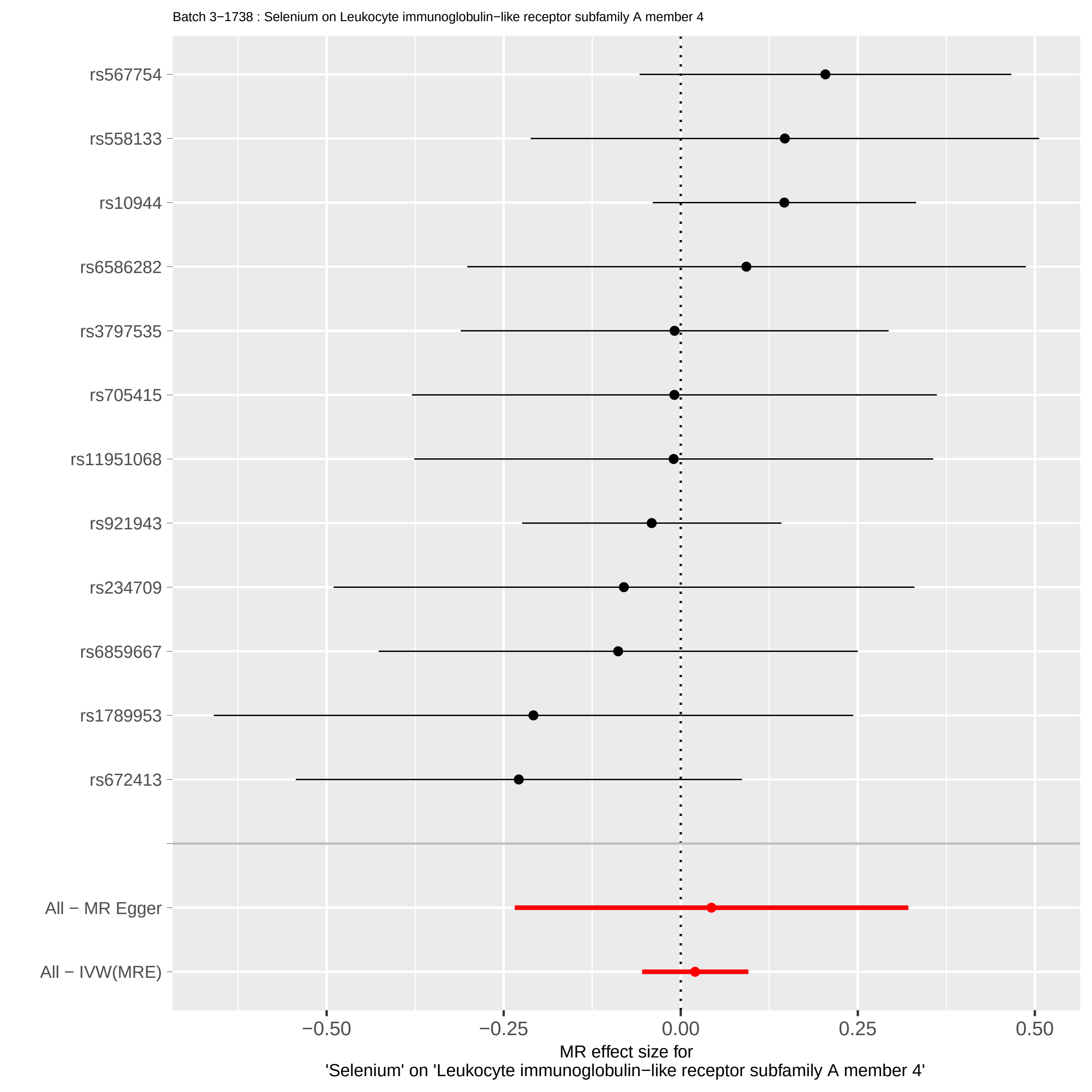

Batch 3−1738 : Selenium on Leukocyte immunoglobulin−like receptor subfamily A member 4
rs567754
rs558133
rs10944
rs6586282
rs3797535
rs705415
rs11951068
rs921943
rs234709
rs6859667
rs1789953
rs672413
All − MR Egger
All − IVW(MRE)
−0.50
−0.25
0.00
0.25
0.50
MR effect size for
'Selenium' on 'Leukocyte immunoglobulin−like receptor subfamily A member 4'

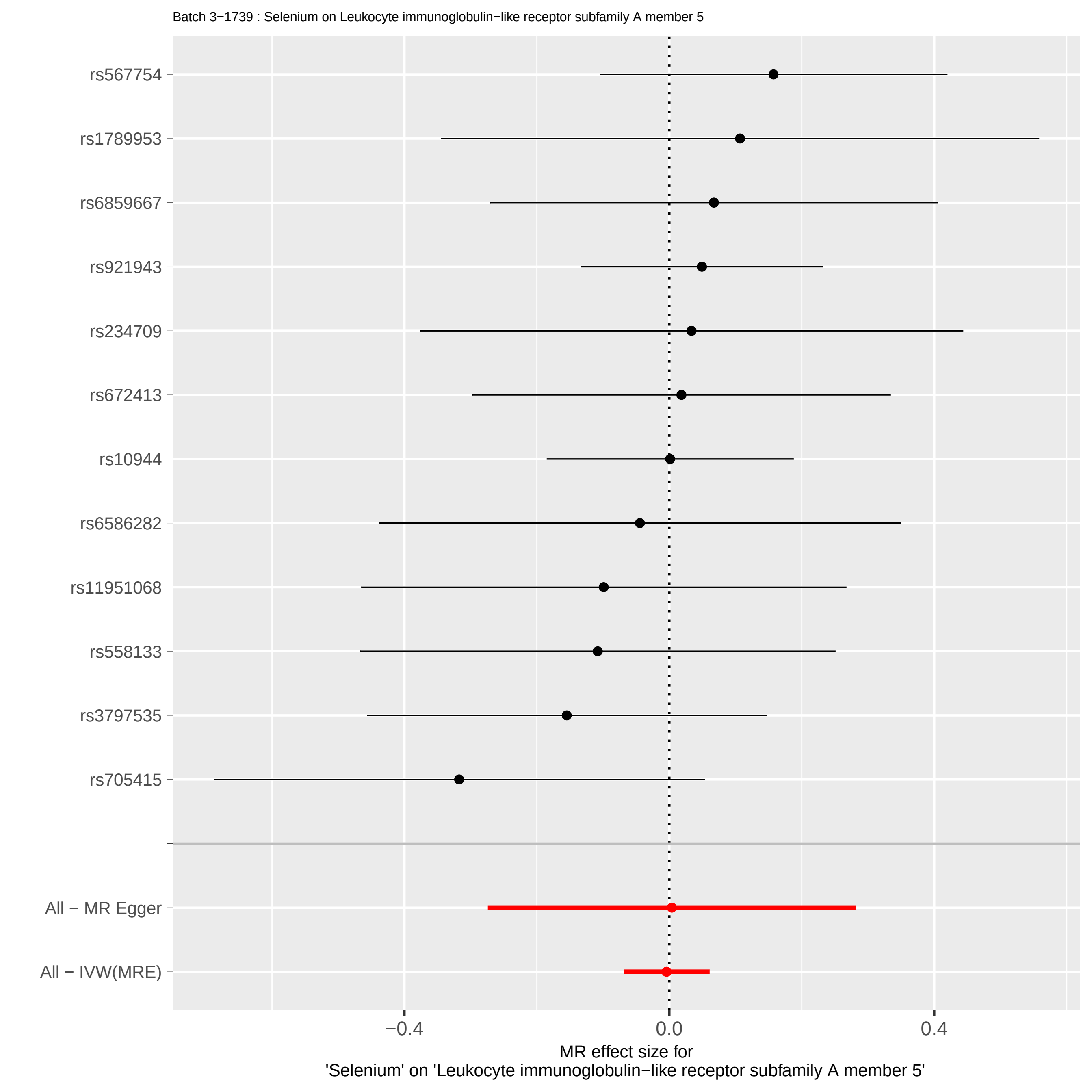
Batch 3−1739 : Selenium on Leukocyte immunoglobulin−like receptor subfamily A member 5
rs567754
rs1789953
rs6859667
rs921943
rs234709
rs672413
rs10944
rs6586282
rs11951068
rs558133
rs3797535
rs705415
All − MR Egger
All − IVW(MRE)
−0.4
0.0
0.4
MR effect size for
'Selenium' on 'Leukocyte immunoglobulin−like receptor subfamily A member 5'

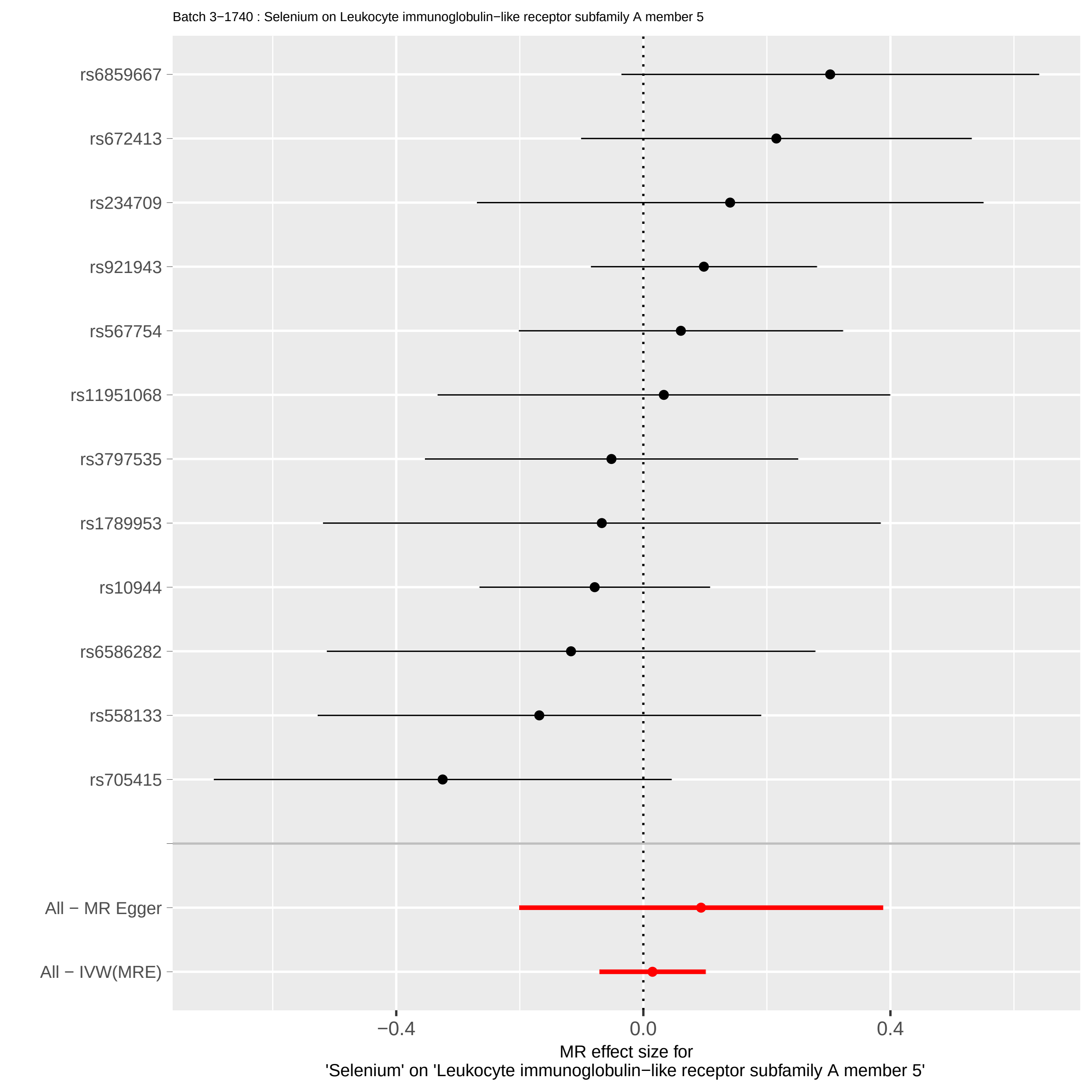

Batch 3−1740 : Selenium on Leukocyte immunoglobulin−like receptor subfamily A member 5
rs6859667
rs672413
rs234709
rs921943
rs567754
rs11951068
rs3797535
rs1789953
rs10944
rs6586282
rs558133
rs705415
All − MR Egger
All − IVW(MRE)
−0.4
0.0
0.4
MR effect size for
'Selenium' on 'Leukocyte immunoglobulin−like receptor subfamily A member 5'

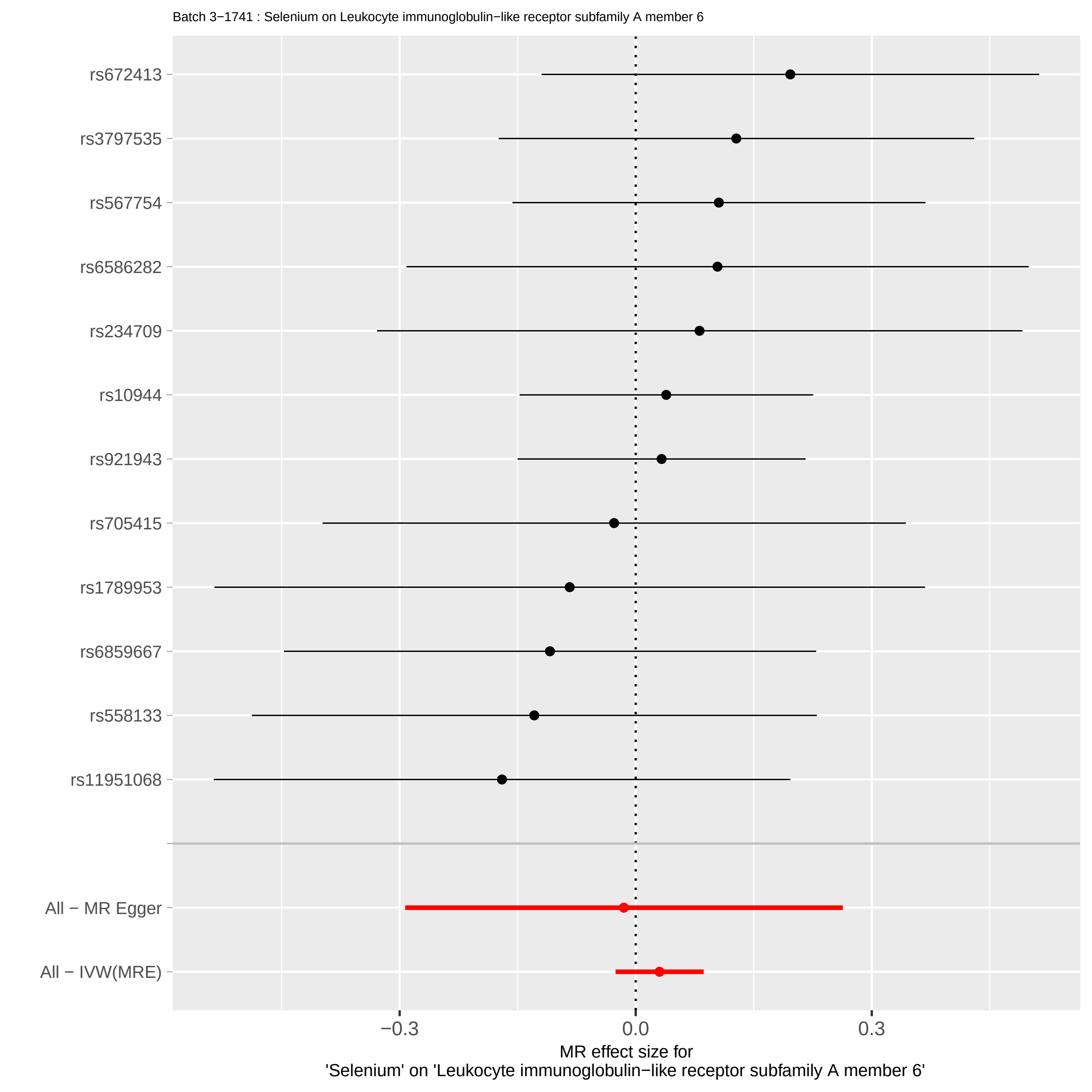
Batch 3−1741 : Selenium on Leukocyte immunoglobulin−like receptor subfamily A member 6
rs672413
rs3797535
rs567754
rs6586282
rs234709
rs10944
rs921943
rs705415
rs1789953
rs6859667
rs558133
rs11951068
All − MR Egger
All − IVW(MRE)
−0.3
0.0
0.3
MR effect size for
'Selenium' on 'Leukocyte immunoglobulin−like receptor subfamily A member 6'

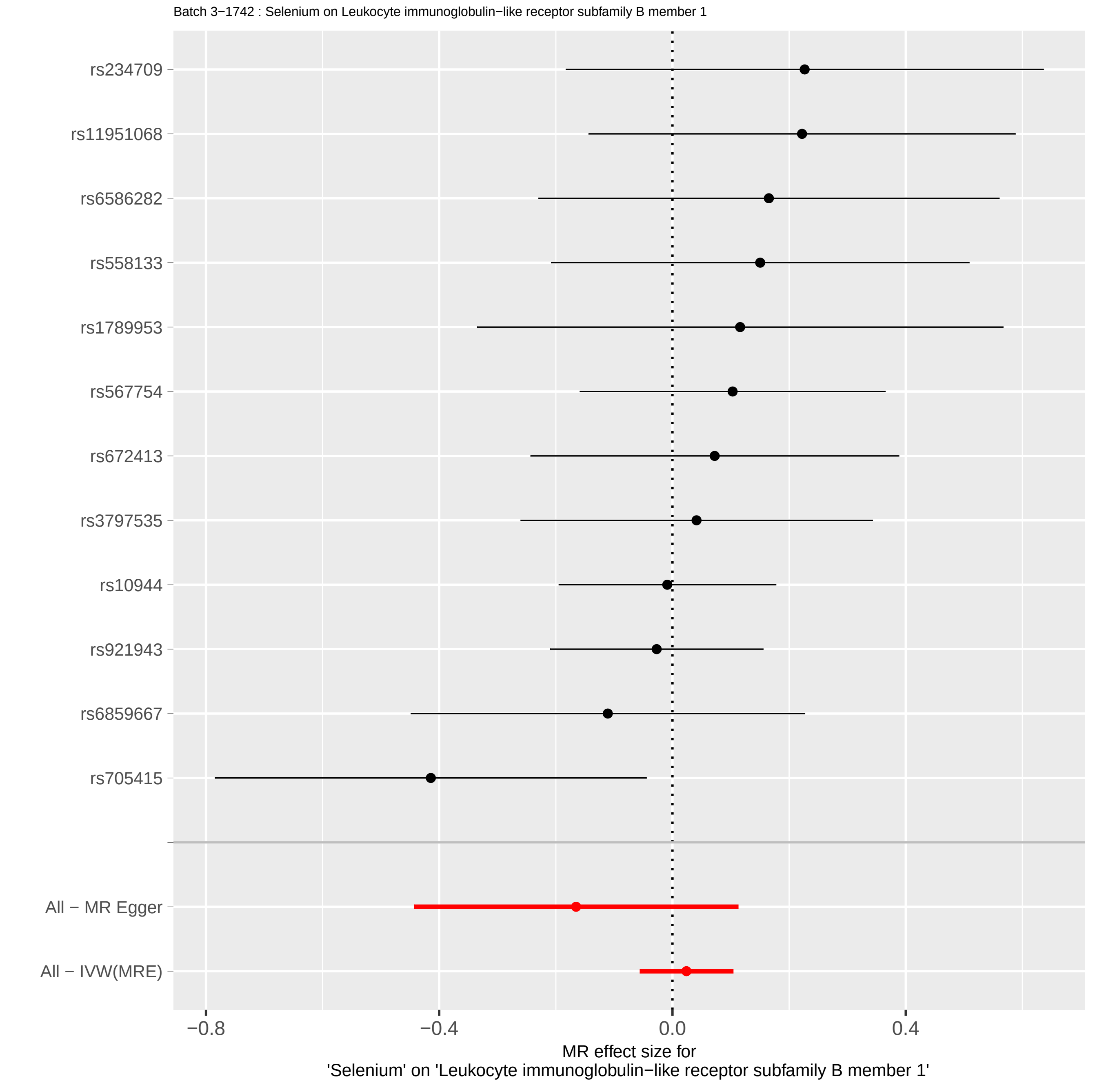

Batch 3−1742 : Selenium on Leukocyte immunoglobulin−like receptor subfamily B member 1
rs234709
rs11951068
rs6586282
rs558133
rs1789953
rs567754
rs672413
rs3797535
rs10944
rs921943
rs6859667
rs705415
All − MR Egger
All − IVW(MRE)
−0.8
−0.4
0.0
0.4
MR effect size for
'Selenium' on 'Leukocyte immunoglobulin−like receptor subfamily B member 1'

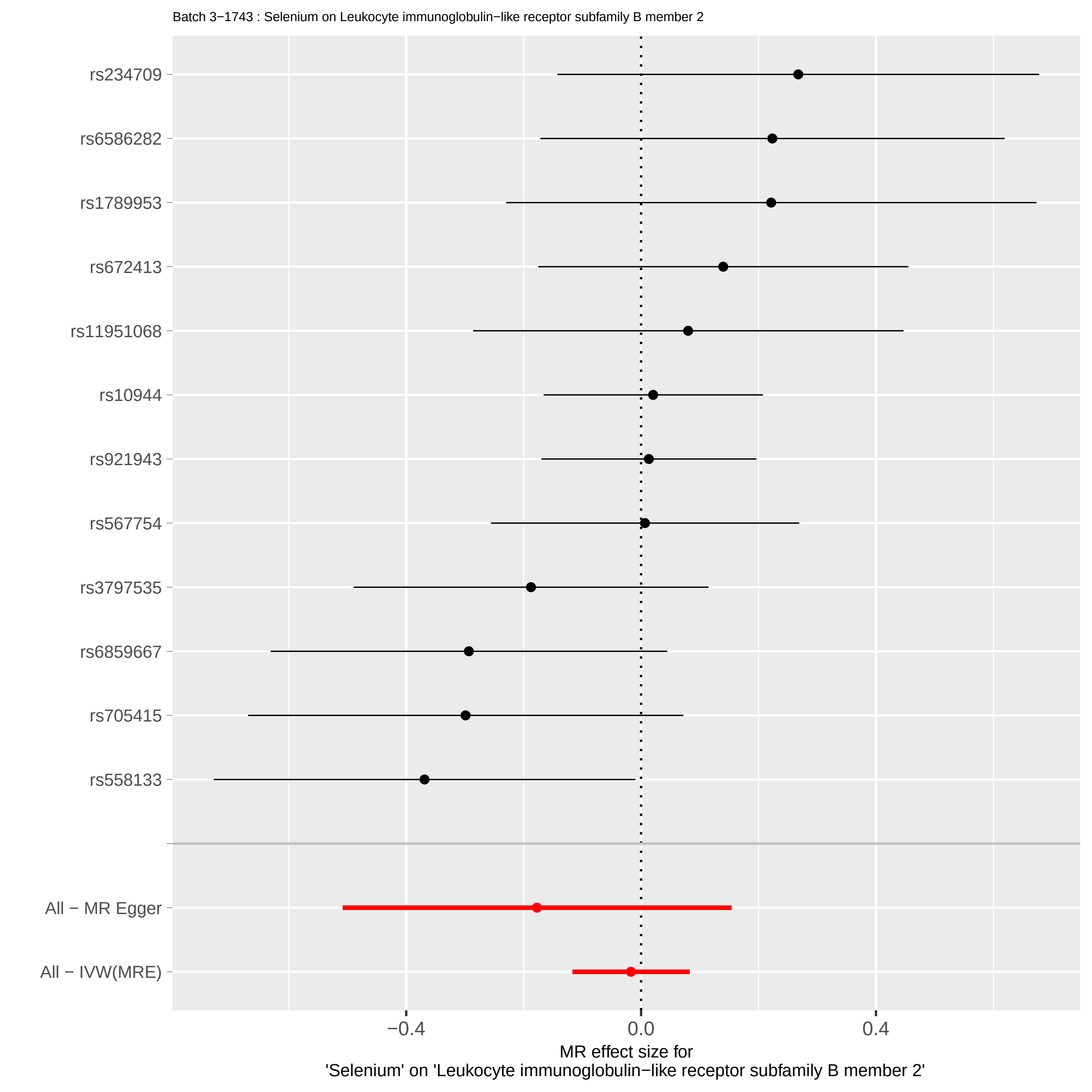

Batch 3−1743 : Selenium on Leukocyte immunoglobulin−like receptor subfamily B member 2
rs234709
rs6586282
rs1789953
rs672413
rs11951068
rs10944
rs921943
rs567754
rs3797535
rs6859667
rs705415
rs558133
All − MR Egger
All − IVW(MRE)
−0.4
0.0
0.4
MR effect size for
'Selenium' on 'Leukocyte immunoglobulin−like receptor subfamily B member 2'

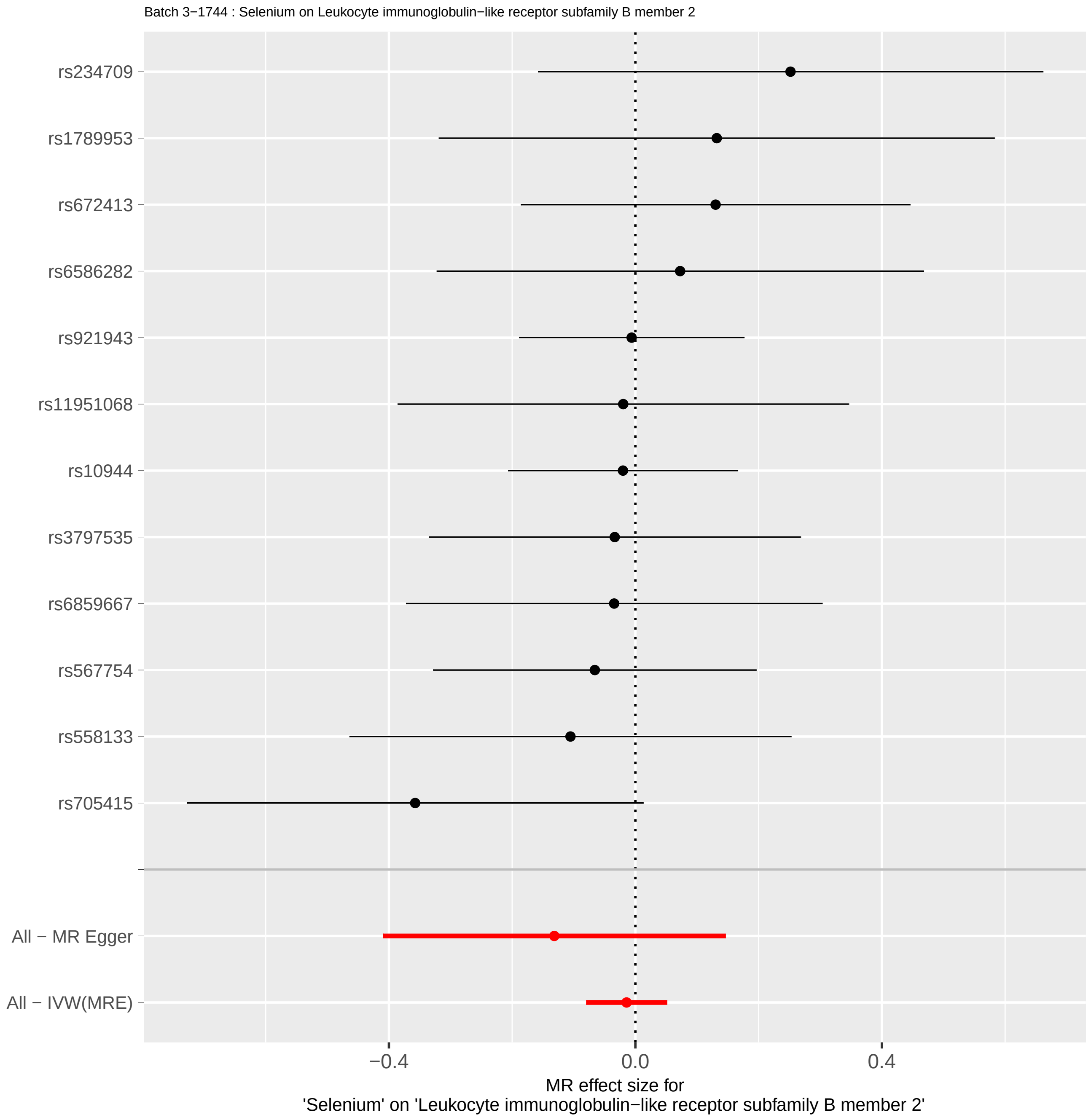

Batch 3−1744 : Selenium on Leukocyte immunoglobulin−like receptor subfamily B member 2
rs234709
rs1789953
rs672413
rs6586282
rs921943
rs11951068
rs10944
rs3797535
rs6859667
rs567754
rs558133
rs705415
All − MR Egger
All − IVW(MRE)
−0.4
0.0
0.4
MR effect size for
'Selenium' on 'Leukocyte immunoglobulin−like receptor subfamily B member 2'

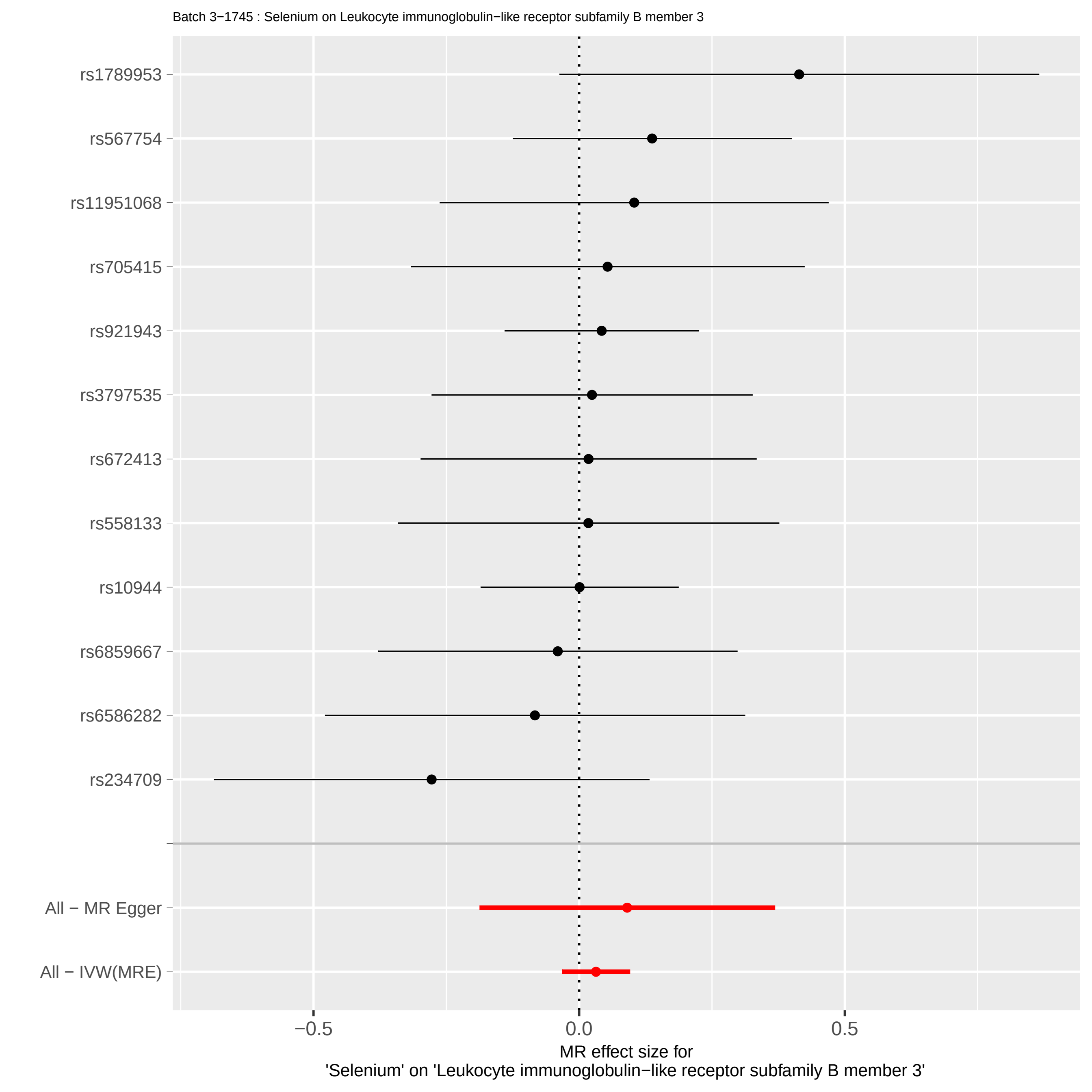

Batch 3−1745 : Selenium on Leukocyte immunoglobulin−like receptor subfamily B member 3
rs1789953
rs567754
rs11951068
rs705415
rs921943
rs3797535
rs672413
rs558133
rs10944
rs6859667
rs6586282
rs234709
All − MR Egger
All − IVW(MRE)
−0.5
0.0
0.5
MR effect size for
'Selenium' on 'Leukocyte immunoglobulin−like receptor subfamily B member 3'

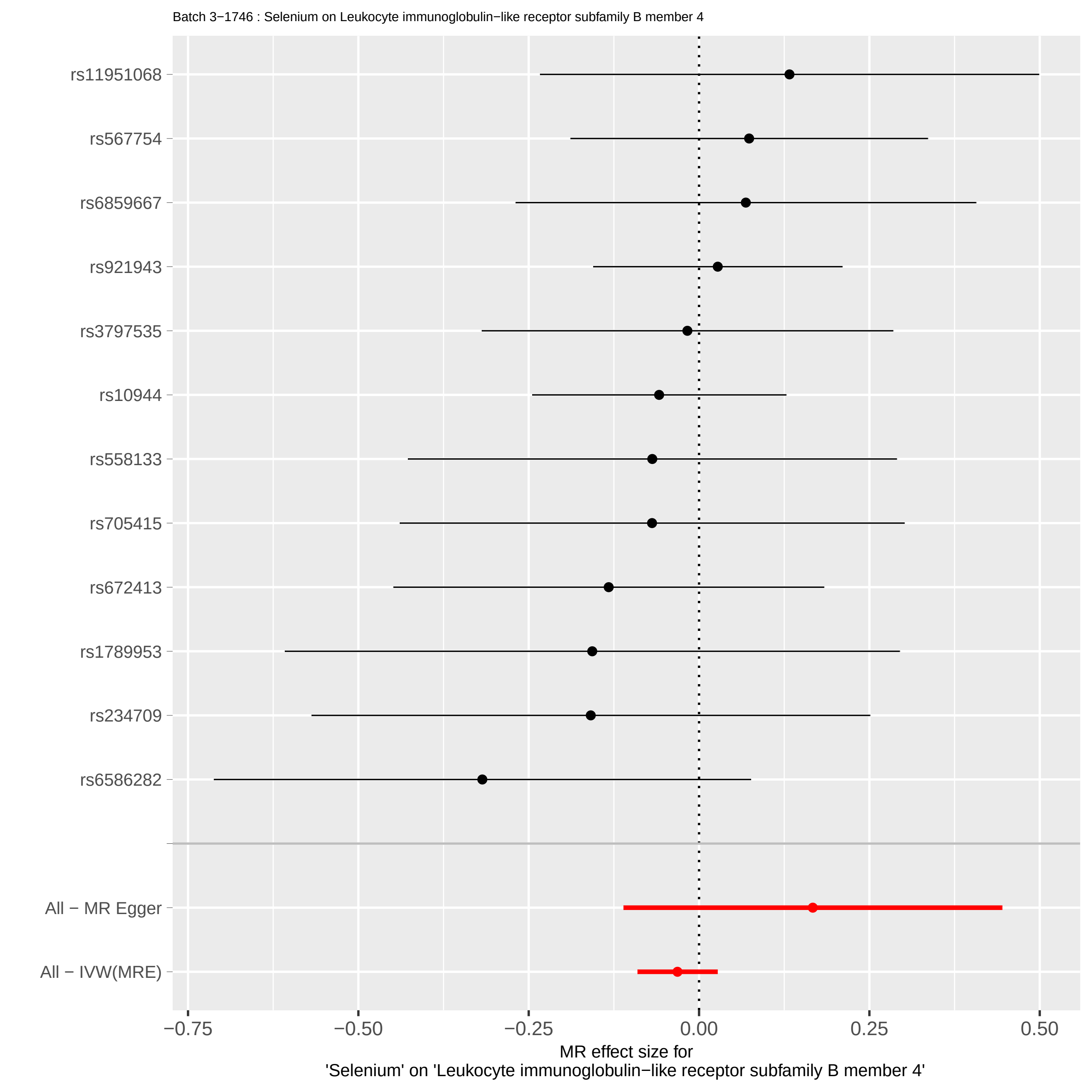

Batch 3−1746 : Selenium on Leukocyte immunoglobulin−like receptor subfamily B member 4
rs11951068
rs567754
rs6859667
rs921943
rs3797535
rs10944
rs558133
rs705415
rs672413
rs1789953
rs234709
rs6586282
All − MR Egger
All − IVW(MRE)
−0.75
−0.50
−0.25
0.00
0.25
0.50
MR effect size for
'Selenium' on 'Leukocyte immunoglobulin−like receptor subfamily B member 4'

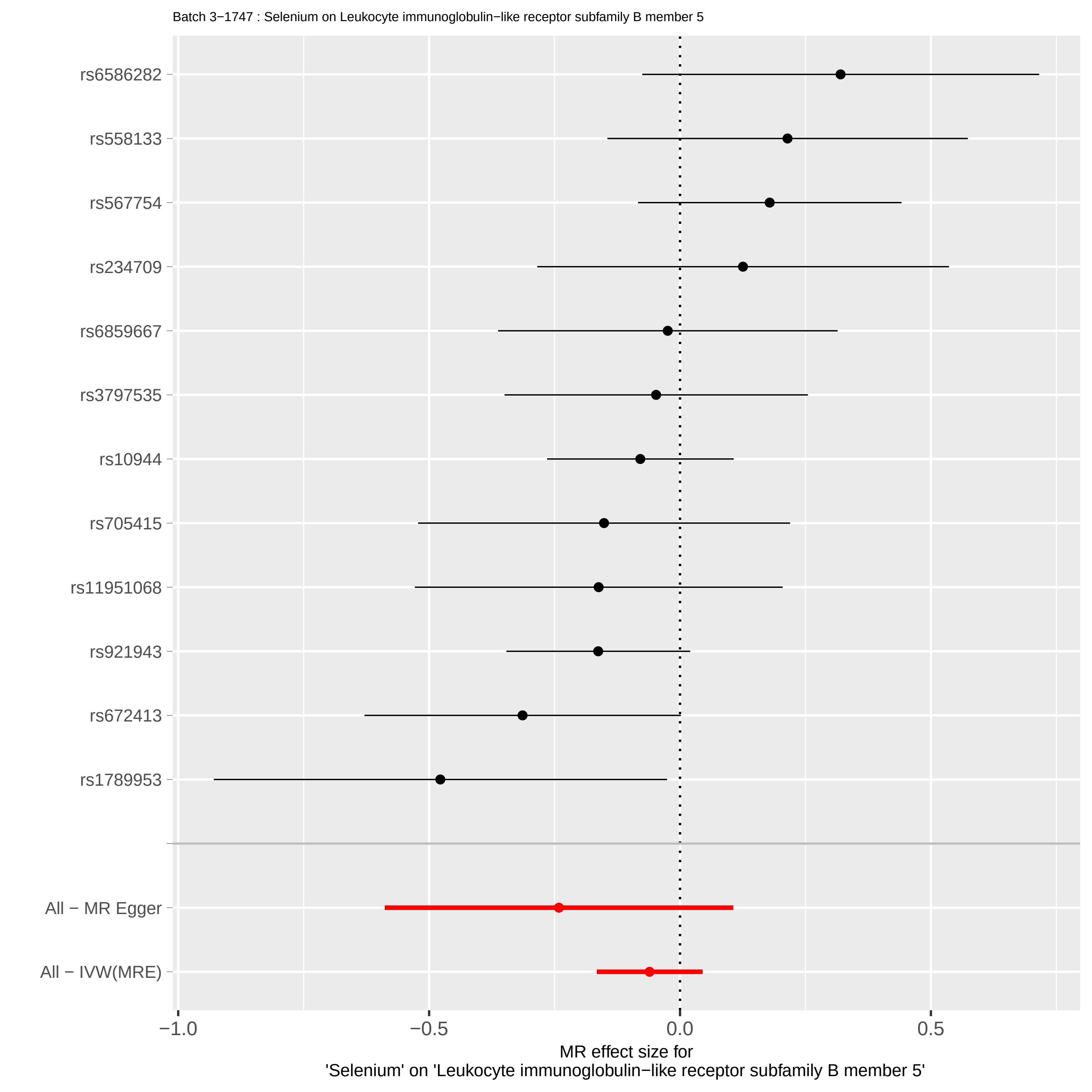

Batch 3−1747 : Selenium on Leukocyte immunoglobulin−like receptor subfamily B member 5
rs6586282
rs558133
rs567754
rs234709
rs6859667
rs3797535
rs10944
rs705415
rs11951068
rs921943
rs672413
rs1789953
All − MR Egger
All − IVW(MRE)
−1.0
−0.5
0.0
0.5
MR effect size for
'Selenium' on 'Leukocyte immunoglobulin−like receptor subfamily B member 5'

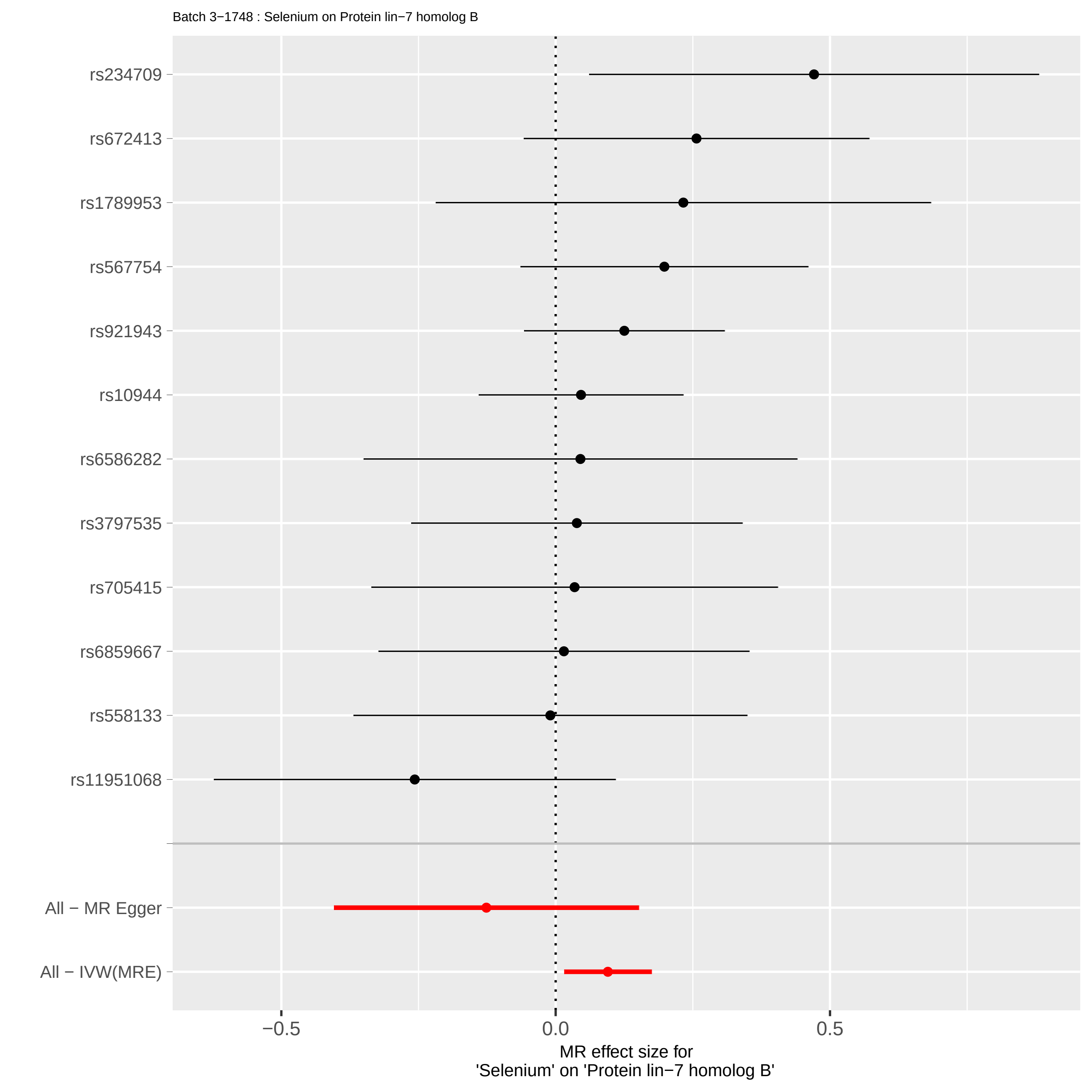

Batch 3−1748 : Selenium on Protein lin−7 homolog B
rs234709
rs672413
rs1789953
rs567754
rs921943
rs10944
rs6586282
rs3797535
rs705415
rs6859667
rs558133
rs11951068
All − MR Egger
All − IVW(MRE)
−0.5
0.0
0.5
MR effect size for
'Selenium' on 'Protein lin−7 homolog B'

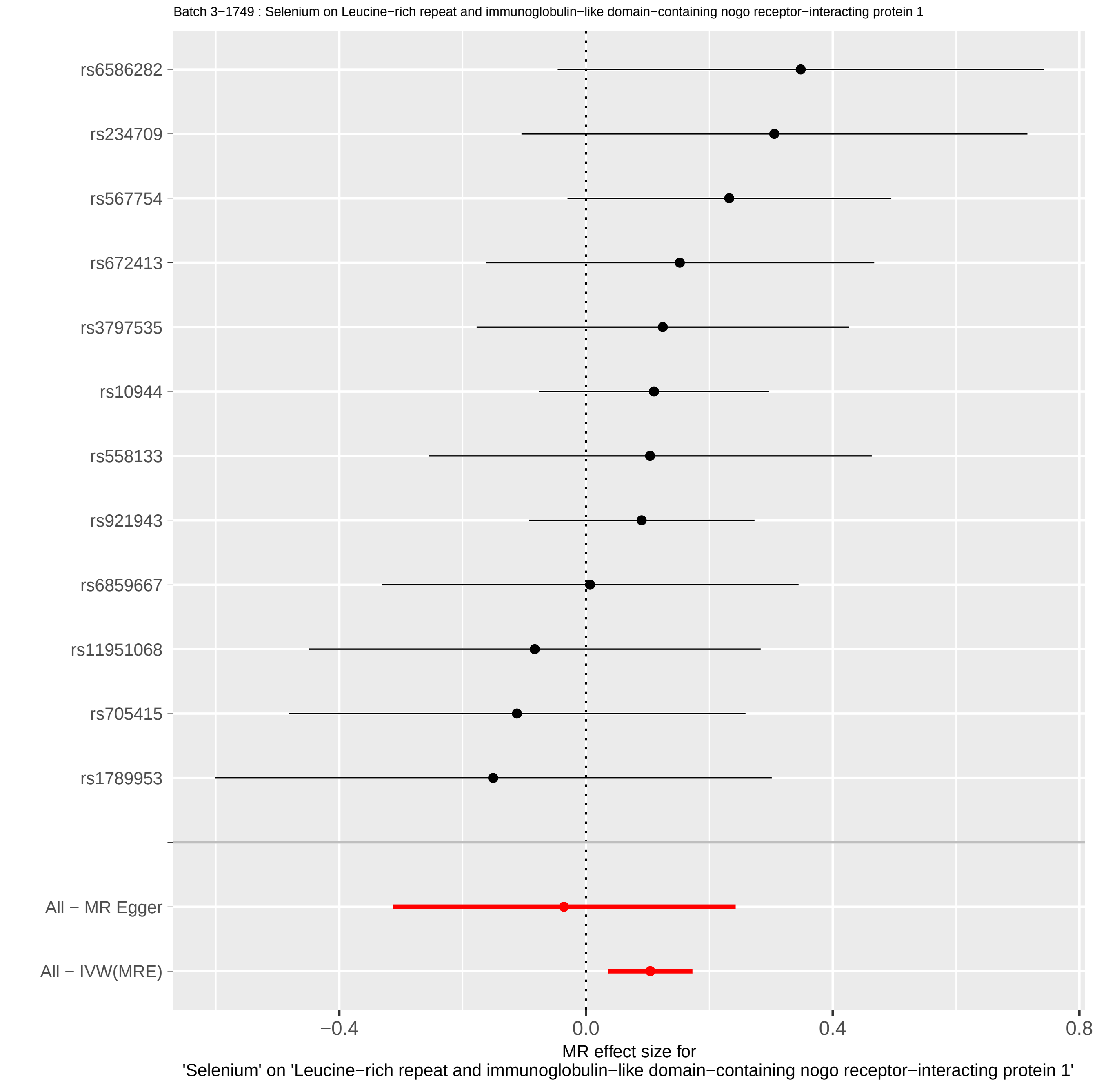
Batch 3−1749 : Selenium on Leucine−rich repeat and immunoglobulin−like domain−containing nogo receptor−interacting protein 1
rs6586282
rs234709
rs567754
rs672413
rs3797535
rs10944
rs558133
rs921943
rs6859667
rs11951068
rs705415
rs1789953
All − MR Egger
All − IVW(MRE)
−0.4
0.0
0.4
0.8
MR effect size for
'Selenium' on 'Leucine−rich repeat and immunoglobulin−like domain−containing nogo receptor−interacting protein 1'

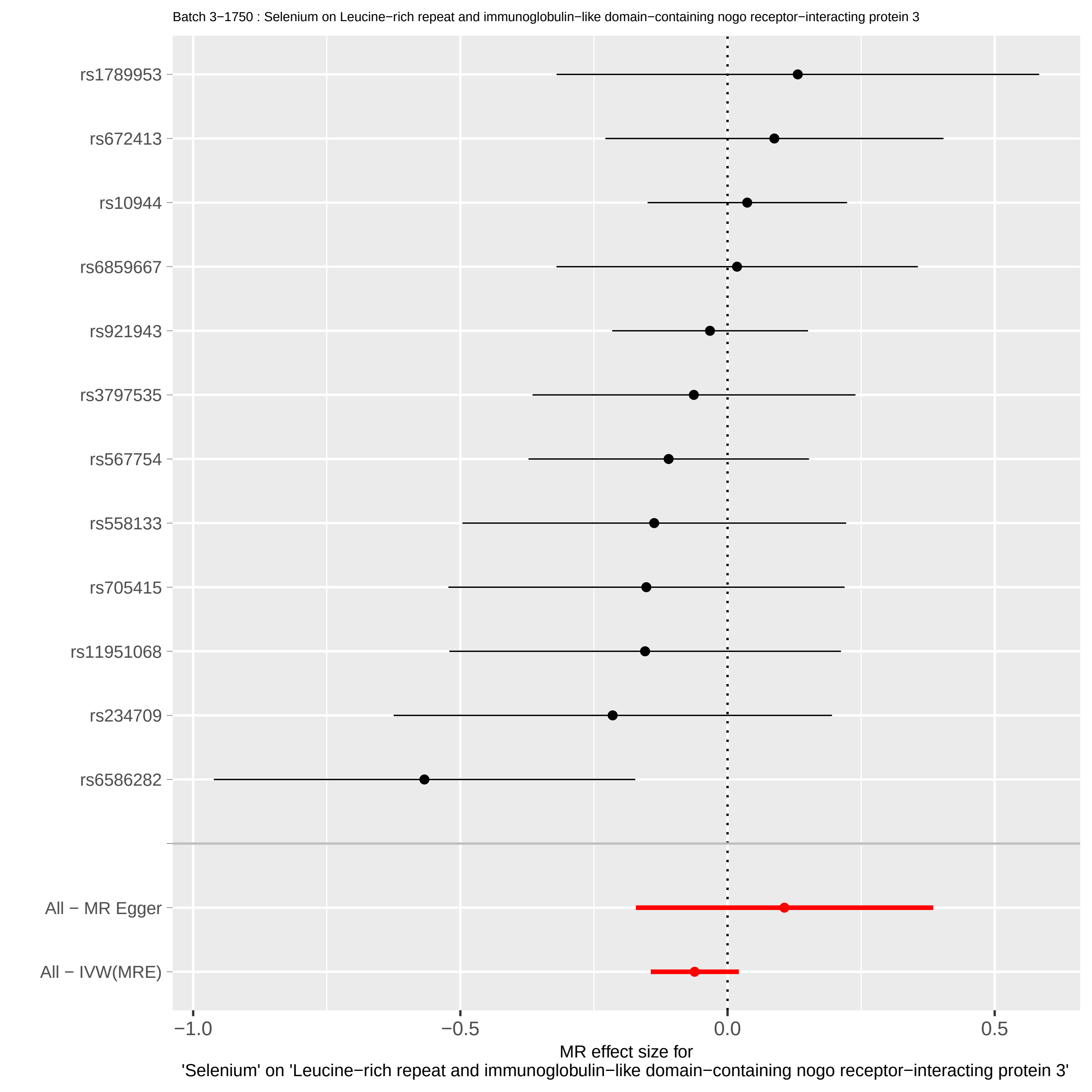

Batch 3−1750 : Selenium on Leucine−rich repeat and immunoglobulin−like domain−containing nogo receptor−interacting protein 3
rs1789953
rs672413
rs10944
rs6859667
rs921943
rs3797535
rs567754
rs558133
rs705415
rs11951068
rs234709
rs6586282
All − MR Egger
All − IVW(MRE)
−1.0
−0.5
0.0
0.5
MR effect size for
'Selenium' on 'Leucine−rich repeat and immunoglobulin−like domain−containing nogo receptor−interacting protein 3'

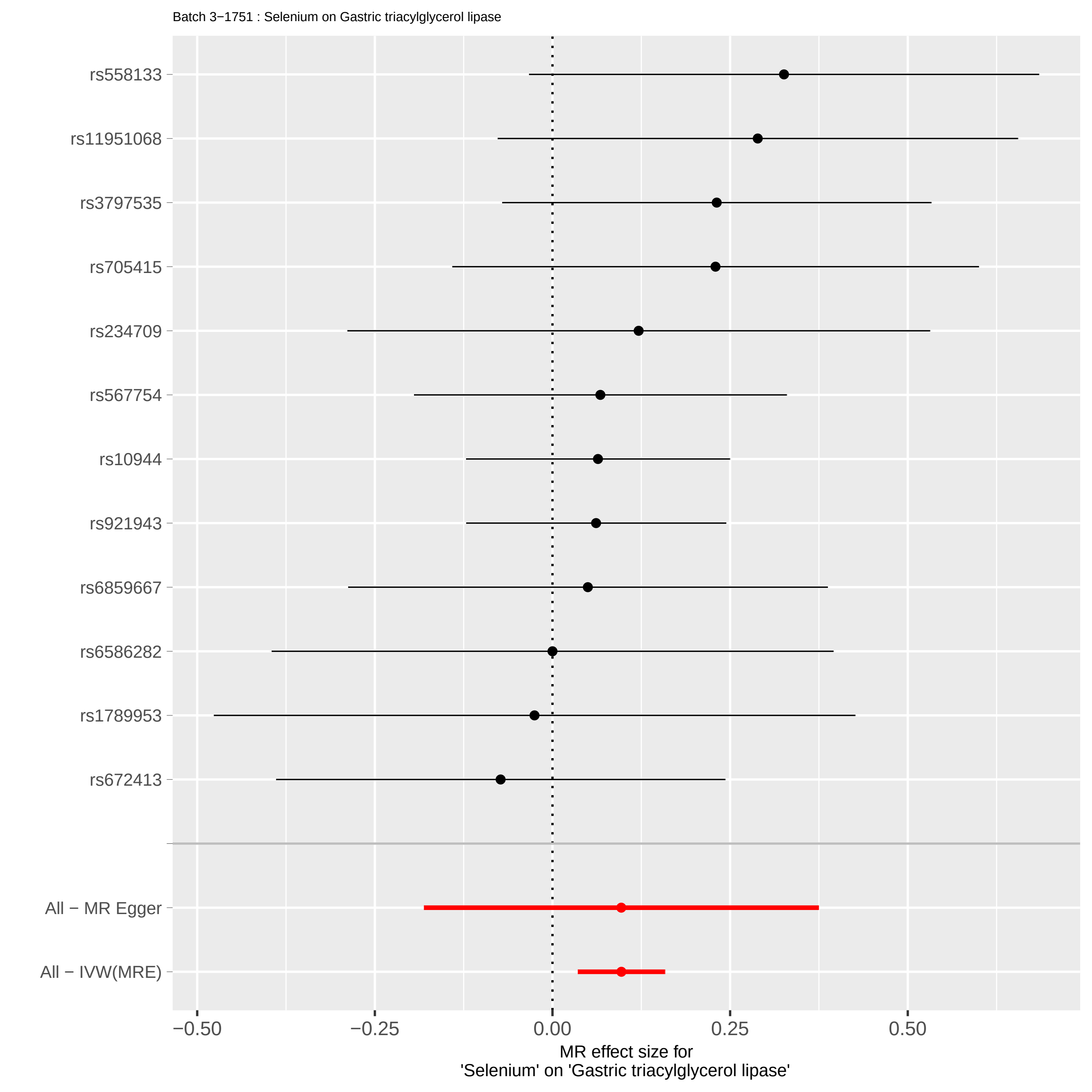

Batch 3−1751 : Selenium on Gastric triacylglycerol lipase
rs558133
rs11951068
rs3797535
rs705415
rs234709
rs567754
rs10944
rs921943
rs6859667
rs6586282
rs1789953
rs672413
All − MR Egger
All − IVW(MRE)
−0.50
−0.25
0.00
0.25
0.50
MR effect size for
'Selenium' on 'Gastric triacylglycerol lipase'

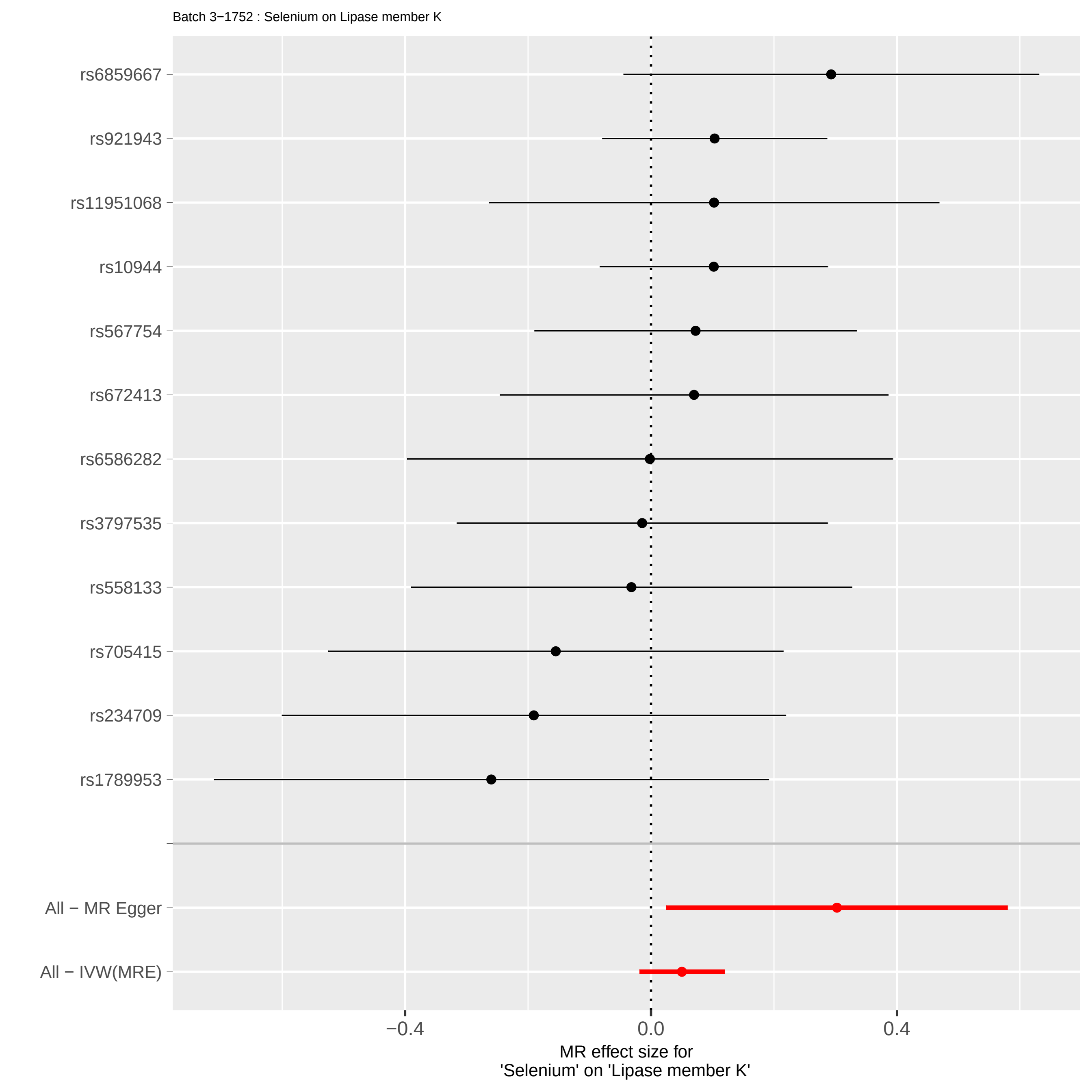

Batch 3−1752 : Selenium on Lipase member K
rs6859667
rs921943
rs11951068
rs10944
rs567754
rs672413
rs6586282
rs3797535
rs558133
rs705415
rs234709
rs1789953
All − MR Egger
All − IVW(MRE)
−0.4
0.0
0.4
MR effect size for
'Selenium' on 'Lipase member K'

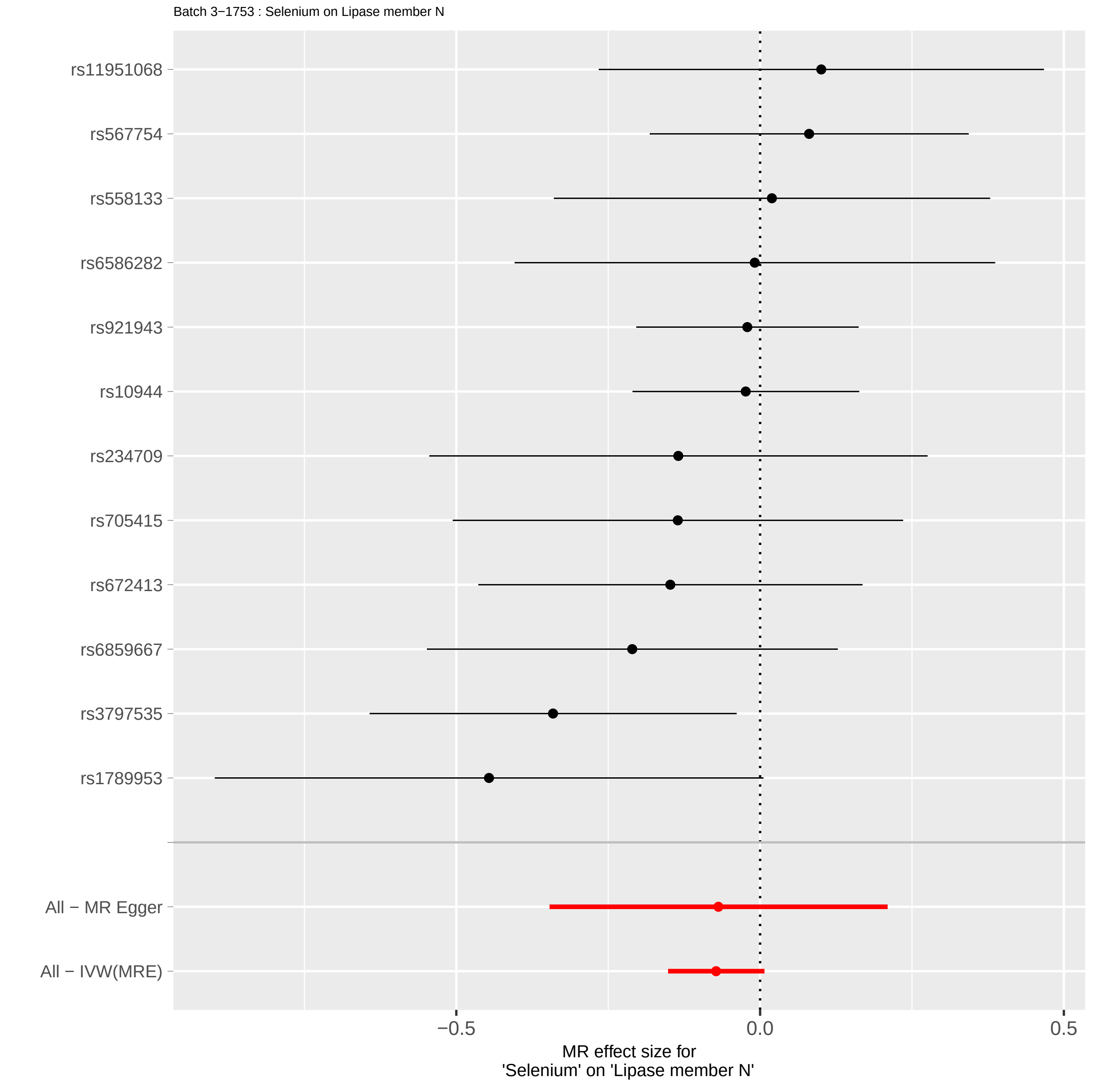

Batch 3−1753 : Selenium on Lipase member N
rs11951068
rs567754
rs558133
rs6586282
rs921943
rs10944
rs234709
rs705415
rs672413
rs6859667
rs3797535
rs1789953
All − MR Egger
All − IVW(MRE)
−0.5
0.0
0.5
MR effect size for
'Selenium' on 'Lipase member N'

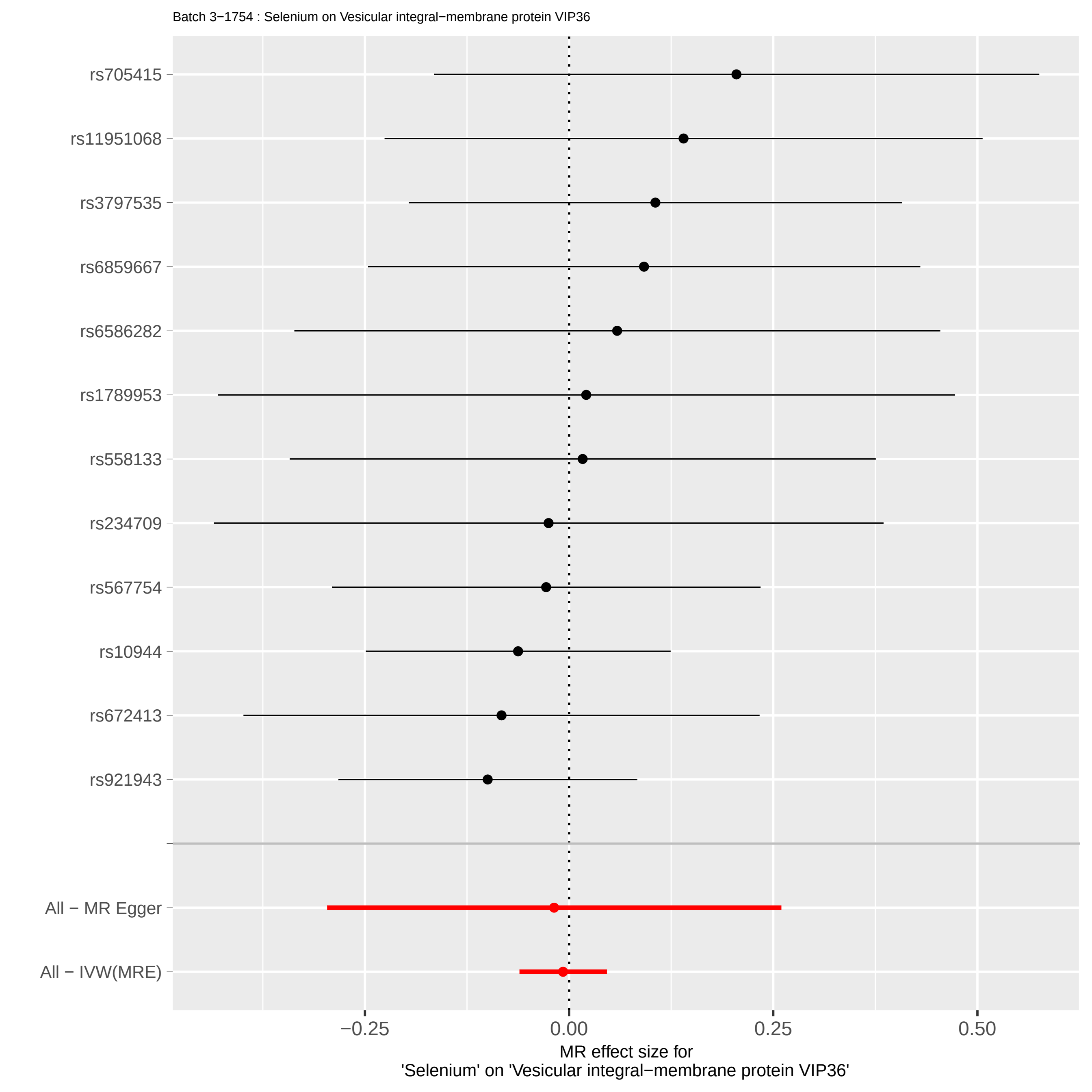

Batch 3−1754 : Selenium on Vesicular integral−membrane protein VIP36
rs705415
rs11951068
rs3797535
rs6859667
rs6586282
rs1789953
rs558133
rs234709
rs567754
rs10944
rs672413
rs921943
All − MR Egger
All − IVW(MRE)
−0.25
0.00
0.25
0.50
MR effect size for
'Selenium' on 'Vesicular integral−membrane protein VIP36'

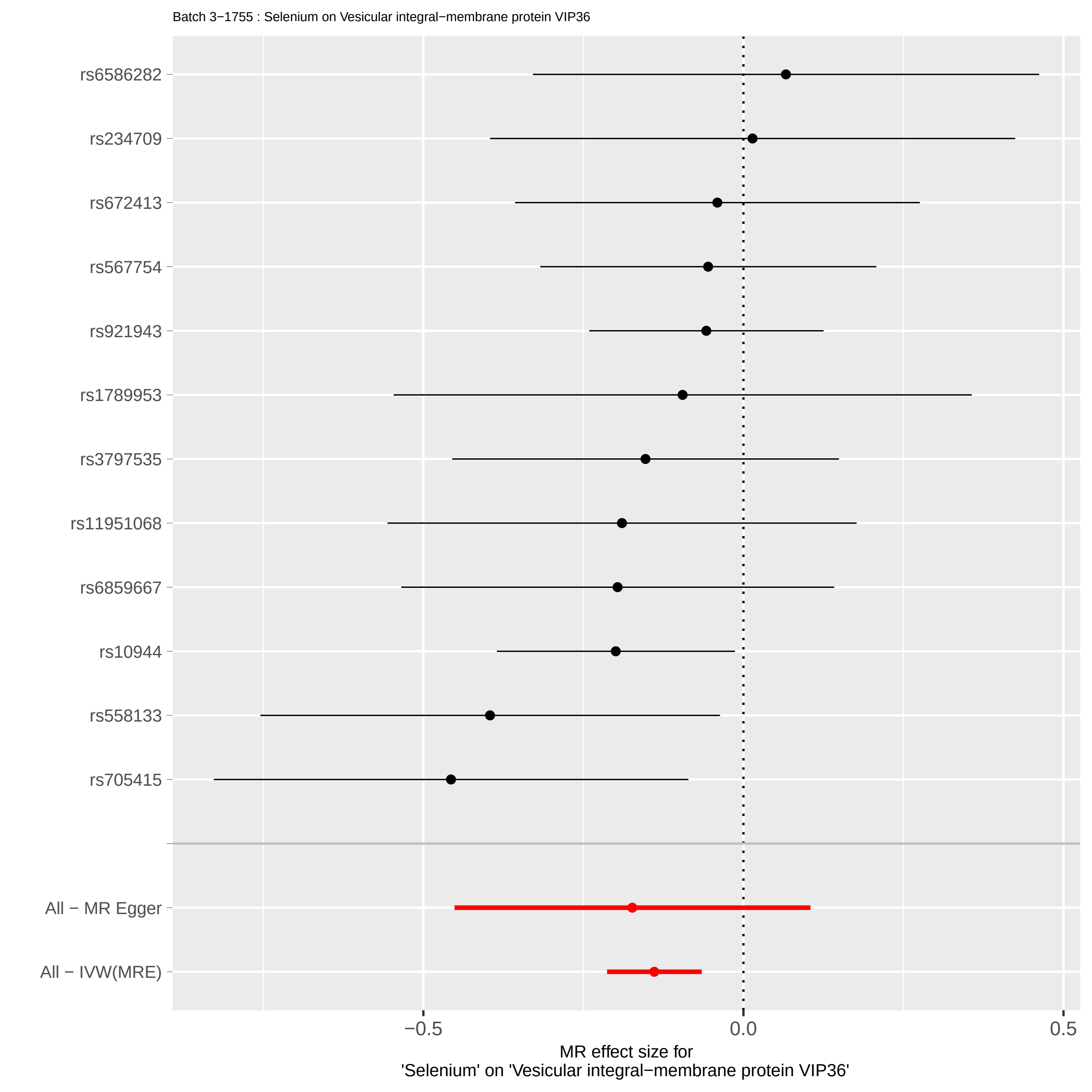

Batch 3−1755 : Selenium on Vesicular integral−membrane protein VIP36
rs6586282
rs234709
rs672413
rs567754
rs921943
rs1789953
rs3797535
rs11951068
rs6859667
rs10944
rs558133
rs705415
All − MR Egger
All − IVW(MRE)
−0.5
0.0
0.5
MR effect size for
'Selenium' on 'Vesicular integral−membrane protein VIP36'

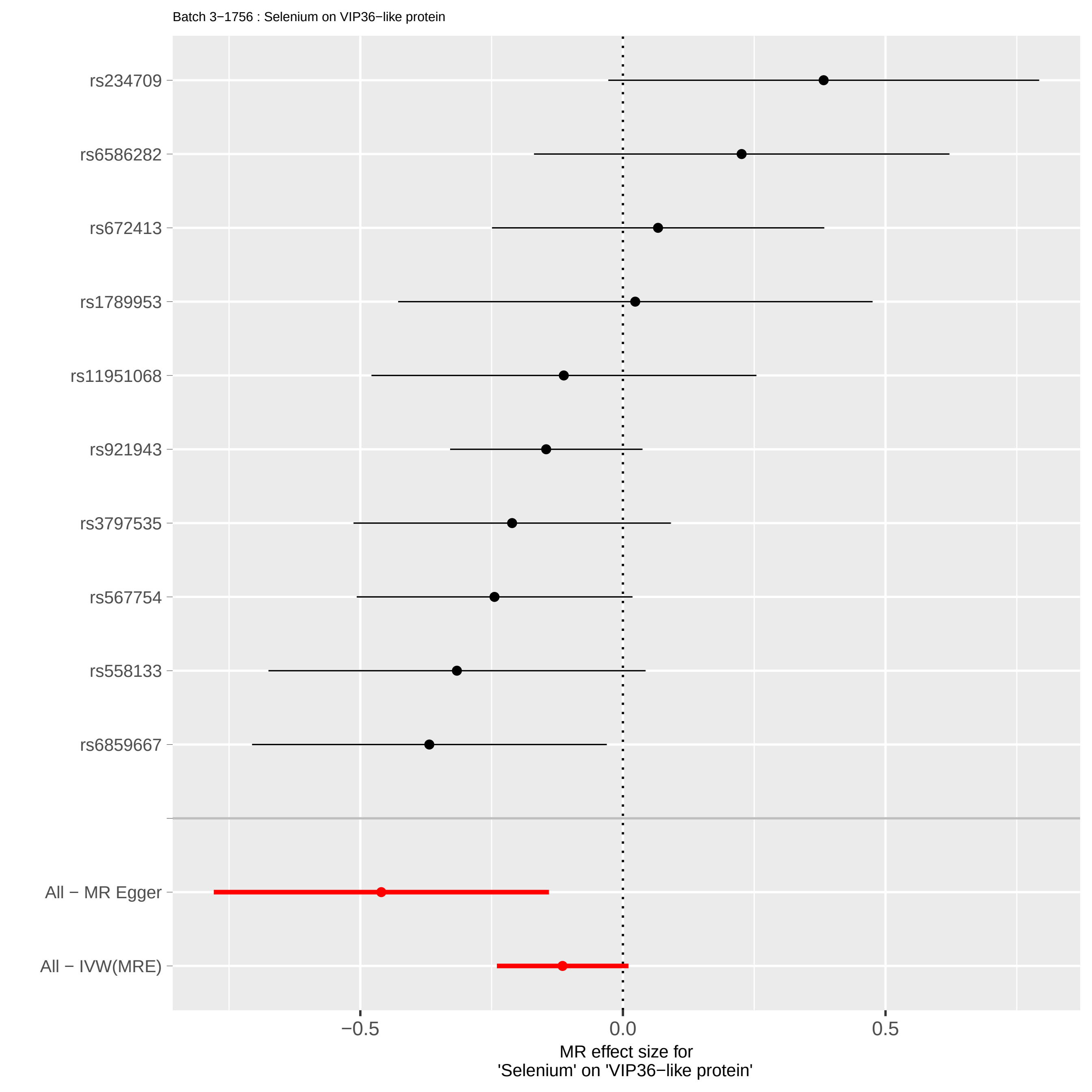
Batch 3−1756 : Selenium on VIP36−like protein
rs234709
rs6586282
rs672413
rs1789953
rs11951068
rs921943
rs3797535
rs567754
rs558133
rs6859667
All − MR Egger
All − IVW(MRE)
−0.5
0.0
0.5
MR effect size for
'Selenium' on 'VIP36−like protein'

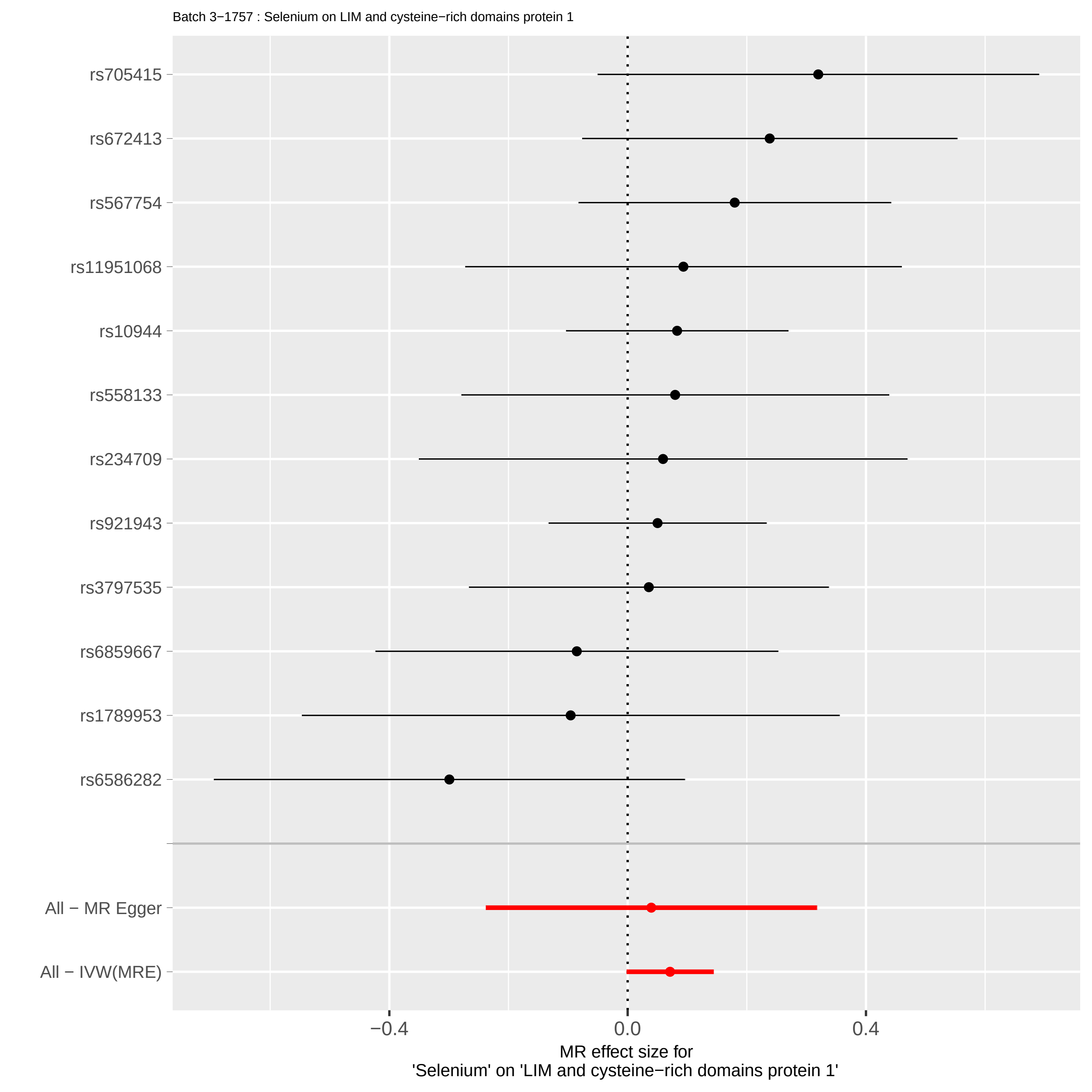

Batch 3−1757 : Selenium on LIM and cysteine−rich domains protein 1
rs705415
rs672413
rs567754
rs11951068
rs10944
rs558133
rs234709
rs921943
rs3797535
rs6859667
rs1789953
rs6586282
All − MR Egger
All − IVW(MRE)
−0.4
0.0
0.4
MR effect size for
'Selenium' on 'LIM and cysteine−rich domains protein 1'

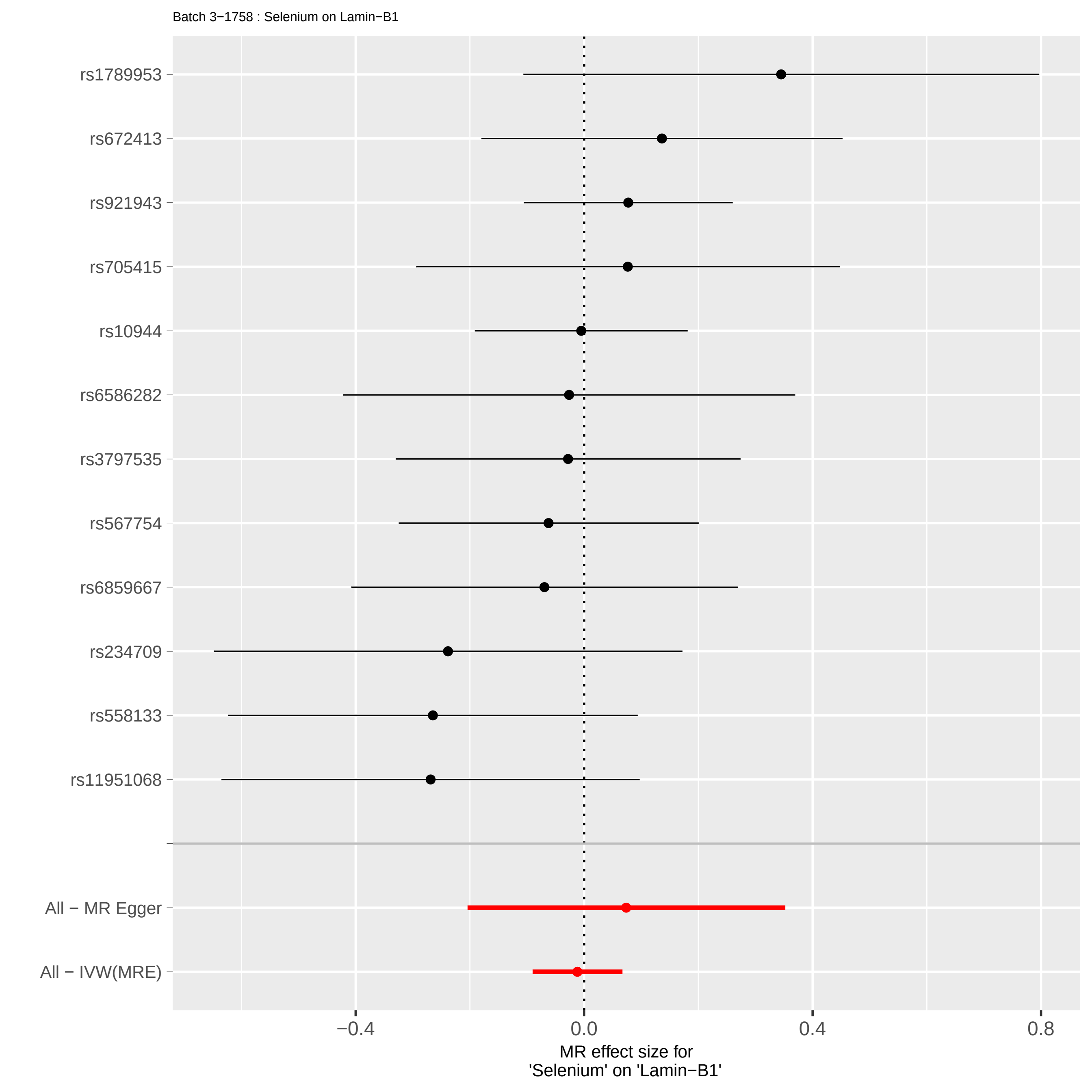

Batch 3−1758 : Selenium on Lamin−B1
rs1789953
rs672413
rs921943
rs705415
rs10944
rs6586282
rs3797535
rs567754
rs6859667
rs234709
rs558133
rs11951068
All − MR Egger
All − IVW(MRE)
−0.4
0.0
0.4
0.8
MR effect size for
'Selenium' on 'Lamin−B1'

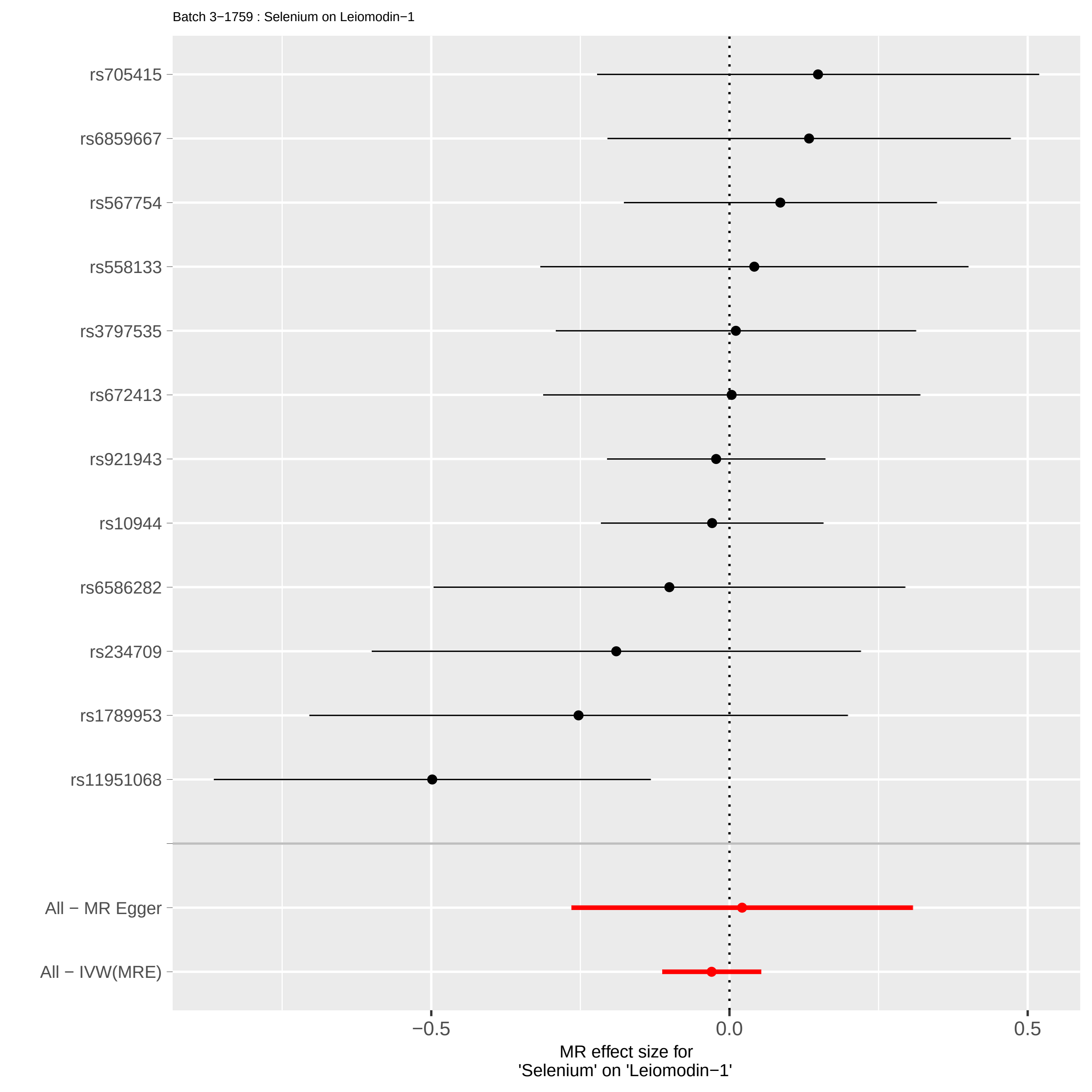

Batch 3−1759 : Selenium on Leiomodin−1
rs705415
rs6859667
rs567754
rs558133
rs3797535
rs672413
rs921943
rs10944
rs6586282
rs234709
rs1789953
rs11951068
All − MR Egger
All − IVW(MRE)
−0.5
0.0
0.5
MR effect size for
'Selenium' on 'Leiomodin−1'

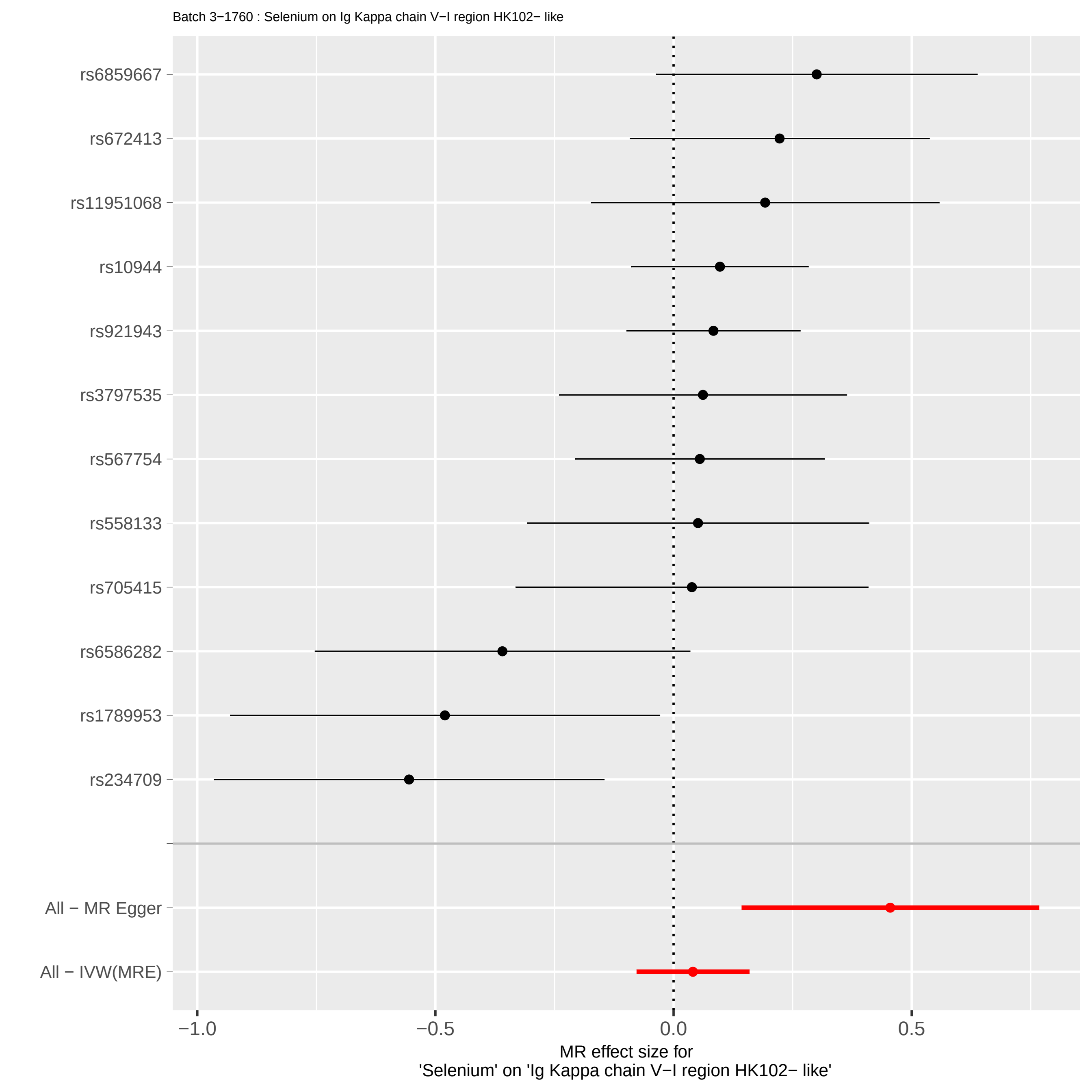
Batch 3−1760 : Selenium on Ig Kappa chain V−I region HK102− like
rs6859667
rs672413
rs11951068
rs10944
rs921943
rs3797535
rs567754
rs558133
rs705415
rs6586282
rs1789953
rs234709
All − MR Egger
All − IVW(MRE)
−1.0
−0.5
0.0
0.5
MR effect size for
'Selenium' on 'Ig Kappa chain V−I region HK102− like'

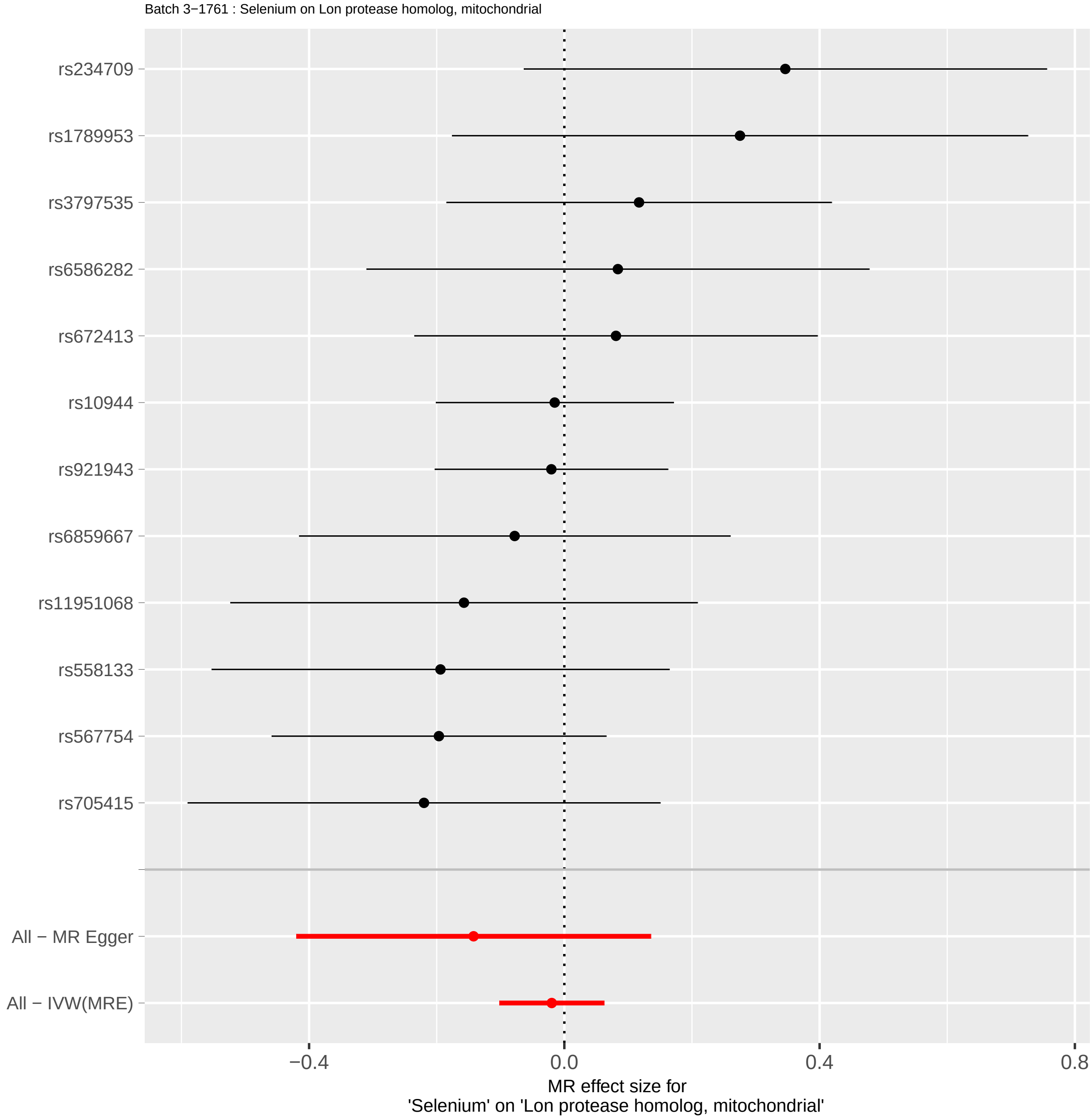
Batch 3−1761 : Selenium on Lon protease homolog, mitochondrial
rs234709
rs1789953
rs3797535
rs6586282
rs672413
rs10944
rs921943
rs6859667
rs11951068
rs558133
rs567754
rs705415
All − MR Egger
All − IVW(MRE)
−0.4
0.0
0.4
0.8
MR effect size for
'Selenium' on 'Lon protease homolog, mitochondrial'

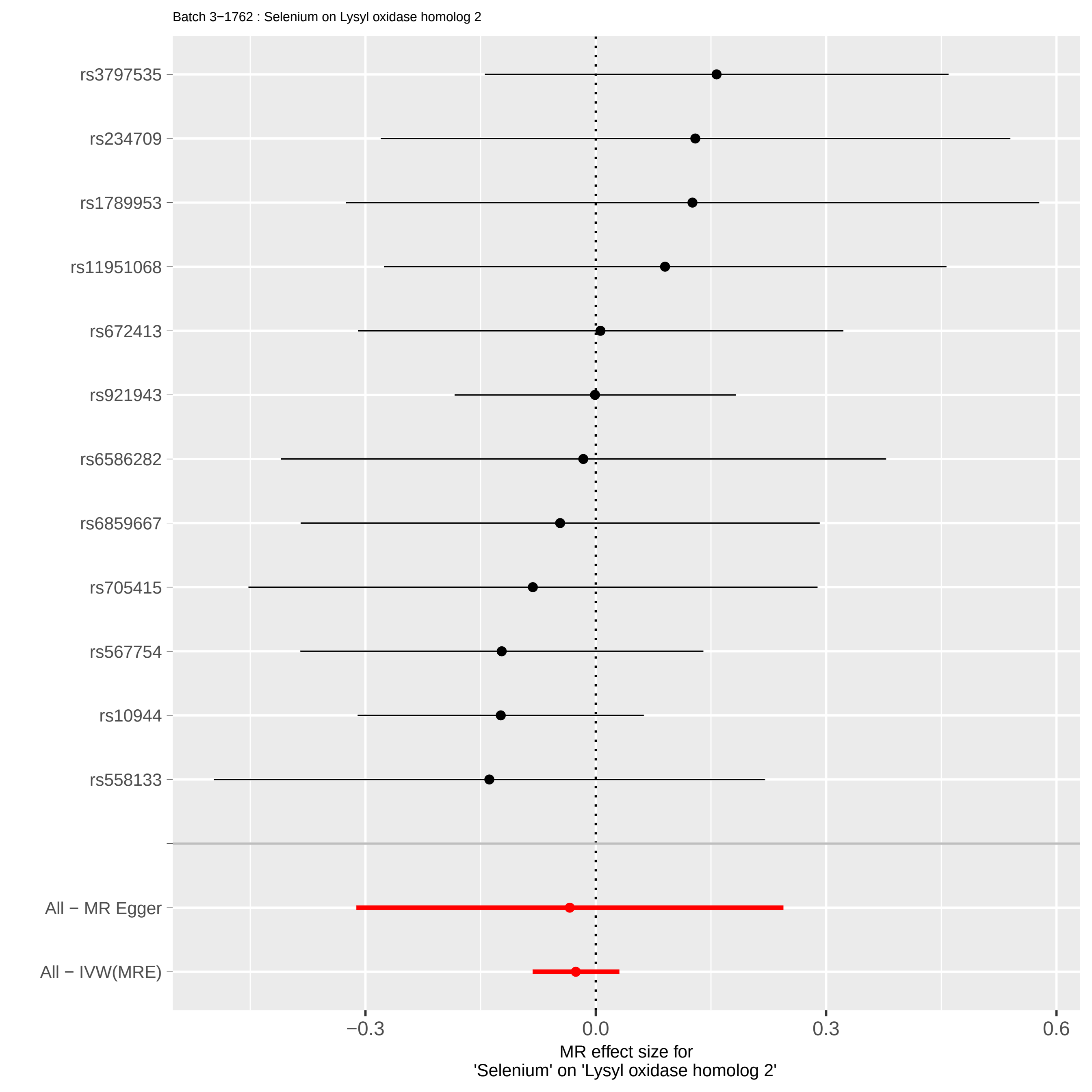
Batch 3−1762 : Selenium on Lysyl oxidase homolog 2
rs3797535
rs234709
rs1789953
rs11951068
rs672413
rs921943
rs6586282
rs6859667
rs705415
rs567754
rs10944
rs558133
All − MR Egger
All − IVW(MRE)
−0.3
0.0
0.3
0.6
MR effect size for
'Selenium' on 'Lysyl oxidase homolog 2'

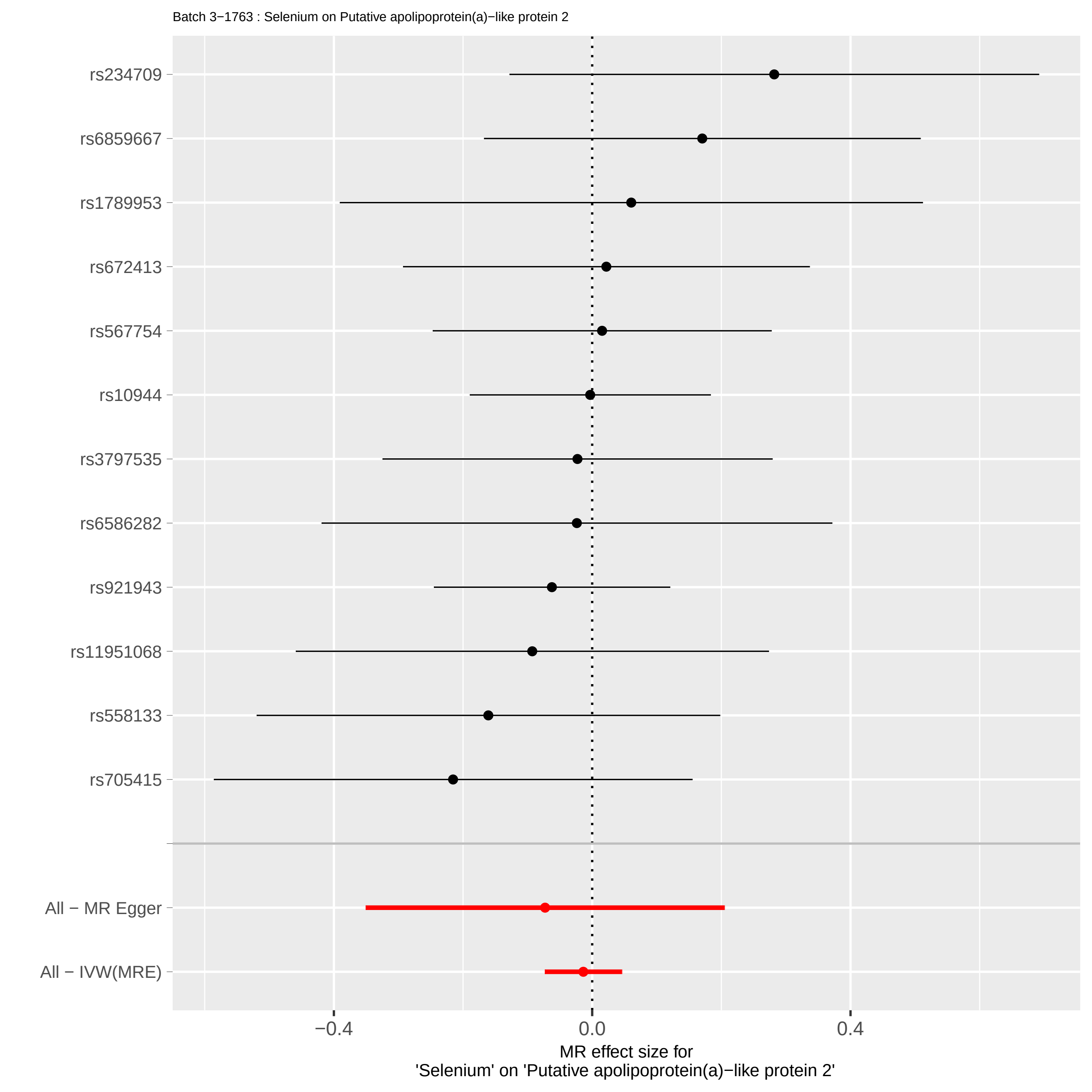

Batch 3−1763 : Selenium on Putative apolipoprotein(a)−like protein 2
rs234709
rs6859667
rs1789953
rs672413
rs567754
rs10944
rs3797535
rs6586282
rs921943
rs11951068
rs558133
rs705415
All − MR Egger
All − IVW(MRE)
−0.4
0.0
0.4
MR effect size for
'Selenium' on 'Putative apolipoprotein(a)−like protein 2'

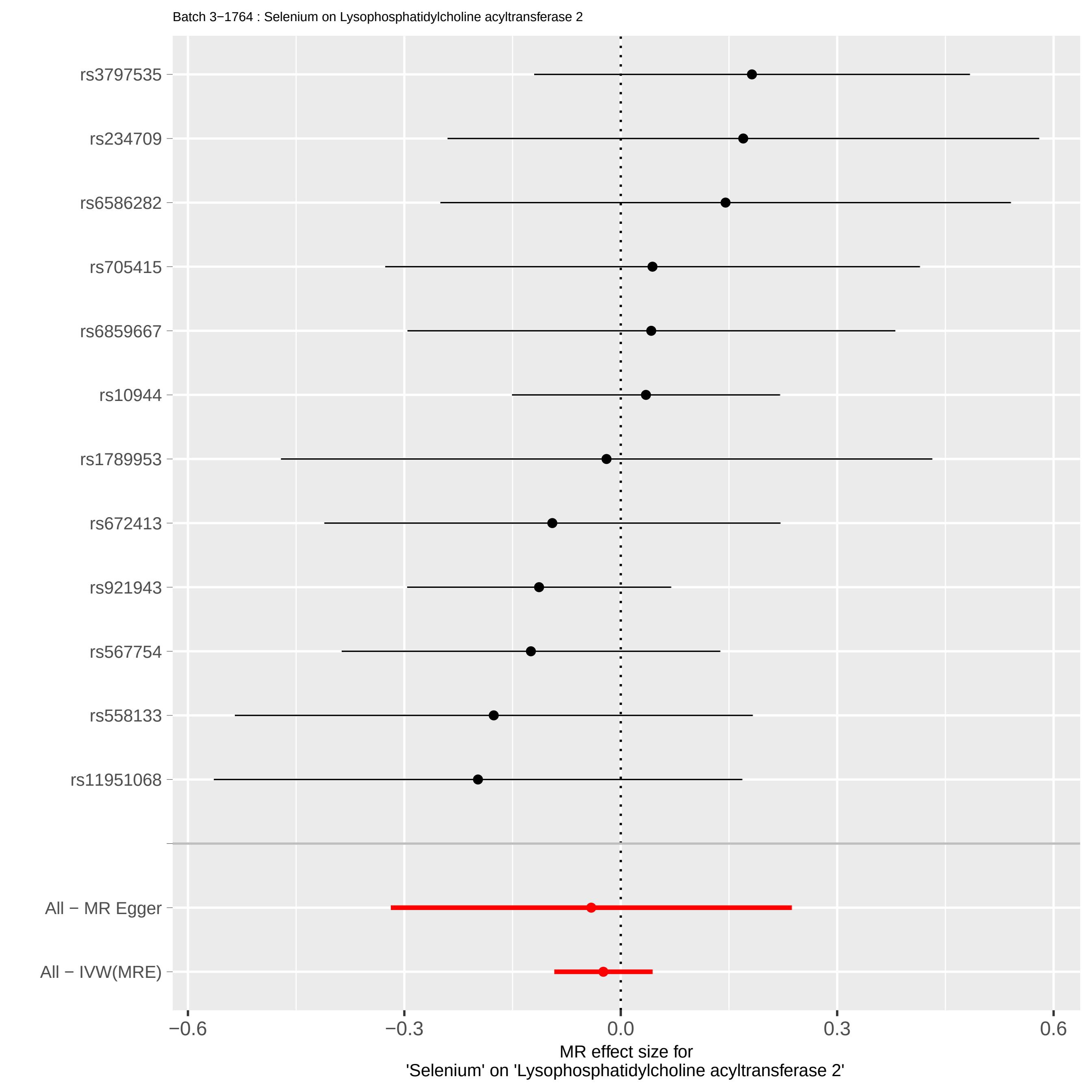

Batch 3−1764 : Selenium on Lysophosphatidylcholine acyltransferase 2
rs3797535
rs234709
rs6586282
rs705415
rs6859667
rs10944
rs1789953
rs672413
rs921943
rs567754
rs558133
rs11951068
All − MR Egger
All − IVW(MRE)
−0.6
−0.3
0.0
0.3
0.6
MR effect size for
'Selenium' on 'Lysophosphatidylcholine acyltransferase 2'

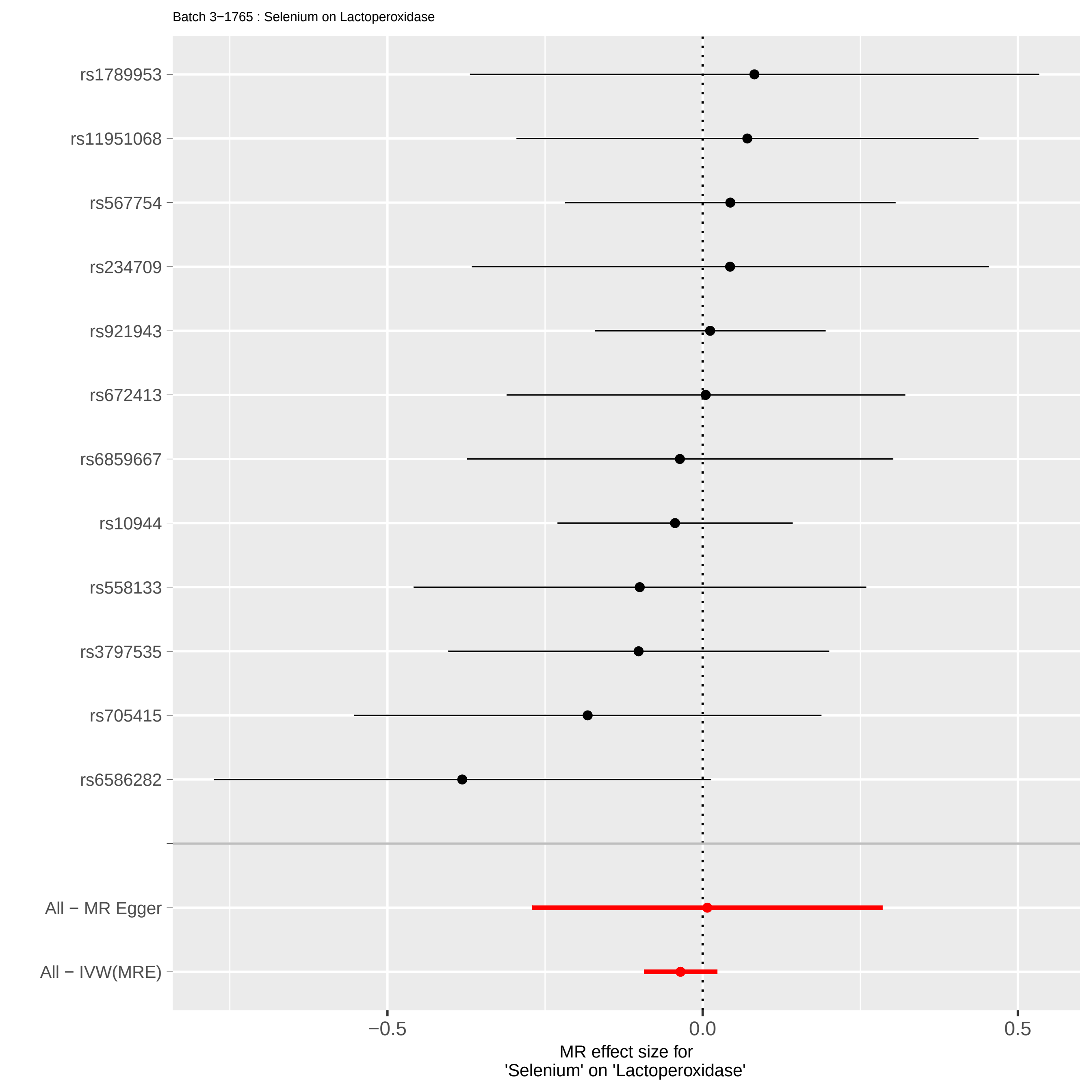

Batch 3−1765 : Selenium on Lactoperoxidase
rs1789953
rs11951068
rs567754
rs234709
rs921943
rs672413
rs6859667
rs10944
rs558133
rs3797535
rs705415
rs6586282
All − MR Egger
All − IVW(MRE)
−0.5
0.0
0.5
MR effect size for
'Selenium' on 'Lactoperoxidase'

Batch 3−1766 : Selenium on Leucine−rich repeat and calponin homology domain−containing protein 4

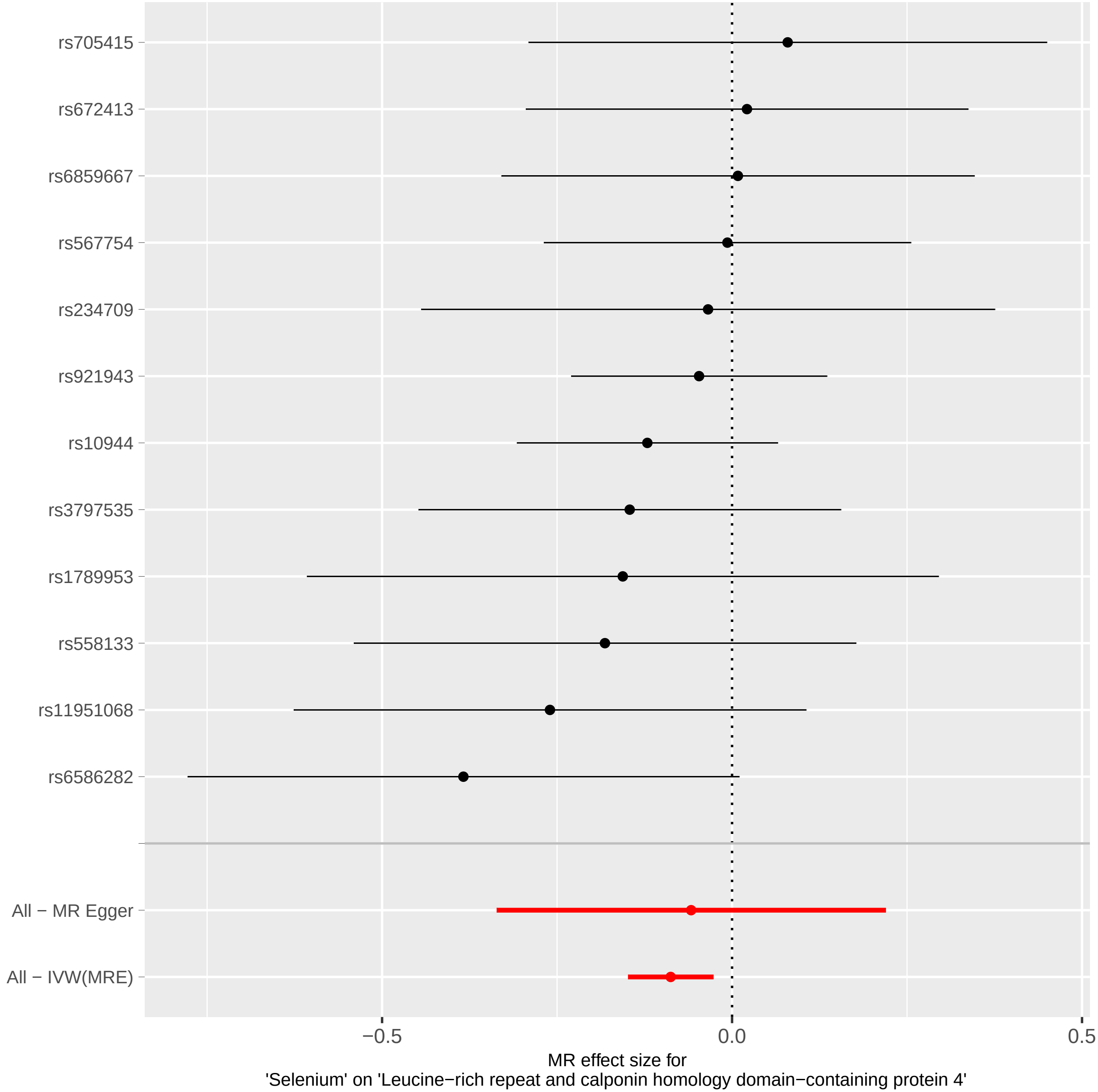

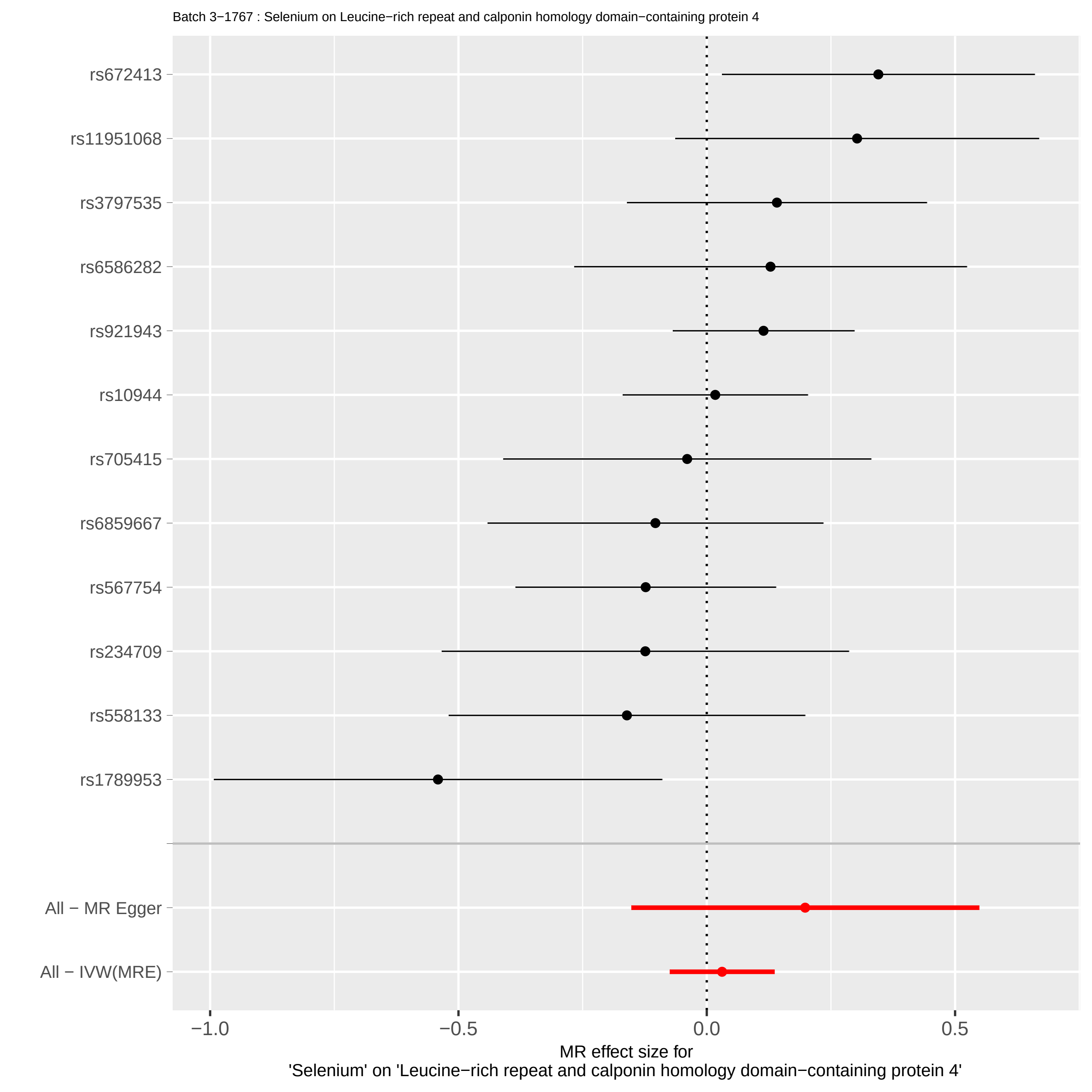
Batch 3−1767 : Selenium on Leucine−rich repeat and calponin homology domain−containing protein 4
rs672413
rs11951068
rs3797535
rs6586282
rs921943
rs10944
rs705415
rs6859667
rs567754
rs234709
rs558133
rs1789953
All − MR Egger
All − IVW(MRE)
−1.0
−0.5
0.0
0.5
MR effect size for
'Selenium' on 'Leucine−rich repeat and calponin homology domain−containing protein 4'

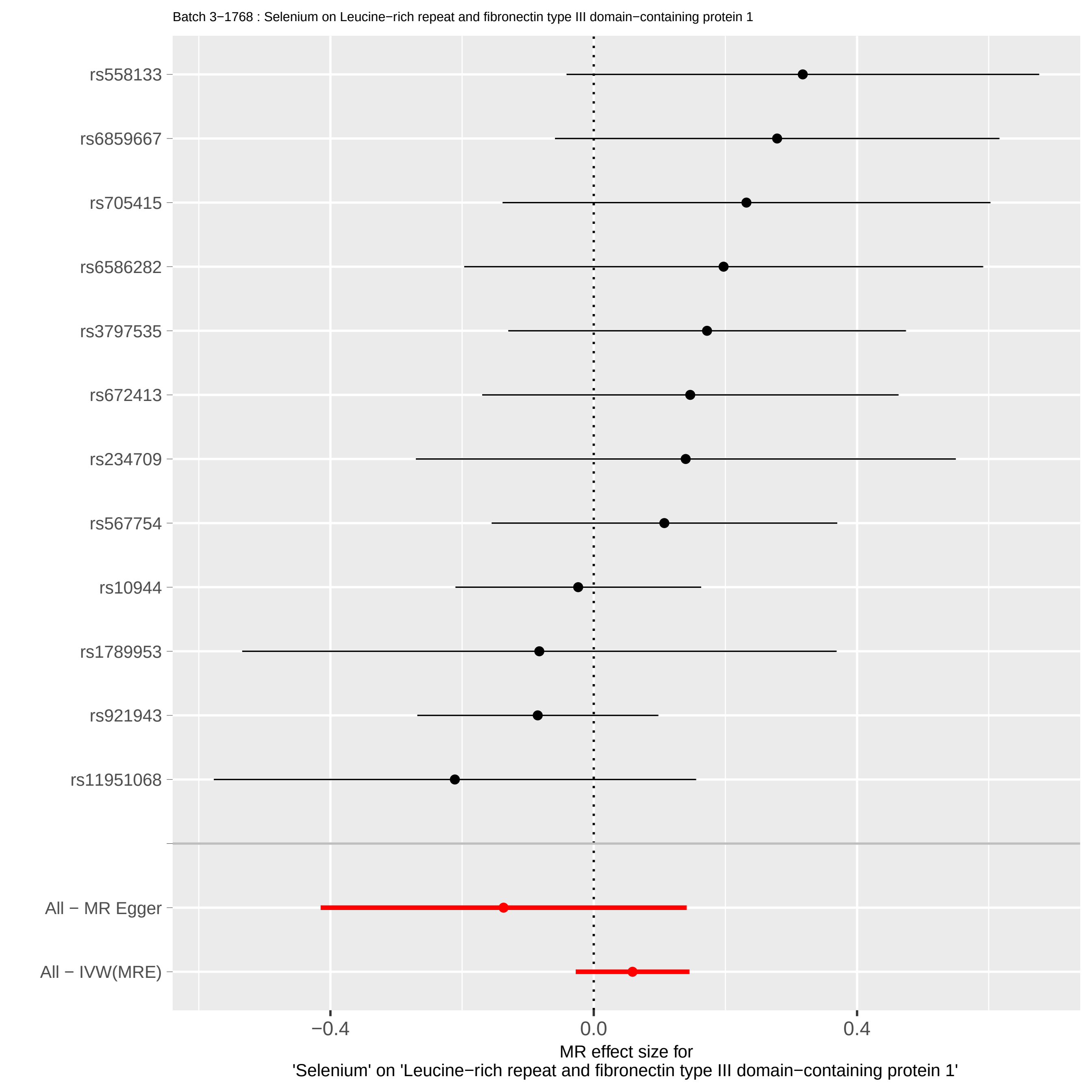

Batch 3−1768 : Selenium on Leucine−rich repeat and fibronectin type III domain−containing protein 1
rs558133
rs6859667
rs705415
rs6586282
rs3797535
rs672413
rs234709
rs567754
rs10944
rs1789953
rs921943
rs11951068
All − MR Egger
All − IVW(MRE)
−0.4
0.0
0.4
MR effect size for
'Selenium' on 'Leucine−rich repeat and fibronectin type III domain−containing protein 1'

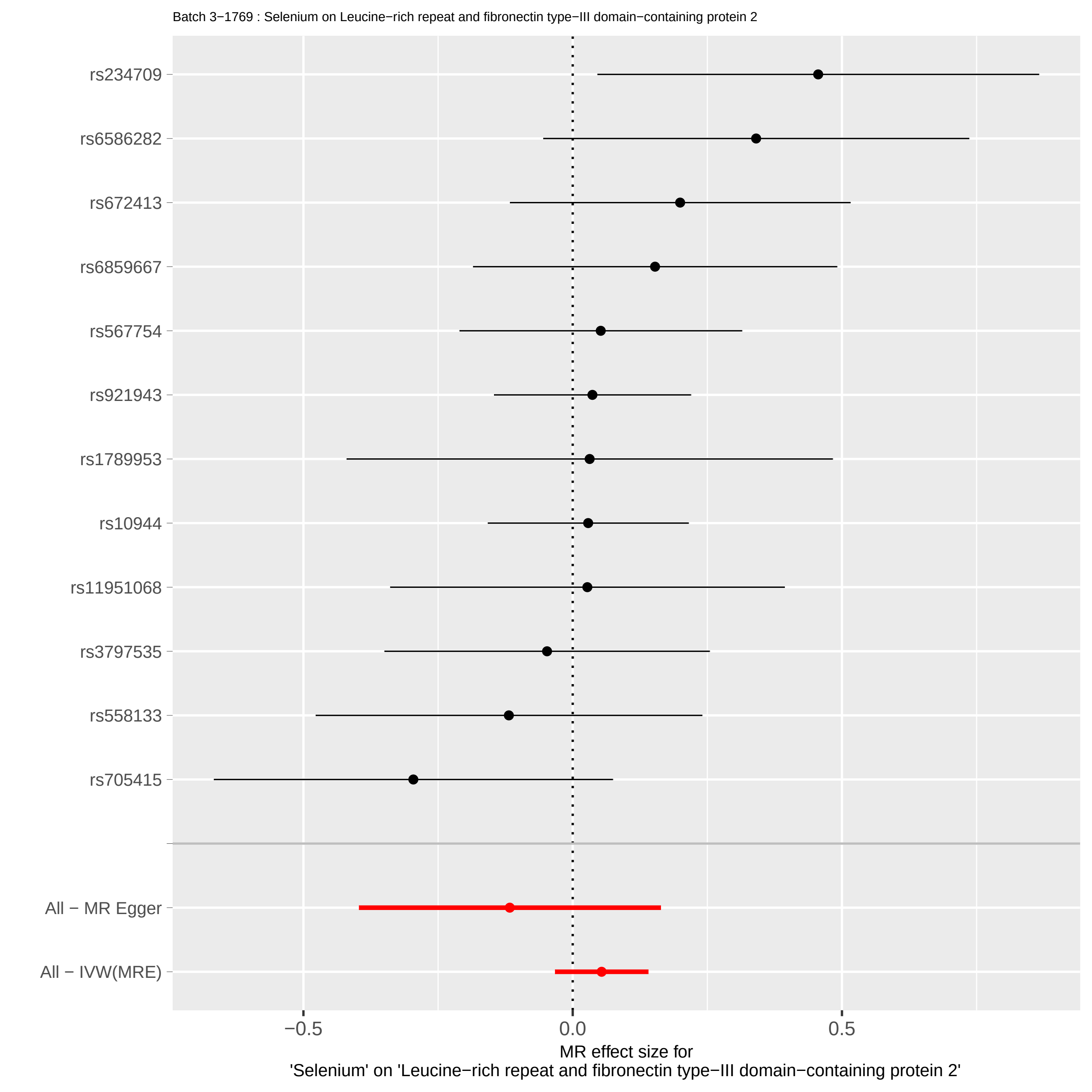

Batch 3−1769 : Selenium on Leucine−rich repeat and fibronectin type−III domain−containing protein 2
rs234709
rs6586282
rs672413
rs6859667
rs567754
rs921943
rs1789953
rs10944
rs11951068
rs3797535
rs558133
rs705415
All − MR Egger
All − IVW(MRE)
−0.5
0.0
0.5
MR effect size for
'Selenium' on 'Leucine−rich repeat and fibronectin type−III domain−containing protein 2'

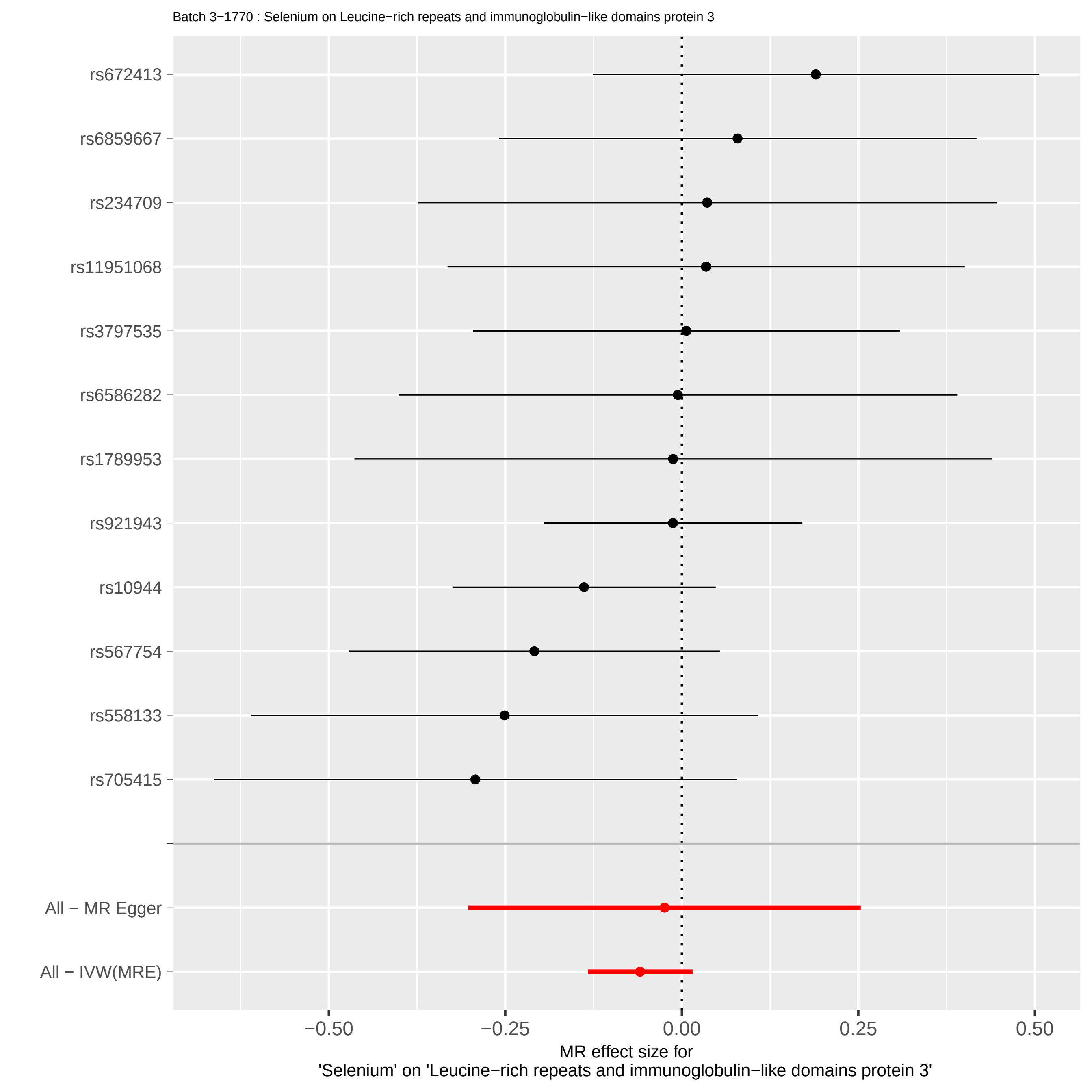

Batch 3−1770 : Selenium on Leucine−rich repeats and immunoglobulin−like domains protein 3
rs672413
rs6859667
rs234709
rs11951068
rs3797535
rs6586282
rs1789953
rs921943
rs10944
rs567754
rs558133
rs705415
All − MR Egger
All − IVW(MRE)
−0.50
−0.25
0.00
0.25
0.50
MR effect size for
'Selenium' on 'Leucine−rich repeats and immunoglobulin−like domains protein 3'

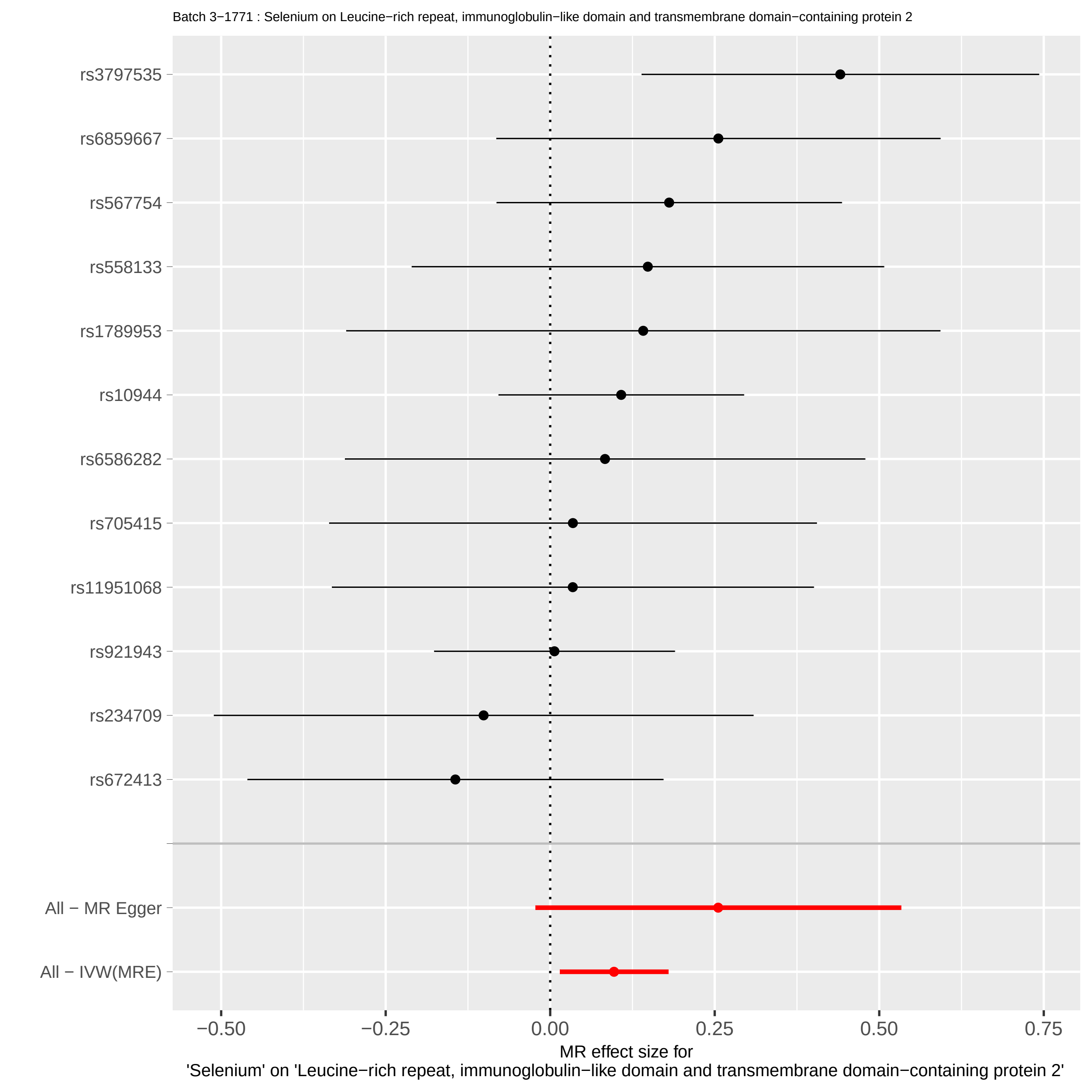

Batch 3−1771 : Selenium on Leucine−rich repeat, immunoglobulin−like domain and transmembrane domain−containing protein 2
rs3797535
rs6859667
rs567754
rs558133
rs1789953
rs10944
rs6586282
rs705415
rs11951068
rs921943
rs234709
rs672413
All − MR Egger
All − IVW(MRE)
−0.50
−0.25
0.00
0.25
0.50
0.75
MR effect size for
'Selenium' on 'Leucine−rich repeat, immunoglobulin−like domain and transmembrane domain−containing protein 2'

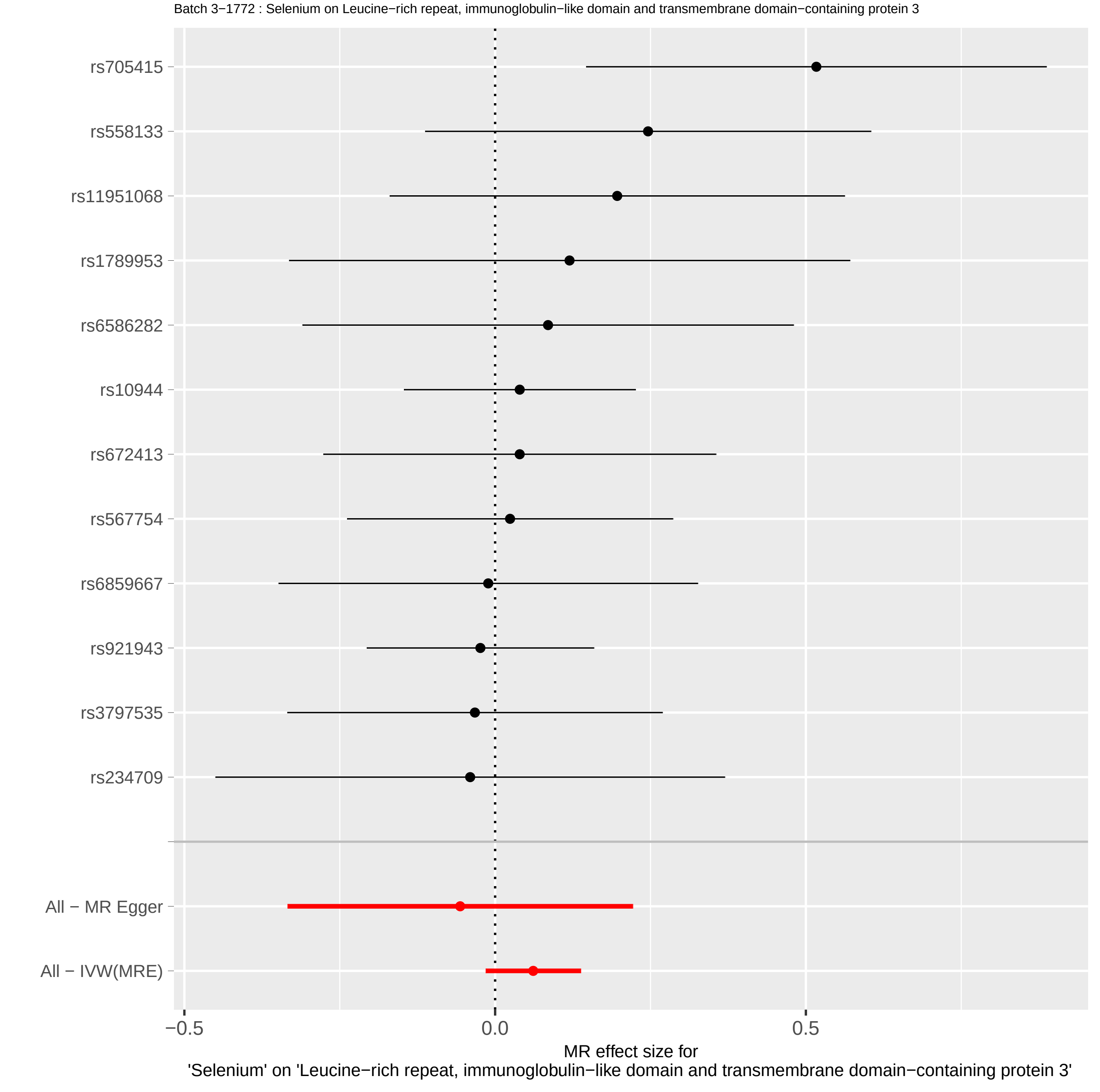
Batch 3−1772 : Selenium on Leucine−rich repeat, immunoglobulin−like domain and transmembrane domain−containing protein 3
rs705415
rs558133
rs11951068
rs1789953
rs6586282
rs10944
rs672413
rs567754
rs6859667
rs921943
rs3797535
rs234709
All − MR Egger
All − IVW(MRE)
−0.5
0.0
0.5
MR effect size for
'Selenium' on 'Leucine−rich repeat, immunoglobulin−like domain and transmembrane domain−containing protein 3'

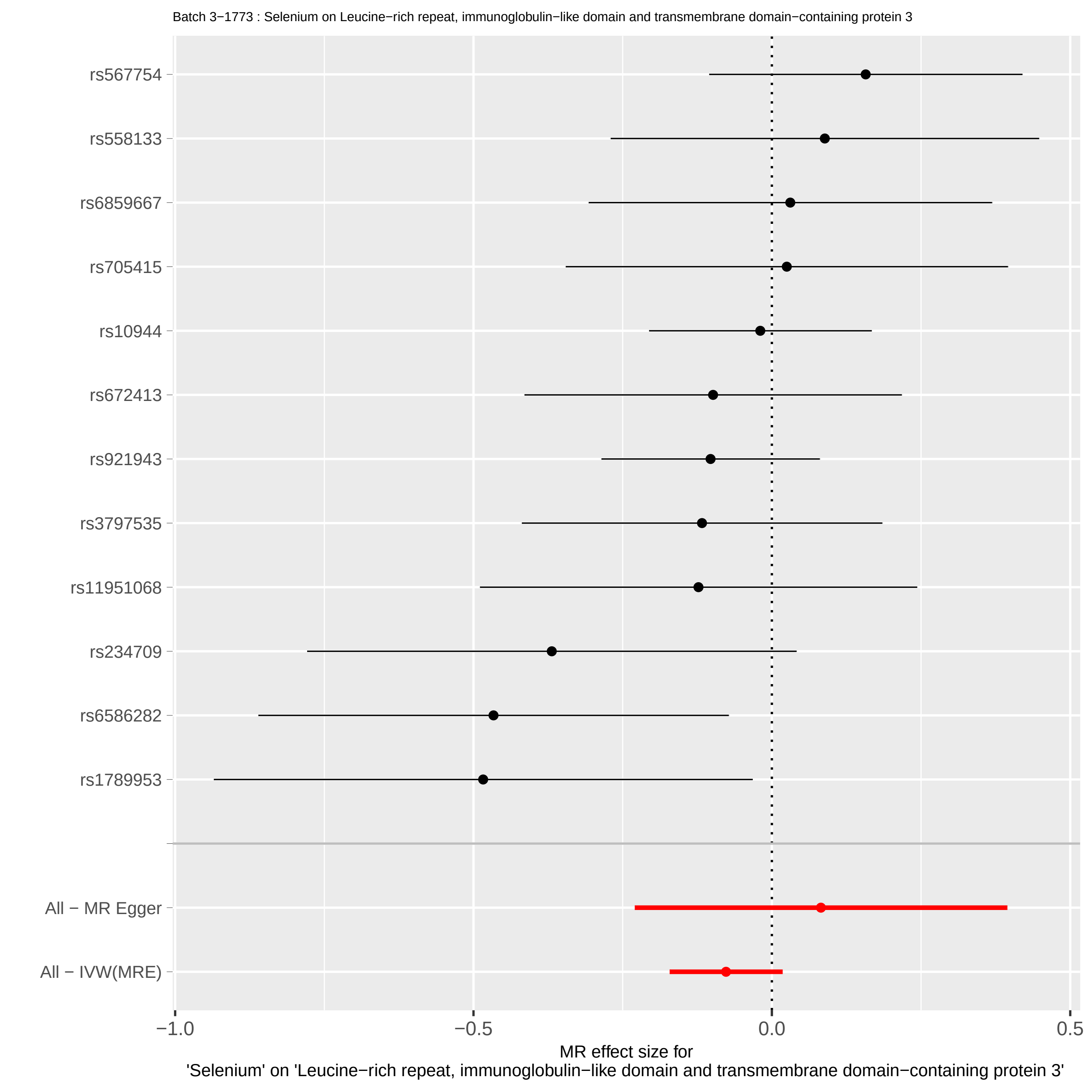
Batch 3−1773 : Selenium on Leucine−rich repeat, immunoglobulin−like domain and transmembrane domain−containing protein 3
rs567754
rs558133
rs6859667
rs705415
rs10944
rs672413
rs921943
rs3797535
rs11951068
rs234709
rs6586282
rs1789953
All − MR Egger
All − IVW(MRE)
−1.0
−0.5
0.0
0.5
MR effect size for
'Selenium' on 'Leucine−rich repeat, immunoglobulin−like domain and transmembrane domain−containing protein 3'

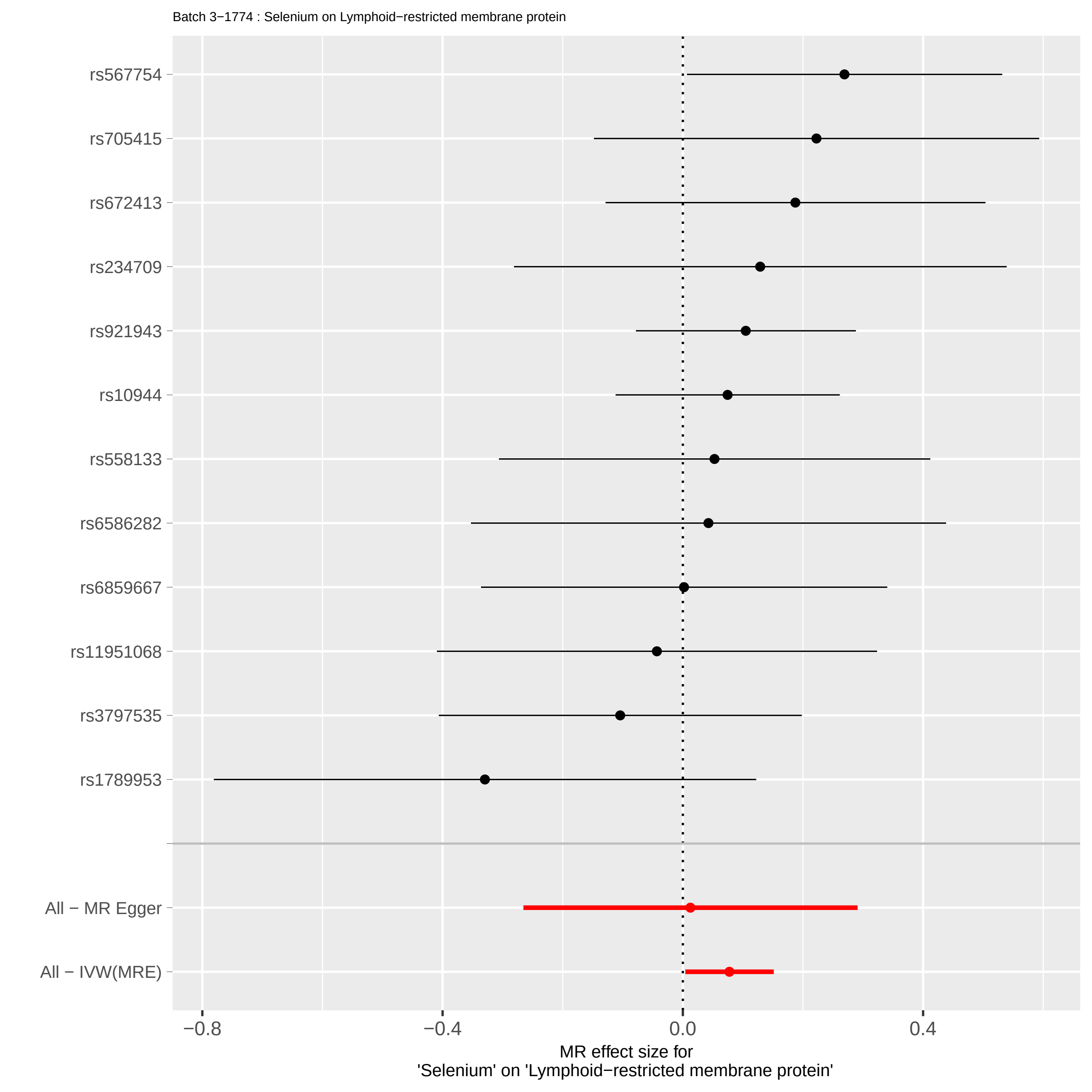

Batch 3−1774 : Selenium on Lymphoid−restricted membrane protein
rs567754
rs705415
rs672413
rs234709
rs921943
rs10944
rs558133
rs6586282
rs6859667
rs11951068
rs3797535
rs1789953
All − MR Egger
All − IVW(MRE)
−0.8
−0.4
0.0
0.4
MR effect size for
'Selenium' on 'Lymphoid−restricted membrane protein'

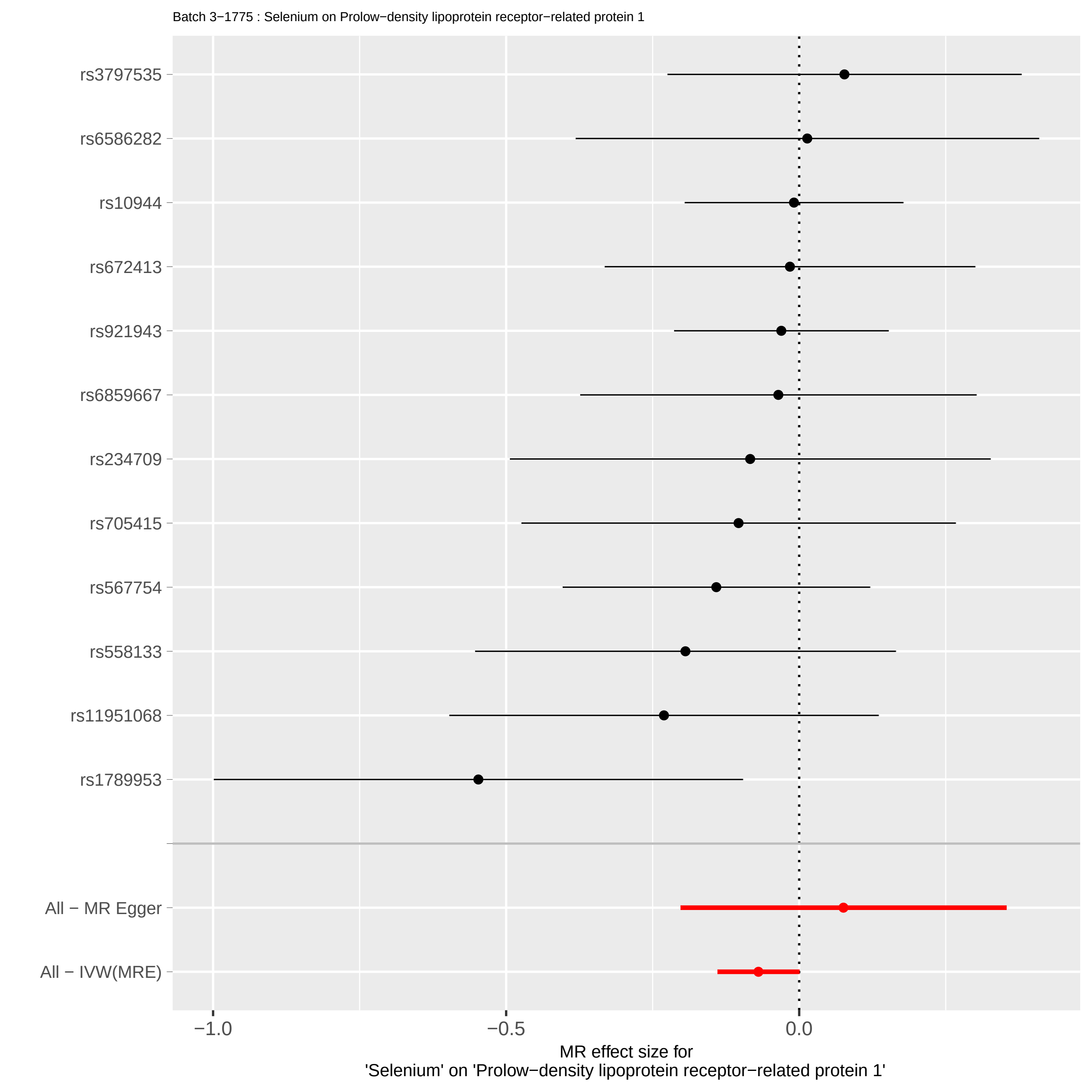

Batch 3−1775 : Selenium on Prolow−density lipoprotein receptor−related protein 1
rs3797535
rs6586282
rs10944
rs672413
rs921943
rs6859667
rs234709
rs705415
rs567754
rs558133
rs11951068
rs1789953
All − MR Egger
All − IVW(MRE)
−1.0
−0.5
0.0
MR effect size for
'Selenium' on 'Prolow−density lipoprotein receptor−related protein 1'

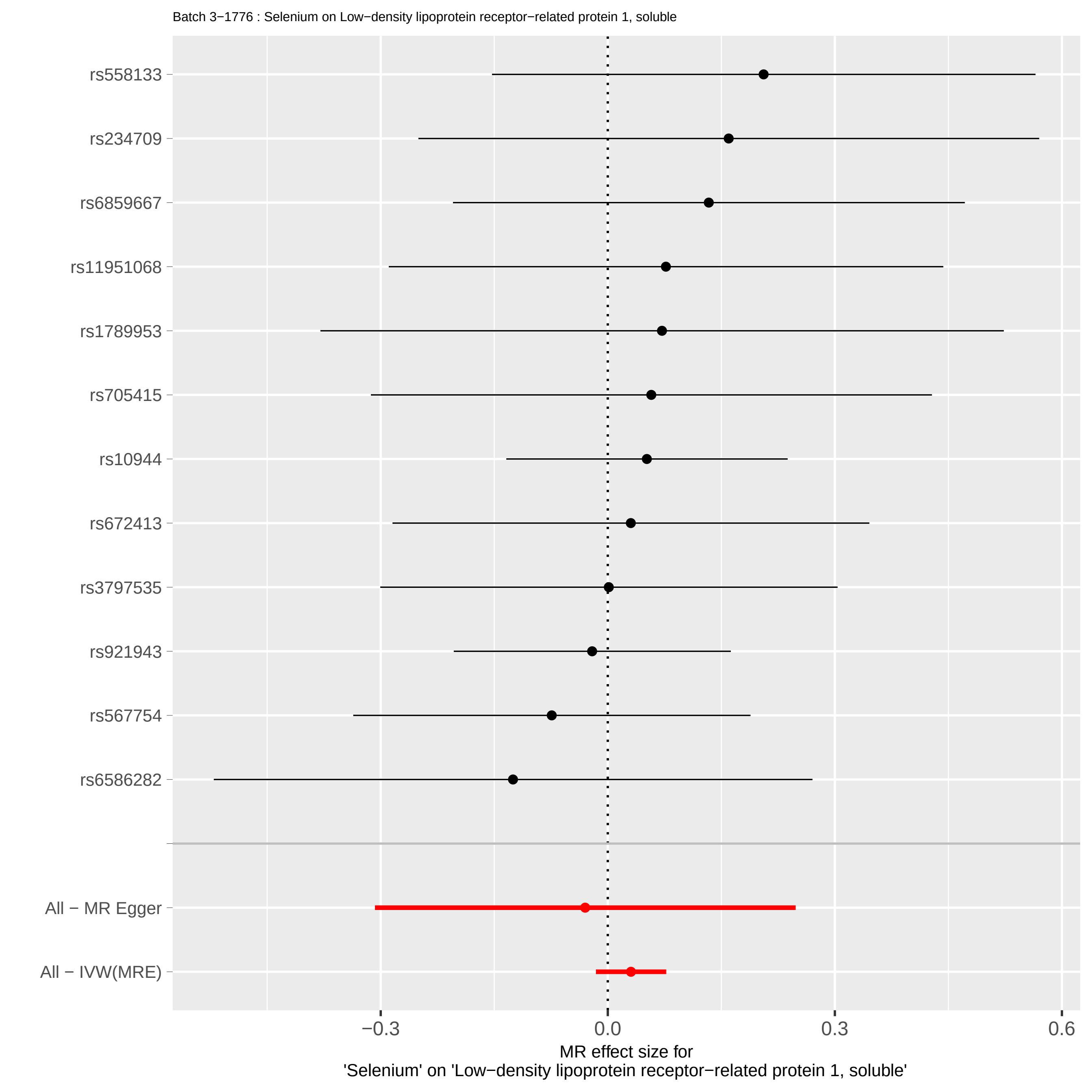

Batch 3−1776 : Selenium on Low−density lipoprotein receptor−related protein 1, soluble
rs558133
rs234709
rs6859667
rs11951068
rs1789953
rs705415
rs10944
rs672413
rs3797535
rs921943
rs567754
rs6586282
All − MR Egger
All − IVW(MRE)
−0.3
0.0
0.3
0.6
MR effect size for
'Selenium' on 'Low−density lipoprotein receptor−related protein 1, soluble'

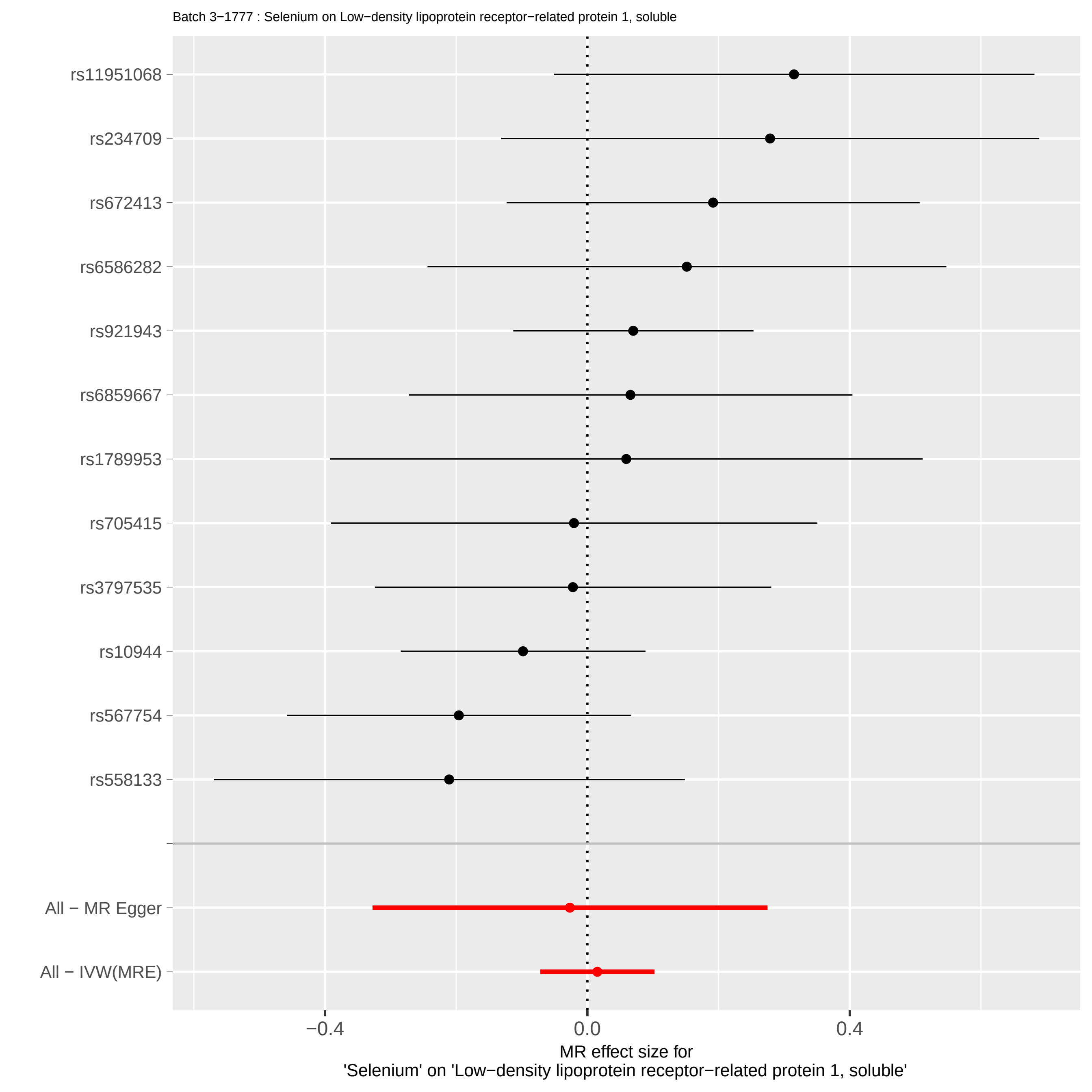

Batch 3−1777 : Selenium on Low−density lipoprotein receptor−related protein 1, soluble
rs11951068
rs234709
rs672413
rs6586282
rs921943
rs6859667
rs1789953
rs705415
rs3797535
rs10944
rs567754
rs558133
All − MR Egger
All − IVW(MRE)
−0.4
0.0
0.4
MR effect size for
'Selenium' on 'Low−density lipoprotein receptor−related protein 1, soluble'

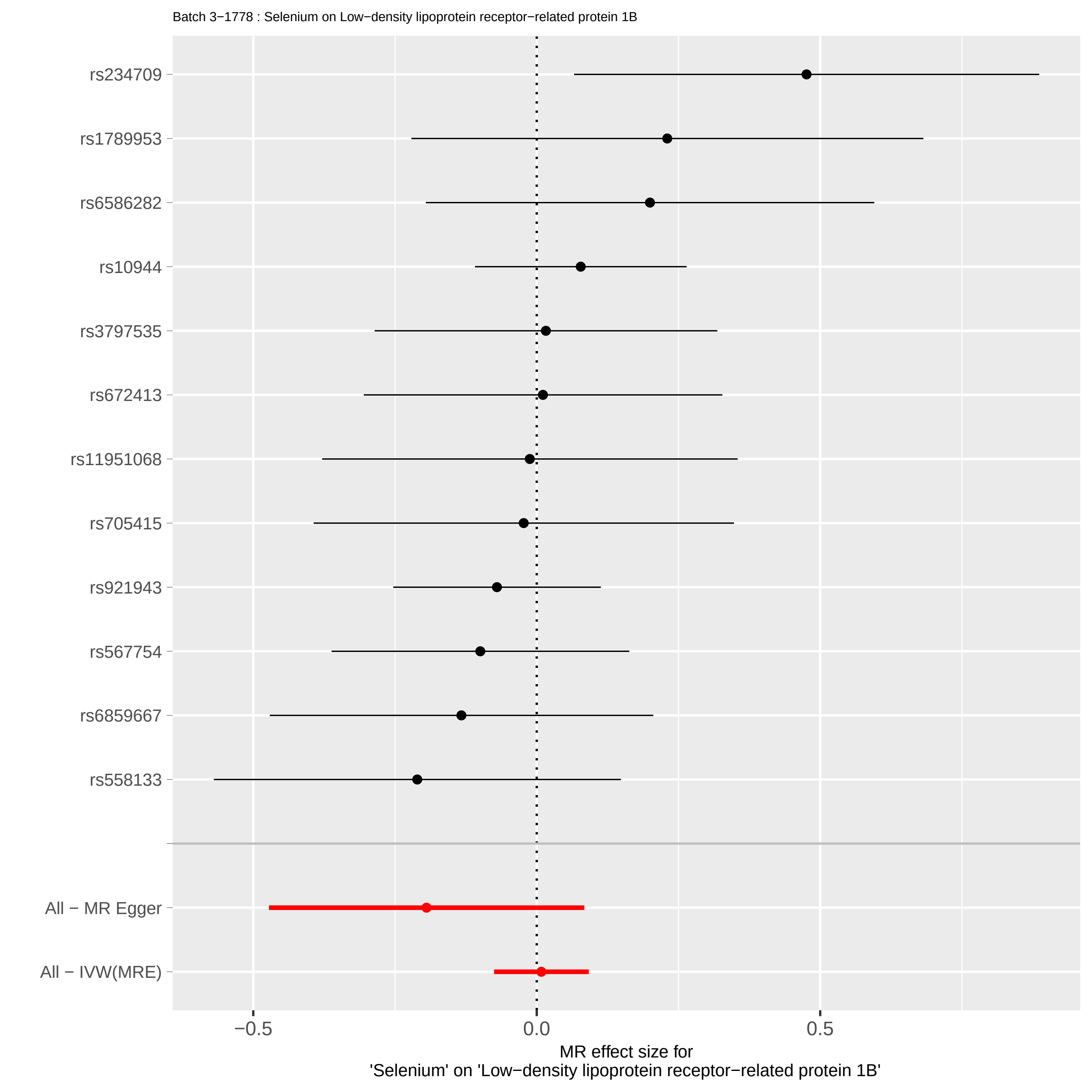

Batch 3−1778 : Selenium on Low−density lipoprotein receptor−related protein 1B
rs234709
rs1789953
rs6586282
rs10944
rs3797535
rs672413
rs11951068
rs705415
rs921943
rs567754
rs6859667
rs558133
All − MR Egger
All − IVW(MRE)
−0.5
0.0
0.5
MR effect size for
'Selenium' on 'Low−density lipoprotein receptor−related protein 1B'

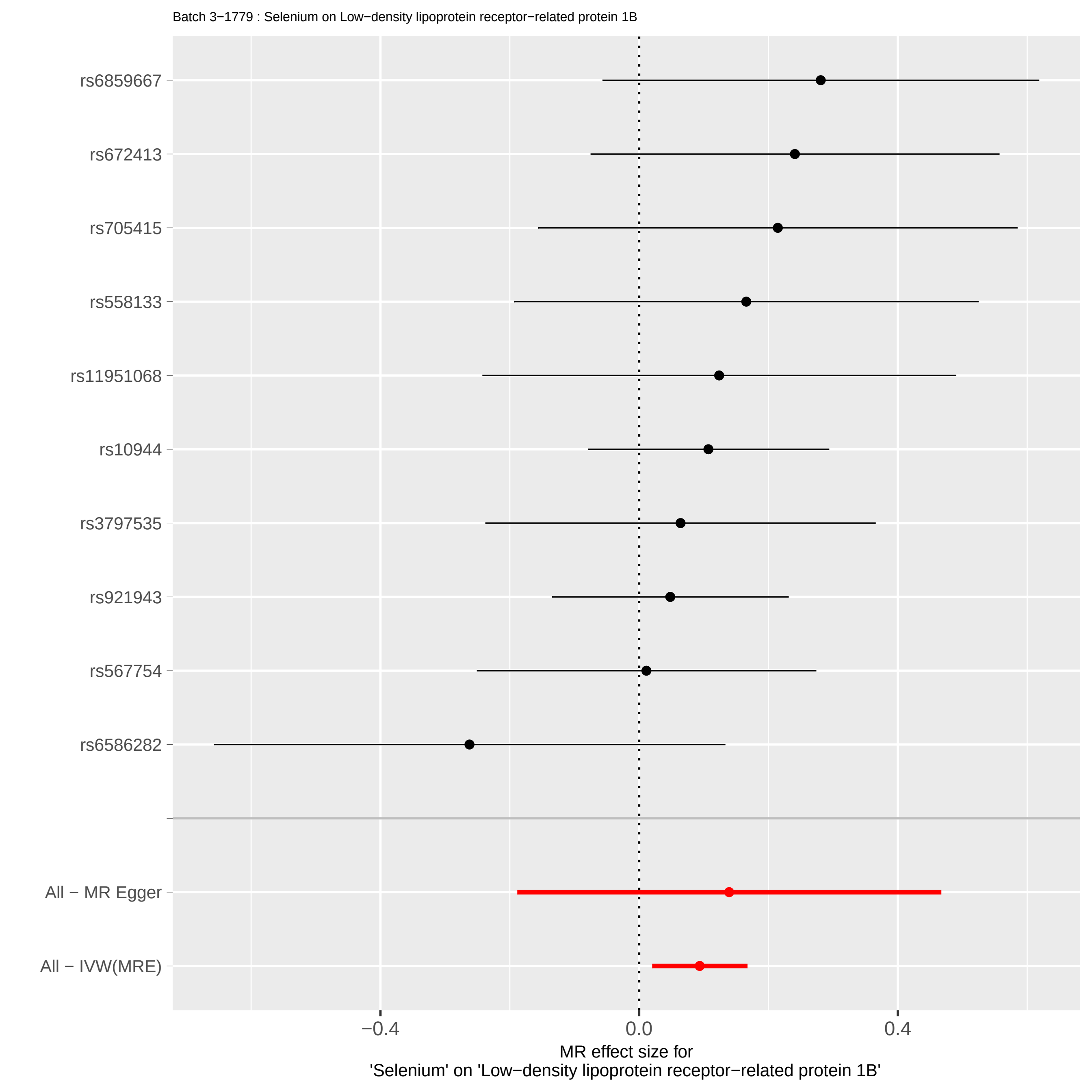

Batch 3−1779 : Selenium on Low−density lipoprotein receptor−related protein 1B
rs6859667
rs672413
rs705415
rs558133
rs11951068
rs10944
rs3797535
rs921943
rs567754
rs6586282
All − MR Egger
All − IVW(MRE)
−0.4
0.0
0.4
MR effect size for
'Selenium' on 'Low−density lipoprotein receptor−related protein 1B'

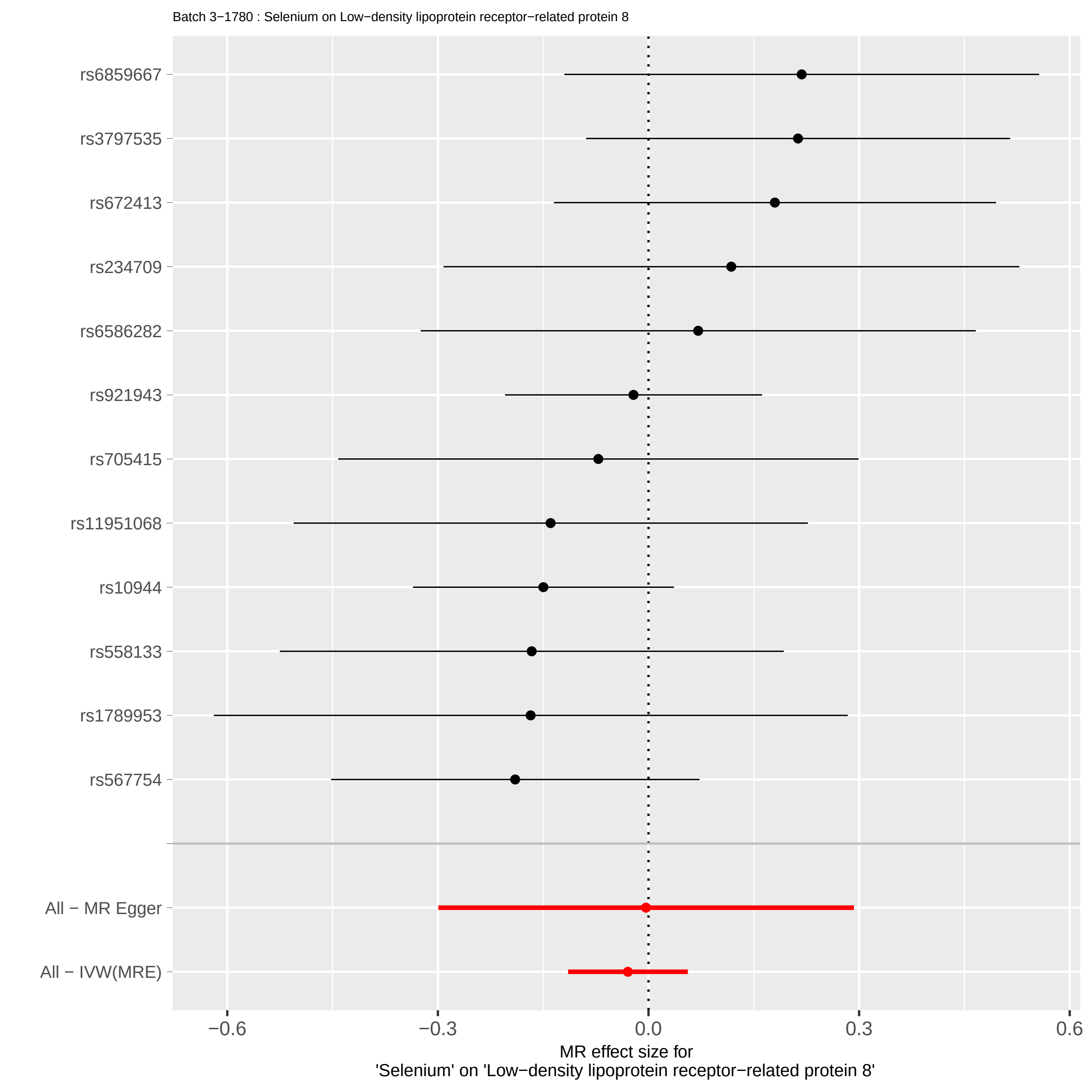
Batch 3−1780 : Selenium on Low−density lipoprotein receptor−related protein 8
rs6859667
rs3797535
rs672413
rs234709
rs6586282
rs921943
rs705415
rs11951068
rs10944
rs558133
rs1789953
rs567754
All − MR Egger
All − IVW(MRE)
−0.6
−0.3
0.0
0.3
0.6
MR effect size for
'Selenium' on 'Low−density lipoprotein receptor−related protein 8'

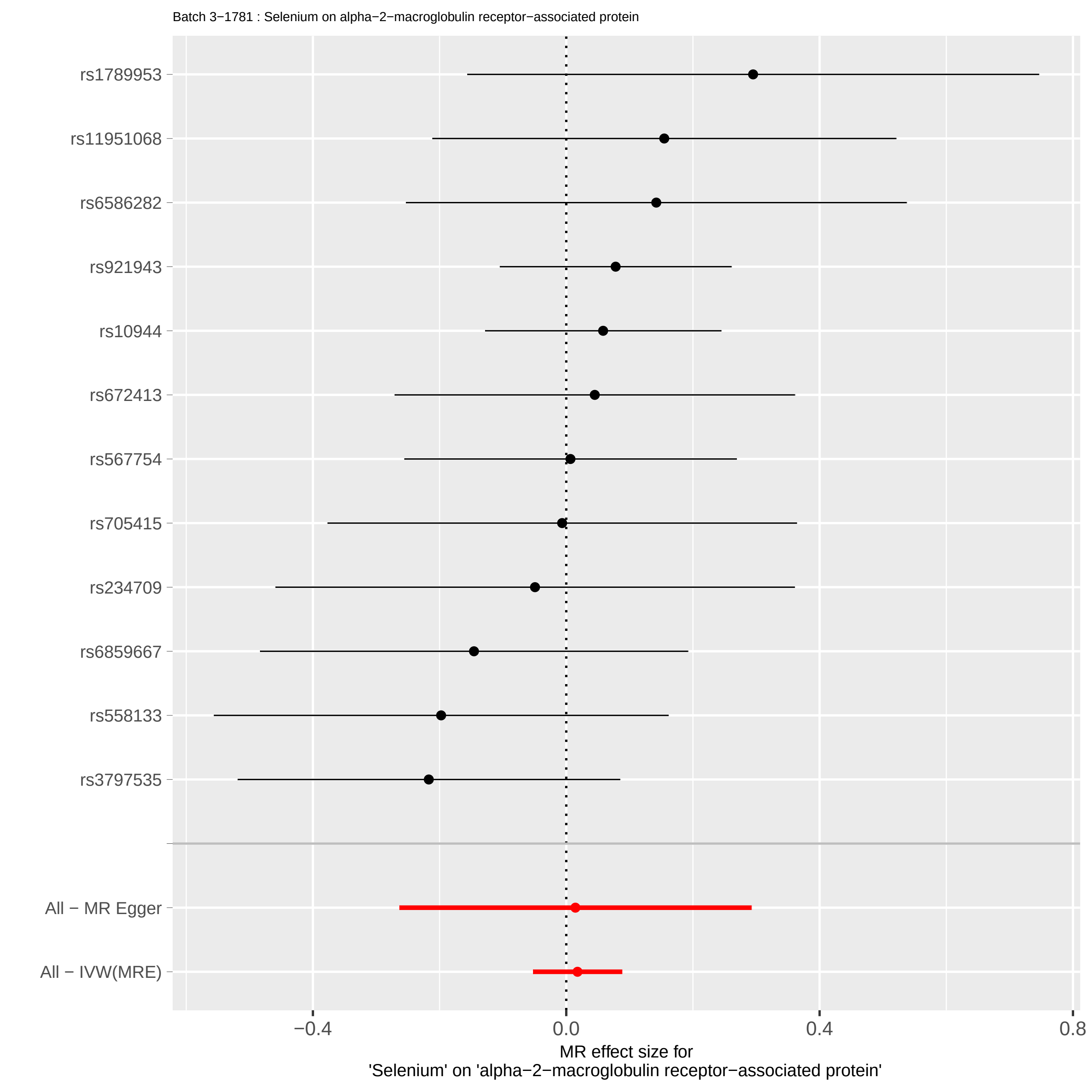
Batch 3−1781 : Selenium on alpha−2−macroglobulin receptor−associated protein
rs1789953
rs11951068
rs6586282
rs921943
rs10944
rs672413
rs567754
rs705415
rs234709
rs6859667
rs558133
rs3797535
All − MR Egger
All − IVW(MRE)
−0.4
0.0
0.4
0.8
MR effect size for
'Selenium' on 'alpha−2−macroglobulin receptor−associated protein'

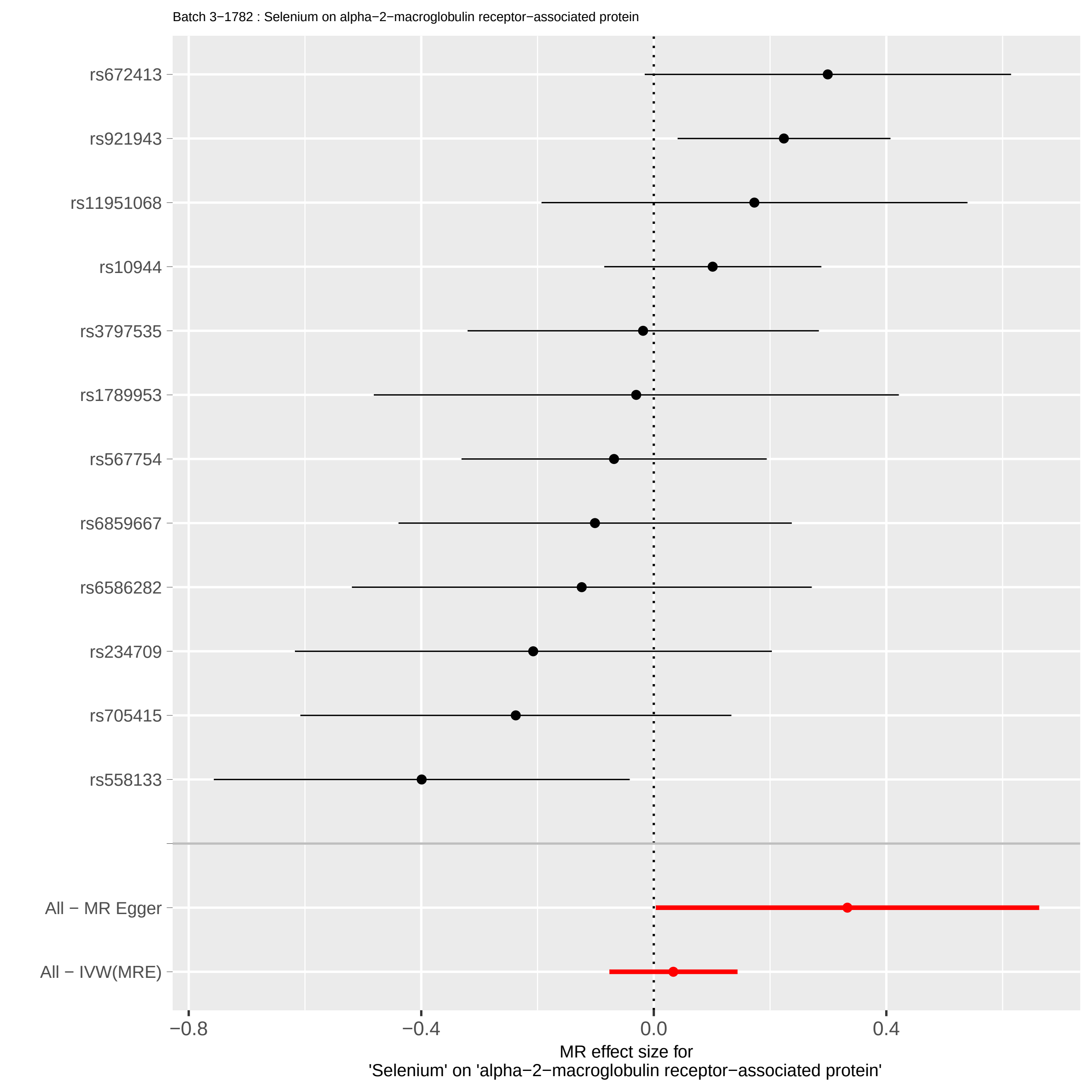

Batch 3−1782 : Selenium on alpha−2−macroglobulin receptor−associated protein
rs672413
rs921943
rs11951068
rs10944
rs3797535
rs1789953
rs567754
rs6859667
rs6586282
rs234709
rs705415
rs558133
All − MR Egger
All − IVW(MRE)
−0.8
−0.4
0.0
0.4
MR effect size for
'Selenium' on 'alpha−2−macroglobulin receptor−associated protein'

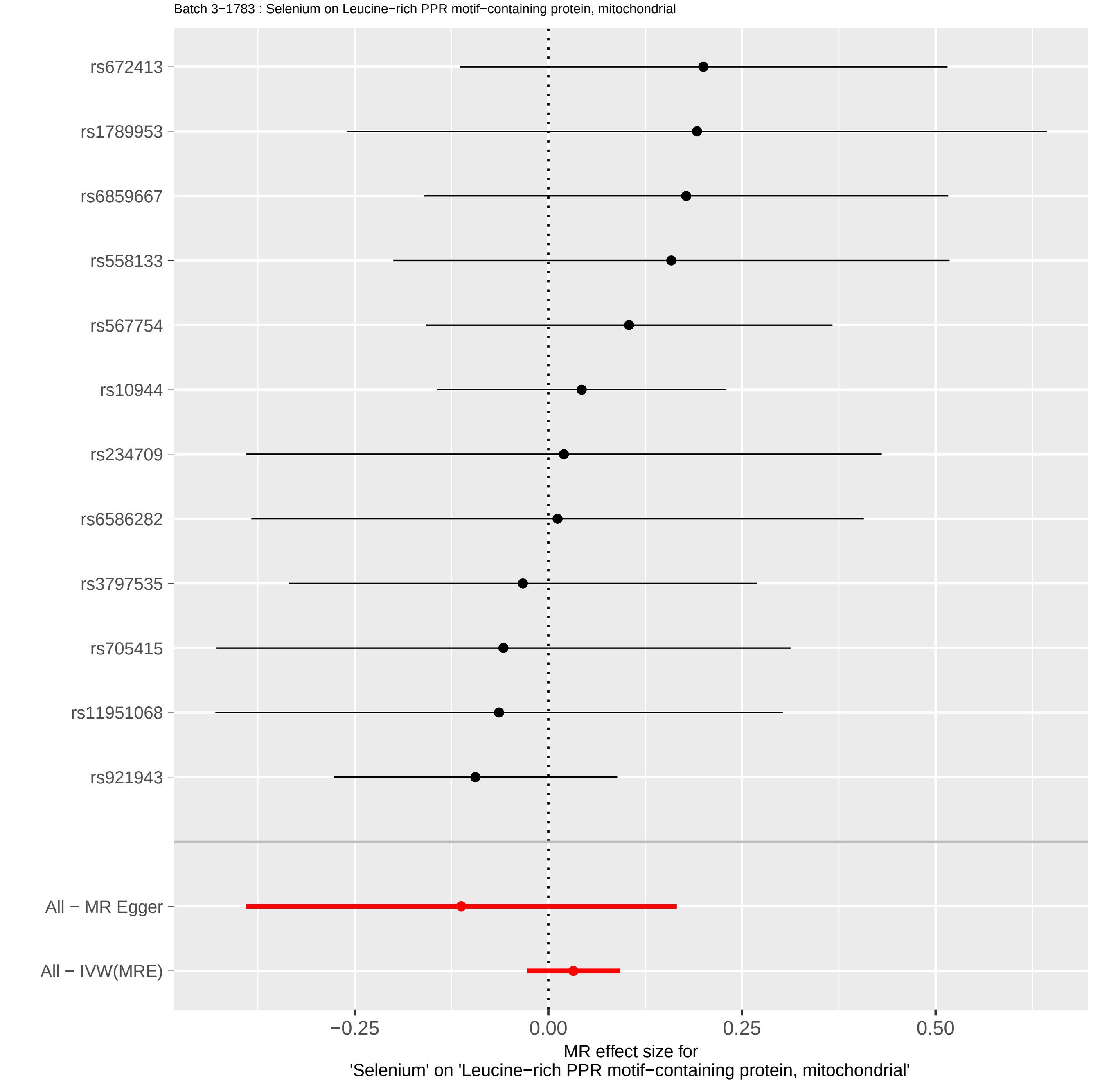

Batch 3−1783 : Selenium on Leucine−rich PPR motif−containing protein, mitochondrial
rs672413
rs1789953
rs6859667
rs558133
rs567754
rs10944
rs234709
rs6586282
rs3797535
rs705415
rs11951068
rs921943
All − MR Egger
All − IVW(MRE)
−0.25
0.00
0.25
0.50
MR effect size for
'Selenium' on 'Leucine−rich PPR motif−containing protein, mitochondrial'

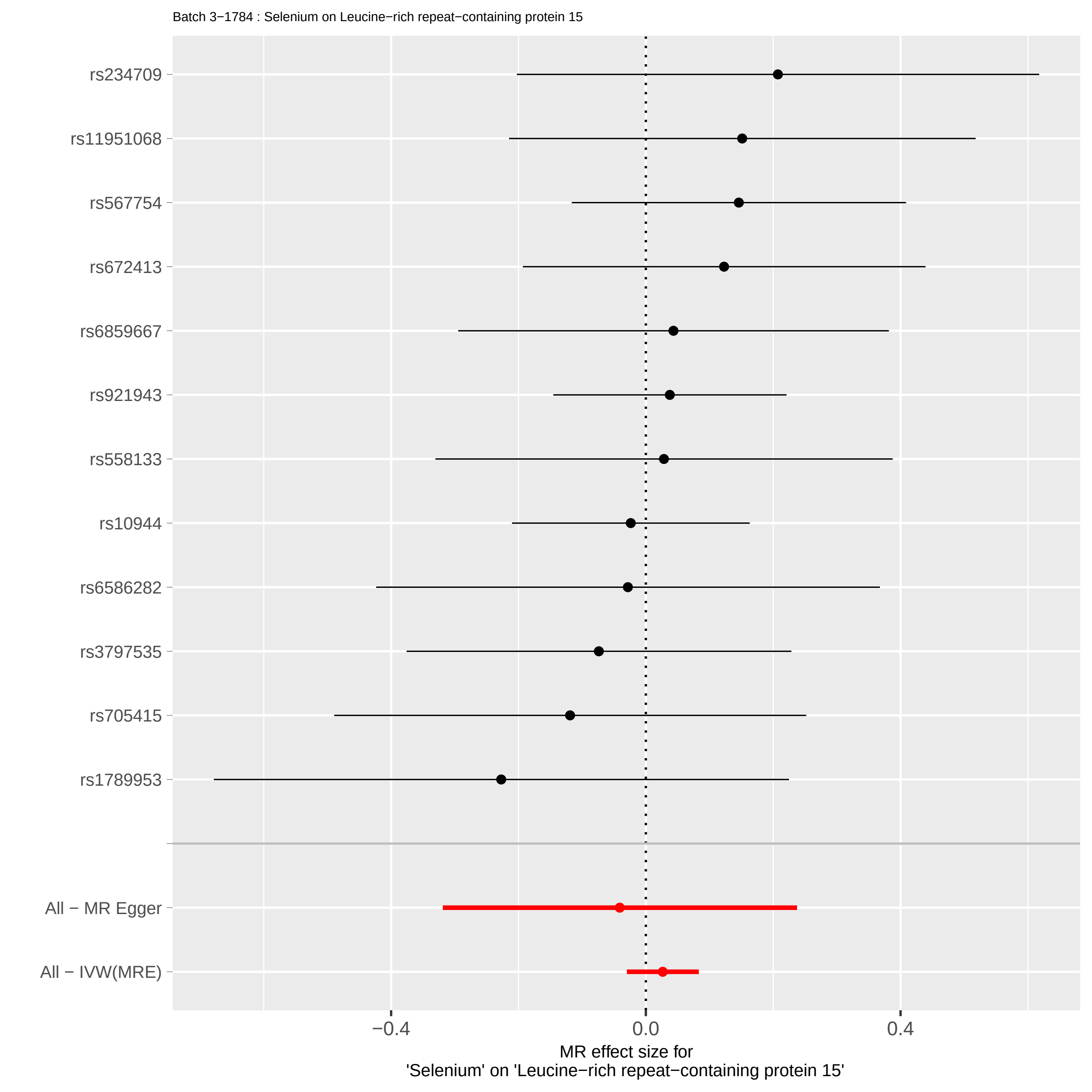
Batch 3−1784 : Selenium on Leucine−rich repeat−containing protein 15
rs234709
rs11951068
rs567754
rs672413
rs6859667
rs921943
rs558133
rs10944
rs6586282
rs3797535
rs705415
rs1789953
All − MR Egger
All − IVW(MRE)
−0.4
0.0
0.4
MR effect size for
'Selenium' on 'Leucine−rich repeat−containing protein 15'

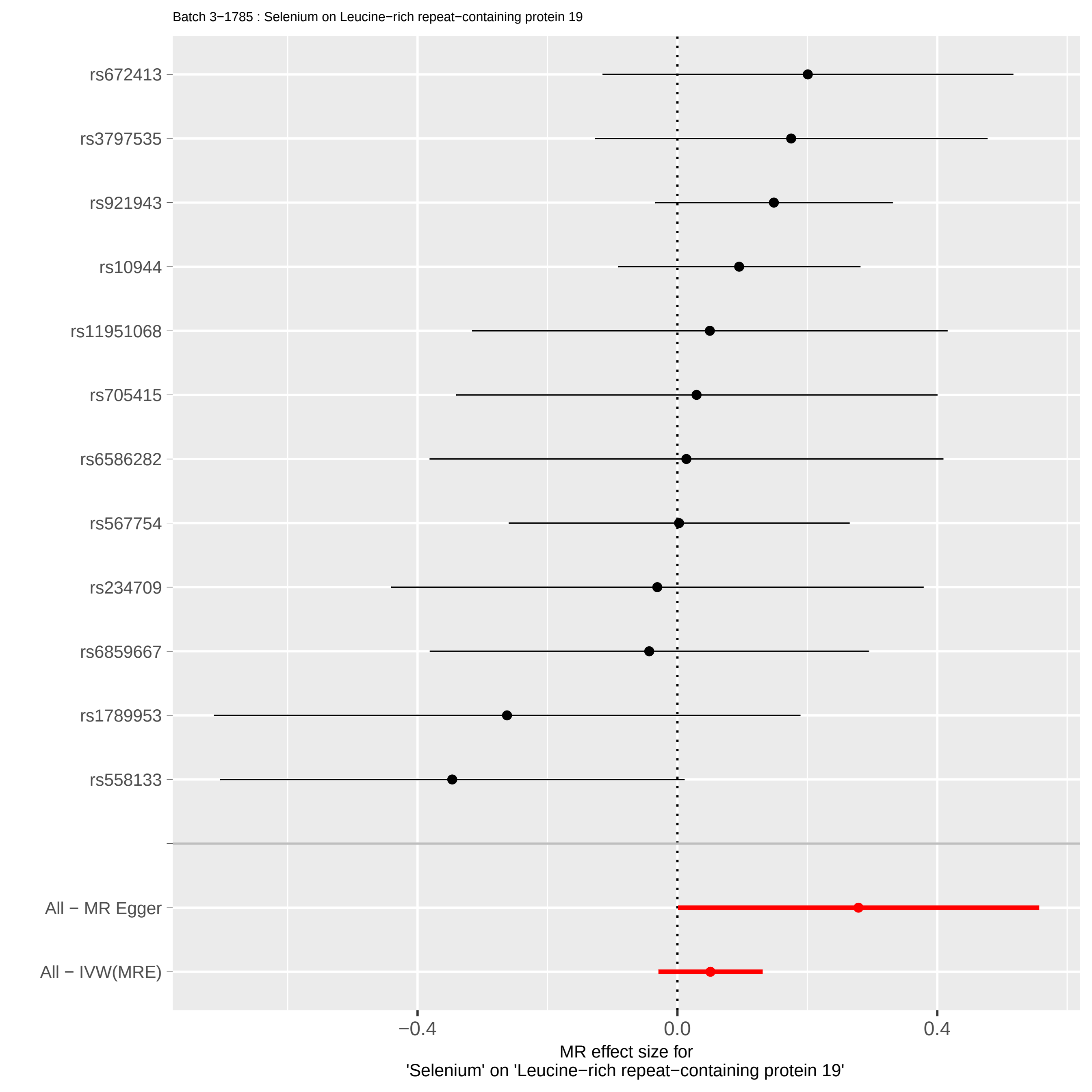

Batch 3−1785 : Selenium on Leucine−rich repeat−containing protein 19
rs672413
rs3797535
rs921943
rs10944
rs11951068
rs705415
rs6586282
rs567754
rs234709
rs6859667
rs1789953
rs558133
All − MR Egger
All − IVW(MRE)
−0.4
0.0
0.4
MR effect size for
'Selenium' on 'Leucine−rich repeat−containing protein 19'

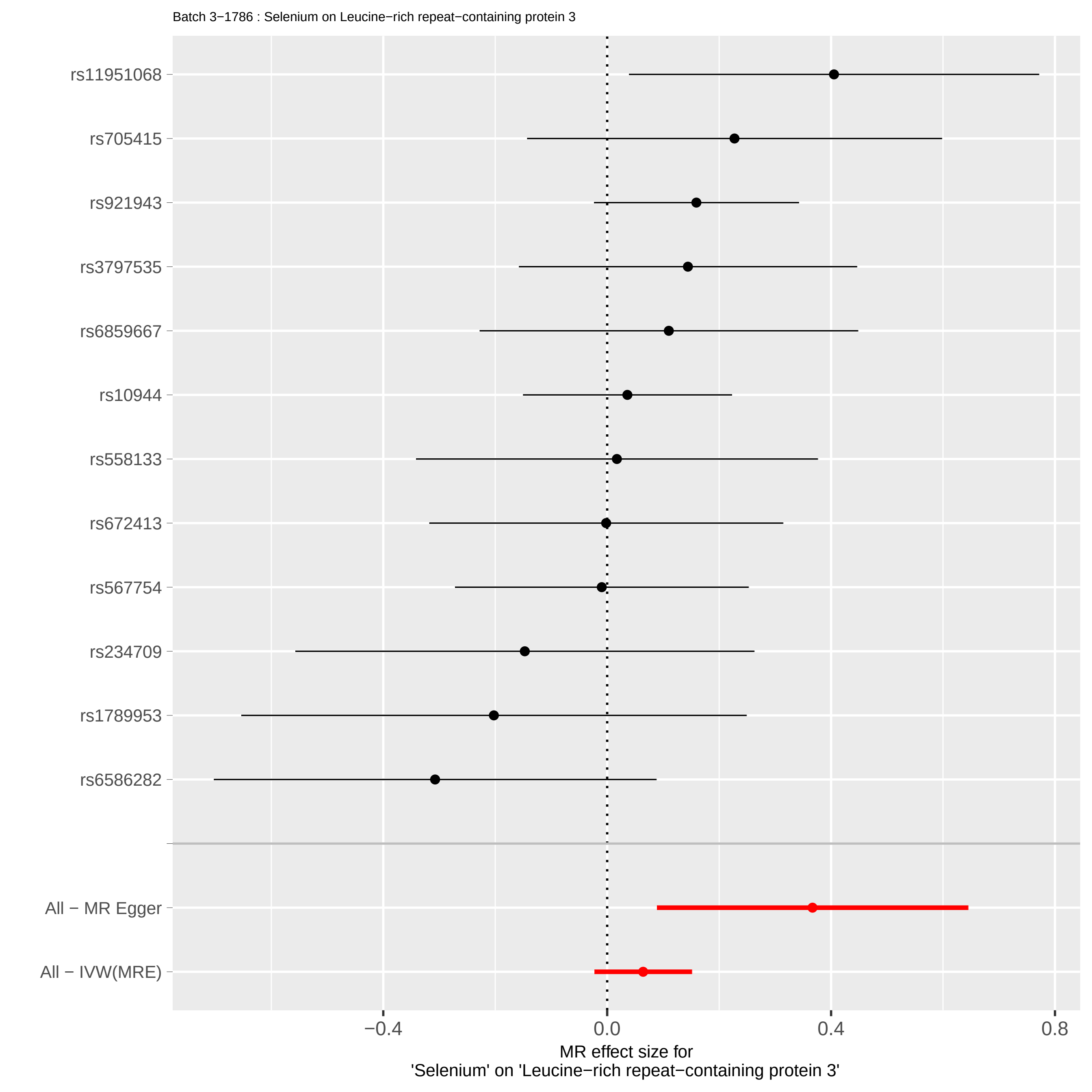

Batch 3−1786 : Selenium on Leucine−rich repeat−containing protein 3
rs11951068
rs705415
rs921943
rs3797535
rs6859667
rs10944
rs558133
rs672413
rs567754
rs234709
rs1789953
rs6586282
All − MR Egger
All − IVW(MRE)
−0.4
0.0
0.4
0.8
MR effect size for
'Selenium' on 'Leucine−rich repeat−containing protein 3'

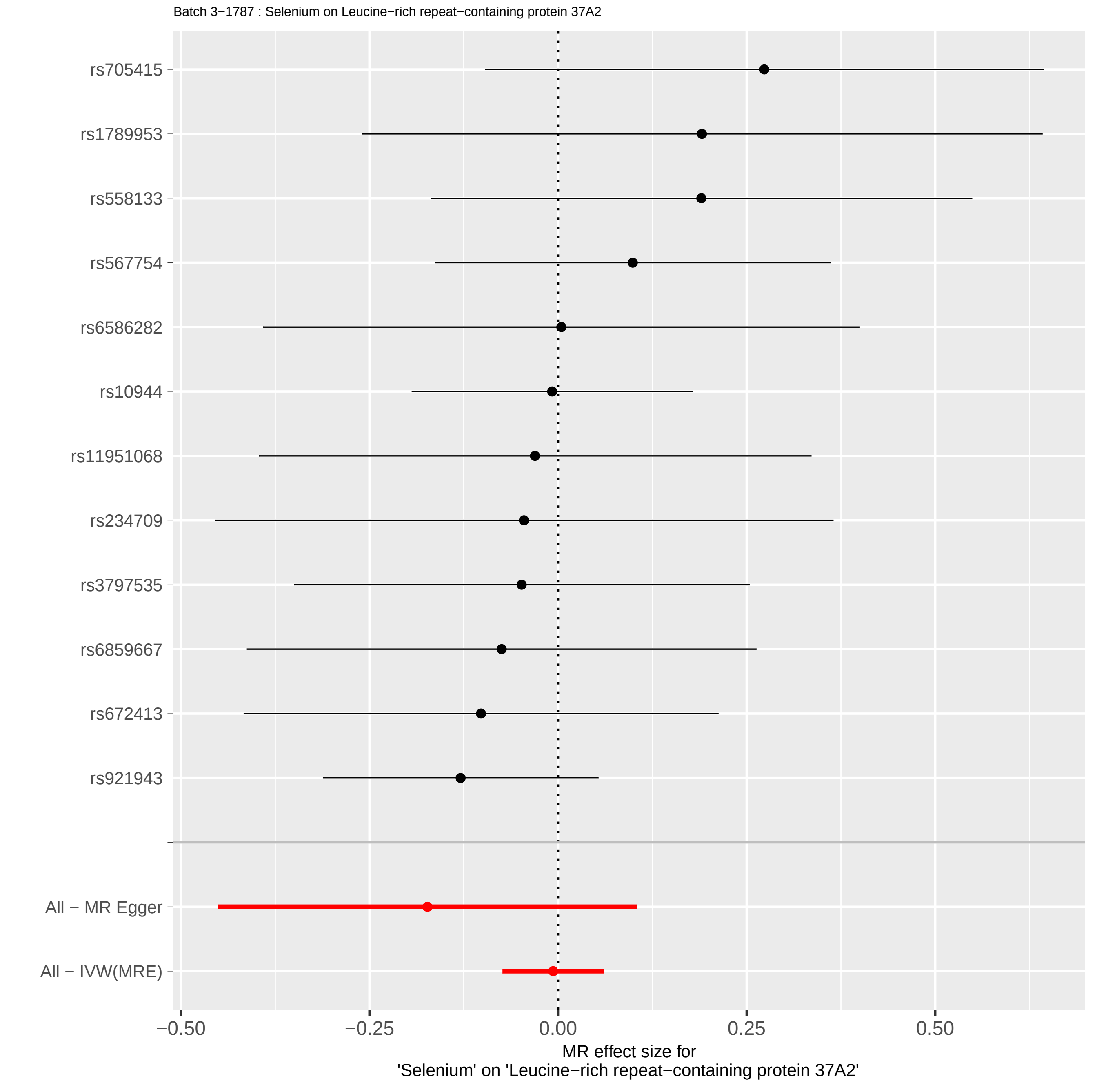

Batch 3−1787 : Selenium on Leucine−rich repeat−containing protein 37A2
rs705415
rs1789953
rs558133
rs567754
rs6586282
rs10944
rs11951068
rs234709
rs3797535
rs6859667
rs672413
rs921943
All − MR Egger
All − IVW(MRE)
−0.50
−0.25
0.00
0.25
0.50
MR effect size for
'Selenium' on 'Leucine−rich repeat−containing protein 37A2'

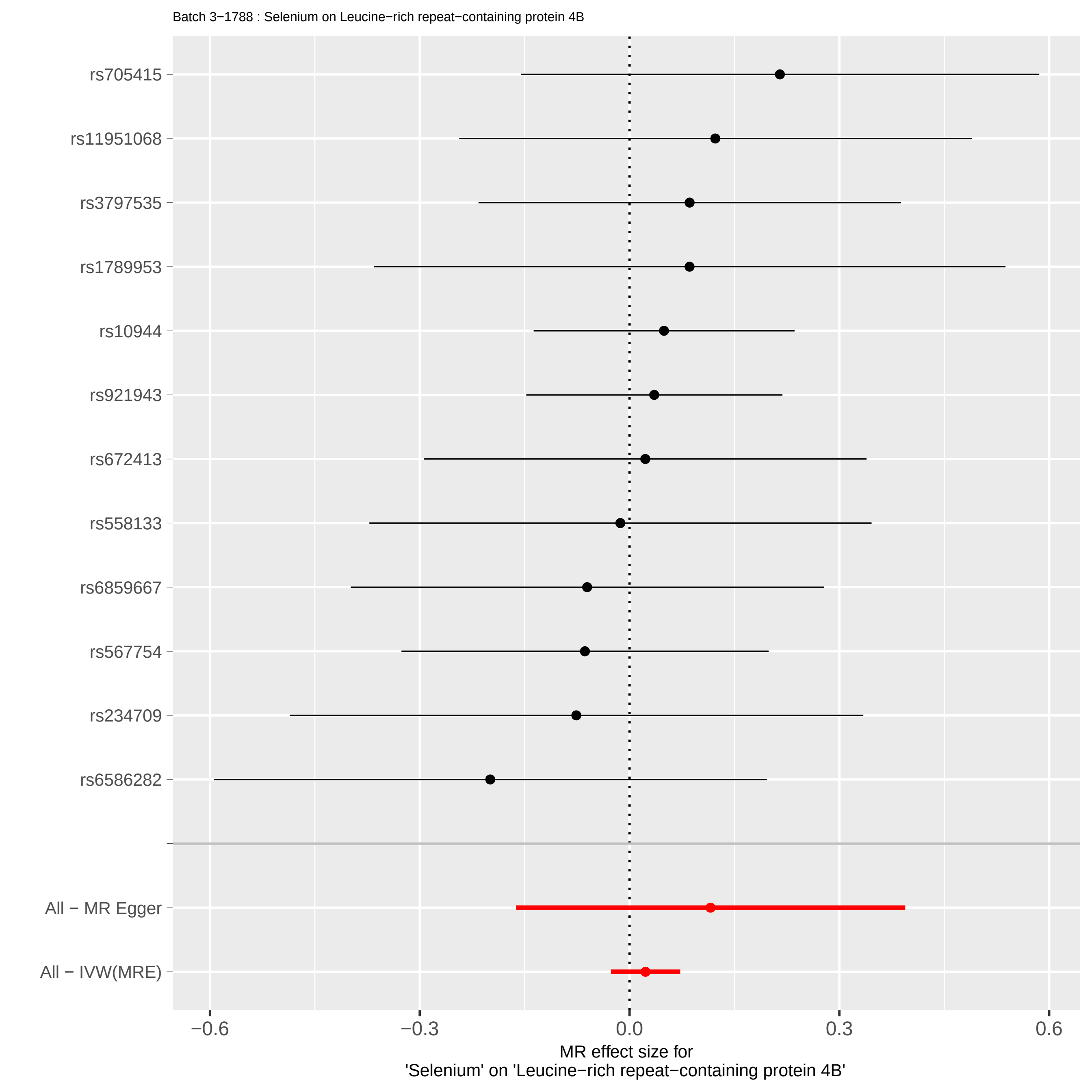
Batch 3−1788 : Selenium on Leucine−rich repeat−containing protein 4B
rs705415
rs11951068
rs3797535
rs1789953
rs10944
rs921943
rs672413
rs558133
rs6859667
rs567754
rs234709
rs6586282
All − MR Egger
All − IVW(MRE)
−0.6
−0.3
0.0
0.3
0.6
MR effect size for
'Selenium' on 'Leucine−rich repeat−containing protein 4B'

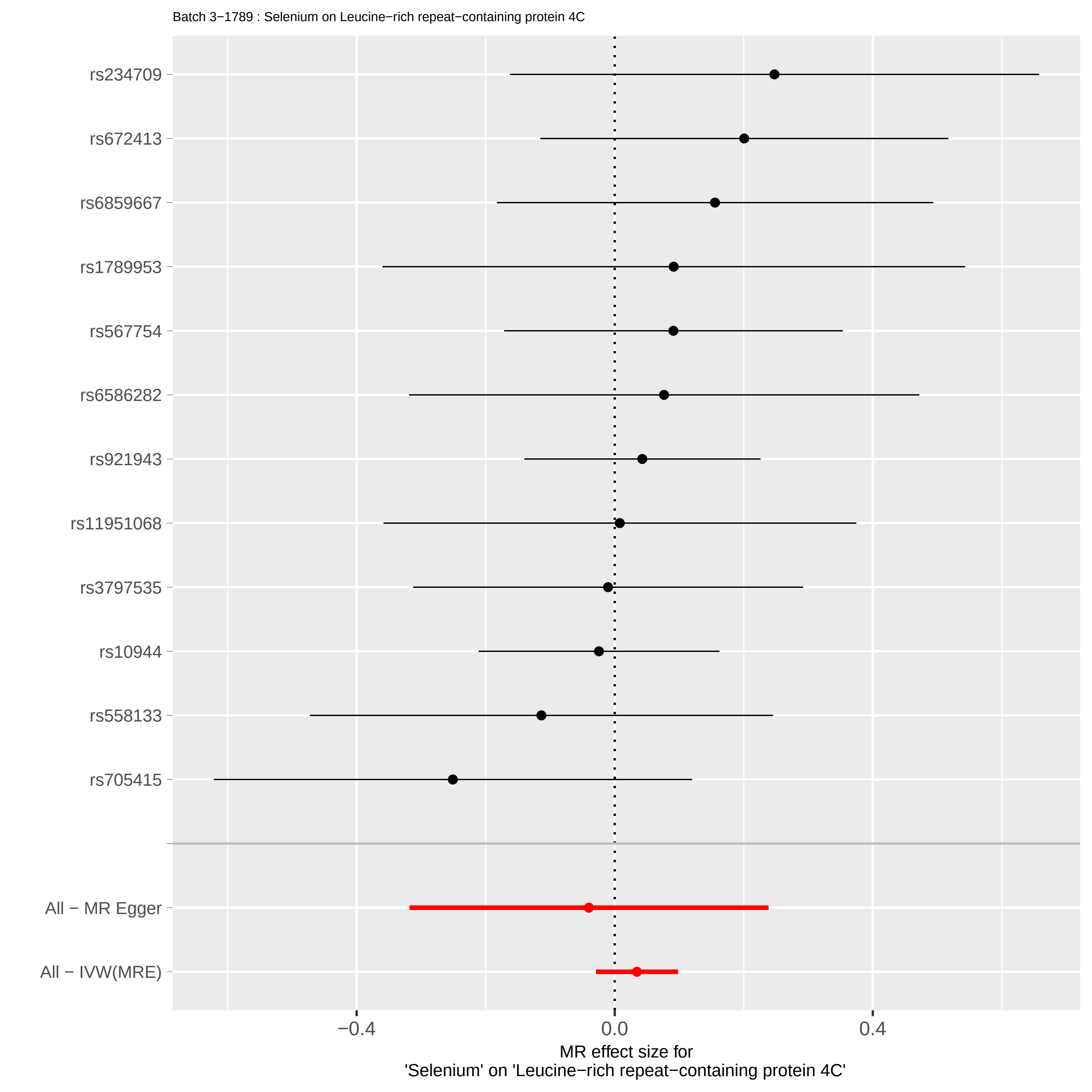

Batch 3−1789 : Selenium on Leucine−rich repeat−containing protein 4C
rs234709
rs672413
rs6859667
rs1789953
rs567754
rs6586282
rs921943
rs11951068
rs3797535
rs10944
rs558133
rs705415
All − MR Egger
All − IVW(MRE)
−0.4
0.0
0.4
MR effect size for
'Selenium' on 'Leucine−rich repeat−containing protein 4C'

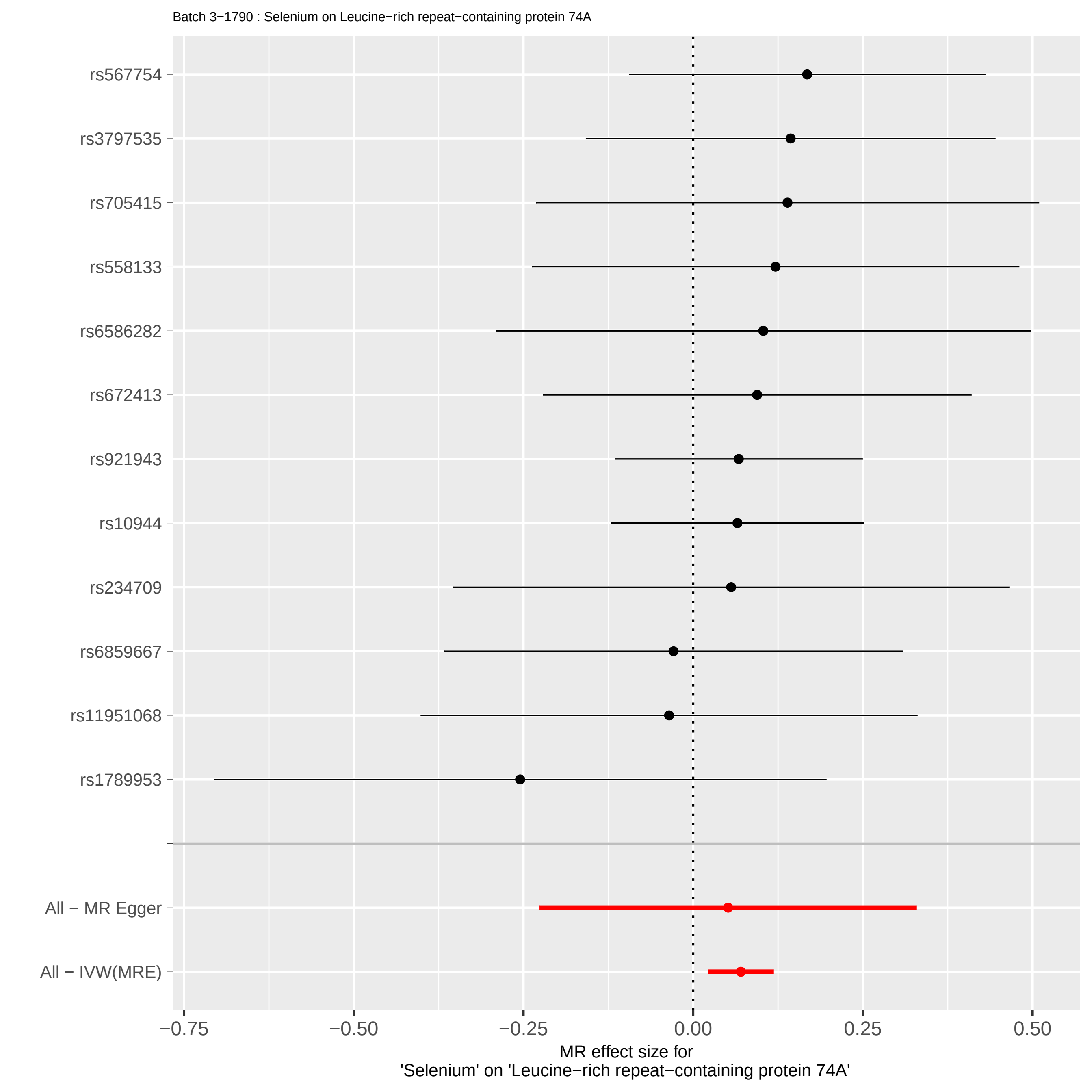

Batch 3−1790 : Selenium on Leucine−rich repeat−containing protein 74A
rs567754
rs3797535
rs705415
rs558133
rs6586282
rs672413
rs921943
rs10944
rs234709
rs6859667
rs11951068
rs1789953
All − MR Egger
All − IVW(MRE)
−0.75
−0.50
−0.25
0.00
0.25
0.50
MR effect size for
'Selenium' on 'Leucine−rich repeat−containing protein 74A'

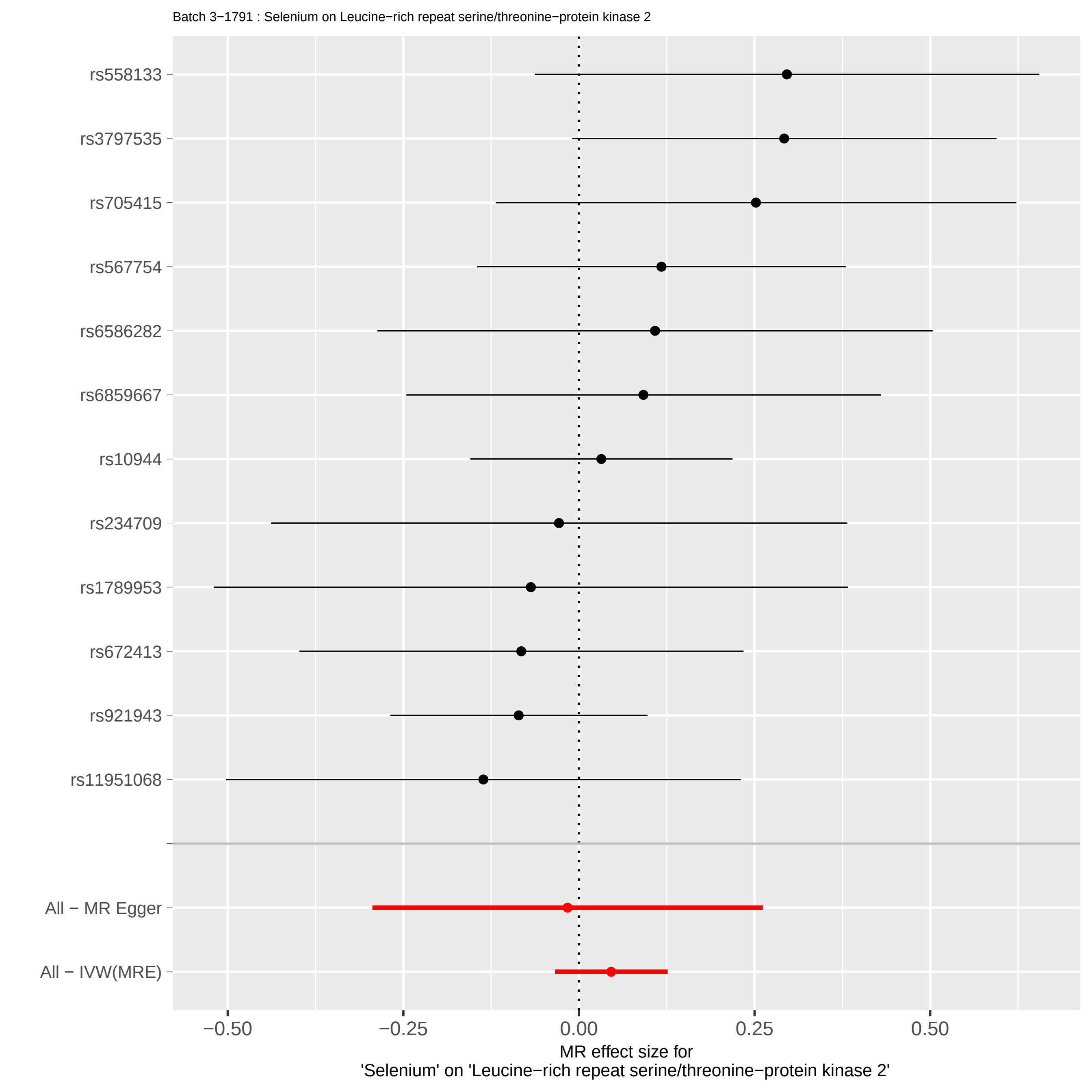

Batch 3−1791 : Selenium on Leucine−rich repeat serine/threonine−protein kinase 2
rs558133
rs3797535
rs705415
rs567754
rs6586282
rs6859667
rs10944
rs234709
rs1789953
rs672413
rs921943
rs11951068
All − MR Egger
All − IVW(MRE)
−0.50
−0.25
0.00
0.25
0.50
MR effect size for
'Selenium' on 'Leucine−rich repeat serine/threonine−protein kinase 2'

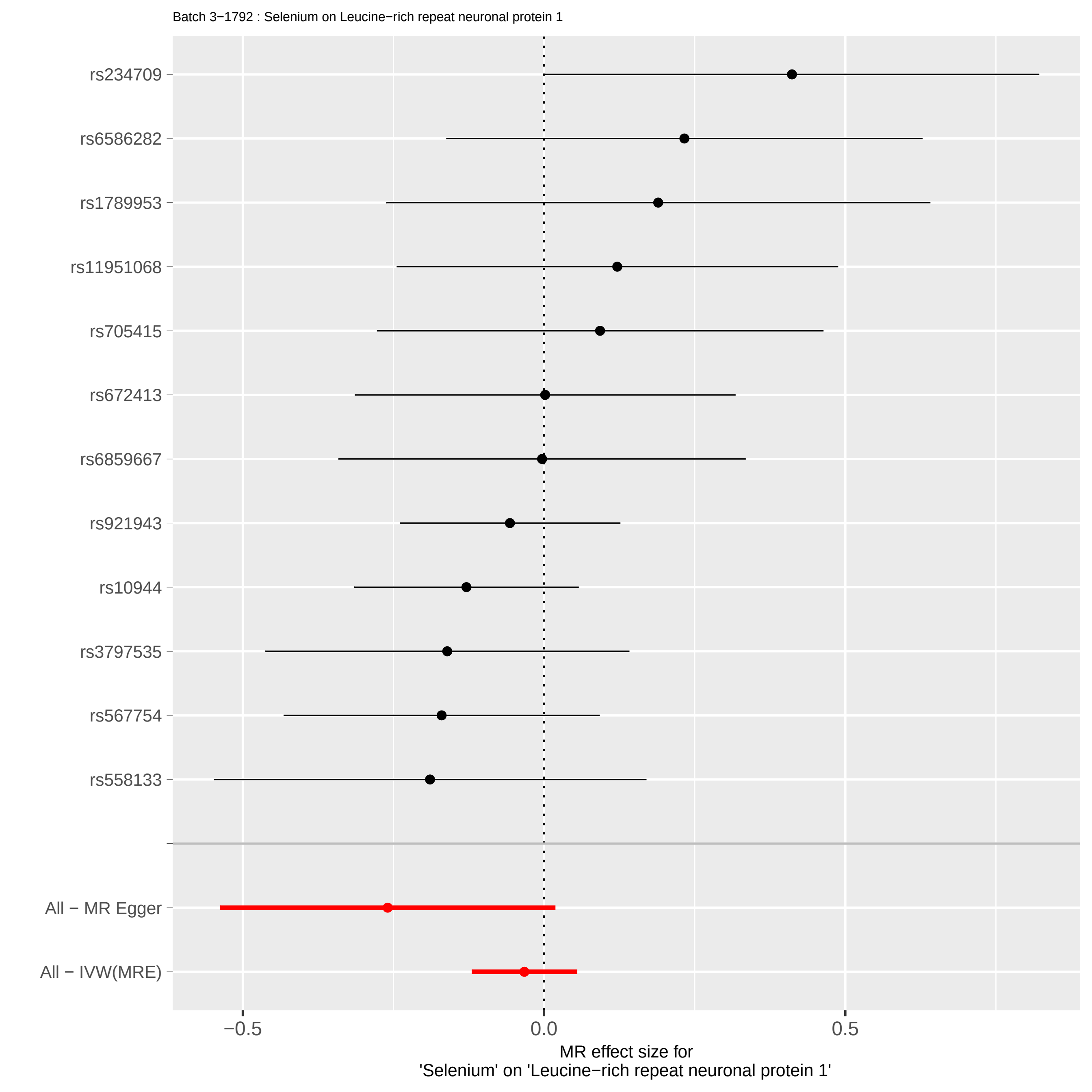

Batch 3−1792 : Selenium on Leucine−rich repeat neuronal protein 1
rs234709
rs6586282
rs1789953
rs11951068
rs705415
rs672413
rs6859667
rs921943
rs10944
rs3797535
rs567754
rs558133
All − MR Egger
All − IVW(MRE)
−0.5
0.0
0.5
MR effect size for
'Selenium' on 'Leucine−rich repeat neuronal protein 1'

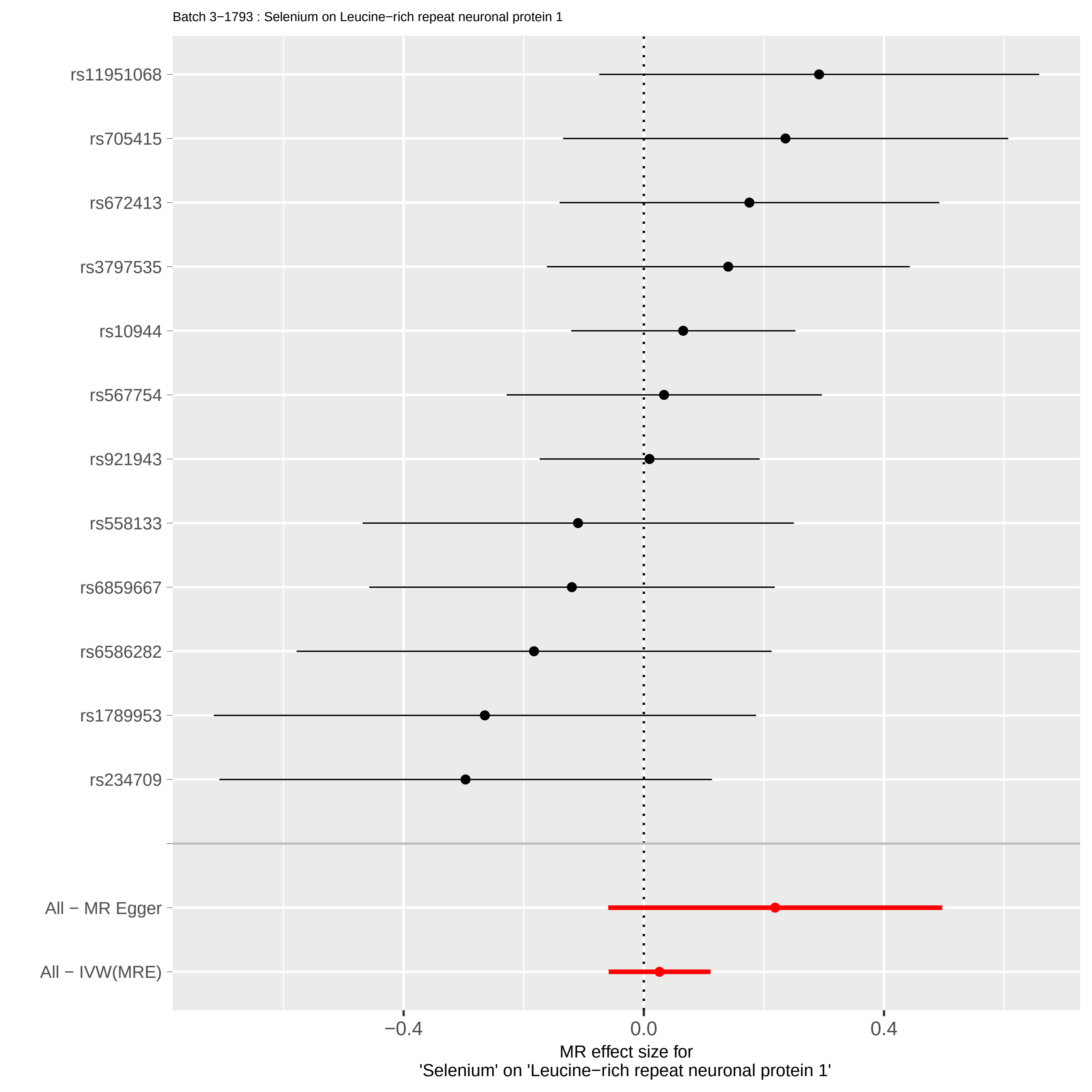
Batch 3−1793 : Selenium on Leucine−rich repeat neuronal protein 1
rs11951068
rs705415
rs672413
rs3797535
rs10944
rs567754
rs921943
rs558133
rs6859667
rs6586282
rs1789953
rs234709
All − MR Egger
All − IVW(MRE)
−0.4
0.0
0.4
MR effect size for
'Selenium' on 'Leucine−rich repeat neuronal protein 1'

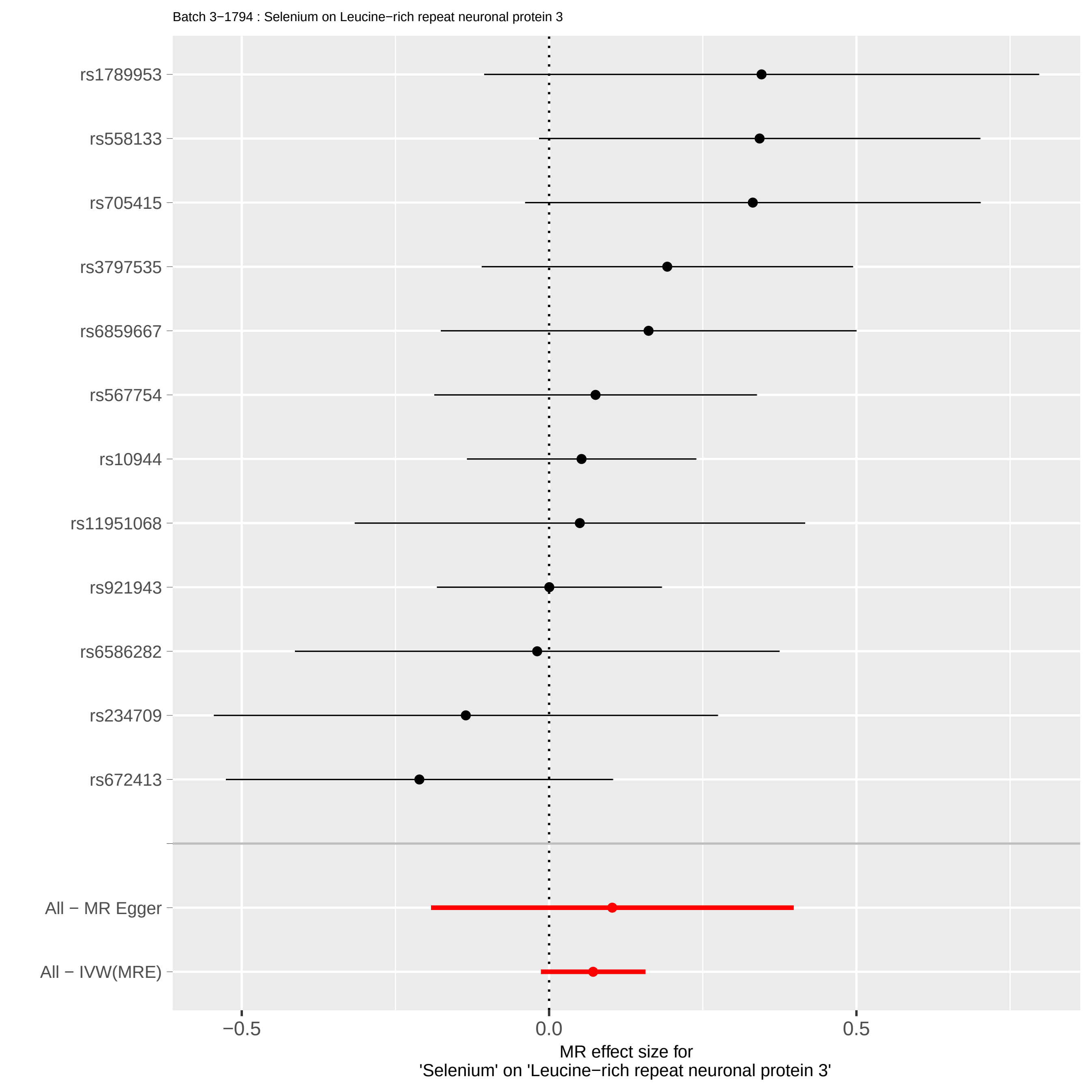

Batch 3−1794 : Selenium on Leucine−rich repeat neuronal protein 3
rs1789953
rs558133
rs705415
rs3797535
rs6859667
rs567754
rs10944
rs11951068
rs921943
rs6586282
rs234709
rs672413
All − MR Egger
All − IVW(MRE)
−0.5
0.0
0.5
MR effect size for
'Selenium' on 'Leucine−rich repeat neuronal protein 3'

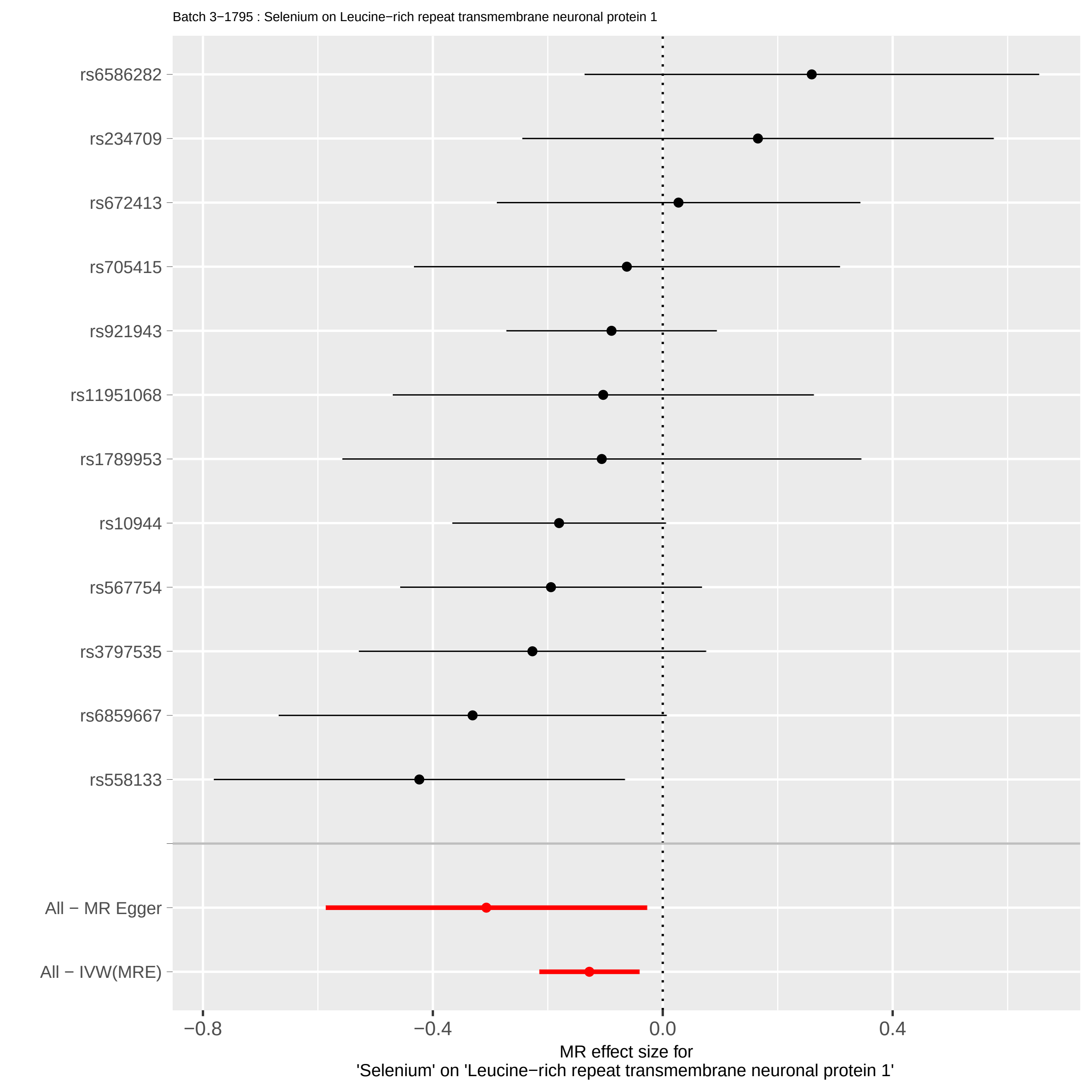
Batch 3−1795 : Selenium on Leucine−rich repeat transmembrane neuronal protein 1
rs6586282
rs234709
rs672413
rs705415
rs921943
rs11951068
rs1789953
rs10944
rs567754
rs3797535
rs6859667
rs558133
All − MR Egger
All − IVW(MRE)
−0.8
−0.4
0.0
0.4
MR effect size for
'Selenium' on 'Leucine−rich repeat transmembrane neuronal protein 1'

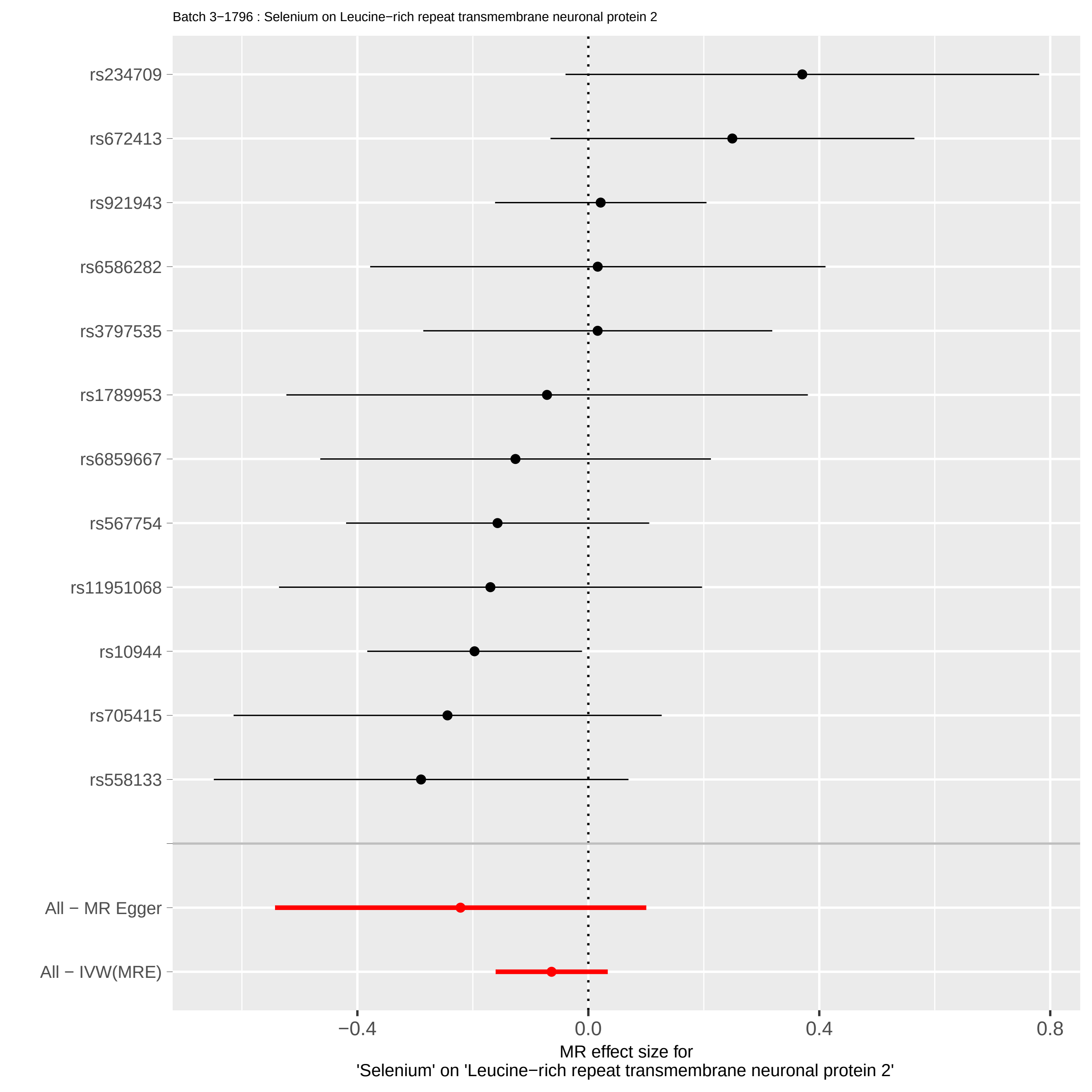
Batch 3−1796 : Selenium on Leucine−rich repeat transmembrane neuronal protein 2
rs234709
rs672413
rs921943
rs6586282
rs3797535
rs1789953
rs6859667
rs567754
rs11951068
rs10944
rs705415
rs558133
All − MR Egger
All − IVW(MRE)
−0.4
0.0
0.4
0.8
MR effect size for
'Selenium' on 'Leucine−rich repeat transmembrane neuronal protein 2'

Batch 3−1797 : Selenium on Leucine−rich repeat transmembrane neuronal protein 4

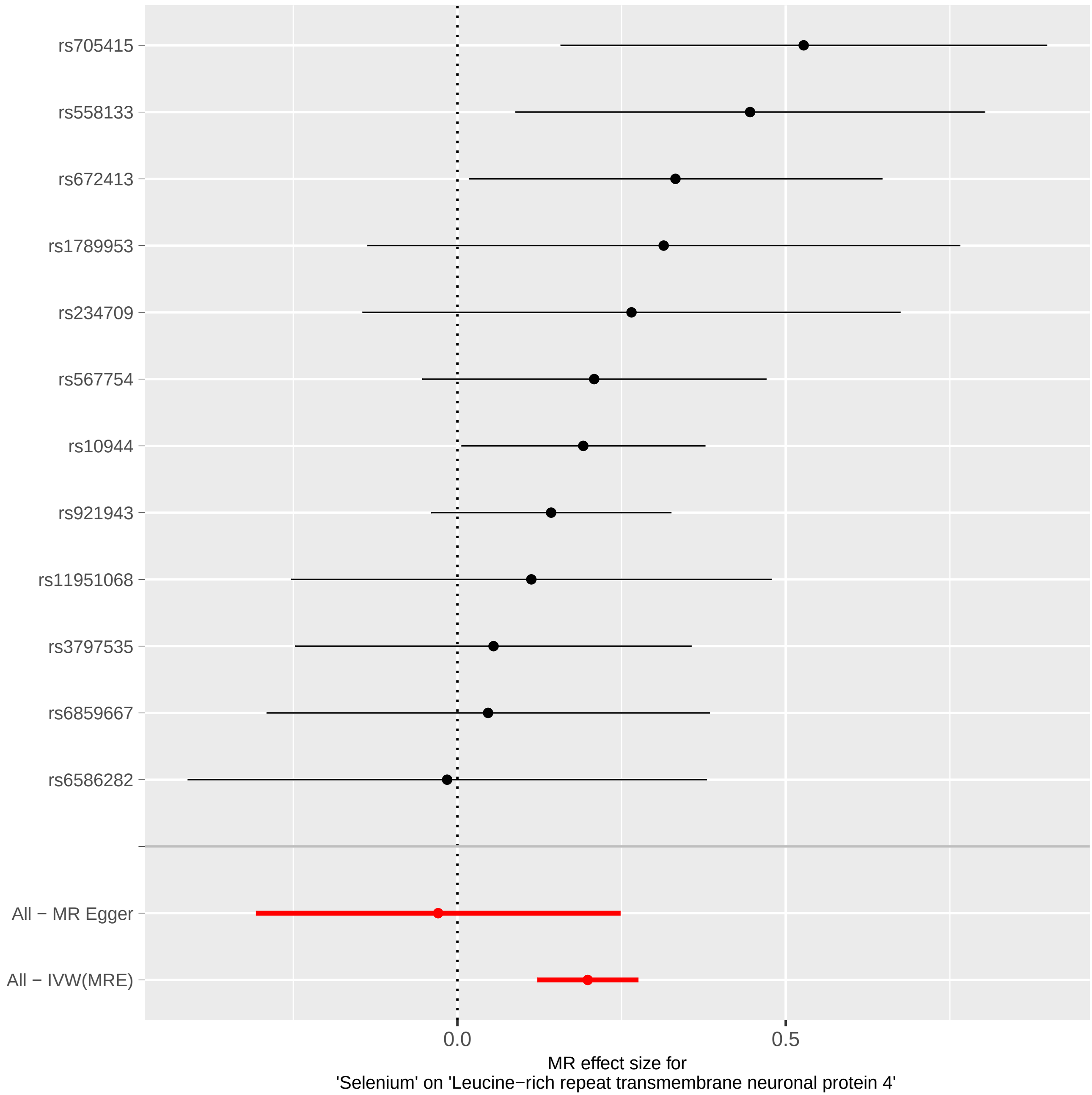

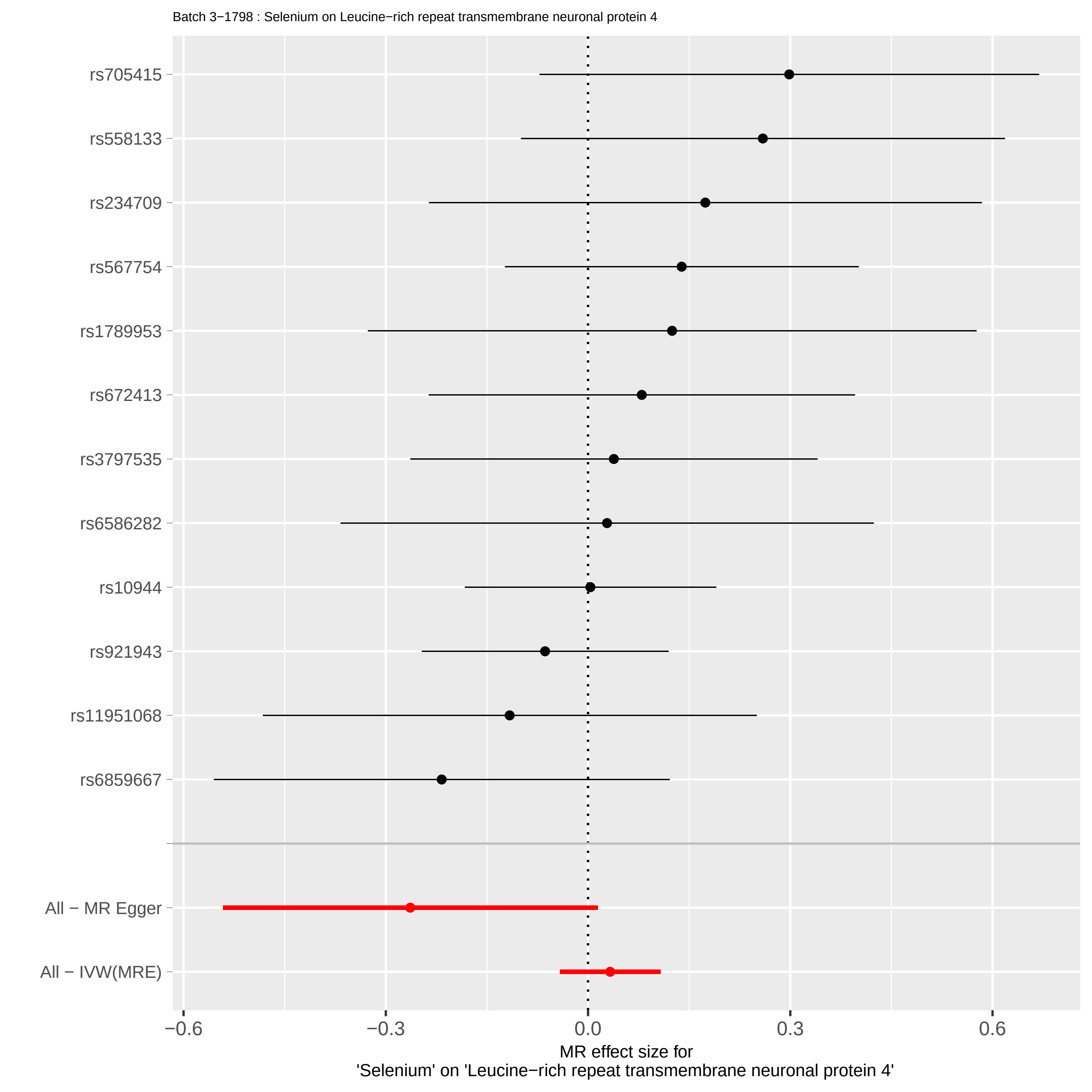

Batch 3−1798 : Selenium on Leucine−rich repeat transmembrane neuronal protein 4
rs705415
rs558133
rs234709
rs567754
rs1789953
rs672413
rs3797535
rs6586282
rs10944
rs921943
rs11951068
rs6859667
All − MR Egger
All − IVW(MRE)
−0.6
−0.3
0.0
0.3
0.6
MR effect size for
'Selenium' on 'Leucine−rich repeat transmembrane neuronal protein 4'

Batch 3−1799 : Selenium on Leucine−rich repeat and transmembrane domain−containing protein 1

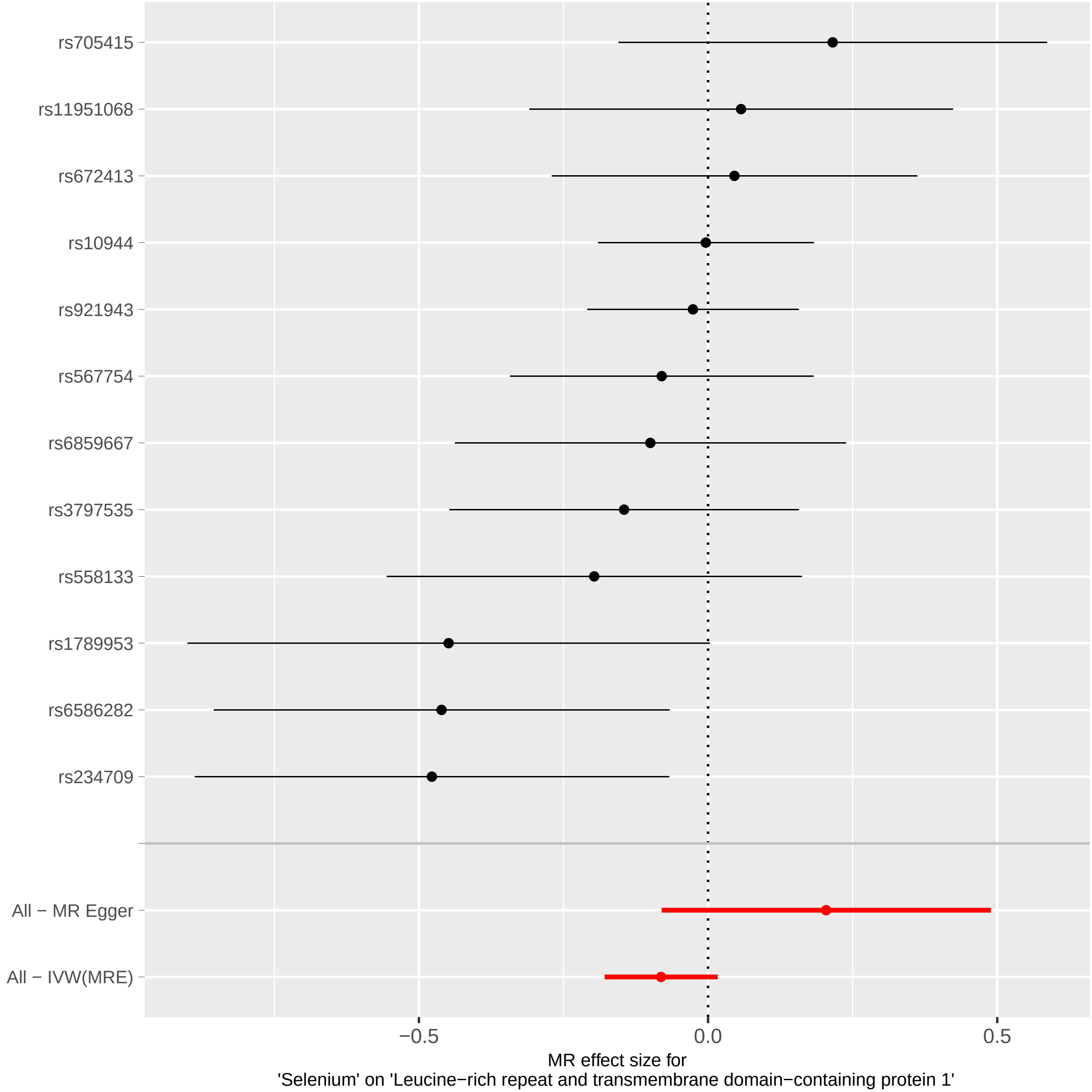

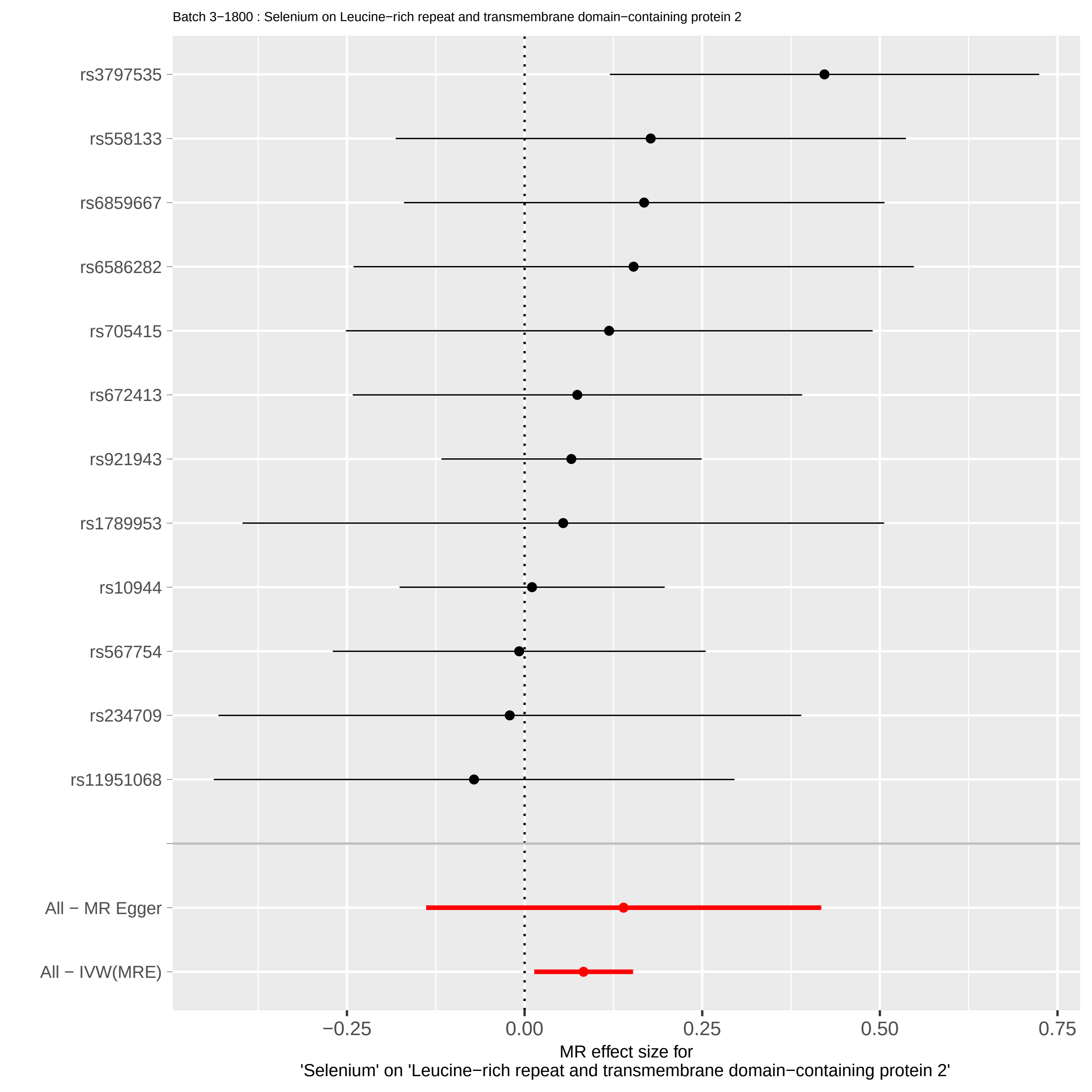

Batch 3−1800 : Selenium on Leucine−rich repeat and transmembrane domain−containing protein 2
rs3797535
rs558133
rs6859667
rs6586282
rs705415
rs672413
rs921943
rs1789953
rs10944
rs567754
rs234709
rs11951068
All − MR Egger
All − IVW(MRE)
−0.25
0.00
0.25
0.50
0.75
MR effect size for
'Selenium' on 'Leucine−rich repeat and transmembrane domain−containing protein 2'

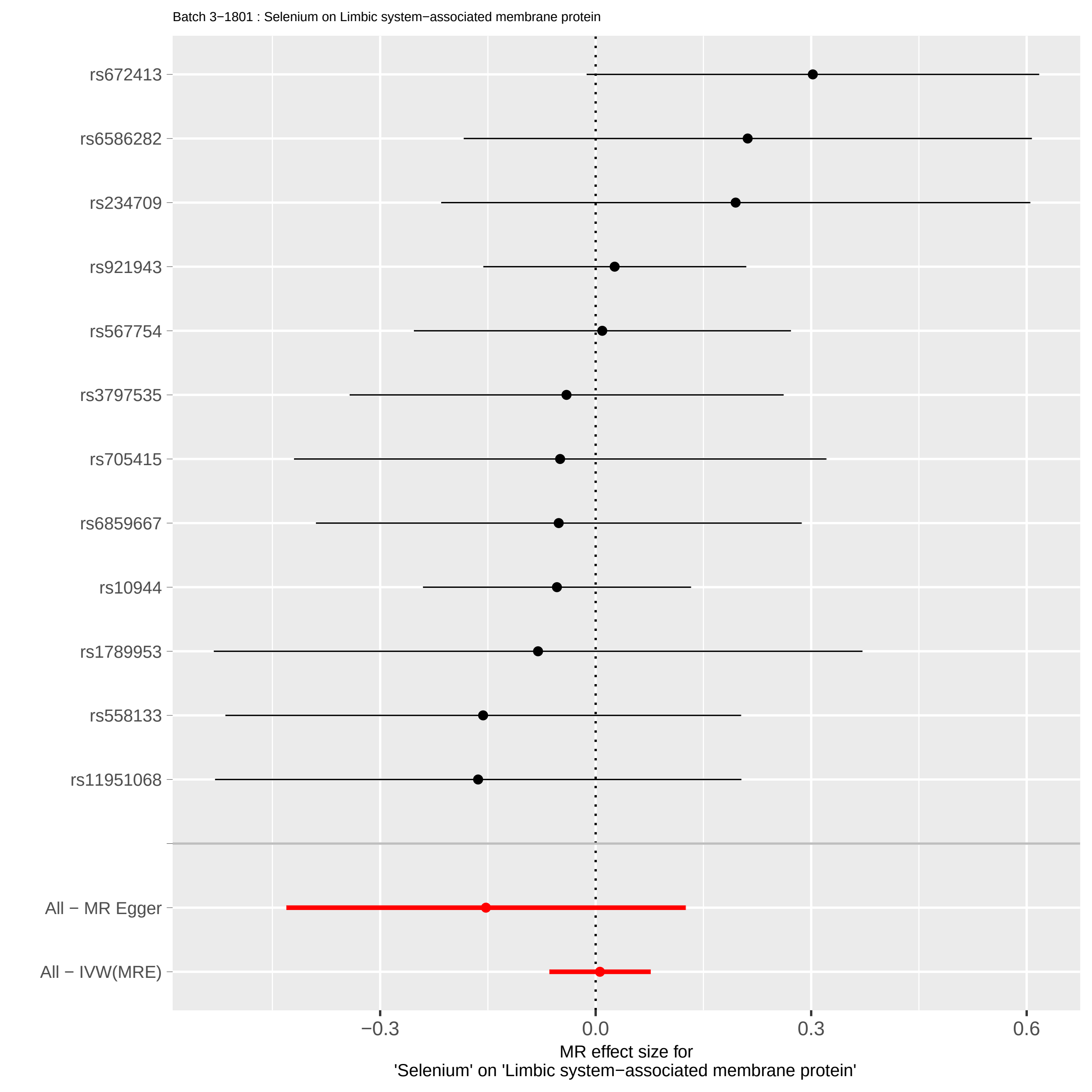
Batch 3−1801 : Selenium on Limbic system−associated membrane protein
rs672413
rs6586282
rs234709
rs921943
rs567754
rs3797535
rs705415
rs6859667
rs10944
rs1789953
rs558133
rs11951068
All − MR Egger
All − IVW(MRE)
−0.3
0.0
0.3
0.6
MR effect size for
'Selenium' on 'Limbic system−associated membrane protein'

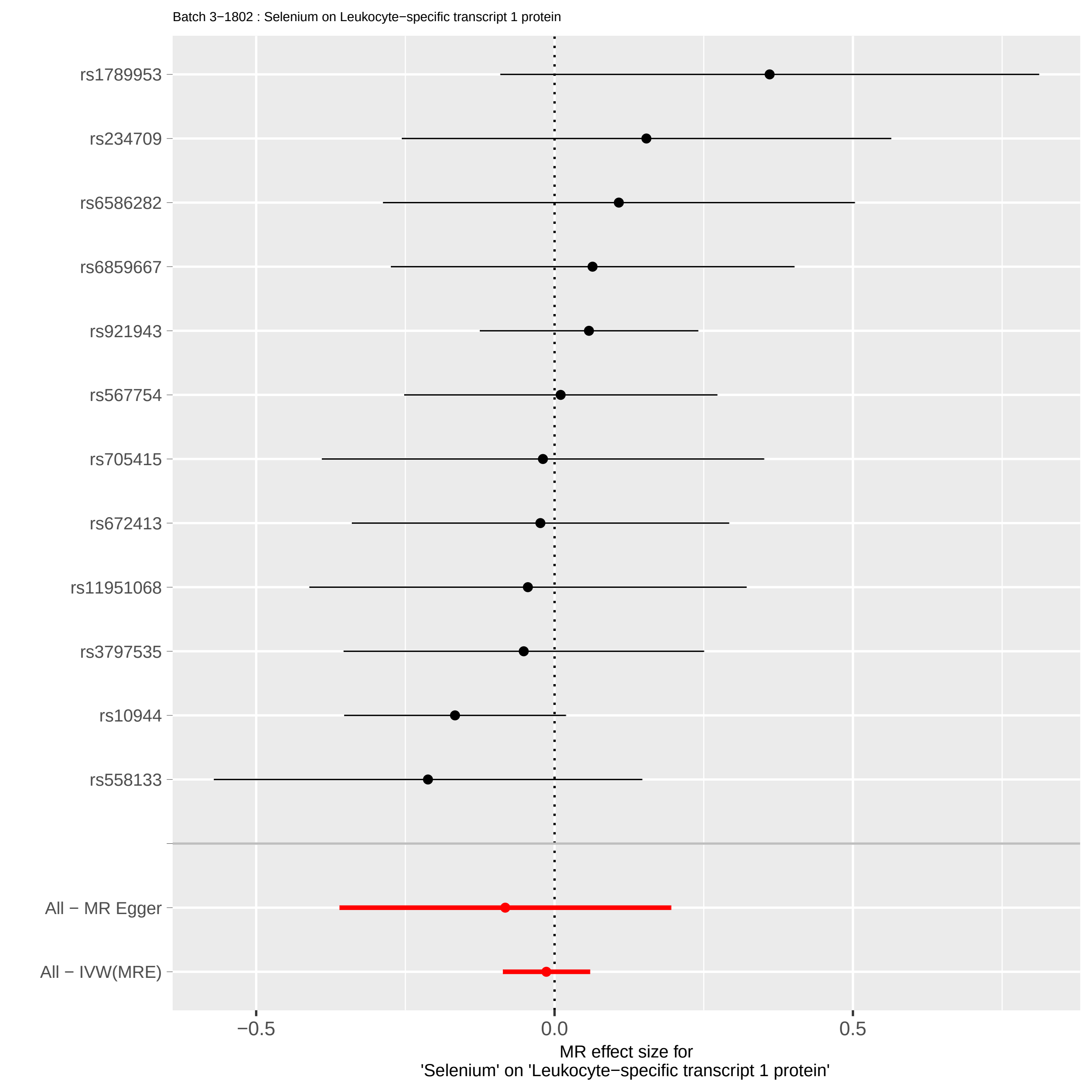

Batch 3−1802 : Selenium on Leukocyte−specific transcript 1 protein
rs1789953
rs234709
rs6586282
rs6859667
rs921943
rs567754
rs705415
rs672413
rs11951068
rs3797535
rs10944
rs558133
All − MR Egger
All − IVW(MRE)
−0.5
0.0
0.5
MR effect size for
'Selenium' on 'Leukocyte−specific transcript 1 protein'

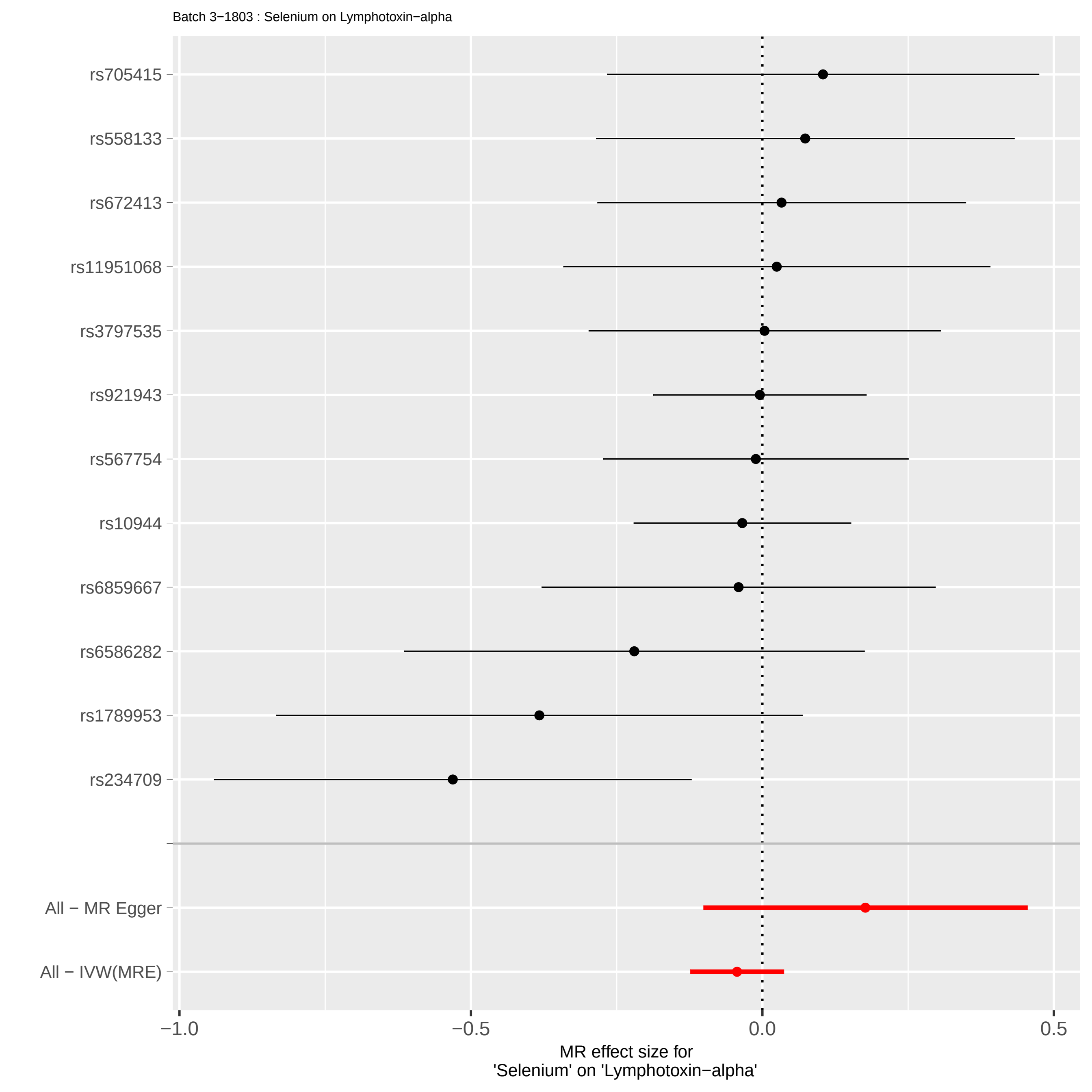

Batch 3−1803 : Selenium on Lymphotoxin−alpha
rs705415
rs558133
rs672413
rs11951068
rs3797535
rs921943
rs567754
rs10944
rs6859667
rs6586282
rs1789953
rs234709
All − MR Egger
All − IVW(MRE)
−1.0
−0.5
0.0
0.5
MR effect size for
'Selenium' on 'Lymphotoxin−alpha'

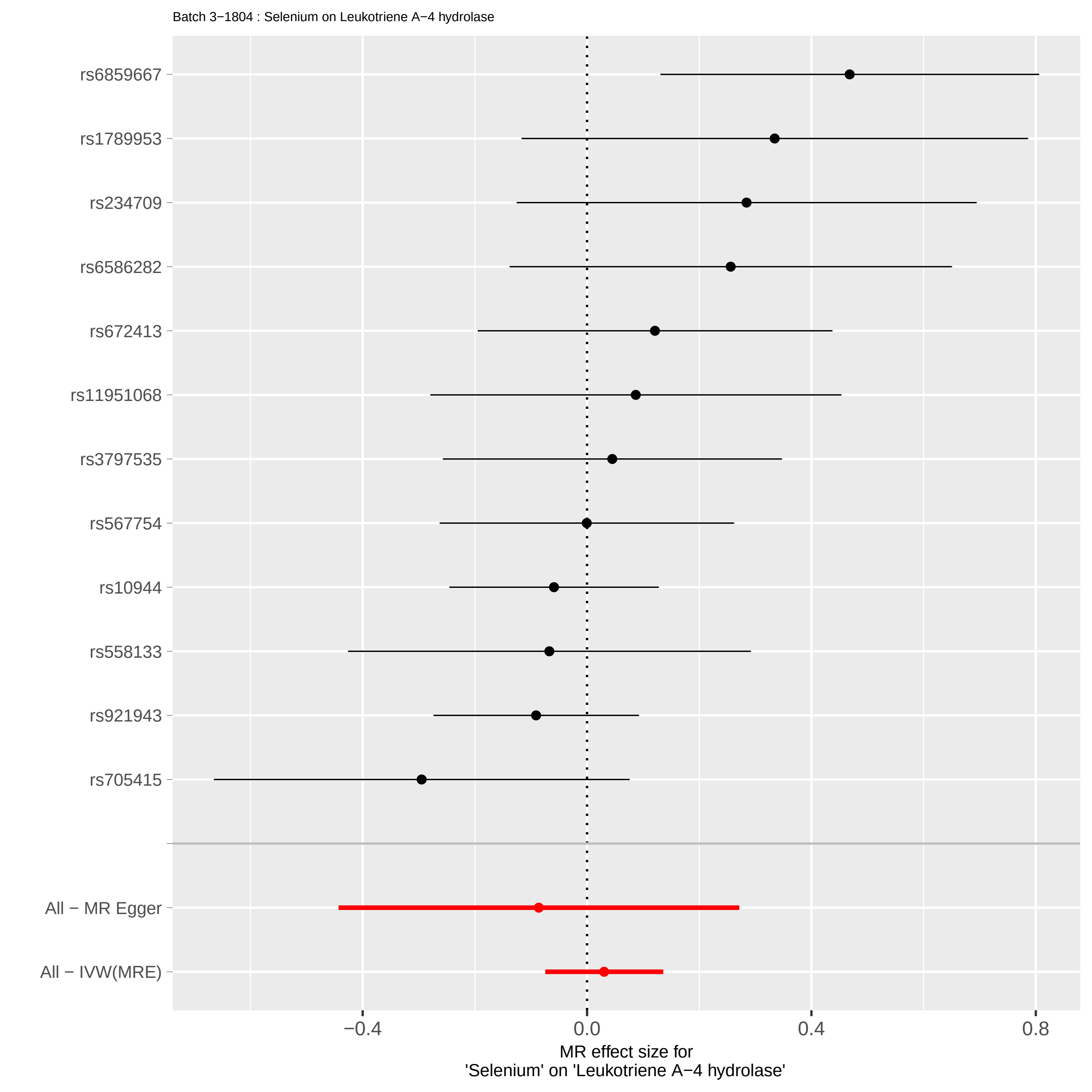
Batch 3−1804 : Selenium on Leukotriene A−4 hydrolase
rs6859667
rs1789953
rs234709
rs6586282
rs672413
rs11951068
rs3797535
rs567754
rs10944
rs558133
rs921943
rs705415
All − MR Egger
All − IVW(MRE)
−0.4
0.0
0.4
0.8
MR effect size for
'Selenium' on 'Leukotriene A−4 hydrolase'

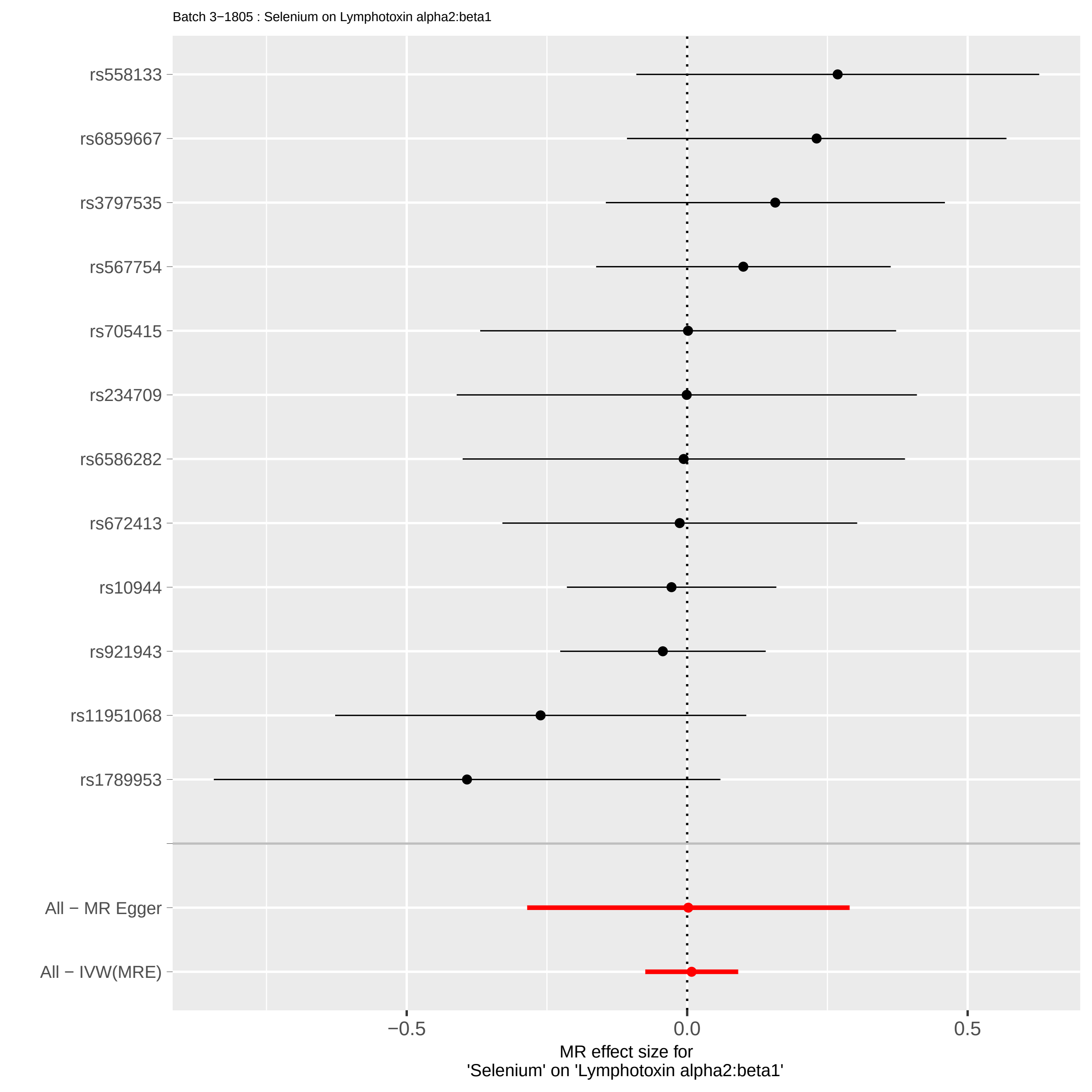

Batch 3−1805 : Selenium on Lymphotoxin alpha2:beta1
rs558133
rs6859667
rs3797535
rs567754
rs705415
rs234709
rs6586282
rs672413
rs10944
rs921943
rs11951068
rs1789953
All − MR Egger
All − IVW(MRE)
−0.5
0.0
0.5
MR effect size for
'Selenium' on 'Lymphotoxin alpha2:beta1'

Batch 3−1806 : Selenium on Latent−transforming growth factor beta−binding protein 4

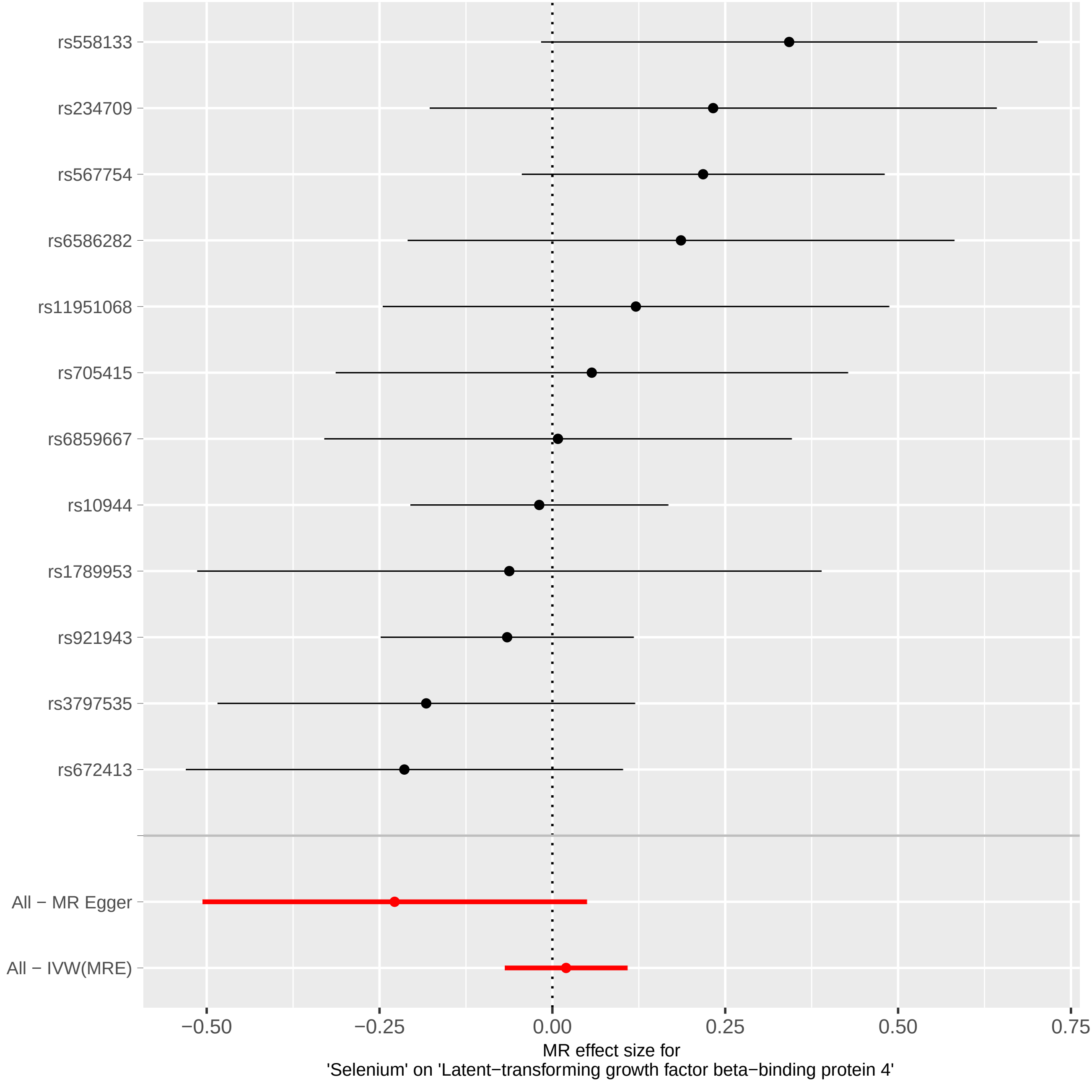

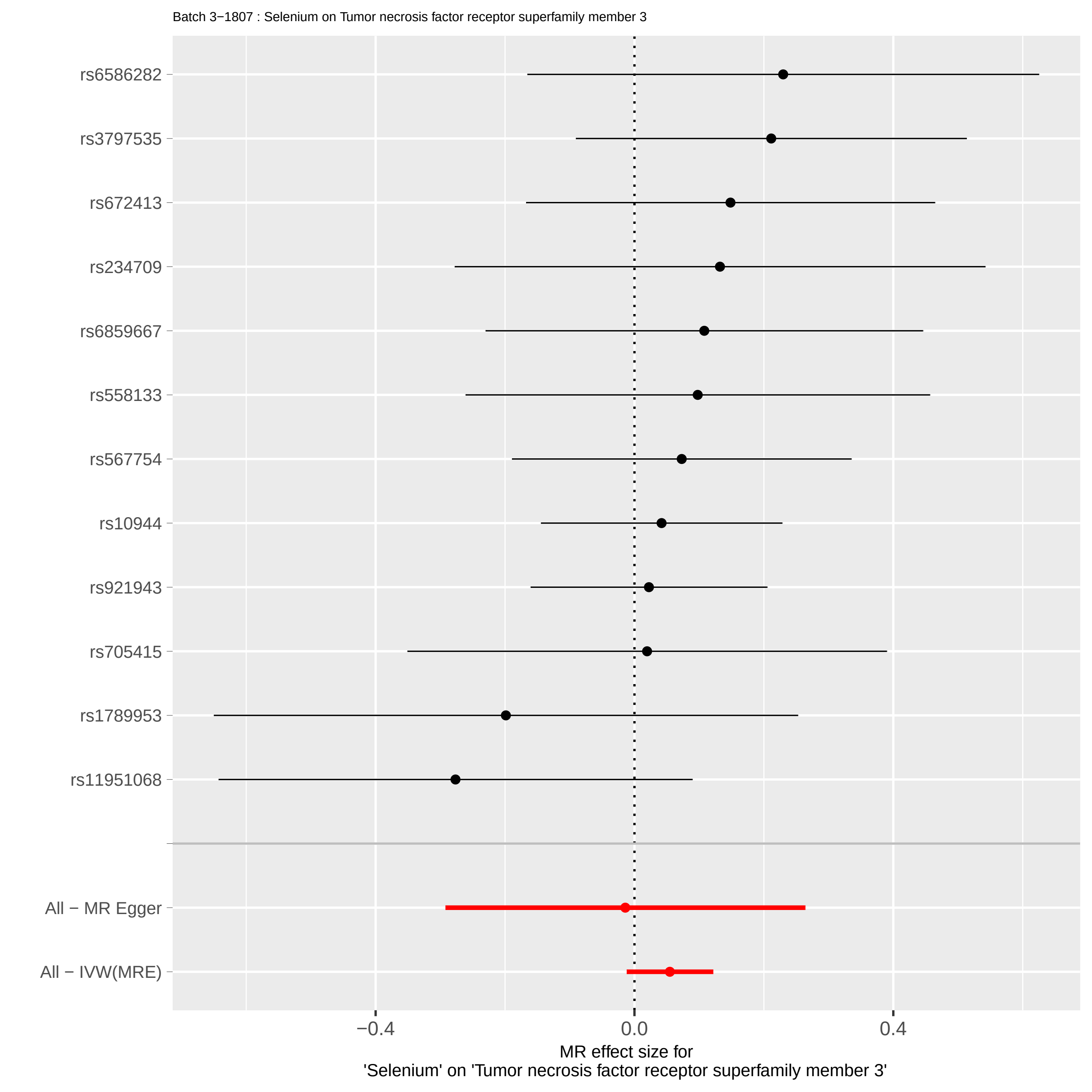

Batch 3−1807 : Selenium on Tumor necrosis factor receptor superfamily member 3
rs6586282
rs3797535
rs672413
rs234709
rs6859667
rs558133
rs567754
rs10944
rs921943
rs705415
rs1789953
rs11951068
All − MR Egger
All − IVW(MRE)
−0.4
0.0
0.4
MR effect size for
'Selenium' on 'Tumor necrosis factor receptor superfamily member 3'

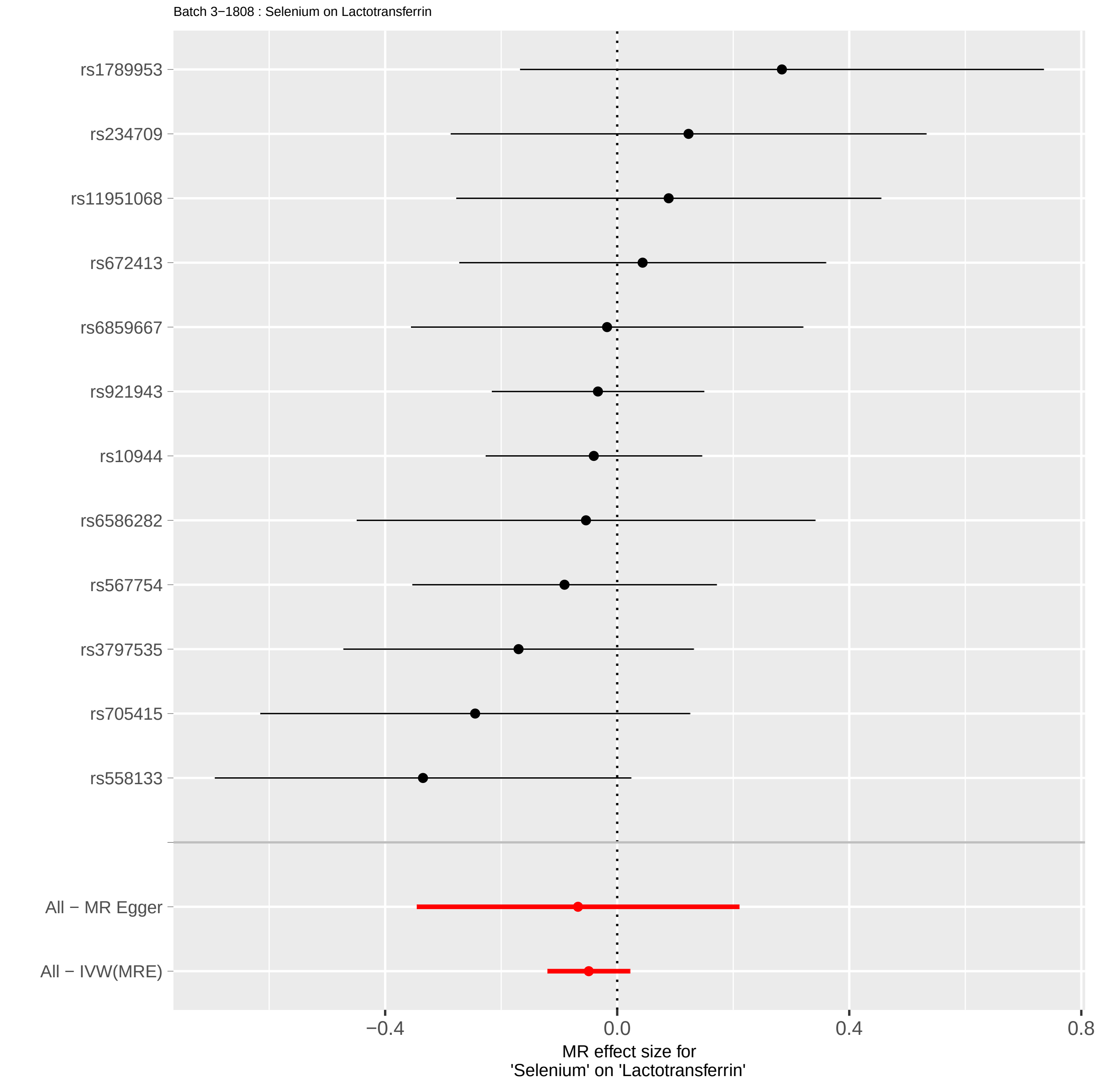

Batch 3−1808 : Selenium on Lactotransferrin
rs1789953
rs234709
rs11951068
rs672413
rs6859667
rs921943
rs10944
rs6586282
rs567754
rs3797535
rs705415
rs558133
All − MR Egger
All − IVW(MRE)
−0.4
0.0
0.4
0.8
MR effect size for
'Selenium' on 'Lactotransferrin'

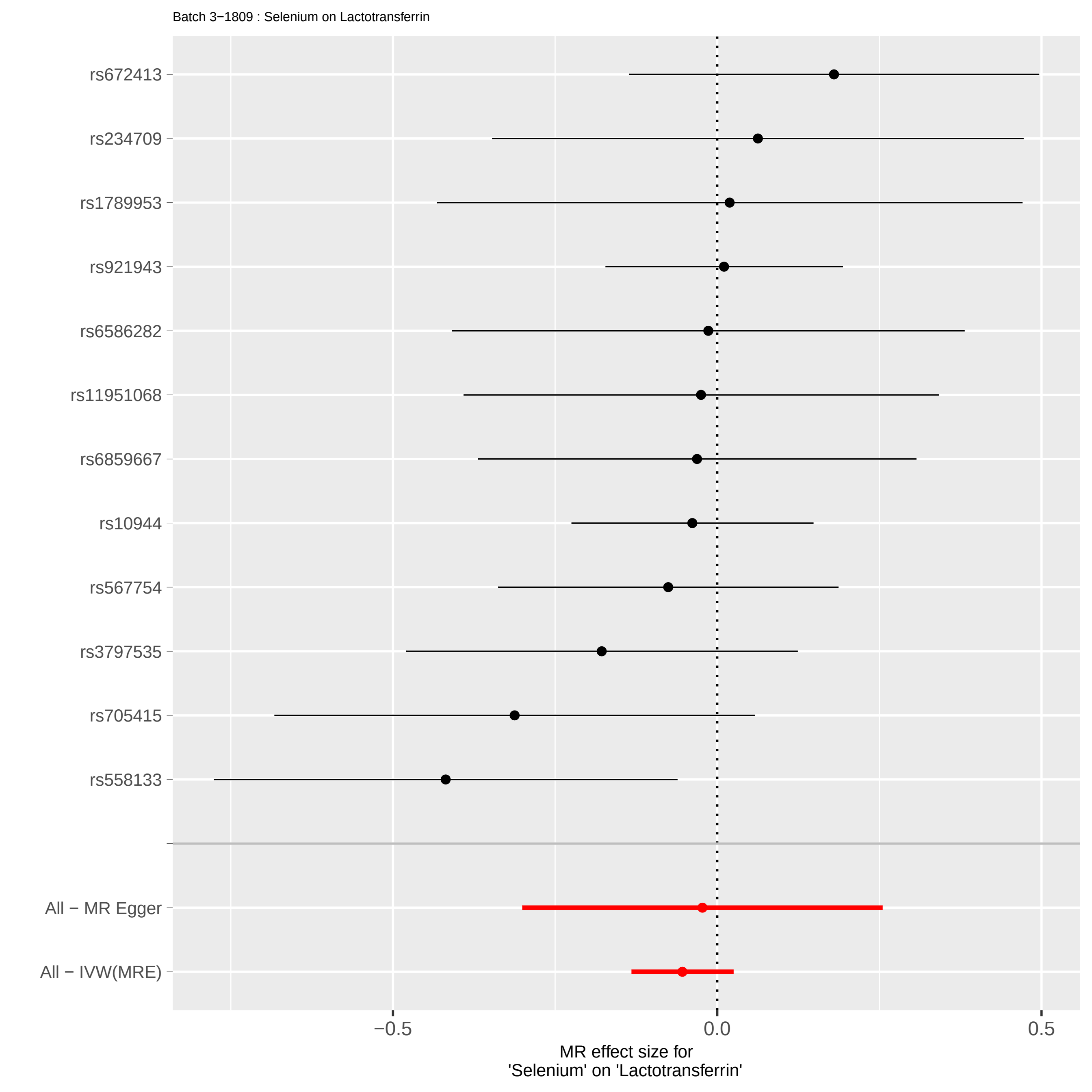

Batch 3−1809 : Selenium on Lactotransferrin
rs672413
rs234709
rs1789953
rs921943
rs6586282
rs11951068
rs6859667
rs10944
rs567754
rs3797535
rs705415
rs558133
All − MR Egger
All − IVW(MRE)
−0.5
0.0
0.5
MR effect size for
'Selenium' on 'Lactotransferrin'

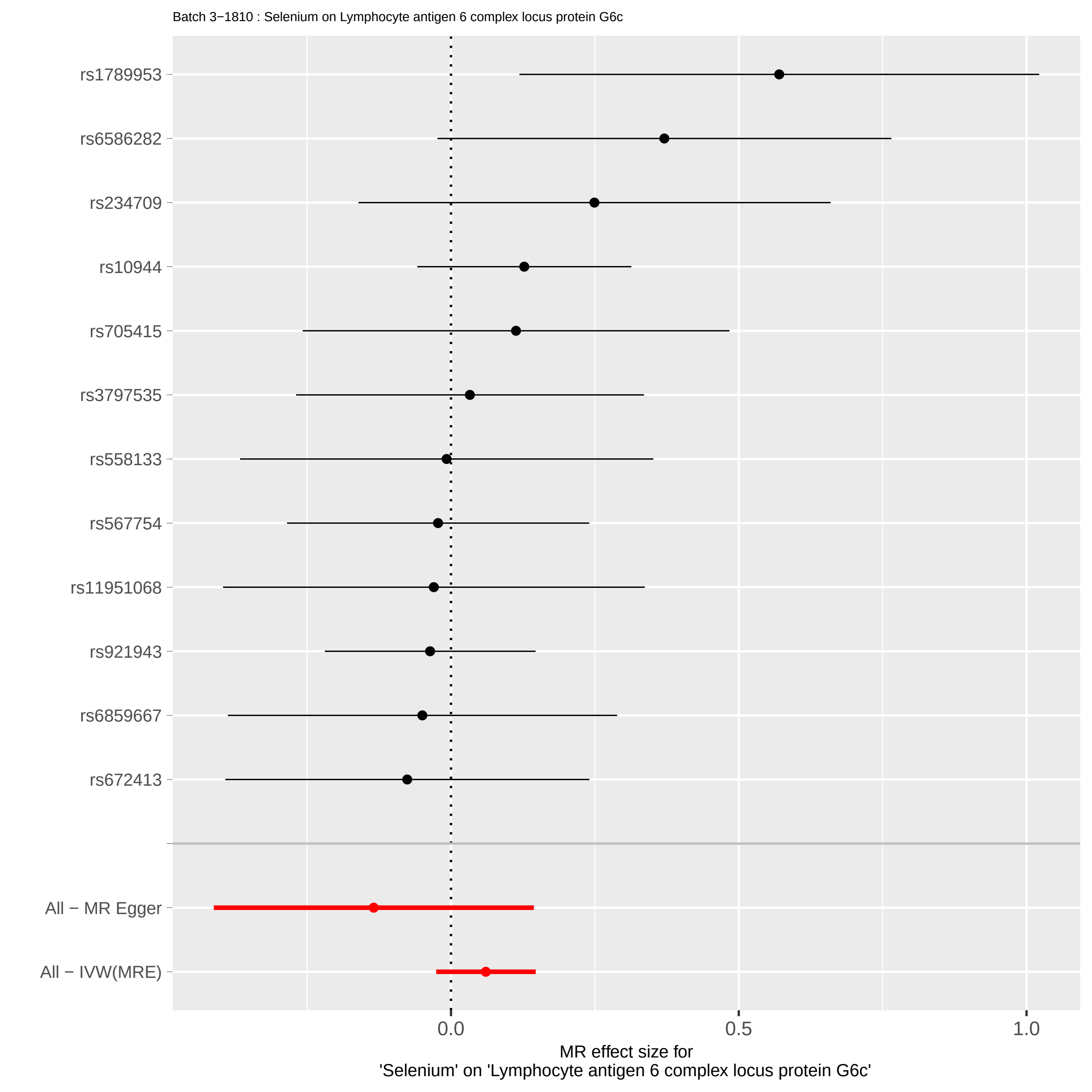

Batch 3−1810 : Selenium on Lymphocyte antigen 6 complex locus protein G6c
rs1789953
rs6586282
rs234709
rs10944
rs705415
rs3797535
rs558133
rs567754
rs11951068
rs921943
rs6859667
rs672413
All − MR Egger
All − IVW(MRE)
0.0
0.5
1.0
MR effect size for
'Selenium' on 'Lymphocyte antigen 6 complex locus protein G6c'

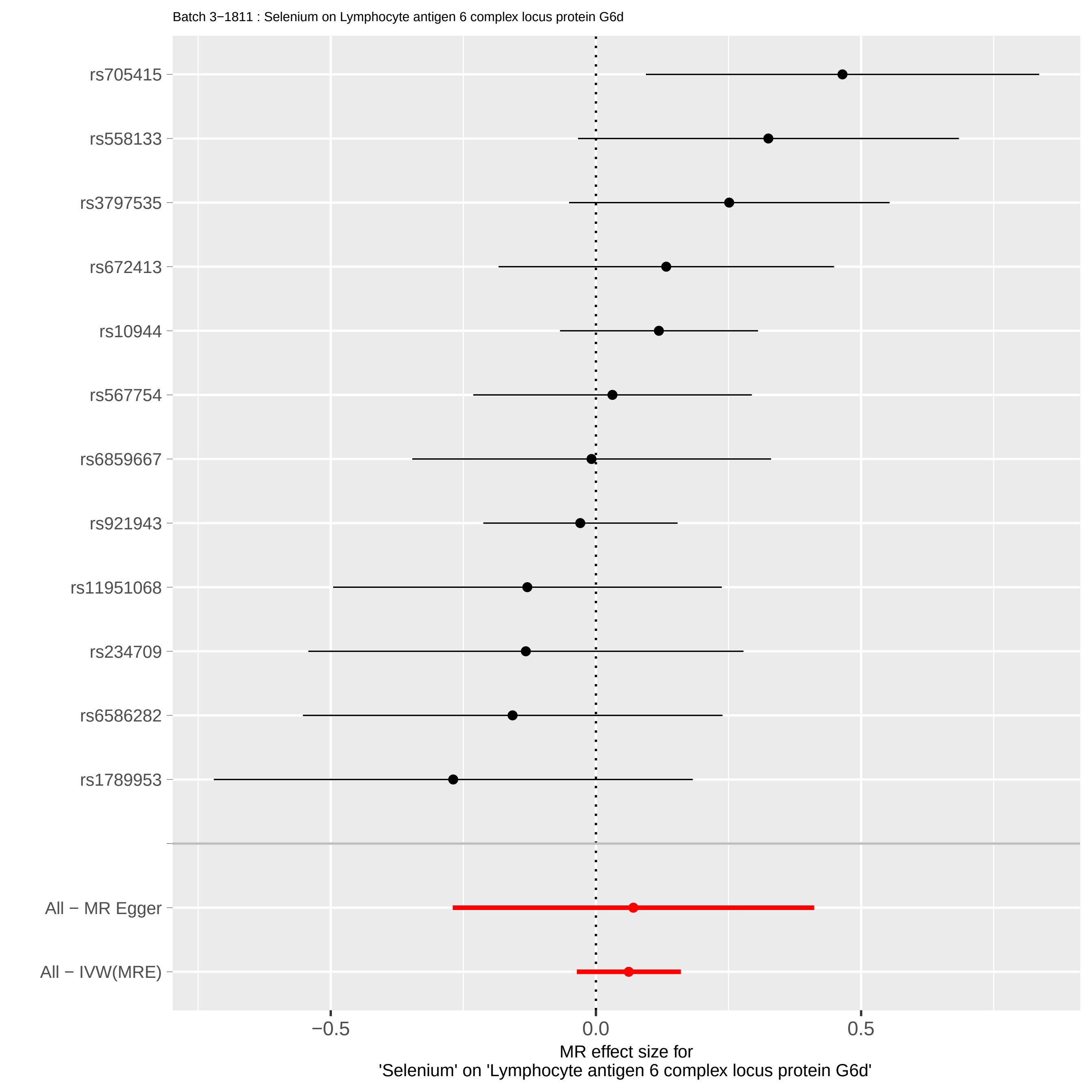

Batch 3−1811 : Selenium on Lymphocyte antigen 6 complex locus protein G6d
rs705415
rs558133
rs3797535
rs672413
rs10944
rs567754
rs6859667
rs921943
rs11951068
rs234709
rs6586282
rs1789953
All − MR Egger
All − IVW(MRE)
−0.5
0.0
0.5
MR effect size for
'Selenium' on 'Lymphocyte antigen 6 complex locus protein G6d'

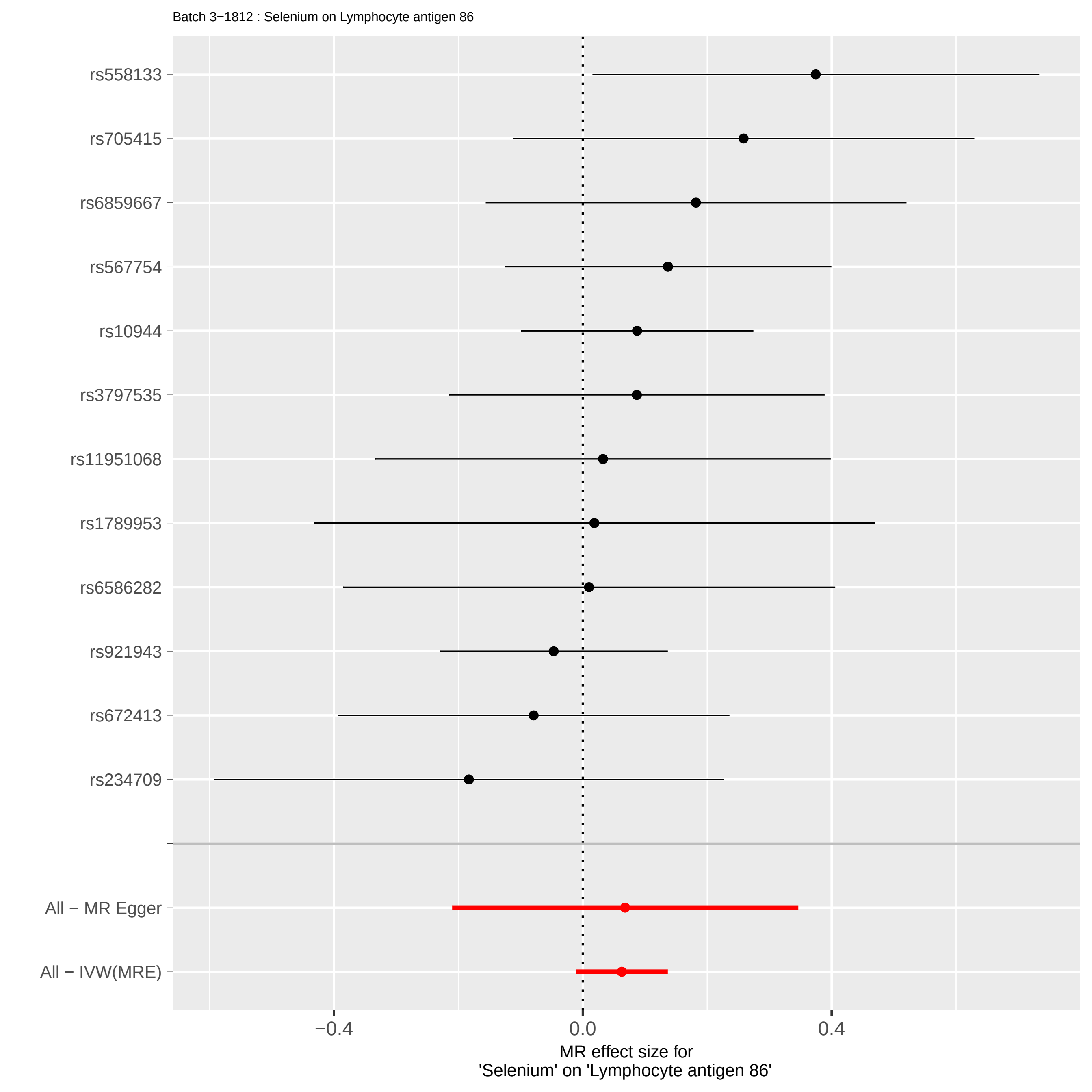

Batch 3−1812 : Selenium on Lymphocyte antigen 86
rs558133
rs705415
rs6859667
rs567754
rs10944
rs3797535
rs11951068
rs1789953
rs6586282
rs921943
rs672413
rs234709
All − MR Egger
All − IVW(MRE)
−0.4
0.0
0.4
MR effect size for
'Selenium' on 'Lymphocyte antigen 86'

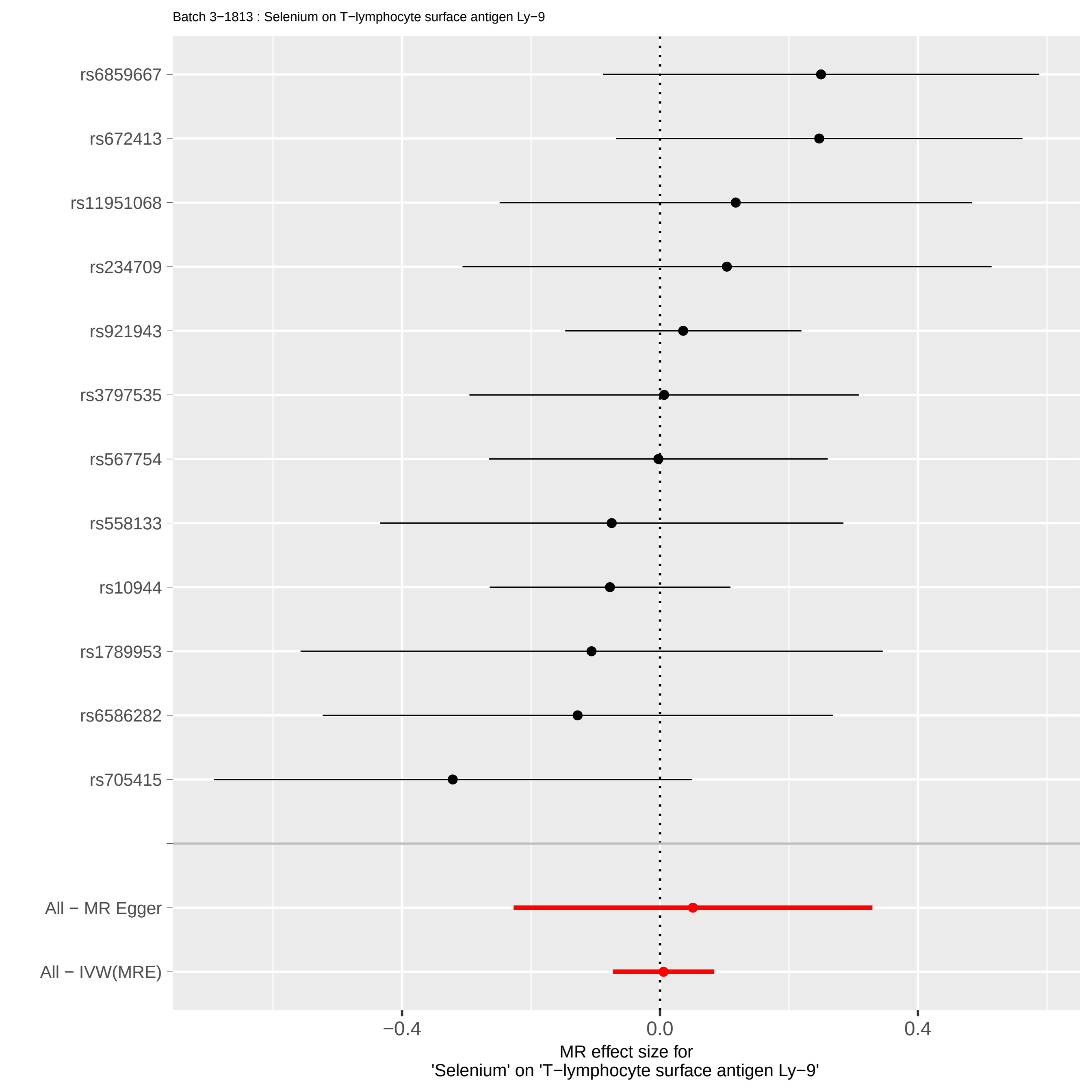

Batch 3−1813 : Selenium on T−lymphocyte surface antigen Ly−9
rs6859667
rs672413
rs11951068
rs234709
rs921943
rs3797535
rs567754
rs558133
rs10944
rs1789953
rs6586282
rs705415
All − MR Egger
All − IVW(MRE)
−0.4
0.0
0.4
MR effect size for
'Selenium' on 'T−lymphocyte surface antigen Ly−9'

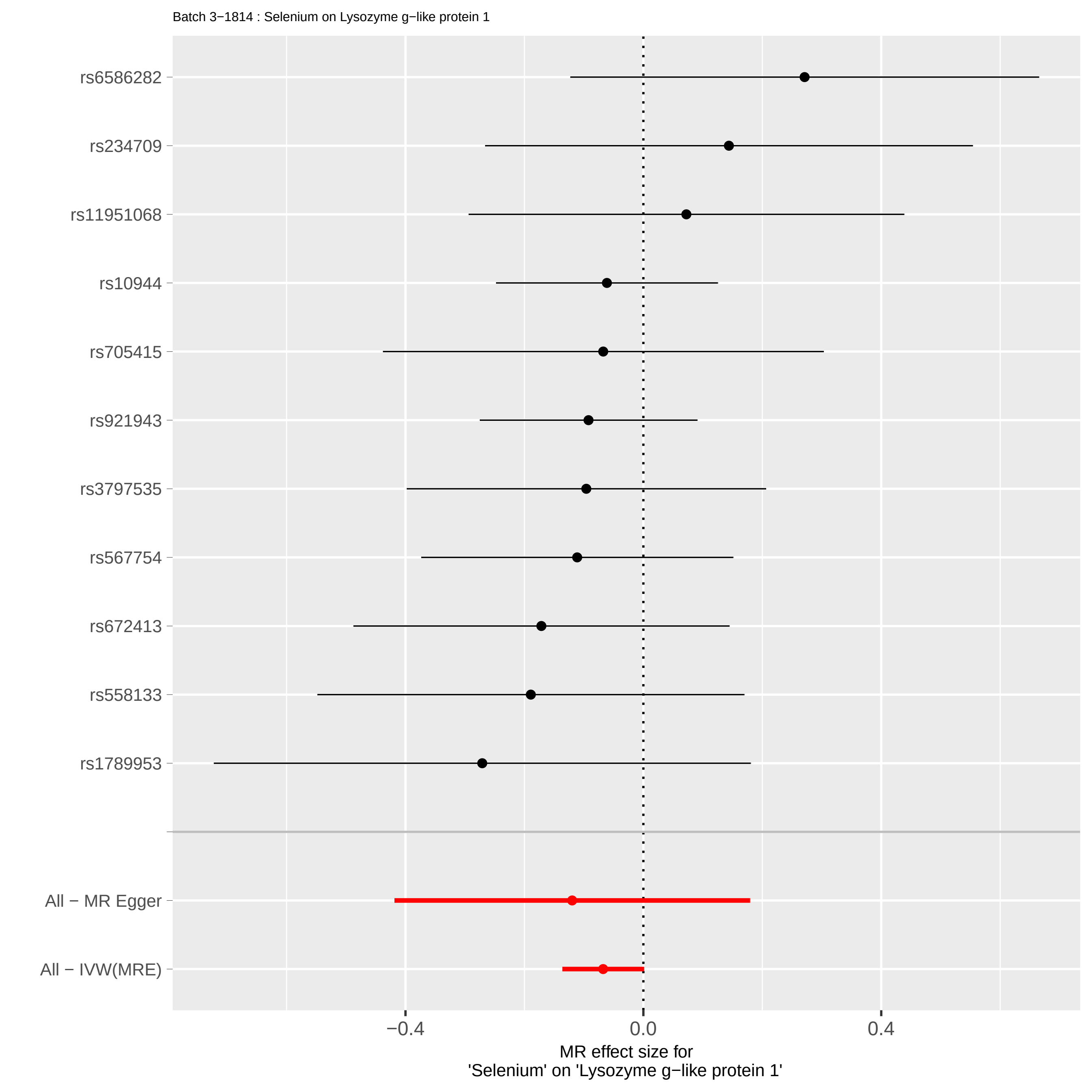

Batch 3−1814 : Selenium on Lysozyme g−like protein 1
rs6586282
rs234709
rs11951068
rs10944
rs705415
rs921943
rs3797535
rs567754
rs672413
rs558133
rs1789953
All − MR Egger
All − IVW(MRE)
−0.4
0.0
0.4
MR effect size for
'Selenium' on 'Lysozyme g−like protein 1'

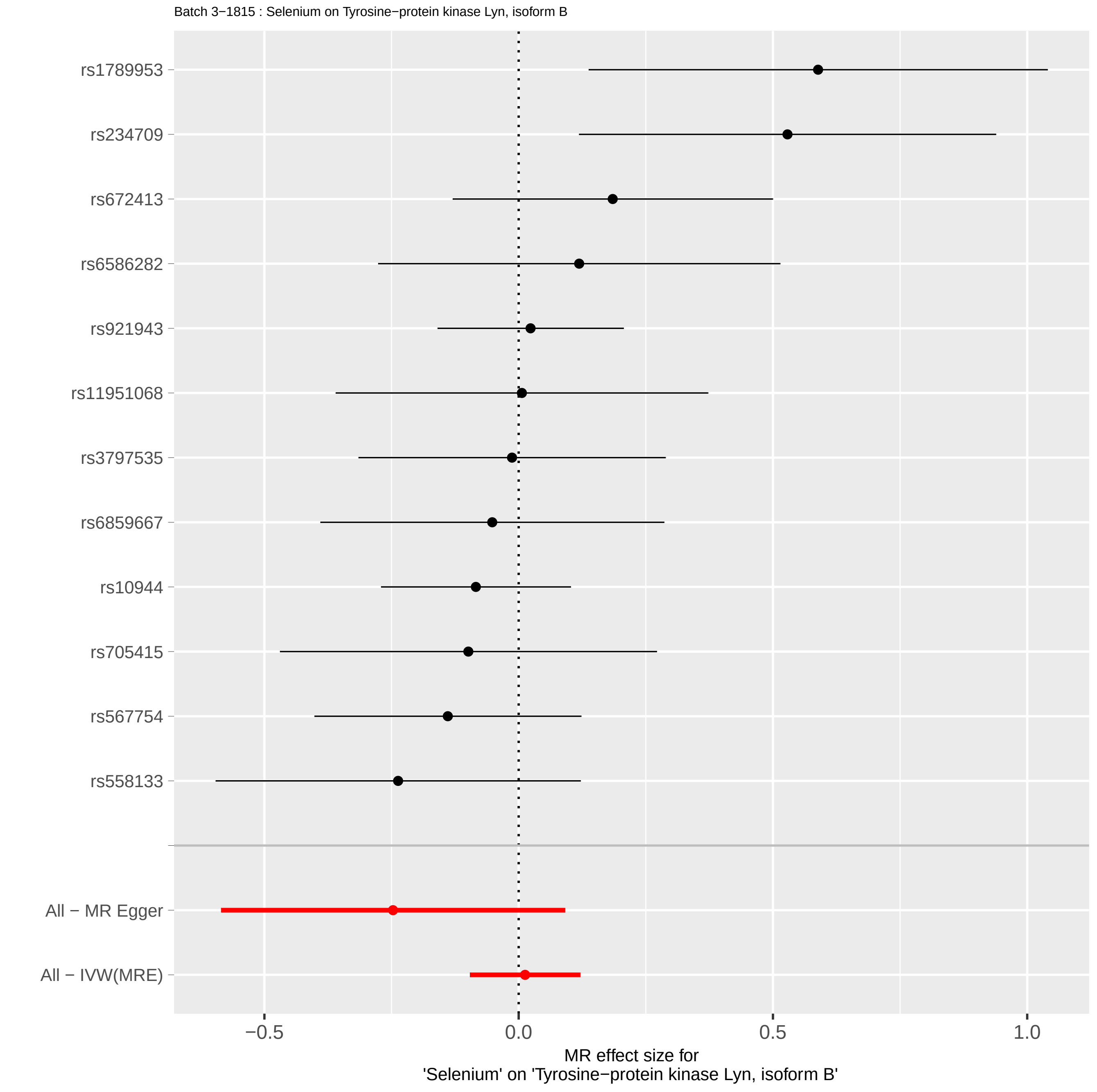

Batch 3−1815 : Selenium on Tyrosine−protein kinase Lyn, isoform B
rs1789953
rs234709
rs672413
rs6586282
rs921943
rs11951068
rs3797535
rs6859667
rs10944
rs705415
rs567754
rs558133
All − MR Egger
All − IVW(MRE)
−0.5
0.0
0.5
1.0
MR effect size for
'Selenium' on 'Tyrosine−protein kinase Lyn, isoform B'

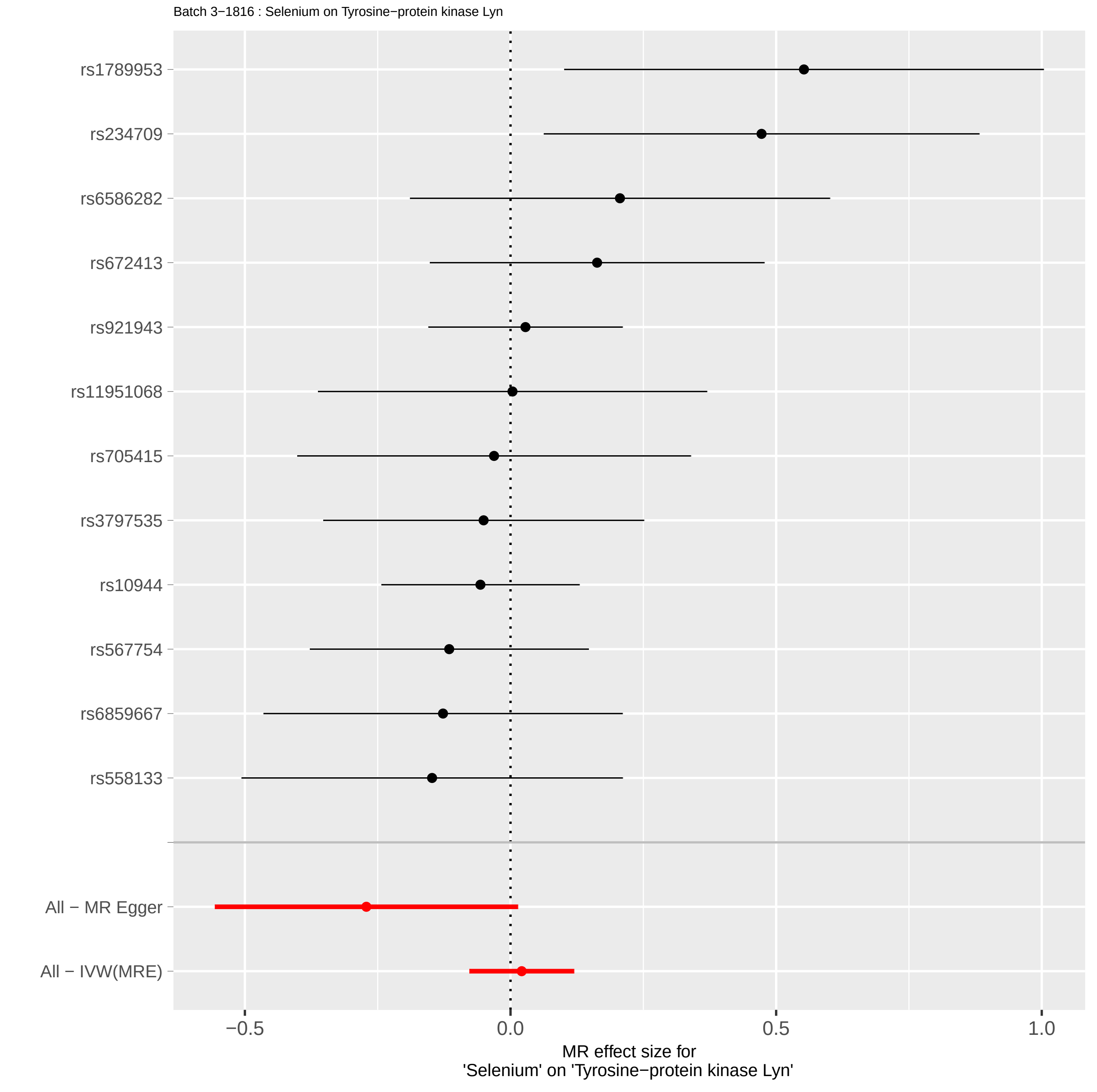
Batch 3−1816 : Selenium on Tyrosine−protein kinase Lyn
rs1789953
rs234709
rs6586282
rs672413
rs921943
rs11951068
rs705415
rs3797535
rs10944
rs567754
rs6859667
rs558133
All − MR Egger
All − IVW(MRE)
−0.5
0.0
0.5
1.0
MR effect size for
'Selenium' on 'Tyrosine−protein kinase Lyn'

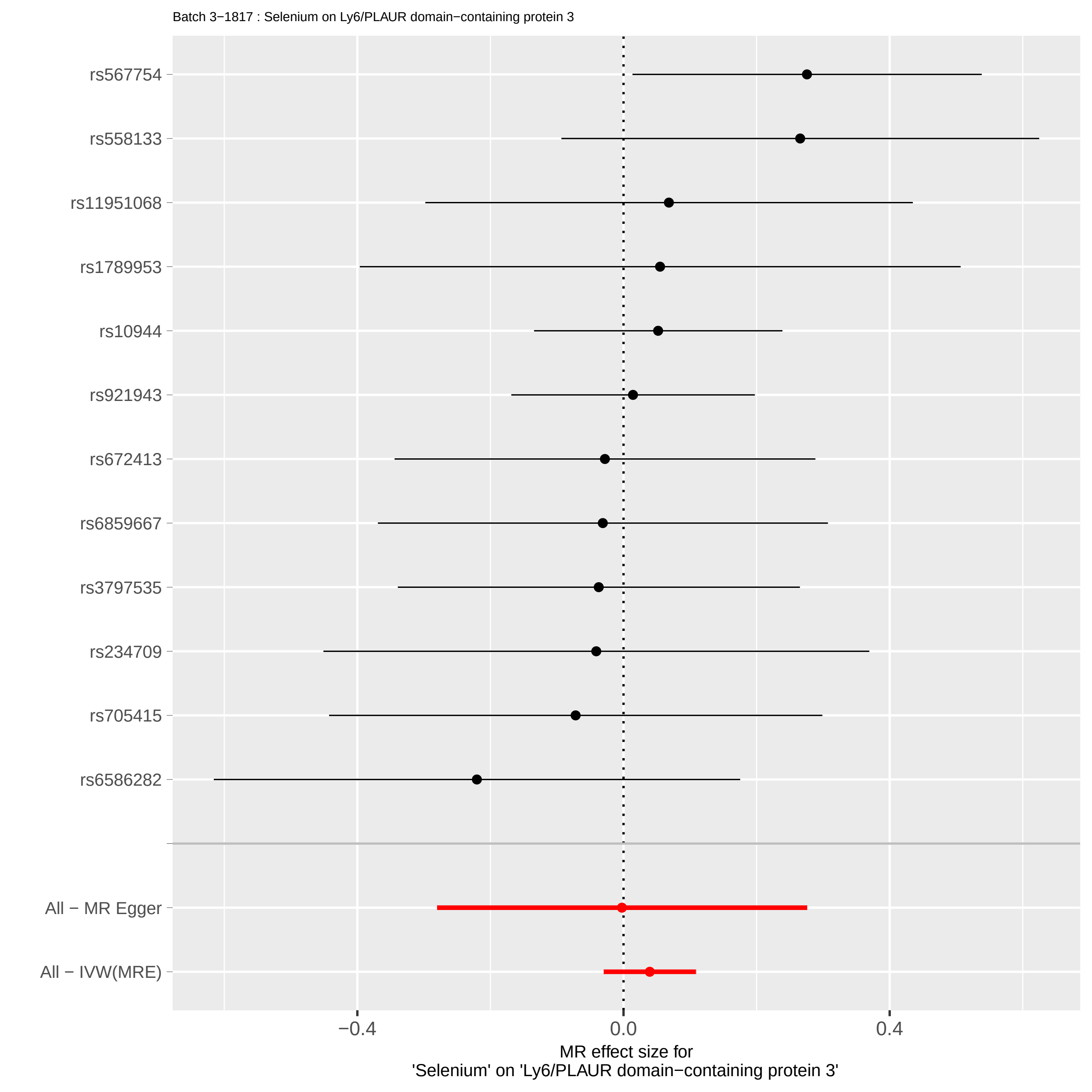

Batch 3−1817 : Selenium on Ly6/PLAUR domain−containing protein 3
rs567754
rs558133
rs11951068
rs1789953
rs10944
rs921943
rs672413
rs6859667
rs3797535
rs234709
rs705415
rs6586282
All − MR Egger
All − IVW(MRE)
−0.4
0.0
0.4
MR effect size for
'Selenium' on 'Ly6/PLAUR domain−containing protein 3'

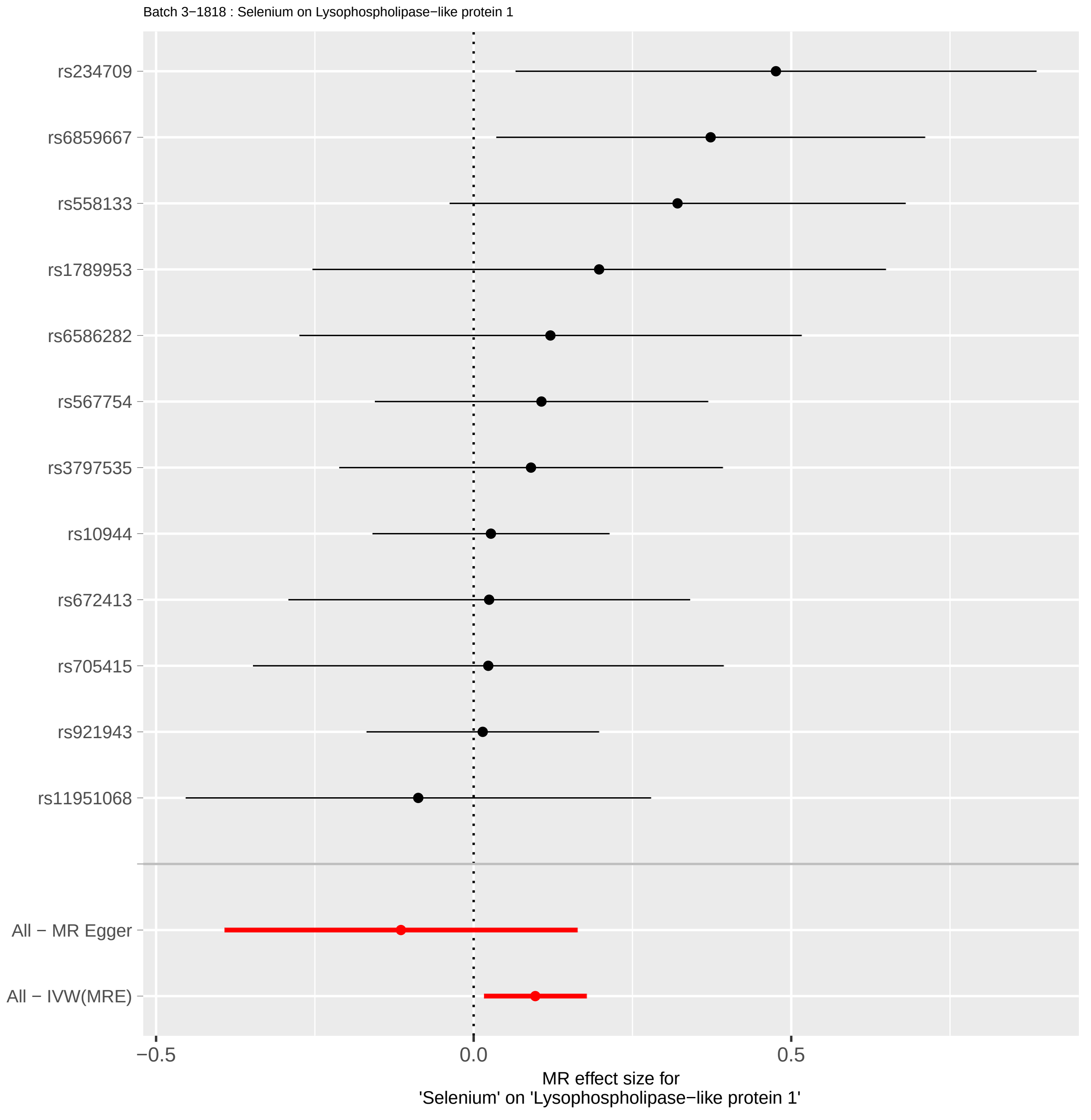

Batch 3−1818 : Selenium on Lysophospholipase−like protein 1
rs234709
rs6859667
rs558133
rs1789953
rs6586282
rs567754
rs3797535
rs10944
rs672413
rs705415
rs921943
rs11951068
All − MR Egger
All − IVW(MRE)
−0.5
0.0
0.5
MR effect size for
'Selenium' on 'Lysophospholipase−like protein 1'

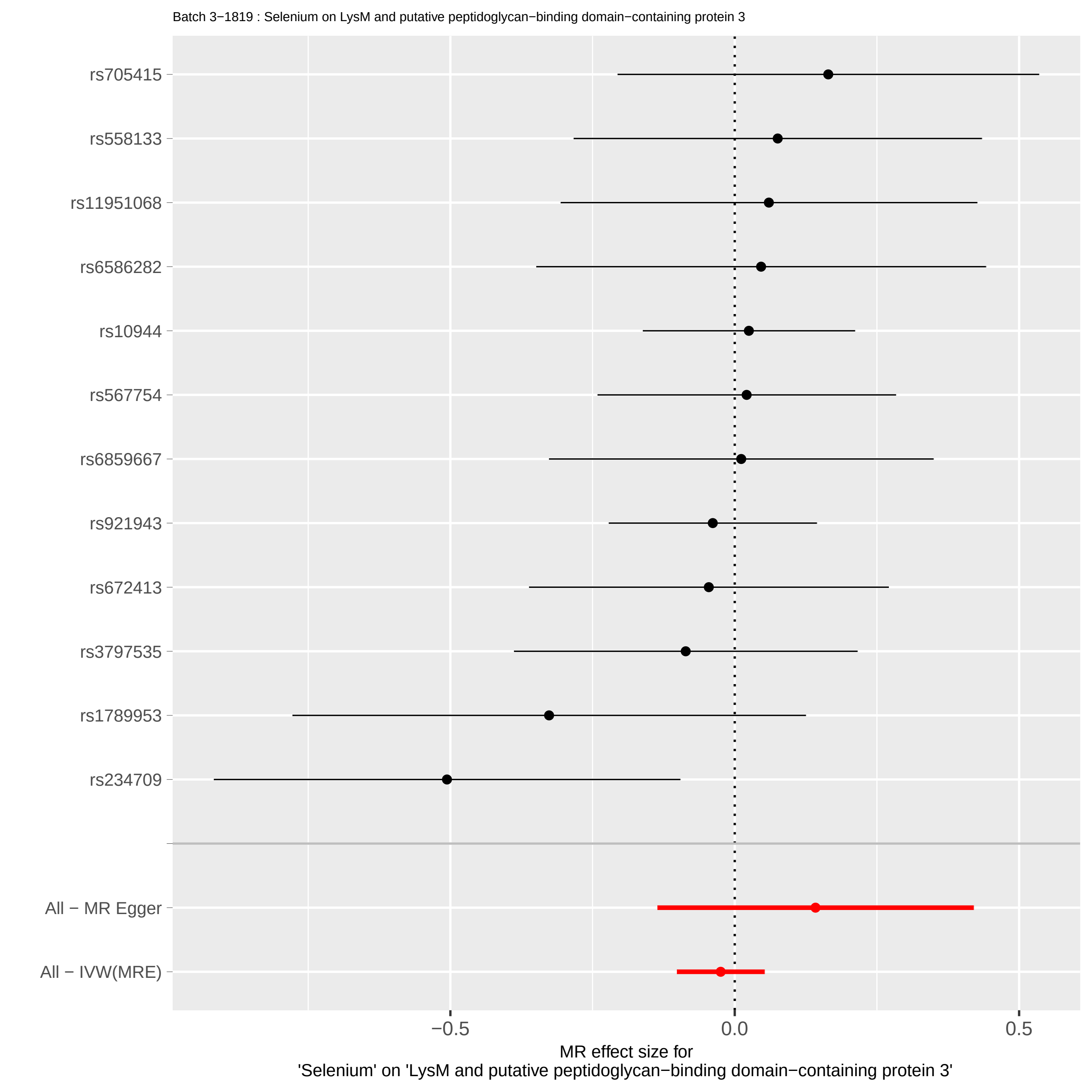

Batch 3−1819 : Selenium on LysM and putative peptidoglycan−binding domain−containing protein 3
rs705415
rs558133
rs11951068
rs6586282
rs10944
rs567754
rs6859667
rs921943
rs672413
rs3797535
rs1789953
rs234709
All − MR Egger
All − IVW(MRE)
−0.5
0.0
0.5
MR effect size for
'Selenium' on 'LysM and putative peptidoglycan−binding domain−containing protein 3'

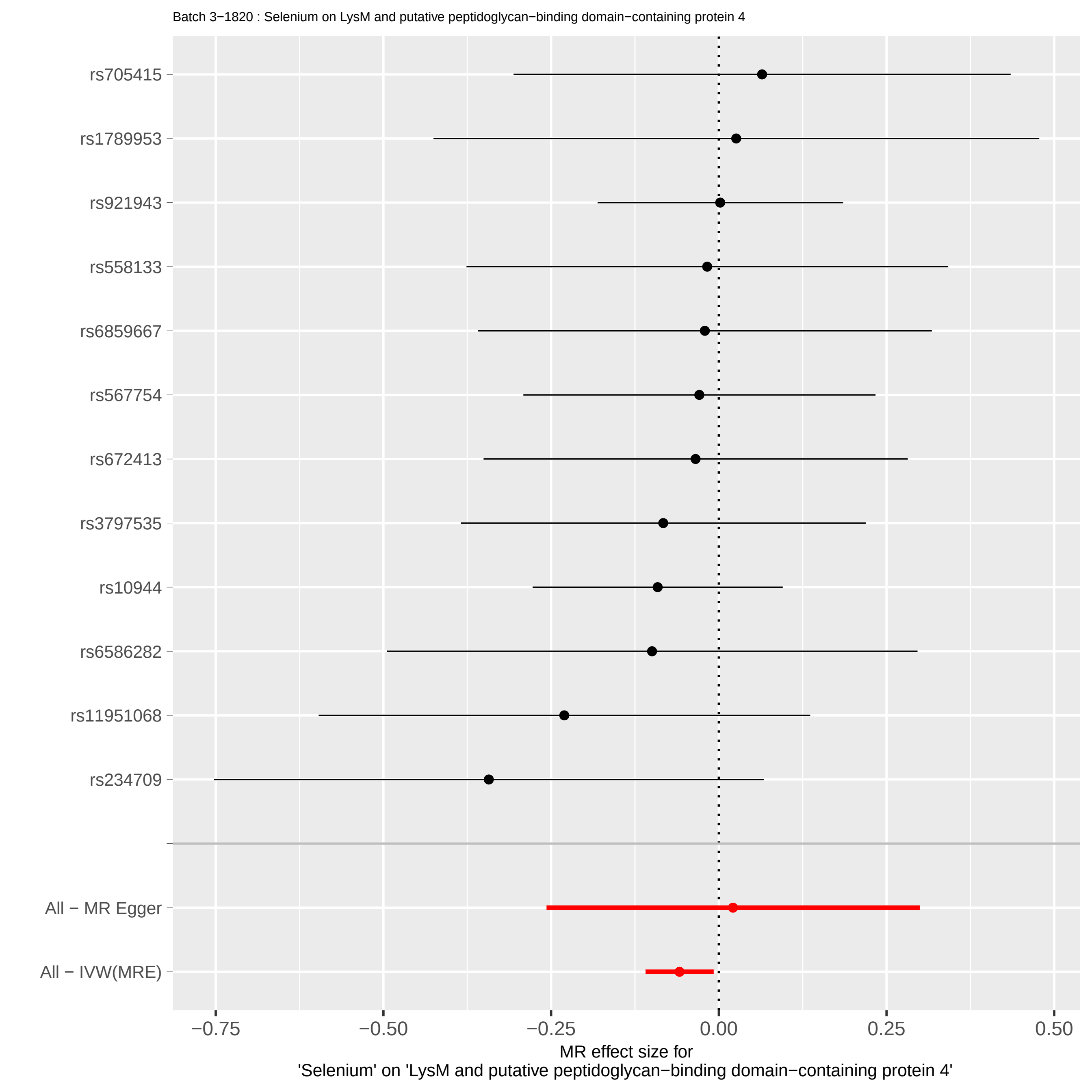

Batch 3−1820 : Selenium on LysM and putative peptidoglycan−binding domain−containing protein 4
rs705415
rs1789953
rs921943
rs558133
rs6859667
rs567754
rs672413
rs3797535
rs10944
rs6586282
rs11951068
rs234709
All − MR Egger
All − IVW(MRE)
−0.75
−0.50
−0.25
0.00
0.25
0.50
MR effect size for
'Selenium' on 'LysM and putative peptidoglycan−binding domain−containing protein 4'

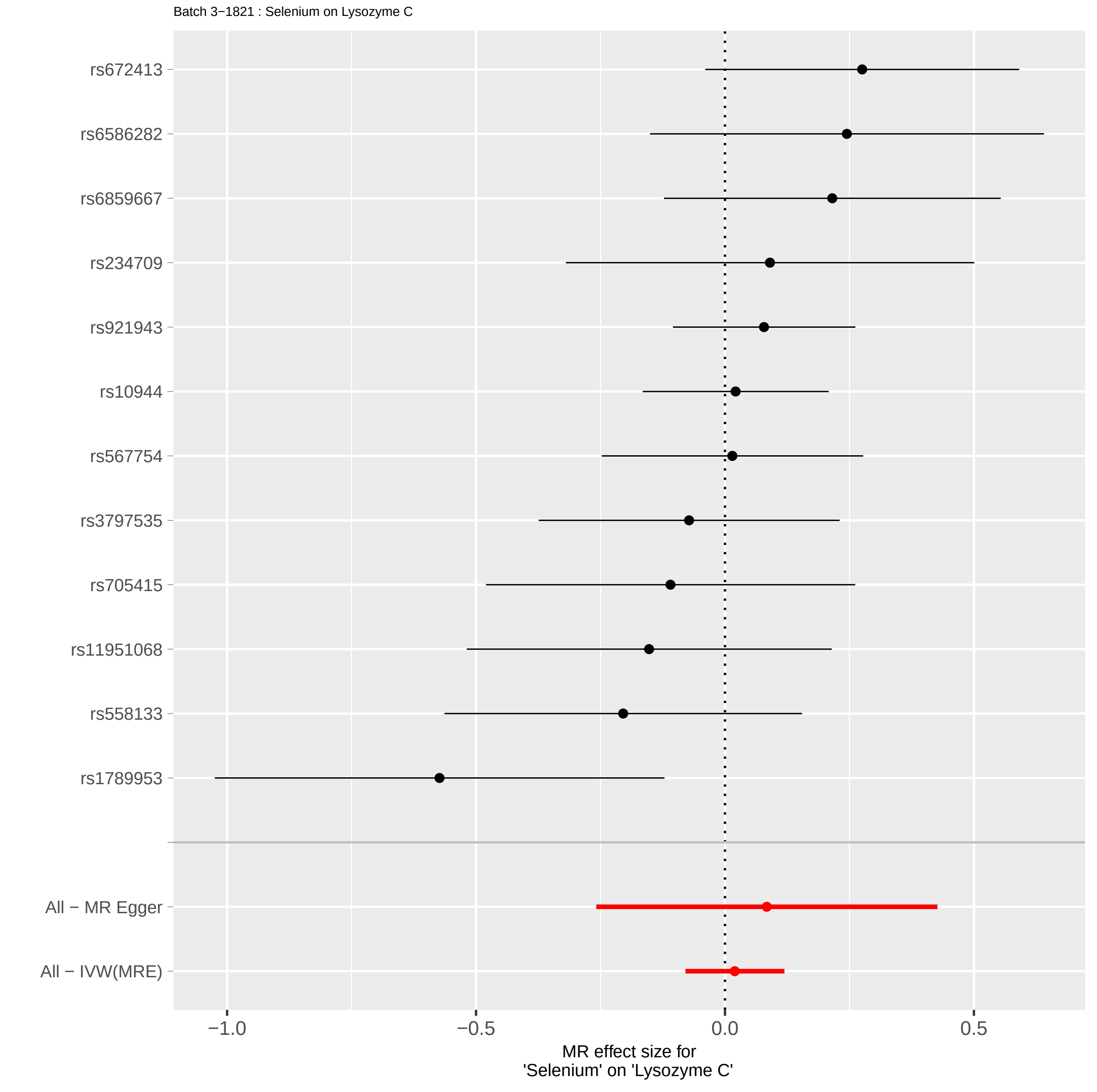

Batch 3−1821 : Selenium on Lysozyme C
rs672413
rs6586282
rs6859667
rs234709
rs921943
rs10944
rs567754
rs3797535
rs705415
rs11951068
rs558133
rs1789953
All − MR Egger
All − IVW(MRE)
−1.0
−0.5
0.0
0.5
MR effect size for
'Selenium' on 'Lysozyme C'

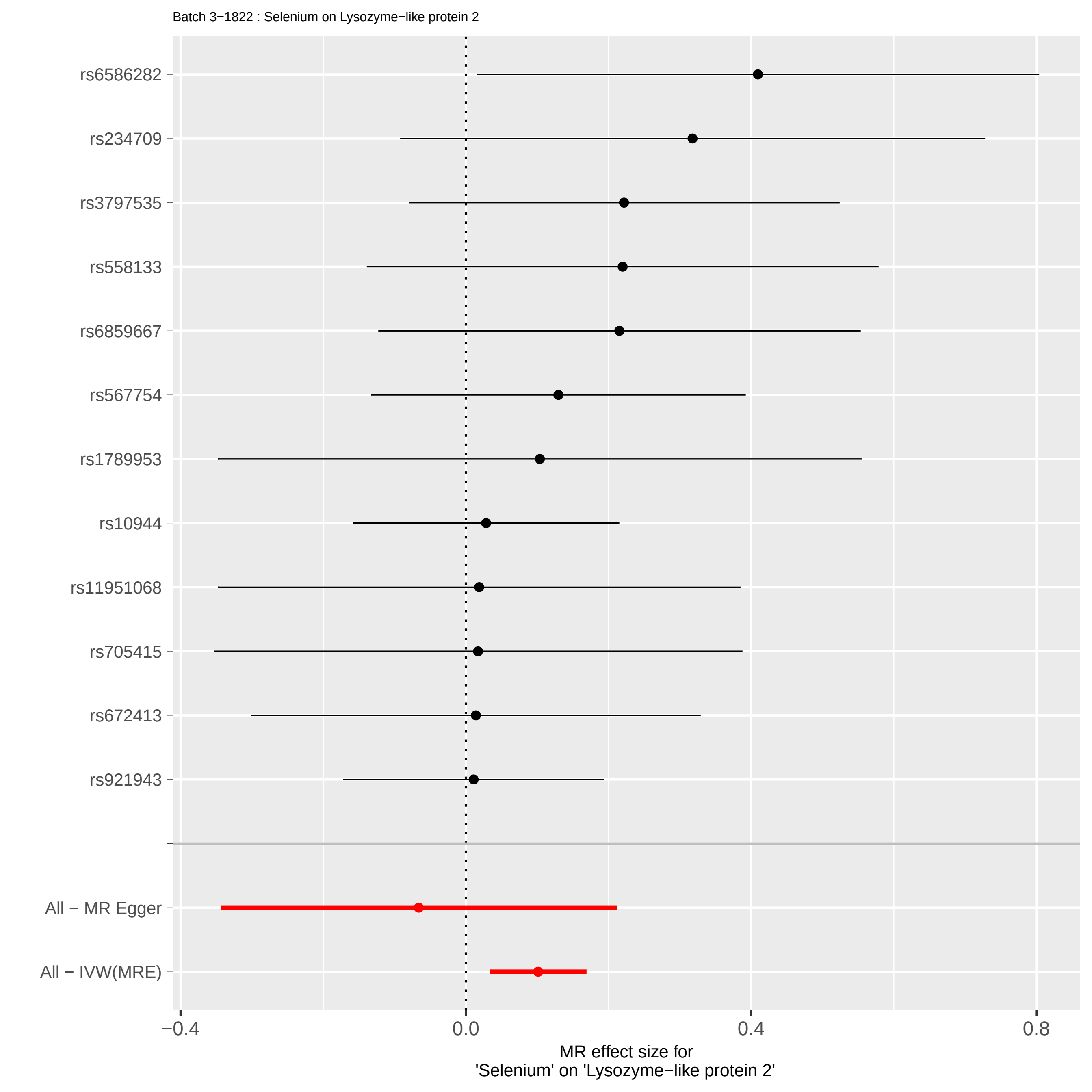

Batch 3−1822 : Selenium on Lysozyme−like protein 2
rs6586282
rs234709
rs3797535
rs558133
rs6859667
rs567754
rs1789953
rs10944
rs11951068
rs705415
rs672413
rs921943
All − MR Egger
All − IVW(MRE)
−0.4
0.0
0.4
0.8
MR effect size for
'Selenium' on 'Lysozyme−like protein 2'

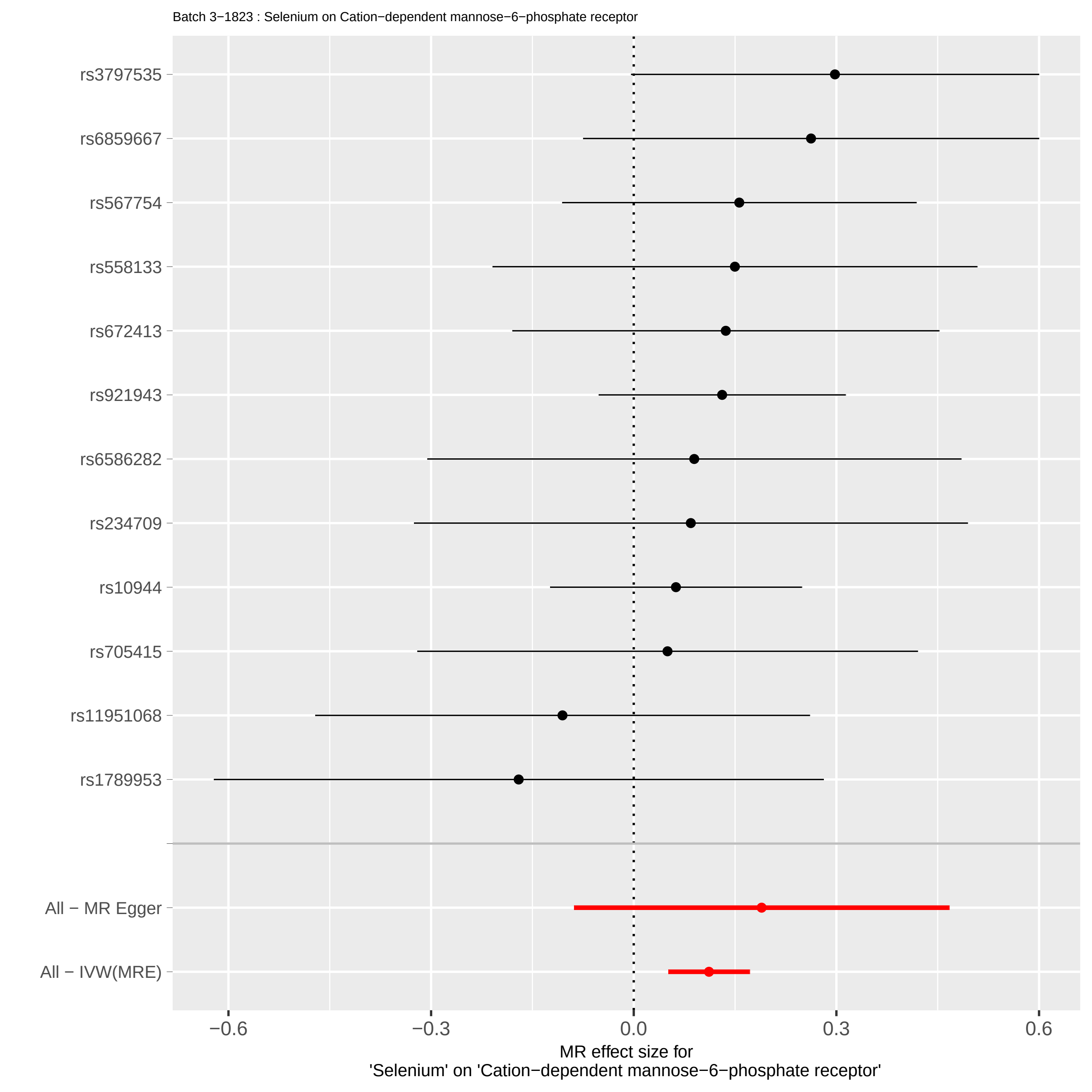
Batch 3−1823 : Selenium on Cation−dependent mannose−6−phosphate receptor
rs3797535
rs6859667
rs567754
rs558133
rs672413
rs921943
rs6586282
rs234709
rs10944
rs705415
rs11951068
rs1789953
All − MR Egger
All − IVW(MRE)
−0.6
−0.3
0.0
0.3
0.6
MR effect size for
'Selenium' on 'Cation−dependent mannose−6−phosphate receptor'

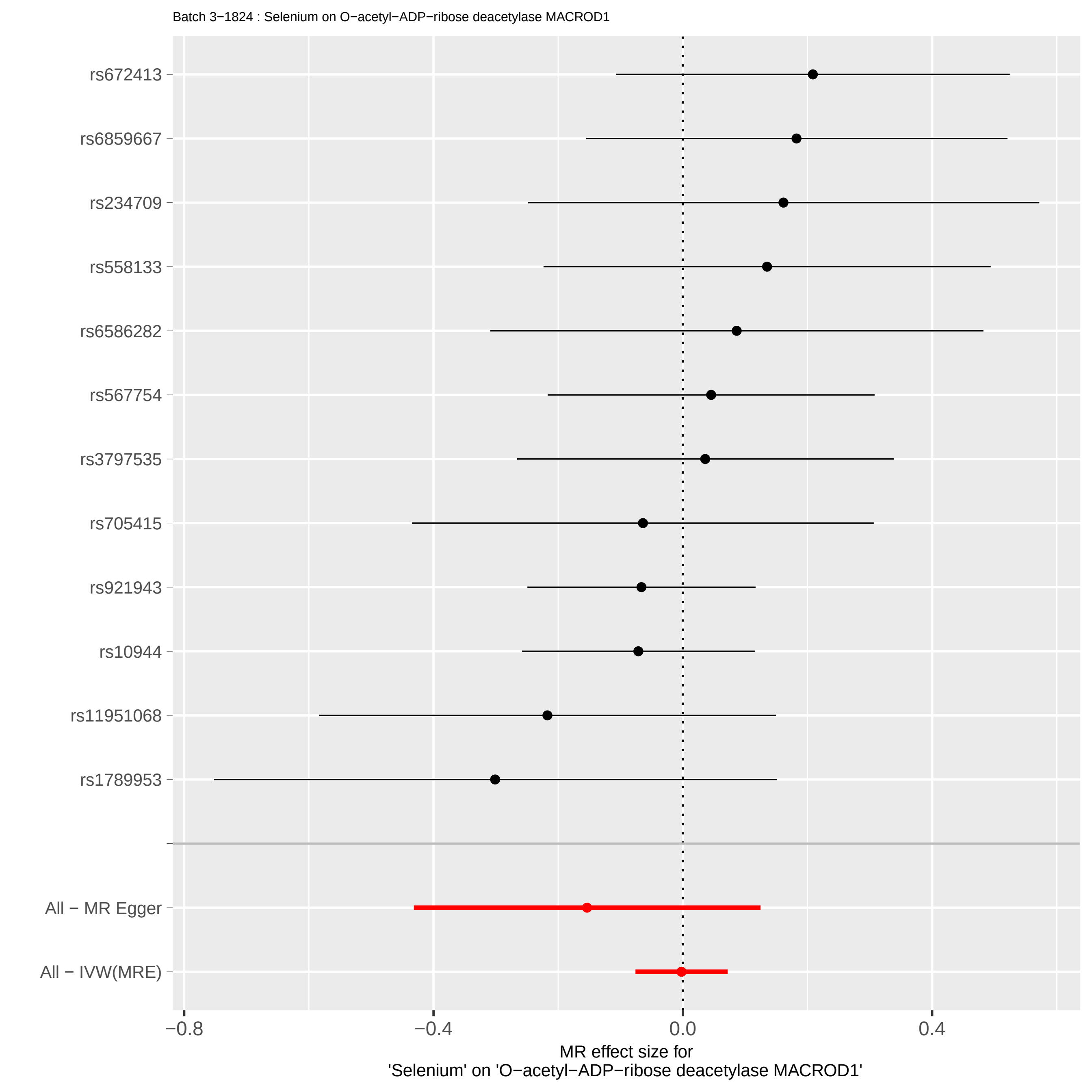
Batch 3−1824 : Selenium on O−acetyl−ADP−ribose deacetylase MACROD1
rs672413
rs6859667
rs234709
rs558133
rs6586282
rs567754
rs3797535
rs705415
rs921943
rs10944
rs11951068
rs1789953
All − MR Egger
All − IVW(MRE)
−0.8
−0.4
0.0
0.4
MR effect size for
'Selenium' on 'O−acetyl−ADP−ribose deacetylase MACROD1'

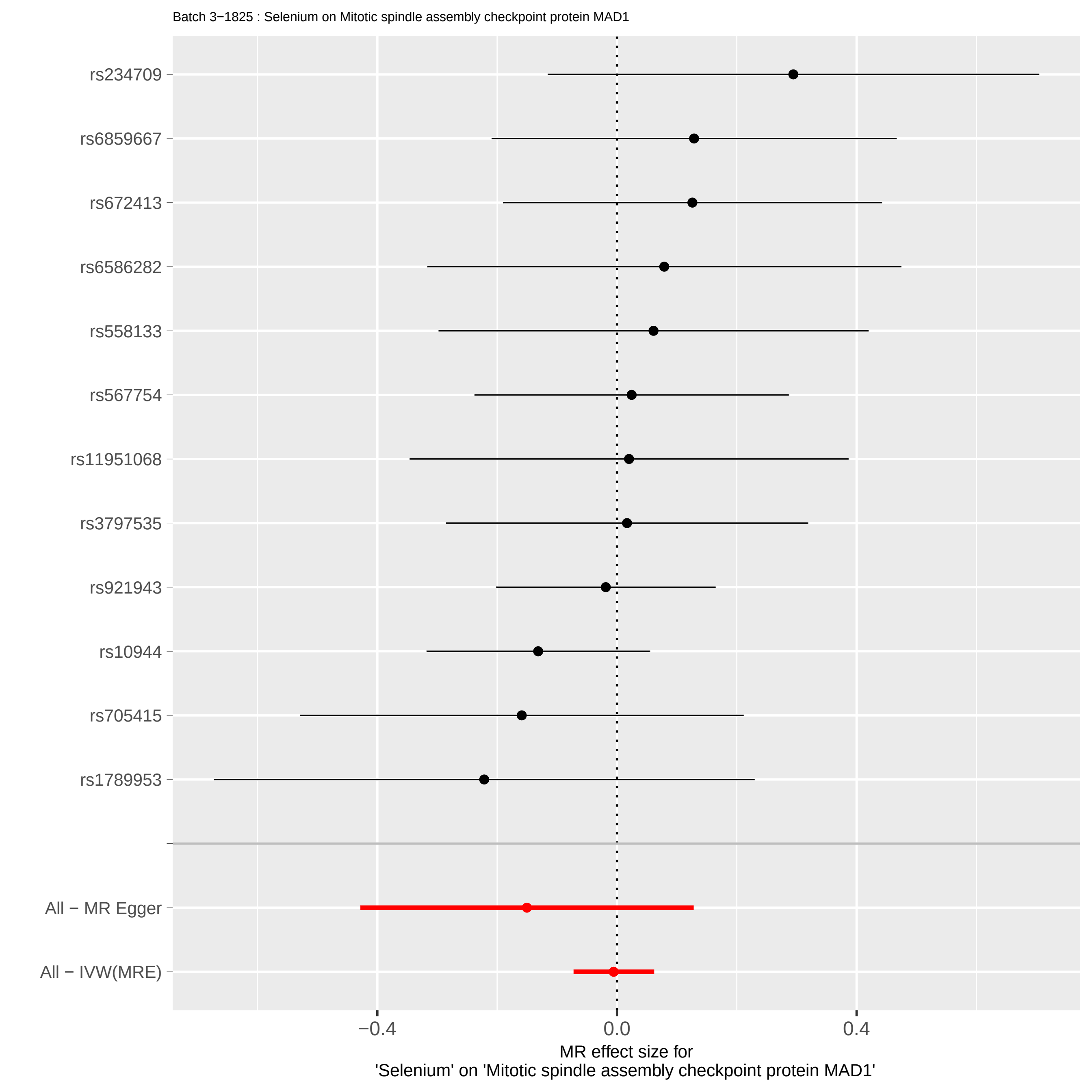
Batch 3−1825 : Selenium on Mitotic spindle assembly checkpoint protein MAD1
rs234709
rs6859667
rs672413
rs6586282
rs558133
rs567754
rs11951068
rs3797535
rs921943
rs10944
rs705415
rs1789953
All − MR Egger
All − IVW(MRE)
−0.4
0.0
0.4
MR effect size for
'Selenium' on 'Mitotic spindle assembly checkpoint protein MAD1'

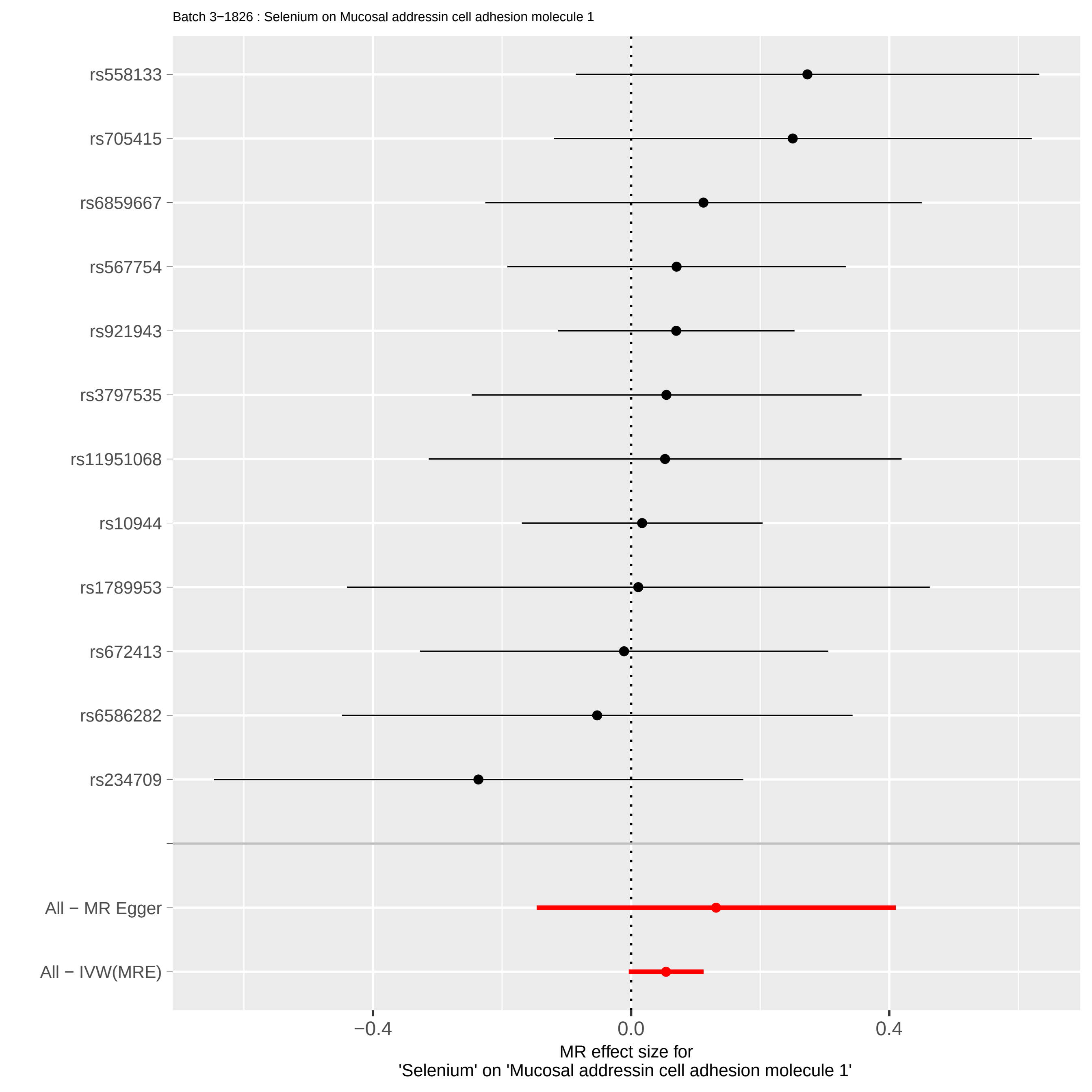

Batch 3−1826 : Selenium on Mucosal addressin cell adhesion molecule 1
rs558133
rs705415
rs6859667
rs567754
rs921943
rs3797535
rs11951068
rs10944
rs1789953
rs672413
rs6586282
rs234709
All − MR Egger
All − IVW(MRE)
−0.4
0.0
0.4
MR effect size for
'Selenium' on 'Mucosal addressin cell adhesion molecule 1'

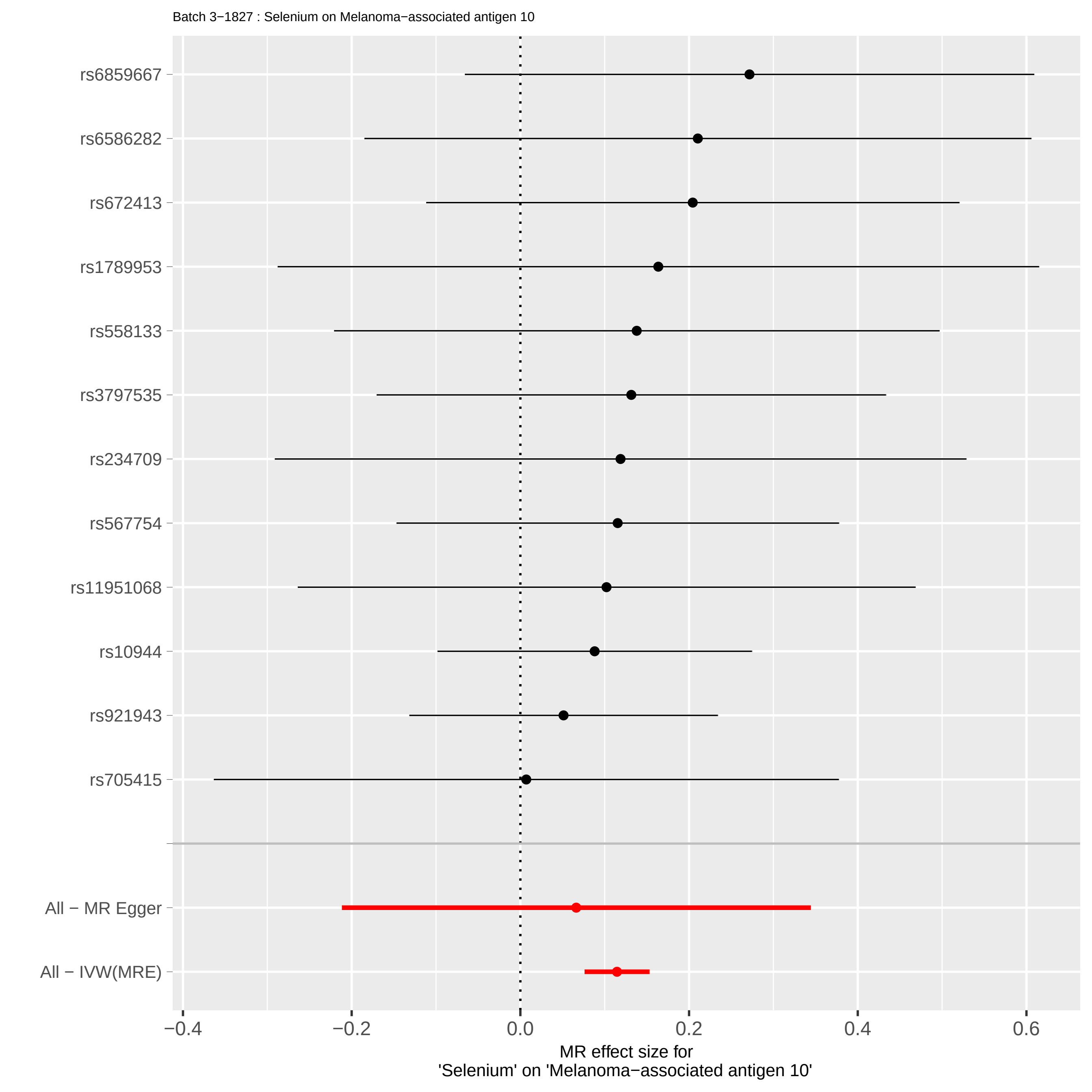

Batch 3−1827 : Selenium on Melanoma−associated antigen 10
rs6859667
rs6586282
rs672413
rs1789953
rs558133
rs3797535
rs234709
rs567754
rs11951068
rs10944
rs921943
rs705415
All − MR Egger
All − IVW(MRE)
−0.4
−0.2
0.0
0.2
0.4
0.6
MR effect size for
'Selenium' on 'Melanoma−associated antigen 10'

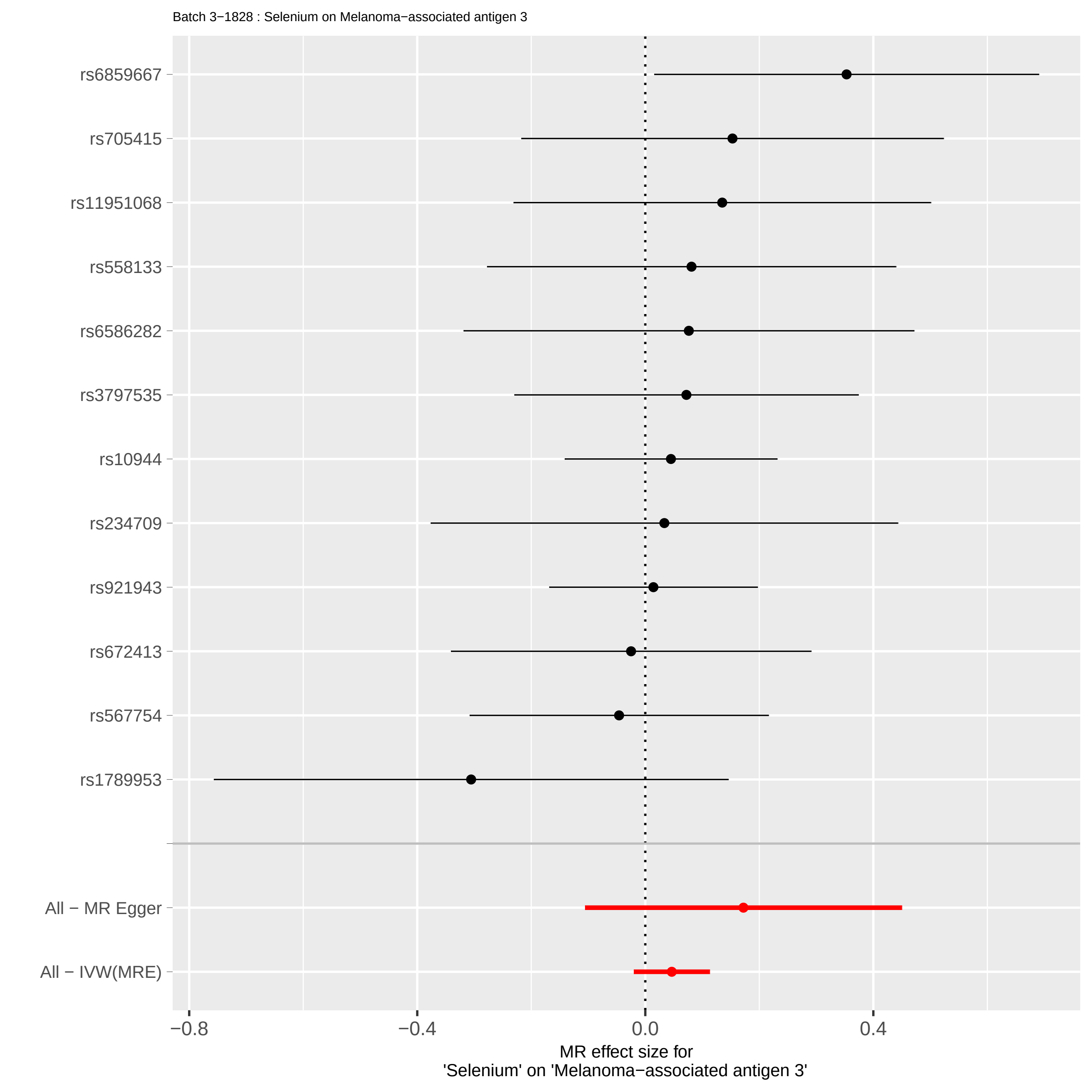

Batch 3−1828 : Selenium on Melanoma−associated antigen 3
rs6859667
rs705415
rs11951068
rs558133
rs6586282
rs3797535
rs10944
rs234709
rs921943
rs672413
rs567754
rs1789953
All − MR Egger
All − IVW(MRE)
−0.8
−0.4
0.0
0.4
MR effect size for
'Selenium' on 'Melanoma−associated antigen 3'

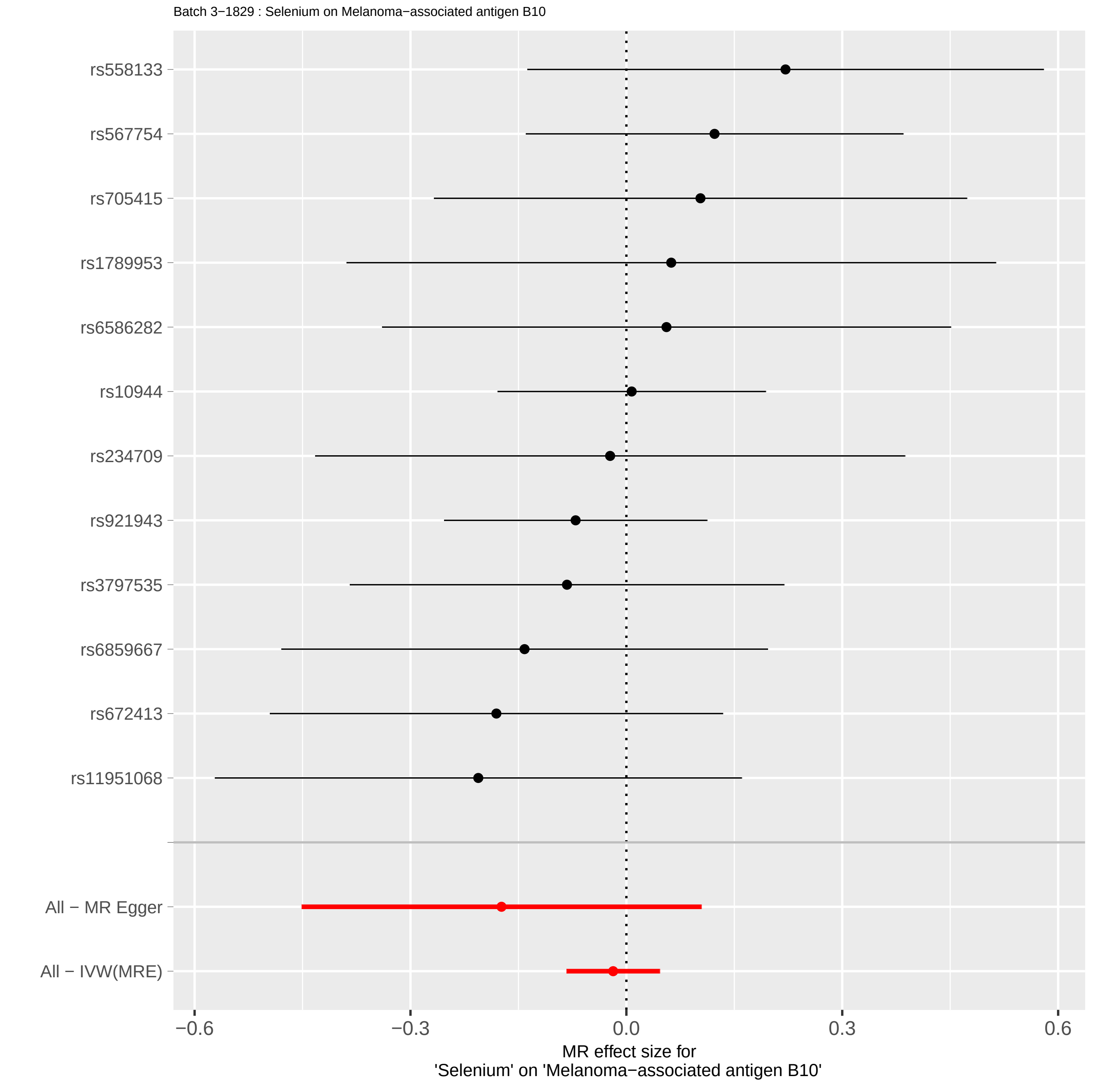

Batch 3−1829 : Selenium on Melanoma−associated antigen B10
rs558133
rs567754
rs705415
rs1789953
rs6586282
rs10944
rs234709
rs921943
rs3797535
rs6859667
rs672413
rs11951068
All − MR Egger
All − IVW(MRE)
−0.6
−0.3
0.0
0.3
0.6
MR effect size for
'Selenium' on 'Melanoma−associated antigen B10'

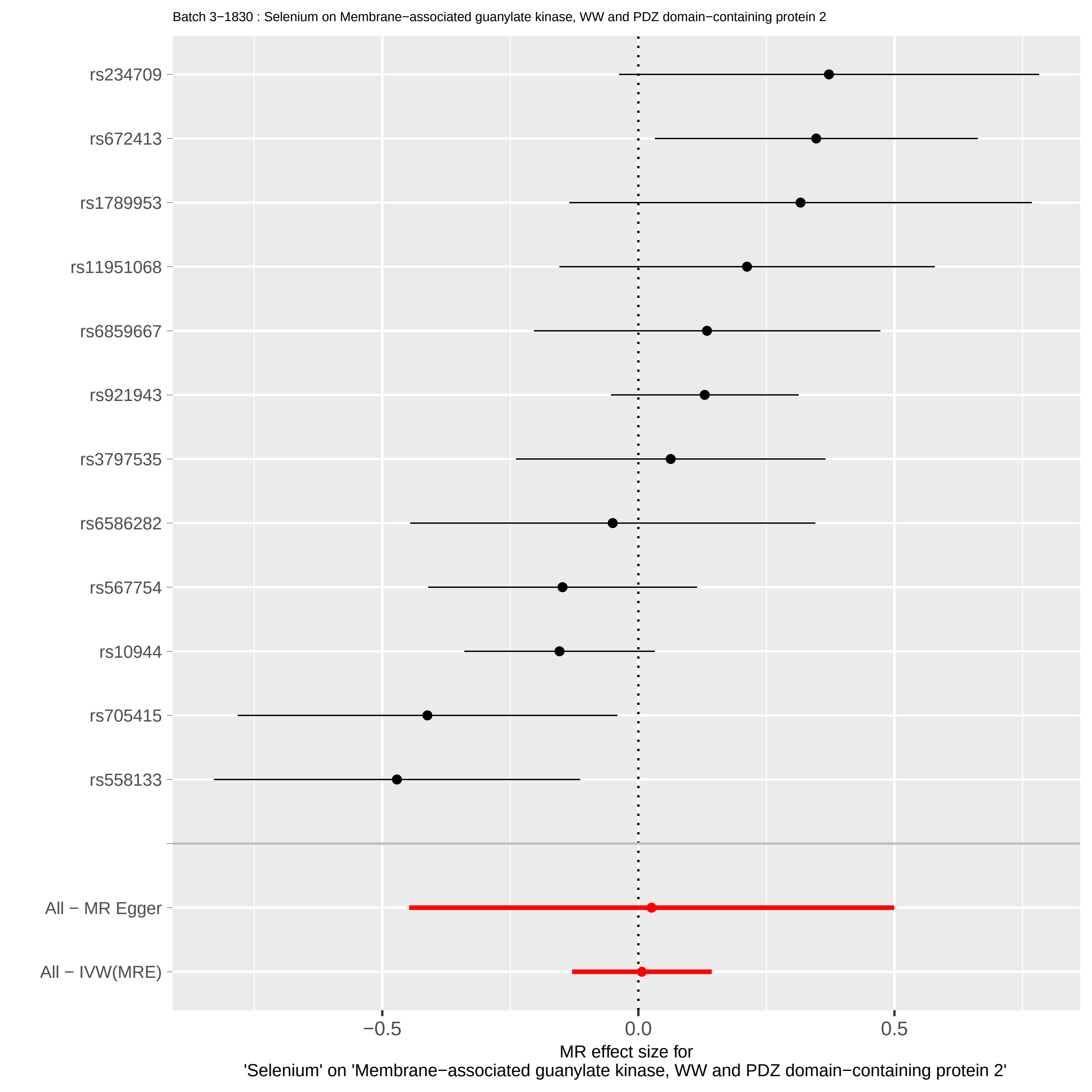
Batch 3−1830 : Selenium on Membrane−associated guanylate kinase, WW and PDZ domain−containing protein 2
rs234709
rs672413
rs1789953
rs11951068
rs6859667
rs921943
rs3797535
rs6586282
rs567754
rs10944
rs705415
rs558133
All − MR Egger
All − IVW(MRE)
−0.5
0.0
0.5
MR effect size for
'Selenium' on 'Membrane−associated guanylate kinase, WW and PDZ domain−containing protein 2'

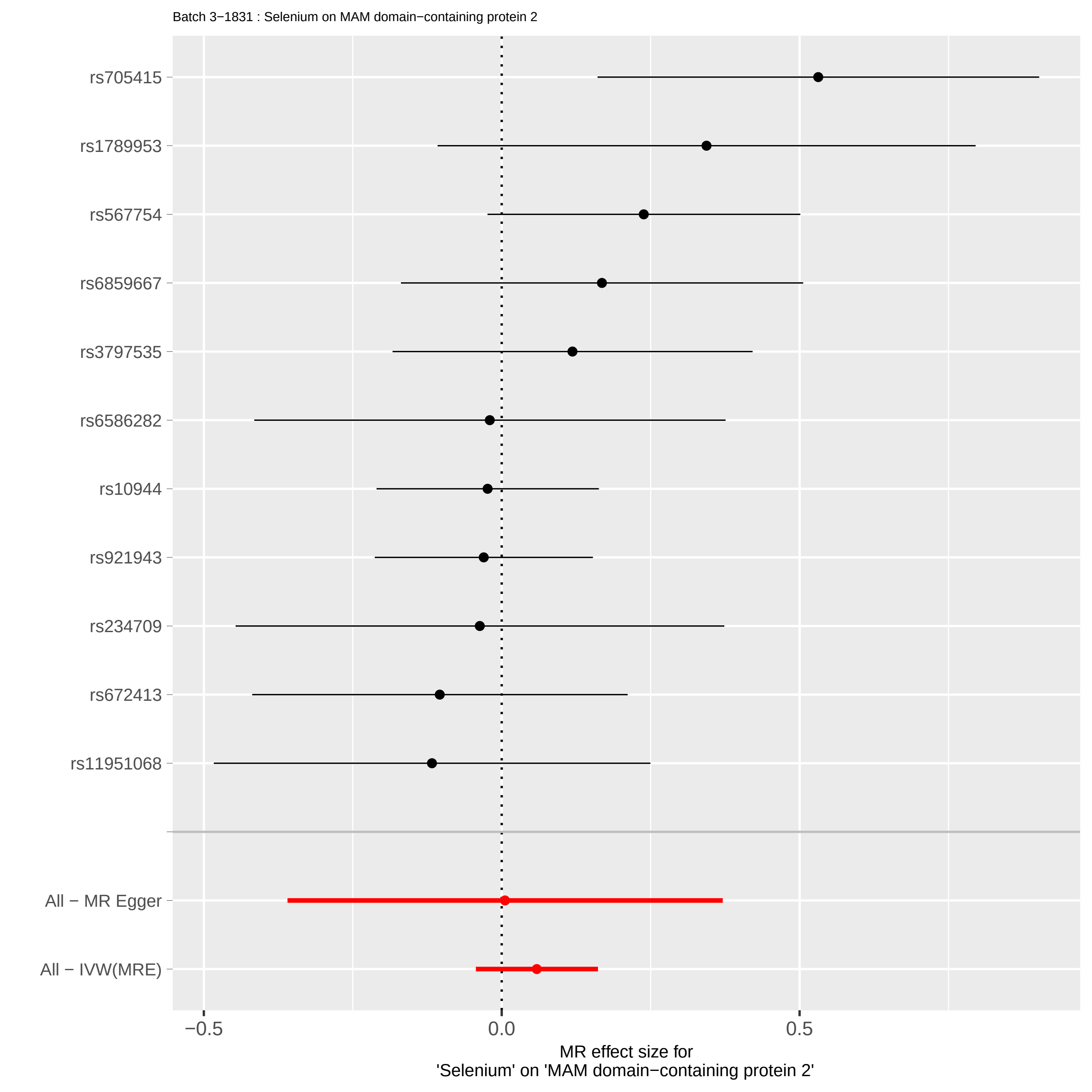

Batch 3−1831 : Selenium on MAM domain−containing protein 2
rs705415
rs1789953
rs567754
rs6859667
rs3797535
rs6586282
rs10944
rs921943
rs234709
rs672413
rs11951068
All − MR Egger
All − IVW(MRE)
−0.5
0.0
0.5
MR effect size for
'Selenium' on 'MAM domain−containing protein 2'

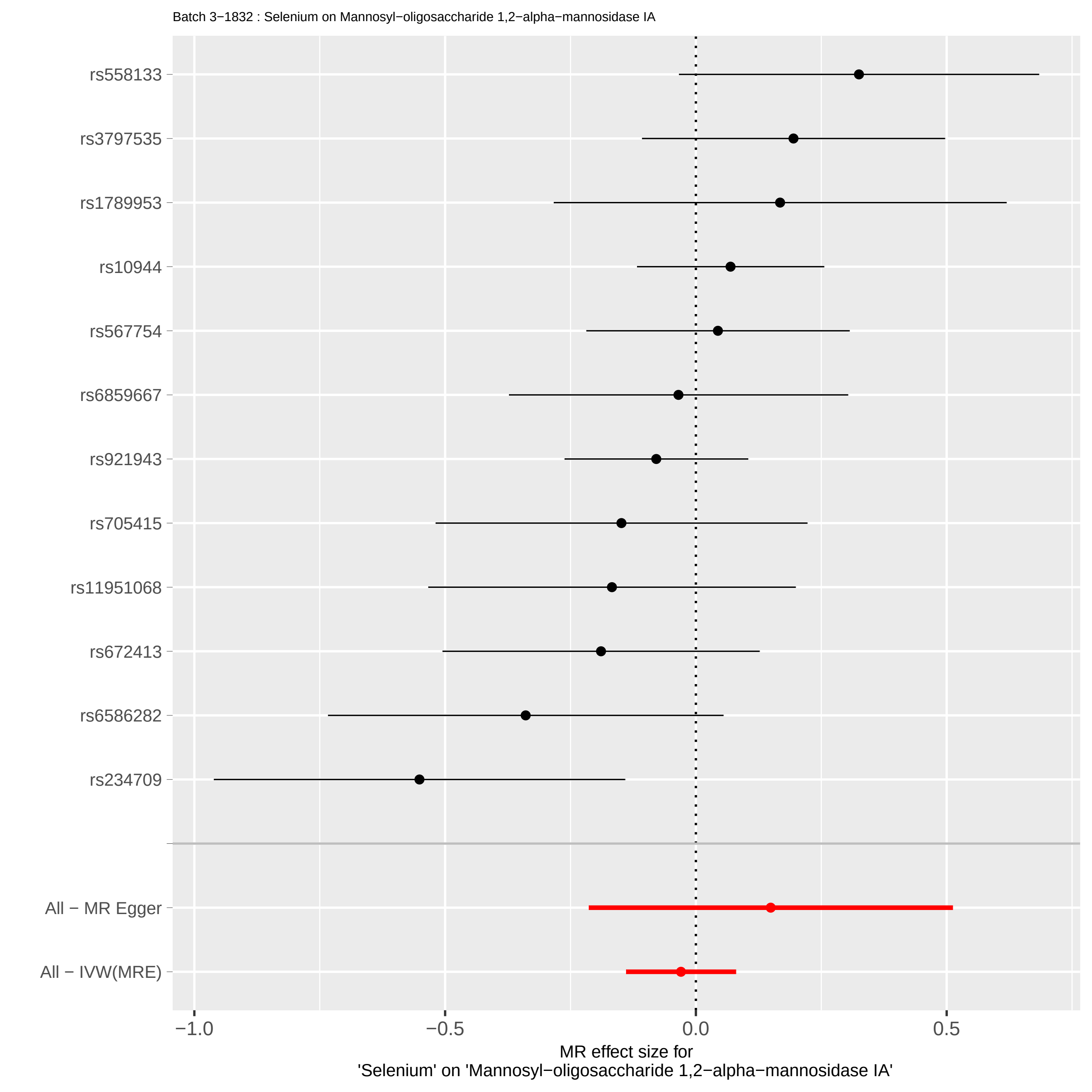

Batch 3−1832 : Selenium on Mannosyl−oligosaccharide 1,2−alpha−mannosidase IA
rs558133
rs3797535
rs1789953
rs10944
rs567754
rs6859667
rs921943
rs705415
rs11951068
rs672413
rs6586282
rs234709
All − MR Egger
All − IVW(MRE)
−1.0
−0.5
0.0
0.5
MR effect size for
'Selenium' on 'Mannosyl−oligosaccharide 1,2−alpha−mannosidase IA'

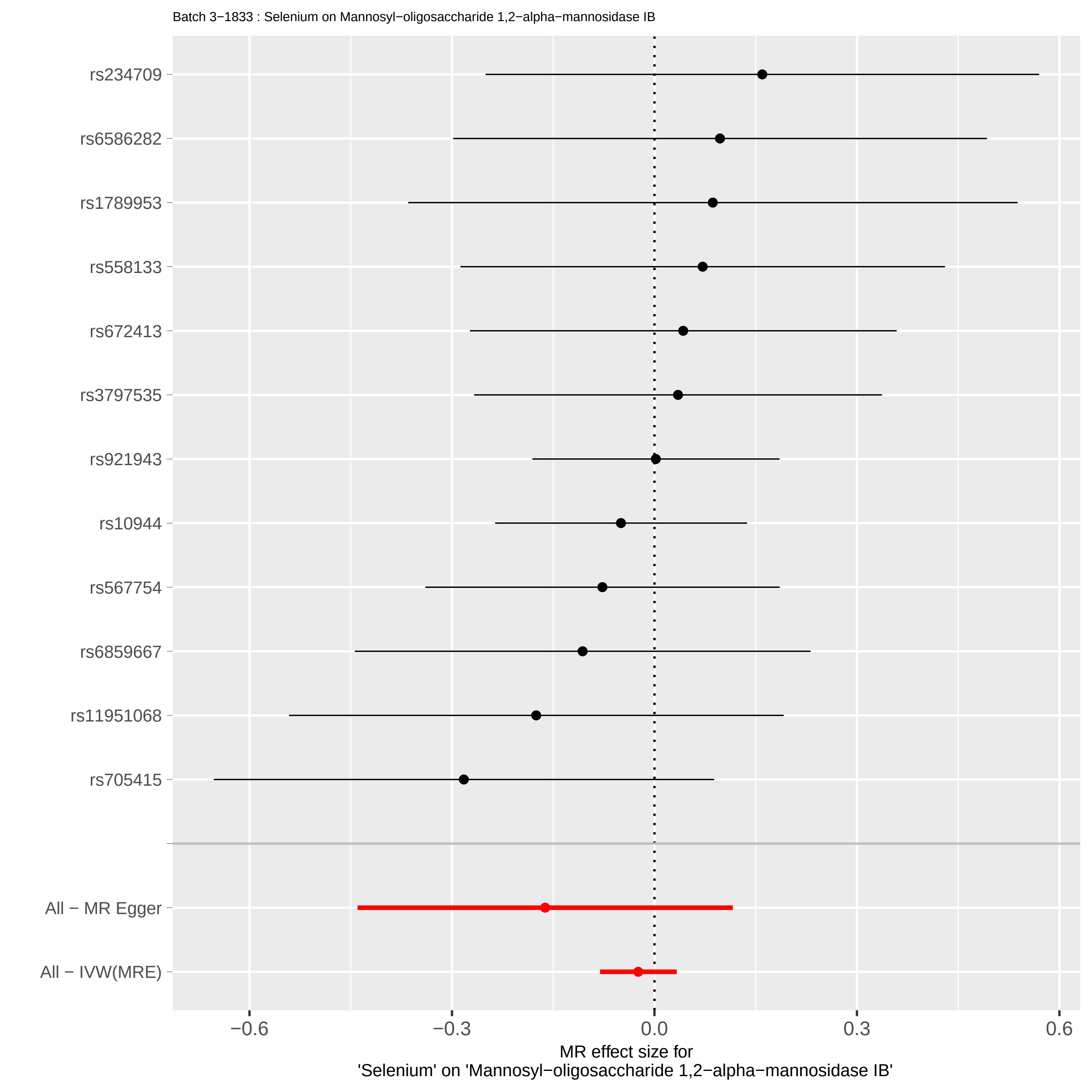
Batch 3−1833 : Selenium on Mannosyl−oligosaccharide 1,2−alpha−mannosidase IB
rs234709
rs6586282
rs1789953
rs558133
rs672413
rs3797535
rs921943
rs10944
rs567754
rs6859667
rs11951068
rs705415
All − MR Egger
All − IVW(MRE)
−0.6
−0.3
0.0
0.3
0.6
MR effect size for
'Selenium' on 'Mannosyl−oligosaccharide 1,2−alpha−mannosidase IB'

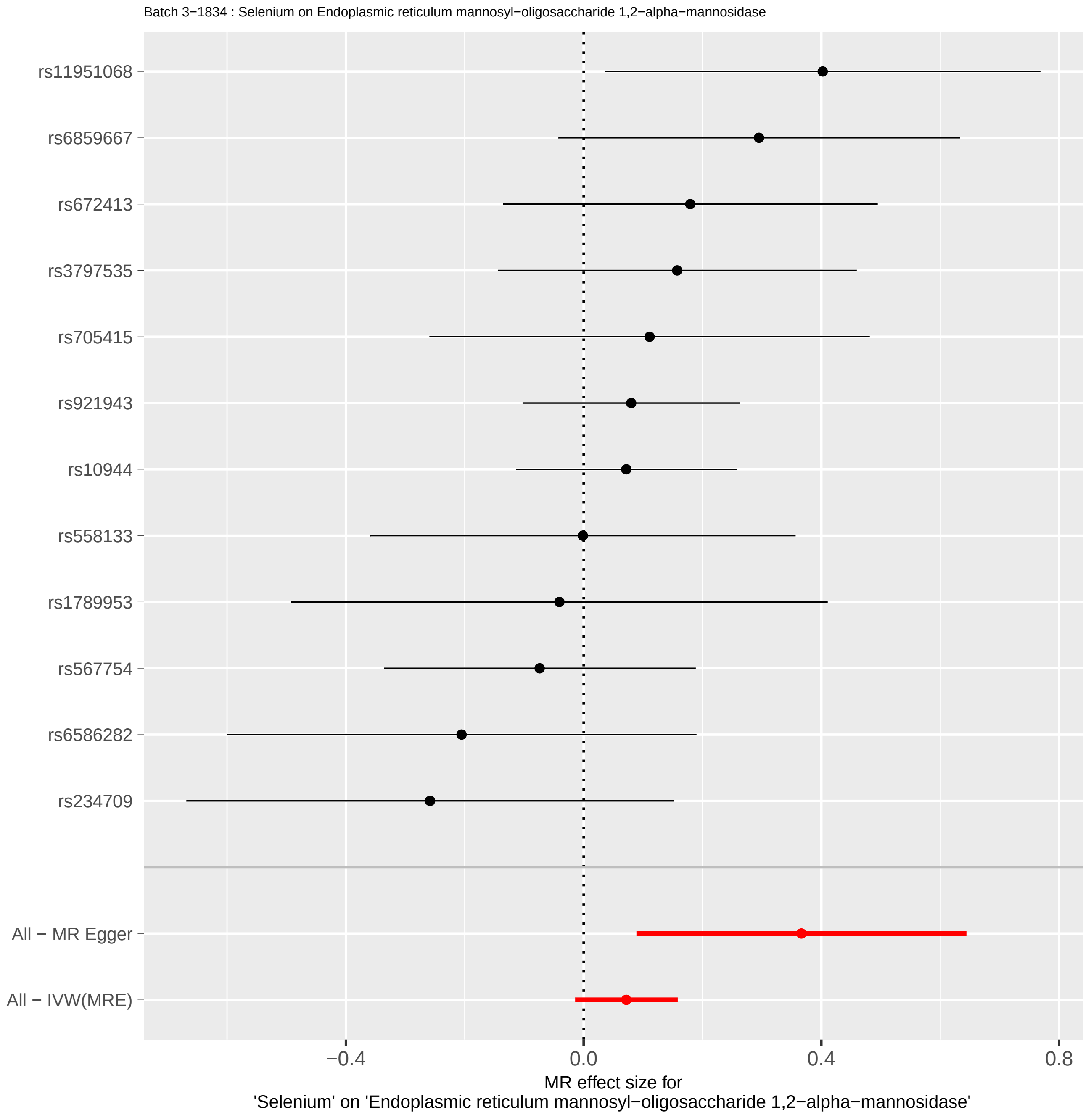

Batch 3−1834 : Selenium on Endoplasmic reticulum mannosyl−oligosaccharide 1,2−alpha−mannosidase
rs11951068
rs6859667
rs672413
rs3797535
rs705415
rs921943
rs10944
rs558133
rs1789953
rs567754
rs6586282
rs234709
All − MR Egger
All − IVW(MRE)
−0.4
0.0
0.4
0.8
MR effect size for
'Selenium' on 'Endoplasmic reticulum mannosyl−oligosaccharide 1,2−alpha−mannosidase'

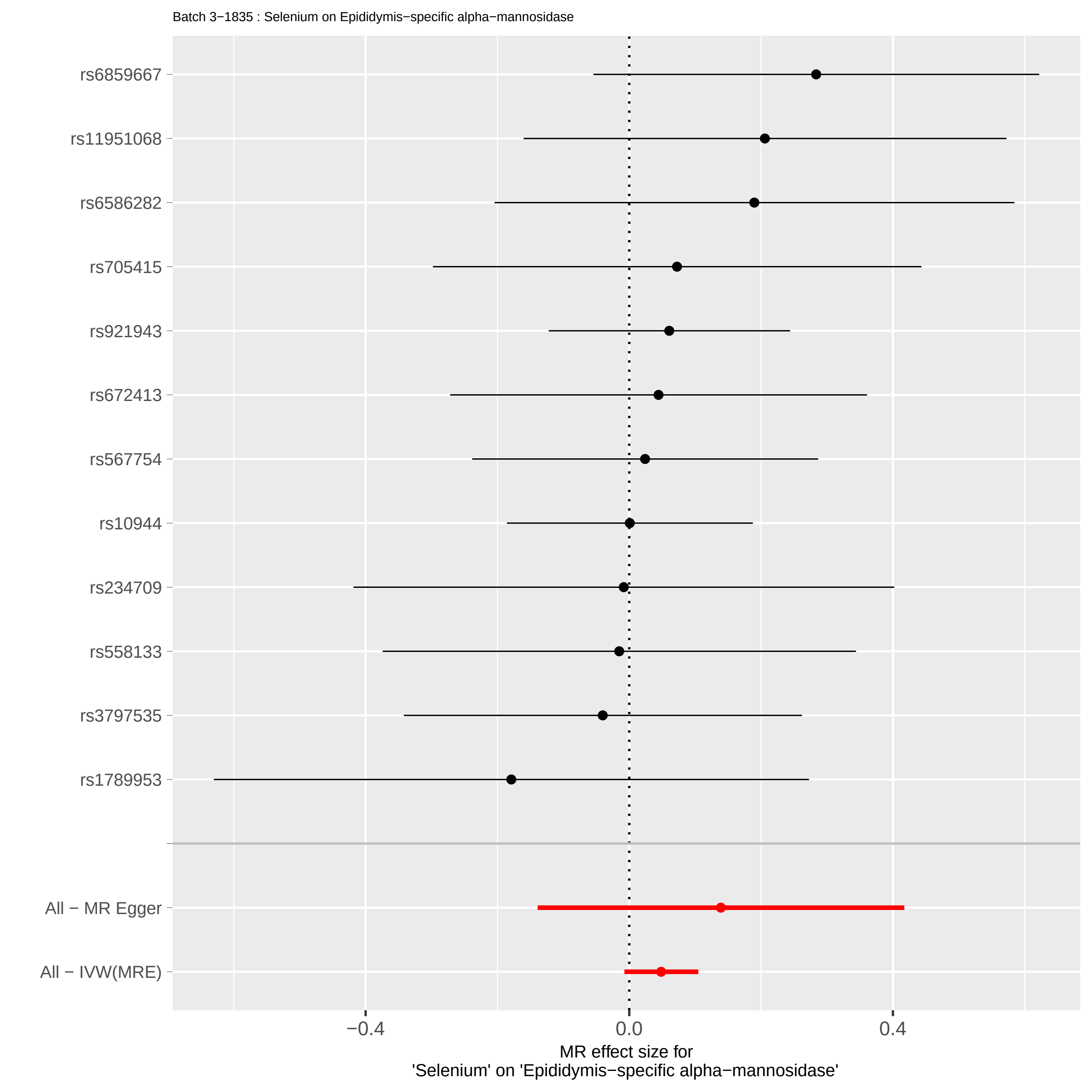

Batch 3−1835 : Selenium on Epididymis−specific alpha−mannosidase
rs6859667
rs11951068
rs6586282
rs705415
rs921943
rs672413
rs567754
rs10944
rs234709
rs558133
rs3797535
rs1789953
All − MR Egger
All − IVW(MRE)
−0.4
0.0
0.4
MR effect size for
'Selenium' on 'Epididymis−specific alpha−mannosidase'

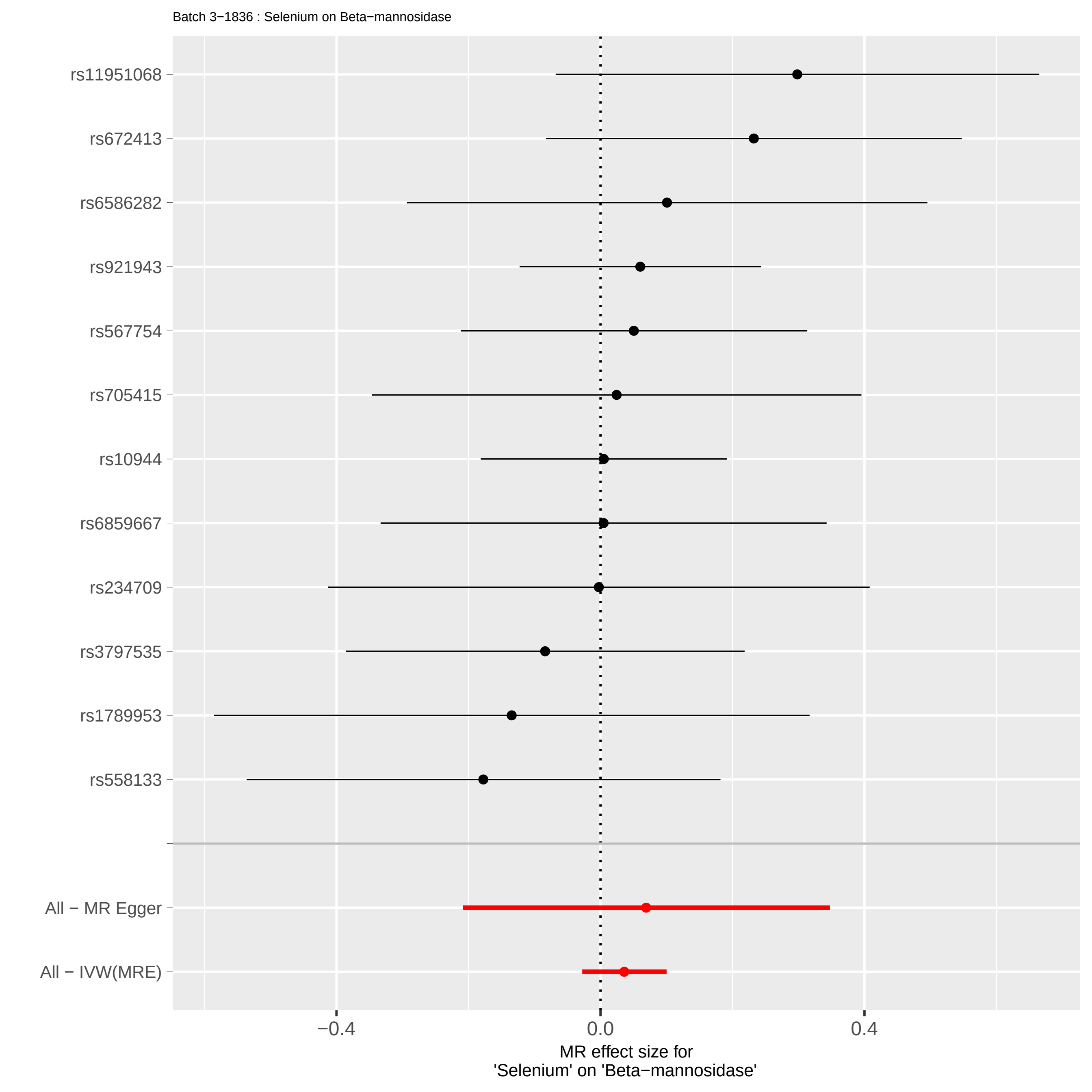

Batch 3−1836 : Selenium on Beta−mannosidase
rs11951068
rs672413
rs6586282
rs921943
rs567754
rs705415
rs10944
rs6859667
rs234709
rs3797535
rs1789953
rs558133
All − MR Egger
All − IVW(MRE)
−0.4
0.0
0.4
MR effect size for
'Selenium' on 'Beta−mannosidase'

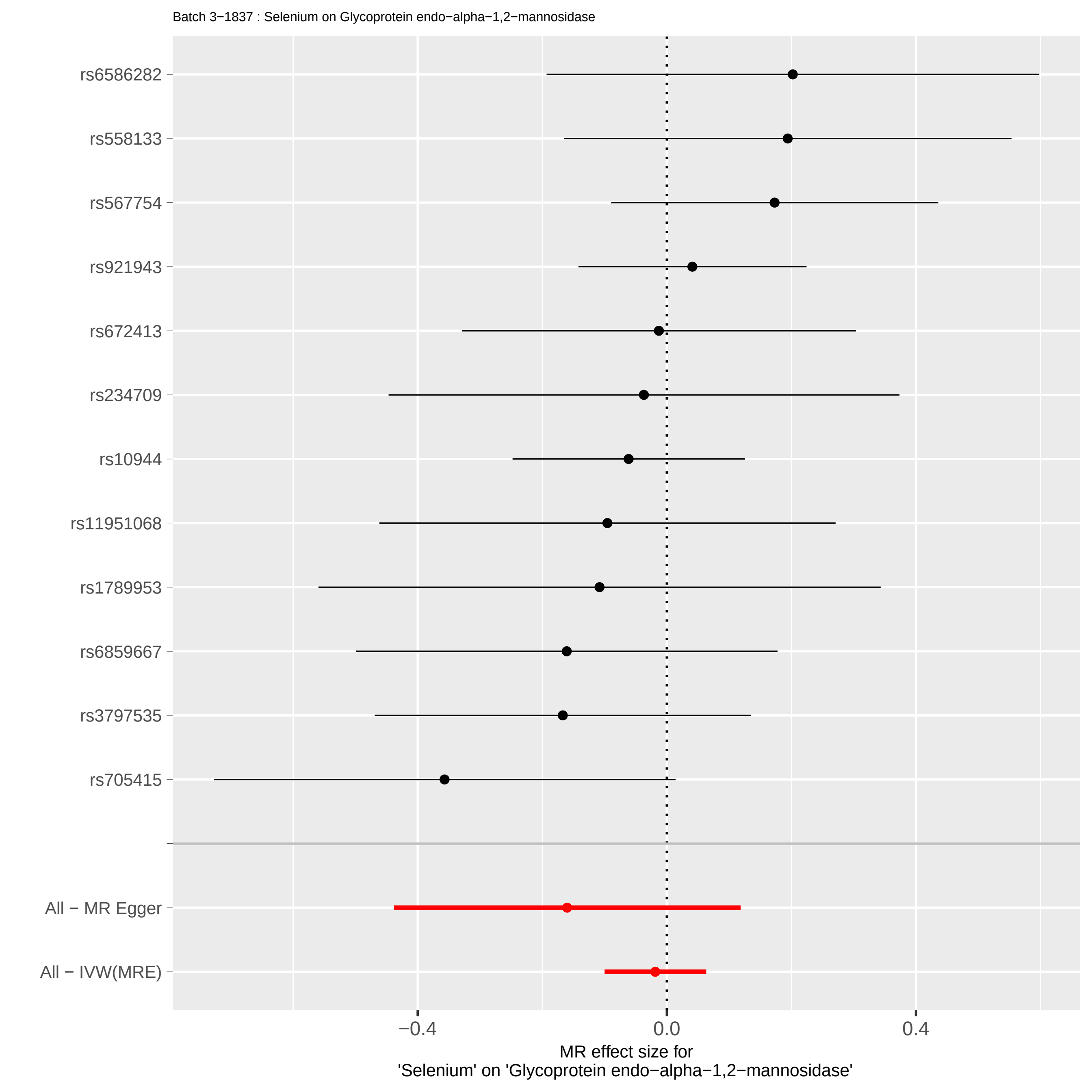

Batch 3−1837 : Selenium on Glycoprotein endo−alpha−1,2−mannosidase
rs6586282
rs558133
rs567754
rs921943
rs672413
rs234709
rs10944
rs11951068
rs1789953
rs6859667
rs3797535
rs705415
All − MR Egger
All − IVW(MRE)
−0.4
0.0
0.4
MR effect size for
'Selenium' on 'Glycoprotein endo−alpha−1,2−mannosidase'

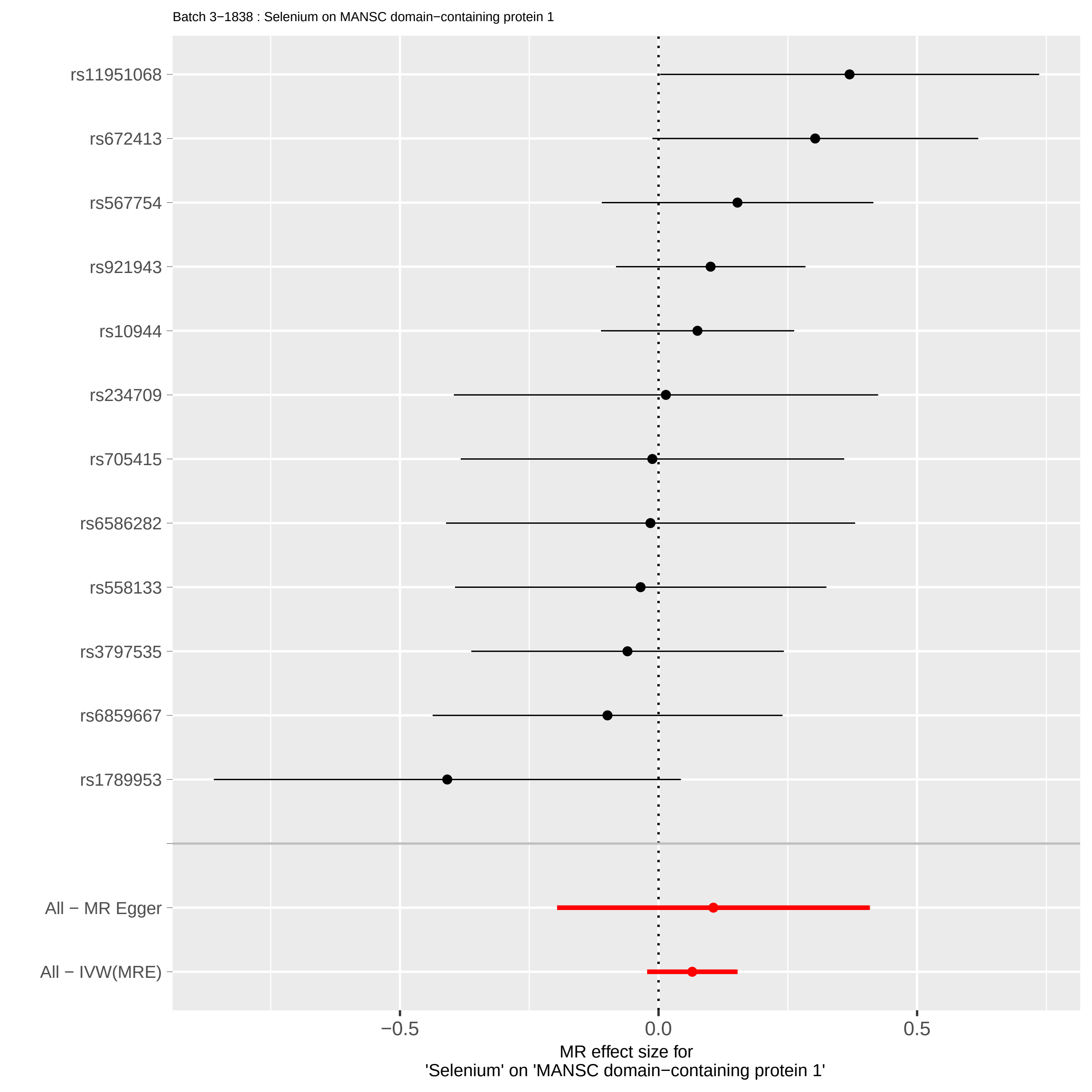
Batch 3−1838 : Selenium on MANSC domain−containing protein 1
rs11951068
rs672413
rs567754
rs921943
rs10944
rs234709
rs705415
rs6586282
rs558133
rs3797535
rs6859667
rs1789953
All − MR Egger
All − IVW(MRE)
−0.5
0.0
0.5
MR effect size for
'Selenium' on 'MANSC domain−containing protein 1'

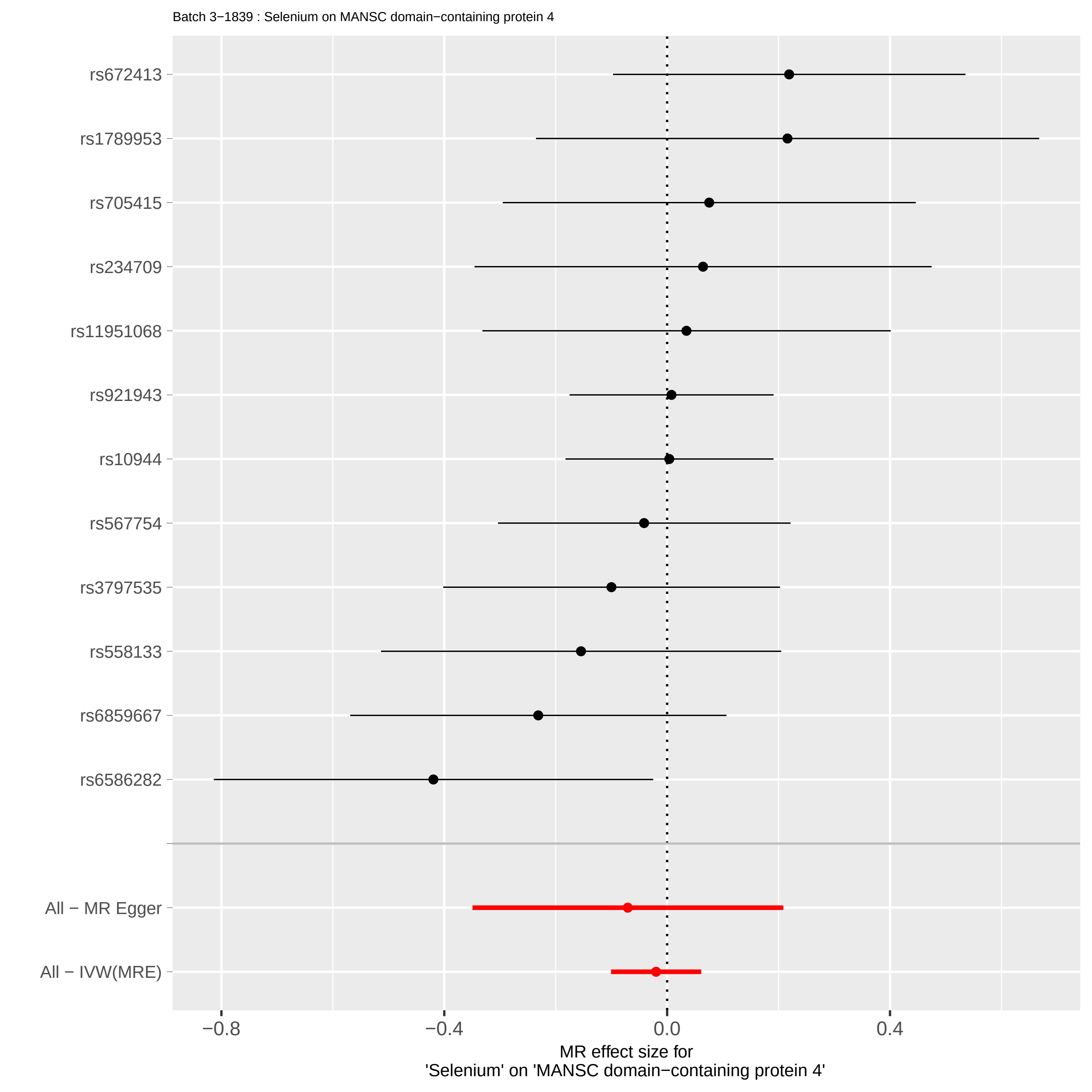

Batch 3−1839 : Selenium on MANSC domain−containing protein 4
rs672413
rs1789953
rs705415
rs234709
rs11951068
rs921943
rs10944
rs567754
rs3797535
rs558133
rs6859667
rs6586282
All − MR Egger
All − IVW(MRE)
−0.8
−0.4
0.0
0.4
MR effect size for
'Selenium' on 'MANSC domain−containing protein 4'

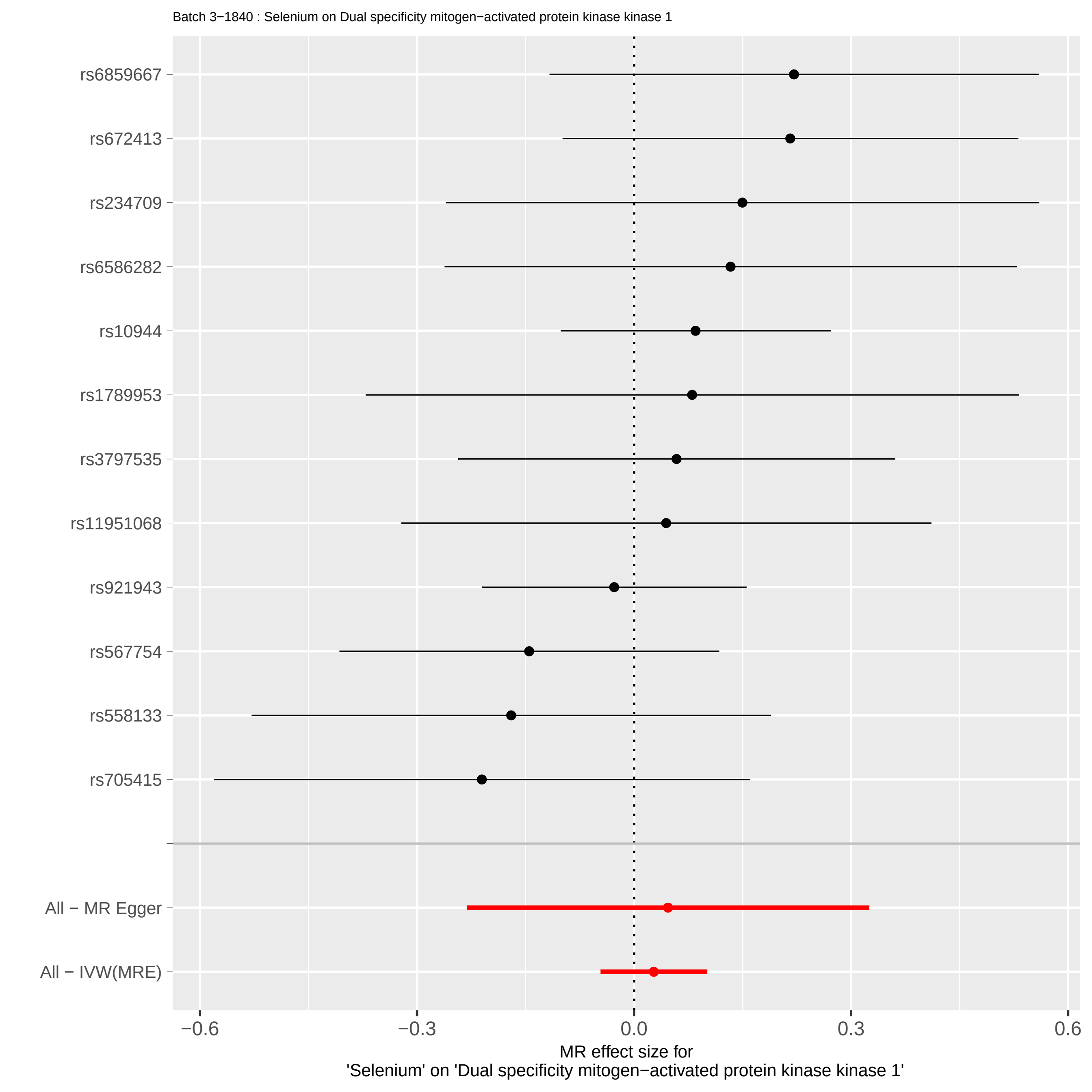
Batch 3−1840 : Selenium on Dual specificity mitogen−activated protein kinase kinase 1
rs6859667
rs672413
rs234709
rs6586282
rs10944
rs1789953
rs3797535
rs11951068
rs921943
rs567754
rs558133
rs705415
All − MR Egger
All − IVW(MRE)
−0.6
−0.3
0.0
0.3
0.6
MR effect size for
'Selenium' on 'Dual specificity mitogen−activated protein kinase kinase 1'

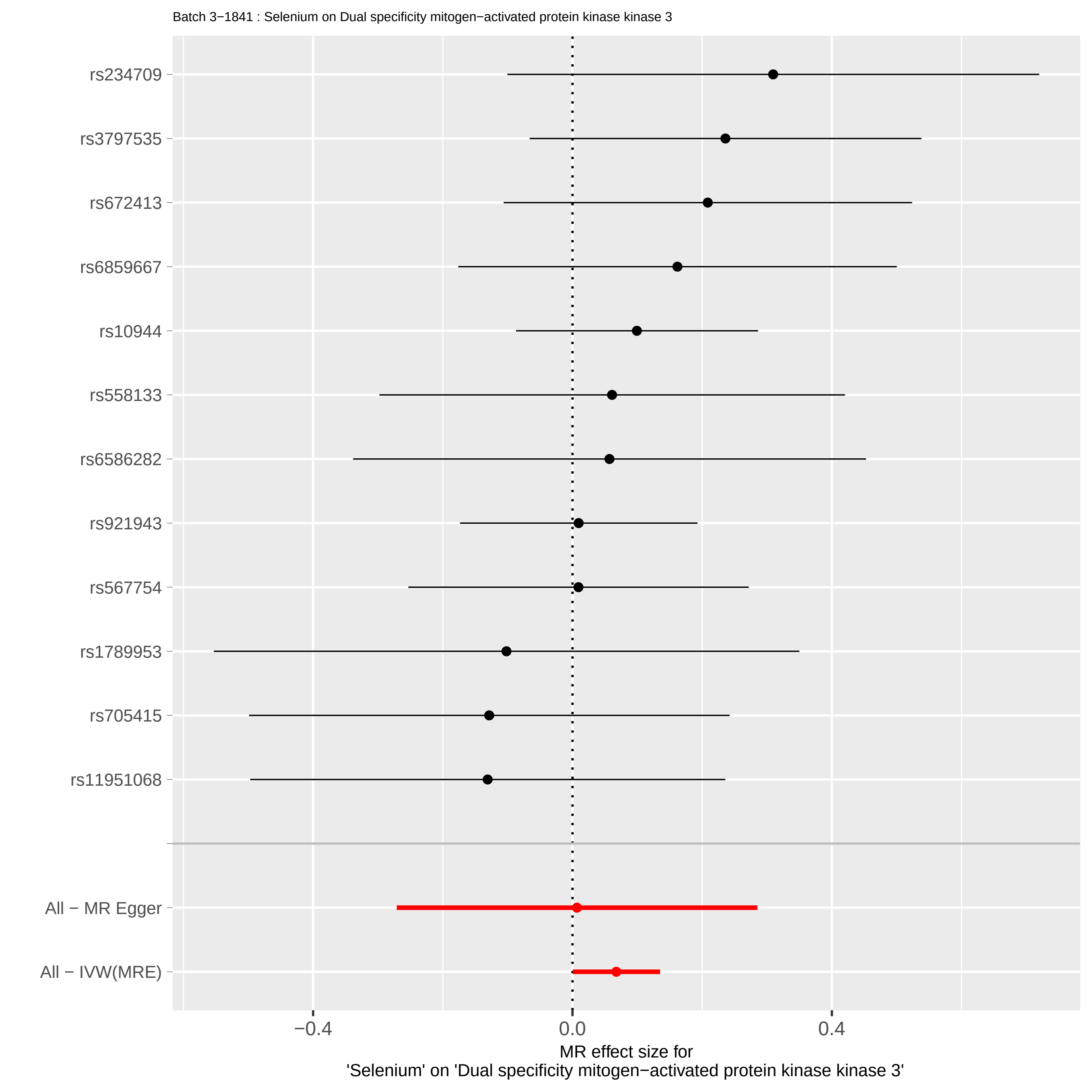

Batch 3−1841 : Selenium on Dual specificity mitogen−activated protein kinase kinase 3
rs234709
rs3797535
rs672413
rs6859667
rs10944
rs558133
rs6586282
rs921943
rs567754
rs1789953
rs705415
rs11951068
All − MR Egger
All − IVW(MRE)
−0.4
0.0
0.4
MR effect size for
'Selenium' on 'Dual specificity mitogen−activated protein kinase kinase 3'

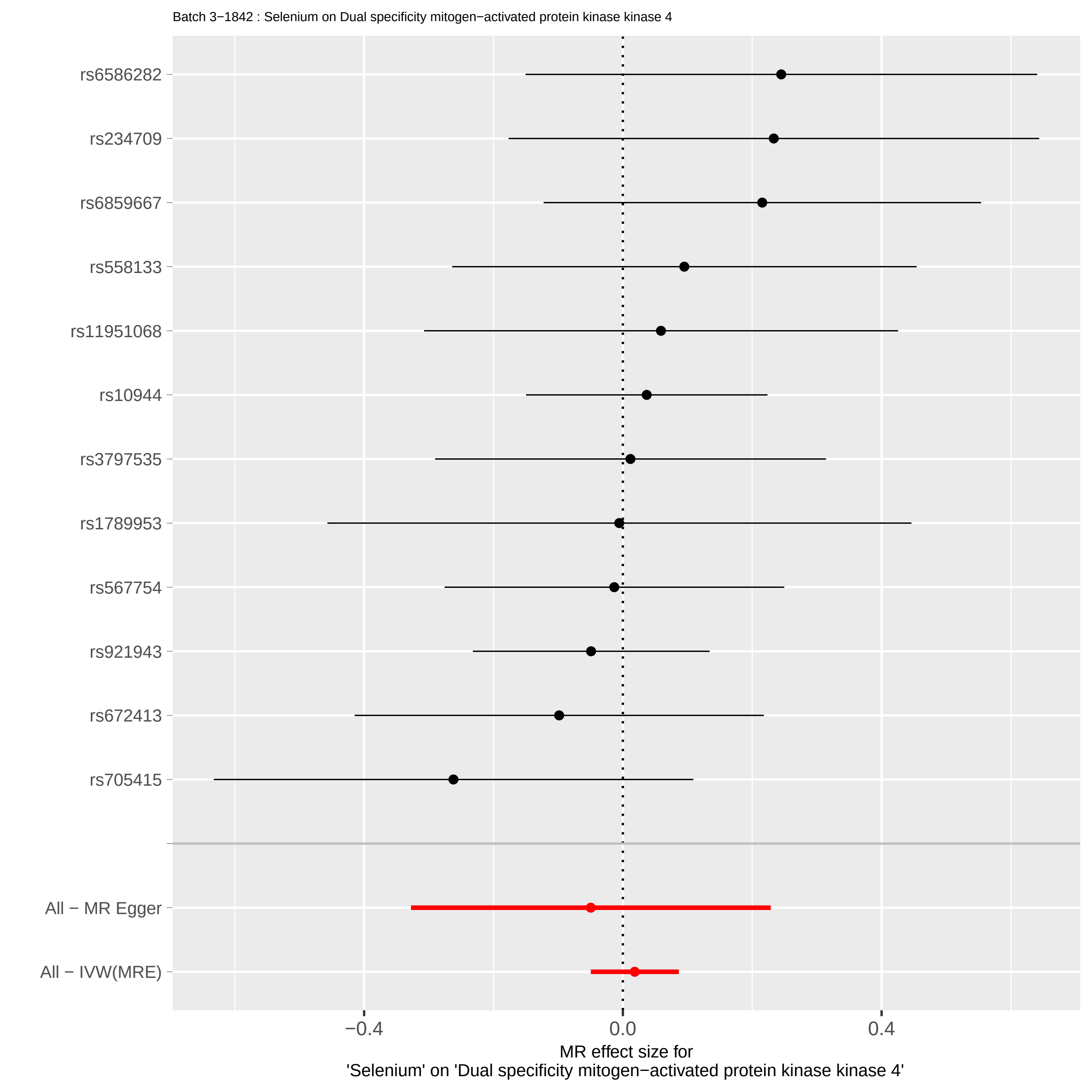
Batch 3−1842 : Selenium on Dual specificity mitogen−activated protein kinase kinase 4
rs6586282
rs234709
rs6859667
rs558133
rs11951068
rs10944
rs3797535
rs1789953
rs567754
rs921943
rs672413
rs705415
All − MR Egger
All − IVW(MRE)
−0.4
0.0
0.4
MR effect size for
'Selenium' on 'Dual specificity mitogen−activated protein kinase kinase 4'

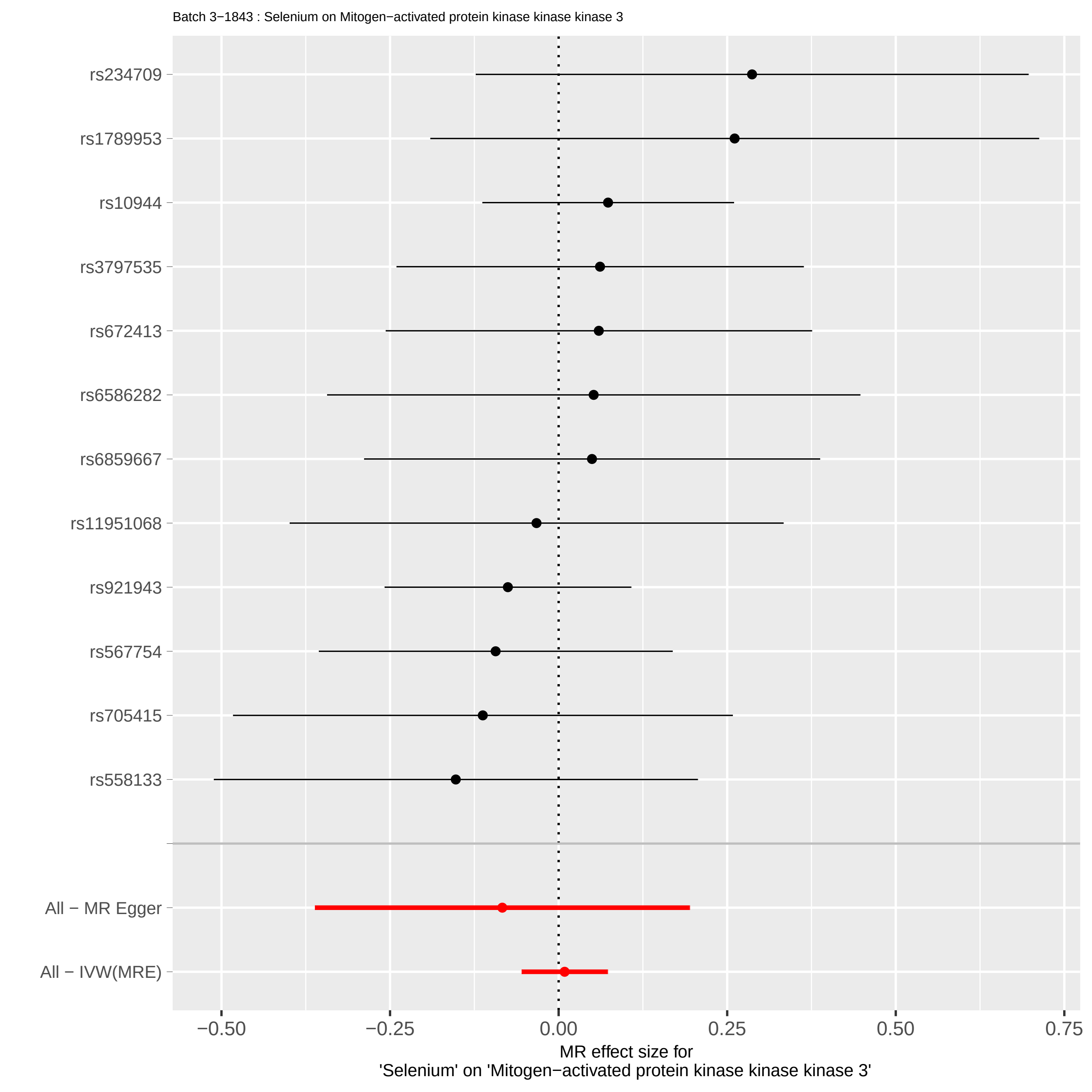
Batch 3−1843 : Selenium on Mitogen−activated protein kinase kinase kinase 3
rs234709
rs1789953
rs10944
rs3797535
rs672413
rs6586282
rs6859667
rs11951068
rs921943
rs567754
rs705415
rs558133
All − MR Egger
All − IVW(MRE)
−0.50
−0.25
0.00
0.25
0.50
0.75
MR effect size for
'Selenium' on 'Mitogen−activated protein kinase kinase kinase 3'

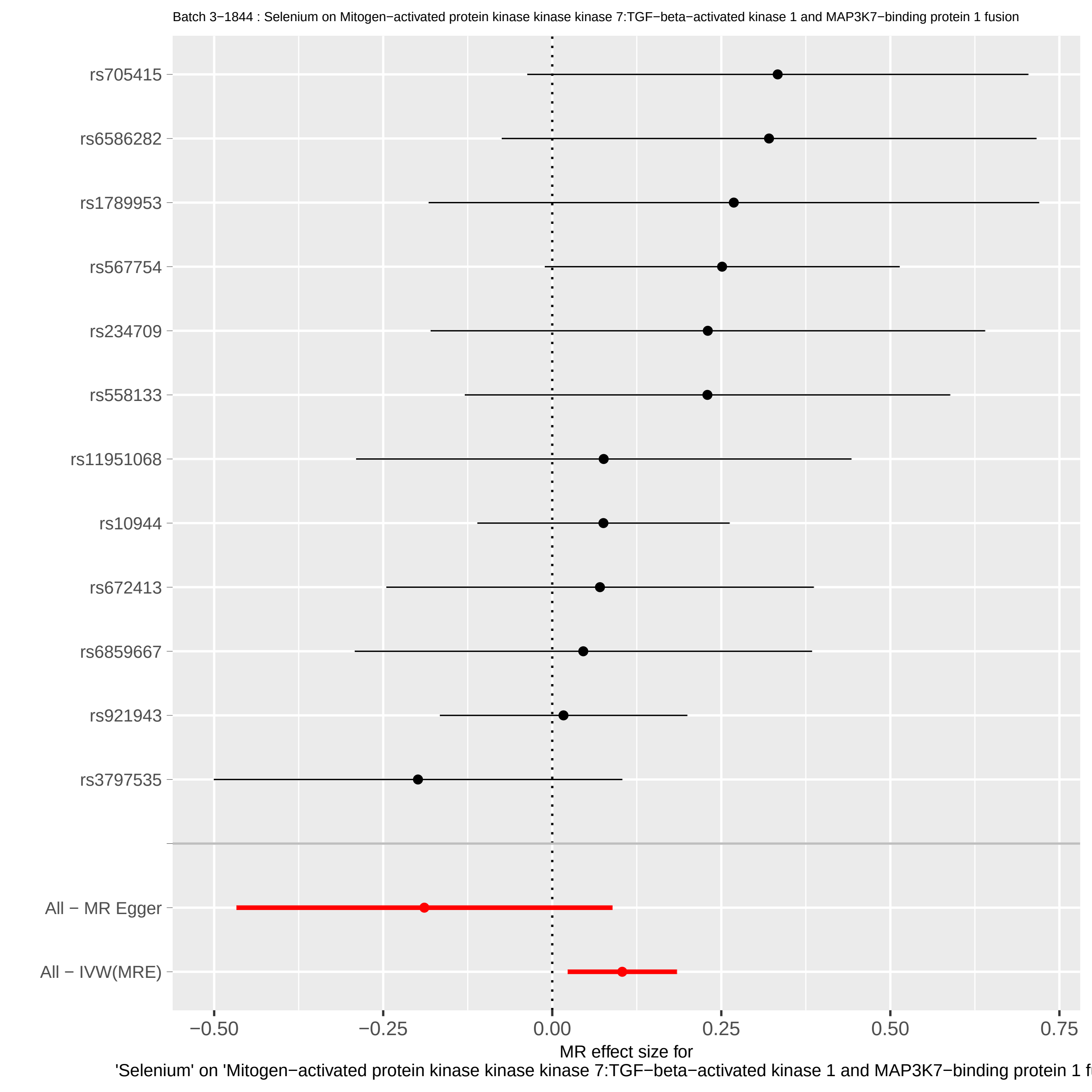
Batch 3−1844 : Selenium on Mitogen−activated protein kinase kinase kinase 7:TGF−beta−activated kinase 1 and MAP3K7−binding protein 1 fusion
rs705415
rs6586282
rs1789953
rs567754
rs234709
rs558133
rs11951068
rs10944
rs672413
rs6859667
rs921943
rs3797535
All − MR Egger
All − IVW(MRE)
−0.50
−0.25
0.00
0.25
0.50
0.75
MR effect size for
'Selenium' on 'Mitogen−activated protein kinase kinase kinase 7:TGF−beta−activated kinase 1 and MAP3K7−binding protein 1 f

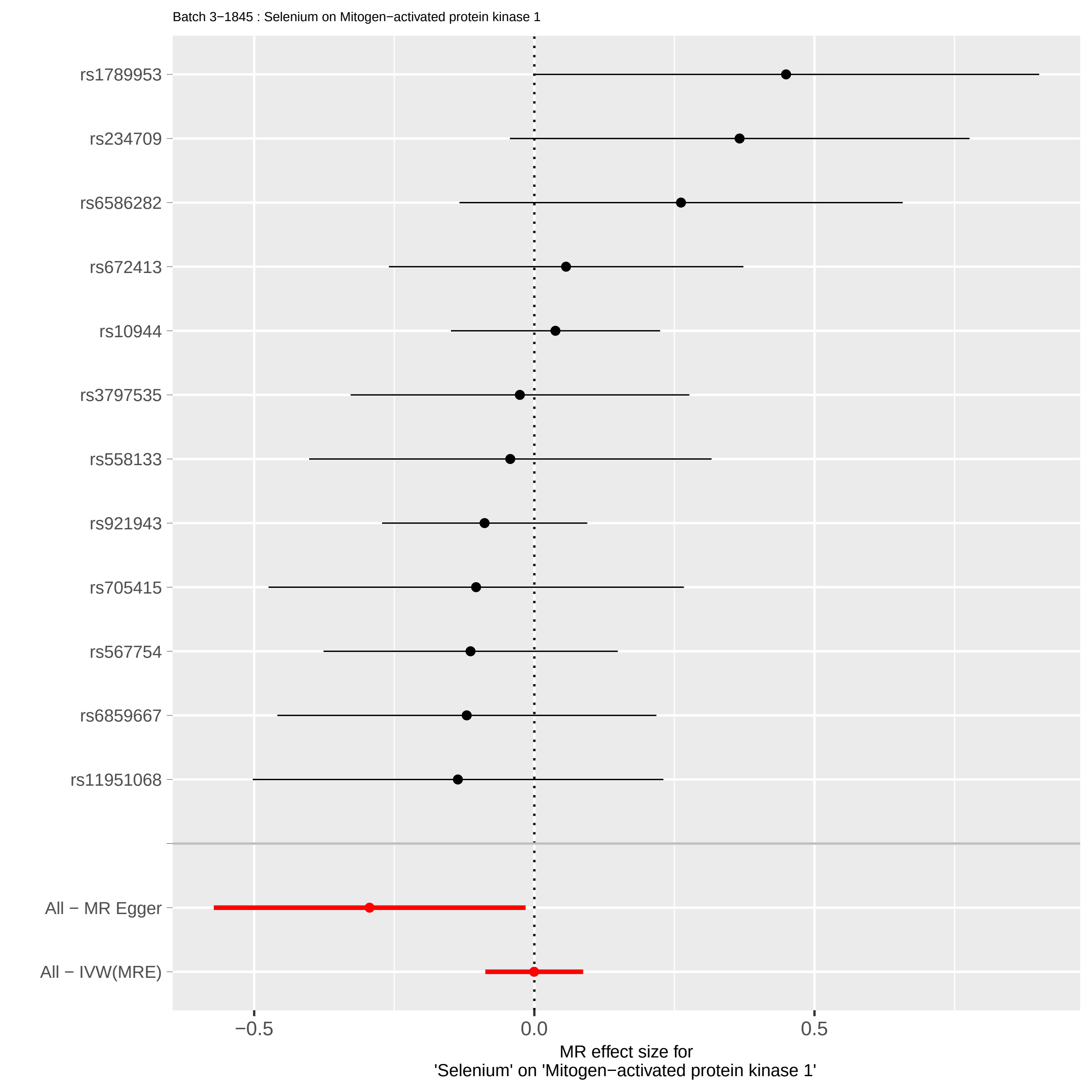
Batch 3−1845 : Selenium on Mitogen−activated protein kinase 1
rs1789953
rs234709
rs6586282
rs672413
rs10944
rs3797535
rs558133
rs921943
rs705415
rs567754
rs6859667
rs11951068
All − MR Egger
All − IVW(MRE)
−0.5
0.0
0.5
MR effect size for
'Selenium' on 'Mitogen−activated protein kinase 1'

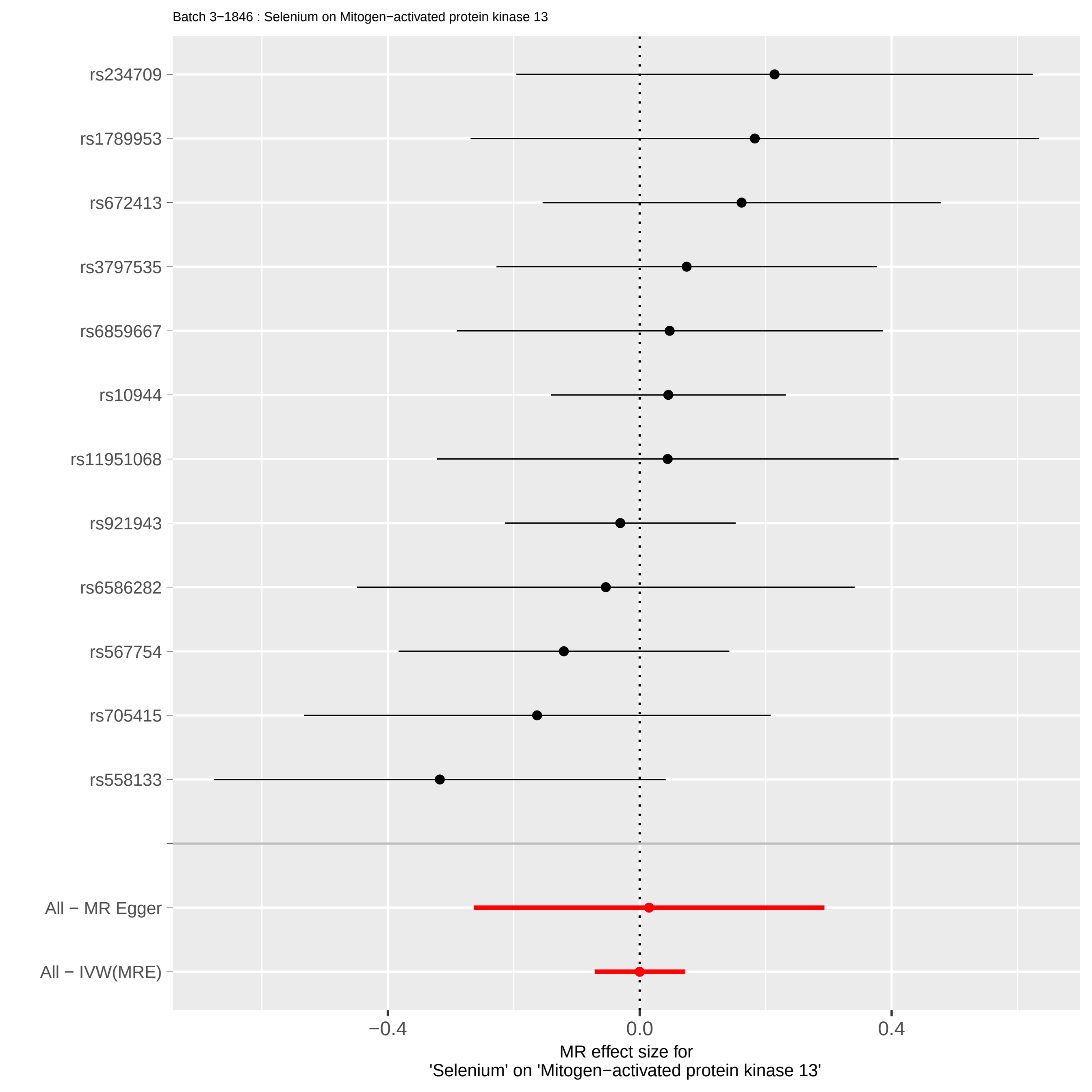

Batch 3−1846 : Selenium on Mitogen−activated protein kinase 13
rs234709
rs1789953
rs672413
rs3797535
rs6859667
rs10944
rs11951068
rs921943
rs6586282
rs567754
rs705415
rs558133
All − MR Egger
All − IVW(MRE)
−0.4
0.0
0.4
MR effect size for
'Selenium' on 'Mitogen−activated protein kinase 13'

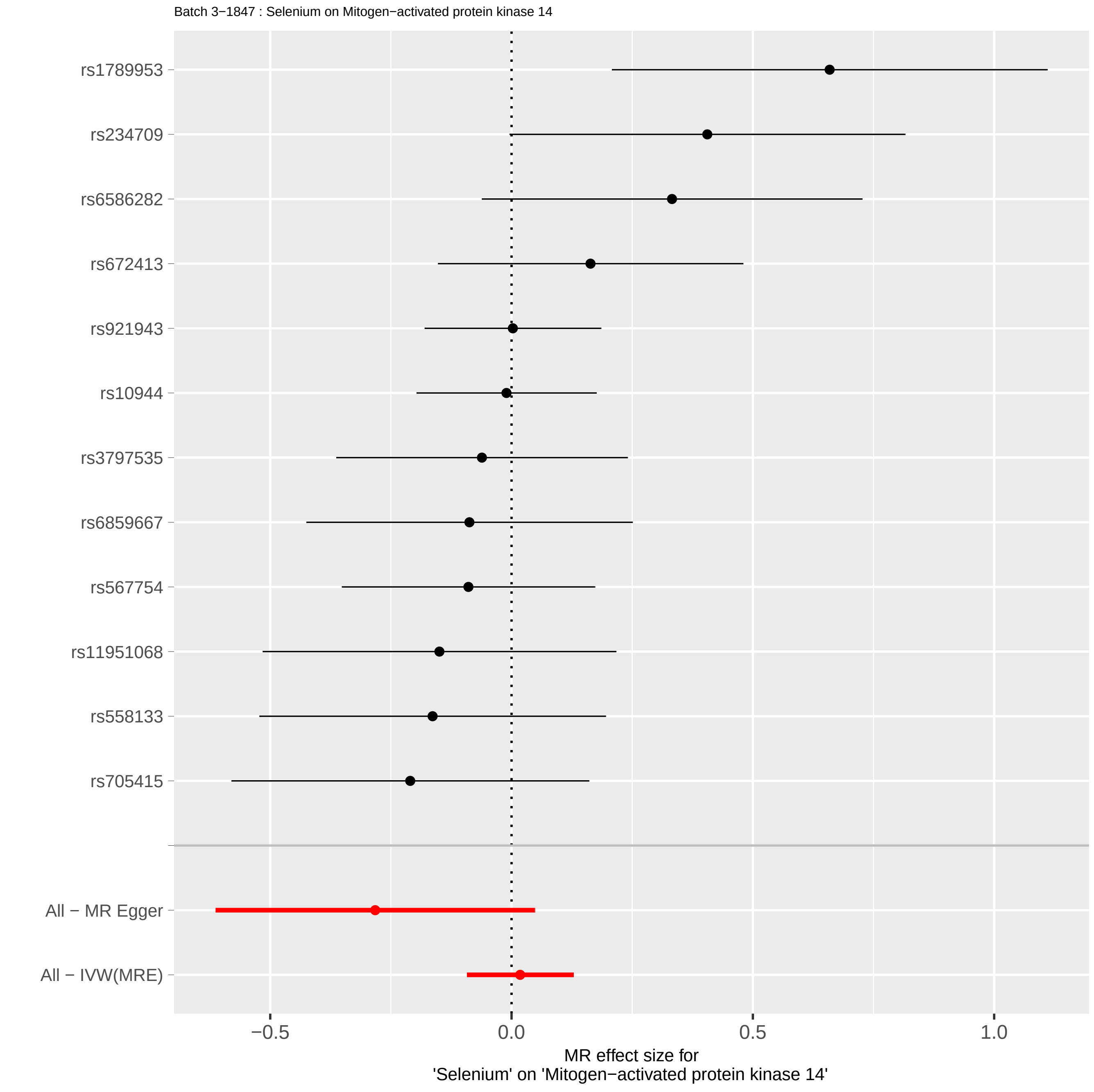

Batch 3−1847 : Selenium on Mitogen−activated protein kinase 14
rs1789953
rs234709
rs6586282
rs672413
rs921943
rs10944
rs3797535
rs6859667
rs567754
rs11951068
rs558133
rs705415
All − MR Egger
All − IVW(MRE)
−0.5
0.0
0.5
1.0
MR effect size for
'Selenium' on 'Mitogen−activated protein kinase 14'

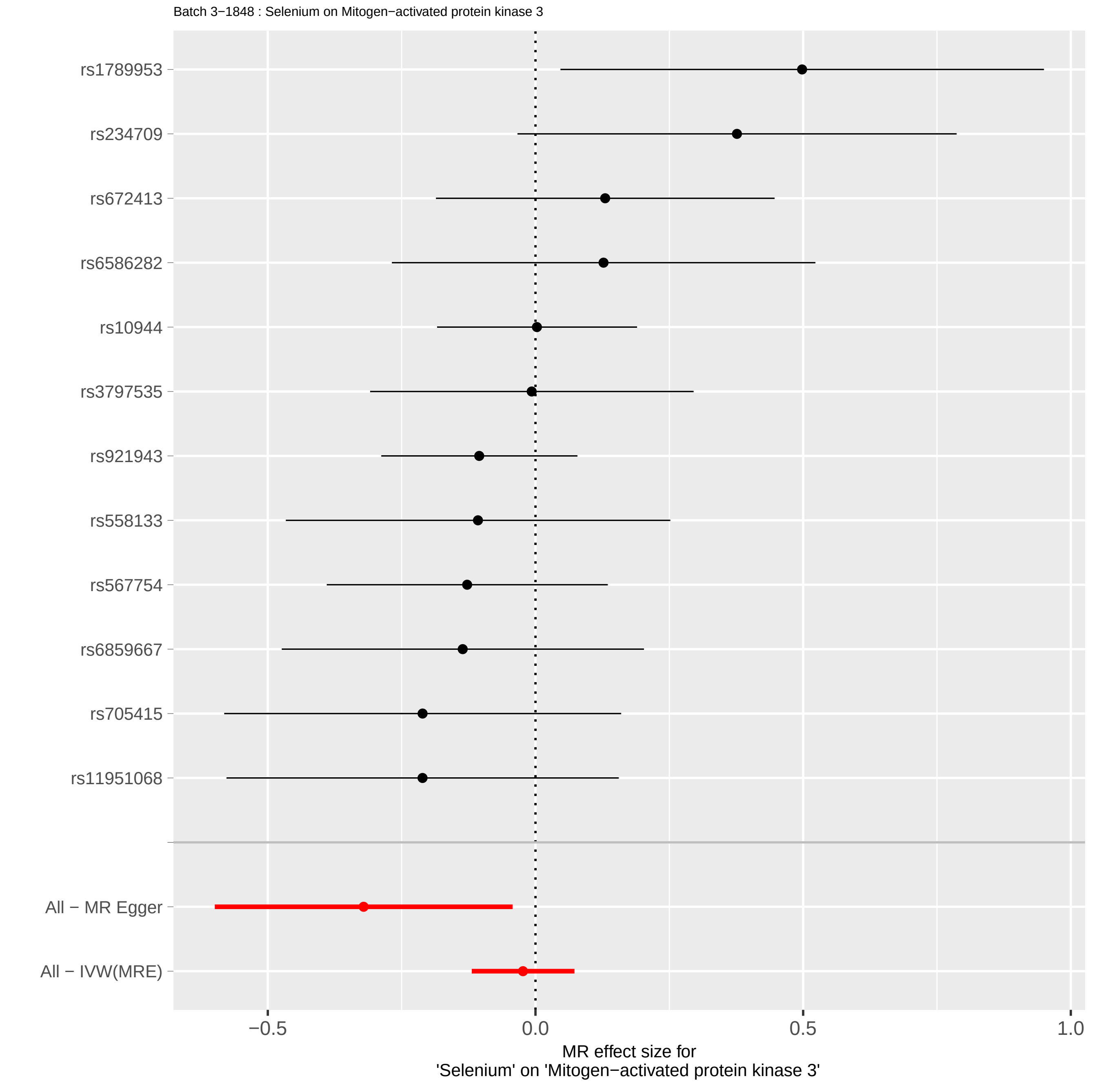

Batch 3−1848 : Selenium on Mitogen−activated protein kinase 3
rs1789953
rs234709
rs672413
rs6586282
rs10944
rs3797535
rs921943
rs558133
rs567754
rs6859667
rs705415
rs11951068
All − MR Egger
All − IVW(MRE)
−0.5
0.0
0.5
1.0
MR effect size for
'Selenium' on 'Mitogen−activated protein kinase 3'

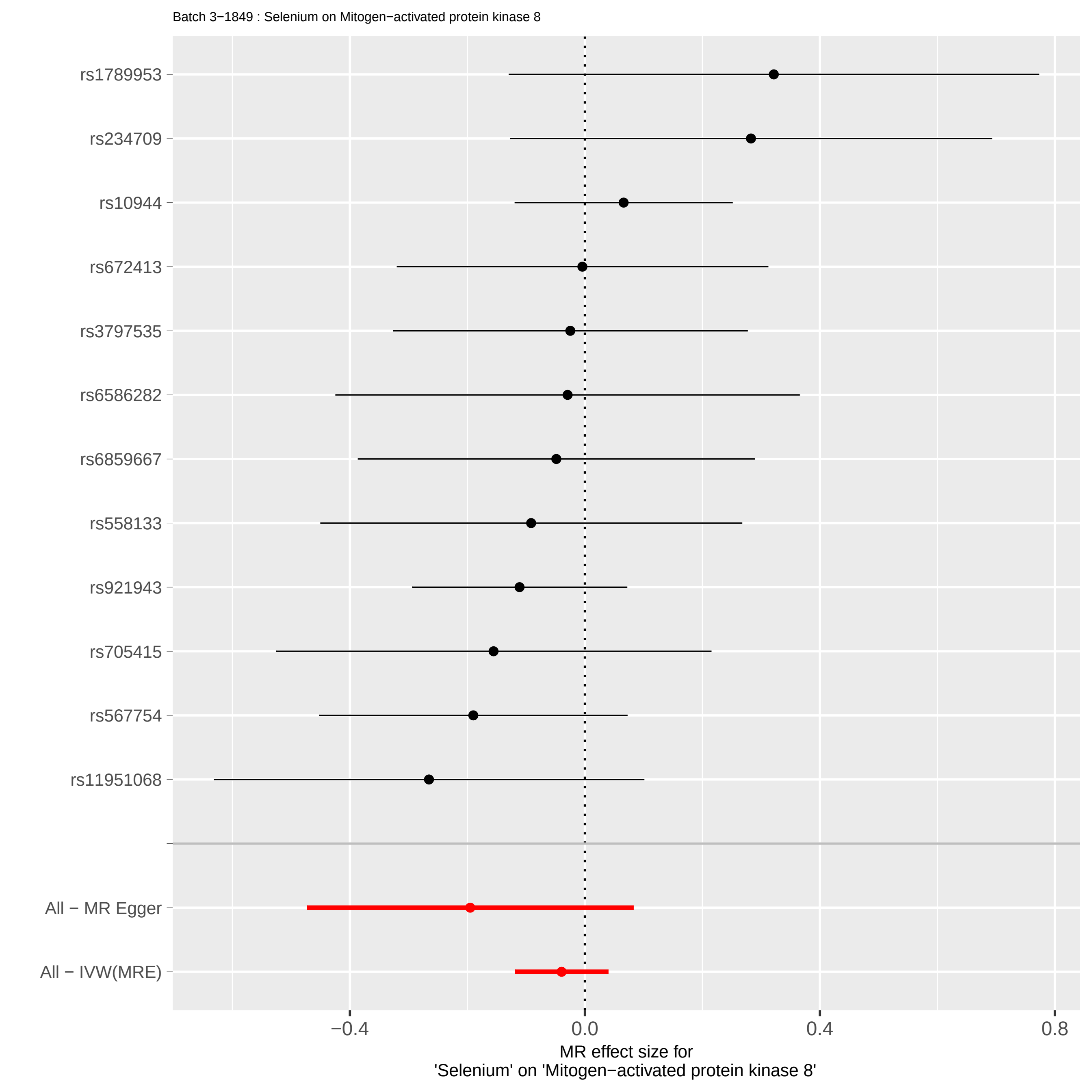

Batch 3−1849 : Selenium on Mitogen−activated protein kinase 8
rs1789953
rs234709
rs10944
rs672413
rs3797535
rs6586282
rs6859667
rs558133
rs921943
rs705415
rs567754
rs11951068
All − MR Egger
All − IVW(MRE)
−0.4
0.0
0.4
0.8
MR effect size for
'Selenium' on 'Mitogen−activated protein kinase 8'

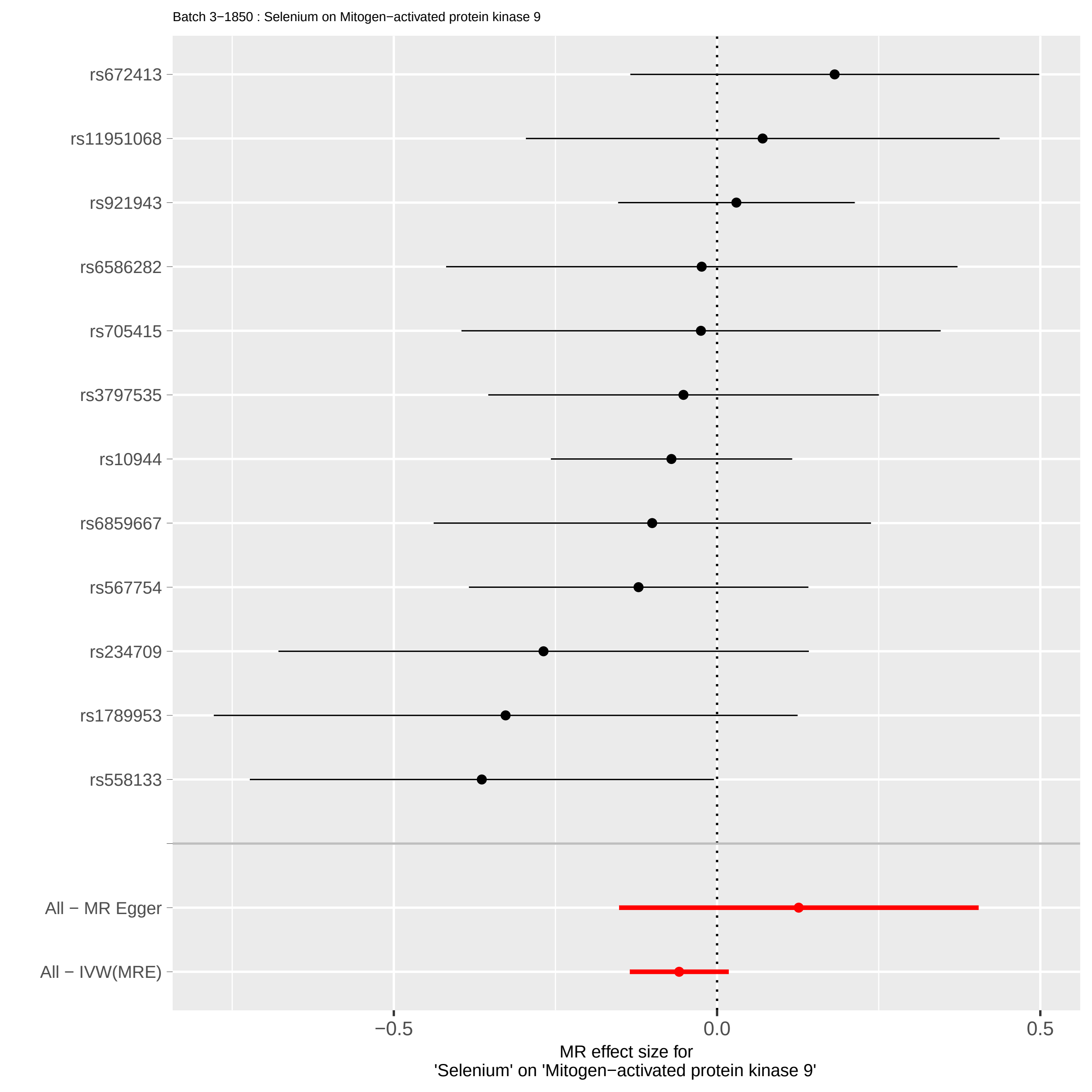
Batch 3−1850 : Selenium on Mitogen−activated protein kinase 9
rs672413
rs11951068
rs921943
rs6586282
rs705415
rs3797535
rs10944
rs6859667
rs567754
rs234709
rs1789953
rs558133
All − MR Egger
All − IVW(MRE)
−0.5
0.0
0.5
MR effect size for
'Selenium' on 'Mitogen−activated protein kinase 9'

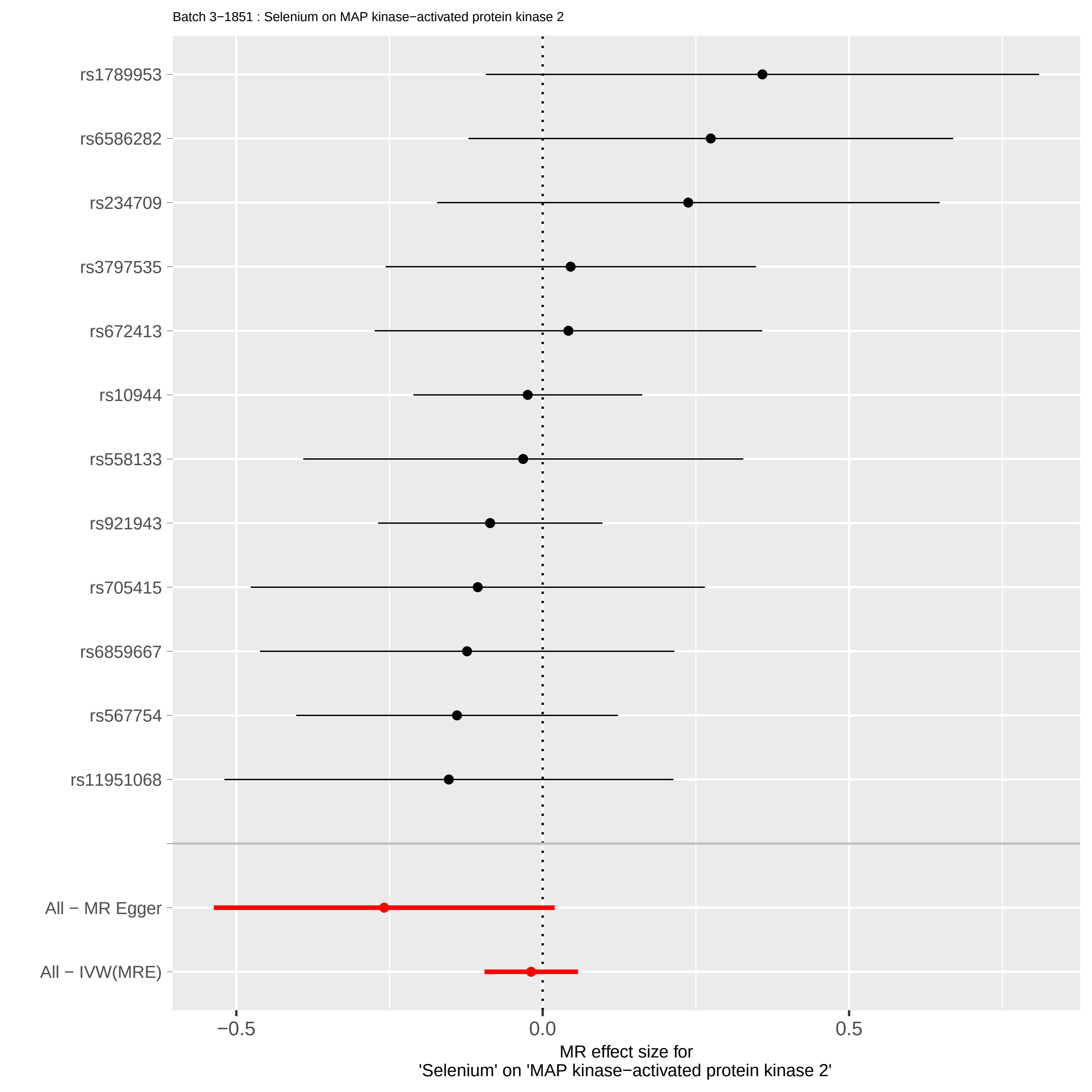

Batch 3−1851 : Selenium on MAP kinase−activated protein kinase 2
rs1789953
rs6586282
rs234709
rs3797535
rs672413
rs10944
rs558133
rs921943
rs705415
rs6859667
rs567754
rs11951068
All − MR Egger
All − IVW(MRE)
−0.5
0.0
0.5
MR effect size for
'Selenium' on 'MAP kinase−activated protein kinase 2'

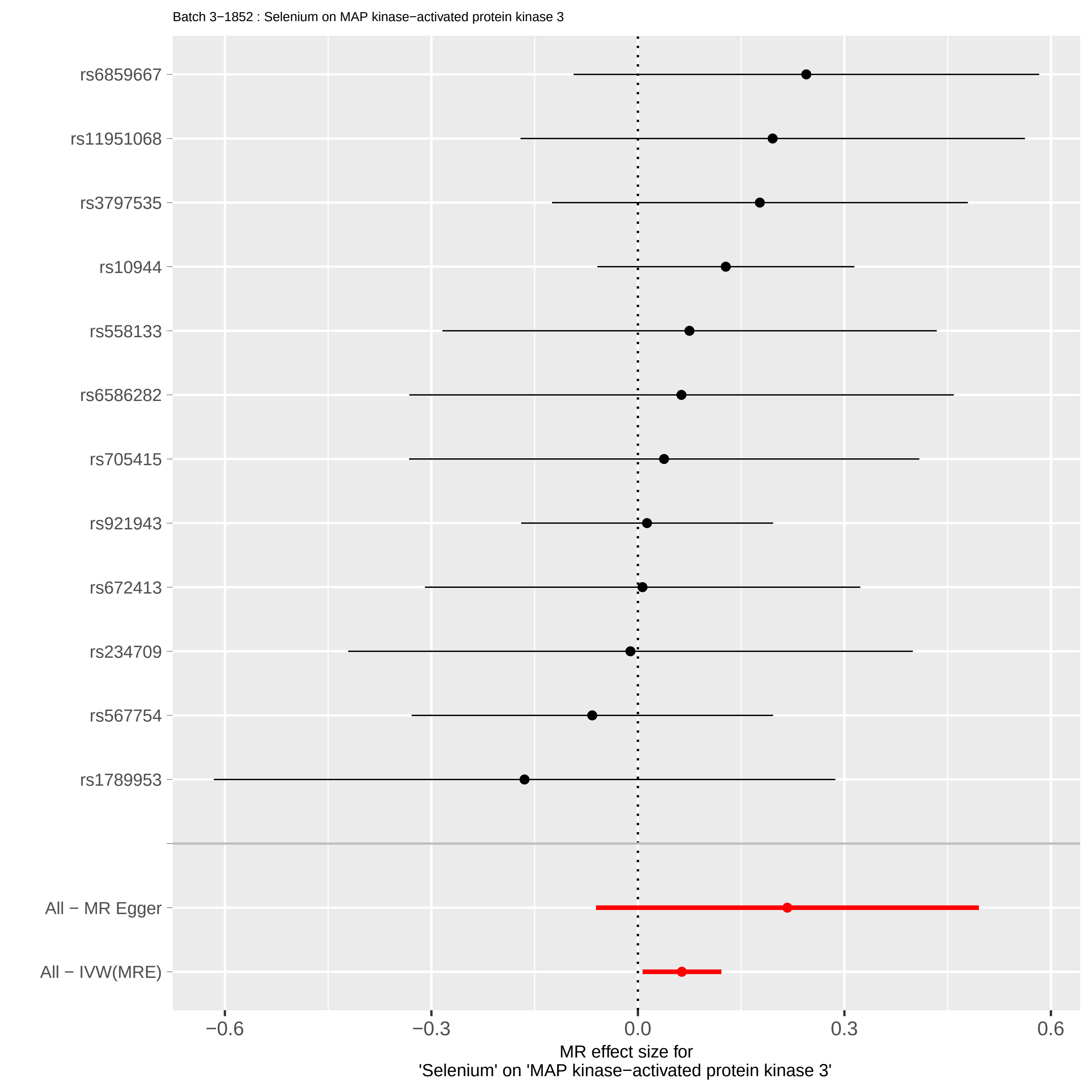

Batch 3−1852 : Selenium on MAP kinase−activated protein kinase 3
rs6859667
rs11951068
rs3797535
rs10944
rs558133
rs6586282
rs705415
rs921943
rs672413
rs234709
rs567754
rs1789953
All − MR Egger
All − IVW(MRE)
−0.6
−0.3
0.0
0.3
0.6
MR effect size for
'Selenium' on 'MAP kinase−activated protein kinase 3'

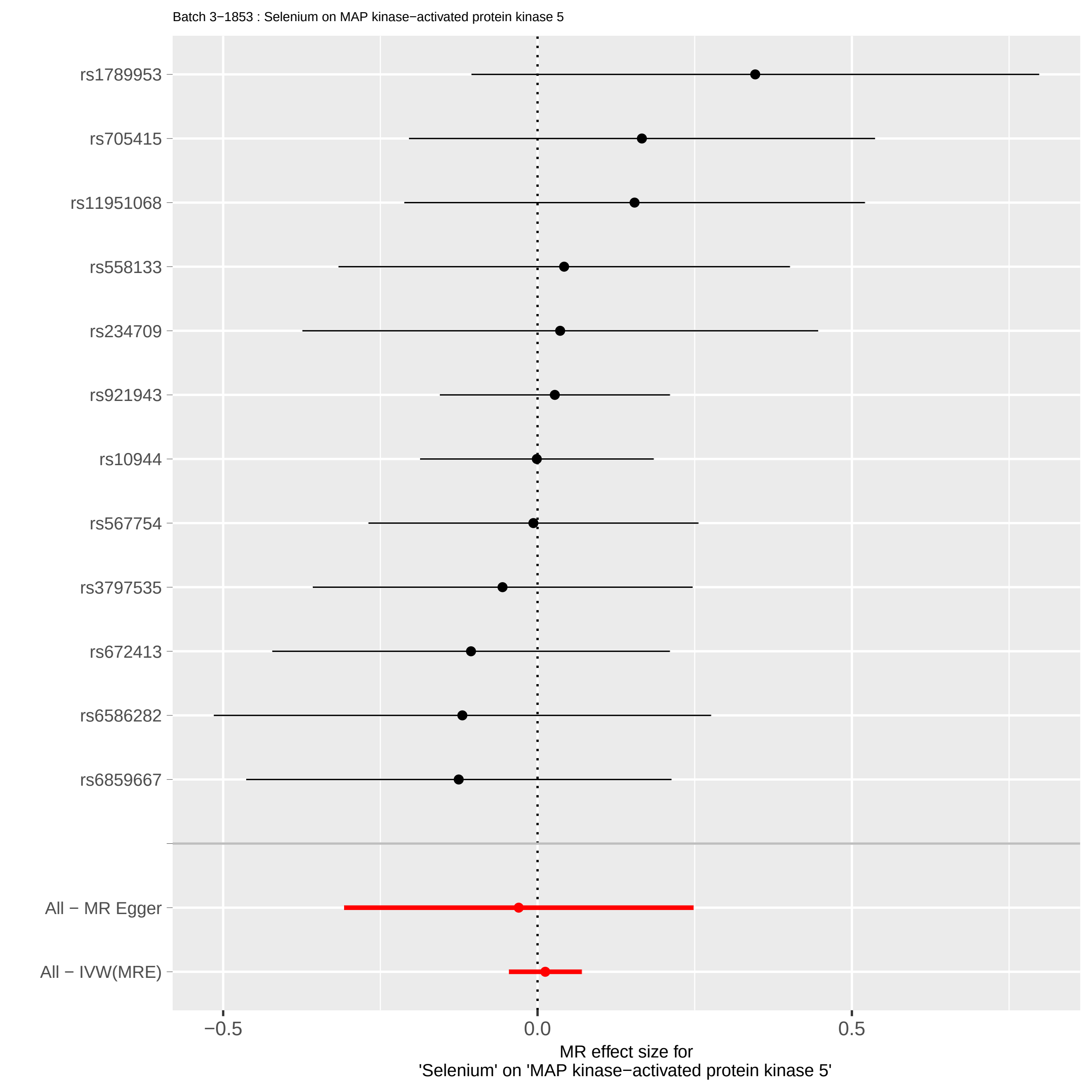

Batch 3−1853 : Selenium on MAP kinase−activated protein kinase 5
rs1789953
rs705415
rs11951068
rs558133
rs234709
rs921943
rs10944
rs567754
rs3797535
rs672413
rs6586282
rs6859667
All − MR Egger
All − IVW(MRE)
−0.5
0.0
0.5
MR effect size for
'Selenium' on 'MAP kinase−activated protein kinase 5'

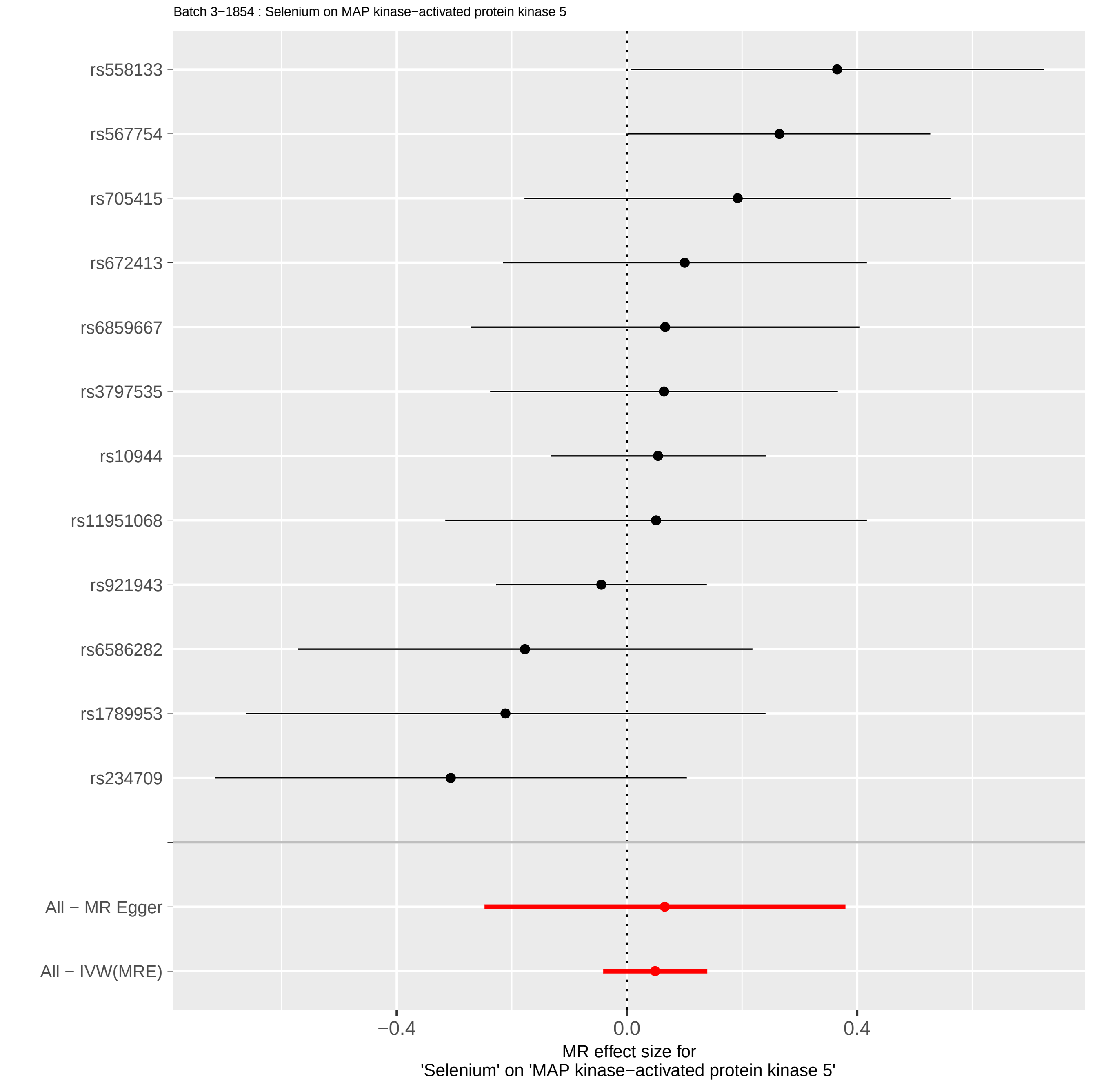
Batch 3−1854 : Selenium on MAP kinase−activated protein kinase 5
rs558133
rs567754
rs705415
rs672413
rs6859667
rs3797535
rs10944
rs11951068
rs921943
rs6586282
rs1789953
rs234709
All − MR Egger
All − IVW(MRE)
−0.4
0.0
0.4
MR effect size for
'Selenium' on 'MAP kinase−activated protein kinase 5'

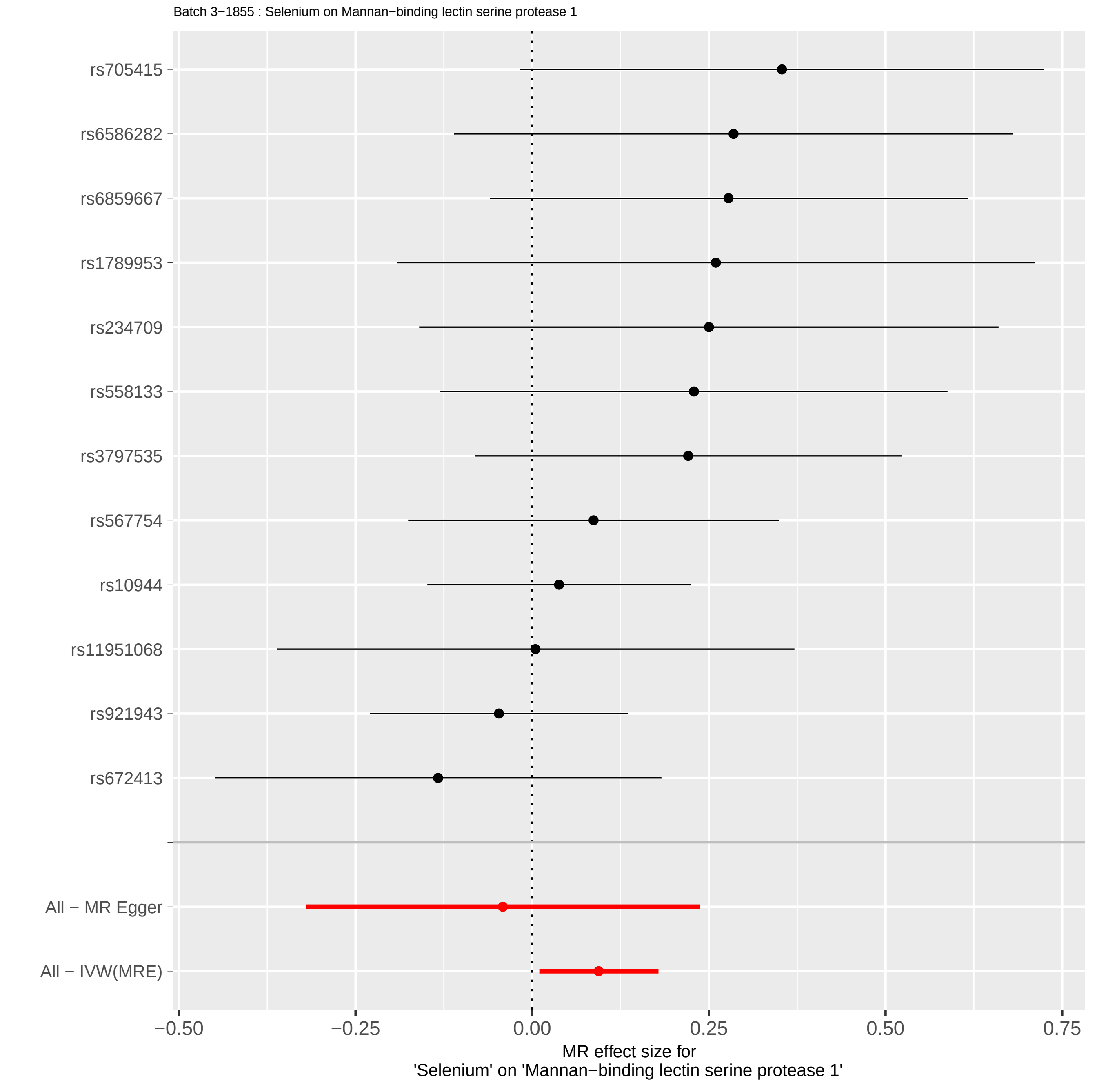

Batch 3−1855 : Selenium on Mannan−binding lectin serine protease 1
rs705415
rs6586282
rs6859667
rs1789953
rs234709
rs558133
rs3797535
rs567754
rs10944
rs11951068
rs921943
rs672413
All − MR Egger
All − IVW(MRE)
−0.50
−0.25
0.00
0.25
0.50
0.75
MR effect size for
'Selenium' on 'Mannan−binding lectin serine protease 1'

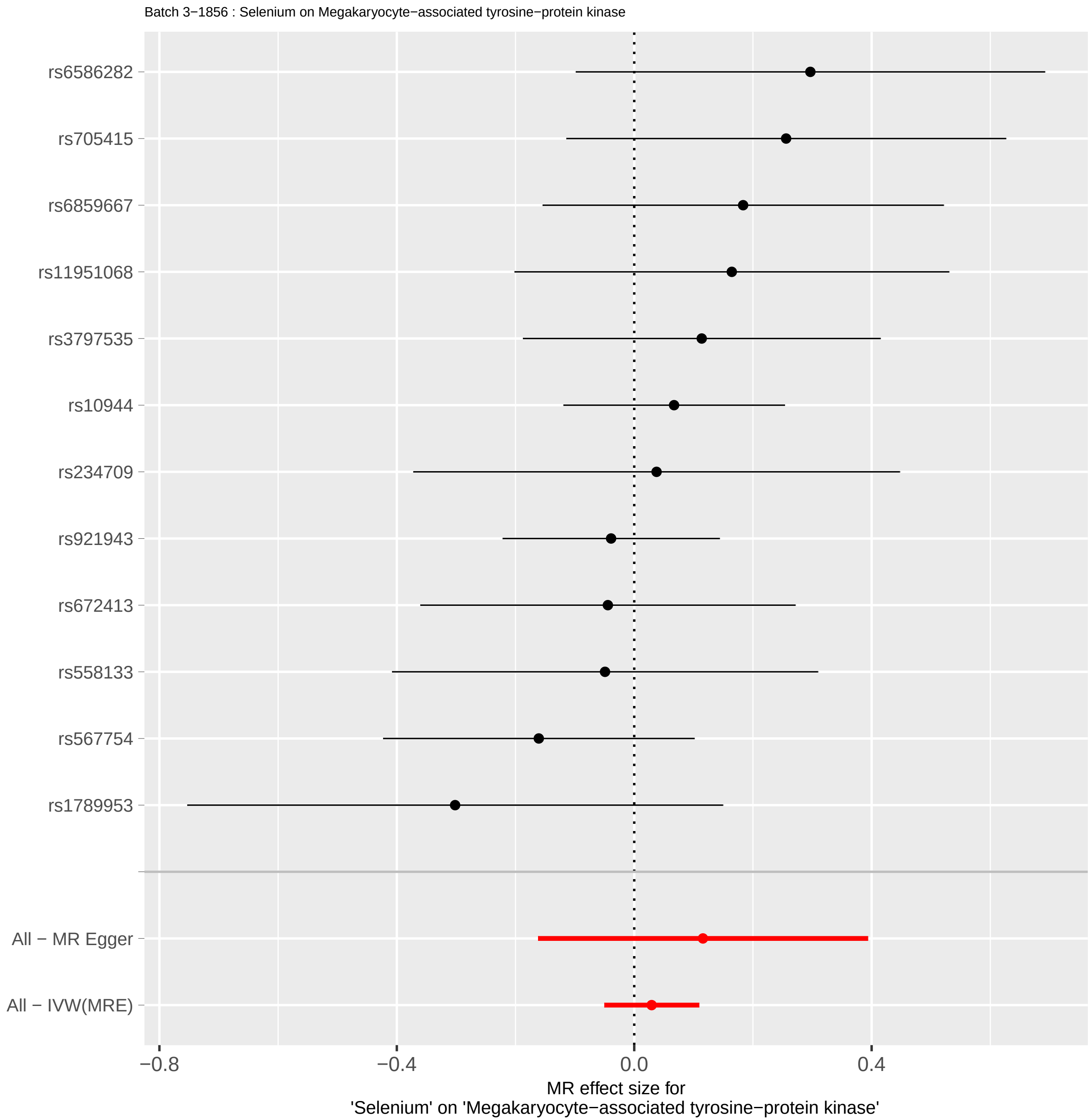

Batch 3−1856 : Selenium on Megakaryocyte−associated tyrosine−protein kinase
rs6586282
rs705415
rs6859667
rs11951068
rs3797535
rs10944
rs234709
rs921943
rs672413
rs558133
rs567754
rs1789953
All − MR Egger
All − IVW(MRE)
−0.8
−0.4
0.0
0.4
MR effect size for
'Selenium' on 'Megakaryocyte−associated tyrosine−protein kinase'

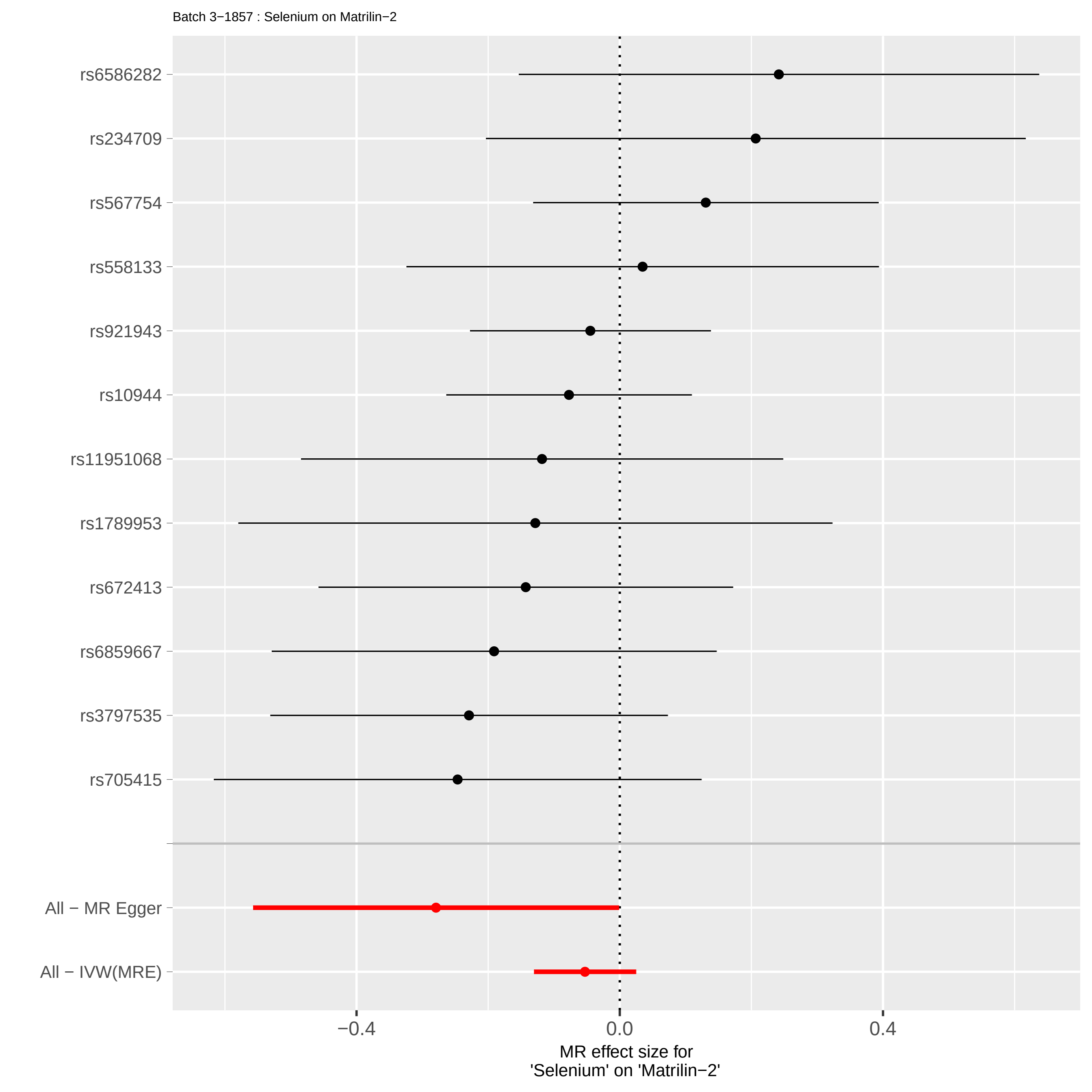
Batch 3−1857 : Selenium on Matrilin−2
rs6586282
rs234709
rs567754
rs558133
rs921943
rs10944
rs11951068
rs1789953
rs672413
rs6859667
rs3797535
rs705415
All − MR Egger
All − IVW(MRE)
−0.4
0.0
0.4
MR effect size for
'Selenium' on 'Matrilin−2'

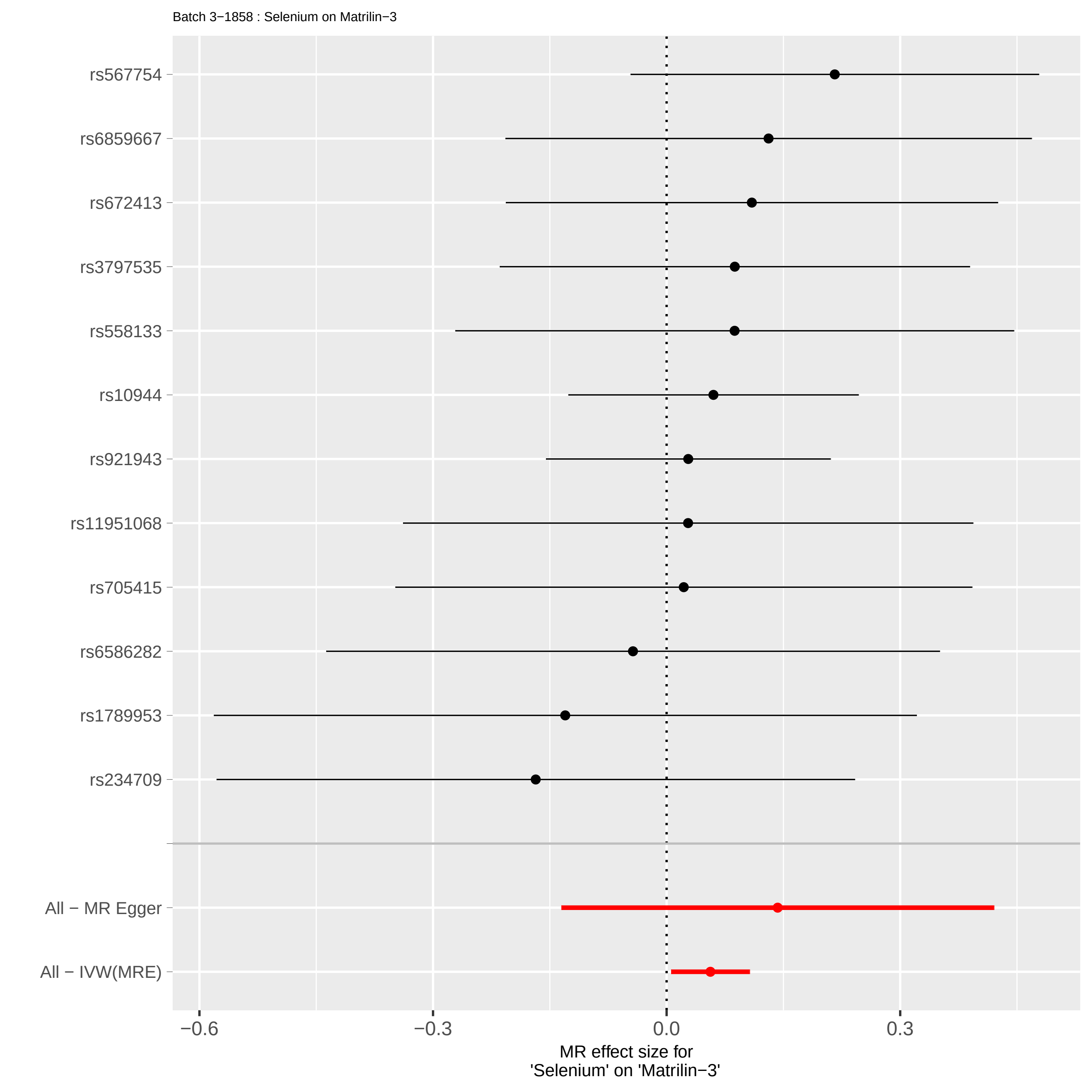

Batch 3−1858 : Selenium on Matrilin−3
rs567754
rs6859667
rs672413
rs3797535
rs558133
rs10944
rs921943
rs11951068
rs705415
rs6586282
rs1789953
rs234709
All − MR Egger
All − IVW(MRE)
−0.6
−0.3
0.0
0.3
MR effect size for
'Selenium' on 'Matrilin−3'

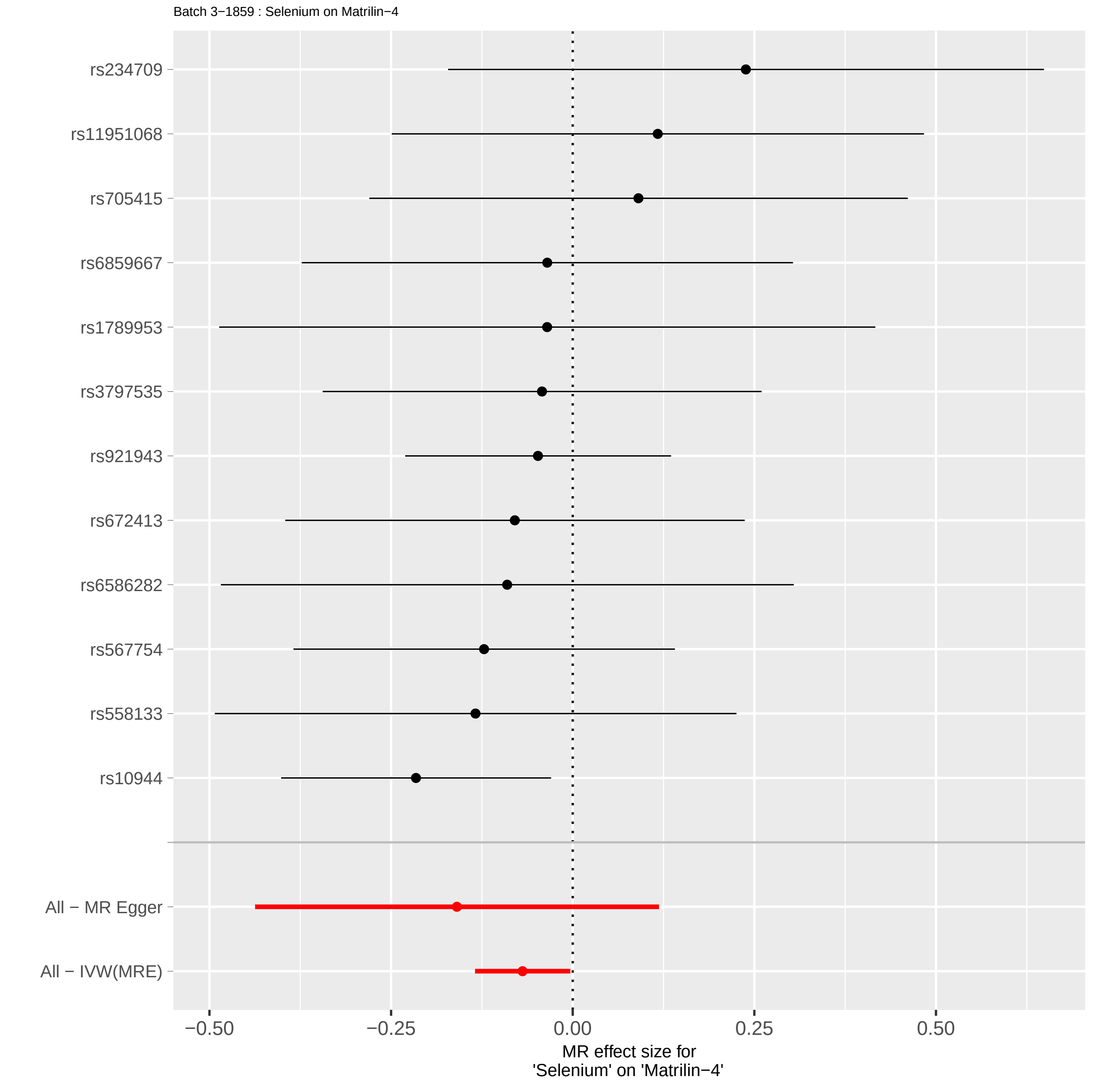
Batch 3−1859 : Selenium on Matrilin−4
rs234709
rs11951068
rs705415
rs6859667
rs1789953
rs3797535
rs921943
rs672413
rs6586282
rs567754
rs558133
rs10944
All − MR Egger
All − IVW(MRE)
−0.50
−0.25
0.00
0.25
0.50
MR effect size for
'Selenium' on 'Matrilin−4'

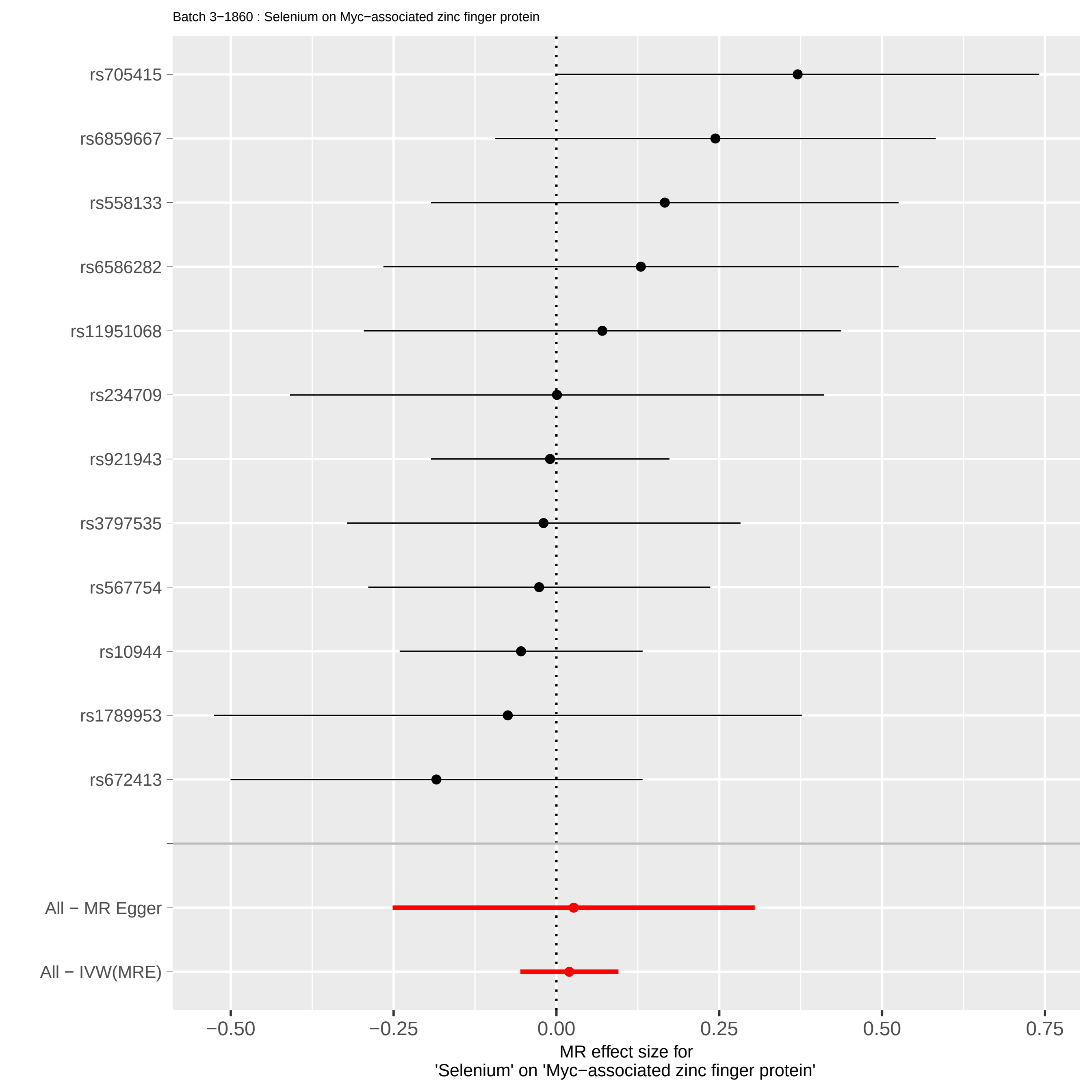
Batch 3−1860 : Selenium on Myc−associated zinc finger protein
rs705415
rs6859667
rs558133
rs6586282
rs11951068
rs234709
rs921943
rs3797535
rs567754
rs10944
rs1789953
rs672413
All − MR Egger
All − IVW(MRE)
−0.50
−0.25
0.00
0.25
0.50
0.75
MR effect size for
'Selenium' on 'Myc−associated zinc finger protein'

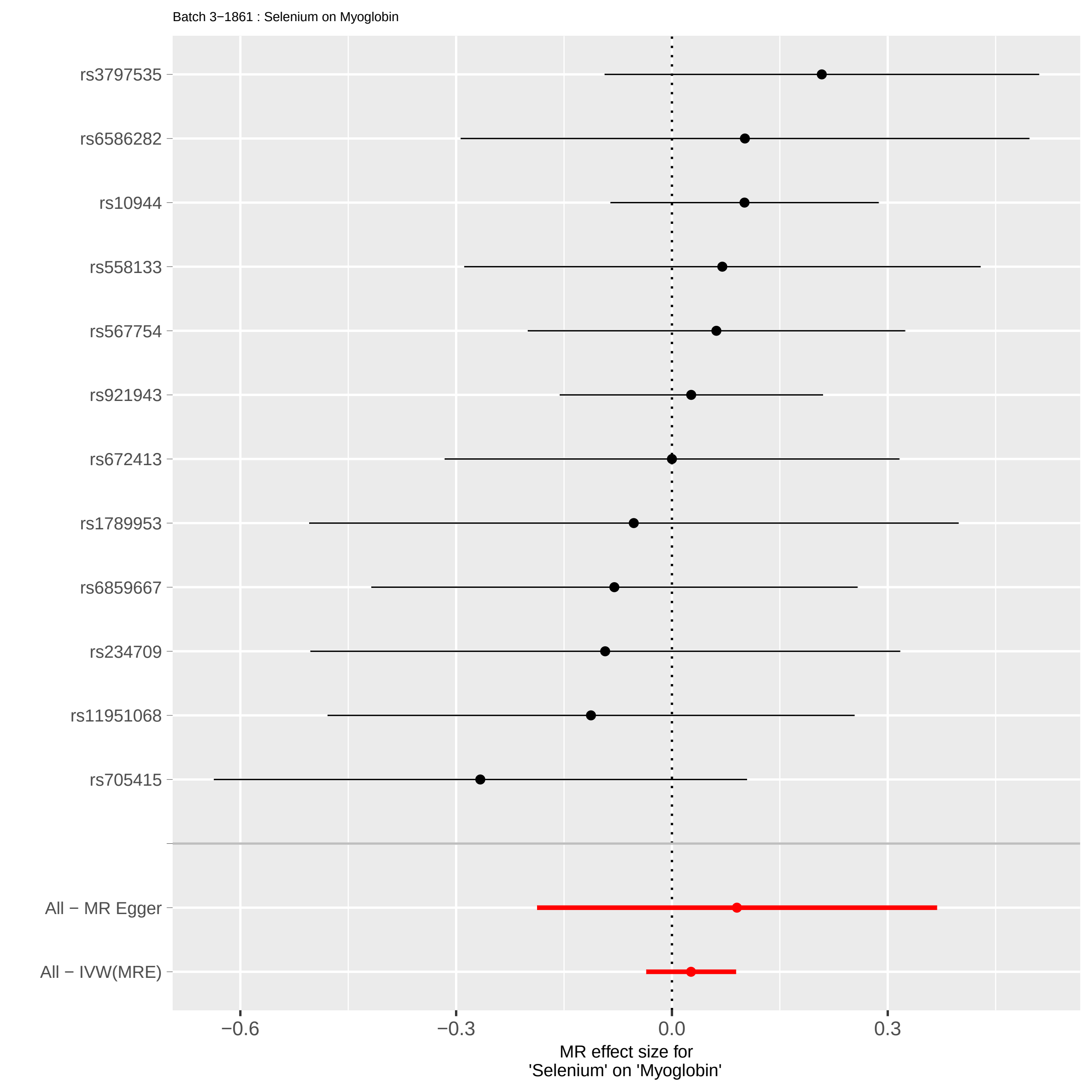

Batch 3−1861 : Selenium on Myoglobin
rs3797535
rs6586282
rs10944
rs558133
rs567754
rs921943
rs672413
rs1789953
rs6859667
rs234709
rs11951068
rs705415
All − MR Egger
All − IVW(MRE)
−0.6
−0.3
0.0
0.3
MR effect size for
'Selenium' on 'Myoglobin'

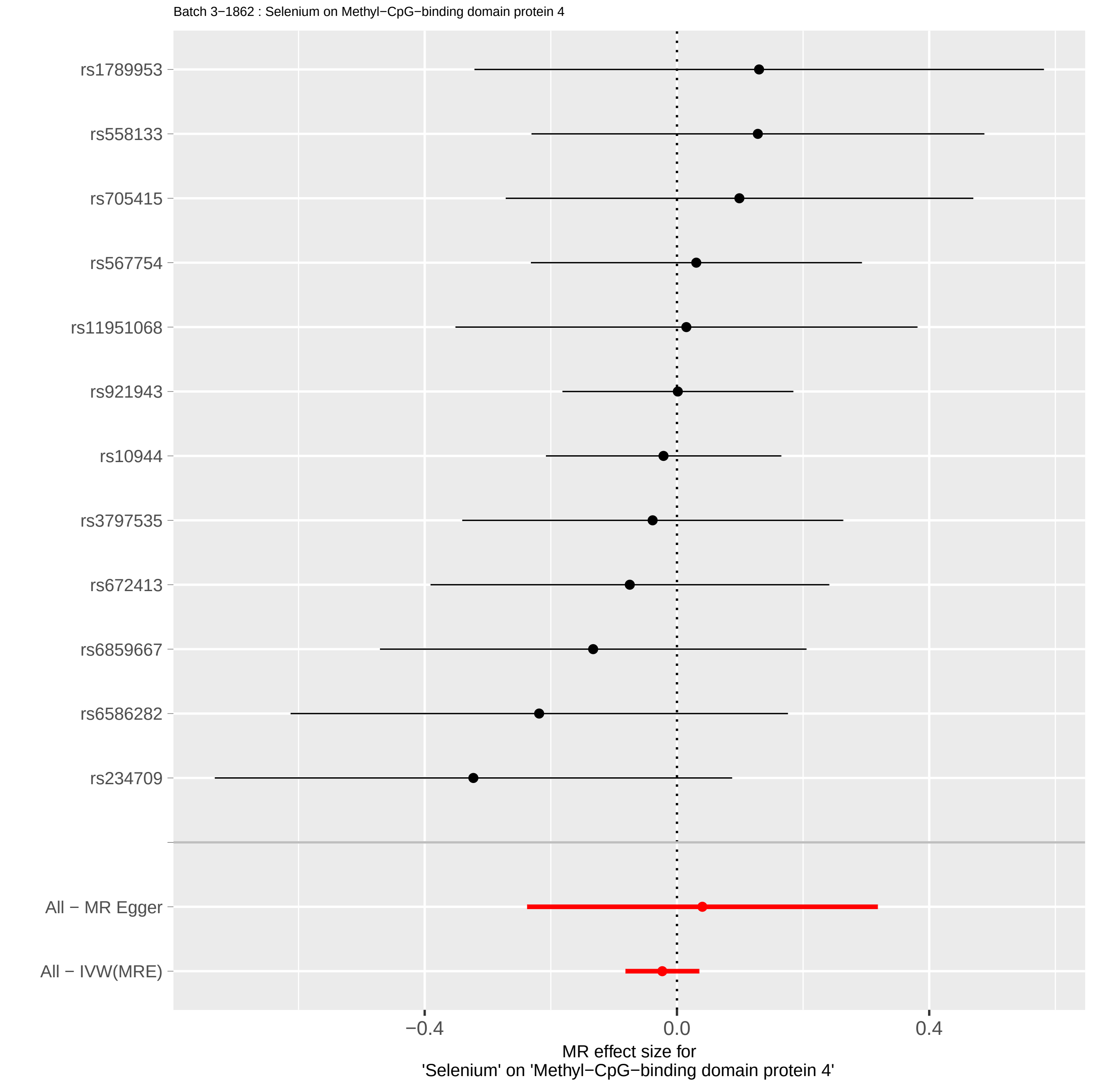

Batch 3−1862 : Selenium on Methyl−CpG−binding domain protein 4
rs1789953
rs558133
rs705415
rs567754
rs11951068
rs921943
rs10944
rs3797535
rs672413
rs6859667
rs6586282
rs234709
All − MR Egger
All − IVW(MRE)
−0.4
0.0
0.4
MR effect size for
'Selenium' on 'Methyl−CpG−binding domain protein 4'

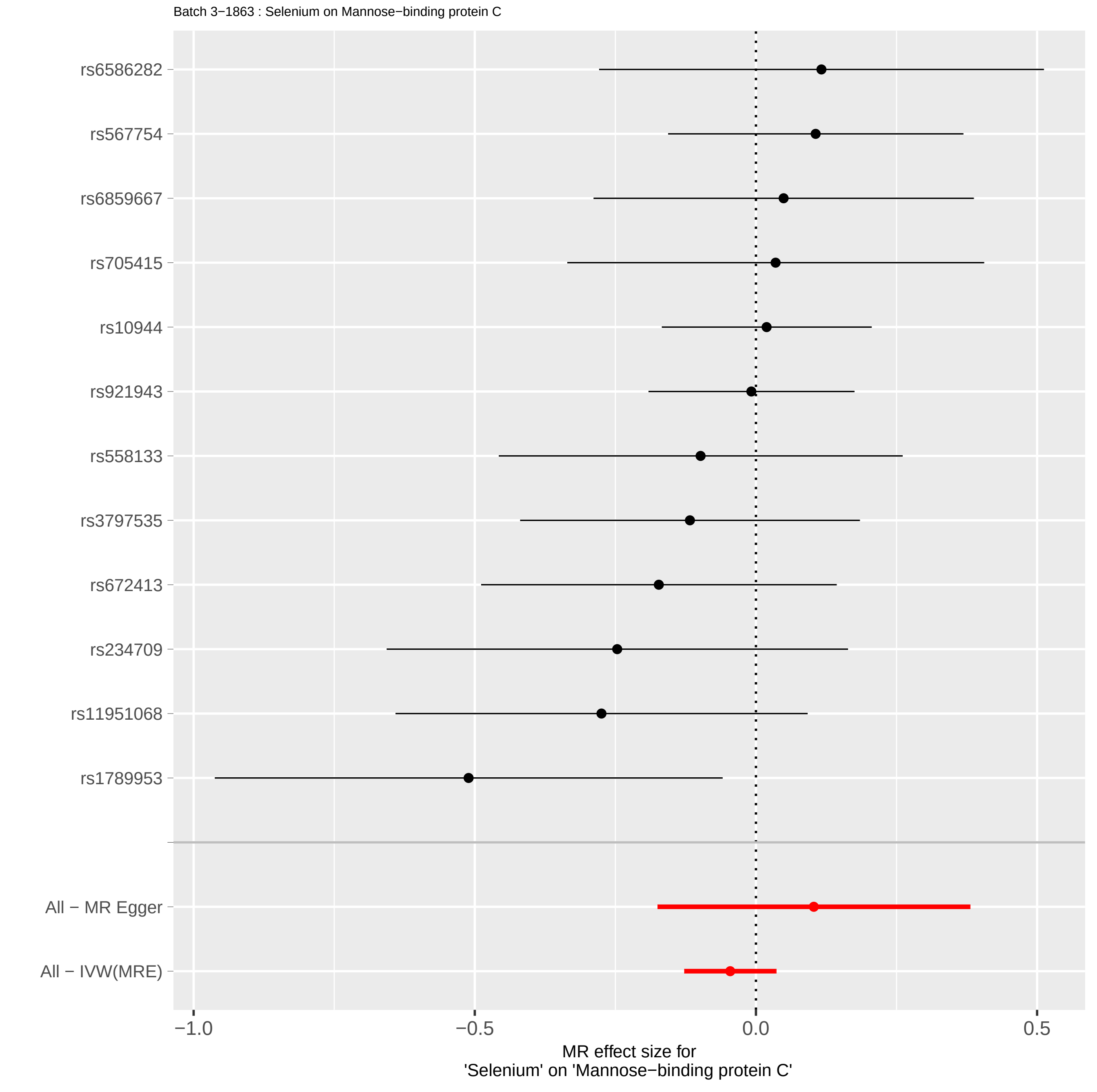

Batch 3−1863 : Selenium on Mannose−binding protein C
rs6586282
rs567754
rs6859667
rs705415
rs10944
rs921943
rs558133
rs3797535
rs672413
rs234709
rs11951068
rs1789953
All − MR Egger
All − IVW(MRE)
−1.0
−0.5
0.0
0.5
MR effect size for
'Selenium' on 'Mannose−binding protein C'

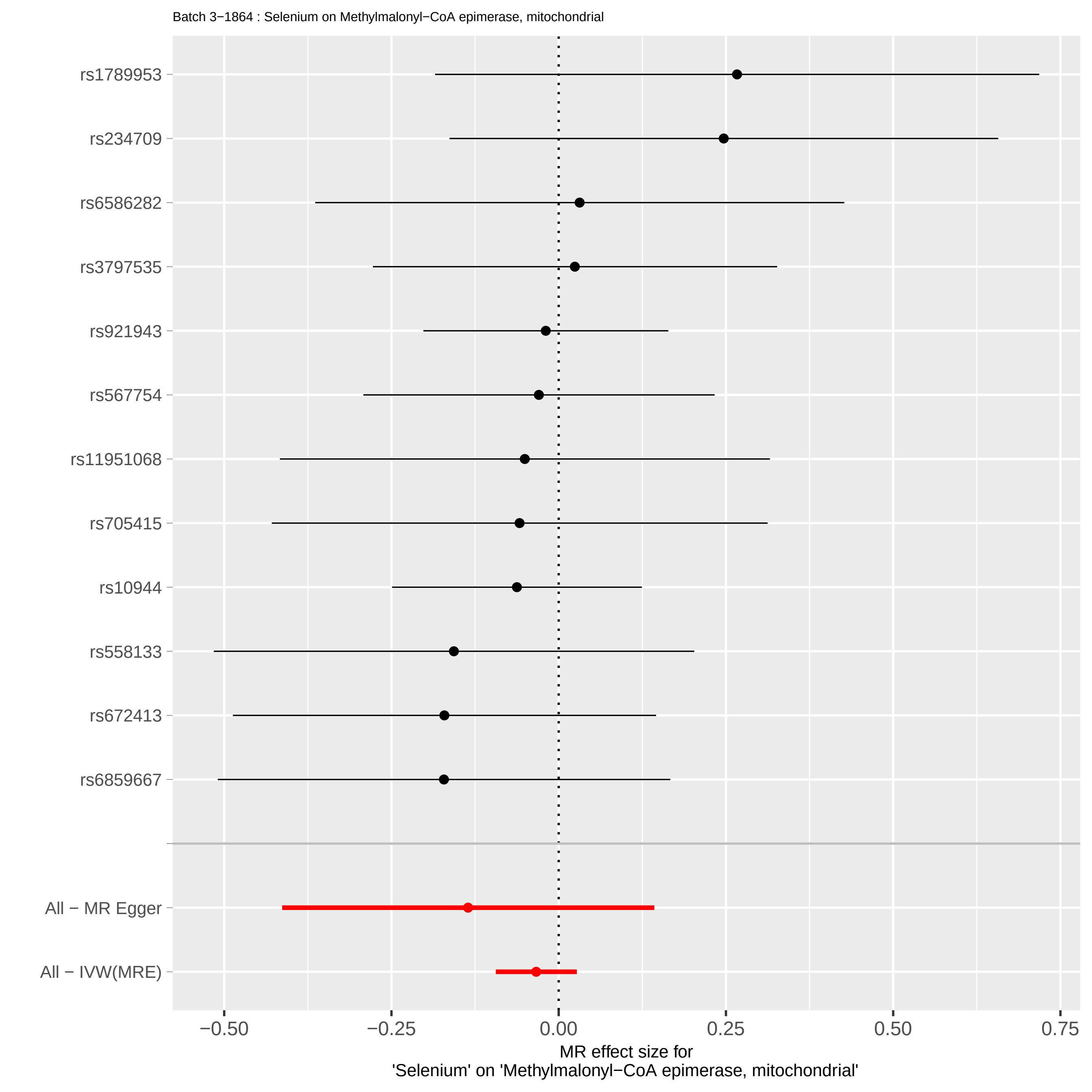

Batch 3−1864 : Selenium on Methylmalonyl−CoA epimerase, mitochondrial
rs1789953
rs234709
rs6586282
rs3797535
rs921943
rs567754
rs11951068
rs705415
rs10944
rs558133
rs672413
rs6859667
All − MR Egger
All − IVW(MRE)
−0.50
−0.25
0.00
0.25
0.50
0.75
MR effect size for
'Selenium' on 'Methylmalonyl−CoA epimerase, mitochondrial'

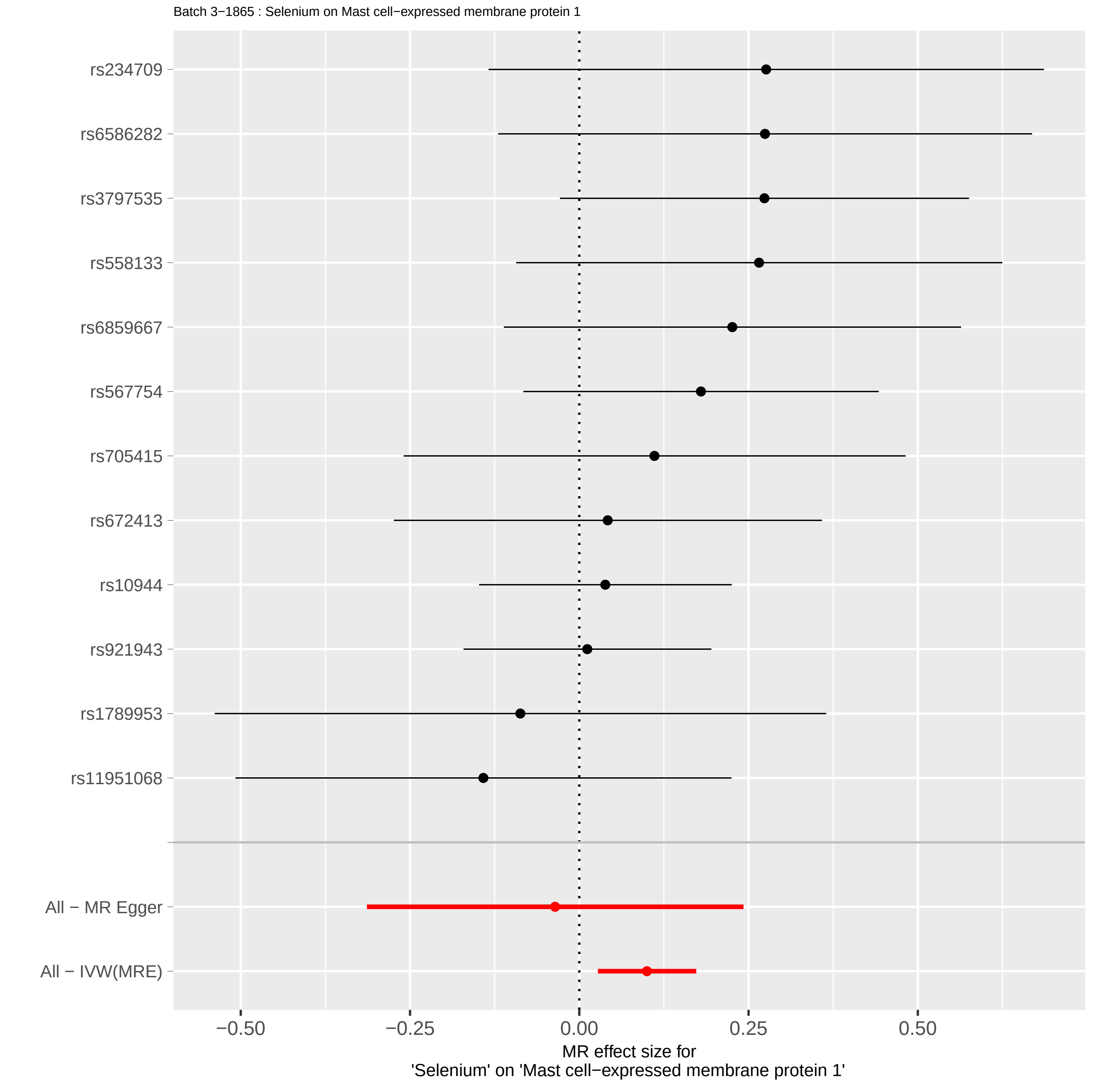

Batch 3−1865 : Selenium on Mast cell−expressed membrane protein 1
rs234709
rs6586282
rs3797535
rs558133
rs6859667
rs567754
rs705415
rs672413
rs10944
rs921943
rs1789953
rs11951068
All − MR Egger
All − IVW(MRE)
−0.50
−0.25
0.00
0.25
0.50
MR effect size for
'Selenium' on 'Mast cell−expressed membrane protein 1'

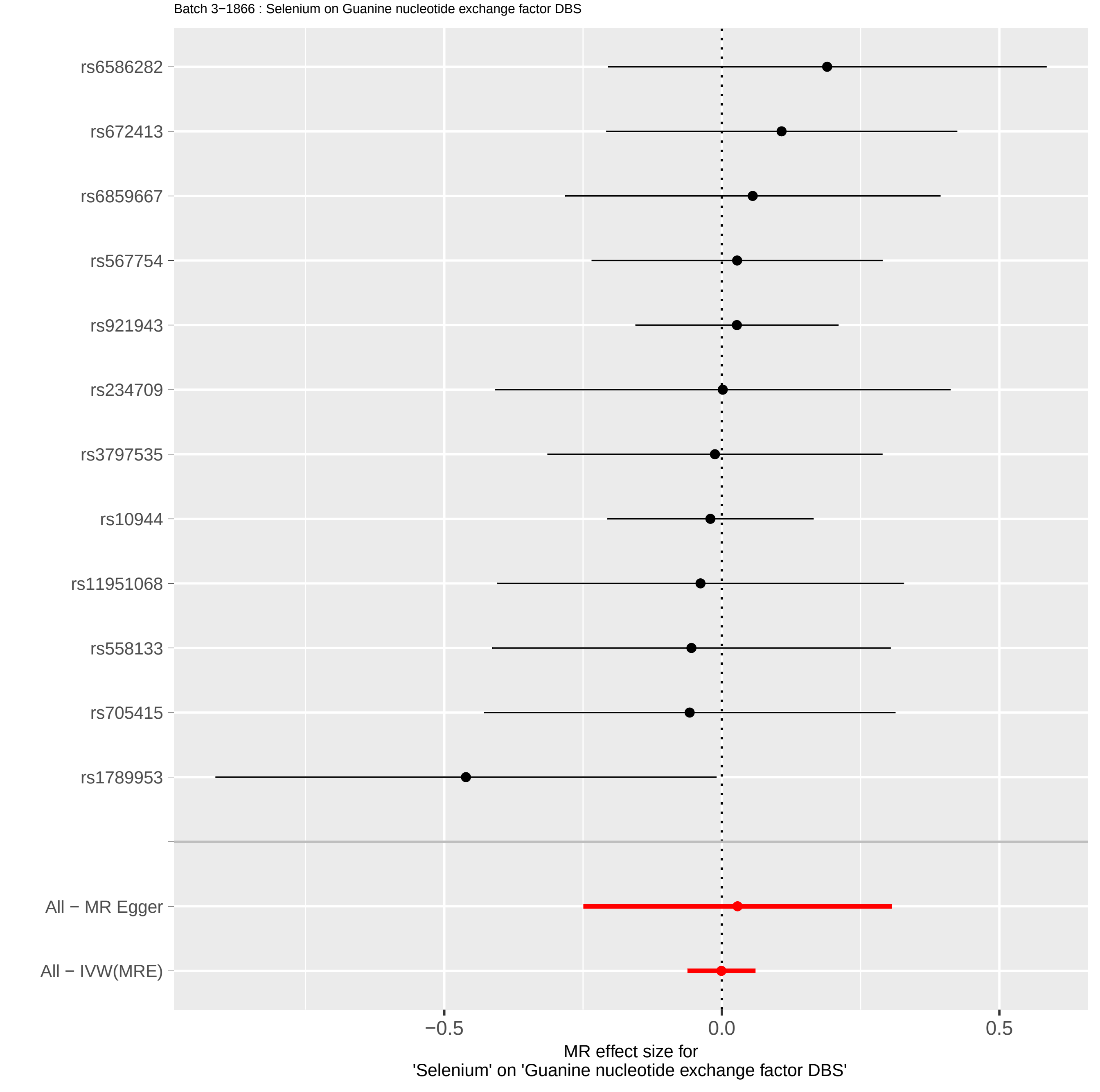

Batch 3−1866 : Selenium on Guanine nucleotide exchange factor DBS
rs6586282
rs672413
rs6859667
rs567754
rs921943
rs234709
rs3797535
rs10944
rs11951068
rs558133
rs705415
rs1789953
All − MR Egger
All − IVW(MRE)
−0.5
0.0
0.5
MR effect size for
'Selenium' on 'Guanine nucleotide exchange factor DBS'

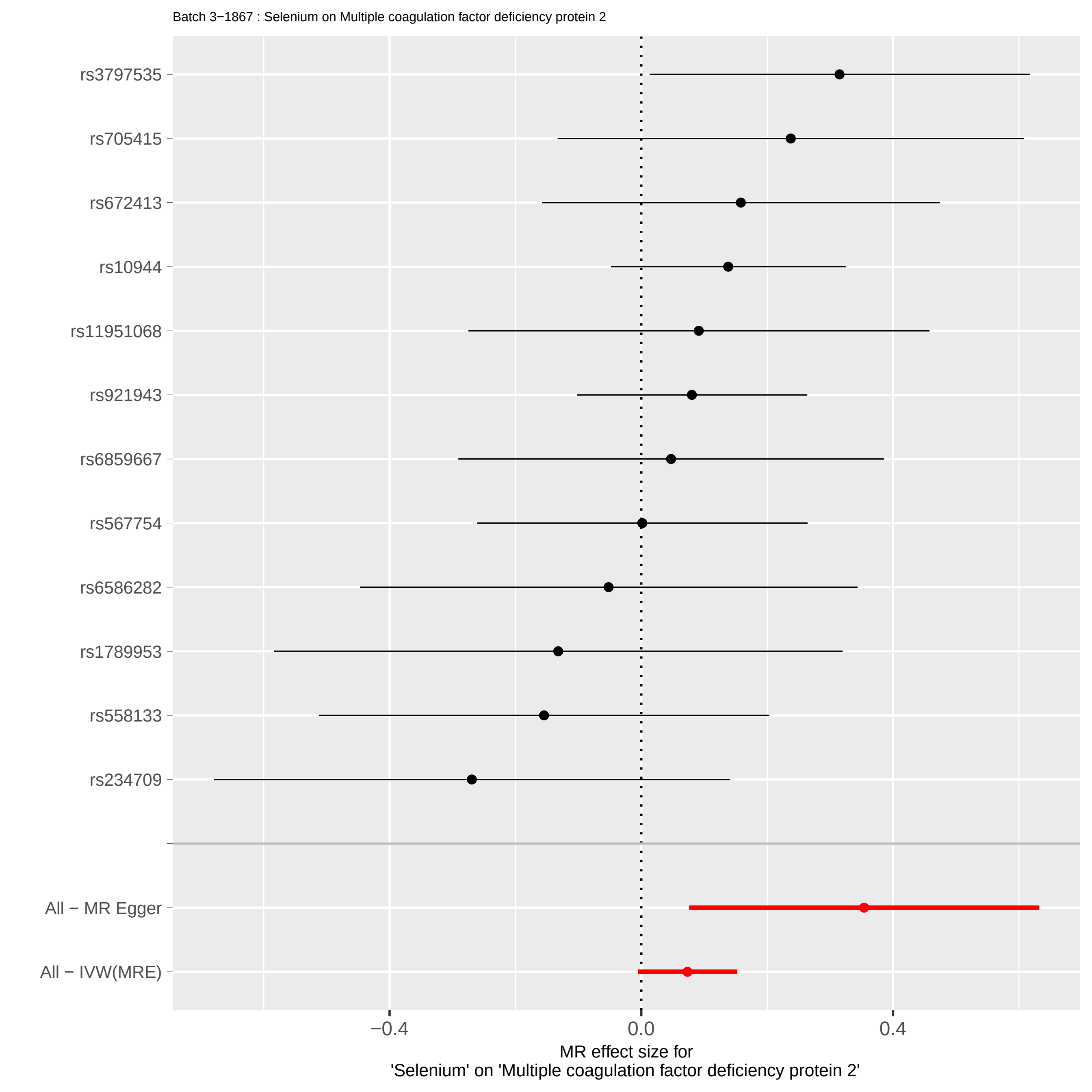
Batch 3−1867 : Selenium on Multiple coagulation factor deficiency protein 2
rs3797535
rs705415
rs672413
rs10944
rs11951068
rs921943
rs6859667
rs567754
rs6586282
rs1789953
rs558133
rs234709
All − MR Egger
All − IVW(MRE)
−0.4
0.0
0.4
MR effect size for
'Selenium' on 'Multiple coagulation factor deficiency protein 2'

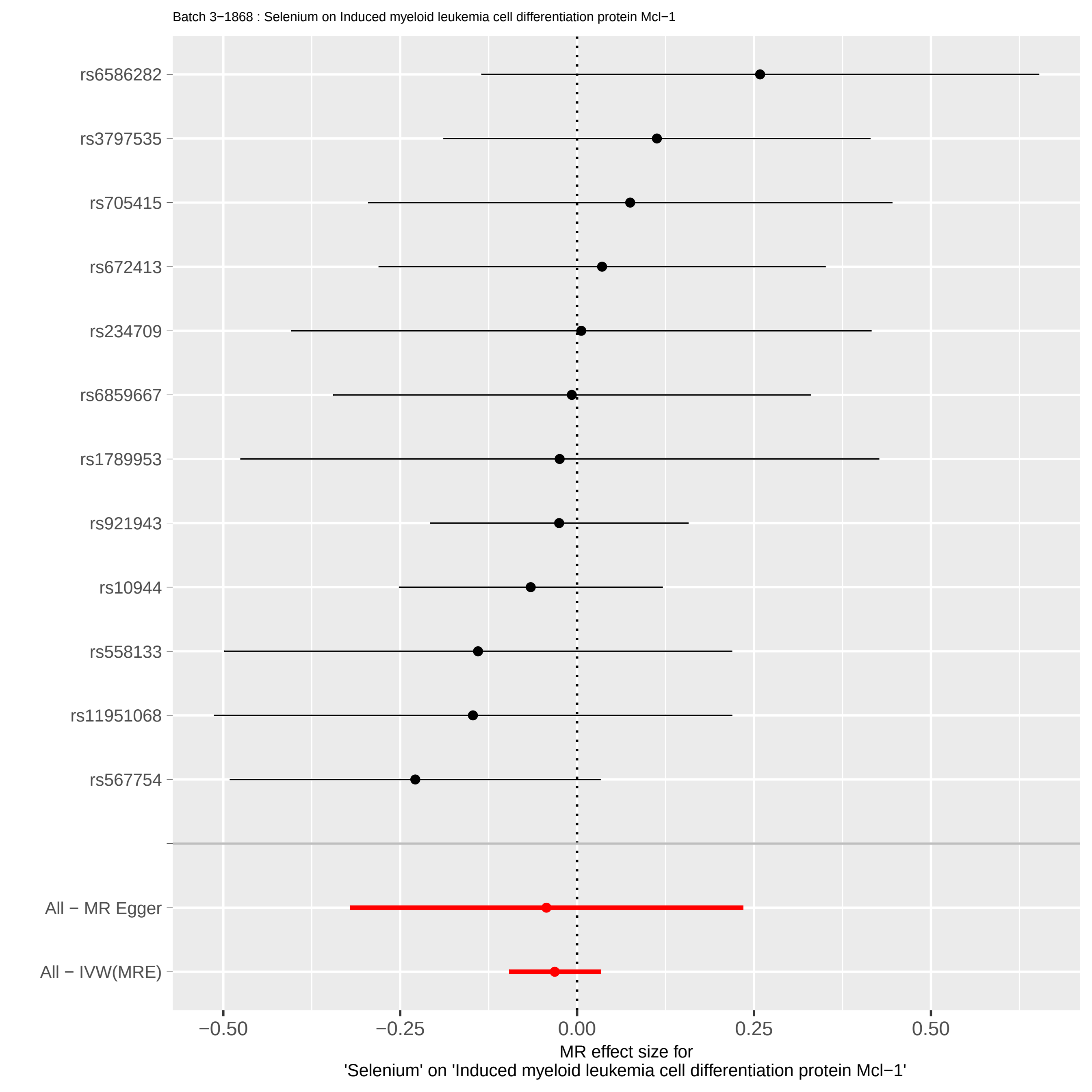
Batch 3−1868 : Selenium on Induced myeloid leukemia cell differentiation protein Mcl−1
rs6586282
rs3797535
rs705415
rs672413
rs234709
rs6859667
rs1789953
rs921943
rs10944
rs558133
rs11951068
rs567754
All − MR Egger
All − IVW(MRE)
−0.50
−0.25
0.00
0.25
0.50
MR effect size for
'Selenium' on 'Induced myeloid leukemia cell differentiation protein Mcl−1'

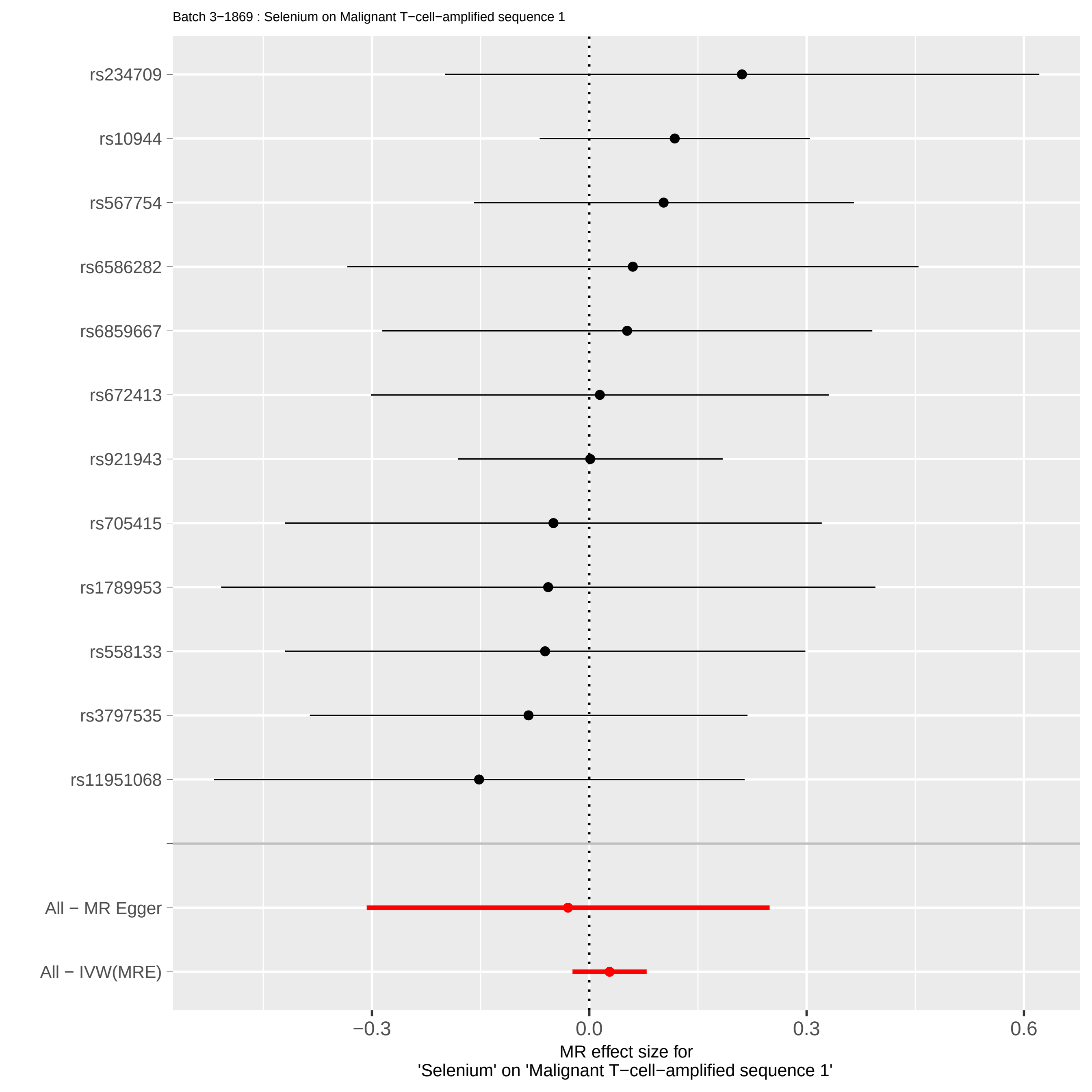
Batch 3−1869 : Selenium on Malignant T−cell−amplified sequence 1
rs234709
rs10944
rs567754
rs6586282
rs6859667
rs672413
rs921943
rs705415
rs1789953
rs558133
rs3797535
rs11951068
All − MR Egger
All − IVW(MRE)
−0.3
0.0
0.3
0.6
MR effect size for
'Selenium' on 'Malignant T−cell−amplified sequence 1'

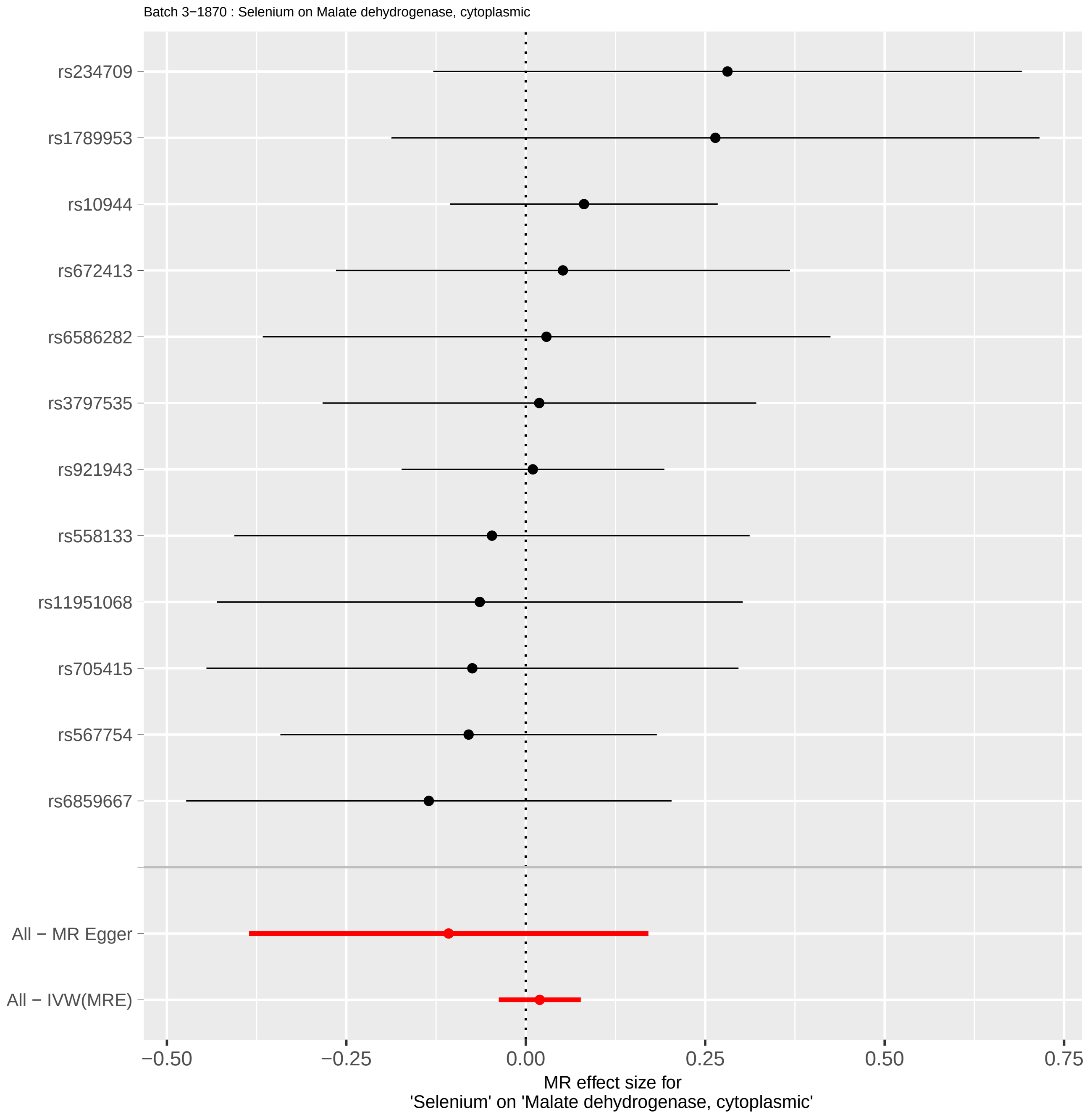

Batch 3−1870 : Selenium on Malate dehydrogenase, cytoplasmic
rs234709
rs1789953
rs10944
rs672413
rs6586282
rs3797535
rs921943
rs558133
rs11951068
rs705415
rs567754
rs6859667
All − MR Egger
All − IVW(MRE)
−0.50
−0.25
0.00
0.25
0.50
0.75
MR effect size for
'Selenium' on 'Malate dehydrogenase, cytoplasmic'

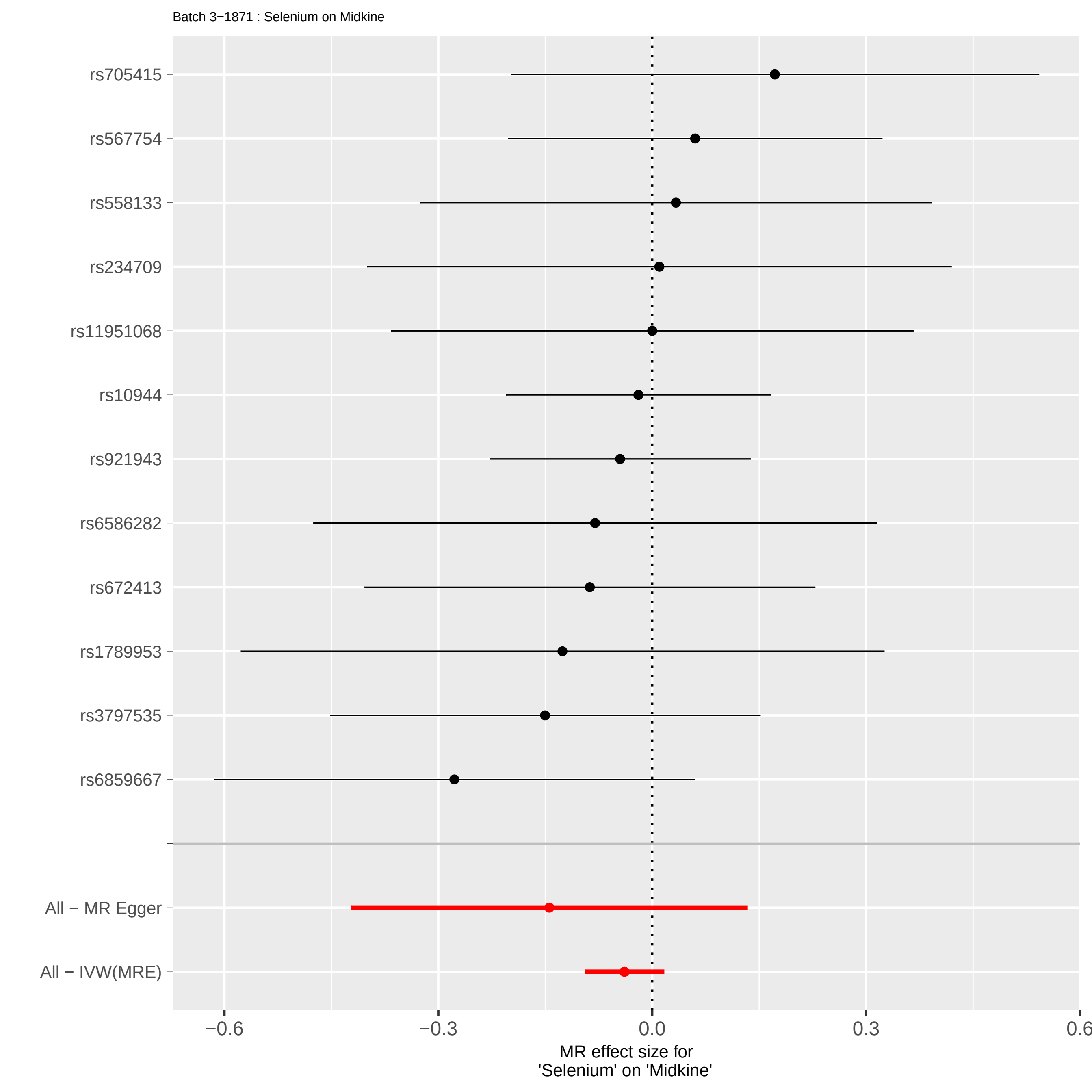

Batch 3−1871 : Selenium on Midkine
rs705415
rs567754
rs558133
rs234709
rs11951068
rs10944
rs921943
rs6586282
rs672413
rs1789953
rs3797535
rs6859667
All − MR Egger
All − IVW(MRE)
−0.6
−0.3
0.0
0.3
0.6
MR effect size for
'Selenium' on 'Midkine'

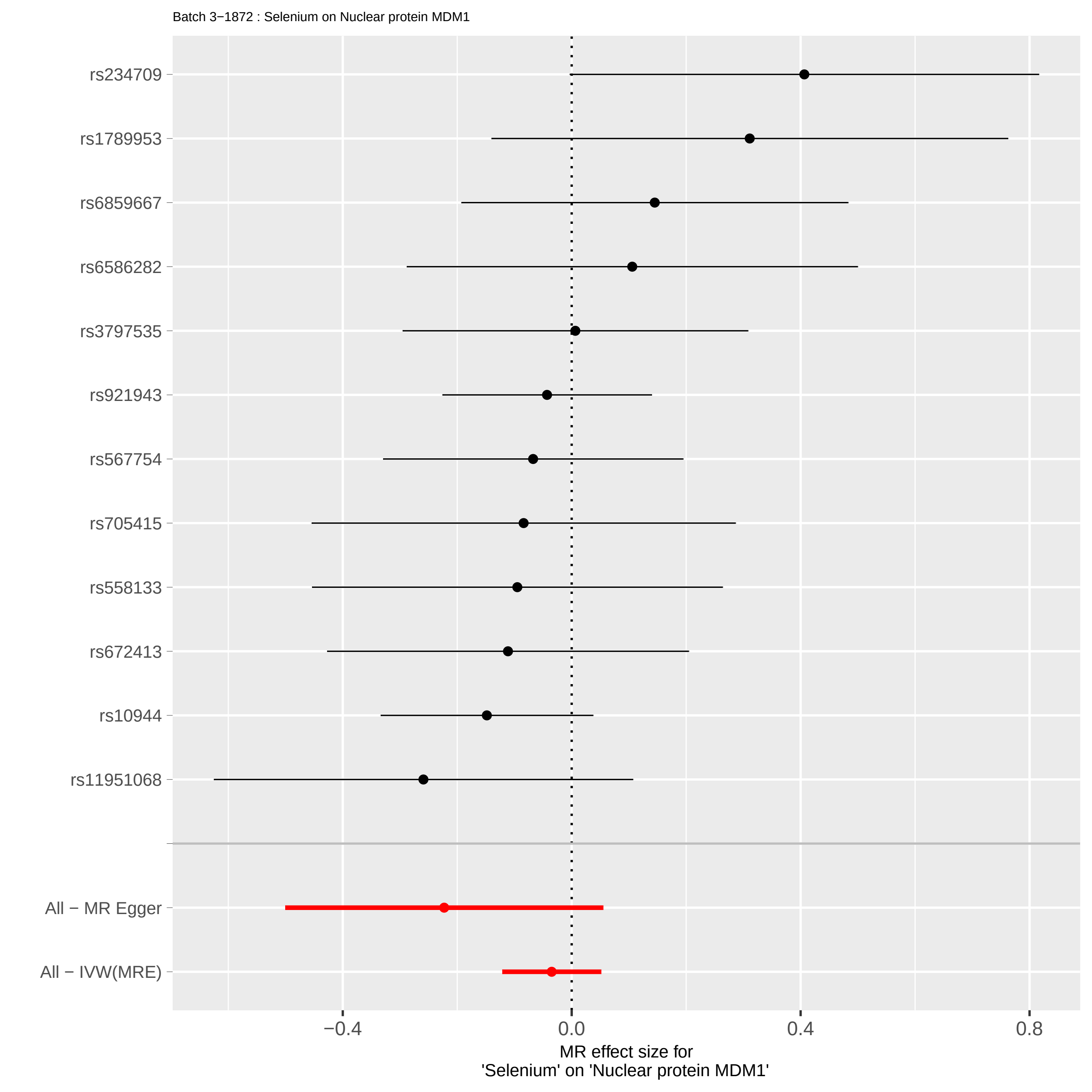
Batch 3−1872 : Selenium on Nuclear protein MDM1
rs234709
rs1789953
rs6859667
rs6586282
rs3797535
rs921943
rs567754
rs705415
rs558133
rs672413
rs10944
rs11951068
All − MR Egger
All − IVW(MRE)
−0.4
0.0
0.4
0.8
MR effect size for
'Selenium' on 'Nuclear protein MDM1'

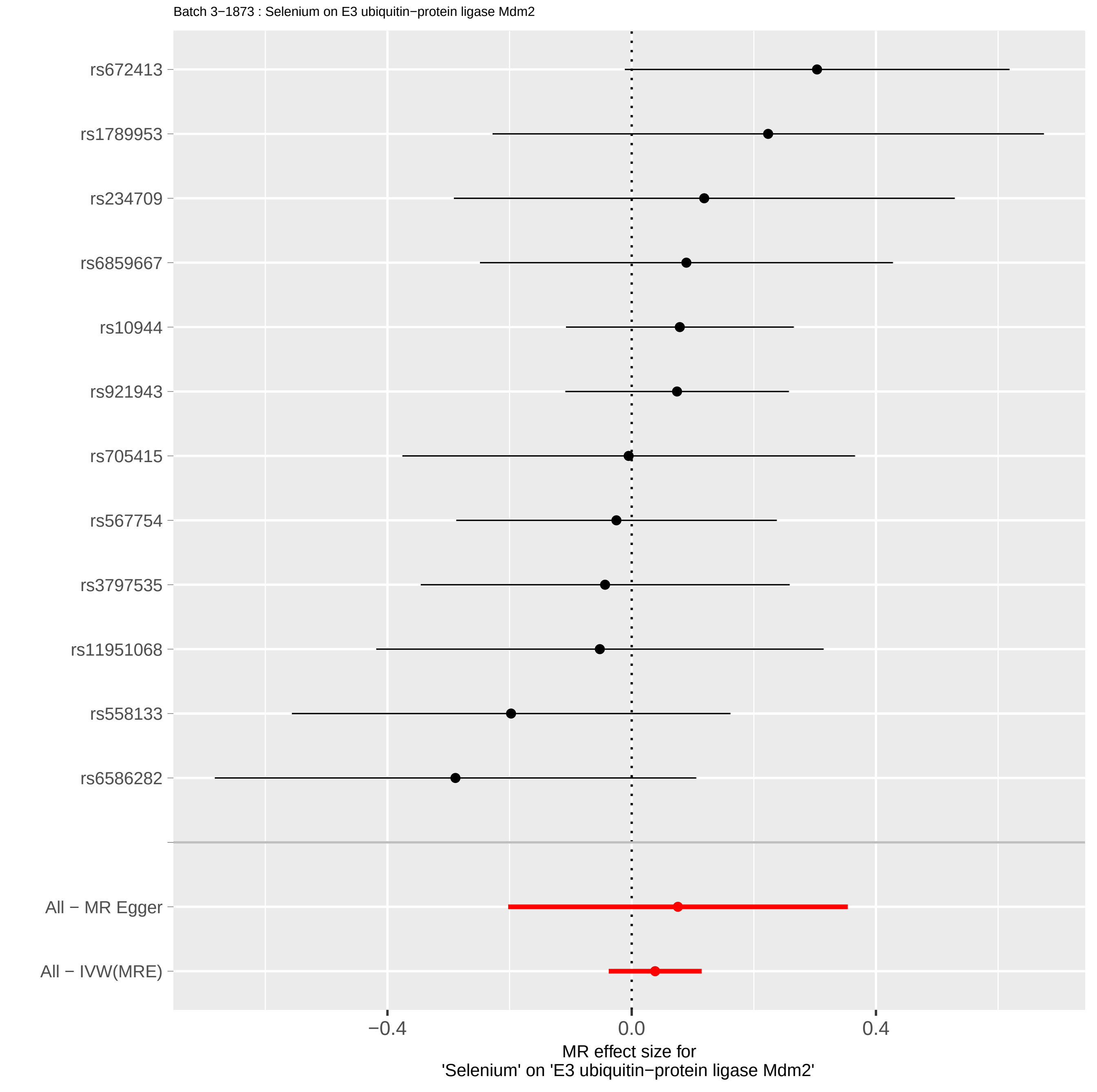

Batch 3−1873 : Selenium on E3 ubiquitin−protein ligase Mdm2
rs672413
rs1789953
rs234709
rs6859667
rs10944
rs921943
rs705415
rs567754
rs3797535
rs11951068
rs558133
rs6586282
All − MR Egger
All − IVW(MRE)
−0.4
0.0
0.4
MR effect size for
'Selenium' on 'E3 ubiquitin−protein ligase Mdm2'

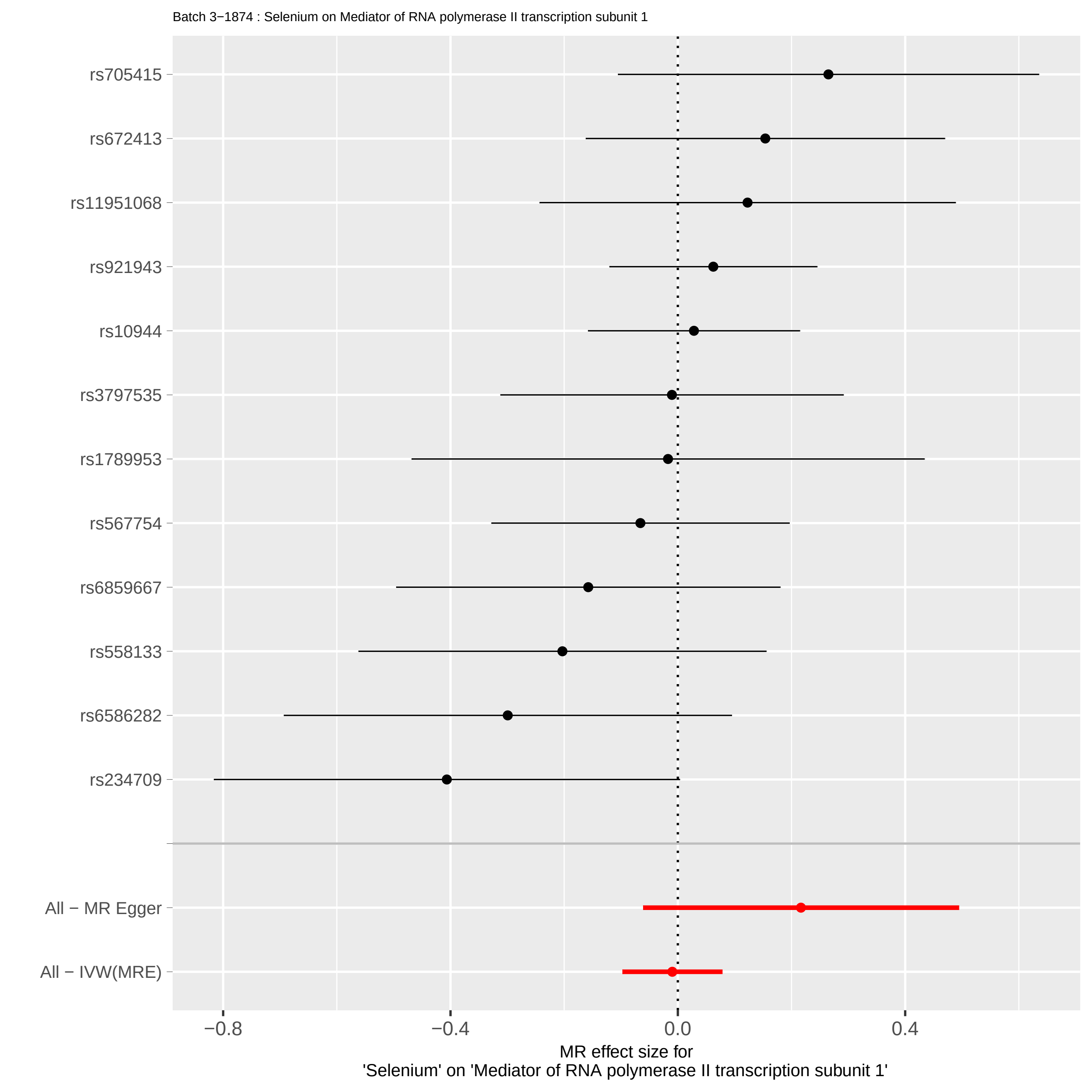

Batch 3−1874 : Selenium on Mediator of RNA polymerase II transcription subunit 1
rs705415
rs672413
rs11951068
rs921943
rs10944
rs3797535
rs1789953
rs567754
rs6859667
rs558133
rs6586282
rs234709
All − MR Egger
All − IVW(MRE)
−0.8
−0.4
0.0
0.4
MR effect size for
'Selenium' on 'Mediator of RNA polymerase II transcription subunit 1'

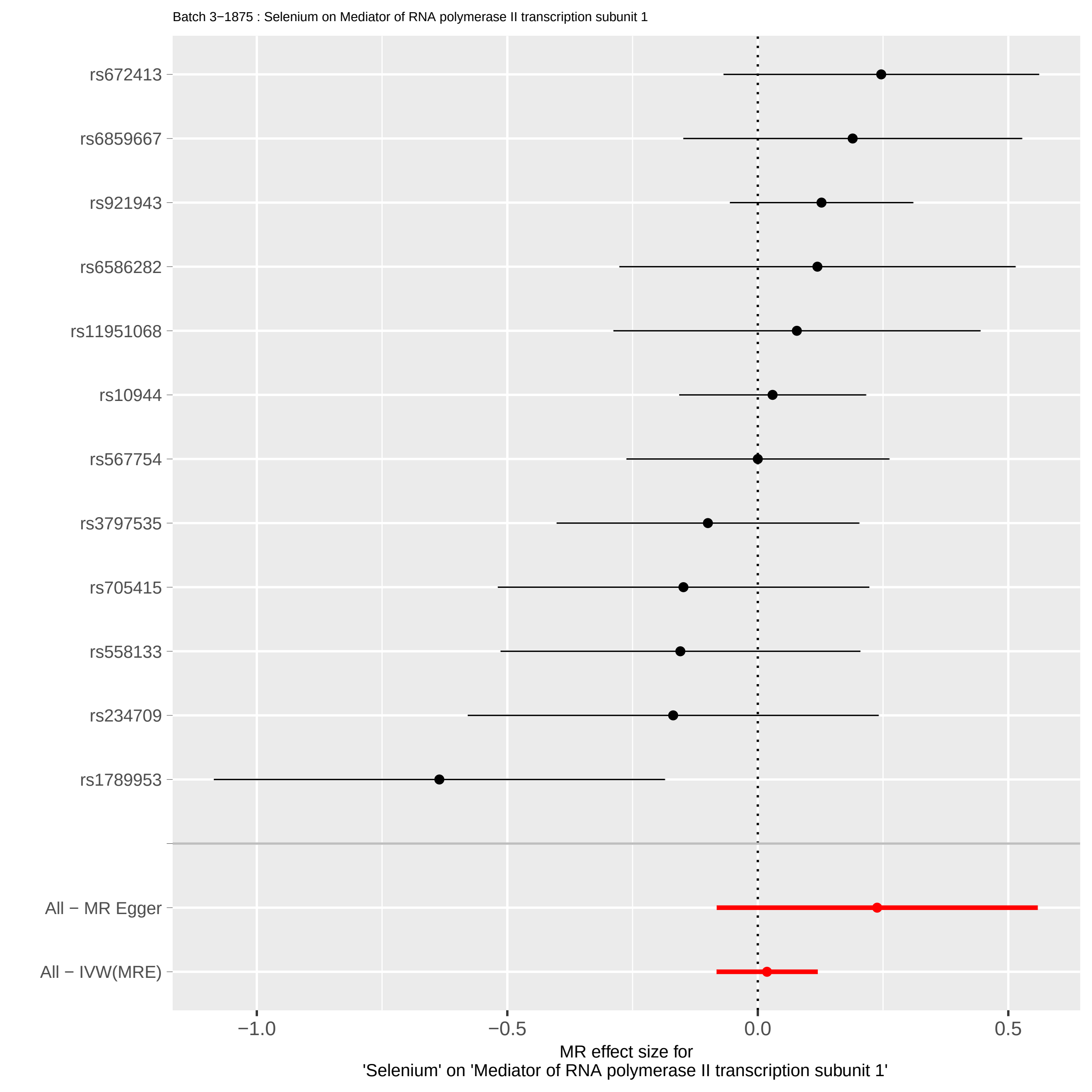

Batch 3−1875 : Selenium on Mediator of RNA polymerase II transcription subunit 1
rs672413
rs6859667
rs921943
rs6586282
rs11951068
rs10944
rs567754
rs3797535
rs705415
rs558133
rs234709
rs1789953
All − MR Egger
All − IVW(MRE)
−1.0
−0.5
0.0
0.5
MR effect size for
'Selenium' on 'Mediator of RNA polymerase II transcription subunit 1'

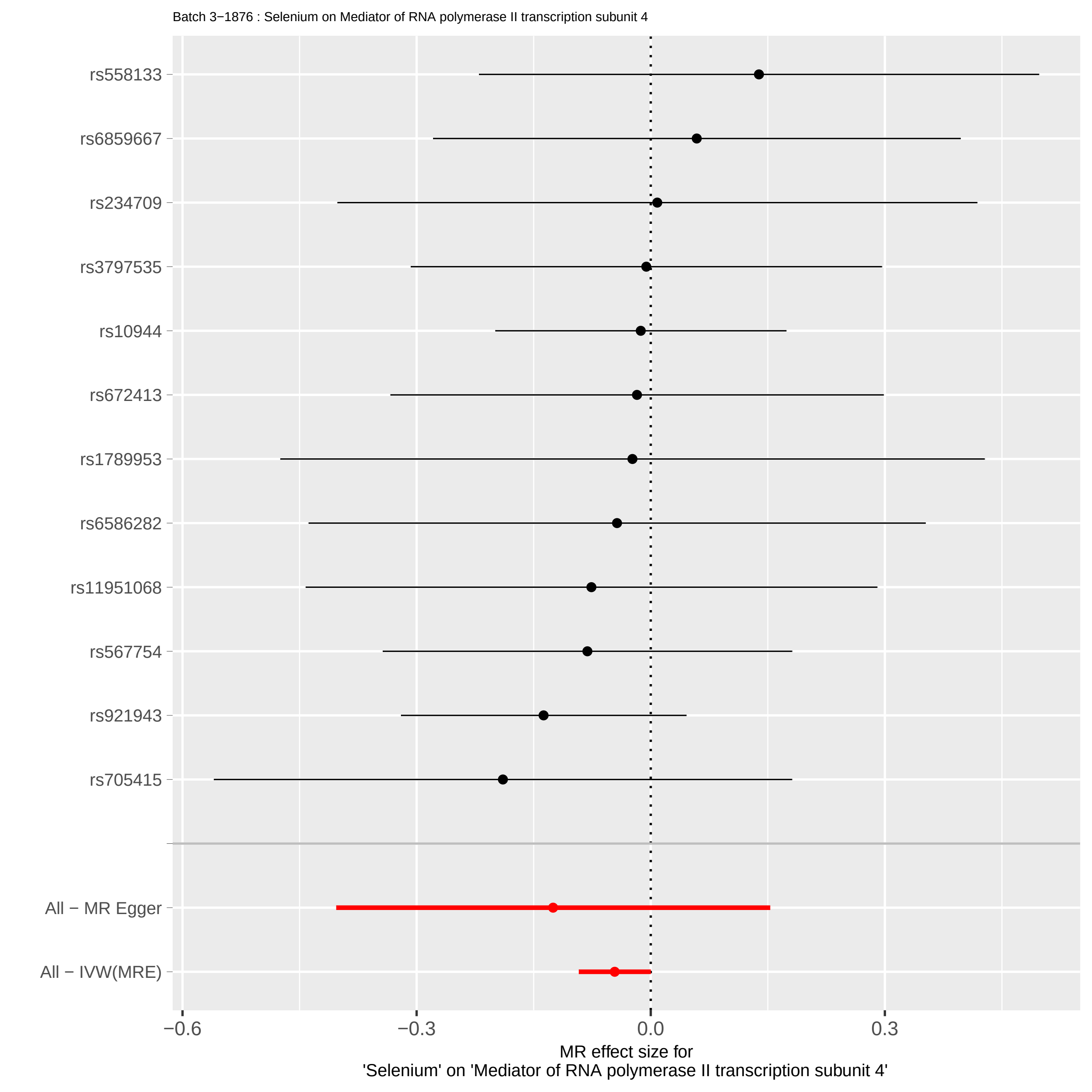

Batch 3−1876 : Selenium on Mediator of RNA polymerase II transcription subunit 4
rs558133
rs6859667
rs234709
rs3797535
rs10944
rs672413
rs1789953
rs6586282
rs11951068
rs567754
rs921943
rs705415
All − MR Egger
All − IVW(MRE)
−0.6
−0.3
0.0
0.3
MR effect size for
'Selenium' on 'Mediator of RNA polymerase II transcription subunit 4'

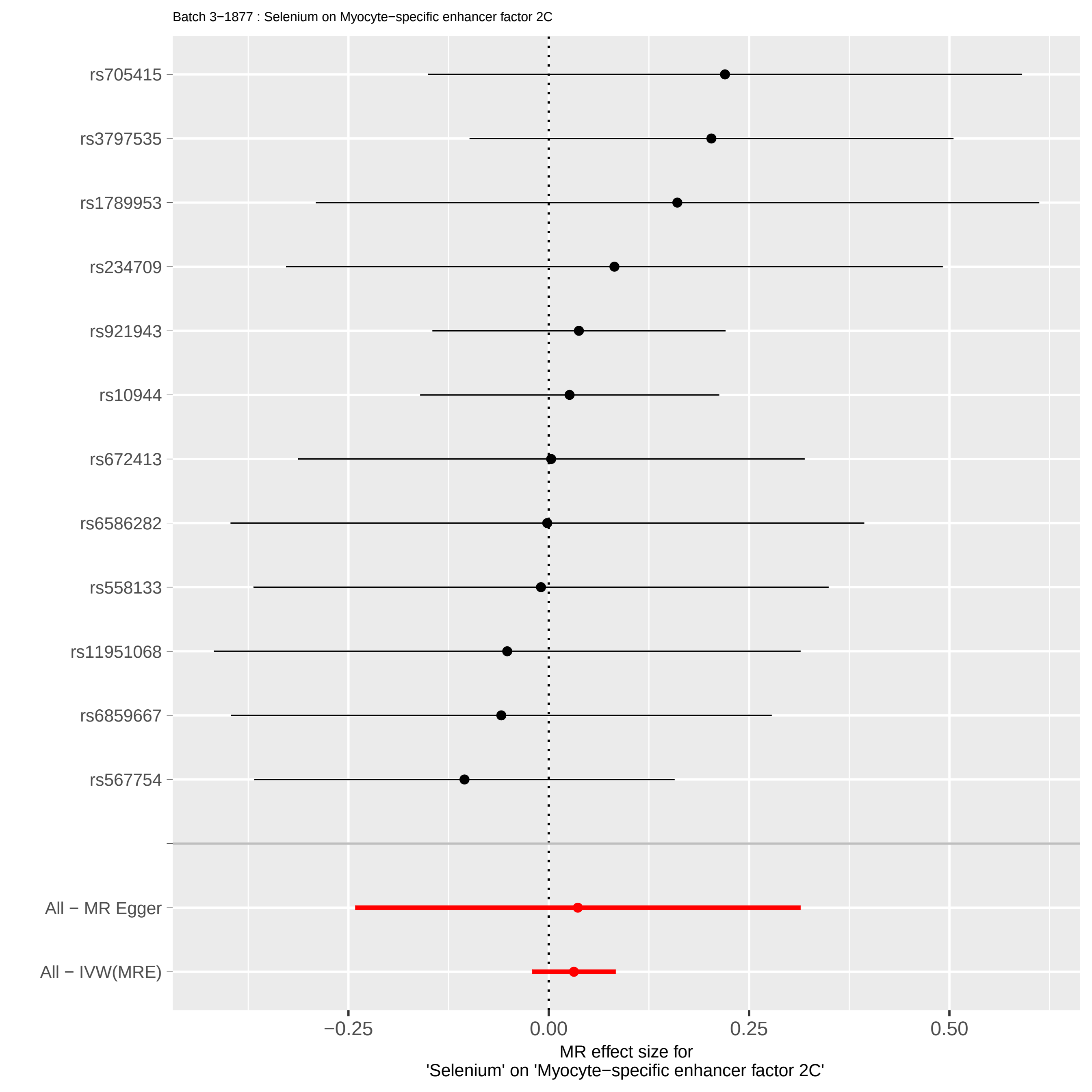

Batch 3−1877 : Selenium on Myocyte−specific enhancer factor 2C
rs705415
rs3797535
rs1789953
rs234709
rs921943
rs10944
rs672413
rs6586282
rs558133
rs11951068
rs6859667
rs567754
All − MR Egger
All − IVW(MRE)
−0.25
0.00
0.25
0.50
MR effect size for
'Selenium' on 'Myocyte−specific enhancer factor 2C'

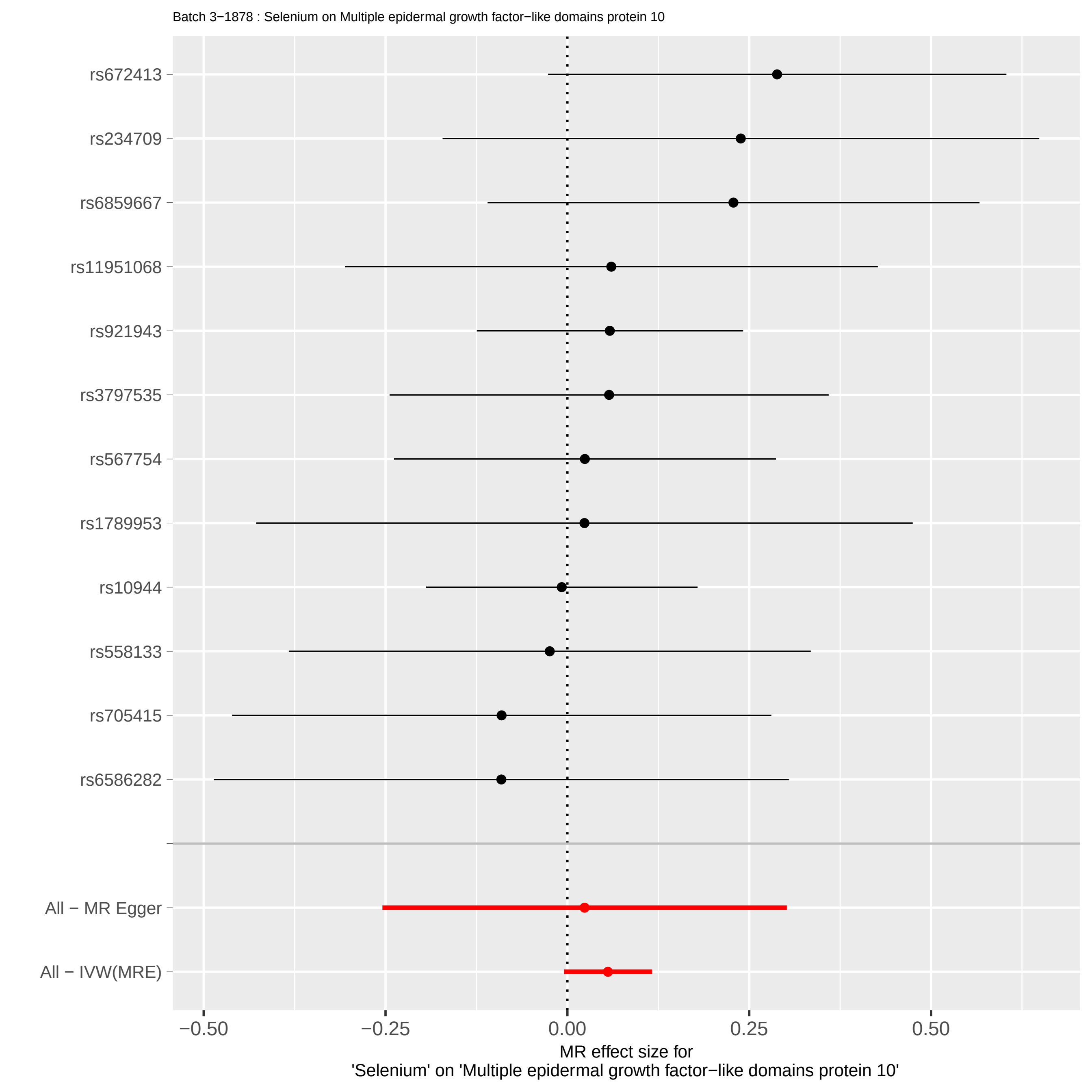

Batch 3−1878 : Selenium on Multiple epidermal growth factor−like domains protein 10
rs672413
rs234709
rs6859667
rs11951068
rs921943
rs3797535
rs567754
rs1789953
rs10944
rs558133
rs705415
rs6586282
All − MR Egger
All − IVW(MRE)
−0.50
−0.25
0.00
0.25
0.50
MR effect size for
'Selenium' on 'Multiple epidermal growth factor−like domains protein 10'

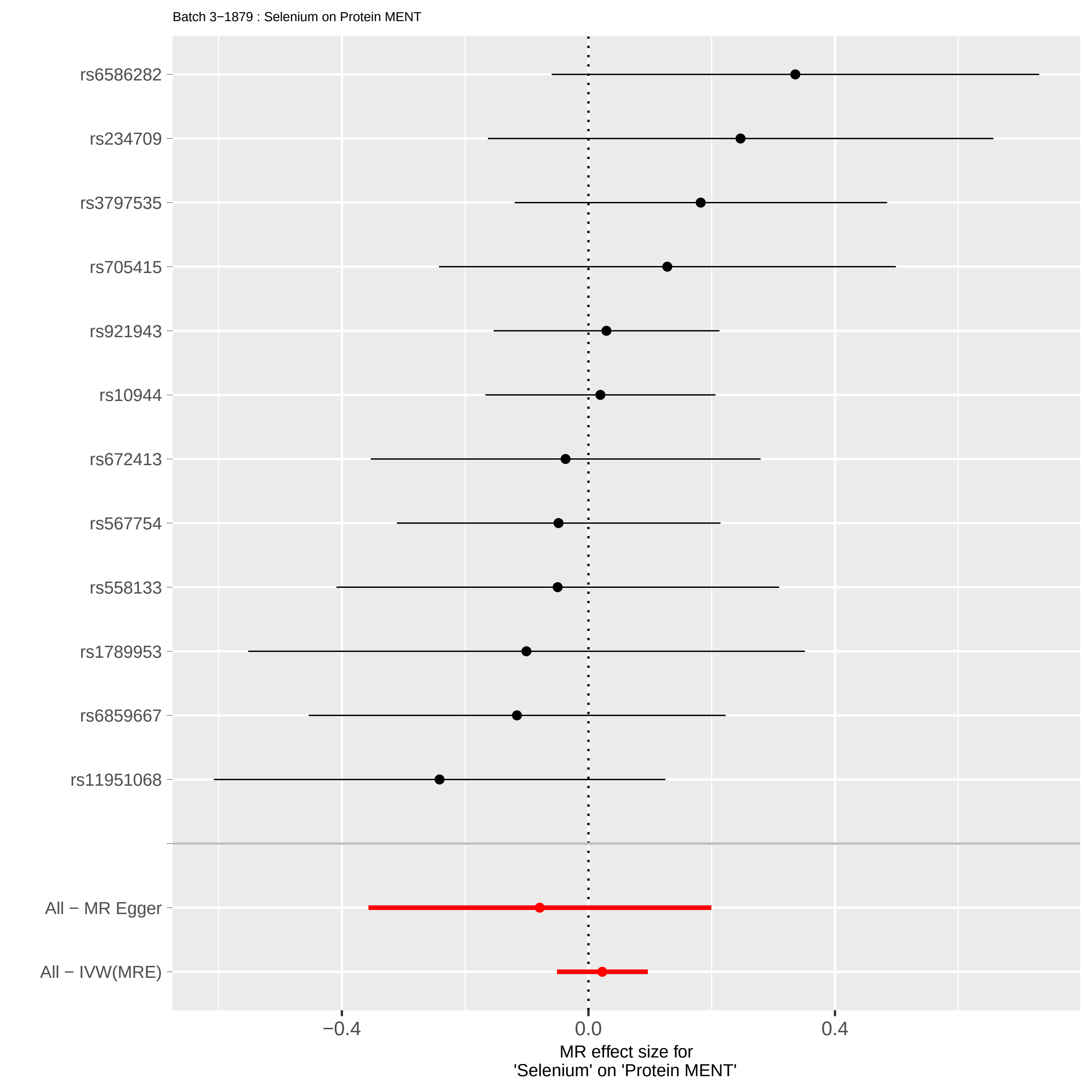

Batch 3−1879 : Selenium on Protein MENT
rs6586282
rs234709
rs3797535
rs705415
rs921943
rs10944
rs672413
rs567754
rs558133
rs1789953
rs6859667
rs11951068
All − MR Egger
All − IVW(MRE)
−0.4
0.0
0.4
MR effect size for
'Selenium' on 'Protein MENT'

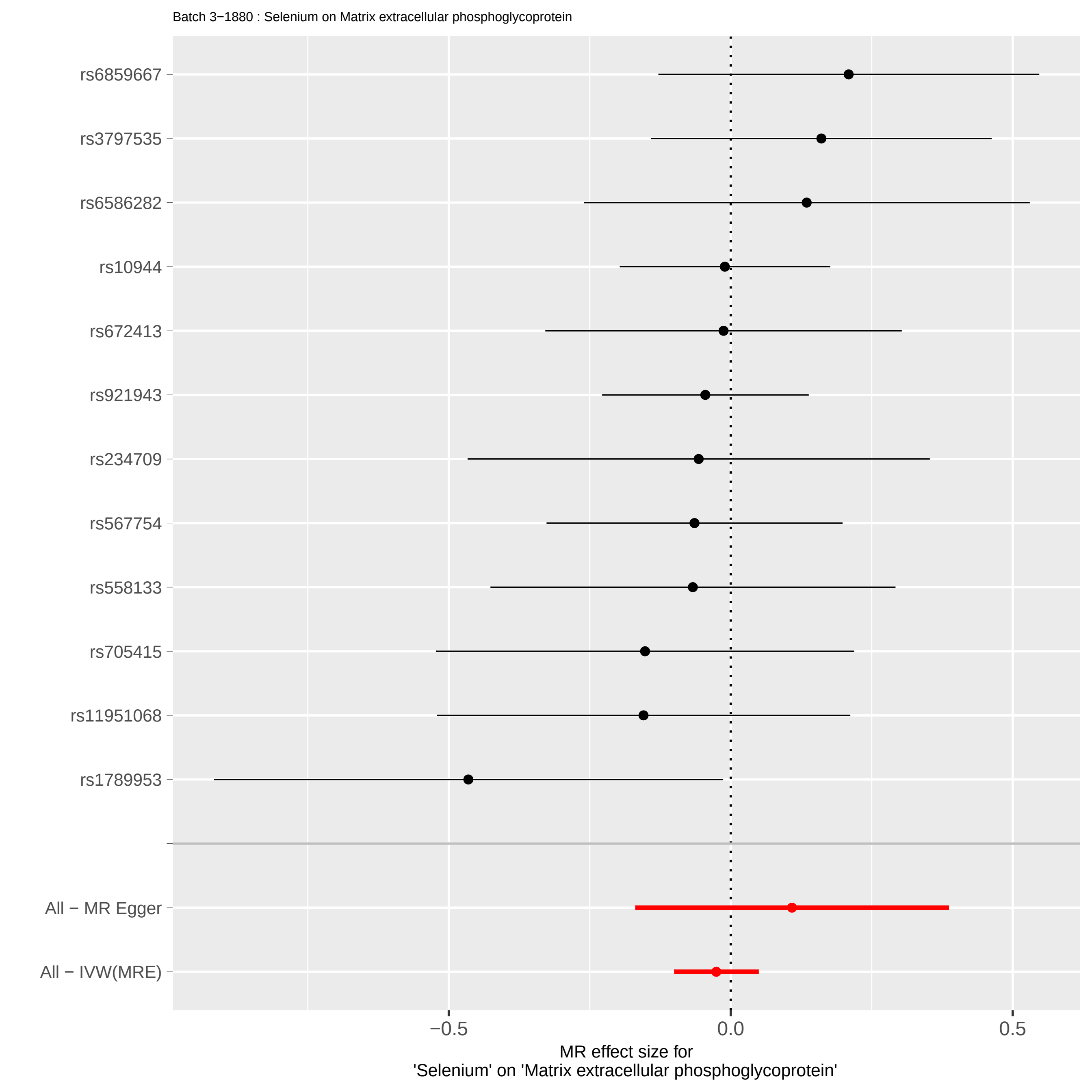

Batch 3−1880 : Selenium on Matrix extracellular phosphoglycoprotein
rs6859667
rs3797535
rs6586282
rs10944
rs672413
rs921943
rs234709
rs567754
rs558133
rs705415
rs11951068
rs1789953
All − MR Egger
All − IVW(MRE)
−0.5
0.0
0.5
MR effect size for
'Selenium' on 'Matrix extracellular phosphoglycoprotein'

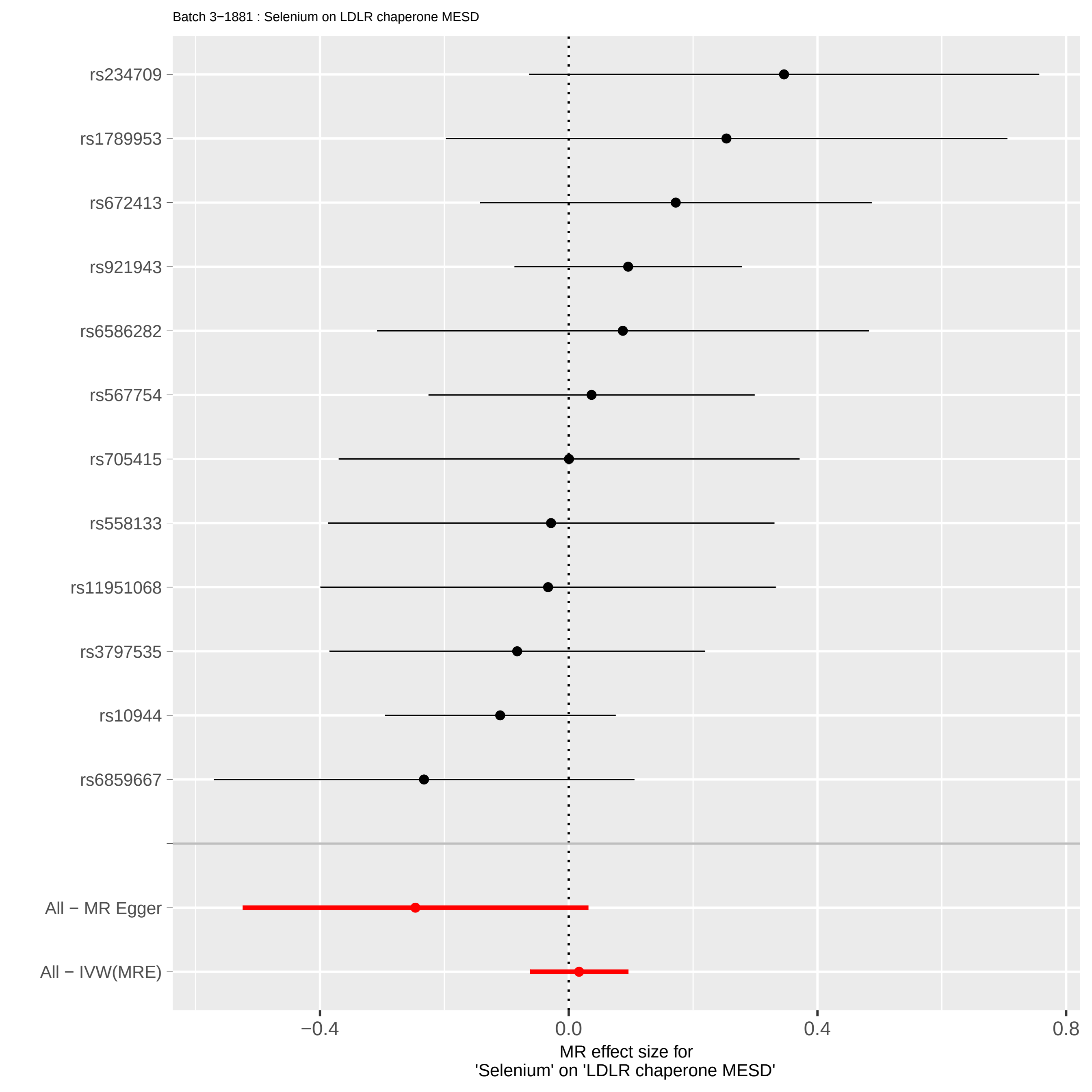
Batch 3−1881 : Selenium on LDLR chaperone MESD
rs234709
rs1789953
rs672413
rs921943
rs6586282
rs567754
rs705415
rs558133
rs11951068
rs3797535
rs10944
rs6859667
All − MR Egger
All − IVW(MRE)
−0.4
0.0
0.4
0.8
MR effect size for
'Selenium' on 'LDLR chaperone MESD'

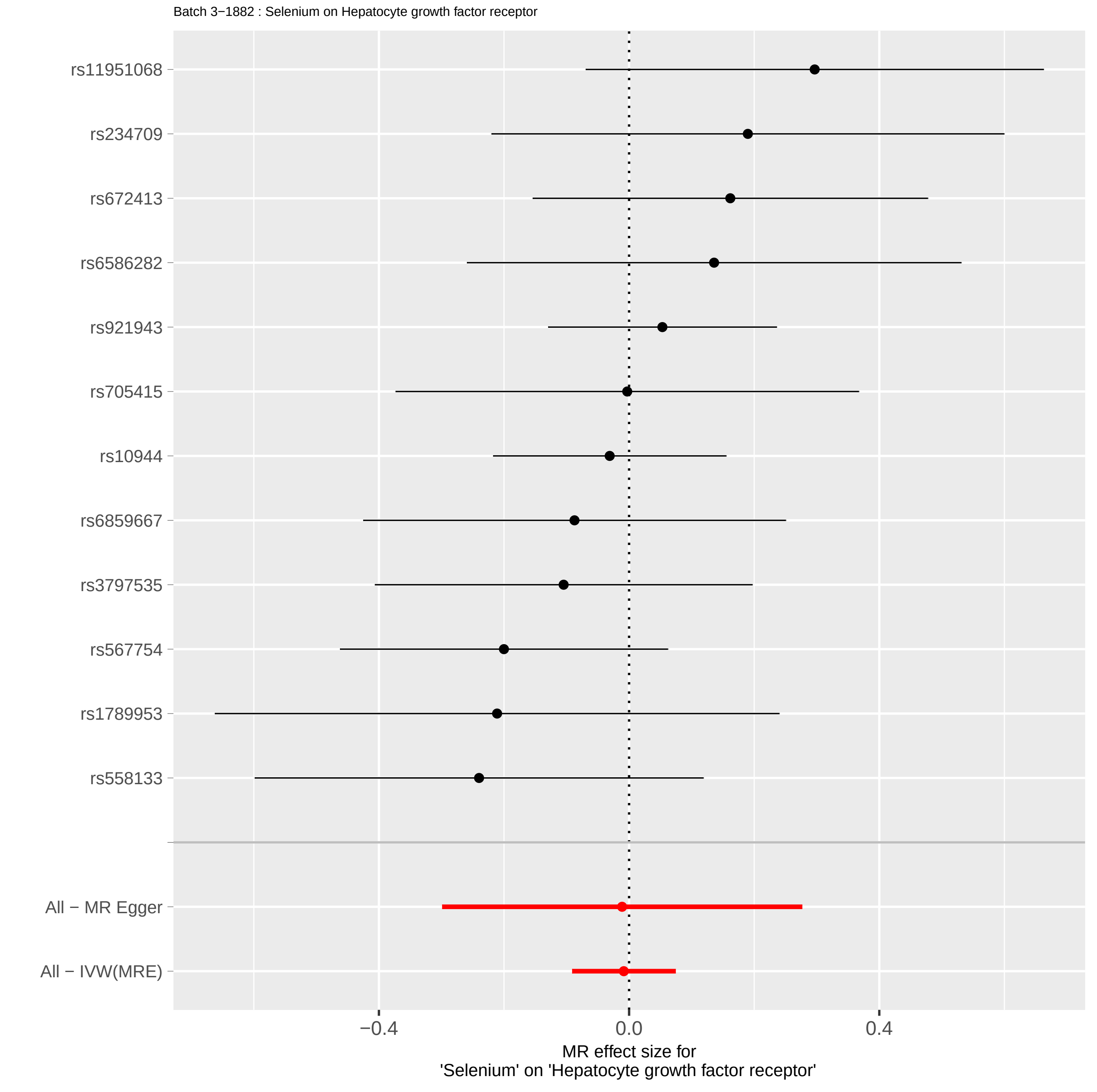

Batch 3−1882 : Selenium on Hepatocyte growth factor receptor
rs11951068
rs234709
rs672413
rs6586282
rs921943
rs705415
rs10944
rs6859667
rs3797535
rs567754
rs1789953
rs558133
All − MR Egger
All − IVW(MRE)
−0.4
0.0
0.4
MR effect size for
'Selenium' on 'Hepatocyte growth factor receptor'

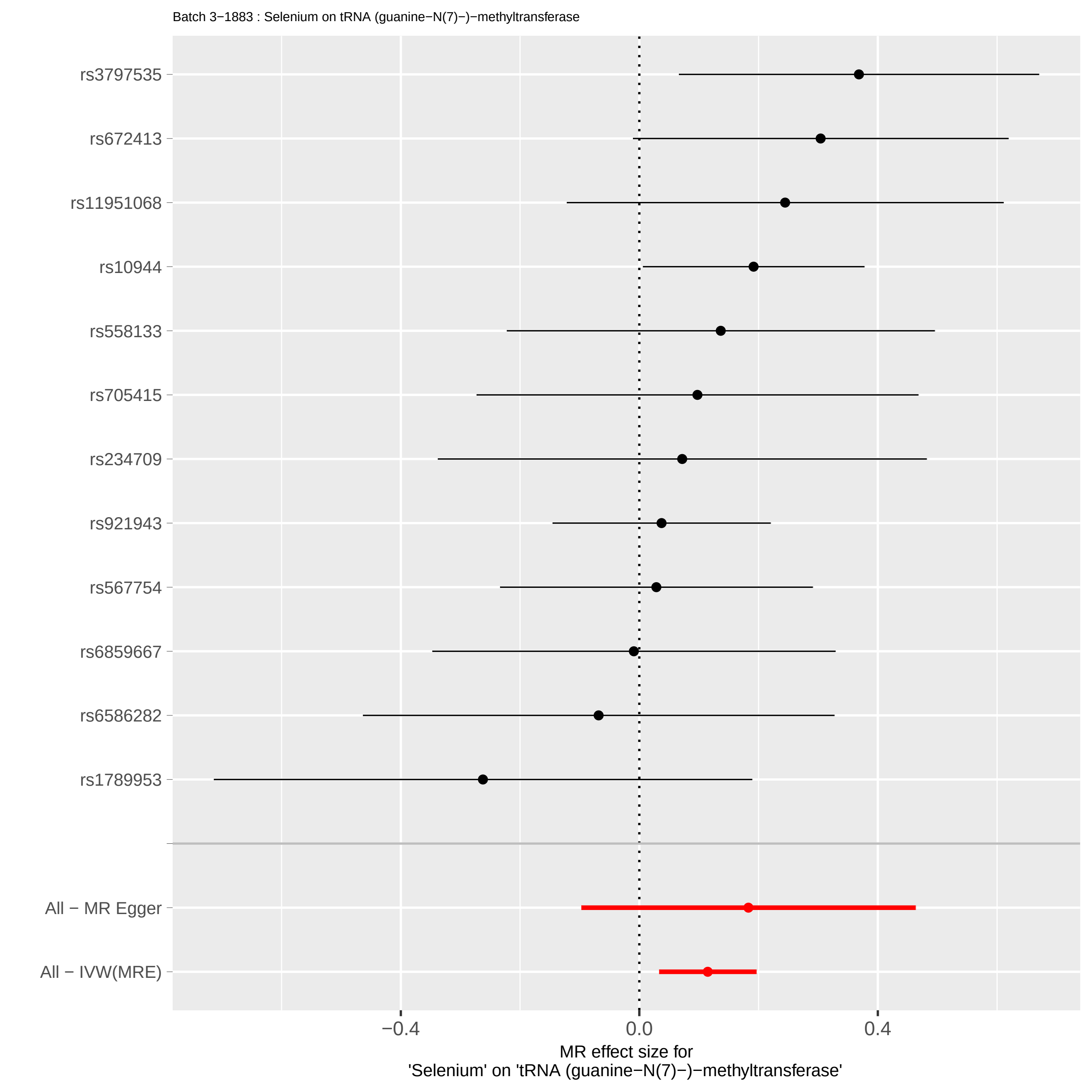
Batch 3−1883 : Selenium on tRNA (guanine−N(7)−)−methyltransferase
rs3797535
rs672413
rs11951068
rs10944
rs558133
rs705415
rs234709
rs921943
rs567754
rs6859667
rs6586282
rs1789953
All − MR Egger
All − IVW(MRE)
−0.4
0.0
0.4
MR effect size for
'Selenium' on 'tRNA (guanine−N(7)−)−methyltransferase'

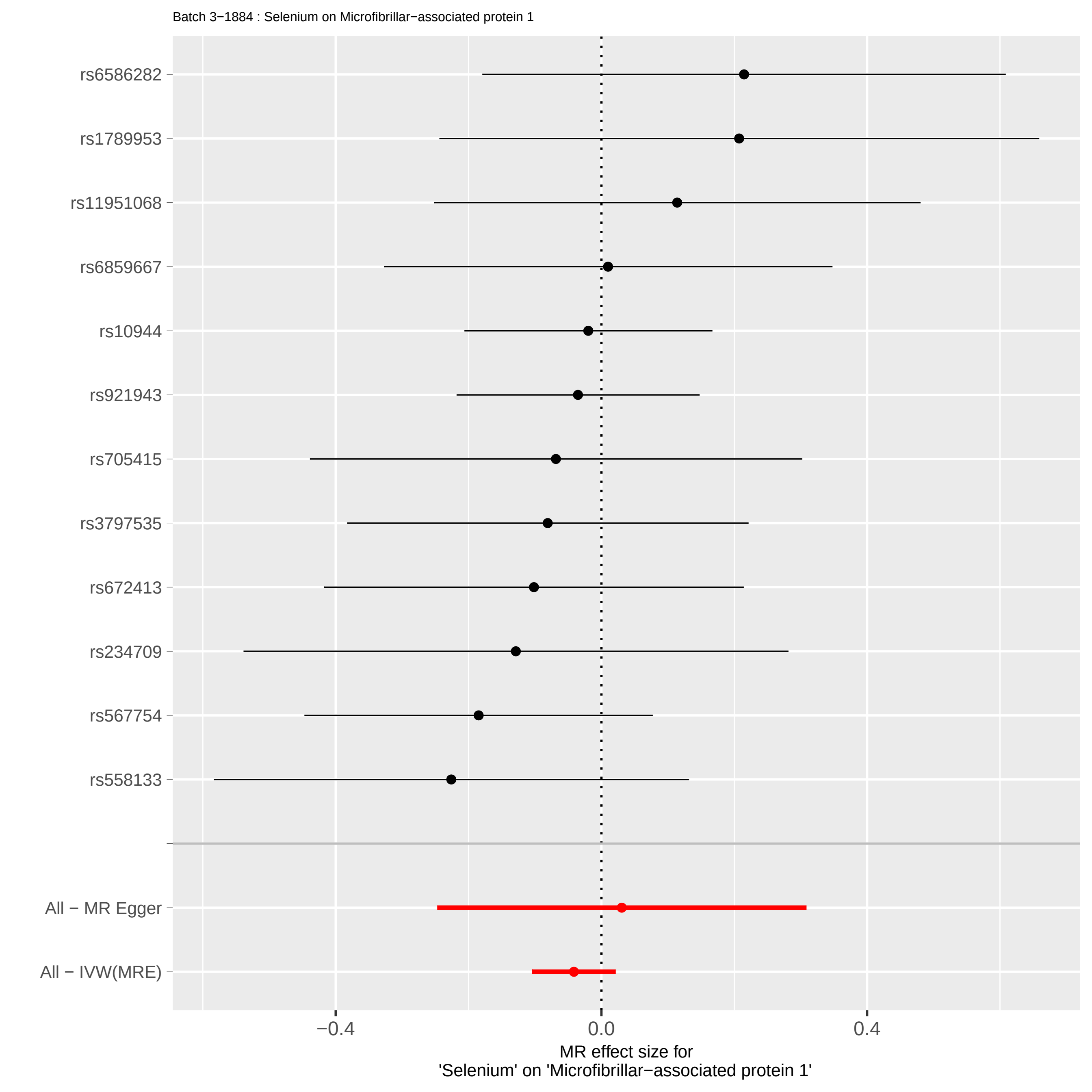

Batch 3−1884 : Selenium on Microfibrillar−associated protein 1
rs6586282
rs1789953
rs11951068
rs6859667
rs10944
rs921943
rs705415
rs3797535
rs672413
rs234709
rs567754
rs558133
All − MR Egger
All − IVW(MRE)
−0.4
0.0
0.4
MR effect size for
'Selenium' on 'Microfibrillar−associated protein 1'

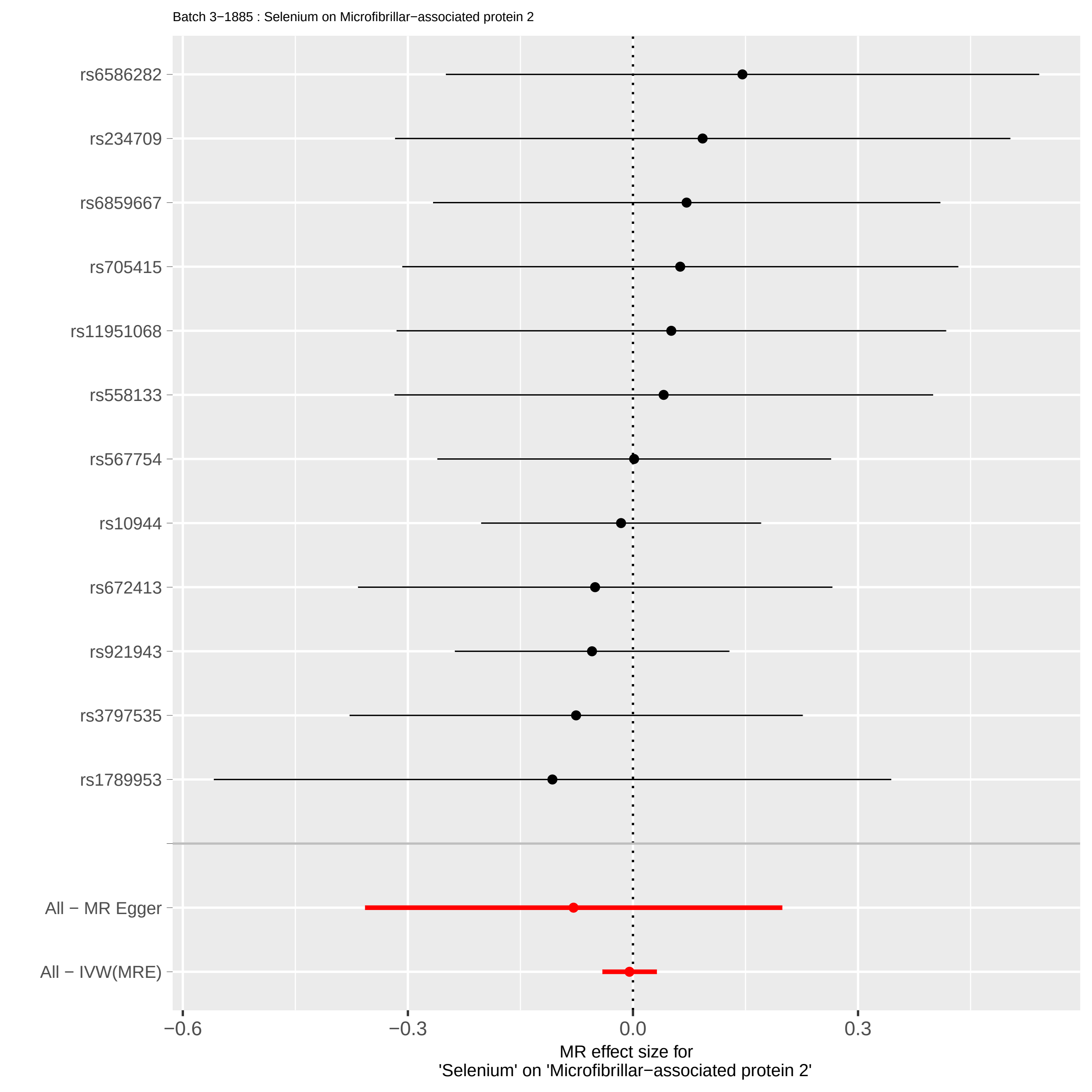
Batch 3−1885 : Selenium on Microfibrillar−associated protein 2
rs6586282
rs234709
rs6859667
rs705415
rs11951068
rs558133
rs567754
rs10944
rs672413
rs921943
rs3797535
rs1789953
All − MR Egger
All − IVW(MRE)
−0.6
−0.3
0.0
0.3
MR effect size for
'Selenium' on 'Microfibrillar−associated protein 2'

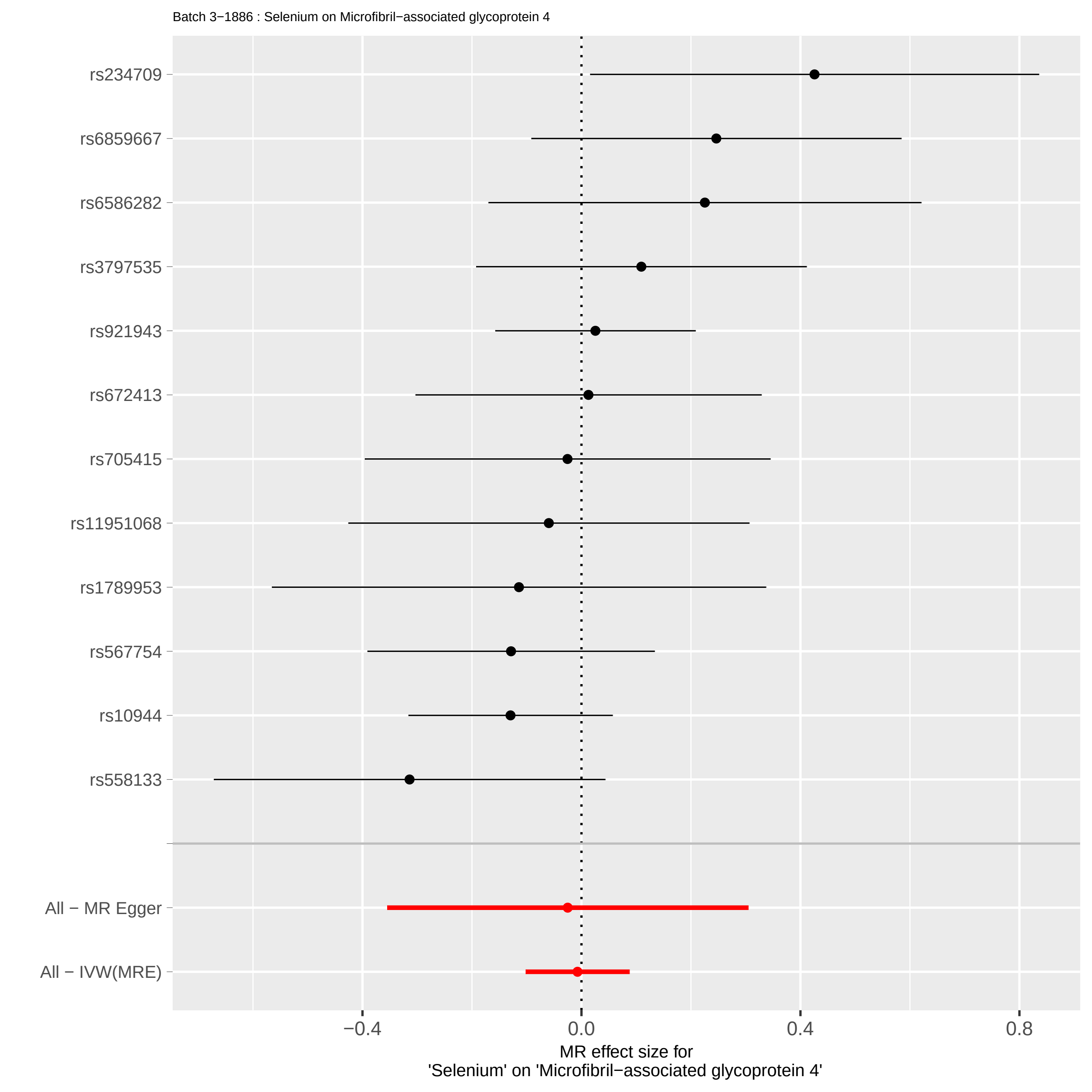

Batch 3−1886 : Selenium on Microfibril−associated glycoprotein 4
rs234709
rs6859667
rs6586282
rs3797535
rs921943
rs672413
rs705415
rs11951068
rs1789953
rs567754
rs10944
rs558133
All − MR Egger
All − IVW(MRE)
−0.4
0.0
0.4
0.8
MR effect size for
'Selenium' on 'Microfibril−associated glycoprotein 4'

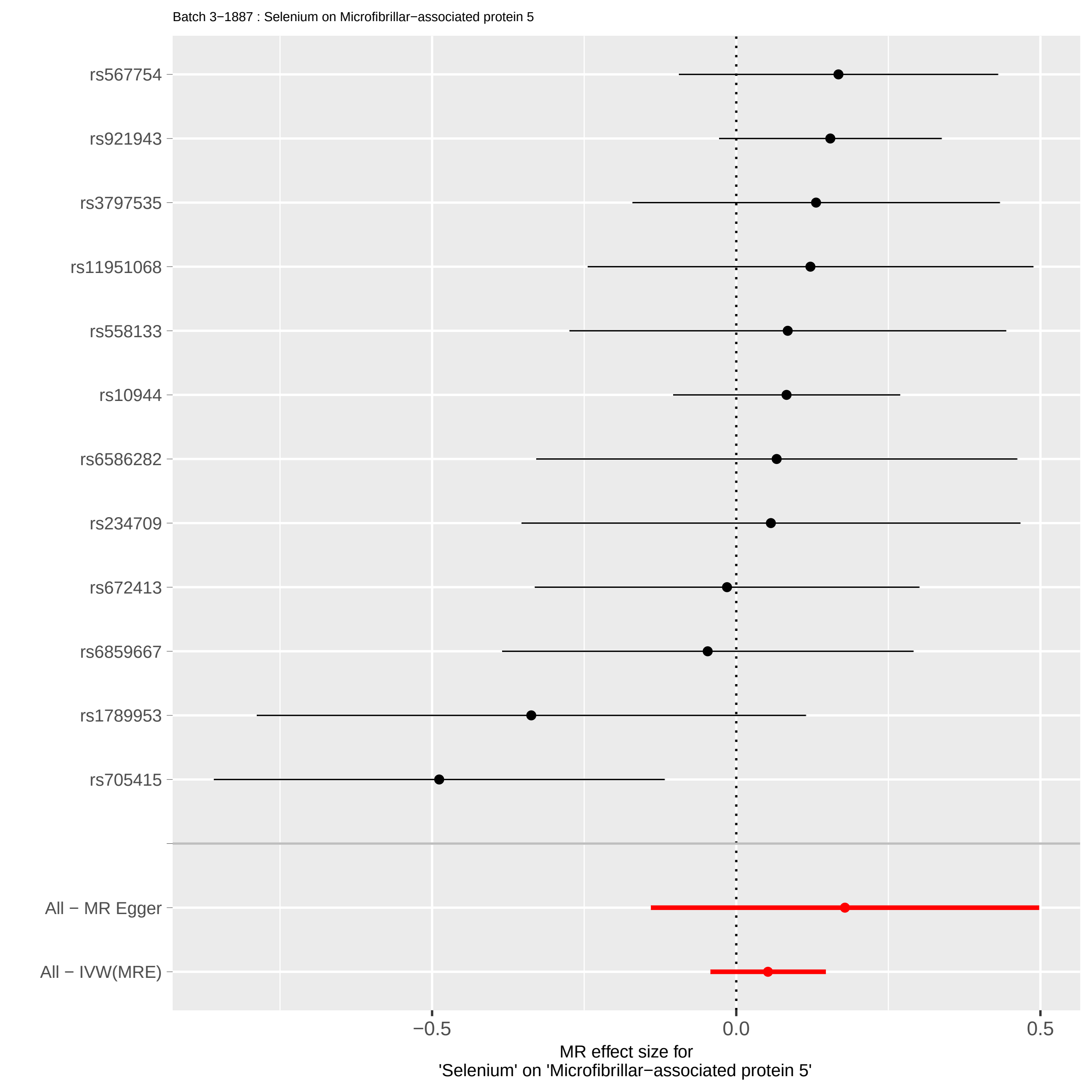

Batch 3−1887 : Selenium on Microfibrillar−associated protein 5
rs567754
rs921943
rs3797535
rs11951068
rs558133
rs10944
rs6586282
rs234709
rs672413
rs6859667
rs1789953
rs705415
All − MR Egger
All − IVW(MRE)
−0.5
0.0
0.5
MR effect size for
'Selenium' on 'Microfibrillar−associated protein 5'

Batch 3−1888 : Selenium on Lactadherin

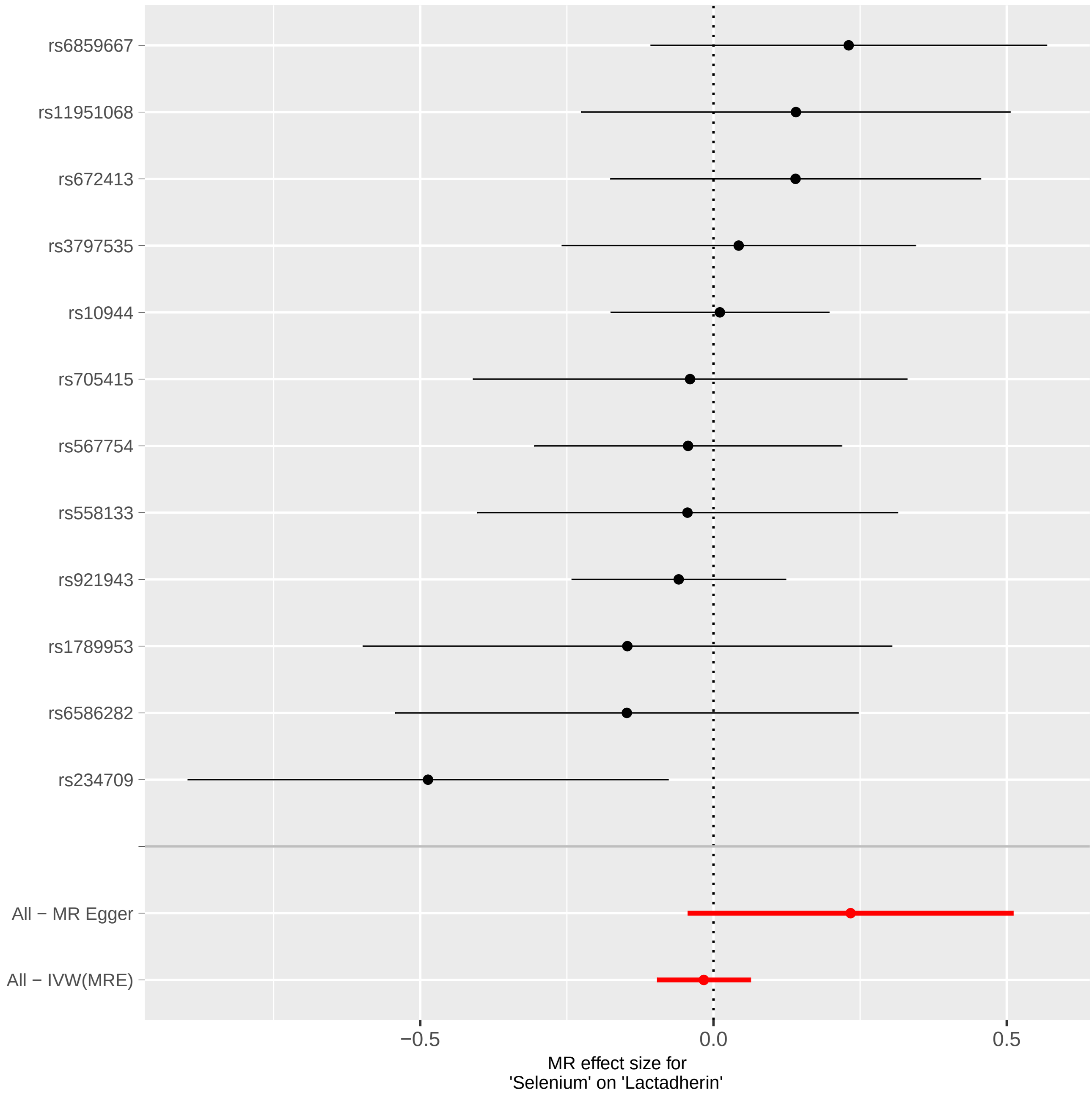

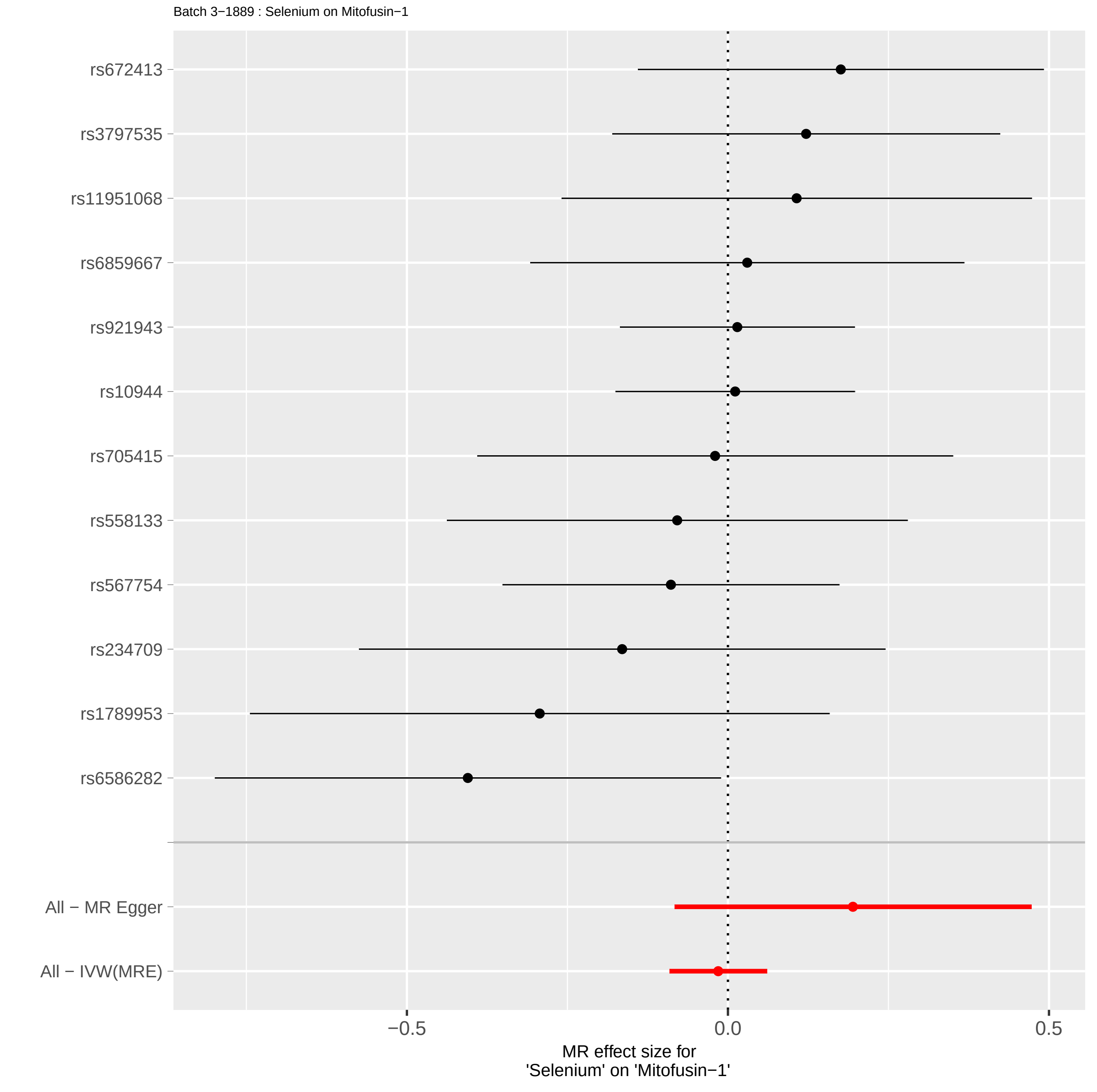

Batch 3−1889 : Selenium on Mitofusin−1
rs672413
rs3797535
rs11951068
rs6859667
rs921943
rs10944
rs705415
rs558133
rs567754
rs234709
rs1789953
rs6586282
All − MR Egger
All − IVW(MRE)
−0.5
0.0
0.5
MR effect size for
'Selenium' on 'Mitofusin−1'

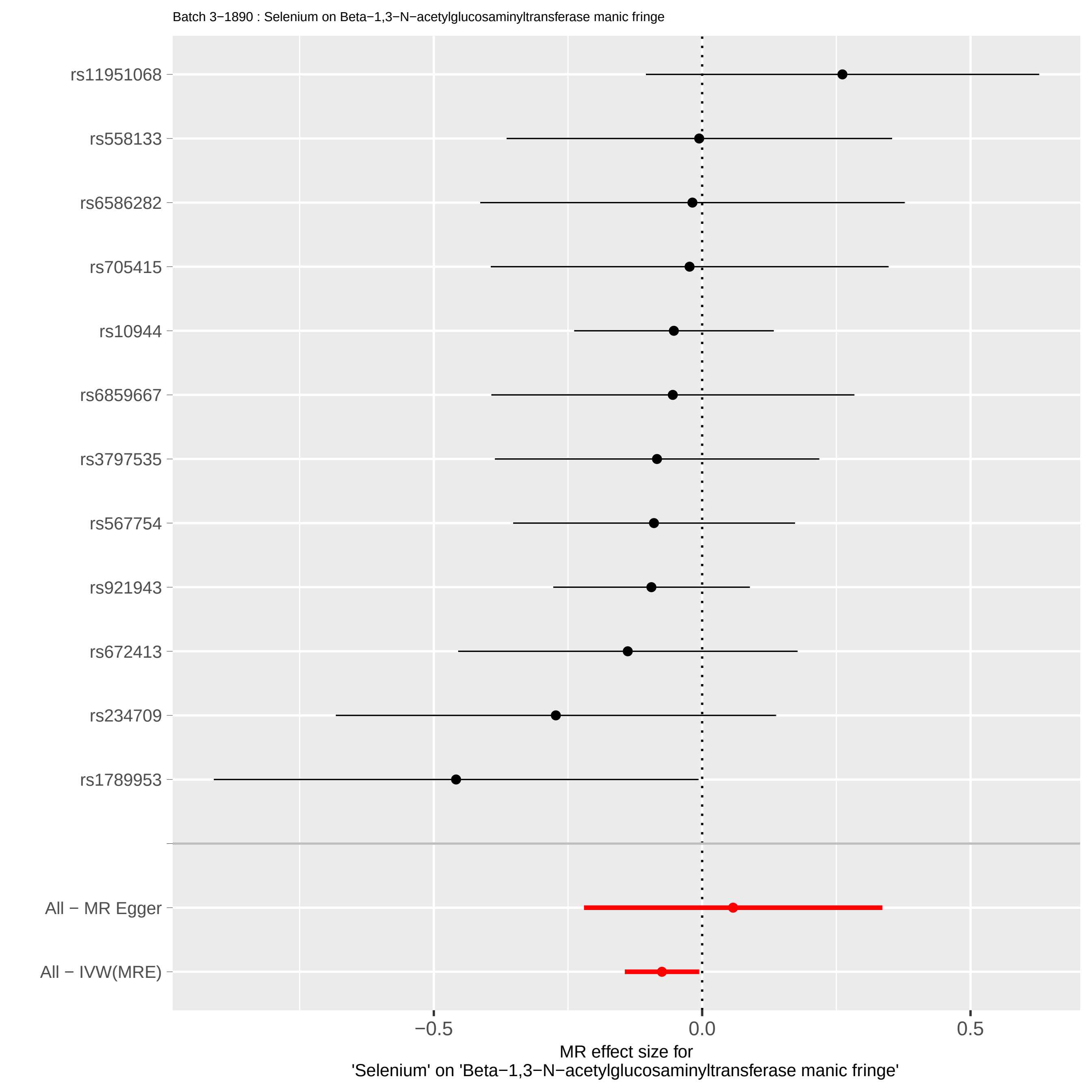

Batch 3−1890 : Selenium on Beta−1,3−N−acetylglucosaminyltransferase manic fringe
rs11951068
rs558133
rs6586282
rs705415
rs10944
rs6859667
rs3797535
rs567754
rs921943
rs672413
rs234709
rs1789953
All − MR Egger
All − IVW(MRE)
−0.5
0.0
0.5
MR effect size for
'Selenium' on 'Beta−1,3−N−acetylglucosaminyltransferase manic fringe'

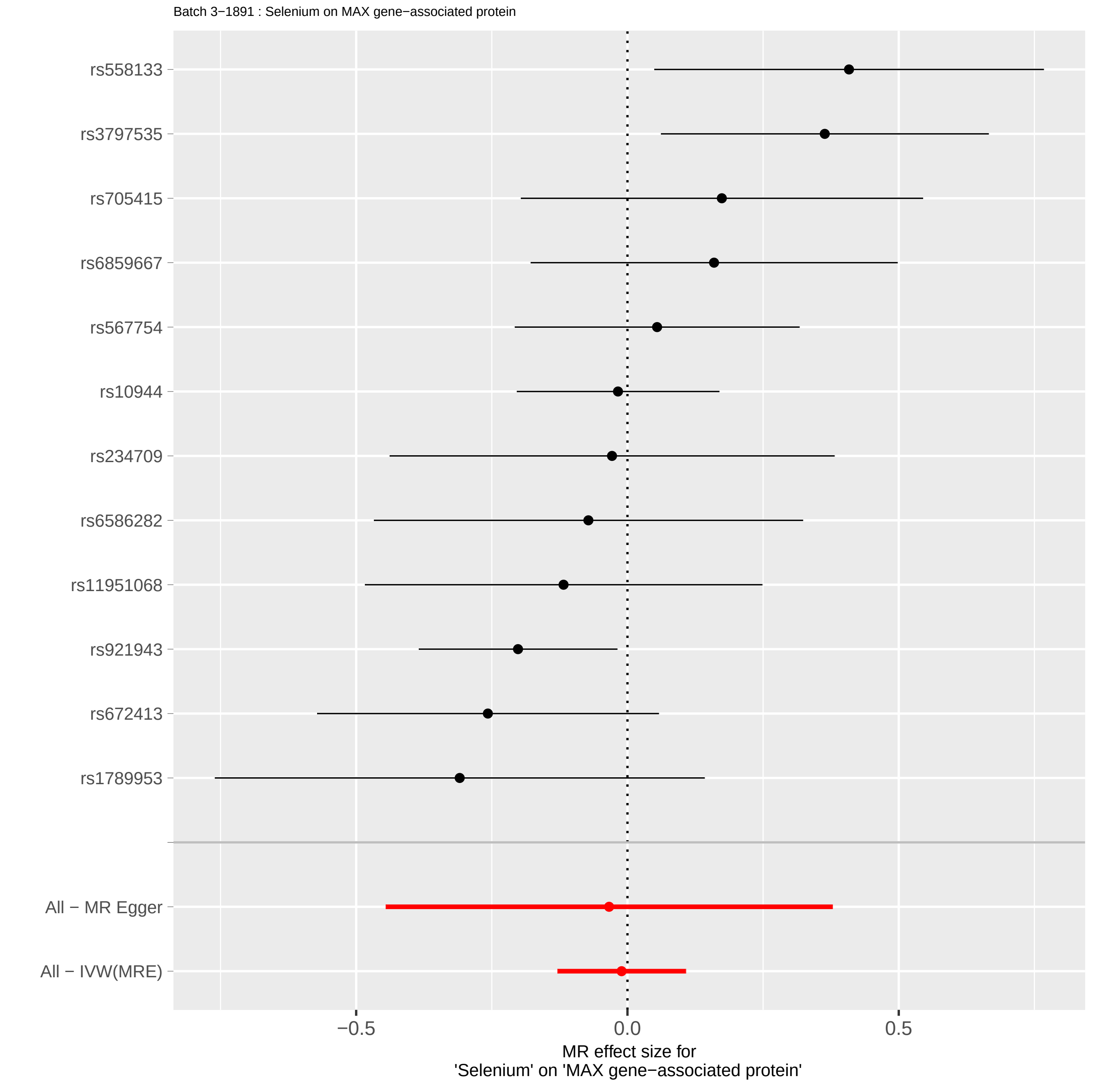

Batch 3−1891 : Selenium on MAX gene−associated protein
rs558133
rs3797535
rs705415
rs6859667
rs567754
rs10944
rs234709
rs6586282
rs11951068
rs921943
rs672413
rs1789953
All − MR Egger
All − IVW(MRE)
−0.5
0.0
0.5
MR effect size for
'Selenium' on 'MAX gene−associated protein'

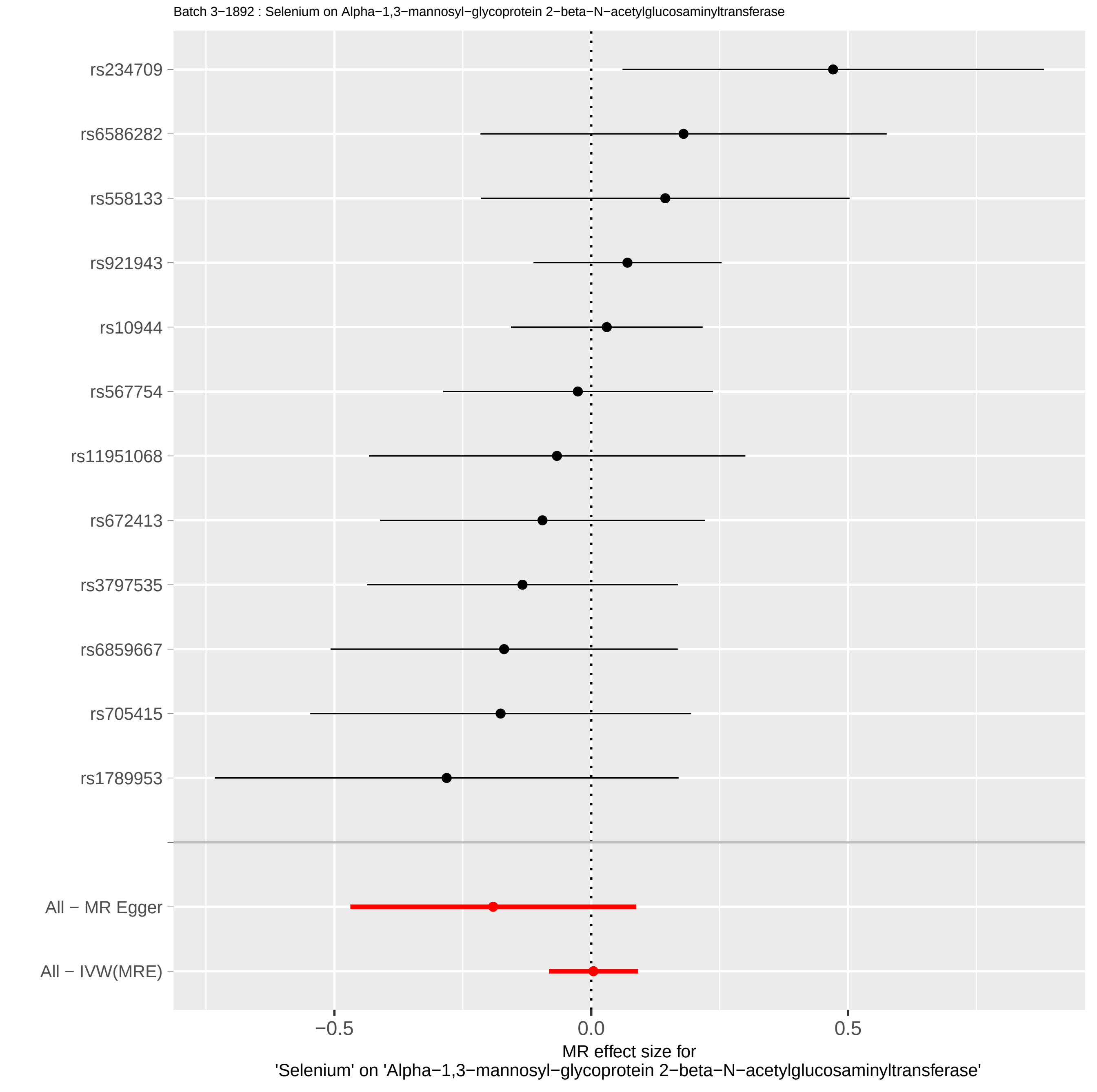

Batch 3−1892 : Selenium on Alpha−1,3−mannosyl−glycoprotein 2−beta−N−acetylglucosaminyltransferase
rs234709
rs6586282
rs558133
rs921943
rs10944
rs567754
rs11951068
rs672413
rs3797535
rs6859667
rs705415
rs1789953
All − MR Egger
All − IVW(MRE)
−0.5
0.0
0.5
MR effect size for
'Selenium' on 'Alpha−1,3−mannosyl−glycoprotein 2−beta−N−acetylglucosaminyltransferase'

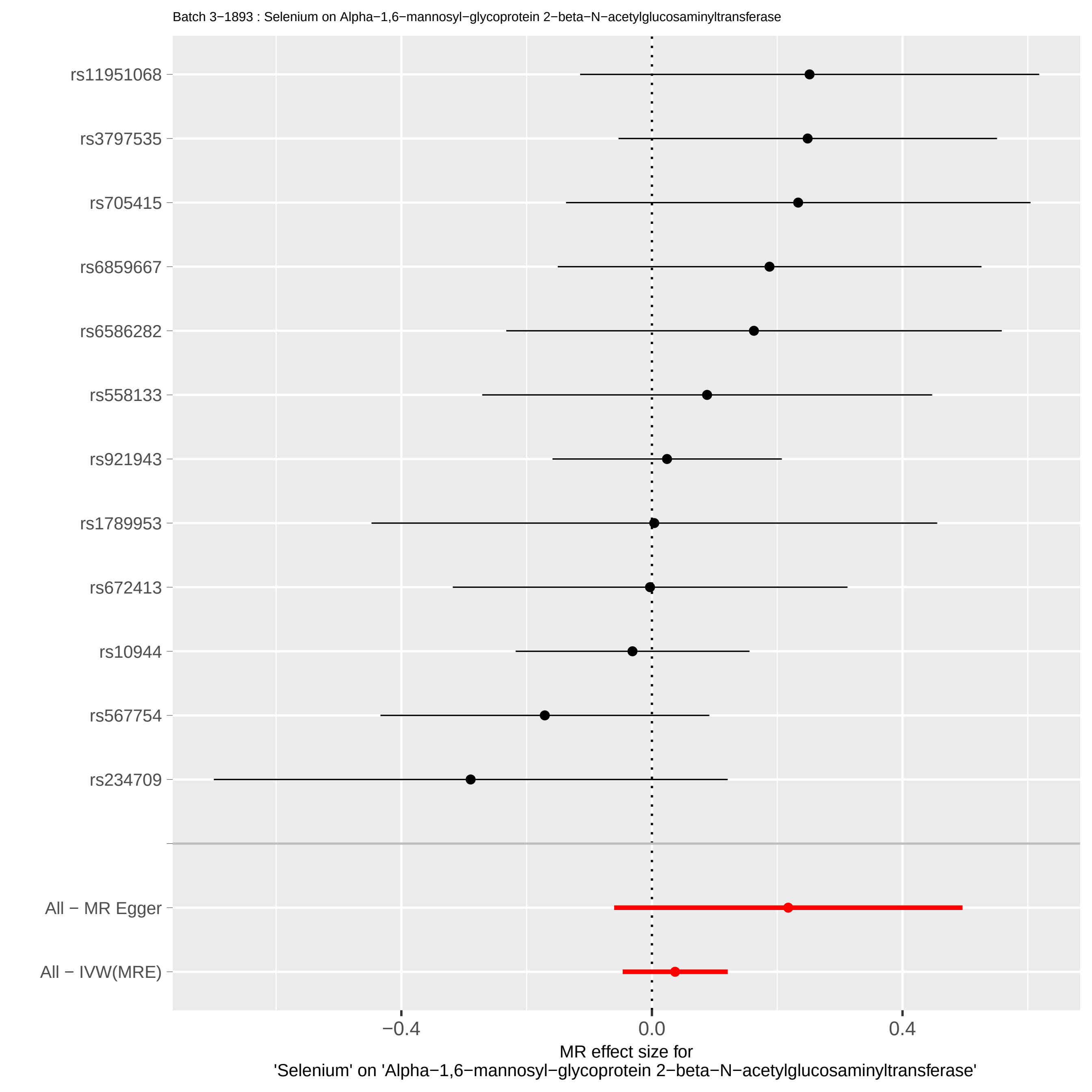
Batch 3−1893 : Selenium on Alpha−1,6−mannosyl−glycoprotein 2−beta−N−acetylglucosaminyltransferase
rs11951068
rs3797535
rs705415
rs6859667
rs6586282
rs558133
rs921943
rs1789953
rs672413
rs10944
rs567754
rs234709
All − MR Egger
All − IVW(MRE)
−0.4
0.0
0.4
MR effect size for
'Selenium' on 'Alpha−1,6−mannosyl−glycoprotein 2−beta−N−acetylglucosaminyltransferase'

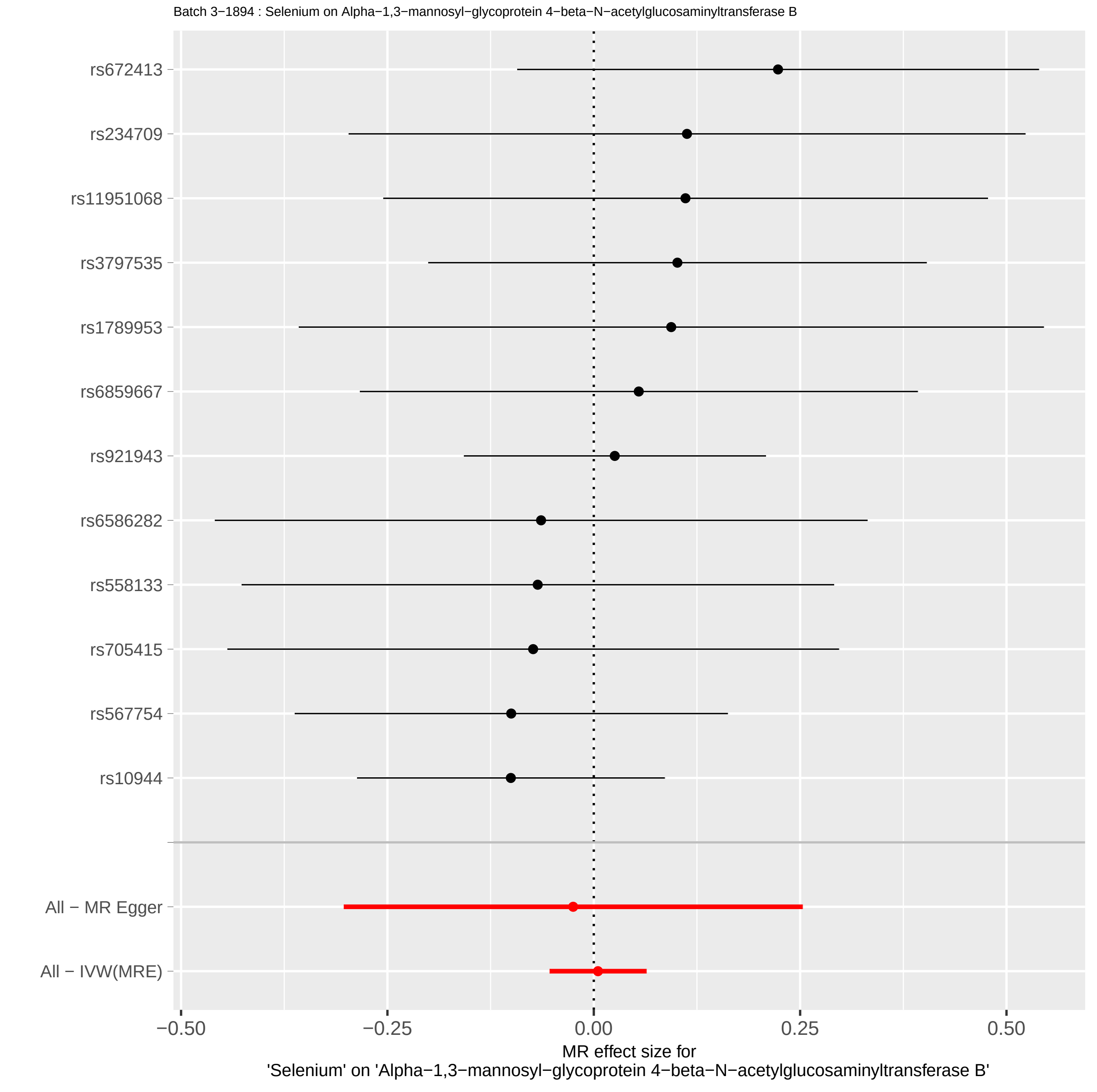
Batch 3−1894 : Selenium on Alpha−1,3−mannosyl−glycoprotein 4−beta−N−acetylglucosaminyltransferase B
rs672413
rs234709
rs11951068
rs3797535
rs1789953
rs6859667
rs921943
rs6586282
rs558133
rs705415
rs567754
rs10944
All − MR Egger
All − IVW(MRE)
−0.50
−0.25
0.00
0.25
0.50
MR effect size for
'Selenium' on 'Alpha−1,3−mannosyl−glycoprotein 4−beta−N−acetylglucosaminyltransferase B'

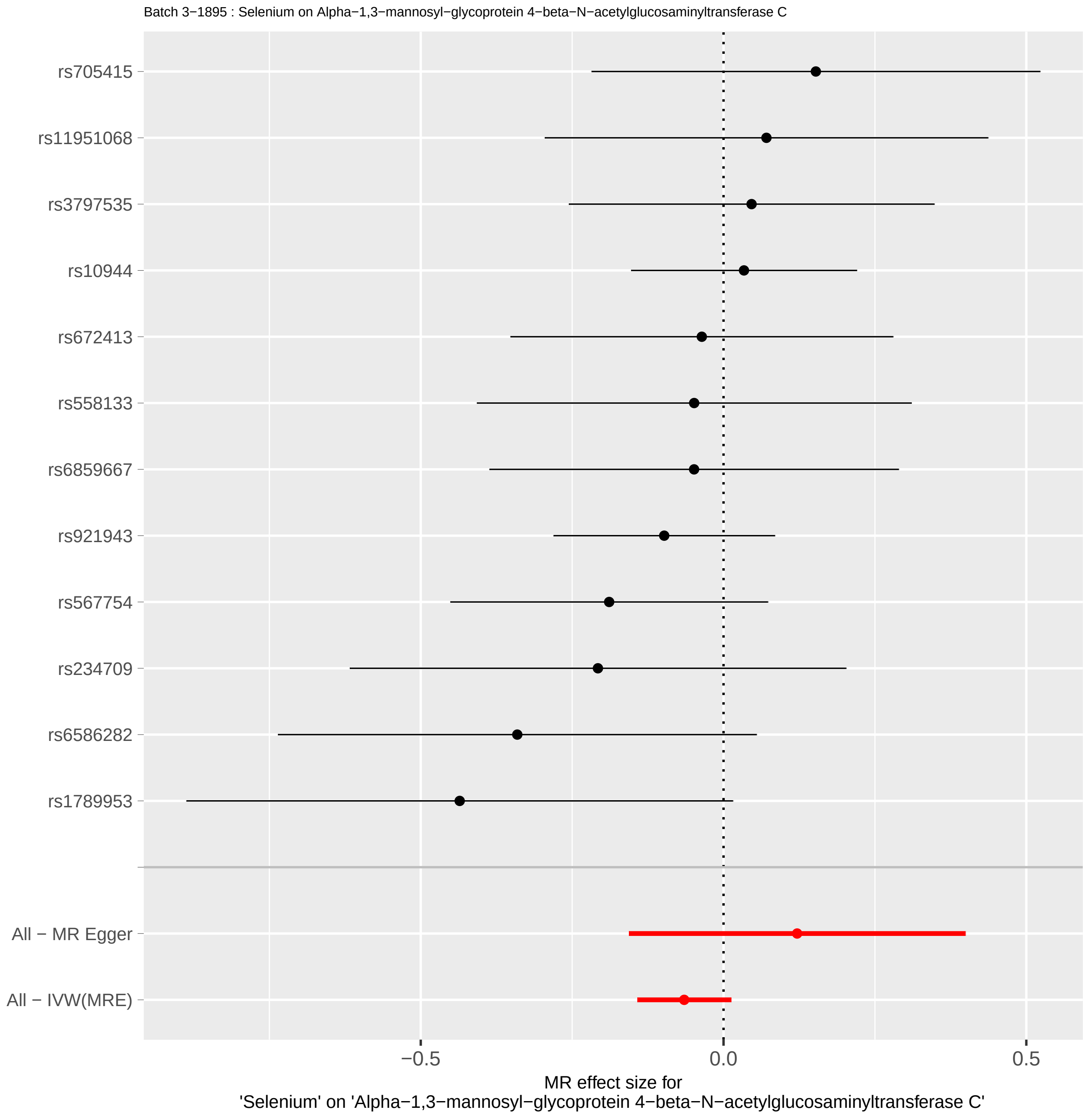

Batch 3−1895 : Selenium on Alpha−1,3−mannosyl−glycoprotein 4−beta−N−acetylglucosaminyltransferase C
rs705415
rs11951068
rs3797535
rs10944
rs672413
rs558133
rs6859667
rs921943
rs567754
rs234709
rs6586282
rs1789953
All − MR Egger
All − IVW(MRE)
−0.5
0.0
0.5
MR effect size for
'Selenium' on 'Alpha−1,3−mannosyl−glycoprotein 4−beta−N−acetylglucosaminyltransferase C'

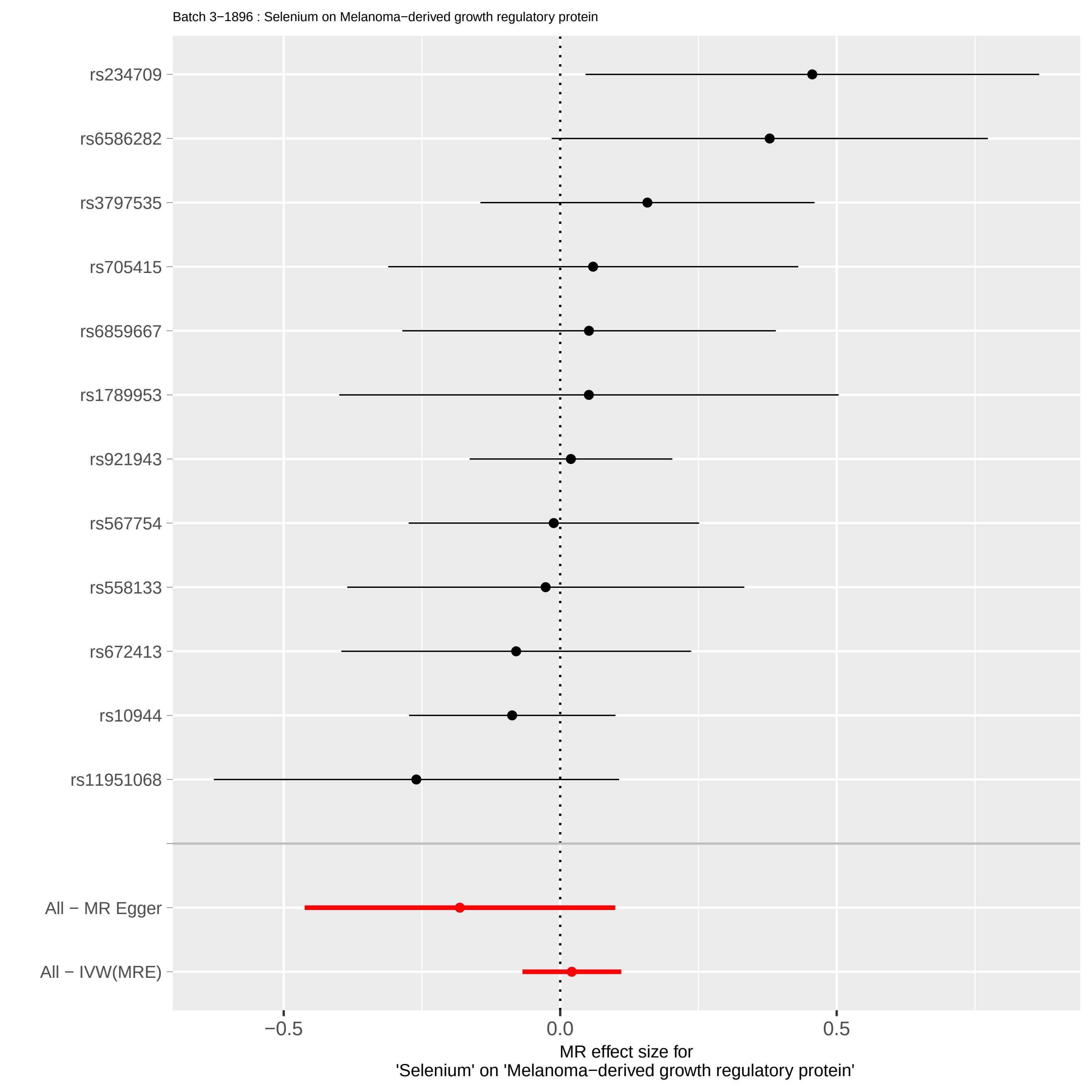

Batch 3−1896 : Selenium on Melanoma−derived growth regulatory protein
rs234709
rs6586282
rs3797535
rs705415
rs6859667
rs1789953
rs921943
rs567754
rs558133
rs672413
rs10944
rs11951068
All − MR Egger
All − IVW(MRE)
−0.5
0.0
0.5
MR effect size for
'Selenium' on 'Melanoma−derived growth regulatory protein'

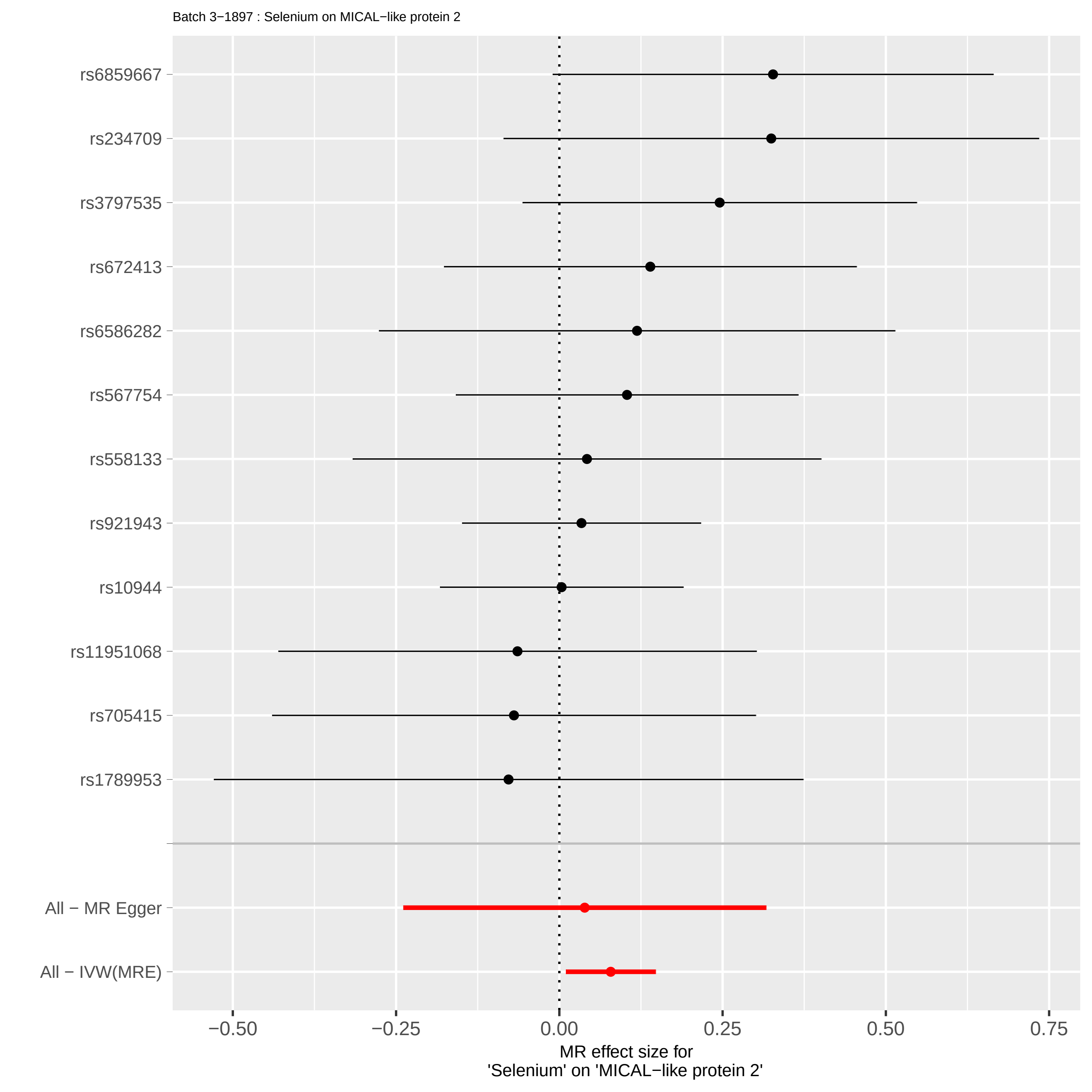
Batch 3−1897 : Selenium on MICAL−like protein 2
rs6859667
rs234709
rs3797535
rs672413
rs6586282
rs567754
rs558133
rs921943
rs10944
rs11951068
rs705415
rs1789953
All − MR Egger
All − IVW(MRE)
−0.50
−0.25
0.00
0.25
0.50
0.75
MR effect size for
'Selenium' on 'MICAL−like protein 2'

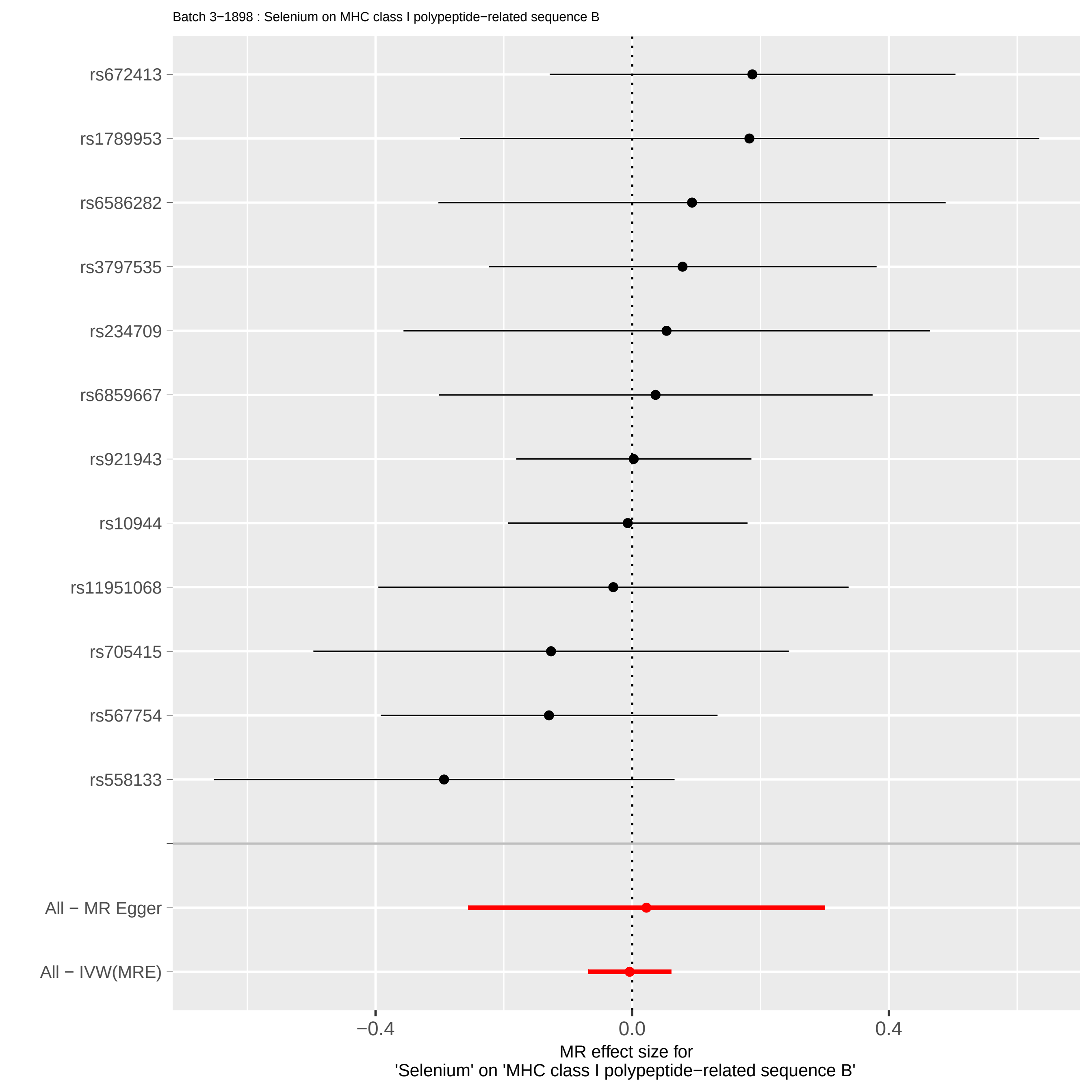
Batch 3−1898 : Selenium on MHC class I polypeptide−related sequence B
rs672413
rs1789953
rs6586282
rs3797535
rs234709
rs6859667
rs921943
rs10944
rs11951068
rs705415
rs567754
rs558133
All − MR Egger
All − IVW(MRE)
−0.4
0.0
0.4
MR effect size for
'Selenium' on 'MHC class I polypeptide−related sequence B'

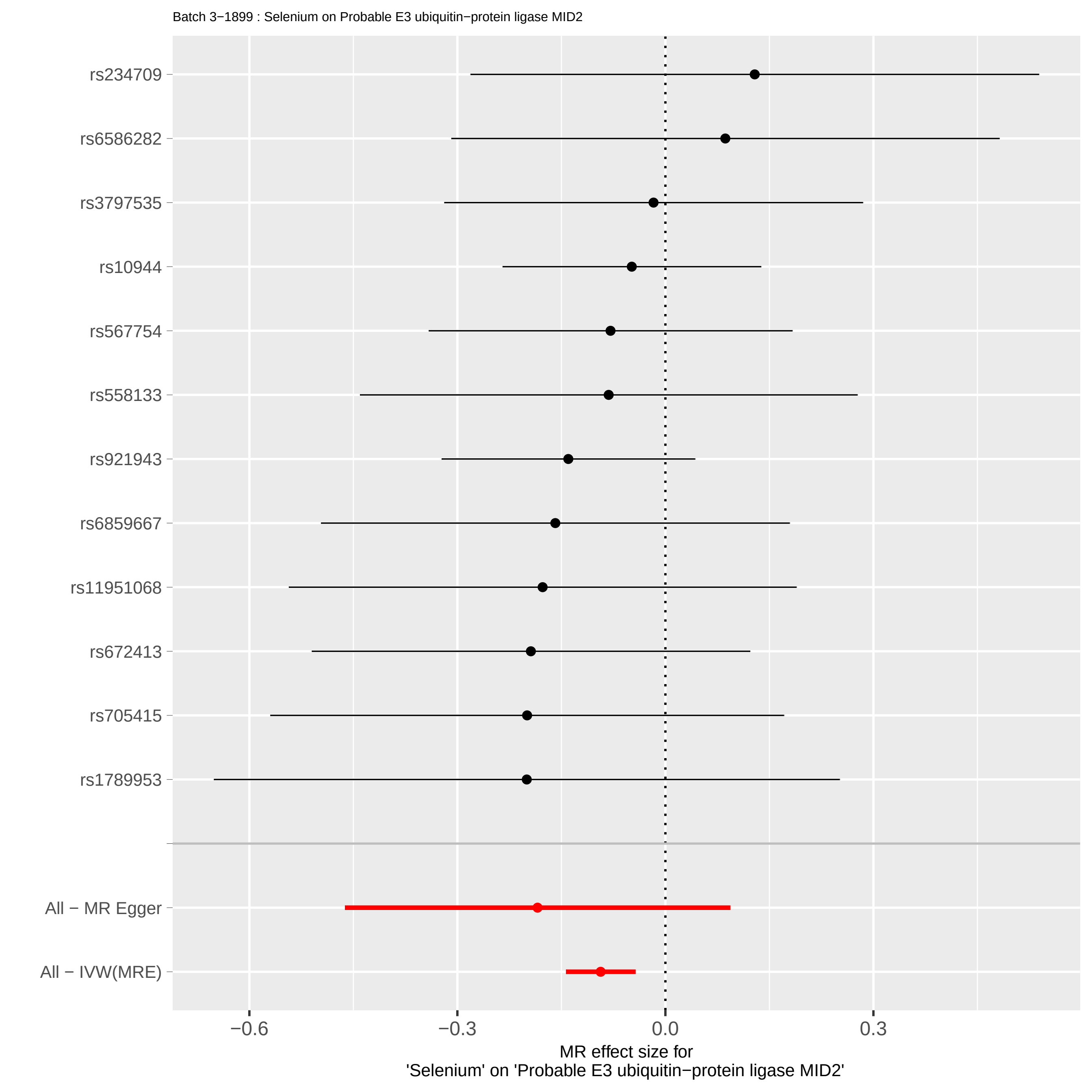

Batch 3−1899 : Selenium on Probable E3 ubiquitin−protein ligase MID2
rs234709
rs6586282
rs3797535
rs10944
rs567754
rs558133
rs921943
rs6859667
rs11951068
rs672413
rs705415
rs1789953
All − MR Egger
All − IVW(MRE)
−0.6
−0.3
0.0
0.3
MR effect size for
'Selenium' on 'Probable E3 ubiquitin−protein ligase MID2'

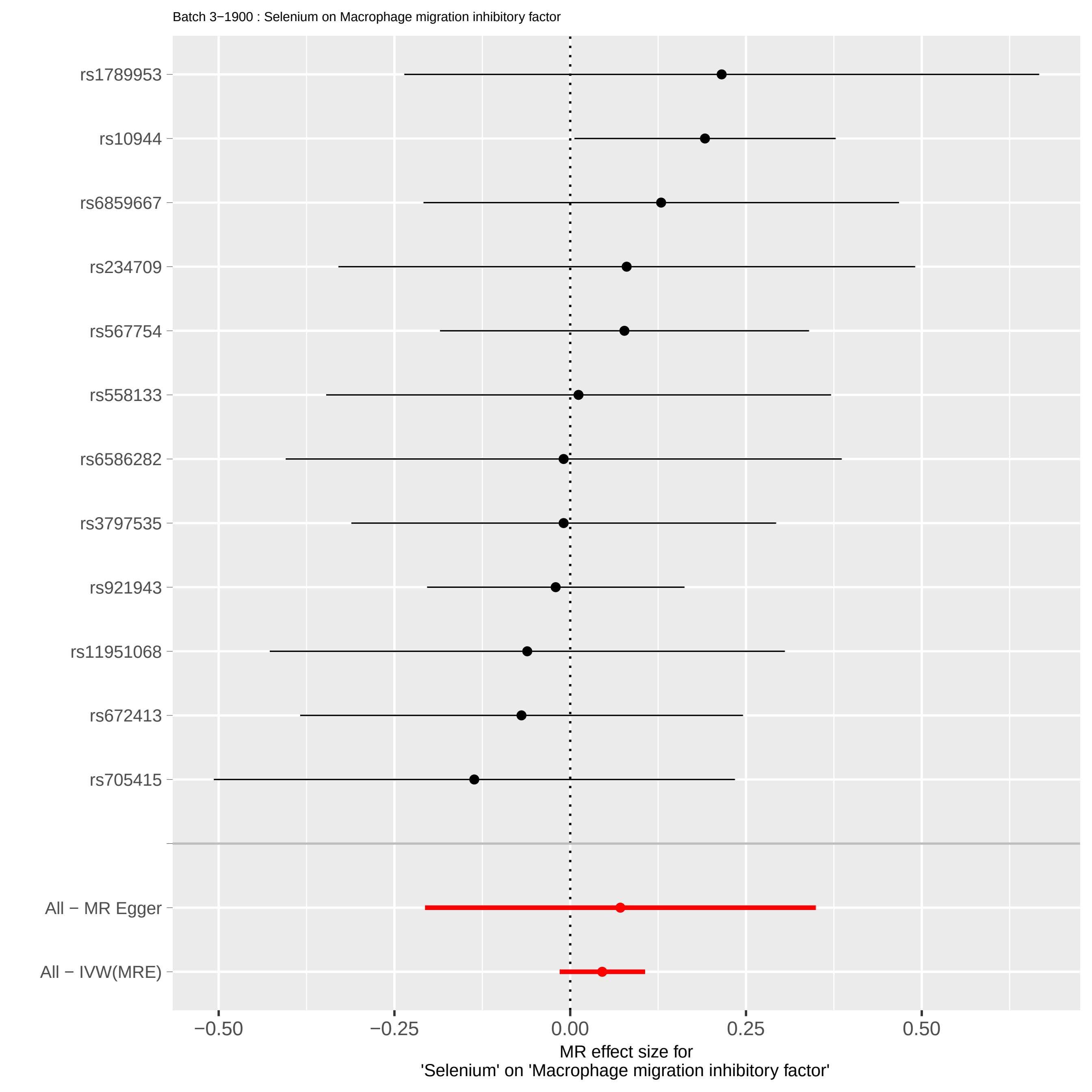

Batch 3−1900 : Selenium on Macrophage migration inhibitory factor
rs1789953
rs10944
rs6859667
rs234709
rs567754
rs558133
rs6586282
rs3797535
rs921943
rs11951068
rs672413
rs705415
All − MR Egger
All − IVW(MRE)
−0.50
−0.25
0.00
0.25
0.50
MR effect size for
'Selenium' on 'Macrophage migration inhibitory factor'

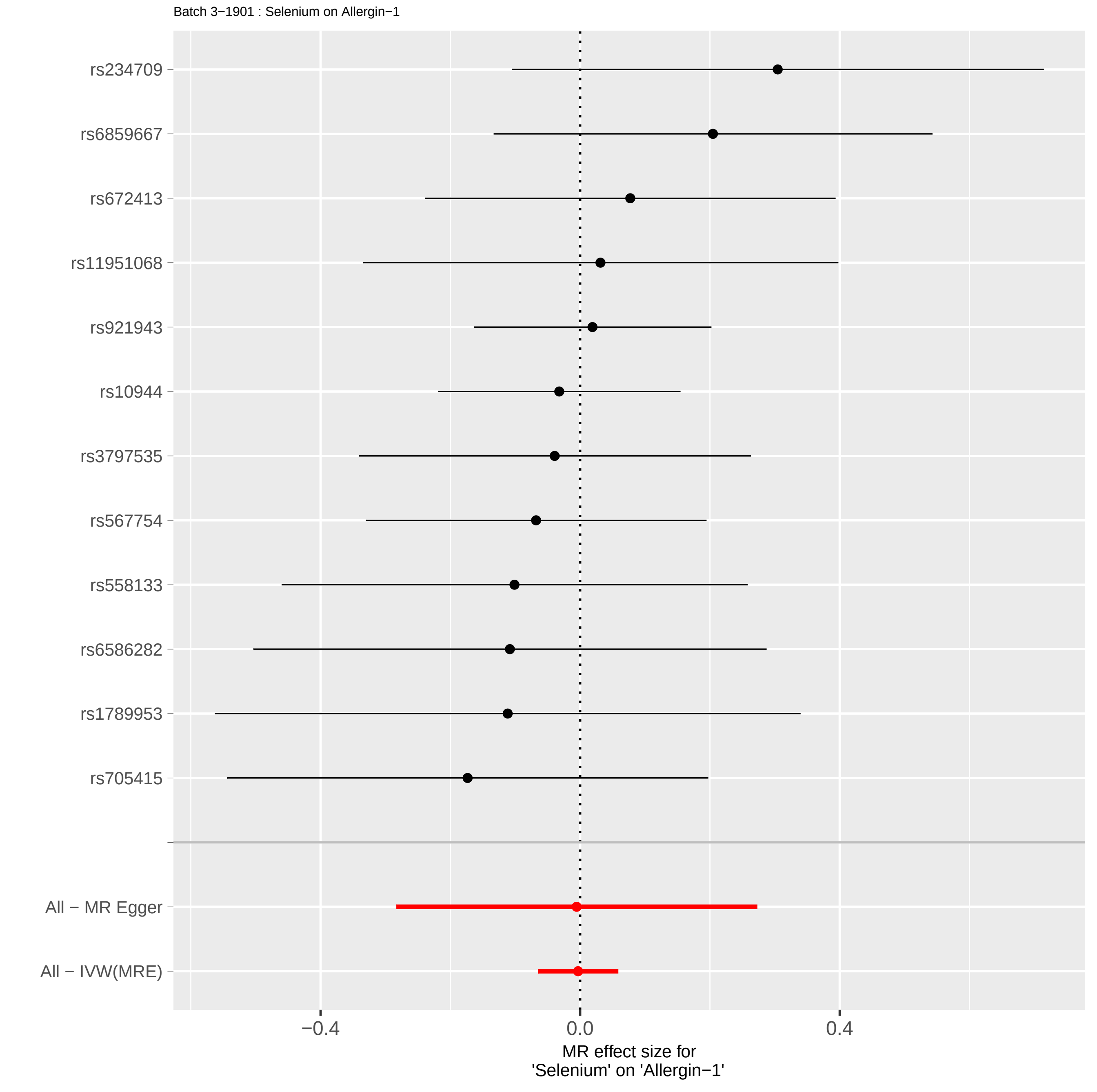

Batch 3−1901 : Selenium on Allergin−1
rs234709
rs6859667
rs672413
rs11951068
rs921943
rs10944
rs3797535
rs567754
rs558133
rs6586282
rs1789953
rs705415
All − MR Egger
All − IVW(MRE)
−0.4
0.0
0.4
MR effect size for
'Selenium' on 'Allergin−1'

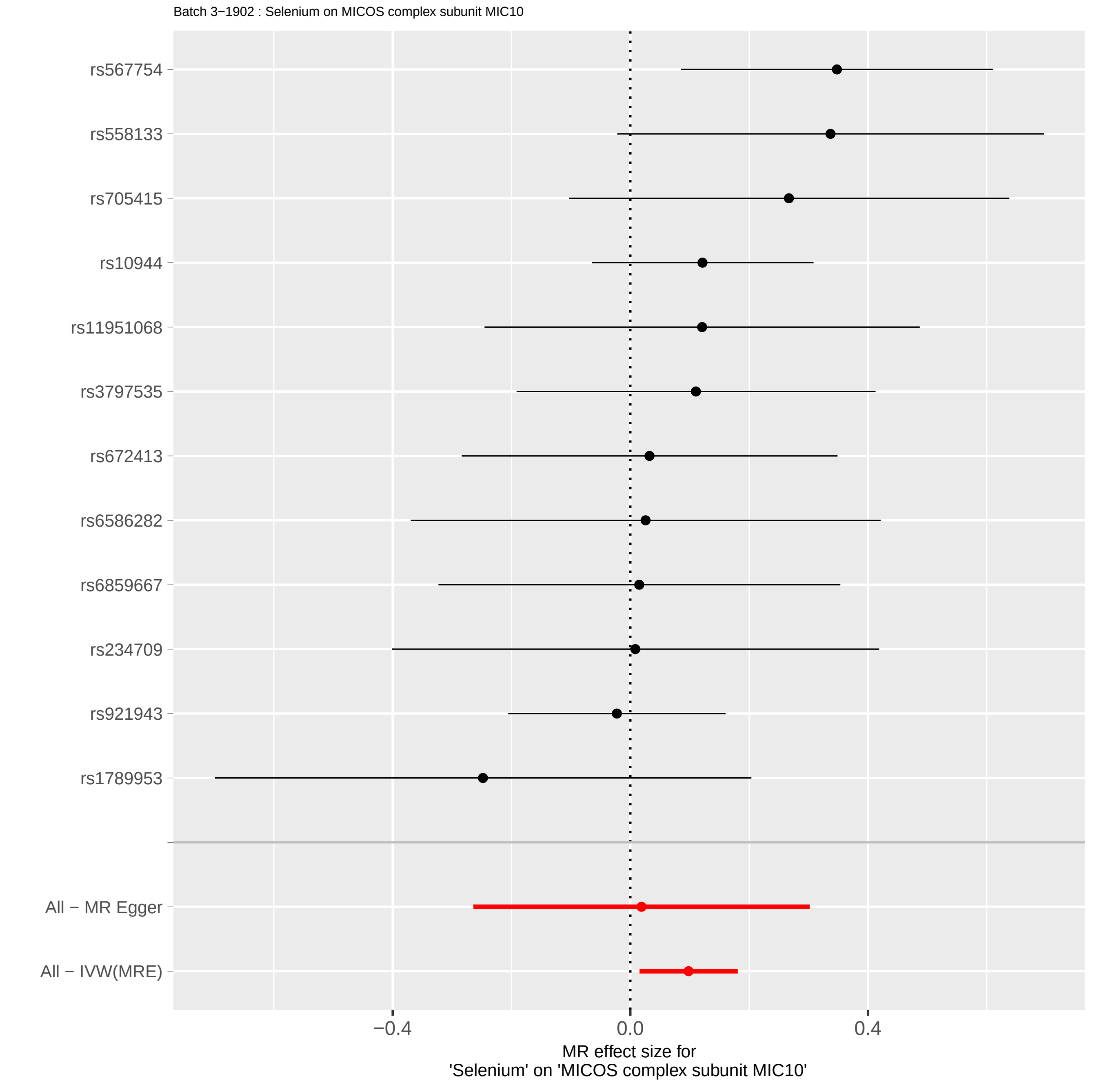

Batch 3−1902 : Selenium on MICOS complex subunit MIC10
rs567754
rs558133
rs705415
rs10944
rs11951068
rs3797535
rs672413
rs6586282
rs6859667
rs234709
rs921943
rs1789953
All − MR Egger
All − IVW(MRE)
−0.4
0.0
0.4
MR effect size for
'Selenium' on 'MICOS complex subunit MIC10'

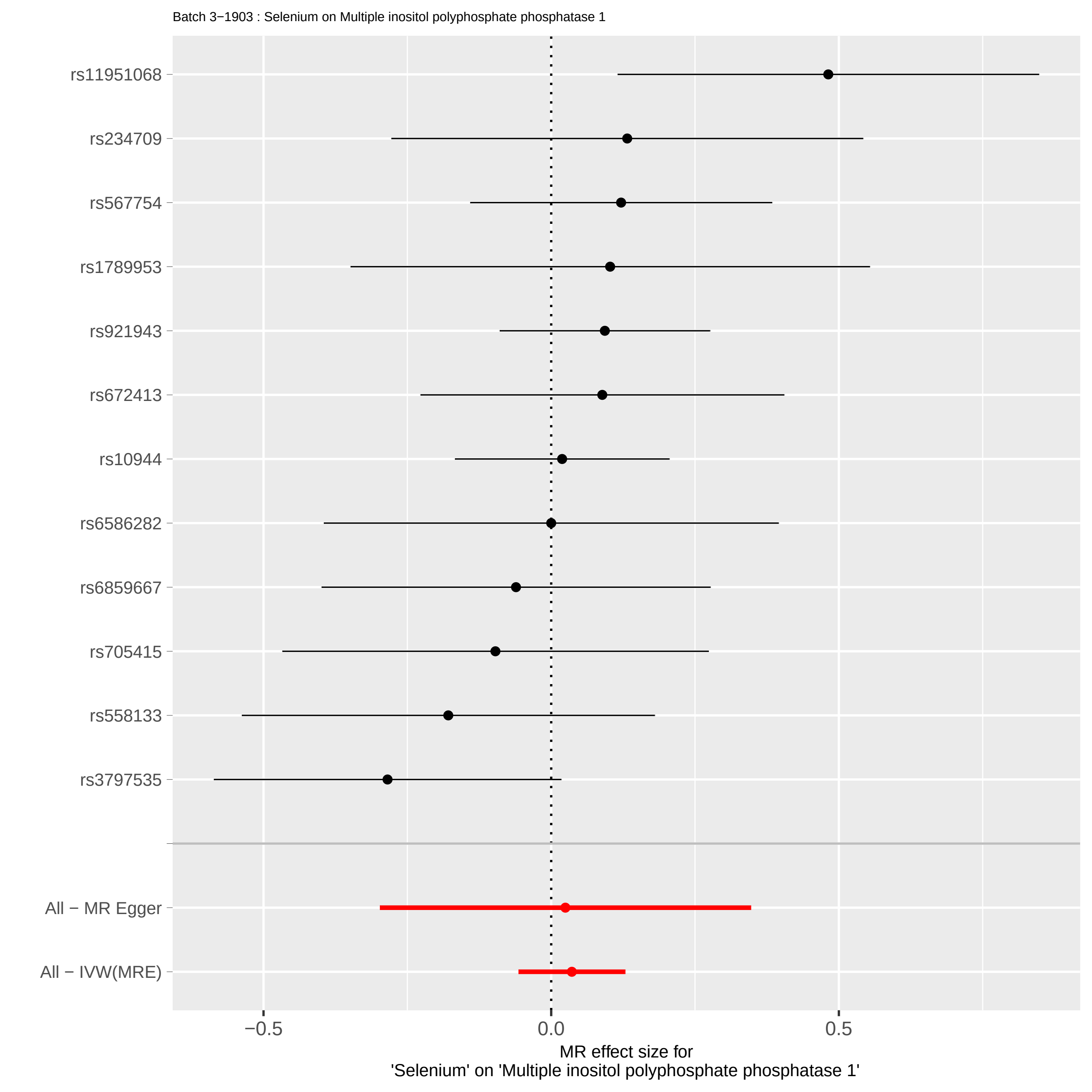
Batch 3−1903 : Selenium on Multiple inositol polyphosphate phosphatase 1
rs11951068
rs234709
rs567754
rs1789953
rs921943
rs672413
rs10944
rs6586282
rs6859667
rs705415
rs558133
rs3797535
All − MR Egger
All − IVW(MRE)
−0.5
0.0
0.5
MR effect size for
'Selenium' on 'Multiple inositol polyphosphate phosphatase 1'

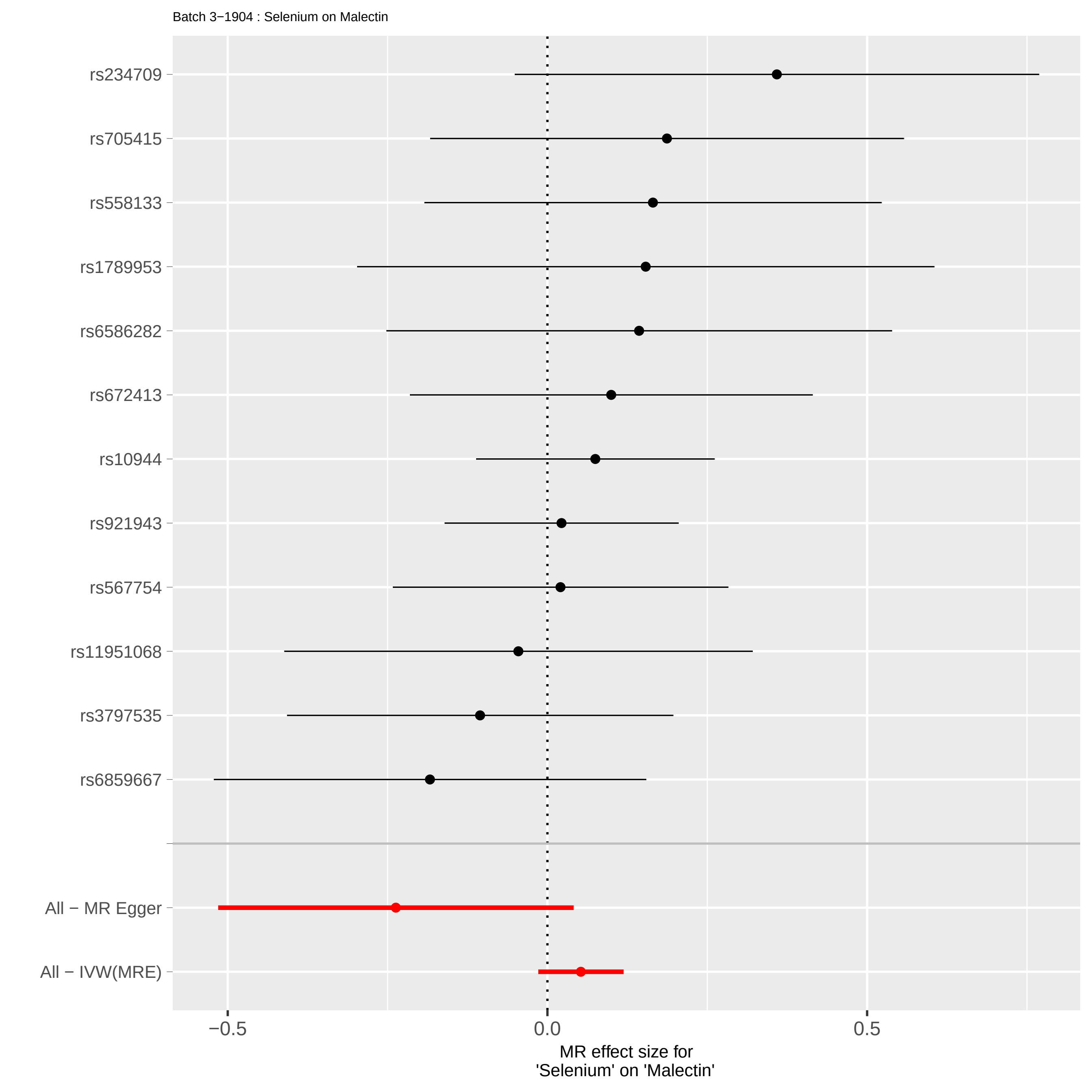

Batch 3−1904 : Selenium on Malectin
rs234709
rs705415
rs558133
rs1789953
rs6586282
rs672413
rs10944
rs921943
rs567754
rs11951068
rs3797535
rs6859667
All − MR Egger
All − IVW(MRE)
−0.5
0.0
0.5
MR effect size for
'Selenium' on 'Malectin'

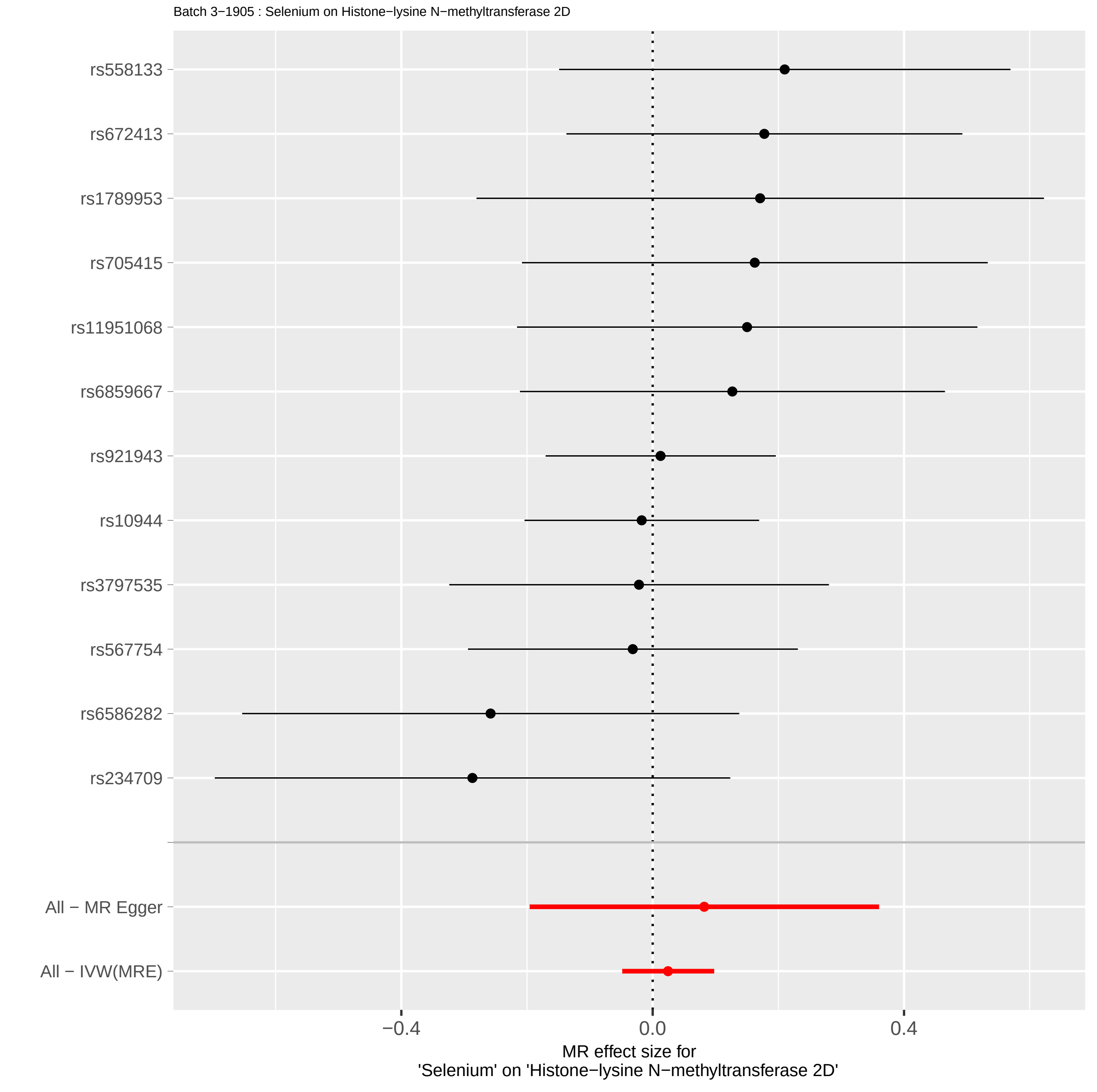
Batch 3−1905 : Selenium on Histone−lysine N−methyltransferase 2D
rs558133
rs672413
rs1789953
rs705415
rs11951068
rs6859667
rs921943
rs10944
rs3797535
rs567754
rs6586282
rs234709
All − MR Egger
All − IVW(MRE)
−0.4
0.0
0.4
MR effect size for
'Selenium' on 'Histone−lysine N−methyltransferase 2D'

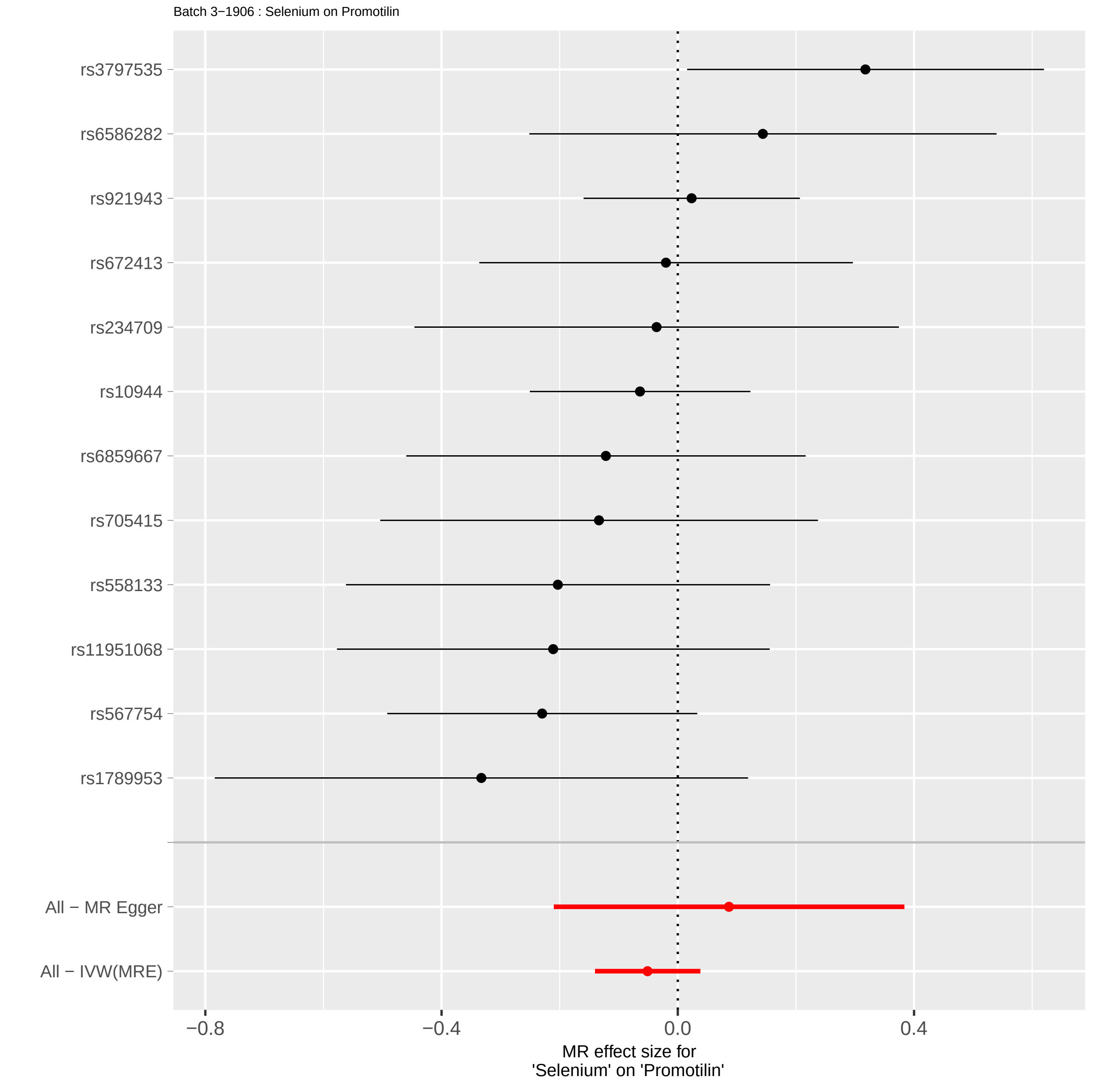

Batch 3−1906 : Selenium on Promotilin
rs3797535
rs6586282
rs921943
rs672413
rs234709
rs10944
rs6859667
rs705415
rs558133
rs11951068
rs567754
rs1789953
All − MR Egger
All − IVW(MRE)
−0.8
−0.4
0.0
0.4
MR effect size for
'Selenium' on 'Promotilin'

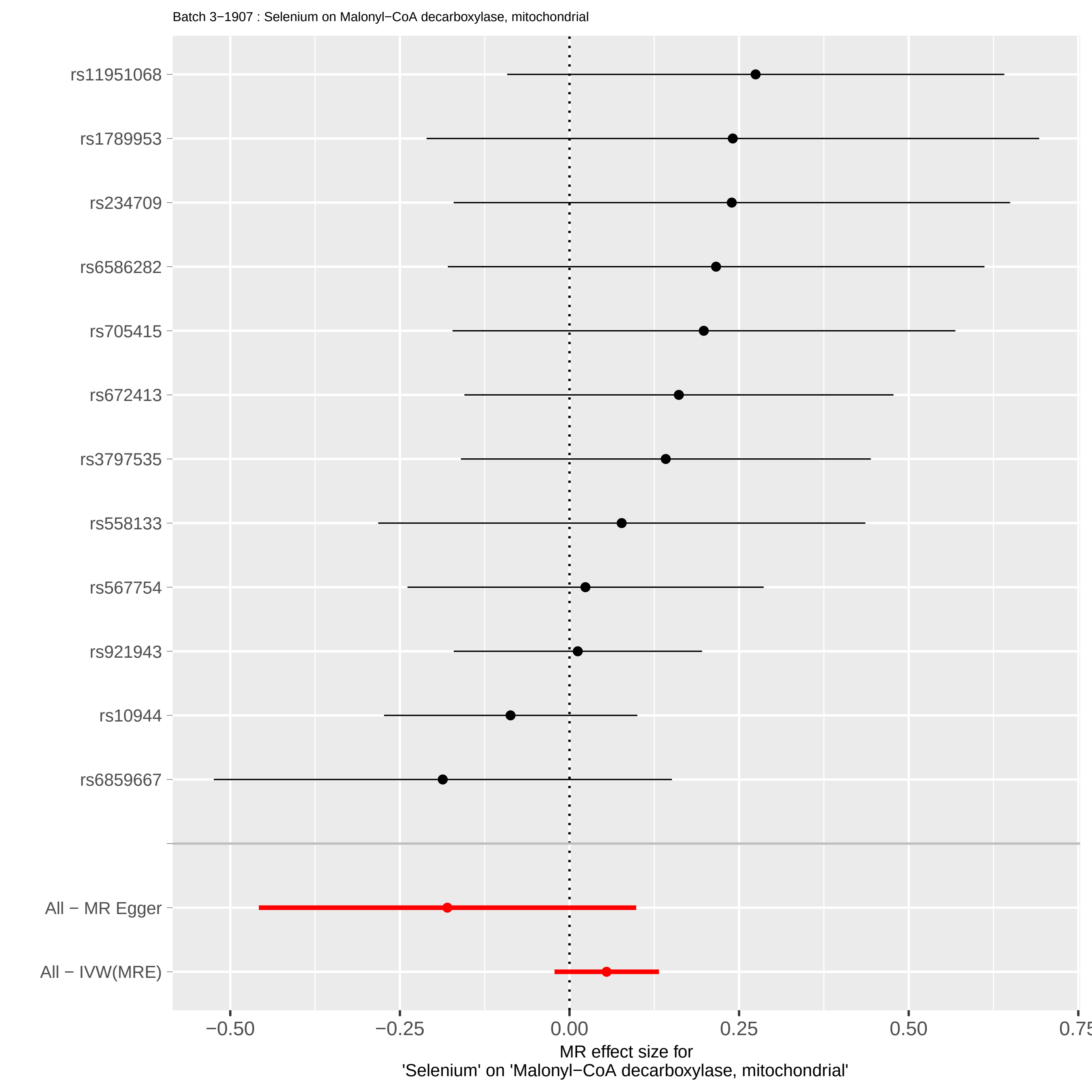

Batch 3−1907 : Selenium on Malonyl−CoA decarboxylase, mitochondrial
rs11951068
rs1789953
rs234709
rs6586282
rs705415
rs672413
rs3797535
rs558133
rs567754
rs921943
rs10944
rs6859667
All − MR Egger
All − IVW(MRE)
−0.50
−0.25
0.00
0.25
0.50
0.75
MR effect size for
'Selenium' on 'Malonyl−CoA decarboxylase, mitochondrial'

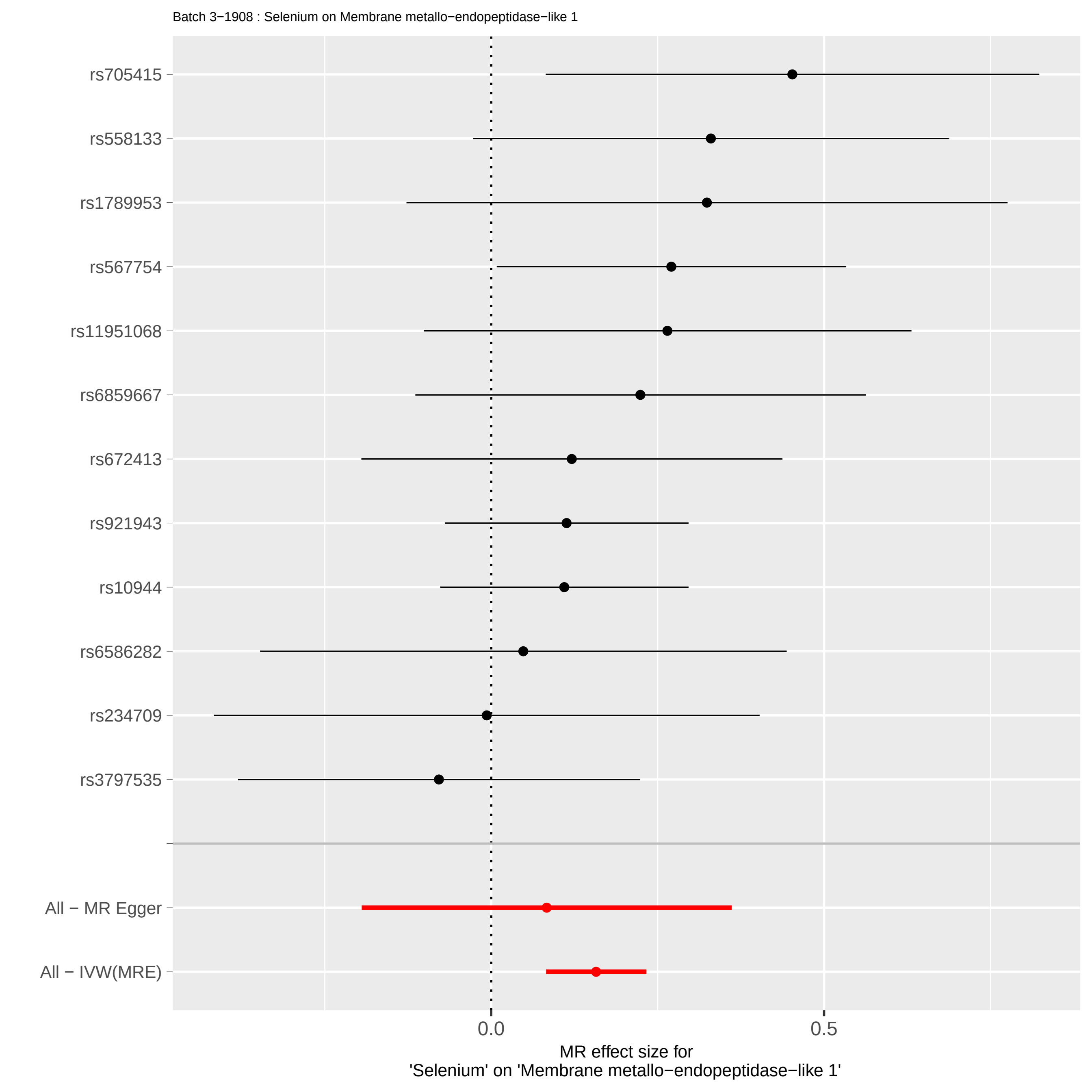
Batch 3−1908 : Selenium on Membrane metallo−endopeptidase−like 1
rs705415
rs558133
rs1789953
rs567754
rs11951068
rs6859667
rs672413
rs921943
rs10944
rs6586282
rs234709
rs3797535
All − MR Egger
All − IVW(MRE)
0.0
0.5
MR effect size for
'Selenium' on 'Membrane metallo−endopeptidase−like 1'

Batch 3−1909 : Selenium on Membrane magnesium transporter 1

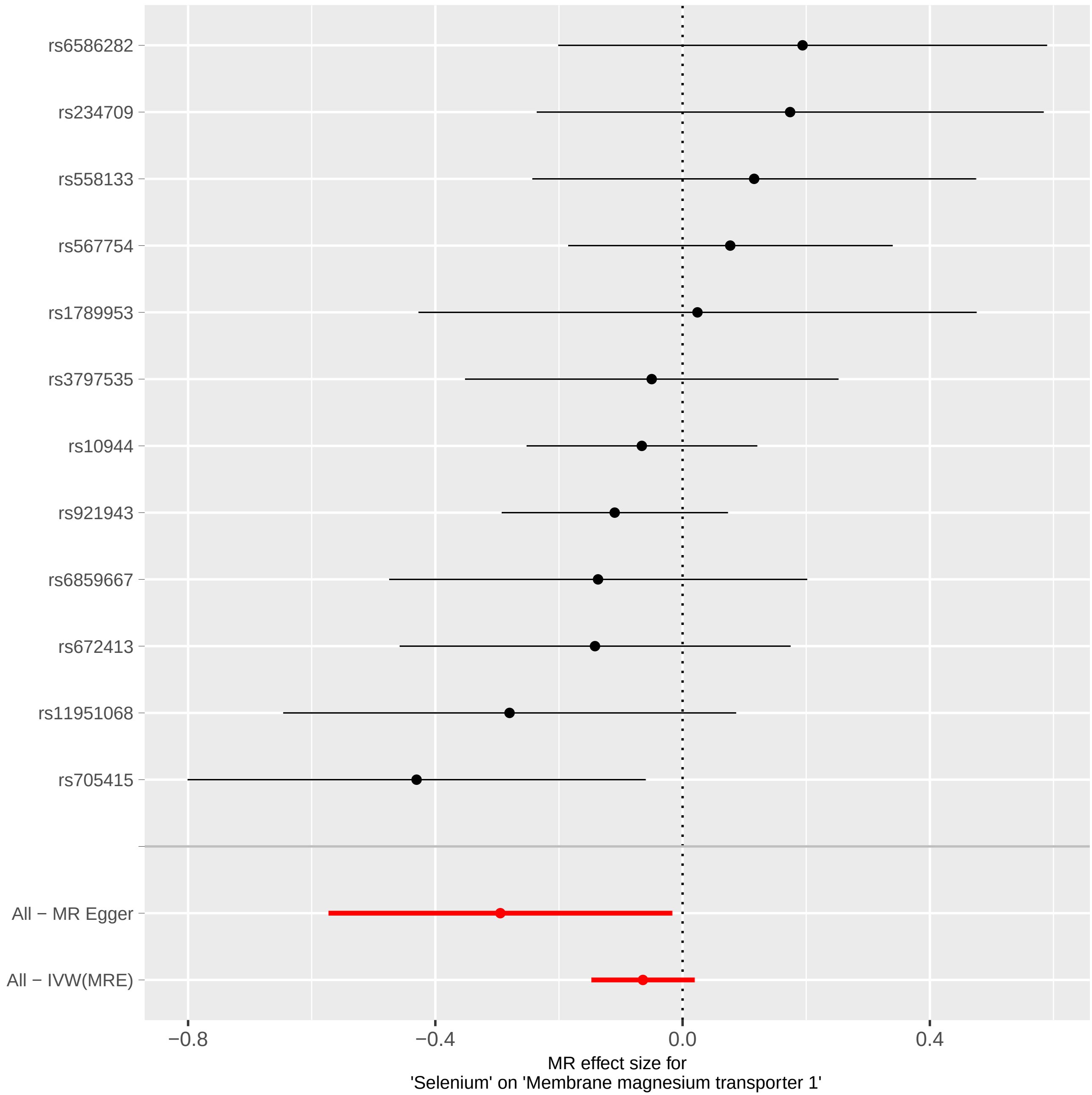

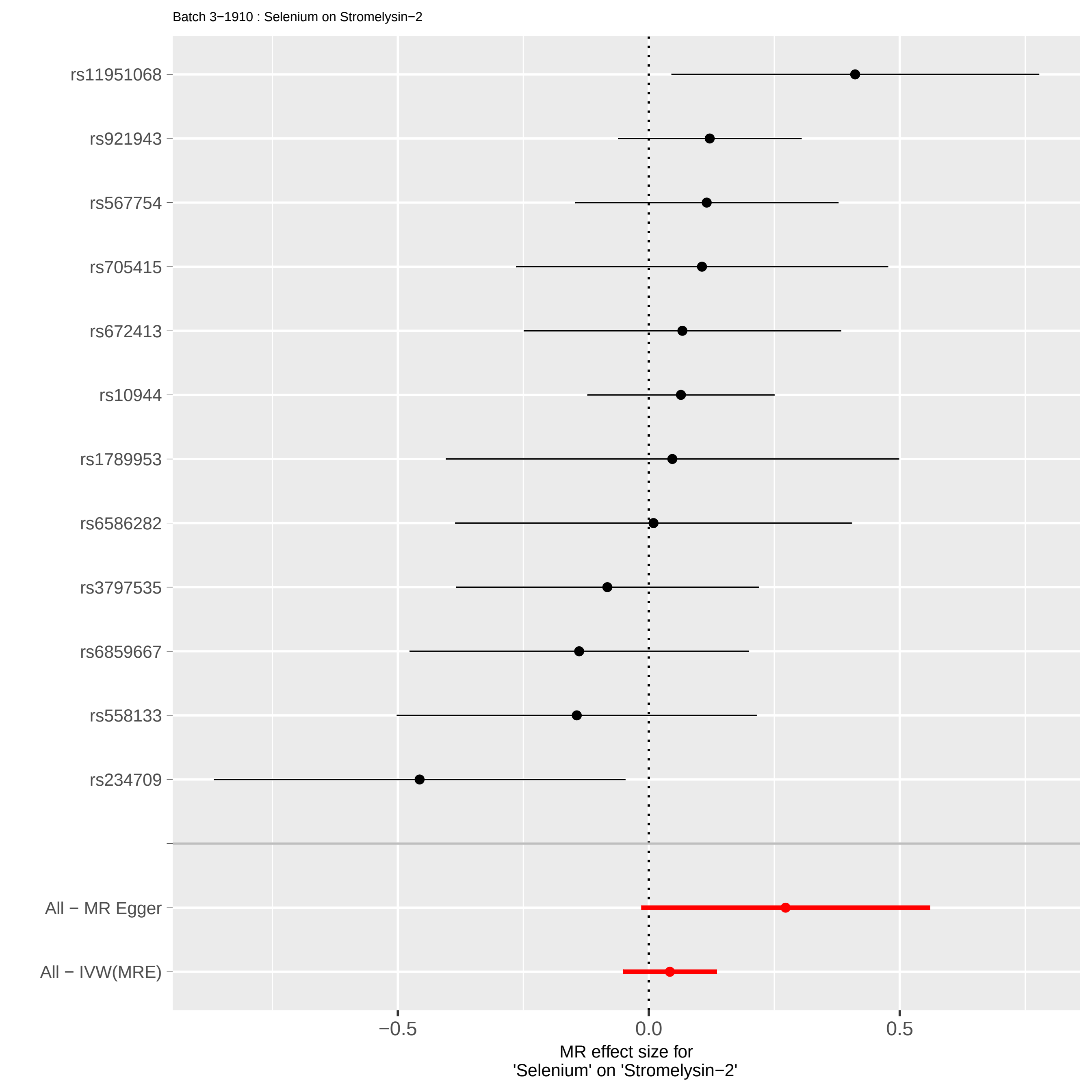
Batch 3−1910 : Selenium on Stromelysin−2
rs11951068
rs921943
rs567754
rs705415
rs672413
rs10944
rs1789953
rs6586282
rs3797535
rs6859667
rs558133
rs234709
All − MR Egger
All − IVW(MRE)
−0.5
0.0
0.5
MR effect size for
'Selenium' on 'Stromelysin−2'

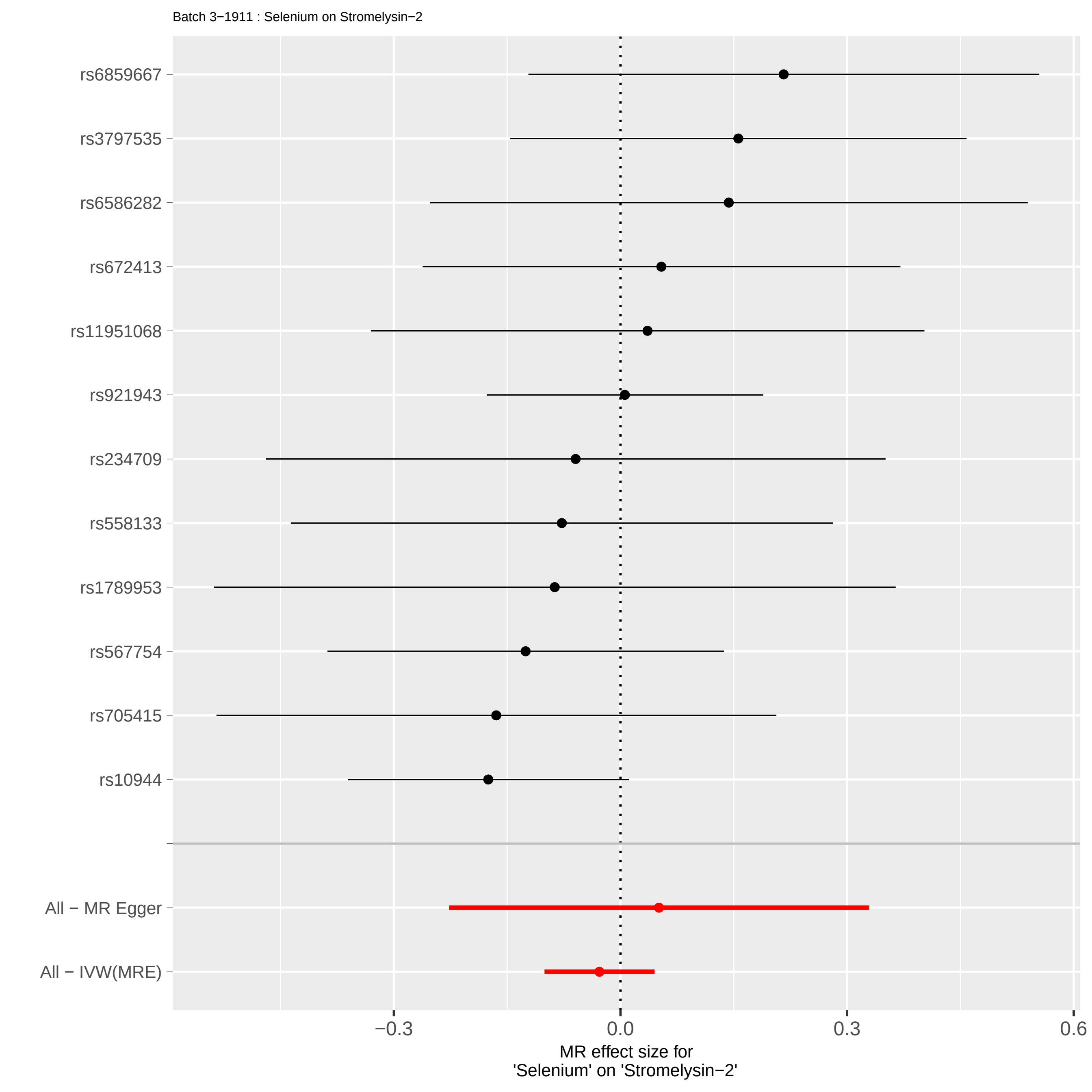

Batch 3−1911 : Selenium on Stromelysin−2
rs6859667
rs3797535
rs6586282
rs672413
rs11951068
rs921943
rs234709
rs558133
rs1789953
rs567754
rs705415
rs10944
All − MR Egger
All − IVW(MRE)
−0.3
0.0
0.3
0.6
MR effect size for
'Selenium' on 'Stromelysin−2'

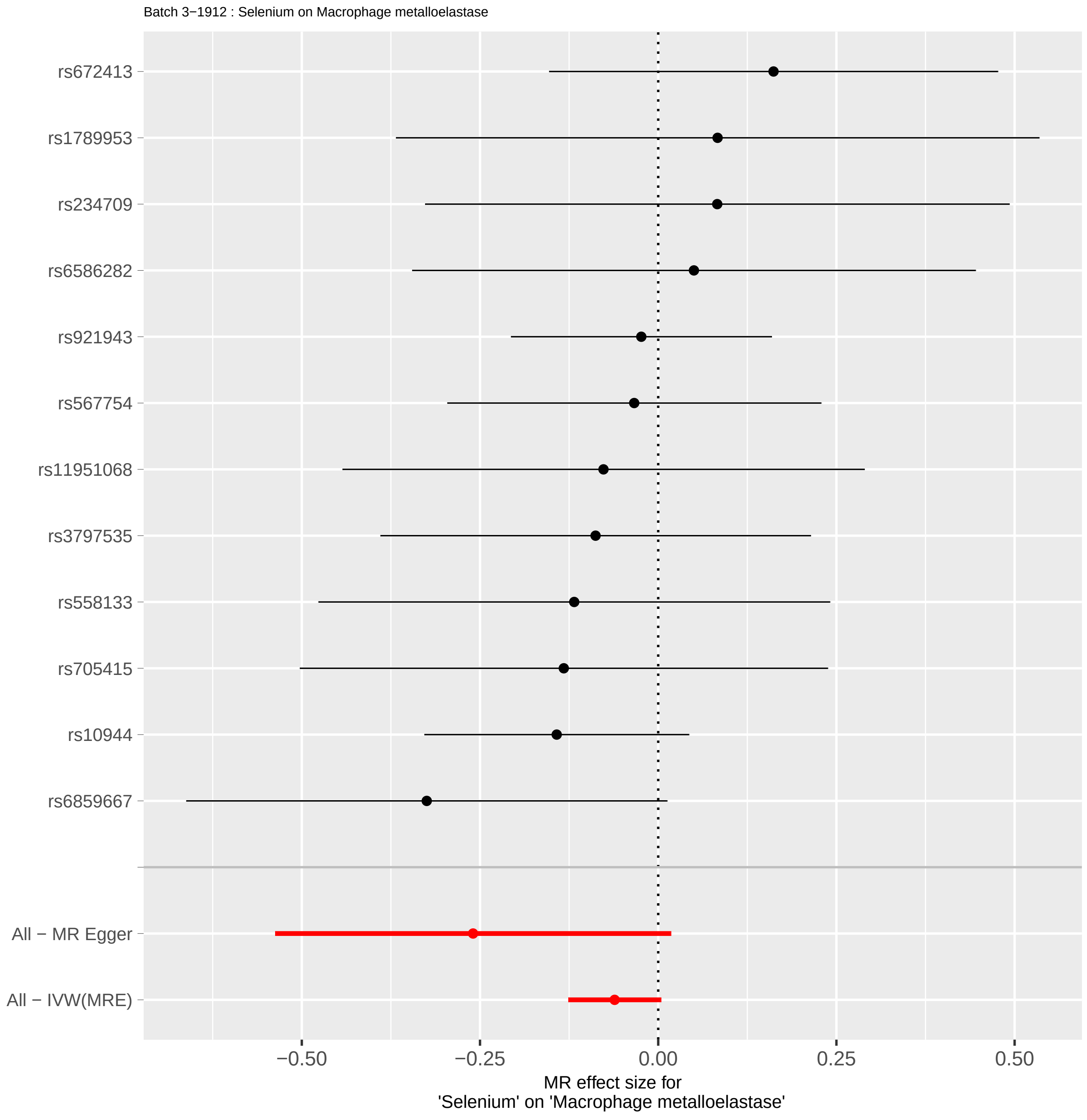
Batch 3−1912 : Selenium on Macrophage metalloelastase
rs672413
rs1789953
rs234709
rs6586282
rs921943
rs567754
rs11951068
rs3797535
rs558133
rs705415
rs10944
rs6859667
All − MR Egger
All − IVW(MRE)
−0.50
−0.25
0.00
0.25
0.50
MR effect size for
'Selenium' on 'Macrophage metalloelastase'

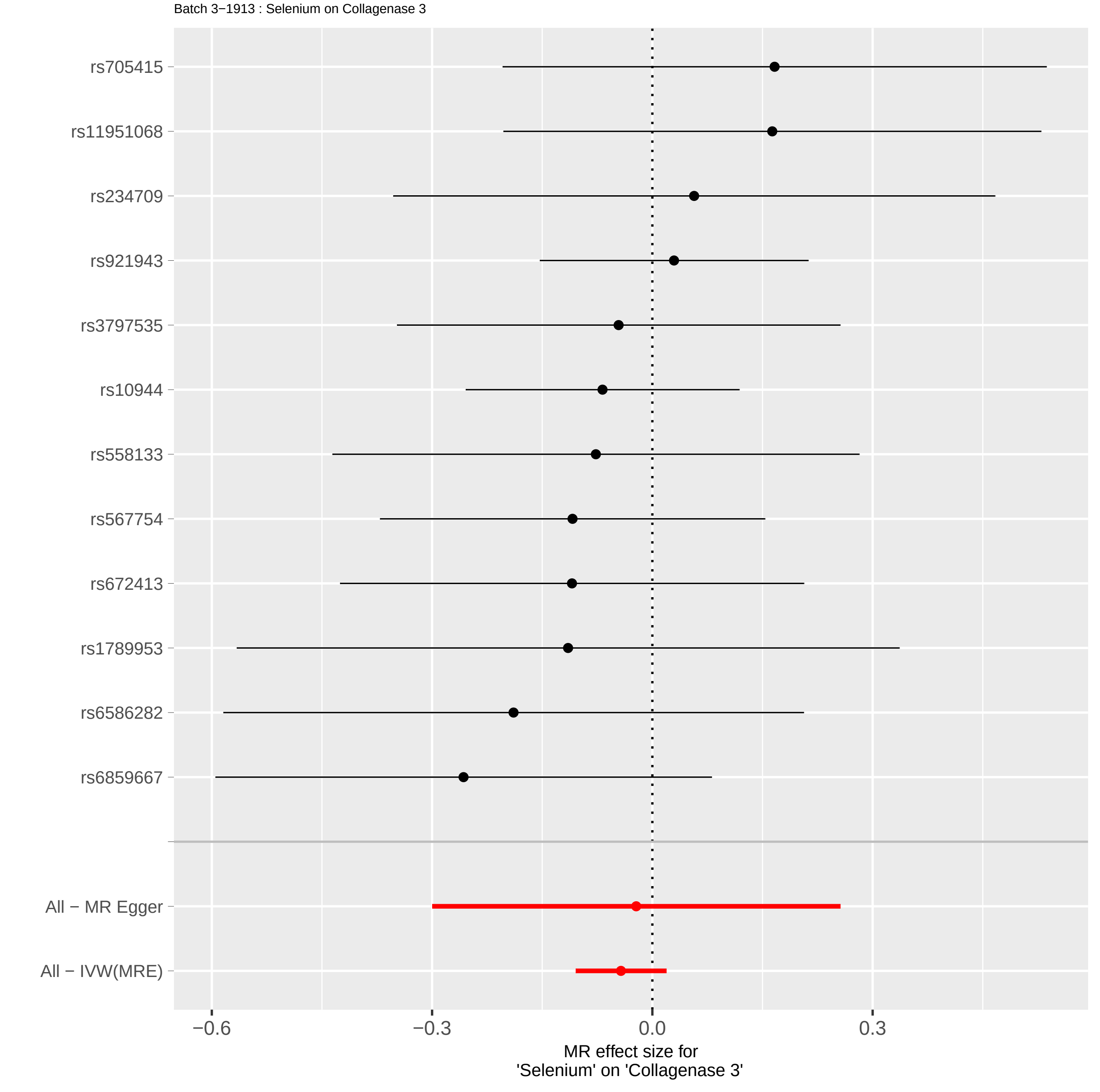

Batch 3−1913 : Selenium on Collagenase 3
rs705415
rs11951068
rs234709
rs921943
rs3797535
rs10944
rs558133
rs567754
rs672413
rs1789953
rs6586282
rs6859667
All − MR Egger
All − IVW(MRE)
−0.6
−0.3
0.0
0.3
MR effect size for
'Selenium' on 'Collagenase 3'

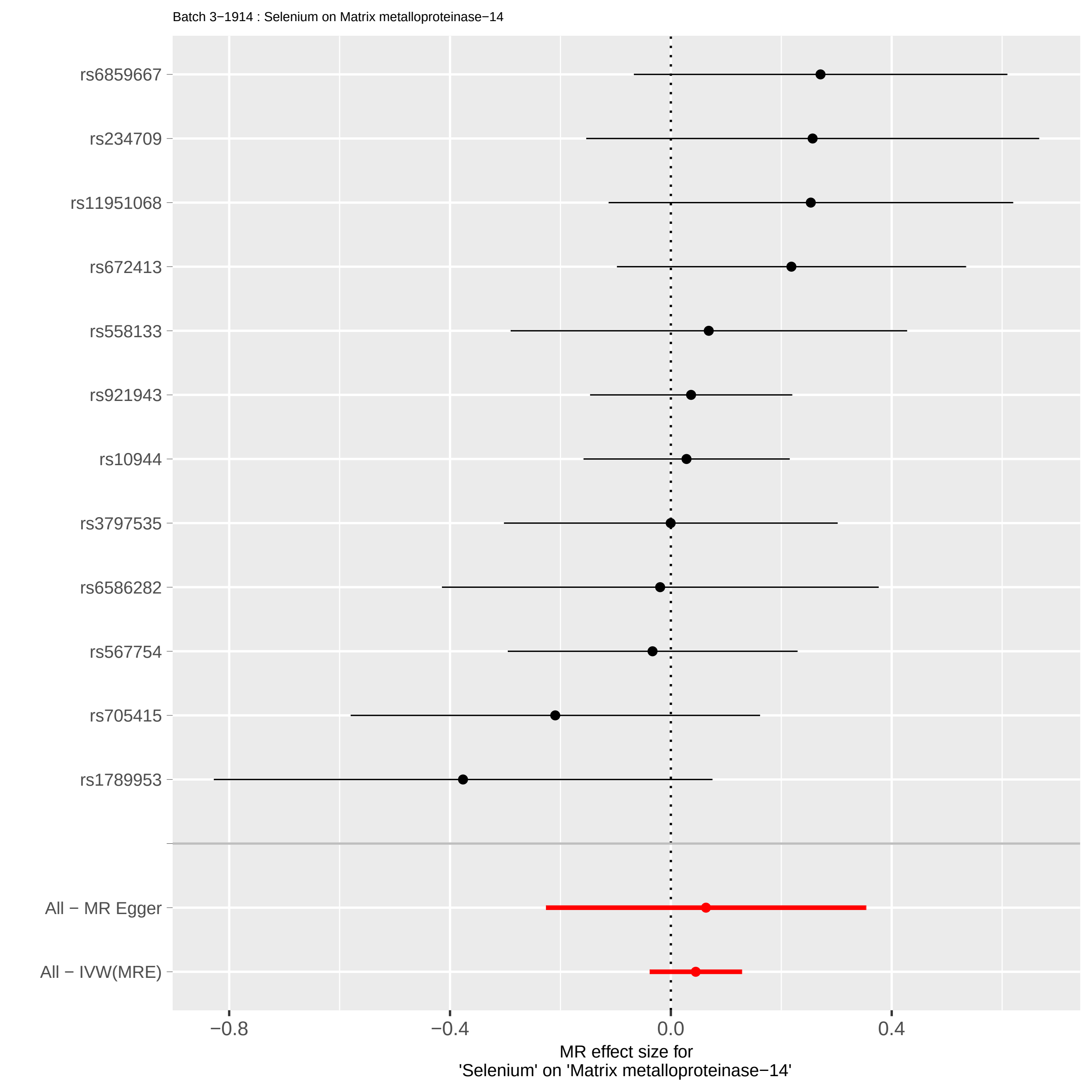
Batch 3−1914 : Selenium on Matrix metalloproteinase−14
rs6859667
rs234709
rs11951068
rs672413
rs558133
rs921943
rs10944
rs3797535
rs6586282
rs567754
rs705415
rs1789953
All − MR Egger
All − IVW(MRE)
−0.8
−0.4
0.0
0.4
MR effect size for
'Selenium' on 'Matrix metalloproteinase−14'

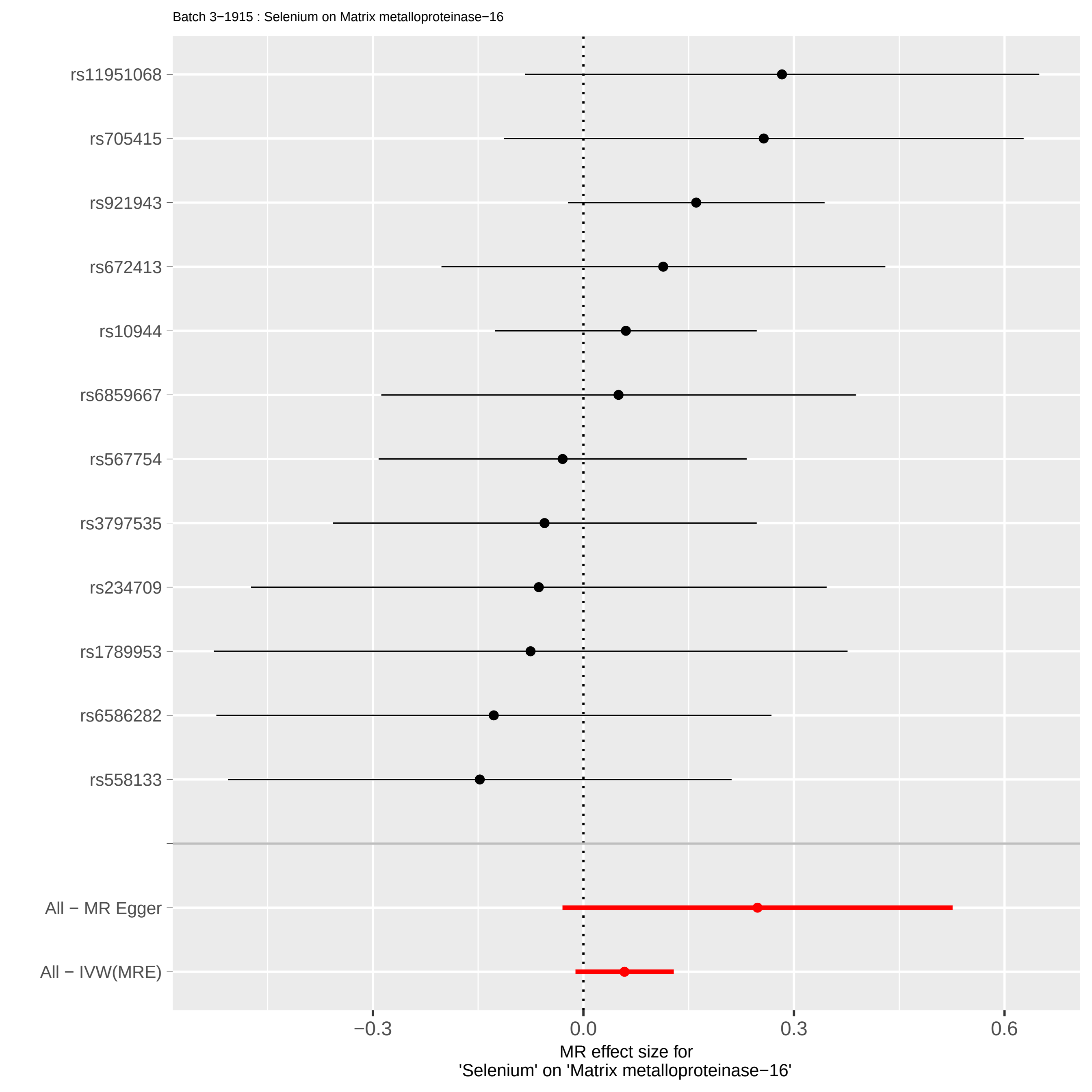
Batch 3−1915 : Selenium on Matrix metalloproteinase−16
rs11951068
rs705415
rs921943
rs672413
rs10944
rs6859667
rs567754
rs3797535
rs234709
rs1789953
rs6586282
rs558133
All − MR Egger
All − IVW(MRE)
−0.3
0.0
0.3
0.6
MR effect size for
'Selenium' on 'Matrix metalloproteinase−16'

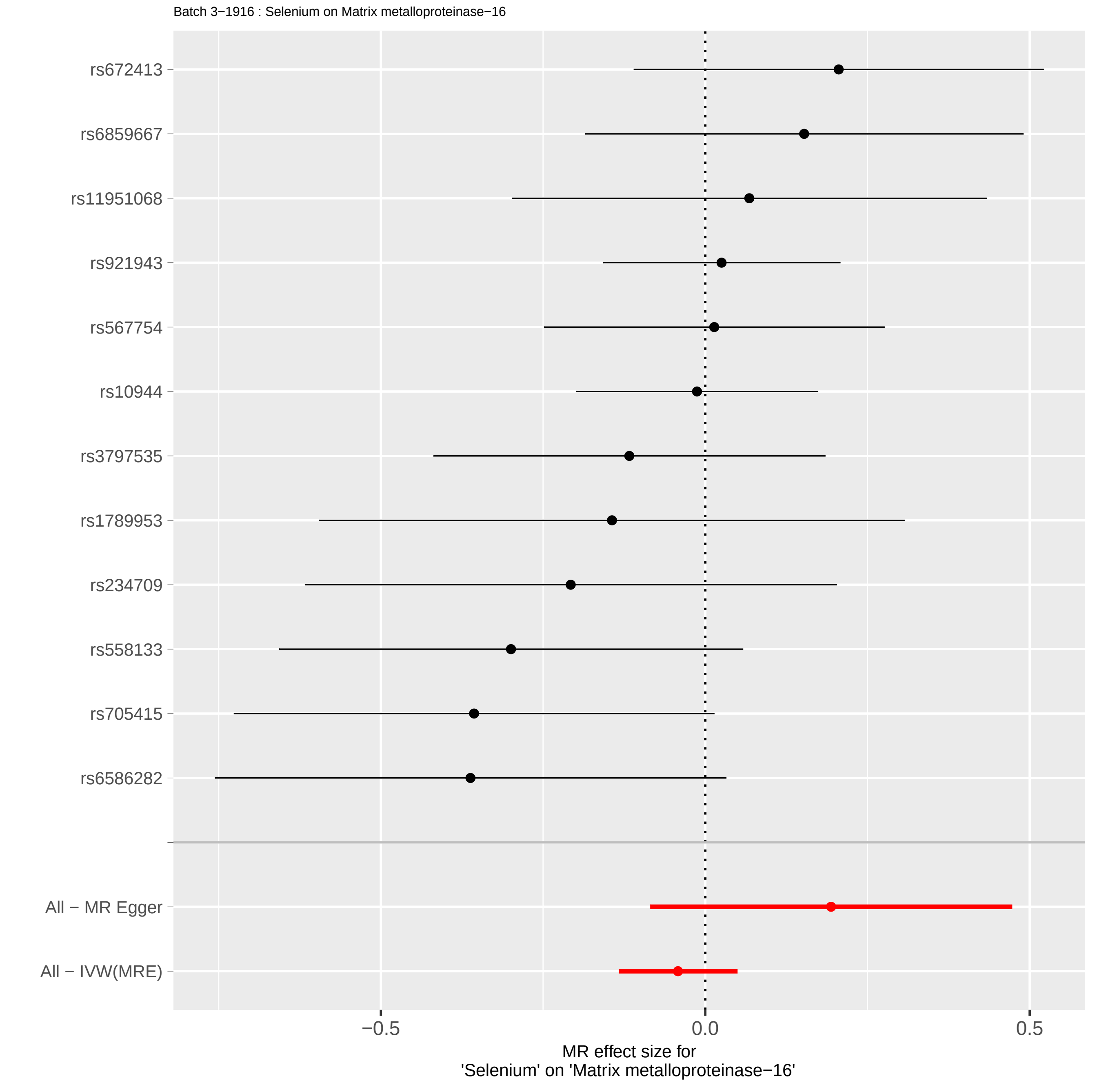

Batch 3−1916 : Selenium on Matrix metalloproteinase−16
rs672413
rs6859667
rs11951068
rs921943
rs567754
rs10944
rs3797535
rs1789953
rs234709
rs558133
rs705415
rs6586282
All − MR Egger
All − IVW(MRE)
−0.5
0.0
0.5
MR effect size for
'Selenium' on 'Matrix metalloproteinase−16'

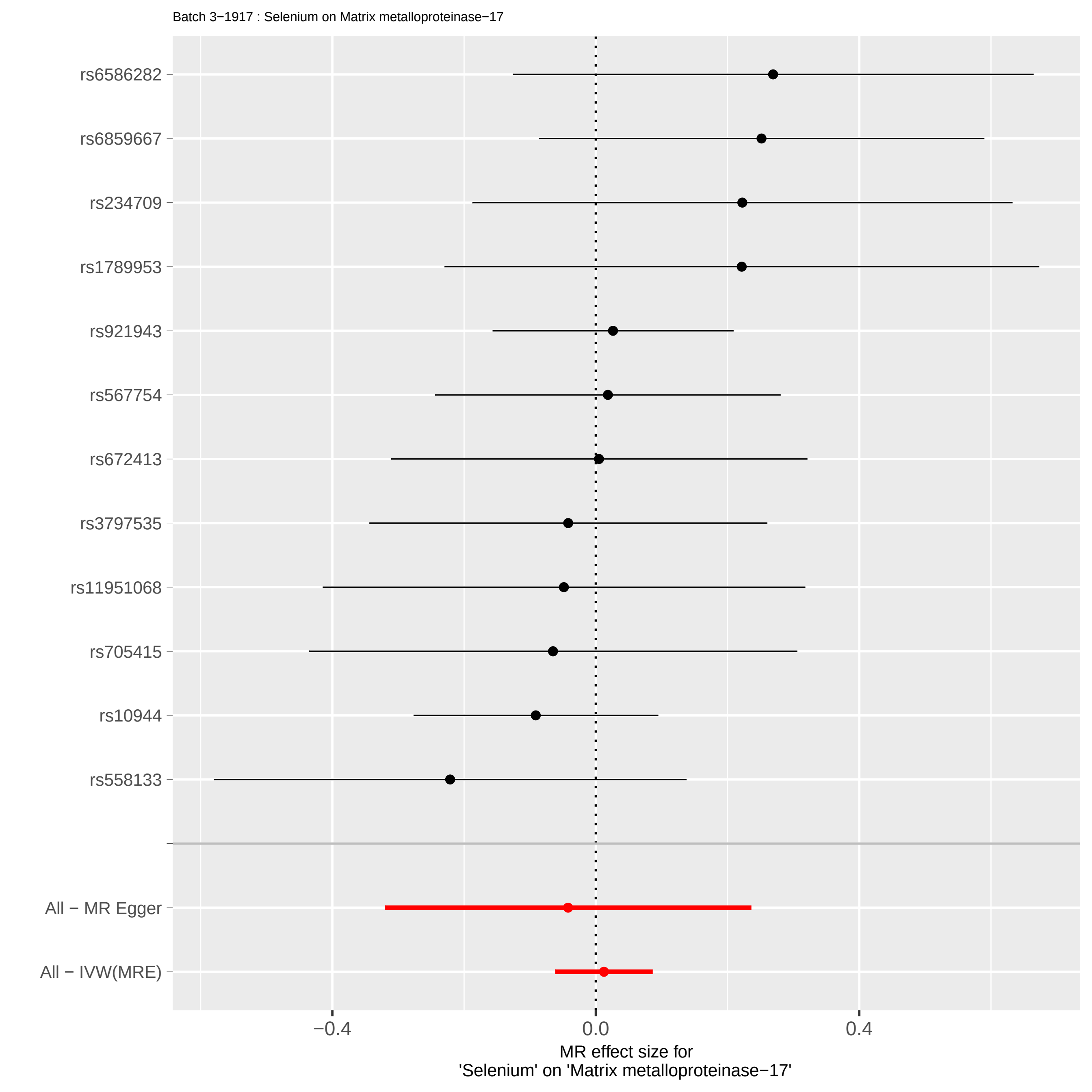
Batch 3−1917 : Selenium on Matrix metalloproteinase−17
rs6586282
rs6859667
rs234709
rs1789953
rs921943
rs567754
rs672413
rs3797535
rs11951068
rs705415
rs10944
rs558133
All − MR Egger
All − IVW(MRE)
−0.4
0.0
0.4
MR effect size for
'Selenium' on 'Matrix metalloproteinase−17'

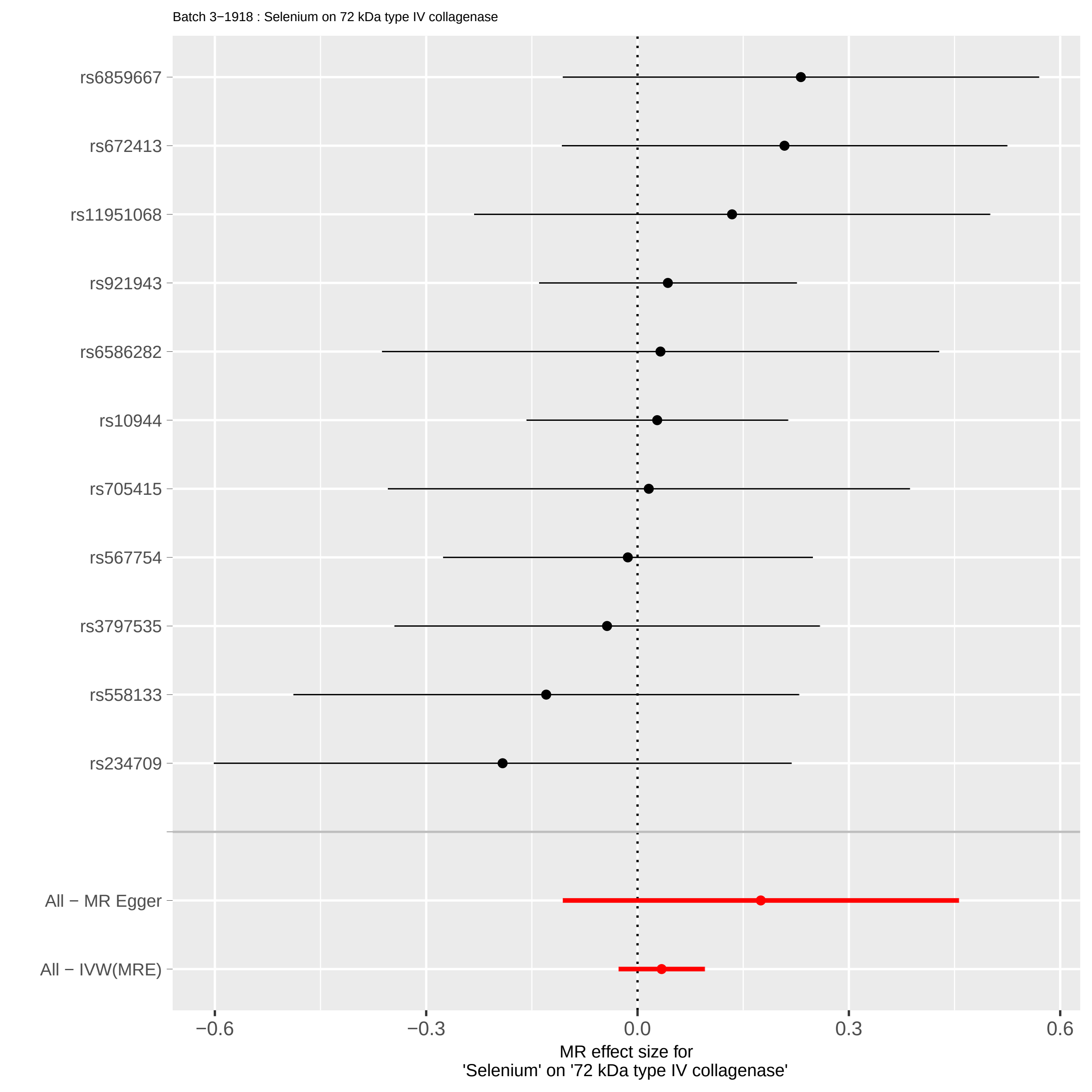

Batch 3−1918 : Selenium on 72 kDa type IV collagenase
rs6859667
rs672413
rs11951068
rs921943
rs6586282
rs10944
rs705415
rs567754
rs3797535
rs558133
rs234709
All − MR Egger
All − IVW(MRE)
−0.6
−0.3
0.0
0.3
0.6
MR effect size for
'Selenium' on '72 kDa type IV collagenase'

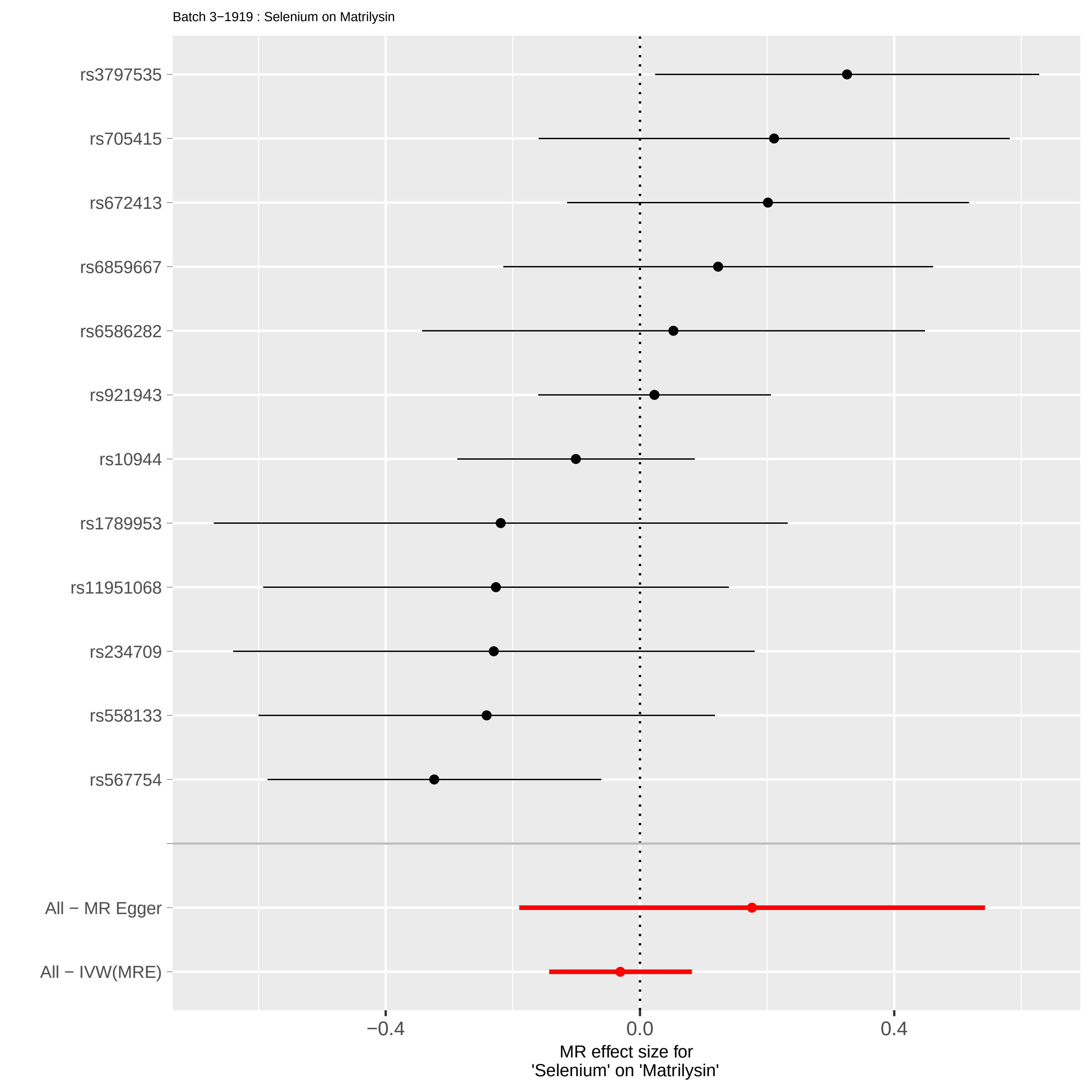

Batch 3−1919 : Selenium on Matrilysin
rs3797535
rs705415
rs672413
rs6859667
rs6586282
rs921943
rs10944
rs1789953
rs11951068
rs234709
rs558133
rs567754
All − MR Egger
All − IVW(MRE)
−0.4
0.0
0.4
MR effect size for
'Selenium' on 'Matrilysin'

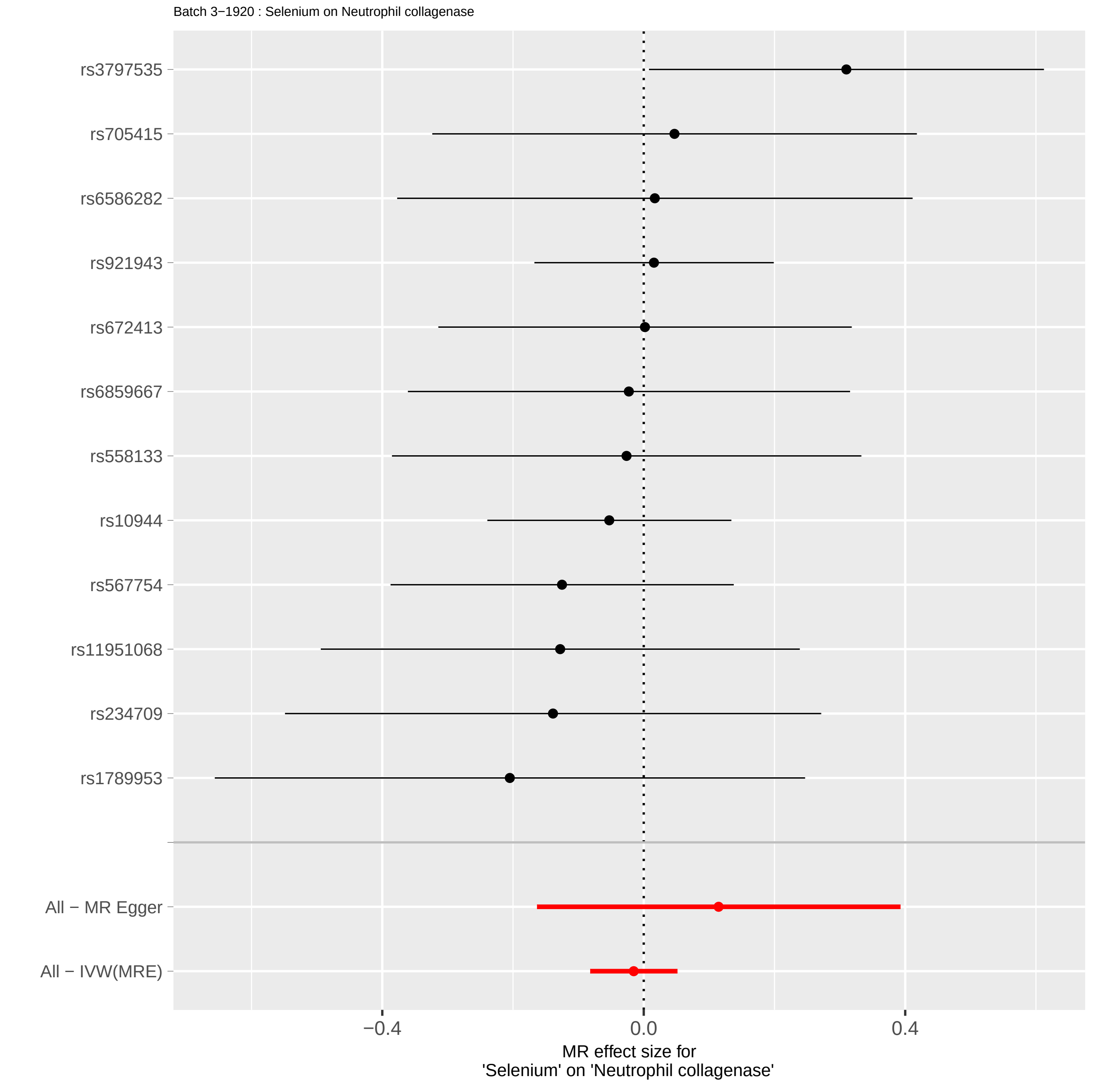

Batch 3−1920 : Selenium on Neutrophil collagenase
rs3797535
rs705415
rs6586282
rs921943
rs672413
rs6859667
rs558133
rs10944
rs567754
rs11951068
rs234709
rs1789953
All − MR Egger
All − IVW(MRE)
−0.4
0.0
0.4
MR effect size for
'Selenium' on 'Neutrophil collagenase'

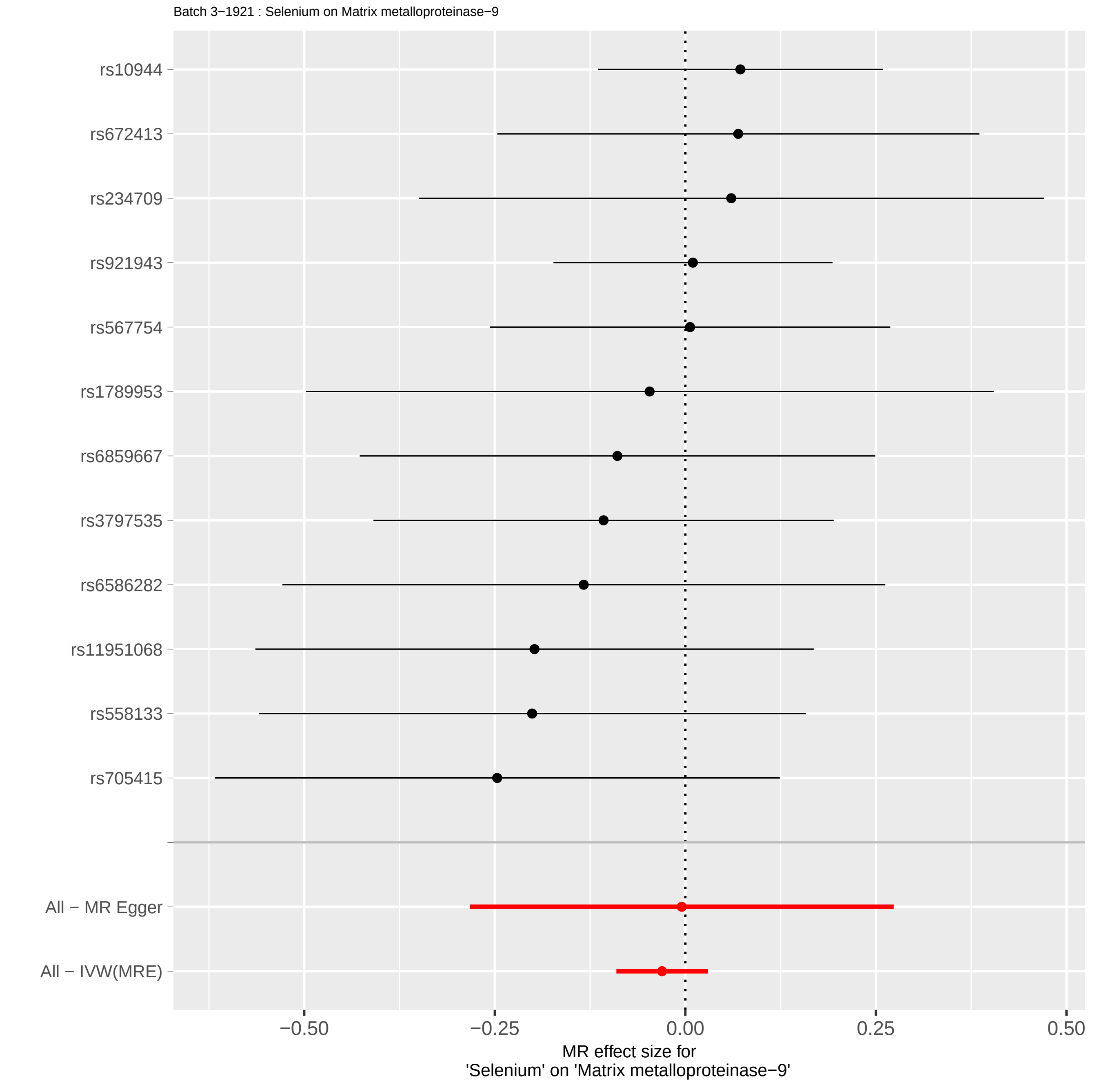

Batch 3−1921 : Selenium on Matrix metalloproteinase−9
rs10944
rs672413
rs234709
rs921943
rs567754
rs1789953
rs6859667
rs3797535
rs6586282
rs11951068
rs558133
rs705415
All − MR Egger
All − IVW(MRE)
−0.50
−0.25
0.00
0.25
0.50
MR effect size for
'Selenium' on 'Matrix metalloproteinase−9'

Batch 3−1922 : Selenium on Multimerin−2

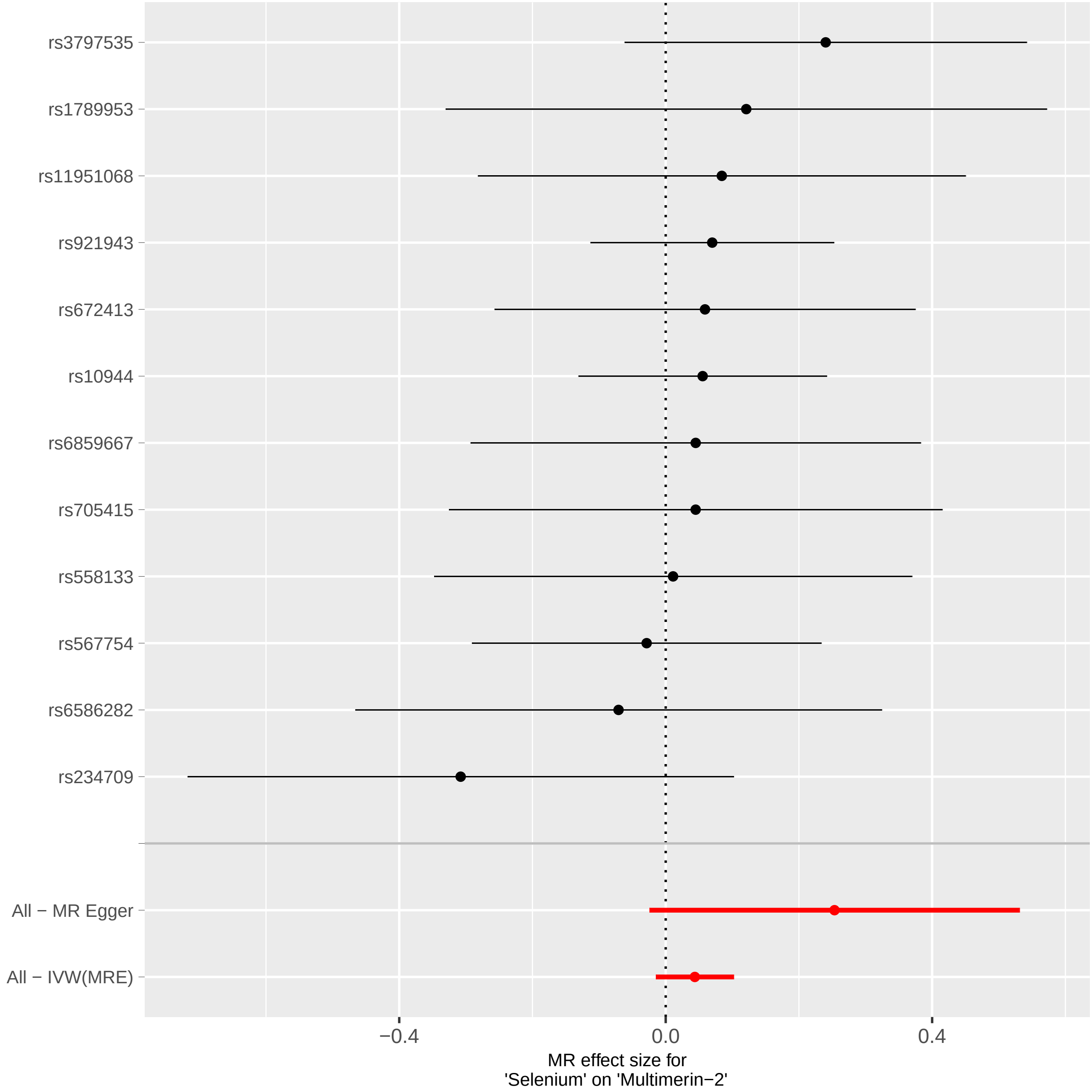

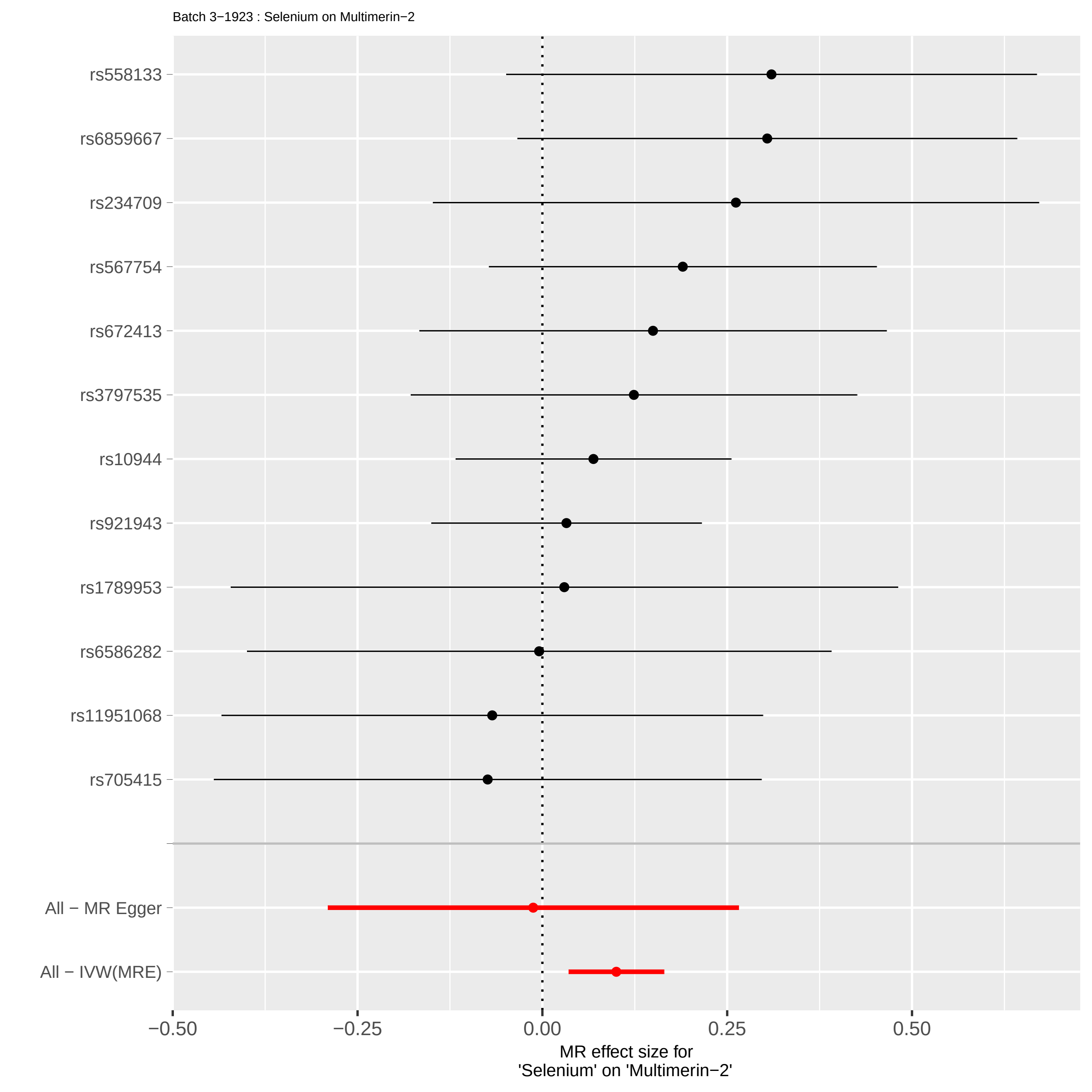

Batch 3−1923 : Selenium on Multimerin−2
rs558133
rs6859667
rs234709
rs567754
rs672413
rs3797535
rs10944
rs921943
rs1789953
rs6586282
rs11951068
rs705415
All − MR Egger
All − IVW(MRE)
−0.50
−0.25
0.00
0.25
0.50
MR effect size for
'Selenium' on 'Multimerin−2'

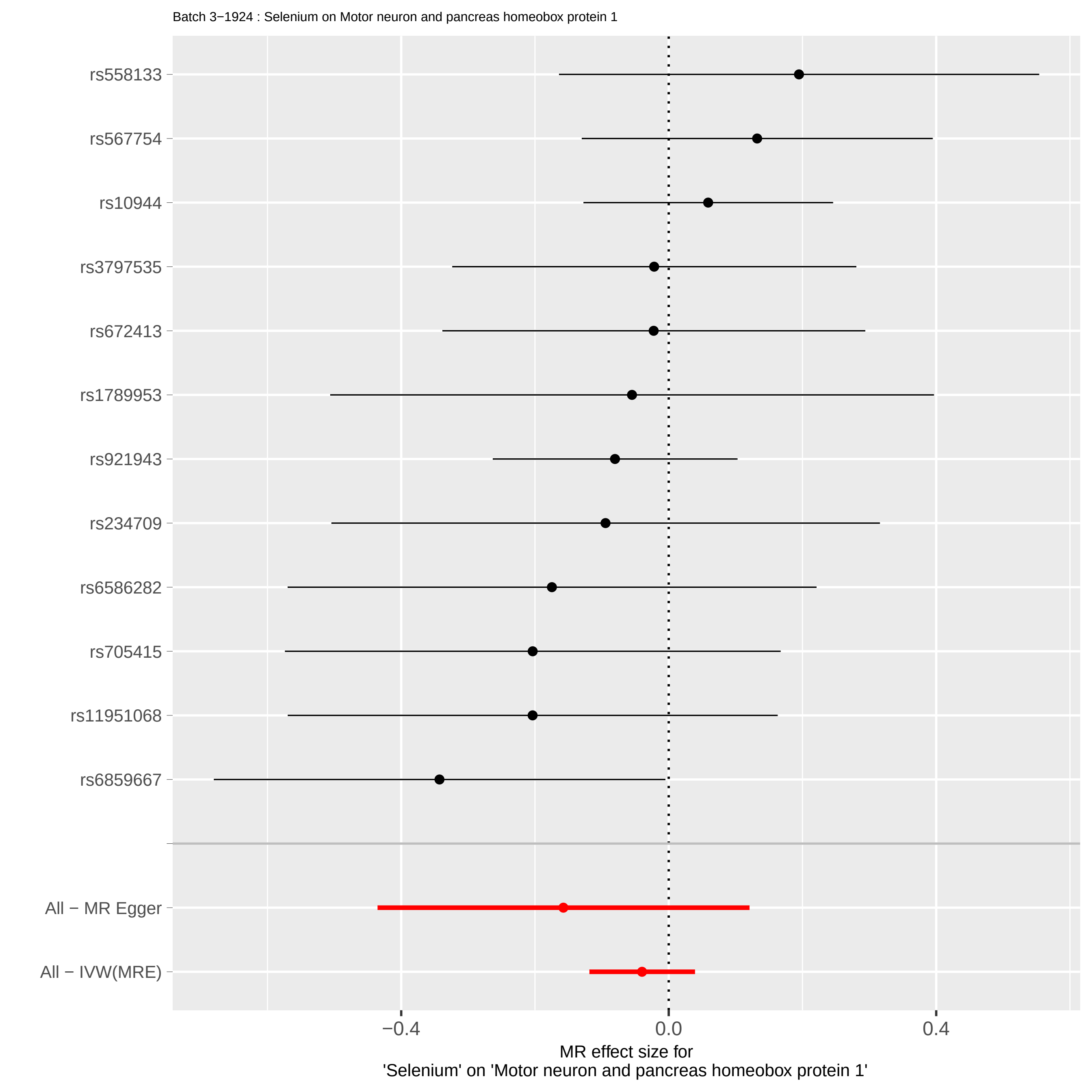

Batch 3−1924 : Selenium on Motor neuron and pancreas homeobox protein 1
rs558133
rs567754
rs10944
rs3797535
rs672413
rs1789953
rs921943
rs234709
rs6586282
rs705415
rs11951068
rs6859667
All − MR Egger
All − IVW(MRE)
−0.4
0.0
0.4
MR effect size for
'Selenium' on 'Motor neuron and pancreas homeobox protein 1'

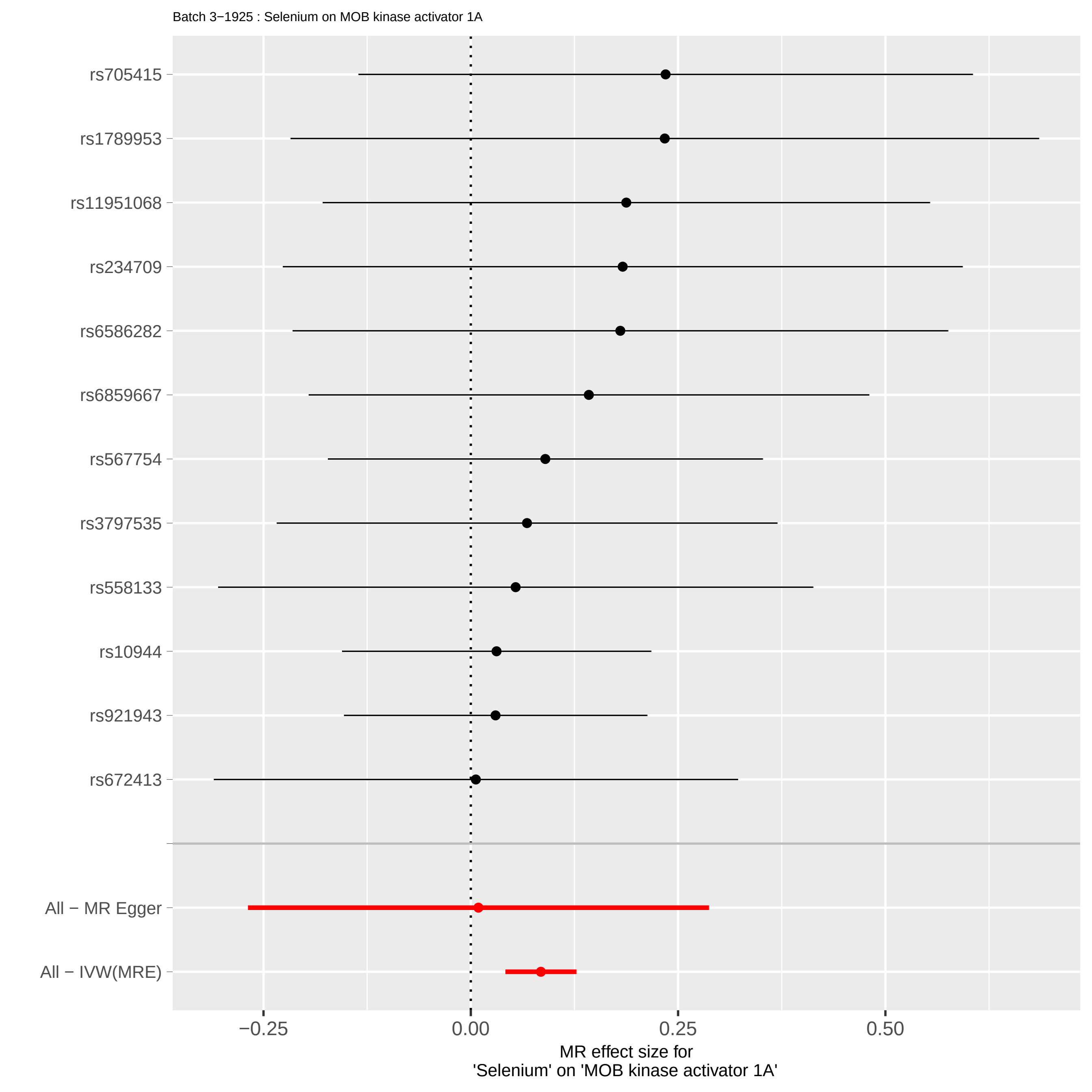
Batch 3−1925 : Selenium on MOB kinase activator 1A
rs705415
rs1789953
rs11951068
rs234709
rs6586282
rs6859667
rs567754
rs3797535
rs558133
rs10944
rs921943
rs672413
All − MR Egger
All − IVW(MRE)
−0.25
0.00
0.25
0.50
MR effect size for
'Selenium' on 'MOB kinase activator 1A'

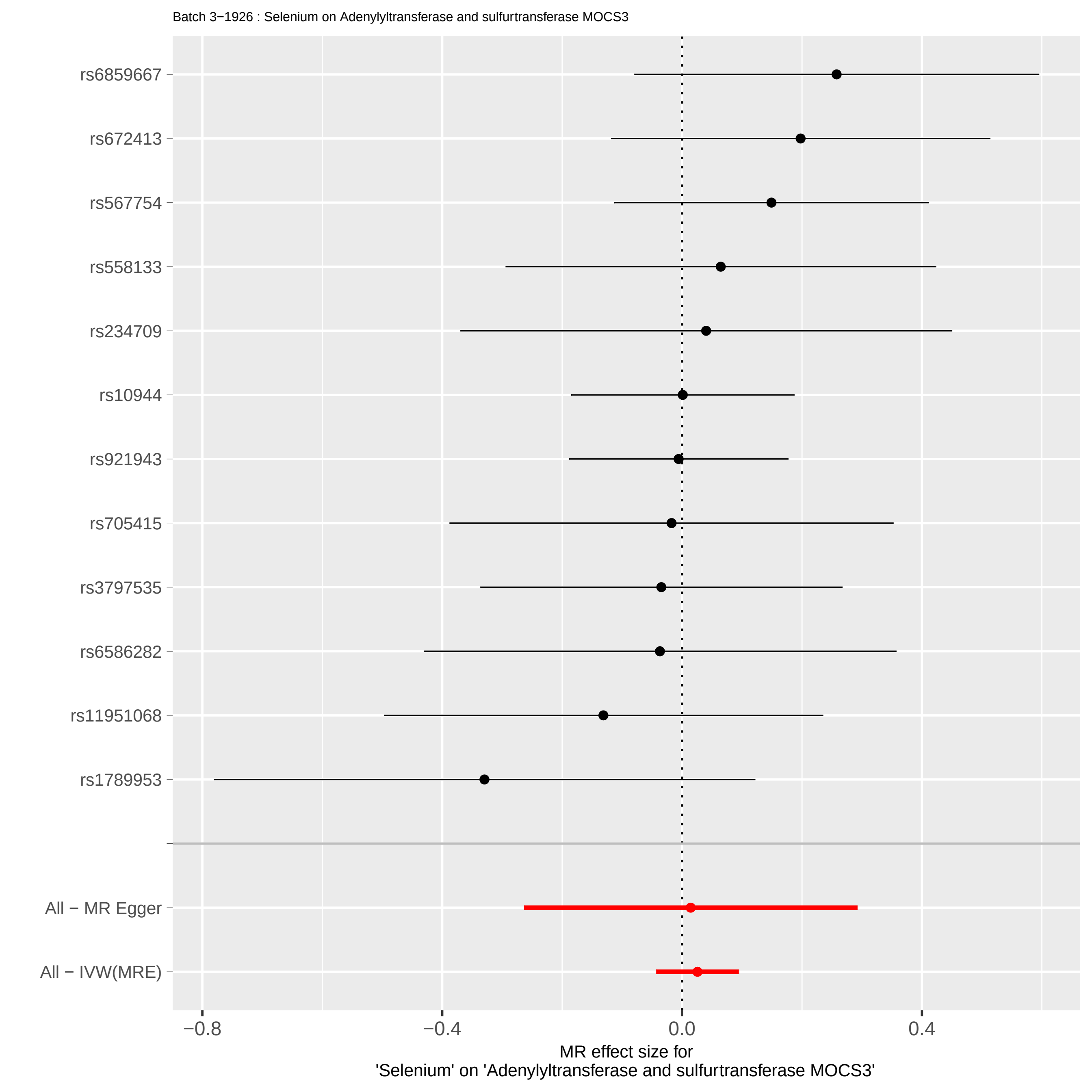

Batch 3−1926 : Selenium on Adenylyltransferase and sulfurtransferase MOCS3
rs6859667
rs672413
rs567754
rs558133
rs234709
rs10944
rs921943
rs705415
rs3797535
rs6586282
rs11951068
rs1789953
All − MR Egger
All − IVW(MRE)
−0.8
−0.4
0.0
0.4
MR effect size for
'Selenium' on 'Adenylyltransferase and sulfurtransferase MOCS3'

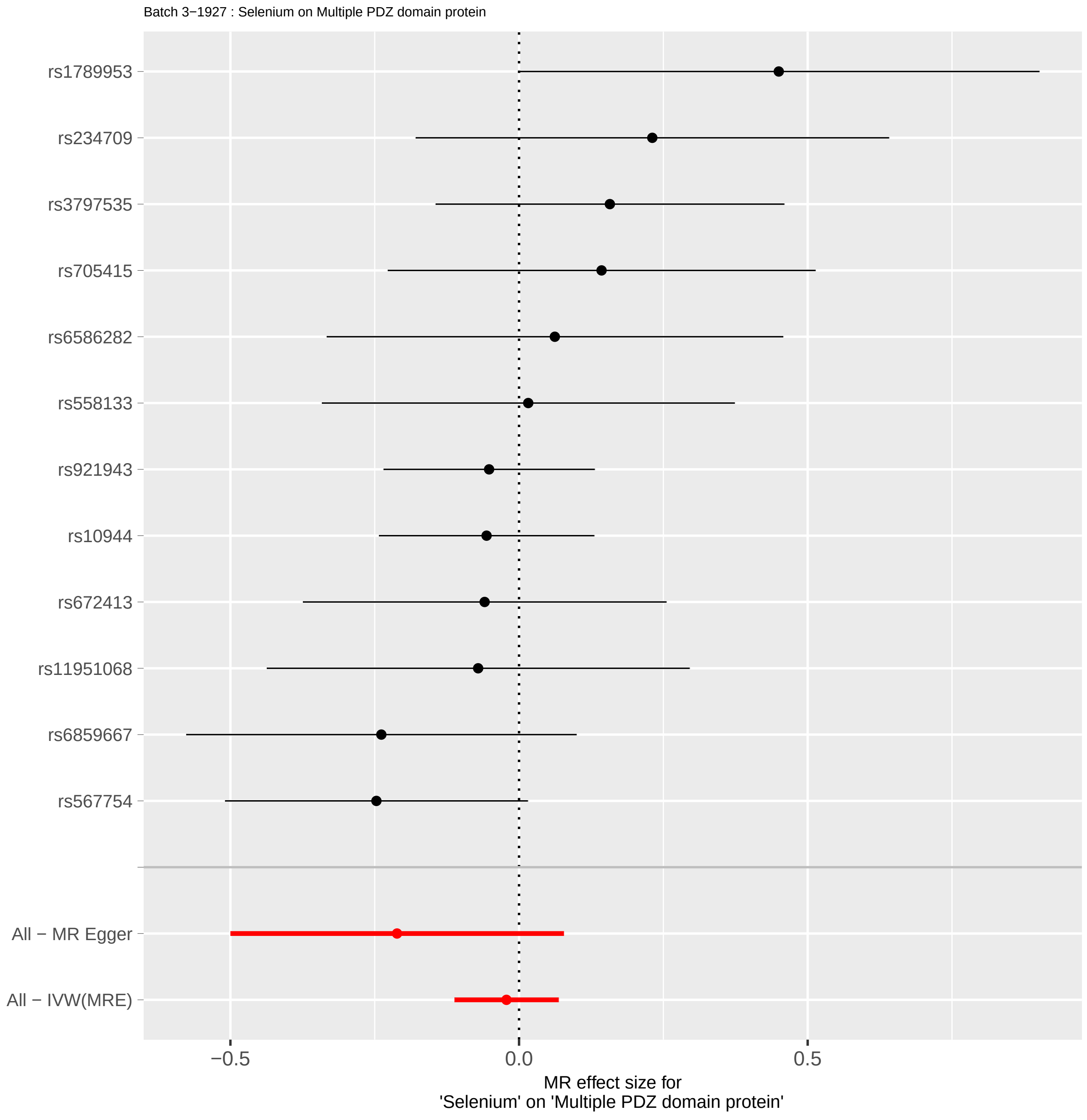

Batch 3−1927 : Selenium on Multiple PDZ domain protein
rs1789953
rs234709
rs3797535
rs705415
rs6586282
rs558133
rs921943
rs10944
rs672413
rs11951068
rs6859667
rs567754
All − MR Egger
All − IVW(MRE)
−0.5
0.0
0.5
MR effect size for
'Selenium' on 'Multiple PDZ domain protein'

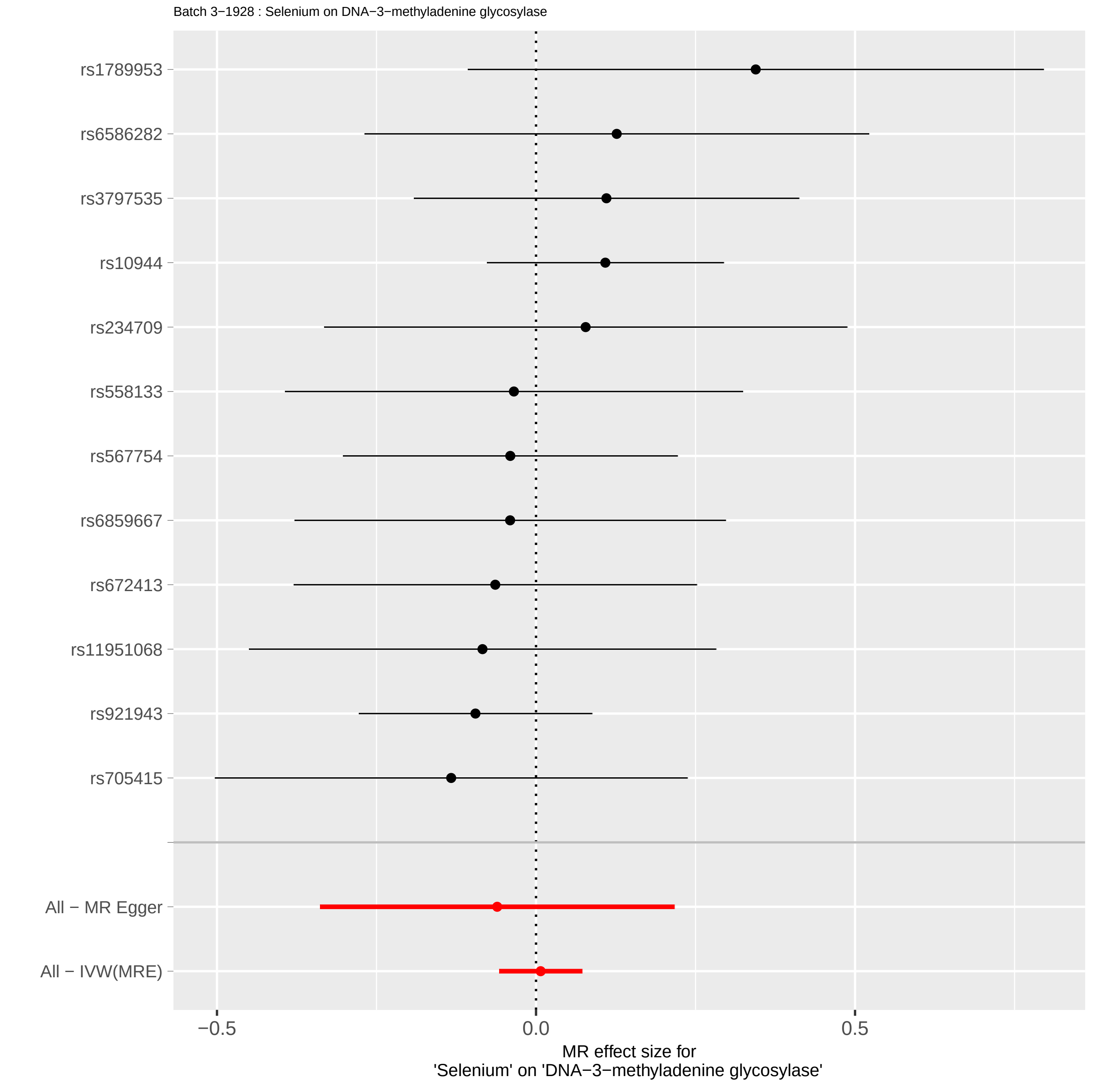

Batch 3−1928 : Selenium on DNA−3−methyladenine glycosylase
rs1789953
rs6586282
rs3797535
rs10944
rs234709
rs558133
rs567754
rs6859667
rs672413
rs11951068
rs921943
rs705415
All − MR Egger
All − IVW(MRE)
−0.5
0.0
0.5
MR effect size for
'Selenium' on 'DNA−3−methyladenine glycosylase'

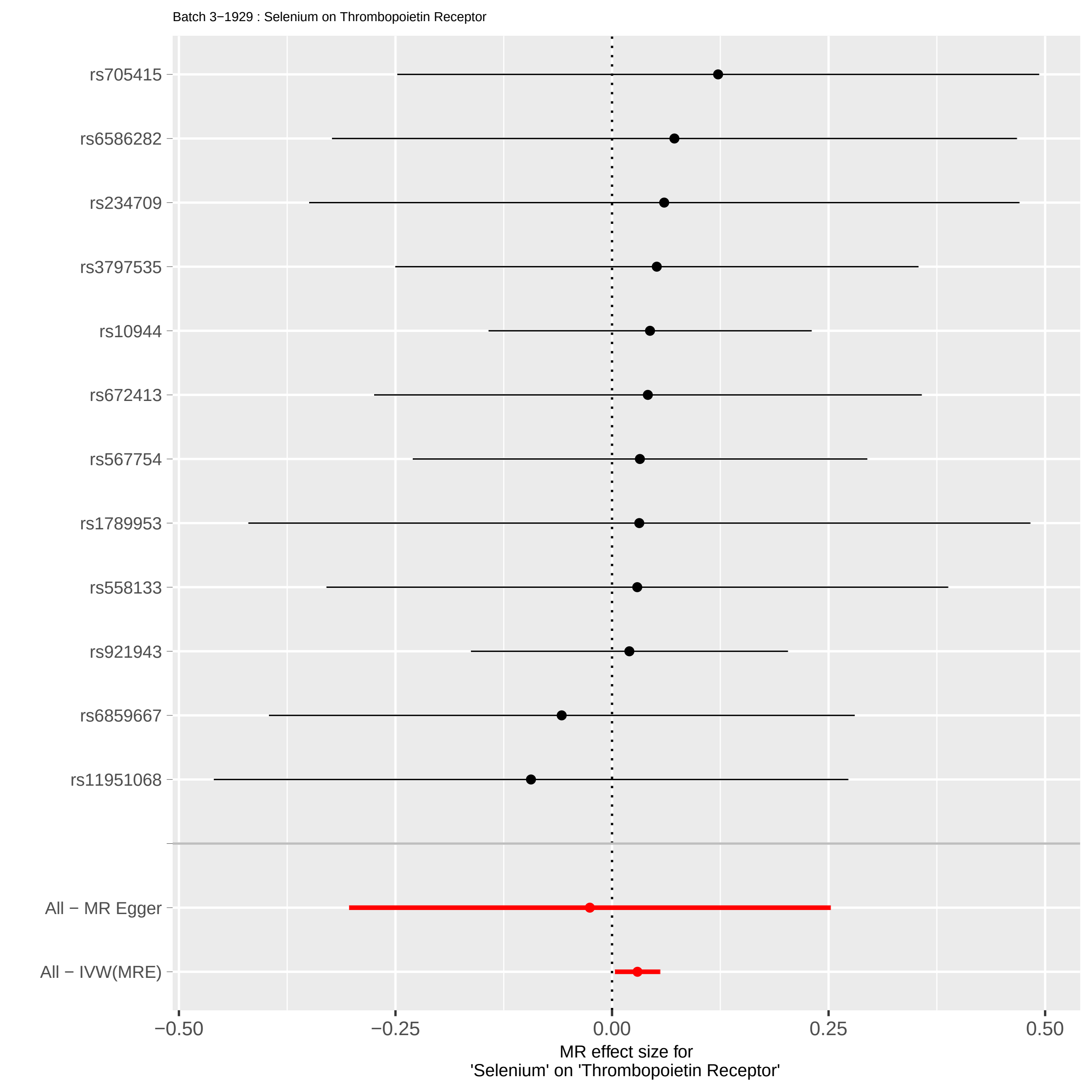

Batch 3−1929 : Selenium on Thrombopoietin Receptor
rs705415
rs6586282
rs234709
rs3797535
rs10944
rs672413
rs567754
rs1789953
rs558133
rs921943
rs6859667
rs11951068
All − MR Egger
All − IVW(MRE)
−0.50
−0.25
0.00
0.25
0.50
MR effect size for
'Selenium' on 'Thrombopoietin Receptor'

Batch 3−1930 : Selenium on Myeloperoxidase

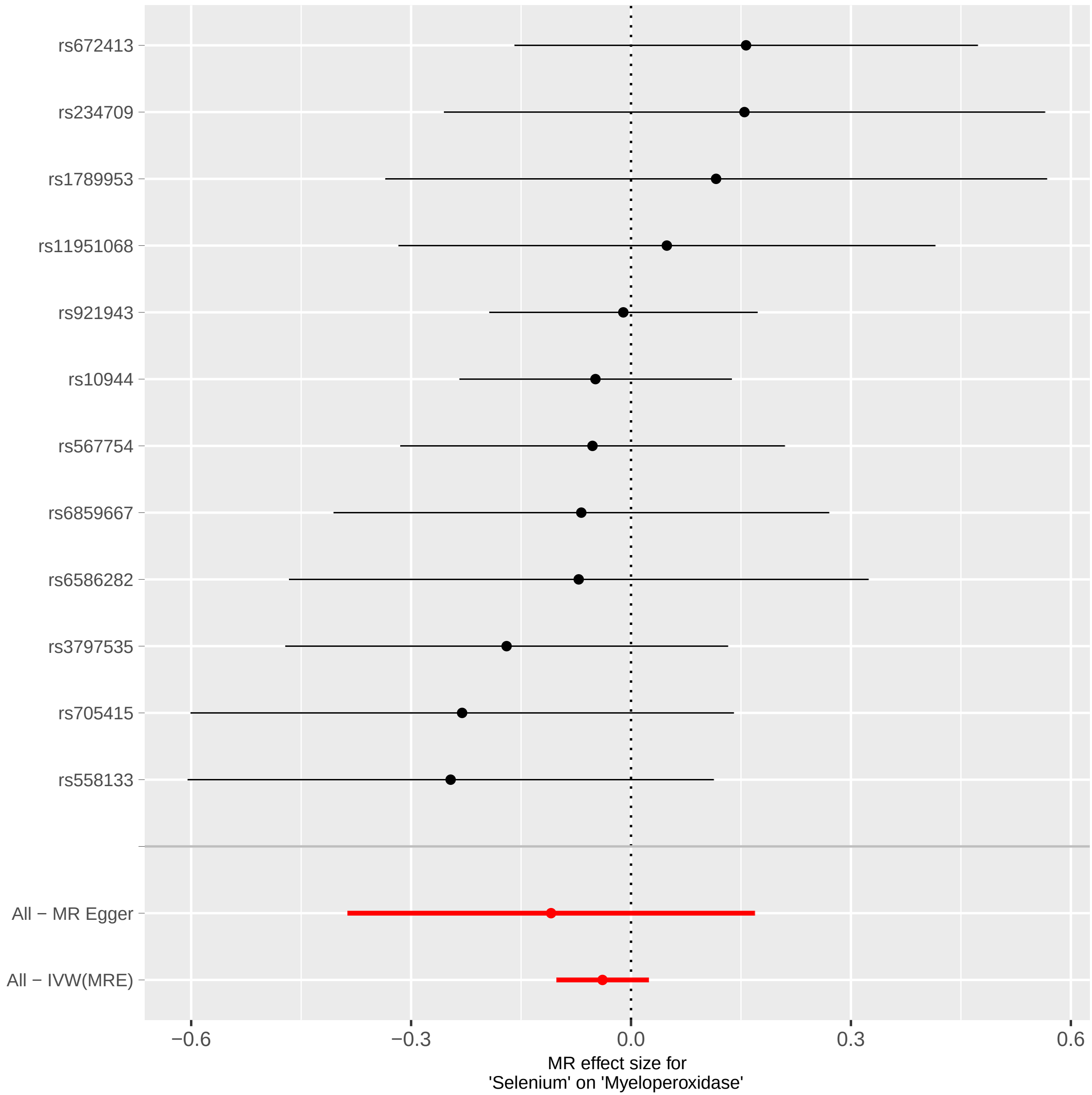

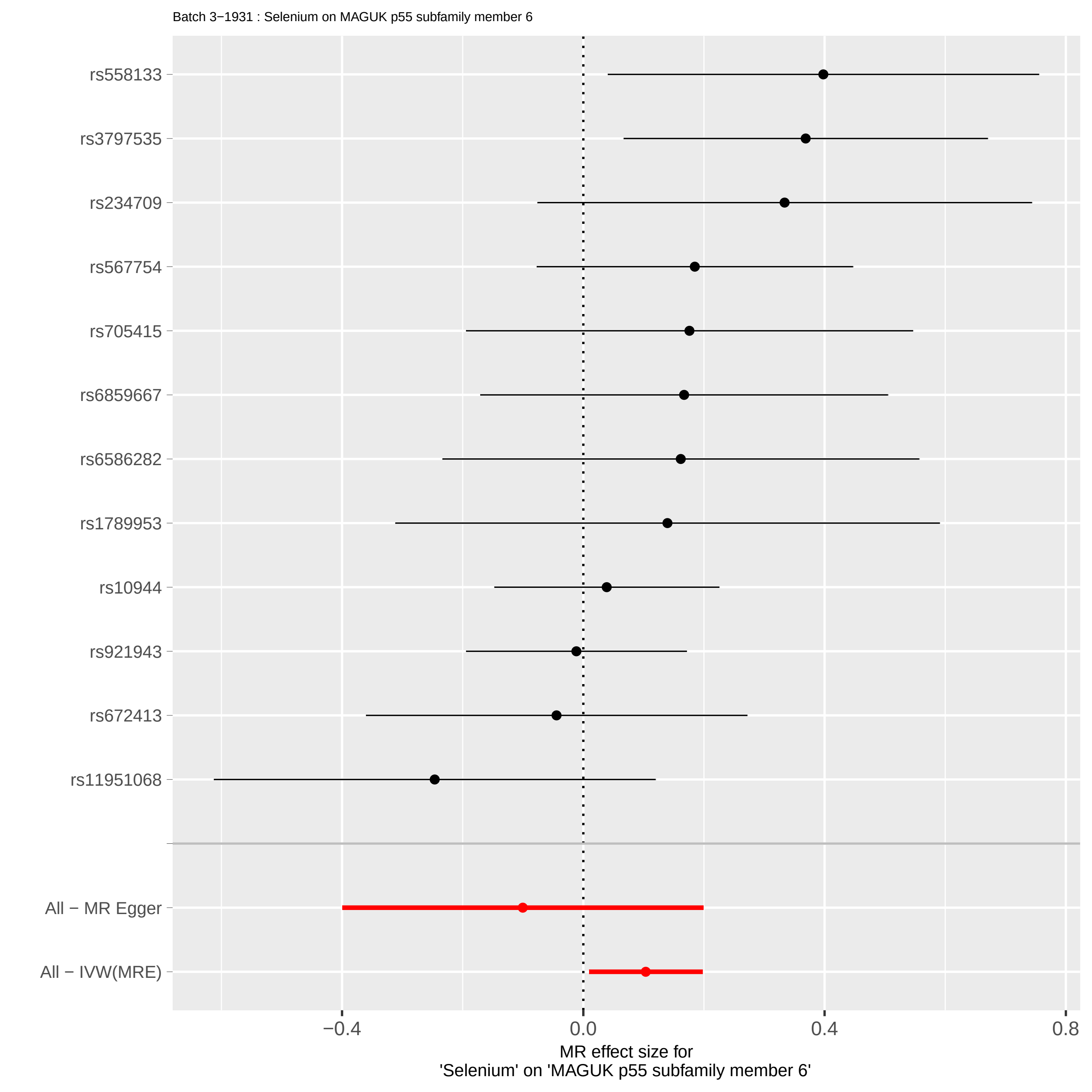
Batch 3−1931 : Selenium on MAGUK p55 subfamily member 6
rs558133
rs3797535
rs234709
rs567754
rs705415
rs6859667
rs6586282
rs1789953
rs10944
rs921943
rs672413
rs11951068
All − MR Egger
All − IVW(MRE)
−0.4
0.0
0.4
0.8
MR effect size for
'Selenium' on 'MAGUK p55 subfamily member 6'

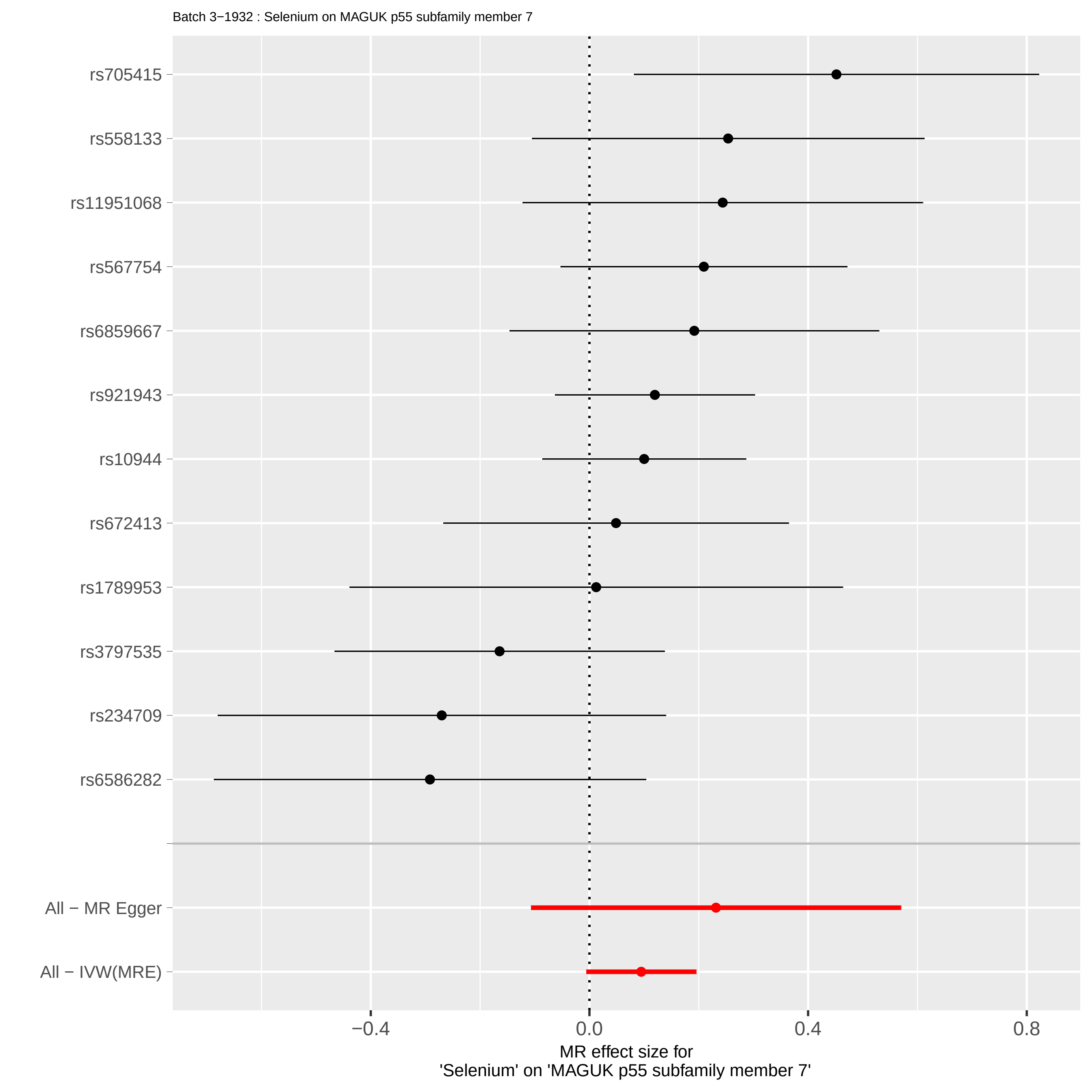
Batch 3−1932 : Selenium on MAGUK p55 subfamily member 7
rs705415
rs558133
rs11951068
rs567754
rs6859667
rs921943
rs10944
rs672413
rs1789953
rs3797535
rs234709
rs6586282
All − MR Egger
All − IVW(MRE)
−0.4
0.0
0.4
0.8
MR effect size for
'Selenium' on 'MAGUK p55 subfamily member 7'

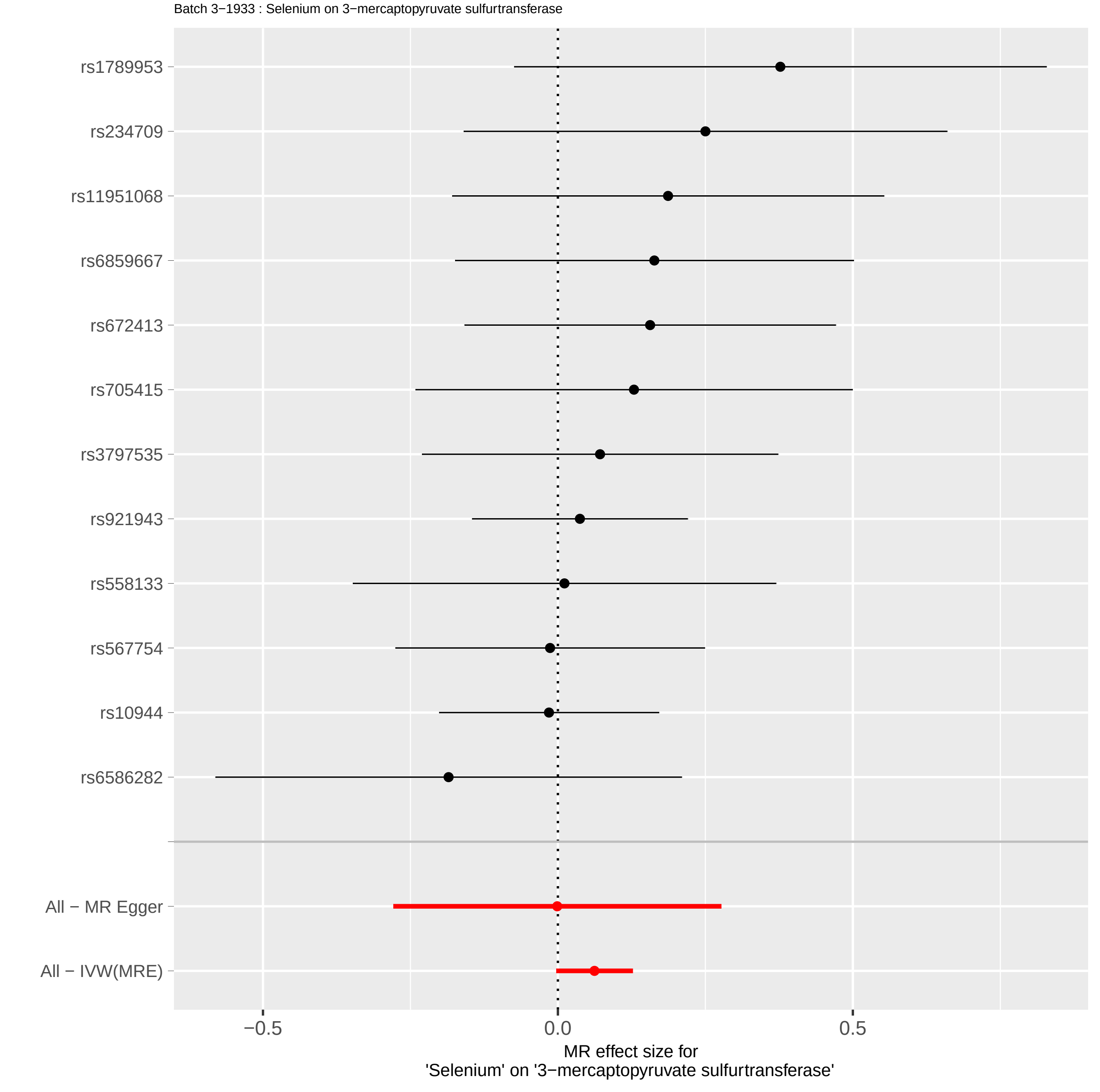
Batch 3−1933 : Selenium on 3−mercaptopyruvate sulfurtransferase
rs1789953
rs234709
rs11951068
rs6859667
rs672413
rs705415
rs3797535
rs921943
rs558133
rs567754
rs10944
rs6586282
All − MR Egger
All − IVW(MRE)
−0.5
0.0
0.5
MR effect size for
'Selenium' on '3−mercaptopyruvate sulfurtransferase'

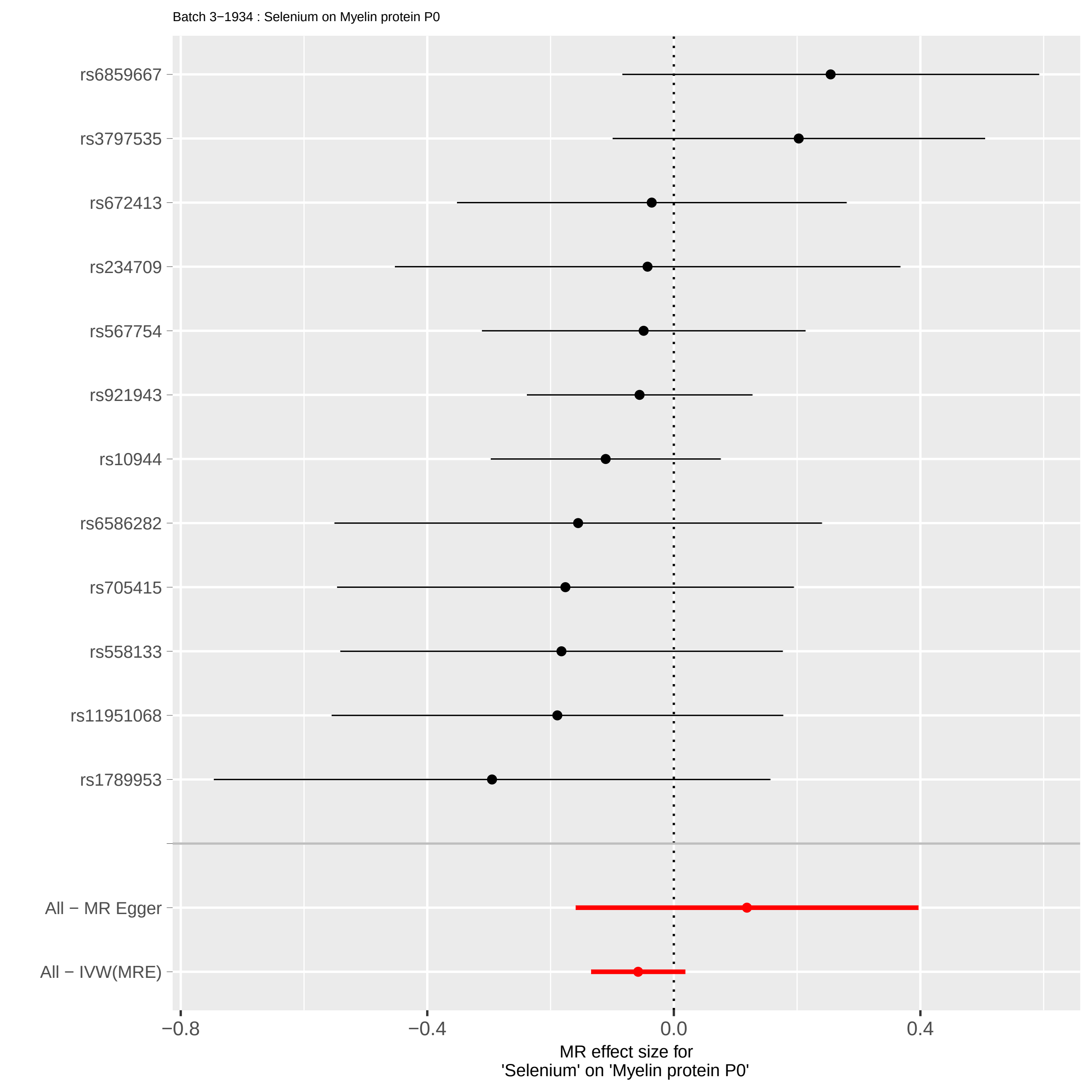

Batch 3−1934 : Selenium on Myelin protein P0
rs6859667
rs3797535
rs672413
rs234709
rs567754
rs921943
rs10944
rs6586282
rs705415
rs558133
rs11951068
rs1789953
All − MR Egger
All − IVW(MRE)
−0.8
−0.4
0.0
0.4
MR effect size for
'Selenium' on 'Myelin protein P0'

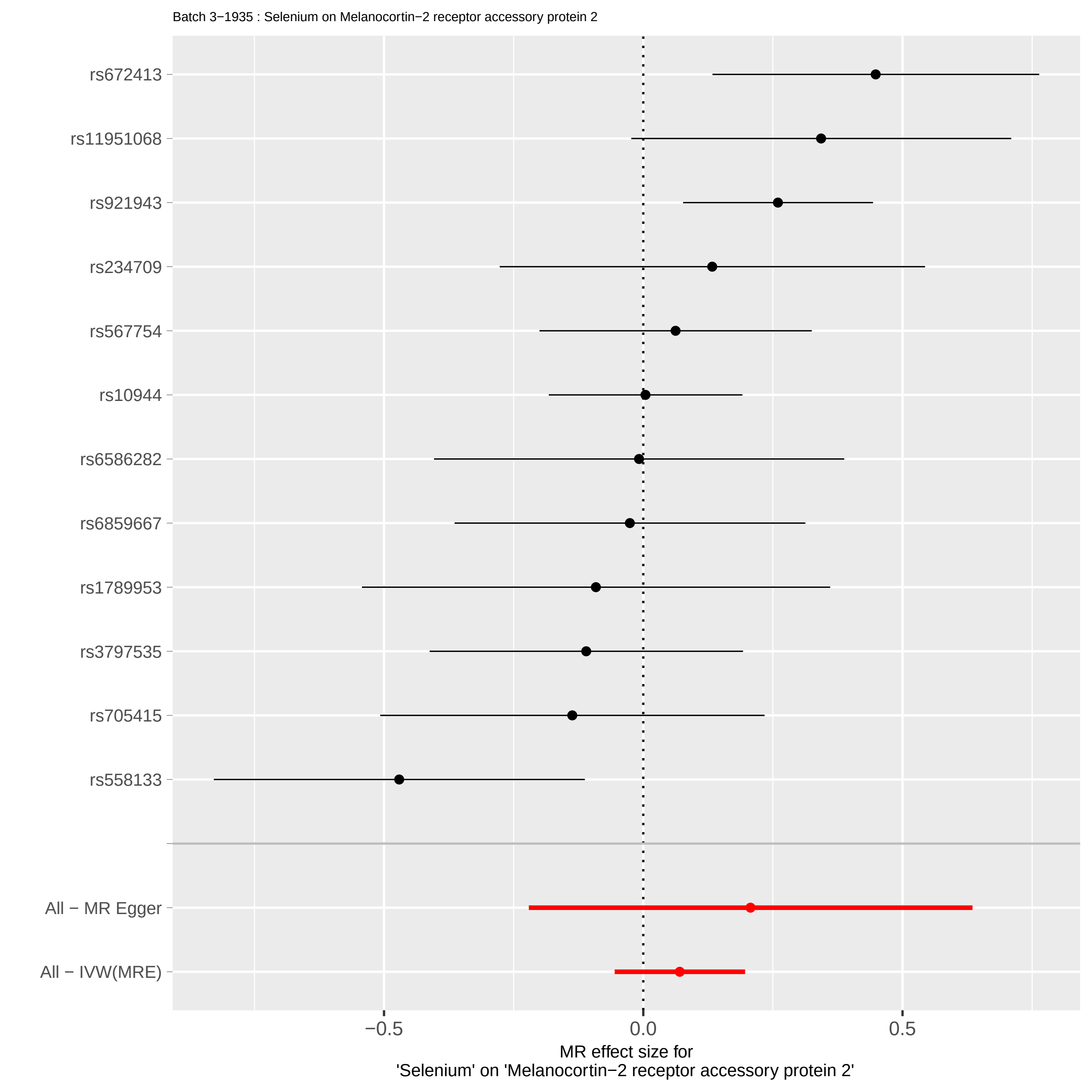

Batch 3−1935 : Selenium on Melanocortin−2 receptor accessory protein 2
rs672413
rs11951068
rs921943
rs234709
rs567754
rs10944
rs6586282
rs6859667
rs1789953
rs3797535
rs705415
rs558133
All − MR Egger
All − IVW(MRE)
−0.5
0.0
0.5
MR effect size for
'Selenium' on 'Melanocortin−2 receptor accessory protein 2'

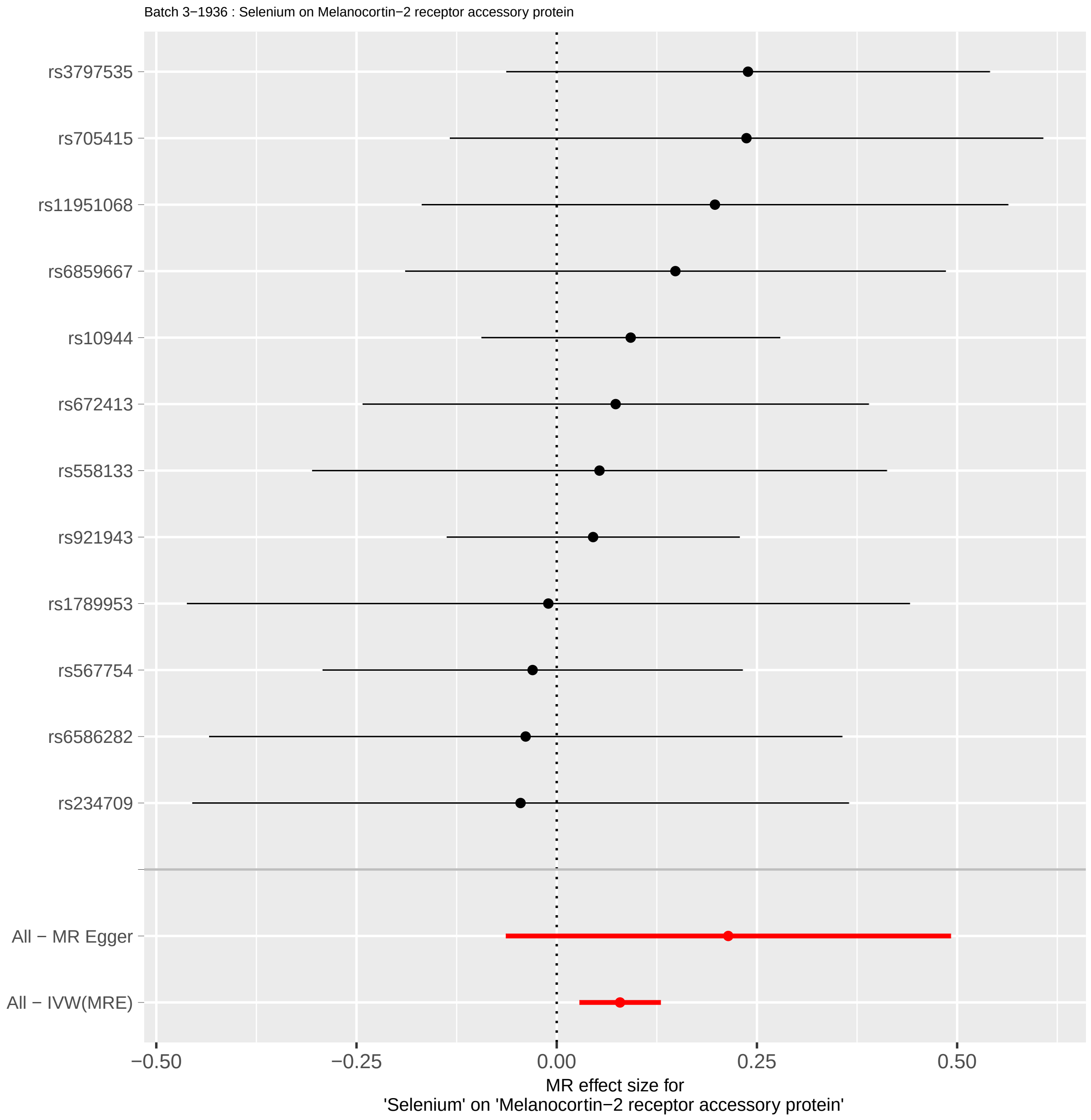

Batch 3−1936 : Selenium on Melanocortin−2 receptor accessory protein
rs3797535
rs705415
rs11951068
rs6859667
rs10944
rs672413
rs558133
rs921943
rs1789953
rs567754
rs6586282
rs234709
All − MR Egger
All − IVW(MRE)
−0.50
−0.25
0.00
0.25
0.50
MR effect size for
'Selenium' on 'Melanocortin−2 receptor accessory protein'

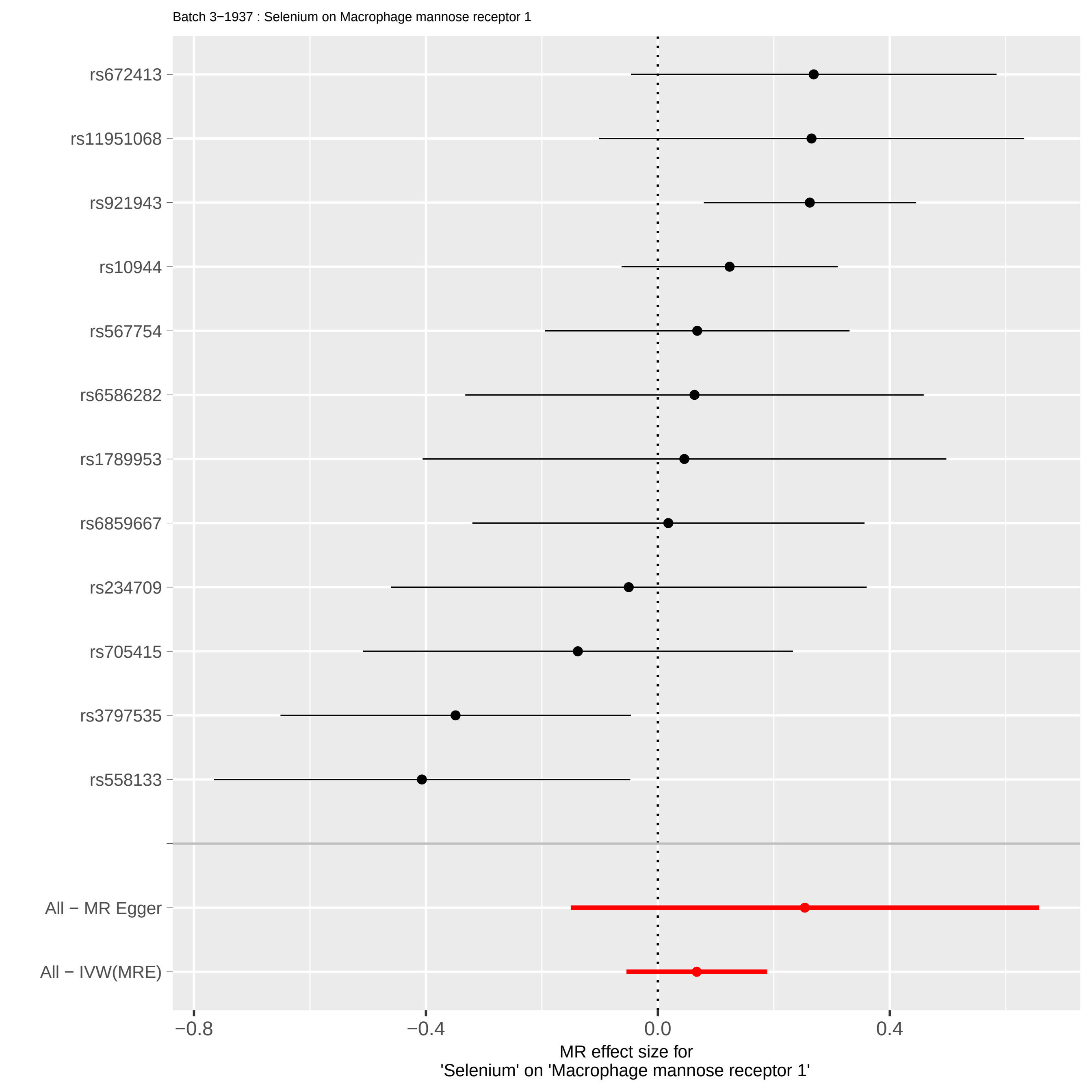

Batch 3−1937 : Selenium on Macrophage mannose receptor 1
rs672413
rs11951068
rs921943
rs10944
rs567754
rs6586282
rs1789953
rs6859667
rs234709
rs705415
rs3797535
rs558133
All − MR Egger
All − IVW(MRE)
−0.8
−0.4
0.0
0.4
MR effect size for
'Selenium' on 'Macrophage mannose receptor 1'

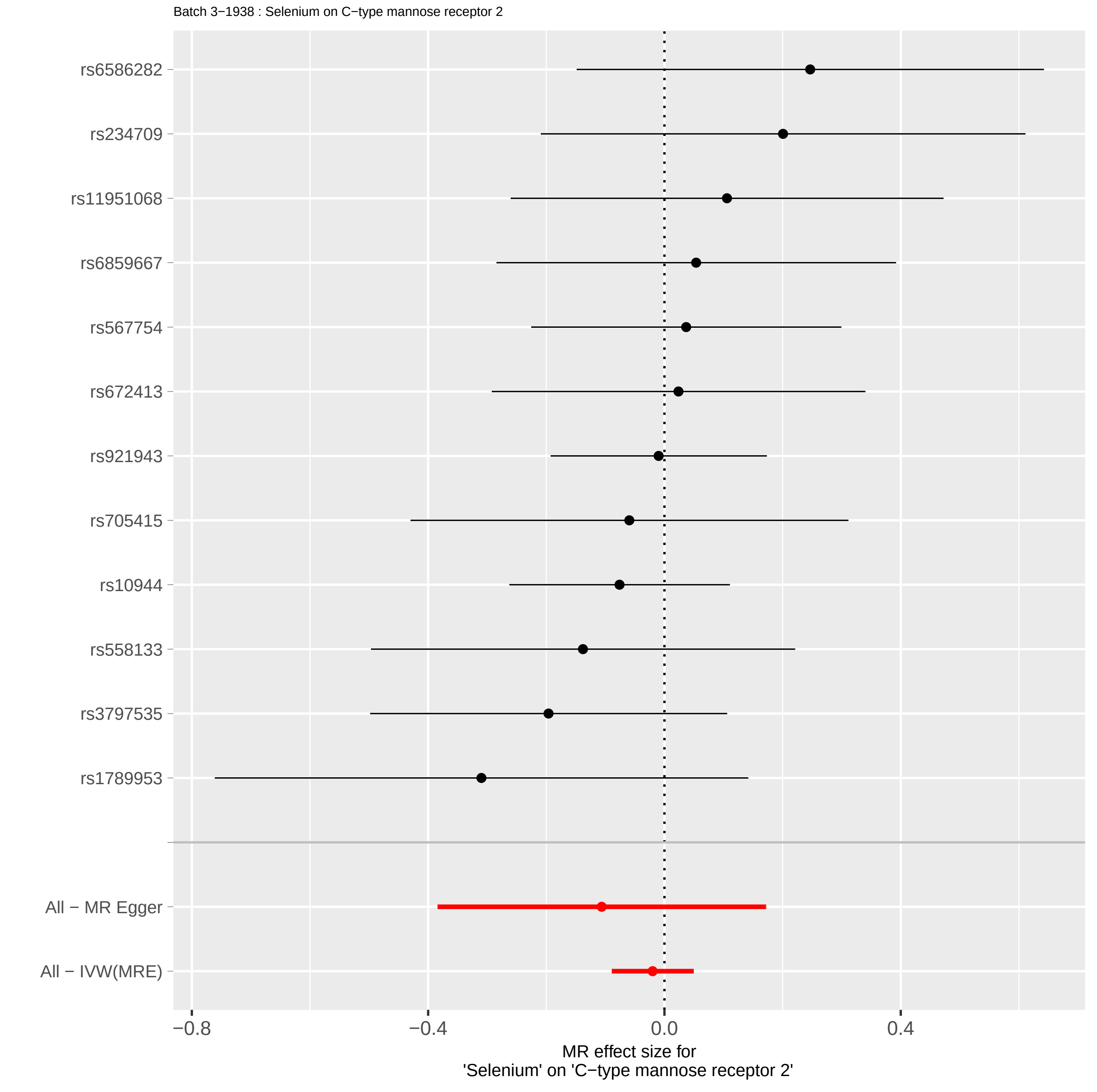

Batch 3−1938 : Selenium on C−type mannose receptor 2
rs6586282
rs234709
rs11951068
rs6859667
rs567754
rs672413
rs921943
rs705415
rs10944
rs558133
rs3797535
rs1789953
All − MR Egger
All − IVW(MRE)
−0.8
−0.4
0.0
0.4
MR effect size for
'Selenium' on 'C−type mannose receptor 2'

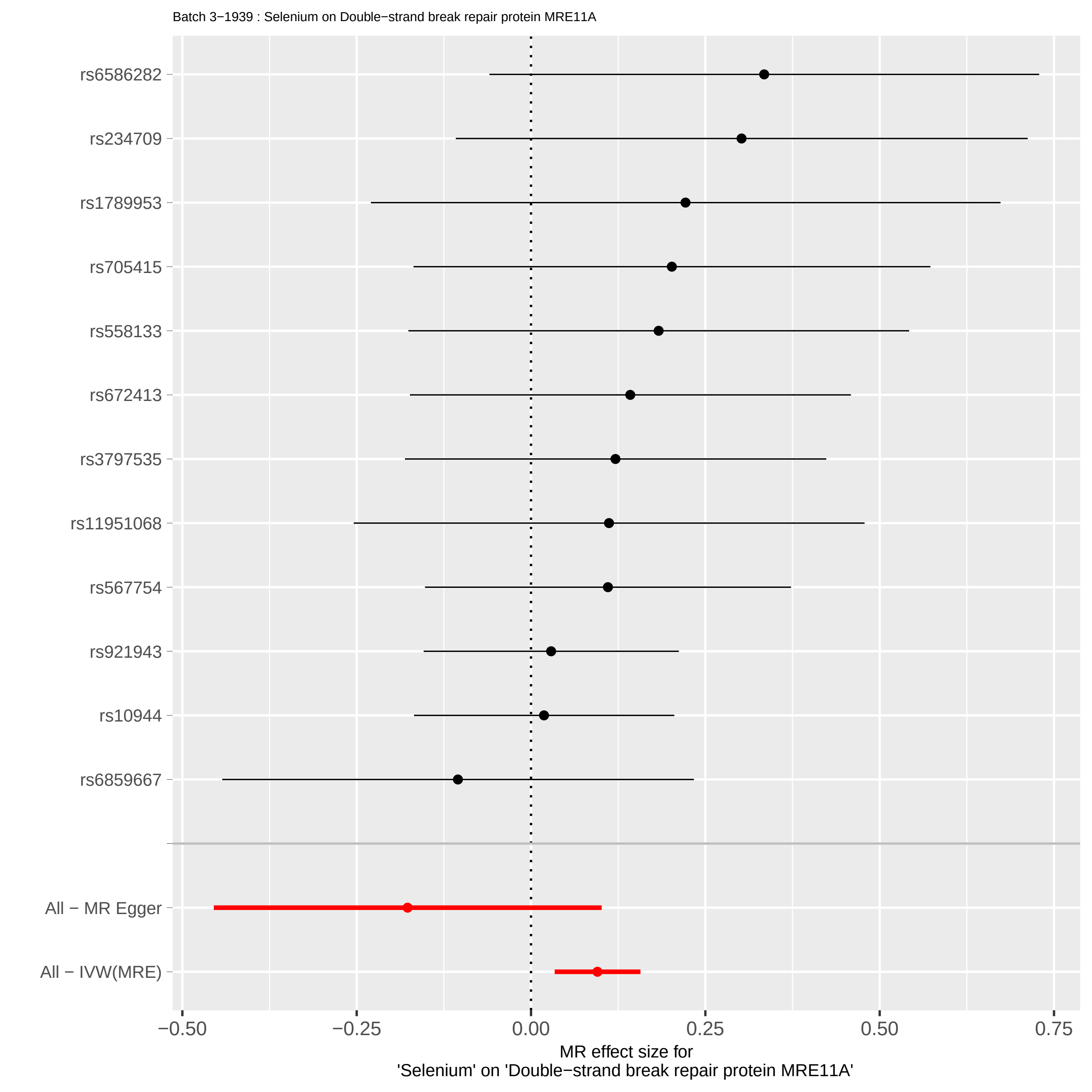

Batch 3−1939 : Selenium on Double−strand break repair protein MRE11A
rs6586282
rs234709
rs1789953
rs705415
rs558133
rs672413
rs3797535
rs11951068
rs567754
rs921943
rs10944
rs6859667
All − MR Egger
All − IVW(MRE)
−0.50
−0.25
0.00
0.25
0.50
0.75
MR effect size for
'Selenium' on 'Double−strand break repair protein MRE11A'

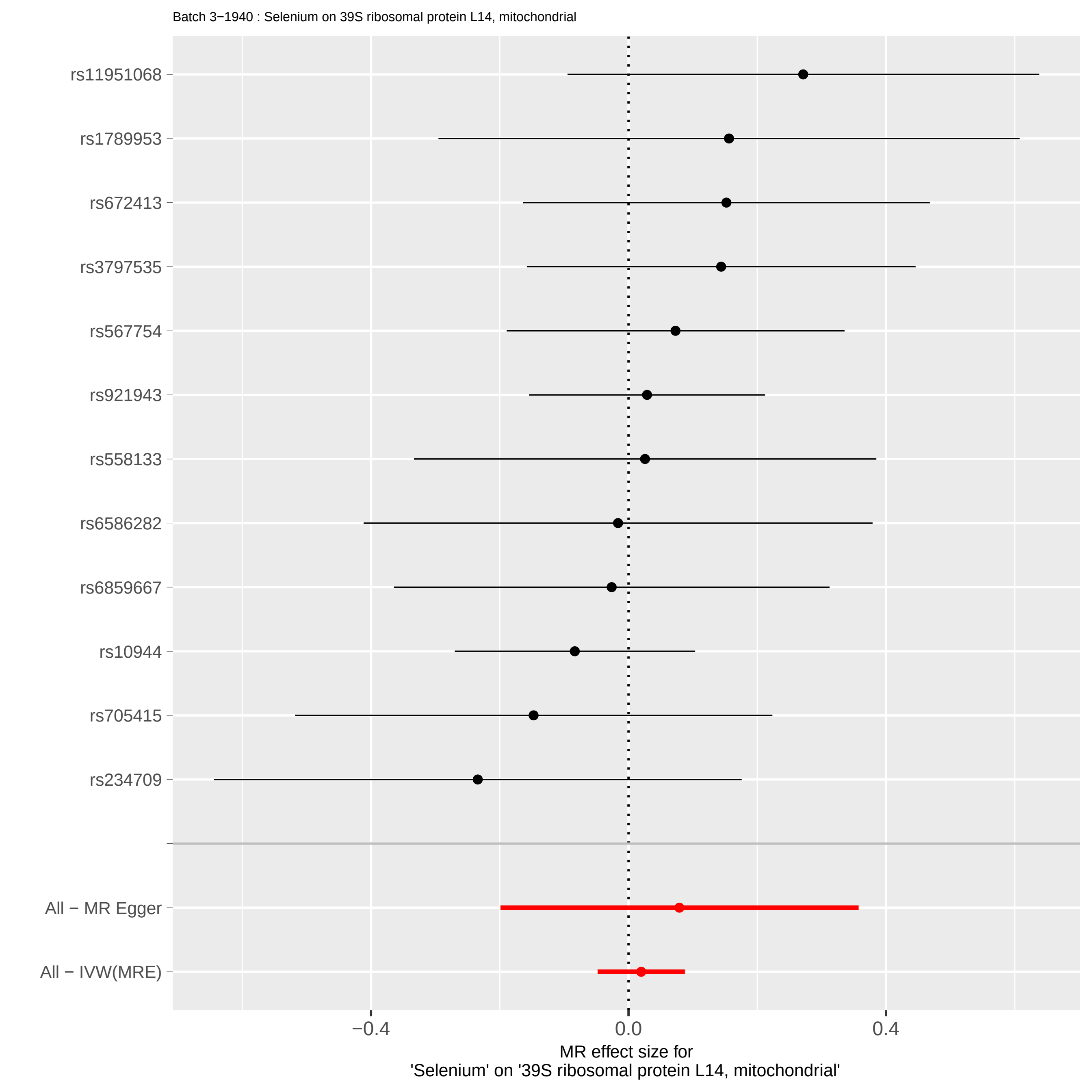
Batch 3−1940 : Selenium on 39S ribosomal protein L14, mitochondrial
rs11951068
rs1789953
rs672413
rs3797535
rs567754
rs921943
rs558133
rs6586282
rs6859667
rs10944
rs705415
rs234709
All − MR Egger
All − IVW(MRE)
−0.4
0.0
0.4
MR effect size for
'Selenium' on '39S ribosomal protein L14, mitochondrial'

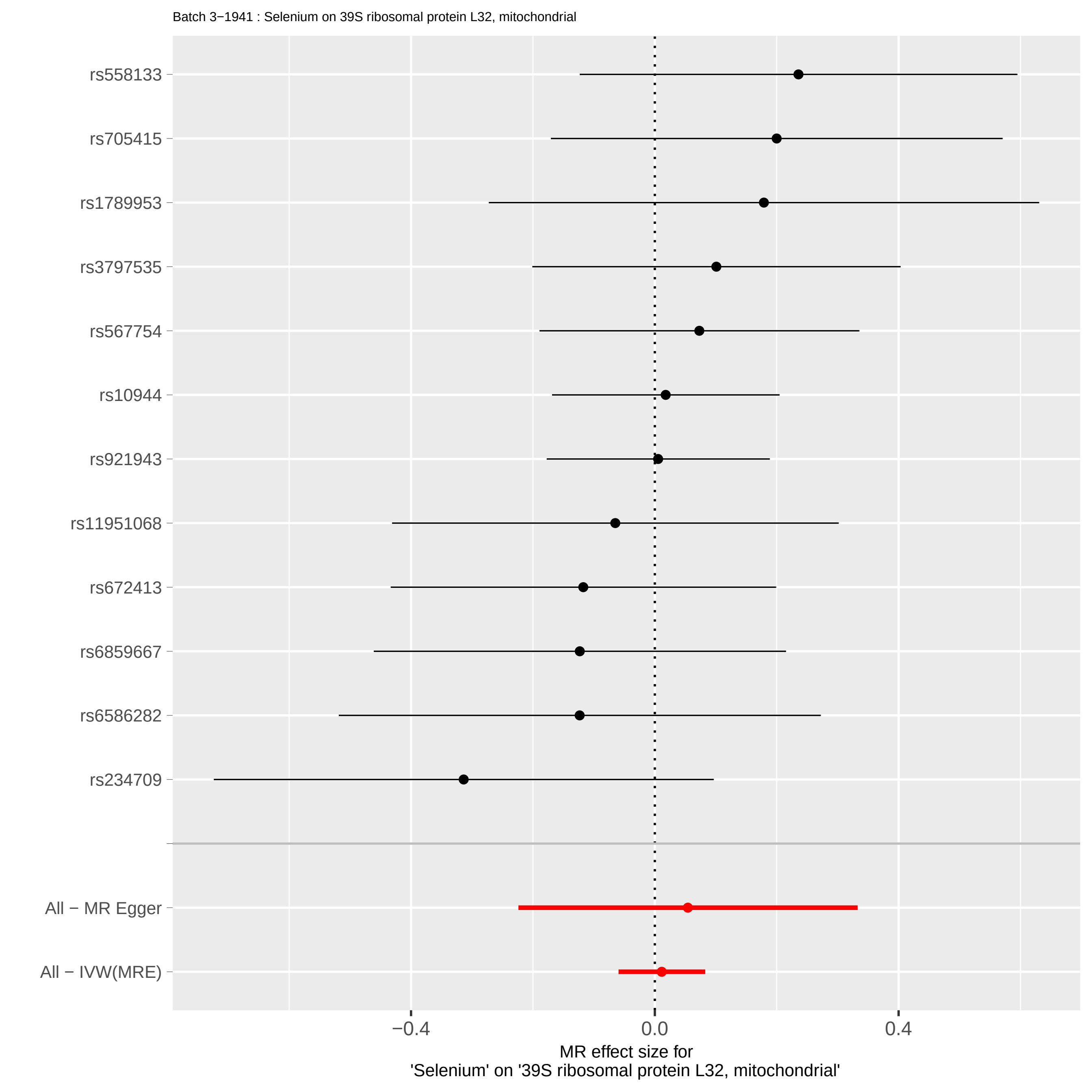

Batch 3−1941 : Selenium on 39S ribosomal protein L32, mitochondrial
rs558133
rs705415
rs1789953
rs3797535
rs567754
rs10944
rs921943
rs11951068
rs672413
rs6859667
rs6586282
rs234709
All − MR Egger
All − IVW(MRE)
−0.4
0.0
0.4
MR effect size for
'Selenium' on '39S ribosomal protein L32, mitochondrial'

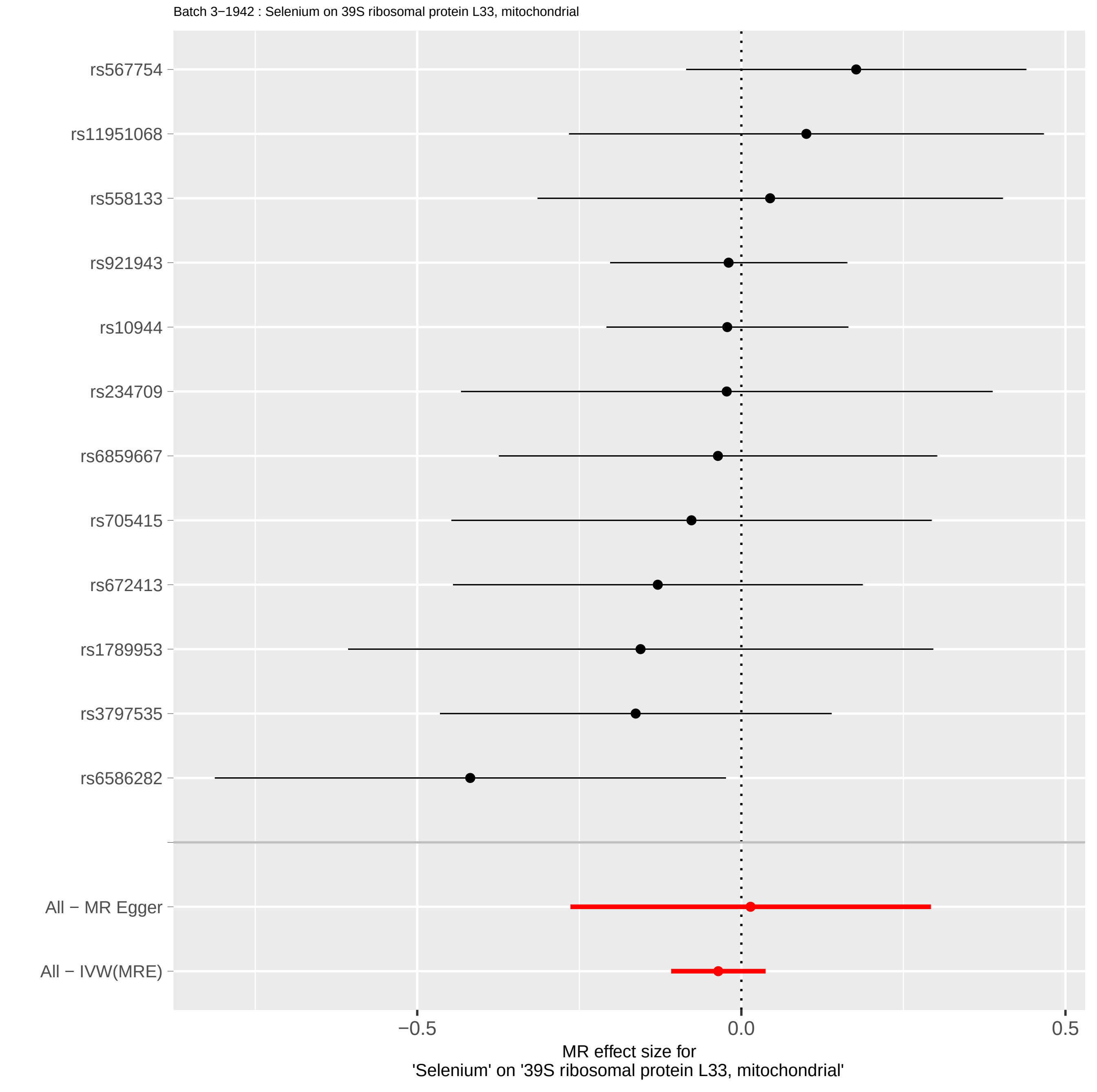

Batch 3−1942 : Selenium on 39S ribosomal protein L33, mitochondrial
rs567754
rs11951068
rs558133
rs921943
rs10944
rs234709
rs6859667
rs705415
rs672413
rs1789953
rs3797535
rs6586282
All − MR Egger
All − IVW(MRE)
−0.5
0.0
0.5
MR effect size for
'Selenium' on '39S ribosomal protein L33, mitochondrial'

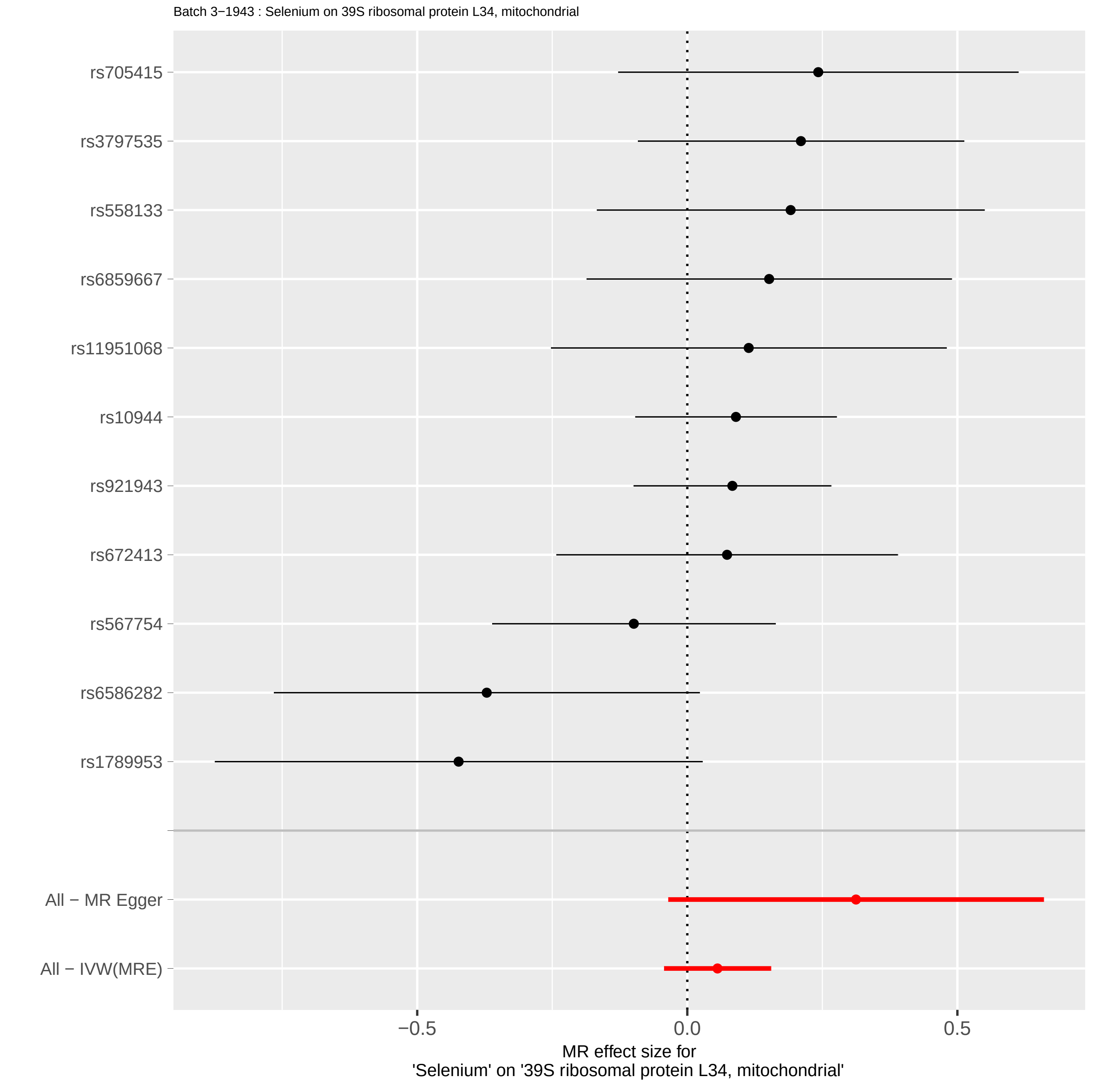
Batch 3−1943 : Selenium on 39S ribosomal protein L34, mitochondrial
rs705415
rs3797535
rs558133
rs6859667
rs11951068
rs10944
rs921943
rs672413
rs567754
rs6586282
rs1789953
All − MR Egger
All − IVW(MRE)
−0.5
0.0
0.5
MR effect size for
'Selenium' on '39S ribosomal protein L34, mitochondrial'

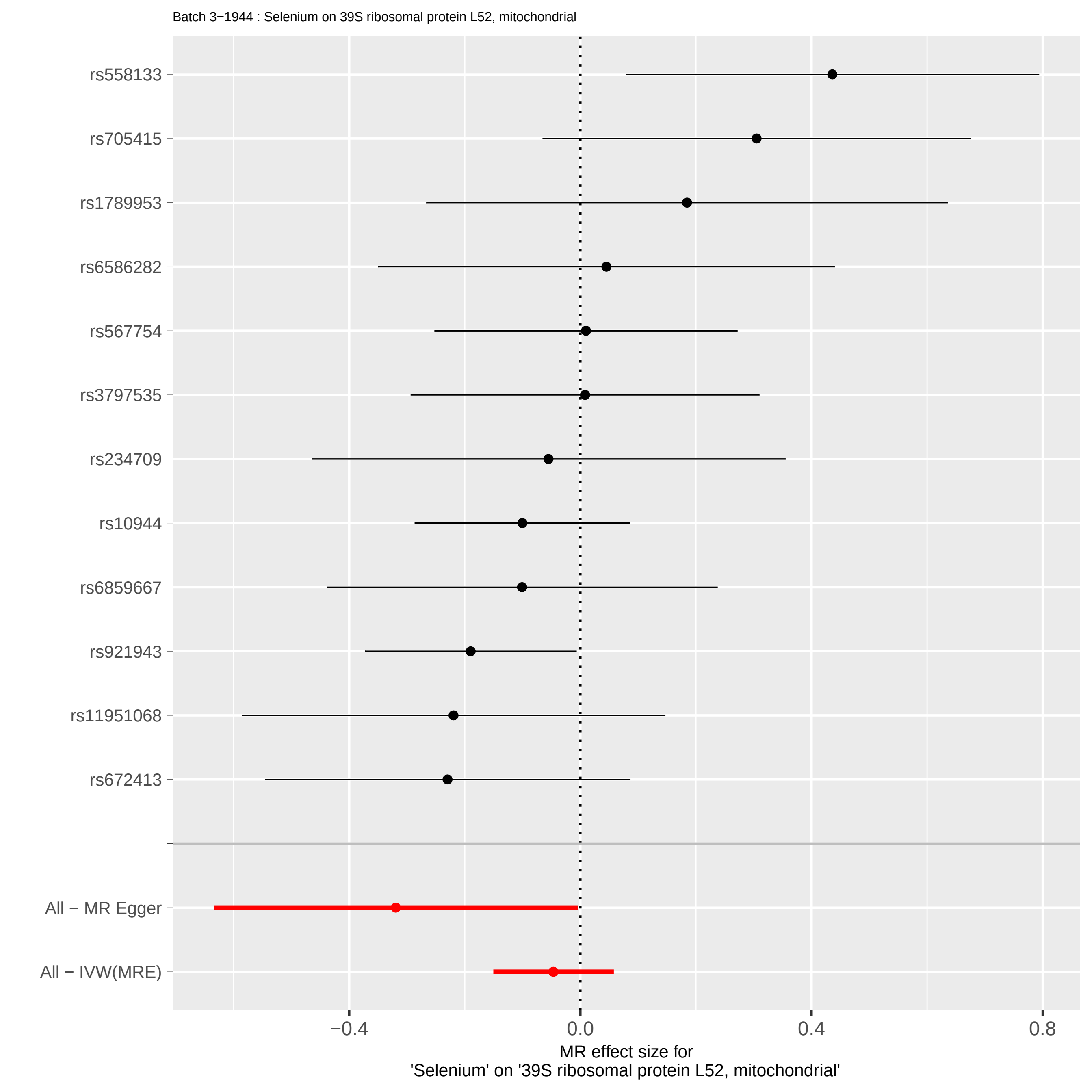
Batch 3−1944 : Selenium on 39S ribosomal protein L52, mitochondrial
rs558133
rs705415
rs1789953
rs6586282
rs567754
rs3797535
rs234709
rs10944
rs6859667
rs921943
rs11951068
rs672413
All − MR Egger
All − IVW(MRE)
−0.4
0.0
0.4
0.8
MR effect size for
'Selenium' on '39S ribosomal protein L52, mitochondrial'

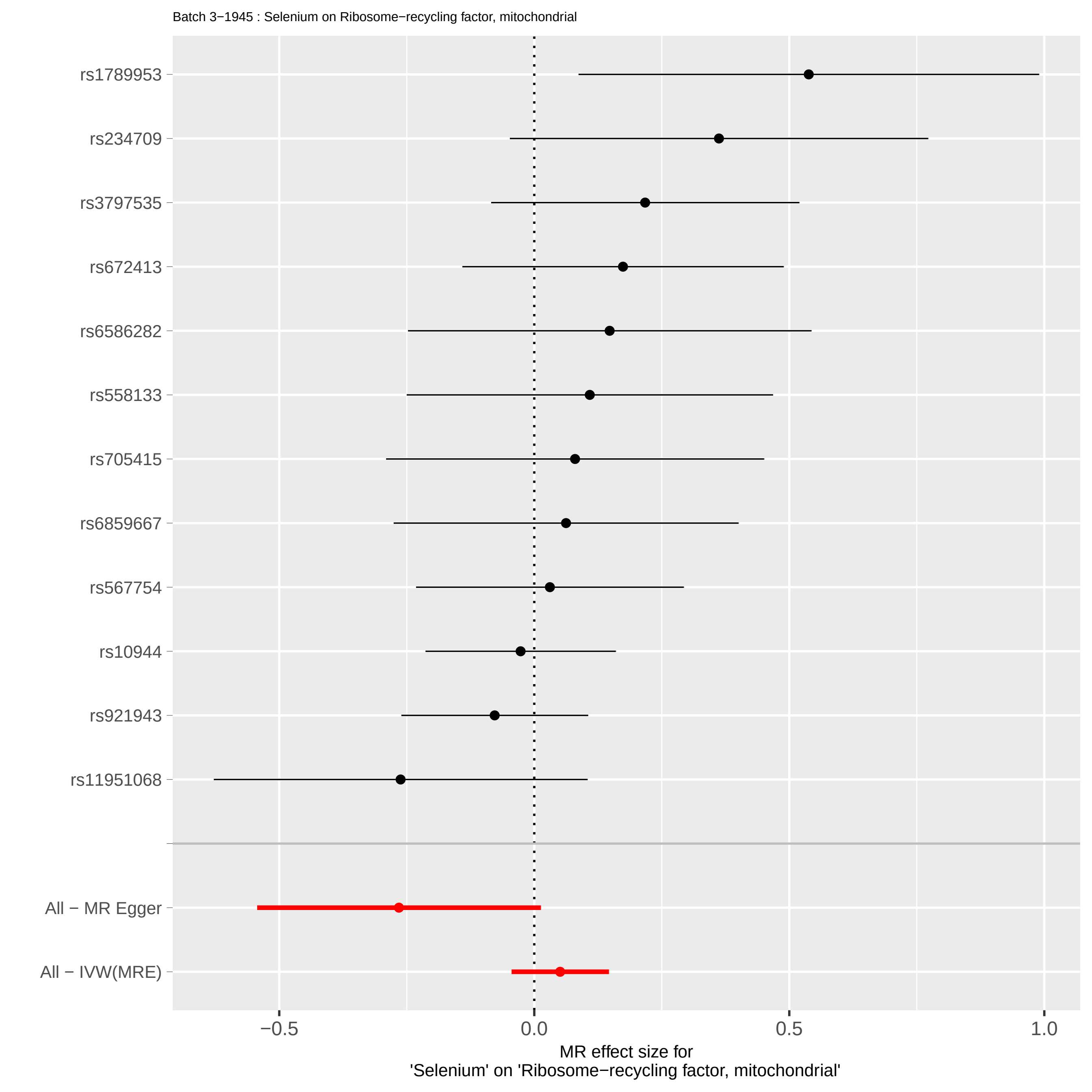

Batch 3−1945 : Selenium on Ribosome−recycling factor, mitochondrial
rs1789953
rs234709
rs3797535
rs672413
rs6586282
rs558133
rs705415
rs6859667
rs567754
rs10944
rs921943
rs11951068
All − MR Egger
All − IVW(MRE)
−0.5
0.0
0.5
1.0
MR effect size for
'Selenium' on 'Ribosome−recycling factor, mitochondrial'

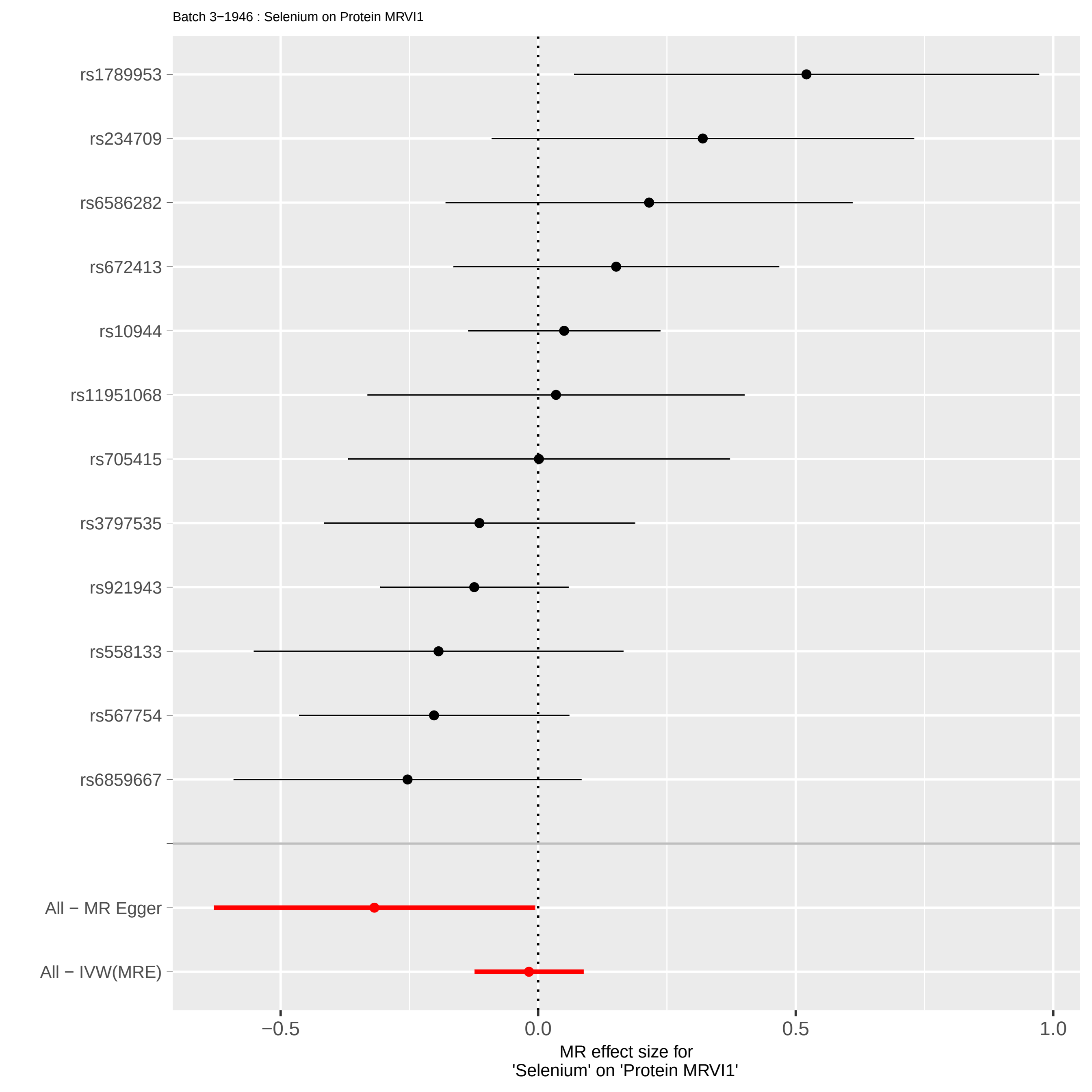

Batch 3−1946 : Selenium on Protein MRVI1
rs1789953
rs234709
rs6586282
rs672413
rs10944
rs11951068
rs705415
rs3797535
rs921943
rs558133
rs567754
rs6859667
All − MR Egger
All − IVW(MRE)
−0.5
0.0
0.5
1.0
MR effect size for
'Selenium' on 'Protein MRVI1'

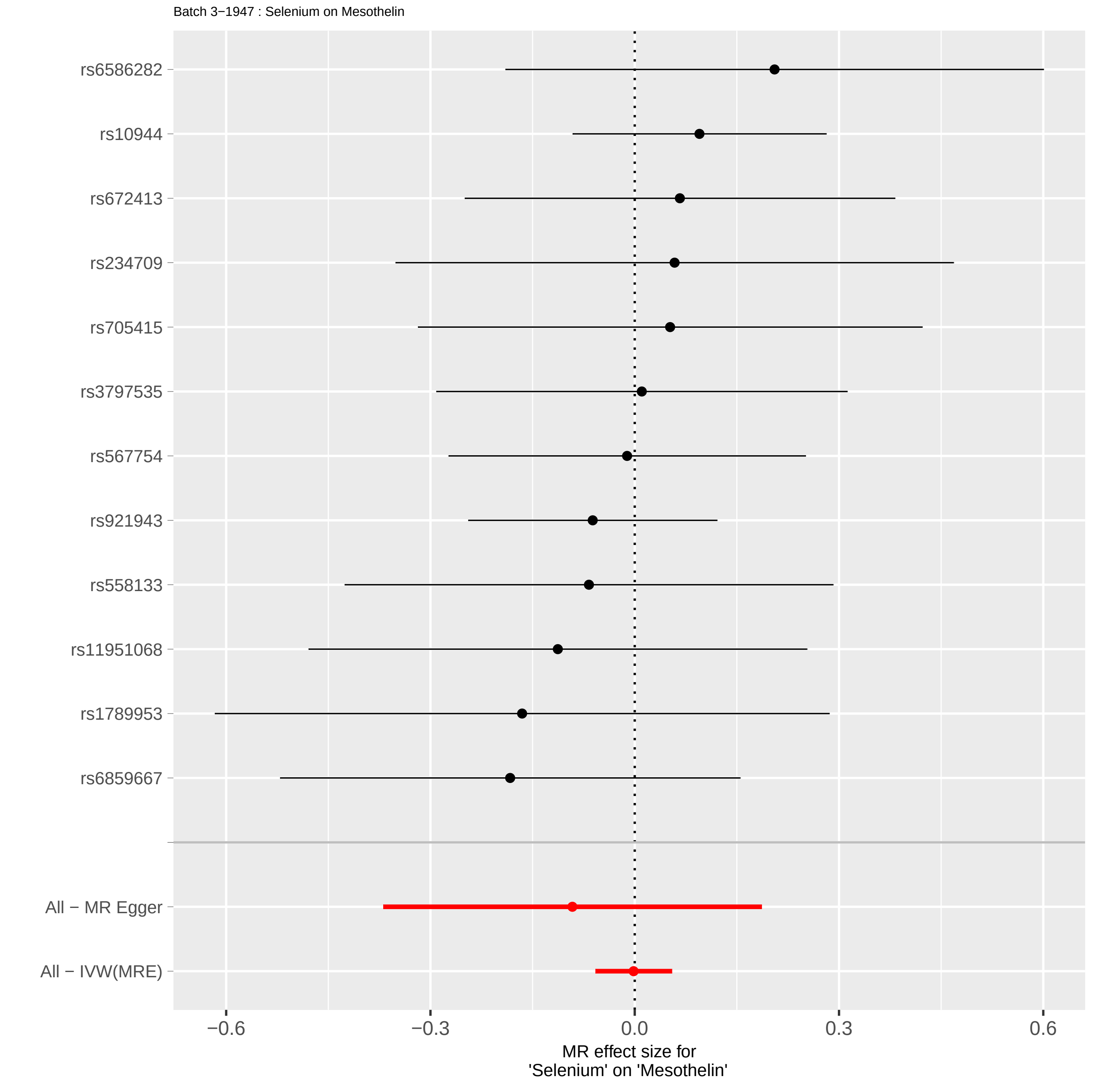
Batch 3−1947 : Selenium on Mesothelin
rs6586282
rs10944
rs672413
rs234709
rs705415
rs3797535
rs567754
rs921943
rs558133
rs11951068
rs1789953
rs6859667
All − MR Egger
All − IVW(MRE)
−0.6
−0.3
0.0
0.3
0.6
MR effect size for
'Selenium' on 'Mesothelin'

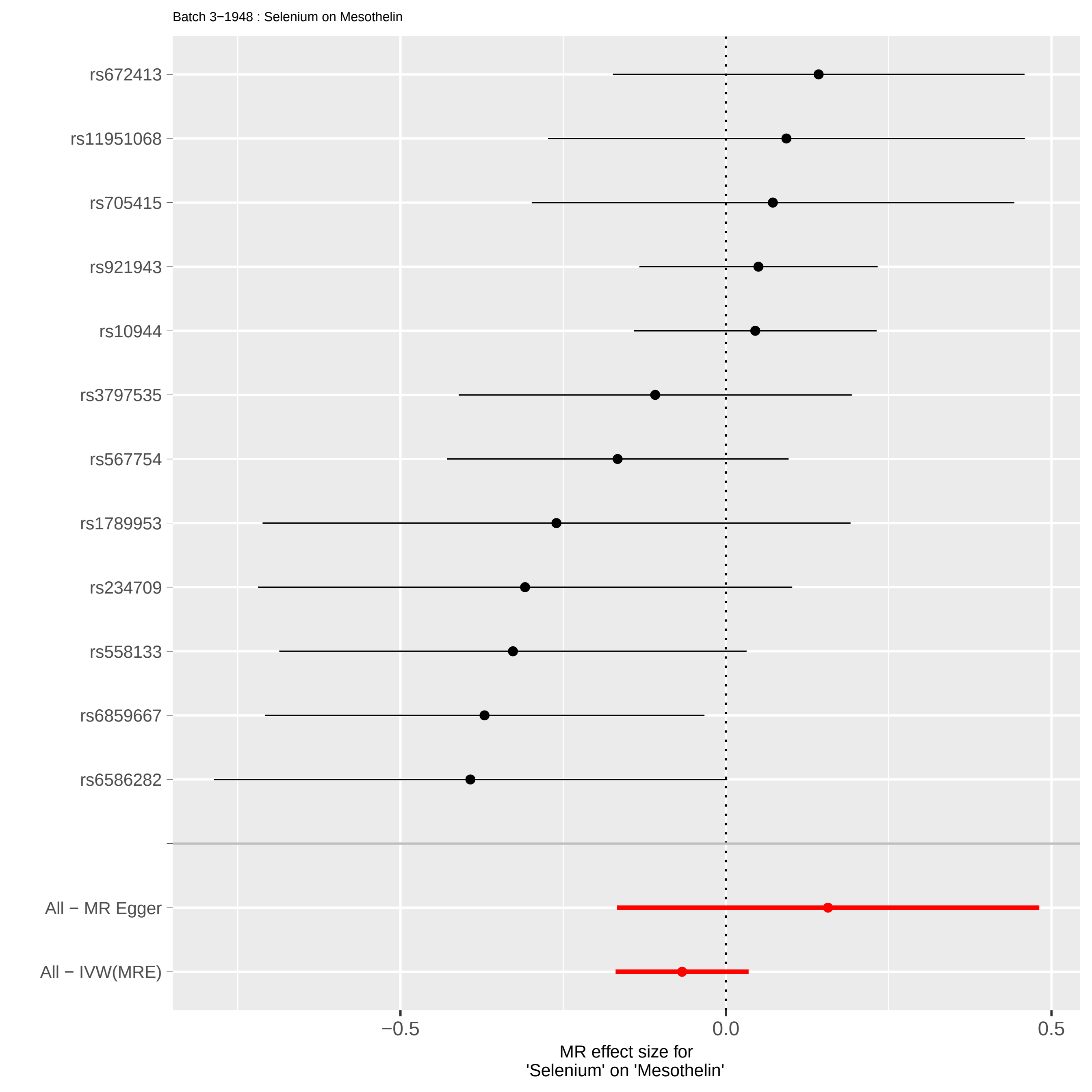

Batch 3−1948 : Selenium on Mesothelin
rs672413
rs11951068
rs705415
rs921943
rs10944
rs3797535
rs567754
rs1789953
rs234709
rs558133
rs6859667
rs6586282
All − MR Egger
All − IVW(MRE)
−0.5
0.0
0.5
MR effect size for
'Selenium' on 'Mesothelin'

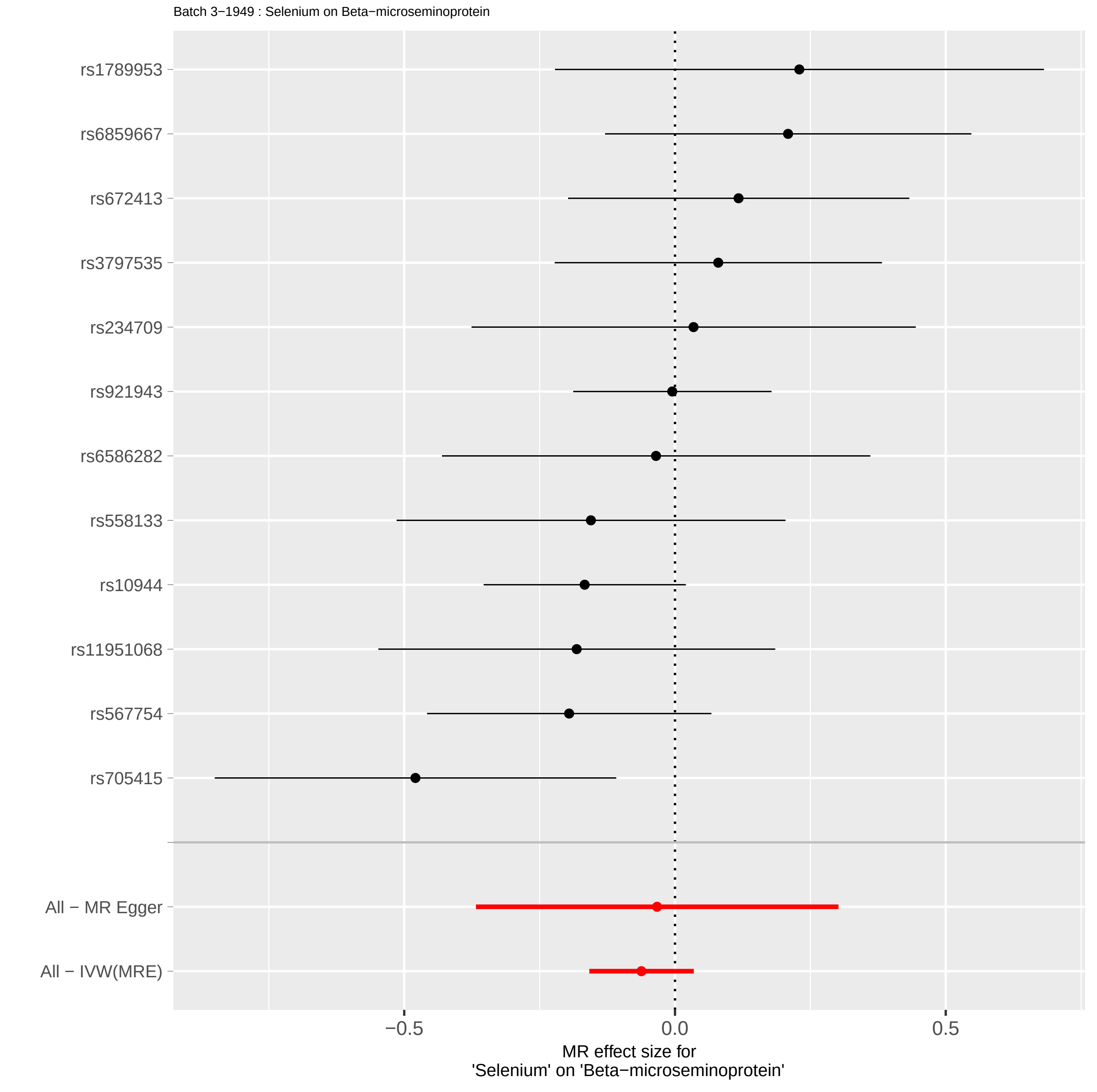

Batch 3−1949 : Selenium on Beta−microseminoprotein
rs1789953
rs6859667
rs672413
rs3797535
rs234709
rs921943
rs6586282
rs558133
rs10944
rs11951068
rs567754
rs705415
All − MR Egger
All − IVW(MRE)
−0.5
0.0
0.5
MR effect size for
'Selenium' on 'Beta−microseminoprotein'

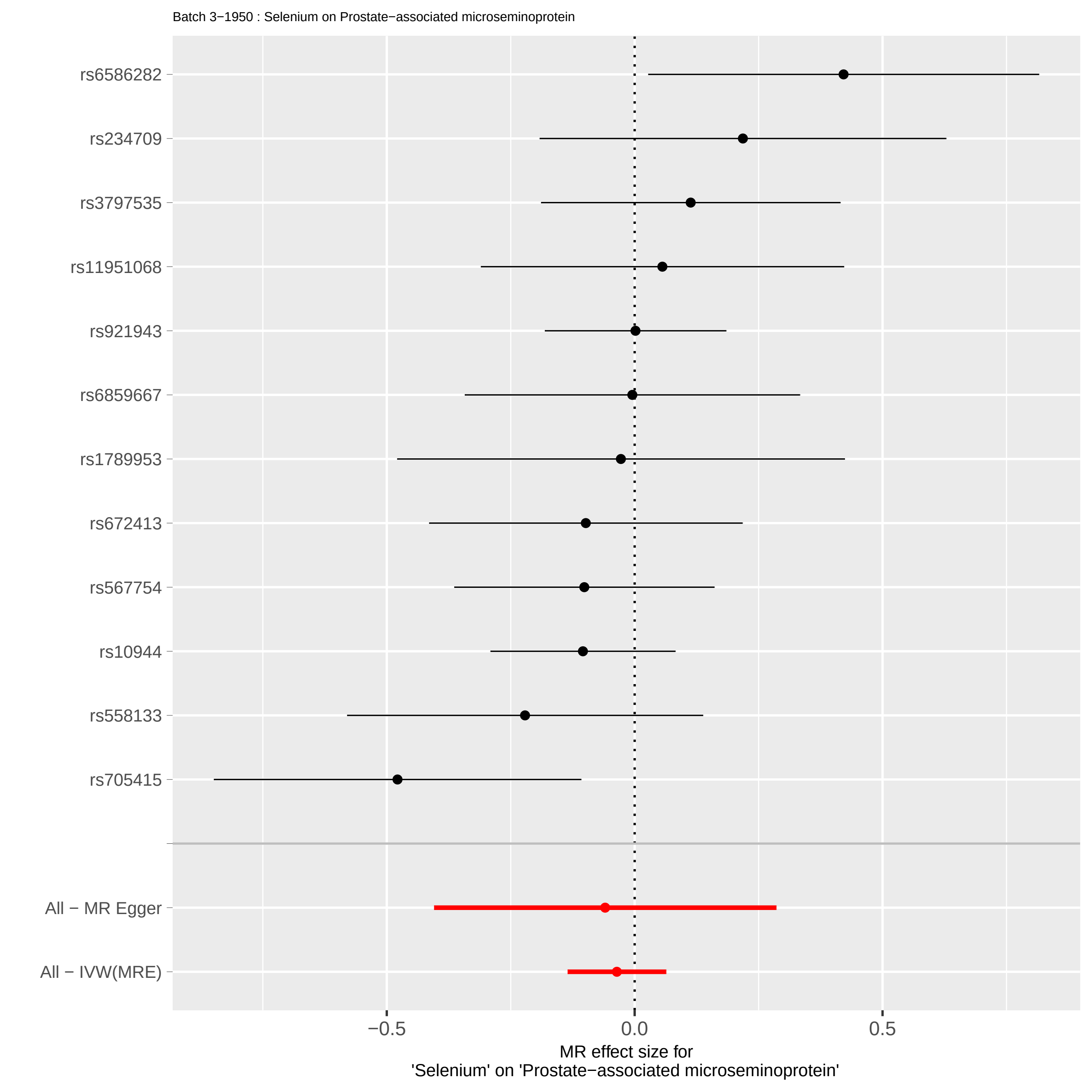

Batch 3−1950 : Selenium on Prostate−associated microseminoprotein
rs6586282
rs234709
rs3797535
rs11951068
rs921943
rs6859667
rs1789953
rs672413
rs567754
rs10944
rs558133
rs705415
All − MR Egger
All − IVW(MRE)
−0.5
0.0
0.5
MR effect size for
'Selenium' on 'Prostate−associated microseminoprotein'

Batch 3−1951 : Selenium on Macrophage scavenger receptor types I and II

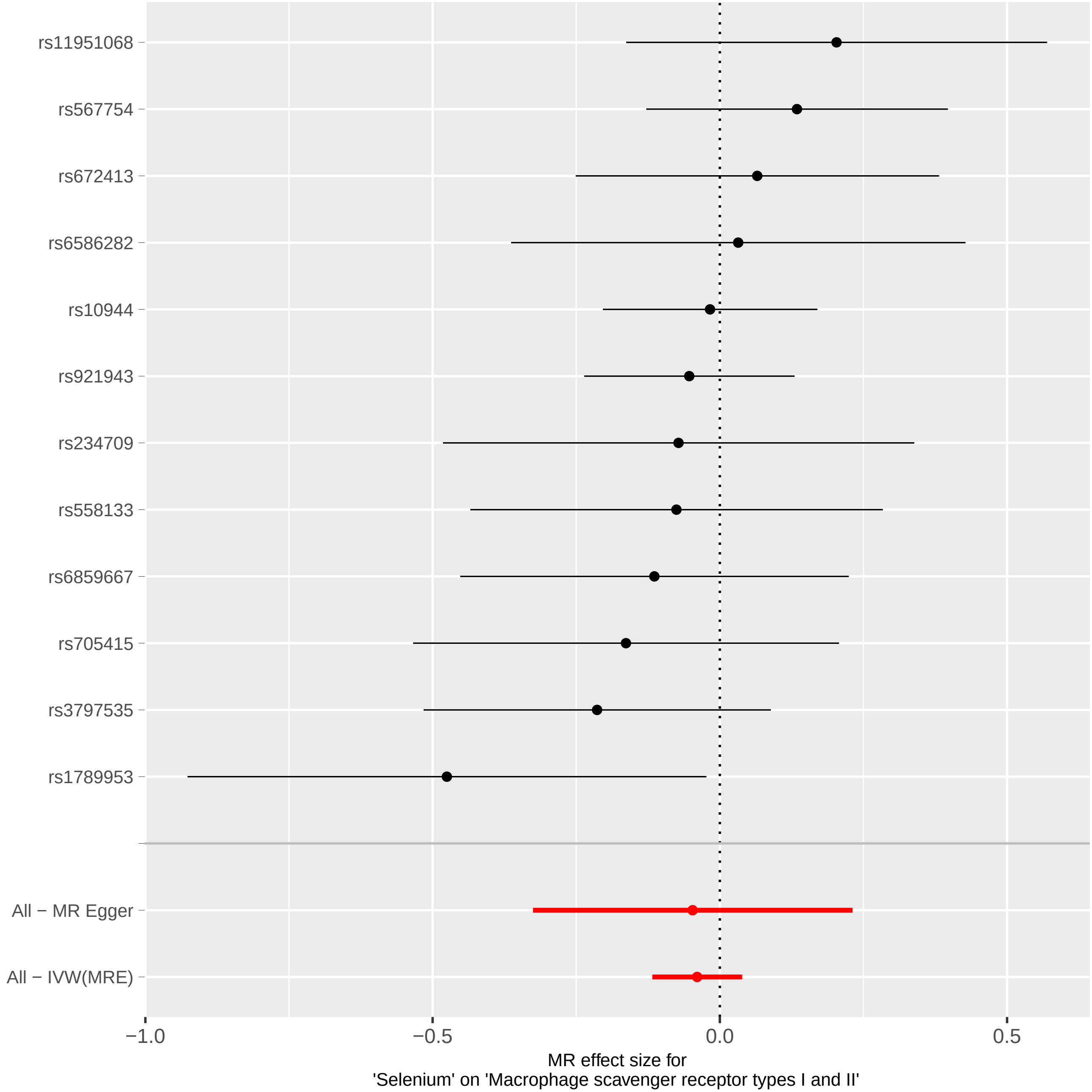

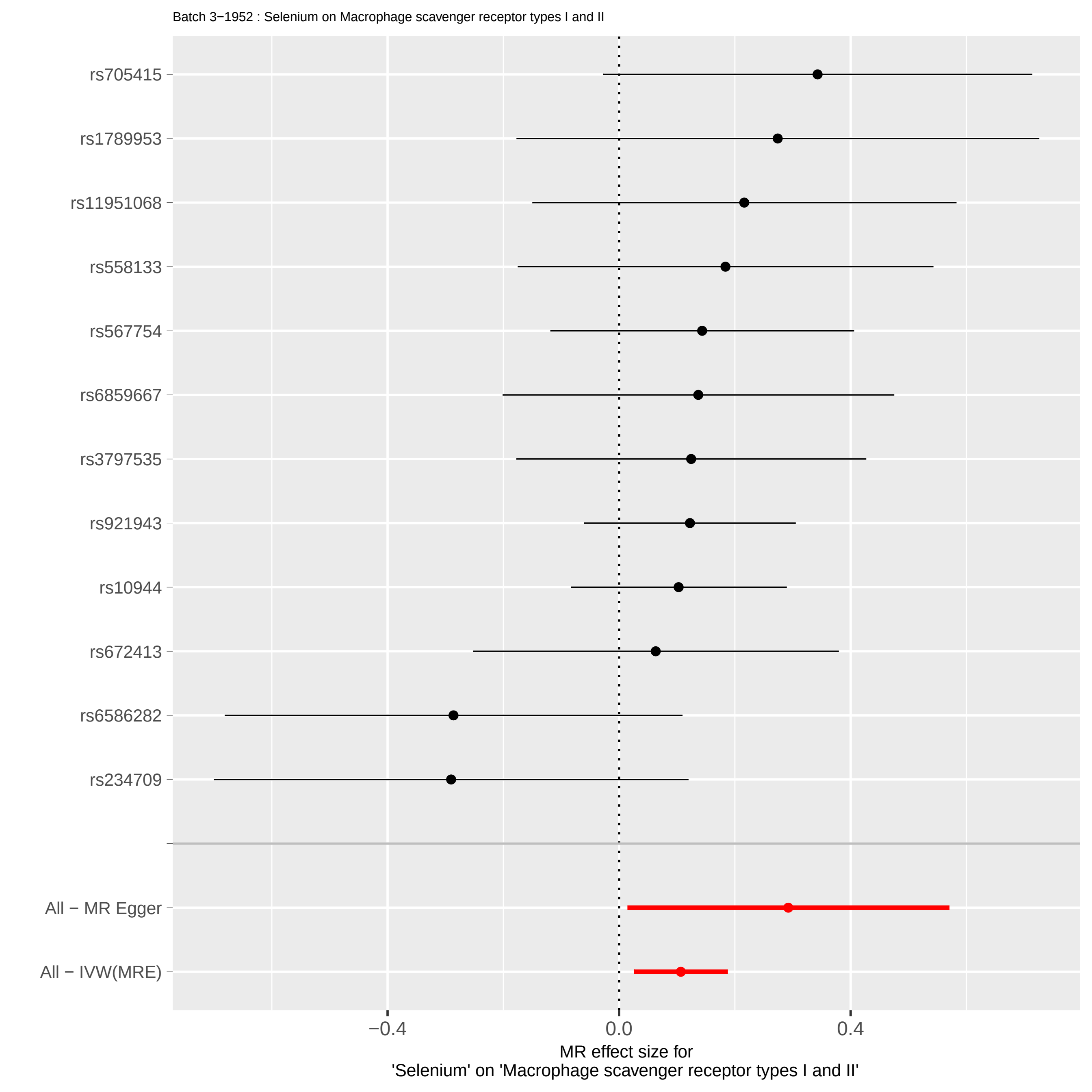

Batch 3−1952 : Selenium on Macrophage scavenger receptor types I and II
rs705415
rs1789953
rs11951068
rs558133
rs567754
rs6859667
rs3797535
rs921943
rs10944
rs672413
rs6586282
rs234709
All − MR Egger
All − IVW(MRE)
−0.4
0.0
0.4
MR effect size for
'Selenium' on 'Macrophage scavenger receptor types I and II'

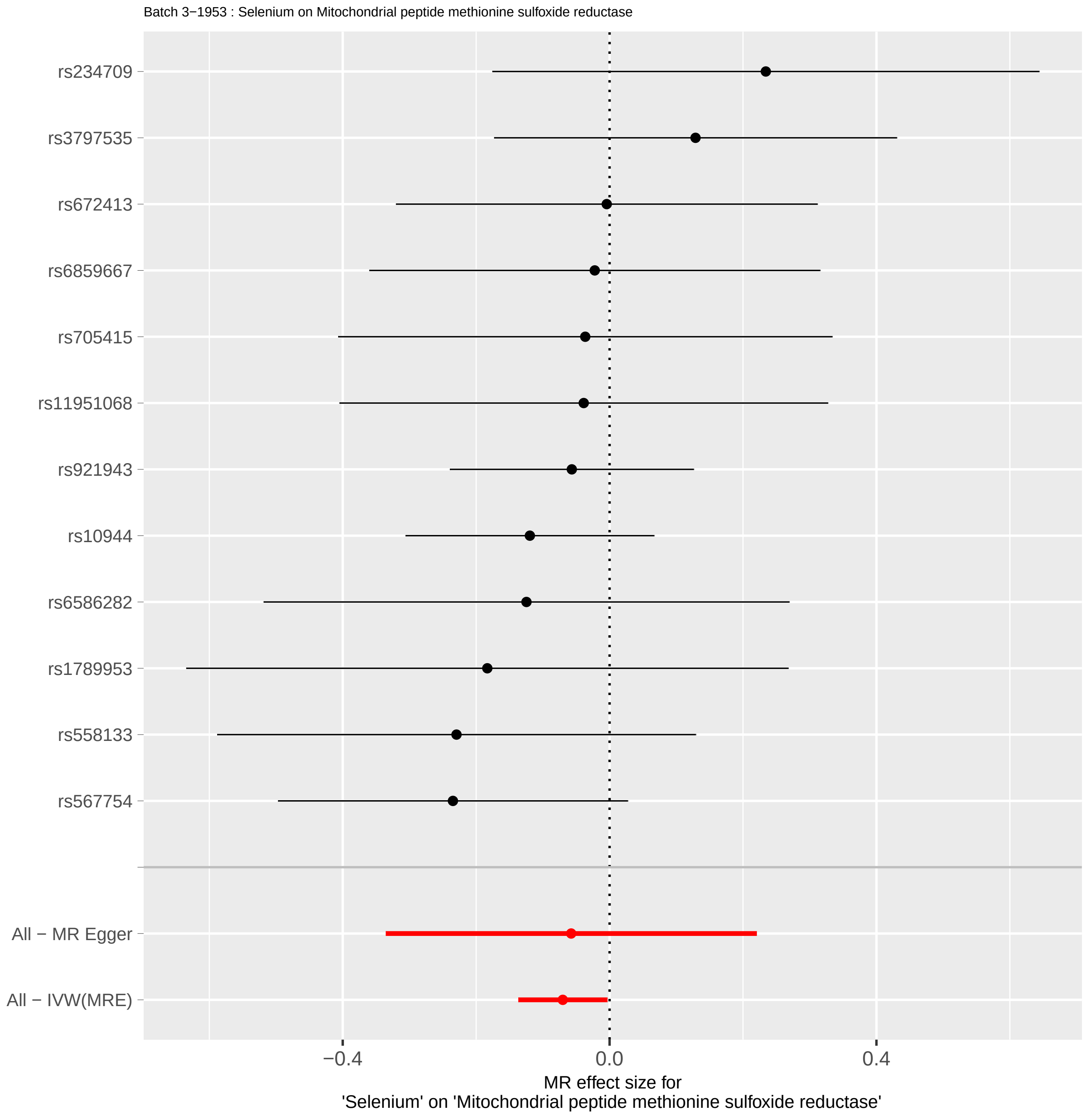

Batch 3−1953 : Selenium on Mitochondrial peptide methionine sulfoxide reductase
rs234709
rs3797535
rs672413
rs6859667
rs705415
rs11951068
rs921943
rs10944
rs6586282
rs1789953
rs558133
rs567754
All − MR Egger
All − IVW(MRE)
−0.4
0.0
0.4
MR effect size for
'Selenium' on 'Mitochondrial peptide methionine sulfoxide reductase'

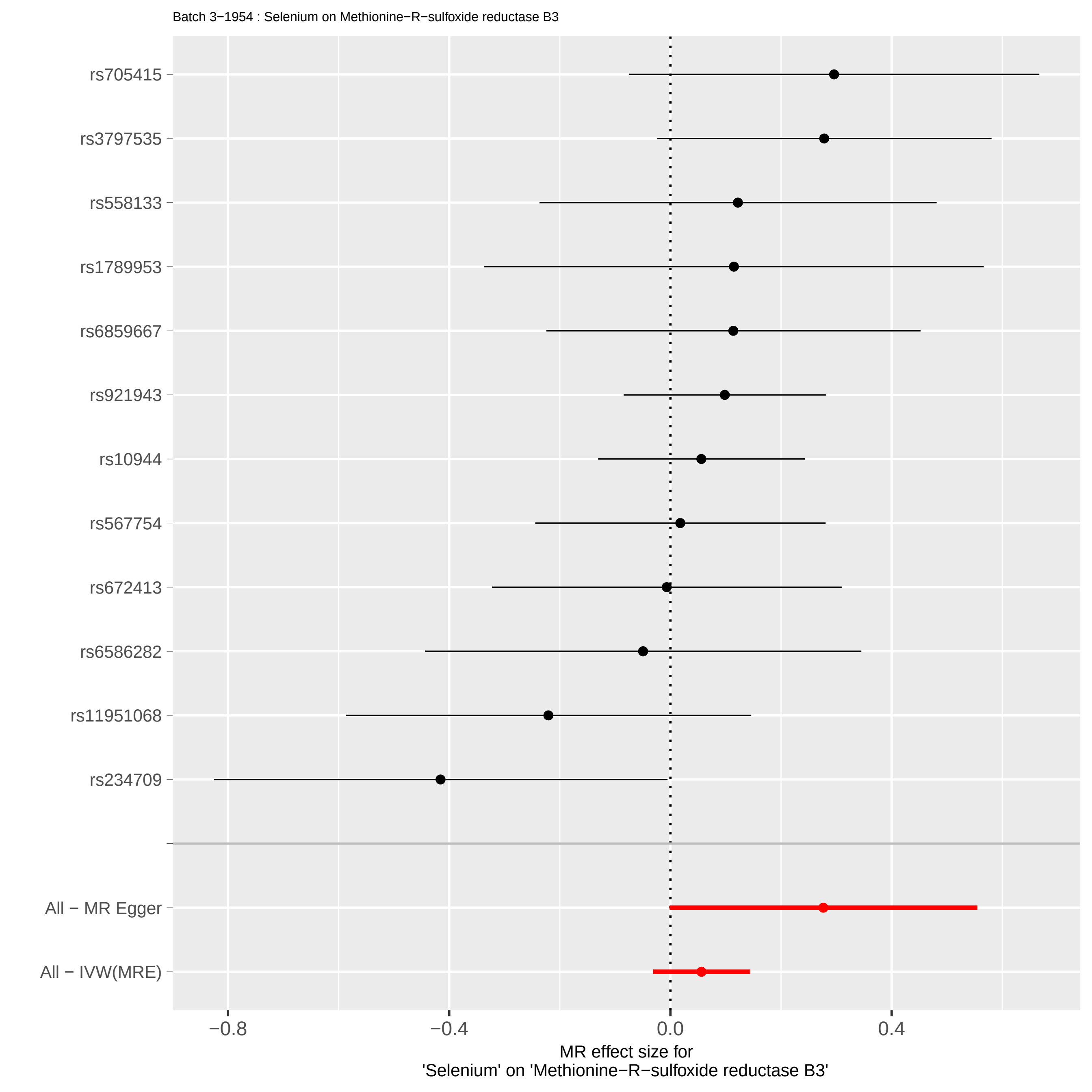

Batch 3−1954 : Selenium on Methionine−R−sulfoxide reductase B3
rs705415
rs3797535
rs558133
rs1789953
rs6859667
rs921943
rs10944
rs567754
rs672413
rs6586282
rs11951068
rs234709
All − MR Egger
All − IVW(MRE)
−0.8
−0.4
0.0
0.4
MR effect size for
'Selenium' on 'Methionine−R−sulfoxide reductase B3'

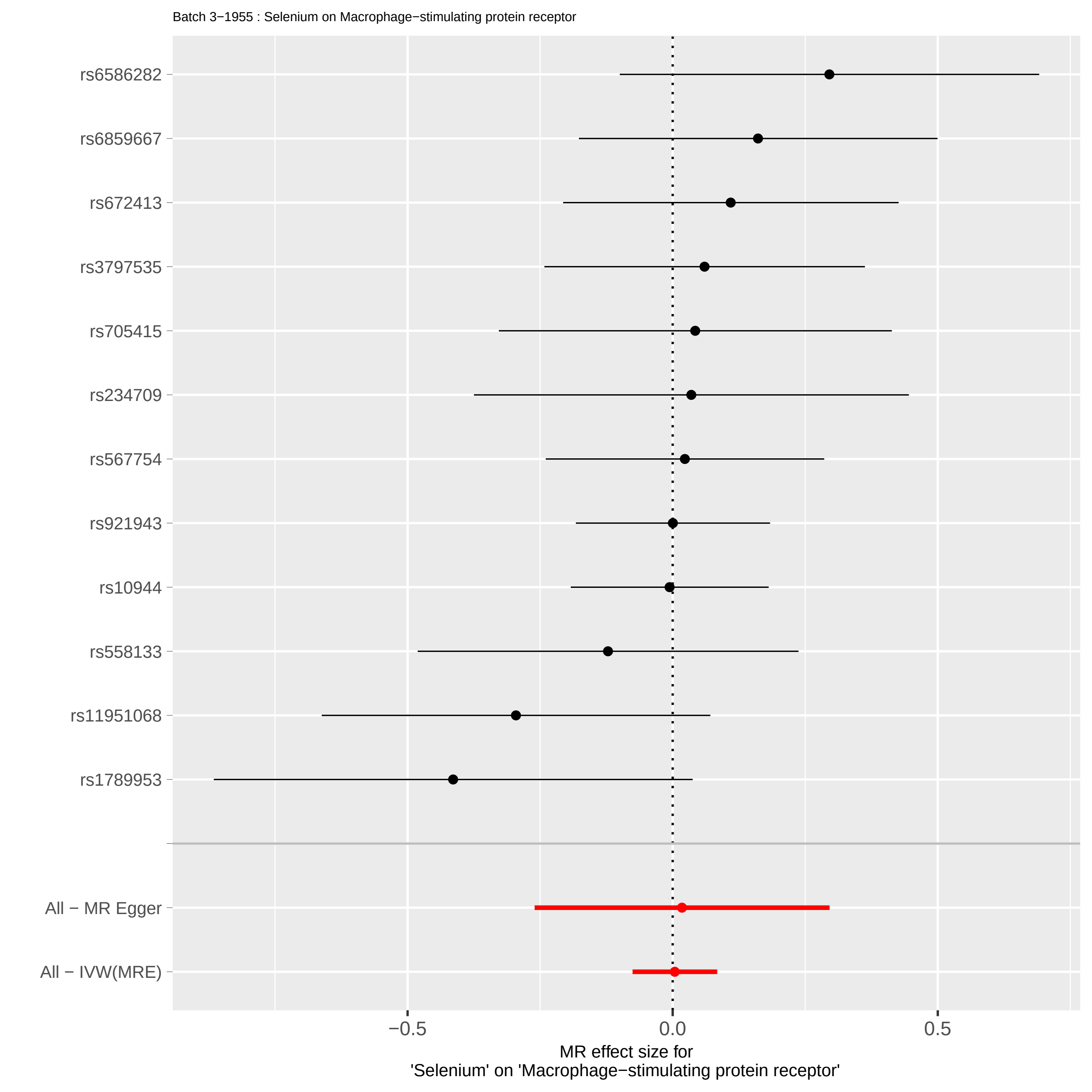
Batch 3−1955 : Selenium on Macrophage−stimulating protein receptor
rs6586282
rs6859667
rs672413
rs3797535
rs705415
rs234709
rs567754
rs921943
rs10944
rs558133
rs11951068
rs1789953
All − MR Egger
All − IVW(MRE)
−0.5
0.0
0.5
MR effect size for
'Selenium' on 'Macrophage−stimulating protein receptor'

Batch 3−1956 : Selenium on Growth/differentiation factor 8

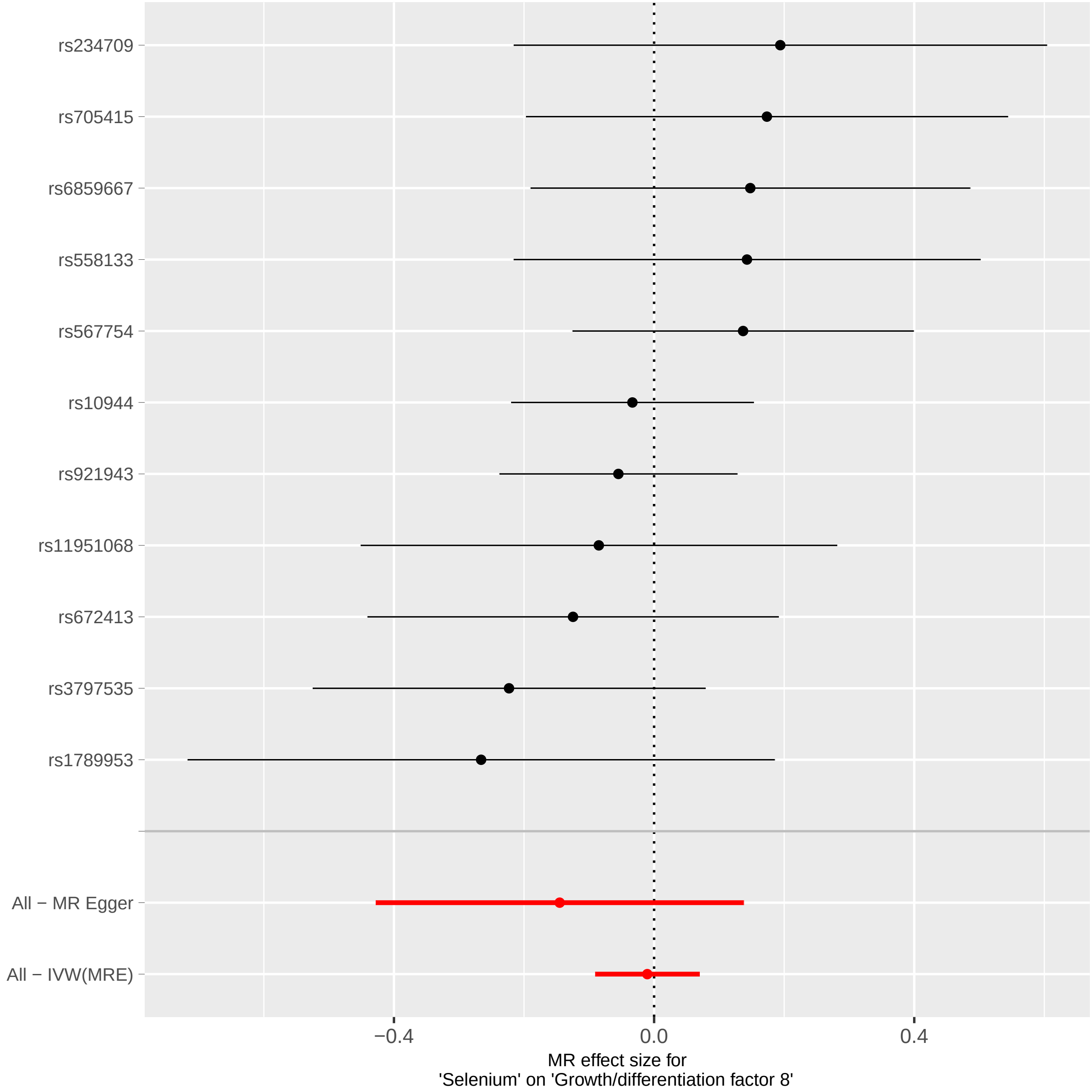

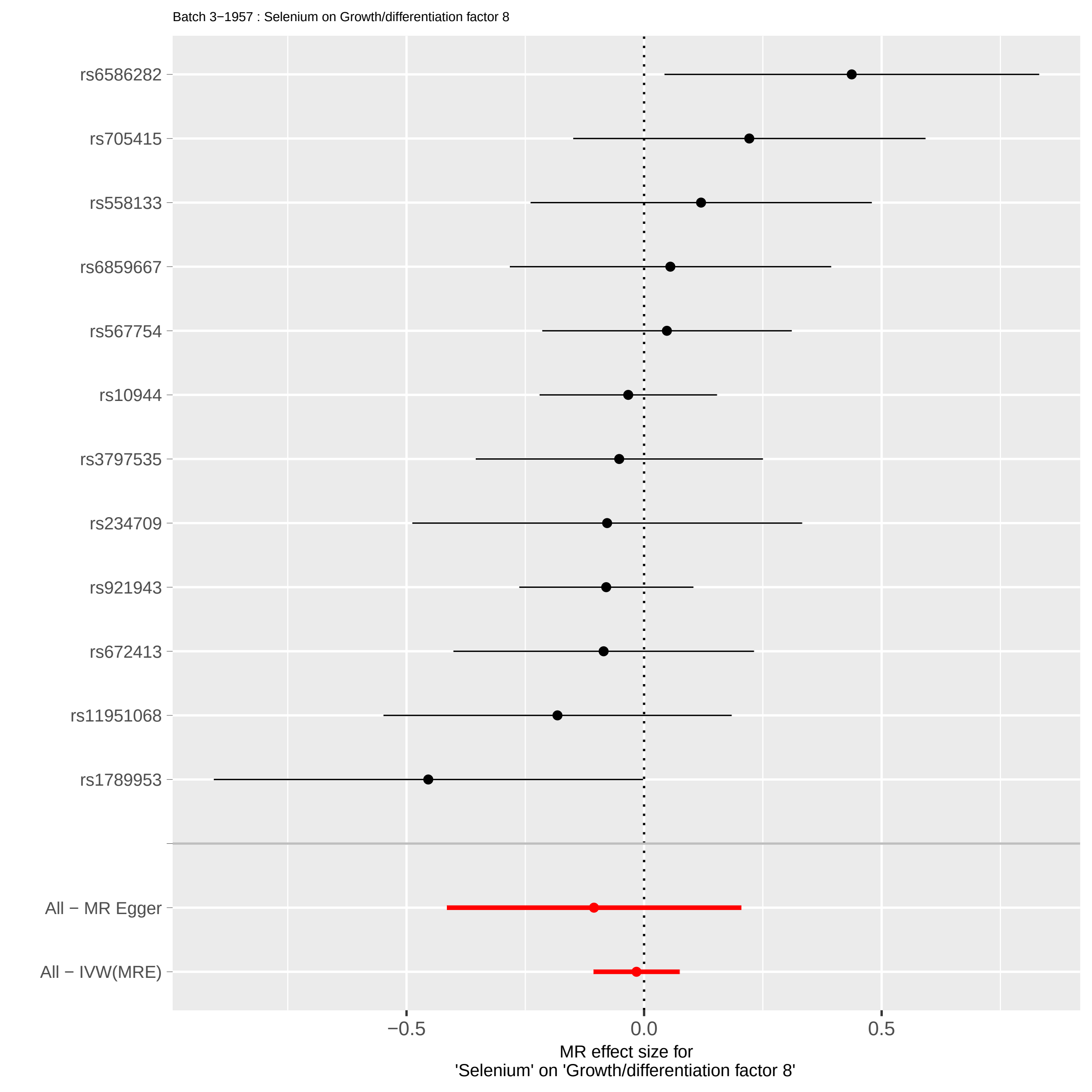

Batch 3−1957 : Selenium on Growth/differentiation factor 8
rs6586282
rs705415
rs558133
rs6859667
rs567754
rs10944
rs3797535
rs234709
rs921943
rs672413
rs11951068
rs1789953
All − MR Egger
All − IVW(MRE)
−0.5
0.0
0.5
MR effect size for
'Selenium' on 'Growth/differentiation factor 8'

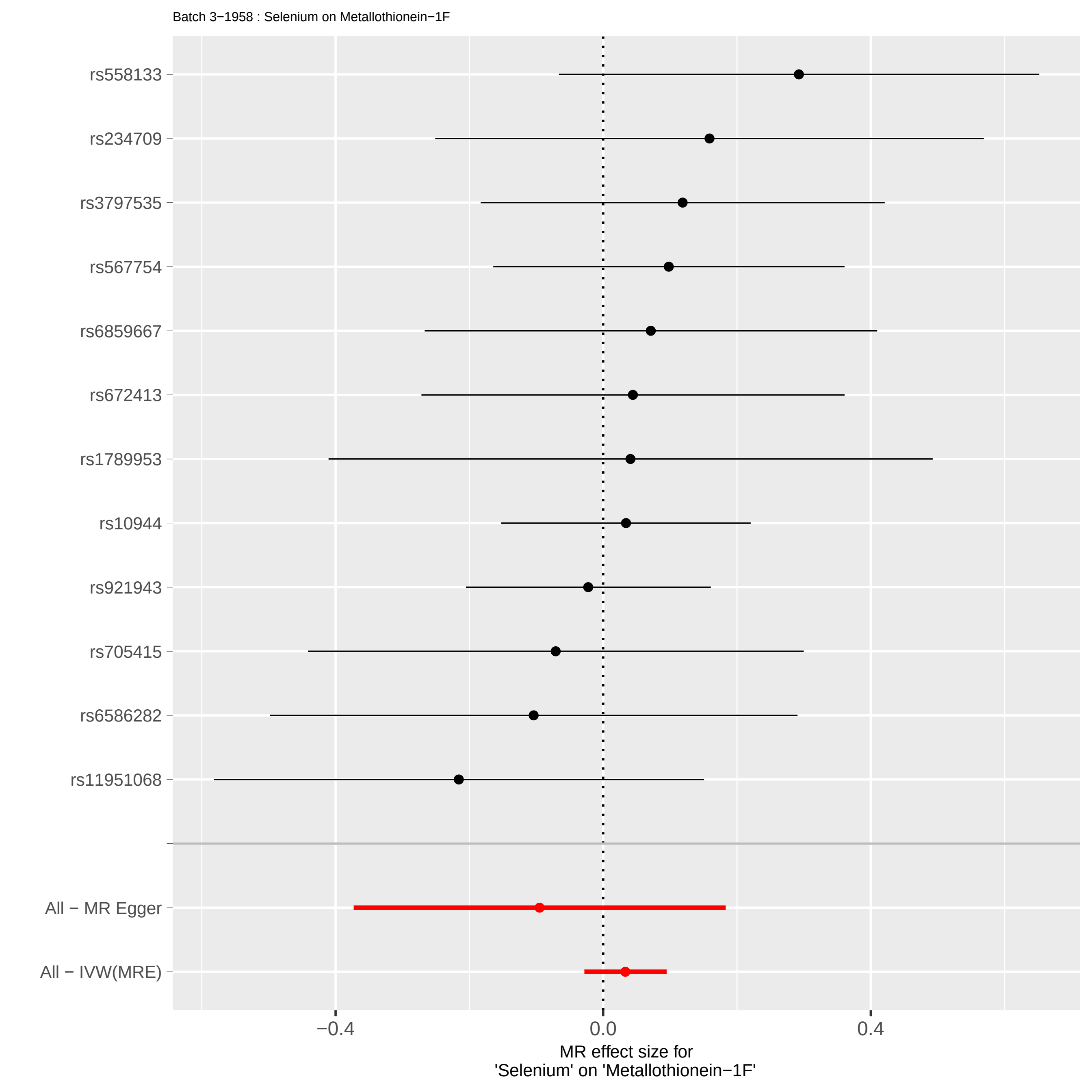

Batch 3−1958 : Selenium on Metallothionein−1F
rs558133
rs234709
rs3797535
rs567754
rs6859667
rs672413
rs1789953
rs10944
rs921943
rs705415
rs6586282
rs11951068
All − MR Egger
All − IVW(MRE)
−0.4
0.0
0.4
MR effect size for
'Selenium' on 'Metallothionein−1F'

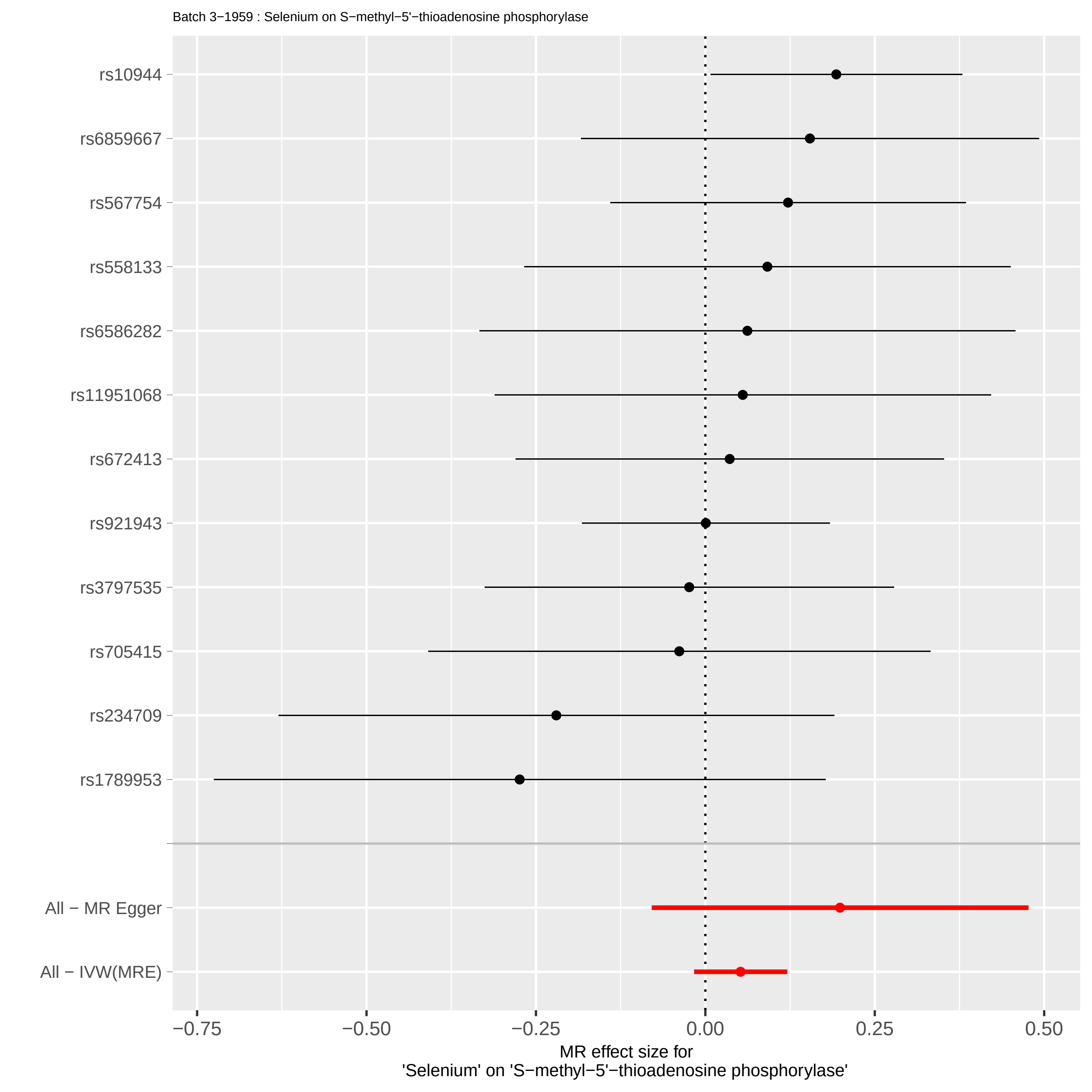

Batch 3−1959 : Selenium on S−methyl−5'−thioadenosine phosphorylase
rs10944
rs6859667
rs567754
rs558133
rs6586282
rs11951068
rs672413
rs921943
rs3797535
rs705415
rs234709
rs1789953
All − MR Egger
All − IVW(MRE)
−0.75
−0.50
−0.25
0.00
0.25
0.50
MR effect size for
'Selenium' on 'S−methyl−5'−thioadenosine phosphorylase'

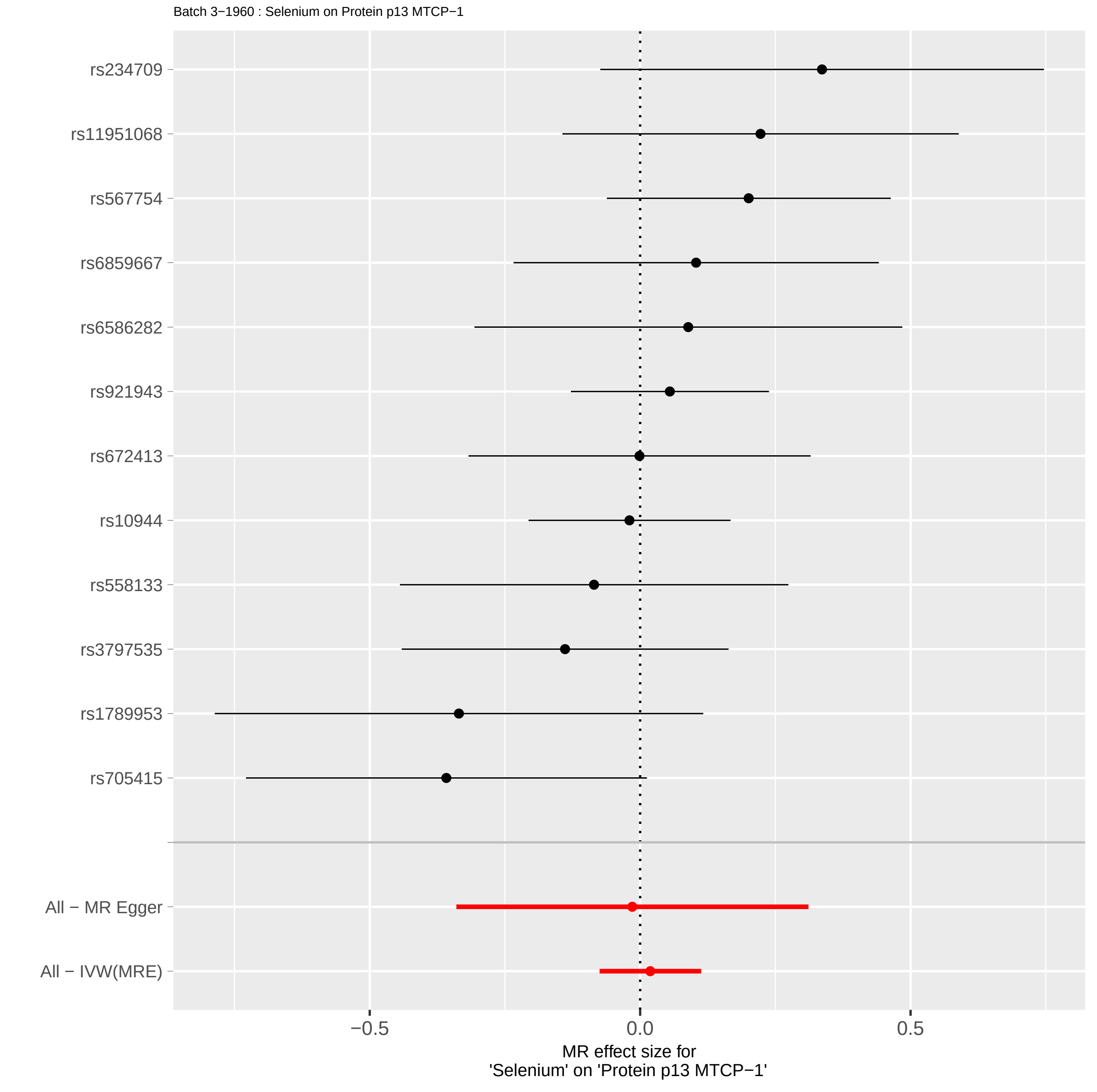

Batch 3−1960 : Selenium on Protein p13 MTCP−1
rs234709
rs11951068
rs567754
rs6859667
rs6586282
rs921943
rs672413
rs10944
rs558133
rs3797535
rs1789953
rs705415
All − MR Egger
All − IVW(MRE)
−0.5
0.0
0.5
MR effect size for
'Selenium' on 'Protein p13 MTCP−1'

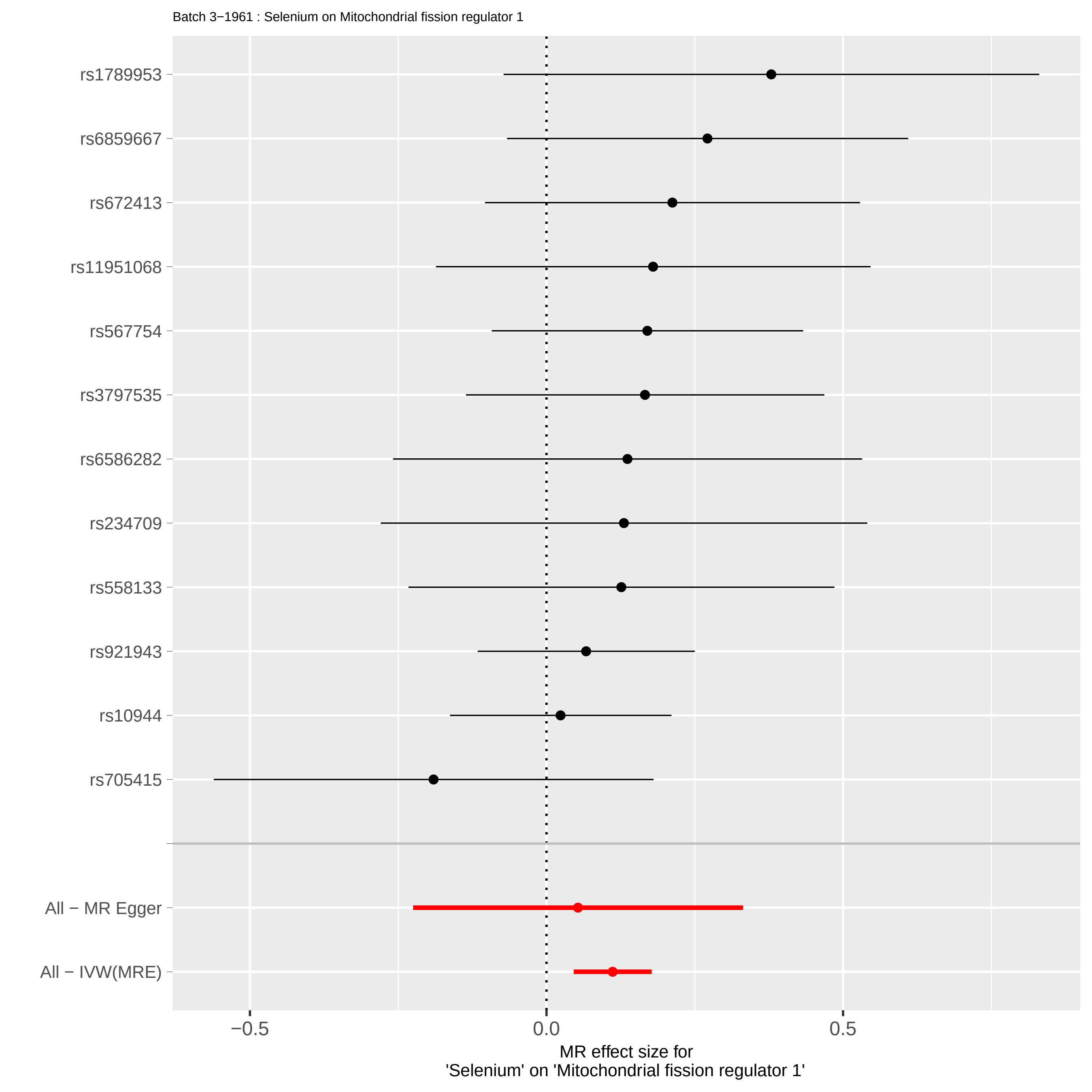
Batch 3−1961 : Selenium on Mitochondrial fission regulator 1
rs1789953
rs6859667
rs672413
rs11951068
rs567754
rs3797535
rs6586282
rs234709
rs558133
rs921943
rs10944
rs705415
All − MR Egger
All − IVW(MRE)
−0.5
0.0
0.5
MR effect size for
'Selenium' on 'Mitochondrial fission regulator 1'

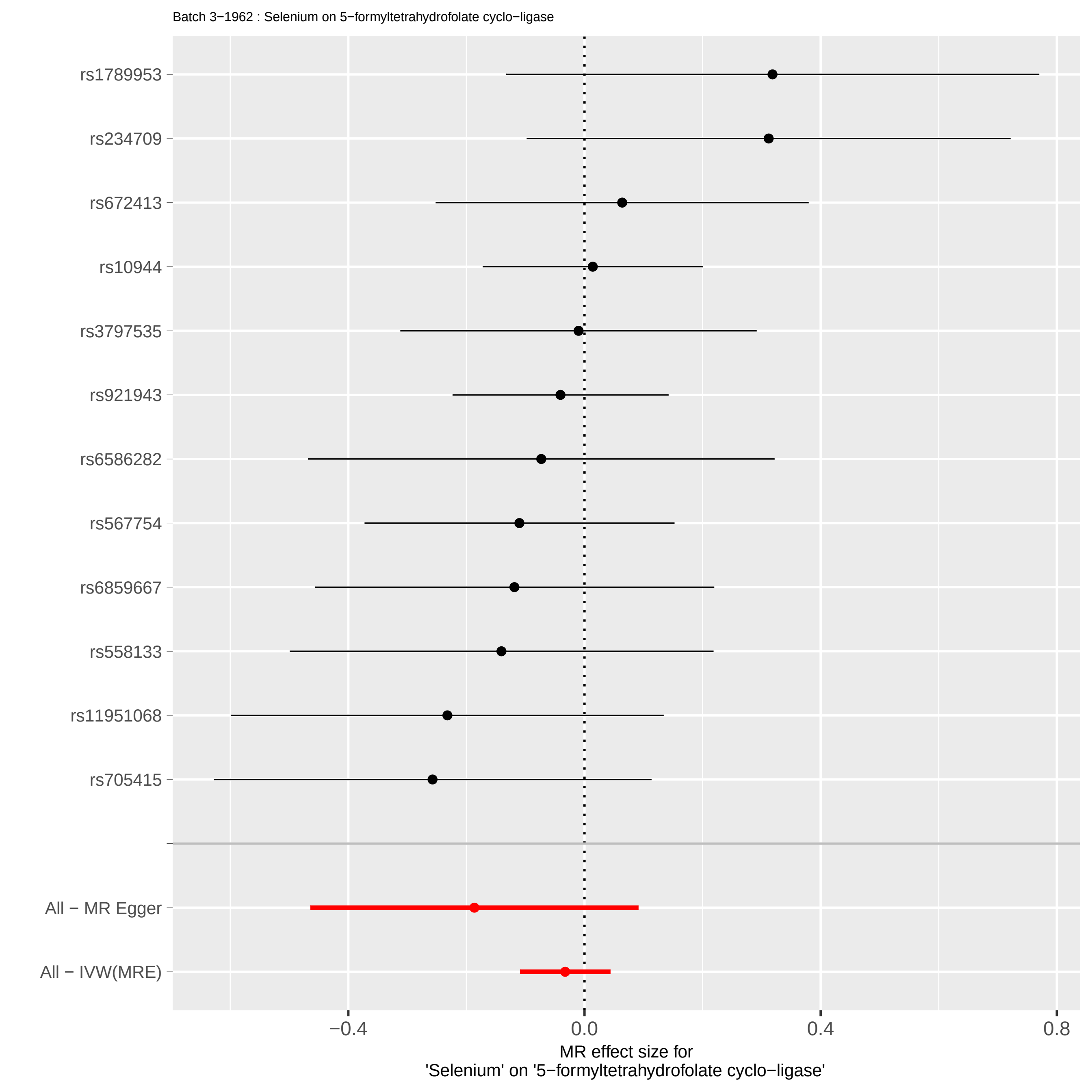
Batch 3−1962 : Selenium on 5−formyltetrahydrofolate cyclo−ligase
rs1789953
rs234709
rs672413
rs10944
rs3797535
rs921943
rs6586282
rs567754
rs6859667
rs558133
rs11951068
rs705415
All − MR Egger
All − IVW(MRE)
−0.4
0.0
0.4
0.8
MR effect size for
'Selenium' on '5−formyltetrahydrofolate cyclo−ligase'

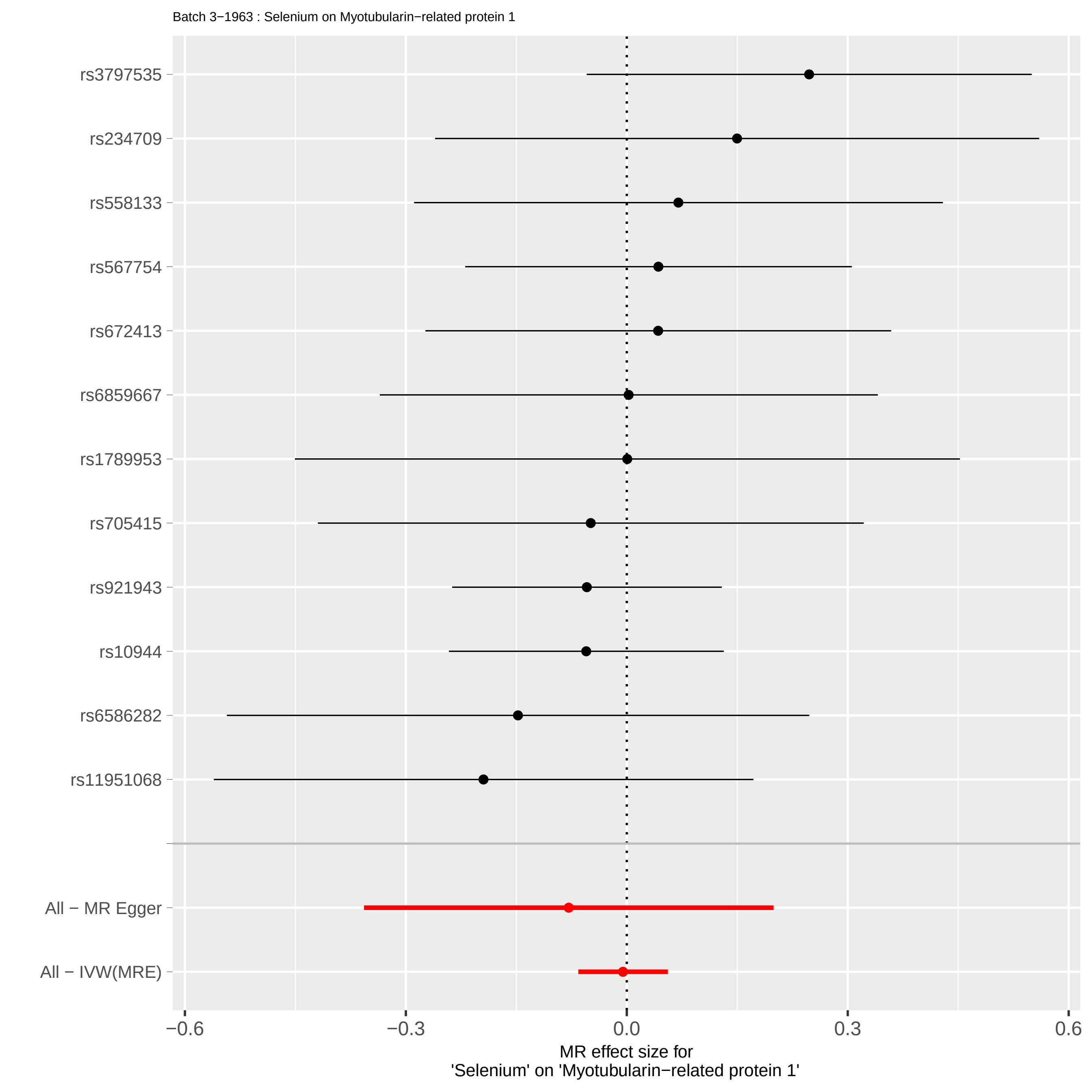

Batch 3−1963 : Selenium on Myotubularin−related protein 1
rs3797535
rs234709
rs558133
rs567754
rs672413
rs6859667
rs1789953
rs705415
rs921943
rs10944
rs6586282
rs11951068
All − MR Egger
All − IVW(MRE)
−0.6
−0.3
0.0
0.3
0.6
MR effect size for
'Selenium' on 'Myotubularin−related protein 1'

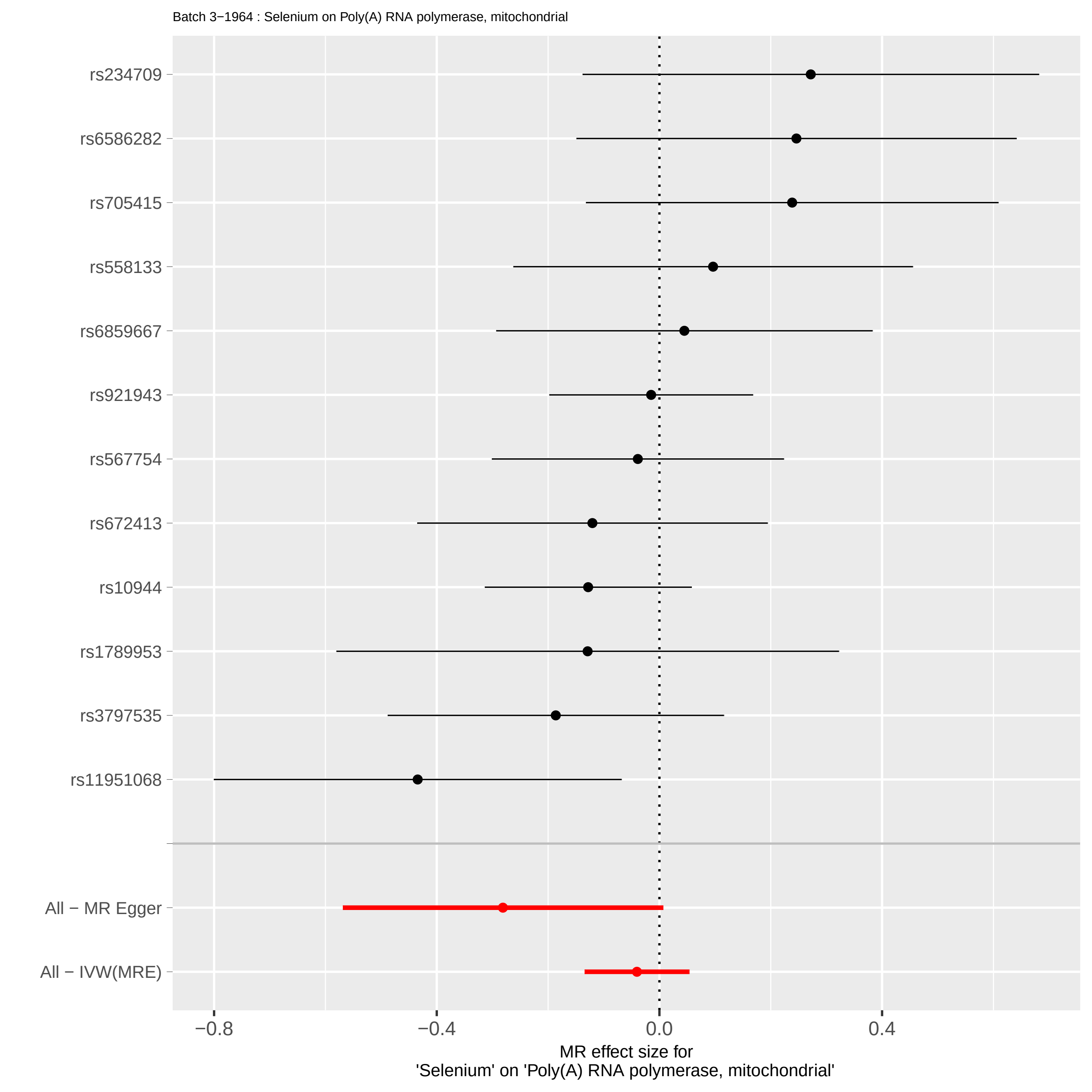

Batch 3−1964 : Selenium on Poly(A) RNA polymerase, mitochondrial
rs234709
rs6586282
rs705415
rs558133
rs6859667
rs921943
rs567754
rs672413
rs10944
rs1789953
rs3797535
rs11951068
All − MR Egger
All − IVW(MRE)
−0.8
−0.4
0.0
0.4
MR effect size for
'Selenium' on 'Poly(A) RNA polymerase, mitochondrial'

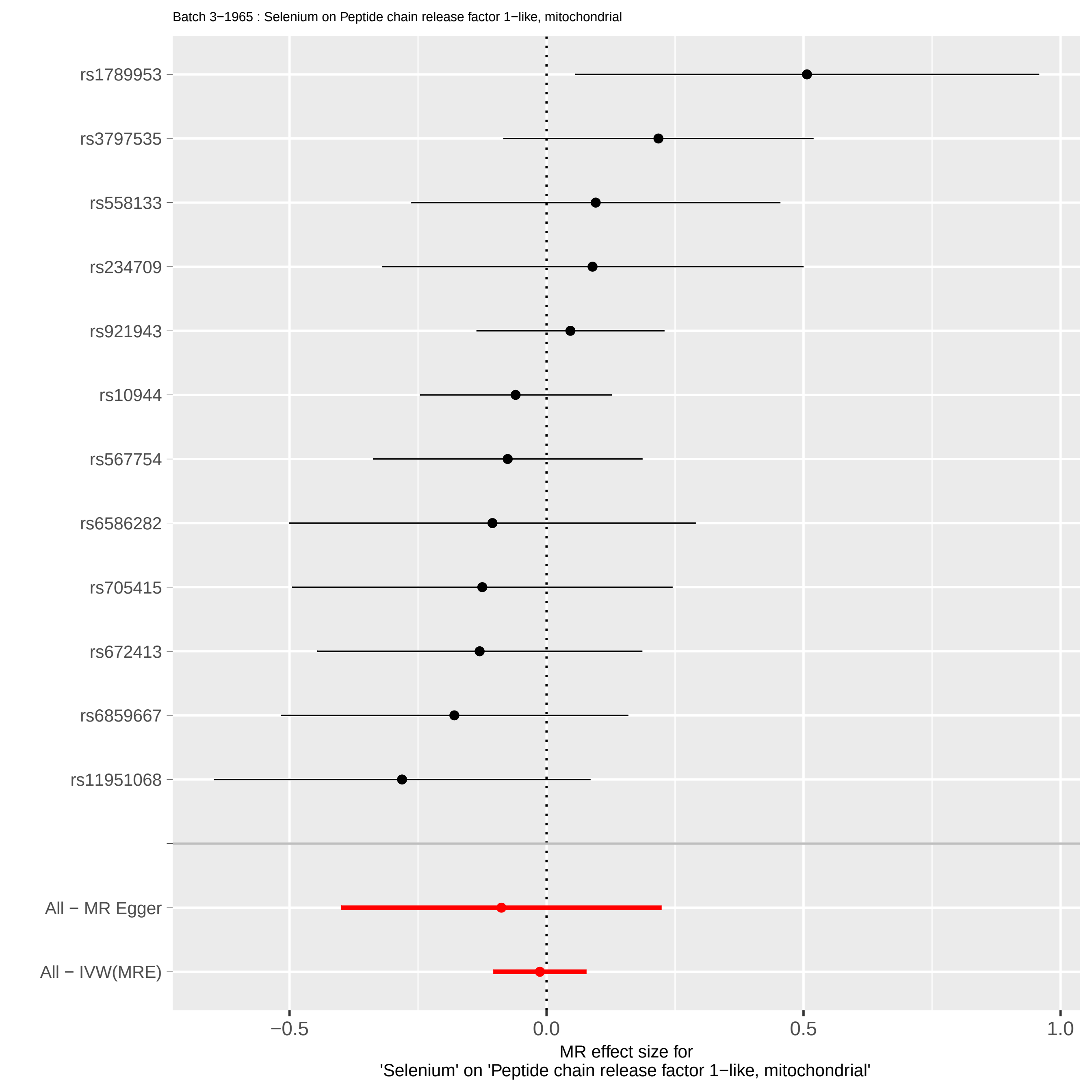

Batch 3−1965 : Selenium on Peptide chain release factor 1−like, mitochondrial
rs1789953
rs3797535
rs558133
rs234709
rs921943
rs10944
rs567754
rs6586282
rs705415
rs672413
rs6859667
rs11951068
All − MR Egger
All − IVW(MRE)
−0.5
0.0
0.5
1.0
MR effect size for
'Selenium' on 'Peptide chain release factor 1−like, mitochondrial'

Batch 3−1966 : Selenium on Mucin−1

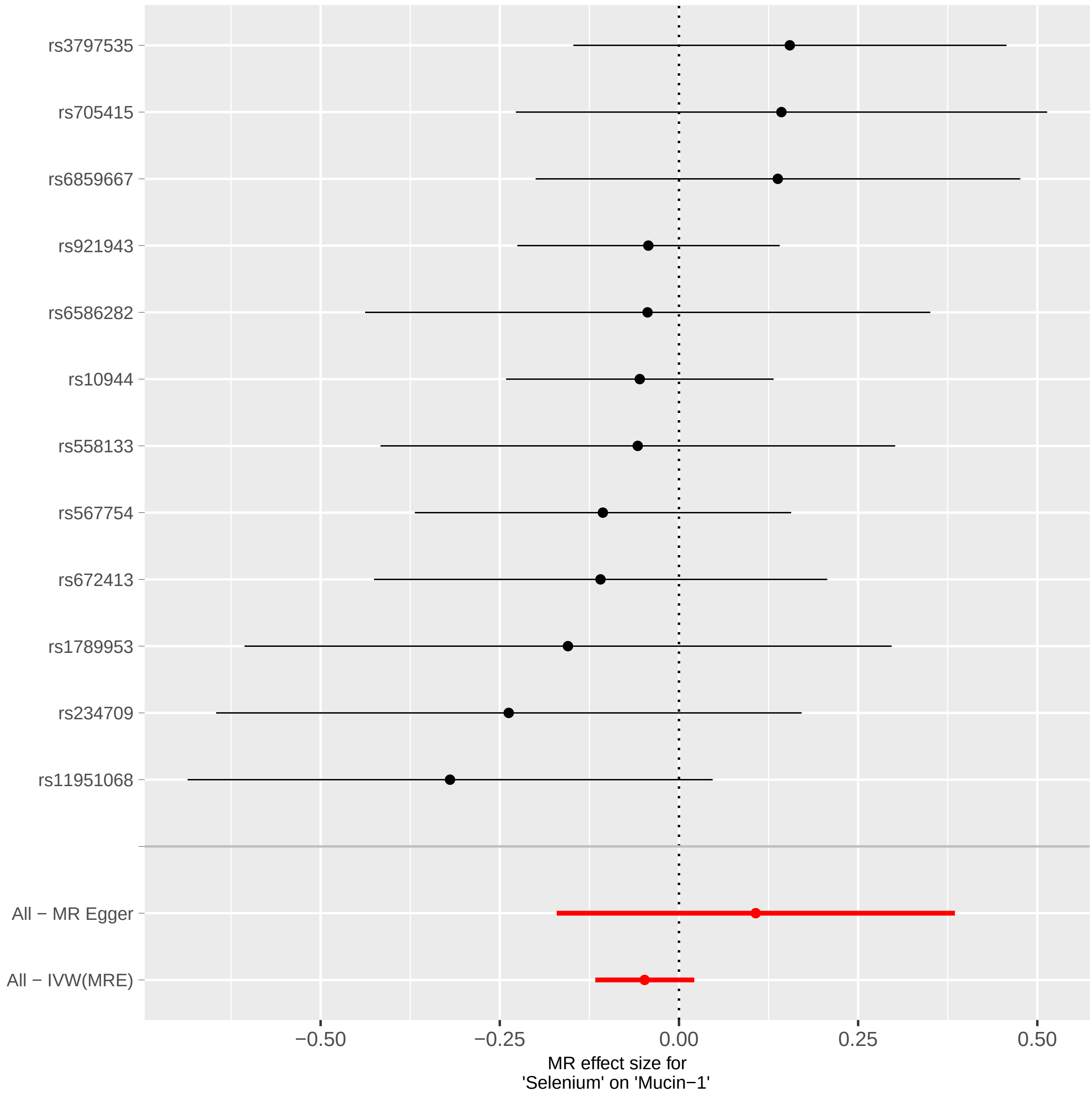

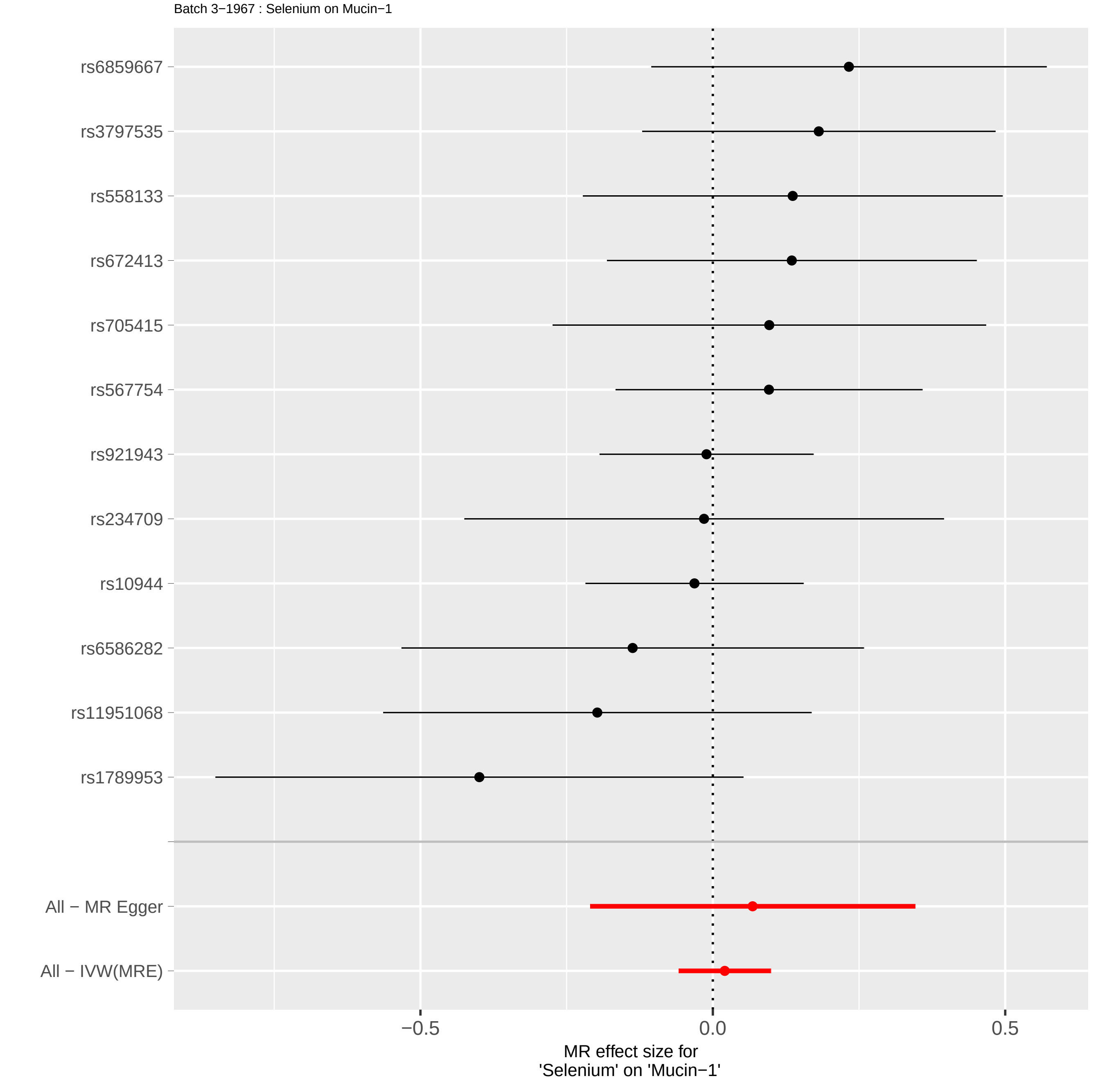
Batch 3−1967 : Selenium on Mucin−1
rs6859667
rs3797535
rs558133
rs672413
rs705415
rs567754
rs921943
rs234709
rs10944
rs6586282
rs11951068
rs1789953
All − MR Egger
All − IVW(MRE)
−0.5
0.0
0.5
MR effect size for
'Selenium' on 'Mucin−1'

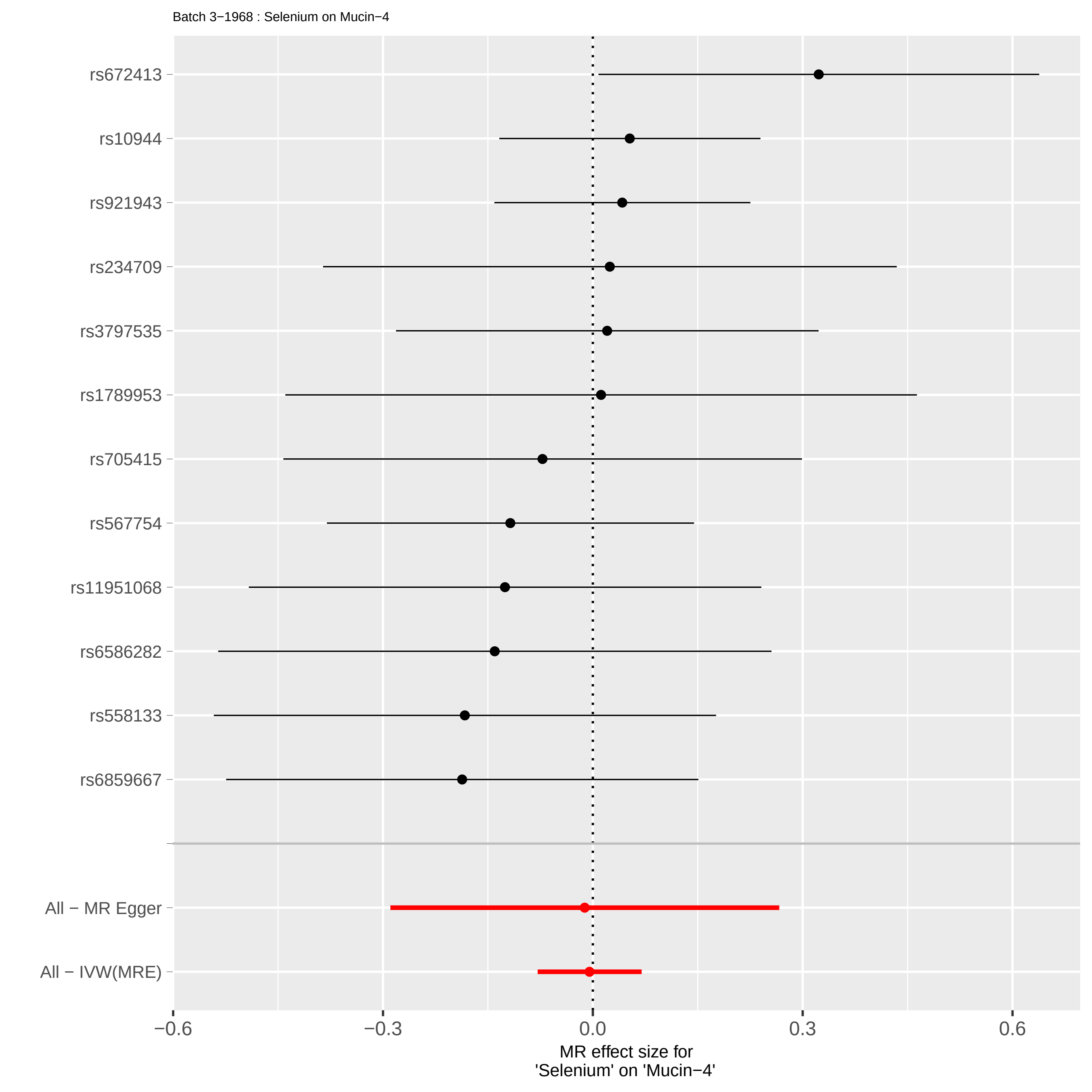

Batch 3−1968 : Selenium on Mucin−4
rs672413
rs10944
rs921943
rs234709
rs3797535
rs1789953
rs705415
rs567754
rs11951068
rs6586282
rs558133
rs6859667
All − MR Egger
All − IVW(MRE)
−0.6
−0.3
0.0
0.3
0.6
MR effect size for
'Selenium' on 'Mucin−4'

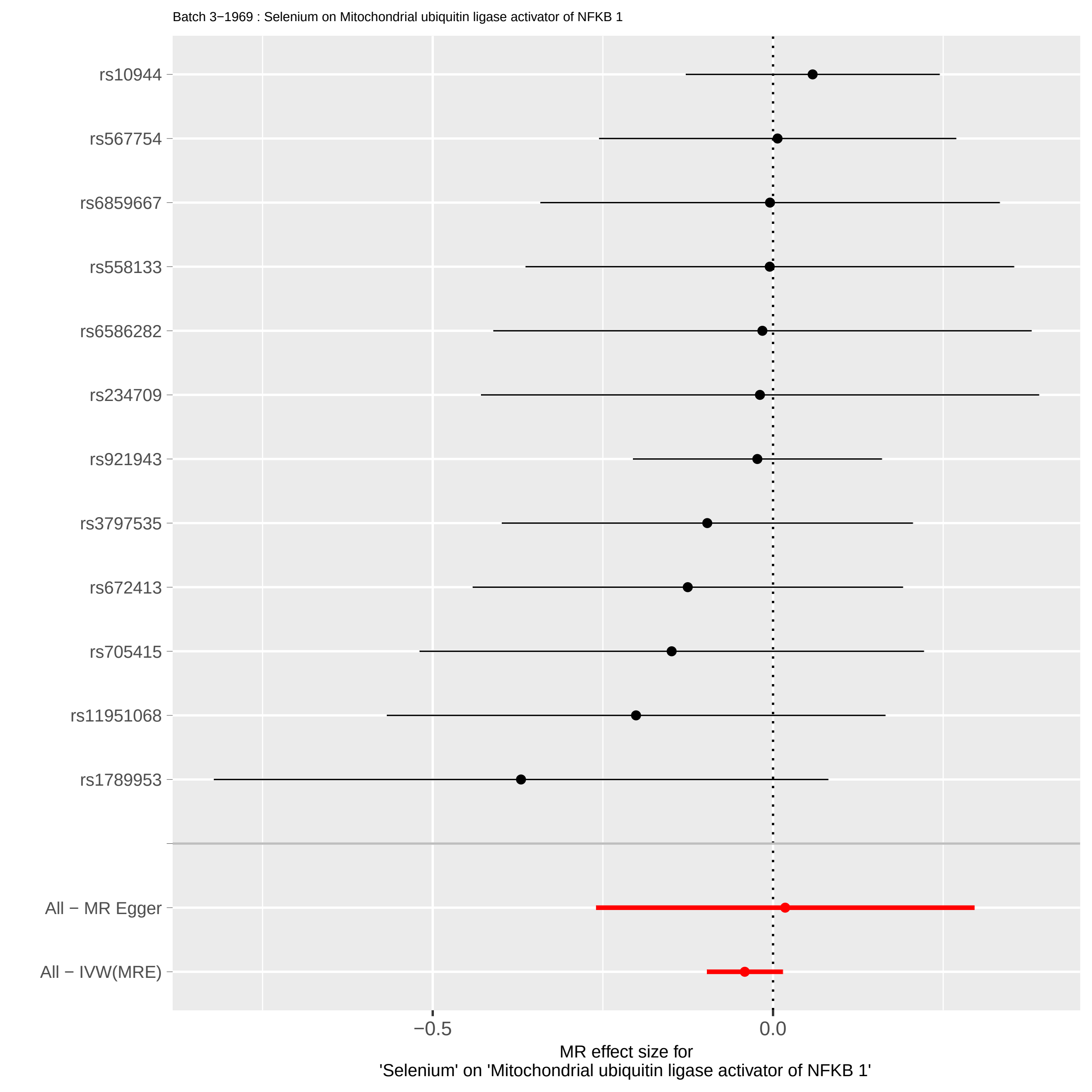

Batch 3−1969 : Selenium on Mitochondrial ubiquitin ligase activator of NFKB 1
rs10944
rs567754
rs6859667
rs558133
rs6586282
rs234709
rs921943
rs3797535
rs672413
rs705415
rs11951068
rs1789953
All − MR Egger
All − IVW(MRE)
−0.5
0.0
MR effect size for
'Selenium' on 'Mitochondrial ubiquitin ligase activator of NFKB 1'

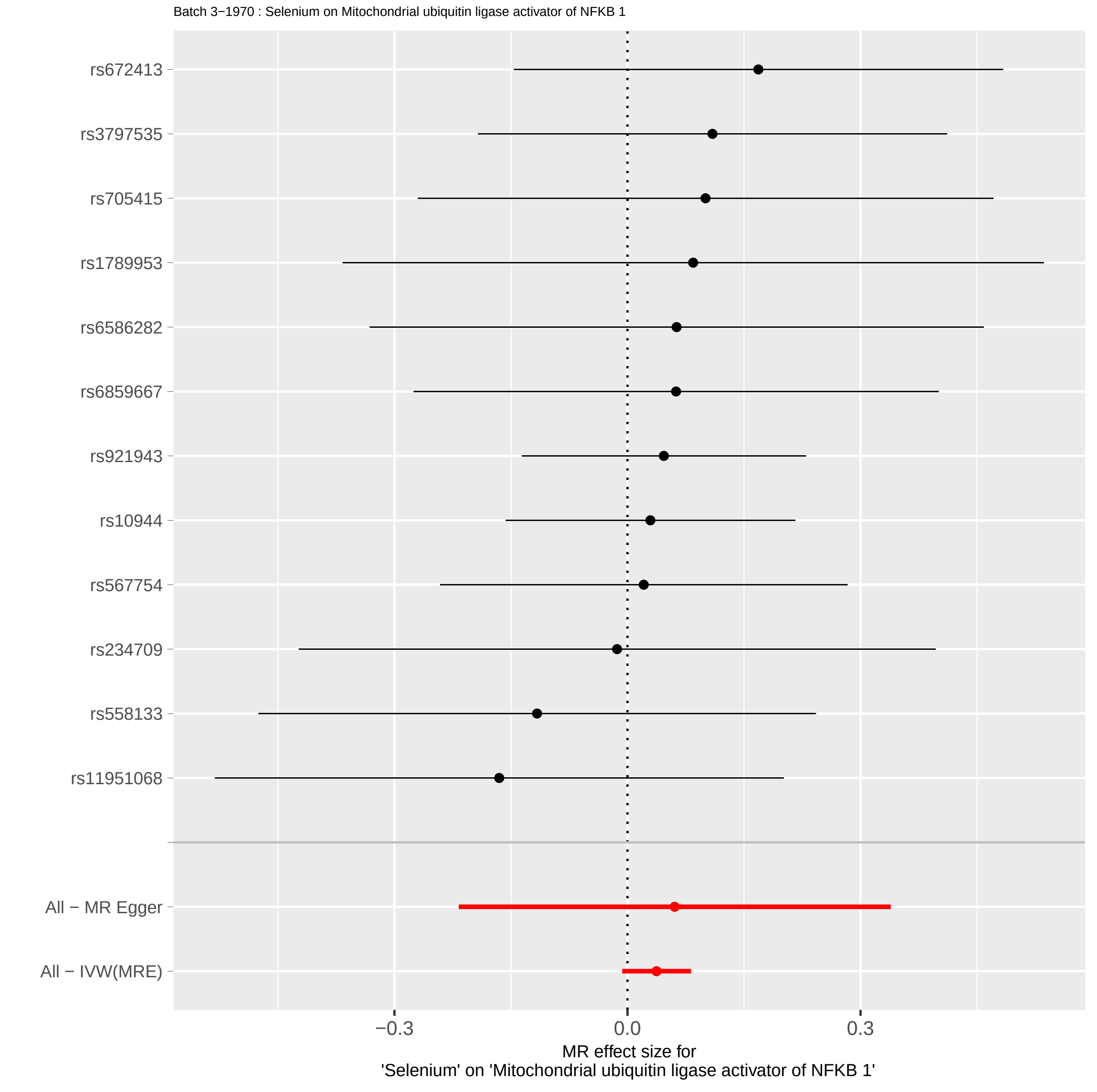

Batch 3−1970 : Selenium on Mitochondrial ubiquitin ligase activator of NFKB 1
rs672413
rs3797535
rs705415
rs1789953
rs6586282
rs6859667
rs921943
rs10944
rs567754
rs234709
rs558133
rs11951068
All − MR Egger
All − IVW(MRE)
−0.3
0.0
0.3
MR effect size for
'Selenium' on 'Mitochondrial ubiquitin ligase activator of NFKB 1'

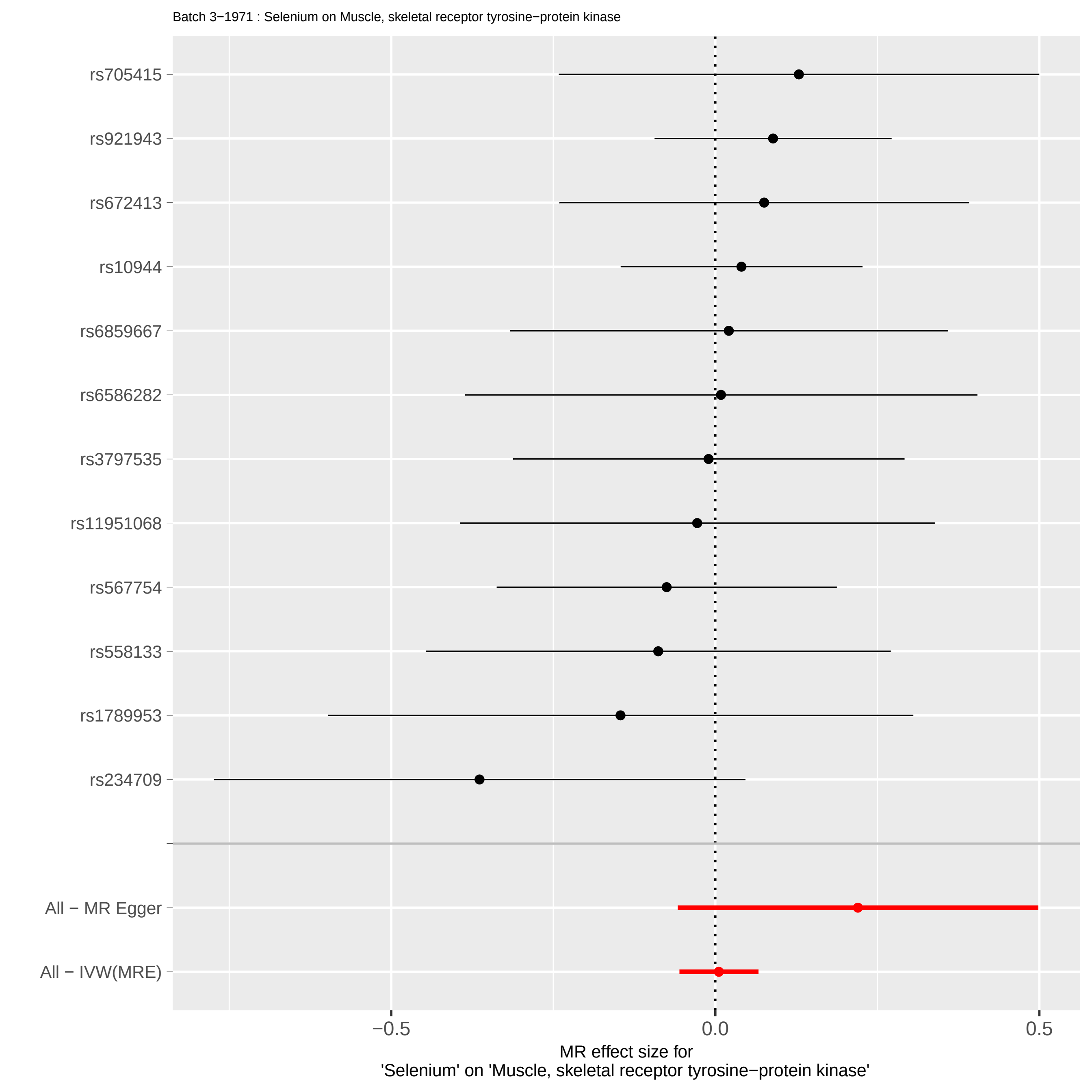

Batch 3−1971 : Selenium on Muscle, skeletal receptor tyrosine−protein kinase
rs705415
rs921943
rs672413
rs10944
rs6859667
rs6586282
rs3797535
rs11951068
rs567754
rs558133
rs1789953
rs234709
All − MR Egger
All − IVW(MRE)
−0.5
0.0
0.5
MR effect size for
'Selenium' on 'Muscle, skeletal receptor tyrosine−protein kinase'

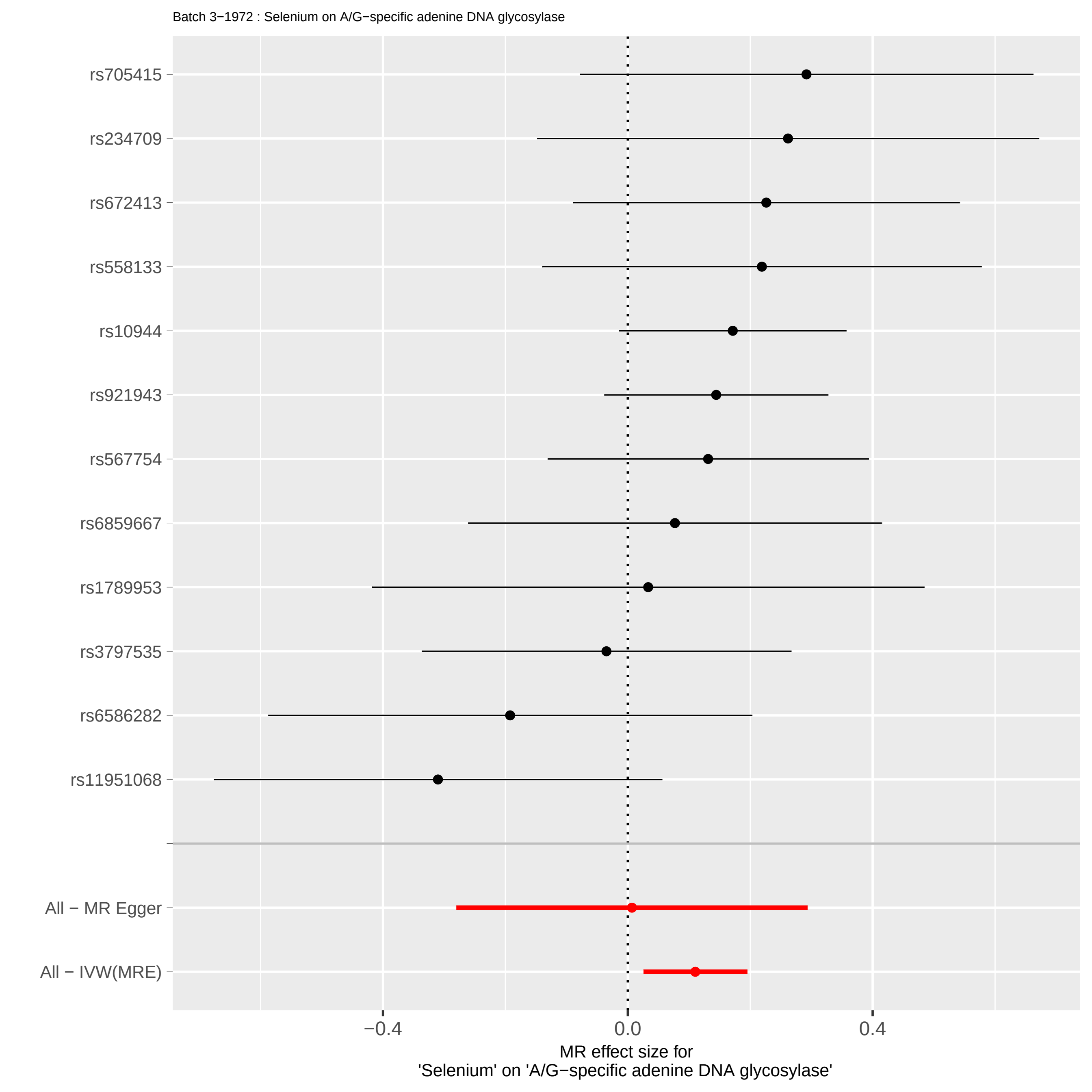
Batch 3−1972 : Selenium on A/G−specific adenine DNA glycosylase
rs705415
rs234709
rs672413
rs558133
rs10944
rs921943
rs567754
rs6859667
rs1789953
rs3797535
rs6586282
rs11951068
All − MR Egger
All − IVW(MRE)
−0.4
0.0
0.4
MR effect size for
'Selenium' on 'A/G−specific adenine DNA glycosylase'

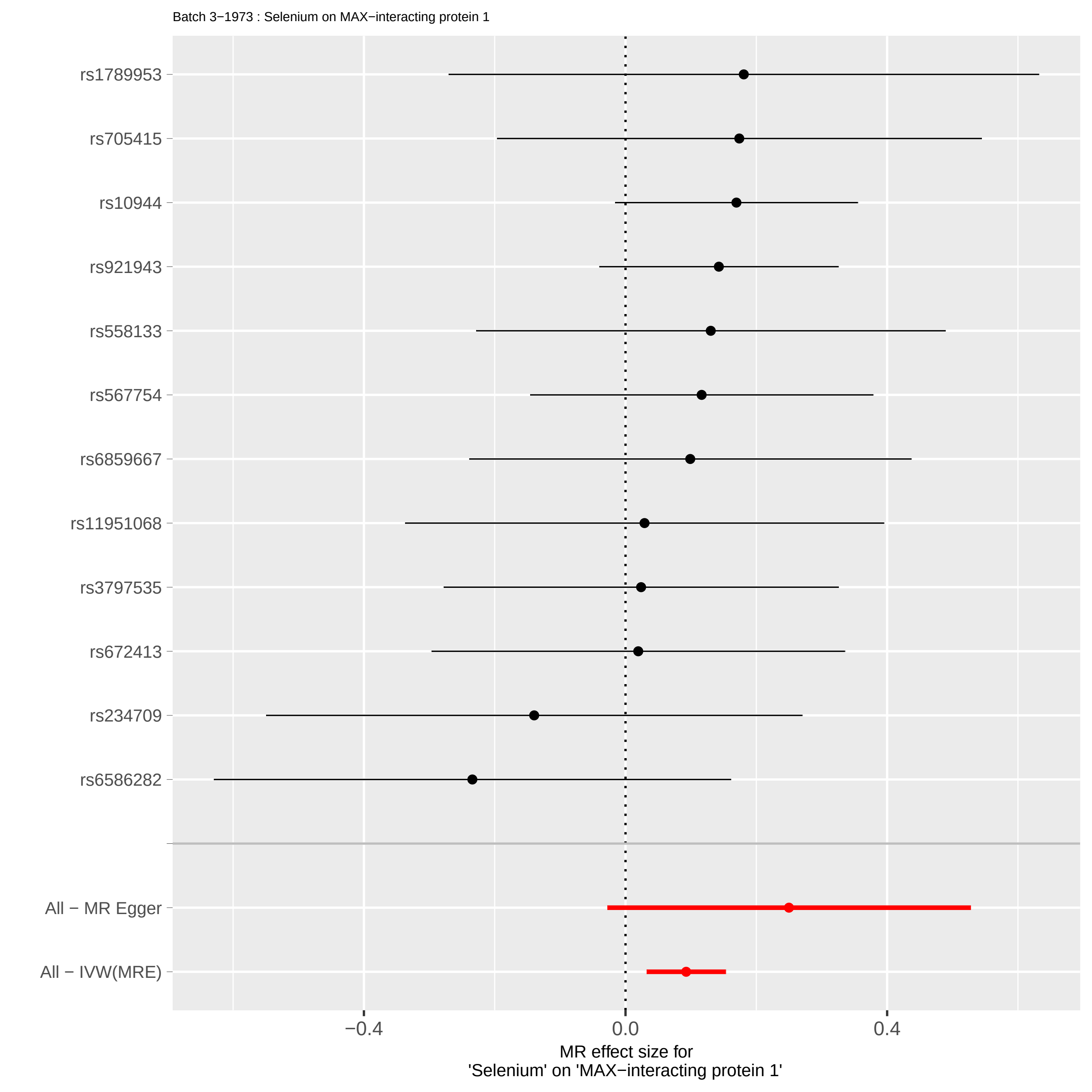

Batch 3−1973 : Selenium on MAX−interacting protein 1
rs1789953
rs705415
rs10944
rs921943
rs558133
rs567754
rs6859667
rs11951068
rs3797535
rs672413
rs234709
rs6586282
All − MR Egger
All − IVW(MRE)
−0.4
0.0
0.4
MR effect size for
'Selenium' on 'MAX−interacting protein 1'

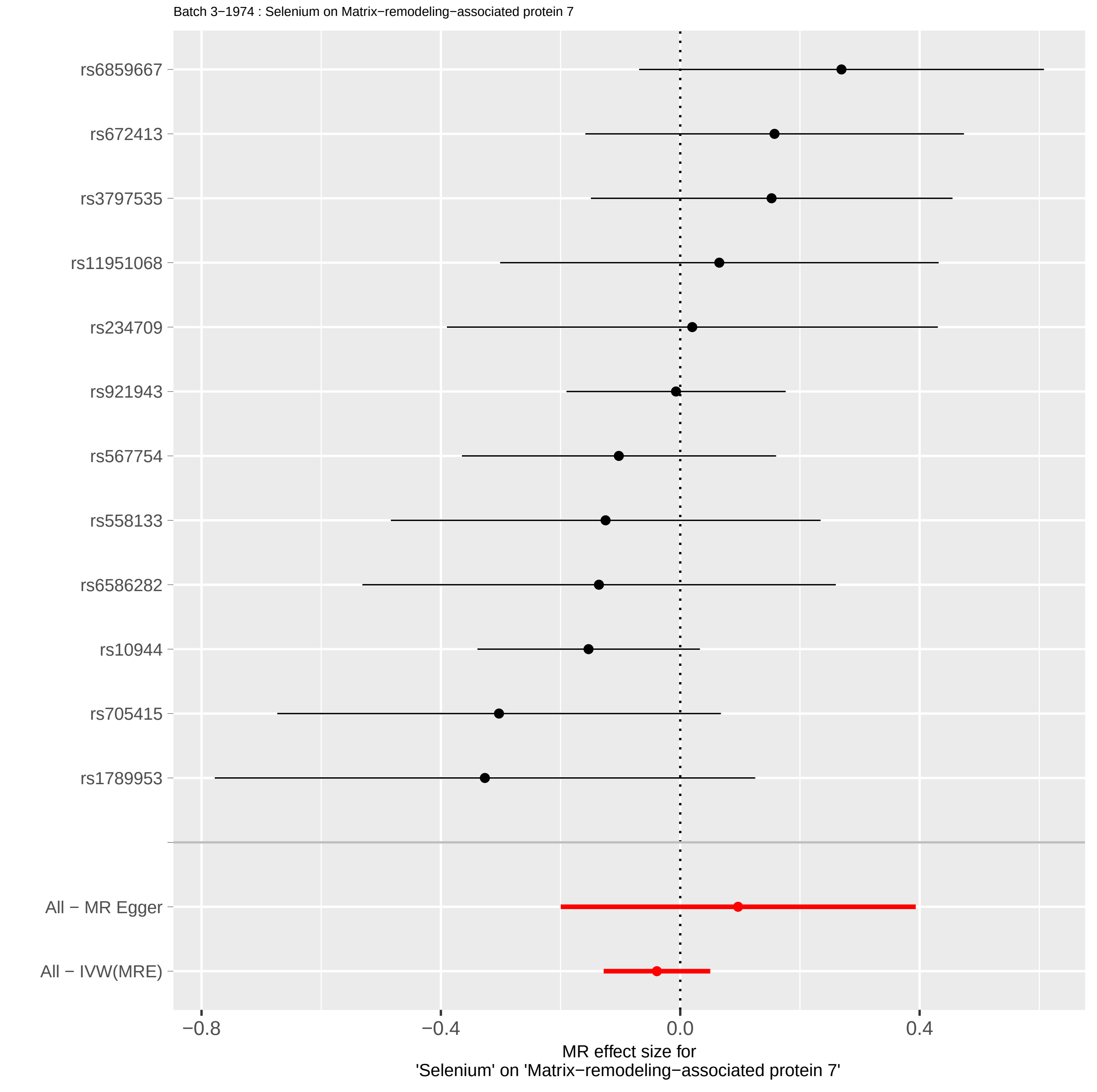

Batch 3−1974 : Selenium on Matrix−remodeling−associated protein 7
rs6859667
rs672413
rs3797535
rs11951068
rs234709
rs921943
rs567754
rs558133
rs6586282
rs10944
rs705415
rs1789953
All − MR Egger
All − IVW(MRE)
−0.8
−0.4
0.0
0.4
MR effect size for
'Selenium' on 'Matrix−remodeling−associated protein 7'

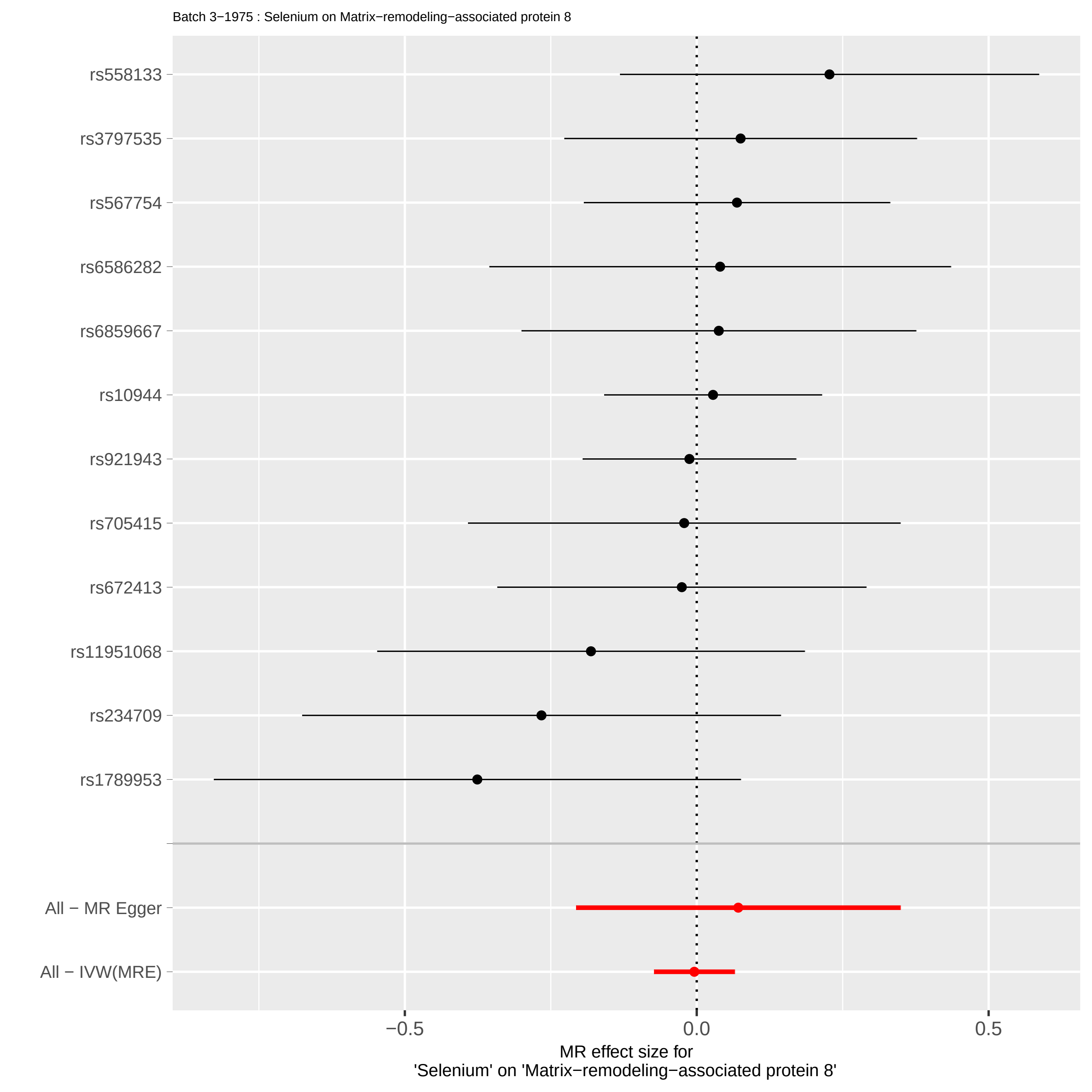
Batch 3−1975 : Selenium on Matrix−remodeling−associated protein 8
rs558133
rs3797535
rs567754
rs6586282
rs6859667
rs10944
rs921943
rs705415
rs672413
rs11951068
rs234709
rs1789953
All − MR Egger
All − IVW(MRE)
−0.5
0.0
0.5
MR effect size for
'Selenium' on 'Matrix−remodeling−associated protein 8'

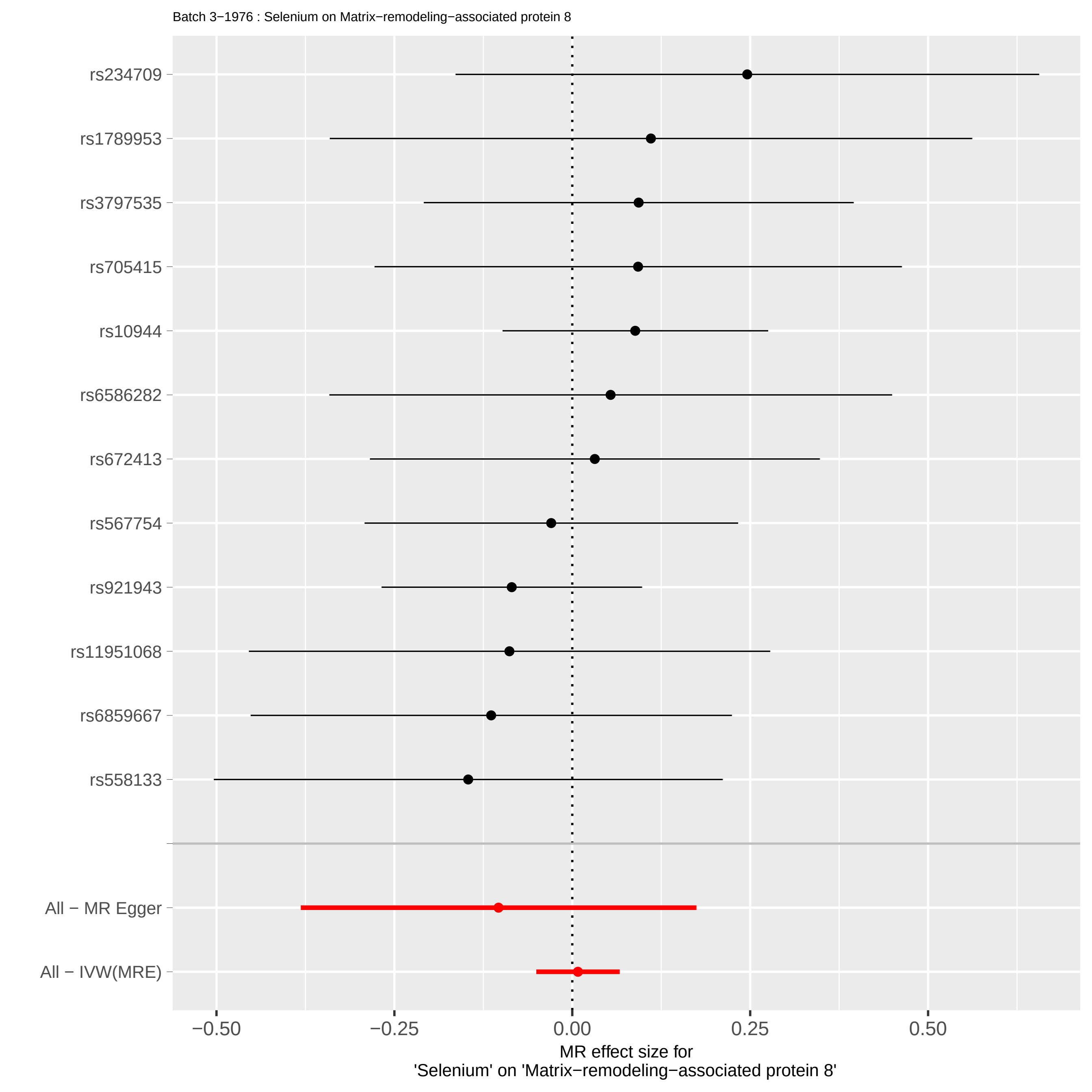
Batch 3−1976 : Selenium on Matrix−remodeling−associated protein 8
rs234709
rs1789953
rs3797535
rs705415
rs10944
rs6586282
rs672413
rs567754
rs921943
rs11951068
rs6859667
rs558133
All − MR Egger
All − IVW(MRE)
−0.50
−0.25
0.00
0.25
0.50
MR effect size for
'Selenium' on 'Matrix−remodeling−associated protein 8'

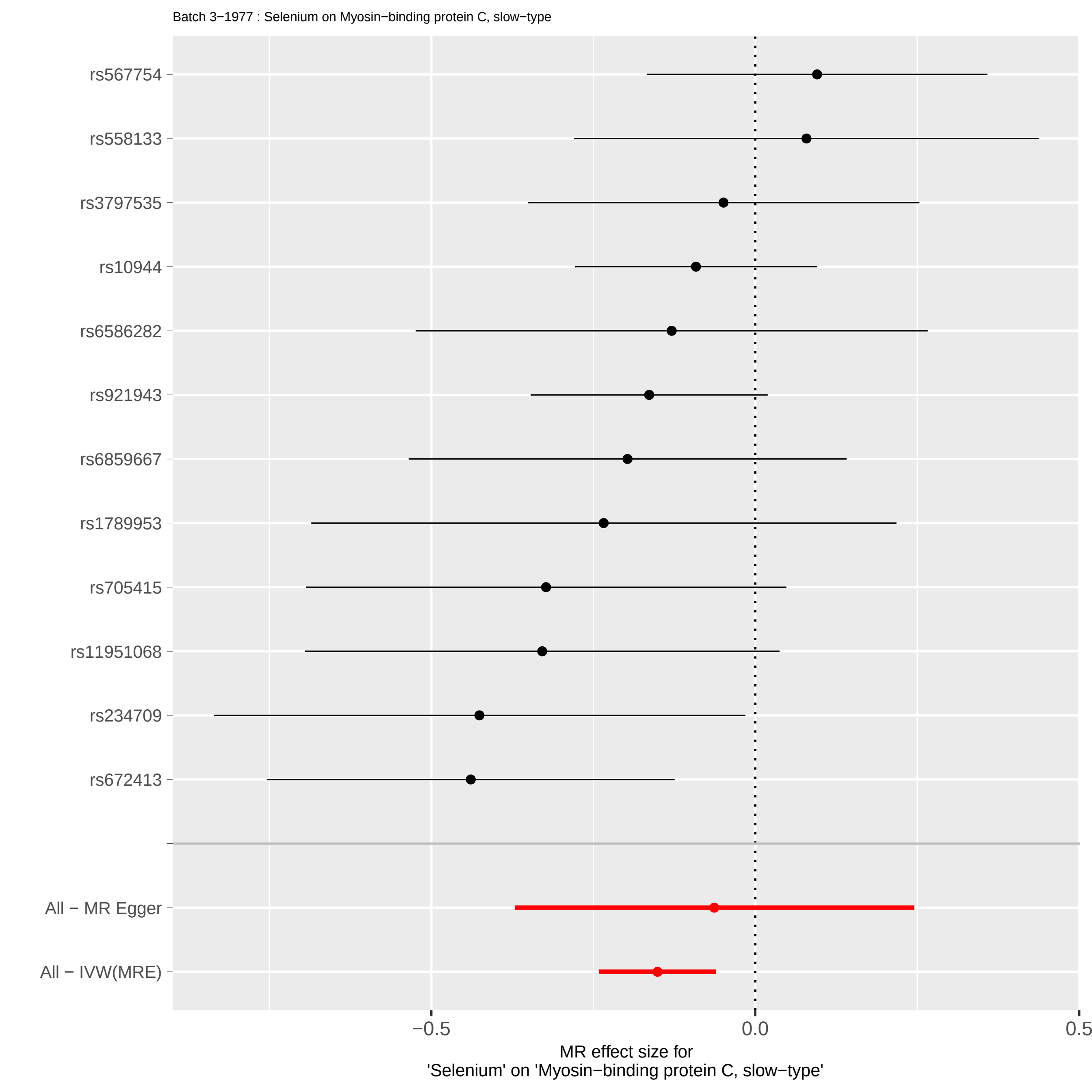

Batch 3−1977 : Selenium on Myosin−binding protein C, slow−type
rs567754
rs558133
rs3797535
rs10944
rs6586282
rs921943
rs6859667
rs1789953
rs705415
rs11951068
rs234709
rs672413
All − MR Egger
All − IVW(MRE)
−0.5
0.0
0.5
MR effect size for
'Selenium' on 'Myosin−binding protein C, slow−type'

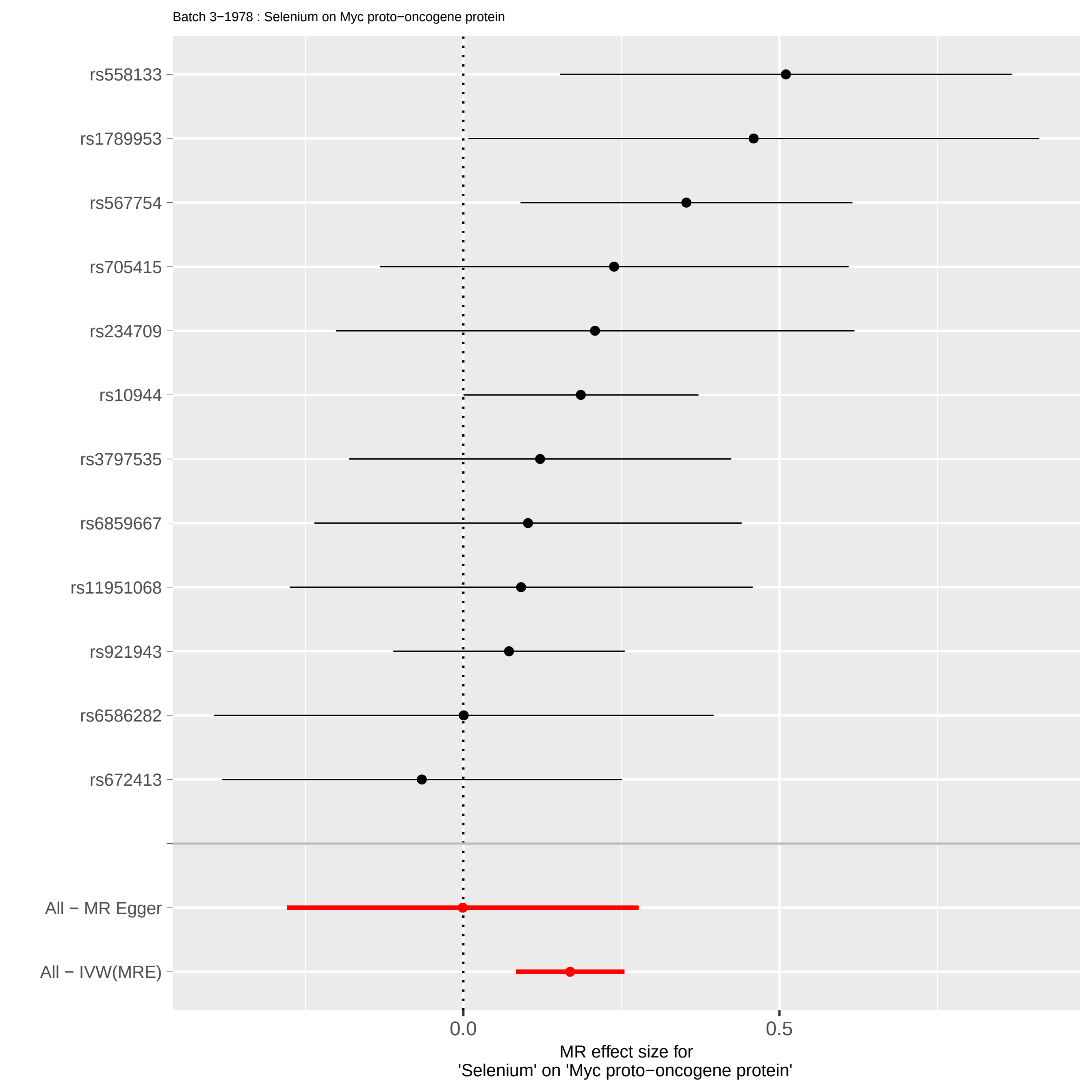

Batch 3−1978 : Selenium on Myc proto−oncogene protein
rs558133
rs1789953
rs567754
rs705415
rs234709
rs10944
rs3797535
rs6859667
rs11951068
rs921943
rs6586282
rs672413
All − MR Egger
All − IVW(MRE)
0.0
0.5
MR effect size for
'Selenium' on 'Myc proto−oncogene protein'

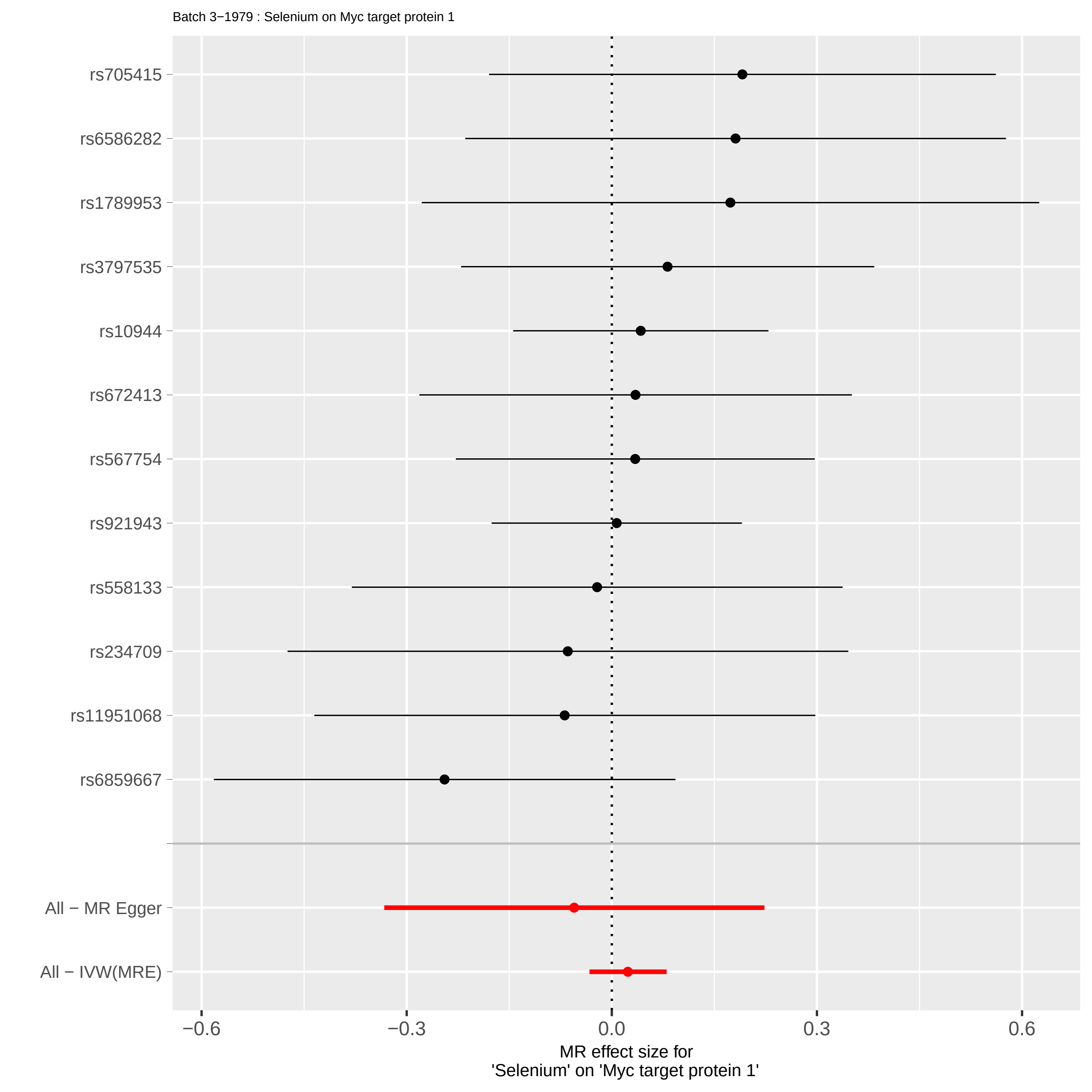

Batch 3−1979 : Selenium on Myc target protein 1
rs705415
rs6586282
rs1789953
rs3797535
rs10944
rs672413
rs567754
rs921943
rs558133
rs234709
rs11951068
rs6859667
All − MR Egger
All − IVW(MRE)
−0.6
−0.3
0.0
0.3
0.6
MR effect size for
'Selenium' on 'Myc target protein 1'

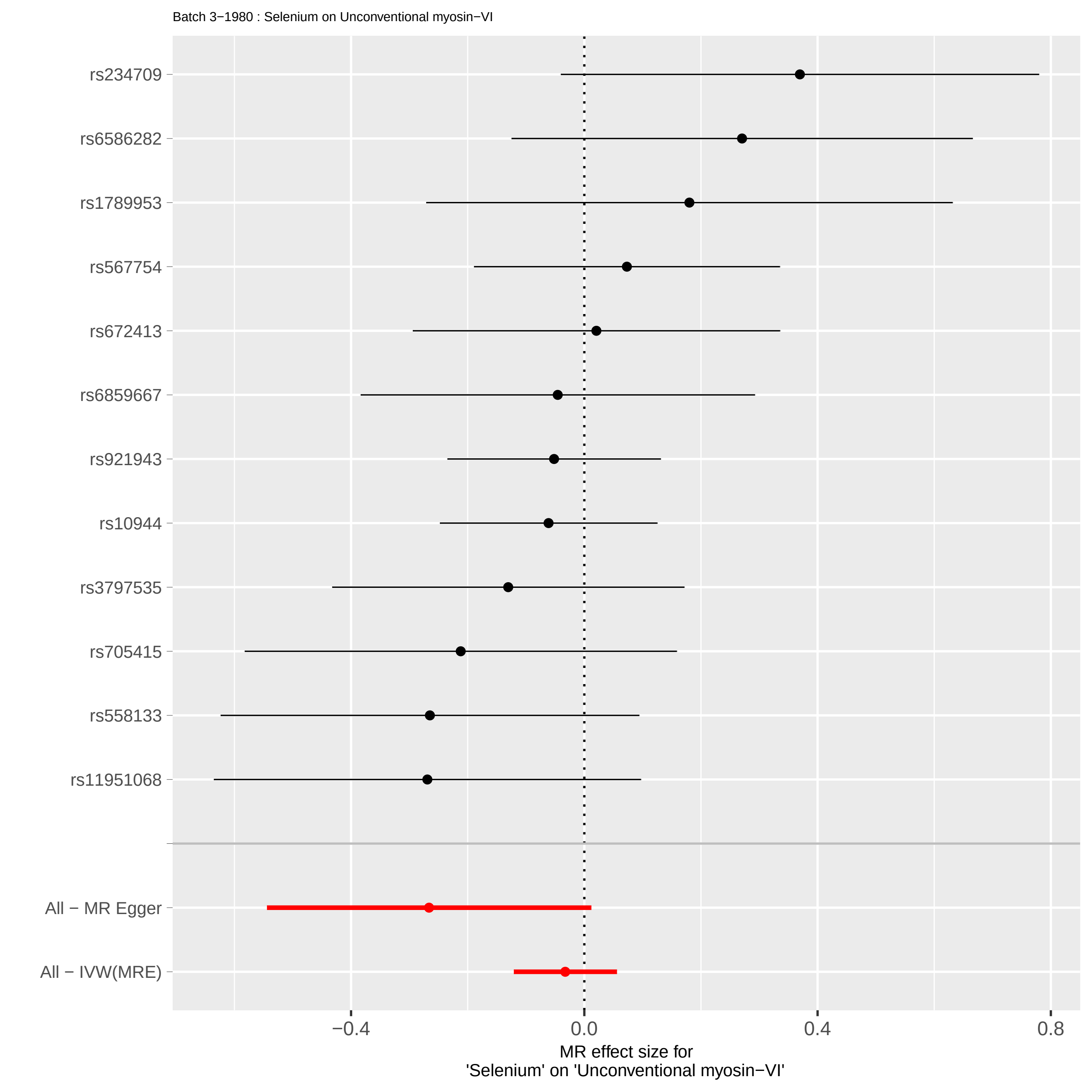

Batch 3−1980 : Selenium on Unconventional myosin−VI
rs234709
rs6586282
rs1789953
rs567754
rs672413
rs6859667
rs921943
rs10944
rs3797535
rs705415
rs558133
rs11951068
All − MR Egger
All − IVW(MRE)
−0.4
0.0
0.4
0.8
MR effect size for
'Selenium' on 'Unconventional myosin−VI'

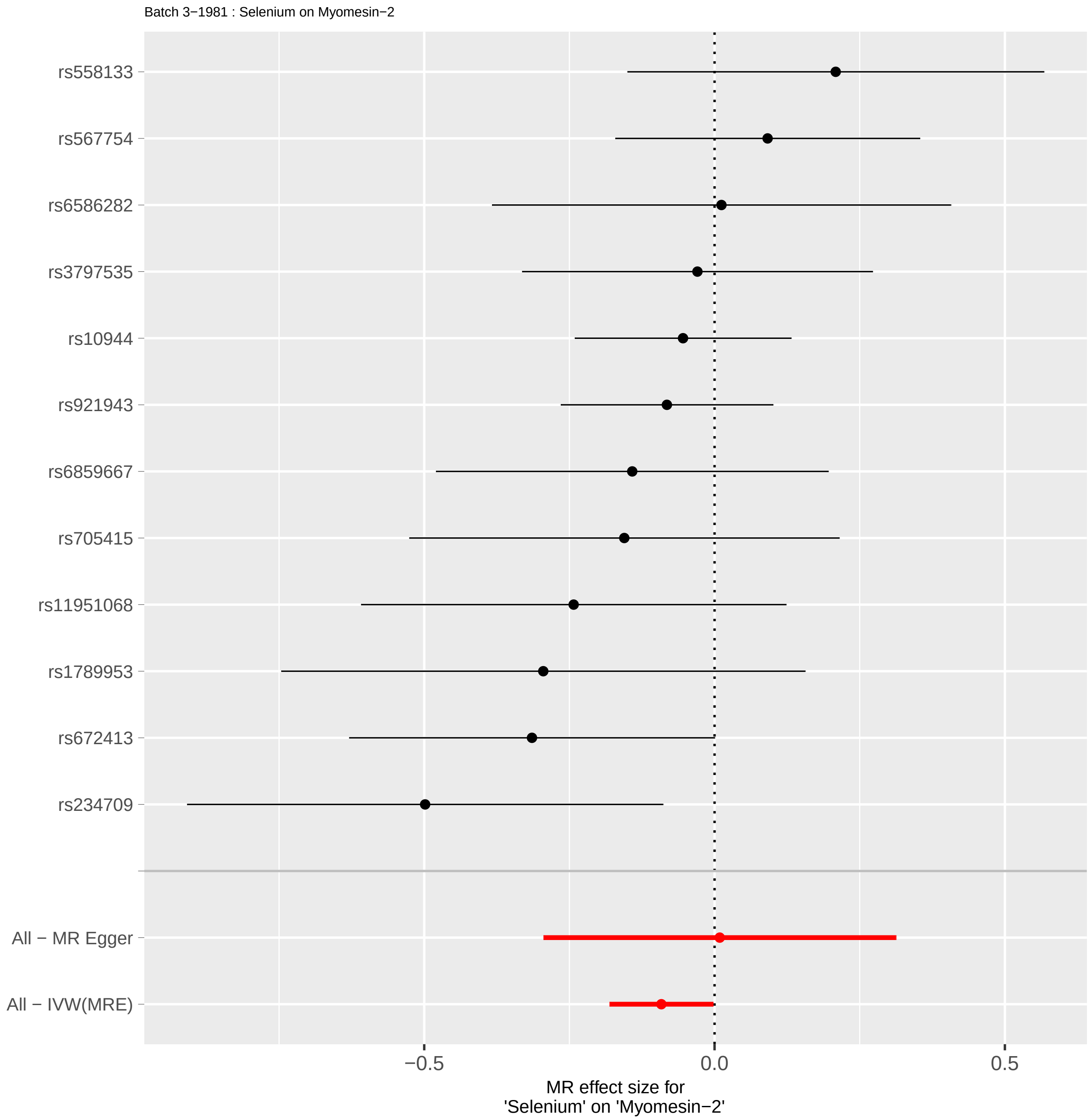

Batch 3−1981 : Selenium on Myomesin−2
rs558133
rs567754
rs6586282
rs3797535
rs10944
rs921943
rs6859667
rs705415
rs11951068
rs1789953
rs672413
rs234709
All − MR Egger
All − IVW(MRE)
−0.5
0.0
0.5
MR effect size for
'Selenium' on 'Myomesin−2'

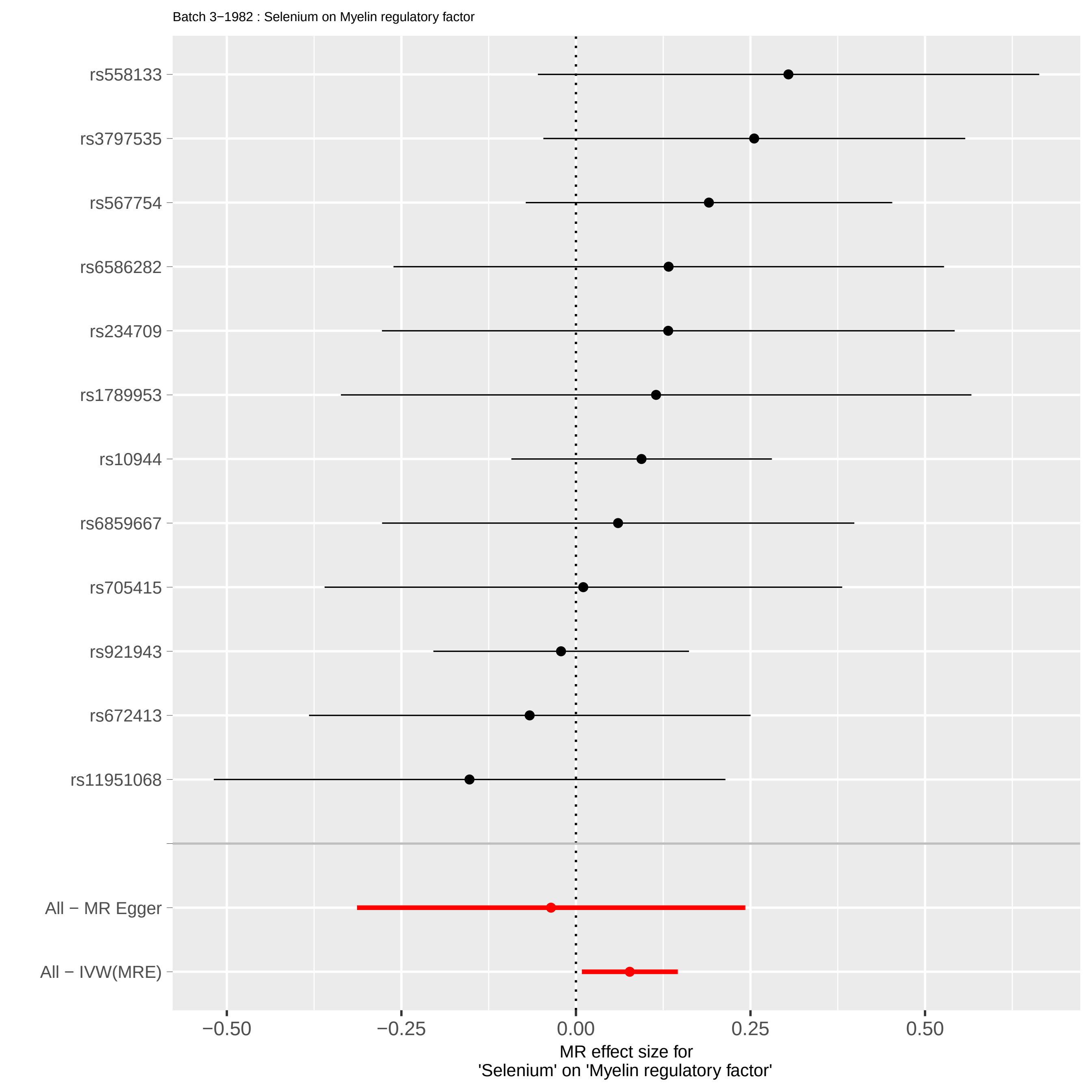

Batch 3−1982 : Selenium on Myelin regulatory factor
rs558133
rs3797535
rs567754
rs6586282
rs234709
rs1789953
rs10944
rs6859667
rs705415
rs921943
rs672413
rs11951068
All − MR Egger
All − IVW(MRE)
−0.50
−0.25
0.00
0.25
0.50
MR effect size for
'Selenium' on 'Myelin regulatory factor'

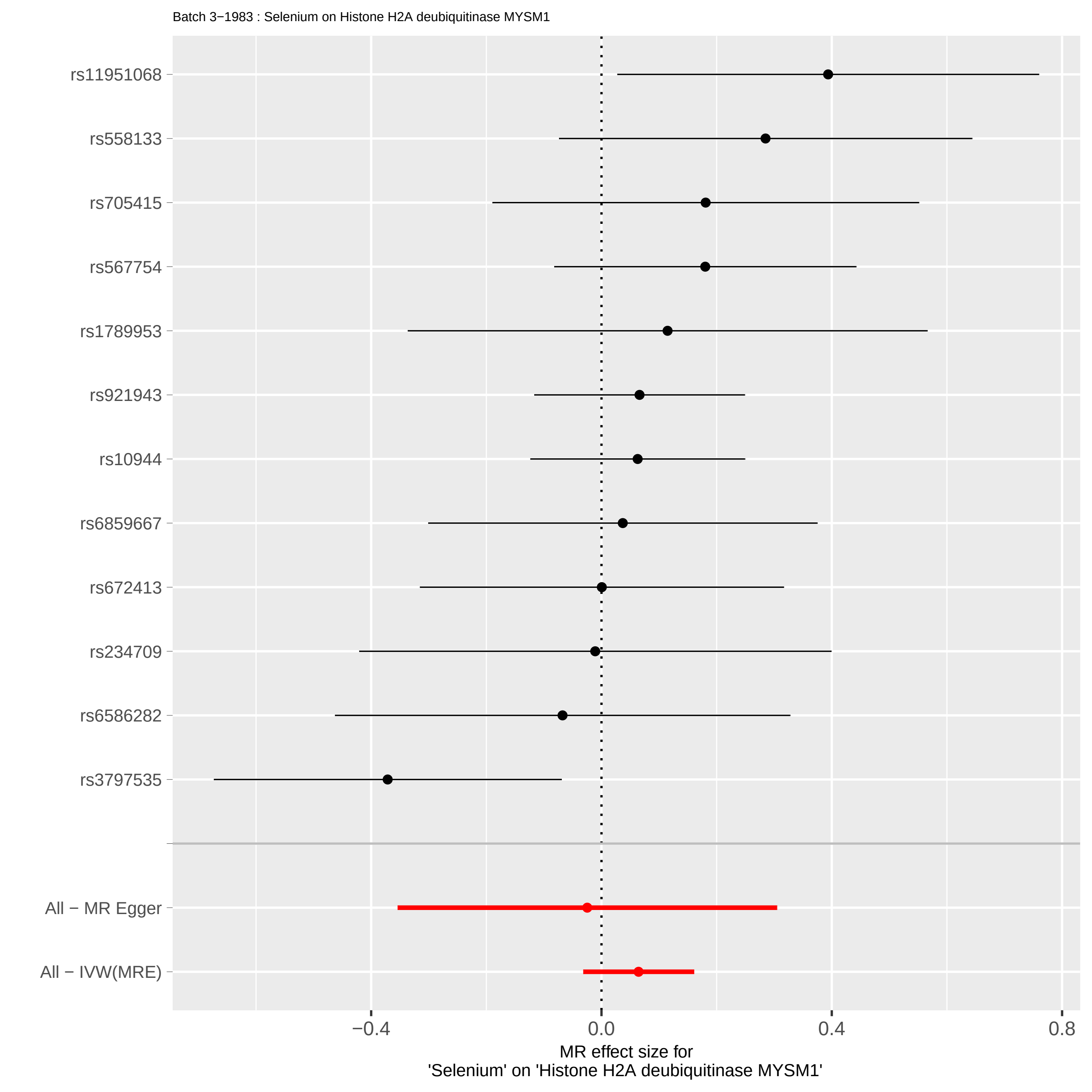

Batch 3−1983 : Selenium on Histone H2A deubiquitinase MYSM1
rs11951068
rs558133
rs705415
rs567754
rs1789953
rs921943
rs10944
rs6859667
rs672413
rs234709
rs6586282
rs3797535
All − MR Egger
All − IVW(MRE)
−0.4
0.0
0.4
0.8
MR effect size for
'Selenium' on 'Histone H2A deubiquitinase MYSM1'

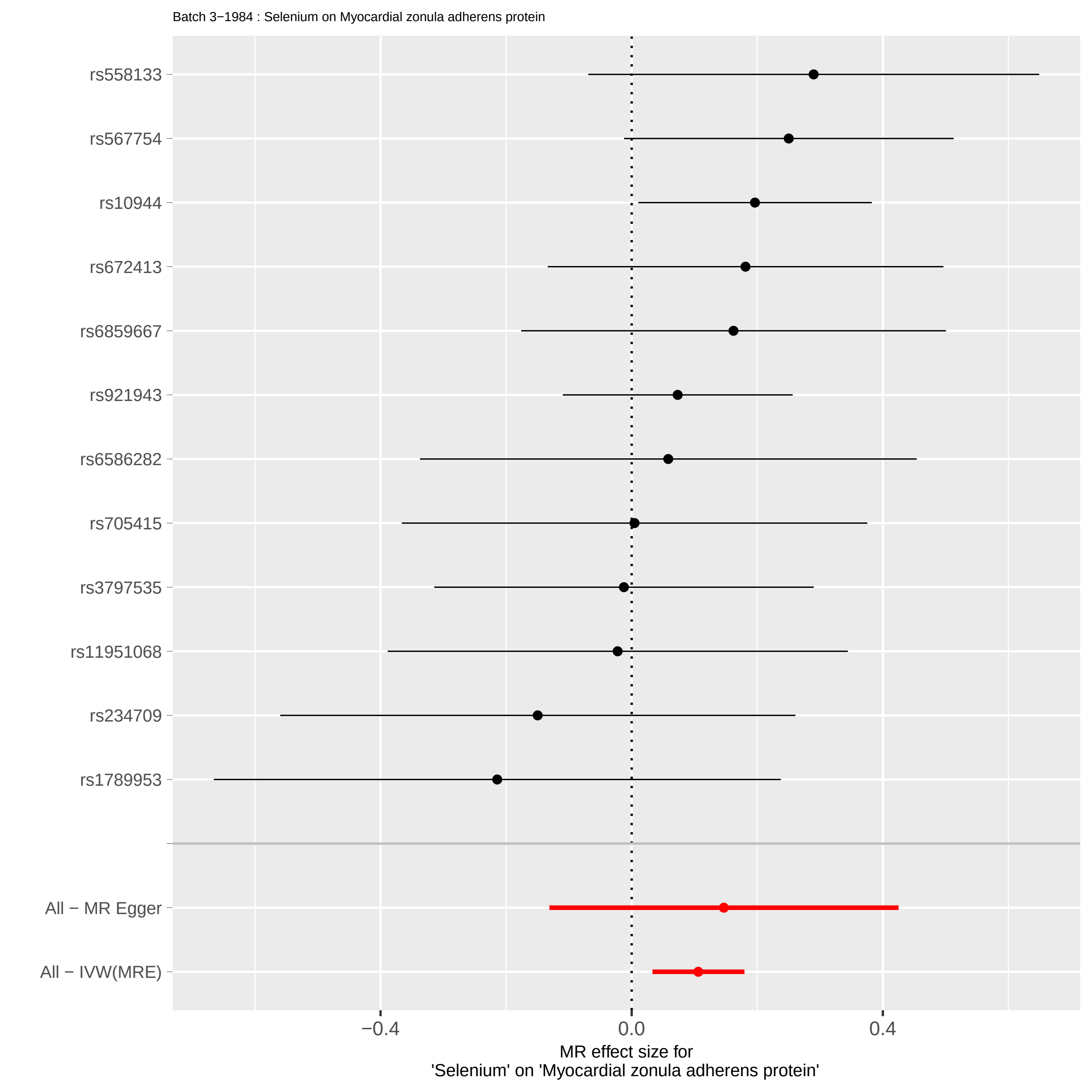

Batch 3−1984 : Selenium on Myocardial zonula adherens protein
rs558133
rs567754
rs10944
rs672413
rs6859667
rs921943
rs6586282
rs705415
rs3797535
rs11951068
rs234709
rs1789953
All − MR Egger
All − IVW(MRE)
−0.4
0.0
0.4
MR effect size for
'Selenium' on 'Myocardial zonula adherens protein'

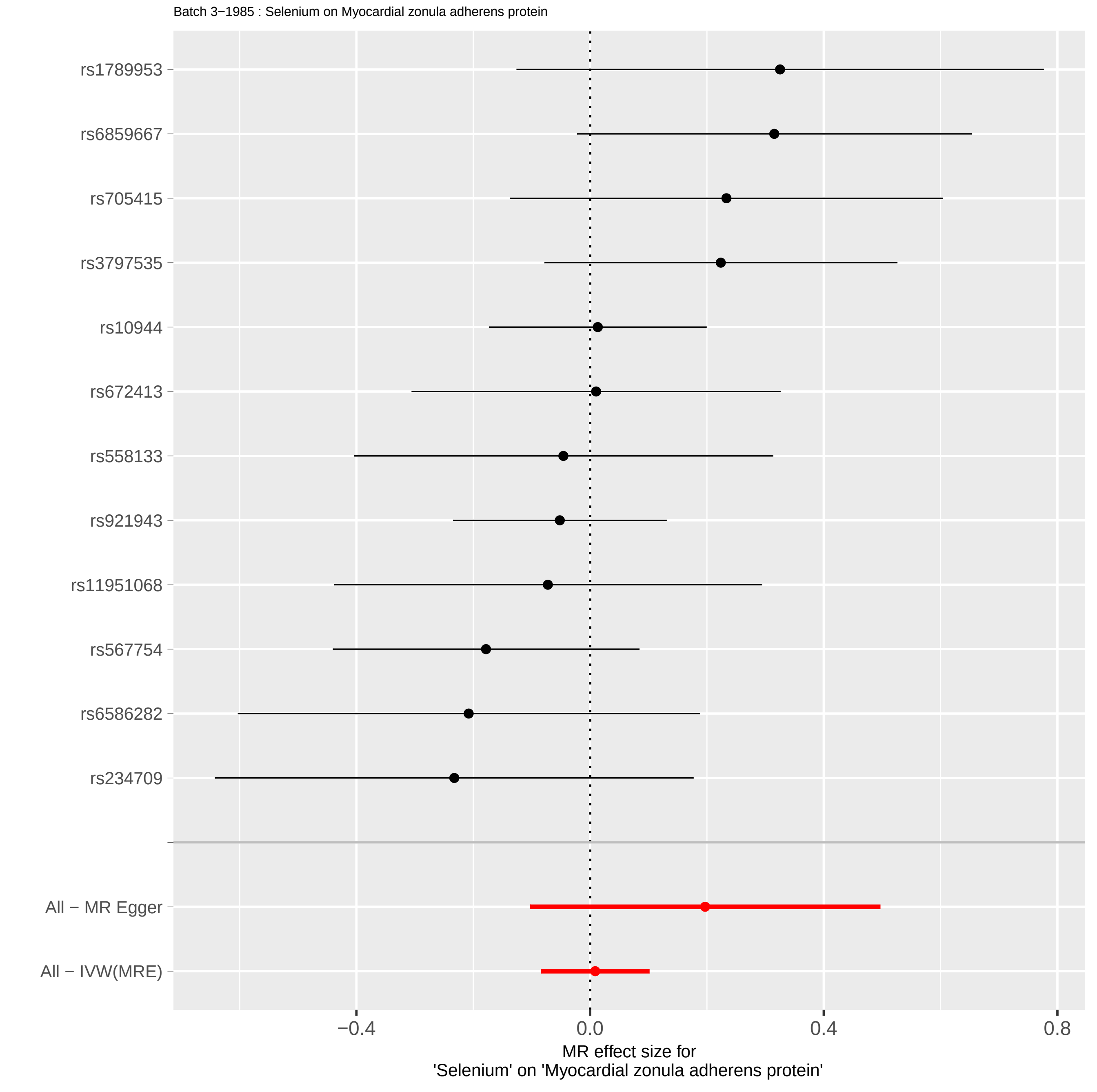
Batch 3−1985 : Selenium on Myocardial zonula adherens protein
rs1789953
rs6859667
rs705415
rs3797535
rs10944
rs672413
rs558133
rs921943
rs11951068
rs567754
rs6586282
rs234709
All − MR Egger
All − IVW(MRE)
−0.4
0.0
0.4
0.8
MR effect size for
'Selenium' on 'Myocardial zonula adherens protein'

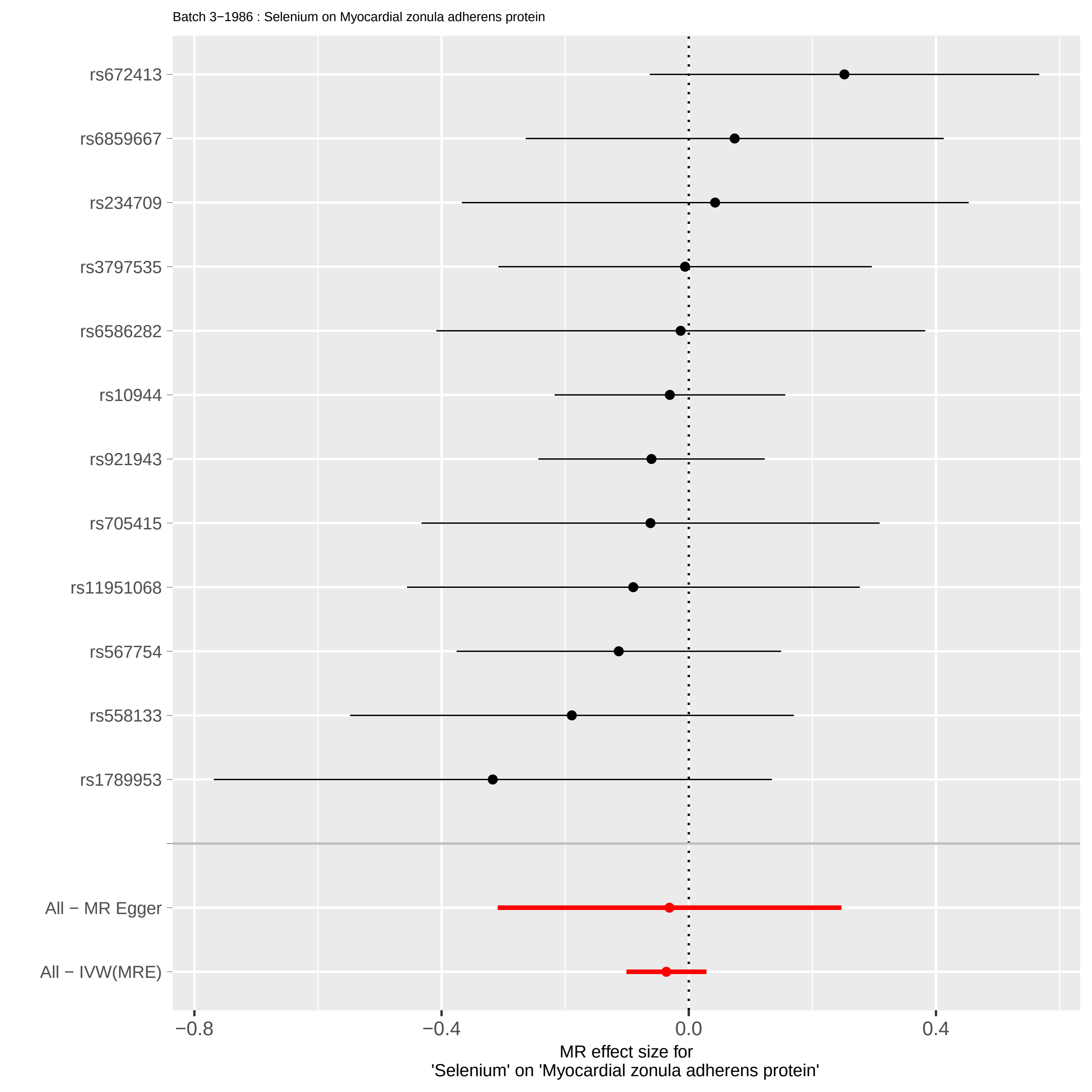

Batch 3−1986 : Selenium on Myocardial zonula adherens protein
rs672413
rs6859667
rs234709
rs3797535
rs6586282
rs10944
rs921943
rs705415
rs11951068
rs567754
rs558133
rs1789953
All − MR Egger
All − IVW(MRE)
−0.8
−0.4
0.0
0.4
MR effect size for
'Selenium' on 'Myocardial zonula adherens protein'

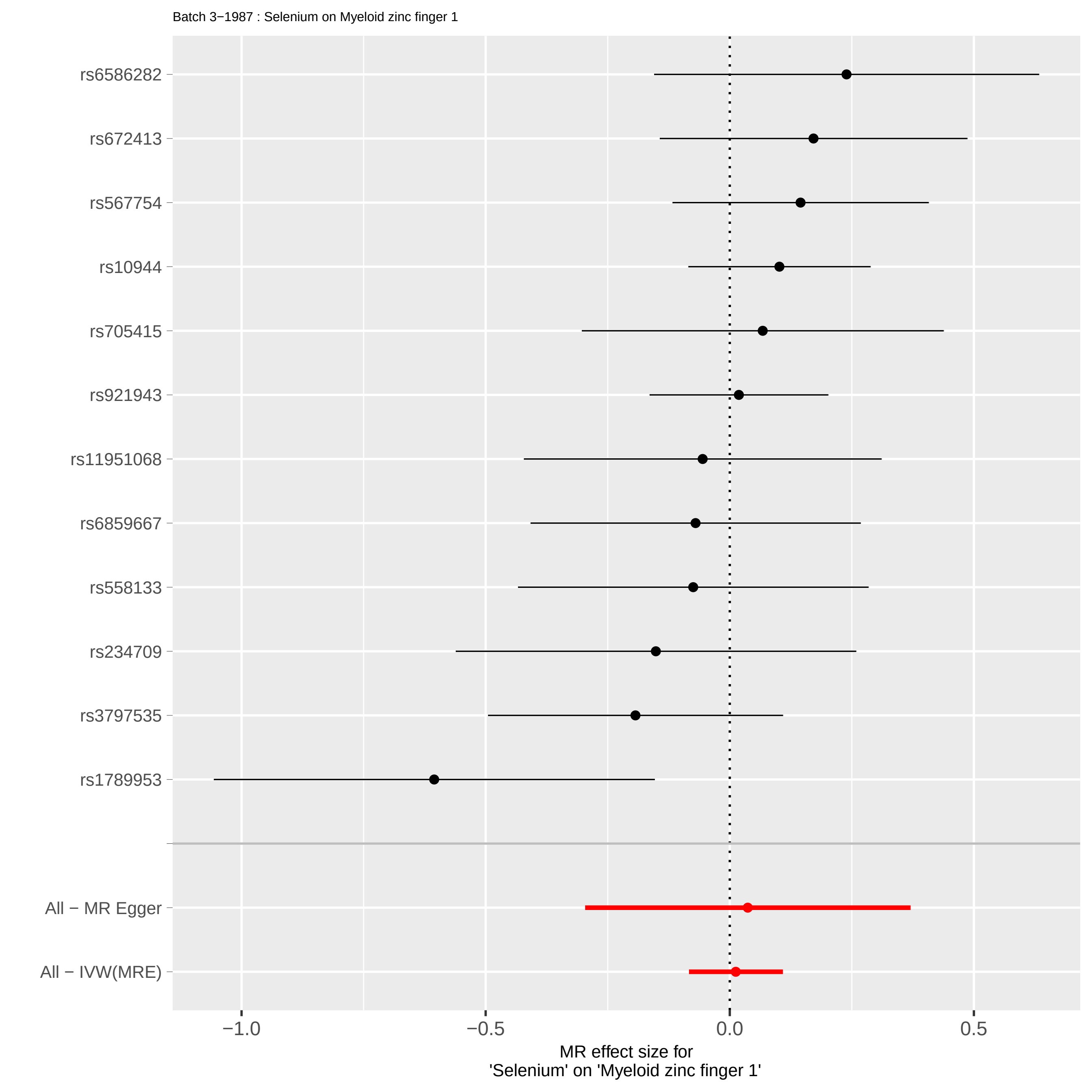

Batch 3−1987 : Selenium on Myeloid zinc finger 1
rs6586282
rs672413
rs567754
rs10944
rs705415
rs921943
rs11951068
rs6859667
rs558133
rs234709
rs3797535
rs1789953
All − MR Egger
All − IVW(MRE)
−1.0
−0.5
0.0
0.5
MR effect size for
'Selenium' on 'Myeloid zinc finger 1'

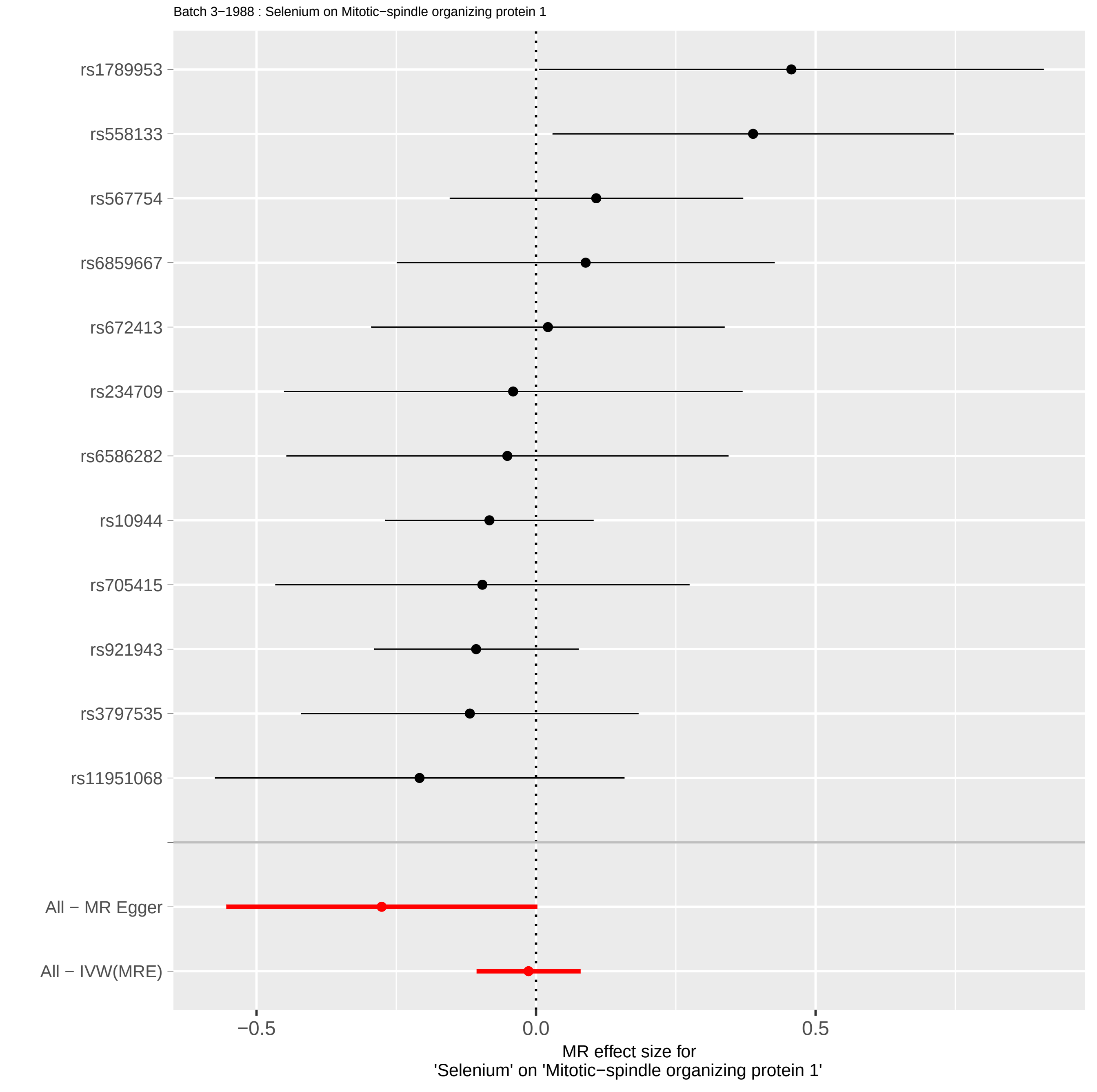

Batch 3−1988 : Selenium on Mitotic−spindle organizing protein 1
rs1789953
rs558133
rs567754
rs6859667
rs672413
rs234709
rs6586282
rs10944
rs705415
rs921943
rs3797535
rs11951068
All − MR Egger
All − IVW(MRE)
−0.5
0.0
0.5
MR effect size for
'Selenium' on 'Mitotic−spindle organizing protein 1'

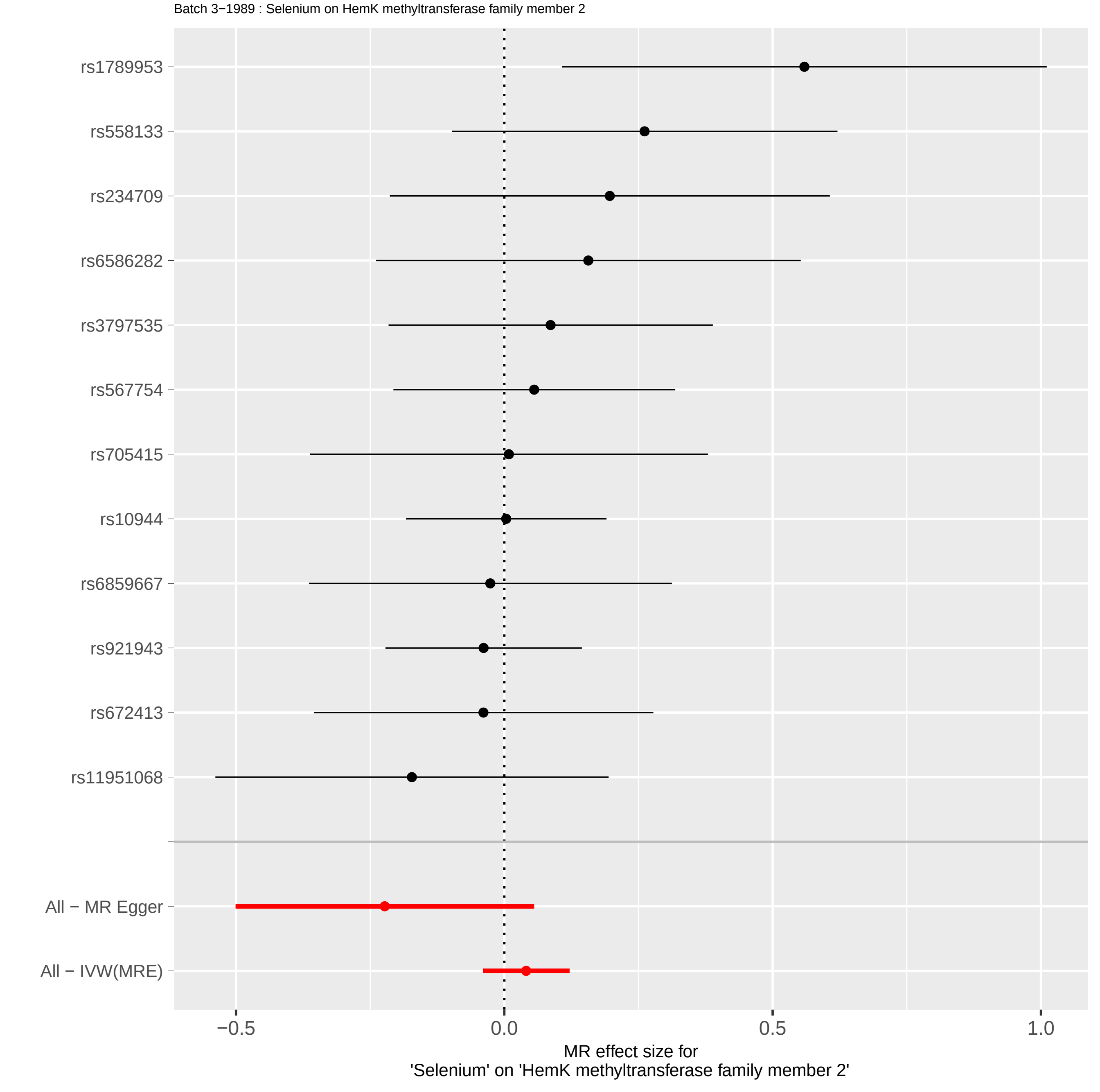

Batch 3−1989 : Selenium on HemK methyltransferase family member 2
rs1789953
rs558133
rs234709
rs6586282
rs3797535
rs567754
rs705415
rs10944
rs6859667
rs921943
rs672413
rs11951068
All − MR Egger
All − IVW(MRE)
−0.5
0.0
0.5
1.0
MR effect size for
'Selenium' on 'HemK methyltransferase family member 2'

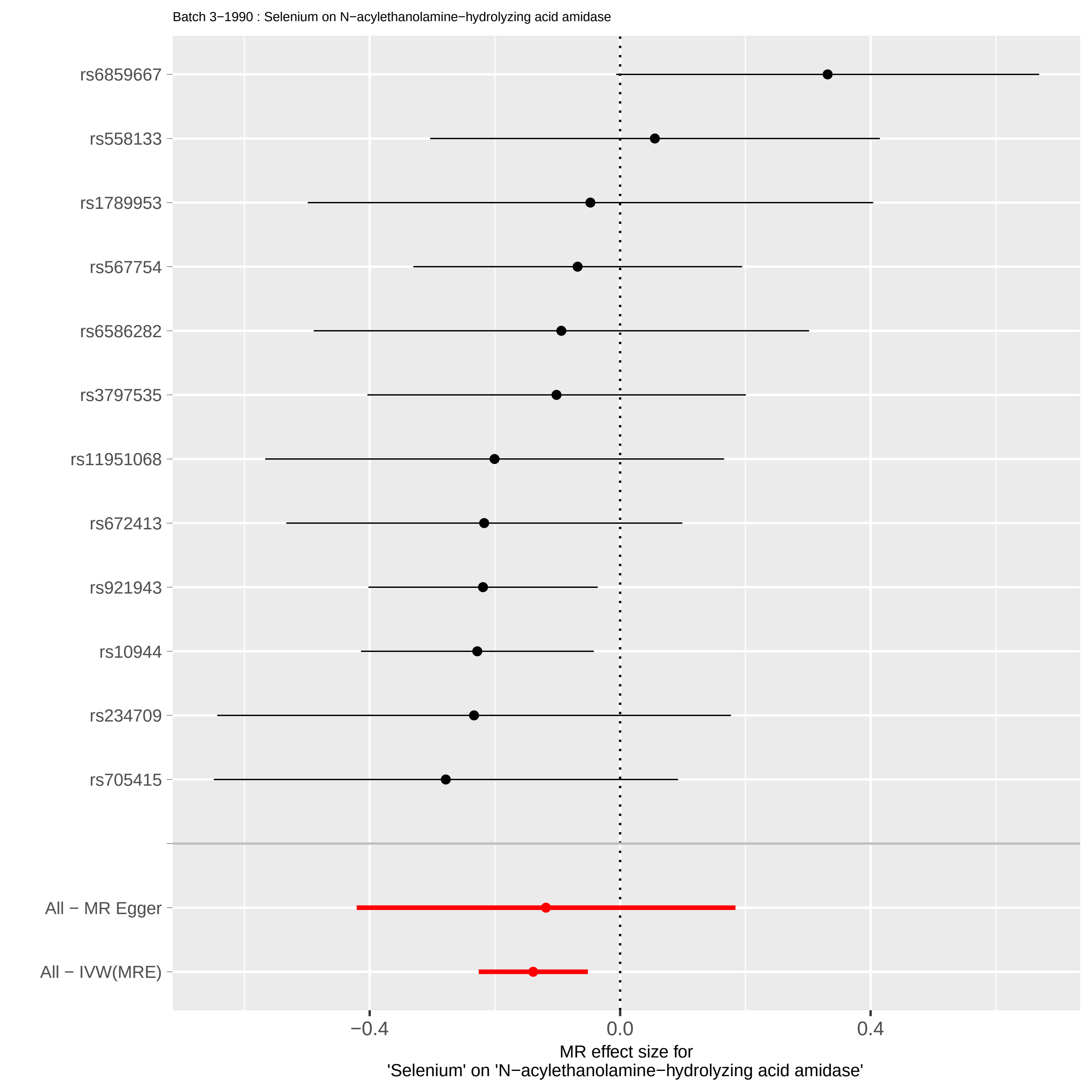
Batch 3−1990 : Selenium on N−acylethanolamine−hydrolyzing acid amidase
rs6859667
rs558133
rs1789953
rs567754
rs6586282
rs3797535
rs11951068
rs672413
rs921943
rs10944
rs234709
rs705415
All − MR Egger
All − IVW(MRE)
−0.4
0.0
0.4
MR effect size for
'Selenium' on 'N−acylethanolamine−hydrolyzing acid amidase'

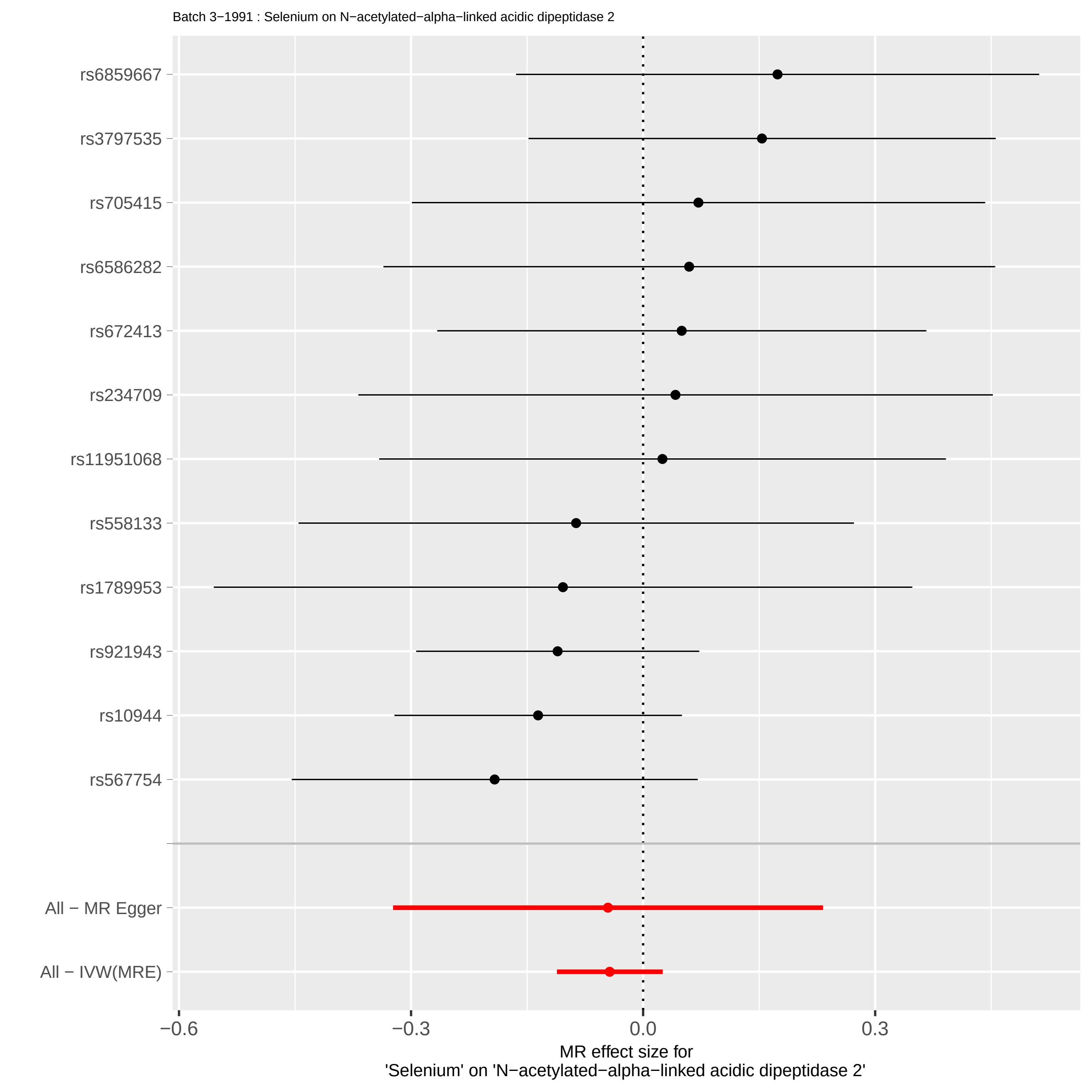
Batch 3−1991 : Selenium on N−acetylated−alpha−linked acidic dipeptidase 2
rs6859667
rs3797535
rs705415
rs6586282
rs672413
rs234709
rs11951068
rs558133
rs1789953
rs921943
rs10944
rs567754
All − MR Egger
All − IVW(MRE)
−0.6
−0.3
0.0
0.3
MR effect size for
'Selenium' on 'N−acetylated−alpha−linked acidic dipeptidase 2'

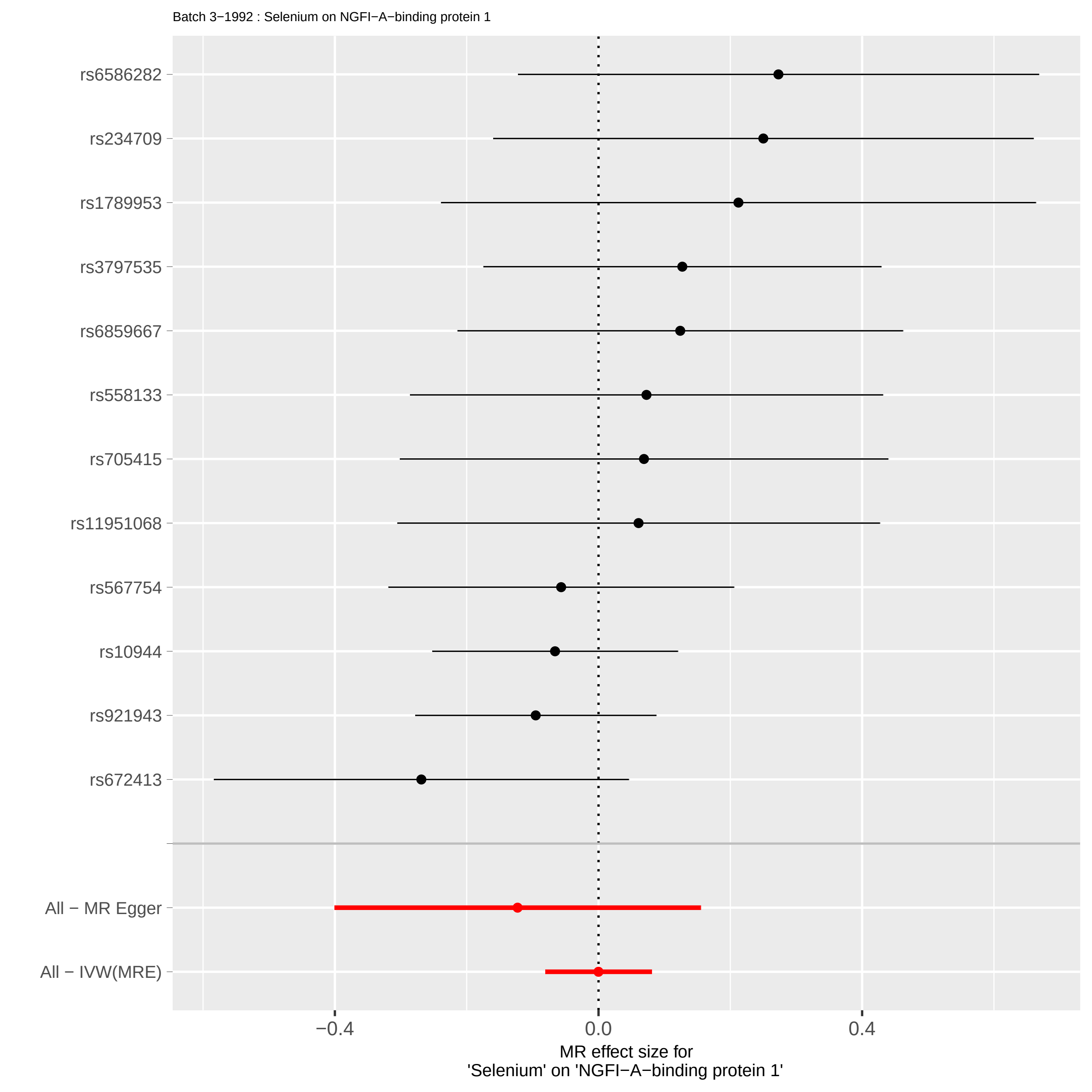
Batch 3−1992 : Selenium on NGFI−A−binding protein 1
rs6586282
rs234709
rs1789953
rs3797535
rs6859667
rs558133
rs705415
rs11951068
rs567754
rs10944
rs921943
rs672413
All − MR Egger
All − IVW(MRE)
−0.4
0.0
0.4
MR effect size for
'Selenium' on 'NGFI−A−binding protein 1'

Batch 3−1993 : Selenium on NGFI−A−binding protein 2

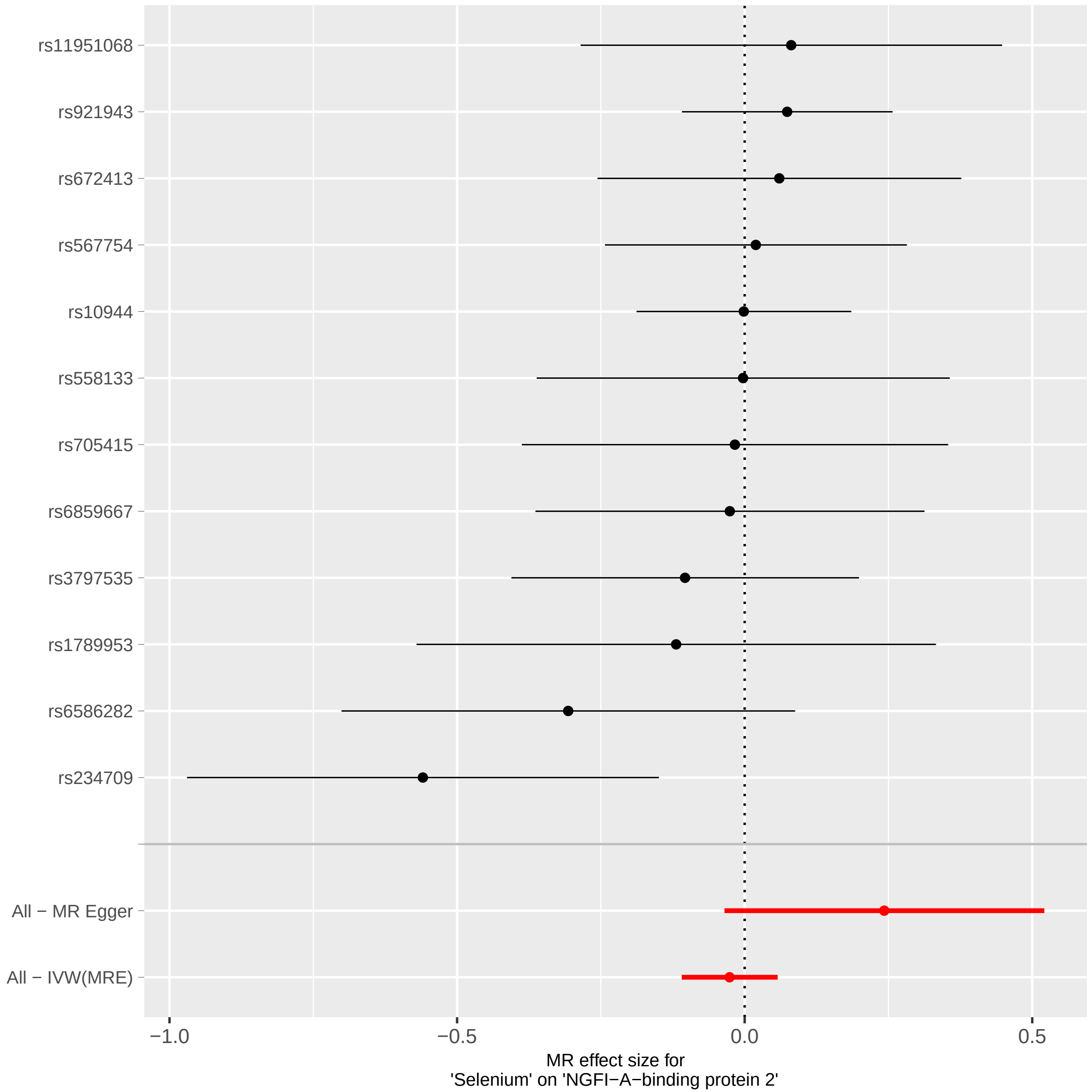

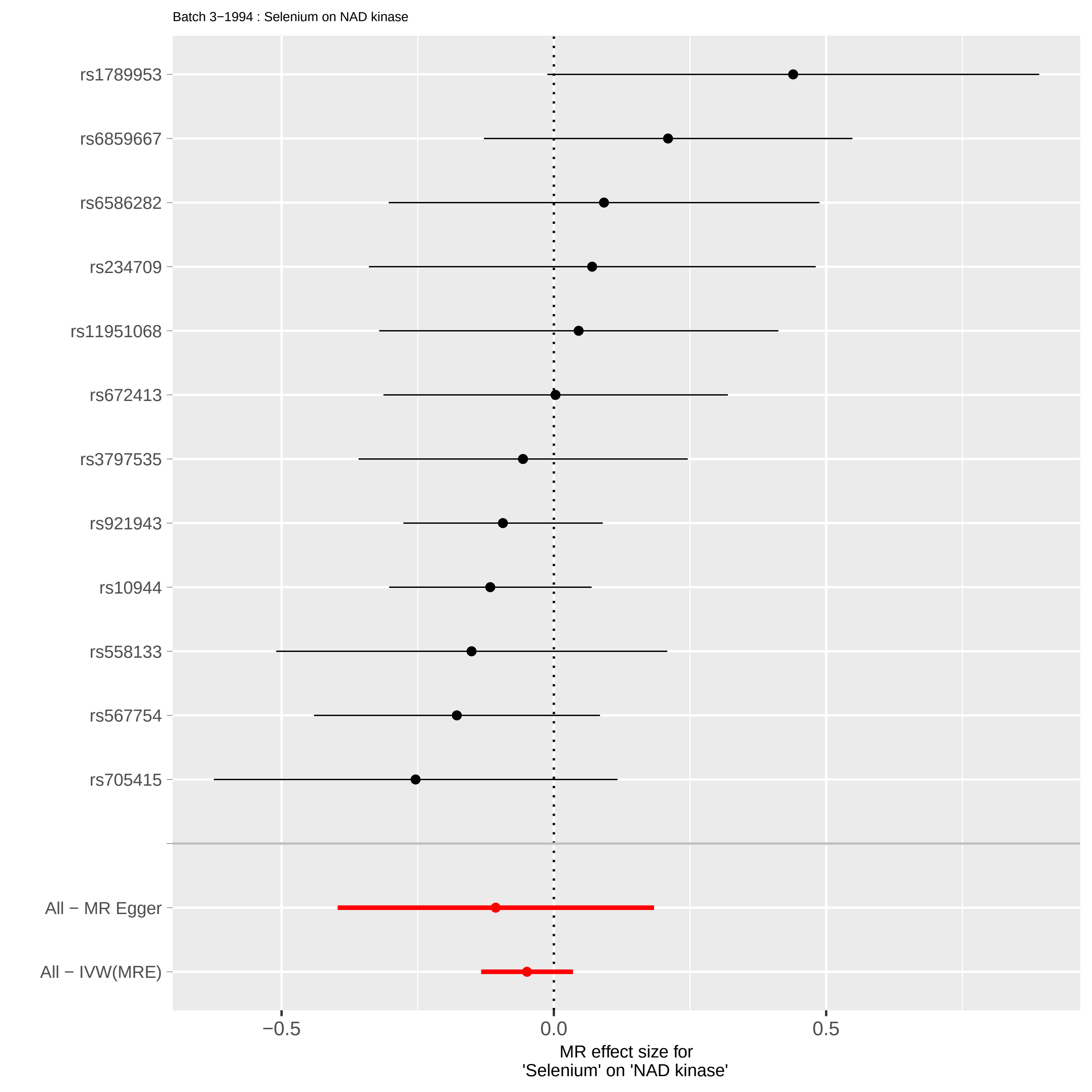

Batch 3−1994 : Selenium on NAD kinase
rs1789953
rs6859667
rs6586282
rs234709
rs11951068
rs672413
rs3797535
rs921943
rs10944
rs558133
rs567754
rs705415
All − MR Egger
All − IVW(MRE)
−0.5
0.0
0.5
MR effect size for
'Selenium' on 'NAD kinase'

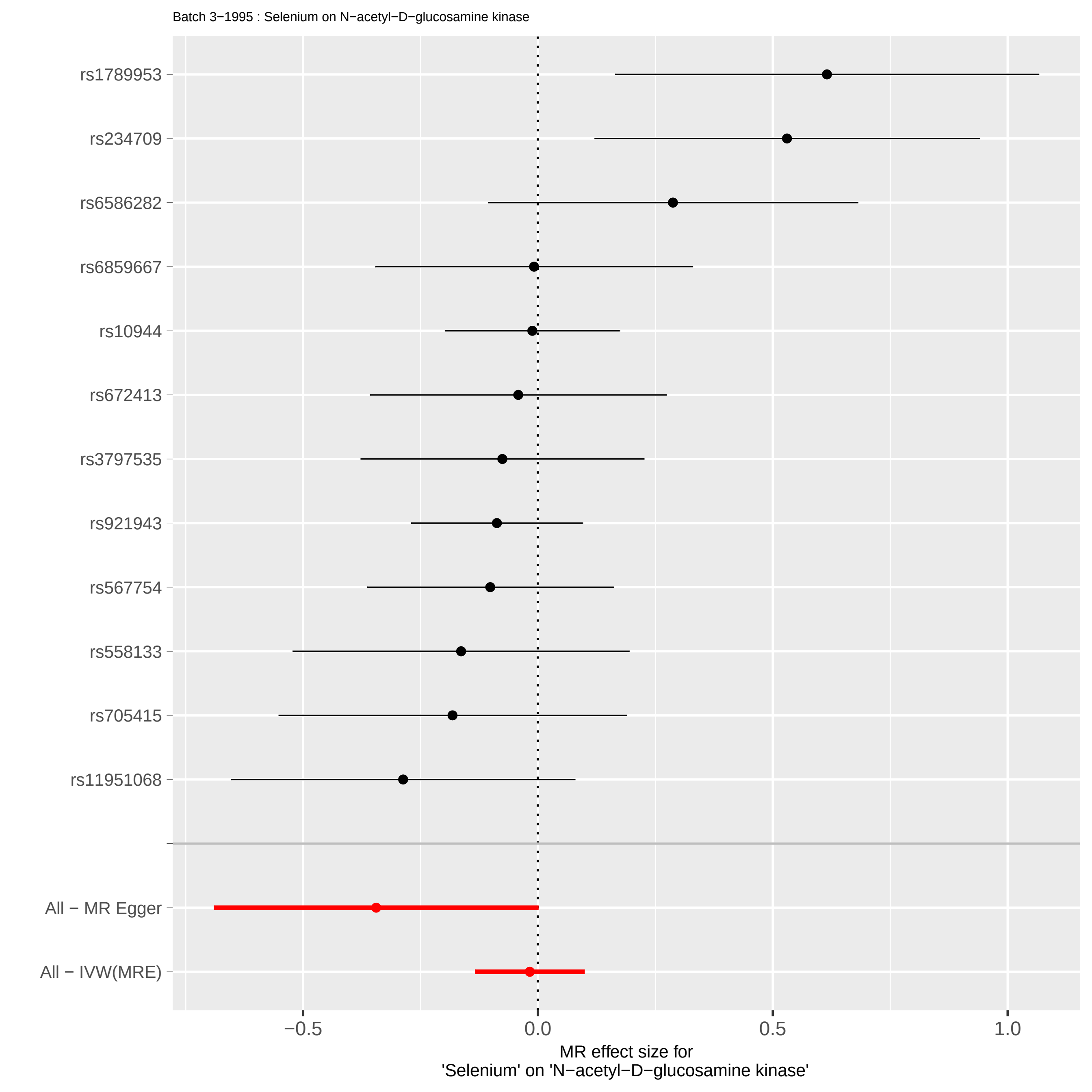

Batch 3−1995 : Selenium on N−acetyl−D−glucosamine kinase
rs1789953
rs234709
rs6586282
rs6859667
rs10944
rs672413
rs3797535
rs921943
rs567754
rs558133
rs705415
rs11951068
All − MR Egger
All − IVW(MRE)
−0.5
0.0
0.5
1.0
MR effect size for
'Selenium' on 'N−acetyl−D−glucosamine kinase'

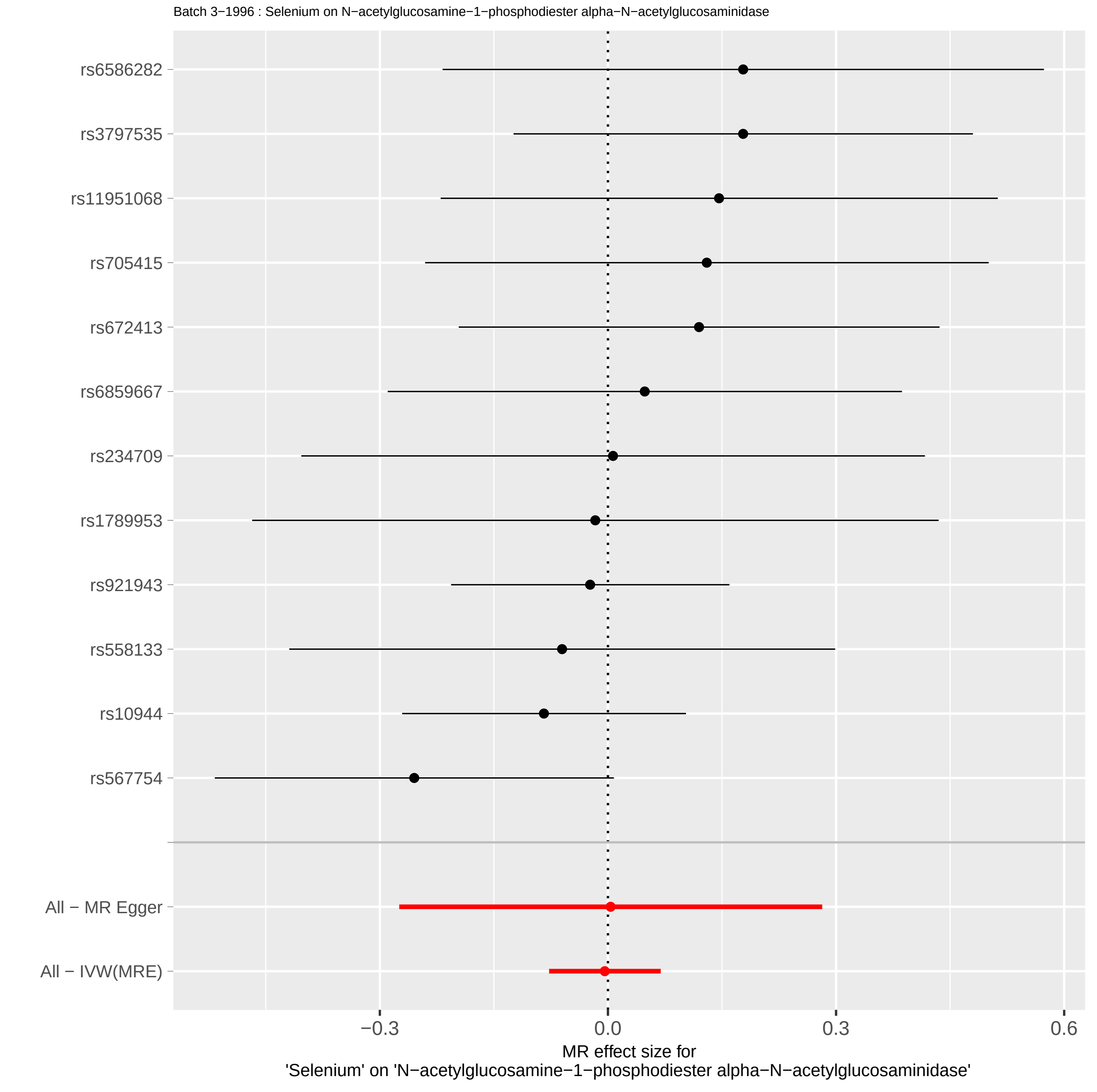

Batch 3−1996 : Selenium on N−acetylglucosamine−1−phosphodiester alpha−N−acetylglucosaminidase
rs6586282
rs3797535
rs11951068
rs705415
rs672413
rs6859667
rs234709
rs1789953
rs921943
rs558133
rs10944
rs567754
All − MR Egger
All − IVW(MRE)
−0.3
0.0
0.3
0.6
MR effect size for
'Selenium' on 'N−acetylglucosamine−1−phosphodiester alpha−N−acetylglucosaminidase'

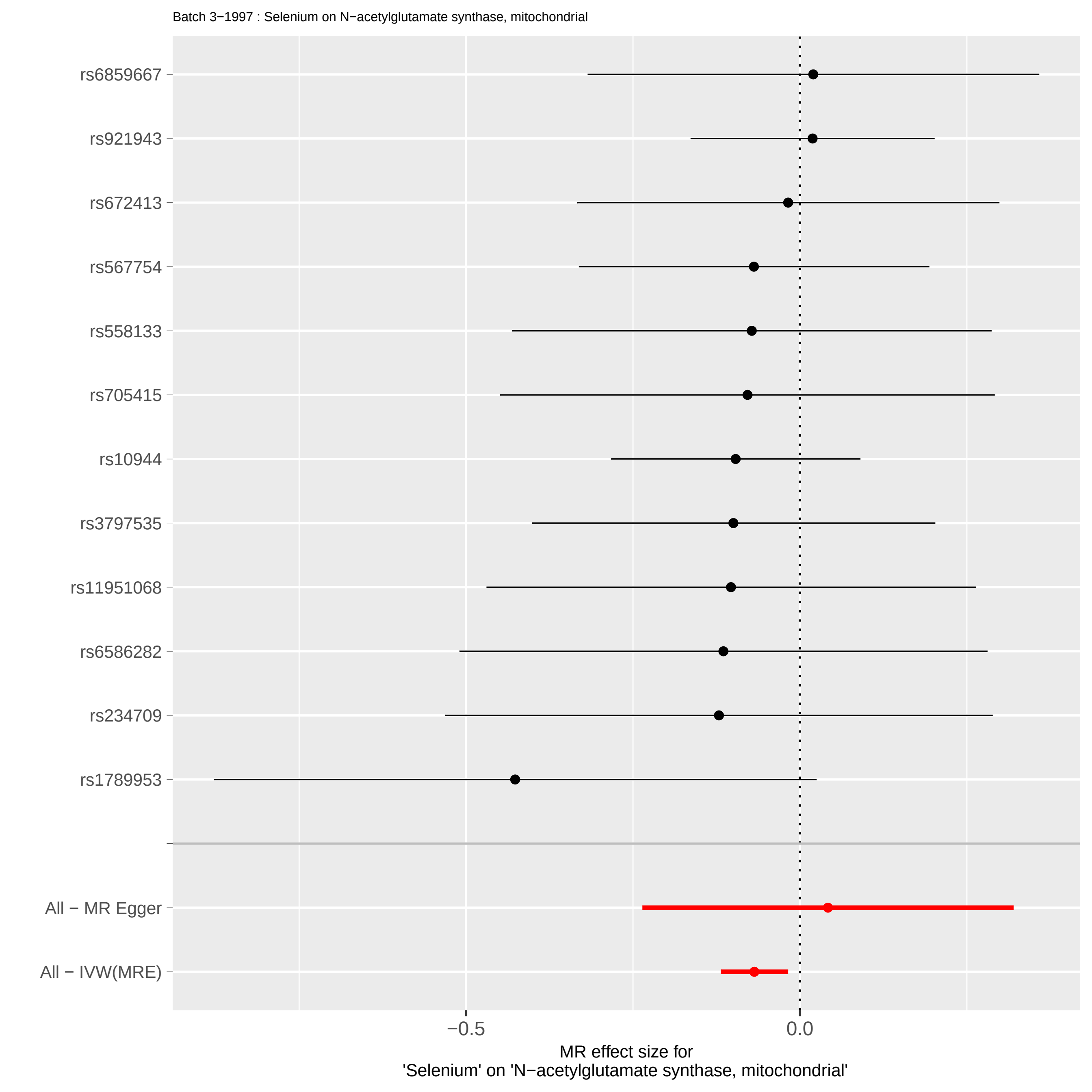

Batch 3−1997 : Selenium on N−acetylglutamate synthase, mitochondrial
rs6859667
rs921943
rs672413
rs567754
rs558133
rs705415
rs10944
rs3797535
rs11951068
rs6586282
rs234709
rs1789953
All − MR Egger
All − IVW(MRE)
−0.5
0.0
MR effect size for
'Selenium' on 'N−acetylglutamate synthase, mitochondrial'

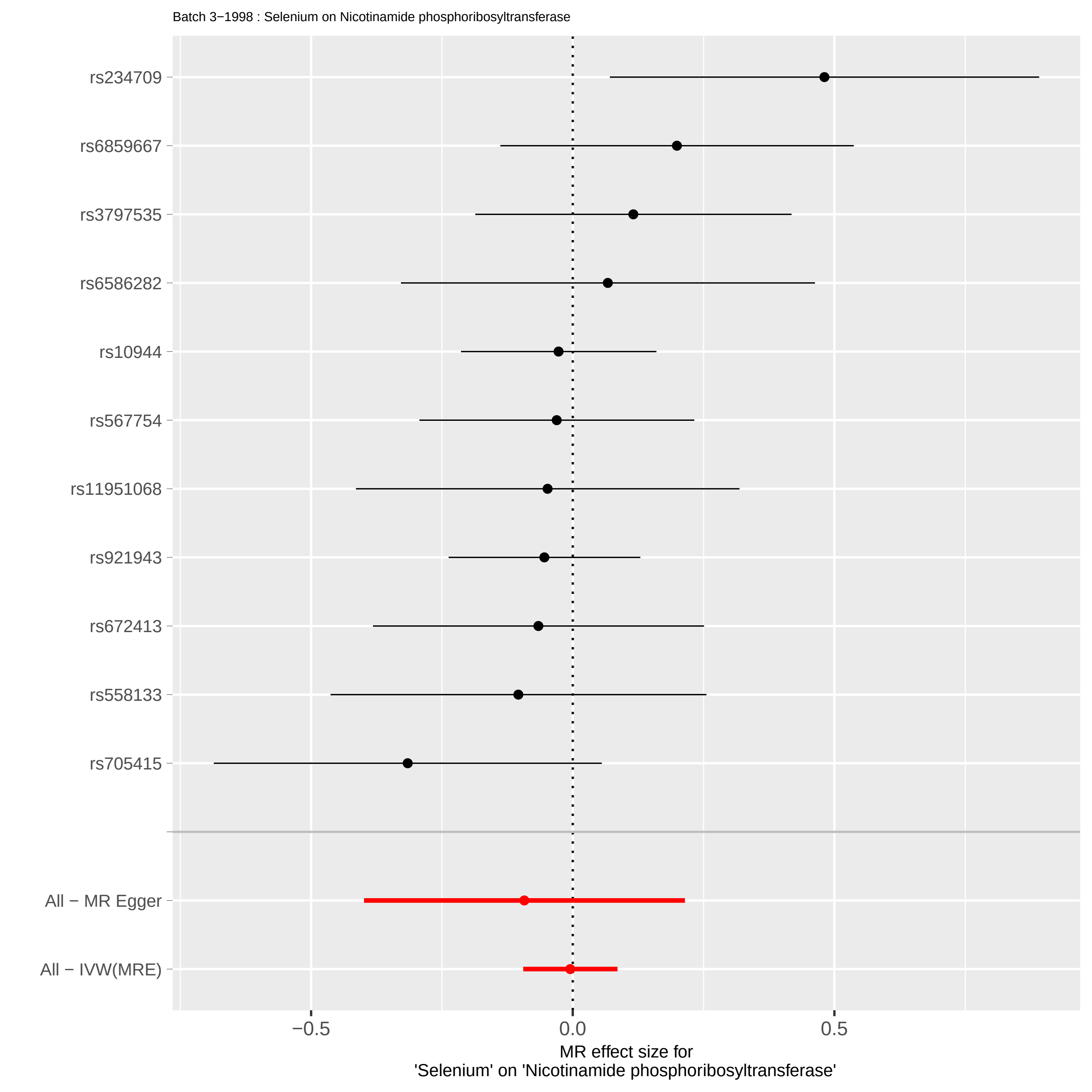

Batch 3−1998 : Selenium on Nicotinamide phosphoribosyltransferase
rs234709
rs6859667
rs3797535
rs6586282
rs10944
rs567754
rs11951068
rs921943
rs672413
rs558133
rs705415
All − MR Egger
All − IVW(MRE)
−0.5
0.0
0.5
MR effect size for
'Selenium' on 'Nicotinamide phosphoribosyltransferase'

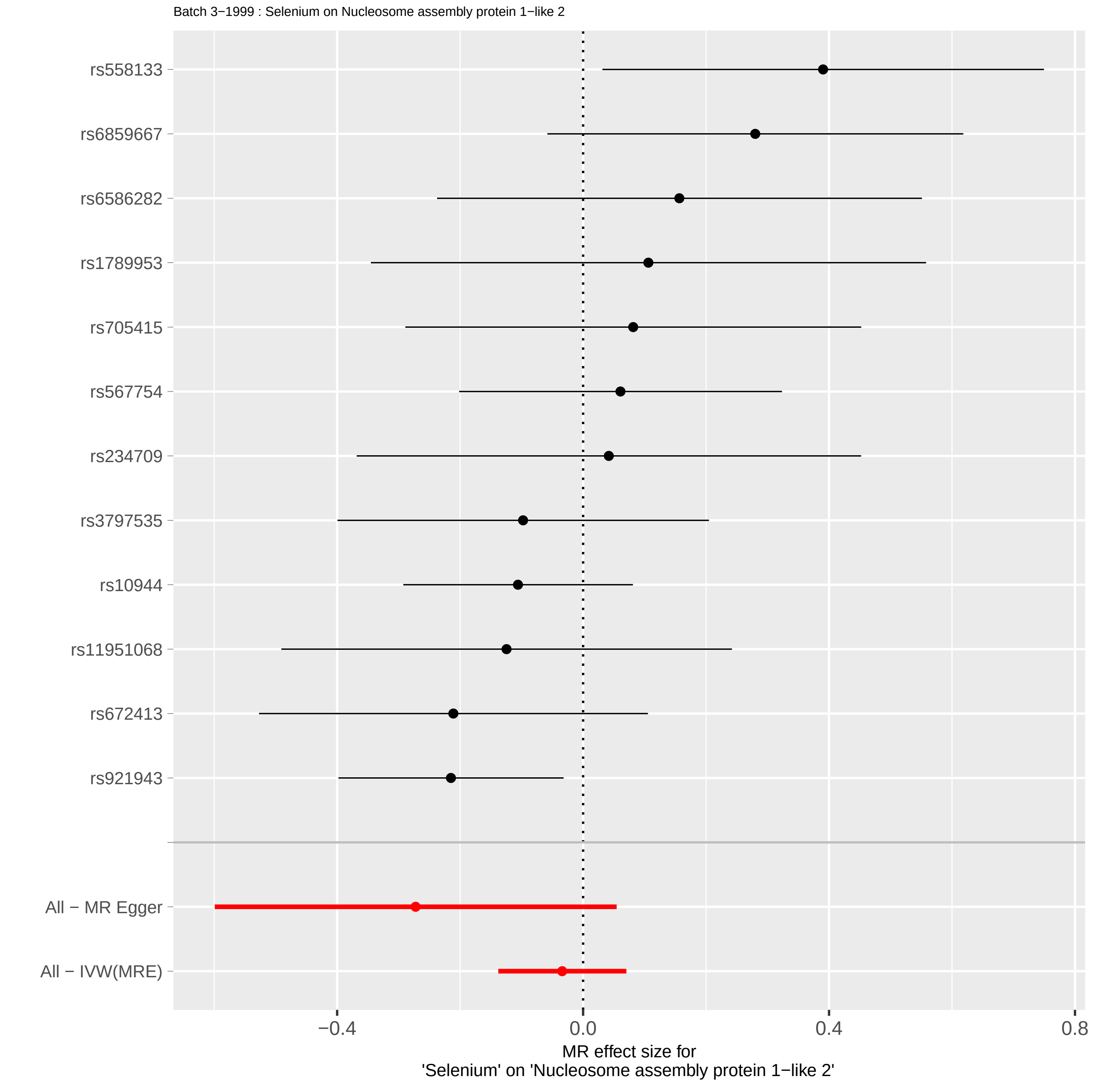
Batch 3−1999 : Selenium on Nucleosome assembly protein 1−like 2
rs558133
rs6859667
rs6586282
rs1789953
rs705415
rs567754
rs234709
rs3797535
rs10944
rs11951068
rs672413
rs921943
All − MR Egger
All − IVW(MRE)
−0.4
0.0
0.4
0.8
MR effect size for
'Selenium' on 'Nucleosome assembly protein 1−like 2'

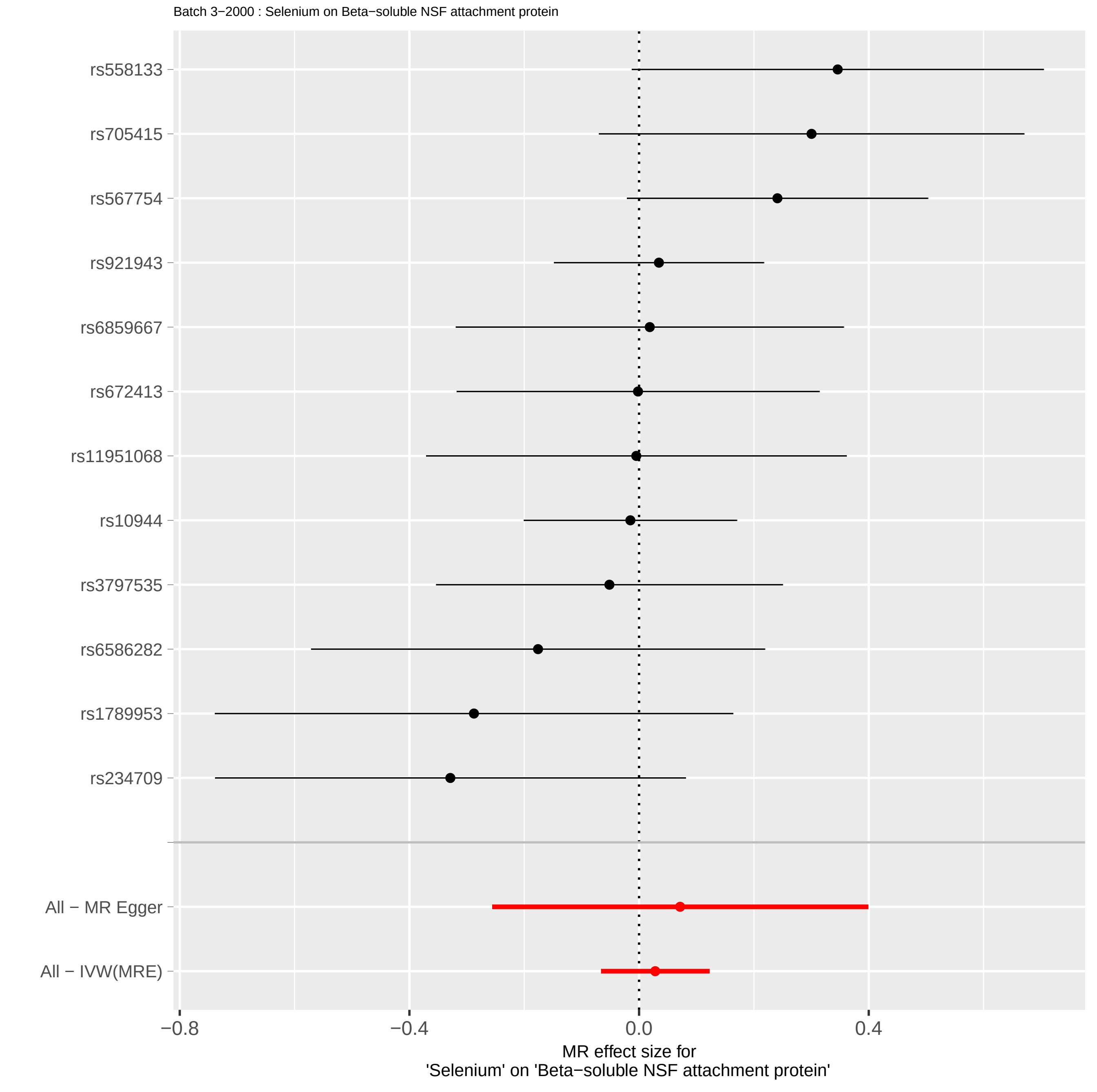
Batch 3−2000 : Selenium on Beta−soluble NSF attachment protein
rs558133
rs705415
rs567754
rs921943
rs6859667
rs672413
rs11951068
rs10944
rs3797535
rs6586282
rs1789953
rs234709
All − MR Egger
All − IVW(MRE)
−0.8
−0.4
0.0
0.4
MR effect size for
'Selenium' on 'Beta−soluble NSF attachment protein'

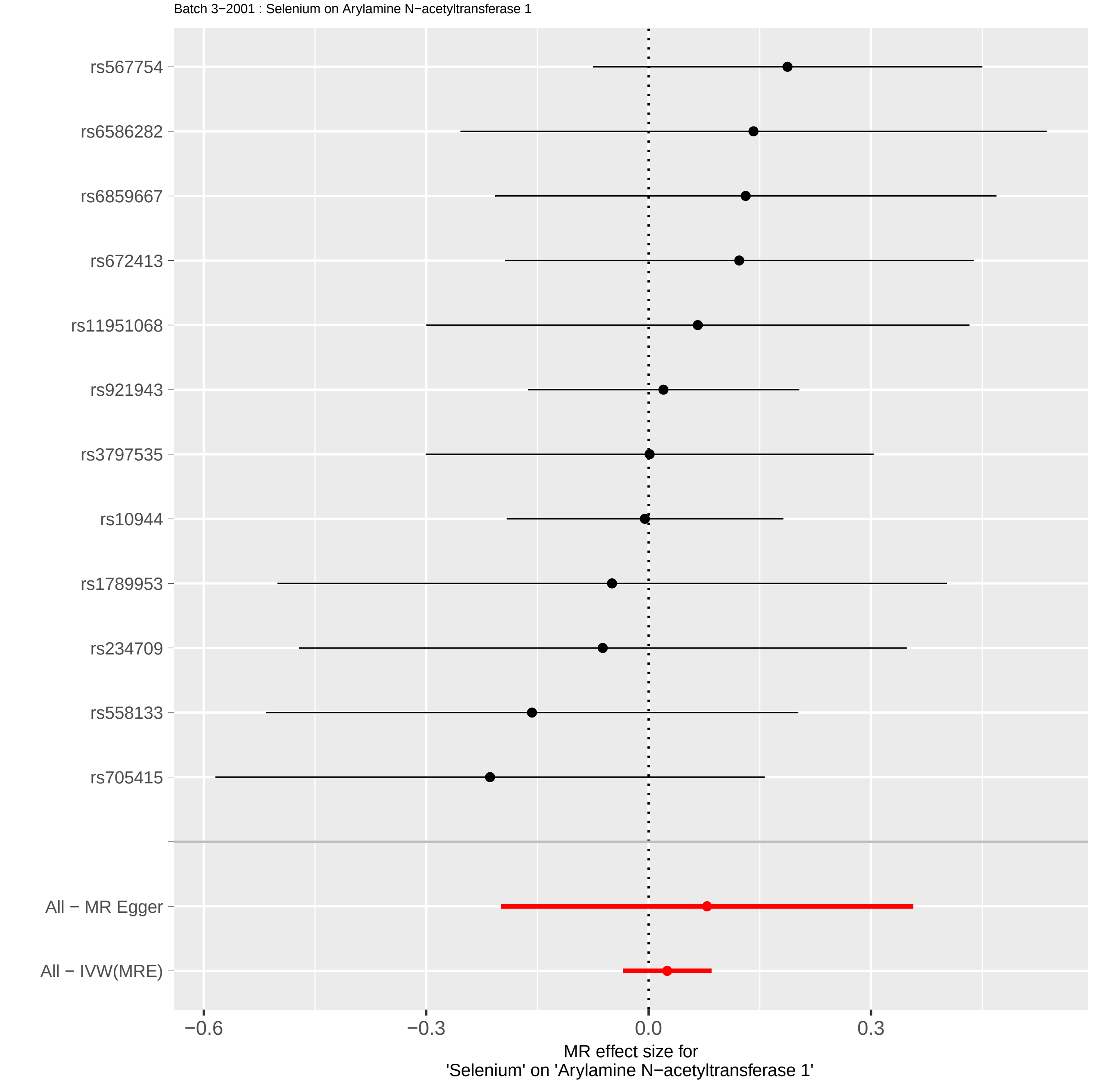

Batch 3−2001 : Selenium on Arylamine N−acetyltransferase 1
rs567754
rs6586282
rs6859667
rs672413
rs11951068
rs921943
rs3797535
rs10944
rs1789953
rs234709
rs558133
rs705415
All − MR Egger
All − IVW(MRE)
−0.6
−0.3
0.0
0.3
MR effect size for
'Selenium' on 'Arylamine N−acetyltransferase 1'

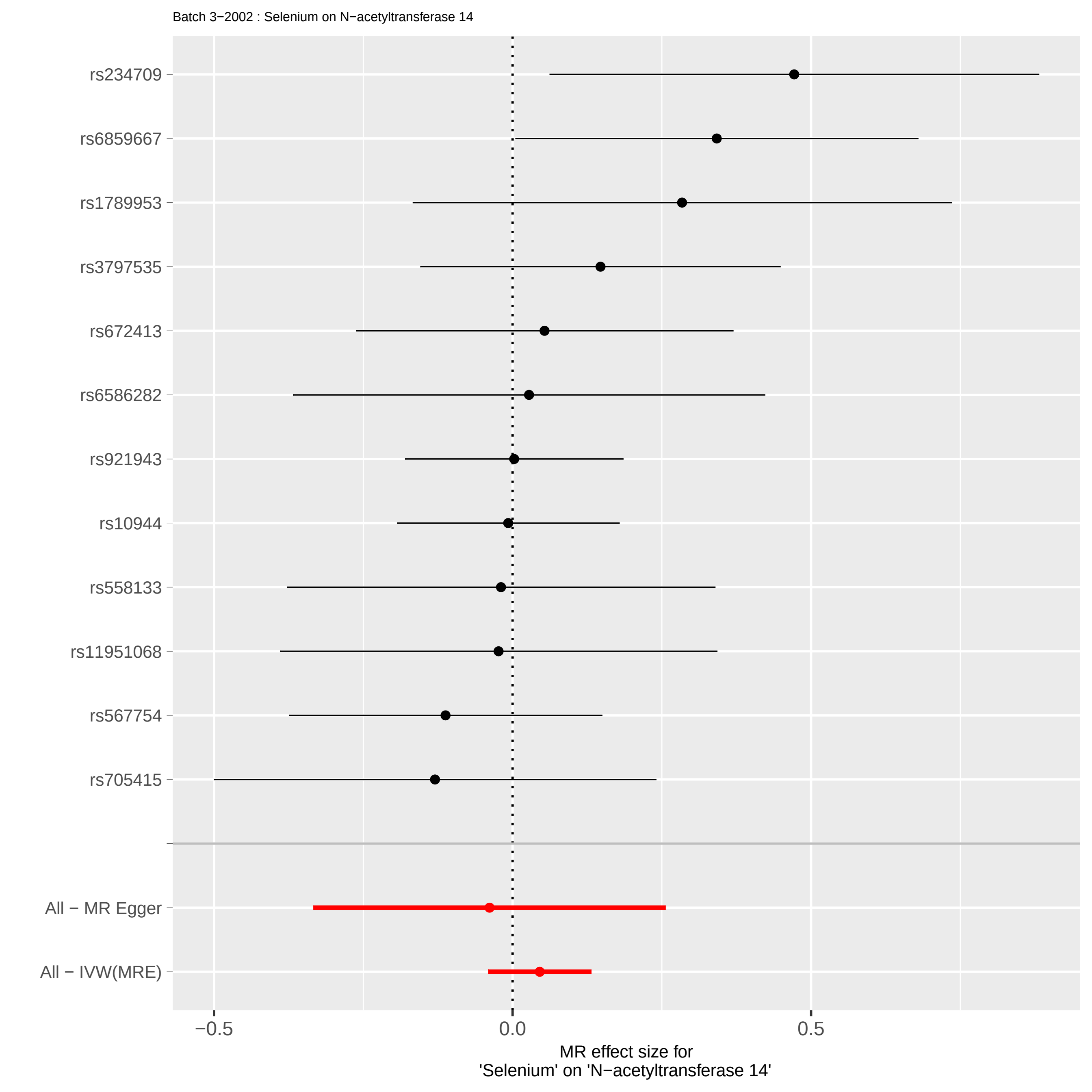

Batch 3−2002 : Selenium on N−acetyltransferase 14
rs234709
rs6859667
rs1789953
rs3797535
rs672413
rs6586282
rs921943
rs10944
rs558133
rs11951068
rs567754
rs705415
All − MR Egger
All − IVW(MRE)
−0.5
0.0
0.5
MR effect size for
'Selenium' on 'N−acetyltransferase 14'

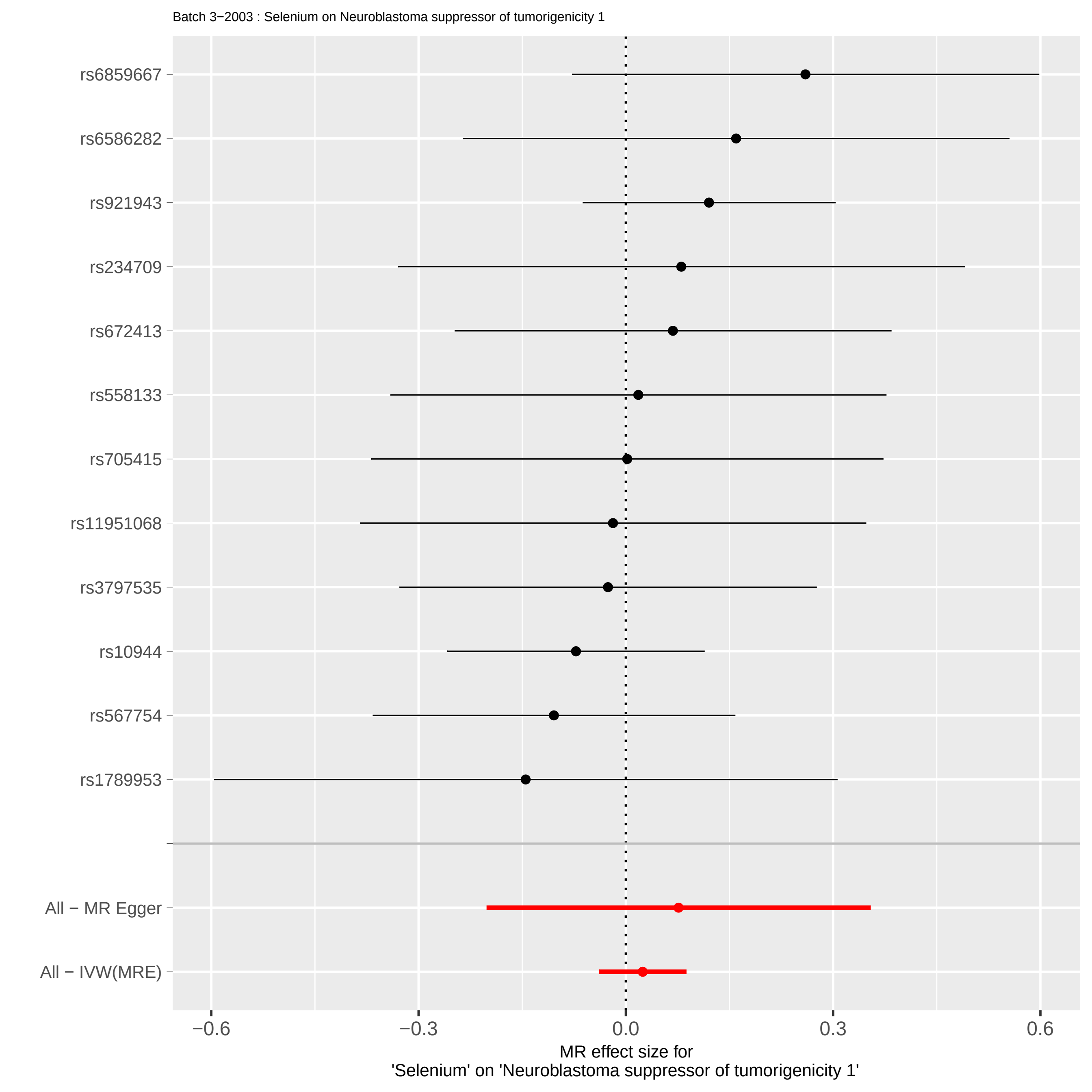
Batch 3−2003 : Selenium on Neuroblastoma suppressor of tumorigenicity 1
rs6859667
rs6586282
rs921943
rs234709
rs672413
rs558133
rs705415
rs11951068
rs3797535
rs10944
rs567754
rs1789953
All − MR Egger
All − IVW(MRE)
−0.6
−0.3
0.0
0.3
0.6
MR effect size for
'Selenium' on 'Neuroblastoma suppressor of tumorigenicity 1'

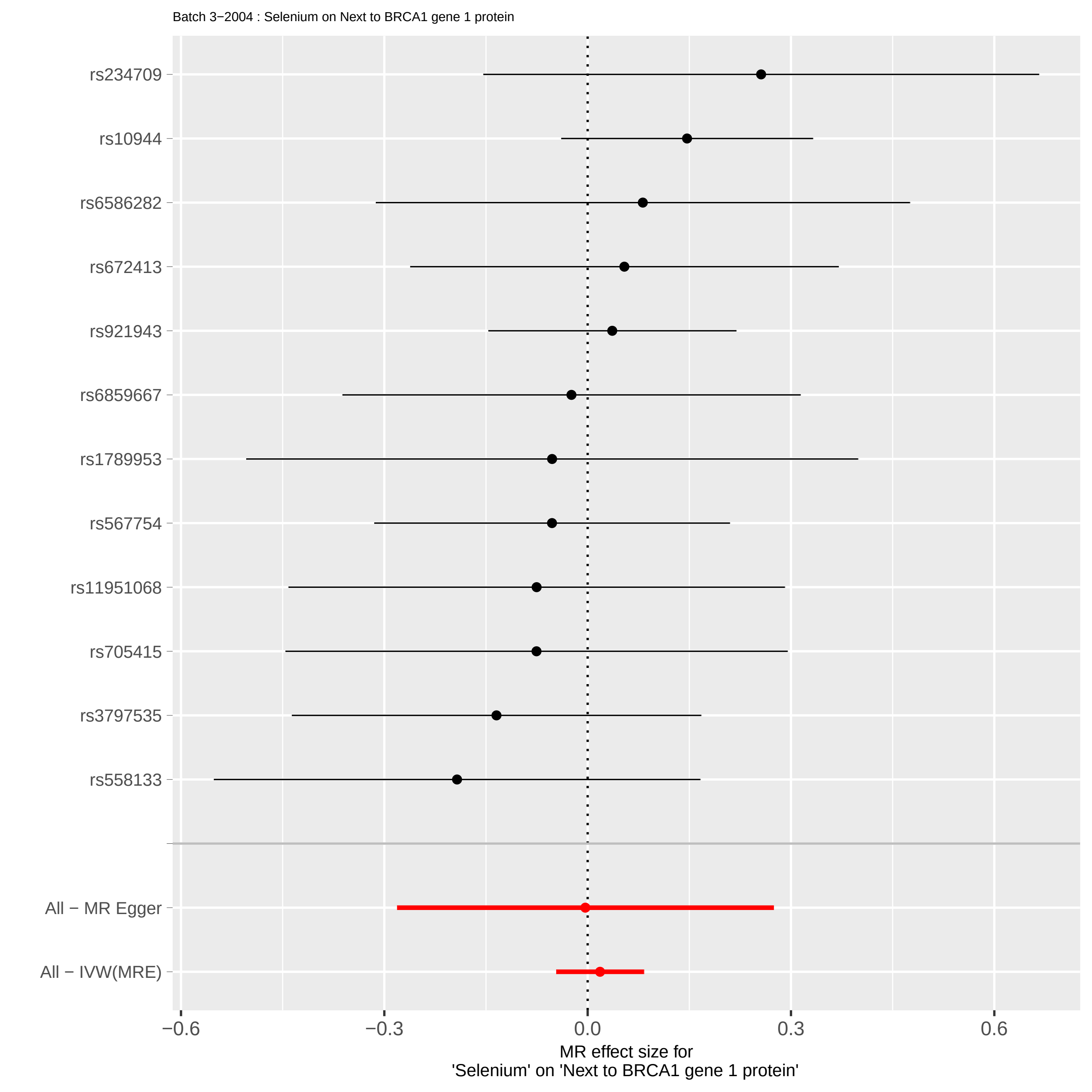

Batch 3−2004 : Selenium on Next to BRCA1 gene 1 protein
rs234709
rs10944
rs6586282
rs672413
rs921943
rs6859667
rs1789953
rs567754
rs11951068
rs705415
rs3797535
rs558133
All − MR Egger
All − IVW(MRE)
−0.6
−0.3
0.0
0.3
0.6
MR effect size for
'Selenium' on 'Next to BRCA1 gene 1 protein'

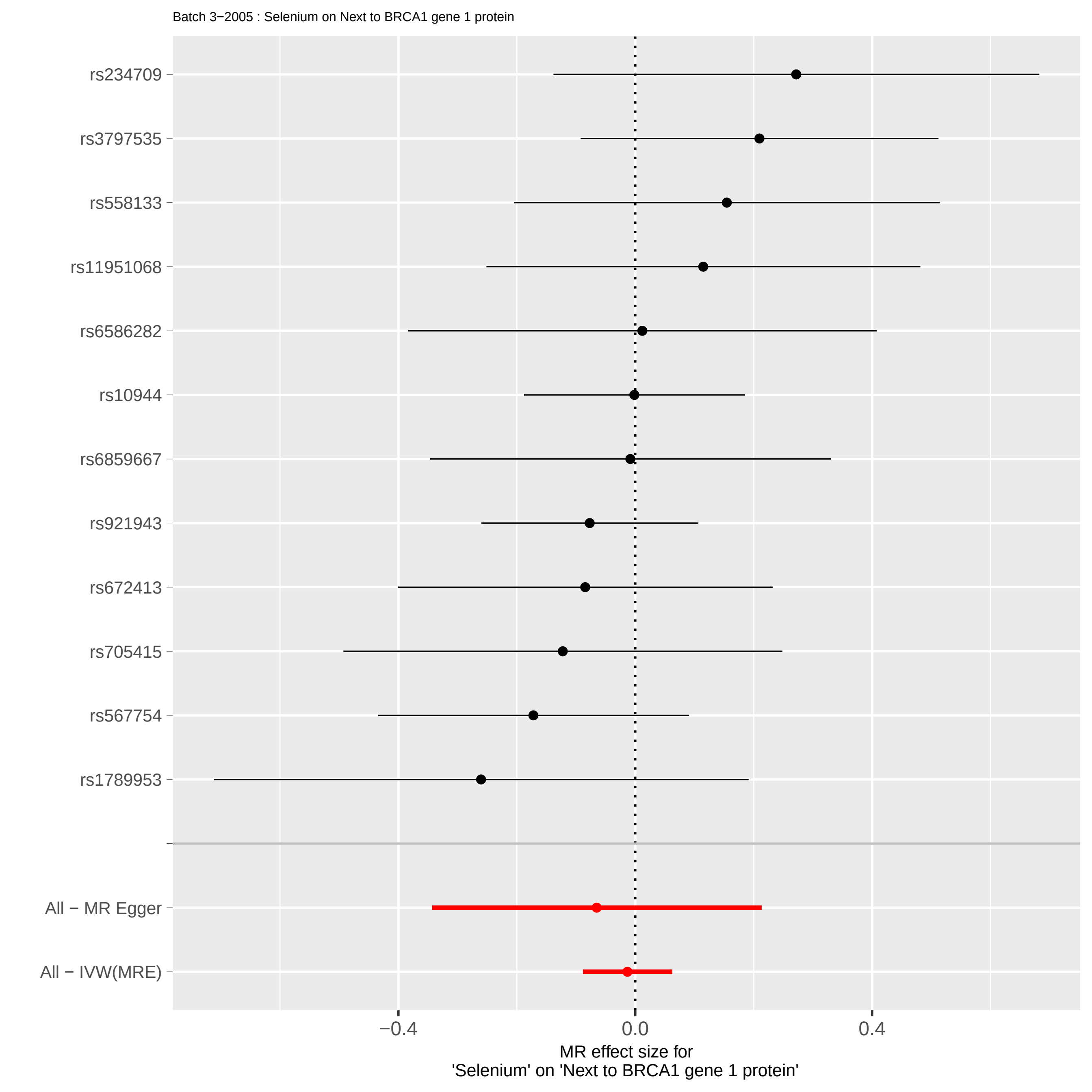
Batch 3−2005 : Selenium on Next to BRCA1 gene 1 protein
rs234709
rs3797535
rs558133
rs11951068
rs6586282
rs10944
rs6859667
rs921943
rs672413
rs705415
rs567754
rs1789953
All − MR Egger
All − IVW(MRE)
−0.4
0.0
0.4
MR effect size for
'Selenium' on 'Next to BRCA1 gene 1 protein'

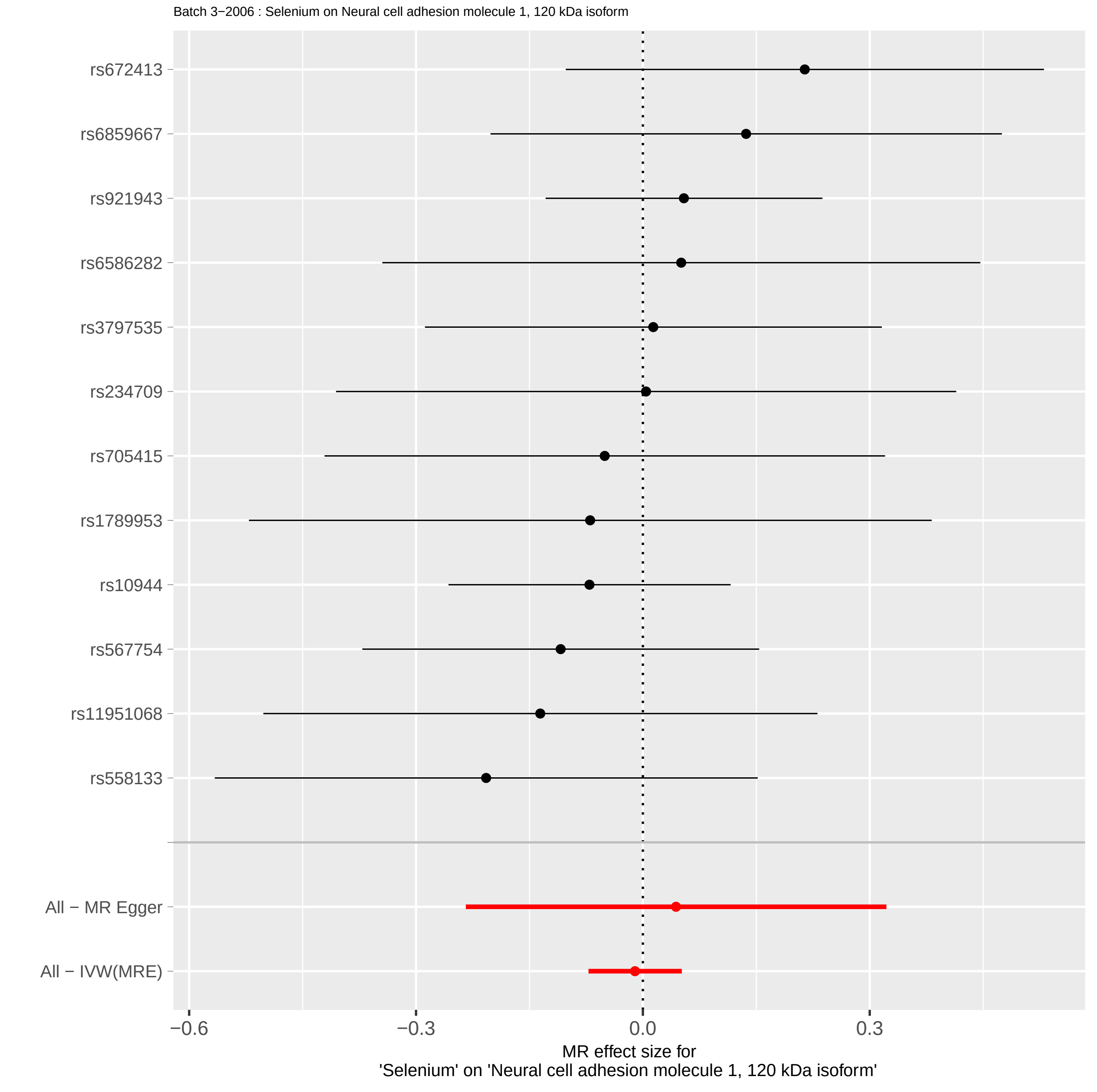
Batch 3−2006 : Selenium on Neural cell adhesion molecule 1, 120 kDa isoform
rs672413
rs6859667
rs921943
rs6586282
rs3797535
rs234709
rs705415
rs1789953
rs10944
rs567754
rs11951068
rs558133
All − MR Egger
All − IVW(MRE)
−0.6
−0.3
0.0
0.3
MR effect size for
'Selenium' on 'Neural cell adhesion molecule 1, 120 kDa isoform'

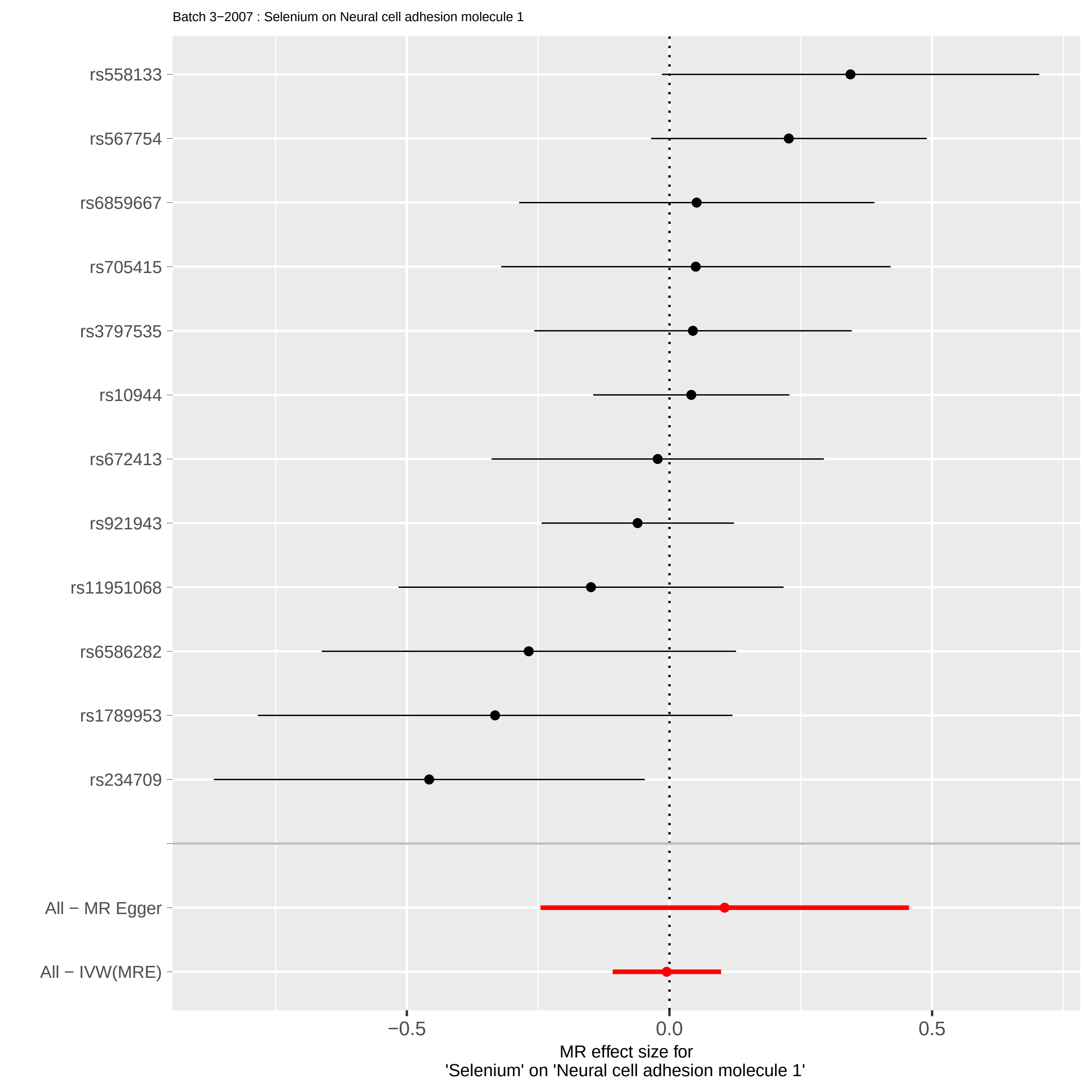
Batch 3−2007 : Selenium on Neural cell adhesion molecule 1
rs558133
rs567754
rs6859667
rs705415
rs3797535
rs10944
rs672413
rs921943
rs11951068
rs6586282
rs1789953
rs234709
All − MR Egger
All − IVW(MRE)
−0.5
0.0
0.5
MR effect size for
'Selenium' on 'Neural cell adhesion molecule 1'

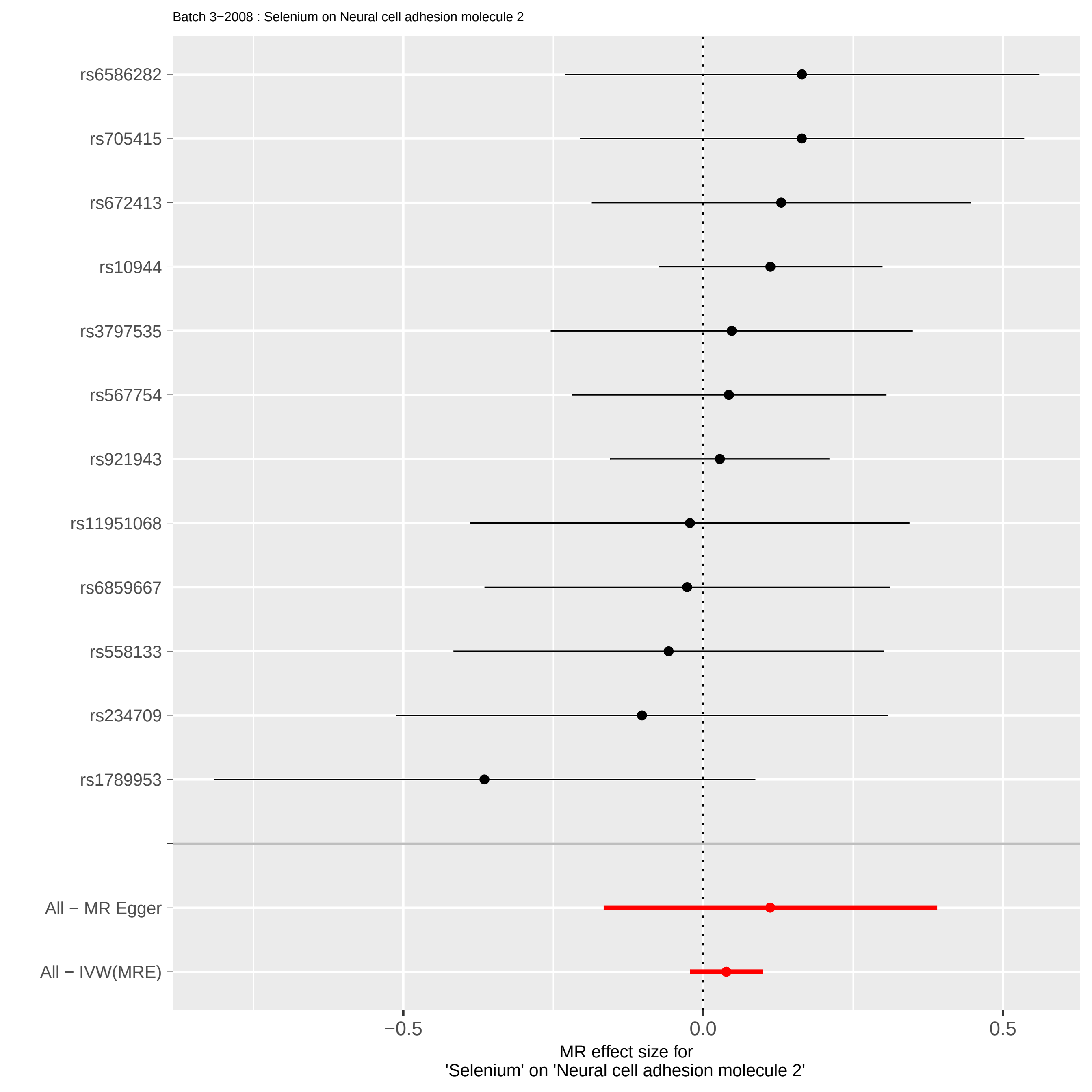

Batch 3−2008 : Selenium on Neural cell adhesion molecule 2
rs6586282
rs705415
rs672413
rs10944
rs3797535
rs567754
rs921943
rs11951068
rs6859667
rs558133
rs234709
rs1789953
All − MR Egger
All − IVW(MRE)
−0.5
0.0
0.5
MR effect size for
'Selenium' on 'Neural cell adhesion molecule 2'

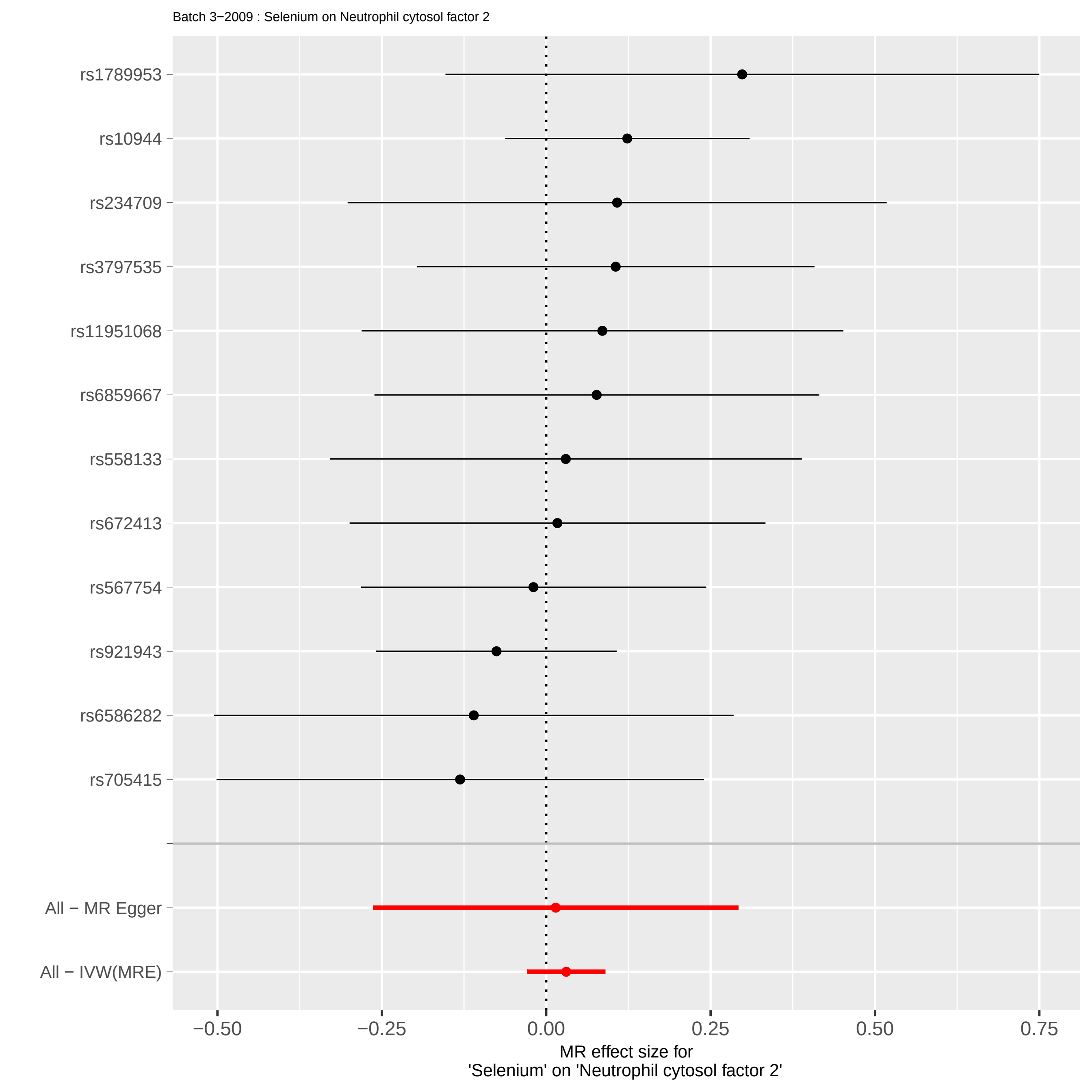

Batch 3−2009 : Selenium on Neutrophil cytosol factor 2
rs1789953
rs10944
rs234709
rs3797535
rs11951068
rs6859667
rs558133
rs672413
rs567754
rs921943
rs6586282
rs705415
All − MR Egger
All − IVW(MRE)
−0.50
−0.25
0.00
0.25
0.50
0.75
MR effect size for
'Selenium' on 'Neutrophil cytosol factor 2'

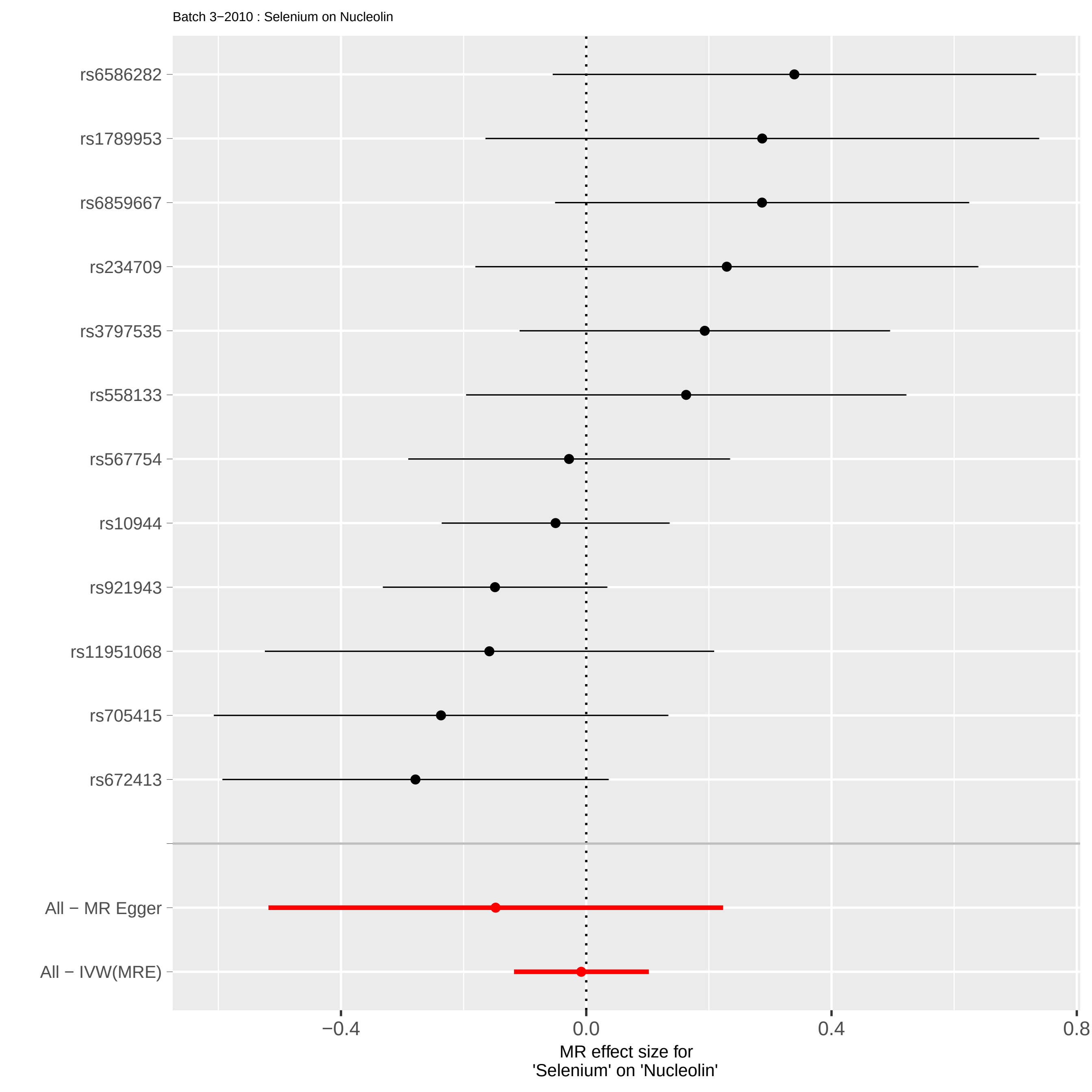

Batch 3−2010 : Selenium on Nucleolin
rs6586282
rs1789953
rs6859667
rs234709
rs3797535
rs558133
rs567754
rs10944
rs921943
rs11951068
rs705415
rs672413
All − MR Egger
All − IVW(MRE)
−0.4
0.0
0.4
0.8
MR effect size for
'Selenium' on 'Nucleolin'

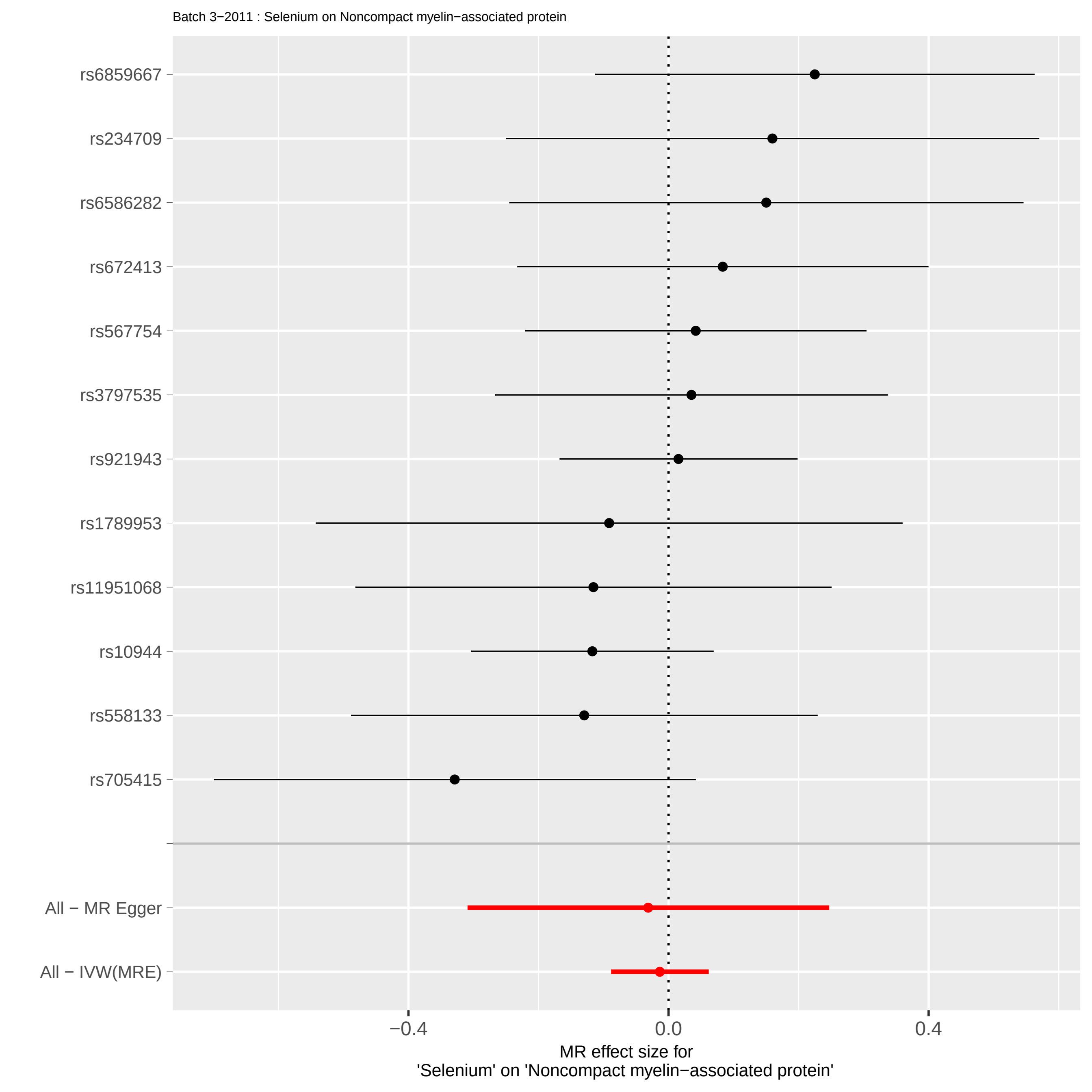
Batch 3−2011 : Selenium on Noncompact myelin−associated protein
rs6859667
rs234709
rs6586282
rs672413
rs567754
rs3797535
rs921943
rs1789953
rs11951068
rs10944
rs558133
rs705415
All − MR Egger
All − IVW(MRE)
−0.4
0.0
0.4
MR effect size for
'Selenium' on 'Noncompact myelin−associated protein'

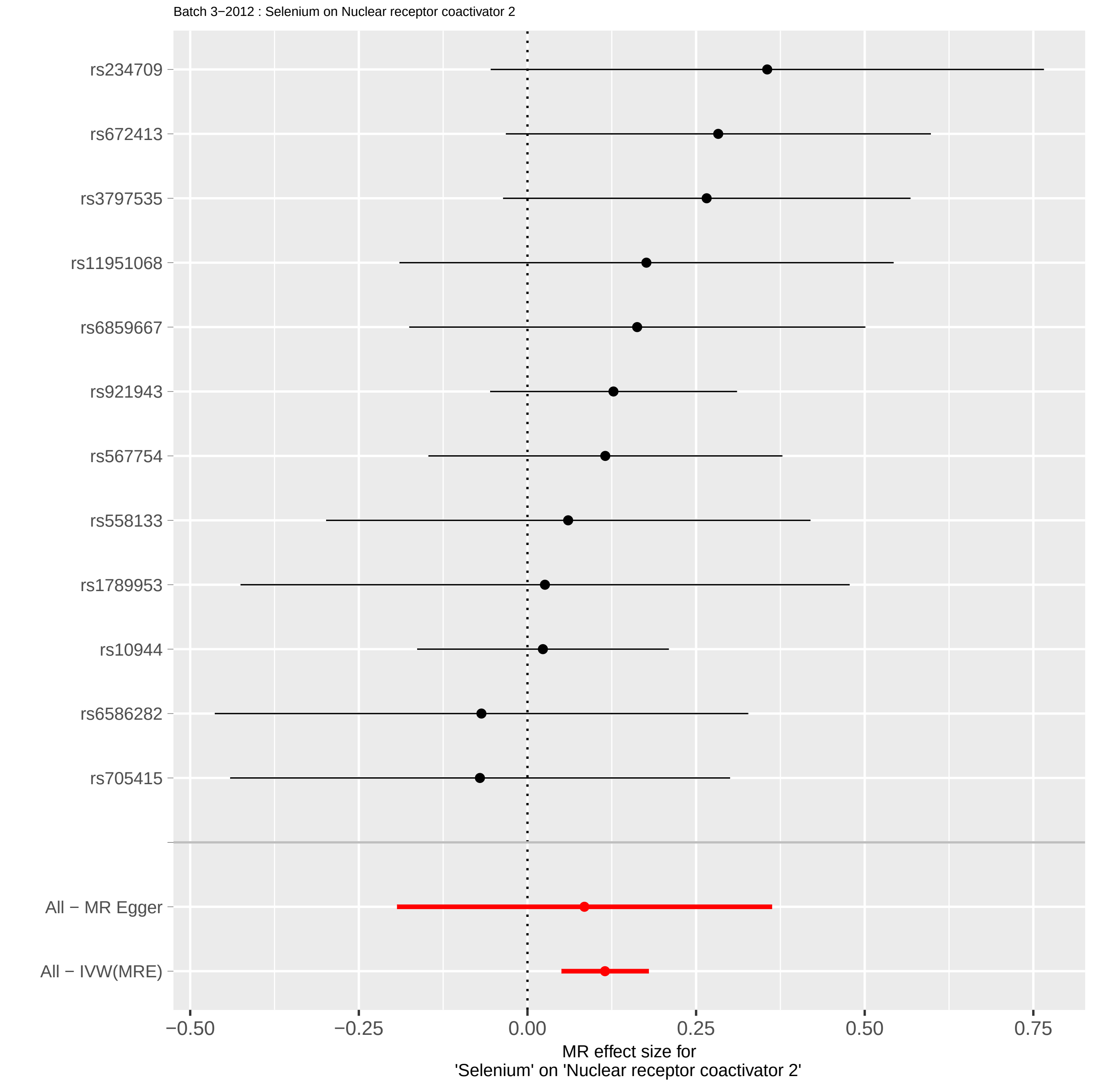

Batch 3−2012 : Selenium on Nuclear receptor coactivator 2
rs234709
rs672413
rs3797535
rs11951068
rs6859667
rs921943
rs567754
rs558133
rs1789953
rs10944
rs6586282
rs705415
All − MR Egger
All − IVW(MRE)
−0.50
−0.25
0.00
0.25
0.50
0.75
MR effect size for
'Selenium' on 'Nuclear receptor coactivator 2'

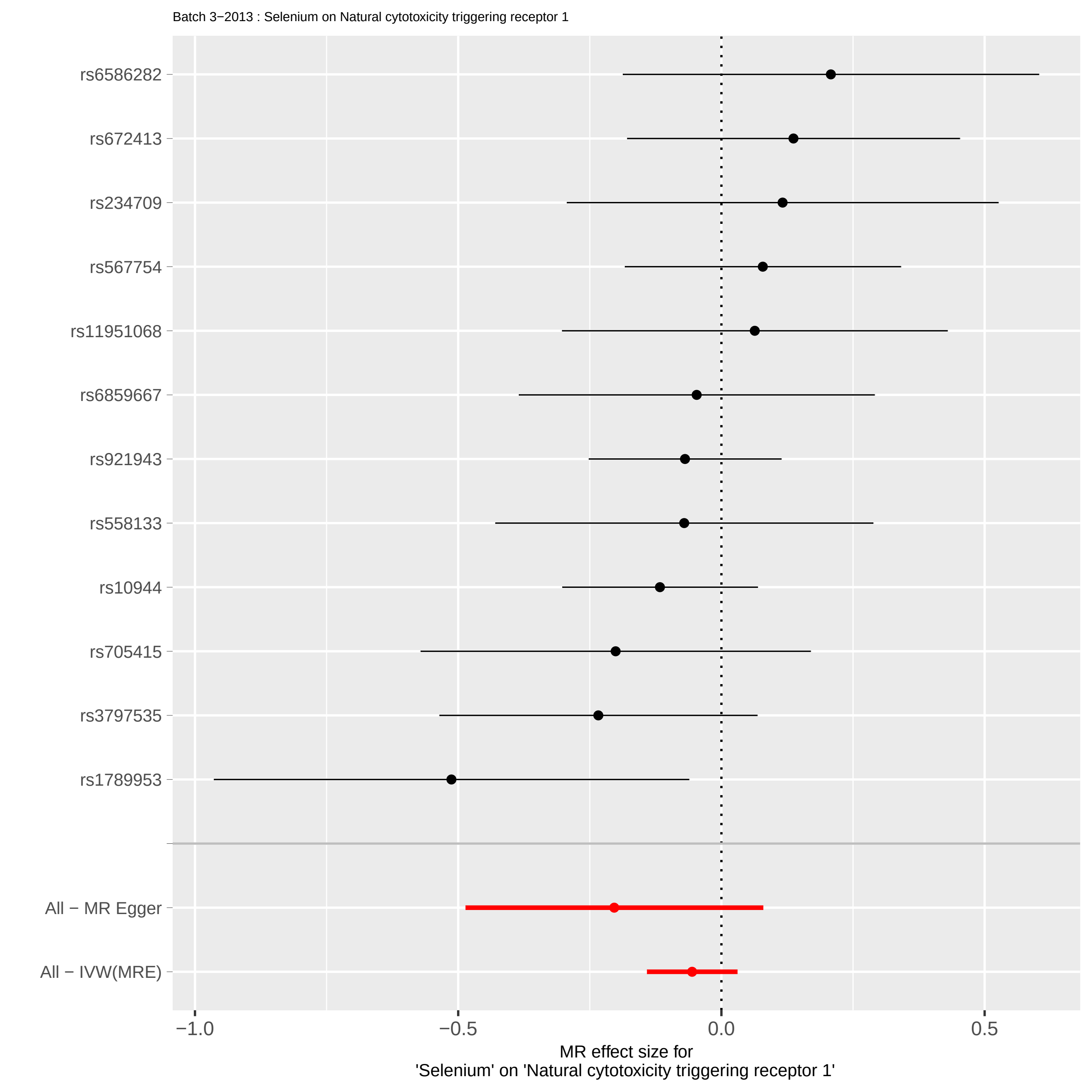
Batch 3−2013 : Selenium on Natural cytotoxicity triggering receptor 1
rs6586282
rs672413
rs234709
rs567754
rs11951068
rs6859667
rs921943
rs558133
rs10944
rs705415
rs3797535
rs1789953
All − MR Egger
All − IVW(MRE)
−1.0
−0.5
0.0
0.5
MR effect size for
'Selenium' on 'Natural cytotoxicity triggering receptor 1'

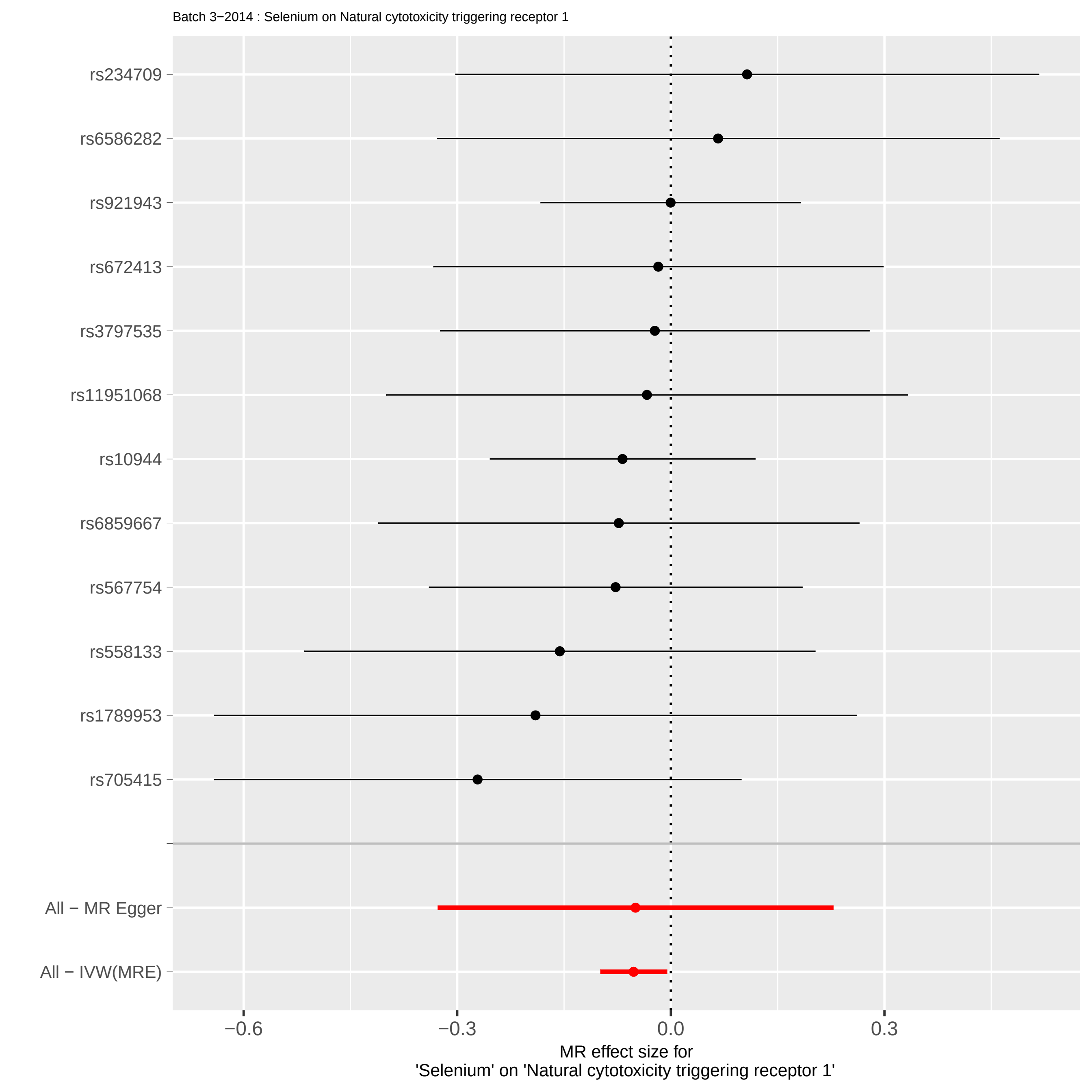

Batch 3−2014 : Selenium on Natural cytotoxicity triggering receptor 1
rs234709
rs6586282
rs921943
rs672413
rs3797535
rs11951068
rs10944
rs6859667
rs567754
rs558133
rs1789953
rs705415
All − MR Egger
All − IVW(MRE)
−0.6
−0.3
0.0
0.3
MR effect size for
'Selenium' on 'Natural cytotoxicity triggering receptor 1'

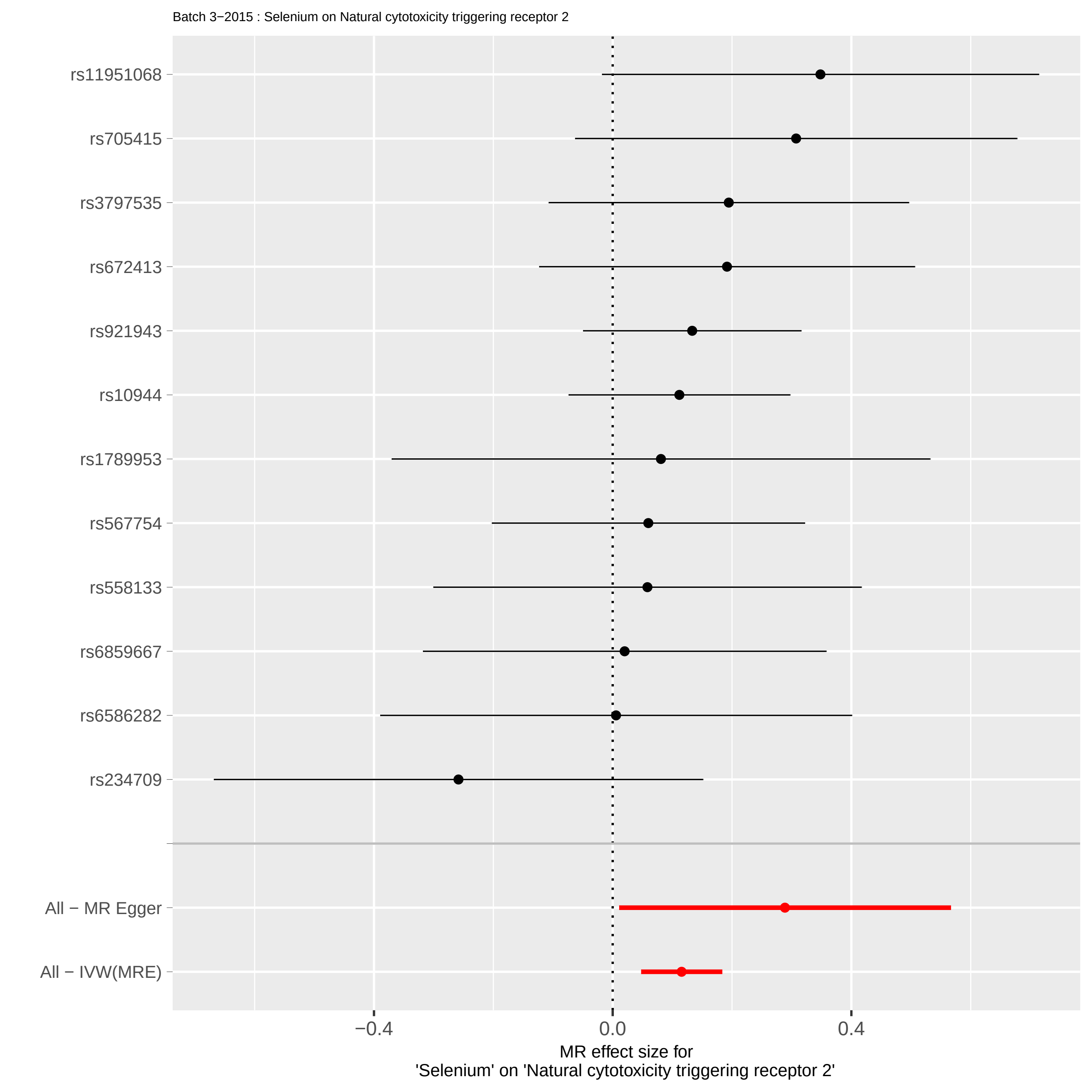

Batch 3−2015 : Selenium on Natural cytotoxicity triggering receptor 2
rs11951068
rs705415
rs3797535
rs672413
rs921943
rs10944
rs1789953
rs567754
rs558133
rs6859667
rs6586282
rs234709
All − MR Egger
All − IVW(MRE)
−0.4
0.0
0.4
MR effect size for
'Selenium' on 'Natural cytotoxicity triggering receptor 2'

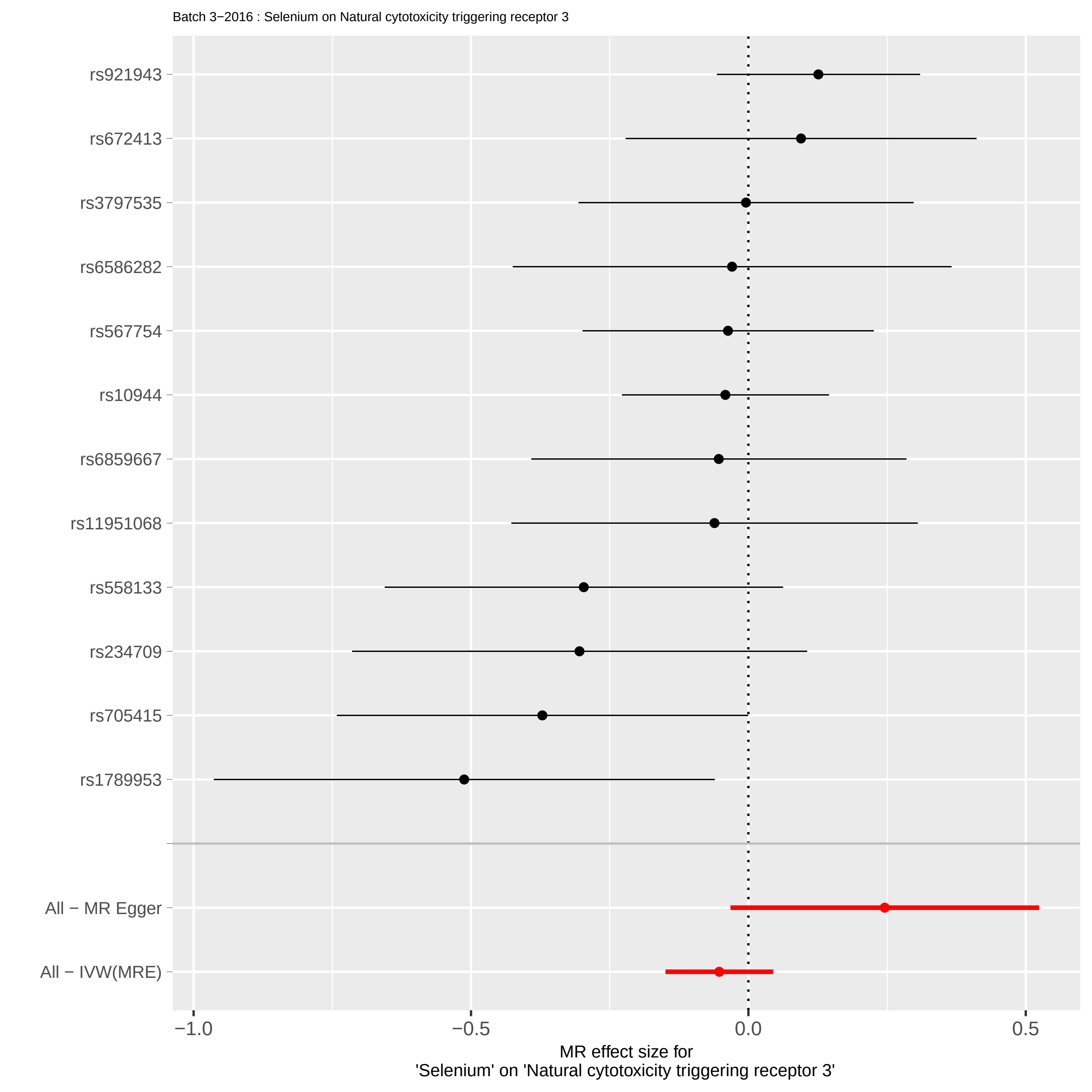

Batch 3−2016 : Selenium on Natural cytotoxicity triggering receptor 3
rs921943
rs672413
rs3797535
rs6586282
rs567754
rs10944
rs6859667
rs11951068
rs558133
rs234709
rs705415
rs1789953
All − MR Egger
All − IVW(MRE)
−1.0
−0.5
0.0
0.5
MR effect size for
'Selenium' on 'Natural cytotoxicity triggering receptor 3'

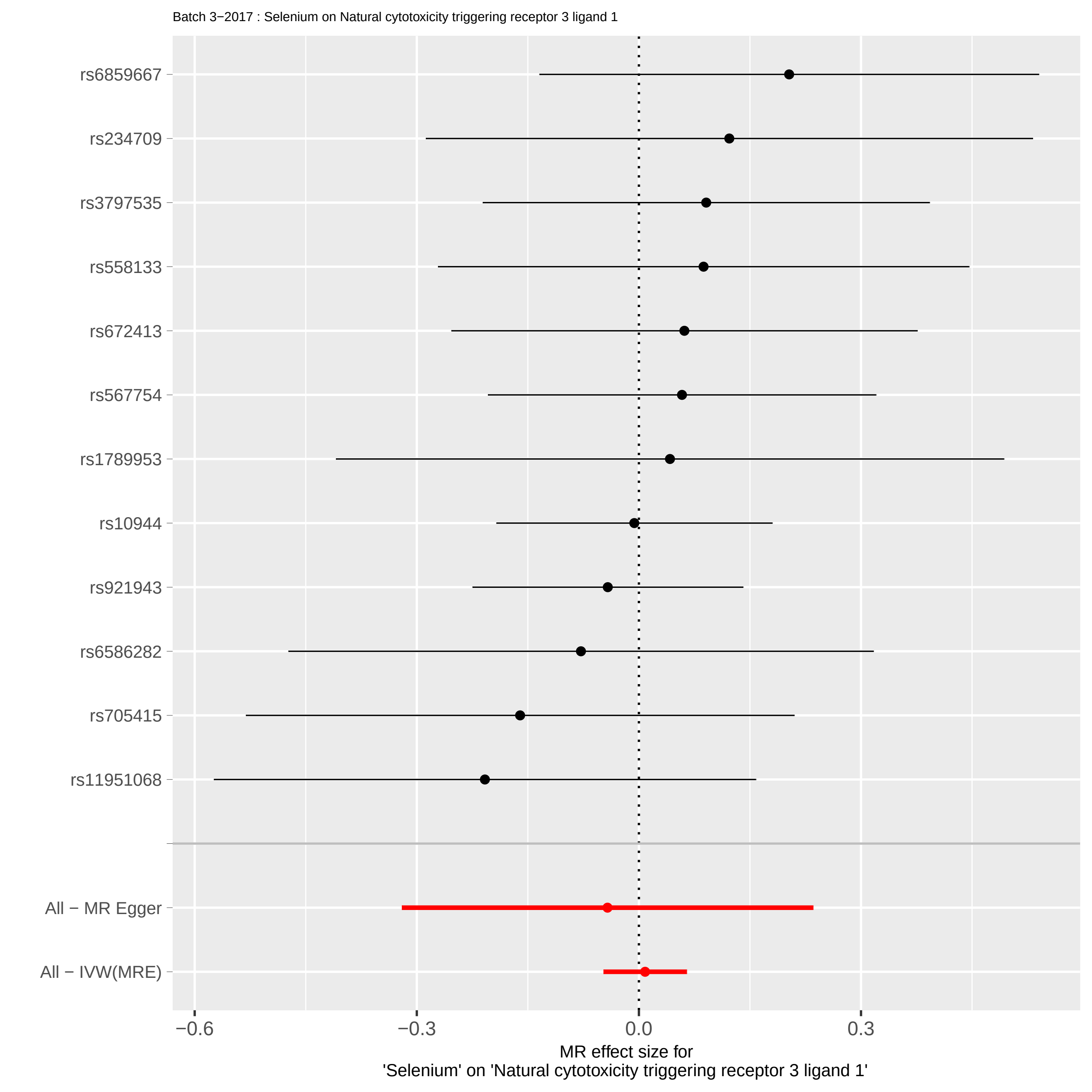

Batch 3−2017 : Selenium on Natural cytotoxicity triggering receptor 3 ligand 1
rs6859667
rs234709
rs3797535
rs558133
rs672413
rs567754
rs1789953
rs10944
rs921943
rs6586282
rs705415
rs11951068
All − MR Egger
All − IVW(MRE)
−0.6
−0.3
0.0
0.3
MR effect size for
'Selenium' on 'Natural cytotoxicity triggering receptor 3 ligand 1'

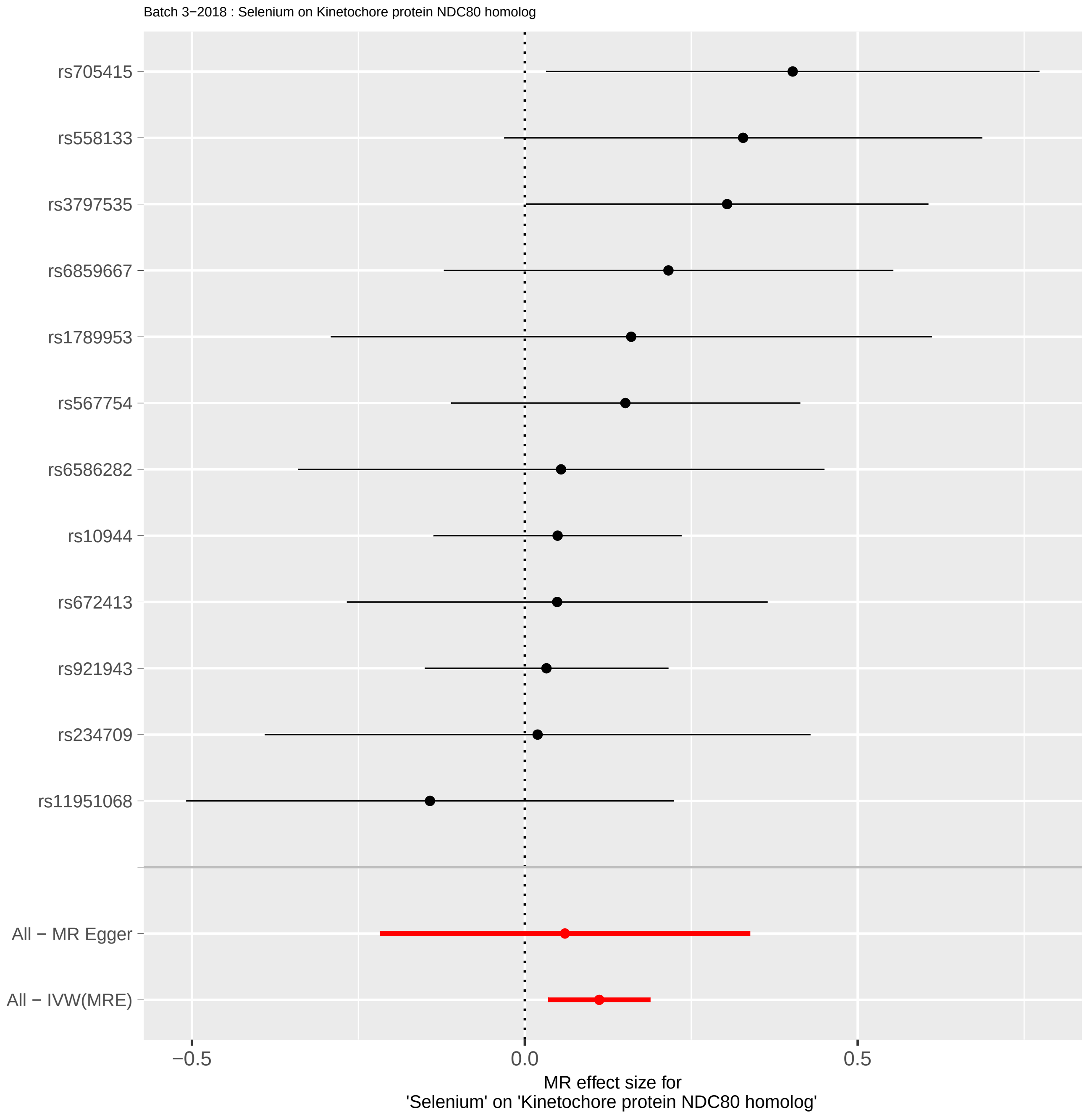

Batch 3−2018 : Selenium on Kinetochore protein NDC80 homolog
rs705415
rs558133
rs3797535
rs6859667
rs1789953
rs567754
rs6586282
rs10944
rs672413
rs921943
rs234709
rs11951068
All − MR Egger
All − IVW(MRE)
−0.5
0.0
0.5
MR effect size for
'Selenium' on 'Kinetochore protein NDC80 homolog'

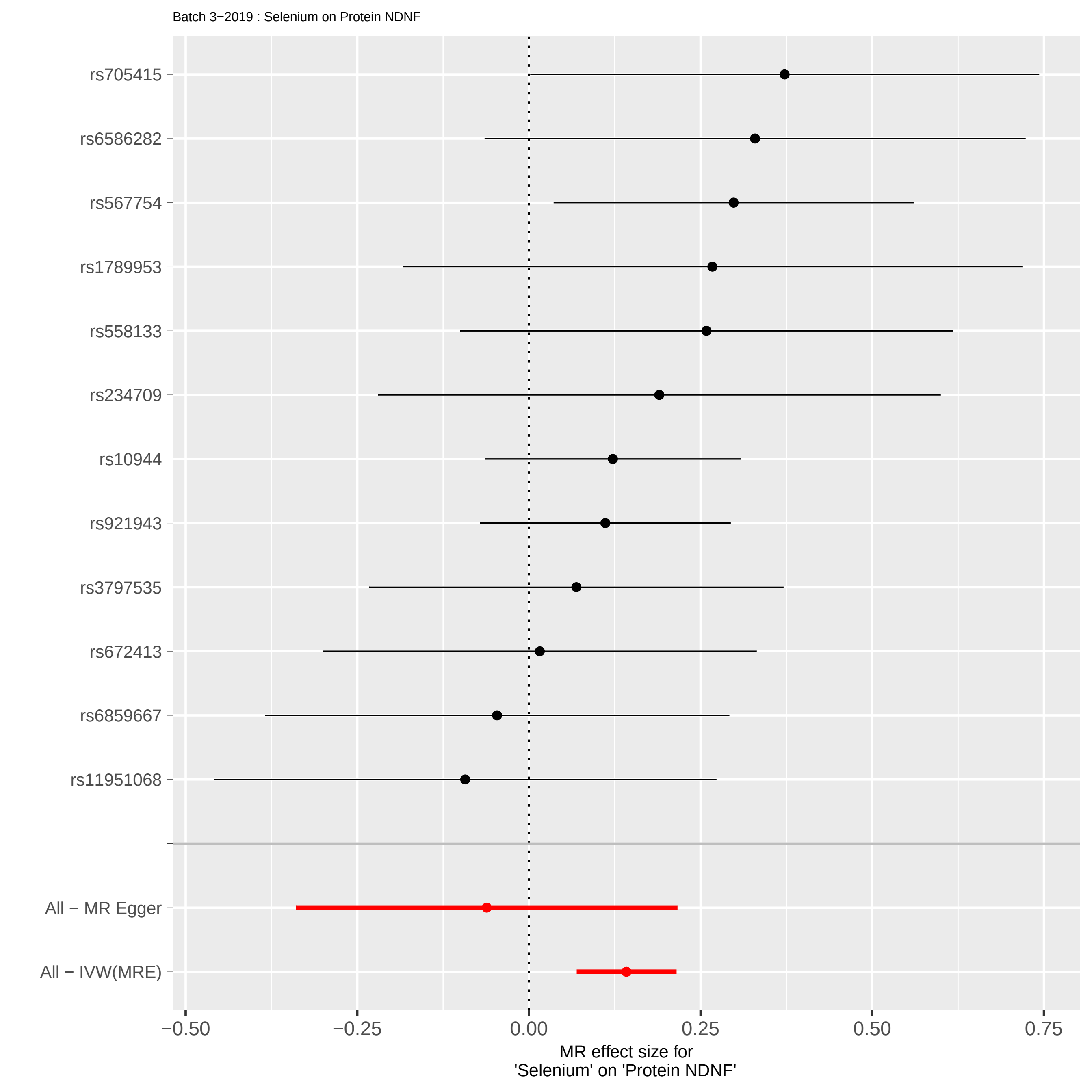

Batch 3−2019 : Selenium on Protein NDNF
rs705415
rs6586282
rs567754
rs1789953
rs558133
rs234709
rs10944
rs921943
rs3797535
rs672413
rs6859667
rs11951068
All − MR Egger
All − IVW(MRE)
−0.50
−0.25
0.00
0.25
0.50
0.75
MR effect size for
'Selenium' on 'Protein NDNF'

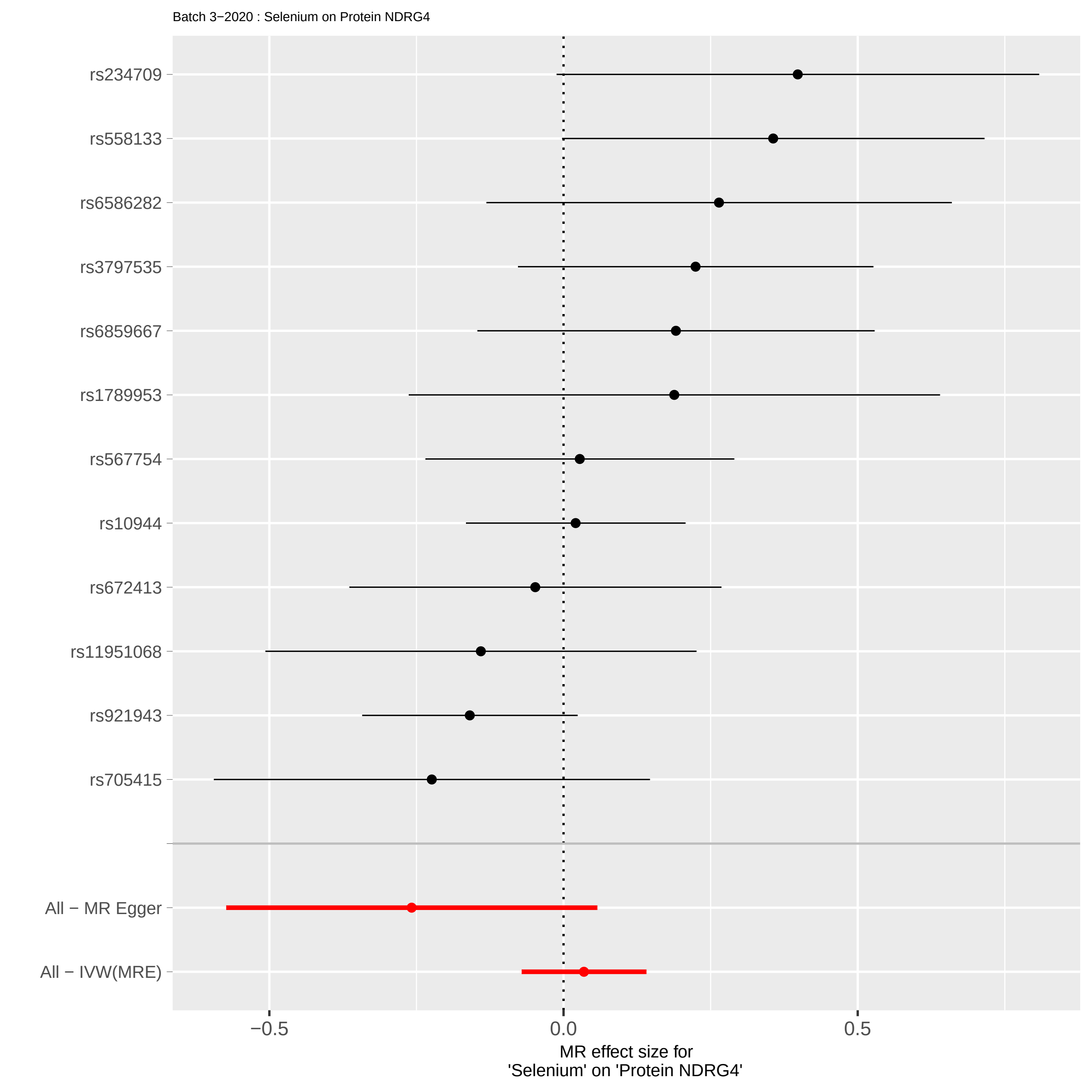

Batch 3−2020 : Selenium on Protein NDRG4
rs234709
rs558133
rs6586282
rs3797535
rs6859667
rs1789953
rs567754
rs10944
rs672413
rs11951068
rs921943
rs705415
All − MR Egger
All − IVW(MRE)
−0.5
0.0
0.5
MR effect size for
'Selenium' on 'Protein NDRG4'

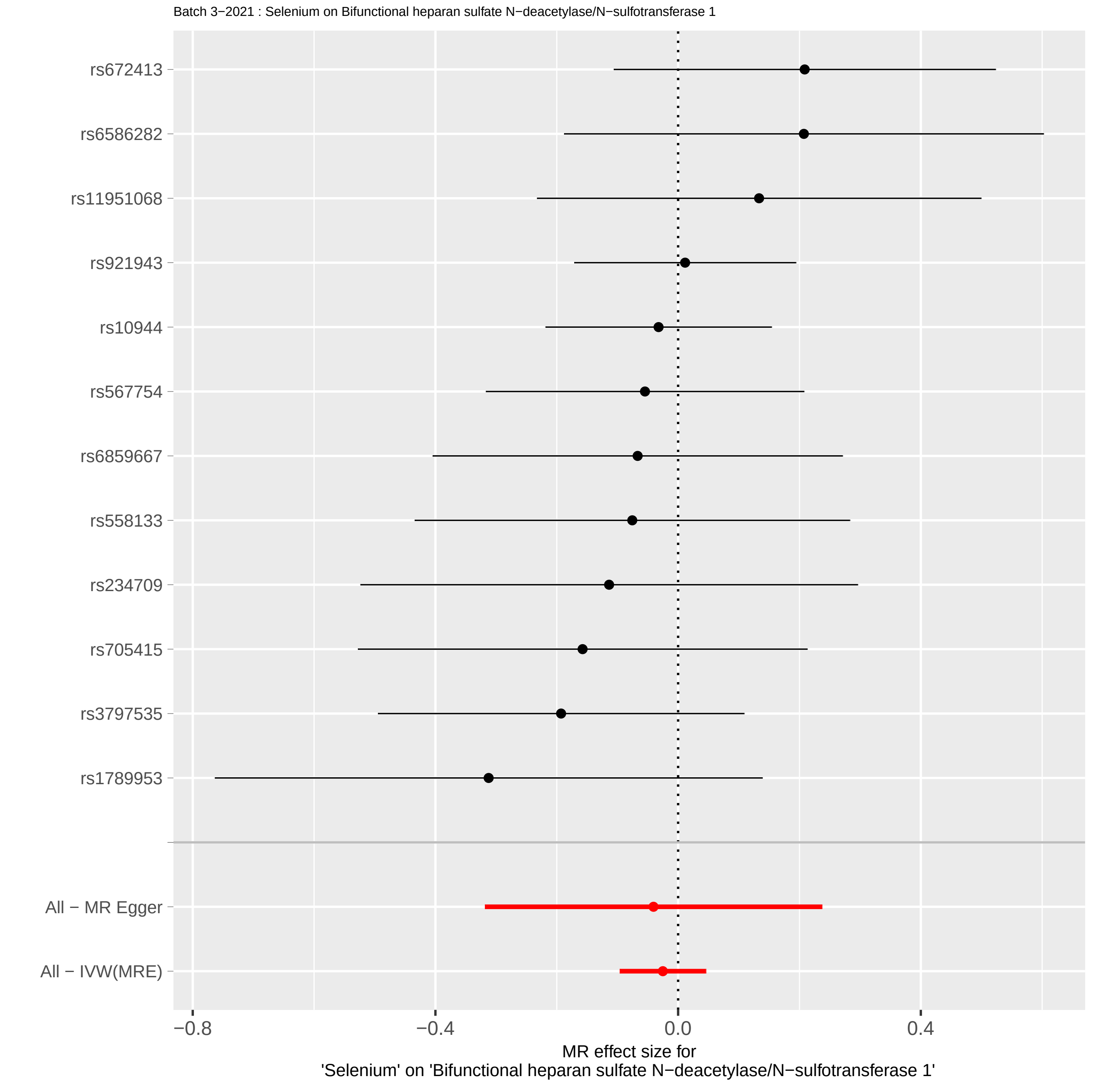

Batch 3−2021 : Selenium on Bifunctional heparan sulfate N−deacetylase/N−sulfotransferase 1
rs672413
rs6586282
rs11951068
rs921943
rs10944
rs567754
rs6859667
rs558133
rs234709
rs705415
rs3797535
rs1789953
All − MR Egger
All − IVW(MRE)
−0.8
−0.4
0.0
0.4
MR effect size for
'Selenium' on 'Bifunctional heparan sulfate N−deacetylase/N−sulfotransferase 1'

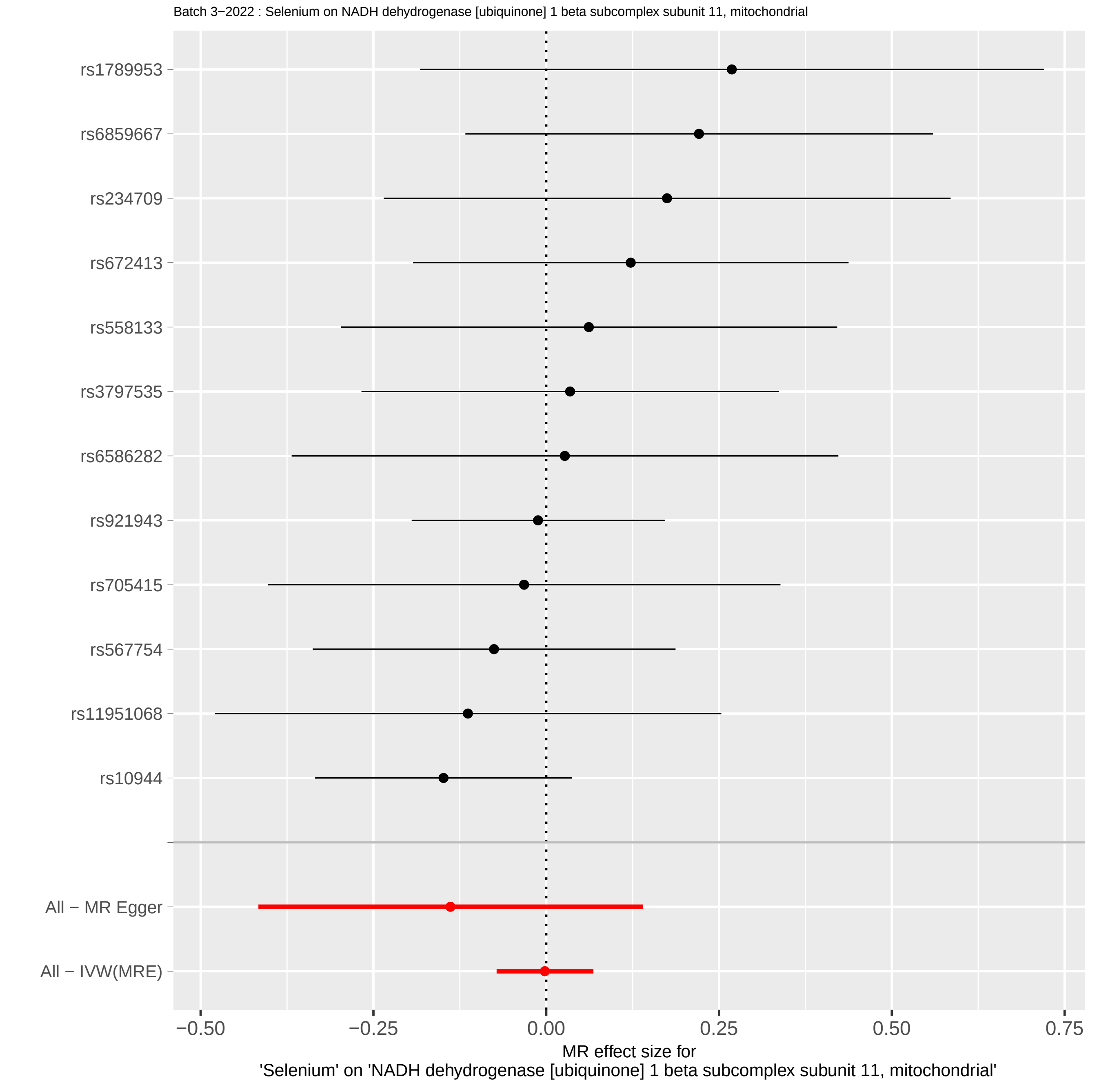

Batch 3−2022 : Selenium on NADH dehydrogenase [ubiquinone] 1 beta subcomplex subunit 11, mitochondrial
rs1789953
rs6859667
rs234709
rs672413
rs558133
rs3797535
rs6586282
rs921943
rs705415
rs567754
rs11951068
rs10944
All − MR Egger
All − IVW(MRE)
−0.50
−0.25
0.00
0.25
0.50
0.75
MR effect size for
'Selenium' on 'NADH dehydrogenase [ubiquinone] 1 beta subcomplex subunit 11, mitochondrial'

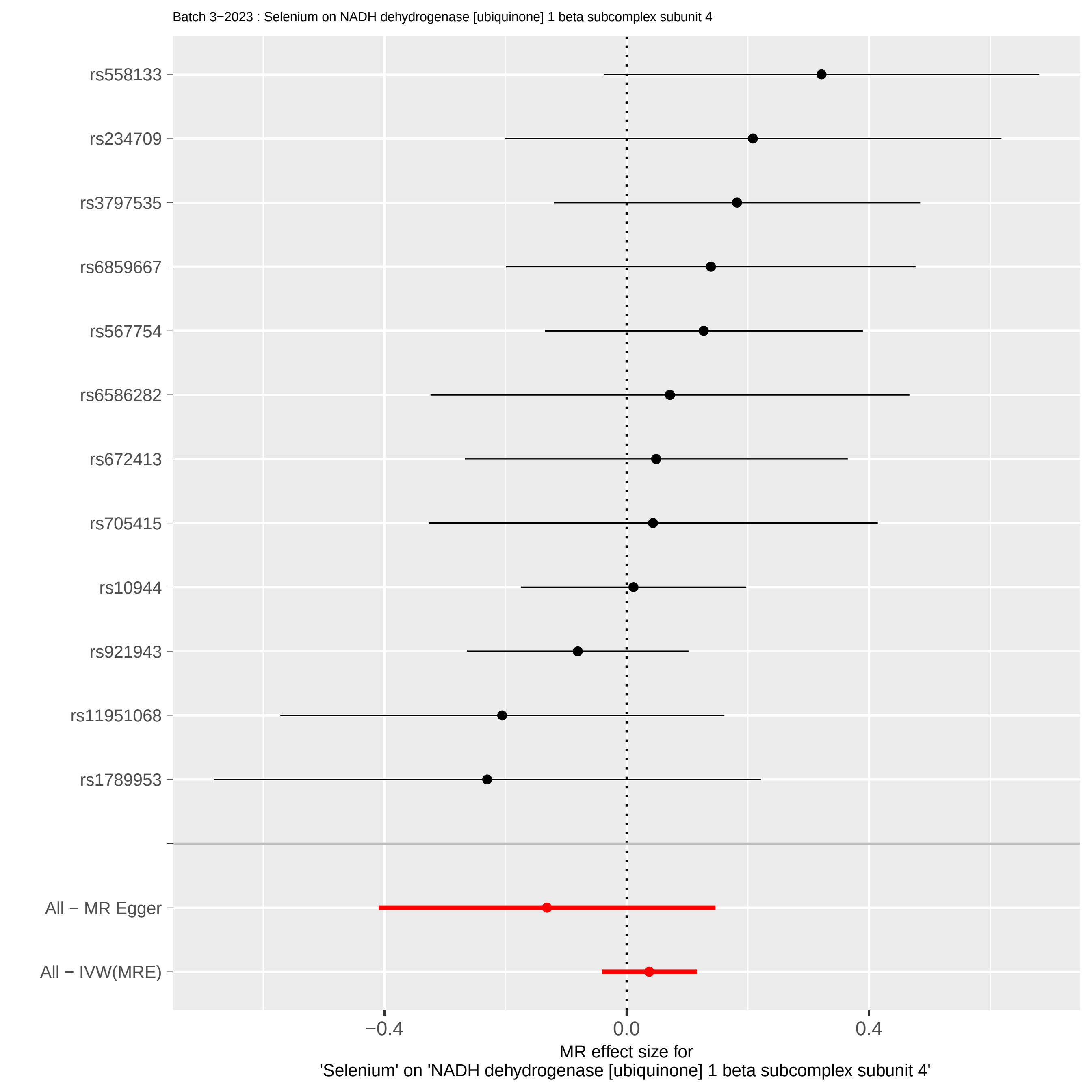

Batch 3−2023 : Selenium on NADH dehydrogenase [ubiquinone] 1 beta subcomplex subunit 4
rs558133
rs234709
rs3797535
rs6859667
rs567754
rs6586282
rs672413
rs705415
rs10944
rs921943
rs11951068
rs1789953
All − MR Egger
All − IVW(MRE)
−0.4
0.0
0.4
MR effect size for
'Selenium' on 'NADH dehydrogenase [ubiquinone] 1 beta subcomplex subunit 4'

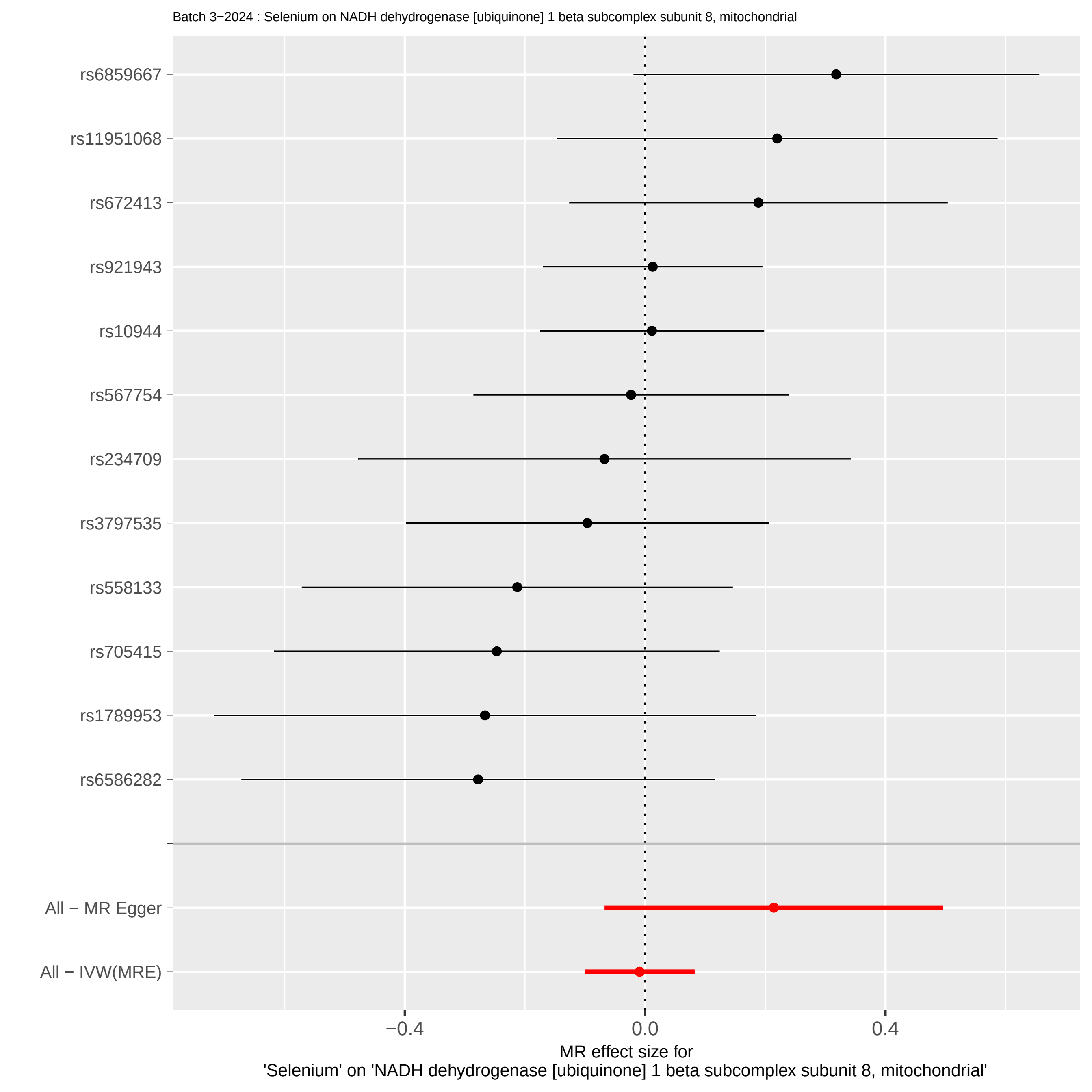

Batch 3−2024 : Selenium on NADH dehydrogenase [ubiquinone] 1 beta subcomplex subunit 8, mitochondrial
rs6859667
rs11951068
rs672413
rs921943
rs10944
rs567754
rs234709
rs3797535
rs558133
rs705415
rs1789953
rs6586282
All − MR Egger
All − IVW(MRE)
−0.4
0.0
0.4
MR effect size for
'Selenium' on 'NADH dehydrogenase [ubiquinone] 1 beta subcomplex subunit 8, mitochondrial'

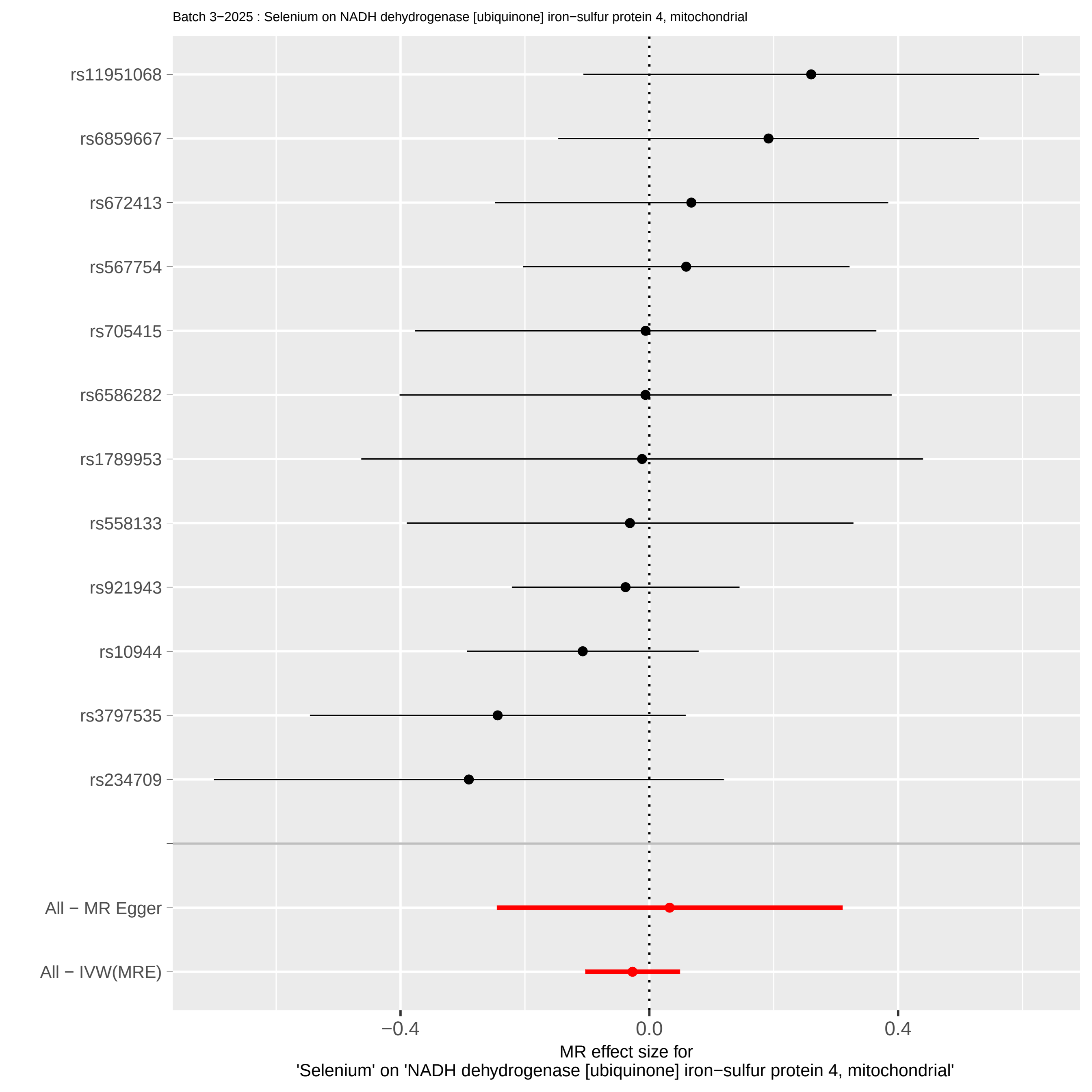

Batch 3−2025 : Selenium on NADH dehydrogenase [ubiquinone] iron−sulfur protein 4, mitochondrial
rs11951068
rs6859667
rs672413
rs567754
rs705415
rs6586282
rs1789953
rs558133
rs921943
rs10944
rs3797535
rs234709
All − MR Egger
All − IVW(MRE)
−0.4
0.0
0.4
MR effect size for
'Selenium' on 'NADH dehydrogenase [ubiquinone] iron−sulfur protein 4, mitochondrial'

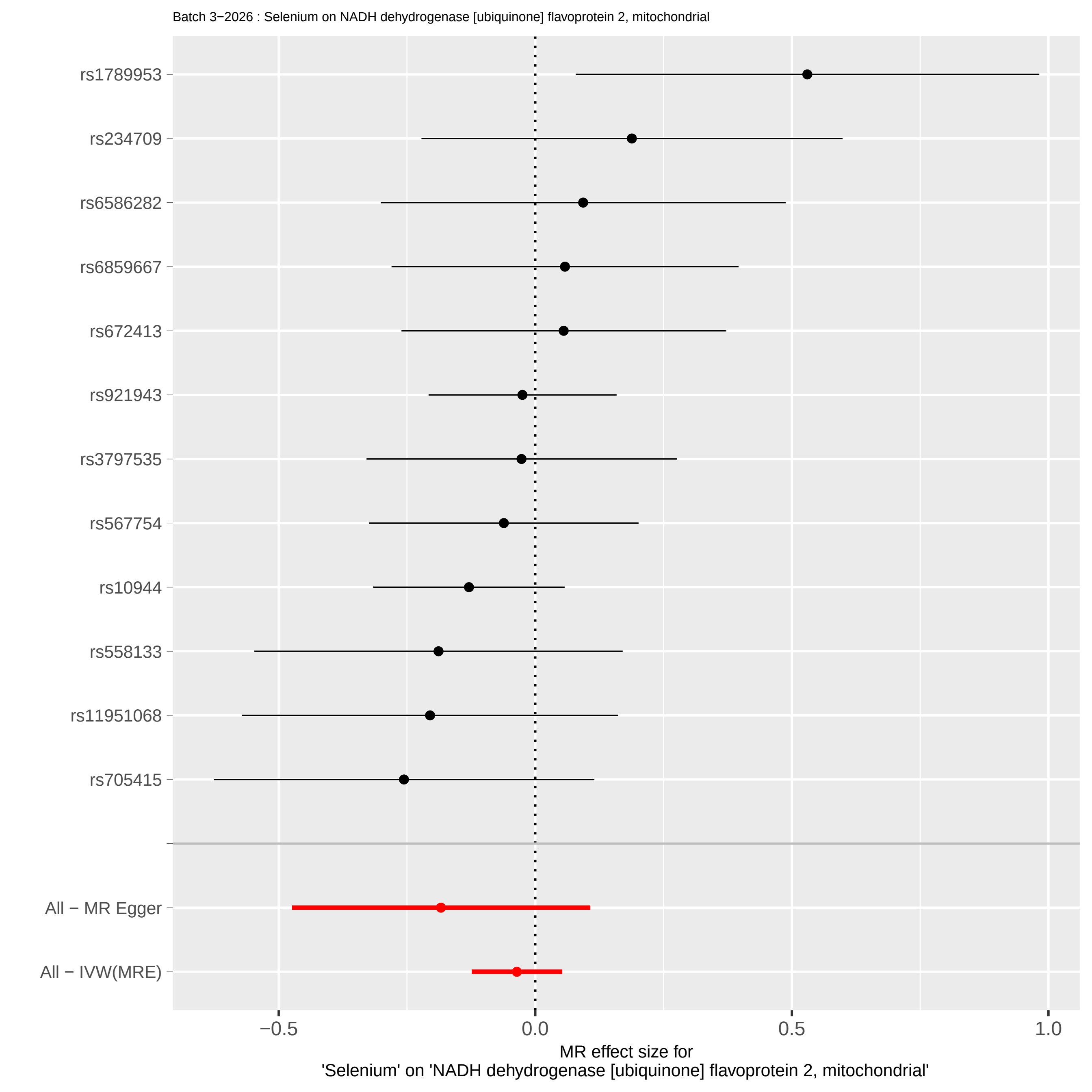

Batch 3−2026 : Selenium on NADH dehydrogenase [ubiquinone] flavoprotein 2, mitochondrial
rs1789953
rs234709
rs6586282
rs6859667
rs672413
rs921943
rs3797535
rs567754
rs10944
rs558133
rs11951068
rs705415
All − MR Egger
All − IVW(MRE)
−0.5
0.0
0.5
1.0
MR effect size for
'Selenium' on 'NADH dehydrogenase [ubiquinone] flavoprotein 2, mitochondrial'

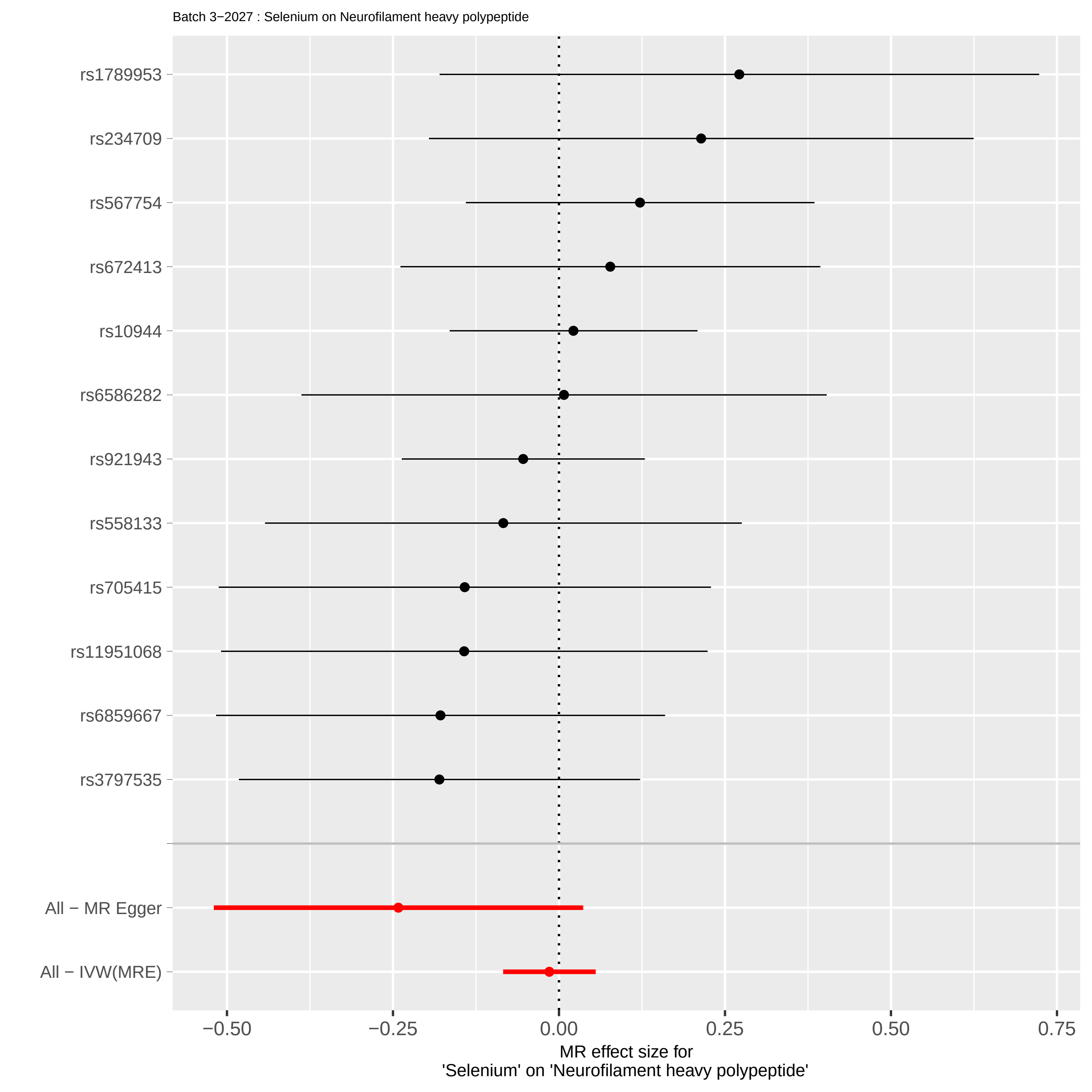

Batch 3−2027 : Selenium on Neurofilament heavy polypeptide
rs1789953
rs234709
rs567754
rs672413
rs10944
rs6586282
rs921943
rs558133
rs705415
rs11951068
rs6859667
rs3797535
All − MR Egger
All − IVW(MRE)
−0.50
−0.25
0.00
0.25
0.50
0.75
MR effect size for
'Selenium' on 'Neurofilament heavy polypeptide'

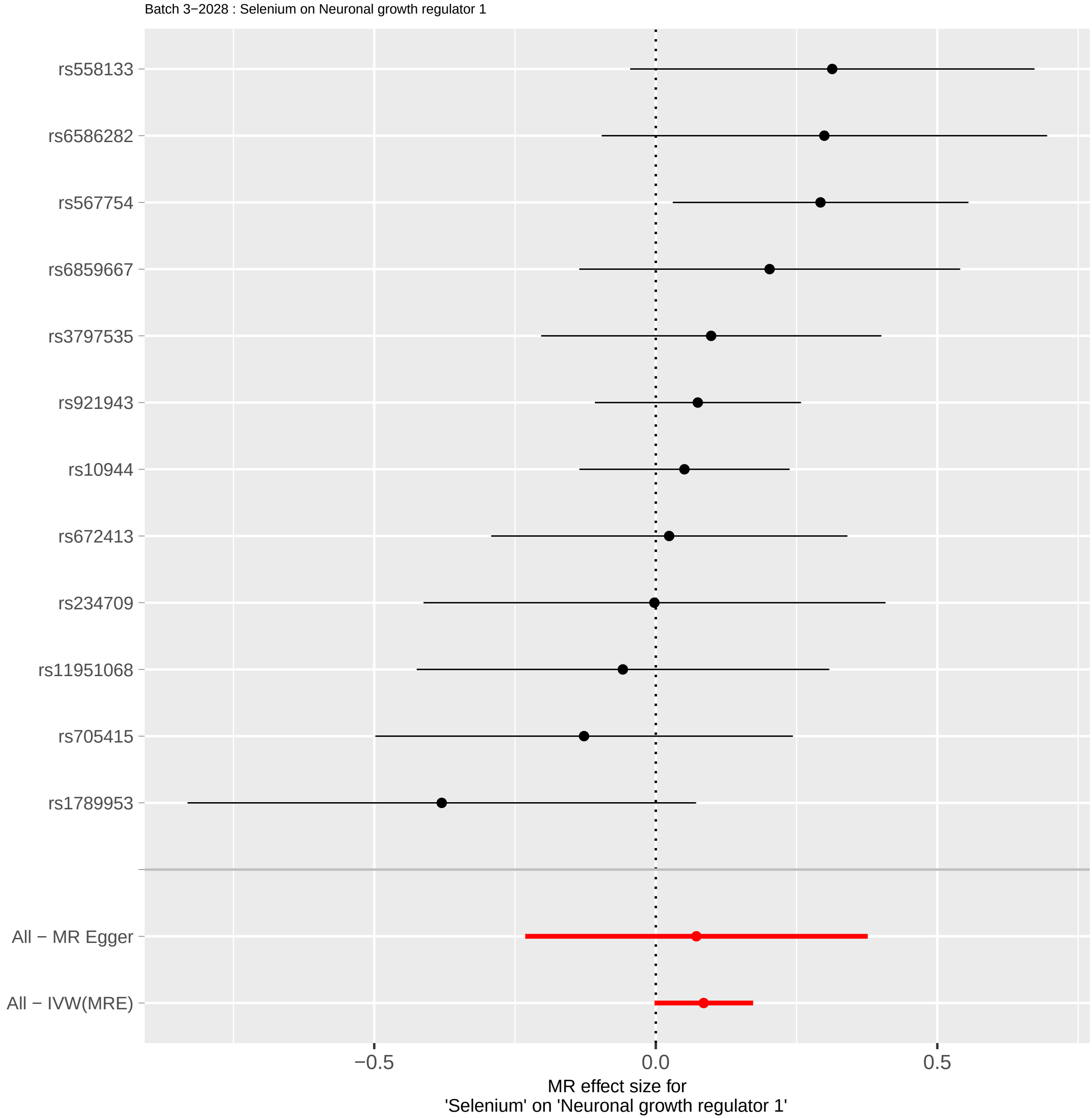
Batch 3−2028 : Selenium on Neuronal growth regulator 1
rs558133
rs6586282
rs567754
rs6859667
rs3797535
rs921943
rs10944
rs672413
rs234709
rs11951068
rs705415
rs1789953
All − MR Egger
All − IVW(MRE)
−0.5
0.0
0.5
MR effect size for
'Selenium' on 'Neuronal growth regulator 1'

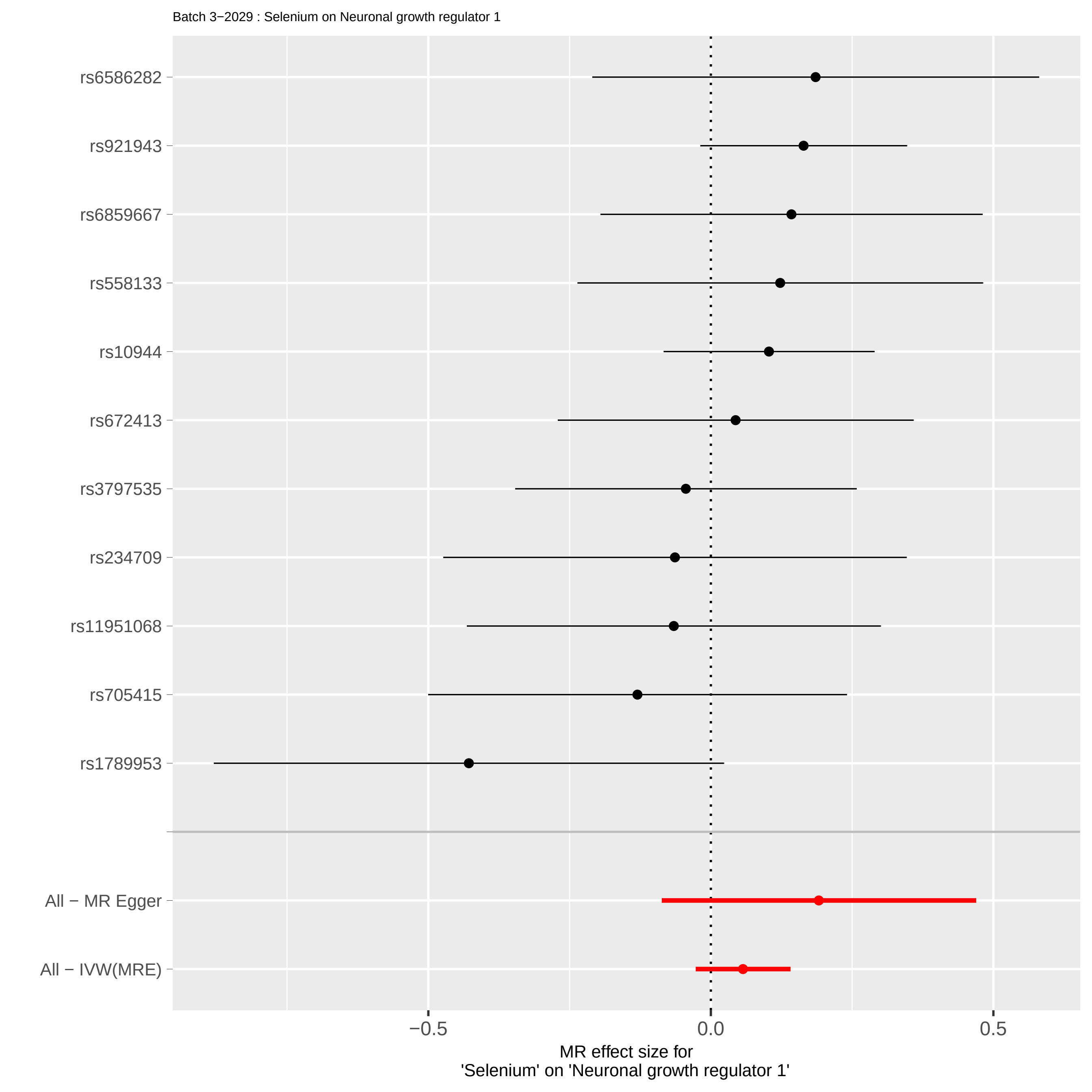
Batch 3−2029 : Selenium on Neuronal growth regulator 1
rs6586282
rs921943
rs6859667
rs558133
rs10944
rs672413
rs3797535
rs234709
rs11951068
rs705415
rs1789953
All − MR Egger
All − IVW(MRE)
−0.5
0.0
0.5
MR effect size for
'Selenium' on 'Neuronal growth regulator 1'

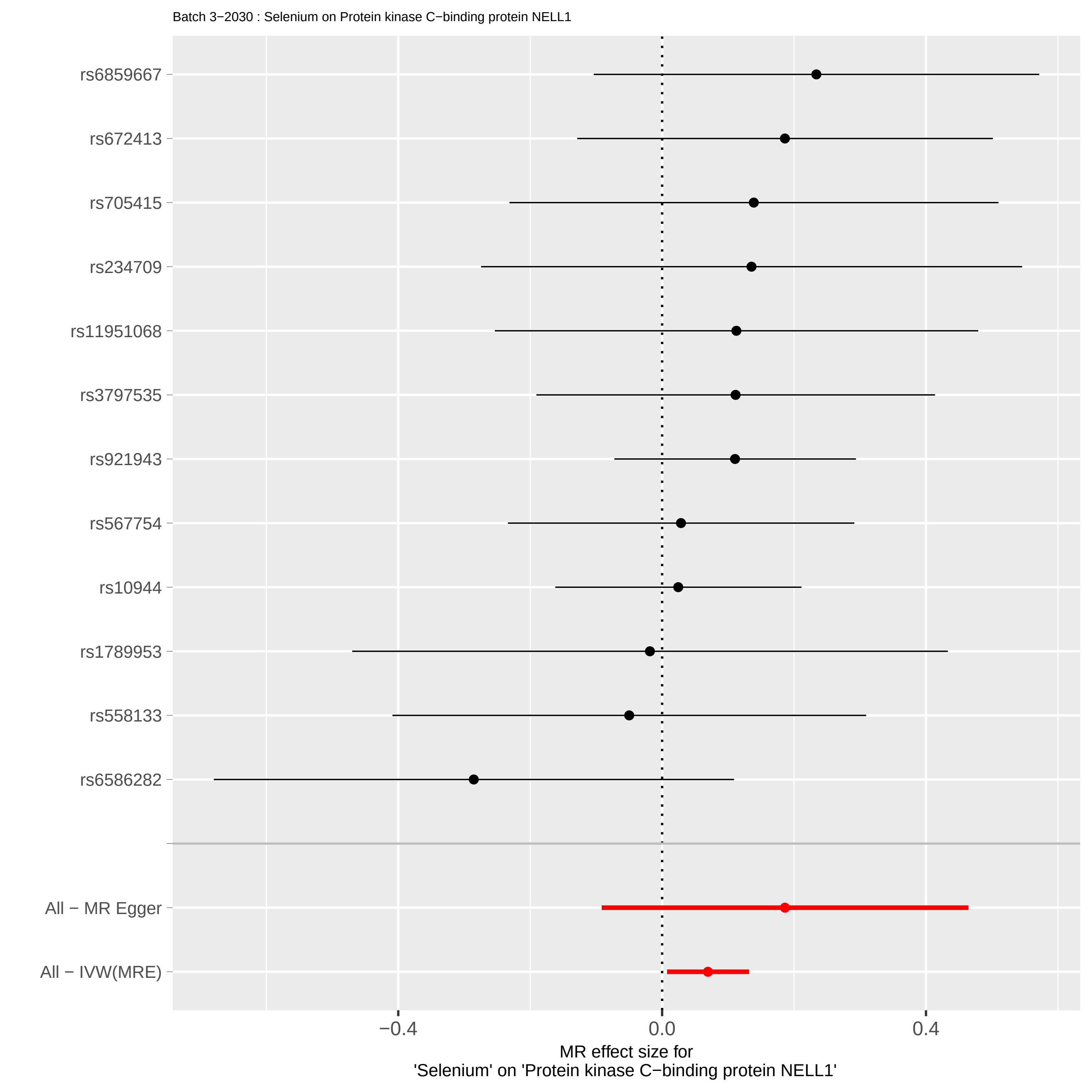

Batch 3−2030 : Selenium on Protein kinase C−binding protein NELL1
rs6859667
rs672413
rs705415
rs234709
rs11951068
rs3797535
rs921943
rs567754
rs10944
rs1789953
rs558133
rs6586282
All − MR Egger
All − IVW(MRE)
−0.4
0.0
0.4
MR effect size for
'Selenium' on 'Protein kinase C−binding protein NELL1'

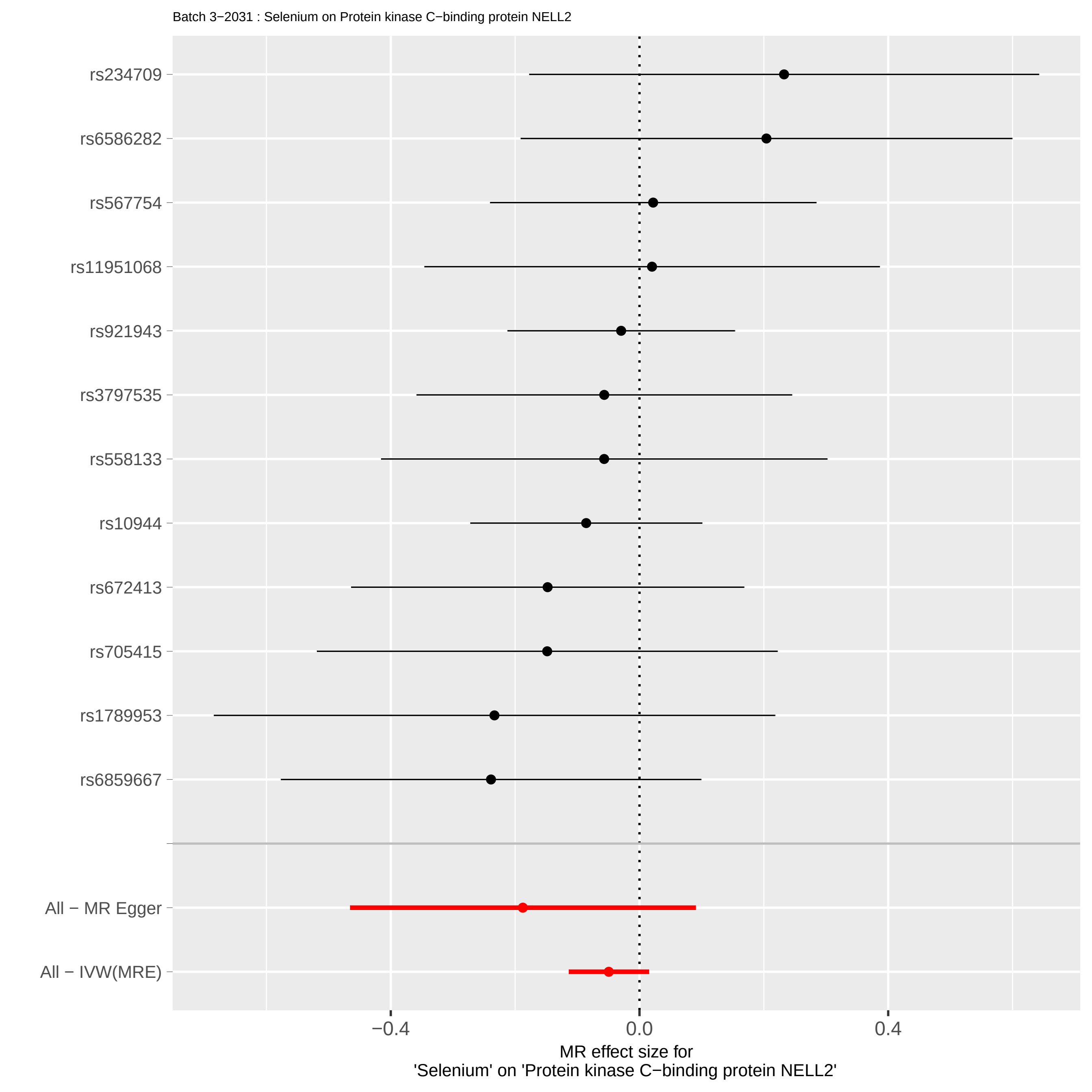
Batch 3−2031 : Selenium on Protein kinase C−binding protein NELL2
rs234709
rs6586282
rs567754
rs11951068
rs921943
rs3797535
rs558133
rs10944
rs672413
rs705415
rs1789953
rs6859667
All − MR Egger
All − IVW(MRE)
−0.4
0.0
0.4
MR effect size for
'Selenium' on 'Protein kinase C−binding protein NELL2'

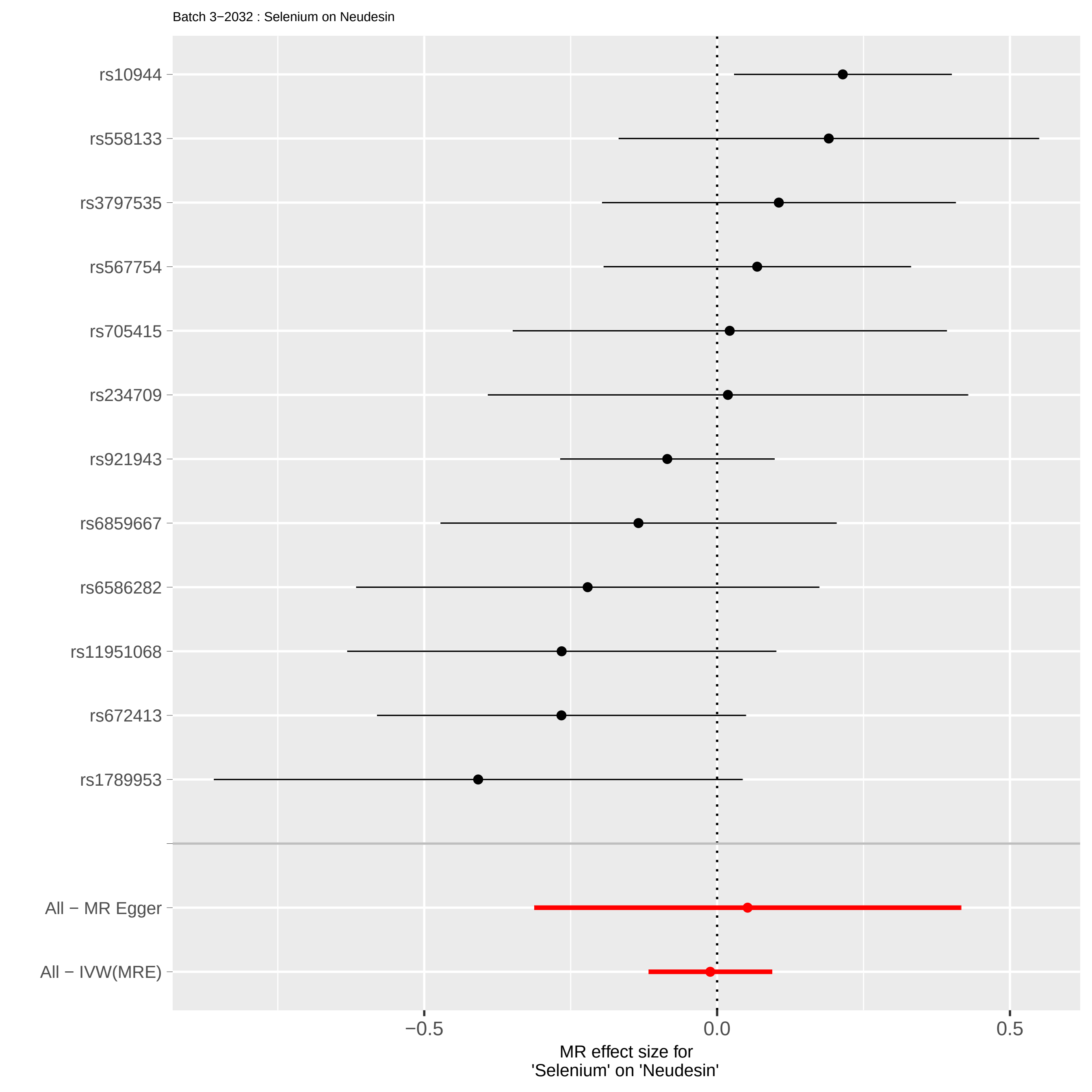

Batch 3−2032 : Selenium on Neudesin
rs10944
rs558133
rs3797535
rs567754
rs705415
rs234709
rs921943
rs6859667
rs6586282
rs11951068
rs672413
rs1789953
All − MR Egger
All − IVW(MRE)
−0.5
0.0
0.5
MR effect size for
'Selenium' on 'Neudesin'

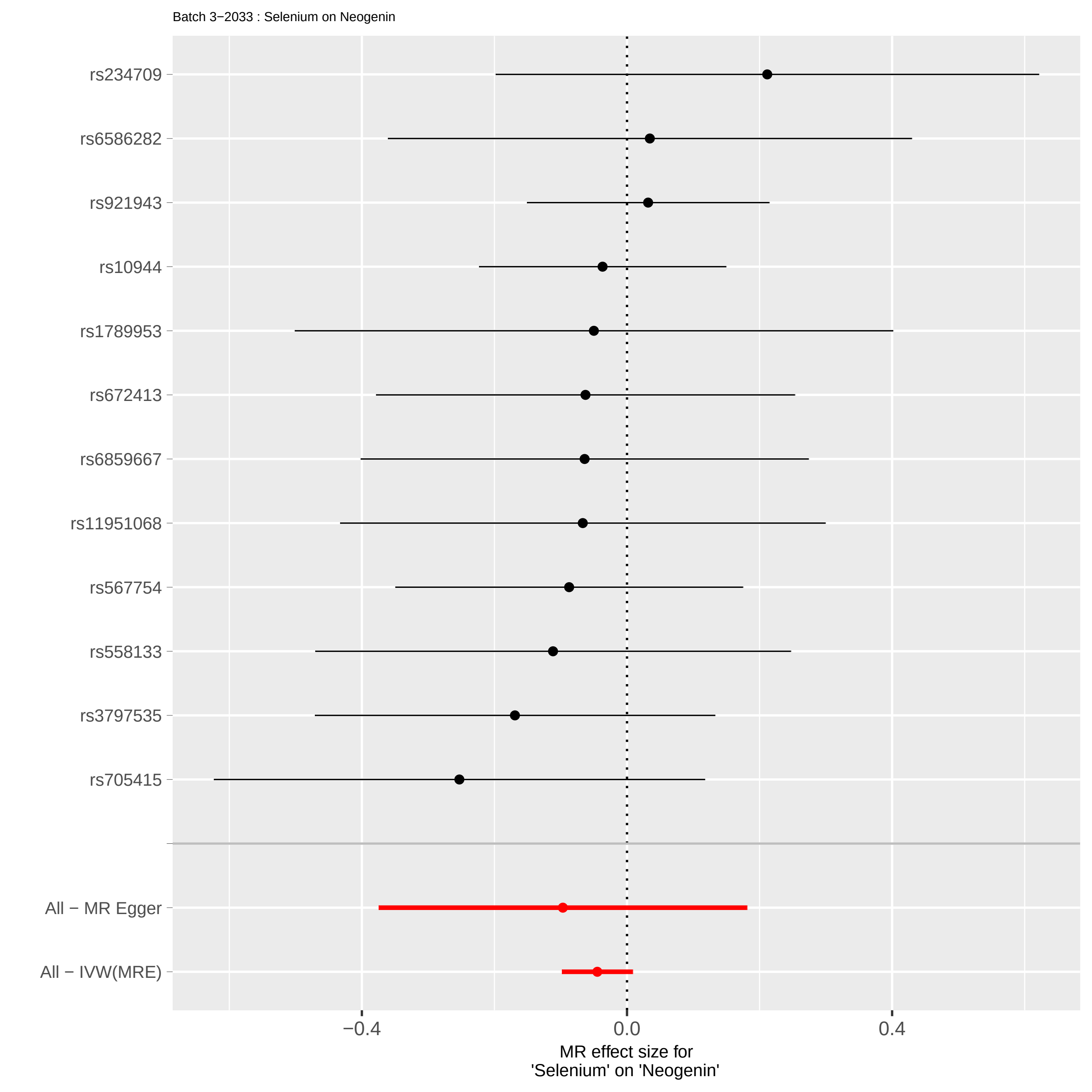
Batch 3−2033 : Selenium on Neogenin
rs234709
rs6586282
rs921943
rs10944
rs1789953
rs672413
rs6859667
rs11951068
rs567754
rs558133
rs3797535
rs705415
All − MR Egger
All − IVW(MRE)
−0.4
0.0
0.4
MR effect size for
'Selenium' on 'Neogenin'

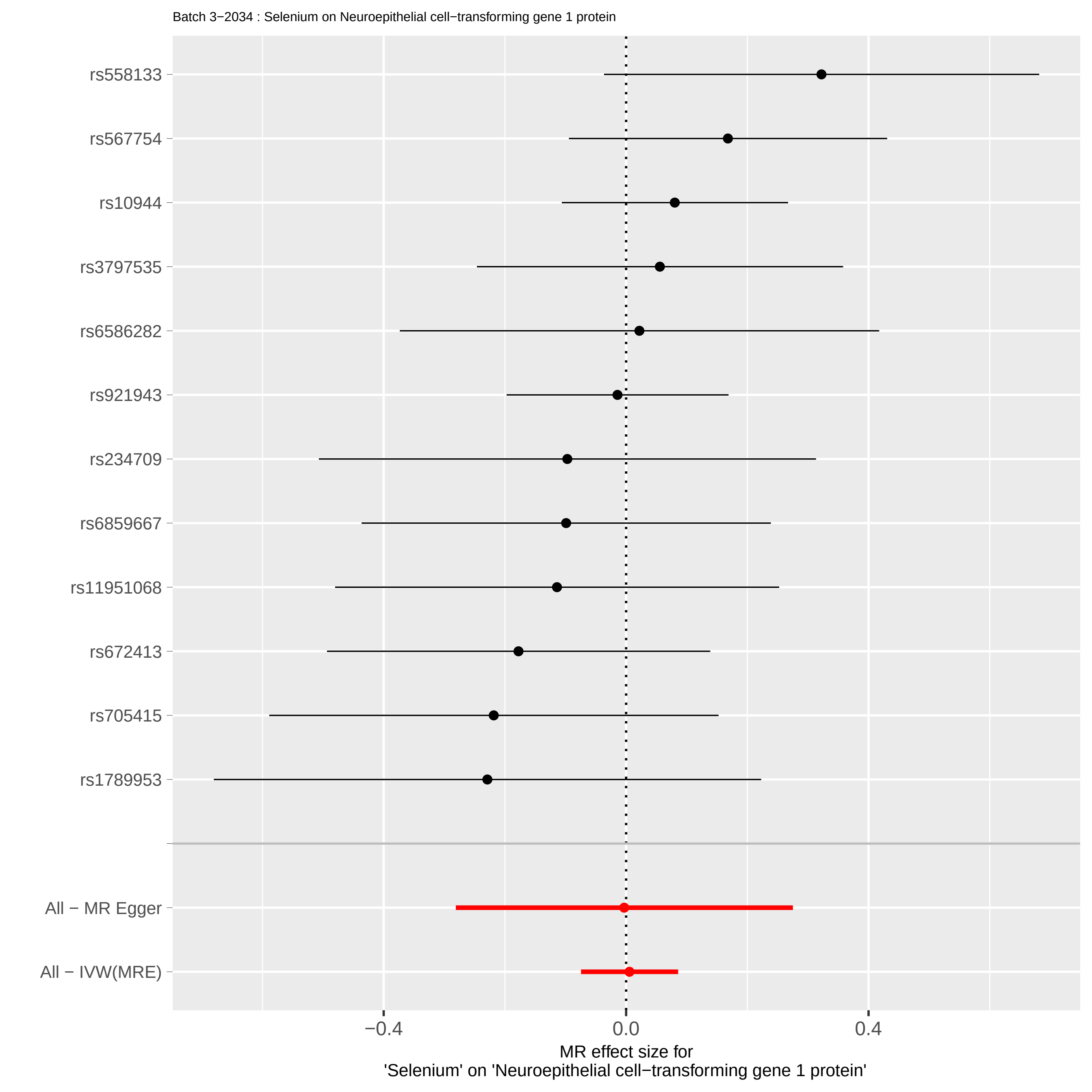

Batch 3−2034 : Selenium on Neuroepithelial cell−transforming gene 1 protein
rs558133
rs567754
rs10944
rs3797535
rs6586282
rs921943
rs234709
rs6859667
rs11951068
rs672413
rs705415
rs1789953
All − MR Egger
All − IVW(MRE)
−0.4
0.0
0.4
MR effect size for
'Selenium' on 'Neuroepithelial cell−transforming gene 1 protein'

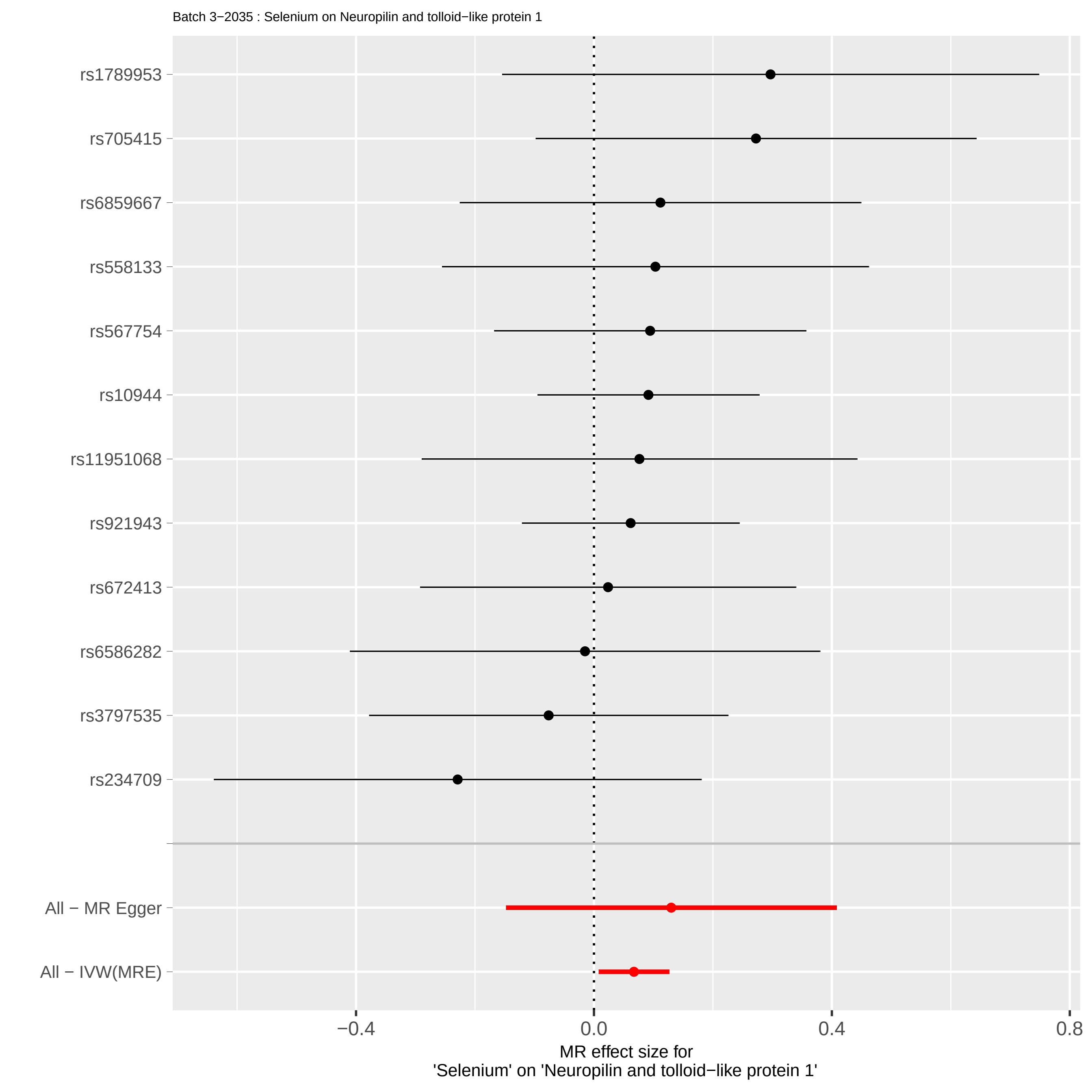

Batch 3−2035 : Selenium on Neuropilin and tolloid−like protein 1
rs1789953
rs705415
rs6859667
rs558133
rs567754
rs10944
rs11951068
rs921943
rs672413
rs6586282
rs3797535
rs234709
All − MR Egger
All − IVW(MRE)
−0.4
0.0
0.4
0.8
MR effect size for
'Selenium' on 'Neuropilin and tolloid−like protein 1'

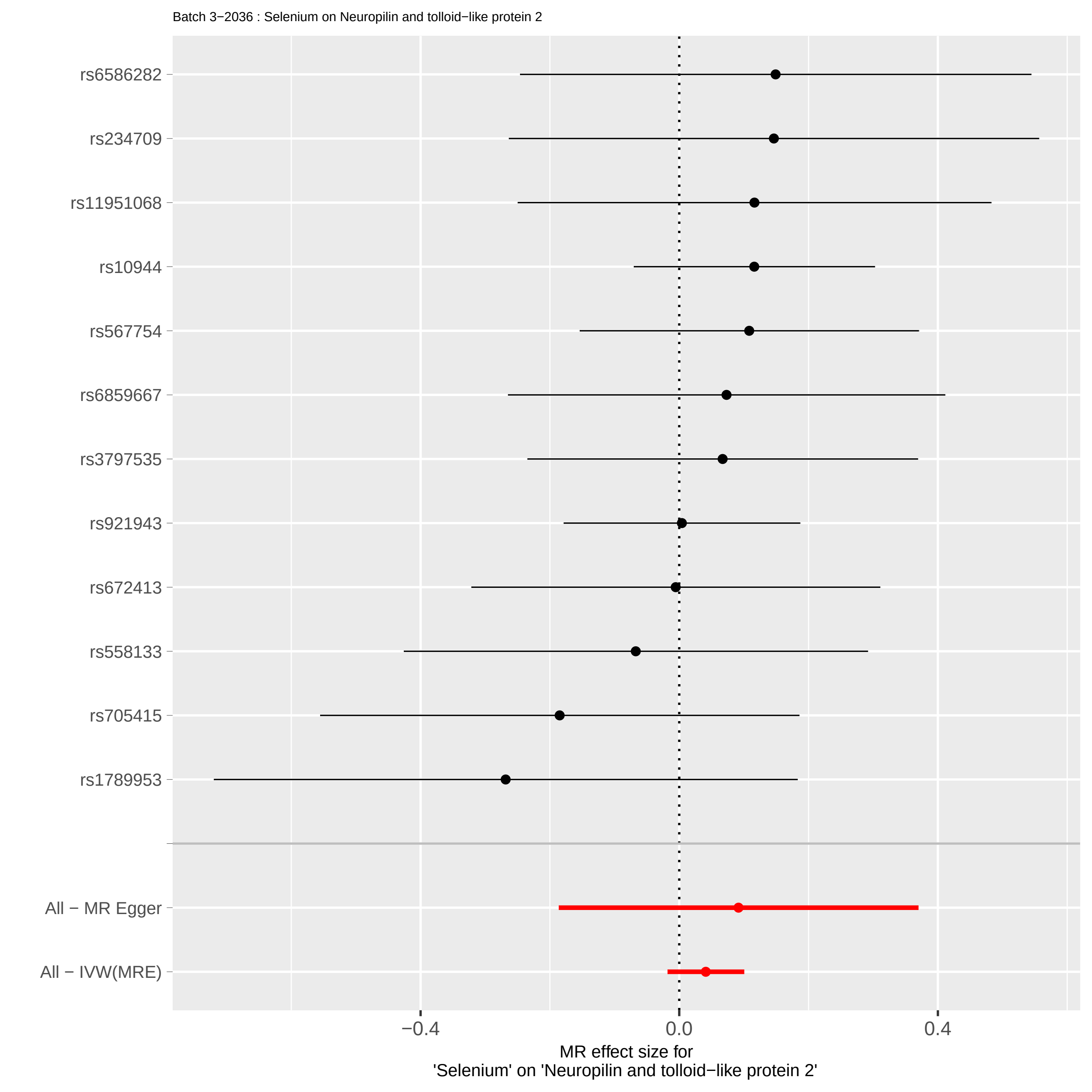
Batch 3−2036 : Selenium on Neuropilin and tolloid−like protein 2
rs6586282
rs234709
rs11951068
rs10944
rs567754
rs6859667
rs3797535
rs921943
rs672413
rs558133
rs705415
rs1789953
All − MR Egger
All − IVW(MRE)
−0.4
0.0
0.4
MR effect size for
'Selenium' on 'Neuropilin and tolloid−like protein 2'

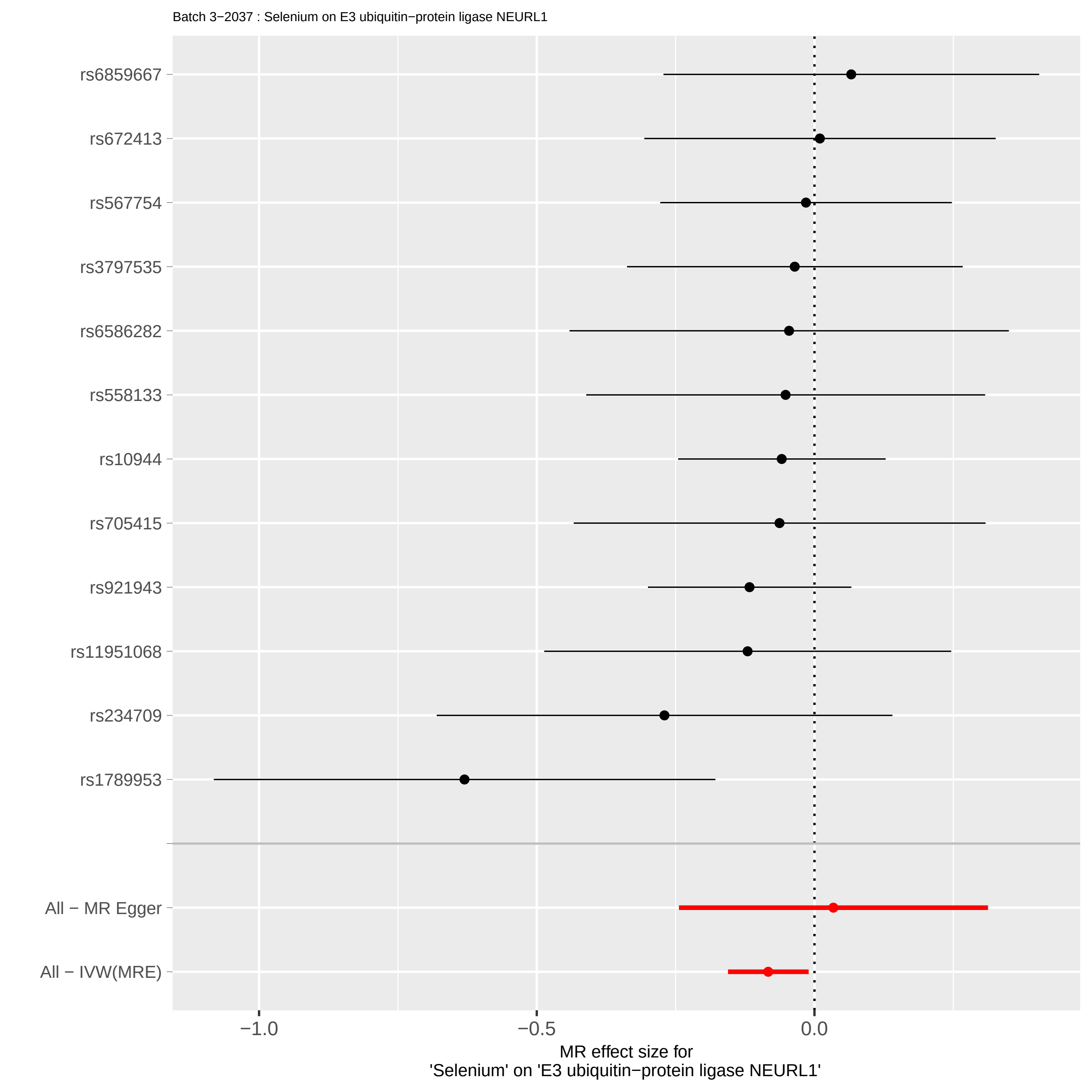

Batch 3−2037 : Selenium on E3 ubiquitin−protein ligase NEURL1
rs6859667
rs672413
rs567754
rs3797535
rs6586282
rs558133
rs10944
rs705415
rs921943
rs11951068
rs234709
rs1789953
All − MR Egger
All − IVW(MRE)
−1.0
−0.5
0.0
MR effect size for
'Selenium' on 'E3 ubiquitin−protein ligase NEURL1'

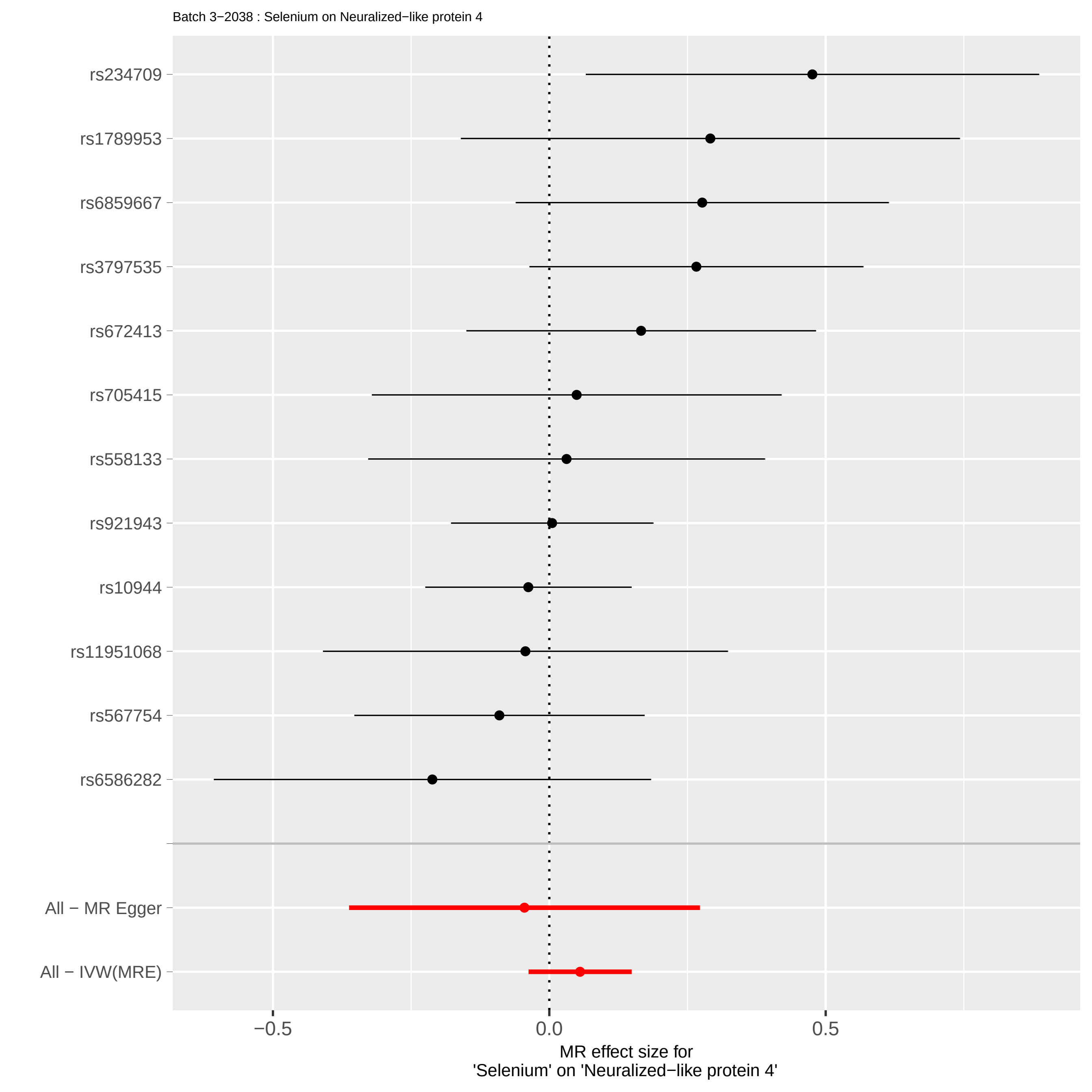

Batch 3−2038 : Selenium on Neuralized−like protein 4
rs234709
rs1789953
rs6859667
rs3797535
rs672413
rs705415
rs558133
rs921943
rs10944
rs11951068
rs567754
rs6586282
All − MR Egger
All − IVW(MRE)
−0.5
0.0
0.5
MR effect size for
'Selenium' on 'Neuralized−like protein 4'

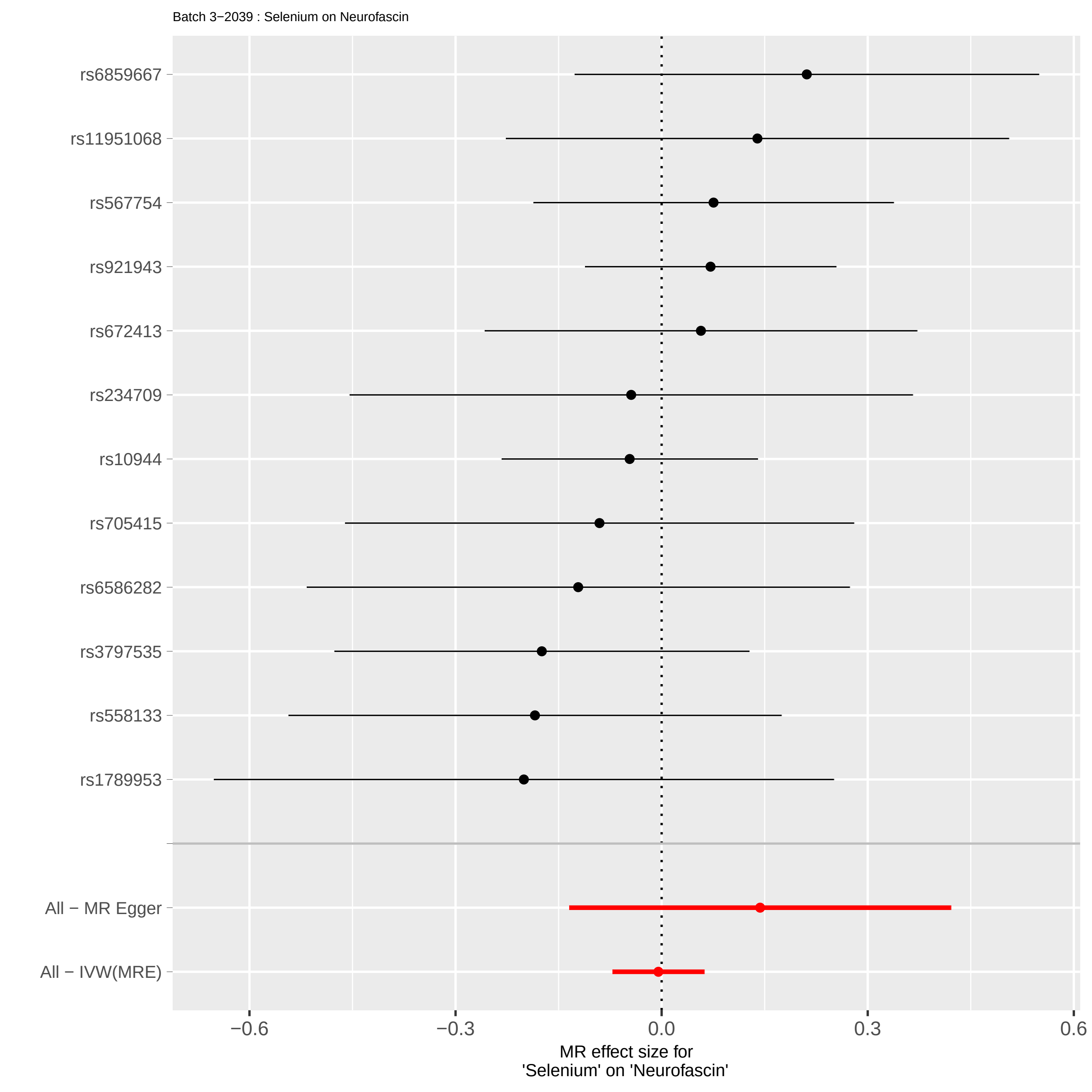

Batch 3−2039 : Selenium on Neurofascin
rs6859667
rs11951068
rs567754
rs921943
rs672413
rs234709
rs10944
rs705415
rs6586282
rs3797535
rs558133
rs1789953
All − MR Egger
All − IVW(MRE)
−0.6
−0.3
0.0
0.3
0.6
MR effect size for
'Selenium' on 'Neurofascin'

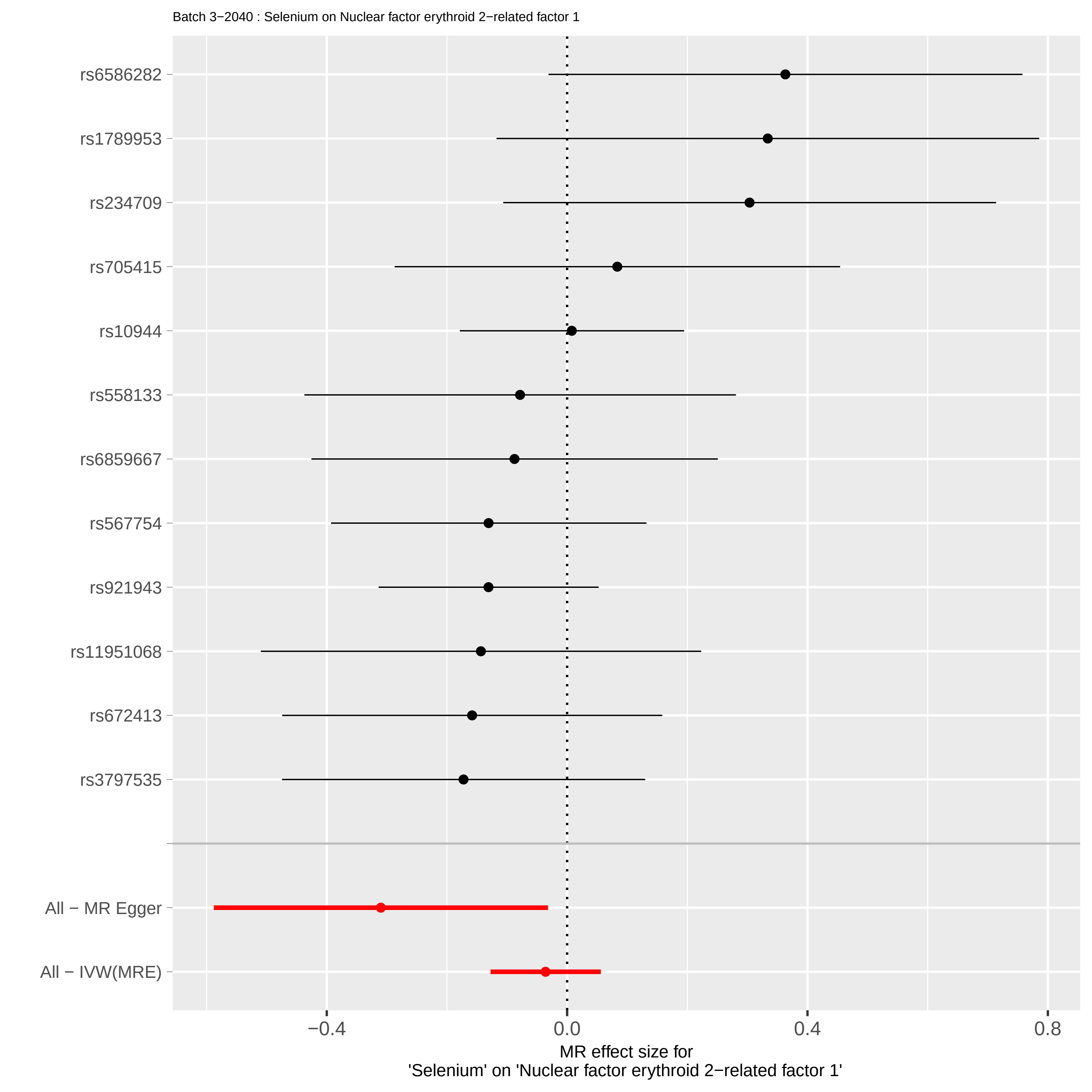

Batch 3−2040 : Selenium on Nuclear factor erythroid 2−related factor 1
rs6586282
rs1789953
rs234709
rs705415
rs10944
rs558133
rs6859667
rs567754
rs921943
rs11951068
rs672413
rs3797535
All − MR Egger
All − IVW(MRE)
−0.4
0.0
0.4
0.8
MR effect size for
'Selenium' on 'Nuclear factor erythroid 2−related factor 1'

Batch 3−2041 : Selenium on NFU1 iron−sulfur cluster scaffold homolog, mitochondrial

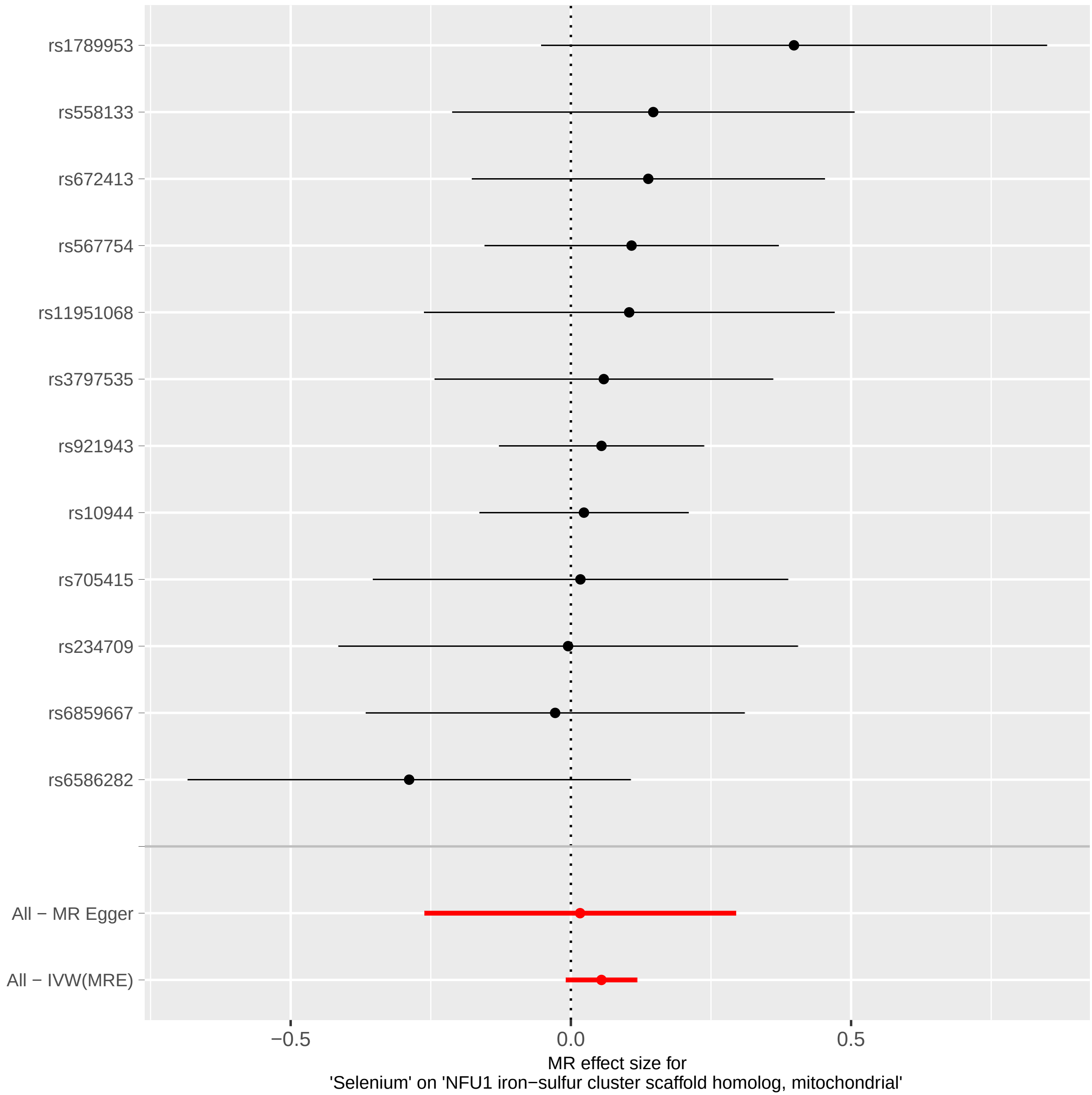

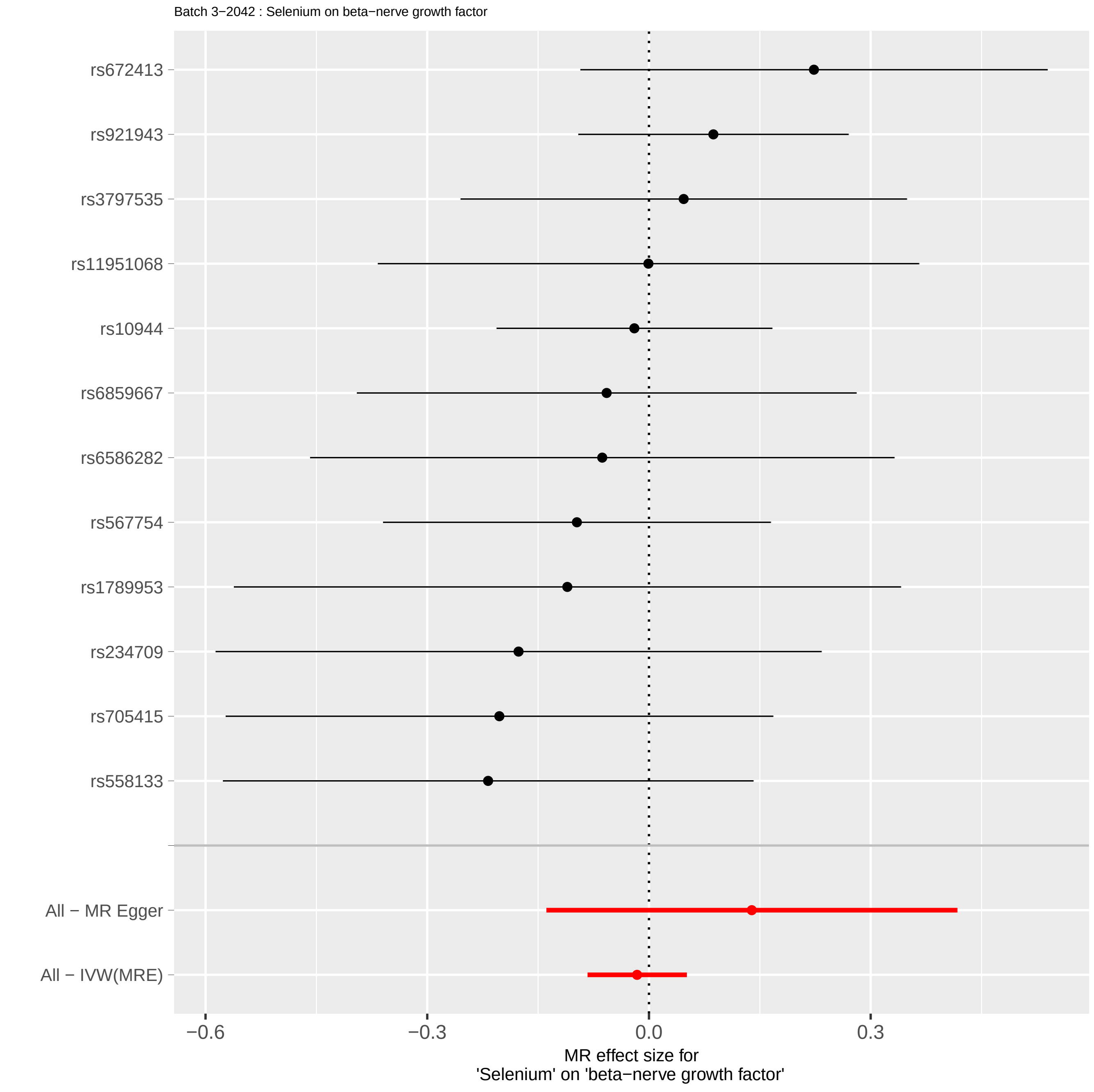
Batch 3−2042 : Selenium on beta−nerve growth factor
rs672413
rs921943
rs3797535
rs11951068
rs10944
rs6859667
rs6586282
rs567754
rs1789953
rs234709
rs705415
rs558133
All − MR Egger
All − IVW(MRE)
−0.6
−0.3
0.0
0.3
MR effect size for
'Selenium' on 'beta−nerve growth factor'

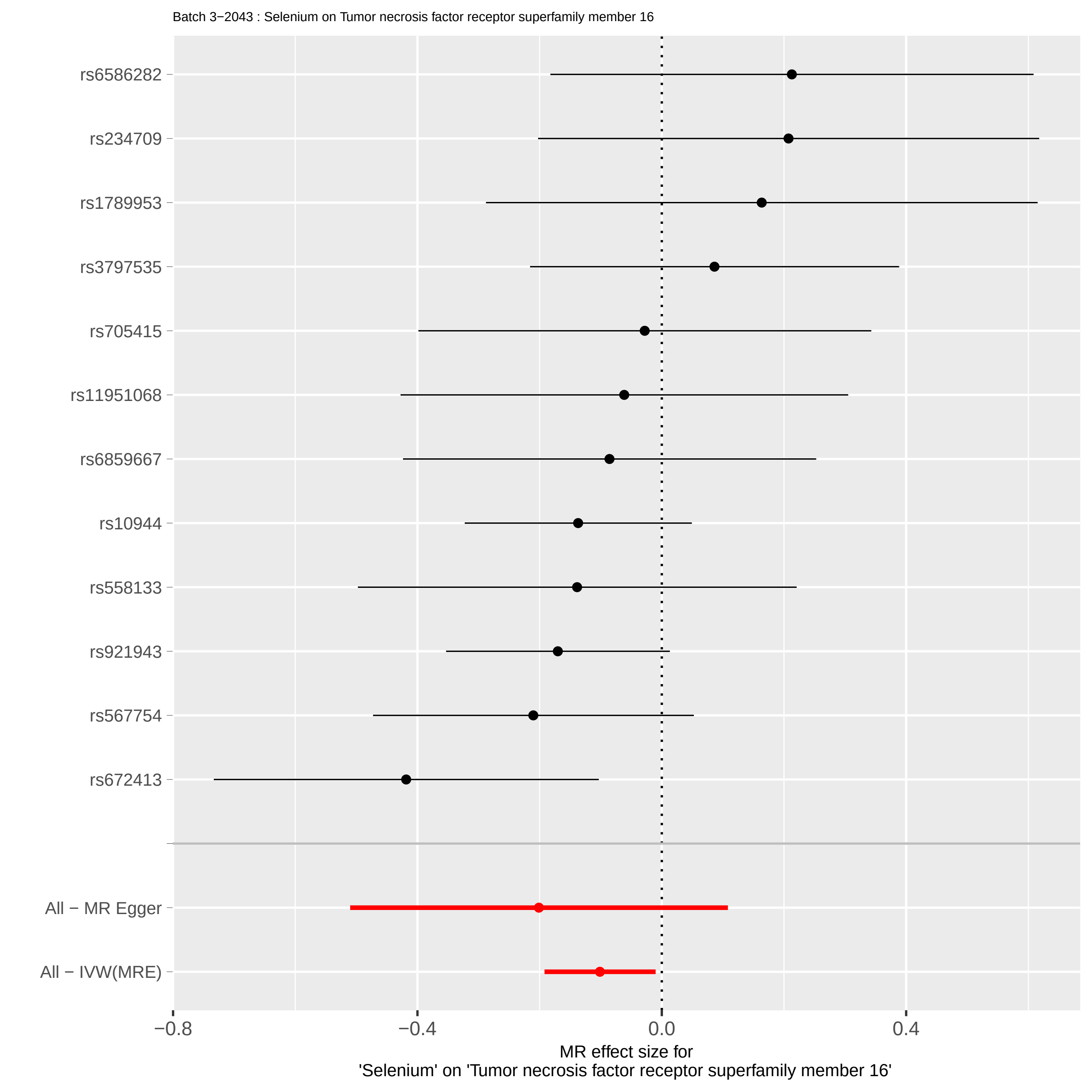

Batch 3−2043 : Selenium on Tumor necrosis factor receptor superfamily member 16
rs6586282
rs234709
rs1789953
rs3797535
rs705415
rs11951068
rs6859667
rs10944
rs558133
rs921943
rs567754
rs672413
All − MR Egger
All − IVW(MRE)
−0.8
−0.4
0.0
0.4
MR effect size for
'Selenium' on 'Tumor necrosis factor receptor superfamily member 16'

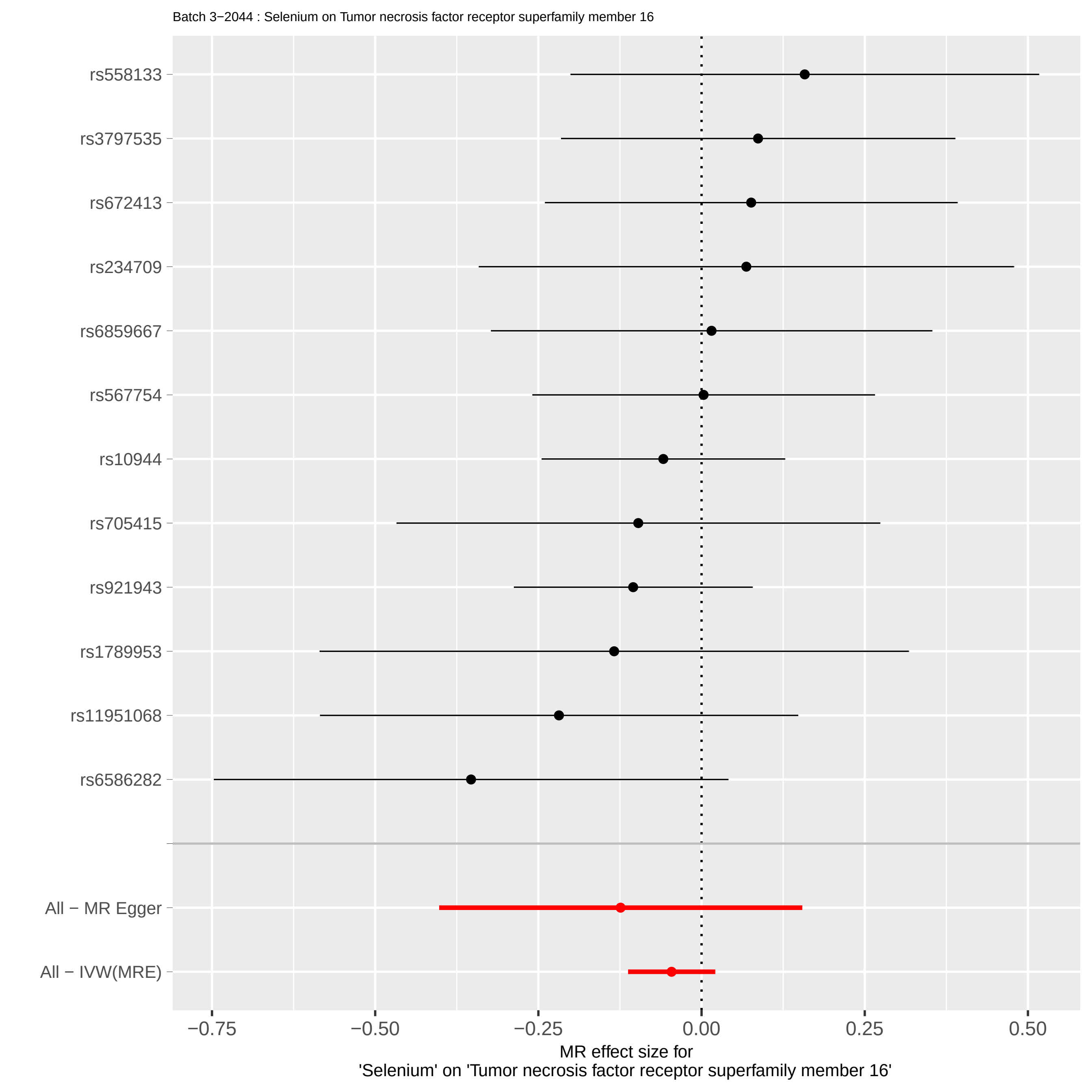

Batch 3−2044 : Selenium on Tumor necrosis factor receptor superfamily member 16
rs558133
rs3797535
rs672413
rs234709
rs6859667
rs567754
rs10944
rs705415
rs921943
rs1789953
rs11951068
rs6586282
All − MR Egger
All − IVW(MRE)
−0.75
−0.50
−0.25
0.00
0.25
0.50
MR effect size for
'Selenium' on 'Tumor necrosis factor receptor superfamily member 16'

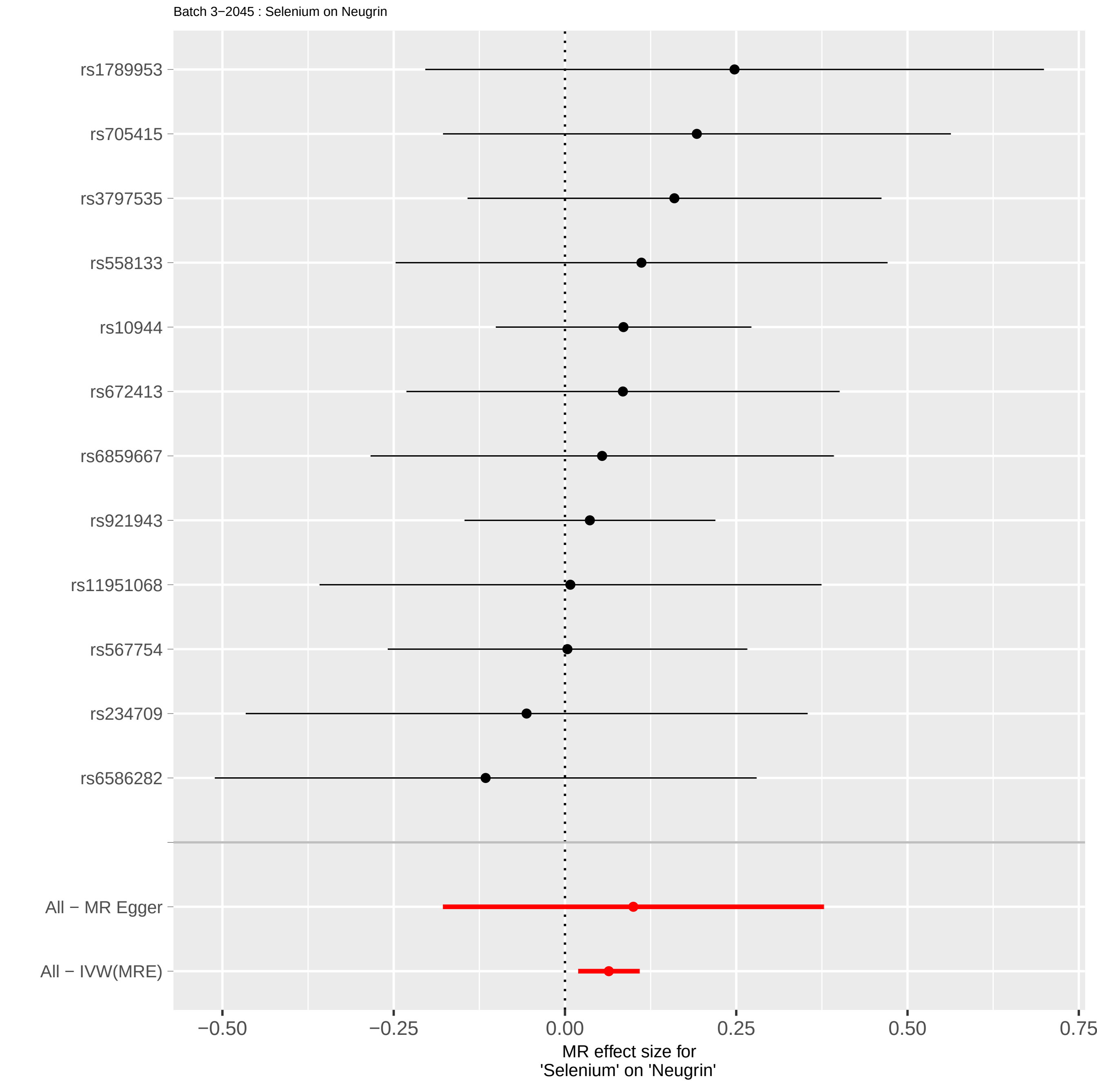

Batch 3−2045 : Selenium on Neugrin
rs1789953
rs705415
rs3797535
rs558133
rs10944
rs672413
rs6859667
rs921943
rs11951068
rs567754
rs234709
rs6586282
All − MR Egger
All − IVW(MRE)
−0.50
−0.25
0.00
0.25
0.50
0.75
MR effect size for
'Selenium' on 'Neugrin'

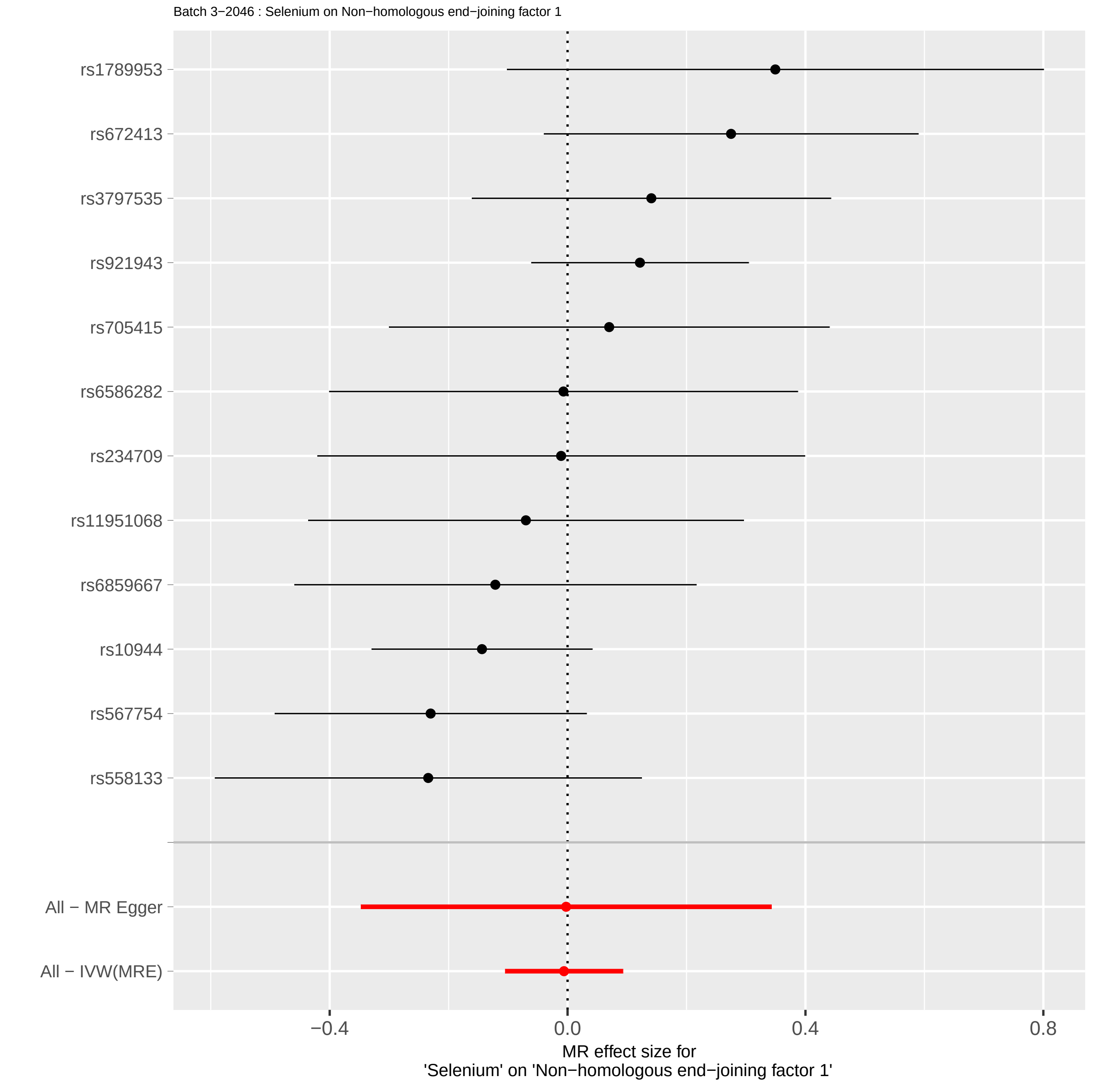
Batch 3−2046 : Selenium on Non−homologous end−joining factor 1
rs1789953
rs672413
rs3797535
rs921943
rs705415
rs6586282
rs234709
rs11951068
rs6859667
rs10944
rs567754
rs558133
All − MR Egger
All − IVW(MRE)
−0.4
0.0
0.4
0.8
MR effect size for
'Selenium' on 'Non−homologous end−joining factor 1'

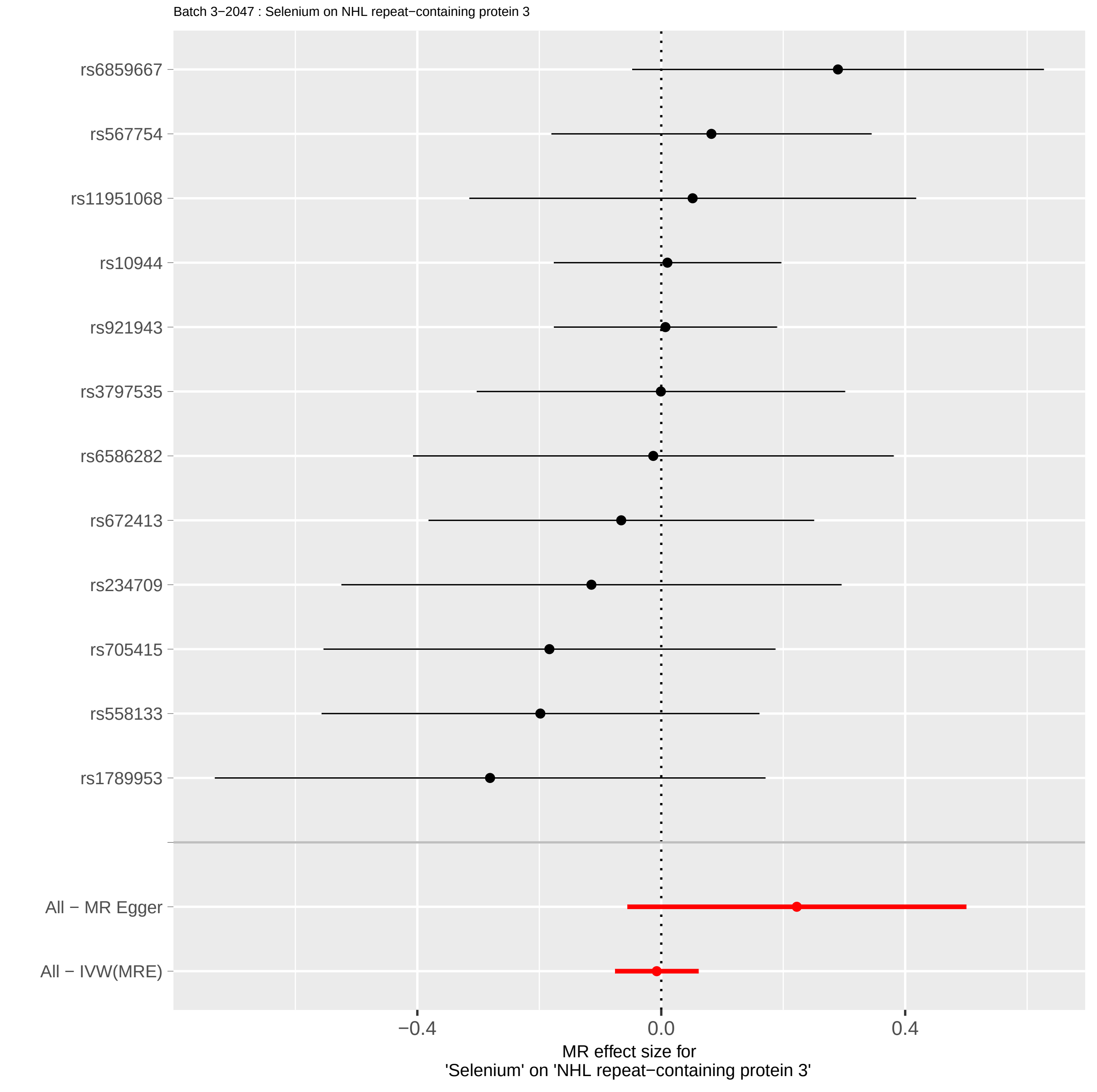
Batch 3−2047 : Selenium on NHL repeat−containing protein 3
rs6859667
rs567754
rs11951068
rs10944
rs921943
rs3797535
rs6586282
rs672413
rs234709
rs705415
rs558133
rs1789953
All − MR Egger
All − IVW(MRE)
−0.4
0.0
0.4
MR effect size for
'Selenium' on 'NHL repeat−containing protein 3'

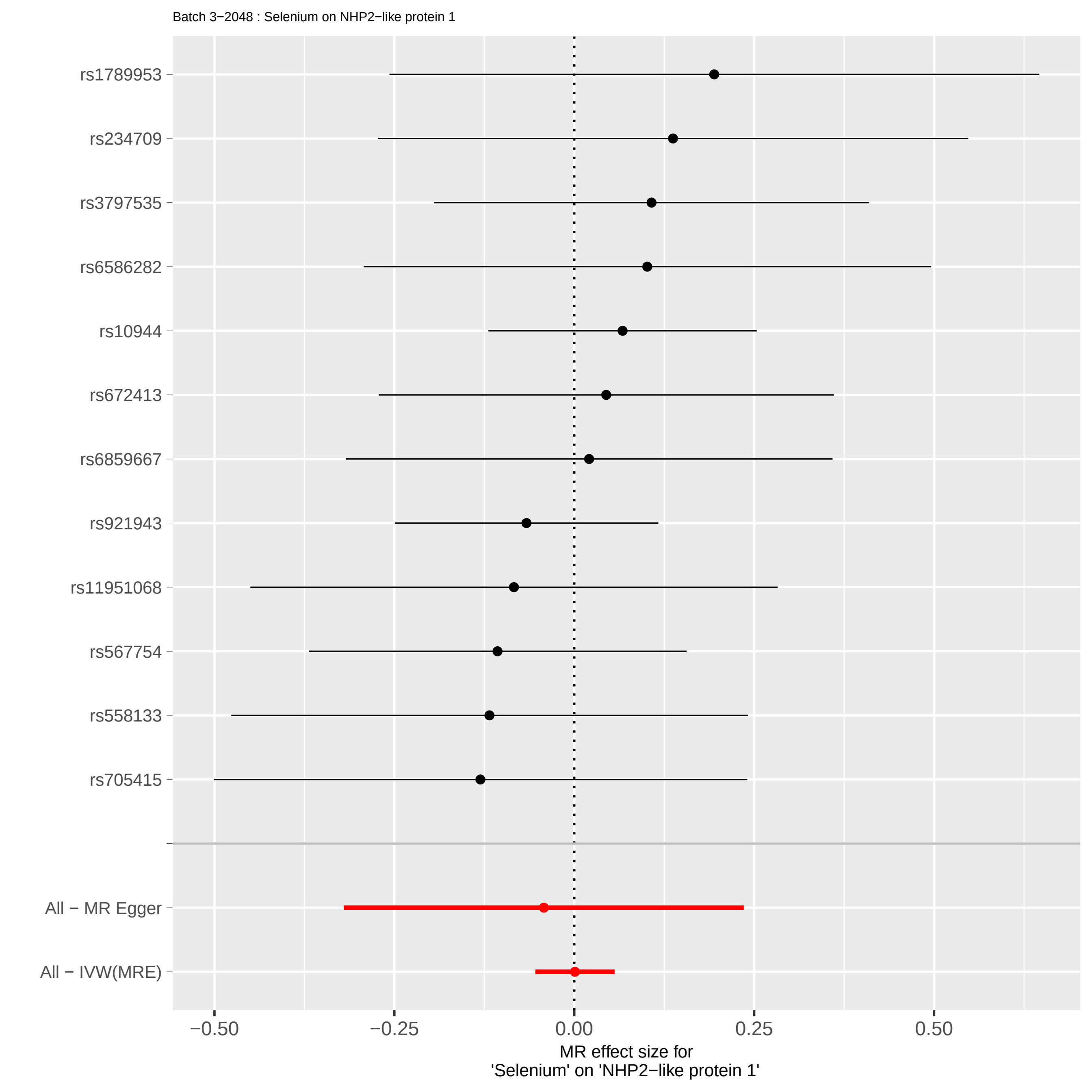

Batch 3−2048 : Selenium on NHP2−like protein 1
rs1789953
rs234709
rs3797535
rs6586282
rs10944
rs672413
rs6859667
rs921943
rs11951068
rs567754
rs558133
rs705415
All − MR Egger
All − IVW(MRE)
−0.50
−0.25
0.00
0.25
0.50
MR effect size for
'Selenium' on 'NHP2−like protein 1'

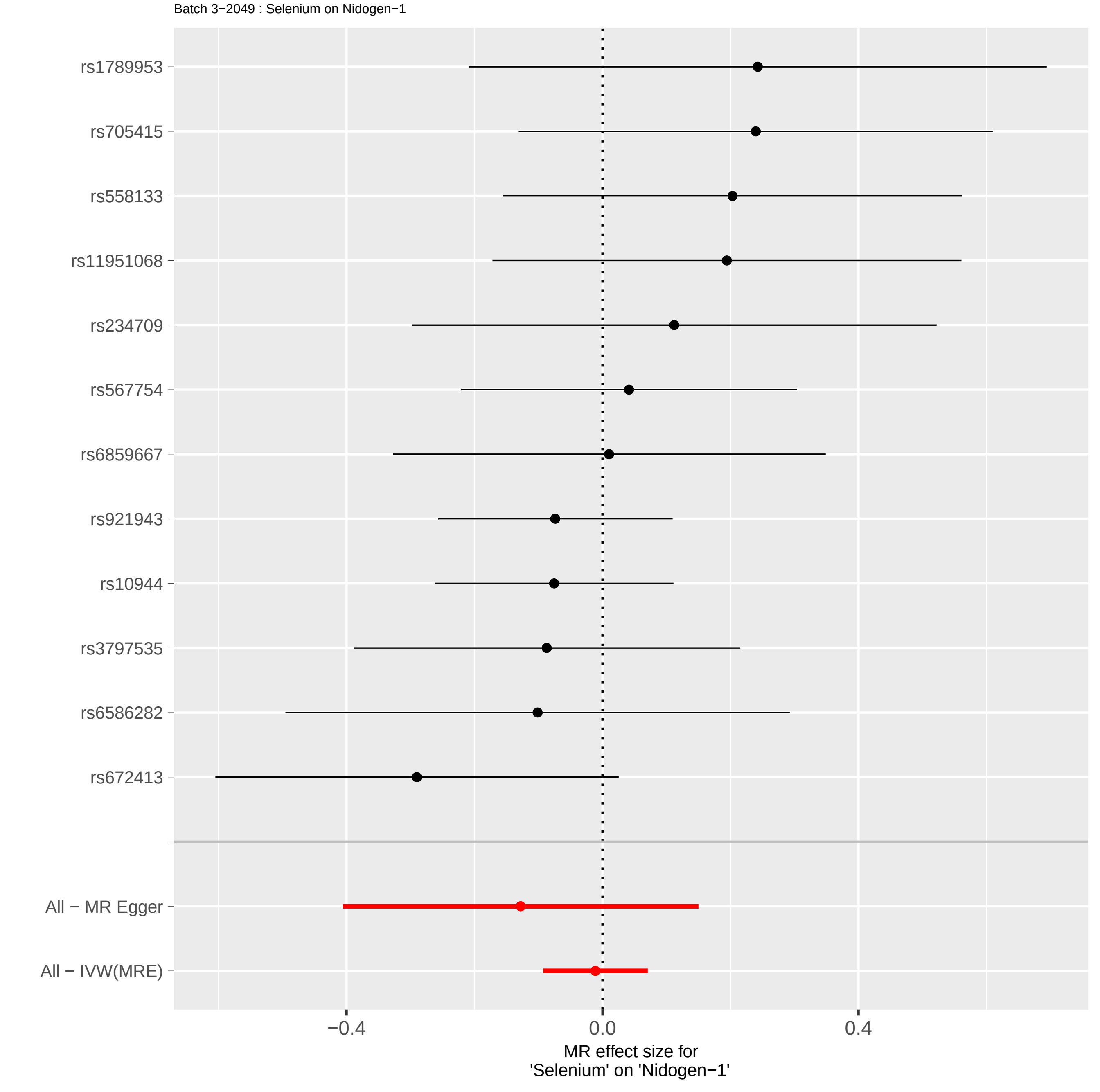

Batch 3−2049 : Selenium on Nidogen−1
rs1789953
rs705415
rs558133
rs11951068
rs234709
rs567754
rs6859667
rs921943
rs10944
rs3797535
rs6586282
rs672413
All − MR Egger
All − IVW(MRE)
−0.4
0.0
0.4
MR effect size for
'Selenium' on 'Nidogen−1'

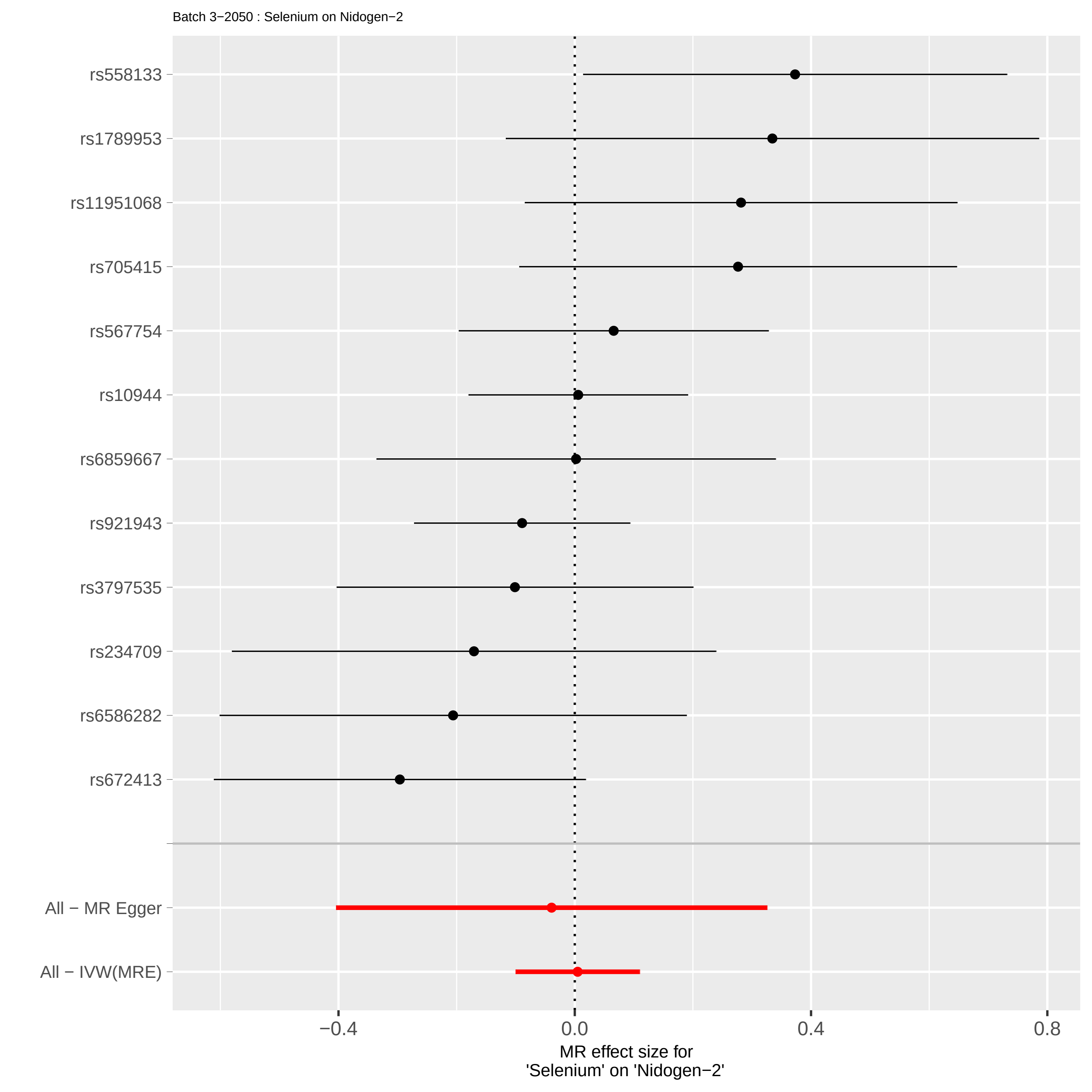

Batch 3−2050 : Selenium on Nidogen−2
rs558133
rs1789953
rs11951068
rs705415
rs567754
rs10944
rs6859667
rs921943
rs3797535
rs234709
rs6586282
rs672413
All − MR Egger
All − IVW(MRE)
−0.4
0.0
0.4
0.8
MR effect size for
'Selenium' on 'Nidogen−2'

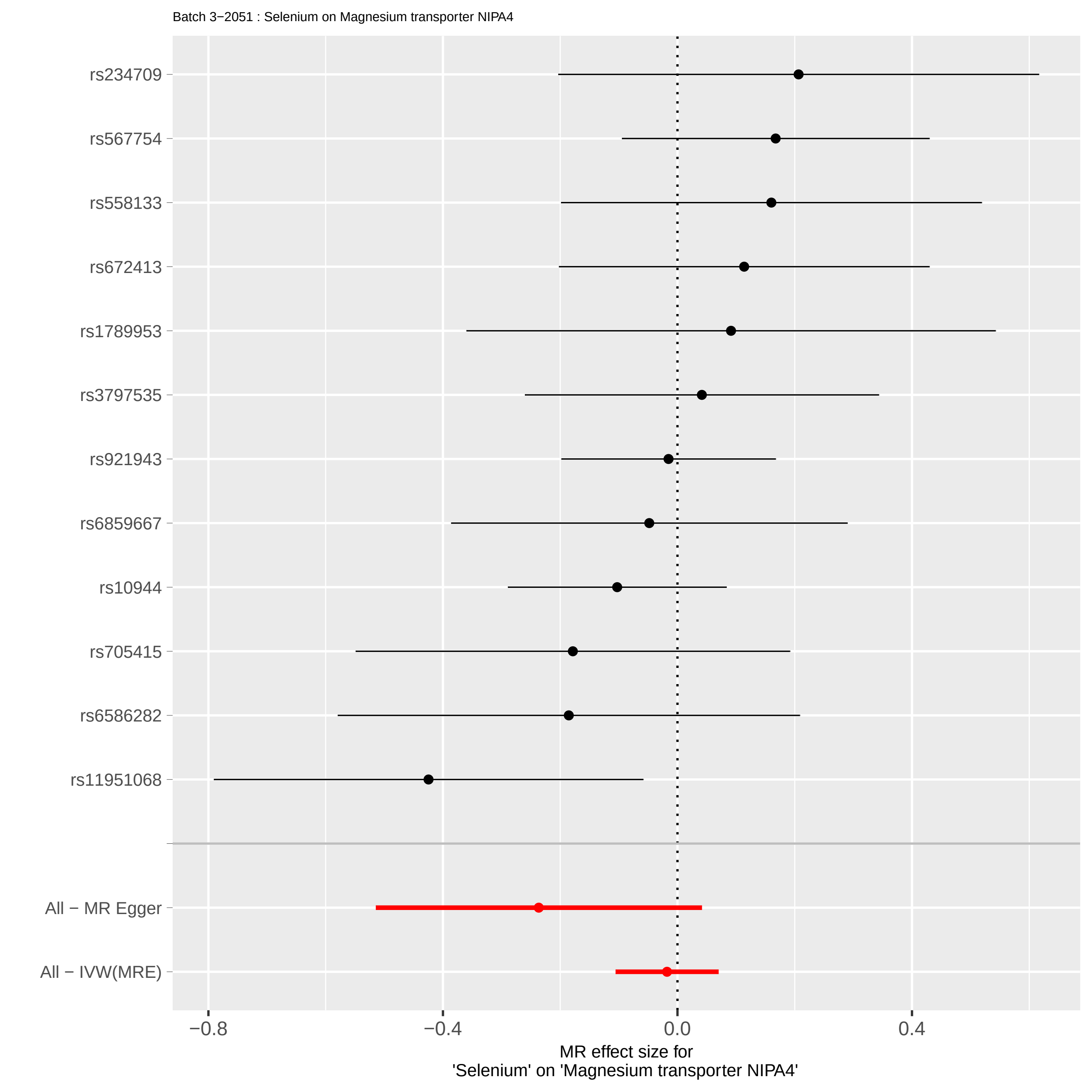

Batch 3−2051 : Selenium on Magnesium transporter NIPA4
rs234709
rs567754
rs558133
rs672413
rs1789953
rs3797535
rs921943
rs6859667
rs10944
rs705415
rs6586282
rs11951068
All − MR Egger
All − IVW(MRE)
−0.8
−0.4
0.0
0.4
MR effect size for
'Selenium' on 'Magnesium transporter NIPA4'

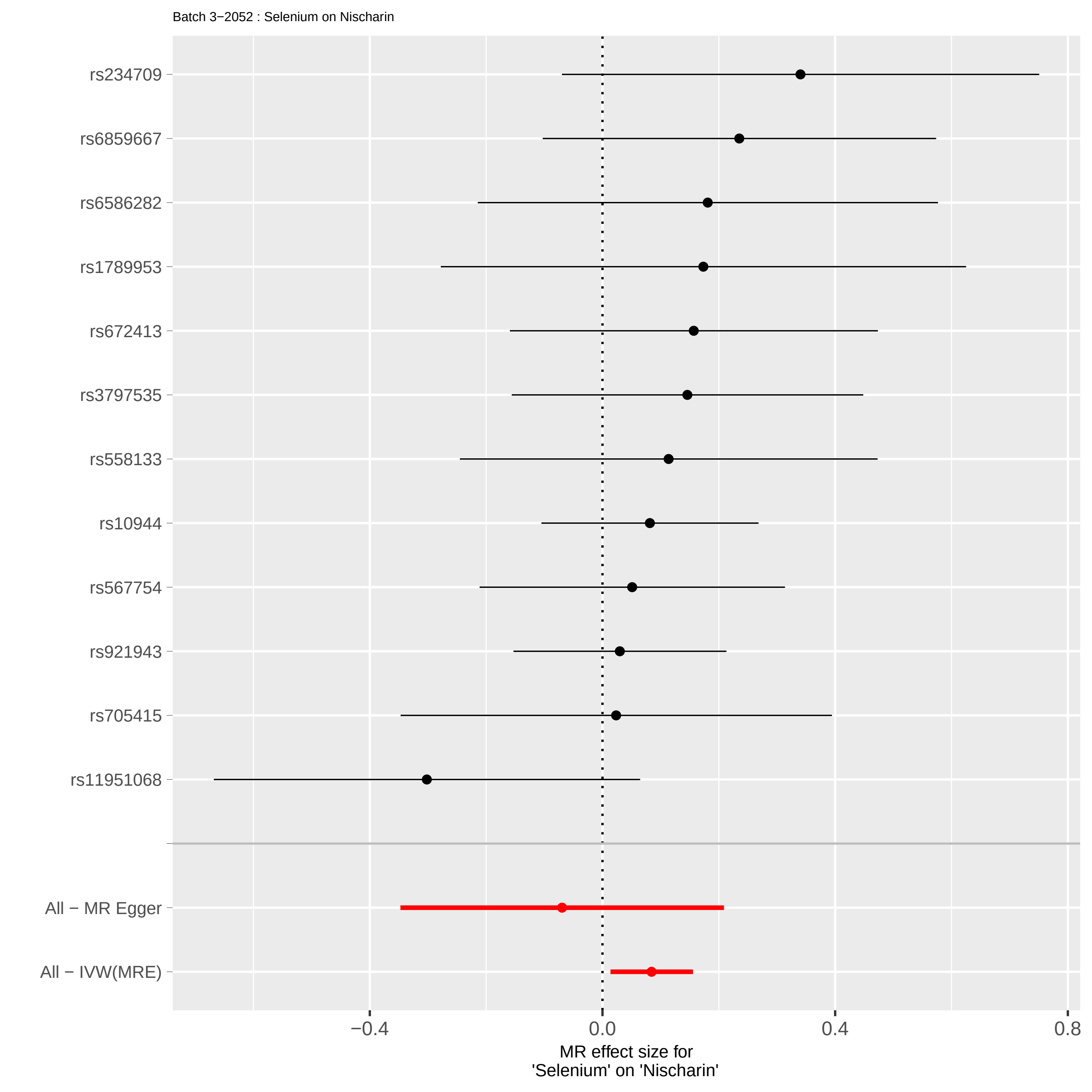

Batch 3−2052 : Selenium on Nischarin
rs234709
rs6859667
rs6586282
rs1789953
rs672413
rs3797535
rs558133
rs10944
rs567754
rs921943
rs705415
rs11951068
All − MR Egger
All − IVW(MRE)
−0.4
0.0
0.4
0.8
MR effect size for
'Selenium' on 'Nischarin'

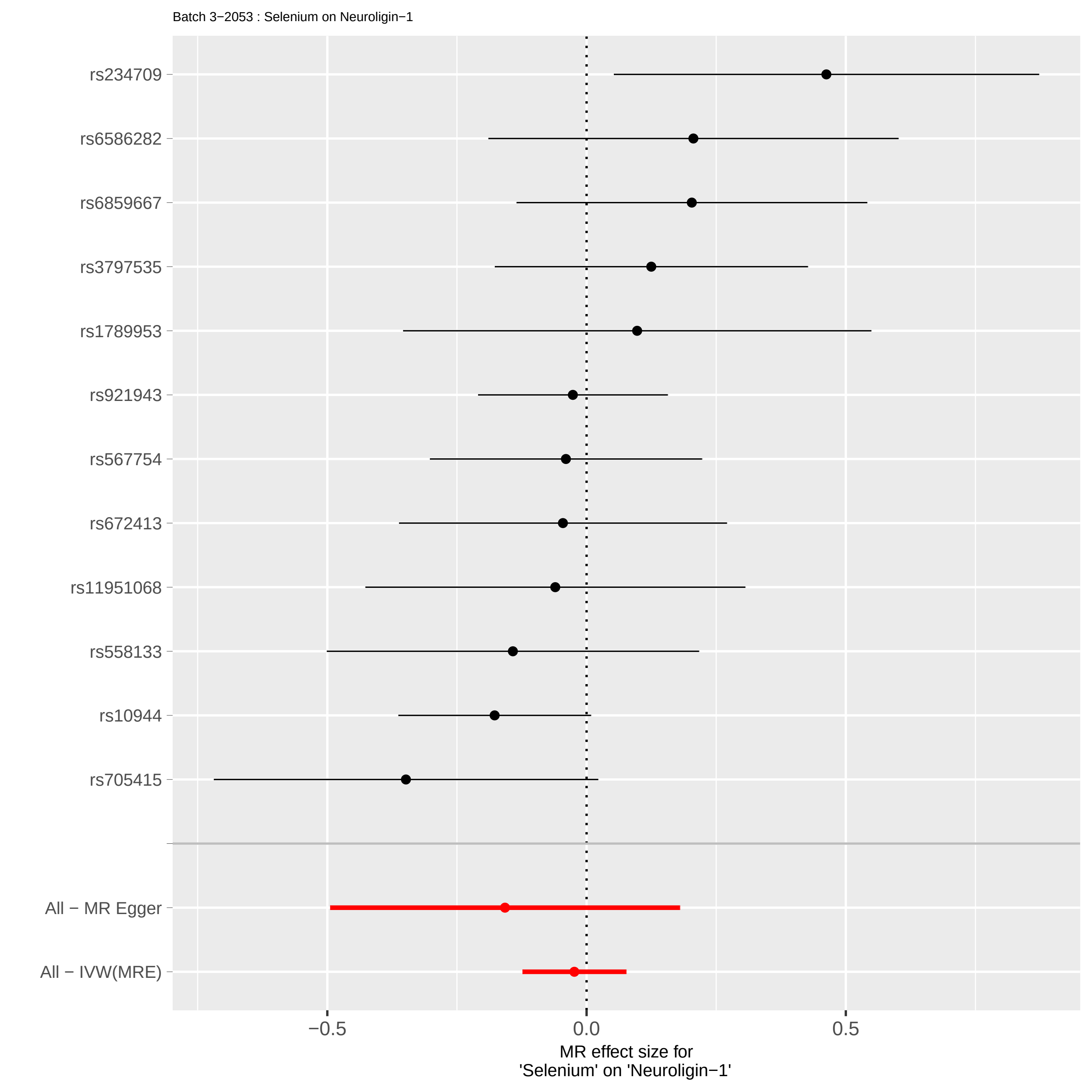

Batch 3−2053 : Selenium on Neuroligin−1
rs234709
rs6586282
rs6859667
rs3797535
rs1789953
rs921943
rs567754
rs672413
rs11951068
rs558133
rs10944
rs705415
All − MR Egger
All − IVW(MRE)
−0.5
0.0
0.5
MR effect size for
'Selenium' on 'Neuroligin−1'

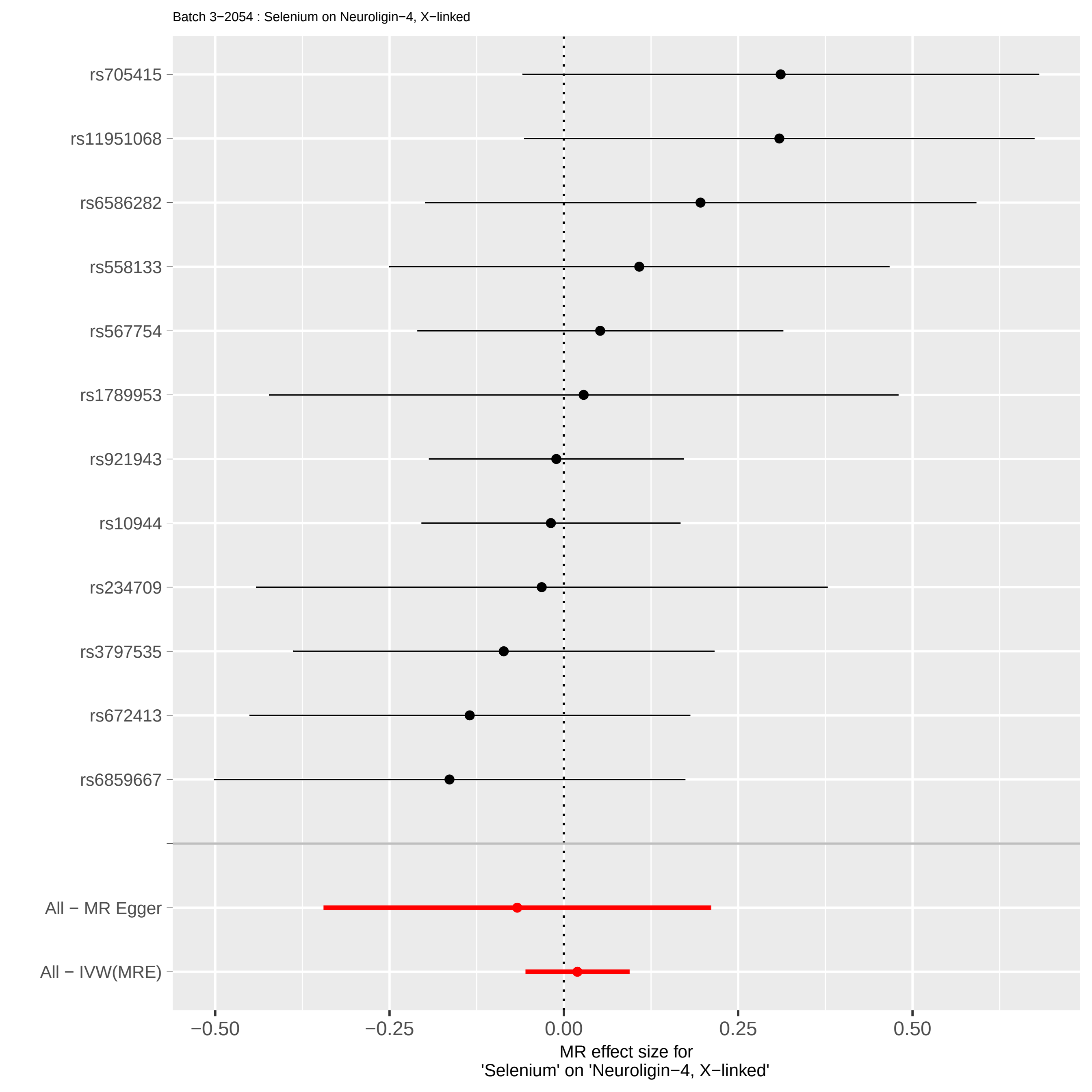
Batch 3−2054 : Selenium on Neuroligin−4, X−linked
rs705415
rs11951068
rs6586282
rs558133
rs567754
rs1789953
rs921943
rs10944
rs234709
rs3797535
rs672413
rs6859667
All − MR Egger
All − IVW(MRE)
−0.50
−0.25
0.00
0.25
0.50
MR effect size for
'Selenium' on 'Neuroligin−4, X−linked'

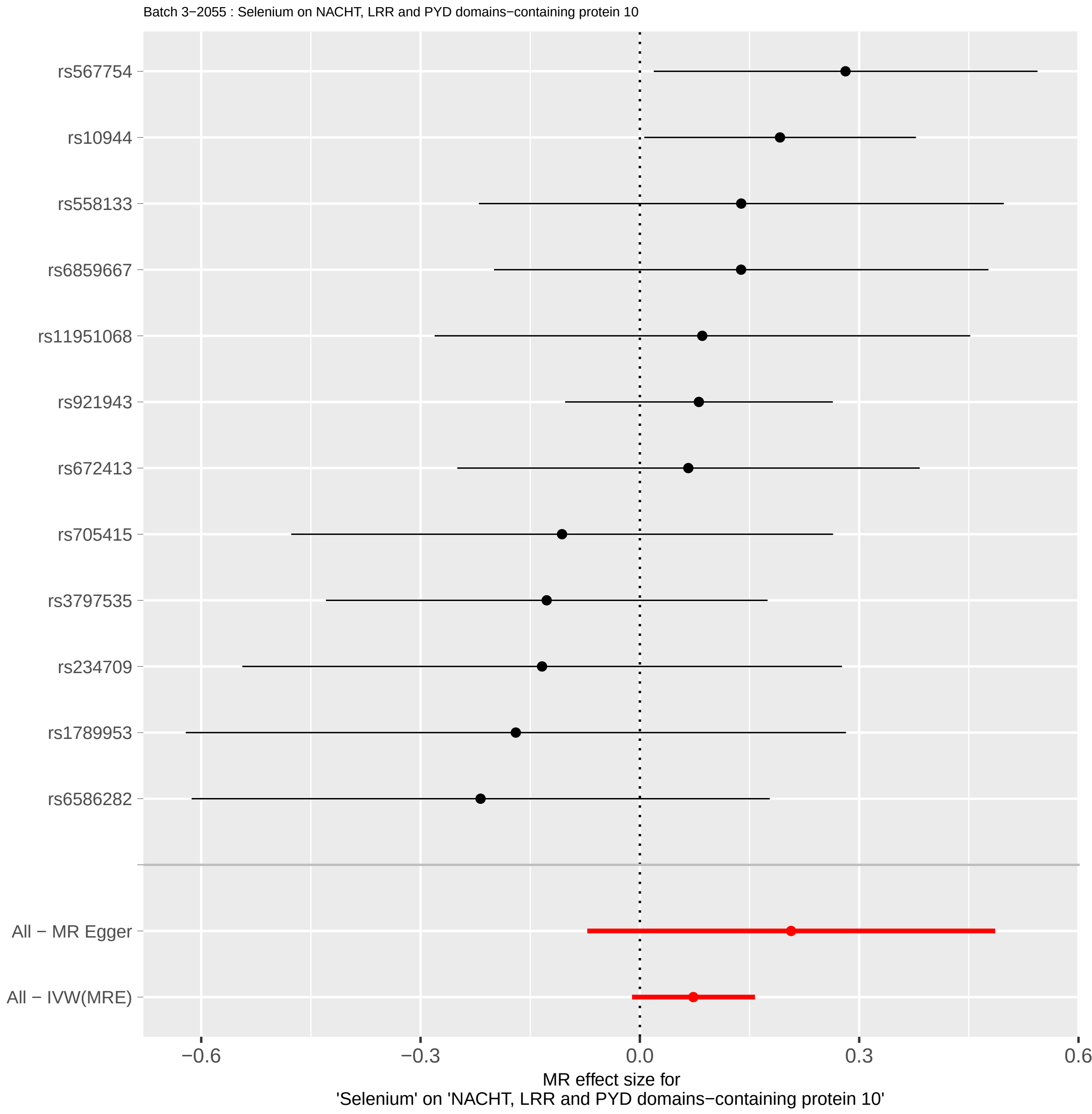

Batch 3−2055 : Selenium on NACHT, LRR and PYD domains−containing protein 10
rs567754
rs10944
rs558133
rs6859667
rs11951068
rs921943
rs672413
rs705415
rs3797535
rs234709
rs1789953
rs6586282
All − MR Egger
All − IVW(MRE)
−0.6
−0.3
0.0
0.3
0.6
MR effect size for
'Selenium' on 'NACHT, LRR and PYD domains−containing protein 10'

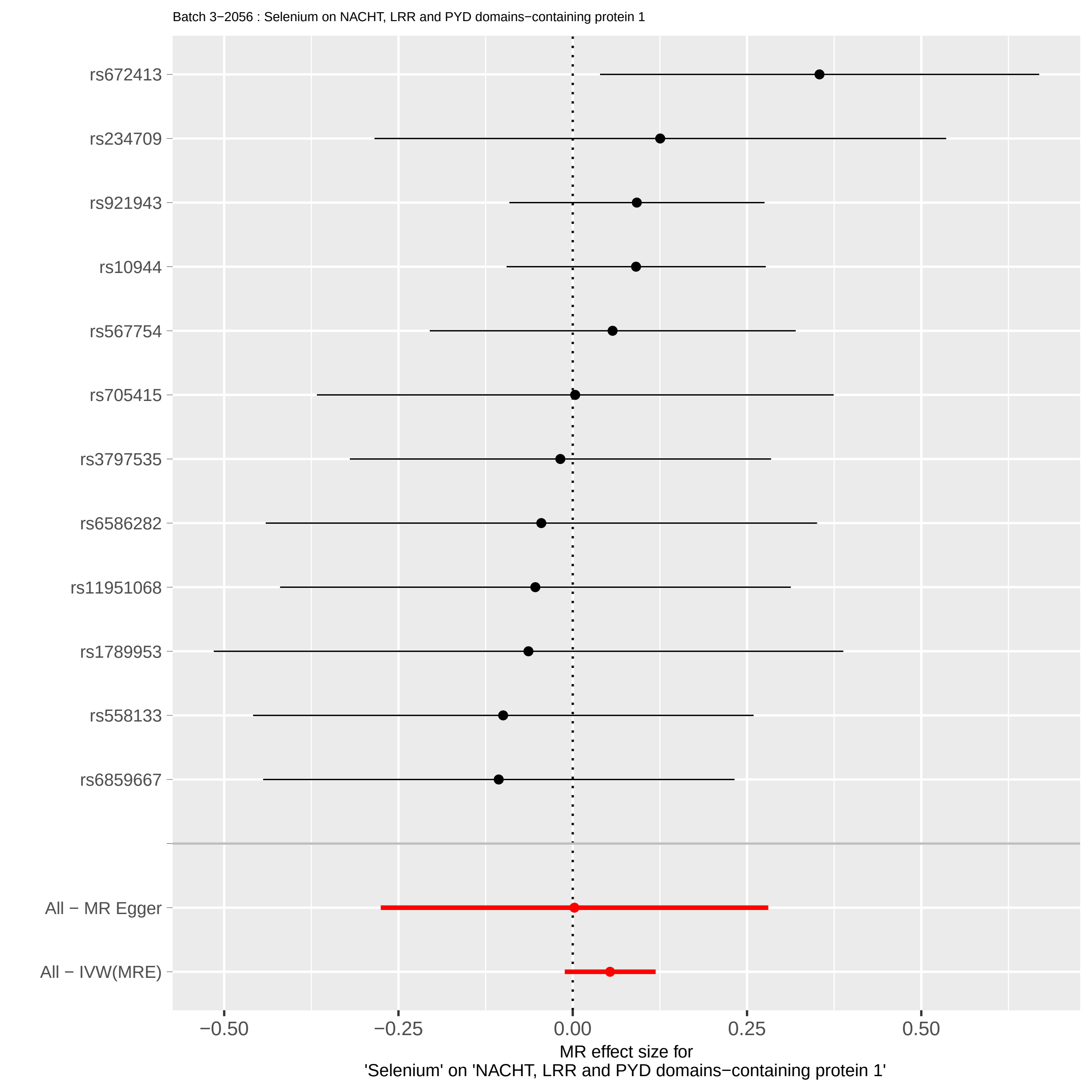
Batch 3−2056 : Selenium on NACHT, LRR and PYD domains−containing protein 1
rs672413
rs234709
rs921943
rs10944
rs567754
rs705415
rs3797535
rs6586282
rs11951068
rs1789953
rs558133
rs6859667
All − MR Egger
All − IVW(MRE)
−0.50
−0.25
0.00
0.25
0.50
MR effect size for
'Selenium' on 'NACHT, LRR and PYD domains−containing protein 1'

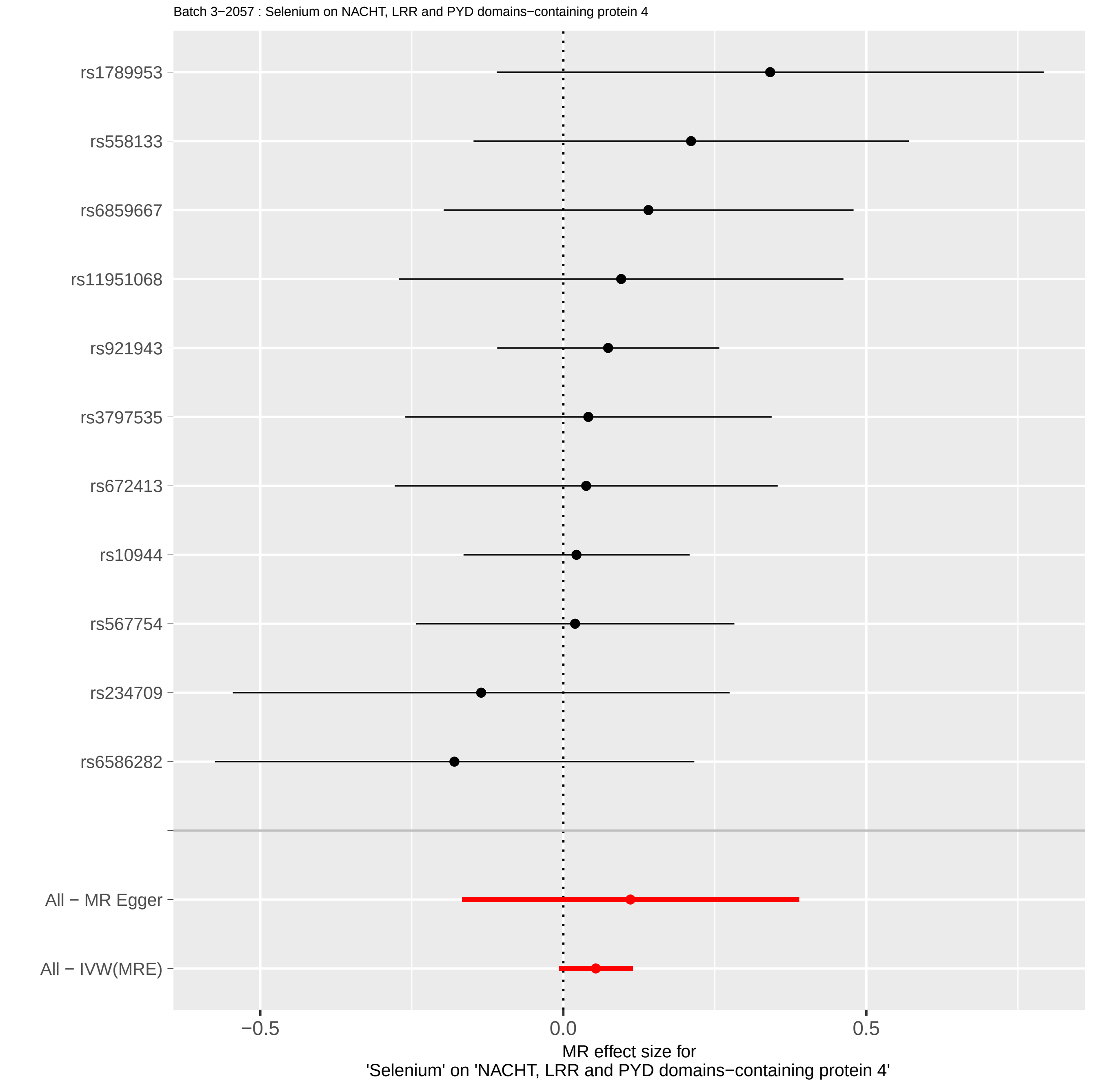

Batch 3−2057 : Selenium on NACHT, LRR and PYD domains−containing protein 4
rs1789953
rs558133
rs6859667
rs11951068
rs921943
rs3797535
rs672413
rs10944
rs567754
rs234709
rs6586282
All − MR Egger
All − IVW(MRE)
−0.5
0.0
0.5
MR effect size for
'Selenium' on 'NACHT, LRR and PYD domains−containing protein 4'

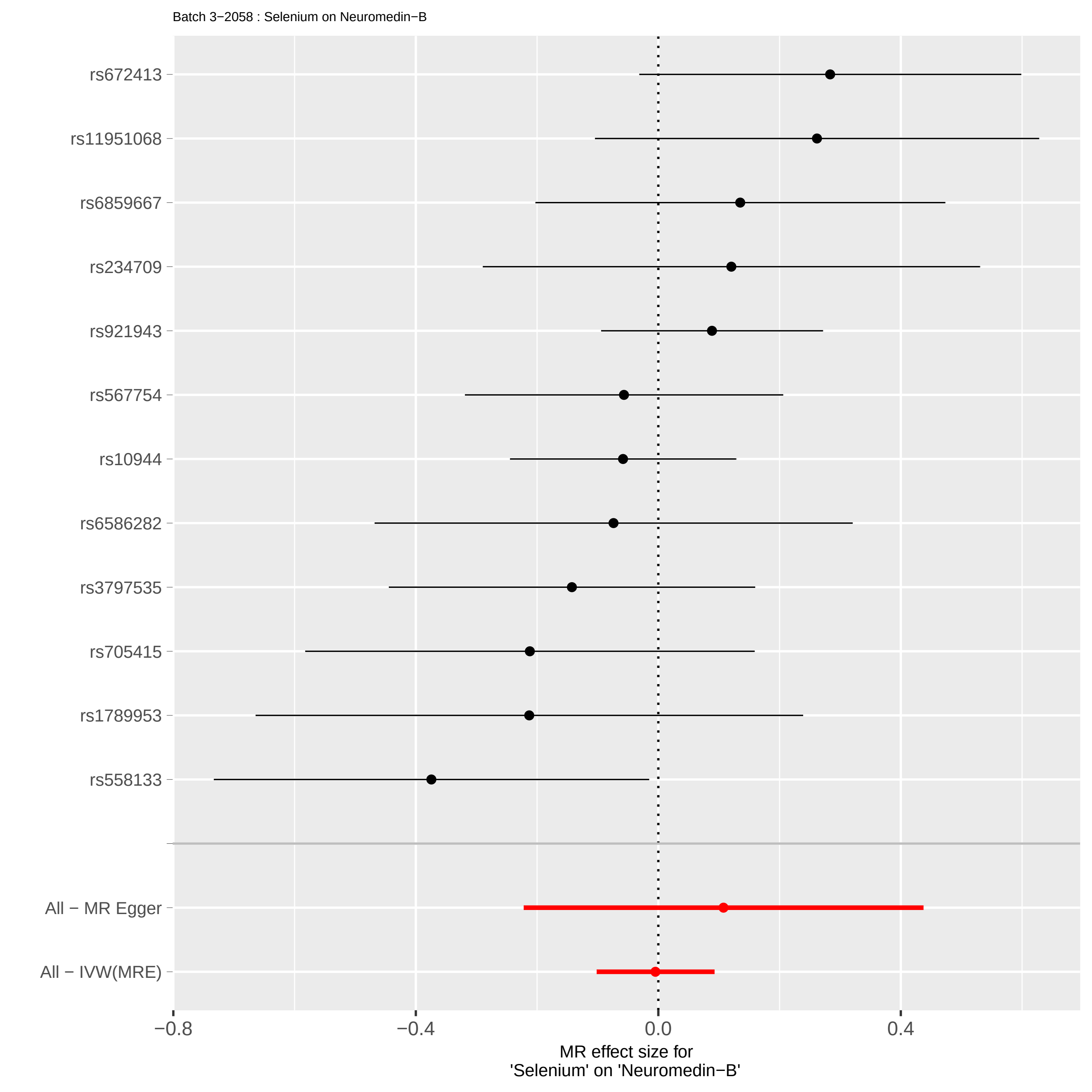

Batch 3−2058 : Selenium on Neuromedin−B
rs672413
rs11951068
rs6859667
rs234709
rs921943
rs567754
rs10944
rs6586282
rs3797535
rs705415
rs1789953
rs558133
All − MR Egger
All − IVW(MRE)
−0.8
−0.4
0.0
0.4
MR effect size for
'Selenium' on 'Neuromedin−B'

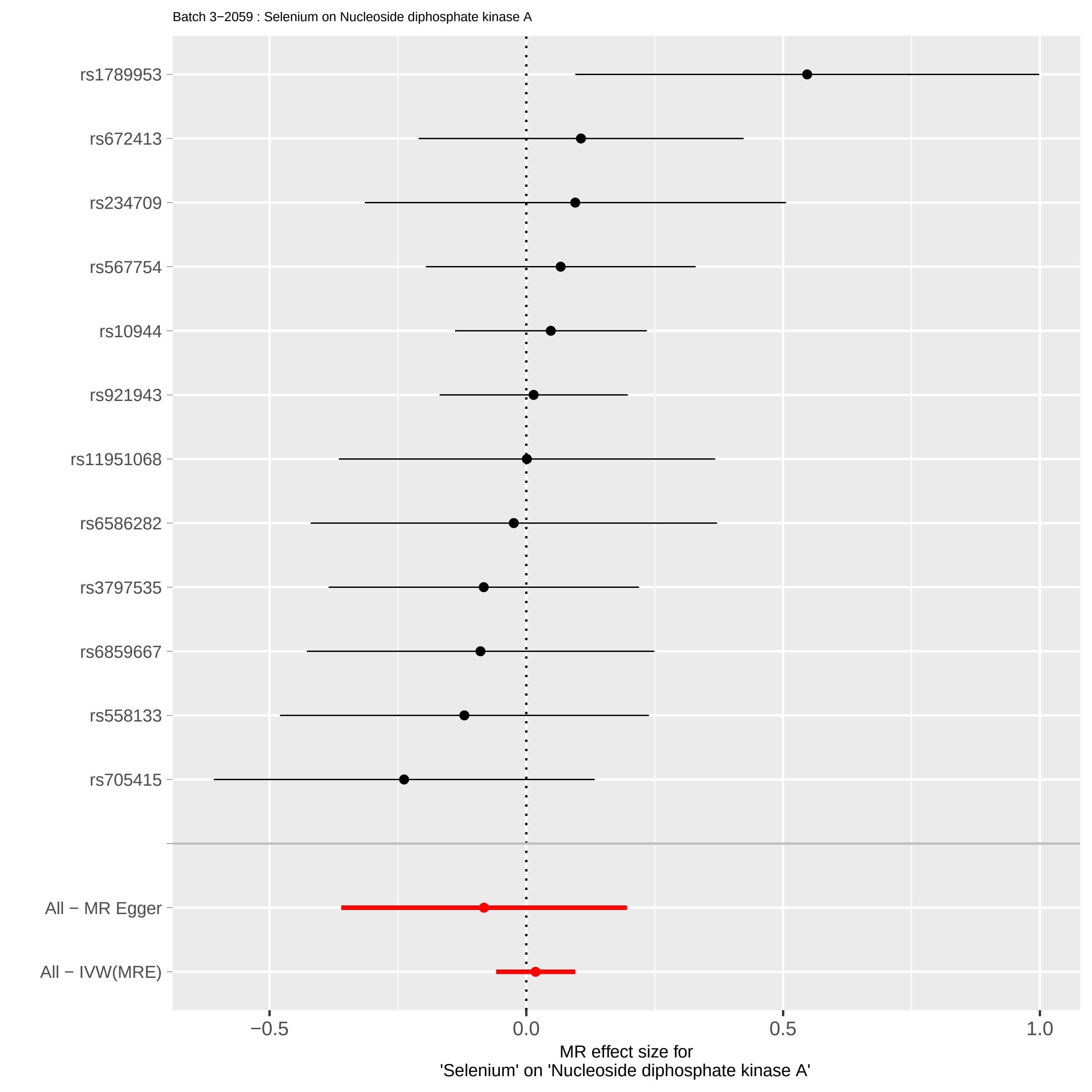

Batch 3−2059 : Selenium on Nucleoside diphosphate kinase A
rs1789953
rs672413
rs234709
rs567754
rs10944
rs921943
rs11951068
rs6586282
rs3797535
rs6859667
rs558133
rs705415
All − MR Egger
All − IVW(MRE)
−0.5
0.0
0.5
1.0
MR effect size for
'Selenium' on 'Nucleoside diphosphate kinase A'

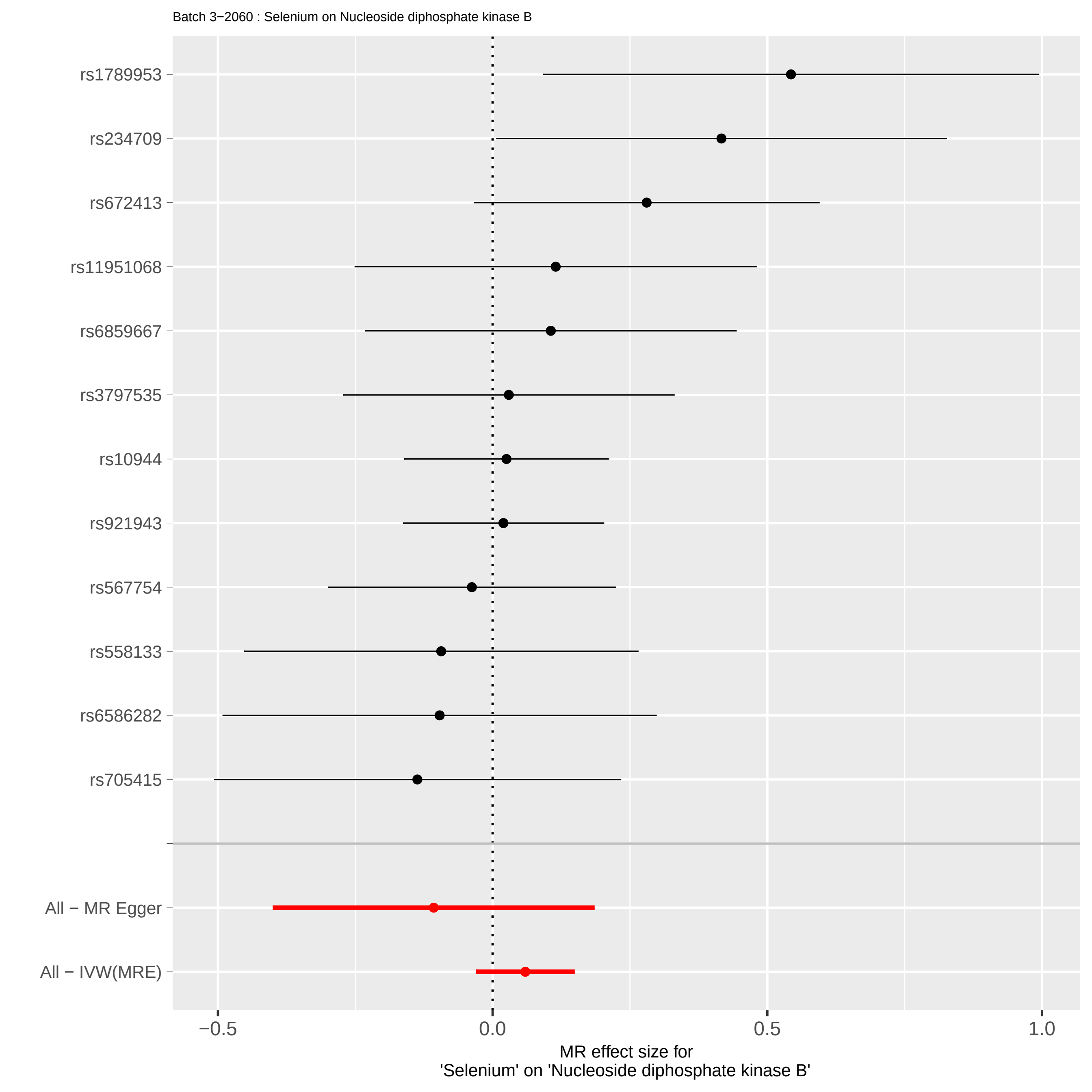

Batch 3−2060 : Selenium on Nucleoside diphosphate kinase B
rs1789953
rs234709
rs672413
rs11951068
rs6859667
rs3797535
rs10944
rs921943
rs567754
rs558133
rs6586282
rs705415
All − MR Egger
All − IVW(MRE)
−0.5
0.0
0.5
1.0
MR effect size for
'Selenium' on 'Nucleoside diphosphate kinase B'

Batch 3−2061 : Selenium on Normal mucosa of esophagus−specific gene 1 protein

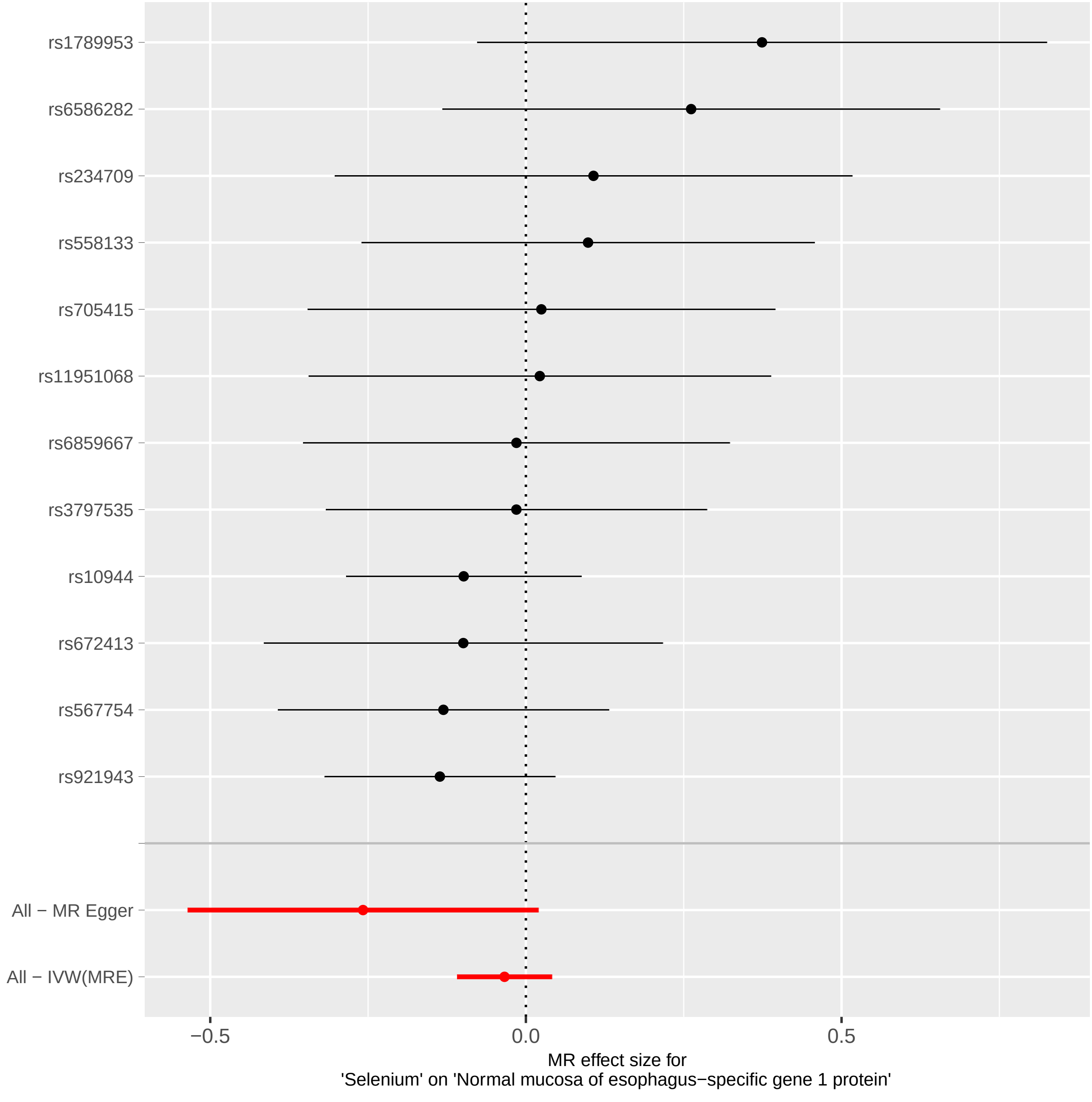

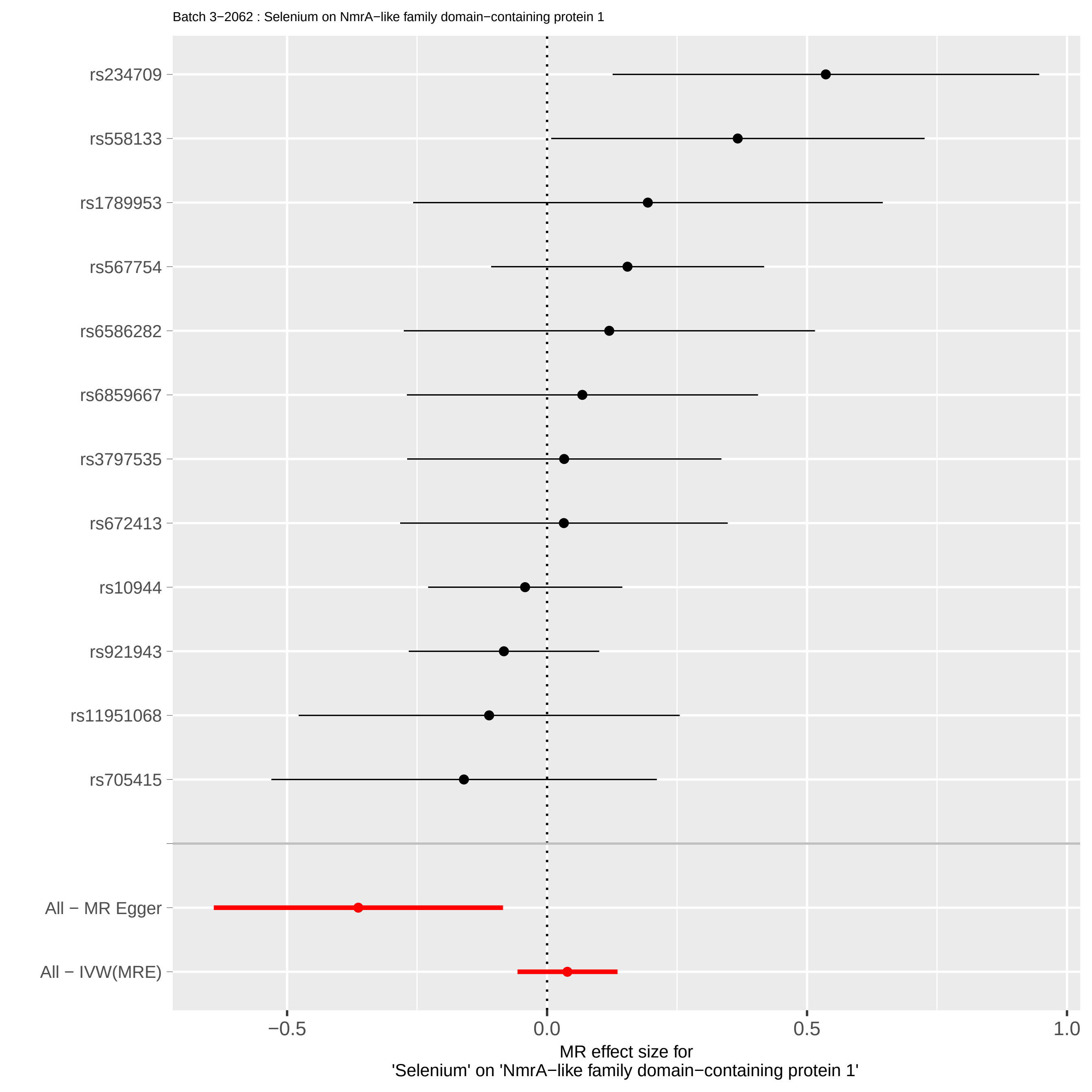

Batch 3−2062 : Selenium on NmrA−like family domain−containing protein 1
rs234709
rs558133
rs1789953
rs567754
rs6586282
rs6859667
rs3797535
rs672413
rs10944
rs921943
rs11951068
rs705415
All − MR Egger
All − IVW(MRE)
−0.5
0.0
0.5
1.0
MR effect size for
'Selenium' on 'NmrA−like family domain−containing protein 1'

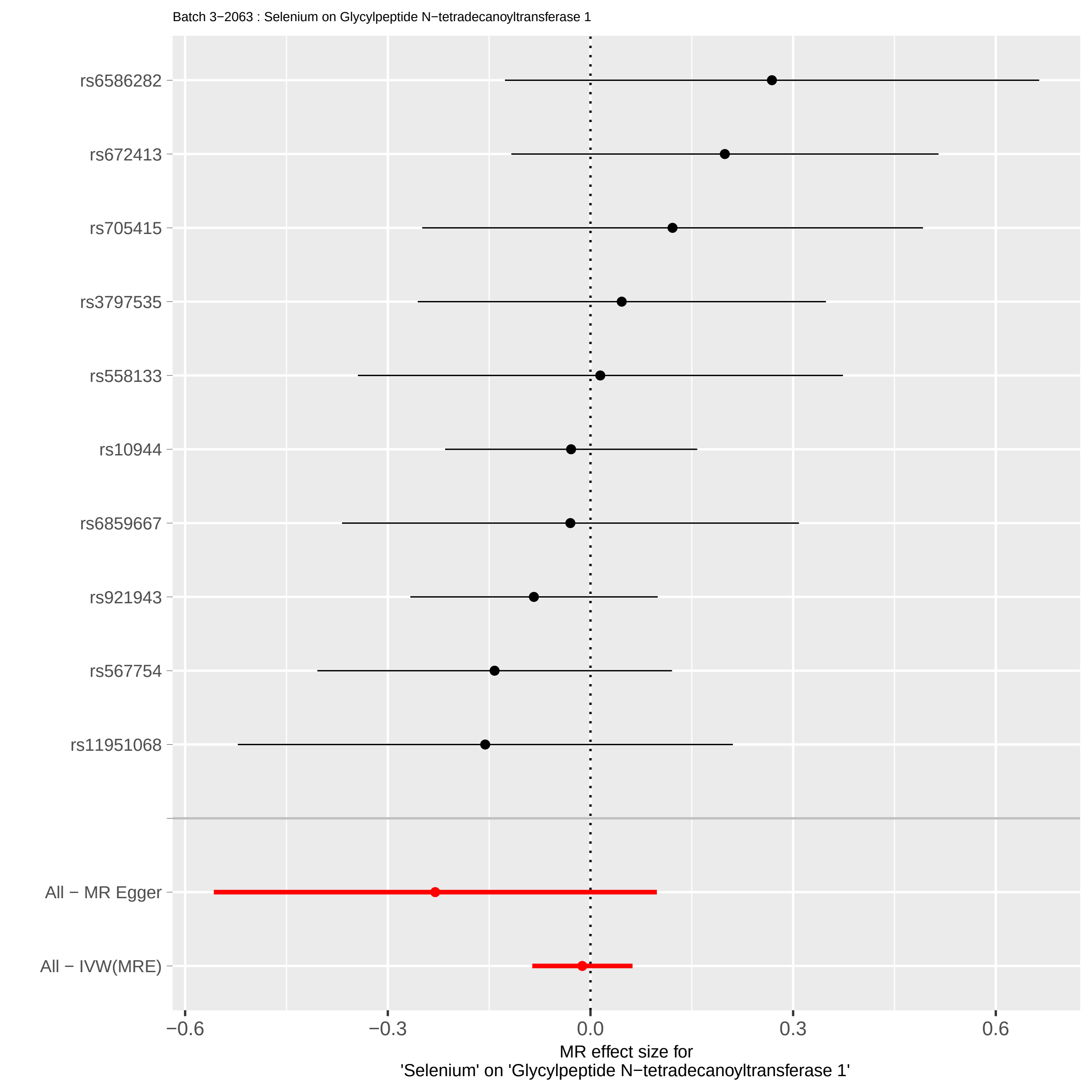

Batch 3−2063 : Selenium on Glycylpeptide N−tetradecanoyltransferase 1
rs6586282
rs672413
rs705415
rs3797535
rs558133
rs10944
rs6859667
rs921943
rs567754
rs11951068
All − MR Egger
All − IVW(MRE)
−0.6
−0.3
0.0
0.3
0.6
MR effect size for
'Selenium' on 'Glycylpeptide N−tetradecanoyltransferase 1'

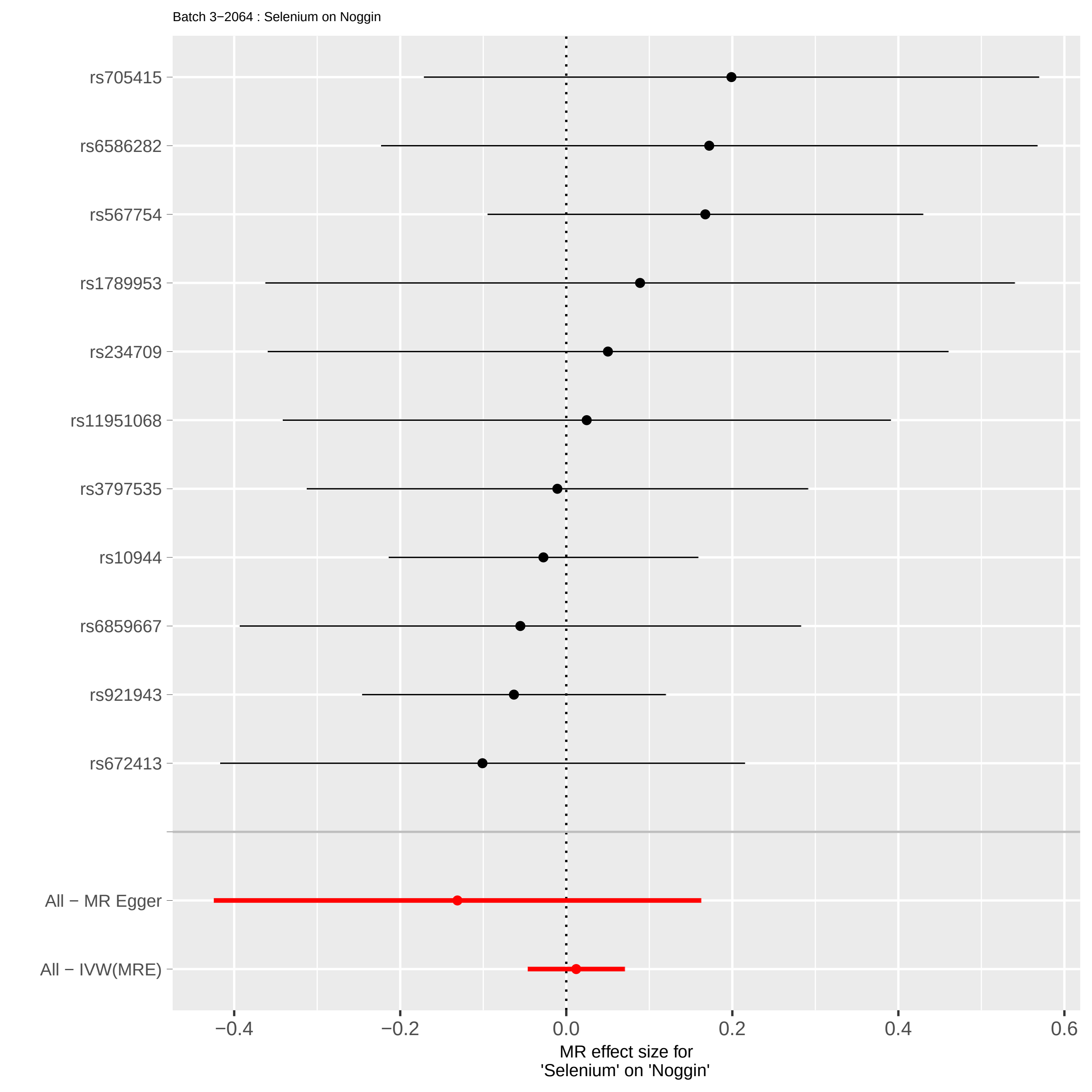

Batch 3−2064 : Selenium on Noggin
rs705415
rs6586282
rs567754
rs1789953
rs234709
rs11951068
rs3797535
rs10944
rs6859667
rs921943
rs672413
All − MR Egger
All − IVW(MRE)
−0.4
−0.2
0.0
0.2
0.4
0.6
MR effect size for
'Selenium' on 'Noggin'

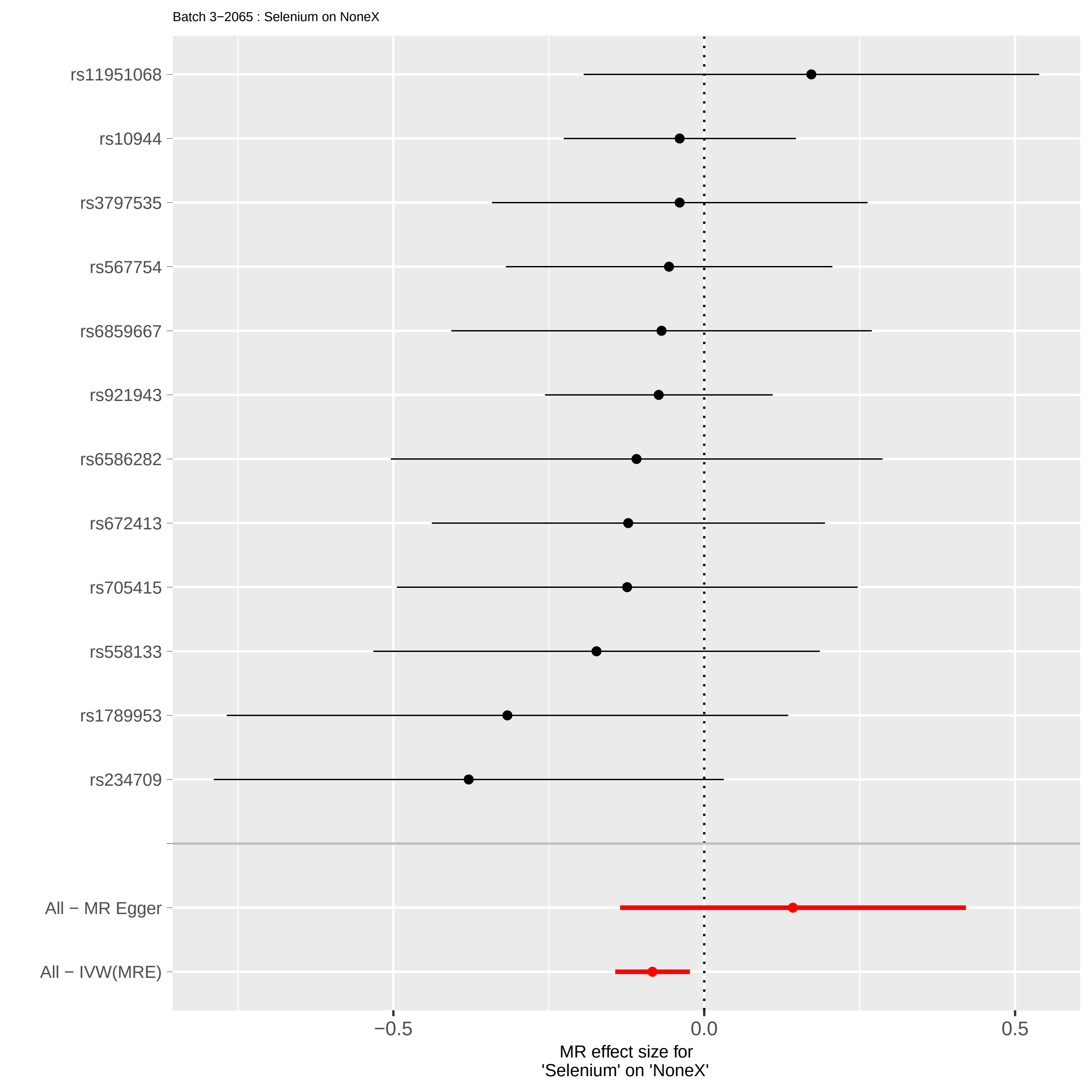
Batch 3−2065 : Selenium on NoneX
rs11951068
rs10944
rs3797535
rs567754
rs6859667
rs921943
rs6586282
rs672413
rs705415
rs558133
rs1789953
rs234709
All − MR Egger
All − IVW(MRE)
−0.5
0.0
0.5
MR effect size for
'Selenium' on 'NoneX'

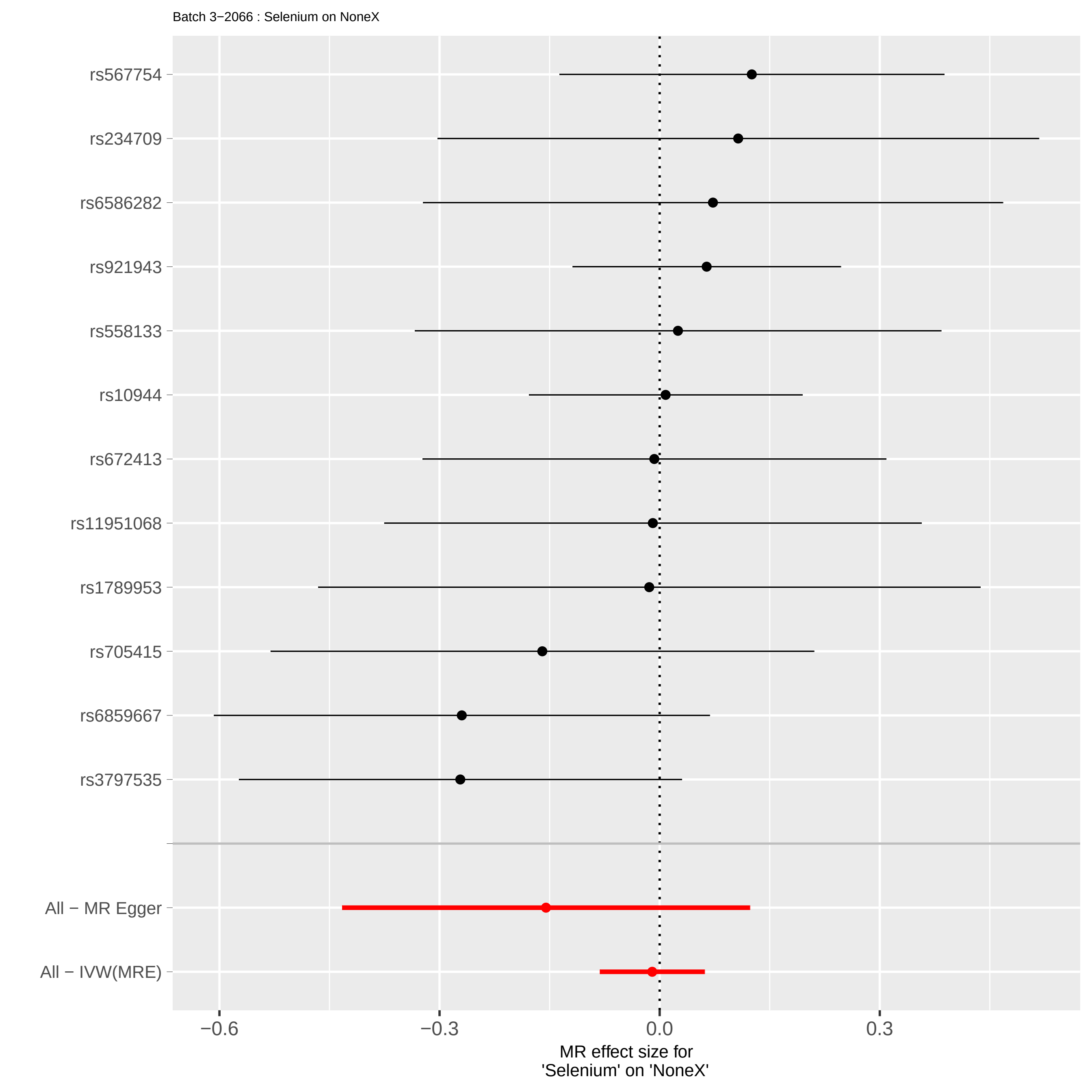

Batch 3−2066 : Selenium on NoneX
rs567754
rs234709
rs6586282
rs921943
rs558133
rs10944
rs672413
rs11951068
rs1789953
rs705415
rs6859667
rs3797535
All − MR Egger
All − IVW(MRE)
−0.6
−0.3
0.0
0.3
MR effect size for
'Selenium' on 'NoneX'

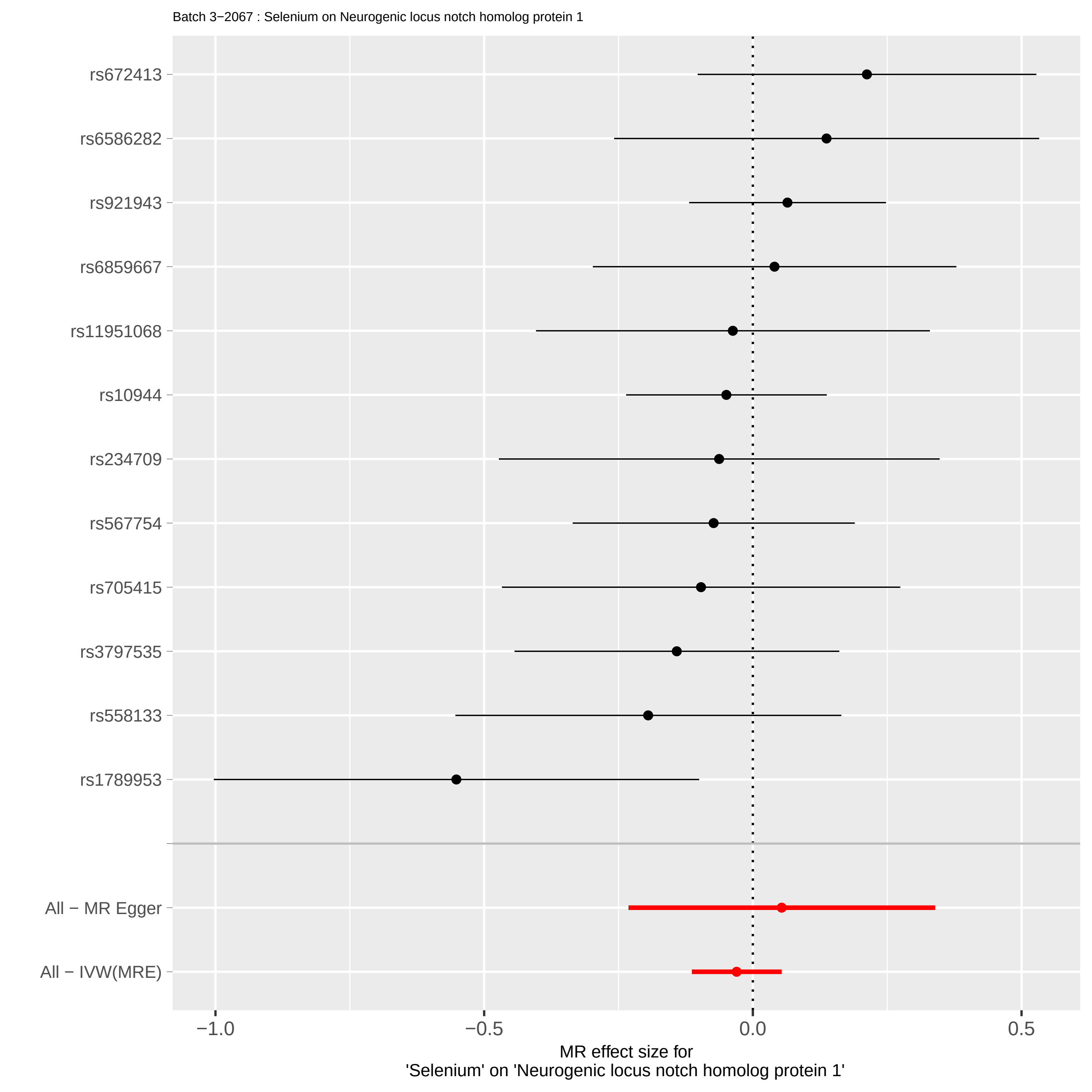

Batch 3−2067 : Selenium on Neurogenic locus notch homolog protein 1
rs672413
rs6586282
rs921943
rs6859667
rs11951068
rs10944
rs234709
rs567754
rs705415
rs3797535
rs558133
rs1789953
All − MR Egger
All − IVW(MRE)
−1.0
−0.5
0.0
0.5
MR effect size for
'Selenium' on 'Neurogenic locus notch homolog protein 1'

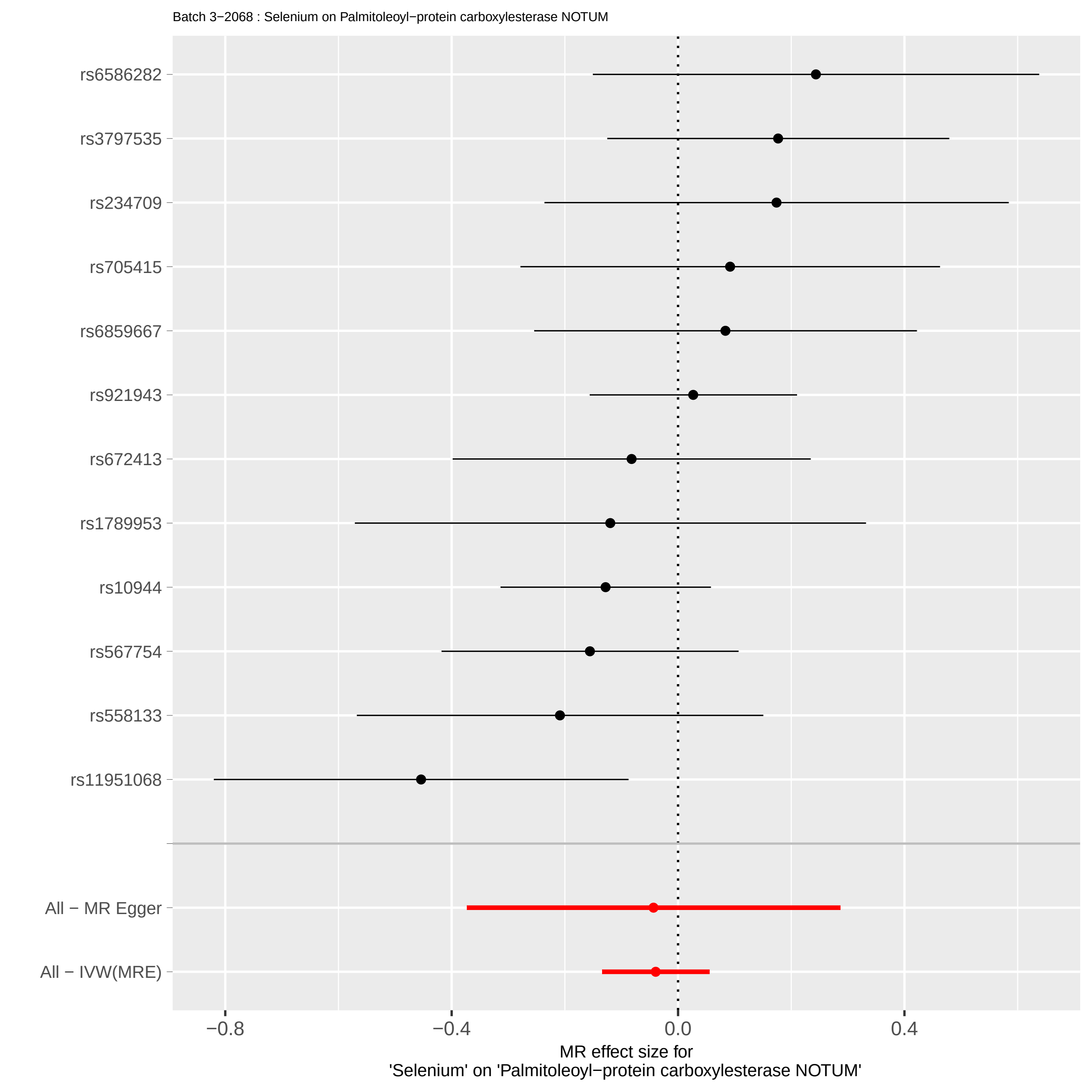

Batch 3−2068 : Selenium on Palmitoleoyl−protein carboxylesterase NOTUM
rs6586282
rs3797535
rs234709
rs705415
rs6859667
rs921943
rs672413
rs1789953
rs10944
rs567754
rs558133
rs11951068
All − MR Egger
All − IVW(MRE)
−0.8
−0.4
0.0
0.4
MR effect size for
'Selenium' on 'Palmitoleoyl−protein carboxylesterase NOTUM'

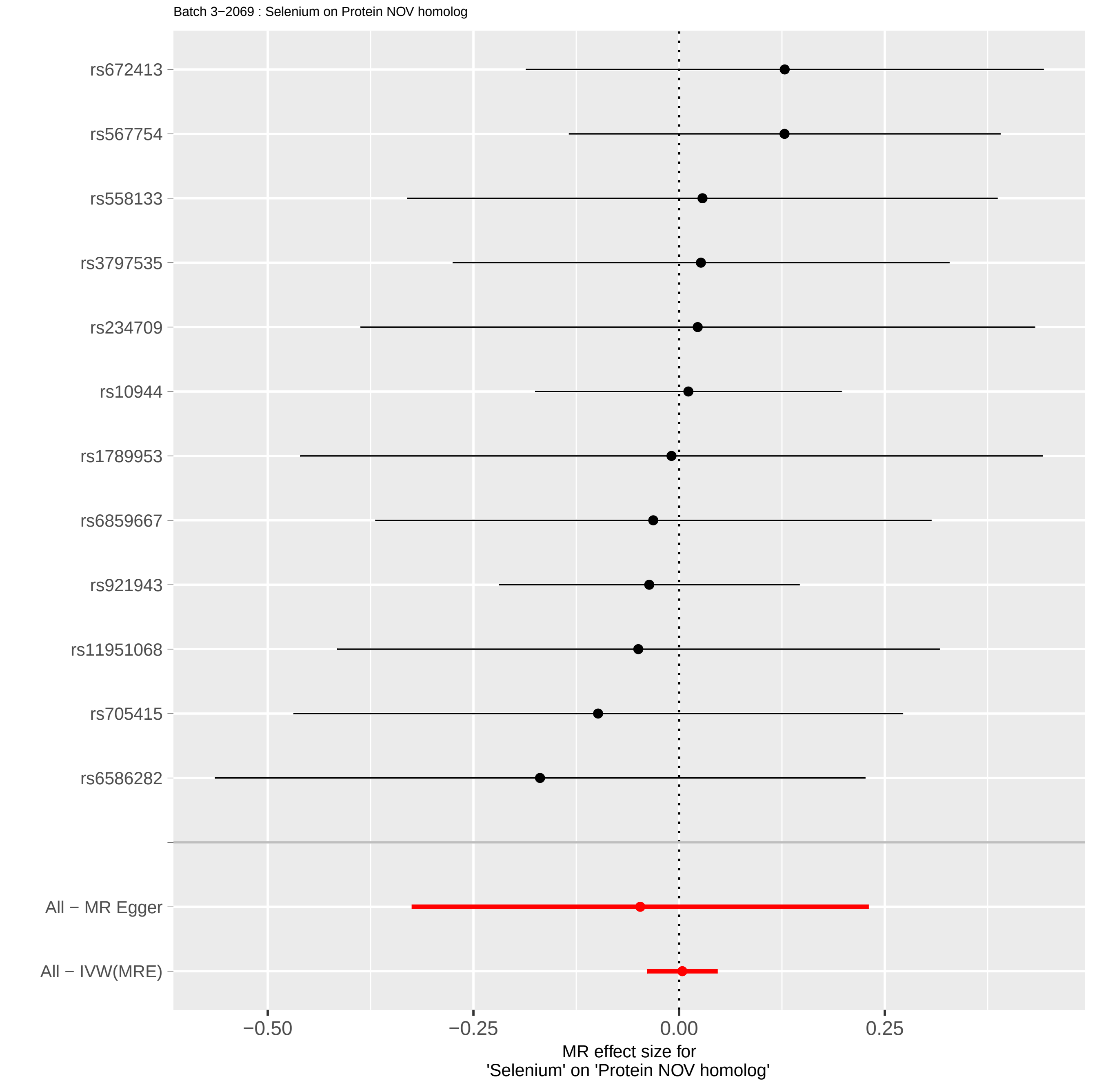

Batch 3−2069 : Selenium on Protein NOV homolog
rs672413
rs567754
rs558133
rs3797535
rs234709
rs10944
rs1789953
rs6859667
rs921943
rs11951068
rs705415
rs6586282
All − MR Egger
All − IVW(MRE)
−0.50
−0.25
0.00
0.25
MR effect size for
'Selenium' on 'Protein NOV homolog'

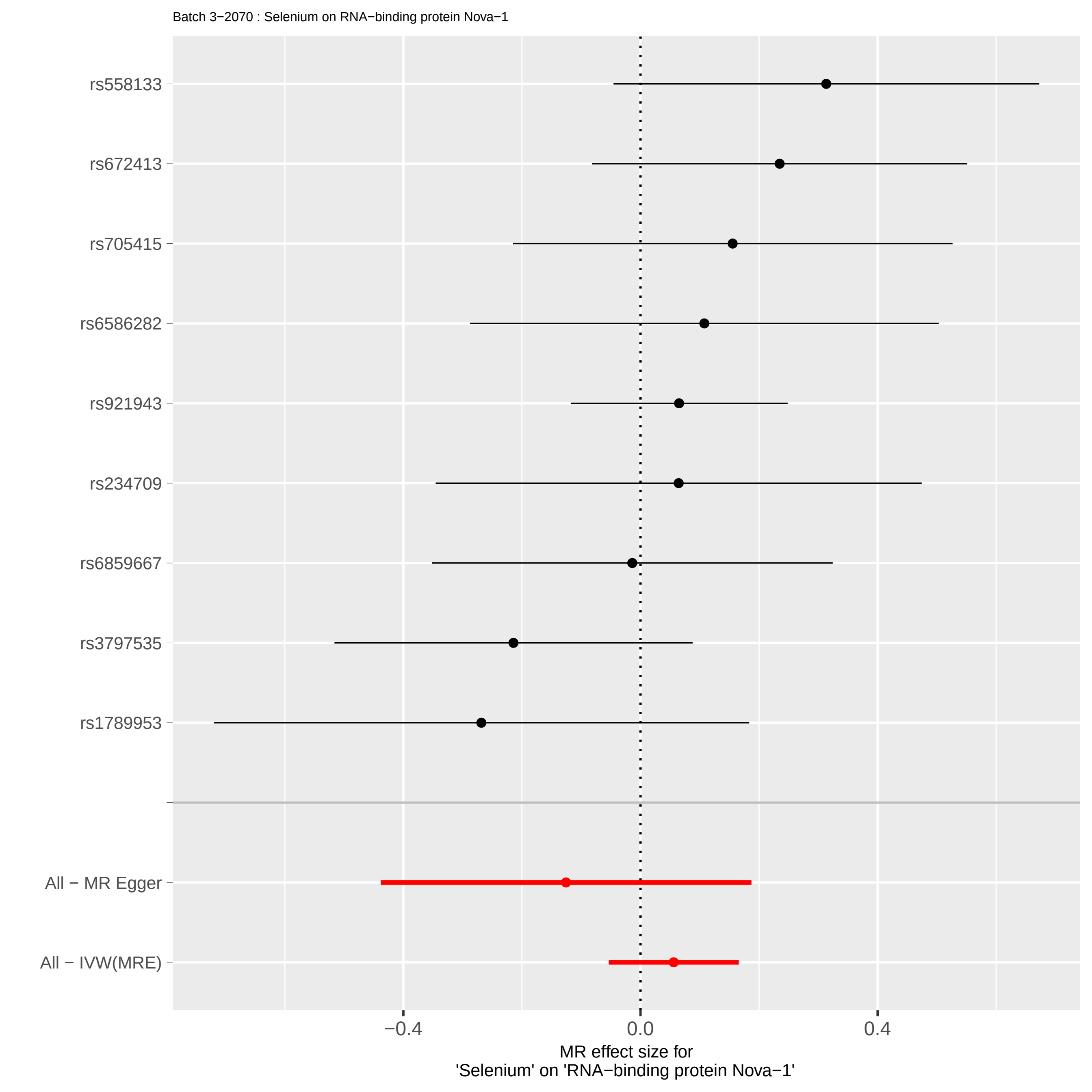
Batch 3−2070 : Selenium on RNA−binding protein Nova−1
rs558133
rs672413
rs705415
rs6586282
rs921943
rs234709
rs6859667
rs3797535
rs1789953
All − MR Egger
All − IVW(MRE)
−0.4
0.0
0.4
MR effect size for
'Selenium' on 'RNA−binding protein Nova−1'

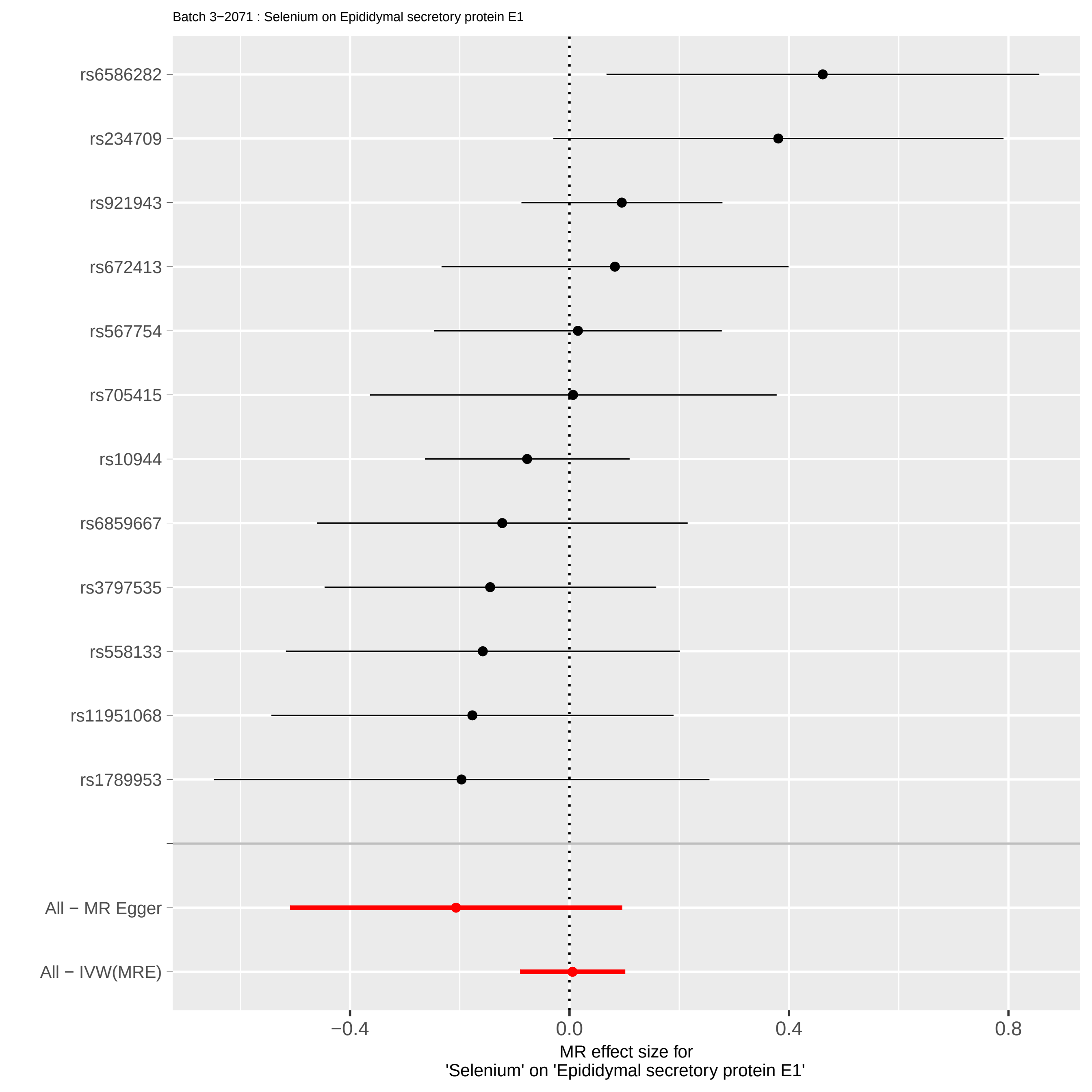

Batch 3−2071 : Selenium on Epididymal secretory protein E1
rs6586282
rs234709
rs921943
rs672413
rs567754
rs705415
rs10944
rs6859667
rs3797535
rs558133
rs11951068
rs1789953
All − MR Egger
All − IVW(MRE)
−0.4
0.0
0.4
0.8
MR effect size for
'Selenium' on 'Epididymal secretory protein E1'

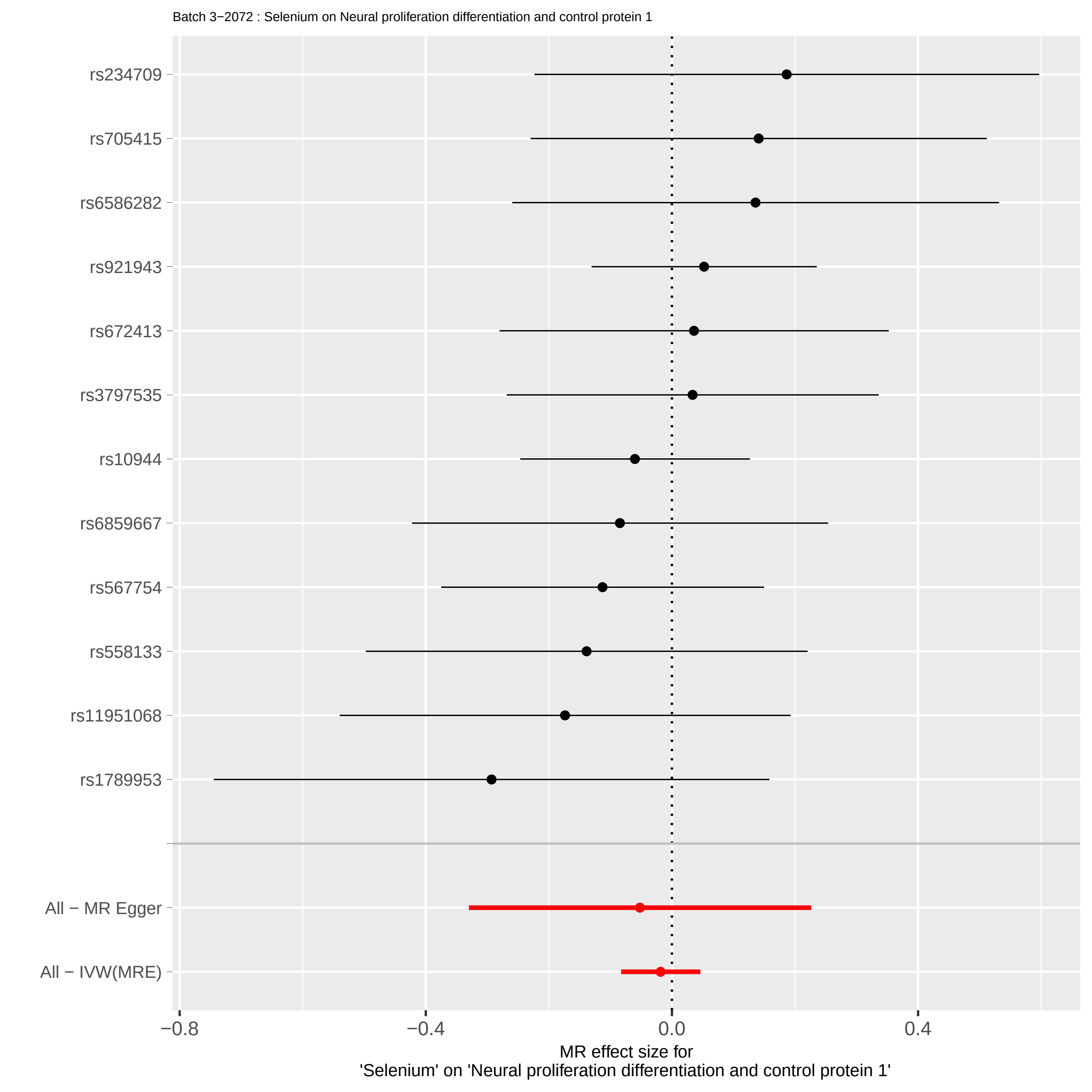

Batch 3−2072 : Selenium on Neural proliferation differentiation and control protein 1
rs234709
rs705415
rs6586282
rs921943
rs672413
rs3797535
rs10944
rs6859667
rs567754
rs558133
rs11951068
rs1789953
All − MR Egger
All − IVW(MRE)
−0.8
−0.4
0.0
0.4
MR effect size for
'Selenium' on 'Neural proliferation differentiation and control protein 1'

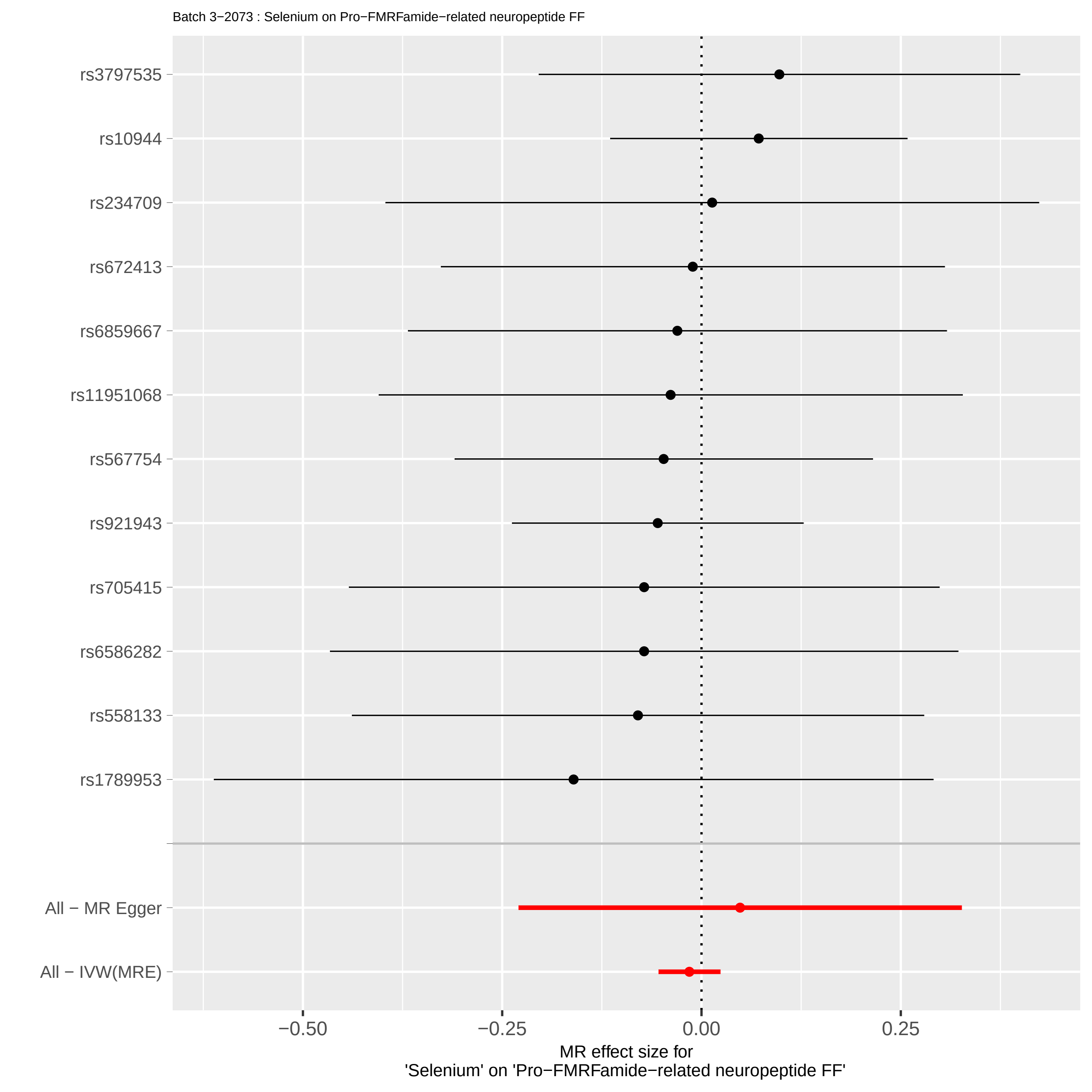
Batch 3−2073 : Selenium on Pro−FMRFamide−related neuropeptide FF
rs3797535
rs10944
rs234709
rs672413
rs6859667
rs11951068
rs567754
rs921943
rs705415
rs6586282
rs558133
rs1789953
All − MR Egger
All − IVW(MRE)
−0.50
−0.25
0.00
0.25
MR effect size for
'Selenium' on 'Pro−FMRFamide−related neuropeptide FF'

Batch 3−2074 : Selenium on Nuclear pore complex−interacting protein family member B3

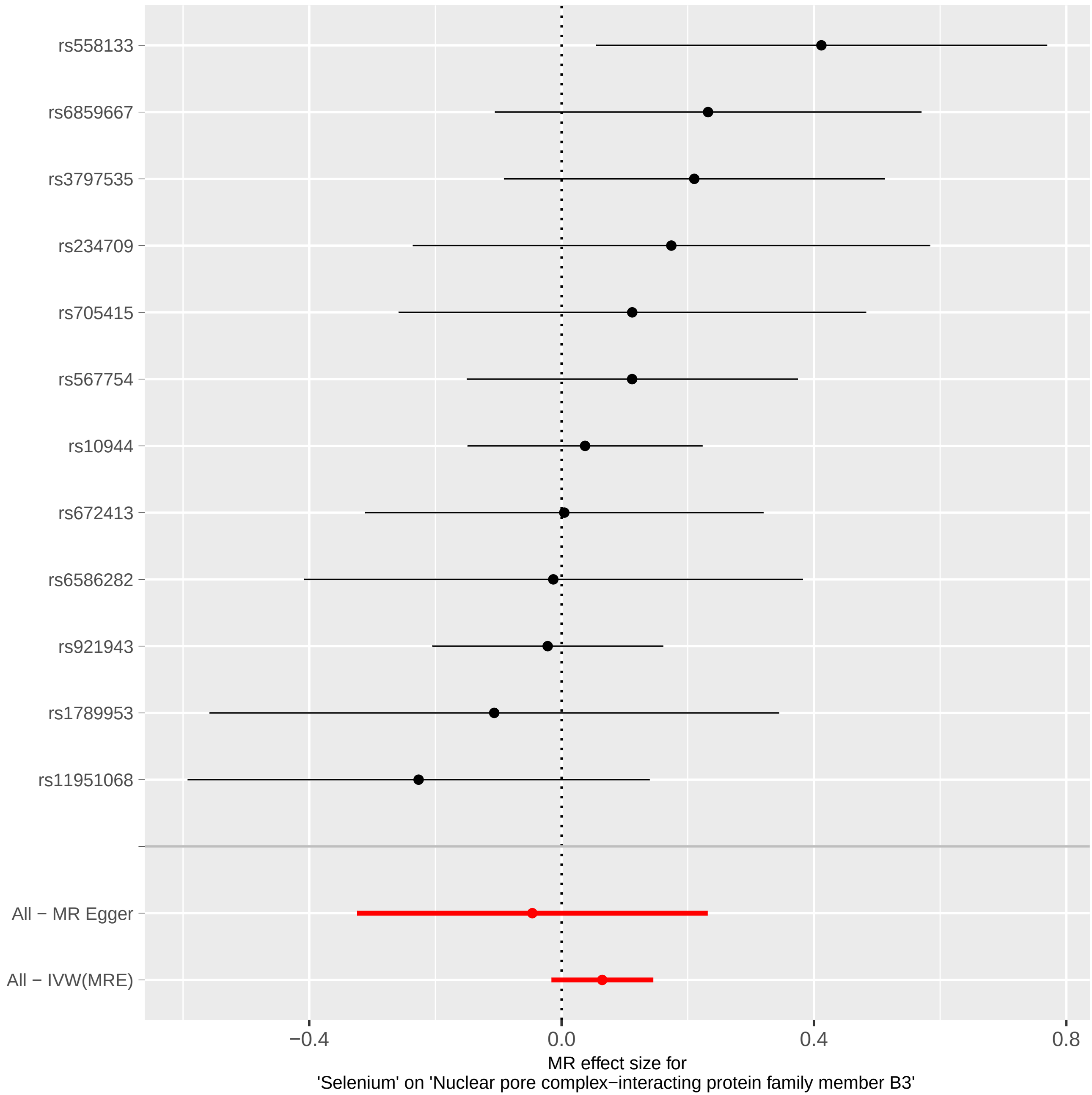

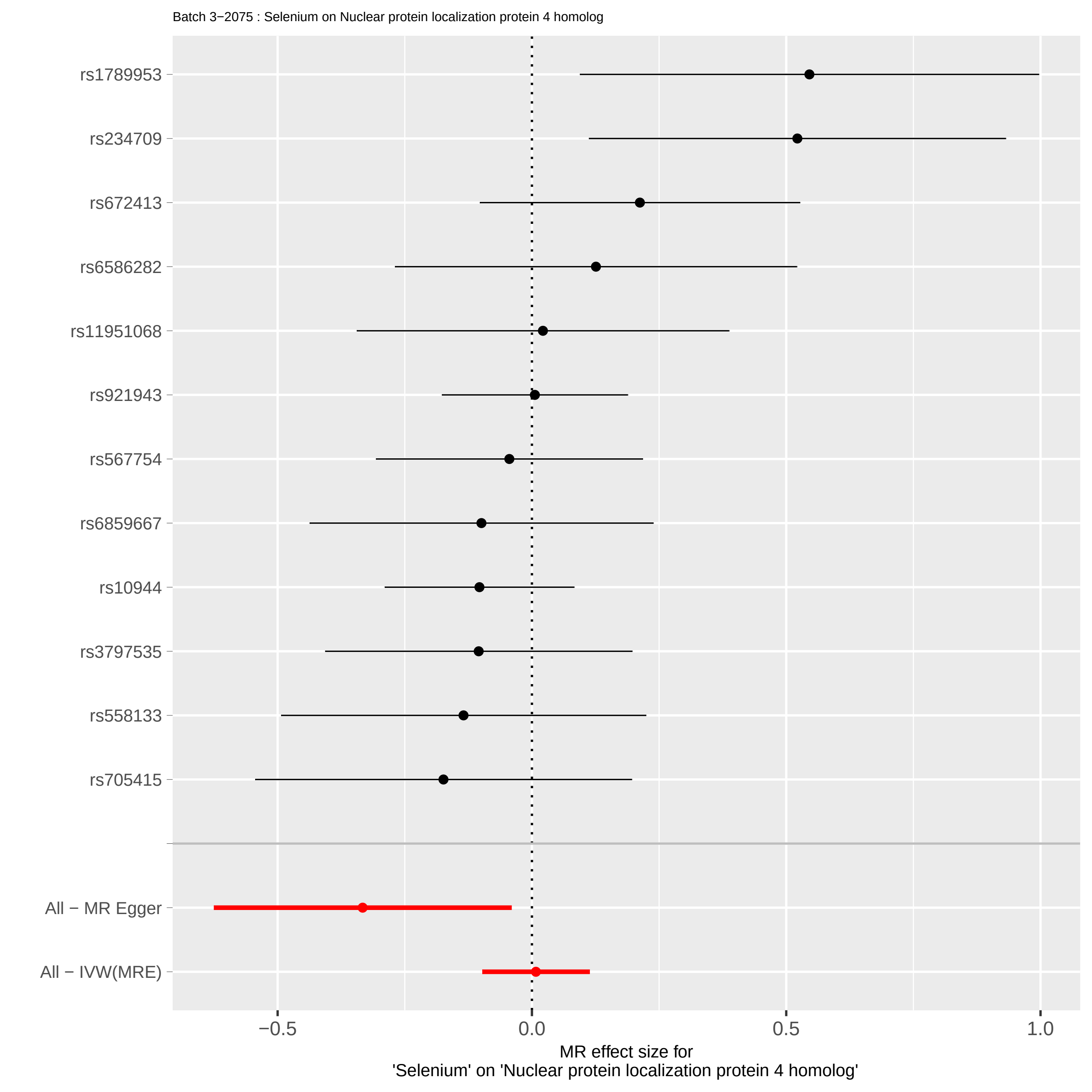

Batch 3−2075 : Selenium on Nuclear protein localization protein 4 homolog
rs1789953
rs234709
rs672413
rs6586282
rs11951068
rs921943
rs567754
rs6859667
rs10944
rs3797535
rs558133
rs705415
All − MR Egger
All − IVW(MRE)
−0.5
0.0
0.5
1.0
MR effect size for
'Selenium' on 'Nuclear protein localization protein 4 homolog'

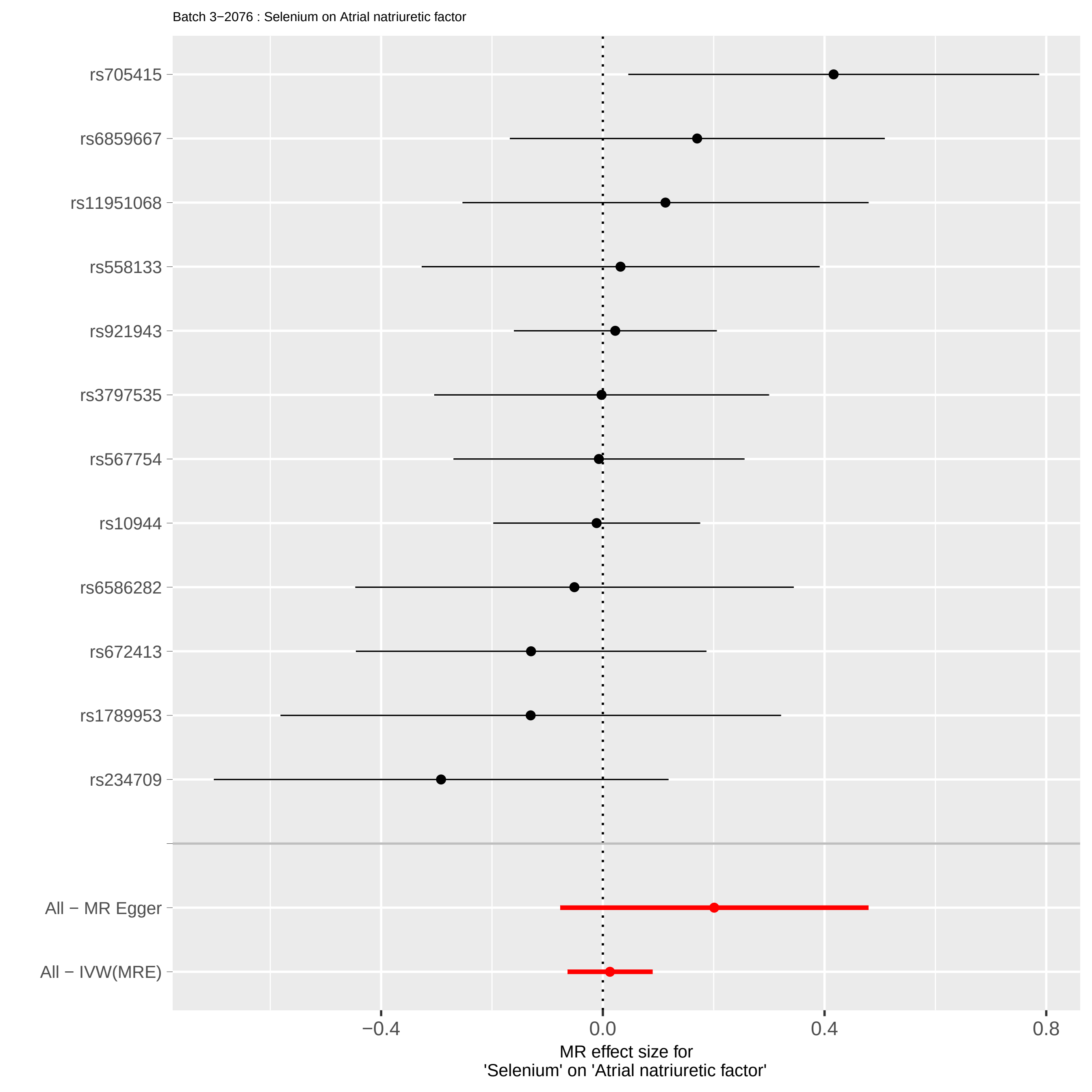

Batch 3−2076 : Selenium on Atrial natriuretic factor
rs705415
rs6859667
rs11951068
rs558133
rs921943
rs3797535
rs567754
rs10944
rs6586282
rs672413
rs1789953
rs234709
All − MR Egger
All − IVW(MRE)
−0.4
0.0
0.4
0.8
MR effect size for
'Selenium' on 'Atrial natriuretic factor'

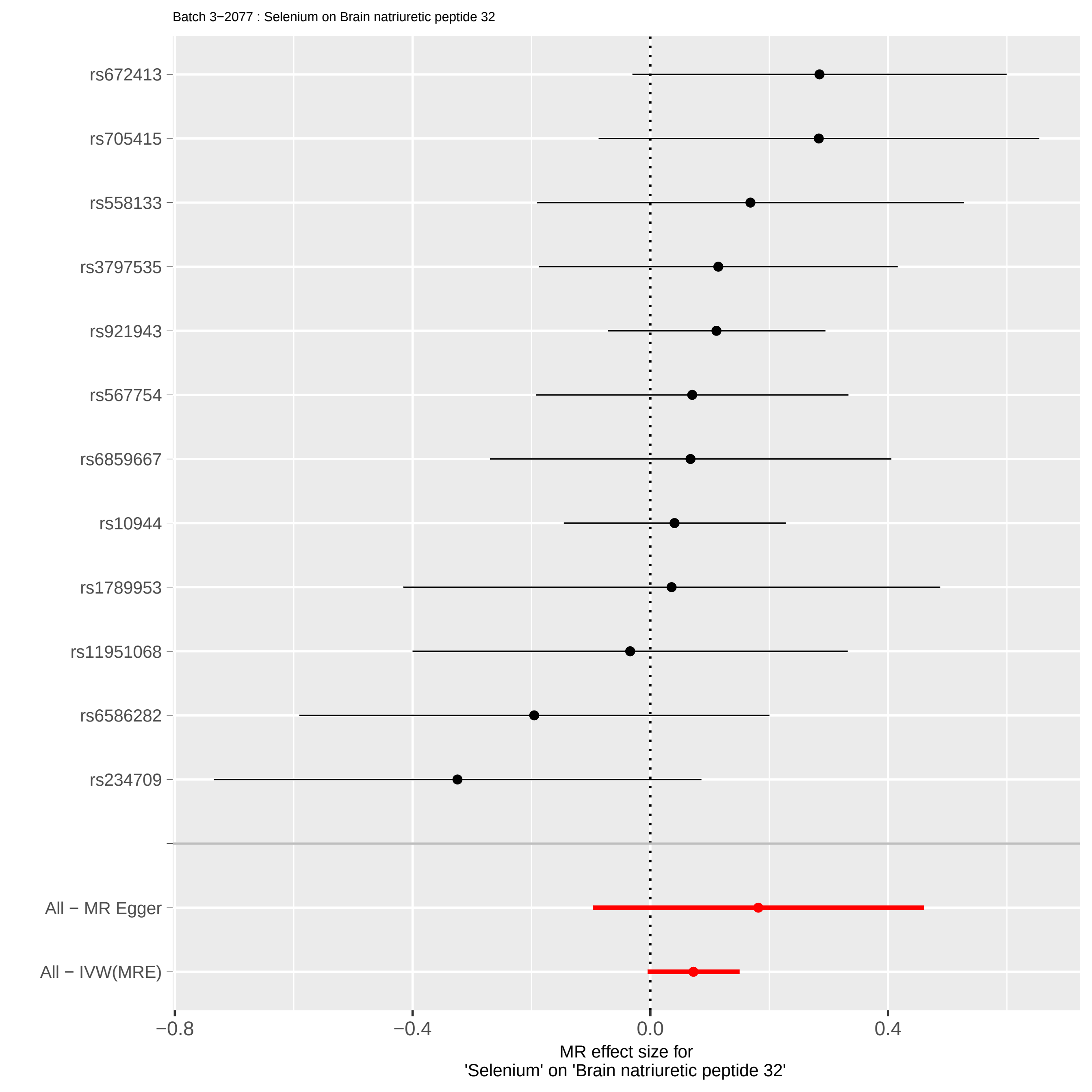
Batch 3−2077 : Selenium on Brain natriuretic peptide 32
rs672413
rs705415
rs558133
rs3797535
rs921943
rs567754
rs6859667
rs10944
rs1789953
rs11951068
rs6586282
rs234709
All − MR Egger
All − IVW(MRE)
−0.8
−0.4
0.0
0.4
MR effect size for
'Selenium' on 'Brain natriuretic peptide 32'

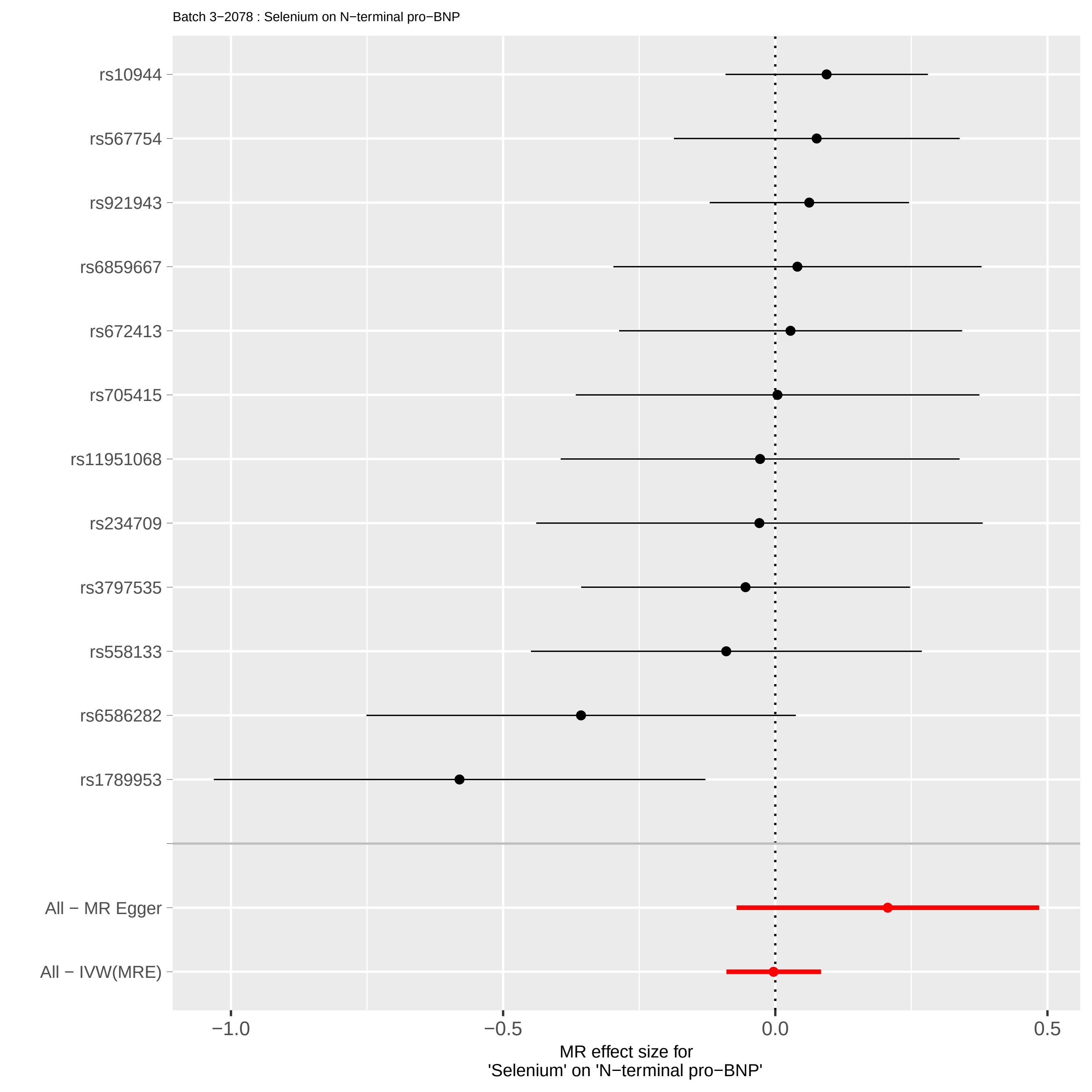

Batch 3−2078 : Selenium on N−terminal pro−BNP
rs10944
rs567754
rs921943
rs6859667
rs672413
rs705415
rs11951068
rs234709
rs3797535
rs558133
rs6586282
rs1789953
All − MR Egger
All − IVW(MRE)
−1.0
−0.5
0.0
0.5
MR effect size for
'Selenium' on 'N−terminal pro−BNP'

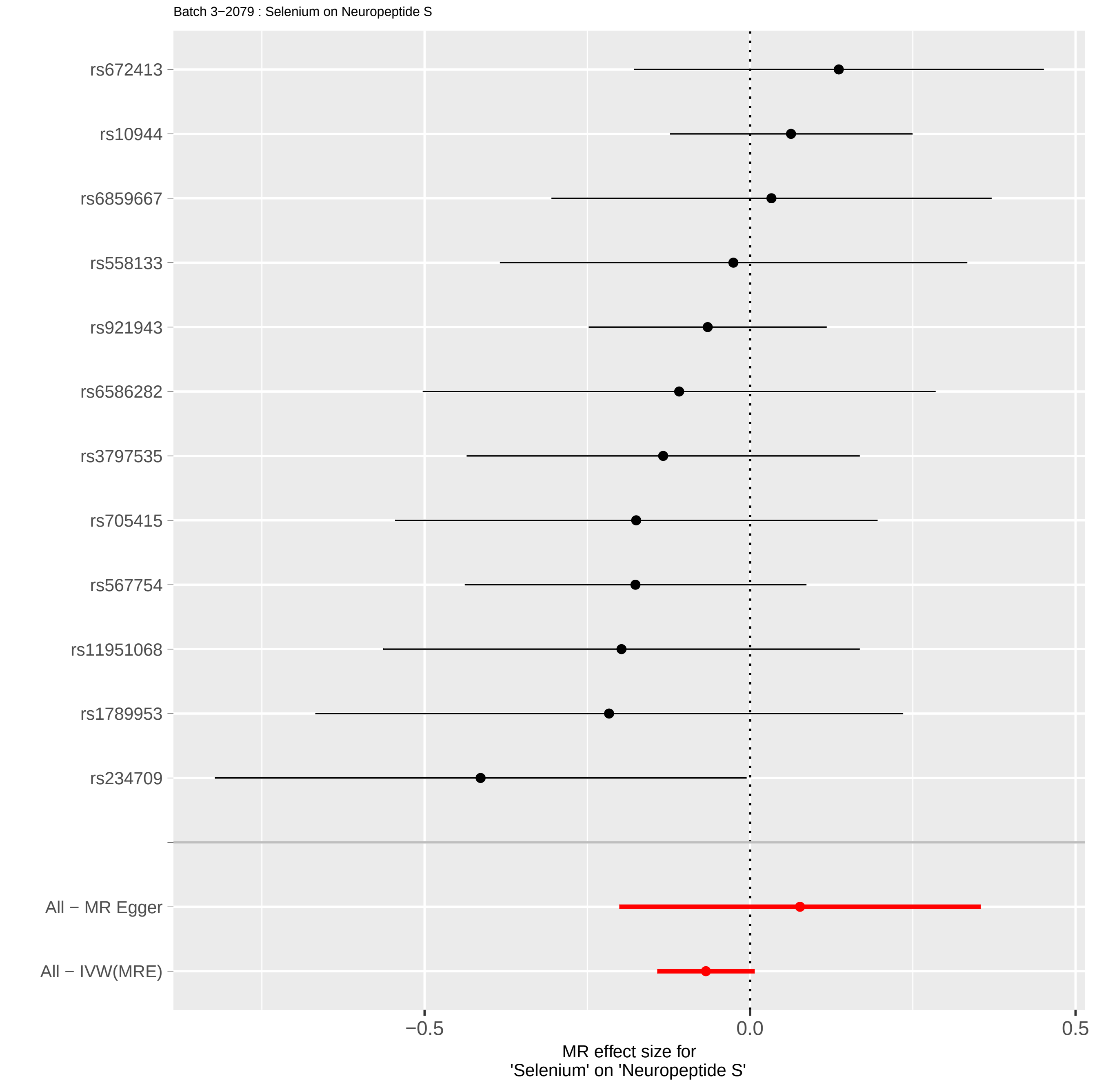

Batch 3−2079 : Selenium on Neuropeptide S
rs672413
rs10944
rs6859667
rs558133
rs921943
rs6586282
rs3797535
rs705415
rs567754
rs11951068
rs1789953
rs234709
All − MR Egger
All − IVW(MRE)
−0.5
0.0
0.5
MR effect size for
'Selenium' on 'Neuropeptide S'

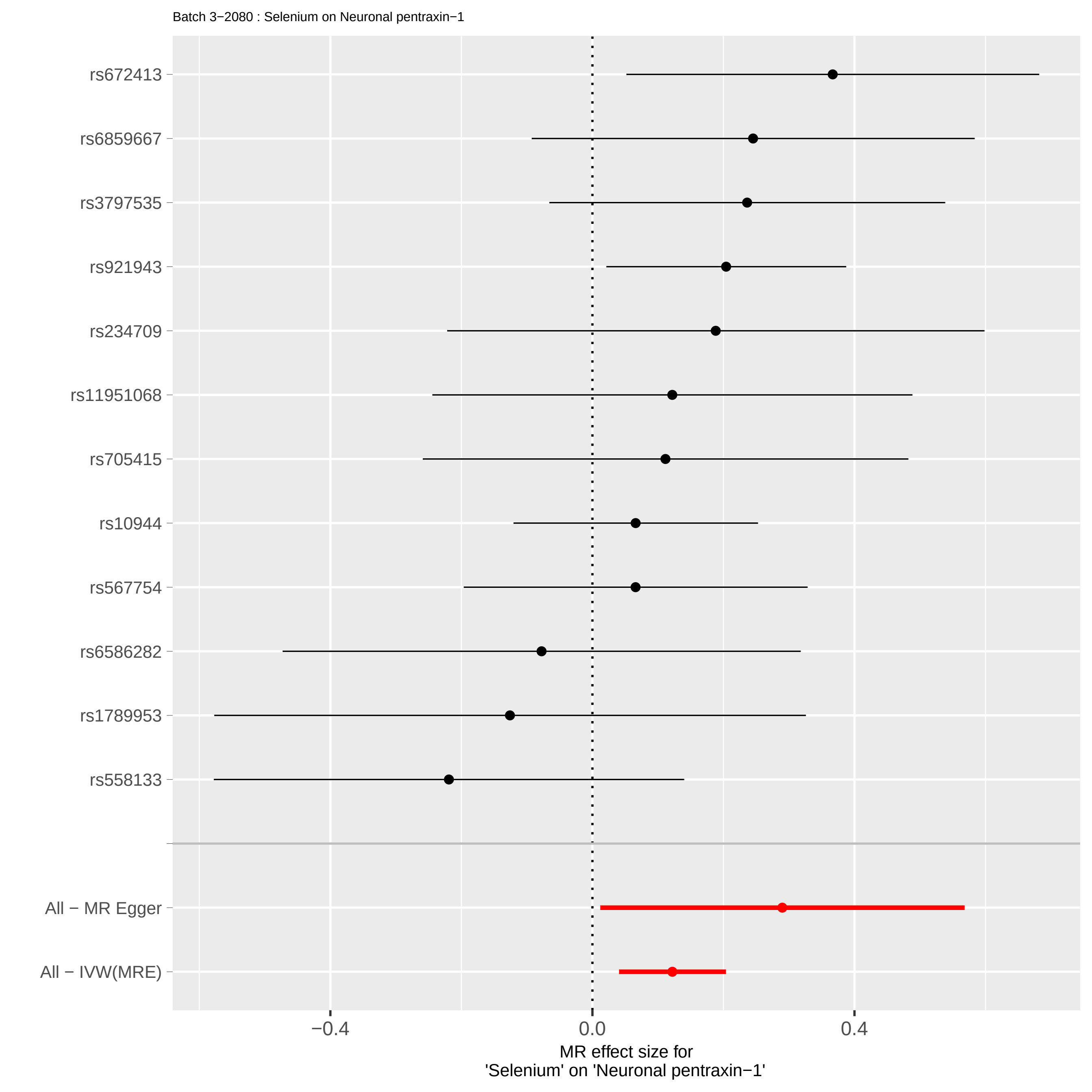

Batch 3−2080 : Selenium on Neuronal pentraxin−1
rs672413
rs6859667
rs3797535
rs921943
rs234709
rs11951068
rs705415
rs10944
rs567754
rs6586282
rs1789953
rs558133
All − MR Egger
All − IVW(MRE)
−0.4
0.0
0.4
MR effect size for
'Selenium' on 'Neuronal pentraxin−1'

Batch 3−2081 : Selenium on Neuronal pentraxin−2

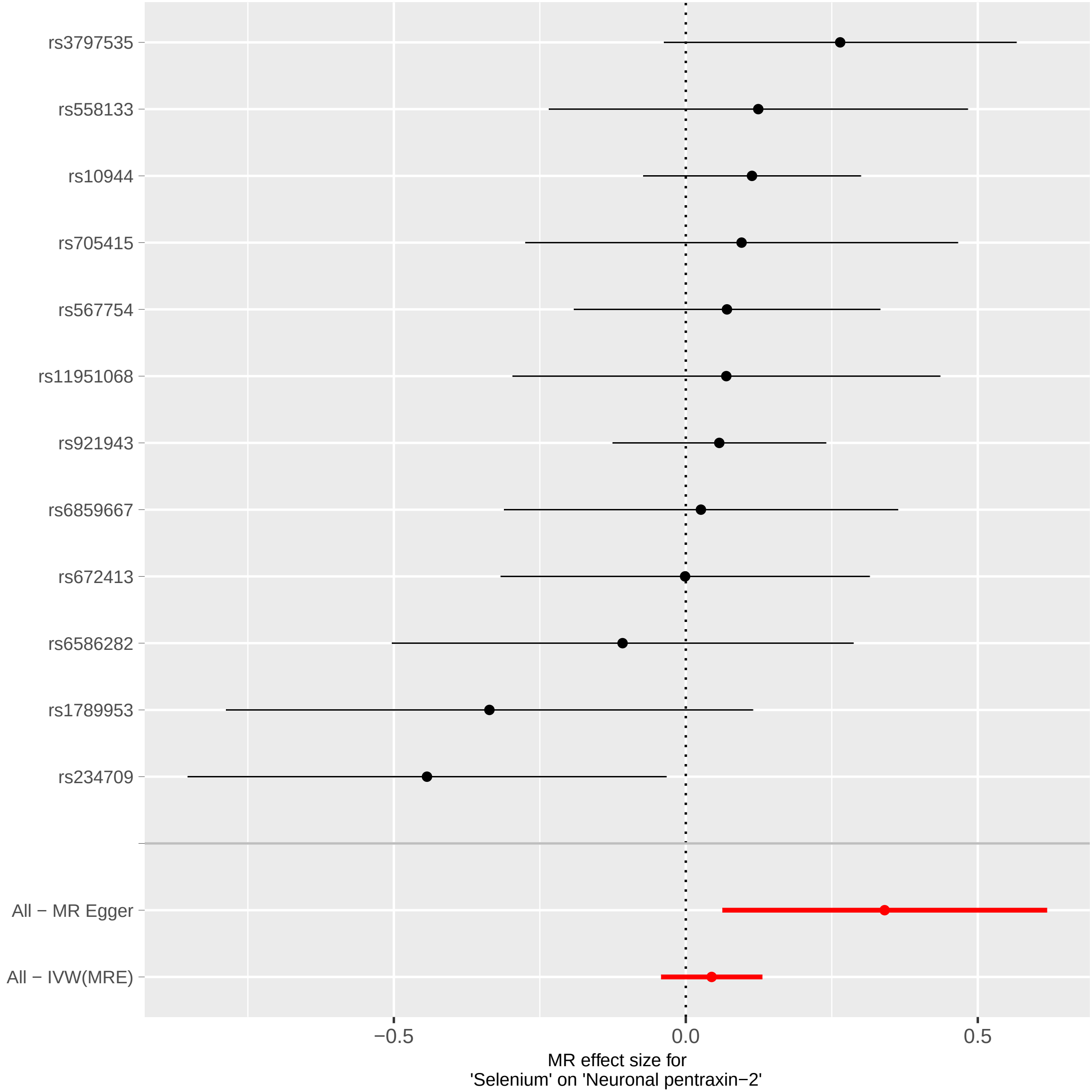

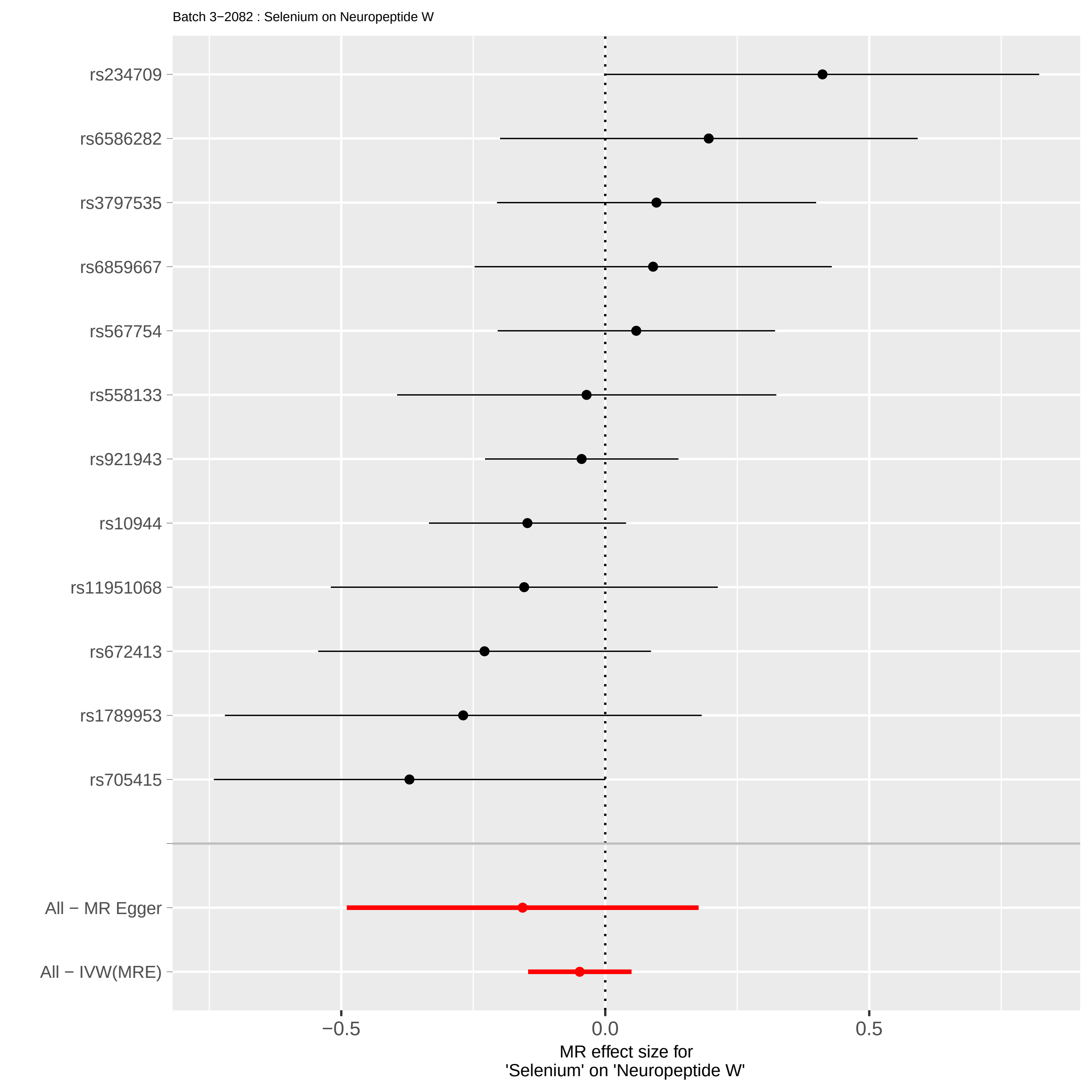

Batch 3−2082 : Selenium on Neuropeptide W
rs234709
rs6586282
rs3797535
rs6859667
rs567754
rs558133
rs921943
rs10944
rs11951068
rs672413
rs1789953
rs705415
All − MR Egger
All − IVW(MRE)
−0.5
0.0
0.5
MR effect size for
'Selenium' on 'Neuropeptide W'

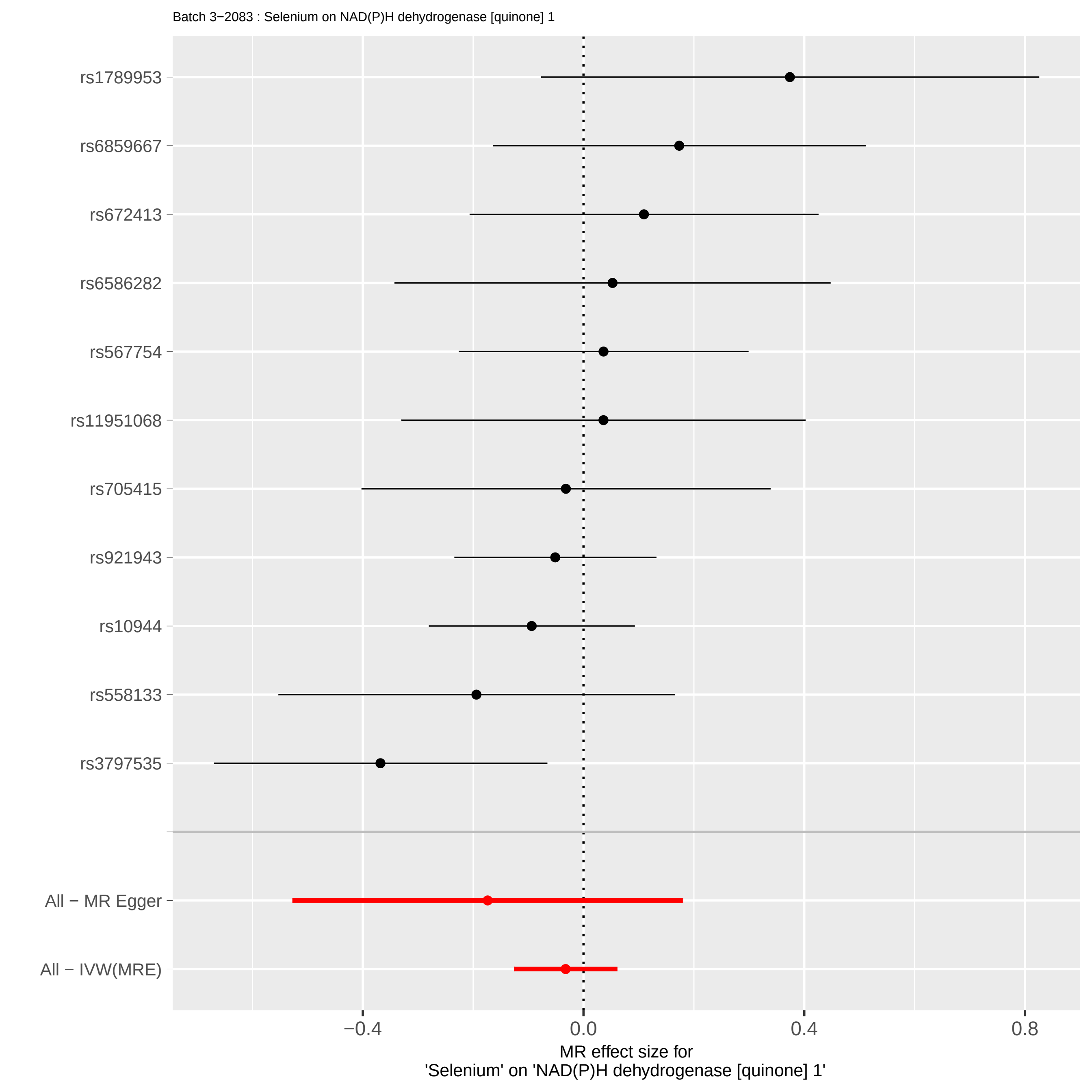
Batch 3−2083 : Selenium on NAD(P)H dehydrogenase [quinone] 1
rs1789953
rs6859667
rs672413
rs6586282
rs567754
rs11951068
rs705415
rs921943
rs10944
rs558133
rs3797535
All − MR Egger
All − IVW(MRE)
−0.4
0.0
0.4
0.8
MR effect size for
'Selenium' on 'NAD(P)H dehydrogenase [quinone] 1'

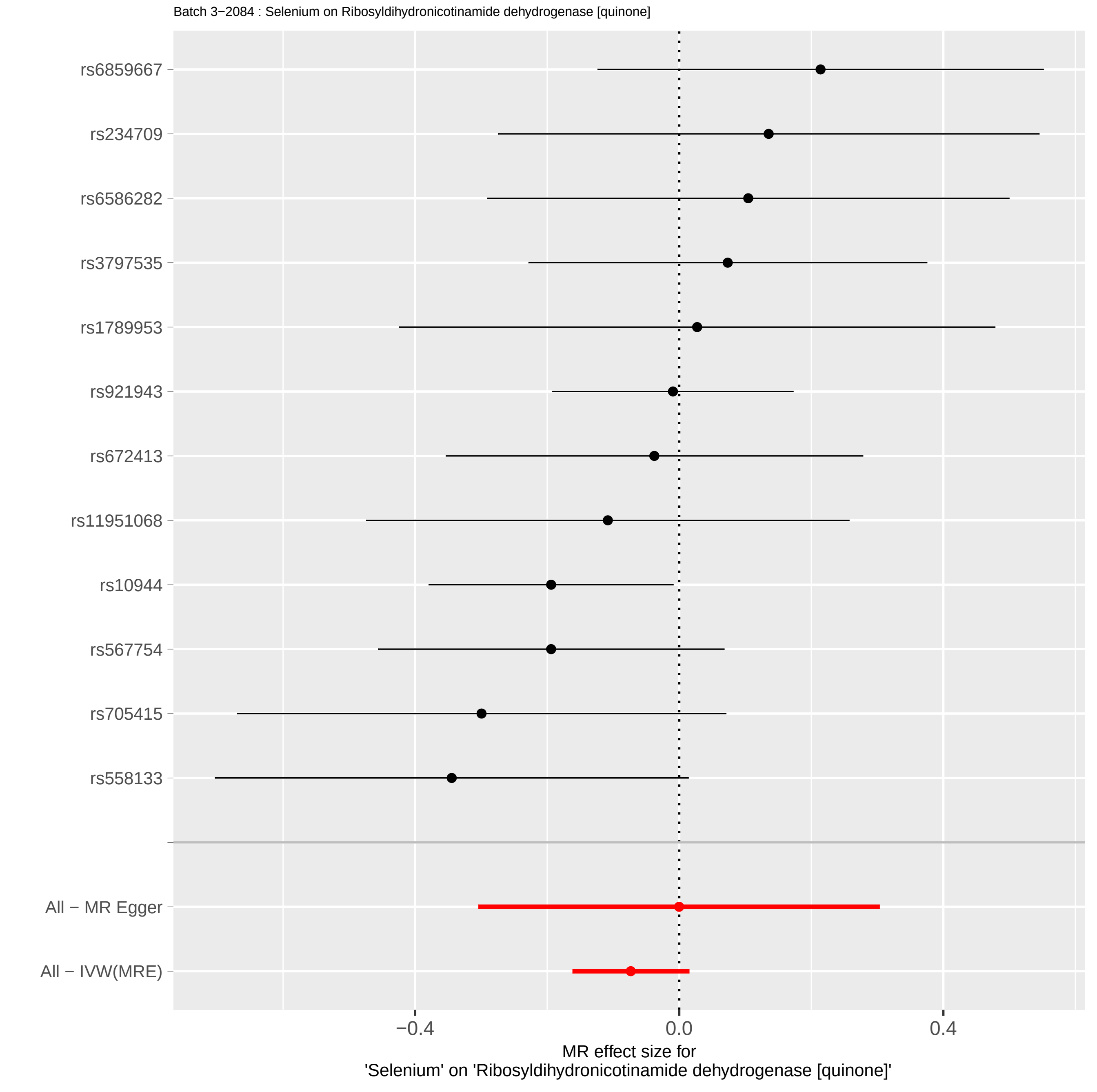

Batch 3−2084 : Selenium on Ribosyldihydronicotinamide dehydrogenase [quinone]
rs6859667
rs234709
rs6586282
rs3797535
rs1789953
rs921943
rs672413
rs11951068
rs10944
rs567754
rs705415
rs558133
All − MR Egger
All − IVW(MRE)
−0.4
0.0
0.4
MR effect size for
'Selenium' on 'Ribosyldihydronicotinamide dehydrogenase [quinone]'

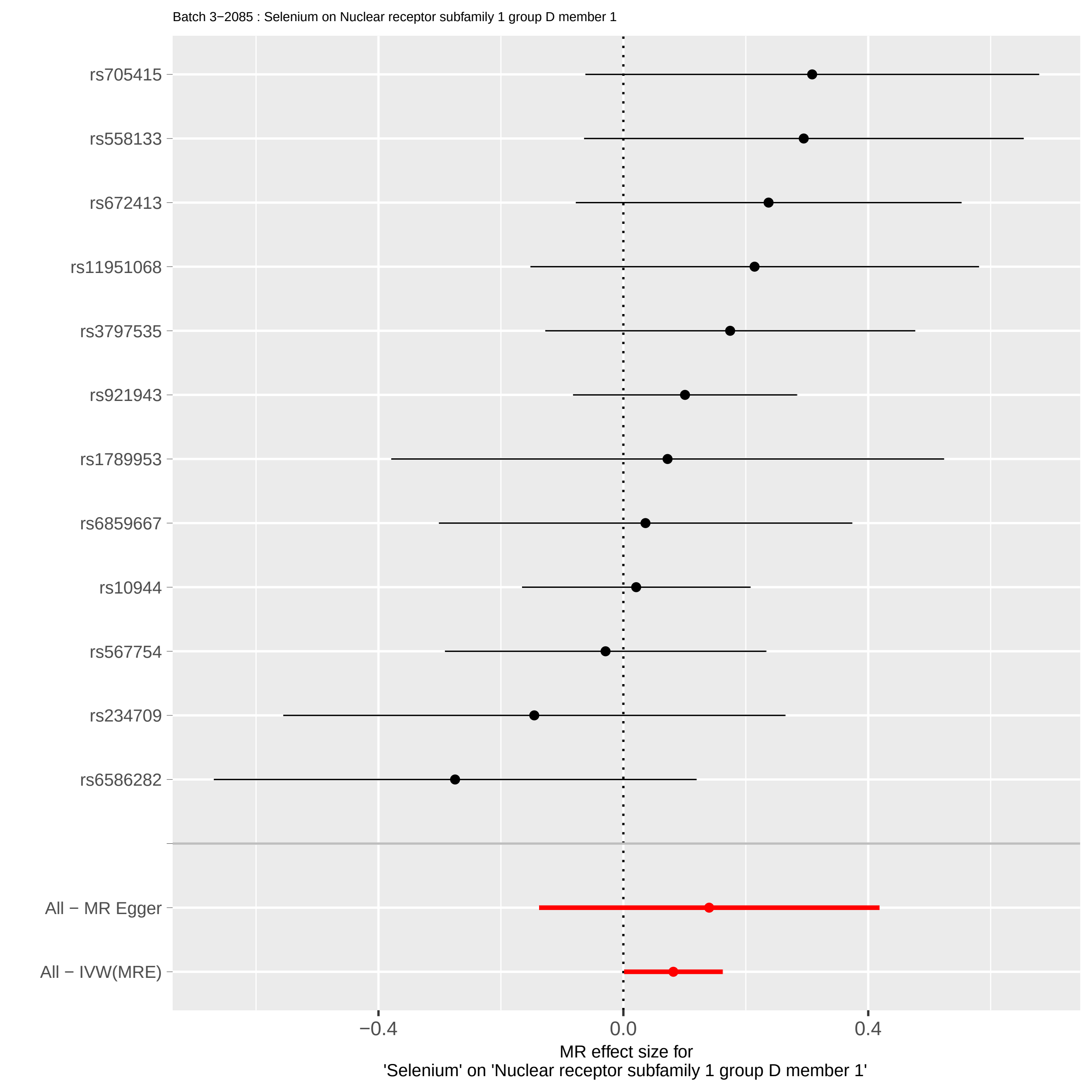

Batch 3−2085 : Selenium on Nuclear receptor subfamily 1 group D member 1
rs705415
rs558133
rs672413
rs11951068
rs3797535
rs921943
rs1789953
rs6859667
rs10944
rs567754
rs234709
rs6586282
All − MR Egger
All − IVW(MRE)
−0.4
0.0
0.4
MR effect size for
'Selenium' on 'Nuclear receptor subfamily 1 group D member 1'

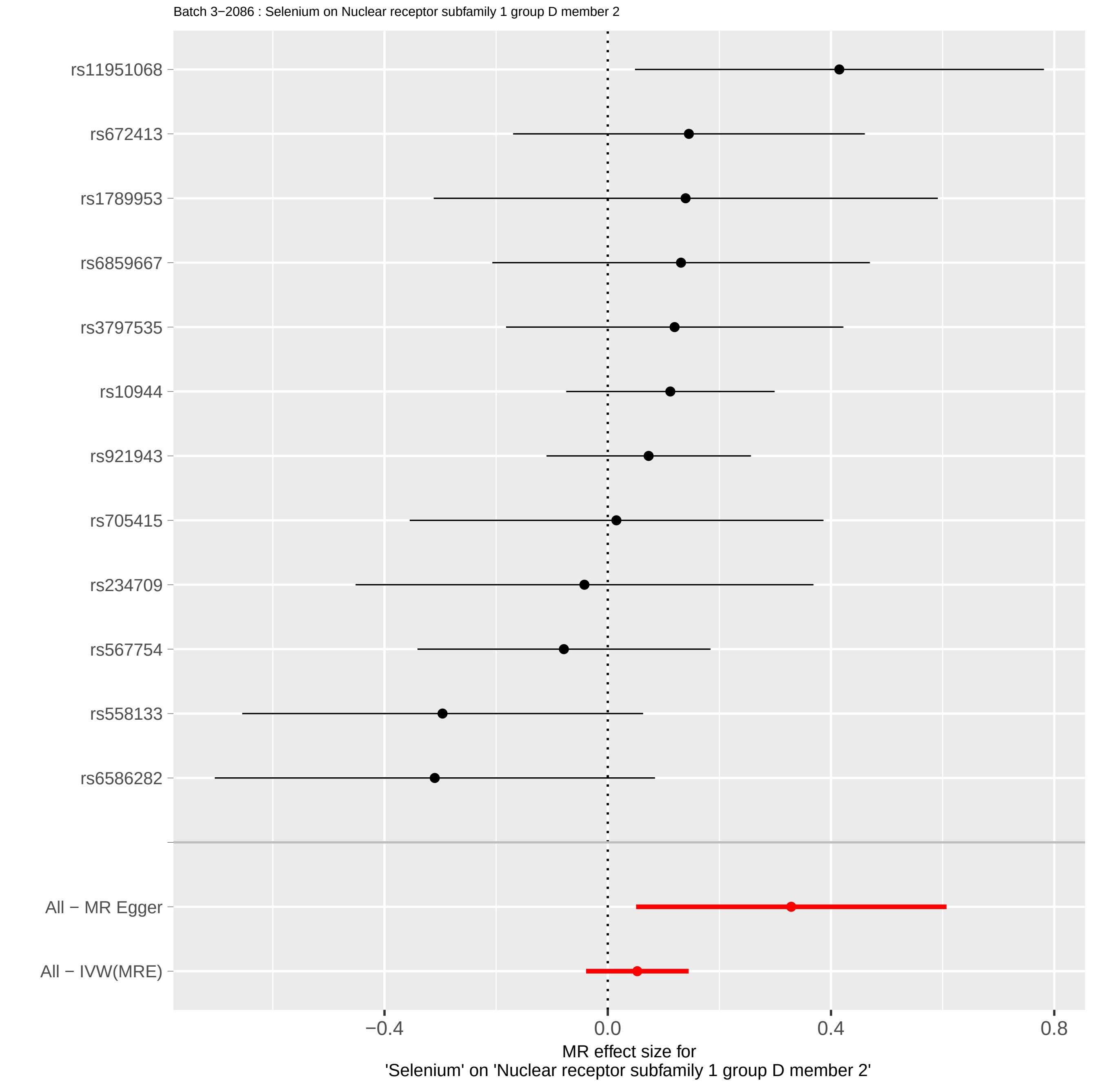
Batch 3−2086 : Selenium on Nuclear receptor subfamily 1 group D member 2
rs11951068
rs672413
rs1789953
rs6859667
rs3797535
rs10944
rs921943
rs705415
rs234709
rs567754
rs558133
rs6586282
All − MR Egger
All − IVW(MRE)
−0.4
0.0
0.4
0.8
MR effect size for
'Selenium' on 'Nuclear receptor subfamily 1 group D member 2'

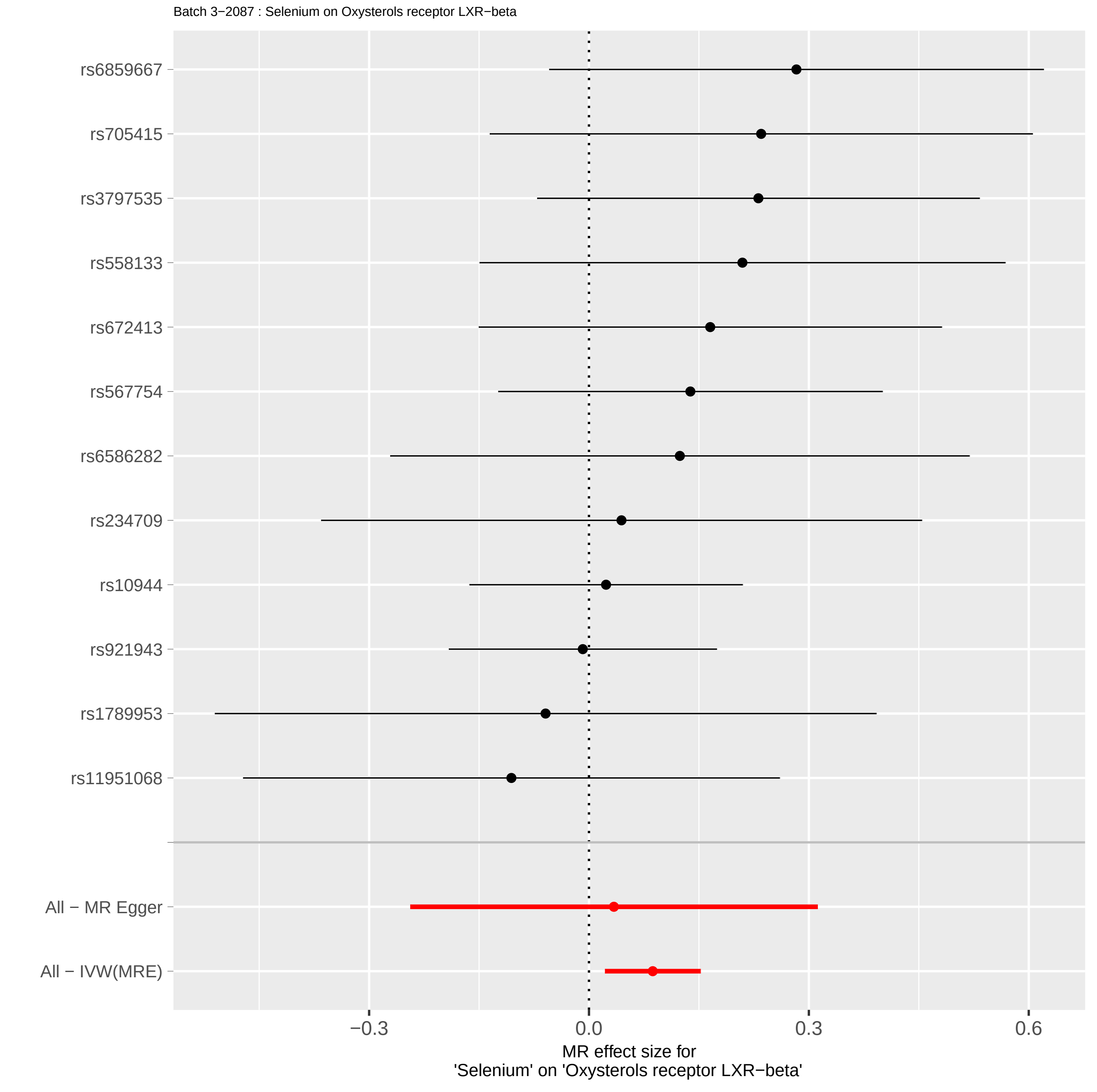

Batch 3−2087 : Selenium on Oxysterols receptor LXR−beta
rs6859667
rs705415
rs3797535
rs558133
rs672413
rs567754
rs6586282
rs234709
rs10944
rs921943
rs1789953
rs11951068
All − MR Egger
All − IVW(MRE)
−0.3
0.0
0.3
0.6
MR effect size for
'Selenium' on 'Oxysterols receptor LXR−beta'

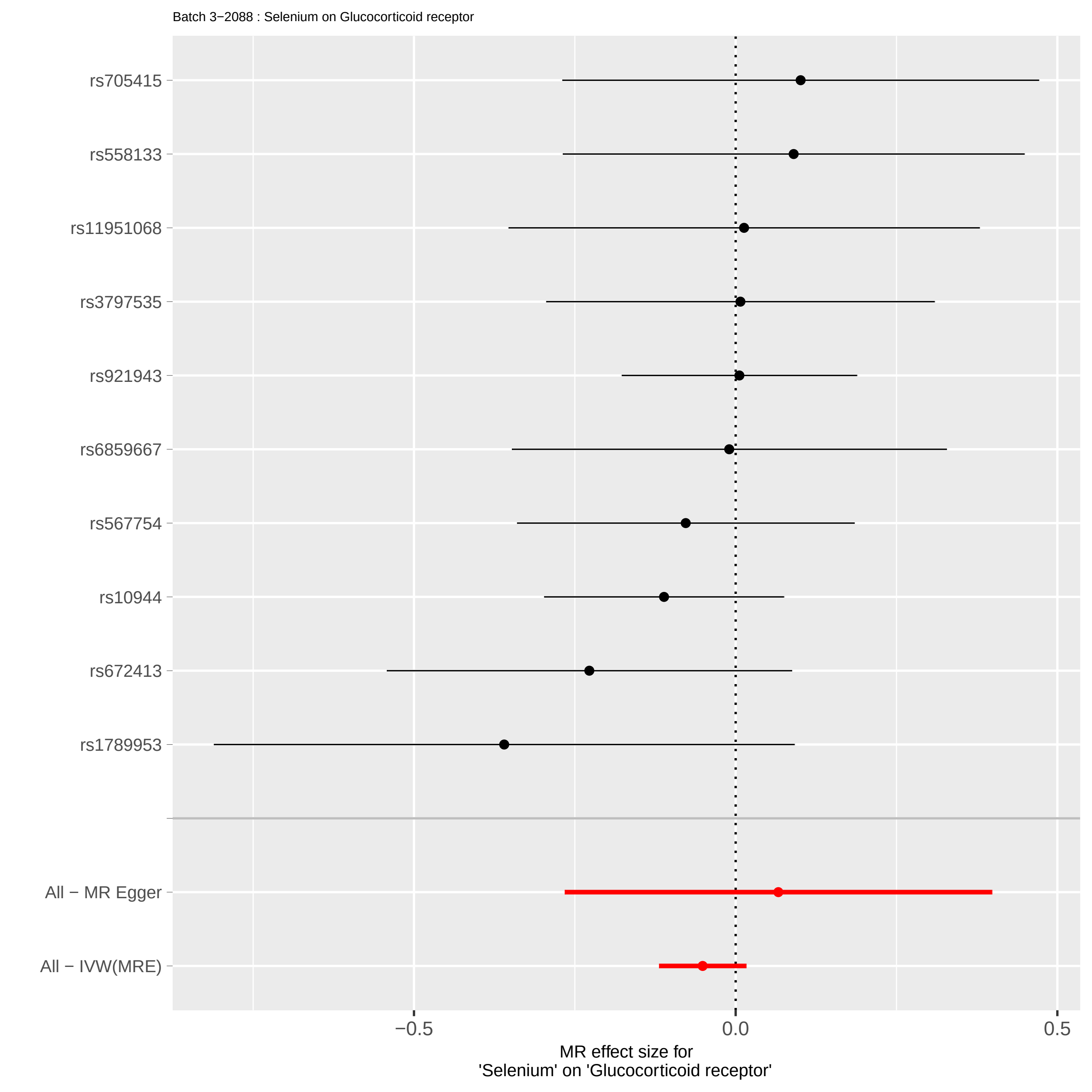

Batch 3−2088 : Selenium on Glucocorticoid receptor
rs705415
rs558133
rs11951068
rs3797535
rs921943
rs6859667
rs567754
rs10944
rs672413
rs1789953
All − MR Egger
All − IVW(MRE)
−0.5
0.0
0.5
MR effect size for
'Selenium' on 'Glucocorticoid receptor'

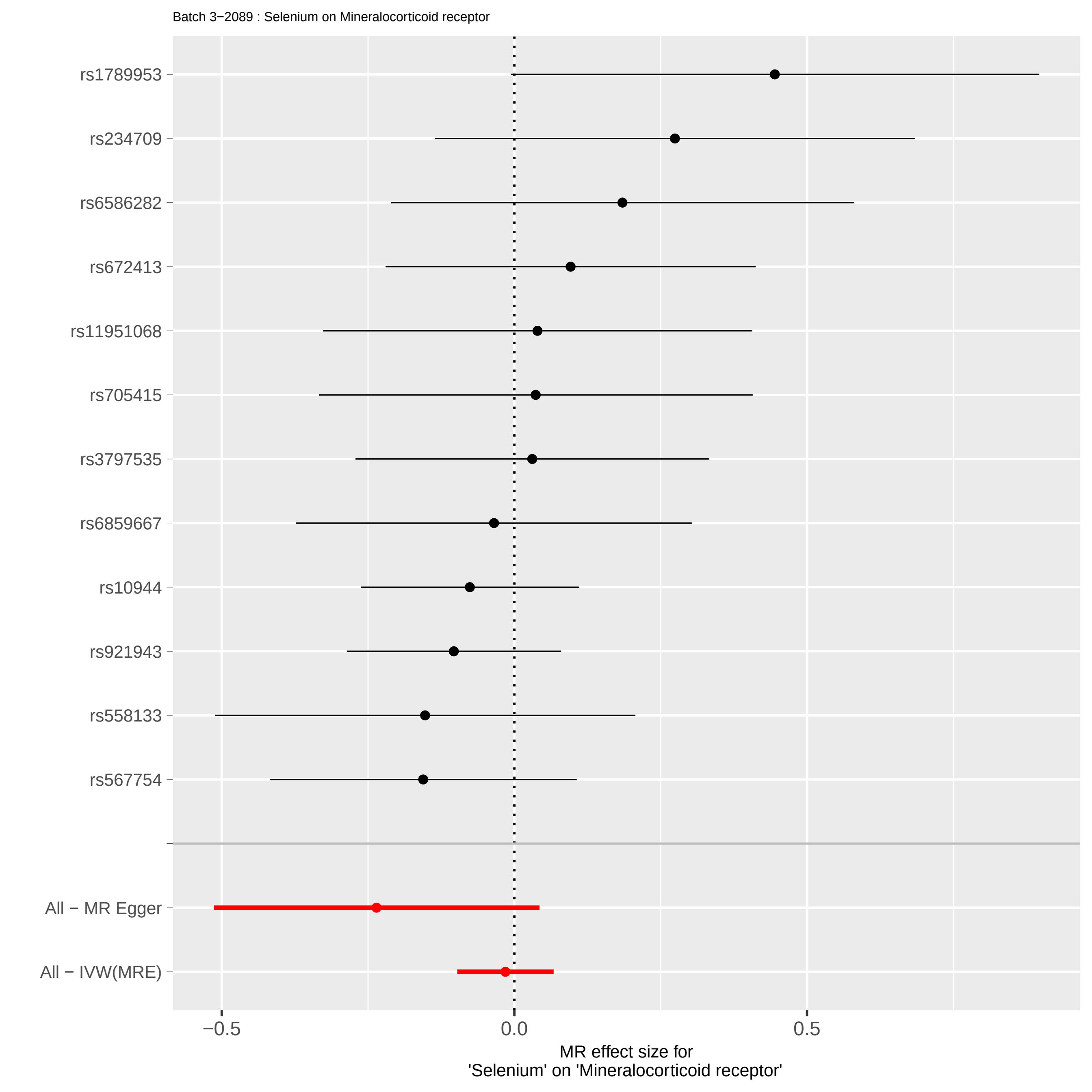
Batch 3−2089 : Selenium on Mineralocorticoid receptor
rs1789953
rs234709
rs6586282
rs672413
rs11951068
rs705415
rs3797535
rs6859667
rs10944
rs921943
rs558133
rs567754
All − MR Egger
All − IVW(MRE)
−0.5
0.0
0.5
MR effect size for
'Selenium' on 'Mineralocorticoid receptor'

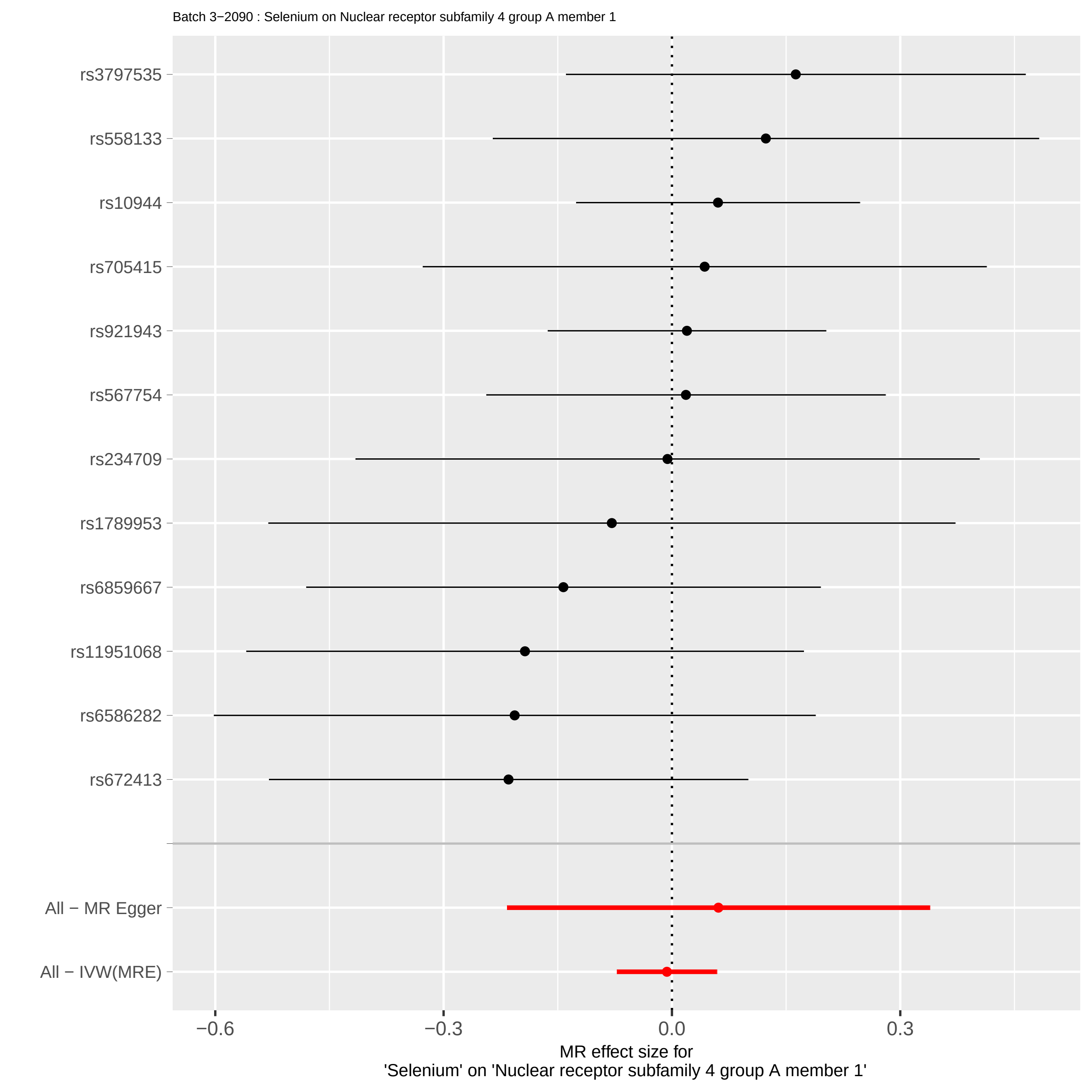

Batch 3−2090 : Selenium on Nuclear receptor subfamily 4 group A member 1
rs3797535
rs558133
rs10944
rs705415
rs921943
rs567754
rs234709
rs1789953
rs6859667
rs11951068
rs6586282
rs672413
All − MR Egger
All − IVW(MRE)
−0.6
−0.3
0.0
0.3
MR effect size for
'Selenium' on 'Nuclear receptor subfamily 4 group A member 1'

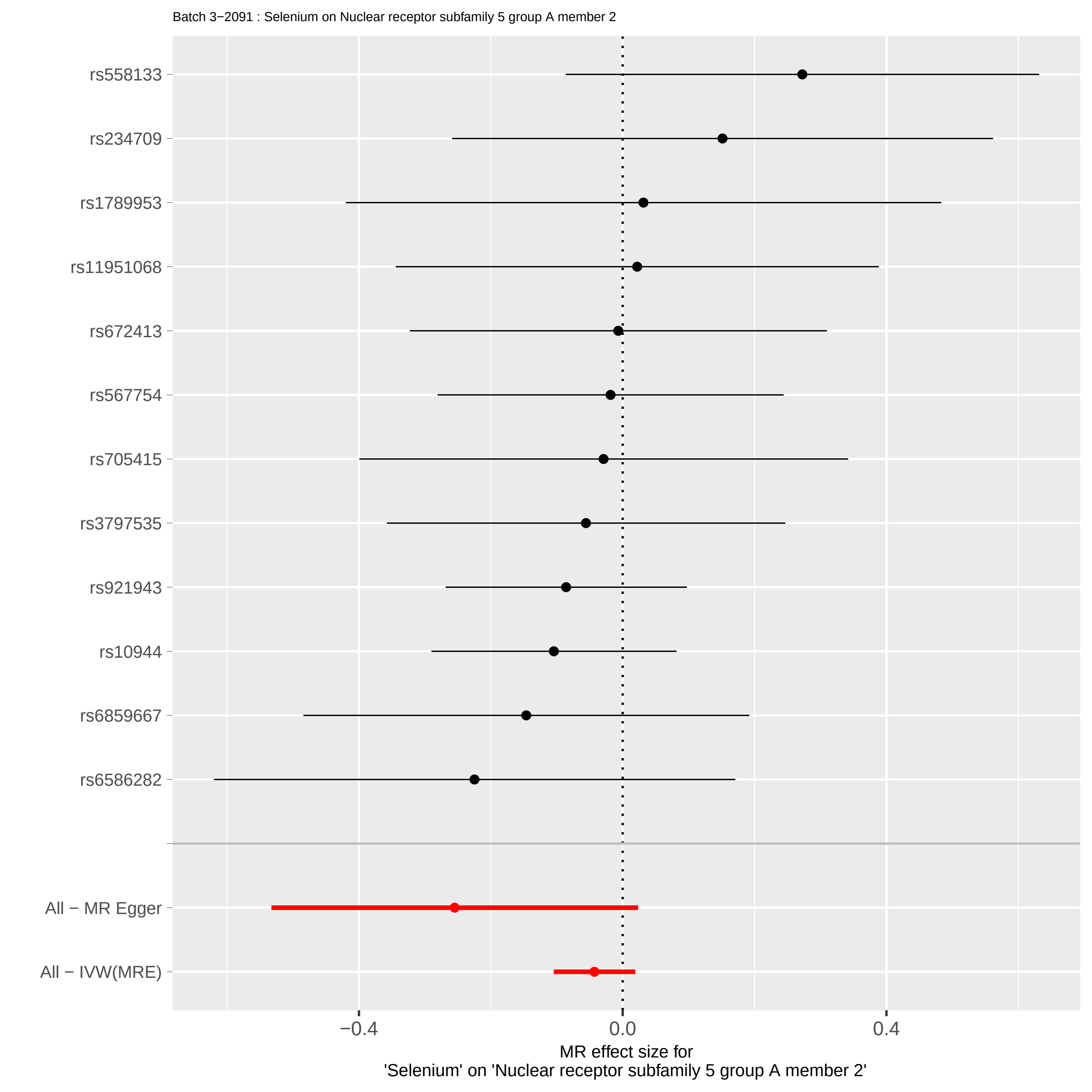

Batch 3−2091 : Selenium on Nuclear receptor subfamily 5 group A member 2
rs558133
rs234709
rs1789953
rs11951068
rs672413
rs567754
rs705415
rs3797535
rs921943
rs10944
rs6859667
rs6586282
All − MR Egger
All − IVW(MRE)
−0.4
0.0
0.4
MR effect size for
'Selenium' on 'Nuclear receptor subfamily 5 group A member 2'

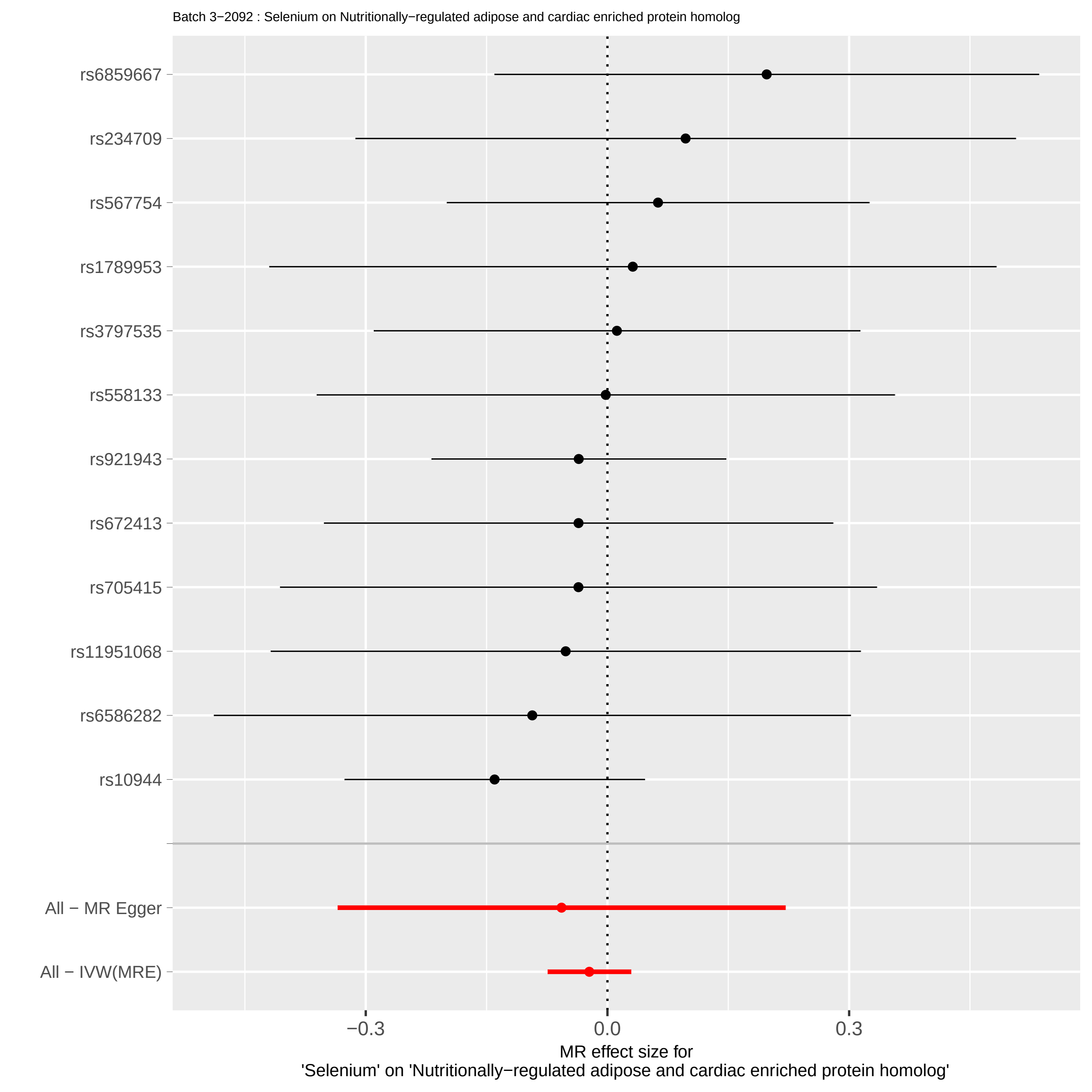

Batch 3−2092 : Selenium on Nutritionally−regulated adipose and cardiac enriched protein homolog
rs6859667
rs234709
rs567754
rs1789953
rs3797535
rs558133
rs921943
rs672413
rs705415
rs11951068
rs6586282
rs10944
All − MR Egger
All − IVW(MRE)
−0.3
0.0
0.3
MR effect size for
'Selenium' on 'Nutritionally−regulated adipose and cardiac enriched protein homolog'

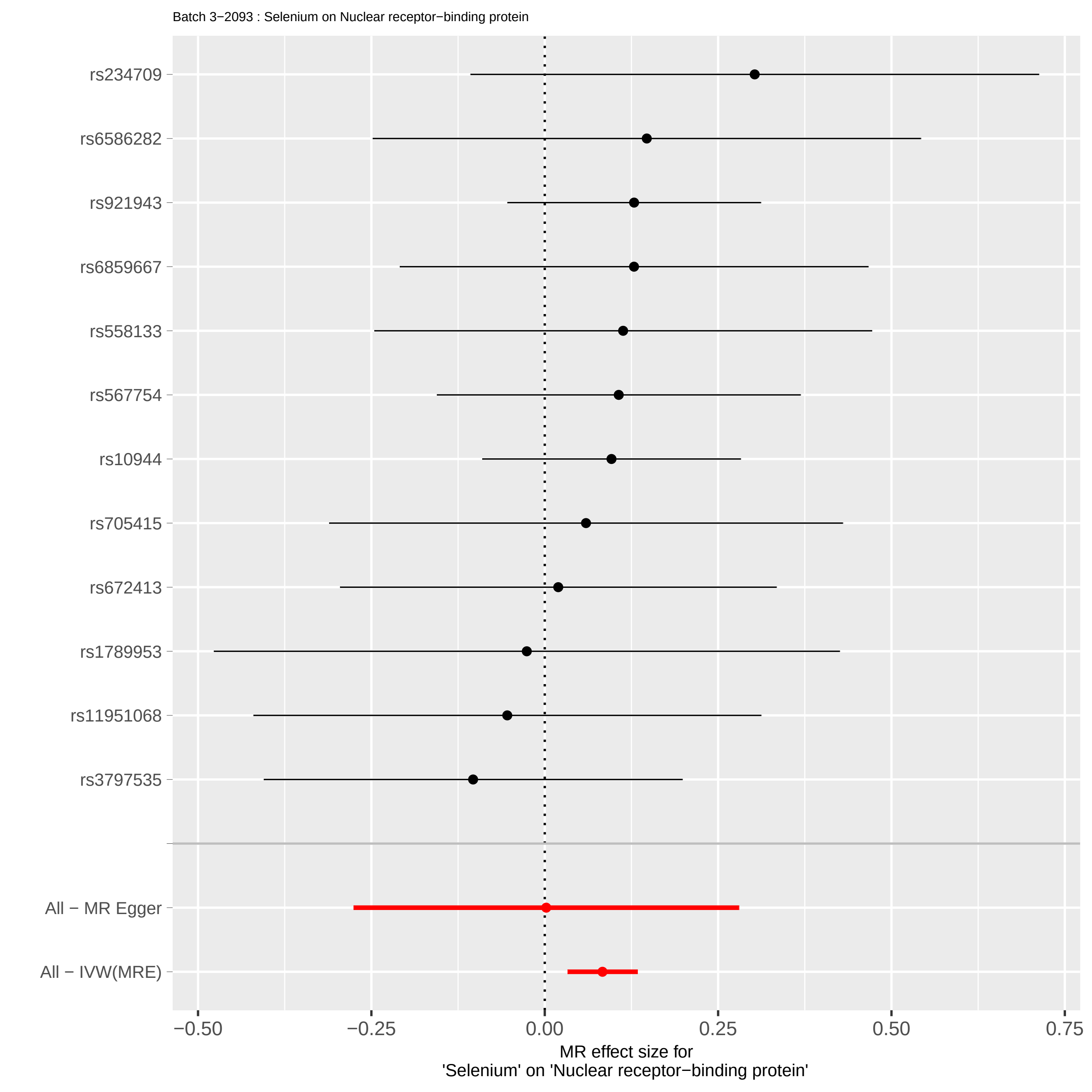

Batch 3−2093 : Selenium on Nuclear receptor−binding protein
rs234709
rs6586282
rs921943
rs6859667
rs558133
rs567754
rs10944
rs705415
rs672413
rs1789953
rs11951068
rs3797535
All − MR Egger
All − IVW(MRE)
−0.50
−0.25
0.00
0.25
0.50
0.75
MR effect size for
'Selenium' on 'Nuclear receptor−binding protein'

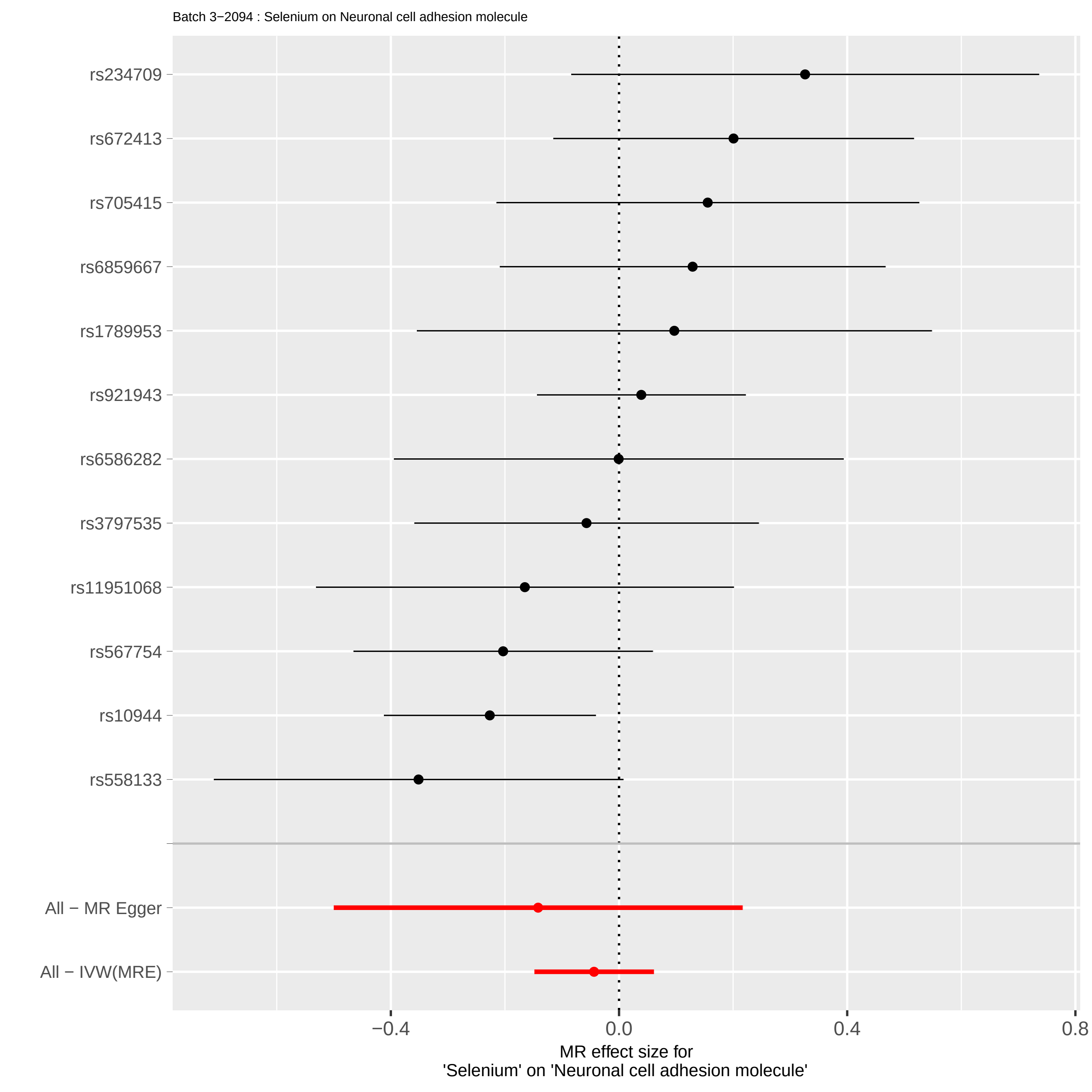

Batch 3−2094 : Selenium on Neuronal cell adhesion molecule
rs234709
rs672413
rs705415
rs6859667
rs1789953
rs921943
rs6586282
rs3797535
rs11951068
rs567754
rs10944
rs558133
All − MR Egger
All − IVW(MRE)
−0.4
0.0
0.4
0.8
MR effect size for
'Selenium' on 'Neuronal cell adhesion molecule'

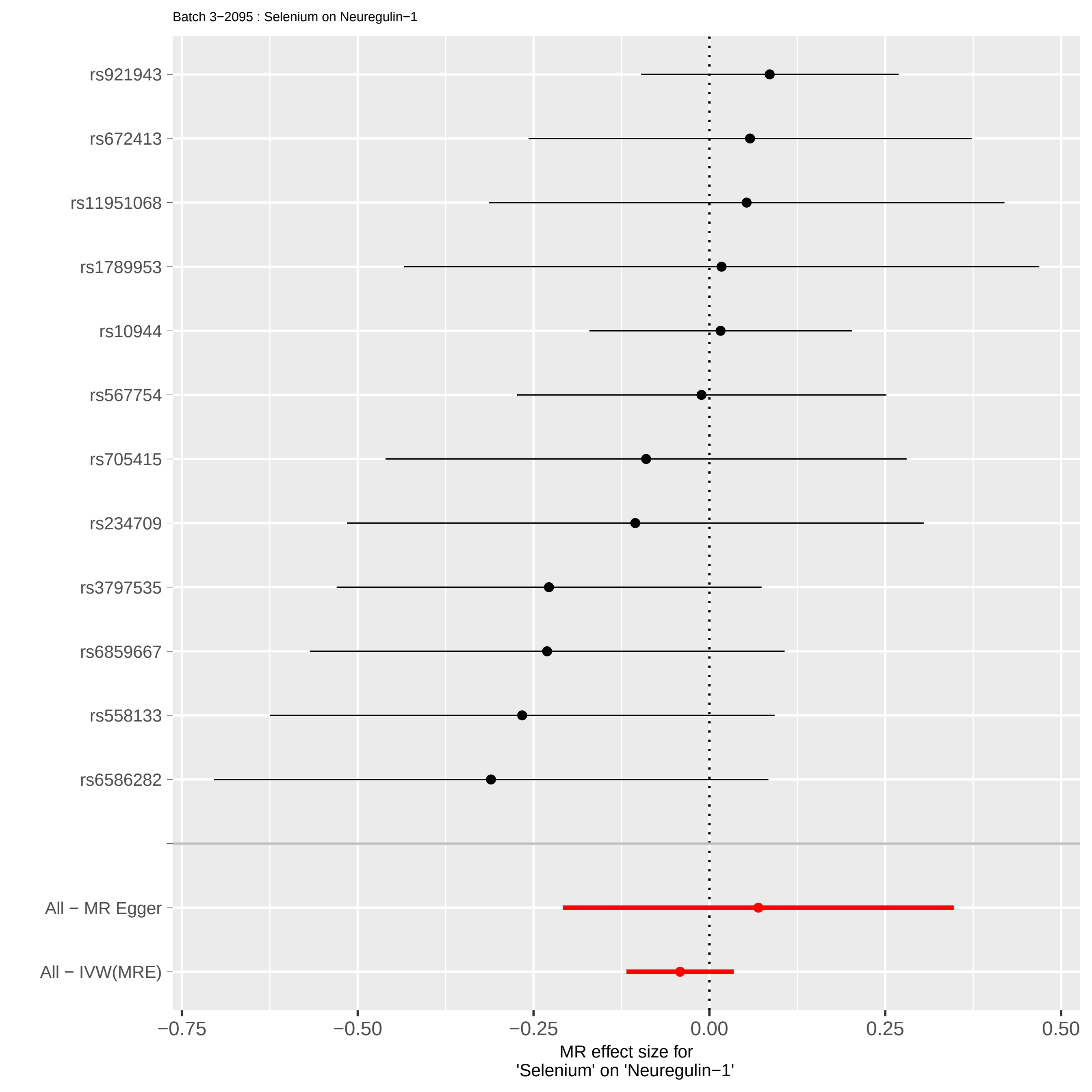

Batch 3−2095 : Selenium on Neuregulin−1
rs921943
rs672413
rs11951068
rs1789953
rs10944
rs567754
rs705415
rs234709
rs3797535
rs6859667
rs558133
rs6586282
All − MR Egger
All − IVW(MRE)
−0.75
−0.50
−0.25
0.00
0.25
0.50
MR effect size for
'Selenium' on 'Neuregulin−1'

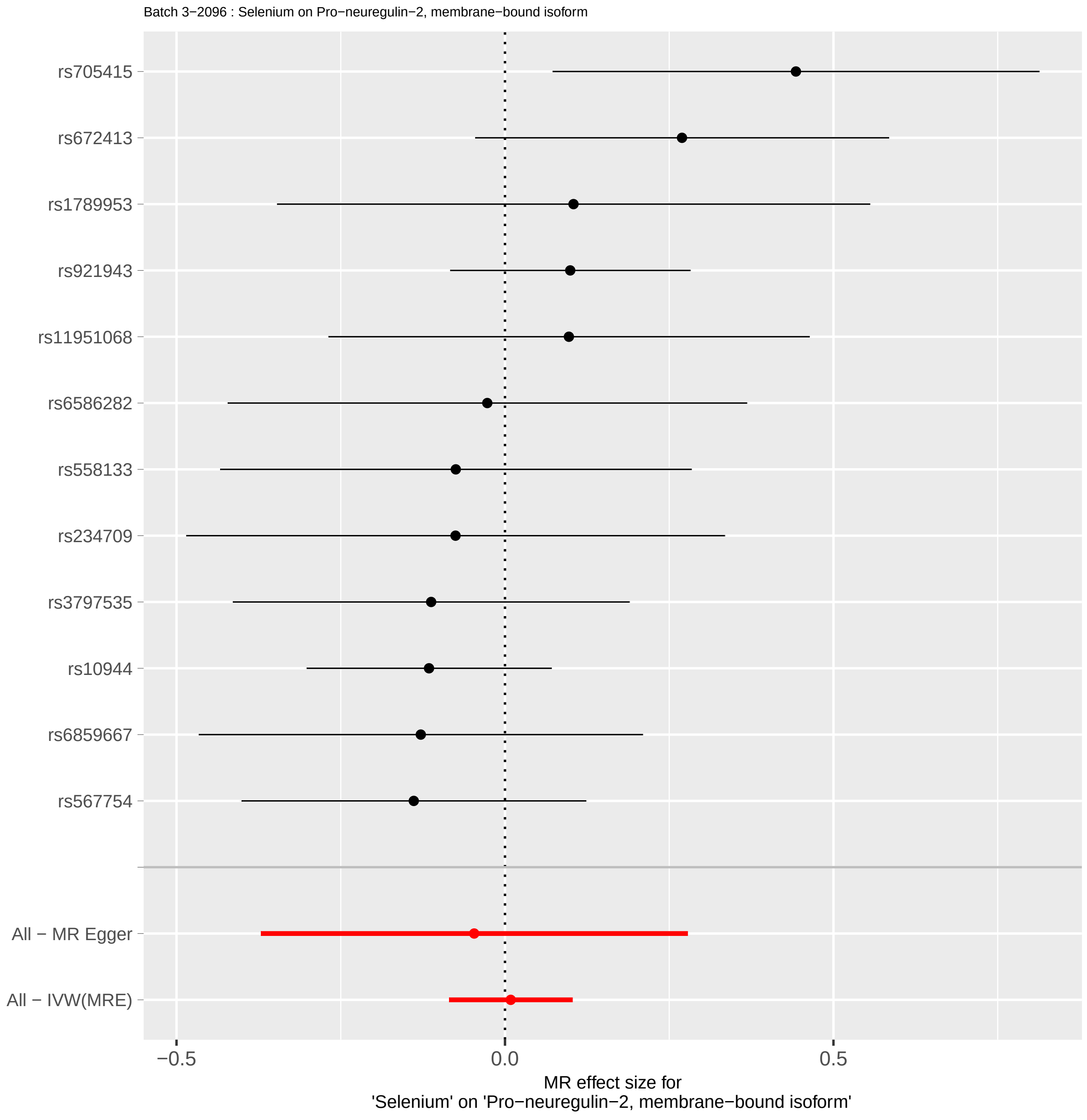
Batch 3−2096 : Selenium on Pro−neuregulin−2, membrane−bound isoform
rs705415
rs672413
rs1789953
rs921943
rs11951068
rs6586282
rs558133
rs234709
rs3797535
rs10944
rs6859667
rs567754
All − MR Egger
All − IVW(MRE)
−0.5
0.0
0.5
MR effect size for
'Selenium' on 'Pro−neuregulin−2, membrane−bound isoform'

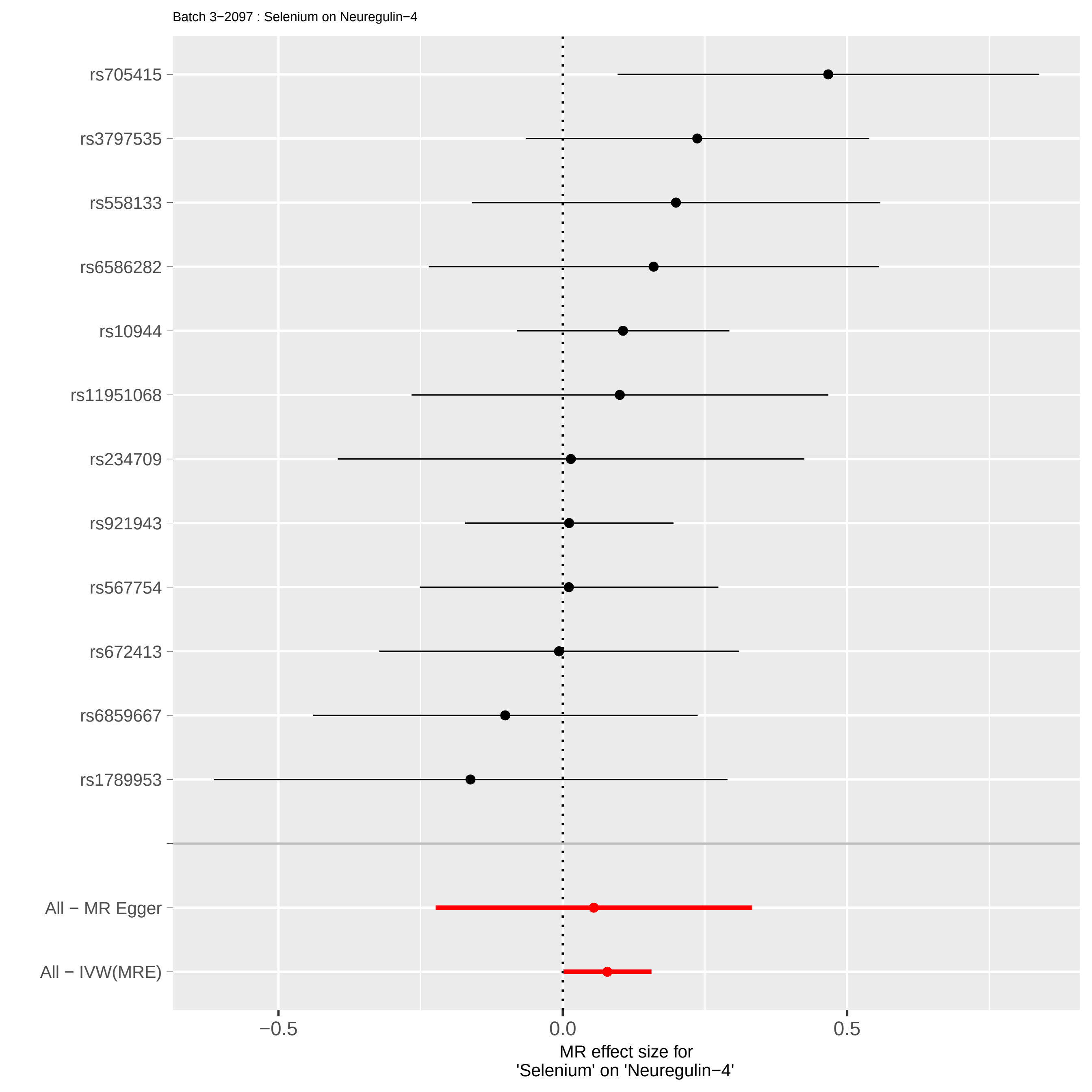

Batch 3−2097 : Selenium on Neuregulin−4
rs705415
rs3797535
rs558133
rs6586282
rs10944
rs11951068
rs234709
rs921943
rs567754
rs672413
rs6859667
rs1789953
All − MR Egger
All − IVW(MRE)
−0.5
0.0
0.5
MR effect size for
'Selenium' on 'Neuregulin−4'

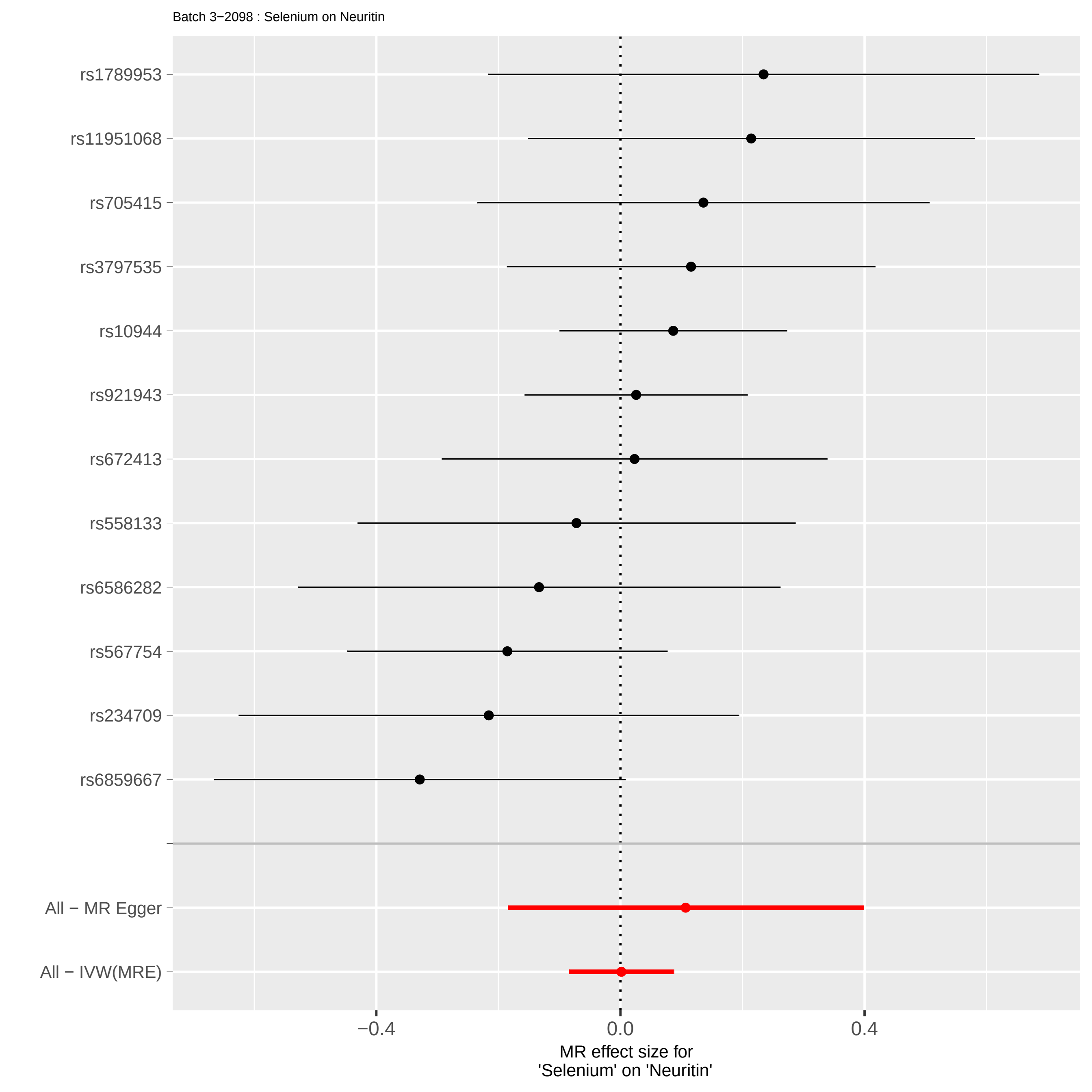

Batch 3−2098 : Selenium on Neuritin
rs1789953
rs11951068
rs705415
rs3797535
rs10944
rs921943
rs672413
rs558133
rs6586282
rs567754
rs234709
rs6859667
All − MR Egger
All − IVW(MRE)
−0.4
0.0
0.4
MR effect size for
'Selenium' on 'Neuritin'

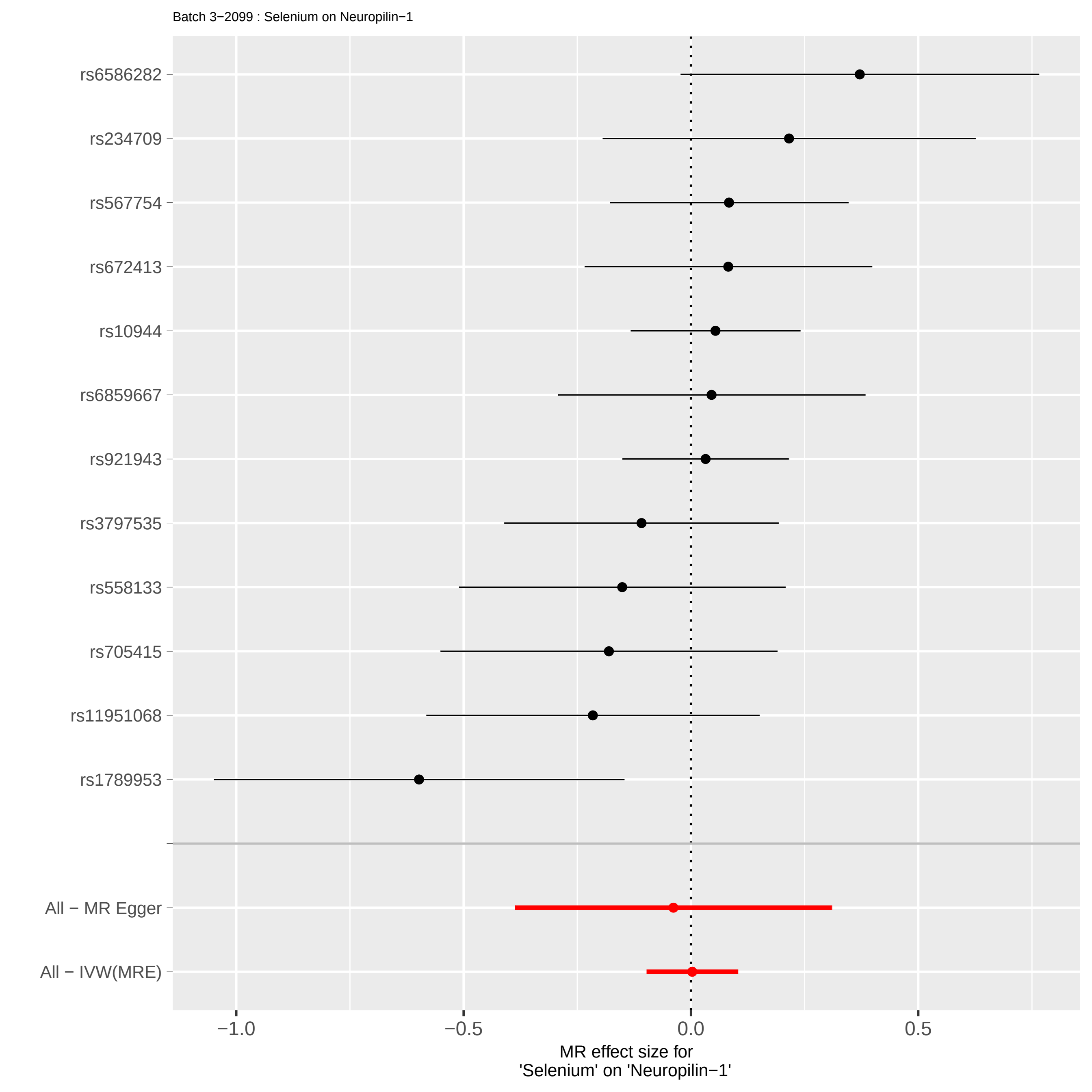
Batch 3−2099 : Selenium on Neuropilin−1
rs6586282
rs234709
rs567754
rs672413
rs10944
rs6859667
rs921943
rs3797535
rs558133
rs705415
rs11951068
rs1789953
All − MR Egger
All − IVW(MRE)
−1.0
−0.5
0.0
0.5
MR effect size for
'Selenium' on 'Neuropilin−1'

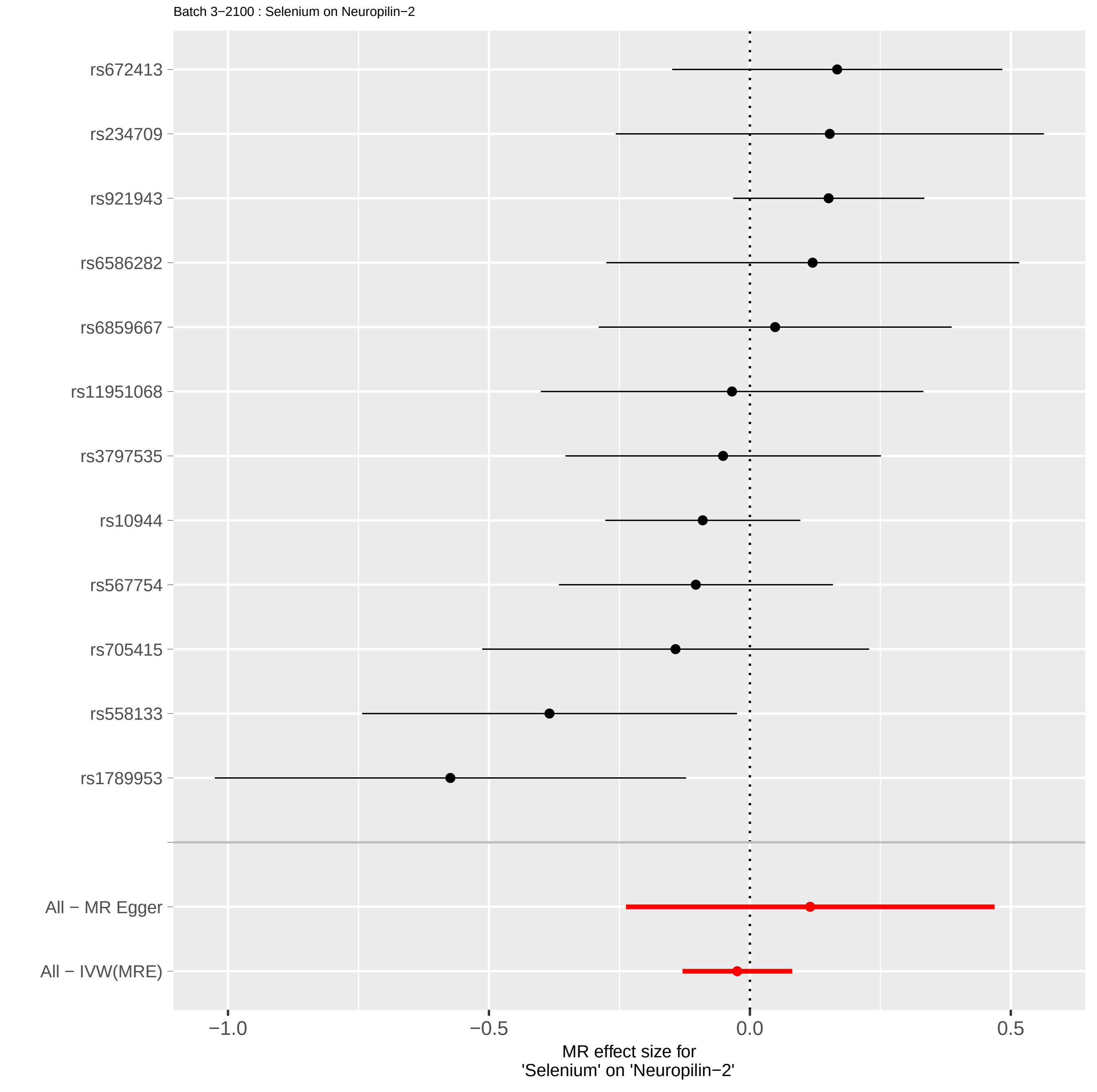

Batch 3−2100 : Selenium on Neuropilin−2
rs672413
rs234709
rs921943
rs6586282
rs6859667
rs11951068
rs3797535
rs10944
rs567754
rs705415
rs558133
rs1789953
All − MR Egger
All − IVW(MRE)
−1.0
−0.5
0.0
0.5
MR effect size for
'Selenium' on 'Neuropilin−2'

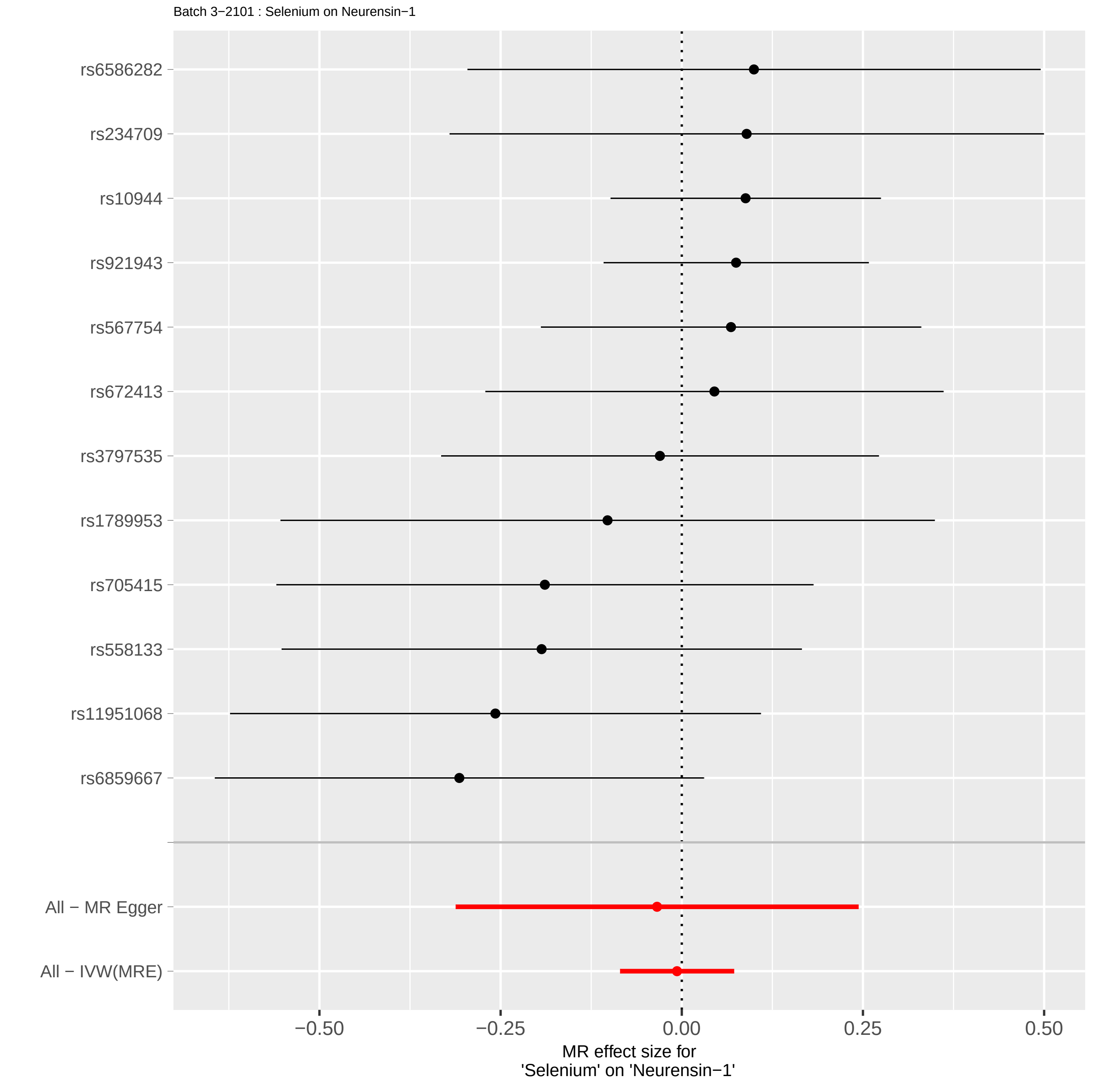
Batch 3−2101 : Selenium on Neurensin−1
rs6586282
rs234709
rs10944
rs921943
rs567754
rs672413
rs3797535
rs1789953
rs705415
rs558133
rs11951068
rs6859667
All − MR Egger
All − IVW(MRE)
−0.50
−0.25
0.00
0.25
0.50
MR effect size for
'Selenium' on 'Neurensin−1'

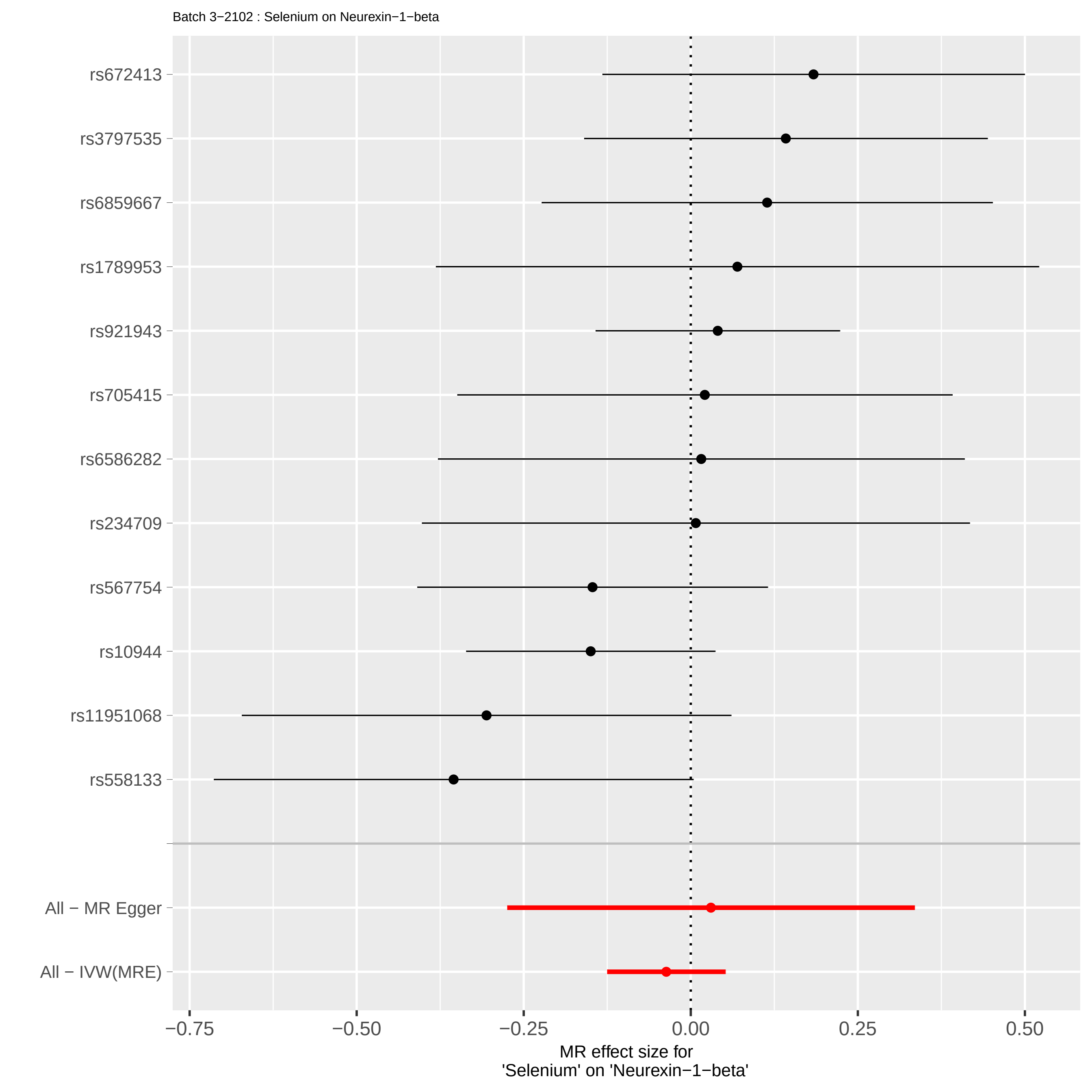

Batch 3−2102 : Selenium on Neurexin−1−beta
rs672413
rs3797535
rs6859667
rs1789953
rs921943
rs705415
rs6586282
rs234709
rs567754
rs10944
rs11951068
rs558133
All − MR Egger
All − IVW(MRE)
−0.75
−0.50
−0.25
0.00
0.25
0.50
MR effect size for
'Selenium' on 'Neurexin−1−beta'

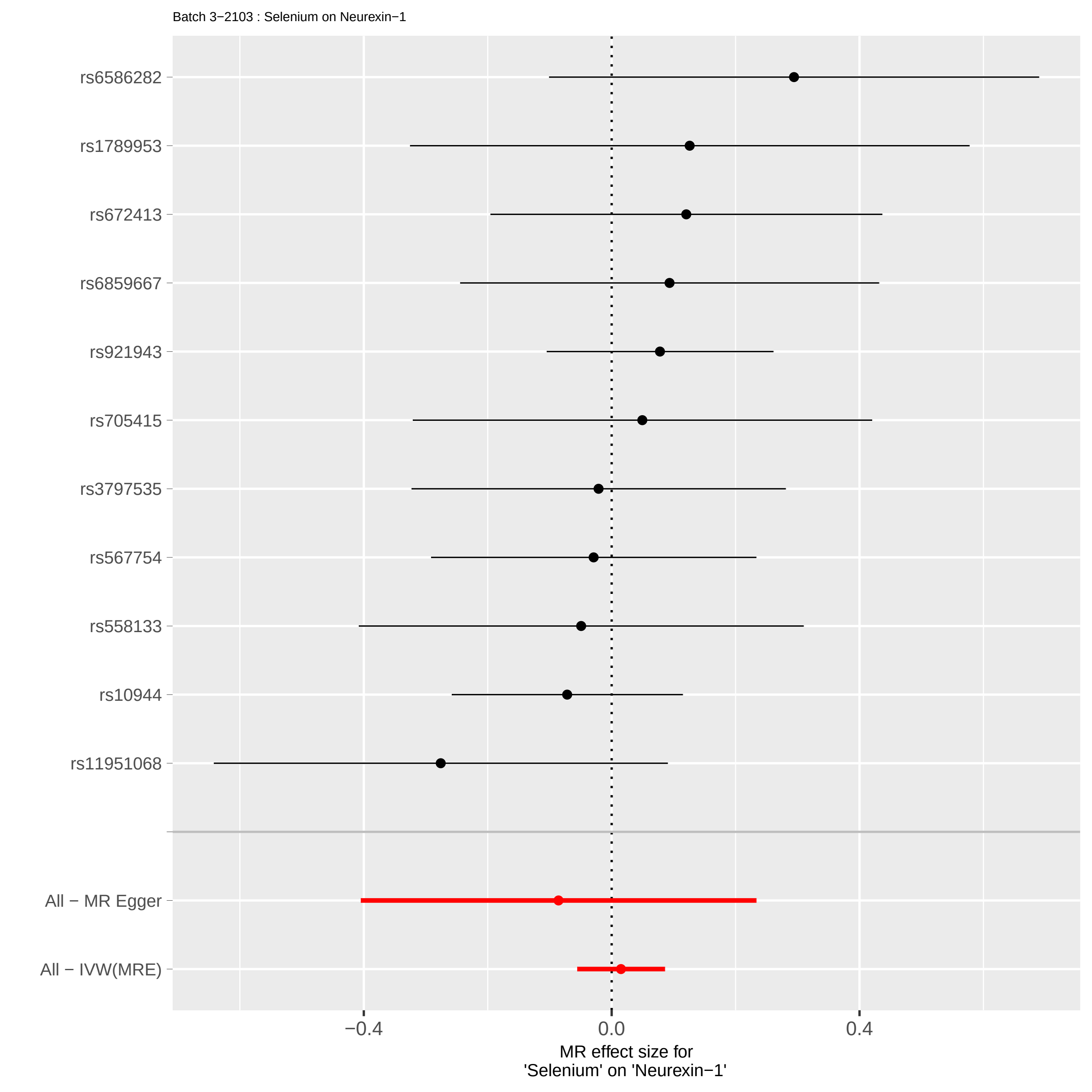
Batch 3−2103 : Selenium on Neurexin−1
rs6586282
rs1789953
rs672413
rs6859667
rs921943
rs705415
rs3797535
rs567754
rs558133
rs10944
rs11951068
All − MR Egger
All − IVW(MRE)
−0.4
0.0
0.4
MR effect size for
'Selenium' on 'Neurexin−1'

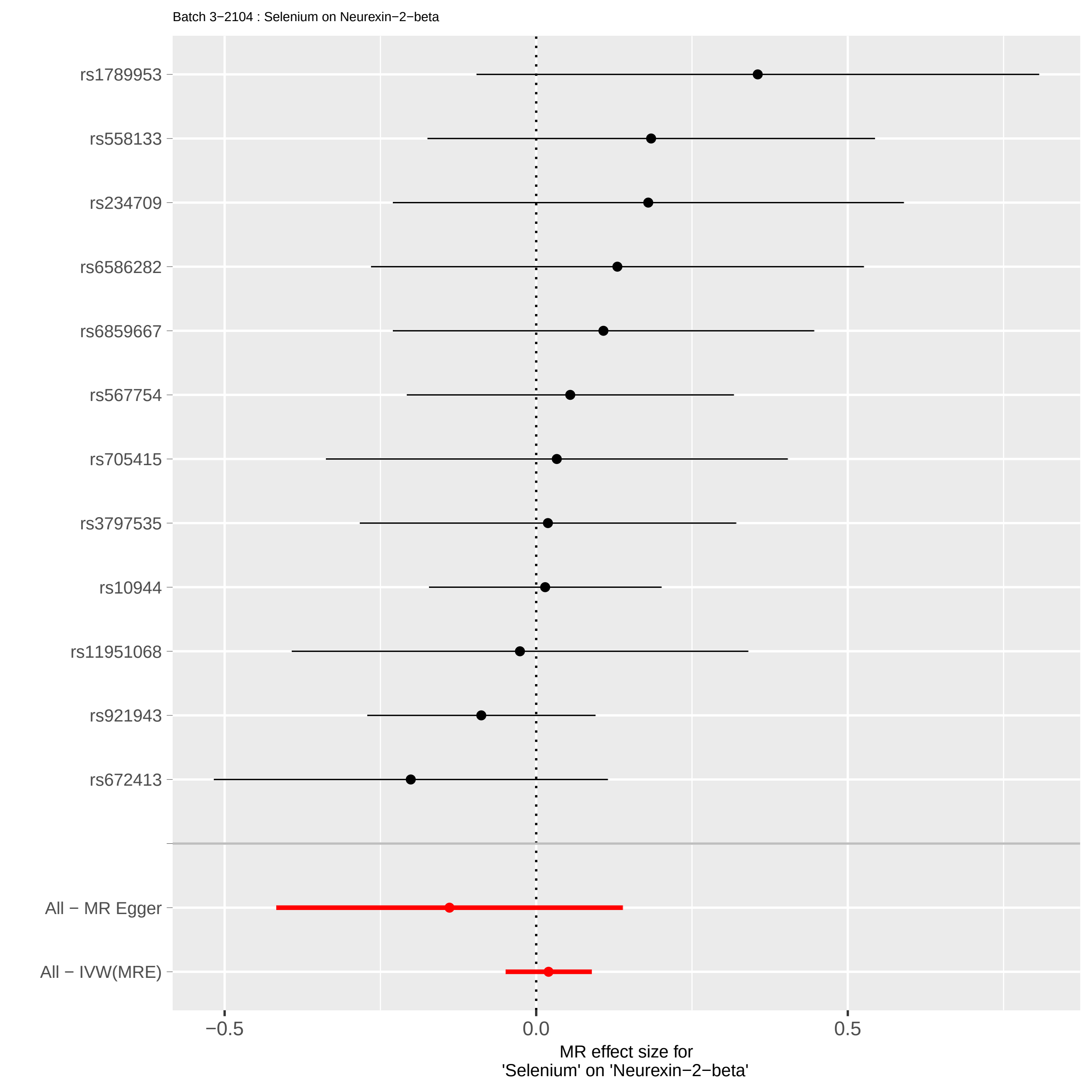

Batch 3−2104 : Selenium on Neurexin−2−beta
rs1789953
rs558133
rs234709
rs6586282
rs6859667
rs567754
rs705415
rs3797535
rs10944
rs11951068
rs921943
rs672413
All − MR Egger
All − IVW(MRE)
−0.5
0.0
0.5
MR effect size for
'Selenium' on 'Neurexin−2−beta'

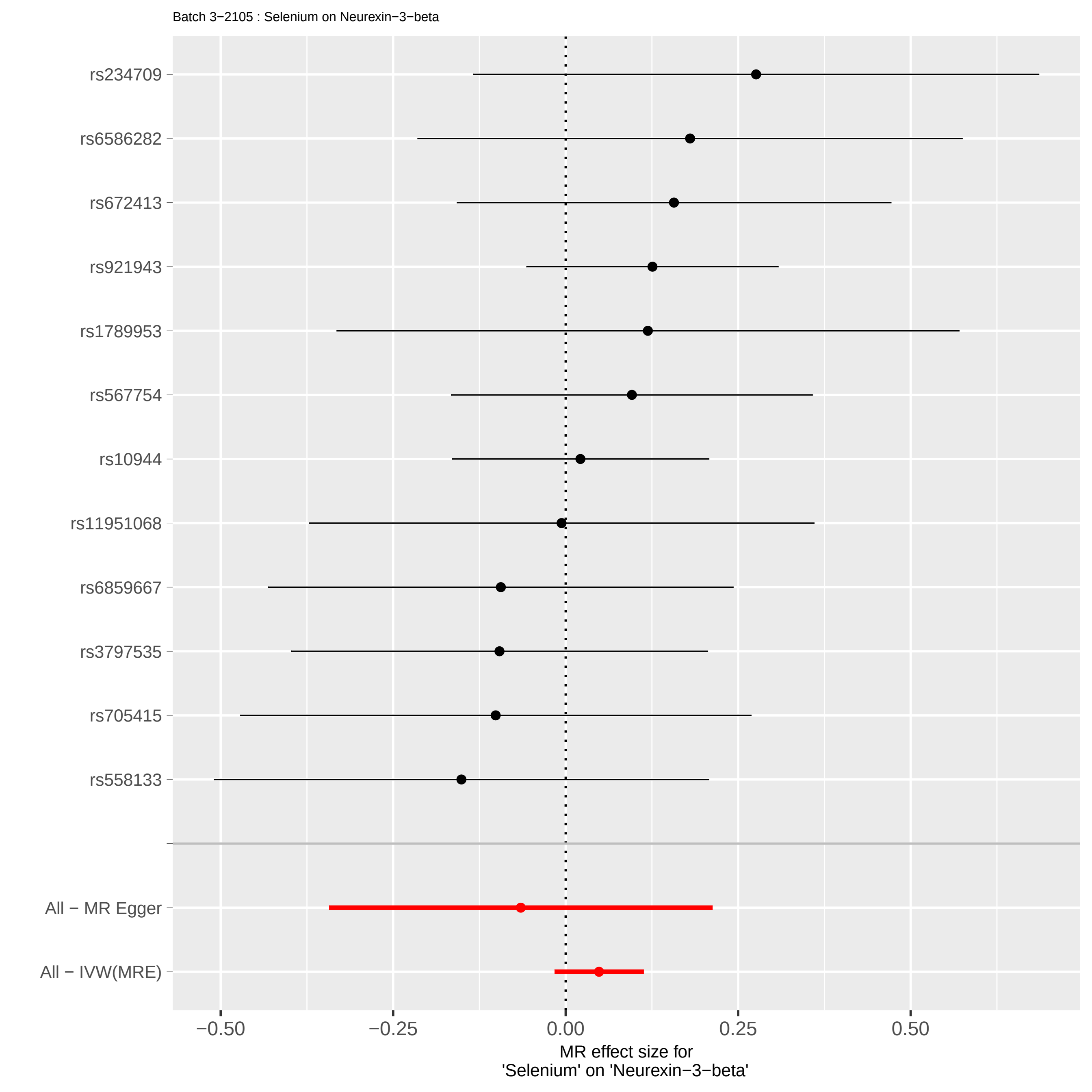

Batch 3−2105 : Selenium on Neurexin−3−beta
rs234709
rs6586282
rs672413
rs921943
rs1789953
rs567754
rs10944
rs11951068
rs6859667
rs3797535
rs705415
rs558133
All − MR Egger
All − IVW(MRE)
−0.50
−0.25
0.00
0.25
0.50
MR effect size for
'Selenium' on 'Neurexin−3−beta'

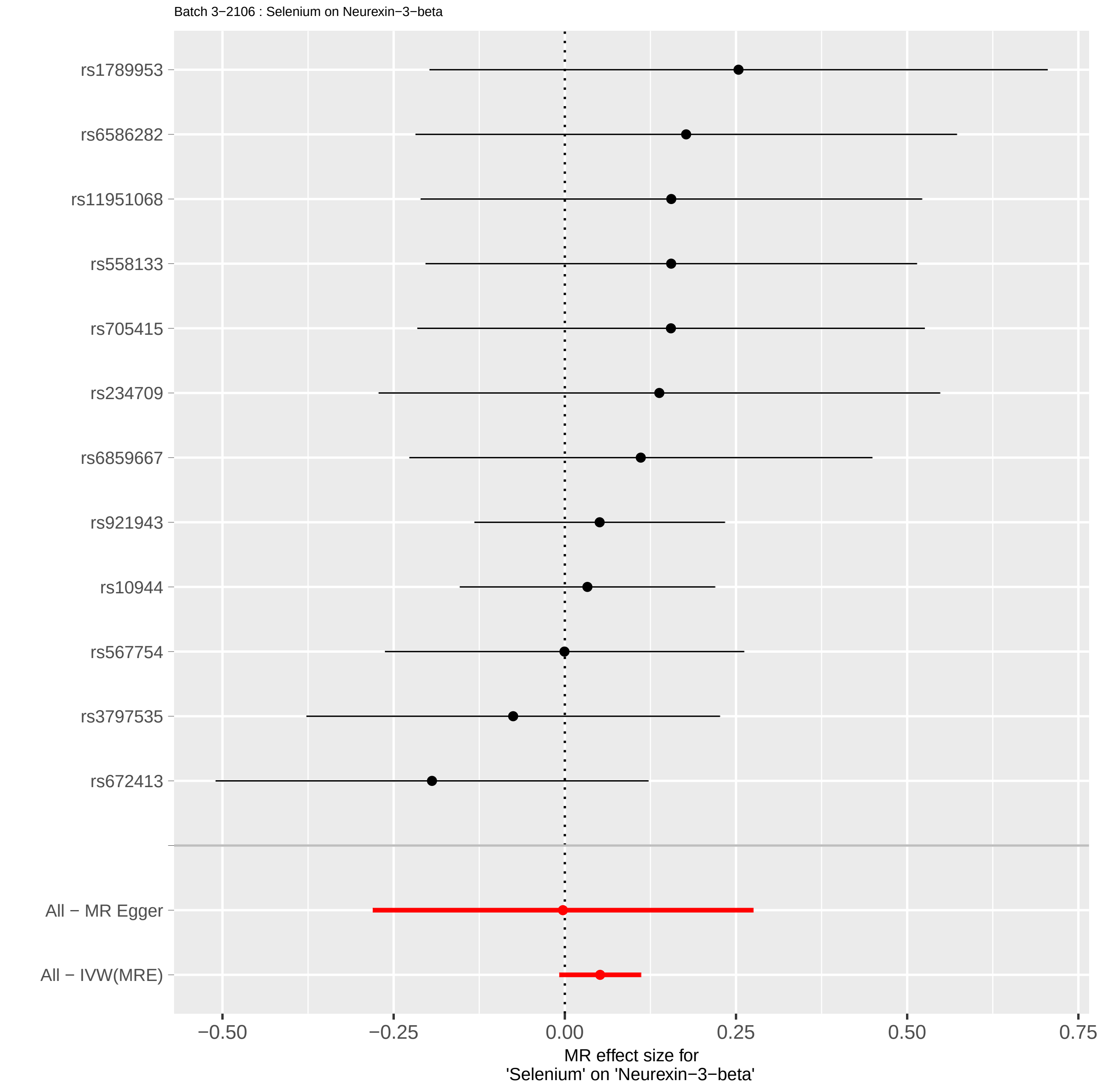

Batch 3−2106 : Selenium on Neurexin−3−beta
rs1789953
rs6586282
rs11951068
rs558133
rs705415
rs234709
rs6859667
rs921943
rs10944
rs567754
rs3797535
rs672413
All − MR Egger
All − IVW(MRE)
−0.50
−0.25
0.00
0.25
0.50
0.75
MR effect size for
'Selenium' on 'Neurexin−3−beta'

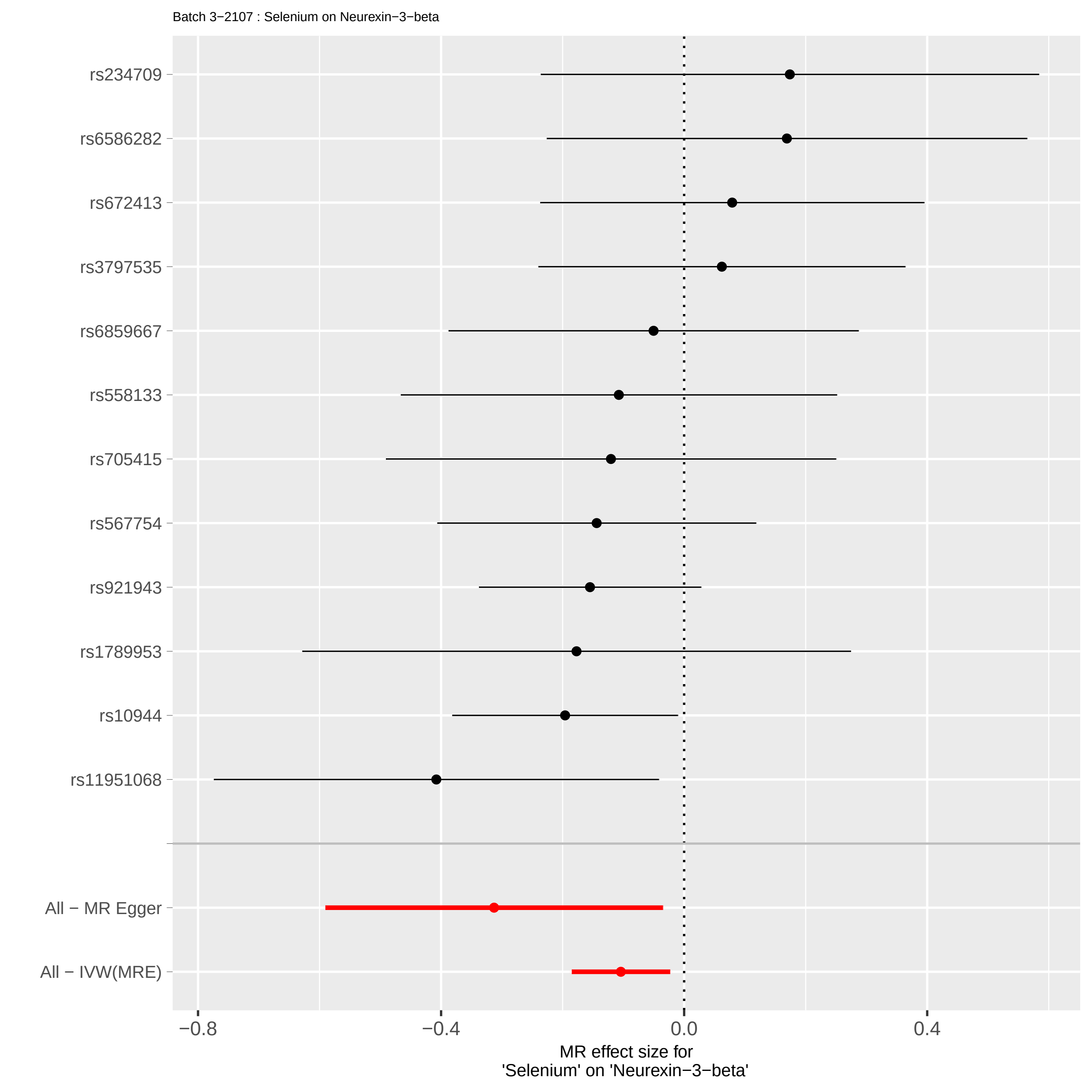
Batch 3−2107 : Selenium on Neurexin−3−beta
rs234709
rs6586282
rs672413
rs3797535
rs6859667
rs558133
rs705415
rs567754
rs921943
rs1789953
rs10944
rs11951068
All − MR Egger
All − IVW(MRE)
−0.8
−0.4
0.0
0.4
MR effect size for
'Selenium' on 'Neurexin−3−beta'

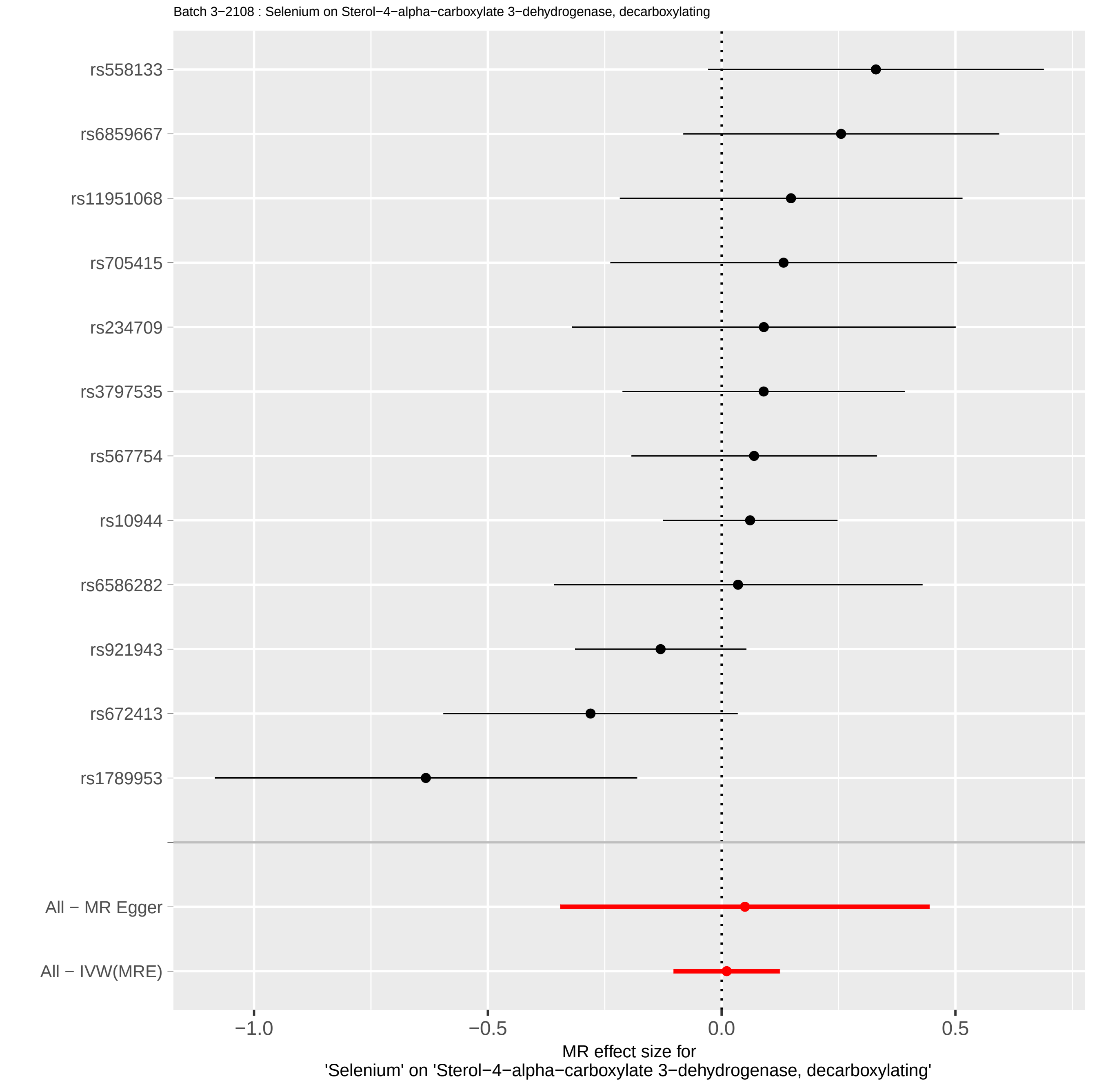

Batch 3−2108 : Selenium on Sterol−4−alpha−carboxylate 3−dehydrogenase, decarboxylating
rs558133
rs6859667
rs11951068
rs705415
rs234709
rs3797535
rs567754
rs10944
rs6586282
rs921943
rs672413
rs1789953
All − MR Egger
All − IVW(MRE)
−1.0
−0.5
0.0
0.5
MR effect size for
'Selenium' on 'Sterol−4−alpha−carboxylate 3−dehydrogenase, decarboxylating'

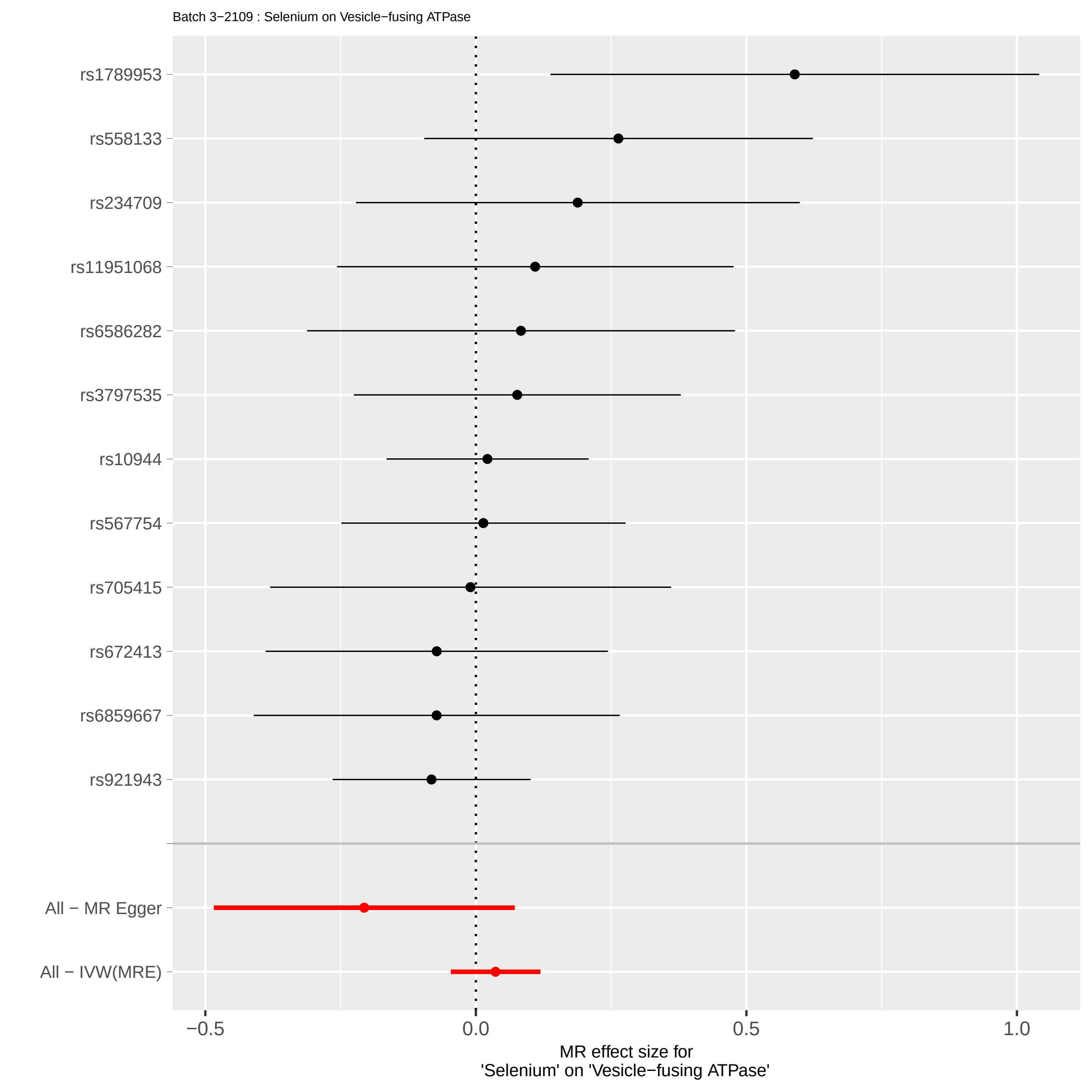

Batch 3−2109 : Selenium on Vesicle−fusing ATPase
rs1789953
rs558133
rs234709
rs11951068
rs6586282
rs3797535
rs10944
rs567754
rs705415
rs672413
rs6859667
rs921943
All − MR Egger
All − IVW(MRE)
−0.5
0.0
0.5
1.0
MR effect size for
'Selenium' on 'Vesicle−fusing ATPase'

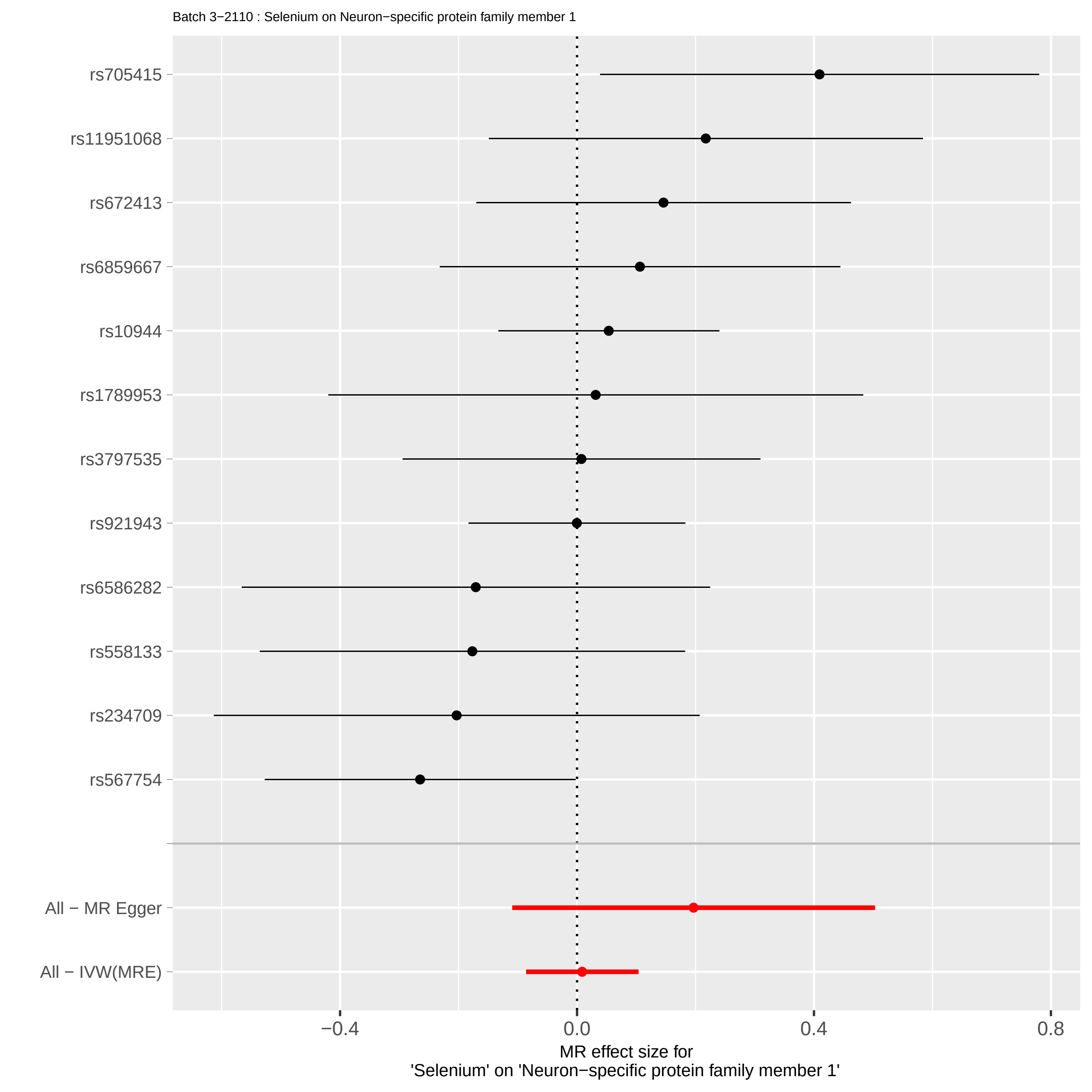

Batch 3−2110 : Selenium on Neuron−specific protein family member 1
rs705415
rs11951068
rs672413
rs6859667
rs10944
rs1789953
rs3797535
rs921943
rs6586282
rs558133
rs234709
rs567754
All − MR Egger
All − IVW(MRE)
−0.4
0.0
0.4
0.8
MR effect size for
'Selenium' on 'Neuron−specific protein family member 1'

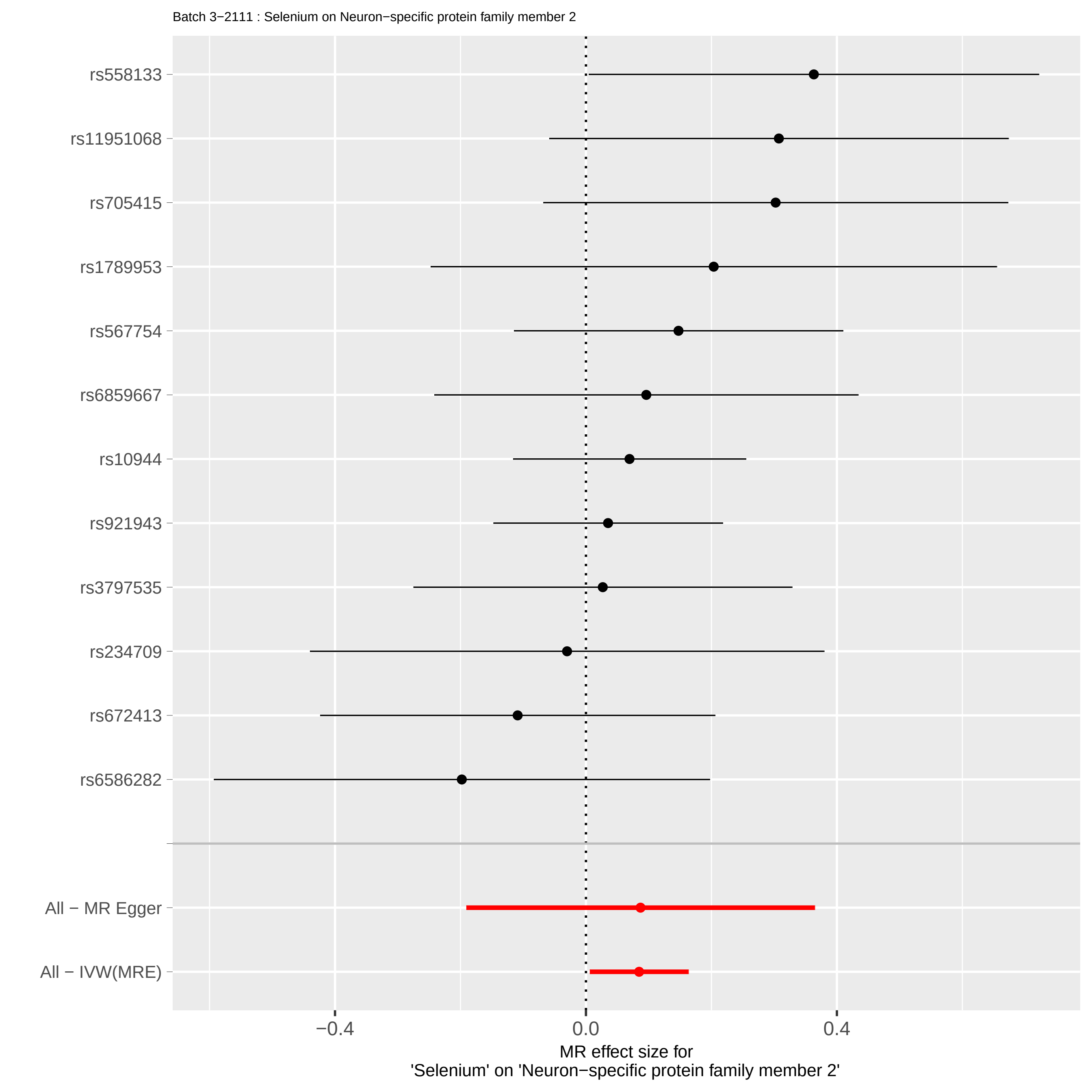

Batch 3−2111 : Selenium on Neuron−specific protein family member 2
rs558133
rs11951068
rs705415
rs1789953
rs567754
rs6859667
rs10944
rs921943
rs3797535
rs234709
rs672413
rs6586282
All − MR Egger
All − IVW(MRE)
−0.4
0.0
0.4
MR effect size for
'Selenium' on 'Neuron−specific protein family member 2'

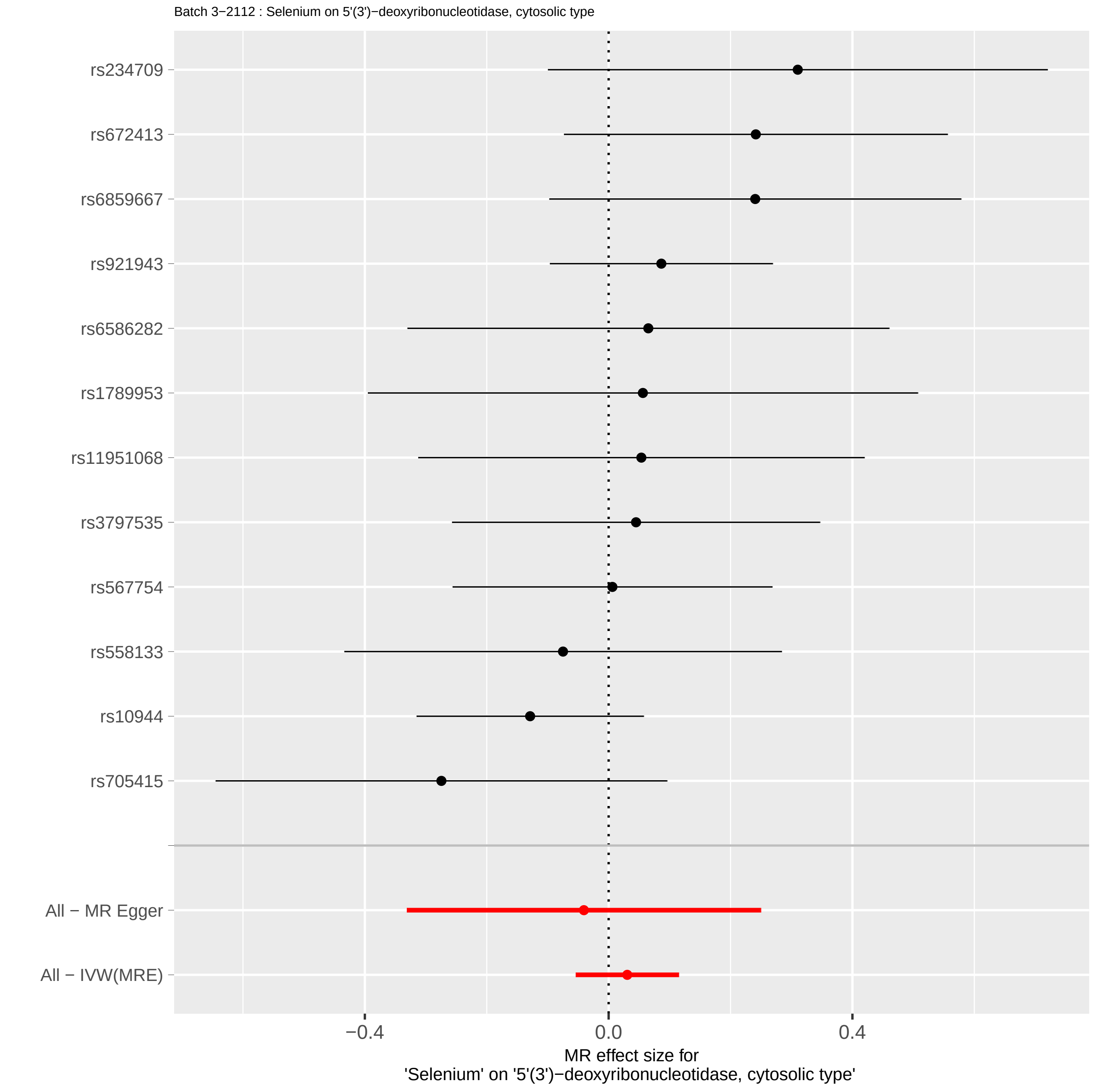
Batch 3−2112 : Selenium on 5'(3')−deoxyribonucleotidase, cytosolic type
rs234709
rs672413
rs6859667
rs921943
rs6586282
rs1789953
rs11951068
rs3797535
rs567754
rs558133
rs10944
rs705415
All − MR Egger
All − IVW(MRE)
−0.4
0.0
0.4
MR effect size for
'Selenium' on '5'(3')−deoxyribonucleotidase, cytosolic type'

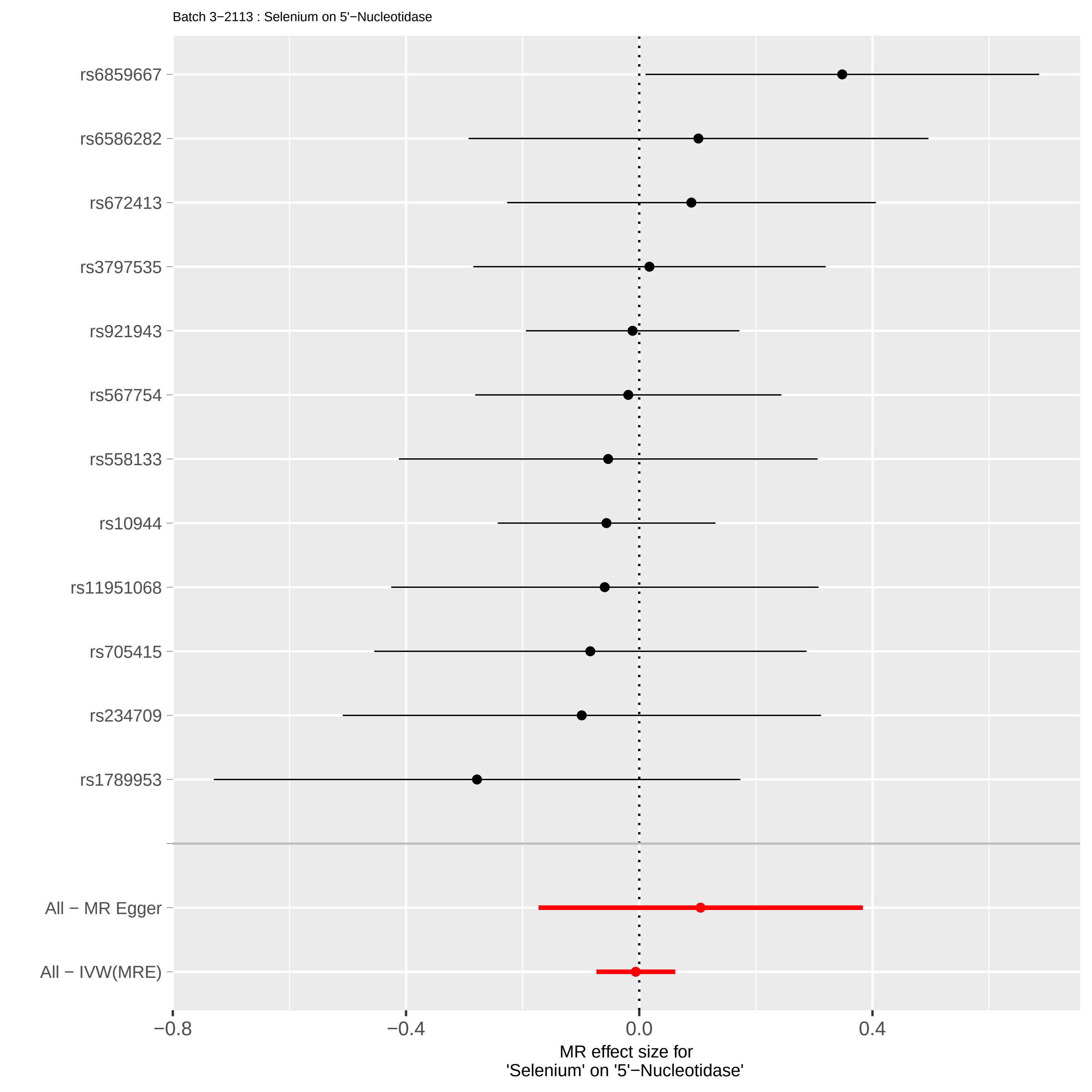

Batch 3−2113 : Selenium on 5'−Nucleotidase
rs6859667
rs6586282
rs672413
rs3797535
rs921943
rs567754
rs558133
rs10944
rs11951068
rs705415
rs234709
rs1789953
All − MR Egger
All − IVW(MRE)
−0.8
−0.4
0.0
0.4
MR effect size for
'Selenium' on '5'−Nucleotidase'

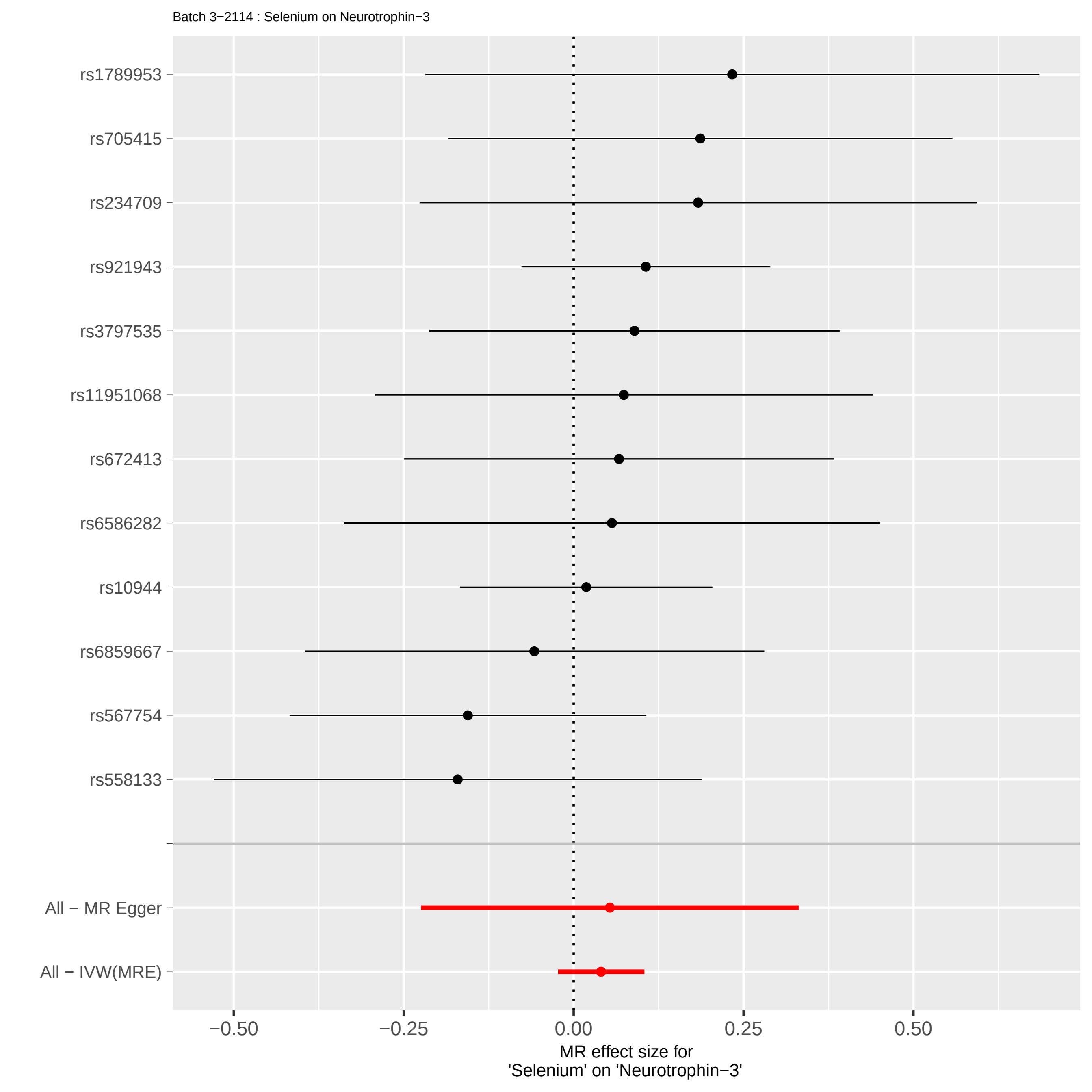

Batch 3−2114 : Selenium on Neurotrophin−3
rs1789953
rs705415
rs234709
rs921943
rs3797535
rs11951068
rs672413
rs6586282
rs10944
rs6859667
rs567754
rs558133
All − MR Egger
All − IVW(MRE)
−0.50
−0.25
0.00
0.25
0.50
MR effect size for
'Selenium' on 'Neurotrophin−3'

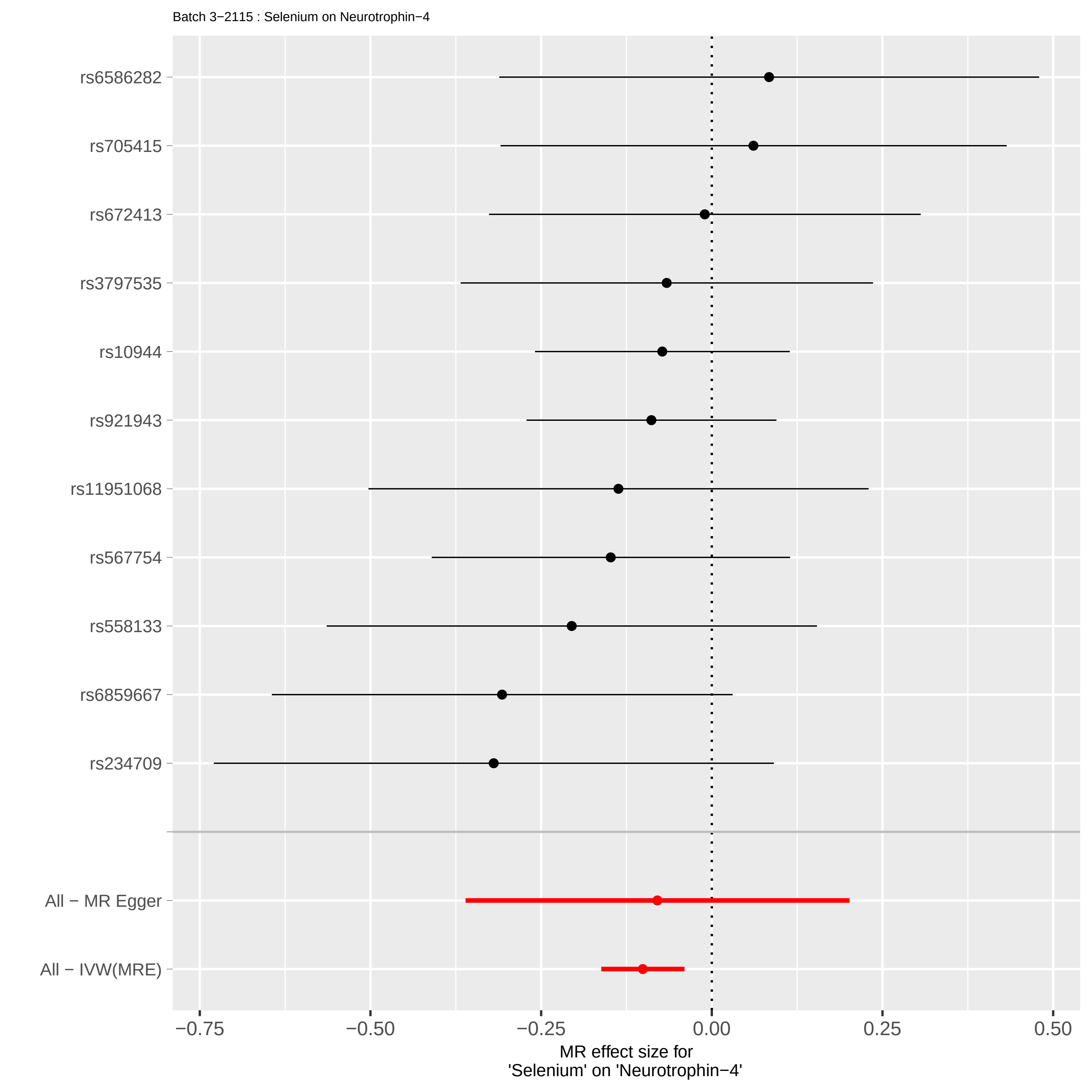

Batch 3−2115 : Selenium on Neurotrophin−4
rs6586282
rs705415
rs672413
rs3797535
rs10944
rs921943
rs11951068
rs567754
rs558133
rs6859667
rs234709
All − MR Egger
All − IVW(MRE)
−0.75
−0.50
−0.25
0.00
0.25
0.50
MR effect size for
'Selenium' on 'Neurotrophin−4'

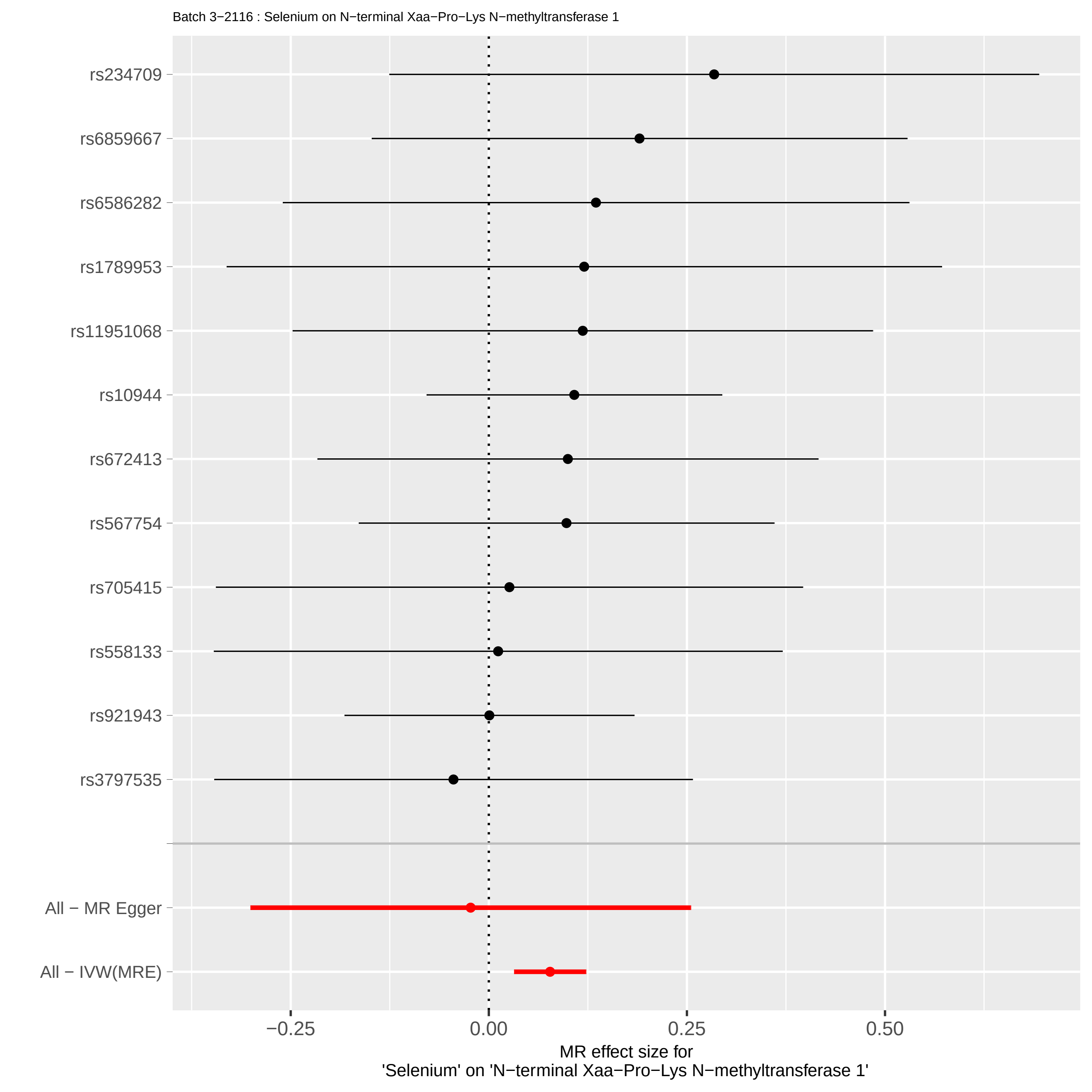

Batch 3−2116 : Selenium on N−terminal Xaa−Pro−Lys N−methyltransferase 1
rs234709
rs6859667
rs6586282
rs1789953
rs11951068
rs10944
rs672413
rs567754
rs705415
rs558133
rs921943
rs3797535
All − MR Egger
All − IVW(MRE)
−0.25
0.00
0.25
0.50
MR effect size for
'Selenium' on 'N−terminal Xaa−Pro−Lys N−methyltransferase 1'

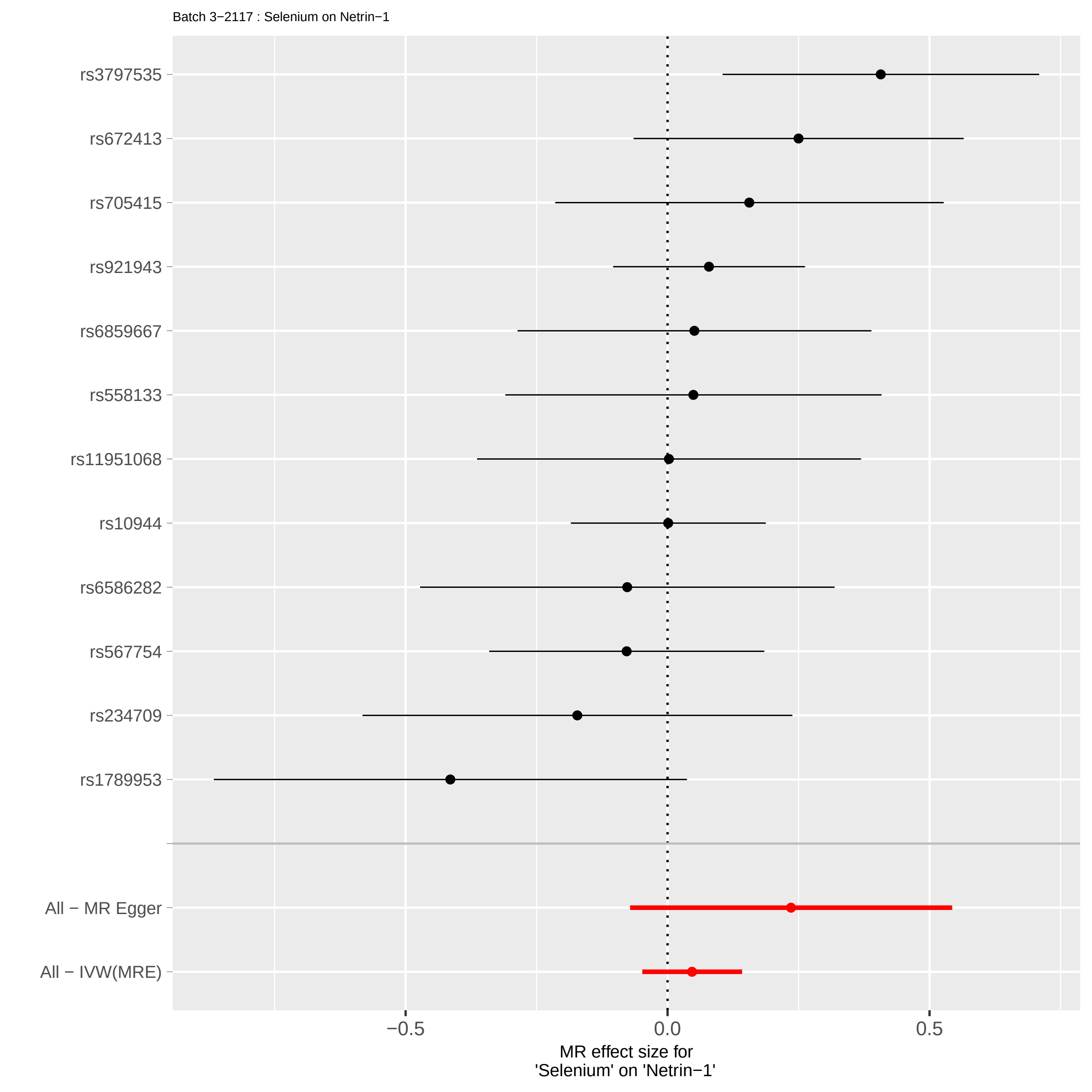

Batch 3−2117 : Selenium on Netrin−1
rs3797535
rs672413
rs705415
rs921943
rs6859667
rs558133
rs11951068
rs10944
rs6586282
rs567754
rs234709
rs1789953
All − MR Egger
All − IVW(MRE)
−0.5
0.0
0.5
MR effect size for
'Selenium' on 'Netrin−1'

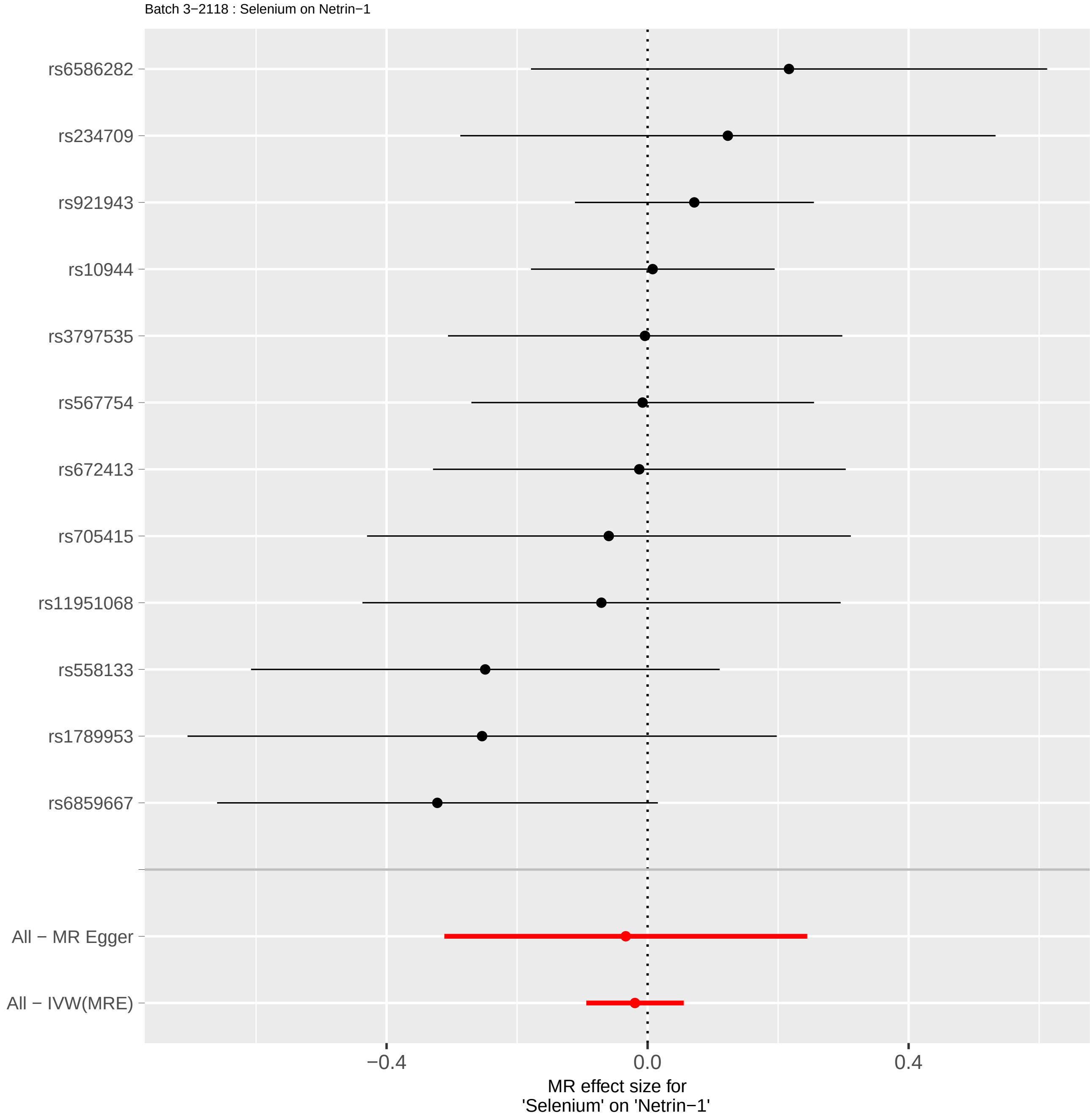

Batch 3−2118 : Selenium on Netrin−1
rs6586282
rs234709
rs921943
rs10944
rs3797535
rs567754
rs672413
rs705415
rs11951068
rs558133
rs1789953
rs6859667
All − MR Egger
All − IVW(MRE)
−0.4
0.0
0.4
MR effect size for
'Selenium' on 'Netrin−1'

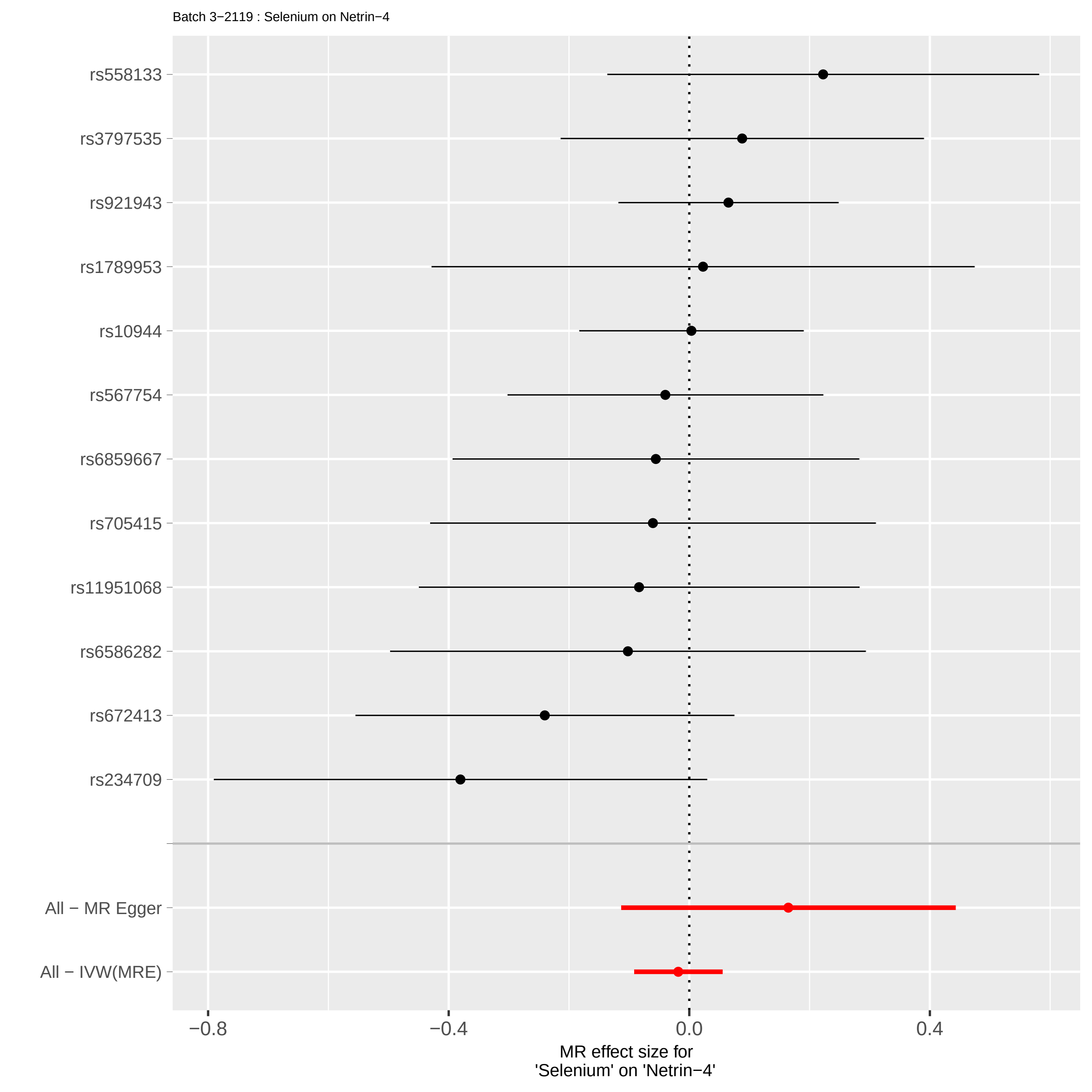
Batch 3−2119 : Selenium on Netrin−4
rs558133
rs3797535
rs921943
rs1789953
rs10944
rs567754
rs6859667
rs705415
rs11951068
rs6586282
rs672413
rs234709
All − MR Egger
All − IVW(MRE)
−0.8
−0.4
0.0
0.4
MR effect size for
'Selenium' on 'Netrin−4'

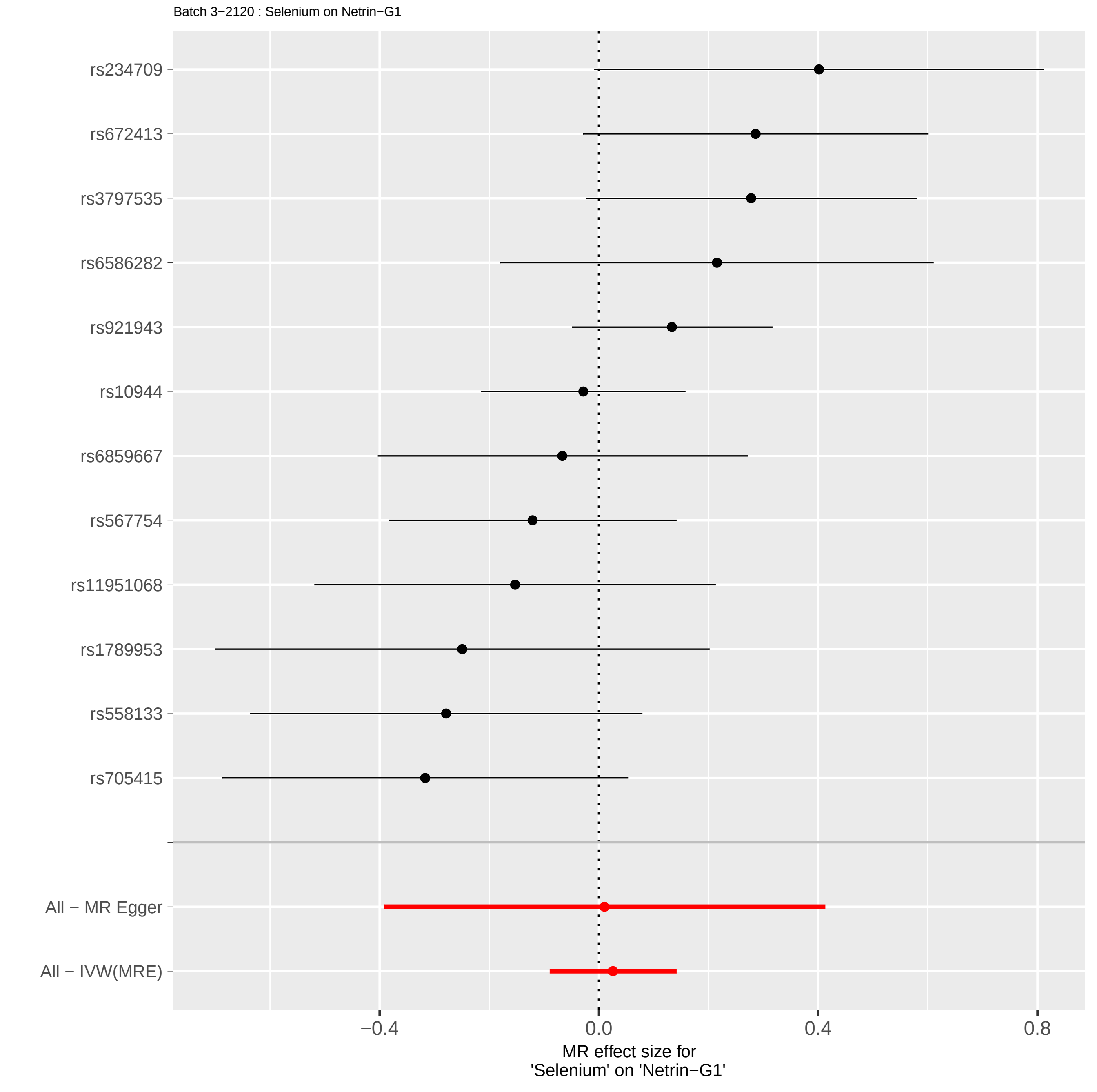

Batch 3−2120 : Selenium on Netrin−G1
rs234709
rs672413
rs3797535
rs6586282
rs921943
rs10944
rs6859667
rs567754
rs11951068
rs1789953
rs558133
rs705415
All − MR Egger
All − IVW(MRE)
−0.4
0.0
0.4
0.8
MR effect size for
'Selenium' on 'Netrin−G1'

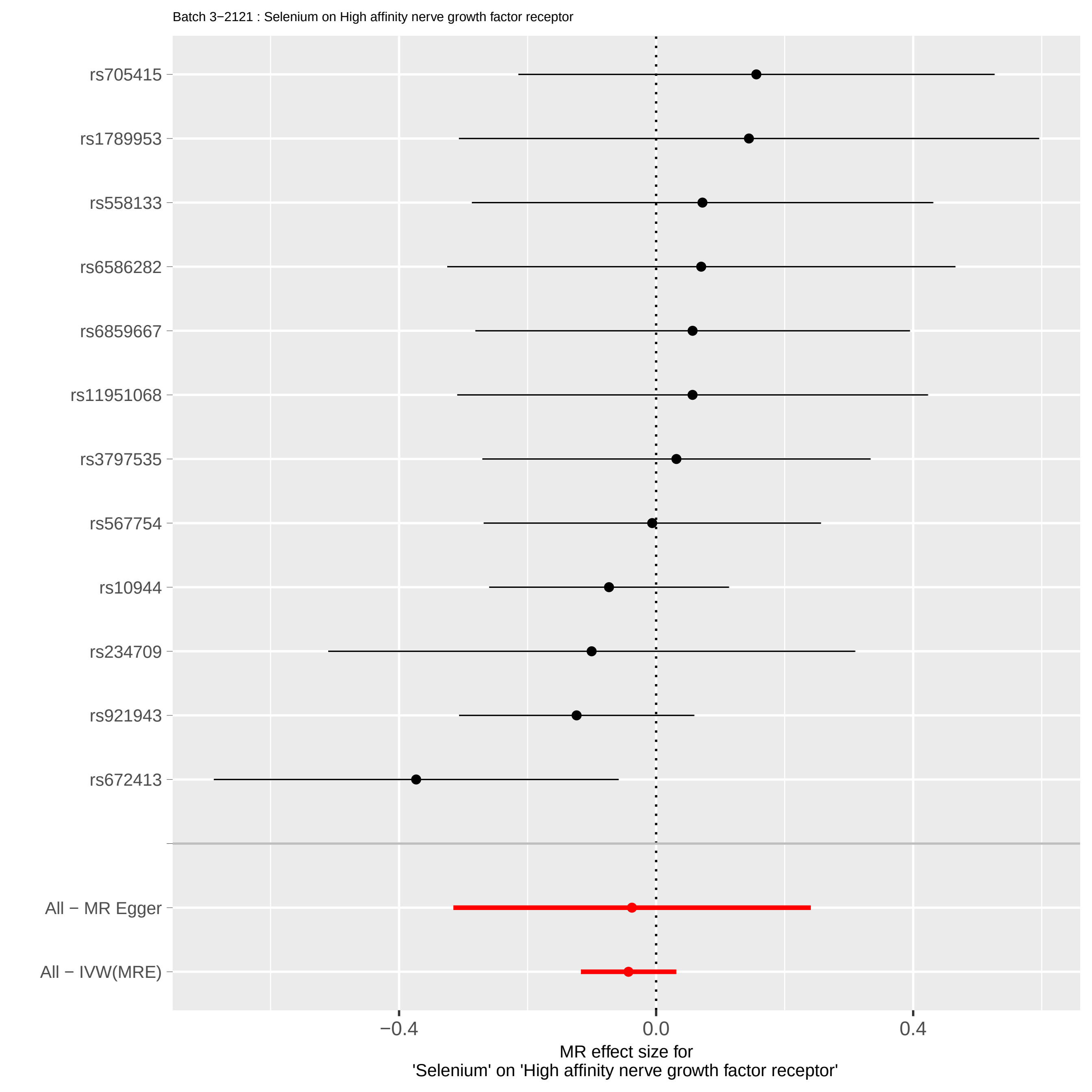

Batch 3−2121 : Selenium on High affinity nerve growth factor receptor
rs705415
rs1789953
rs558133
rs6586282
rs6859667
rs11951068
rs3797535
rs567754
rs10944
rs234709
rs921943
rs672413
All − MR Egger
All − IVW(MRE)
−0.4
0.0
0.4
MR effect size for
'Selenium' on 'High affinity nerve growth factor receptor'

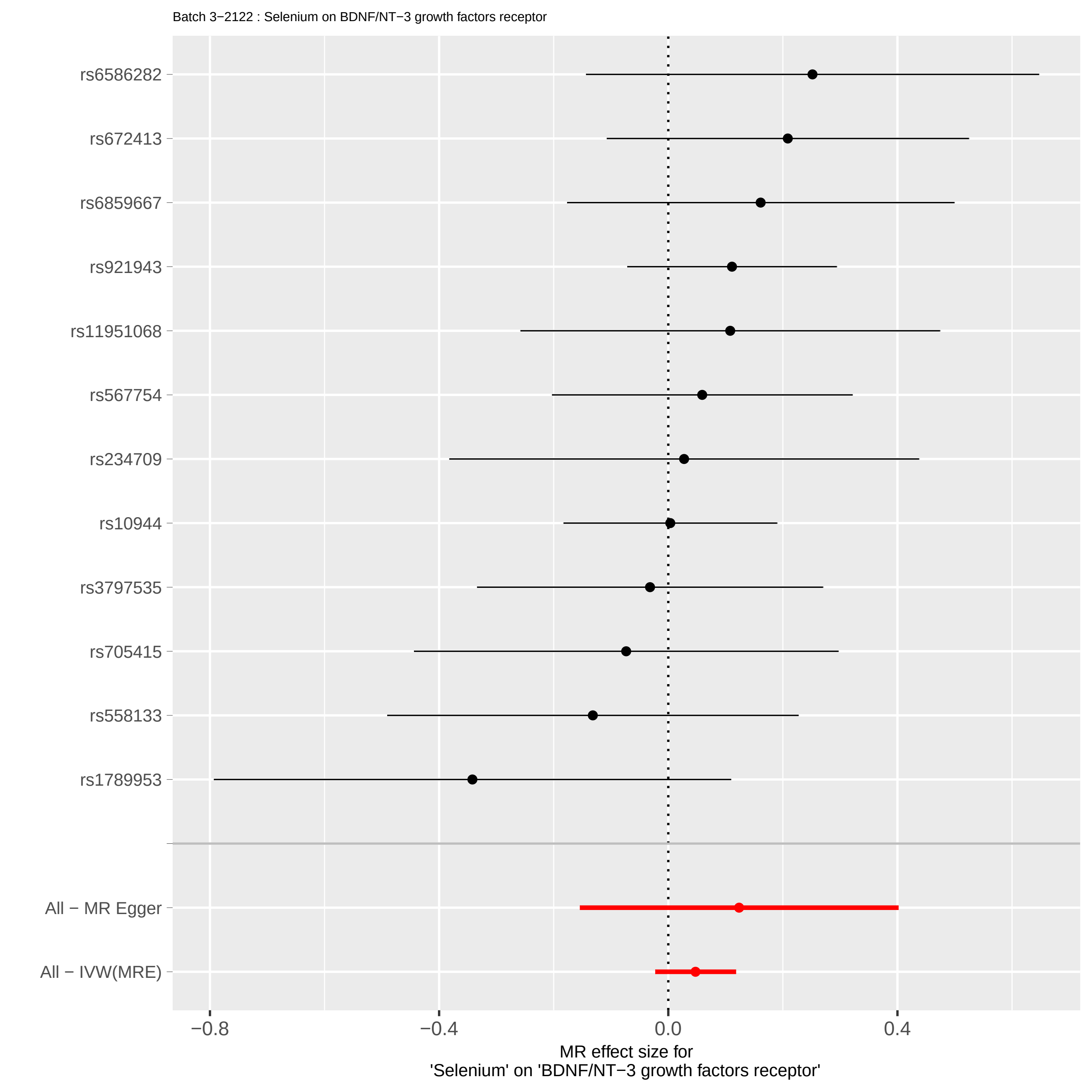

Batch 3−2122 : Selenium on BDNF/NT−3 growth factors receptor
rs6586282
rs672413
rs6859667
rs921943
rs11951068
rs567754
rs234709
rs10944
rs3797535
rs705415
rs558133
rs1789953
All − MR Egger
All − IVW(MRE)
−0.8
−0.4
0.0
0.4
MR effect size for
'Selenium' on 'BDNF/NT−3 growth factors receptor'

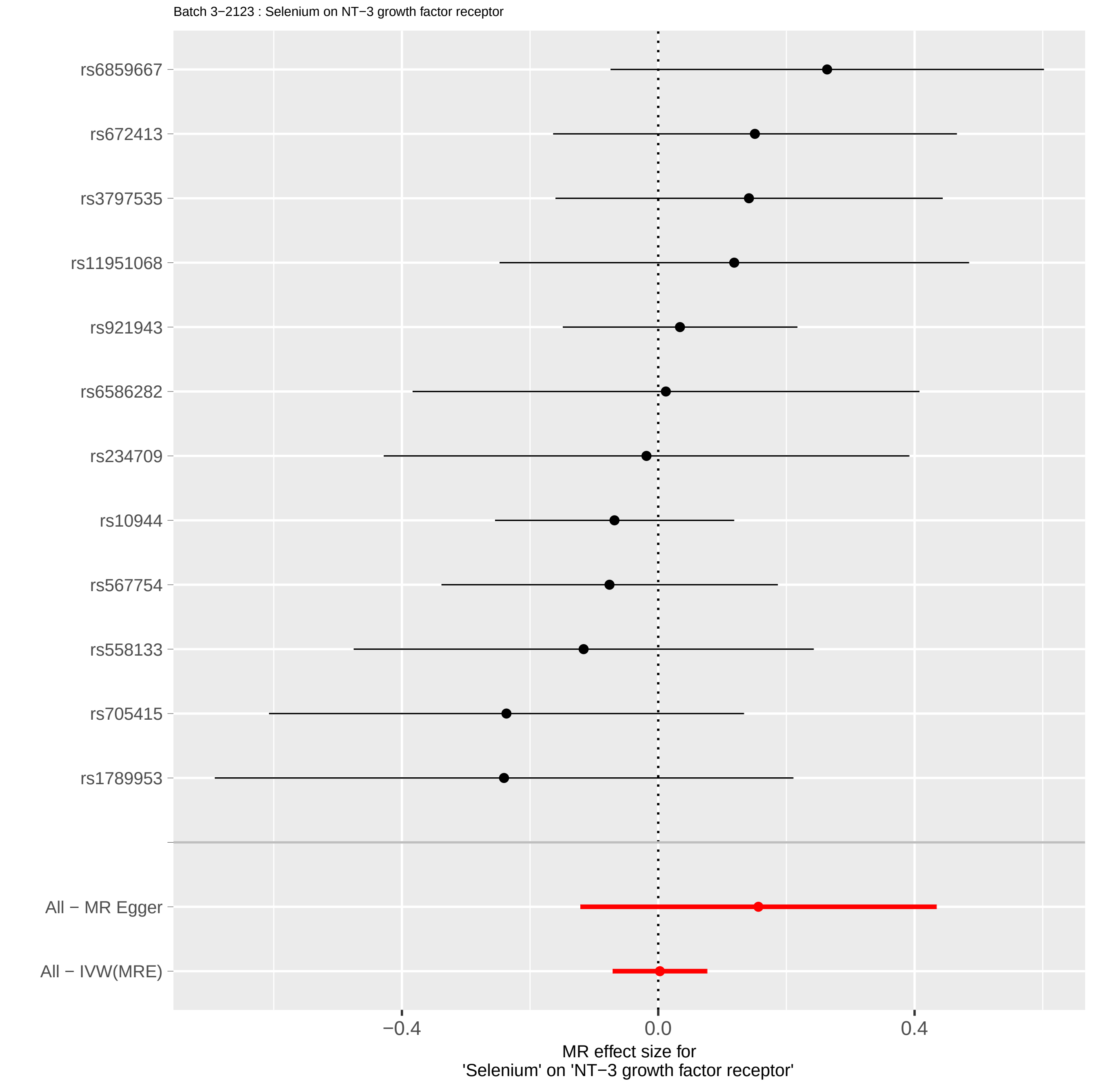

Batch 3−2123 : Selenium on NT−3 growth factor receptor
rs6859667
rs672413
rs3797535
rs11951068
rs921943
rs6586282
rs234709
rs10944
rs567754
rs558133
rs705415
rs1789953
All − MR Egger
All − IVW(MRE)
−0.4
0.0
0.4
MR effect size for
'Selenium' on 'NT−3 growth factor receptor'

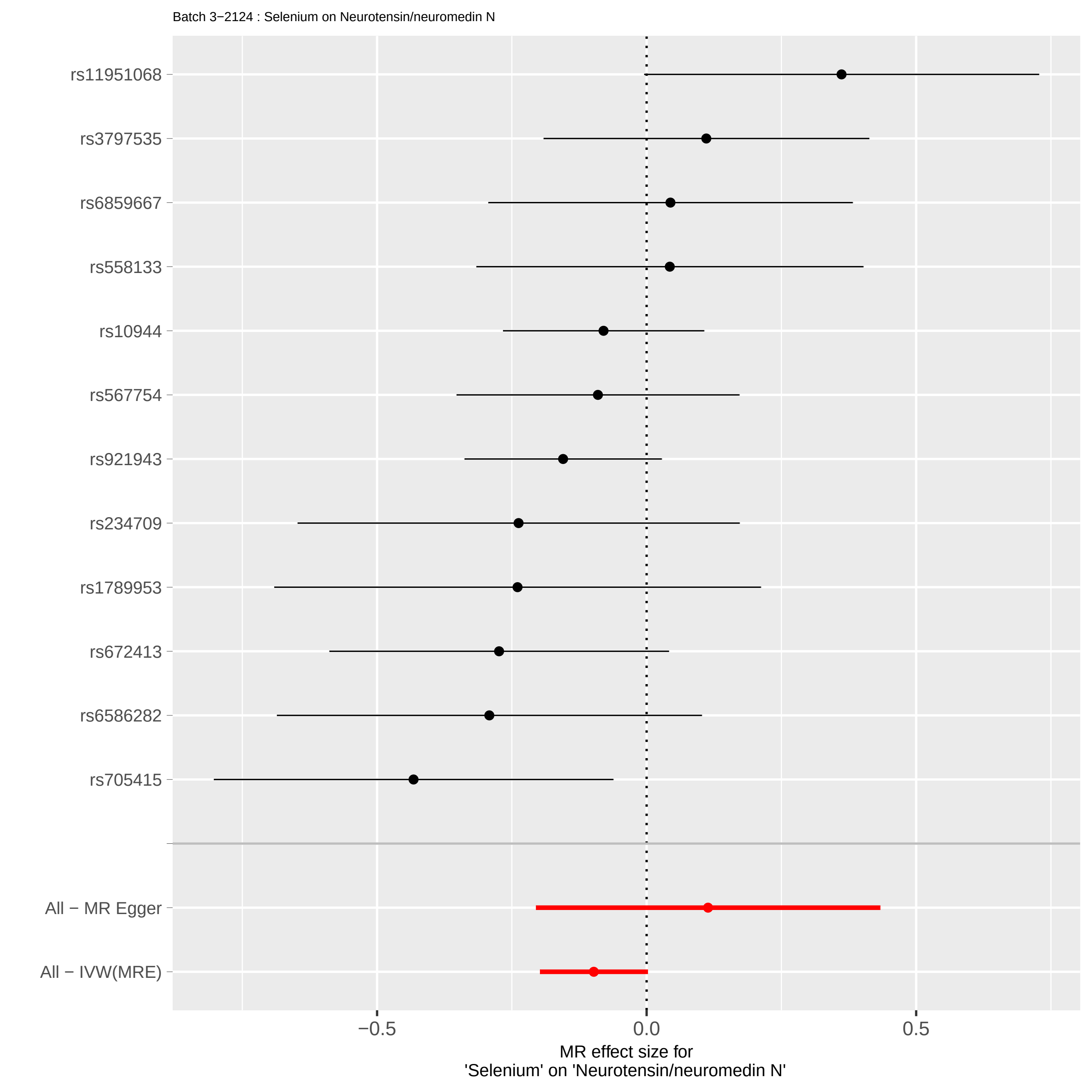
Batch 3−2124 : Selenium on Neurotensin/neuromedin N
rs11951068
rs3797535
rs6859667
rs558133
rs10944
rs567754
rs921943
rs234709
rs1789953
rs672413
rs6586282
rs705415
All − MR Egger
All − IVW(MRE)
−0.5
0.0
0.5
MR effect size for
'Selenium' on 'Neurotensin/neuromedin N'

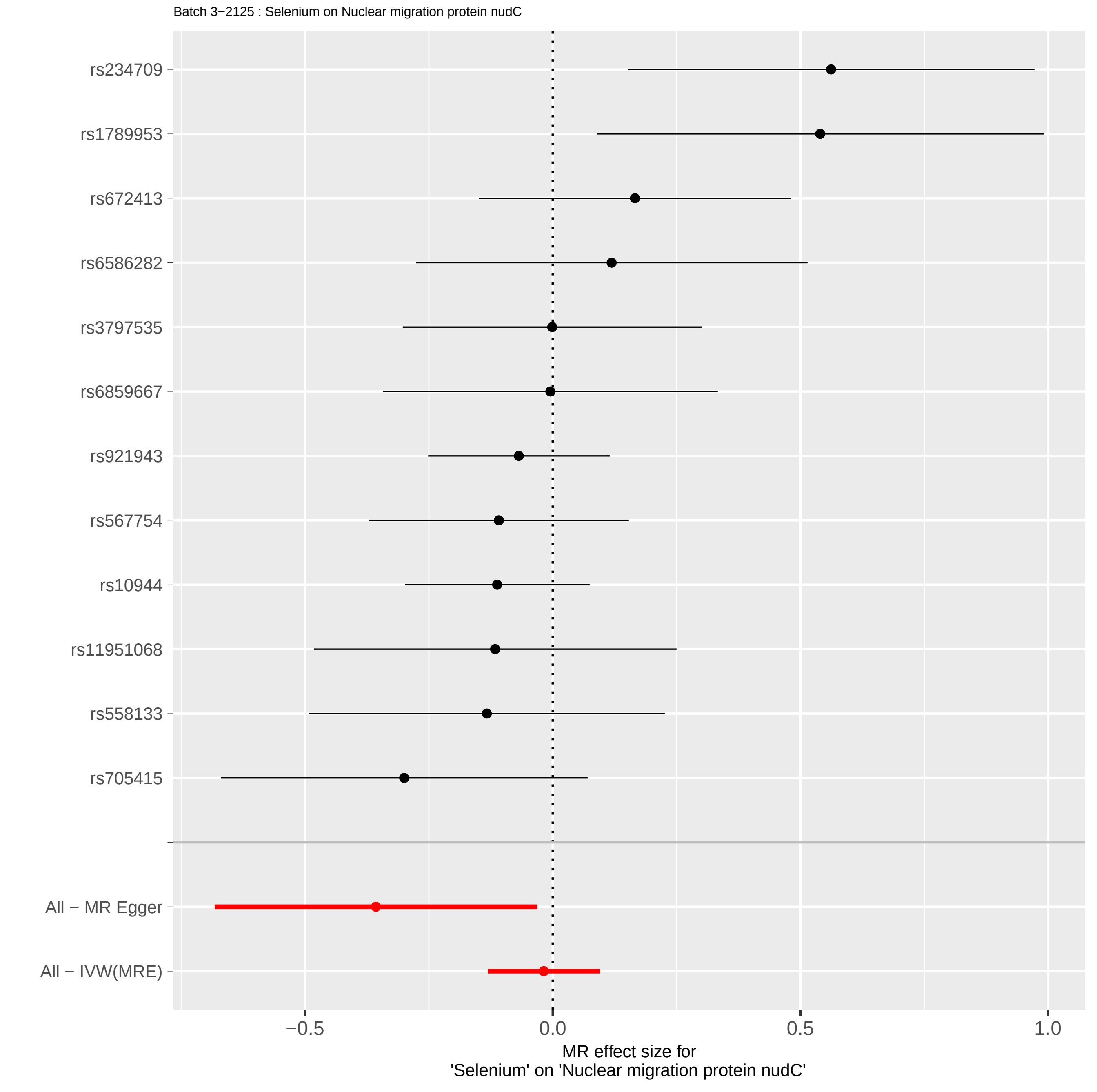
Batch 3−2125 : Selenium on Nuclear migration protein nudC
rs234709
rs1789953
rs672413
rs6586282
rs3797535
rs6859667
rs921943
rs567754
rs10944
rs11951068
rs558133
rs705415
All − MR Egger
All − IVW(MRE)
−0.5
0.0
0.5
1.0
MR effect size for
'Selenium' on 'Nuclear migration protein nudC'

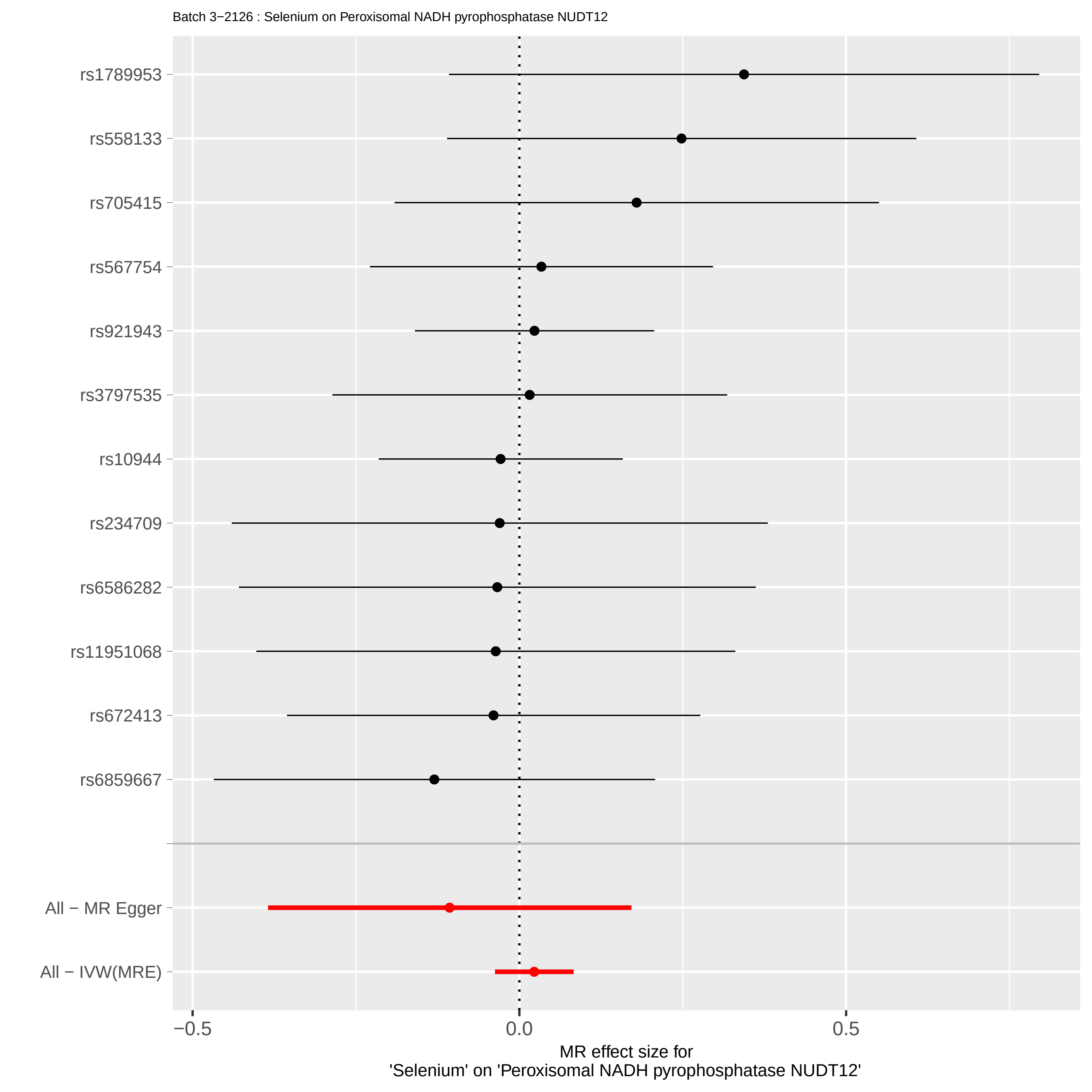

Batch 3−2126 : Selenium on Peroxisomal NADH pyrophosphatase NUDT12
rs1789953
rs558133
rs705415
rs567754
rs921943
rs3797535
rs10944
rs234709
rs6586282
rs11951068
rs672413
rs6859667
All − MR Egger
All − IVW(MRE)
−0.5
0.0
0.5
MR effect size for
'Selenium' on 'Peroxisomal NADH pyrophosphatase NUDT12'

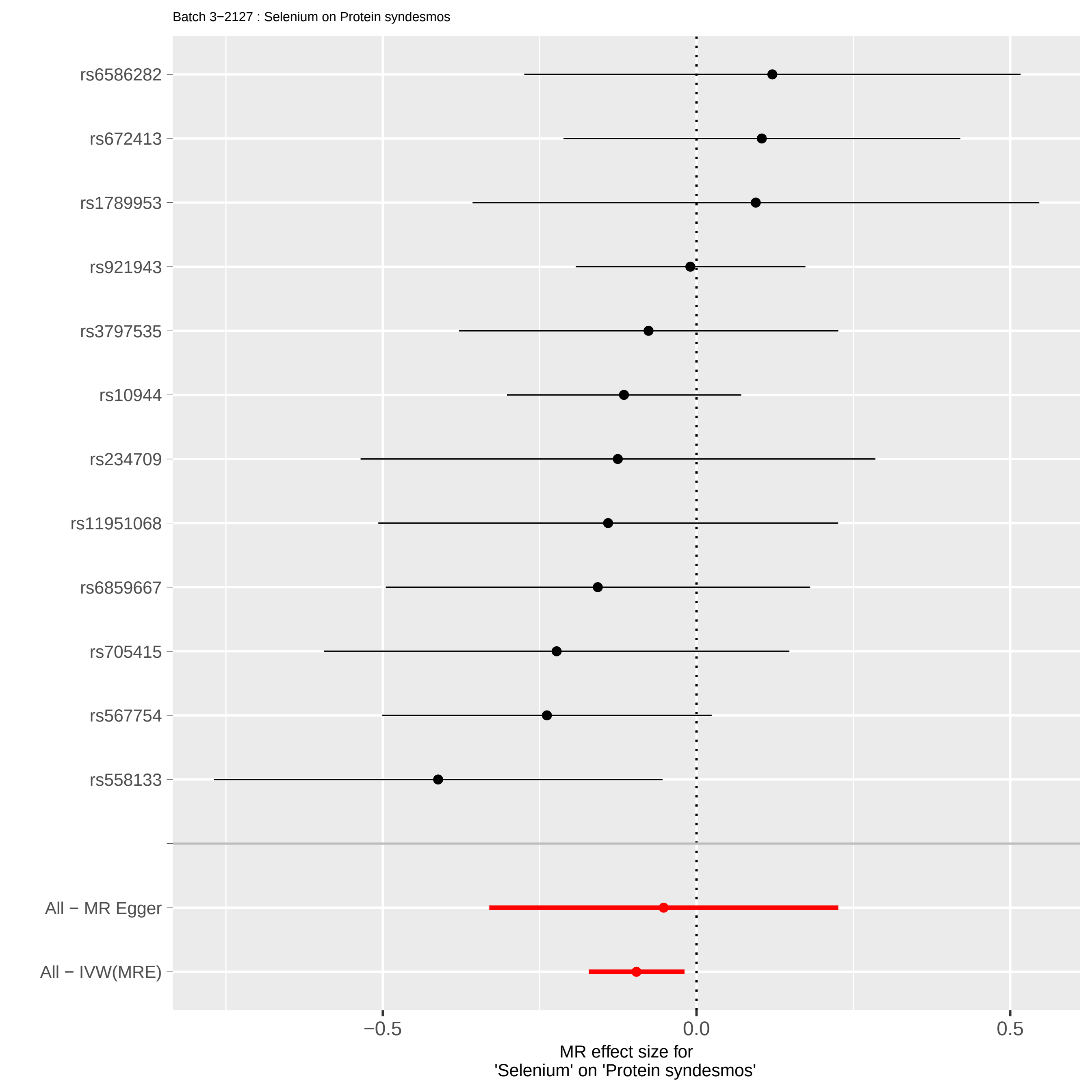
Batch 3−2127 : Selenium on Protein syndesmos
rs6586282
rs672413
rs1789953
rs921943
rs3797535
rs10944
rs234709
rs11951068
rs6859667
rs705415
rs567754
rs558133
All − MR Egger
All − IVW(MRE)
−0.5
0.0
0.5
MR effect size for
'Selenium' on 'Protein syndesmos'

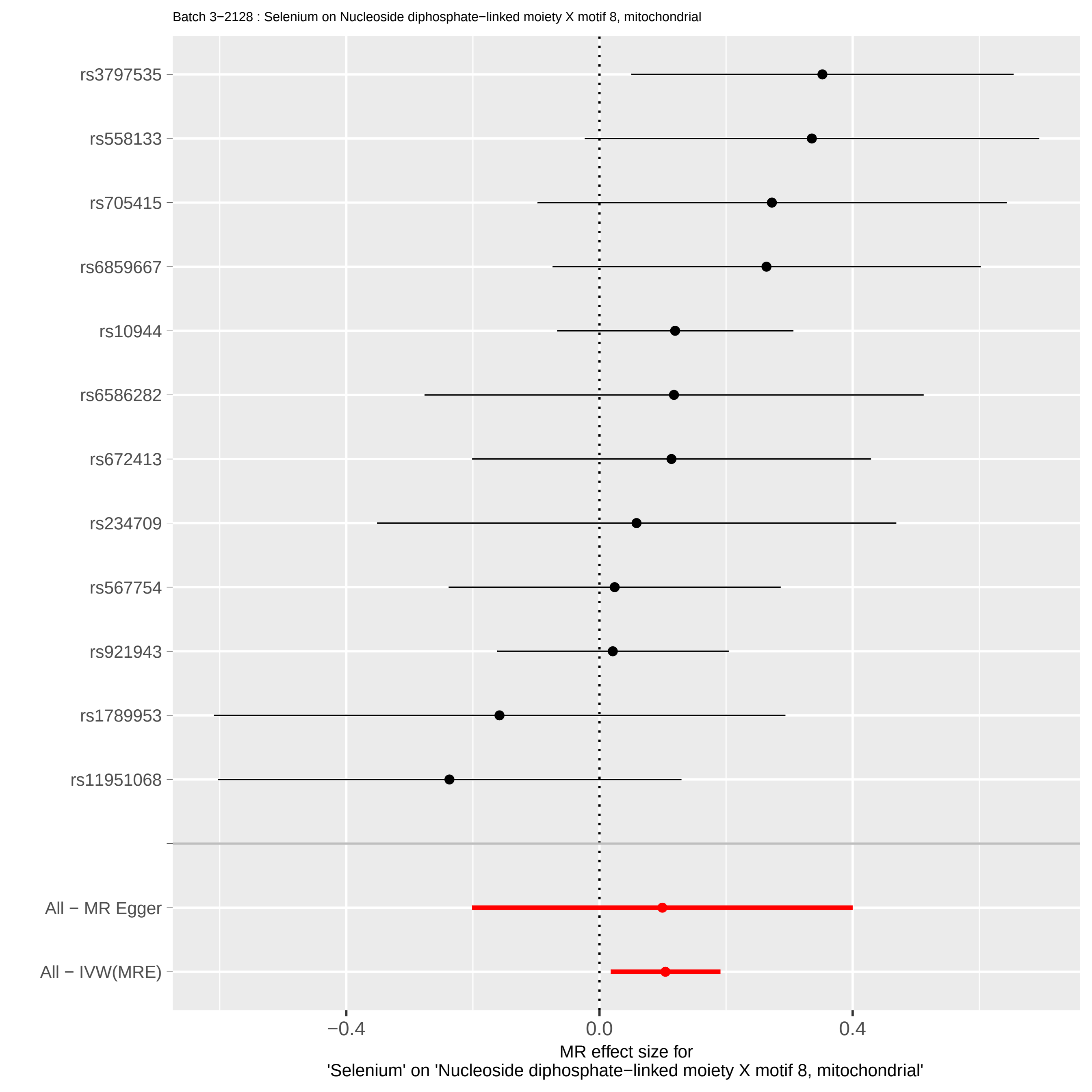

Batch 3−2128 : Selenium on Nucleoside diphosphate−linked moiety X motif 8, mitochondrial
rs3797535
rs558133
rs705415
rs6859667
rs10944
rs6586282
rs672413
rs234709
rs567754
rs921943
rs1789953
rs11951068
All − MR Egger
All − IVW(MRE)
−0.4
0.0
0.4
MR effect size for
'Selenium' on 'Nucleoside diphosphate−linked moiety X motif 8, mitochondrial'

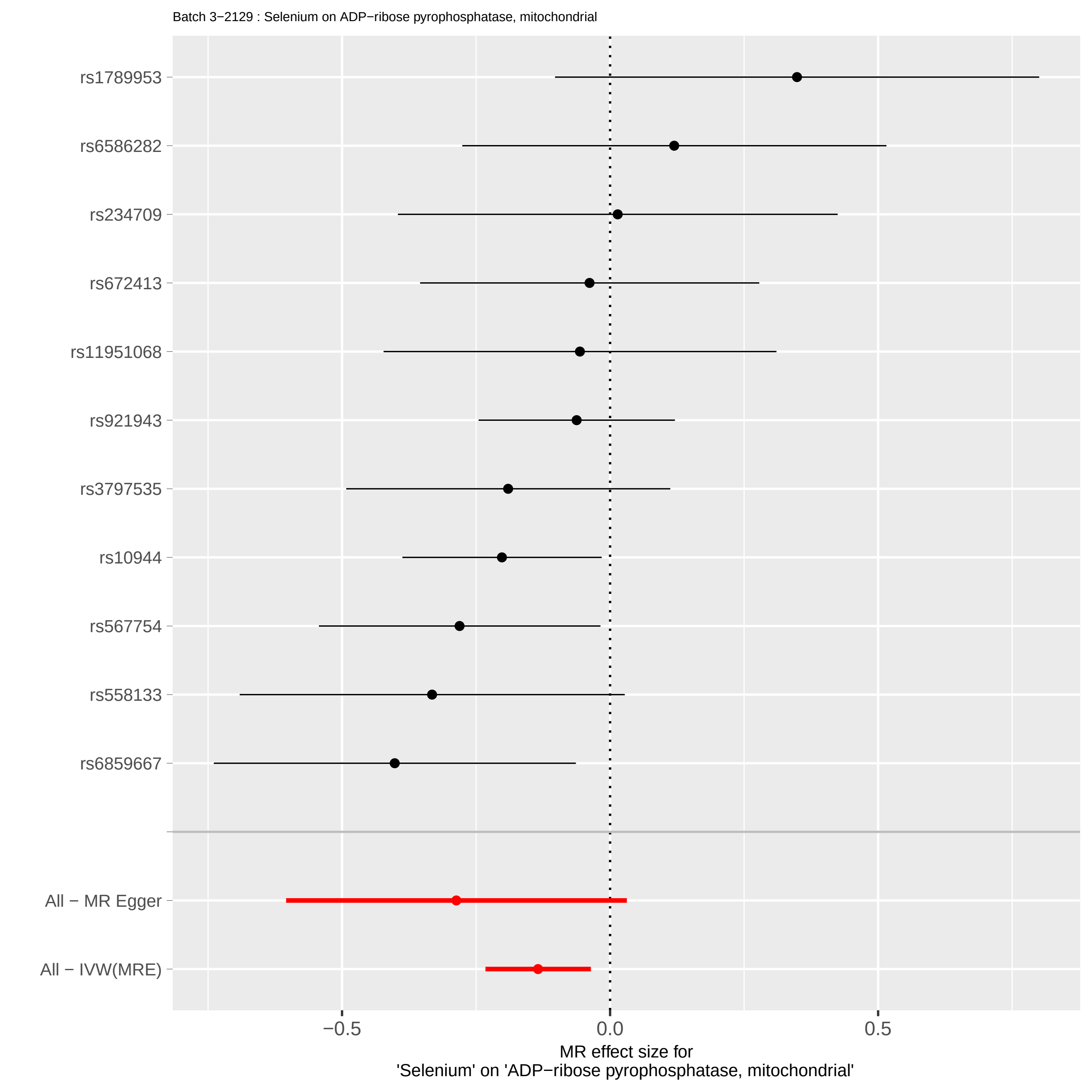

Batch 3−2129 : Selenium on ADP−ribose pyrophosphatase, mitochondrial
rs1789953
rs6586282
rs234709
rs672413
rs11951068
rs921943
rs3797535
rs10944
rs567754
rs558133
rs6859667
All − MR Egger
All − IVW(MRE)
−0.5
0.0
0.5
MR effect size for
'Selenium' on 'ADP−ribose pyrophosphatase, mitochondrial'

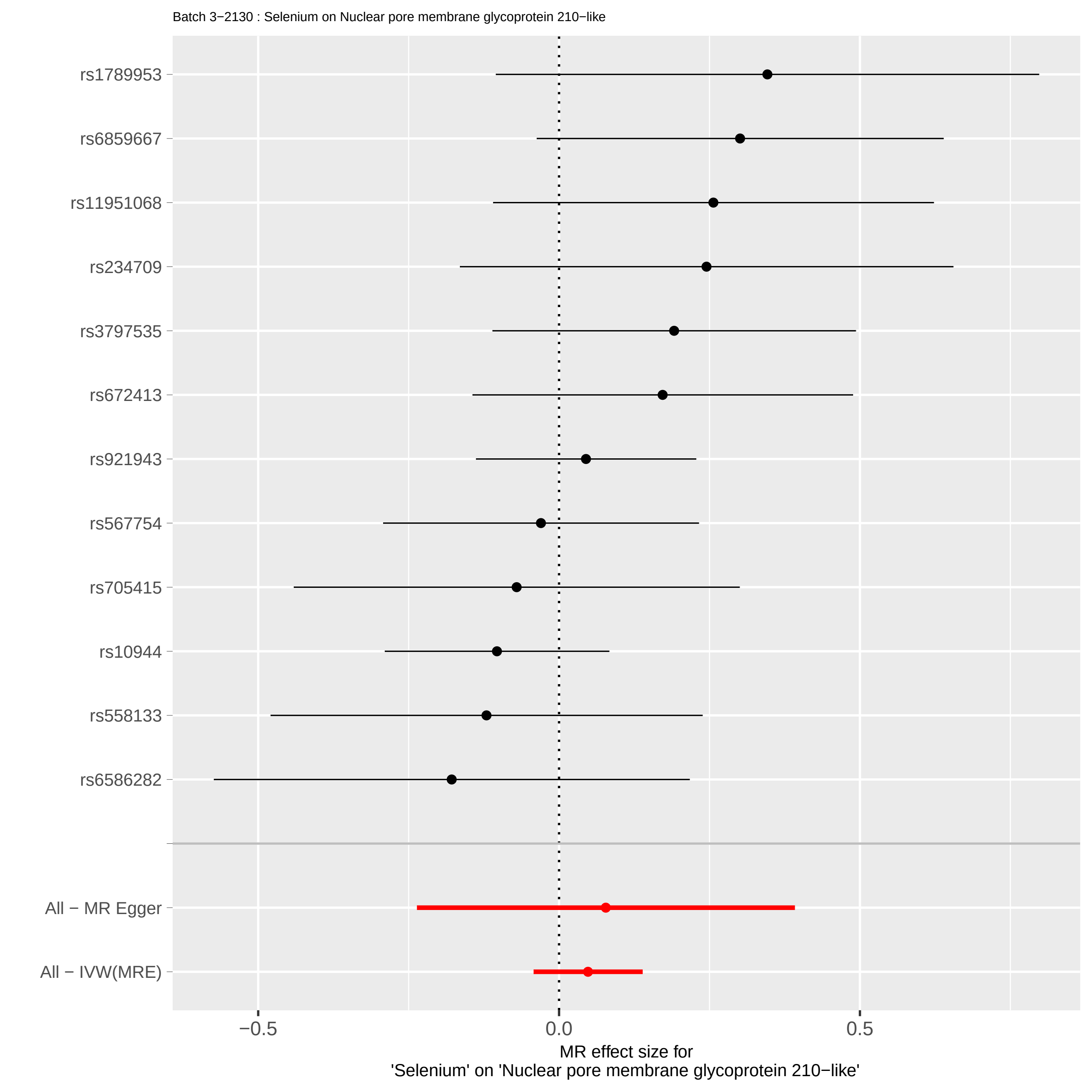

Batch 3−2130 : Selenium on Nuclear pore membrane glycoprotein 210−like
rs1789953
rs6859667
rs11951068
rs234709
rs3797535
rs672413
rs921943
rs567754
rs705415
rs10944
rs558133
rs6586282
All − MR Egger
All − IVW(MRE)
−0.5
0.0
0.5
MR effect size for
'Selenium' on 'Nuclear pore membrane glycoprotein 210−like'

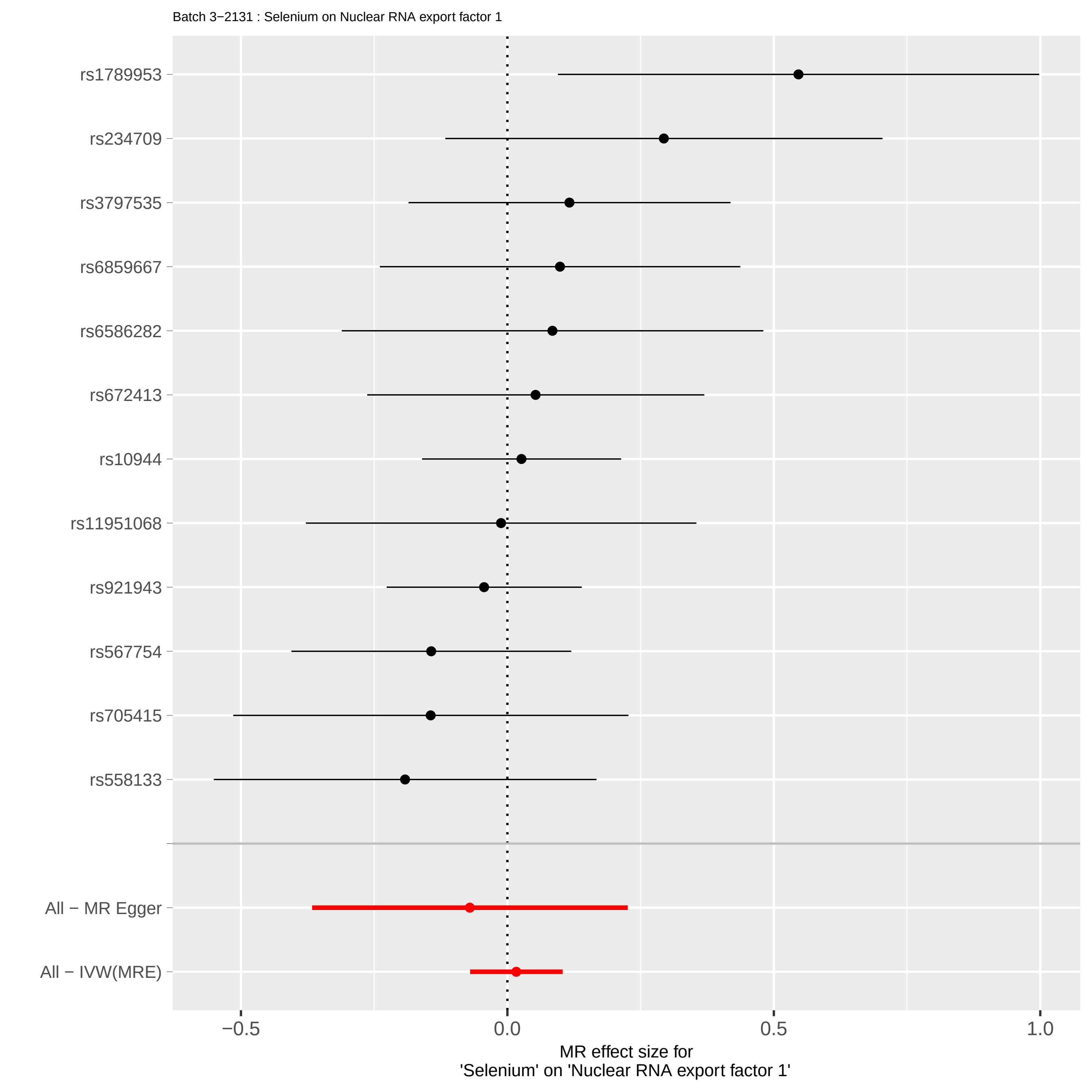

Batch 3−2131 : Selenium on Nuclear RNA export factor 1
rs1789953
rs234709
rs3797535
rs6859667
rs6586282
rs672413
rs10944
rs11951068
rs921943
rs567754
rs705415
rs558133
All − MR Egger
All − IVW(MRE)
−0.5
0.0
0.5
1.0
MR effect size for
'Selenium' on 'Nuclear RNA export factor 1'

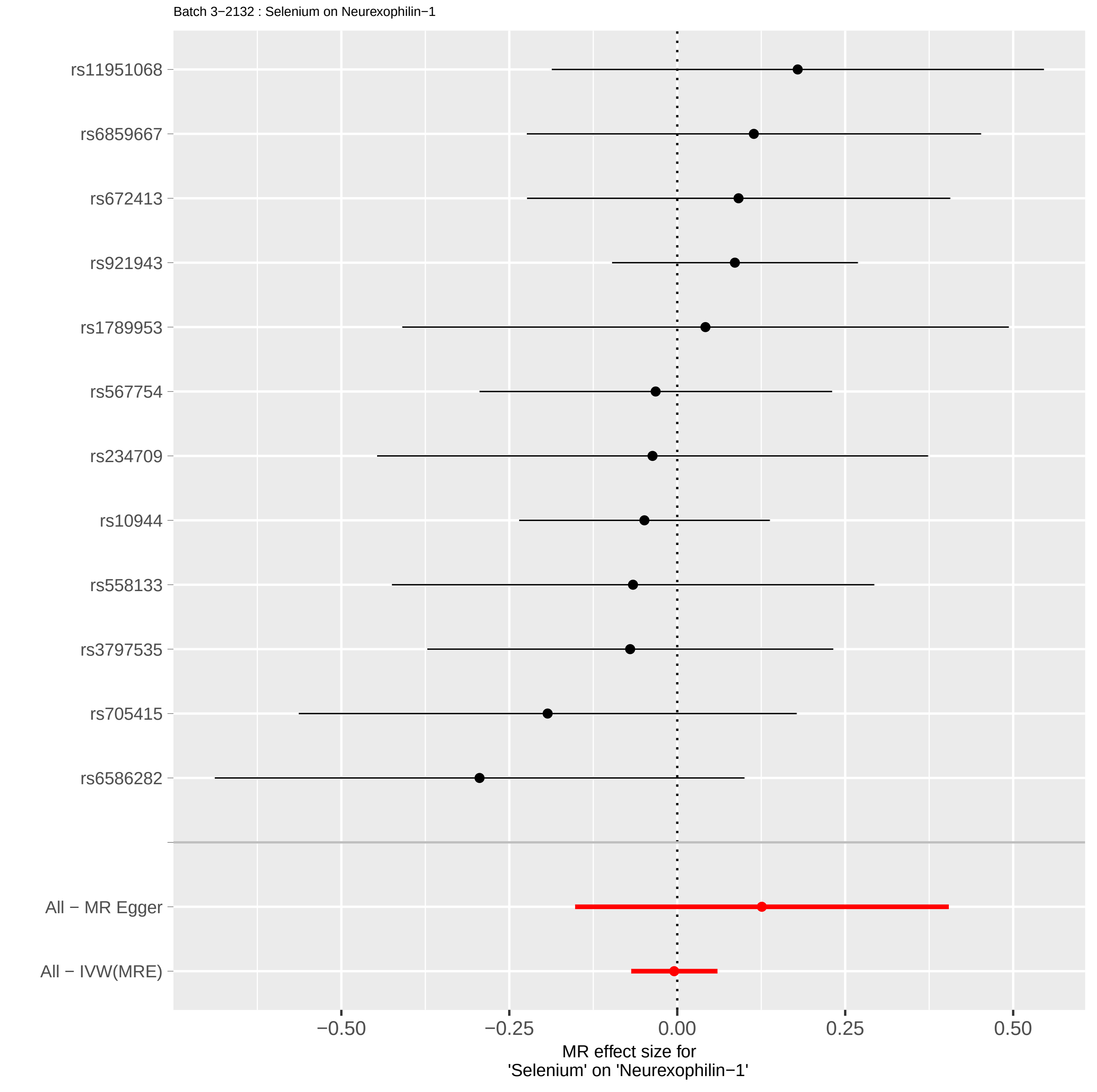
Batch 3−2132 : Selenium on Neurexophilin−1
rs11951068
rs6859667
rs672413
rs921943
rs1789953
rs567754
rs234709
rs10944
rs558133
rs3797535
rs705415
rs6586282
All − MR Egger
All − IVW(MRE)
−0.50
−0.25
0.00
0.25
0.50
MR effect size for
'Selenium' on 'Neurexophilin−1'

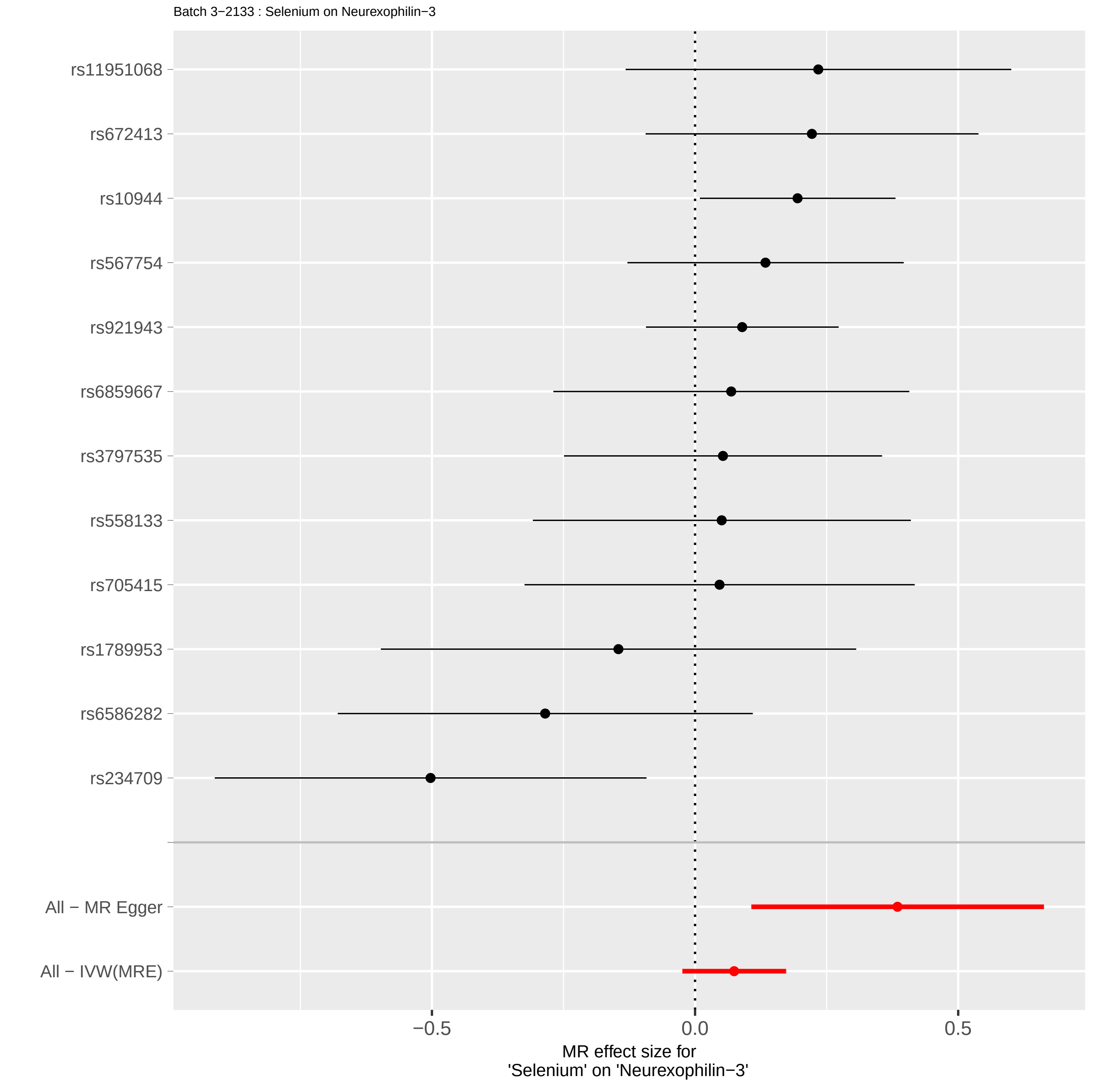
Batch 3−2133 : Selenium on Neurexophilin−3
rs11951068
rs672413
rs10944
rs567754
rs921943
rs6859667
rs3797535
rs558133
rs705415
rs1789953
rs6586282
rs234709
All − MR Egger
All − IVW(MRE)
−0.5
0.0
0.5
MR effect size for
'Selenium' on 'Neurexophilin−3'

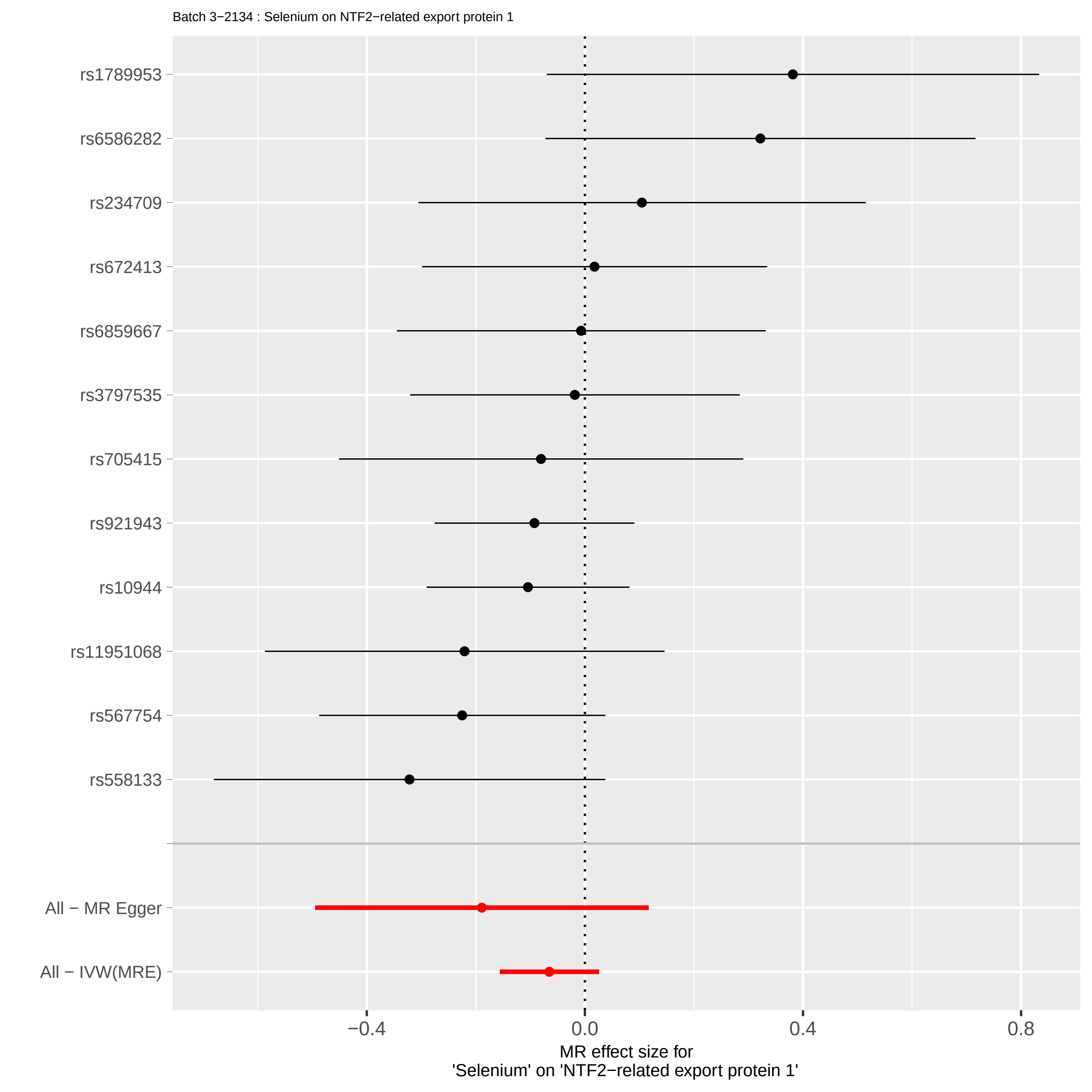

Batch 3−2134 : Selenium on NTF2−related export protein 1
rs1789953
rs6586282
rs234709
rs672413
rs6859667
rs3797535
rs705415
rs921943
rs10944
rs11951068
rs567754
rs558133
All − MR Egger
All − IVW(MRE)
−0.4
0.0
0.4
0.8
MR effect size for
'Selenium' on 'NTF2−related export protein 1'

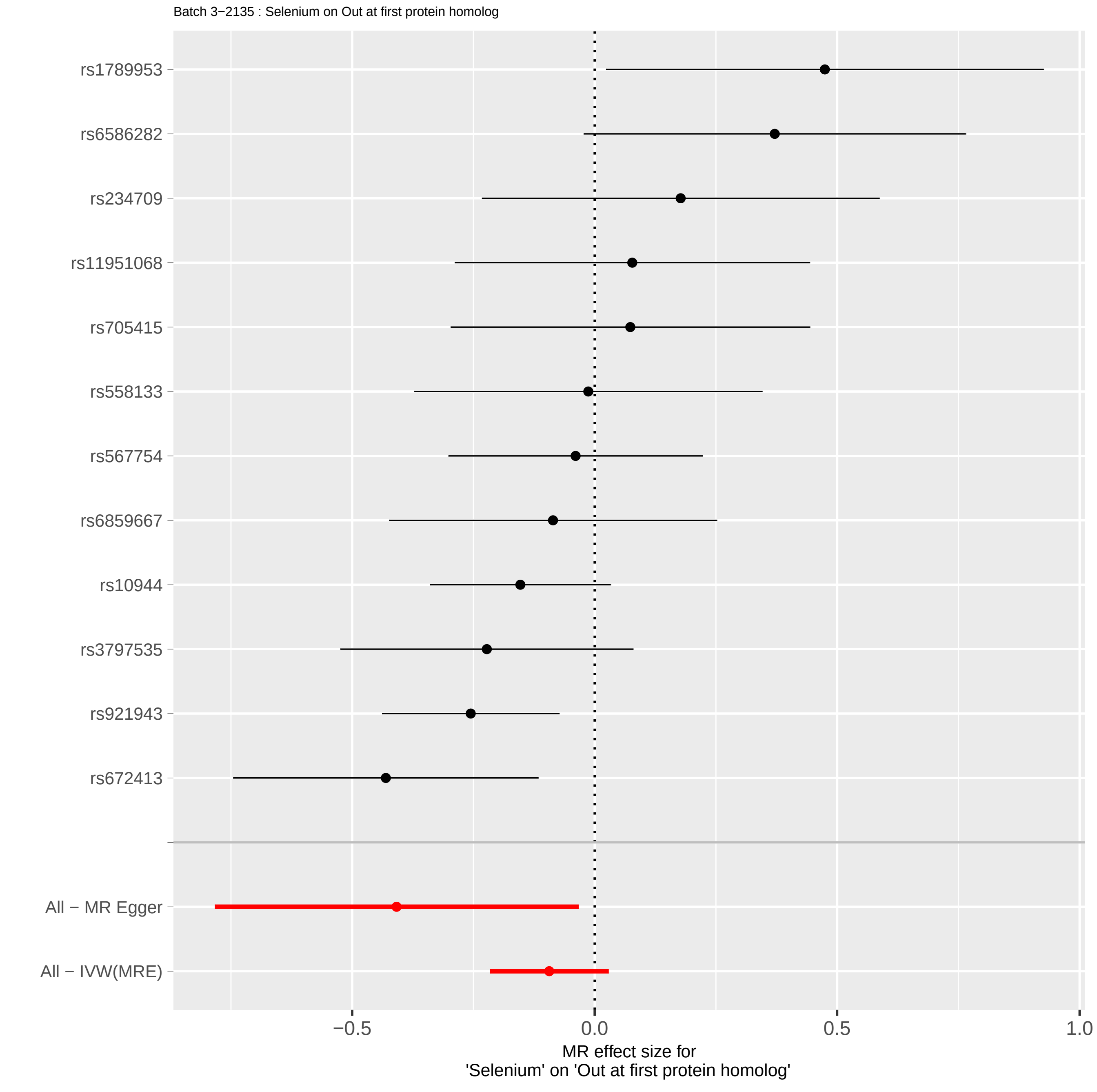

Batch 3−2135 : Selenium on Out at first protein homolog
rs1789953
rs6586282
rs234709
rs11951068
rs705415
rs558133
rs567754
rs6859667
rs10944
rs3797535
rs921943
rs672413
All − MR Egger
All − IVW(MRE)
−0.5
0.0
0.5
1.0
MR effect size for
'Selenium' on 'Out at first protein homolog'

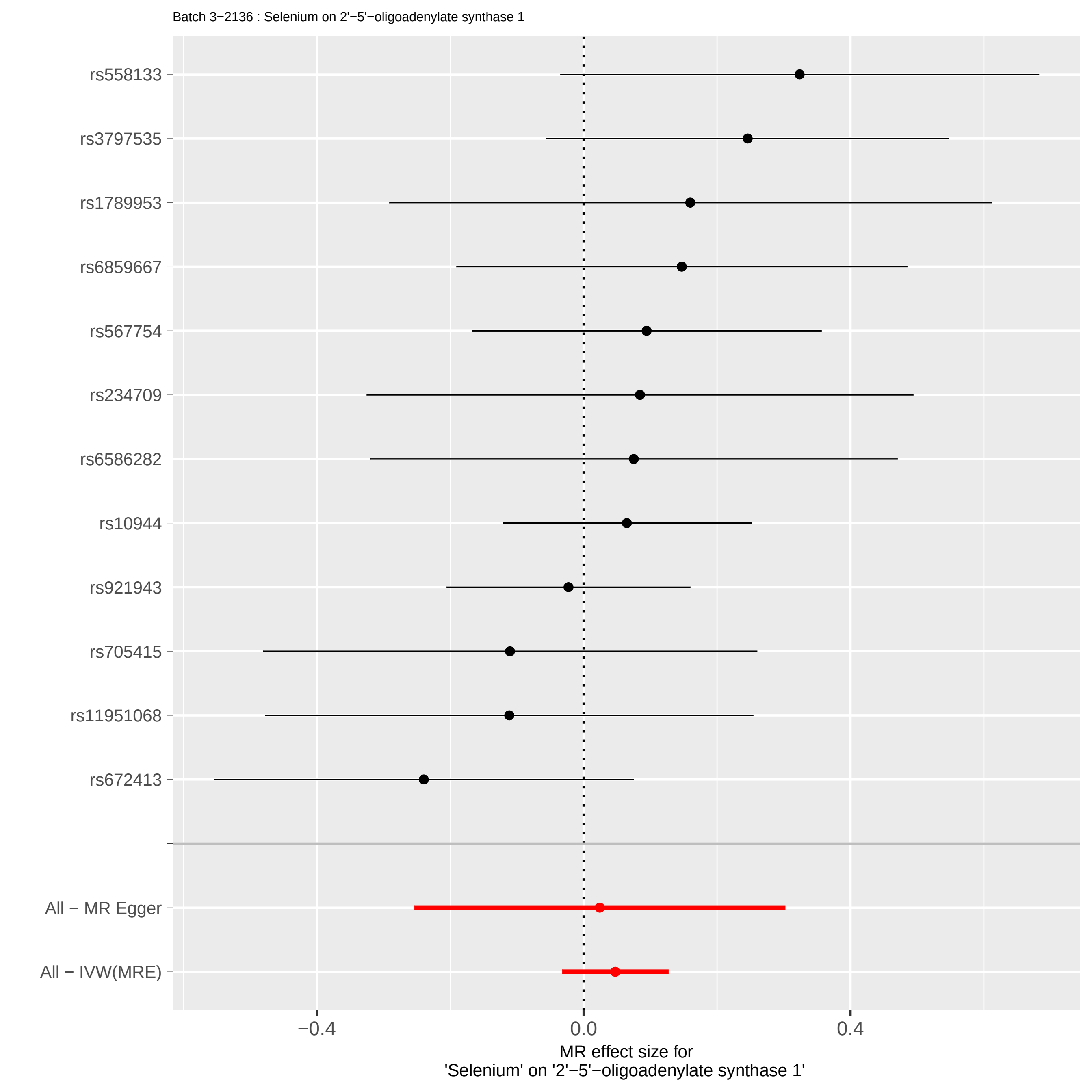

Batch 3−2136 : Selenium on 2'−5'−oligoadenylate synthase 1
rs558133
rs3797535
rs1789953
rs6859667
rs567754
rs234709
rs6586282
rs10944
rs921943
rs705415
rs11951068
rs672413
All − MR Egger
All − IVW(MRE)
−0.4
0.0
0.4
MR effect size for
'Selenium' on '2'−5'−oligoadenylate synthase 1'

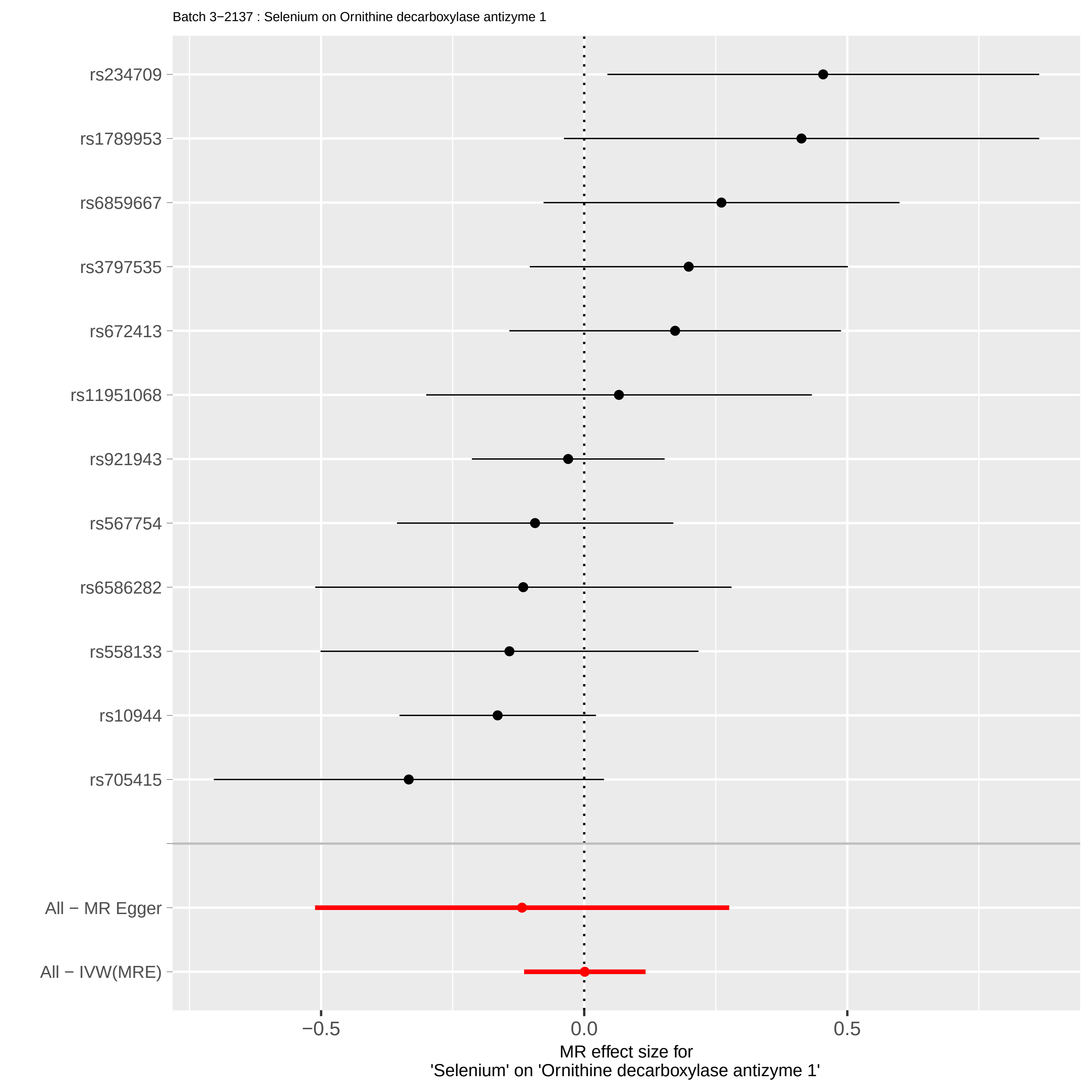
Batch 3−2137 : Selenium on Ornithine decarboxylase antizyme 1
rs234709
rs1789953
rs6859667
rs3797535
rs672413
rs11951068
rs921943
rs567754
rs6586282
rs558133
rs10944
rs705415
All − MR Egger
All − IVW(MRE)
−0.5
0.0
0.5
MR effect size for
'Selenium' on 'Ornithine decarboxylase antizyme 1'

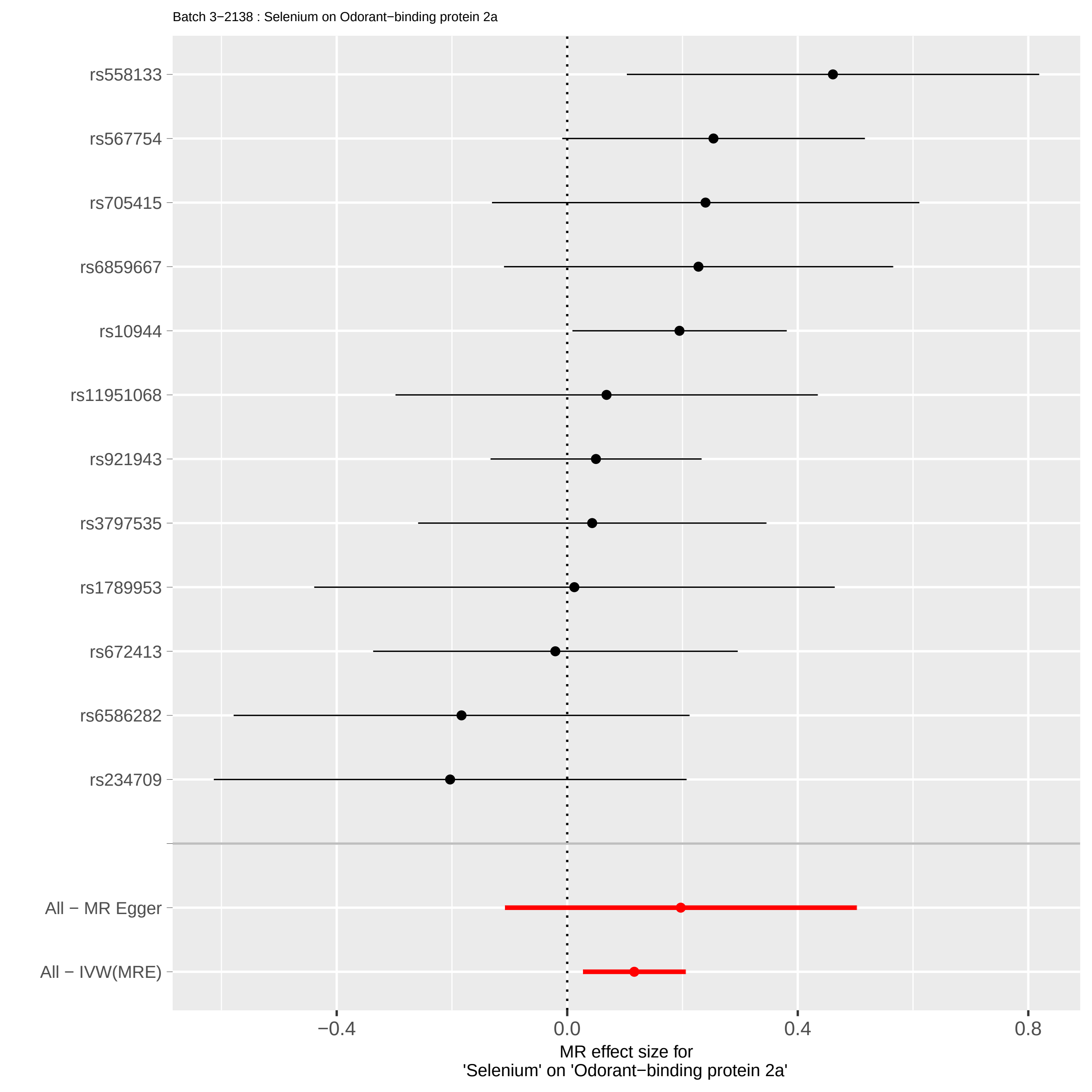

Batch 3−2138 : Selenium on Odorant−binding protein 2a
rs558133
rs567754
rs705415
rs6859667
rs10944
rs11951068
rs921943
rs3797535
rs1789953
rs672413
rs6586282
rs234709
All − MR Egger
All − IVW(MRE)
−0.4
0.0
0.4
0.8
MR effect size for
'Selenium' on 'Odorant−binding protein 2a'

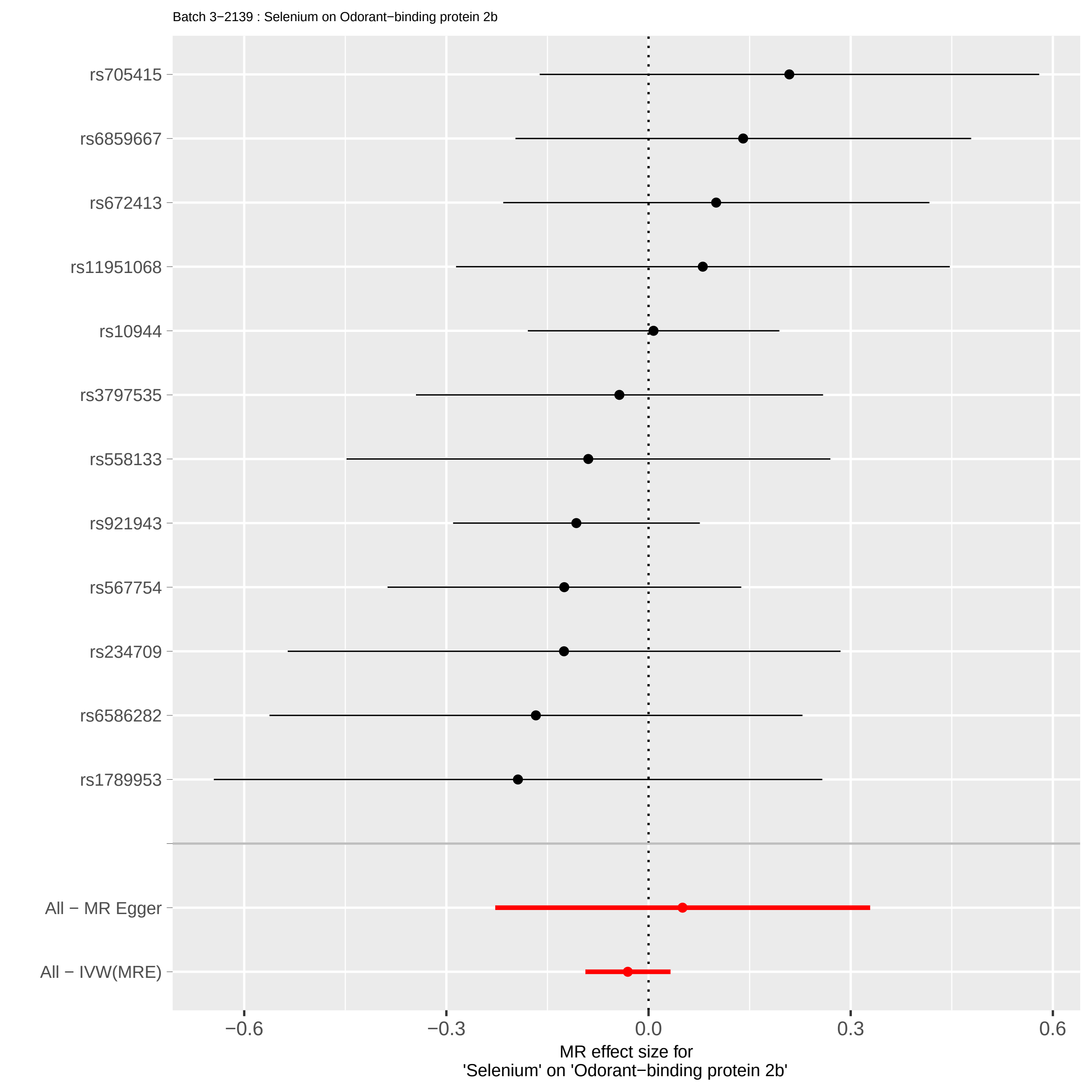
Batch 3−2139 : Selenium on Odorant−binding protein 2b
rs705415
rs6859667
rs672413
rs11951068
rs10944
rs3797535
rs558133
rs921943
rs567754
rs234709
rs6586282
rs1789953
All − MR Egger
All − IVW(MRE)
−0.6
−0.3
0.0
0.3
0.6
MR effect size for
'Selenium' on 'Odorant−binding protein 2b'

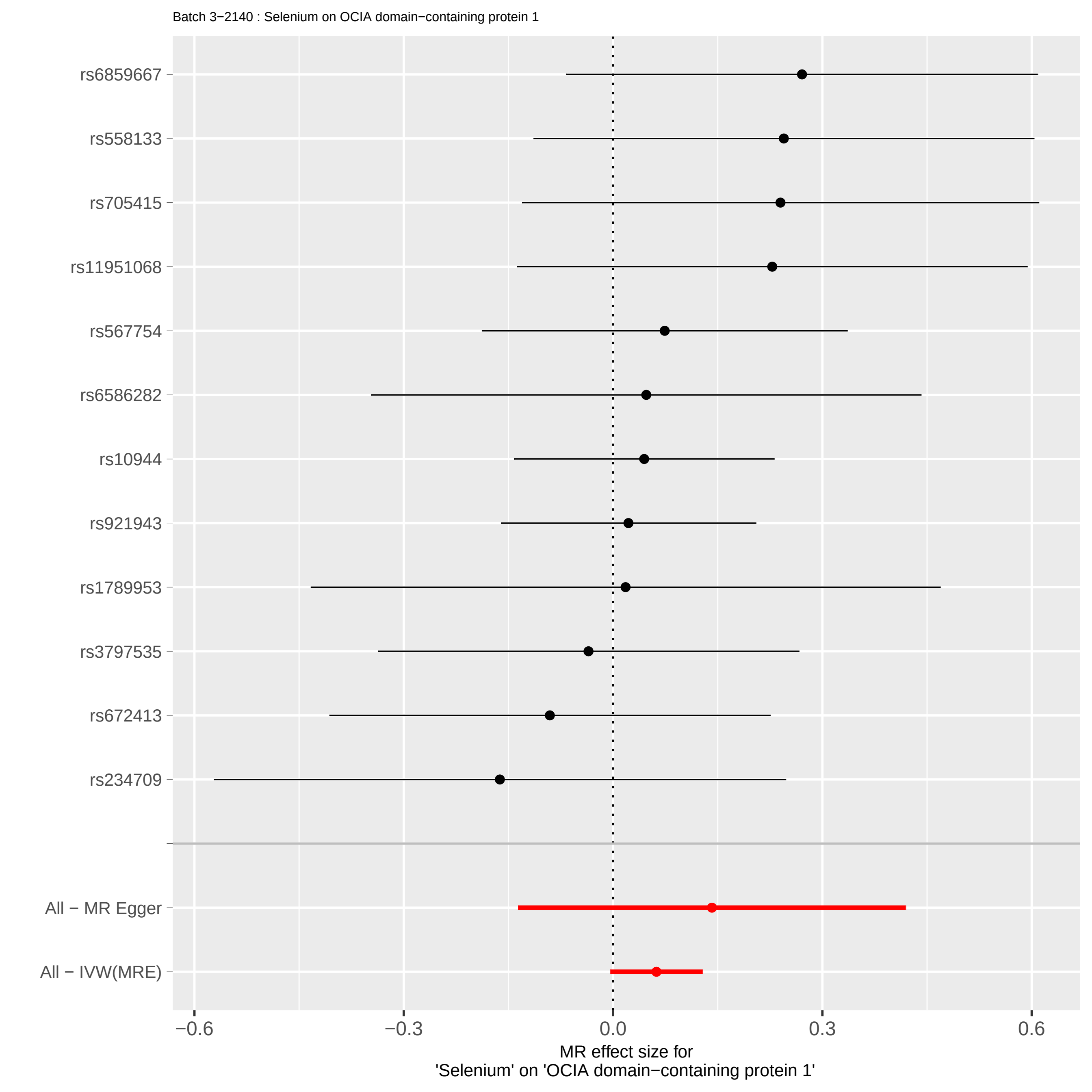

Batch 3−2140 : Selenium on OCIA domain−containing protein 1
rs6859667
rs558133
rs705415
rs11951068
rs567754
rs6586282
rs10944
rs921943
rs1789953
rs3797535
rs672413
rs234709
All − MR Egger
All − IVW(MRE)
−0.6
−0.3
0.0
0.3
0.6
MR effect size for
'Selenium' on 'OCIA domain−containing protein 1'

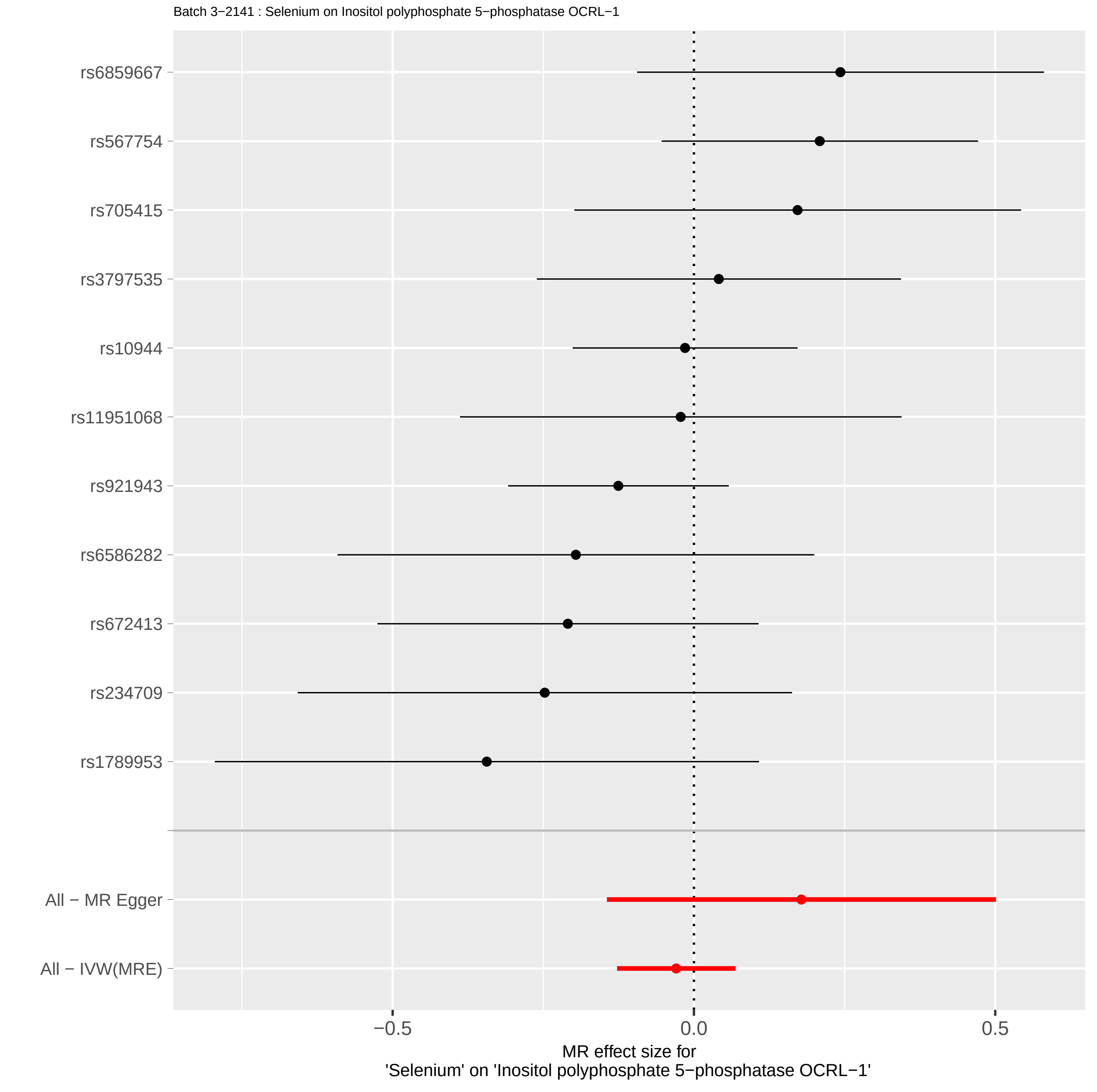

Batch 3−2141 : Selenium on Inositol polyphosphate 5−phosphatase OCRL−1
rs6859667
rs567754
rs705415
rs3797535
rs10944
rs11951068
rs921943
rs6586282
rs672413
rs234709
rs1789953
All − MR Egger
All − IVW(MRE)
−0.5
0.0
0.5
MR effect size for
'Selenium' on 'Inositol polyphosphate 5−phosphatase OCRL−1'

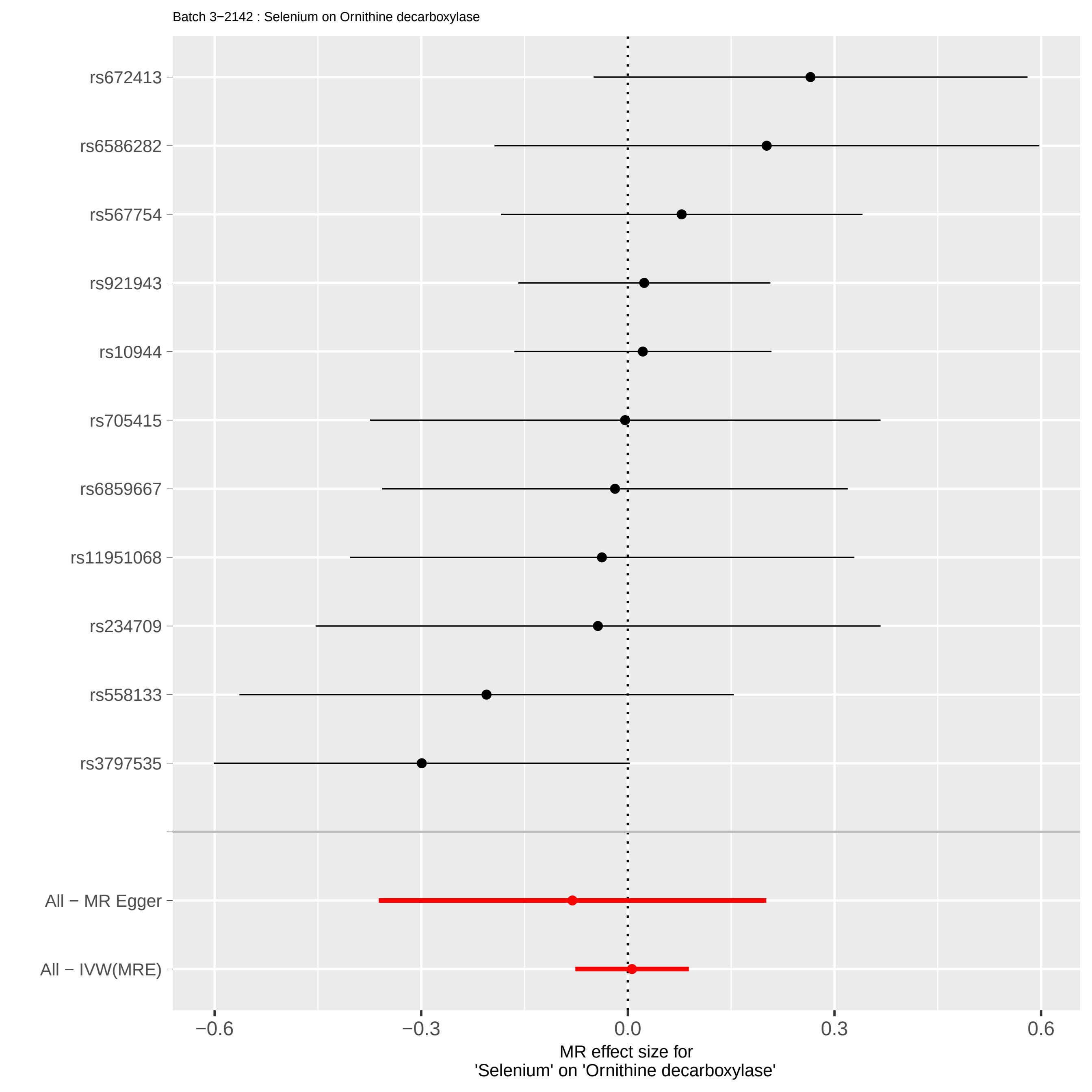

Batch 3−2142 : Selenium on Ornithine decarboxylase
rs672413
rs6586282
rs567754
rs921943
rs10944
rs705415
rs6859667
rs11951068
rs234709
rs558133
rs3797535
All − MR Egger
All − IVW(MRE)
−0.6
−0.3
0.0
0.3
0.6
MR effect size for
'Selenium' on 'Ornithine decarboxylase'

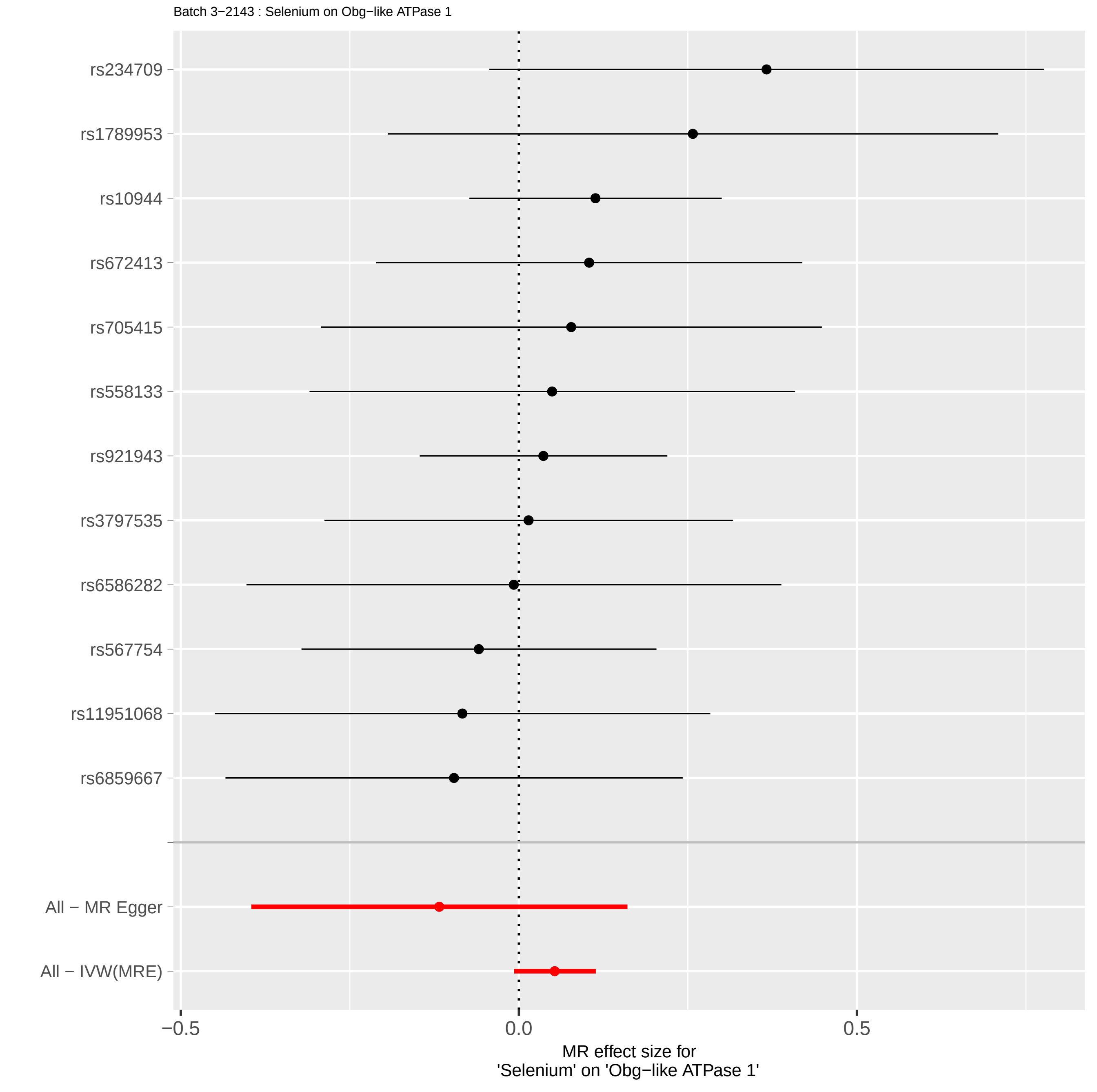
Batch 3−2143 : Selenium on Obg−like ATPase 1
rs234709
rs1789953
rs10944
rs672413
rs705415
rs558133
rs921943
rs3797535
rs6586282
rs567754
rs11951068
rs6859667
All − MR Egger
All − IVW(MRE)
−0.5
0.0
0.5
MR effect size for
'Selenium' on 'Obg−like ATPase 1'

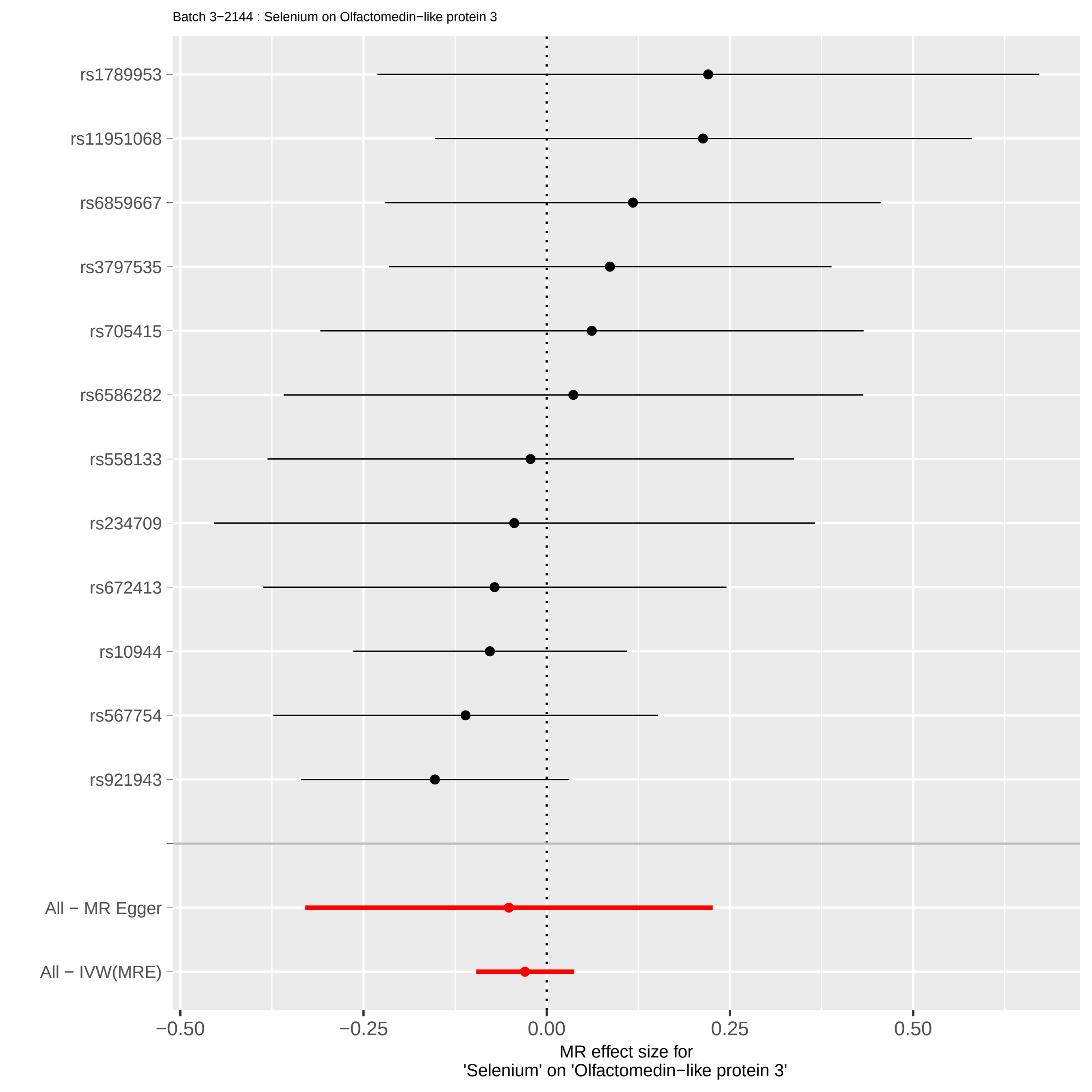

Batch 3−2144 : Selenium on Olfactomedin−like protein 3
rs1789953
rs11951068
rs6859667
rs3797535
rs705415
rs6586282
rs558133
rs234709
rs672413
rs10944
rs567754
rs921943
All − MR Egger
All − IVW(MRE)
−0.50
−0.25
0.00
0.25
0.50
MR effect size for
'Selenium' on 'Olfactomedin−like protein 3'

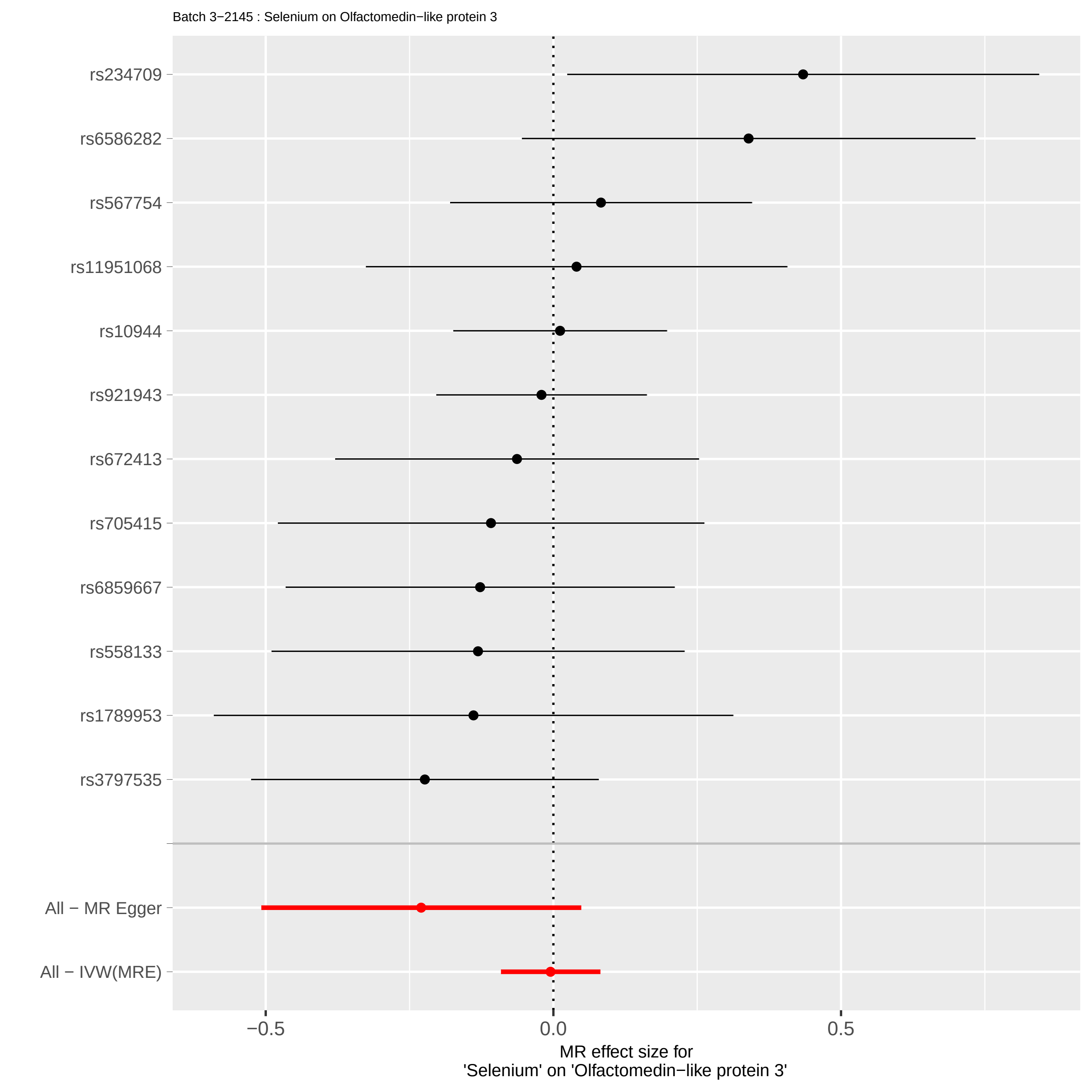

Batch 3−2145 : Selenium on Olfactomedin−like protein 3
rs234709
rs6586282
rs567754
rs11951068
rs10944
rs921943
rs672413
rs705415
rs6859667
rs558133
rs1789953
rs3797535
All − MR Egger
All − IVW(MRE)
−0.5
0.0
0.5
MR effect size for
'Selenium' on 'Olfactomedin−like protein 3'

Batch 3−2146 : Selenium on Oxidized low−density lipoprotein receptor 1

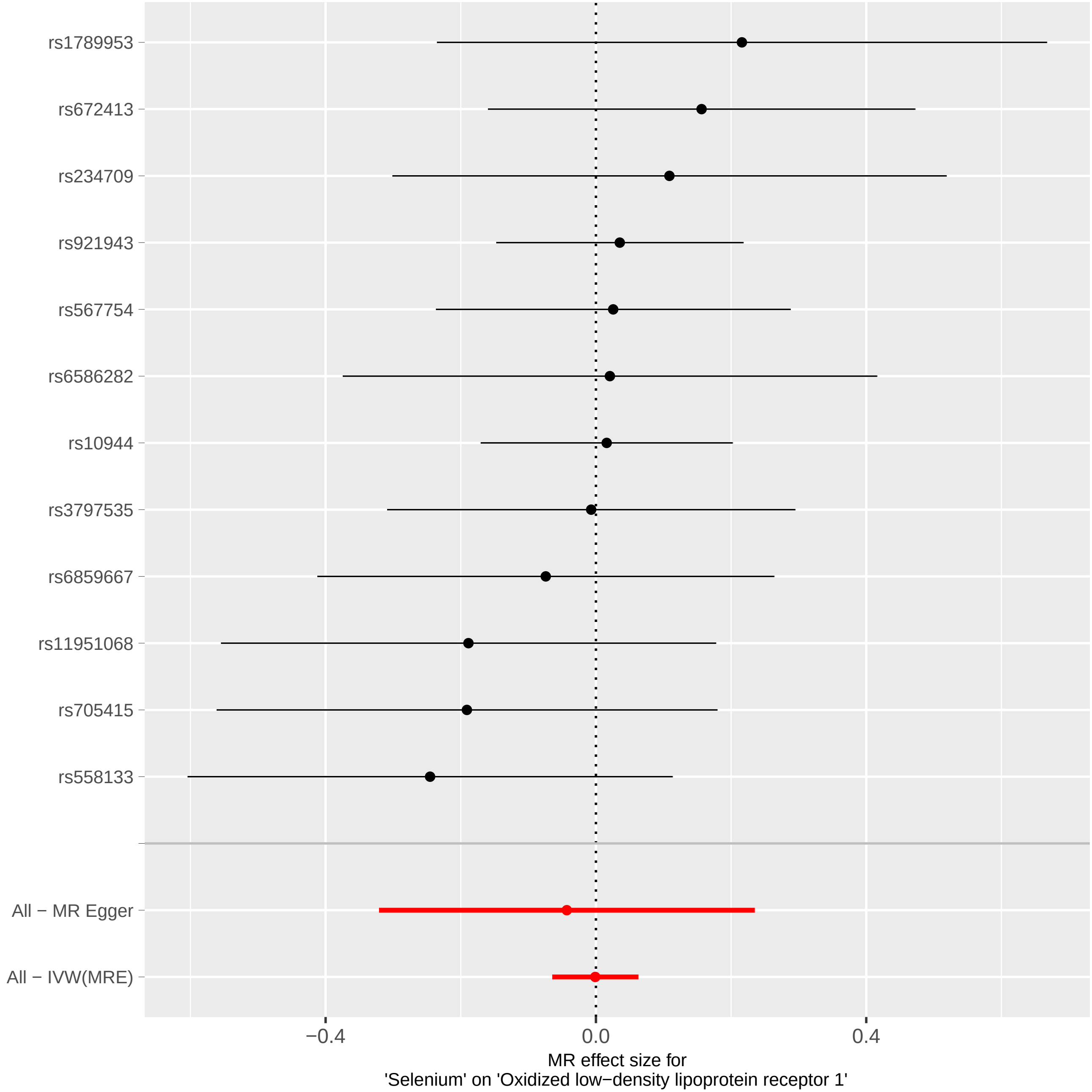

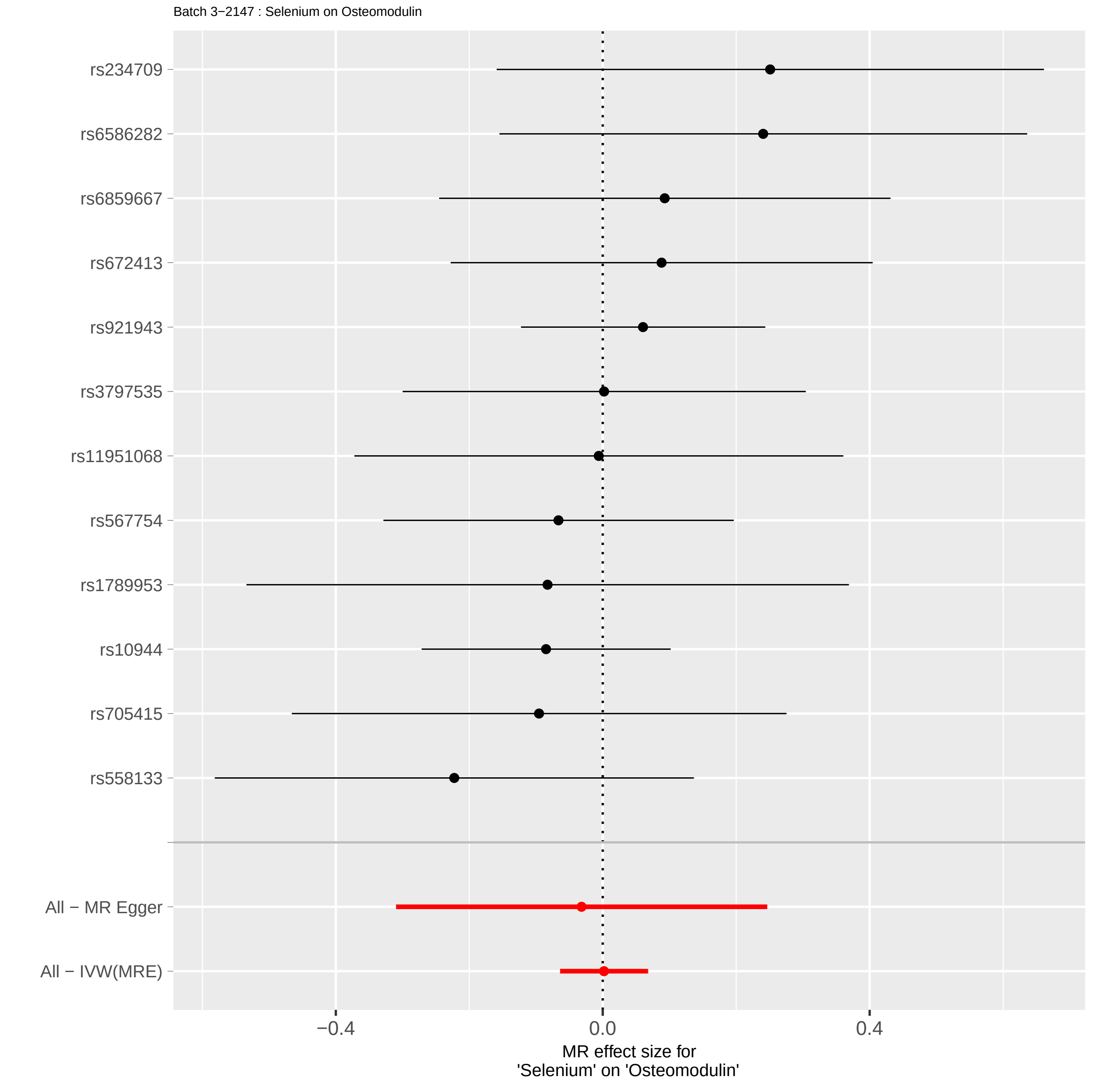

Batch 3−2147 : Selenium on Osteomodulin
rs234709
rs6586282
rs6859667
rs672413
rs921943
rs3797535
rs11951068
rs567754
rs1789953
rs10944
rs705415
rs558133
All − MR Egger
All − IVW(MRE)
−0.4
0.0
0.4
MR effect size for
'Selenium' on 'Osteomodulin'

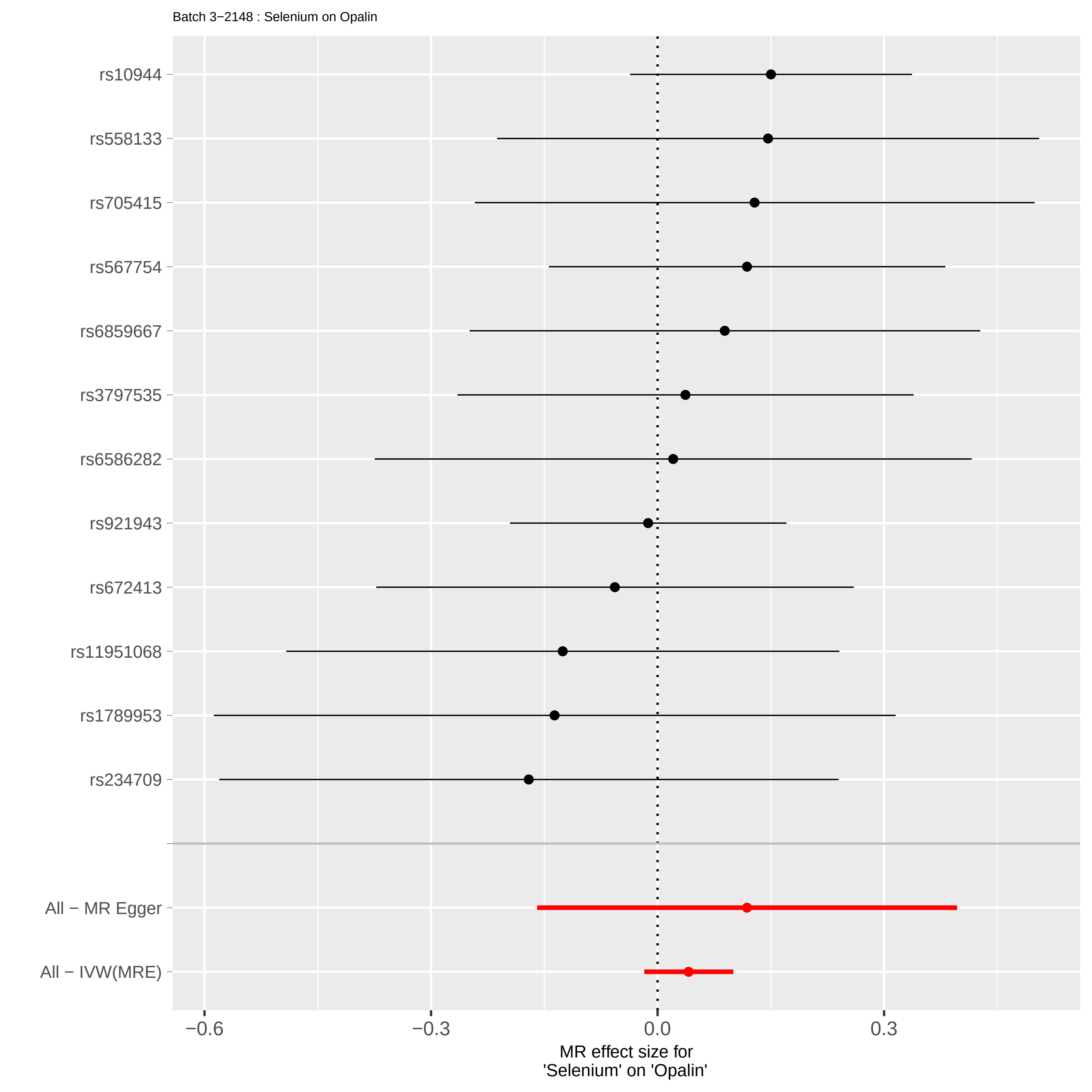
Batch 3−2148 : Selenium on Opalin
rs10944
rs558133
rs705415
rs567754
rs6859667
rs3797535
rs6586282
rs921943
rs672413
rs11951068
rs1789953
rs234709
All − MR Egger
All − IVW(MRE)
−0.6
−0.3
0.0
0.3
MR effect size for
'Selenium' on 'Opalin'

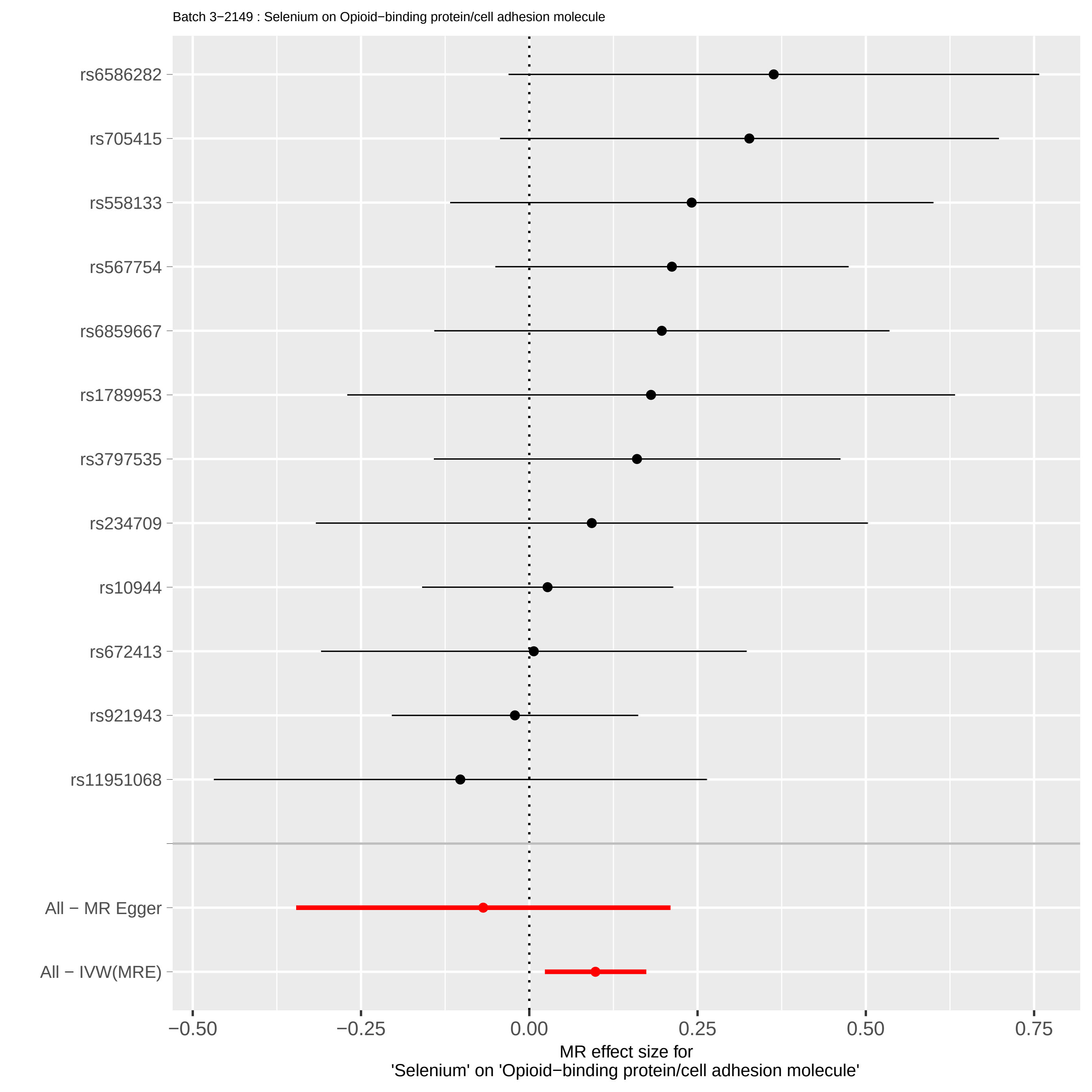

Batch 3−2149 : Selenium on Opioid−binding protein/cell adhesion molecule
rs6586282
rs705415
rs558133
rs567754
rs6859667
rs1789953
rs3797535
rs234709
rs10944
rs672413
rs921943
rs11951068
All − MR Egger
All − IVW(MRE)
−0.50
−0.25
0.00
0.25
0.50
0.75
MR effect size for
'Selenium' on 'Opioid−binding protein/cell adhesion molecule'

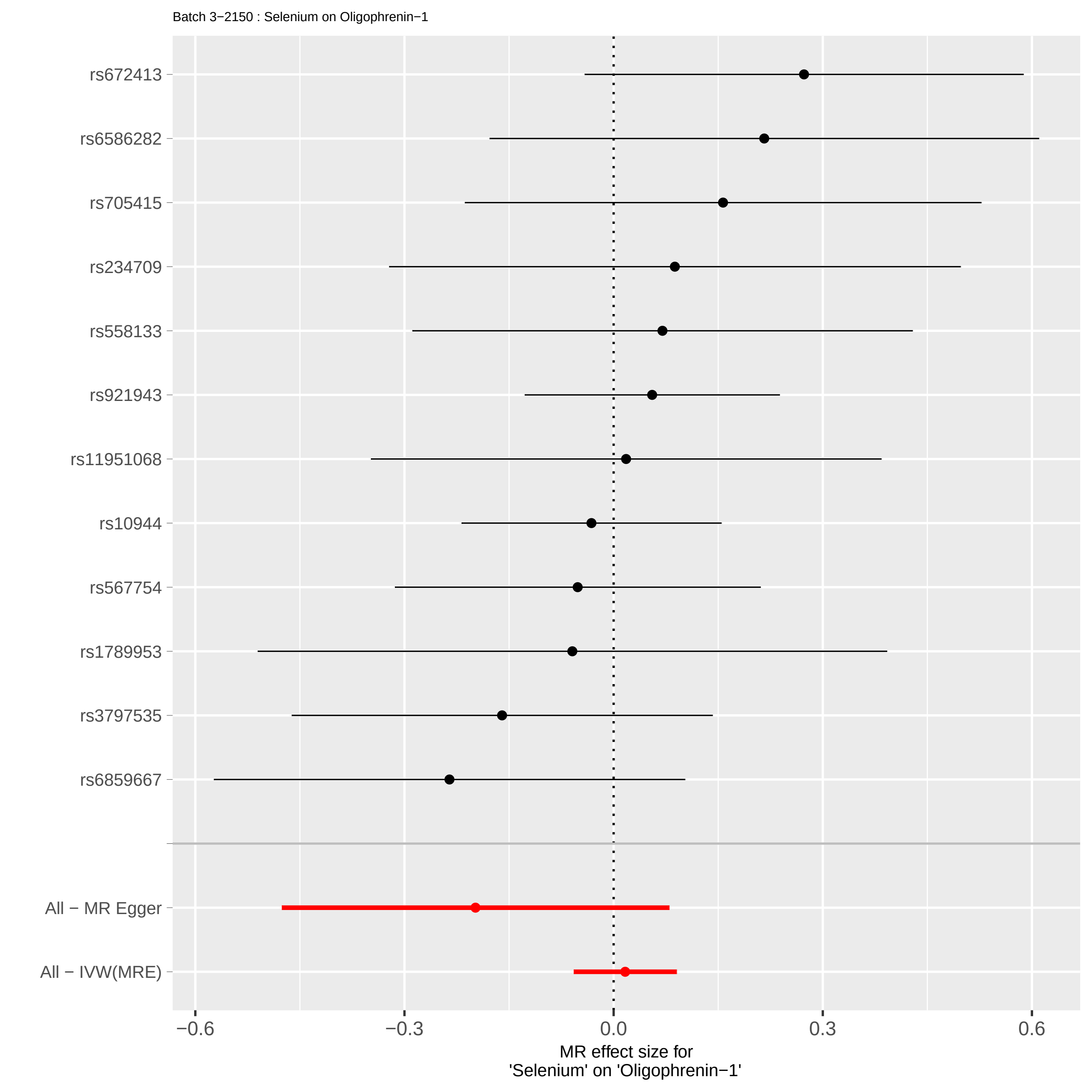

Batch 3−2150 : Selenium on Oligophrenin−1
rs672413
rs6586282
rs705415
rs234709
rs558133
rs921943
rs11951068
rs10944
rs567754
rs1789953
rs3797535
rs6859667
All − MR Egger
All − IVW(MRE)
−0.6
−0.3
0.0
0.3
0.6
MR effect size for
'Selenium' on 'Oligophrenin−1'

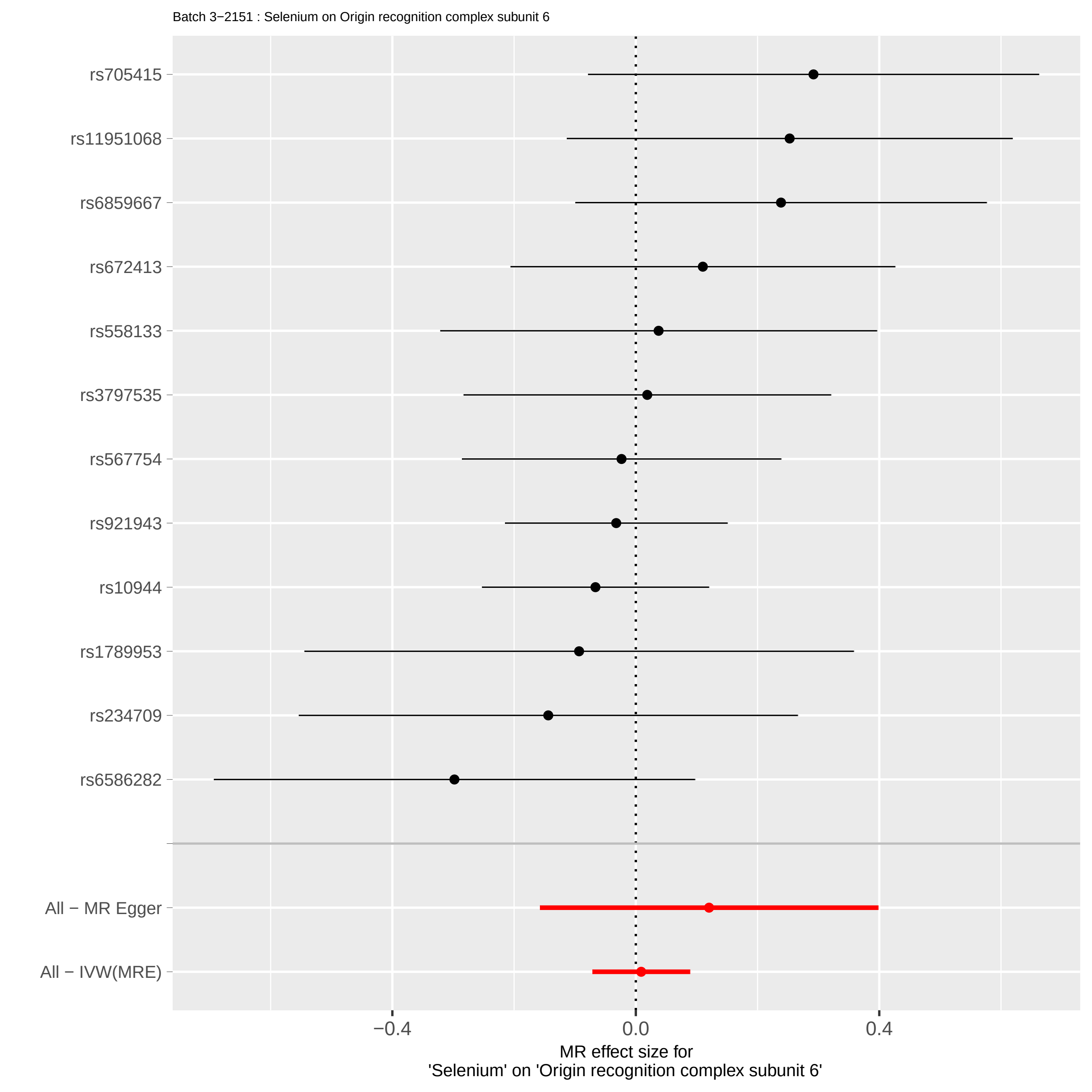

Batch 3−2151 : Selenium on Origin recognition complex subunit 6
rs705415
rs11951068
rs6859667
rs672413
rs558133
rs3797535
rs567754
rs921943
rs10944
rs1789953
rs234709
rs6586282
All − MR Egger
All − IVW(MRE)
−0.4
0.0
0.4
MR effect size for
'Selenium' on 'Origin recognition complex subunit 6'

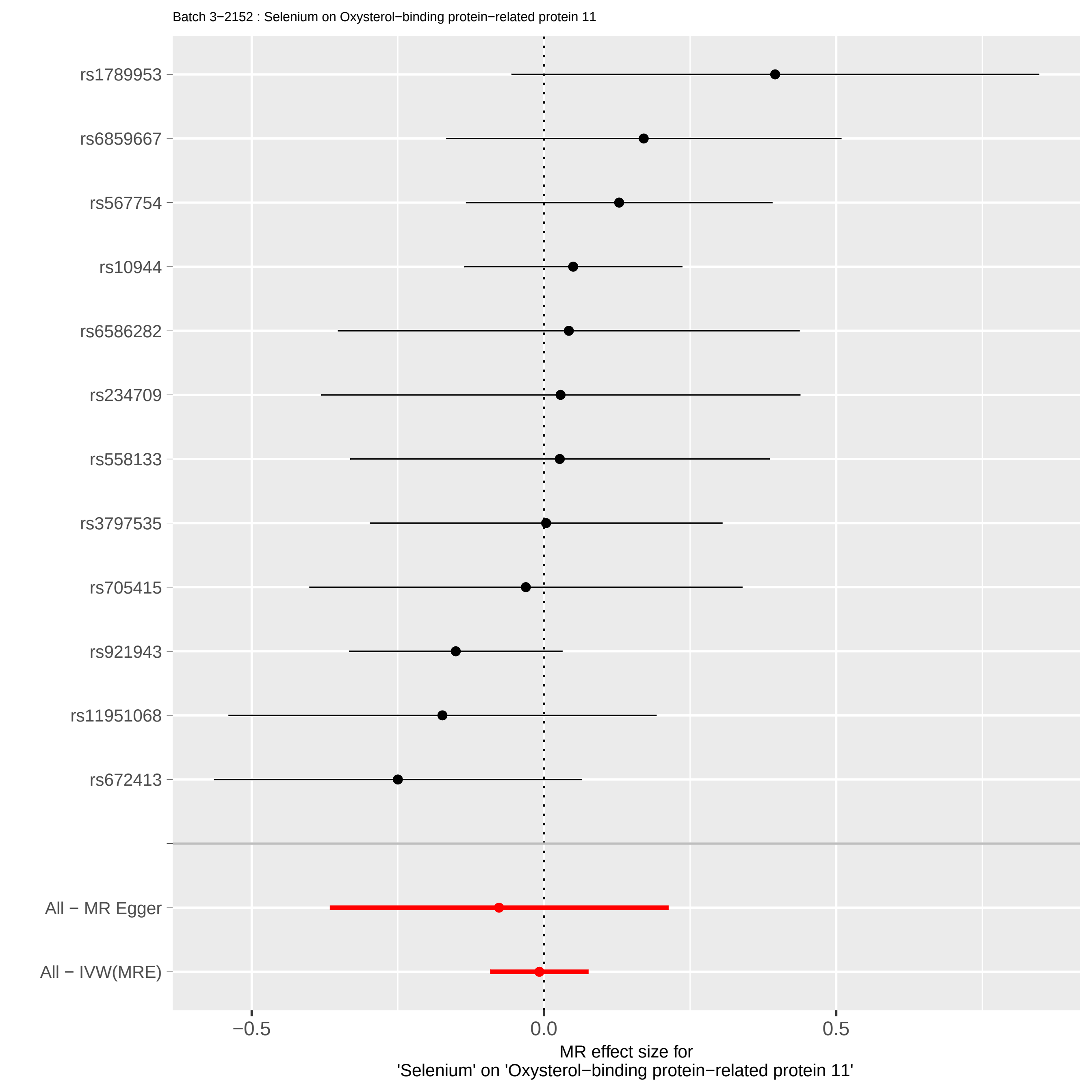

Batch 3−2152 : Selenium on Oxysterol−binding protein−related protein 11
rs1789953
rs6859667
rs567754
rs10944
rs6586282
rs234709
rs558133
rs3797535
rs705415
rs921943
rs11951068
rs672413
All − MR Egger
All − IVW(MRE)
−0.5
0.0
0.5
MR effect size for
'Selenium' on 'Oxysterol−binding protein−related protein 11'

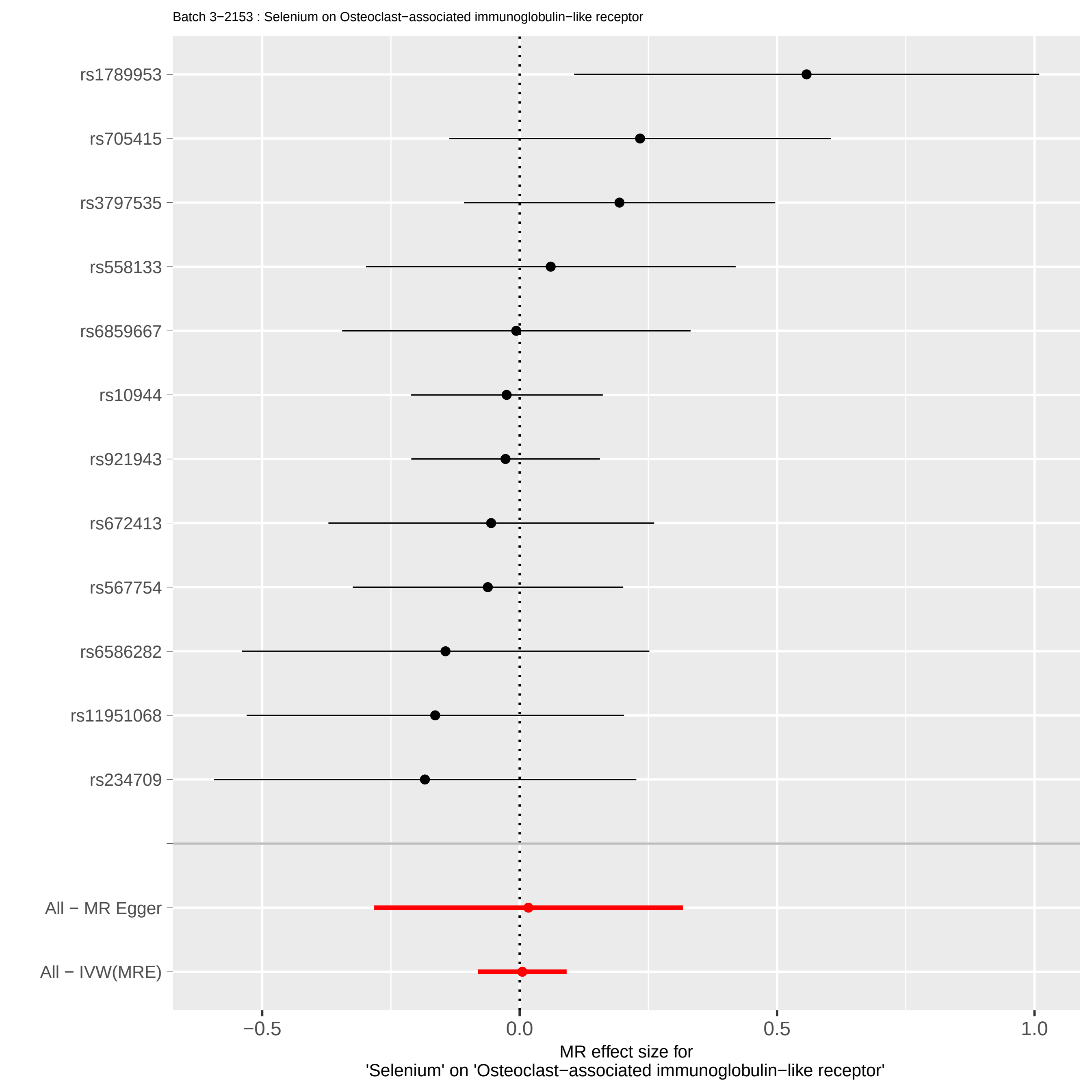
Batch 3−2153 : Selenium on Osteoclast−associated immunoglobulin−like receptor
rs1789953
rs705415
rs3797535
rs558133
rs6859667
rs10944
rs921943
rs672413
rs567754
rs6586282
rs11951068
rs234709
All − MR Egger
All − IVW(MRE)
−0.5
0.0
0.5
1.0
MR effect size for
'Selenium' on 'Osteoclast−associated immunoglobulin−like receptor'

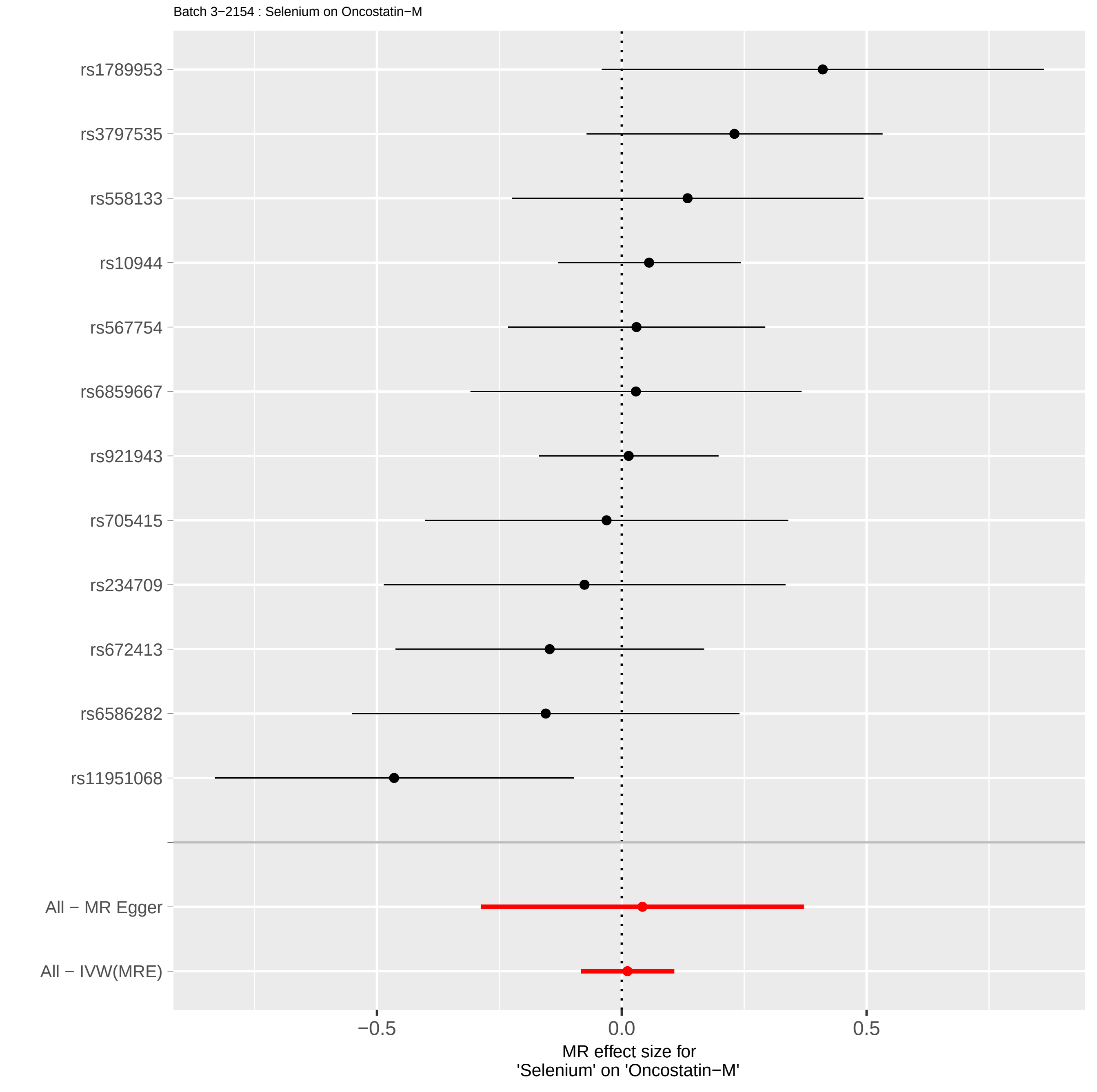

Batch 3−2154 : Selenium on Oncostatin−M
rs1789953
rs3797535
rs558133
rs10944
rs567754
rs6859667
rs921943
rs705415
rs234709
rs672413
rs6586282
rs11951068
All − MR Egger
All − IVW(MRE)
−0.5
0.0
0.5
MR effect size for
'Selenium' on 'Oncostatin−M'

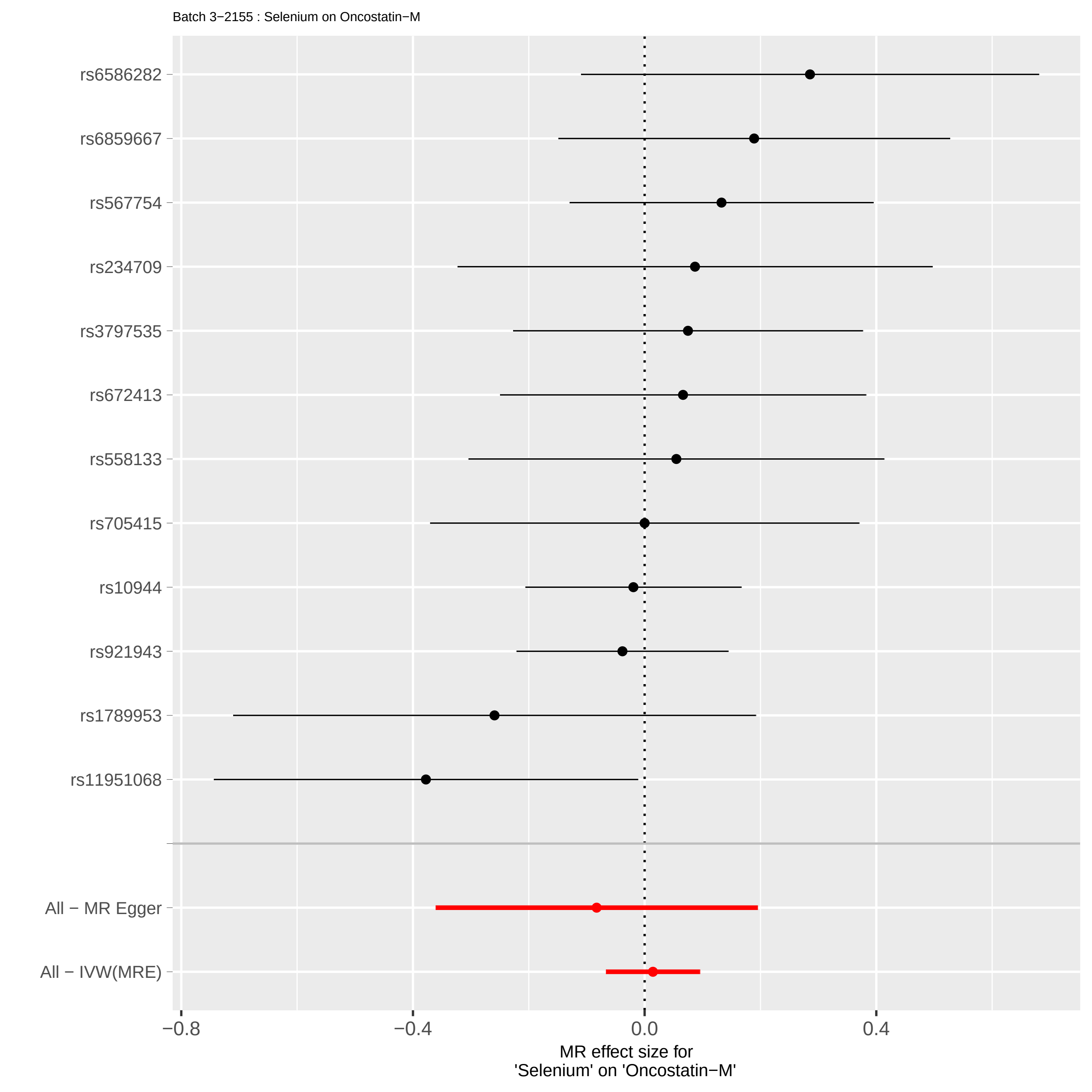

Batch 3−2155 : Selenium on Oncostatin−M
rs6586282
rs6859667
rs567754
rs234709
rs3797535
rs672413
rs558133
rs705415
rs10944
rs921943
rs1789953
rs11951068
All − MR Egger
All − IVW(MRE)
−0.8
−0.4
0.0
0.4
MR effect size for
'Selenium' on 'Oncostatin−M'

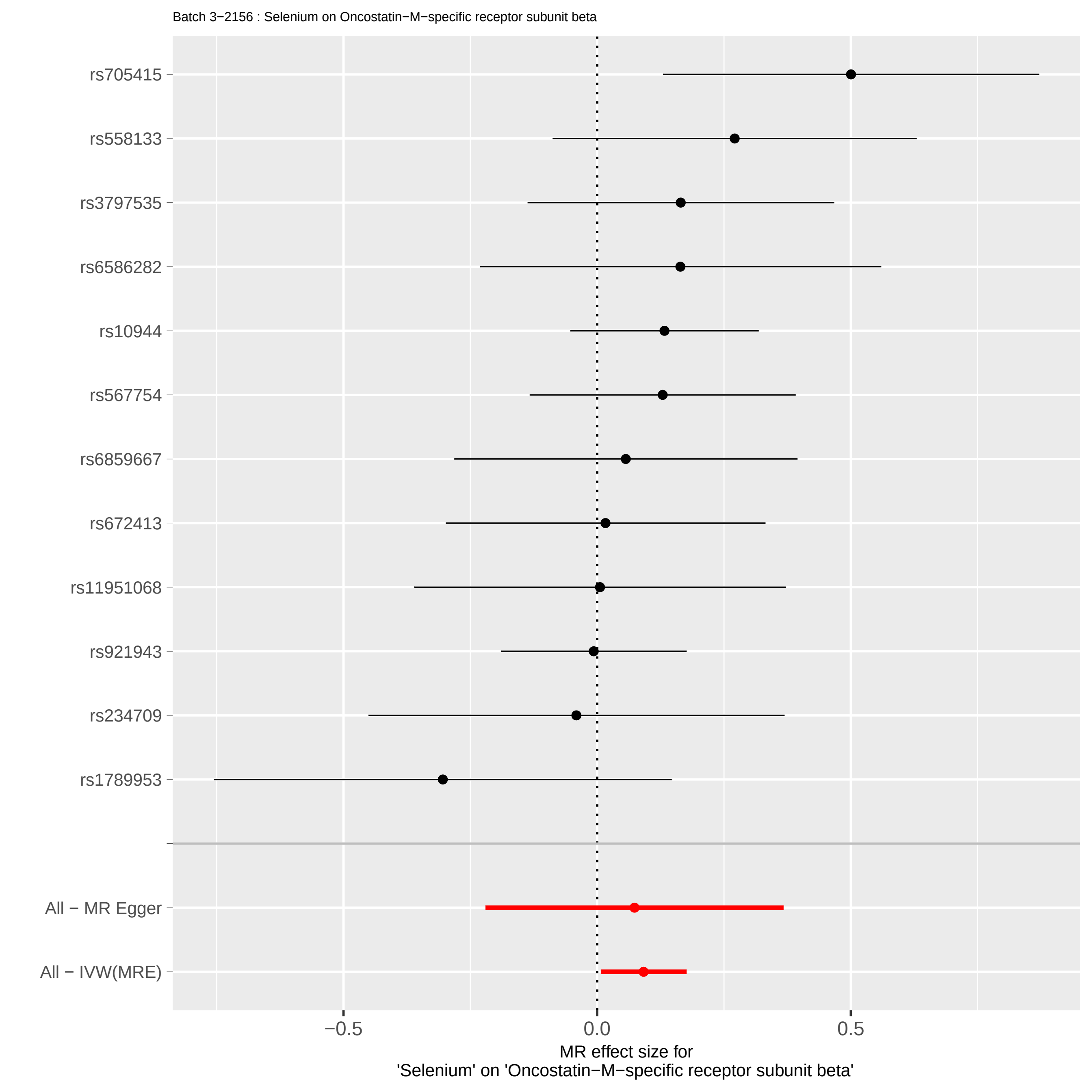
Batch 3−2156 : Selenium on Oncostatin−M−specific receptor subunit beta
rs705415
rs558133
rs3797535
rs6586282
rs10944
rs567754
rs6859667
rs672413
rs11951068
rs921943
rs234709
rs1789953
All − MR Egger
All − IVW(MRE)
−0.5
0.0
0.5
MR effect size for
'Selenium' on 'Oncostatin−M−specific receptor subunit beta'

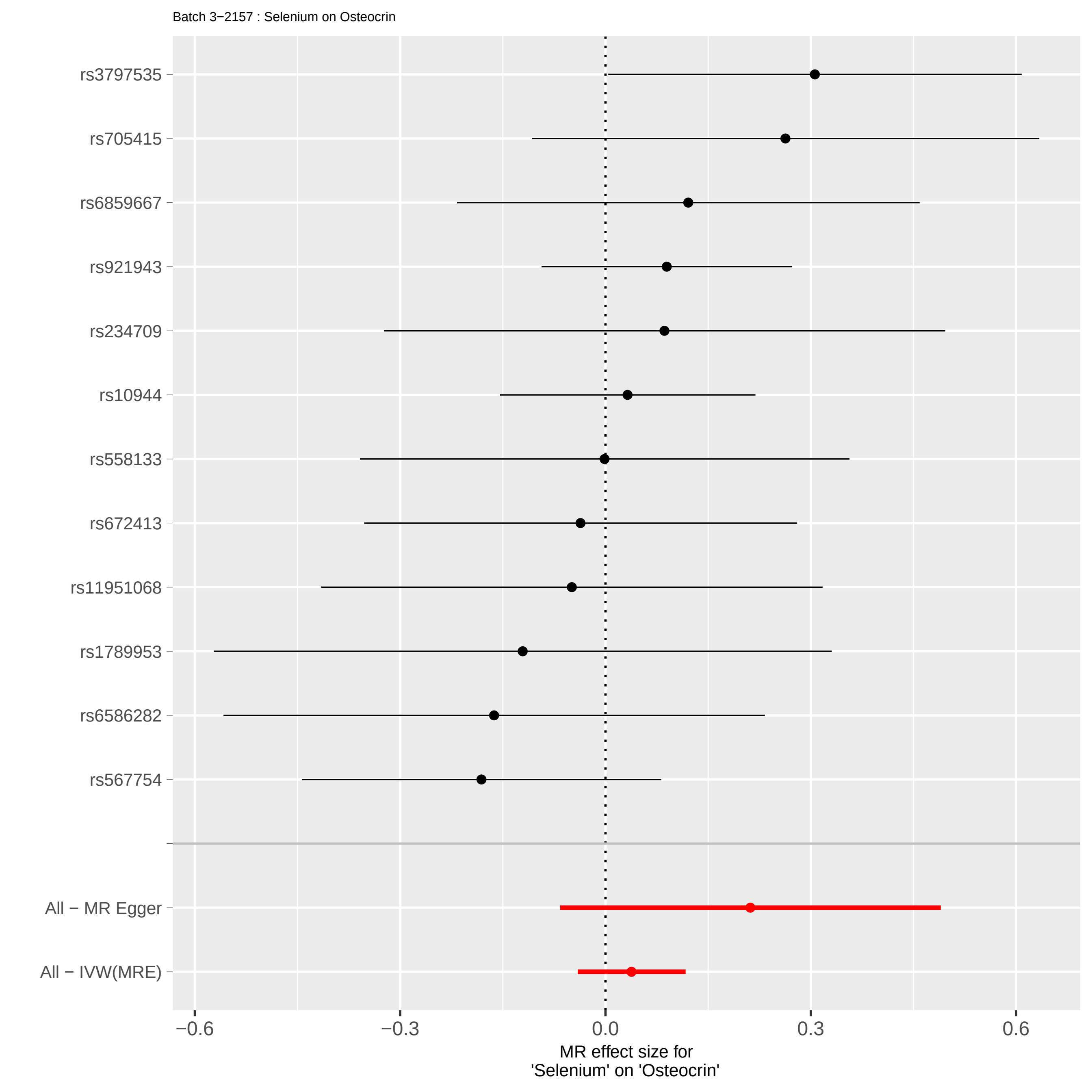

Batch 3−2157 : Selenium on Osteocrin
rs3797535
rs705415
rs6859667
rs921943
rs234709
rs10944
rs558133
rs672413
rs11951068
rs1789953
rs6586282
rs567754
All − MR Egger
All − IVW(MRE)
−0.6
−0.3
0.0
0.3
0.6
MR effect size for
'Selenium' on 'Osteocrin'

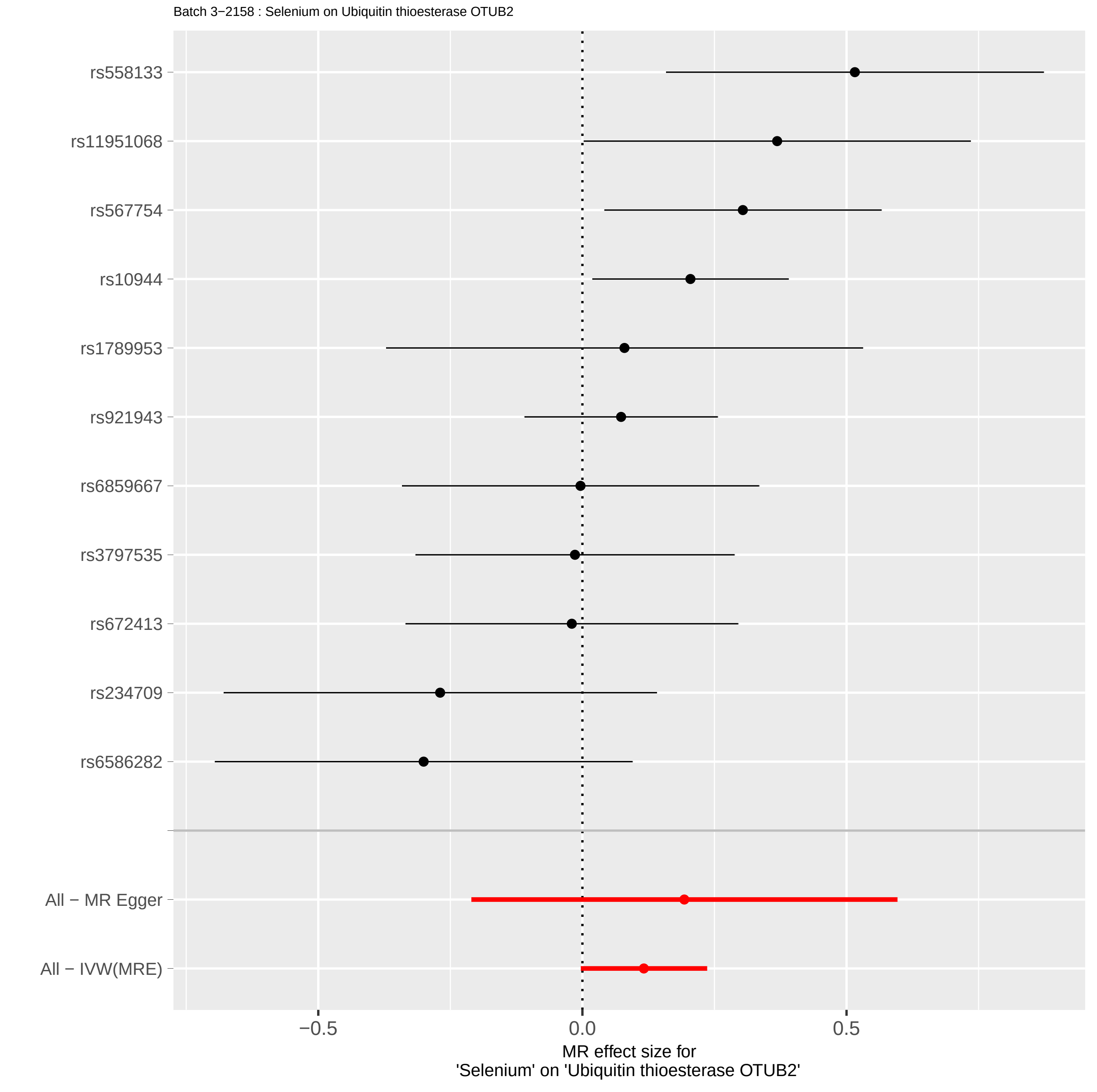

Batch 3−2158 : Selenium on Ubiquitin thioesterase OTUB2
rs558133
rs11951068
rs567754
rs10944
rs1789953
rs921943
rs6859667
rs3797535
rs672413
rs234709
rs6586282
All − MR Egger
All − IVW(MRE)
−0.5
0.0
0.5
MR effect size for
'Selenium' on 'Ubiquitin thioesterase OTUB2'

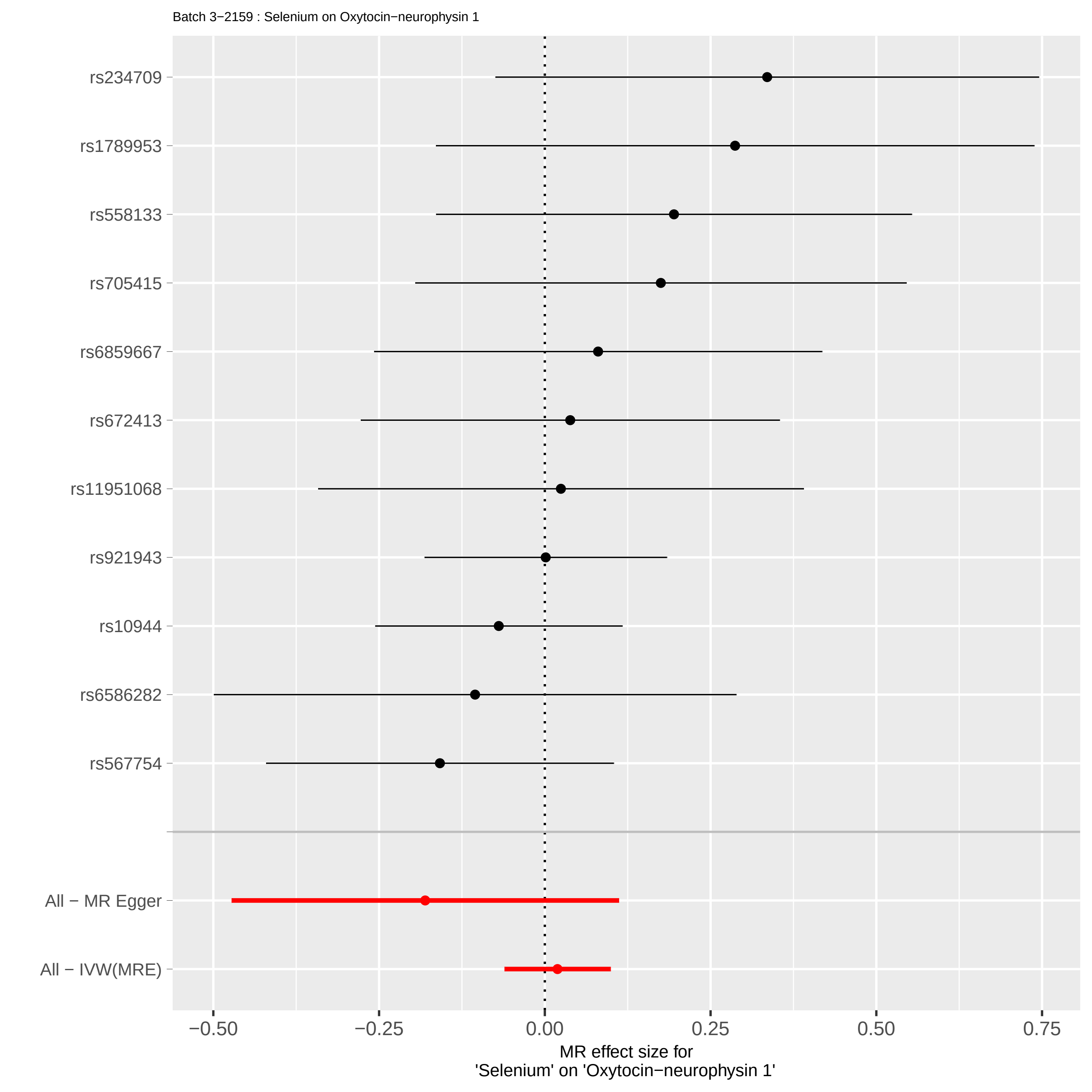

Batch 3−2159 : Selenium on Oxytocin−neurophysin 1
rs234709
rs1789953
rs558133
rs705415
rs6859667
rs672413
rs11951068
rs921943
rs10944
rs6586282
rs567754
All − MR Egger
All − IVW(MRE)
−0.50
−0.25
0.00
0.25
0.50
0.75
MR effect size for
'Selenium' on 'Oxytocin−neurophysin 1'

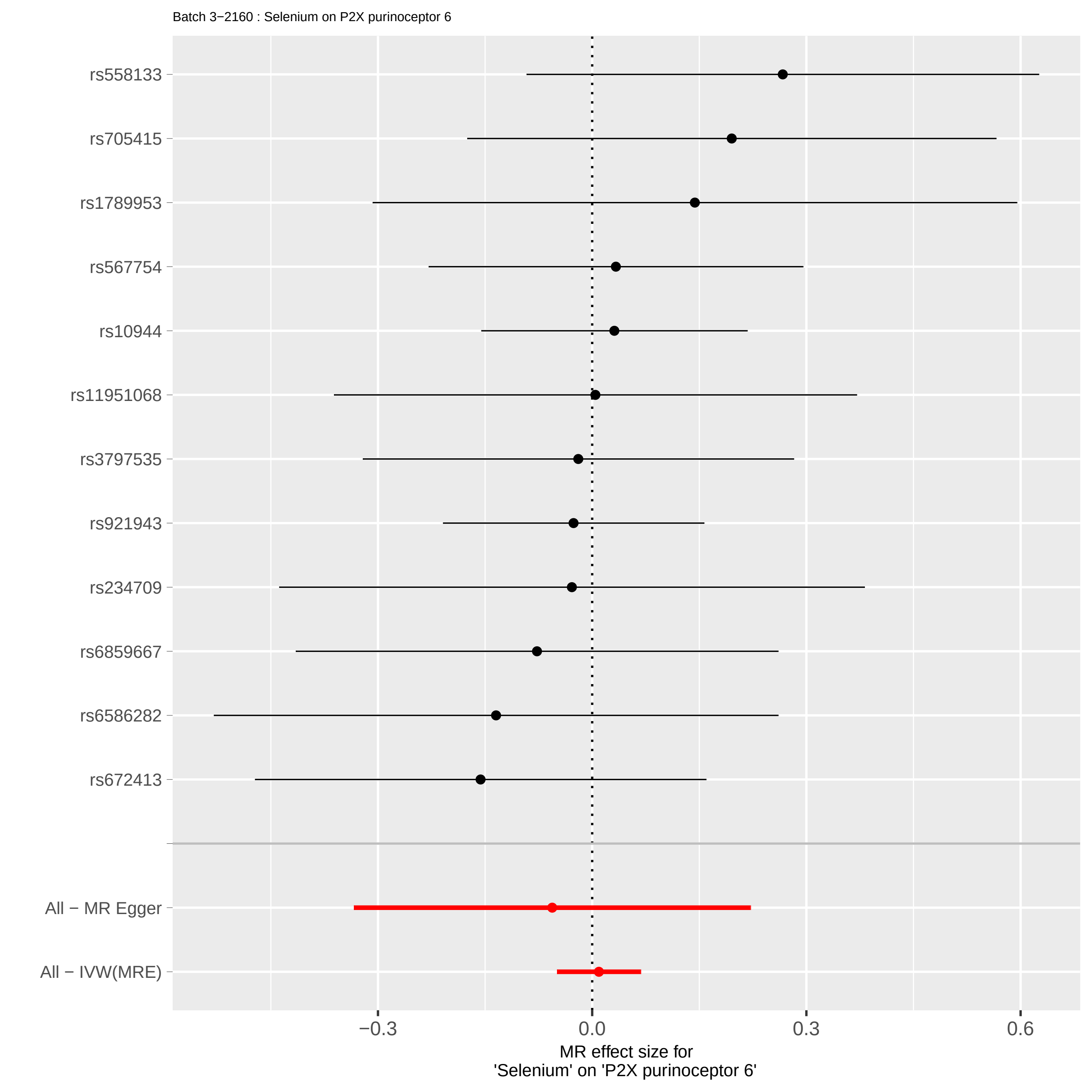

Batch 3−2160 : Selenium on P2X purinoceptor 6
rs558133
rs705415
rs1789953
rs567754
rs10944
rs11951068
rs3797535
rs921943
rs234709
rs6859667
rs6586282
rs672413
All − MR Egger
All − IVW(MRE)
−0.3
0.0
0.3
0.6
MR effect size for
'Selenium' on 'P2X purinoceptor 6'

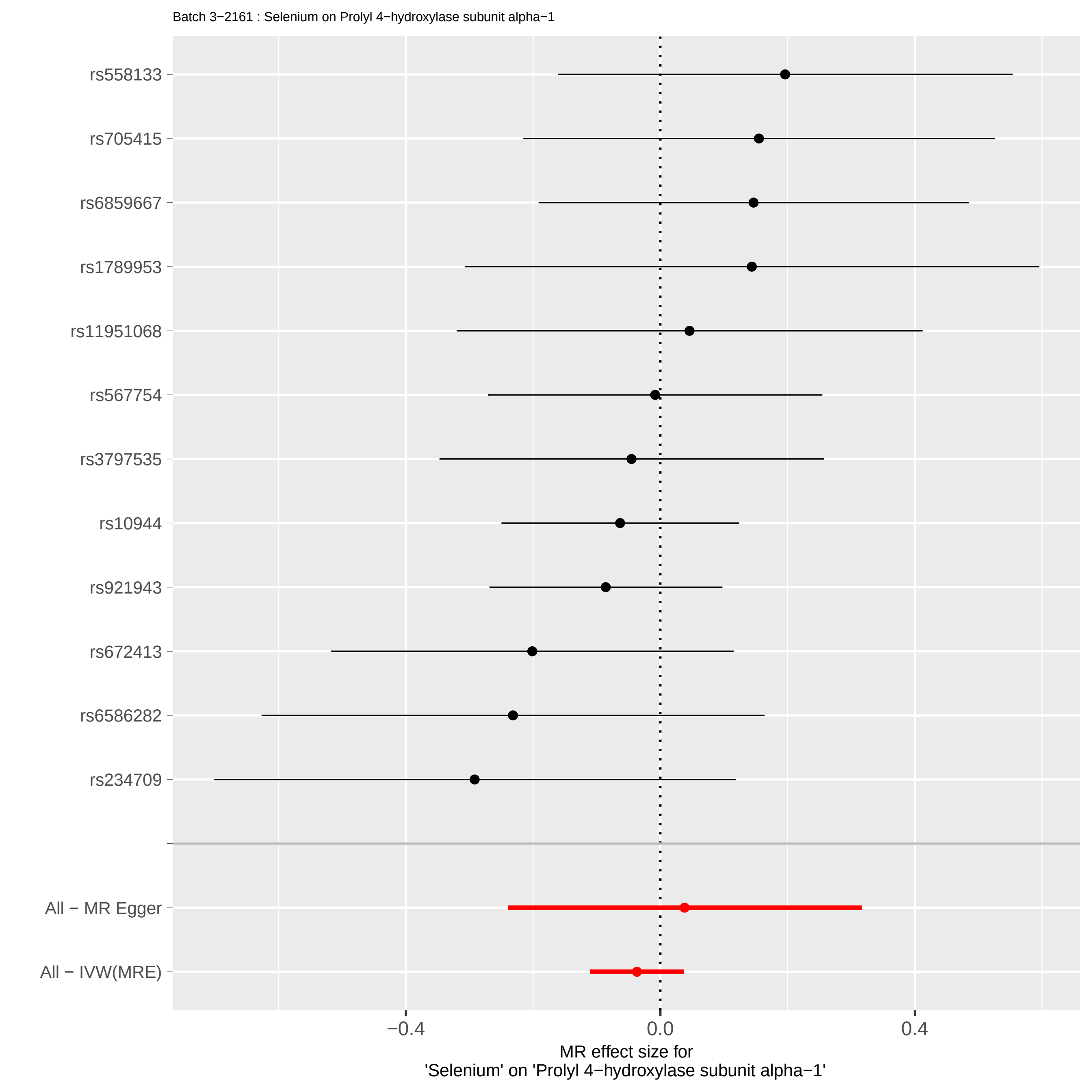

Batch 3−2161 : Selenium on Prolyl 4−hydroxylase subunit alpha−1
rs558133
rs705415
rs6859667
rs1789953
rs11951068
rs567754
rs3797535
rs10944
rs921943
rs672413
rs6586282
rs234709
All − MR Egger
All − IVW(MRE)
−0.4
0.0
0.4
MR effect size for
'Selenium' on 'Prolyl 4−hydroxylase subunit alpha−1'

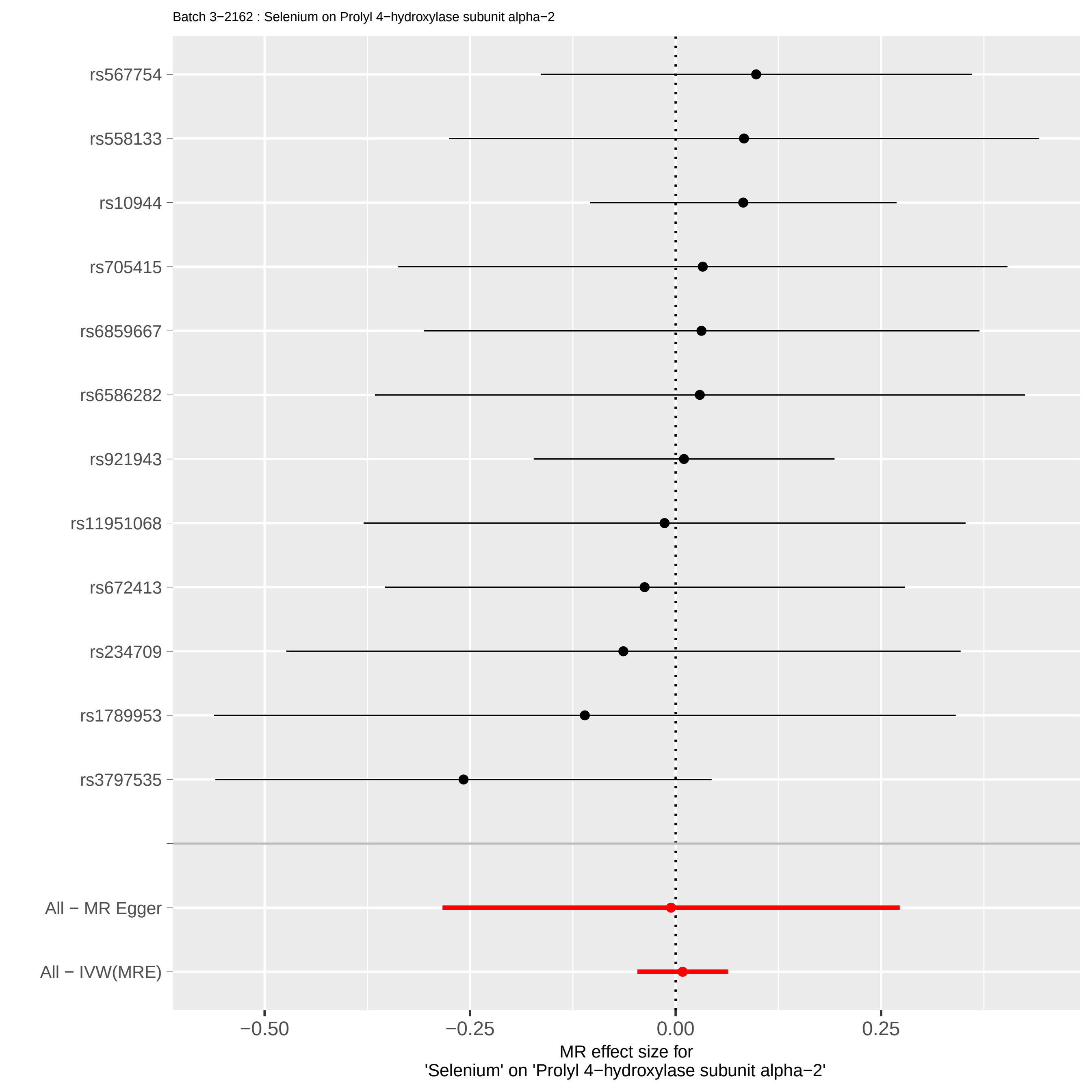

Batch 3−2162 : Selenium on Prolyl 4−hydroxylase subunit alpha−2
rs567754
rs558133
rs10944
rs705415
rs6859667
rs6586282
rs921943
rs11951068
rs672413
rs234709
rs1789953
rs3797535
All − MR Egger
All − IVW(MRE)
−0.50
−0.25
0.00
0.25
MR effect size for
'Selenium' on 'Prolyl 4−hydroxylase subunit alpha−2'

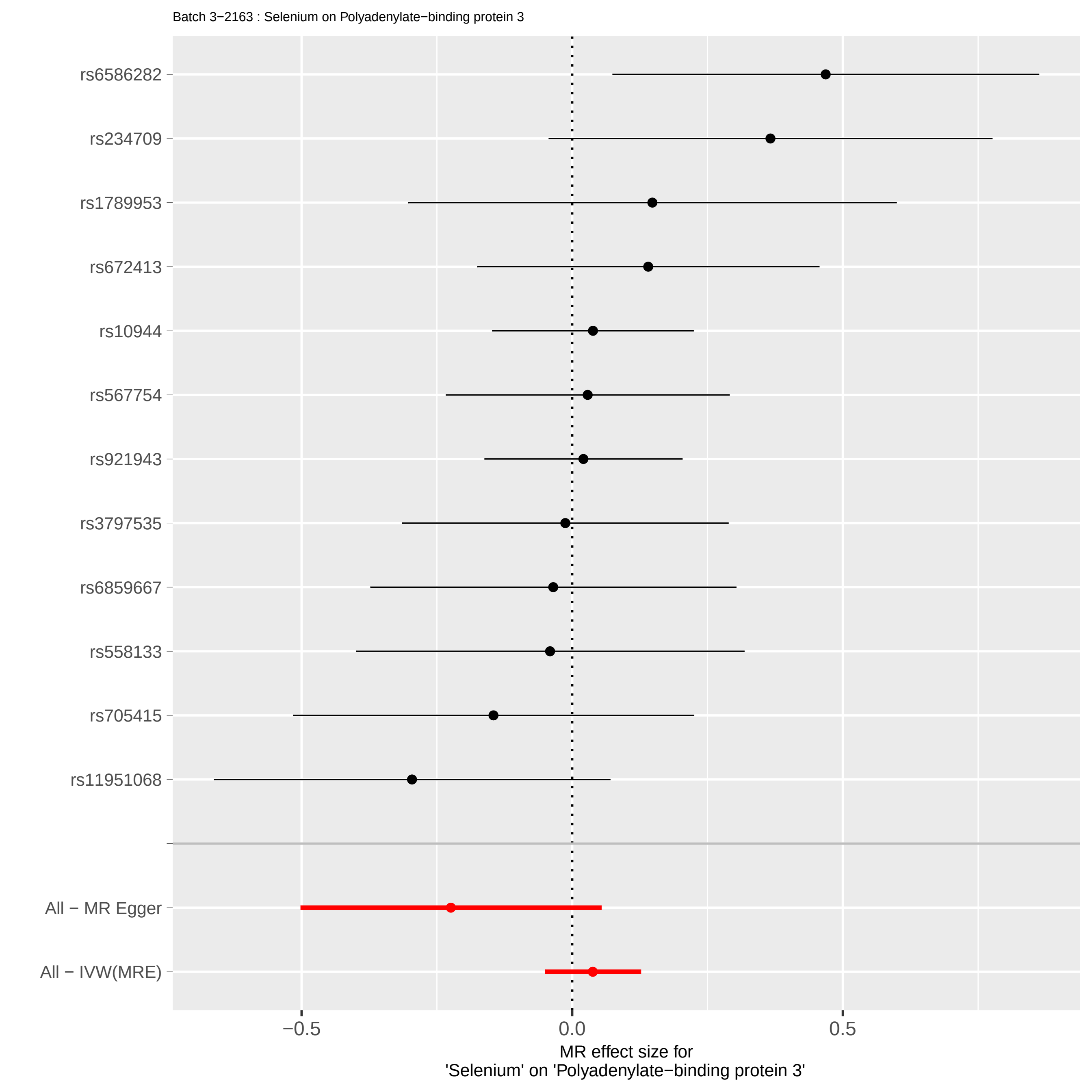
Batch 3−2163 : Selenium on Polyadenylate−binding protein 3
rs6586282
rs234709
rs1789953
rs672413
rs10944
rs567754
rs921943
rs3797535
rs6859667
rs558133
rs705415
rs11951068
All − MR Egger
All − IVW(MRE)
−0.5
0.0
0.5
MR effect size for
'Selenium' on 'Polyadenylate−binding protein 3'

Batch 3−2164 : Selenium on Polyadenylate−binding protein 4

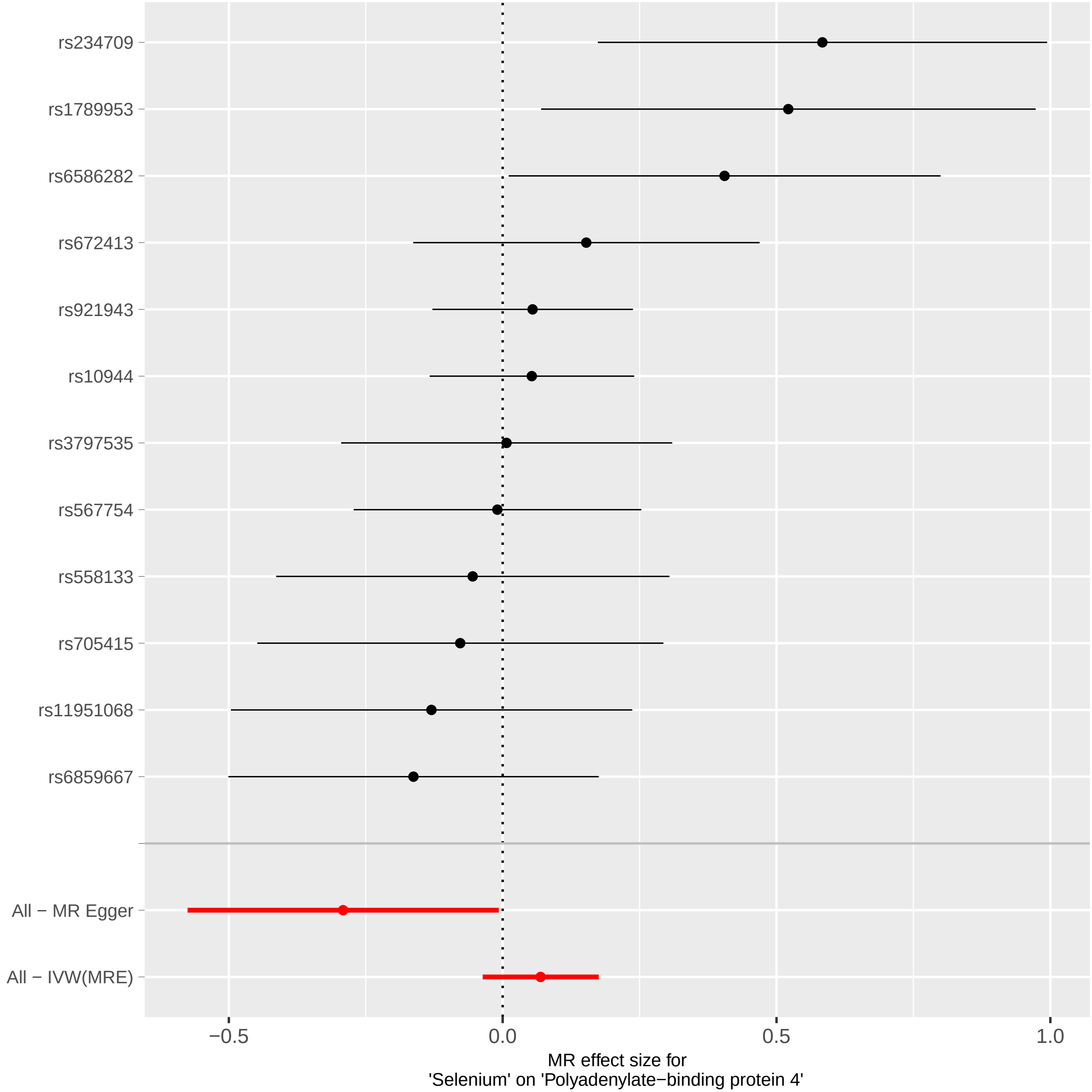

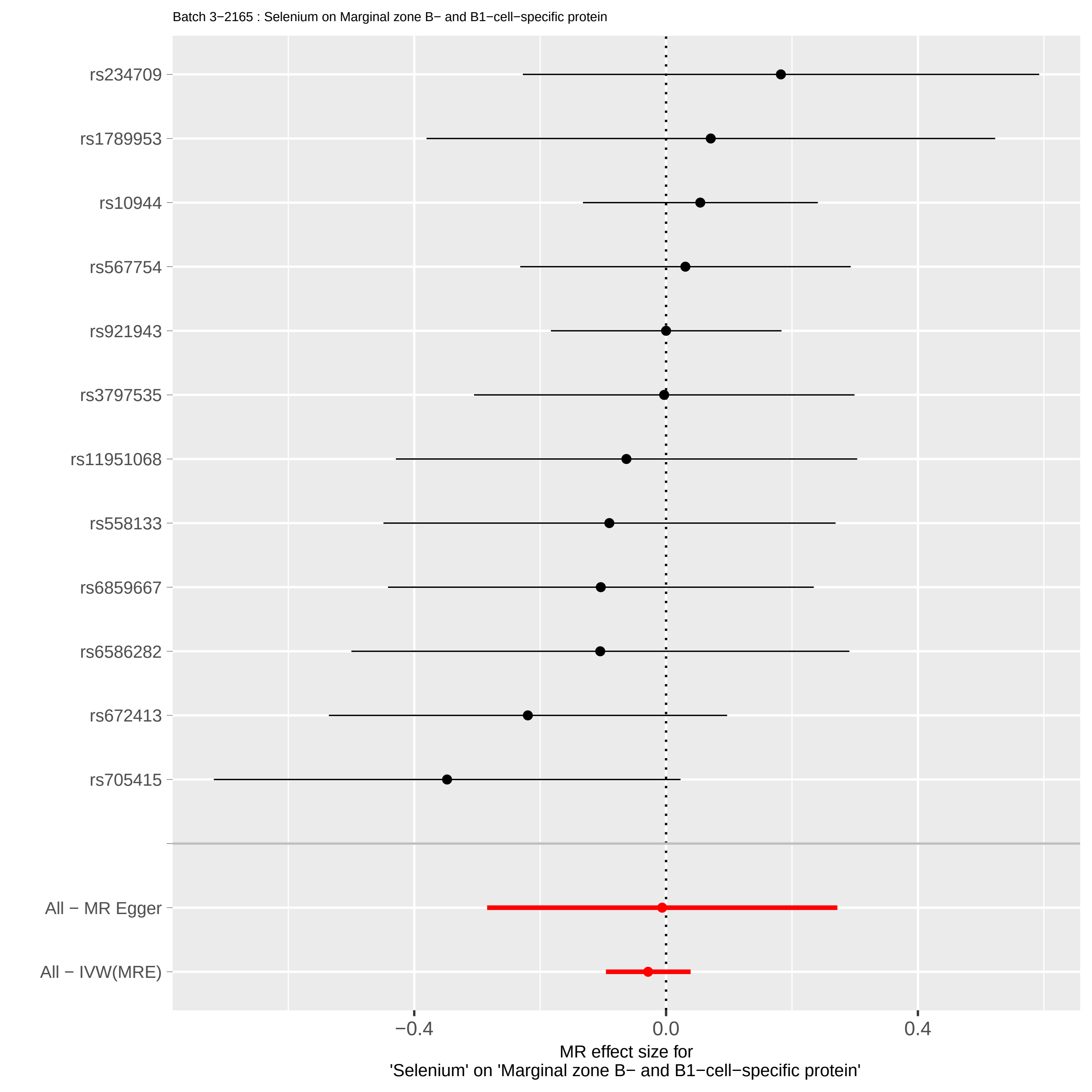

Batch 3−2165 : Selenium on Marginal zone B− and B1−cell−specific protein
rs234709
rs1789953
rs10944
rs567754
rs921943
rs3797535
rs11951068
rs558133
rs6859667
rs6586282
rs672413
rs705415
All − MR Egger
All − IVW(MRE)
−0.4
0.0
0.4
MR effect size for
'Selenium' on 'Marginal zone B− and B1−cell−specific protein'

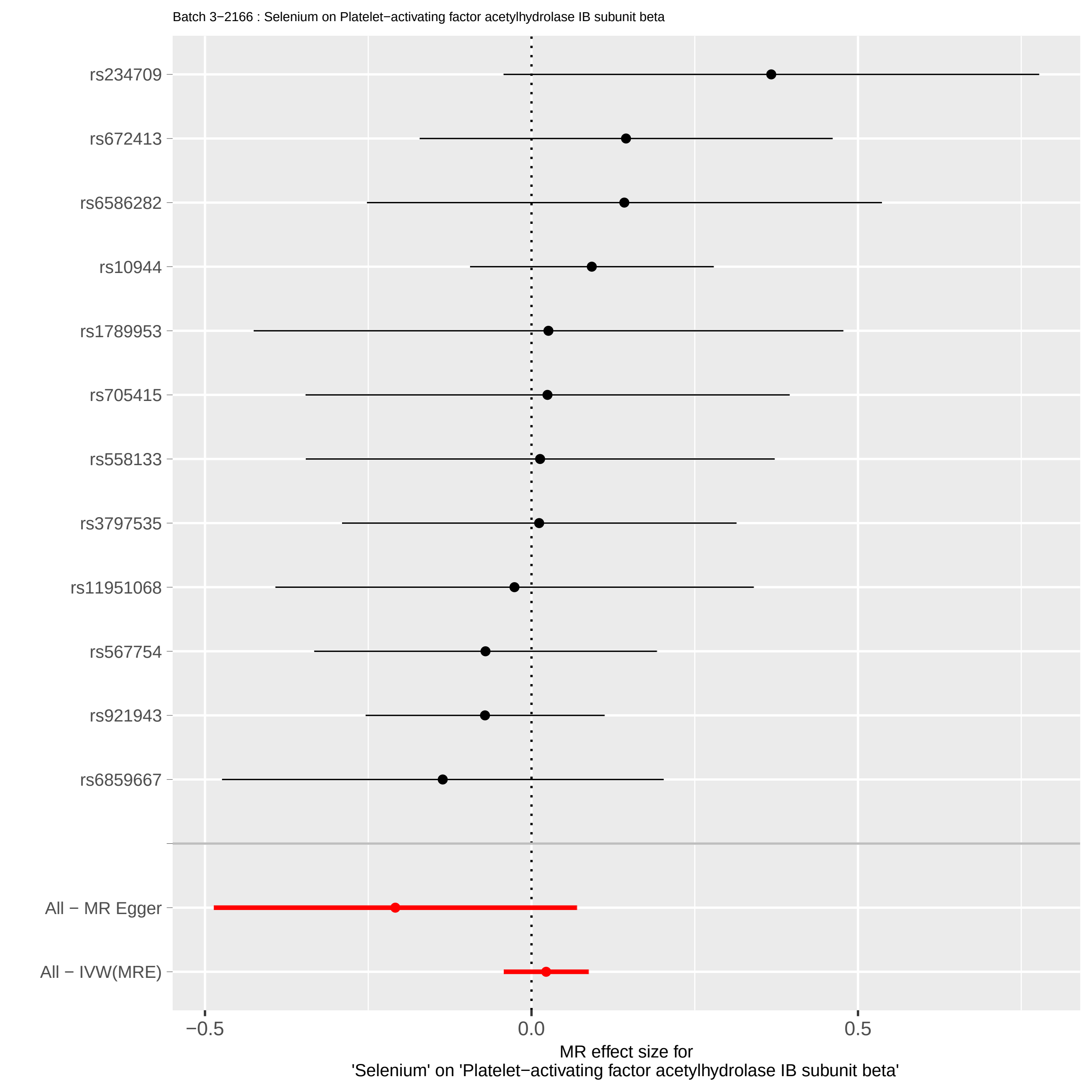

Batch 3−2166 : Selenium on Platelet−activating factor acetylhydrolase IB subunit beta
rs234709
rs672413
rs6586282
rs10944
rs1789953
rs705415
rs558133
rs3797535
rs11951068
rs567754
rs921943
rs6859667
All − MR Egger
All − IVW(MRE)
−0.5
0.0
0.5
MR effect size for
'Selenium' on 'Platelet−activating factor acetylhydrolase IB subunit beta'

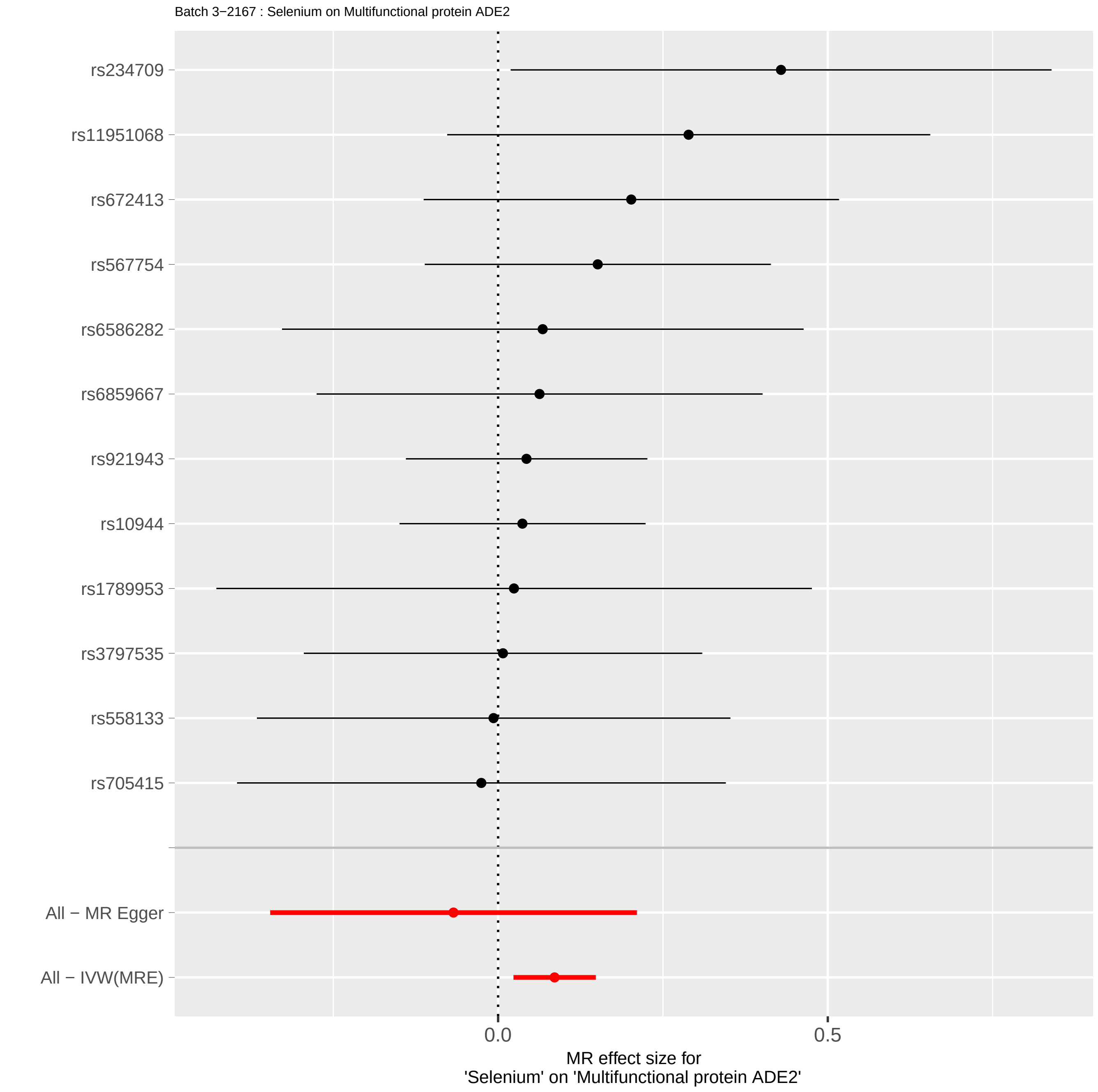
Batch 3−2167 : Selenium on Multifunctional protein ADE2
rs234709
rs11951068
rs672413
rs567754
rs6586282
rs6859667
rs921943
rs10944
rs1789953
rs3797535
rs558133
rs705415
All − MR Egger
All − IVW(MRE)
0.0
0.5
MR effect size for
'Selenium' on 'Multifunctional protein ADE2'

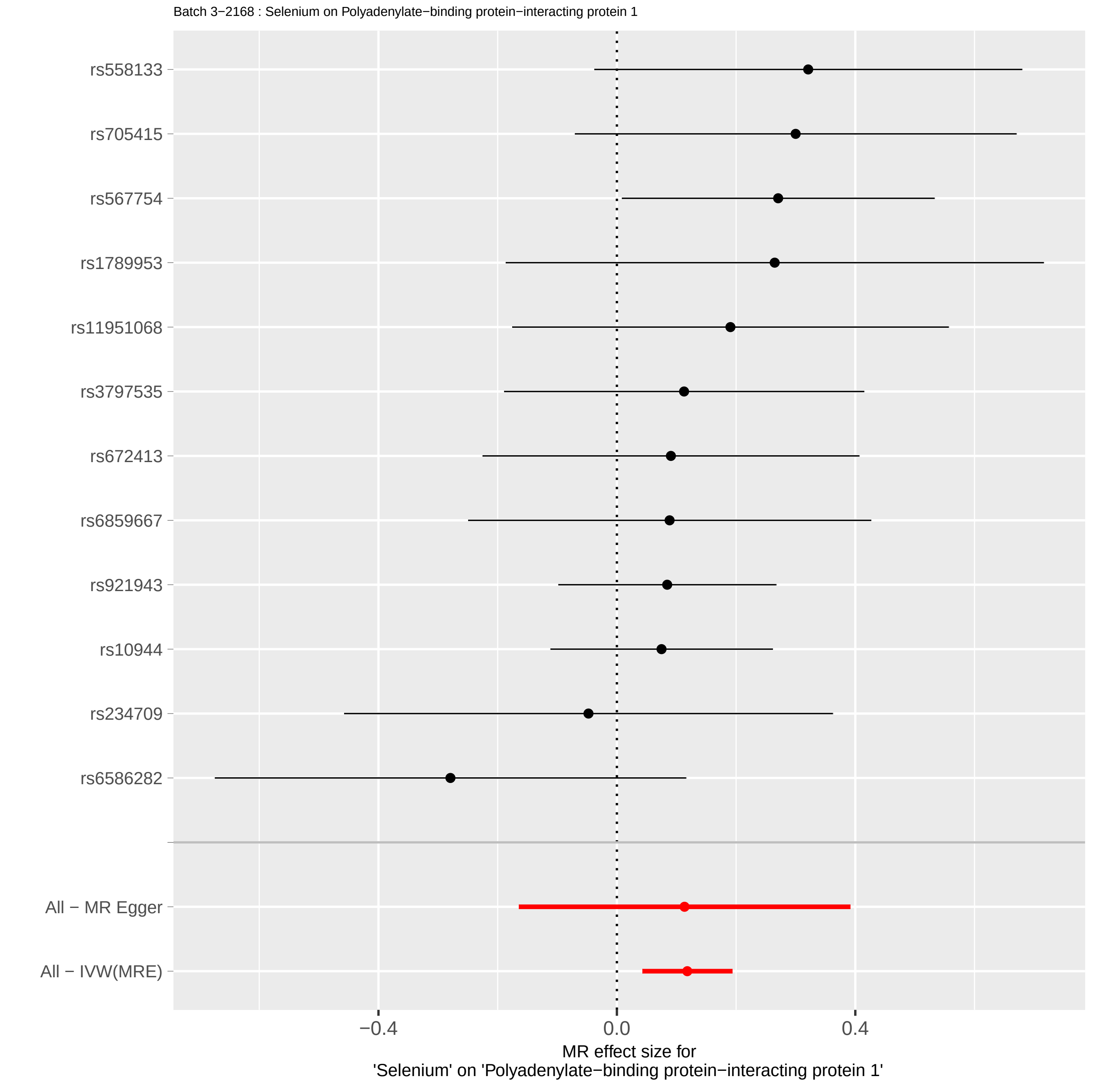
Batch 3−2168 : Selenium on Polyadenylate−binding protein−interacting protein 1
rs558133
rs705415
rs567754
rs1789953
rs11951068
rs3797535
rs672413
rs6859667
rs921943
rs10944
rs234709
rs6586282
All − MR Egger
All − IVW(MRE)
−0.4
0.0
0.4
MR effect size for
'Selenium' on 'Polyadenylate−binding protein−interacting protein 1'

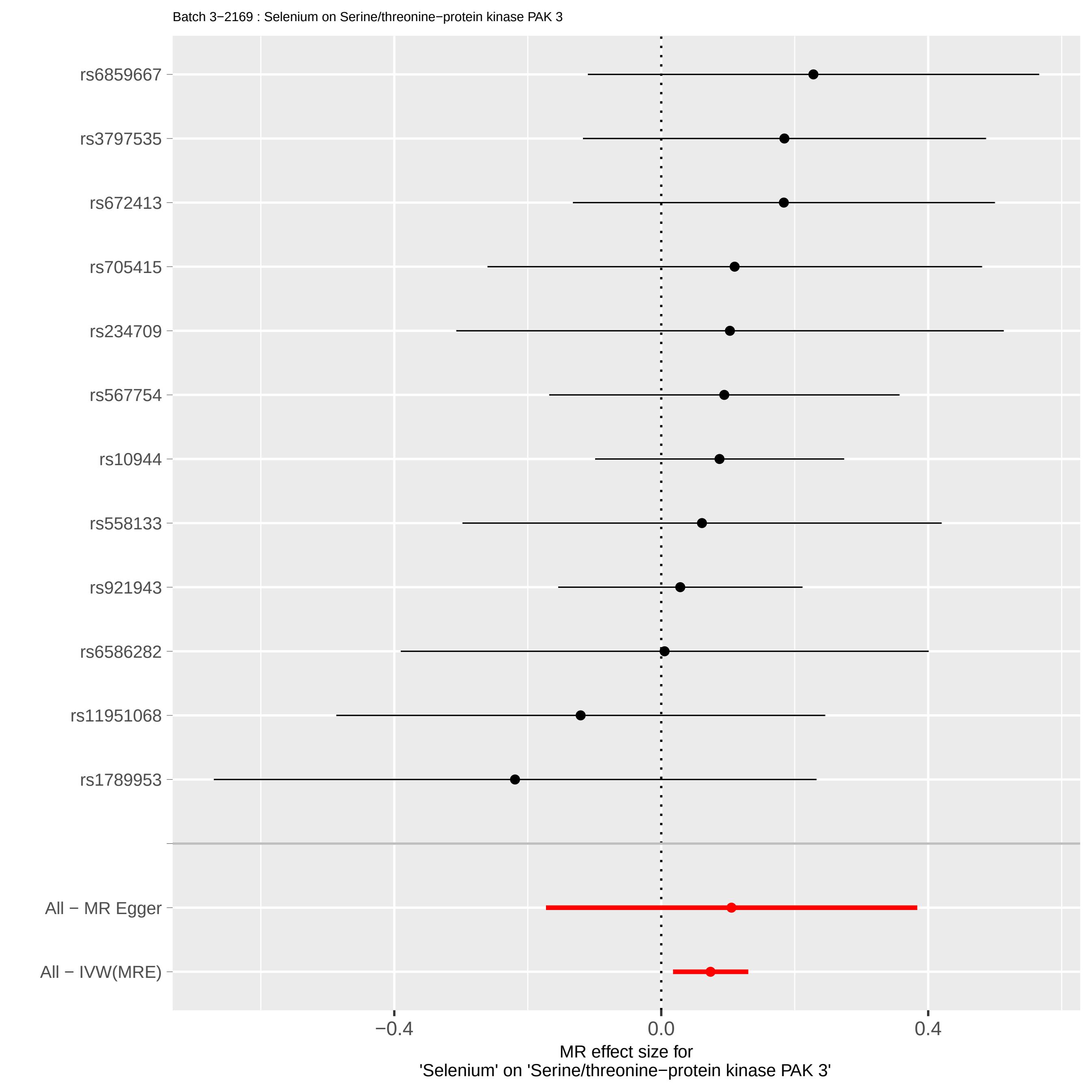
Batch 3−2169 : Selenium on Serine/threonine−protein kinase PAK 3
rs6859667
rs3797535
rs672413
rs705415
rs234709
rs567754
rs10944
rs558133
rs921943
rs6586282
rs11951068
rs1789953
All − MR Egger
All − IVW(MRE)
−0.4
0.0
0.4
MR effect size for
'Selenium' on 'Serine/threonine−protein kinase PAK 3'

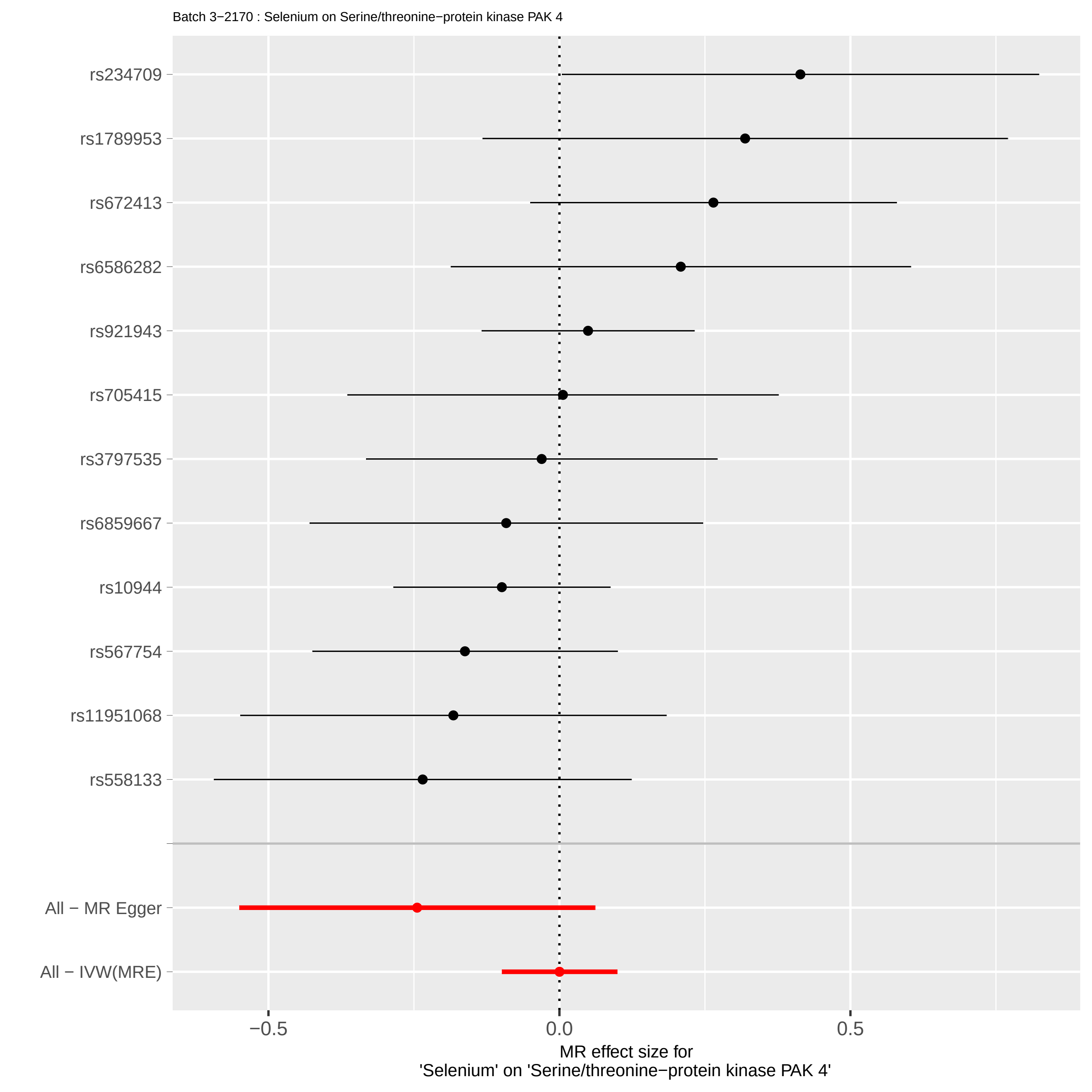

Batch 3−2170 : Selenium on Serine/threonine−protein kinase PAK 4
rs234709
rs1789953
rs672413
rs6586282
rs921943
rs705415
rs3797535
rs6859667
rs10944
rs567754
rs11951068
rs558133
All − MR Egger
All − IVW(MRE)
−0.5
0.0
0.5
MR effect size for
'Selenium' on 'Serine/threonine−protein kinase PAK 4'

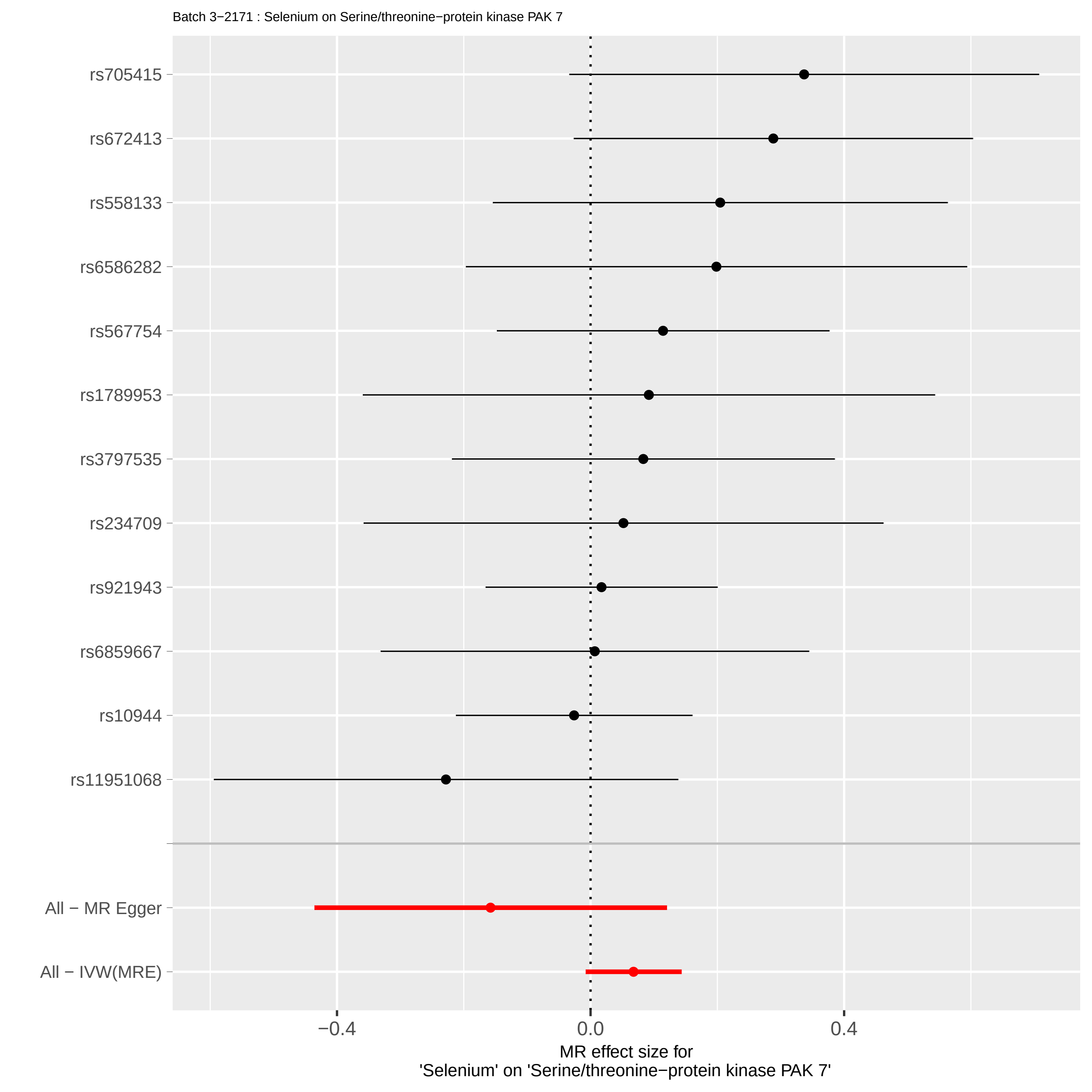

Batch 3−2171 : Selenium on Serine/threonine−protein kinase PAK 7
rs705415
rs672413
rs558133
rs6586282
rs567754
rs1789953
rs3797535
rs234709
rs921943
rs6859667
rs10944
rs11951068
All − MR Egger
All − IVW(MRE)
−0.4
0.0
0.4
MR effect size for
'Selenium' on 'Serine/threonine−protein kinase PAK 7'

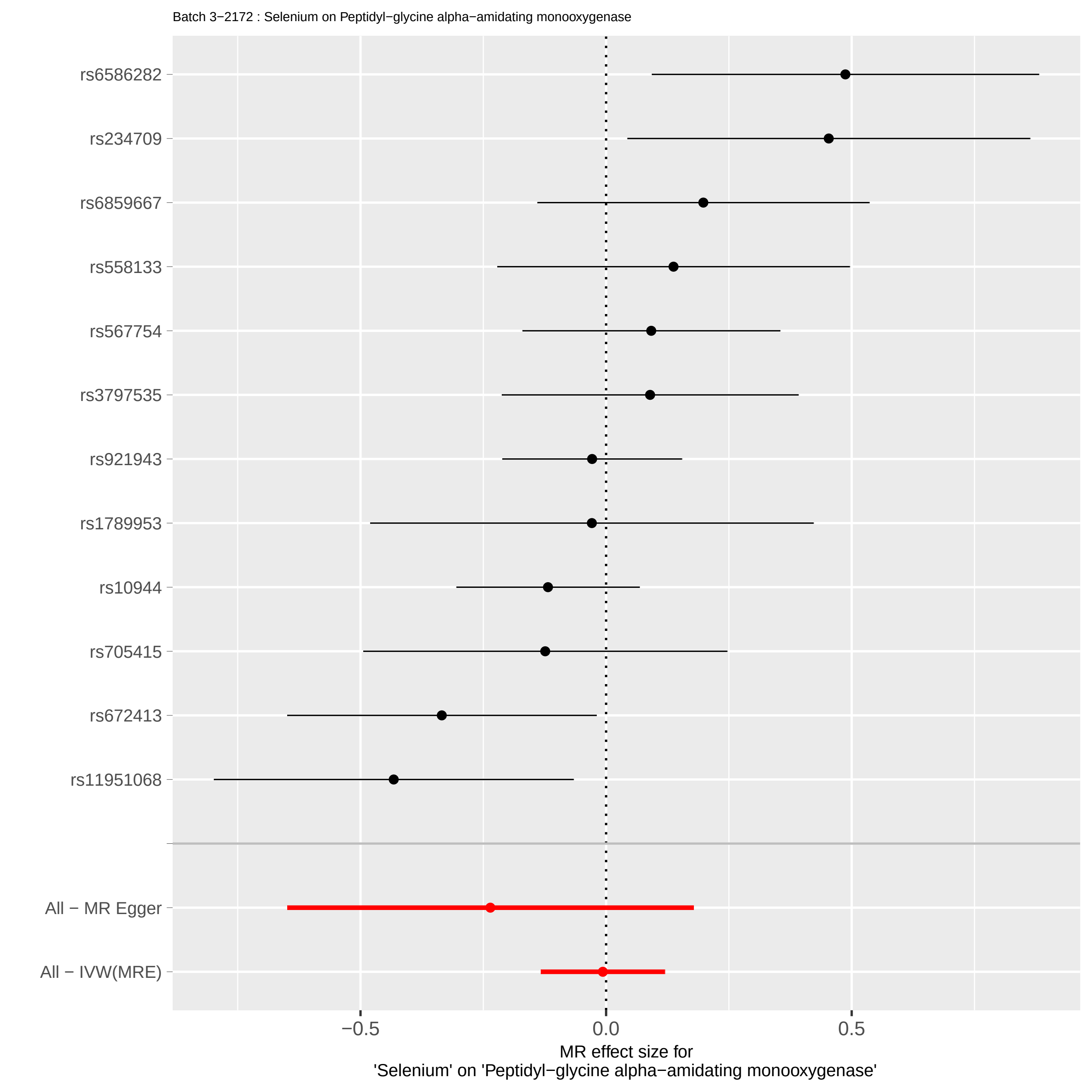

Batch 3−2172 : Selenium on Peptidyl−glycine alpha−amidating monooxygenase
rs6586282
rs234709
rs6859667
rs558133
rs567754
rs3797535
rs921943
rs1789953
rs10944
rs705415
rs672413
rs11951068
All − MR Egger
All − IVW(MRE)
−0.5
0.0
0.5
MR effect size for
'Selenium' on 'Peptidyl−glycine alpha−amidating monooxygenase'

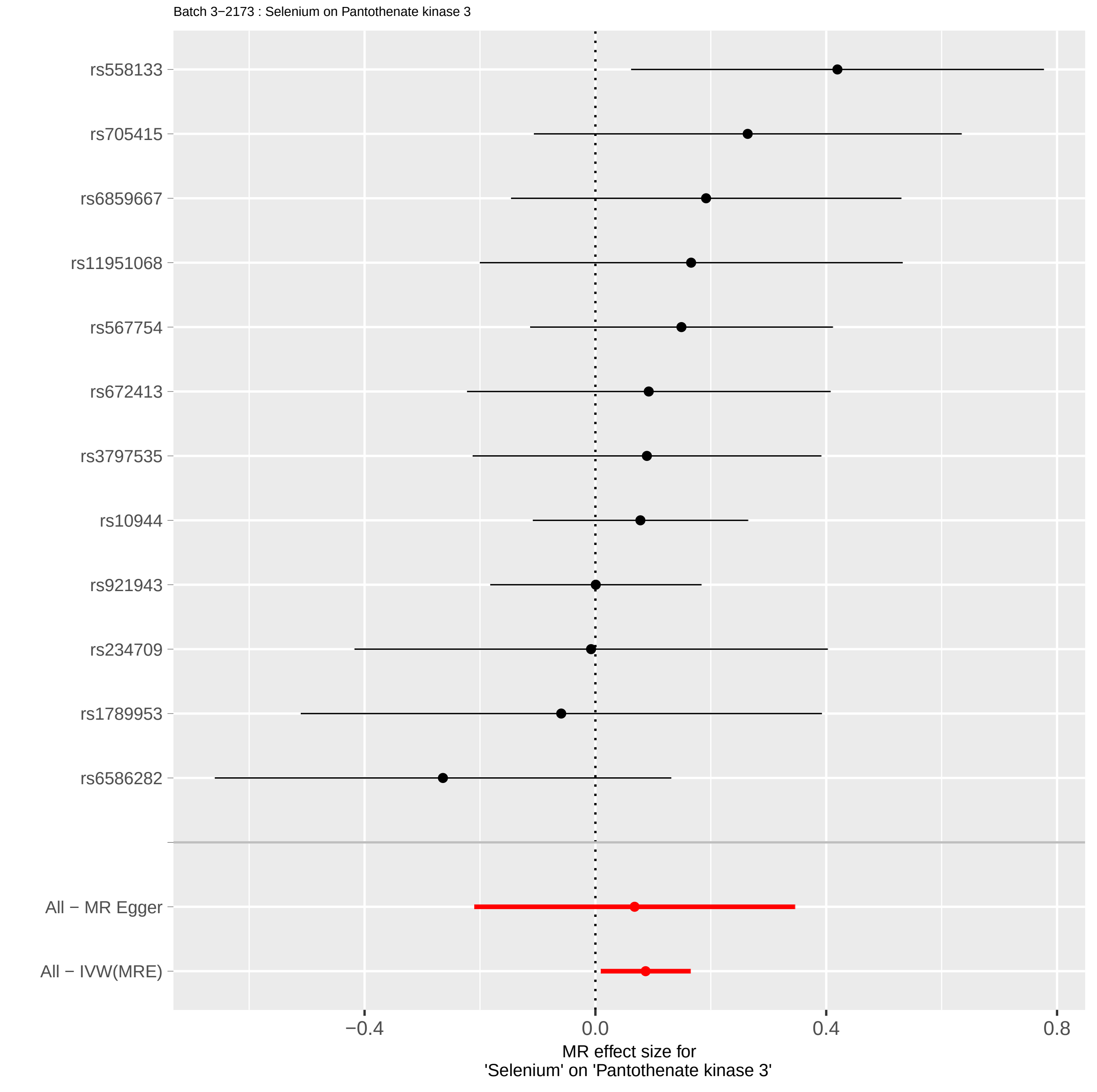

Batch 3−2173 : Selenium on Pantothenate kinase 3
rs558133
rs705415
rs6859667
rs11951068
rs567754
rs672413
rs3797535
rs10944
rs921943
rs234709
rs1789953
rs6586282
All − MR Egger
All − IVW(MRE)
−0.4
0.0
0.4
0.8
MR effect size for
'Selenium' on 'Pantothenate kinase 3'

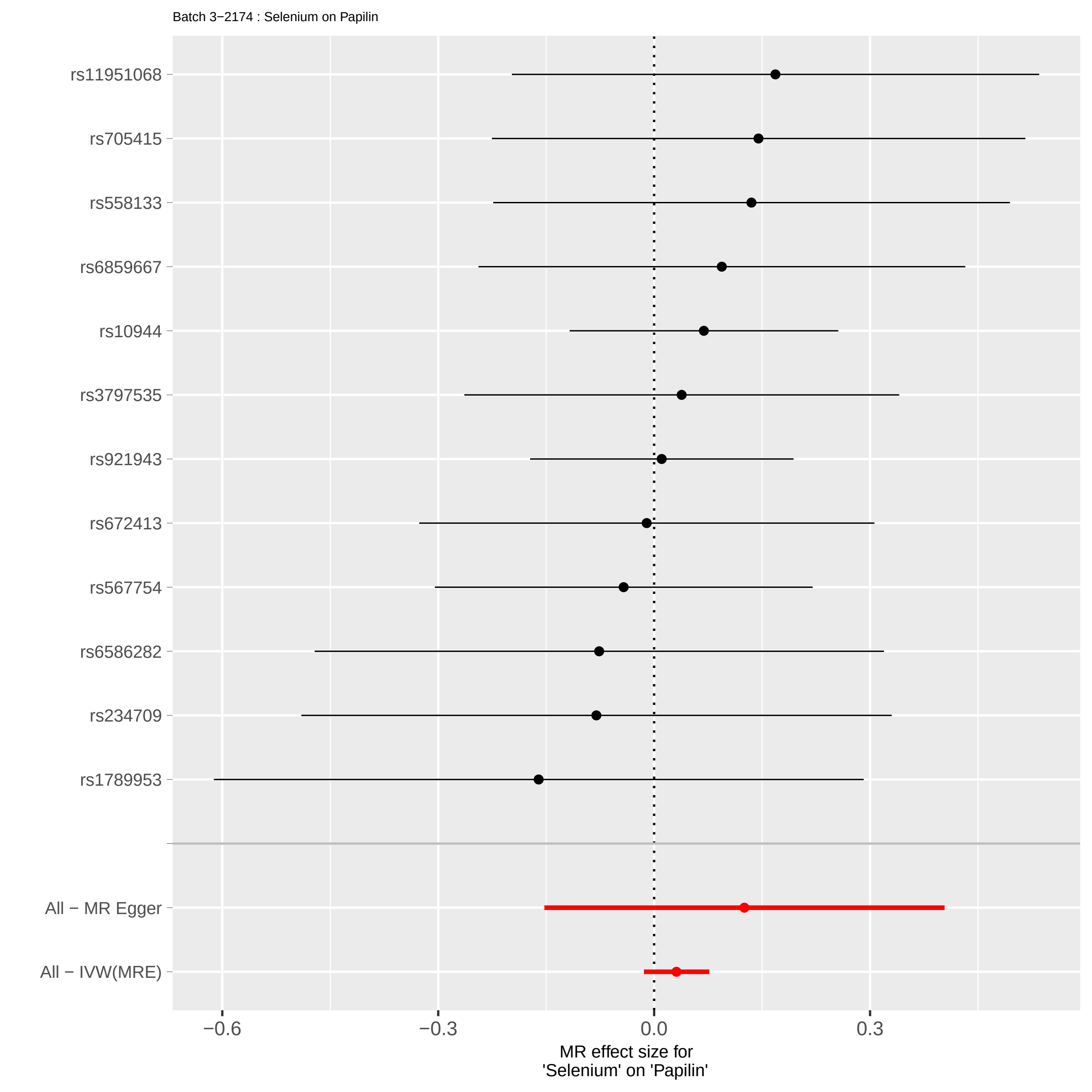
Batch 3−2174 : Selenium on Papilin
rs11951068
rs705415
rs558133
rs6859667
rs10944
rs3797535
rs921943
rs672413
rs567754
rs6586282
rs234709
rs1789953
All − MR Egger
All − IVW(MRE)
−0.6
−0.3
0.0
0.3
MR effect size for
'Selenium' on 'Papilin'

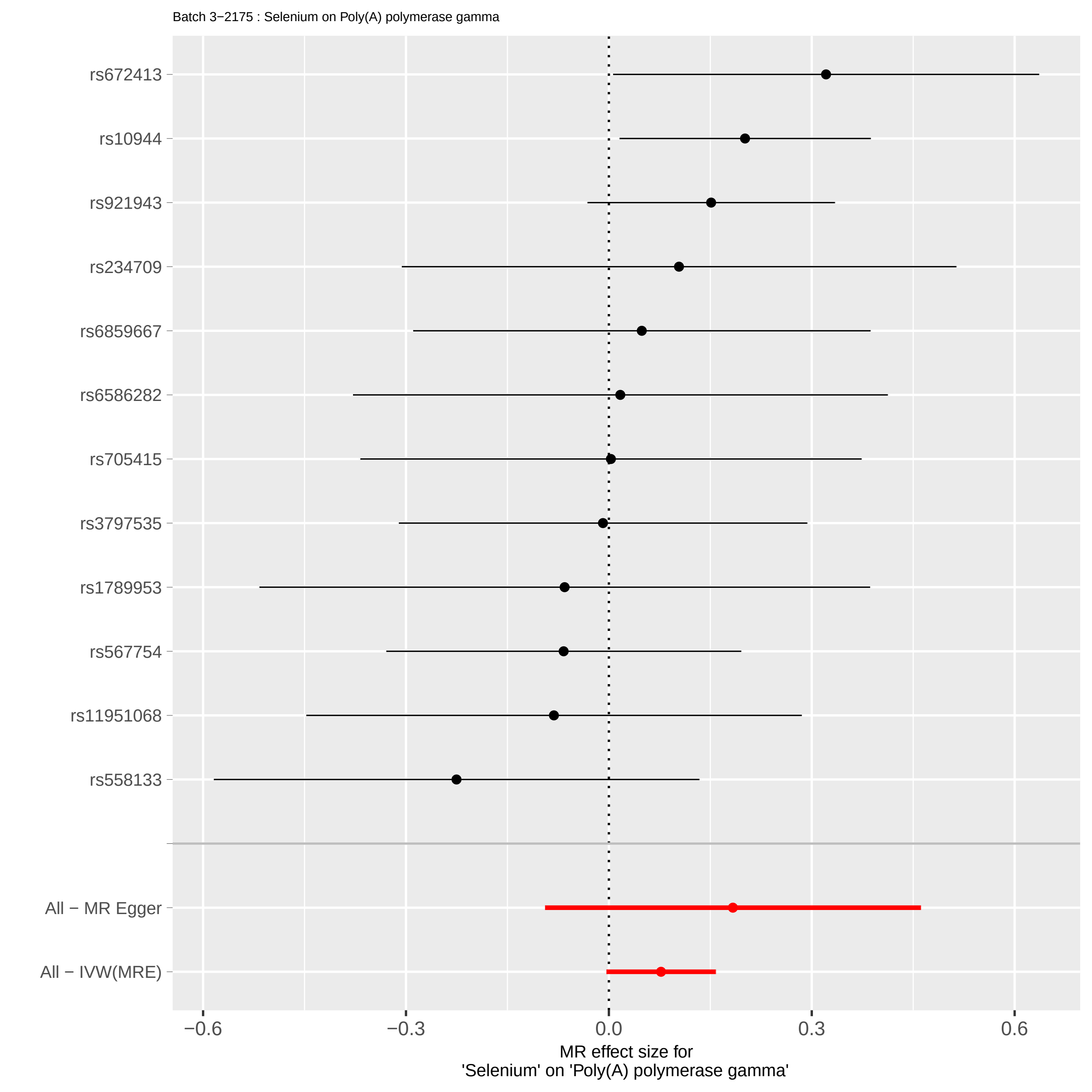
Batch 3−2175 : Selenium on Poly(A) polymerase gamma
rs672413
rs10944
rs921943
rs234709
rs6859667
rs6586282
rs705415
rs3797535
rs1789953
rs567754
rs11951068
rs558133
All − MR Egger
All − IVW(MRE)
−0.6
−0.3
0.0
0.3
0.6
MR effect size for
'Selenium' on 'Poly(A) polymerase gamma'

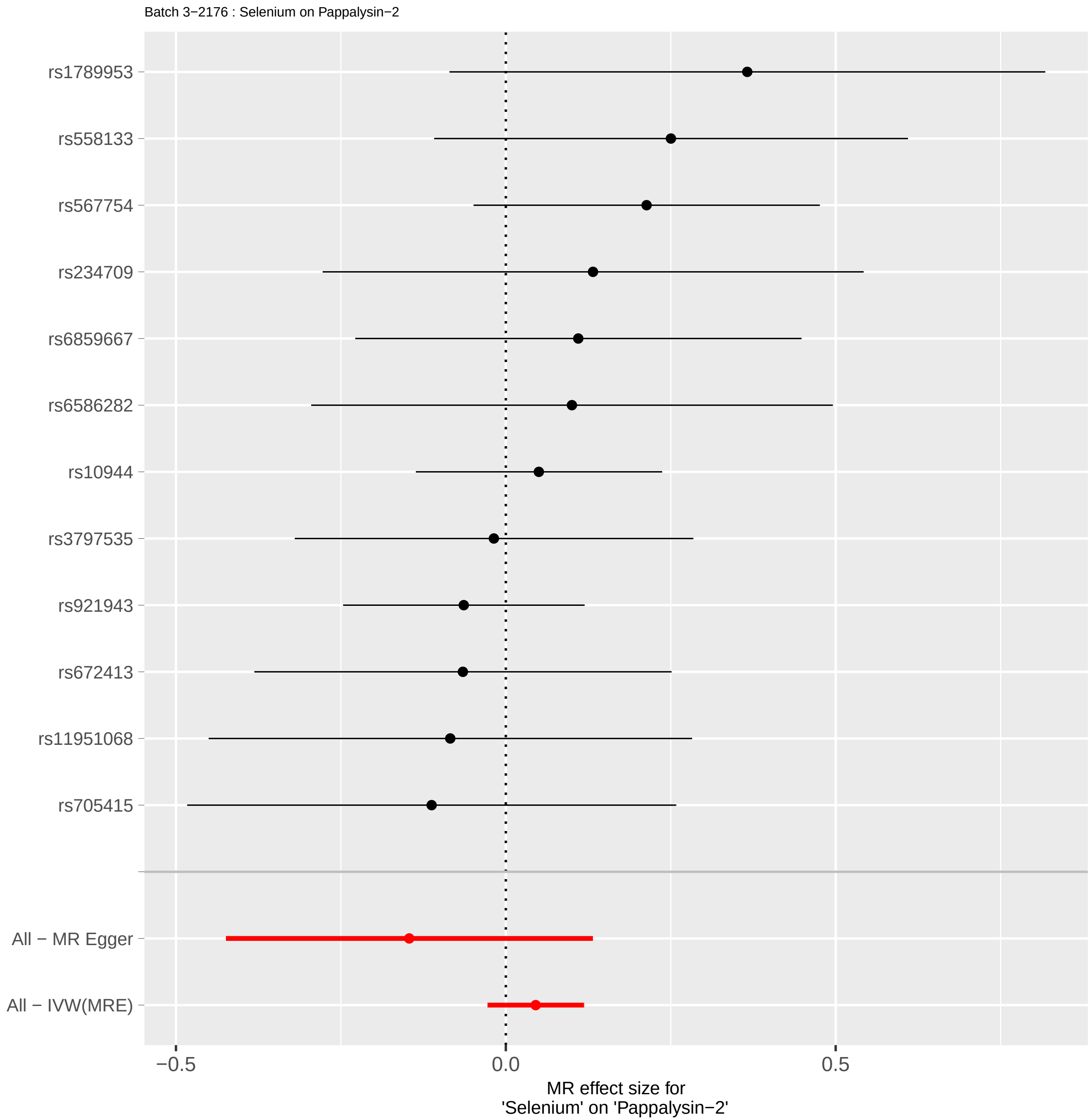
Batch 3−2176 : Selenium on Pappalysin−2
rs1789953
rs558133
rs567754
rs234709
rs6859667
rs6586282
rs10944
rs3797535
rs921943
rs672413
rs11951068
rs705415
All − MR Egger
All − IVW(MRE)
−0.5
0.0
0.5
MR effect size for
'Selenium' on 'Pappalysin−2'

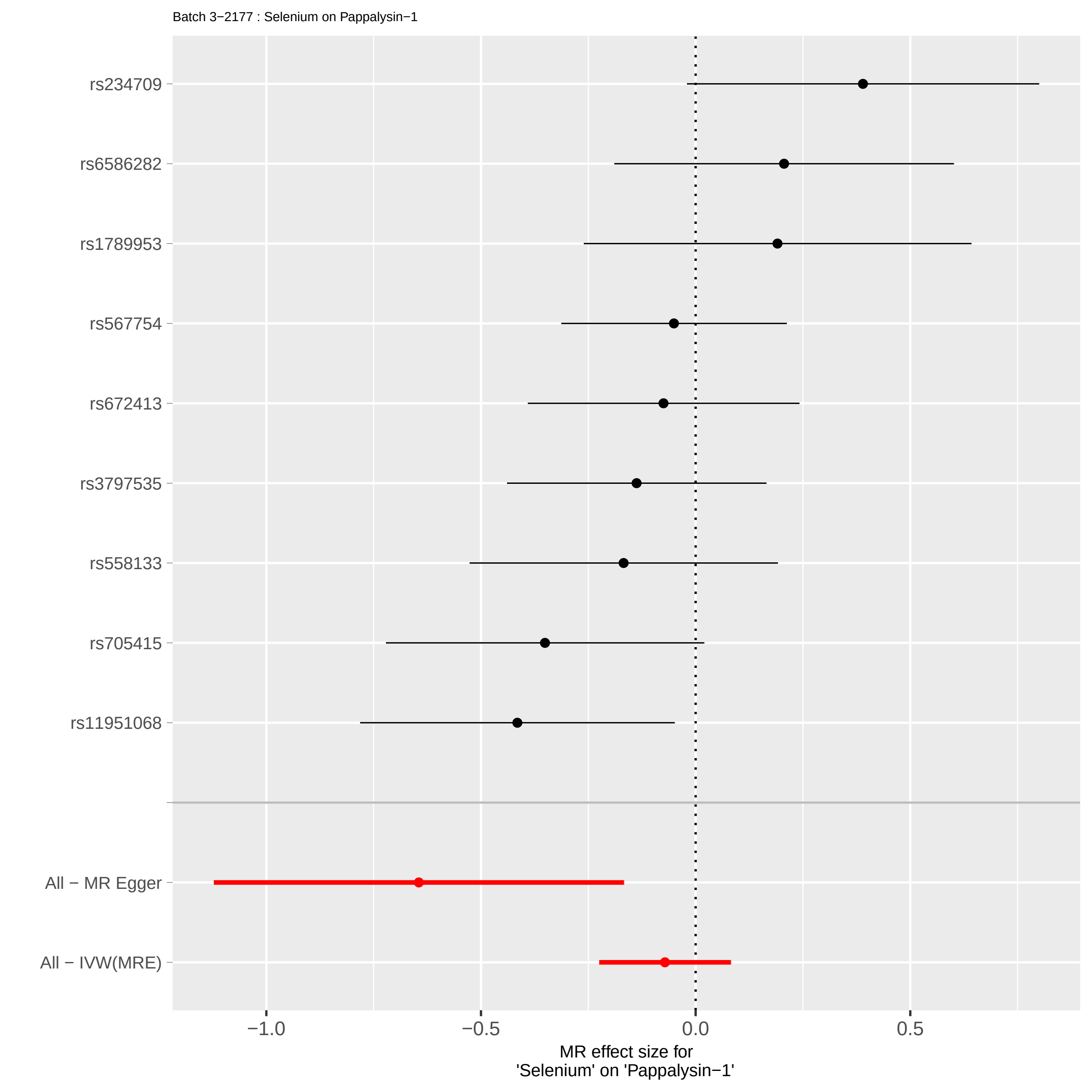

Batch 3−2177 : Selenium on Pappalysin−1
rs234709
rs6586282
rs1789953
rs567754
rs672413
rs3797535
rs558133
rs705415
rs11951068
All − MR Egger
All − IVW(MRE)
−1.0
−0.5
0.0
0.5
MR effect size for
'Selenium' on 'Pappalysin−1'

Batch 3−2178 : Selenium on Bifunctional 3'−phosphoadenosine 5'−phosphosulfate synthase 1

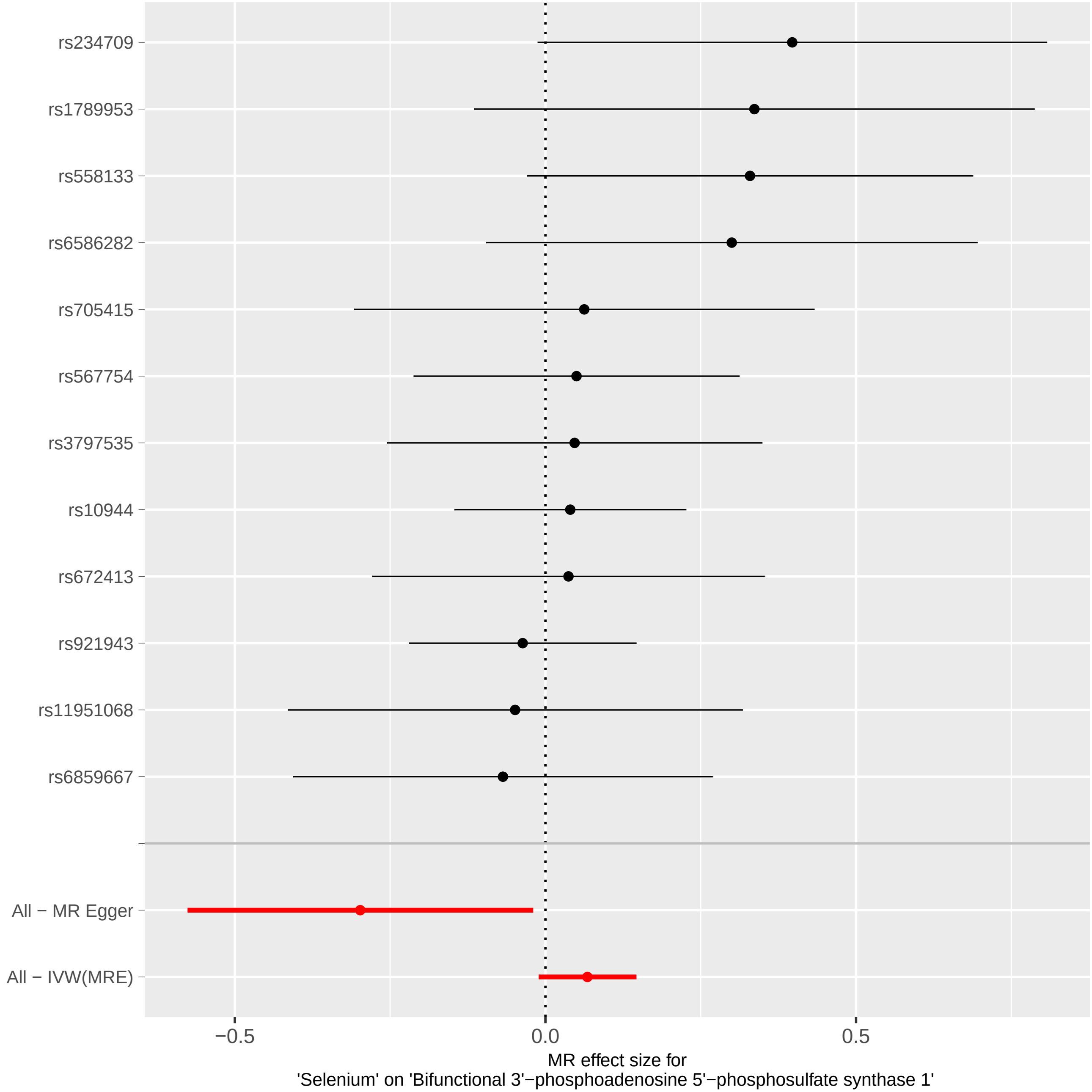

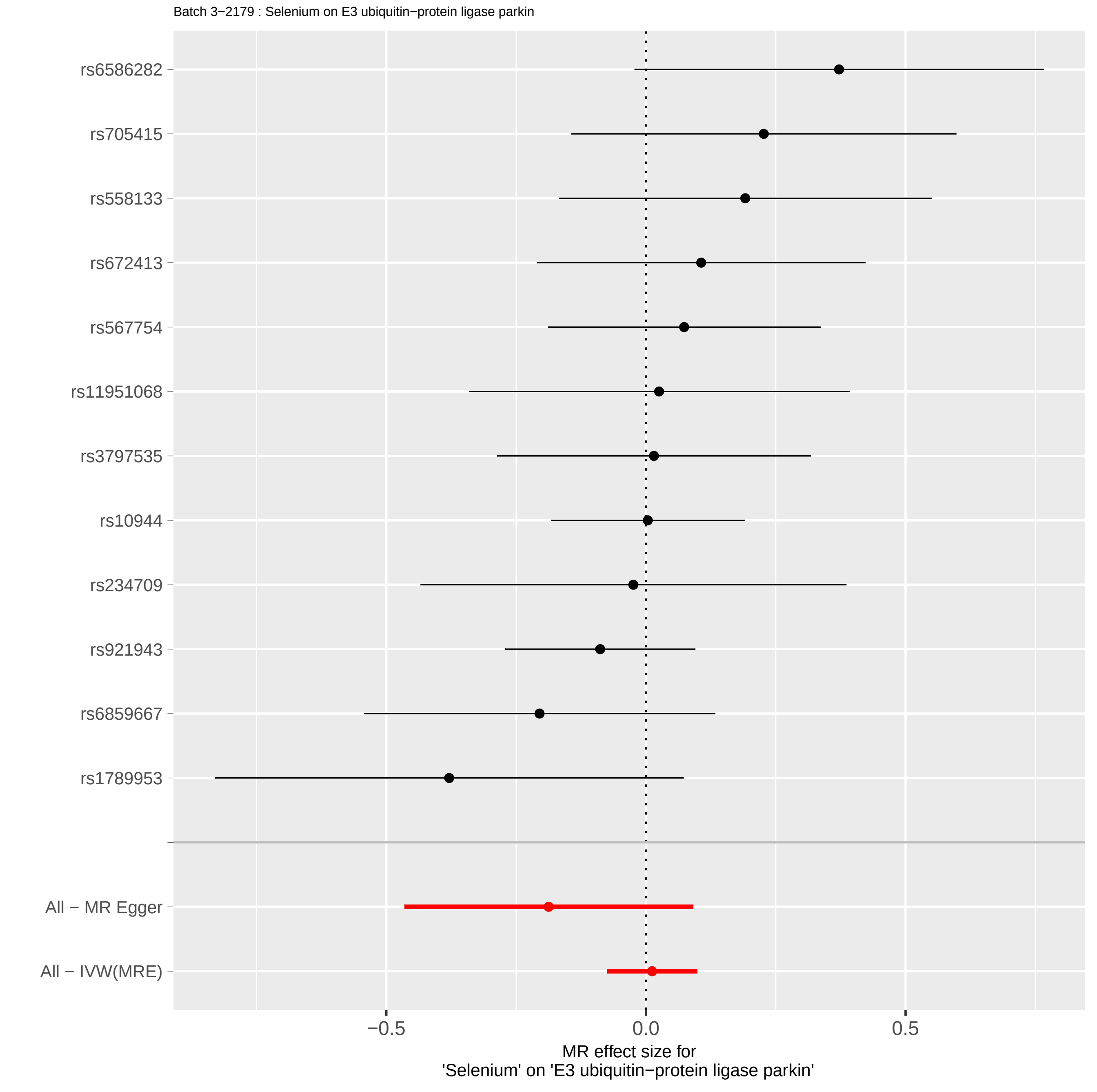
Batch 3−2179 : Selenium on E3 ubiquitin−protein ligase parkin
rs6586282
rs705415
rs558133
rs672413
rs567754
rs11951068
rs3797535
rs10944
rs234709
rs921943
rs6859667
rs1789953
All − MR Egger
All − IVW(MRE)
−0.5
0.0
0.5
MR effect size for
'Selenium' on 'E3 ubiquitin−protein ligase parkin'

Batch 3−2180 : Selenium on Protein deglycase DJ−1

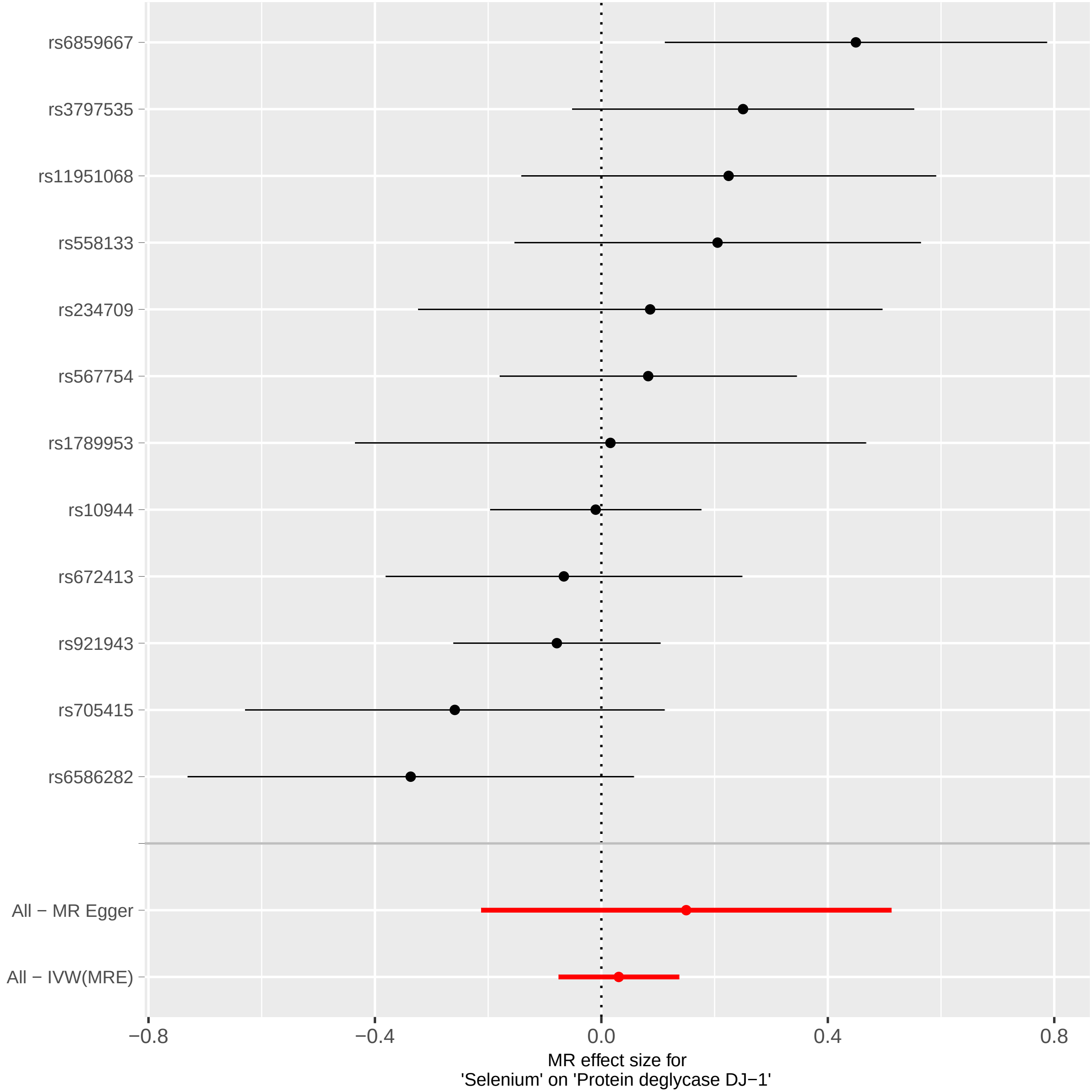

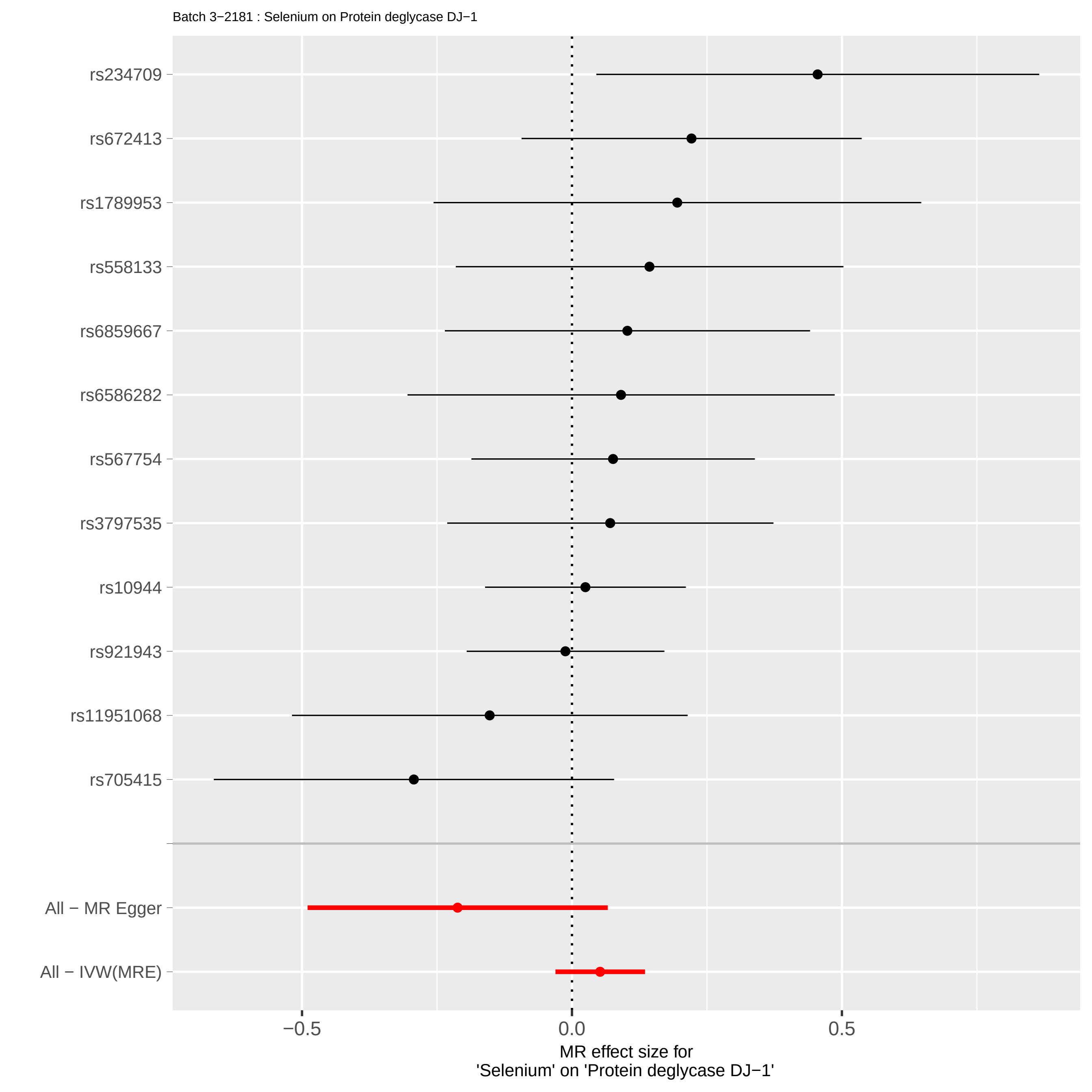
Batch 3−2181 : Selenium on Protein deglycase DJ−1
rs234709
rs672413
rs1789953
rs558133
rs6859667
rs6586282
rs567754
rs3797535
rs10944
rs921943
rs11951068
rs705415
All − MR Egger
All − IVW(MRE)
−0.5
0.0
0.5
MR effect size for
'Selenium' on 'Protein deglycase DJ−1'

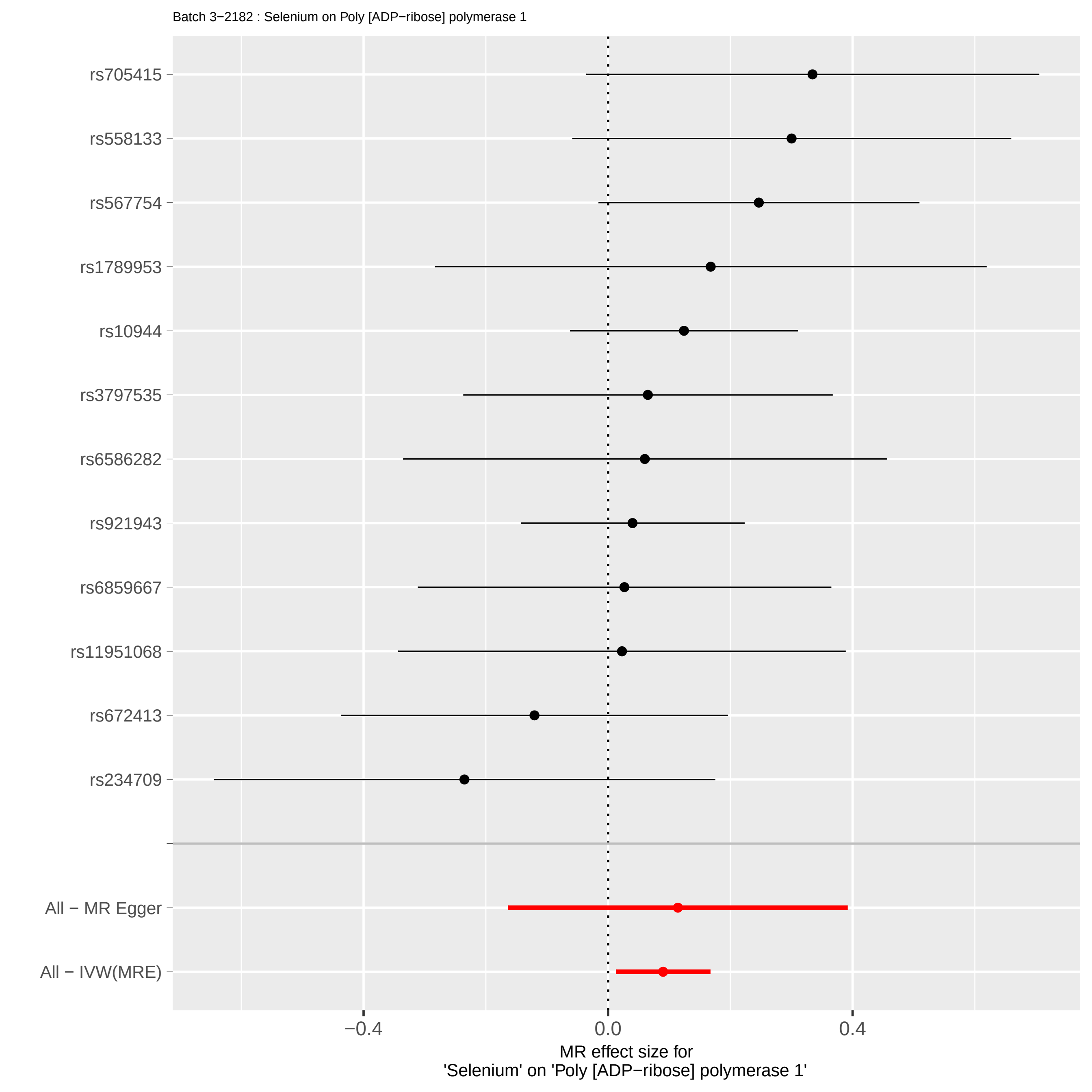
Batch 3−2182 : Selenium on Poly [ADP−ribose] polymerase 1
rs705415
rs558133
rs567754
rs1789953
rs10944
rs3797535
rs6586282
rs921943
rs6859667
rs11951068
rs672413
rs234709
All − MR Egger
All − IVW(MRE)
−0.4
0.0
0.4
MR effect size for
'Selenium' on 'Poly [ADP−ribose] polymerase 1'

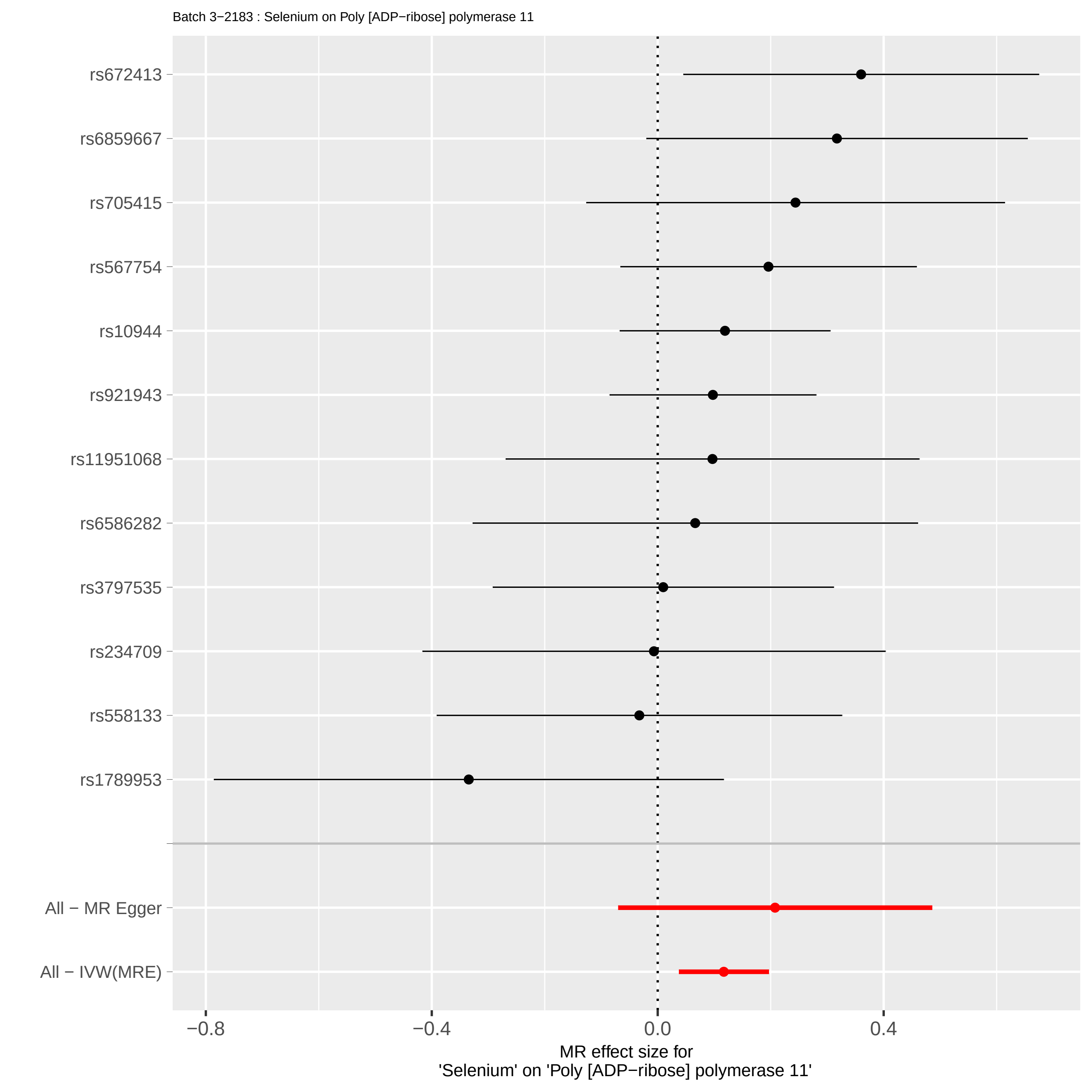

Batch 3−2183 : Selenium on Poly [ADP−ribose] polymerase 11
rs672413
rs6859667
rs705415
rs567754
rs10944
rs921943
rs11951068
rs6586282
rs3797535
rs234709
rs558133
rs1789953
All − MR Egger
All − IVW(MRE)
−0.8
−0.4
0.0
0.4
MR effect size for
'Selenium' on 'Poly [ADP−ribose] polymerase 11'

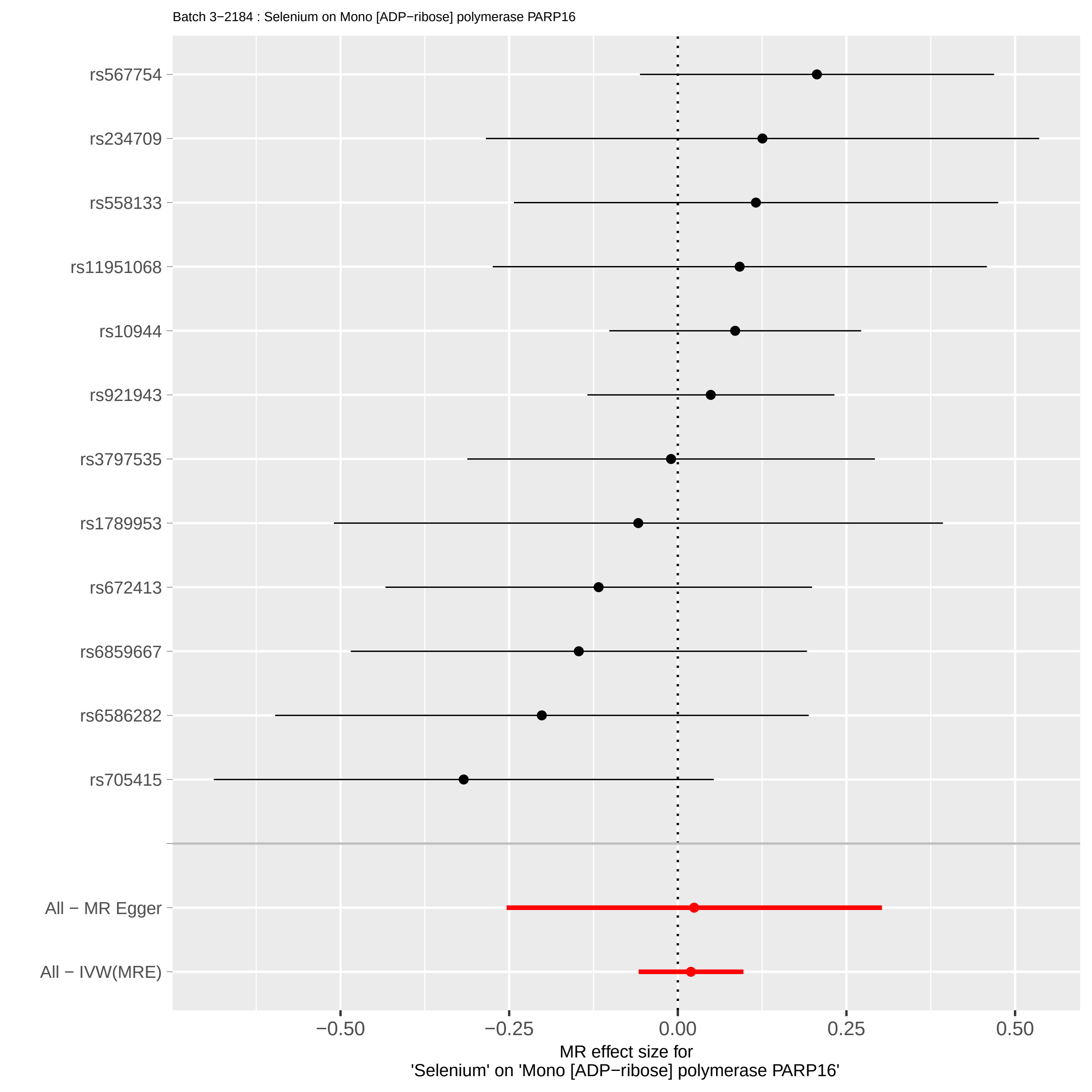

Batch 3−2184 : Selenium on Mono [ADP−ribose] polymerase PARP16
rs567754
rs234709
rs558133
rs11951068
rs10944
rs921943
rs3797535
rs1789953
rs672413
rs6859667
rs6586282
rs705415
All − MR Egger
All − IVW(MRE)
−0.50
−0.25
0.00
0.25
0.50
MR effect size for
'Selenium' on 'Mono [ADP−ribose] polymerase PARP16'

Batch 3−2185 : Selenium on Alpha−parvin

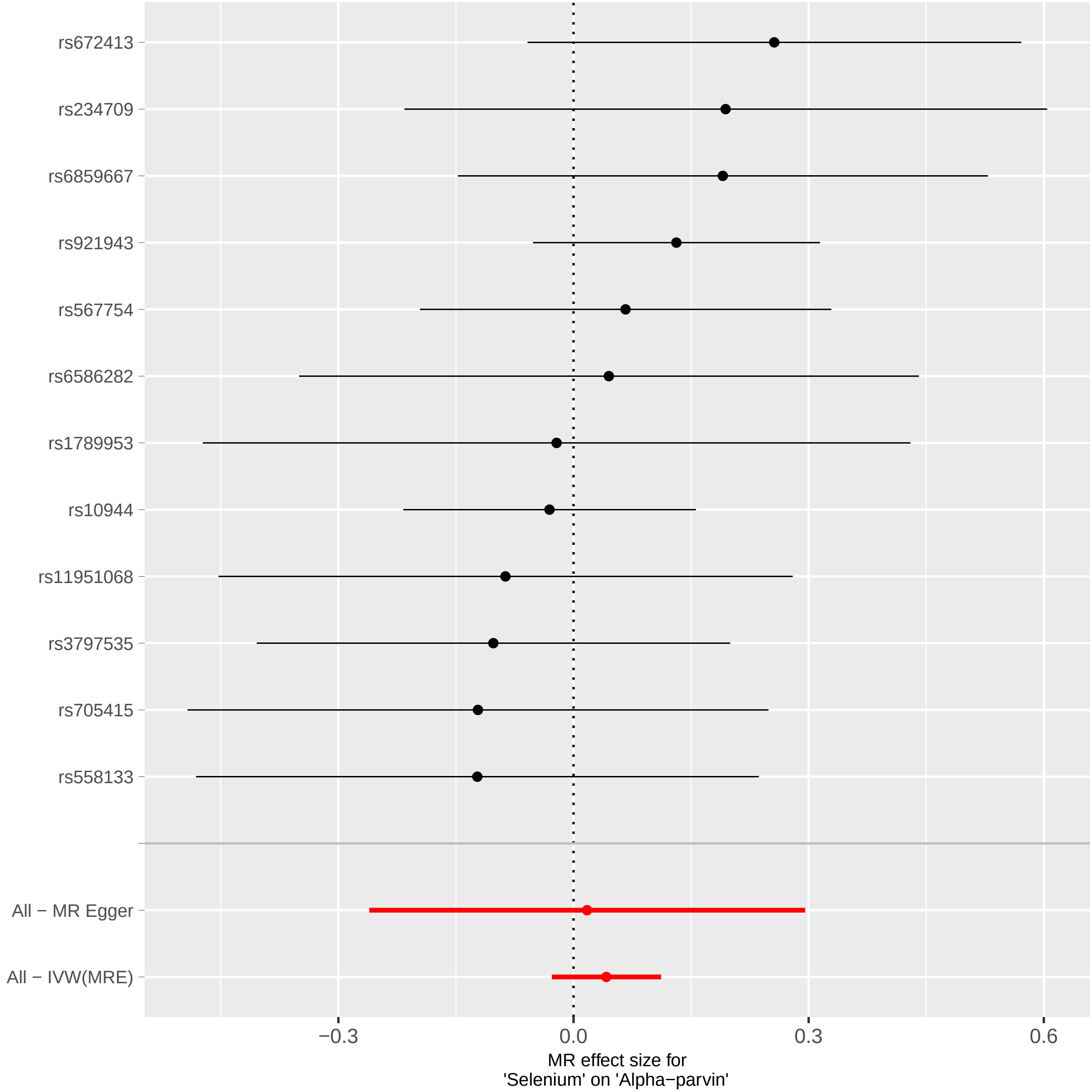

Batch 3−2186 : Selenium on Prostate and testis expressed protein 1

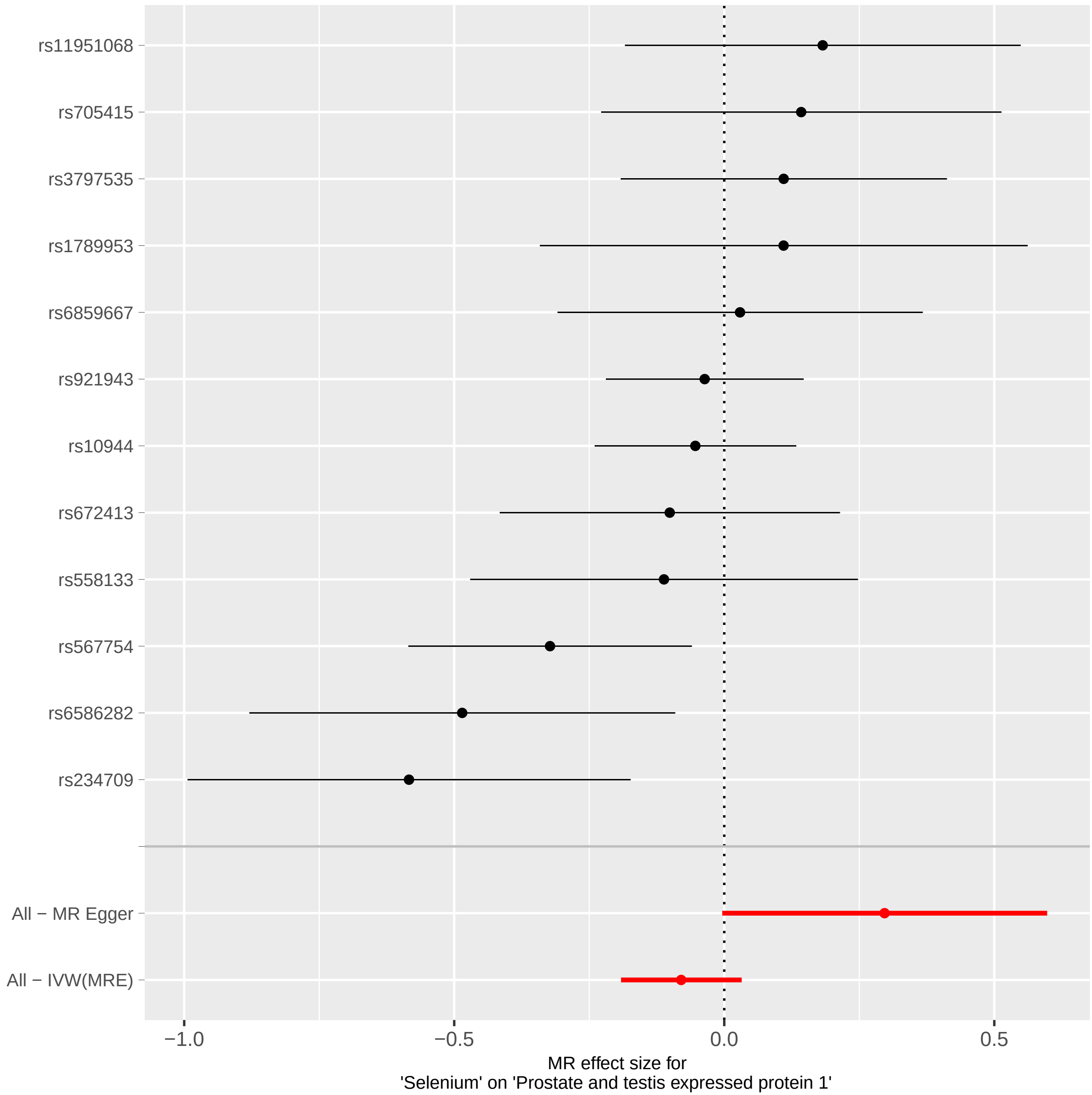

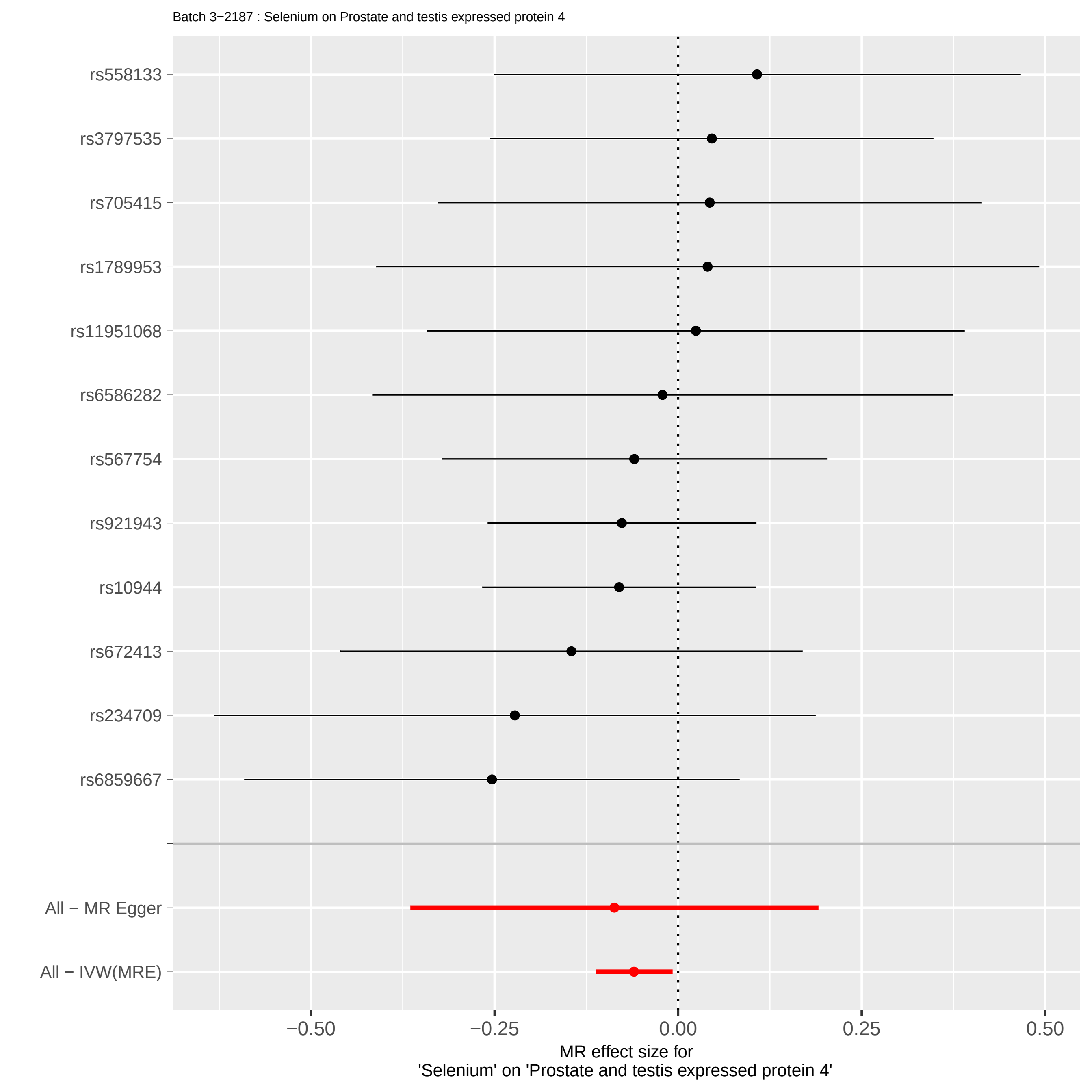

Batch 3−2187 : Selenium on Prostate and testis expressed protein 4
rs558133
rs3797535
rs705415
rs1789953
rs11951068
rs6586282
rs567754
rs921943
rs10944
rs672413
rs234709
rs6859667
All − MR Egger
All − IVW(MRE)
−0.50
−0.25
0.00
0.25
0.50
MR effect size for
'Selenium' on 'Prostate and testis expressed protein 4'

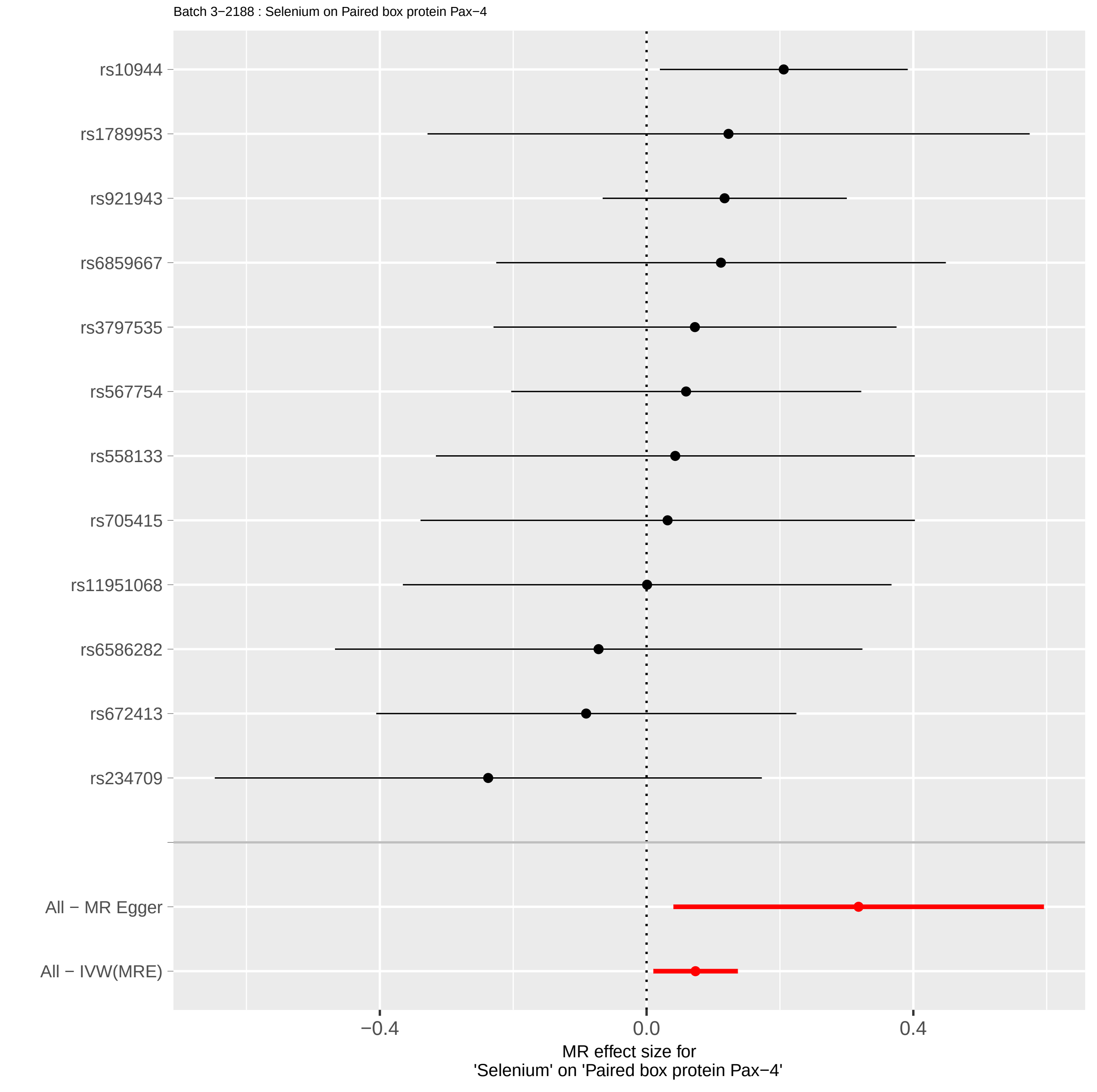

Batch 3−2188 : Selenium on Paired box protein Pax−4
rs10944
rs1789953
rs921943
rs6859667
rs3797535
rs567754
rs558133
rs705415
rs11951068
rs6586282
rs672413
rs234709
All − MR Egger
All − IVW(MRE)
−0.4
0.0
0.4
MR effect size for
'Selenium' on 'Paired box protein Pax−4'

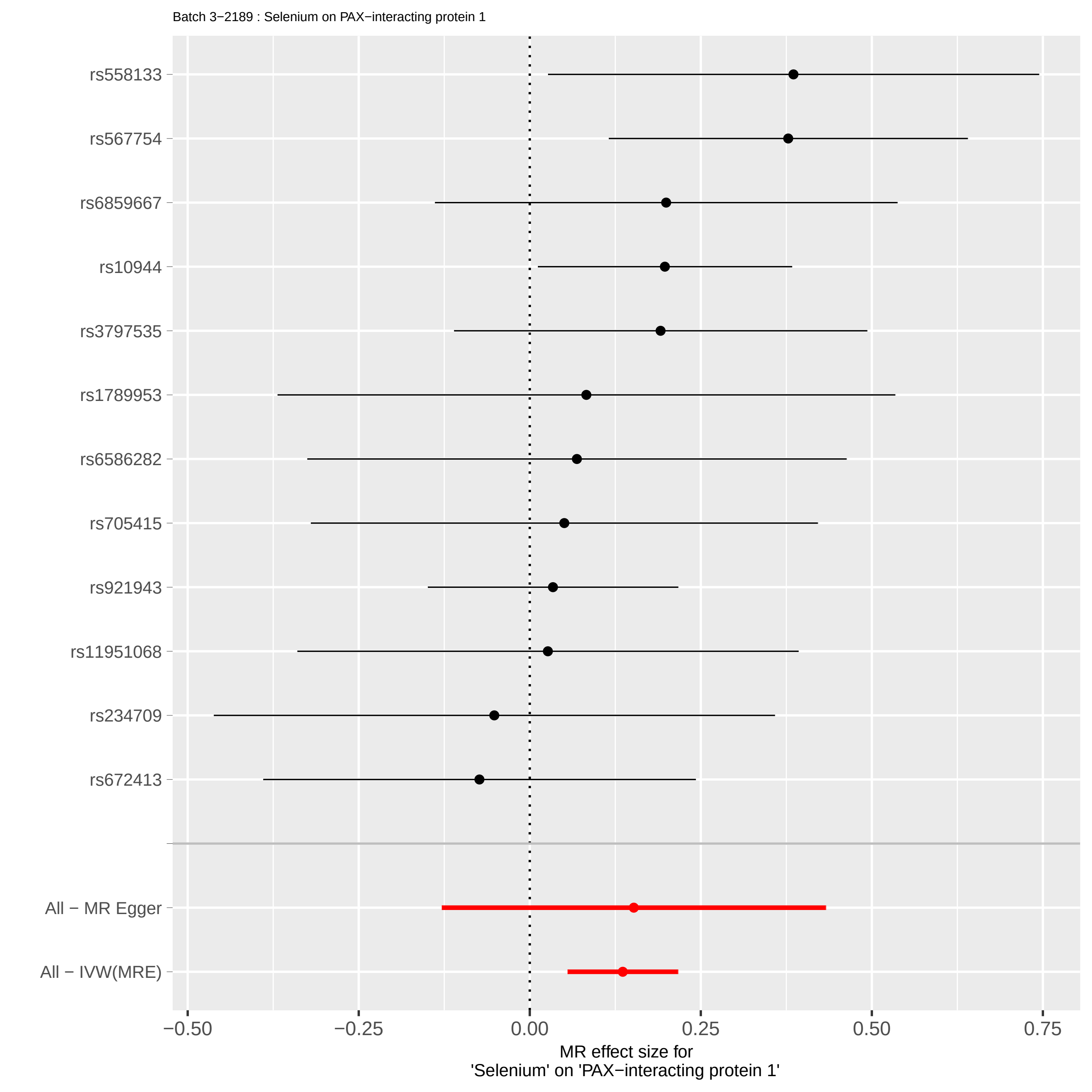

Batch 3−2189 : Selenium on PAX−interacting protein 1
rs558133
rs567754
rs6859667
rs10944
rs3797535
rs1789953
rs6586282
rs705415
rs921943
rs11951068
rs234709
rs672413
All − MR Egger
All − IVW(MRE)
−0.50
−0.25
0.00
0.25
0.50
0.75
MR effect size for
'Selenium' on 'PAX−interacting protein 1'

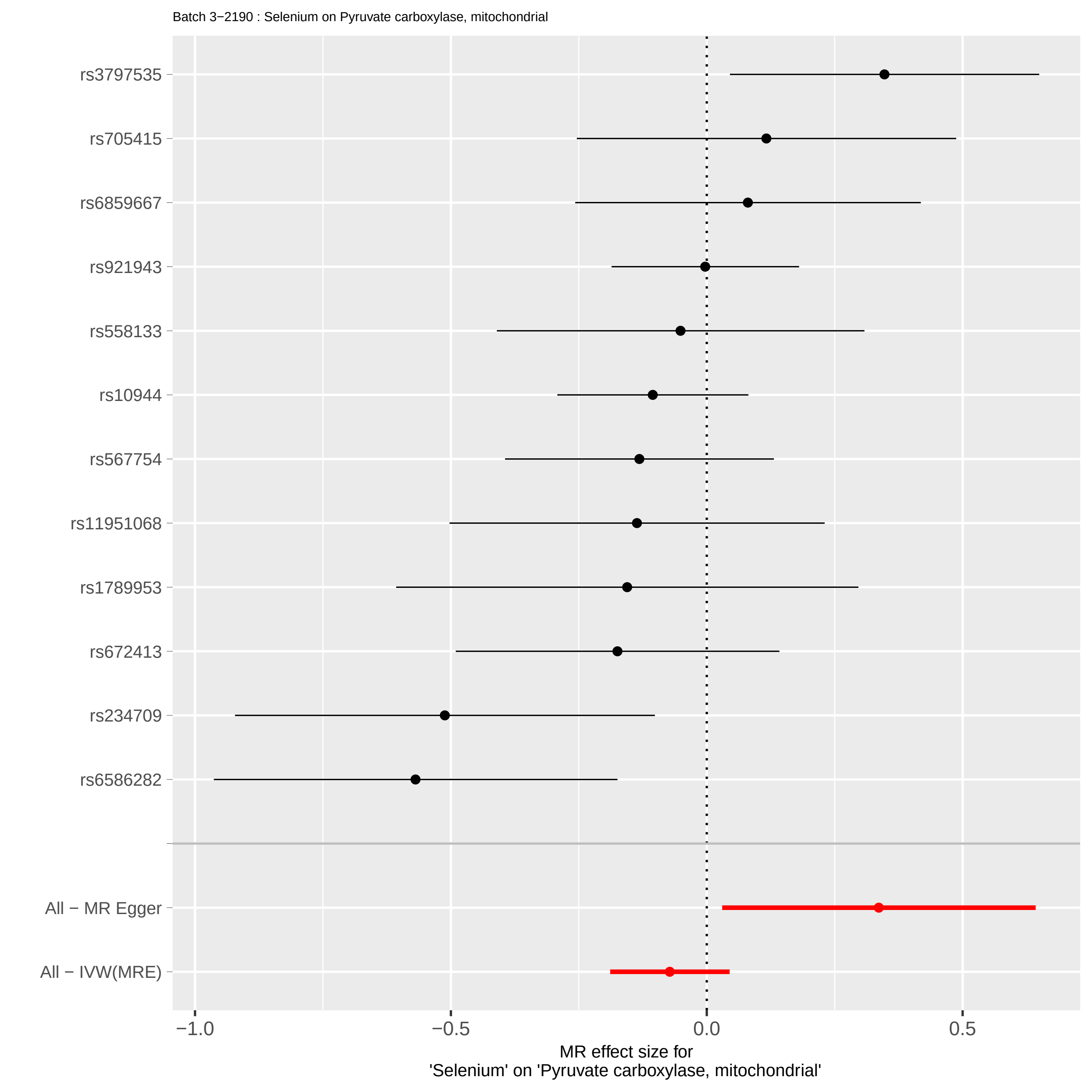
Batch 3−2190 : Selenium on Pyruvate carboxylase, mitochondrial
rs3797535
rs705415
rs6859667
rs921943
rs558133
rs10944
rs567754
rs11951068
rs1789953
rs672413
rs234709
rs6586282
All − MR Egger
All − IVW(MRE)
−1.0
−0.5
0.0
0.5
MR effect size for
'Selenium' on 'Pyruvate carboxylase, mitochondrial'

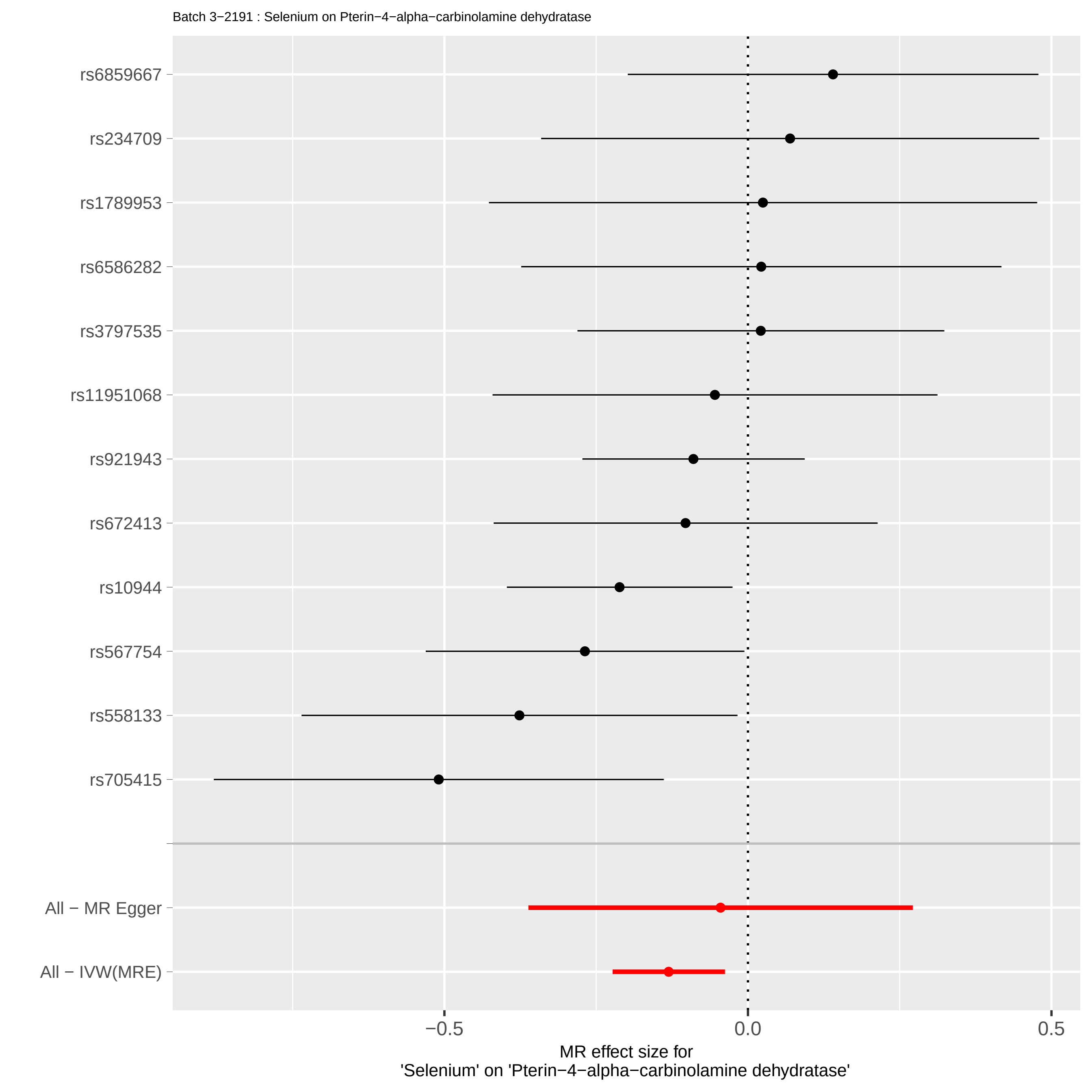
Batch 3−2191 : Selenium on Pterin−4−alpha−carbinolamine dehydratase
rs6859667
rs234709
rs1789953
rs6586282
rs3797535
rs11951068
rs921943
rs672413
rs10944
rs567754
rs558133
rs705415
All − MR Egger
All − IVW(MRE)
−0.5
0.0
0.5
MR effect size for
'Selenium' on 'Pterin−4−alpha−carbinolamine dehydratase'

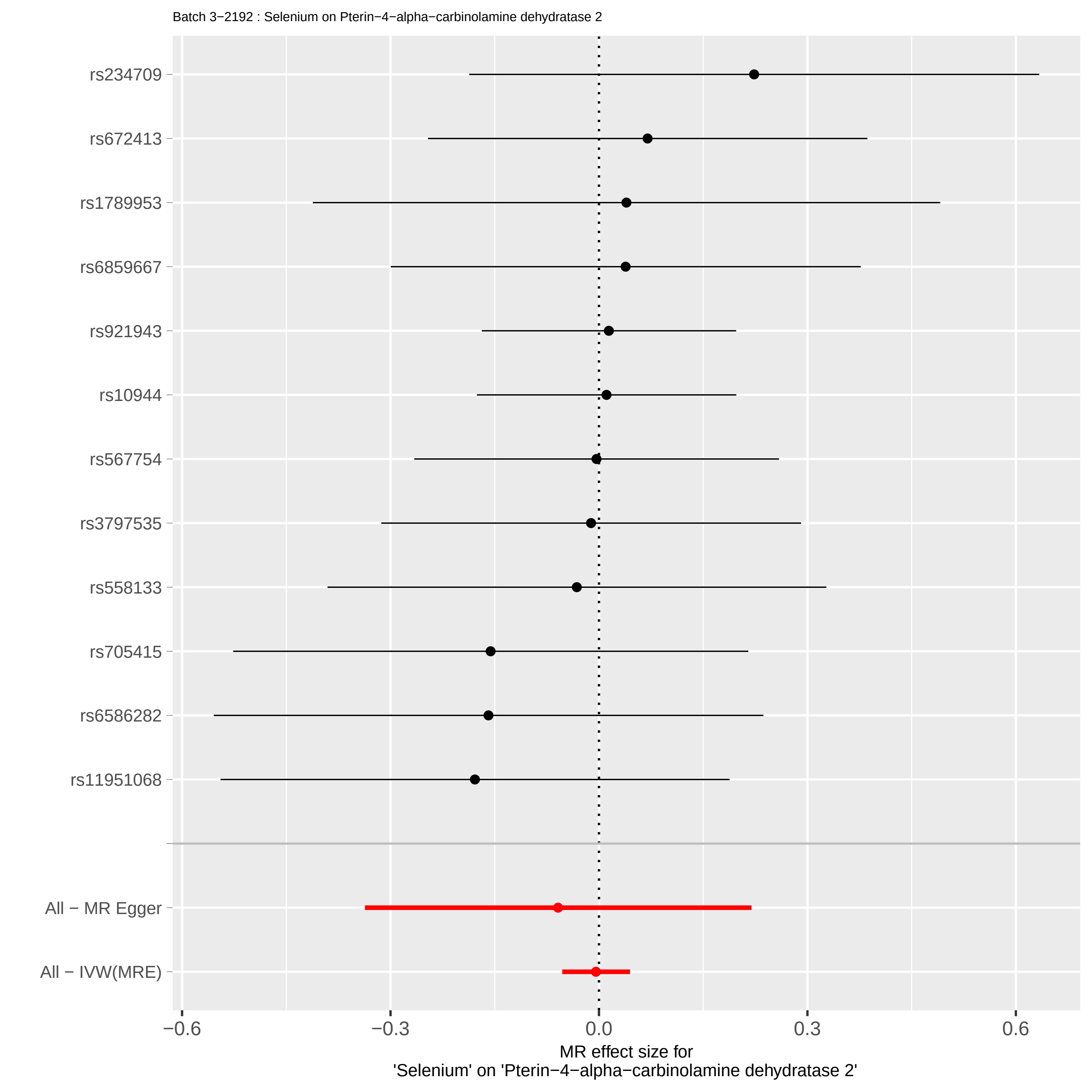

Batch 3−2192 : Selenium on Pterin−4−alpha−carbinolamine dehydratase 2
rs234709
rs672413
rs1789953
rs6859667
rs921943
rs10944
rs567754
rs3797535
rs558133
rs705415
rs6586282
rs11951068
All − MR Egger
All − IVW(MRE)
−0.6
−0.3
0.0
0.3
0.6
MR effect size for
'Selenium' on 'Pterin−4−alpha−carbinolamine dehydratase 2'

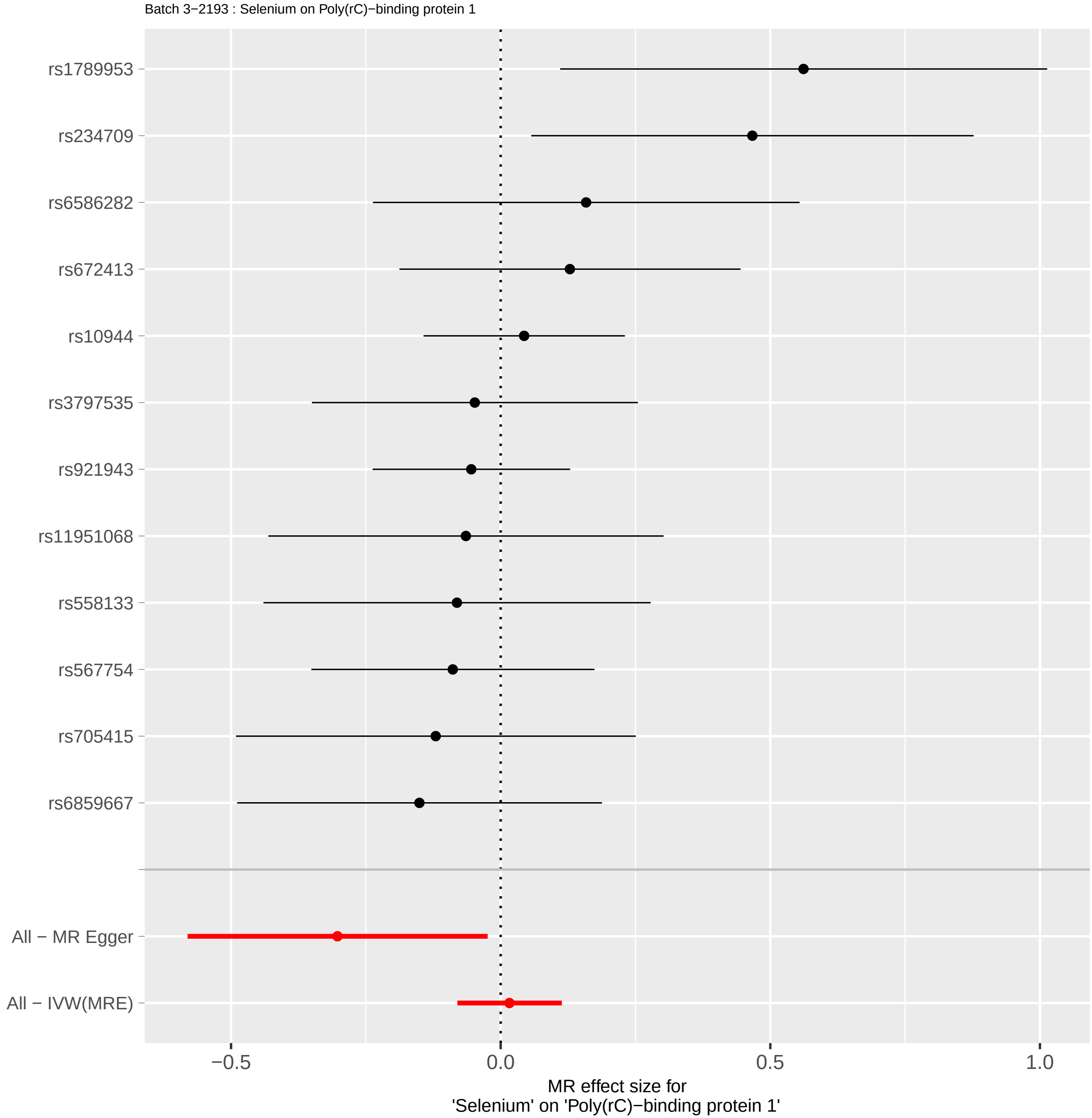

Batch 3−2193 : Selenium on Poly(rC)−binding protein 1
rs1789953
rs234709
rs6586282
rs672413
rs10944
rs3797535
rs921943
rs11951068
rs558133
rs567754
rs705415
rs6859667
All − MR Egger
All − IVW(MRE)
−0.5
0.0
0.5
1.0
MR effect size for
'Selenium' on 'Poly(rC)−binding protein 1'

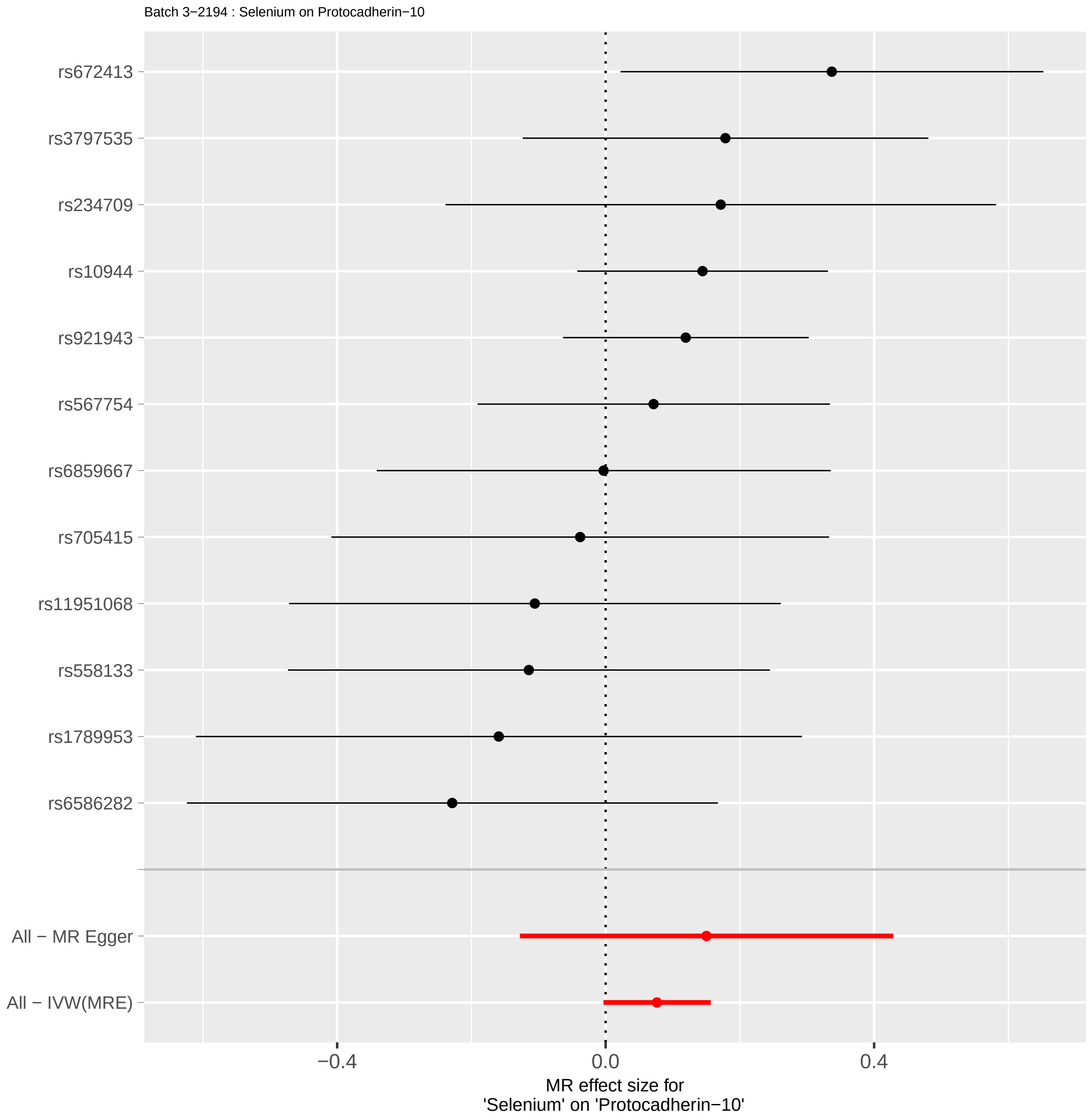
Batch 3−2194 : Selenium on Protocadherin−10
rs672413
rs3797535
rs234709
rs10944
rs921943
rs567754
rs6859667
rs705415
rs11951068
rs558133
rs1789953
rs6586282
All − MR Egger
All − IVW(MRE)
−0.4
0.0
0.4
MR effect size for
'Selenium' on 'Protocadherin−10'

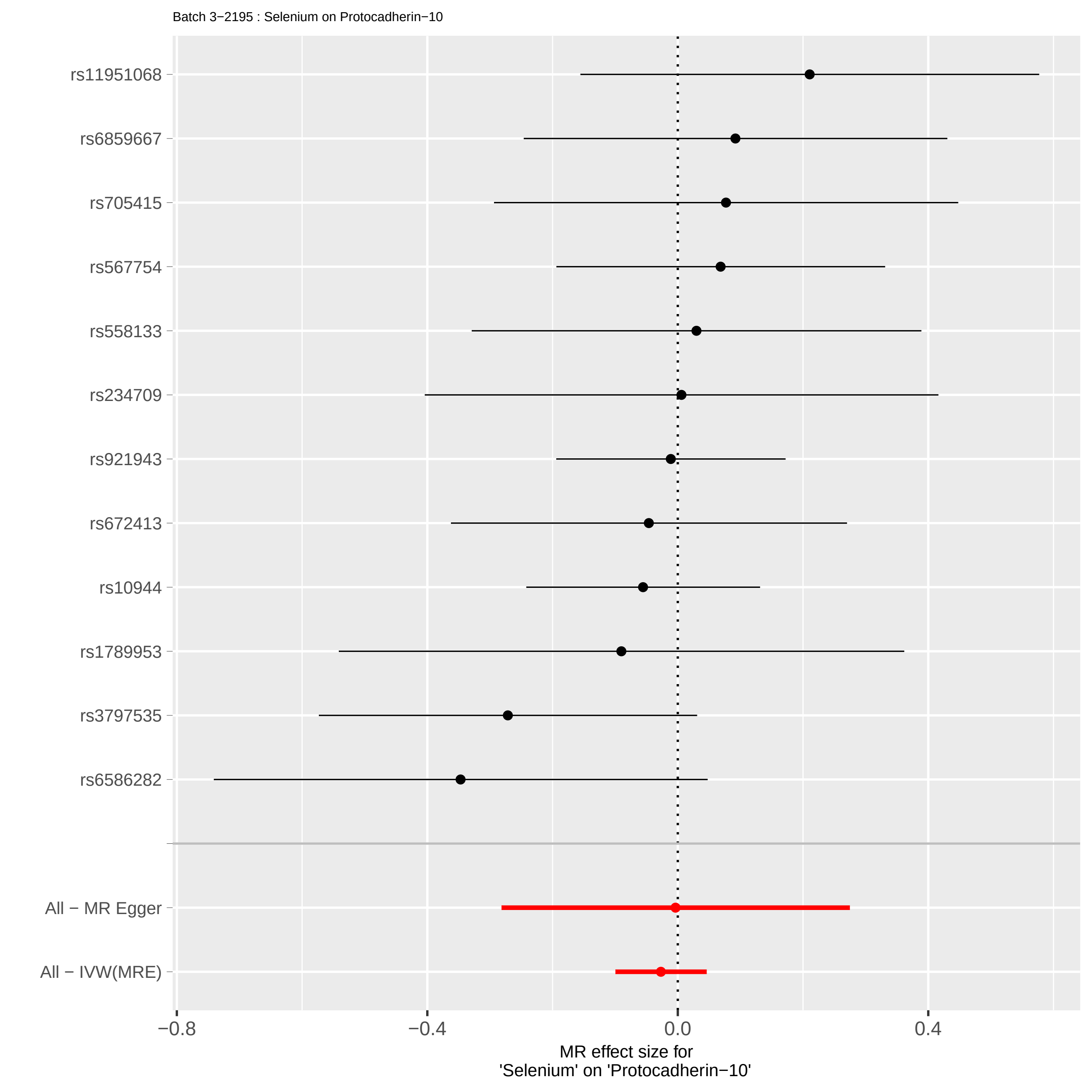

Batch 3−2195 : Selenium on Protocadherin−10
rs11951068
rs6859667
rs705415
rs567754
rs558133
rs234709
rs921943
rs672413
rs10944
rs1789953
rs3797535
rs6586282
All − MR Egger
All − IVW(MRE)
−0.8
−0.4
0.0
0.4
MR effect size for
'Selenium' on 'Protocadherin−10'

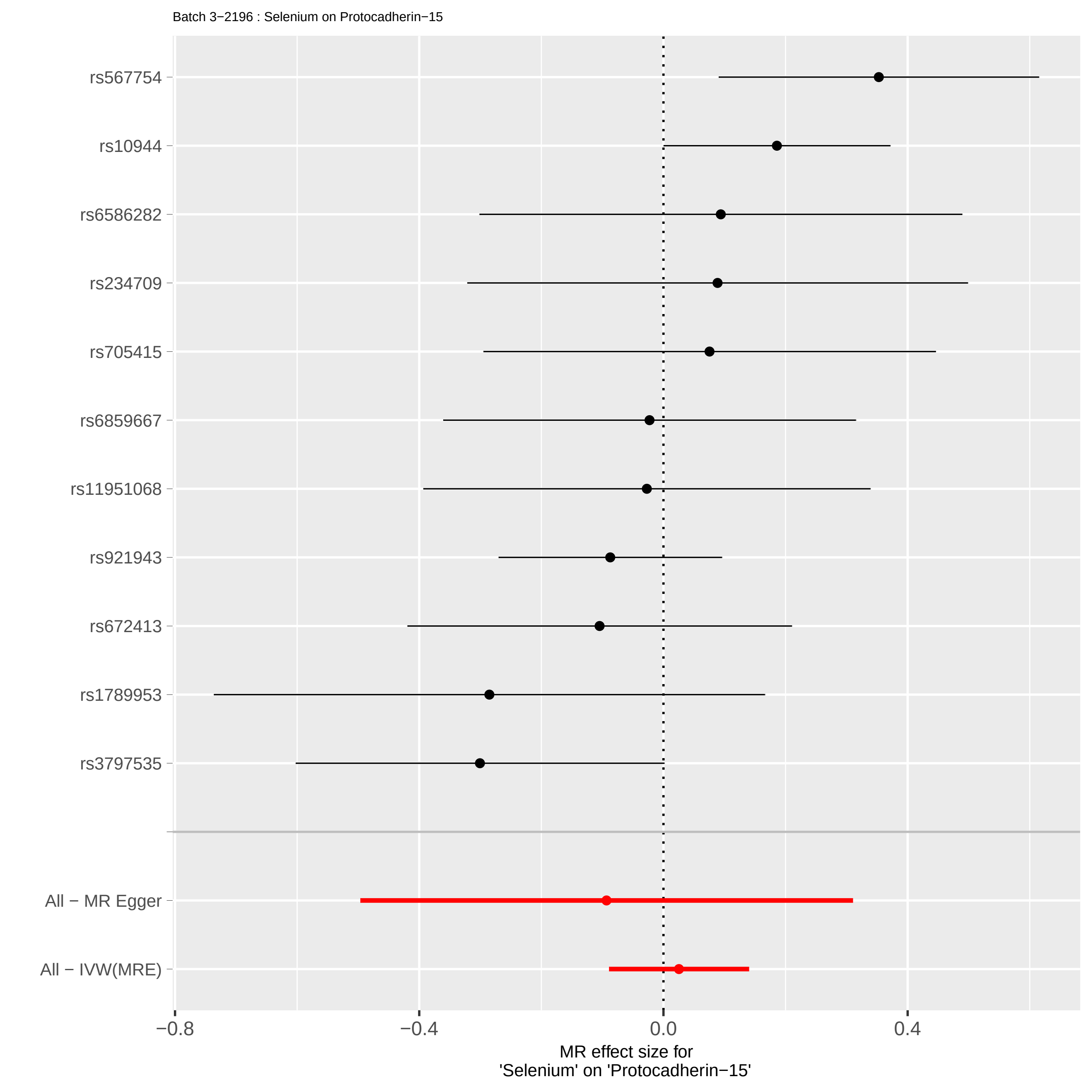

Batch 3−2196 : Selenium on Protocadherin−15
rs567754
rs10944
rs6586282
rs234709
rs705415
rs6859667
rs11951068
rs921943
rs672413
rs1789953
rs3797535
All − MR Egger
All − IVW(MRE)
−0.8
−0.4
0.0
0.4
MR effect size for
'Selenium' on 'Protocadherin−15'

Batch 3−2197 : Selenium on Protocadherin−9

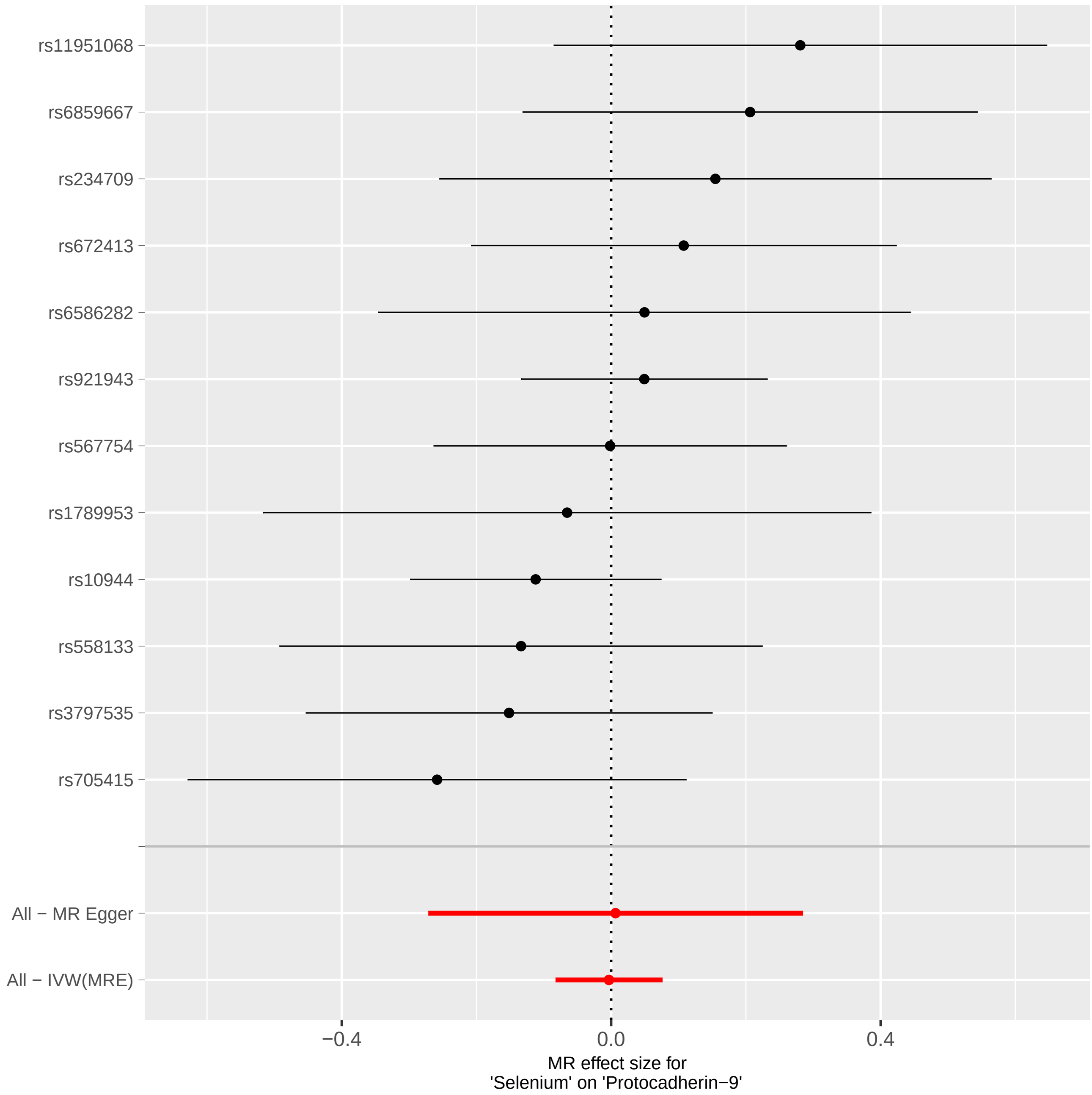

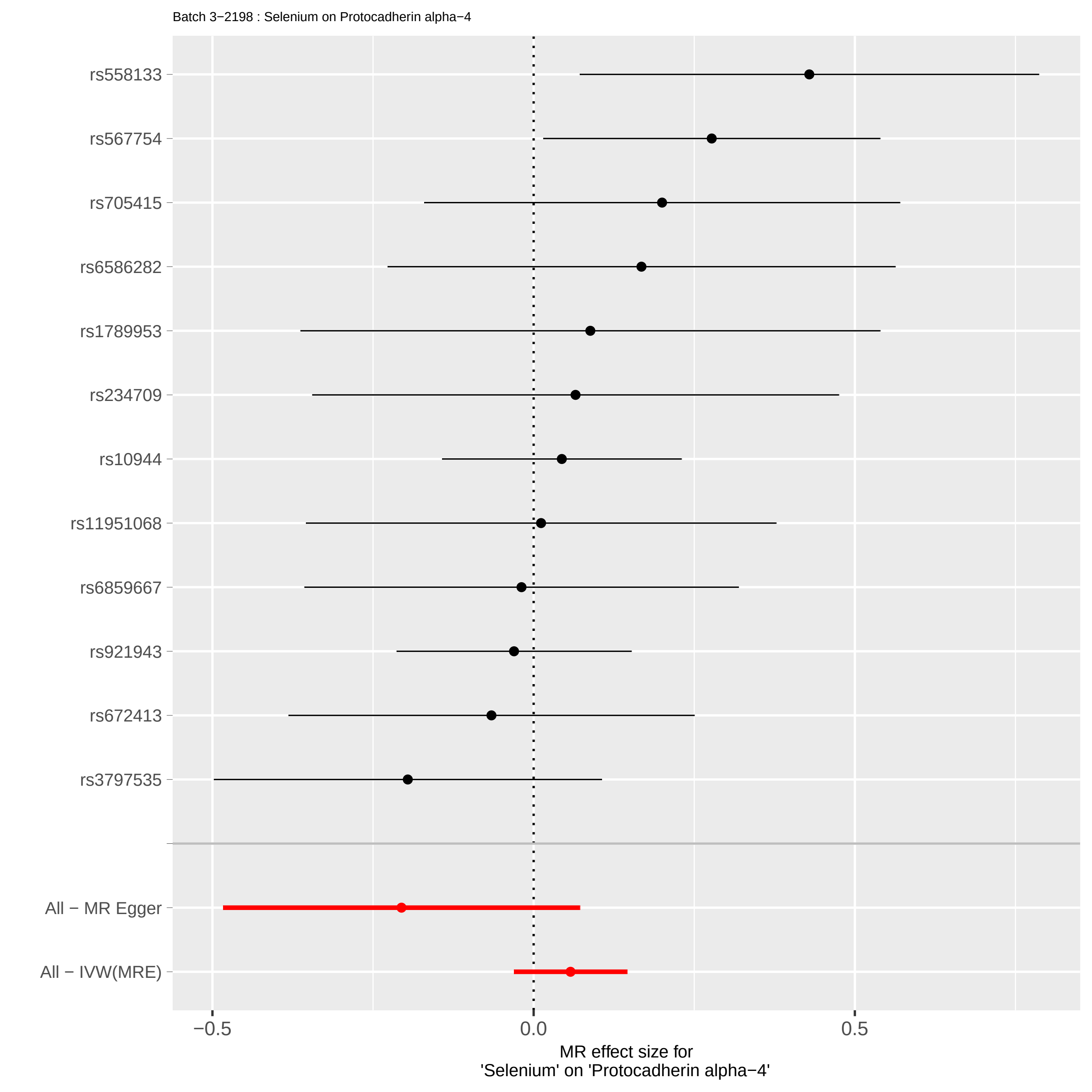

Batch 3−2198 : Selenium on Protocadherin alpha−4
rs558133
rs567754
rs705415
rs6586282
rs1789953
rs234709
rs10944
rs11951068
rs6859667
rs921943
rs672413
rs3797535
All − MR Egger
All − IVW(MRE)
−0.5
0.0
0.5
MR effect size for
'Selenium' on 'Protocadherin alpha−4'

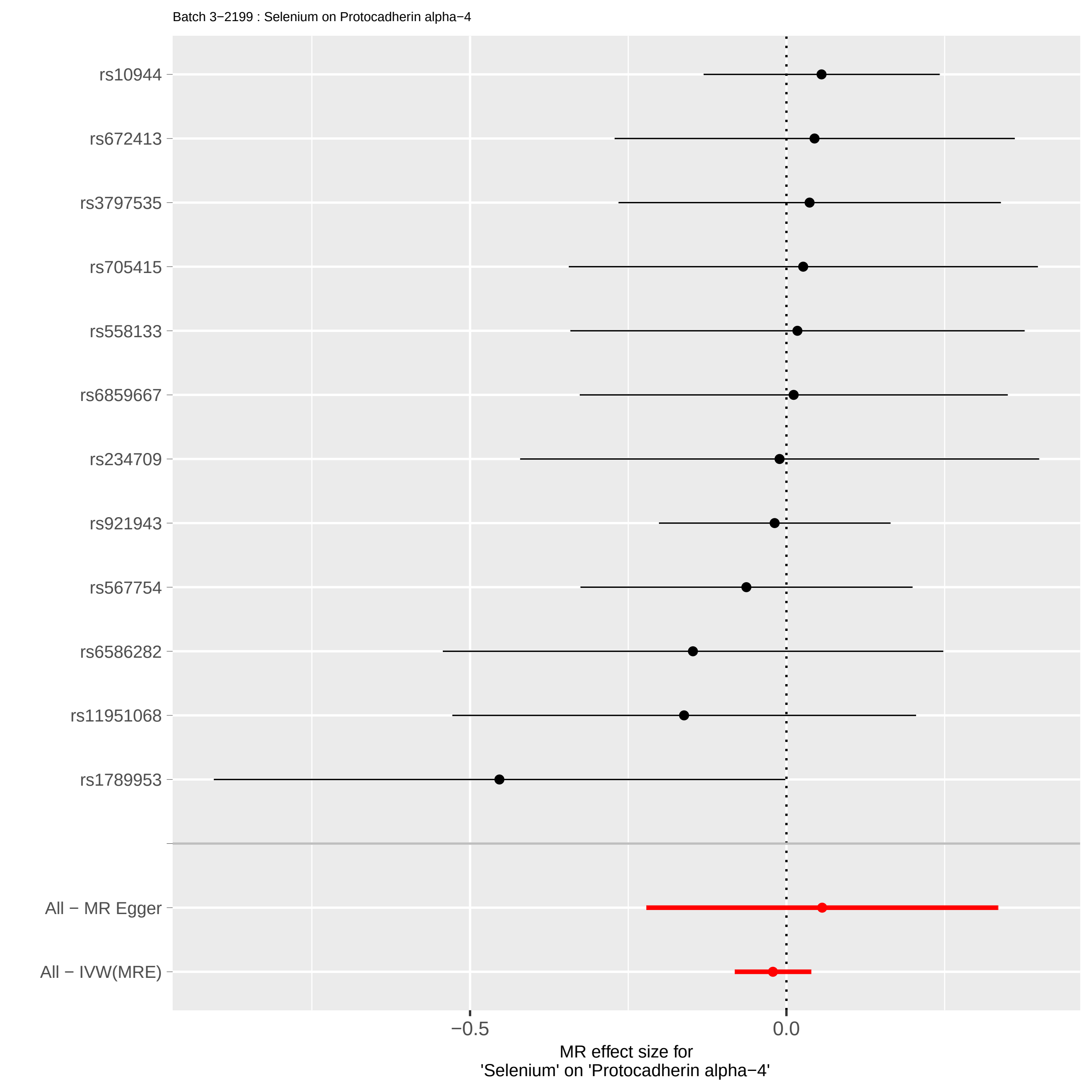

Batch 3−2199 : Selenium on Protocadherin alpha−4
rs10944
rs672413
rs3797535
rs705415
rs558133
rs6859667
rs234709
rs921943
rs567754
rs6586282
rs11951068
rs1789953
All − MR Egger
All − IVW(MRE)
−0.5
0.0
MR effect size for
'Selenium' on 'Protocadherin alpha−4'

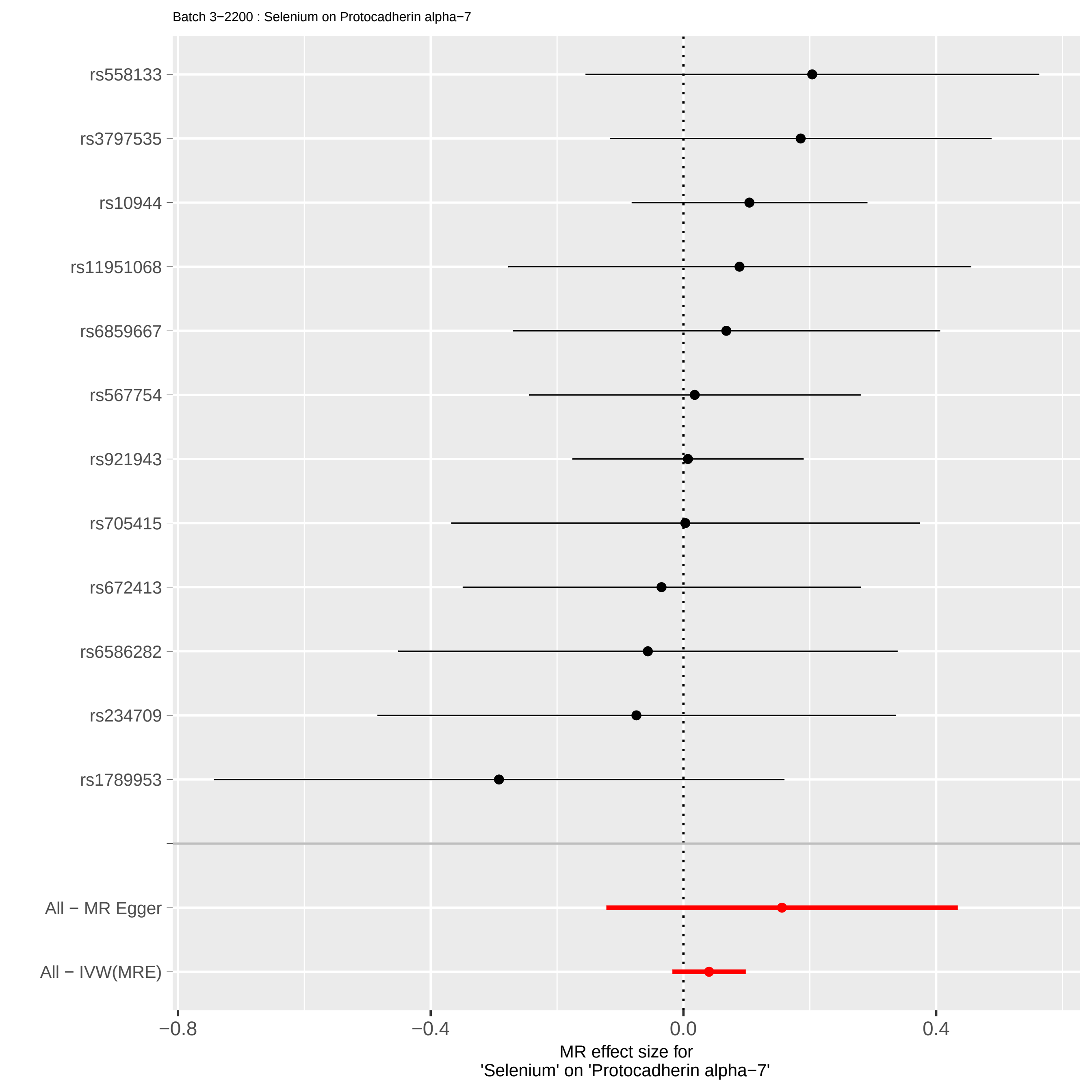

Batch 3−2200 : Selenium on Protocadherin alpha−7
rs558133
rs3797535
rs10944
rs11951068
rs6859667
rs567754
rs921943
rs705415
rs672413
rs6586282
rs234709
rs1789953
All − MR Egger
All − IVW(MRE)
−0.8
−0.4
0.0
0.4
MR effect size for
'Selenium' on 'Protocadherin alpha−7'

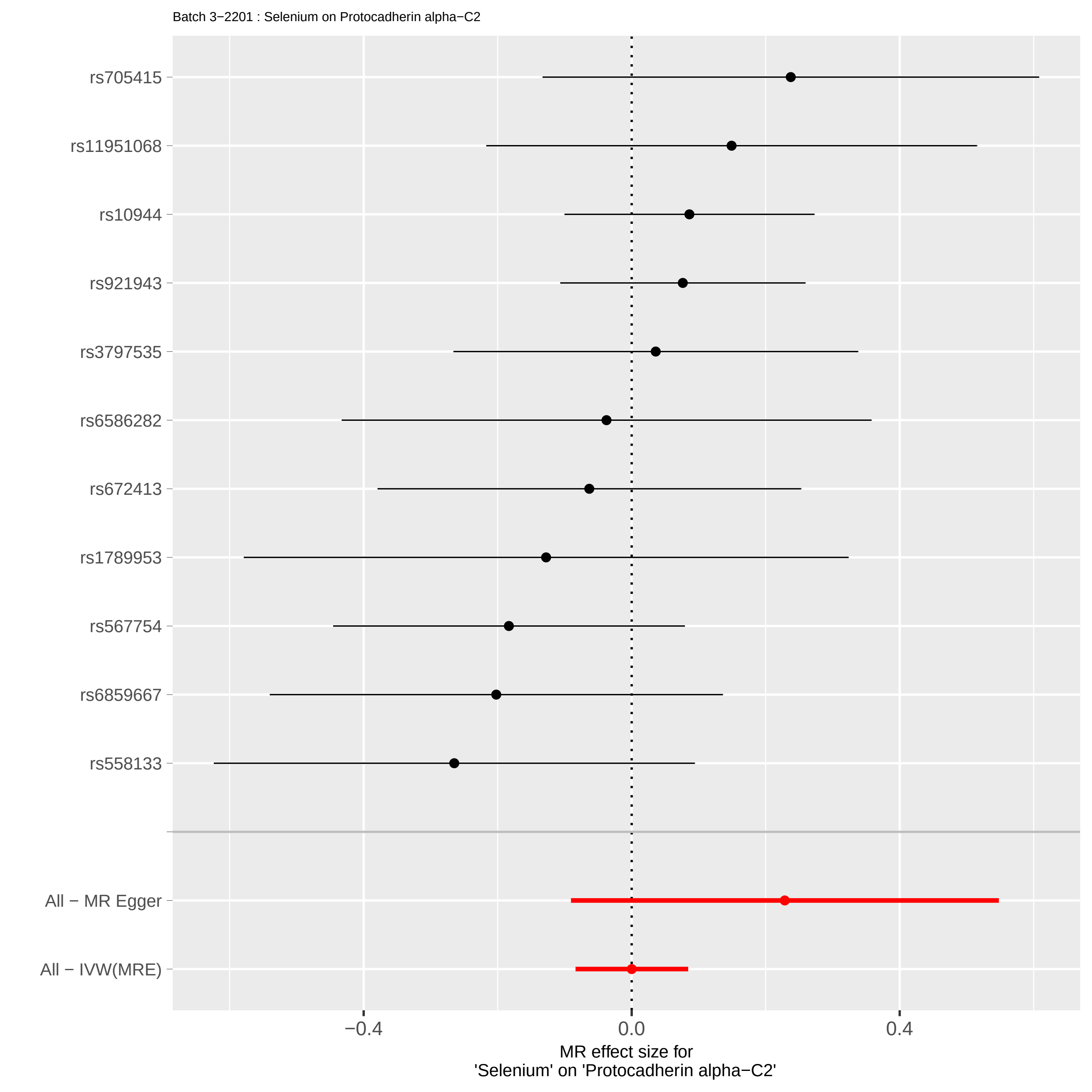

Batch 3−2201 : Selenium on Protocadherin alpha−C2
rs705415
rs11951068
rs10944
rs921943
rs3797535
rs6586282
rs672413
rs1789953
rs567754
rs6859667
rs558133
All − MR Egger
All − IVW(MRE)
−0.4
0.0
0.4
MR effect size for
'Selenium' on 'Protocadherin alpha−C2'

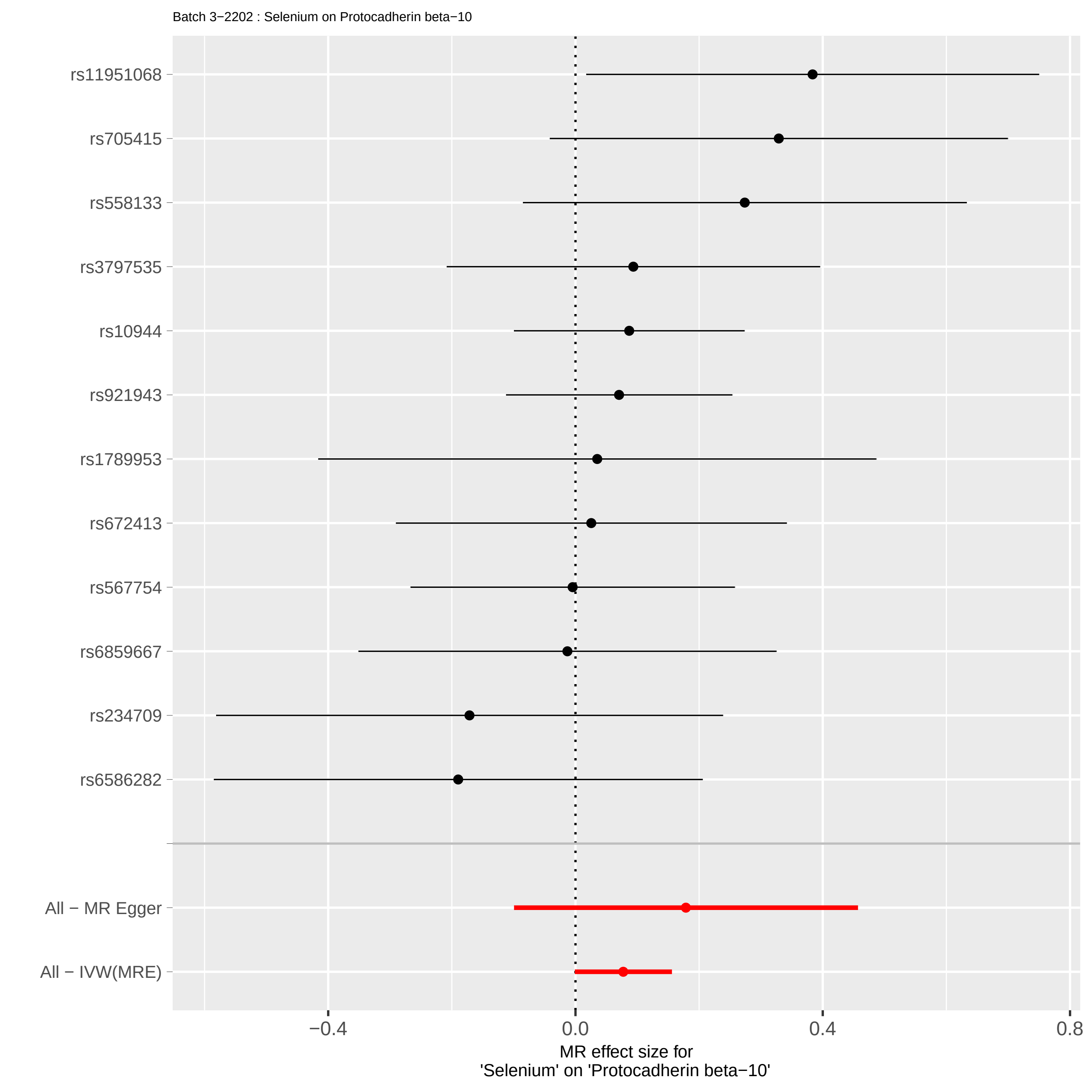

Batch 3−2202 : Selenium on Protocadherin beta−10
rs11951068
rs705415
rs558133
rs3797535
rs10944
rs921943
rs1789953
rs672413
rs567754
rs6859667
rs234709
rs6586282
All − MR Egger
All − IVW(MRE)
−0.4
0.0
0.4
0.8
MR effect size for
'Selenium' on 'Protocadherin beta−10'

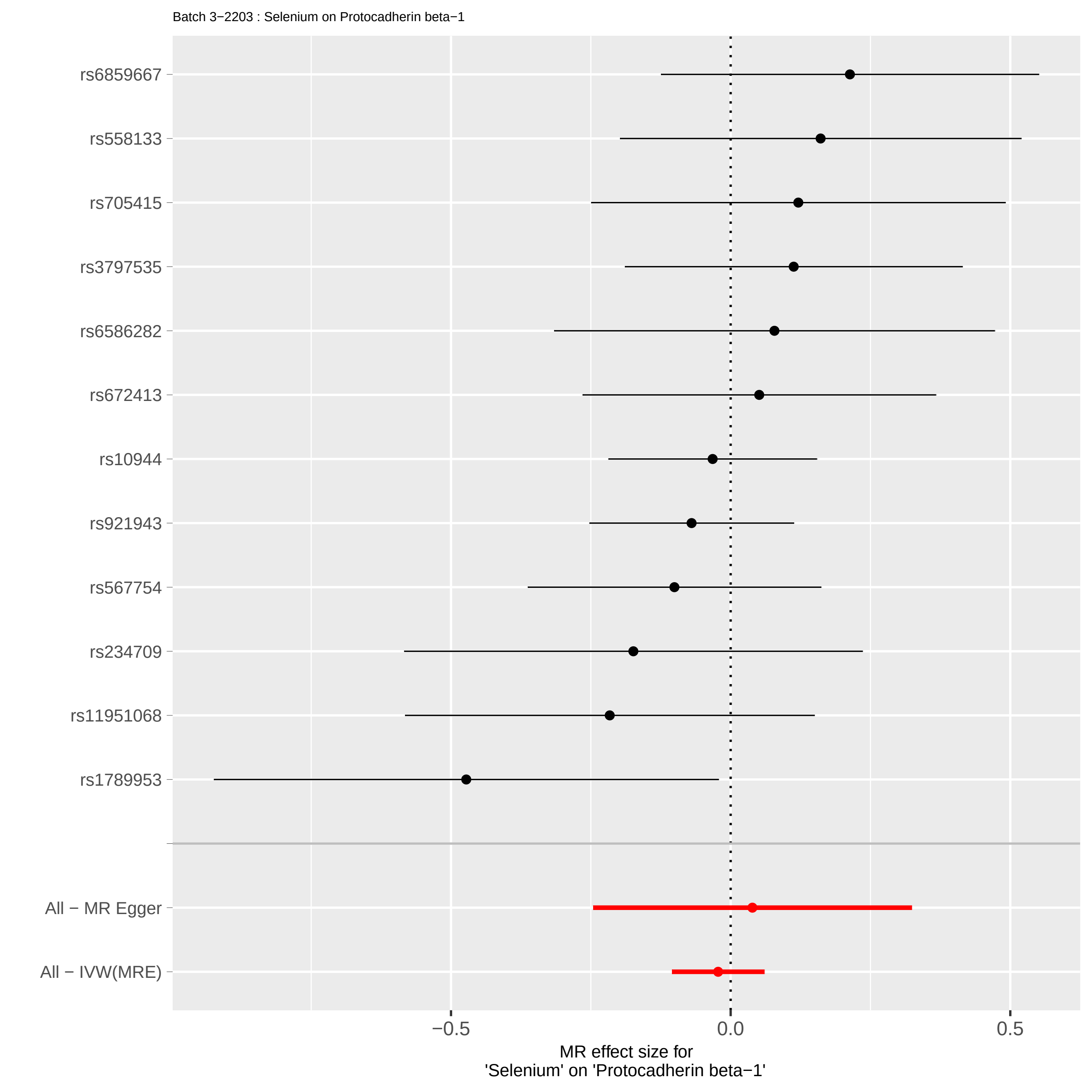
Batch 3−2203 : Selenium on Protocadherin beta−1
rs6859667
rs558133
rs705415
rs3797535
rs6586282
rs672413
rs10944
rs921943
rs567754
rs234709
rs11951068
rs1789953
All − MR Egger
All − IVW(MRE)
−0.5
0.0
0.5
MR effect size for
'Selenium' on 'Protocadherin beta−1'

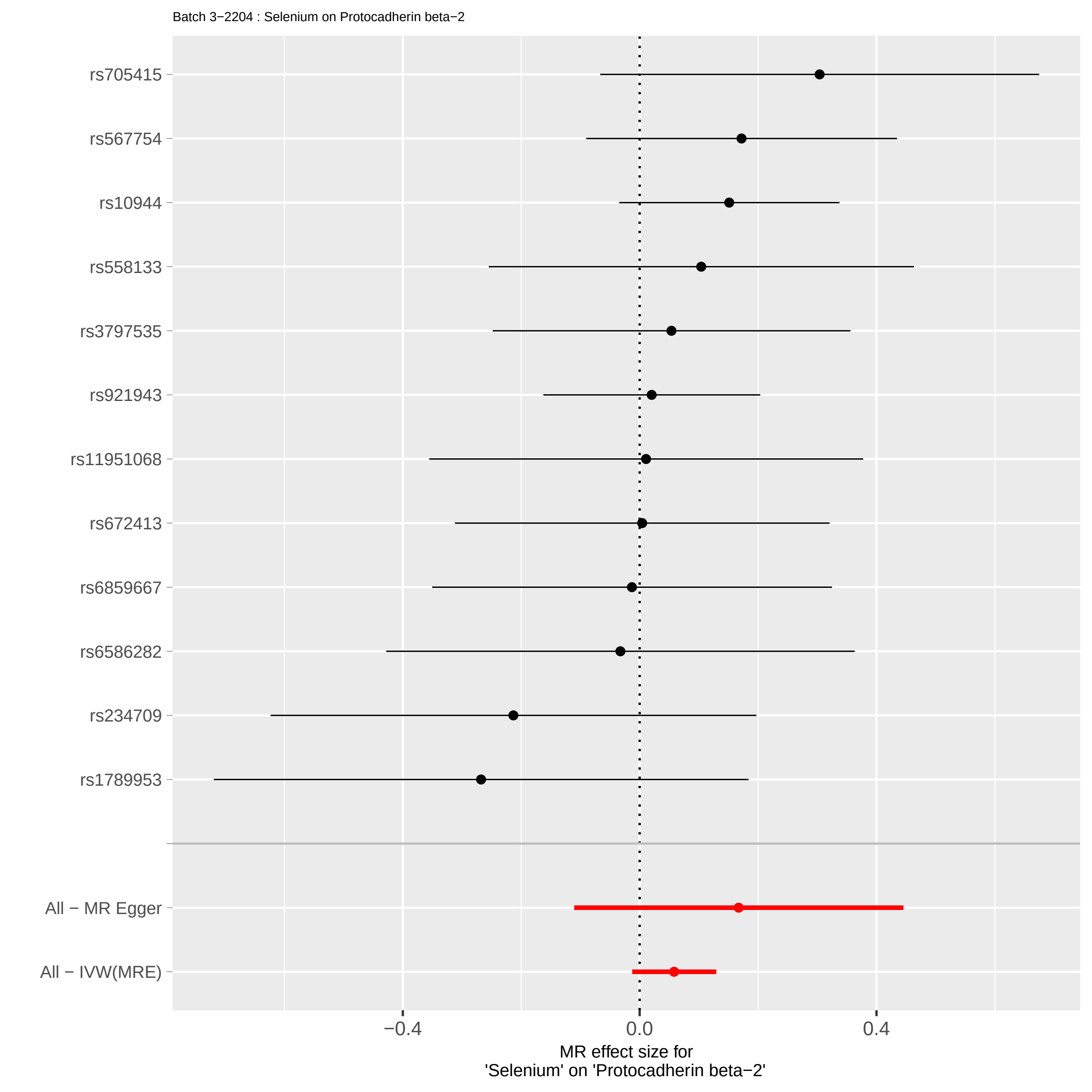
Batch 3−2204 : Selenium on Protocadherin beta−2
rs705415
rs567754
rs10944
rs558133
rs3797535
rs921943
rs11951068
rs672413
rs6859667
rs6586282
rs234709
rs1789953
All − MR Egger
All − IVW(MRE)
−0.4
0.0
0.4
MR effect size for
'Selenium' on 'Protocadherin beta−2'

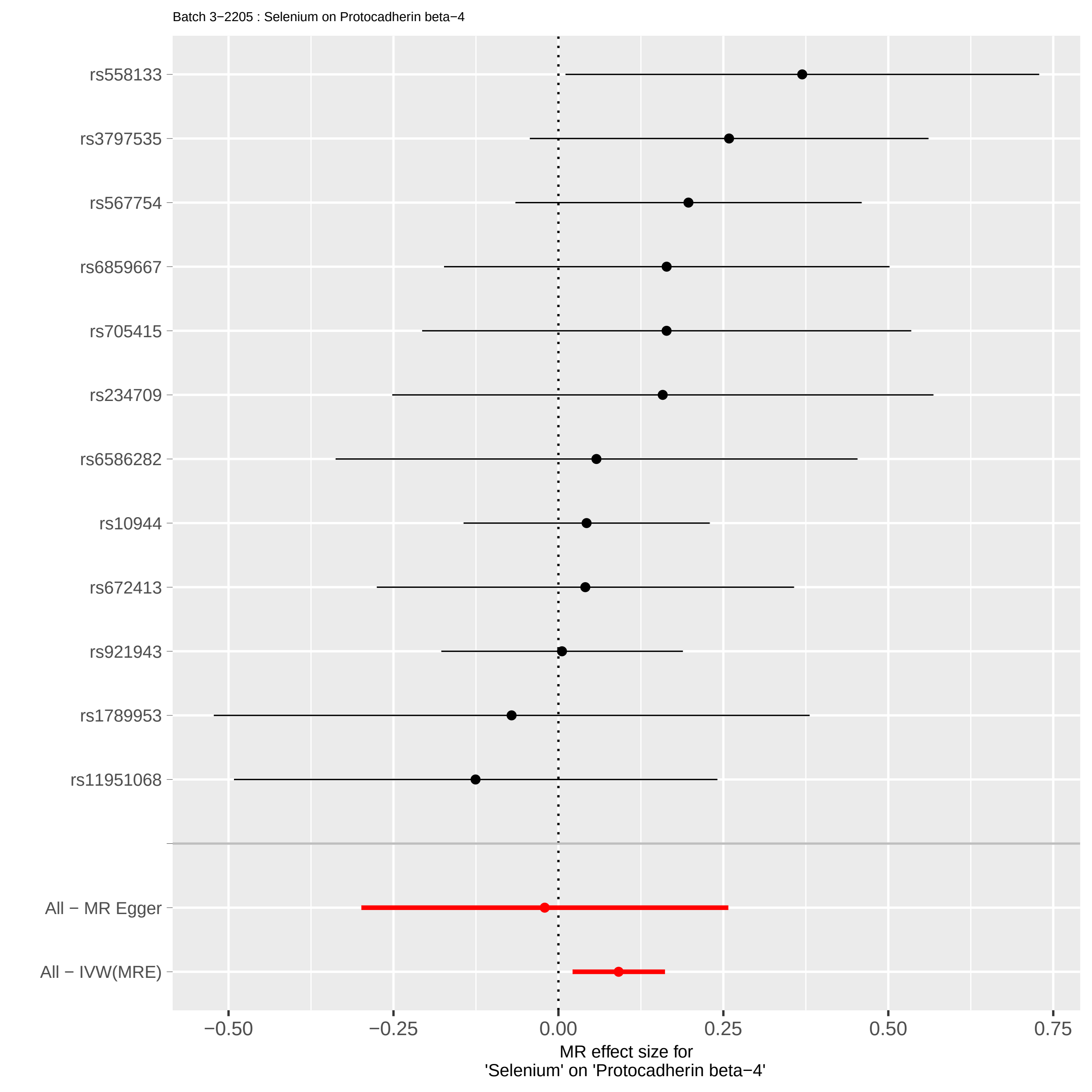
Batch 3−2205 : Selenium on Protocadherin beta−4
rs558133
rs3797535
rs567754
rs6859667
rs705415
rs234709
rs6586282
rs10944
rs672413
rs921943
rs1789953
rs11951068
All − MR Egger
All − IVW(MRE)
−0.50
−0.25
0.00
0.25
0.50
0.75
MR effect size for
'Selenium' on 'Protocadherin beta−4'

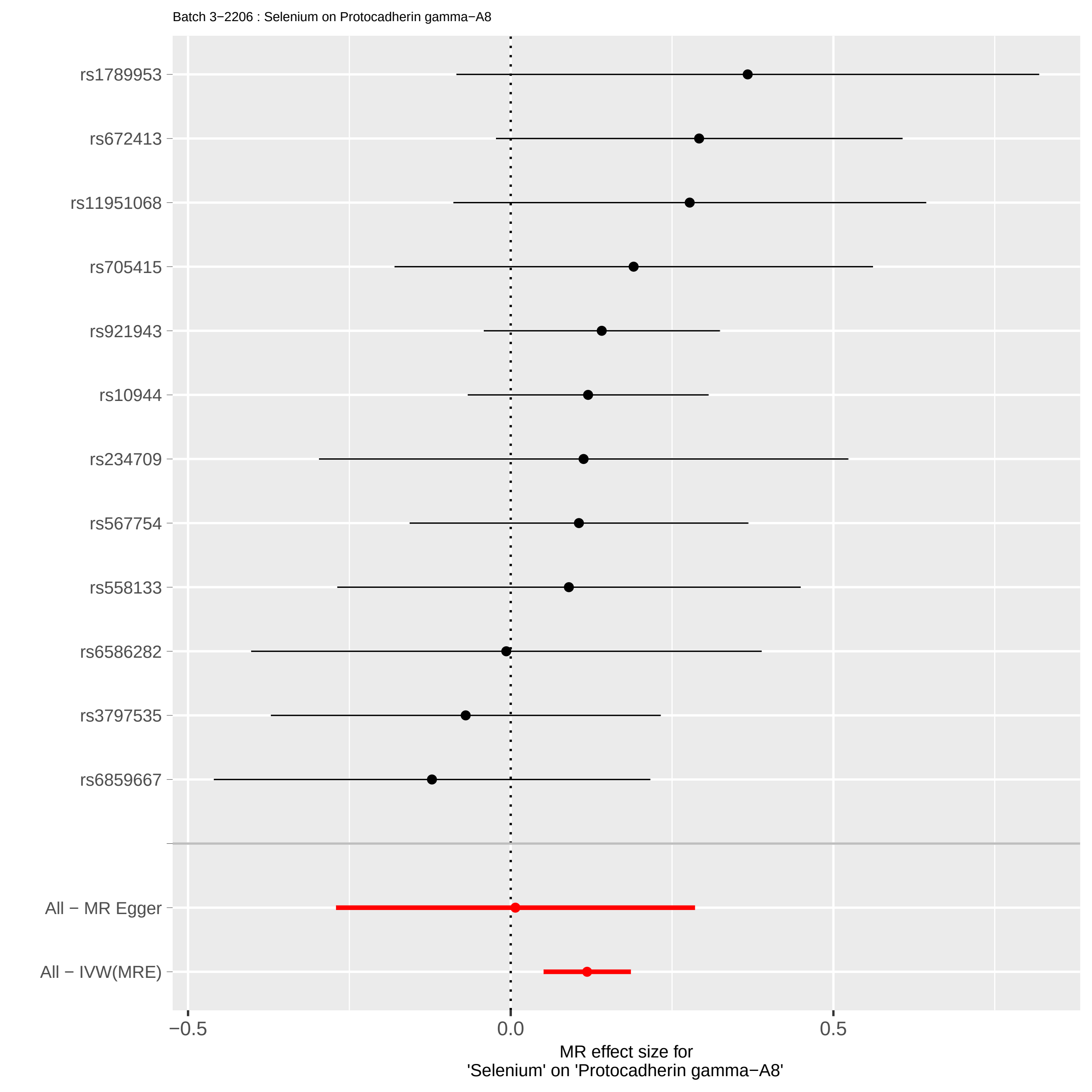

Batch 3−2206 : Selenium on Protocadherin gamma−A8
rs1789953
rs672413
rs11951068
rs705415
rs921943
rs10944
rs234709
rs567754
rs558133
rs6586282
rs3797535
rs6859667
All − MR Egger
All − IVW(MRE)
−0.5
0.0
0.5
MR effect size for
'Selenium' on 'Protocadherin gamma−A8'

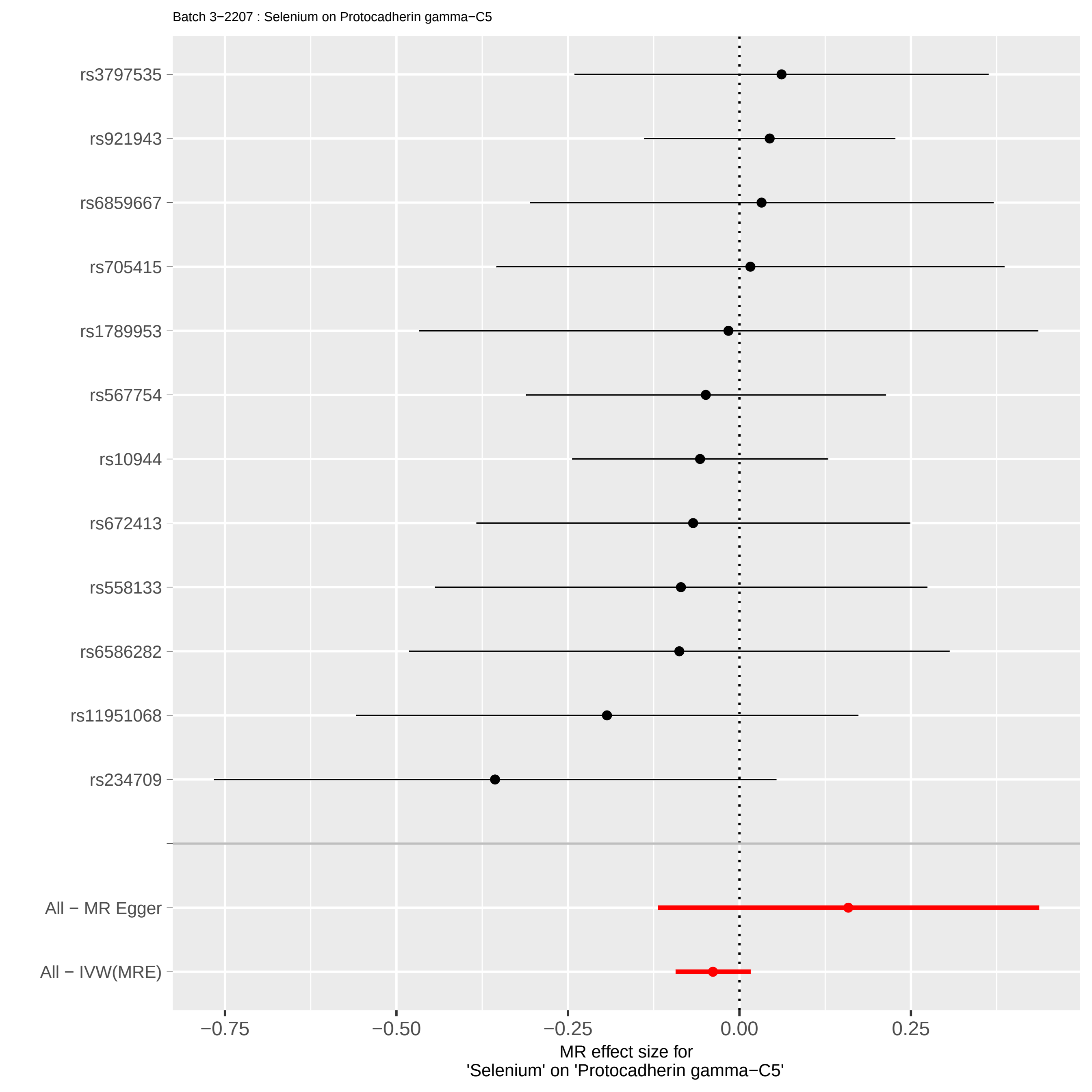

Batch 3−2207 : Selenium on Protocadherin gamma−C5
rs3797535
rs921943
rs6859667
rs705415
rs1789953
rs567754
rs10944
rs672413
rs558133
rs6586282
rs11951068
rs234709
All − MR Egger
All − IVW(MRE)
−0.75
−0.50
−0.25
0.00
0.25
MR effect size for
'Selenium' on 'Protocadherin gamma−C5'

Batch 3−2208 : Selenium on Proliferating cell nuclear antigen

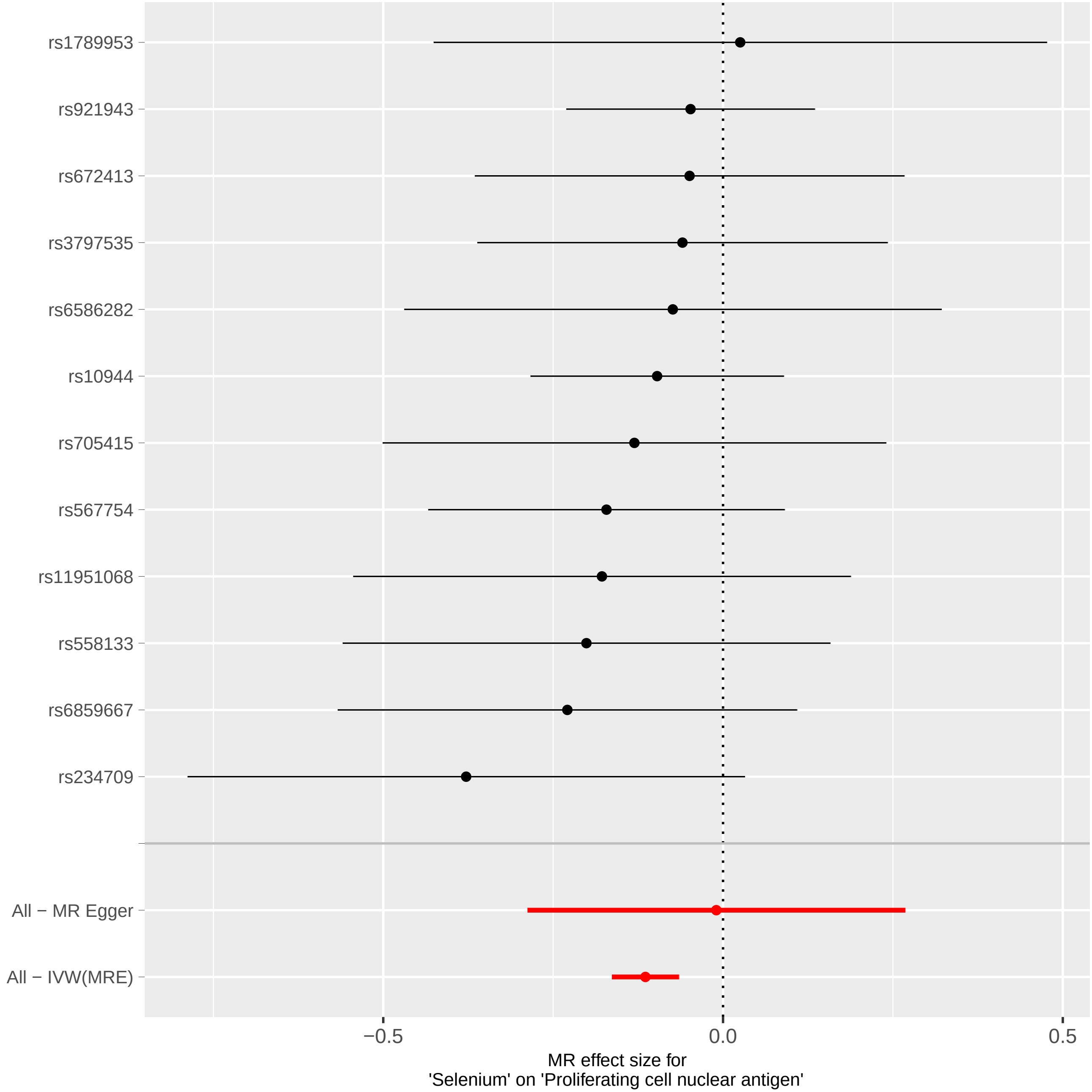

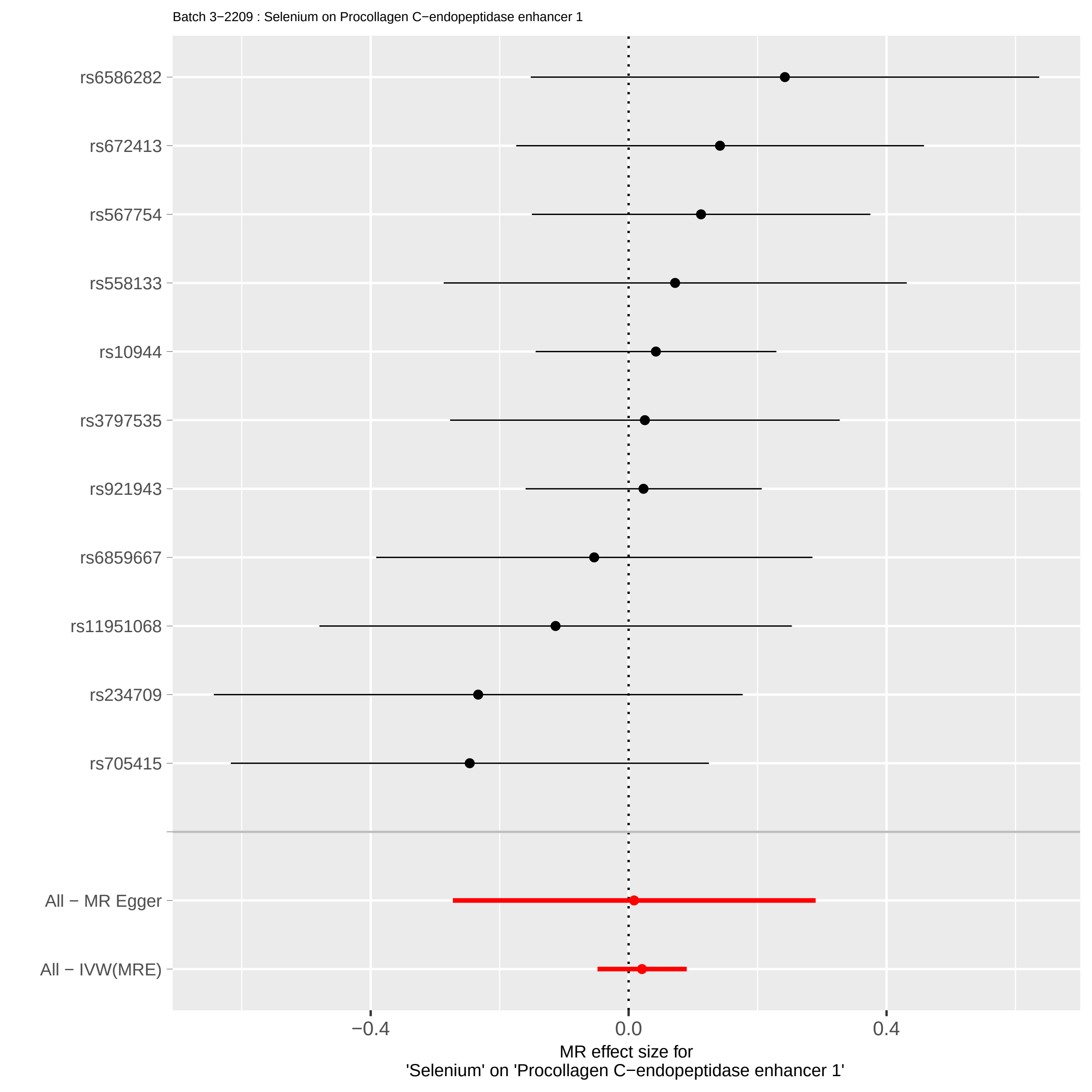

Batch 3−2209 : Selenium on Procollagen C−endopeptidase enhancer 1
rs6586282
rs672413
rs567754
rs558133
rs10944
rs3797535
rs921943
rs6859667
rs11951068
rs234709
rs705415
All − MR Egger
All − IVW(MRE)
−0.4
0.0
0.4
MR effect size for
'Selenium' on 'Procollagen C−endopeptidase enhancer 1'

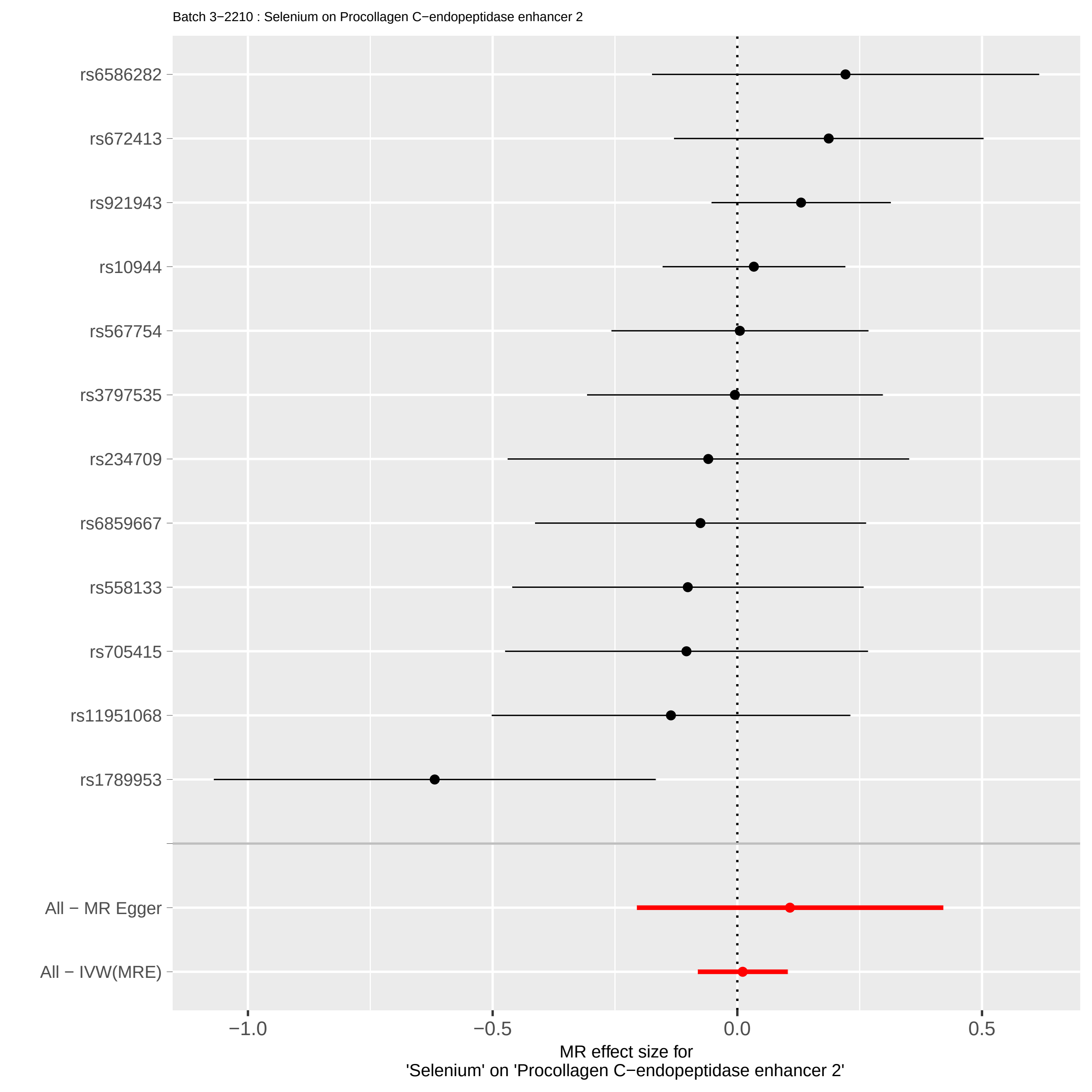

Batch 3−2210 : Selenium on Procollagen C−endopeptidase enhancer 2
rs6586282
rs672413
rs921943
rs10944
rs567754
rs3797535
rs234709
rs6859667
rs558133
rs705415
rs11951068
rs1789953
All − MR Egger
All − IVW(MRE)
−1.0
−0.5
0.0
0.5
MR effect size for
'Selenium' on 'Procollagen C−endopeptidase enhancer 2'

Batch 3−2211 : Selenium on Neuroendocrine convertase 1

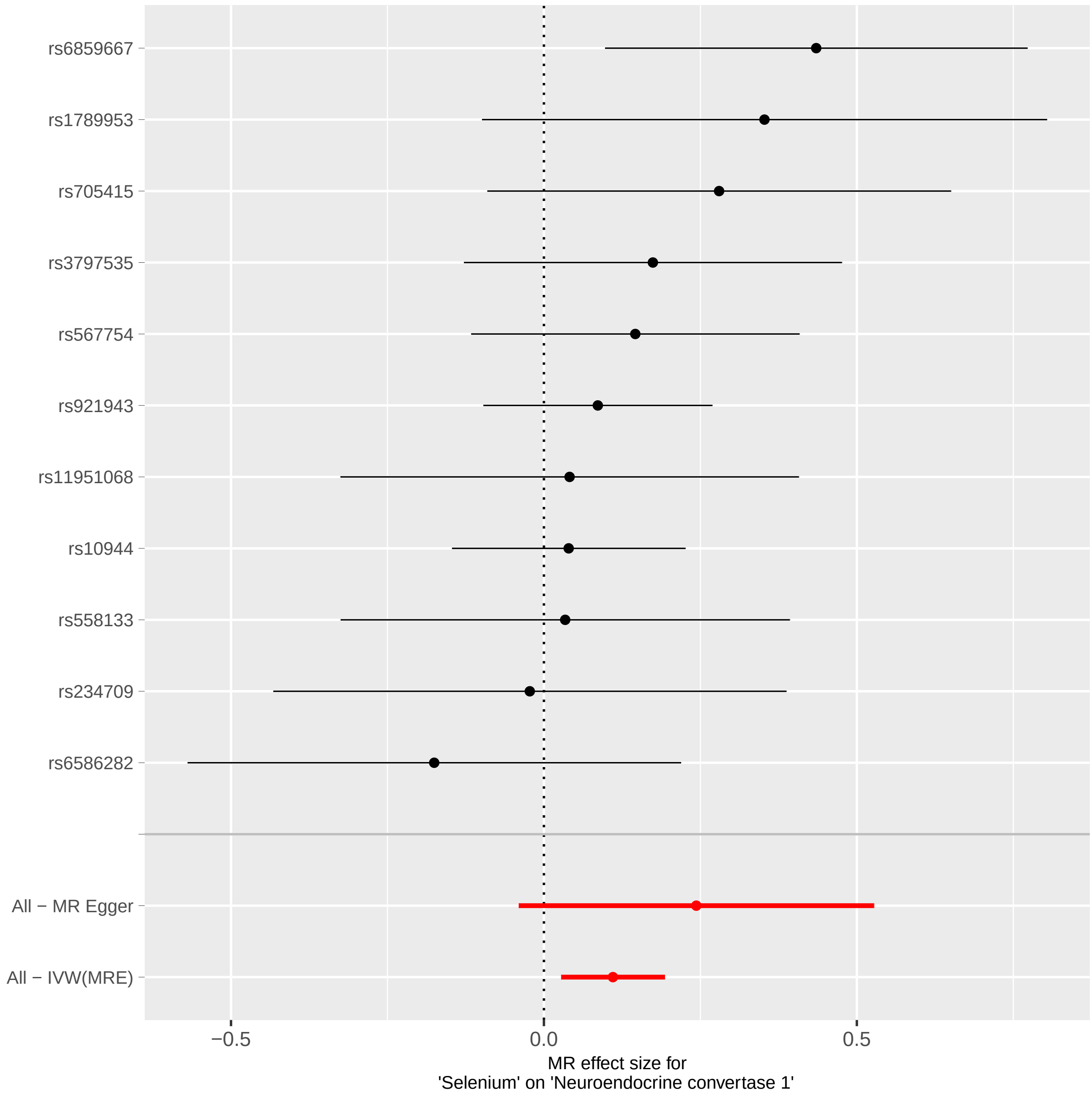

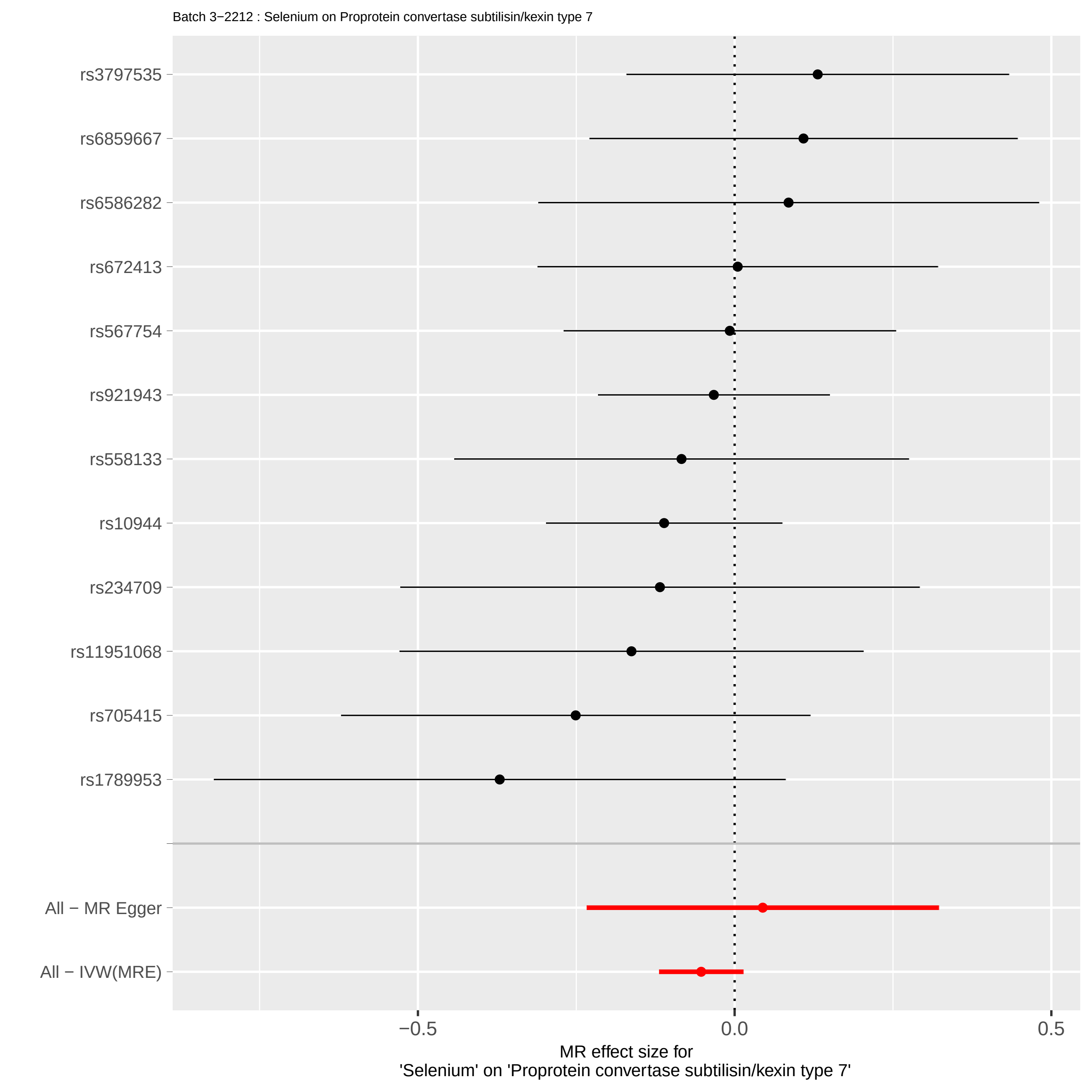

Batch 3−2212 : Selenium on Proprotein convertase subtilisin/kexin type 7
rs3797535
rs6859667
rs6586282
rs672413
rs567754
rs921943
rs558133
rs10944
rs234709
rs11951068
rs705415
rs1789953
All − MR Egger
All − IVW(MRE)
−0.5
0.0
0.5
MR effect size for
'Selenium' on 'Proprotein convertase subtilisin/kexin type 7'

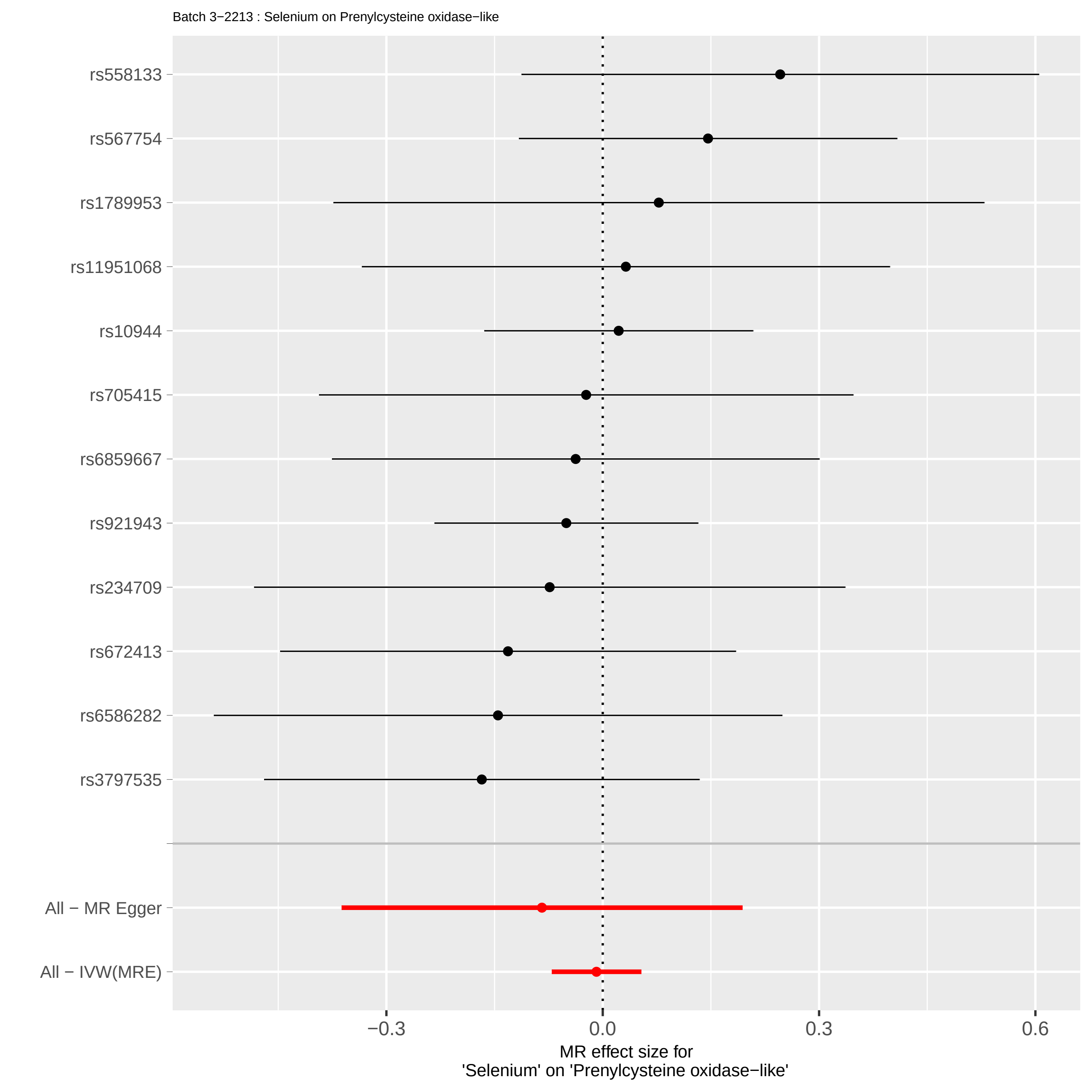

Batch 3−2213 : Selenium on Prenylcysteine oxidase−like
rs558133
rs567754
rs1789953
rs11951068
rs10944
rs705415
rs6859667
rs921943
rs234709
rs672413
rs6586282
rs3797535
All − MR Egger
All − IVW(MRE)
−0.3
0.0
0.3
0.6
MR effect size for
'Selenium' on 'Prenylcysteine oxidase−like'

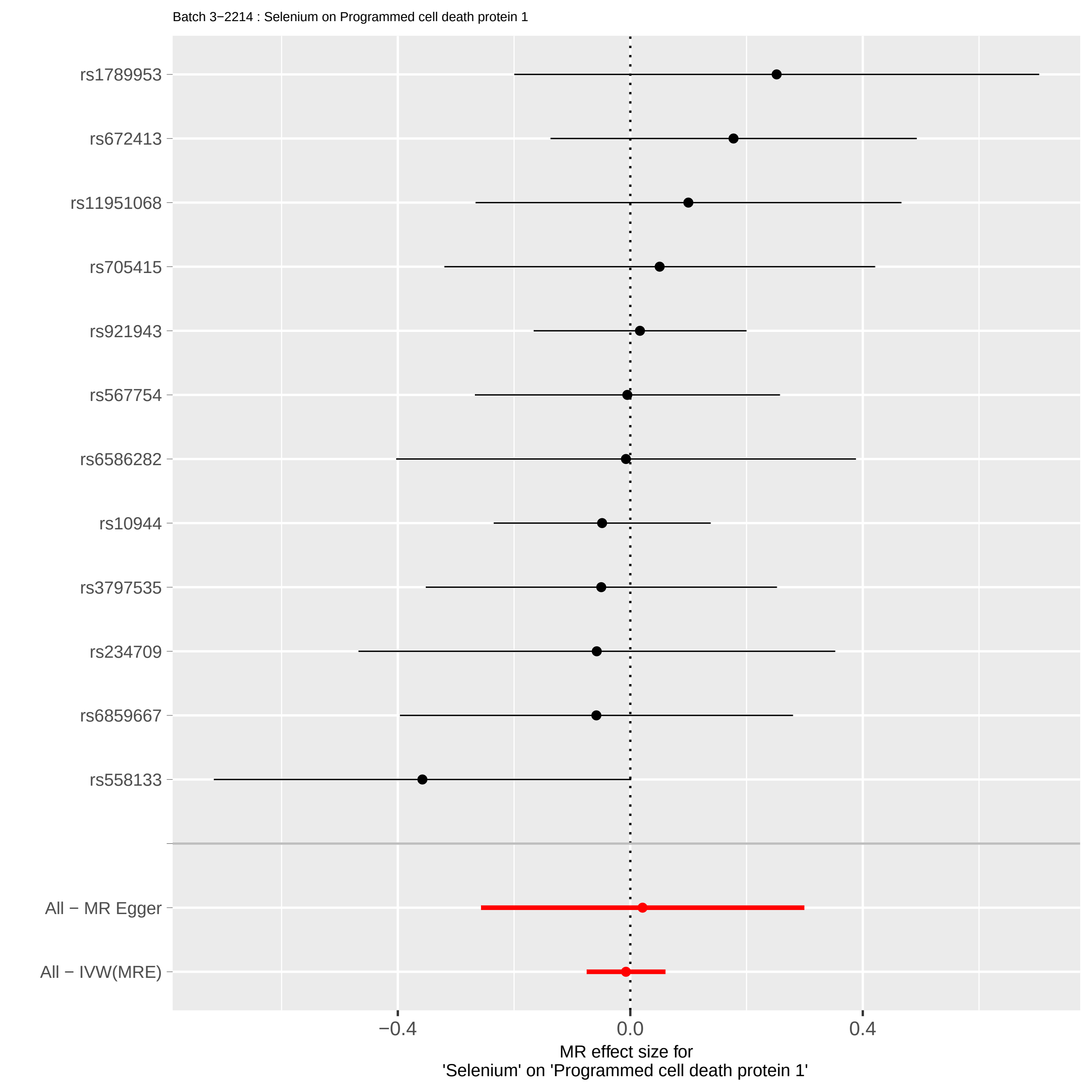

Batch 3−2214 : Selenium on Programmed cell death protein 1
rs1789953
rs672413
rs11951068
rs705415
rs921943
rs567754
rs6586282
rs10944
rs3797535
rs234709
rs6859667
rs558133
All − MR Egger
All − IVW(MRE)
−0.4
0.0
0.4
MR effect size for
'Selenium' on 'Programmed cell death protein 1'

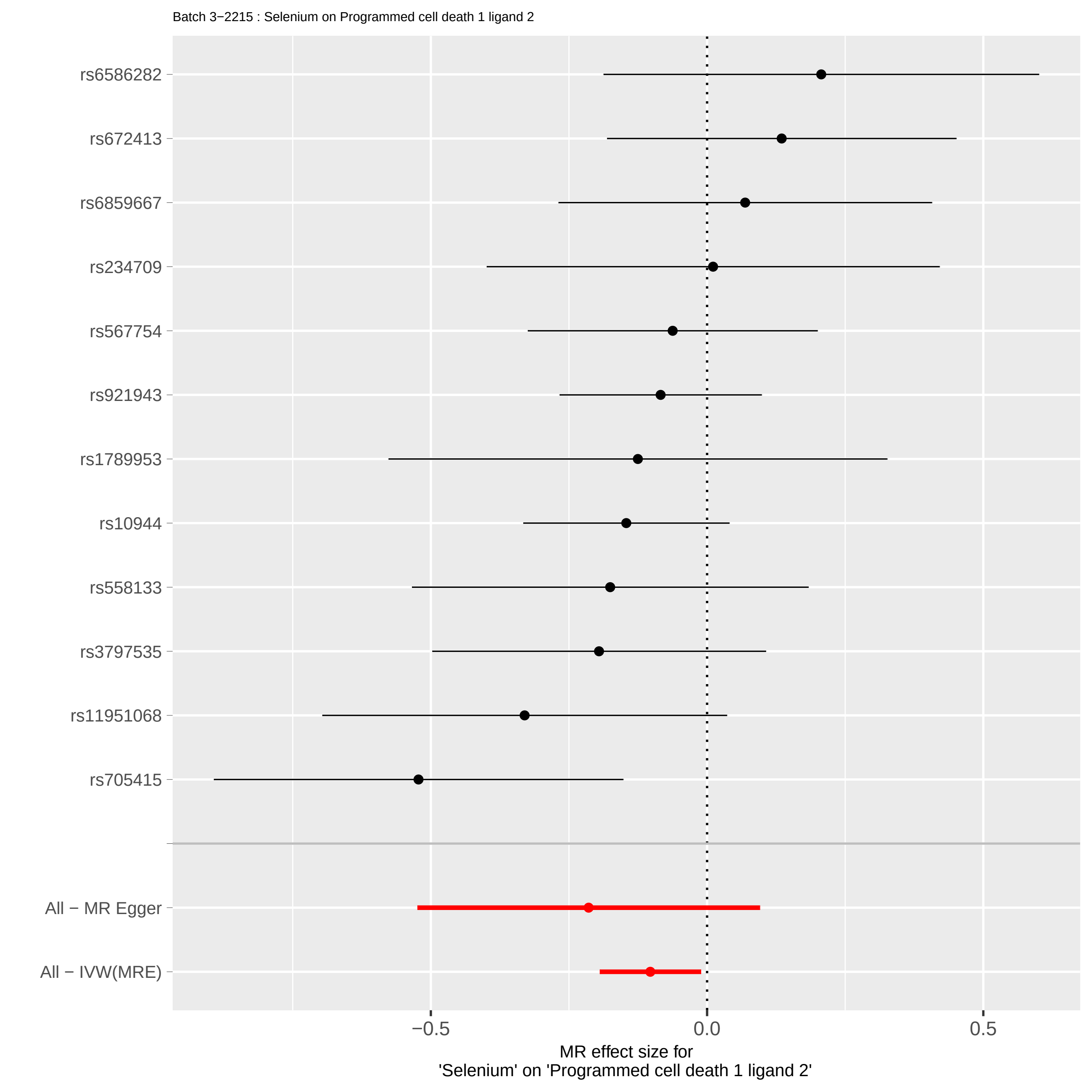

Batch 3−2215 : Selenium on Programmed cell death 1 ligand 2
rs6586282
rs672413
rs6859667
rs234709
rs567754
rs921943
rs1789953
rs10944
rs558133
rs3797535
rs11951068
rs705415
All − MR Egger
All − IVW(MRE)
−0.5
0.0
0.5
MR effect size for
'Selenium' on 'Programmed cell death 1 ligand 2'

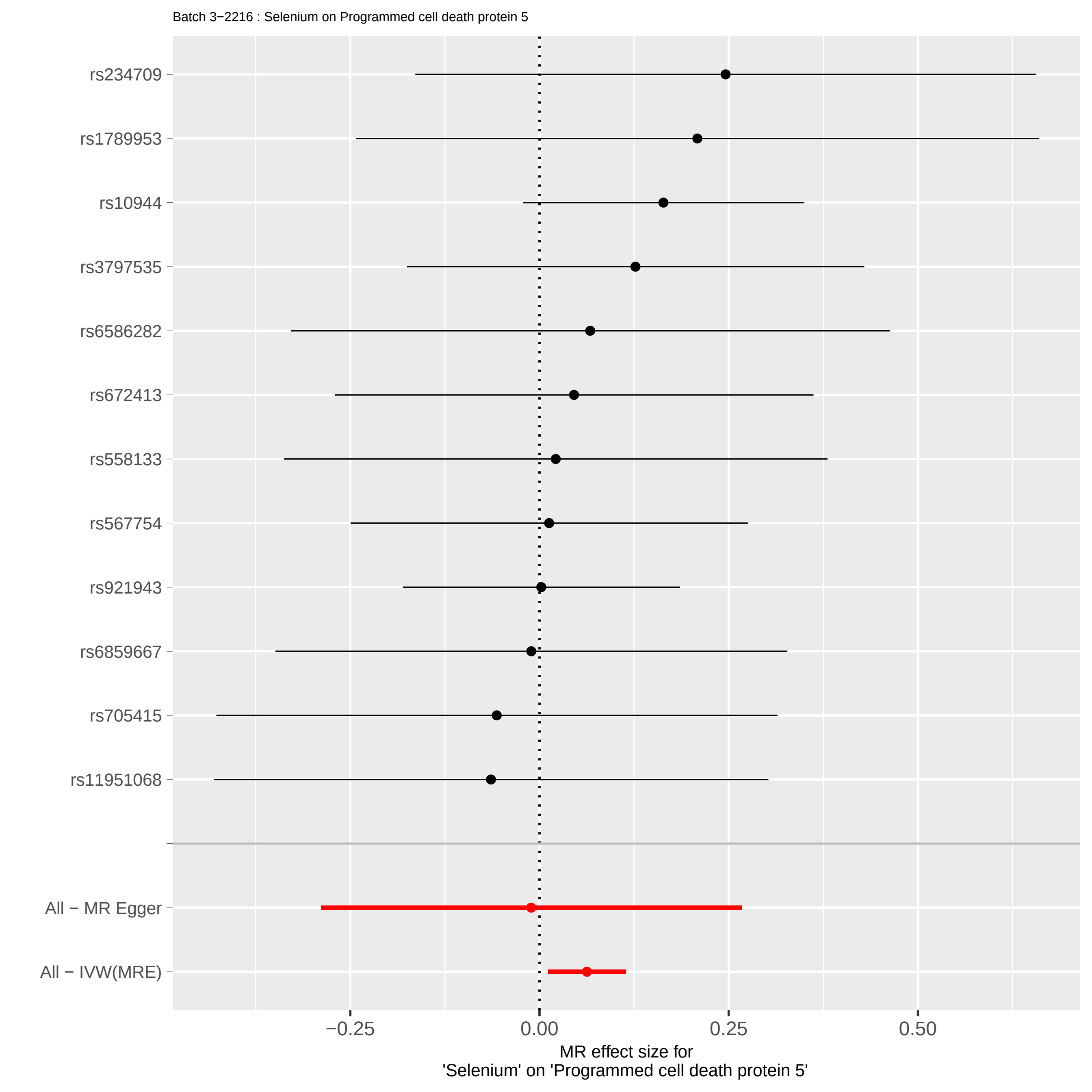

Batch 3−2216 : Selenium on Programmed cell death protein 5
rs234709
rs1789953
rs10944
rs3797535
rs6586282
rs672413
rs558133
rs567754
rs921943
rs6859667
rs705415
rs11951068
All − MR Egger
All − IVW(MRE)
−0.25
0.00
0.25
0.50
MR effect size for
'Selenium' on 'Programmed cell death protein 5'

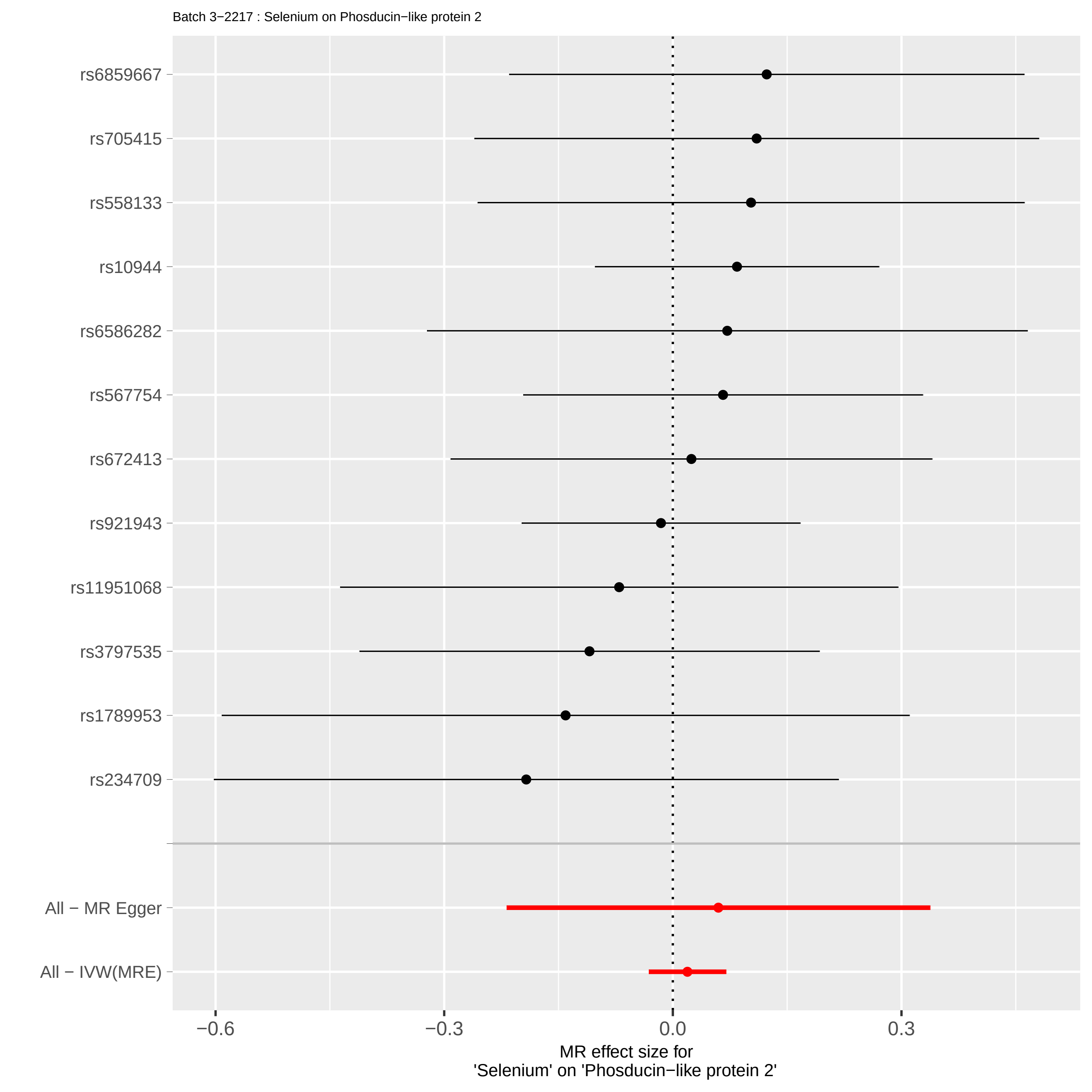

Batch 3−2217 : Selenium on Phosducin−like protein 2
rs6859667
rs705415
rs558133
rs10944
rs6586282
rs567754
rs672413
rs921943
rs11951068
rs3797535
rs1789953
rs234709
All − MR Egger
All − IVW(MRE)
−0.6
−0.3
0.0
0.3
MR effect size for
'Selenium' on 'Phosducin−like protein 2'

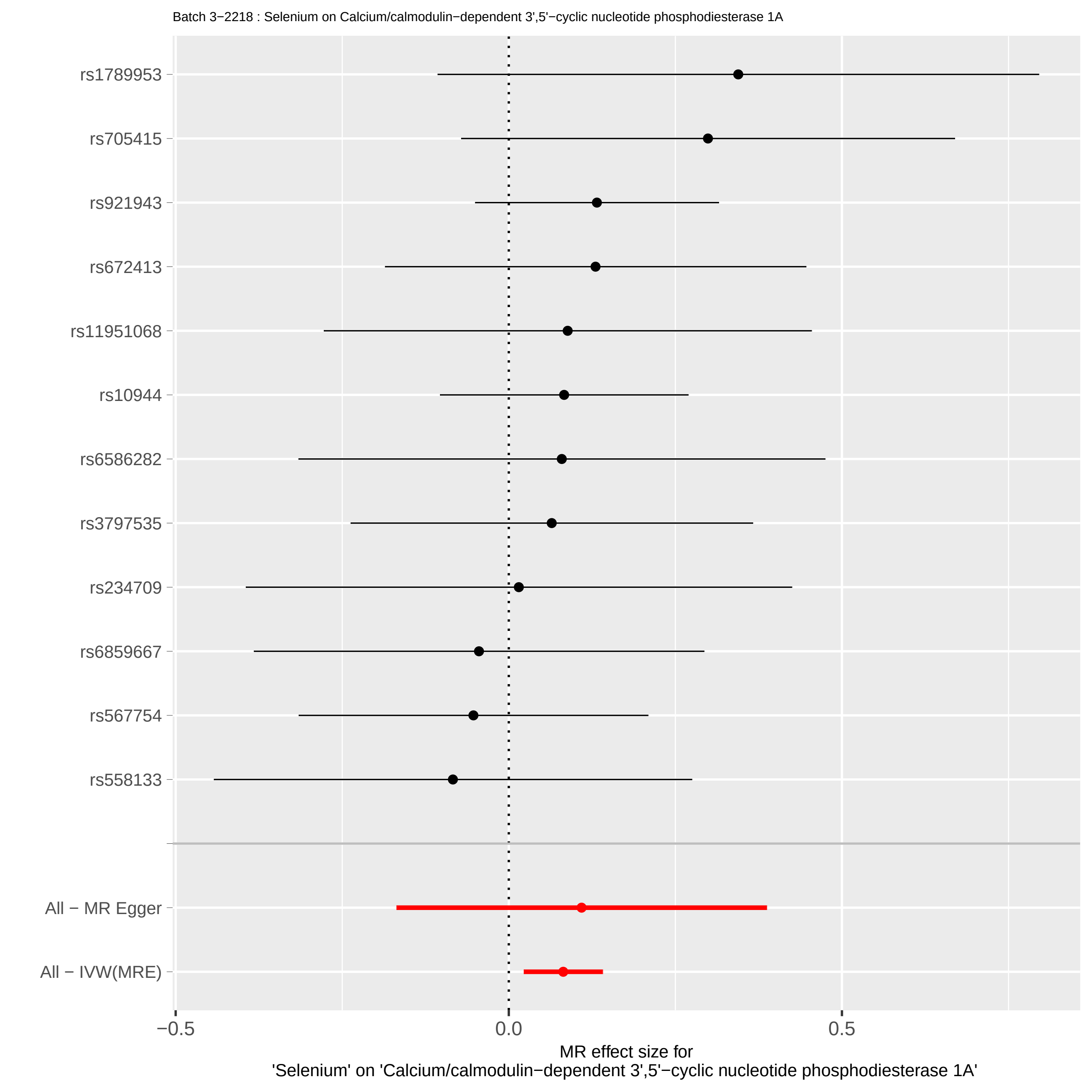

Batch 3−2218 : Selenium on Calcium/calmodulin−dependent 3',5'−cyclic nucleotide phosphodiesterase 1A
rs1789953
rs705415
rs921943
rs672413
rs11951068
rs10944
rs6586282
rs3797535
rs234709
rs6859667
rs567754
rs558133
All − MR Egger
All − IVW(MRE)
−0.5
0.0
0.5
MR effect size for
'Selenium' on 'Calcium/calmodulin−dependent 3',5'−cyclic nucleotide phosphodiesterase 1A'

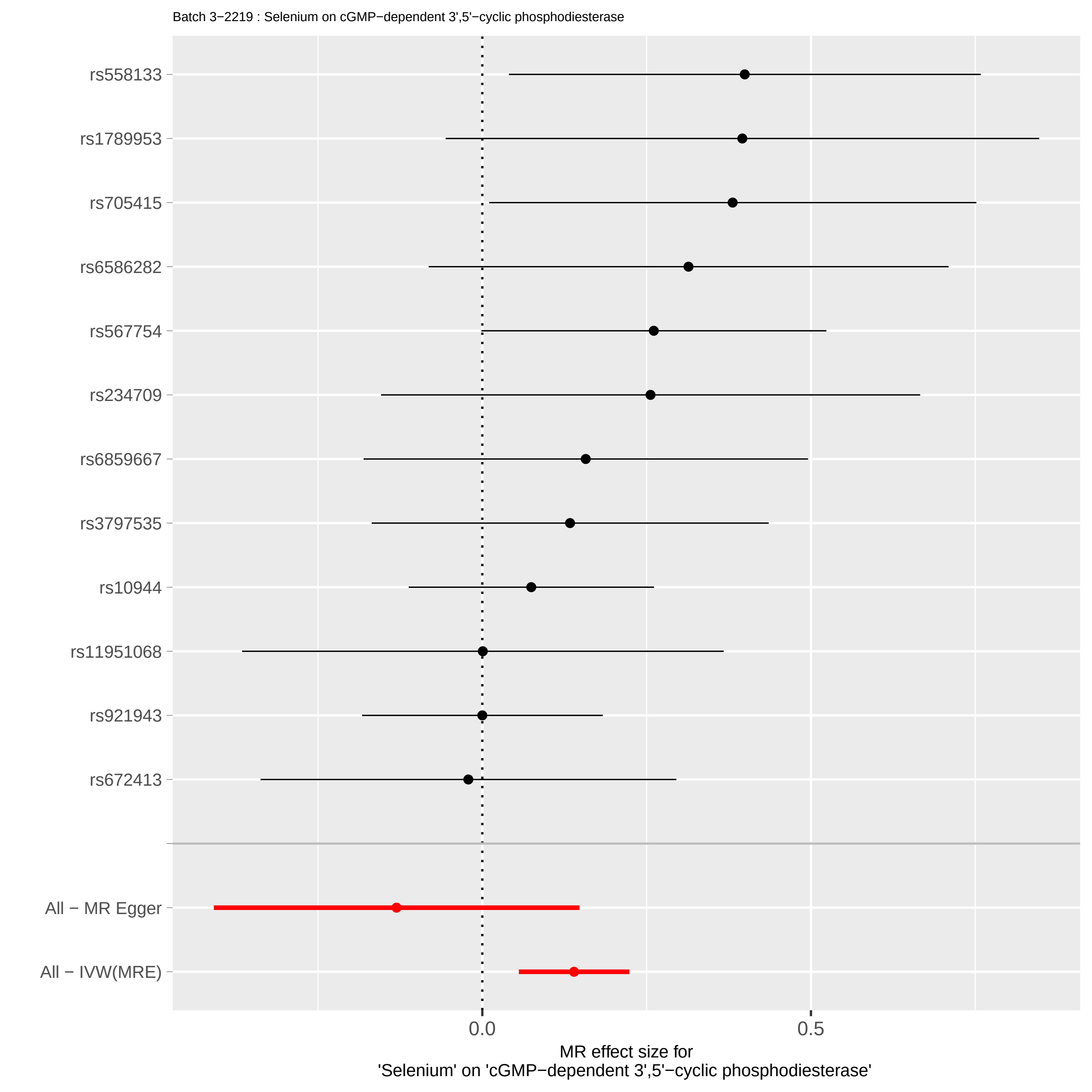

Batch 3−2219 : Selenium on cGMP−dependent 3',5'−cyclic phosphodiesterase
rs558133
rs1789953
rs705415
rs6586282
rs567754
rs234709
rs6859667
rs3797535
rs10944
rs11951068
rs921943
rs672413
All − MR Egger
All − IVW(MRE)
0.0
0.5
MR effect size for
'Selenium' on 'cGMP−dependent 3',5'−cyclic phosphodiesterase'

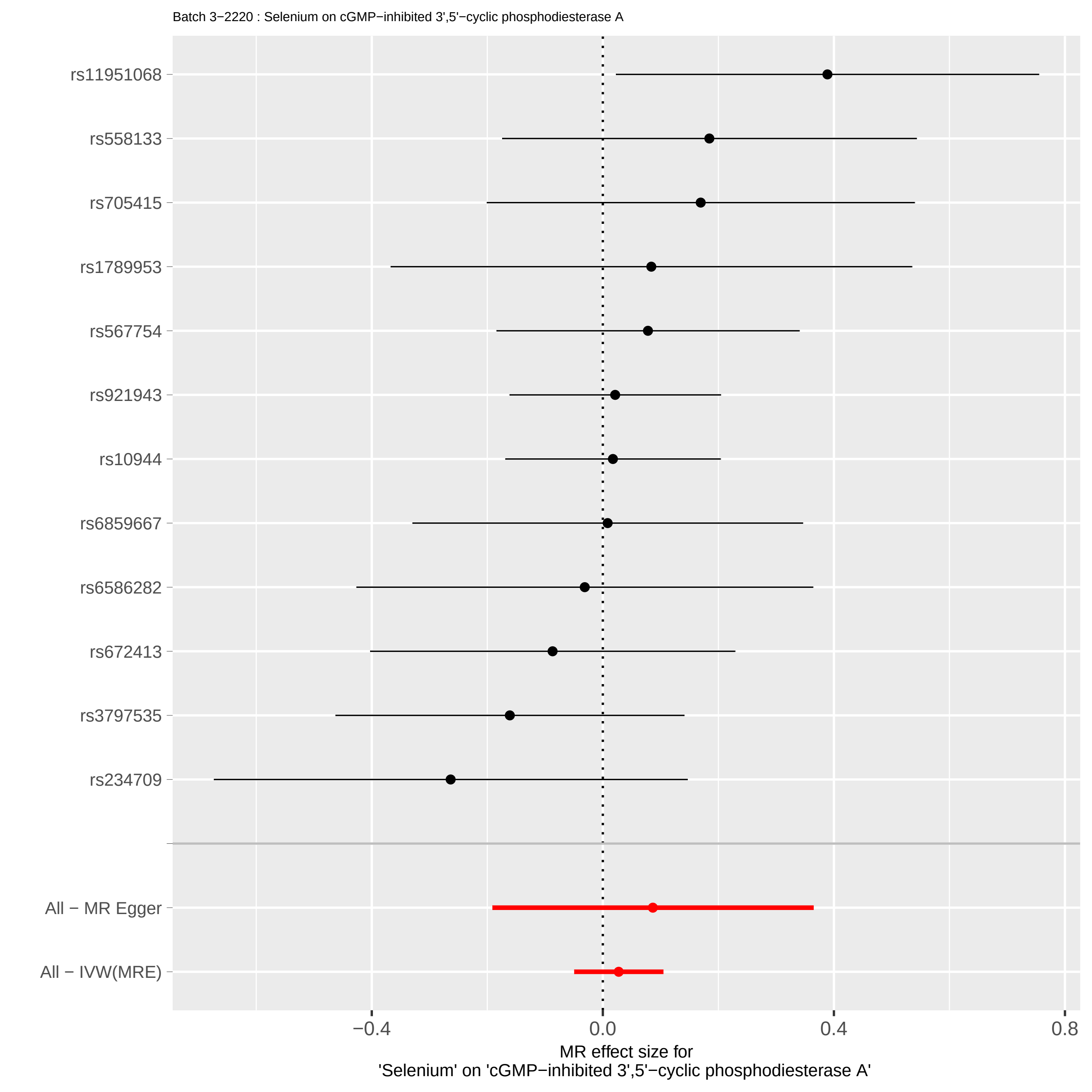
Batch 3−2220 : Selenium on cGMP−inhibited 3',5'−cyclic phosphodiesterase A
rs11951068
rs558133
rs705415
rs1789953
rs567754
rs921943
rs10944
rs6859667
rs6586282
rs672413
rs3797535
rs234709
All − MR Egger
All − IVW(MRE)
−0.4
0.0
0.4
0.8
MR effect size for
'Selenium' on 'cGMP−inhibited 3',5'−cyclic phosphodiesterase A'

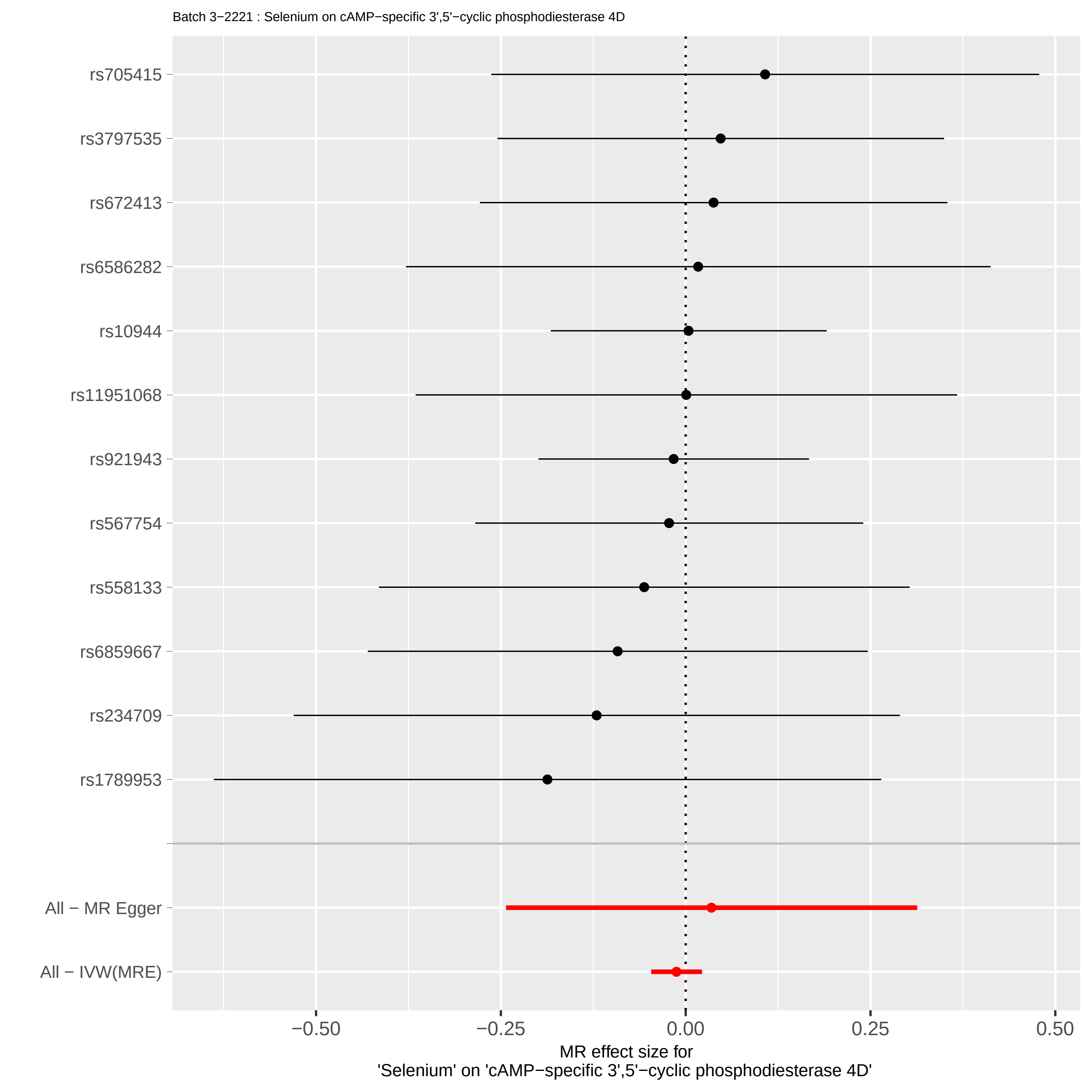
Batch 3−2221 : Selenium on cAMP−specific 3',5'−cyclic phosphodiesterase 4D
rs705415
rs3797535
rs672413
rs6586282
rs10944
rs11951068
rs921943
rs567754
rs558133
rs6859667
rs234709
rs1789953
All − MR Egger
All − IVW(MRE)
−0.50
−0.25
0.00
0.25
0.50
MR effect size for
'Selenium' on 'cAMP−specific 3',5'−cyclic phosphodiesterase 4D'

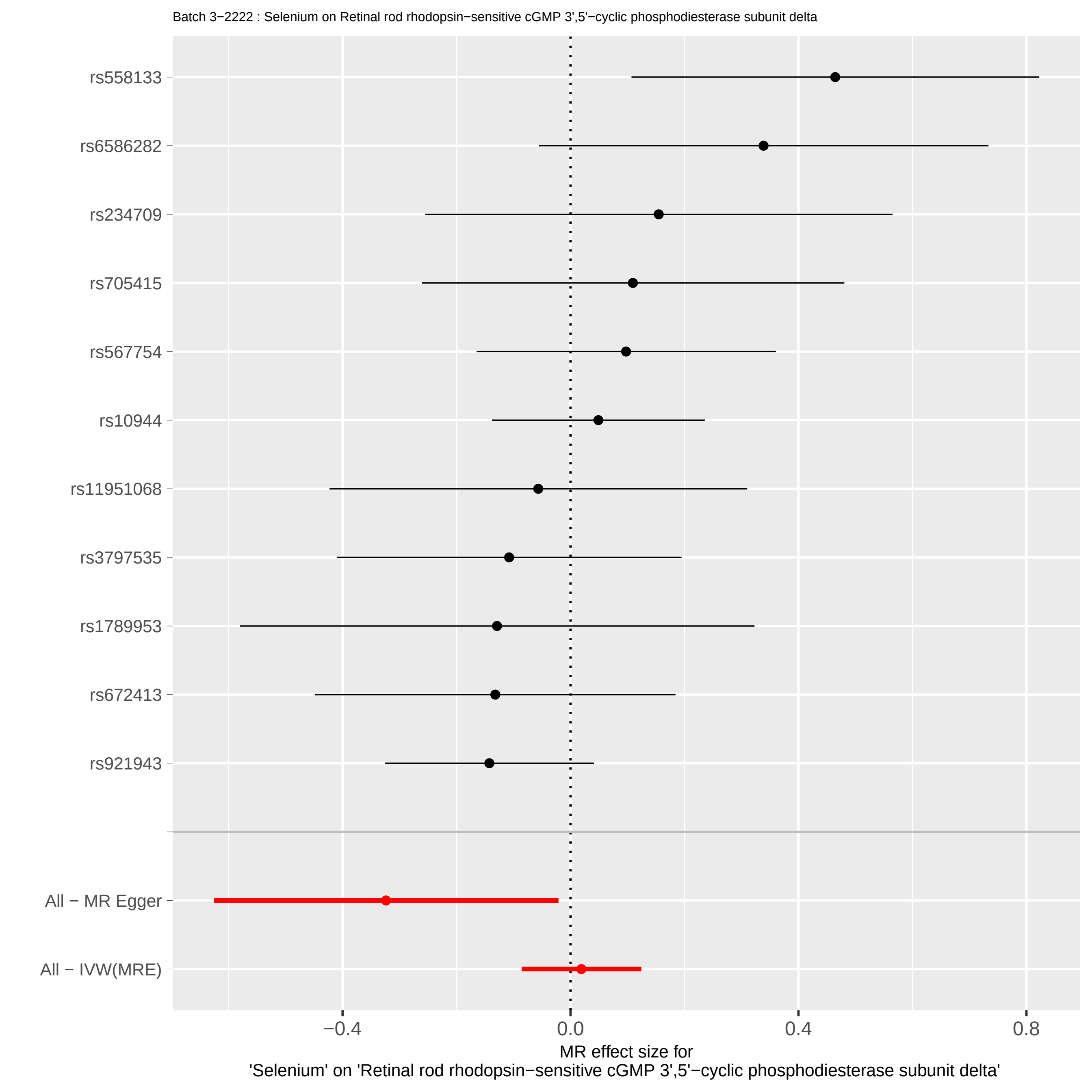
Batch 3−2222 : Selenium on Retinal rod rhodopsin−sensitive cGMP 3',5'−cyclic phosphodiesterase subunit delta
rs558133
rs6586282
rs234709
rs705415
rs567754
rs10944
rs11951068
rs3797535
rs1789953
rs672413
rs921943
All − MR Egger
All − IVW(MRE)
−0.4
0.0
0.4
0.8
MR effect size for
'Selenium' on 'Retinal rod rhodopsin−sensitive cGMP 3',5'−cyclic phosphodiesterase subunit delta'

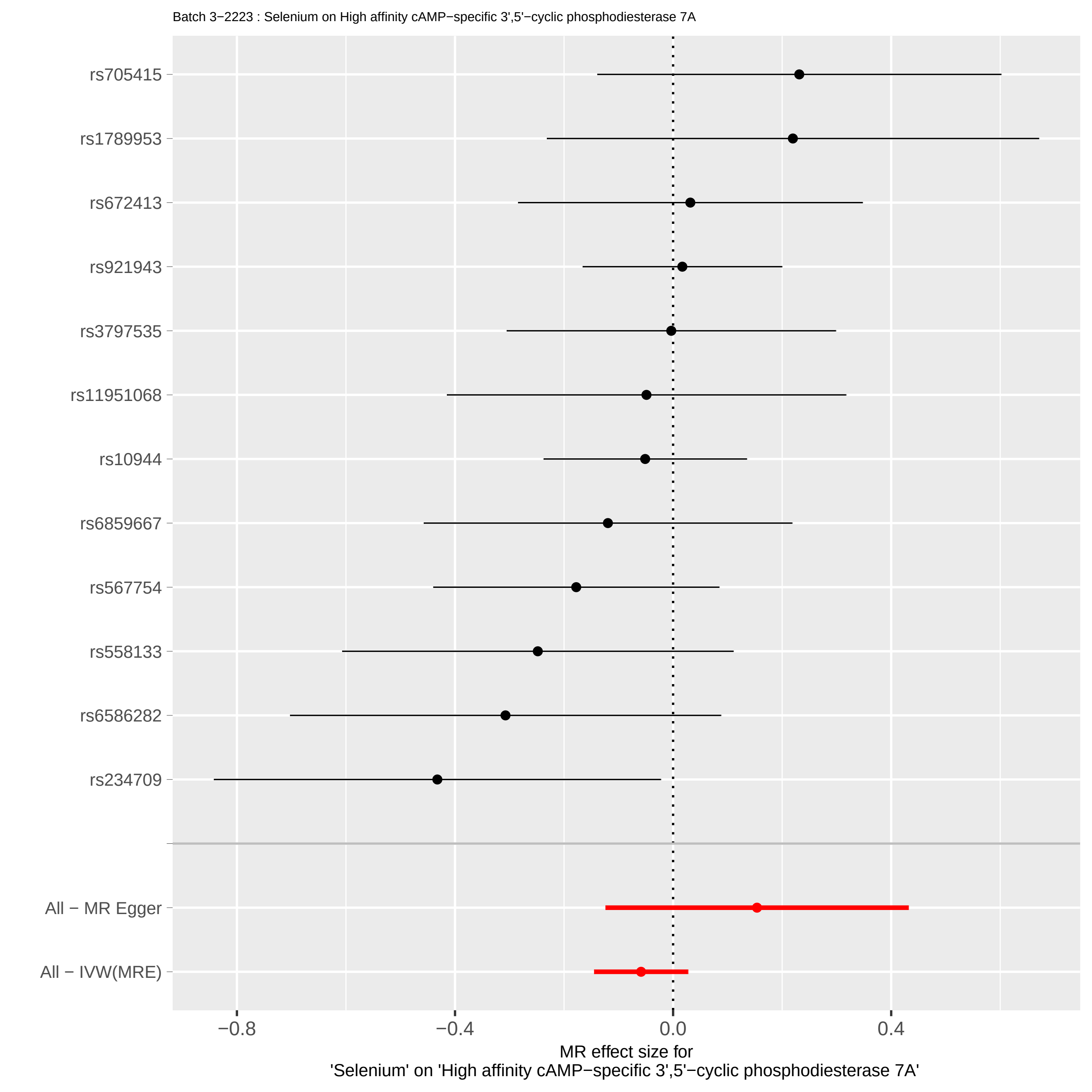

Batch 3−2223 : Selenium on High affinity cAMP−specific 3',5'−cyclic phosphodiesterase 7A
rs705415
rs1789953
rs672413
rs921943
rs3797535
rs11951068
rs10944
rs6859667
rs567754
rs558133
rs6586282
rs234709
All − MR Egger
All − IVW(MRE)
−0.8
−0.4
0.0
0.4
MR effect size for
'Selenium' on 'High affinity cAMP−specific 3',5'−cyclic phosphodiesterase 7A'

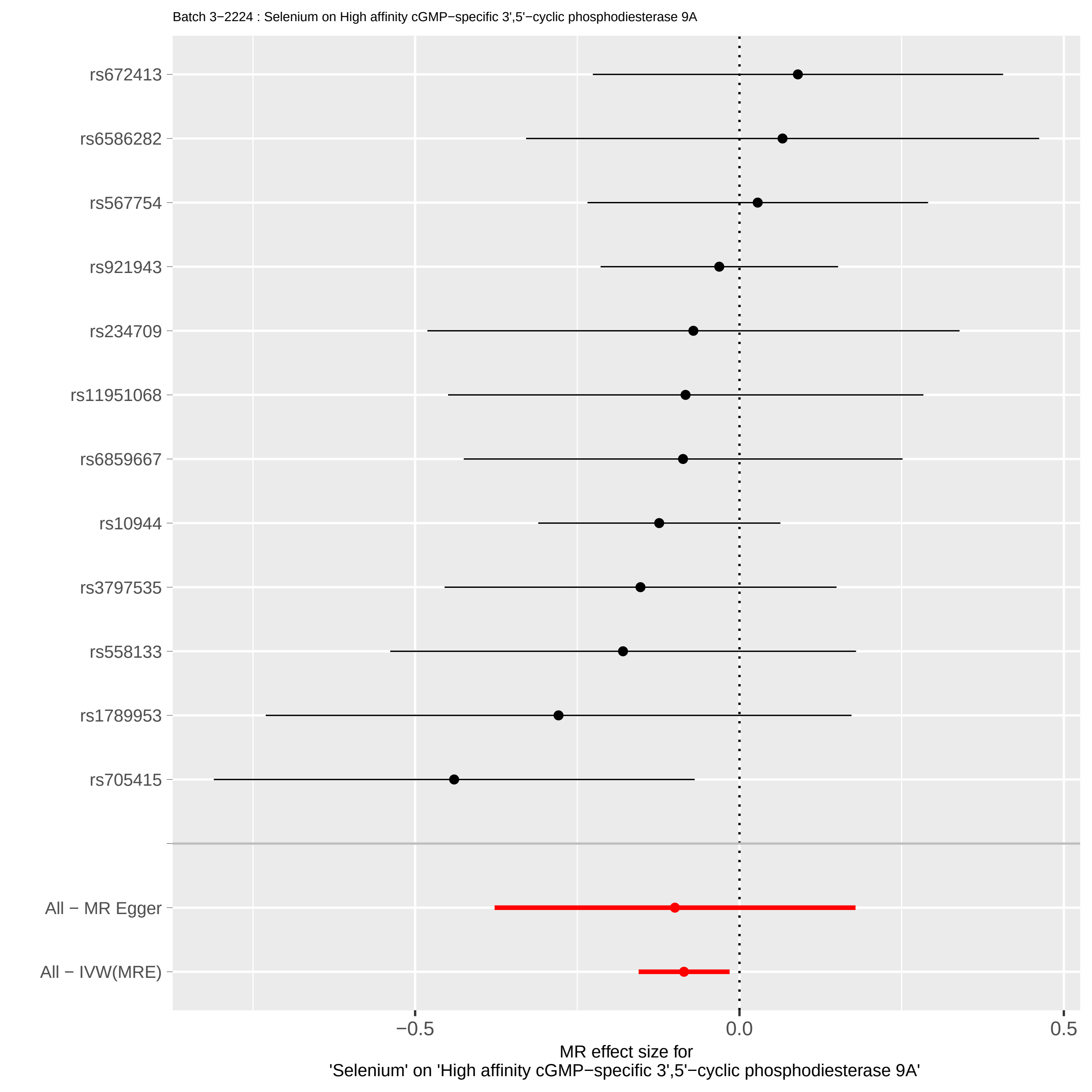

Batch 3−2224 : Selenium on High affinity cGMP−specific 3',5'−cyclic phosphodiesterase 9A
rs672413
rs6586282
rs567754
rs921943
rs234709
rs11951068
rs6859667
rs10944
rs3797535
rs558133
rs1789953
rs705415
All − MR Egger
All − IVW(MRE)
−0.5
0.0
0.5
MR effect size for
'Selenium' on 'High affinity cGMP−specific 3',5'−cyclic phosphodiesterase 9A'

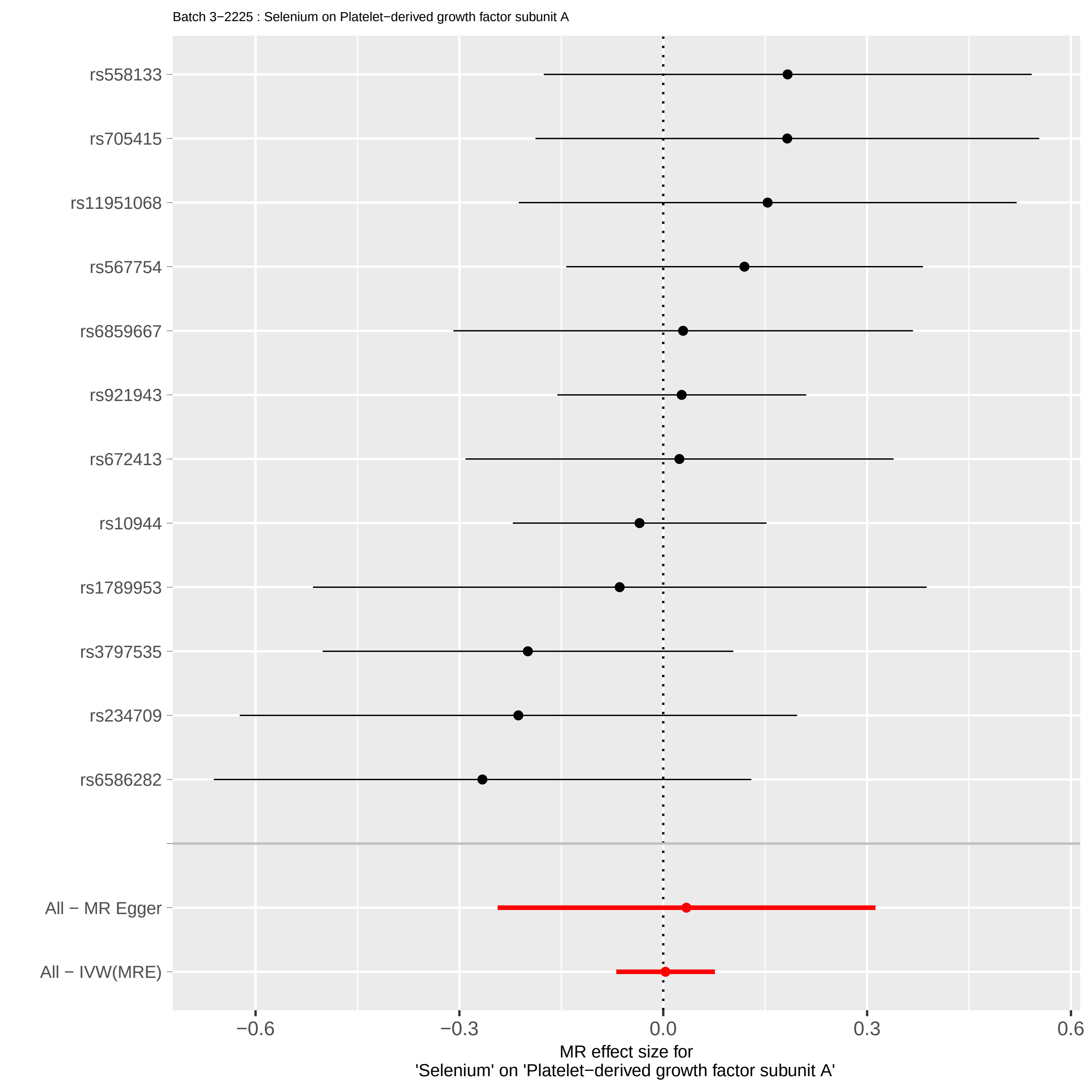

Batch 3−2225 : Selenium on Platelet−derived growth factor subunit A
rs558133
rs705415
rs11951068
rs567754
rs6859667
rs921943
rs672413
rs10944
rs1789953
rs3797535
rs234709
rs6586282
All − MR Egger
All − IVW(MRE)
−0.6
−0.3
0.0
0.3
0.6
MR effect size for
'Selenium' on 'Platelet−derived growth factor subunit A'

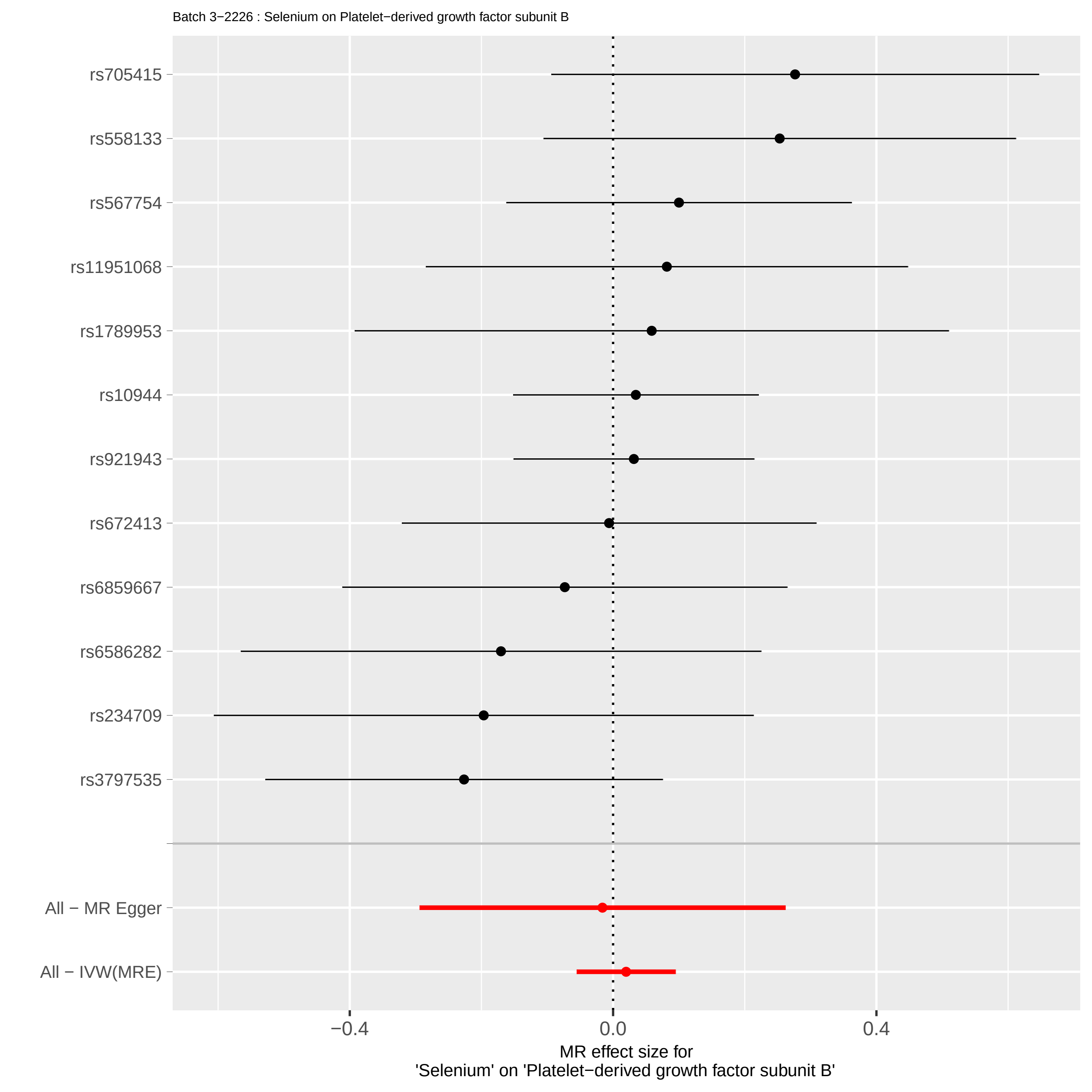

Batch 3−2226 : Selenium on Platelet−derived growth factor subunit B
rs705415
rs558133
rs567754
rs11951068
rs1789953
rs10944
rs921943
rs672413
rs6859667
rs6586282
rs234709
rs3797535
All − MR Egger
All − IVW(MRE)
−0.4
0.0
0.4
MR effect size for
'Selenium' on 'Platelet−derived growth factor subunit B'

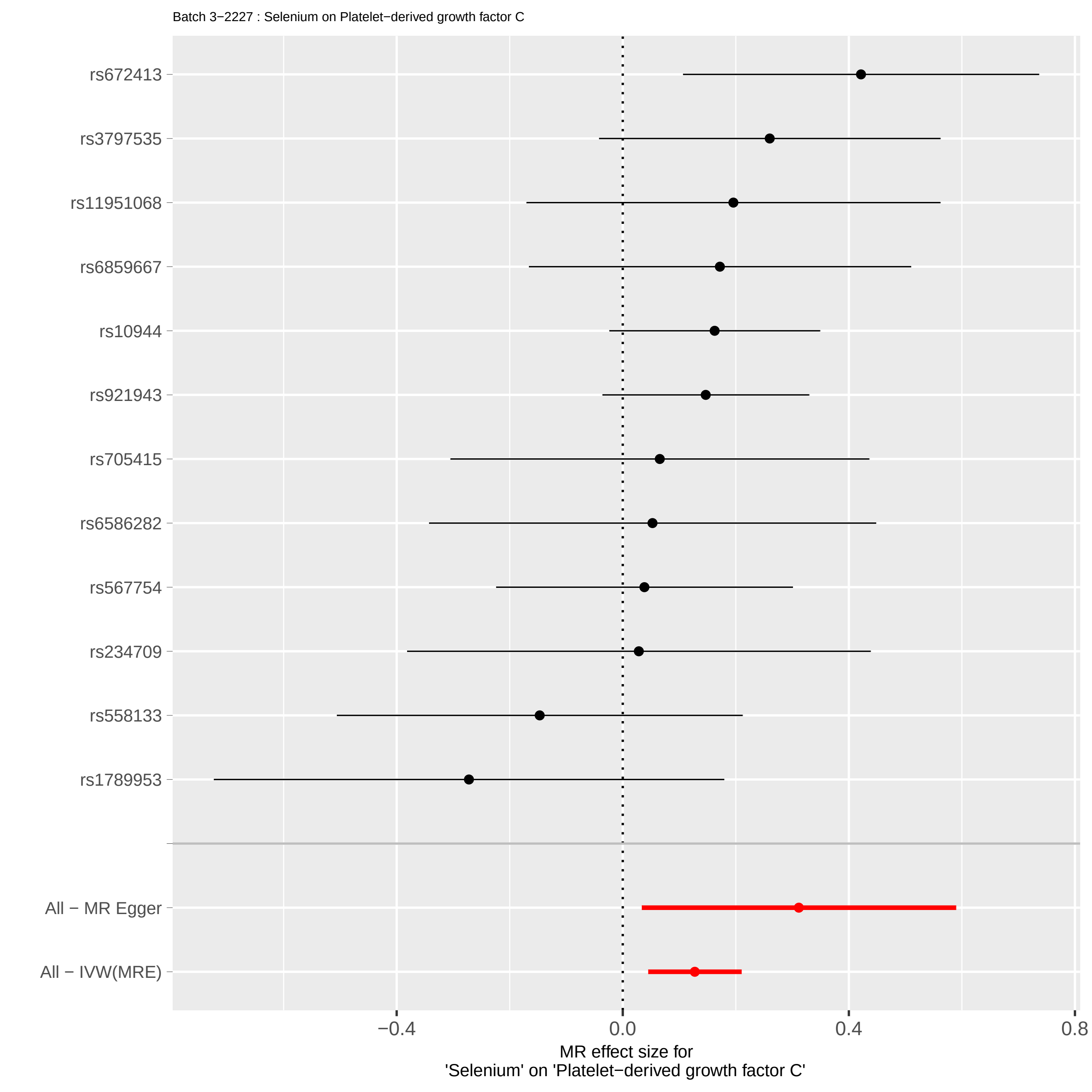
Batch 3−2227 : Selenium on Platelet−derived growth factor C
rs672413
rs3797535
rs11951068
rs6859667
rs10944
rs921943
rs705415
rs6586282
rs567754
rs234709
rs558133
rs1789953
All − MR Egger
All − IVW(MRE)
−0.4
0.0
0.4
0.8
MR effect size for
'Selenium' on 'Platelet−derived growth factor C'

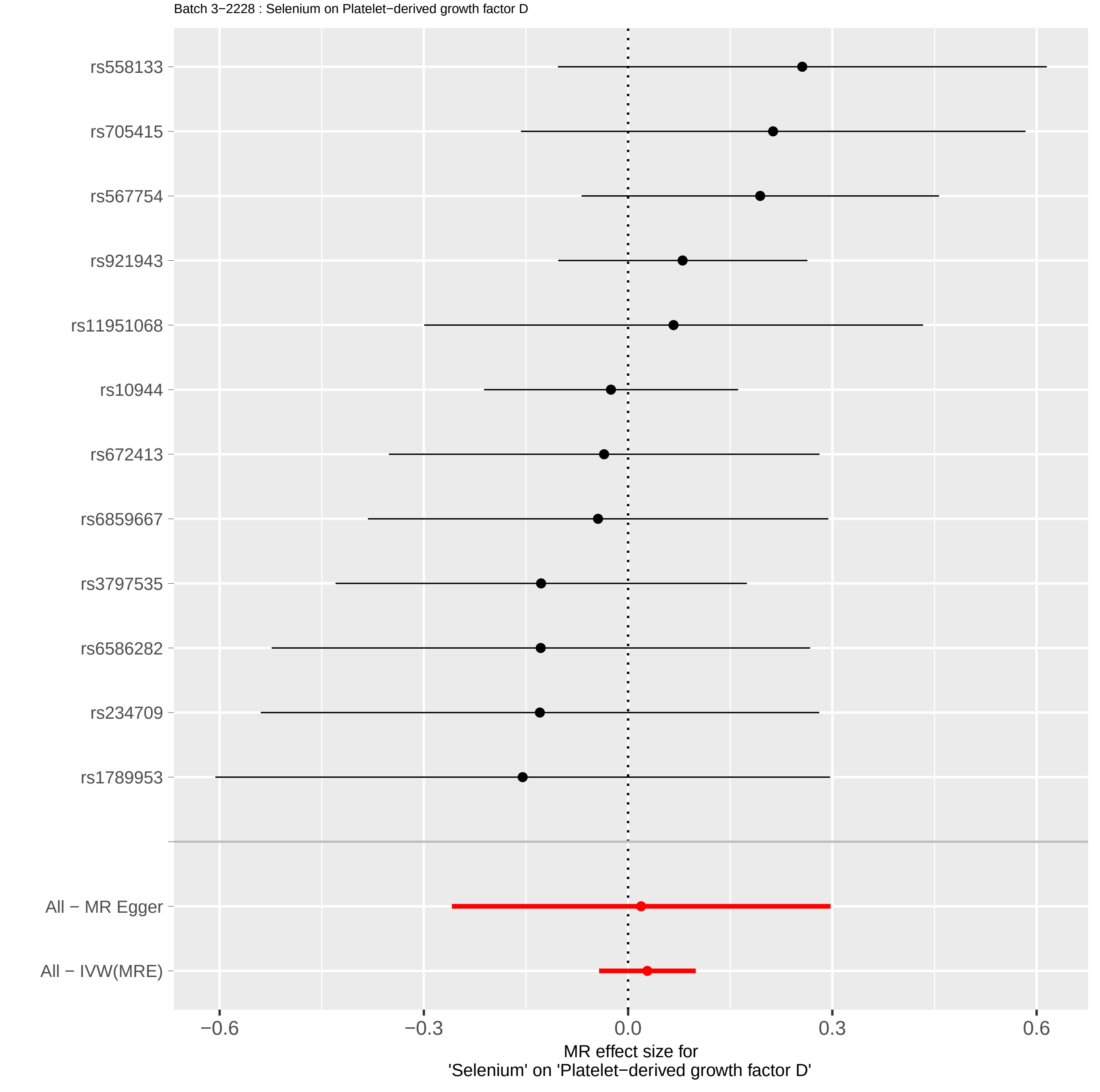

Batch 3−2228 : Selenium on Platelet−derived growth factor D
rs558133
rs705415
rs567754
rs921943
rs11951068
rs10944
rs672413
rs6859667
rs3797535
rs6586282
rs234709
rs1789953
All − MR Egger
All − IVW(MRE)
−0.6
−0.3
0.0
0.3
0.6
MR effect size for
'Selenium' on 'Platelet−derived growth factor D'

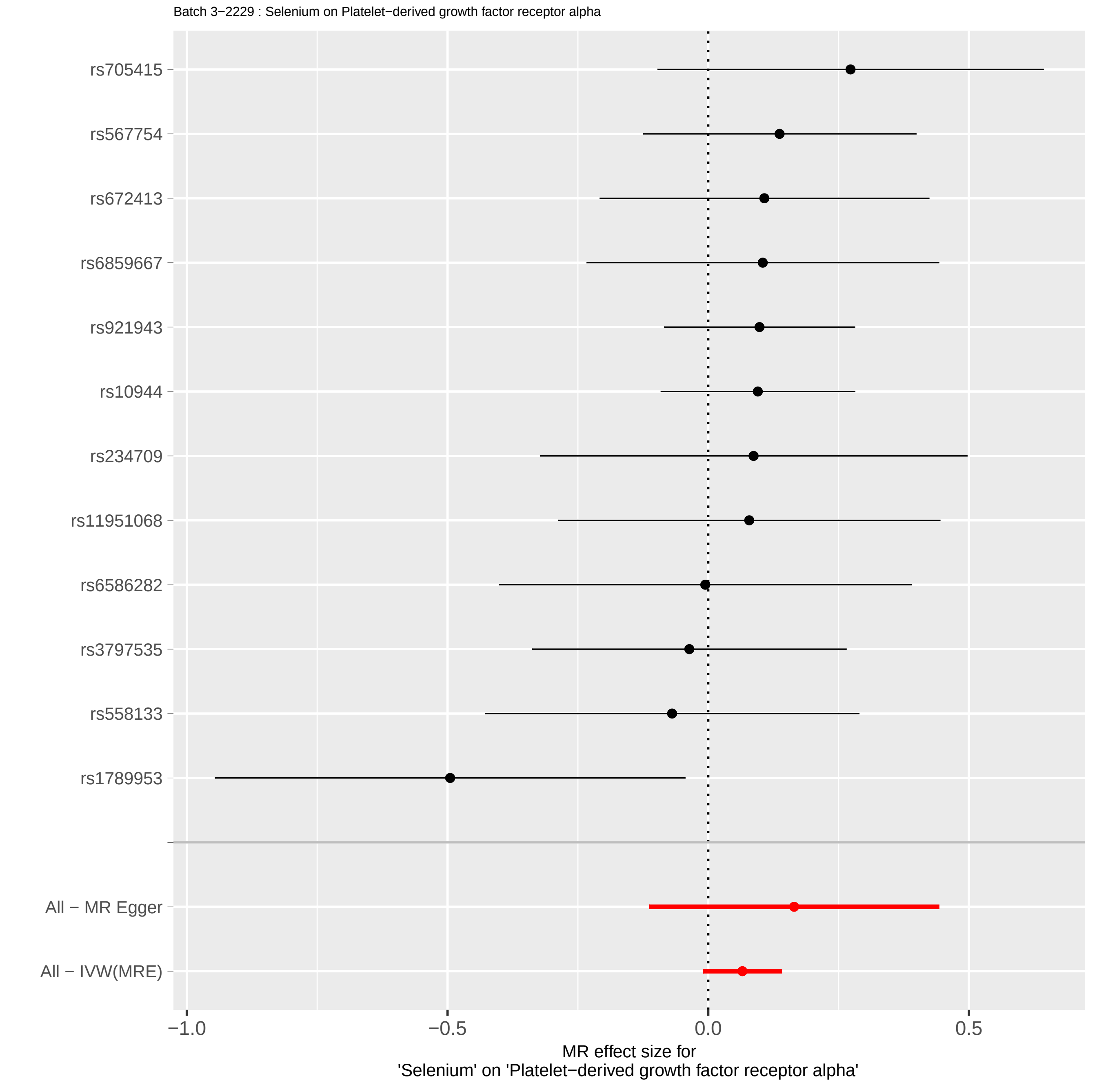

Batch 3−2229 : Selenium on Platelet−derived growth factor receptor alpha
rs705415
rs567754
rs672413
rs6859667
rs921943
rs10944
rs234709
rs11951068
rs6586282
rs3797535
rs558133
rs1789953
All − MR Egger
All − IVW(MRE)
−1.0
−0.5
0.0
0.5
MR effect size for
'Selenium' on 'Platelet−derived growth factor receptor alpha'

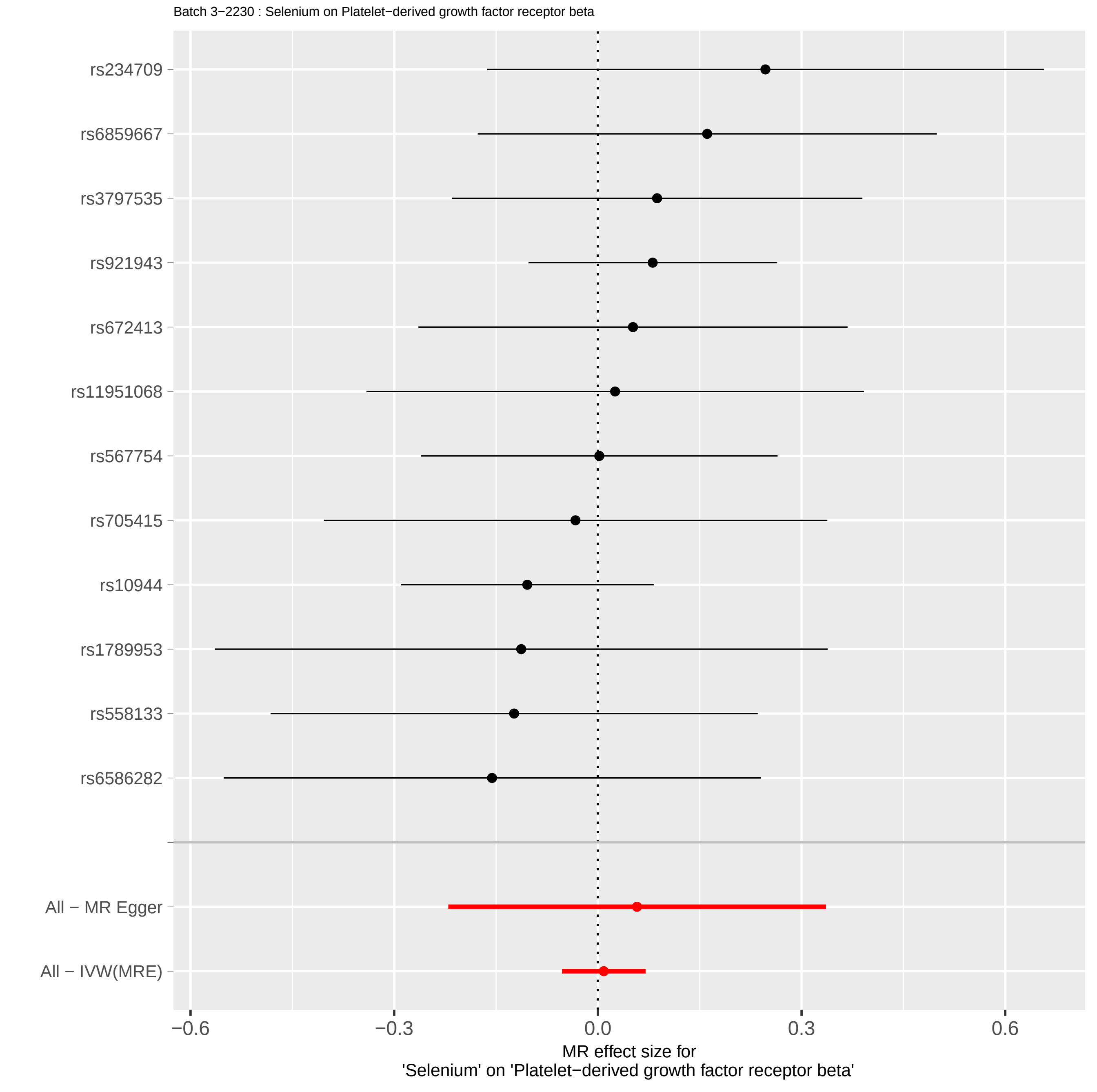

Batch 3−2230 : Selenium on Platelet−derived growth factor receptor beta
rs234709
rs6859667
rs3797535
rs921943
rs672413
rs11951068
rs567754
rs705415
rs10944
rs1789953
rs558133
rs6586282
All − MR Egger
All − IVW(MRE)
−0.6
−0.3
0.0
0.3
0.6
MR effect size for
'Selenium' on 'Platelet−derived growth factor receptor beta'

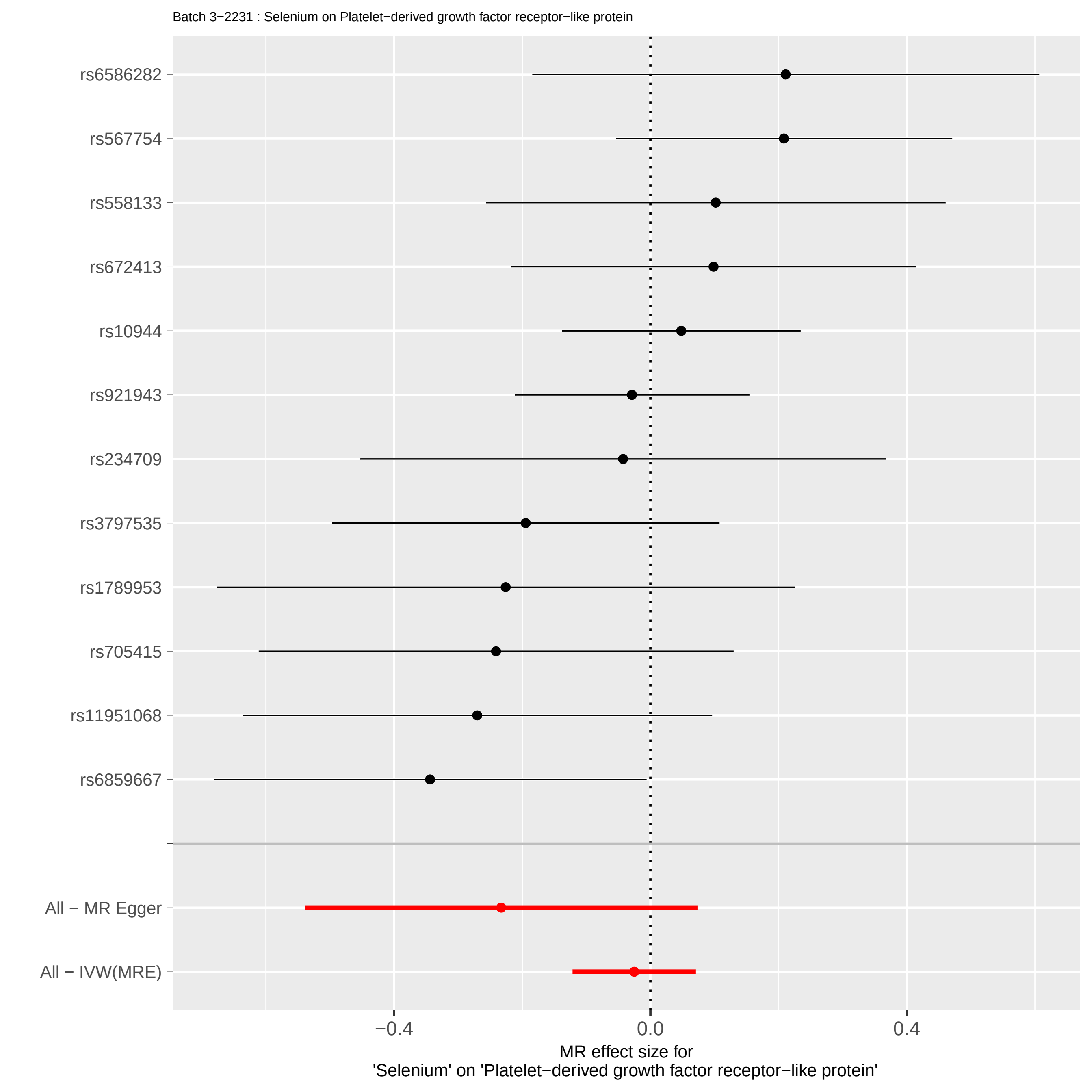

Batch 3−2231 : Selenium on Platelet−derived growth factor receptor−like protein
rs6586282
rs567754
rs558133
rs672413
rs10944
rs921943
rs234709
rs3797535
rs1789953
rs705415
rs11951068
rs6859667
All − MR Egger
All − IVW(MRE)
−0.4
0.0
0.4
MR effect size for
'Selenium' on 'Platelet−derived growth factor receptor−like protein'

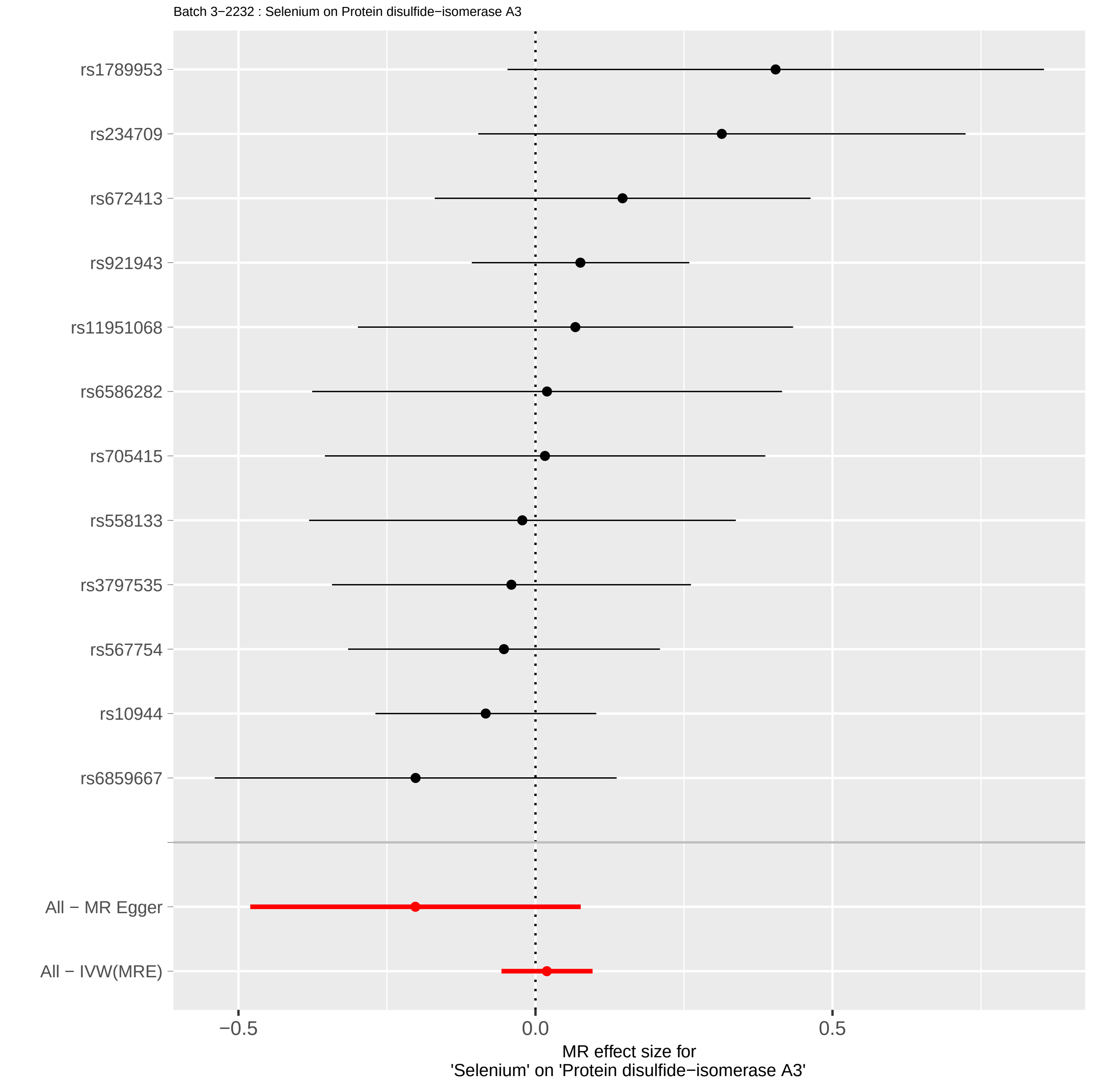

Batch 3−2232 : Selenium on Protein disulfide−isomerase A3
rs1789953
rs234709
rs672413
rs921943
rs11951068
rs6586282
rs705415
rs558133
rs3797535
rs567754
rs10944
rs6859667
All − MR Egger
All − IVW(MRE)
−0.5
0.0
0.5
MR effect size for
'Selenium' on 'Protein disulfide−isomerase A3'

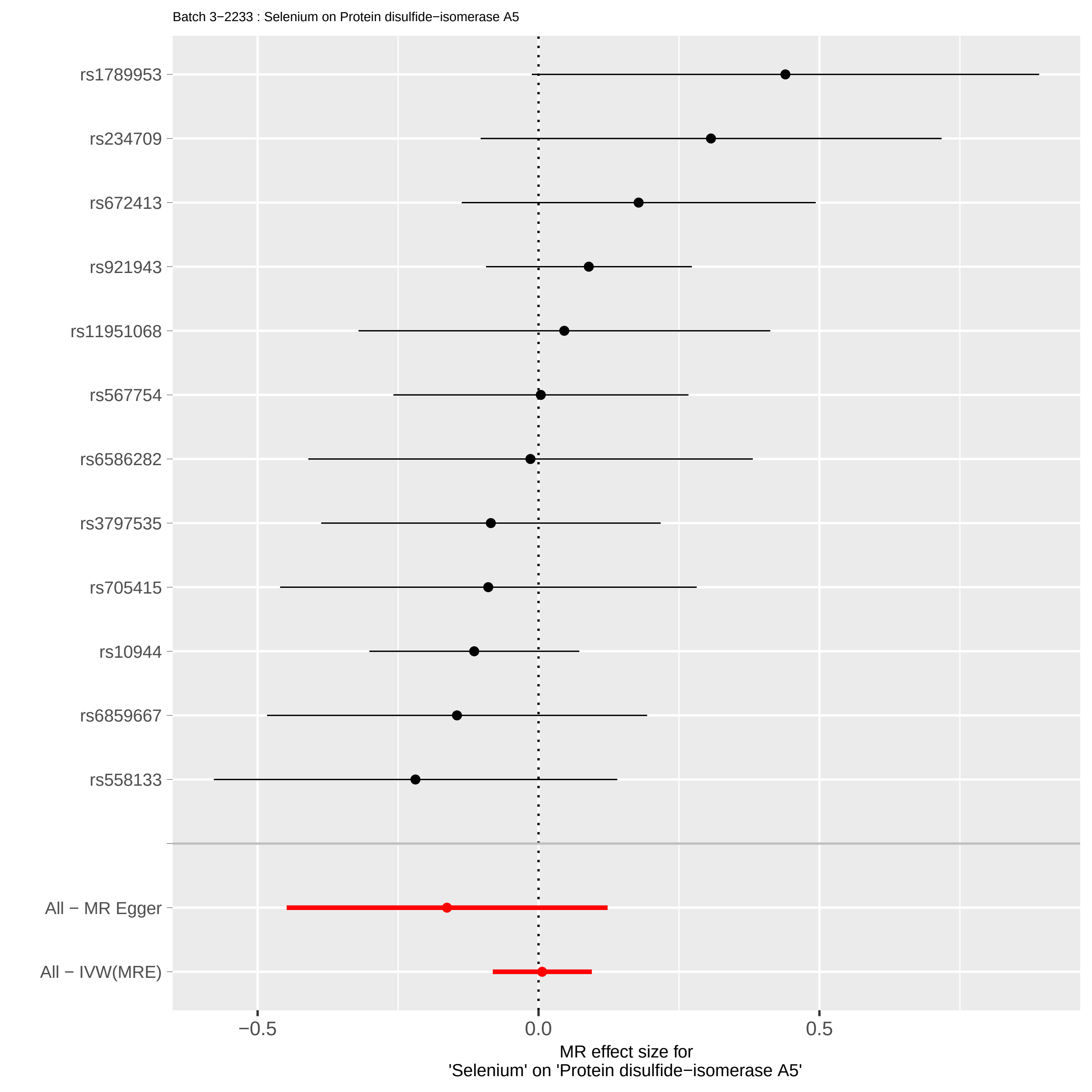

Batch 3−2233 : Selenium on Protein disulfide−isomerase A5
rs1789953
rs234709
rs672413
rs921943
rs11951068
rs567754
rs6586282
rs3797535
rs705415
rs10944
rs6859667
rs558133
All − MR Egger
All − IVW(MRE)
−0.5
0.0
0.5
MR effect size for
'Selenium' on 'Protein disulfide−isomerase A5'

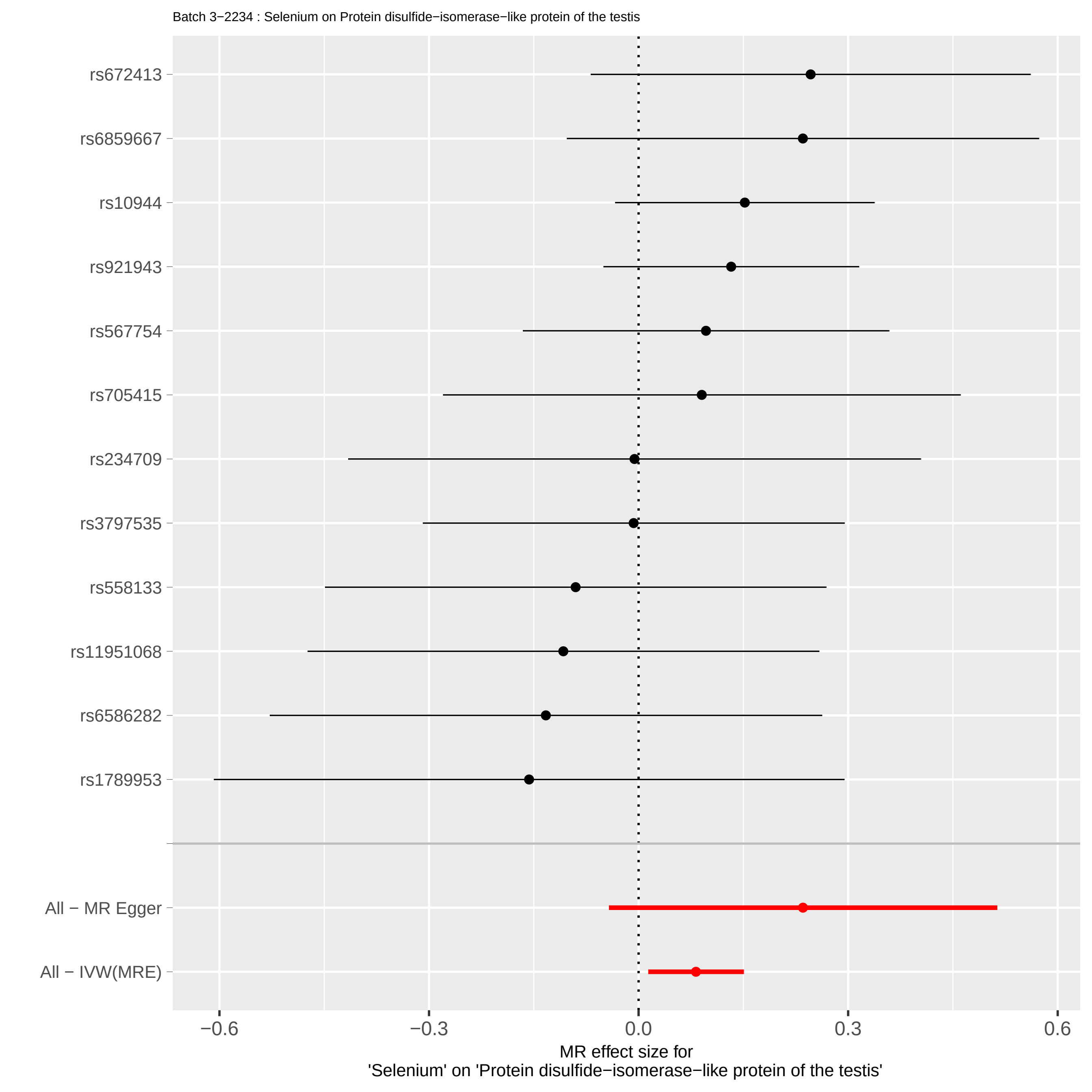

Batch 3−2234 : Selenium on Protein disulfide−isomerase−like protein of the testis
rs672413
rs6859667
rs10944
rs921943
rs567754
rs705415
rs234709
rs3797535
rs558133
rs11951068
rs6586282
rs1789953
All − MR Egger
All − IVW(MRE)
−0.6
−0.3
0.0
0.3
0.6
MR effect size for
'Selenium' on 'Protein disulfide−isomerase−like protein of the testis'

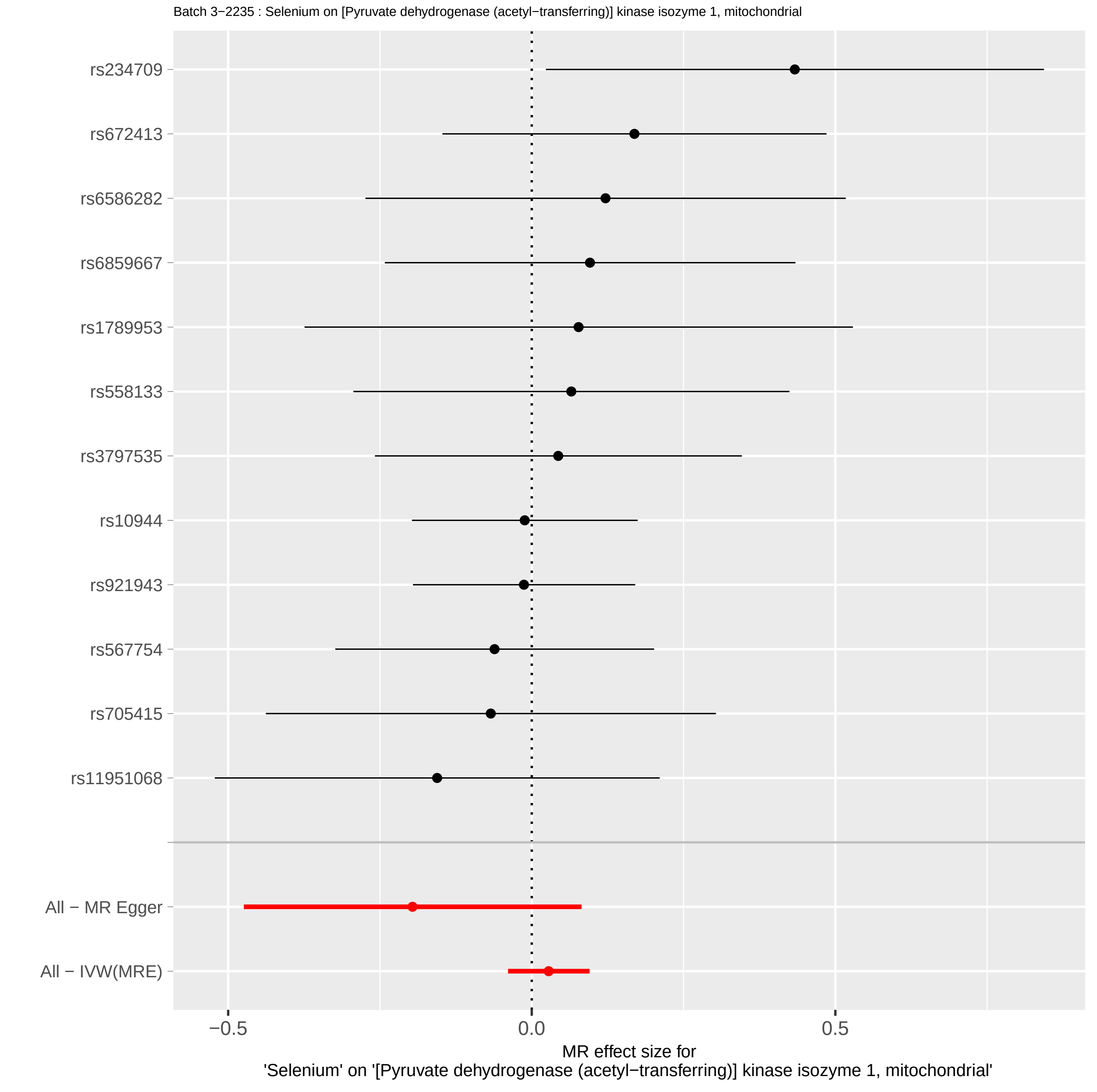

Batch 3−2235 : Selenium on [Pyruvate dehydrogenase (acetyl−transferring)] kinase isozyme 1, mitochondrial
rs234709
rs672413
rs6586282
rs6859667
rs1789953
rs558133
rs3797535
rs10944
rs921943
rs567754
rs705415
rs11951068
All − MR Egger
All − IVW(MRE)
−0.5
0.0
0.5
MR effect size for
'Selenium' on '[Pyruvate dehydrogenase (acetyl−transferring)] kinase isozyme 1, mitochondrial'

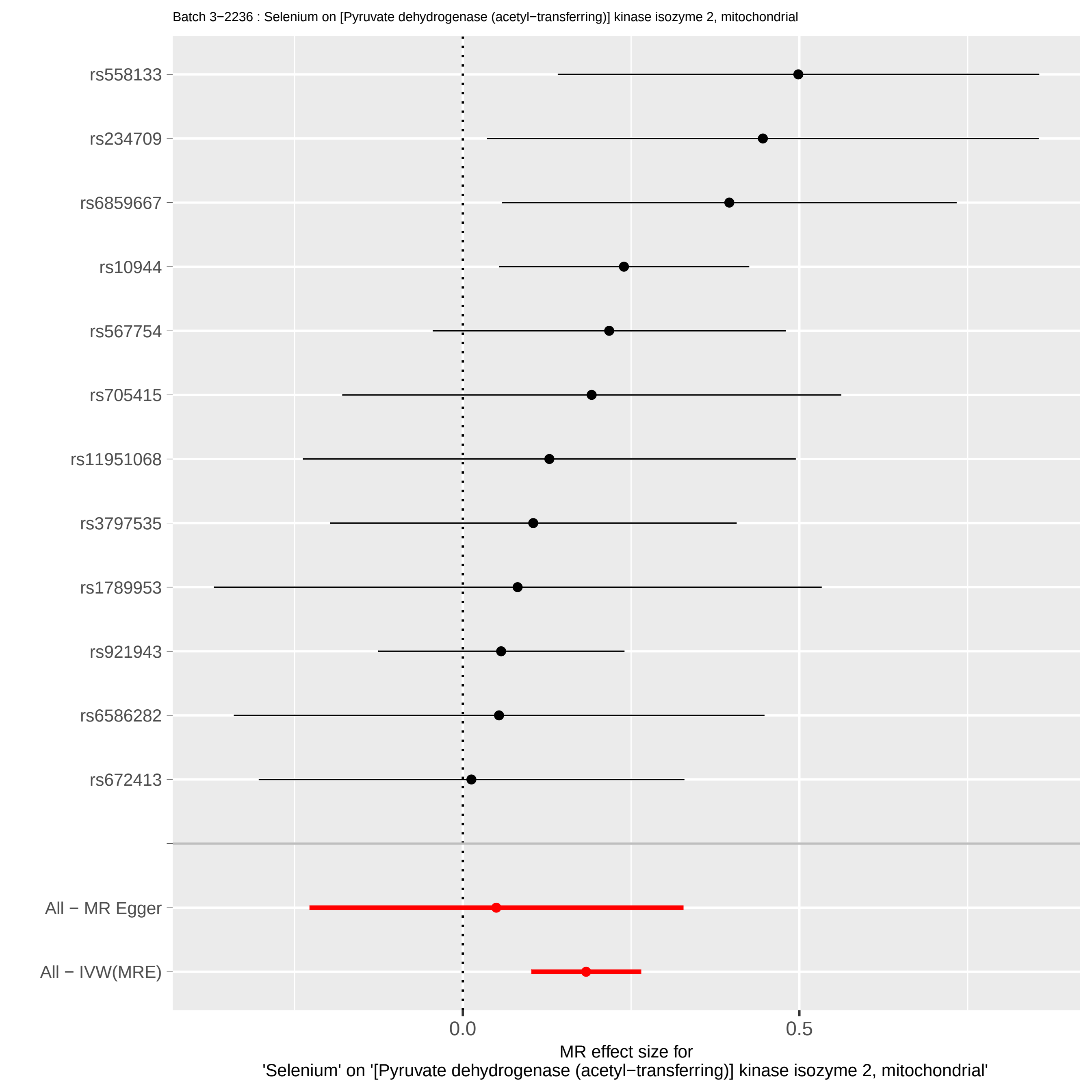

Batch 3−2236 : Selenium on [Pyruvate dehydrogenase (acetyl−transferring)] kinase isozyme 2, mitochondrial
rs558133
rs234709
rs6859667
rs10944
rs567754
rs705415
rs11951068
rs3797535
rs1789953
rs921943
rs6586282
rs672413
All − MR Egger
All − IVW(MRE)
0.0
0.5
MR effect size for
'Selenium' on '[Pyruvate dehydrogenase (acetyl−transferring)] kinase isozyme 2, mitochondrial'

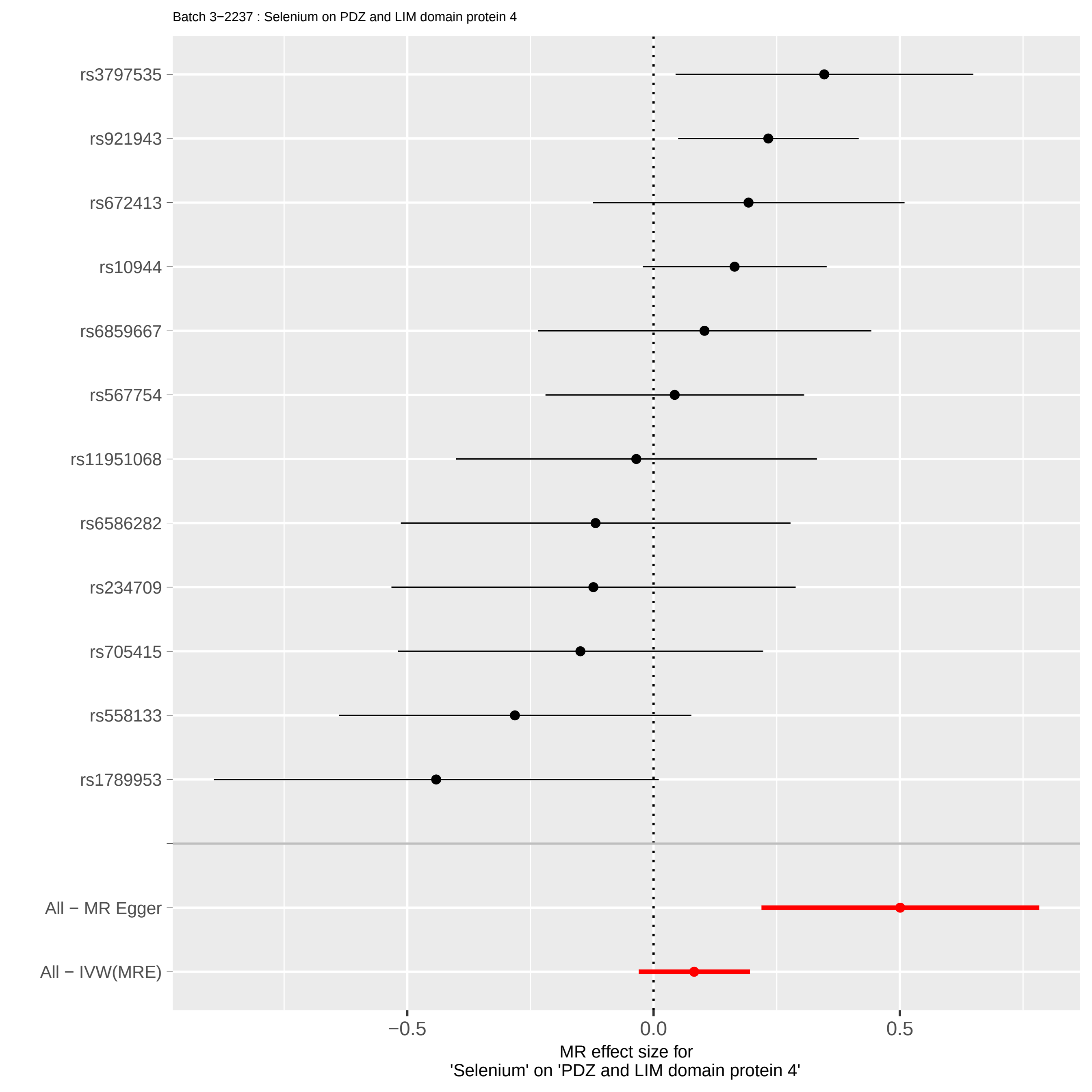

Batch 3−2237 : Selenium on PDZ and LIM domain protein 4
rs3797535
rs921943
rs672413
rs10944
rs6859667
rs567754
rs11951068
rs6586282
rs234709
rs705415
rs558133
rs1789953
All − MR Egger
All − IVW(MRE)
−0.5
0.0
0.5
MR effect size for
'Selenium' on 'PDZ and LIM domain protein 4'

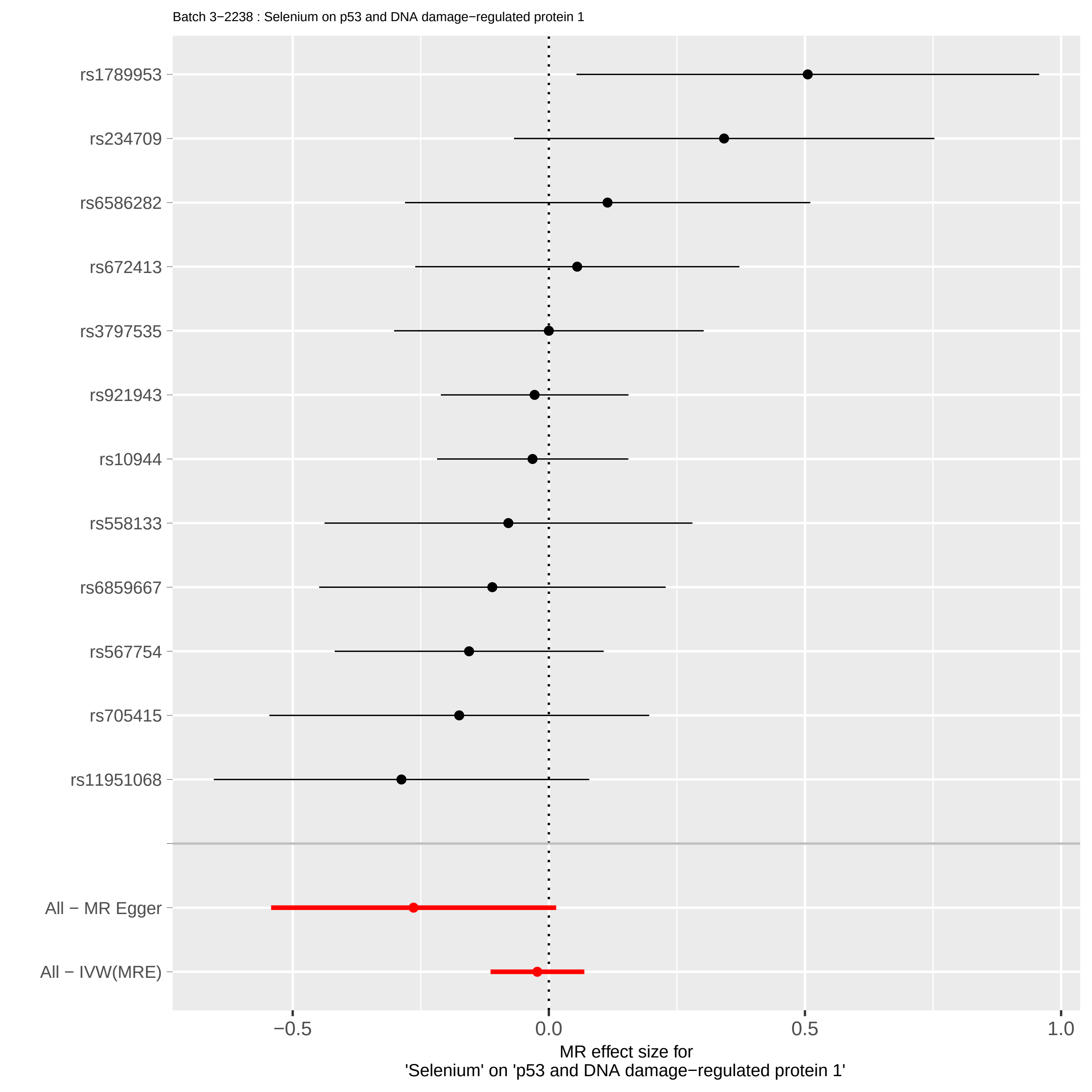

Batch 3−2238 : Selenium on p53 and DNA damage−regulated protein 1
rs1789953
rs234709
rs6586282
rs672413
rs3797535
rs921943
rs10944
rs558133
rs6859667
rs567754
rs705415
rs11951068
All − MR Egger
All − IVW(MRE)
−0.5
0.0
0.5
1.0
MR effect size for
'Selenium' on 'p53 and DNA damage−regulated protein 1'

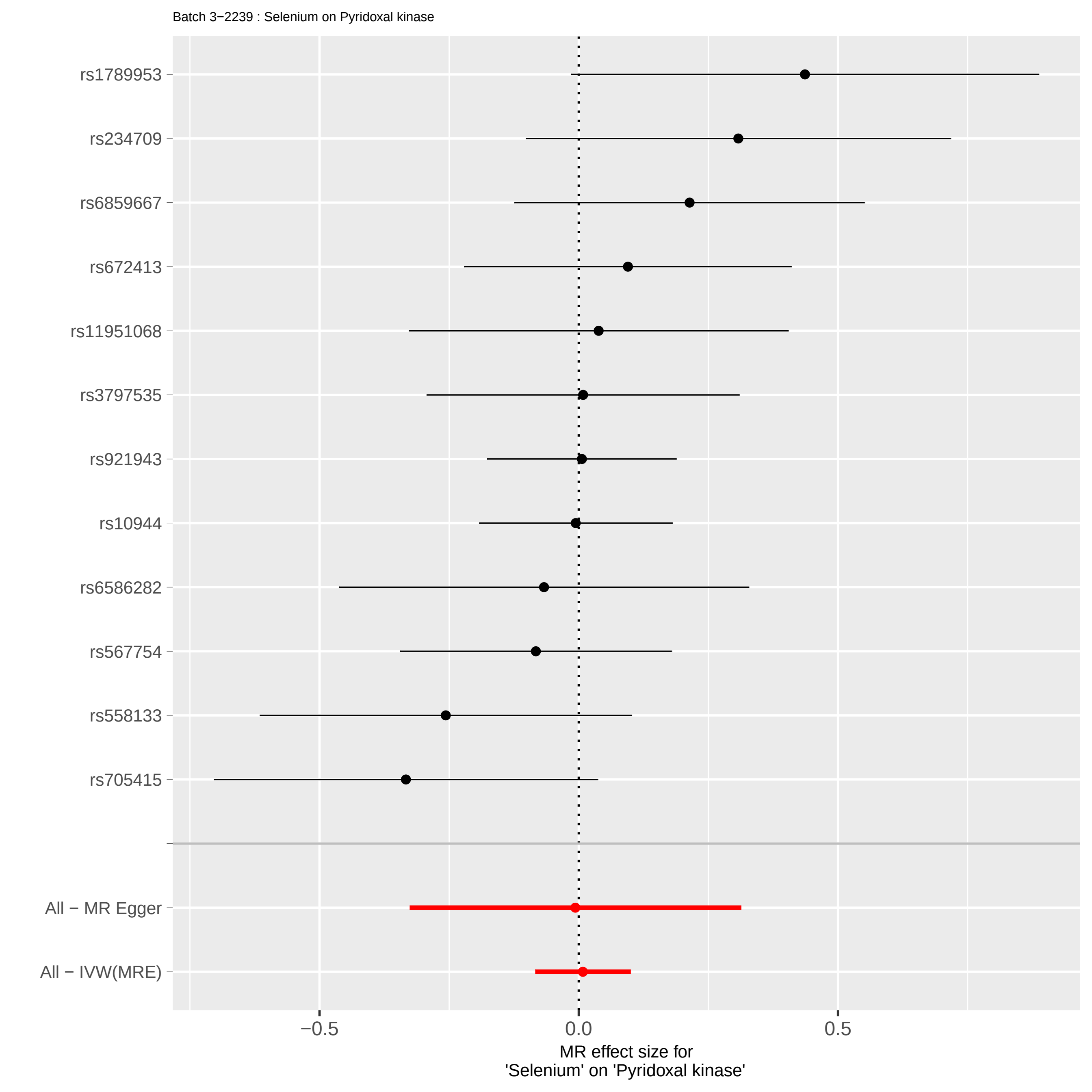

Batch 3−2239 : Selenium on Pyridoxal kinase
rs1789953
rs234709
rs6859667
rs672413
rs11951068
rs3797535
rs921943
rs10944
rs6586282
rs567754
rs558133
rs705415
All − MR Egger
All − IVW(MRE)
−0.5
0.0
0.5
MR effect size for
'Selenium' on 'Pyridoxal kinase'

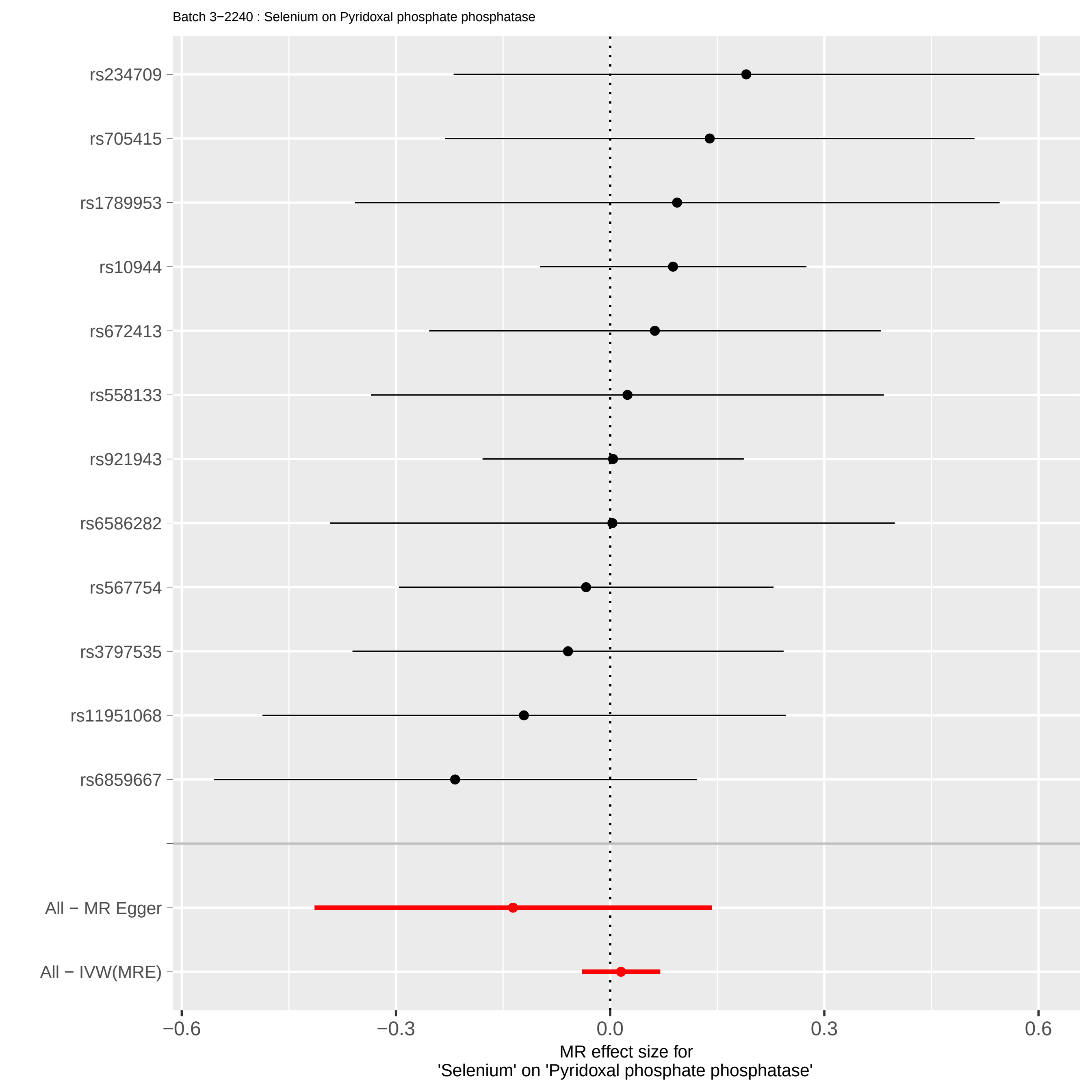

Batch 3−2240 : Selenium on Pyridoxal phosphate phosphatase
rs234709
rs705415
rs1789953
rs10944
rs672413
rs558133
rs921943
rs6586282
rs567754
rs3797535
rs11951068
rs6859667
All − MR Egger
All − IVW(MRE)
−0.6
−0.3
0.0
0.3
0.6
MR effect size for
'Selenium' on 'Pyridoxal phosphate phosphatase'

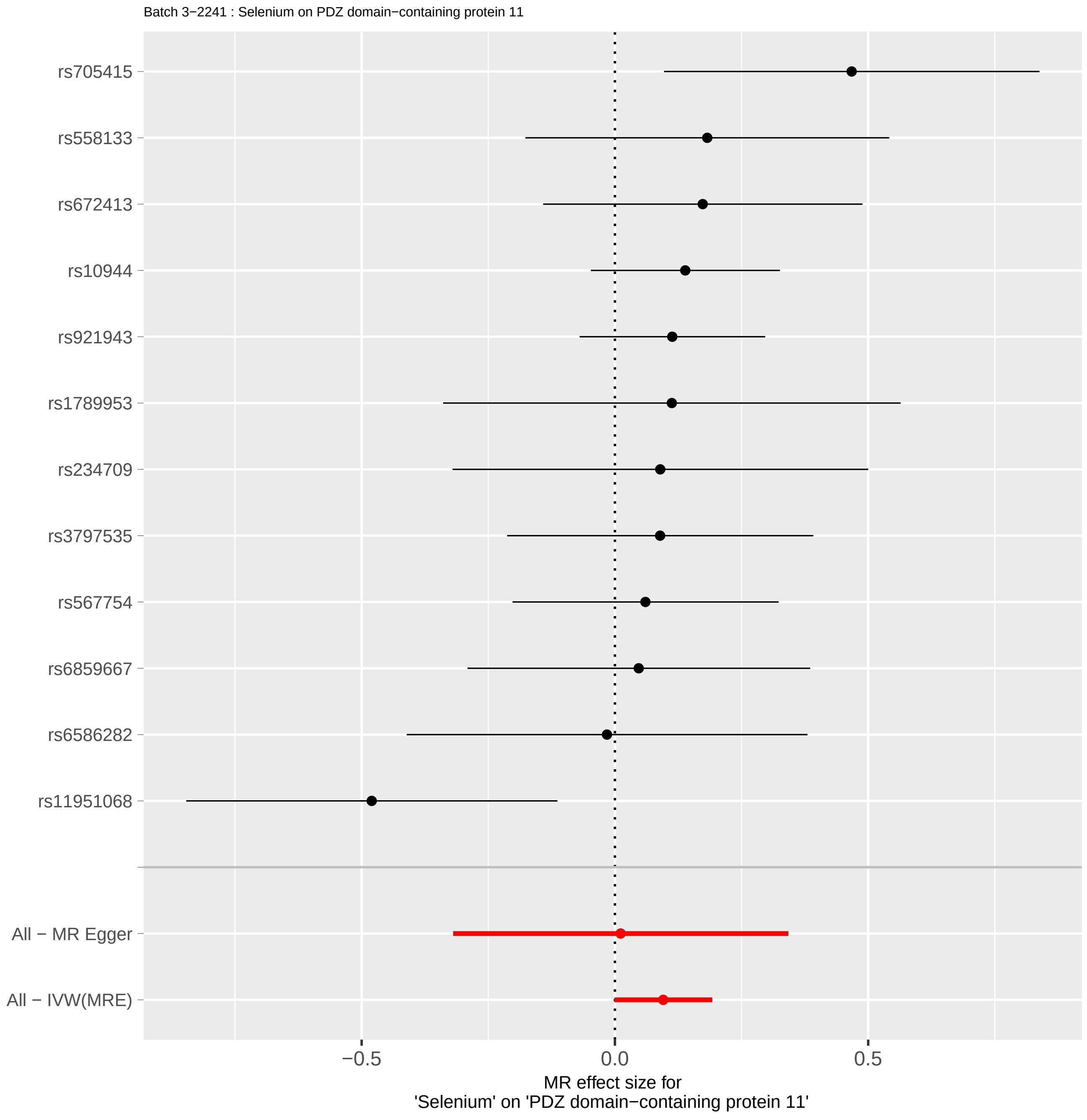
Batch 3−2241 : Selenium on PDZ domain−containing protein 11
rs705415
rs558133
rs672413
rs10944
rs921943
rs1789953
rs234709
rs3797535
rs567754
rs6859667
rs6586282
rs11951068
All − MR Egger
All − IVW(MRE)
−0.5
0.0
0.5
MR effect size for
'Selenium' on 'PDZ domain−containing protein 11'

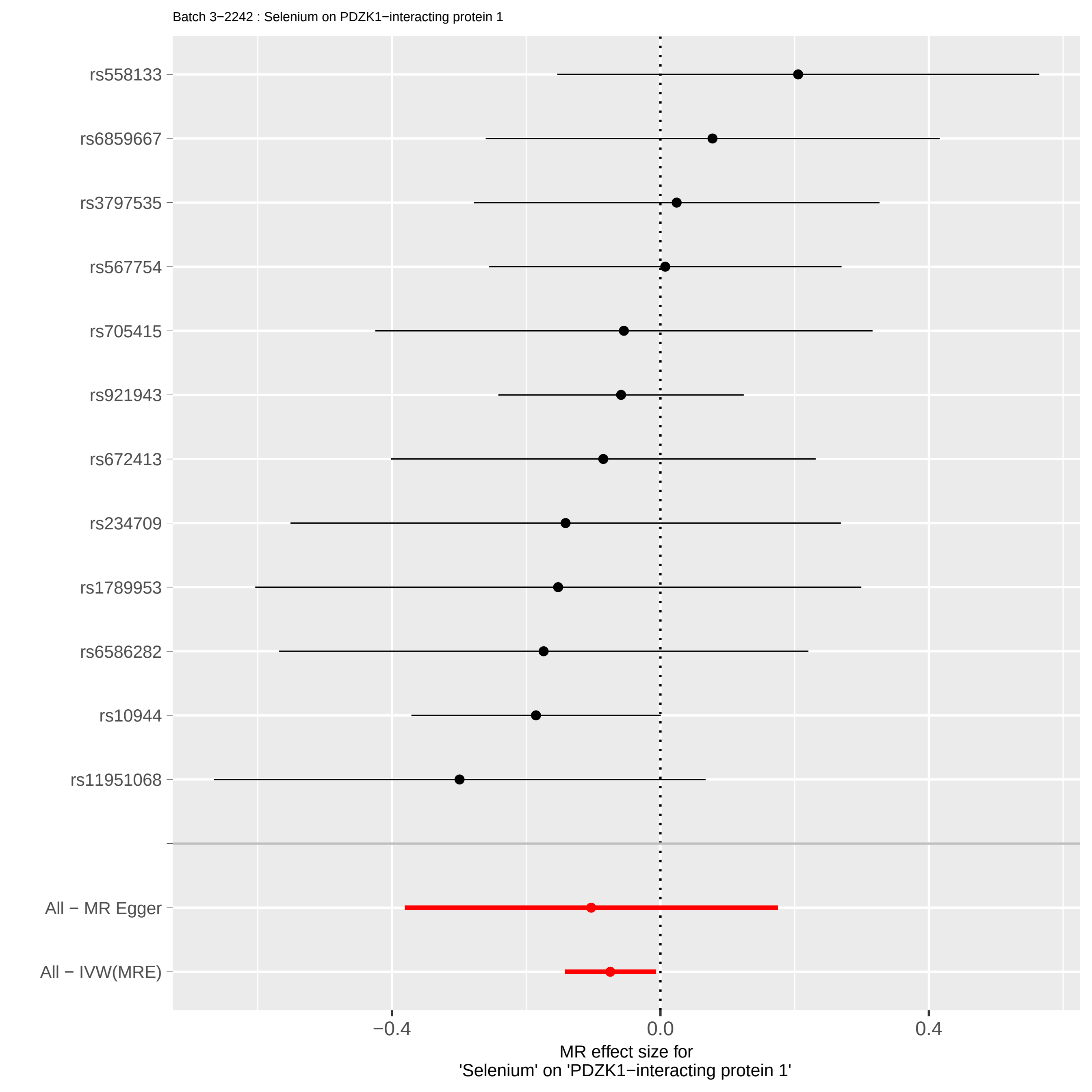
Batch 3−2242 : Selenium on PDZK1−interacting protein 1
rs558133
rs6859667
rs3797535
rs567754
rs705415
rs921943
rs672413
rs234709
rs1789953
rs6586282
rs10944
rs11951068
All − MR Egger
All − IVW(MRE)
−0.4
0.0
0.4
MR effect size for
'Selenium' on 'PDZK1−interacting protein 1'

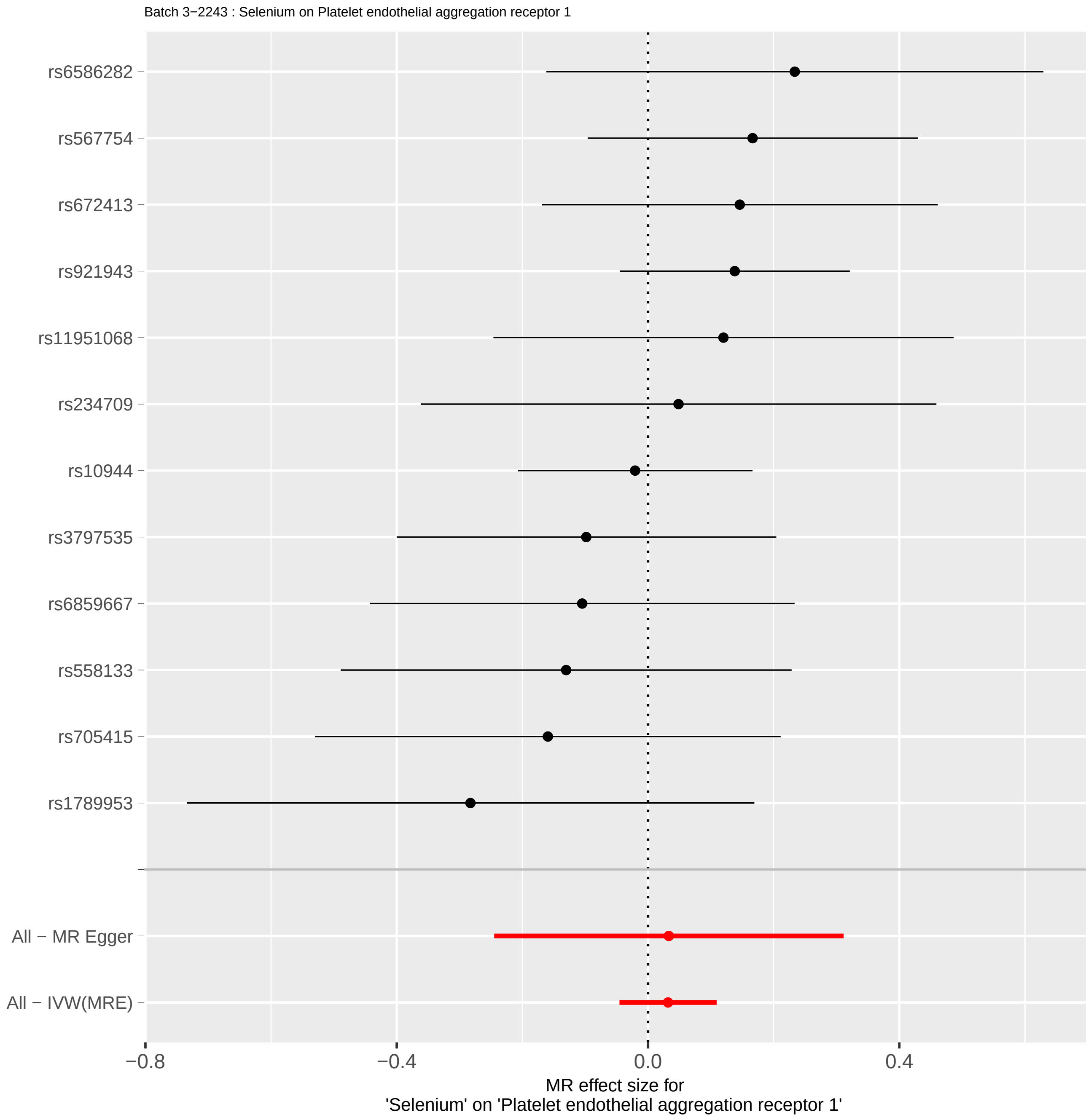

Batch 3−2243 : Selenium on Platelet endothelial aggregation receptor 1
rs6586282
rs567754
rs672413
rs921943
rs11951068
rs234709
rs10944
rs3797535
rs6859667
rs558133
rs705415
rs1789953
All − MR Egger
All − IVW(MRE)
−0.8
−0.4
0.0
0.4
MR effect size for
'Selenium' on 'Platelet endothelial aggregation receptor 1'

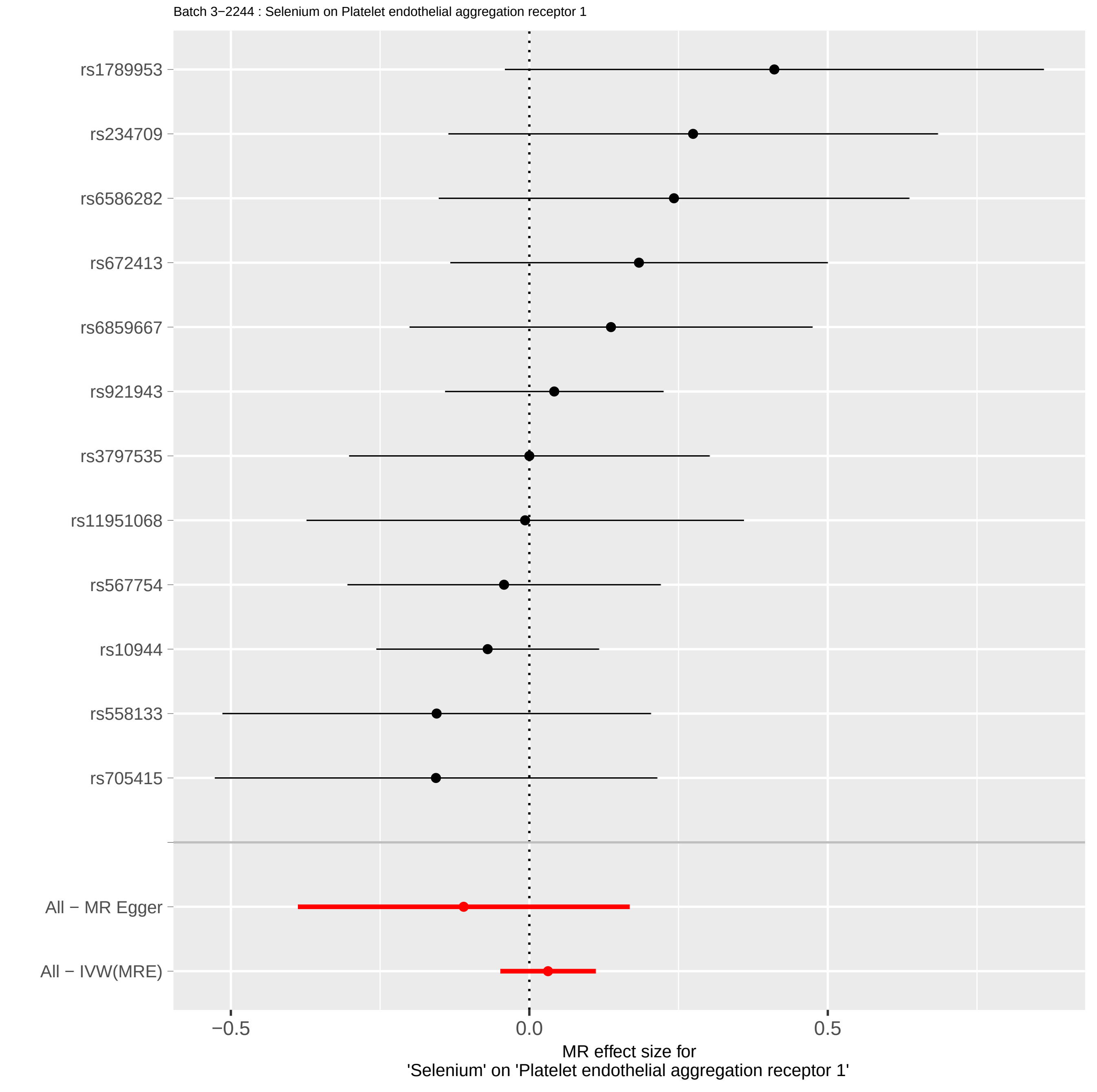

Batch 3−2244 : Selenium on Platelet endothelial aggregation receptor 1
rs1789953
rs234709
rs6586282
rs672413
rs6859667
rs921943
rs3797535
rs11951068
rs567754
rs10944
rs558133
rs705415
All − MR Egger
All − IVW(MRE)
−0.5
0.0
0.5
MR effect size for
'Selenium' on 'Platelet endothelial aggregation receptor 1'

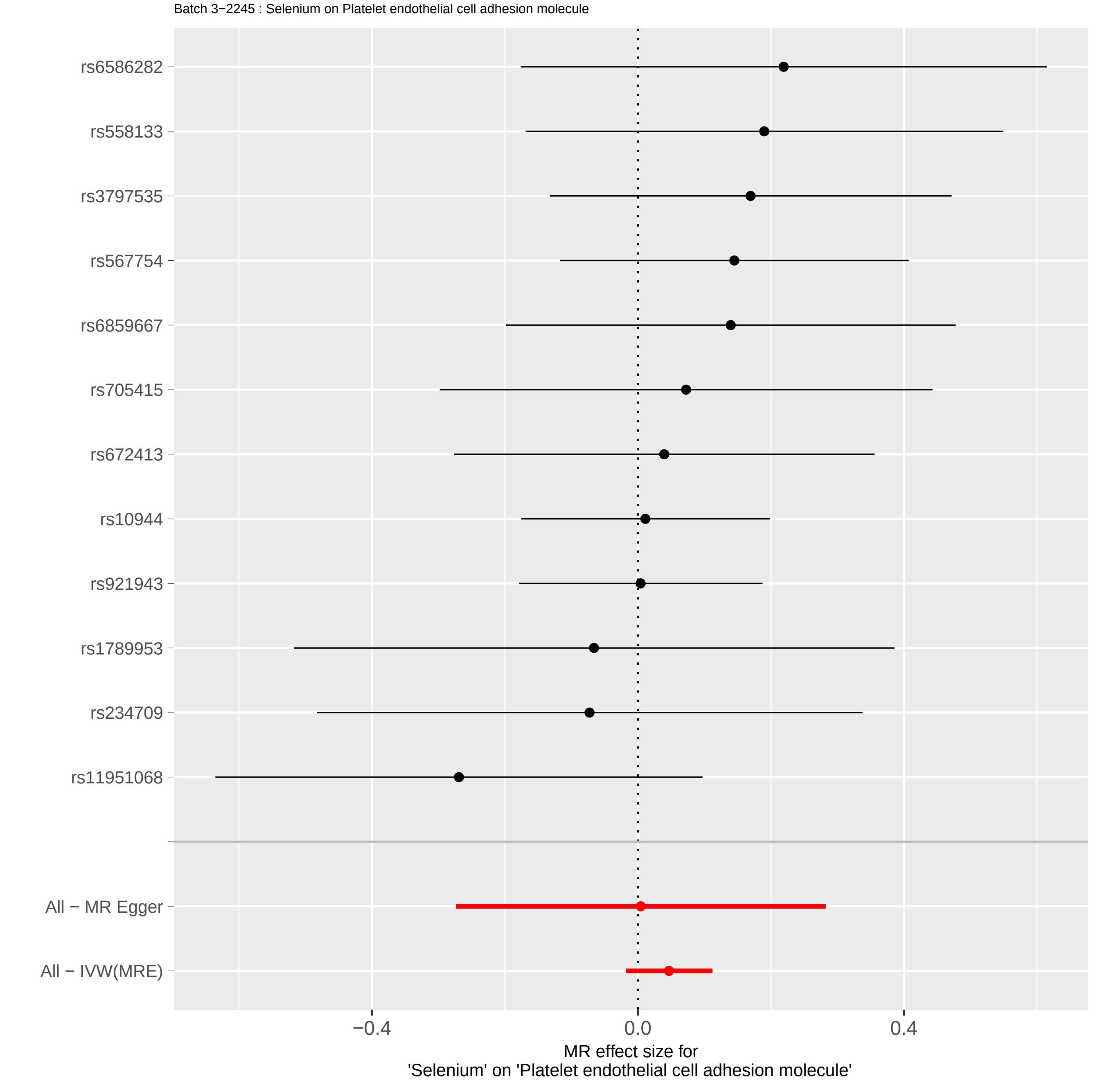

Batch 3−2245 : Selenium on Platelet endothelial cell adhesion molecule
rs6586282
rs558133
rs3797535
rs567754
rs6859667
rs705415
rs672413
rs10944
rs921943
rs1789953
rs234709
rs11951068
All − MR Egger
All − IVW(MRE)
−0.4
0.0
0.4
MR effect size for
'Selenium' on 'Platelet endothelial cell adhesion molecule'

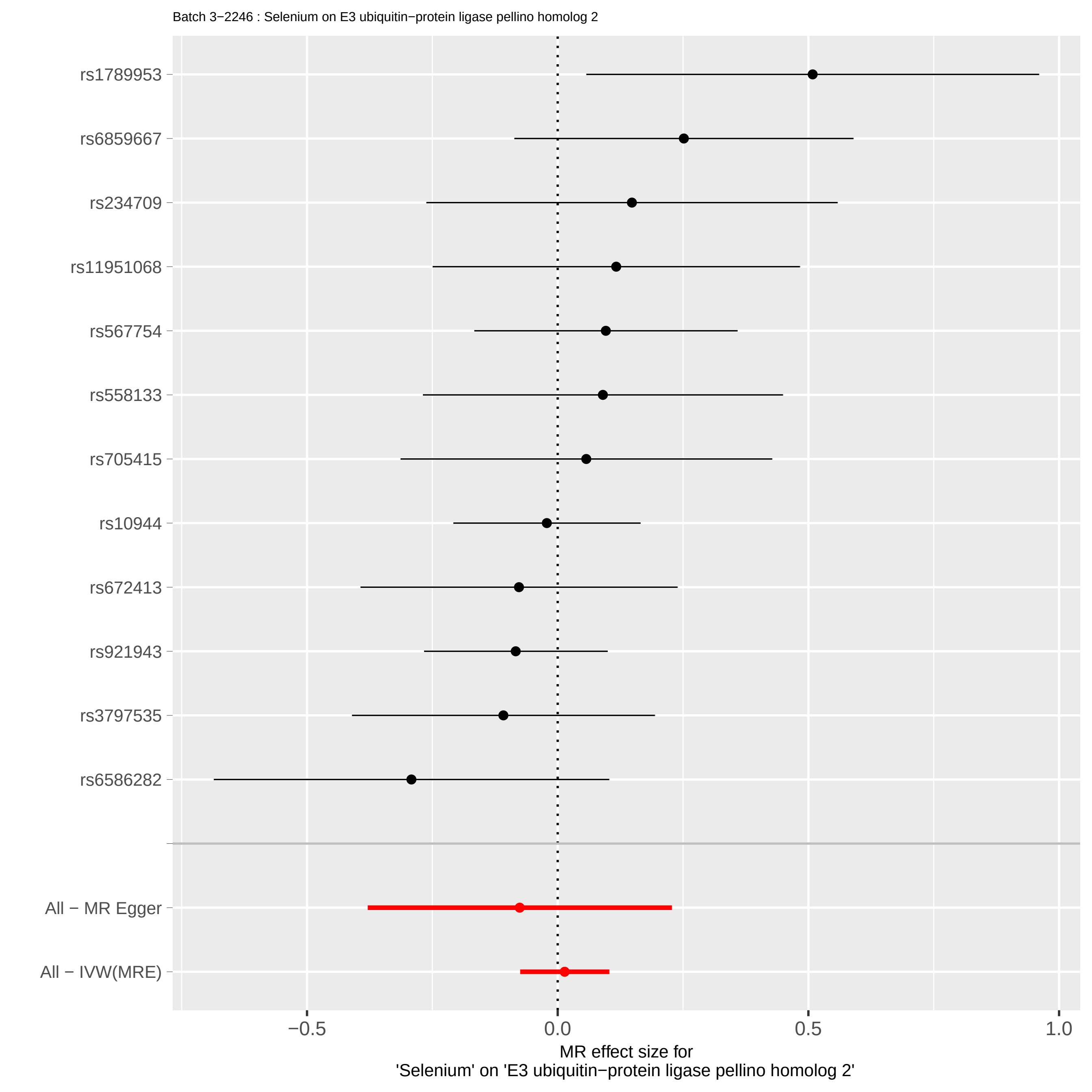
Batch 3−2246 : Selenium on E3 ubiquitin−protein ligase pellino homolog 2
rs1789953
rs6859667
rs234709
rs11951068
rs567754
rs558133
rs705415
rs10944
rs672413
rs921943
rs3797535
rs6586282
All − MR Egger
All − IVW(MRE)
−0.5
0.0
0.5
1.0
MR effect size for
'Selenium' on 'E3 ubiquitin−protein ligase pellino homolog 2'

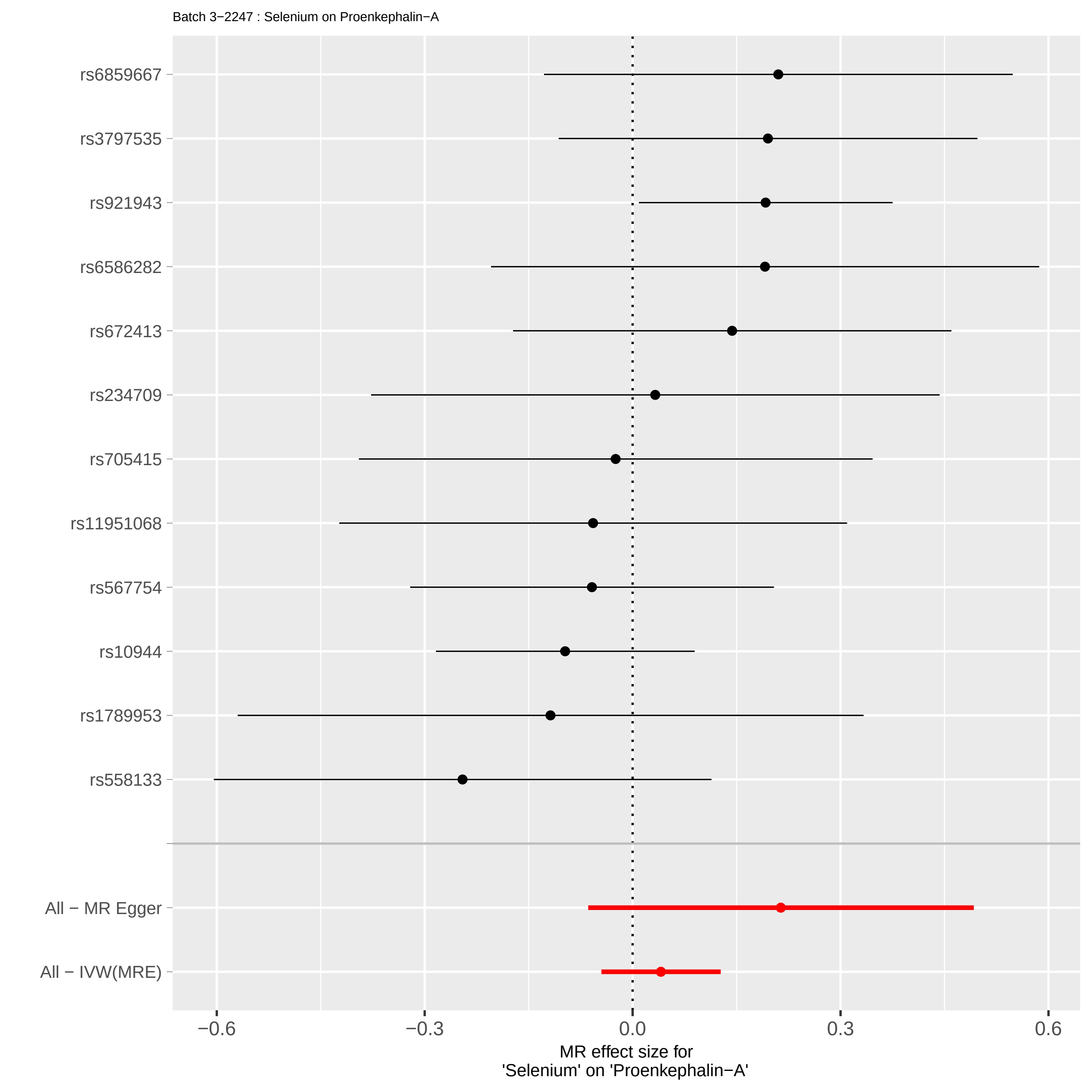

Batch 3−2247 : Selenium on Proenkephalin−A
rs6859667
rs3797535
rs921943
rs6586282
rs672413
rs234709
rs705415
rs11951068
rs567754
rs10944
rs1789953
rs558133
All − MR Egger
All − IVW(MRE)
−0.6
−0.3
0.0
0.3
0.6
MR effect size for
'Selenium' on 'Proenkephalin−A'

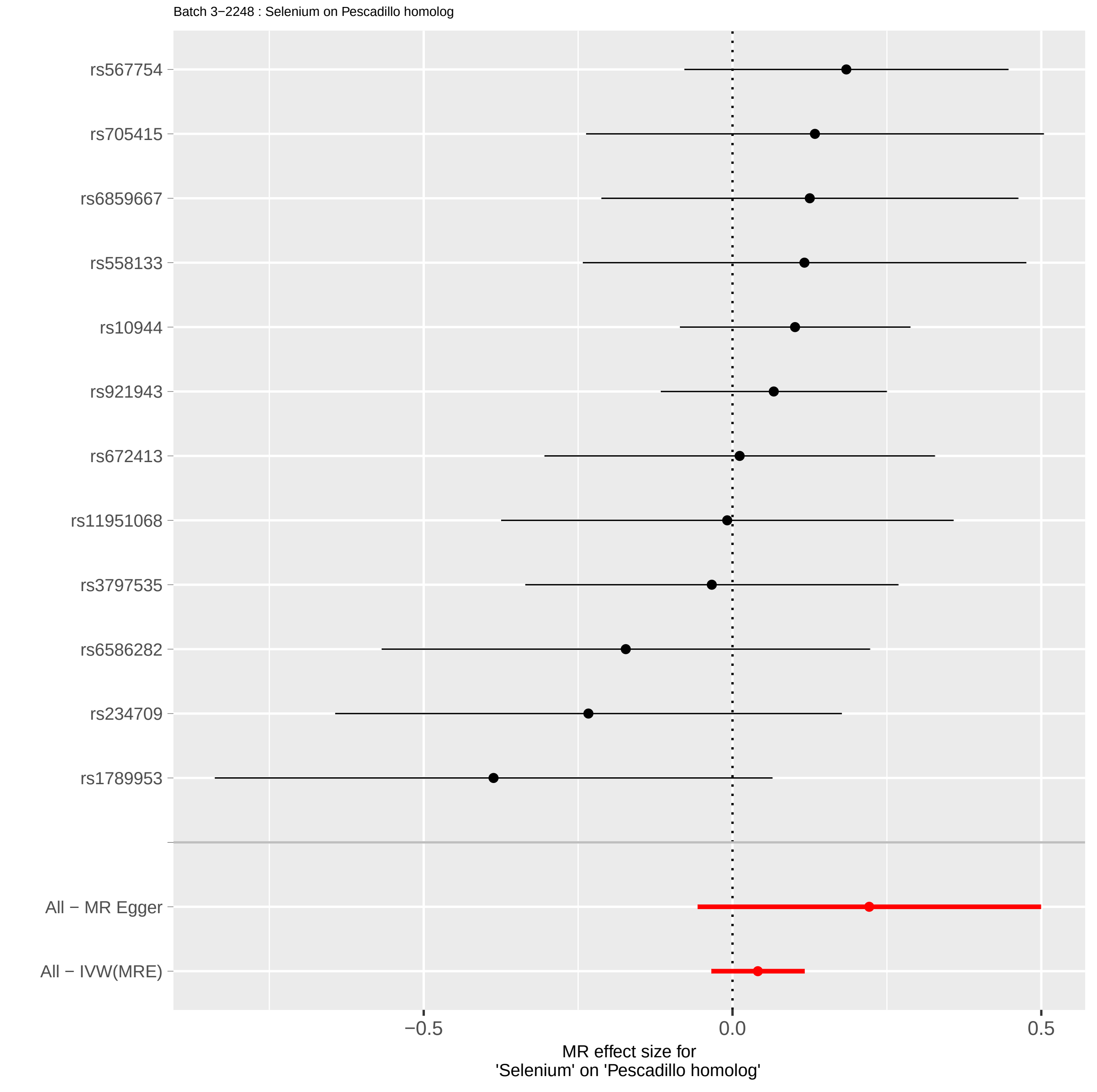

Batch 3−2248 : Selenium on Pescadillo homolog
rs567754
rs705415
rs6859667
rs558133
rs10944
rs921943
rs672413
rs11951068
rs3797535
rs6586282
rs234709
rs1789953
All − MR Egger
All − IVW(MRE)
−0.5
0.0
0.5
MR effect size for
'Selenium' on 'Pescadillo homolog'

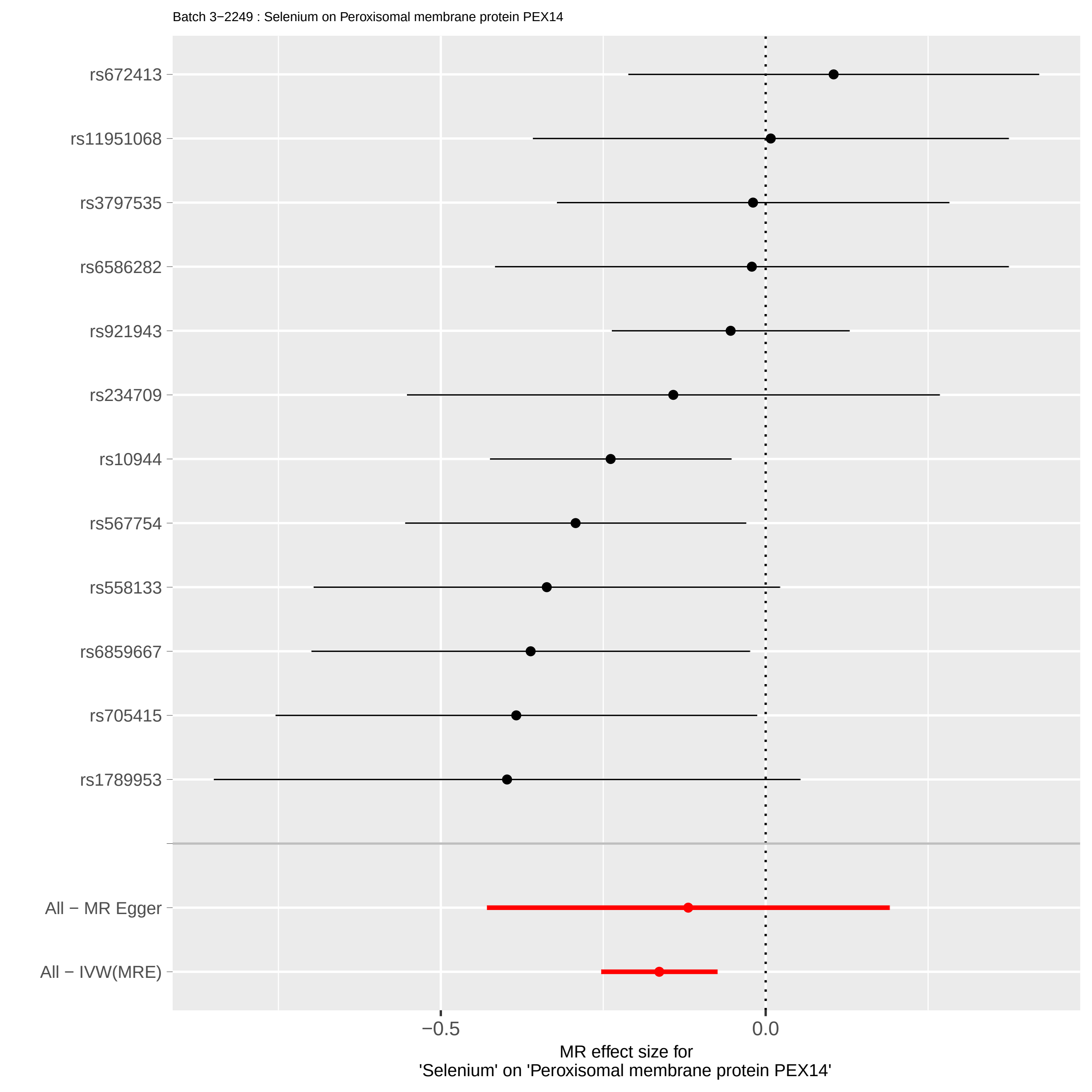

Batch 3−2249 : Selenium on Peroxisomal membrane protein PEX14
rs672413
rs11951068
rs3797535
rs6586282
rs921943
rs234709
rs10944
rs567754
rs558133
rs6859667
rs705415
rs1789953
All − MR Egger
All − IVW(MRE)
−0.5
0.0
MR effect size for
'Selenium' on 'Peroxisomal membrane protein PEX14'

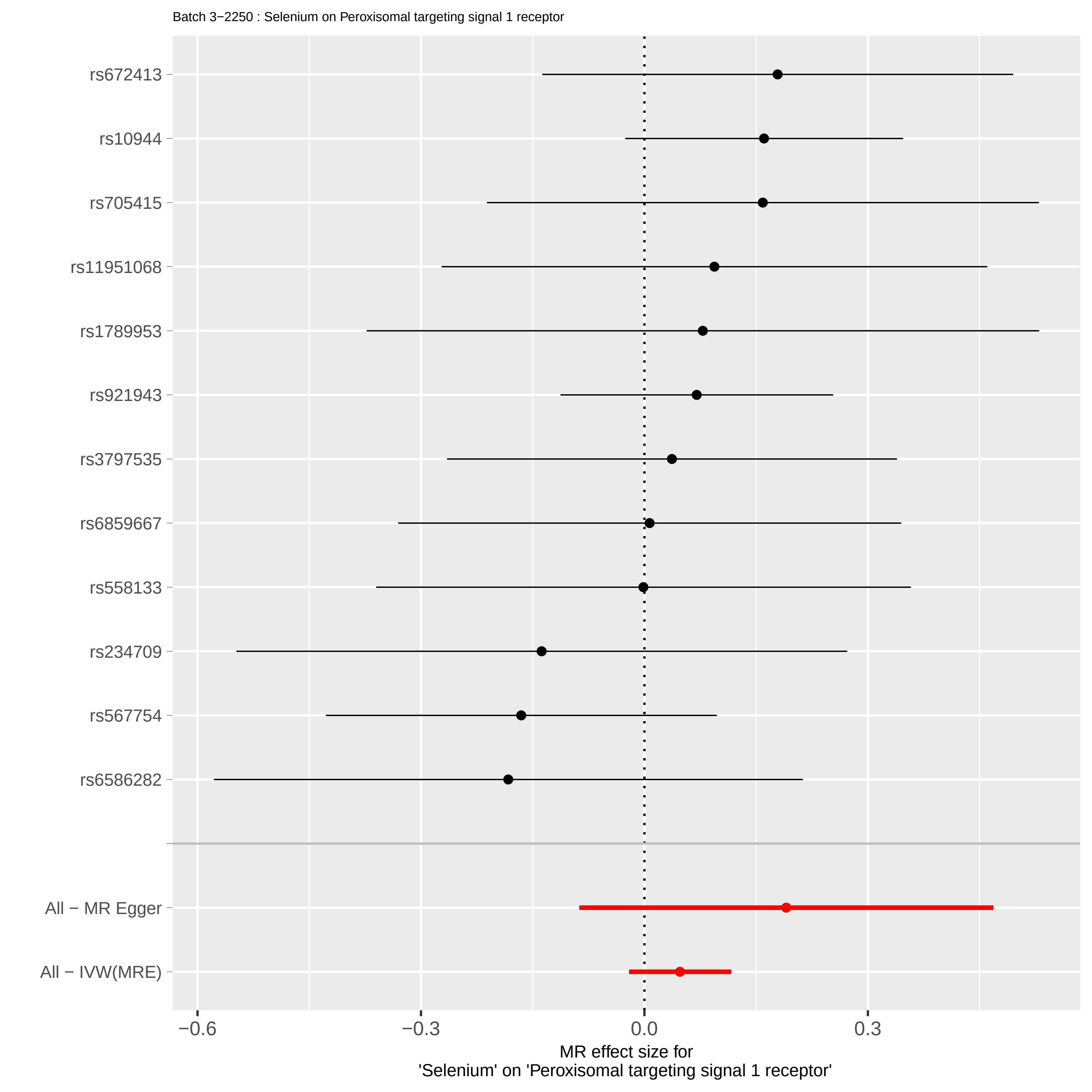

Batch 3−2250 : Selenium on Peroxisomal targeting signal 1 receptor
rs672413
rs10944
rs705415
rs11951068
rs1789953
rs921943
rs3797535
rs6859667
rs558133
rs234709
rs567754
rs6586282
All − MR Egger
All − IVW(MRE)
−0.6
−0.3
0.0
0.3
MR effect size for
'Selenium' on 'Peroxisomal targeting signal 1 receptor'

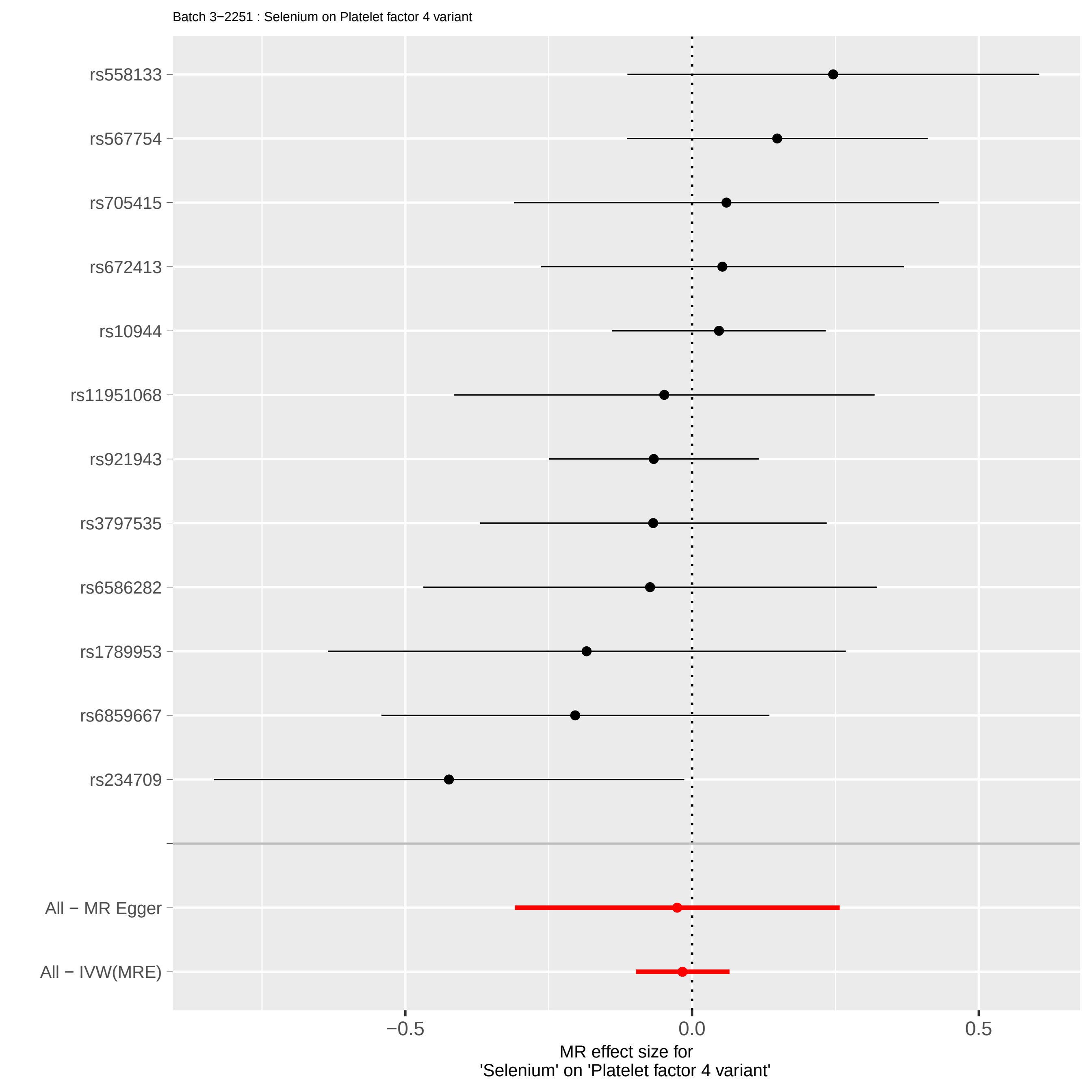

Batch 3−2251 : Selenium on Platelet factor 4 variant
rs558133
rs567754
rs705415
rs672413
rs10944
rs11951068
rs921943
rs3797535
rs6586282
rs1789953
rs6859667
rs234709
All − MR Egger
All − IVW(MRE)
−0.5
0.0
0.5
MR effect size for
'Selenium' on 'Platelet factor 4 variant'

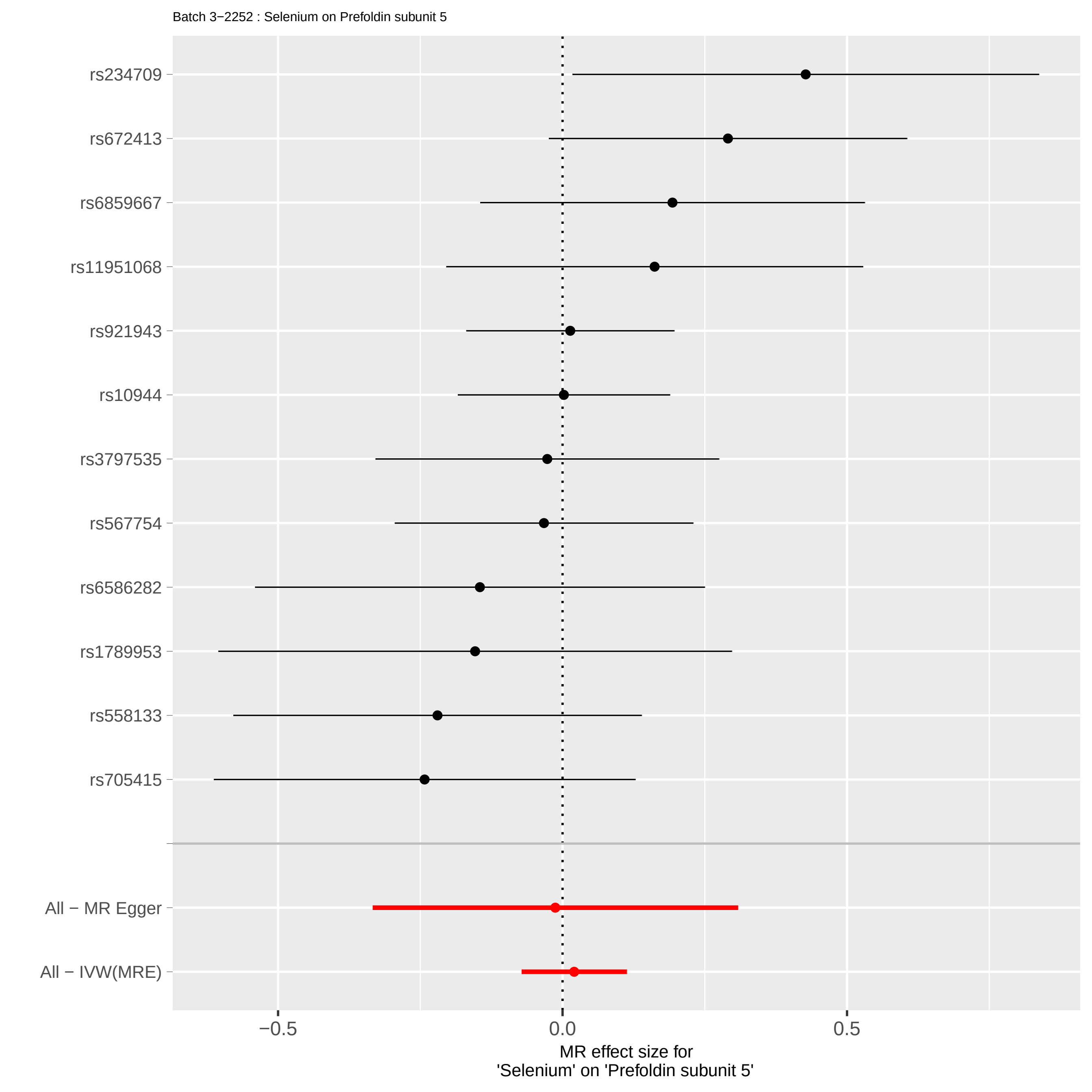

Batch 3−2252 : Selenium on Prefoldin subunit 5
rs234709
rs672413
rs6859667
rs11951068
rs921943
rs10944
rs3797535
rs567754
rs6586282
rs1789953
rs558133
rs705415
All − MR Egger
All − IVW(MRE)
−0.5
0.0
0.5
MR effect size for
'Selenium' on 'Prefoldin subunit 5'

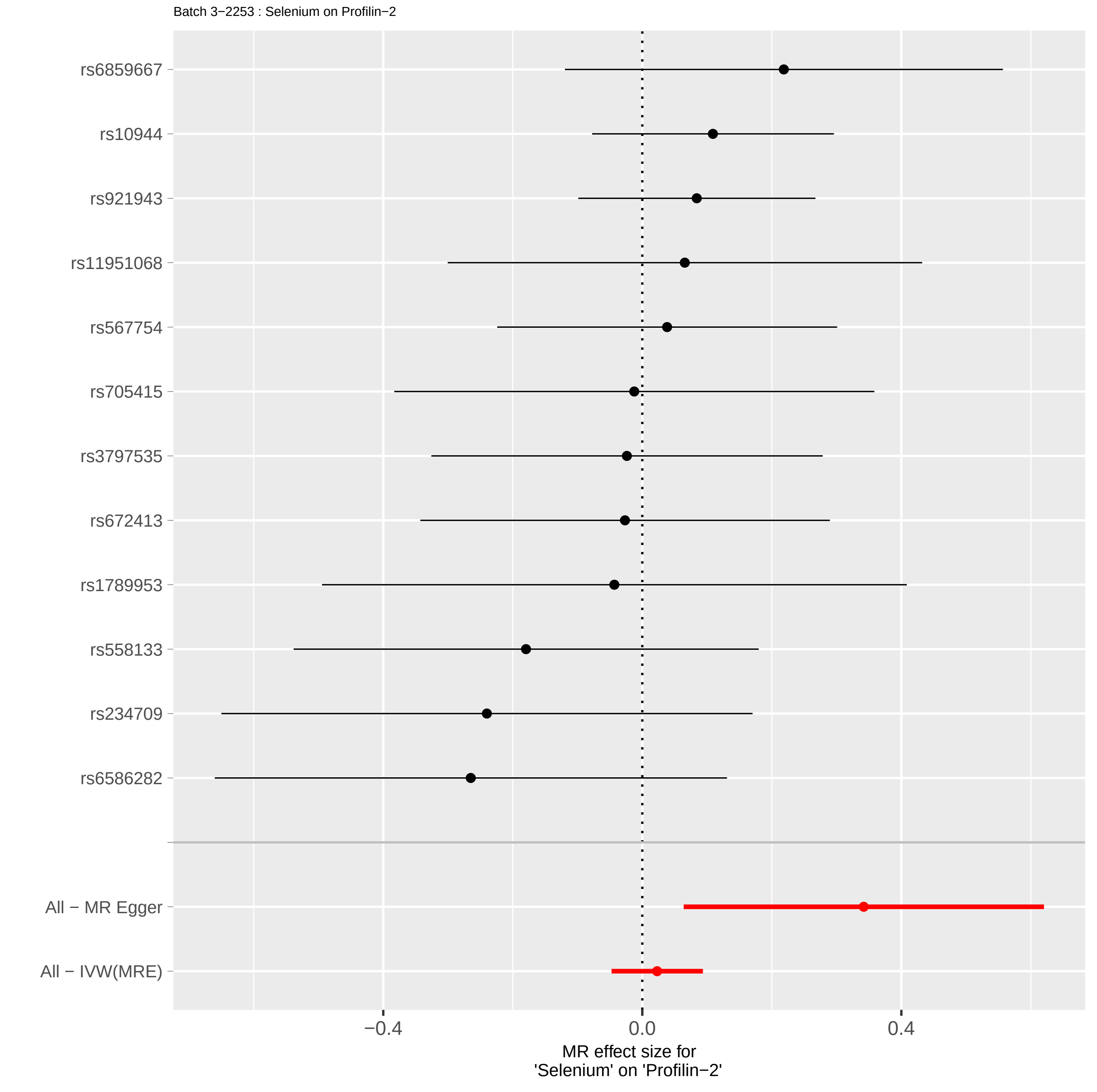
Batch 3−2253 : Selenium on Profilin−2
rs6859667
rs10944
rs921943
rs11951068
rs567754
rs705415
rs3797535
rs672413
rs1789953
rs558133
rs234709
rs6586282
All − MR Egger
All − IVW(MRE)
−0.4
0.0
0.4
MR effect size for
'Selenium' on 'Profilin−2'

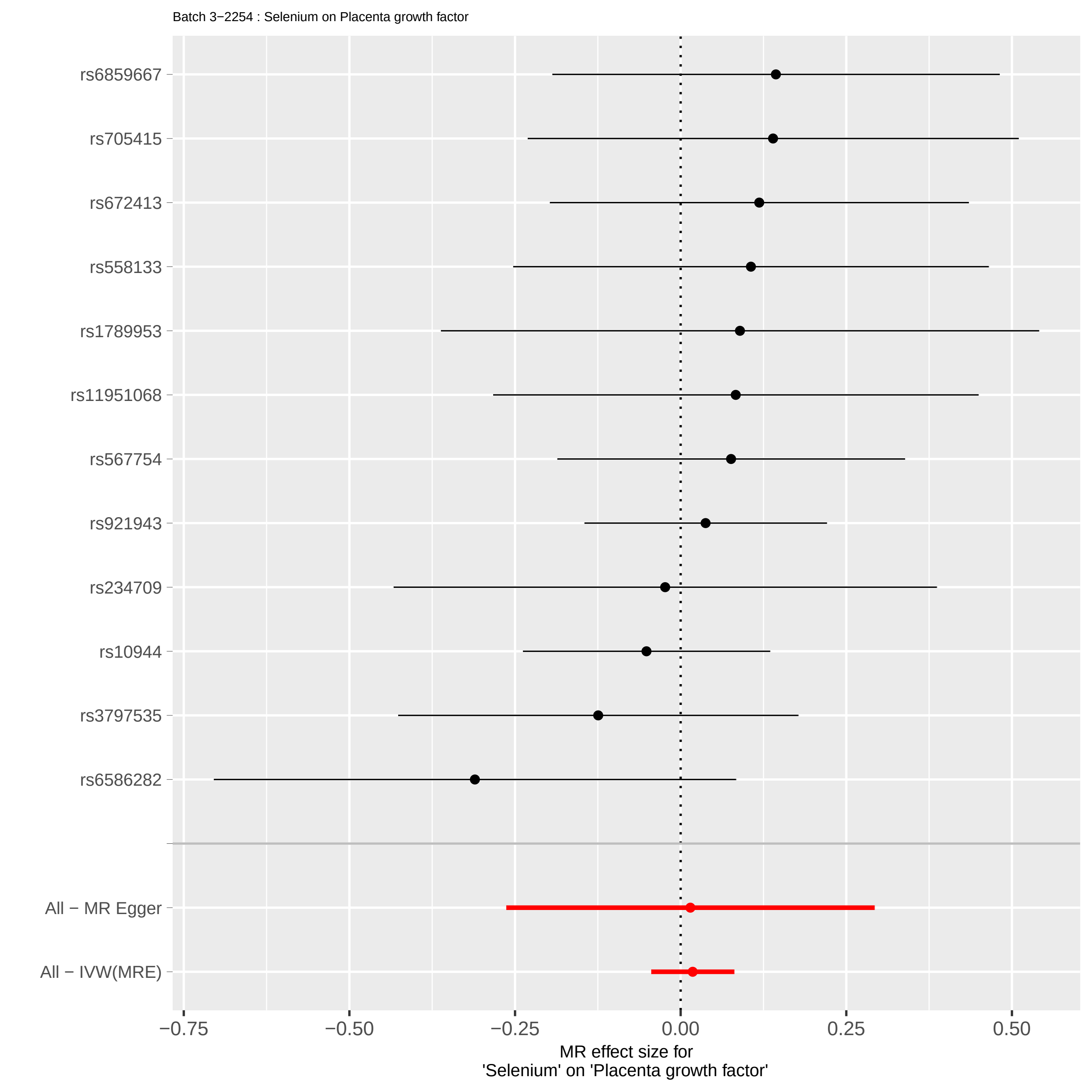

Batch 3−2254 : Selenium on Placenta growth factor
rs6859667
rs705415
rs672413
rs558133
rs1789953
rs11951068
rs567754
rs921943
rs234709
rs10944
rs3797535
rs6586282
All − MR Egger
All − IVW(MRE)
−0.75
−0.50
−0.25
0.00
0.25
0.50
MR effect size for
'Selenium' on 'Placenta growth factor'

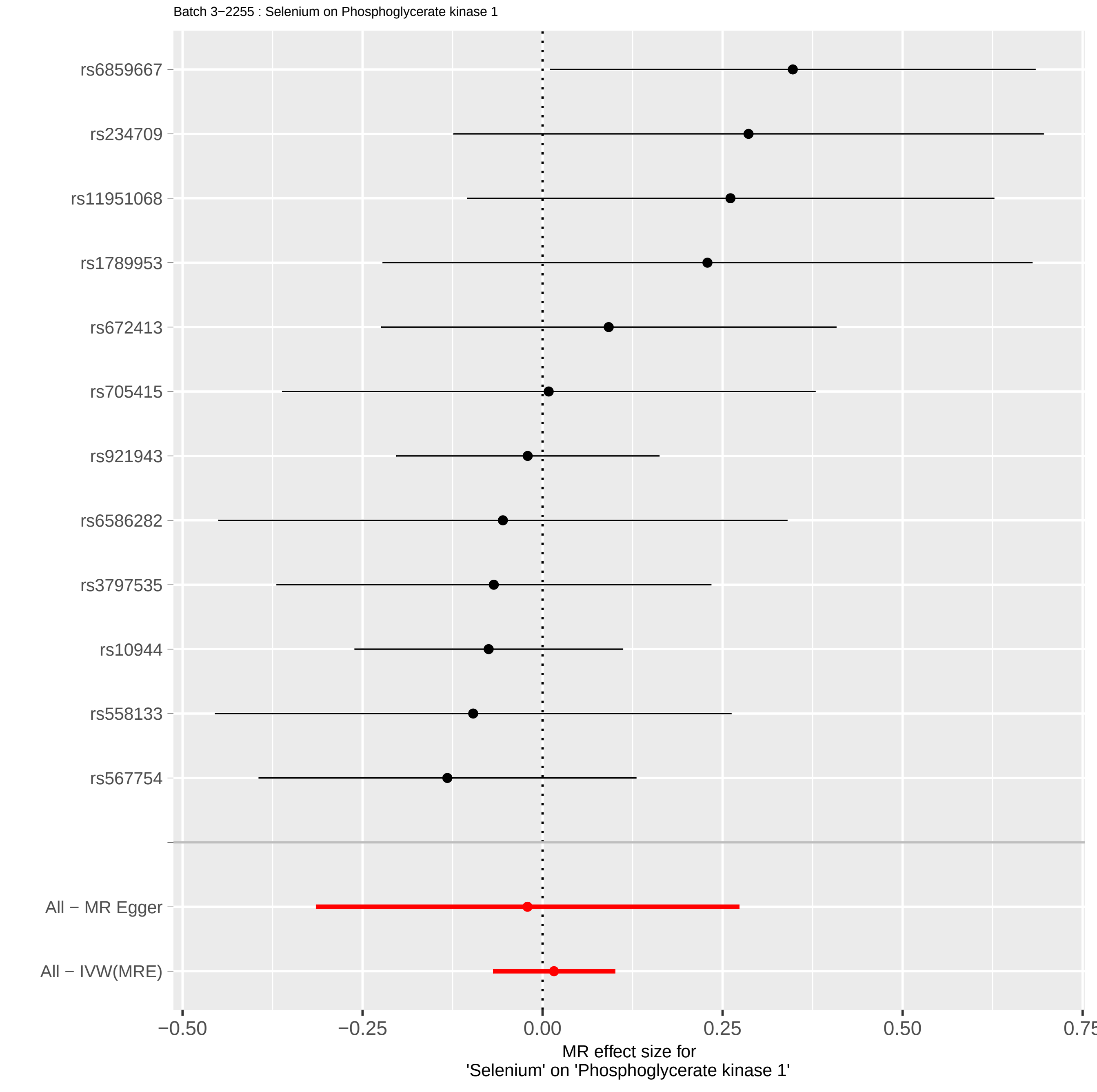

Batch 3−2255 : Selenium on Phosphoglycerate kinase 1
rs6859667
rs234709
rs11951068
rs1789953
rs672413
rs705415
rs921943
rs6586282
rs3797535
rs10944
rs558133
rs567754
All − MR Egger
All − IVW(MRE)
−0.50
−0.25
0.00
0.25
0.50
0.75
MR effect size for
'Selenium' on 'Phosphoglycerate kinase 1'

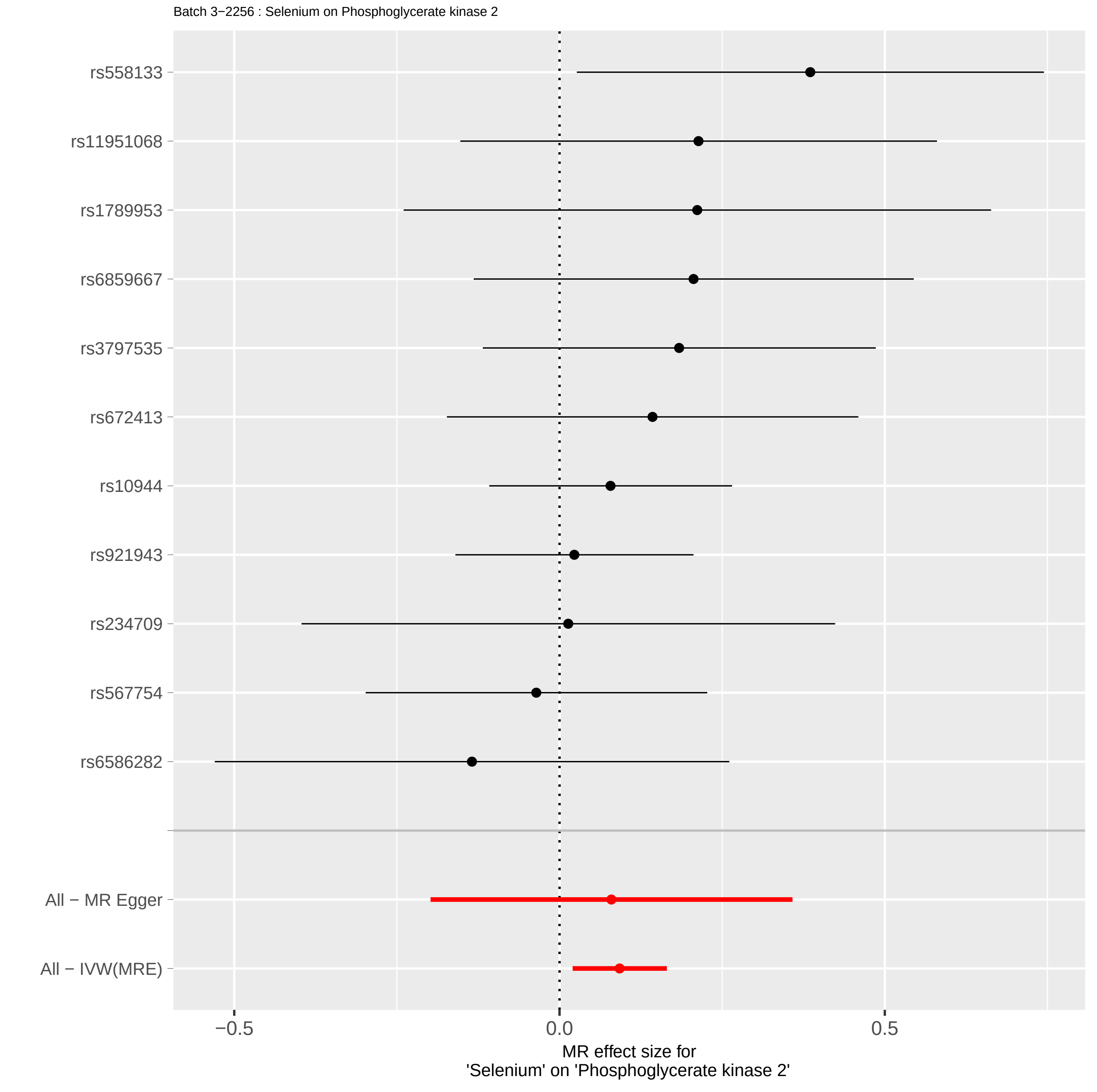
Batch 3−2256 : Selenium on Phosphoglycerate kinase 2
rs558133
rs11951068
rs1789953
rs6859667
rs3797535
rs672413
rs10944
rs921943
rs234709
rs567754
rs6586282
All − MR Egger
All − IVW(MRE)
−0.5
0.0
0.5
MR effect size for
'Selenium' on 'Phosphoglycerate kinase 2'

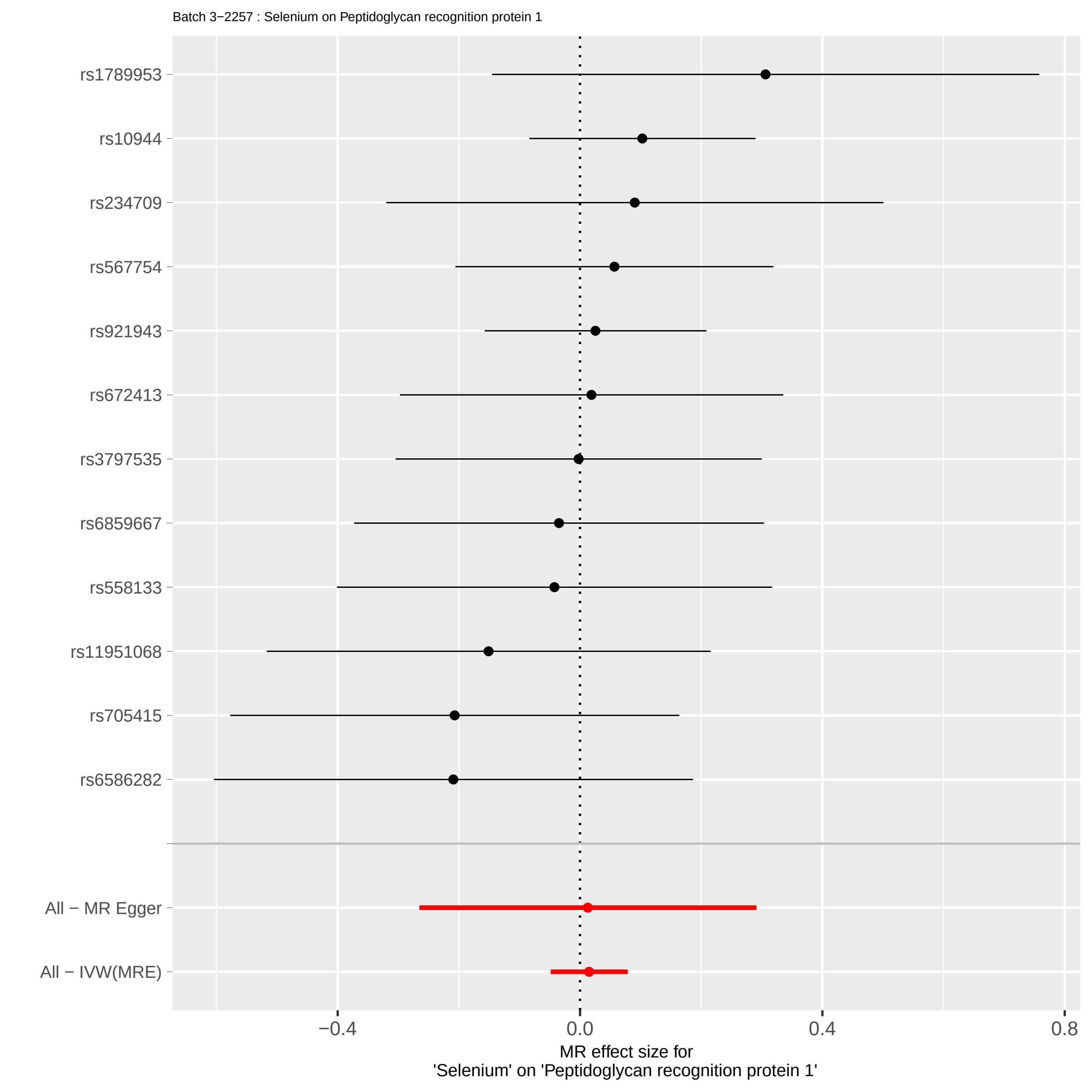

Batch 3−2257 : Selenium on Peptidoglycan recognition protein 1
rs1789953
rs10944
rs234709
rs567754
rs921943
rs672413
rs3797535
rs6859667
rs558133
rs11951068
rs705415
rs6586282
All − MR Egger
All − IVW(MRE)
−0.4
0.0
0.4
0.8
MR effect size for
'Selenium' on 'Peptidoglycan recognition protein 1'

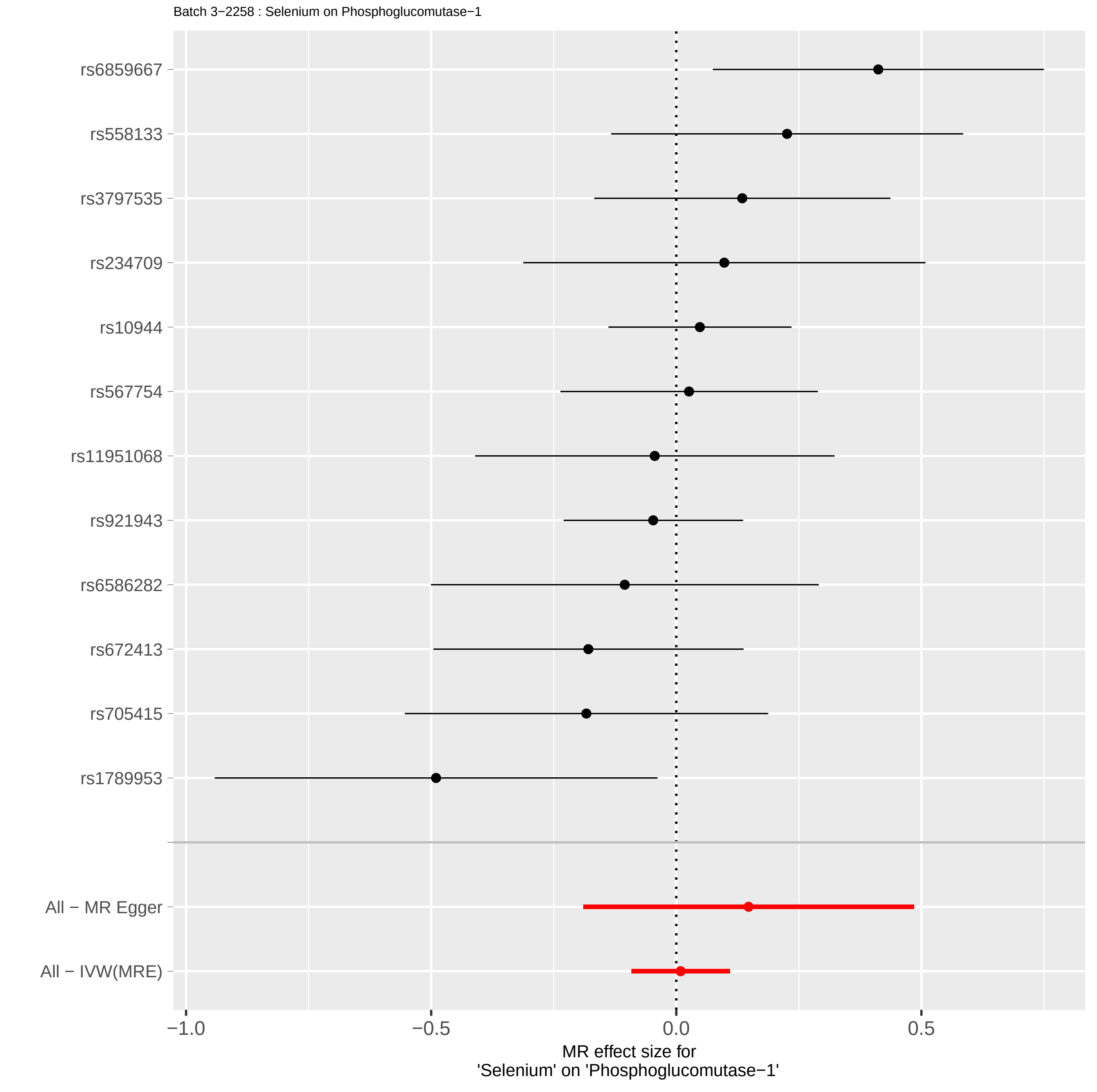
Batch 3−2258 : Selenium on Phosphoglucomutase−1
rs6859667
rs558133
rs3797535
rs234709
rs10944
rs567754
rs11951068
rs921943
rs6586282
rs672413
rs705415
rs1789953
All − MR Egger
All − IVW(MRE)
−1.0
−0.5
0.0
0.5
MR effect size for
'Selenium' on 'Phosphoglucomutase−1'

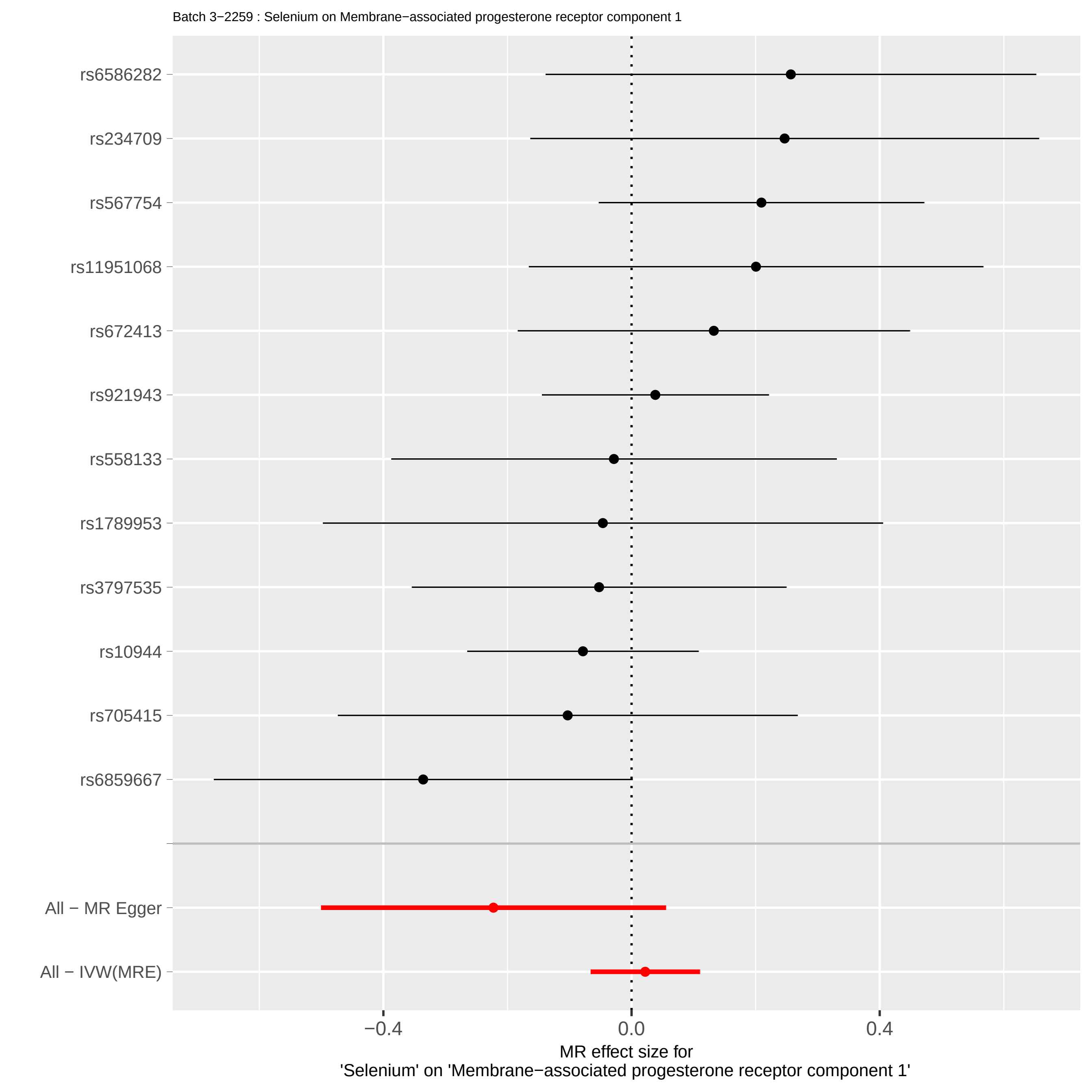

Batch 3−2259 : Selenium on Membrane−associated progesterone receptor component 1
rs6586282
rs234709
rs567754
rs11951068
rs672413
rs921943
rs558133
rs1789953
rs3797535
rs10944
rs705415
rs6859667
All − MR Egger
All − IVW(MRE)
−0.4
0.0
0.4
MR effect size for
'Selenium' on 'Membrane−associated progesterone receptor component 1'

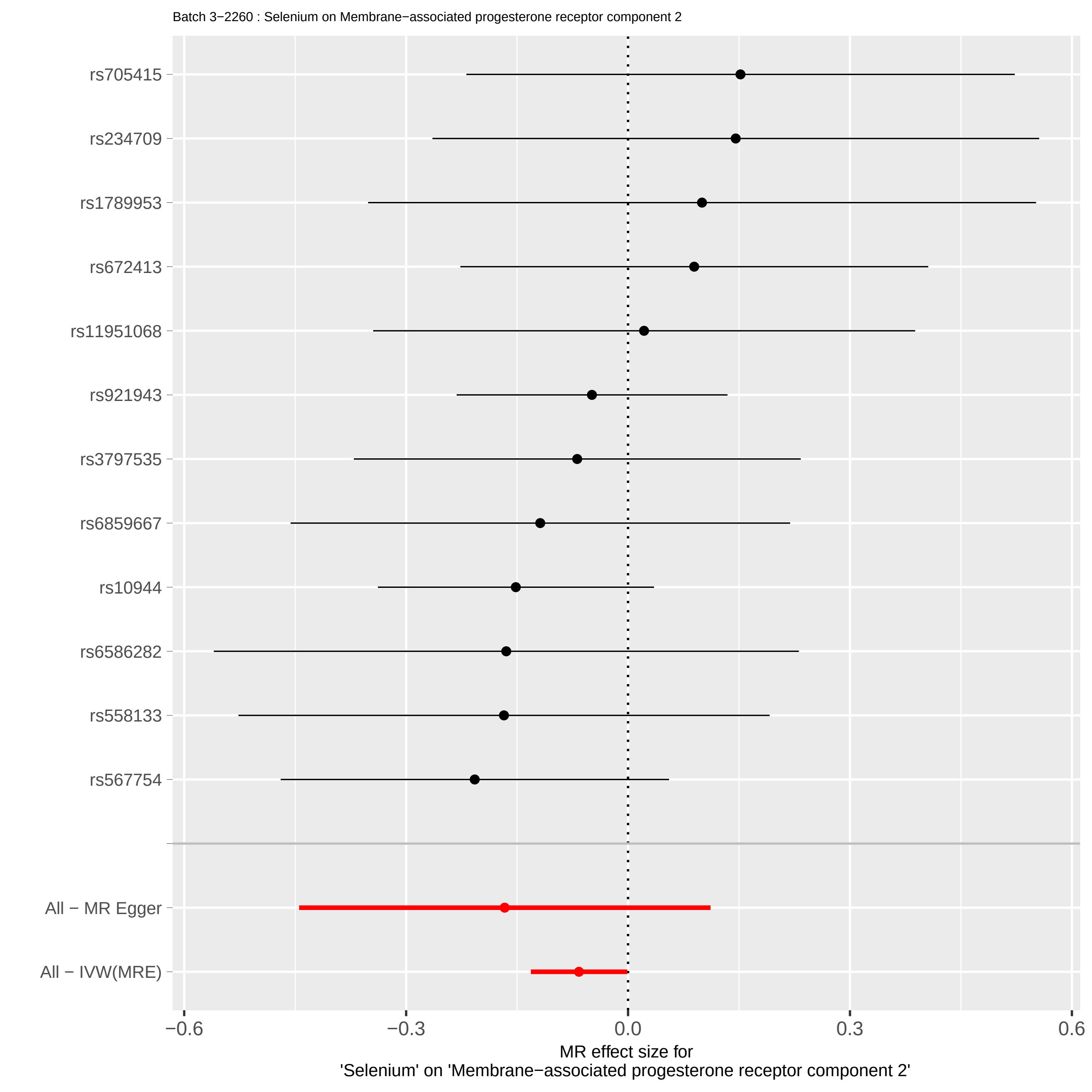

Batch 3−2260 : Selenium on Membrane−associated progesterone receptor component 2
rs705415
rs234709
rs1789953
rs672413
rs11951068
rs921943
rs3797535
rs6859667
rs10944
rs6586282
rs558133
rs567754
All − MR Egger
All − IVW(MRE)
−0.6
−0.3
0.0
0.3
0.6
MR effect size for
'Selenium' on 'Membrane−associated progesterone receptor component 2'

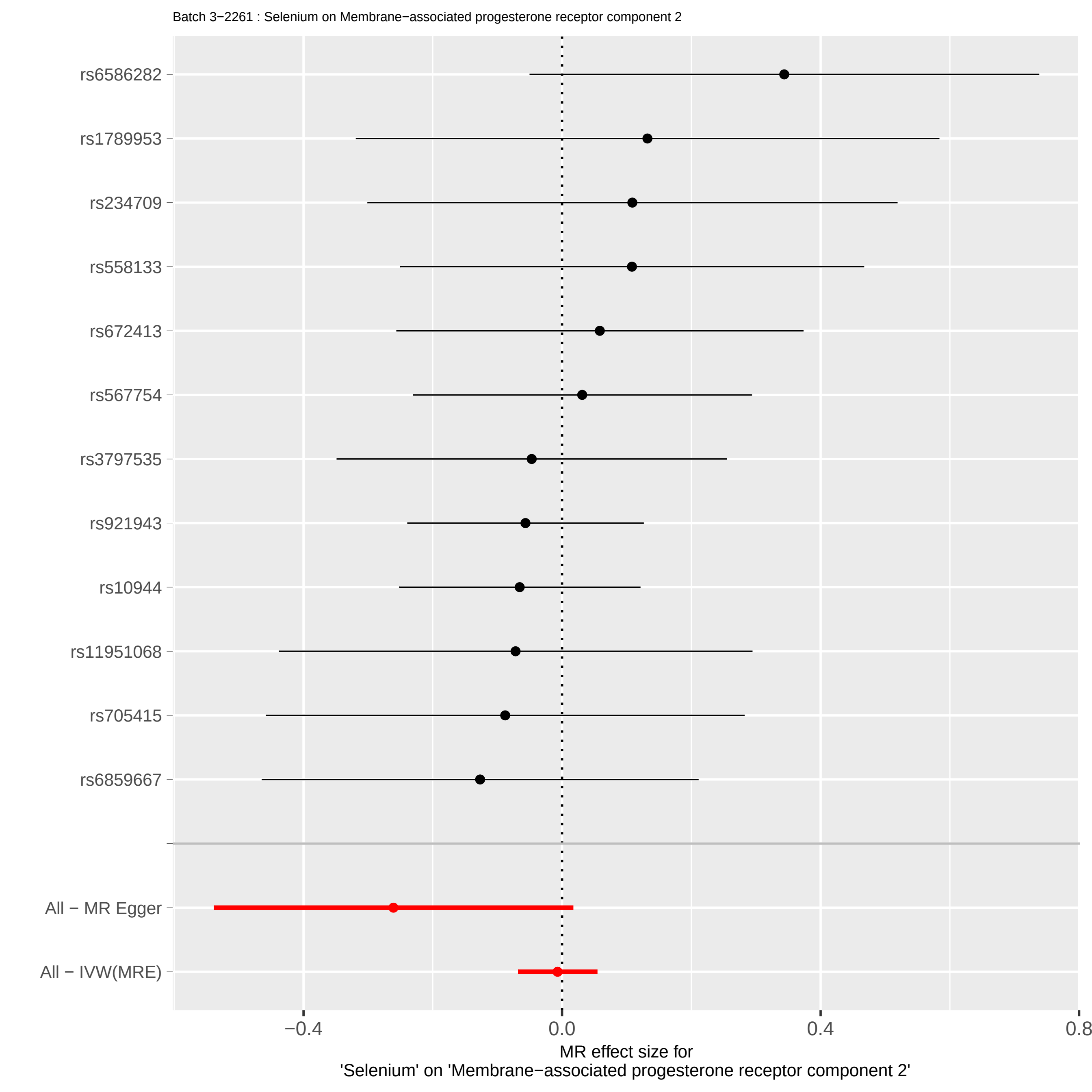

Batch 3−2261 : Selenium on Membrane−associated progesterone receptor component 2
rs6586282
rs1789953
rs234709
rs558133
rs672413
rs567754
rs3797535
rs921943
rs10944
rs11951068
rs705415
rs6859667
All − MR Egger
All − IVW(MRE)
−0.4
0.0
0.4
0.8
MR effect size for
'Selenium' on 'Membrane−associated progesterone receptor component 2'

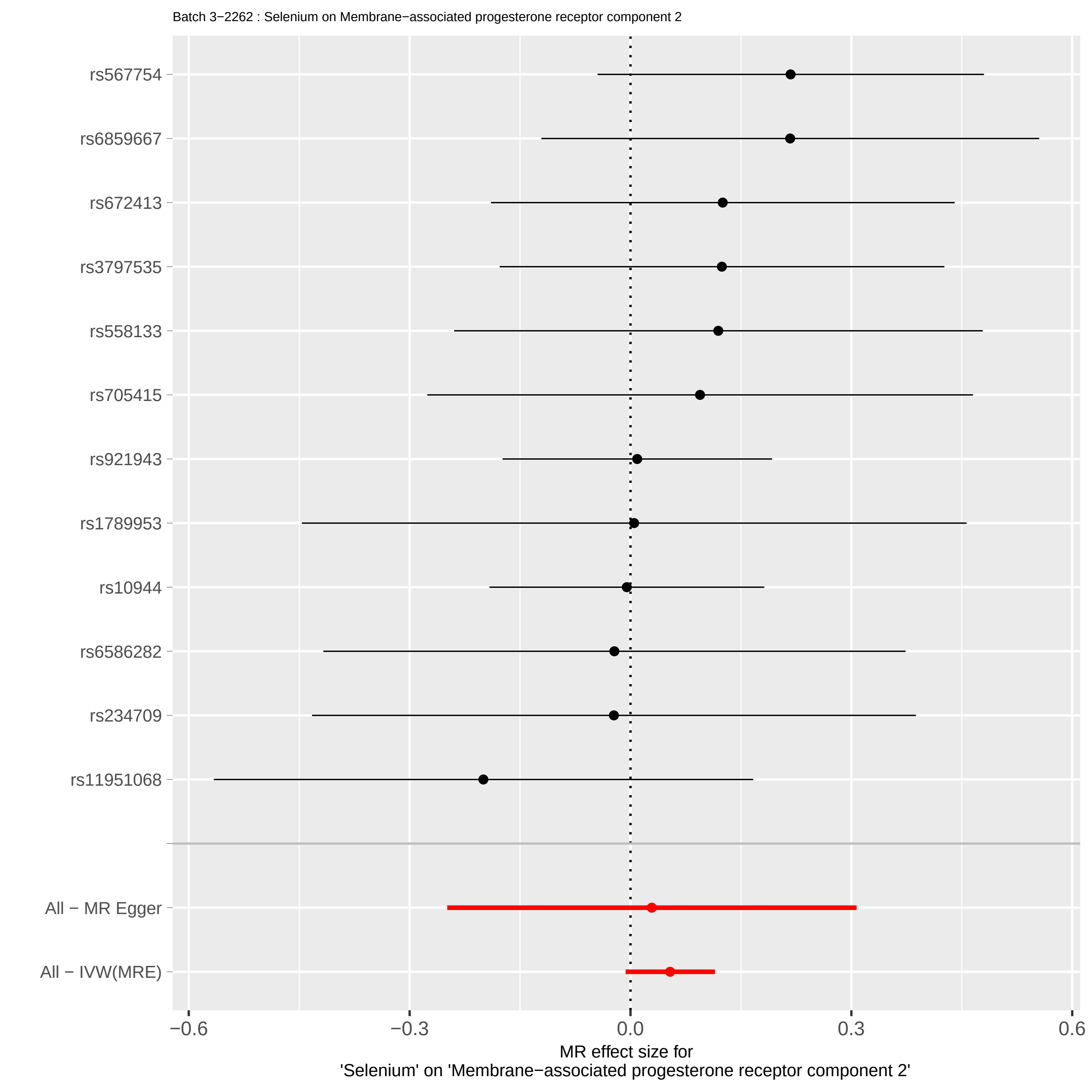

Batch 3−2262 : Selenium on Membrane−associated progesterone receptor component 2
rs567754
rs6859667
rs672413
rs3797535
rs558133
rs705415
rs921943
rs1789953
rs10944
rs6586282
rs234709
rs11951068
All − MR Egger
All − IVW(MRE)
−0.6
−0.3
0.0
0.3
0.6
MR effect size for
'Selenium' on 'Membrane−associated progesterone receptor component 2'

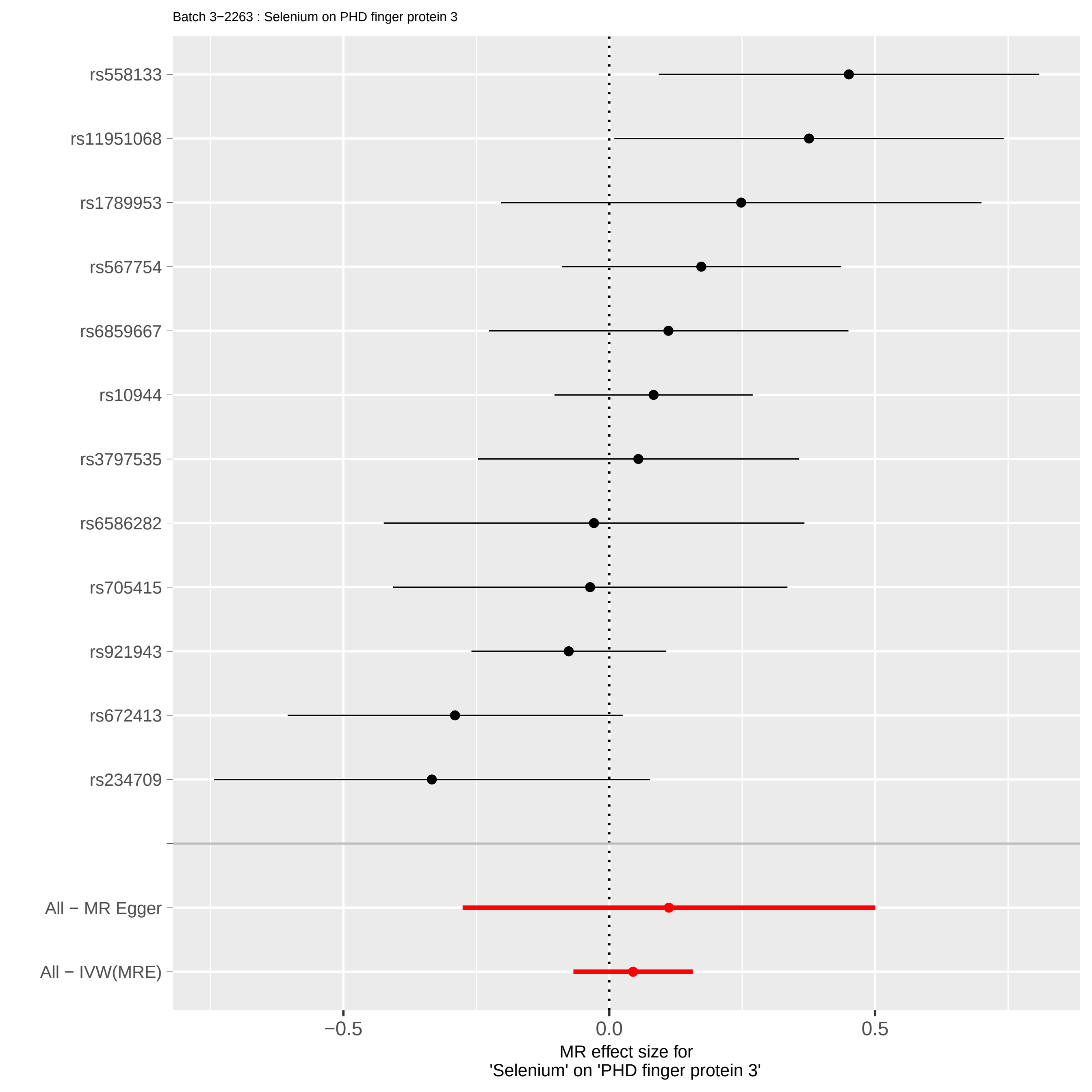

Batch 3−2263 : Selenium on PHD finger protein 3
rs558133
rs11951068
rs1789953
rs567754
rs6859667
rs10944
rs3797535
rs6586282
rs705415
rs921943
rs672413
rs234709
All − MR Egger
All − IVW(MRE)
−0.5
0.0
0.5
MR effect size for
'Selenium' on 'PHD finger protein 3'

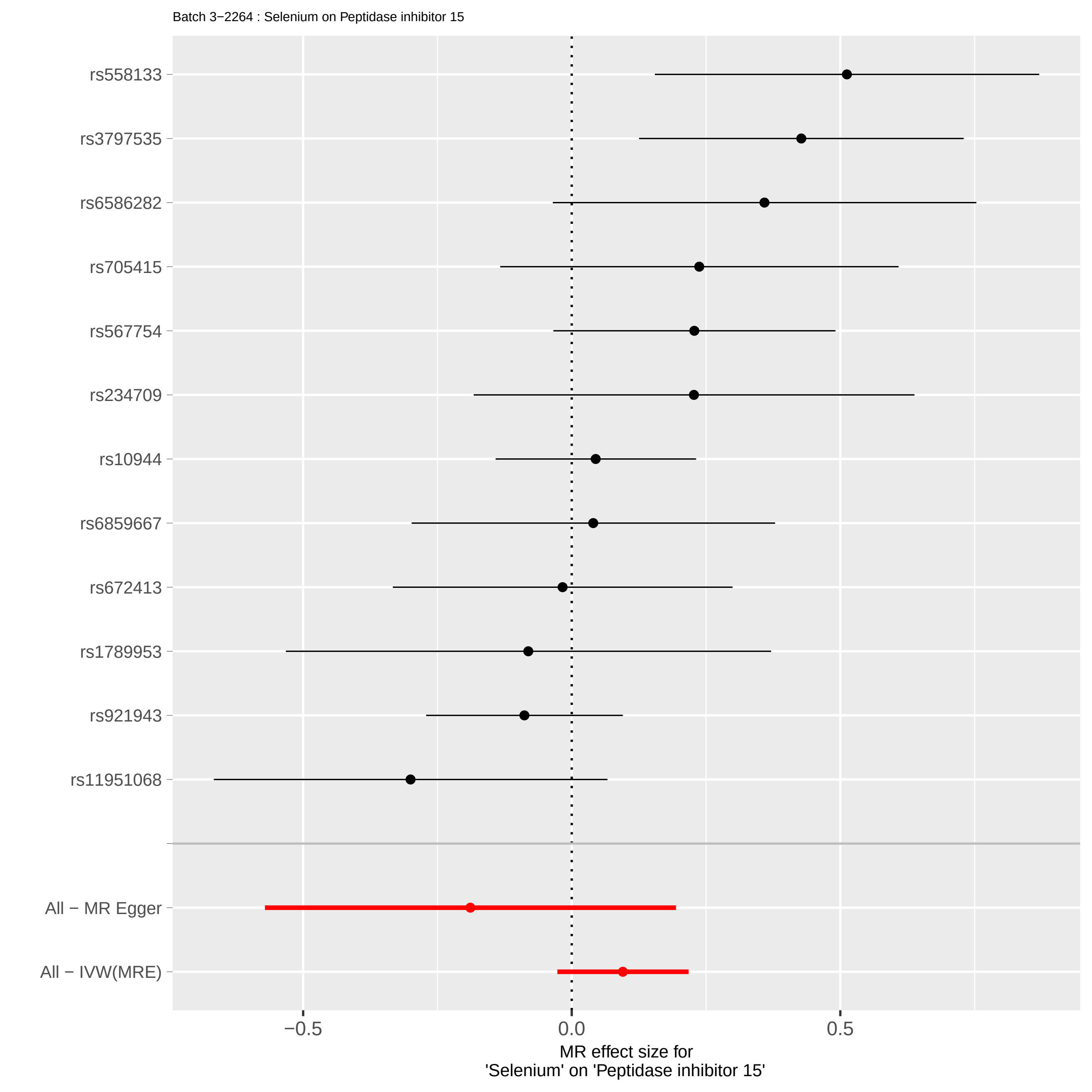

Batch 3−2264 : Selenium on Peptidase inhibitor 15
rs558133
rs3797535
rs6586282
rs705415
rs567754
rs234709
rs10944
rs6859667
rs672413
rs1789953
rs921943
rs11951068
All − MR Egger
All − IVW(MRE)
−0.5
0.0
0.5
MR effect size for
'Selenium' on 'Peptidase inhibitor 15'

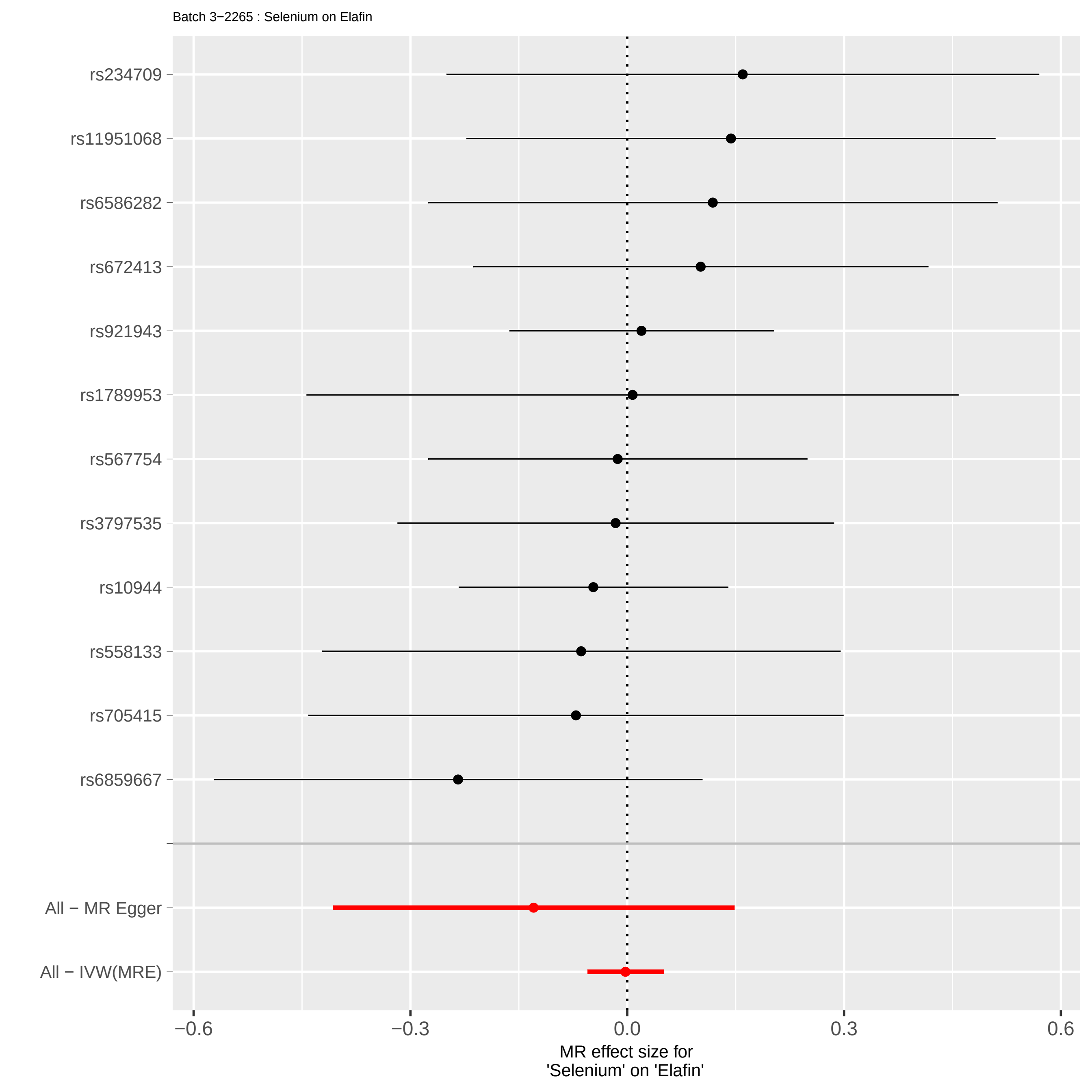
Batch 3−2265 : Selenium on Elafin
rs234709
rs11951068
rs6586282
rs672413
rs921943
rs1789953
rs567754
rs3797535
rs10944
rs558133
rs705415
rs6859667
All − MR Egger
All − IVW(MRE)
−0.6
−0.3
0.0
0.3
0.6
MR effect size for
'Selenium' on 'Elafin'

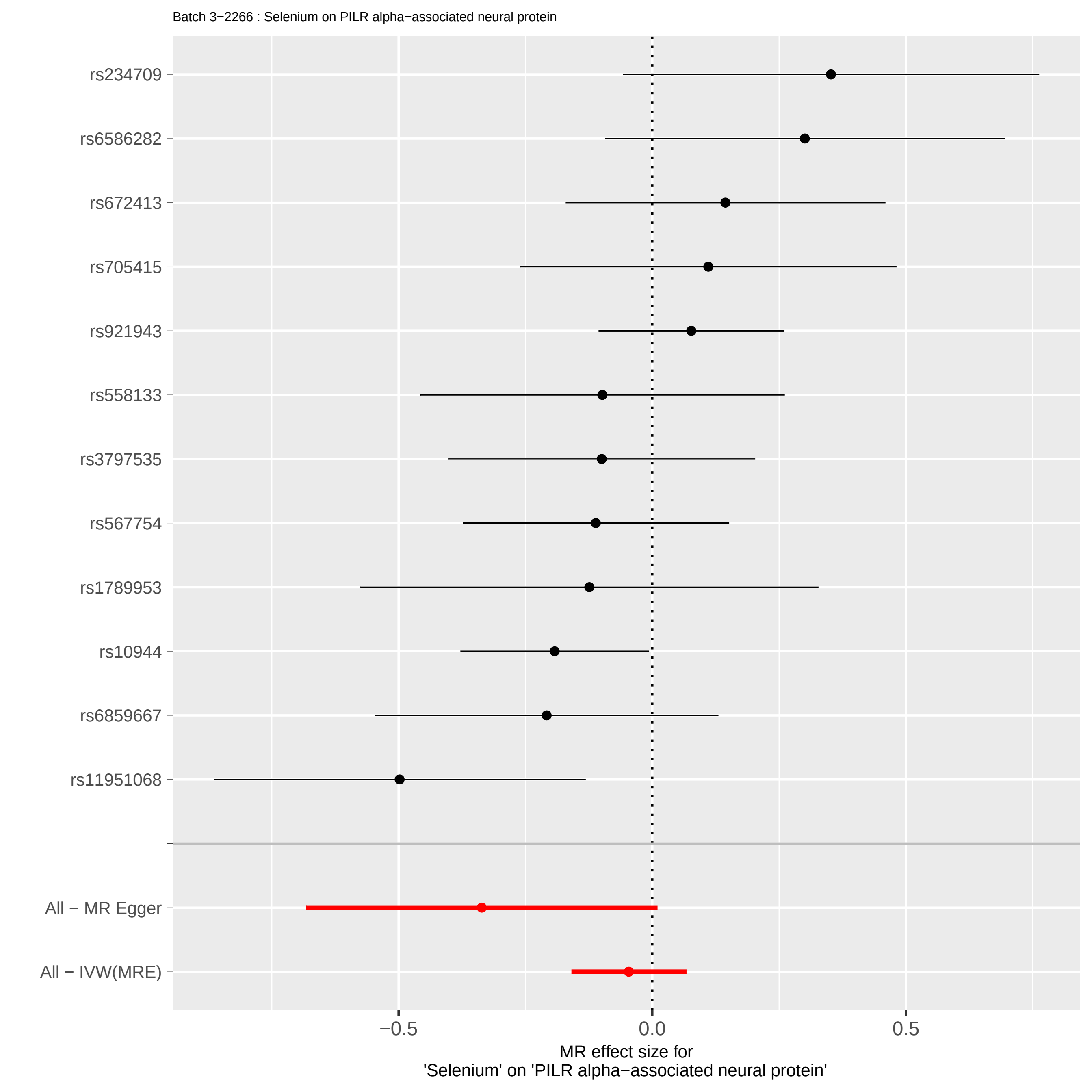

Batch 3−2266 : Selenium on PILR alpha−associated neural protein
rs234709
rs6586282
rs672413
rs705415
rs921943
rs558133
rs3797535
rs567754
rs1789953
rs10944
rs6859667
rs11951068
All − MR Egger
All − IVW(MRE)
−0.5
0.0
0.5
MR effect size for
'Selenium' on 'PILR alpha−associated neural protein'

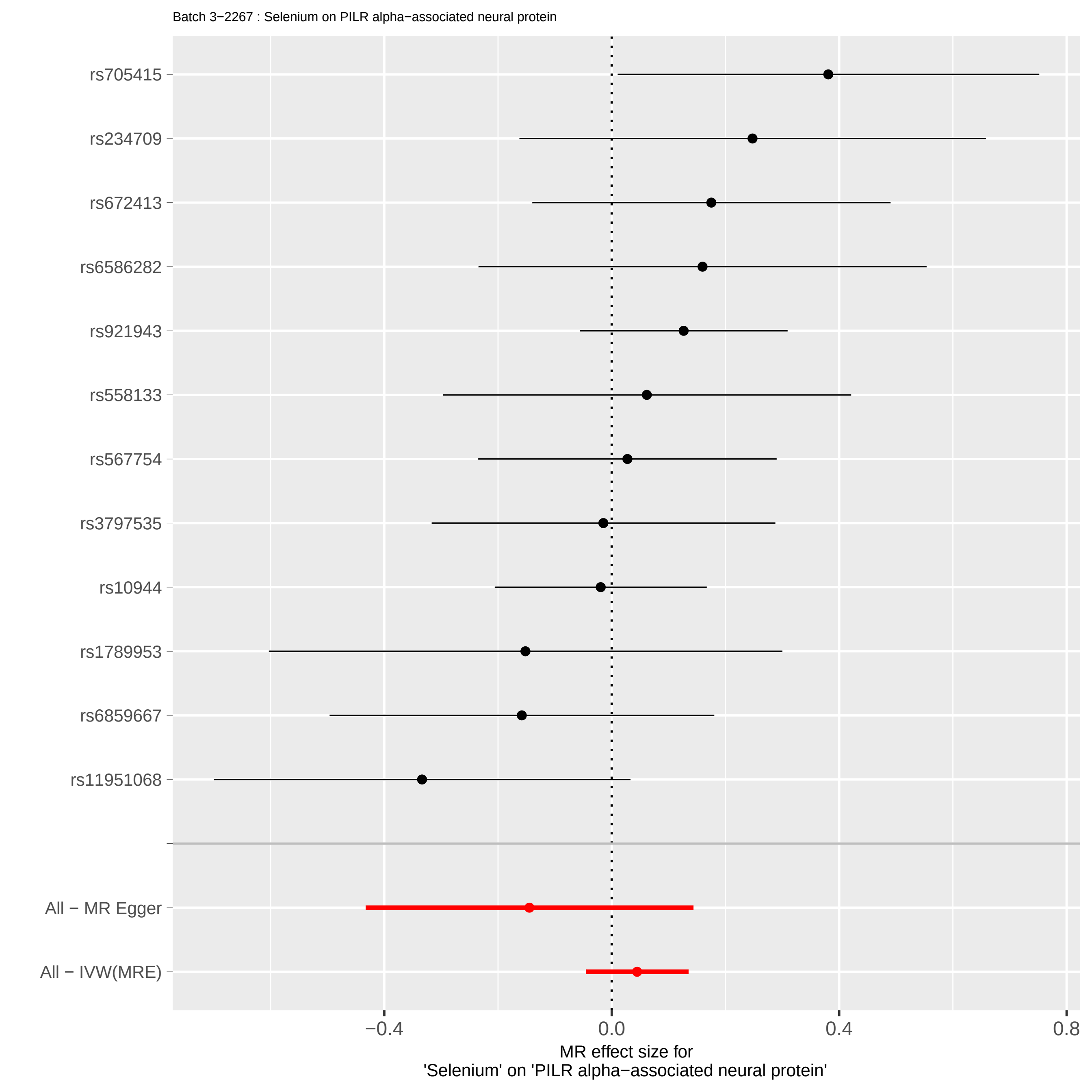

Batch 3−2267 : Selenium on PILR alpha−associated neural protein
rs705415
rs234709
rs672413
rs6586282
rs921943
rs558133
rs567754
rs3797535
rs10944
rs1789953
rs6859667
rs11951068
All − MR Egger
All − IVW(MRE)
−0.4
0.0
0.4
0.8
MR effect size for
'Selenium' on 'PILR alpha−associated neural protein'

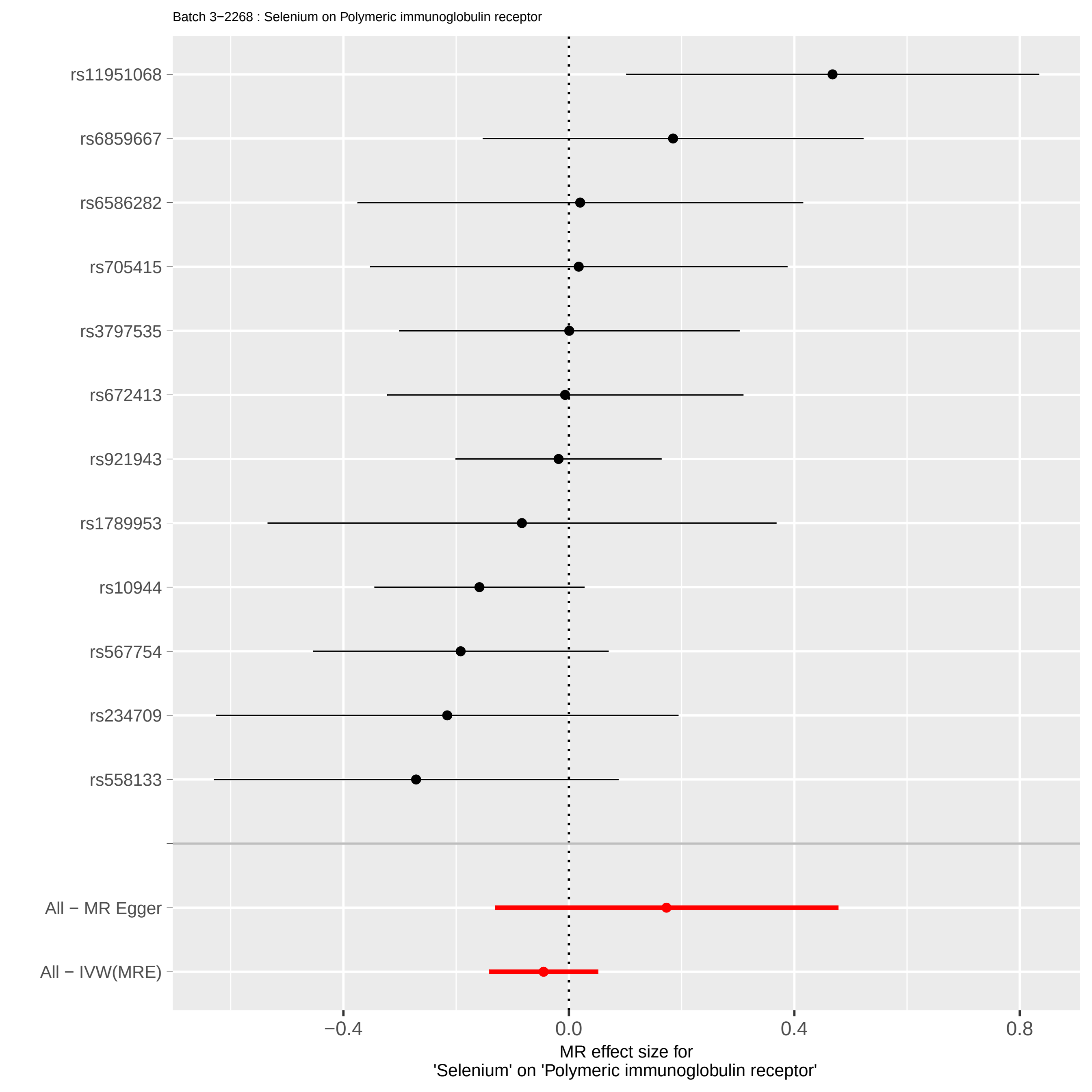

Batch 3−2268 : Selenium on Polymeric immunoglobulin receptor
rs11951068
rs6859667
rs6586282
rs705415
rs3797535
rs672413
rs921943
rs1789953
rs10944
rs567754
rs234709
rs558133
All − MR Egger
All − IVW(MRE)
−0.4
0.0
0.4
0.8
MR effect size for
'Selenium' on 'Polymeric immunoglobulin receptor'

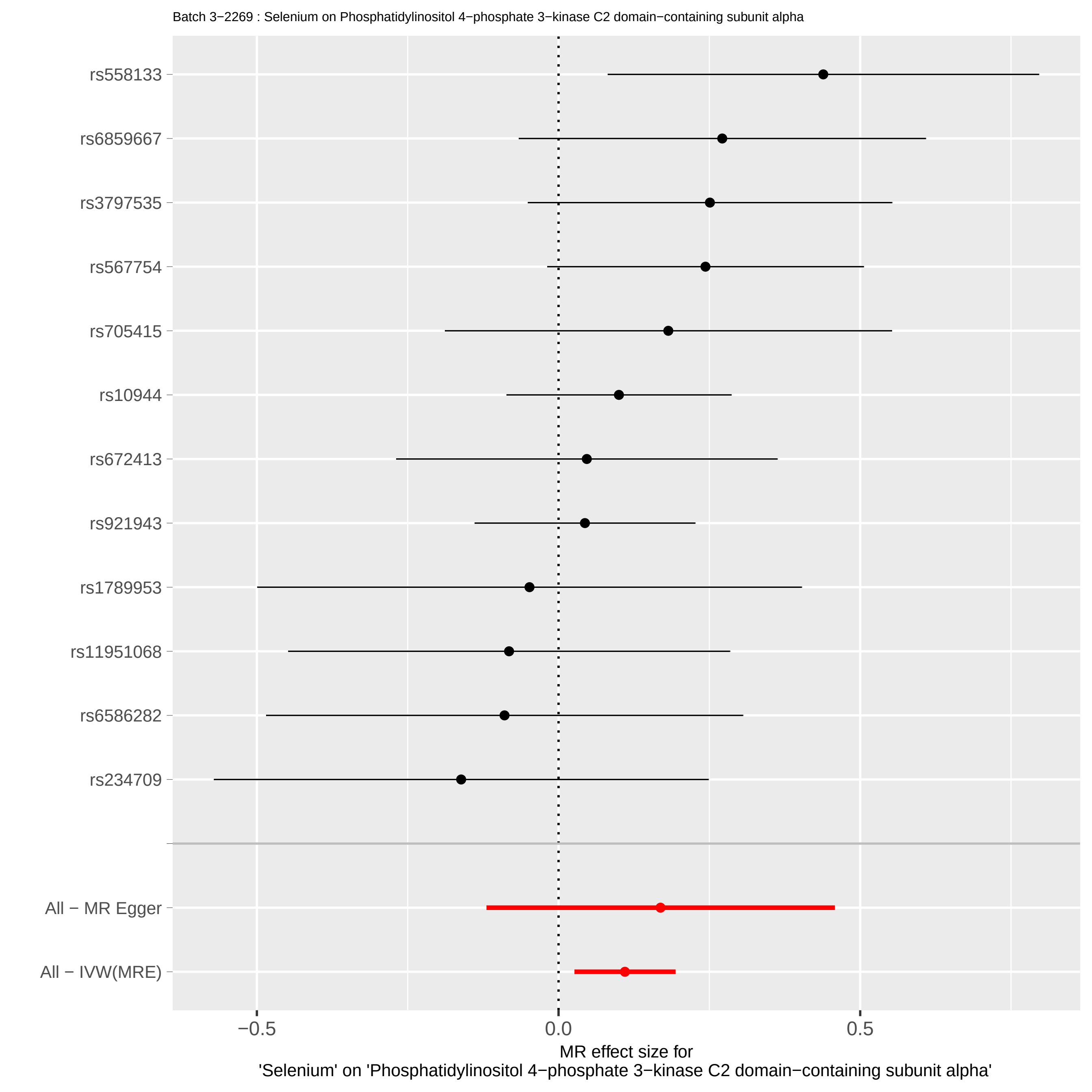
Batch 3−2269 : Selenium on Phosphatidylinositol 4−phosphate 3−kinase C2 domain−containing subunit alpha
rs558133
rs6859667
rs3797535
rs567754
rs705415
rs10944
rs672413
rs921943
rs1789953
rs11951068
rs6586282
rs234709
All − MR Egger
All − IVW(MRE)
−0.5
0.0
0.5
MR effect size for
'Selenium' on 'Phosphatidylinositol 4−phosphate 3−kinase C2 domain−containing subunit alpha'

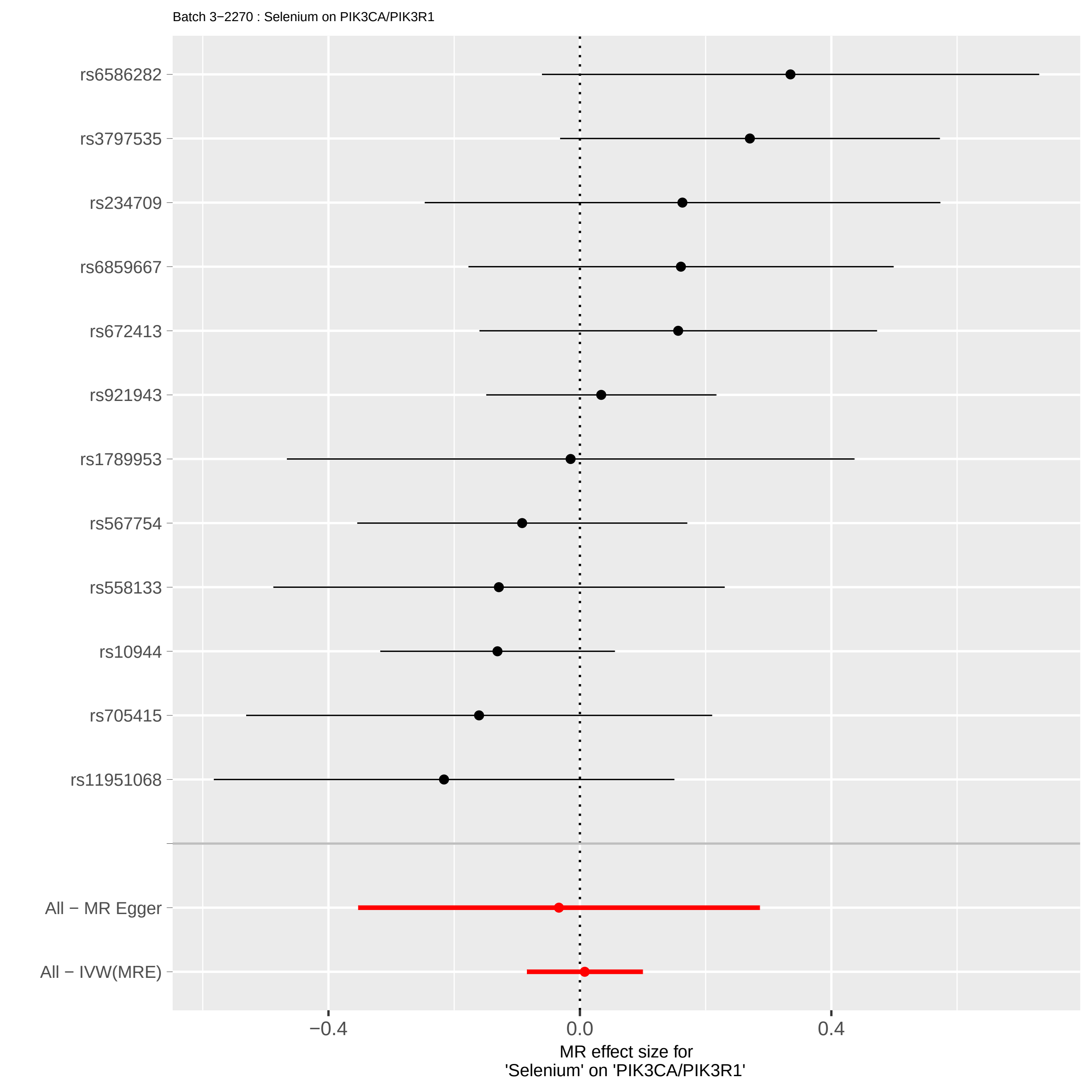

Batch 3−2270 : Selenium on PIK3CA/PIK3R1
rs6586282
rs3797535
rs234709
rs6859667
rs672413
rs921943
rs1789953
rs567754
rs558133
rs10944
rs705415
rs11951068
All − MR Egger
All − IVW(MRE)
−0.4
0.0
0.4
MR effect size for
'Selenium' on 'PIK3CA/PIK3R1'

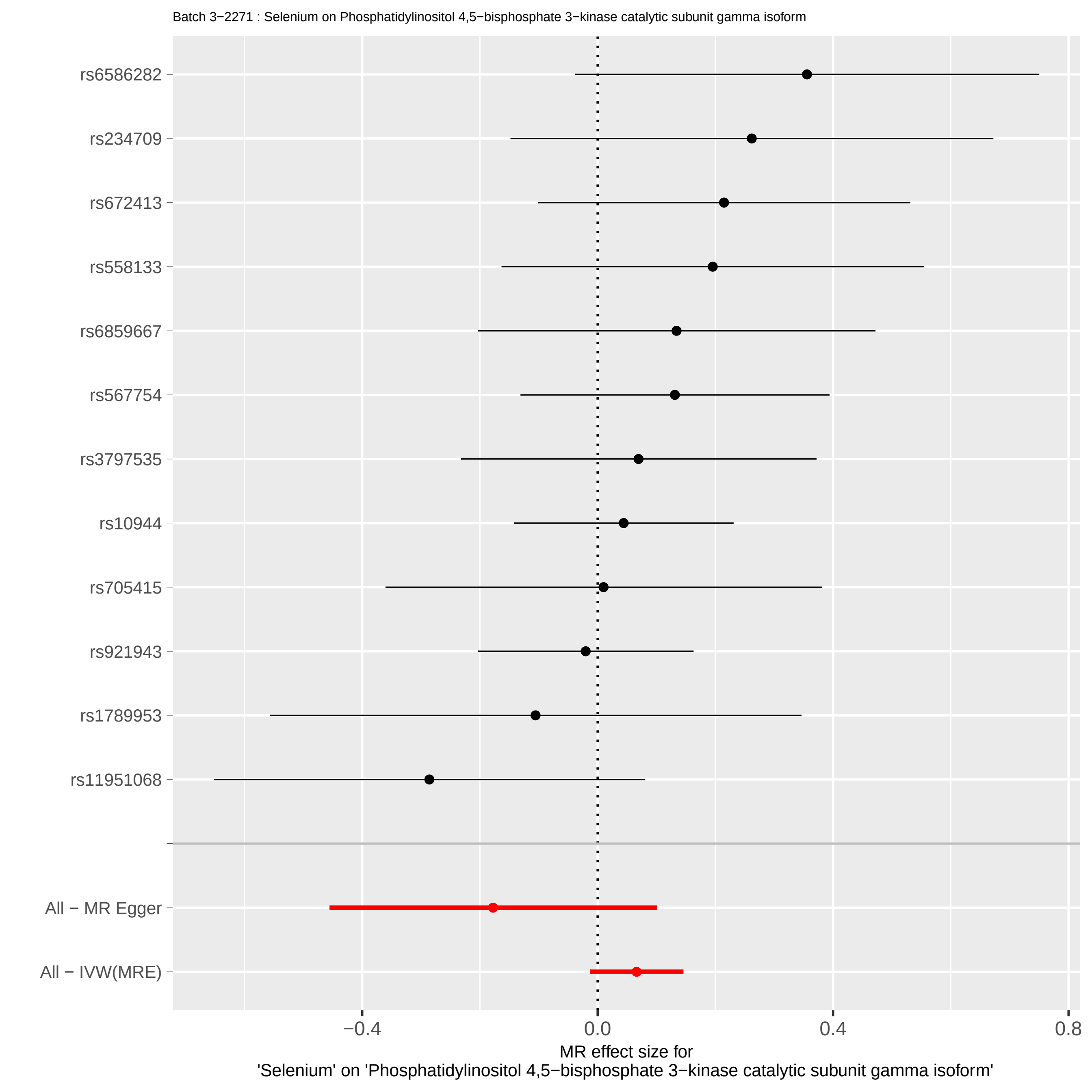

Batch 3−2271 : Selenium on Phosphatidylinositol 4,5−bisphosphate 3−kinase catalytic subunit gamma isoform
rs6586282
rs234709
rs672413
rs558133
rs6859667
rs567754
rs3797535
rs10944
rs705415
rs921943
rs1789953
rs11951068
All − MR Egger
All − IVW(MRE)
−0.4
0.0
0.4
0.8
MR effect size for
'Selenium' on 'Phosphatidylinositol 4,5−bisphosphate 3−kinase catalytic subunit gamma isoform'

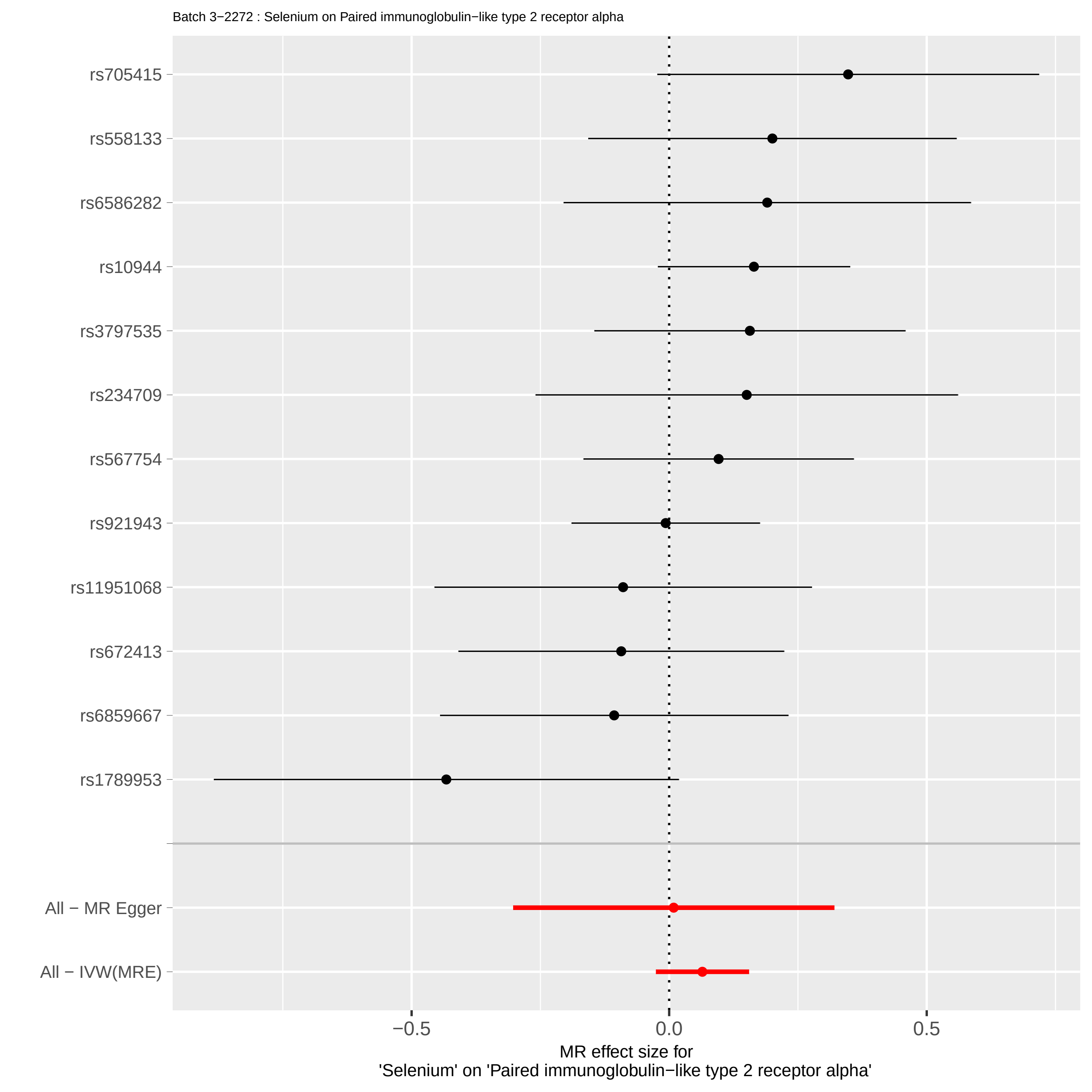

Batch 3−2272 : Selenium on Paired immunoglobulin−like type 2 receptor alpha
rs705415
rs558133
rs6586282
rs10944
rs3797535
rs234709
rs567754
rs921943
rs11951068
rs672413
rs6859667
rs1789953
All − MR Egger
All − IVW(MRE)
−0.5
0.0
0.5
MR effect size for
'Selenium' on 'Paired immunoglobulin−like type 2 receptor alpha'

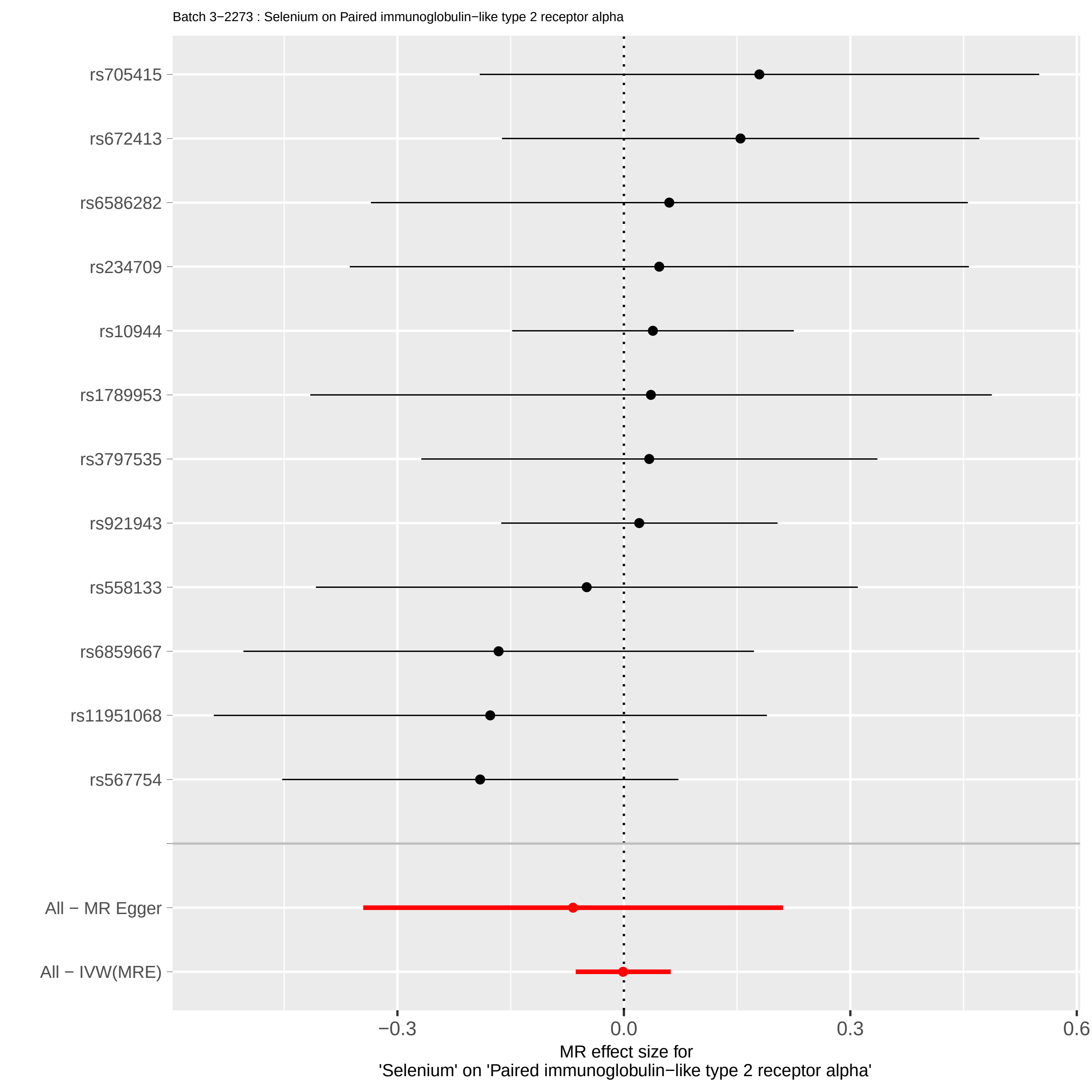

Batch 3−2273 : Selenium on Paired immunoglobulin−like type 2 receptor alpha
rs705415
rs672413
rs6586282
rs234709
rs10944
rs1789953
rs3797535
rs921943
rs558133
rs6859667
rs11951068
rs567754
All − MR Egger
All − IVW(MRE)
−0.3
0.0
0.3
0.6
MR effect size for
'Selenium' on 'Paired immunoglobulin−like type 2 receptor alpha'

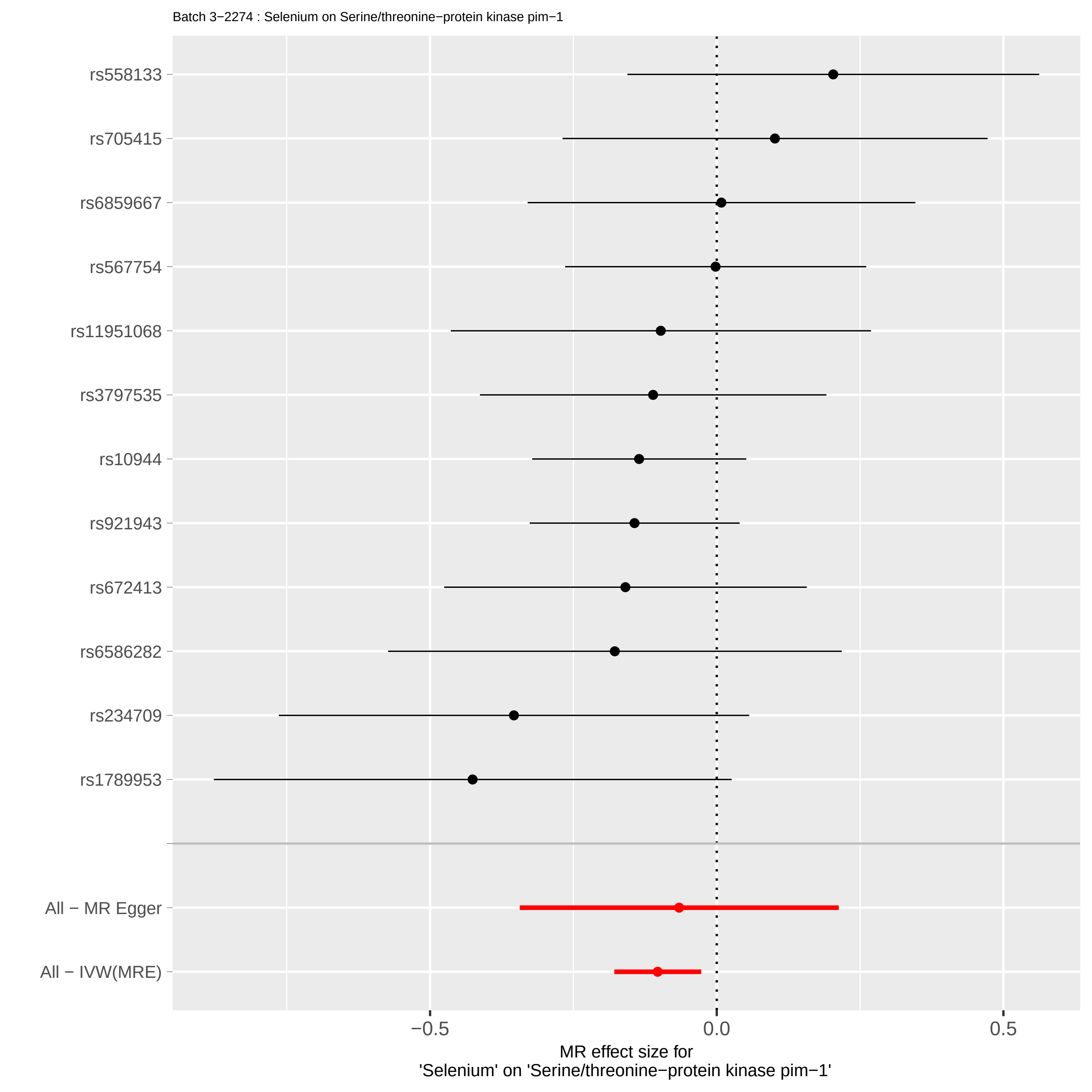

Batch 3−2274 : Selenium on Serine/threonine−protein kinase pim−1
rs558133
rs705415
rs6859667
rs567754
rs11951068
rs3797535
rs10944
rs921943
rs672413
rs6586282
rs234709
rs1789953
All − MR Egger
All − IVW(MRE)
−0.5
0.0
0.5
MR effect size for
'Selenium' on 'Serine/threonine−protein kinase pim−1'

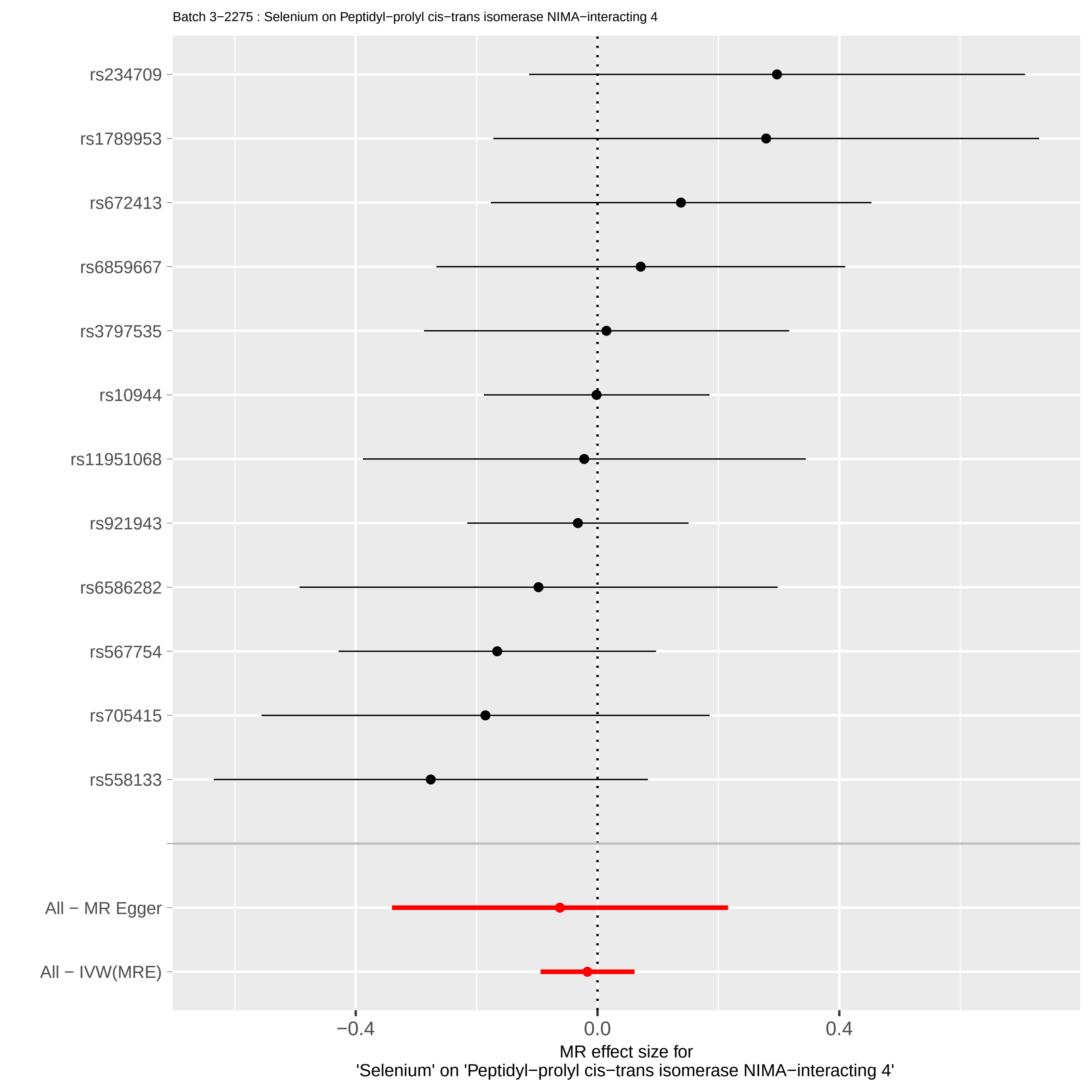

Batch 3−2275 : Selenium on Peptidyl−prolyl cis−trans isomerase NIMA−interacting 4
rs234709
rs1789953
rs672413
rs6859667
rs3797535
rs10944
rs11951068
rs921943
rs6586282
rs567754
rs705415
rs558133
All − MR Egger
All − IVW(MRE)
−0.4
0.0
0.4
MR effect size for
'Selenium' on 'Peptidyl−prolyl cis−trans isomerase NIMA−interacting 4'

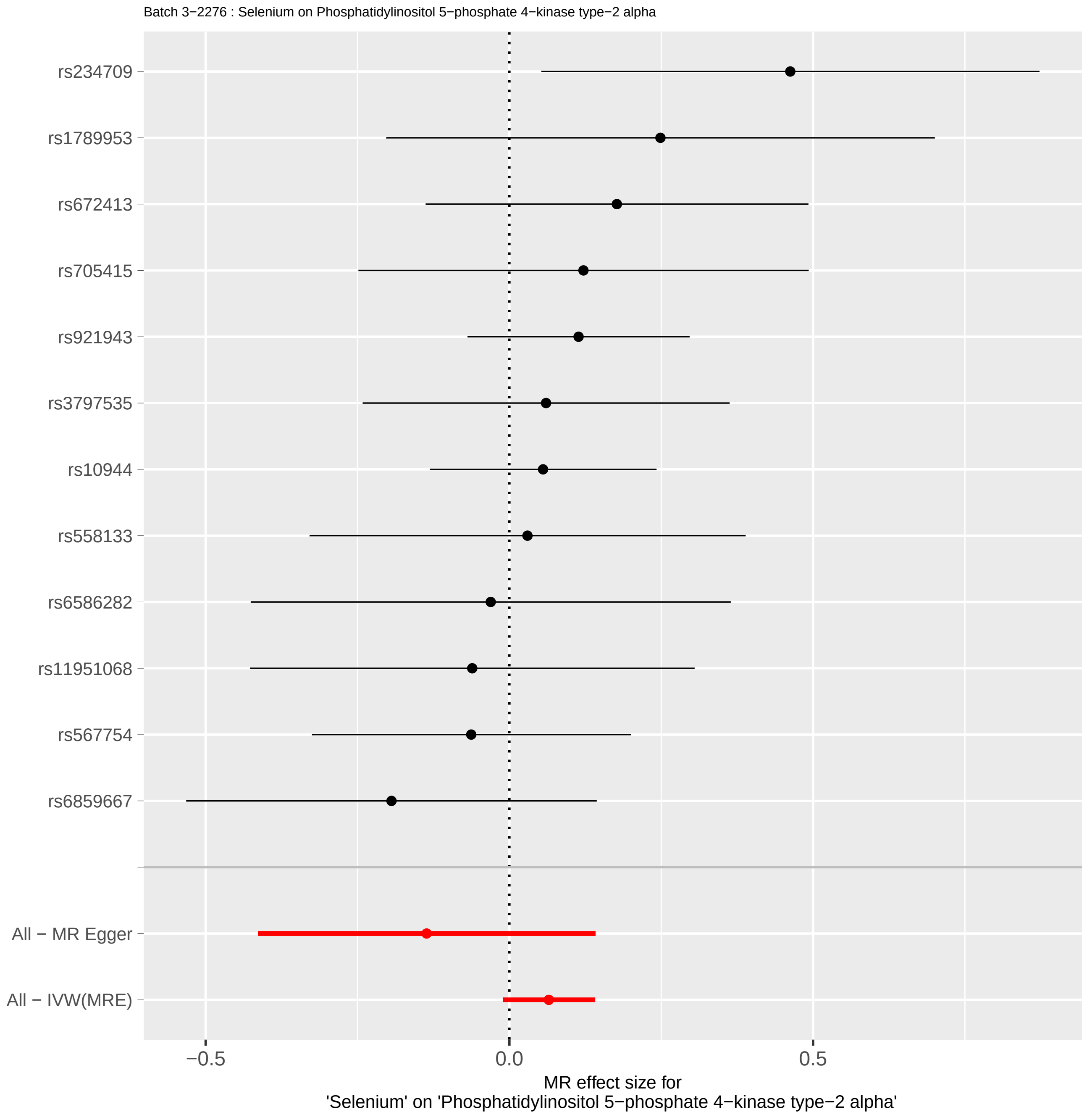
Batch 3−2276 : Selenium on Phosphatidylinositol 5−phosphate 4−kinase type−2 alpha
rs234709
rs1789953
rs672413
rs705415
rs921943
rs3797535
rs10944
rs558133
rs6586282
rs11951068
rs567754
rs6859667
All − MR Egger
All − IVW(MRE)
−0.5
0.0
0.5
MR effect size for
'Selenium' on 'Phosphatidylinositol 5−phosphate 4−kinase type−2 alpha'

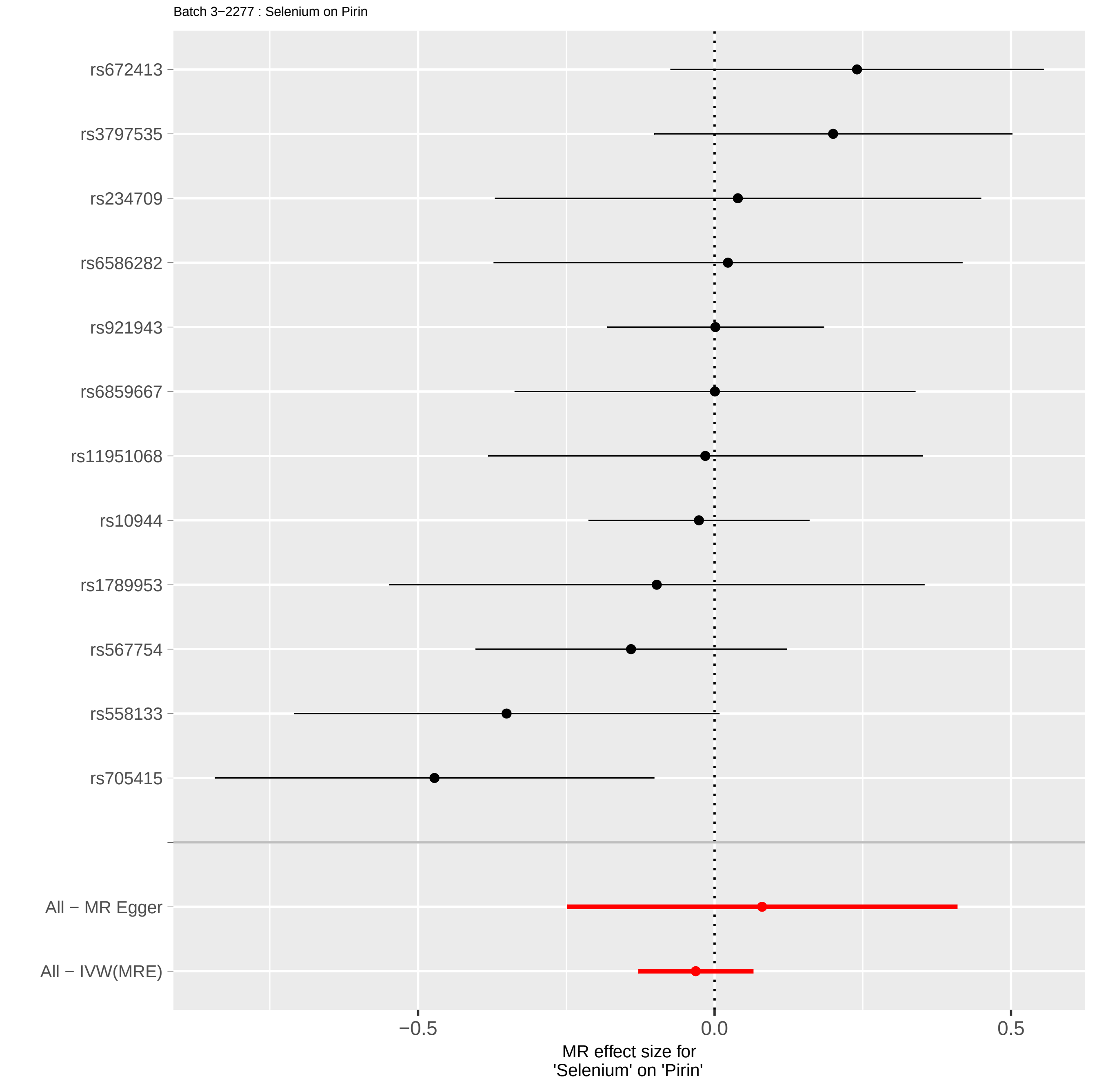

Batch 3−2277 : Selenium on Pirin
rs672413
rs3797535
rs234709
rs6586282
rs921943
rs6859667
rs11951068
rs10944
rs1789953
rs567754
rs558133
rs705415
All − MR Egger
All − IVW(MRE)
−0.5
0.0
0.5
MR effect size for
'Selenium' on 'Pirin'

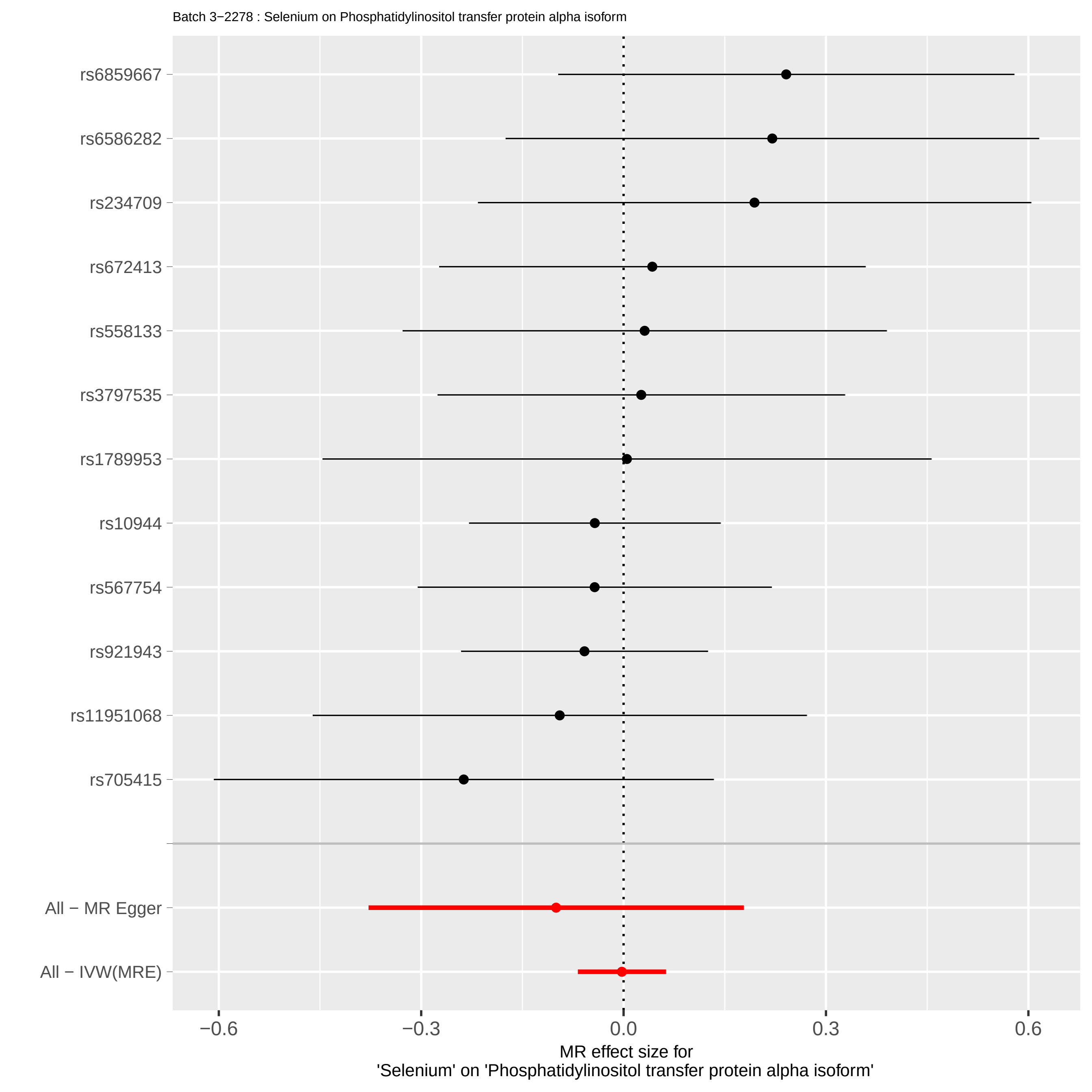

Batch 3−2278 : Selenium on Phosphatidylinositol transfer protein alpha isoform
rs6859667
rs6586282
rs234709
rs672413
rs558133
rs3797535
rs1789953
rs10944
rs567754
rs921943
rs11951068
rs705415
All − MR Egger
All − IVW(MRE)
−0.6
−0.3
0.0
0.3
0.6
MR effect size for
'Selenium' on 'Phosphatidylinositol transfer protein alpha isoform'

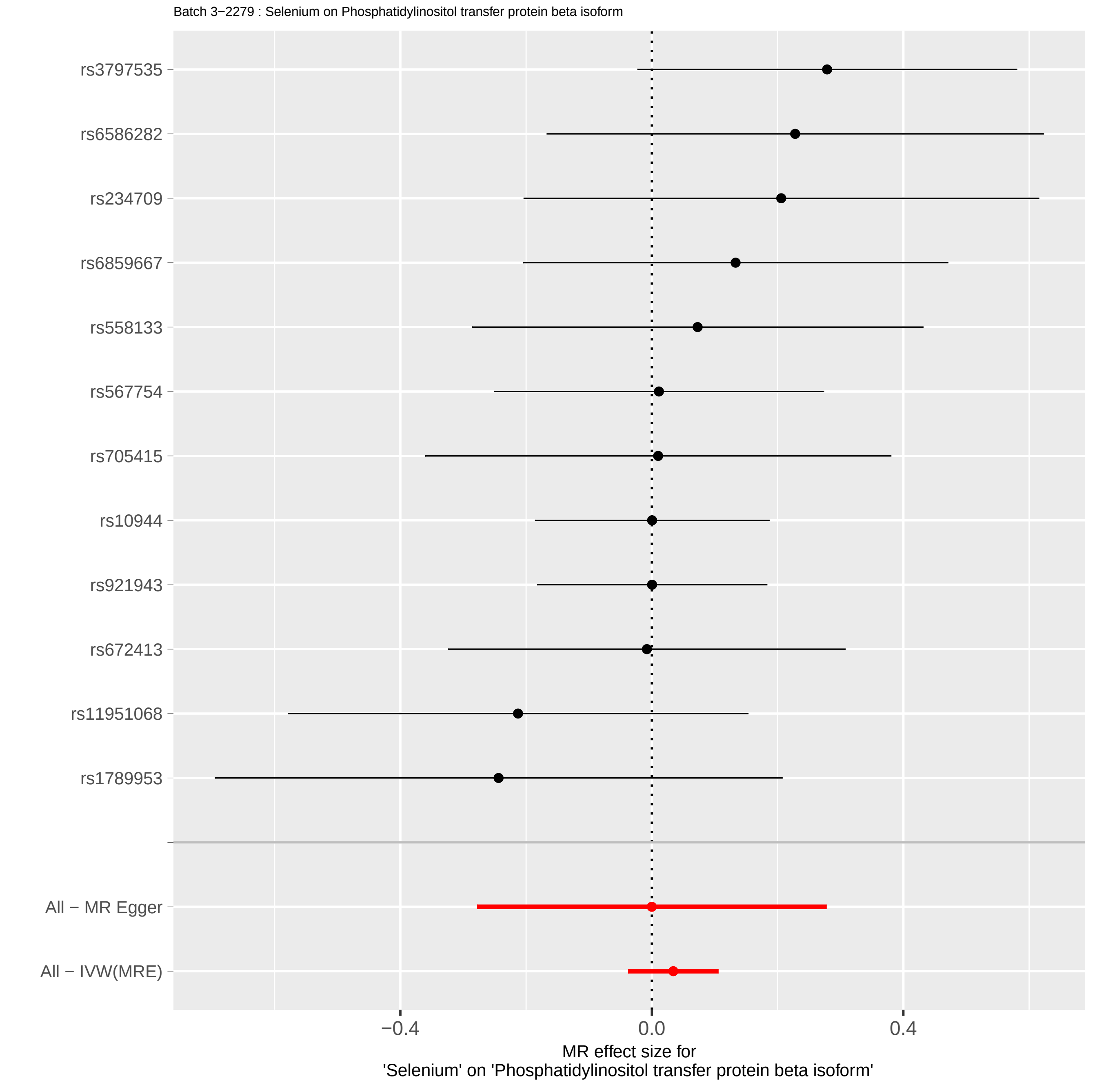

Batch 3−2279 : Selenium on Phosphatidylinositol transfer protein beta isoform
rs3797535
rs6586282
rs234709
rs6859667
rs558133
rs567754
rs705415
rs10944
rs921943
rs672413
rs11951068
rs1789953
All − MR Egger
All − IVW(MRE)
−0.4
0.0
0.4
MR effect size for
'Selenium' on 'Phosphatidylinositol transfer protein beta isoform'

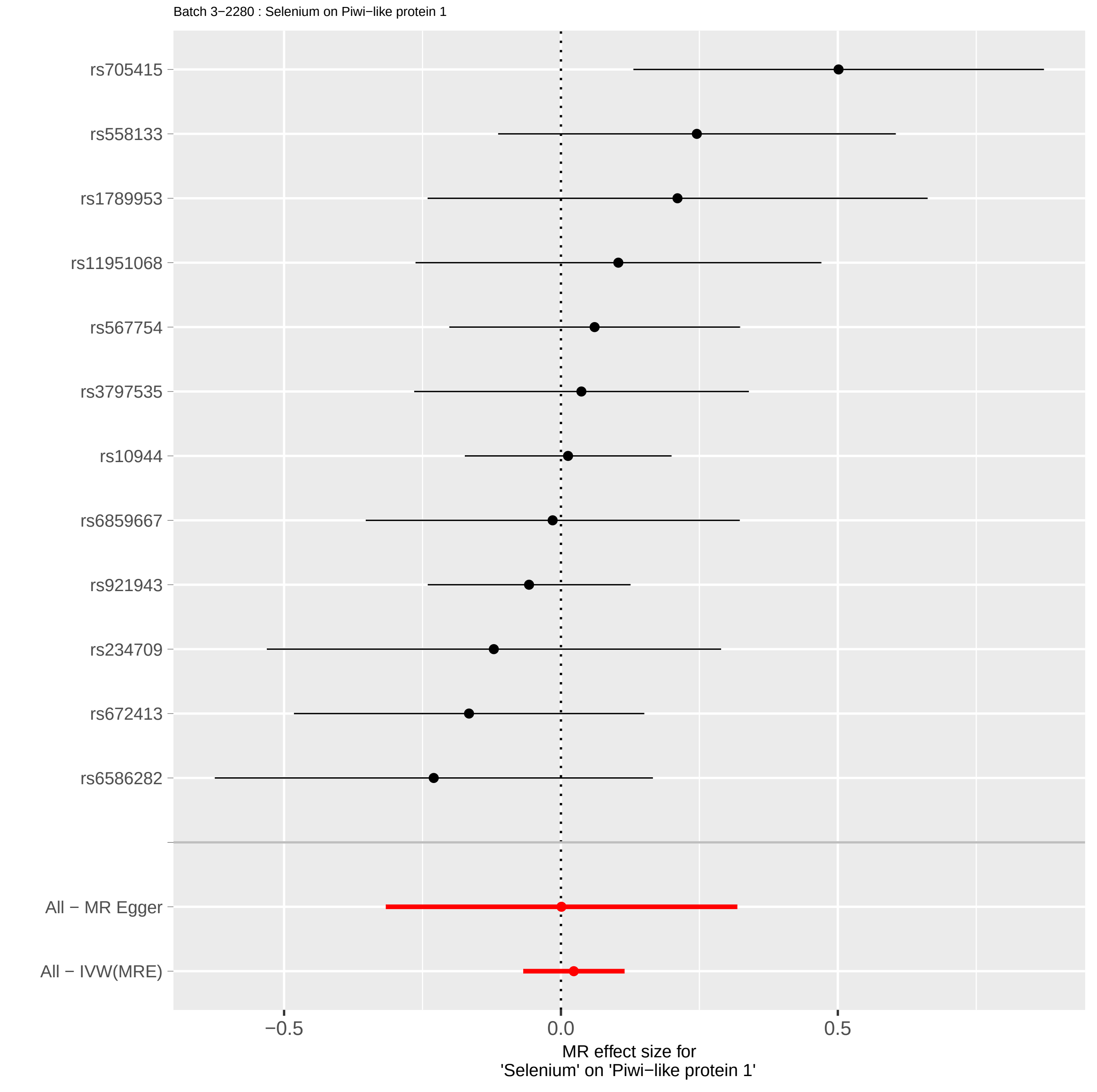

Batch 3−2280 : Selenium on Piwi−like protein 1
rs705415
rs558133
rs1789953
rs11951068
rs567754
rs3797535
rs10944
rs6859667
rs921943
rs234709
rs672413
rs6586282
All − MR Egger
All − IVW(MRE)
−0.5
0.0
0.5
MR effect size for
'Selenium' on 'Piwi−like protein 1'

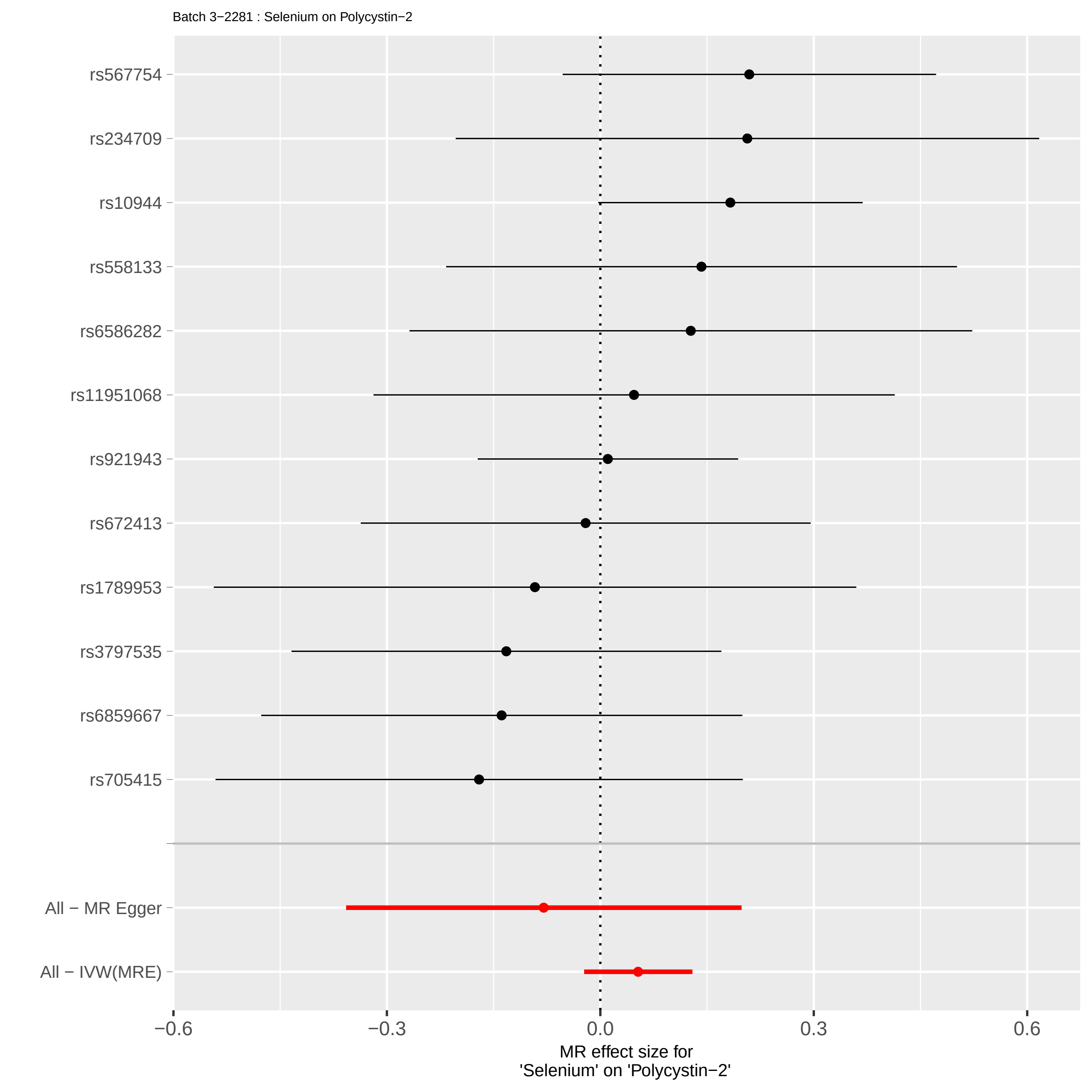

Batch 3−2281 : Selenium on Polycystin−2
rs567754
rs234709
rs10944
rs558133
rs6586282
rs11951068
rs921943
rs672413
rs1789953
rs3797535
rs6859667
rs705415
All − MR Egger
All − IVW(MRE)
−0.6
−0.3
0.0
0.3
0.6
MR effect size for
'Selenium' on 'Polycystin−2'

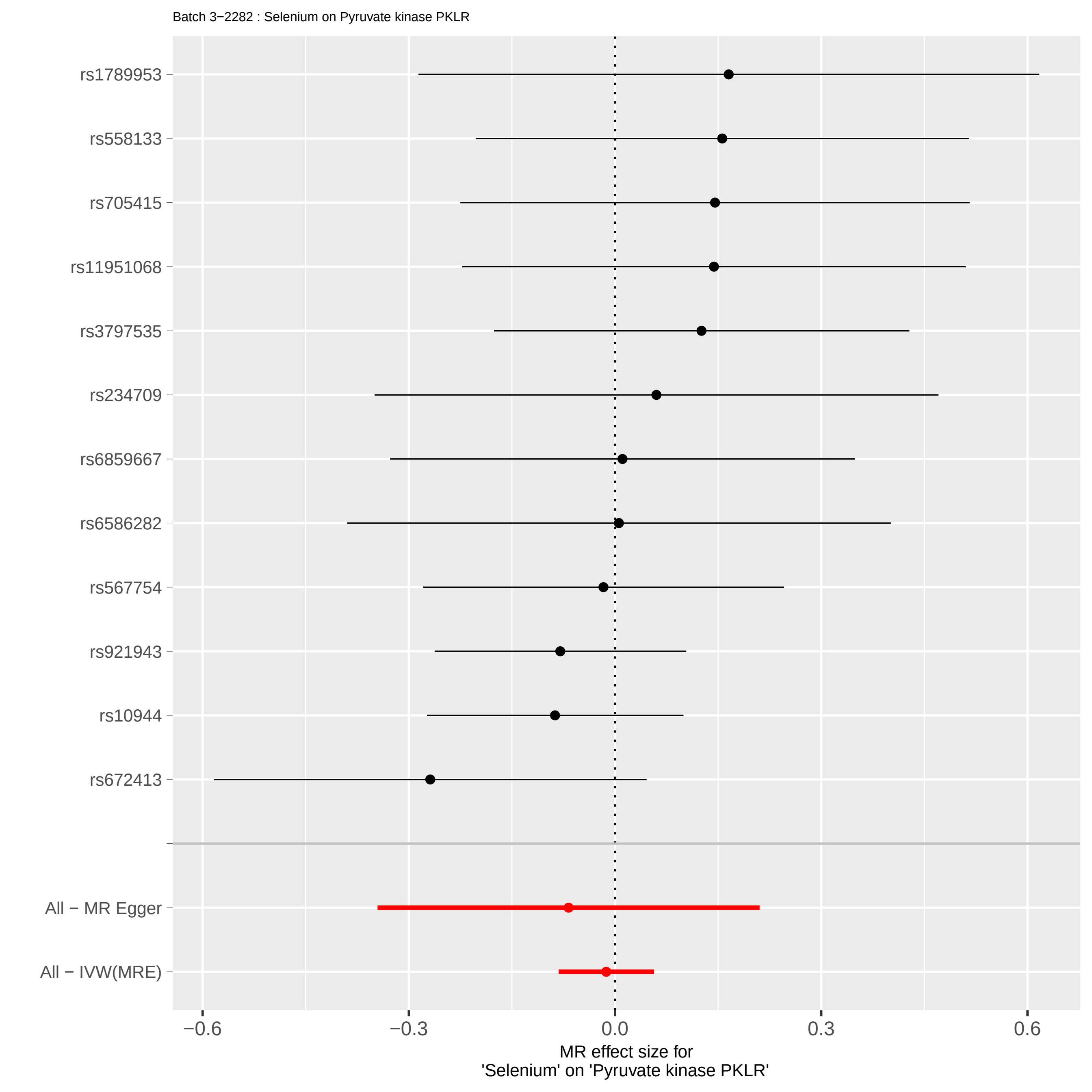

Batch 3−2282 : Selenium on Pyruvate kinase PKLR
rs1789953
rs558133
rs705415
rs11951068
rs3797535
rs234709
rs6859667
rs6586282
rs567754
rs921943
rs10944
rs672413
All − MR Egger
All − IVW(MRE)
−0.6
−0.3
0.0
0.3
0.6
MR effect size for
'Selenium' on 'Pyruvate kinase PKLR'

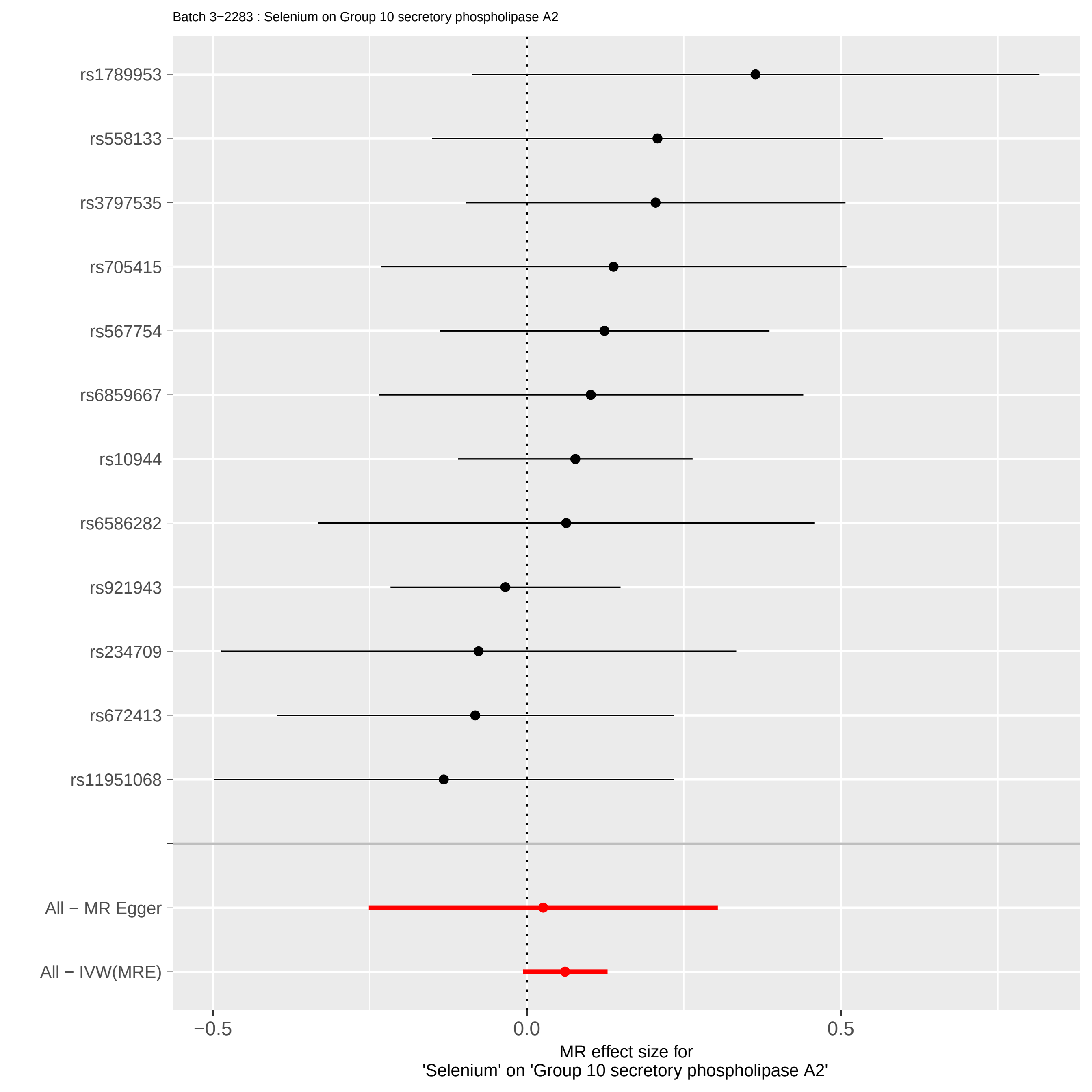
Batch 3−2283 : Selenium on Group 10 secretory phospholipase A2
rs1789953
rs558133
rs3797535
rs705415
rs567754
rs6859667
rs10944
rs6586282
rs921943
rs234709
rs672413
rs11951068
All − MR Egger
All − IVW(MRE)
−0.5
0.0
0.5
MR effect size for
'Selenium' on 'Group 10 secretory phospholipase A2'

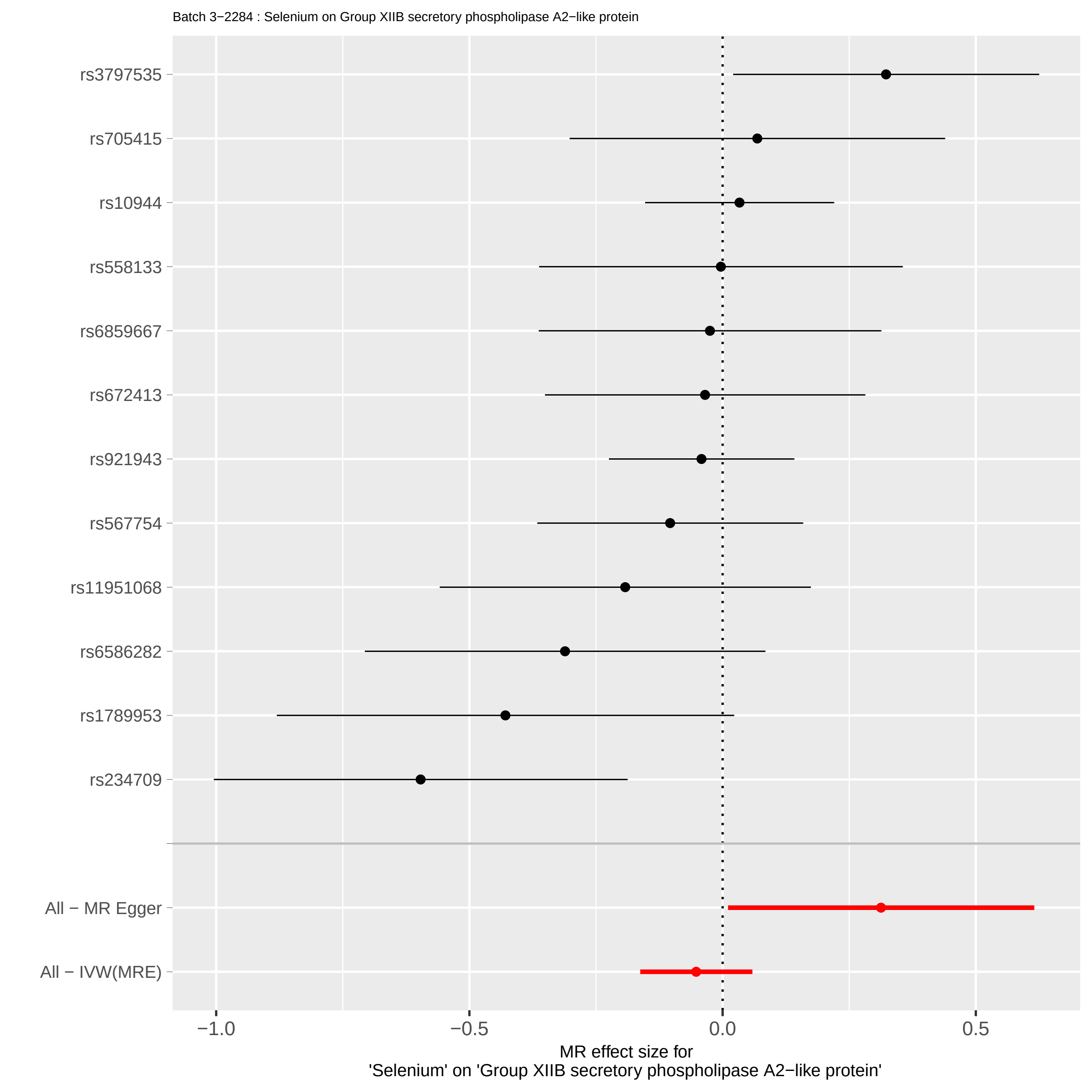

Batch 3−2284 : Selenium on Group XIIB secretory phospholipase A2−like protein
rs3797535
rs705415
rs10944
rs558133
rs6859667
rs672413
rs921943
rs567754
rs11951068
rs6586282
rs1789953
rs234709
All − MR Egger
All − IVW(MRE)
−1.0
−0.5
0.0
0.5
MR effect size for
'Selenium' on 'Group XIIB secretory phospholipase A2−like protein'

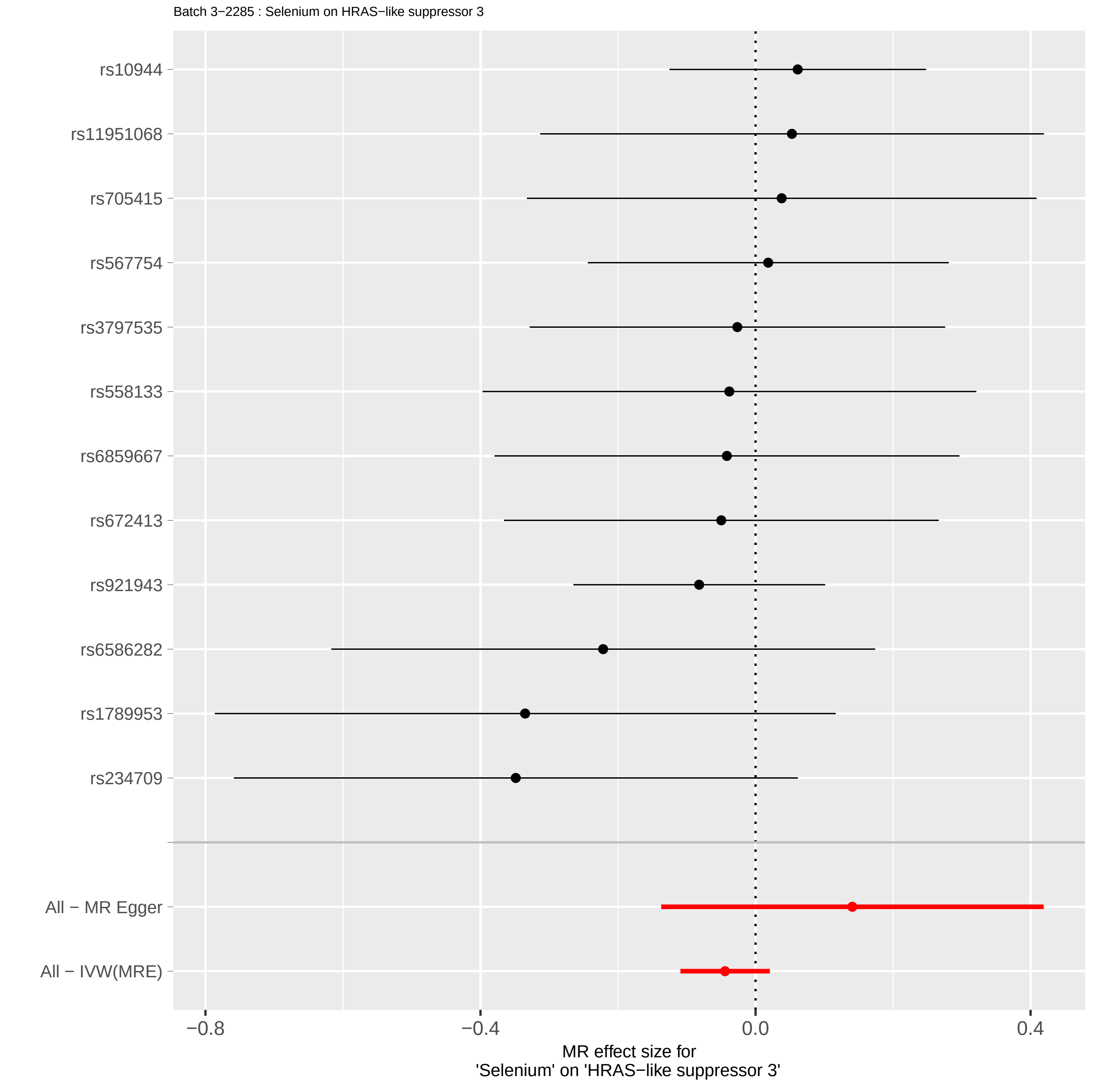

Batch 3−2285 : Selenium on HRAS−like suppressor 3
rs10944
rs11951068
rs705415
rs567754
rs3797535
rs558133
rs6859667
rs672413
rs921943
rs6586282
rs1789953
rs234709
All − MR Egger
All − IVW(MRE)
−0.8
−0.4
0.0
0.4
MR effect size for
'Selenium' on 'HRAS−like suppressor 3'

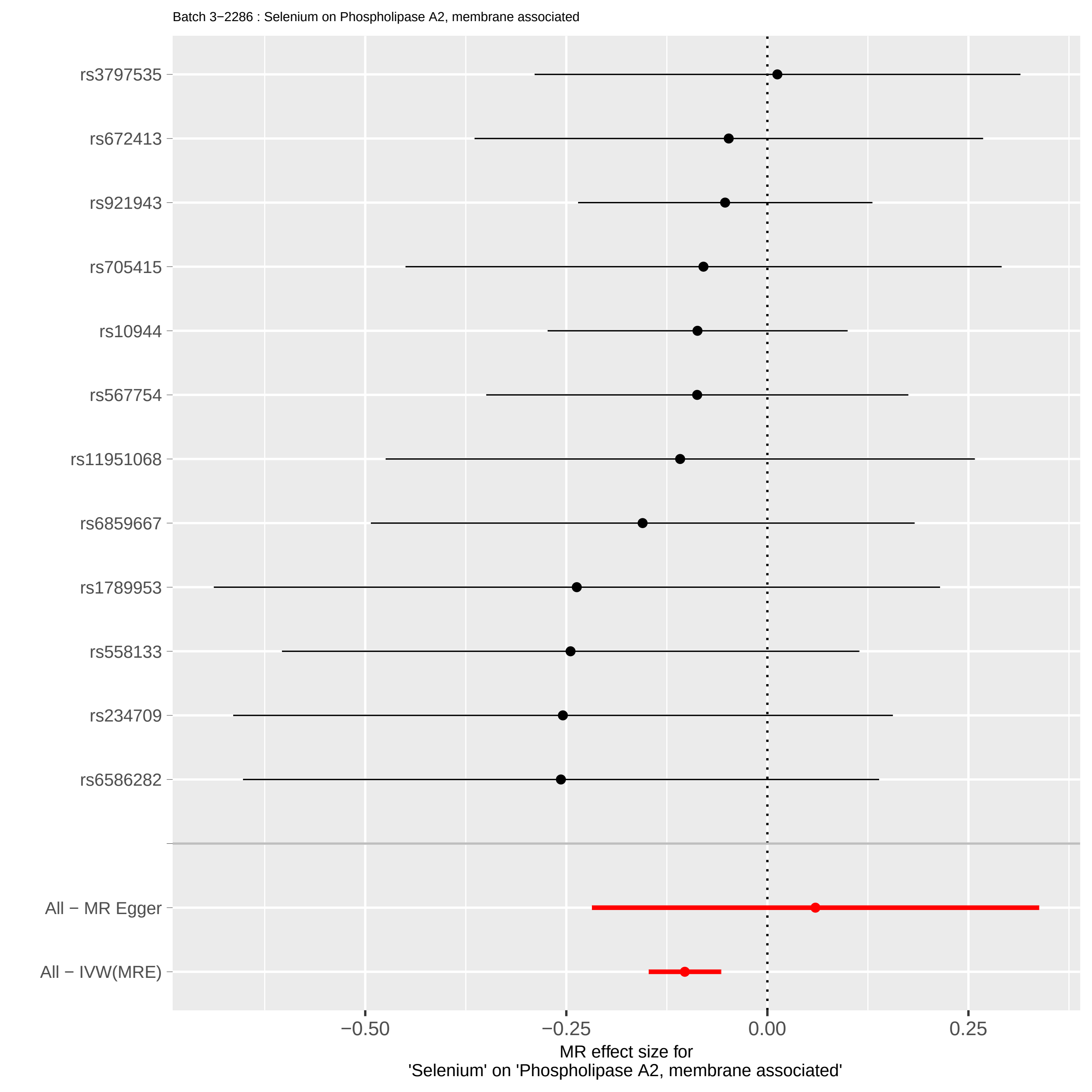

Batch 3−2286 : Selenium on Phospholipase A2, membrane associated
rs3797535
rs672413
rs921943
rs705415
rs10944
rs567754
rs11951068
rs6859667
rs1789953
rs558133
rs234709
rs6586282
All − MR Egger
All − IVW(MRE)
−0.50
−0.25
0.00
0.25
MR effect size for
'Selenium' on 'Phospholipase A2, membrane associated'

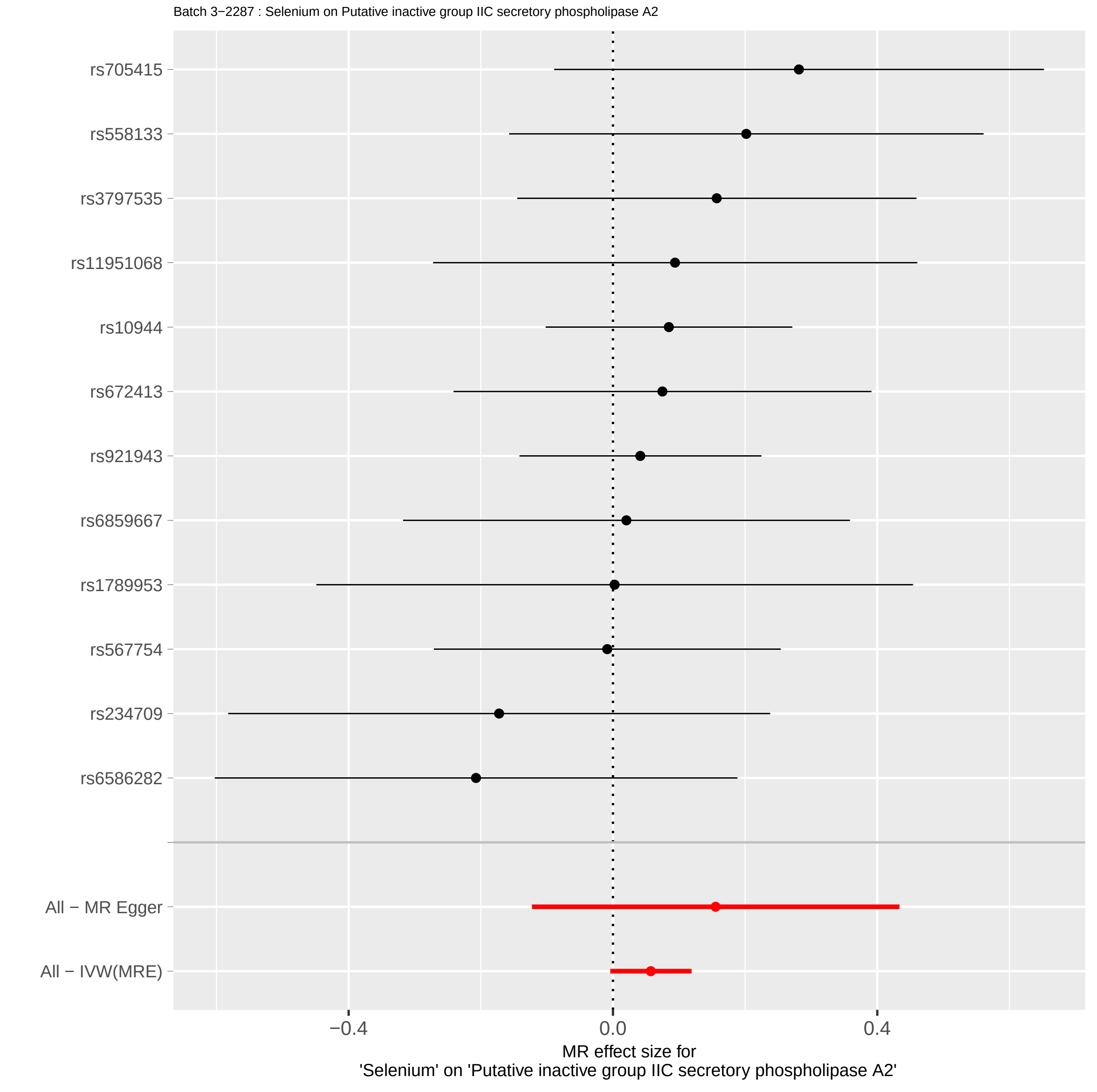

Batch 3−2287 : Selenium on Putative inactive group IIC secretory phospholipase A2
rs705415
rs558133
rs3797535
rs11951068
rs10944
rs672413
rs921943
rs6859667
rs1789953
rs567754
rs234709
rs6586282
All − MR Egger
All − IVW(MRE)
−0.4
0.0
0.4
MR effect size for
'Selenium' on 'Putative inactive group IIC secretory phospholipase A2'

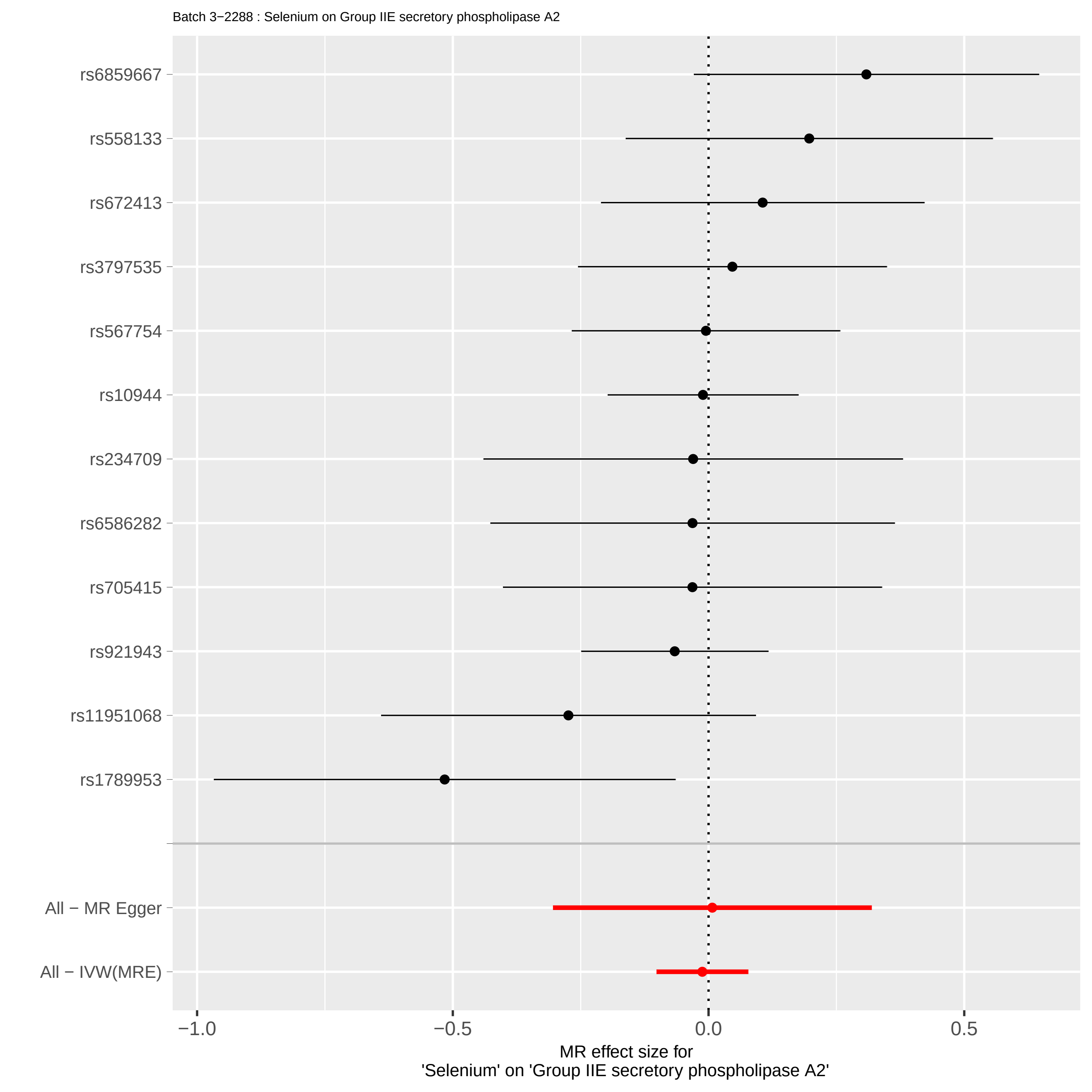

Batch 3−2288 : Selenium on Group IIE secretory phospholipase A2
rs6859667
rs558133
rs672413
rs3797535
rs567754
rs10944
rs234709
rs6586282
rs705415
rs921943
rs11951068
rs1789953
All − MR Egger
All − IVW(MRE)
−1.0
−0.5
0.0
0.5
MR effect size for
'Selenium' on 'Group IIE secretory phospholipase A2'

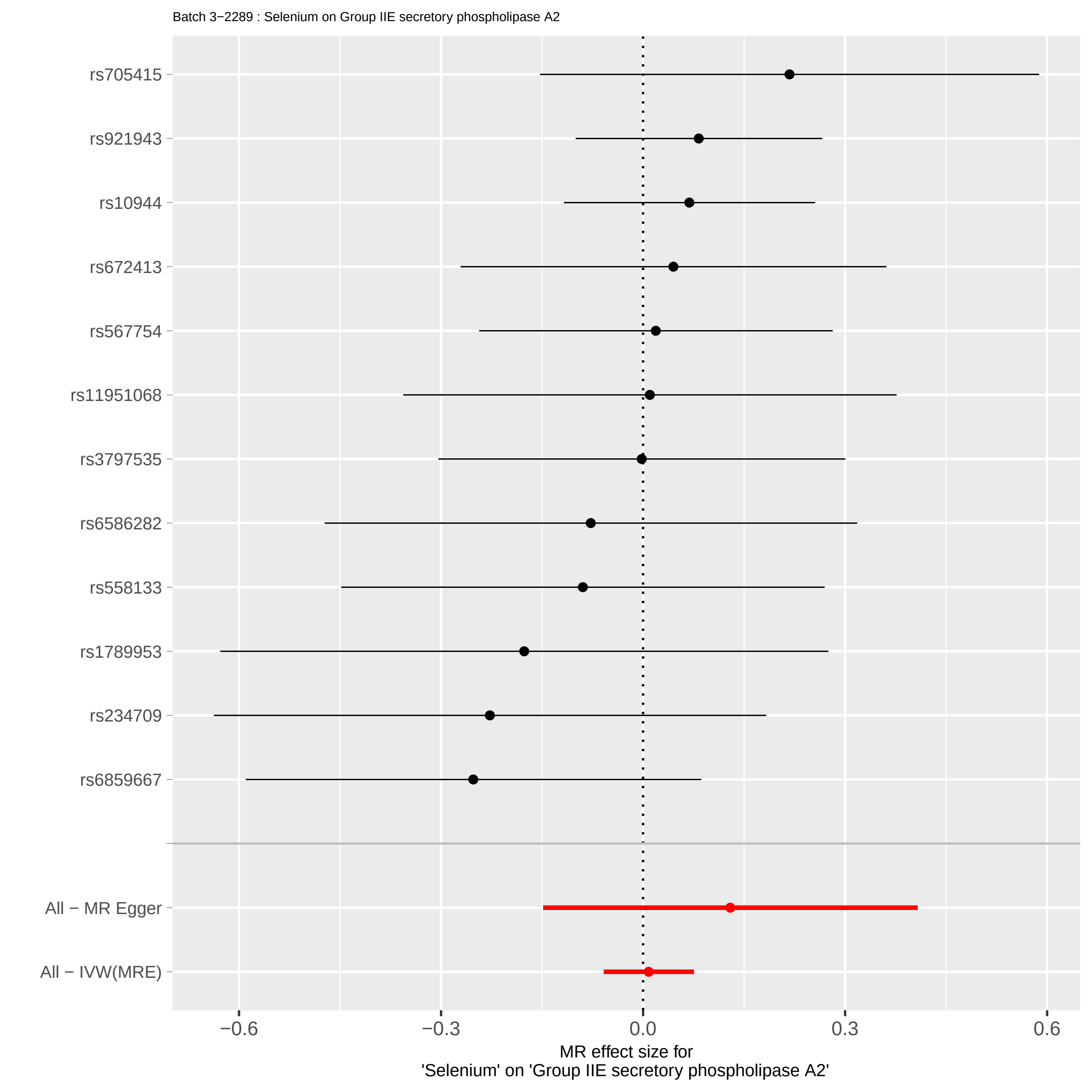

Batch 3−2289 : Selenium on Group IIE secretory phospholipase A2
rs705415
rs921943
rs10944
rs672413
rs567754
rs11951068
rs3797535
rs6586282
rs558133
rs1789953
rs234709
rs6859667
All − MR Egger
All − IVW(MRE)
−0.6
−0.3
0.0
0.3
0.6
MR effect size for
'Selenium' on 'Group IIE secretory phospholipase A2'

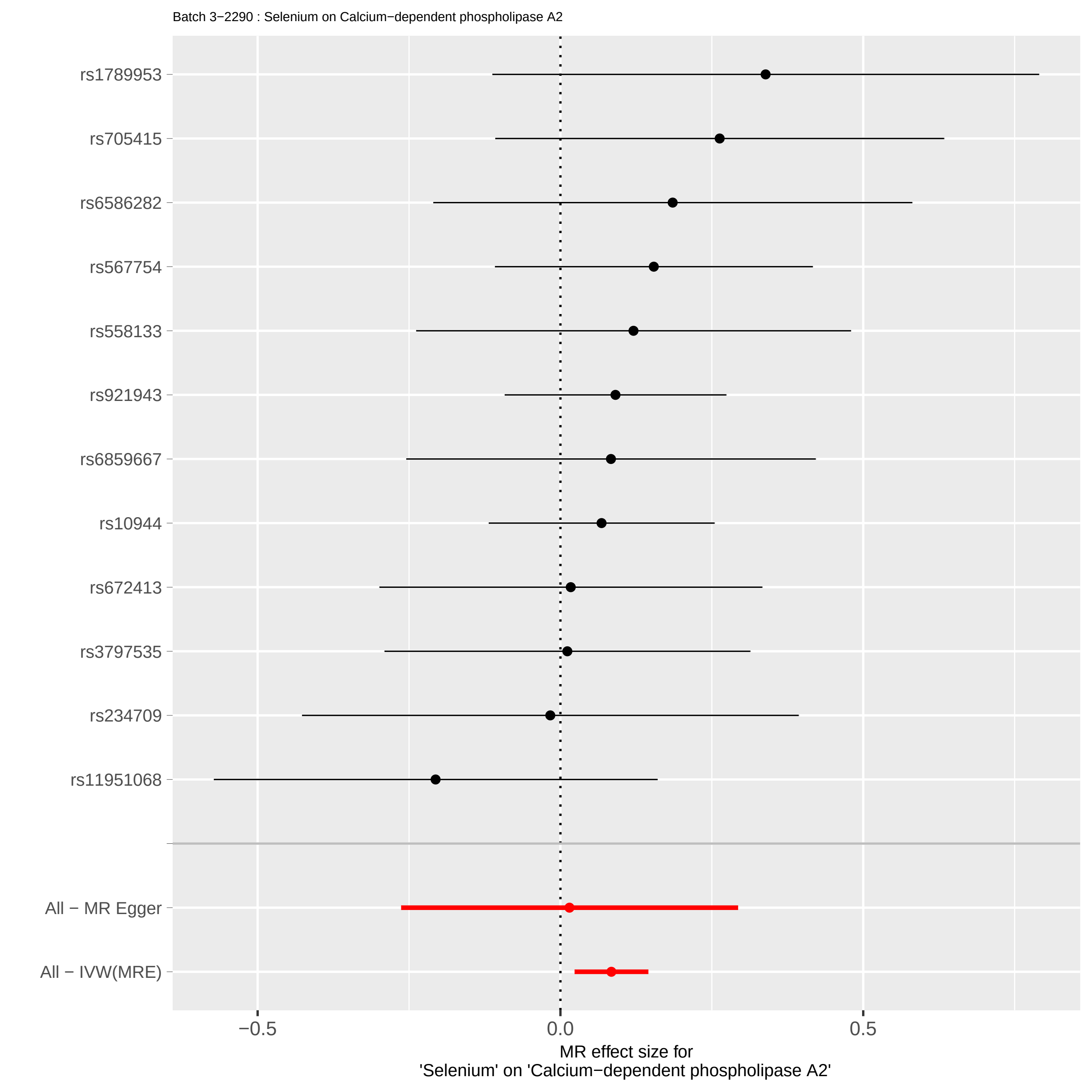

Batch 3−2290 : Selenium on Calcium−dependent phospholipase A2
rs1789953
rs705415
rs6586282
rs567754
rs558133
rs921943
rs6859667
rs10944
rs672413
rs3797535
rs234709
rs11951068
All − MR Egger
All − IVW(MRE)
−0.5
0.0
0.5
MR effect size for
'Selenium' on 'Calcium−dependent phospholipase A2'

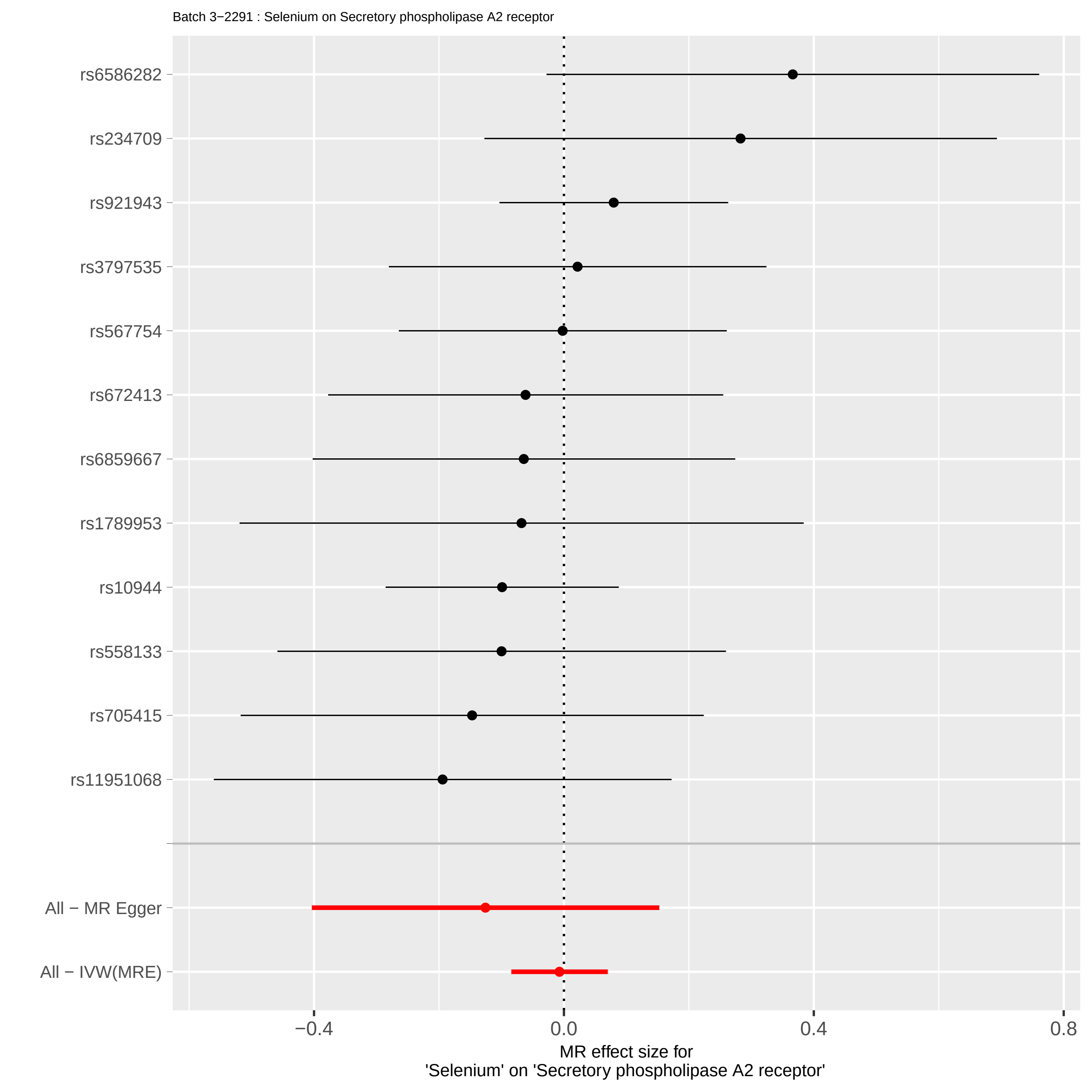

Batch 3−2291 : Selenium on Secretory phospholipase A2 receptor
rs6586282
rs234709
rs921943
rs3797535
rs567754
rs672413
rs6859667
rs1789953
rs10944
rs558133
rs705415
rs11951068
All − MR Egger
All − IVW(MRE)
−0.4
0.0
0.4
0.8
MR effect size for
'Selenium' on 'Secretory phospholipase A2 receptor'

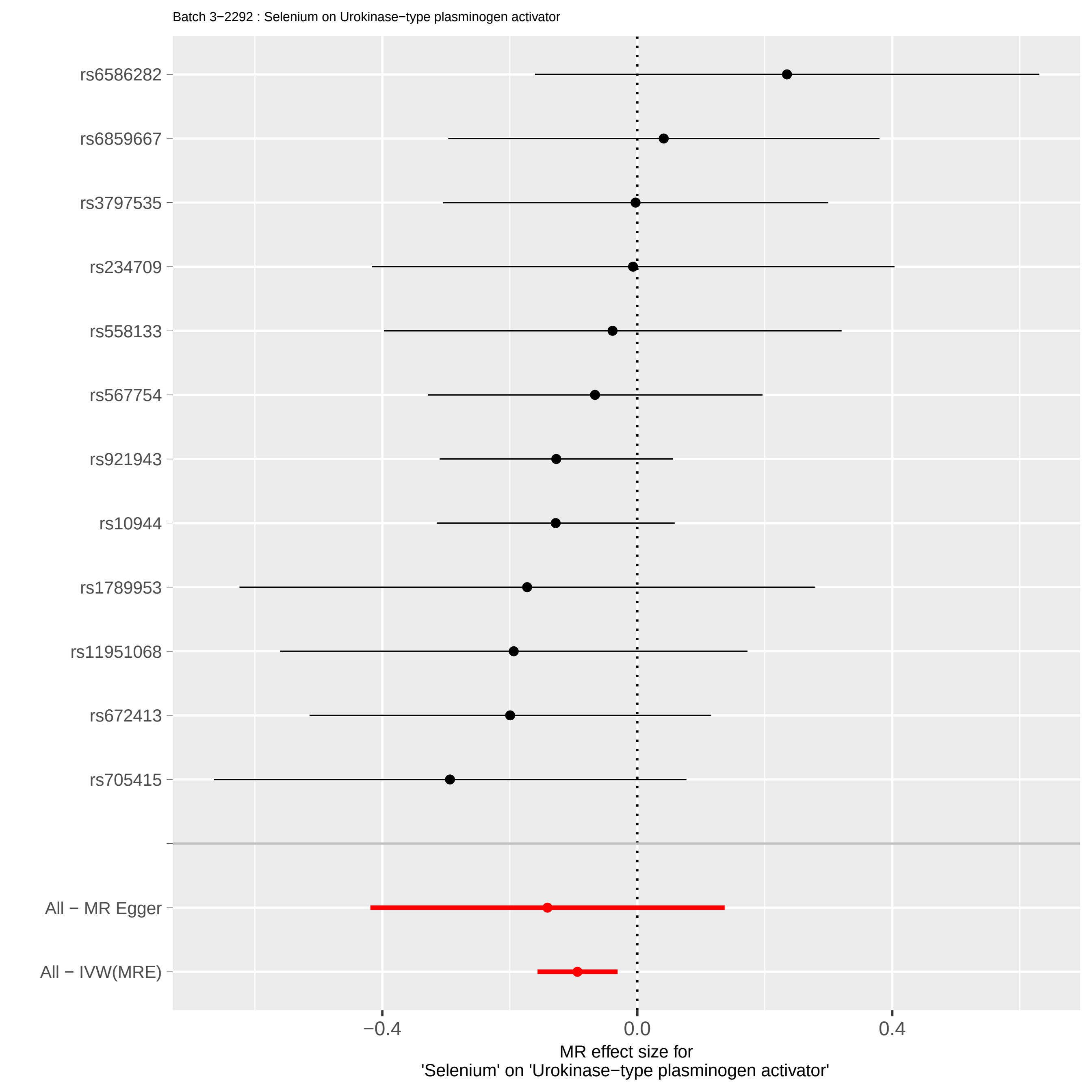

Batch 3−2292 : Selenium on Urokinase−type plasminogen activator
rs6586282
rs6859667
rs3797535
rs234709
rs558133
rs567754
rs921943
rs10944
rs1789953
rs11951068
rs672413
rs705415
All − MR Egger
All − IVW(MRE)
−0.4
0.0
0.4
MR effect size for
'Selenium' on 'Urokinase−type plasminogen activator'

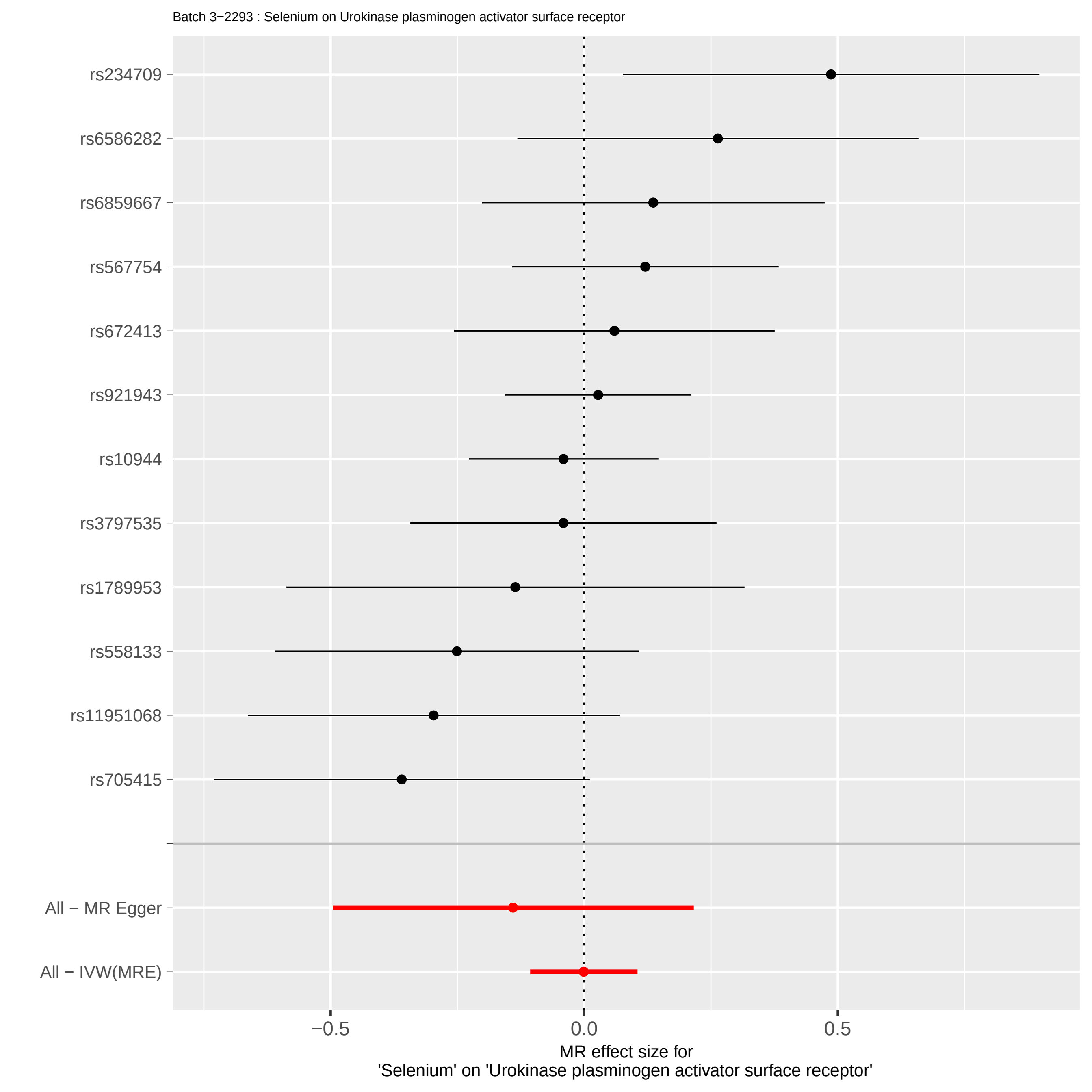

Batch 3−2293 : Selenium on Urokinase plasminogen activator surface receptor
rs234709
rs6586282
rs6859667
rs567754
rs672413
rs921943
rs10944
rs3797535
rs1789953
rs558133
rs11951068
rs705415
All − MR Egger
All − IVW(MRE)
−0.5
0.0
0.5
MR effect size for
'Selenium' on 'Urokinase plasminogen activator surface receptor'

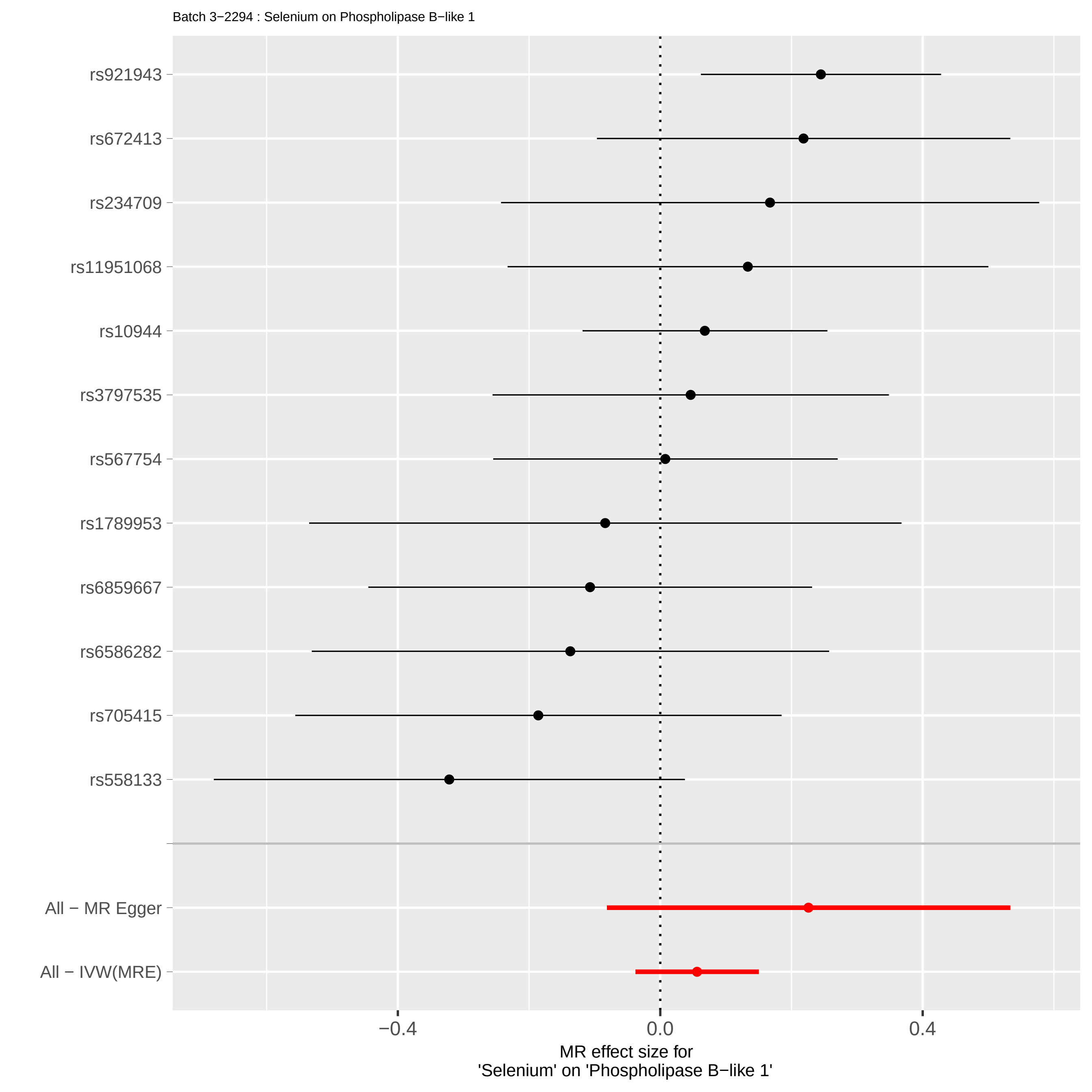

Batch 3−2294 : Selenium on Phospholipase B−like 1
rs921943
rs672413
rs234709
rs11951068
rs10944
rs3797535
rs567754
rs1789953
rs6859667
rs6586282
rs705415
rs558133
All − MR Egger
All − IVW(MRE)
−0.4
0.0
0.4
MR effect size for
'Selenium' on 'Phospholipase B−like 1'

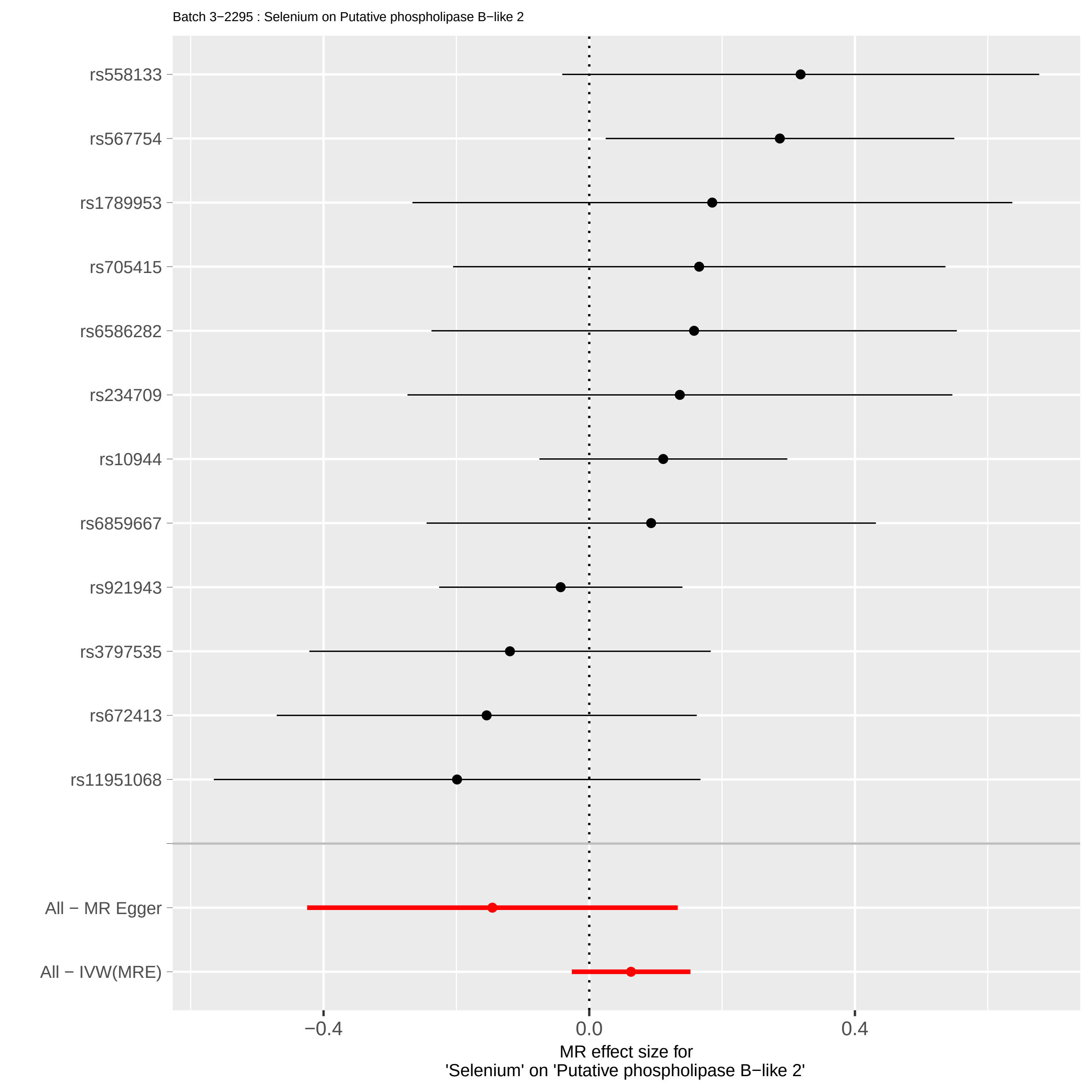

Batch 3−2295 : Selenium on Putative phospholipase B−like 2
rs558133
rs567754
rs1789953
rs705415
rs6586282
rs234709
rs10944
rs6859667
rs921943
rs3797535
rs672413
rs11951068
All − MR Egger
All − IVW(MRE)
−0.4
0.0
0.4
MR effect size for
'Selenium' on 'Putative phospholipase B−like 2'

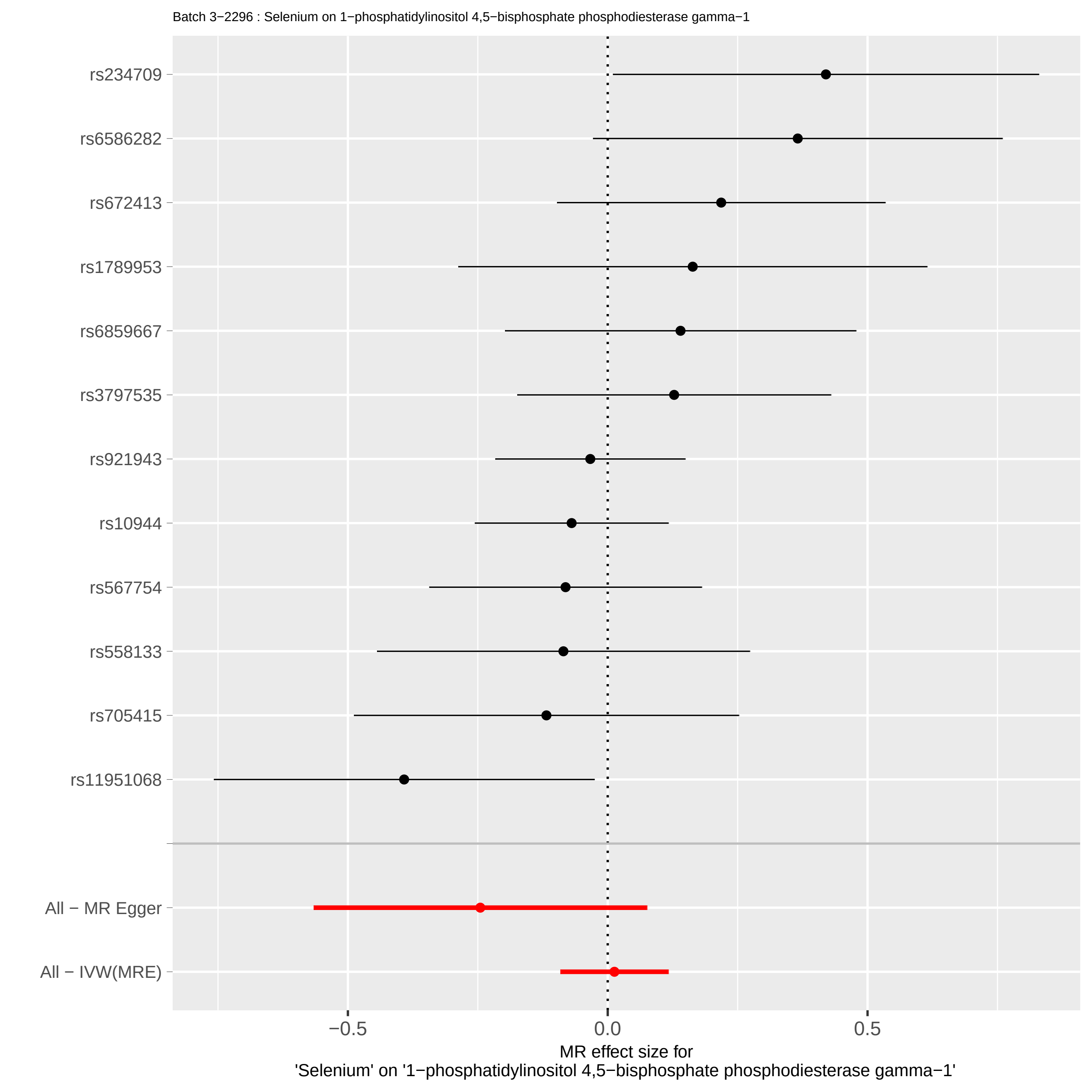
Batch 3−2296 : Selenium on 1−phosphatidylinositol 4,5−bisphosphate phosphodiesterase gamma−1
rs234709
rs6586282
rs672413
rs1789953
rs6859667
rs3797535
rs921943
rs10944
rs567754
rs558133
rs705415
rs11951068
All − MR Egger
All − IVW(MRE)
−0.5
0.0
0.5
MR effect size for
'Selenium' on '1−phosphatidylinositol 4,5−bisphosphate phosphodiesterase gamma−1'

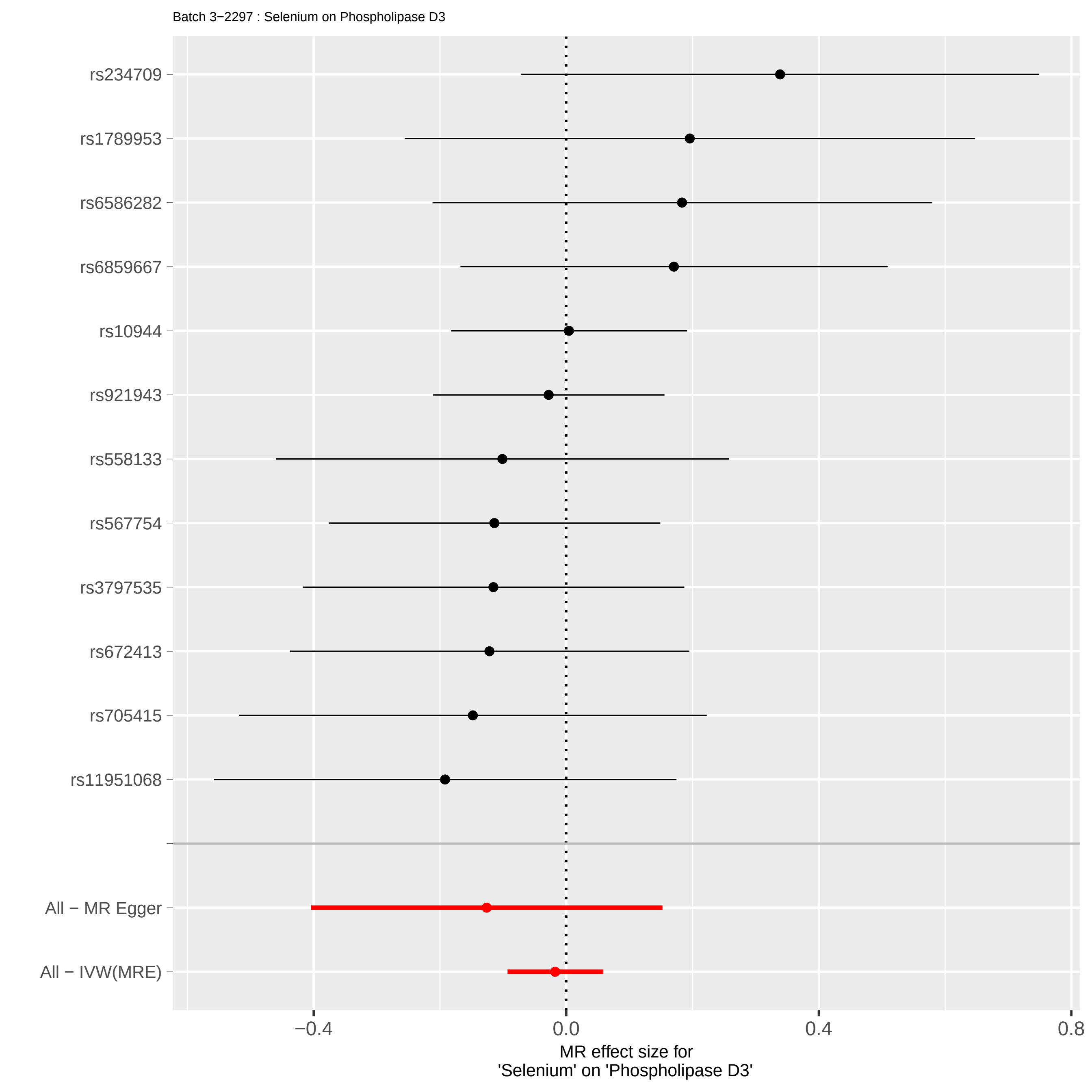

Batch 3−2297 : Selenium on Phospholipase D3
rs234709
rs1789953
rs6586282
rs6859667
rs10944
rs921943
rs558133
rs567754
rs3797535
rs672413
rs705415
rs11951068
All − MR Egger
All − IVW(MRE)
−0.4
0.0
0.4
0.8
MR effect size for
'Selenium' on 'Phospholipase D3'

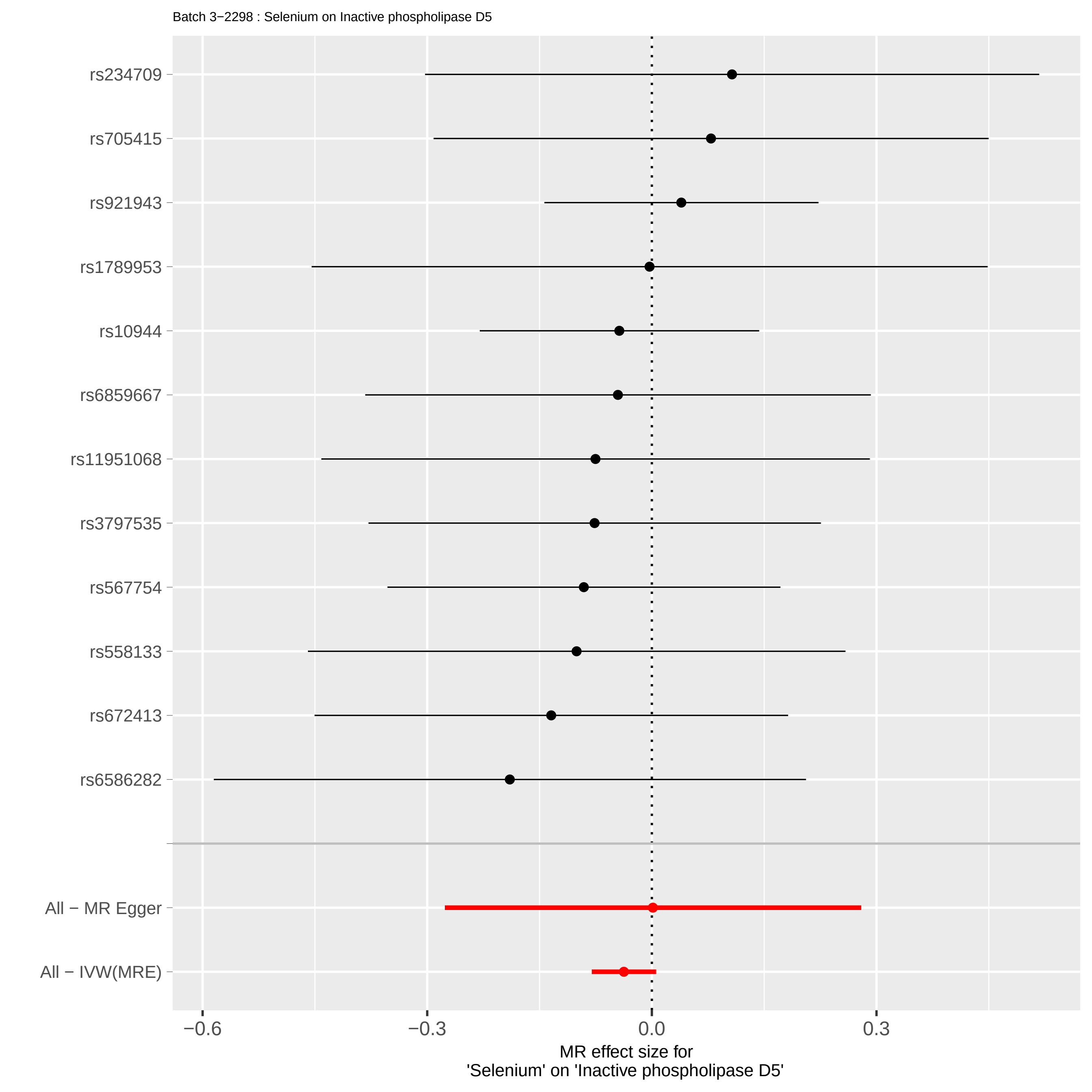

Batch 3−2298 : Selenium on Inactive phospholipase D5
rs234709
rs705415
rs921943
rs1789953
rs10944
rs6859667
rs11951068
rs3797535
rs567754
rs558133
rs672413
rs6586282
All − MR Egger
All − IVW(MRE)
−0.6
−0.3
0.0
0.3
MR effect size for
'Selenium' on 'Inactive phospholipase D5'

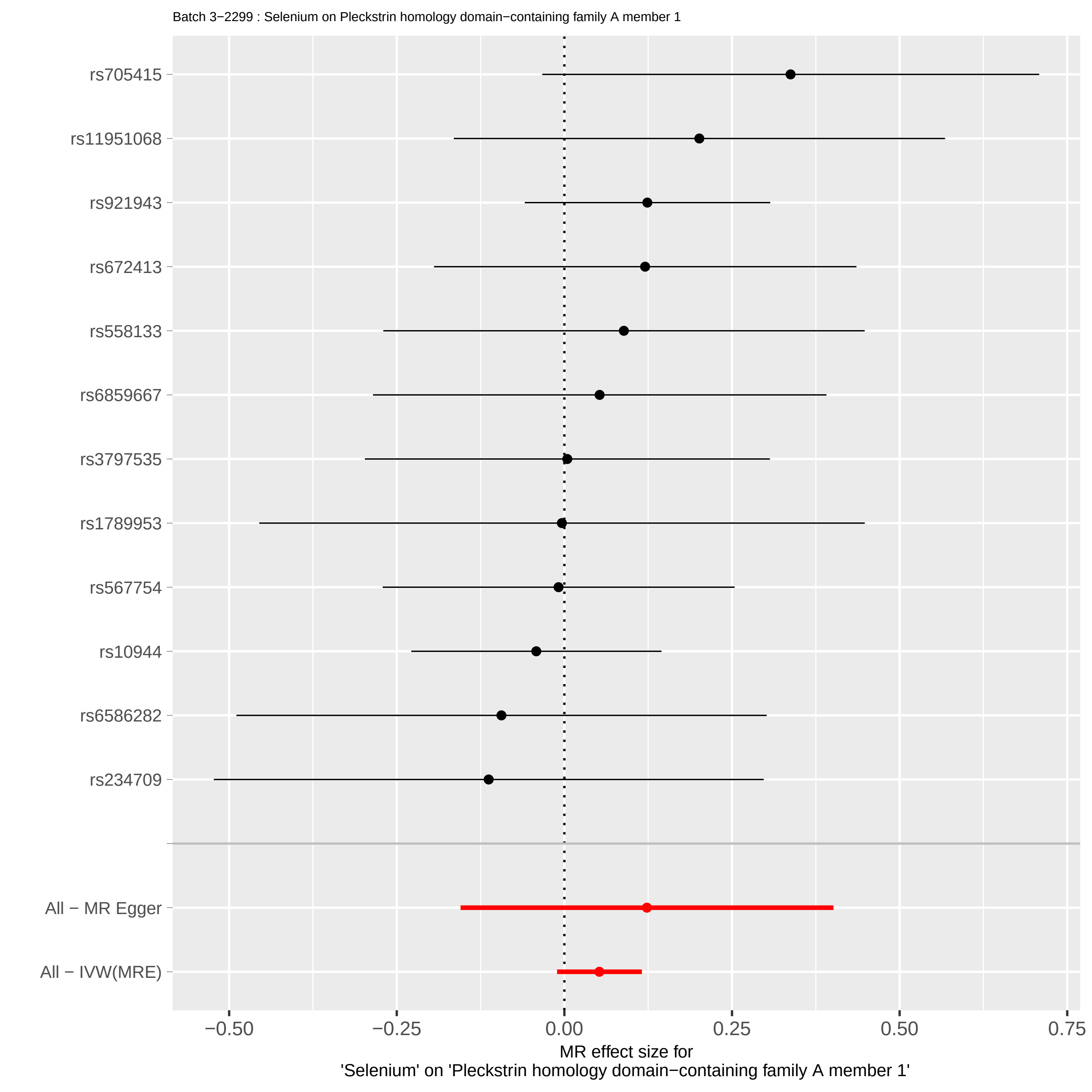

Batch 3−2299 : Selenium on Pleckstrin homology domain−containing family A member 1
rs705415
rs11951068
rs921943
rs672413
rs558133
rs6859667
rs3797535
rs1789953
rs567754
rs10944
rs6586282
rs234709
All − MR Egger
All − IVW(MRE)
−0.50
−0.25
0.00
0.25
0.50
0.75
MR effect size for
'Selenium' on 'Pleckstrin homology domain−containing family A member 1'

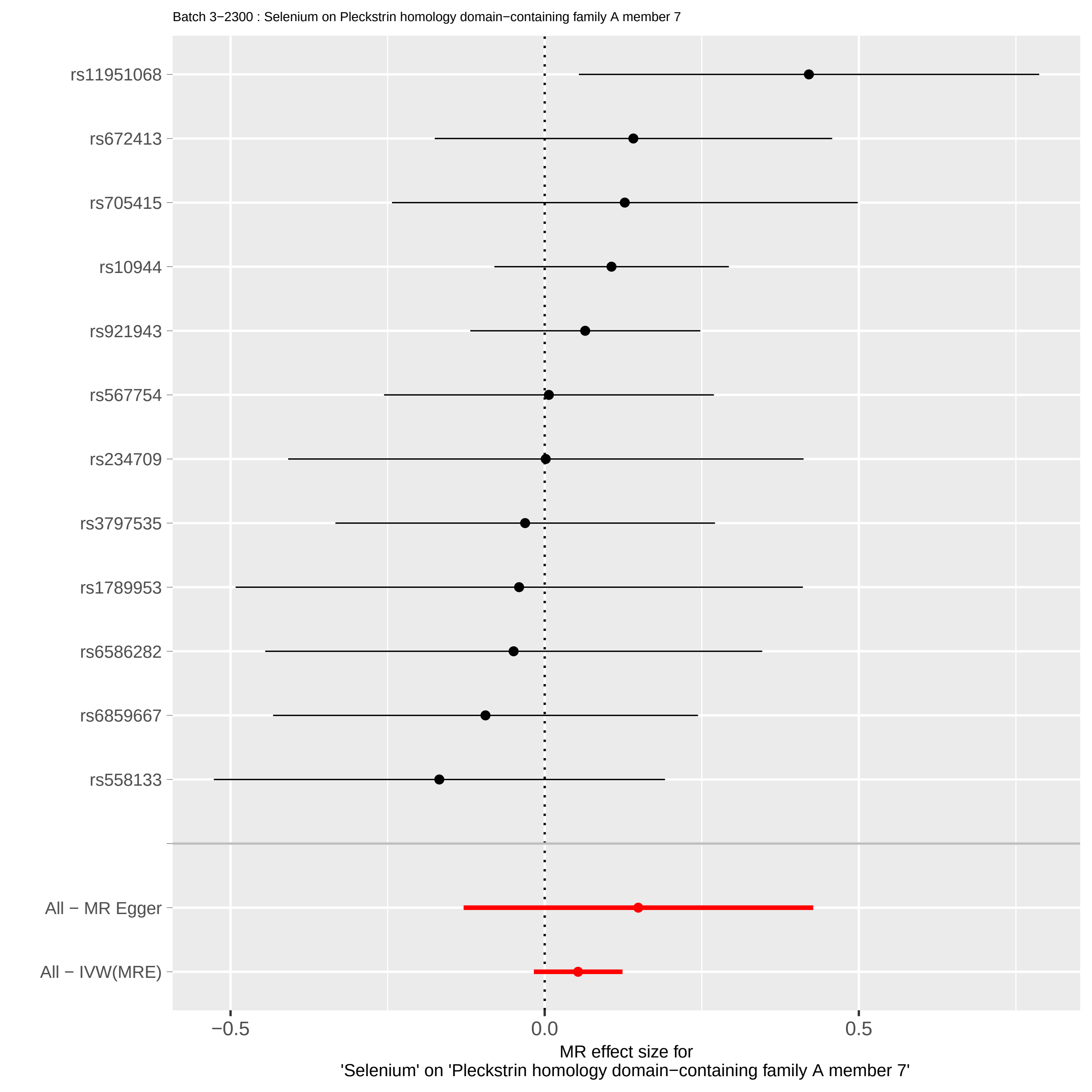

Batch 3−2300 : Selenium on Pleckstrin homology domain−containing family A member 7
rs11951068
rs672413
rs705415
rs10944
rs921943
rs567754
rs234709
rs3797535
rs1789953
rs6586282
rs6859667
rs558133
All − MR Egger
All − IVW(MRE)
−0.5
0.0
0.5
MR effect size for
'Selenium' on 'Pleckstrin homology domain−containing family A member 7'

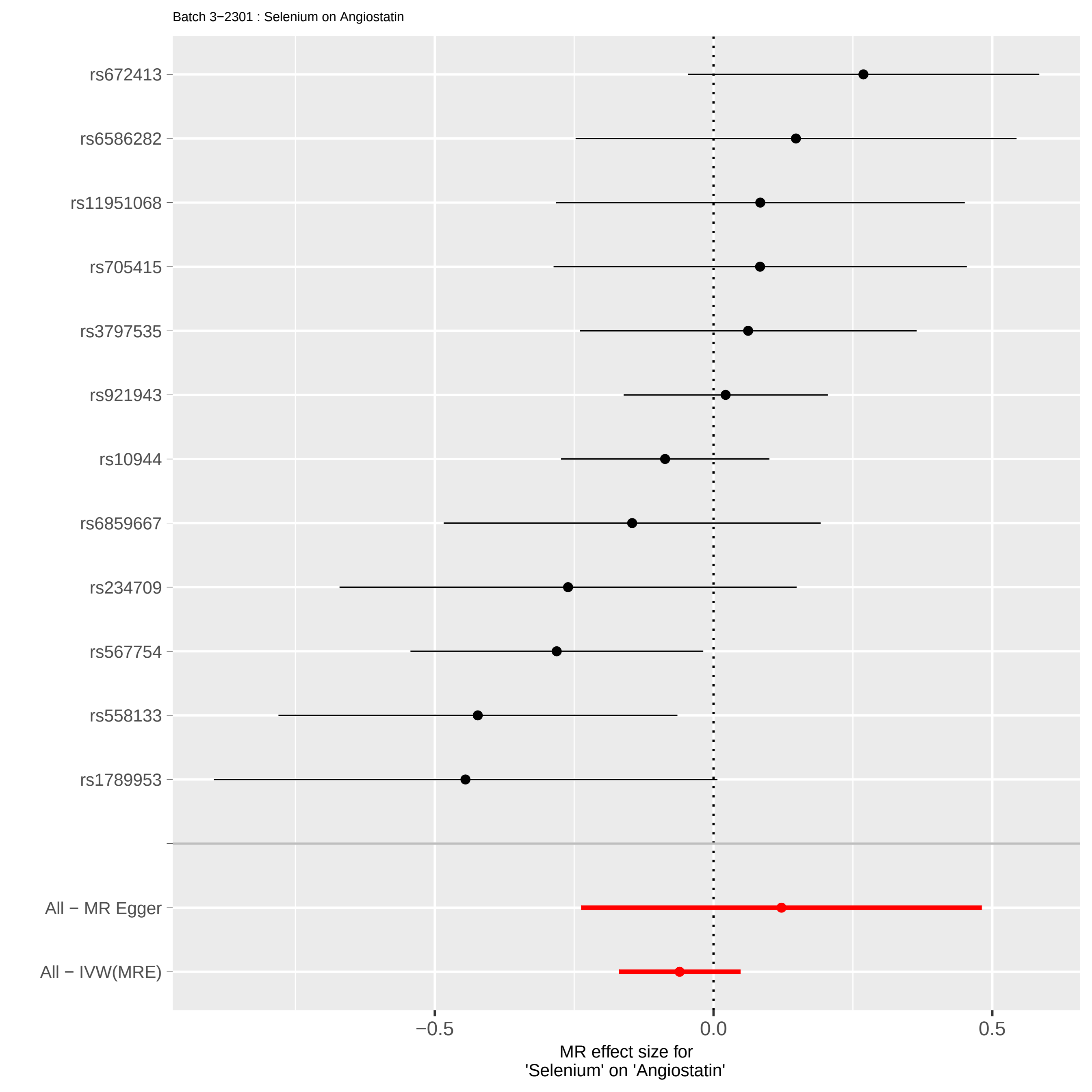

Batch 3−2301 : Selenium on Angiostatin
rs672413
rs6586282
rs11951068
rs705415
rs3797535
rs921943
rs10944
rs6859667
rs234709
rs567754
rs558133
rs1789953
All − MR Egger
All − IVW(MRE)
−0.5
0.0
0.5
MR effect size for
'Selenium' on 'Angiostatin'

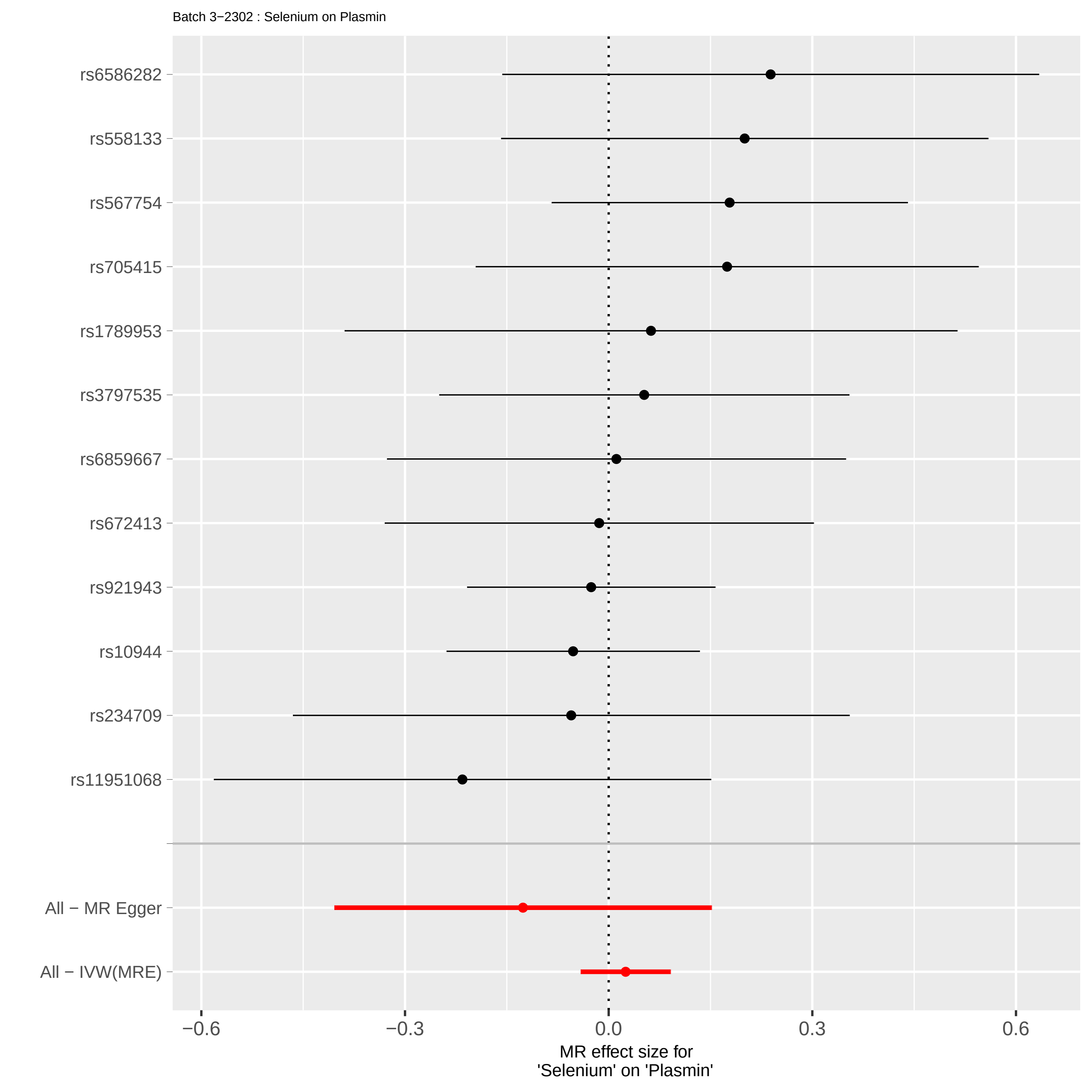

Batch 3−2302 : Selenium on Plasmin
rs6586282
rs558133
rs567754
rs705415
rs1789953
rs3797535
rs6859667
rs672413
rs921943
rs10944
rs234709
rs11951068
All − MR Egger
All − IVW(MRE)
−0.6
−0.3
0.0
0.3
0.6
MR effect size for
'Selenium' on 'Plasmin'

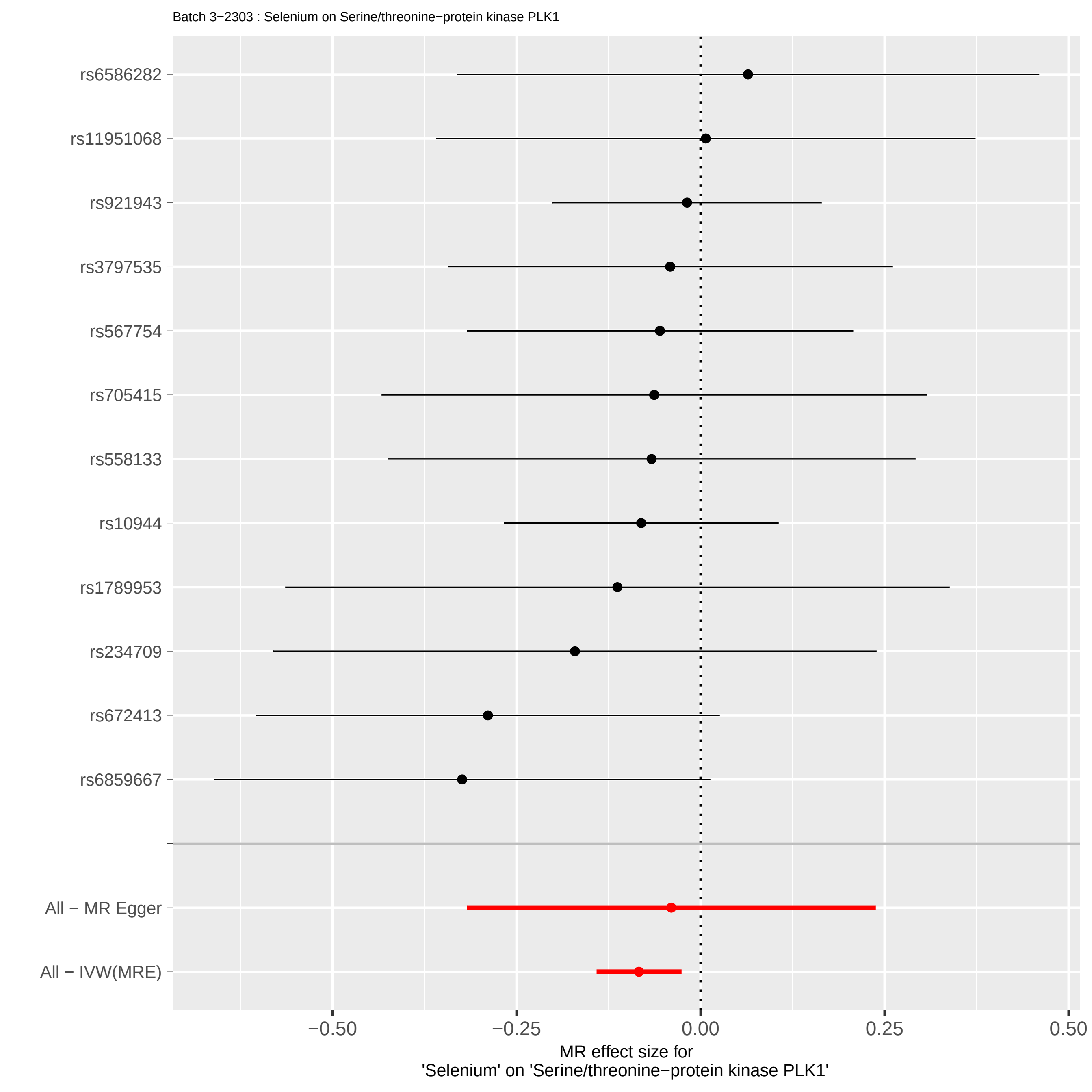

Batch 3−2303 : Selenium on Serine/threonine−protein kinase PLK1
rs6586282
rs11951068
rs921943
rs3797535
rs567754
rs705415
rs558133
rs10944
rs1789953
rs234709
rs672413
rs6859667
All − MR Egger
All − IVW(MRE)
−0.50
−0.25
0.00
0.25
0.50
MR effect size for
'Selenium' on 'Serine/threonine−protein kinase PLK1'

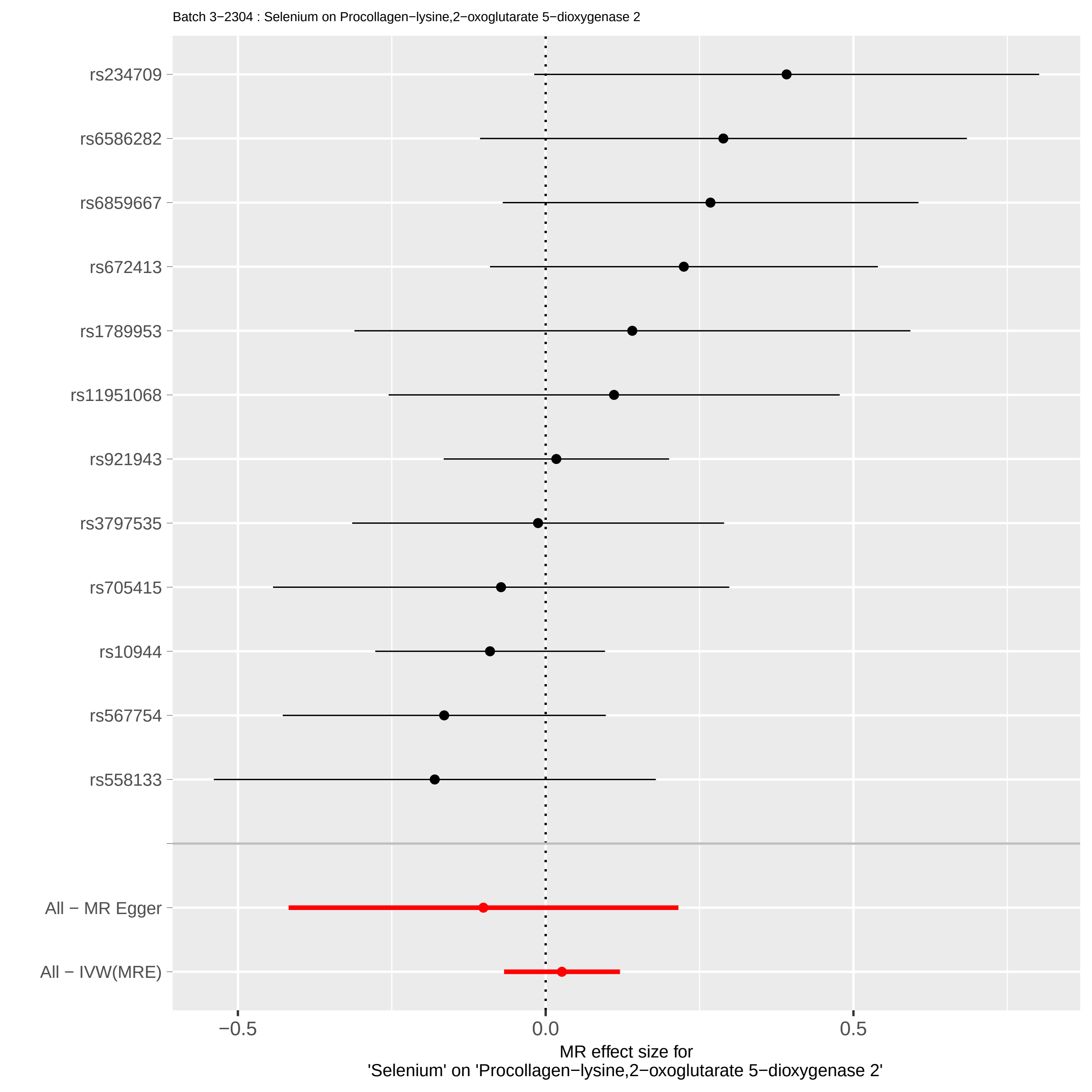

Batch 3−2304 : Selenium on Procollagen−lysine,2−oxoglutarate 5−dioxygenase 2
rs234709
rs6586282
rs6859667
rs672413
rs1789953
rs11951068
rs921943
rs3797535
rs705415
rs10944
rs567754
rs558133
All − MR Egger
All − IVW(MRE)
−0.5
0.0
0.5
MR effect size for
'Selenium' on 'Procollagen−lysine,2−oxoglutarate 5−dioxygenase 2'

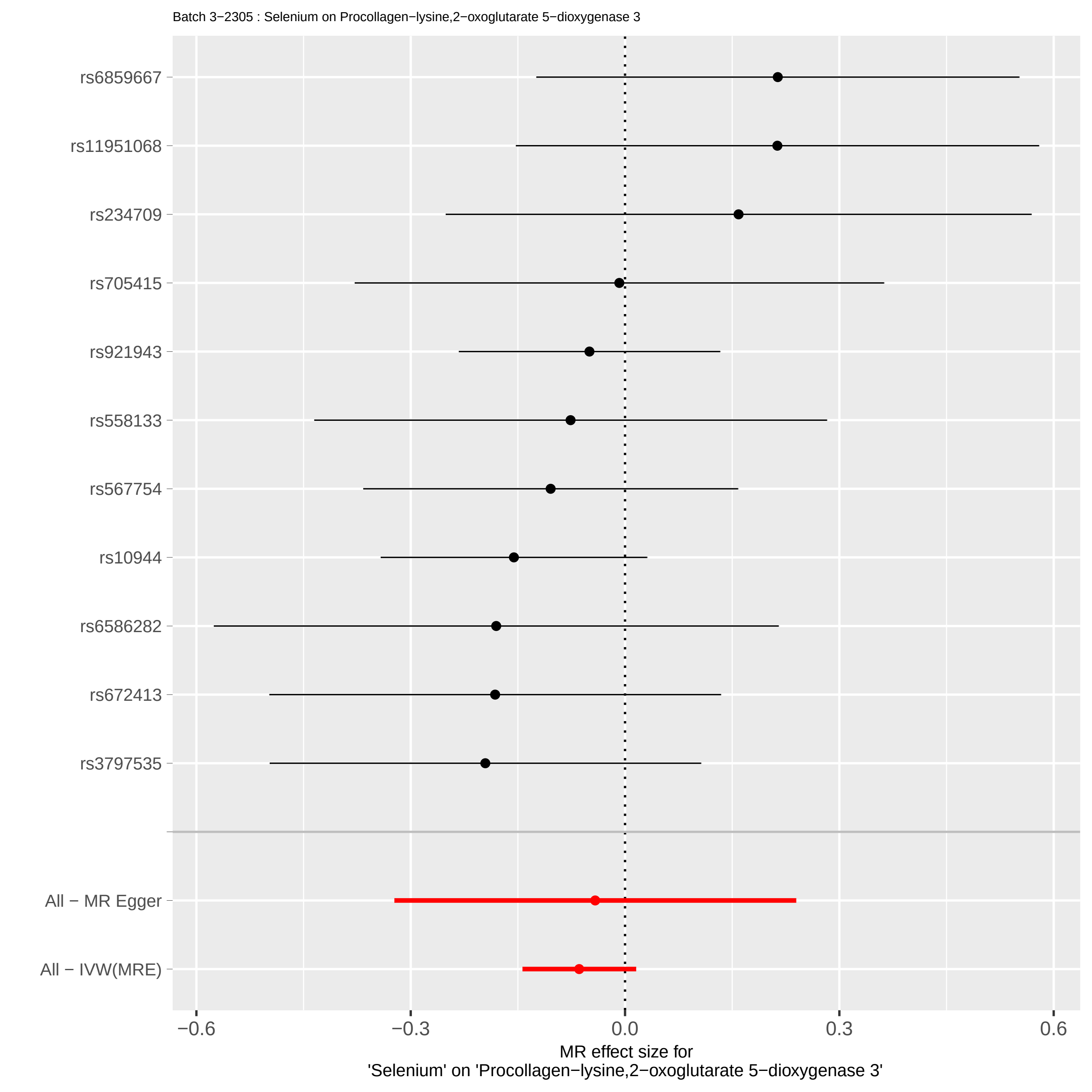
Batch 3−2305 : Selenium on Procollagen−lysine,2−oxoglutarate 5−dioxygenase 3
rs6859667
rs11951068
rs234709
rs705415
rs921943
rs558133
rs567754
rs10944
rs6586282
rs672413
rs3797535
All − MR Egger
All − IVW(MRE)
−0.6
−0.3
0.0
0.3
0.6
MR effect size for
'Selenium' on 'Procollagen−lysine,2−oxoglutarate 5−dioxygenase 3'

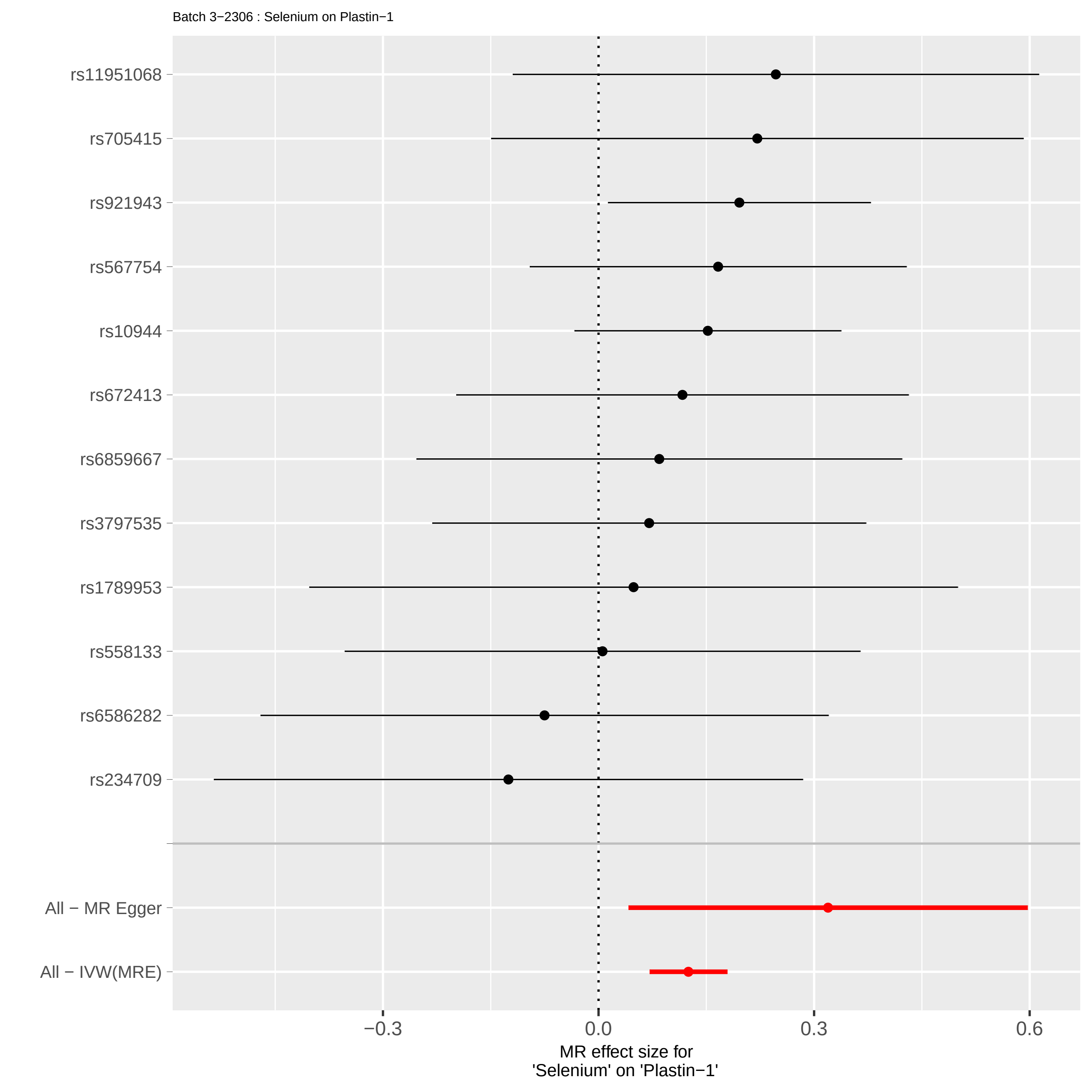

Batch 3−2306 : Selenium on Plastin−1
rs11951068
rs705415
rs921943
rs567754
rs10944
rs672413
rs6859667
rs3797535
rs1789953
rs558133
rs6586282
rs234709
All − MR Egger
All − IVW(MRE)
−0.3
0.0
0.3
0.6
MR effect size for
'Selenium' on 'Plastin−1'

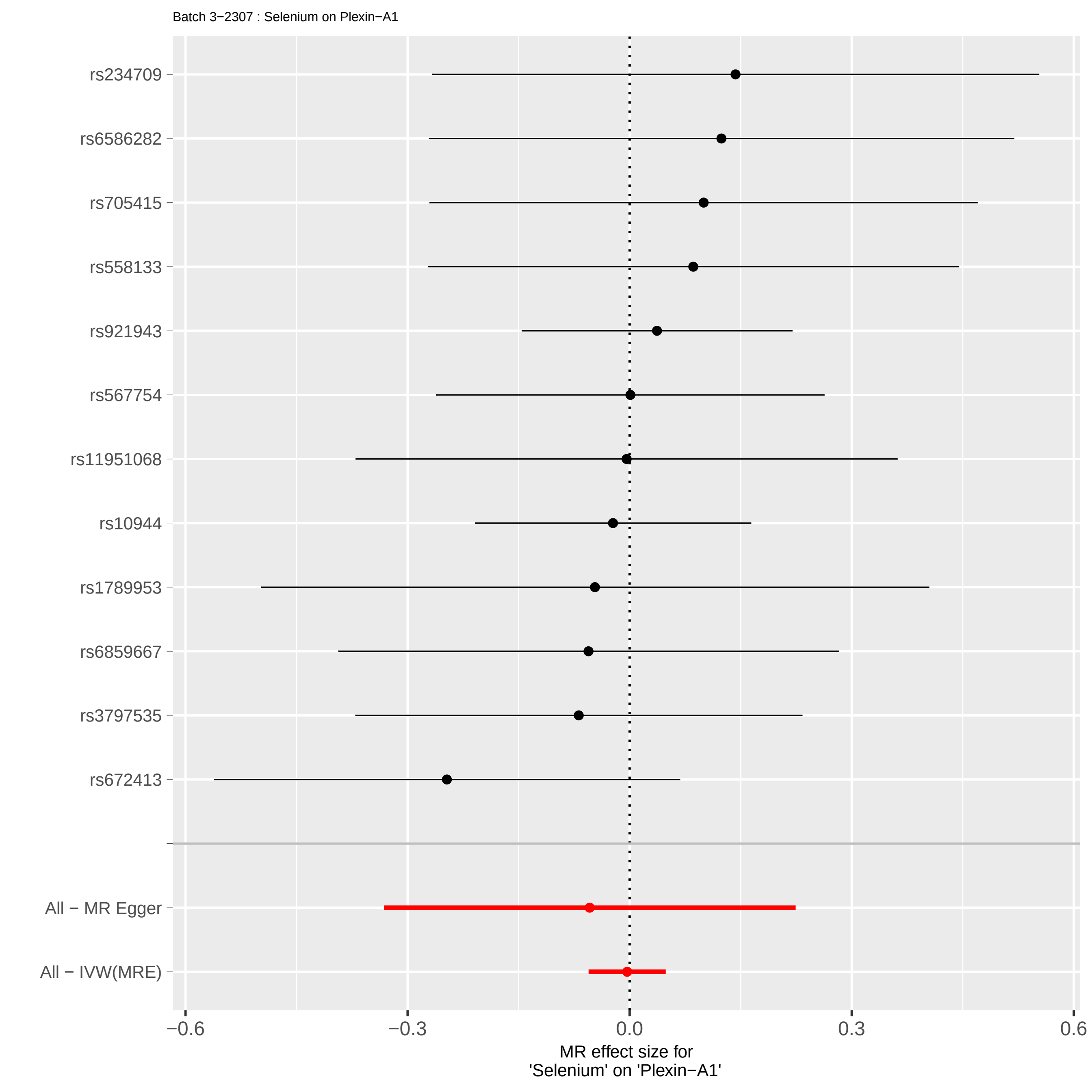

Batch 3−2307 : Selenium on Plexin−A1
rs234709
rs6586282
rs705415
rs558133
rs921943
rs567754
rs11951068
rs10944
rs1789953
rs6859667
rs3797535
rs672413
All − MR Egger
All − IVW(MRE)
−0.6
−0.3
0.0
0.3
0.6
MR effect size for
'Selenium' on 'Plexin−A1'

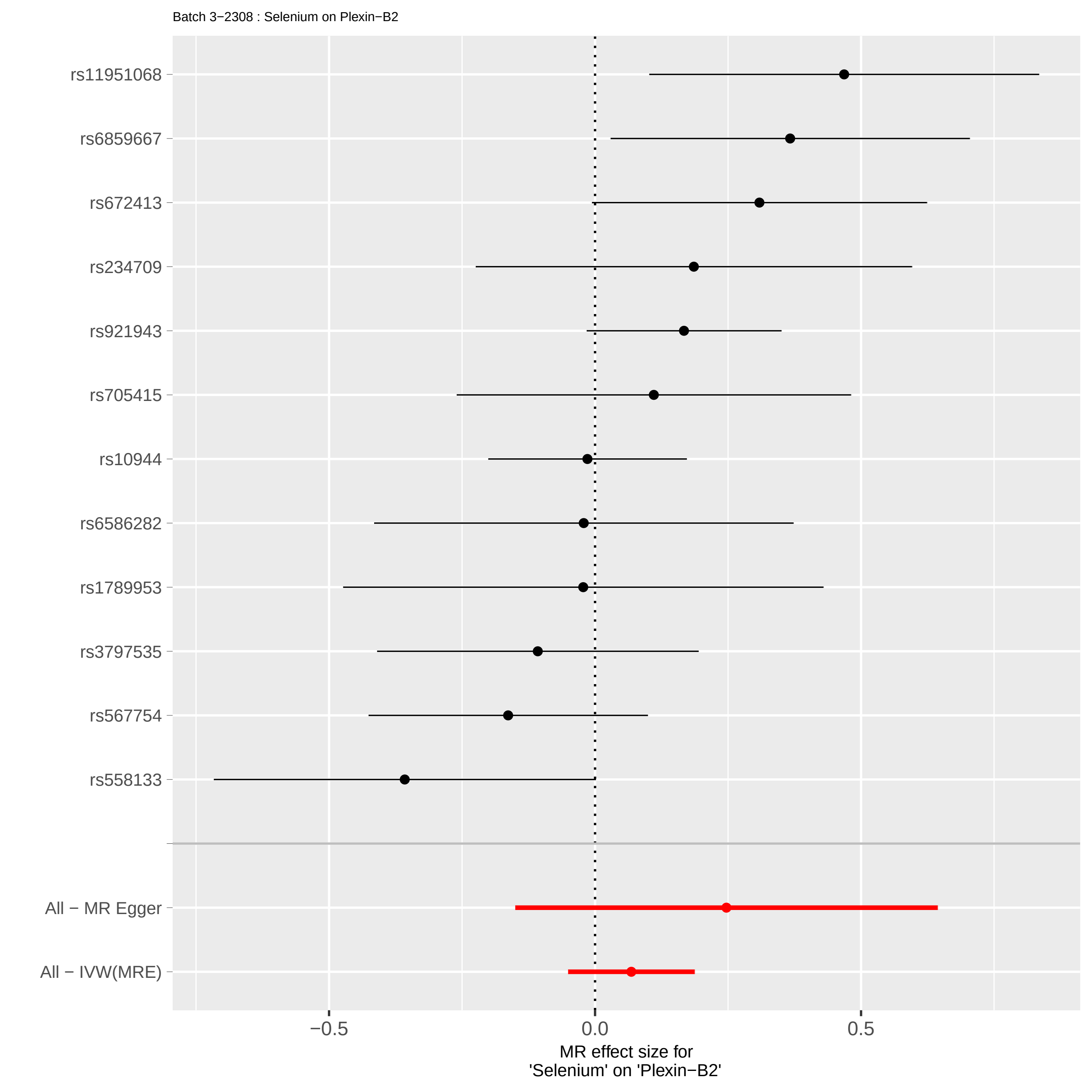

Batch 3−2308 : Selenium on Plexin−B2
rs11951068
rs6859667
rs672413
rs234709
rs921943
rs705415
rs10944
rs6586282
rs1789953
rs3797535
rs567754
rs558133
All − MR Egger
All − IVW(MRE)
−0.5
0.0
0.5
MR effect size for
'Selenium' on 'Plexin−B2'

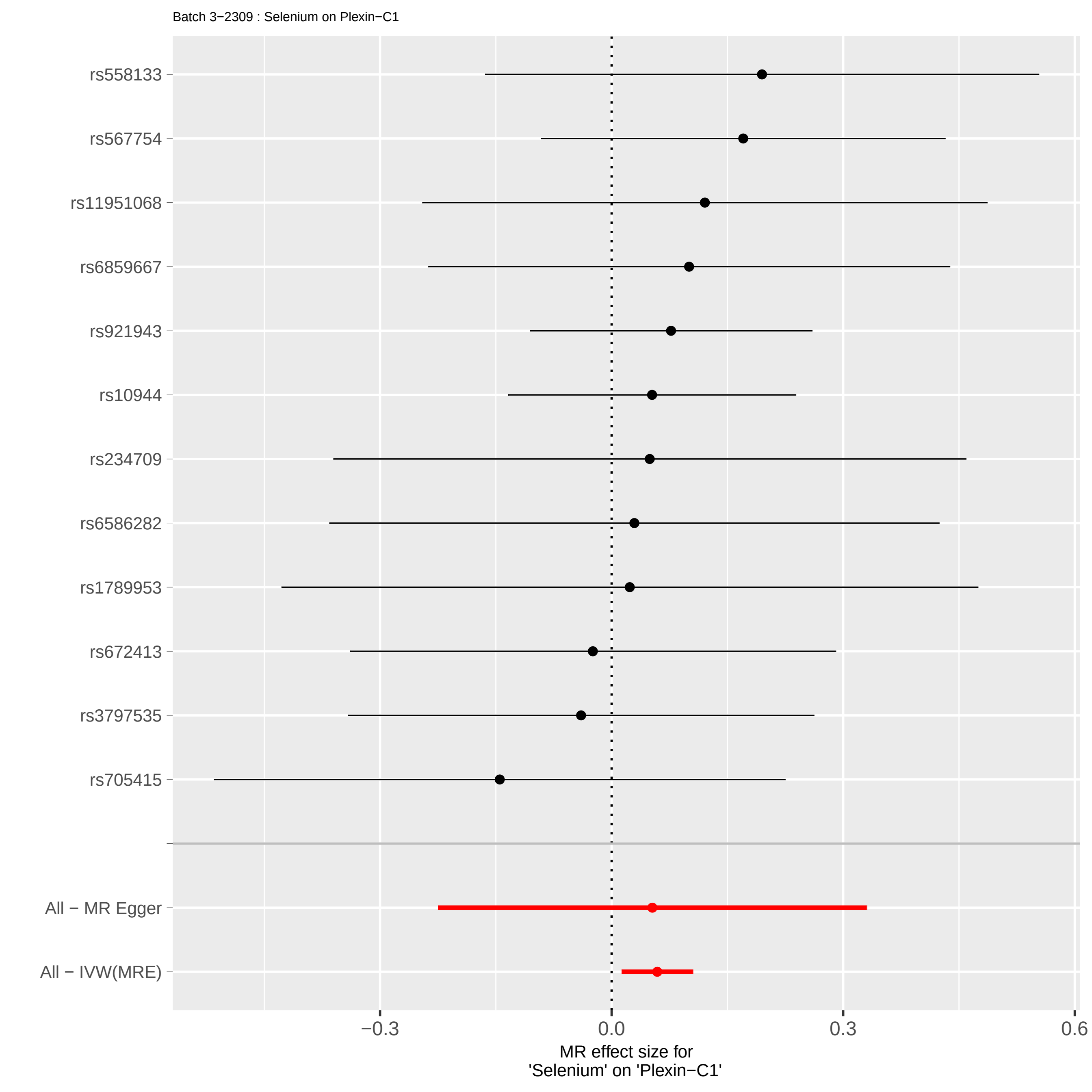

Batch 3−2309 : Selenium on Plexin−C1
rs558133
rs567754
rs11951068
rs6859667
rs921943
rs10944
rs234709
rs6586282
rs1789953
rs672413
rs3797535
rs705415
All − MR Egger
All − IVW(MRE)
−0.3
0.0
0.3
0.6
MR effect size for
'Selenium' on 'Plexin−C1'

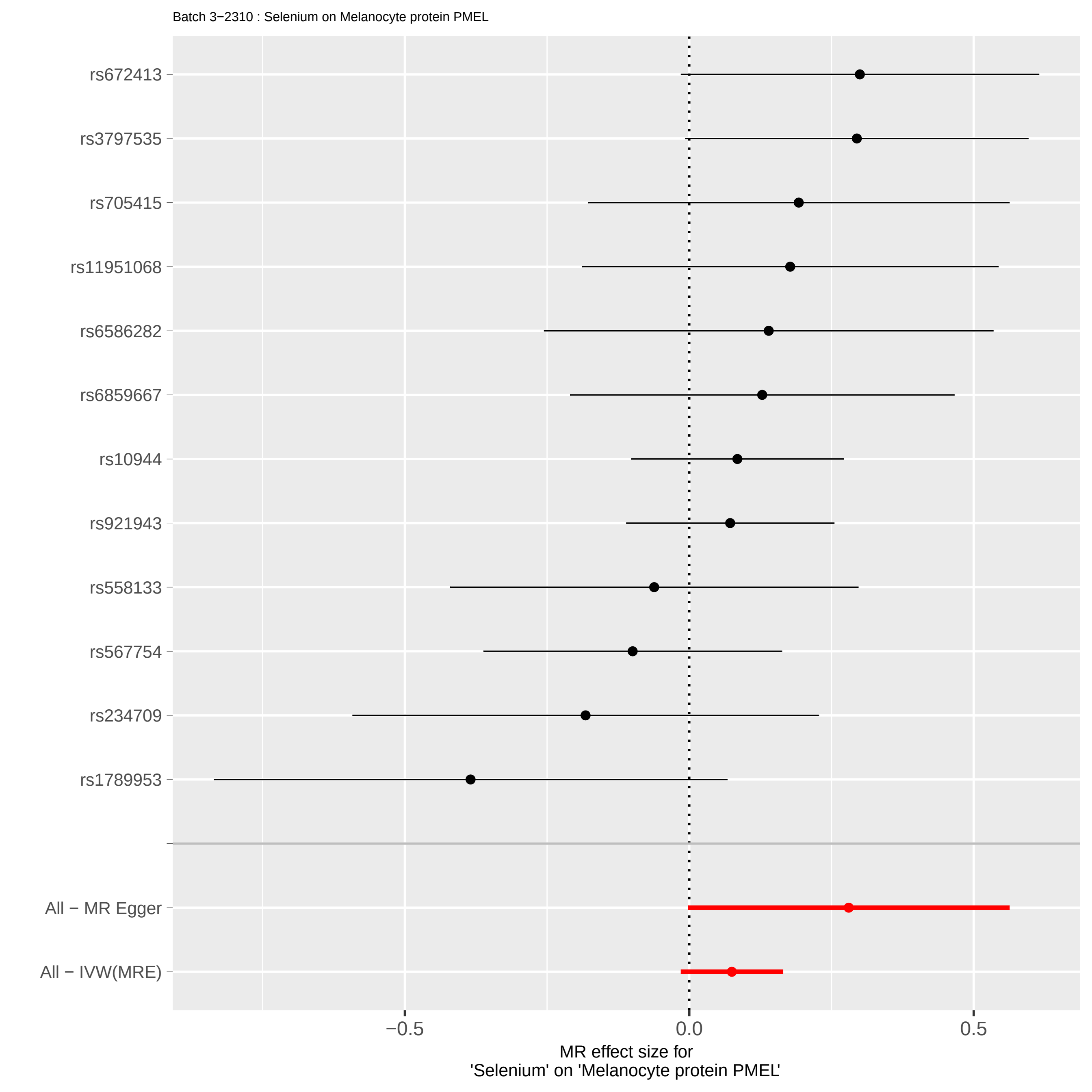

Batch 3−2310 : Selenium on Melanocyte protein PMEL
rs672413
rs3797535
rs705415
rs11951068
rs6586282
rs6859667
rs10944
rs921943
rs558133
rs567754
rs234709
rs1789953
All − MR Egger
All − IVW(MRE)
−0.5
0.0
0.5
MR effect size for
'Selenium' on 'Melanocyte protein PMEL'

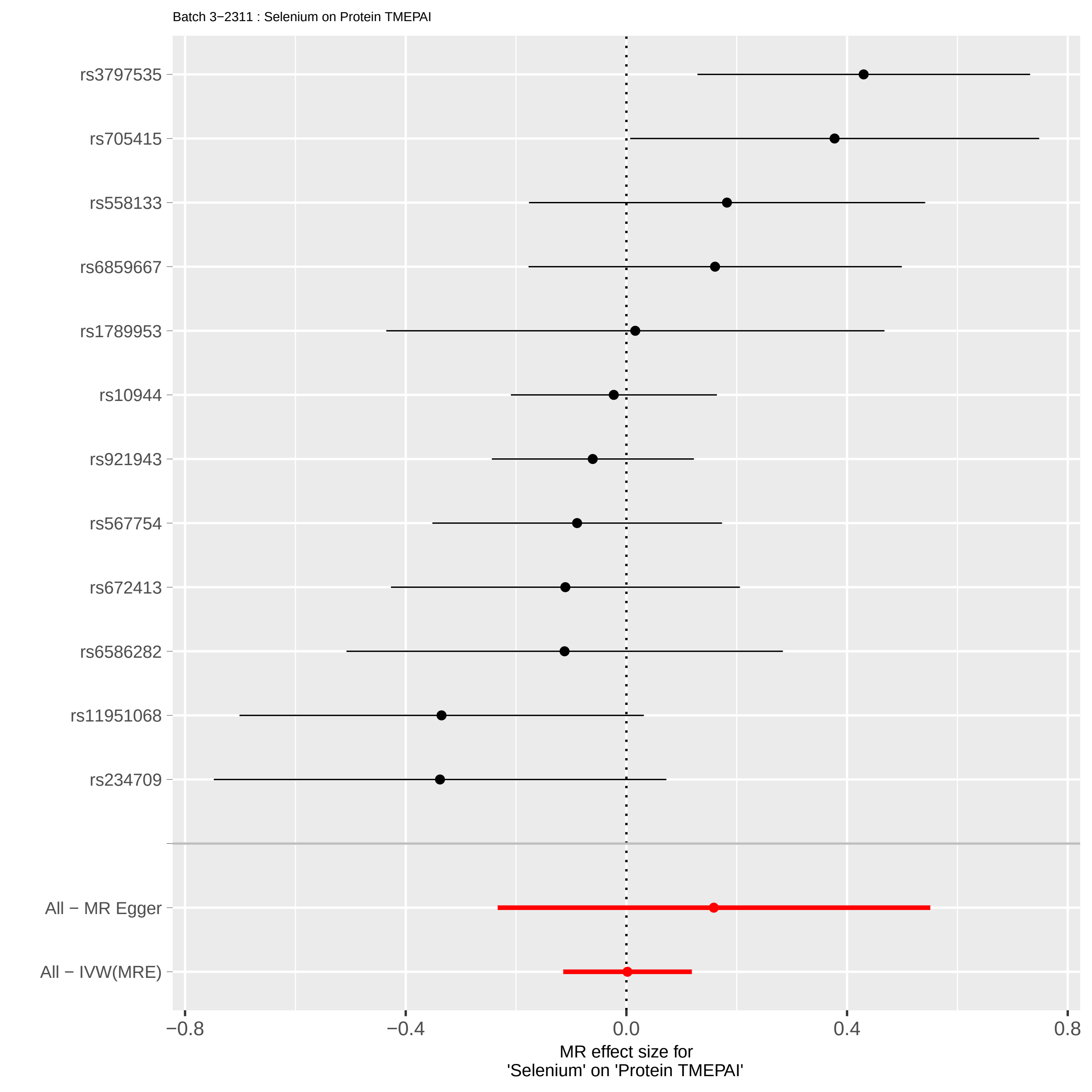

Batch 3−2311 : Selenium on Protein TMEPAI
rs3797535
rs705415
rs558133
rs6859667
rs1789953
rs10944
rs921943
rs567754
rs672413
rs6586282
rs11951068
rs234709
All − MR Egger
All − IVW(MRE)
−0.8
−0.4
0.0
0.4
0.8
MR effect size for
'Selenium' on 'Protein TMEPAI'

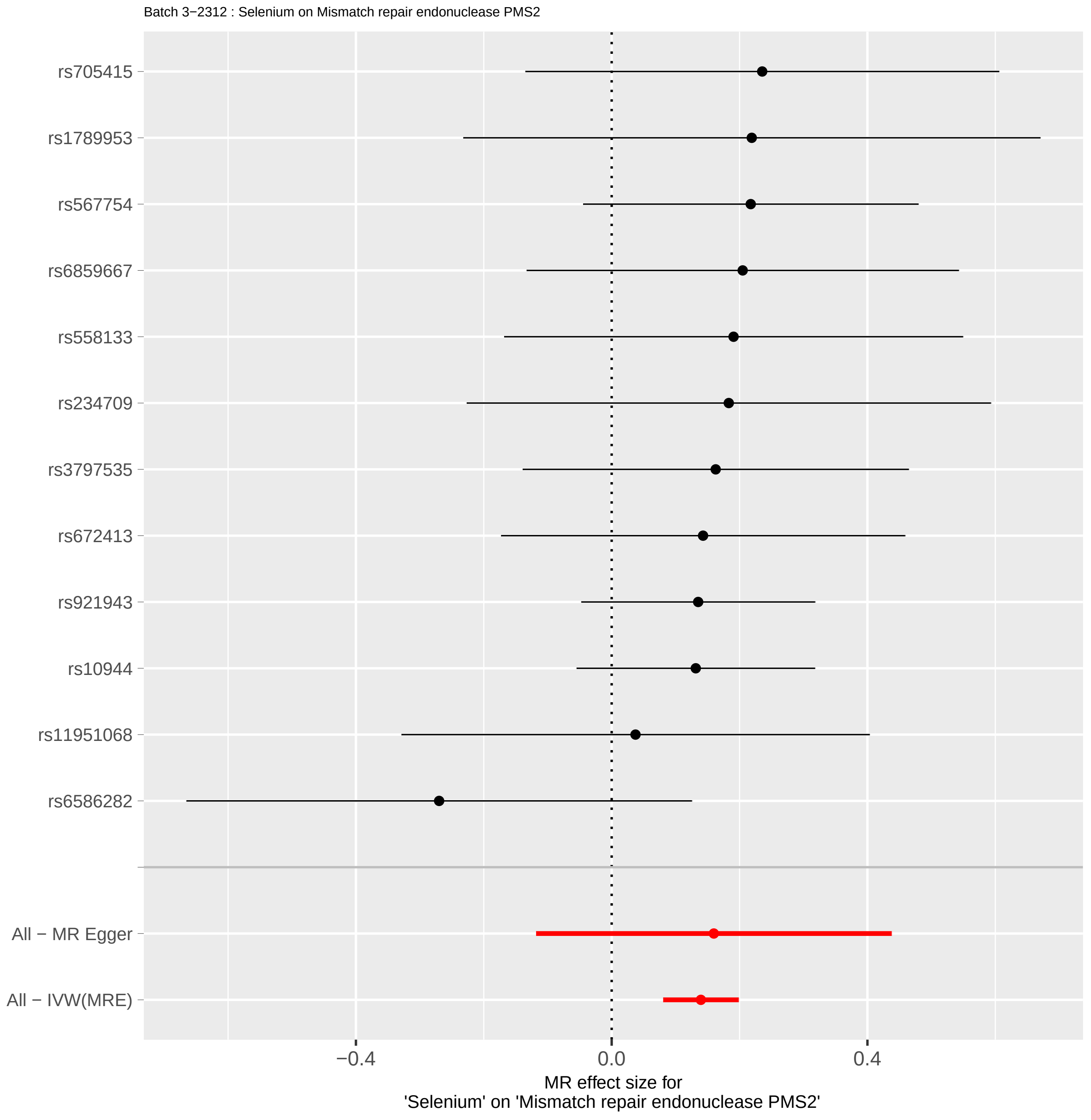
Batch 3−2312 : Selenium on Mismatch repair endonuclease PMS2
rs705415
rs1789953
rs567754
rs6859667
rs558133
rs234709
rs3797535
rs672413
rs921943
rs10944
rs11951068
rs6586282
All − MR Egger
All − IVW(MRE)
−0.4
0.0
0.4
MR effect size for
'Selenium' on 'Mismatch repair endonuclease PMS2'

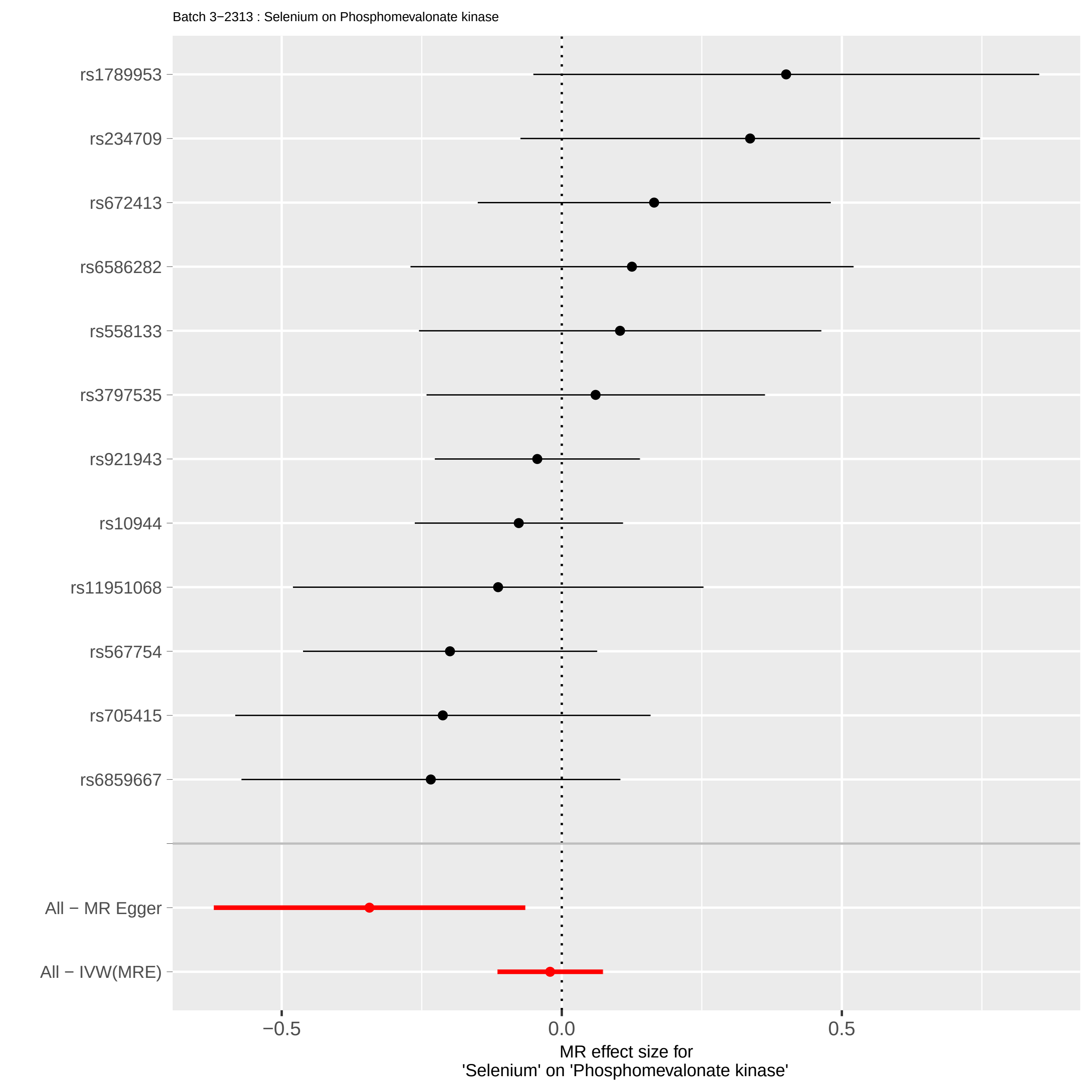
Batch 3−2313 : Selenium on Phosphomevalonate kinase
rs1789953
rs234709
rs672413
rs6586282
rs558133
rs3797535
rs921943
rs10944
rs11951068
rs567754
rs705415
rs6859667
All − MR Egger
All − IVW(MRE)
−0.5
0.0
0.5
MR effect size for
'Selenium' on 'Phosphomevalonate kinase'

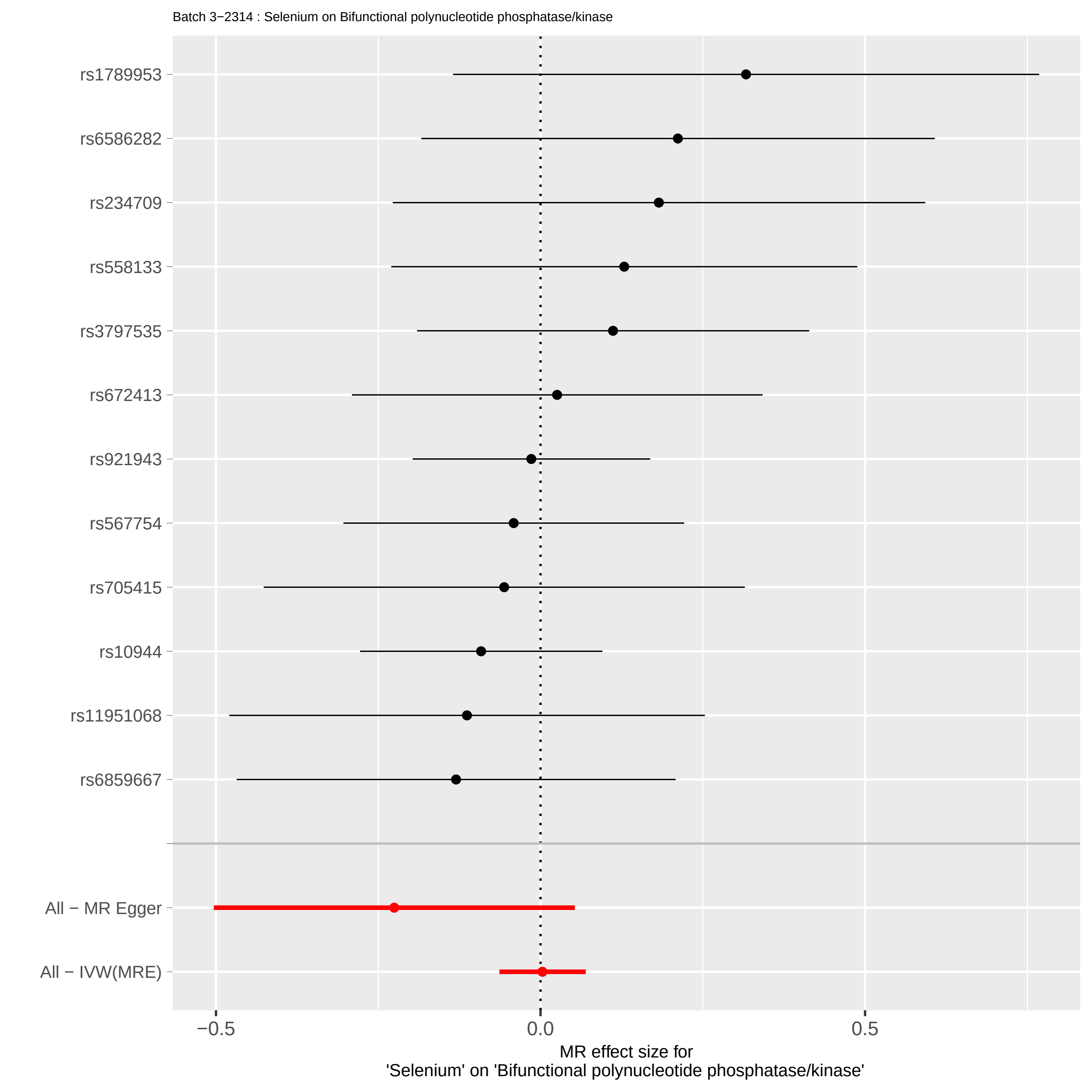
Batch 3−2314 : Selenium on Bifunctional polynucleotide phosphatase/kinase
rs1789953
rs6586282
rs234709
rs558133
rs3797535
rs672413
rs921943
rs567754
rs705415
rs10944
rs11951068
rs6859667
All − MR Egger
All − IVW(MRE)
−0.5
0.0
0.5
MR effect size for
'Selenium' on 'Bifunctional polynucleotide phosphatase/kinase'

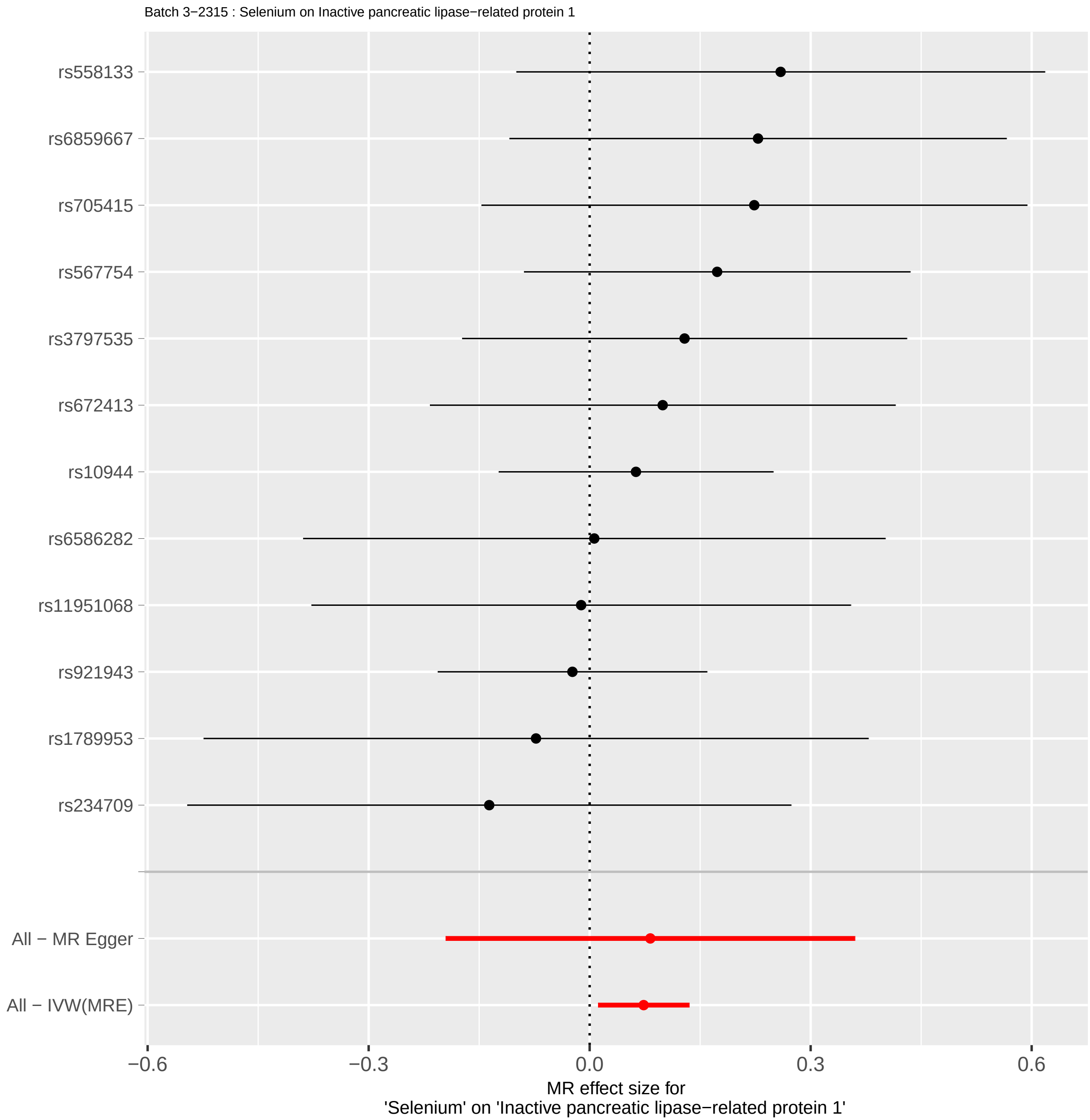

Batch 3−2315 : Selenium on Inactive pancreatic lipase−related protein 1
rs558133
rs6859667
rs705415
rs567754
rs3797535
rs672413
rs10944
rs6586282
rs11951068
rs921943
rs1789953
rs234709
All − MR Egger
All − IVW(MRE)
−0.6
−0.3
0.0
0.3
0.6
MR effect size for
'Selenium' on 'Inactive pancreatic lipase−related protein 1'

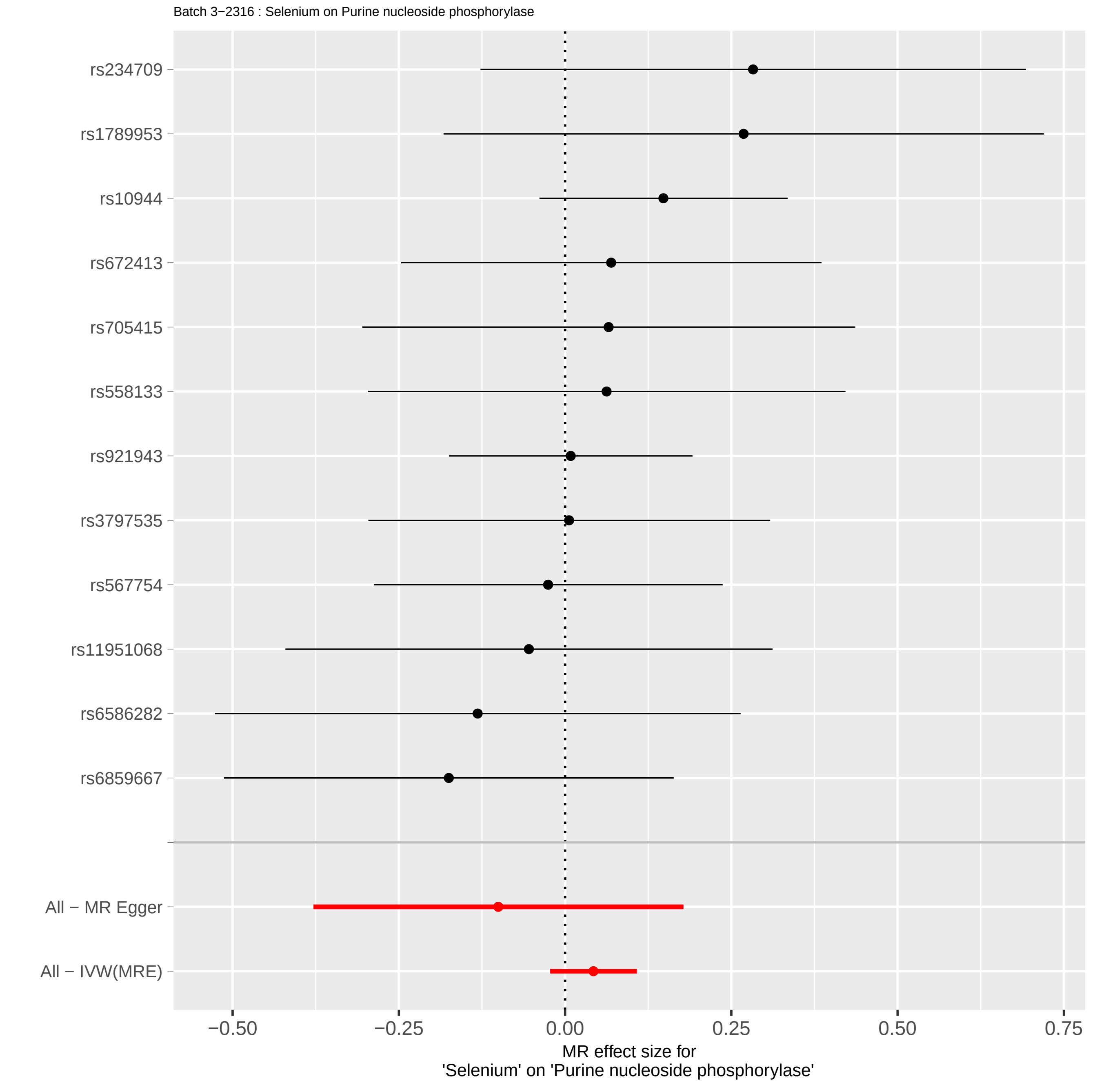

Batch 3−2316 : Selenium on Purine nucleoside phosphorylase
rs234709
rs1789953
rs10944
rs672413
rs705415
rs558133
rs921943
rs3797535
rs567754
rs11951068
rs6586282
rs6859667
All − MR Egger
All − IVW(MRE)
−0.50
−0.25
0.00
0.25
0.50
0.75
MR effect size for
'Selenium' on 'Purine nucleoside phosphorylase'

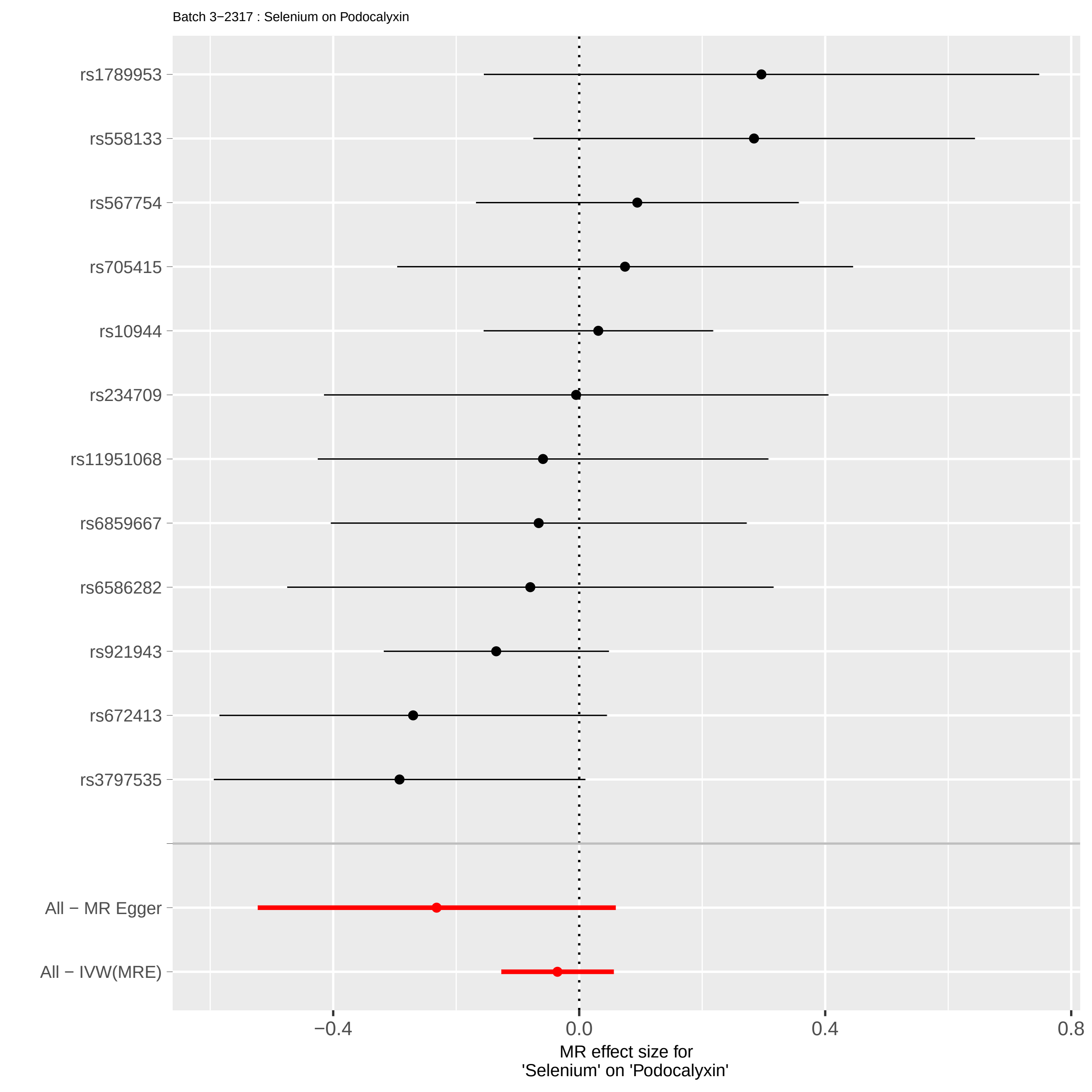
Batch 3−2317 : Selenium on Podocalyxin
rs1789953
rs558133
rs567754
rs705415
rs10944
rs234709
rs11951068
rs6859667
rs6586282
rs921943
rs672413
rs3797535
All − MR Egger
All − IVW(MRE)
−0.4
0.0
0.4
0.8
MR effect size for
'Selenium' on 'Podocalyxin'

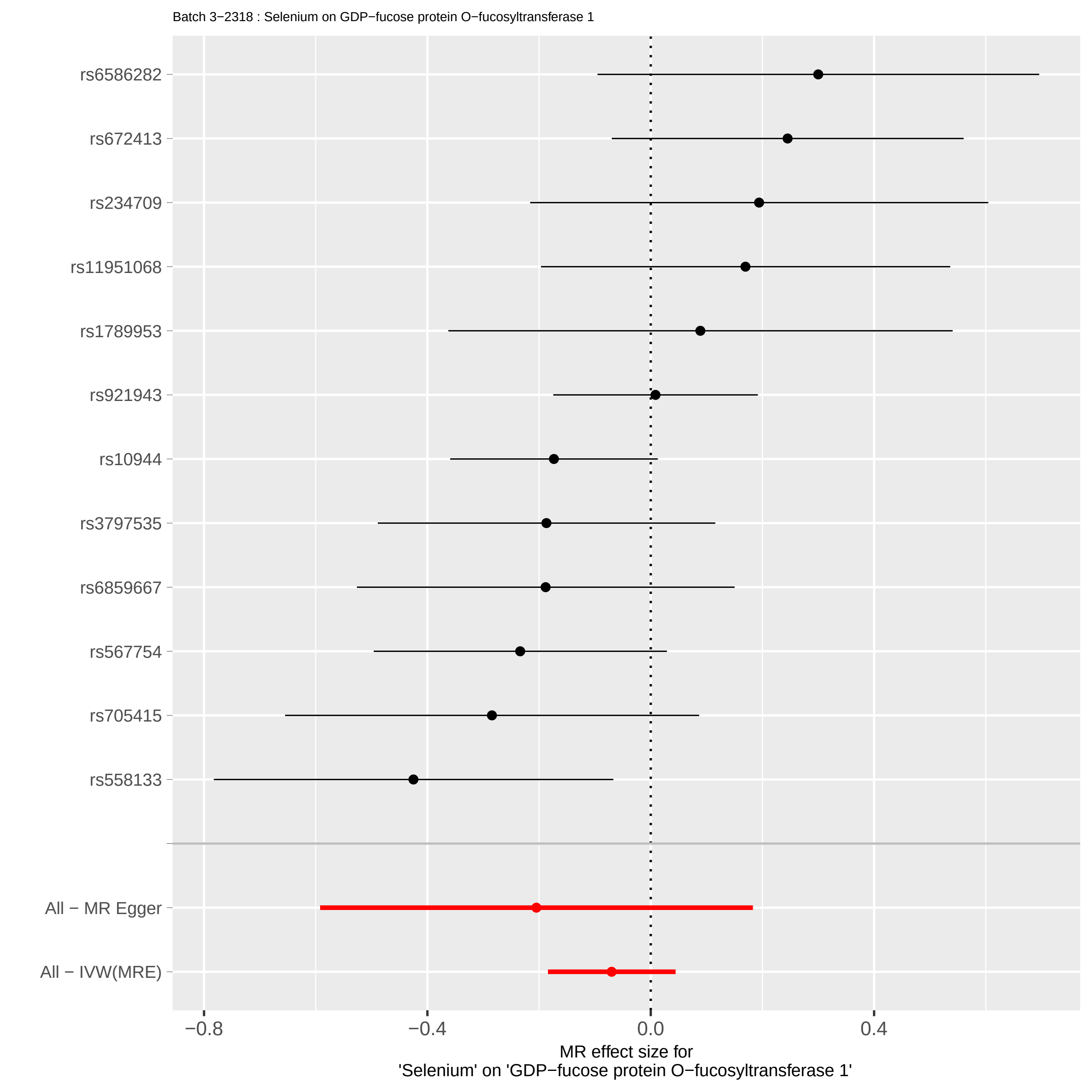

Batch 3−2318 : Selenium on GDP−fucose protein O−fucosyltransferase 1
rs6586282
rs672413
rs234709
rs11951068
rs1789953
rs921943
rs10944
rs3797535
rs6859667
rs567754
rs705415
rs558133
All − MR Egger
All − IVW(MRE)
−0.8
−0.4
0.0
0.4
MR effect size for
'Selenium' on 'GDP−fucose protein O−fucosyltransferase 1'

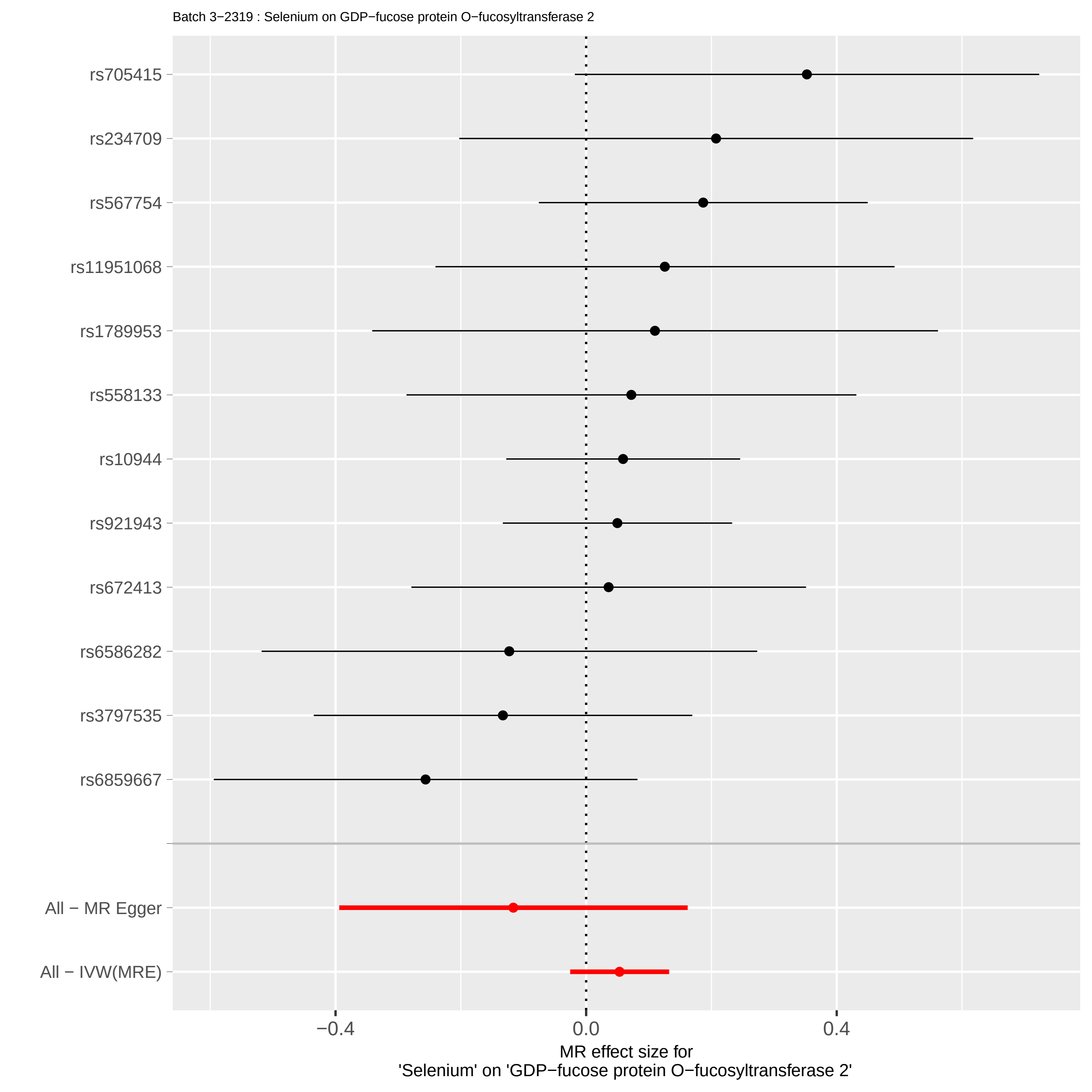
Batch 3−2319 : Selenium on GDP−fucose protein O−fucosyltransferase 2
rs705415
rs234709
rs567754
rs11951068
rs1789953
rs558133
rs10944
rs921943
rs672413
rs6586282
rs3797535
rs6859667
All − MR Egger
All − IVW(MRE)
−0.4
0.0
0.4
MR effect size for
'Selenium' on 'GDP−fucose protein O−fucosyltransferase 2'

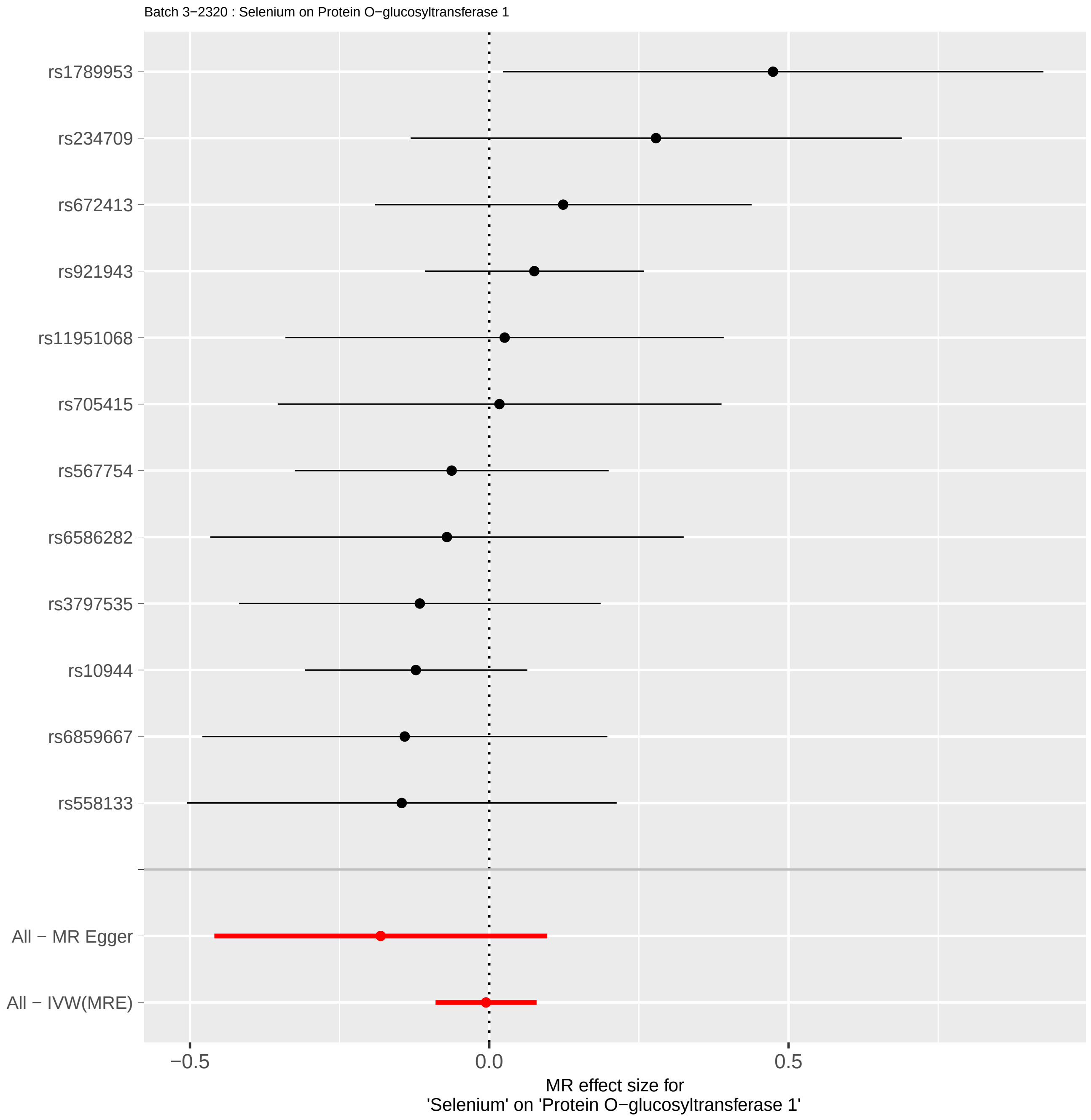

Batch 3−2320 : Selenium on Protein O−glucosyltransferase 1
rs1789953
rs234709
rs672413
rs921943
rs11951068
rs705415
rs567754
rs6586282
rs3797535
rs10944
rs6859667
rs558133
All − MR Egger
All − IVW(MRE)
−0.5
0.0
0.5
MR effect size for
'Selenium' on 'Protein O−glucosyltransferase 1'

Batch 3−2321 : Selenium on DNA polymerase eta

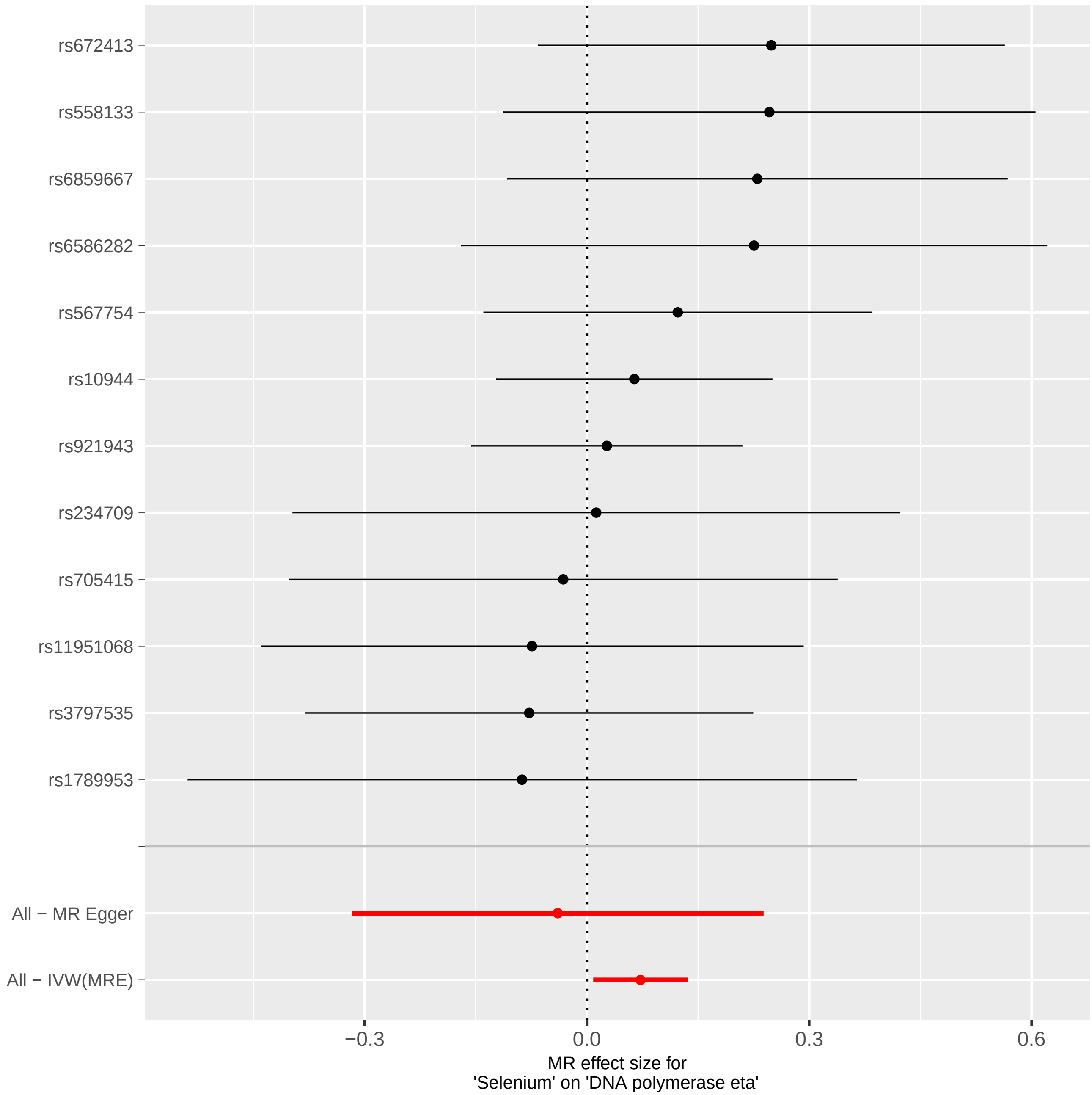

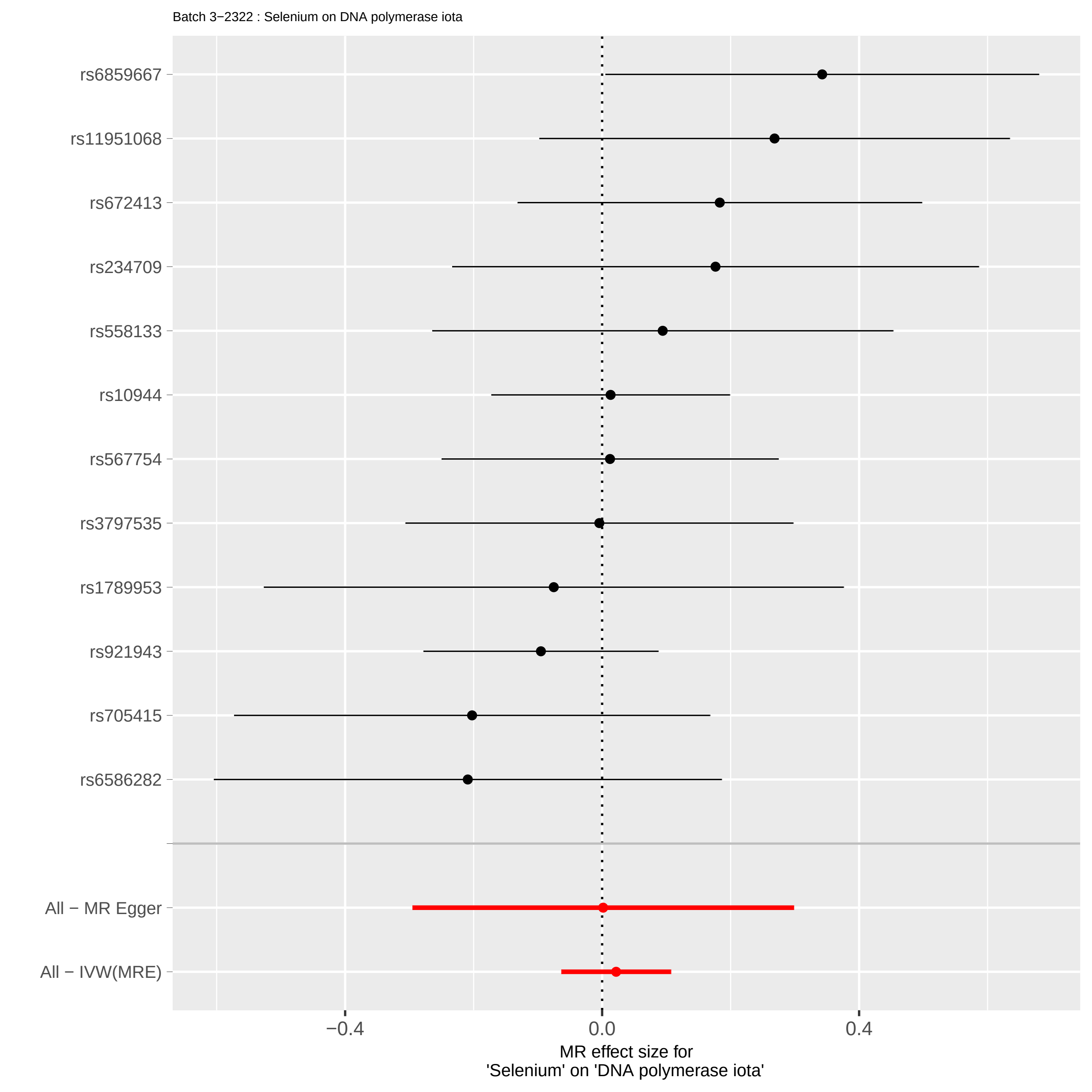

Batch 3−2322 : Selenium on DNA polymerase iota
rs6859667
rs11951068
rs672413
rs234709
rs558133
rs10944
rs567754
rs3797535
rs1789953
rs921943
rs705415
rs6586282
All − MR Egger
All − IVW(MRE)
−0.4
0.0
0.4
MR effect size for
'Selenium' on 'DNA polymerase iota'

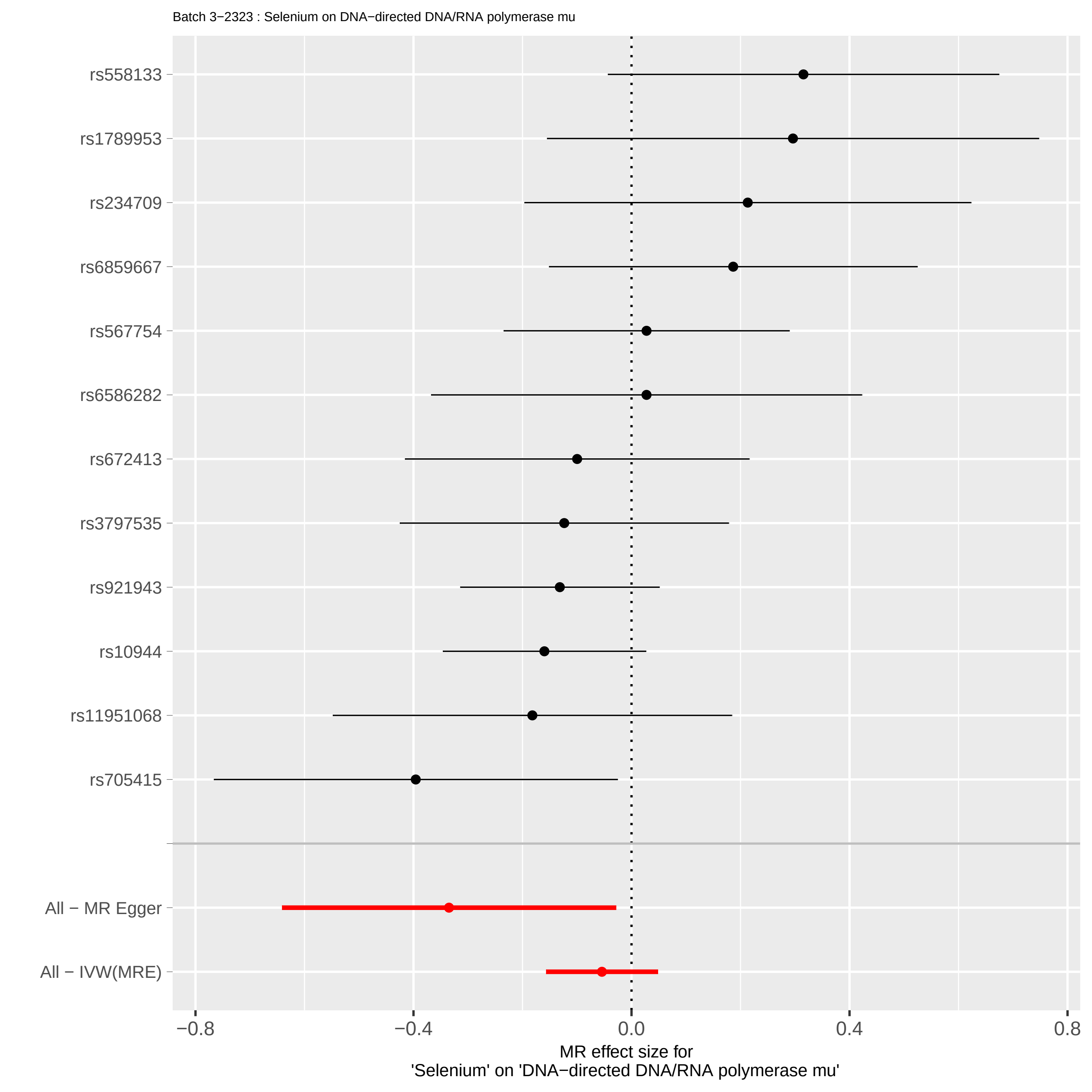

Batch 3−2323 : Selenium on DNA−directed DNA/RNA polymerase mu
rs558133
rs1789953
rs234709
rs6859667
rs567754
rs6586282
rs672413
rs3797535
rs921943
rs10944
rs11951068
rs705415
All − MR Egger
All − IVW(MRE)
−0.8
−0.4
0.0
0.4
0.8
MR effect size for
'Selenium' on 'DNA−directed DNA/RNA polymerase mu'

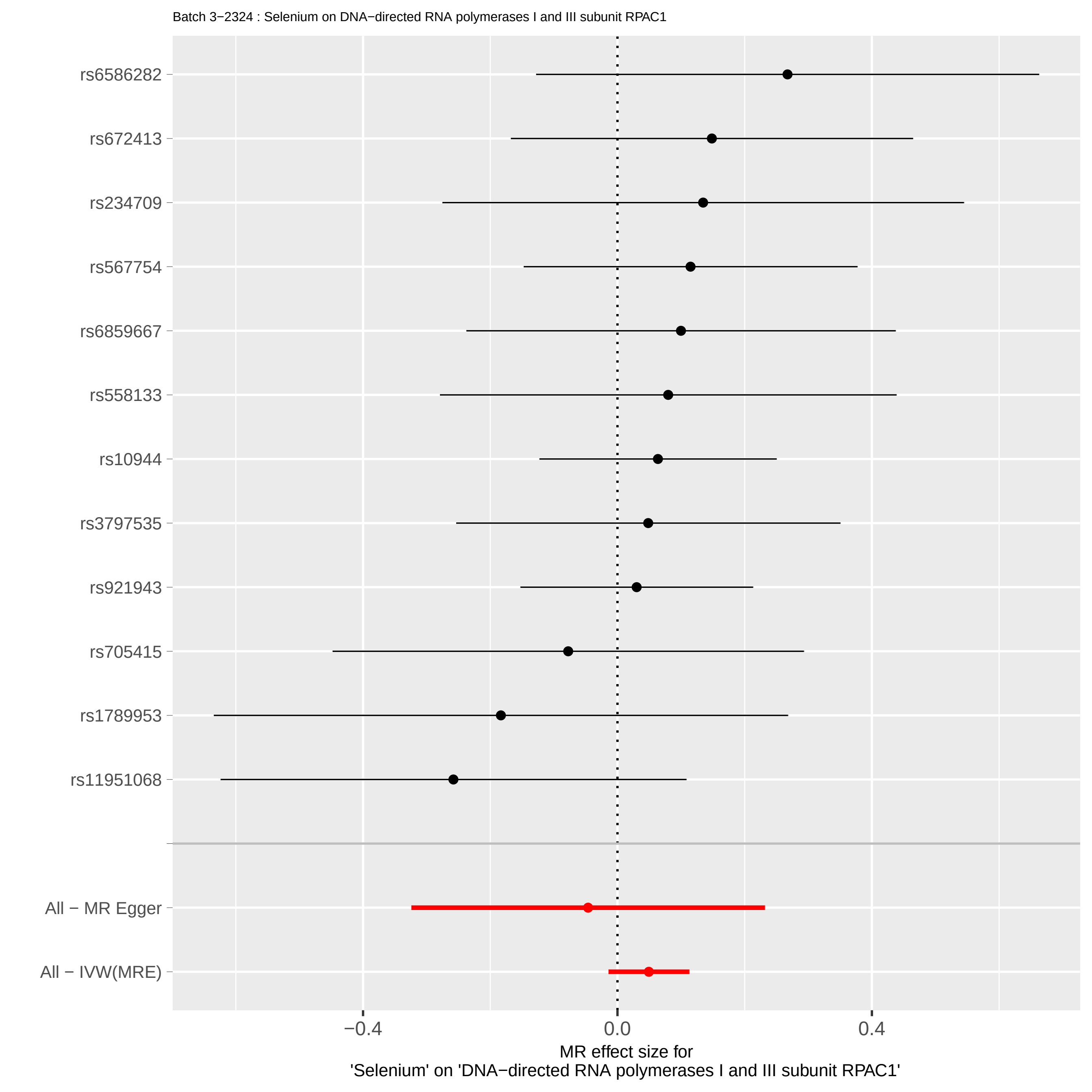

Batch 3−2324 : Selenium on DNA−directed RNA polymerases I and III subunit RPAC1
rs6586282
rs672413
rs234709
rs567754
rs6859667
rs558133
rs10944
rs3797535
rs921943
rs705415
rs1789953
rs11951068
All − MR Egger
All − IVW(MRE)
−0.4
0.0
0.4
MR effect size for
'Selenium' on 'DNA−directed RNA polymerases I and III subunit RPAC1'

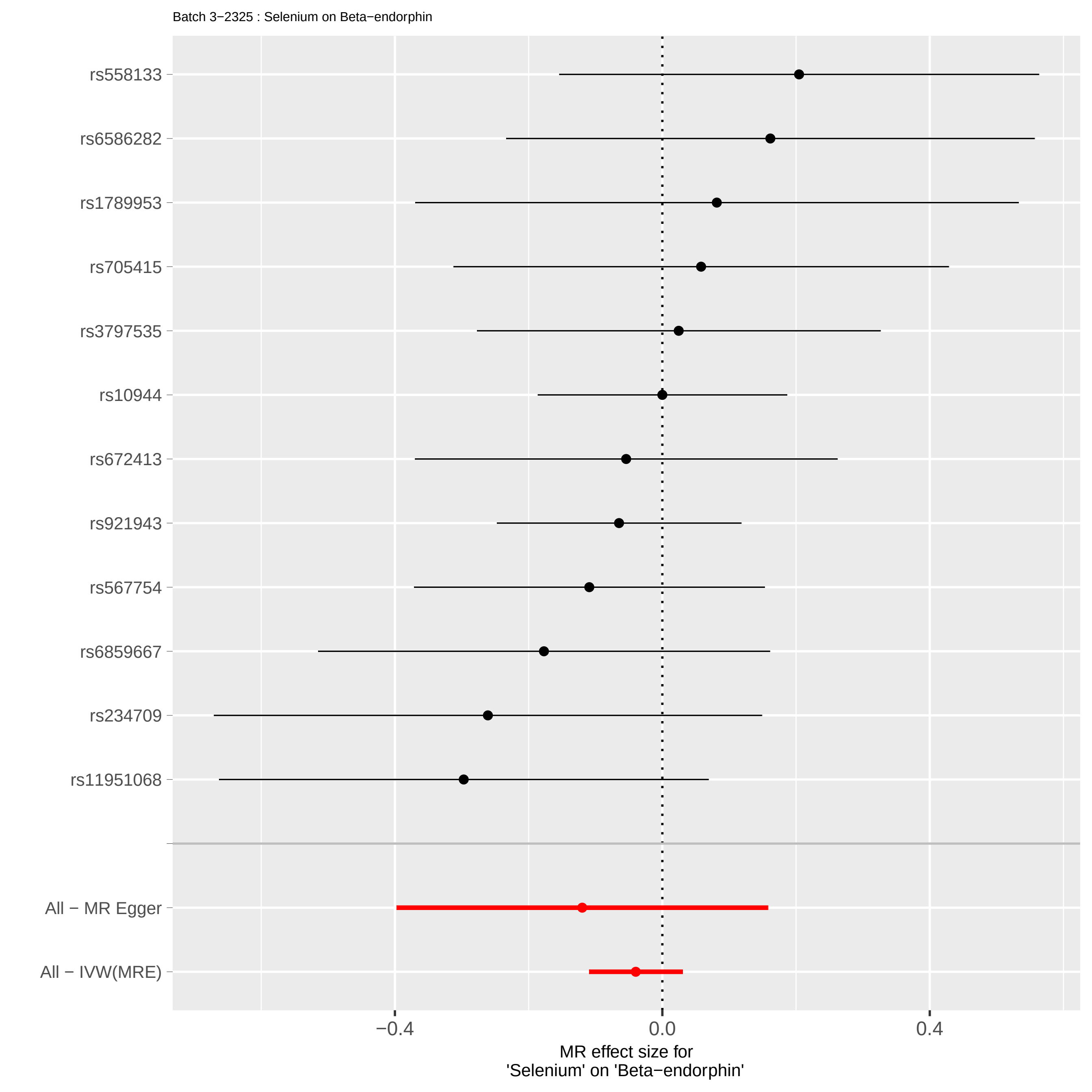

Batch 3−2325 : Selenium on Beta−endorphin
rs558133
rs6586282
rs1789953
rs705415
rs3797535
rs10944
rs672413
rs921943
rs567754
rs6859667
rs234709
rs11951068
All − MR Egger
All − IVW(MRE)
−0.4
0.0
0.4
MR effect size for
'Selenium' on 'Beta−endorphin'

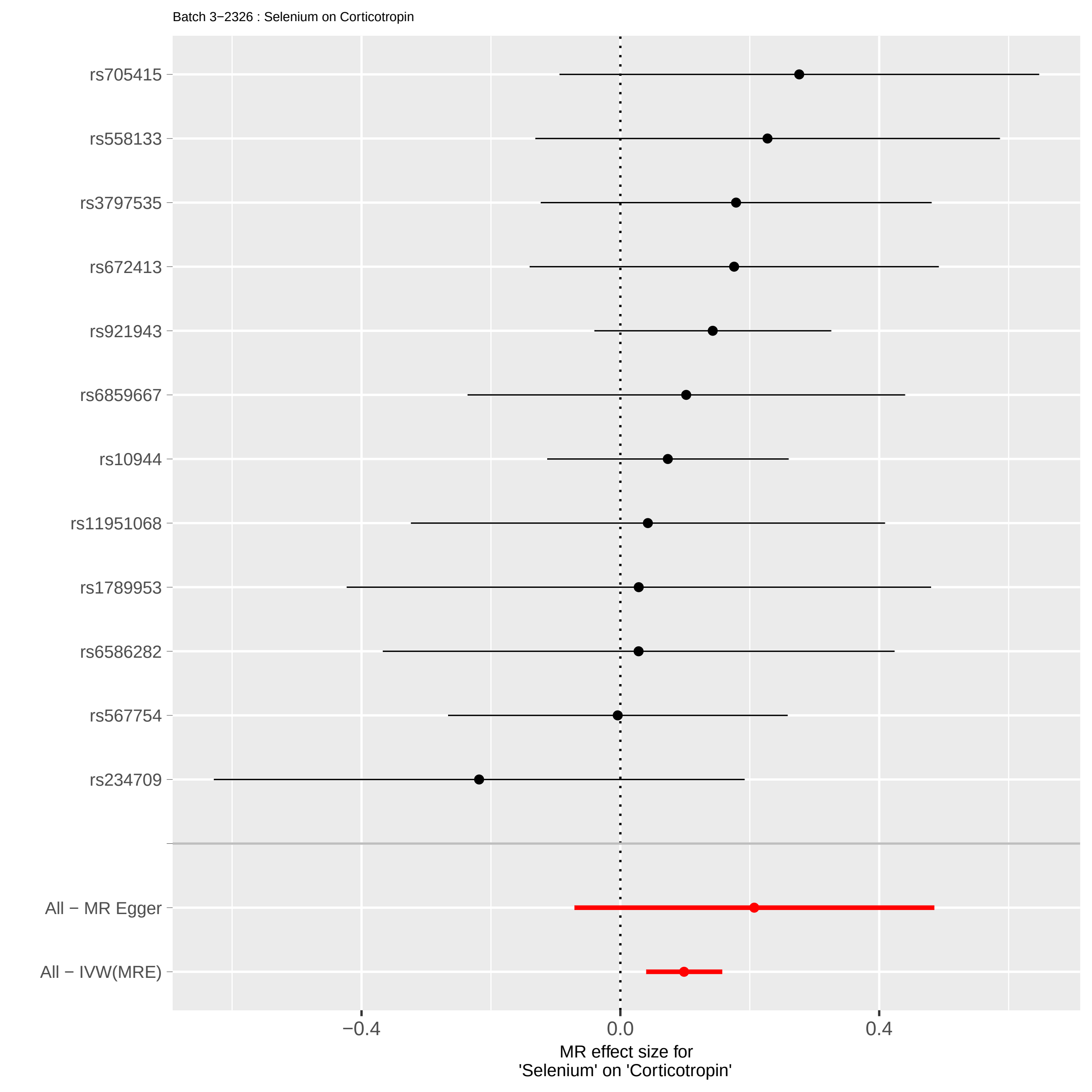

Batch 3−2326 : Selenium on Corticotropin
rs705415
rs558133
rs3797535
rs672413
rs921943
rs6859667
rs10944
rs11951068
rs1789953
rs6586282
rs567754
rs234709
All − MR Egger
All − IVW(MRE)
−0.4
0.0
0.4
MR effect size for
'Selenium' on 'Corticotropin'

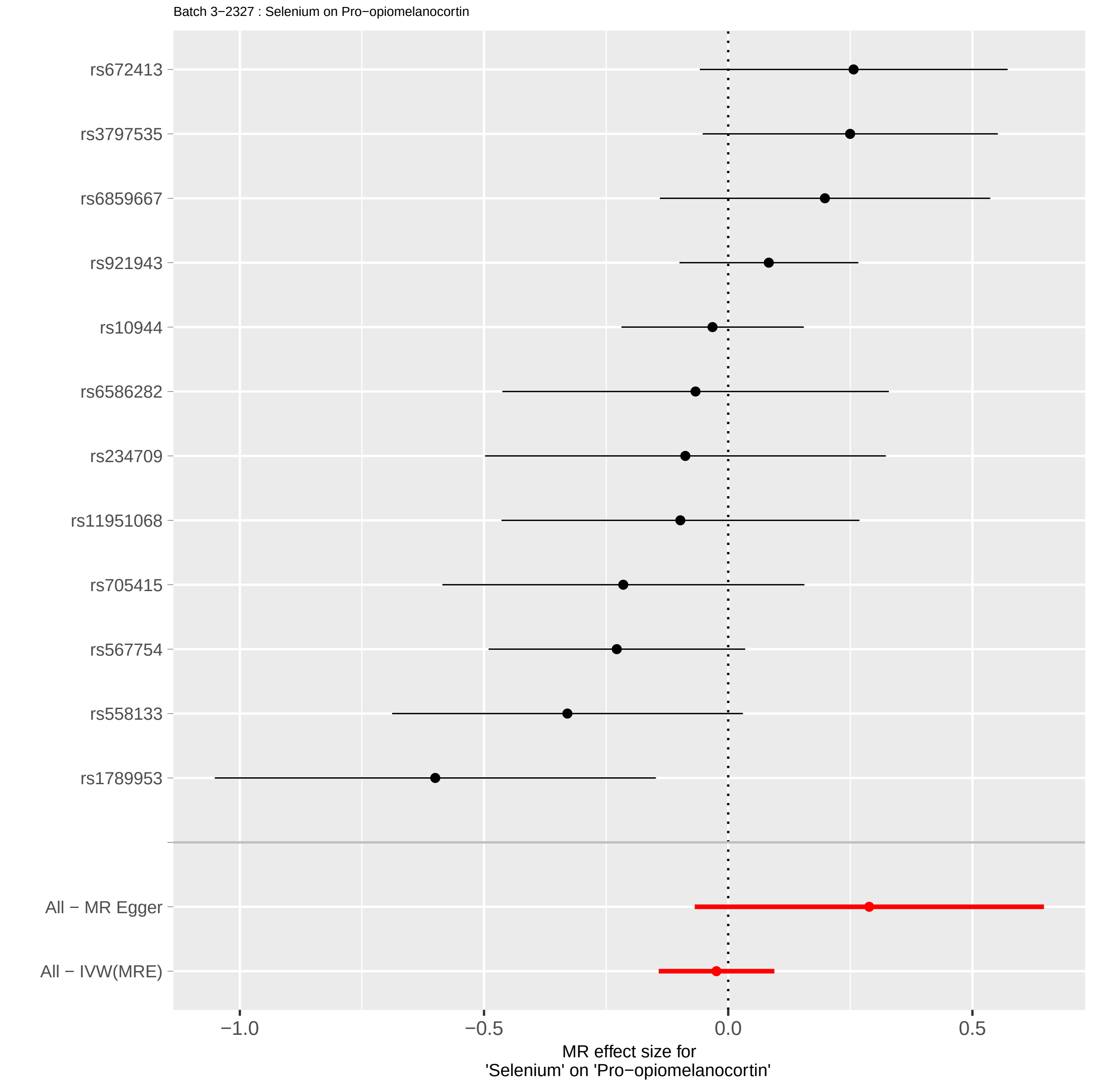

Batch 3−2327 : Selenium on Pro−opiomelanocortin
rs672413
rs3797535
rs6859667
rs921943
rs10944
rs6586282
rs234709
rs11951068
rs705415
rs567754
rs558133
rs1789953
All − MR Egger
All − IVW(MRE)
−1.0
−0.5
0.0
0.5
MR effect size for
'Selenium' on 'Pro−opiomelanocortin'

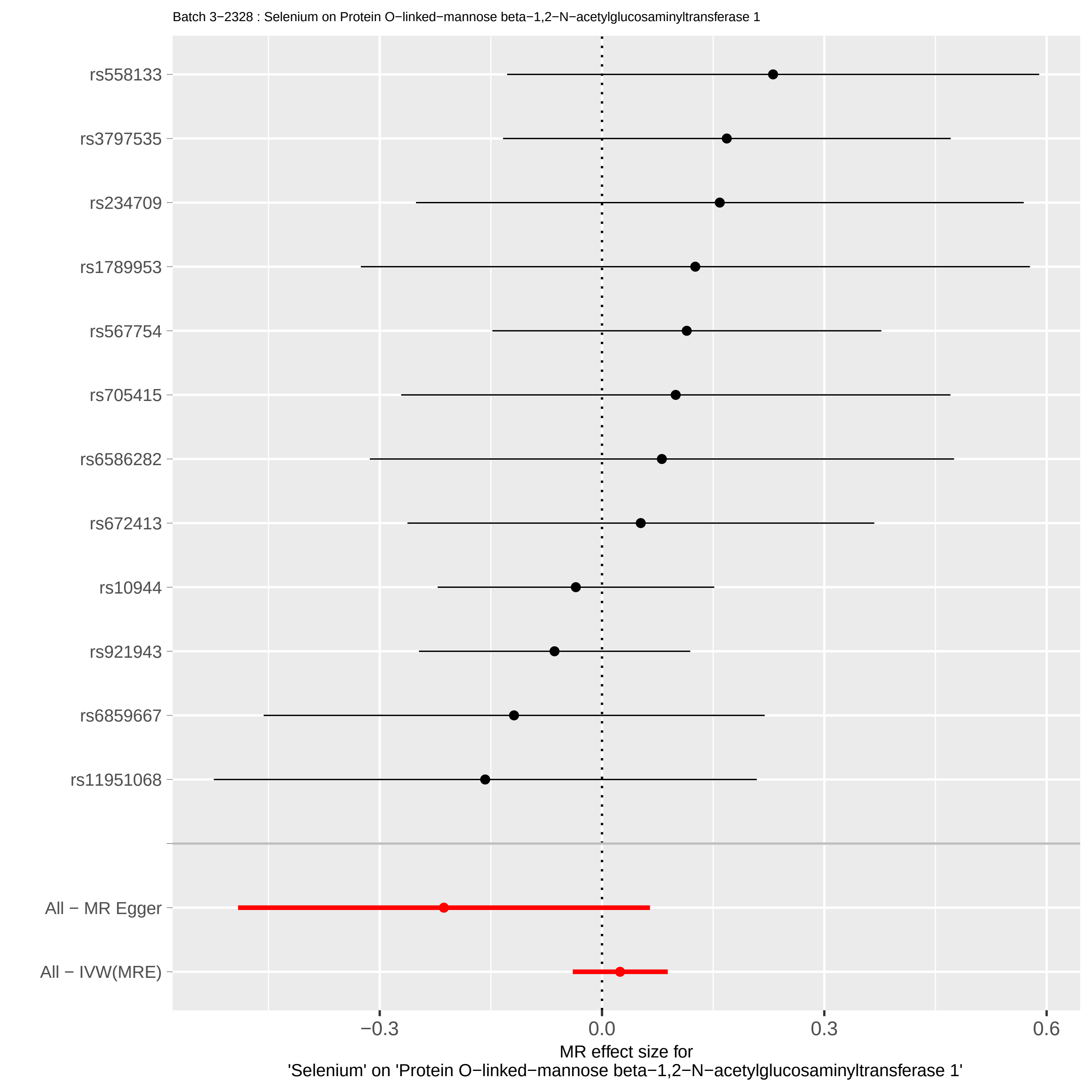

Batch 3−2328 : Selenium on Protein O−linked−mannose beta−1,2−N−acetylglucosaminyltransferase 1
rs558133
rs3797535
rs234709
rs1789953
rs567754
rs705415
rs6586282
rs672413
rs10944
rs921943
rs6859667
rs11951068
All − MR Egger
All − IVW(MRE)
−0.3
0.0
0.3
0.6
MR effect size for
'Selenium' on 'Protein O−linked−mannose beta−1,2−N−acetylglucosaminyltransferase 1'

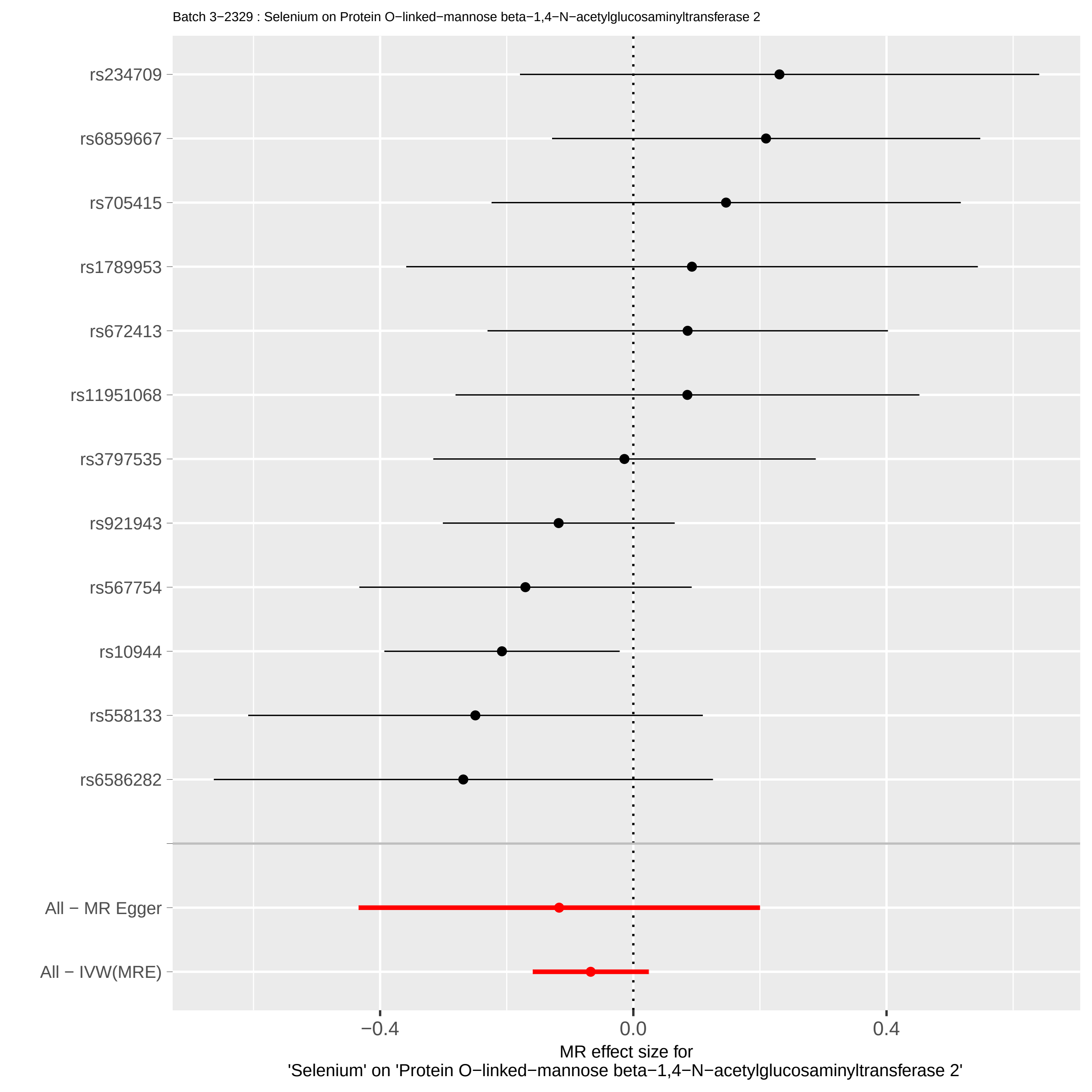

Batch 3−2329 : Selenium on Protein O−linked−mannose beta−1,4−N−acetylglucosaminyltransferase 2
rs234709
rs6859667
rs705415
rs1789953
rs672413
rs11951068
rs3797535
rs921943
rs567754
rs10944
rs558133
rs6586282
All − MR Egger
All − IVW(MRE)
−0.4
0.0
0.4
MR effect size for
'Selenium' on 'Protein O−linked−mannose beta−1,4−N−acetylglucosaminyltransferase 2'

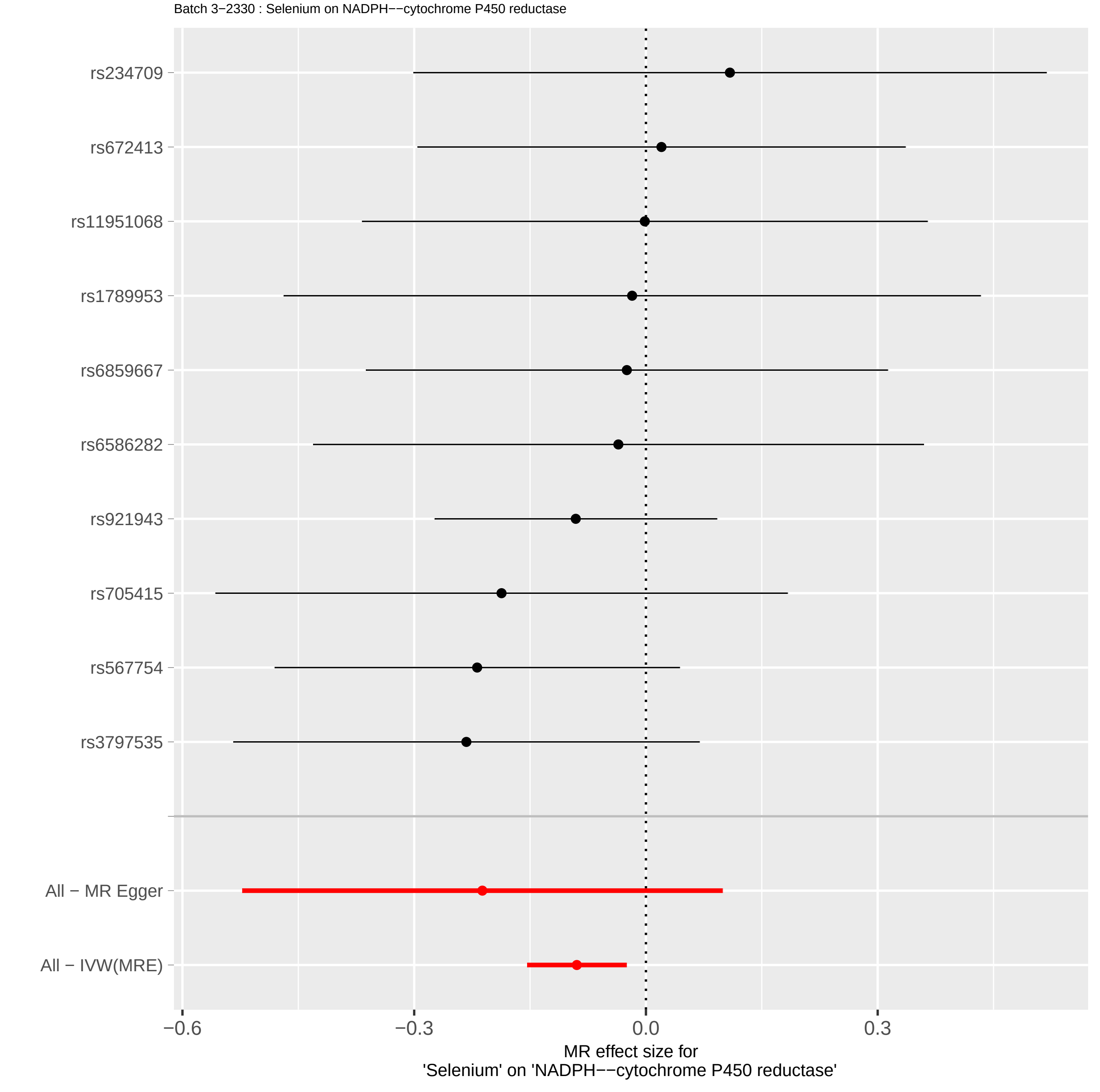

Batch 3−2330 : Selenium on NADPH−−cytochrome P450 reductase
rs234709
rs672413
rs11951068
rs1789953
rs6859667
rs6586282
rs921943
rs705415
rs567754
rs3797535
All − MR Egger
All − IVW(MRE)
−0.6
−0.3
0.0
0.3
MR effect size for
'Selenium' on 'NADPH−−cytochrome P450 reductase'

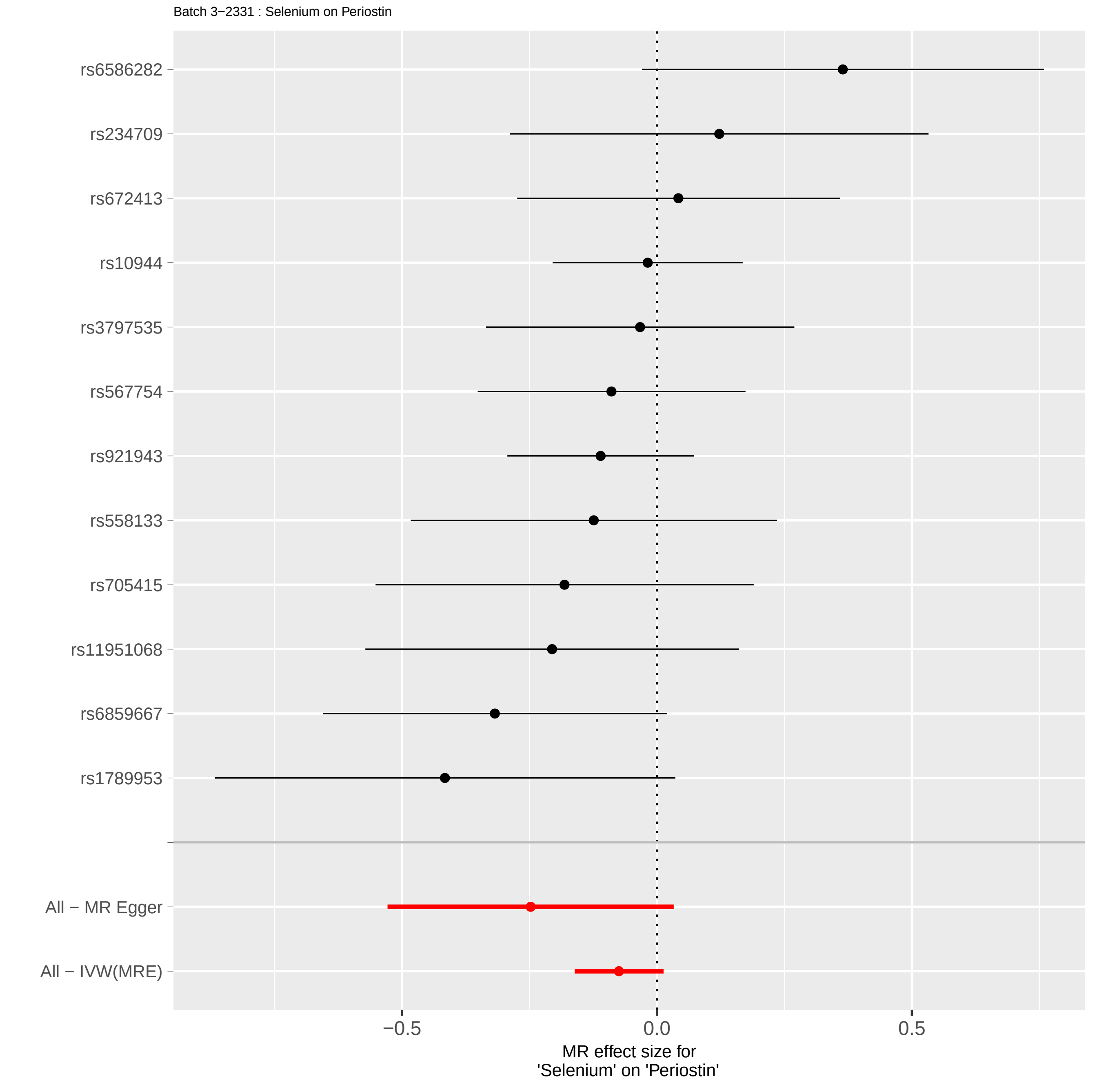

Batch 3−2331 : Selenium on Periostin
rs6586282
rs234709
rs672413
rs10944
rs3797535
rs567754
rs921943
rs558133
rs705415
rs11951068
rs6859667
rs1789953
All − MR Egger
All − IVW(MRE)
−0.5
0.0
0.5
MR effect size for
'Selenium' on 'Periostin'

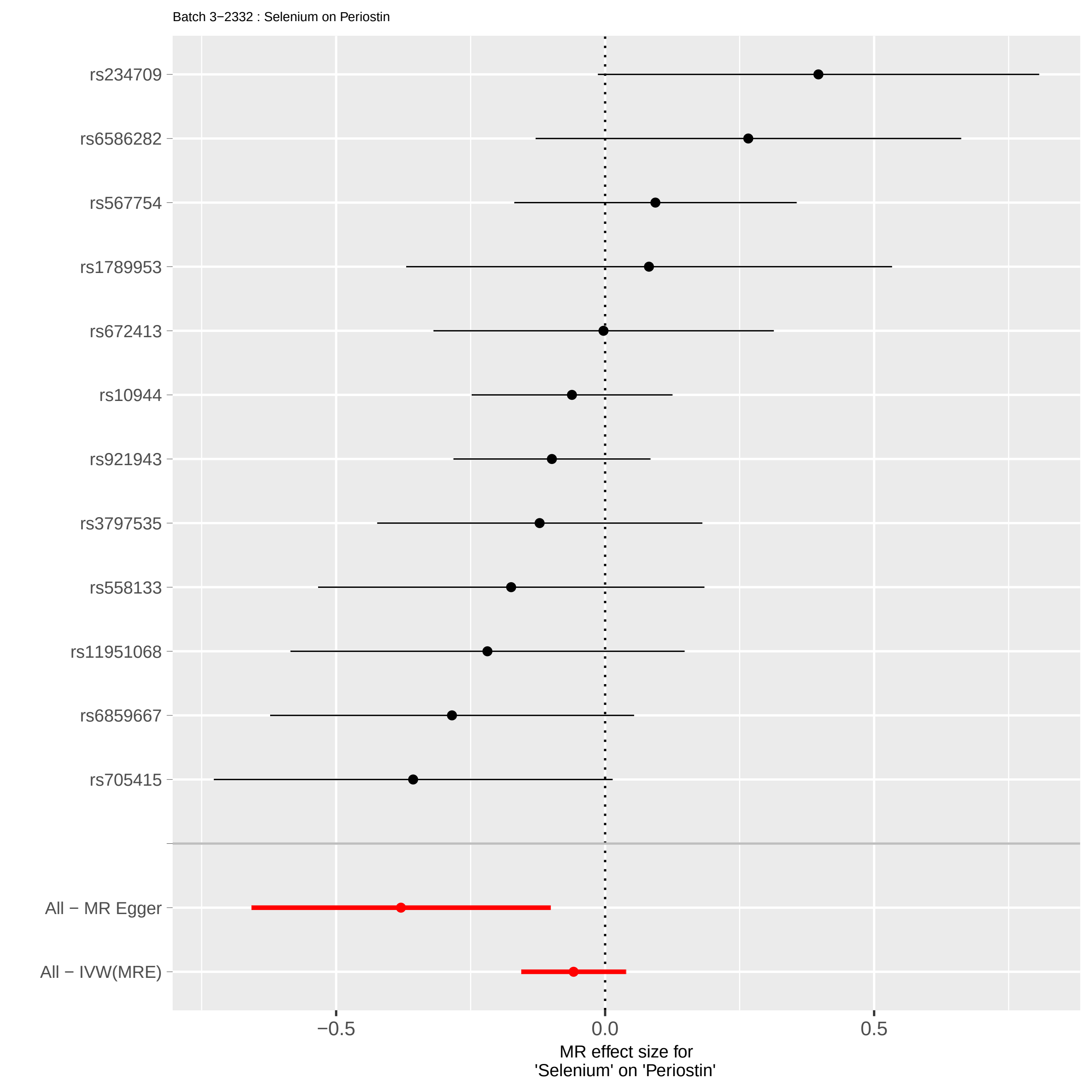

Batch 3−2332 : Selenium on Periostin
rs234709
rs6586282
rs567754
rs1789953
rs672413
rs10944
rs921943
rs3797535
rs558133
rs11951068
rs6859667
rs705415
All − MR Egger
All − IVW(MRE)
−0.5
0.0
0.5
MR effect size for
'Selenium' on 'Periostin'

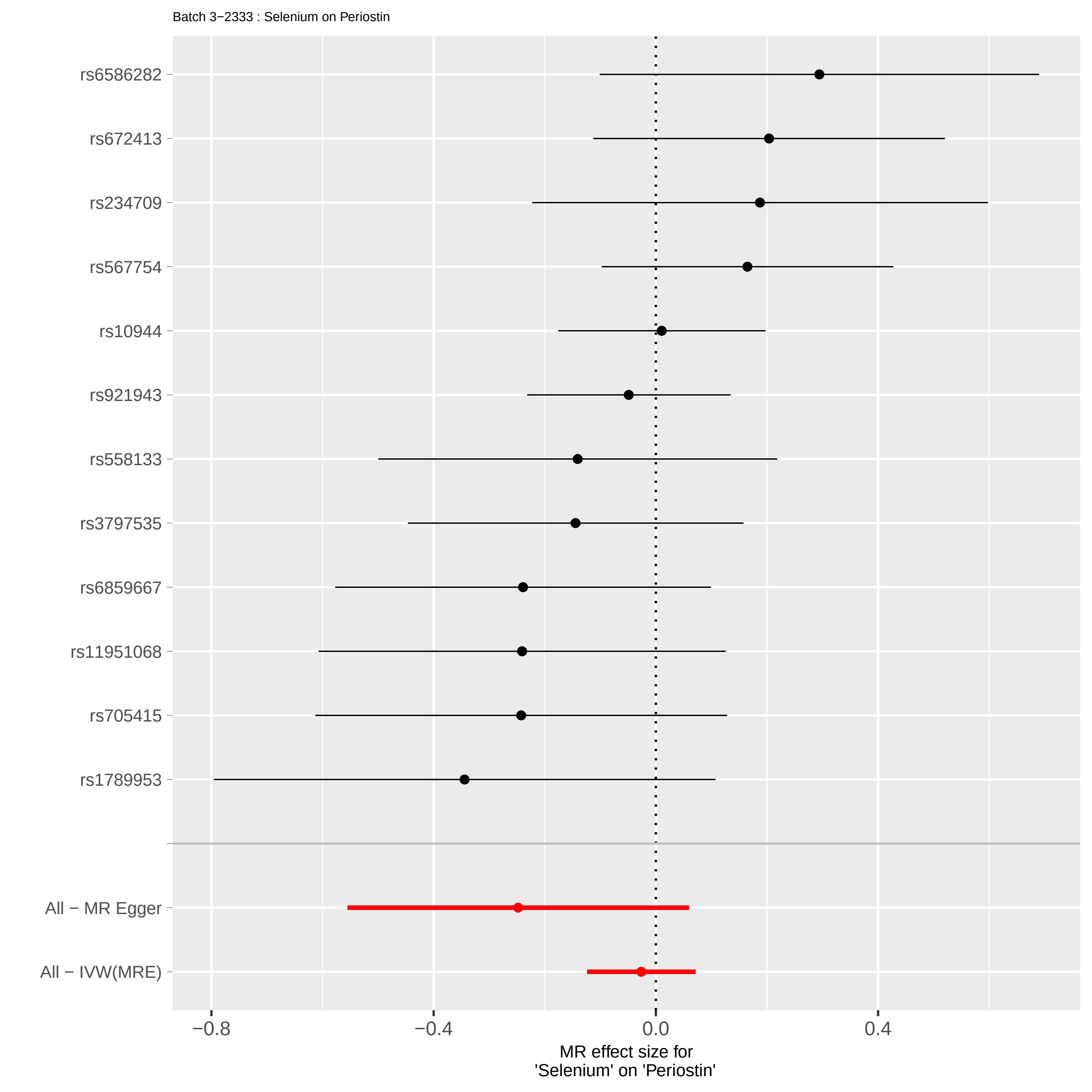
Batch 3−2333 : Selenium on Periostin
rs6586282
rs672413
rs234709
rs567754
rs10944
rs921943
rs558133
rs3797535
rs6859667
rs11951068
rs705415
rs1789953
All − MR Egger
All − IVW(MRE)
−0.8
−0.4
0.0
0.4
MR effect size for
'Selenium' on 'Periostin'

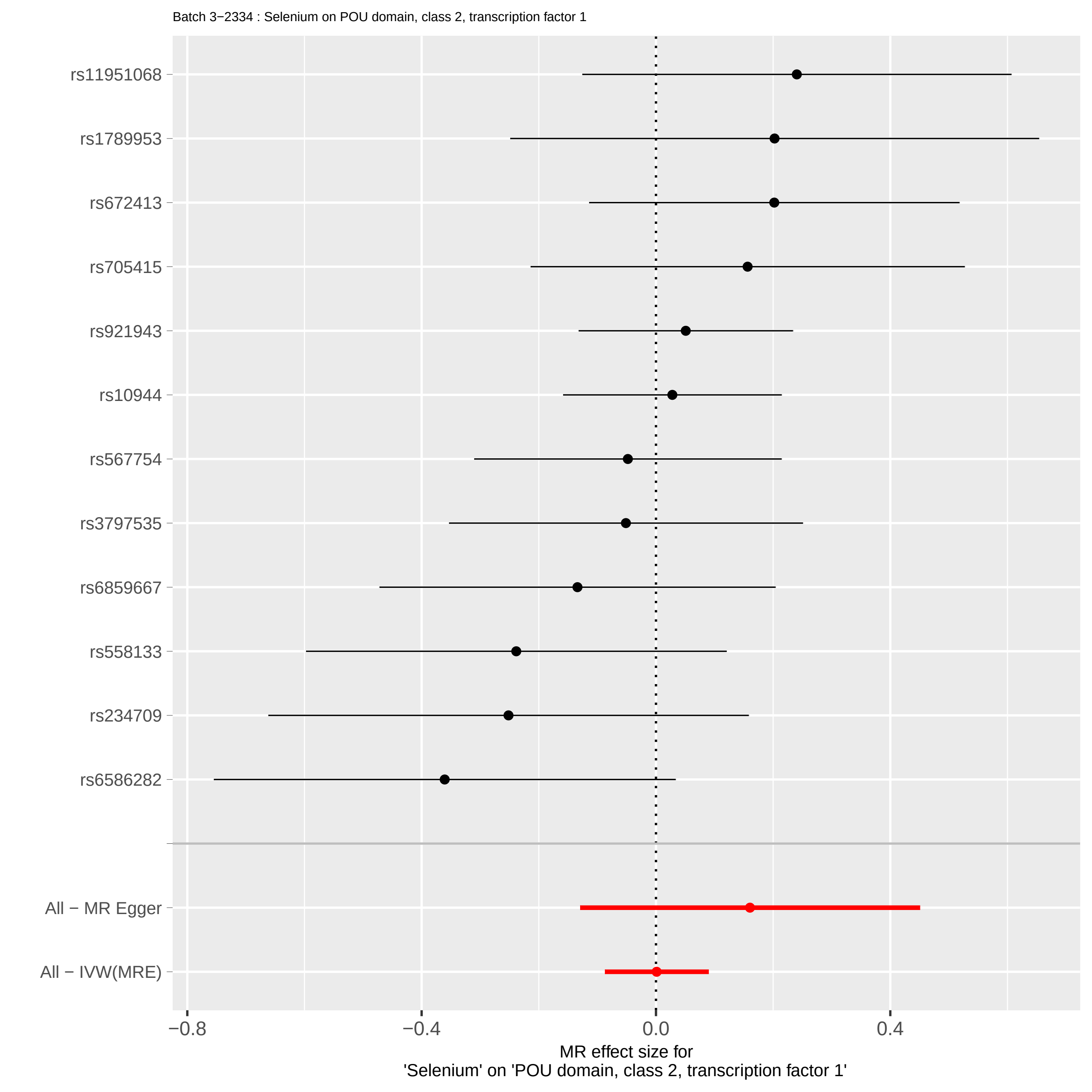
Batch 3−2334 : Selenium on POU domain, class 2, transcription factor 1
rs11951068
rs1789953
rs672413
rs705415
rs921943
rs10944
rs567754
rs3797535
rs6859667
rs558133
rs234709
rs6586282
All − MR Egger
All − IVW(MRE)
−0.8
−0.4
0.0
0.4
MR effect size for
'Selenium' on 'POU domain, class 2, transcription factor 1'

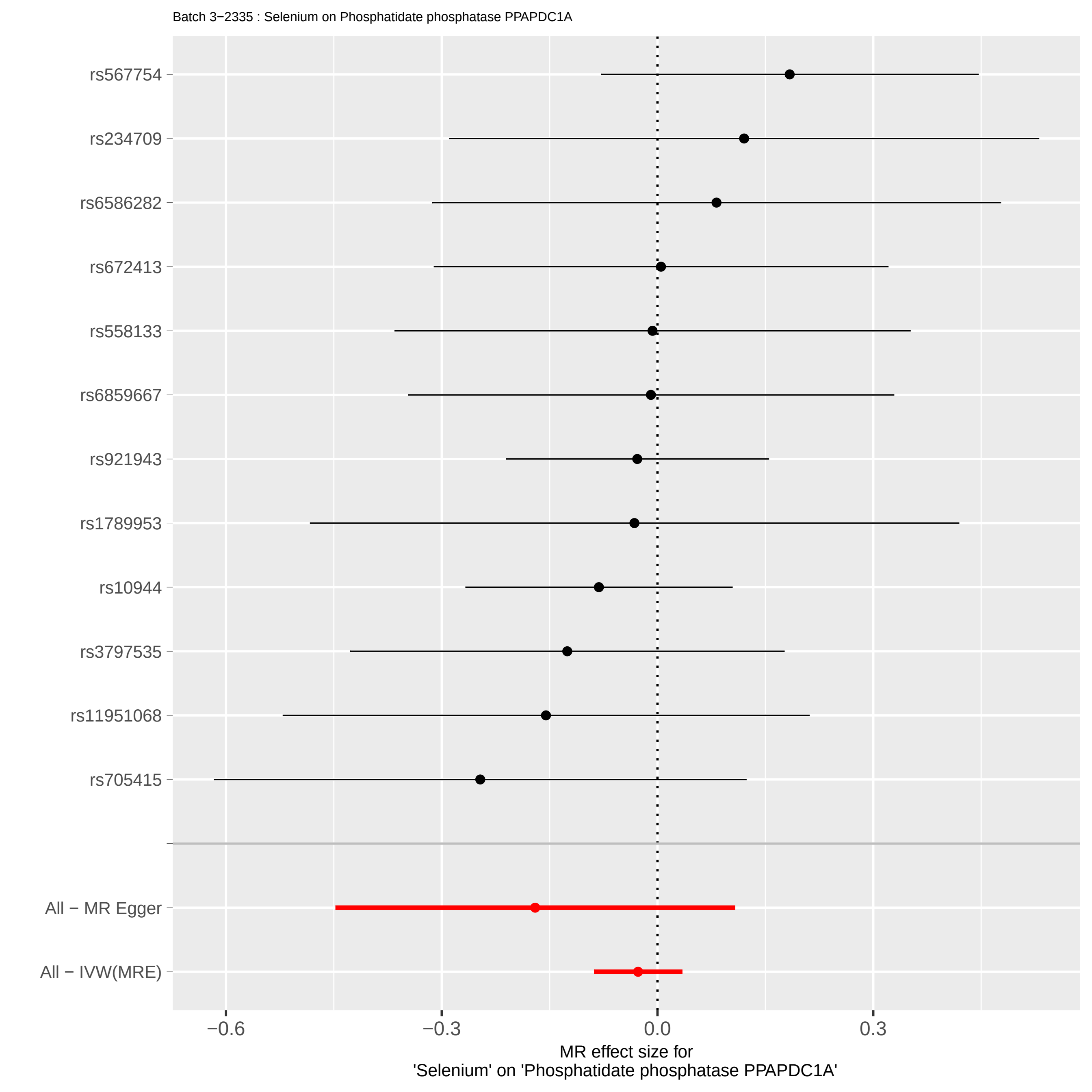

Batch 3−2335 : Selenium on Phosphatidate phosphatase PPAPDC1A
rs567754
rs234709
rs6586282
rs672413
rs558133
rs6859667
rs921943
rs1789953
rs10944
rs3797535
rs11951068
rs705415
All − MR Egger
All − IVW(MRE)
−0.6
−0.3
0.0
0.3
MR effect size for
'Selenium' on 'Phosphatidate phosphatase PPAPDC1A'

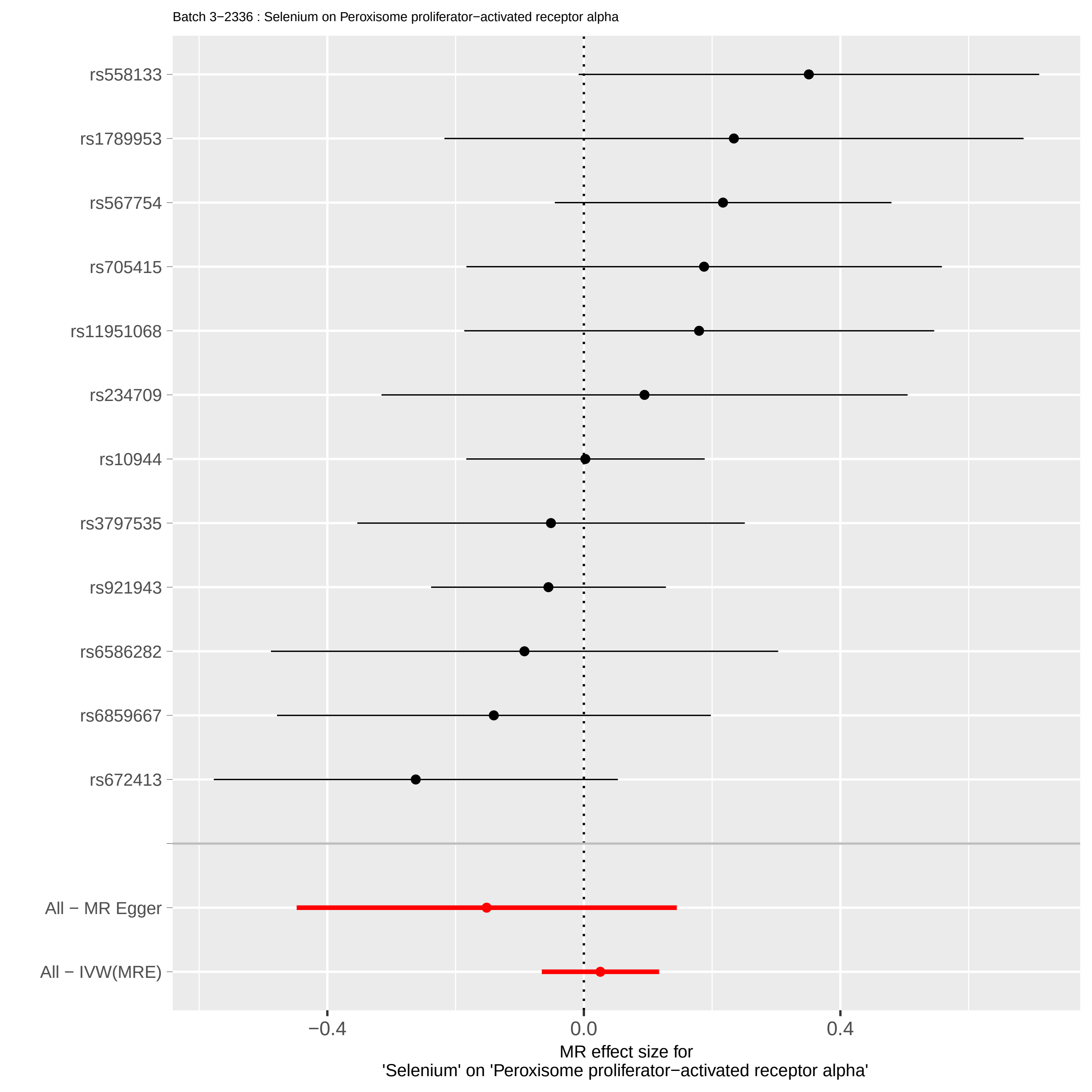
Batch 3−2336 : Selenium on Peroxisome proliferator−activated receptor alpha
rs558133
rs1789953
rs567754
rs705415
rs11951068
rs234709
rs10944
rs3797535
rs921943
rs6586282
rs6859667
rs672413
All − MR Egger
All − IVW(MRE)
−0.4
0.0
0.4
MR effect size for
'Selenium' on 'Peroxisome proliferator−activated receptor alpha'

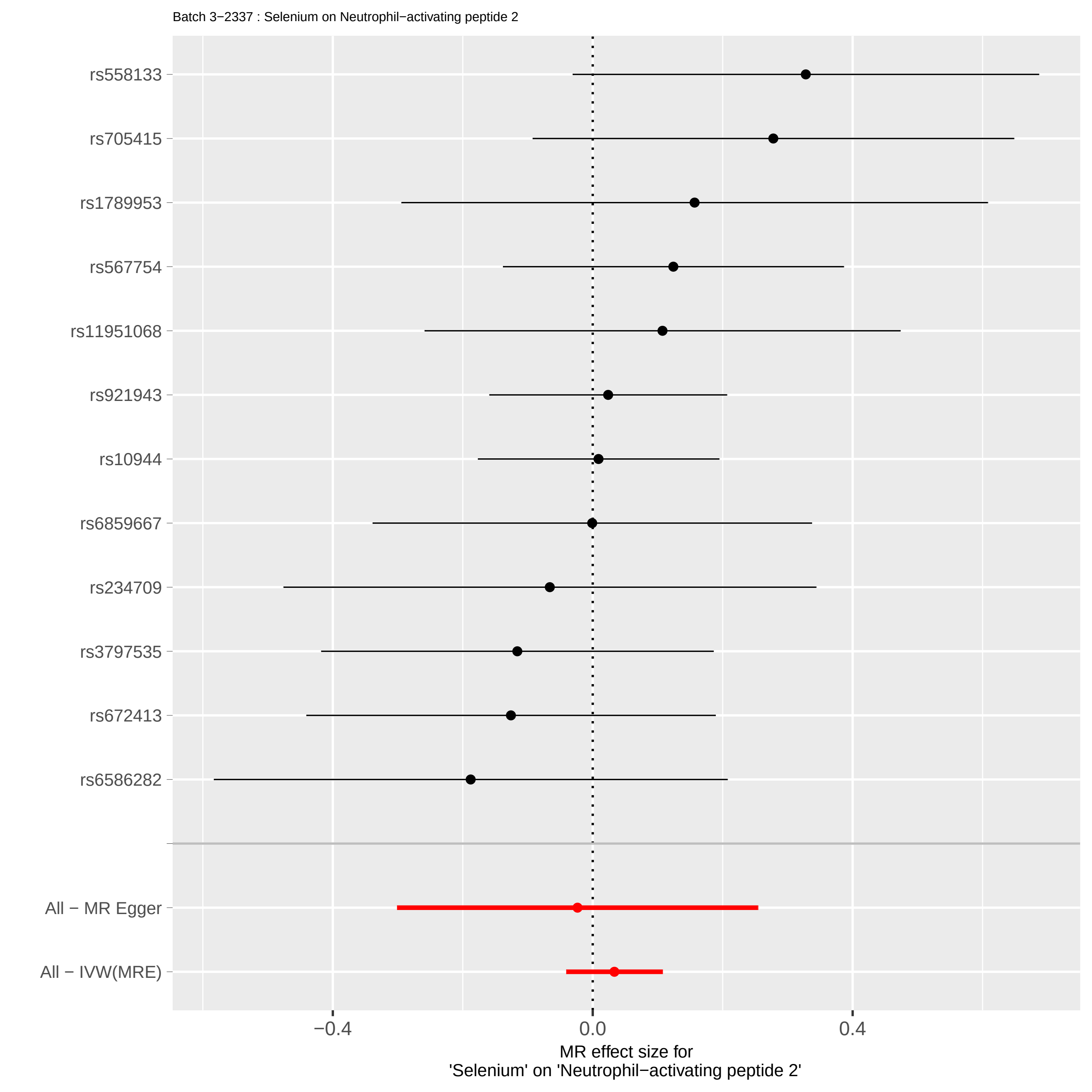
Batch 3−2337 : Selenium on Neutrophil−activating peptide 2
rs558133
rs705415
rs1789953
rs567754
rs11951068
rs921943
rs10944
rs6859667
rs234709
rs3797535
rs672413
rs6586282
All − MR Egger
All − IVW(MRE)
−0.4
0.0
0.4
MR effect size for
'Selenium' on 'Neutrophil−activating peptide 2'

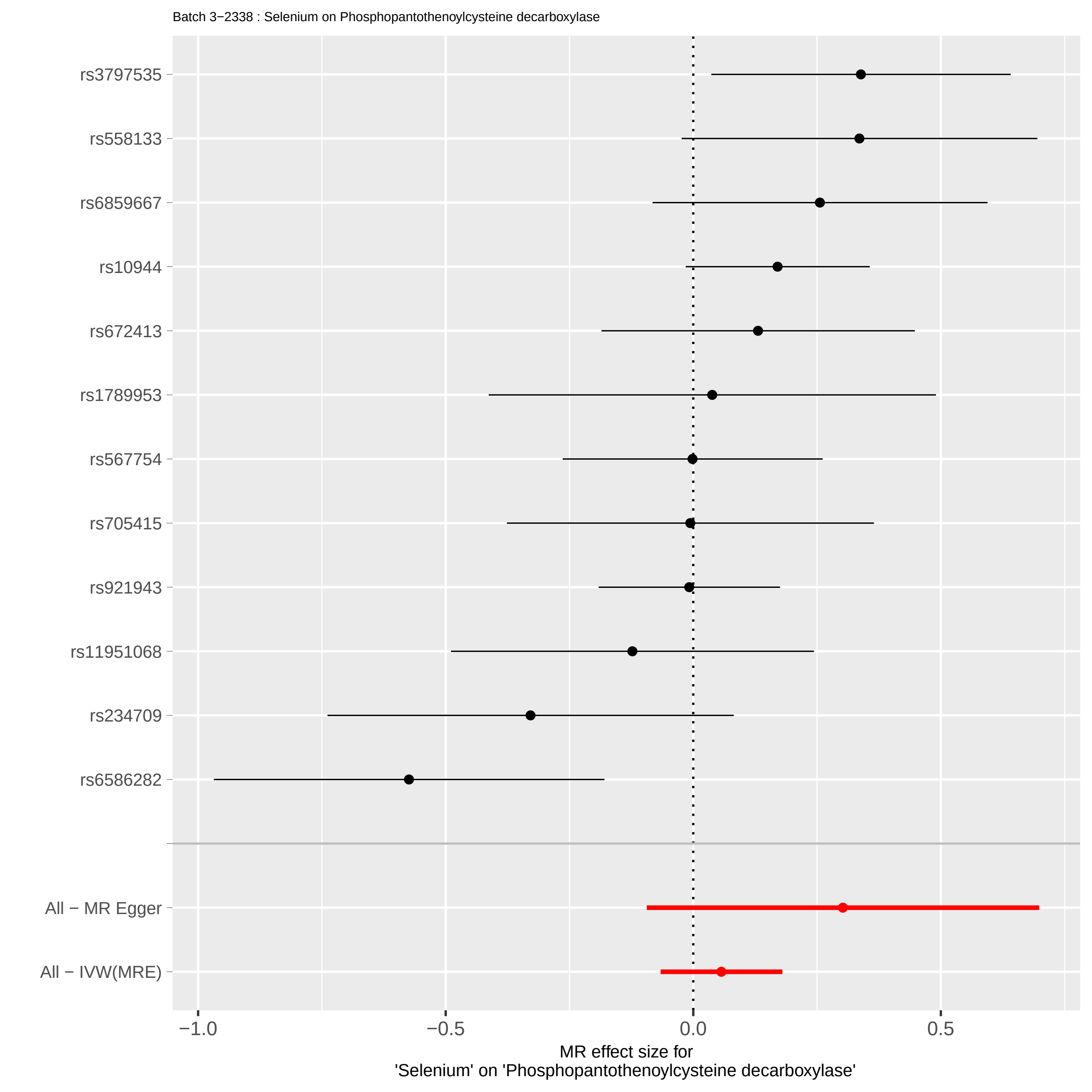

Batch 3−2338 : Selenium on Phosphopantothenoylcysteine decarboxylase
rs3797535
rs558133
rs6859667
rs10944
rs672413
rs1789953
rs567754
rs705415
rs921943
rs11951068
rs234709
rs6586282
All − MR Egger
All − IVW(MRE)
−1.0
−0.5
0.0
0.5
MR effect size for
'Selenium' on 'Phosphopantothenoylcysteine decarboxylase'

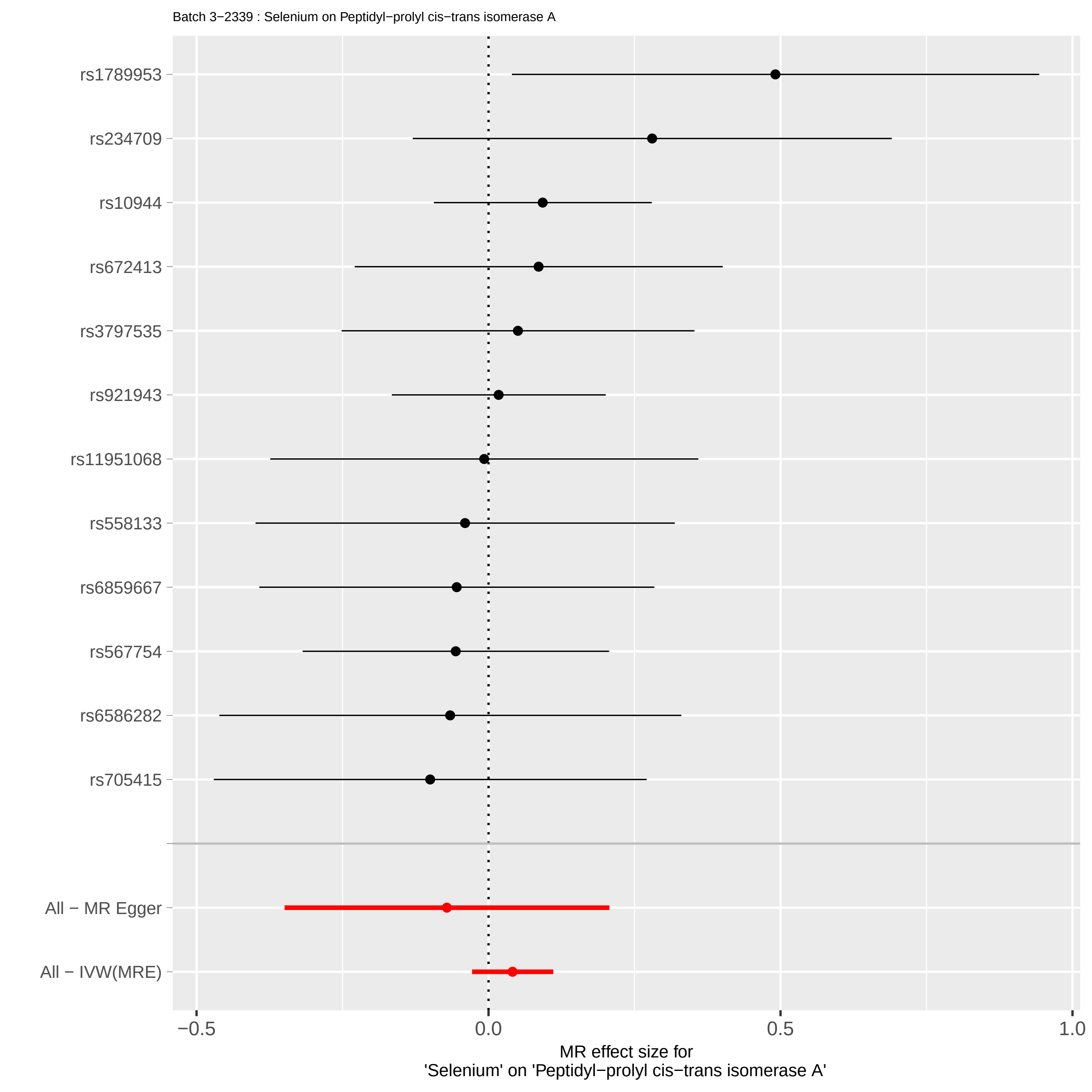

Batch 3−2339 : Selenium on Peptidyl−prolyl cis−trans isomerase A
rs1789953
rs234709
rs10944
rs672413
rs3797535
rs921943
rs11951068
rs558133
rs6859667
rs567754
rs6586282
rs705415
All − MR Egger
All − IVW(MRE)
−0.5
0.0
0.5
1.0
MR effect size for
'Selenium' on 'Peptidyl−prolyl cis−trans isomerase A'

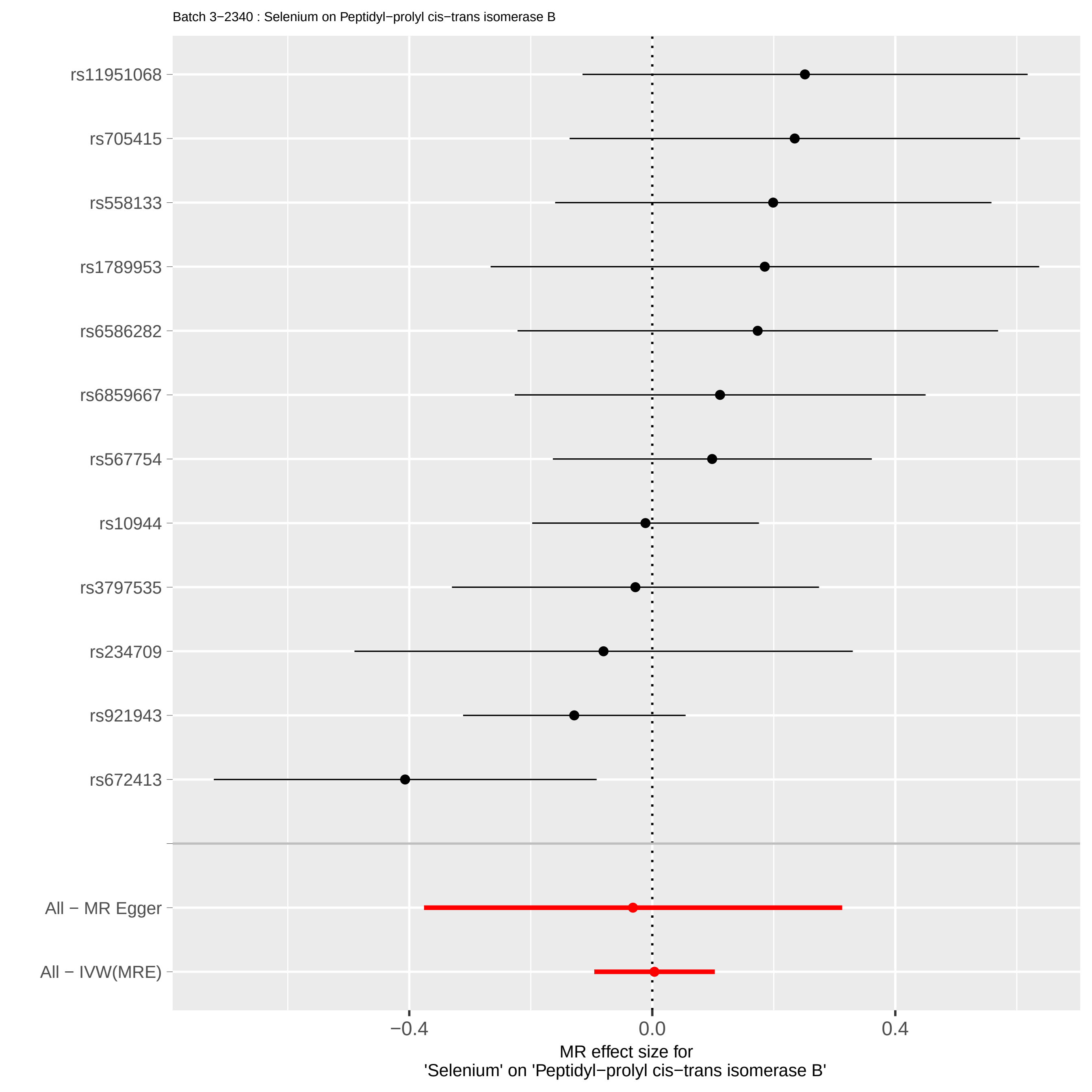
Batch 3−2340 : Selenium on Peptidyl−prolyl cis−trans isomerase B
rs11951068
rs705415
rs558133
rs1789953
rs6586282
rs6859667
rs567754
rs10944
rs3797535
rs234709
rs921943
rs672413
All − MR Egger
All − IVW(MRE)
−0.4
0.0
0.4
MR effect size for
'Selenium' on 'Peptidyl−prolyl cis−trans isomerase B'

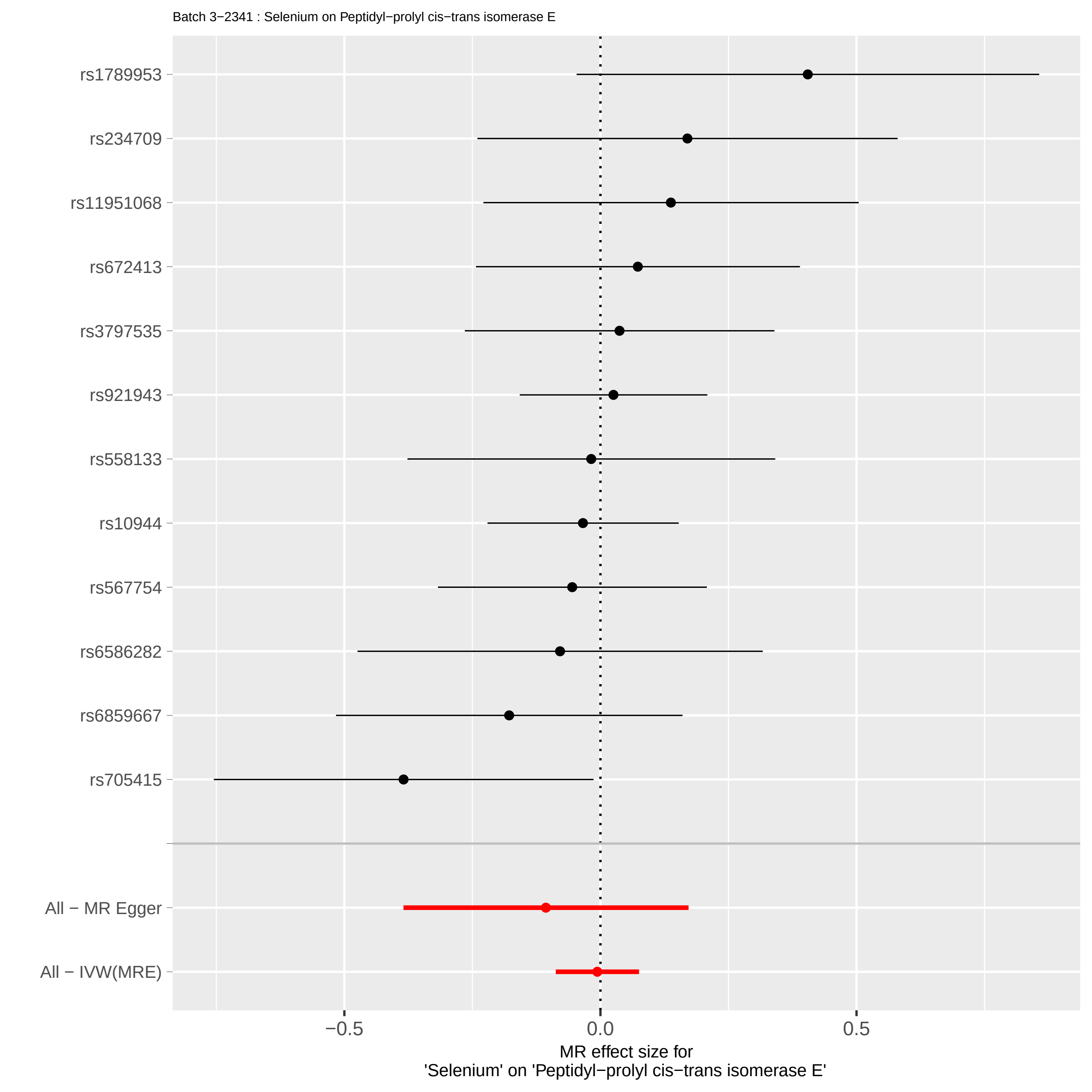
Batch 3−2341 : Selenium on Peptidyl−prolyl cis−trans isomerase E
rs1789953
rs234709
rs11951068
rs672413
rs3797535
rs921943
rs558133
rs10944
rs567754
rs6586282
rs6859667
rs705415
All − MR Egger
All − IVW(MRE)
−0.5
0.0
0.5
MR effect size for
'Selenium' on 'Peptidyl−prolyl cis−trans isomerase E'

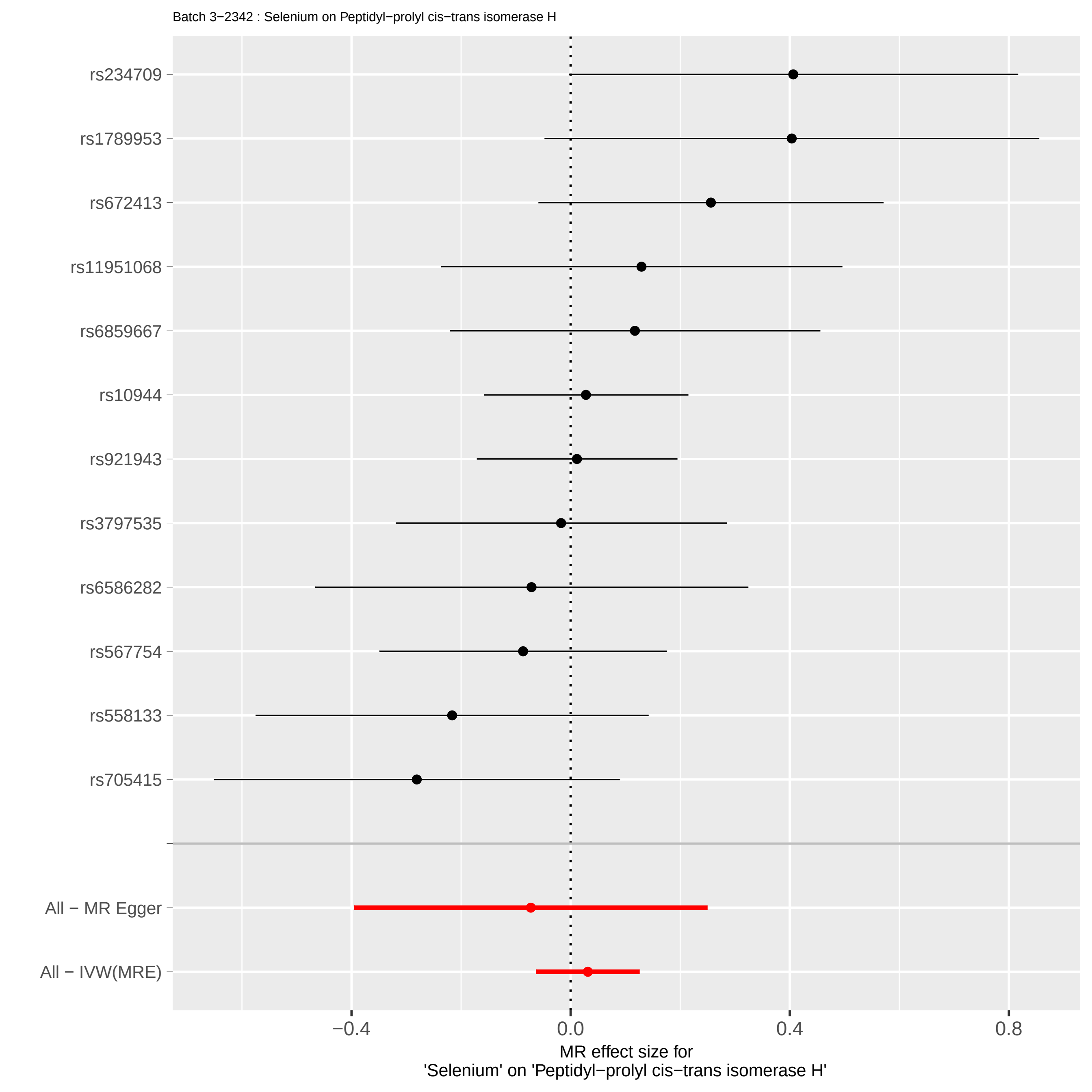

Batch 3−2342 : Selenium on Peptidyl−prolyl cis−trans isomerase H
rs234709
rs1789953
rs672413
rs11951068
rs6859667
rs10944
rs921943
rs3797535
rs6586282
rs567754
rs558133
rs705415
All − MR Egger
All − IVW(MRE)
−0.4
0.0
0.4
0.8
MR effect size for
'Selenium' on 'Peptidyl−prolyl cis−trans isomerase H'

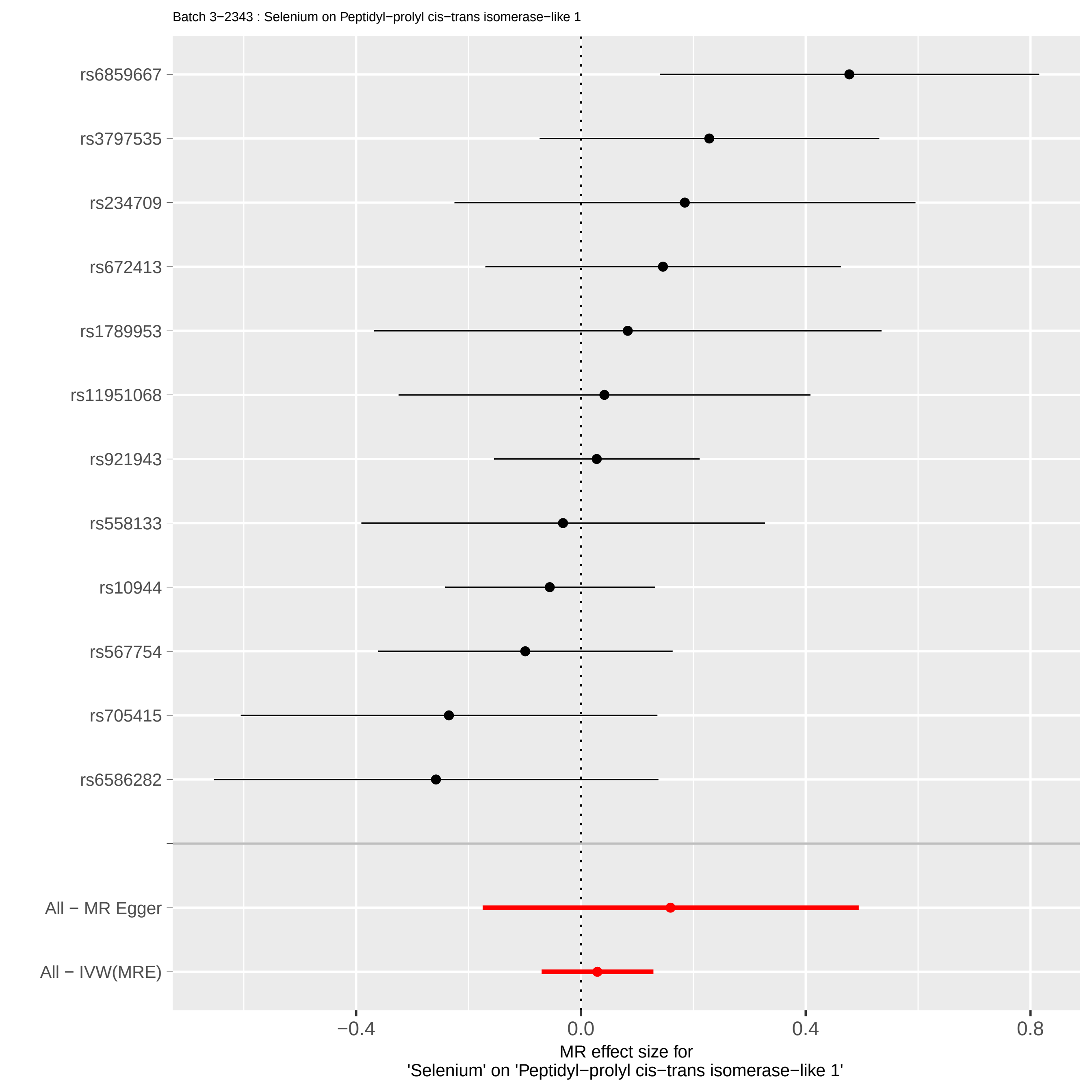

Batch 3−2343 : Selenium on Peptidyl−prolyl cis−trans isomerase−like 1
rs6859667
rs3797535
rs234709
rs672413
rs1789953
rs11951068
rs921943
rs558133
rs10944
rs567754
rs705415
rs6586282
All − MR Egger
All − IVW(MRE)
−0.4
0.0
0.4
0.8
MR effect size for
'Selenium' on 'Peptidyl−prolyl cis−trans isomerase−like 1'

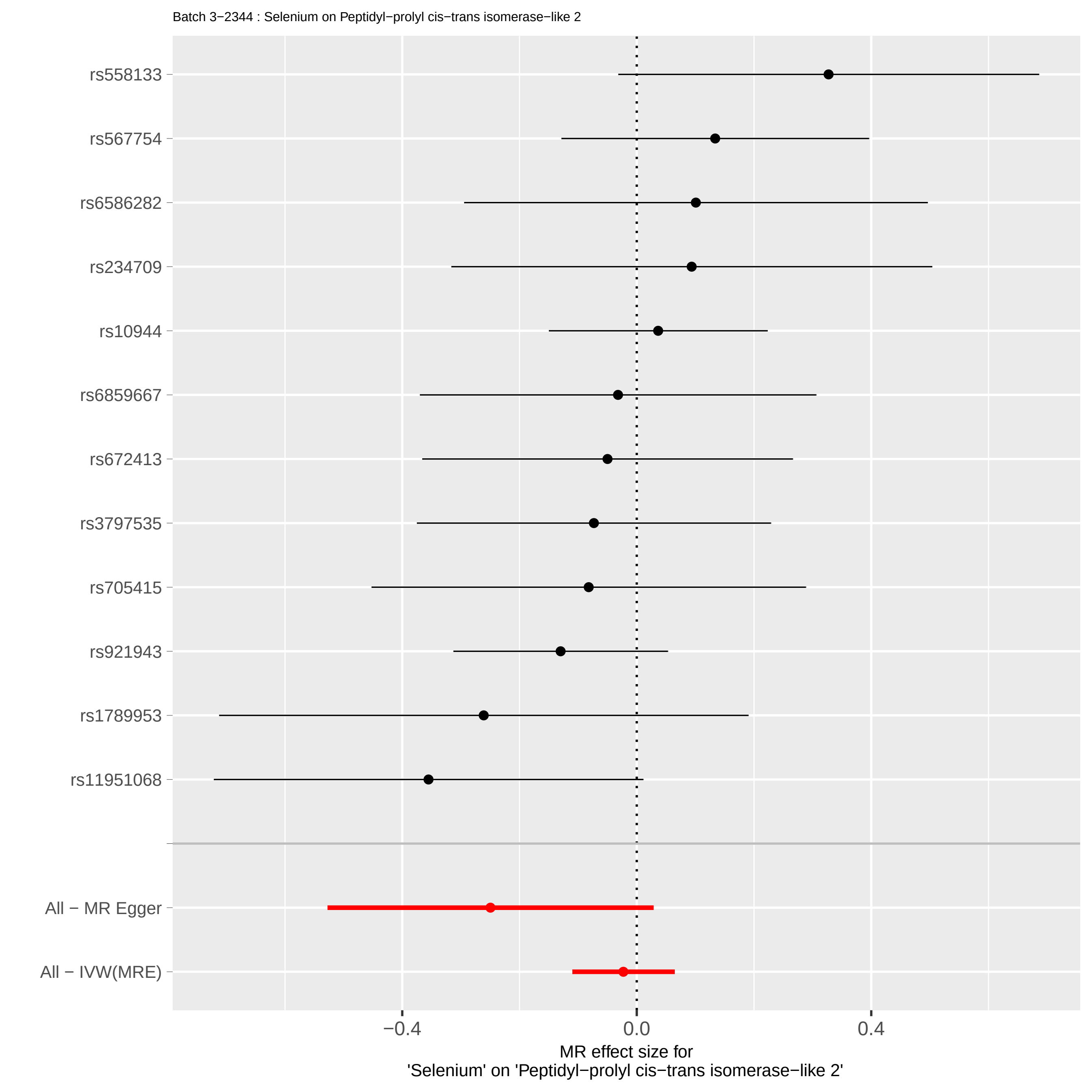

Batch 3−2344 : Selenium on Peptidyl−prolyl cis−trans isomerase−like 2
rs558133
rs567754
rs6586282
rs234709
rs10944
rs6859667
rs672413
rs3797535
rs705415
rs921943
rs1789953
rs11951068
All − MR Egger
All − IVW(MRE)
−0.4
0.0
0.4
MR effect size for
'Selenium' on 'Peptidyl−prolyl cis−trans isomerase−like 2'

Batch 3−2345 : Selenium on Protein phosphatase 1A

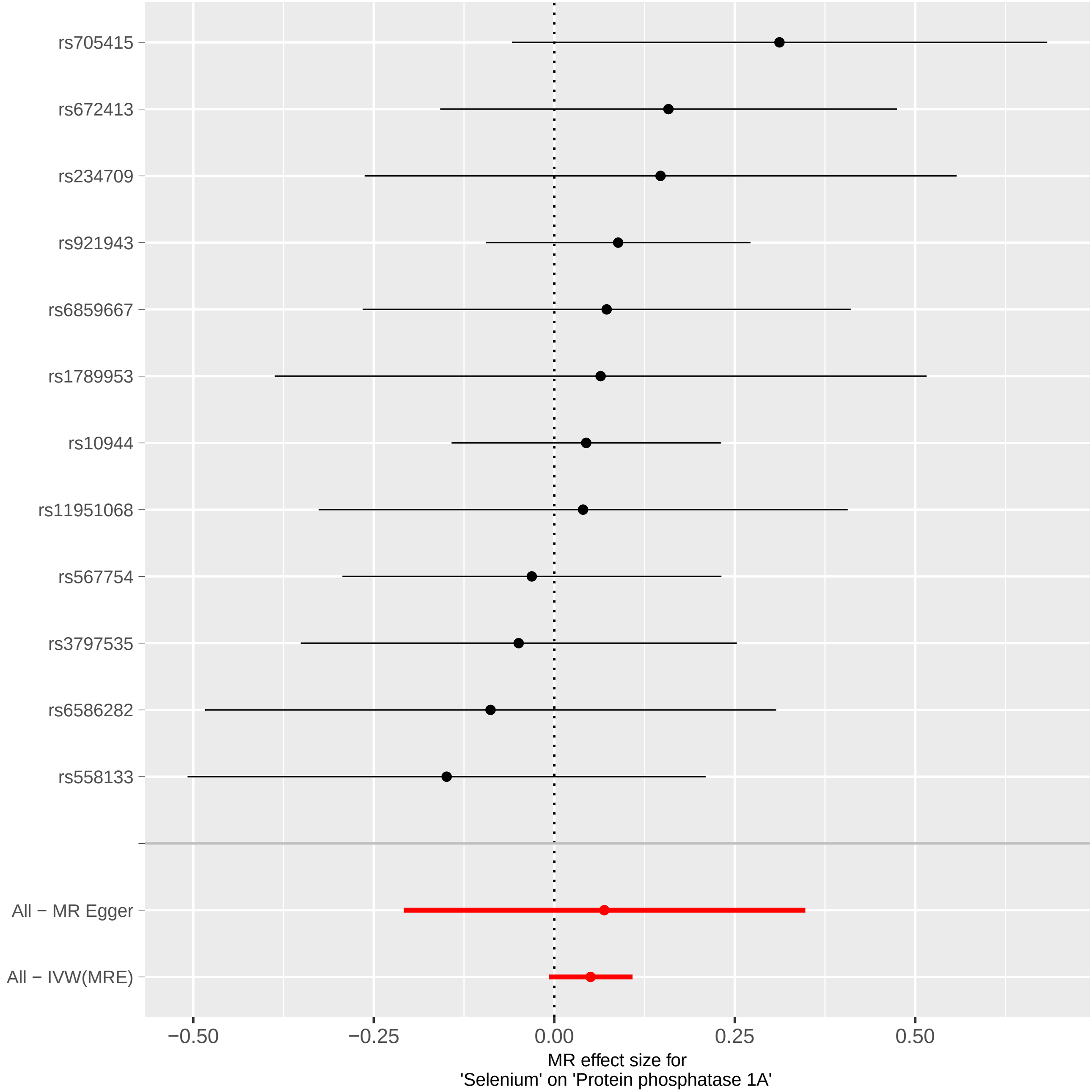

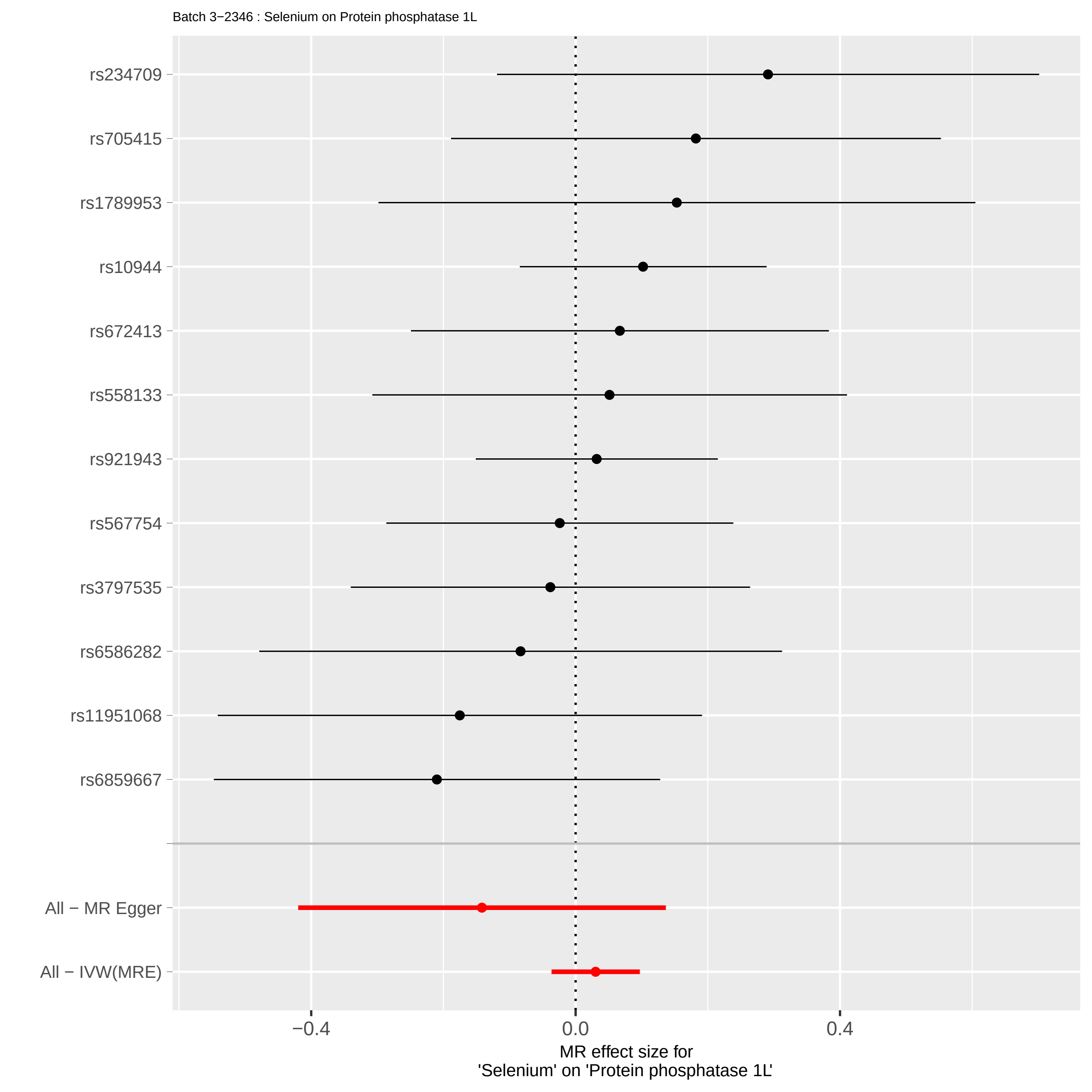

Batch 3−2346 : Selenium on Protein phosphatase 1L
rs234709
rs705415
rs1789953
rs10944
rs672413
rs558133
rs921943
rs567754
rs3797535
rs6586282
rs11951068
rs6859667
All − MR Egger
All − IVW(MRE)
−0.4
0.0
0.4
MR effect size for
'Selenium' on 'Protein phosphatase 1L'

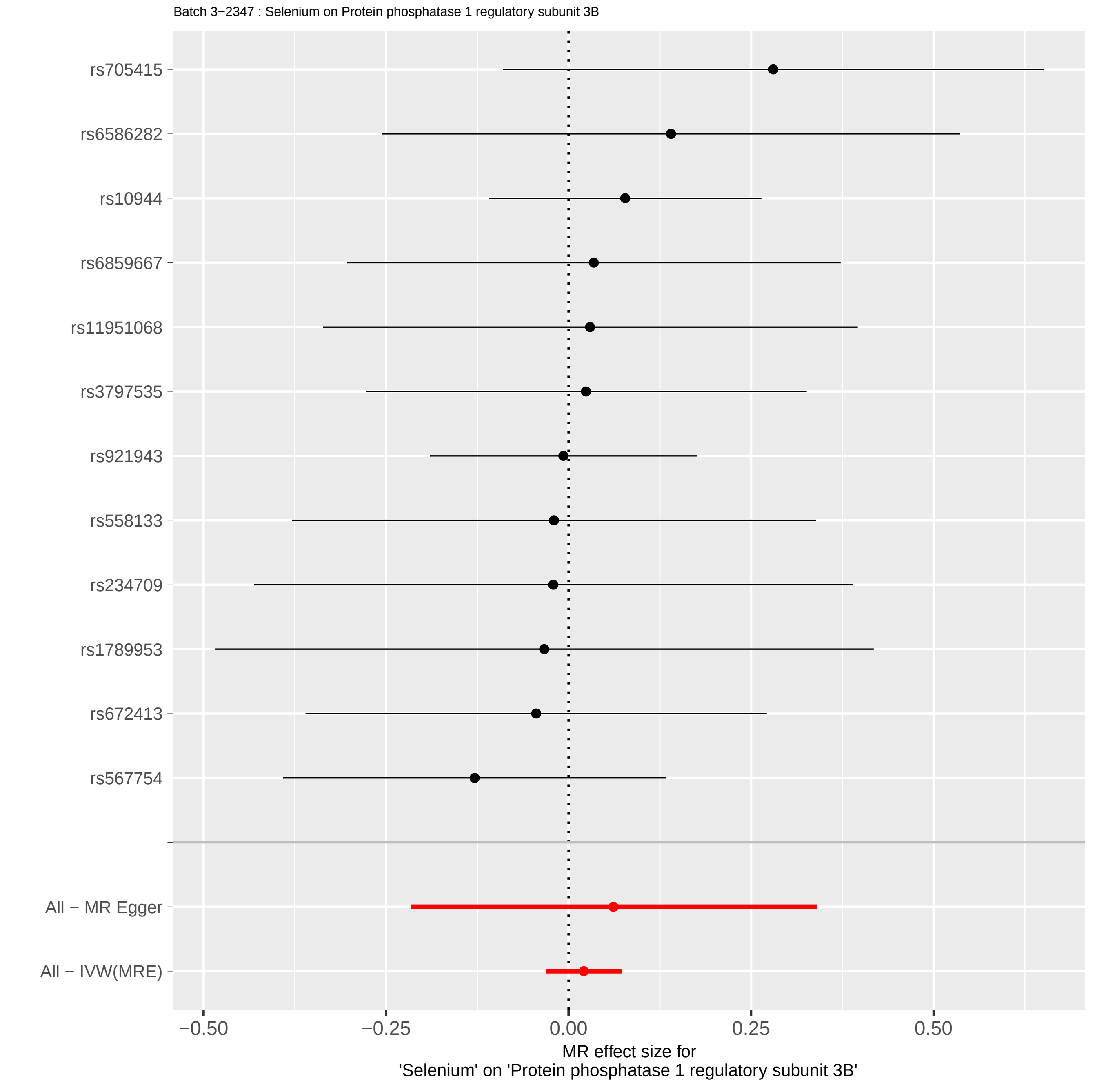

Batch 3−2347 : Selenium on Protein phosphatase 1 regulatory subunit 3B
rs705415
rs6586282
rs10944
rs6859667
rs11951068
rs3797535
rs921943
rs558133
rs234709
rs1789953
rs672413
rs567754
All − MR Egger
All − IVW(MRE)
−0.50
−0.25
0.00
0.25
0.50
MR effect size for
'Selenium' on 'Protein phosphatase 1 regulatory subunit 3B'

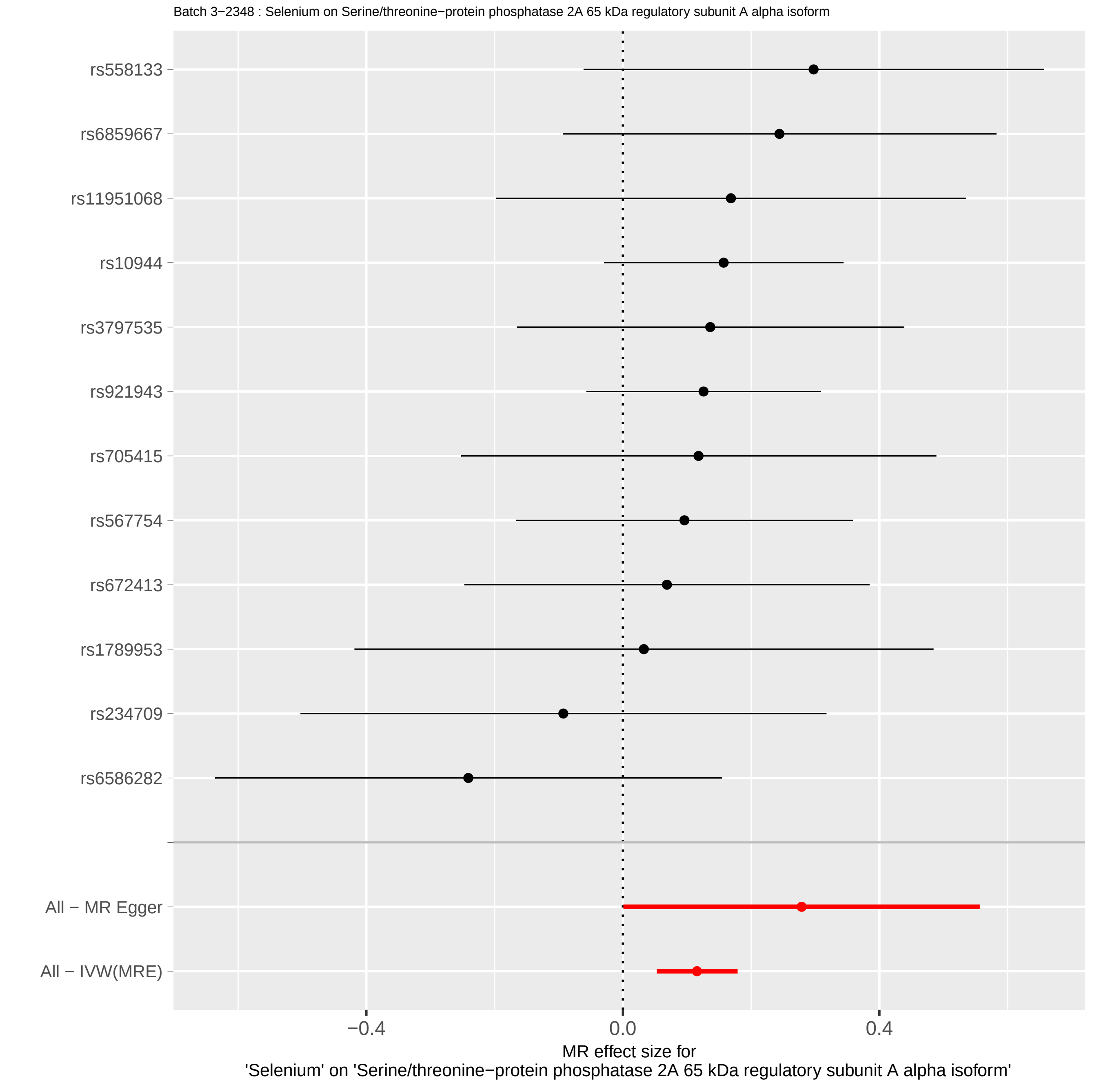

Batch 3−2348 : Selenium on Serine/threonine−protein phosphatase 2A 65 kDa regulatory subunit A alpha isoform
rs558133
rs6859667
rs11951068
rs10944
rs3797535
rs921943
rs705415
rs567754
rs672413
rs1789953
rs234709
rs6586282
All − MR Egger
All − IVW(MRE)
−0.4
0.0
0.4
MR effect size for
'Selenium' on 'Serine/threonine−protein phosphatase 2A 65 kDa regulatory subunit A alpha isoform'

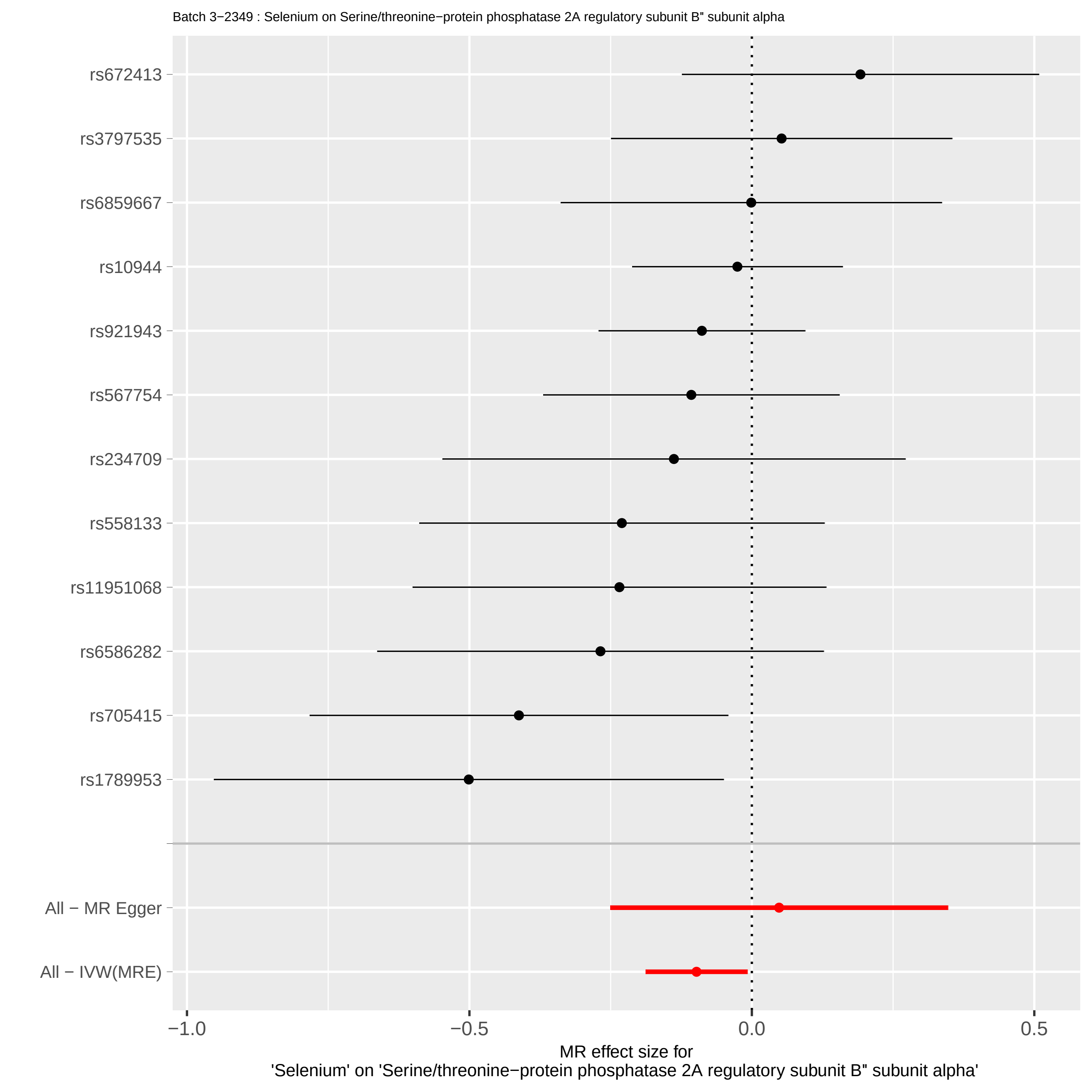

Batch 3−2349 : Selenium on Serine/threonine−protein phosphatase 2A regulatory subunit B" subunit alpha
rs672413
rs3797535
rs6859667
rs10944
rs921943
rs567754
rs234709
rs558133
rs11951068
rs6586282
rs705415
rs1789953
All − MR Egger
All − IVW(MRE)
−1.0
−0.5
0.0
0.5
MR effect size for
'Selenium' on 'Serine/threonine−protein phosphatase 2A regulatory subunit B" subunit alpha'

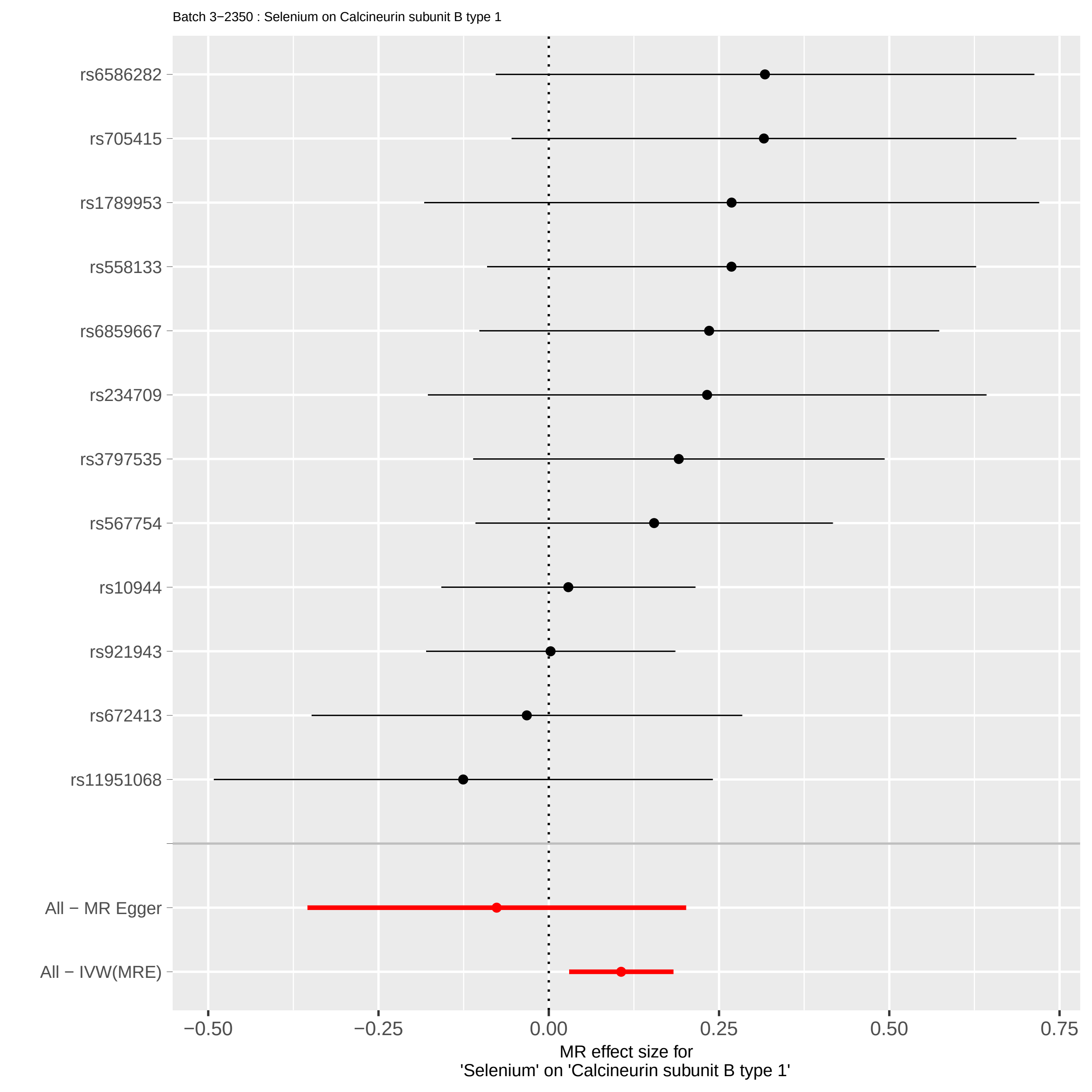

Batch 3−2350 : Selenium on Calcineurin subunit B type 1
rs6586282
rs705415
rs1789953
rs558133
rs6859667
rs234709
rs3797535
rs567754
rs10944
rs921943
rs672413
rs11951068
All − MR Egger
All − IVW(MRE)
−0.50
−0.25
0.00
0.25
0.50
0.75
MR effect size for
'Selenium' on 'Calcineurin subunit B type 1'

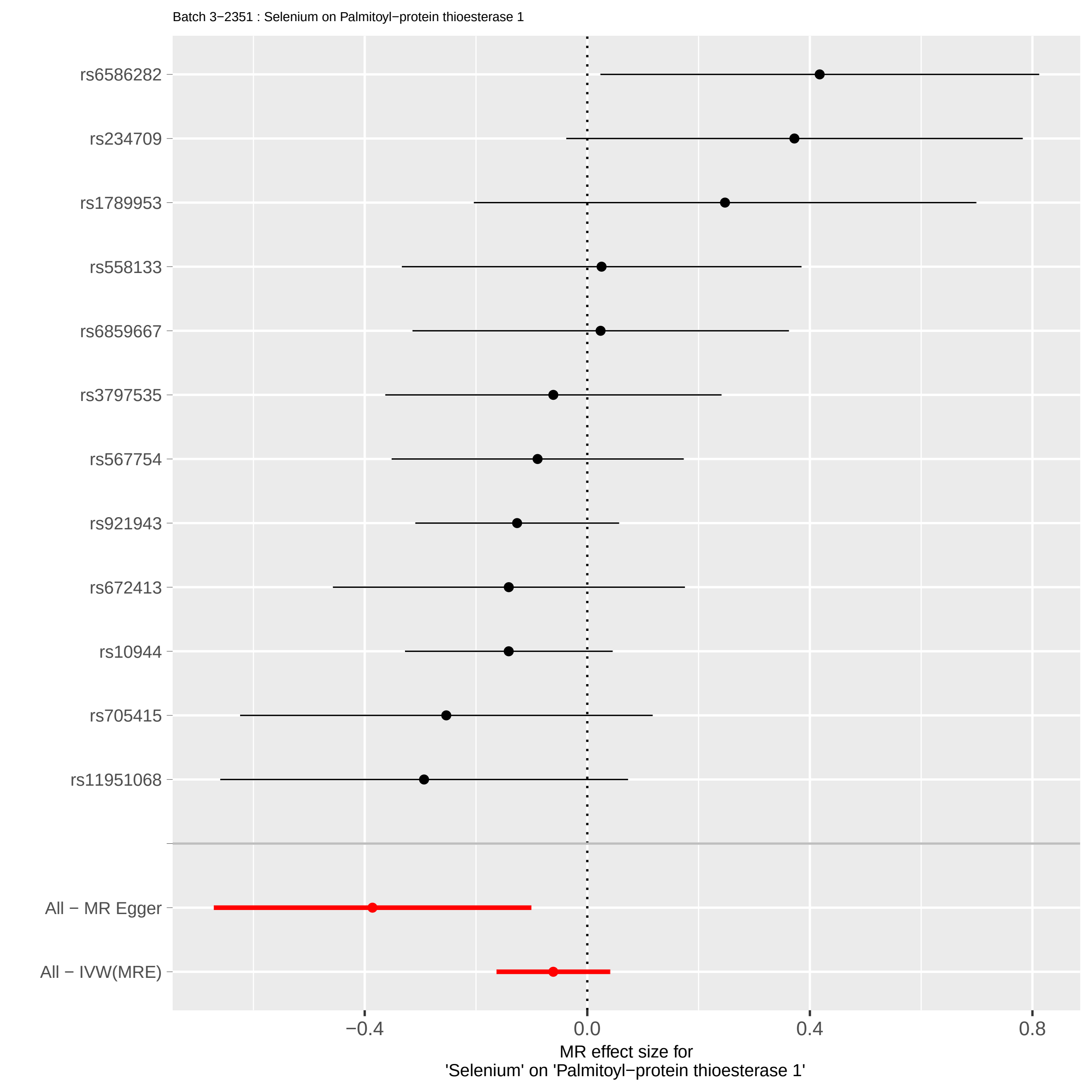

Batch 3−2351 : Selenium on Palmitoyl−protein thioesterase 1
rs6586282
rs234709
rs1789953
rs558133
rs6859667
rs3797535
rs567754
rs921943
rs672413
rs10944
rs705415
rs11951068
All − MR Egger
All − IVW(MRE)
−0.4
0.0
0.4
0.8
MR effect size for
'Selenium' on 'Palmitoyl−protein thioesterase 1'

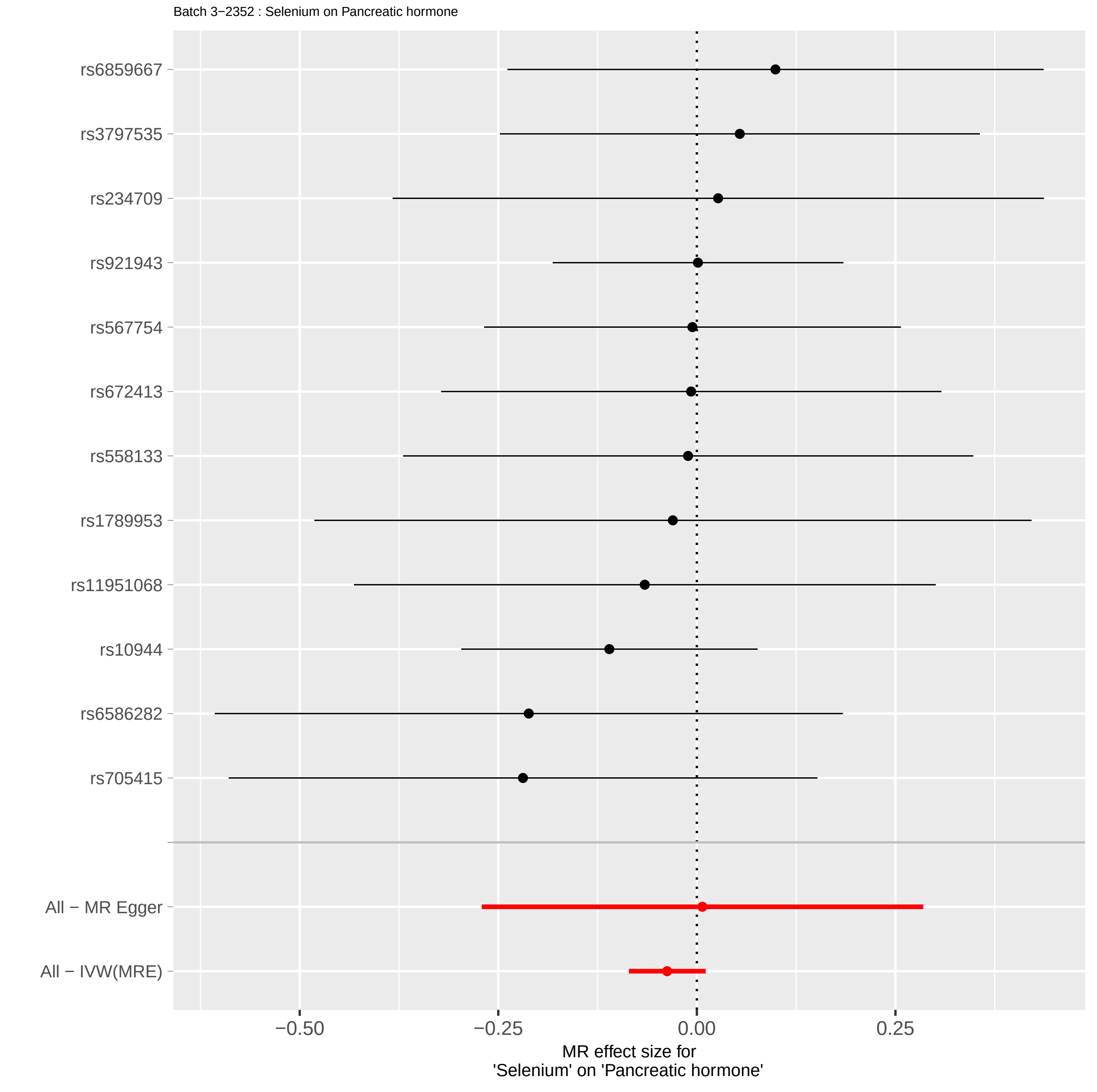
Batch 3−2352 : Selenium on Pancreatic hormone
rs6859667
rs3797535
rs234709
rs921943
rs567754
rs672413
rs558133
rs1789953
rs11951068
rs10944
rs6586282
rs705415
All − MR Egger
All − IVW(MRE)
−0.50
−0.25
0.00
0.25
MR effect size for
'Selenium' on 'Pancreatic hormone'

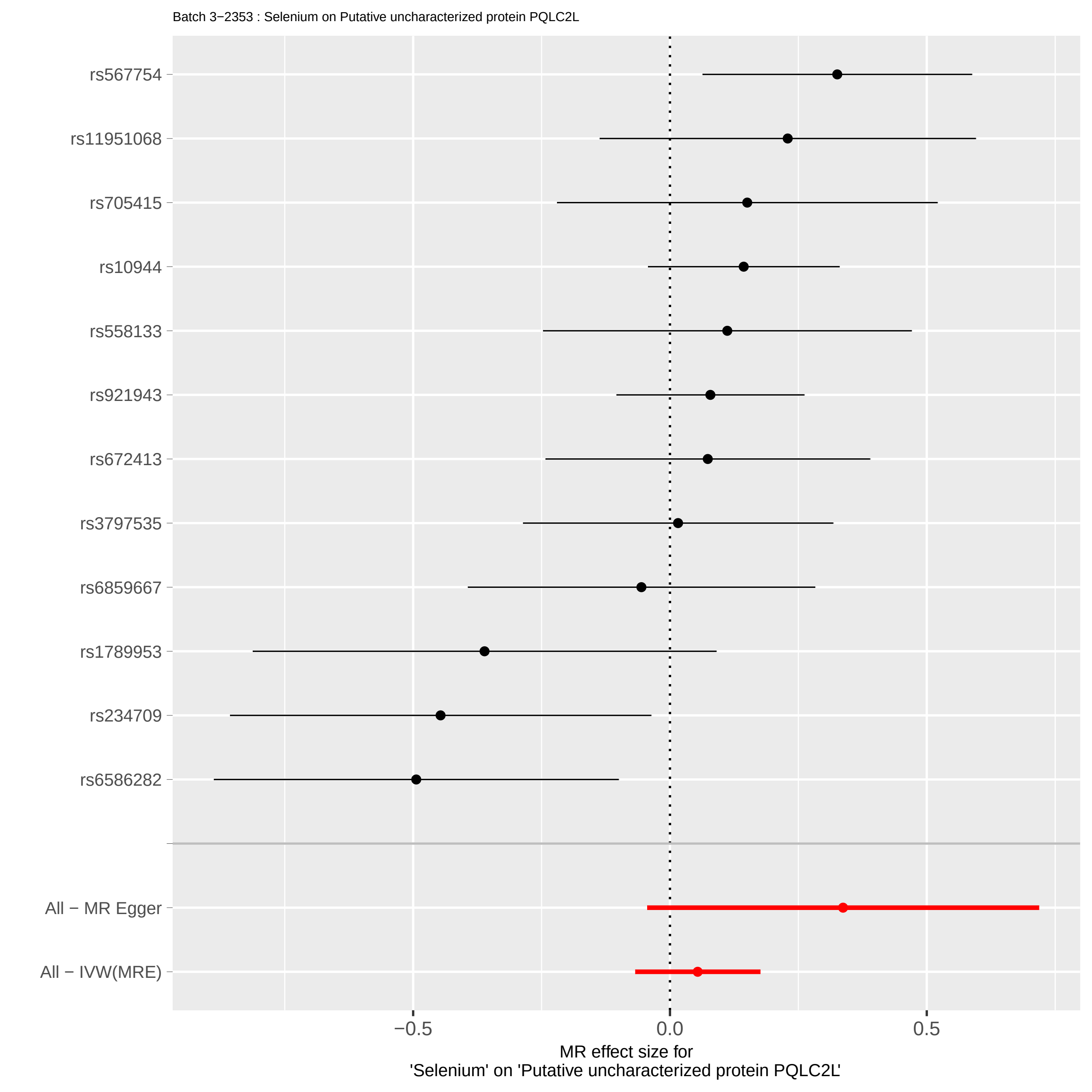

Batch 3−2353 : Selenium on Putative uncharacterized protein PQLC2L
rs567754
rs11951068
rs705415
rs10944
rs558133
rs921943
rs672413
rs3797535
rs6859667
rs1789953
rs234709
rs6586282
All − MR Egger
All − IVW(MRE)
−0.5
0.0
0.5
MR effect size for
'Selenium' on 'Putative uncharacterized protein PQLC2L'

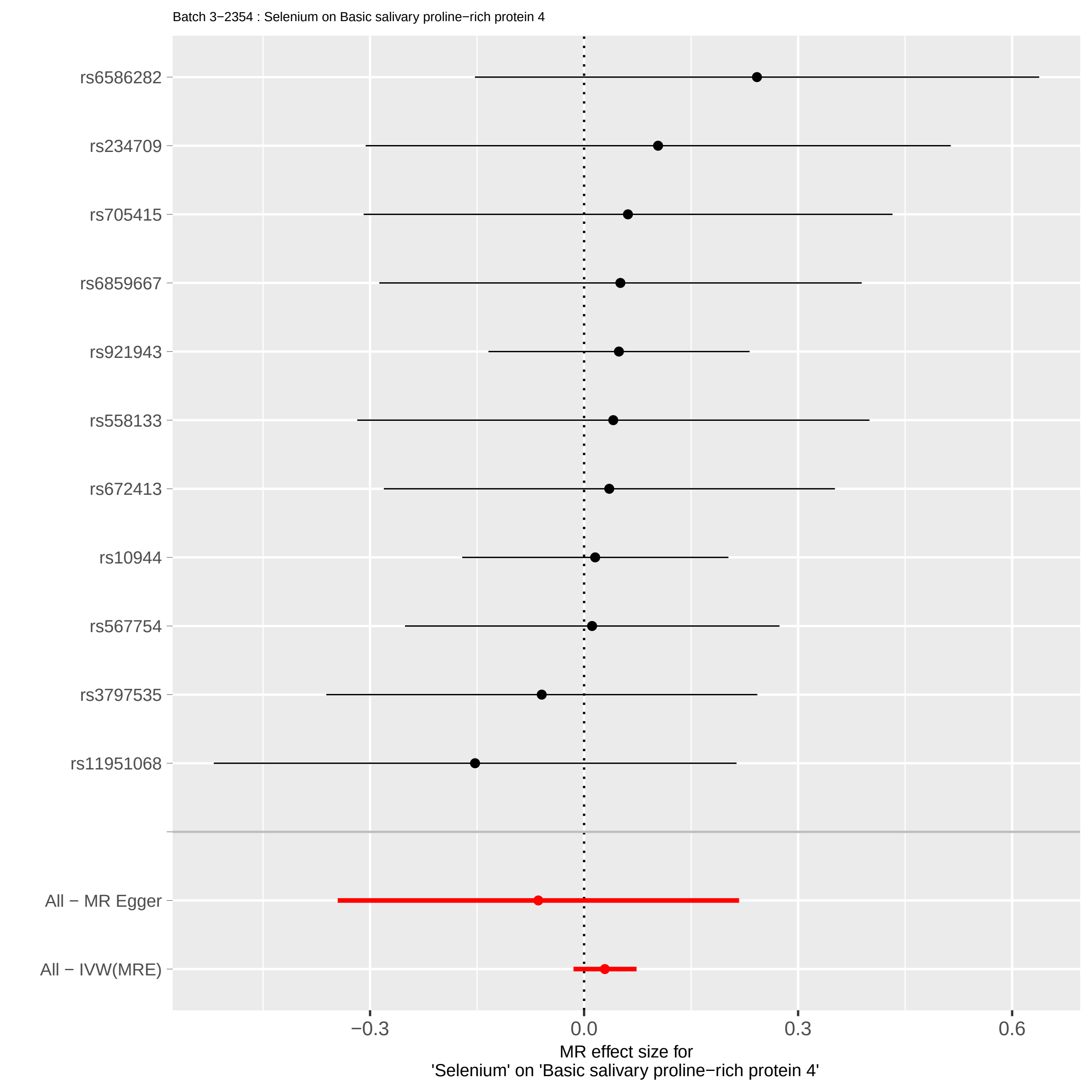

Batch 3−2354 : Selenium on Basic salivary proline−rich protein 4
rs6586282
rs234709
rs705415
rs6859667
rs921943
rs558133
rs672413
rs10944
rs567754
rs3797535
rs11951068
All − MR Egger
All − IVW(MRE)
−0.3
0.0
0.3
0.6
MR effect size for
'Selenium' on 'Basic salivary proline−rich protein 4'

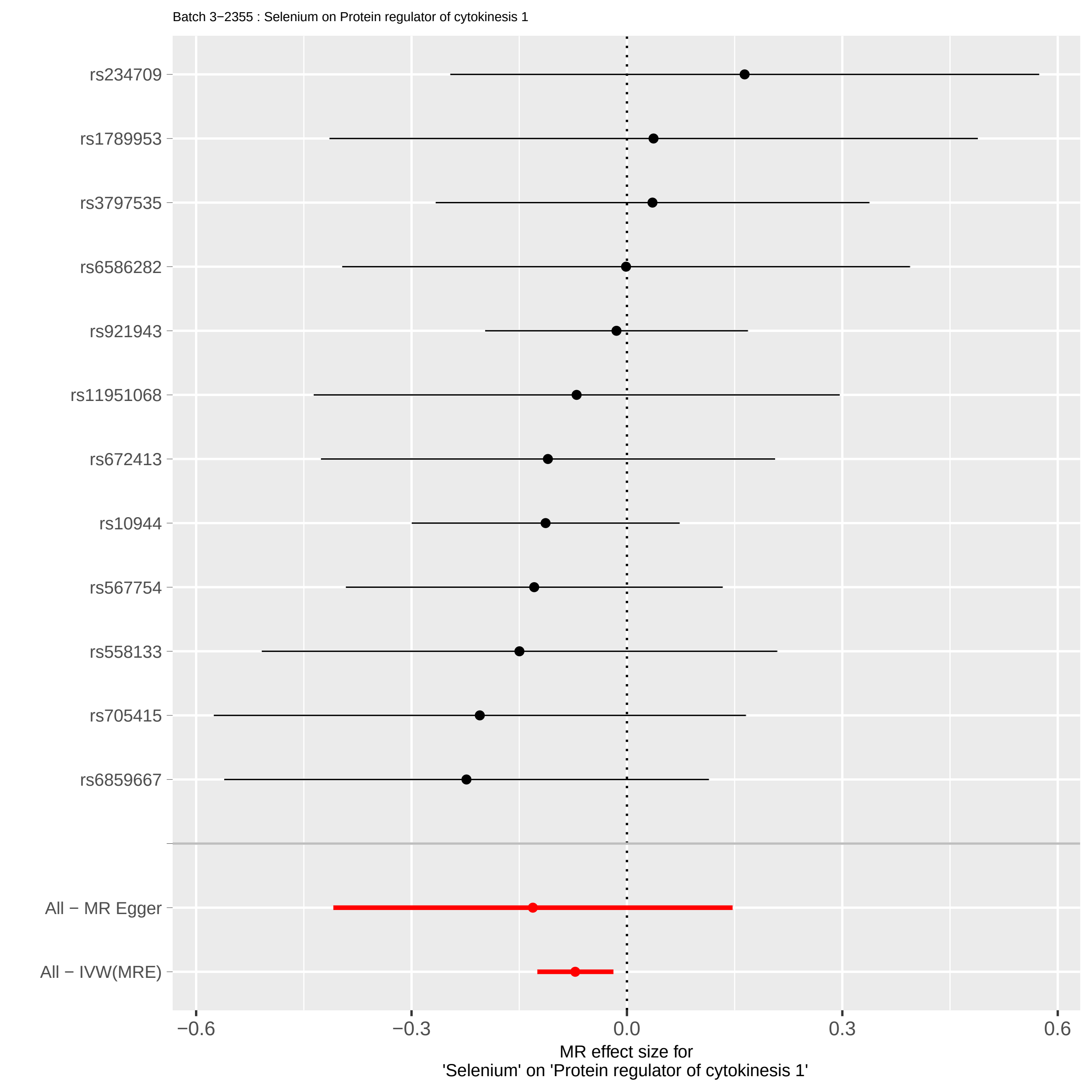
Batch 3−2355 : Selenium on Protein regulator of cytokinesis 1
rs234709
rs1789953
rs3797535
rs6586282
rs921943
rs11951068
rs672413
rs10944
rs567754
rs558133
rs705415
rs6859667
All − MR Egger
All − IVW(MRE)
−0.6
−0.3
0.0
0.3
0.6
MR effect size for
'Selenium' on 'Protein regulator of cytokinesis 1'

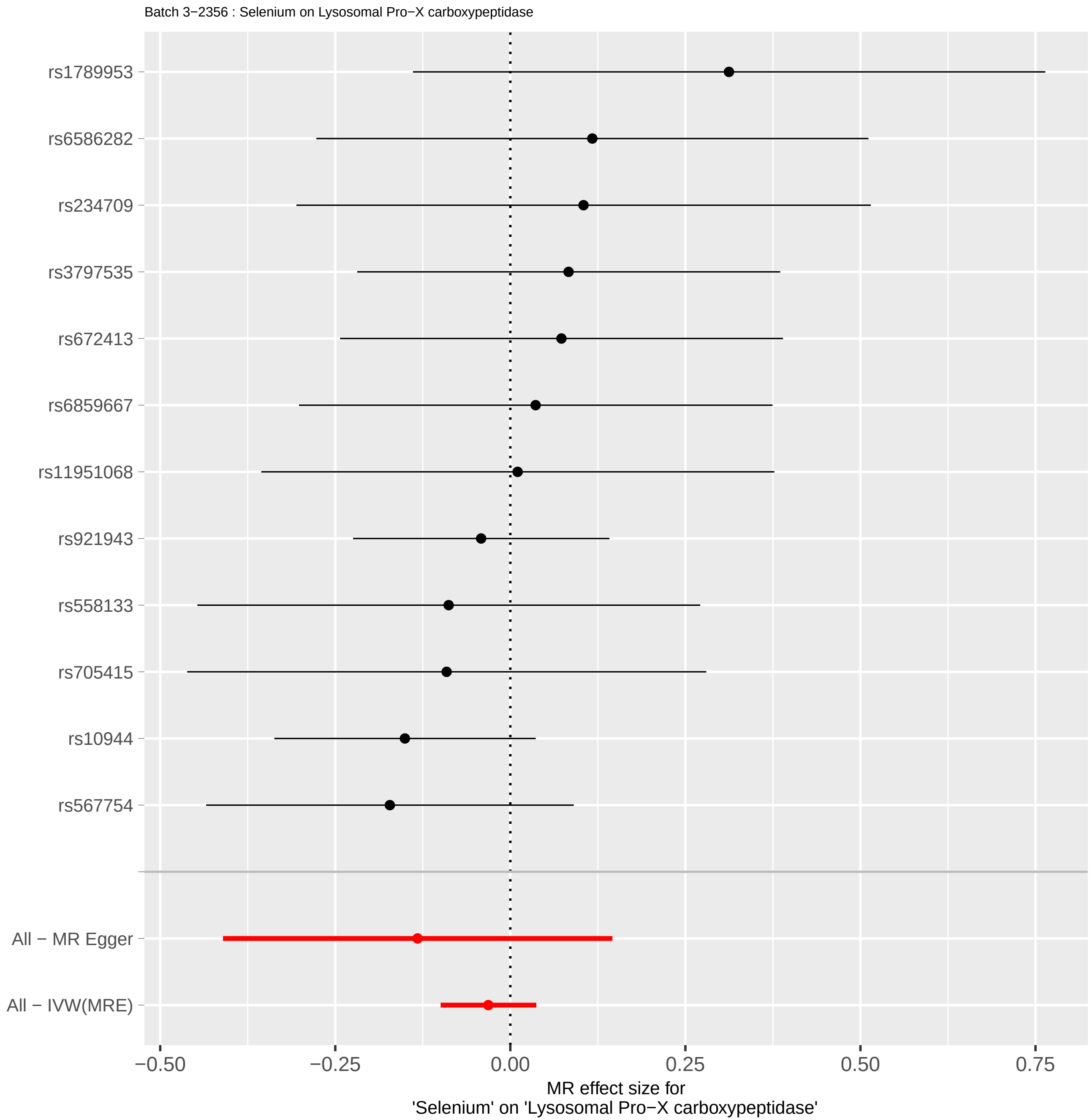
Batch 3−2356 : Selenium on Lysosomal Pro−X carboxypeptidase
rs1789953
rs6586282
rs234709
rs3797535
rs672413
rs6859667
rs11951068
rs921943
rs558133
rs705415
rs10944
rs567754
All − MR Egger
All − IVW(MRE)
−0.50
−0.25
0.00
0.25
0.50
0.75
MR effect size for
'Selenium' on 'Lysosomal Pro−X carboxypeptidase'

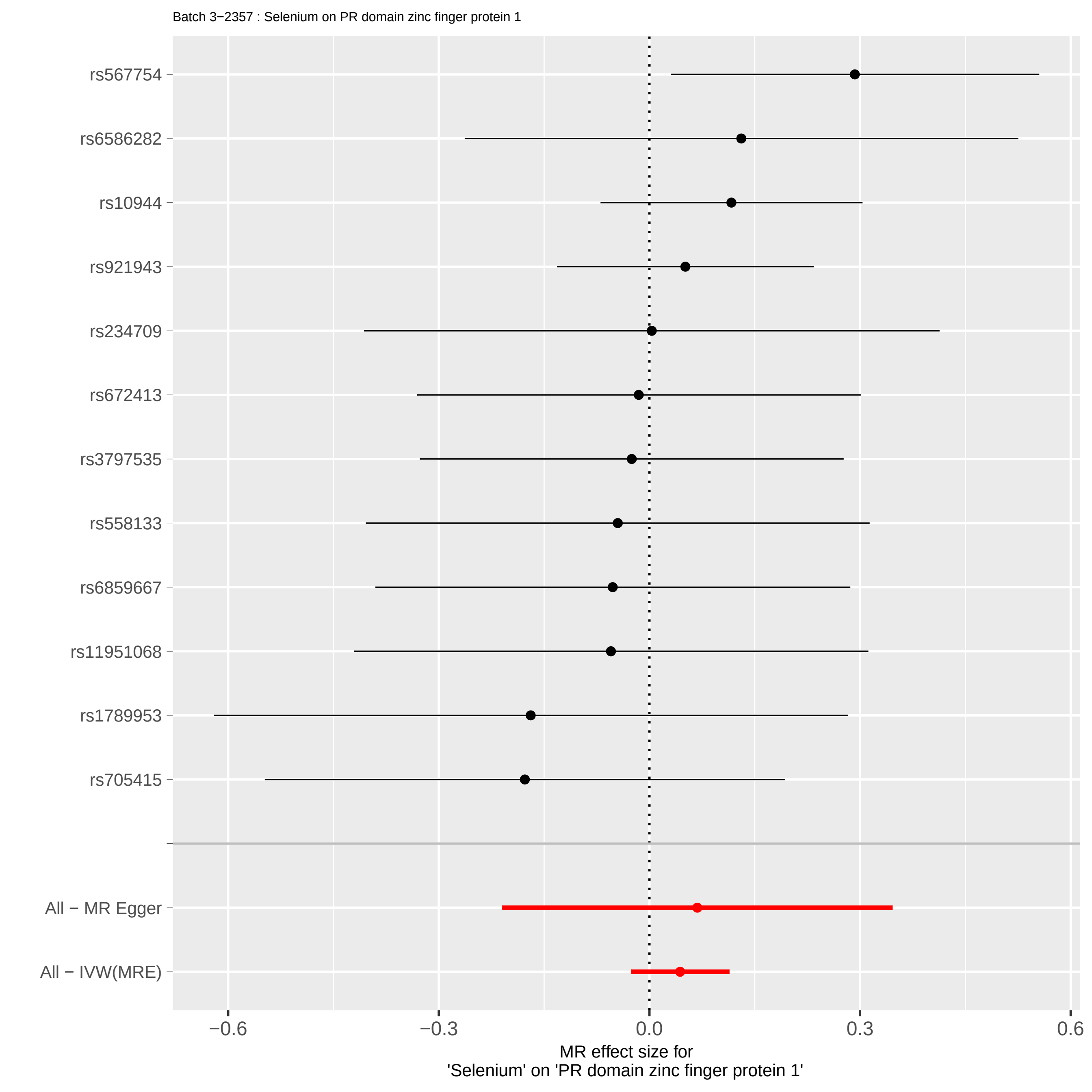

Batch 3−2357 : Selenium on PR domain zinc finger protein 1
rs567754
rs6586282
rs10944
rs921943
rs234709
rs672413
rs3797535
rs558133
rs6859667
rs11951068
rs1789953
rs705415
All − MR Egger
All − IVW(MRE)
−0.6
−0.3
0.0
0.3
0.6
MR effect size for
'Selenium' on 'PR domain zinc finger protein 1'

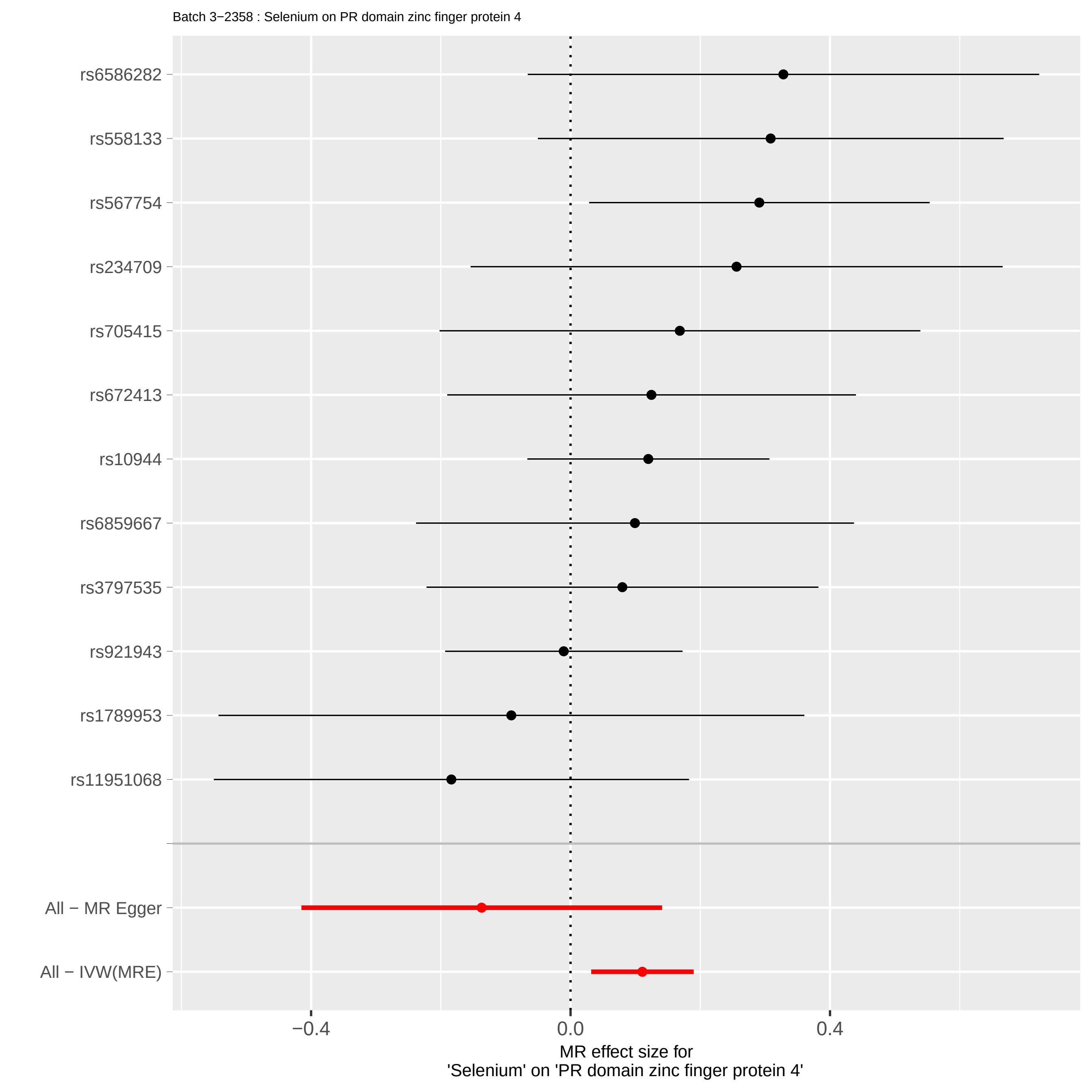

Batch 3−2358 : Selenium on PR domain zinc finger protein 4
rs6586282
rs558133
rs567754
rs234709
rs705415
rs672413
rs10944
rs6859667
rs3797535
rs921943
rs1789953
rs11951068
All − MR Egger
All − IVW(MRE)
−0.4
0.0
0.4
MR effect size for
'Selenium' on 'PR domain zinc finger protein 4'

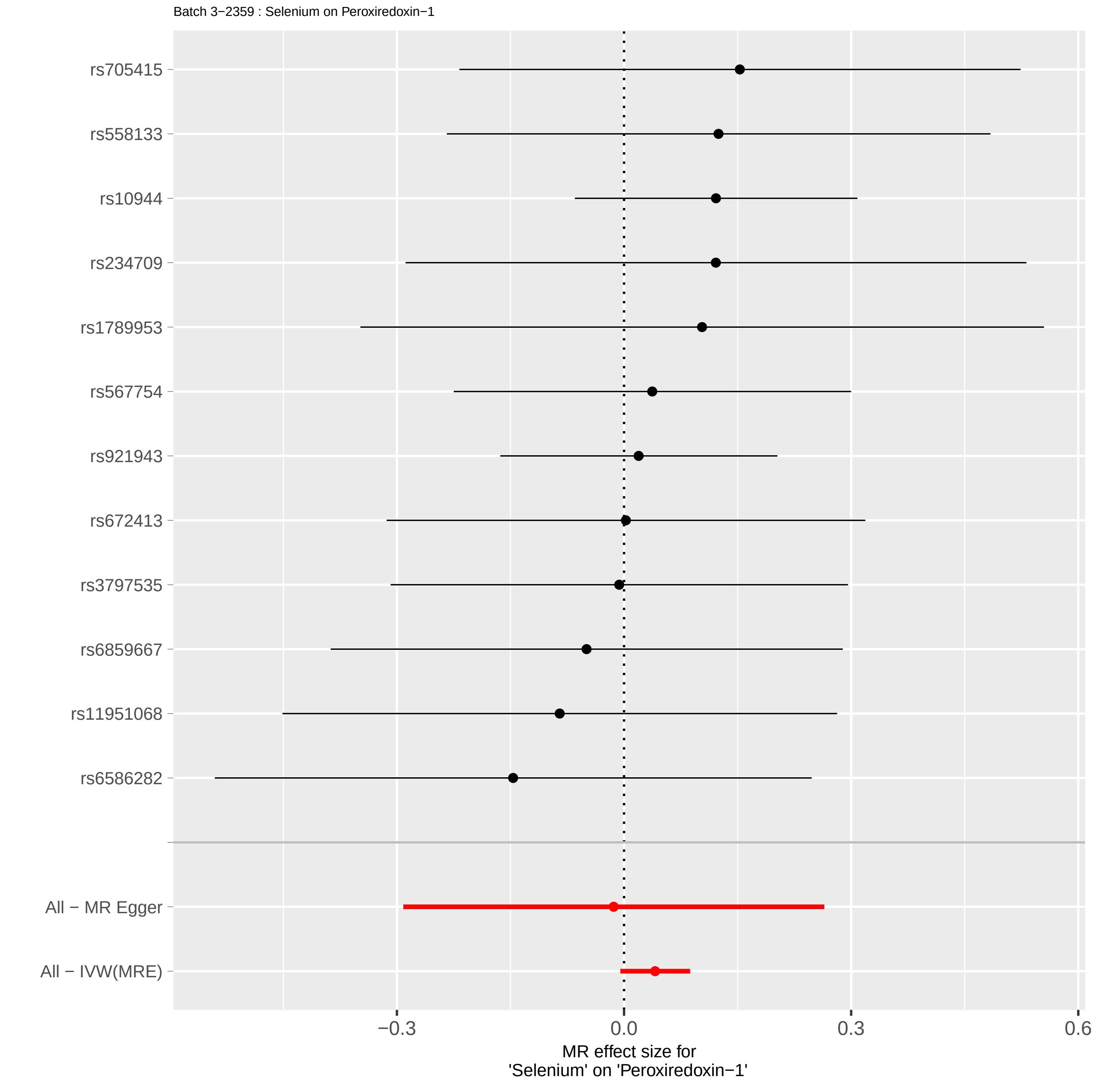

Batch 3−2359 : Selenium on Peroxiredoxin−1
rs705415
rs558133
rs10944
rs234709
rs1789953
rs567754
rs921943
rs672413
rs3797535
rs6859667
rs11951068
rs6586282
All − MR Egger
All − IVW(MRE)
−0.3
0.0
0.3
0.6
MR effect size for
'Selenium' on 'Peroxiredoxin−1'

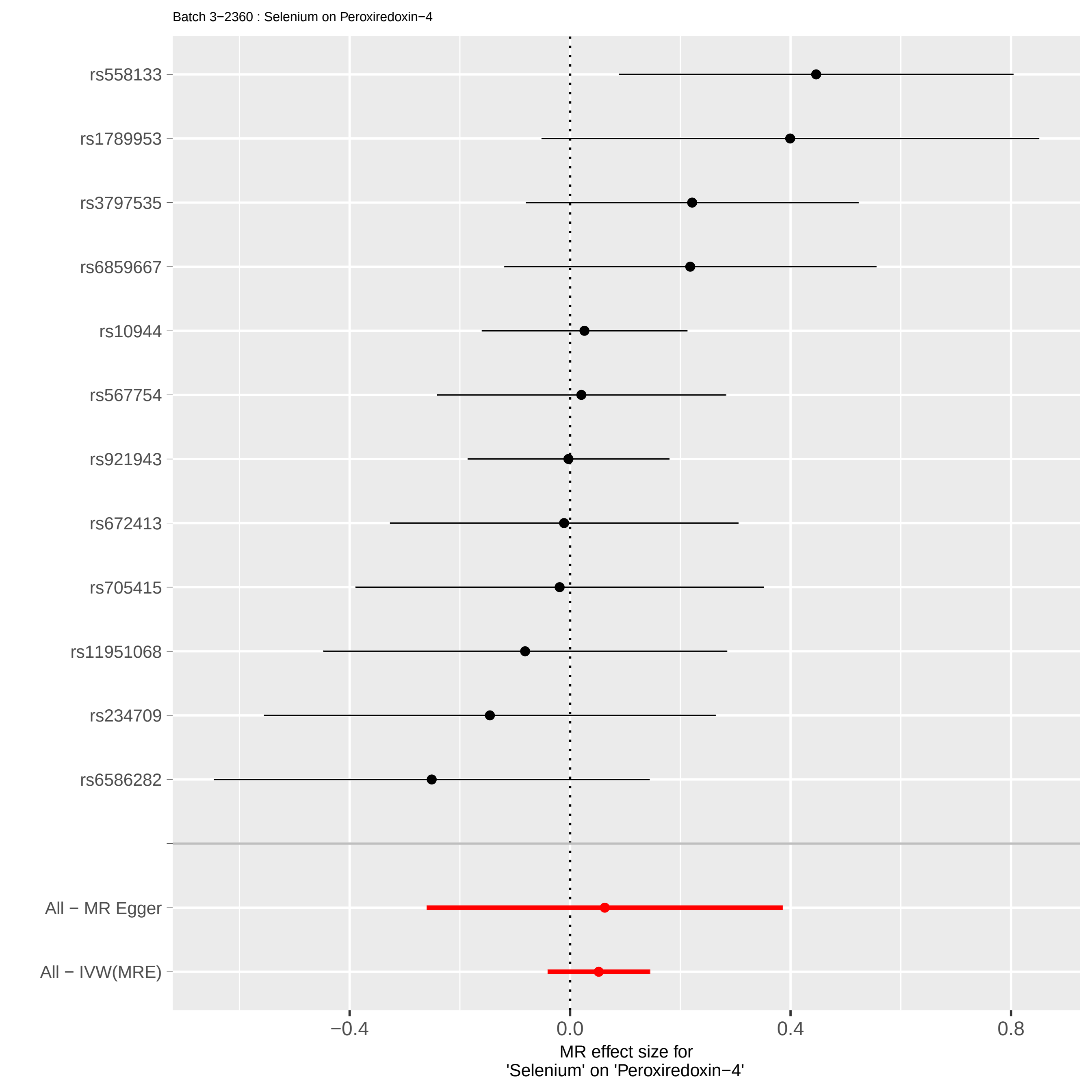

Batch 3−2360 : Selenium on Peroxiredoxin−4
rs558133
rs1789953
rs3797535
rs6859667
rs10944
rs567754
rs921943
rs672413
rs705415
rs11951068
rs234709
rs6586282
All − MR Egger
All − IVW(MRE)
−0.4
0.0
0.4
0.8
MR effect size for
'Selenium' on 'Peroxiredoxin−4'

Batch 3−2361 : Selenium on Prolargin

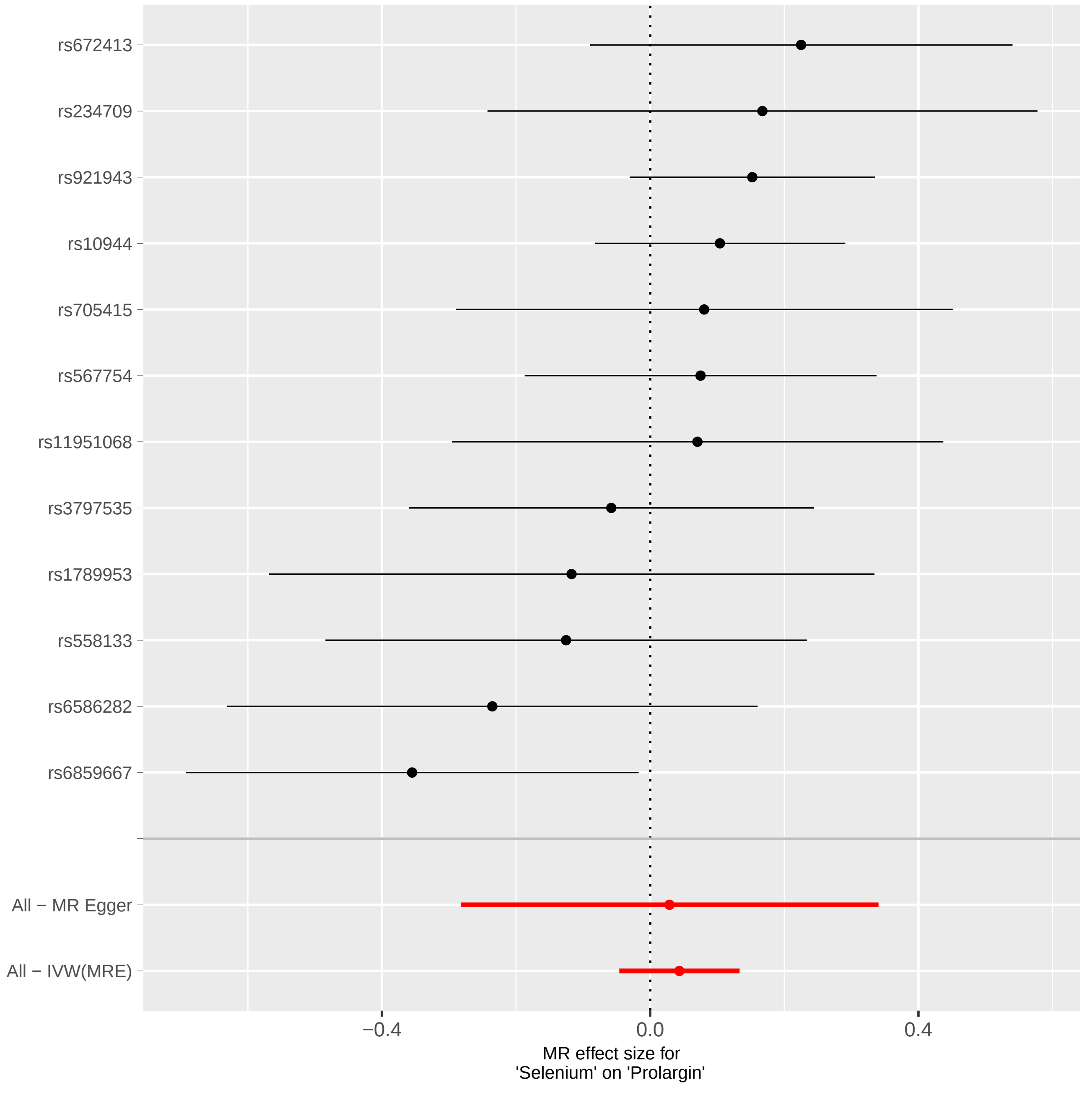

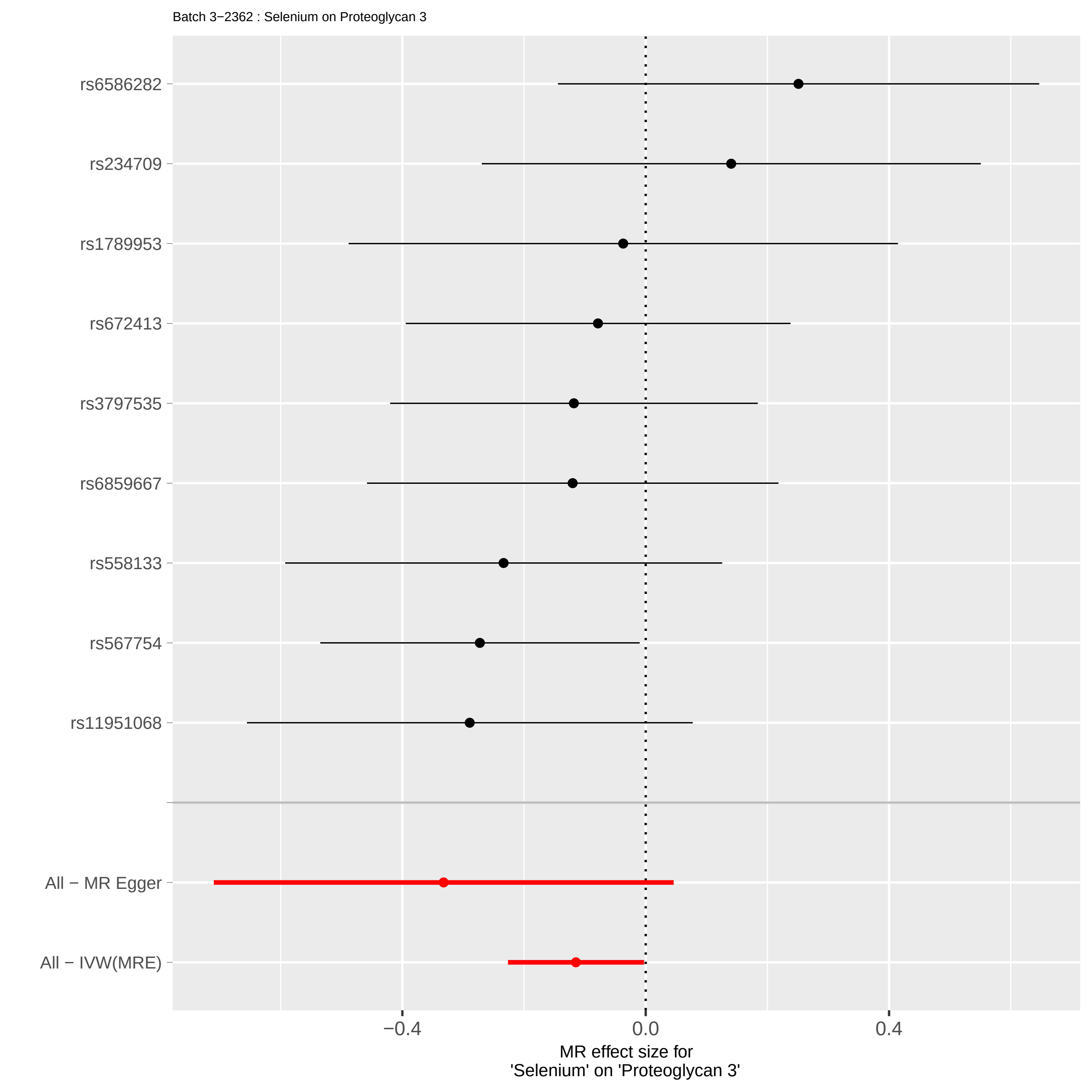

Batch 3−2362 : Selenium on Proteoglycan 3
rs6586282
rs234709
rs1789953
rs672413
rs3797535
rs6859667
rs558133
rs567754
rs11951068
All − MR Egger
All − IVW(MRE)
−0.4
0.0
0.4
MR effect size for
'Selenium' on 'Proteoglycan 3'

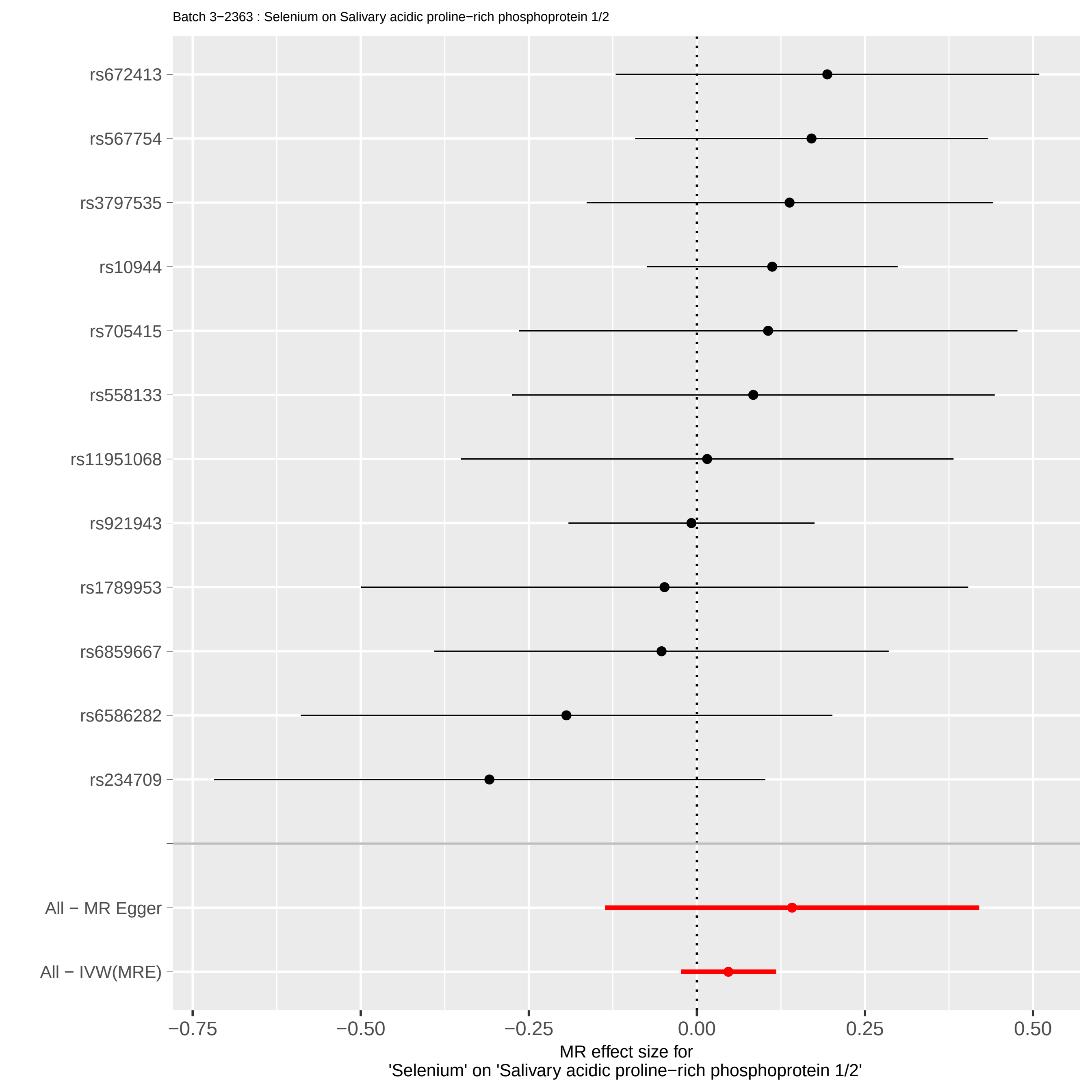

Batch 3−2363 : Selenium on Salivary acidic proline−rich phosphoprotein 1/2
rs672413
rs567754
rs3797535
rs10944
rs705415
rs558133
rs11951068
rs921943
rs1789953
rs6859667
rs6586282
rs234709
All − MR Egger
All − IVW(MRE)
−0.75
−0.50
−0.25
0.00
0.25
0.50
MR effect size for
'Selenium' on 'Salivary acidic proline−rich phosphoprotein 1/2'

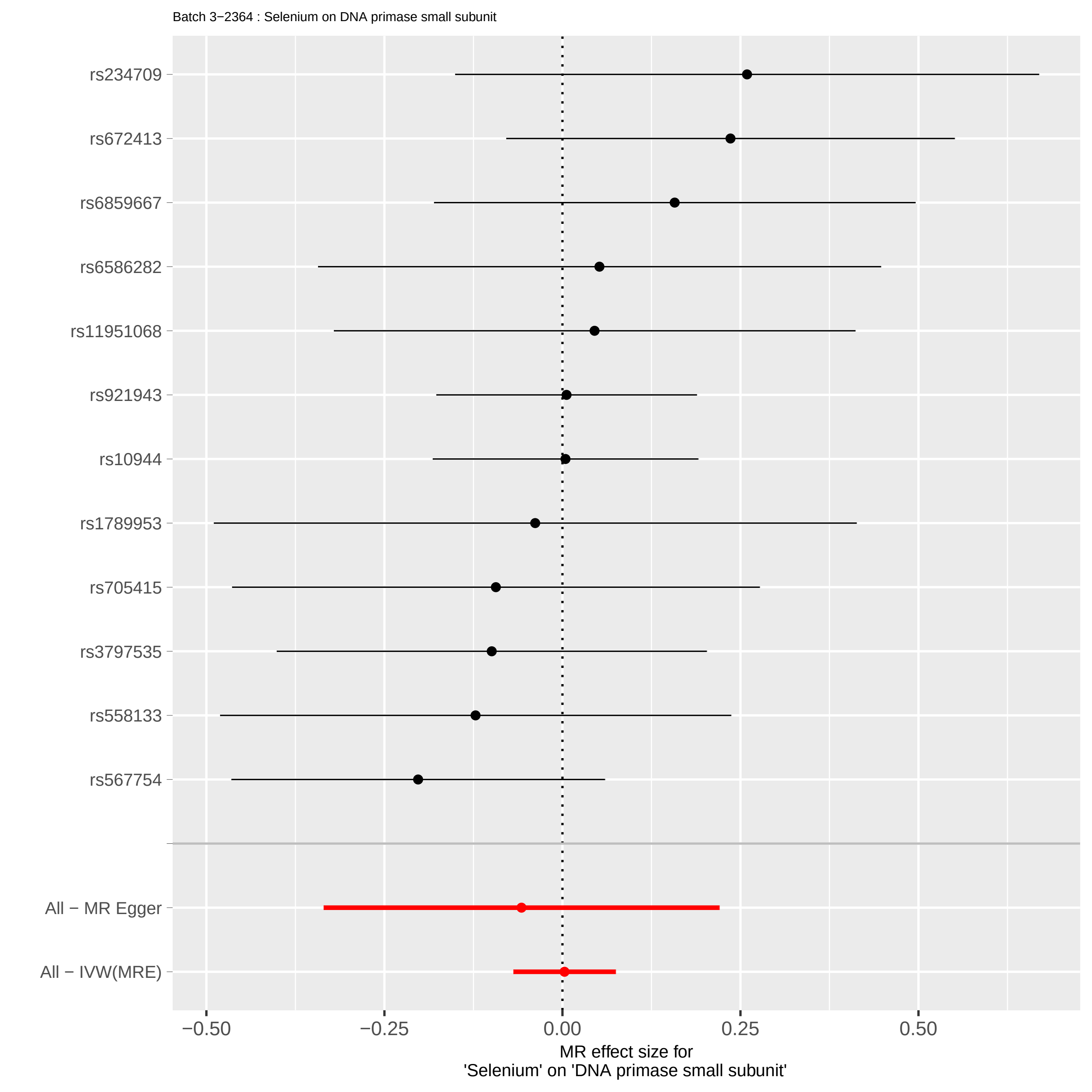

Batch 3−2364 : Selenium on DNA primase small subunit
rs234709
rs672413
rs6859667
rs6586282
rs11951068
rs921943
rs10944
rs1789953
rs705415
rs3797535
rs558133
rs567754
All − MR Egger
All − IVW(MRE)
−0.50
−0.25
0.00
0.25
0.50
MR effect size for
'Selenium' on 'DNA primase small subunit'

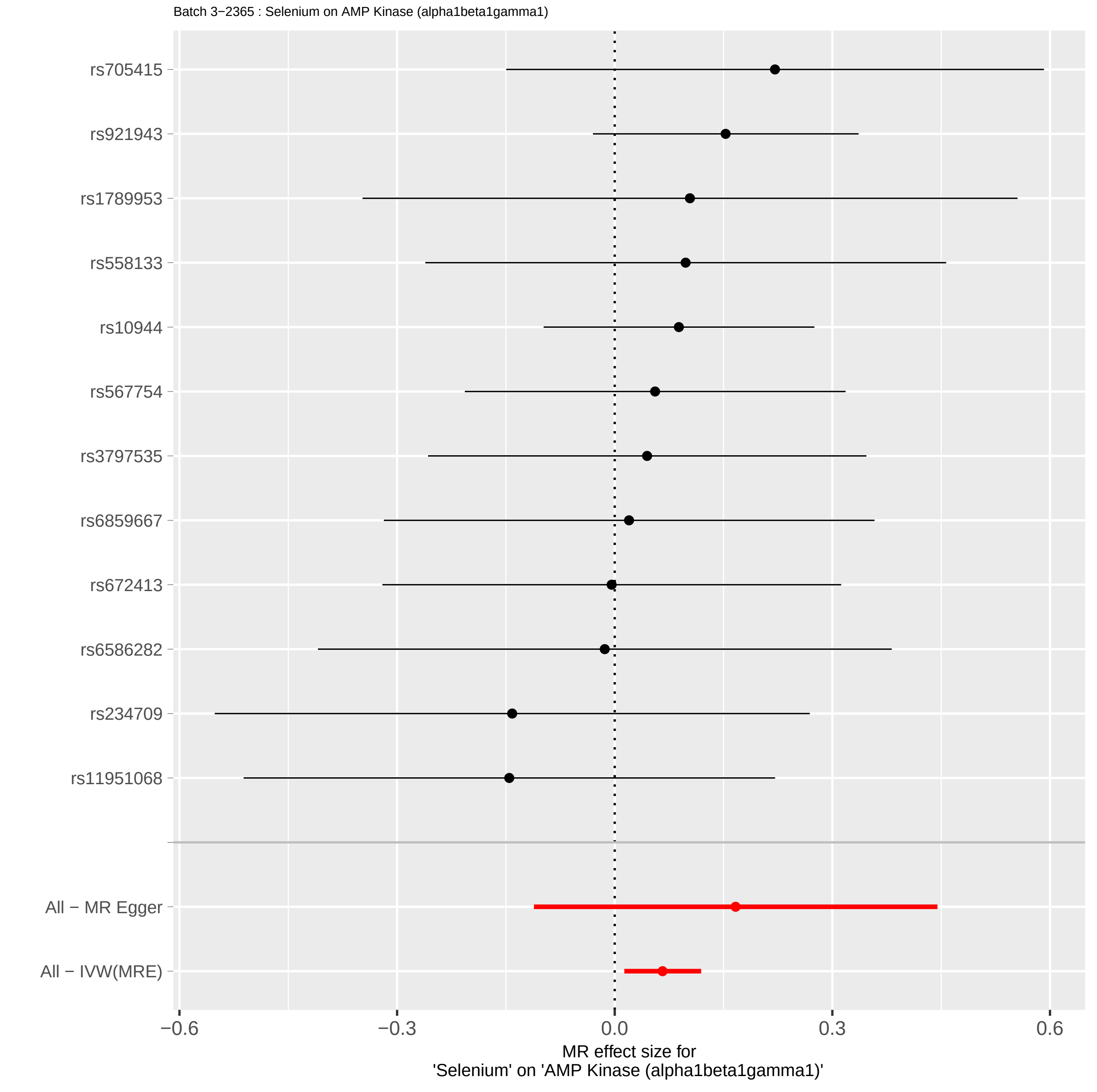

Batch 3−2365 : Selenium on AMP Kinase (alpha1beta1gamma1)
rs705415
rs921943
rs1789953
rs558133
rs10944
rs567754
rs3797535
rs6859667
rs672413
rs6586282
rs234709
rs11951068
All − MR Egger
All − IVW(MRE)
−0.6
−0.3
0.0
0.3
0.6
MR effect size for
'Selenium' on 'AMP Kinase (alpha1beta1gamma1)'

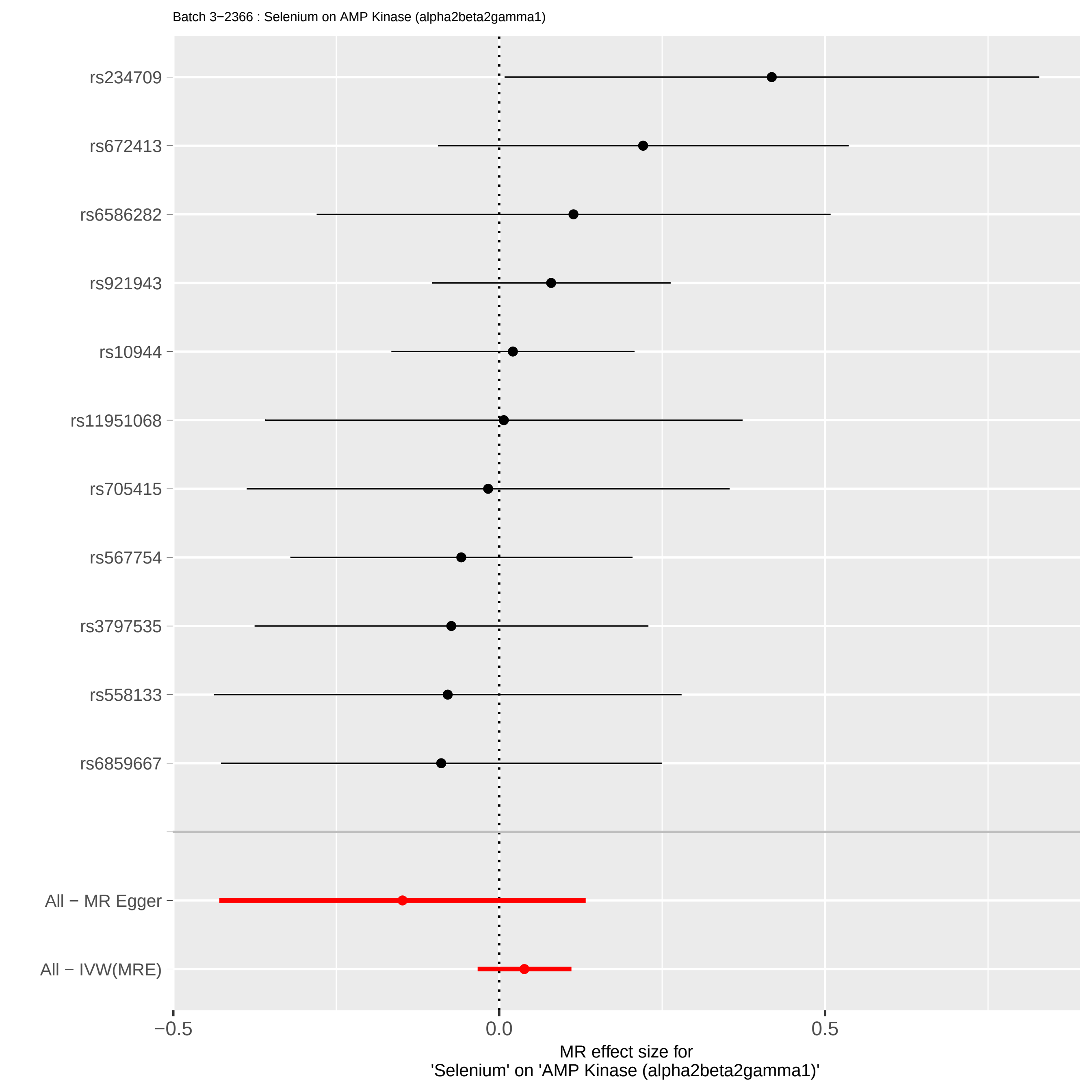
Batch 3−2366 : Selenium on AMP Kinase (alpha2beta2gamma1)
rs234709
rs672413
rs6586282
rs921943
rs10944
rs11951068
rs705415
rs567754
rs3797535
rs558133
rs6859667
All − MR Egger
All − IVW(MRE)
−0.5
0.0
0.5
MR effect size for
'Selenium' on 'AMP Kinase (alpha2beta2gamma1)'

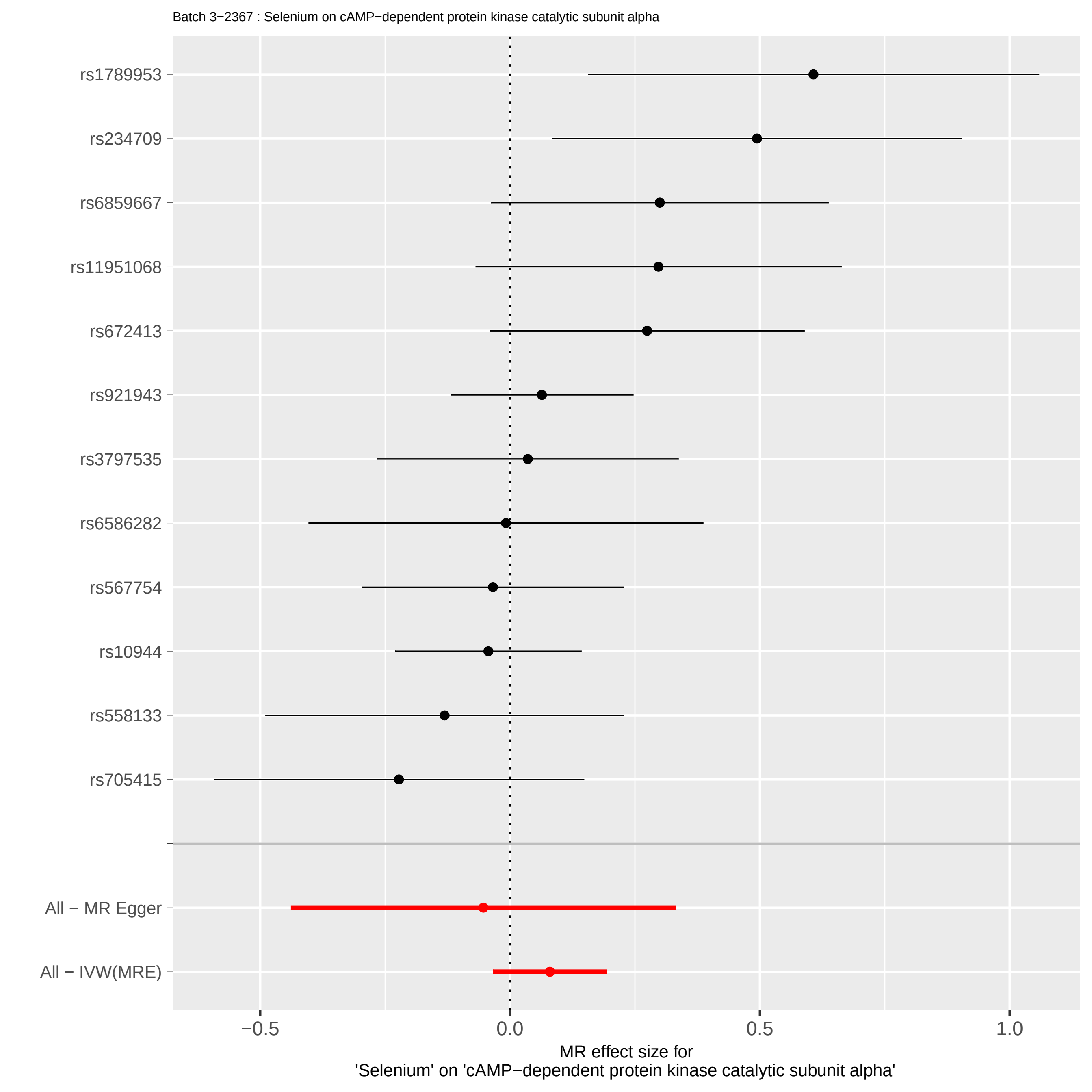

Batch 3−2367 : Selenium on cAMP−dependent protein kinase catalytic subunit alpha
rs1789953
rs234709
rs6859667
rs11951068
rs672413
rs921943
rs3797535
rs6586282
rs567754
rs10944
rs558133
rs705415
All − MR Egger
All − IVW(MRE)
−0.5
0.0
0.5
1.0
MR effect size for
'Selenium' on 'cAMP−dependent protein kinase catalytic subunit alpha'

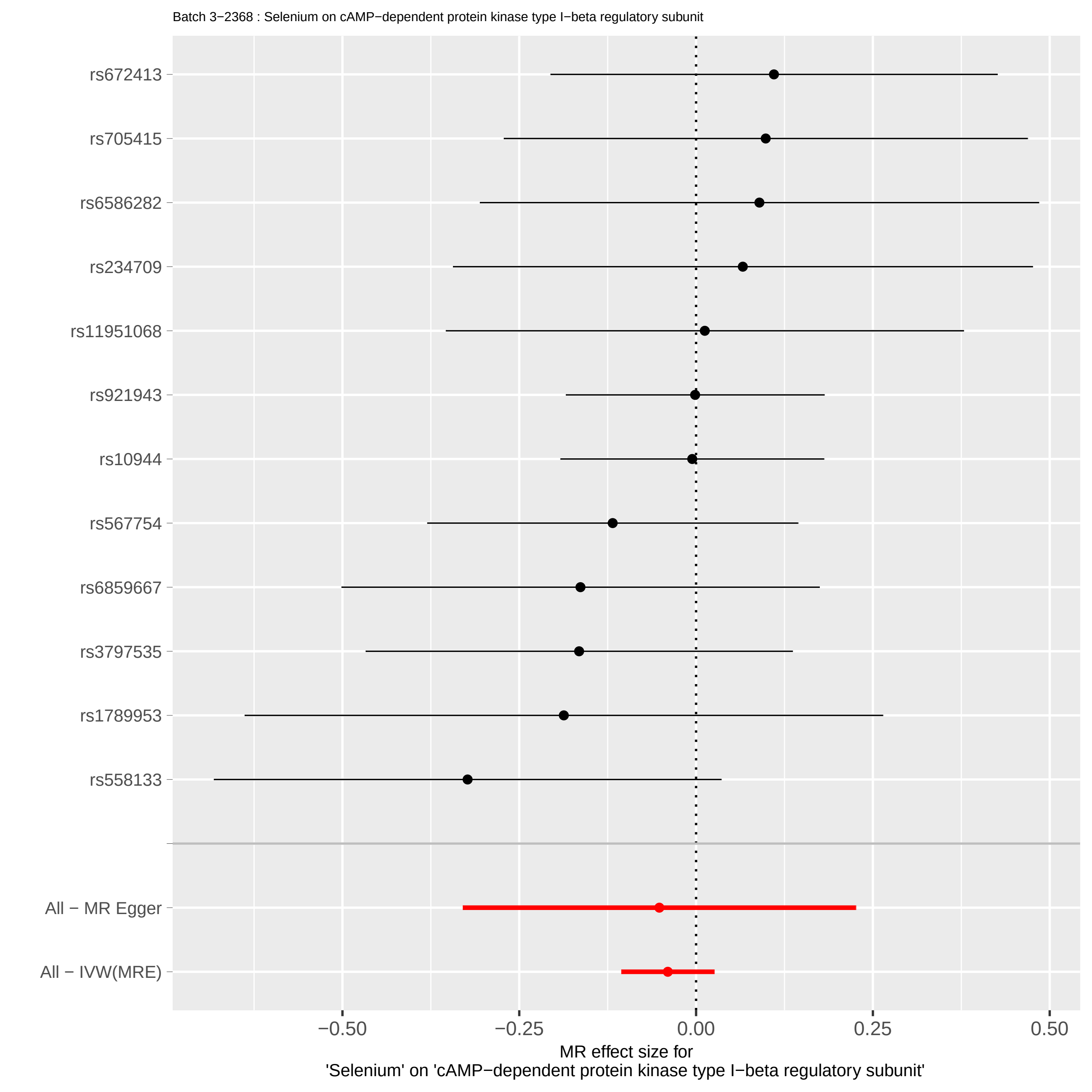
Batch 3−2368 : Selenium on cAMP−dependent protein kinase type I−beta regulatory subunit
rs672413
rs705415
rs6586282
rs234709
rs11951068
rs921943
rs10944
rs567754
rs6859667
rs3797535
rs1789953
rs558133
All − MR Egger
All − IVW(MRE)
−0.50
−0.25
0.00
0.25
0.50
MR effect size for
'Selenium' on 'cAMP−dependent protein kinase type I−beta regulatory subunit'

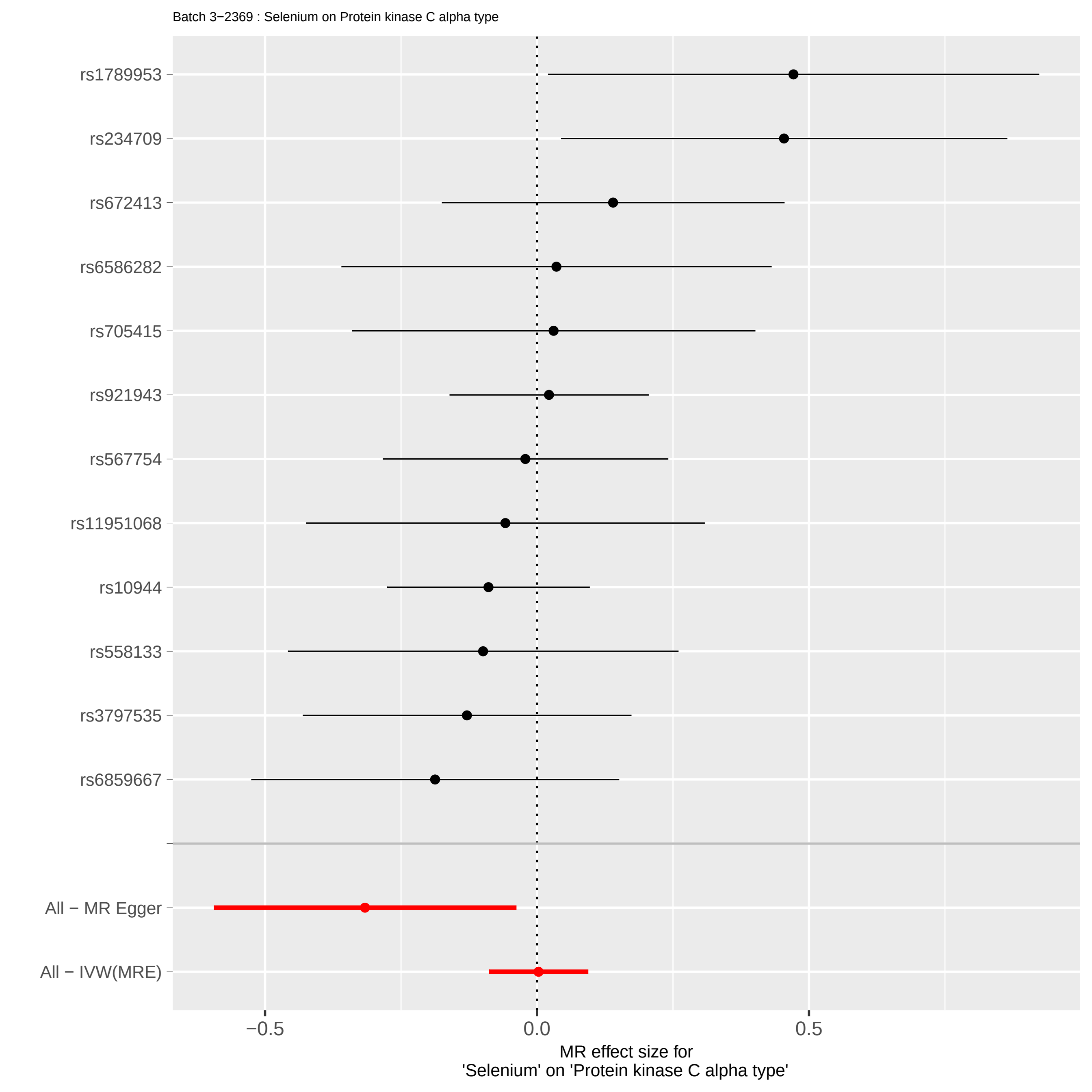

Batch 3−2369 : Selenium on Protein kinase C alpha type
rs1789953
rs234709
rs672413
rs6586282
rs705415
rs921943
rs567754
rs11951068
rs10944
rs558133
rs3797535
rs6859667
All − MR Egger
All − IVW(MRE)
−0.5
0.0
0.5
MR effect size for
'Selenium' on 'Protein kinase C alpha type'

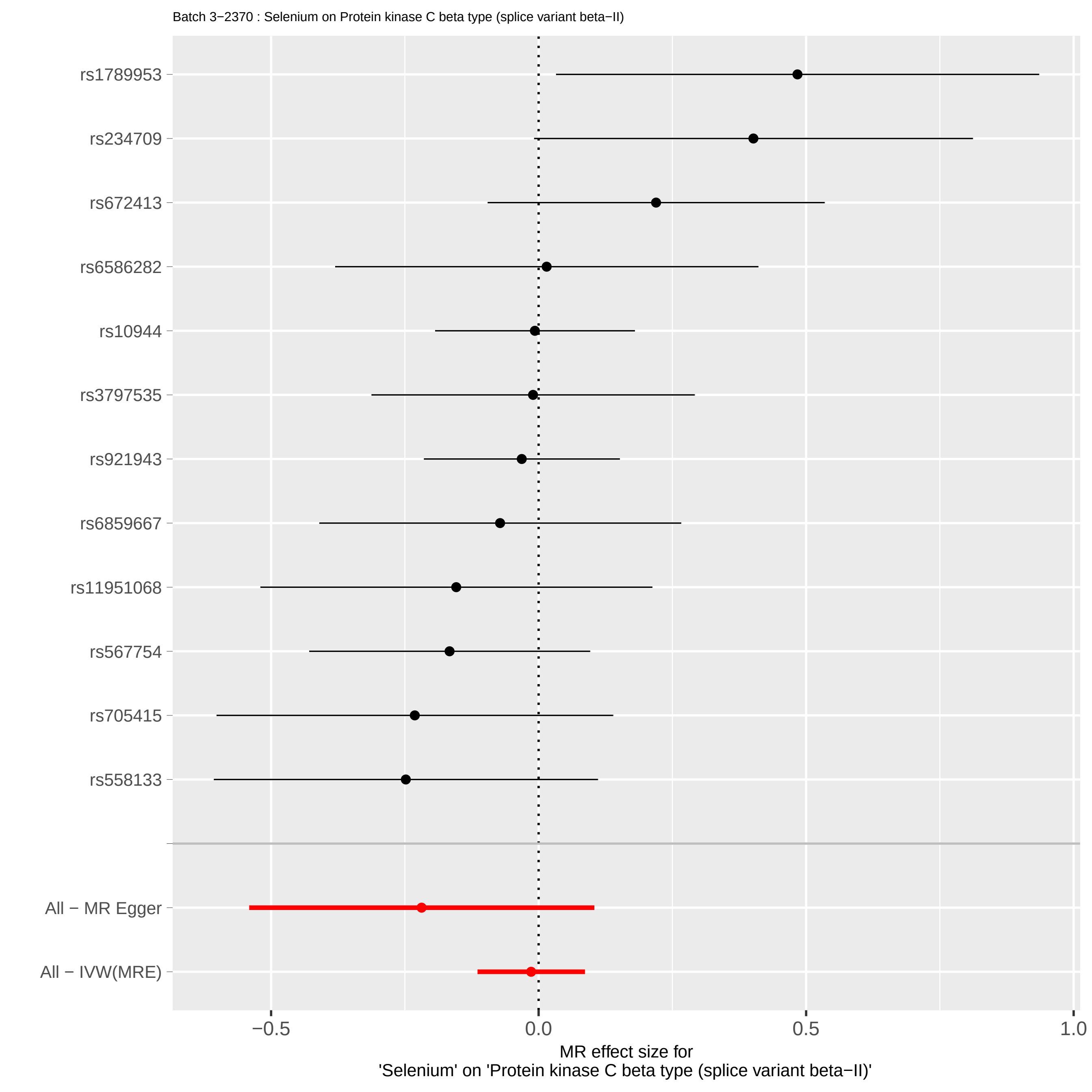

Batch 3−2370 : Selenium on Protein kinase C beta type (splice variant beta−II)
rs1789953
rs234709
rs672413
rs6586282
rs10944
rs3797535
rs921943
rs6859667
rs11951068
rs567754
rs705415
rs558133
All − MR Egger
All − IVW(MRE)
−0.5
0.0
0.5
1.0
MR effect size for
'Selenium' on 'Protein kinase C beta type (splice variant beta−II)'

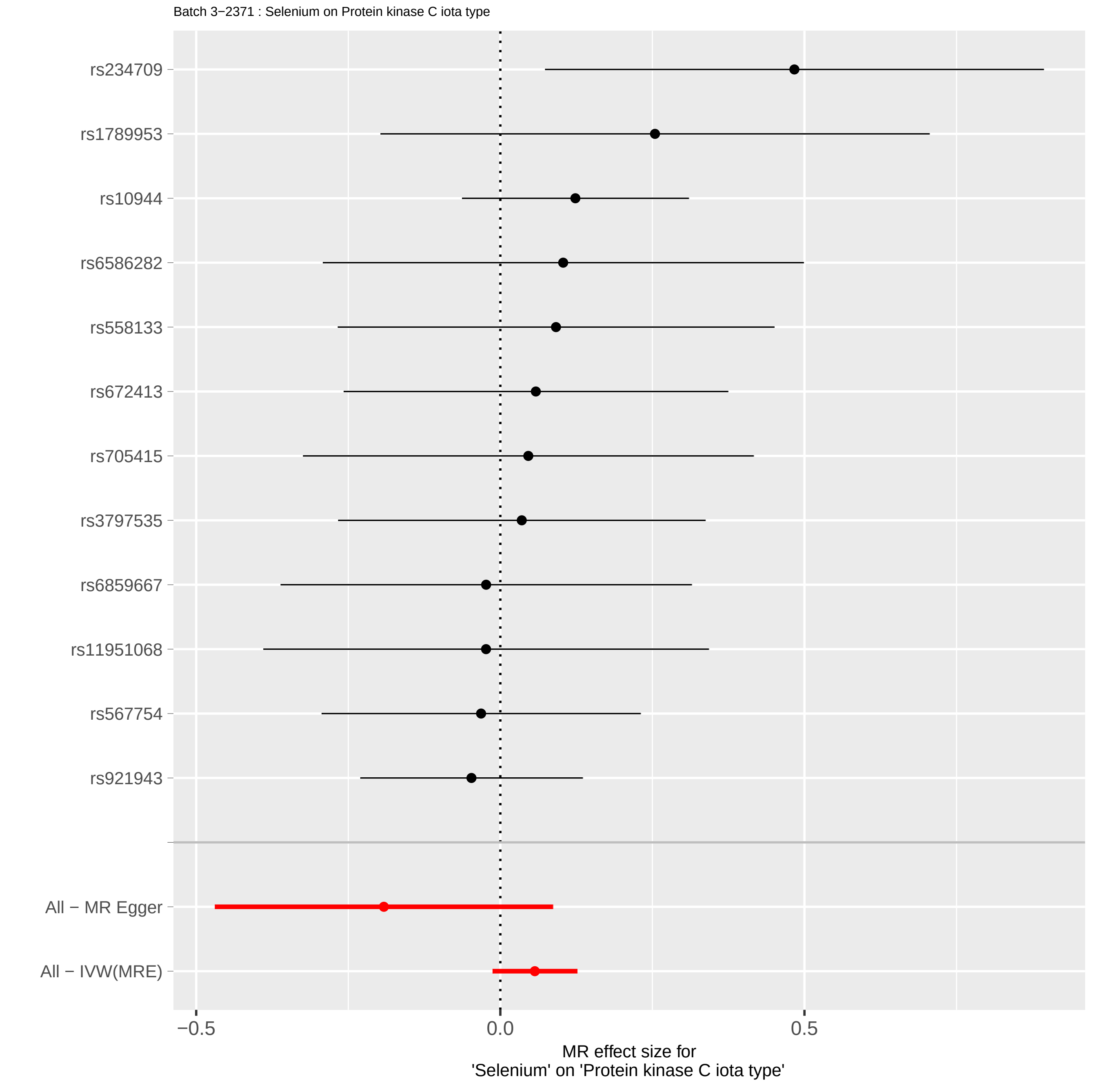
Batch 3−2371 : Selenium on Protein kinase C iota type
rs234709
rs1789953
rs10944
rs6586282
rs558133
rs672413
rs705415
rs3797535
rs6859667
rs11951068
rs567754
rs921943
All − MR Egger
All − IVW(MRE)
−0.5
0.0
0.5
MR effect size for
'Selenium' on 'Protein kinase C iota type'

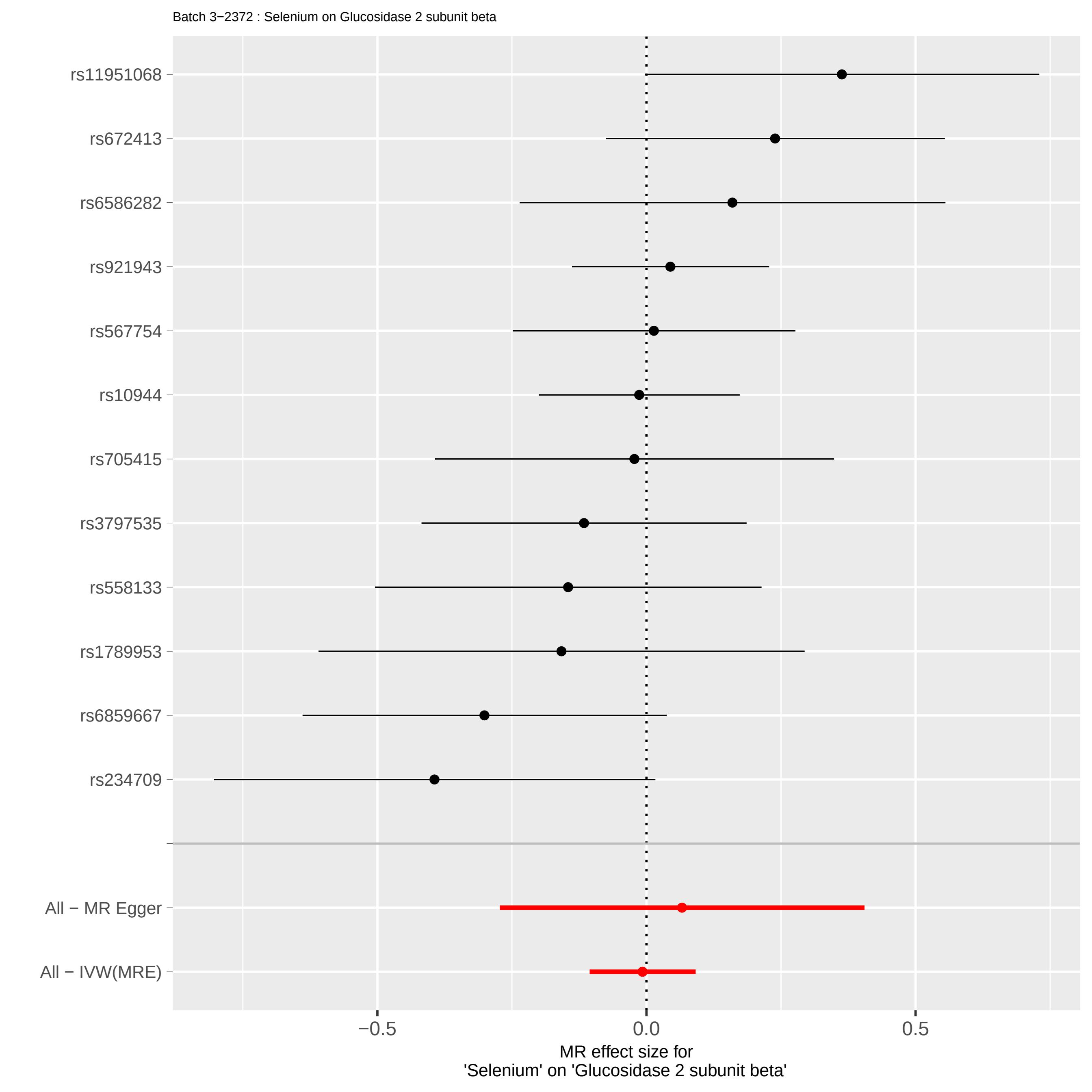

Batch 3−2372 : Selenium on Glucosidase 2 subunit beta
rs11951068
rs672413
rs6586282
rs921943
rs567754
rs10944
rs705415
rs3797535
rs558133
rs1789953
rs6859667
rs234709
All − MR Egger
All − IVW(MRE)
−0.5
0.0
0.5
MR effect size for
'Selenium' on 'Glucosidase 2 subunit beta'

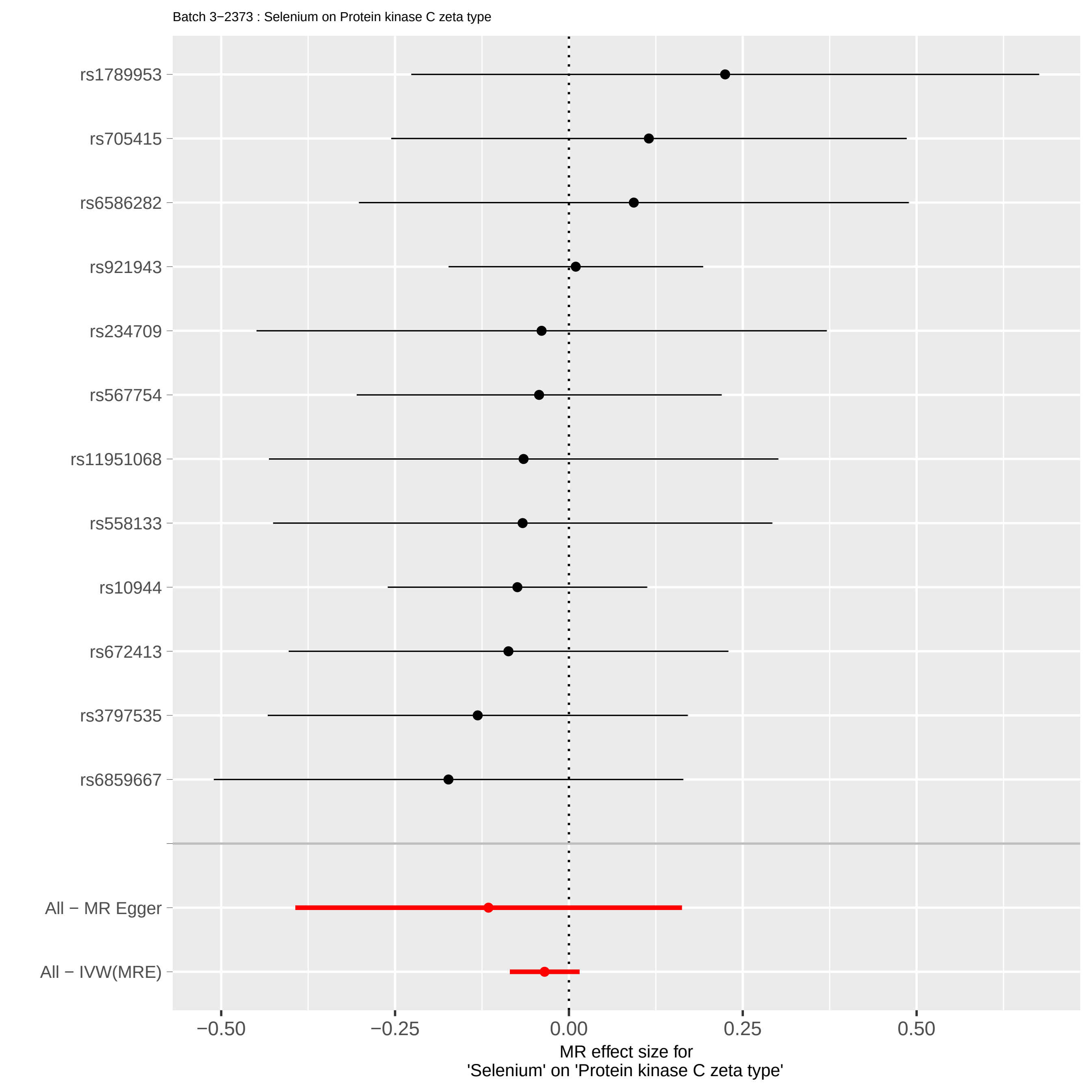

Batch 3−2373 : Selenium on Protein kinase C zeta type
rs1789953
rs705415
rs6586282
rs921943
rs234709
rs567754
rs11951068
rs558133
rs10944
rs672413
rs3797535
rs6859667
All − MR Egger
All − IVW(MRE)
−0.50
−0.25
0.00
0.25
0.50
MR effect size for
'Selenium' on 'Protein kinase C zeta type'

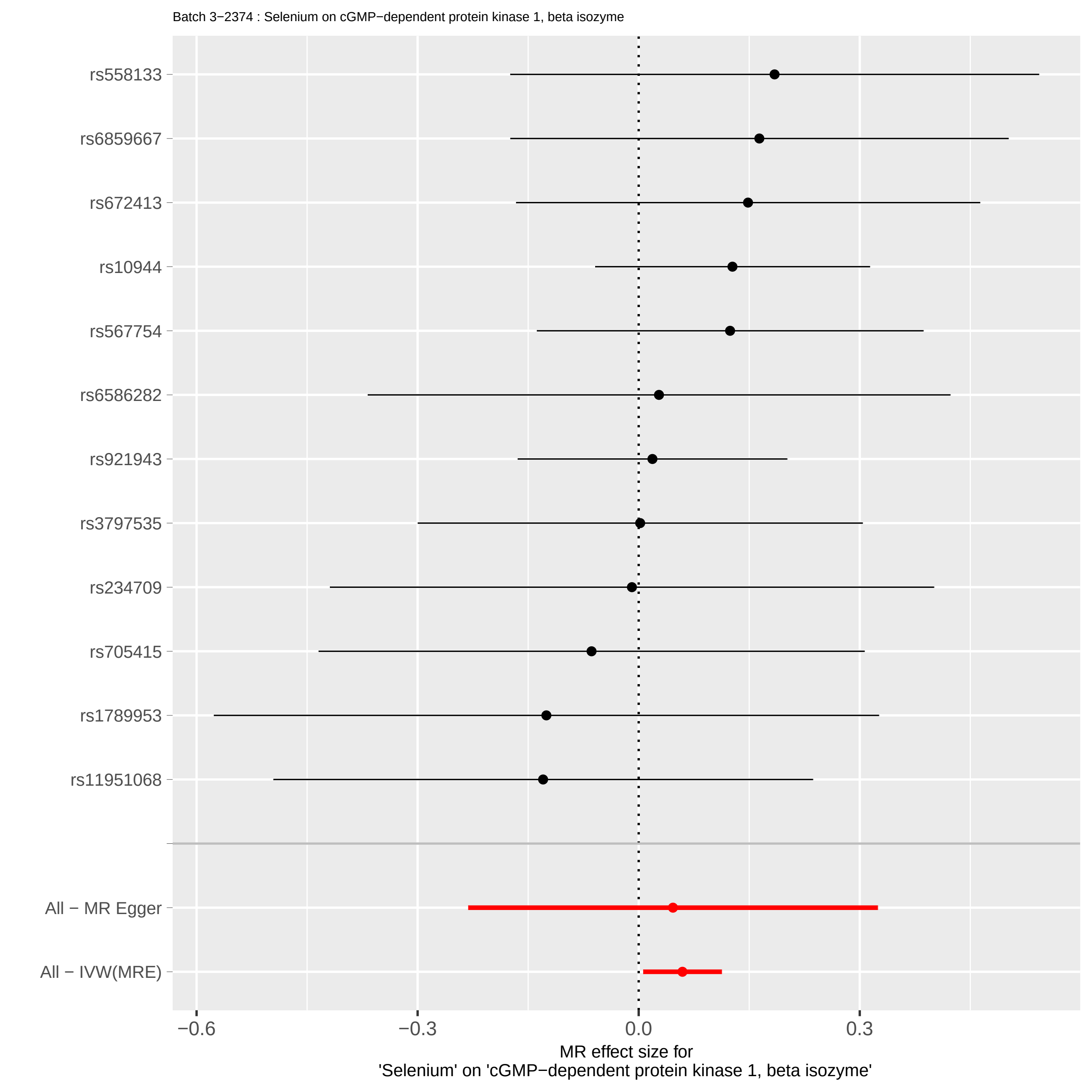
Batch 3−2374 : Selenium on cGMP−dependent protein kinase 1, beta isozyme
rs558133
rs6859667
rs672413
rs10944
rs567754
rs6586282
rs921943
rs3797535
rs234709
rs705415
rs1789953
rs11951068
All − MR Egger
All − IVW(MRE)
−0.6
−0.3
0.0
0.3
MR effect size for
'Selenium' on 'cGMP−dependent protein kinase 1, beta isozyme'

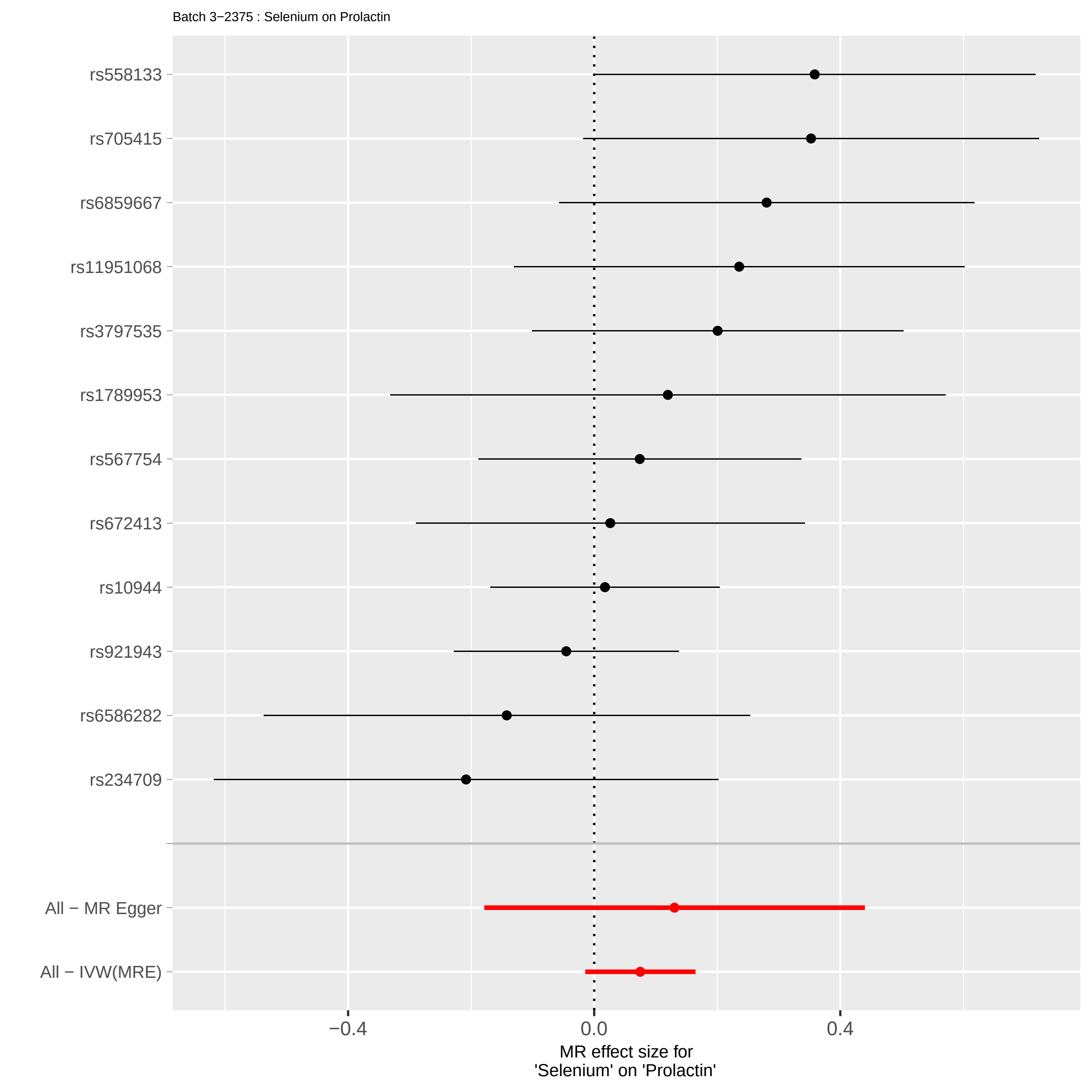

Batch 3−2375 : Selenium on Prolactin
rs558133
rs705415
rs6859667
rs11951068
rs3797535
rs1789953
rs567754
rs672413
rs10944
rs921943
rs6586282
rs234709
All − MR Egger
All − IVW(MRE)
−0.4
0.0
0.4
MR effect size for
'Selenium' on 'Prolactin'

Batch 3−2376 : Selenium on Prolactin−releasing peptide

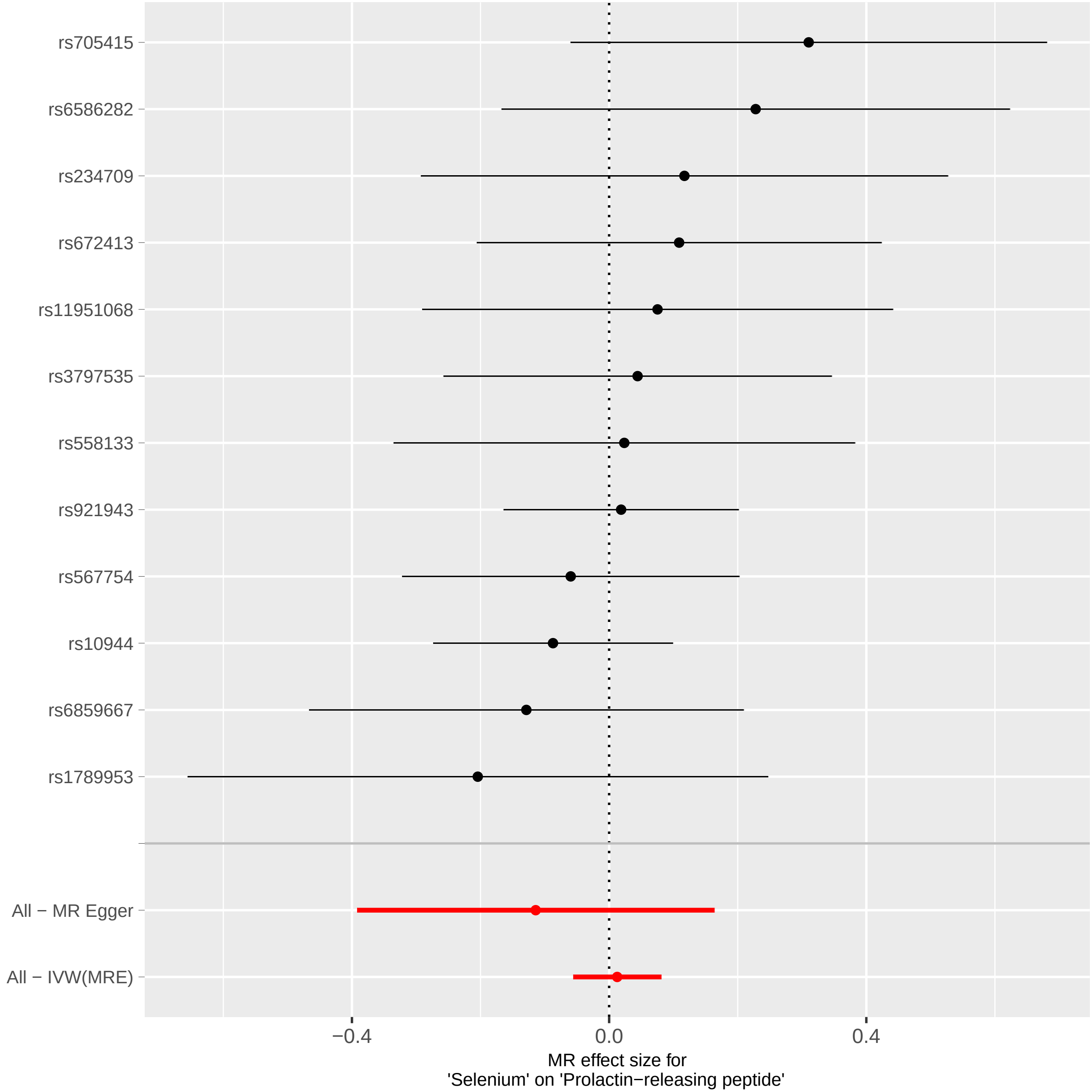

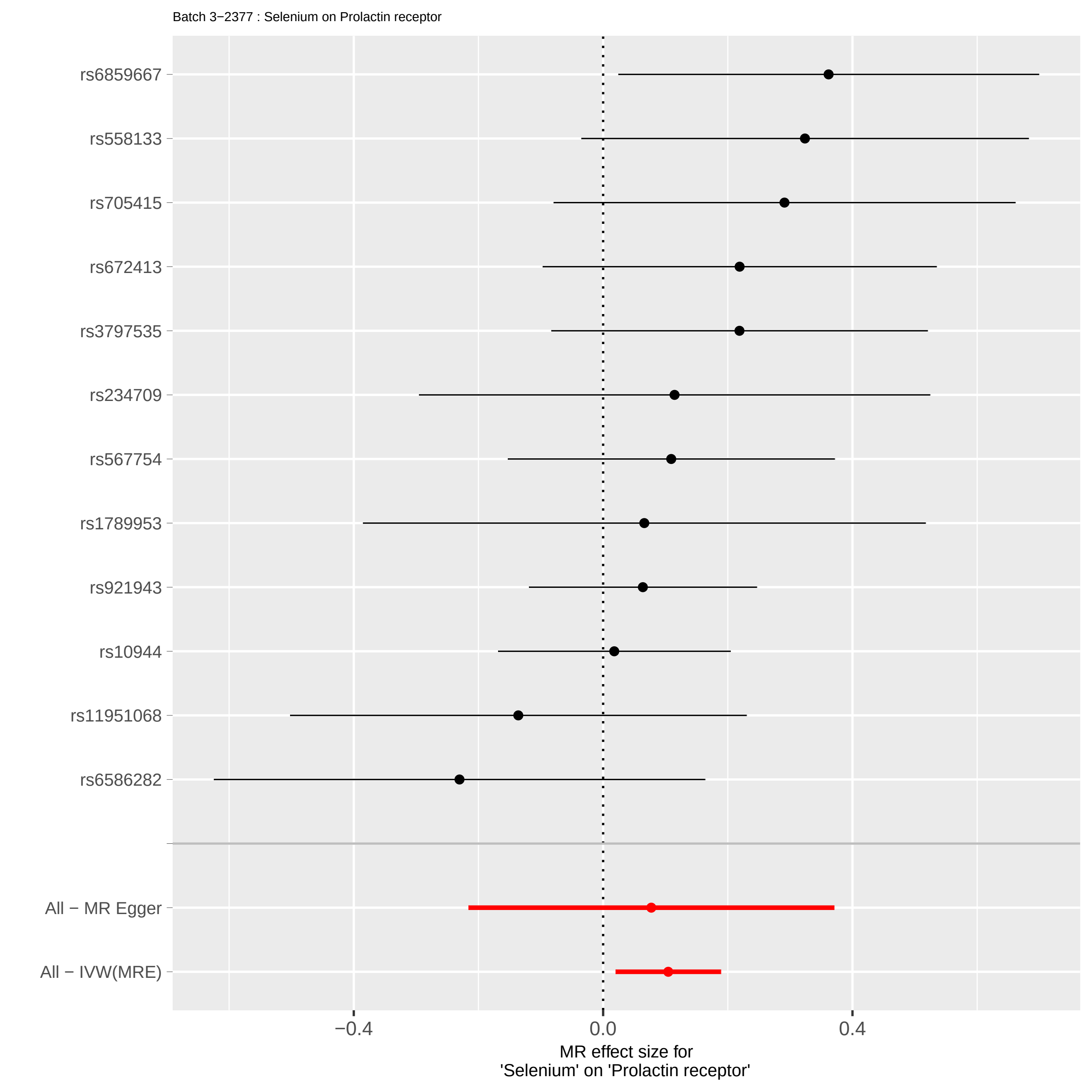
Batch 3−2377 : Selenium on Prolactin receptor
rs6859667
rs558133
rs705415
rs672413
rs3797535
rs234709
rs567754
rs1789953
rs921943
rs10944
rs11951068
rs6586282
All − MR Egger
All − IVW(MRE)
−0.4
0.0
0.4
MR effect size for
'Selenium' on 'Prolactin receptor'

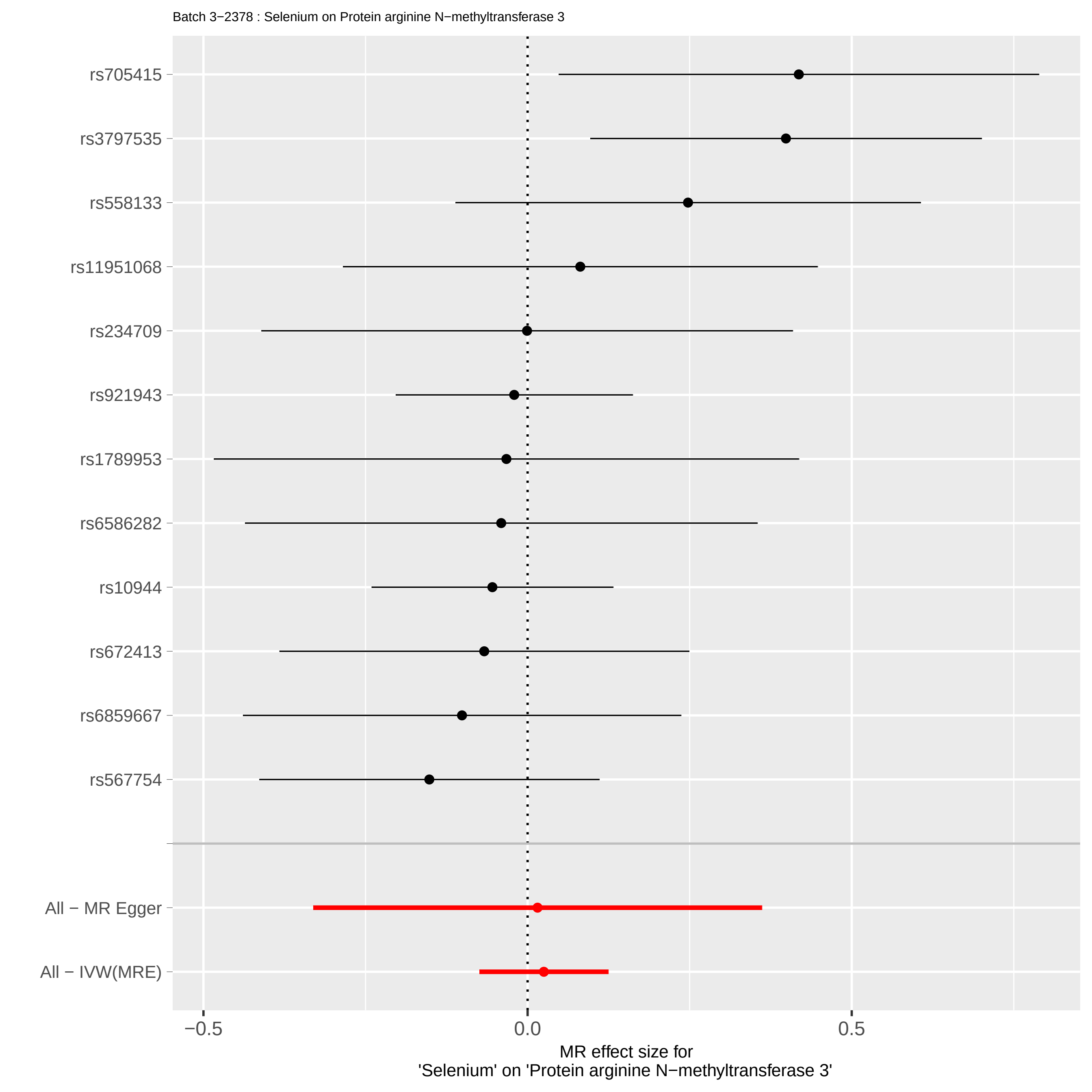

Batch 3−2378 : Selenium on Protein arginine N−methyltransferase 3
rs705415
rs3797535
rs558133
rs11951068
rs234709
rs921943
rs1789953
rs6586282
rs10944
rs672413
rs6859667
rs567754
All − MR Egger
All − IVW(MRE)
−0.5
0.0
0.5
MR effect size for
'Selenium' on 'Protein arginine N−methyltransferase 3'

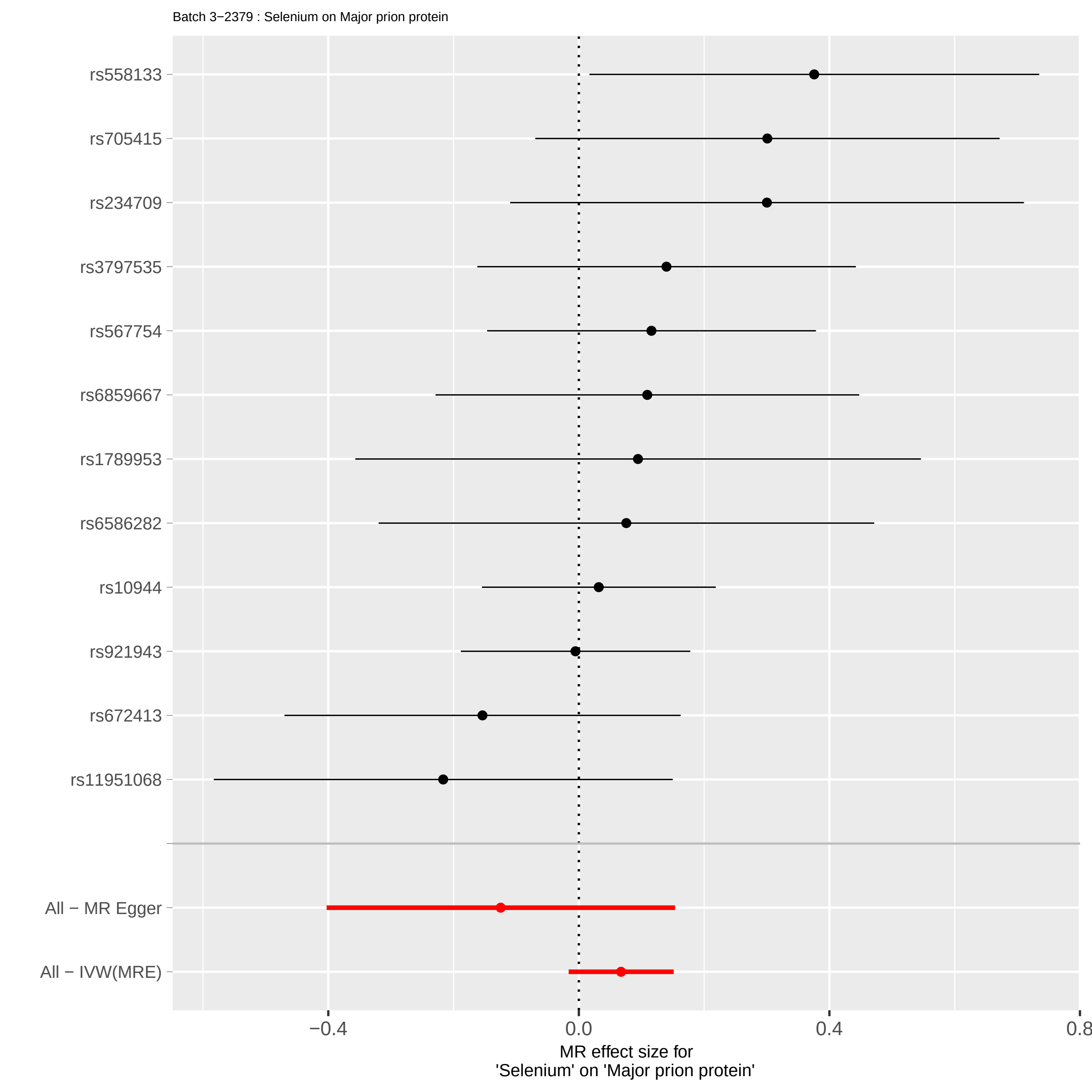

Batch 3−2379 : Selenium on Major prion protein
rs558133
rs705415
rs234709
rs3797535
rs567754
rs6859667
rs1789953
rs6586282
rs10944
rs921943
rs672413
rs11951068
All − MR Egger
All − IVW(MRE)
−0.4
0.0
0.4
0.8
MR effect size for
'Selenium' on 'Major prion protein'

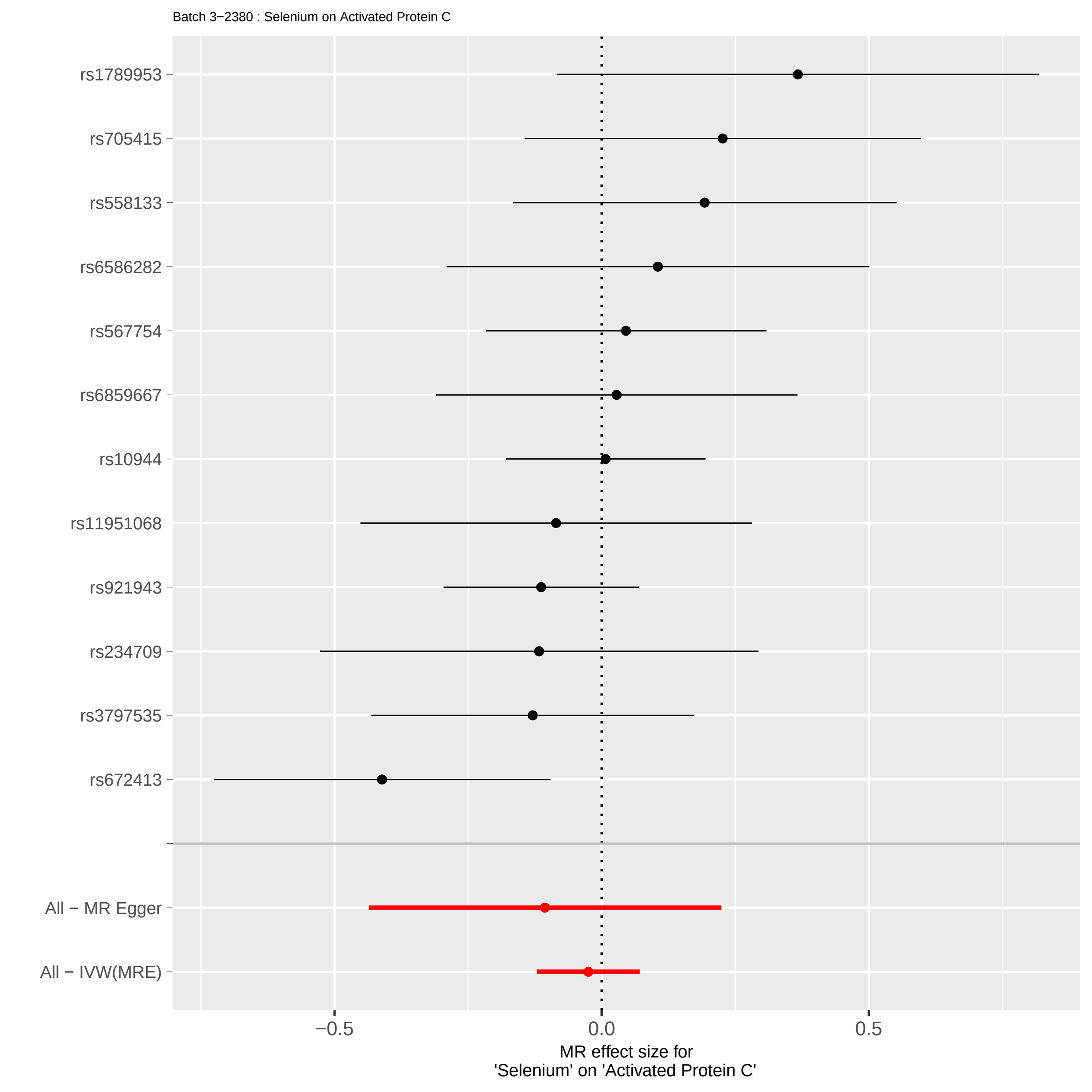

Batch 3−2380 : Selenium on Activated Protein C
rs1789953
rs705415
rs558133
rs6586282
rs567754
rs6859667
rs10944
rs11951068
rs921943
rs234709
rs3797535
rs672413
All − MR Egger
All − IVW(MRE)
−0.5
0.0
0.5
MR effect size for
'Selenium' on 'Activated Protein C'

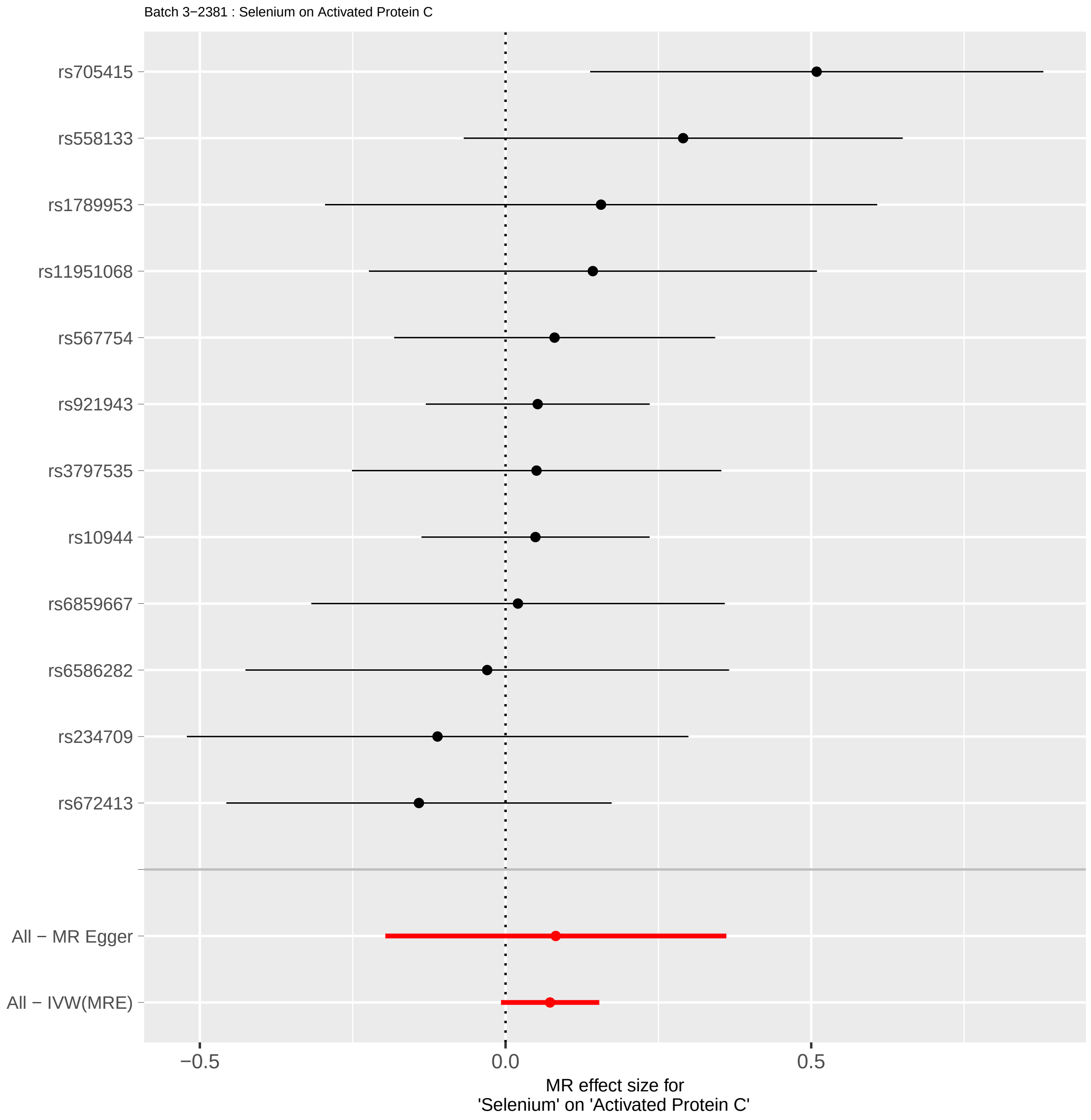
Batch 3−2381 : Selenium on Activated Protein C
rs705415
rs558133
rs1789953
rs11951068
rs567754
rs921943
rs3797535
rs10944
rs6859667
rs6586282
rs234709
rs672413
All − MR Egger
All − IVW(MRE)
−0.5
0.0
0.5
MR effect size for
'Selenium' on 'Activated Protein C'

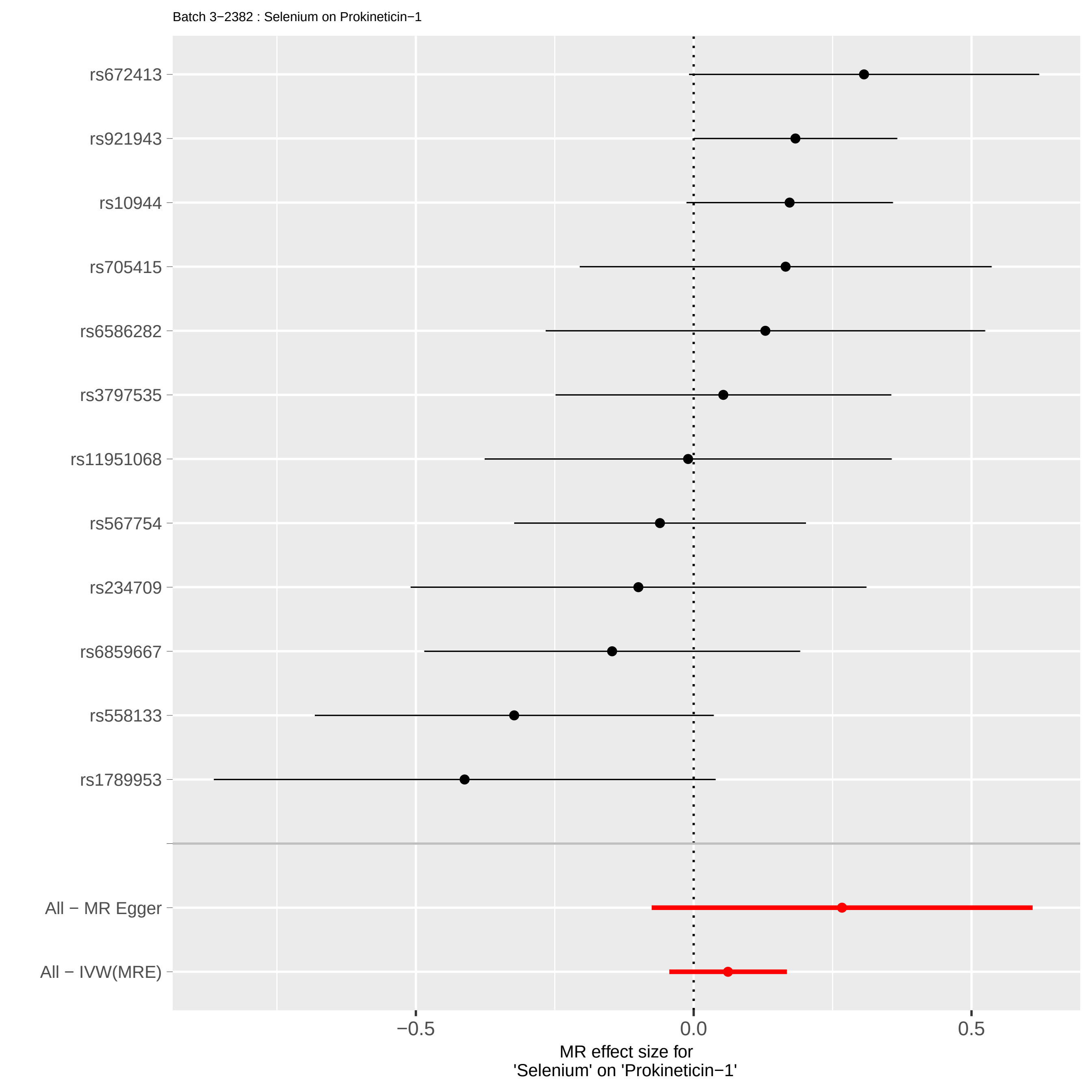
Batch 3−2382 : Selenium on Prokineticin−1
rs672413
rs921943
rs10944
rs705415
rs6586282
rs3797535
rs11951068
rs567754
rs234709
rs6859667
rs558133
rs1789953
All − MR Egger
All − IVW(MRE)
−0.5
0.0
0.5
MR effect size for
'Selenium' on 'Prokineticin−1'

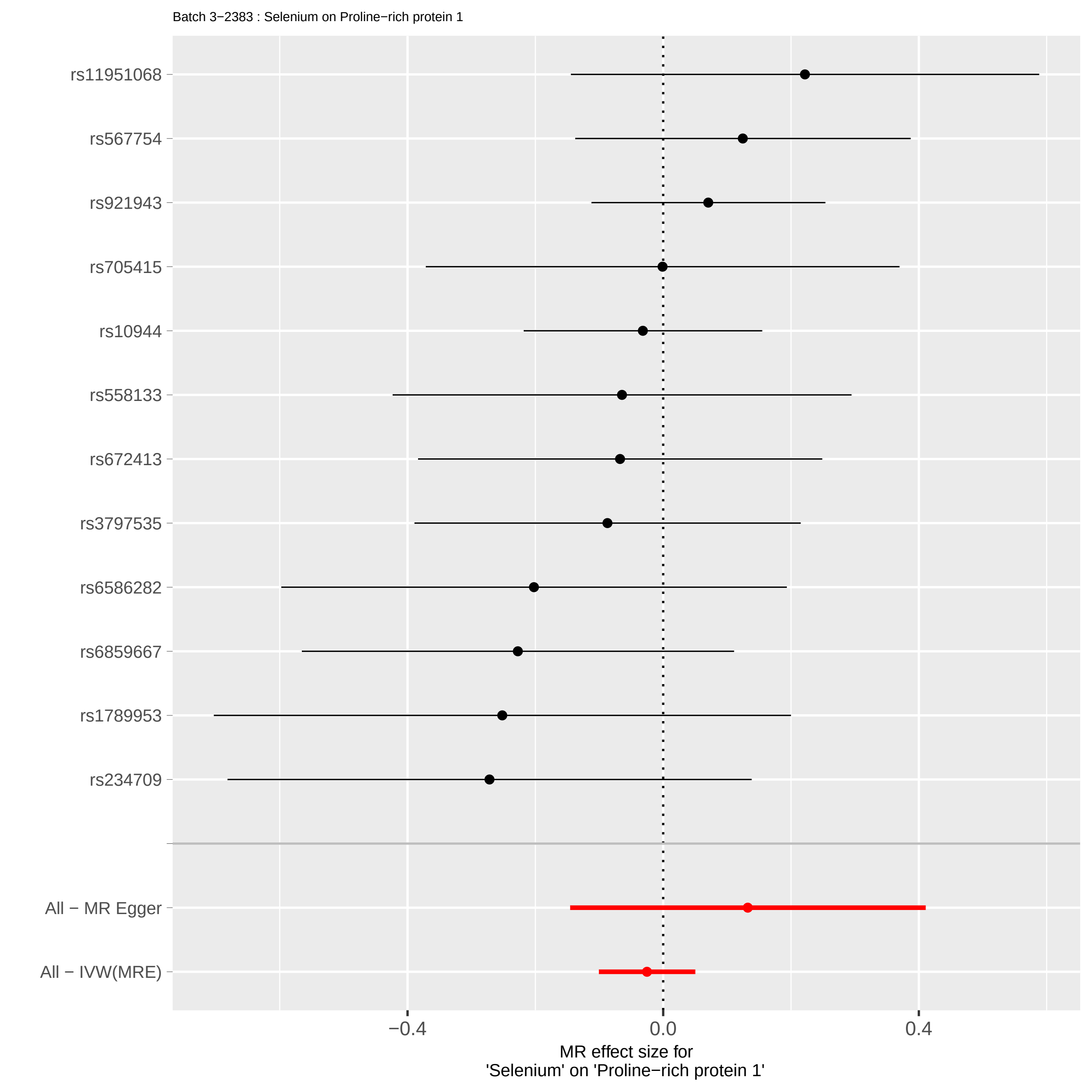

Batch 3−2383 : Selenium on Proline−rich protein 1
rs11951068
rs567754
rs921943
rs705415
rs10944
rs558133
rs672413
rs3797535
rs6586282
rs6859667
rs1789953
rs234709
All − MR Egger
All − IVW(MRE)
−0.4
0.0
0.4
MR effect size for
'Selenium' on 'Proline−rich protein 1'

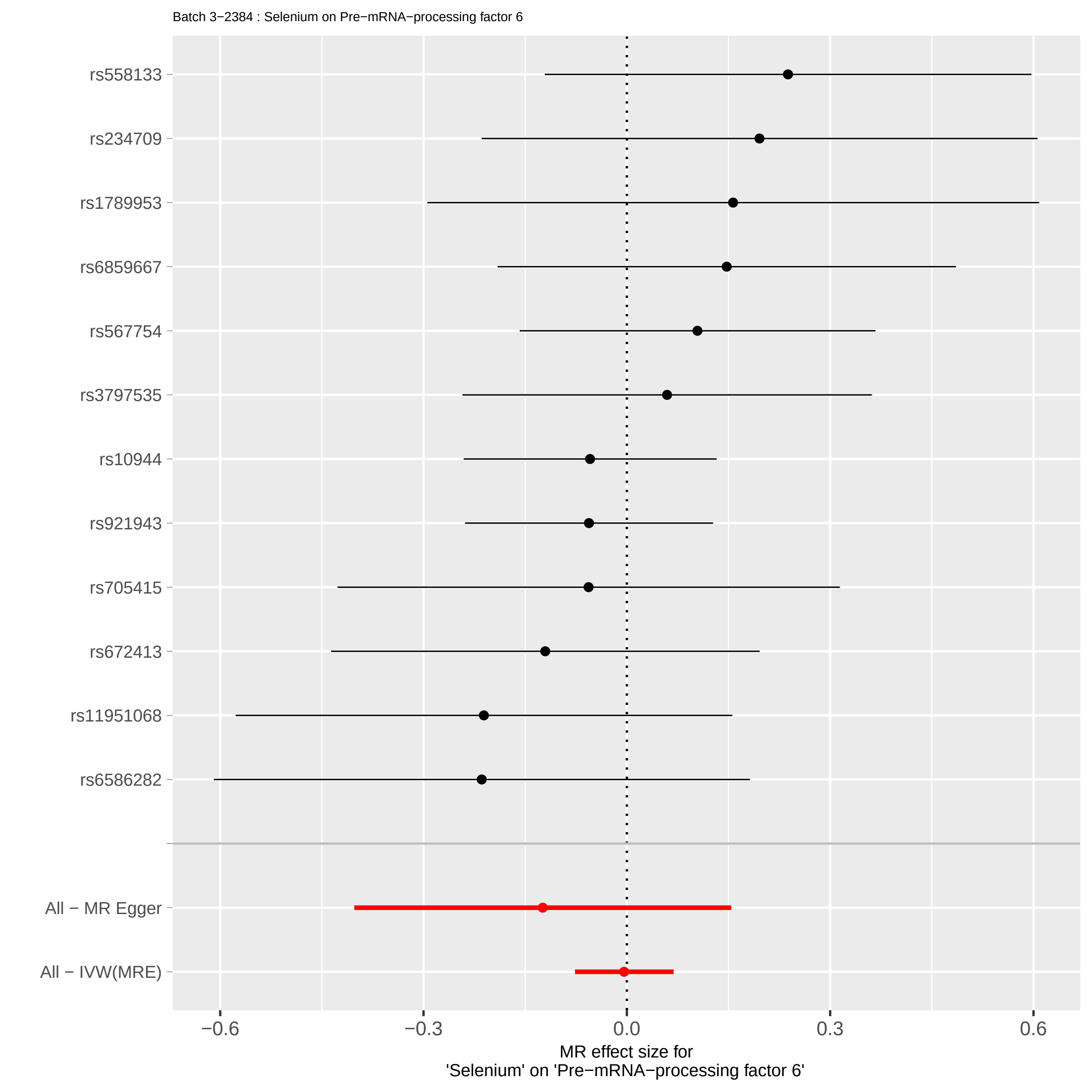

Batch 3−2384 : Selenium on Pre−mRNA−processing factor 6
rs558133
rs234709
rs1789953
rs6859667
rs567754
rs3797535
rs10944
rs921943
rs705415
rs672413
rs11951068
rs6586282
All − MR Egger
All − IVW(MRE)
−0.6
−0.3
0.0
0.3
0.6
MR effect size for
'Selenium' on 'Pre−mRNA−processing factor 6'

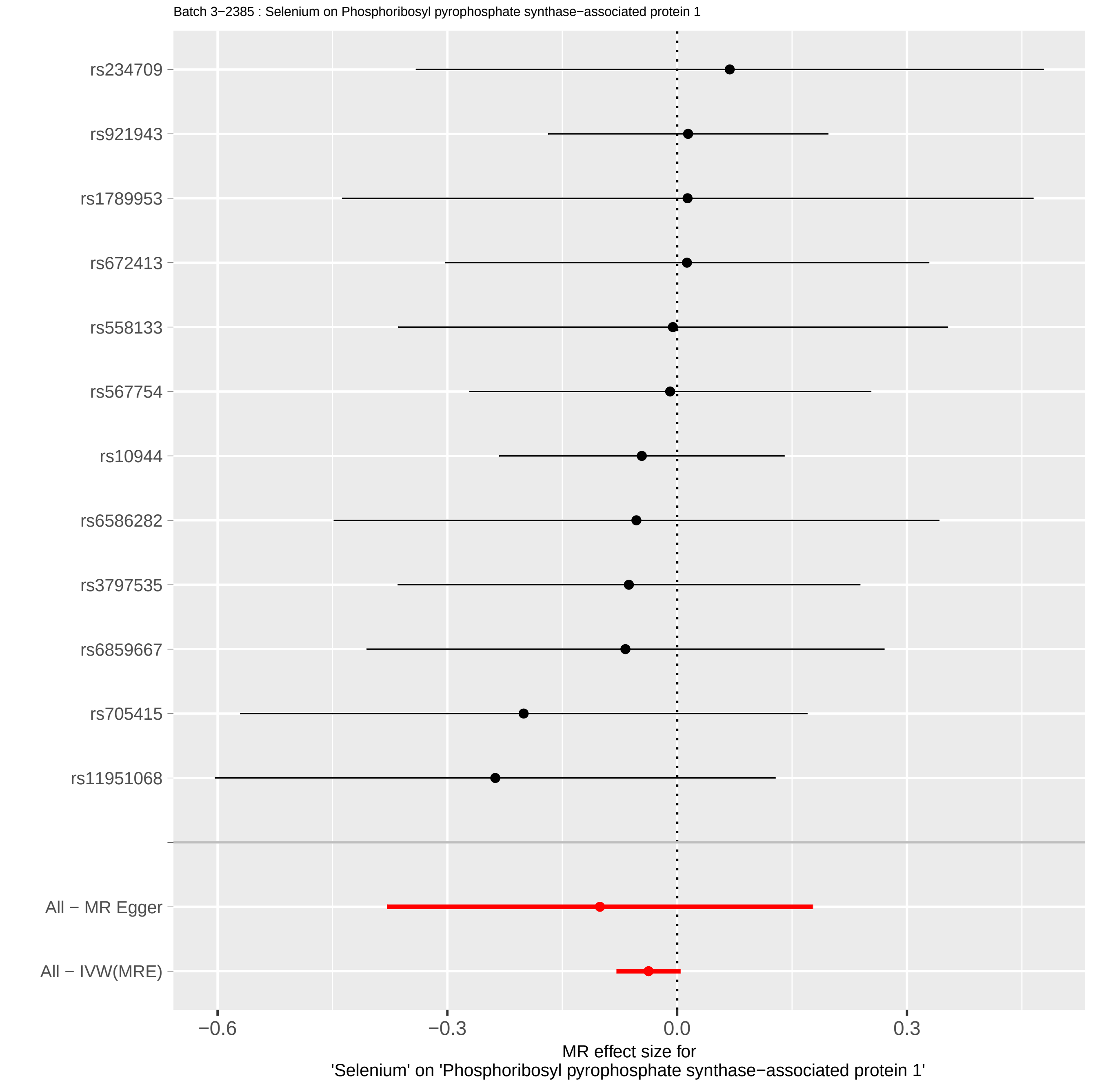
Batch 3−2385 : Selenium on Phosphoribosyl pyrophosphate synthase−associated protein 1
rs234709
rs921943
rs1789953
rs672413
rs558133
rs567754
rs10944
rs6586282
rs3797535
rs6859667
rs705415
rs11951068
All − MR Egger
All − IVW(MRE)
−0.6
−0.3
0.0
0.3
MR effect size for
'Selenium' on 'Phosphoribosyl pyrophosphate synthase−associated protein 1'

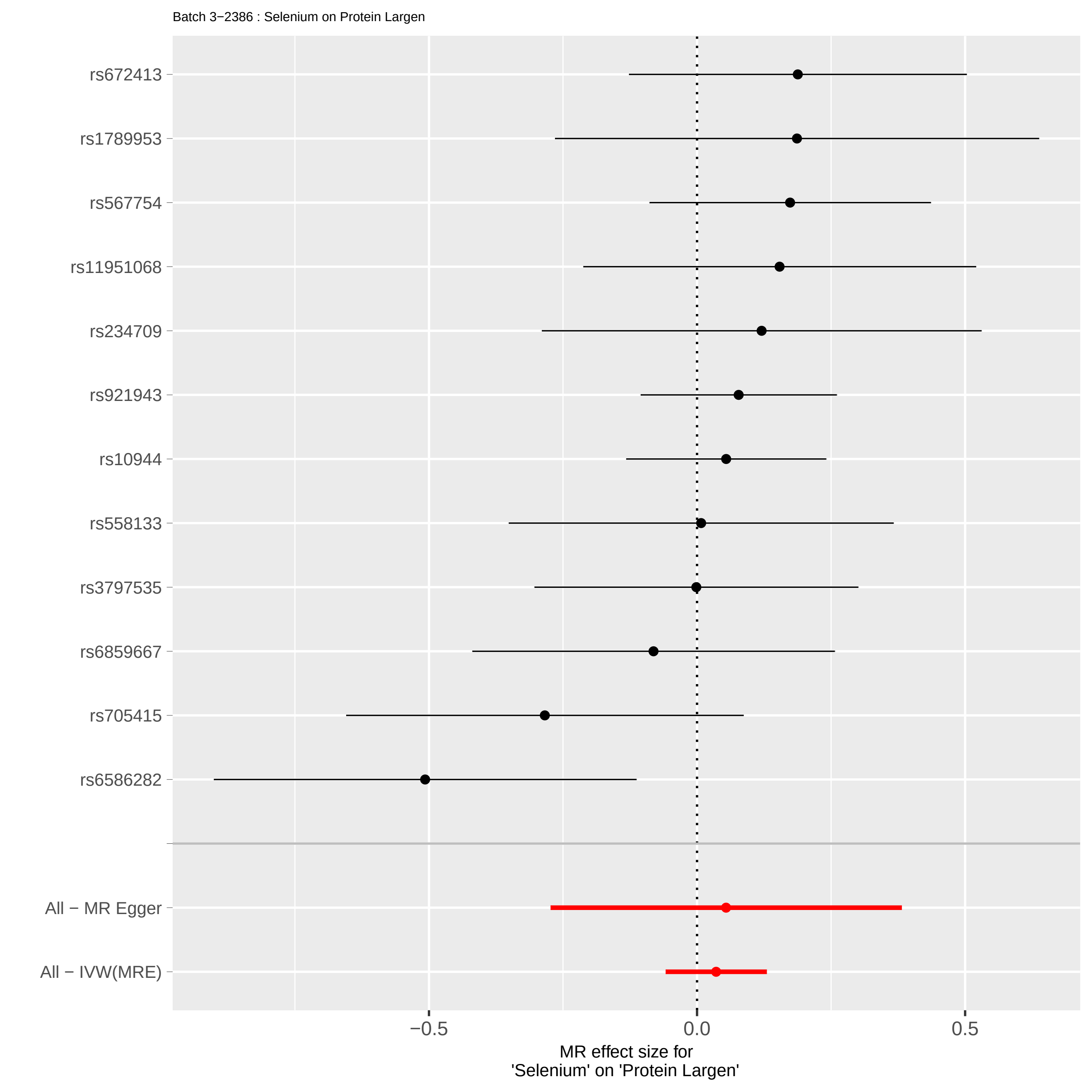

Batch 3−2386 : Selenium on Protein Largen
rs672413
rs1789953
rs567754
rs11951068
rs234709
rs921943
rs10944
rs558133
rs3797535
rs6859667
rs705415
rs6586282
All − MR Egger
All − IVW(MRE)
−0.5
0.0
0.5
MR effect size for
'Selenium' on 'Protein Largen'

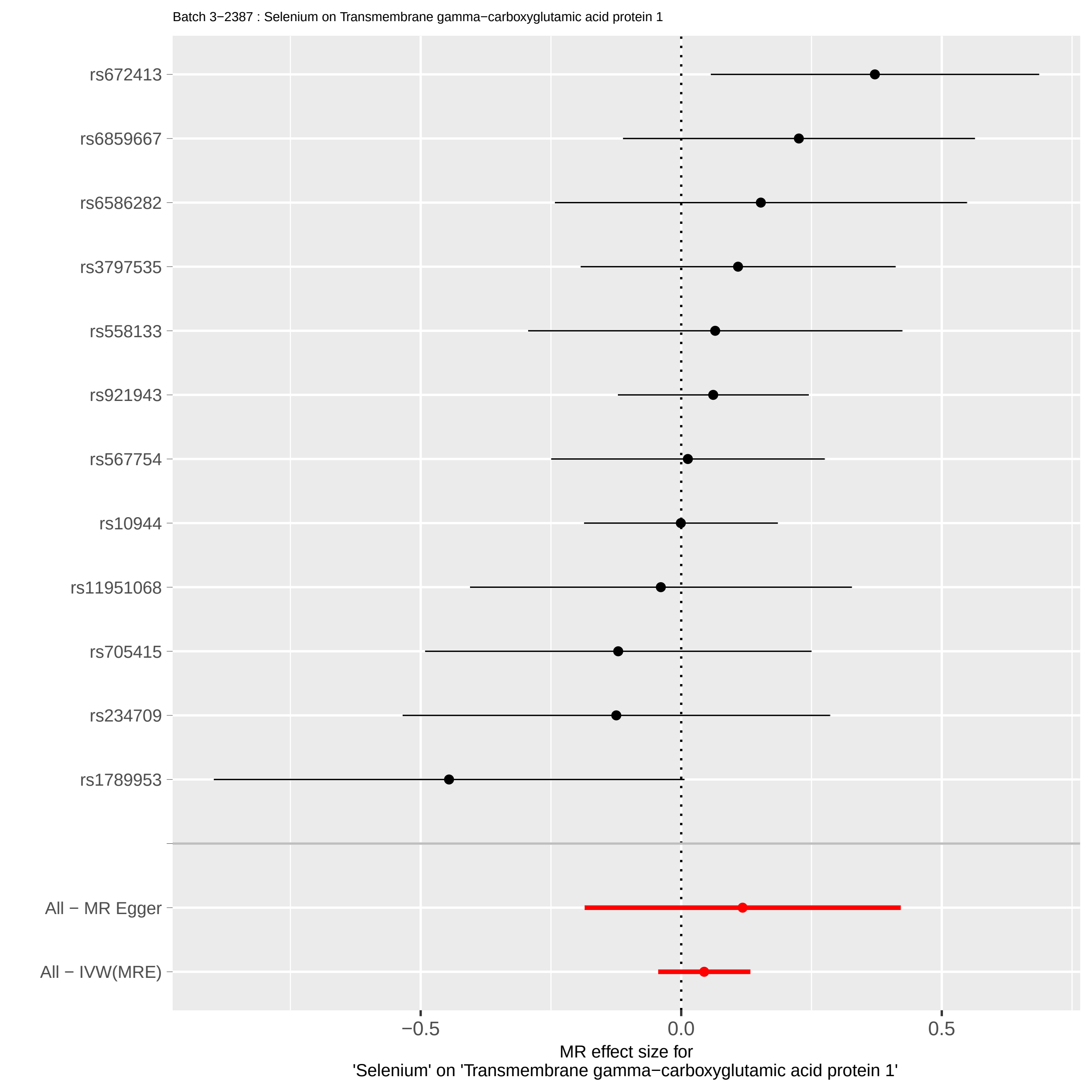
Batch 3−2387 : Selenium on Transmembrane gamma−carboxyglutamic acid protein 1
rs672413
rs6859667
rs6586282
rs3797535
rs558133
rs921943
rs567754
rs10944
rs11951068
rs705415
rs234709
rs1789953
All − MR Egger
All − IVW(MRE)
−0.5
0.0
0.5
MR effect size for
'Selenium' on 'Transmembrane gamma−carboxyglutamic acid protein 1'

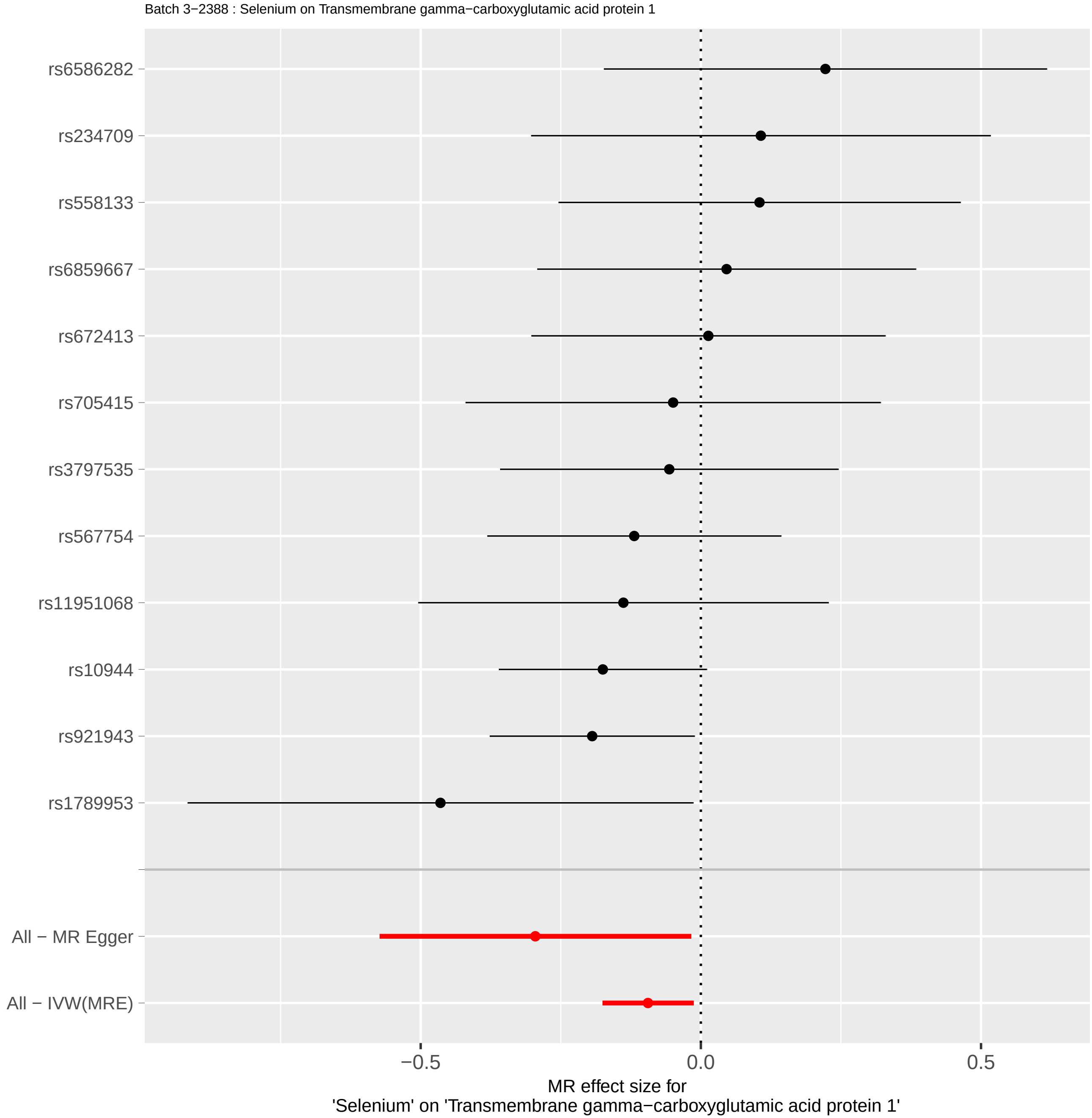

Batch 3−2388 : Selenium on Transmembrane gamma−carboxyglutamic acid protein 1
rs6586282
rs234709
rs558133
rs6859667
rs672413
rs705415
rs3797535
rs567754
rs11951068
rs10944
rs921943
rs1789953
All − MR Egger
All − IVW(MRE)
−0.5
0.0
0.5
MR effect size for
'Selenium' on 'Transmembrane gamma−carboxyglutamic acid protein 1'

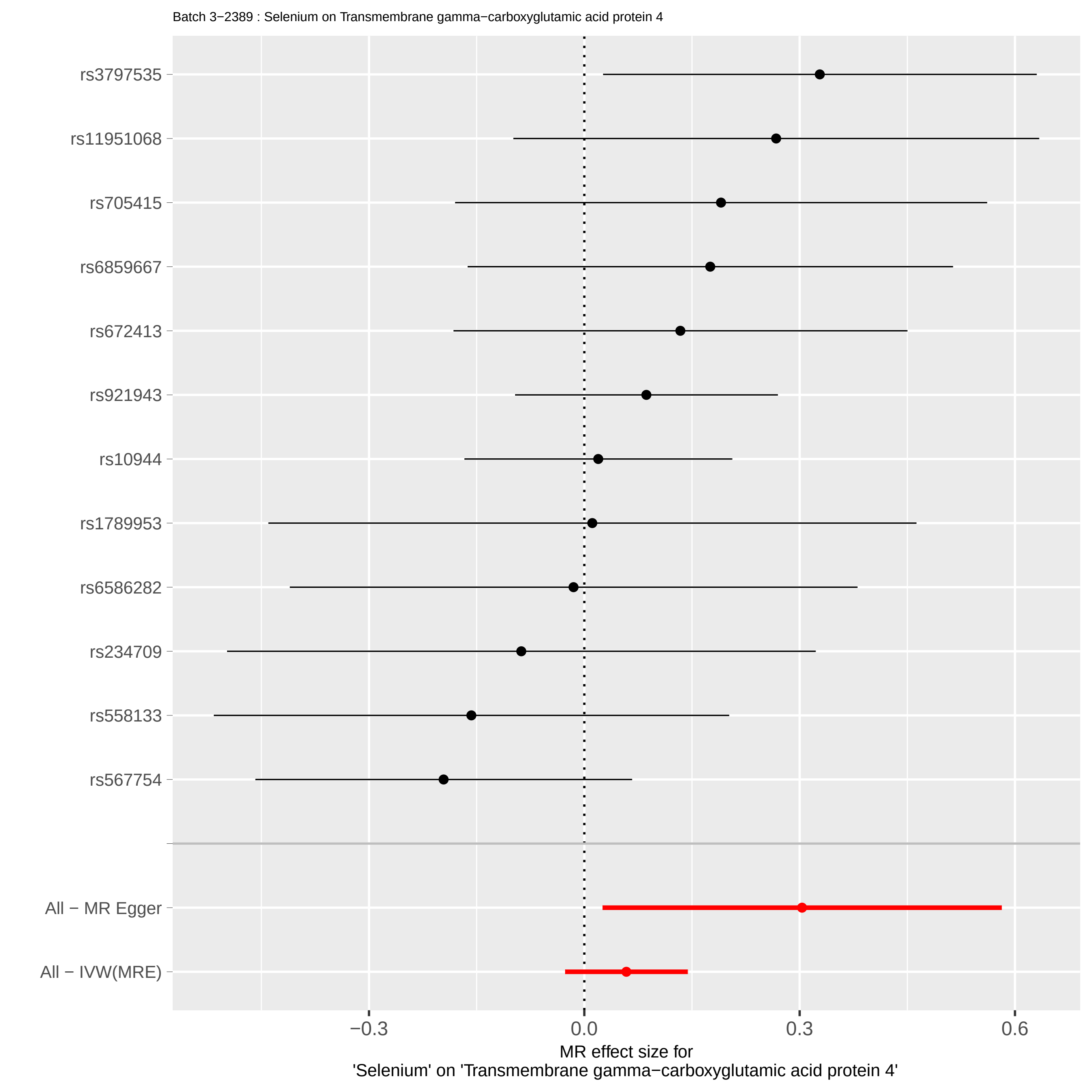

Batch 3−2389 : Selenium on Transmembrane gamma−carboxyglutamic acid protein 4
rs3797535
rs11951068
rs705415
rs6859667
rs672413
rs921943
rs10944
rs1789953
rs6586282
rs234709
rs558133
rs567754
All − MR Egger
All − IVW(MRE)
−0.3
0.0
0.3
0.6
MR effect size for
'Selenium' on 'Transmembrane gamma−carboxyglutamic acid protein 4'

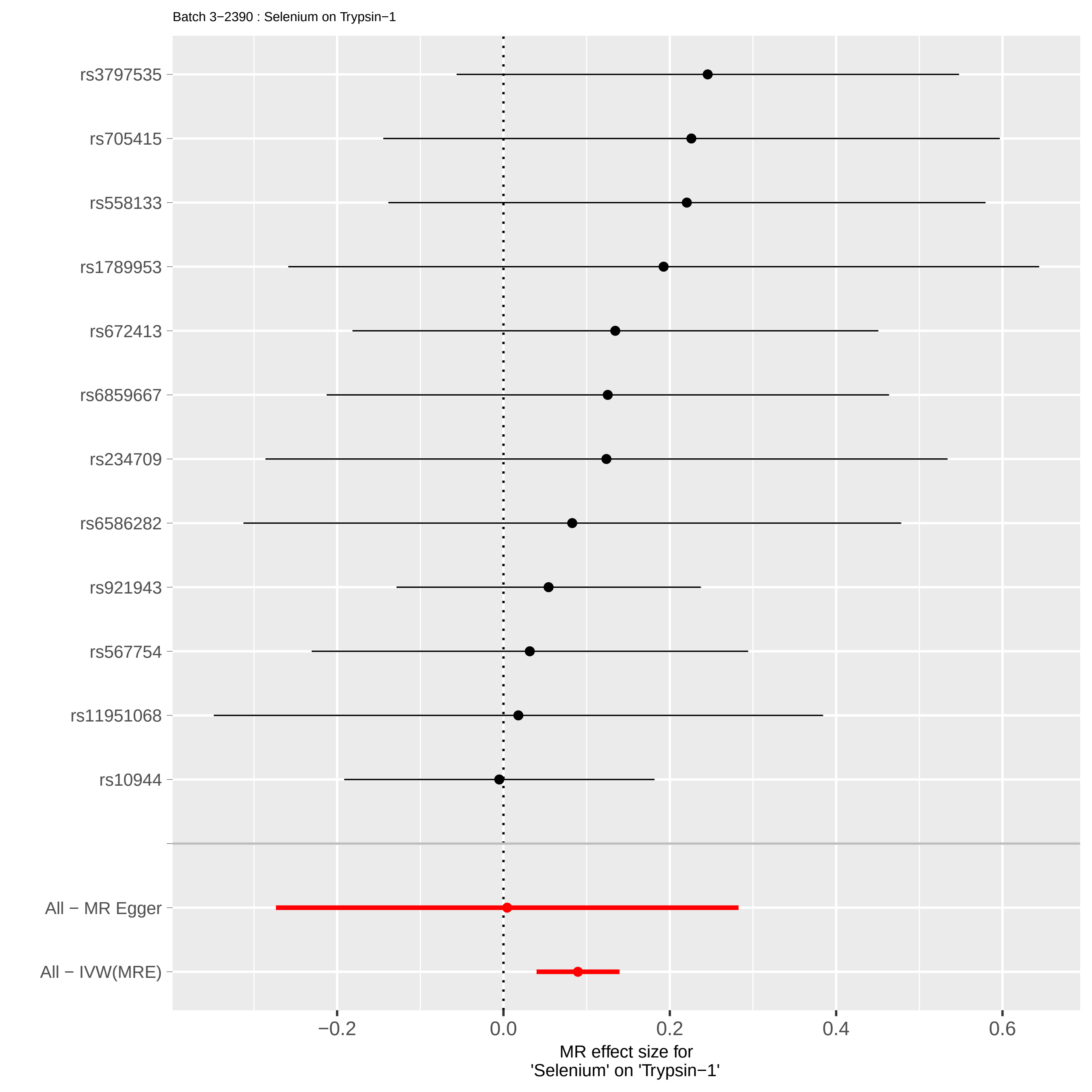

Batch 3−2390 : Selenium on Trypsin−1
rs3797535
rs705415
rs558133
rs1789953
rs672413
rs6859667
rs234709
rs6586282
rs921943
rs567754
rs11951068
rs10944
All − MR Egger
All − IVW(MRE)
−0.2
0.0
0.2
0.4
0.6
MR effect size for
'Selenium' on 'Trypsin−1'

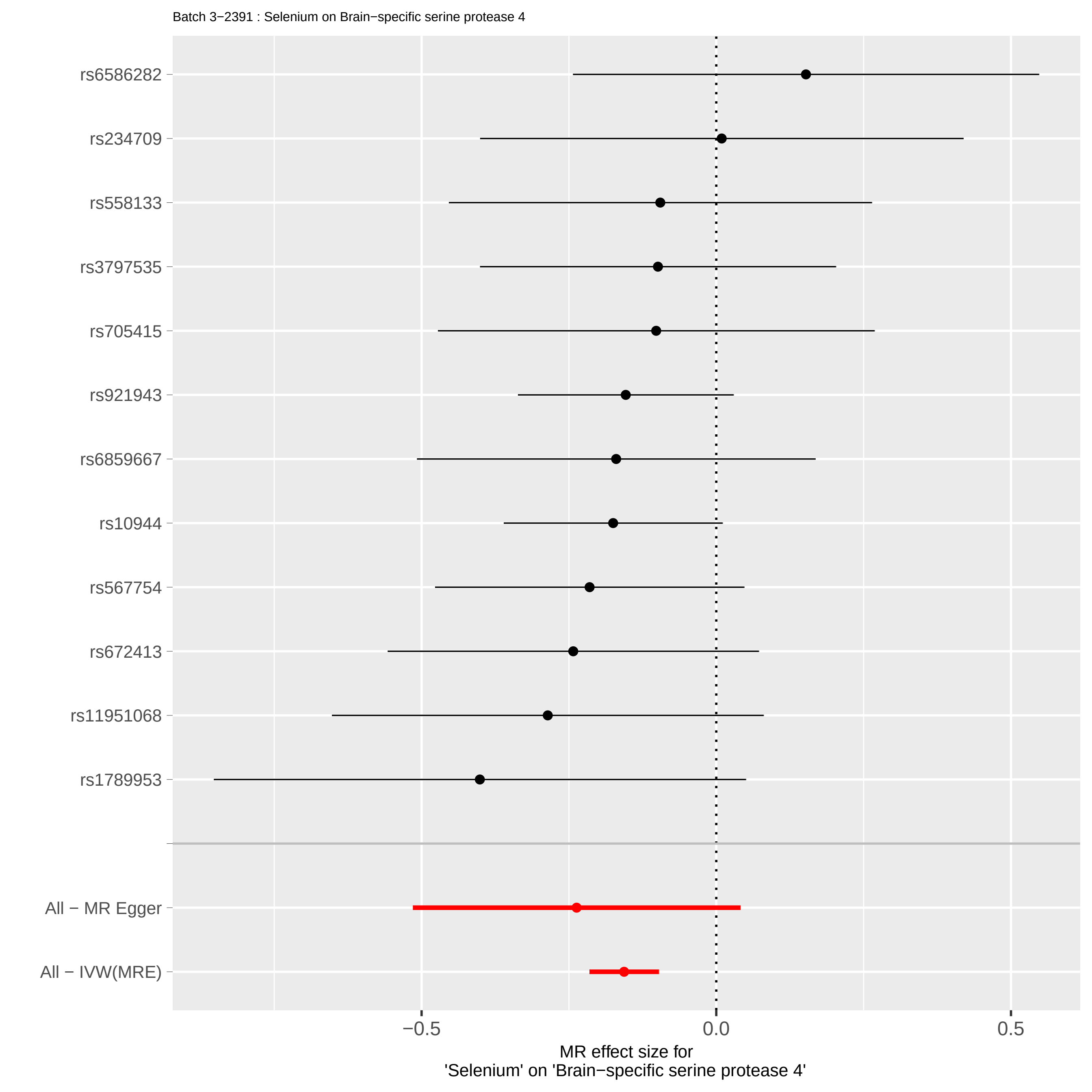

Batch 3−2391 : Selenium on Brain−specific serine protease 4
rs6586282
rs234709
rs558133
rs3797535
rs705415
rs921943
rs6859667
rs10944
rs567754
rs672413
rs11951068
rs1789953
All − MR Egger
All − IVW(MRE)
−0.5
0.0
0.5
MR effect size for
'Selenium' on 'Brain−specific serine protease 4'

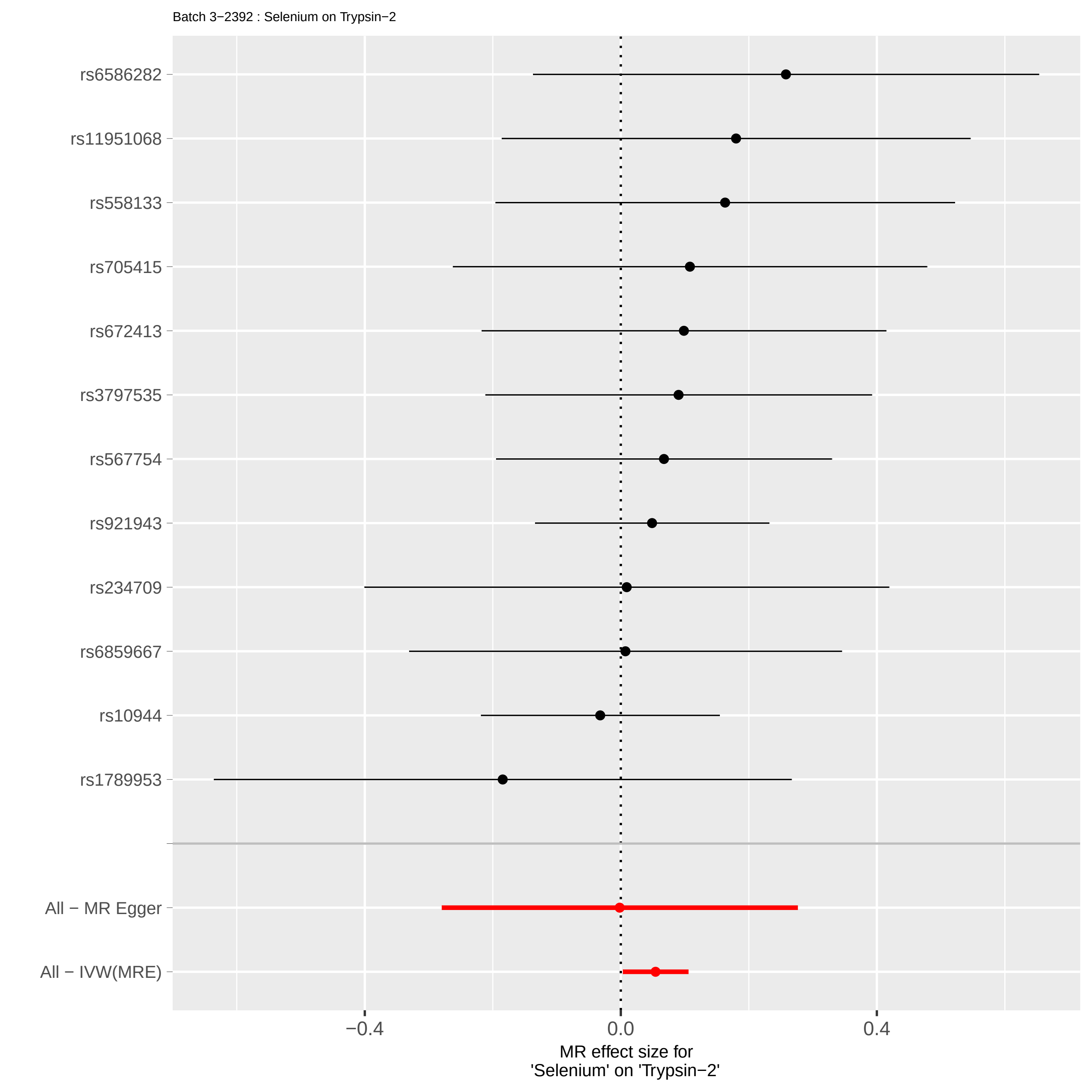

Batch 3−2392 : Selenium on Trypsin−2
rs6586282
rs11951068
rs558133
rs705415
rs672413
rs3797535
rs567754
rs921943
rs234709
rs6859667
rs10944
rs1789953
All − MR Egger
All − IVW(MRE)
−0.4
0.0
0.4
MR effect size for
'Selenium' on 'Trypsin−2'

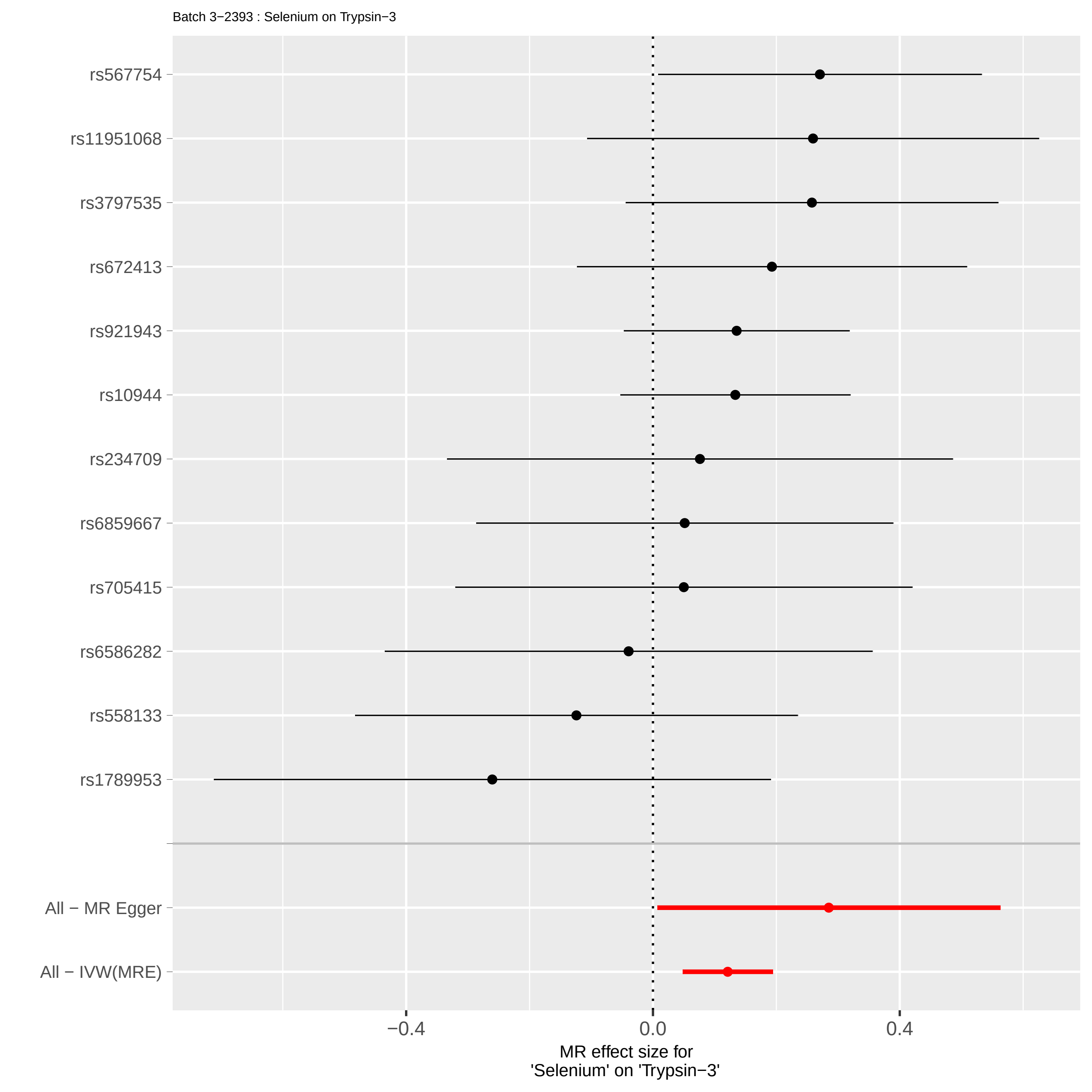

Batch 3−2393 : Selenium on Trypsin−3
rs567754
rs11951068
rs3797535
rs672413
rs921943
rs10944
rs234709
rs6859667
rs705415
rs6586282
rs558133
rs1789953
All − MR Egger
All − IVW(MRE)
−0.4
0.0
0.4
MR effect size for
'Selenium' on 'Trypsin−3'

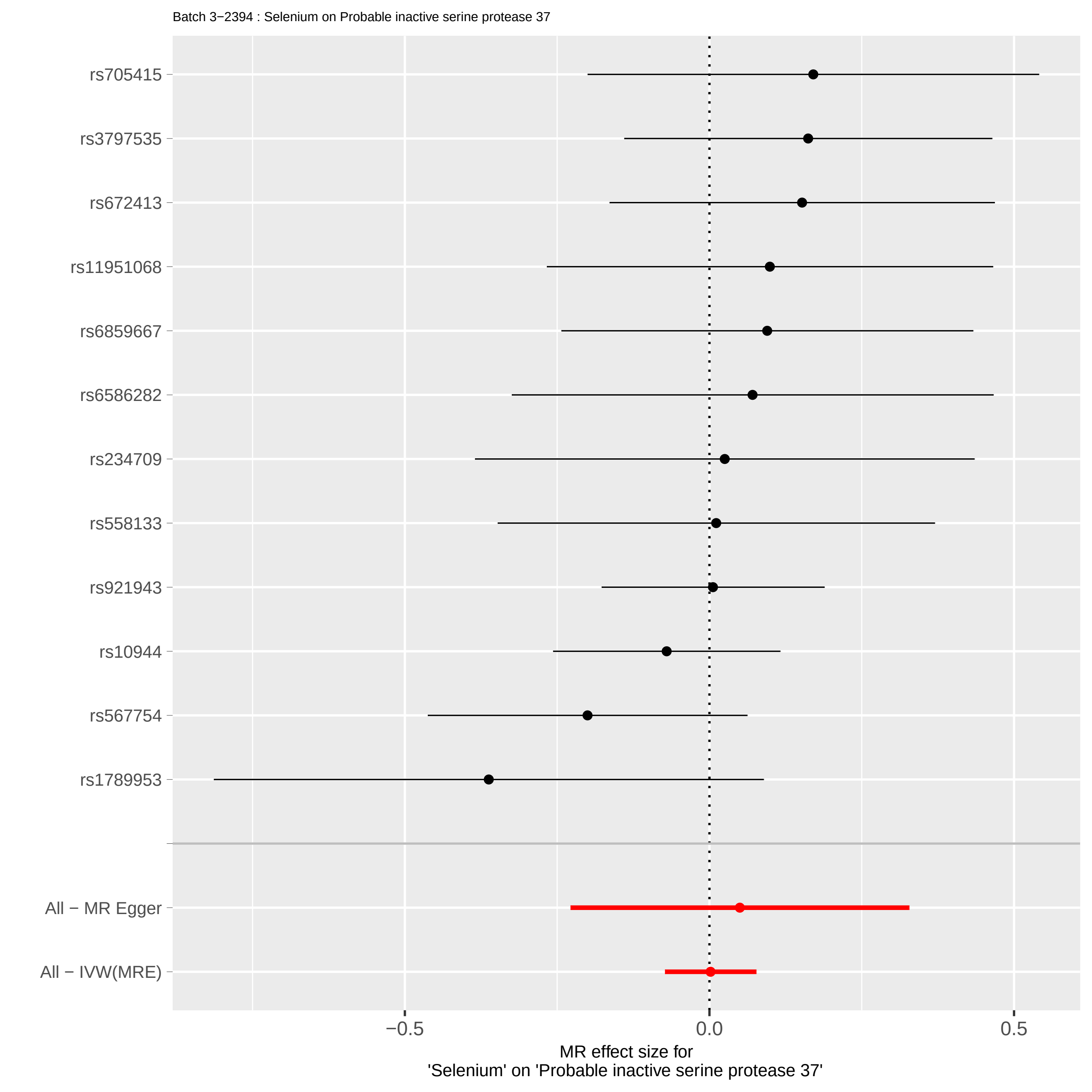

Batch 3−2394 : Selenium on Probable inactive serine protease 37
rs705415
rs3797535
rs672413
rs11951068
rs6859667
rs6586282
rs234709
rs558133
rs921943
rs10944
rs567754
rs1789953
All − MR Egger
All − IVW(MRE)
−0.5
0.0
0.5
MR effect size for
'Selenium' on 'Probable inactive serine protease 37'

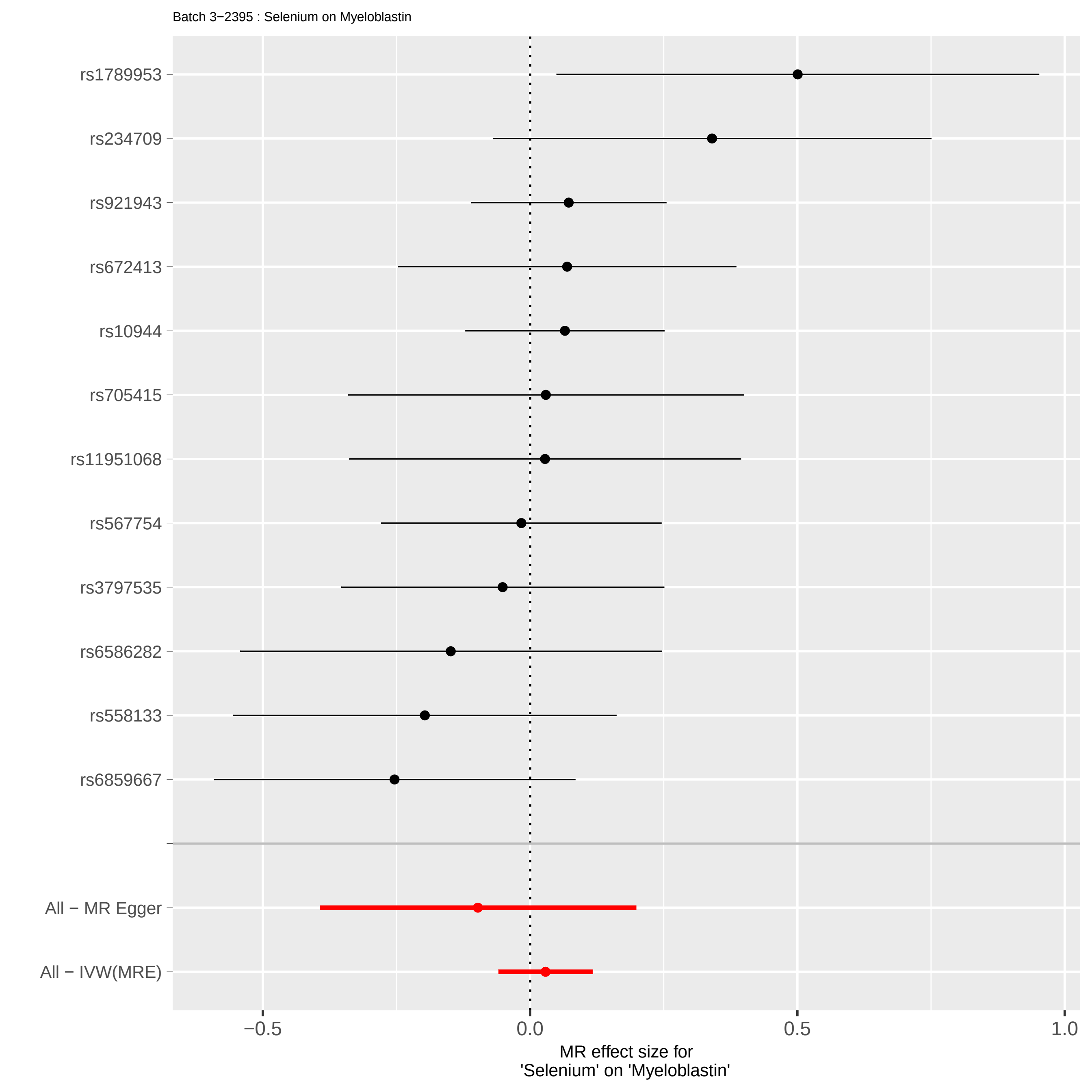

Batch 3−2395 : Selenium on Myeloblastin
rs1789953
rs234709
rs921943
rs672413
rs10944
rs705415
rs11951068
rs567754
rs3797535
rs6586282
rs558133
rs6859667
All − MR Egger
All − IVW(MRE)
−0.5
0.0
0.5
1.0
MR effect size for
'Selenium' on 'Myeloblastin'

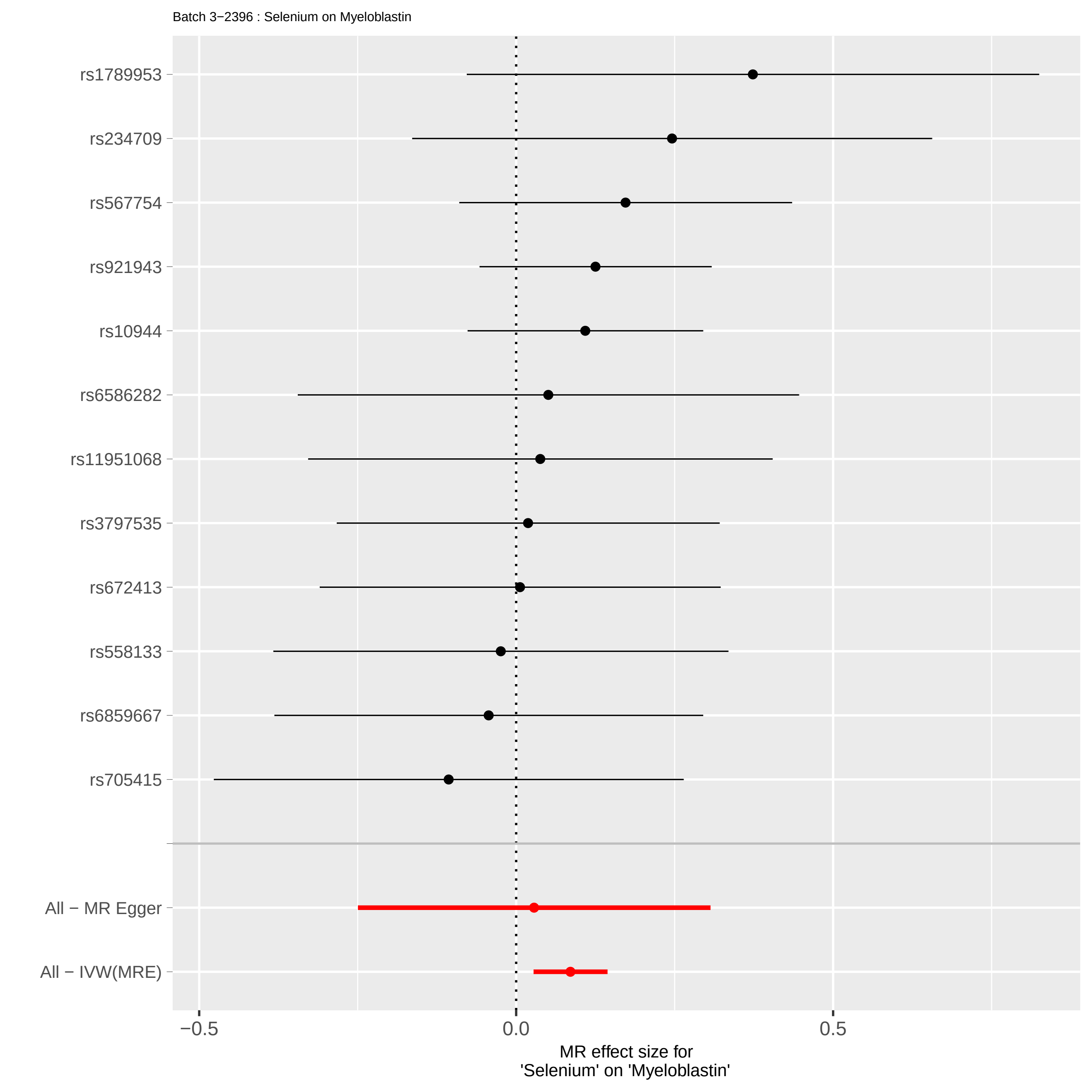

Batch 3−2396 : Selenium on Myeloblastin
rs1789953
rs234709
rs567754
rs921943
rs10944
rs6586282
rs11951068
rs3797535
rs672413
rs558133
rs6859667
rs705415
All − MR Egger
All − IVW(MRE)
−0.5
0.0
0.5
MR effect size for
'Selenium' on 'Myeloblastin'

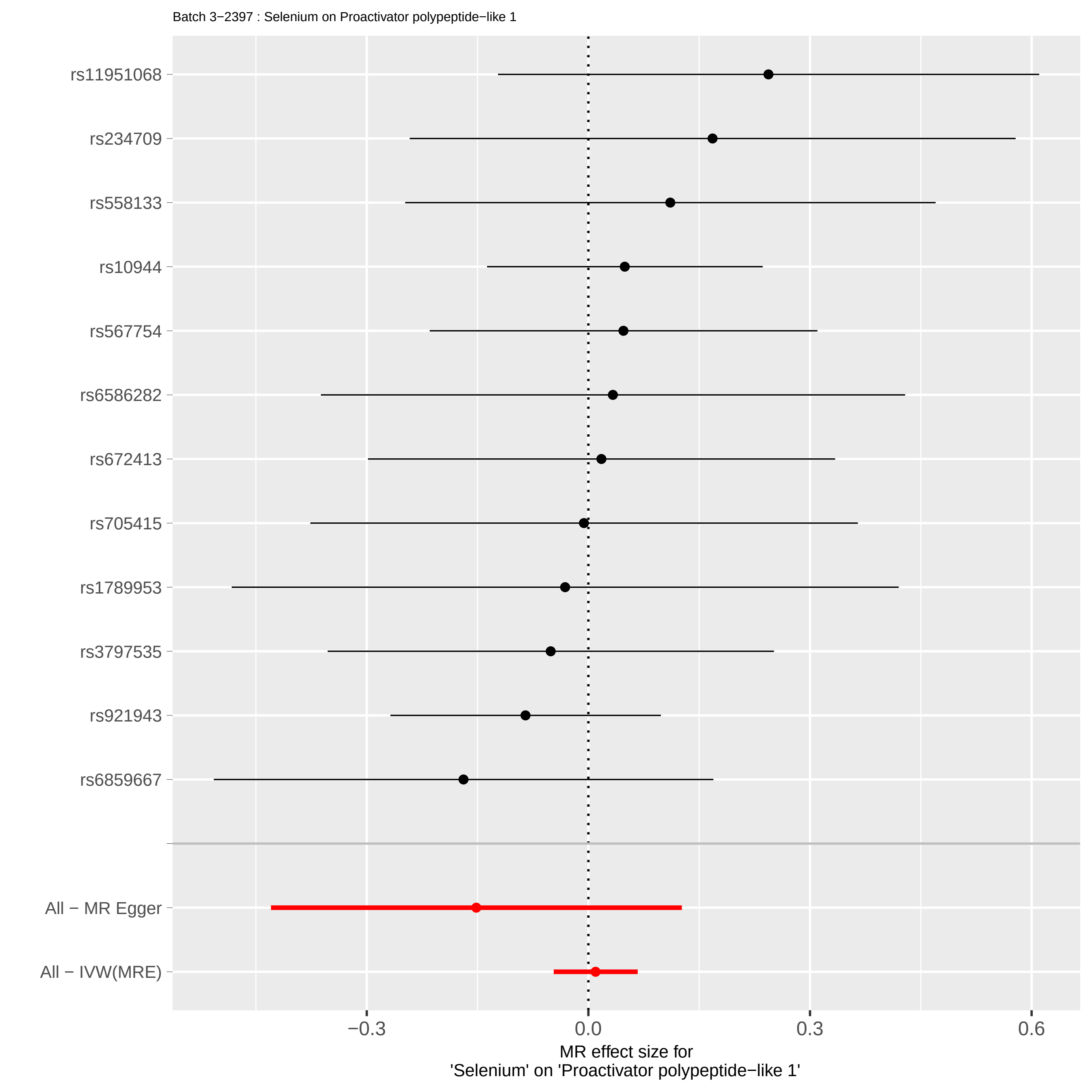

Batch 3−2397 : Selenium on Proactivator polypeptide−like 1
rs11951068
rs234709
rs558133
rs10944
rs567754
rs6586282
rs672413
rs705415
rs1789953
rs3797535
rs921943
rs6859667
All − MR Egger
All − IVW(MRE)
−0.3
0.0
0.3
0.6
MR effect size for
'Selenium' on 'Proactivator polypeptide−like 1'

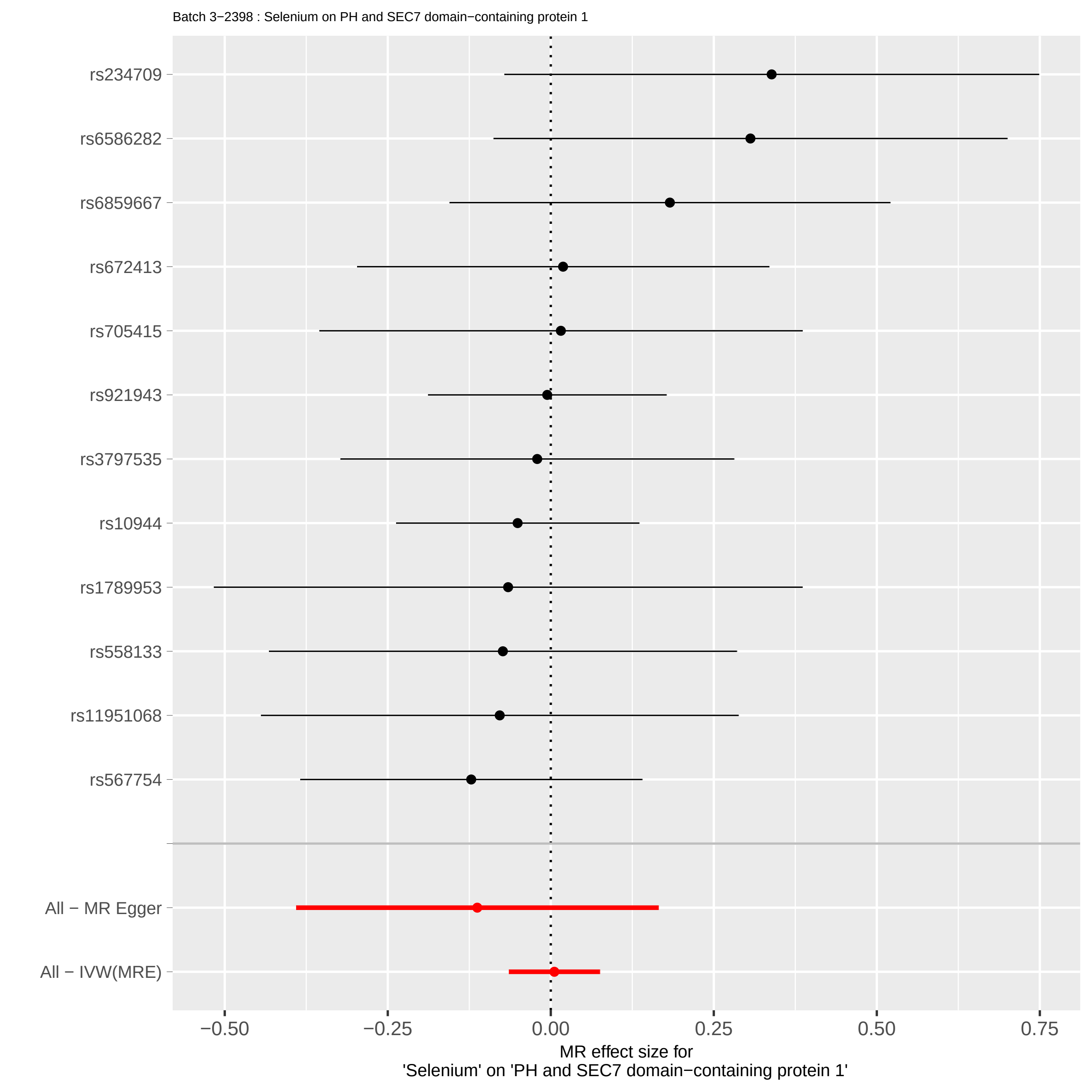

Batch 3−2398 : Selenium on PH and SEC7 domain−containing protein 1
rs234709
rs6586282
rs6859667
rs672413
rs705415
rs921943
rs3797535
rs10944
rs1789953
rs558133
rs11951068
rs567754
All − MR Egger
All − IVW(MRE)
−0.50
−0.25
0.00
0.25
0.50
0.75
MR effect size for
'Selenium' on 'PH and SEC7 domain−containing protein 1'

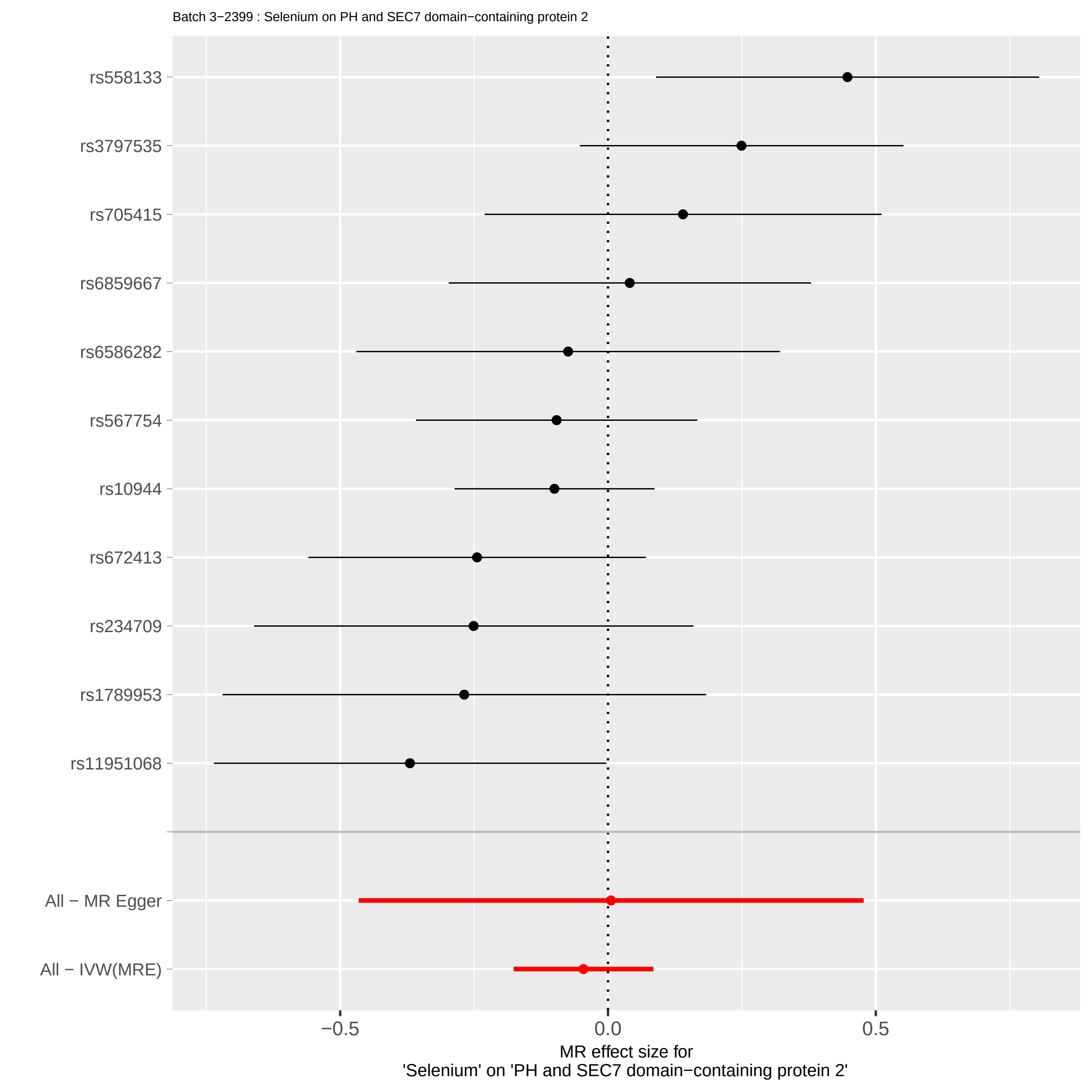

Batch 3−2399 : Selenium on PH and SEC7 domain−containing protein 2
rs558133
rs3797535
rs705415
rs6859667
rs6586282
rs567754
rs10944
rs672413
rs234709
rs1789953
rs11951068
All − MR Egger
All − IVW(MRE)
−0.5
0.0
0.5
MR effect size for
'Selenium' on 'PH and SEC7 domain−containing protein 2'

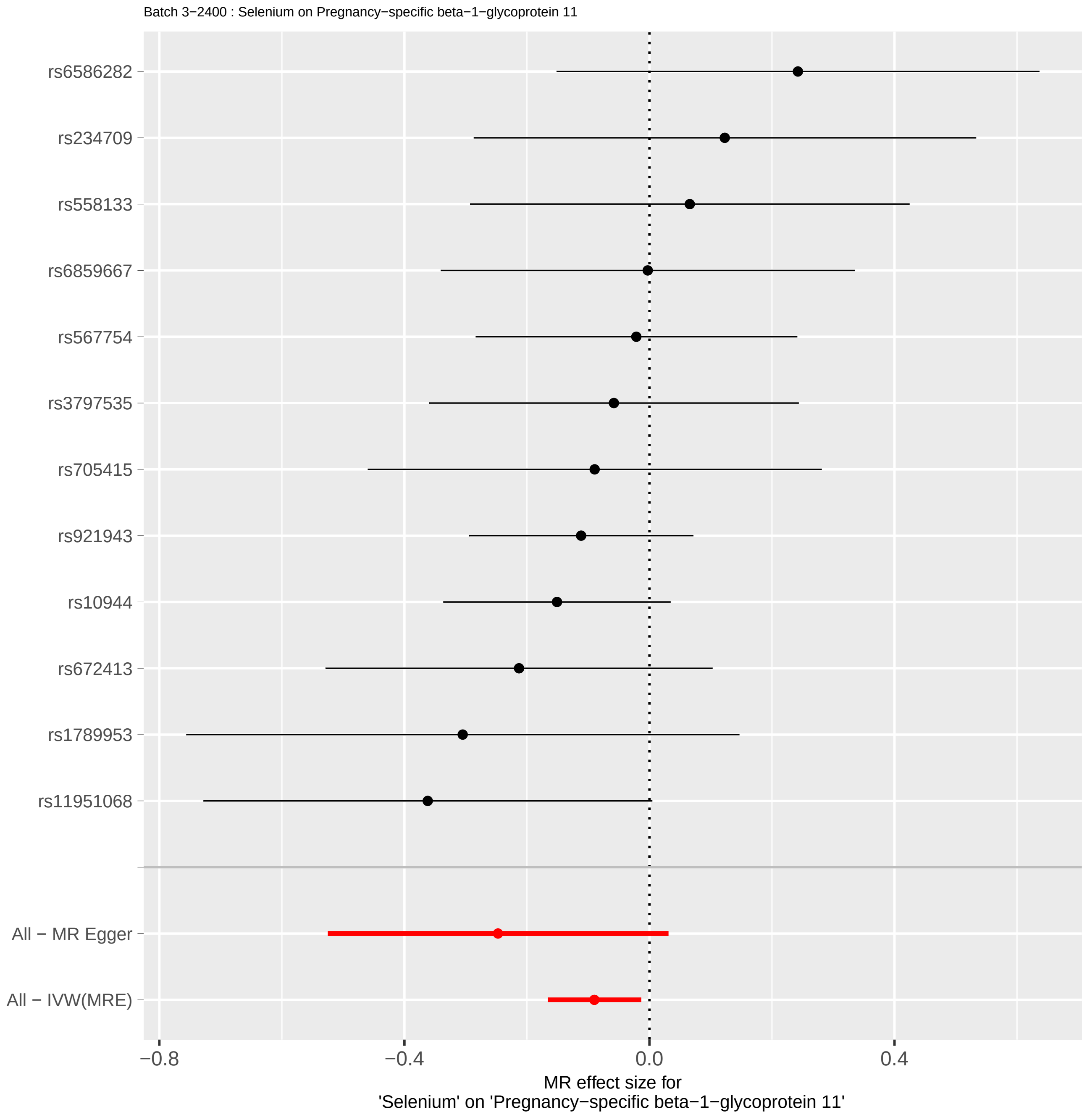

Batch 3−2400 : Selenium on Pregnancy−specific beta−1−glycoprotein 11
rs6586282
rs234709
rs558133
rs6859667
rs567754
rs3797535
rs705415
rs921943
rs10944
rs672413
rs1789953
rs11951068
All − MR Egger
All − IVW(MRE)
−0.8
−0.4
0.0
0.4
MR effect size for
'Selenium' on 'Pregnancy−specific beta−1−glycoprotein 11'

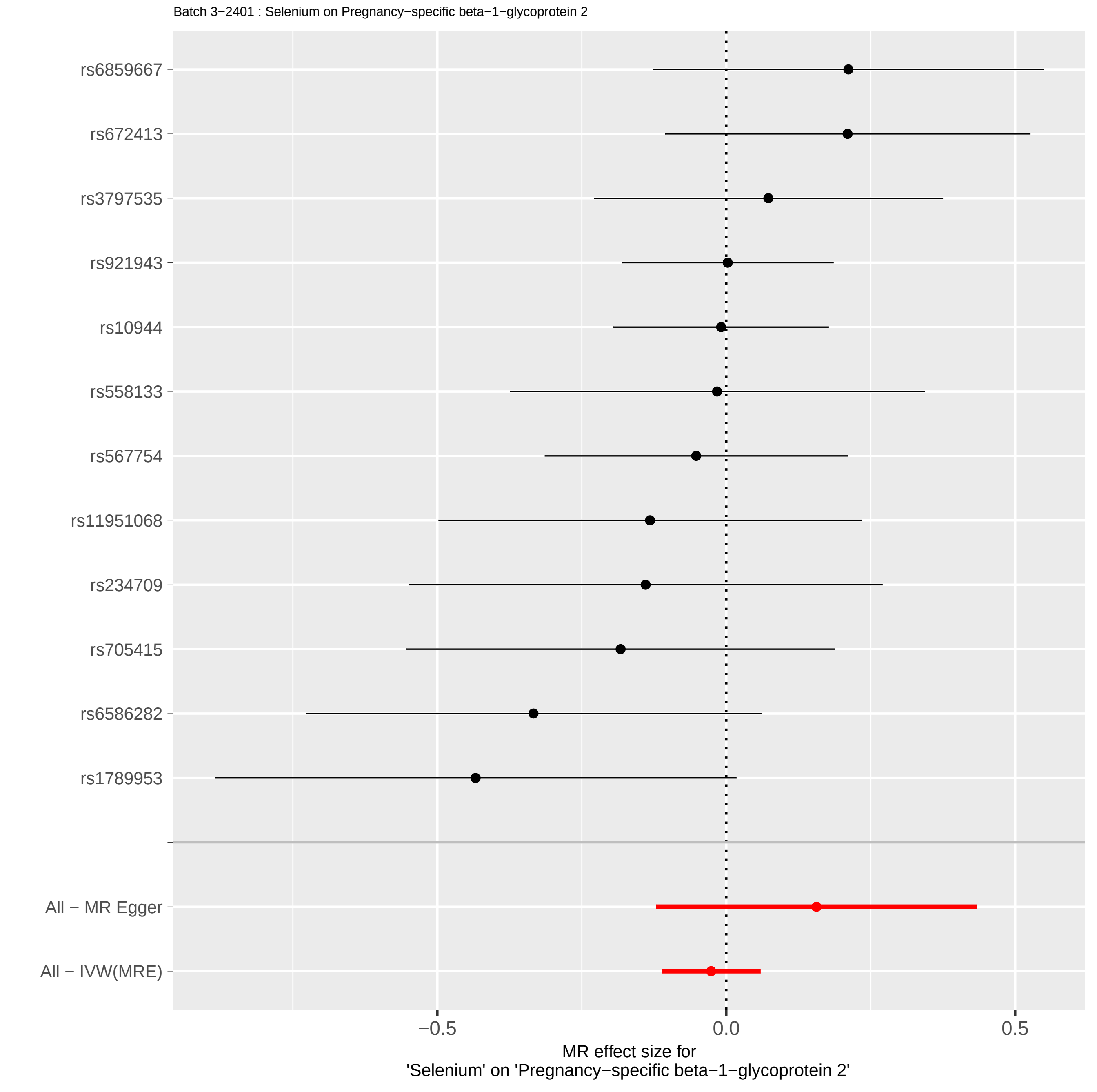

Batch 3−2401 : Selenium on Pregnancy−specific beta−1−glycoprotein 2
rs6859667
rs672413
rs3797535
rs921943
rs10944
rs558133
rs567754
rs11951068
rs234709
rs705415
rs6586282
rs1789953
All − MR Egger
All − IVW(MRE)
−0.5
0.0
0.5
MR effect size for
'Selenium' on 'Pregnancy−specific beta−1−glycoprotein 2'

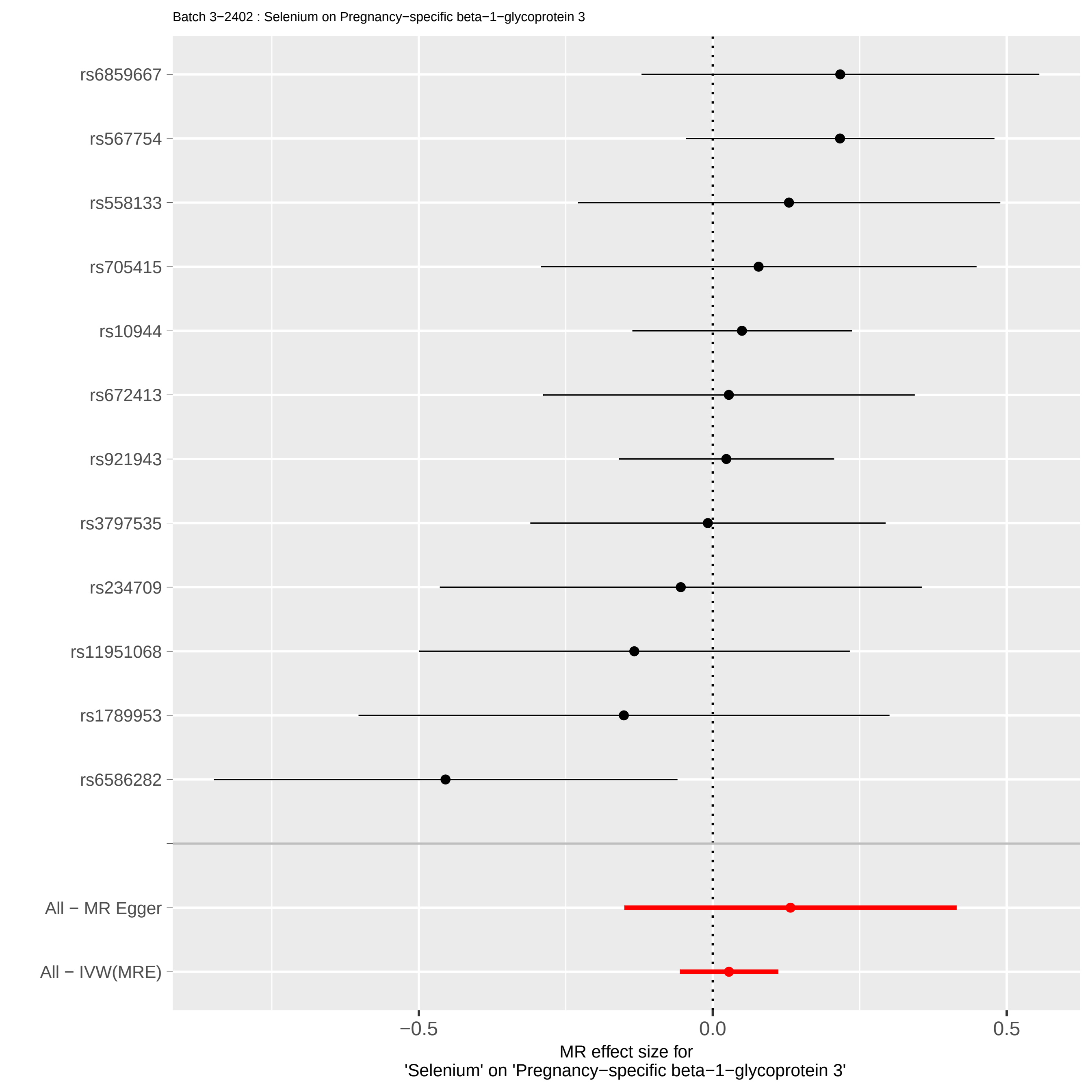

Batch 3−2402 : Selenium on Pregnancy−specific beta−1−glycoprotein 3
rs6859667
rs567754
rs558133
rs705415
rs10944
rs672413
rs921943
rs3797535
rs234709
rs11951068
rs1789953
rs6586282
All − MR Egger
All − IVW(MRE)
−0.5
0.0
0.5
MR effect size for
'Selenium' on 'Pregnancy−specific beta−1−glycoprotein 3'

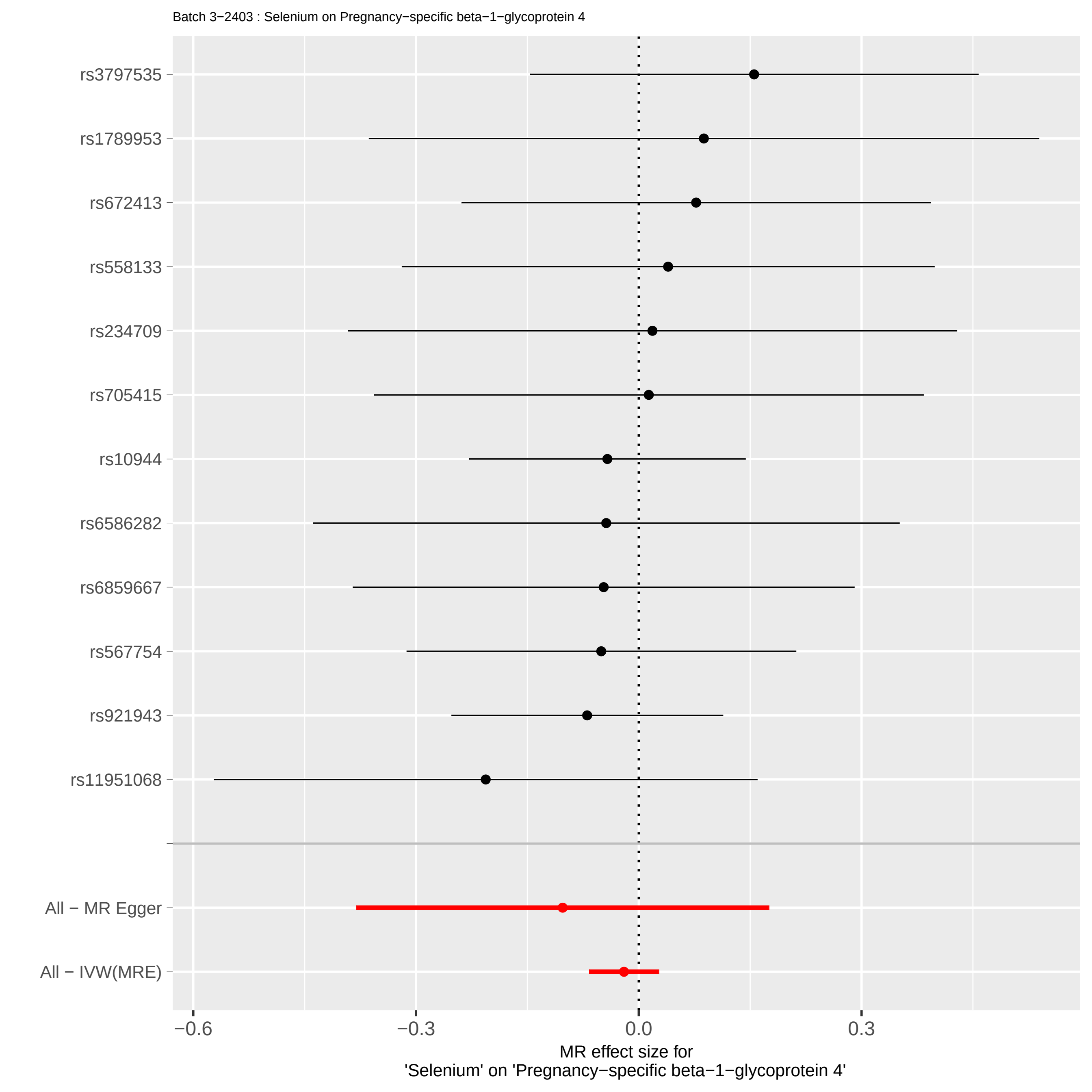

Batch 3−2403 : Selenium on Pregnancy−specific beta−1−glycoprotein 4
rs3797535
rs1789953
rs672413
rs558133
rs234709
rs705415
rs10944
rs6586282
rs6859667
rs567754
rs921943
rs11951068
All − MR Egger
All − IVW(MRE)
−0.6
−0.3
0.0
0.3
MR effect size for
'Selenium' on 'Pregnancy−specific beta−1−glycoprotein 4'

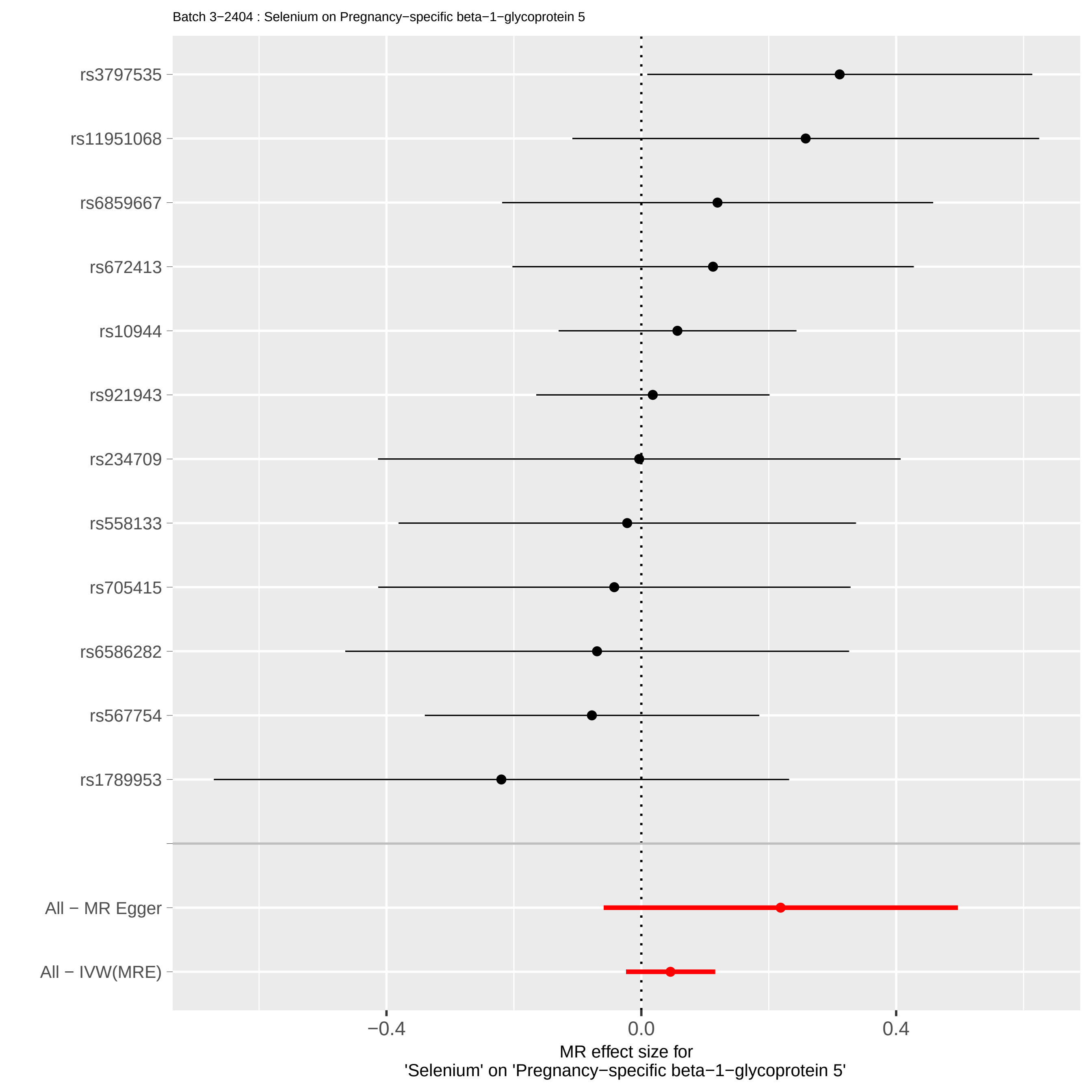
Batch 3−2404 : Selenium on Pregnancy−specific beta−1−glycoprotein 5
rs3797535
rs11951068
rs6859667
rs672413
rs10944
rs921943
rs234709
rs558133
rs705415
rs6586282
rs567754
rs1789953
All − MR Egger
All − IVW(MRE)
−0.4
0.0
0.4
MR effect size for
'Selenium' on 'Pregnancy−specific beta−1−glycoprotein 5'

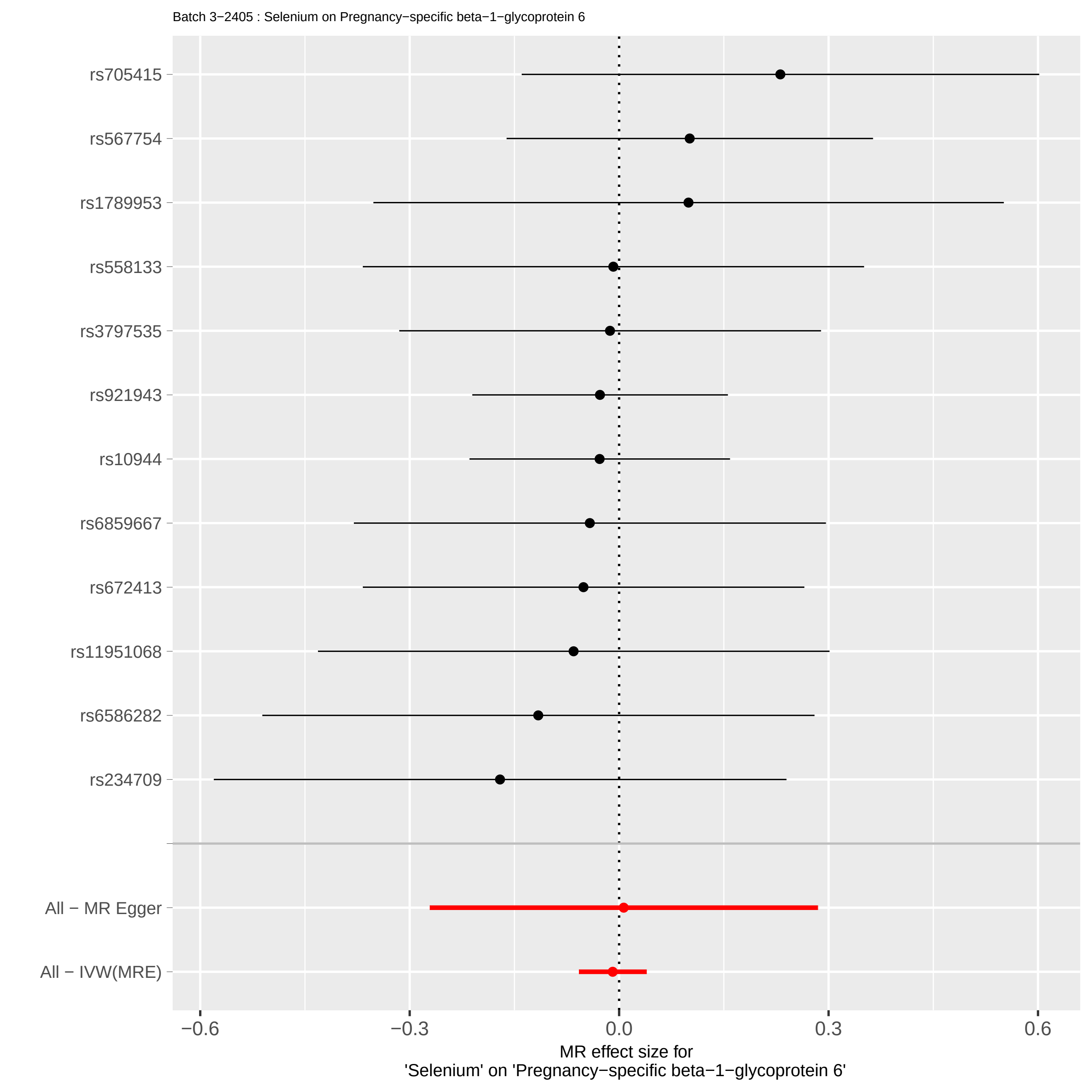

Batch 3−2405 : Selenium on Pregnancy−specific beta−1−glycoprotein 6
rs705415
rs567754
rs1789953
rs558133
rs3797535
rs921943
rs10944
rs6859667
rs672413
rs11951068
rs6586282
rs234709
All − MR Egger
All − IVW(MRE)
−0.6
−0.3
0.0
0.3
0.6
MR effect size for
'Selenium' on 'Pregnancy−specific beta−1−glycoprotein 6'

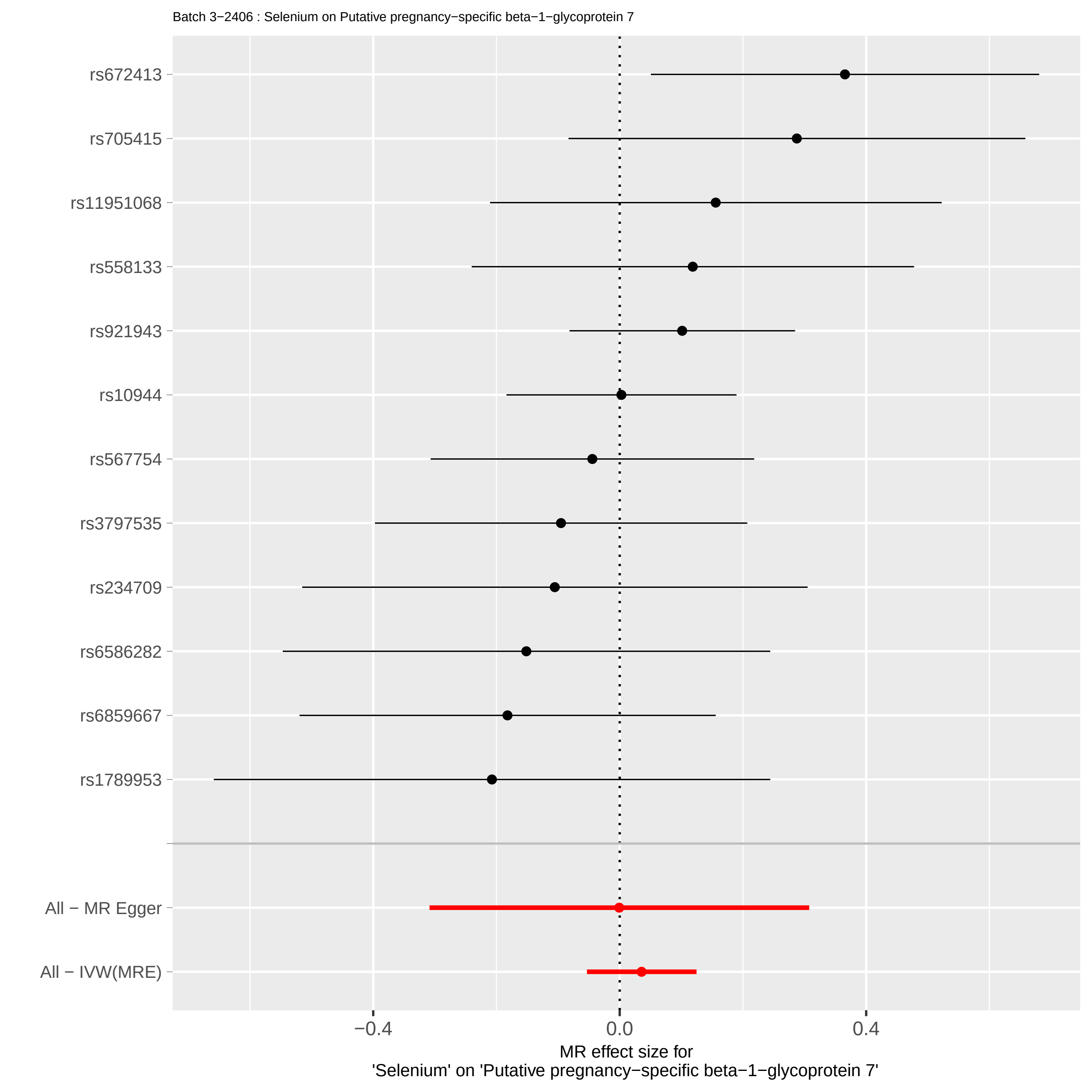
Batch 3−2406 : Selenium on Putative pregnancy−specific beta−1−glycoprotein 7
rs672413
rs705415
rs11951068
rs558133
rs921943
rs10944
rs567754
rs3797535
rs234709
rs6586282
rs6859667
rs1789953
All − MR Egger
All − IVW(MRE)
−0.4
0.0
0.4
MR effect size for
'Selenium' on 'Putative pregnancy−specific beta−1−glycoprotein 7'

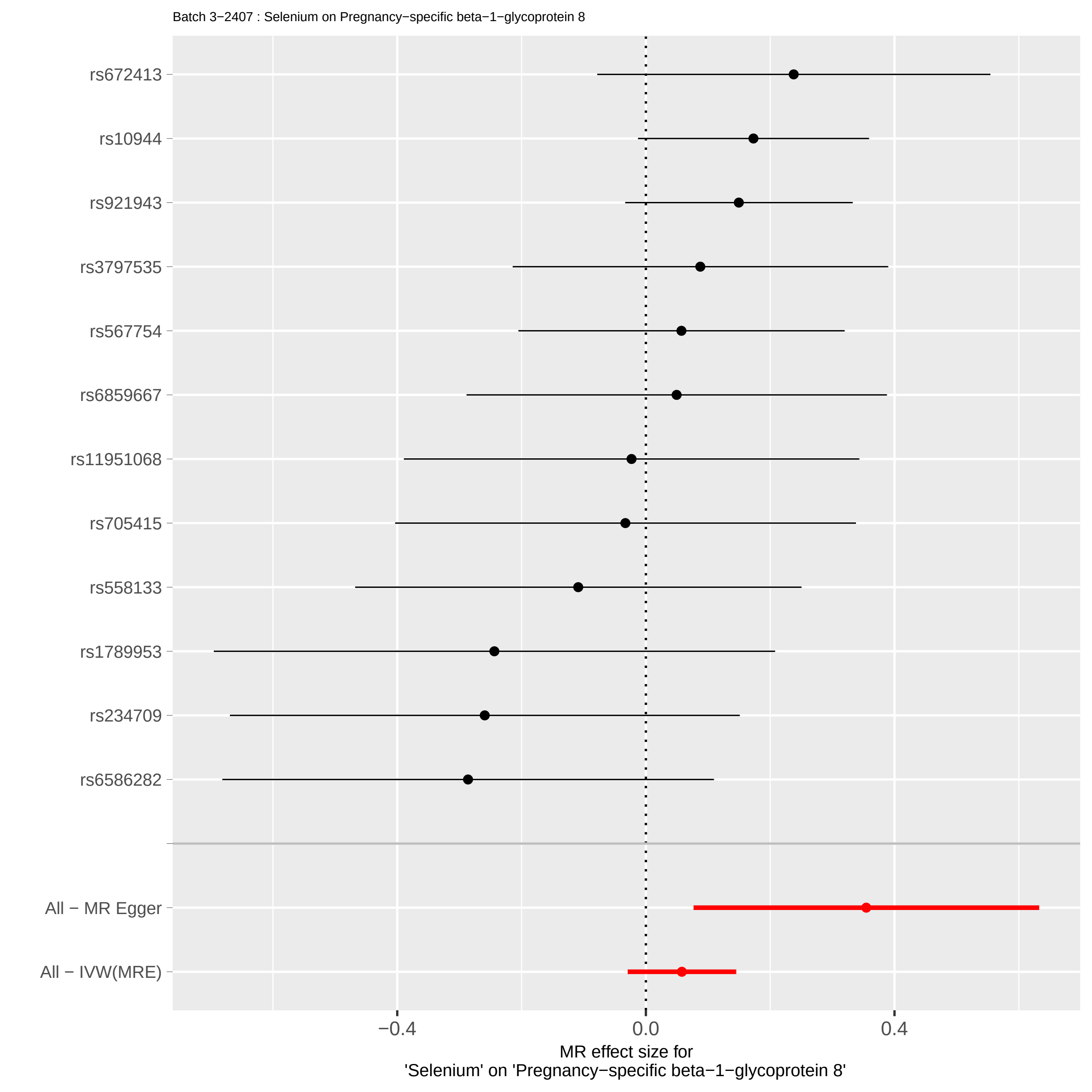

Batch 3−2407 : Selenium on Pregnancy−specific beta−1−glycoprotein 8
rs672413
rs10944
rs921943
rs3797535
rs567754
rs6859667
rs11951068
rs705415
rs558133
rs1789953
rs234709
rs6586282
All − MR Egger
All − IVW(MRE)
−0.4
0.0
0.4
MR effect size for
'Selenium' on 'Pregnancy−specific beta−1−glycoprotein 8'

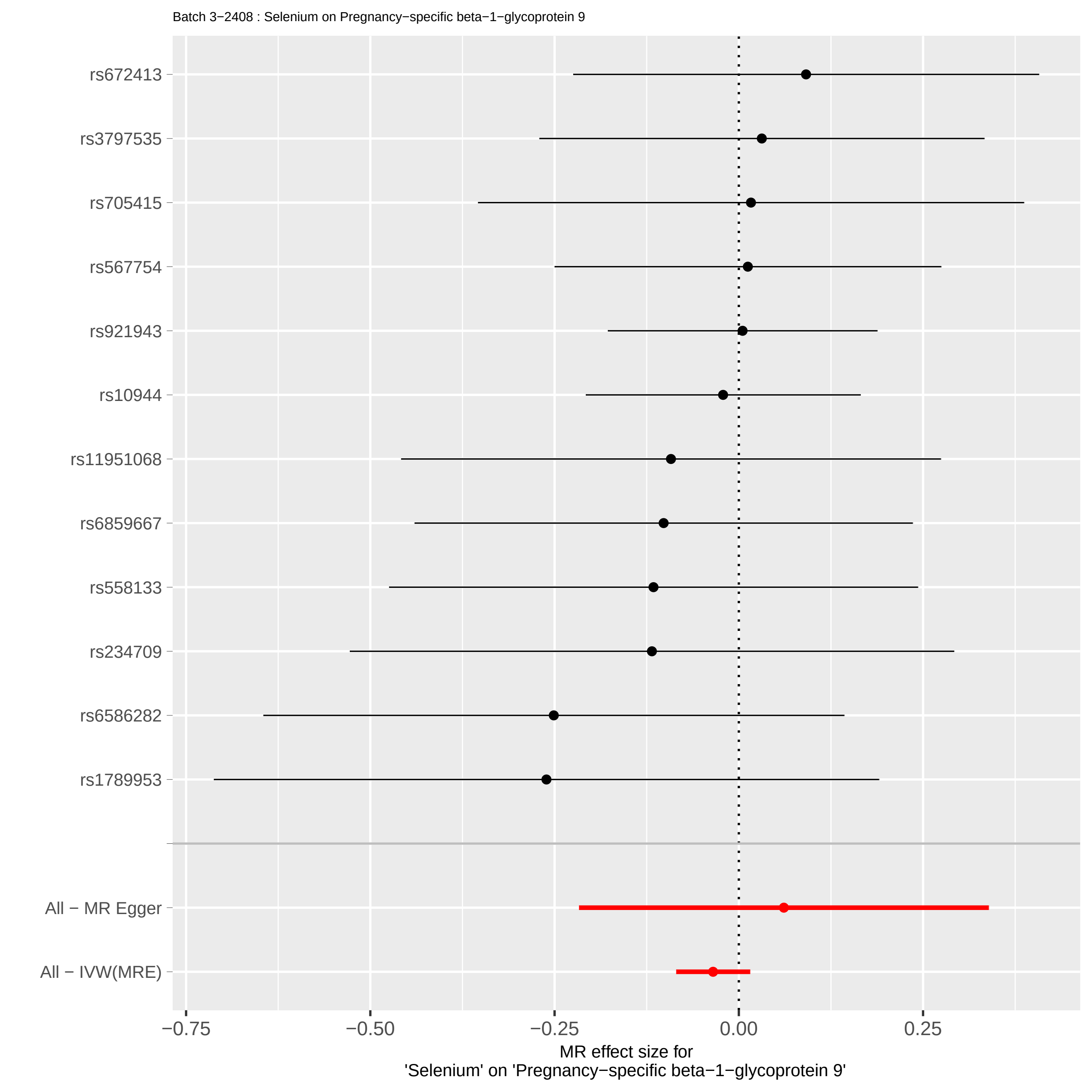

Batch 3−2408 : Selenium on Pregnancy−specific beta−1−glycoprotein 9
rs672413
rs3797535
rs705415
rs567754
rs921943
rs10944
rs11951068
rs6859667
rs558133
rs234709
rs6586282
rs1789953
All − MR Egger
All − IVW(MRE)
−0.75
−0.50
−0.25
0.00
0.25
MR effect size for
'Selenium' on 'Pregnancy−specific beta−1−glycoprotein 9'

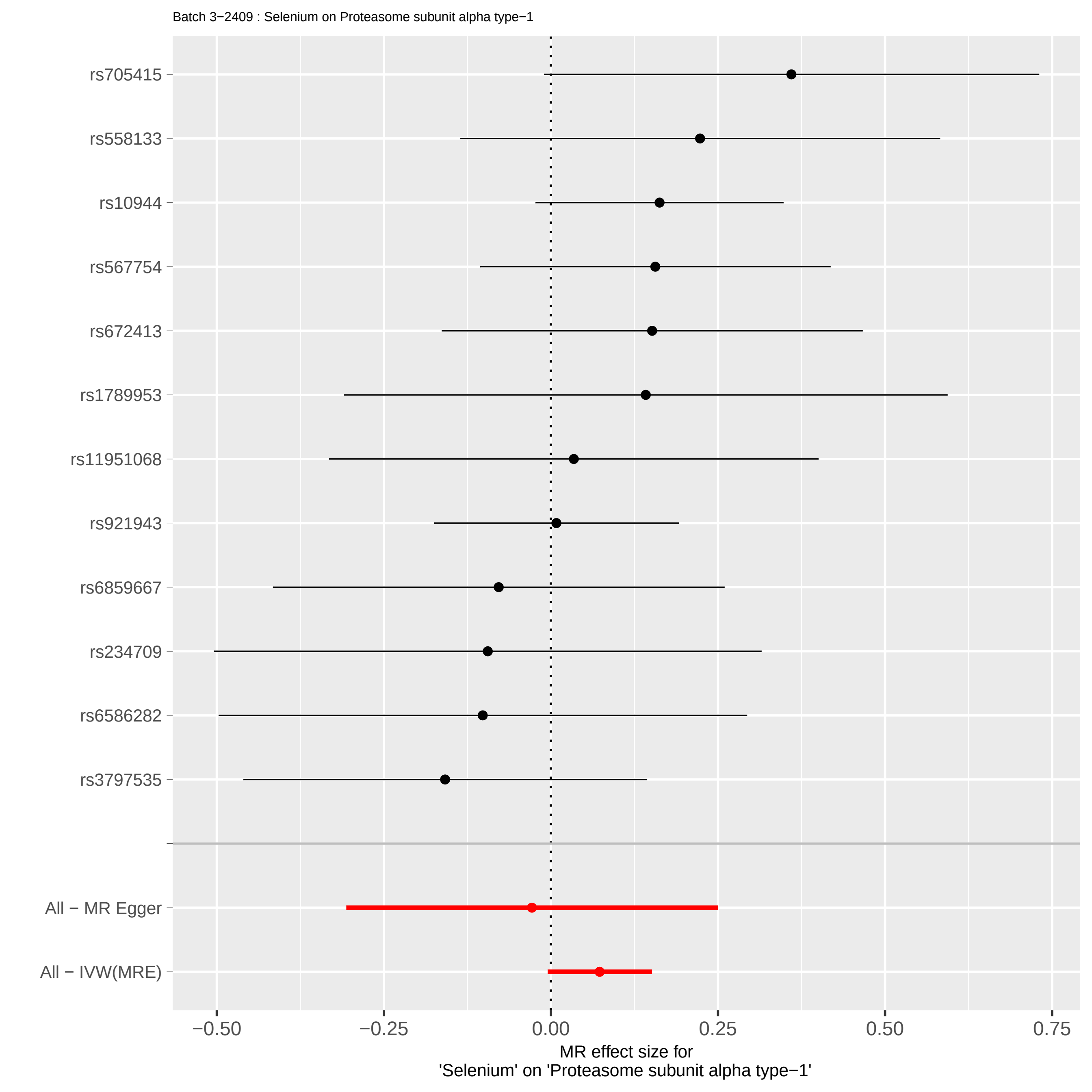
Batch 3−2409 : Selenium on Proteasome subunit alpha type−1
rs705415
rs558133
rs10944
rs567754
rs672413
rs1789953
rs11951068
rs921943
rs6859667
rs234709
rs6586282
rs3797535
All − MR Egger
All − IVW(MRE)
−0.50
−0.25
0.00
0.25
0.50
0.75
MR effect size for
'Selenium' on 'Proteasome subunit alpha type−1'

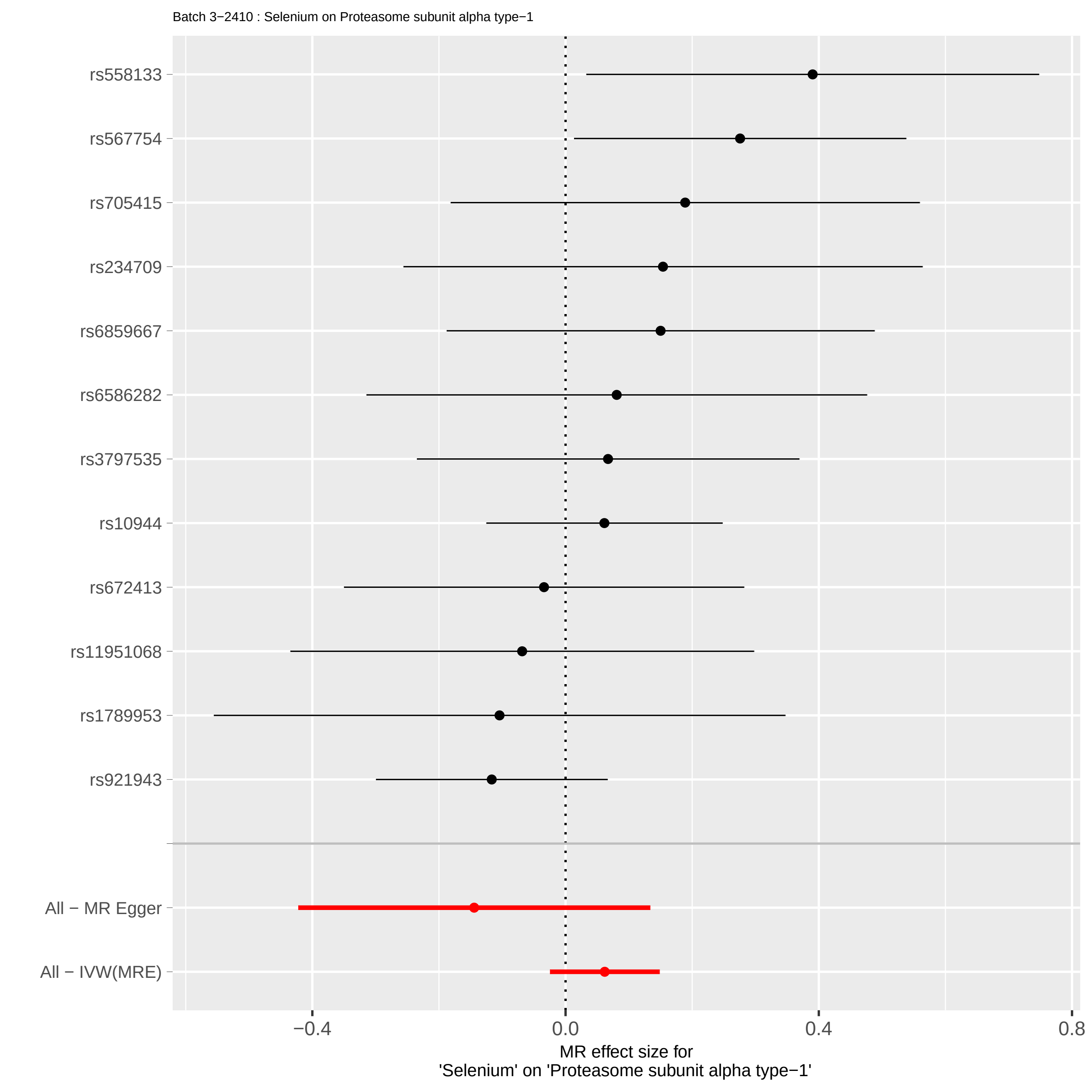

Batch 3−2410 : Selenium on Proteasome subunit alpha type−1
rs558133
rs567754
rs705415
rs234709
rs6859667
rs6586282
rs3797535
rs10944
rs672413
rs11951068
rs1789953
rs921943
All − MR Egger
All − IVW(MRE)
−0.4
0.0
0.4
0.8
MR effect size for
'Selenium' on 'Proteasome subunit alpha type−1'

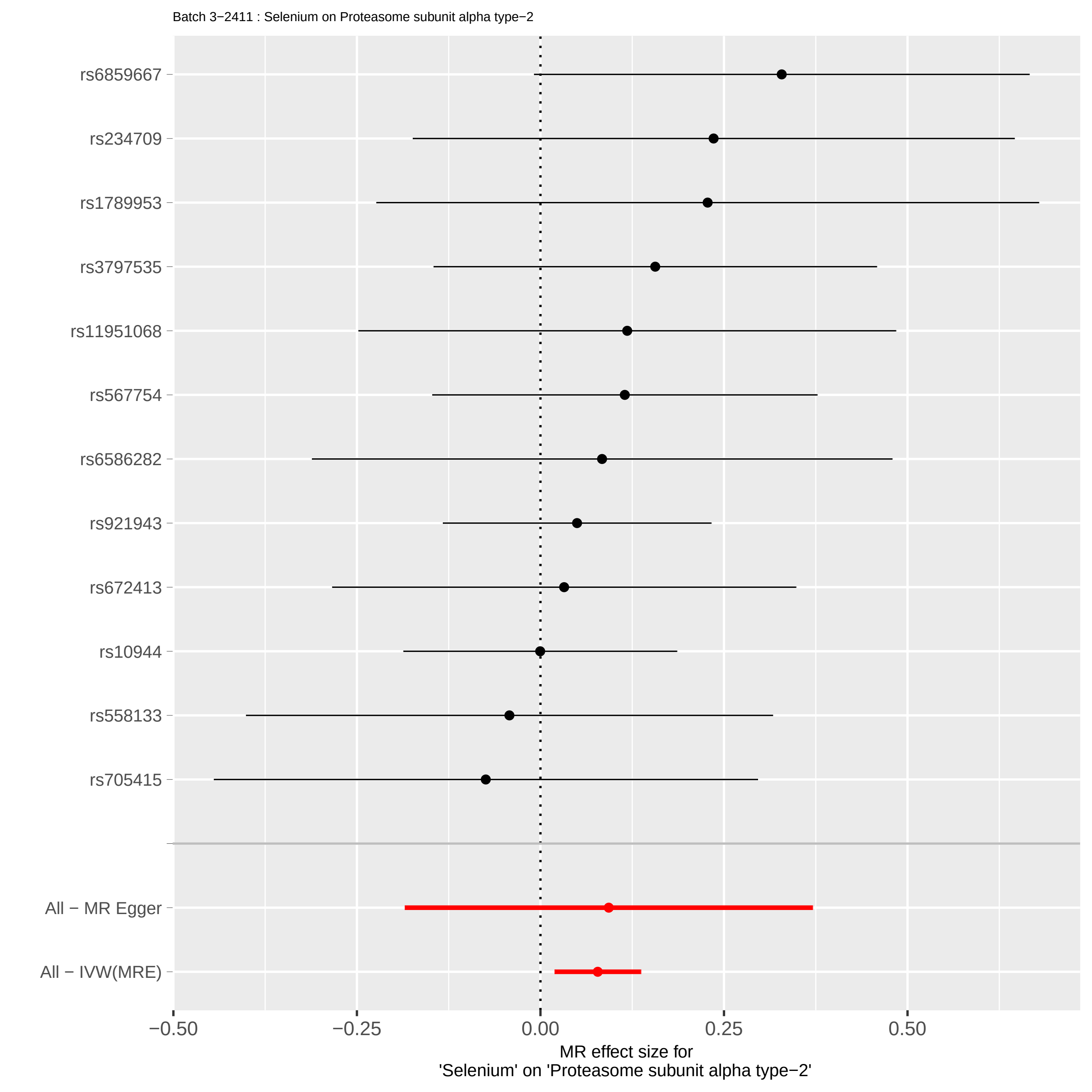
Batch 3−2411 : Selenium on Proteasome subunit alpha type−2
rs6859667
rs234709
rs1789953
rs3797535
rs11951068
rs567754
rs6586282
rs921943
rs672413
rs10944
rs558133
rs705415
All − MR Egger
All − IVW(MRE)
−0.50
−0.25
0.00
0.25
0.50
MR effect size for
'Selenium' on 'Proteasome subunit alpha type−2'

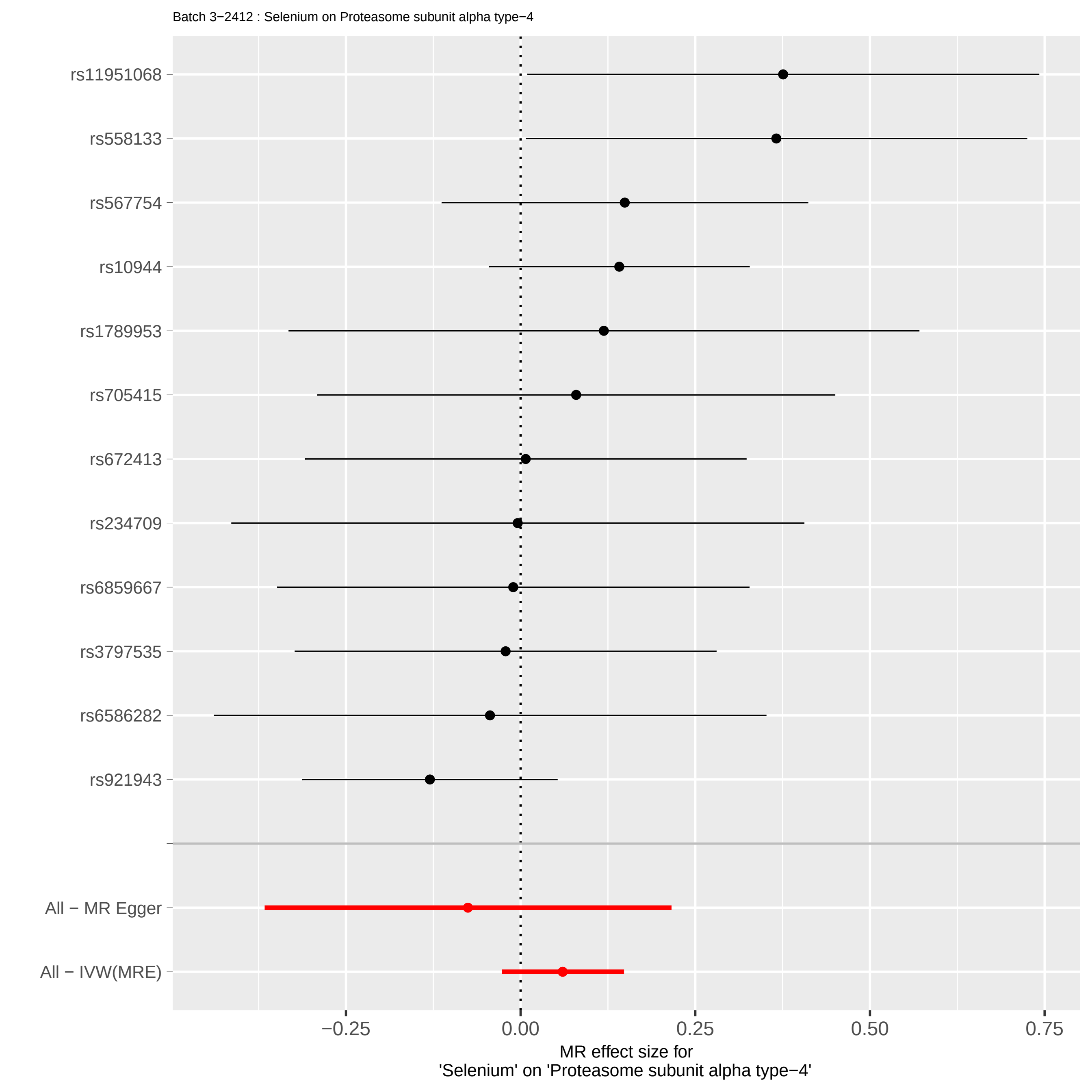
Batch 3−2412 : Selenium on Proteasome subunit alpha type−4
rs11951068
rs558133
rs567754
rs10944
rs1789953
rs705415
rs672413
rs234709
rs6859667
rs3797535
rs6586282
rs921943
All − MR Egger
All − IVW(MRE)
−0.25
0.00
0.25
0.50
0.75
MR effect size for
'Selenium' on 'Proteasome subunit alpha type−4'

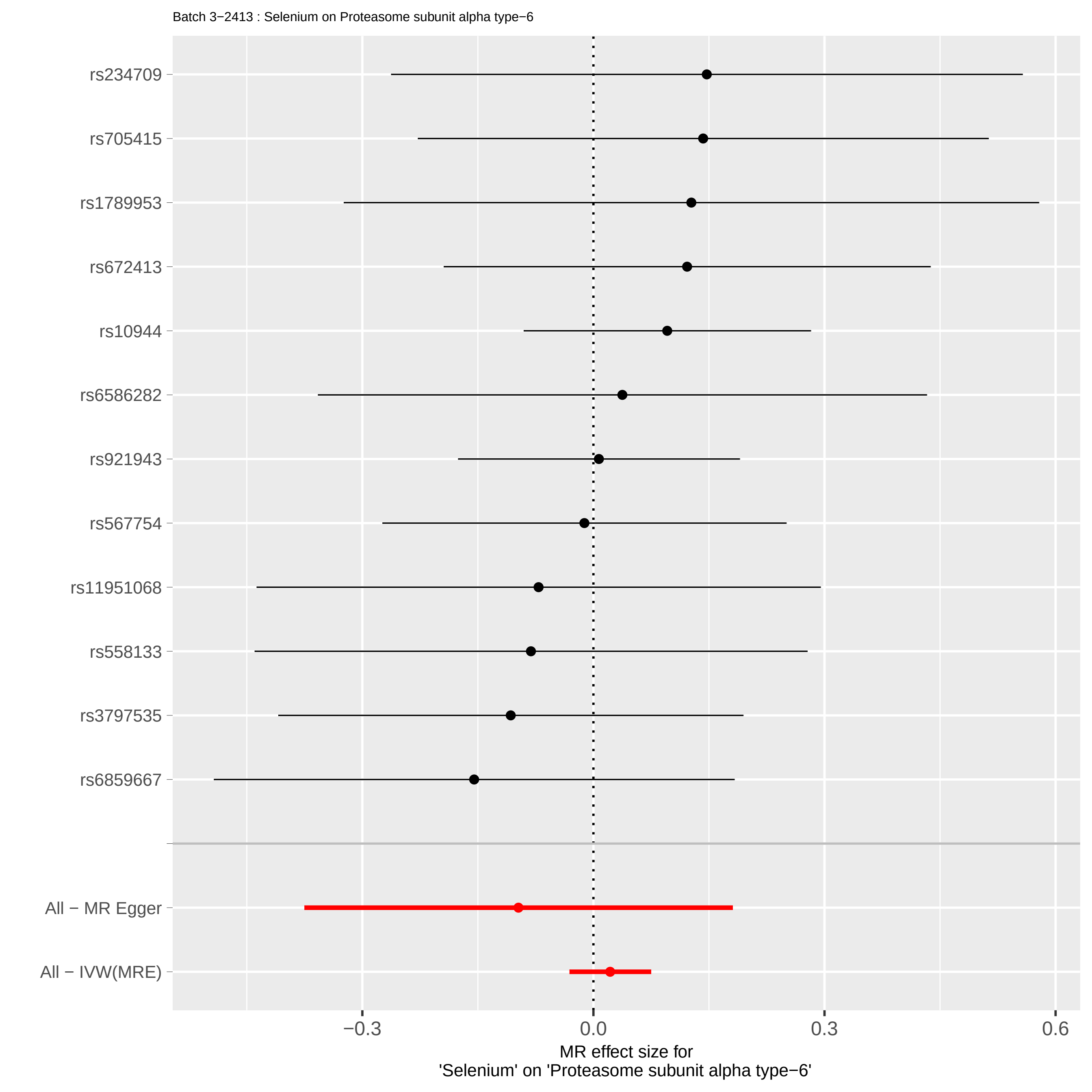
Batch 3−2413 : Selenium on Proteasome subunit alpha type−6
rs234709
rs705415
rs1789953
rs672413
rs10944
rs6586282
rs921943
rs567754
rs11951068
rs558133
rs3797535
rs6859667
All − MR Egger
All − IVW(MRE)
−0.3
0.0
0.3
0.6
MR effect size for
'Selenium' on 'Proteasome subunit alpha type−6'

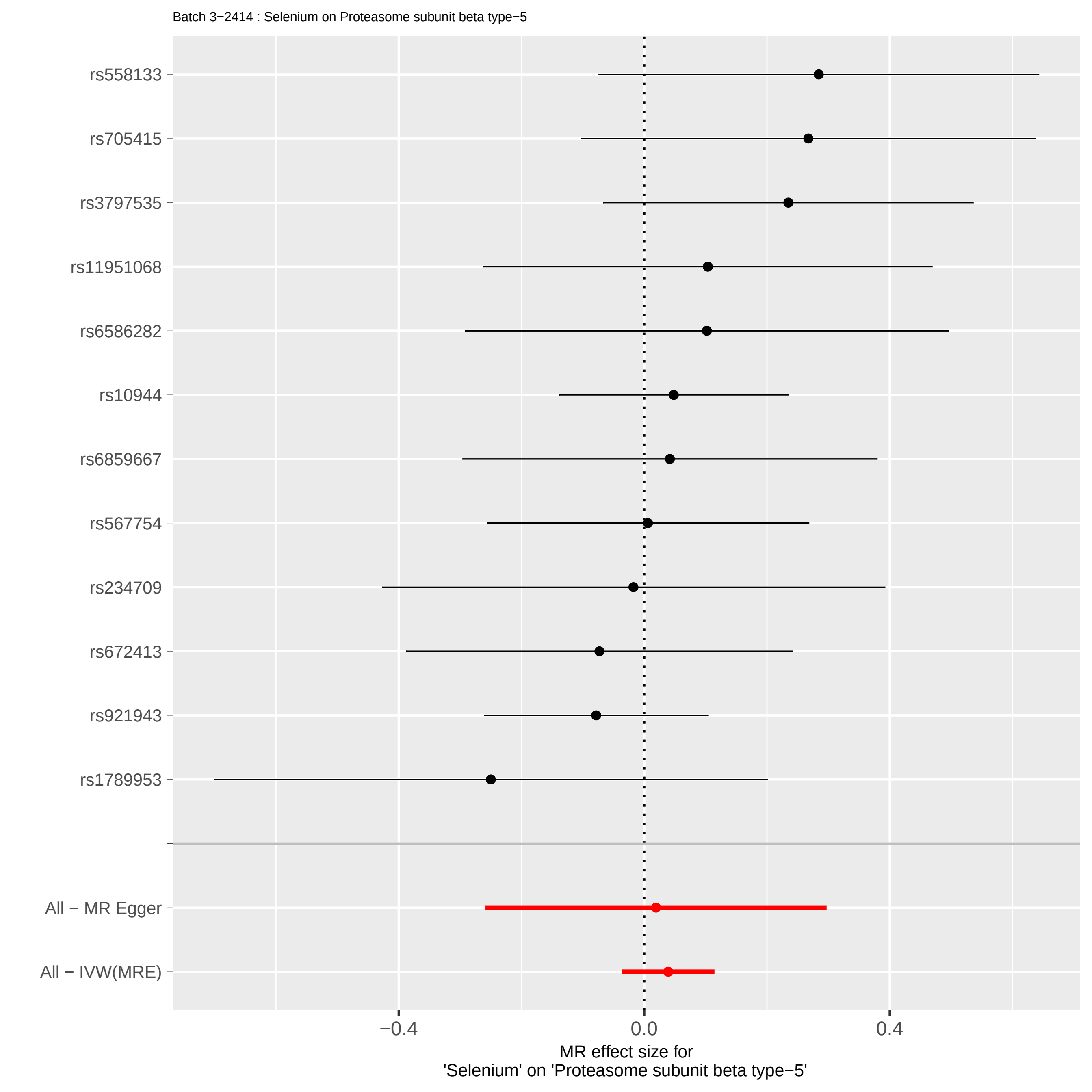

Batch 3−2414 : Selenium on Proteasome subunit beta type−5
rs558133
rs705415
rs3797535
rs11951068
rs6586282
rs10944
rs6859667
rs567754
rs234709
rs672413
rs921943
rs1789953
All − MR Egger
All − IVW(MRE)
−0.4
0.0
0.4
MR effect size for
'Selenium' on 'Proteasome subunit beta type−5'

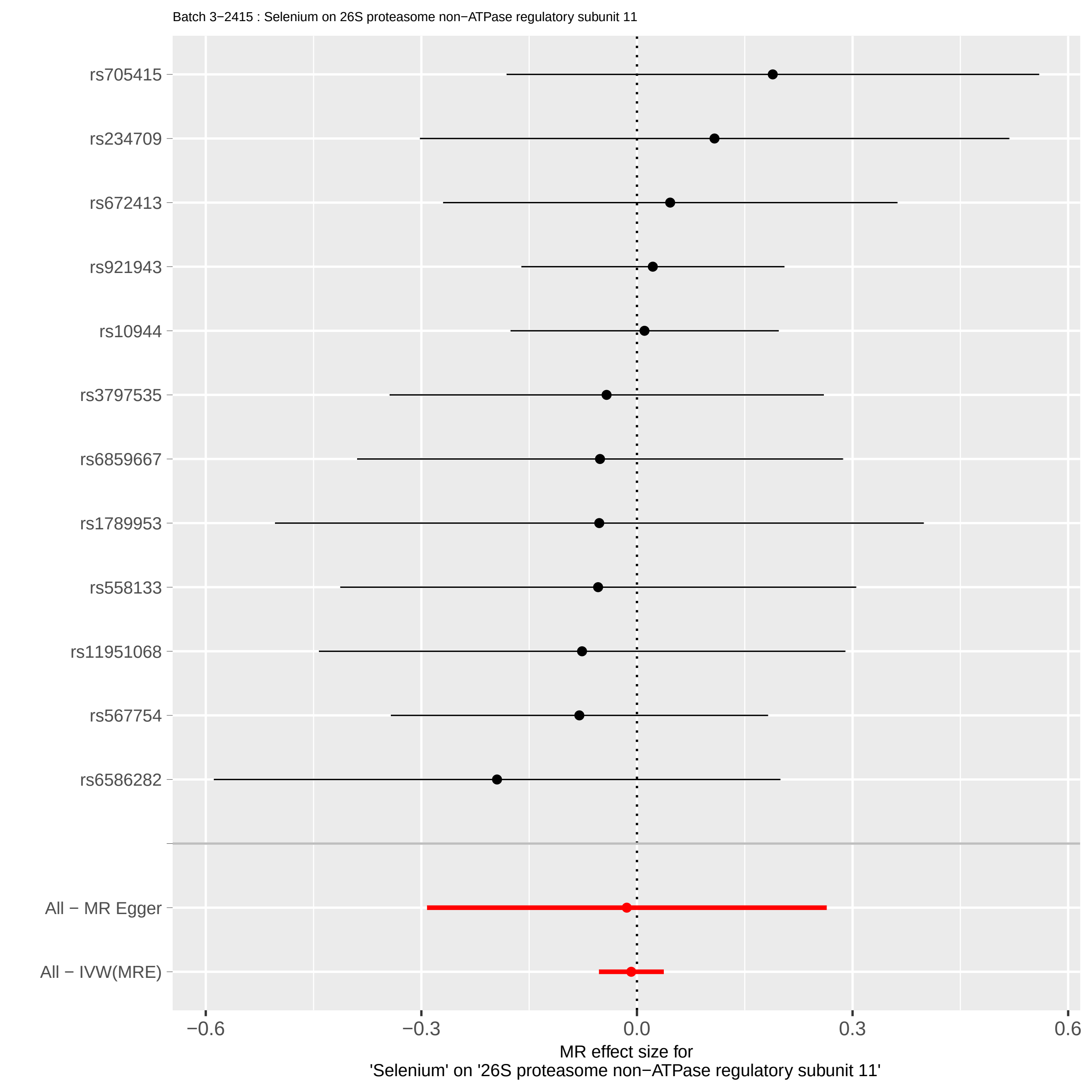

Batch 3−2415 : Selenium on 26S proteasome non−ATPase regulatory subunit 11
rs705415
rs234709
rs672413
rs921943
rs10944
rs3797535
rs6859667
rs1789953
rs558133
rs11951068
rs567754
rs6586282
All − MR Egger
All − IVW(MRE)
−0.6
−0.3
0.0
0.3
0.6
MR effect size for
'Selenium' on '26S proteasome non−ATPase regulatory subunit 11'

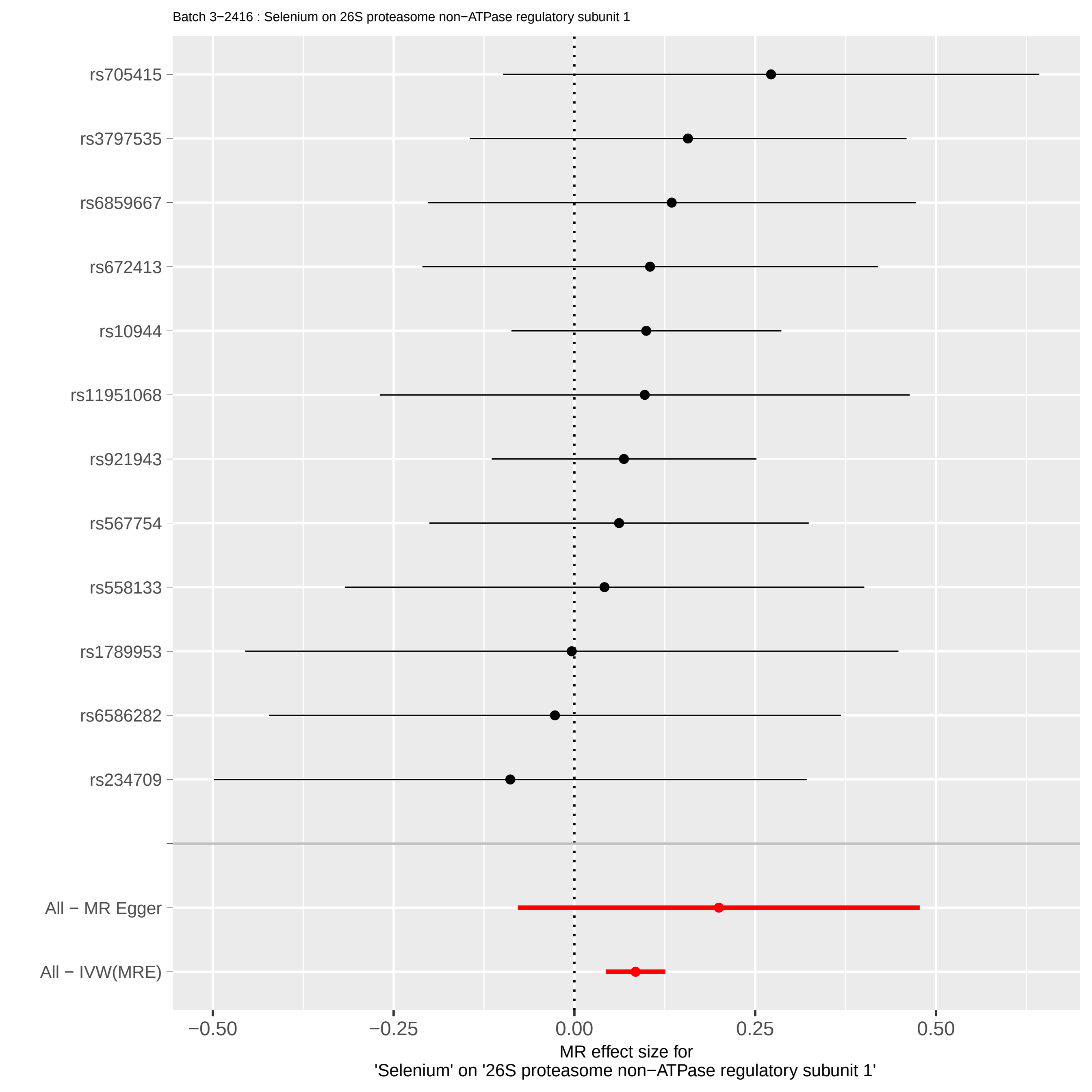

Batch 3−2416 : Selenium on 26S proteasome non−ATPase regulatory subunit 1
rs705415
rs3797535
rs6859667
rs672413
rs10944
rs11951068
rs921943
rs567754
rs558133
rs1789953
rs6586282
rs234709
All − MR Egger
All − IVW(MRE)
−0.50
−0.25
0.00
0.25
0.50
MR effect size for
'Selenium' on '26S proteasome non−ATPase regulatory subunit 1'

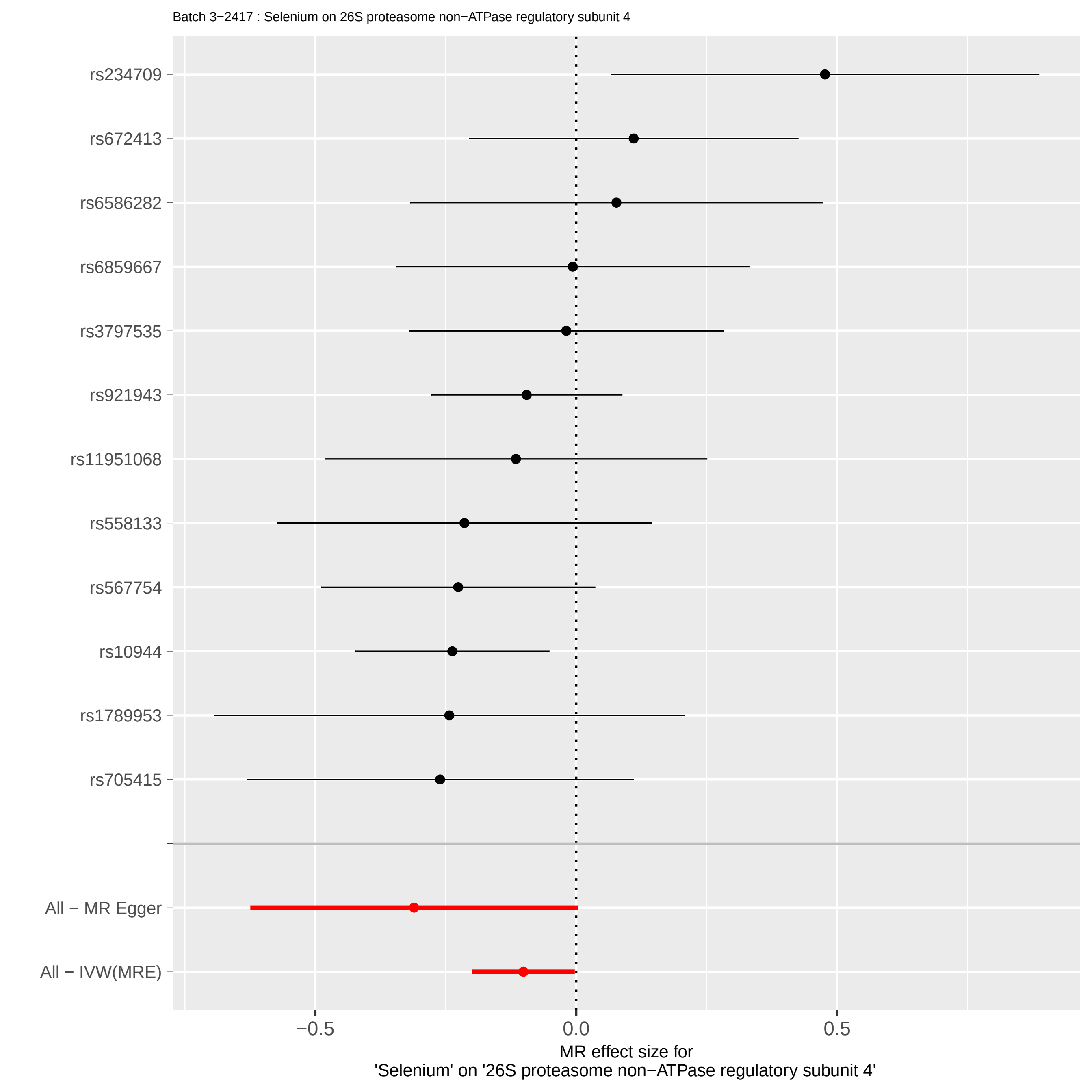

Batch 3−2417 : Selenium on 26S proteasome non−ATPase regulatory subunit 4
rs234709
rs672413
rs6586282
rs6859667
rs3797535
rs921943
rs11951068
rs558133
rs567754
rs10944
rs1789953
rs705415
All − MR Egger
All − IVW(MRE)
−0.5
0.0
0.5
MR effect size for
'Selenium' on '26S proteasome non−ATPase regulatory subunit 4'

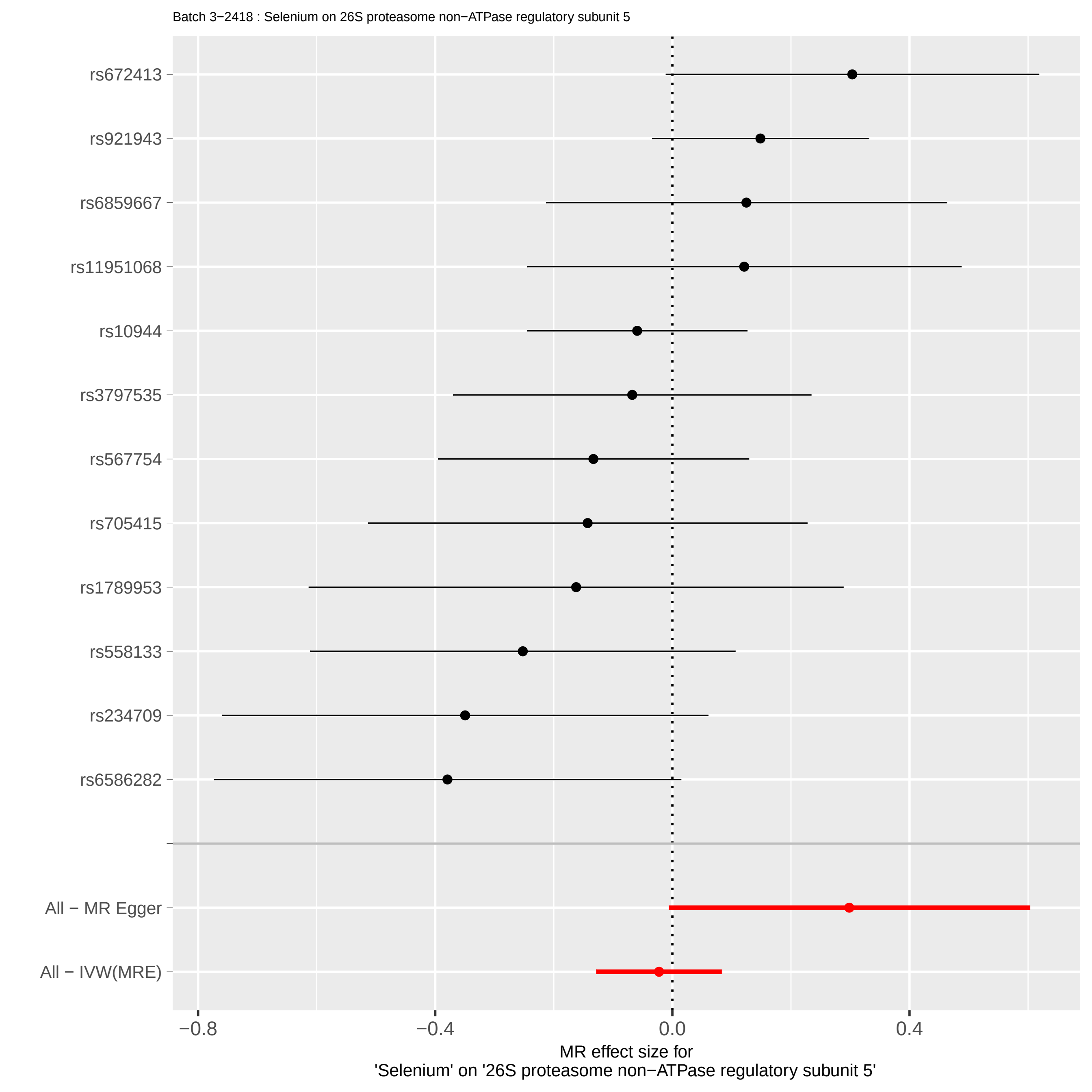

Batch 3−2418 : Selenium on 26S proteasome non−ATPase regulatory subunit 5
rs672413
rs921943
rs6859667
rs11951068
rs10944
rs3797535
rs567754
rs705415
rs1789953
rs558133
rs234709
rs6586282
All − MR Egger
All − IVW(MRE)
−0.8
−0.4
0.0
0.4
MR effect size for
'Selenium' on '26S proteasome non−ATPase regulatory subunit 5'

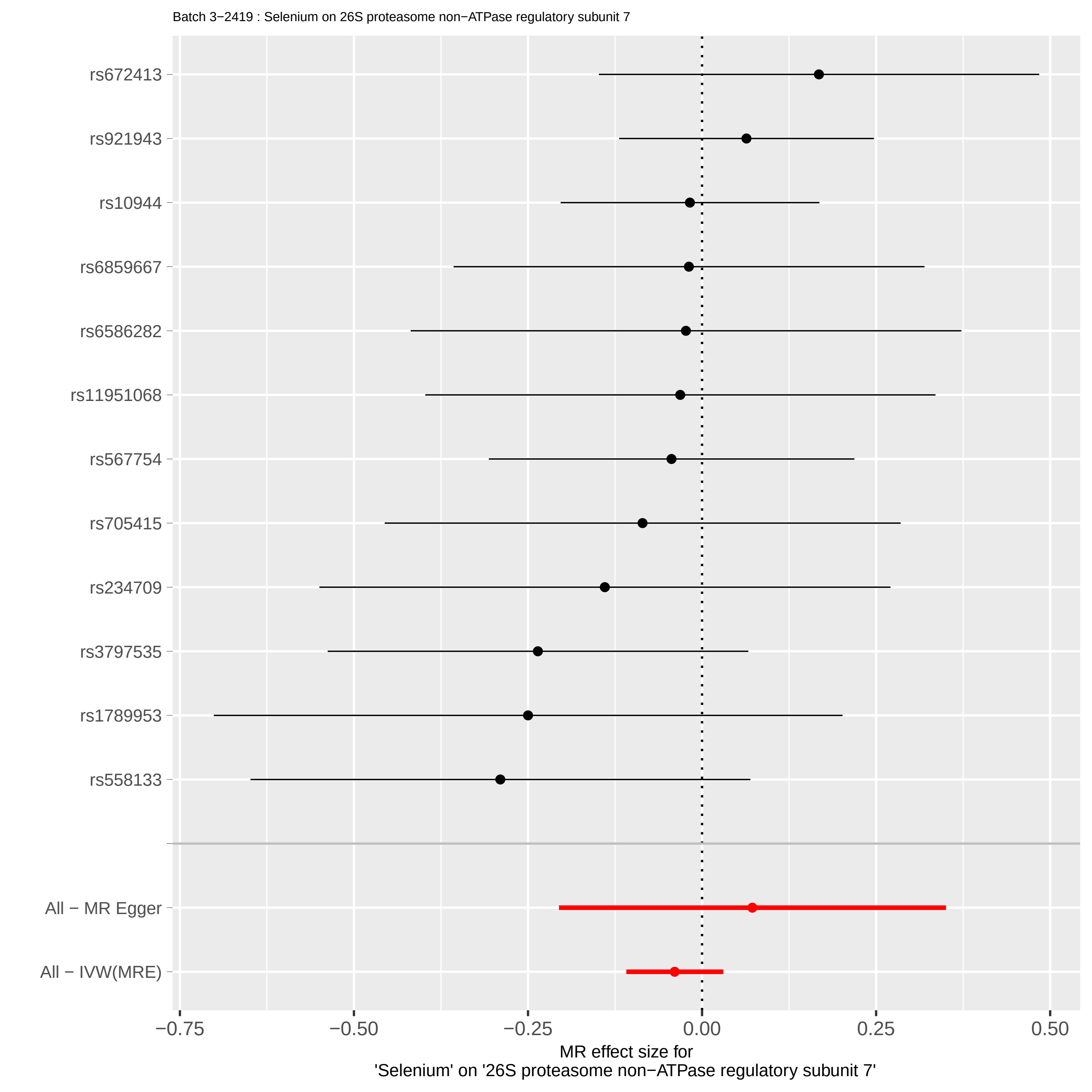

Batch 3−2419 : Selenium on 26S proteasome non−ATPase regulatory subunit 7
rs672413
rs921943
rs10944
rs6859667
rs6586282
rs11951068
rs567754
rs705415
rs234709
rs3797535
rs1789953
rs558133
All − MR Egger
All − IVW(MRE)
−0.75
−0.50
−0.25
0.00
0.25
0.50
MR effect size for
'Selenium' on '26S proteasome non−ATPase regulatory subunit 7'

Batch 3−2420 : Selenium on Proteasome activator complex subunit 1

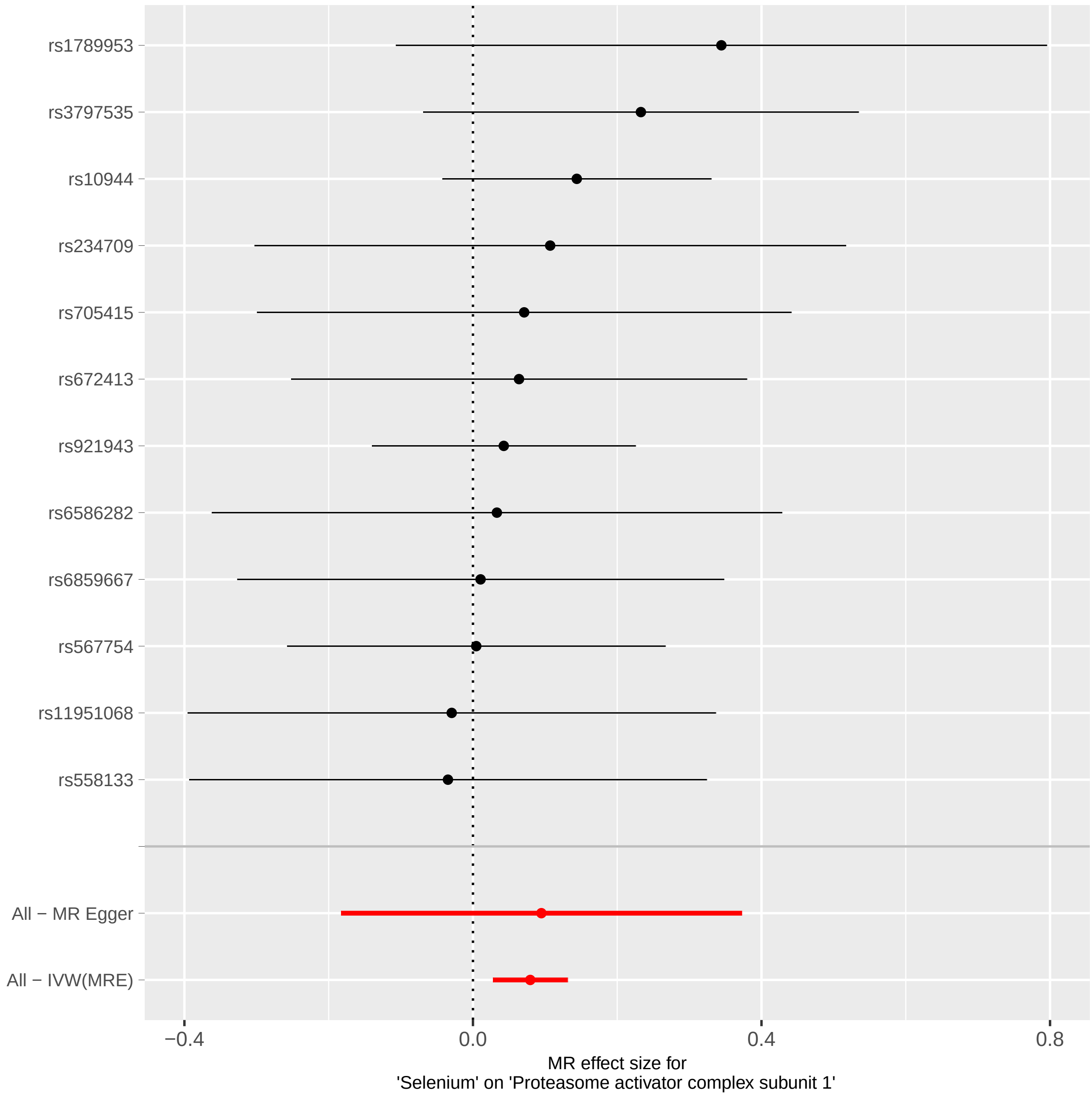

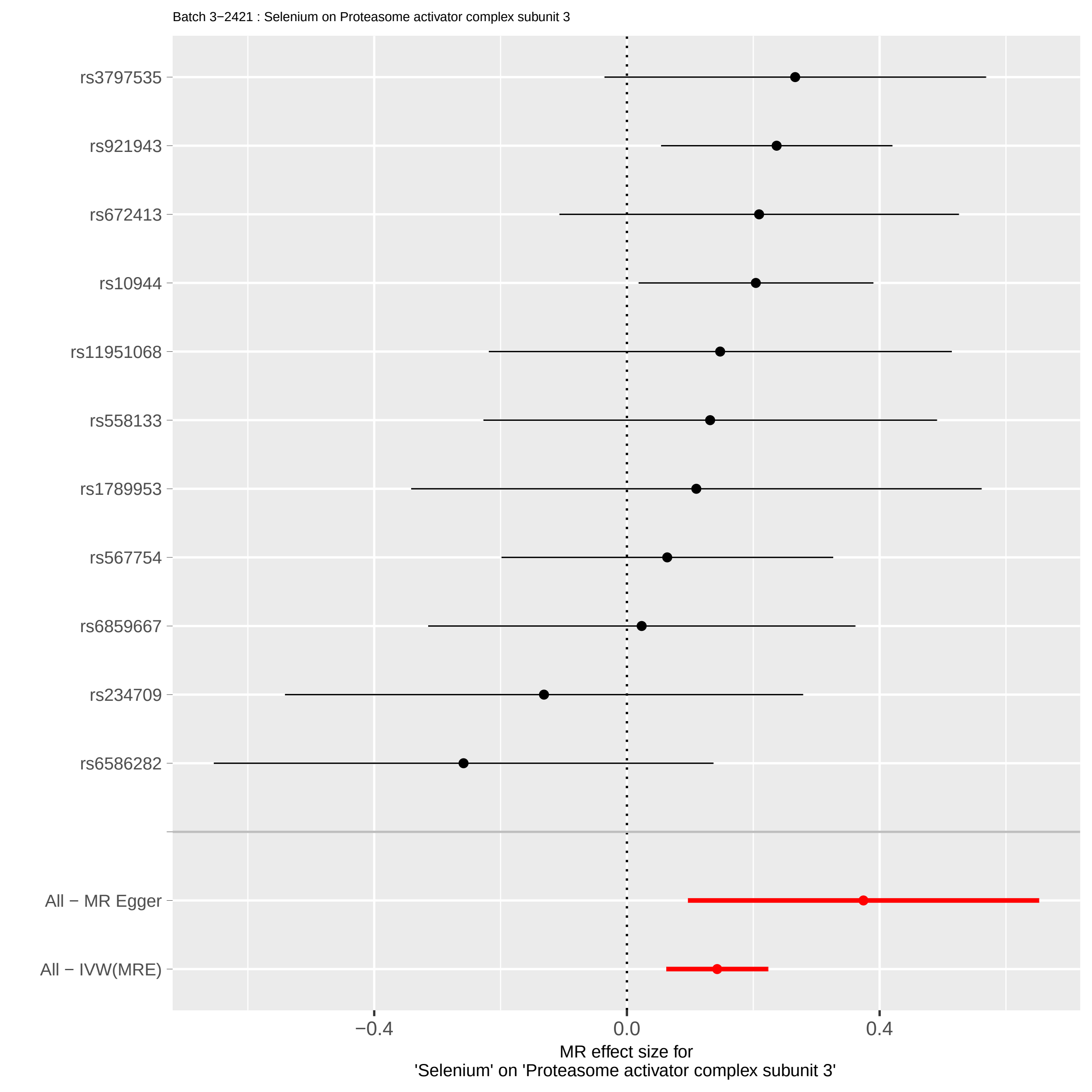

Batch 3−2421 : Selenium on Proteasome activator complex subunit 3
rs3797535
rs921943
rs672413
rs10944
rs11951068
rs558133
rs1789953
rs567754
rs6859667
rs234709
rs6586282
All − MR Egger
All − IVW(MRE)
−0.4
0.0
0.4
MR effect size for
'Selenium' on 'Proteasome activator complex subunit 3'

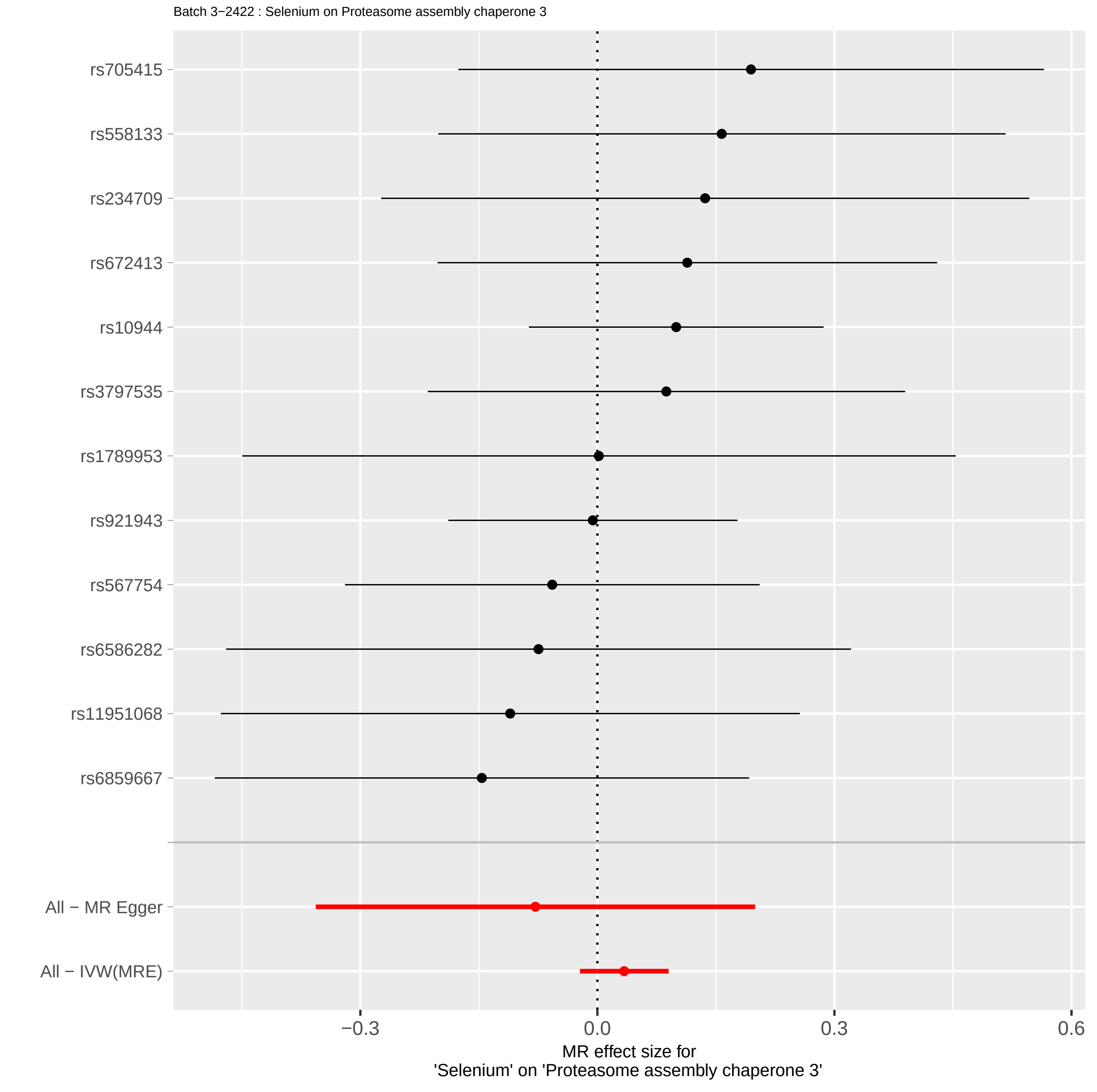

Batch 3−2422 : Selenium on Proteasome assembly chaperone 3
rs705415
rs558133
rs234709
rs672413
rs10944
rs3797535
rs1789953
rs921943
rs567754
rs6586282
rs11951068
rs6859667
All − MR Egger
All − IVW(MRE)
−0.3
0.0
0.3
0.6
MR effect size for
'Selenium' on 'Proteasome assembly chaperone 3'

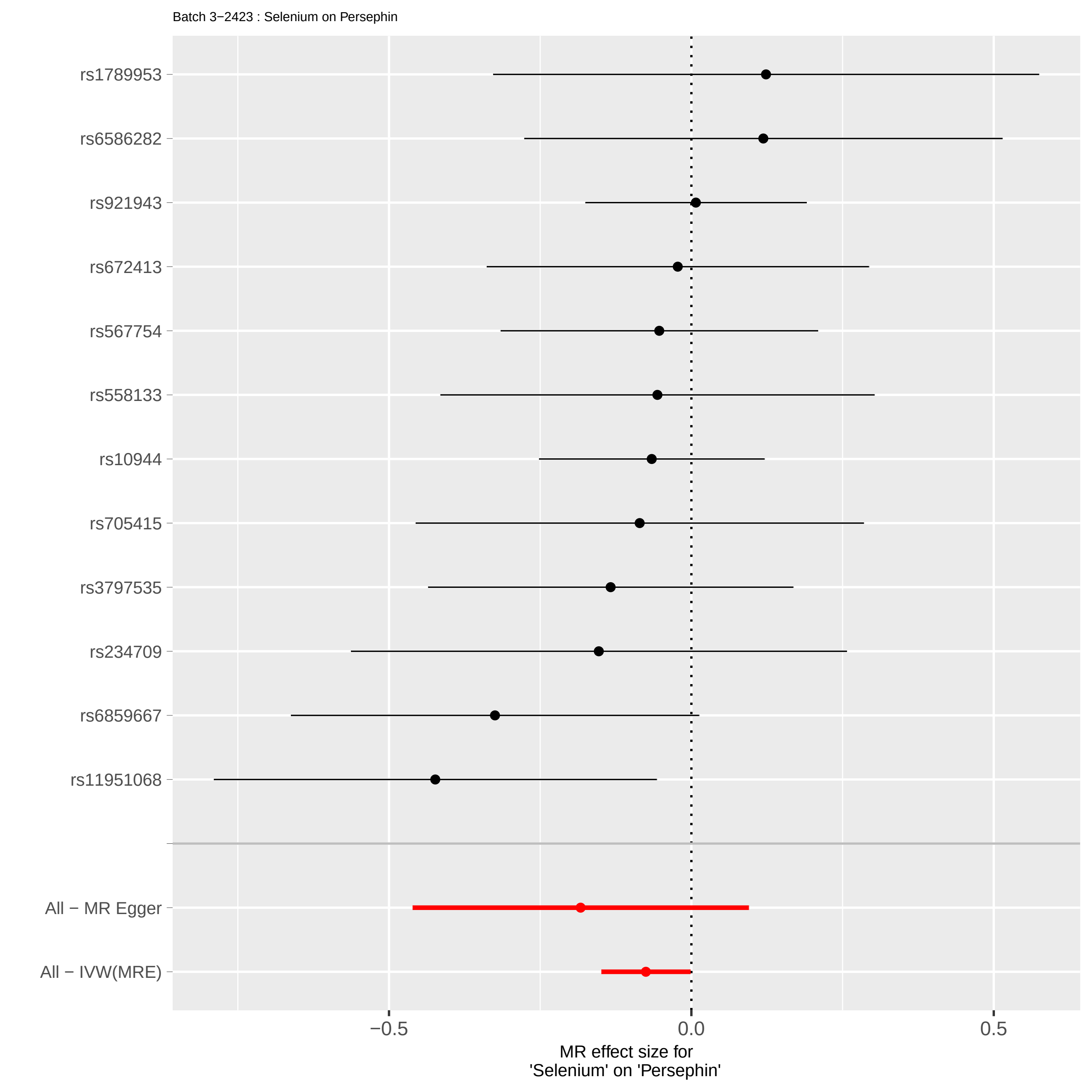

Batch 3−2423 : Selenium on Persephin
rs1789953
rs6586282
rs921943
rs672413
rs567754
rs558133
rs10944
rs705415
rs3797535
rs234709
rs6859667
rs11951068
All − MR Egger
All − IVW(MRE)
−0.5
0.0
0.5
MR effect size for
'Selenium' on 'Persephin'

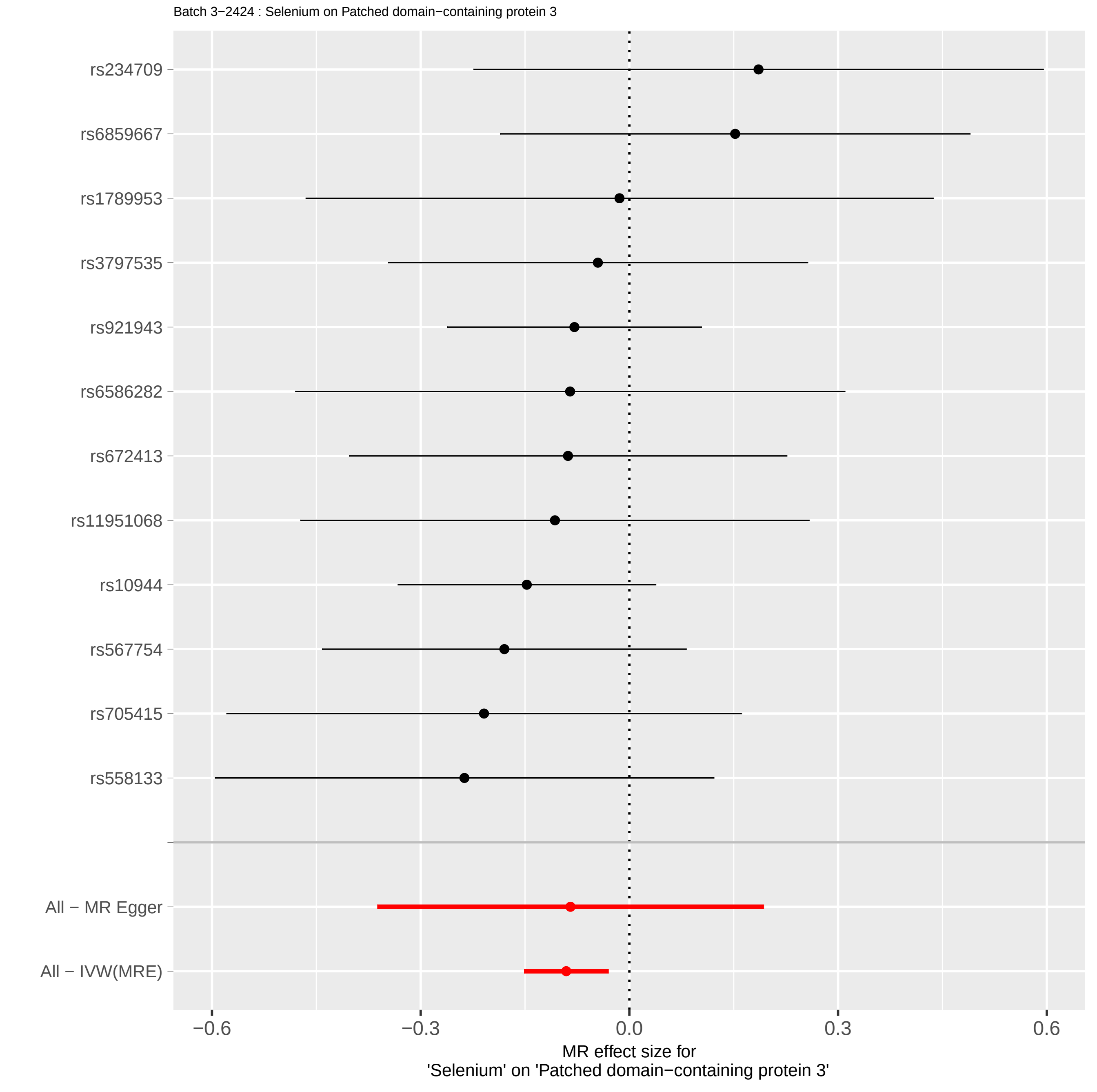

Batch 3−2424 : Selenium on Patched domain−containing protein 3
rs234709
rs6859667
rs1789953
rs3797535
rs921943
rs6586282
rs672413
rs11951068
rs10944
rs567754
rs705415
rs558133
All − MR Egger
All − IVW(MRE)
−0.6
−0.3
0.0
0.3
0.6
MR effect size for
'Selenium' on 'Patched domain−containing protein 3'

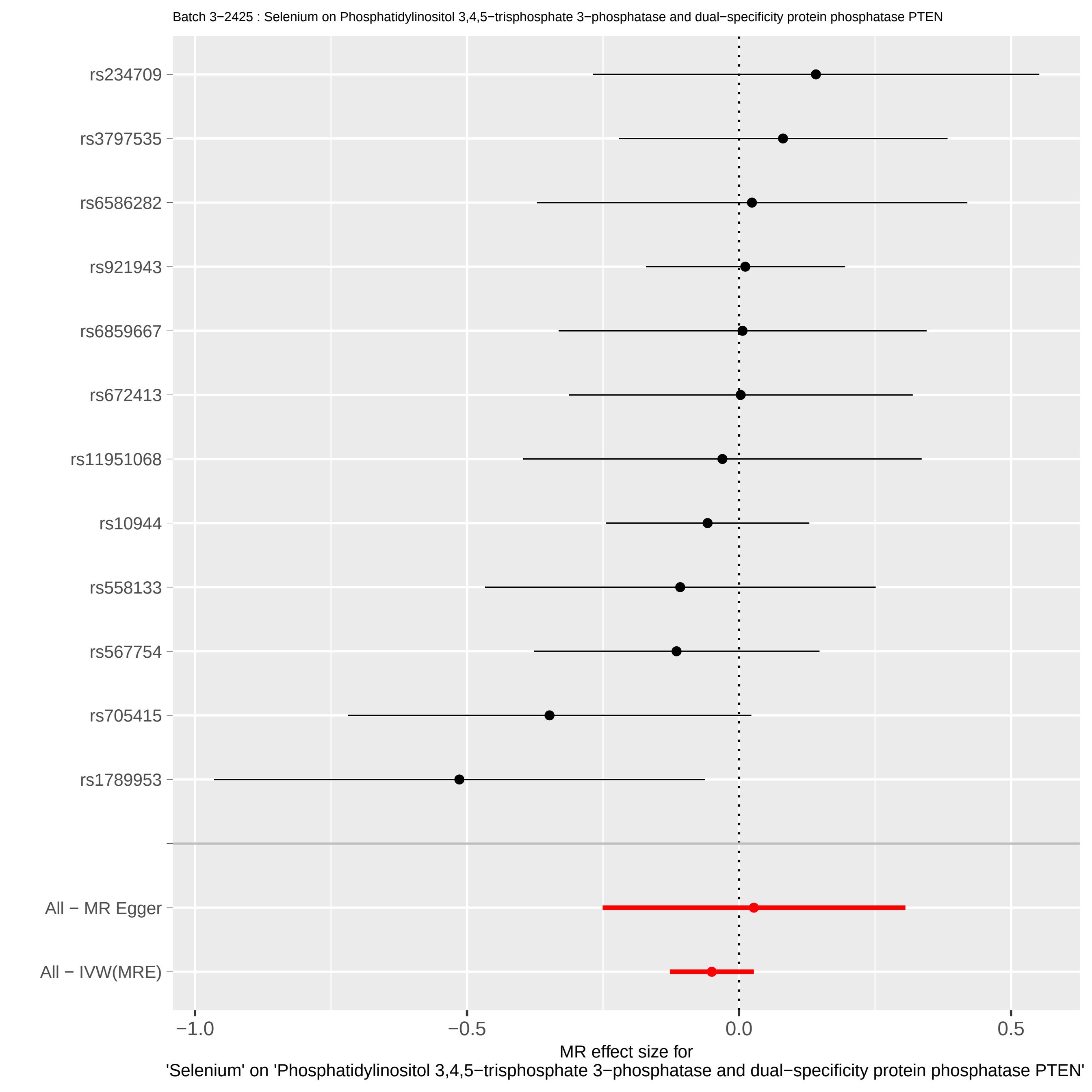

Batch 3−2425 : Selenium on Phosphatidylinositol 3,4,5−trisphosphate 3−phosphatase and dual−specificity protein phosphatase PTEN
rs234709
rs3797535
rs6586282
rs921943
rs6859667
rs672413
rs11951068
rs10944
rs558133
rs567754
rs705415
rs1789953
All − MR Egger
All − IVW(MRE)
−1.0
−0.5
0.0
0.5
MR effect size for
'Selenium' on 'Phosphatidylinositol 3,4,5−trisphosphate 3−phosphatase and dual−specificity protein phosphatase PTEN'

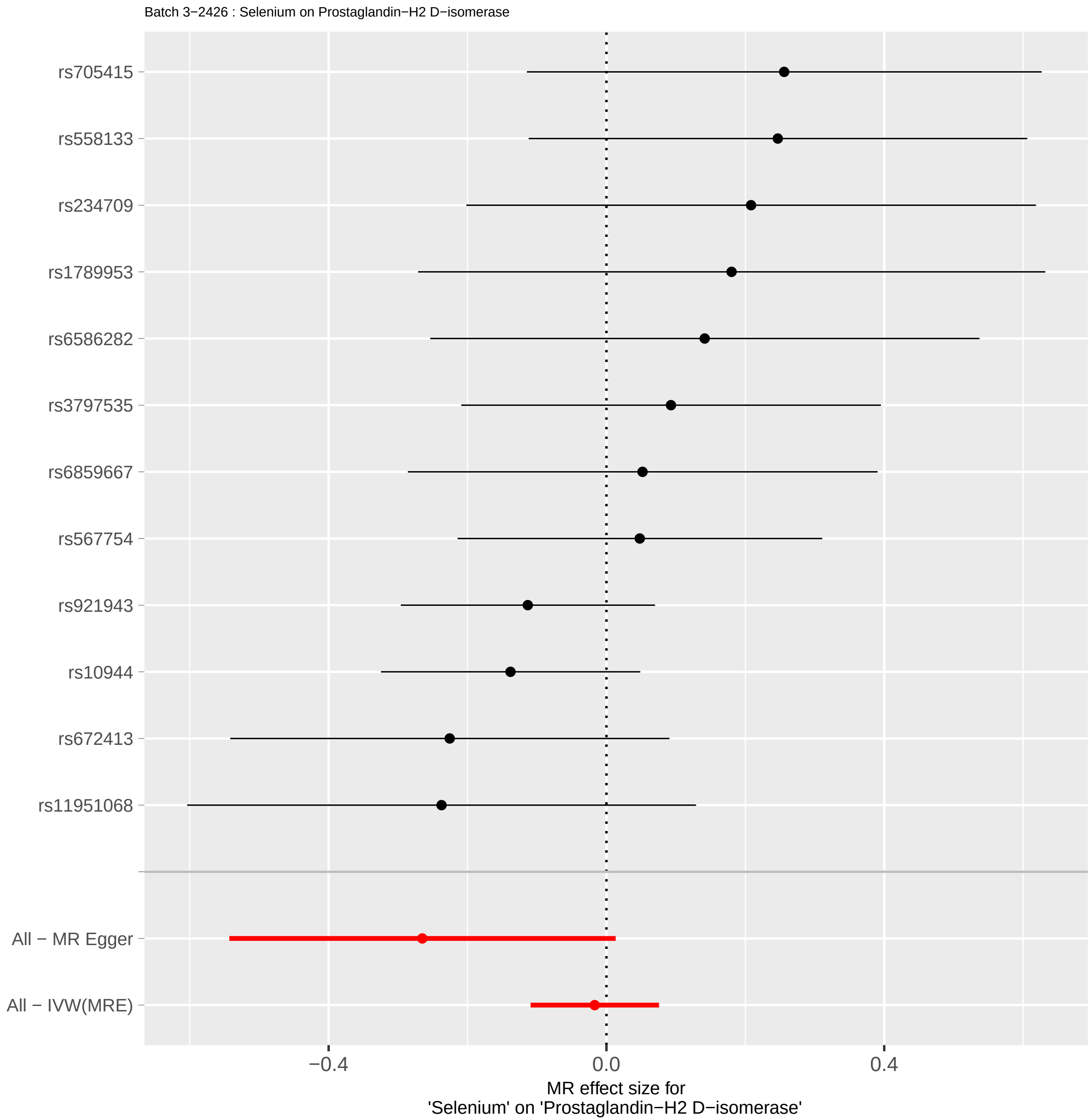

Batch 3−2426 : Selenium on Prostaglandin−H2 D−isomerase
rs705415
rs558133
rs234709
rs1789953
rs6586282
rs3797535
rs6859667
rs567754
rs921943
rs10944
rs672413
rs11951068
All − MR Egger
All − IVW(MRE)
−0.4
0.0
0.4
MR effect size for
'Selenium' on 'Prostaglandin−H2 D−isomerase'

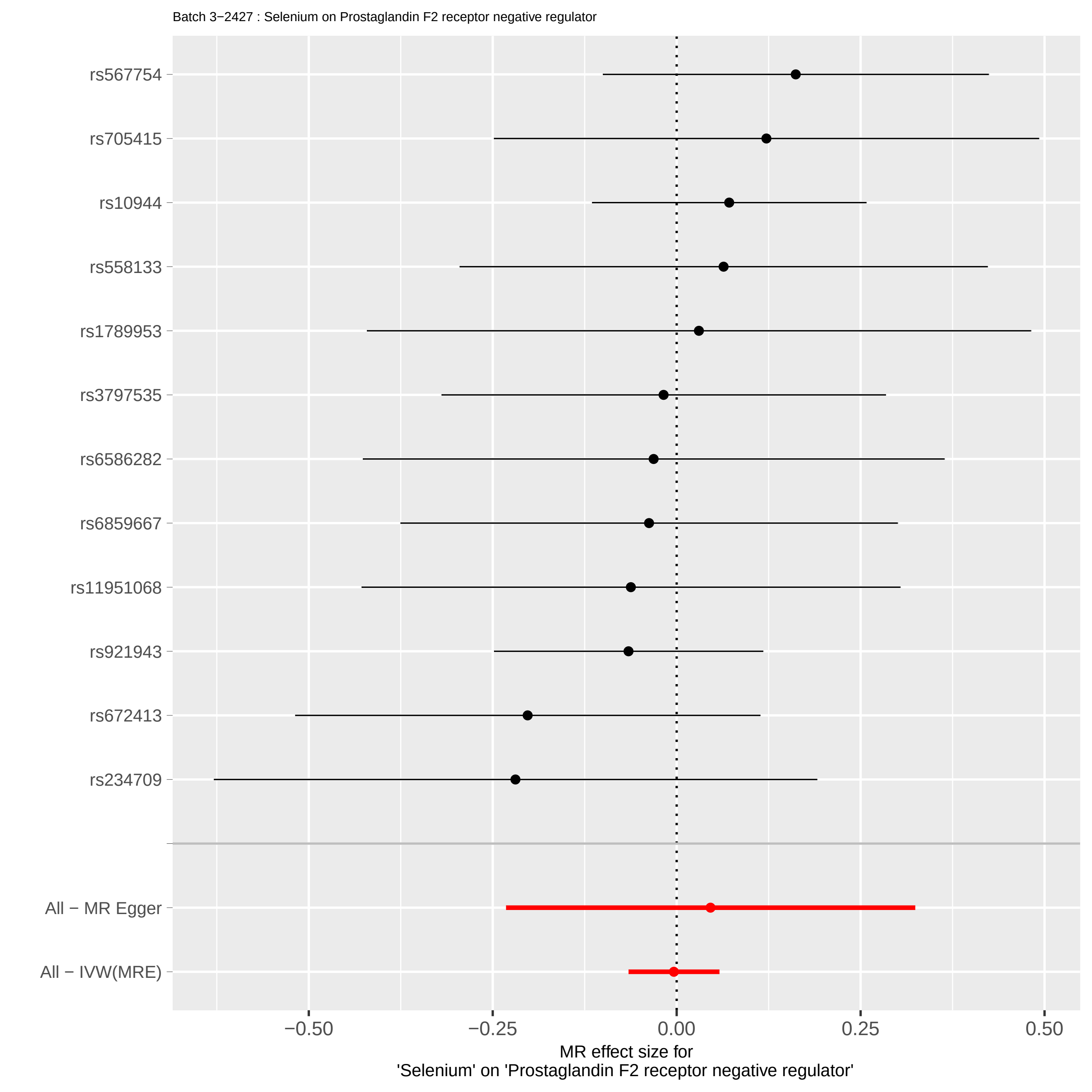

Batch 3−2427 : Selenium on Prostaglandin F2 receptor negative regulator
rs567754
rs705415
rs10944
rs558133
rs1789953
rs3797535
rs6586282
rs6859667
rs11951068
rs921943
rs672413
rs234709
All − MR Egger
All − IVW(MRE)
−0.50
−0.25
0.00
0.25
0.50
MR effect size for
'Selenium' on 'Prostaglandin F2 receptor negative regulator'

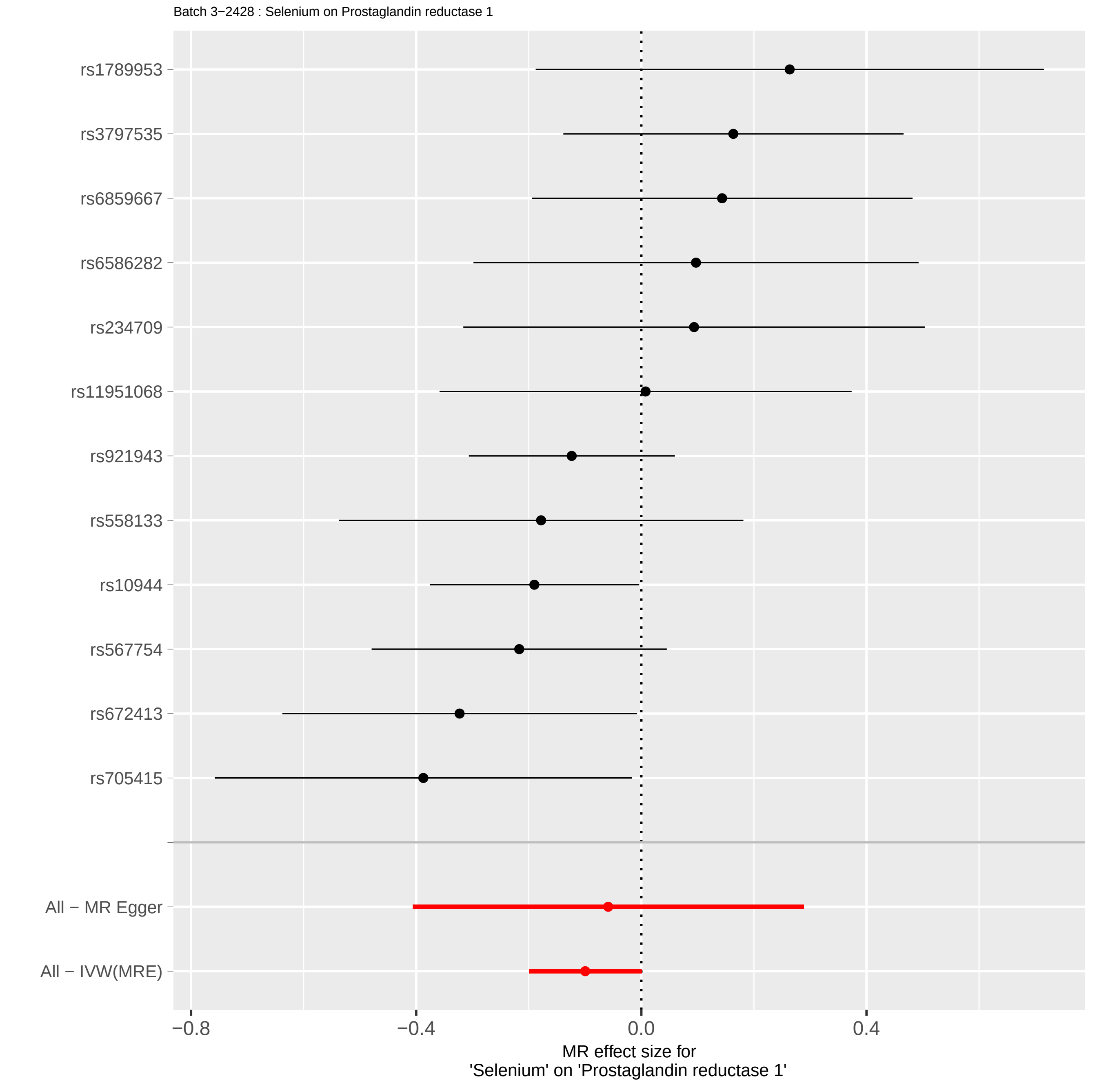

Batch 3−2428 : Selenium on Prostaglandin reductase 1
rs1789953
rs3797535
rs6859667
rs6586282
rs234709
rs11951068
rs921943
rs558133
rs10944
rs567754
rs672413
rs705415
All − MR Egger
All − IVW(MRE)
−0.8
−0.4
0.0
0.4
MR effect size for
'Selenium' on 'Prostaglandin reductase 1'

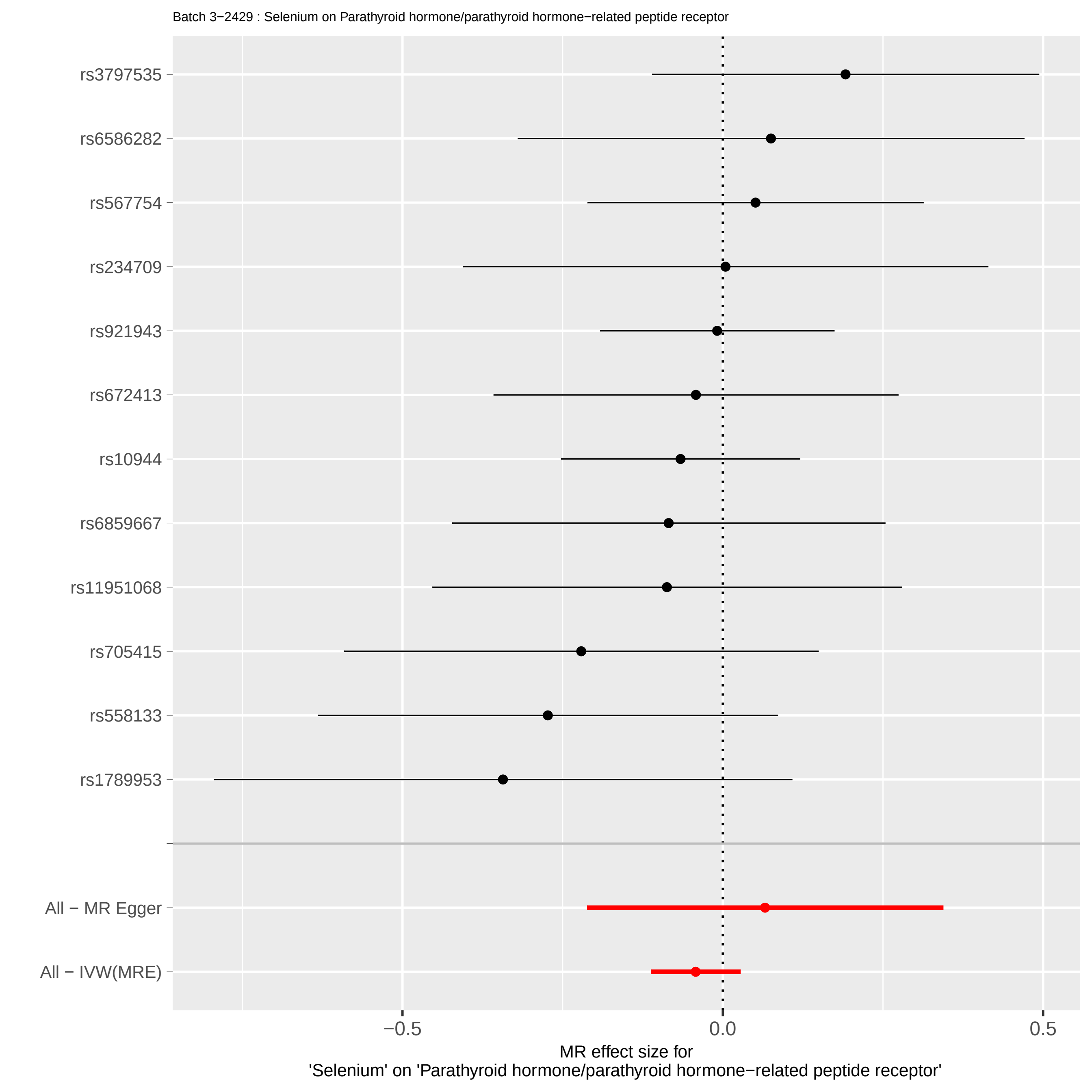
Batch 3−2429 : Selenium on Parathyroid hormone/parathyroid hormone−related peptide receptor
rs3797535
rs6586282
rs567754
rs234709
rs921943
rs672413
rs10944
rs6859667
rs11951068
rs705415
rs558133
rs1789953
All − MR Egger
All − IVW(MRE)
−0.5
0.0
0.5
MR effect size for
'Selenium' on 'Parathyroid hormone/parathyroid hormone−related peptide receptor'

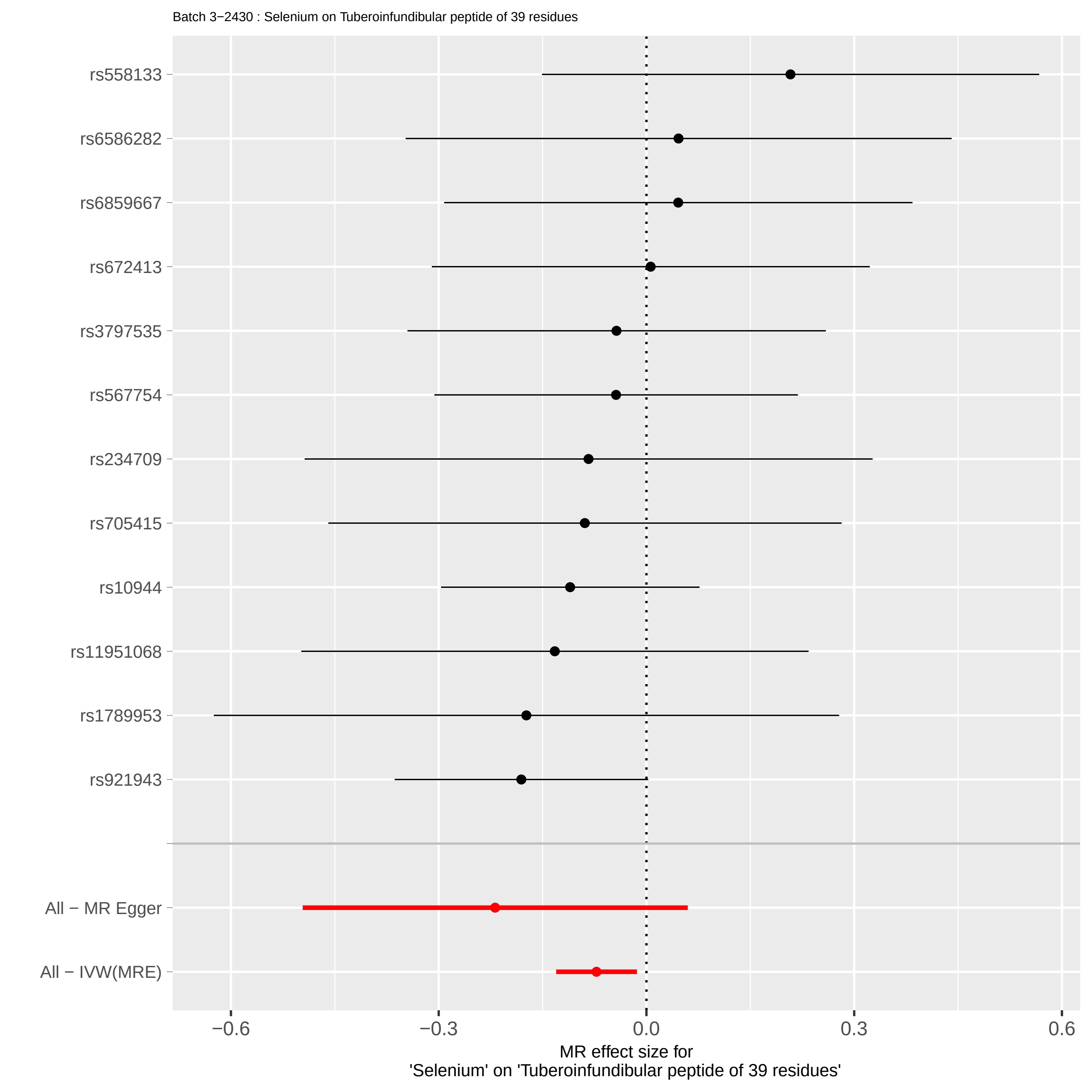

Batch 3−2430 : Selenium on Tuberoinfundibular peptide of 39 residues
rs558133
rs6586282
rs6859667
rs672413
rs3797535
rs567754
rs234709
rs705415
rs10944
rs11951068
rs1789953
rs921943
All − MR Egger
All − IVW(MRE)
−0.6
−0.3
0.0
0.3
0.6
MR effect size for
'Selenium' on 'Tuberoinfundibular peptide of 39 residues'

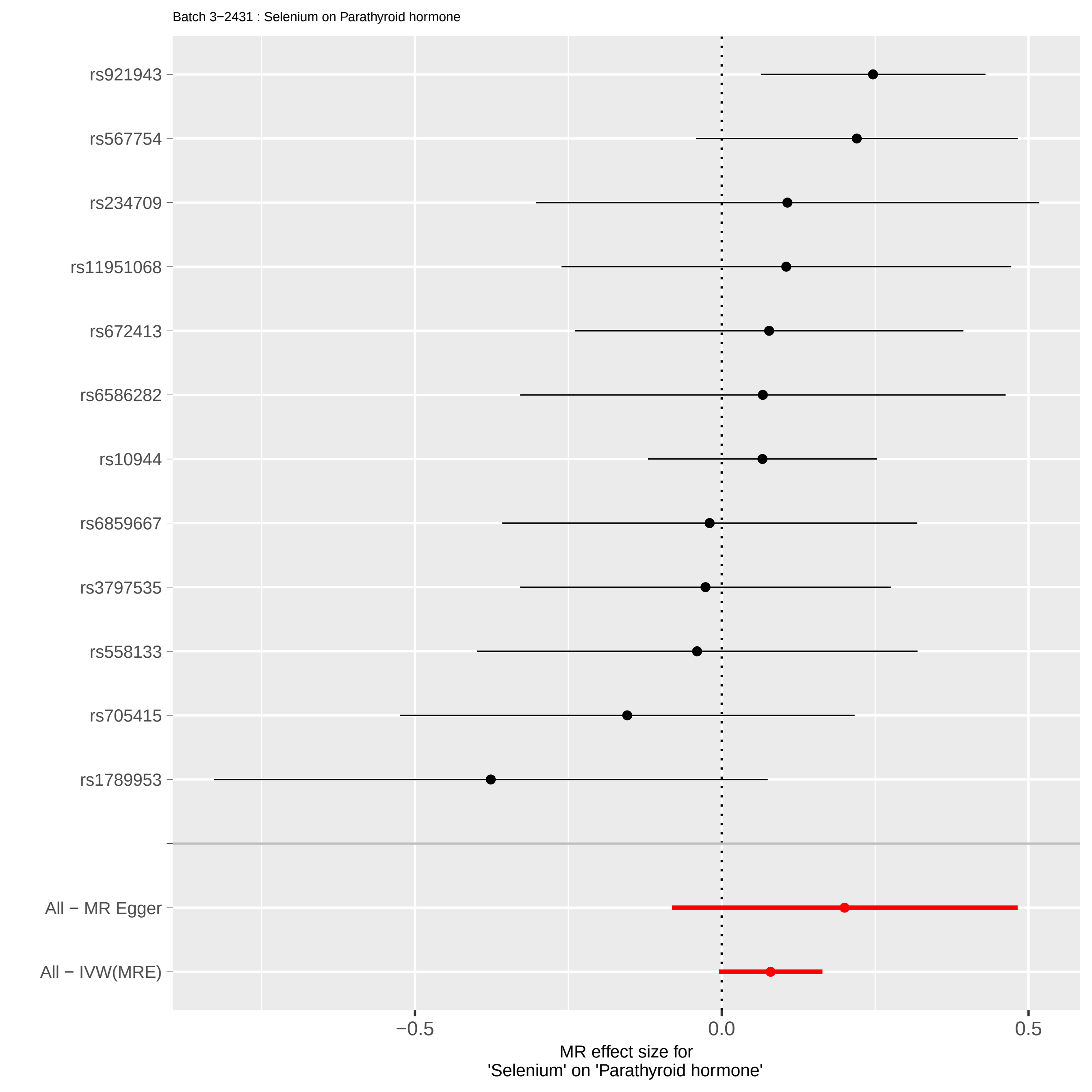
Batch 3−2431 : Selenium on Parathyroid hormone
rs921943
rs567754
rs234709
rs11951068
rs672413
rs6586282
rs10944
rs6859667
rs3797535
rs558133
rs705415
rs1789953
All − MR Egger
All − IVW(MRE)
−0.5
0.0
0.5
MR effect size for
'Selenium' on 'Parathyroid hormone'

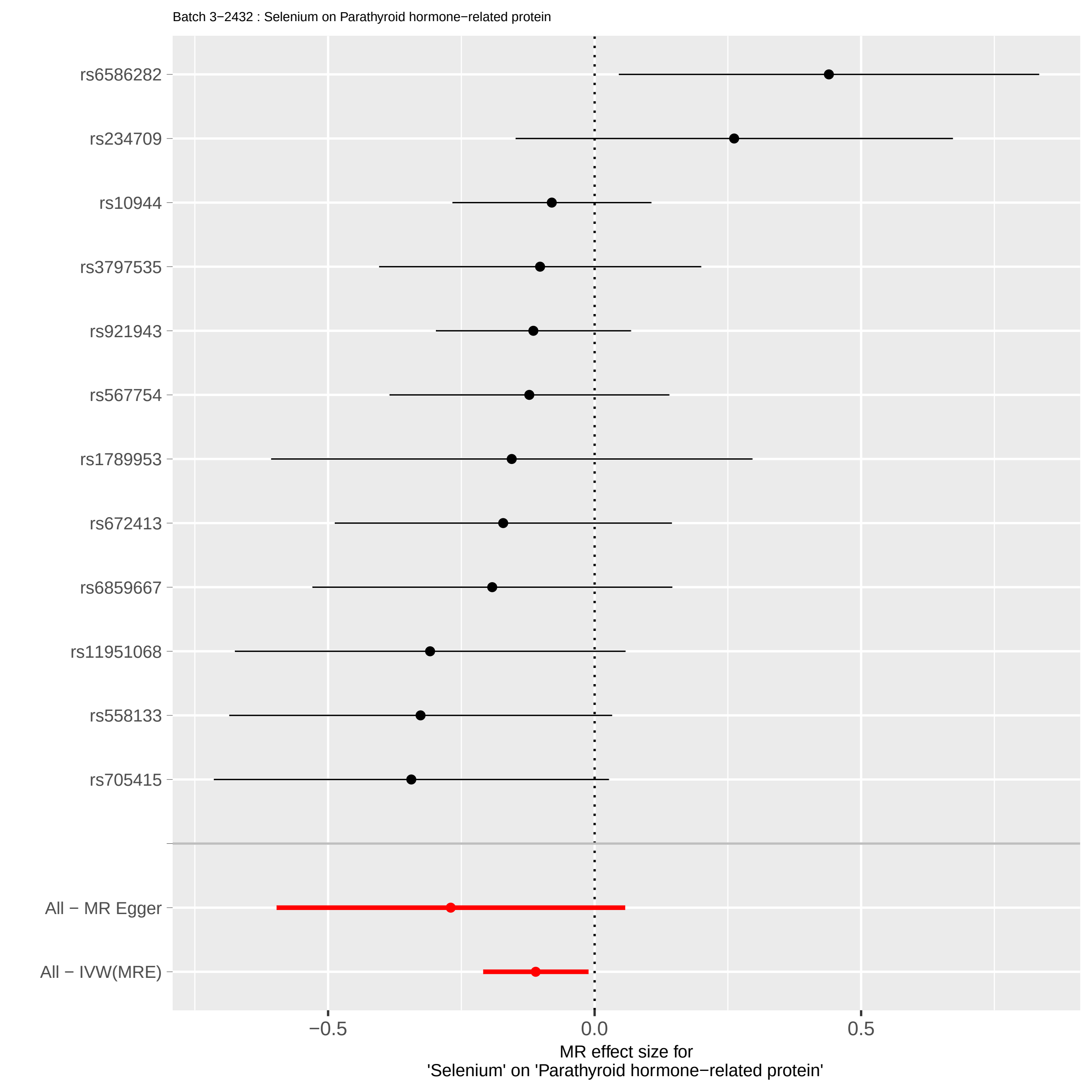

Batch 3−2432 : Selenium on Parathyroid hormone−related protein
rs6586282
rs234709
rs10944
rs3797535
rs921943
rs567754
rs1789953
rs672413
rs6859667
rs11951068
rs558133
rs705415
All − MR Egger
All − IVW(MRE)
−0.5
0.0
0.5
MR effect size for
'Selenium' on 'Parathyroid hormone−related protein'

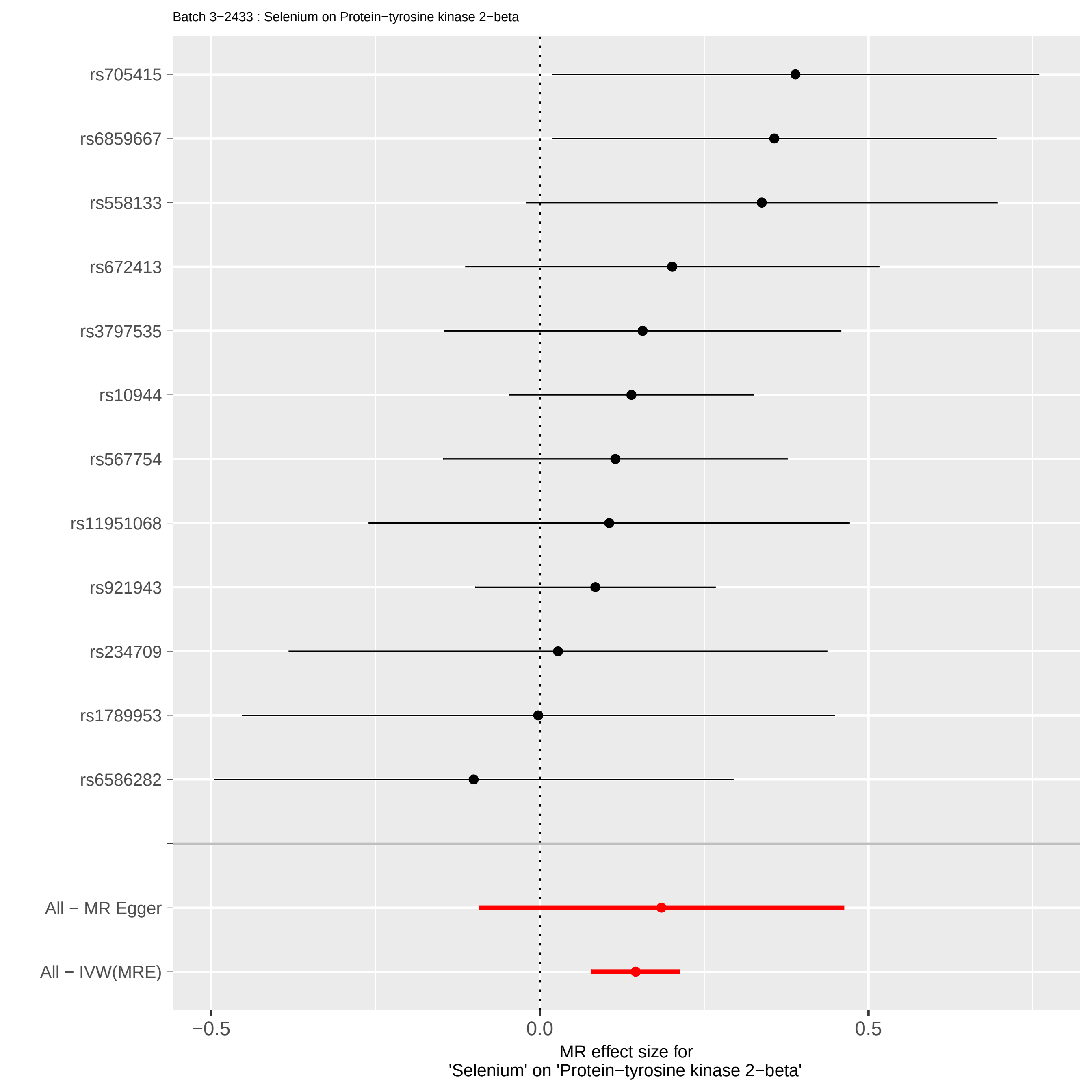

Batch 3−2433 : Selenium on Protein−tyrosine kinase 2−beta
rs705415
rs6859667
rs558133
rs672413
rs3797535
rs10944
rs567754
rs11951068
rs921943
rs234709
rs1789953
rs6586282
All − MR Egger
All − IVW(MRE)
−0.5
0.0
0.5
MR effect size for
'Selenium' on 'Protein−tyrosine kinase 2−beta'

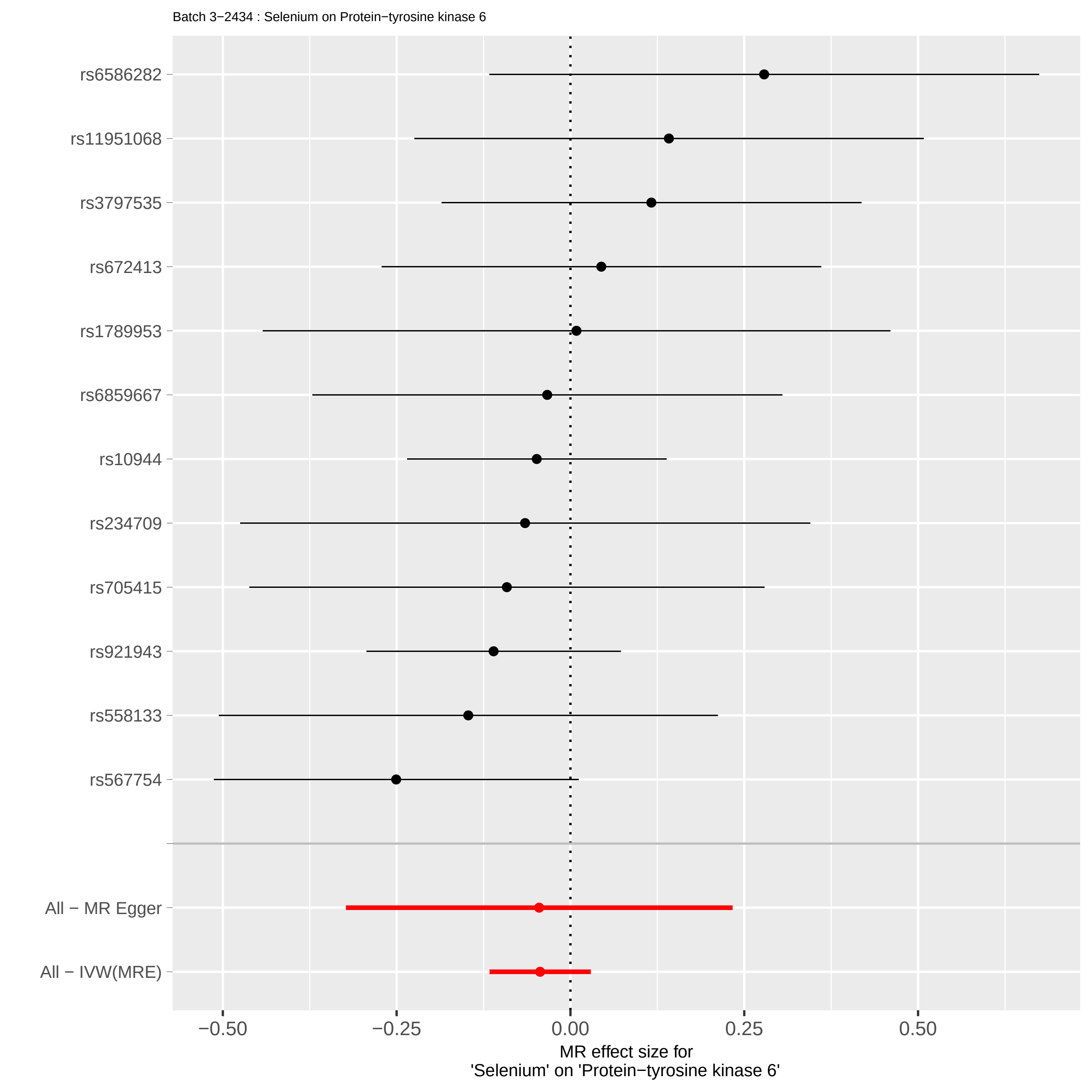

Batch 3−2434 : Selenium on Protein−tyrosine kinase 6
rs6586282
rs11951068
rs3797535
rs672413
rs1789953
rs6859667
rs10944
rs234709
rs705415
rs921943
rs558133
rs567754
All − MR Egger
All − IVW(MRE)
−0.50
−0.25
0.00
0.25
0.50
MR effect size for
'Selenium' on 'Protein−tyrosine kinase 6'

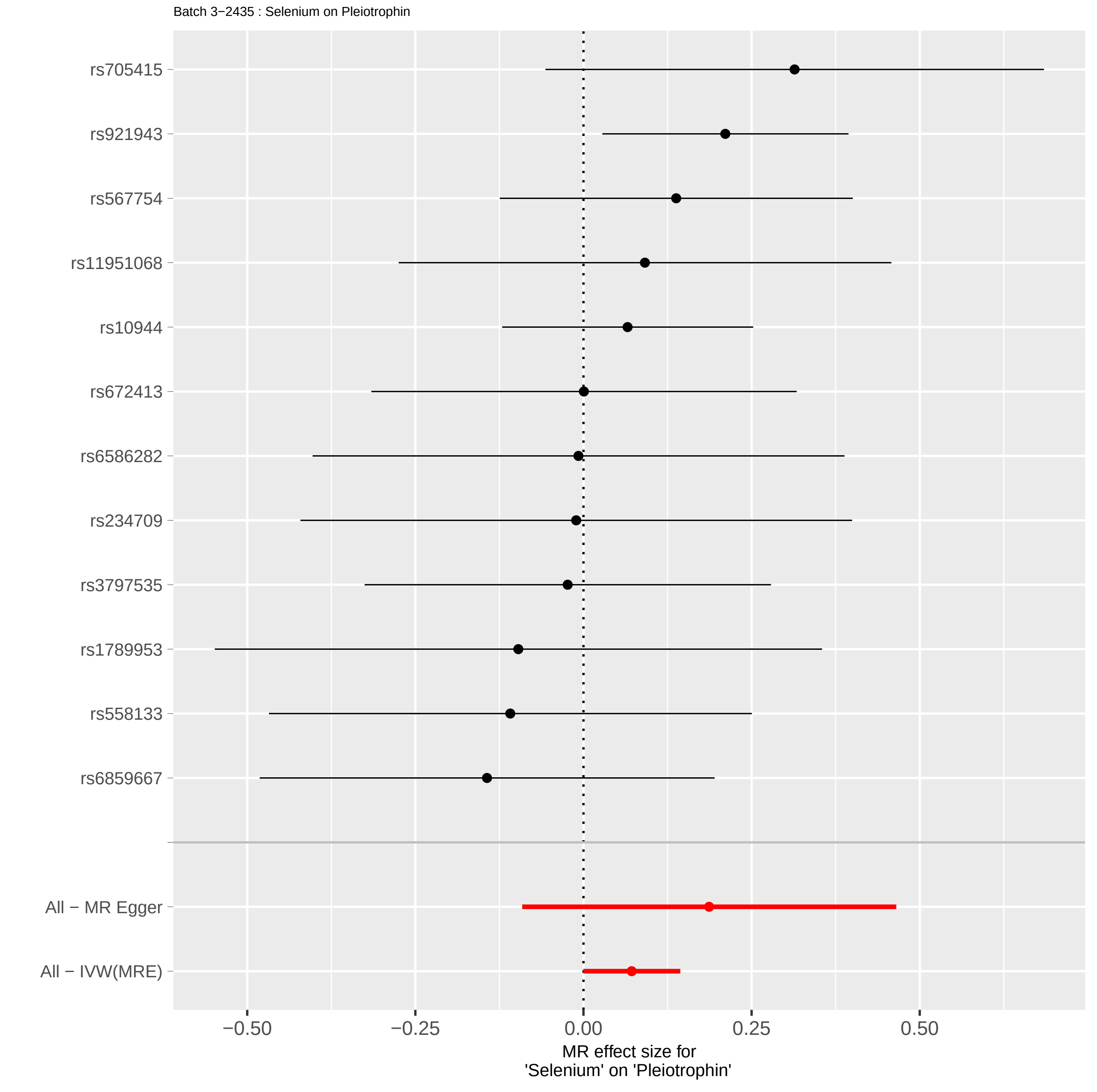

Batch 3−2435 : Selenium on Pleiotrophin
rs705415
rs921943
rs567754
rs11951068
rs10944
rs672413
rs6586282
rs234709
rs3797535
rs1789953
rs558133
rs6859667
All − MR Egger
All − IVW(MRE)
−0.50
−0.25
0.00
0.25
0.50
MR effect size for
'Selenium' on 'Pleiotrophin'

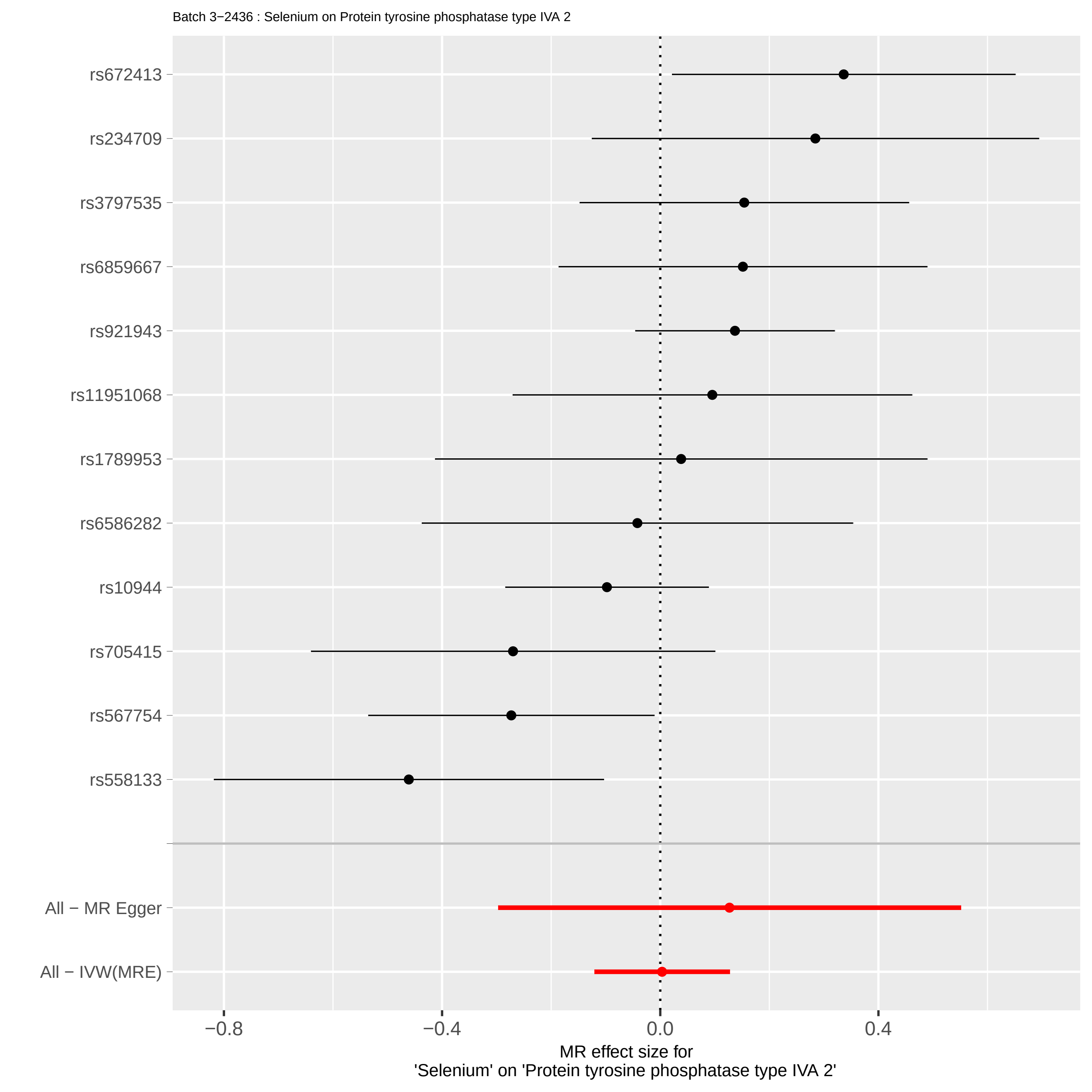

Batch 3−2436 : Selenium on Protein tyrosine phosphatase type IVA 2
rs672413
rs234709
rs3797535
rs6859667
rs921943
rs11951068
rs1789953
rs6586282
rs10944
rs705415
rs567754
rs558133
All − MR Egger
All − IVW(MRE)
−0.8
−0.4
0.0
0.4
MR effect size for
'Selenium' on 'Protein tyrosine phosphatase type IVA 2'

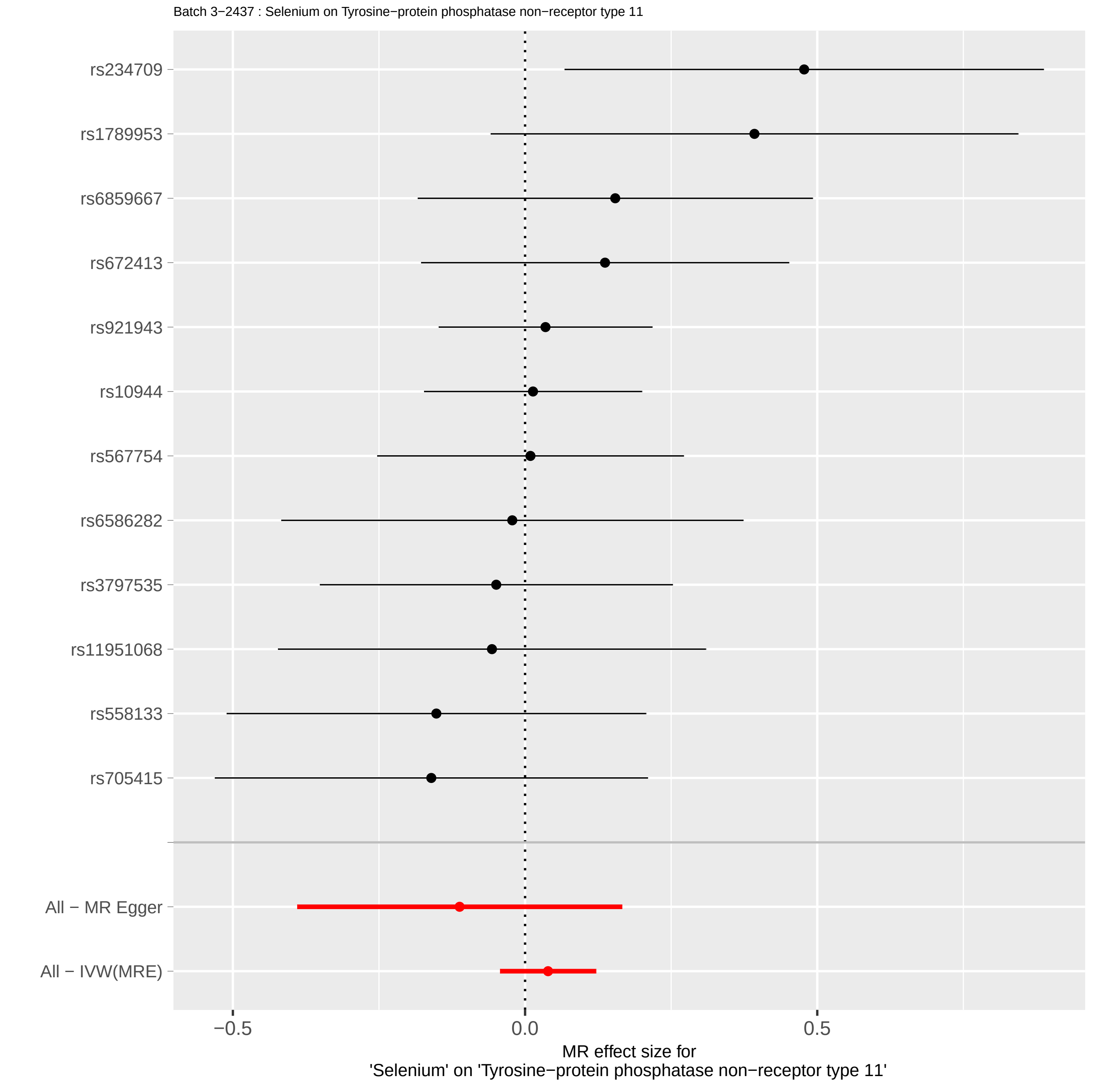
Batch 3−2437 : Selenium on Tyrosine−protein phosphatase non−receptor type 11
rs234709
rs1789953
rs6859667
rs672413
rs921943
rs10944
rs567754
rs6586282
rs3797535
rs11951068
rs558133
rs705415
All − MR Egger
All − IVW(MRE)
−0.5
0.0
0.5
MR effect size for
'Selenium' on 'Tyrosine−protein phosphatase non−receptor type 11'

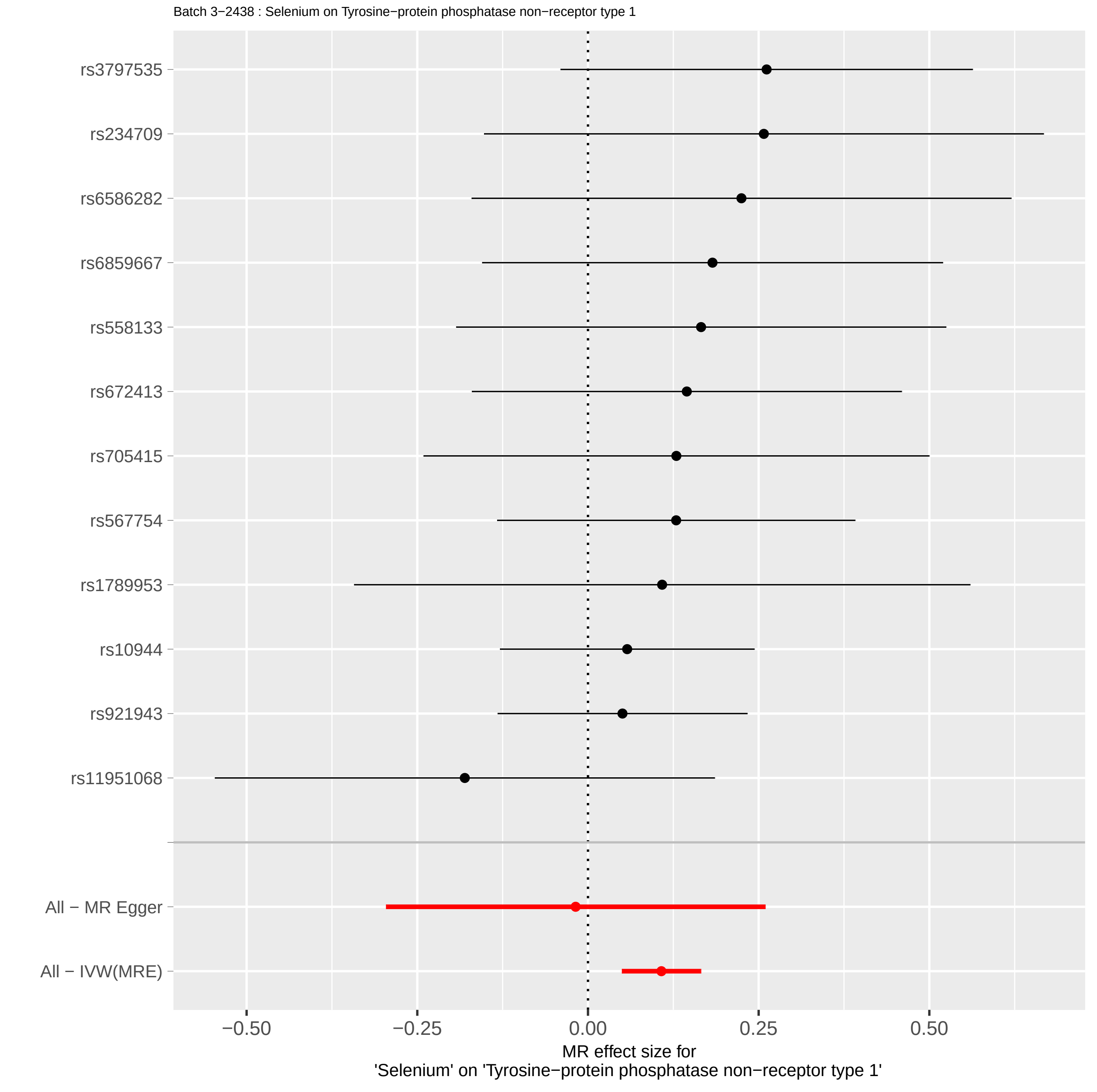
Batch 3−2438 : Selenium on Tyrosine−protein phosphatase non−receptor type 1
rs3797535
rs234709
rs6586282
rs6859667
rs558133
rs672413
rs705415
rs567754
rs1789953
rs10944
rs921943
rs11951068
All − MR Egger
All − IVW(MRE)
−0.50
−0.25
0.00
0.25
0.50
MR effect size for
'Selenium' on 'Tyrosine−protein phosphatase non−receptor type 1'

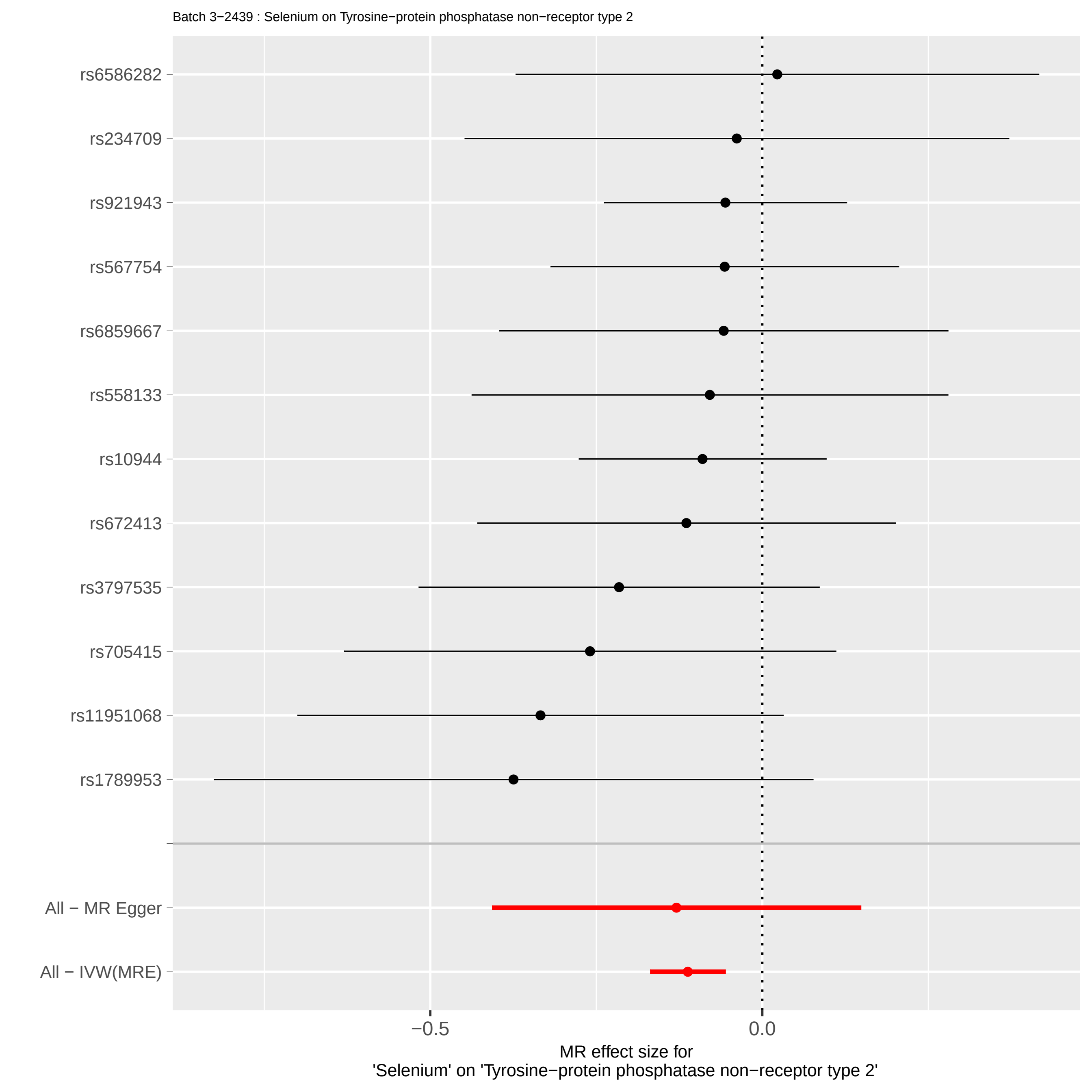

Batch 3−2439 : Selenium on Tyrosine−protein phosphatase non−receptor type 2
rs6586282
rs234709
rs921943
rs567754
rs6859667
rs558133
rs10944
rs672413
rs3797535
rs705415
rs11951068
rs1789953
All − MR Egger
All − IVW(MRE)
−0.5
0.0
MR effect size for
'Selenium' on 'Tyrosine−protein phosphatase non−receptor type 2'

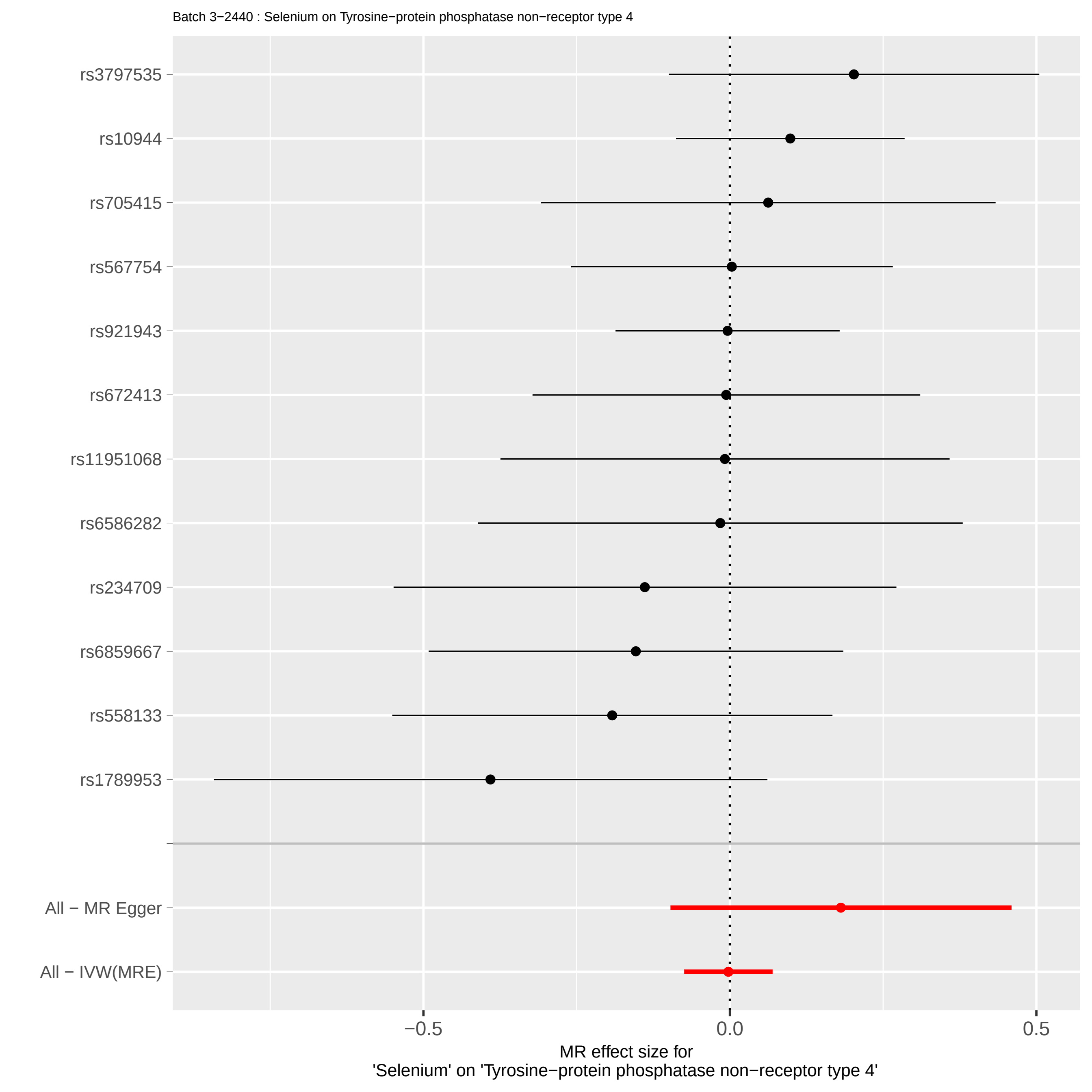
Batch 3−2440 : Selenium on Tyrosine−protein phosphatase non−receptor type 4
rs3797535
rs10944
rs705415
rs567754
rs921943
rs672413
rs11951068
rs6586282
rs234709
rs6859667
rs558133
rs1789953
All − MR Egger
All − IVW(MRE)
−0.5
0.0
0.5
MR effect size for
'Selenium' on 'Tyrosine−protein phosphatase non−receptor type 4'

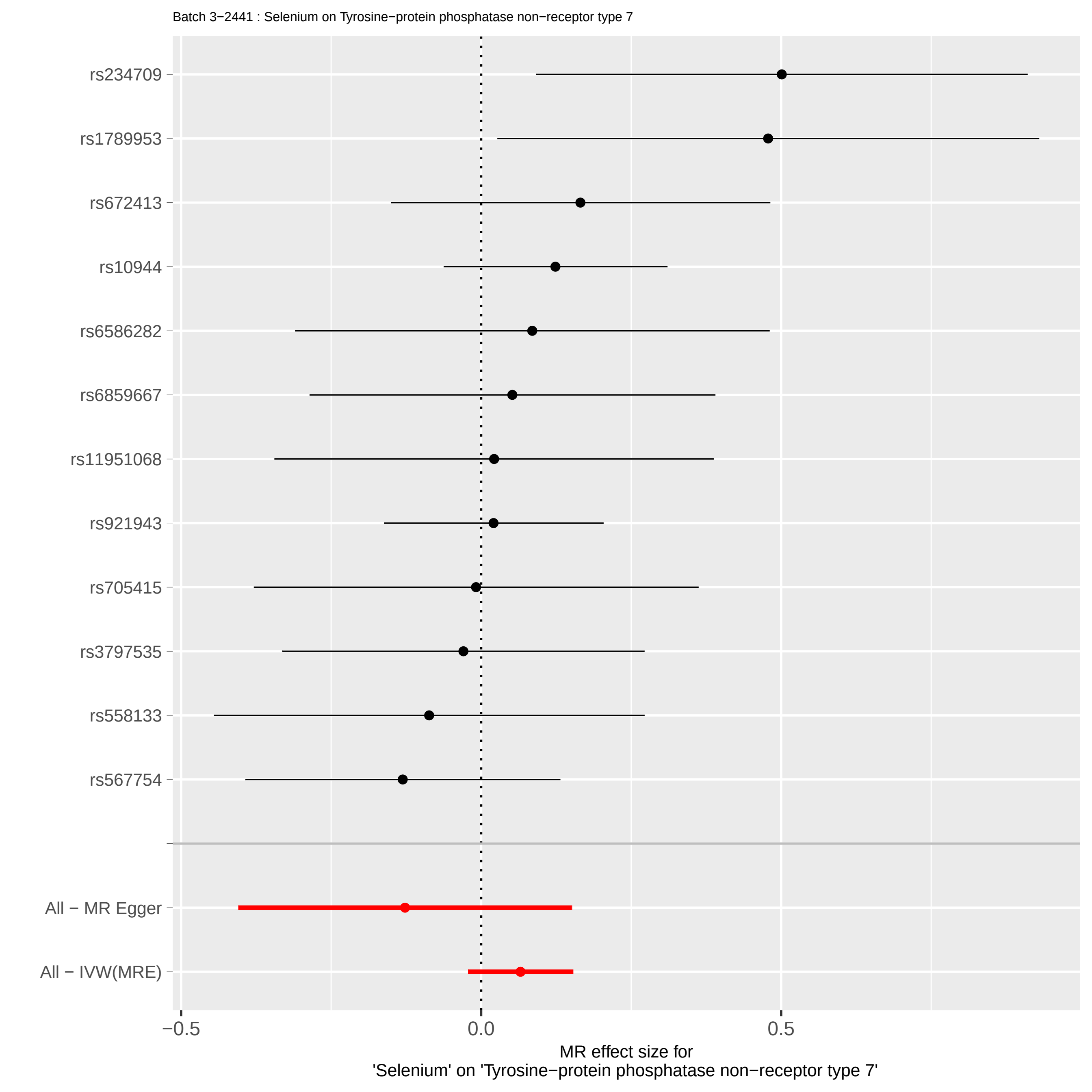

Batch 3−2441 : Selenium on Tyrosine−protein phosphatase non−receptor type 7
rs234709
rs1789953
rs672413
rs10944
rs6586282
rs6859667
rs11951068
rs921943
rs705415
rs3797535
rs558133
rs567754
All − MR Egger
All − IVW(MRE)
−0.5
0.0
0.5
MR effect size for
'Selenium' on 'Tyrosine−protein phosphatase non−receptor type 7'

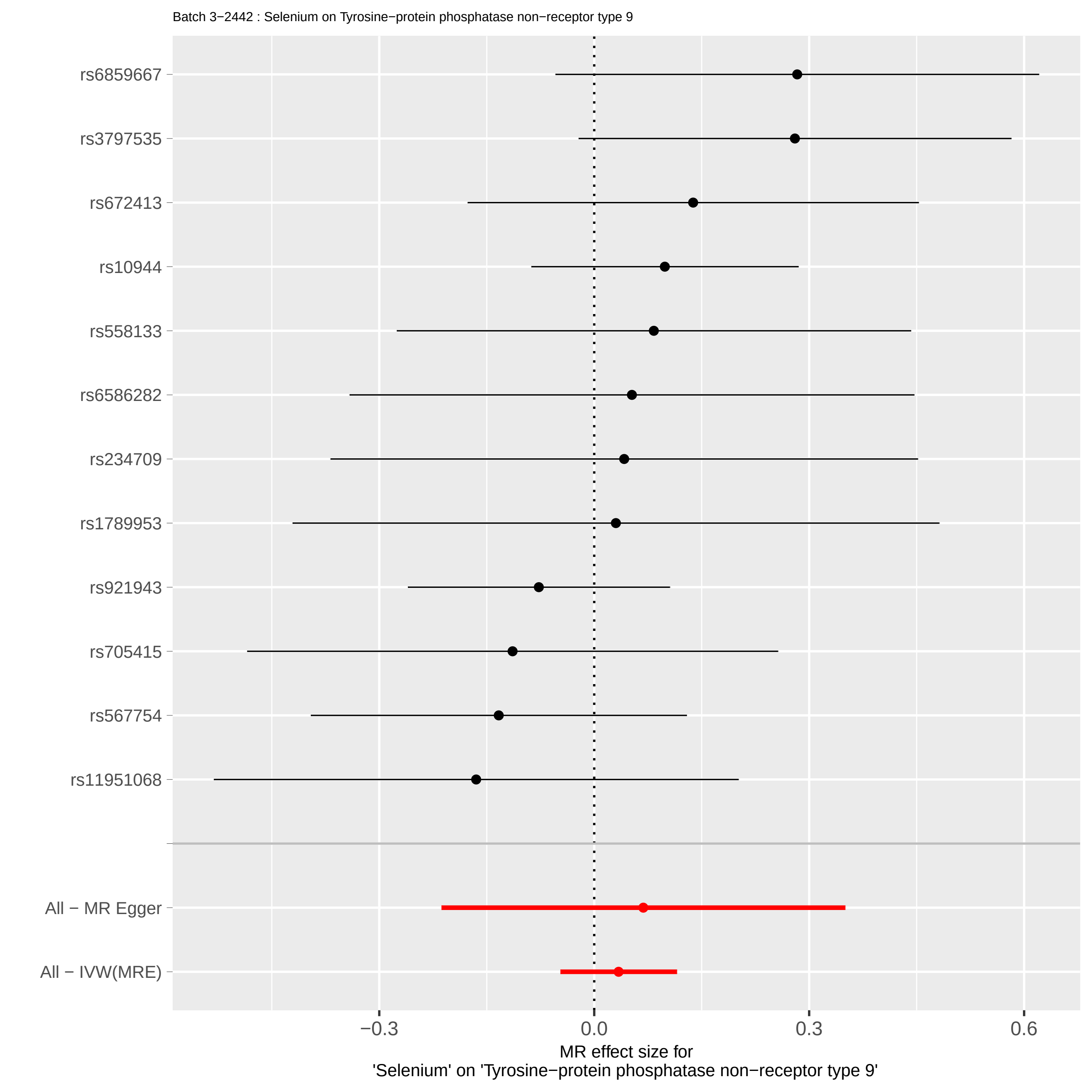

Batch 3−2442 : Selenium on Tyrosine−protein phosphatase non−receptor type 9
rs6859667
rs3797535
rs672413
rs10944
rs558133
rs6586282
rs234709
rs1789953
rs921943
rs705415
rs567754
rs11951068
All − MR Egger
All − IVW(MRE)
−0.3
0.0
0.3
0.6
MR effect size for
'Selenium' on 'Tyrosine−protein phosphatase non−receptor type 9'

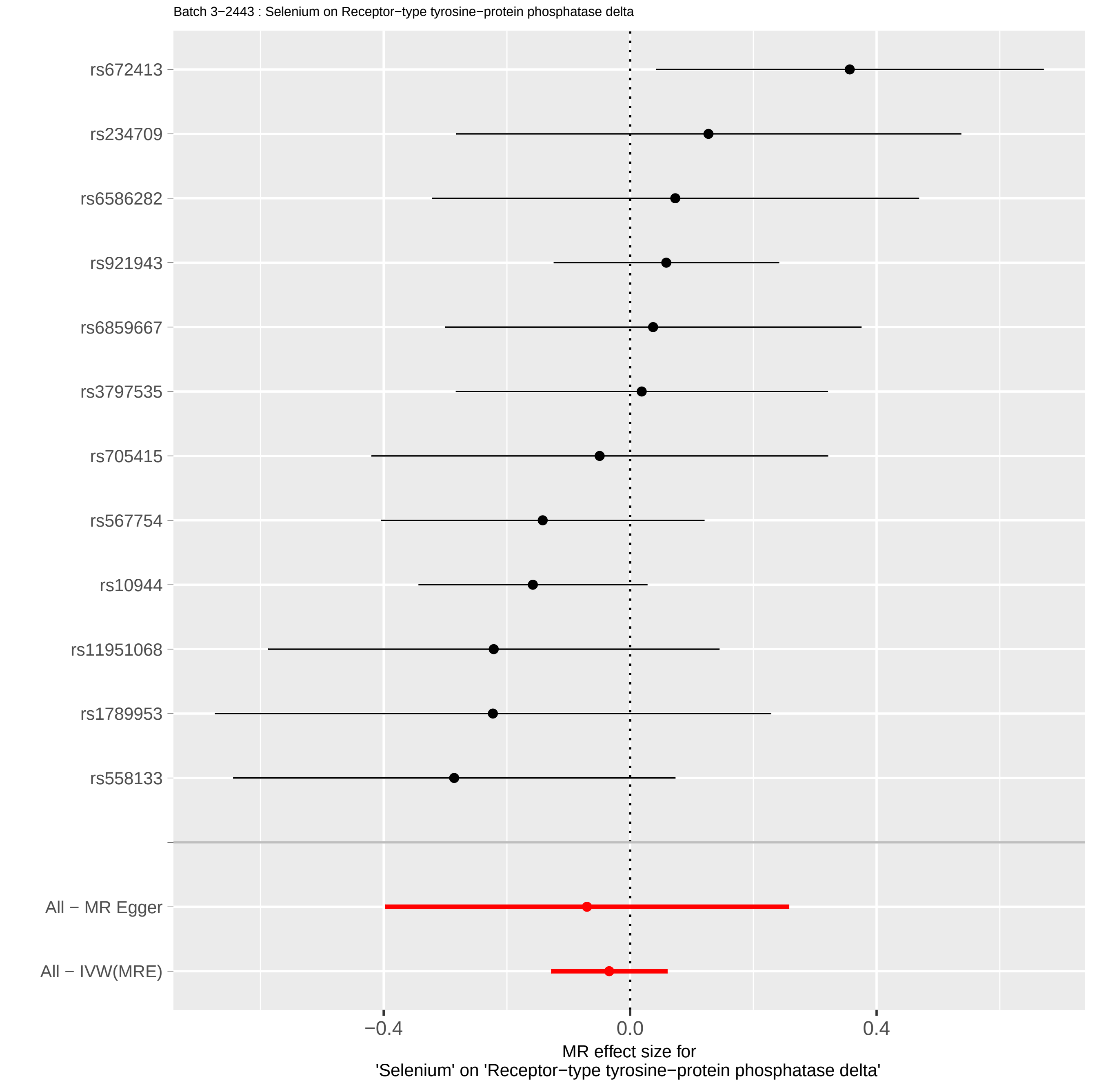
Batch 3−2443 : Selenium on Receptor−type tyrosine−protein phosphatase delta
rs672413
rs234709
rs6586282
rs921943
rs6859667
rs3797535
rs705415
rs567754
rs10944
rs11951068
rs1789953
rs558133
All − MR Egger
All − IVW(MRE)
−0.4
0.0
0.4
MR effect size for
'Selenium' on 'Receptor−type tyrosine−protein phosphatase delta'

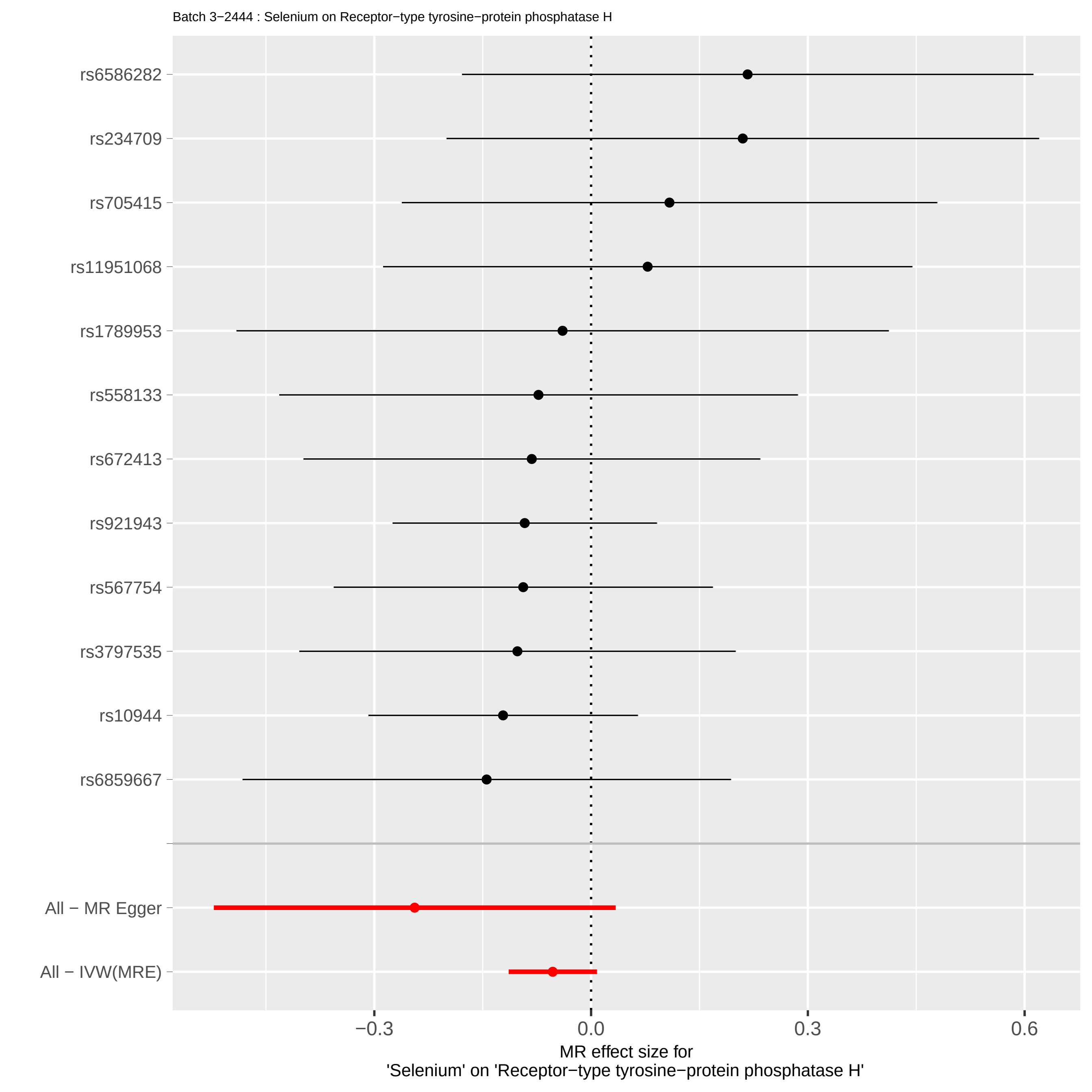
Batch 3−2444 : Selenium on Receptor−type tyrosine−protein phosphatase H
rs6586282
rs234709
rs705415
rs11951068
rs1789953
rs558133
rs672413
rs921943
rs567754
rs3797535
rs10944
rs6859667
All − MR Egger
All − IVW(MRE)
−0.3
0.0
0.3
0.6
MR effect size for
'Selenium' on 'Receptor−type tyrosine−protein phosphatase H'

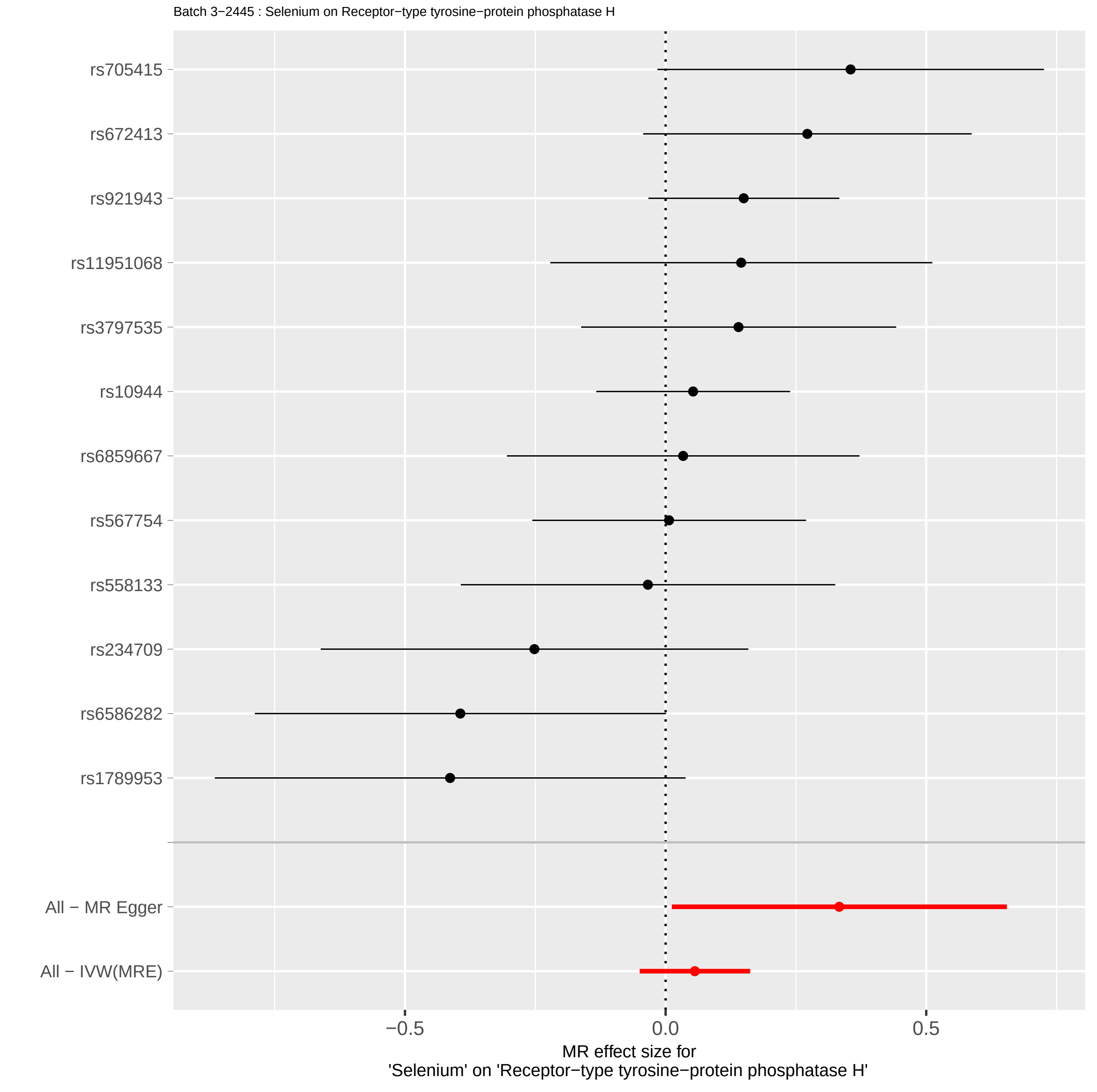

Batch 3−2445 : Selenium on Receptor−type tyrosine−protein phosphatase H
rs705415
rs672413
rs921943
rs11951068
rs3797535
rs10944
rs6859667
rs567754
rs558133
rs234709
rs6586282
rs1789953
All − MR Egger
All − IVW(MRE)
−0.5
0.0
0.5
MR effect size for
'Selenium' on 'Receptor−type tyrosine−protein phosphatase H'

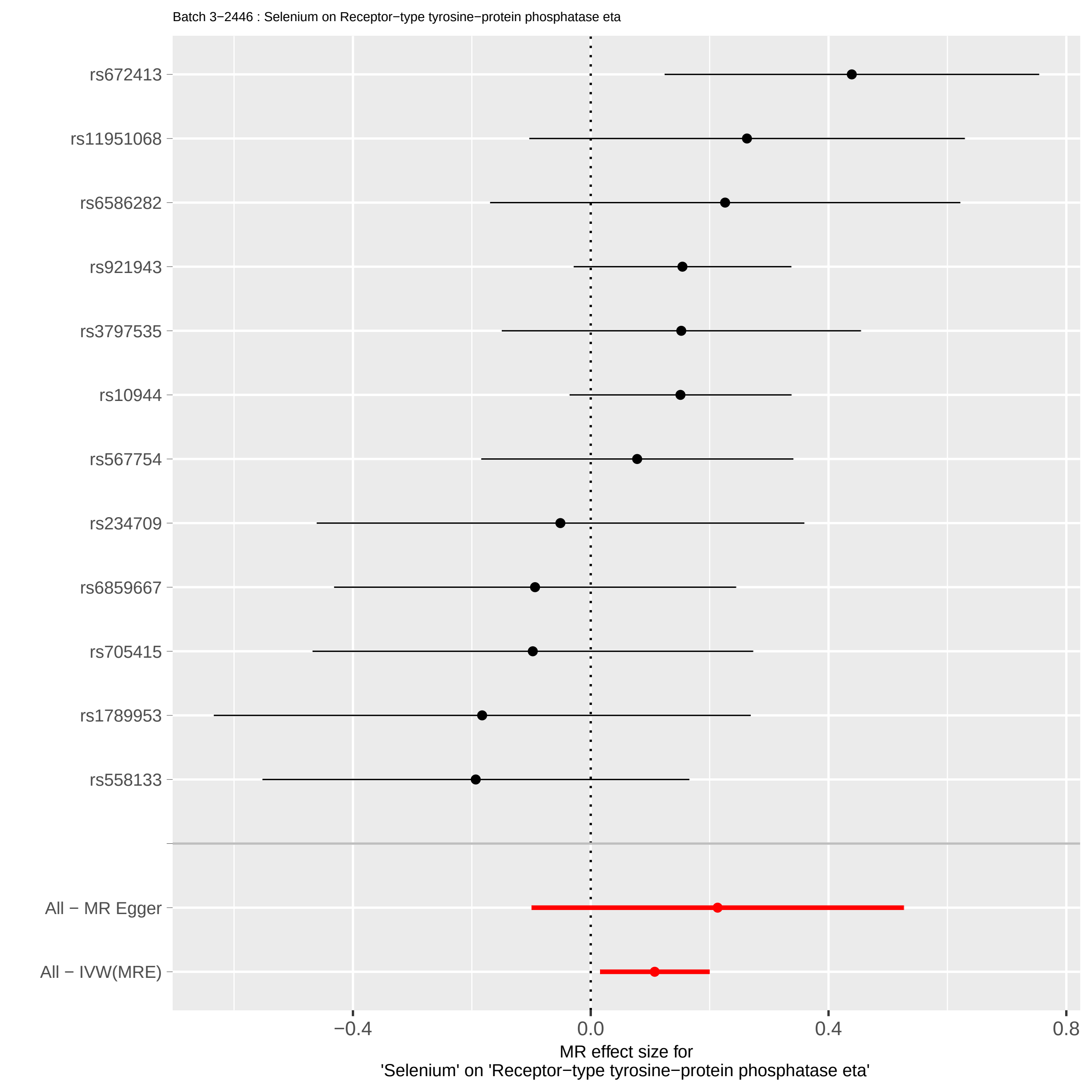

Batch 3−2446 : Selenium on Receptor−type tyrosine−protein phosphatase eta
rs672413
rs11951068
rs6586282
rs921943
rs3797535
rs10944
rs567754
rs234709
rs6859667
rs705415
rs1789953
rs558133
All − MR Egger
All − IVW(MRE)
−0.4
0.0
0.4
0.8
MR effect size for
'Selenium' on 'Receptor−type tyrosine−protein phosphatase eta'

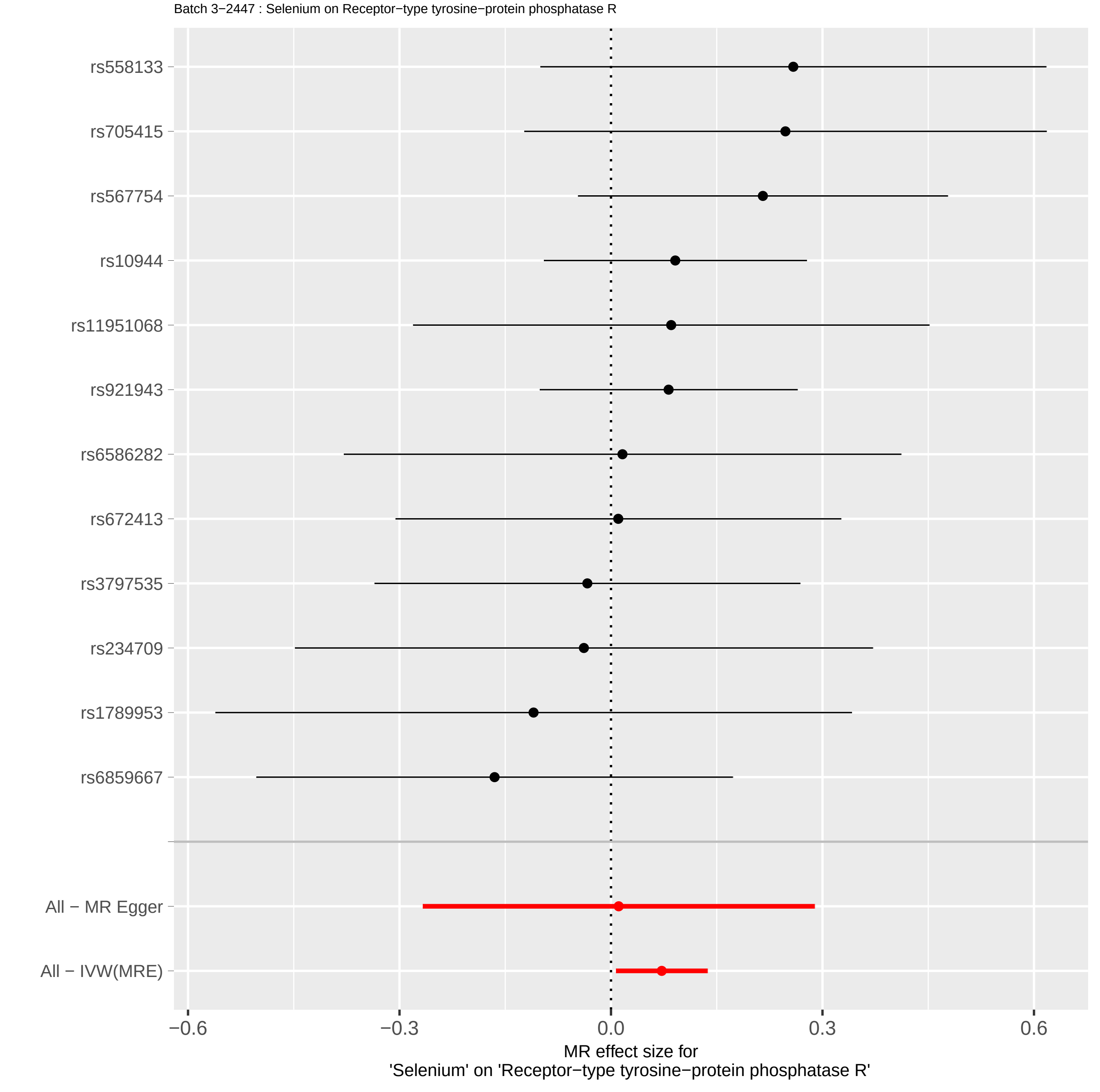

Batch 3−2447 : Selenium on Receptor−type tyrosine−protein phosphatase R
rs558133
rs705415
rs567754
rs10944
rs11951068
rs921943
rs6586282
rs672413
rs3797535
rs234709
rs1789953
rs6859667
All − MR Egger
All − IVW(MRE)
−0.6
−0.3
0.0
0.3
0.6
MR effect size for
'Selenium' on 'Receptor−type tyrosine−protein phosphatase R'

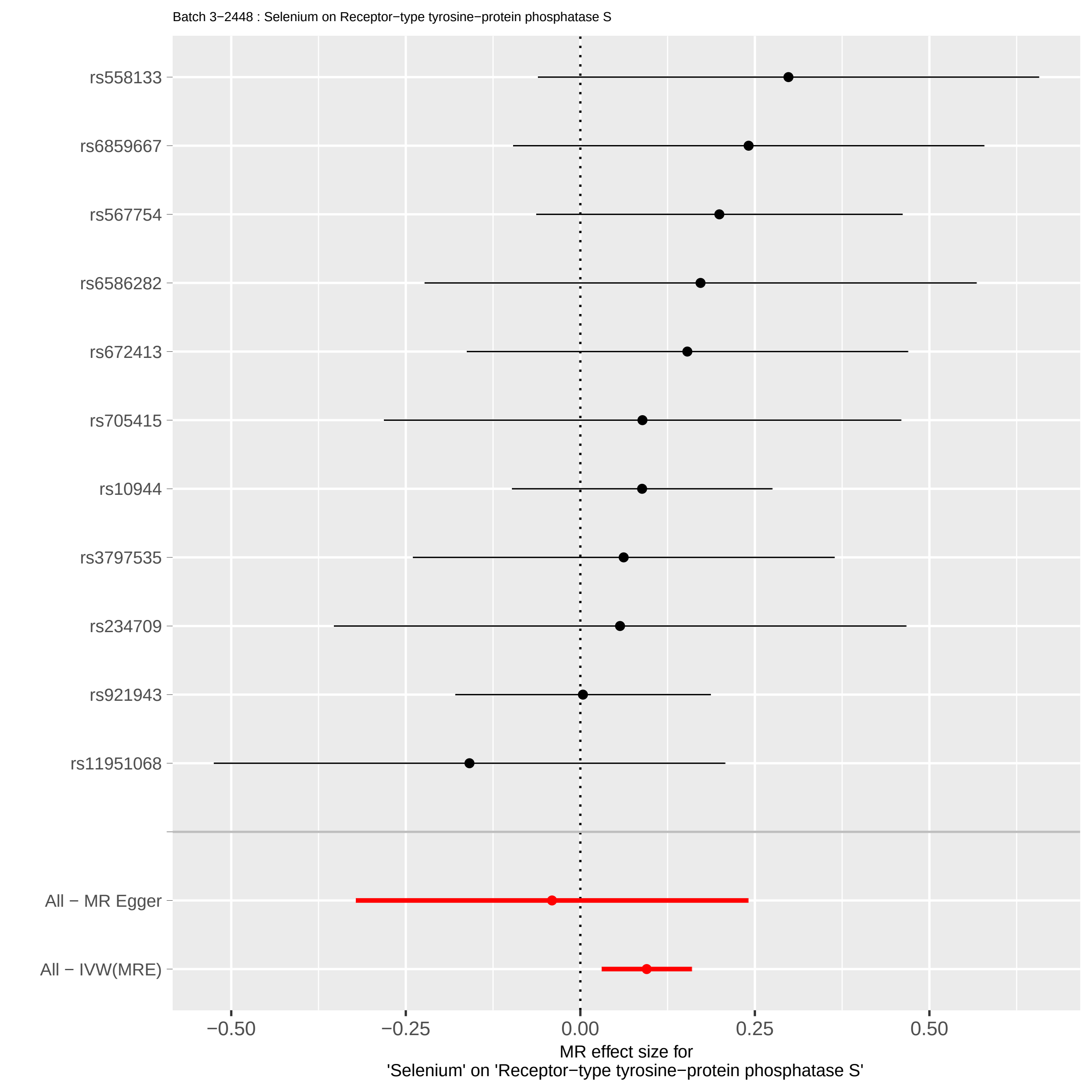

Batch 3−2448 : Selenium on Receptor−type tyrosine−protein phosphatase S
rs558133
rs6859667
rs567754
rs6586282
rs672413
rs705415
rs10944
rs3797535
rs234709
rs921943
rs11951068
All − MR Egger
All − IVW(MRE)
−0.50
−0.25
0.00
0.25
0.50
MR effect size for
'Selenium' on 'Receptor−type tyrosine−protein phosphatase S'

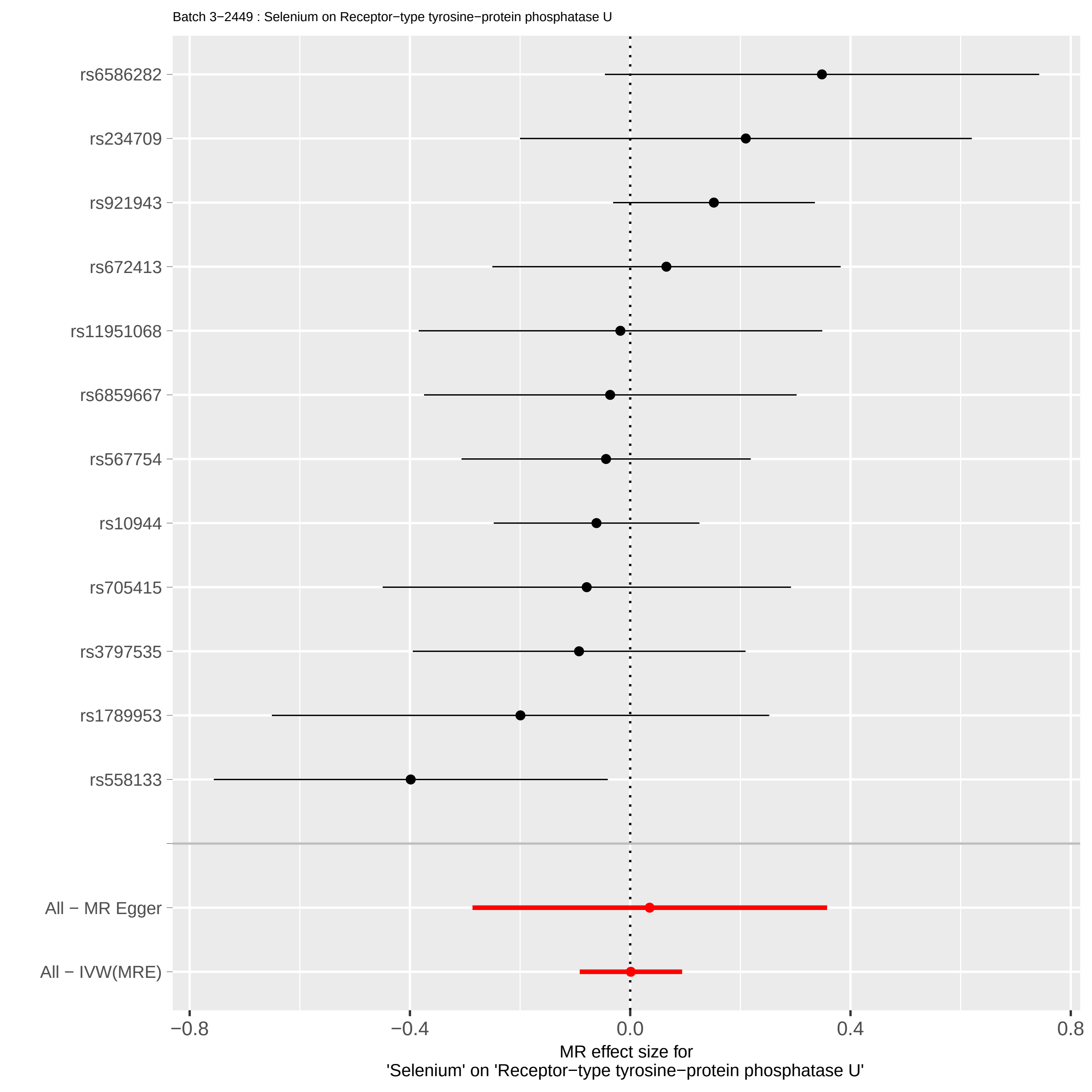

Batch 3−2449 : Selenium on Receptor−type tyrosine−protein phosphatase U
rs6586282
rs234709
rs921943
rs672413
rs11951068
rs6859667
rs567754
rs10944
rs705415
rs3797535
rs1789953
rs558133
All − MR Egger
All − IVW(MRE)
−0.8
−0.4
0.0
0.4
0.8
MR effect size for
'Selenium' on 'Receptor−type tyrosine−protein phosphatase U'

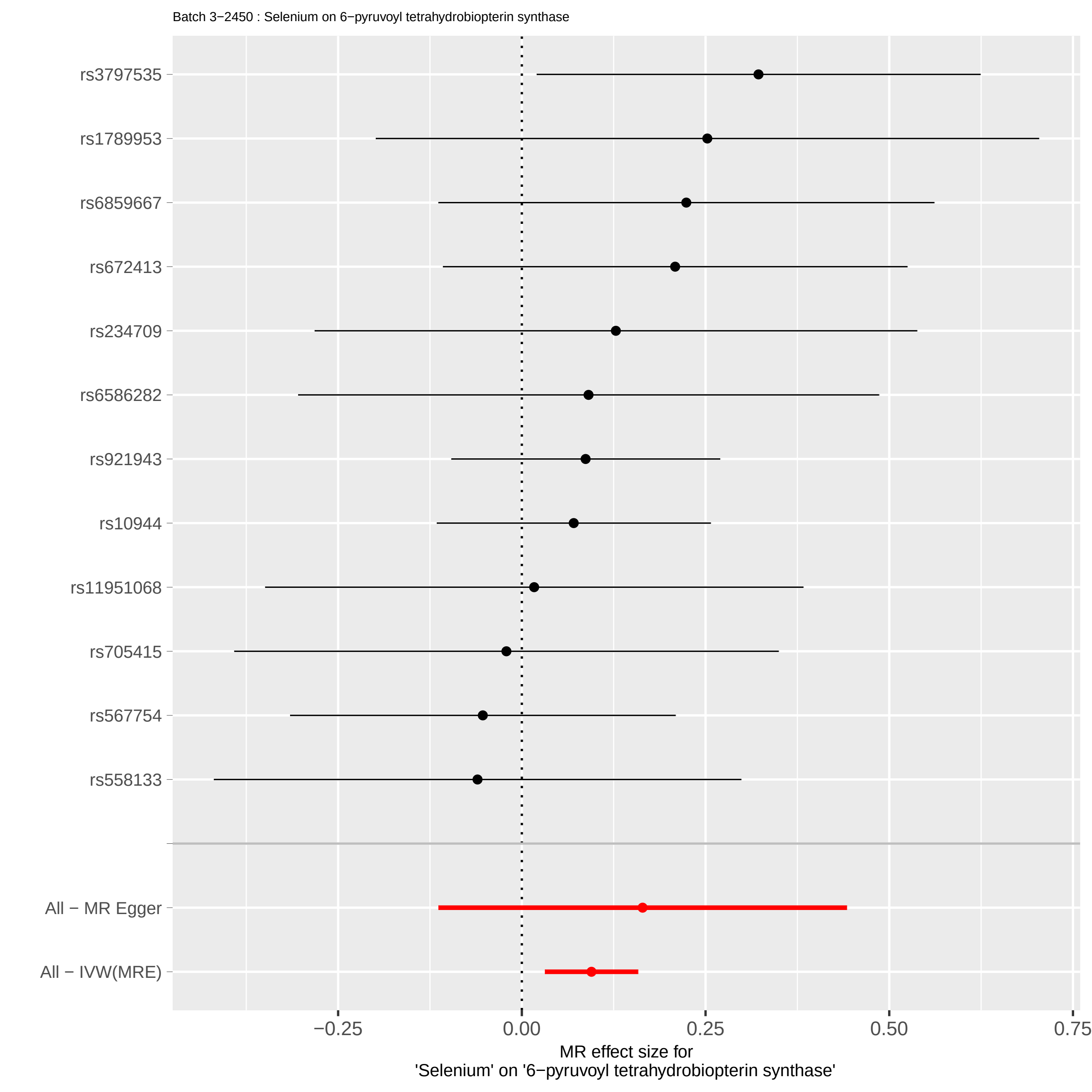
Batch 3−2450 : Selenium on 6−pyruvoyl tetrahydrobiopterin synthase
rs3797535
rs1789953
rs6859667
rs672413
rs234709
rs6586282
rs921943
rs10944
rs11951068
rs705415
rs567754
rs558133
All − MR Egger
All − IVW(MRE)
−0.25
0.00
0.25
0.50
0.75
MR effect size for
'Selenium' on '6−pyruvoyl tetrahydrobiopterin synthase'

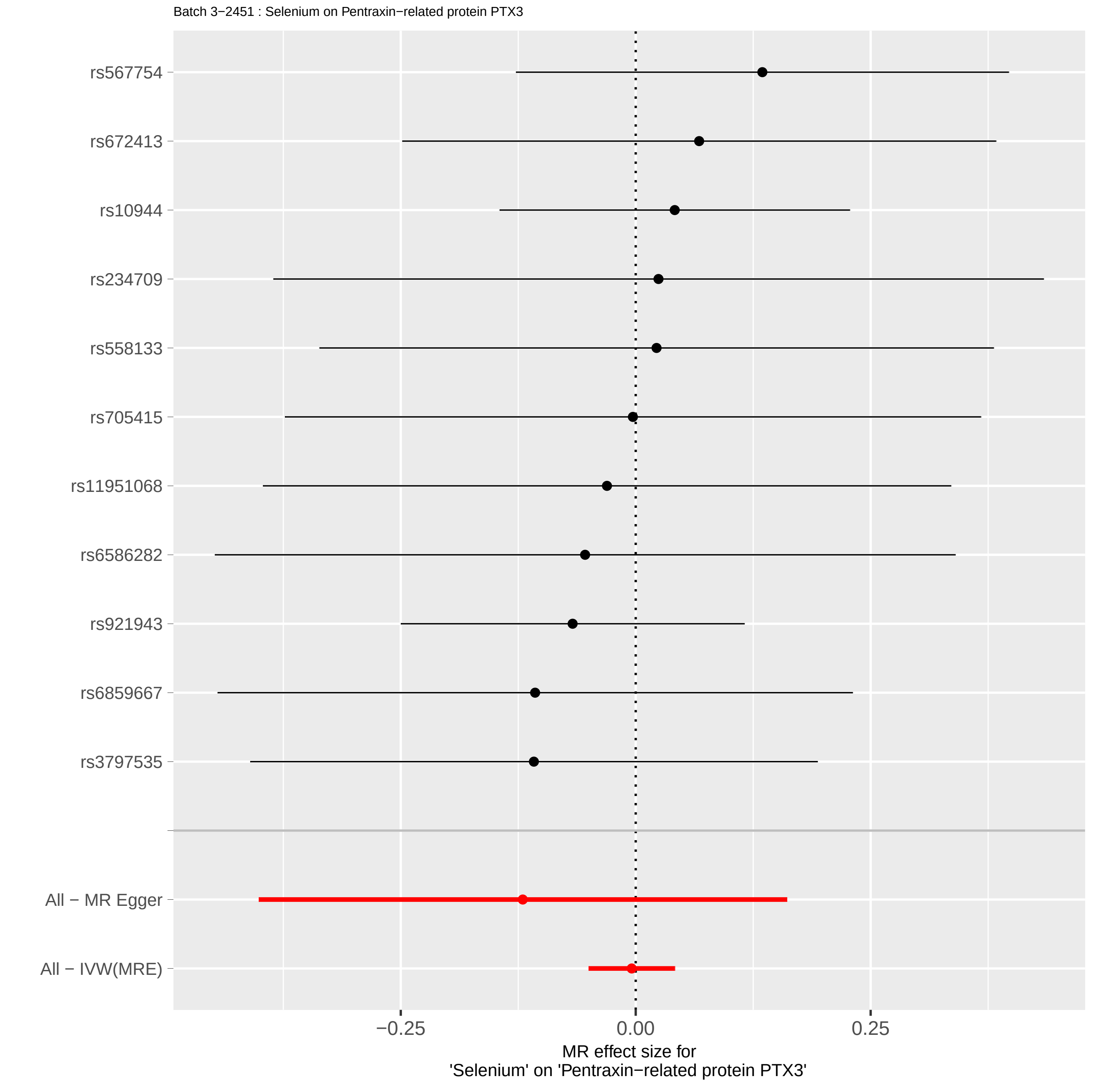

Batch 3−2451 : Selenium on Pentraxin−related protein PTX3
rs567754
rs672413
rs10944
rs234709
rs558133
rs705415
rs11951068
rs6586282
rs921943
rs6859667
rs3797535
All − MR Egger
All − IVW(MRE)
−0.25
0.00
0.25
MR effect size for
'Selenium' on 'Pentraxin−related protein PTX3'

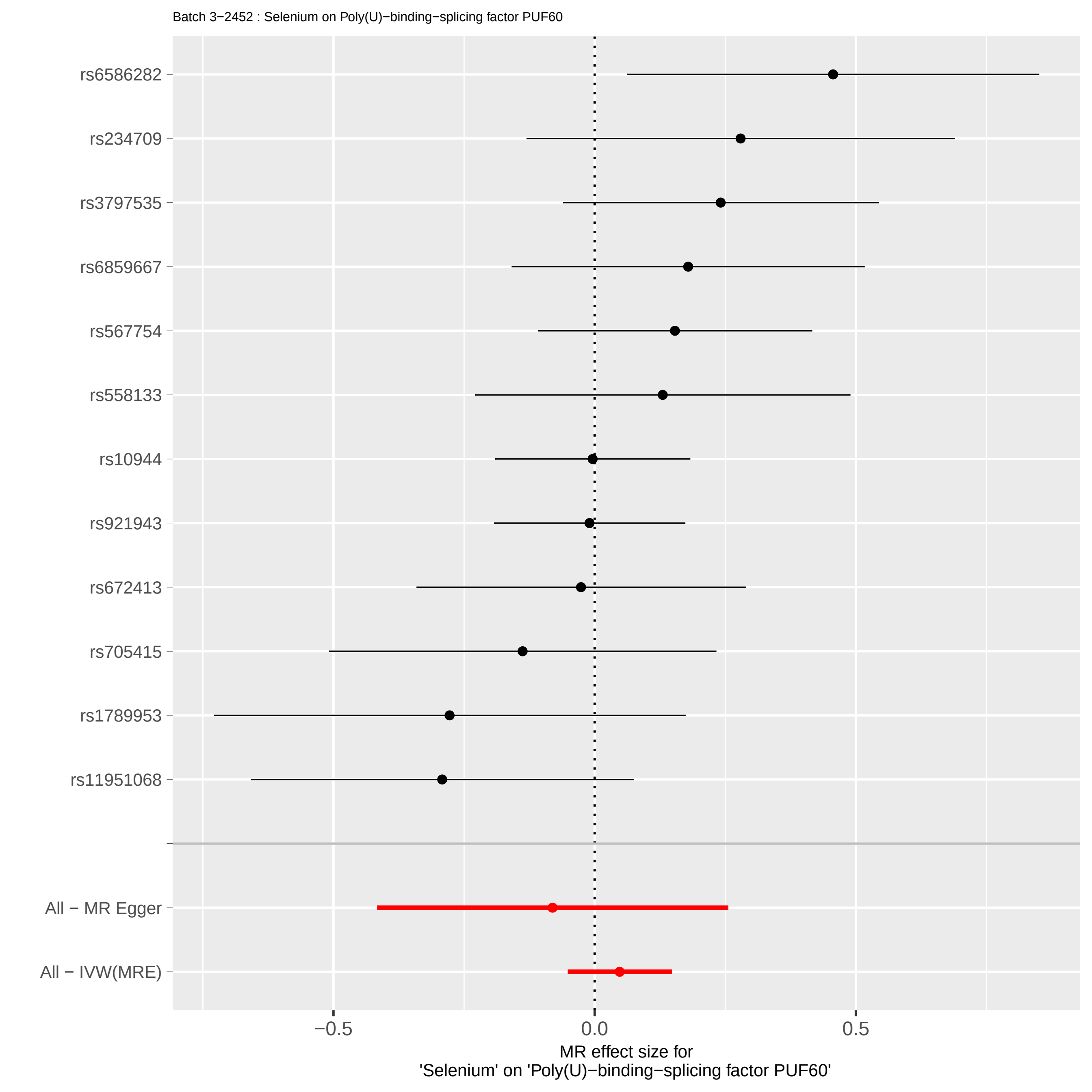
Batch 3−2452 : Selenium on Poly(U)−binding−splicing factor PUF60
rs6586282
rs234709
rs3797535
rs6859667
rs567754
rs558133
rs10944
rs921943
rs672413
rs705415
rs1789953
rs11951068
All − MR Egger
All − IVW(MRE)
−0.5
0.0
0.5
MR effect size for
'Selenium' on 'Poly(U)−binding−splicing factor PUF60'

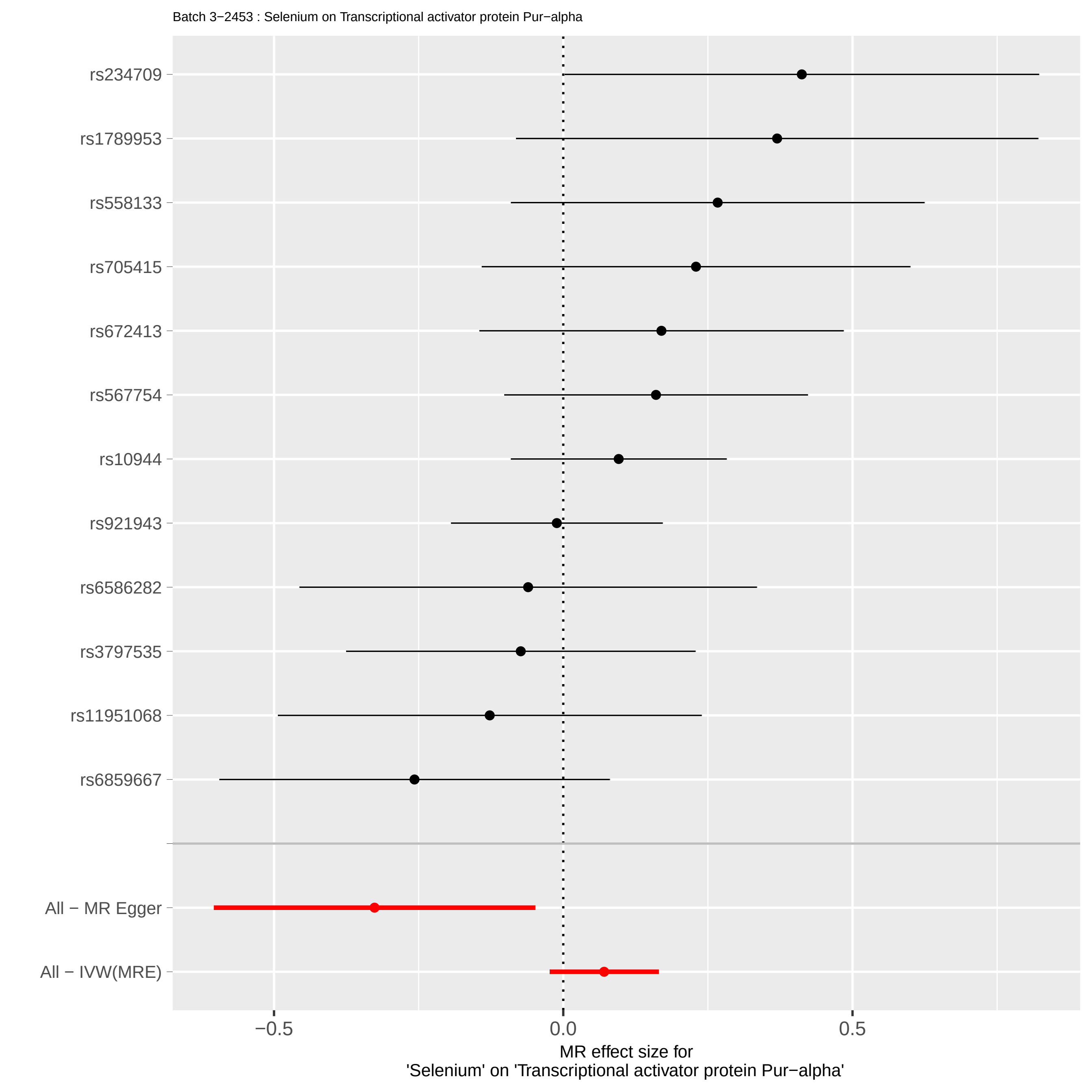
Batch 3−2453 : Selenium on Transcriptional activator protein Pur−alpha
rs234709
rs1789953
rs558133
rs705415
rs672413
rs567754
rs10944
rs921943
rs6586282
rs3797535
rs11951068
rs6859667
All − MR Egger
All − IVW(MRE)
−0.5
0.0
0.5
MR effect size for
'Selenium' on 'Transcriptional activator protein Pur−alpha'

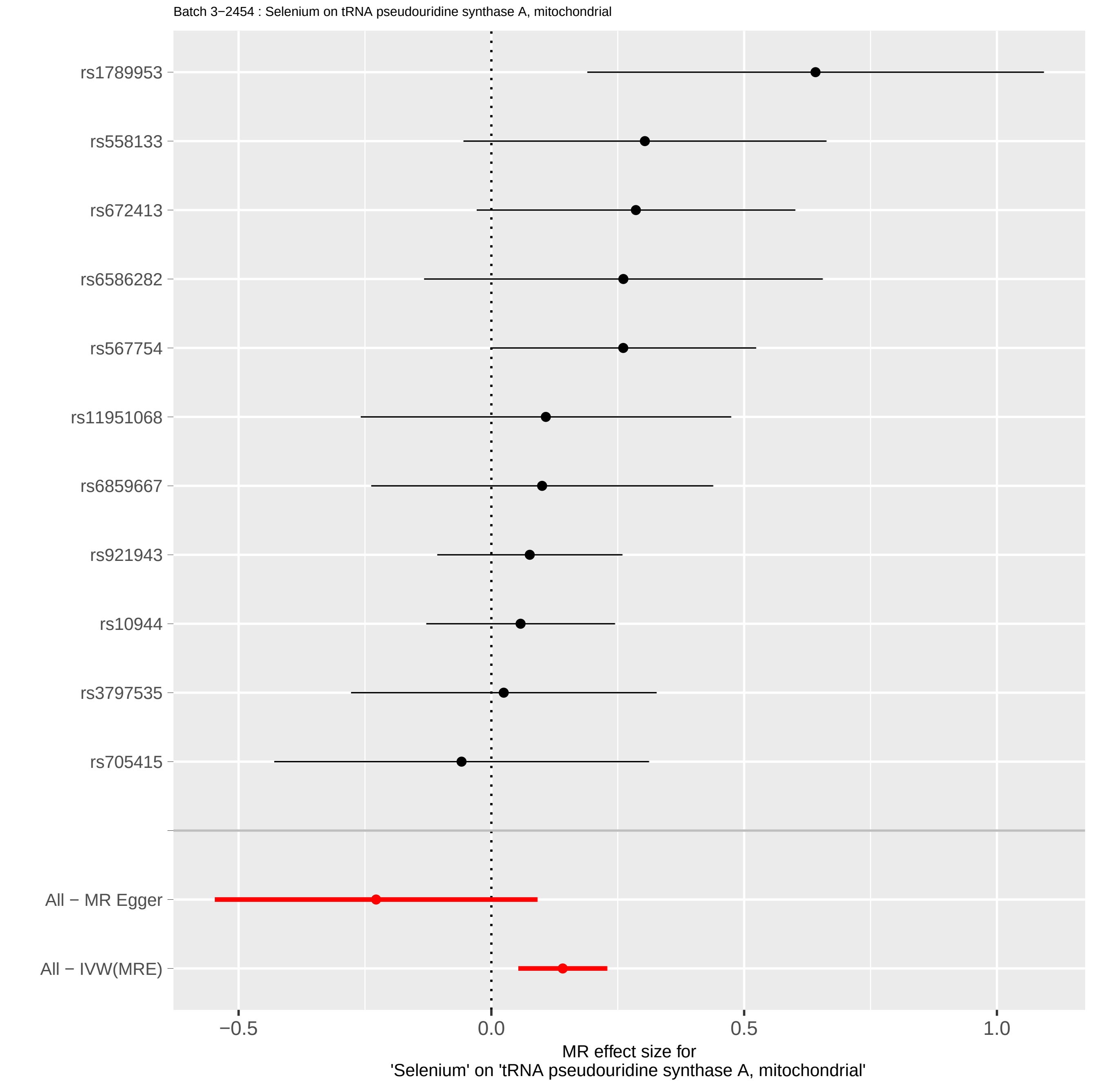

Batch 3−2454 : Selenium on tRNA pseudouridine synthase A, mitochondrial
rs1789953
rs558133
rs672413
rs6586282
rs567754
rs11951068
rs6859667
rs921943
rs10944
rs3797535
rs705415
All − MR Egger
All − IVW(MRE)
−0.5
0.0
0.5
1.0
MR effect size for
'Selenium' on 'tRNA pseudouridine synthase A, mitochondrial'

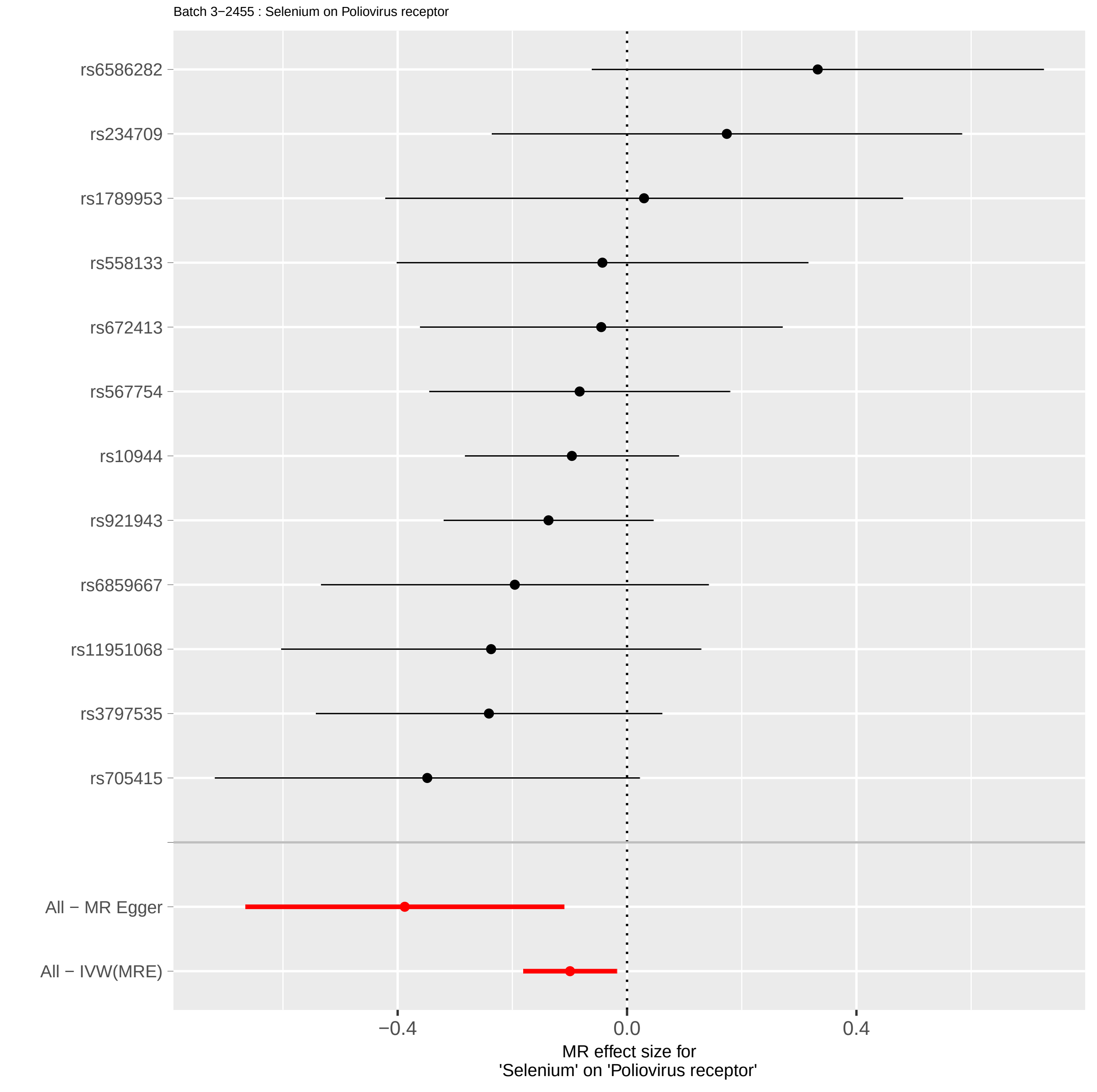

Batch 3−2455 : Selenium on Poliovirus receptor
rs6586282
rs234709
rs1789953
rs558133
rs672413
rs567754
rs10944
rs921943
rs6859667
rs11951068
rs3797535
rs705415
All − MR Egger
All − IVW(MRE)
−0.4
0.0
0.4
MR effect size for
'Selenium' on 'Poliovirus receptor'

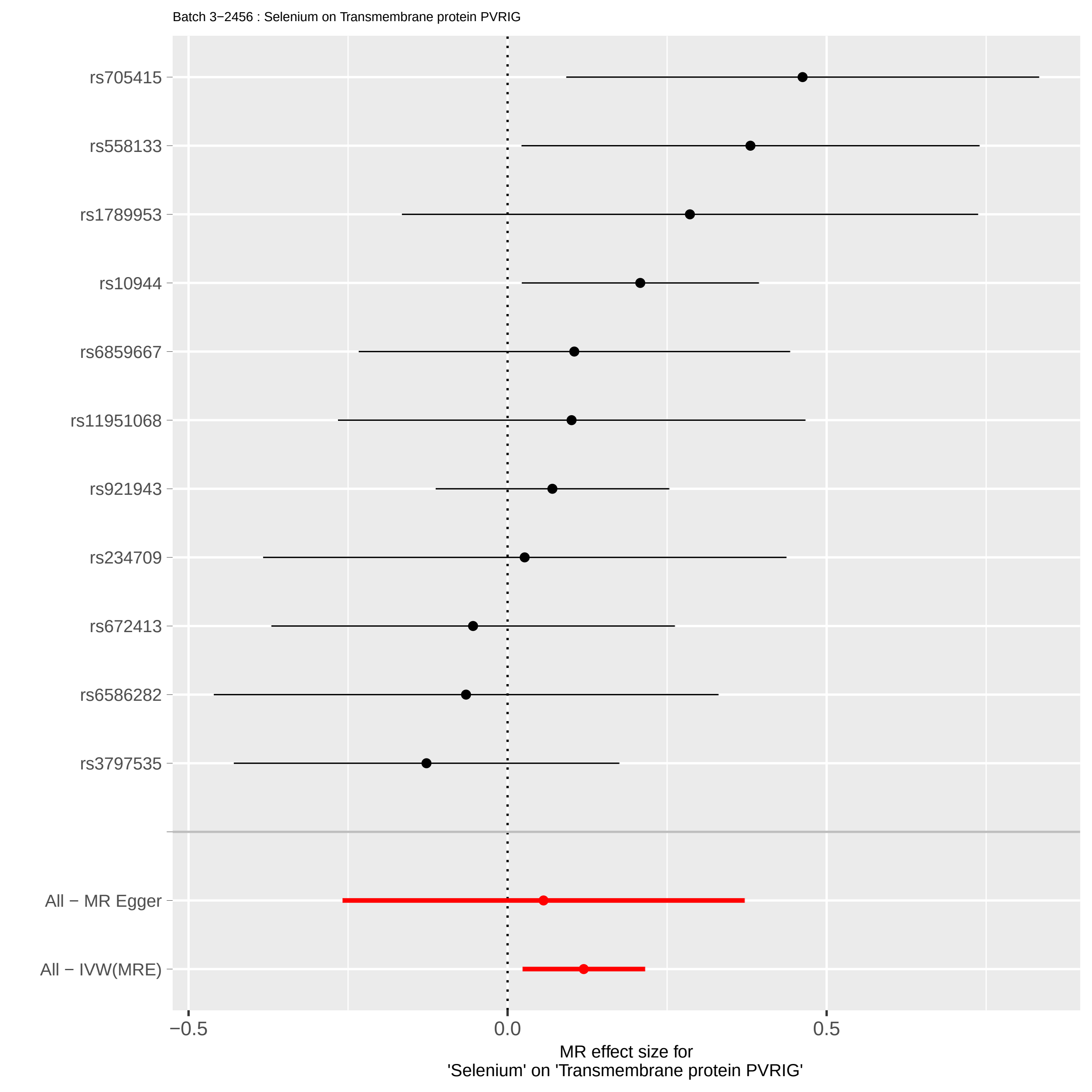
Batch 3−2456 : Selenium on Transmembrane protein PVRIG
rs705415
rs558133
rs1789953
rs10944
rs6859667
rs11951068
rs921943
rs234709
rs672413
rs6586282
rs3797535
All − MR Egger
All − IVW(MRE)
−0.5
0.0
0.5
MR effect size for
'Selenium' on 'Transmembrane protein PVRIG'

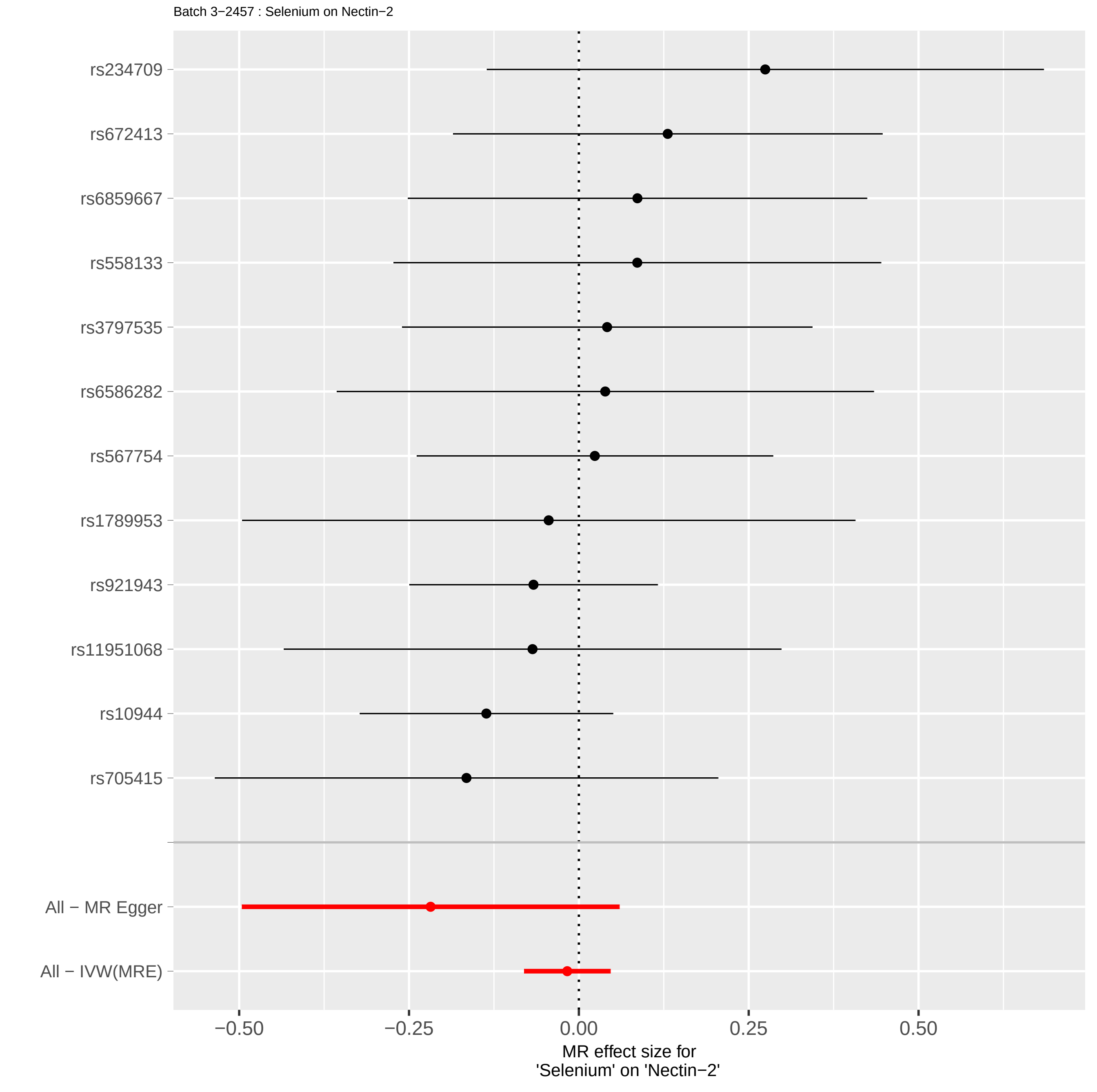

Batch 3−2457 : Selenium on Nectin−2
rs234709
rs672413
rs6859667
rs558133
rs3797535
rs6586282
rs567754
rs1789953
rs921943
rs11951068
rs10944
rs705415
All − MR Egger
All − IVW(MRE)
−0.50
−0.25
0.00
0.25
0.50
MR effect size for
'Selenium' on 'Nectin−2'

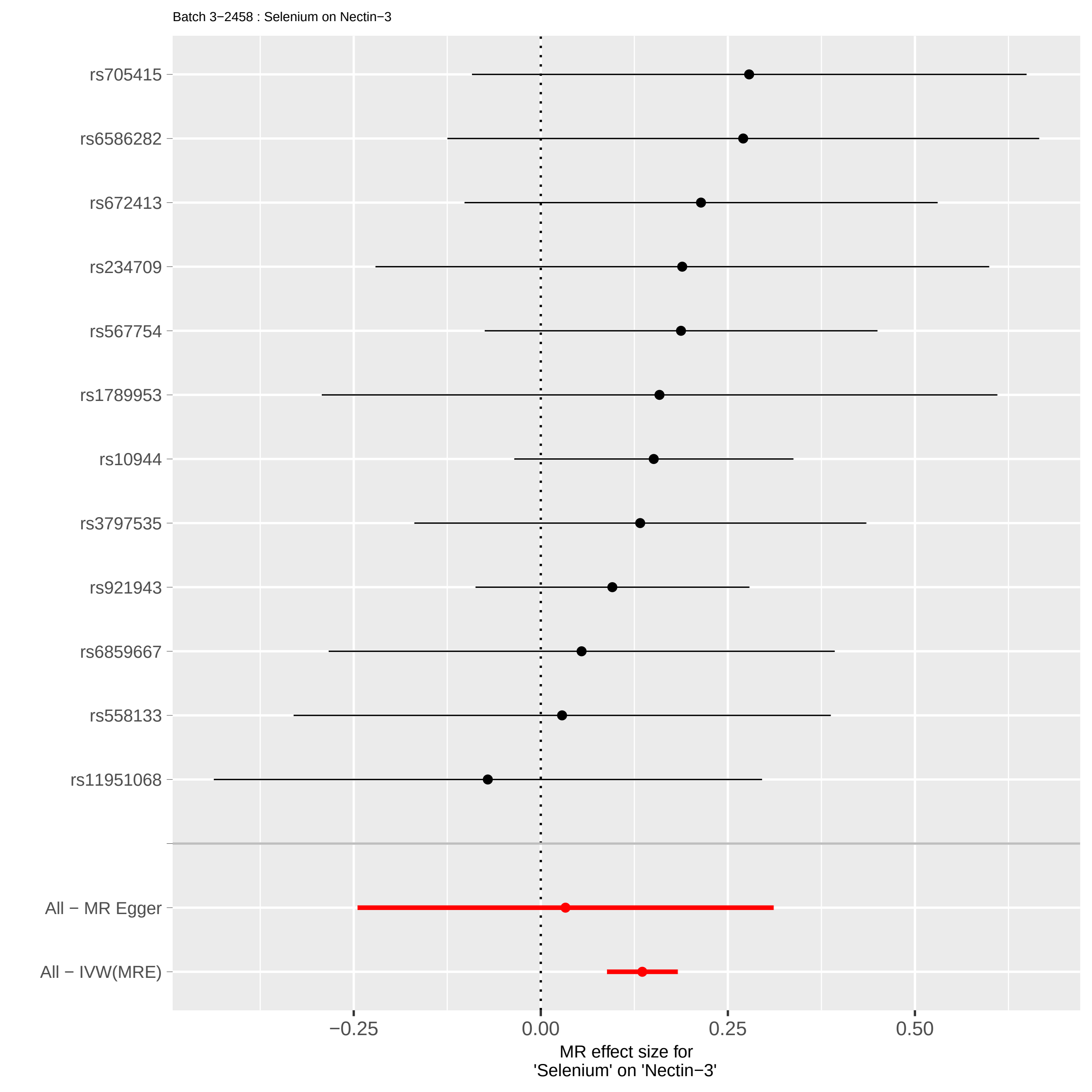

Batch 3−2458 : Selenium on Nectin−3
rs705415
rs6586282
rs672413
rs234709
rs567754
rs1789953
rs10944
rs3797535
rs921943
rs6859667
rs558133
rs11951068
All − MR Egger
All − IVW(MRE)
−0.25
0.00
0.25
0.50
MR effect size for
'Selenium' on 'Nectin−3'

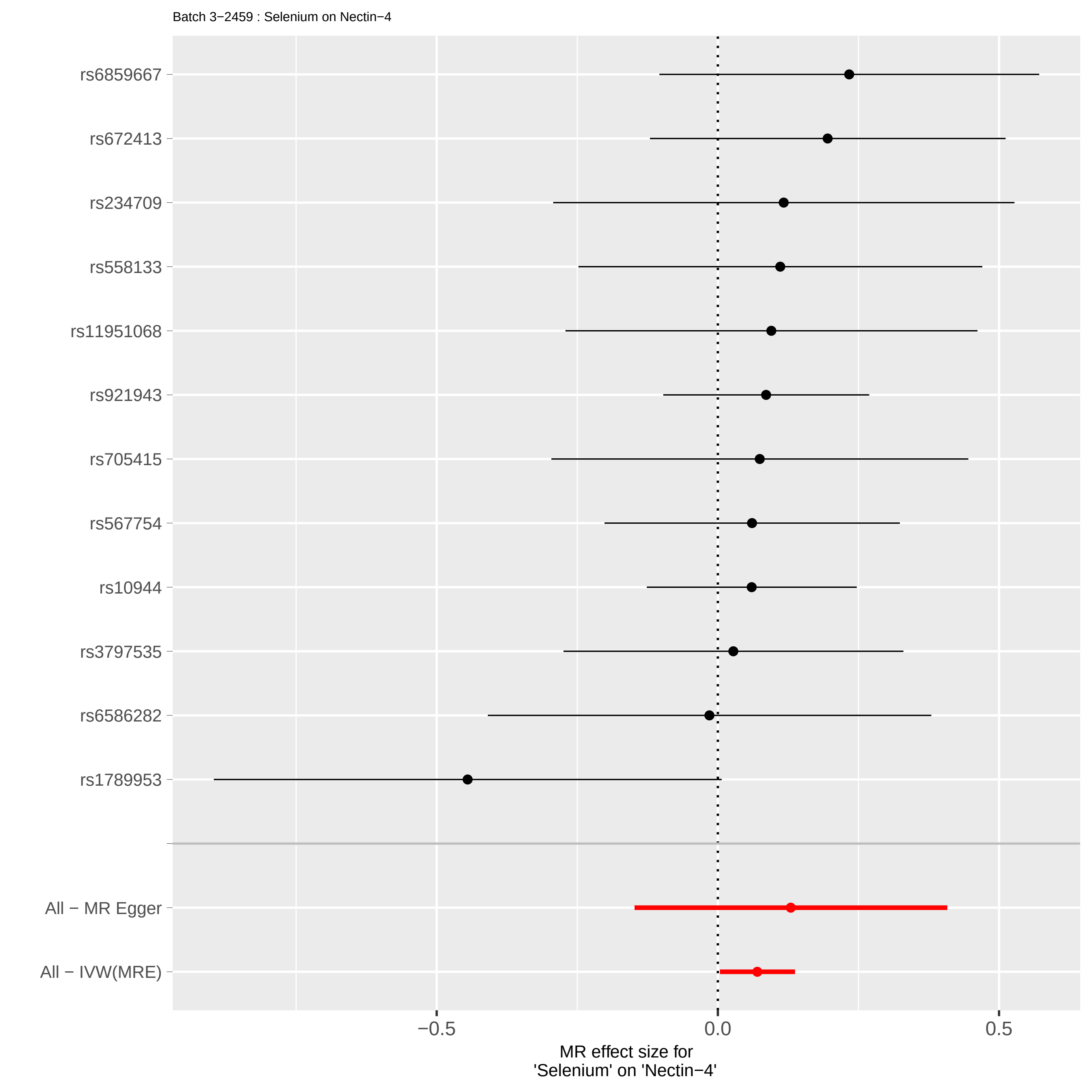

Batch 3−2459 : Selenium on Nectin−4
rs6859667
rs672413
rs234709
rs558133
rs11951068
rs921943
rs705415
rs567754
rs10944
rs3797535
rs6586282
rs1789953
All − MR Egger
All − IVW(MRE)
−0.5
0.0
0.5
MR effect size for
'Selenium' on 'Nectin−4'

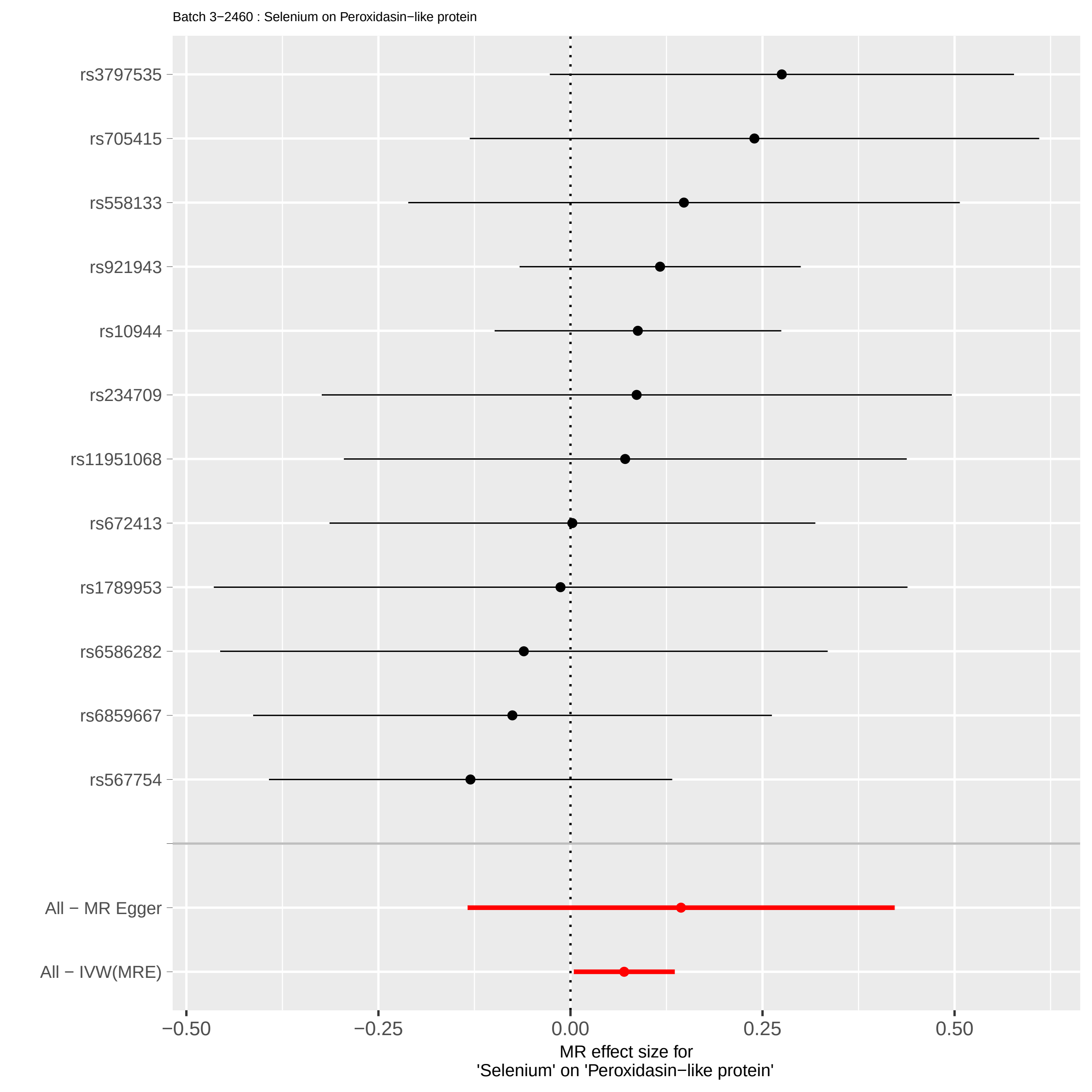

Batch 3−2460 : Selenium on Peroxidasin−like protein
rs3797535
rs705415
rs558133
rs921943
rs10944
rs234709
rs11951068
rs672413
rs1789953
rs6586282
rs6859667
rs567754
All − MR Egger
All − IVW(MRE)
−0.50
−0.25
0.00
0.25
0.50
MR effect size for
'Selenium' on 'Peroxidasin−like protein'

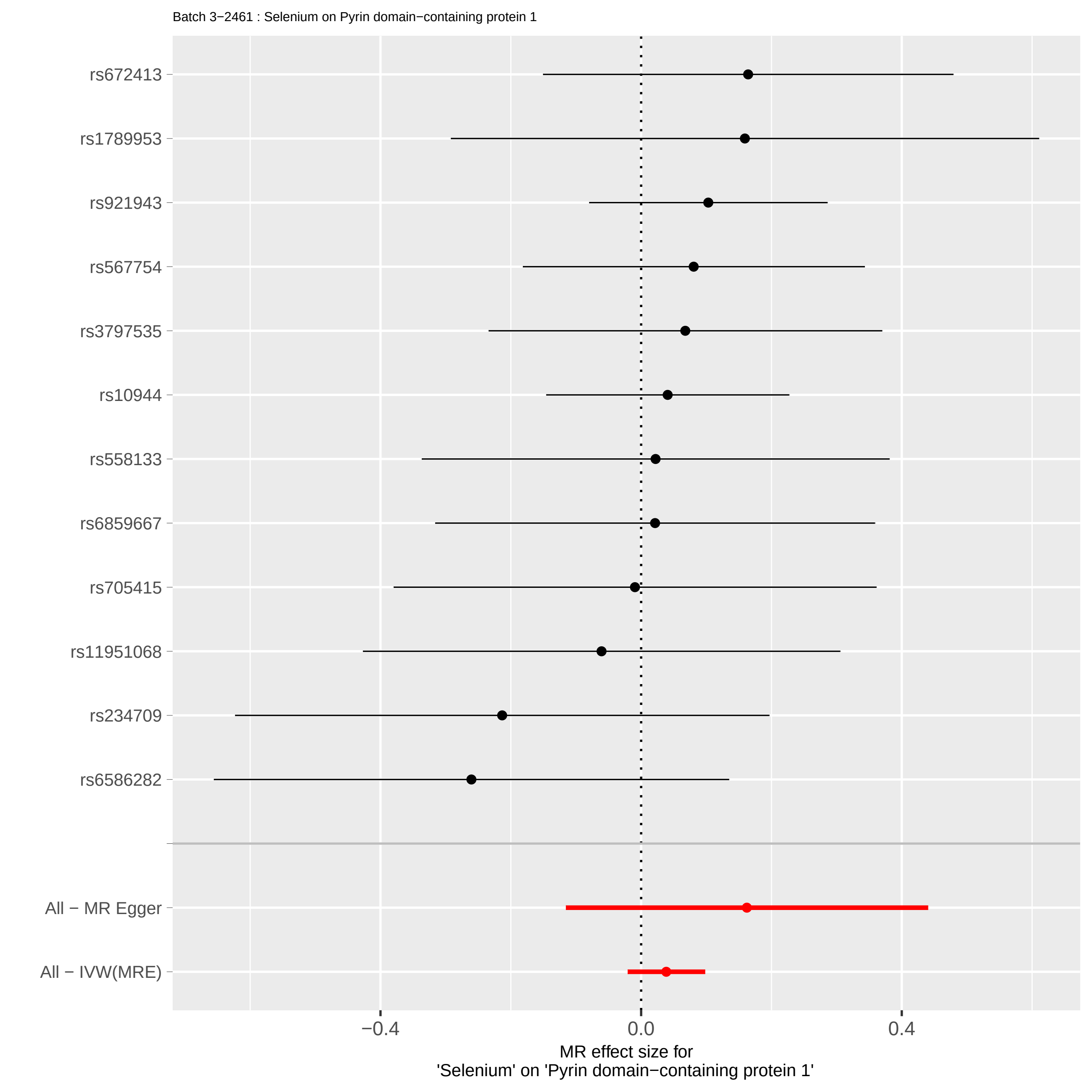

Batch 3−2461 : Selenium on Pyrin domain−containing protein 1
rs672413
rs1789953
rs921943
rs567754
rs3797535
rs10944
rs558133
rs6859667
rs705415
rs11951068
rs234709
rs6586282
All − MR Egger
All − IVW(MRE)
−0.4
0.0
0.4
MR effect size for
'Selenium' on 'Pyrin domain−containing protein 1'

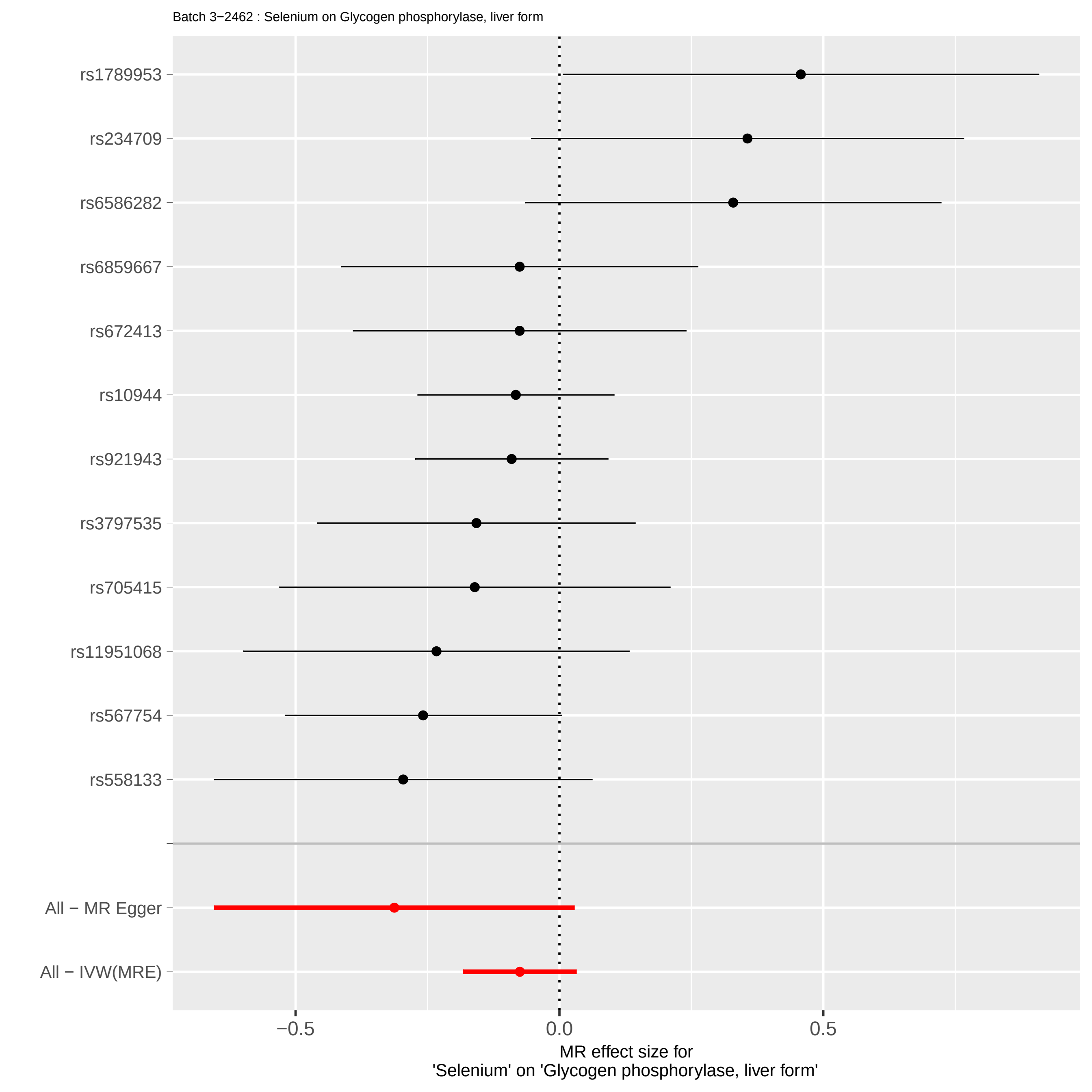

Batch 3−2462 : Selenium on Glycogen phosphorylase, liver form
rs1789953
rs234709
rs6586282
rs6859667
rs672413
rs10944
rs921943
rs3797535
rs705415
rs11951068
rs567754
rs558133
All − MR Egger
All − IVW(MRE)
−0.5
0.0
0.5
MR effect size for
'Selenium' on 'Glycogen phosphorylase, liver form'

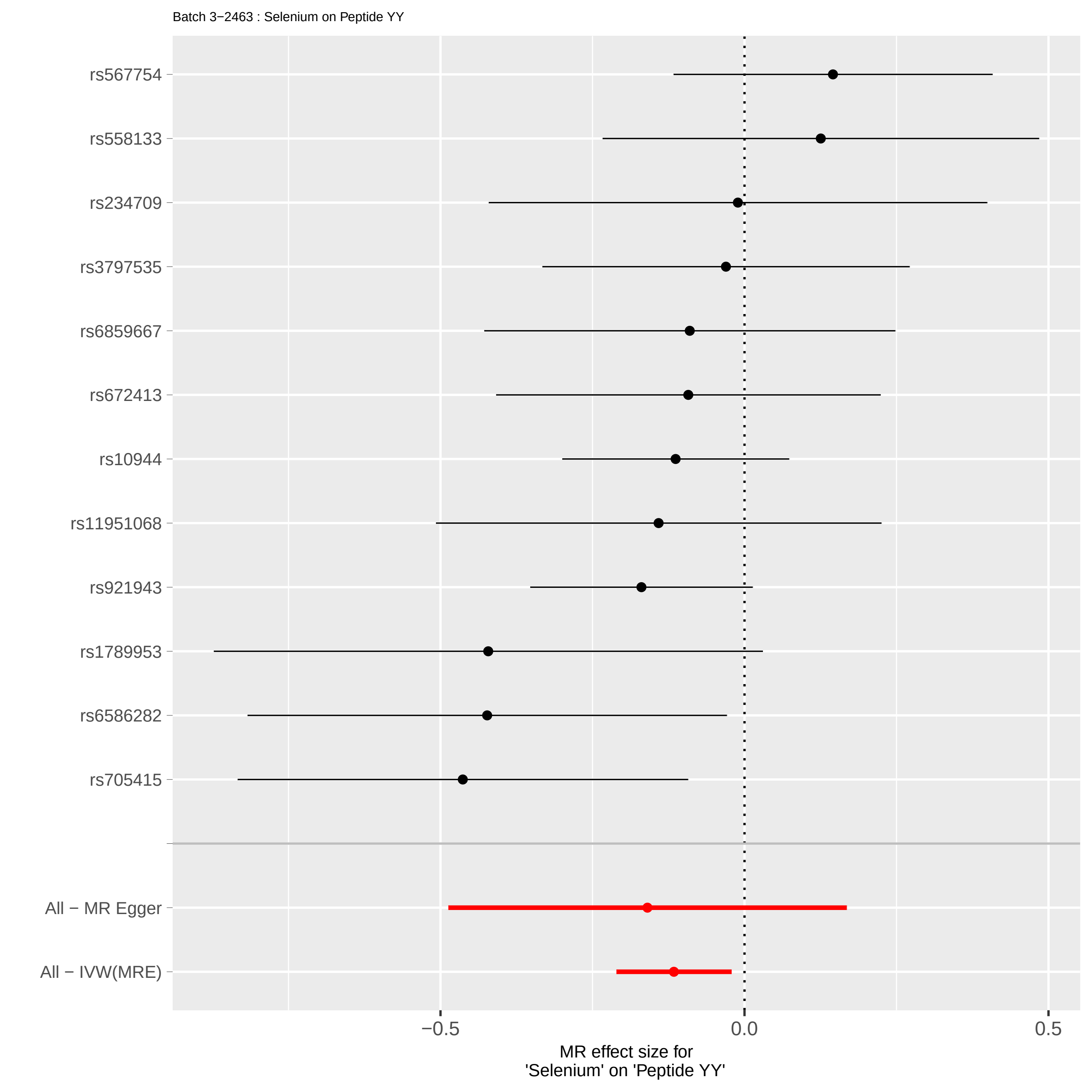
Batch 3−2463 : Selenium on Peptide YY
rs567754
rs558133
rs234709
rs3797535
rs6859667
rs672413
rs10944
rs11951068
rs921943
rs1789953
rs6586282
rs705415
All − MR Egger
All − IVW(MRE)
−0.5
0.0
0.5
MR effect size for
'Selenium' on 'Peptide YY'

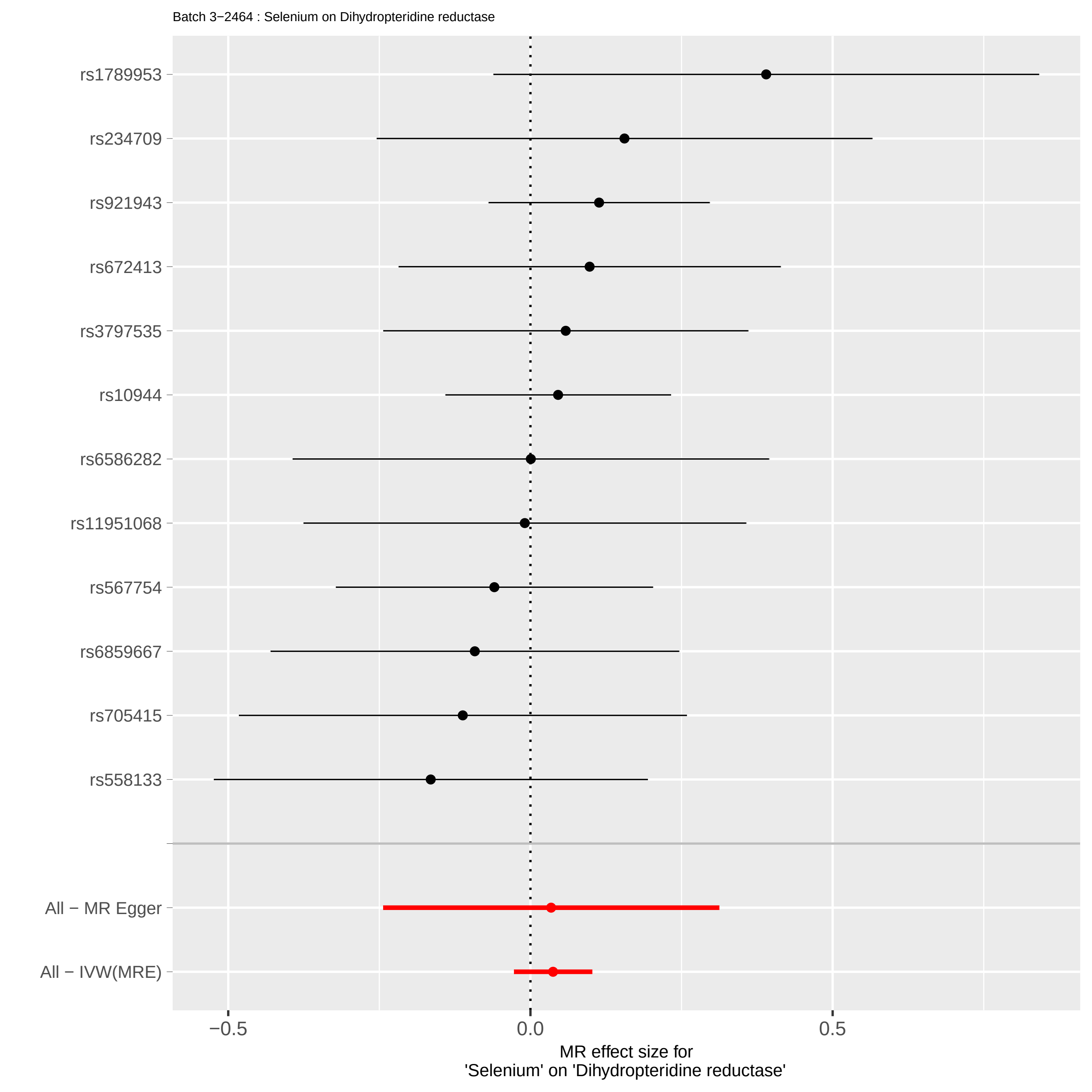

Batch 3−2464 : Selenium on Dihydropteridine reductase
rs1789953
rs234709
rs921943
rs672413
rs3797535
rs10944
rs6586282
rs11951068
rs567754
rs6859667
rs705415
rs558133
All − MR Egger
All − IVW(MRE)
−0.5
0.0
0.5
MR effect size for
'Selenium' on 'Dihydropteridine reductase'

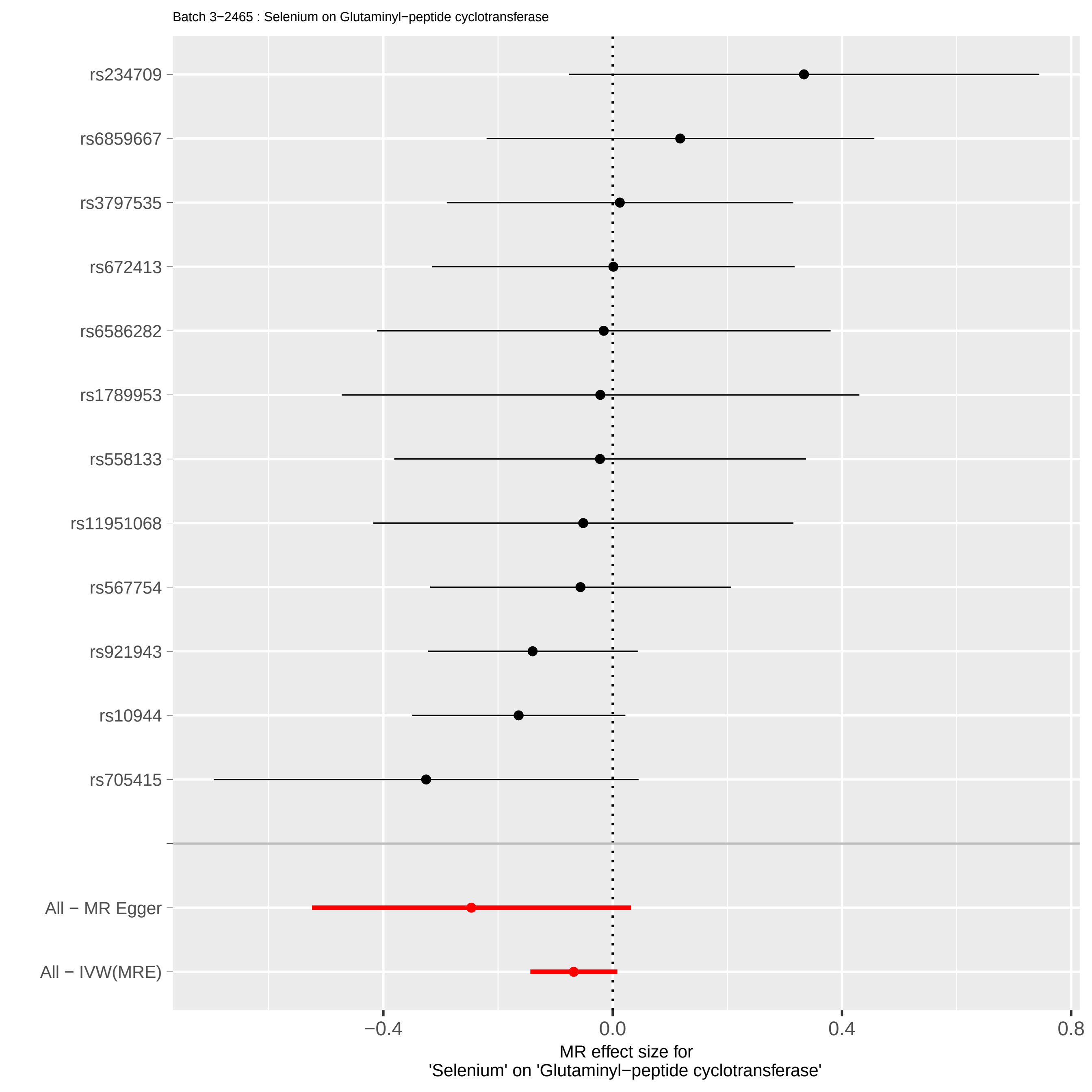

Batch 3−2465 : Selenium on Glutaminyl−peptide cyclotransferase
rs234709
rs6859667
rs3797535
rs672413
rs6586282
rs1789953
rs558133
rs11951068
rs567754
rs921943
rs10944
rs705415
All − MR Egger
All − IVW(MRE)
−0.4
0.0
0.4
0.8
MR effect size for
'Selenium' on 'Glutaminyl−peptide cyclotransferase'

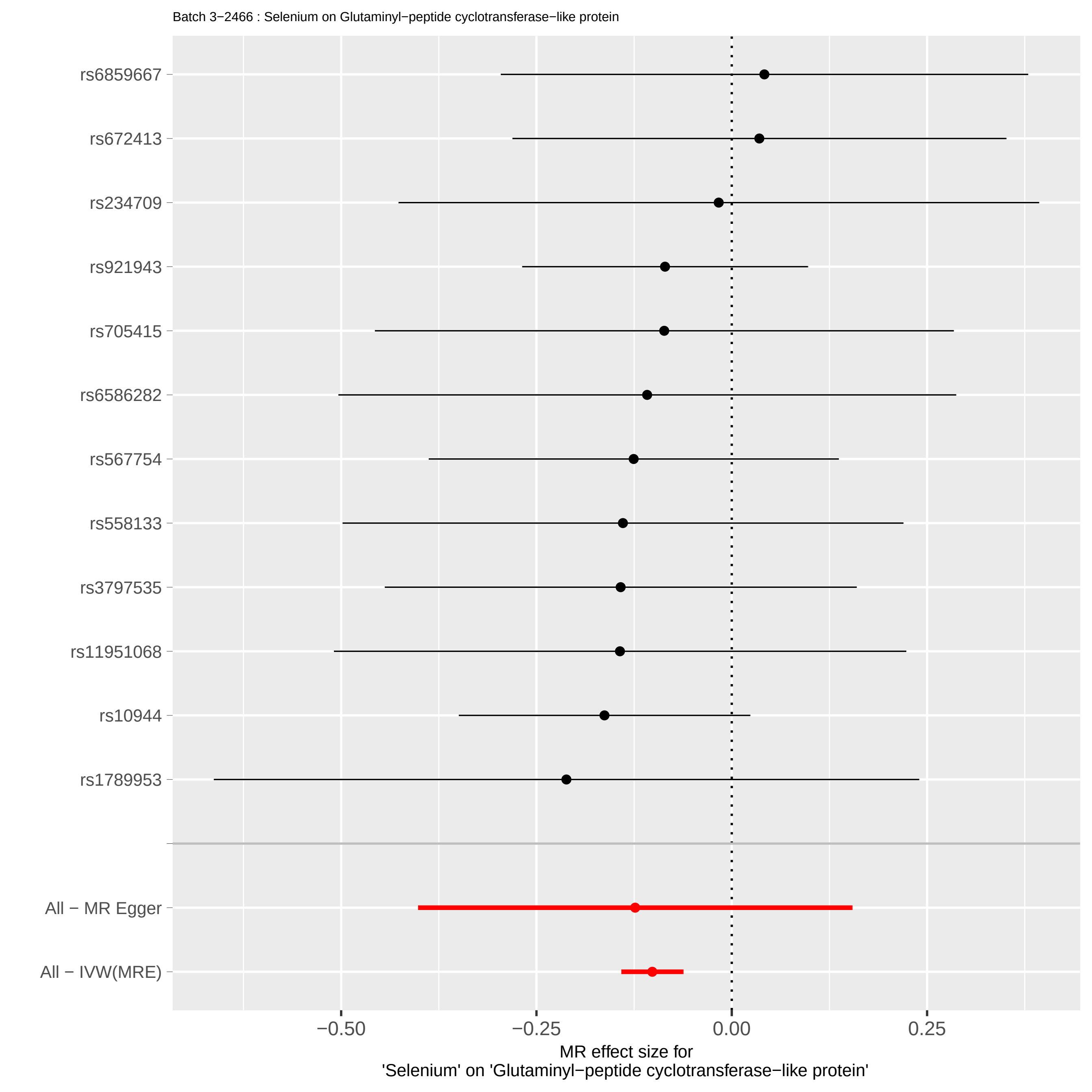
Batch 3−2466 : Selenium on Glutaminyl−peptide cyclotransferase−like protein
rs6859667
rs672413
rs234709
rs921943
rs705415
rs6586282
rs567754
rs558133
rs3797535
rs11951068
rs10944
rs1789953
All − MR Egger
All − IVW(MRE)
−0.50
−0.25
0.00
0.25
MR effect size for
'Selenium' on 'Glutaminyl−peptide cyclotransferase−like protein'

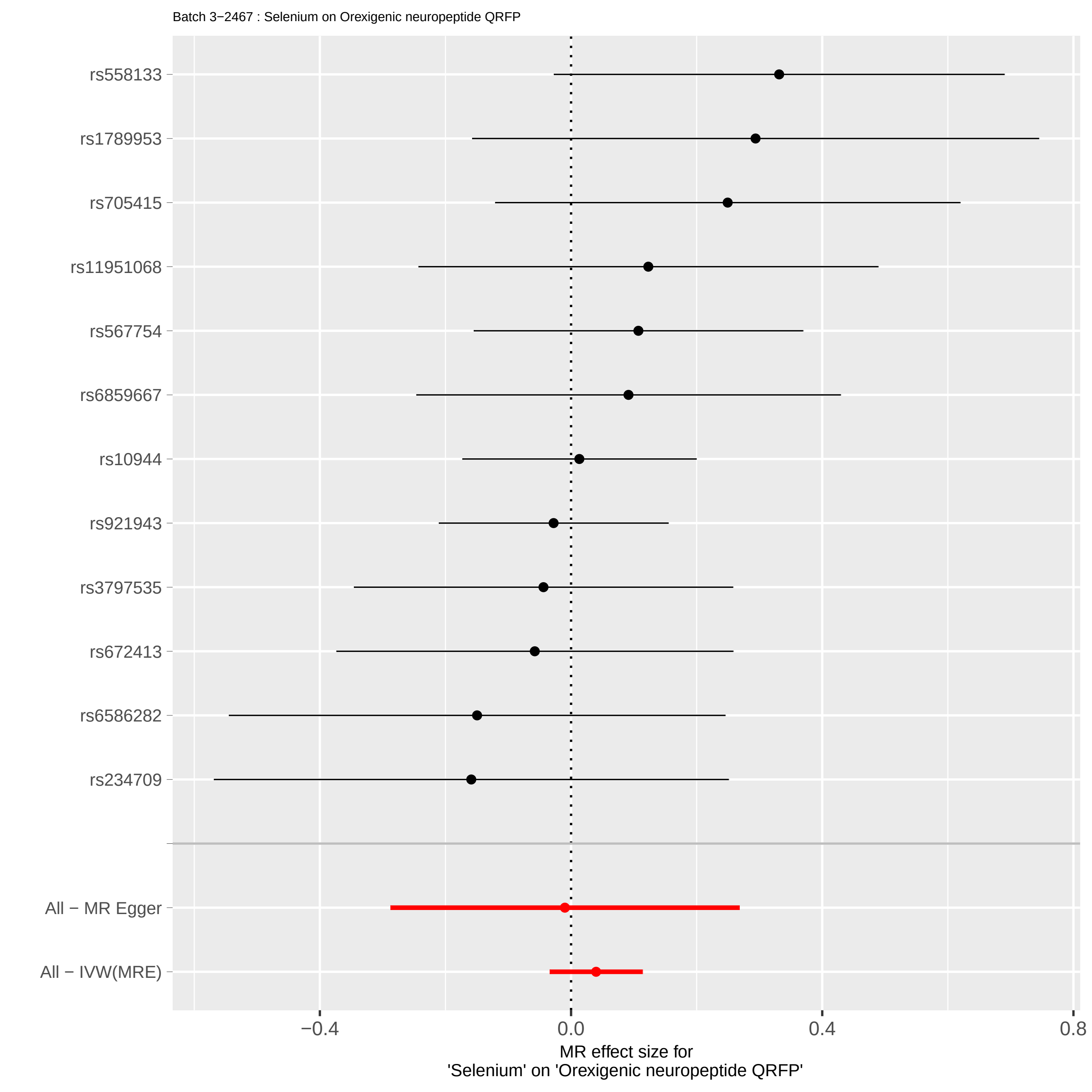

Batch 3−2467 : Selenium on Orexigenic neuropeptide QRFP
rs558133
rs1789953
rs705415
rs11951068
rs567754
rs6859667
rs10944
rs921943
rs3797535
rs672413
rs6586282
rs234709
All − MR Egger
All − IVW(MRE)
−0.4
0.0
0.4
0.8
MR effect size for
'Selenium' on 'Orexigenic neuropeptide QRFP'

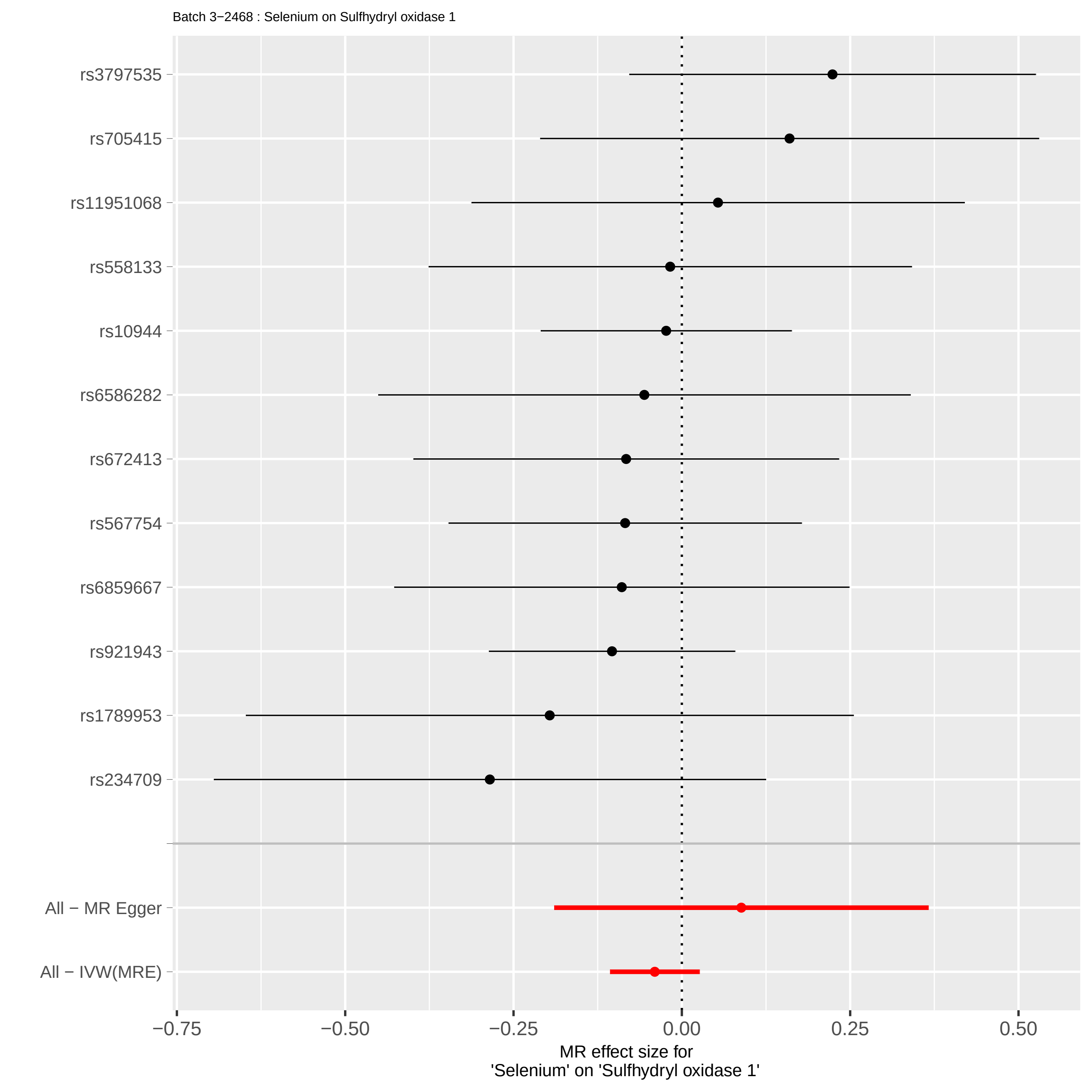

Batch 3−2468 : Selenium on Sulfhydryl oxidase 1
rs3797535
rs705415
rs11951068
rs558133
rs10944
rs6586282
rs672413
rs567754
rs6859667
rs921943
rs1789953
rs234709
All − MR Egger
All − IVW(MRE)
−0.75
−0.50
−0.25
0.00
0.25
0.50
MR effect size for
'Selenium' on 'Sulfhydryl oxidase 1'

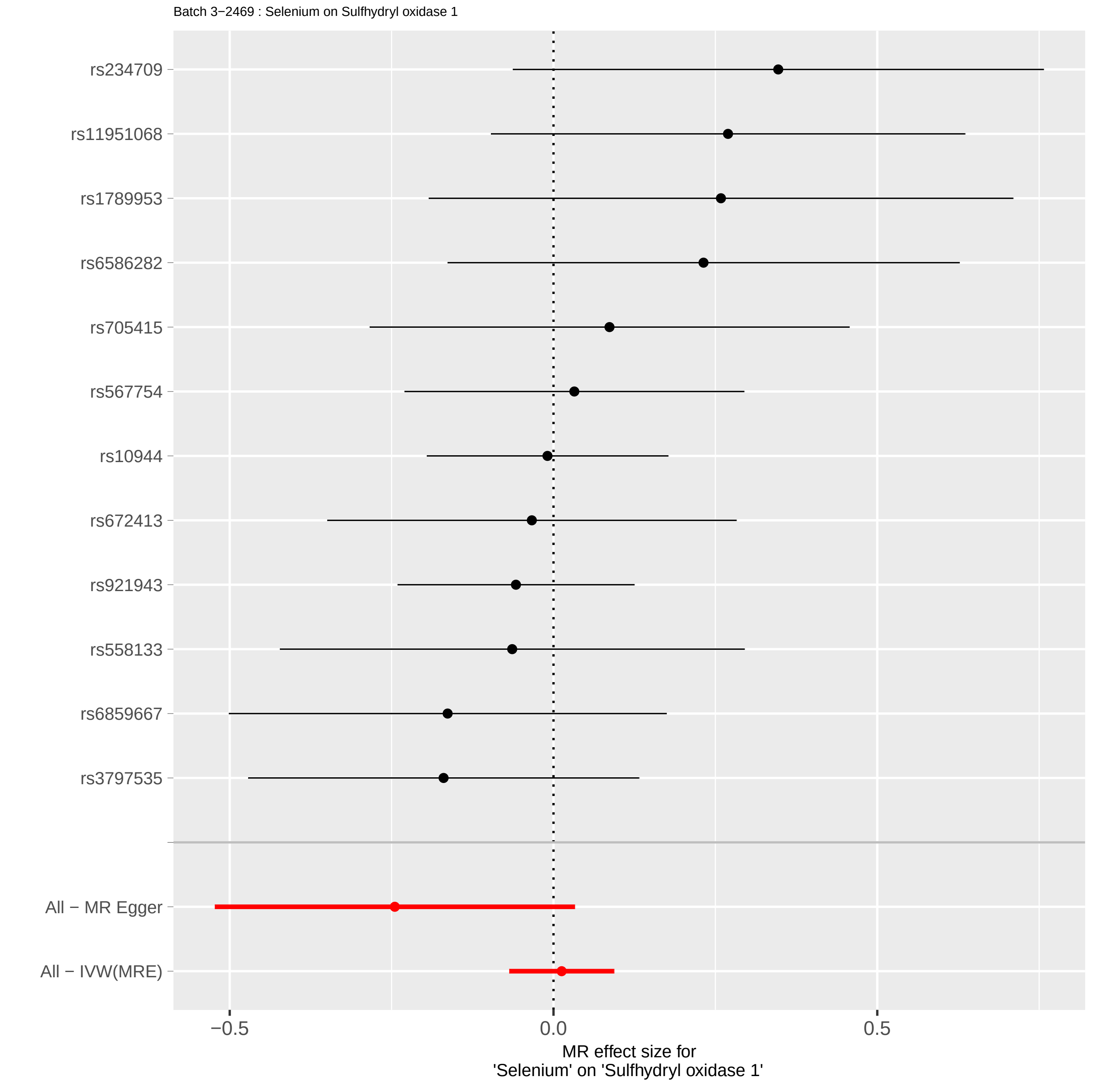

Batch 3−2469 : Selenium on Sulfhydryl oxidase 1
rs234709
rs11951068
rs1789953
rs6586282
rs705415
rs567754
rs10944
rs672413
rs921943
rs558133
rs6859667
rs3797535
All − MR Egger
All − IVW(MRE)
−0.5
0.0
0.5
MR effect size for
'Selenium' on 'Sulfhydryl oxidase 1'

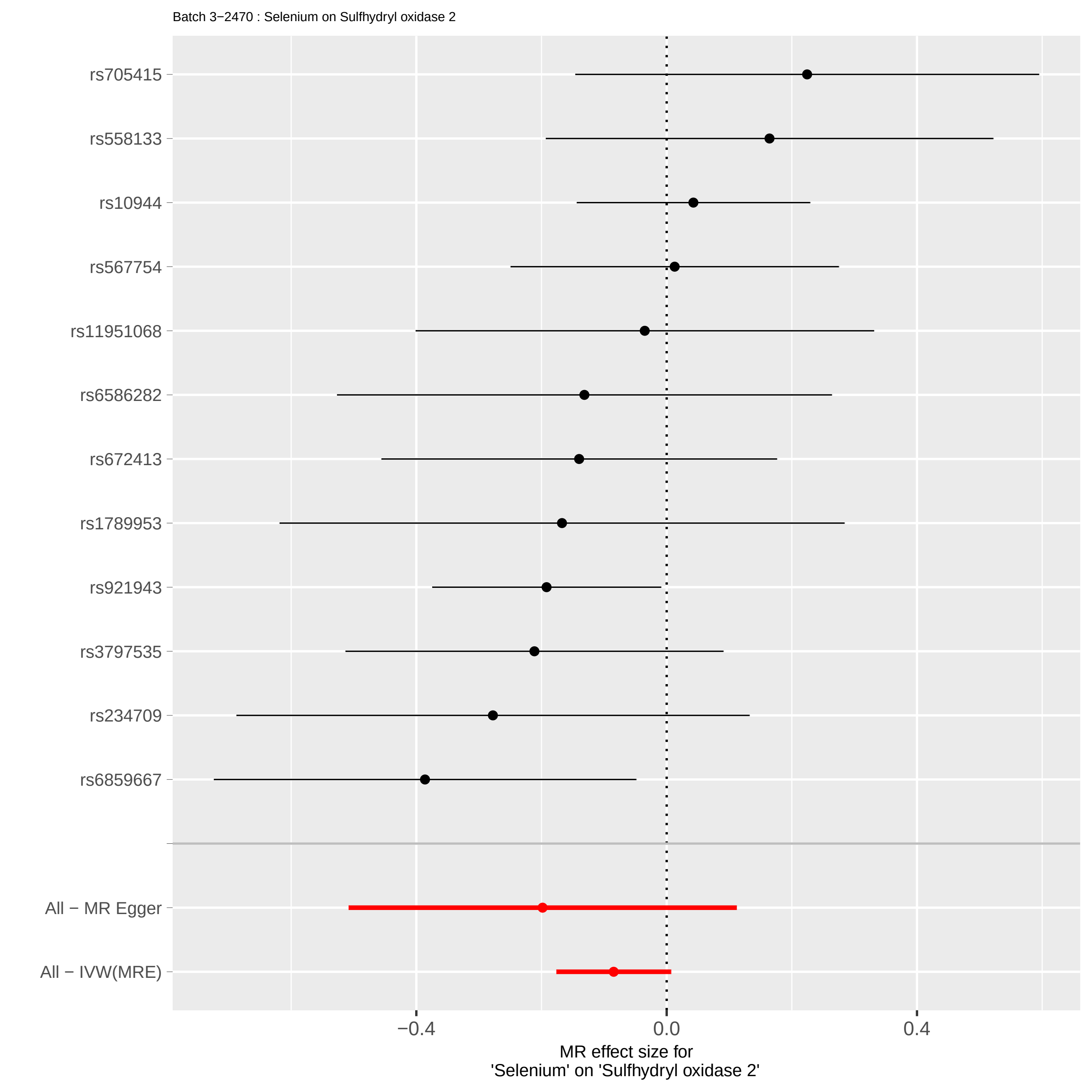

Batch 3−2470 : Selenium on Sulfhydryl oxidase 2
rs705415
rs558133
rs10944
rs567754
rs11951068
rs6586282
rs672413
rs1789953
rs921943
rs3797535
rs234709
rs6859667
All − MR Egger
All − IVW(MRE)
−0.4
0.0
0.4
MR effect size for
'Selenium' on 'Sulfhydryl oxidase 2'

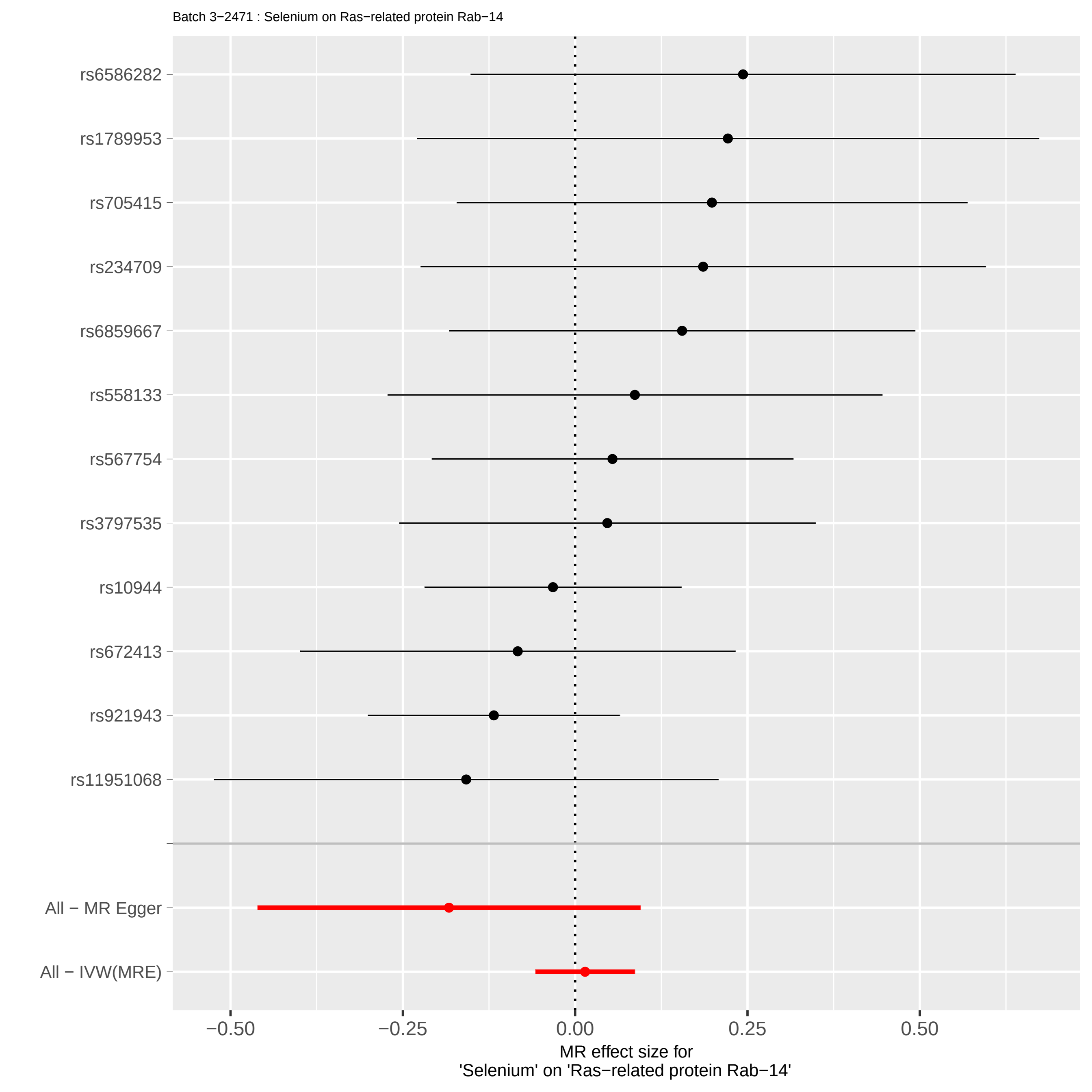

Batch 3−2471 : Selenium on Ras−related protein Rab−14
rs6586282
rs1789953
rs705415
rs234709
rs6859667
rs558133
rs567754
rs3797535
rs10944
rs672413
rs921943
rs11951068
All − MR Egger
All − IVW(MRE)
−0.50
−0.25
0.00
0.25
0.50
MR effect size for
'Selenium' on 'Ras−related protein Rab−14'

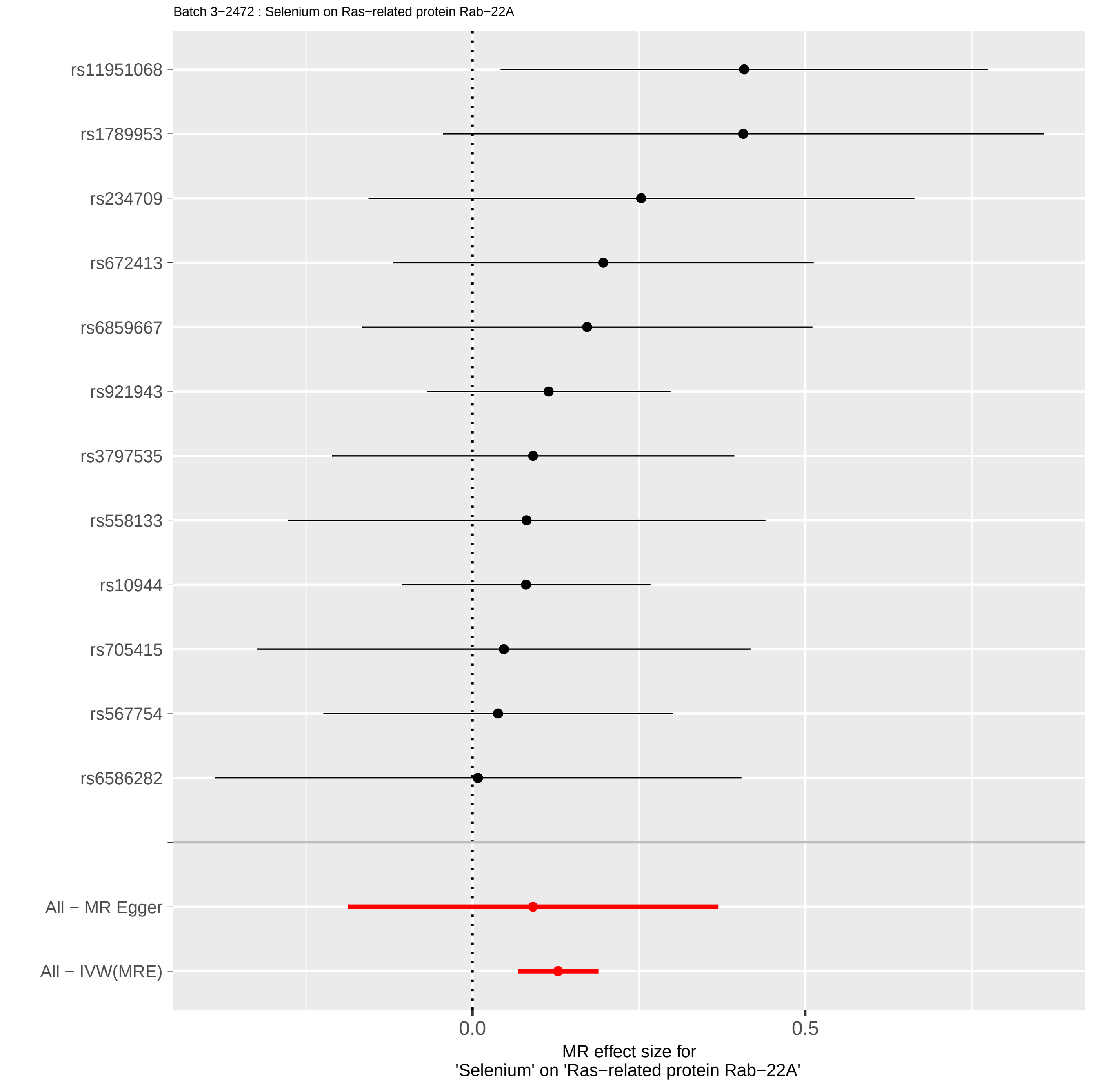

Batch 3−2472 : Selenium on Ras−related protein Rab−22A
rs11951068
rs1789953
rs234709
rs672413
rs6859667
rs921943
rs3797535
rs558133
rs10944
rs705415
rs567754
rs6586282
All − MR Egger
All − IVW(MRE)
0.0
0.5
MR effect size for
'Selenium' on 'Ras−related protein Rab−22A'

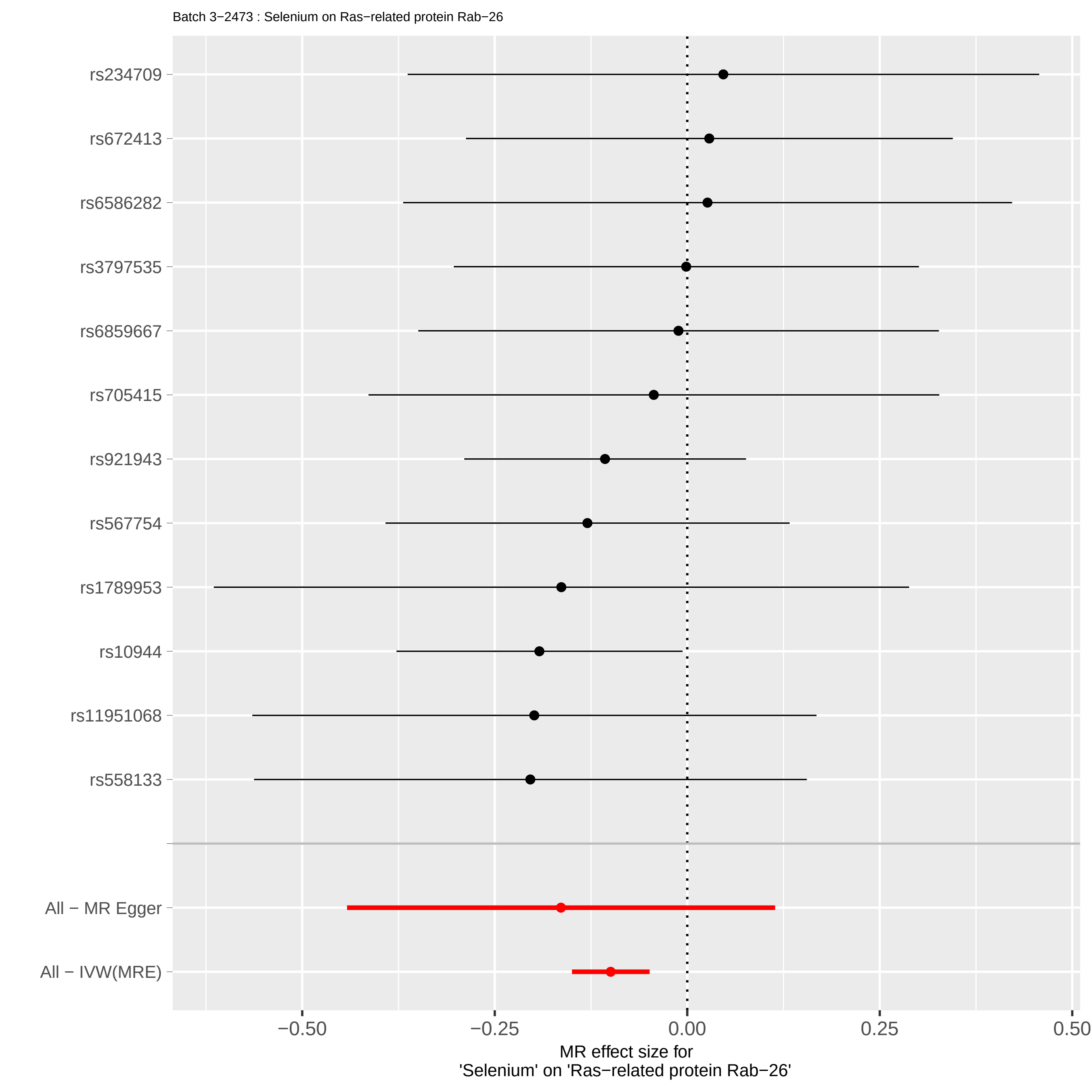
Batch 3−2473 : Selenium on Ras−related protein Rab−26
rs234709
rs672413
rs6586282
rs3797535
rs6859667
rs705415
rs921943
rs567754
rs1789953
rs10944
rs11951068
rs558133
All − MR Egger
All − IVW(MRE)
−0.50
−0.25
0.00
0.25
0.50
MR effect size for
'Selenium' on 'Ras−related protein Rab−26'

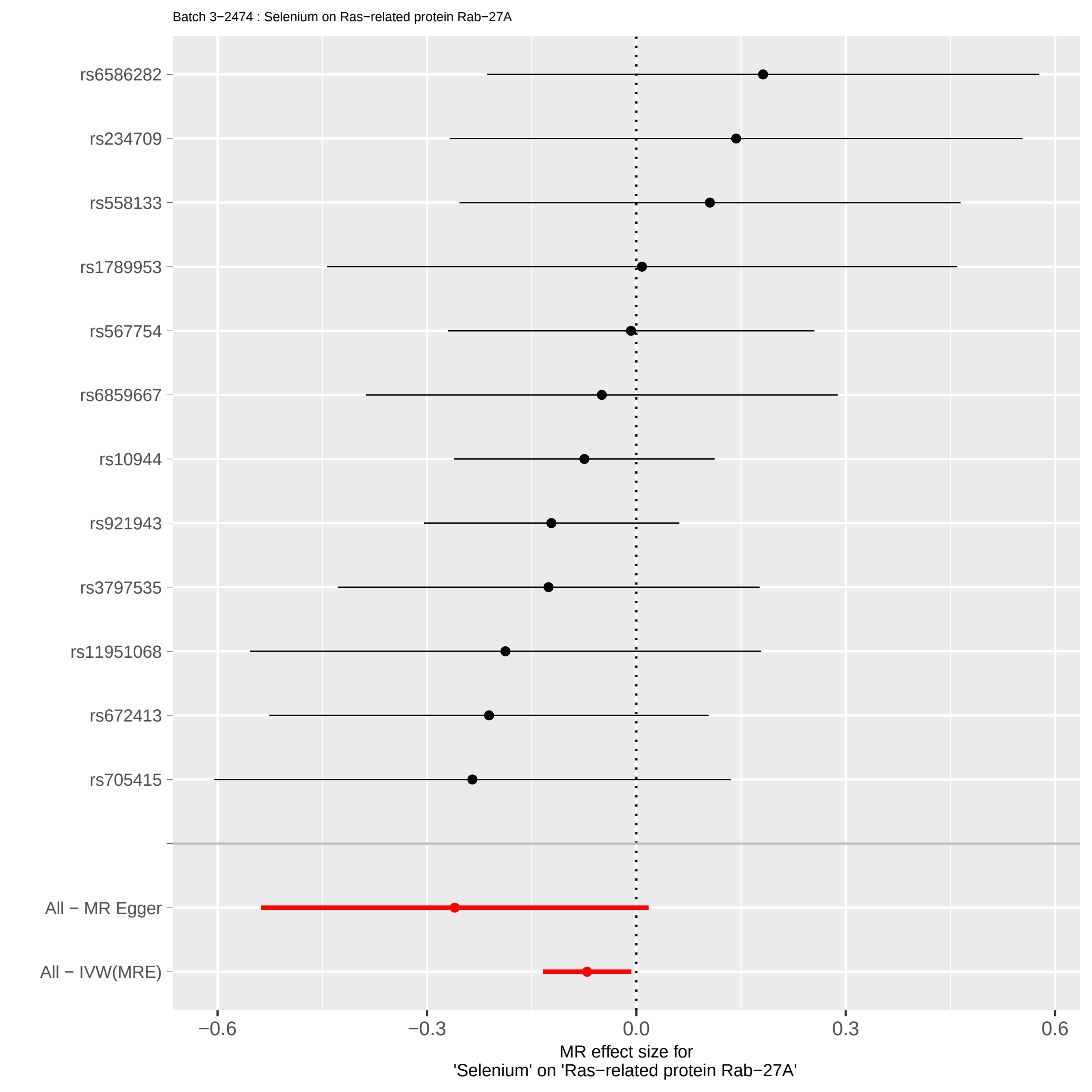

Batch 3−2474 : Selenium on Ras−related protein Rab−27A
rs6586282
rs234709
rs558133
rs1789953
rs567754
rs6859667
rs10944
rs921943
rs3797535
rs11951068
rs672413
rs705415
All − MR Egger
All − IVW(MRE)
−0.6
−0.3
0.0
0.3
0.6
MR effect size for
'Selenium' on 'Ras−related protein Rab−27A'

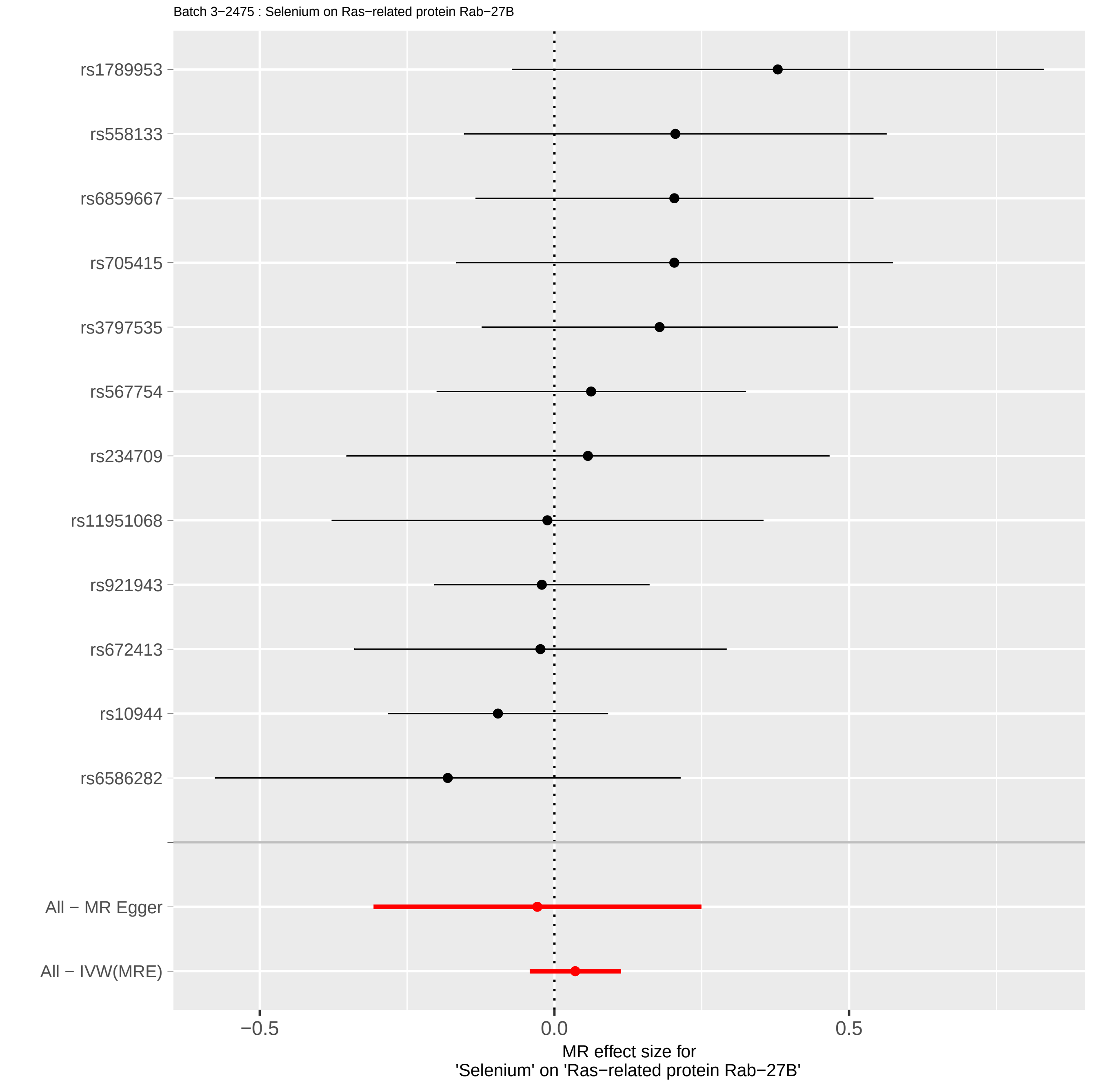

Batch 3−2475 : Selenium on Ras−related protein Rab−27B
rs1789953
rs558133
rs6859667
rs705415
rs3797535
rs567754
rs234709
rs11951068
rs921943
rs672413
rs10944
rs6586282
All − MR Egger
All − IVW(MRE)
−0.5
0.0
0.5
MR effect size for
'Selenium' on 'Ras−related protein Rab−27B'

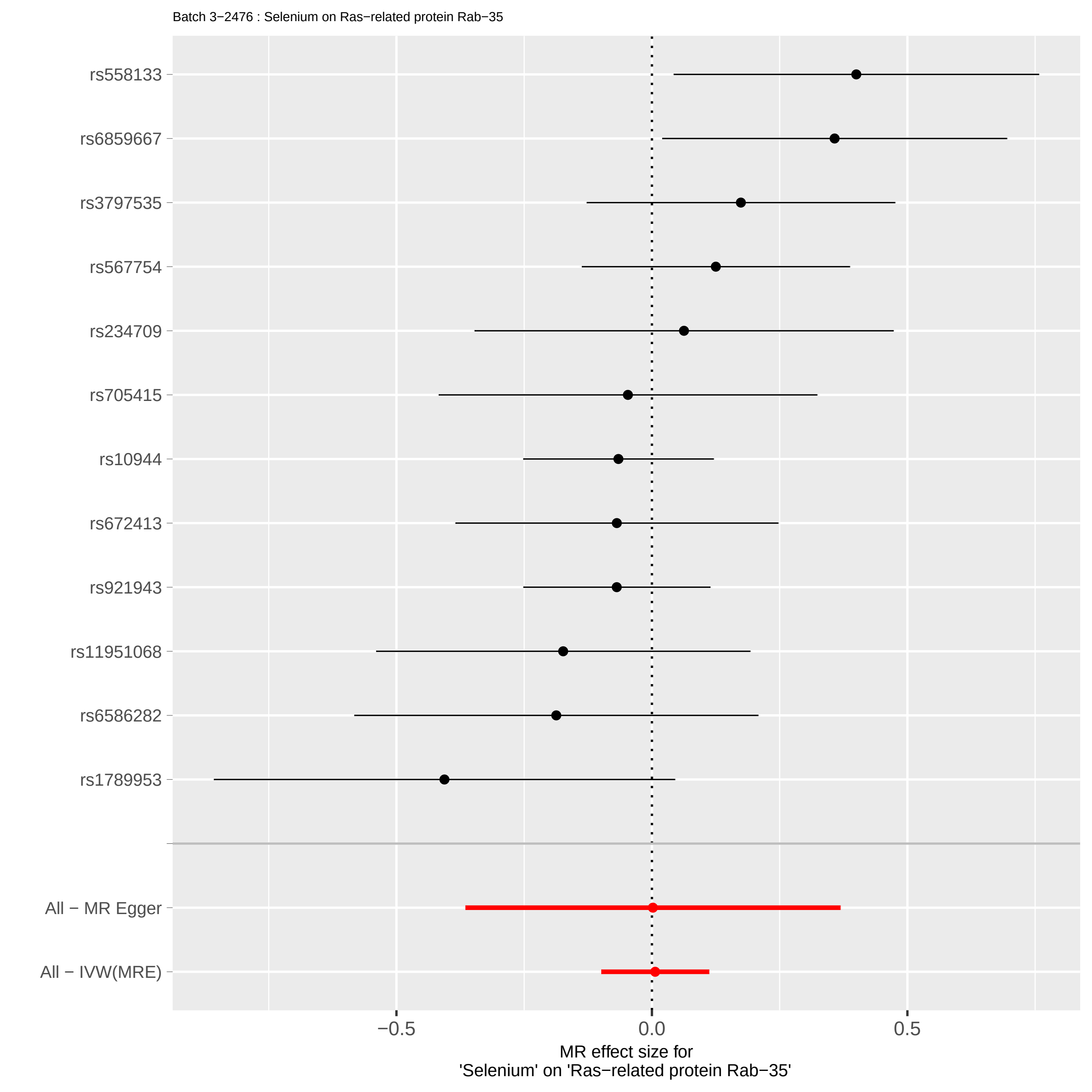

Batch 3−2476 : Selenium on Ras−related protein Rab−35
rs558133
rs6859667
rs3797535
rs567754
rs234709
rs705415
rs10944
rs672413
rs921943
rs11951068
rs6586282
rs1789953
All − MR Egger
All − IVW(MRE)
−0.5
0.0
0.5
MR effect size for
'Selenium' on 'Ras−related protein Rab−35'

Batch 3−2477 : Selenium on Ras−related protein Rab−39B

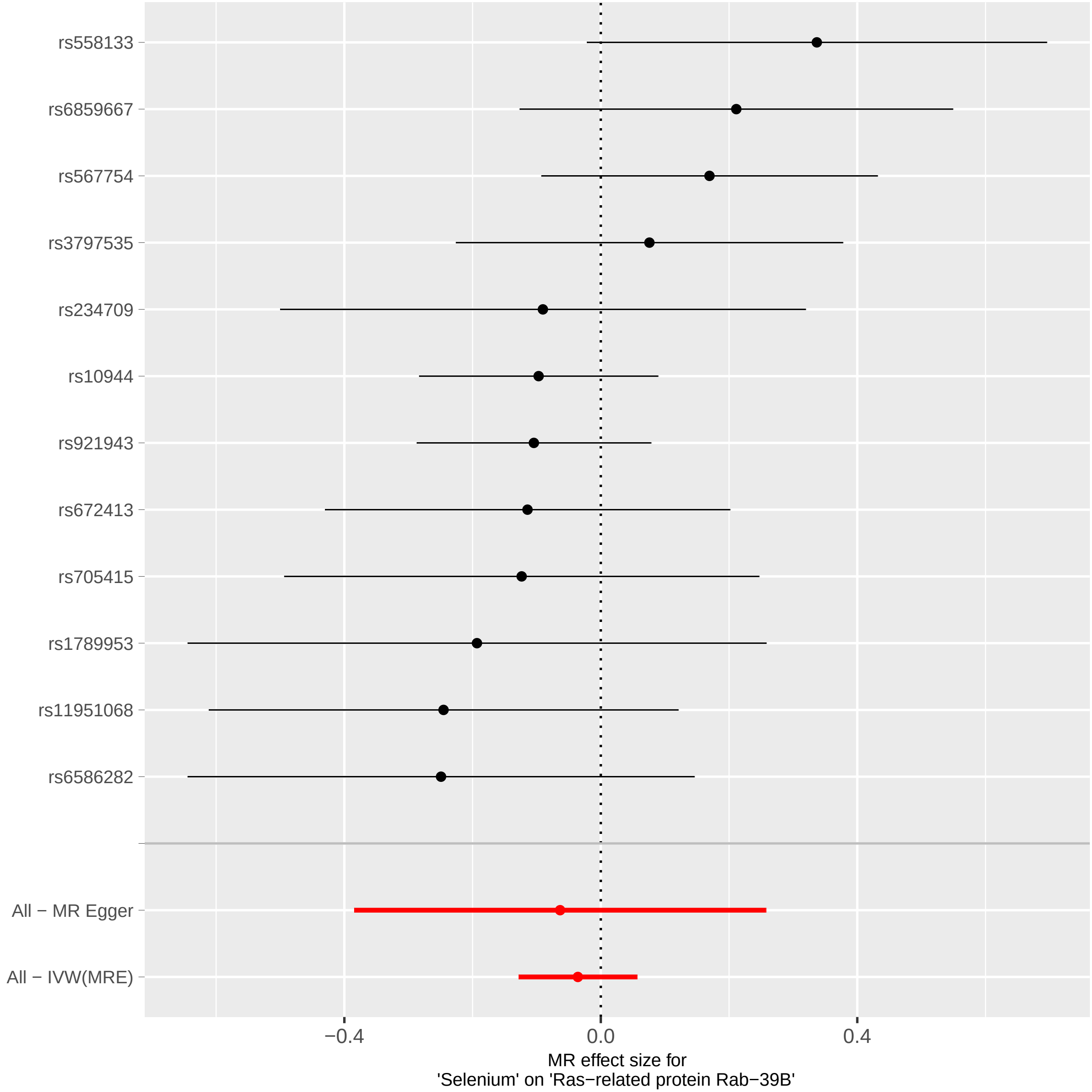

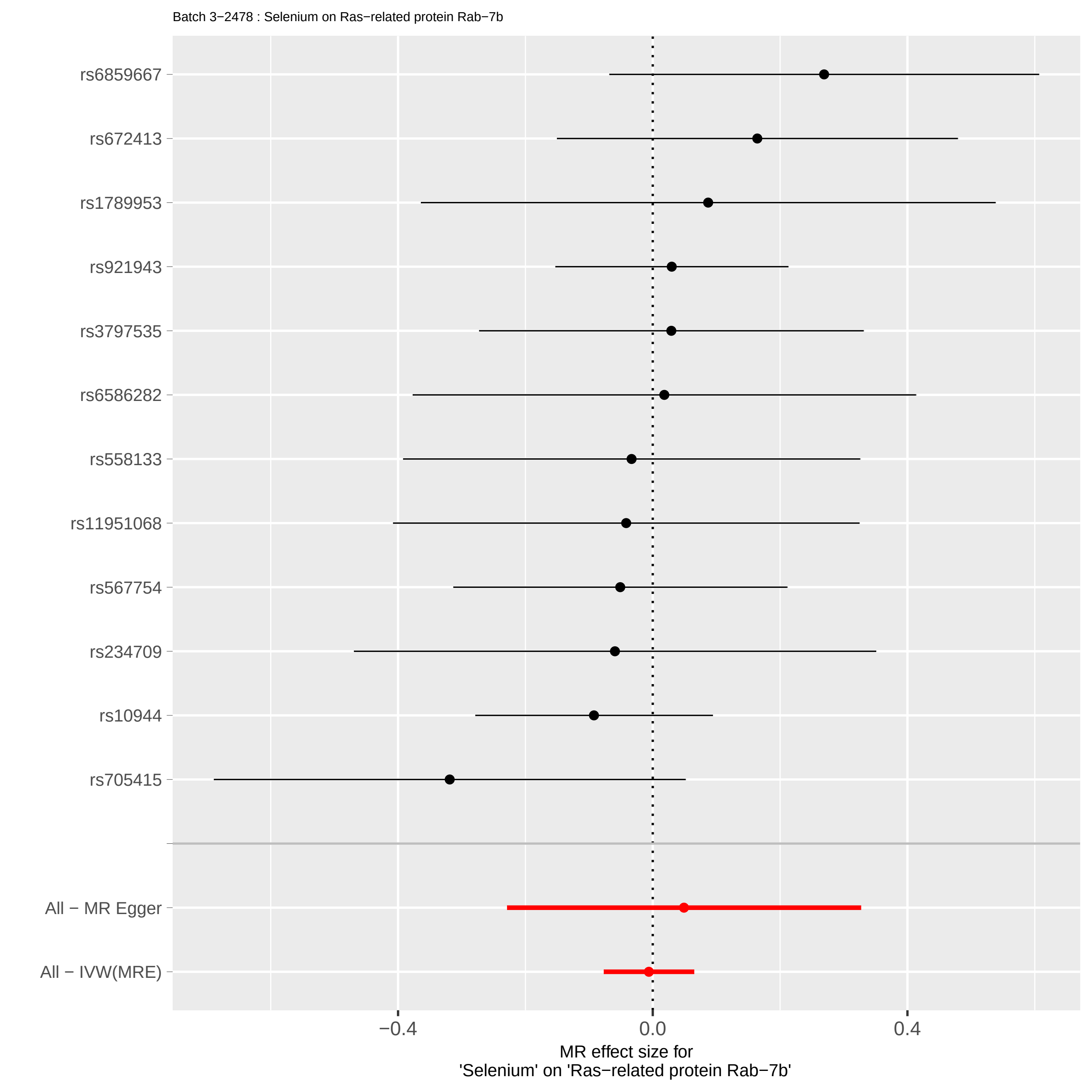
Batch 3−2478 : Selenium on Ras−related protein Rab−7b
rs6859667
rs672413
rs1789953
rs921943
rs3797535
rs6586282
rs558133
rs11951068
rs567754
rs234709
rs10944
rs705415
All − MR Egger
All − IVW(MRE)
−0.4
0.0
0.4
MR effect size for
'Selenium' on 'Ras−related protein Rab−7b'

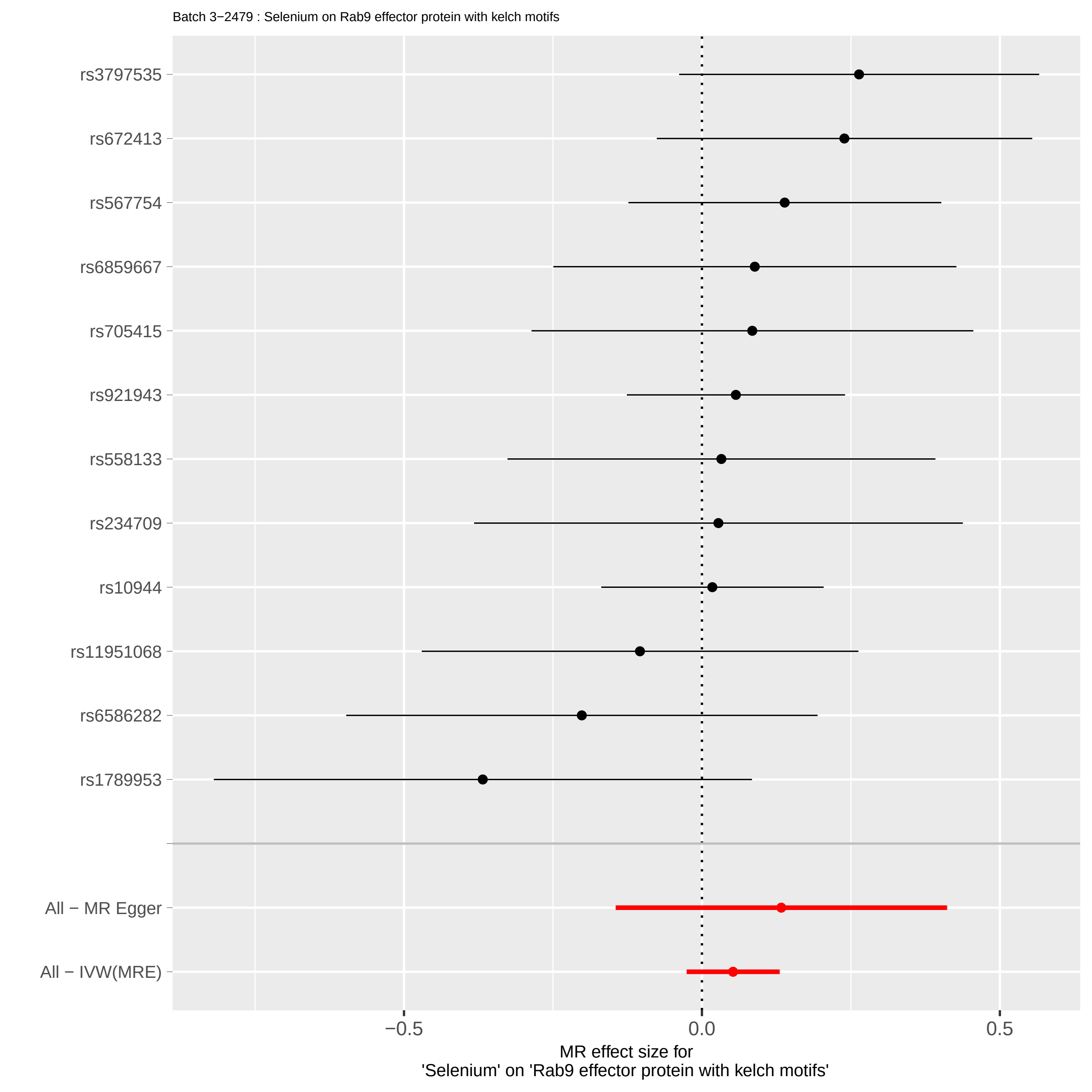

Batch 3−2479 : Selenium on Rab9 effector protein with kelch motifs
rs3797535
rs672413
rs567754
rs6859667
rs705415
rs921943
rs558133
rs234709
rs10944
rs11951068
rs6586282
rs1789953
All − MR Egger
All − IVW(MRE)
−0.5
0.0
0.5
MR effect size for
'Selenium' on 'Rab9 effector protein with kelch motifs'

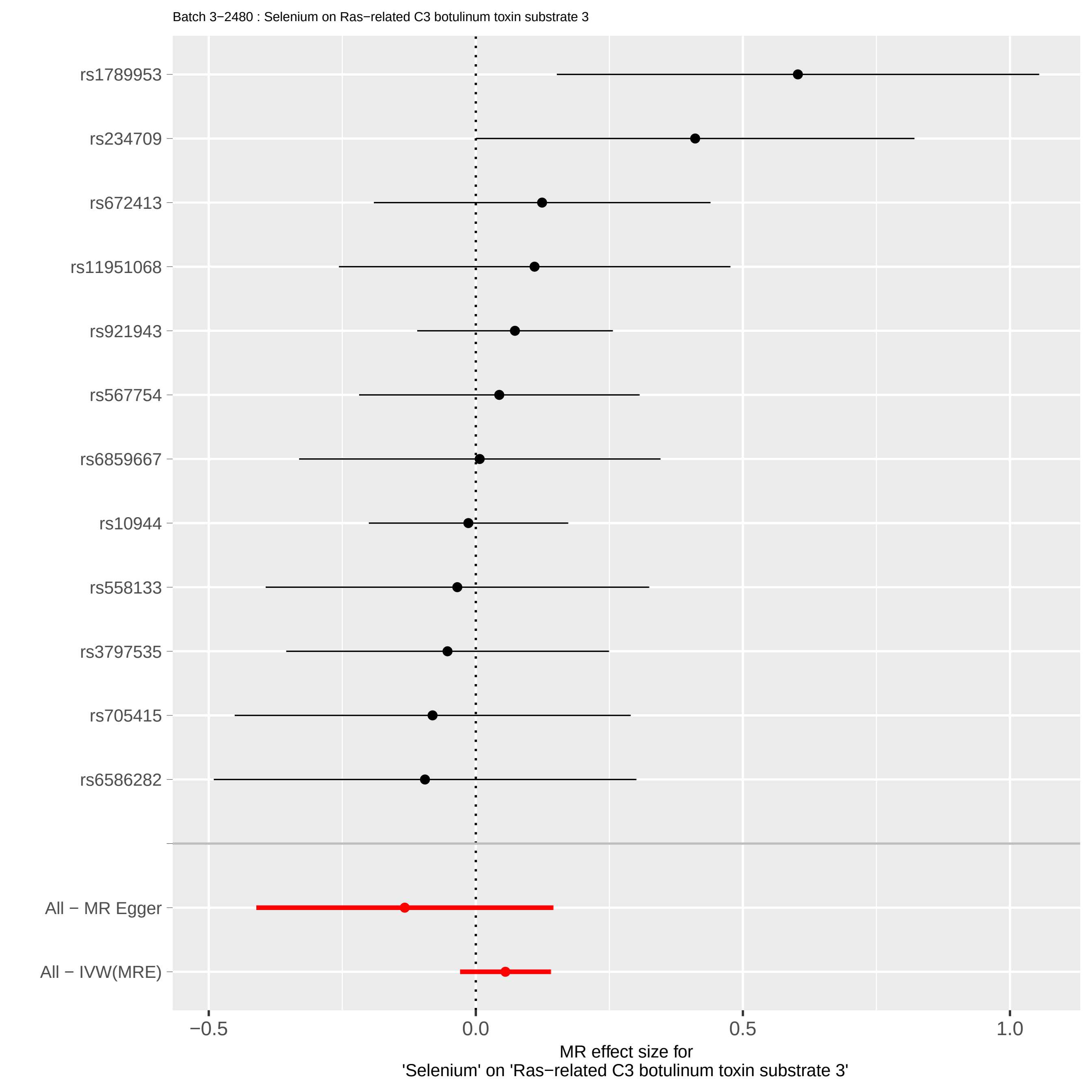

Batch 3−2480 : Selenium on Ras−related C3 botulinum toxin substrate 3
rs1789953
rs234709
rs672413
rs11951068
rs921943
rs567754
rs6859667
rs10944
rs558133
rs3797535
rs705415
rs6586282
All − MR Egger
All − IVW(MRE)
−0.5
0.0
0.5
1.0
MR effect size for
'Selenium' on 'Ras−related C3 botulinum toxin substrate 3'

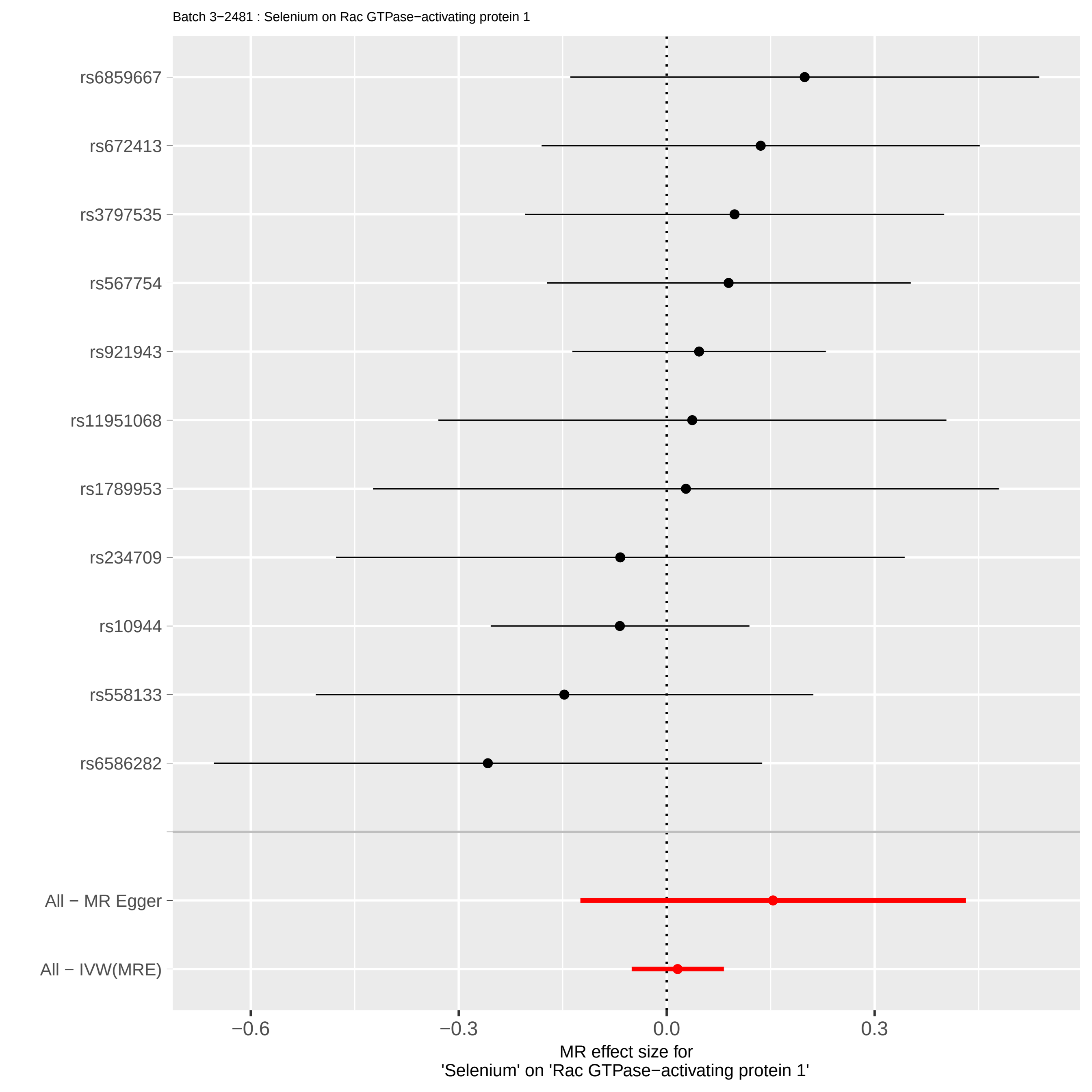
Batch 3−2481 : Selenium on Rac GTPase−activating protein 1
rs6859667
rs672413
rs3797535
rs567754
rs921943
rs11951068
rs1789953
rs234709
rs10944
rs558133
rs6586282
All − MR Egger
All − IVW(MRE)
−0.6
−0.3
0.0
0.3
MR effect size for
'Selenium' on 'Rac GTPase−activating protein 1'

Batch 3−2482 : Selenium on Cell cycle checkpoint protein RAD1

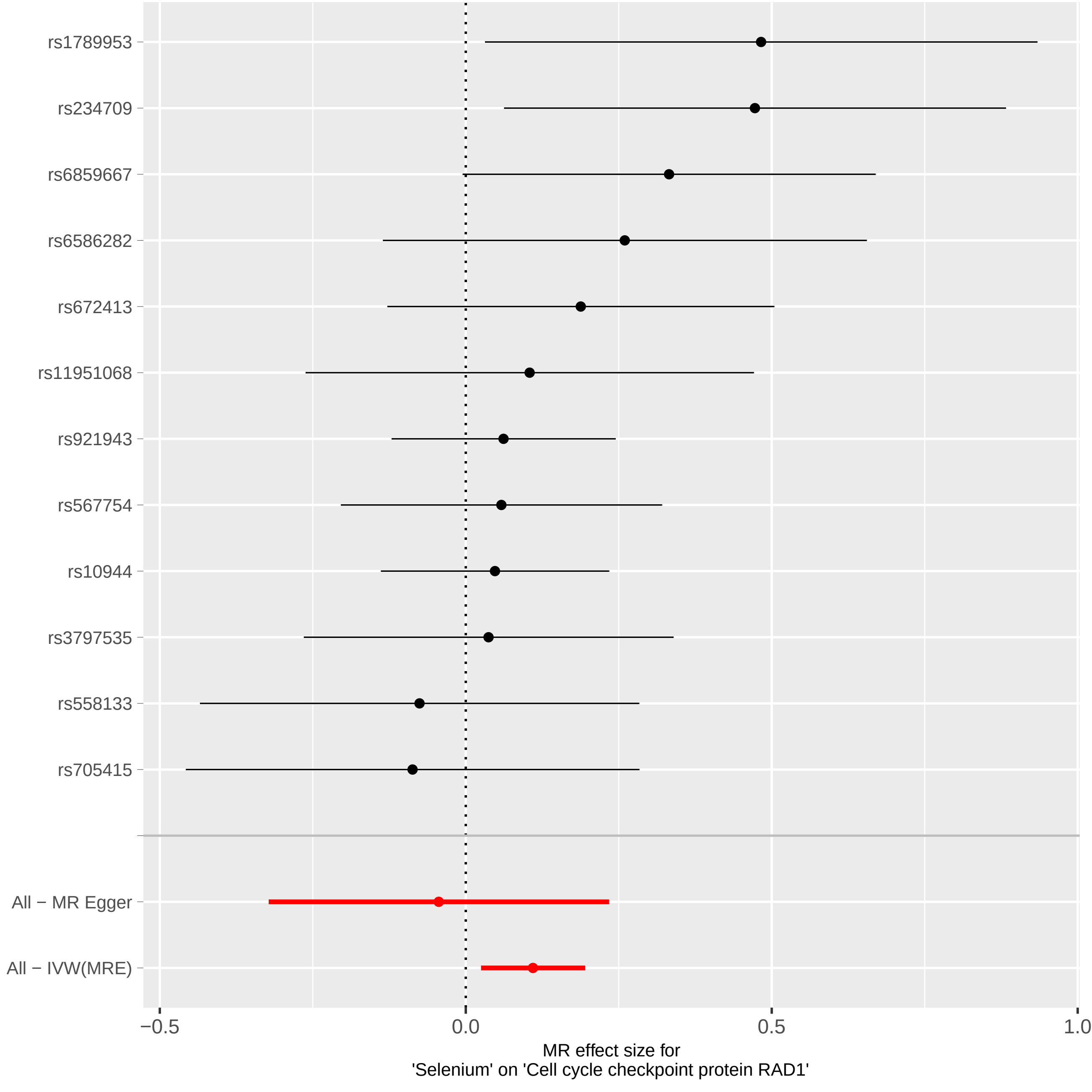

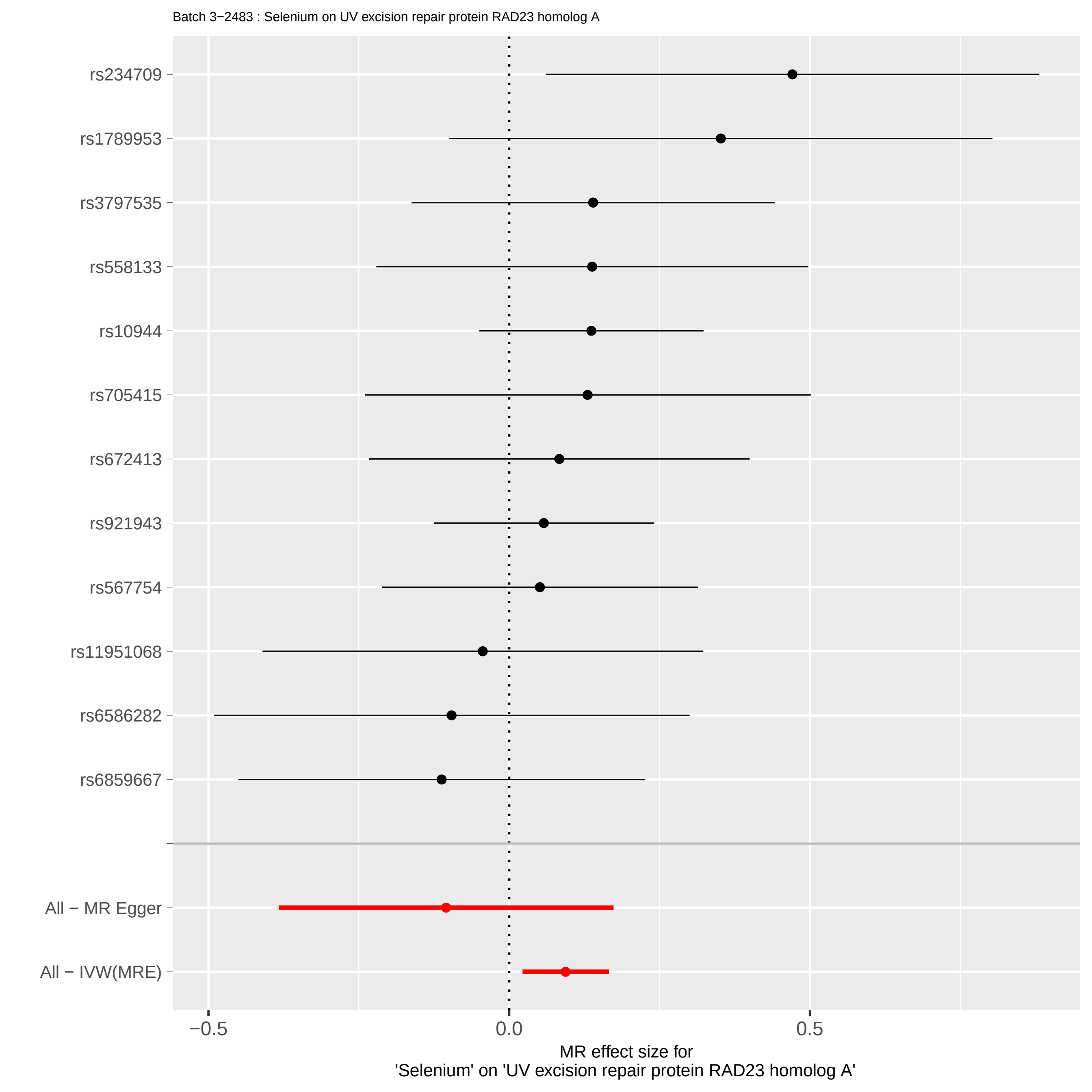

Batch 3−2483 : Selenium on UV excision repair protein RAD23 homolog A
rs234709
rs1789953
rs3797535
rs558133
rs10944
rs705415
rs672413
rs921943
rs567754
rs11951068
rs6586282
rs6859667
All − MR Egger
All − IVW(MRE)
−0.5
0.0
0.5
MR effect size for
'Selenium' on 'UV excision repair protein RAD23 homolog A'

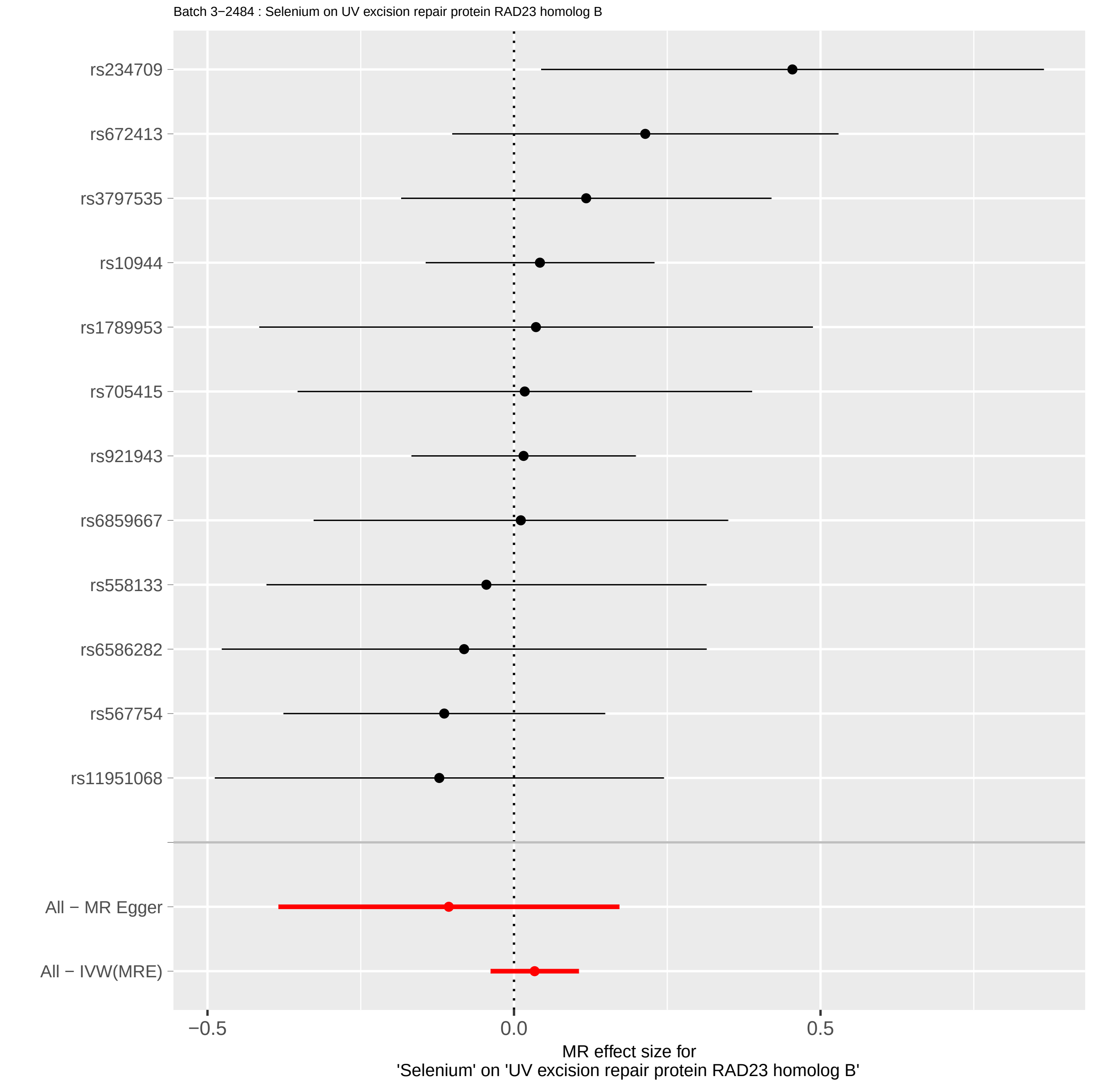

Batch 3−2484 : Selenium on UV excision repair protein RAD23 homolog B
rs234709
rs672413
rs3797535
rs10944
rs1789953
rs705415
rs921943
rs6859667
rs558133
rs6586282
rs567754
rs11951068
All − MR Egger
All − IVW(MRE)
−0.5
0.0
0.5
MR effect size for
'Selenium' on 'UV excision repair protein RAD23 homolog B'

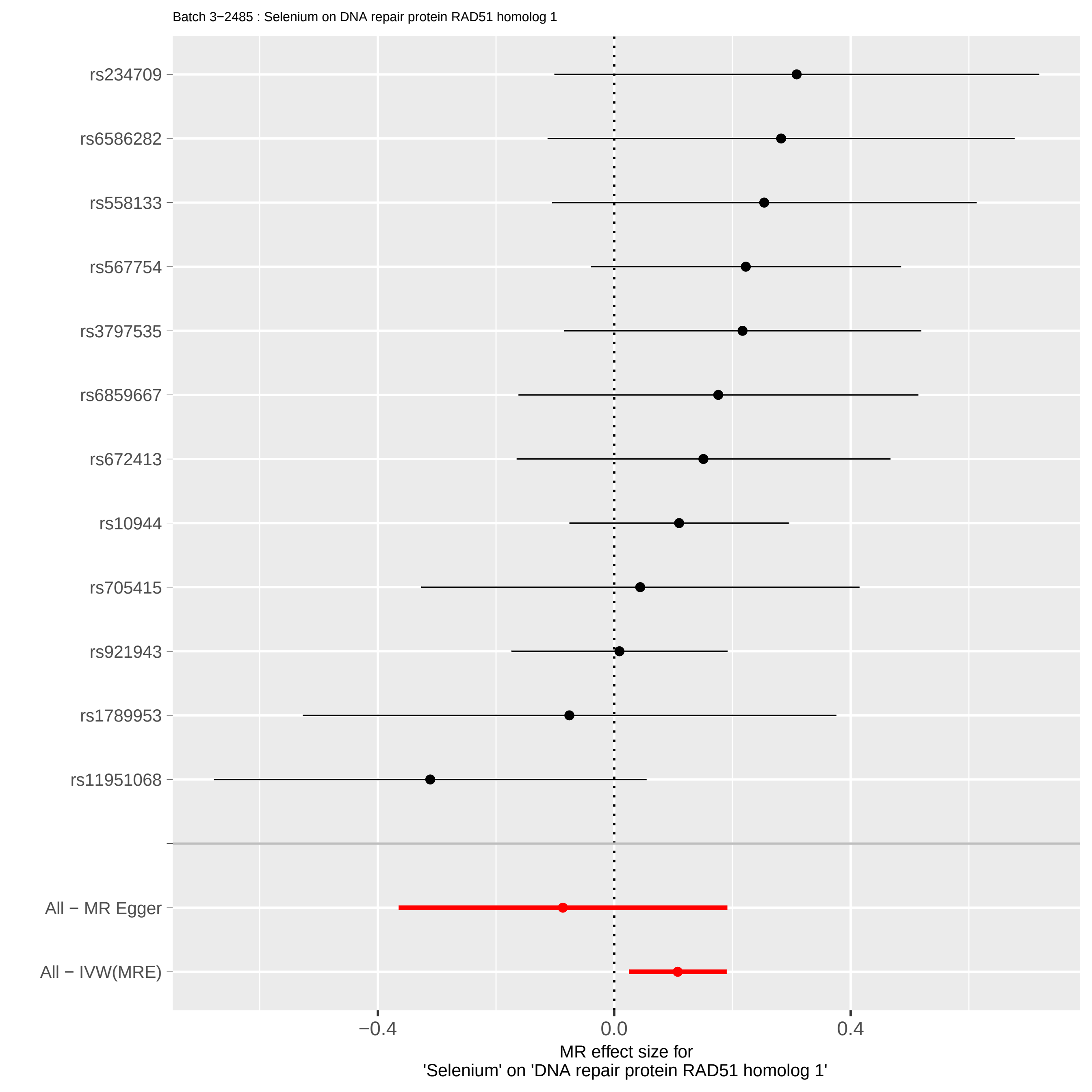

Batch 3−2485 : Selenium on DNA repair protein RAD51 homolog 1
rs234709
rs6586282
rs558133
rs567754
rs3797535
rs6859667
rs672413
rs10944
rs705415
rs921943
rs1789953
rs11951068
All − MR Egger
All − IVW(MRE)
−0.4
0.0
0.4
MR effect size for
'Selenium' on 'DNA repair protein RAD51 homolog 1'

Batch 3−2486 : Selenium on DNA repair protein RAD51 homolog 4

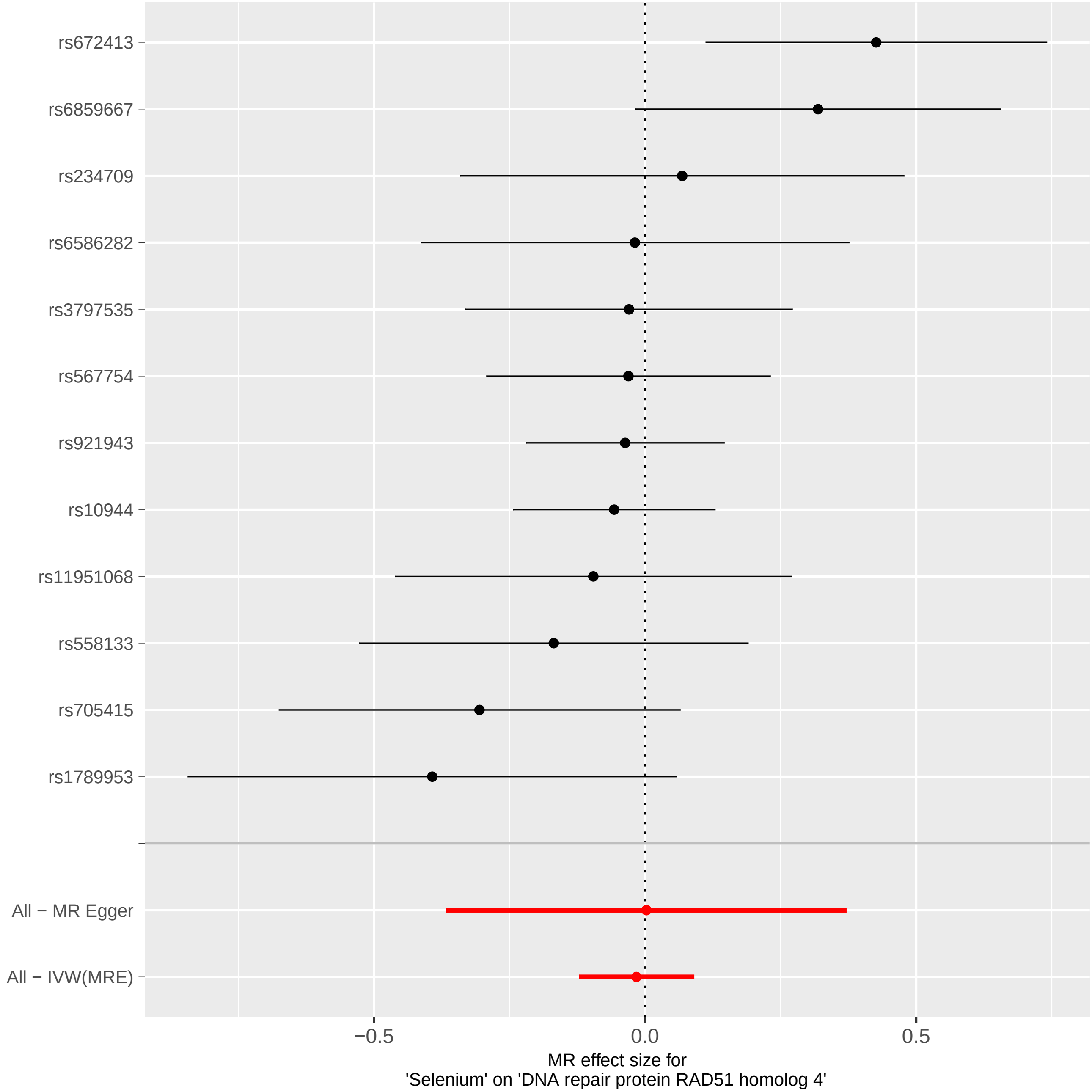

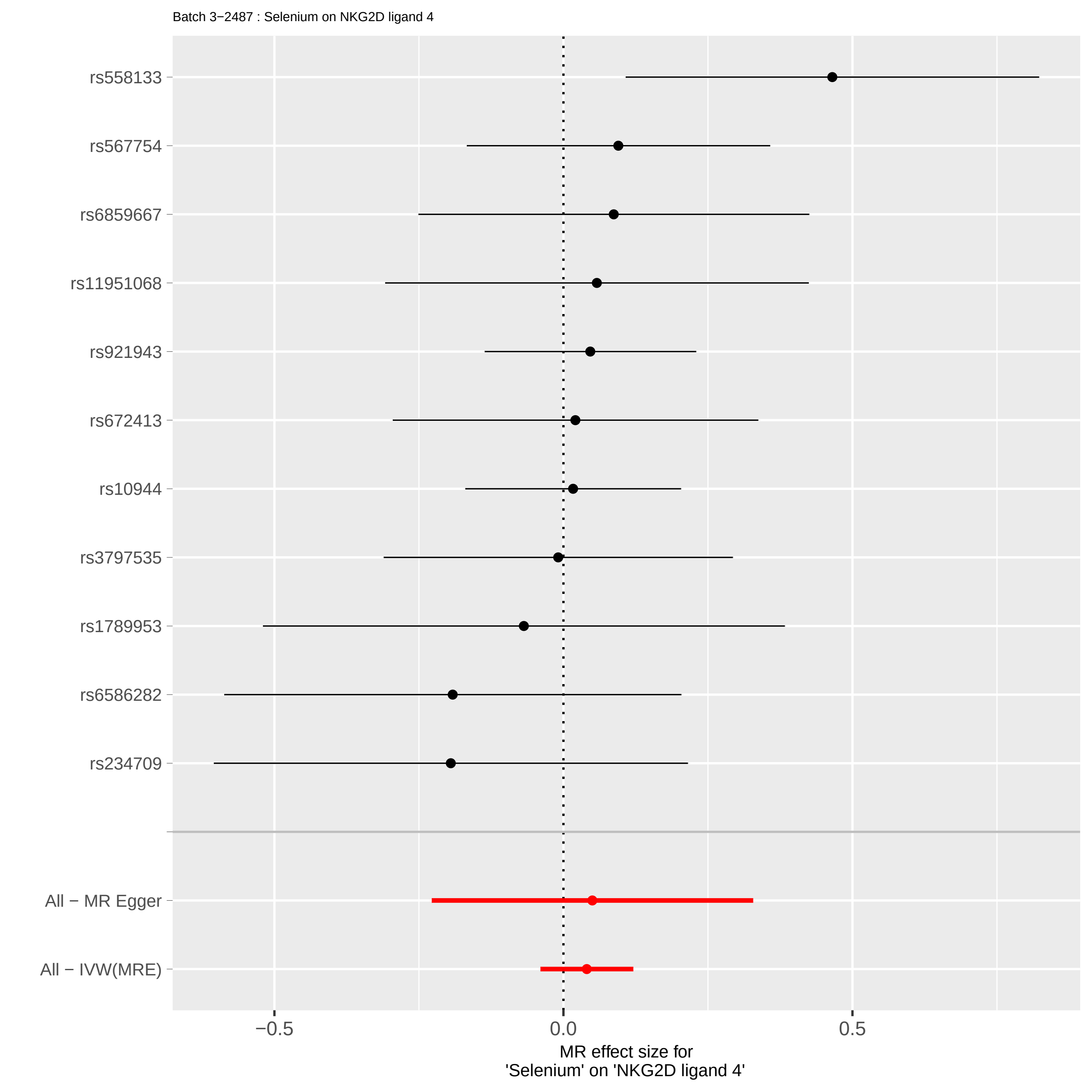
Batch 3−2487 : Selenium on NKG2D ligand 4
rs558133
rs567754
rs6859667
rs11951068
rs921943
rs672413
rs10944
rs3797535
rs1789953
rs6586282
rs234709
All − MR Egger
All − IVW(MRE)
−0.5
0.0
0.5
MR effect size for
'Selenium' on 'NKG2D ligand 4'

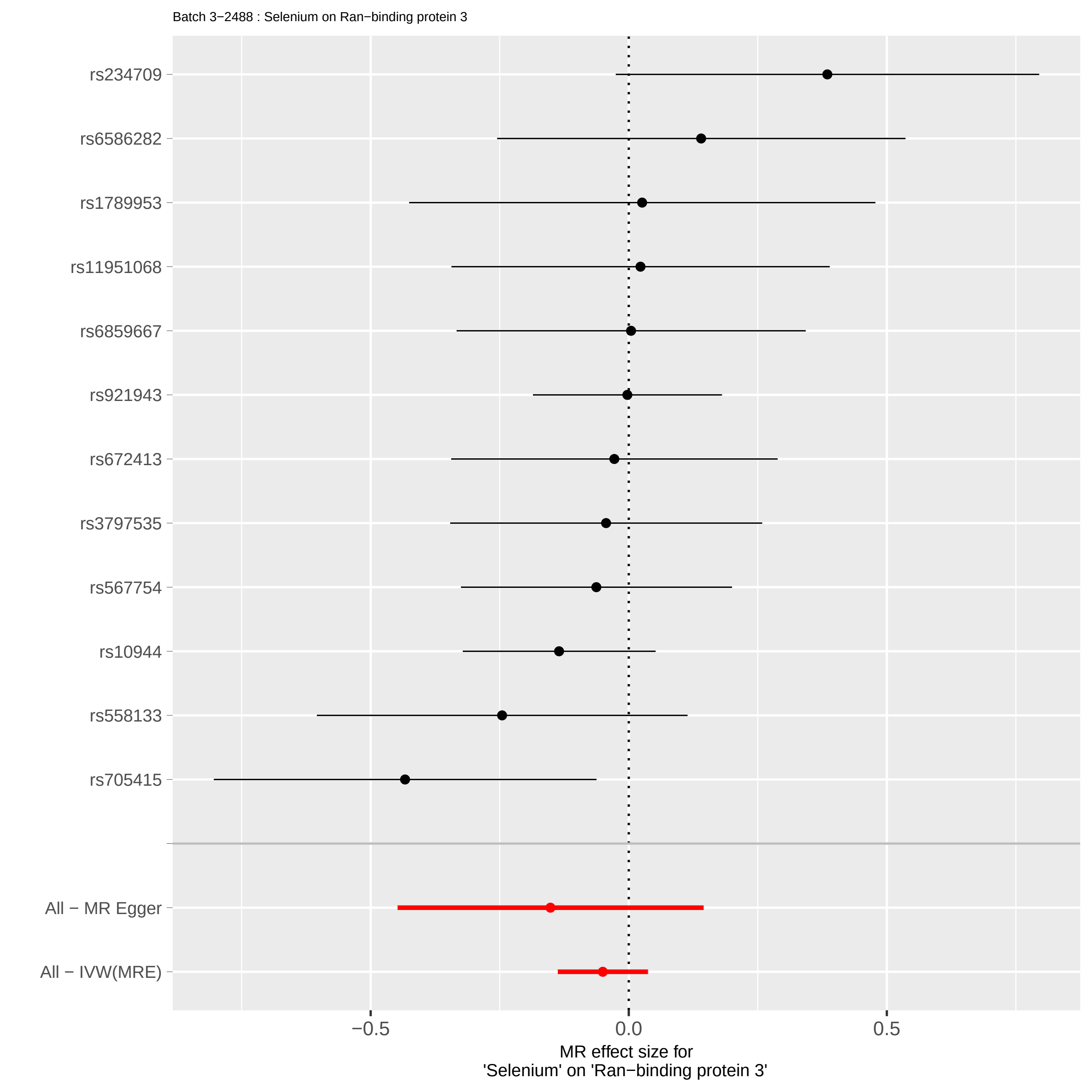

Batch 3−2488 : Selenium on Ran−binding protein 3
rs234709
rs6586282
rs1789953
rs11951068
rs6859667
rs921943
rs672413
rs3797535
rs567754
rs10944
rs558133
rs705415
All − MR Egger
All − IVW(MRE)
−0.5
0.0
0.5
MR effect size for
'Selenium' on 'Ran−binding protein 3'

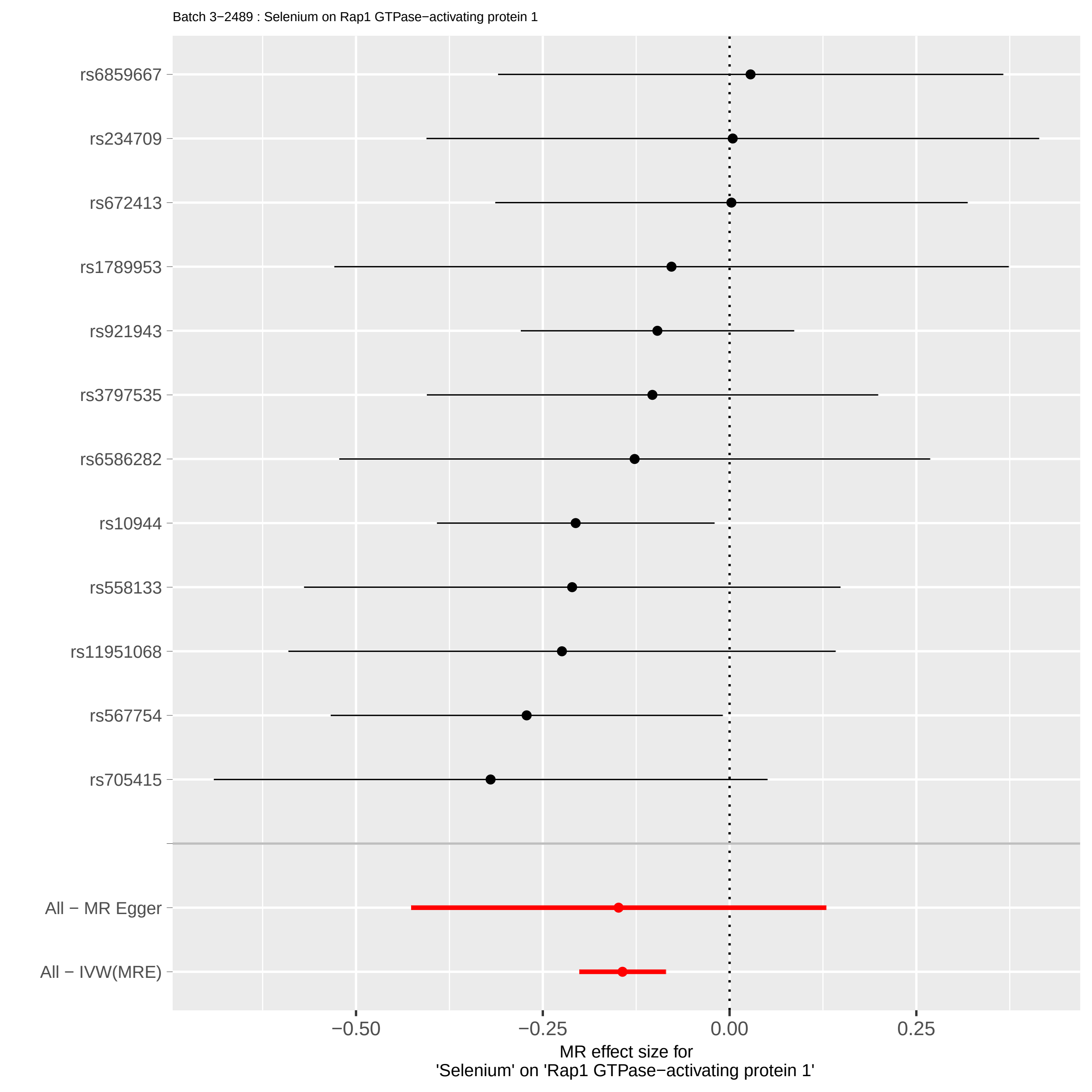

Batch 3−2489 : Selenium on Rap1 GTPase−activating protein 1
rs6859667
rs234709
rs672413
rs1789953
rs921943
rs3797535
rs6586282
rs10944
rs558133
rs11951068
rs567754
rs705415
All − MR Egger
All − IVW(MRE)
−0.50
−0.25
0.00
0.25
MR effect size for
'Selenium' on 'Rap1 GTPase−activating protein 1'

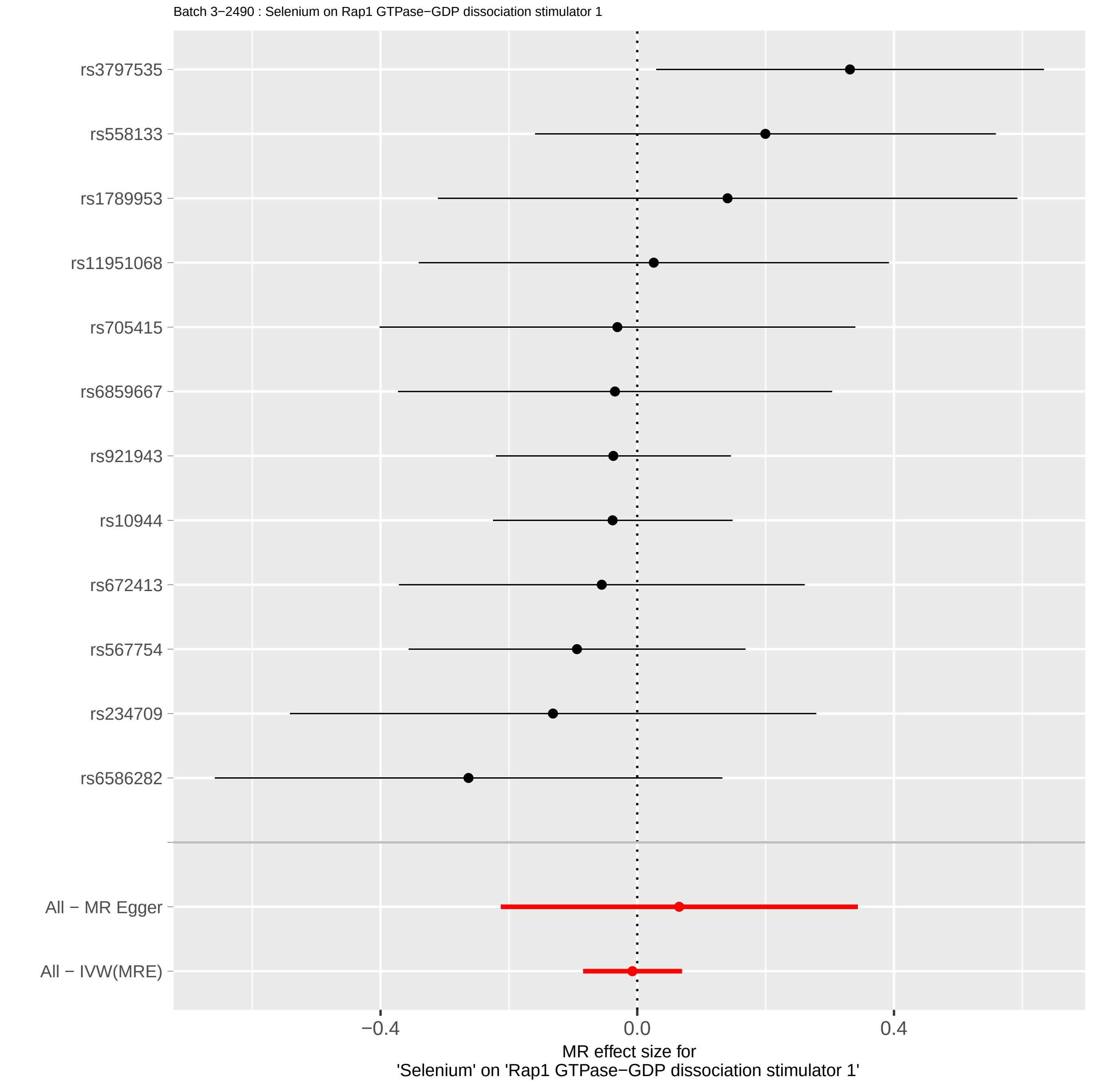

Batch 3−2490 : Selenium on Rap1 GTPase−GDP dissociation stimulator 1
rs3797535
rs558133
rs1789953
rs11951068
rs705415
rs6859667
rs921943
rs10944
rs672413
rs567754
rs234709
rs6586282
All − MR Egger
All − IVW(MRE)
−0.4
0.0
0.4
MR effect size for
'Selenium' on 'Rap1 GTPase−GDP dissociation stimulator 1'

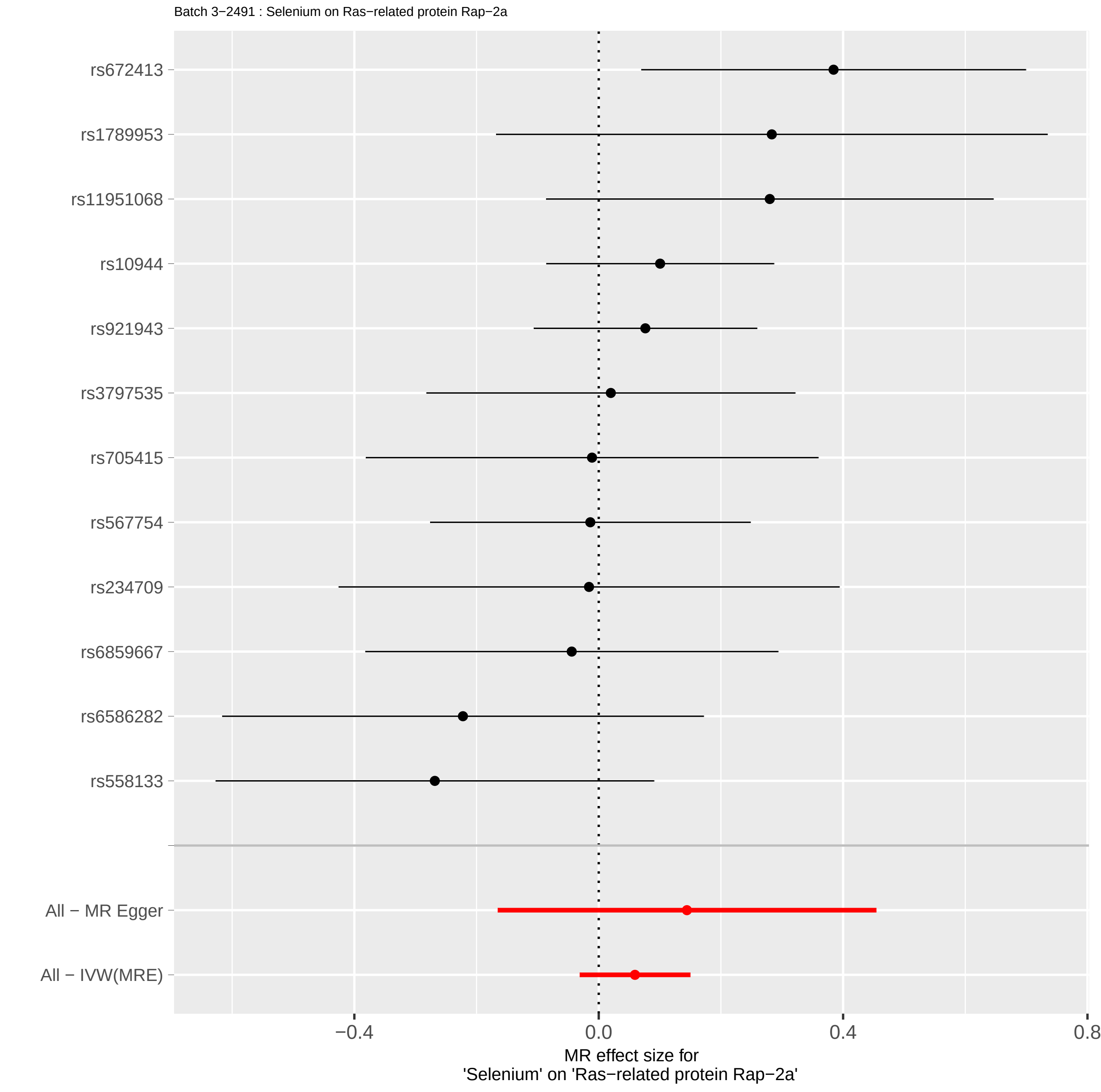
Batch 3−2491 : Selenium on Ras−related protein Rap−2a
rs672413
rs1789953
rs11951068
rs10944
rs921943
rs3797535
rs705415
rs567754
rs234709
rs6859667
rs6586282
rs558133
All − MR Egger
All − IVW(MRE)
−0.4
0.0
0.4
0.8
MR effect size for
'Selenium' on 'Ras−related protein Rap−2a'

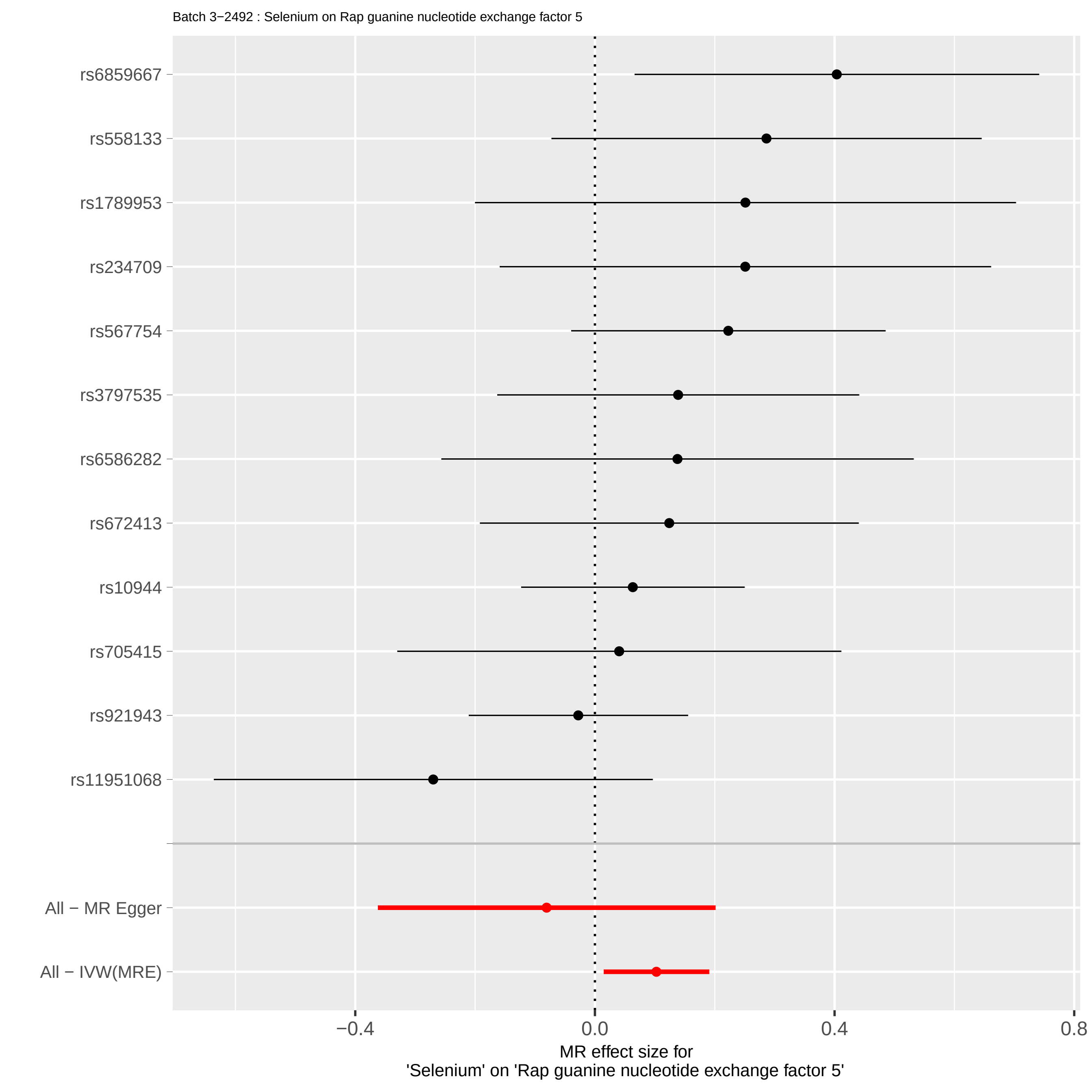

Batch 3−2492 : Selenium on Rap guanine nucleotide exchange factor 5
rs6859667
rs558133
rs1789953
rs234709
rs567754
rs3797535
rs6586282
rs672413
rs10944
rs705415
rs921943
rs11951068
All − MR Egger
All − IVW(MRE)
−0.4
0.0
0.4
0.8
MR effect size for
'Selenium' on 'Rap guanine nucleotide exchange factor 5'

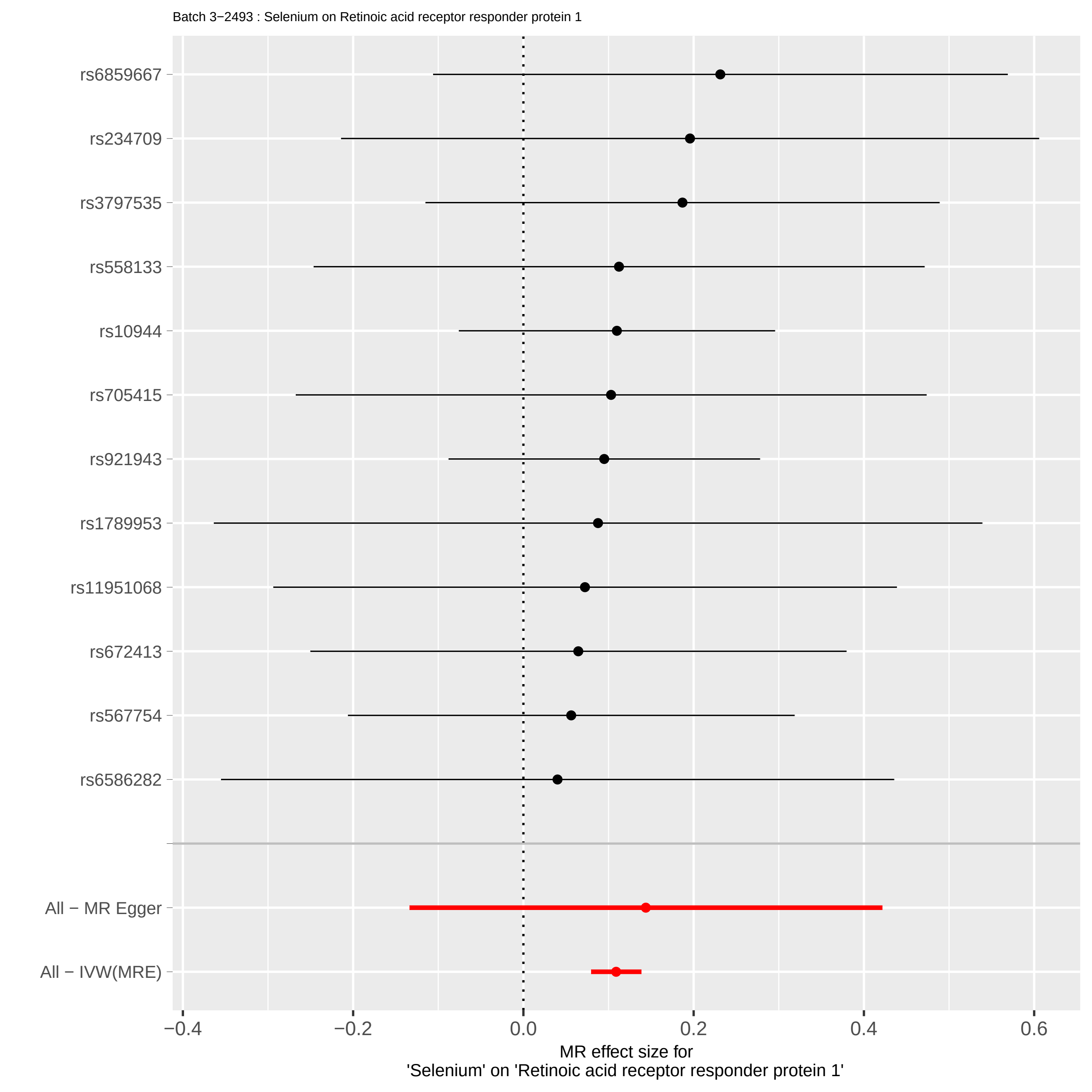

Batch 3−2493 : Selenium on Retinoic acid receptor responder protein 1
rs6859667
rs234709
rs3797535
rs558133
rs10944
rs705415
rs921943
rs1789953
rs11951068
rs672413
rs567754
rs6586282
All − MR Egger
All − IVW(MRE)
−0.4
−0.2
0.0
0.2
0.4
0.6
MR effect size for
'Selenium' on 'Retinoic acid receptor responder protein 1'

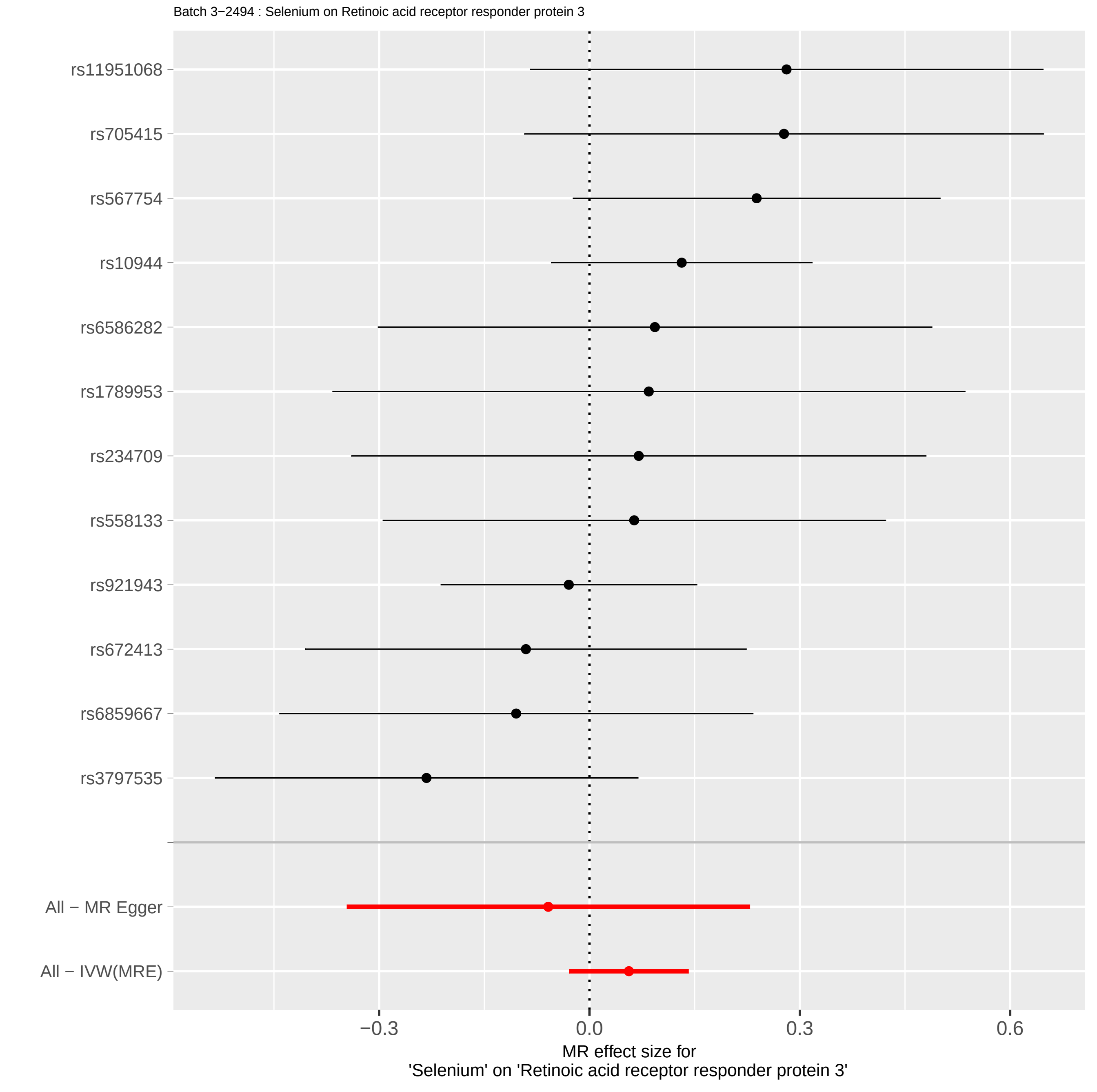
Batch 3−2494 : Selenium on Retinoic acid receptor responder protein 3
rs11951068
rs705415
rs567754
rs10944
rs6586282
rs1789953
rs234709
rs558133
rs921943
rs672413
rs6859667
rs3797535
All − MR Egger
All − IVW(MRE)
−0.3
0.0
0.3
0.6
MR effect size for
'Selenium' on 'Retinoic acid receptor responder protein 3'

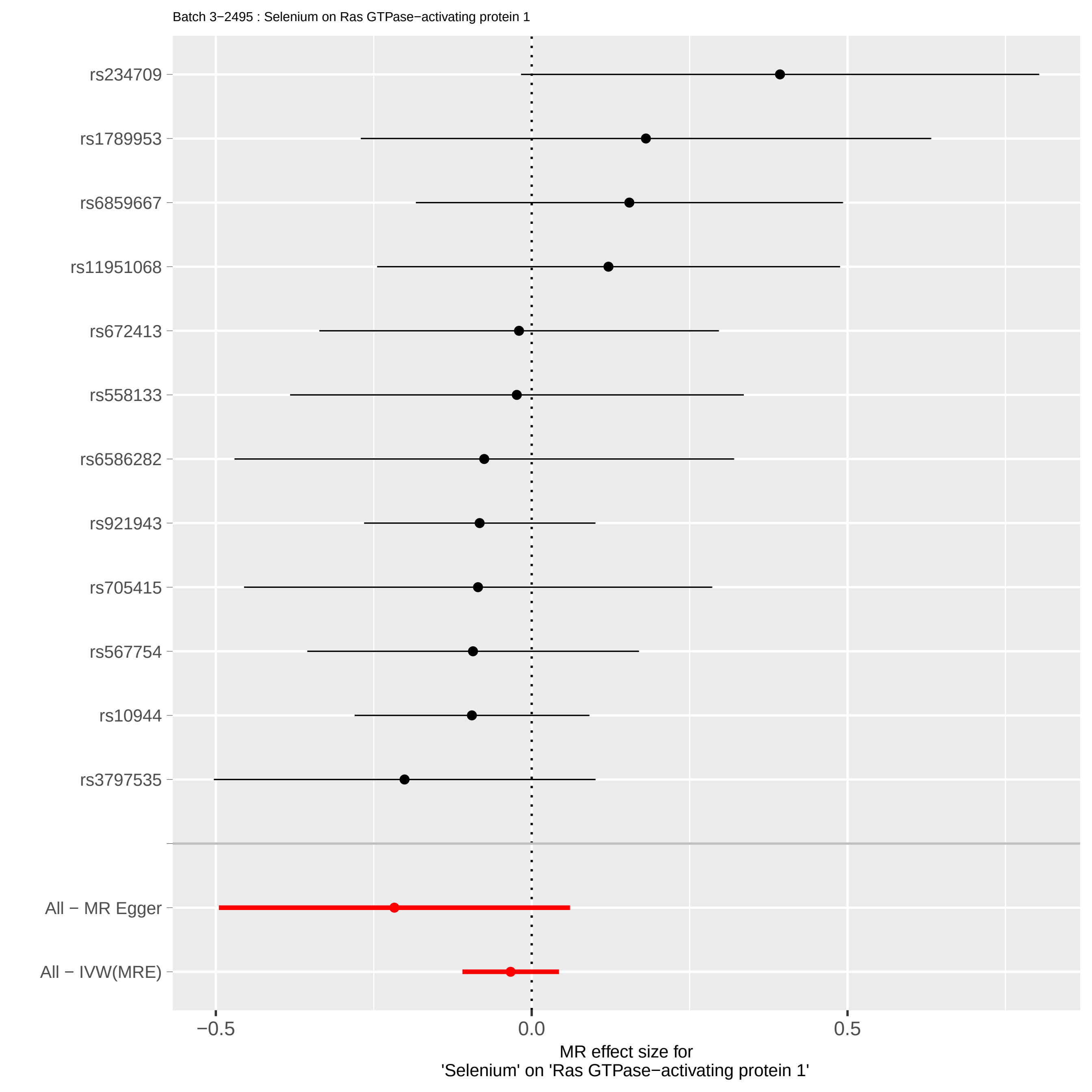

Batch 3−2495 : Selenium on Ras GTPase−activating protein 1
rs234709
rs1789953
rs6859667
rs11951068
rs672413
rs558133
rs6586282
rs921943
rs705415
rs567754
rs10944
rs3797535
All − MR Egger
All − IVW(MRE)
−0.5
0.0
0.5
MR effect size for
'Selenium' on 'Ras GTPase−activating protein 1'

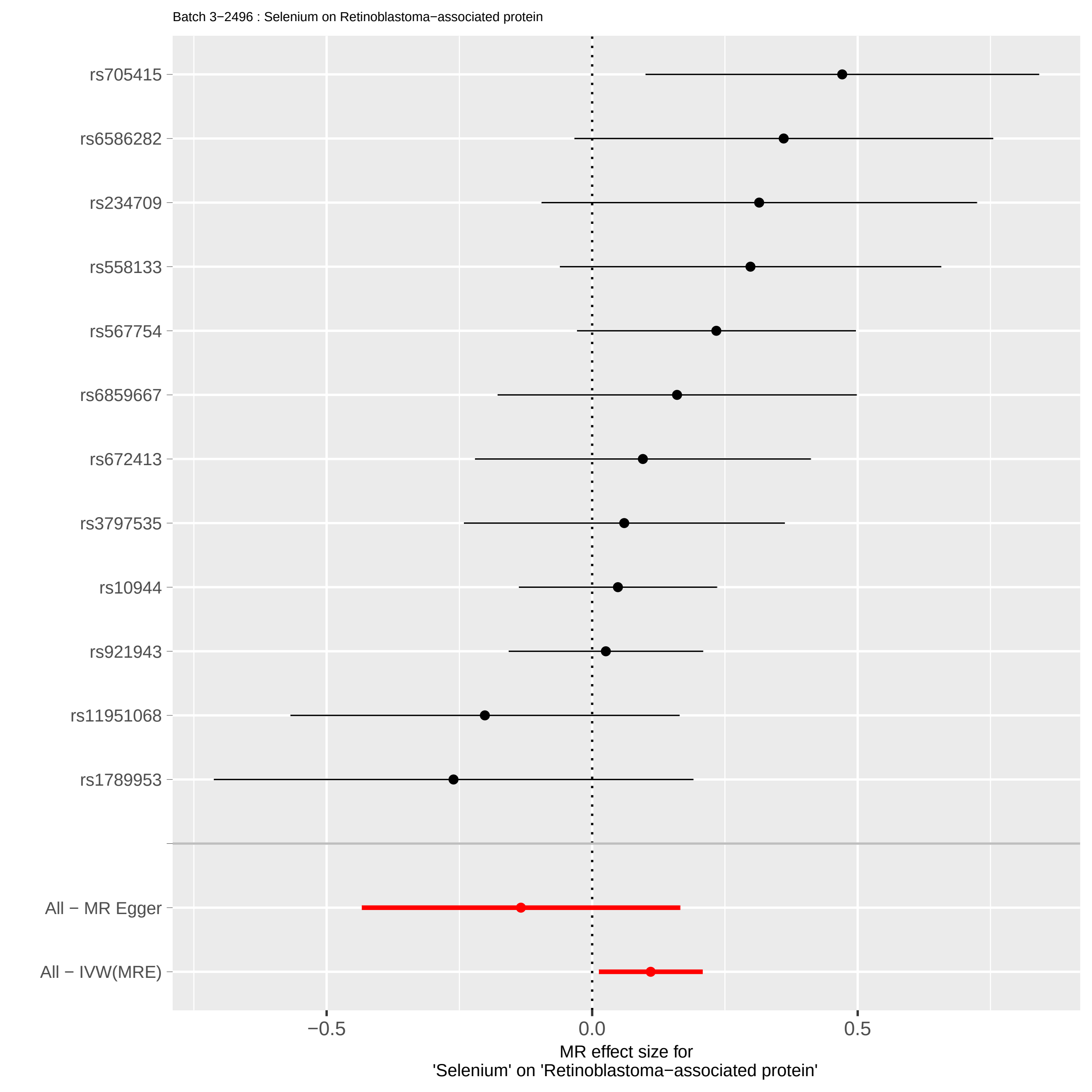

Batch 3−2496 : Selenium on Retinoblastoma−associated protein
rs705415
rs6586282
rs234709
rs558133
rs567754
rs6859667
rs672413
rs3797535
rs10944
rs921943
rs11951068
rs1789953
All − MR Egger
All − IVW(MRE)
−0.5
0.0
0.5
MR effect size for
'Selenium' on 'Retinoblastoma−associated protein'

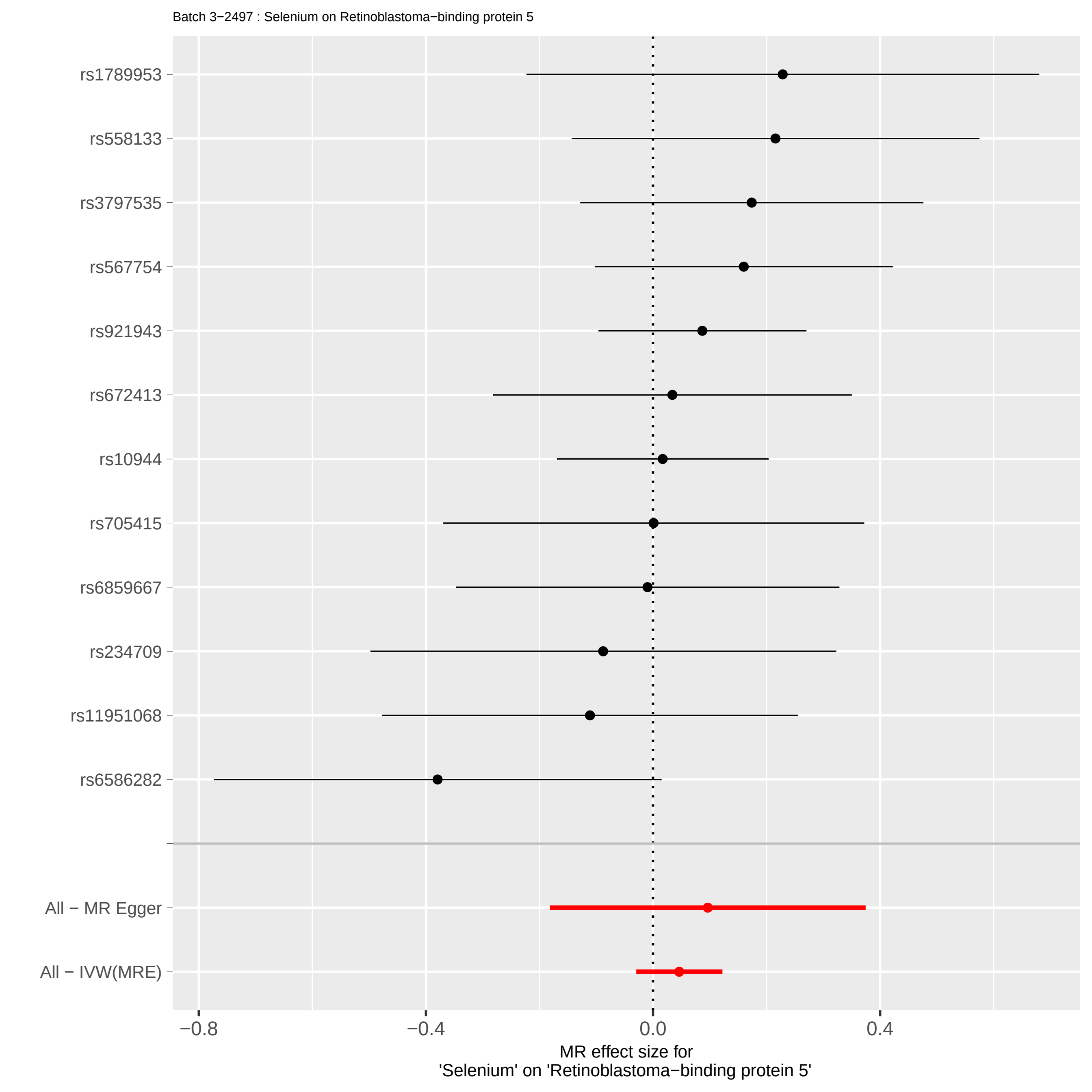

Batch 3−2497 : Selenium on Retinoblastoma−binding protein 5
rs1789953
rs558133
rs3797535
rs567754
rs921943
rs672413
rs10944
rs705415
rs6859667
rs234709
rs11951068
rs6586282
All − MR Egger
All − IVW(MRE)
−0.8
−0.4
0.0
0.4
MR effect size for
'Selenium' on 'Retinoblastoma−binding protein 5'

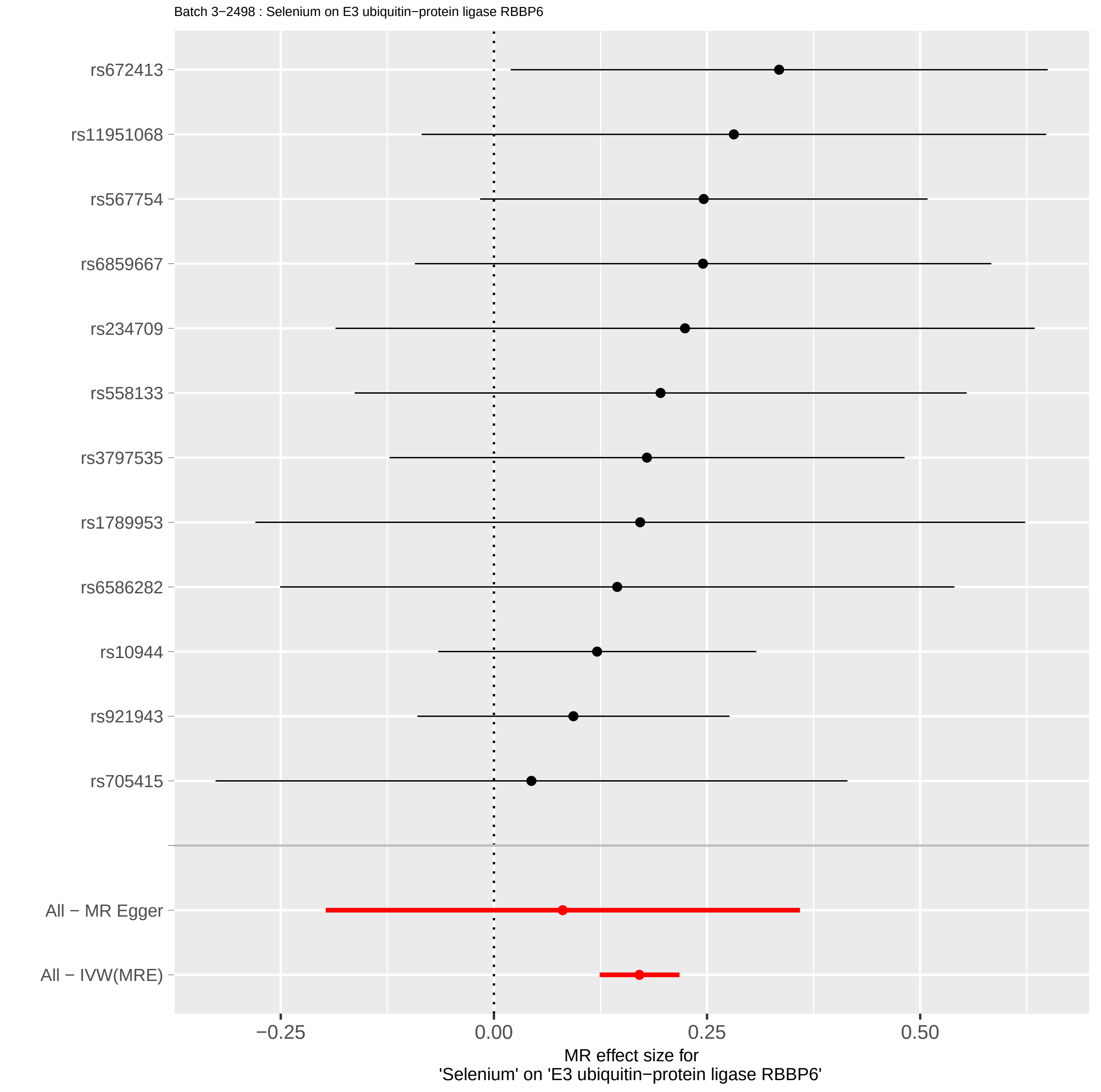

Batch 3−2498 : Selenium on E3 ubiquitin−protein ligase RBBP6
rs672413
rs11951068
rs567754
rs6859667
rs234709
rs558133
rs3797535
rs1789953
rs6586282
rs10944
rs921943
rs705415
All − MR Egger
All − IVW(MRE)
−0.25
0.00
0.25
0.50
MR effect size for
'Selenium' on 'E3 ubiquitin−protein ligase RBBP6'

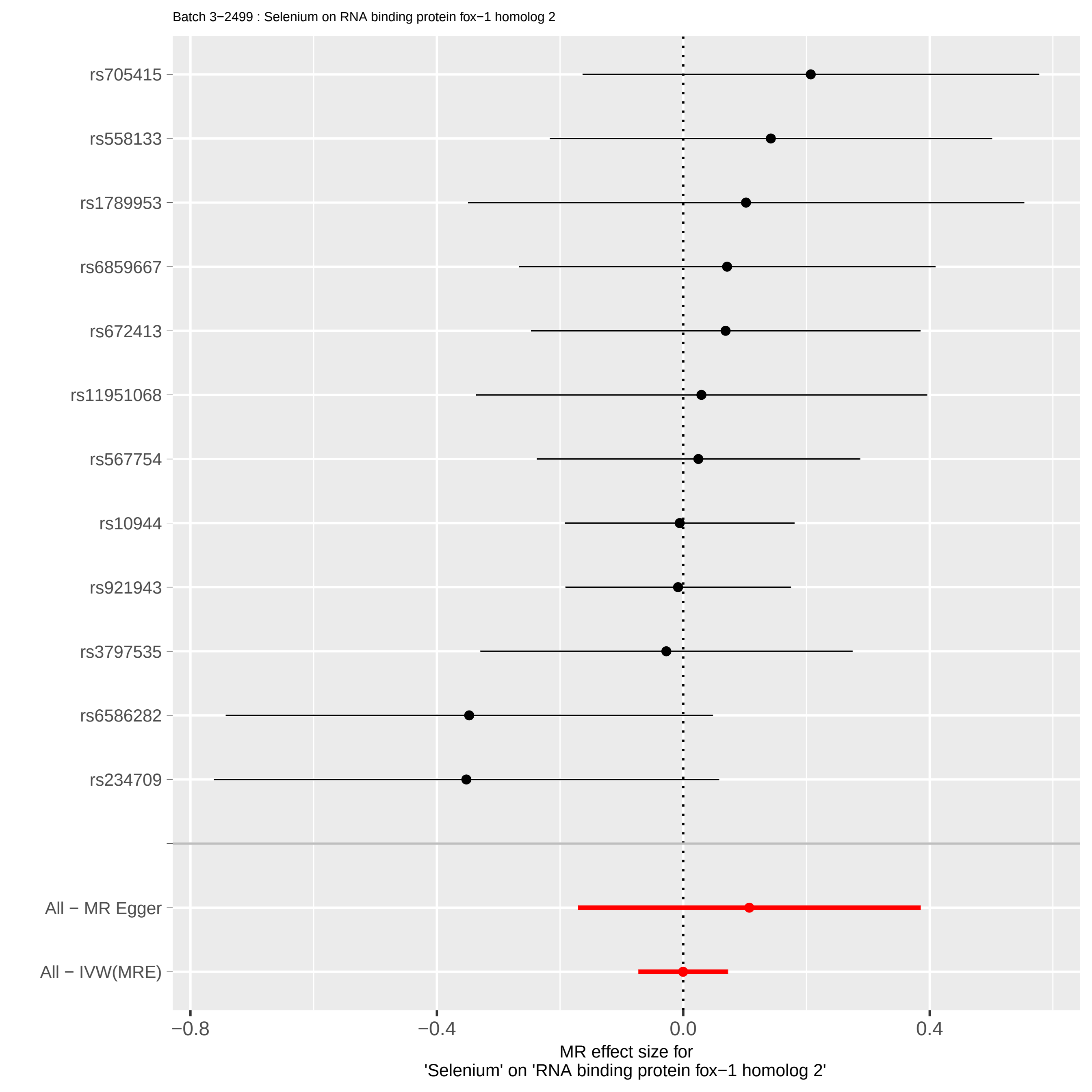
Batch 3−2499 : Selenium on RNA binding protein fox−1 homolog 2
rs705415
rs558133
rs1789953
rs6859667
rs672413
rs11951068
rs567754
rs10944
rs921943
rs3797535
rs6586282
rs234709
All − MR Egger
All − IVW(MRE)
−0.8
−0.4
0.0
0.4
MR effect size for
'Selenium' on 'RNA binding protein fox−1 homolog 2'

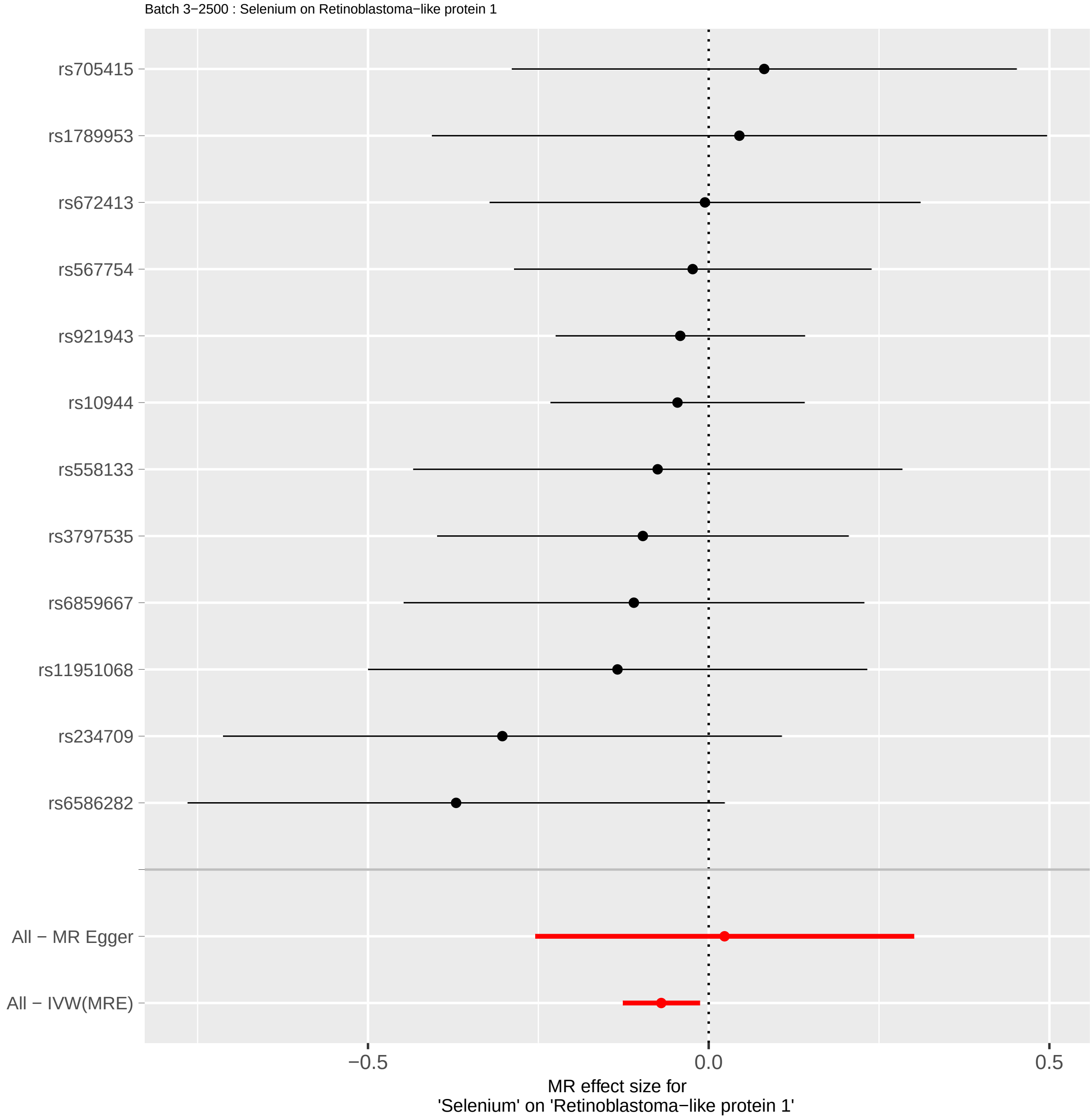
Batch 3−2500 : Selenium on Retinoblastoma−like protein 1
rs705415
rs1789953
rs672413
rs567754
rs921943
rs10944
rs558133
rs3797535
rs6859667
rs11951068
rs234709
rs6586282
All − MR Egger
All − IVW(MRE)
−0.5
0.0
0.5
MR effect size for
'Selenium' on 'Retinoblastoma−like protein 1'

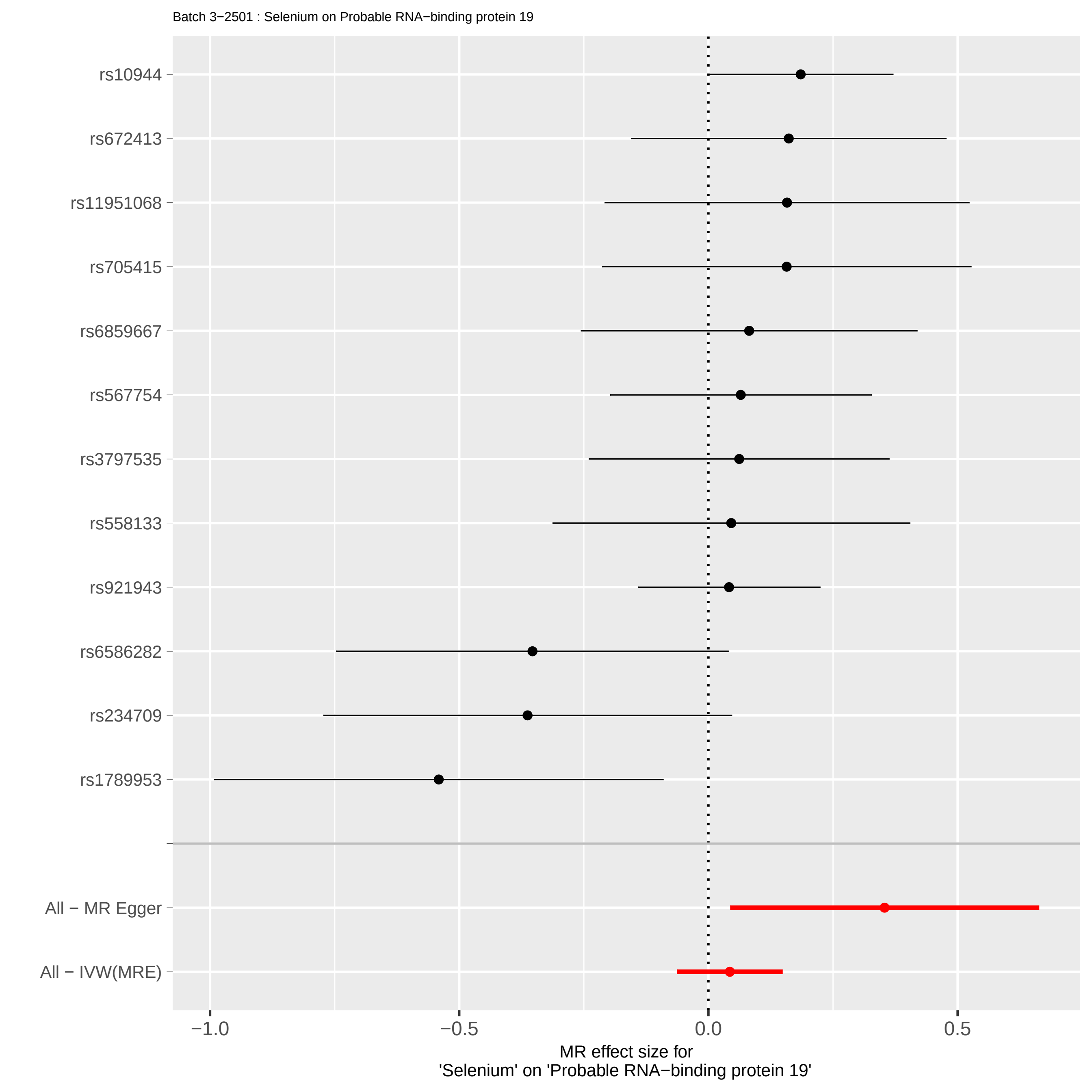

Batch 3−2501 : Selenium on Probable RNA−binding protein 19
rs10944
rs672413
rs11951068
rs705415
rs6859667
rs567754
rs3797535
rs558133
rs921943
rs6586282
rs234709
rs1789953
All − MR Egger
All − IVW(MRE)
−1.0
−0.5
0.0
0.5
MR effect size for
'Selenium' on 'Probable RNA−binding protein 19'

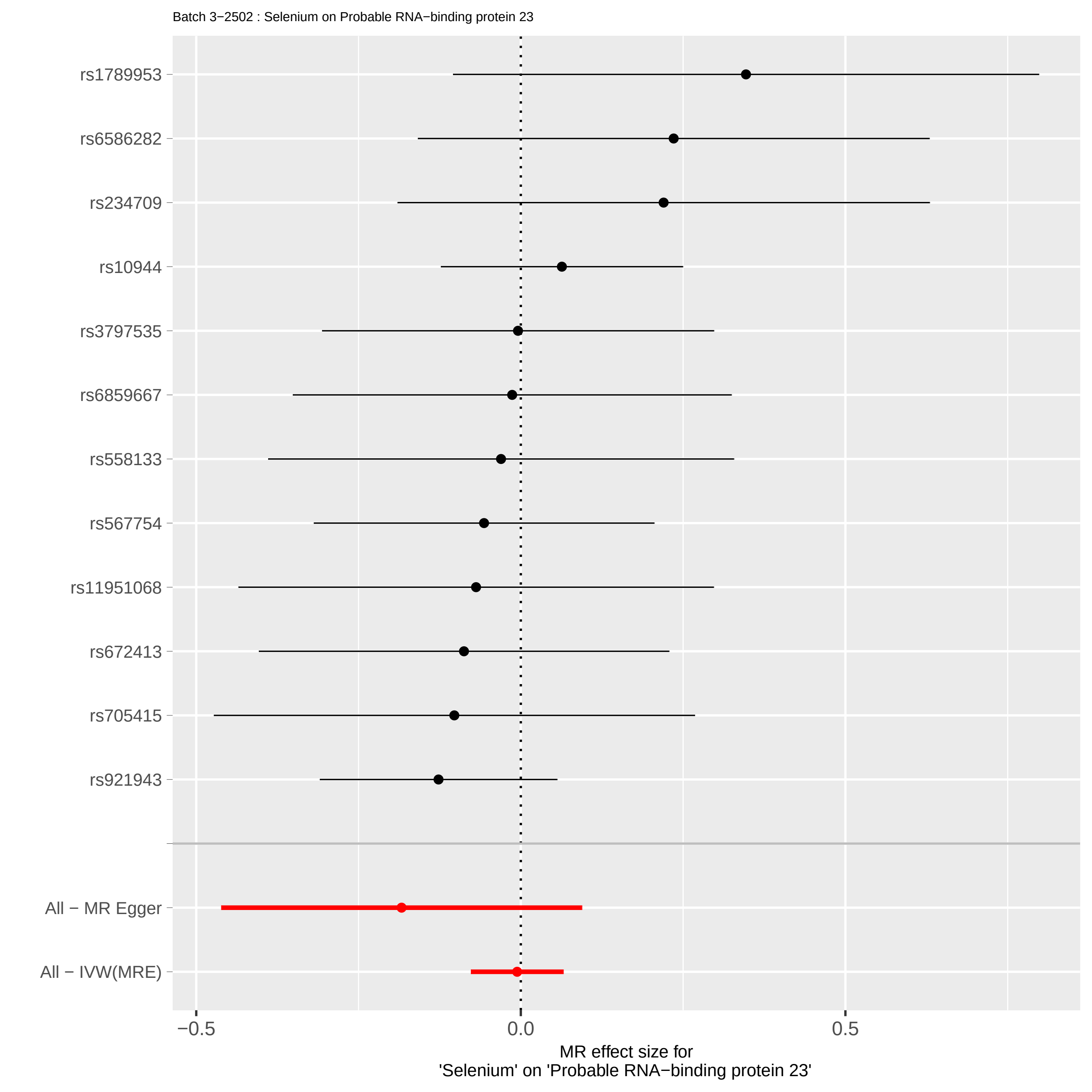
Batch 3−2502 : Selenium on Probable RNA−binding protein 23
rs1789953
rs6586282
rs234709
rs10944
rs3797535
rs6859667
rs558133
rs567754
rs11951068
rs672413
rs705415
rs921943
All − MR Egger
All − IVW(MRE)
−0.5
0.0
0.5
MR effect size for
'Selenium' on 'Probable RNA−binding protein 23'

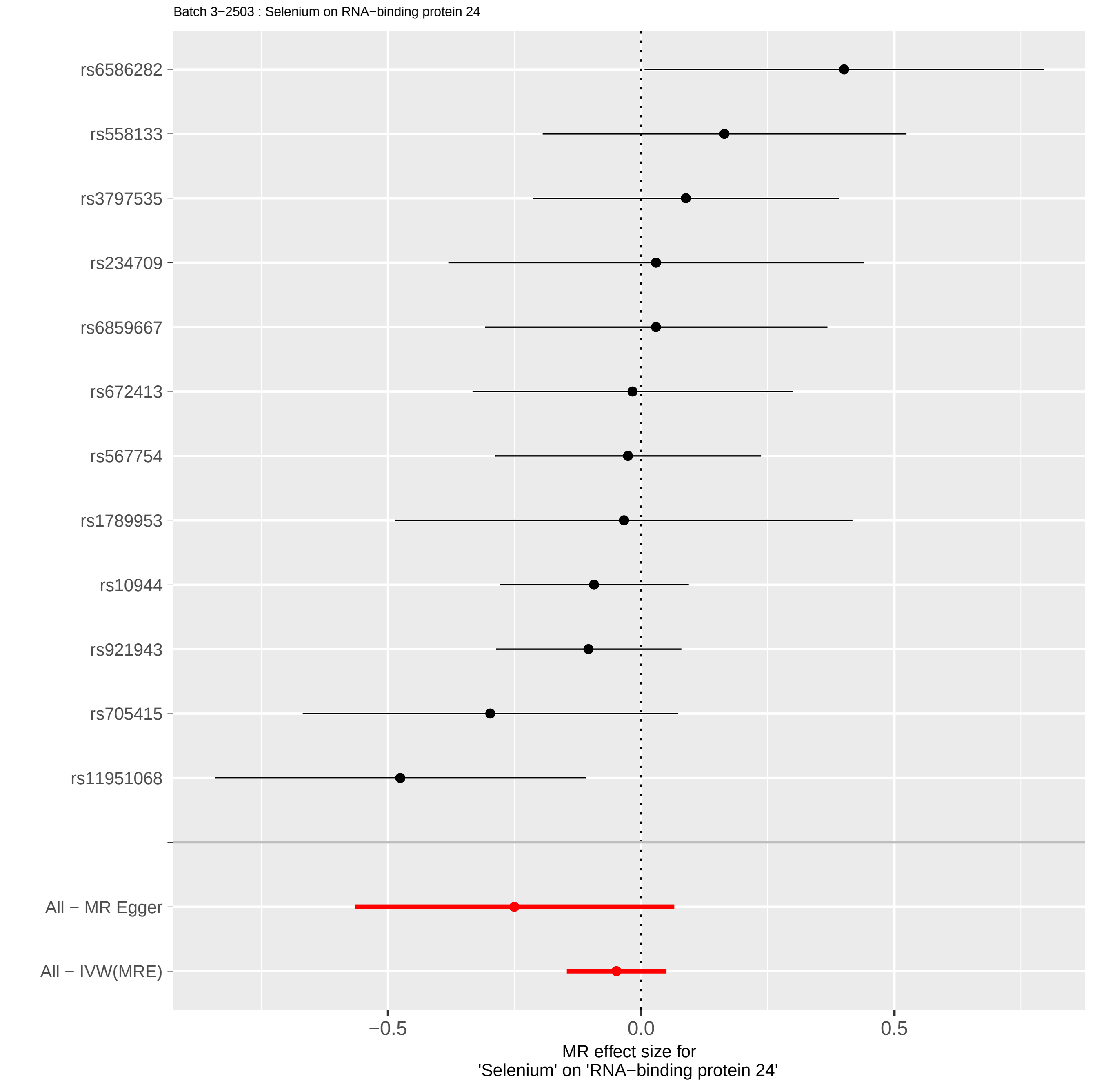

Batch 3−2503 : Selenium on RNA−binding protein 24
rs6586282
rs558133
rs3797535
rs234709
rs6859667
rs672413
rs567754
rs1789953
rs10944
rs921943
rs705415
rs11951068
All − MR Egger
All − IVW(MRE)
−0.5
0.0
0.5
MR effect size for
'Selenium' on 'RNA−binding protein 24'

Batch 3−2504 : Selenium on RNA−binding protein 28

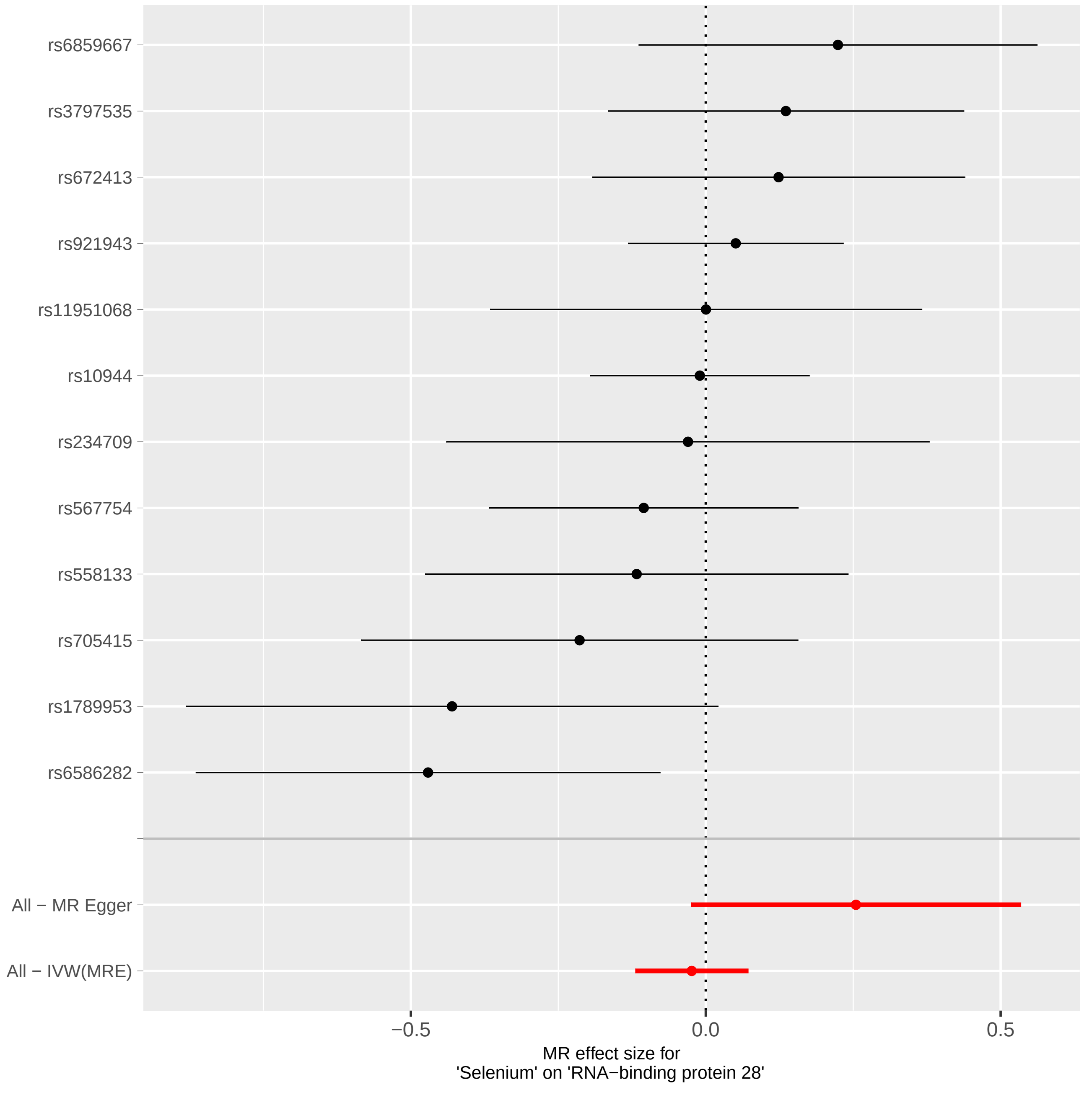

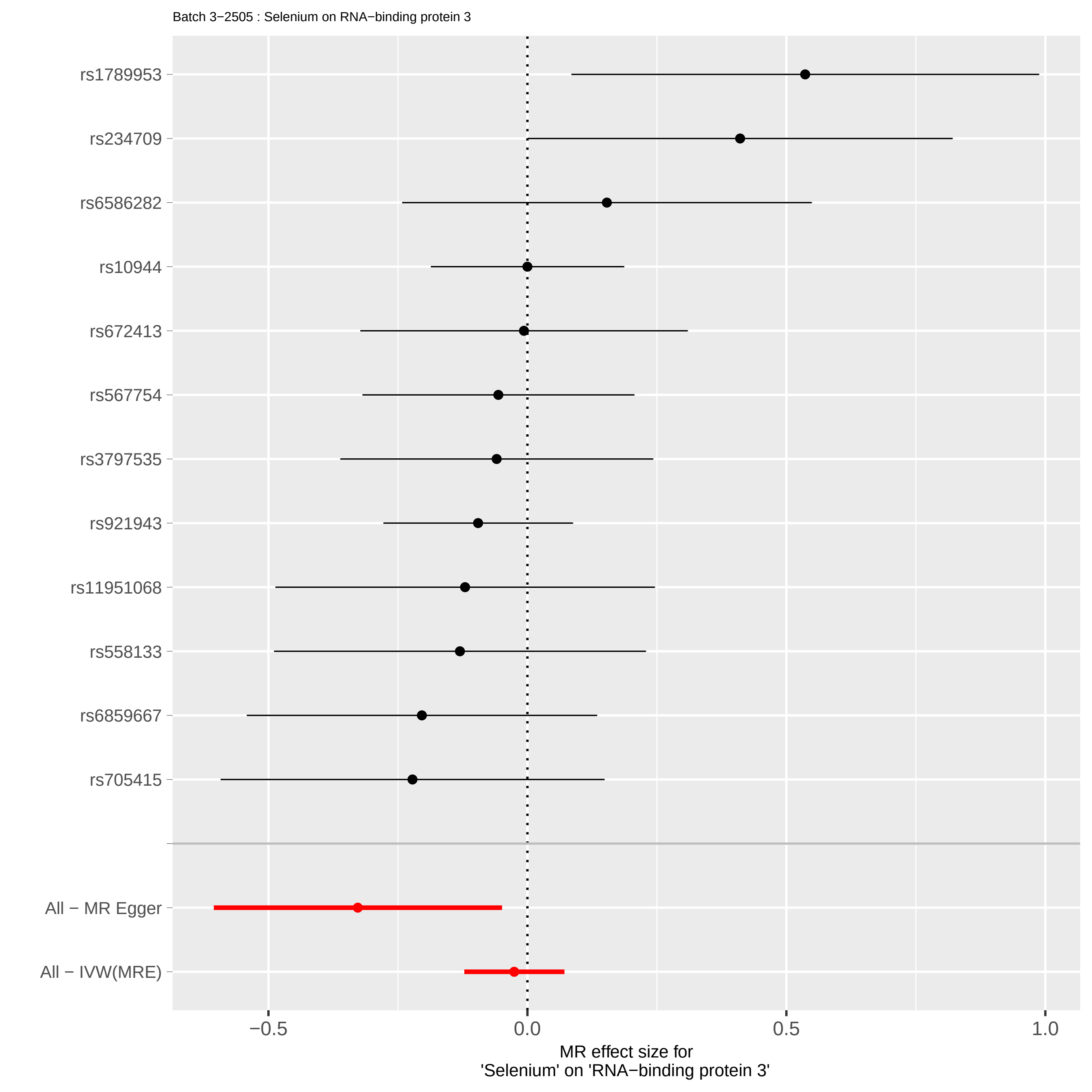

Batch 3−2505 : Selenium on RNA−binding protein 3
rs1789953
rs234709
rs6586282
rs10944
rs672413
rs567754
rs3797535
rs921943
rs11951068
rs558133
rs6859667
rs705415
All − MR Egger
All − IVW(MRE)
−0.5
0.0
0.5
1.0
MR effect size for
'Selenium' on 'RNA−binding protein 3'

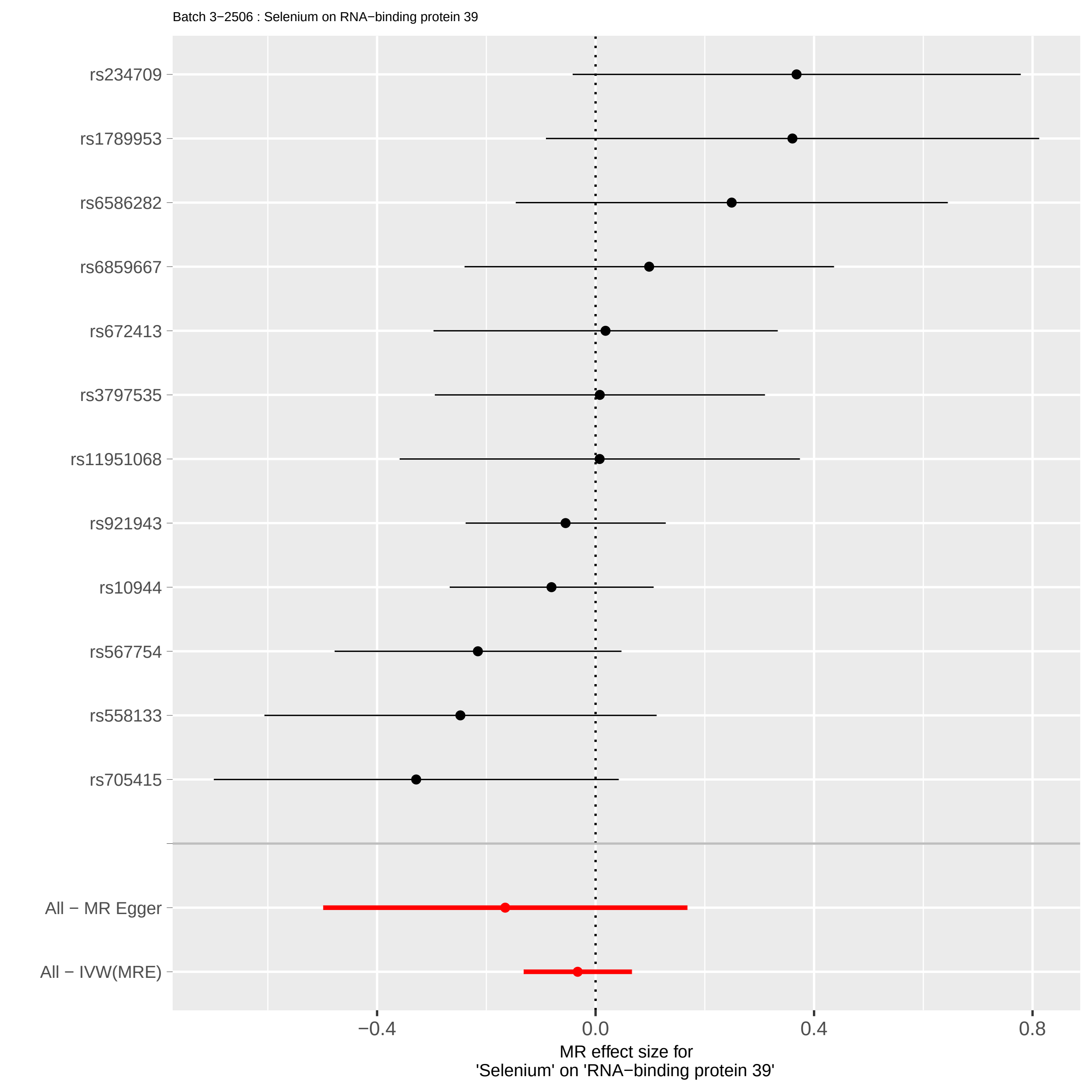

Batch 3−2506 : Selenium on RNA−binding protein 39
rs234709
rs1789953
rs6586282
rs6859667
rs672413
rs3797535
rs11951068
rs921943
rs10944
rs567754
rs558133
rs705415
All − MR Egger
All − IVW(MRE)
−0.4
0.0
0.4
0.8
MR effect size for
'Selenium' on 'RNA−binding protein 39'

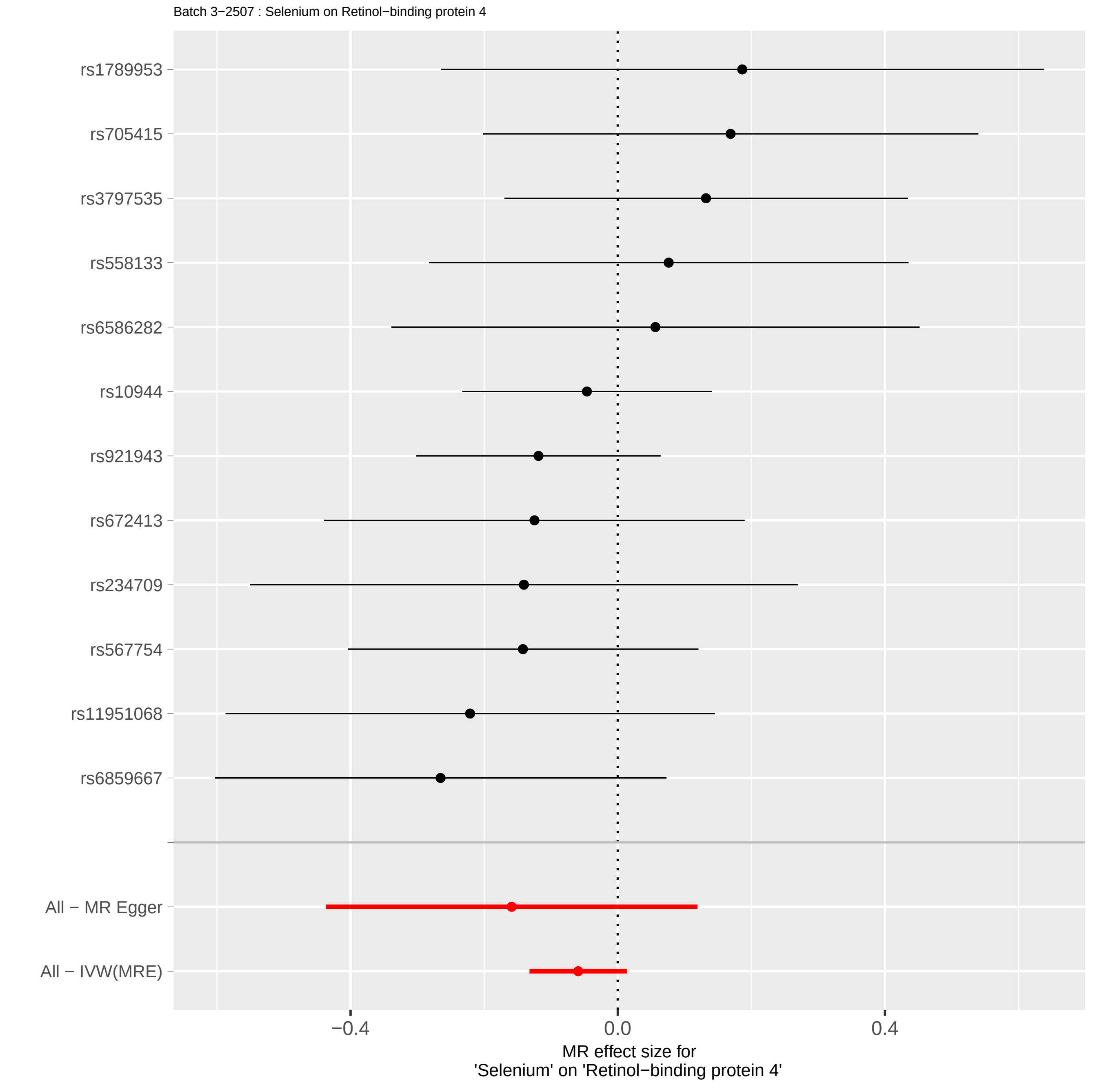

Batch 3−2507 : Selenium on Retinol−binding protein 4
rs1789953
rs705415
rs3797535
rs558133
rs6586282
rs10944
rs921943
rs672413
rs234709
rs567754
rs11951068
rs6859667
All − MR Egger
All − IVW(MRE)
−0.4
0.0
0.4
MR effect size for
'Selenium' on 'Retinol−binding protein 4'

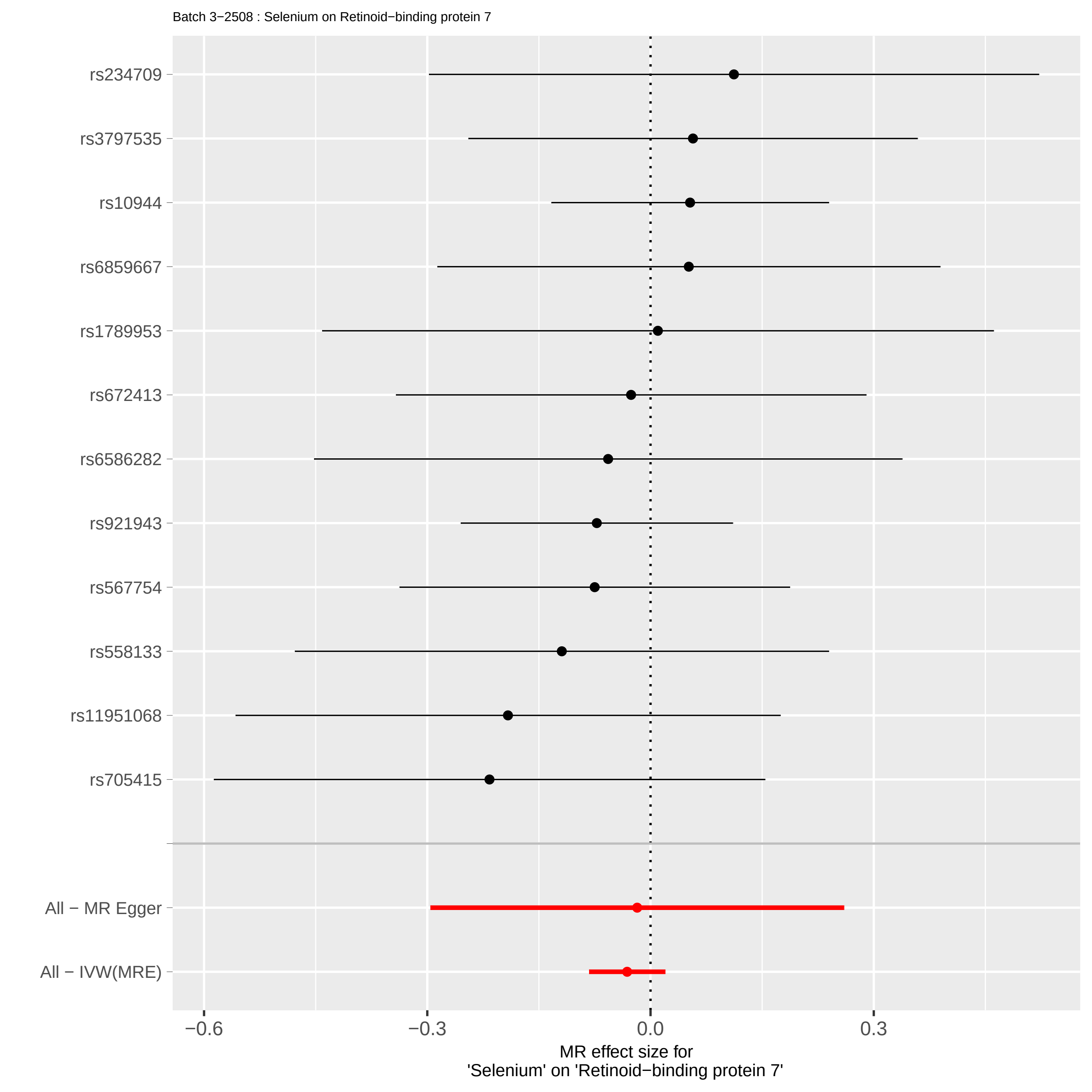

Batch 3−2508 : Selenium on Retinoid−binding protein 7
rs234709
rs3797535
rs10944
rs6859667
rs1789953
rs672413
rs6586282
rs921943
rs567754
rs558133
rs11951068
rs705415
All − MR Egger
All − IVW(MRE)
−0.6
−0.3
0.0
0.3
MR effect size for
'Selenium' on 'Retinoid−binding protein 7'

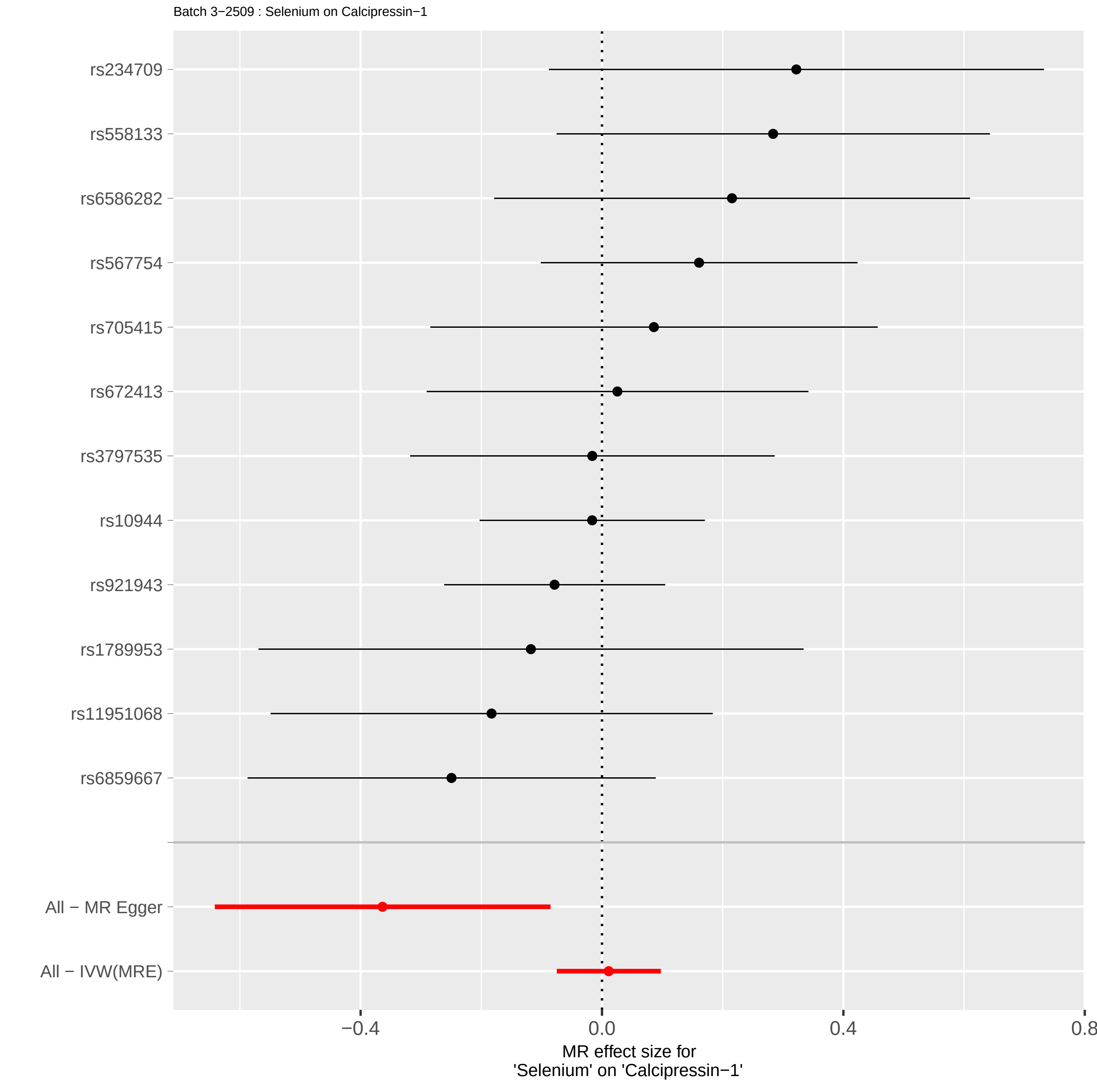

Batch 3−2509 : Selenium on Calcipressin−1
rs234709
rs558133
rs6586282
rs567754
rs705415
rs672413
rs3797535
rs10944
rs921943
rs1789953
rs11951068
rs6859667
All − MR Egger
All − IVW(MRE)
−0.4
0.0
0.4
0.8
MR effect size for
'Selenium' on 'Calcipressin−1'

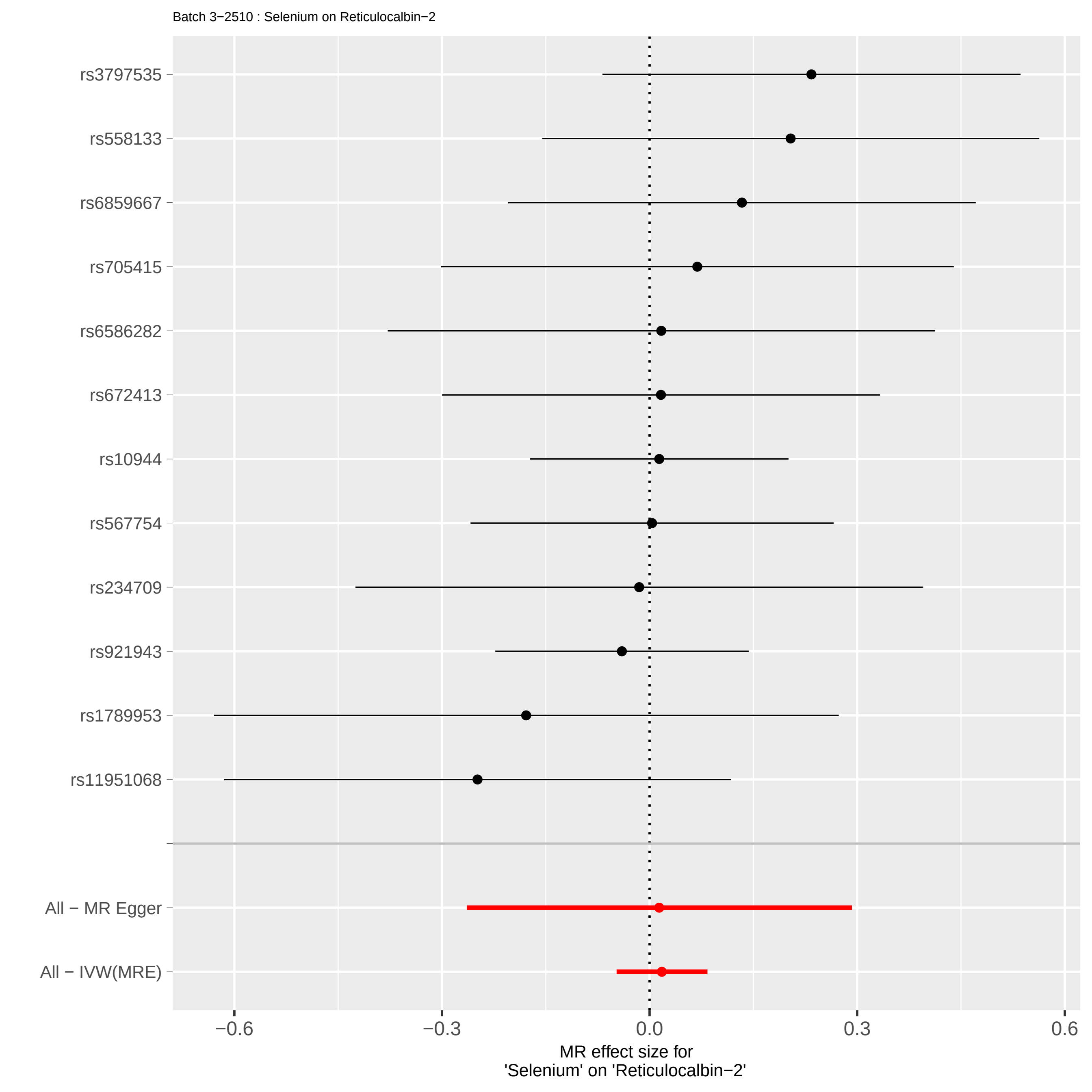
Batch 3−2510 : Selenium on Reticulocalbin−2
rs3797535
rs558133
rs6859667
rs705415
rs6586282
rs672413
rs10944
rs567754
rs234709
rs921943
rs1789953
rs11951068
All − MR Egger
All − IVW(MRE)
−0.6
−0.3
0.0
0.3
0.6
MR effect size for
'Selenium' on 'Reticulocalbin−2'

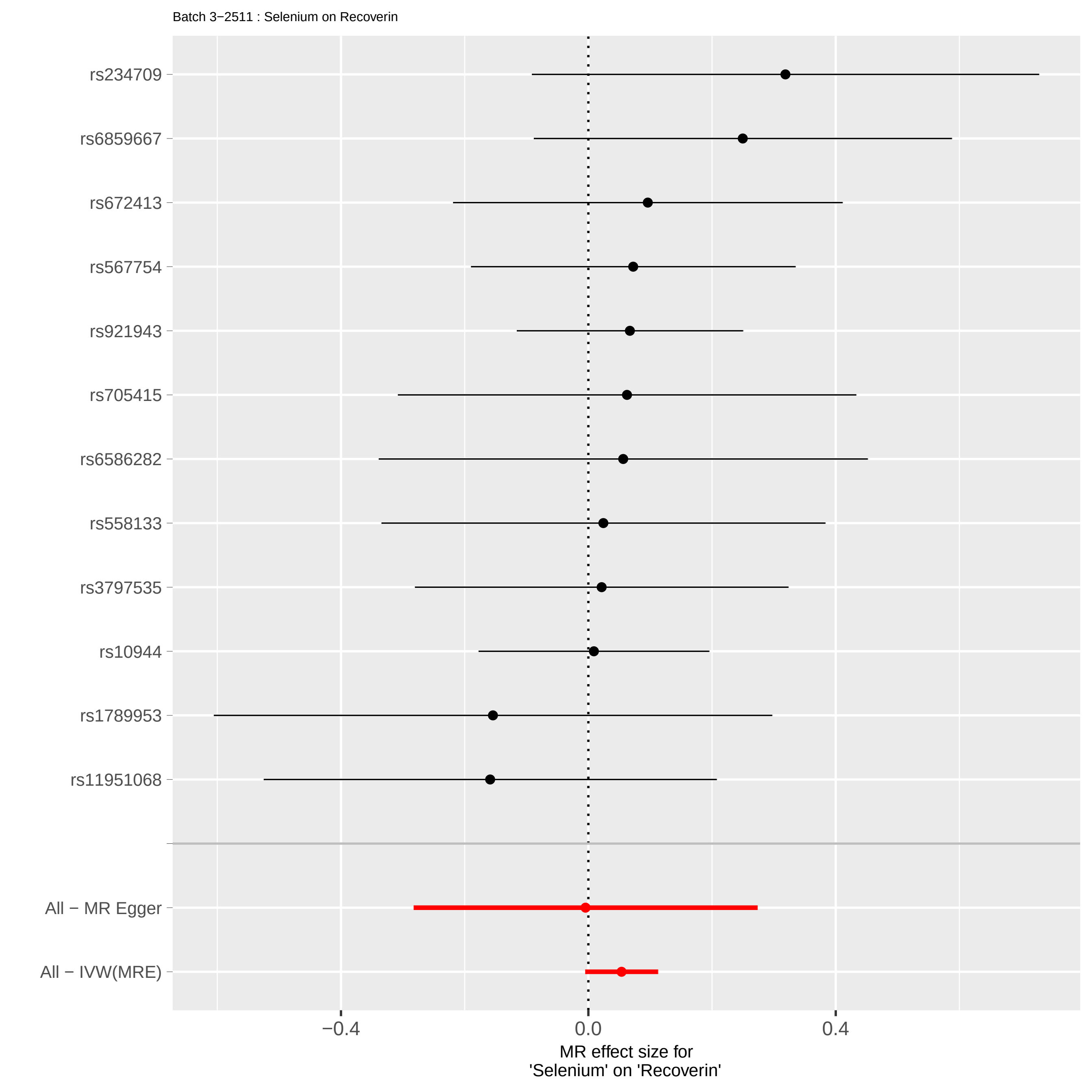

Batch 3−2511 : Selenium on Recoverin
rs234709
rs6859667
rs672413
rs567754
rs921943
rs705415
rs6586282
rs558133
rs3797535
rs10944
rs1789953
rs11951068
All − MR Egger
All − IVW(MRE)
−0.4
0.0
0.4
MR effect size for
'Selenium' on 'Recoverin'

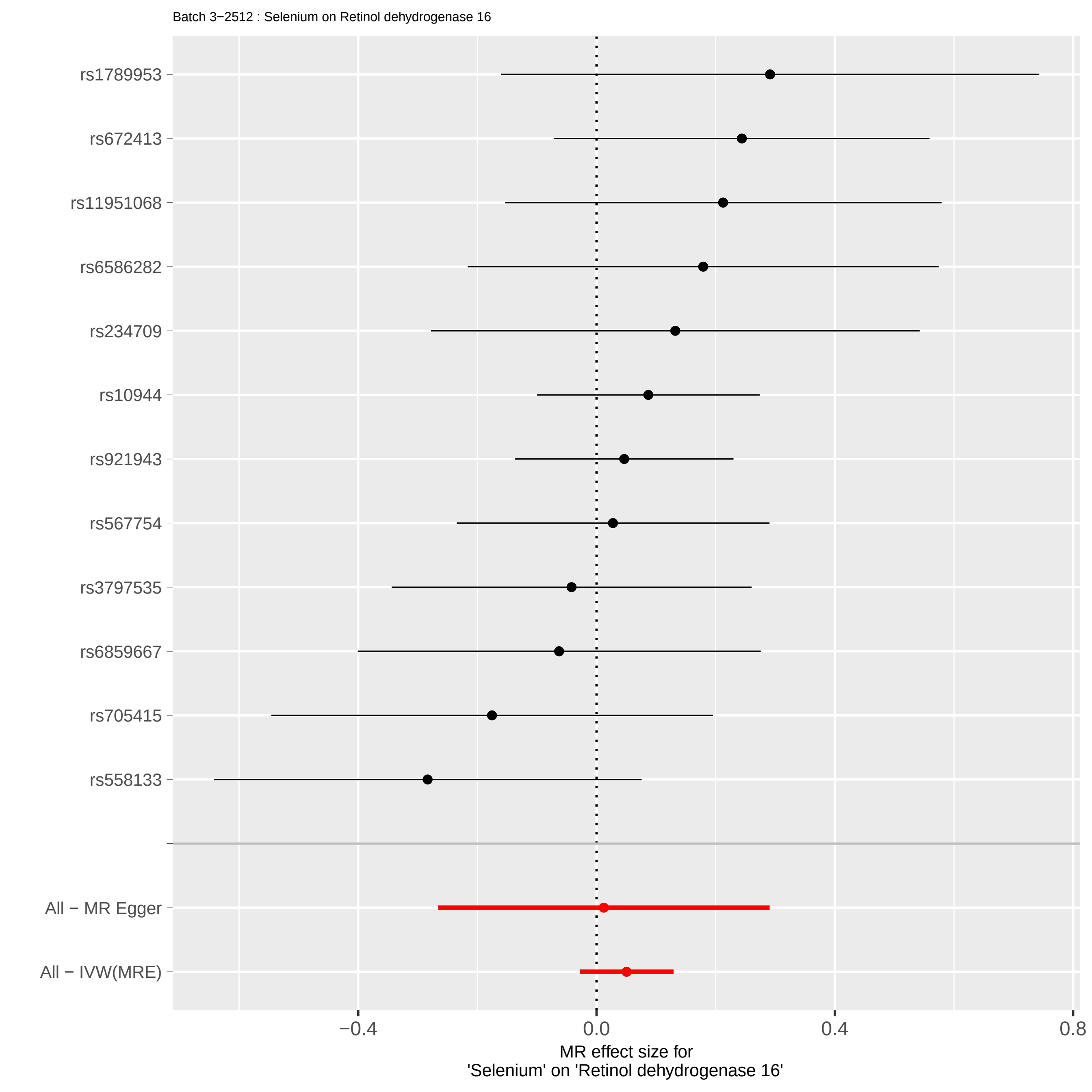

Batch 3−2512 : Selenium on Retinol dehydrogenase 16
rs1789953
rs672413
rs11951068
rs6586282
rs234709
rs10944
rs921943
rs567754
rs3797535
rs6859667
rs705415
rs558133
All − MR Egger
All − IVW(MRE)
−0.4
0.0
0.4
0.8
MR effect size for
'Selenium' on 'Retinol dehydrogenase 16'

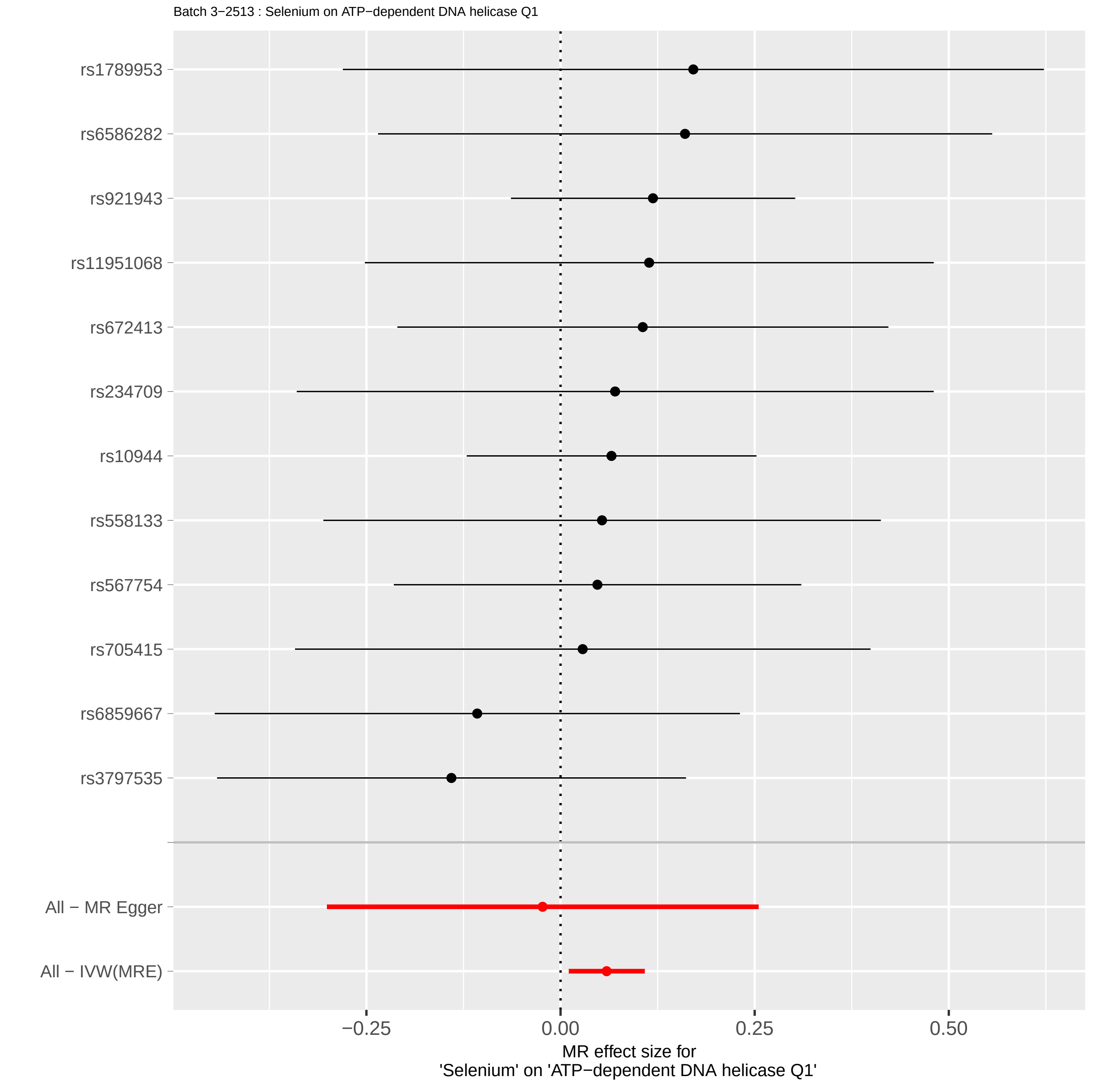

Batch 3−2513 : Selenium on ATP−dependent DNA helicase Q1
rs1789953
rs6586282
rs921943
rs11951068
rs672413
rs234709
rs10944
rs558133
rs567754
rs705415
rs6859667
rs3797535
All − MR Egger
All − IVW(MRE)
−0.25
0.00
0.25
0.50
MR effect size for
'Selenium' on 'ATP−dependent DNA helicase Q1'

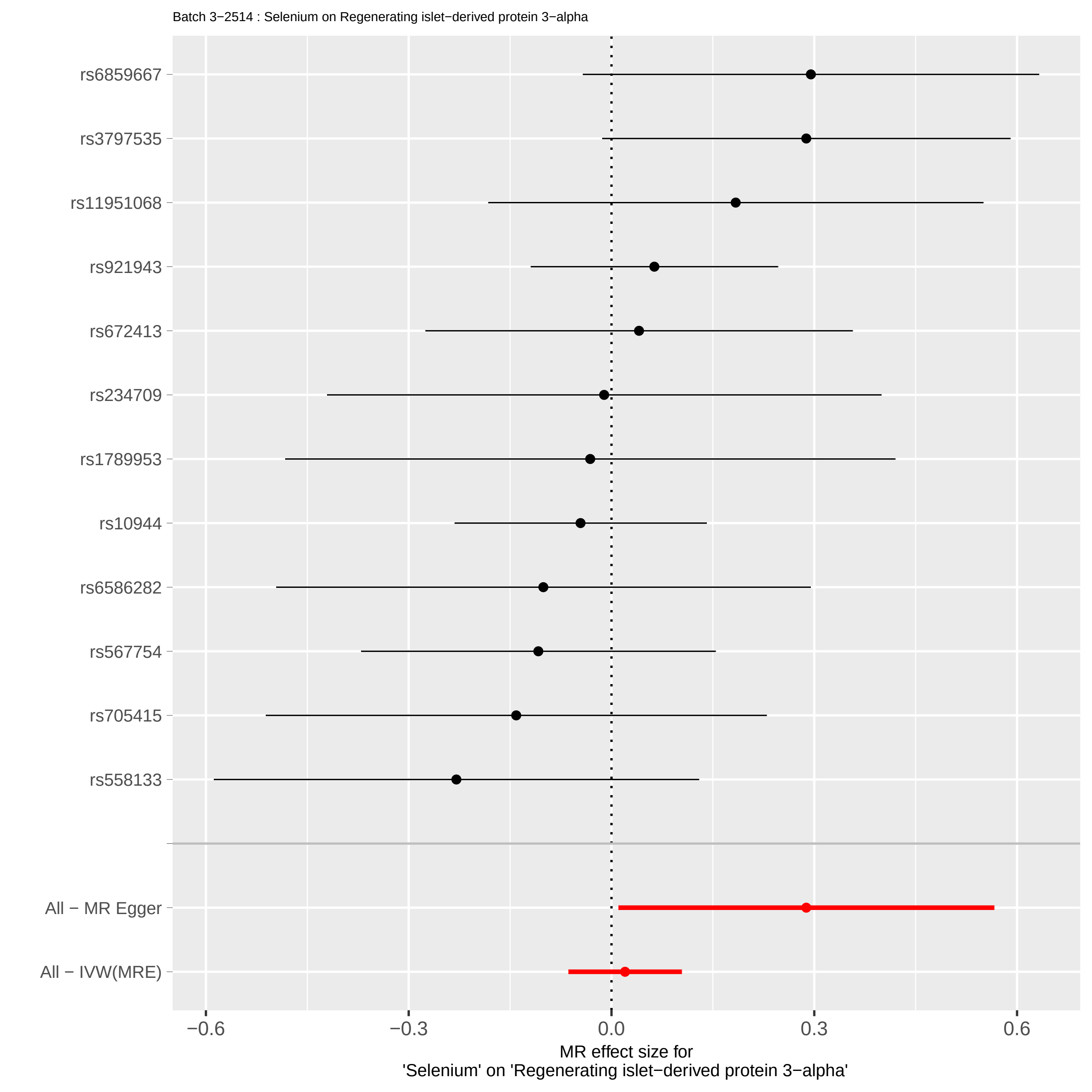
Batch 3−2514 : Selenium on Regenerating islet−derived protein 3−alpha
rs6859667
rs3797535
rs11951068
rs921943
rs672413
rs234709
rs1789953
rs10944
rs6586282
rs567754
rs705415
rs558133
All − MR Egger
All − IVW(MRE)
−0.6
−0.3
0.0
0.3
0.6
MR effect size for
'Selenium' on 'Regenerating islet−derived protein 3−alpha'

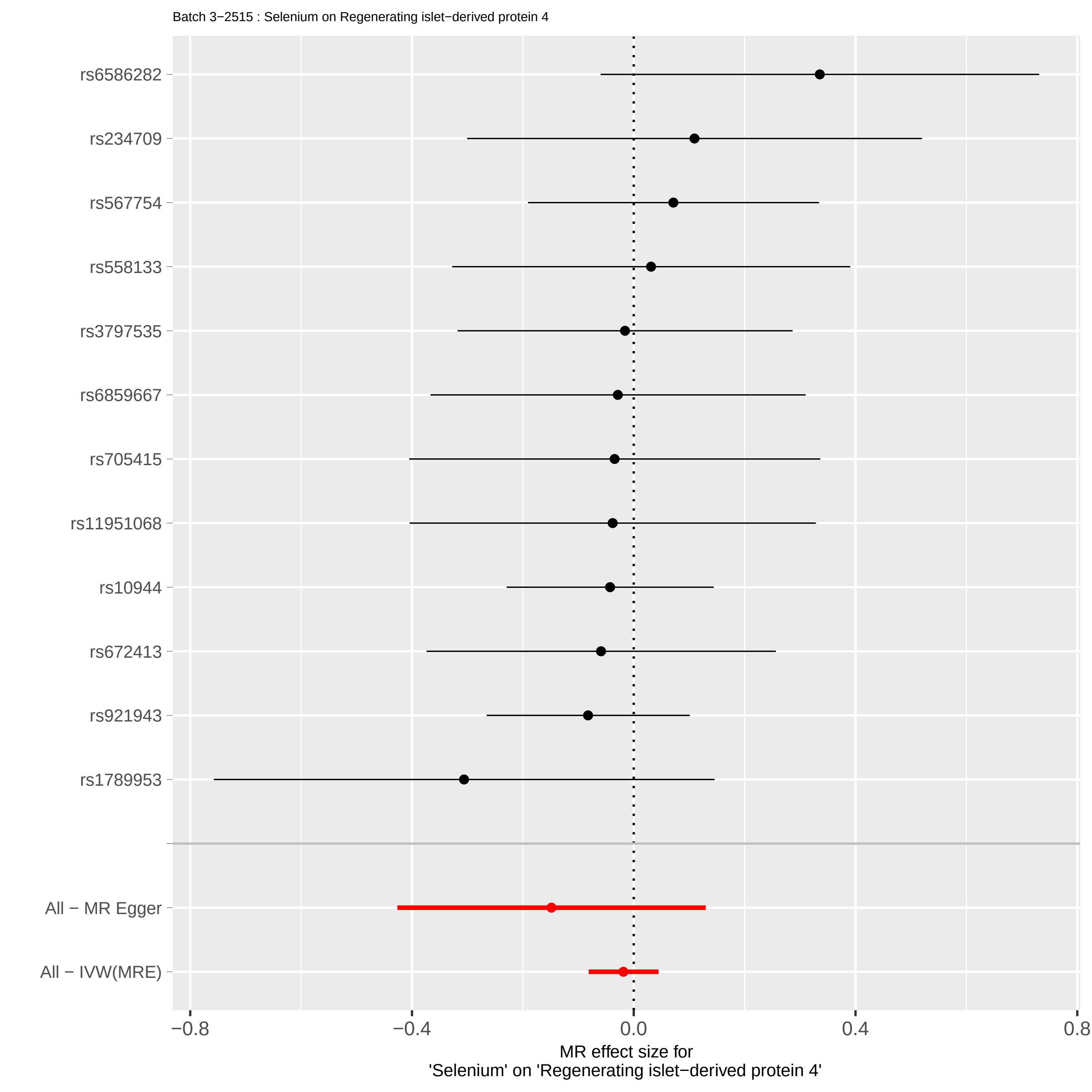

Batch 3−2515 : Selenium on Regenerating islet−derived protein 4
rs6586282
rs234709
rs567754
rs558133
rs3797535
rs6859667
rs705415
rs11951068
rs10944
rs672413
rs921943
rs1789953
All − MR Egger
All − IVW(MRE)
−0.8
−0.4
0.0
0.4
0.8
MR effect size for
'Selenium' on 'Regenerating islet−derived protein 4'

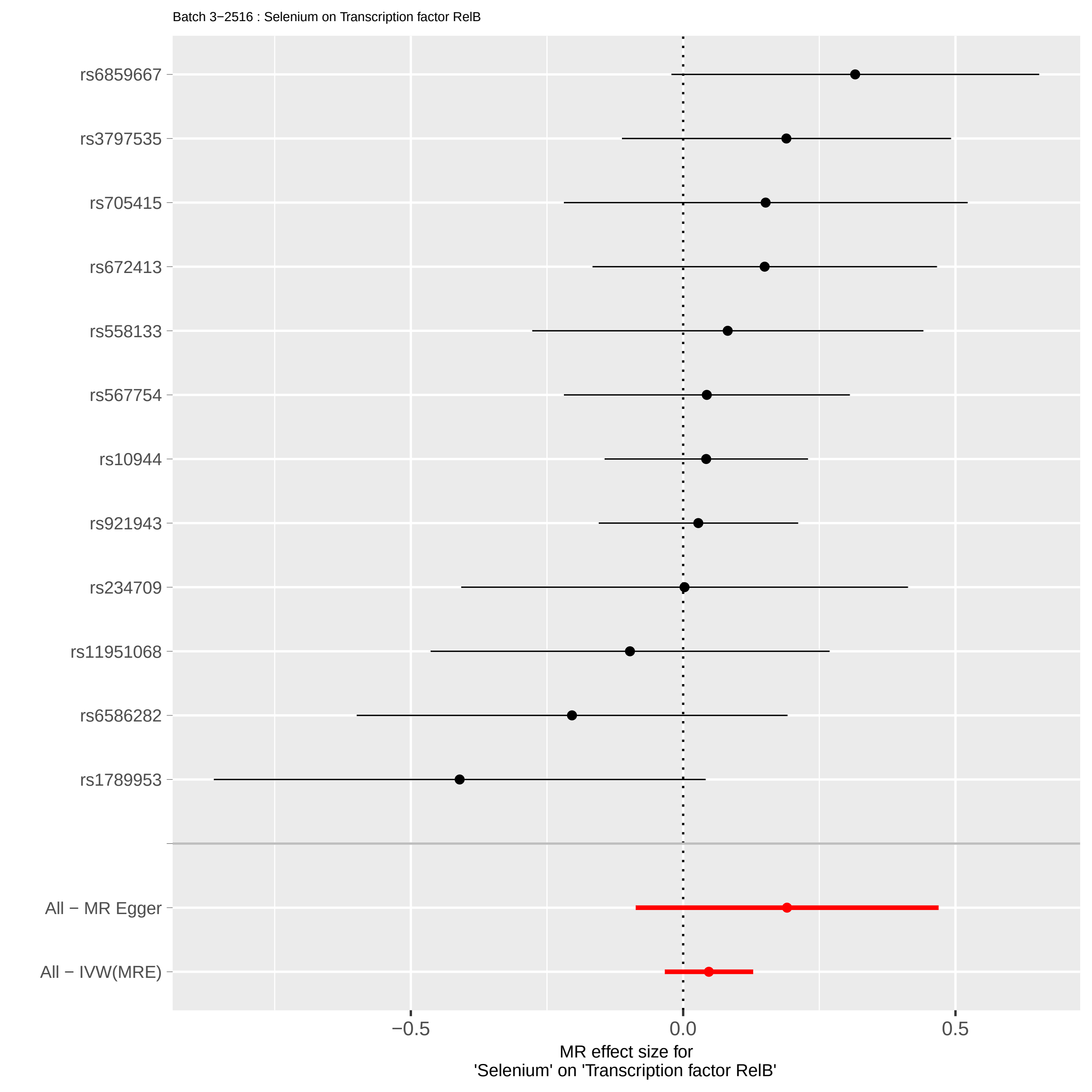

Batch 3−2516 : Selenium on Transcription factor RelB
rs6859667
rs3797535
rs705415
rs672413
rs558133
rs567754
rs10944
rs921943
rs234709
rs11951068
rs6586282
rs1789953
All − MR Egger
All − IVW(MRE)
−0.5
0.0
0.5
MR effect size for
'Selenium' on 'Transcription factor RelB'

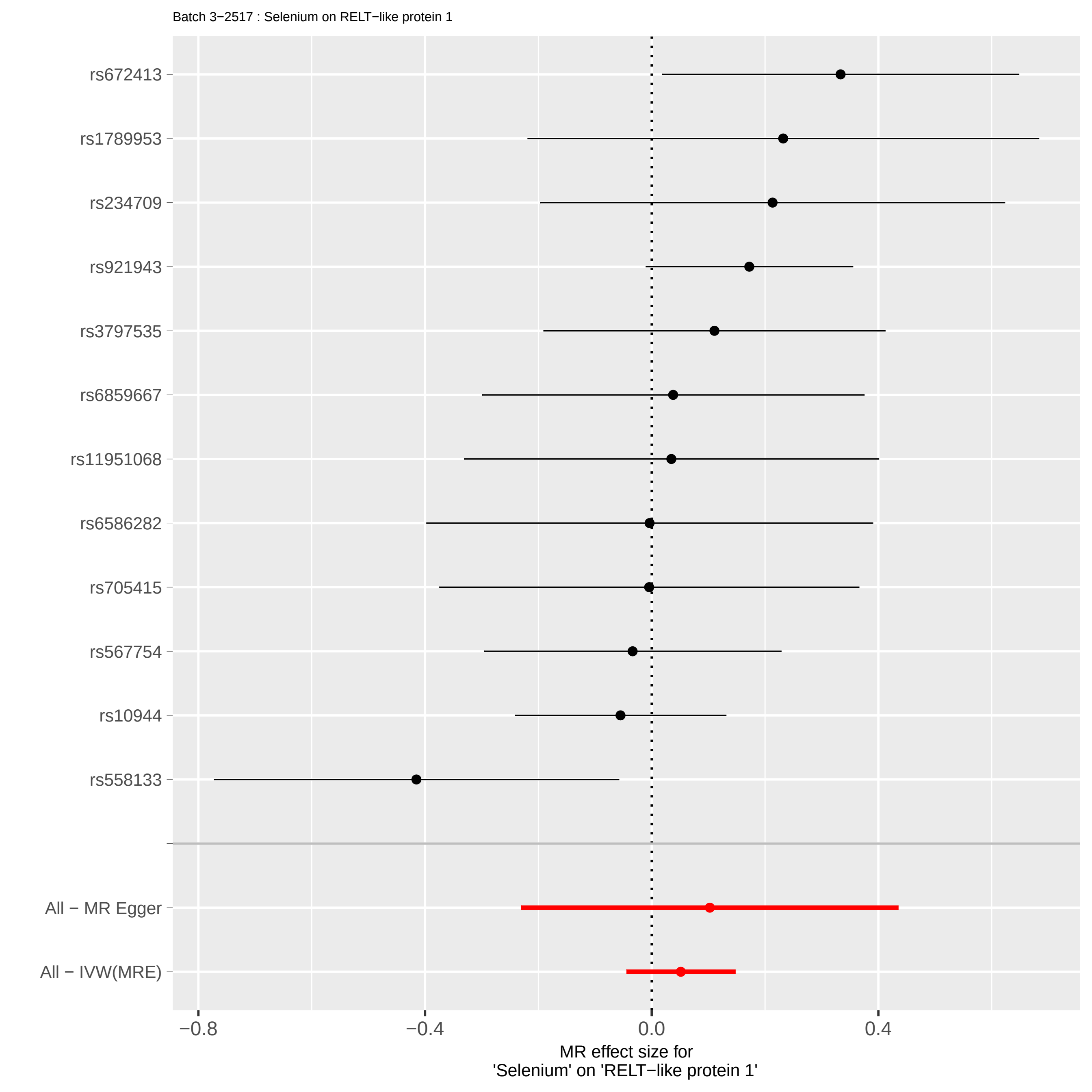
Batch 3−2517 : Selenium on RELT−like protein 1
rs672413
rs1789953
rs234709
rs921943
rs3797535
rs6859667
rs11951068
rs6586282
rs705415
rs567754
rs10944
rs558133
All − MR Egger
All − IVW(MRE)
−0.8
−0.4
0.0
0.4
MR effect size for
'Selenium' on 'RELT−like protein 1'

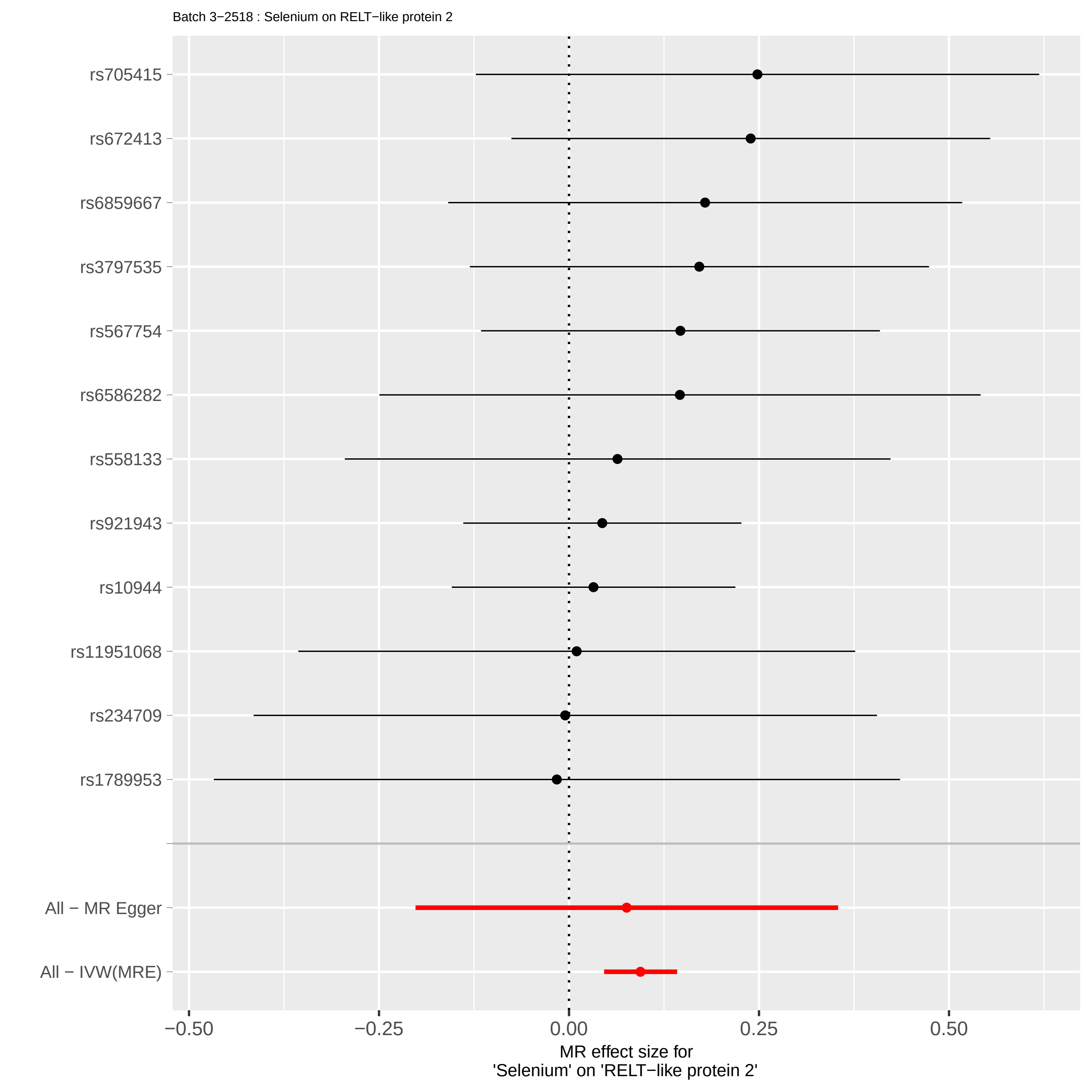

Batch 3−2518 : Selenium on RELT−like protein 2
rs705415
rs672413
rs6859667
rs3797535
rs567754
rs6586282
rs558133
rs921943
rs10944
rs11951068
rs234709
rs1789953
All − MR Egger
All − IVW(MRE)
−0.50
−0.25
0.00
0.25
0.50
MR effect size for
'Selenium' on 'RELT−like protein 2'

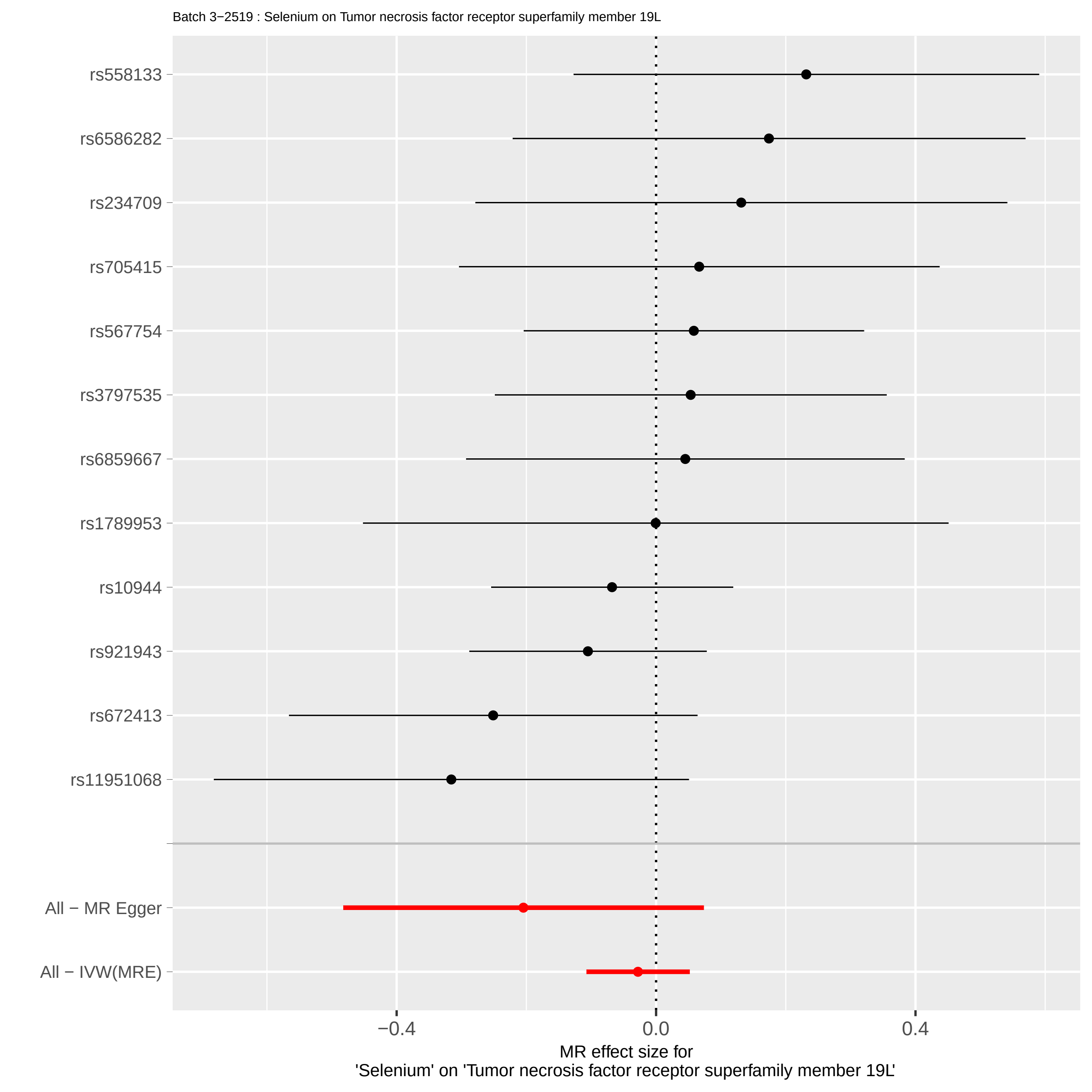

Batch 3−2519 : Selenium on Tumor necrosis factor receptor superfamily member 19L
rs558133
rs6586282
rs234709
rs705415
rs567754
rs3797535
rs6859667
rs1789953
rs10944
rs921943
rs672413
rs11951068
All − MR Egger
All − IVW(MRE)
−0.4
0.0
0.4
MR effect size for
'Selenium' on 'Tumor necrosis factor receptor superfamily member 19L'

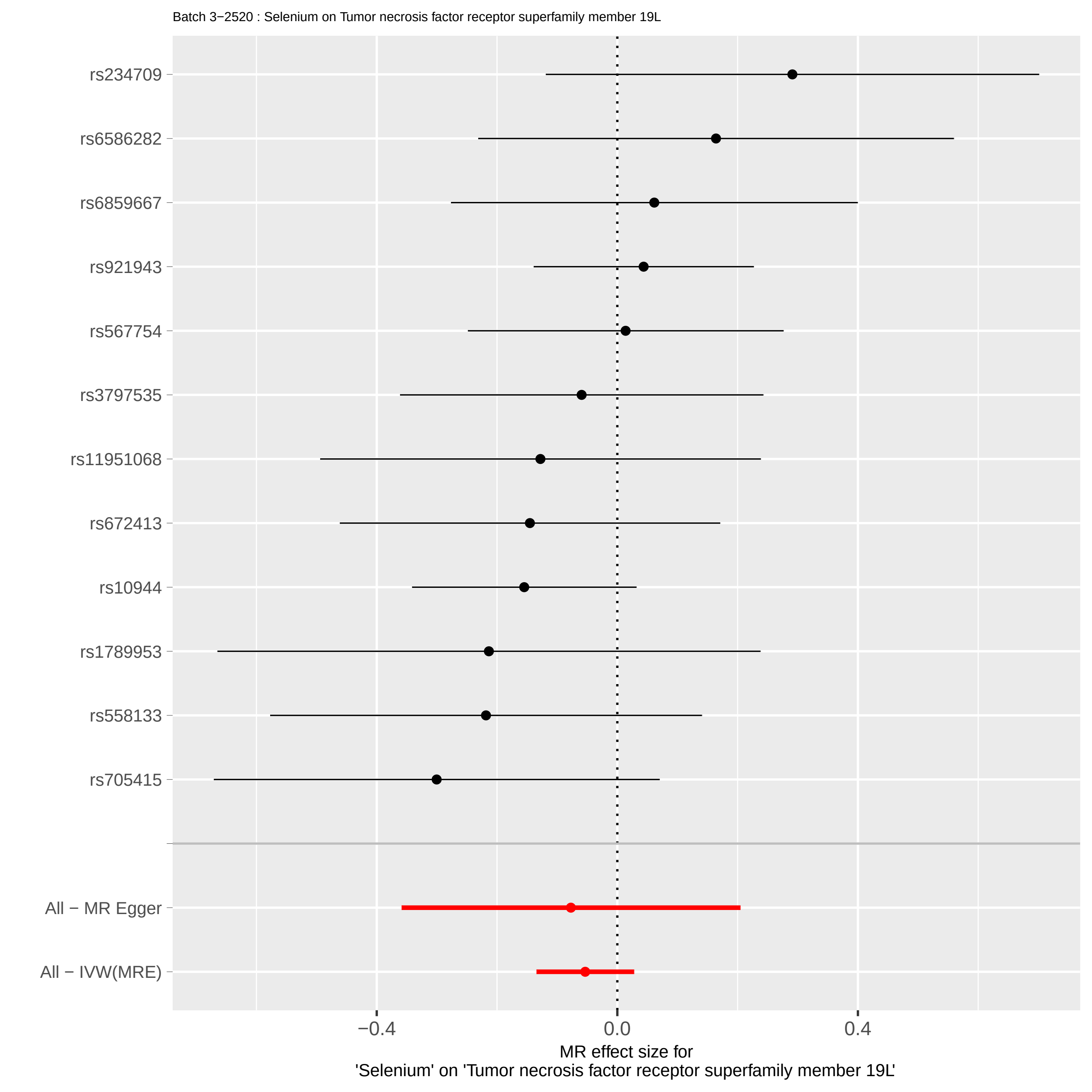
Batch 3−2520 : Selenium on Tumor necrosis factor receptor superfamily member 19L
rs234709
rs6586282
rs6859667
rs921943
rs567754
rs3797535
rs11951068
rs672413
rs10944
rs1789953
rs558133
rs705415
All − MR Egger
All − IVW(MRE)
−0.4
0.0
0.4
MR effect size for
'Selenium' on 'Tumor necrosis factor receptor superfamily member 19L'

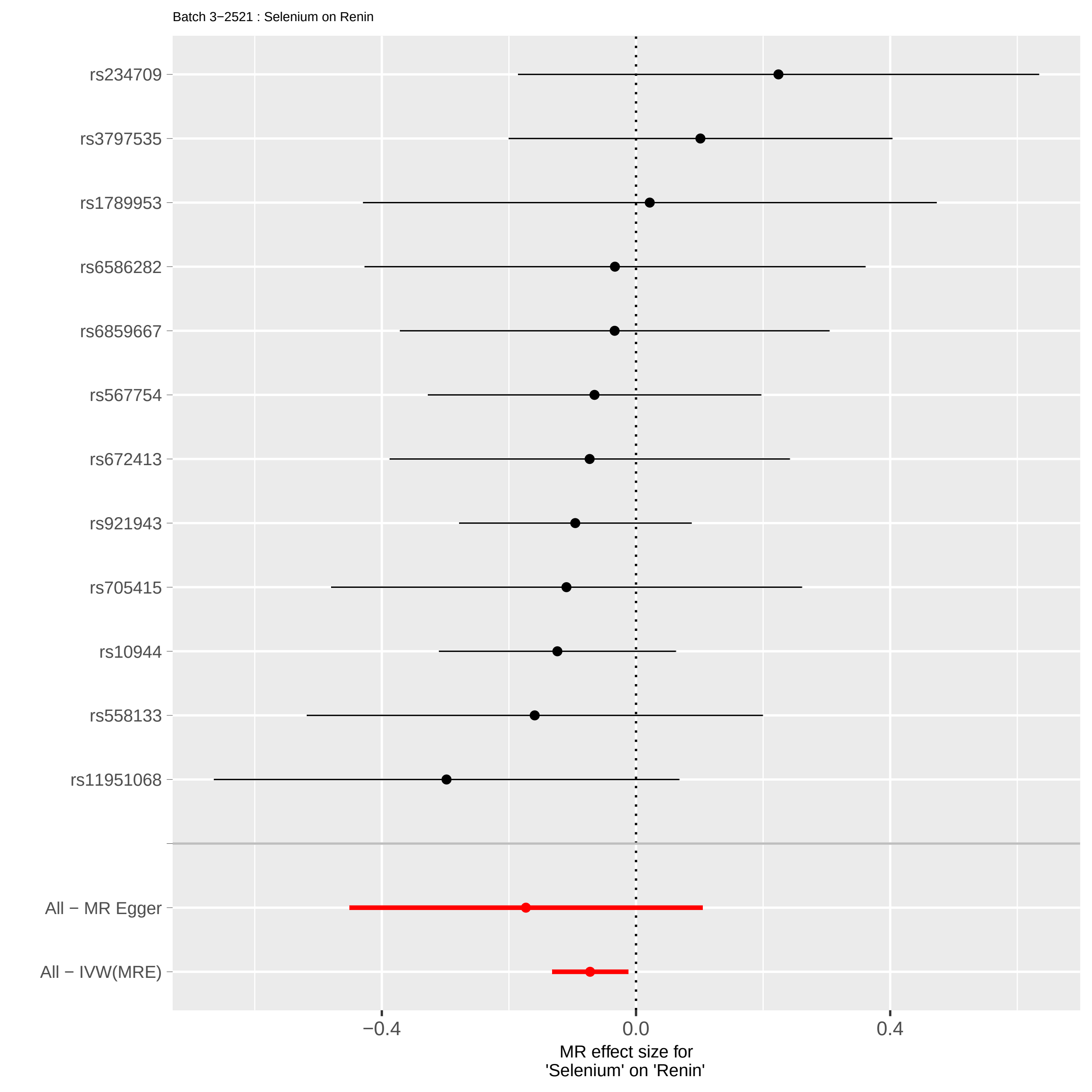

Batch 3−2521 : Selenium on Renin
rs234709
rs3797535
rs1789953
rs6586282
rs6859667
rs567754
rs672413
rs921943
rs705415
rs10944
rs558133
rs11951068
All − MR Egger
All − IVW(MRE)
−0.4
0.0
0.4
MR effect size for
'Selenium' on 'Renin'

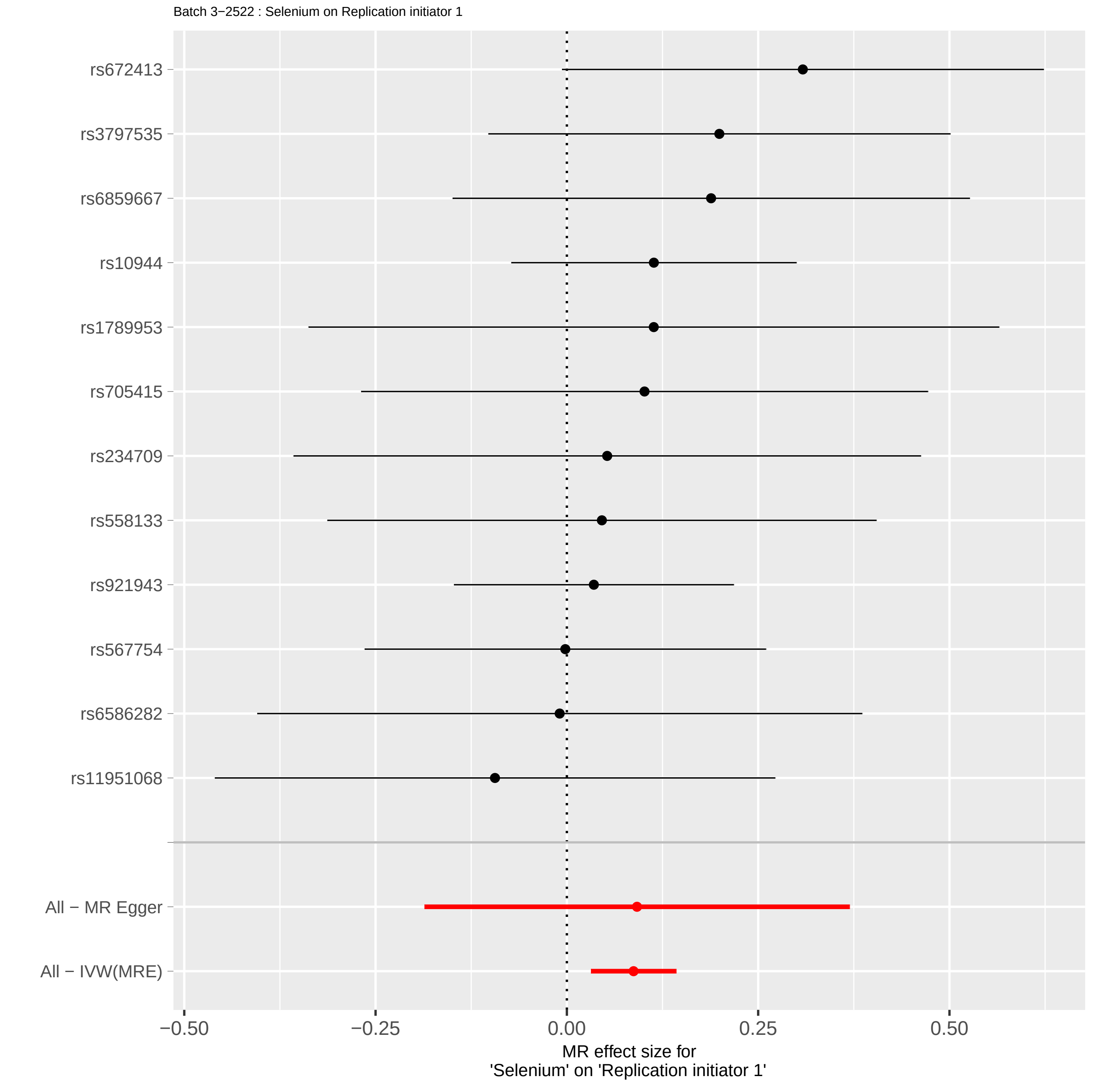

Batch 3−2522 : Selenium on Replication initiator 1
rs672413
rs3797535
rs6859667
rs10944
rs1789953
rs705415
rs234709
rs558133
rs921943
rs567754
rs6586282
rs11951068
All − MR Egger
All − IVW(MRE)
−0.50
−0.25
0.00
0.25
0.50
MR effect size for
'Selenium' on 'Replication initiator 1'

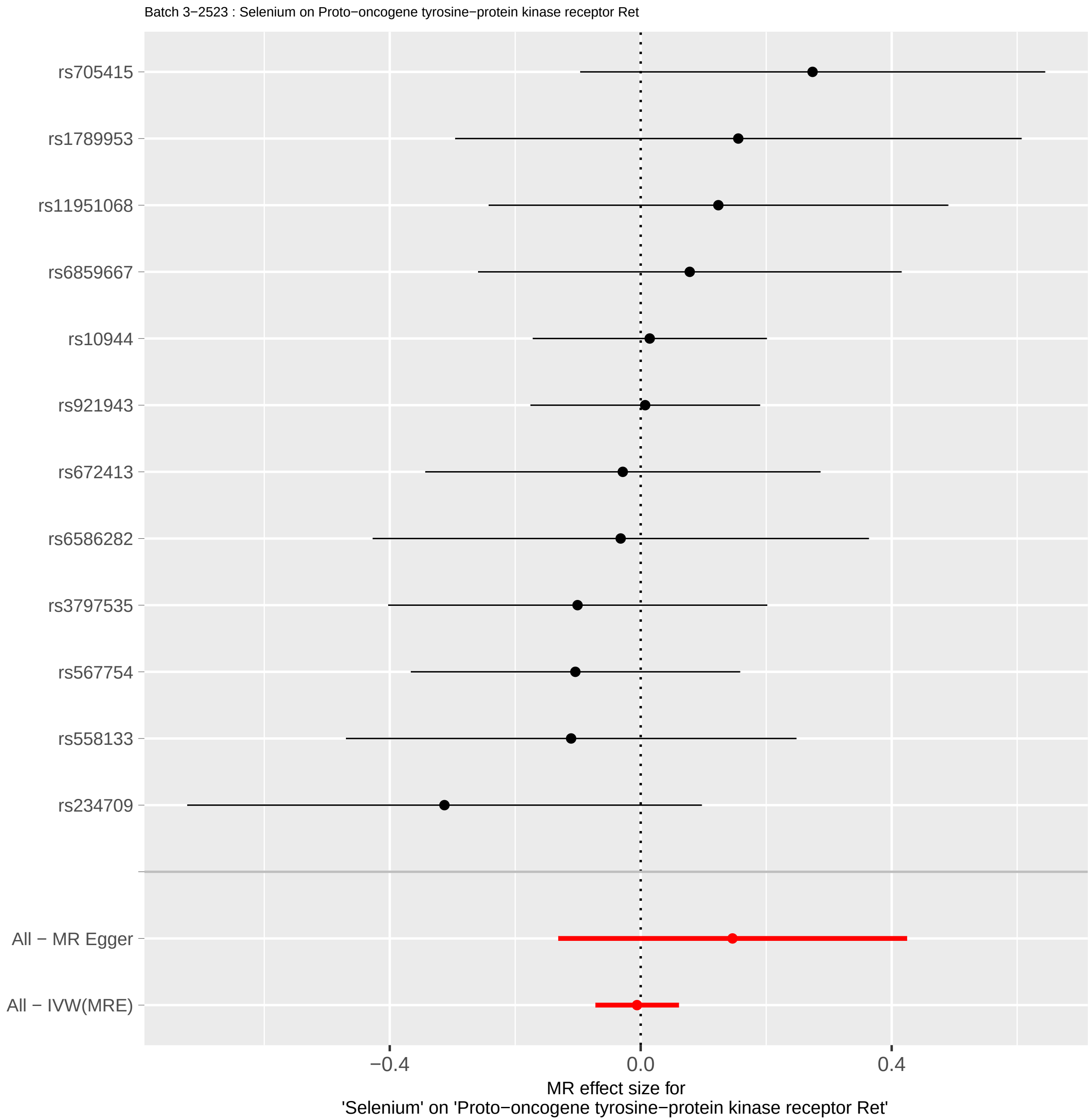
Batch 3−2523 : Selenium on Proto−oncogene tyrosine−protein kinase receptor Ret
rs705415
rs1789953
rs11951068
rs6859667
rs10944
rs921943
rs672413
rs6586282
rs3797535
rs567754
rs558133
rs234709
All − MR Egger
All − IVW(MRE)
−0.4
0.0
0.4
MR effect size for
'Selenium' on 'Proto−oncogene tyrosine−protein kinase receptor Ret'

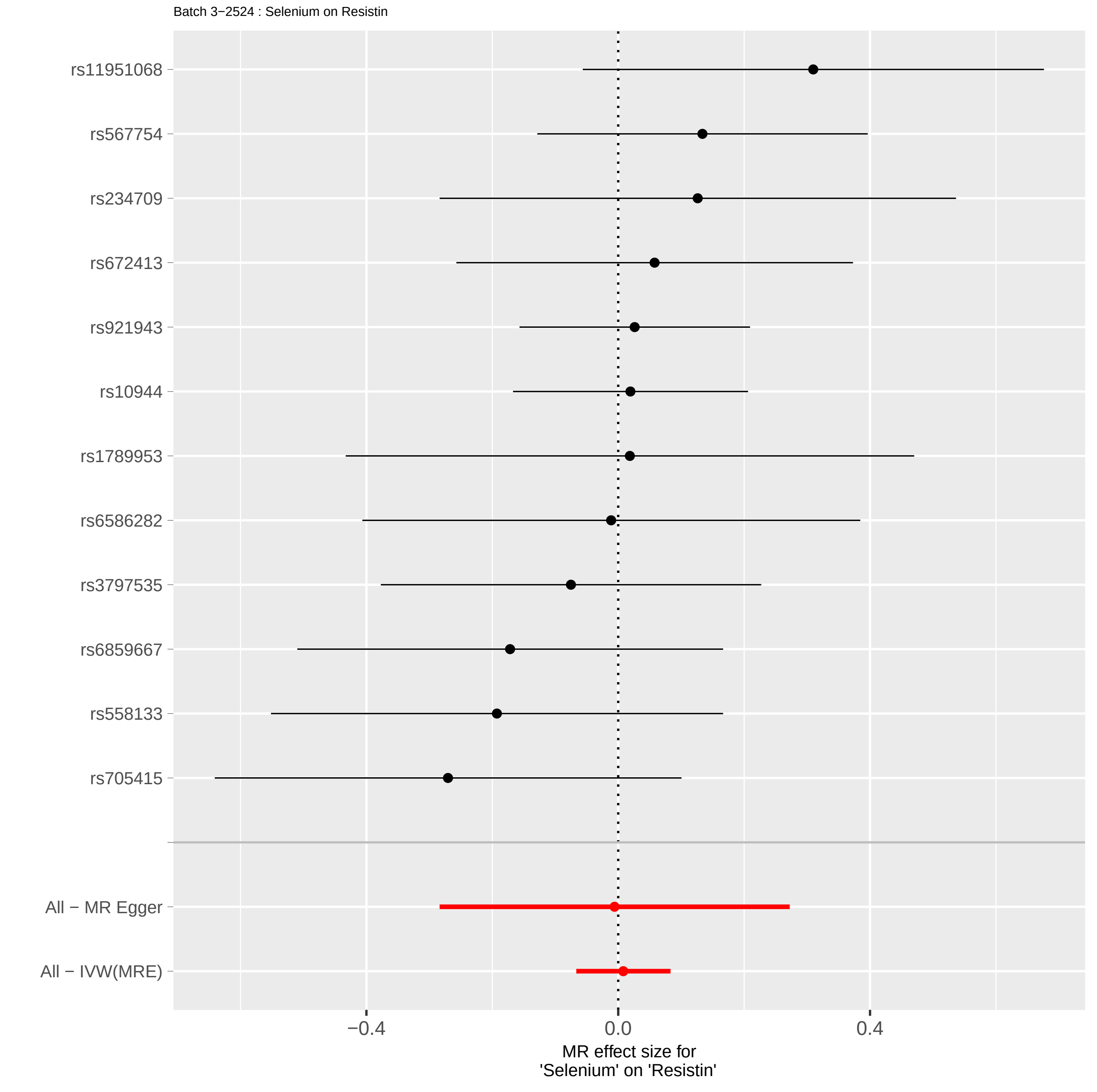

Batch 3−2524 : Selenium on Resistin
rs11951068
rs567754
rs234709
rs672413
rs921943
rs10944
rs1789953
rs6586282
rs3797535
rs6859667
rs558133
rs705415
All − MR Egger
All − IVW(MRE)
−0.4
0.0
0.4
MR effect size for
'Selenium' on 'Resistin'

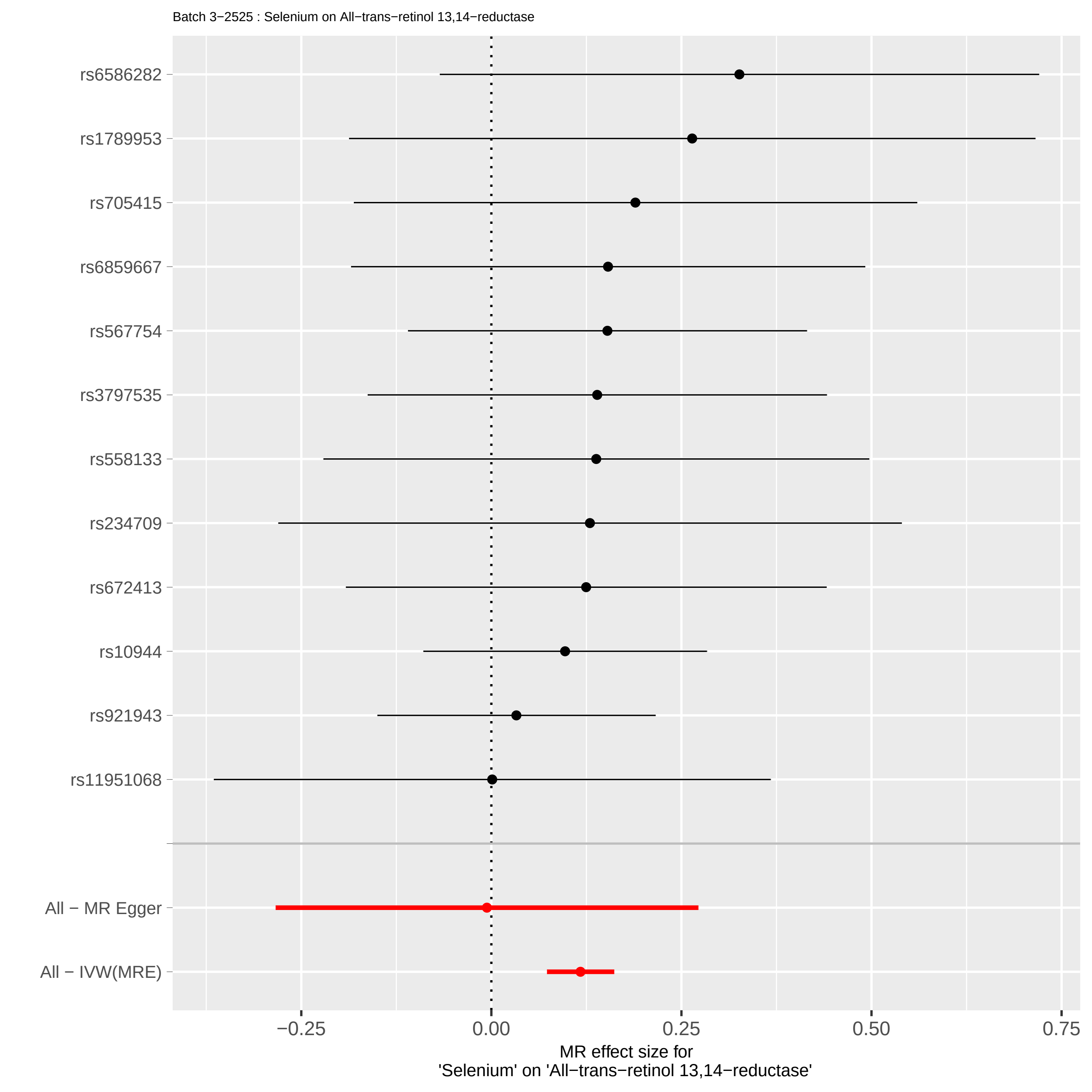

Batch 3−2525 : Selenium on All−trans−retinol 13,14−reductase
rs6586282
rs1789953
rs705415
rs6859667
rs567754
rs3797535
rs558133
rs234709
rs672413
rs10944
rs921943
rs11951068
All − MR Egger
All − IVW(MRE)
−0.25
0.00
0.25
0.50
0.75
MR effect size for
'Selenium' on 'All−trans−retinol 13,14−reductase'

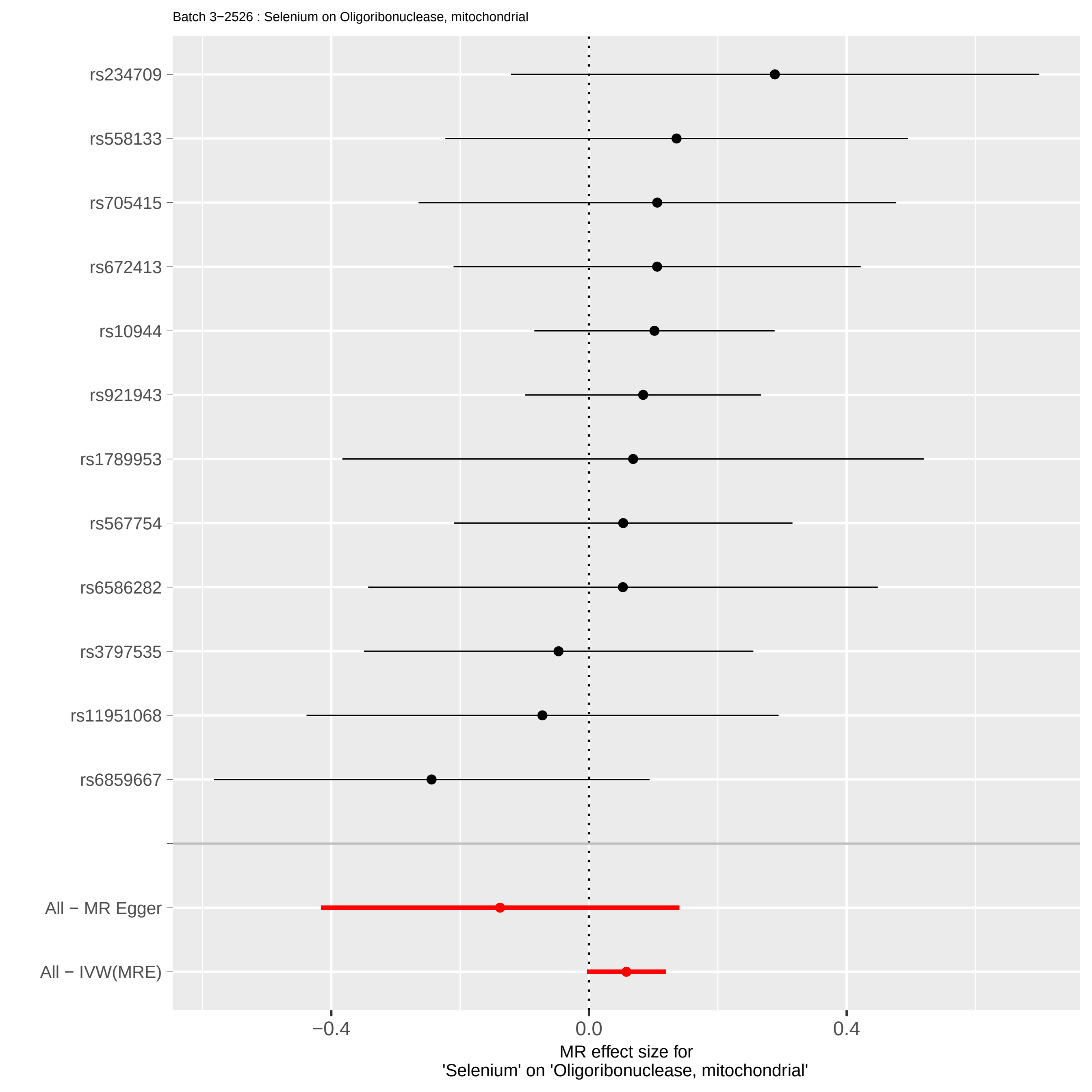

Batch 3−2526 : Selenium on Oligoribonuclease, mitochondrial
rs234709
rs558133
rs705415
rs672413
rs10944
rs921943
rs1789953
rs567754
rs6586282
rs3797535
rs11951068
rs6859667
All − MR Egger
All − IVW(MRE)
−0.4
0.0
0.4
MR effect size for
'Selenium' on 'Oligoribonuclease, mitochondrial'

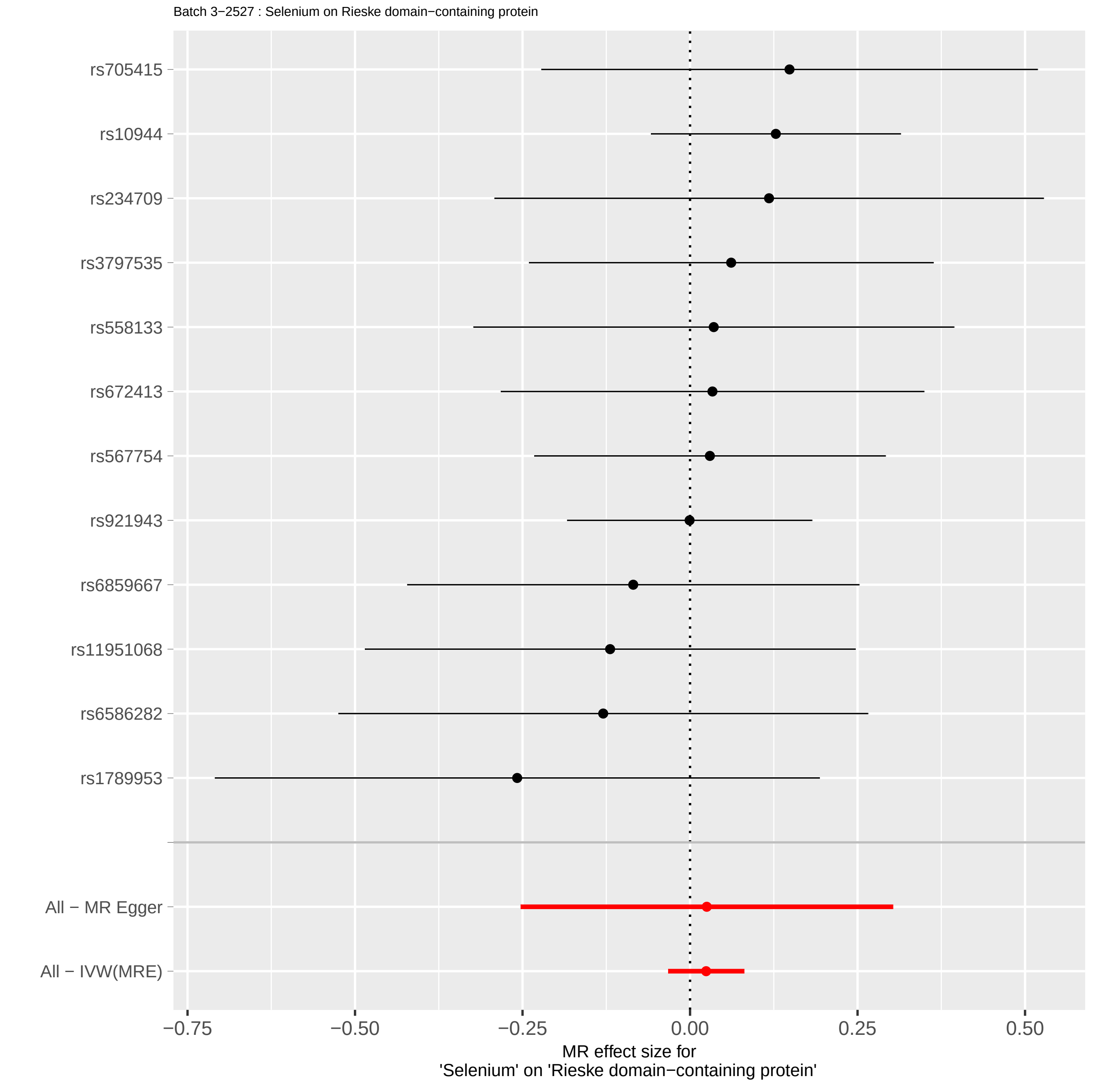

Batch 3−2527 : Selenium on Rieske domain−containing protein
rs705415
rs10944
rs234709
rs3797535
rs558133
rs672413
rs567754
rs921943
rs6859667
rs11951068
rs6586282
rs1789953
All − MR Egger
All − IVW(MRE)
−0.75
−0.50
−0.25
0.00
0.25
0.50
MR effect size for
'Selenium' on 'Rieske domain−containing protein'

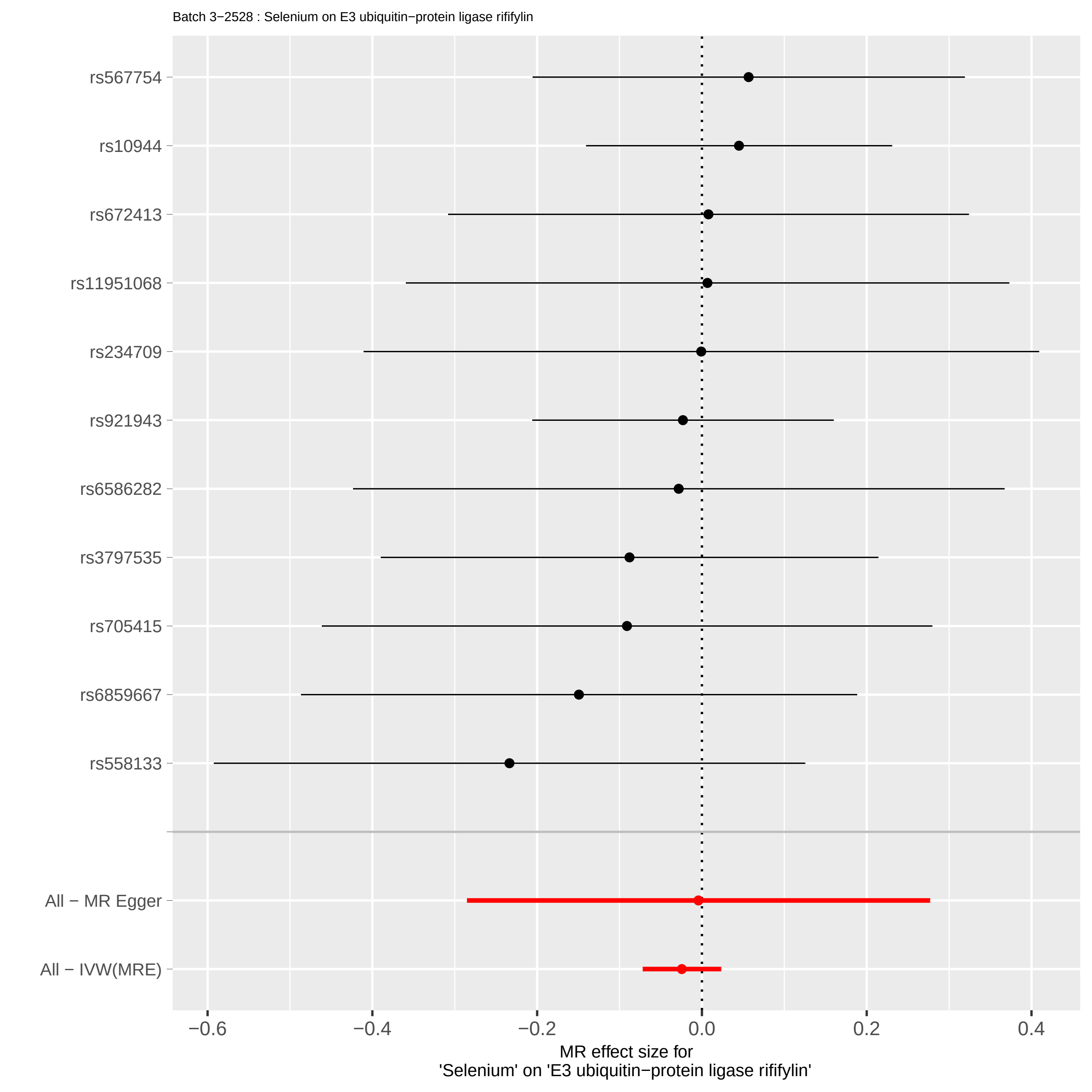

Batch 3−2528 : Selenium on E3 ubiquitin−protein ligase rififylin
rs567754
rs10944
rs672413
rs11951068
rs234709
rs921943
rs6586282
rs3797535
rs705415
rs6859667
rs558133
All − MR Egger
All − IVW(MRE)
−0.6
−0.4
−0.2
0.0
0.2
0.4
MR effect size for
'Selenium' on 'E3 ubiquitin−protein ligase rififylin'

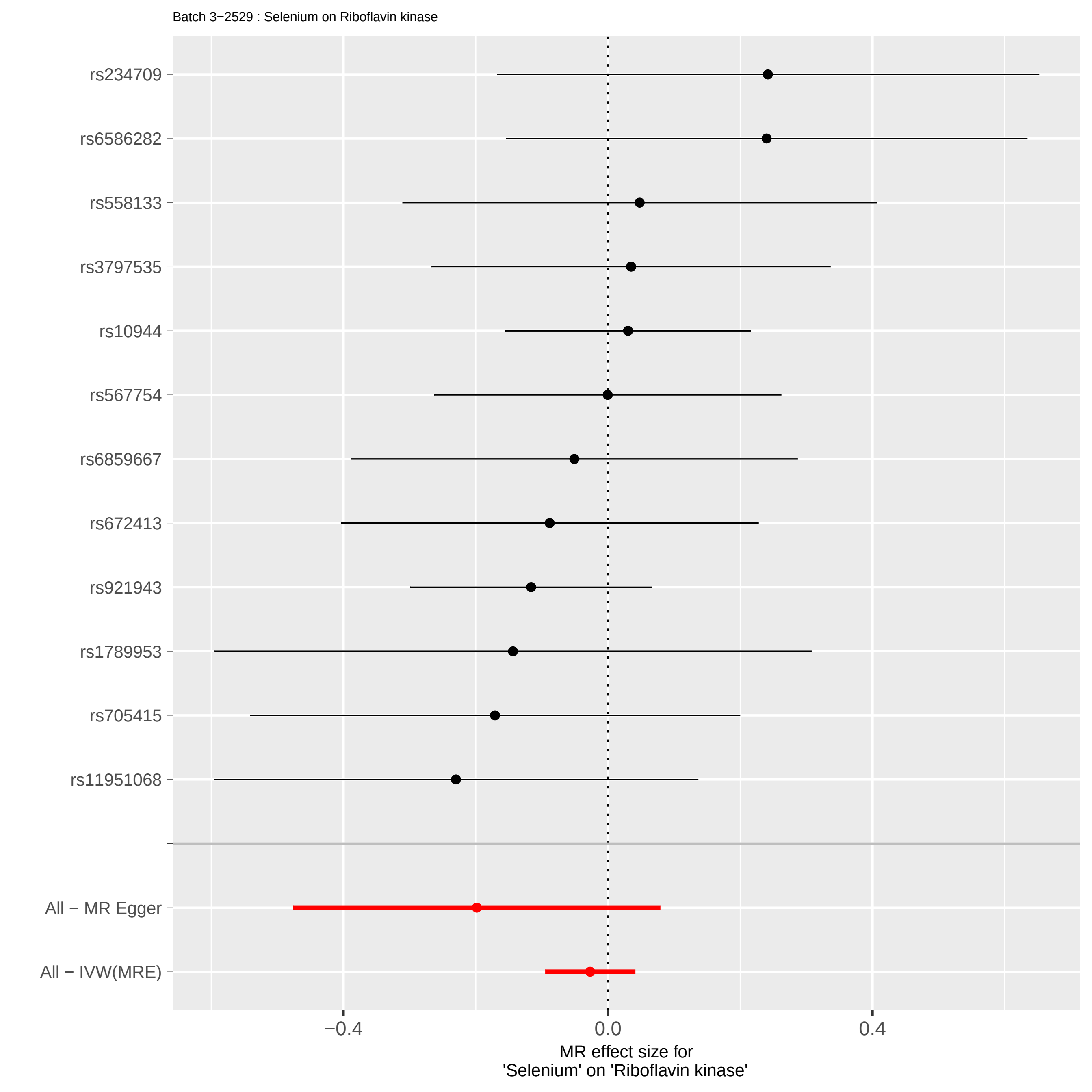

Batch 3−2529 : Selenium on Riboflavin kinase
rs234709
rs6586282
rs558133
rs3797535
rs10944
rs567754
rs6859667
rs672413
rs921943
rs1789953
rs705415
rs11951068
All − MR Egger
All − IVW(MRE)
−0.4
0.0
0.4
MR effect size for
'Selenium' on 'Riboflavin kinase'

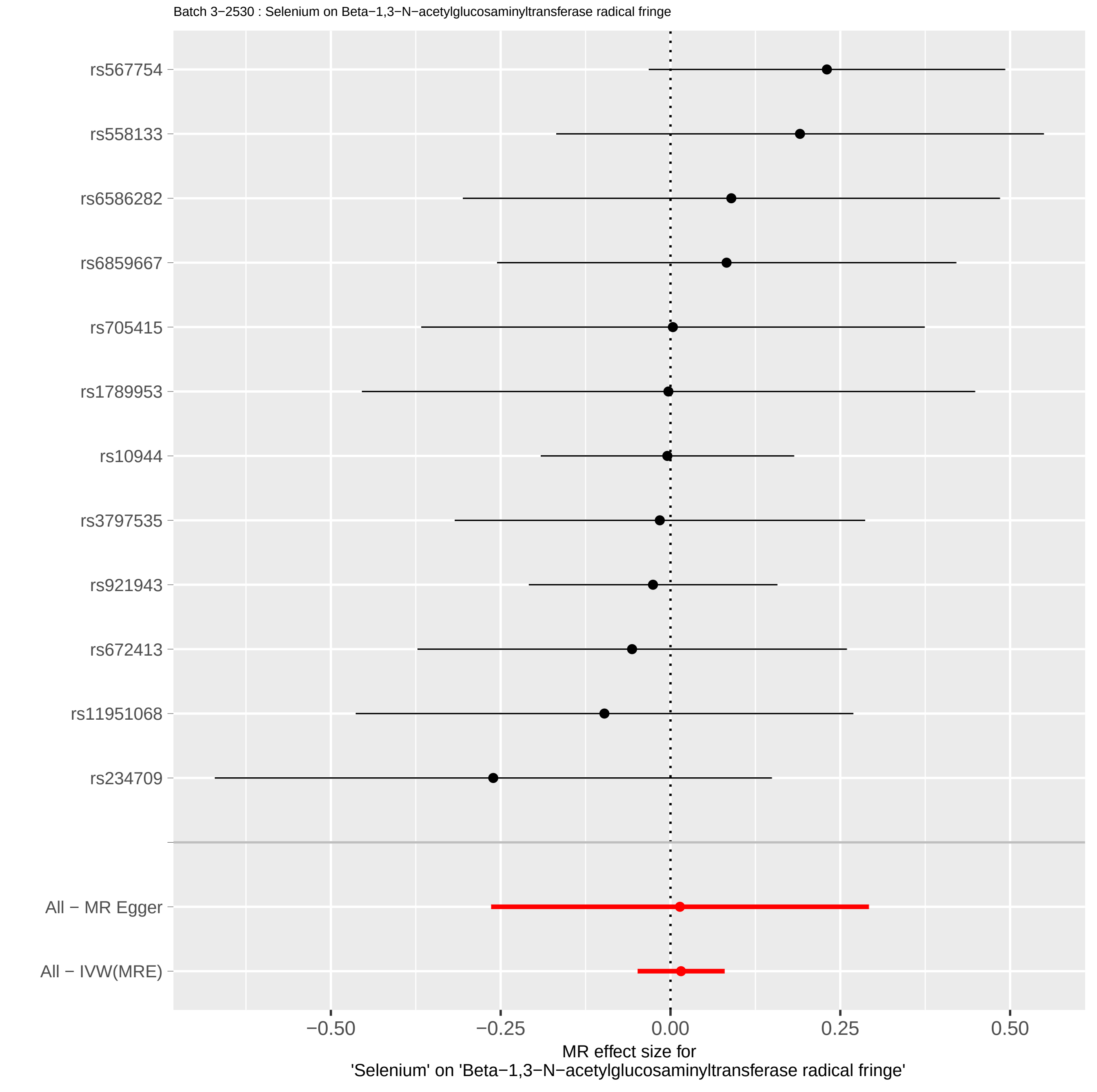
Batch 3−2530 : Selenium on Beta−1,3−N−acetylglucosaminyltransferase radical fringe
rs567754
rs558133
rs6586282
rs6859667
rs705415
rs1789953
rs10944
rs3797535
rs921943
rs672413
rs11951068
rs234709
All − MR Egger
All − IVW(MRE)
−0.50
−0.25
0.00
0.25
0.50
MR effect size for
'Selenium' on 'Beta−1,3−N−acetylglucosaminyltransferase radical fringe'

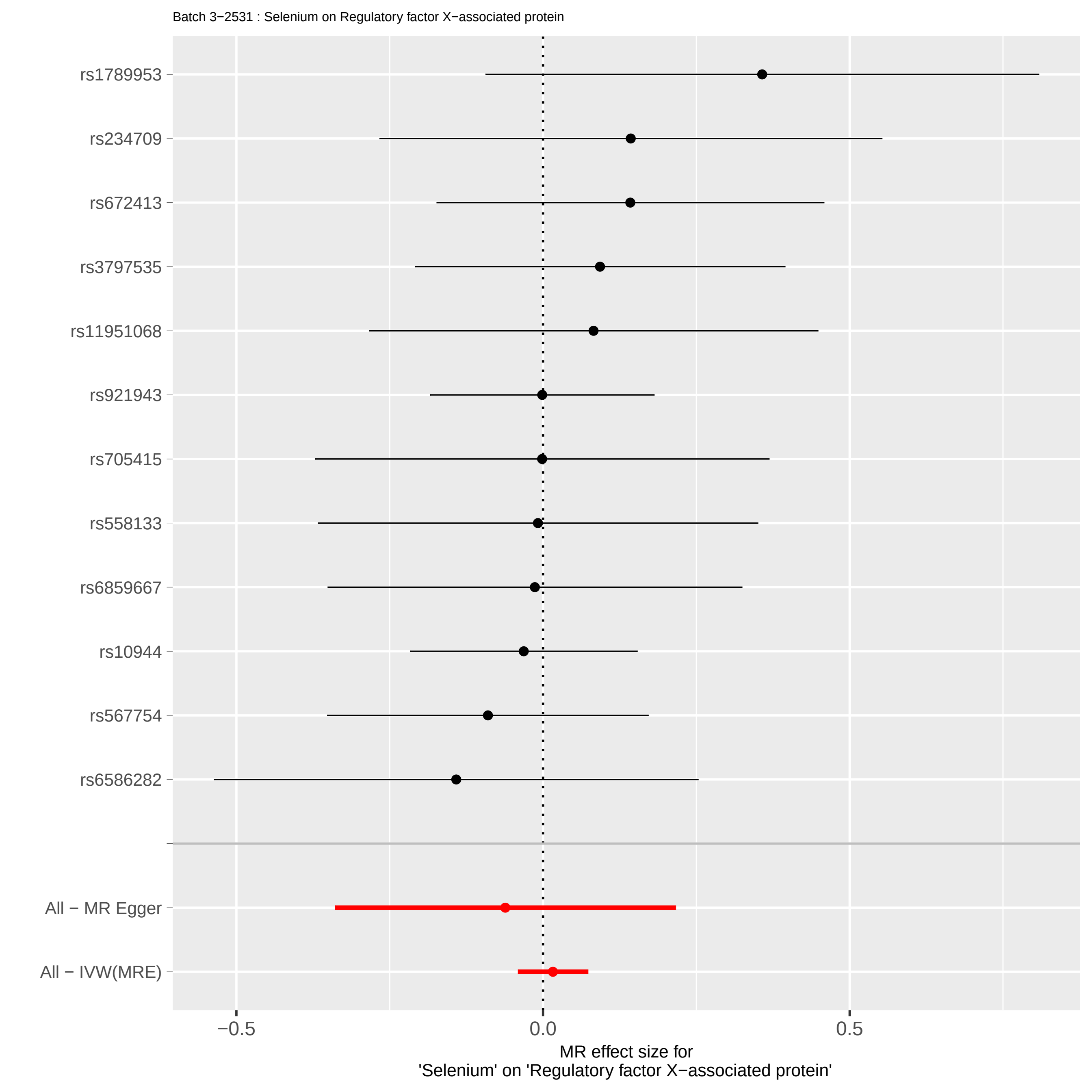

Batch 3−2531 : Selenium on Regulatory factor X−associated protein
rs1789953
rs234709
rs672413
rs3797535
rs11951068
rs921943
rs705415
rs558133
rs6859667
rs10944
rs567754
rs6586282
All − MR Egger
All − IVW(MRE)
−0.5
0.0
0.5
MR effect size for
'Selenium' on 'Regulatory factor X−associated protein'

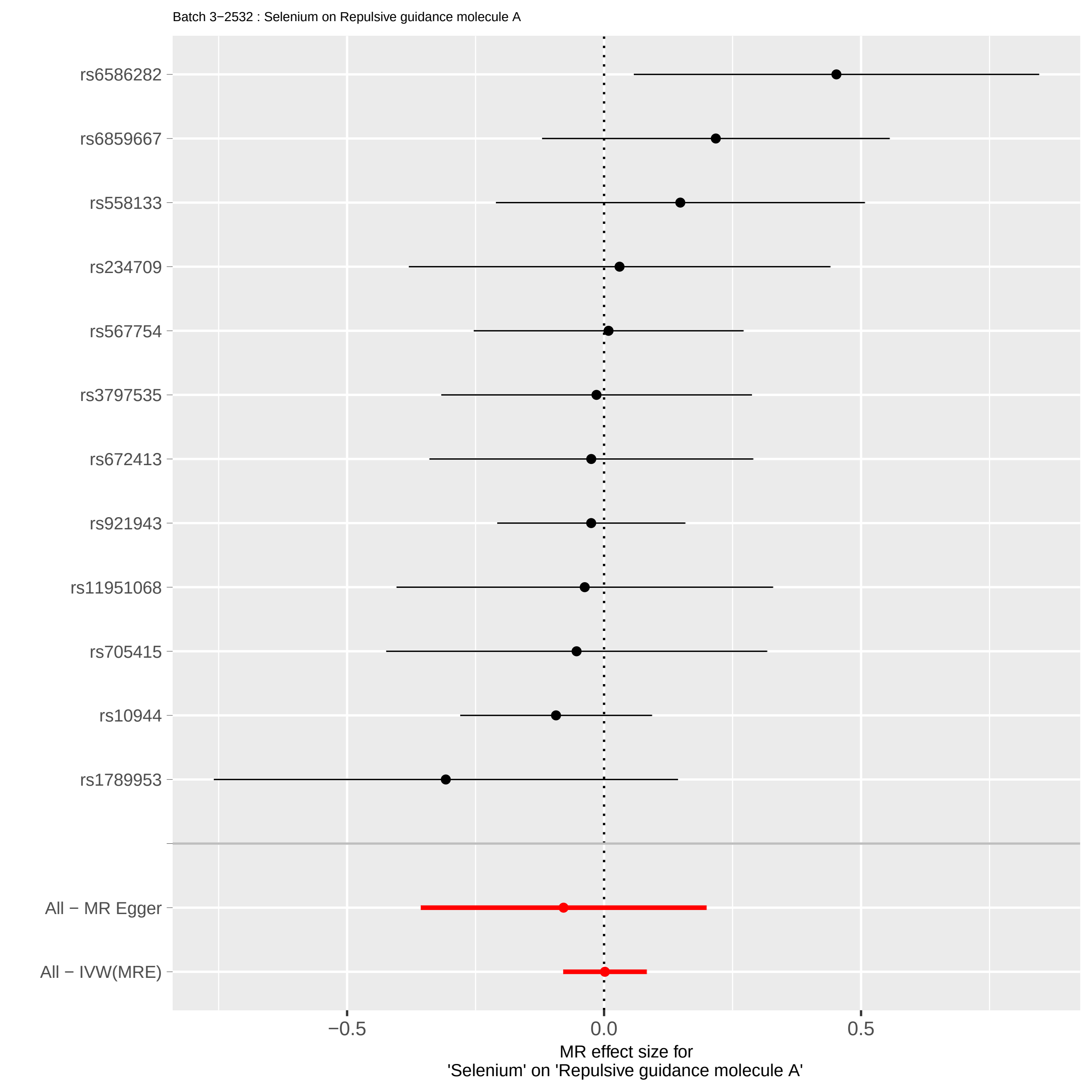

Batch 3−2532 : Selenium on Repulsive guidance molecule A
rs6586282
rs6859667
rs558133
rs234709
rs567754
rs3797535
rs672413
rs921943
rs11951068
rs705415
rs10944
rs1789953
All − MR Egger
All − IVW(MRE)
−0.5
0.0
0.5
MR effect size for
'Selenium' on 'Repulsive guidance molecule A'

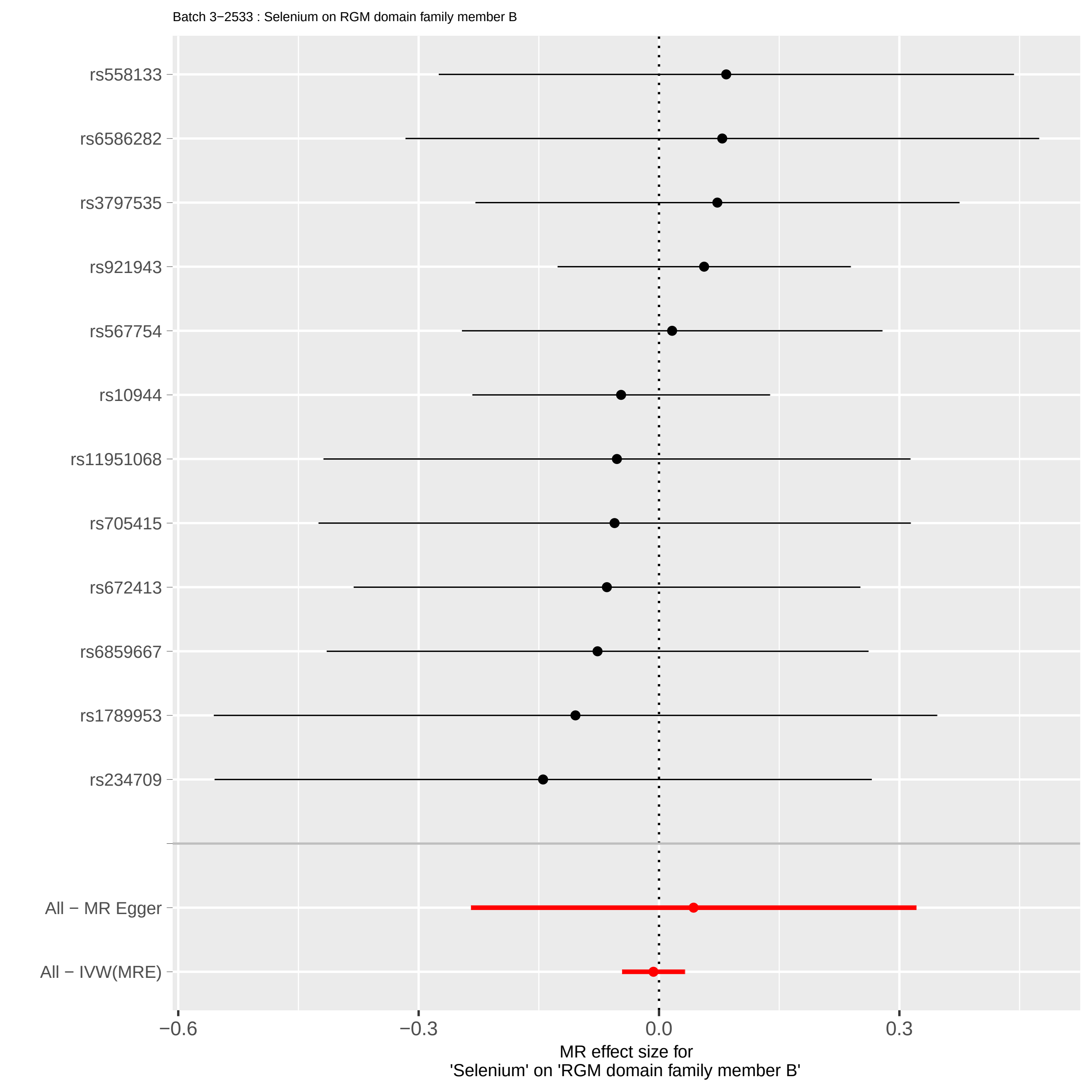

Batch 3−2533 : Selenium on RGM domain family member B
rs558133
rs6586282
rs3797535
rs921943
rs567754
rs10944
rs11951068
rs705415
rs672413
rs6859667
rs1789953
rs234709
All − MR Egger
All − IVW(MRE)
−0.6
−0.3
0.0
0.3
MR effect size for
'Selenium' on 'RGM domain family member B'

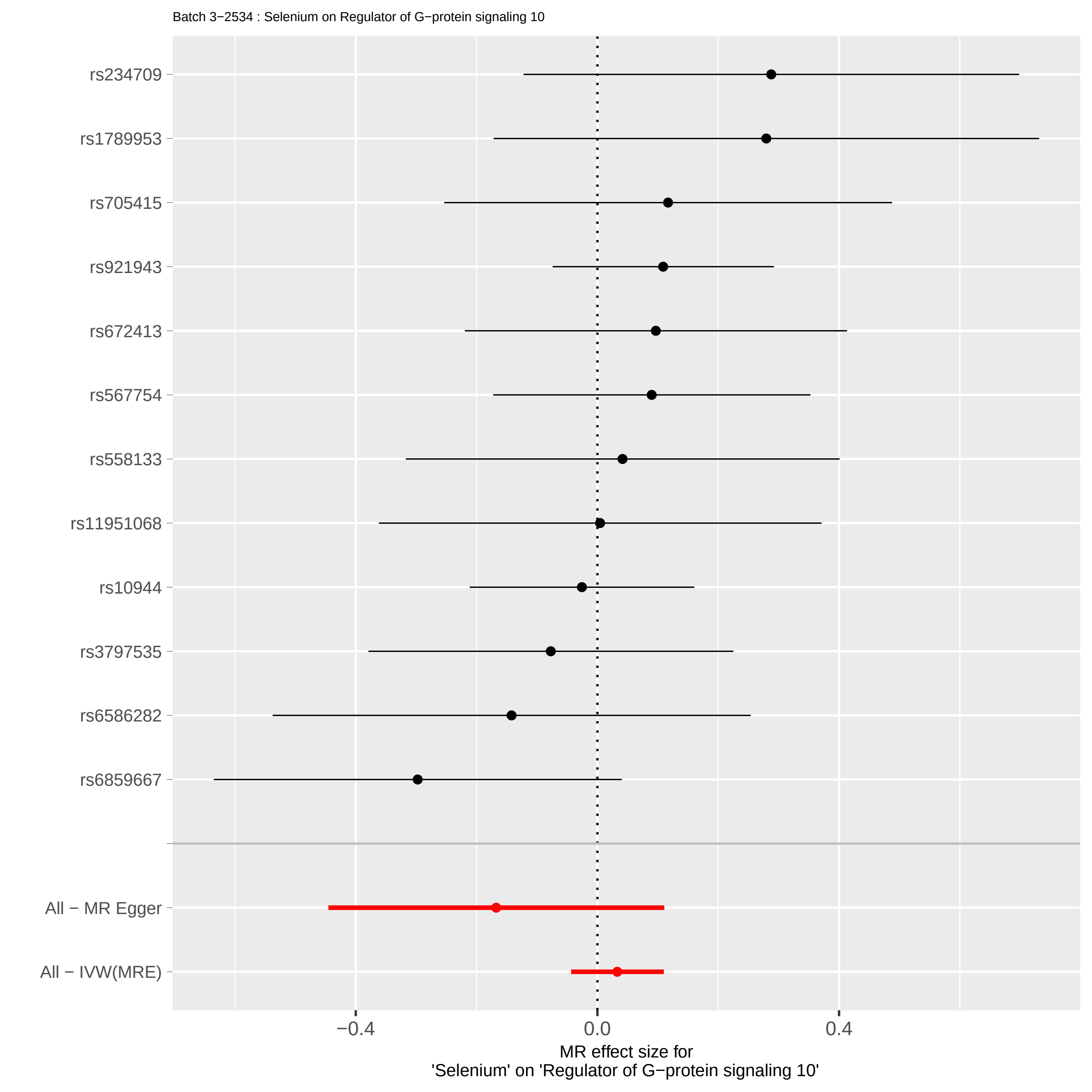

Batch 3−2534 : Selenium on Regulator of G−protein signaling 10
rs234709
rs1789953
rs705415
rs921943
rs672413
rs567754
rs558133
rs11951068
rs10944
rs3797535
rs6586282
rs6859667
All − MR Egger
All − IVW(MRE)
−0.4
0.0
0.4
MR effect size for
'Selenium' on 'Regulator of G−protein signaling 10'

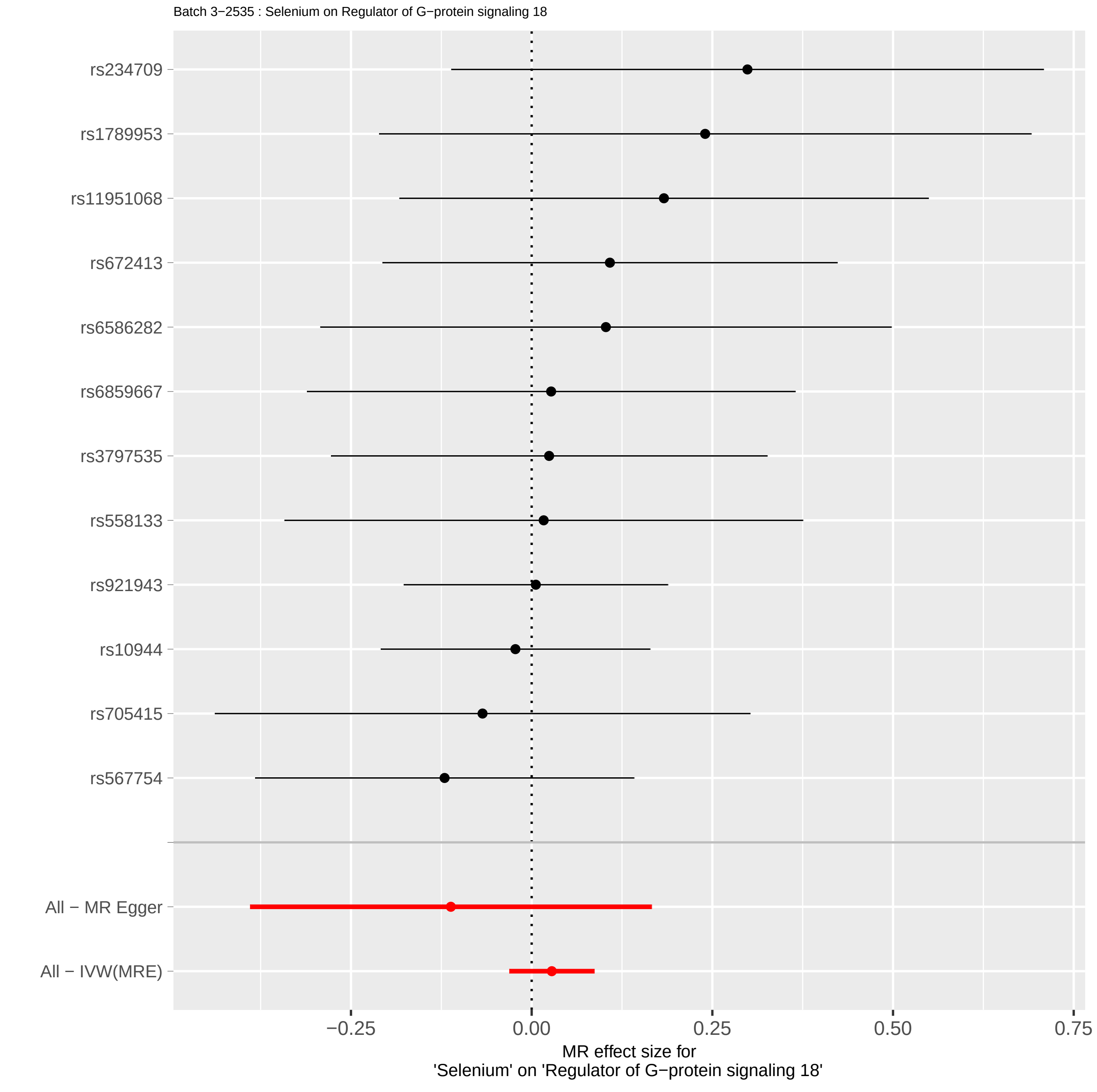

Batch 3−2535 : Selenium on Regulator of G−protein signaling 18
rs234709
rs1789953
rs11951068
rs672413
rs6586282
rs6859667
rs3797535
rs558133
rs921943
rs10944
rs705415
rs567754
All − MR Egger
All − IVW(MRE)
−0.25
0.00
0.25
0.50
0.75
MR effect size for
'Selenium' on 'Regulator of G−protein signaling 18'

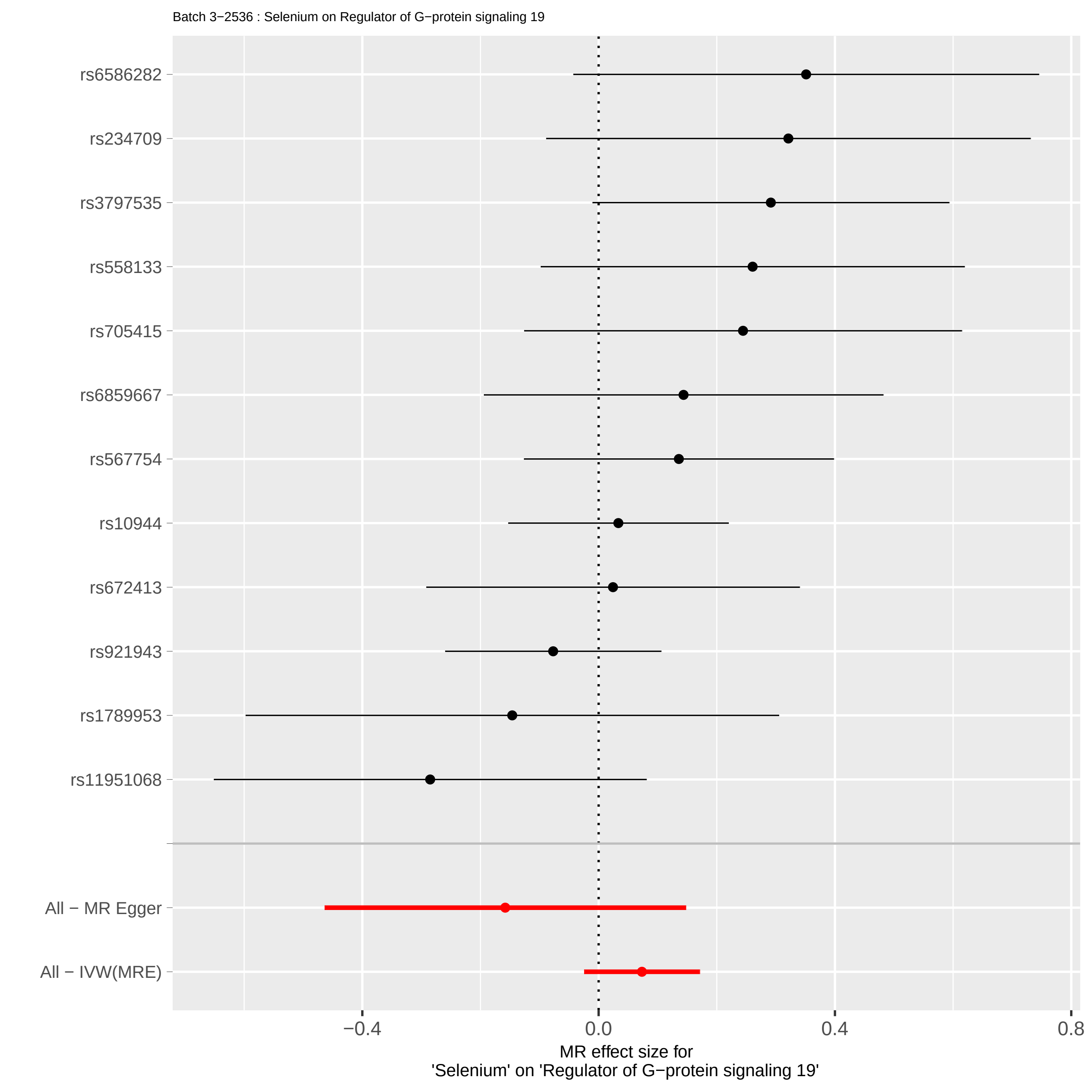
Batch 3−2536 : Selenium on Regulator of G−protein signaling 19
rs6586282
rs234709
rs3797535
rs558133
rs705415
rs6859667
rs567754
rs10944
rs672413
rs921943
rs1789953
rs11951068
All − MR Egger
All − IVW(MRE)
−0.4
0.0
0.4
0.8
MR effect size for
'Selenium' on 'Regulator of G−protein signaling 19'

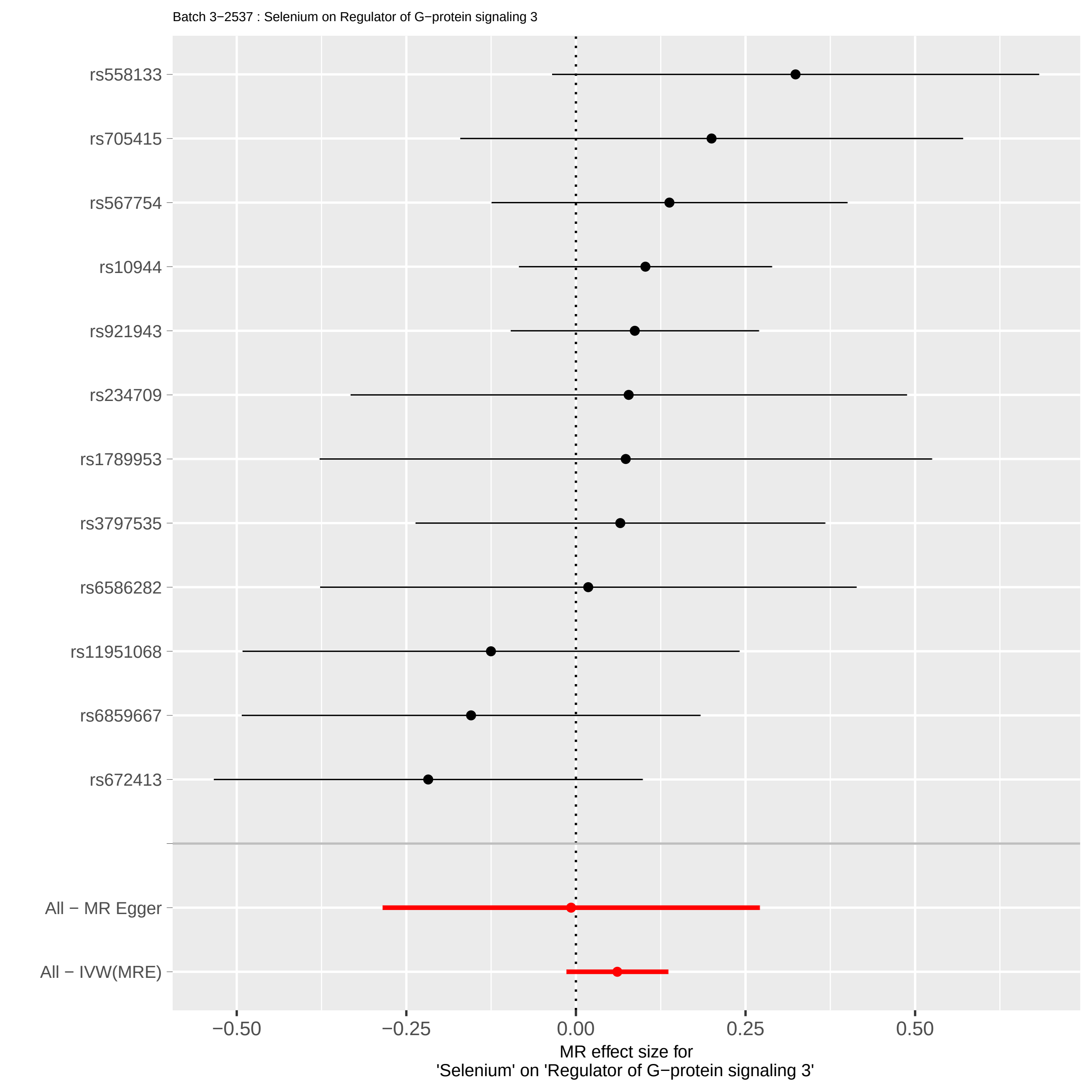

Batch 3−2537 : Selenium on Regulator of G−protein signaling 3
rs558133
rs705415
rs567754
rs10944
rs921943
rs234709
rs1789953
rs3797535
rs6586282
rs11951068
rs6859667
rs672413
All − MR Egger
All − IVW(MRE)
−0.50
−0.25
0.00
0.25
0.50
MR effect size for
'Selenium' on 'Regulator of G−protein signaling 3'

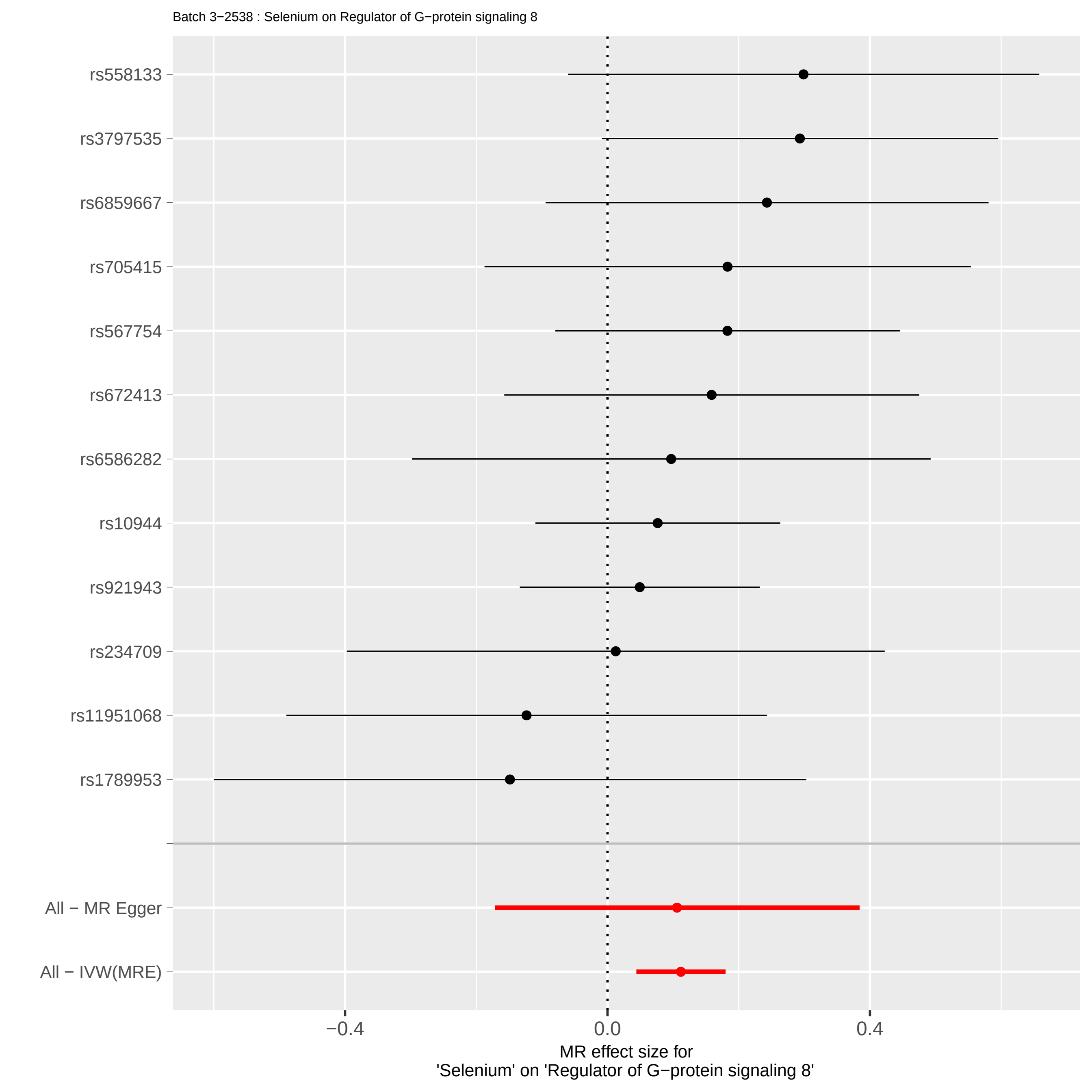

Batch 3−2538 : Selenium on Regulator of G−protein signaling 8
rs558133
rs3797535
rs6859667
rs705415
rs567754
rs672413
rs6586282
rs10944
rs921943
rs234709
rs11951068
rs1789953
All − MR Egger
All − IVW(MRE)
−0.4
0.0
0.4
MR effect size for
'Selenium' on 'Regulator of G−protein signaling 8'

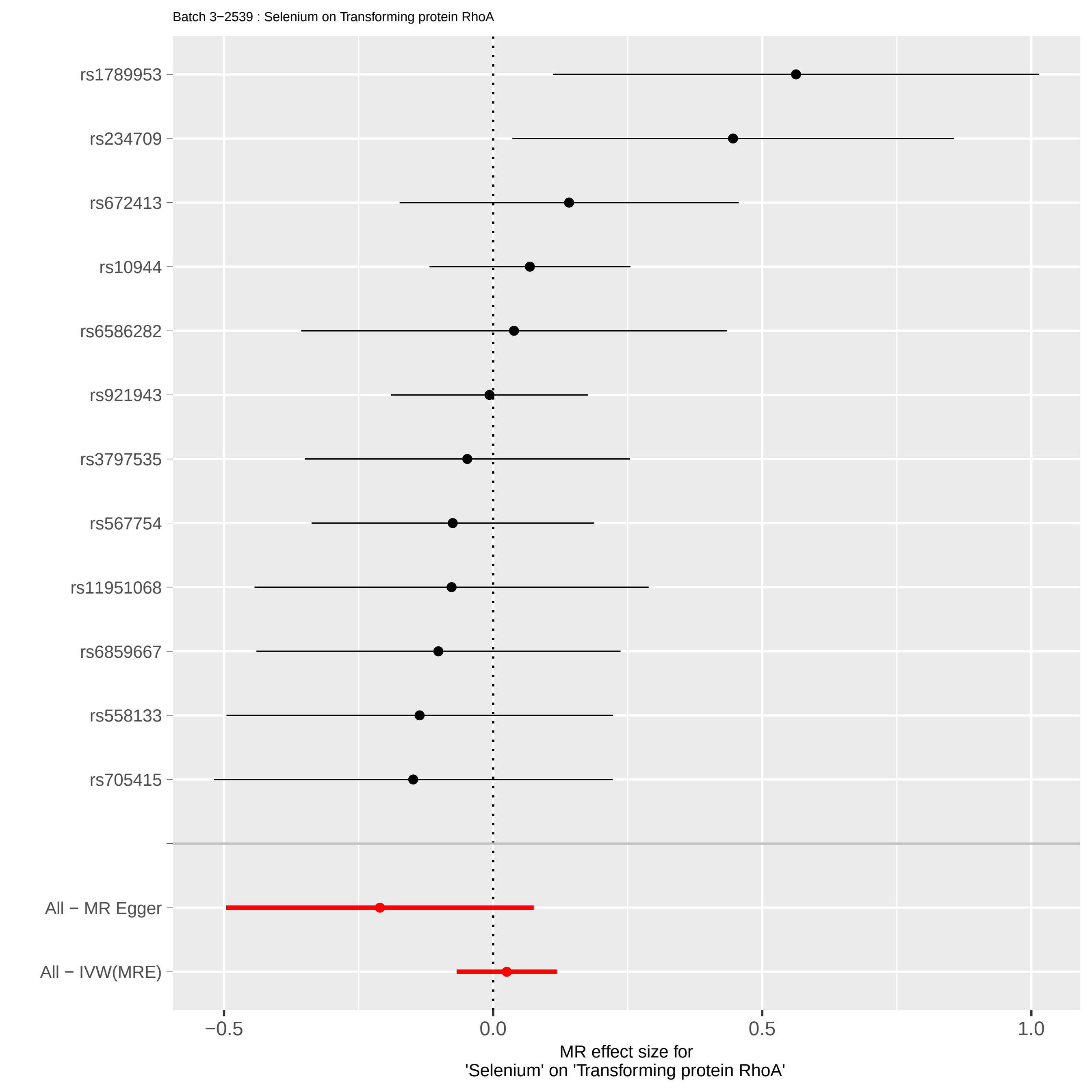
Batch 3−2539 : Selenium on Transforming protein RhoA
rs1789953
rs234709
rs672413
rs10944
rs6586282
rs921943
rs3797535
rs567754
rs11951068
rs6859667
rs558133
rs705415
All − MR Egger
All − IVW(MRE)
−0.5
0.0
0.5
1.0
MR effect size for
'Selenium' on 'Transforming protein RhoA'

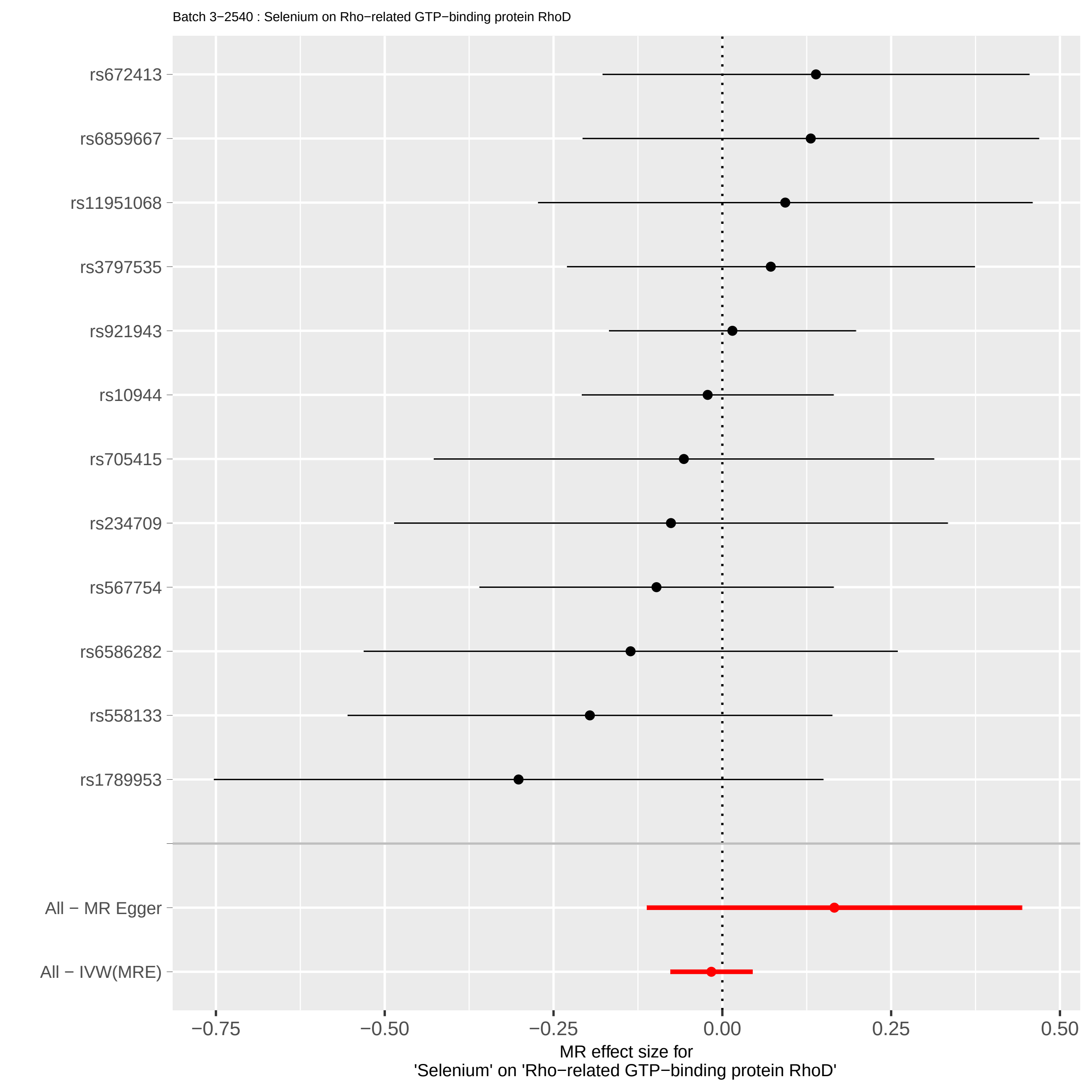
Batch 3−2540 : Selenium on Rho−related GTP−binding protein RhoD
rs672413
rs6859667
rs11951068
rs3797535
rs921943
rs10944
rs705415
rs234709
rs567754
rs6586282
rs558133
rs1789953
All − MR Egger
All − IVW(MRE)
−0.75
−0.50
−0.25
0.00
0.25
0.50
MR effect size for
'Selenium' on 'Rho−related GTP−binding protein RhoD'

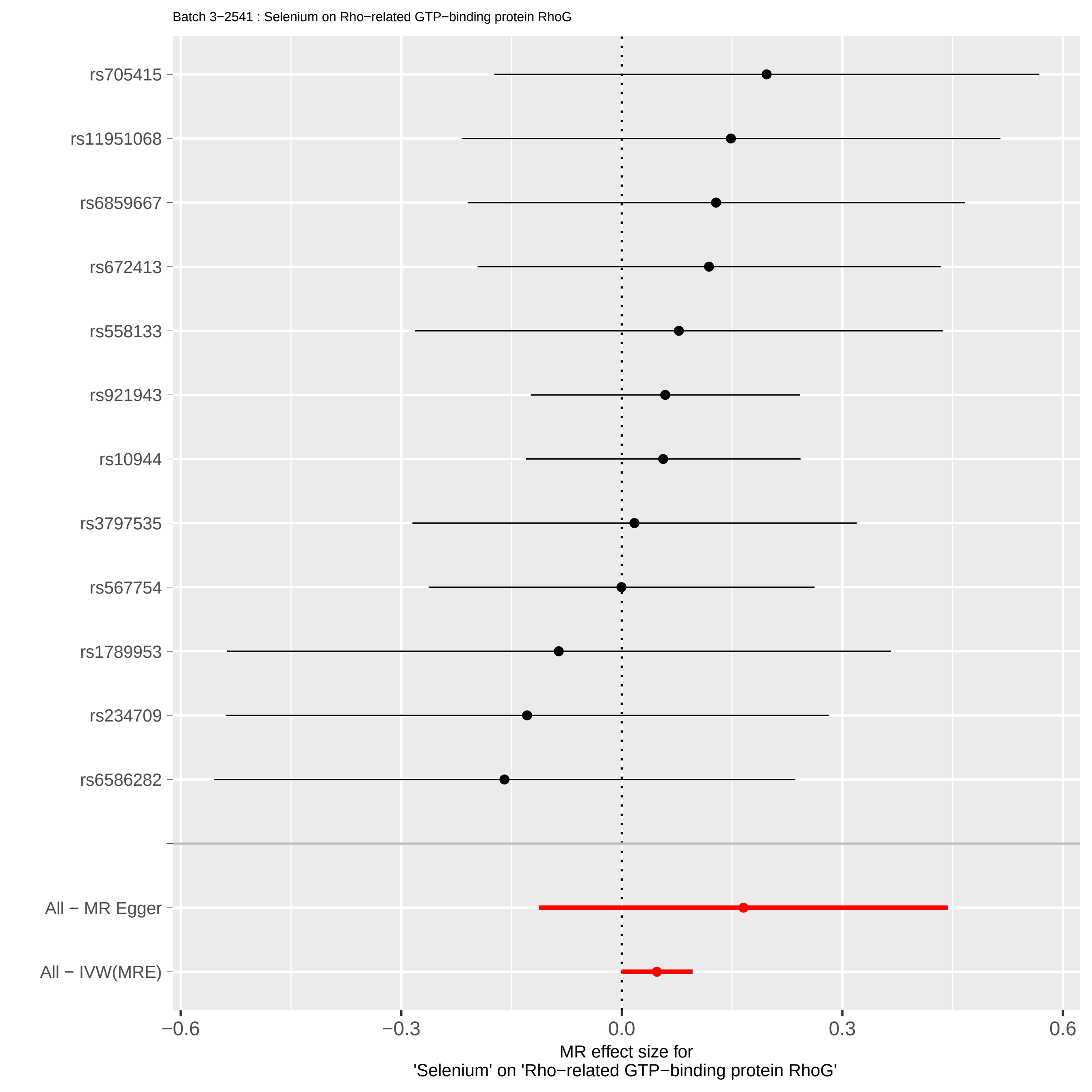

Batch 3−2541 : Selenium on Rho−related GTP−binding protein RhoG
rs705415
rs11951068
rs6859667
rs672413
rs558133
rs921943
rs10944
rs3797535
rs567754
rs1789953
rs234709
rs6586282
All − MR Egger
All − IVW(MRE)
−0.6
−0.3
0.0
0.3
0.6
MR effect size for
'Selenium' on 'Rho−related GTP−binding protein RhoG'

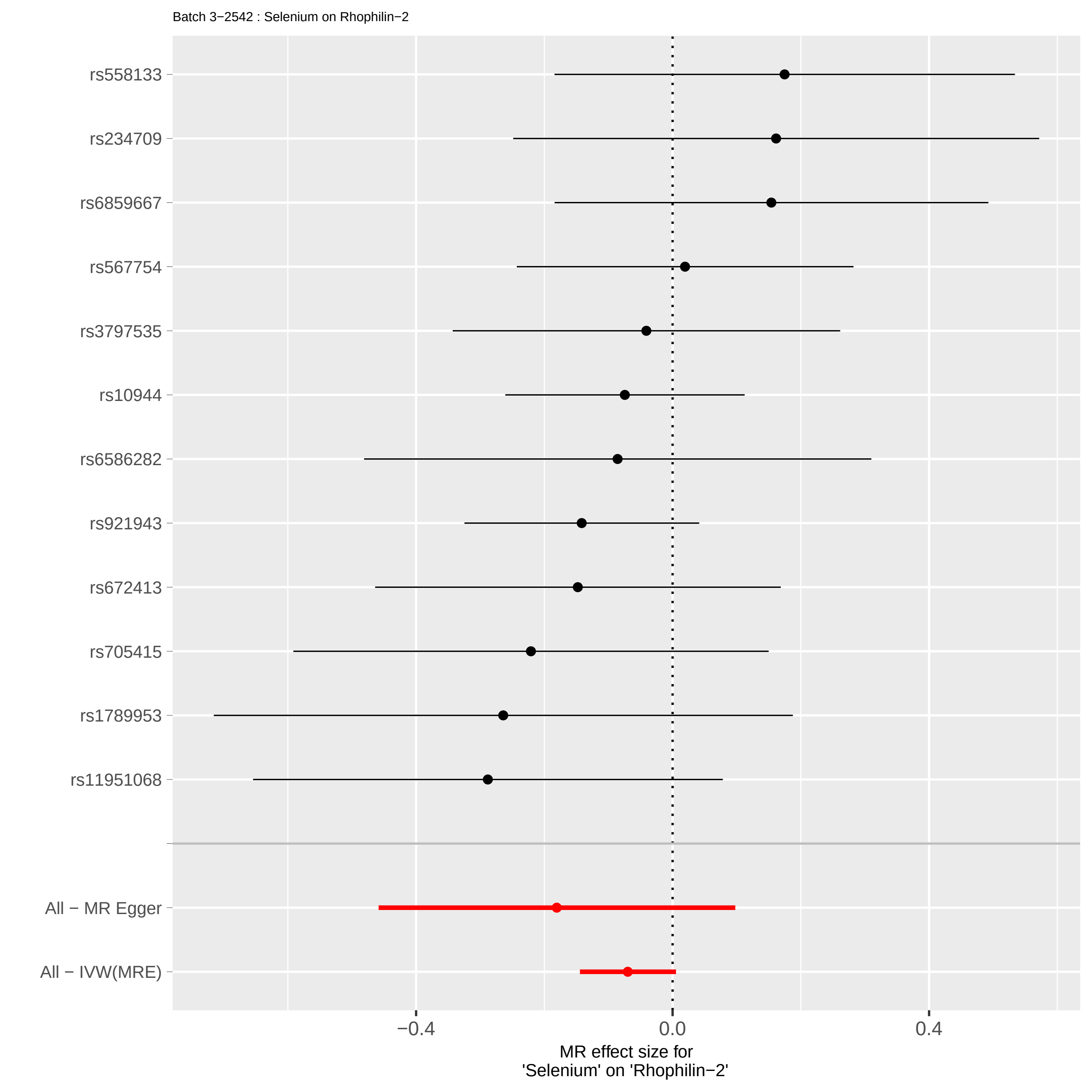
Batch 3−2542 : Selenium on Rhophilin−2
rs558133
rs234709
rs6859667
rs567754
rs3797535
rs10944
rs6586282
rs921943
rs672413
rs705415
rs1789953
rs11951068
All − MR Egger
All − IVW(MRE)
−0.4
0.0
0.4
MR effect size for
'Selenium' on 'Rhophilin−2'

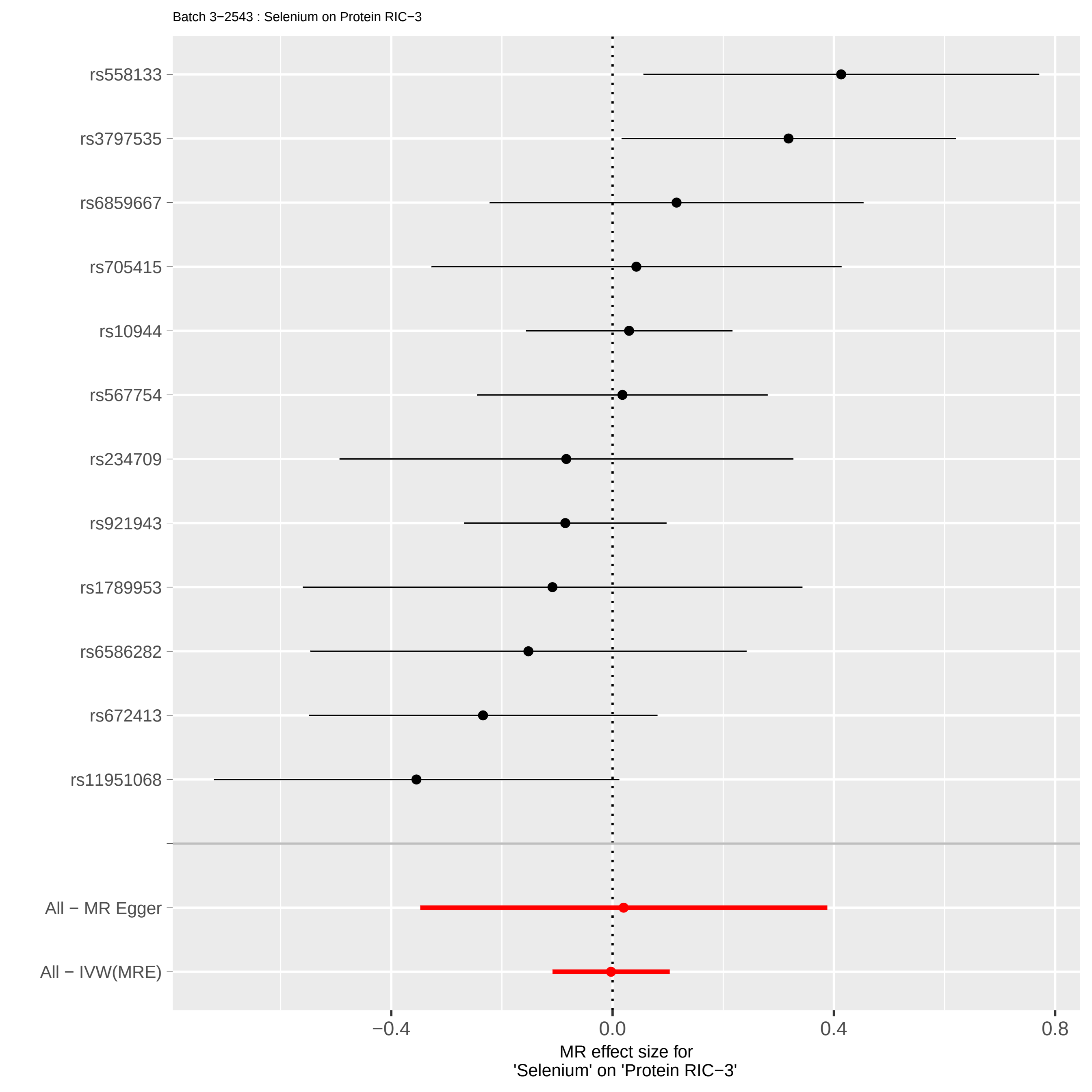

Batch 3−2543 : Selenium on Protein RIC−3
rs558133
rs3797535
rs6859667
rs705415
rs10944
rs567754
rs234709
rs921943
rs1789953
rs6586282
rs672413
rs11951068
All − MR Egger
All − IVW(MRE)
−0.4
0.0
0.4
0.8
MR effect size for
'Selenium' on 'Protein RIC−3'

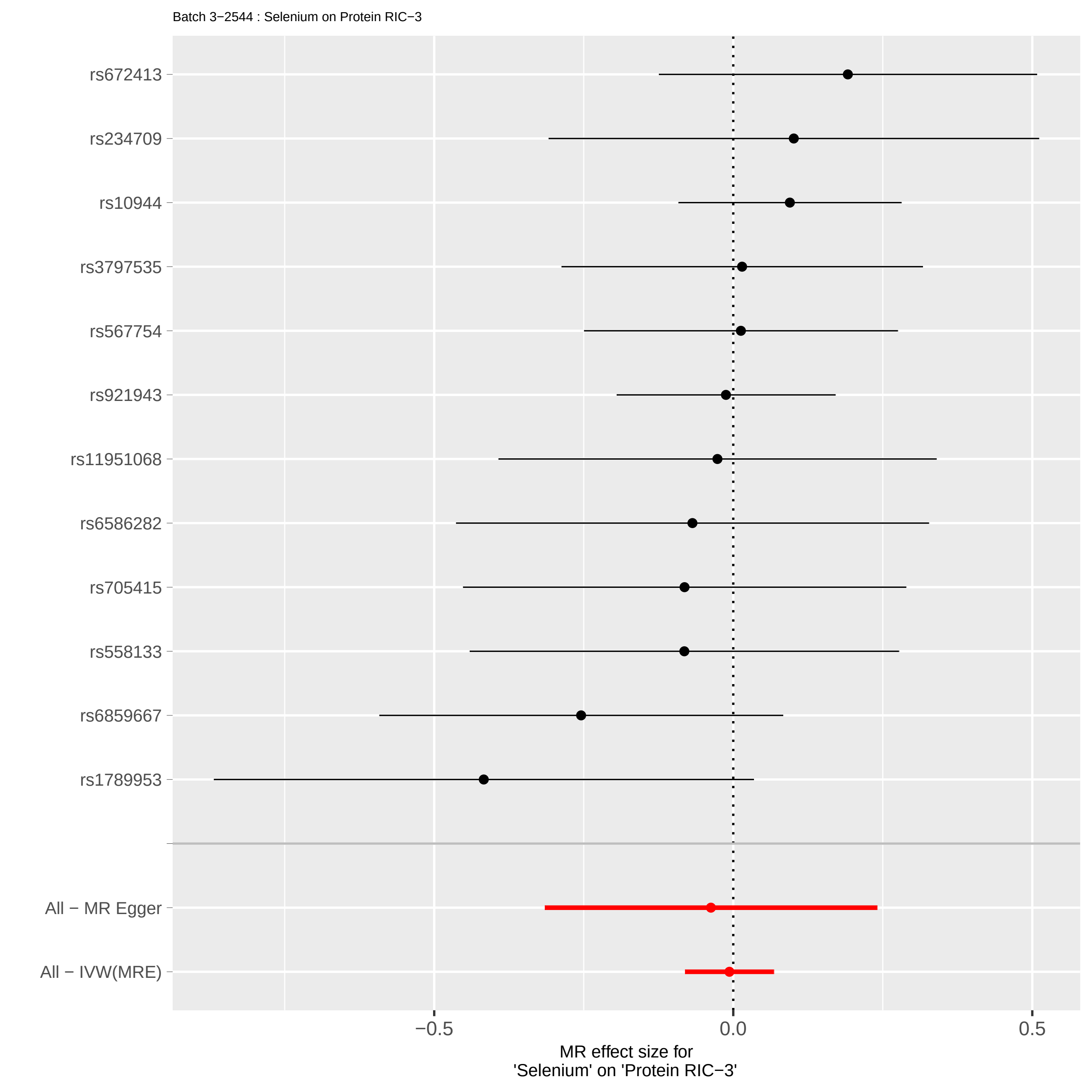

Batch 3−2544 : Selenium on Protein RIC−3
rs672413
rs234709
rs10944
rs3797535
rs567754
rs921943
rs11951068
rs6586282
rs705415
rs558133
rs6859667
rs1789953
All − MR Egger
All − IVW(MRE)
−0.5
0.0
0.5
MR effect size for
'Selenium' on 'Protein RIC−3'

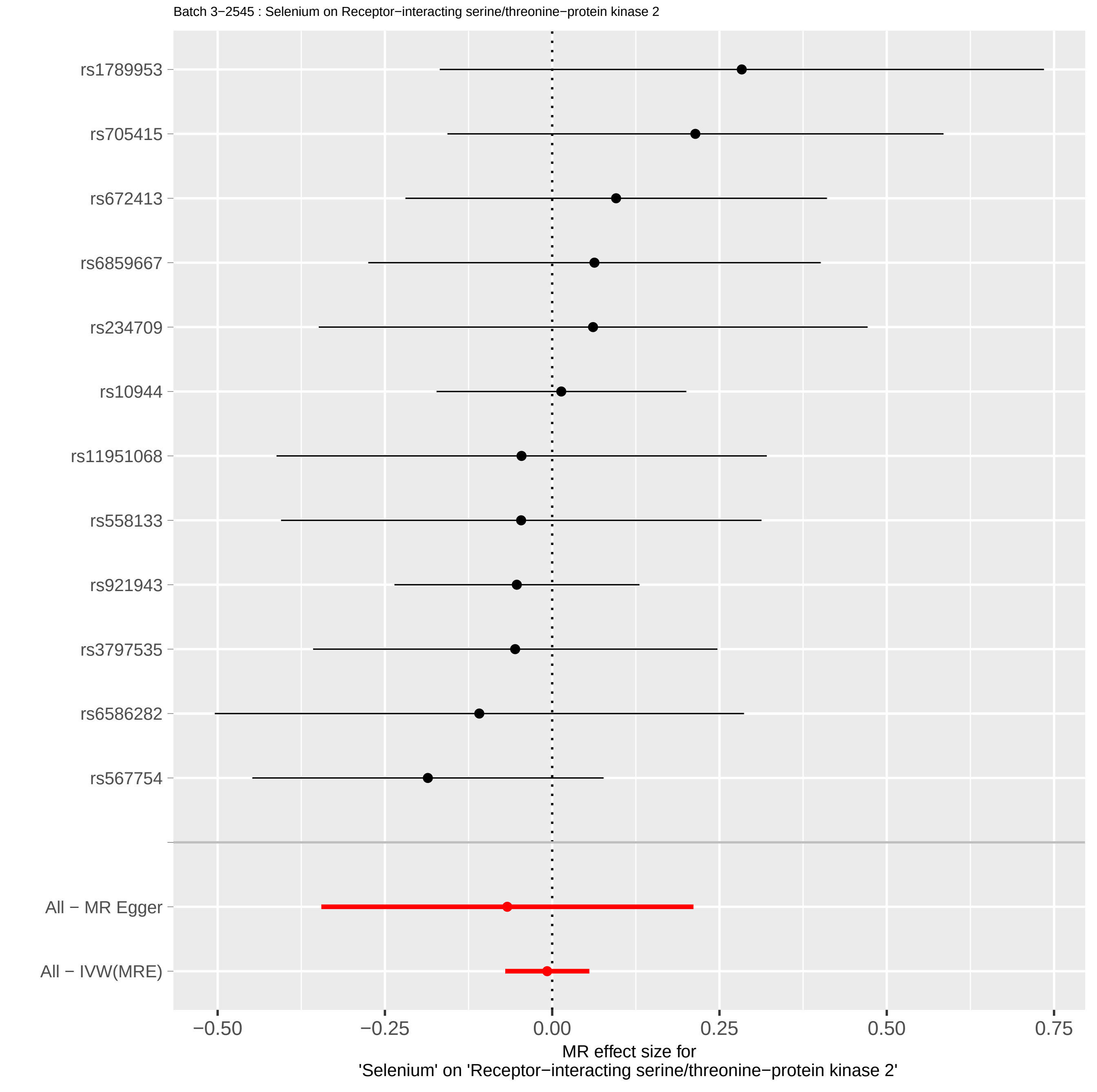

Batch 3−2545 : Selenium on Receptor−interacting serine/threonine−protein kinase 2
rs1789953
rs705415
rs672413
rs6859667
rs234709
rs10944
rs11951068
rs558133
rs921943
rs3797535
rs6586282
rs567754
All − MR Egger
All − IVW(MRE)
−0.50
−0.25
0.00
0.25
0.50
0.75
MR effect size for
'Selenium' on 'Receptor−interacting serine/threonine−protein kinase 2'

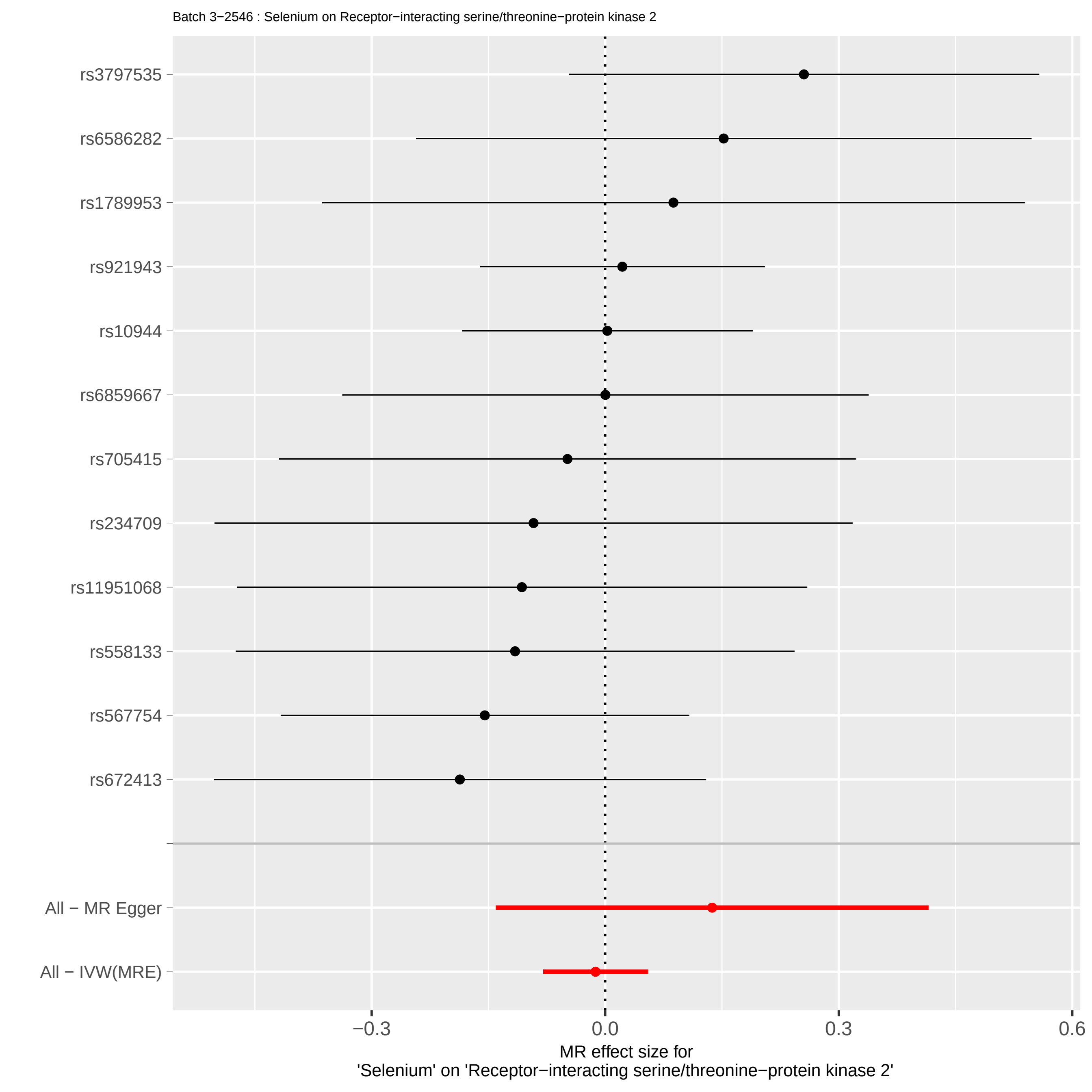

Batch 3−2546 : Selenium on Receptor−interacting serine/threonine−protein kinase 2
rs3797535
rs6586282
rs1789953
rs921943
rs10944
rs6859667
rs705415
rs234709
rs11951068
rs558133
rs567754
rs672413
All − MR Egger
All − IVW(MRE)
−0.3
0.0
0.3
0.6
MR effect size for
'Selenium' on 'Receptor−interacting serine/threonine−protein kinase 2'

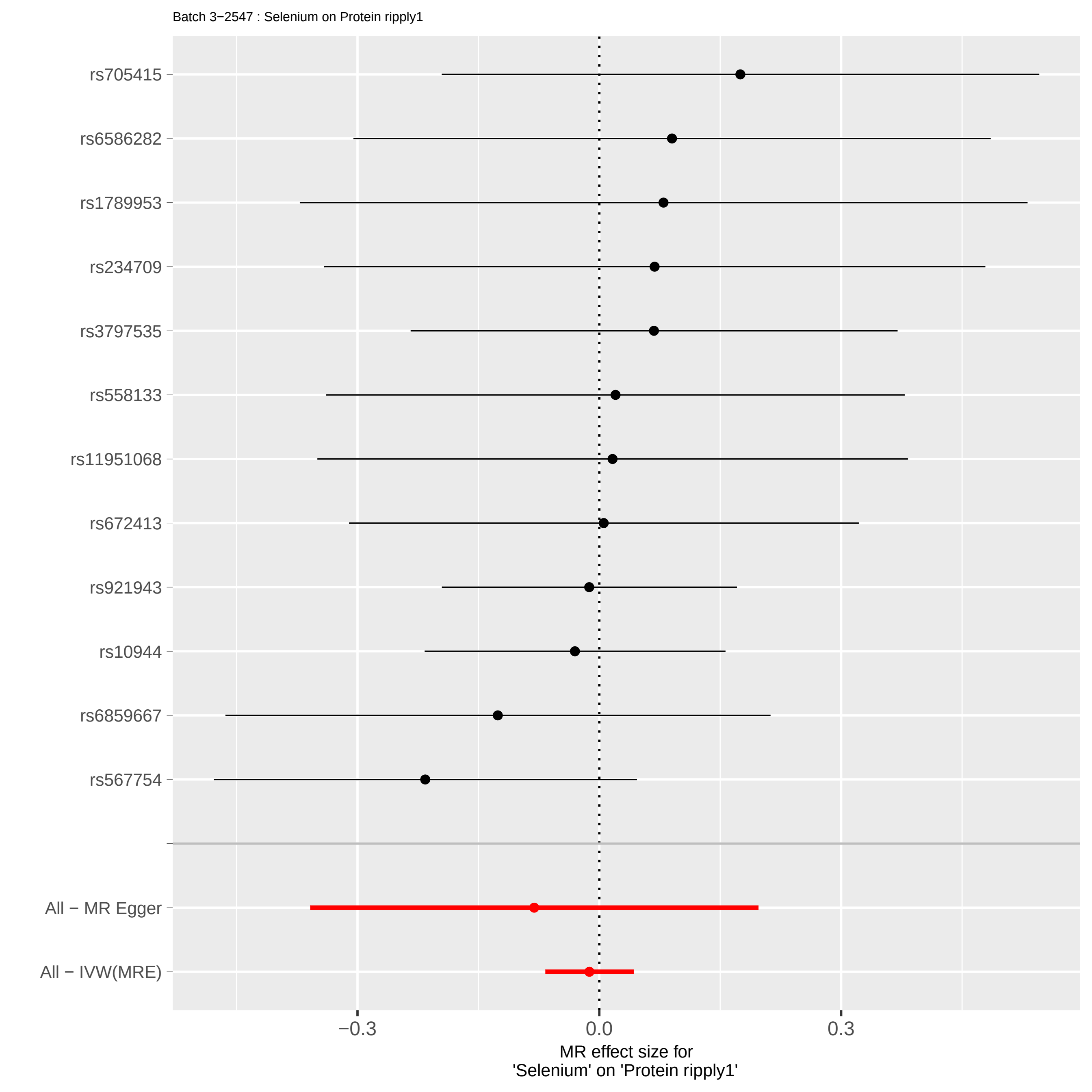
Batch 3−2547 : Selenium on Protein ripply1
rs705415
rs6586282
rs1789953
rs234709
rs3797535
rs558133
rs11951068
rs672413
rs921943
rs10944
rs6859667
rs567754
All − MR Egger
All − IVW(MRE)
−0.3
0.0
0.3
MR effect size for
'Selenium' on 'Protein ripply1'

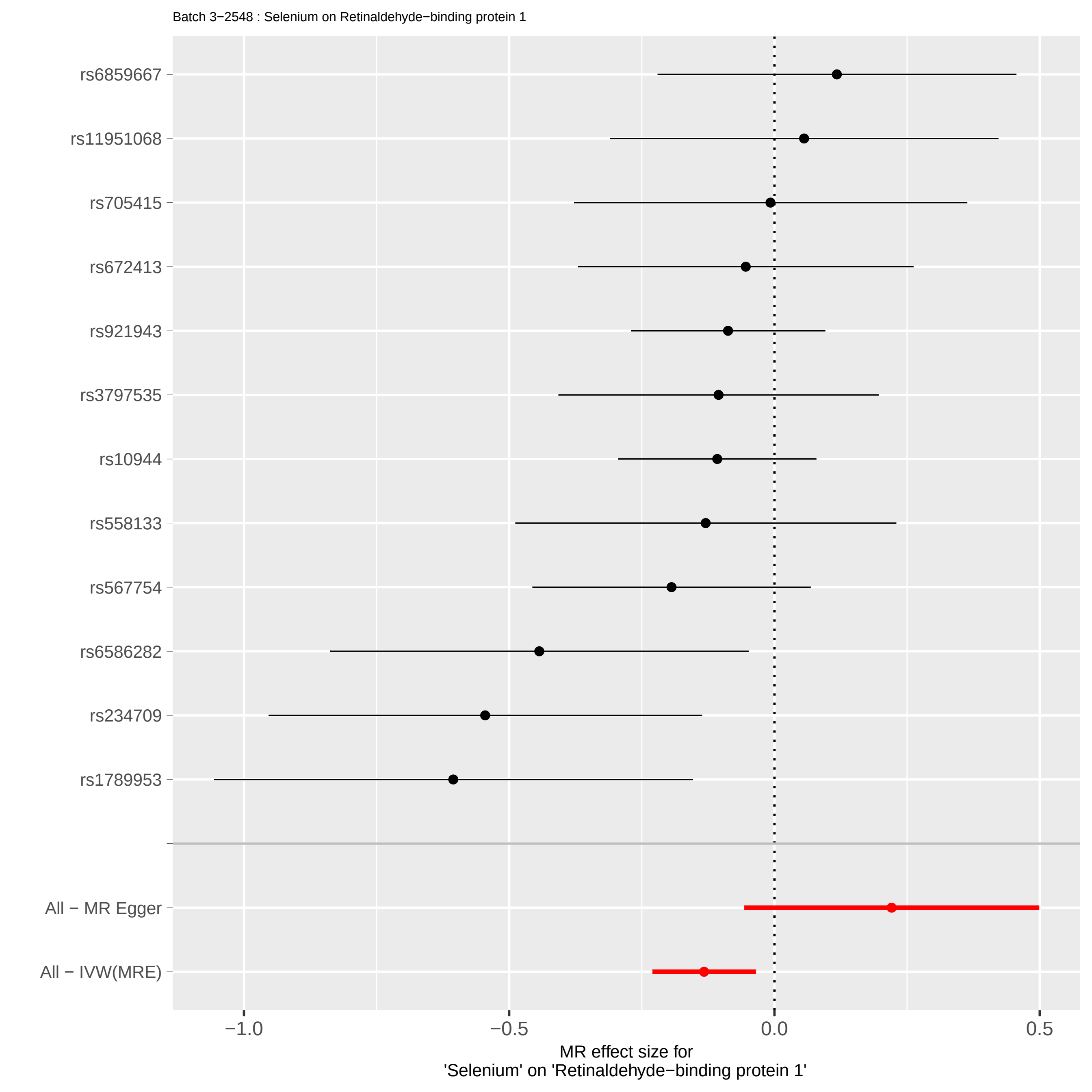

Batch 3−2548 : Selenium on Retinaldehyde−binding protein 1
rs6859667
rs11951068
rs705415
rs672413
rs921943
rs3797535
rs10944
rs558133
rs567754
rs6586282
rs234709
rs1789953
All − MR Egger
All − IVW(MRE)
−1.0
−0.5
0.0
0.5
MR effect size for
'Selenium' on 'Retinaldehyde−binding protein 1'

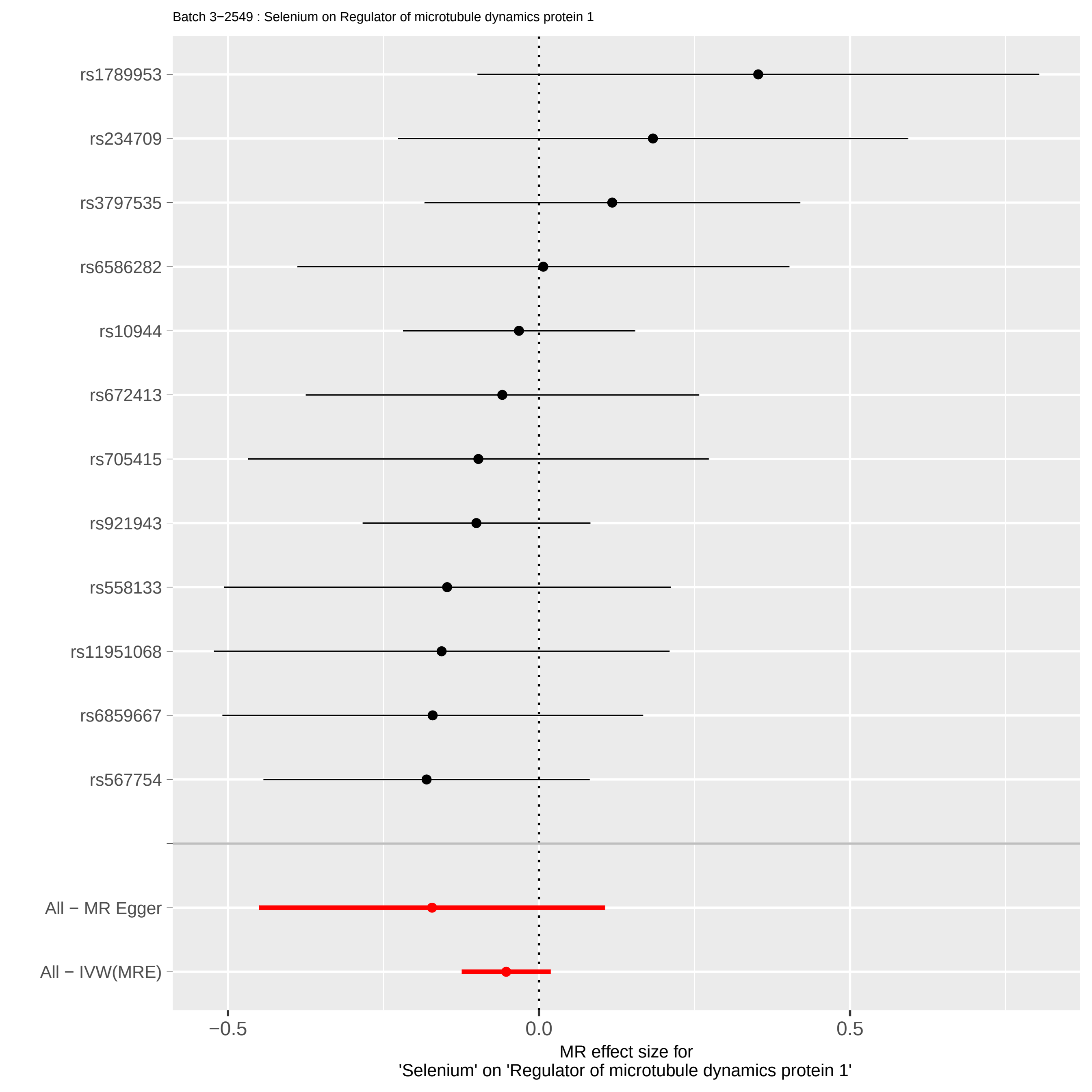

Batch 3−2549 : Selenium on Regulator of microtubule dynamics protein 1
rs1789953
rs234709
rs3797535
rs6586282
rs10944
rs672413
rs705415
rs921943
rs558133
rs11951068
rs6859667
rs567754
All − MR Egger
All − IVW(MRE)
−0.5
0.0
0.5
MR effect size for
'Selenium' on 'Regulator of microtubule dynamics protein 1'

Batch 3−2550 : Selenium on Regulator of microtubule dynamics protein 3

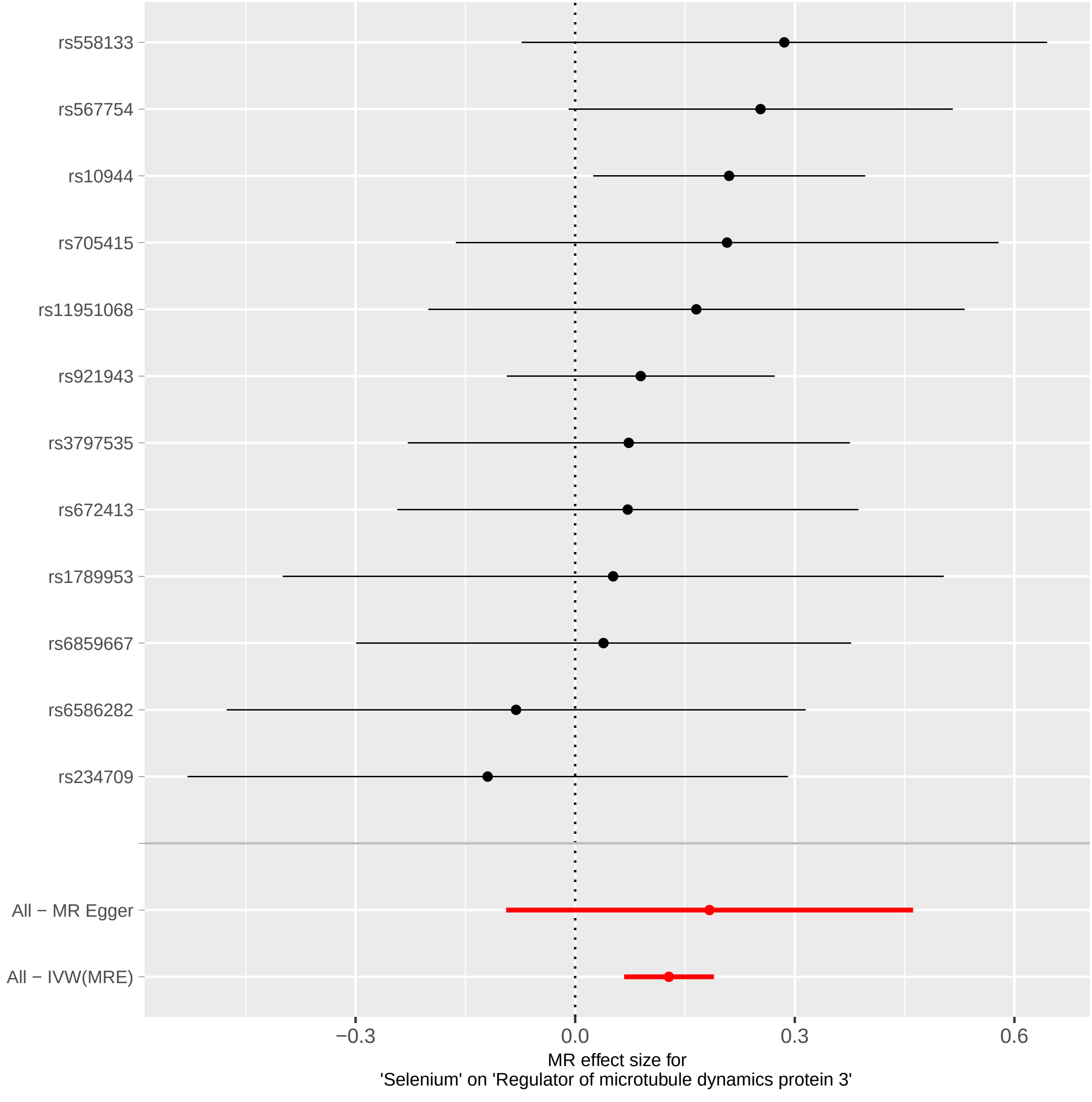

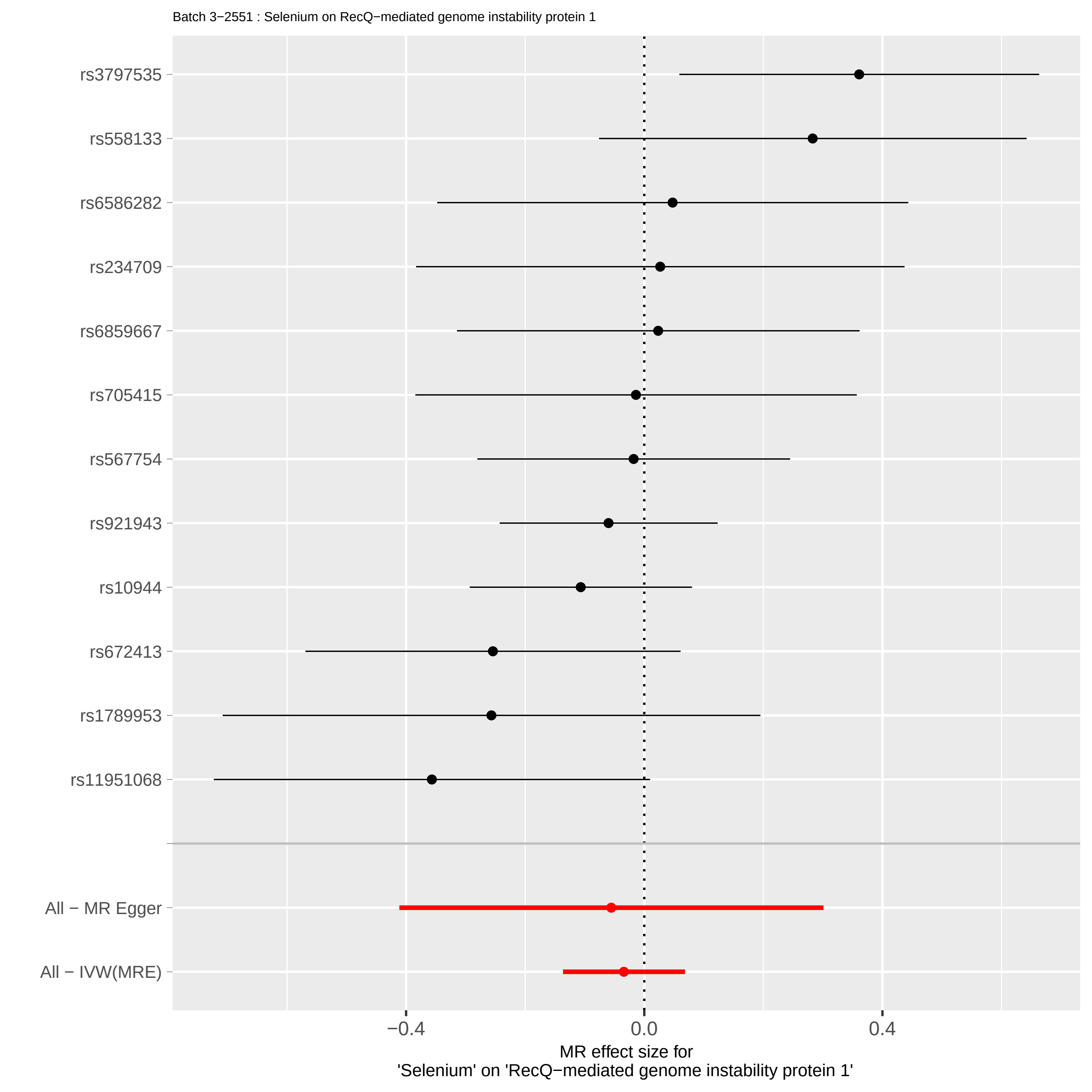
Batch 3−2551 : Selenium on RecQ−mediated genome instability protein 1
rs3797535
rs558133
rs6586282
rs234709
rs6859667
rs705415
rs567754
rs921943
rs10944
rs672413
rs1789953
rs11951068
All − MR Egger
All − IVW(MRE)
−0.4
0.0
0.4
MR effect size for
'Selenium' on 'RecQ−mediated genome instability protein 1'

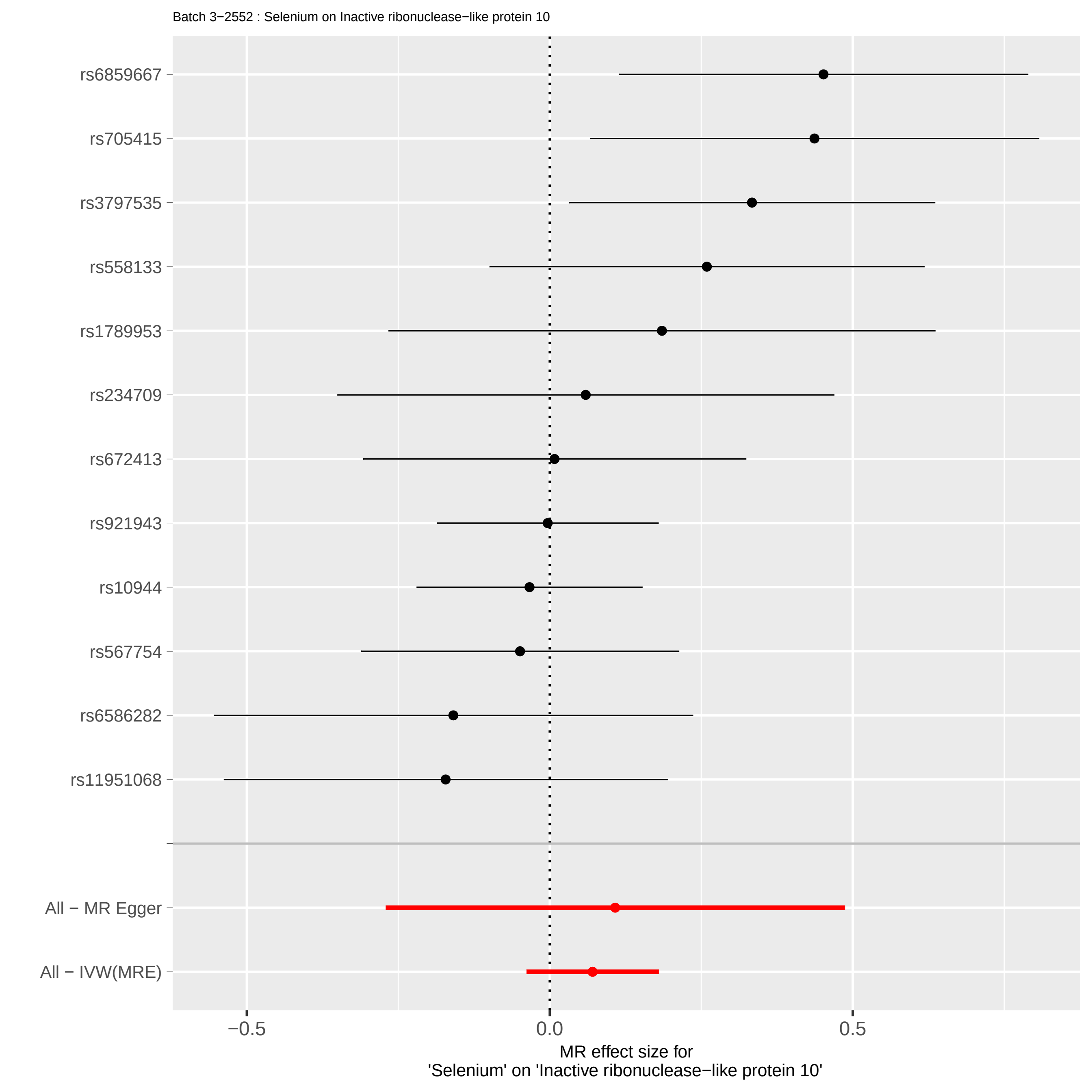

Batch 3−2552 : Selenium on Inactive ribonuclease−like protein 10
rs6859667
rs705415
rs3797535
rs558133
rs1789953
rs234709
rs672413
rs921943
rs10944
rs567754
rs6586282
rs11951068
All − MR Egger
All − IVW(MRE)
−0.5
0.0
0.5
MR effect size for
'Selenium' on 'Inactive ribonuclease−like protein 10'

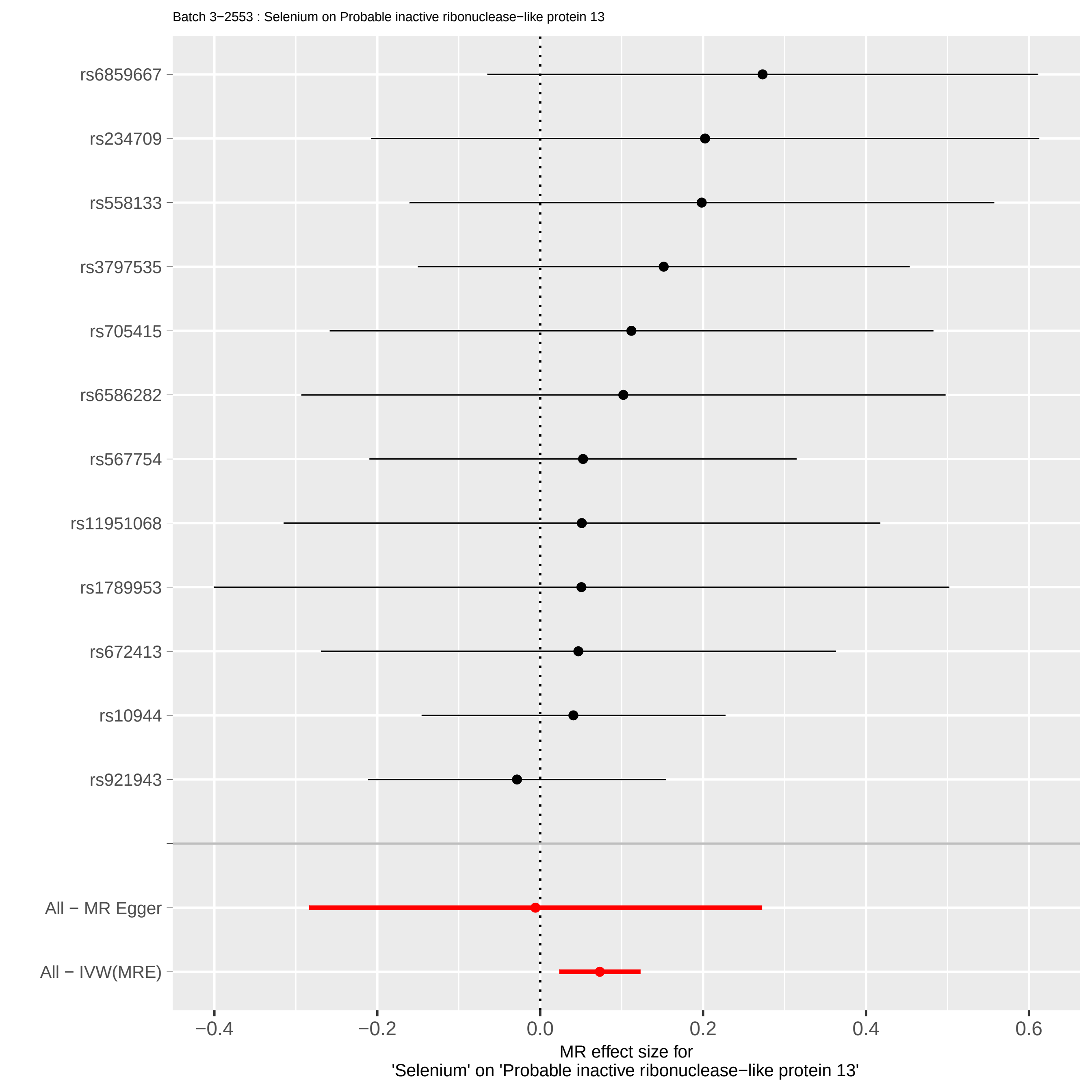

Batch 3−2553 : Selenium on Probable inactive ribonuclease−like protein 13
rs6859667
rs234709
rs558133
rs3797535
rs705415
rs6586282
rs567754
rs11951068
rs1789953
rs672413
rs10944
rs921943
All − MR Egger
All − IVW(MRE)
−0.4
−0.2
0.0
0.2
0.4
0.6
MR effect size for
'Selenium' on 'Probable inactive ribonuclease−like protein 13'

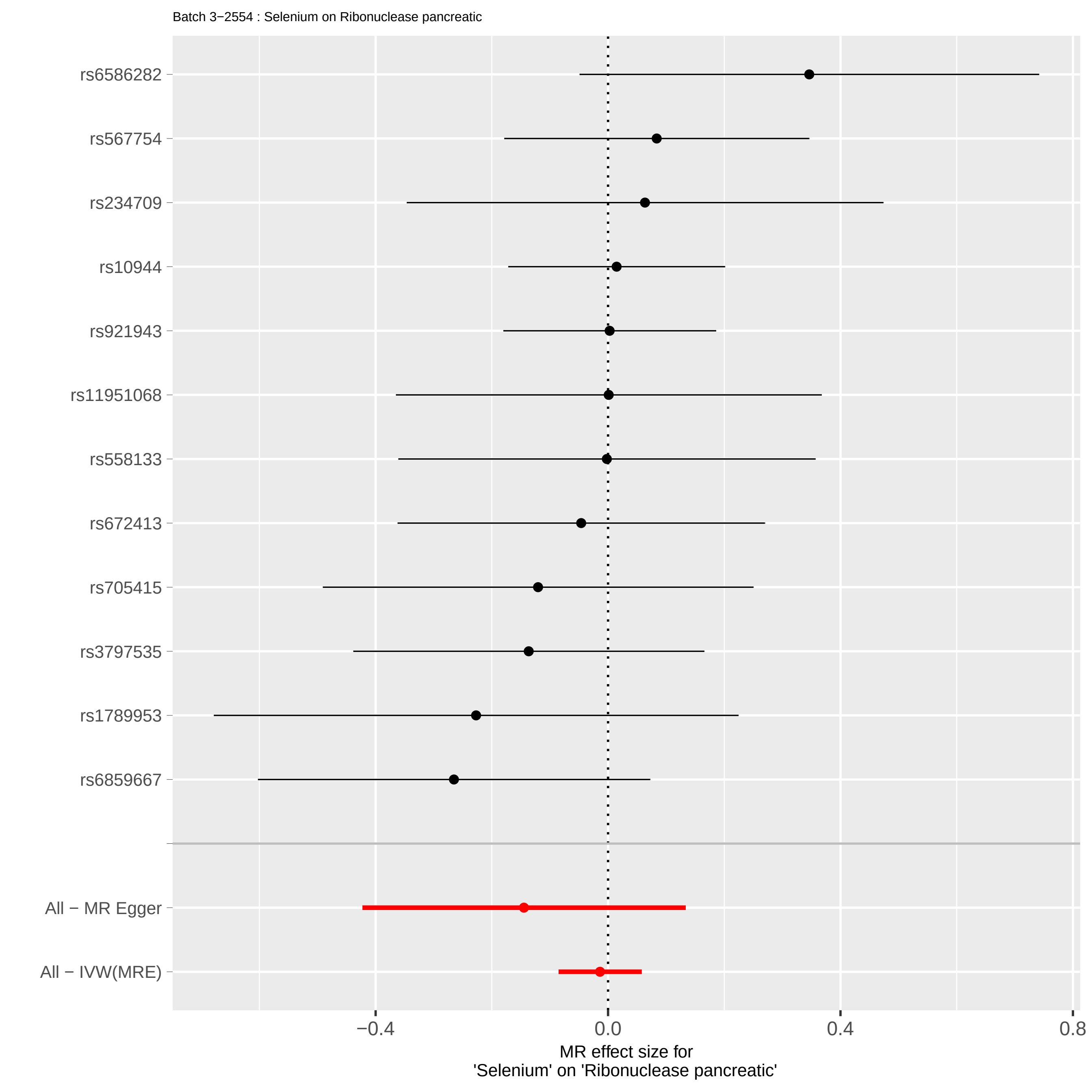

Batch 3−2554 : Selenium on Ribonuclease pancreatic
rs6586282
rs567754
rs234709
rs10944
rs921943
rs11951068
rs558133
rs672413
rs705415
rs3797535
rs1789953
rs6859667
All − MR Egger
All − IVW(MRE)
−0.4
0.0
0.4
0.8
MR effect size for
'Selenium' on 'Ribonuclease pancreatic'

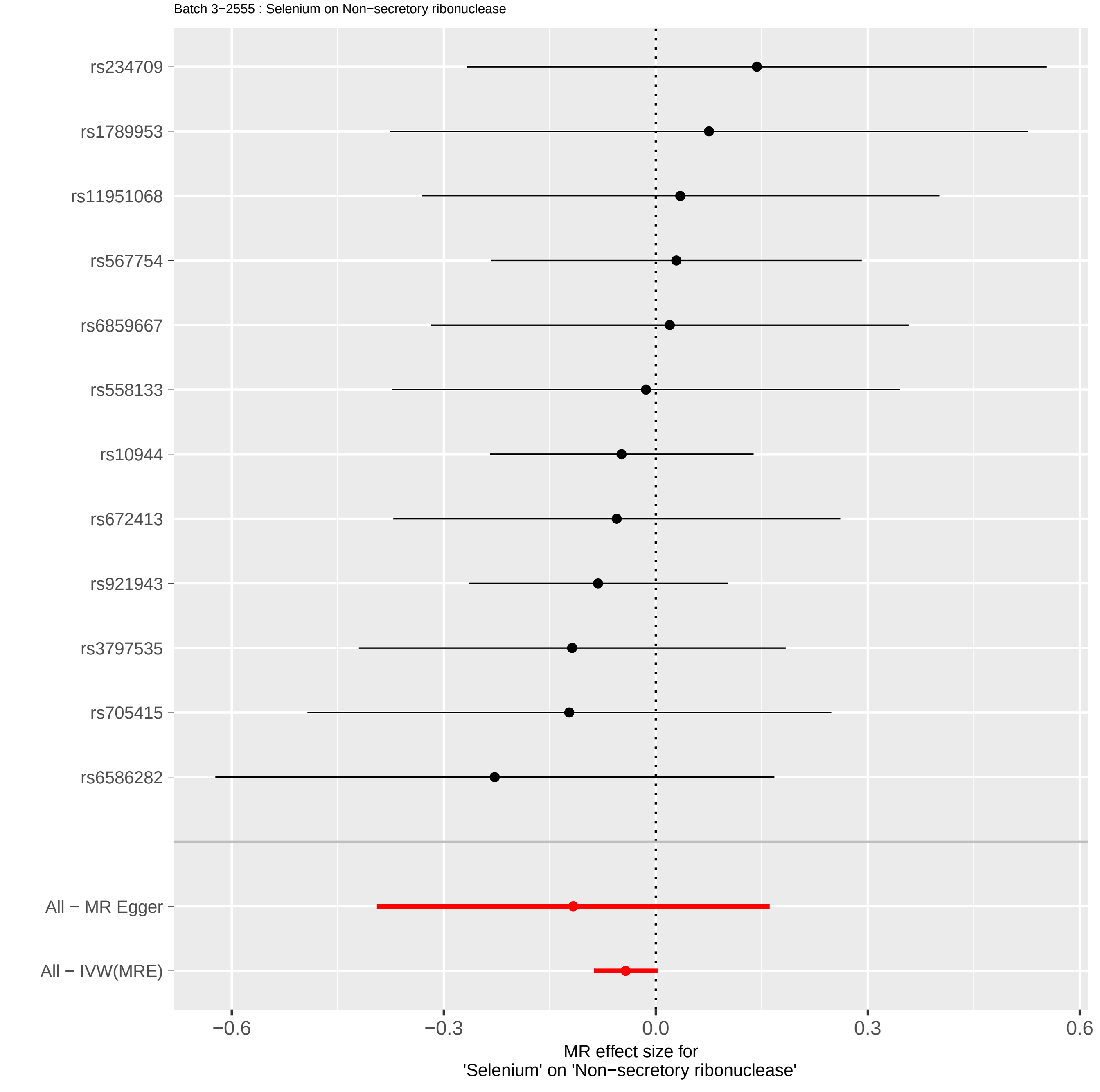
Batch 3−2555 : Selenium on Non−secretory ribonuclease
rs234709
rs1789953
rs11951068
rs567754
rs6859667
rs558133
rs10944
rs672413
rs921943
rs3797535
rs705415
rs6586282
All − MR Egger
All − IVW(MRE)
−0.6
−0.3
0.0
0.3
0.6
MR effect size for
'Selenium' on 'Non−secretory ribonuclease'

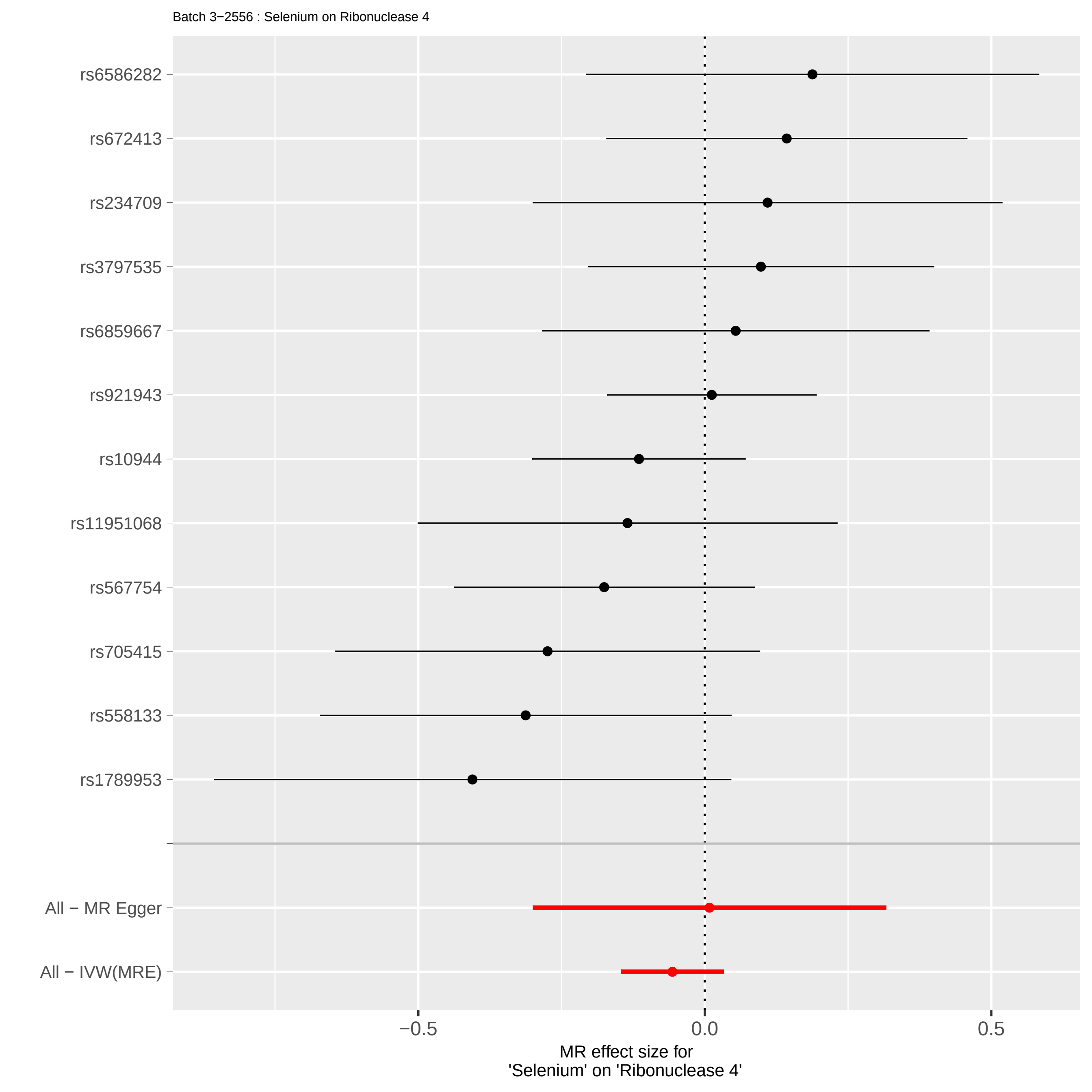

Batch 3−2556 : Selenium on Ribonuclease 4
rs6586282
rs672413
rs234709
rs3797535
rs6859667
rs921943
rs10944
rs11951068
rs567754
rs705415
rs558133
rs1789953
All − MR Egger
All − IVW(MRE)
−0.5
0.0
0.5
MR effect size for
'Selenium' on 'Ribonuclease 4'

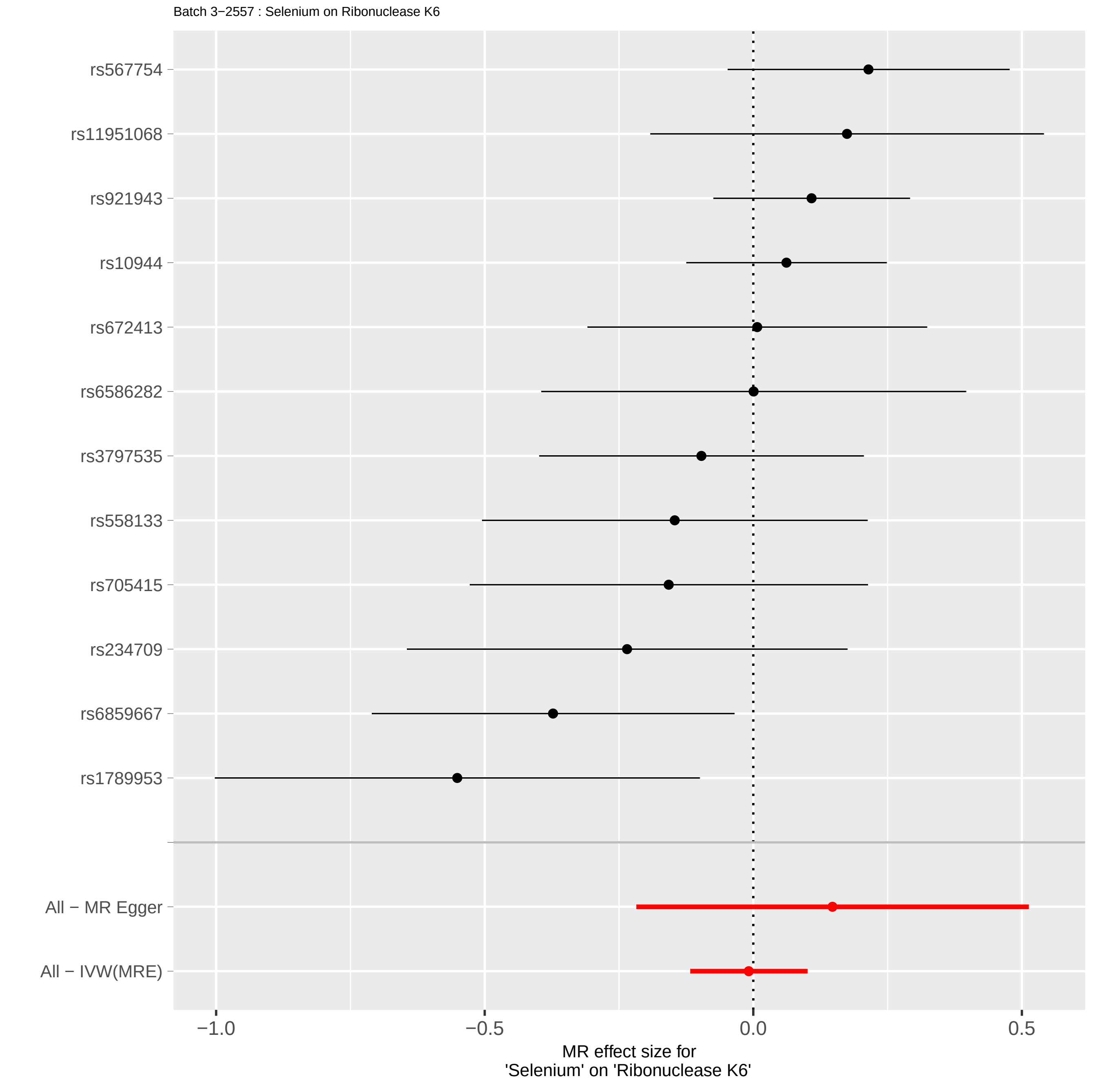

Batch 3−2557 : Selenium on Ribonuclease K6
rs567754
rs11951068
rs921943
rs10944
rs672413
rs6586282
rs3797535
rs558133
rs705415
rs234709
rs6859667
rs1789953
All − MR Egger
All − IVW(MRE)
−1.0
−0.5
0.0
0.5
MR effect size for
'Selenium' on 'Ribonuclease K6'

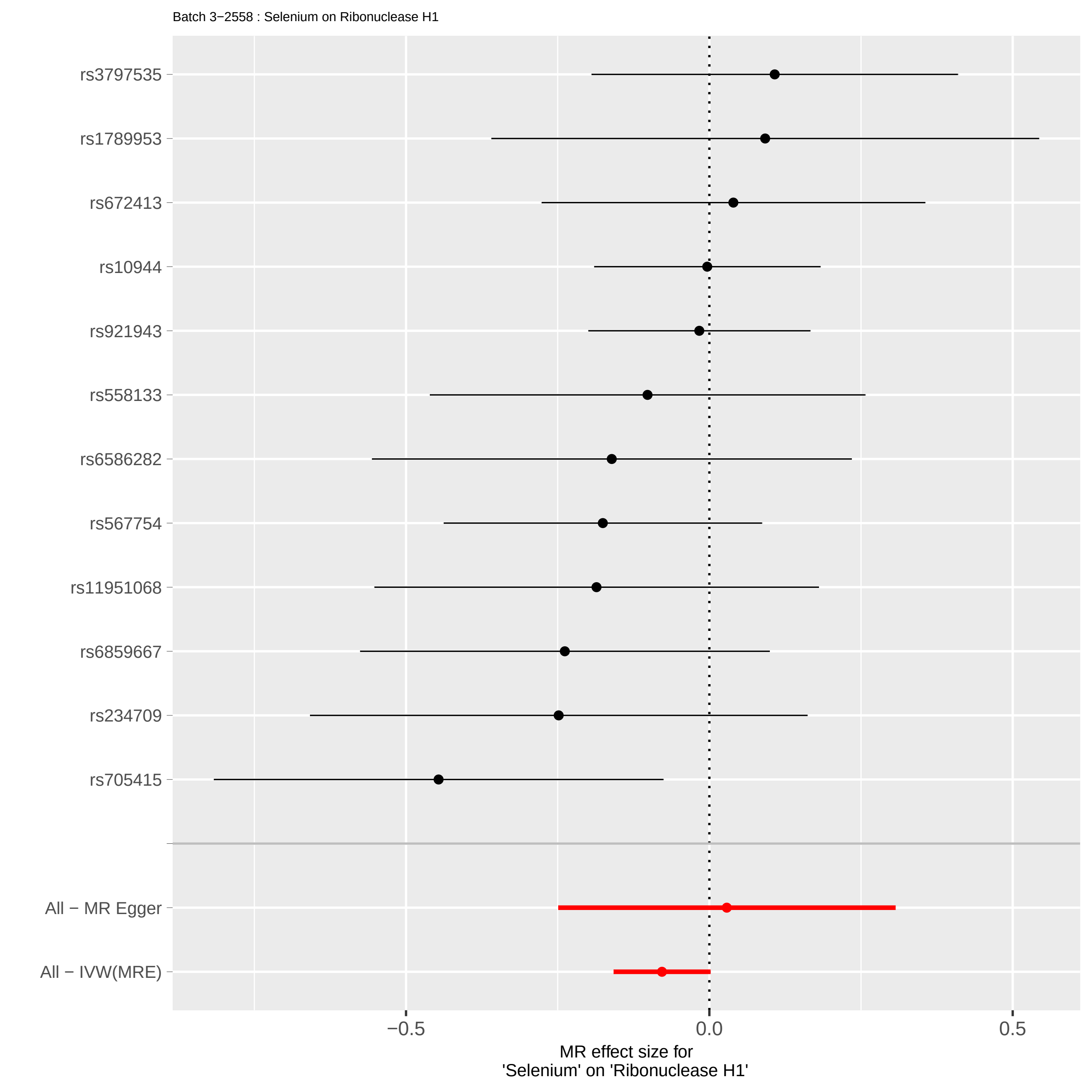
Batch 3−2558 : Selenium on Ribonuclease H1
rs3797535
rs1789953
rs672413
rs10944
rs921943
rs558133
rs6586282
rs567754
rs11951068
rs6859667
rs234709
rs705415
All − MR Egger
All − IVW(MRE)
−0.5
0.0
0.5
MR effect size for
'Selenium' on 'Ribonuclease H1'

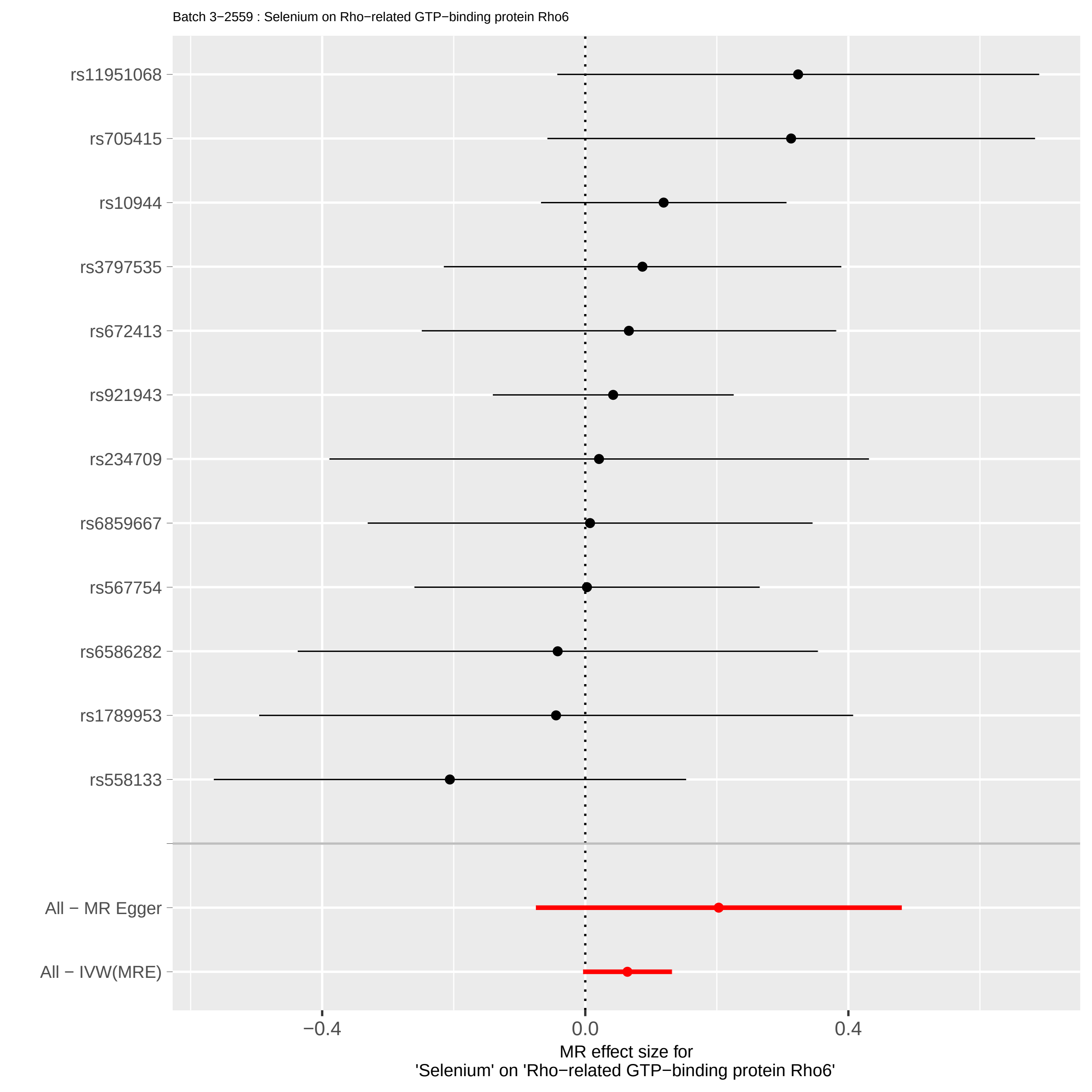

Batch 3−2559 : Selenium on Rho−related GTP−binding protein Rho6
rs11951068
rs705415
rs10944
rs3797535
rs672413
rs921943
rs234709
rs6859667
rs567754
rs6586282
rs1789953
rs558133
All − MR Egger
All − IVW(MRE)
−0.4
0.0
0.4
MR effect size for
'Selenium' on 'Rho−related GTP−binding protein Rho6'

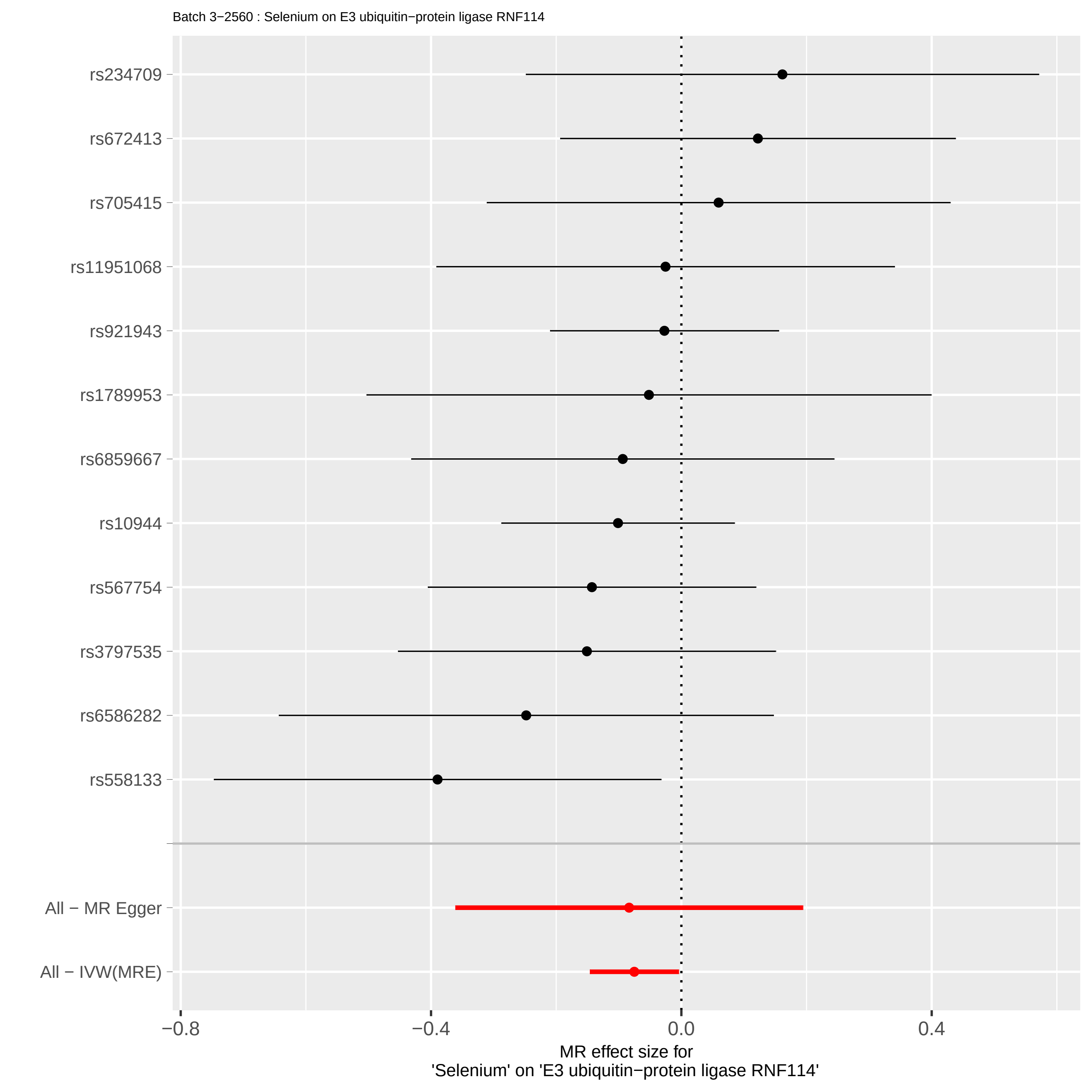
Batch 3−2560 : Selenium on E3 ubiquitin−protein ligase RNF114
rs234709
rs672413
rs705415
rs11951068
rs921943
rs1789953
rs6859667
rs10944
rs567754
rs3797535
rs6586282
rs558133
All − MR Egger
All − IVW(MRE)
−0.8
−0.4
0.0
0.4
MR effect size for
'Selenium' on 'E3 ubiquitin−protein ligase RNF114'

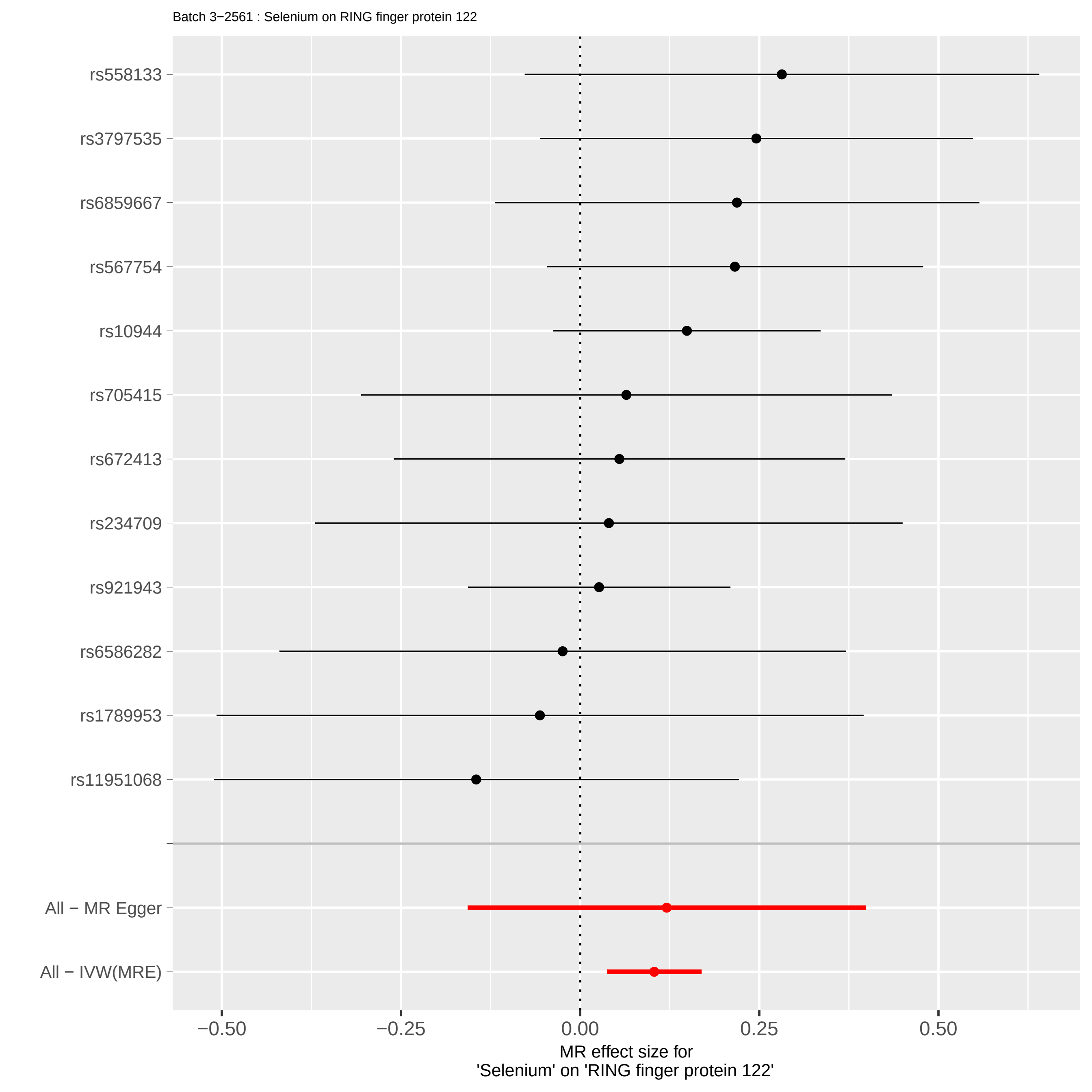

Batch 3−2561 : Selenium on RING finger protein 122
rs558133
rs3797535
rs6859667
rs567754
rs10944
rs705415
rs672413
rs234709
rs921943
rs6586282
rs1789953
rs11951068
All − MR Egger
All − IVW(MRE)
−0.50
−0.25
0.00
0.25
0.50
MR effect size for
'Selenium' on 'RING finger protein 122'

Batch 3−2562 : Selenium on E3 ubiquitin−protein ligase RNF128

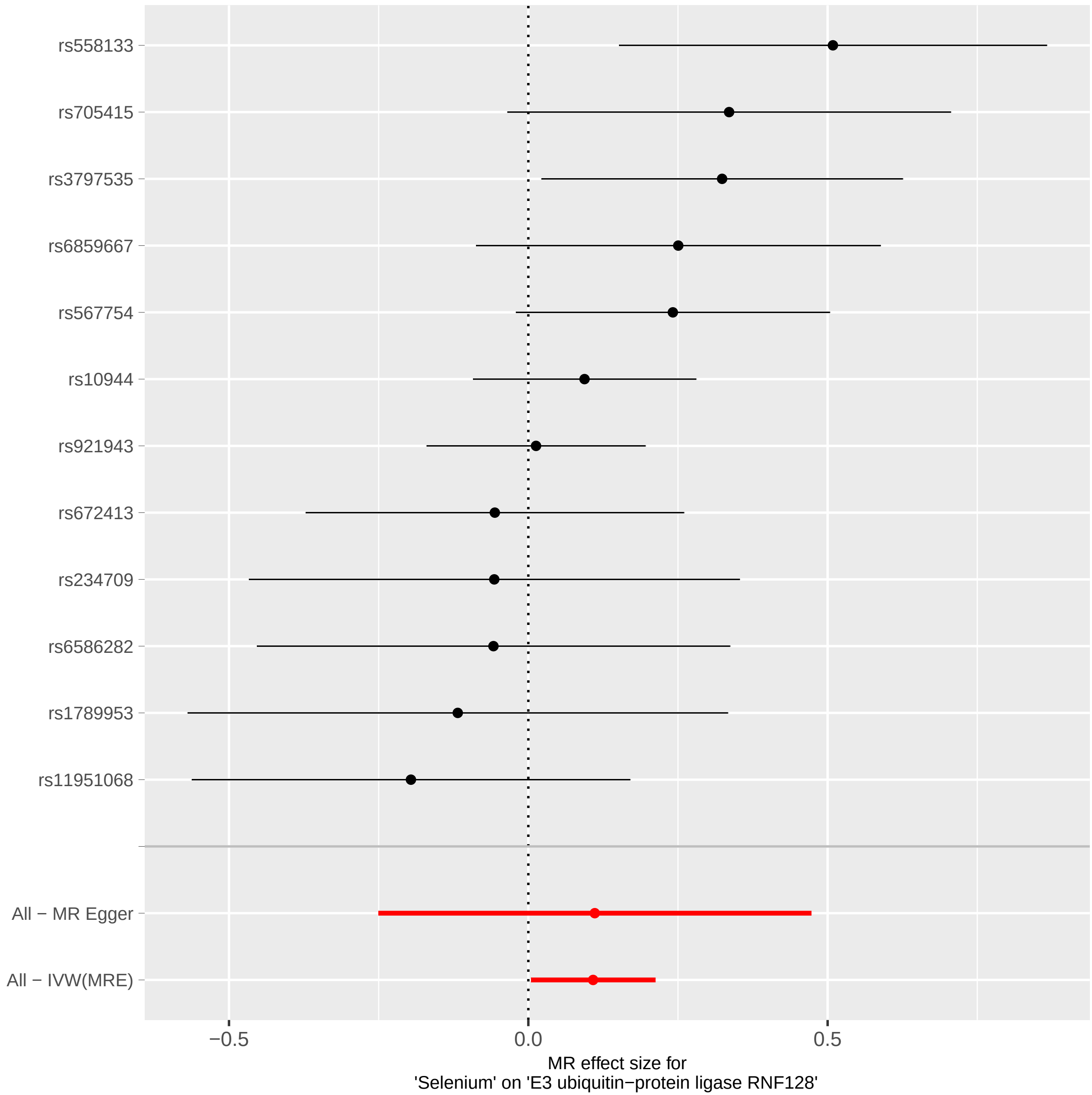

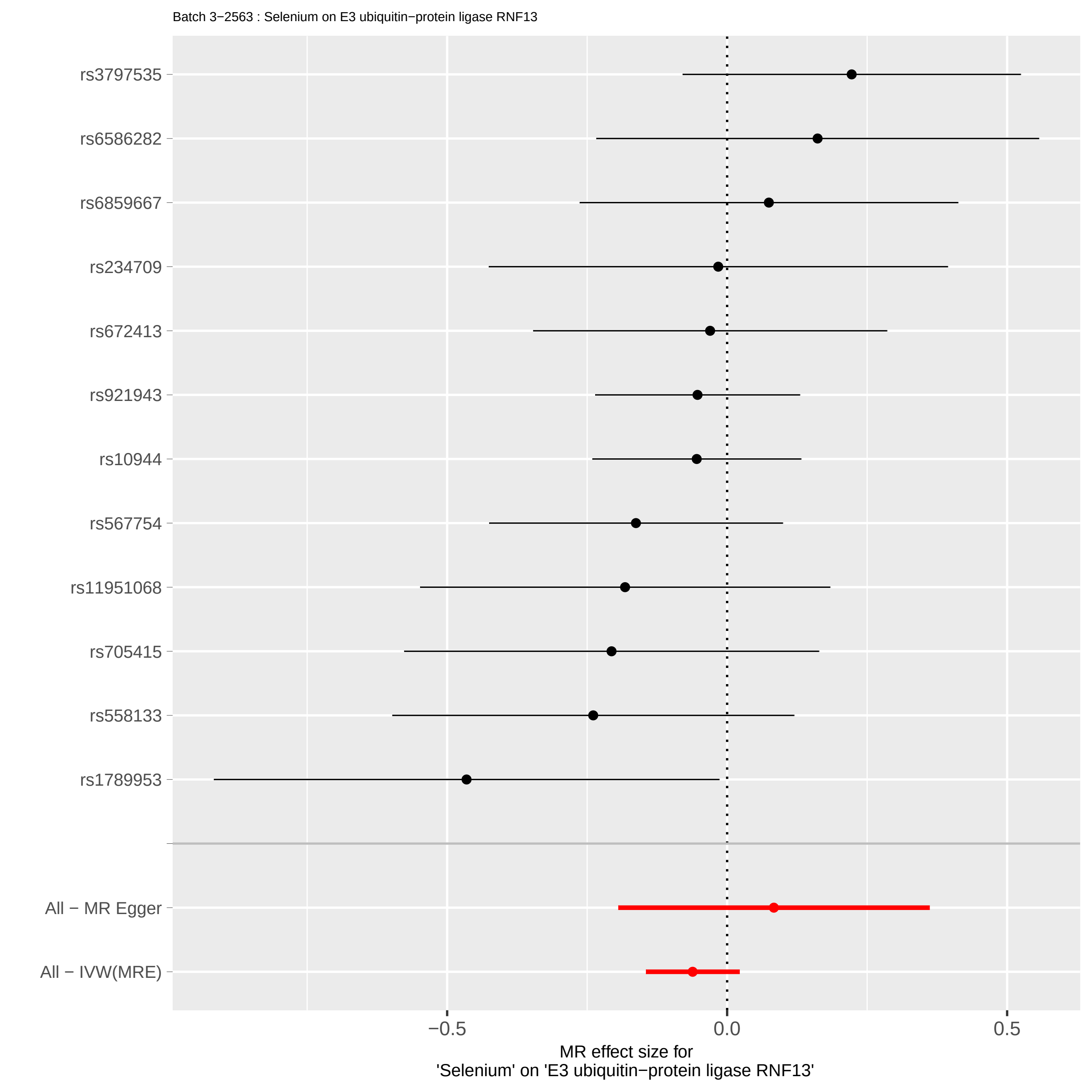

Batch 3−2563 : Selenium on E3 ubiquitin−protein ligase RNF13
rs3797535
rs6586282
rs6859667
rs234709
rs672413
rs921943
rs10944
rs567754
rs11951068
rs705415
rs558133
rs1789953
All − MR Egger
All − IVW(MRE)
−0.5
0.0
0.5
MR effect size for
'Selenium' on 'E3 ubiquitin−protein ligase RNF13'

Batch 3−2564 : Selenium on RING finger protein 148

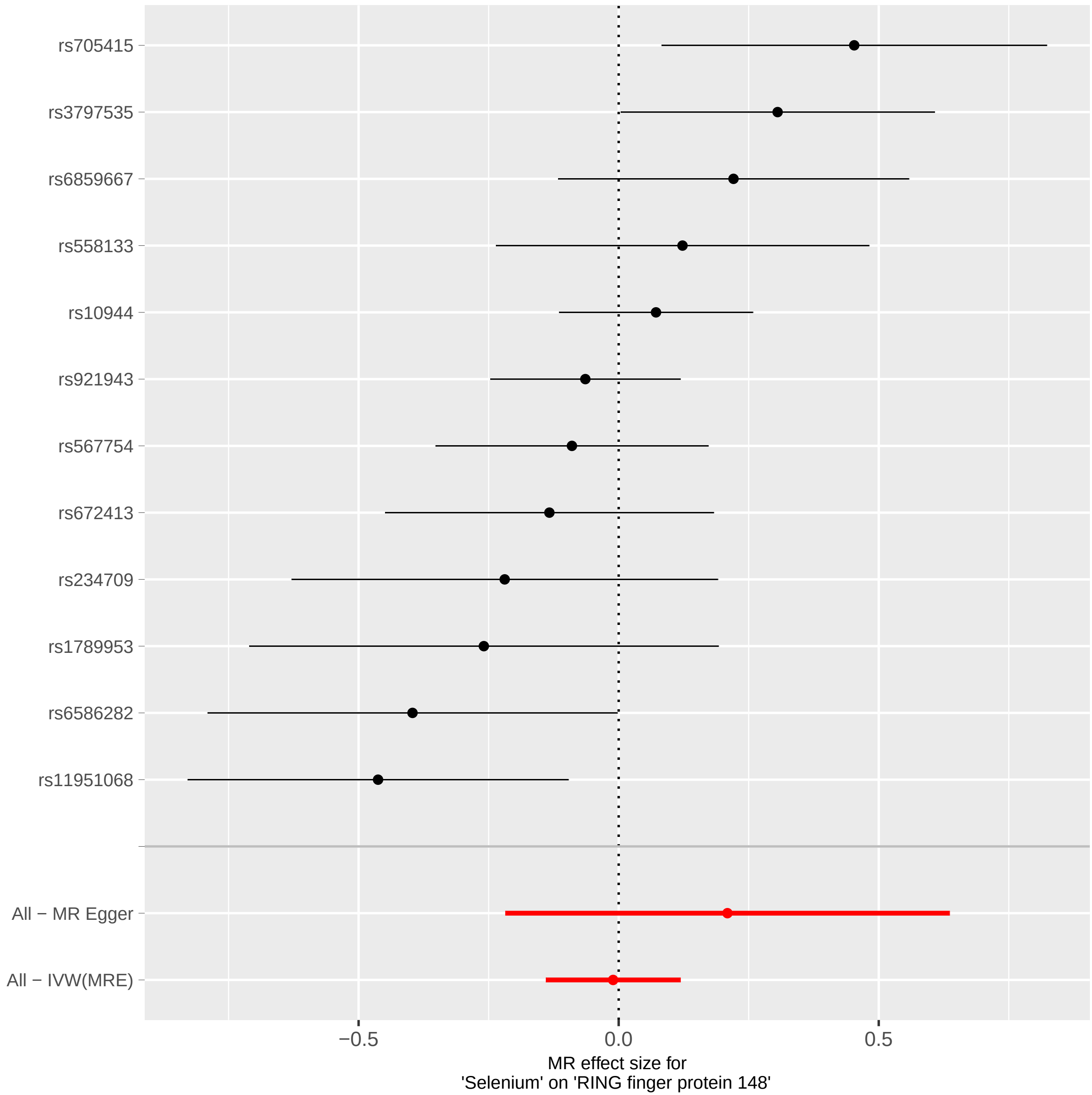

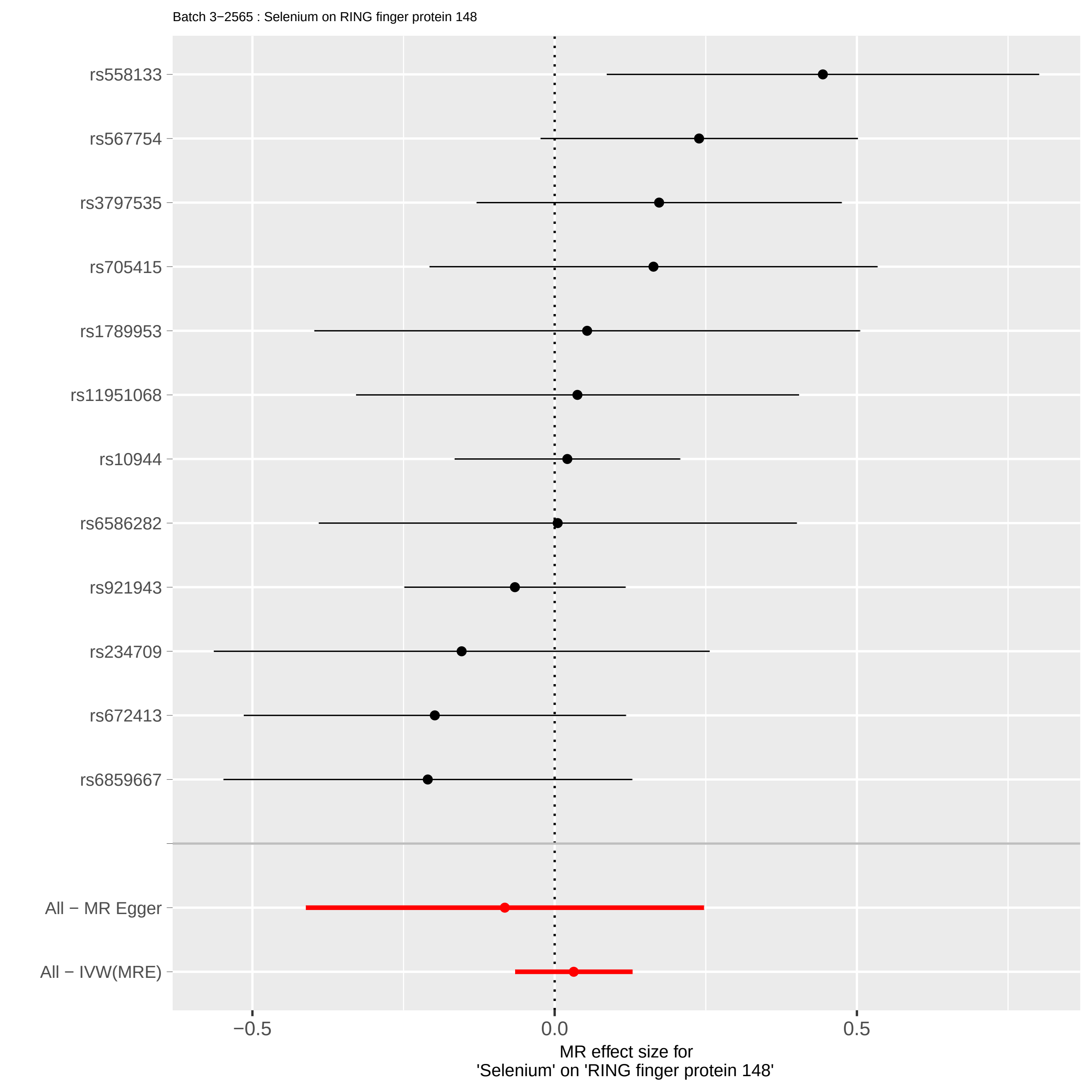

Batch 3−2565 : Selenium on RING finger protein 148
rs558133
rs567754
rs3797535
rs705415
rs1789953
rs11951068
rs10944
rs6586282
rs921943
rs234709
rs672413
rs6859667
All − MR Egger
All − IVW(MRE)
−0.5
0.0
0.5
MR effect size for
'Selenium' on 'RING finger protein 148'

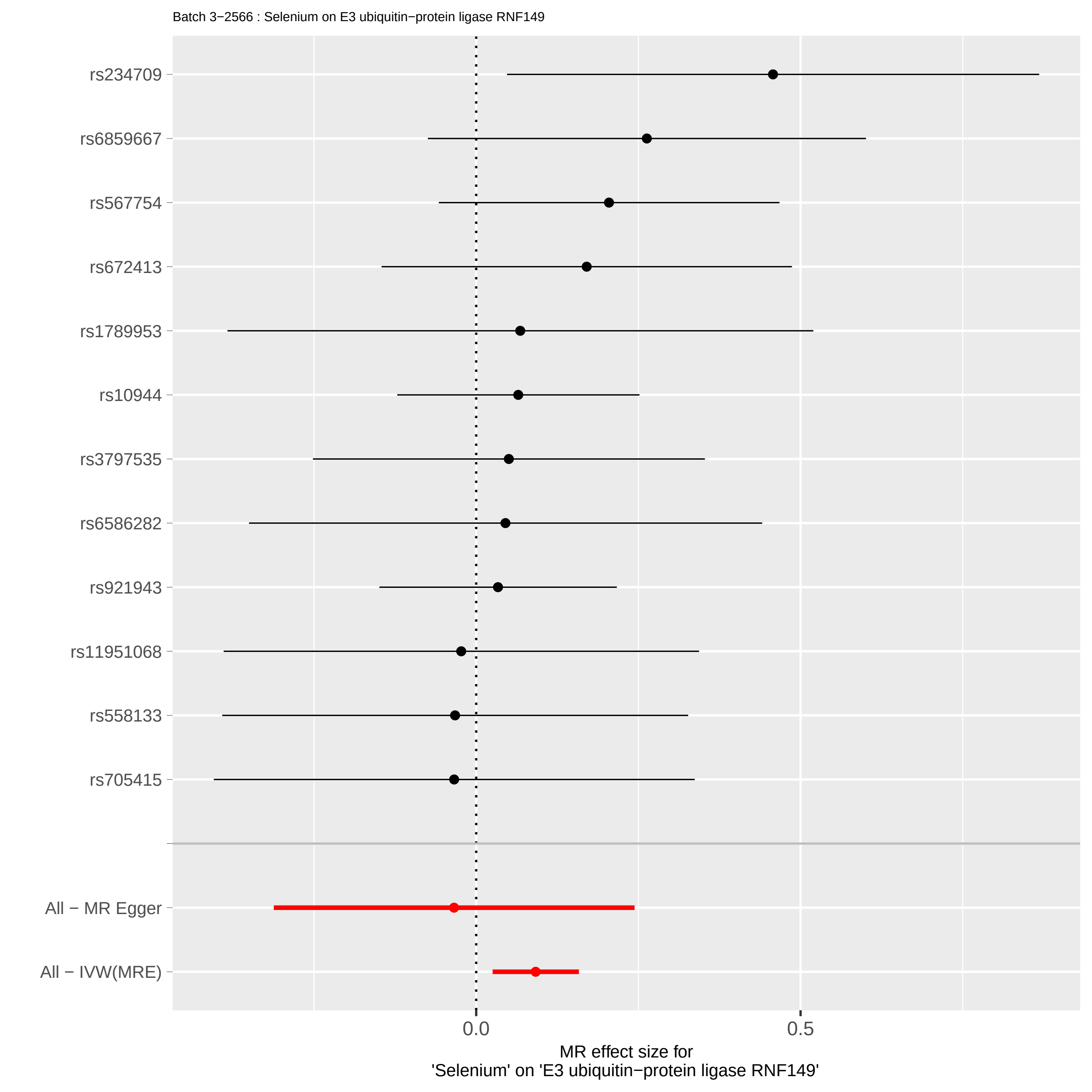

Batch 3−2566 : Selenium on E3 ubiquitin−protein ligase RNF149
rs234709
rs6859667
rs567754
rs672413
rs1789953
rs10944
rs3797535
rs6586282
rs921943
rs11951068
rs558133
rs705415
All − MR Egger
All − IVW(MRE)
0.0
0.5
MR effect size for
'Selenium' on 'E3 ubiquitin−protein ligase RNF149'

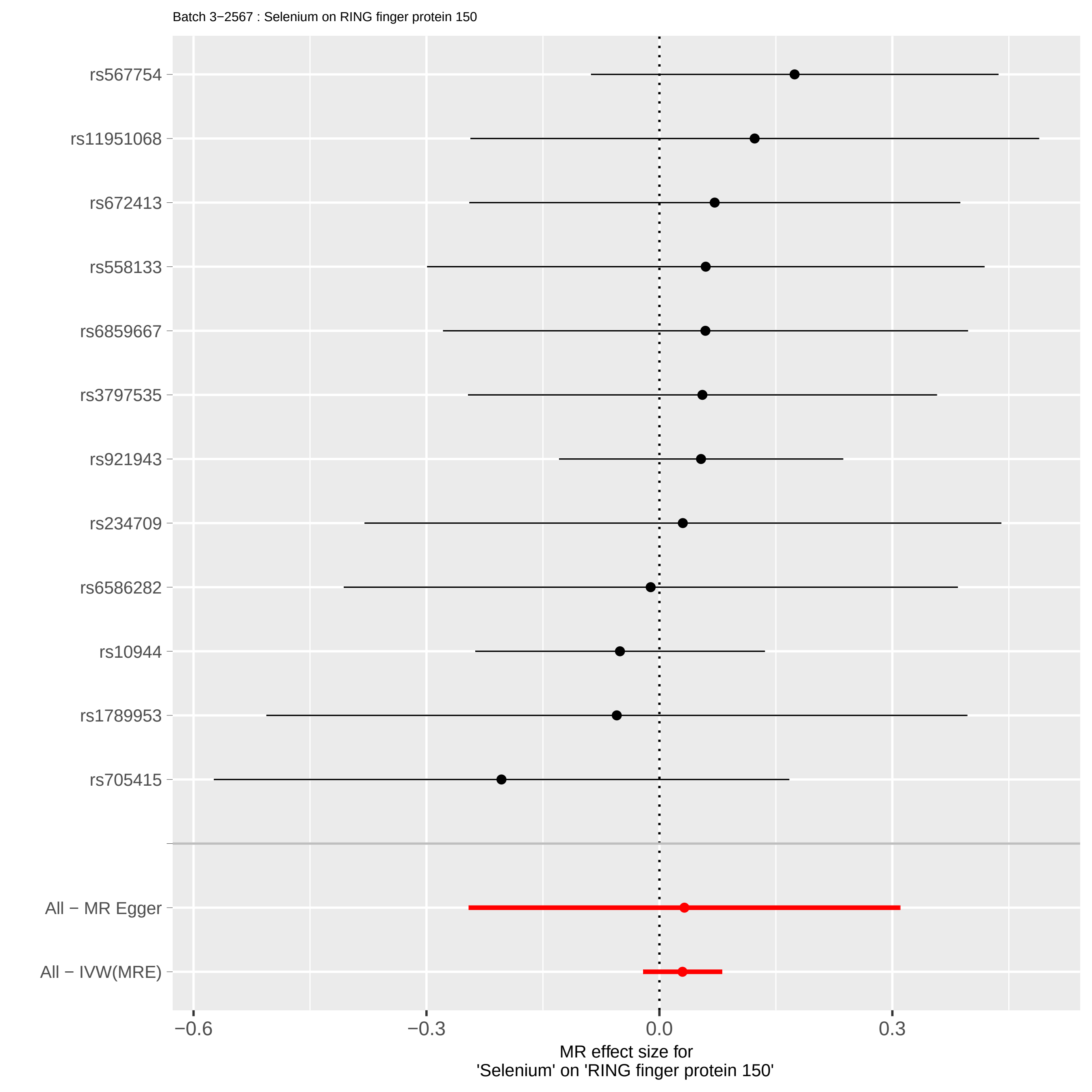

Batch 3−2567 : Selenium on RING finger protein 150
rs567754
rs11951068
rs672413
rs558133
rs6859667
rs3797535
rs921943
rs234709
rs6586282
rs10944
rs1789953
rs705415
All − MR Egger
All − IVW(MRE)
−0.6
−0.3
0.0
0.3
MR effect size for
'Selenium' on 'RING finger protein 150'

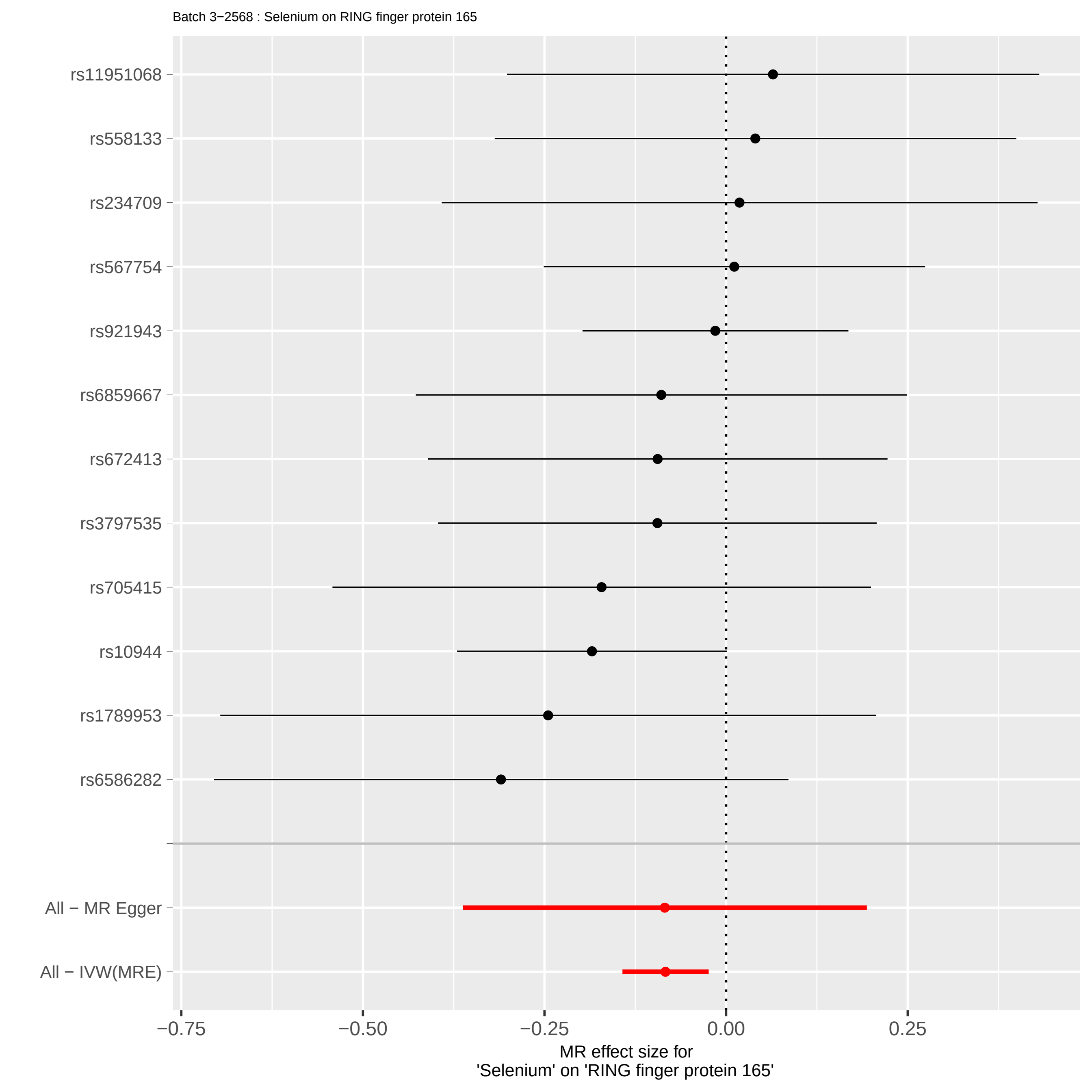
Batch 3−2568 : Selenium on RING finger protein 165
rs11951068
rs558133
rs234709
rs567754
rs921943
rs6859667
rs672413
rs3797535
rs705415
rs10944
rs1789953
rs6586282
All − MR Egger
All − IVW(MRE)
−0.75
−0.50
−0.25
0.00
0.25
MR effect size for
'Selenium' on 'RING finger protein 165'

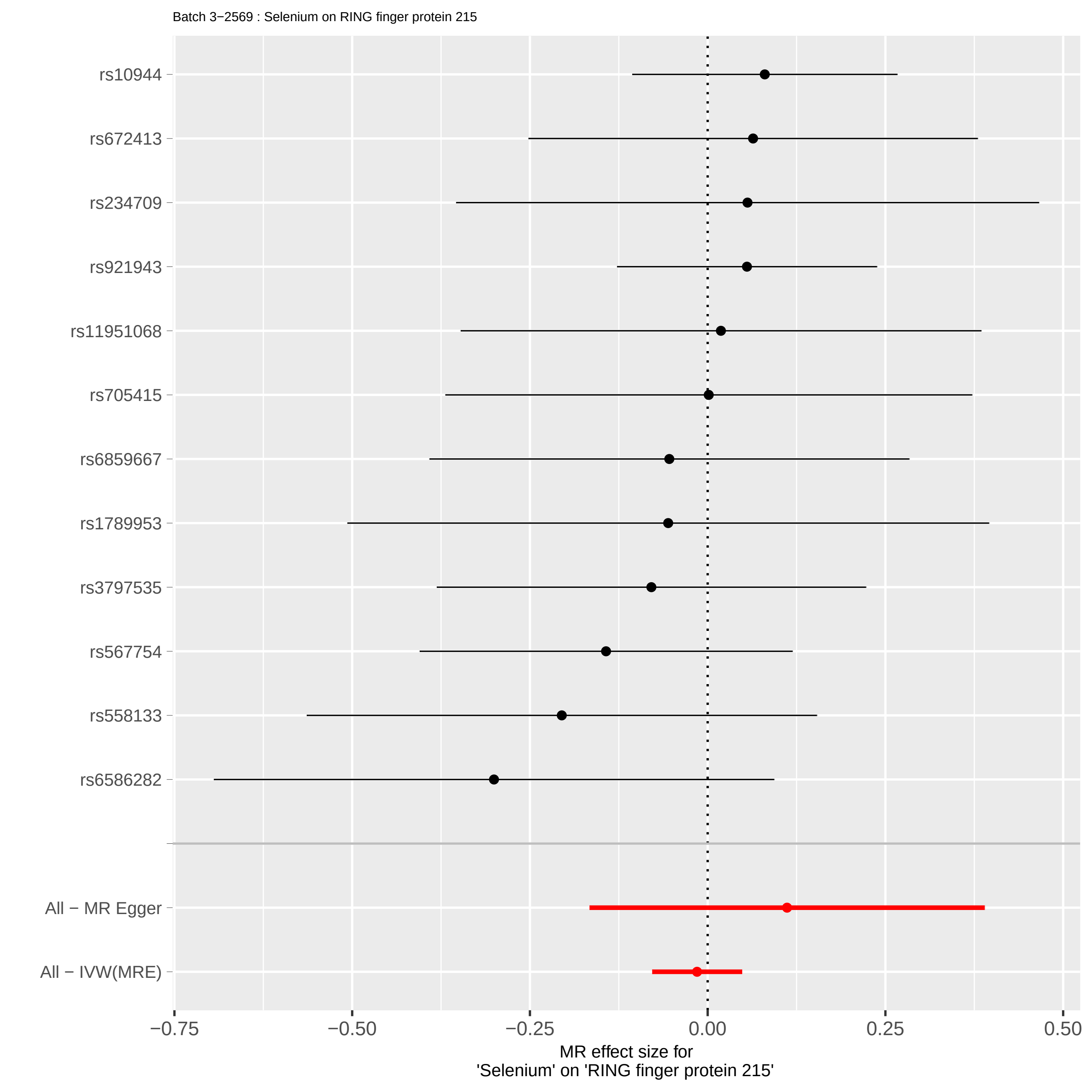
Batch 3−2569 : Selenium on RING finger protein 215
rs10944
rs672413
rs234709
rs921943
rs11951068
rs705415
rs6859667
rs1789953
rs3797535
rs567754
rs558133
rs6586282
All − MR Egger
All − IVW(MRE)
−0.75
−0.50
−0.25
0.00
0.25
0.50
MR effect size for
'Selenium' on 'RING finger protein 215'

Batch 3−2570 : Selenium on RING finger protein 219

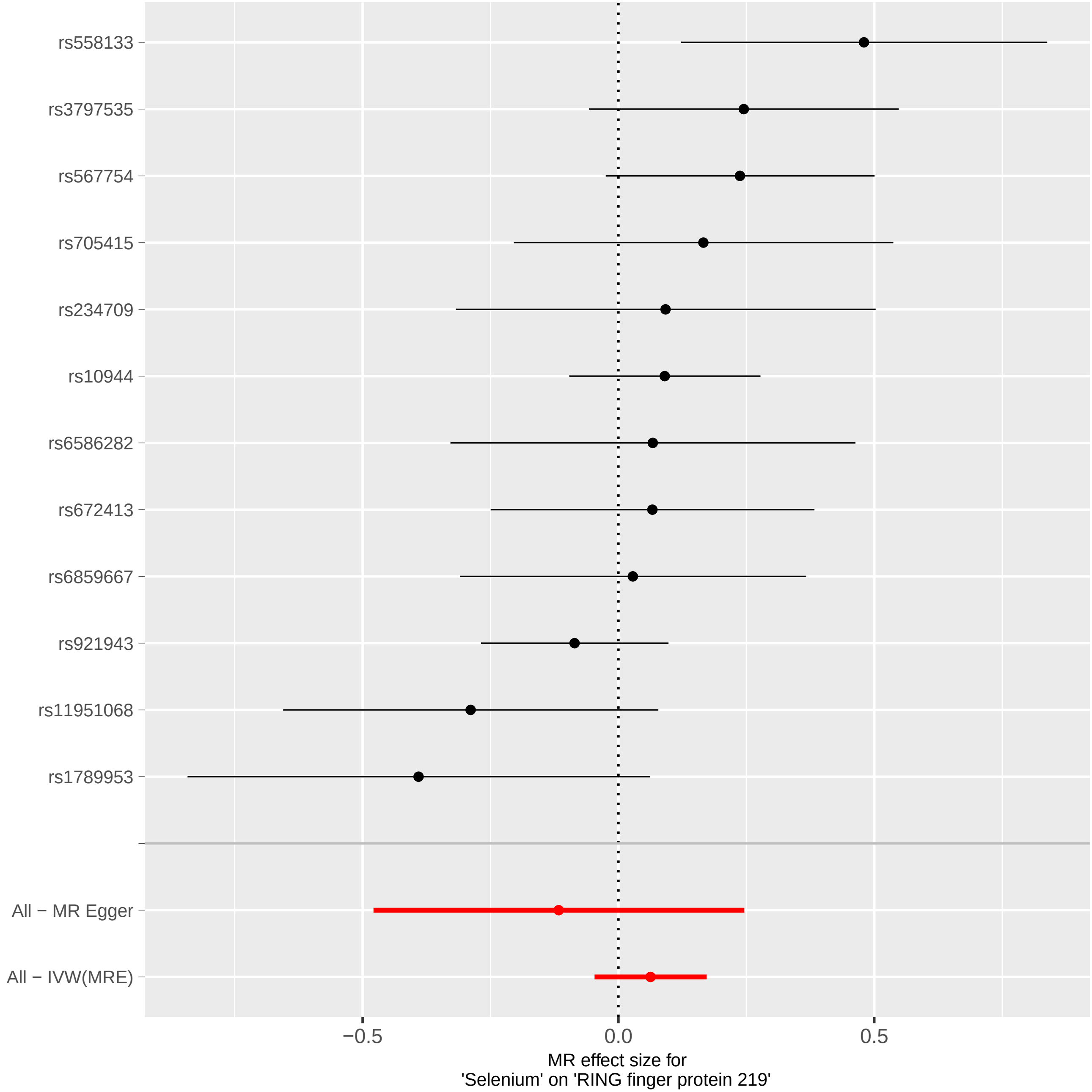

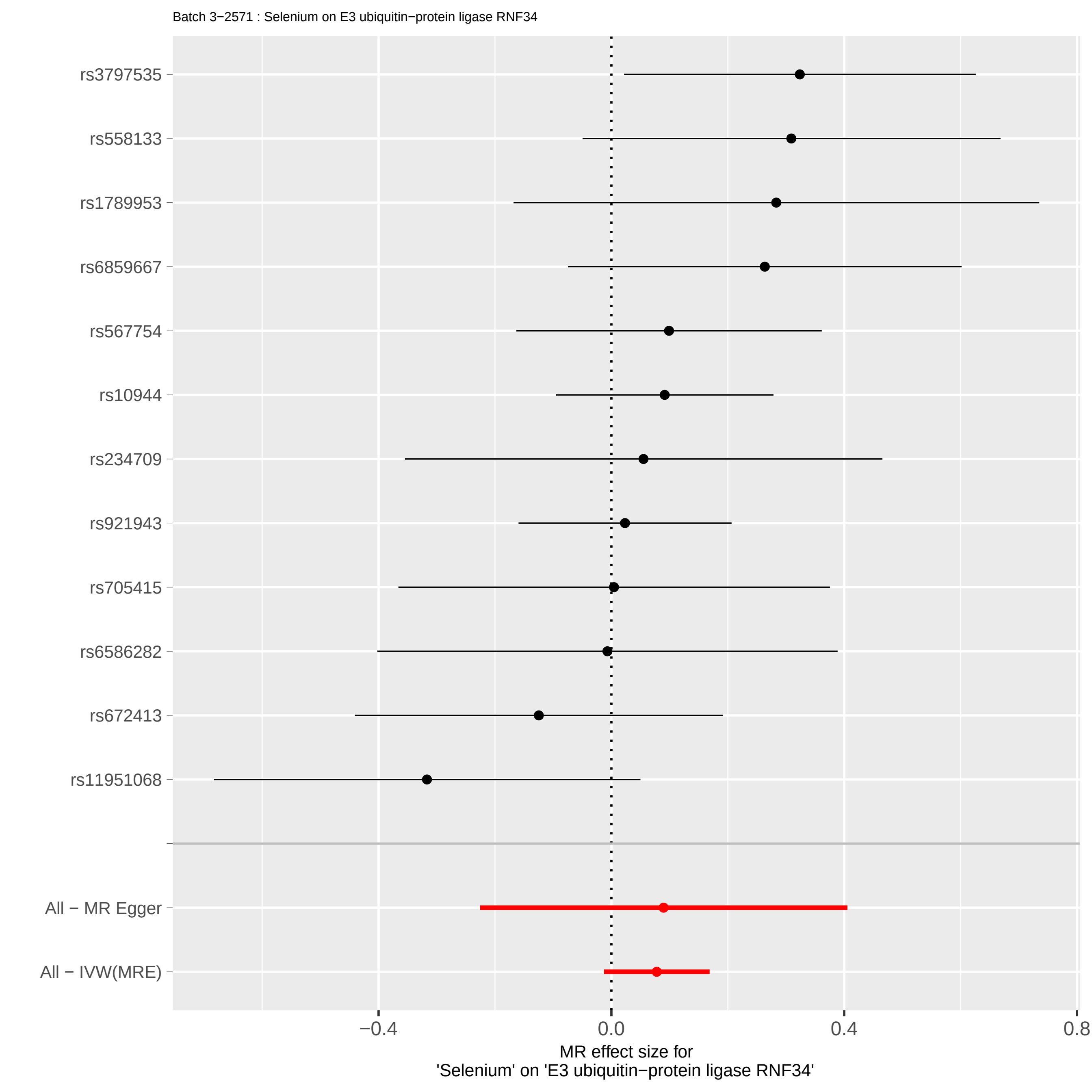

Batch 3−2571 : Selenium on E3 ubiquitin−protein ligase RNF34
rs3797535
rs558133
rs1789953
rs6859667
rs567754
rs10944
rs234709
rs921943
rs705415
rs6586282
rs672413
rs11951068
All − MR Egger
All − IVW(MRE)
−0.4
0.0
0.4
0.8
MR effect size for
'Selenium' on 'E3 ubiquitin−protein ligase RNF34'

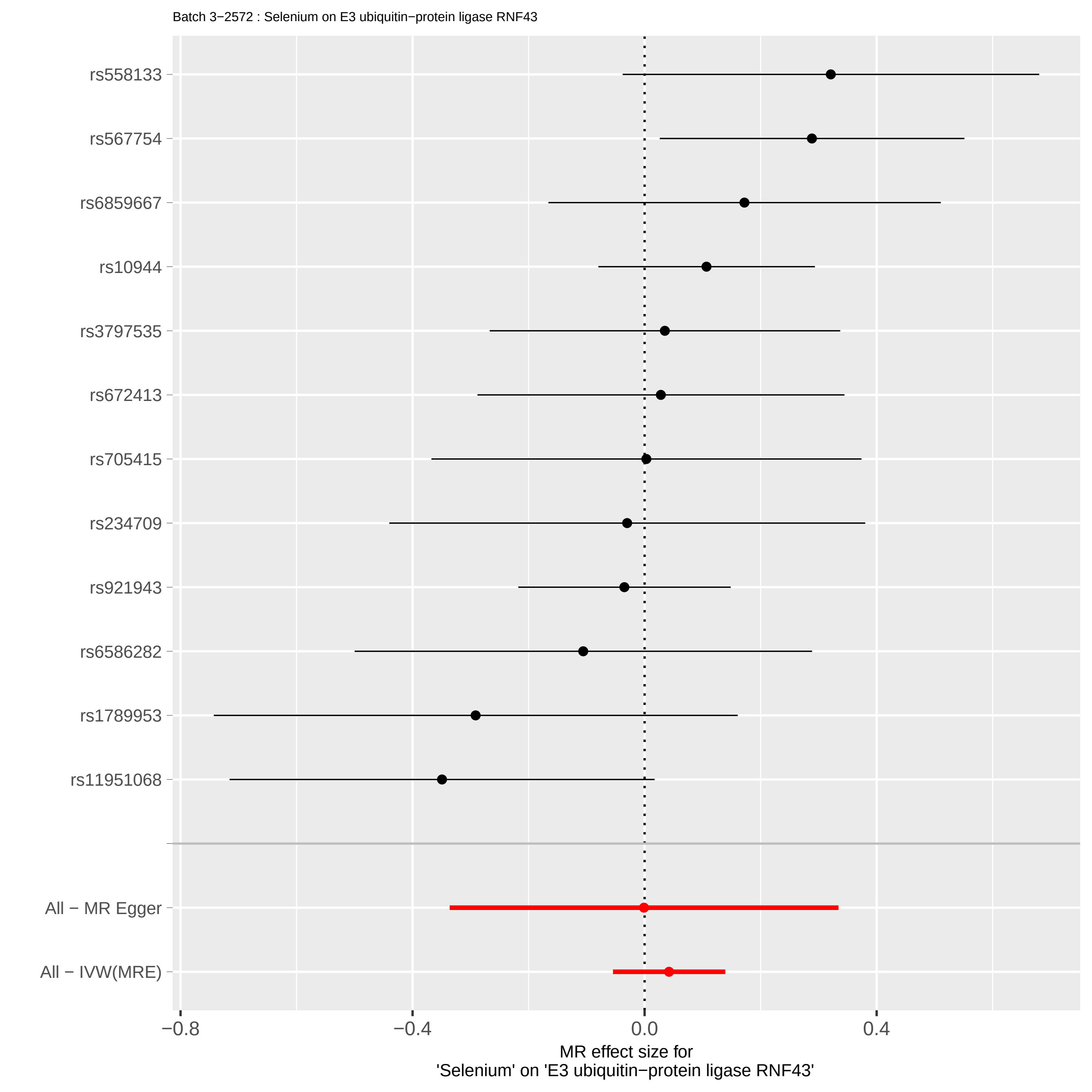

Batch 3−2572 : Selenium on E3 ubiquitin−protein ligase RNF43
rs558133
rs567754
rs6859667
rs10944
rs3797535
rs672413
rs705415
rs234709
rs921943
rs6586282
rs1789953
rs11951068
All − MR Egger
All − IVW(MRE)
−0.8
−0.4
0.0
0.4
MR effect size for
'Selenium' on 'E3 ubiquitin−protein ligase RNF43'

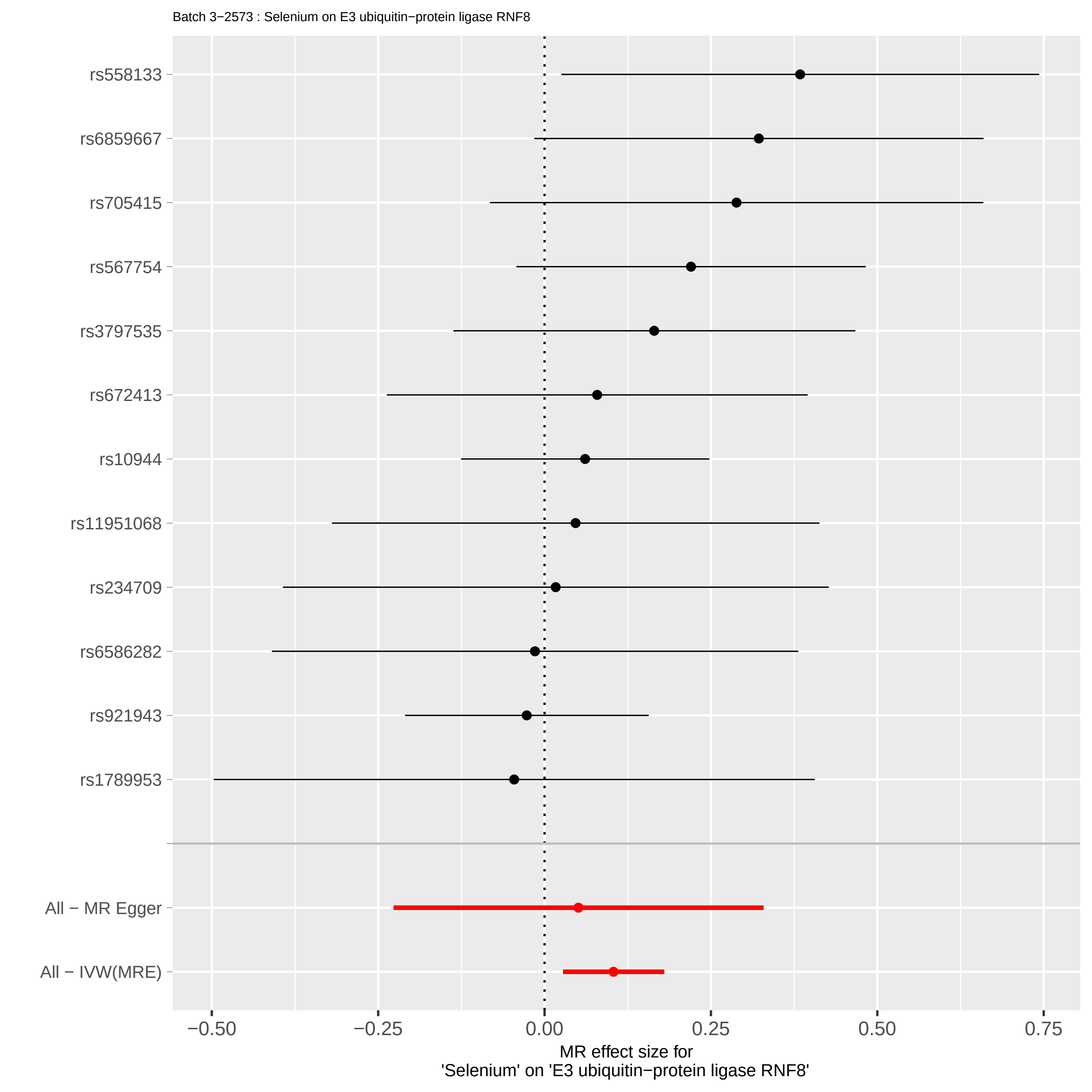

Batch 3−2573 : Selenium on E3 ubiquitin−protein ligase RNF8
rs558133
rs6859667
rs705415
rs567754
rs3797535
rs672413
rs10944
rs11951068
rs234709
rs6586282
rs921943
rs1789953
All − MR Egger
All − IVW(MRE)
−0.50
−0.25
0.00
0.25
0.50
0.75
MR effect size for
'Selenium' on 'E3 ubiquitin−protein ligase RNF8'

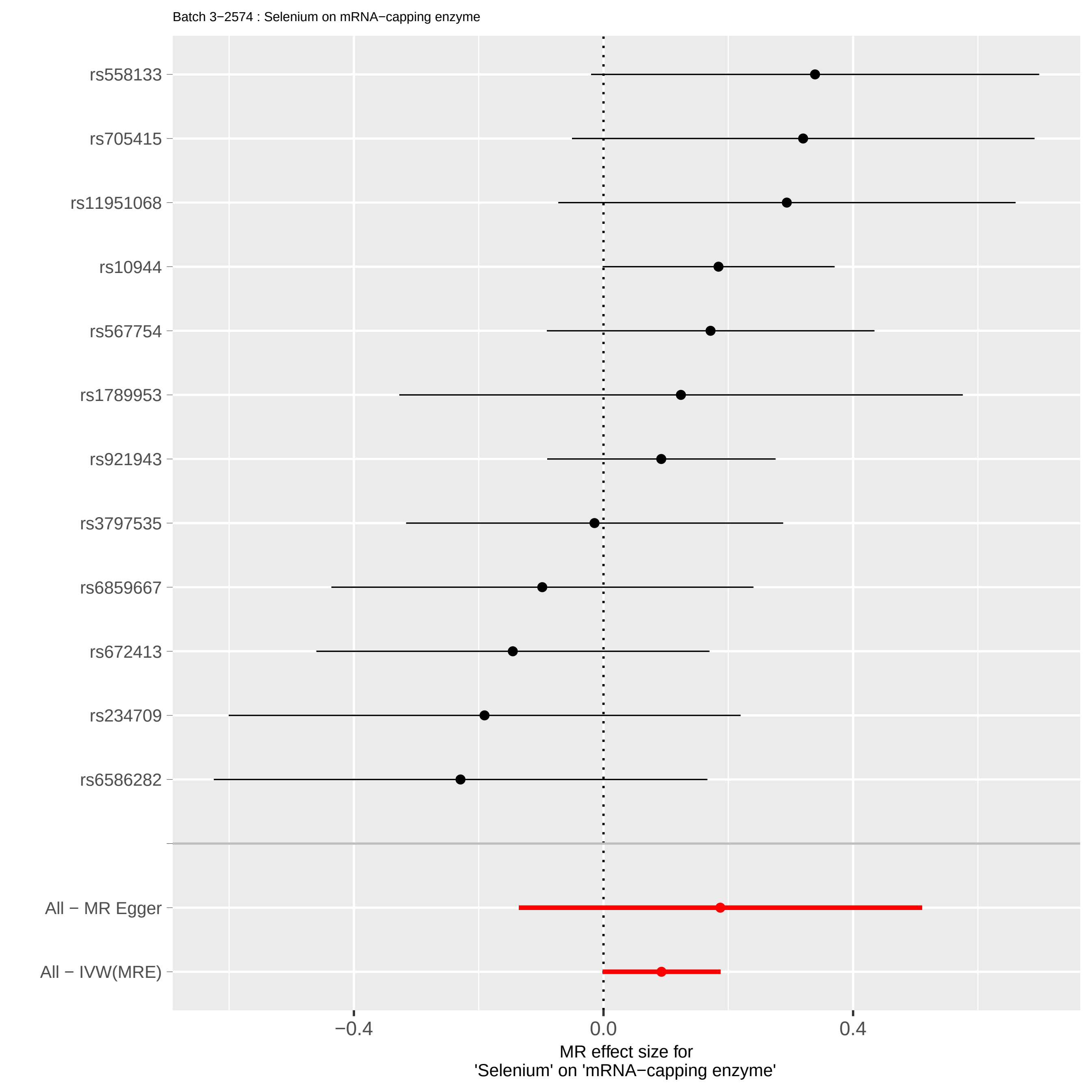
Batch 3−2574 : Selenium on mRNA−capping enzyme
rs558133
rs705415
rs11951068
rs10944
rs567754
rs1789953
rs921943
rs3797535
rs6859667
rs672413
rs234709
rs6586282
All − MR Egger
All − IVW(MRE)
−0.4
0.0
0.4
MR effect size for
'Selenium' on 'mRNA−capping enzyme'

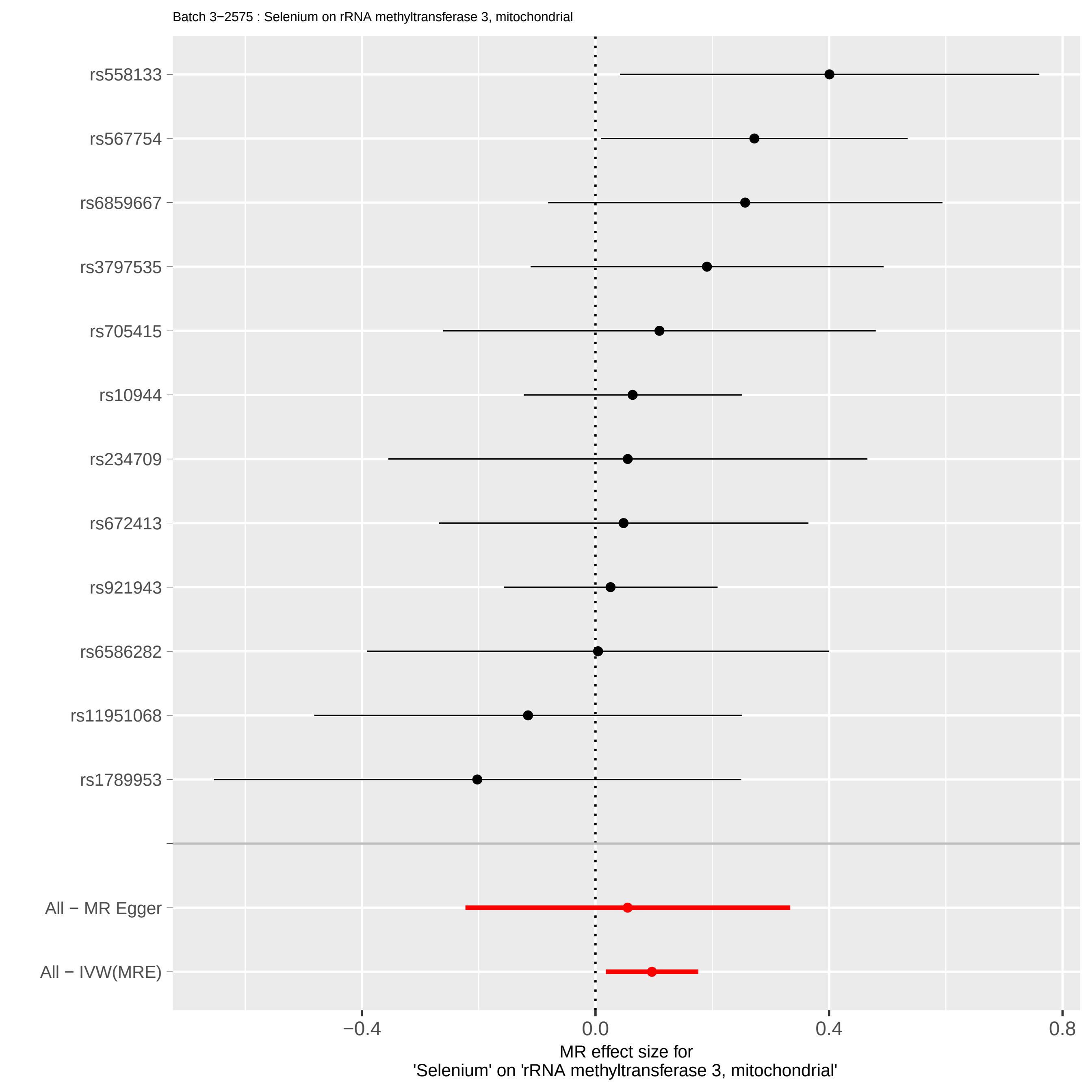

Batch 3−2575 : Selenium on rRNA methyltransferase 3, mitochondrial
rs558133
rs567754
rs6859667
rs3797535
rs705415
rs10944
rs234709
rs672413
rs921943
rs6586282
rs11951068
rs1789953
All − MR Egger
All − IVW(MRE)
−0.4
0.0
0.4
0.8
MR effect size for
'Selenium' on 'rRNA methyltransferase 3, mitochondrial'

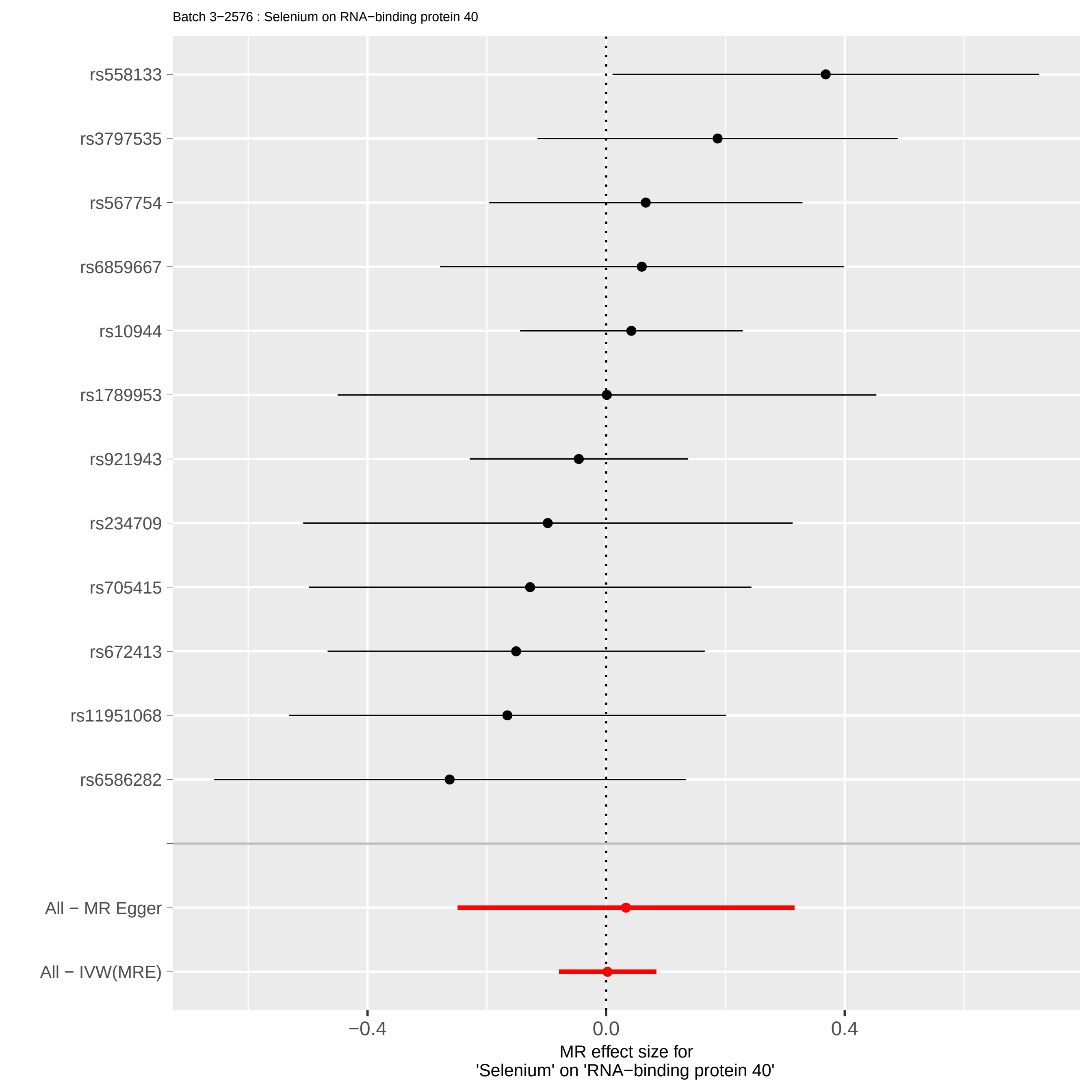

Batch 3−2576 : Selenium on RNA−binding protein 40
rs558133
rs3797535
rs567754
rs6859667
rs10944
rs1789953
rs921943
rs234709
rs705415
rs672413
rs11951068
rs6586282
All − MR Egger
All − IVW(MRE)
−0.4
0.0
0.4
MR effect size for
'Selenium' on 'RNA−binding protein 40'

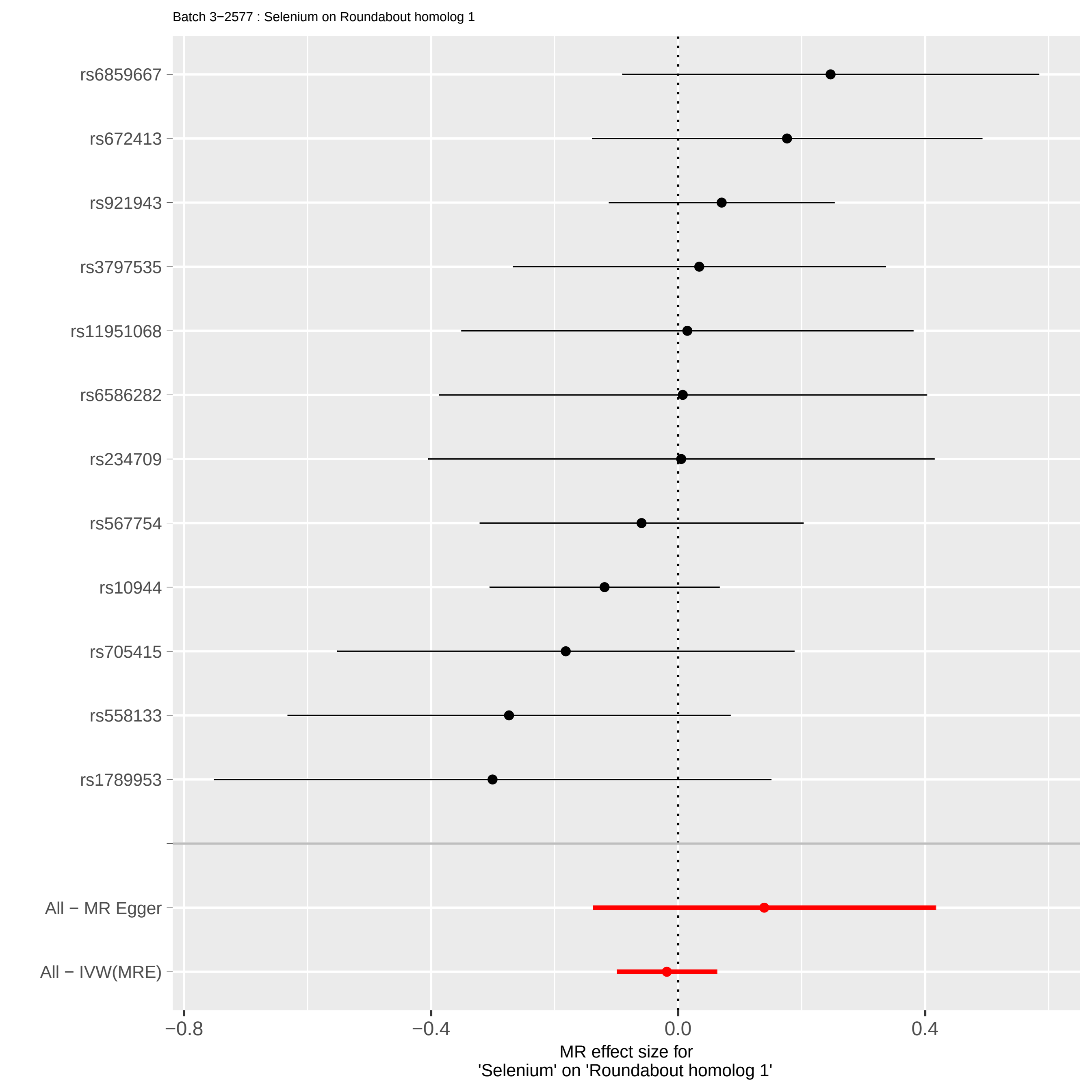

Batch 3−2577 : Selenium on Roundabout homolog 1
rs6859667
rs672413
rs921943
rs3797535
rs11951068
rs6586282
rs234709
rs567754
rs10944
rs705415
rs558133
rs1789953
All − MR Egger
All − IVW(MRE)
−0.8
−0.4
0.0
0.4
MR effect size for
'Selenium' on 'Roundabout homolog 1'

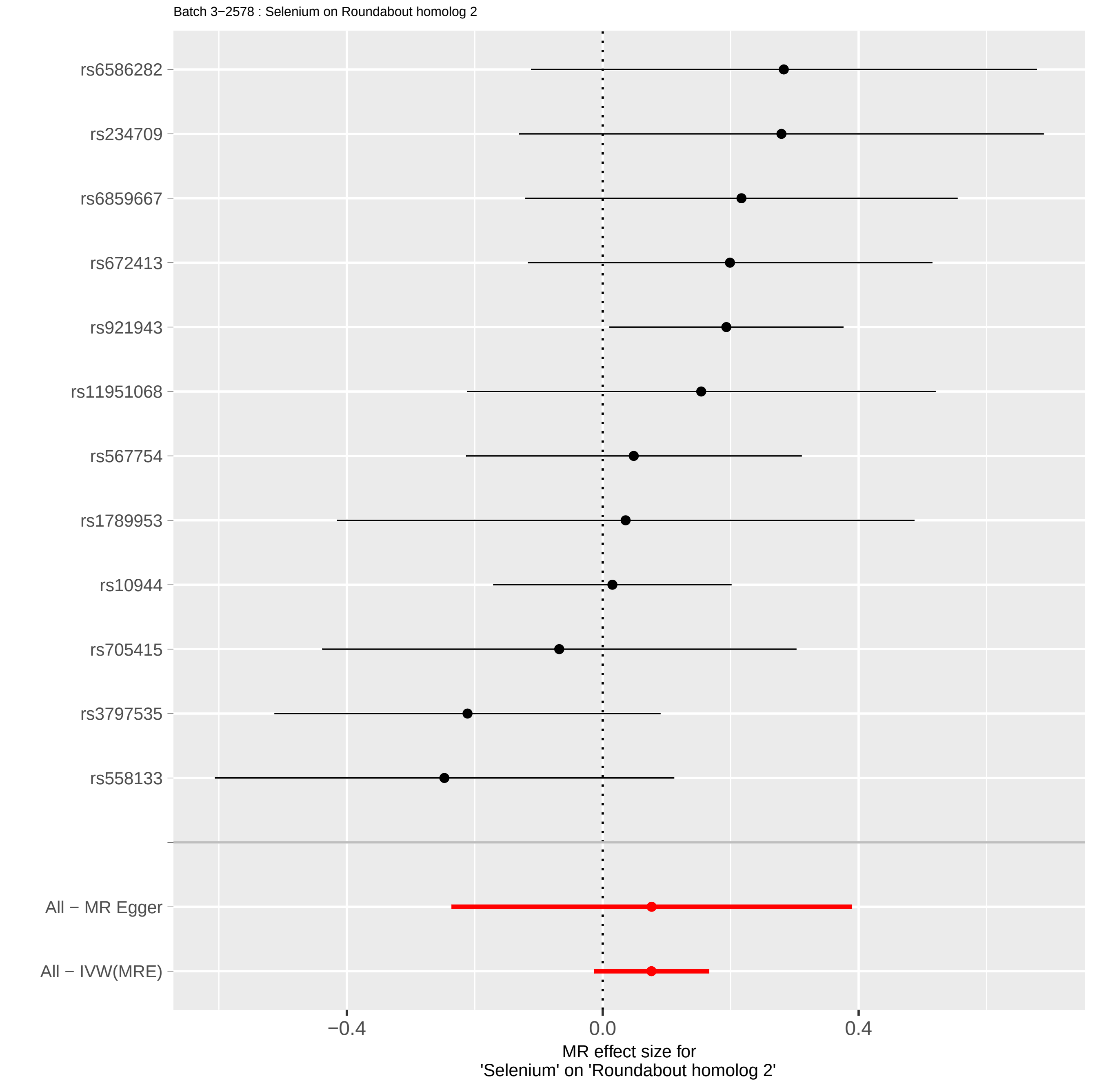

Batch 3−2578 : Selenium on Roundabout homolog 2
rs6586282
rs234709
rs6859667
rs672413
rs921943
rs11951068
rs567754
rs1789953
rs10944
rs705415
rs3797535
rs558133
All − MR Egger
All − IVW(MRE)
−0.4
0.0
0.4
MR effect size for
'Selenium' on 'Roundabout homolog 2'

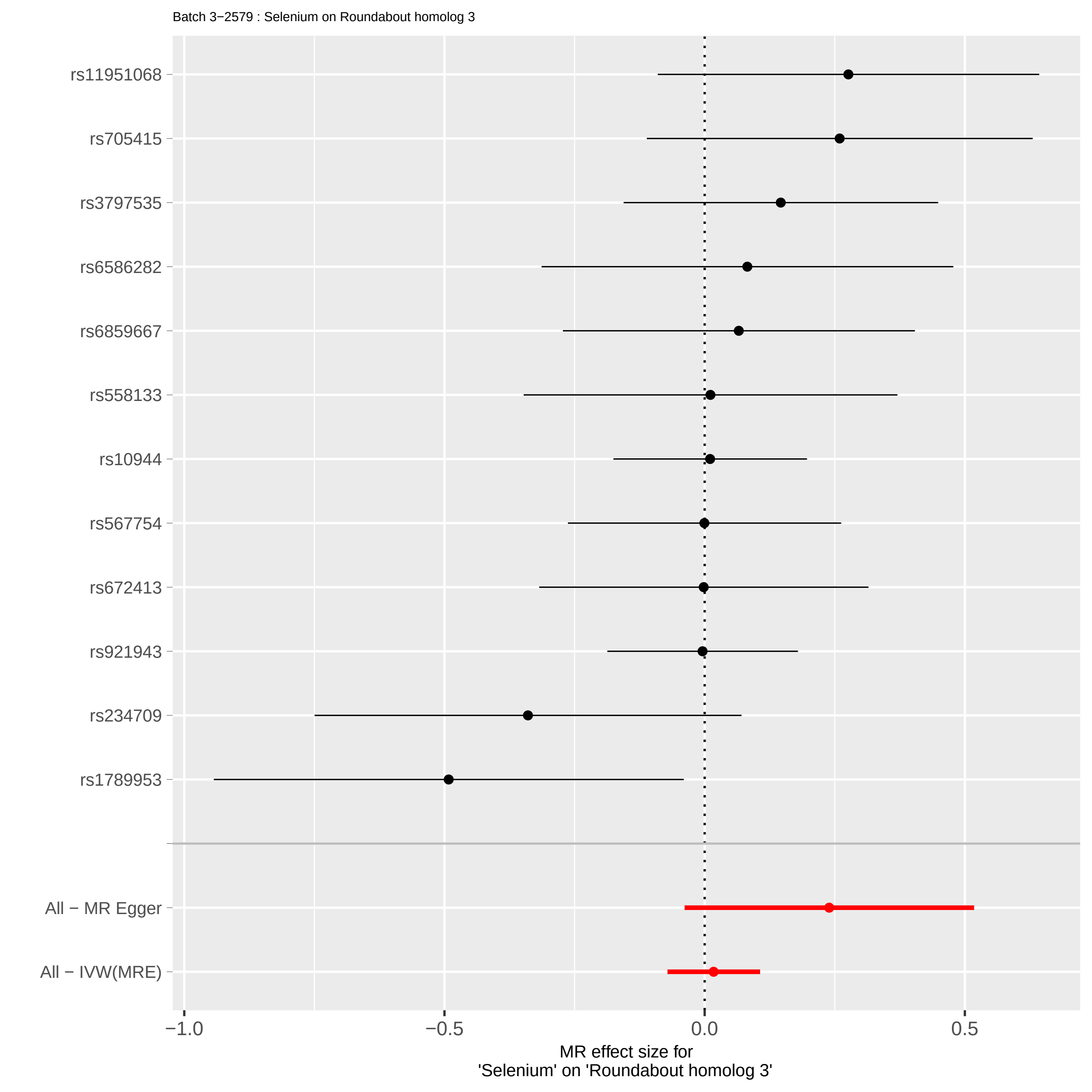

Batch 3−2579 : Selenium on Roundabout homolog 3
rs11951068
rs705415
rs3797535
rs6586282
rs6859667
rs558133
rs10944
rs567754
rs672413
rs921943
rs234709
rs1789953
All − MR Egger
All − IVW(MRE)
−1.0
−0.5
0.0
0.5
MR effect size for
'Selenium' on 'Roundabout homolog 3'

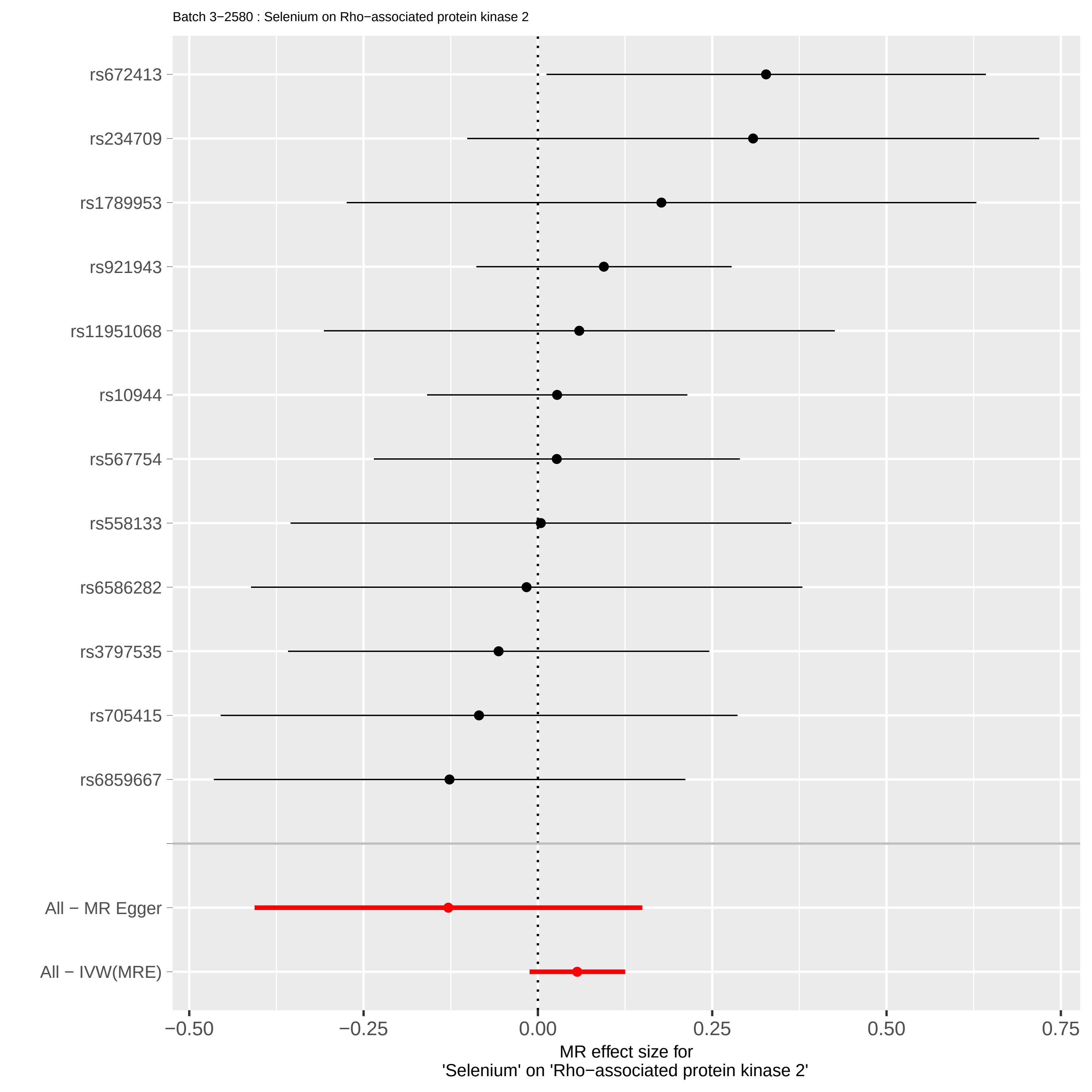

Batch 3−2580 : Selenium on Rho−associated protein kinase 2
rs672413
rs234709
rs1789953
rs921943
rs11951068
rs10944
rs567754
rs558133
rs6586282
rs3797535
rs705415
rs6859667
All − MR Egger
All − IVW(MRE)
−0.50
−0.25
0.00
0.25
0.50
0.75
MR effect size for
'Selenium' on 'Rho−associated protein kinase 2'

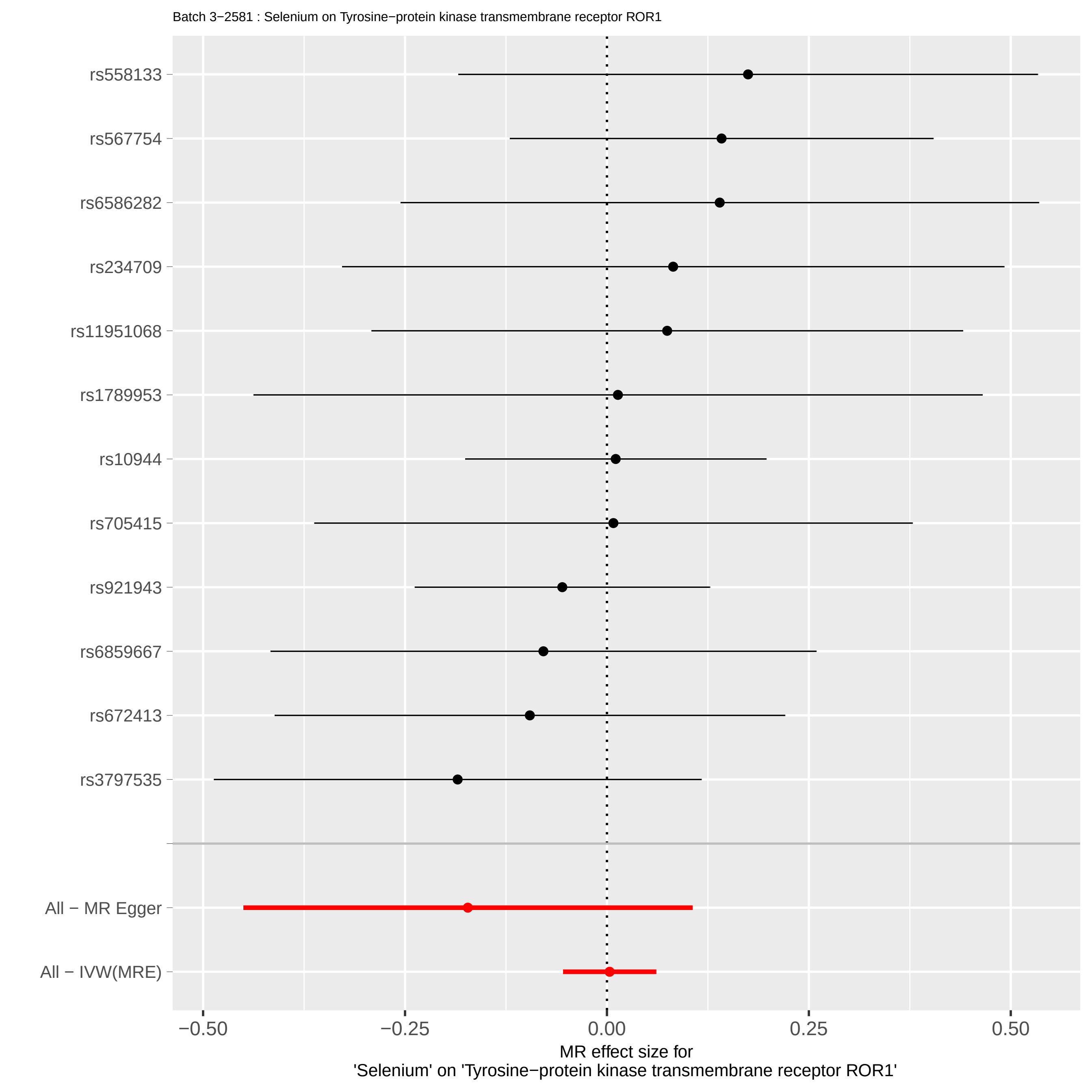

Batch 3−2581 : Selenium on Tyrosine−protein kinase transmembrane receptor ROR1
rs558133
rs567754
rs6586282
rs234709
rs11951068
rs1789953
rs10944
rs705415
rs921943
rs6859667
rs672413
rs3797535
All − MR Egger
All − IVW(MRE)
−0.50
−0.25
0.00
0.25
0.50
MR effect size for
'Selenium' on 'Tyrosine−protein kinase transmembrane receptor ROR1'

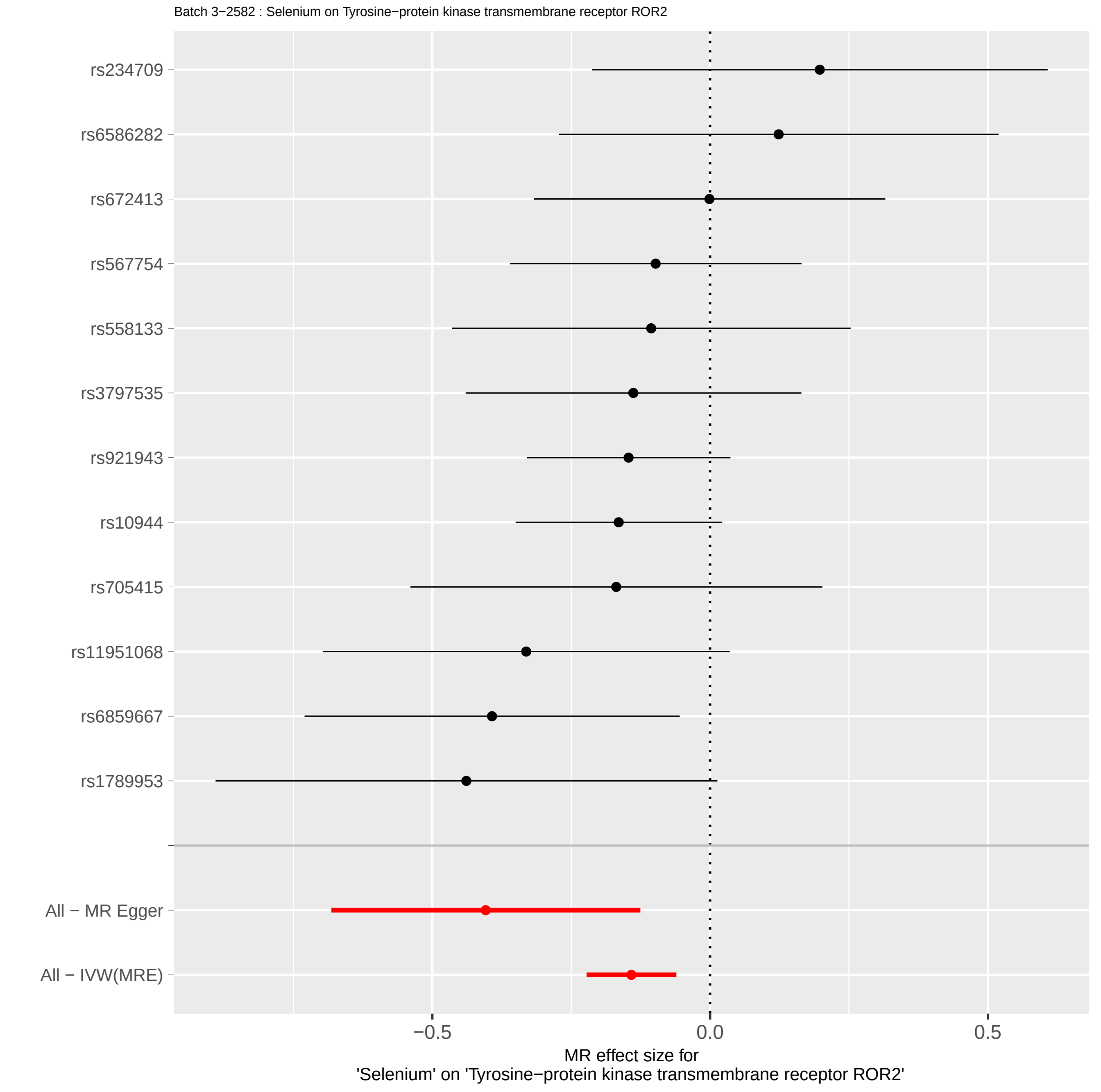

Batch 3−2582 : Selenium on Tyrosine−protein kinase transmembrane receptor ROR2
rs234709
rs6586282
rs672413
rs567754
rs558133
rs3797535
rs921943
rs10944
rs705415
rs11951068
rs6859667
rs1789953
All − MR Egger
All − IVW(MRE)
−0.5
0.0
0.5
MR effect size for
'Selenium' on 'Tyrosine−protein kinase transmembrane receptor ROR2'

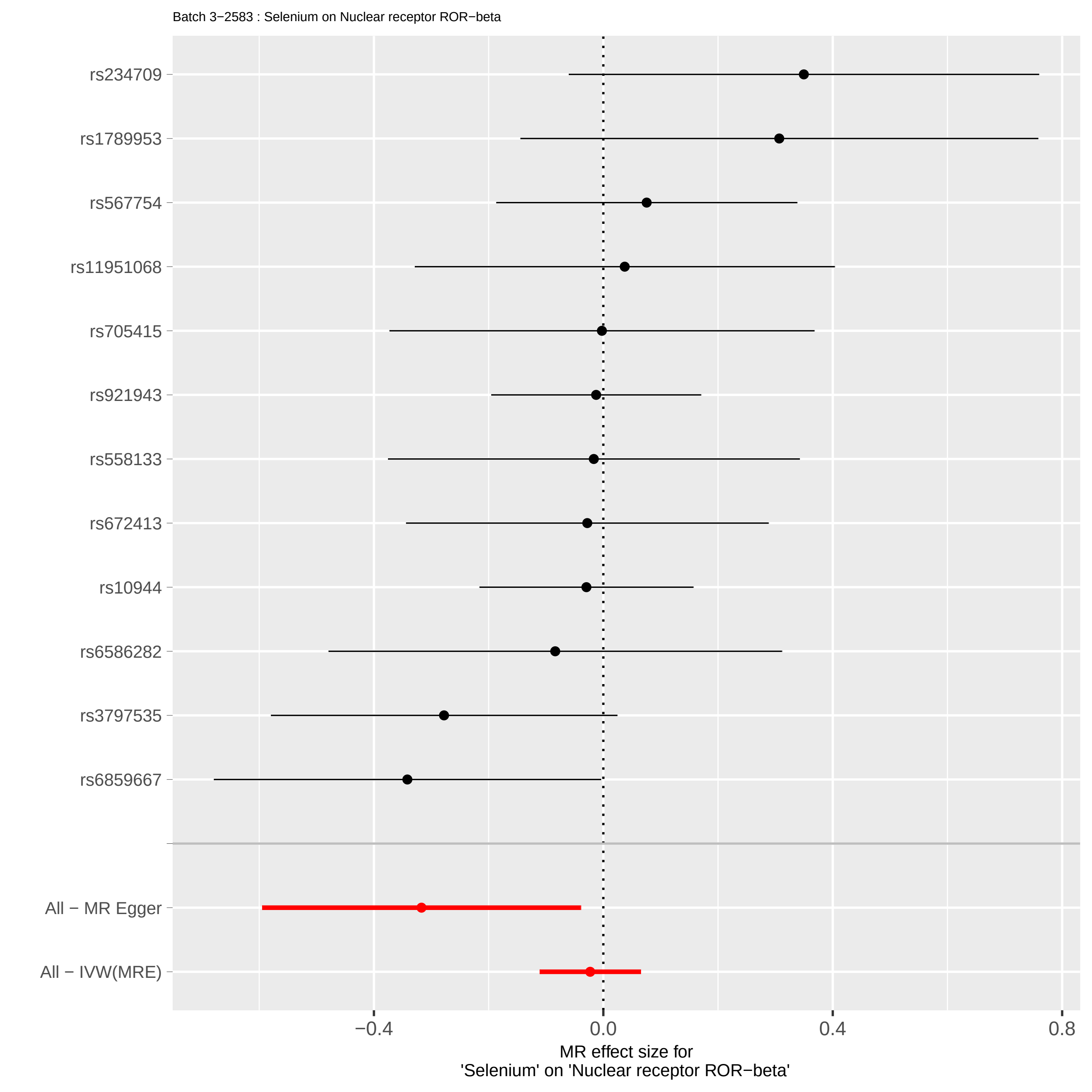

Batch 3−2583 : Selenium on Nuclear receptor ROR−beta
rs234709
rs1789953
rs567754
rs11951068
rs705415
rs921943
rs558133
rs672413
rs10944
rs6586282
rs3797535
rs6859667
All − MR Egger
All − IVW(MRE)
−0.4
0.0
0.4
0.8
MR effect size for
'Selenium' on 'Nuclear receptor ROR−beta'

Batch 3−2584 : Selenium on Ribulose−phosphate 3−epimerase

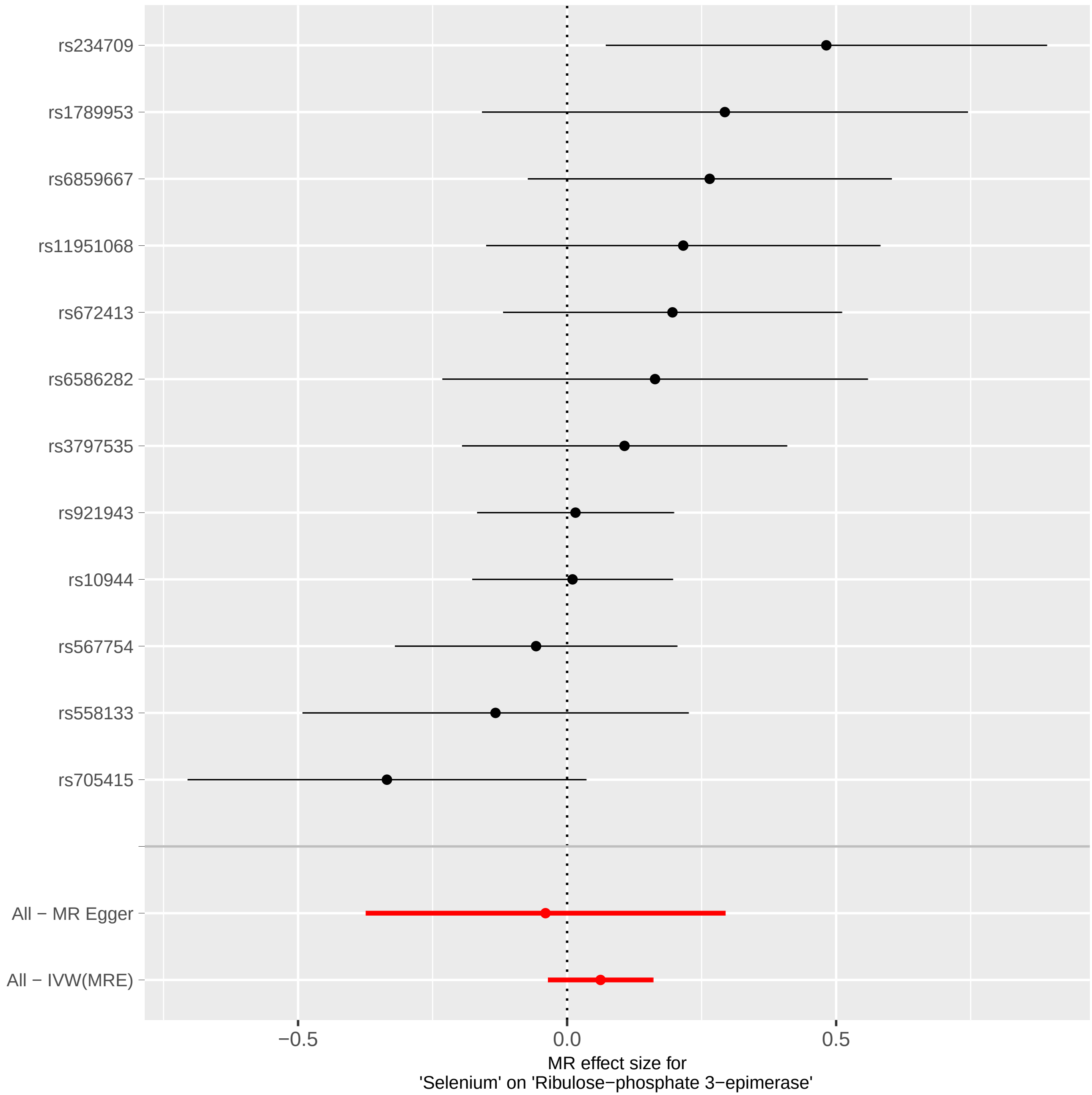

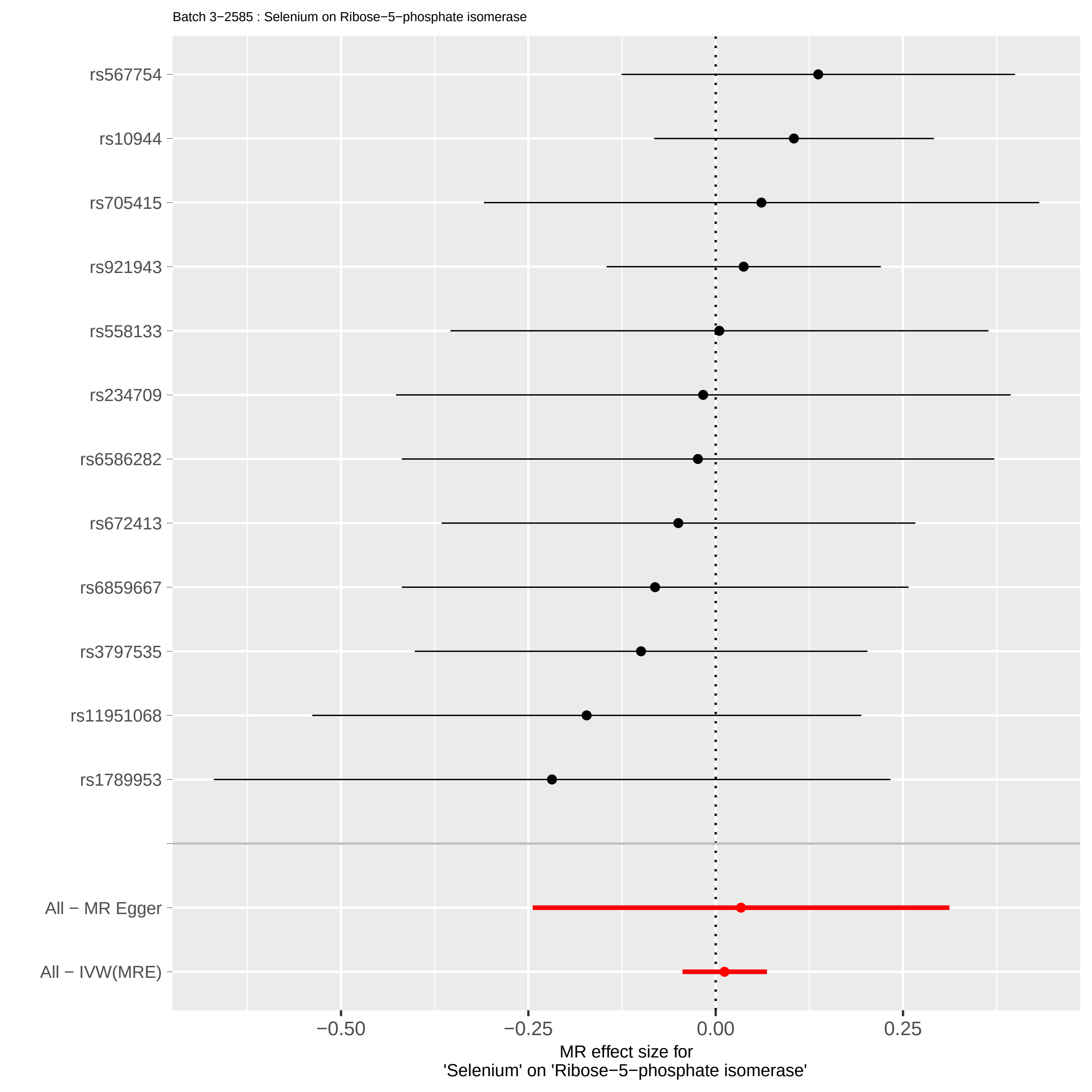

Batch 3−2585 : Selenium on Ribose−5−phosphate isomerase
rs567754
rs10944
rs705415
rs921943
rs558133
rs234709
rs6586282
rs672413
rs6859667
rs3797535
rs11951068
rs1789953
All − MR Egger
All − IVW(MRE)
−0.50
−0.25
0.00
0.25
MR effect size for
'Selenium' on 'Ribose−5−phosphate isomerase'

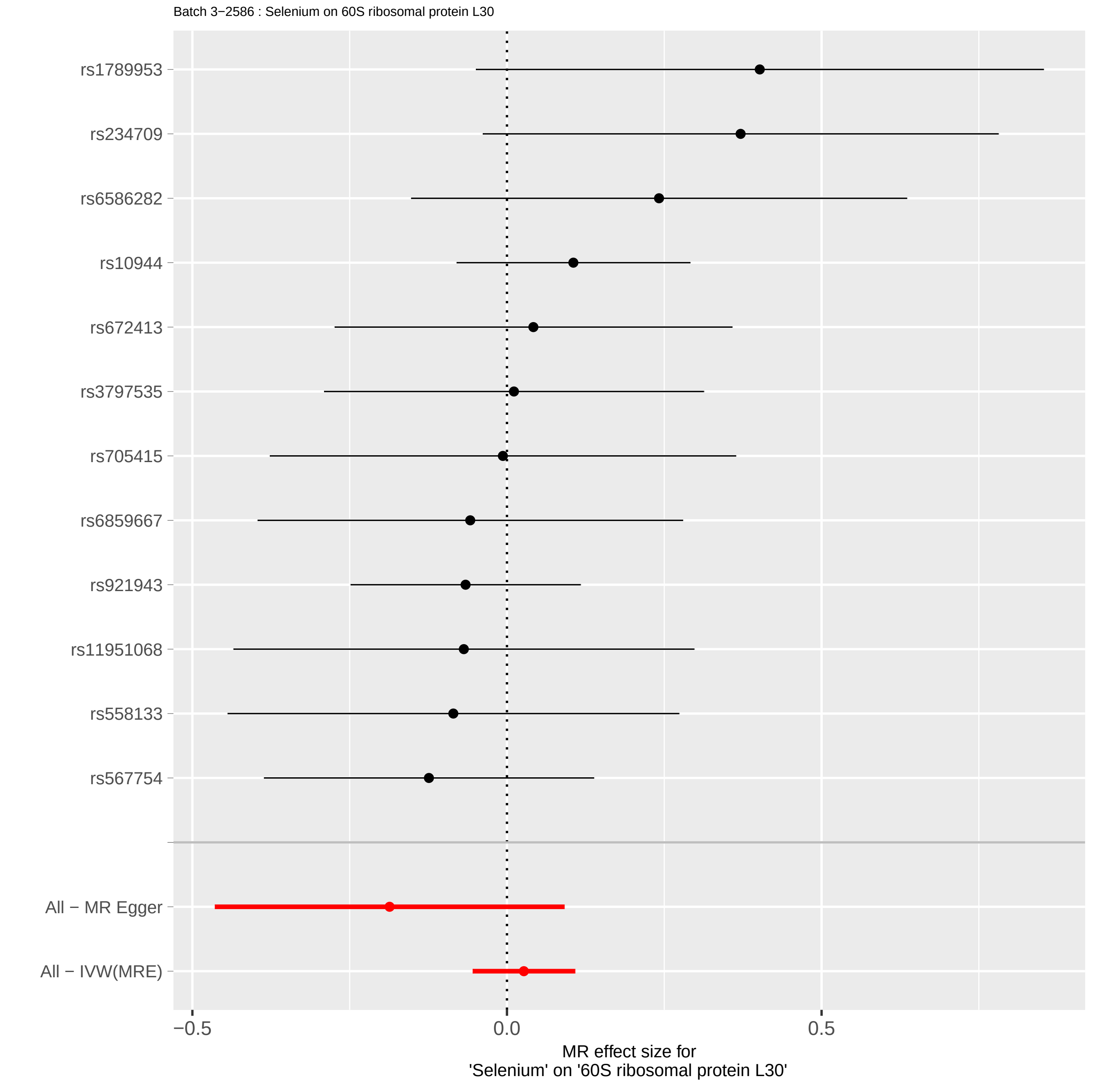

Batch 3−2586 : Selenium on 60S ribosomal protein L30
rs1789953
rs234709
rs6586282
rs10944
rs672413
rs3797535
rs705415
rs6859667
rs921943
rs11951068
rs558133
rs567754
All − MR Egger
All − IVW(MRE)
−0.5
0.0
0.5
MR effect size for
'Selenium' on '60S ribosomal protein L30'

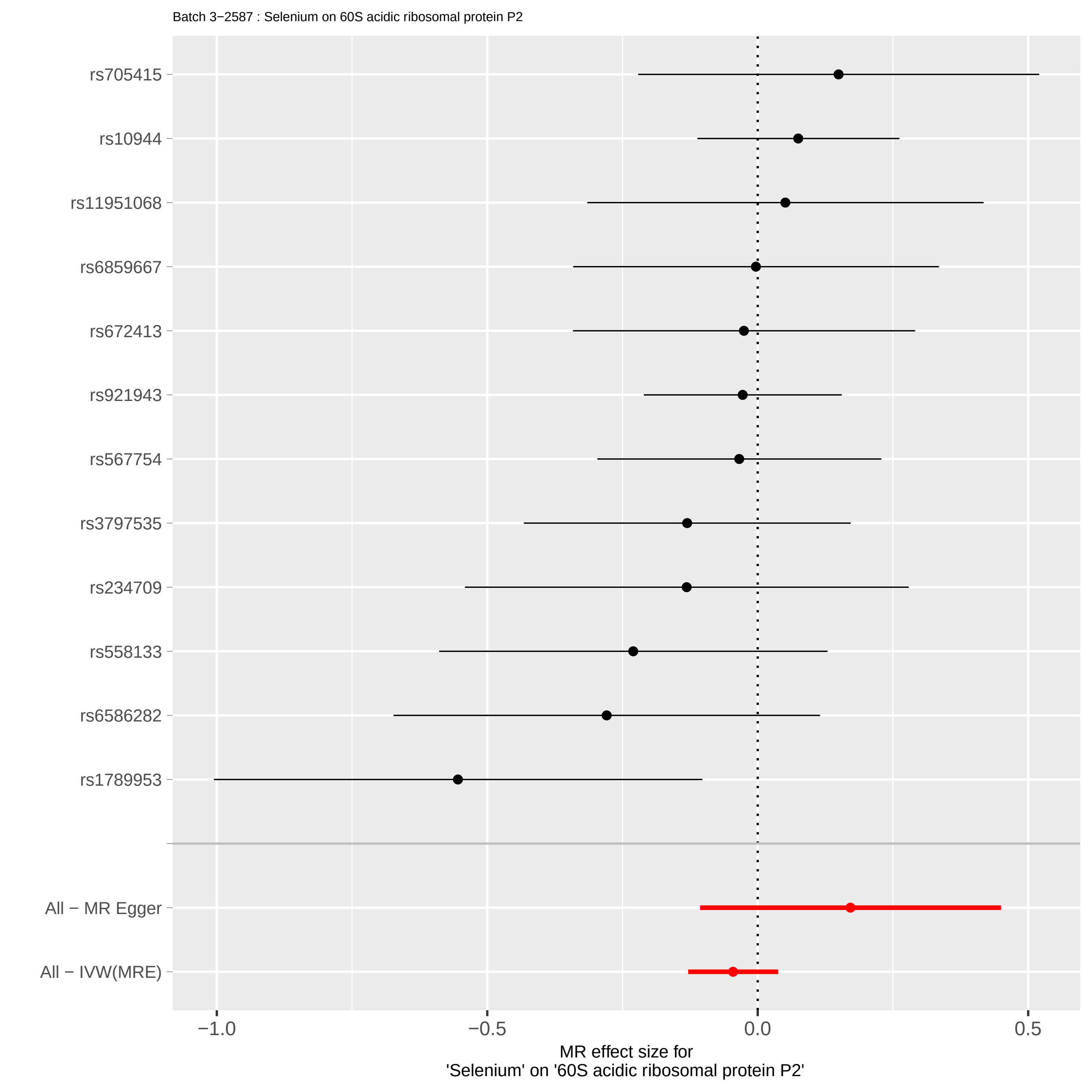

Batch 3−2587 : Selenium on 60S acidic ribosomal protein P2
rs705415
rs10944
rs11951068
rs6859667
rs672413
rs921943
rs567754
rs3797535
rs234709
rs558133
rs6586282
rs1789953
All − MR Egger
All − IVW(MRE)
−1.0
−0.5
0.0
0.5
MR effect size for
'Selenium' on '60S acidic ribosomal protein P2'

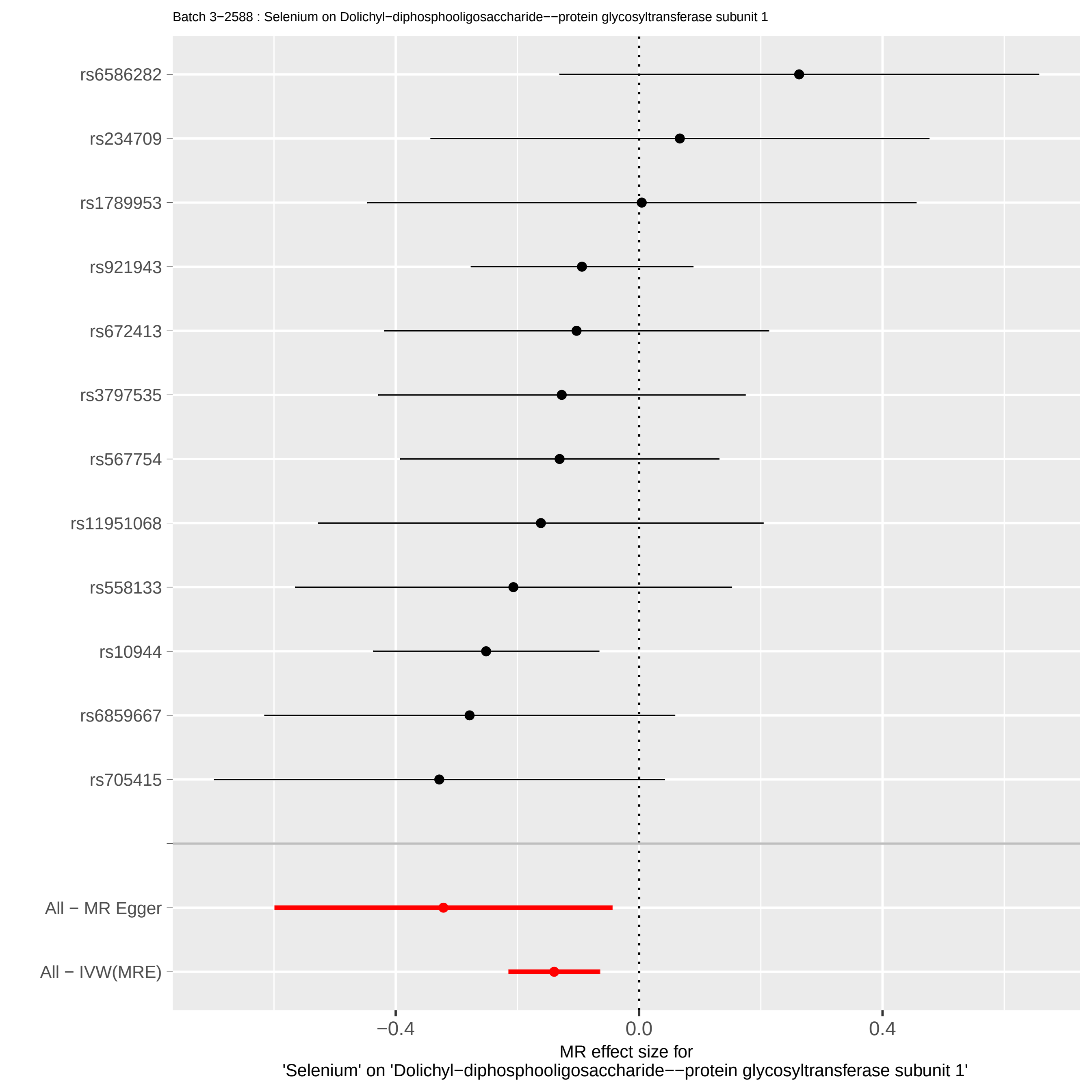
Batch 3−2588 : Selenium on Dolichyl−diphosphooligosaccharide−−protein glycosyltransferase subunit 1
rs6586282
rs234709
rs1789953
rs921943
rs672413
rs3797535
rs567754
rs11951068
rs558133
rs10944
rs6859667
rs705415
All − MR Egger
All − IVW(MRE)
−0.4
0.0
0.4
MR effect size for
'Selenium' on 'Dolichyl−diphosphooligosaccharide−−protein glycosyltransferase subunit 1'

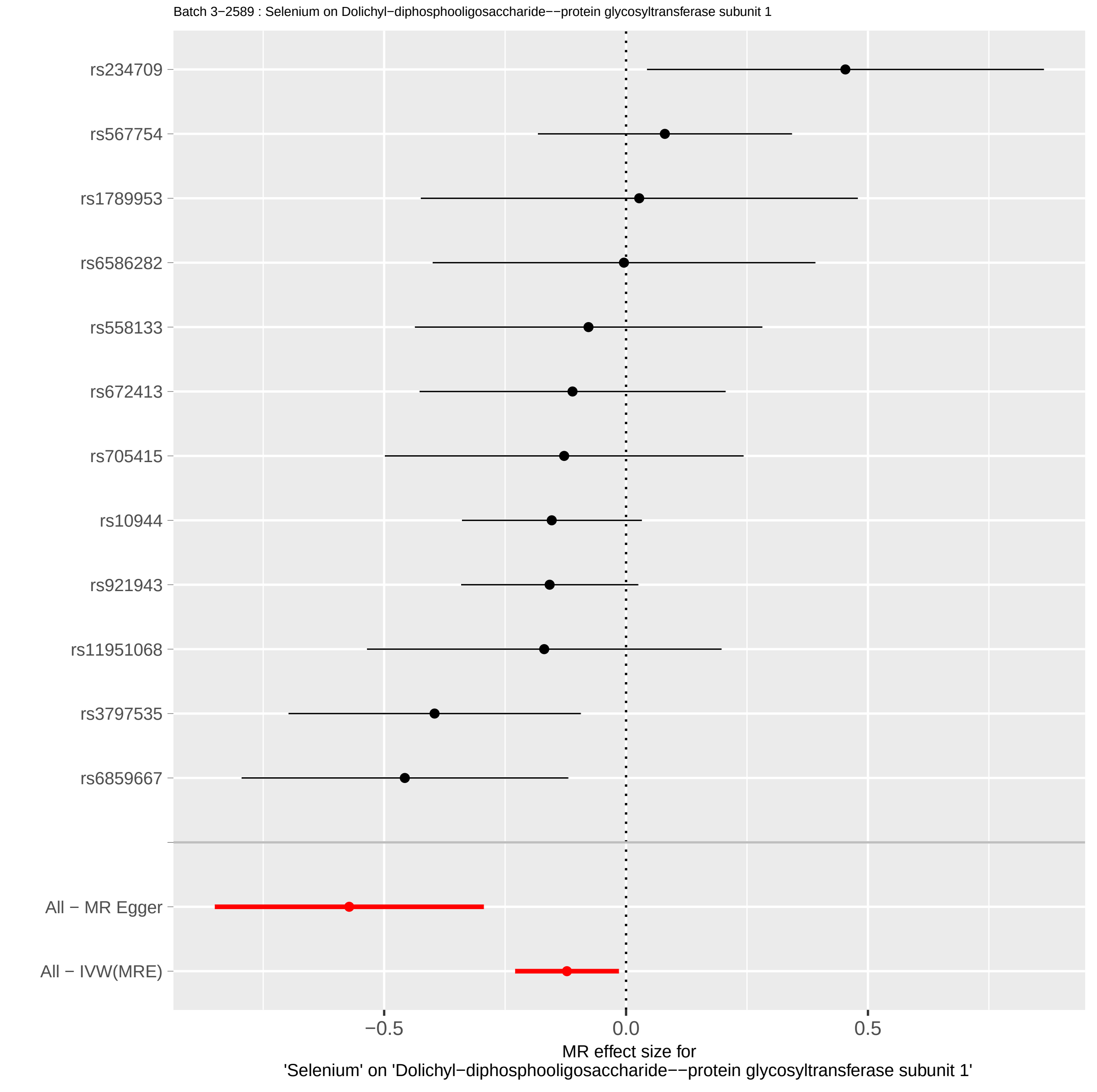

Batch 3−2589 : Selenium on Dolichyl−diphosphooligosaccharide−−protein glycosyltransferase subunit 1
rs234709
rs567754
rs1789953
rs6586282
rs558133
rs672413
rs705415
rs10944
rs921943
rs11951068
rs3797535
rs6859667
All − MR Egger
All − IVW(MRE)
−0.5
0.0
0.5
MR effect size for
'Selenium' on 'Dolichyl−diphosphooligosaccharide−−protein glycosyltransferase subunit 1'

Batch 3−2590 : Selenium on Regulation of nuclear pre−mRNA domain−containing protein 1A

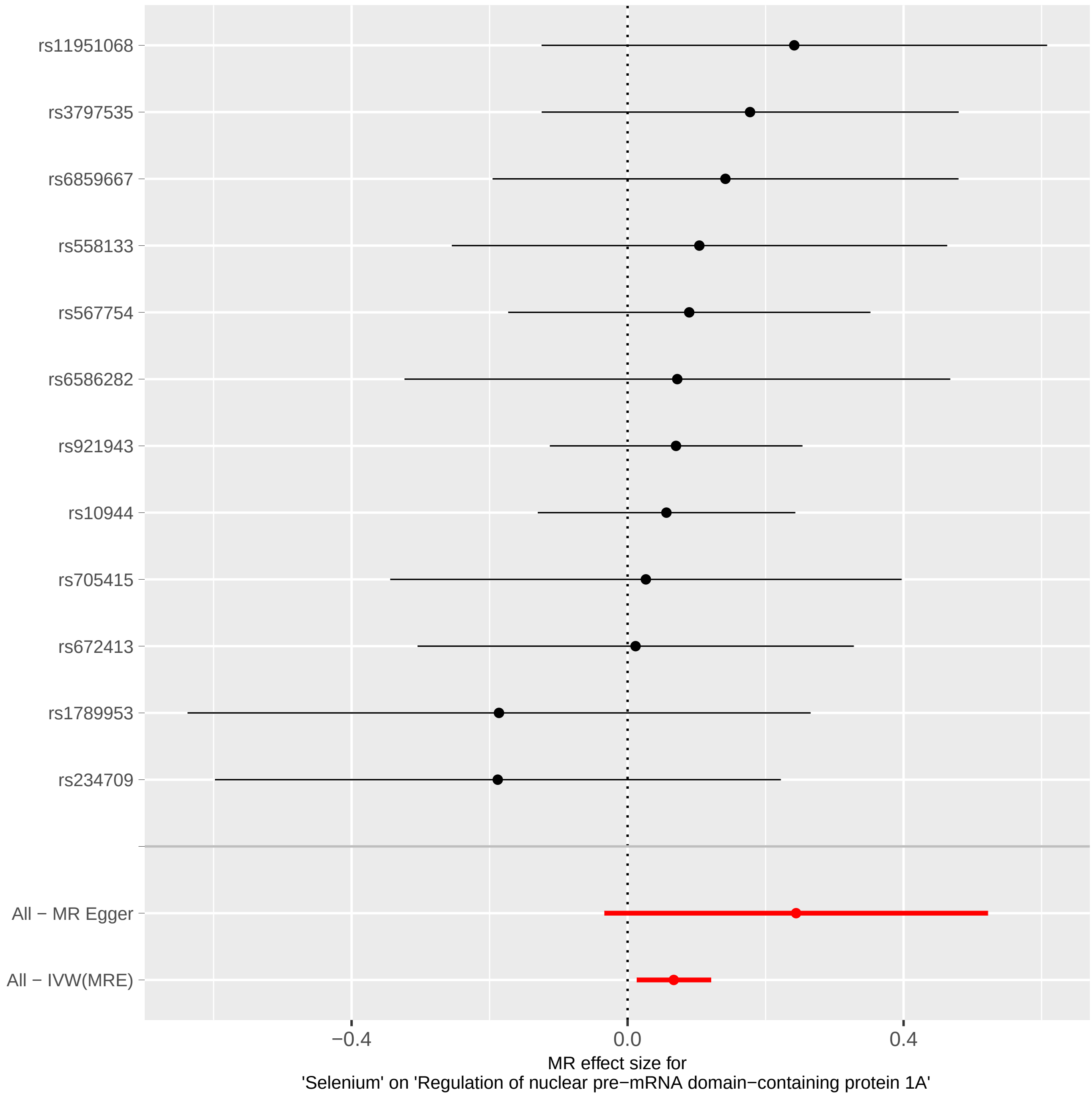

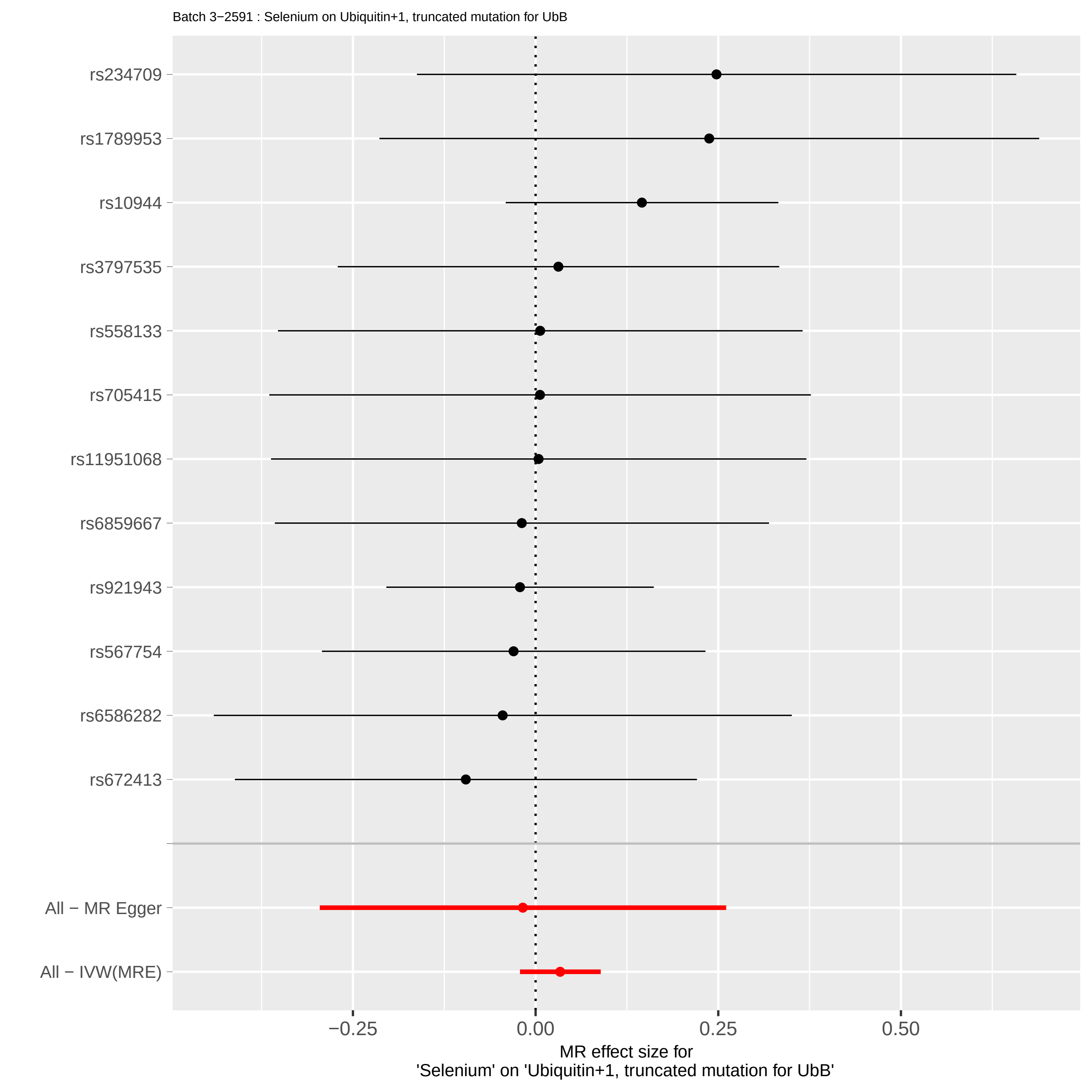

Batch 3−2591 : Selenium on Ubiquitin+1, truncated mutation for UbB
rs234709
rs1789953
rs10944
rs3797535
rs558133
rs705415
rs11951068
rs6859667
rs921943
rs567754
rs6586282
rs672413
All − MR Egger
All − IVW(MRE)
−0.25
0.00
0.25
0.50
MR effect size for
'Selenium' on 'Ubiquitin+1, truncated mutation for UbB'

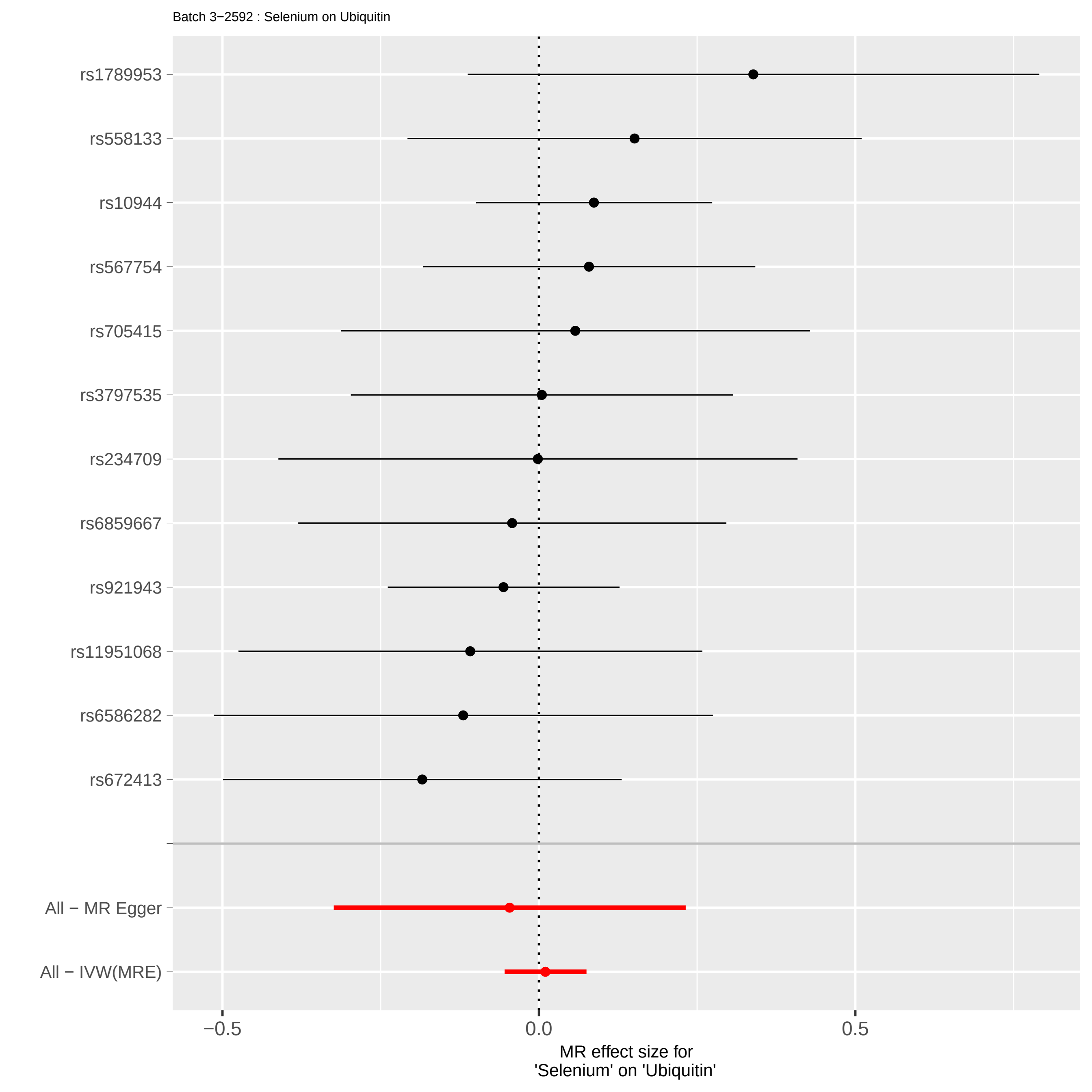

Batch 3−2592 : Selenium on Ubiquitin
rs1789953
rs558133
rs10944
rs567754
rs705415
rs3797535
rs234709
rs6859667
rs921943
rs11951068
rs6586282
rs672413
All − MR Egger
All − IVW(MRE)
−0.5
0.0
0.5
MR effect size for
'Selenium' on 'Ubiquitin'

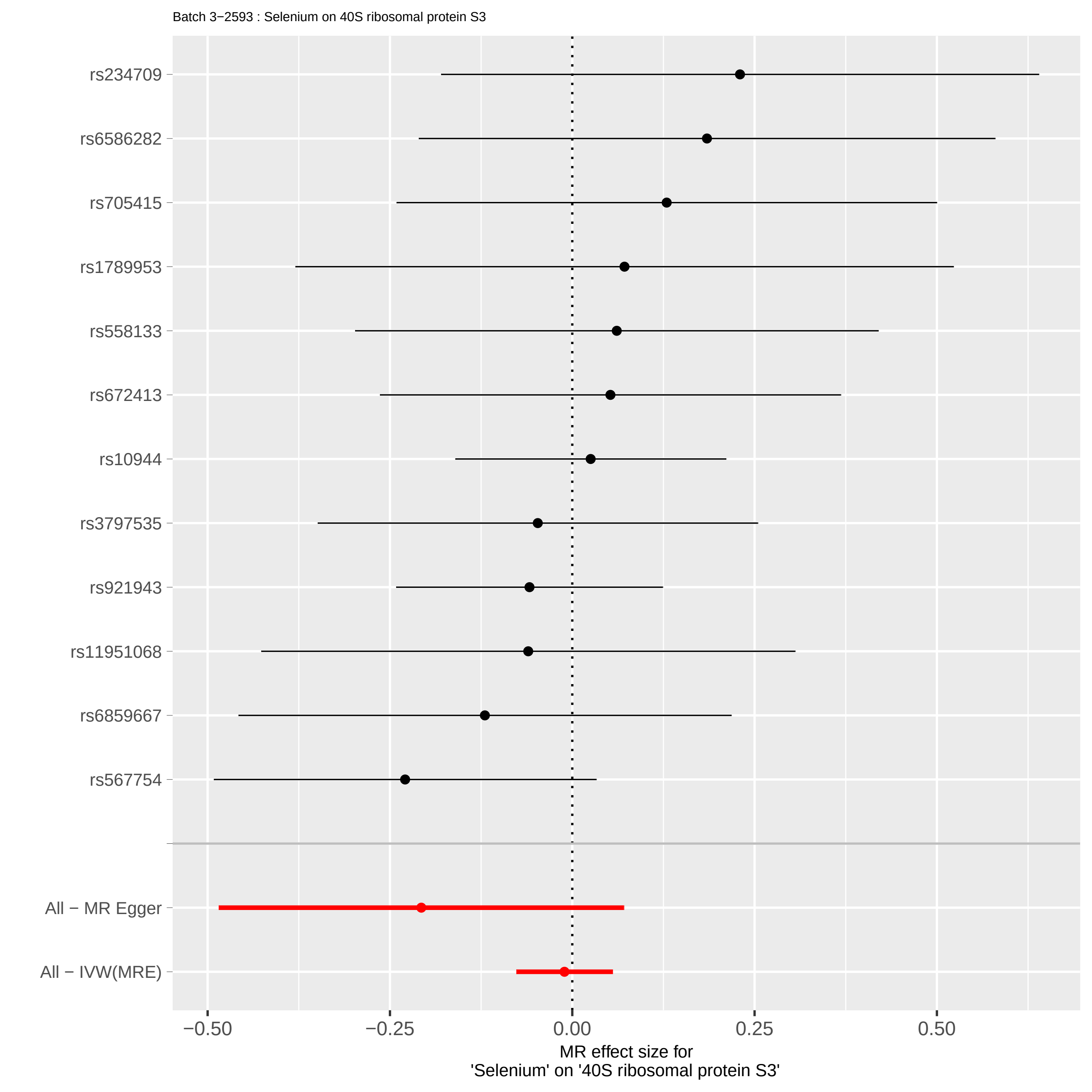

Batch 3−2593 : Selenium on 40S ribosomal protein S3
rs234709
rs6586282
rs705415
rs1789953
rs558133
rs672413
rs10944
rs3797535
rs921943
rs11951068
rs6859667
rs567754
All − MR Egger
All − IVW(MRE)
−0.50
−0.25
0.00
0.25
0.50
MR effect size for
'Selenium' on '40S ribosomal protein S3'

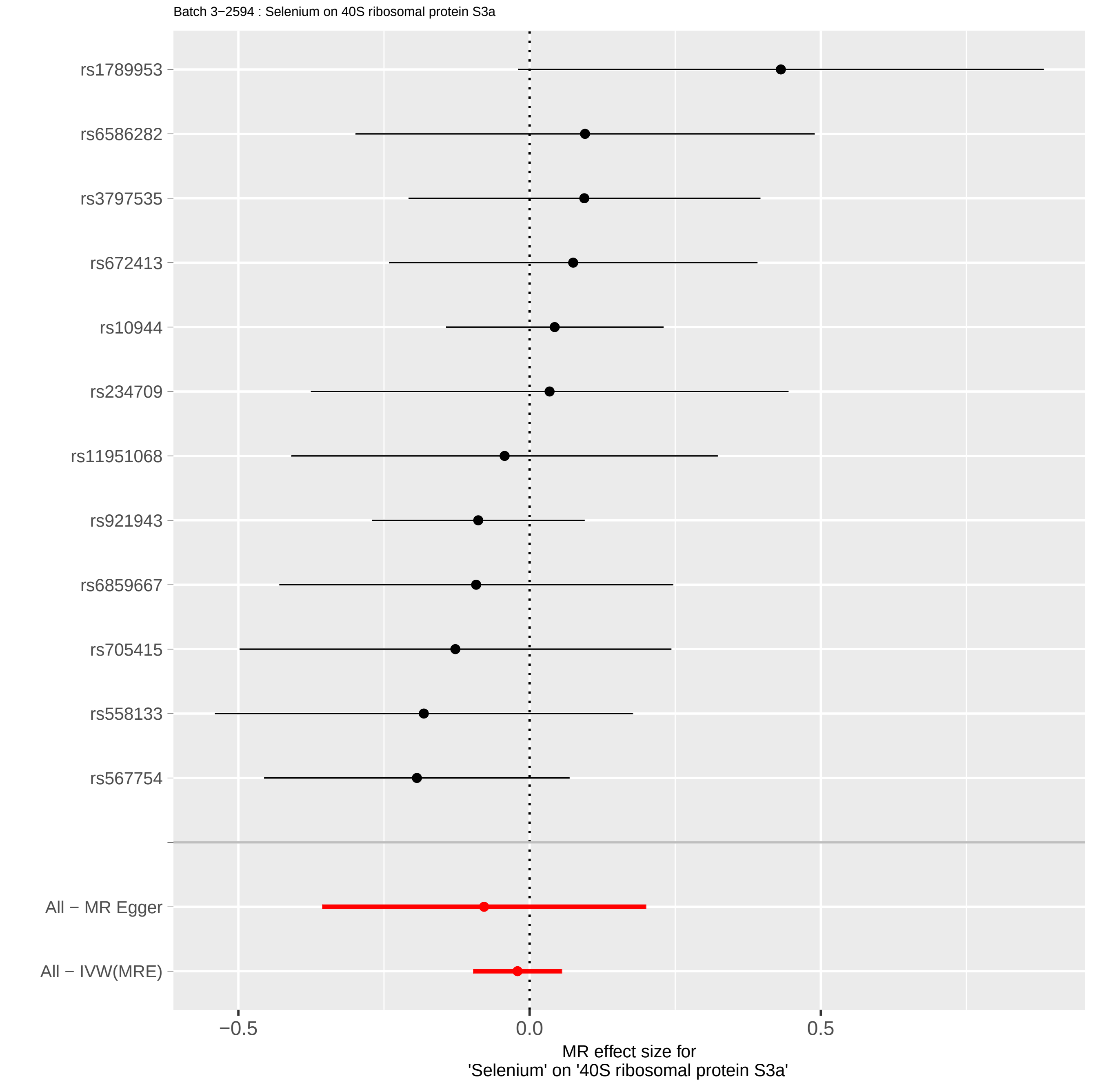

Batch 3−2594 : Selenium on 40S ribosomal protein S3a
rs1789953
rs6586282
rs3797535
rs672413
rs10944
rs234709
rs11951068
rs921943
rs6859667
rs705415
rs558133
rs567754
All − MR Egger
All − IVW(MRE)
−0.5
0.0
0.5
MR effect size for
'Selenium' on '40S ribosomal protein S3a'

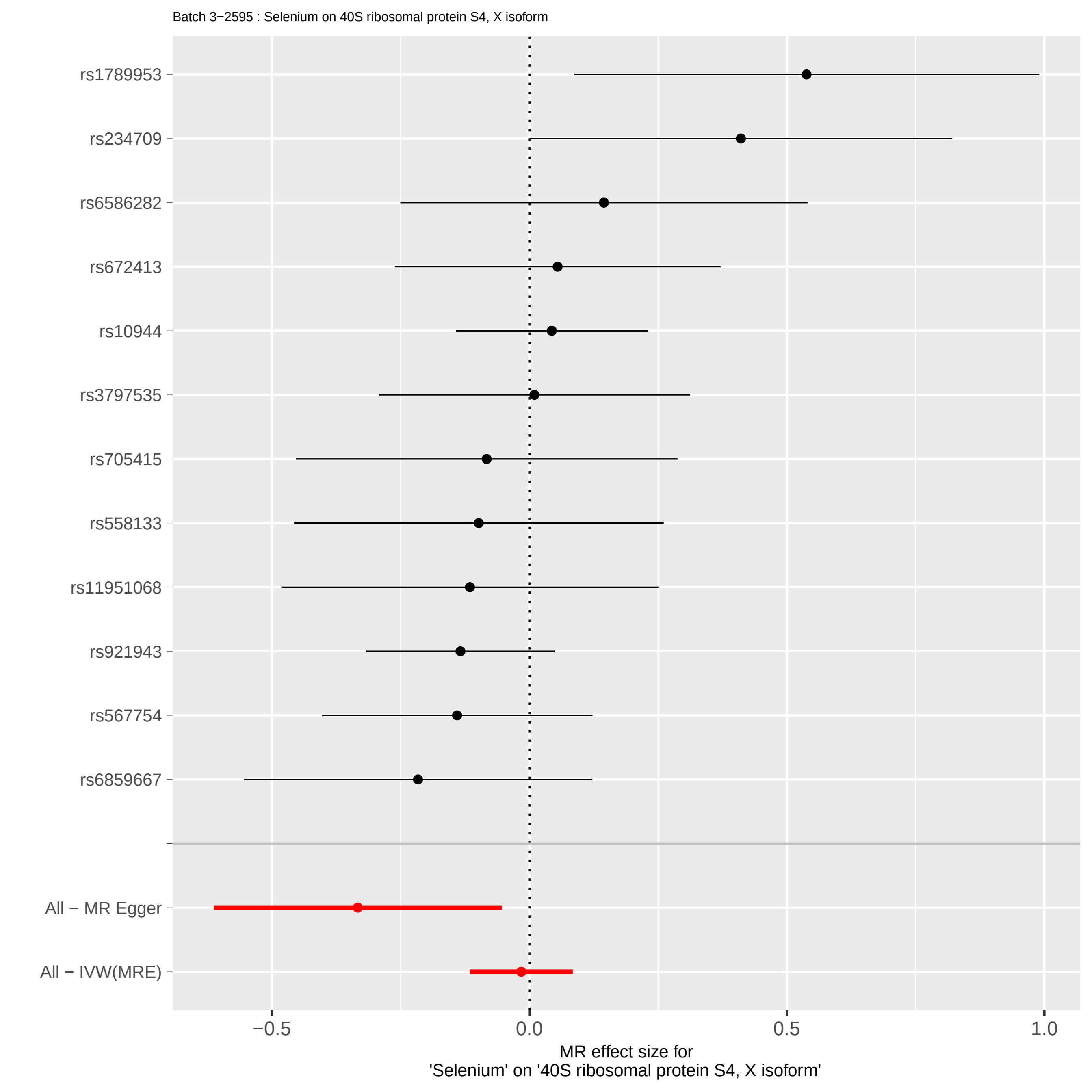

Batch 3−2595 : Selenium on 40S ribosomal protein S4, X isoform
rs1789953
rs234709
rs6586282
rs672413
rs10944
rs3797535
rs705415
rs558133
rs11951068
rs921943
rs567754
rs6859667
All − MR Egger
All − IVW(MRE)
−0.5
0.0
0.5
1.0
MR effect size for
'Selenium' on '40S ribosomal protein S4, X isoform'

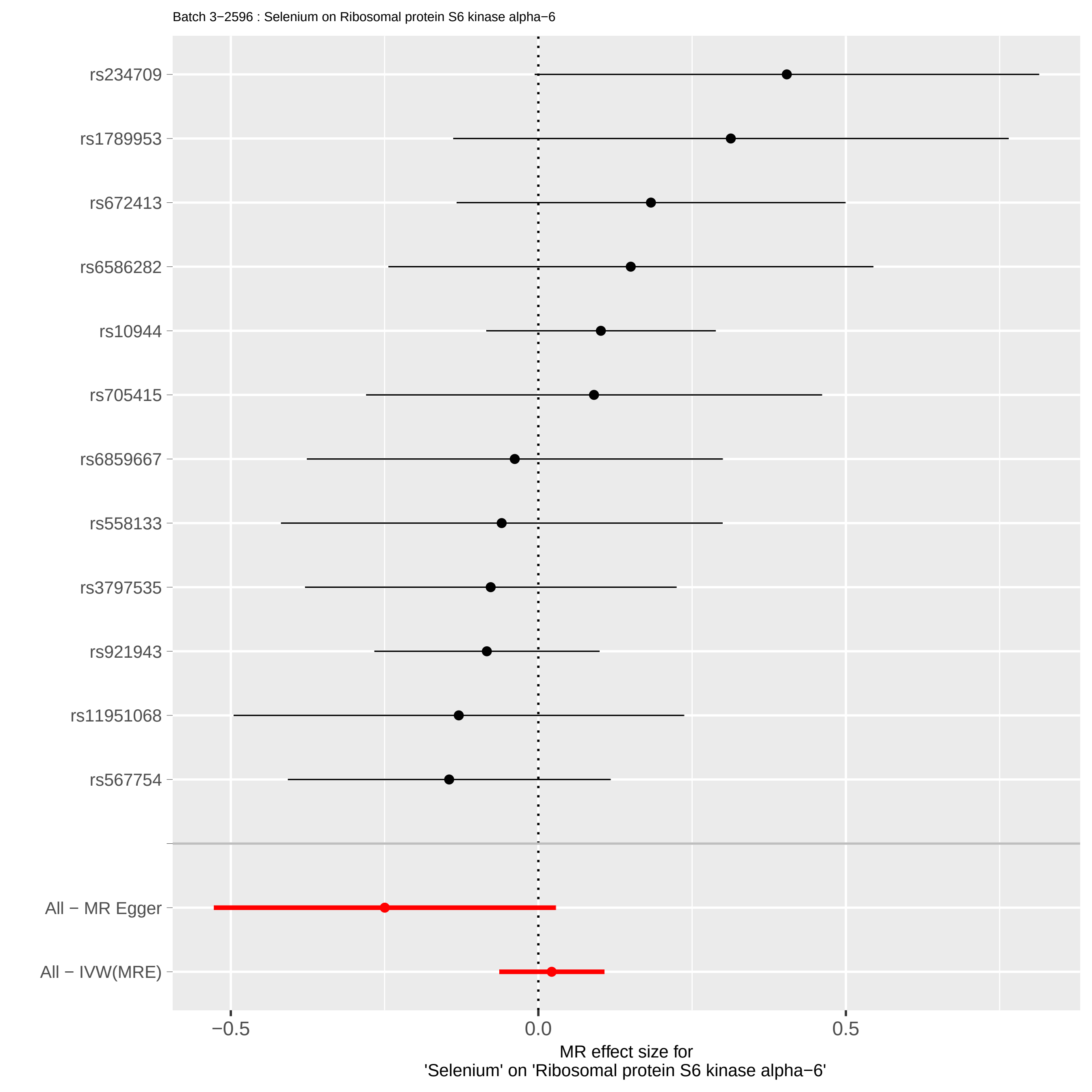

Batch 3−2596 : Selenium on Ribosomal protein S6 kinase alpha−6
rs234709
rs1789953
rs672413
rs6586282
rs10944
rs705415
rs6859667
rs558133
rs3797535
rs921943
rs11951068
rs567754
All − MR Egger
All − IVW(MRE)
−0.5
0.0
0.5
MR effect size for
'Selenium' on 'Ribosomal protein S6 kinase alpha−6'

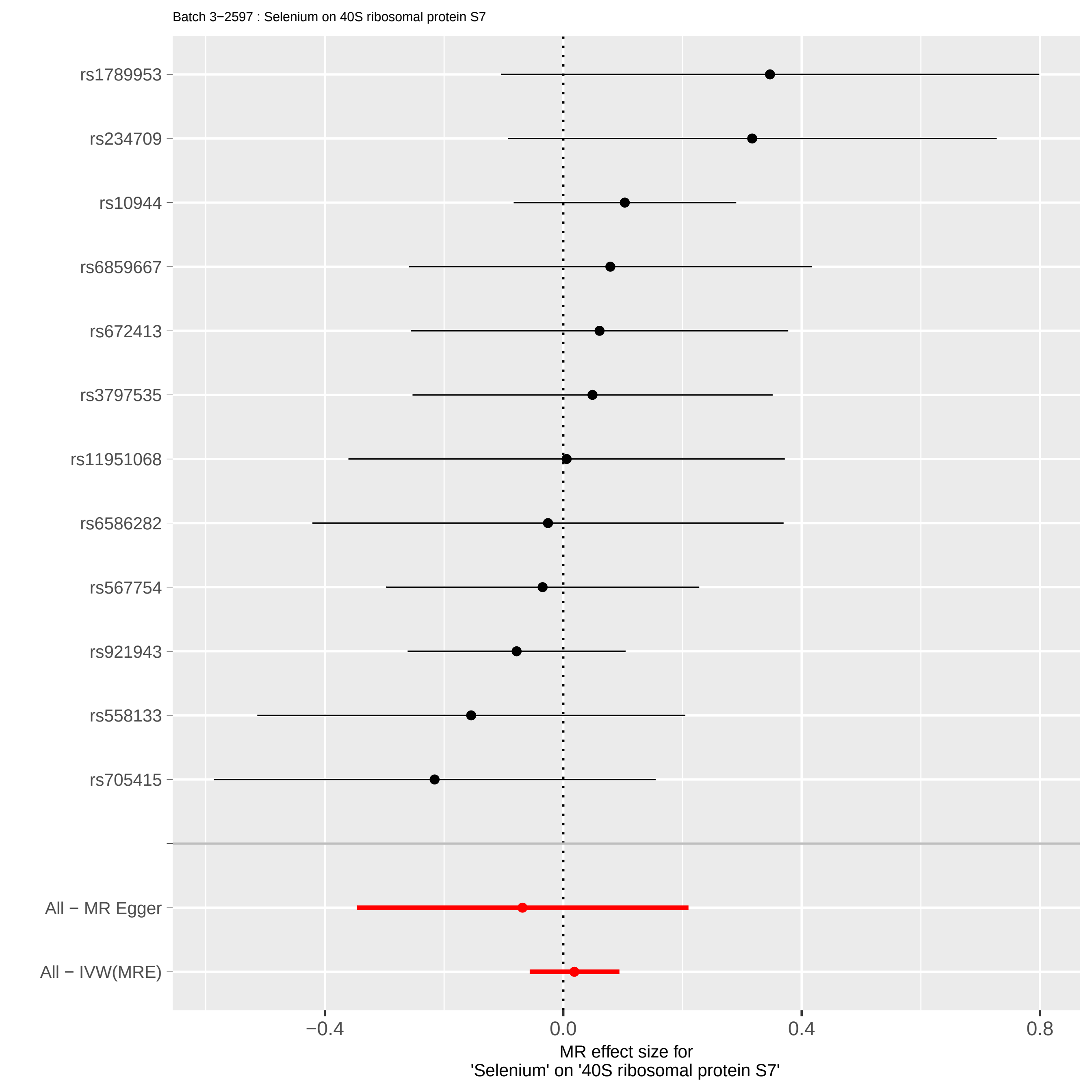

Batch 3−2597 : Selenium on 40S ribosomal protein S7
rs1789953
rs234709
rs10944
rs6859667
rs672413
rs3797535
rs11951068
rs6586282
rs567754
rs921943
rs558133
rs705415
All − MR Egger
All − IVW(MRE)
−0.4
0.0
0.4
0.8
MR effect size for
'Selenium' on '40S ribosomal protein S7'

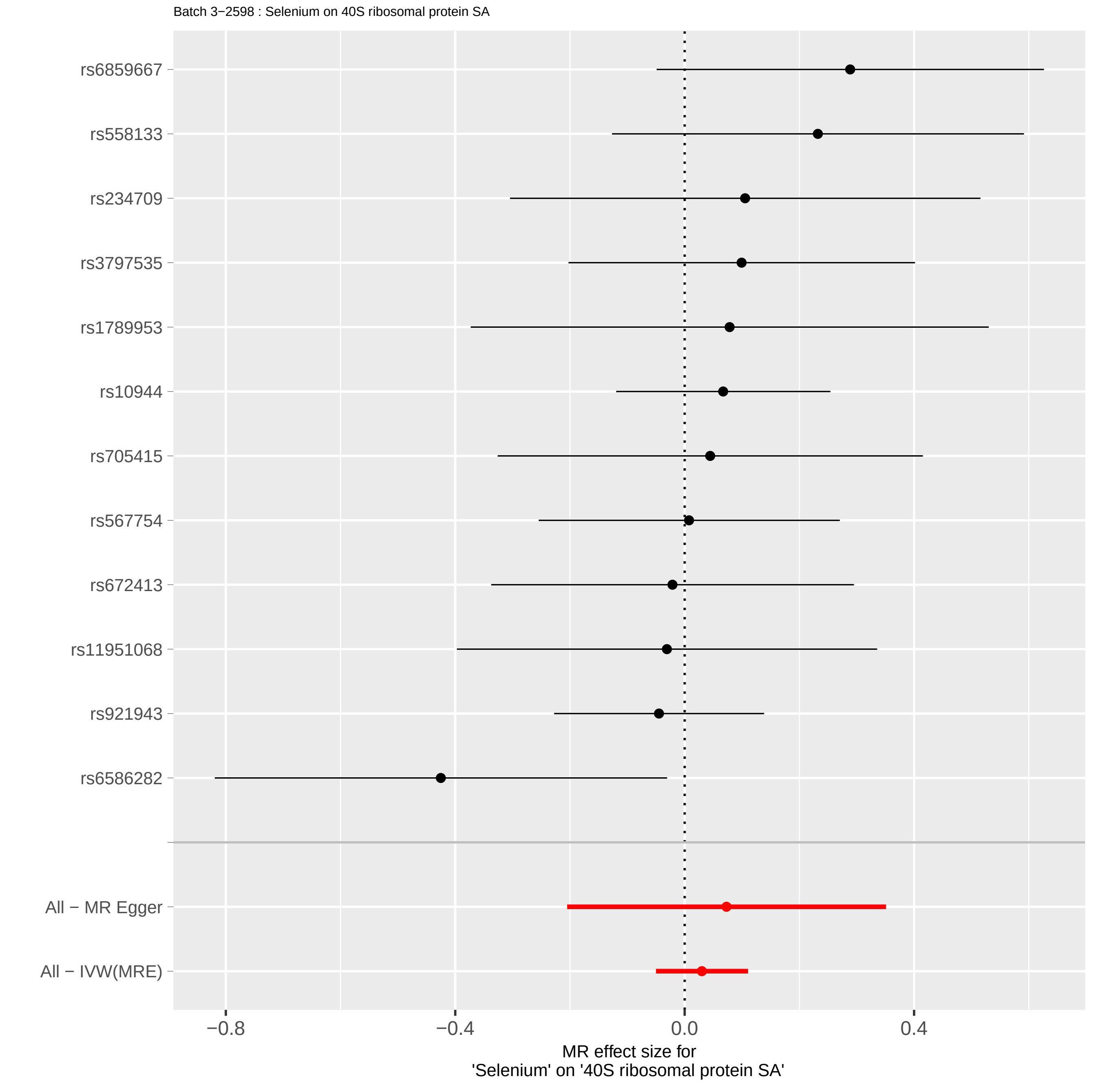

Batch 3−2598 : Selenium on 40S ribosomal protein SA
rs6859667
rs558133
rs234709
rs3797535
rs1789953
rs10944
rs705415
rs567754
rs672413
rs11951068
rs921943
rs6586282
All − MR Egger
All − IVW(MRE)
−0.8
−0.4
0.0
0.4
MR effect size for
'Selenium' on '40S ribosomal protein SA'

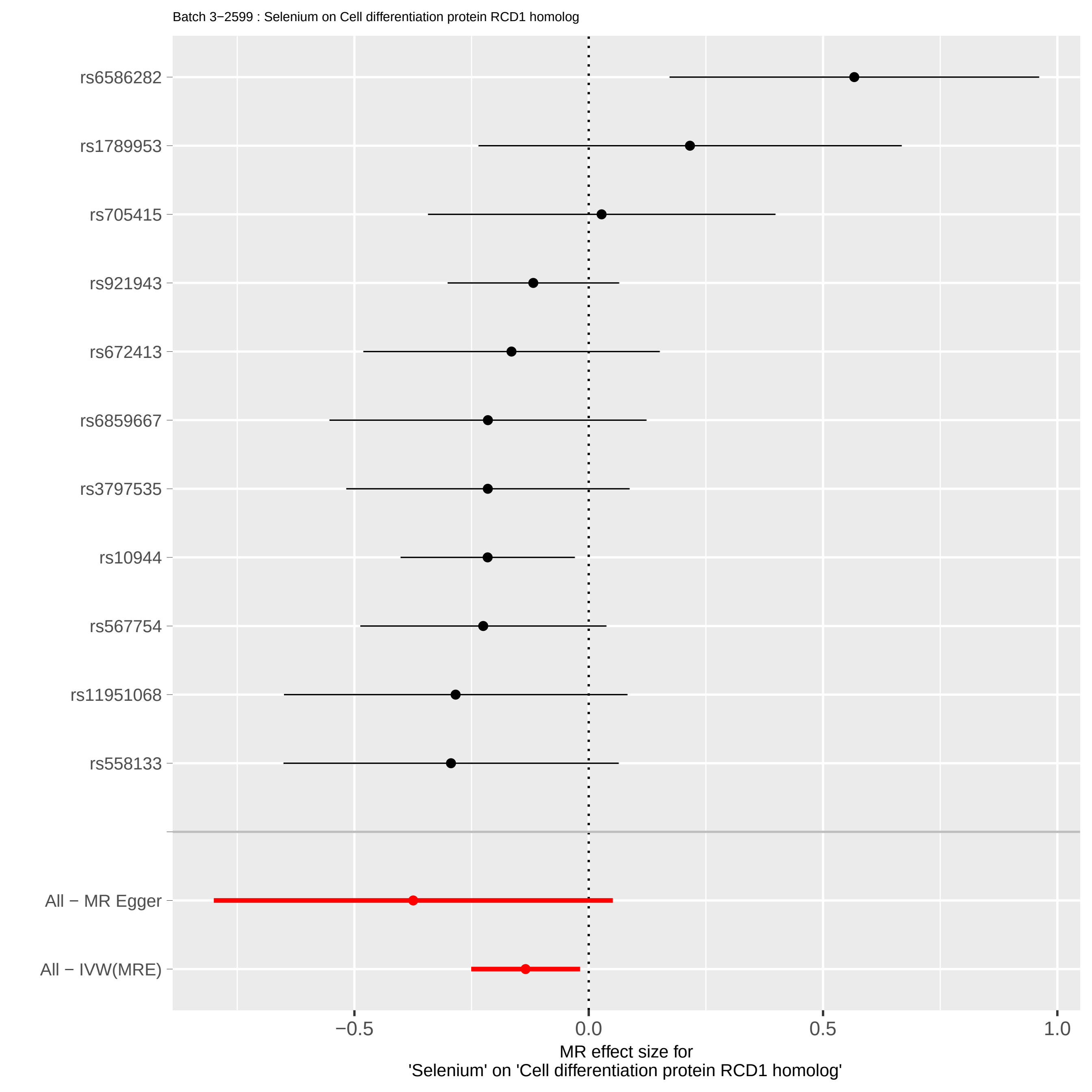

Batch 3−2599 : Selenium on Cell differentiation protein RCD1 homolog
rs6586282
rs1789953
rs705415
rs921943
rs672413
rs6859667
rs3797535
rs10944
rs567754
rs11951068
rs558133
All − MR Egger
All − IVW(MRE)
−0.5
0.0
0.5
1.0
MR effect size for
'Selenium' on 'Cell differentiation protein RCD1 homolog'

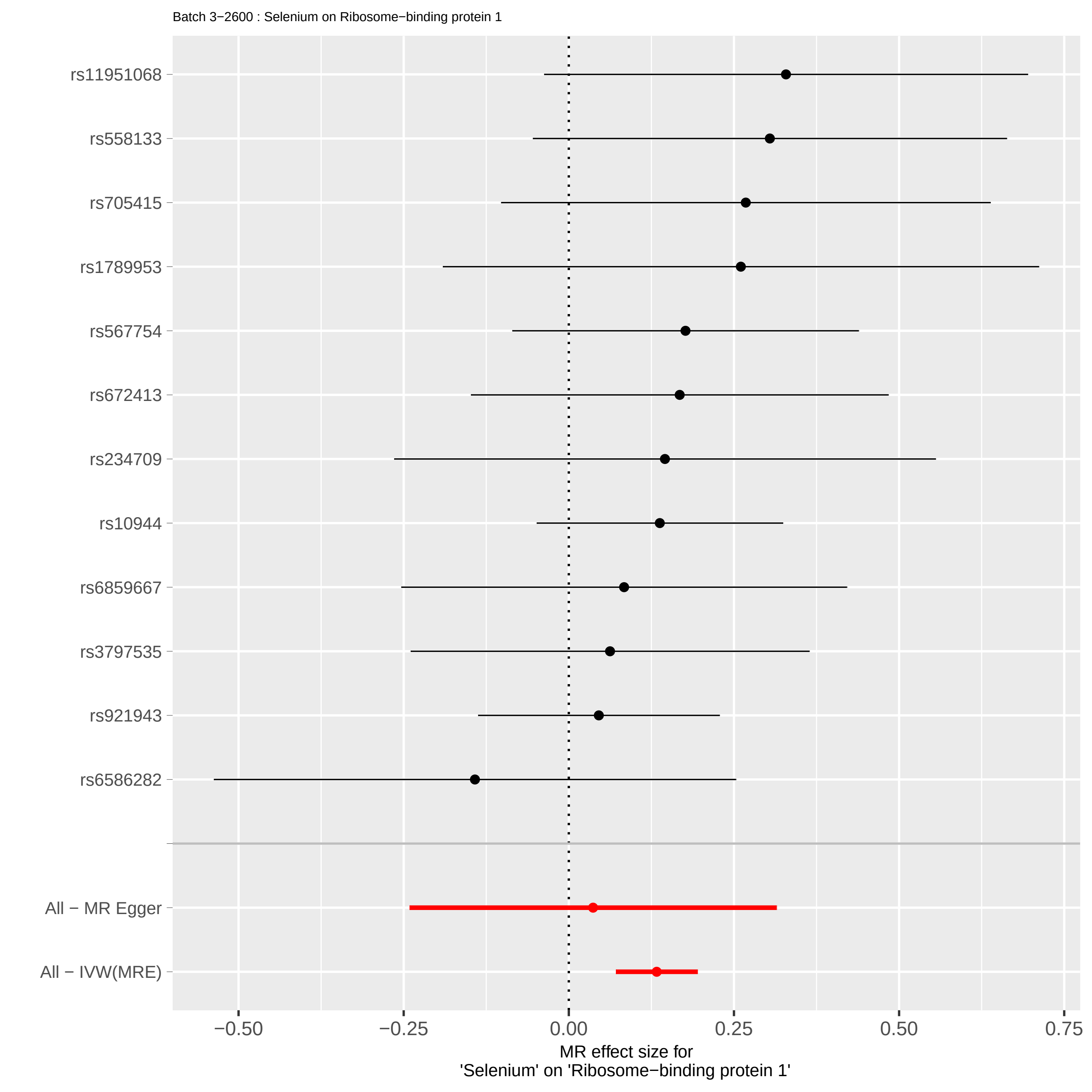

Batch 3−2600 : Selenium on Ribosome−binding protein 1
rs11951068
rs558133
rs705415
rs1789953
rs567754
rs672413
rs234709
rs10944
rs6859667
rs3797535
rs921943
rs6586282
All − MR Egger
All − IVW(MRE)
−0.50
−0.25
0.00
0.25
0.50
0.75
MR effect size for
'Selenium' on 'Ribosome−binding protein 1'

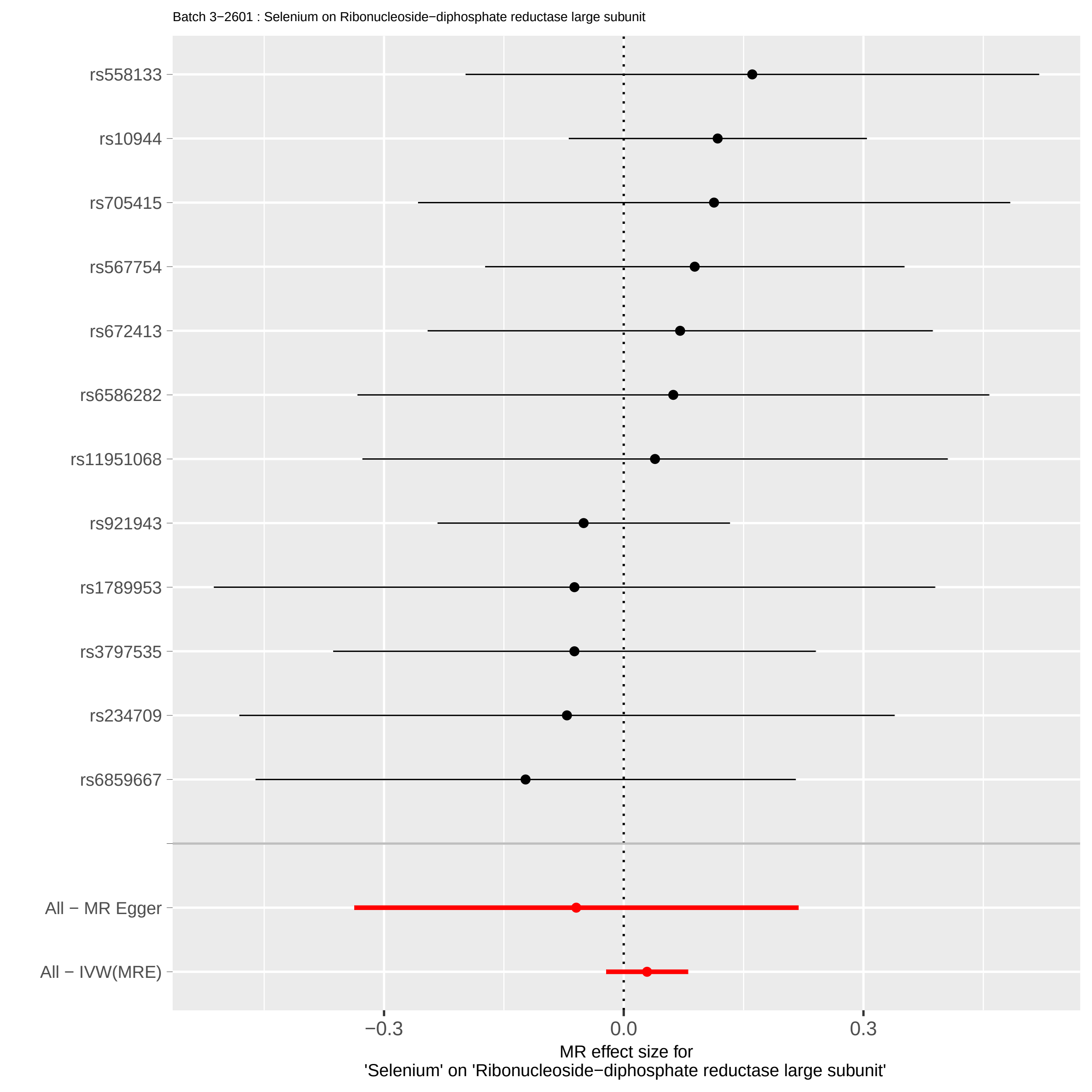

Batch 3−2601 : Selenium on Ribonucleoside−diphosphate reductase large subunit
rs558133
rs10944
rs705415
rs567754
rs672413
rs6586282
rs11951068
rs921943
rs1789953
rs3797535
rs234709
rs6859667
All − MR Egger
All − IVW(MRE)
−0.3
0.0
0.3
MR effect size for
'Selenium' on 'Ribonucleoside−diphosphate reductase large subunit'

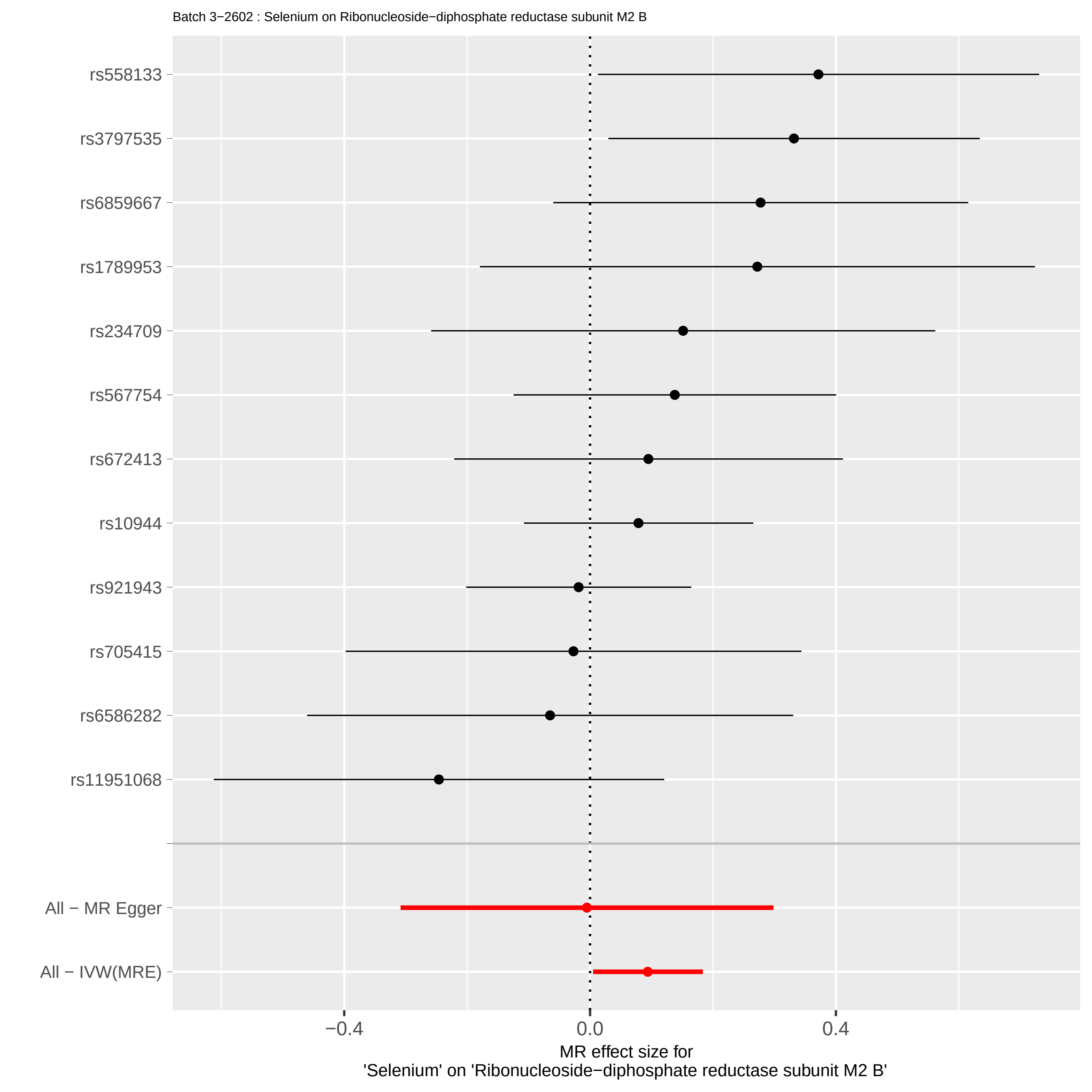

Batch 3−2602 : Selenium on Ribonucleoside−diphosphate reductase subunit M2 B
rs558133
rs3797535
rs6859667
rs1789953
rs234709
rs567754
rs672413
rs10944
rs921943
rs705415
rs6586282
rs11951068
All − MR Egger
All − IVW(MRE)
−0.4
0.0
0.4
MR effect size for
'Selenium' on 'Ribonucleoside−diphosphate reductase subunit M2 B'

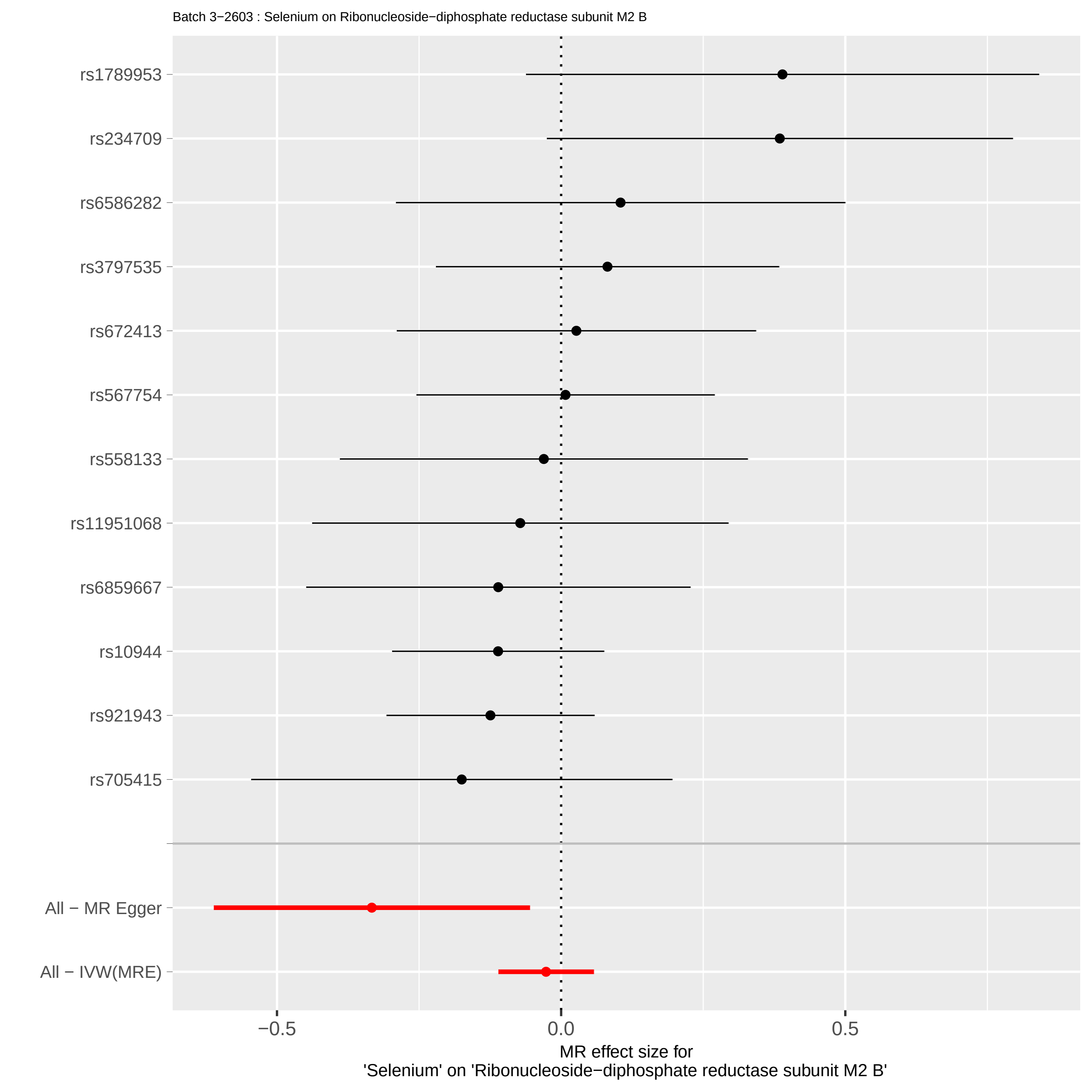

Batch 3−2603 : Selenium on Ribonucleoside−diphosphate reductase subunit M2 B
rs1789953
rs234709
rs6586282
rs3797535
rs672413
rs567754
rs558133
rs11951068
rs6859667
rs10944
rs921943
rs705415
All − MR Egger
All − IVW(MRE)
−0.5
0.0
0.5
MR effect size for
'Selenium' on 'Ribonucleoside−diphosphate reductase subunit M2 B'

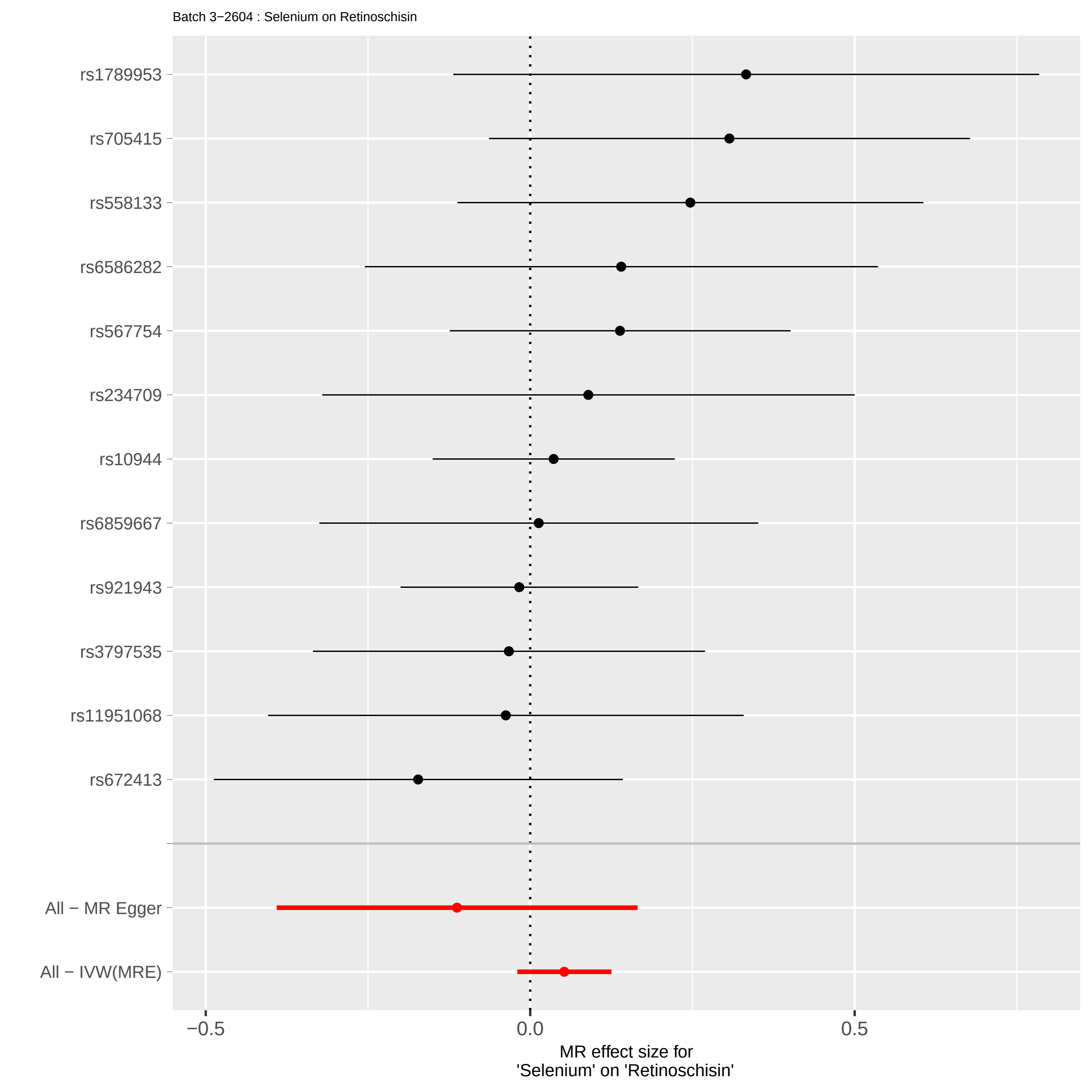

Batch 3−2604 : Selenium on Retinoschisin
rs1789953
rs705415
rs558133
rs6586282
rs567754
rs234709
rs10944
rs6859667
rs921943
rs3797535
rs11951068
rs672413
All − MR Egger
All − IVW(MRE)
−0.5
0.0
0.5
MR effect size for
'Selenium' on 'Retinoschisin'

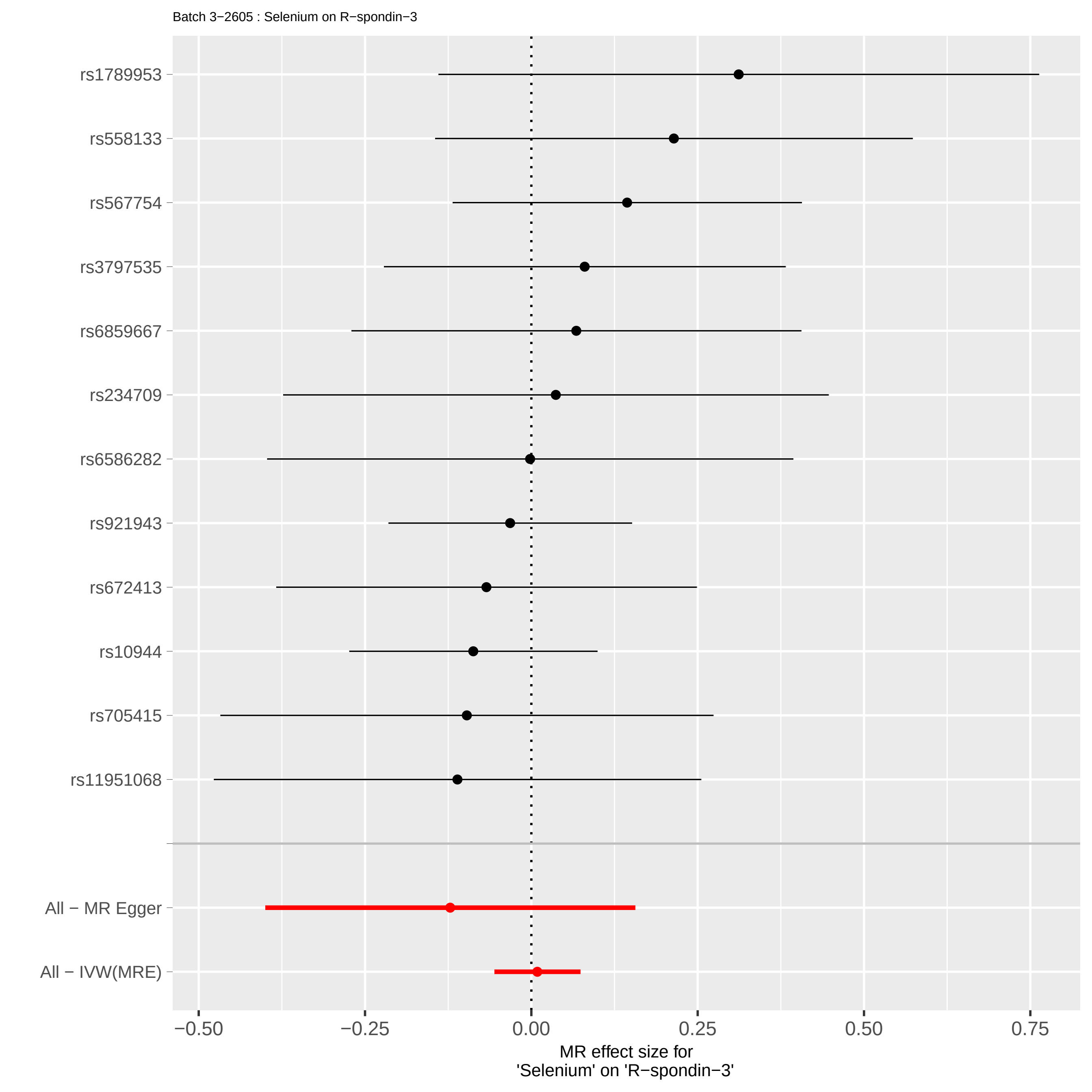

Batch 3−2605 : Selenium on R−spondin−3
rs1789953
rs558133
rs567754
rs3797535
rs6859667
rs234709
rs6586282
rs921943
rs672413
rs10944
rs705415
rs11951068
All − MR Egger
All − IVW(MRE)
−0.50
−0.25
0.00
0.25
0.50
0.75
MR effect size for
'Selenium' on 'R−spondin−3'

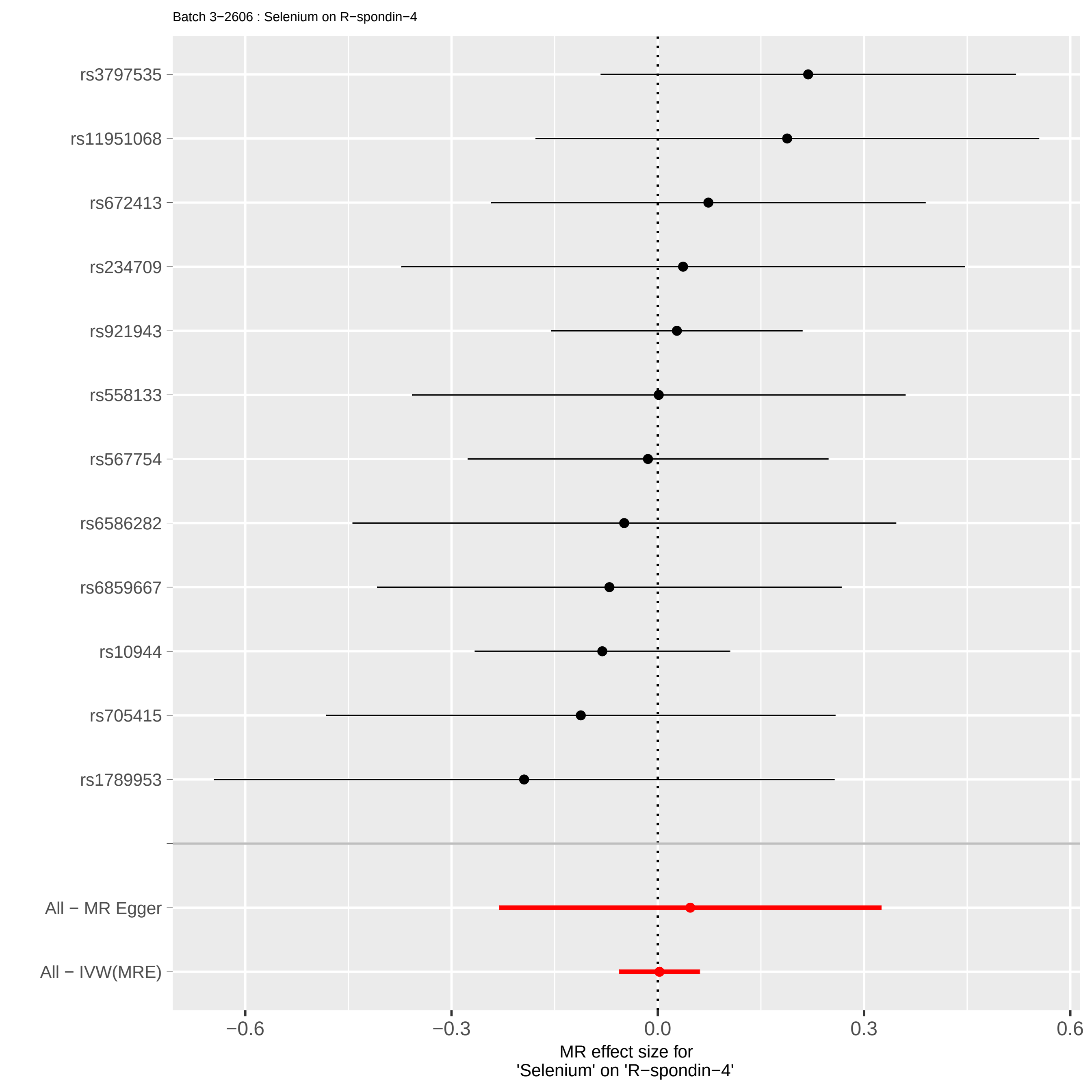

Batch 3−2606 : Selenium on R−spondin−4
rs3797535
rs11951068
rs672413
rs234709
rs921943
rs558133
rs567754
rs6586282
rs6859667
rs10944
rs705415
rs1789953
All − MR Egger
All − IVW(MRE)
−0.6
−0.3
0.0
0.3
0.6
MR effect size for
'Selenium' on 'R−spondin−4'

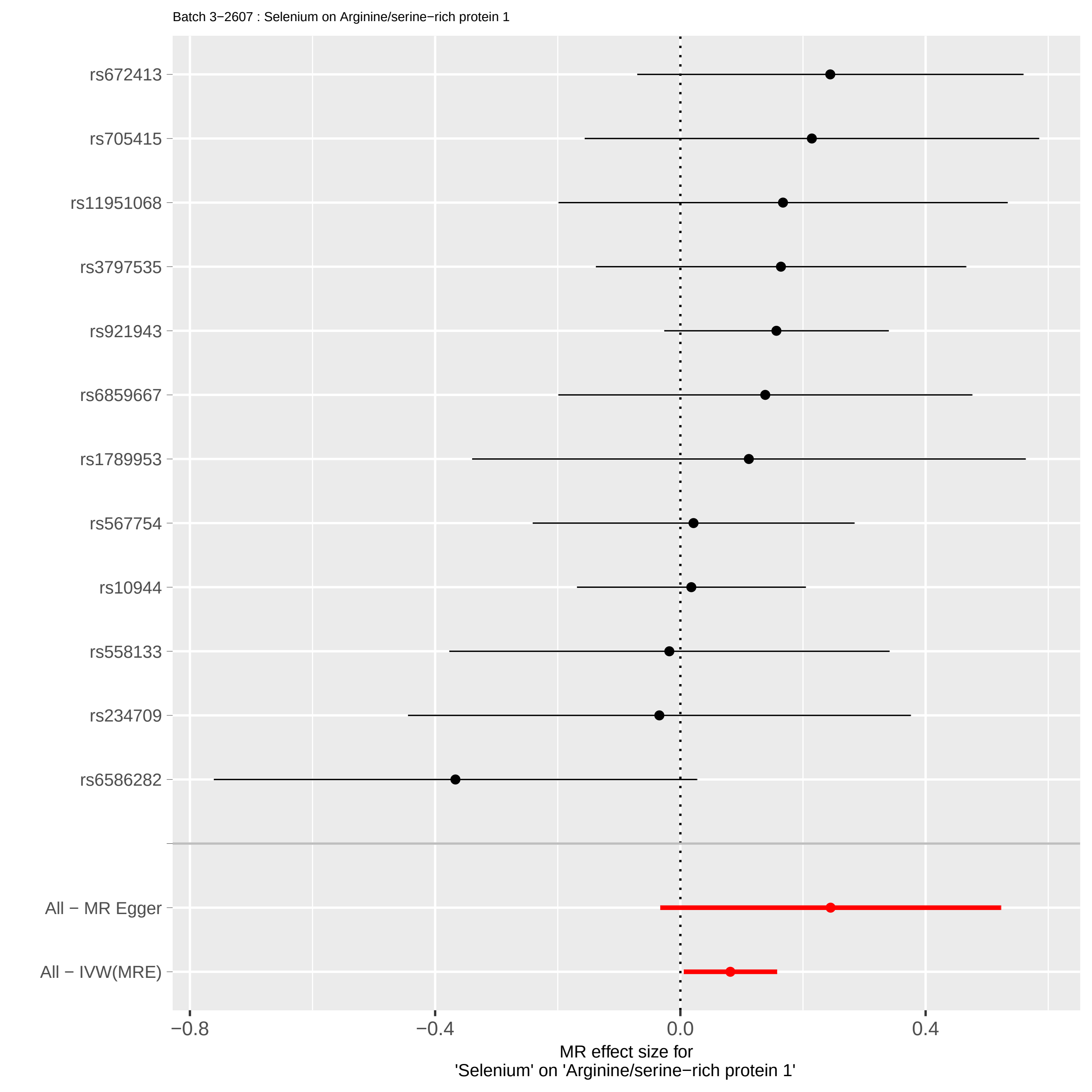

Batch 3−2607 : Selenium on Arginine/serine−rich protein 1
rs672413
rs705415
rs11951068
rs3797535
rs921943
rs6859667
rs1789953
rs567754
rs10944
rs558133
rs234709
rs6586282
All − MR Egger
All − IVW(MRE)
−0.8
−0.4
0.0
0.4
MR effect size for
'Selenium' on 'Arginine/serine−rich protein 1'

Batch 3−2608 : Selenium on Retbindin

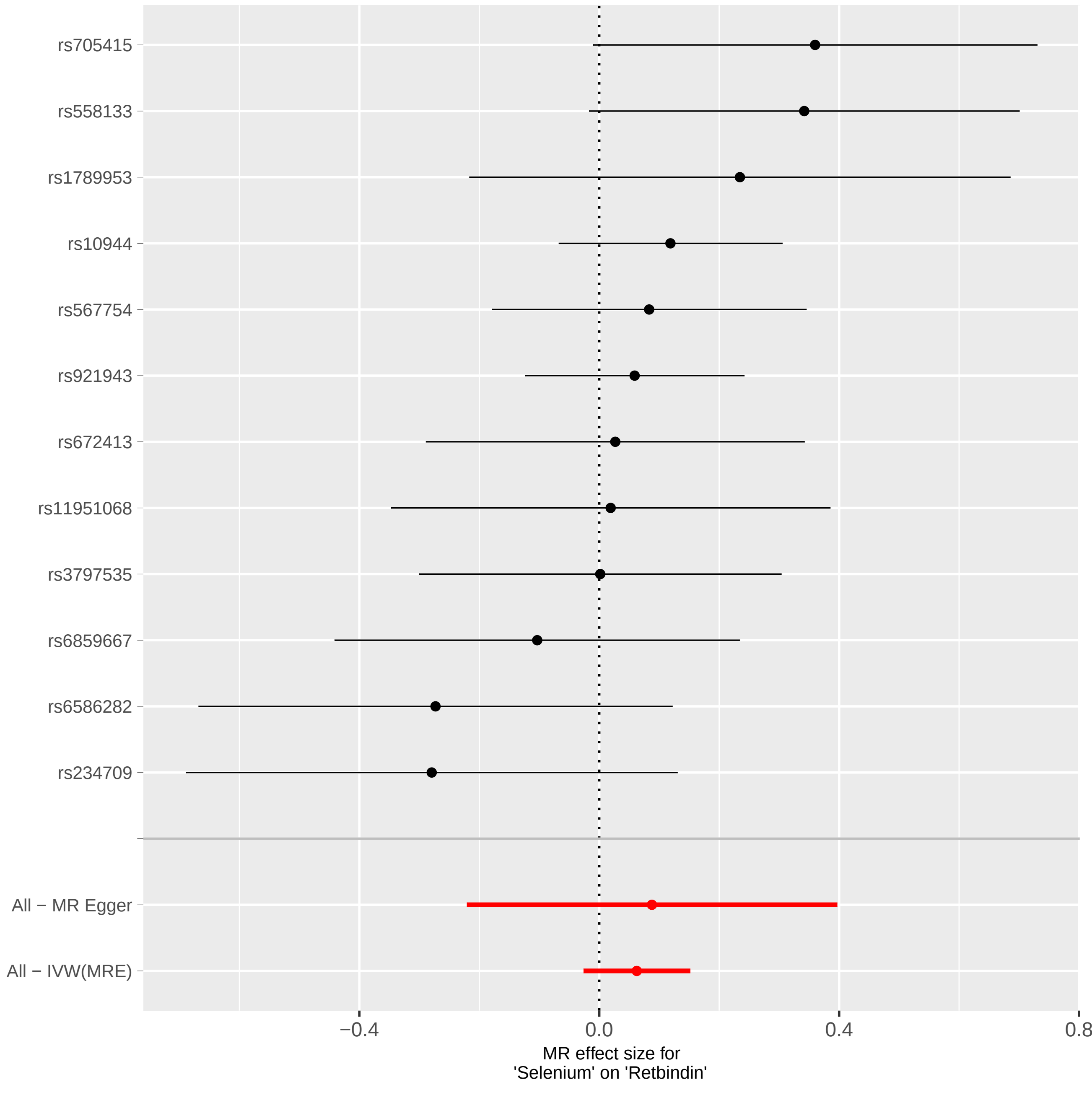

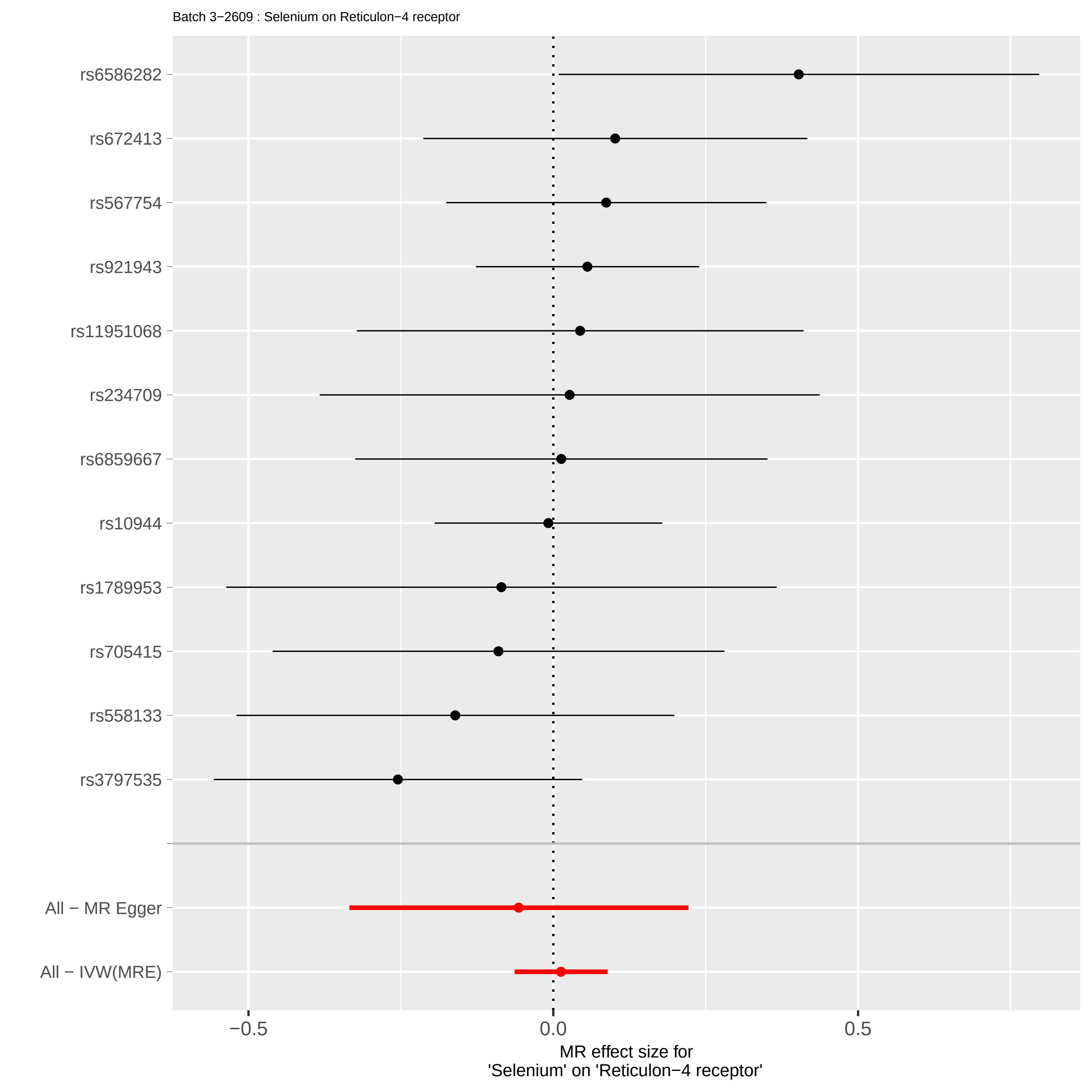

Batch 3−2609 : Selenium on Reticulon−4 receptor
rs6586282
rs672413
rs567754
rs921943
rs11951068
rs234709
rs6859667
rs10944
rs1789953
rs705415
rs558133
rs3797535
All − MR Egger
All − IVW(MRE)
−0.5
0.0
0.5
MR effect size for
'Selenium' on 'Reticulon−4 receptor'

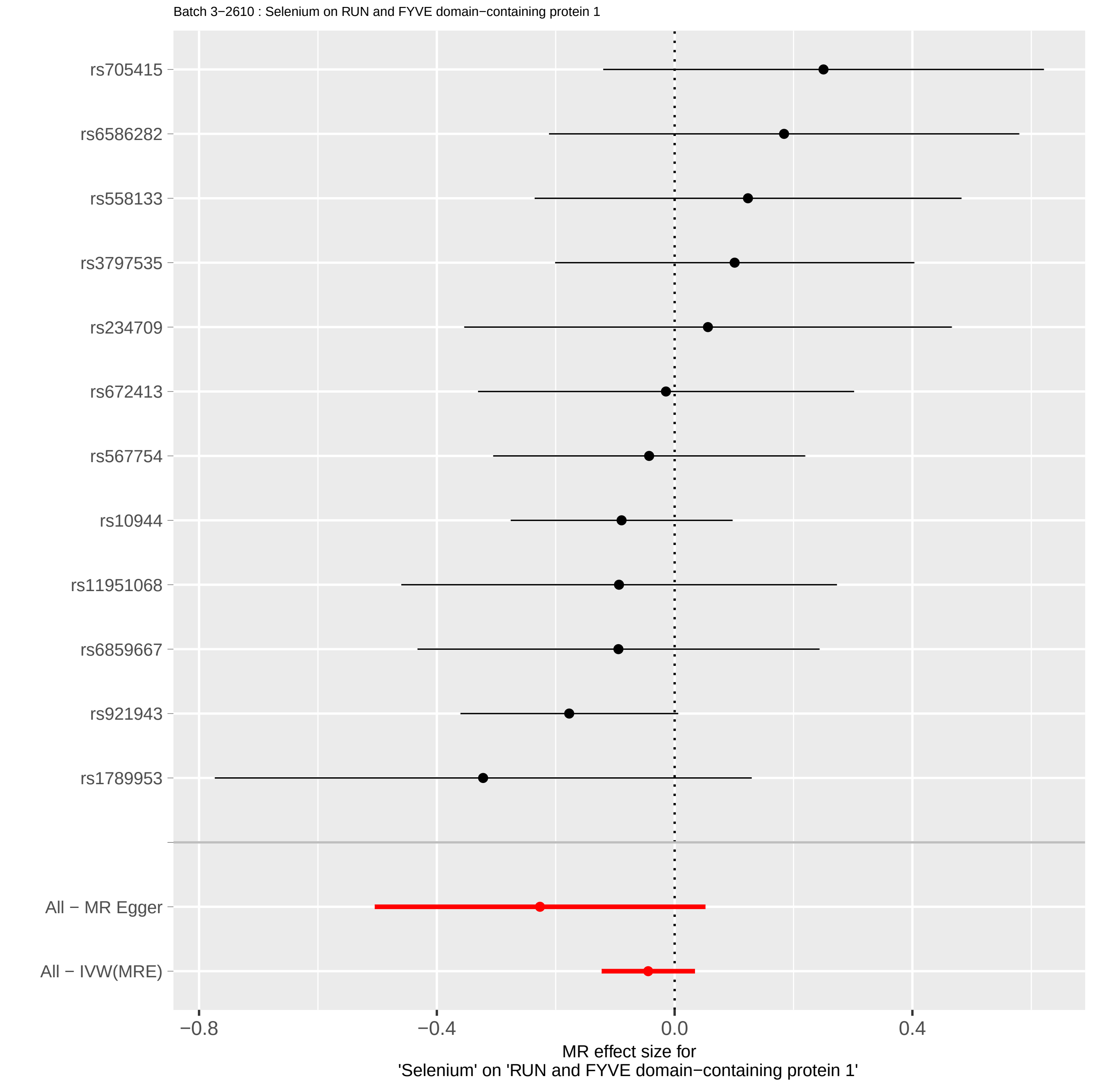

Batch 3−2610 : Selenium on RUN and FYVE domain−containing protein 1
rs705415
rs6586282
rs558133
rs3797535
rs234709
rs672413
rs567754
rs10944
rs11951068
rs6859667
rs921943
rs1789953
All − MR Egger
All − IVW(MRE)
−0.8
−0.4
0.0
0.4
MR effect size for
'Selenium' on 'RUN and FYVE domain−containing protein 1'

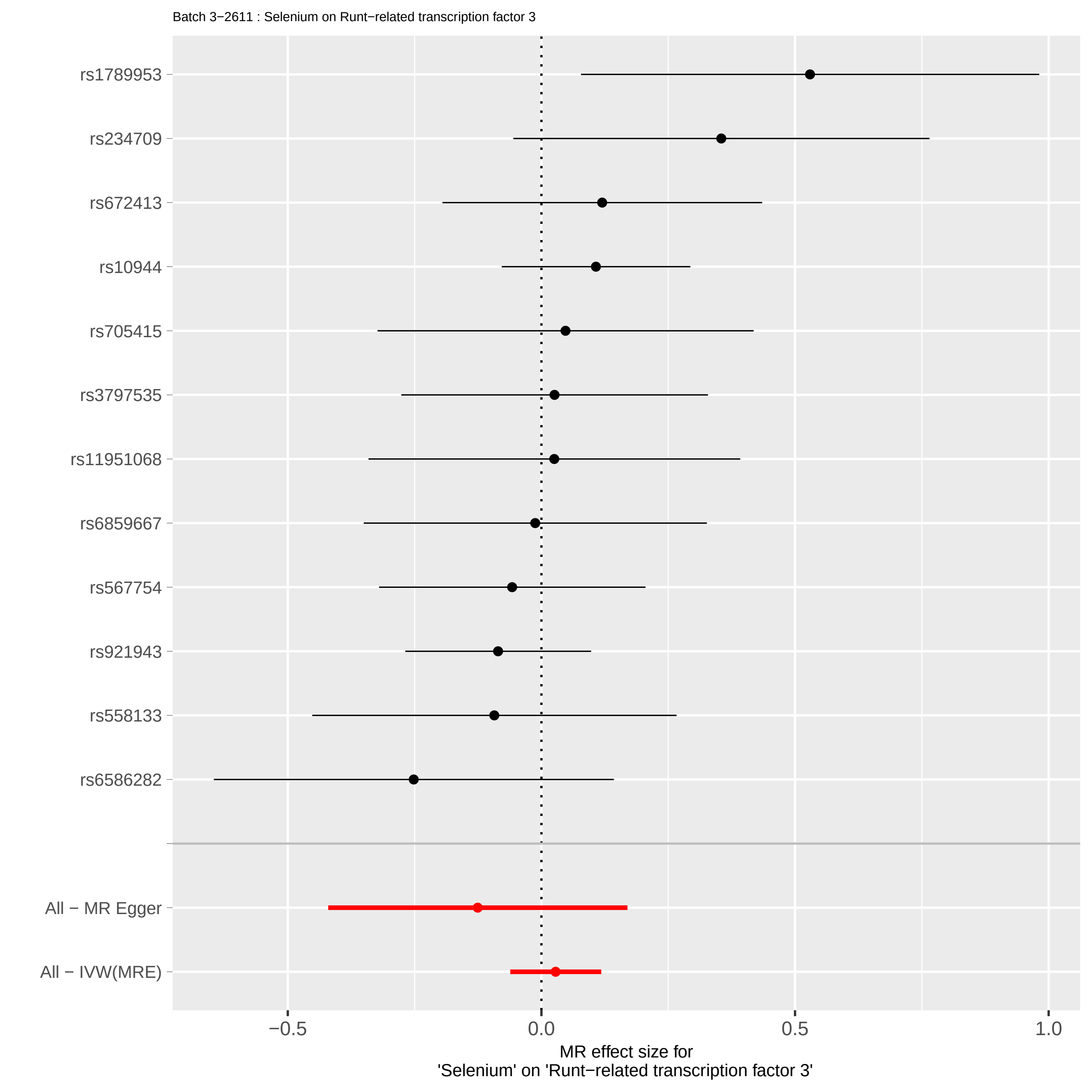

Batch 3−2611 : Selenium on Runt−related transcription factor 3
rs1789953
rs234709
rs672413
rs10944
rs705415
rs3797535
rs11951068
rs6859667
rs567754
rs921943
rs558133
rs6586282
All − MR Egger
All − IVW(MRE)
−0.5
0.0
0.5
1.0
MR effect size for
'Selenium' on 'Runt−related transcription factor 3'

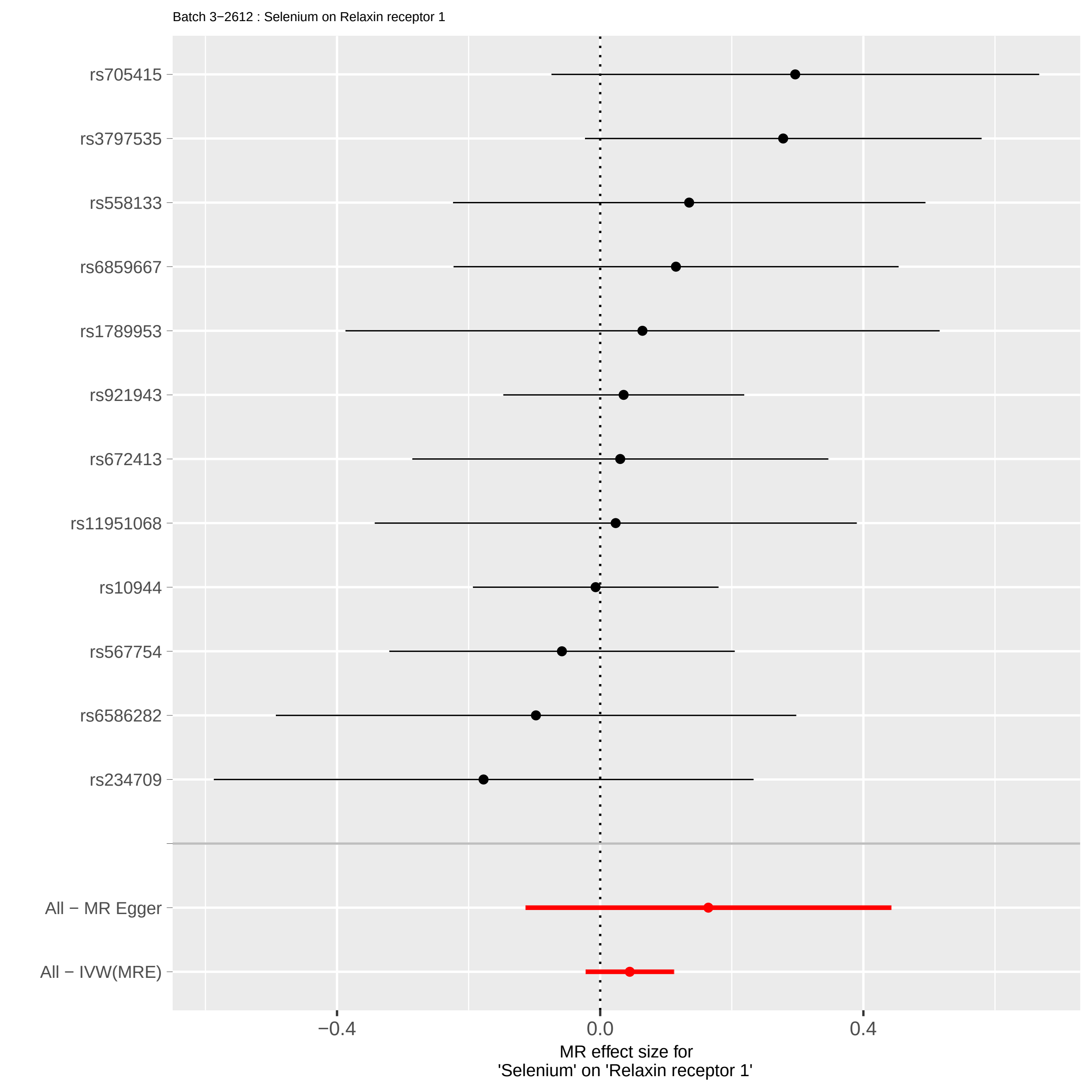
Batch 3−2612 : Selenium on Relaxin receptor 1
rs705415
rs3797535
rs558133
rs6859667
rs1789953
rs921943
rs672413
rs11951068
rs10944
rs567754
rs6586282
rs234709
All − MR Egger
All − IVW(MRE)
−0.4
0.0
0.4
MR effect size for
'Selenium' on 'Relaxin receptor 1'

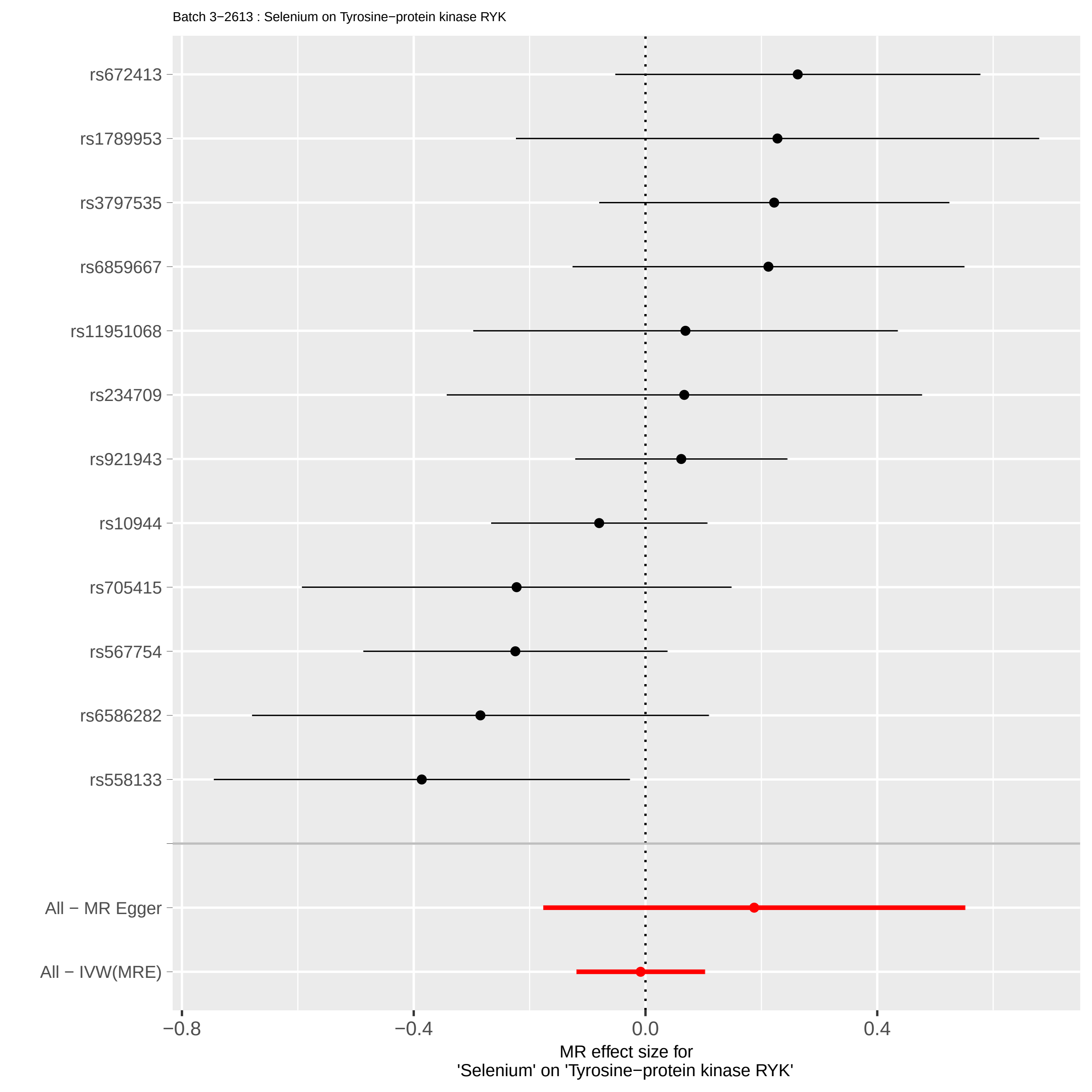

Batch 3−2613 : Selenium on Tyrosine−protein kinase RYK
rs672413
rs1789953
rs3797535
rs6859667
rs11951068
rs234709
rs921943
rs10944
rs705415
rs567754
rs6586282
rs558133
All − MR Egger
All − IVW(MRE)
−0.8
−0.4
0.0
0.4
MR effect size for
'Selenium' on 'Tyrosine−protein kinase RYK'

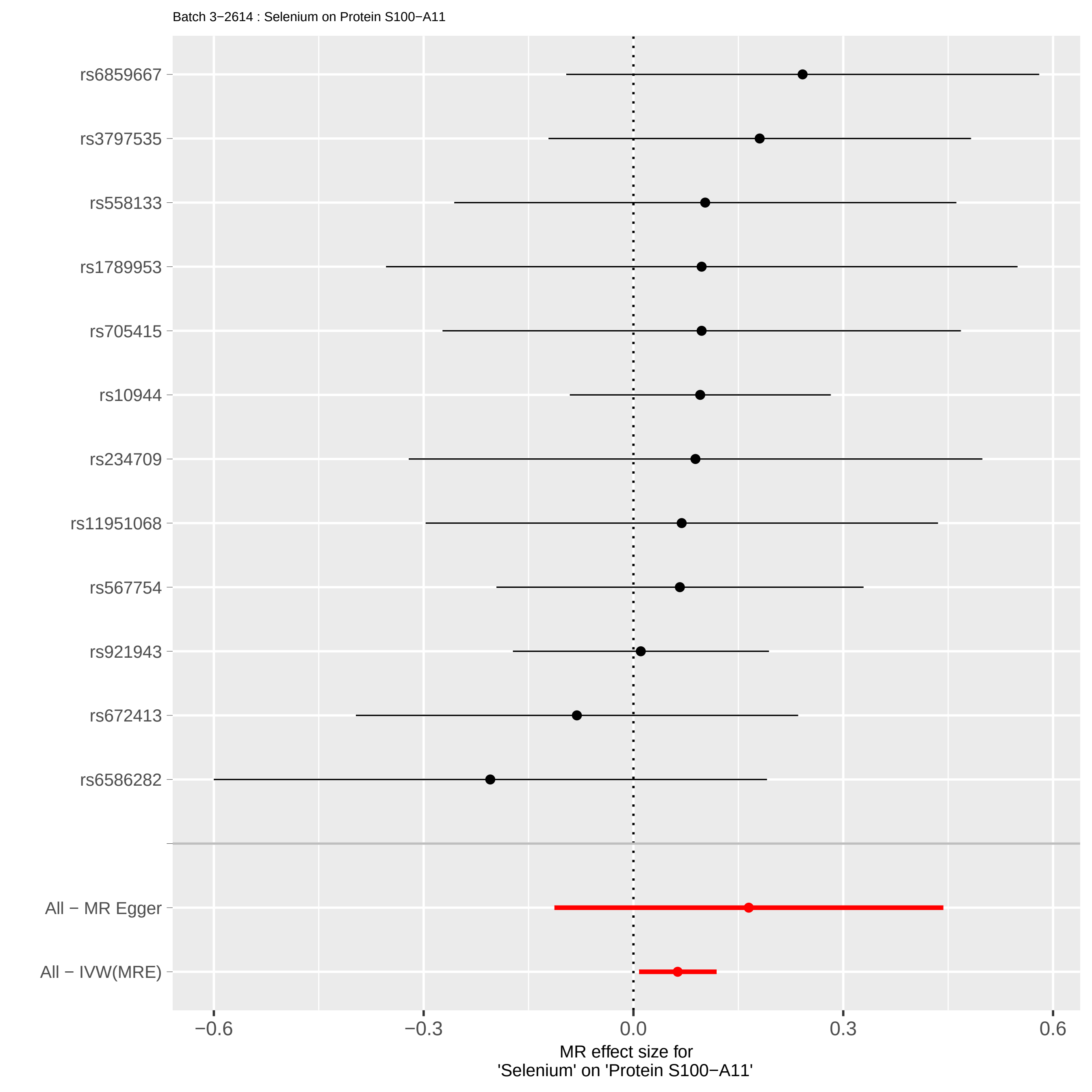
Batch 3−2614 : Selenium on Protein S100−A11
rs6859667
rs3797535
rs558133
rs1789953
rs705415
rs10944
rs234709
rs11951068
rs567754
rs921943
rs672413
rs6586282
All − MR Egger
All − IVW(MRE)
−0.6
−0.3
0.0
0.3
0.6
MR effect size for
'Selenium' on 'Protein S100−A11'

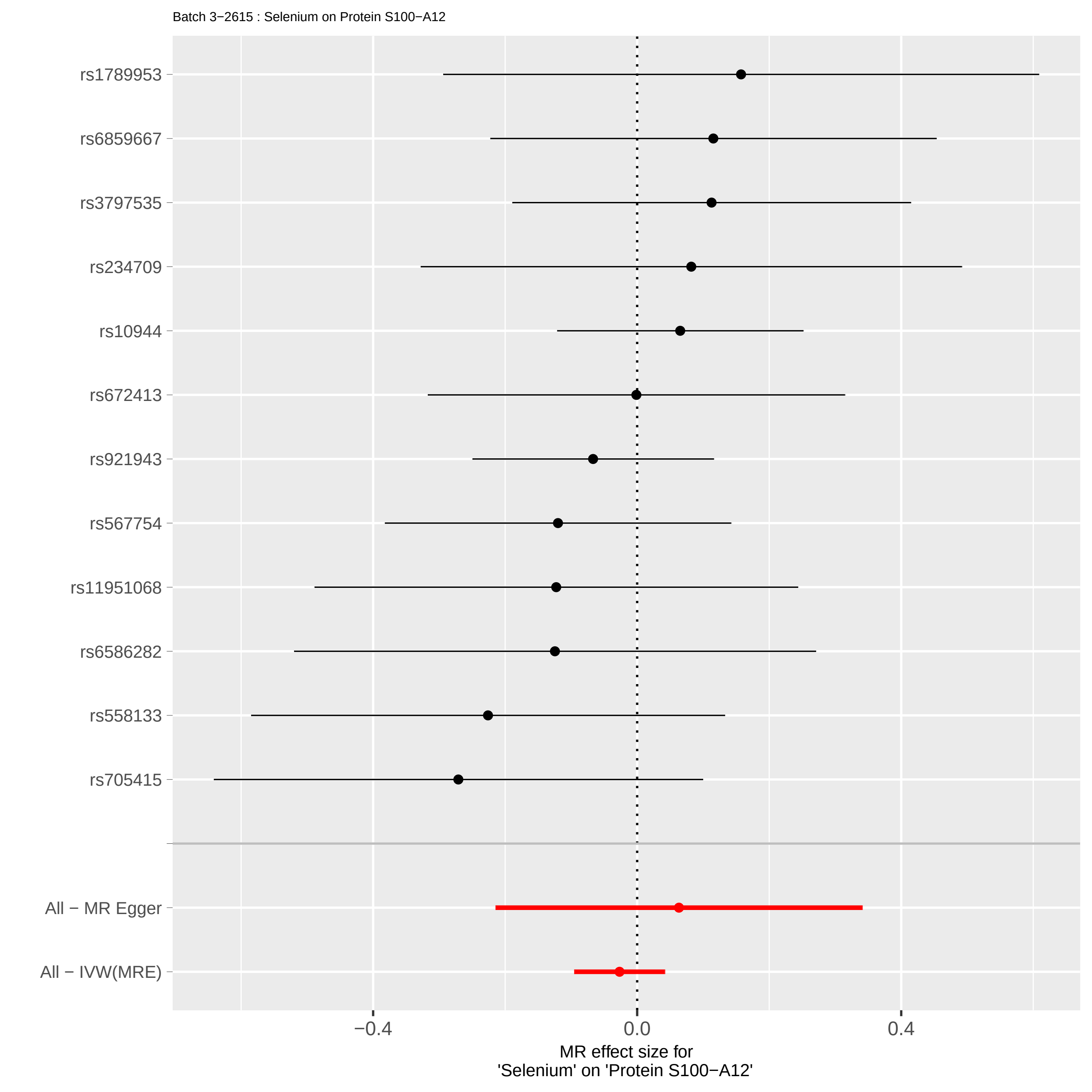
Batch 3−2615 : Selenium on Protein S100−A12
rs1789953
rs6859667
rs3797535
rs234709
rs10944
rs672413
rs921943
rs567754
rs11951068
rs6586282
rs558133
rs705415
All − MR Egger
All − IVW(MRE)
−0.4
0.0
0.4
MR effect size for
'Selenium' on 'Protein S100−A12'

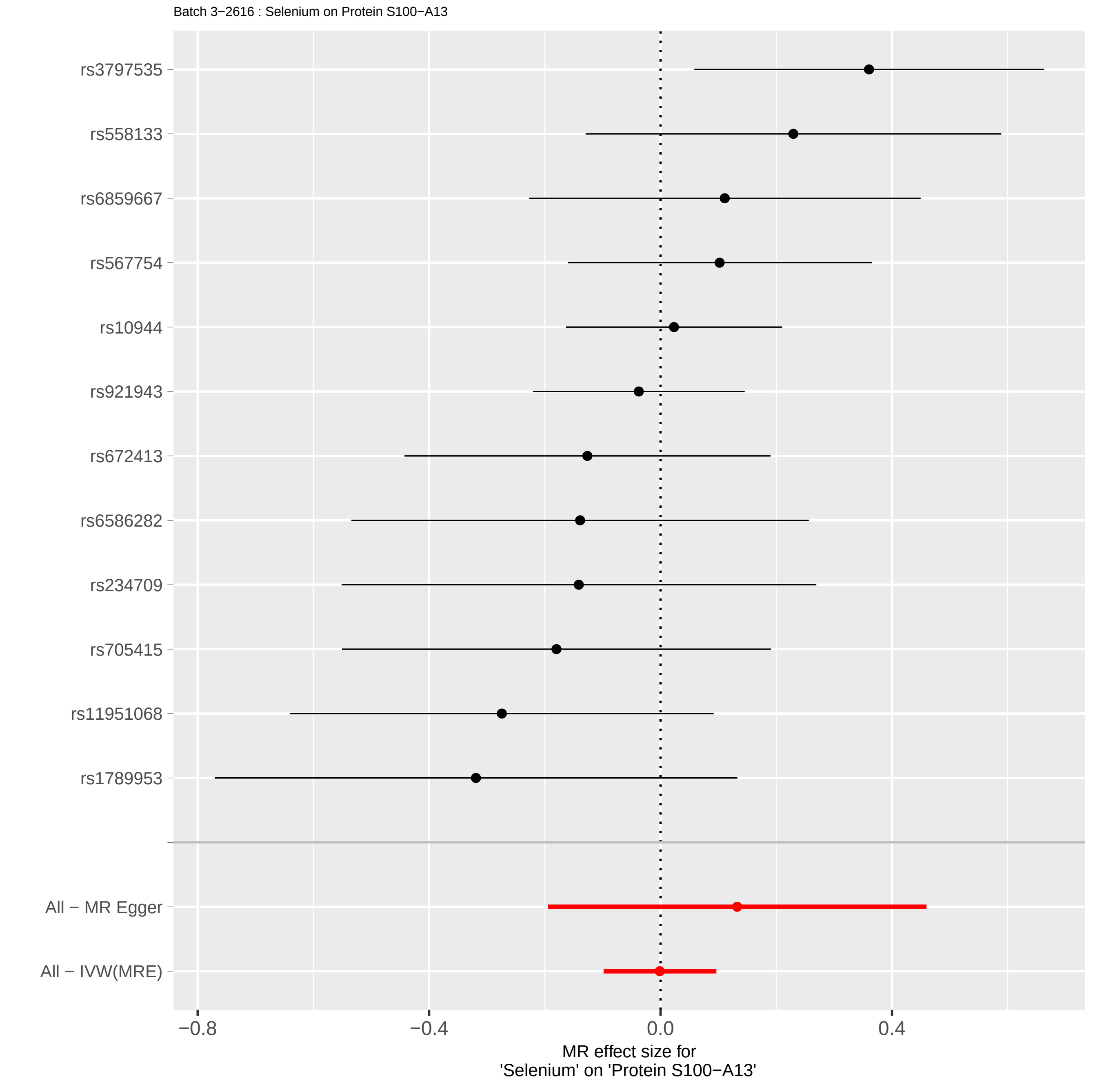

Batch 3−2616 : Selenium on Protein S100−A13
rs3797535
rs558133
rs6859667
rs567754
rs10944
rs921943
rs672413
rs6586282
rs234709
rs705415
rs11951068
rs1789953
All − MR Egger
All − IVW(MRE)
−0.8
−0.4
0.0
0.4
MR effect size for
'Selenium' on 'Protein S100−A13'

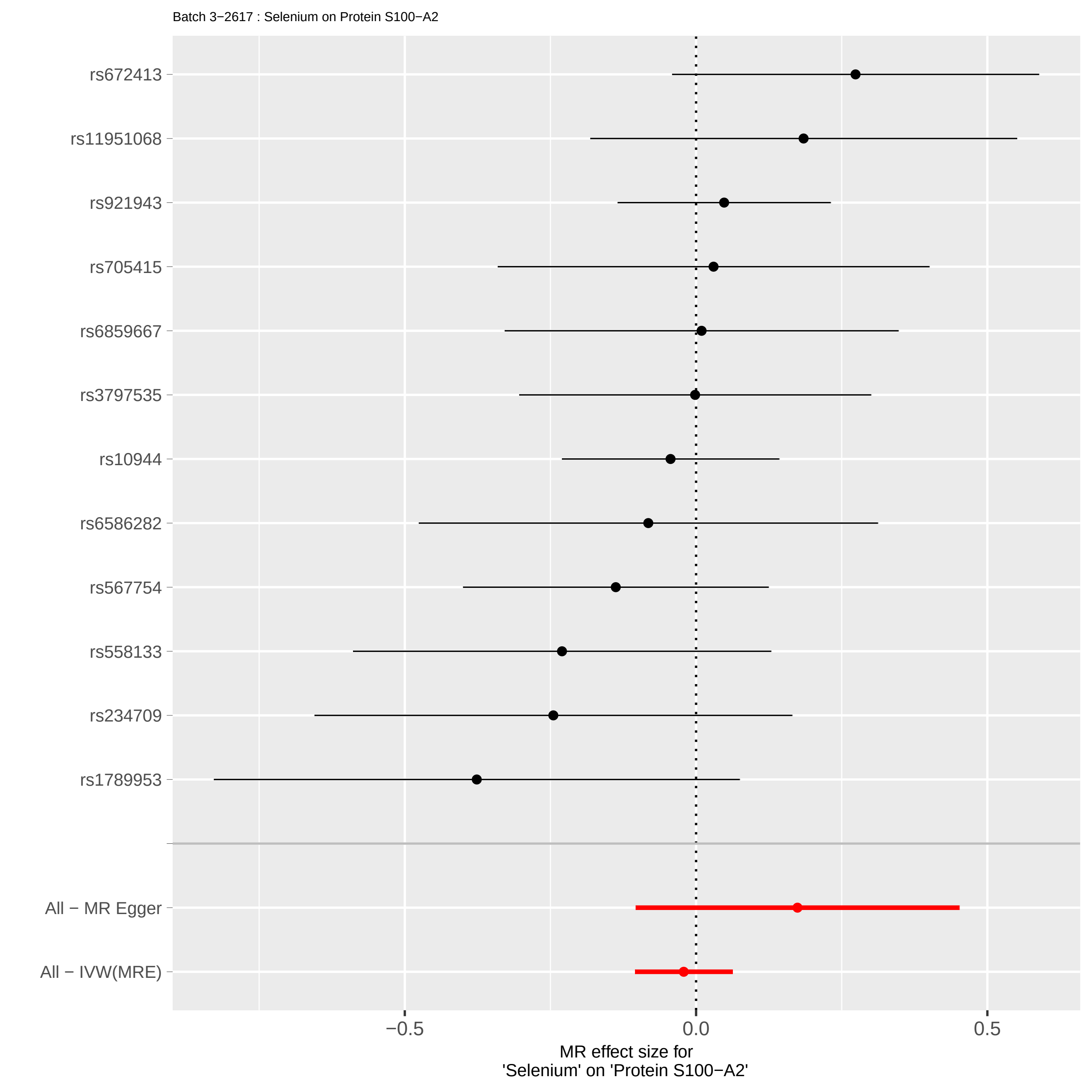

Batch 3−2617 : Selenium on Protein S100−A2
rs672413
rs11951068
rs921943
rs705415
rs6859667
rs3797535
rs10944
rs6586282
rs567754
rs558133
rs234709
rs1789953
All − MR Egger
All − IVW(MRE)
−0.5
0.0
0.5
MR effect size for
'Selenium' on 'Protein S100−A2'

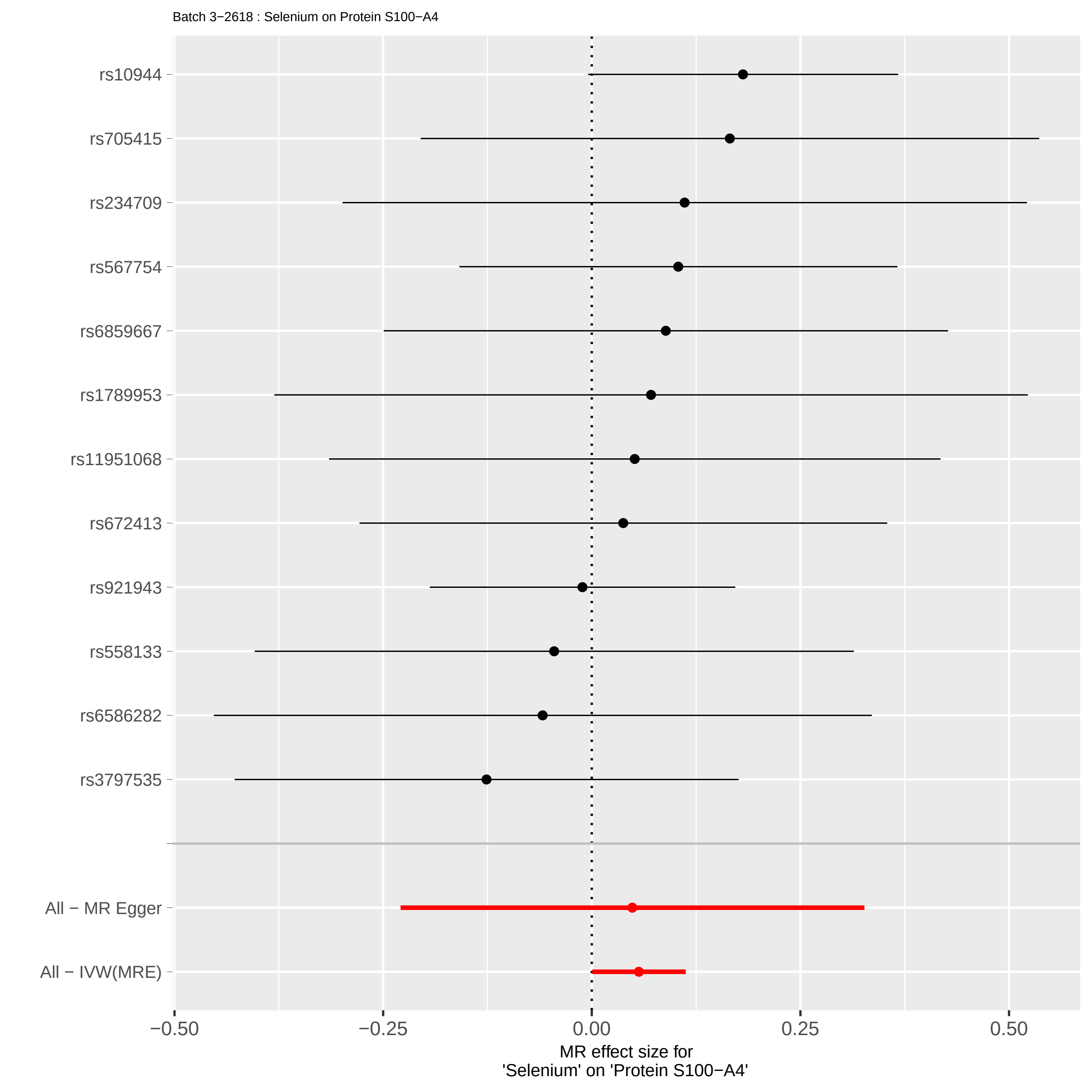

Batch 3−2618 : Selenium on Protein S100−A4
rs10944
rs705415
rs234709
rs567754
rs6859667
rs1789953
rs11951068
rs672413
rs921943
rs558133
rs6586282
rs3797535
All − MR Egger
All − IVW(MRE)
−0.50
−0.25
0.00
0.25
0.50
MR effect size for
'Selenium' on 'Protein S100−A4'

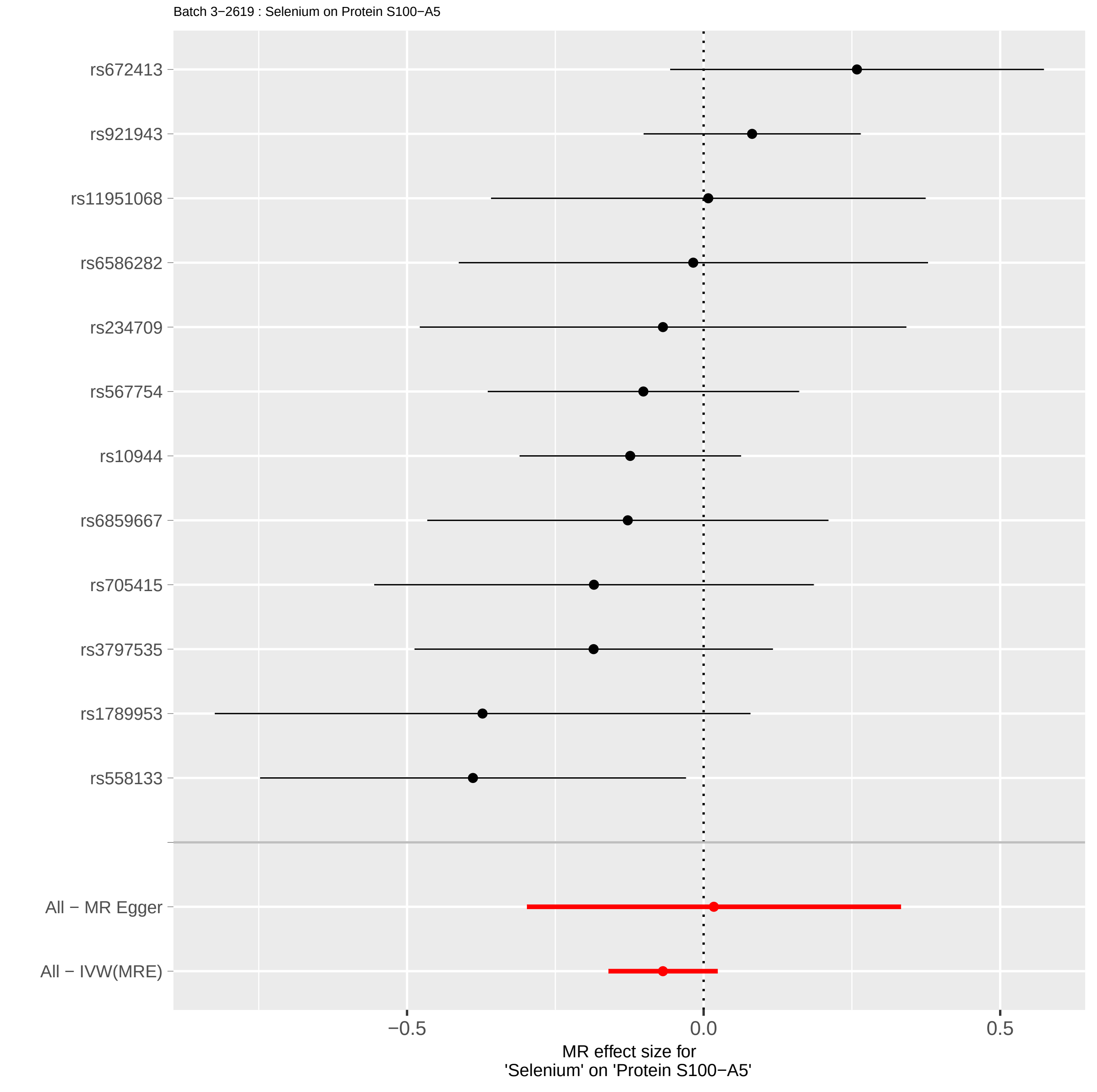

Batch 3−2619 : Selenium on Protein S100−A5
rs672413
rs921943
rs11951068
rs6586282
rs234709
rs567754
rs10944
rs6859667
rs705415
rs3797535
rs1789953
rs558133
All − MR Egger
All − IVW(MRE)
−0.5
0.0
0.5
MR effect size for
'Selenium' on 'Protein S100−A5'

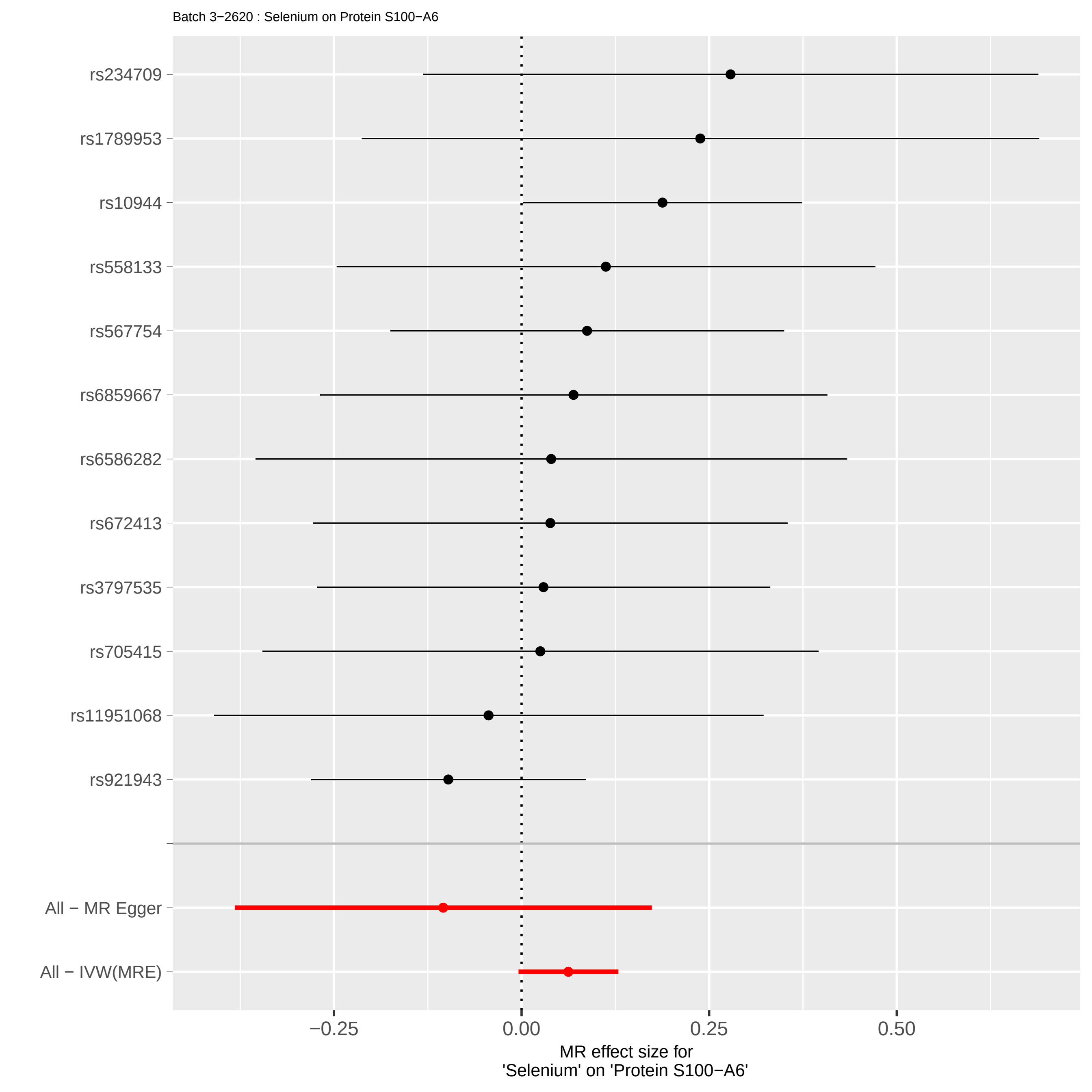

Batch 3−2620 : Selenium on Protein S100−A6
rs234709
rs1789953
rs10944
rs558133
rs567754
rs6859667
rs6586282
rs672413
rs3797535
rs705415
rs11951068
rs921943
All − MR Egger
All − IVW(MRE)
−0.25
0.00
0.25
0.50
MR effect size for
'Selenium' on 'Protein S100−A6'

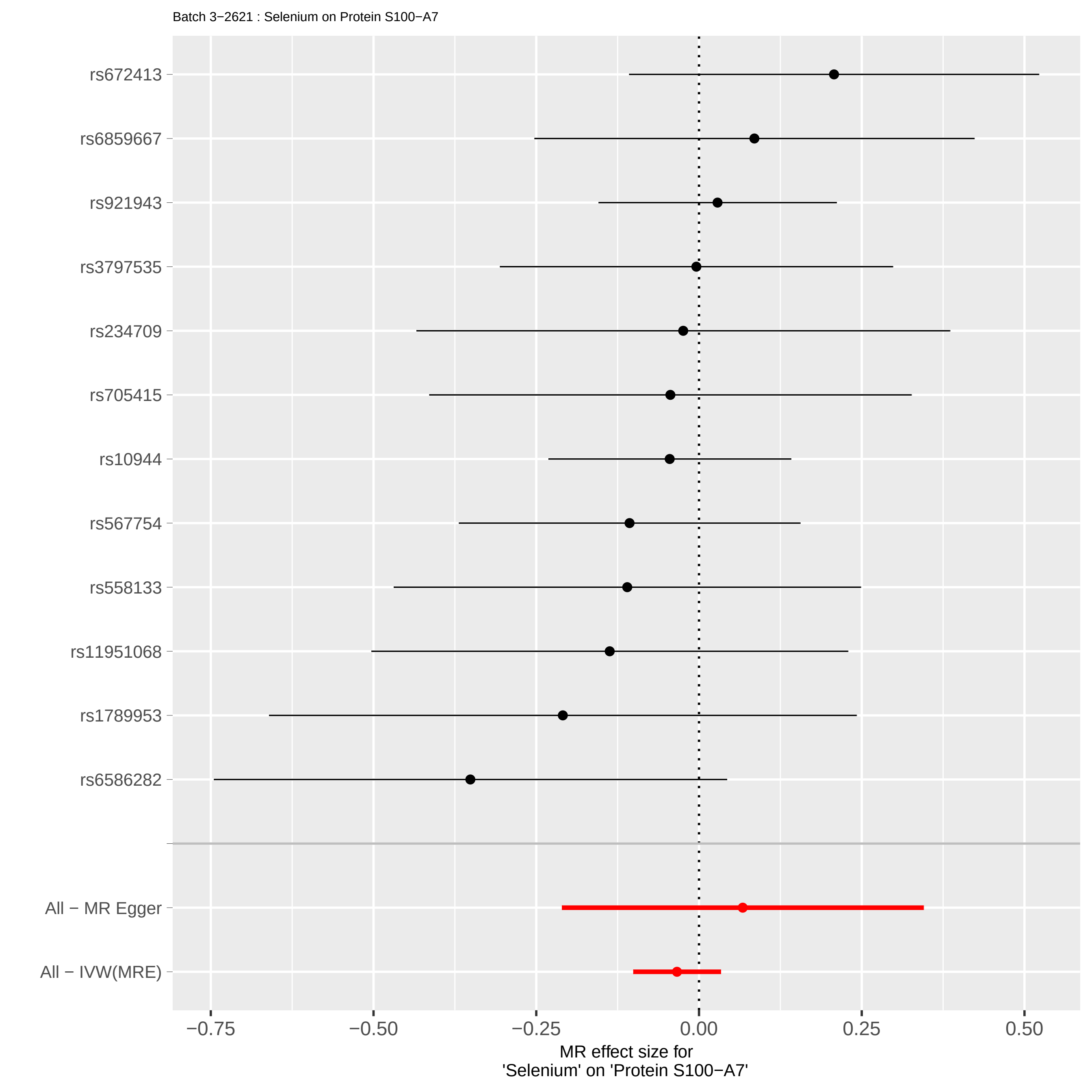
Batch 3−2621 : Selenium on Protein S100−A7
rs672413
rs6859667
rs921943
rs3797535
rs234709
rs705415
rs10944
rs567754
rs558133
rs11951068
rs1789953
rs6586282
All − MR Egger
All − IVW(MRE)
−0.75
−0.50
−0.25
0.00
0.25
0.50
MR effect size for
'Selenium' on 'Protein S100−A7'

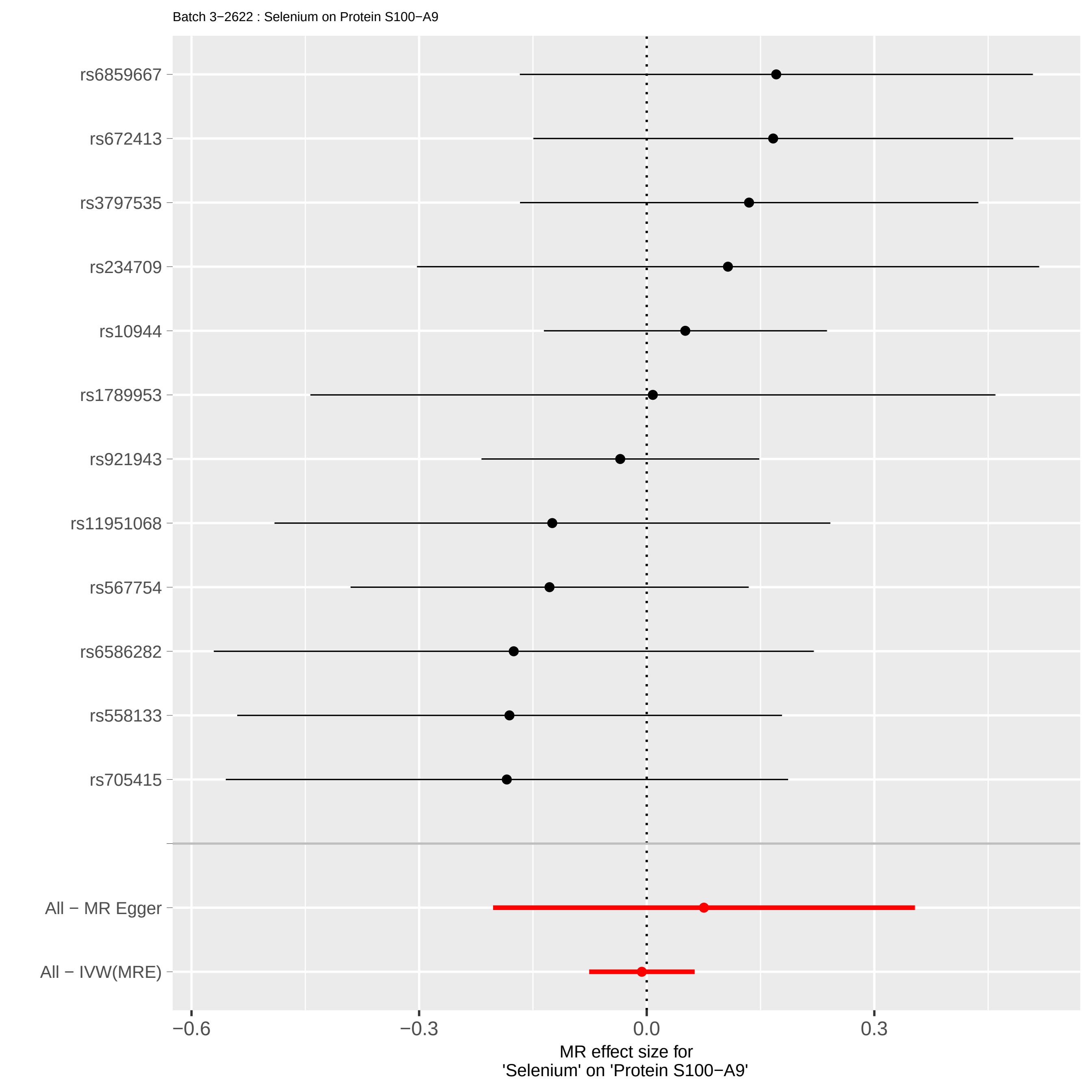

Batch 3−2622 : Selenium on Protein S100−A9
rs6859667
rs672413
rs3797535
rs234709
rs10944
rs1789953
rs921943
rs11951068
rs567754
rs6586282
rs558133
rs705415
All − MR Egger
All − IVW(MRE)
−0.6
−0.3
0.0
0.3
MR effect size for
'Selenium' on 'Protein S100−A9'

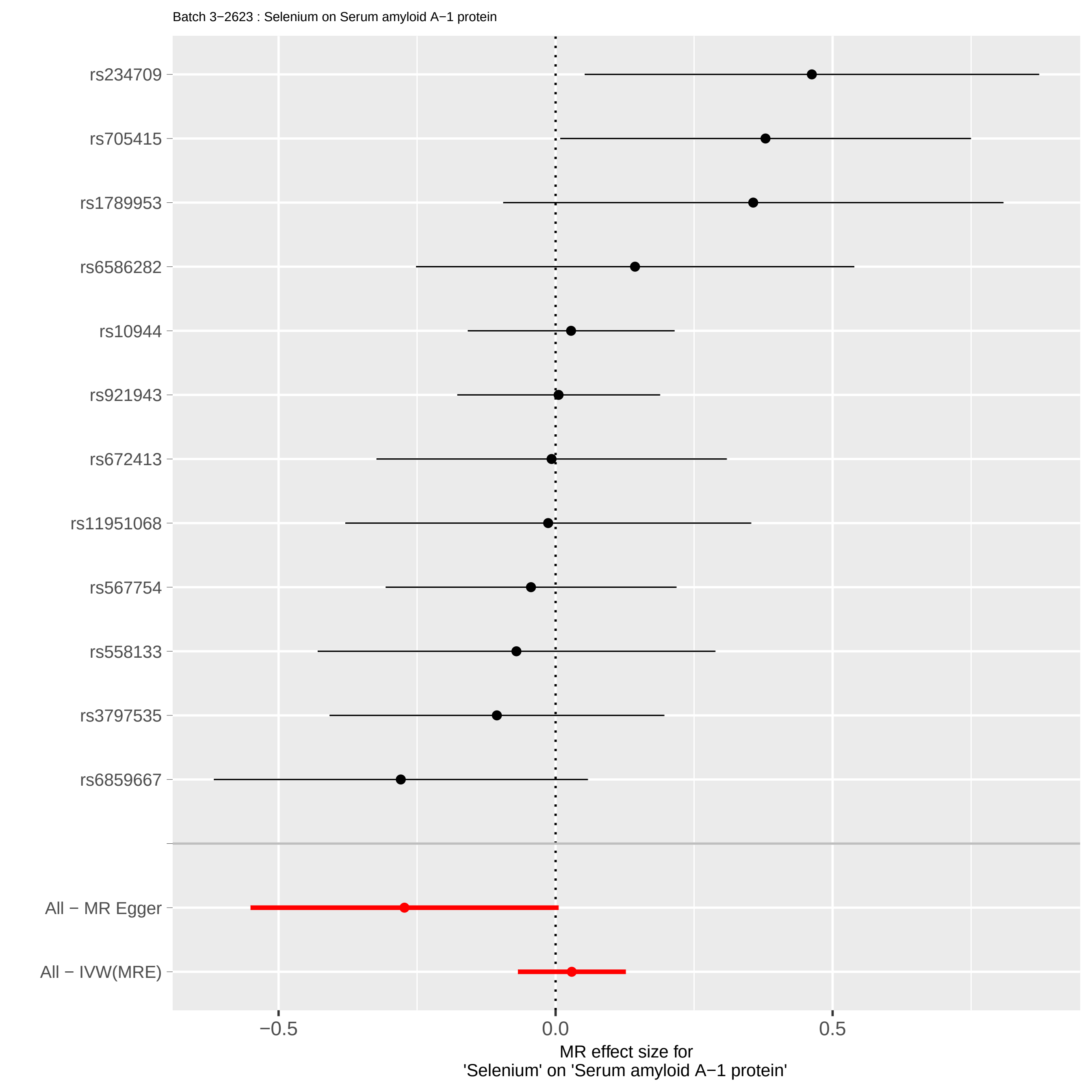
Batch 3−2623 : Selenium on Serum amyloid A−1 protein
rs234709
rs705415
rs1789953
rs6586282
rs10944
rs921943
rs672413
rs11951068
rs567754
rs558133
rs3797535
rs6859667
All − MR Egger
All − IVW(MRE)
−0.5
0.0
0.5
MR effect size for
'Selenium' on 'Serum amyloid A−1 protein'

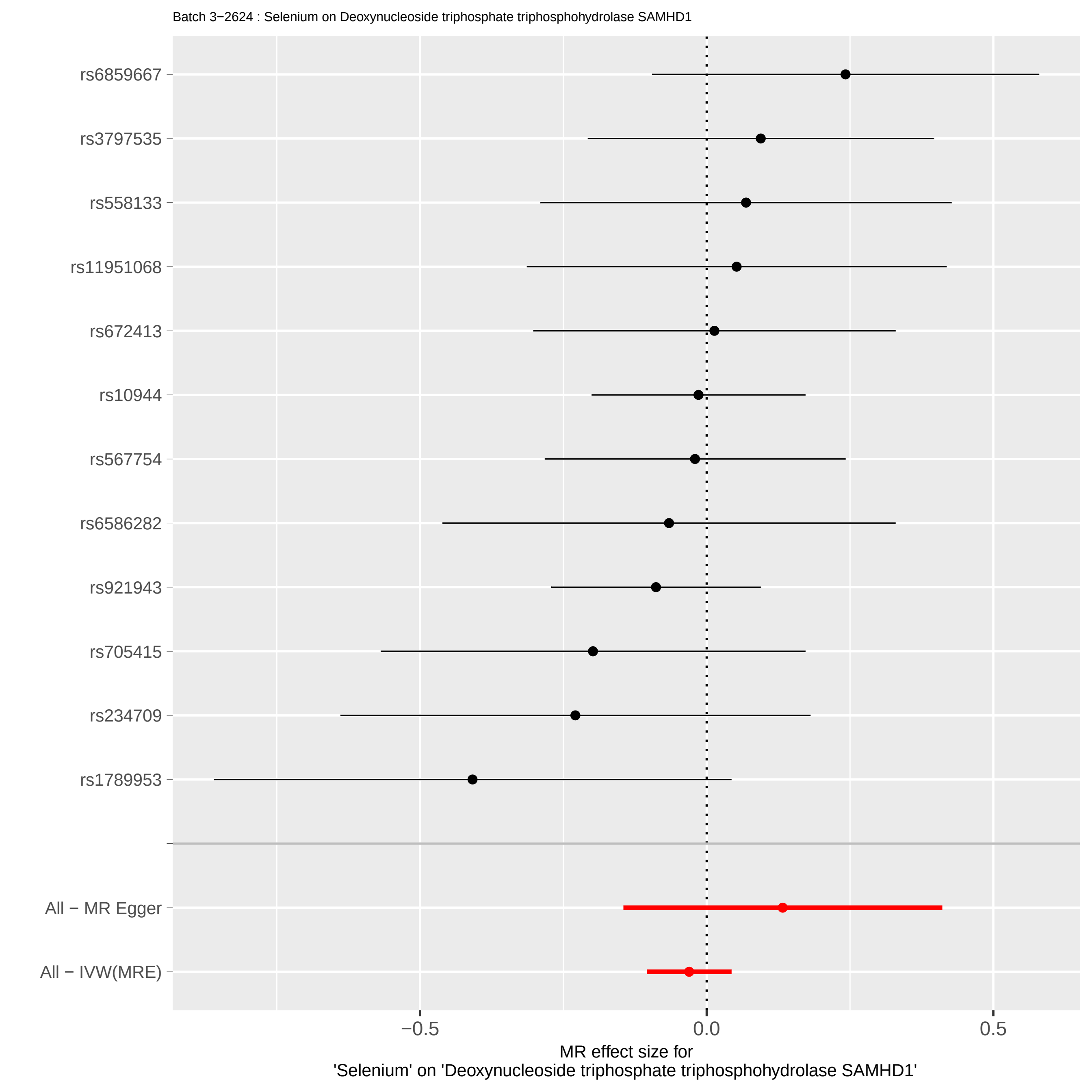
Batch 3−2624 : Selenium on Deoxynucleoside triphosphate triphosphohydrolase SAMHD1
rs6859667
rs3797535
rs558133
rs11951068
rs672413
rs10944
rs567754
rs6586282
rs921943
rs705415
rs234709
rs1789953
All − MR Egger
All − IVW(MRE)
−0.5
0.0
0.5
MR effect size for
'Selenium' on 'Deoxynucleoside triphosphate triphosphohydrolase SAMHD1'

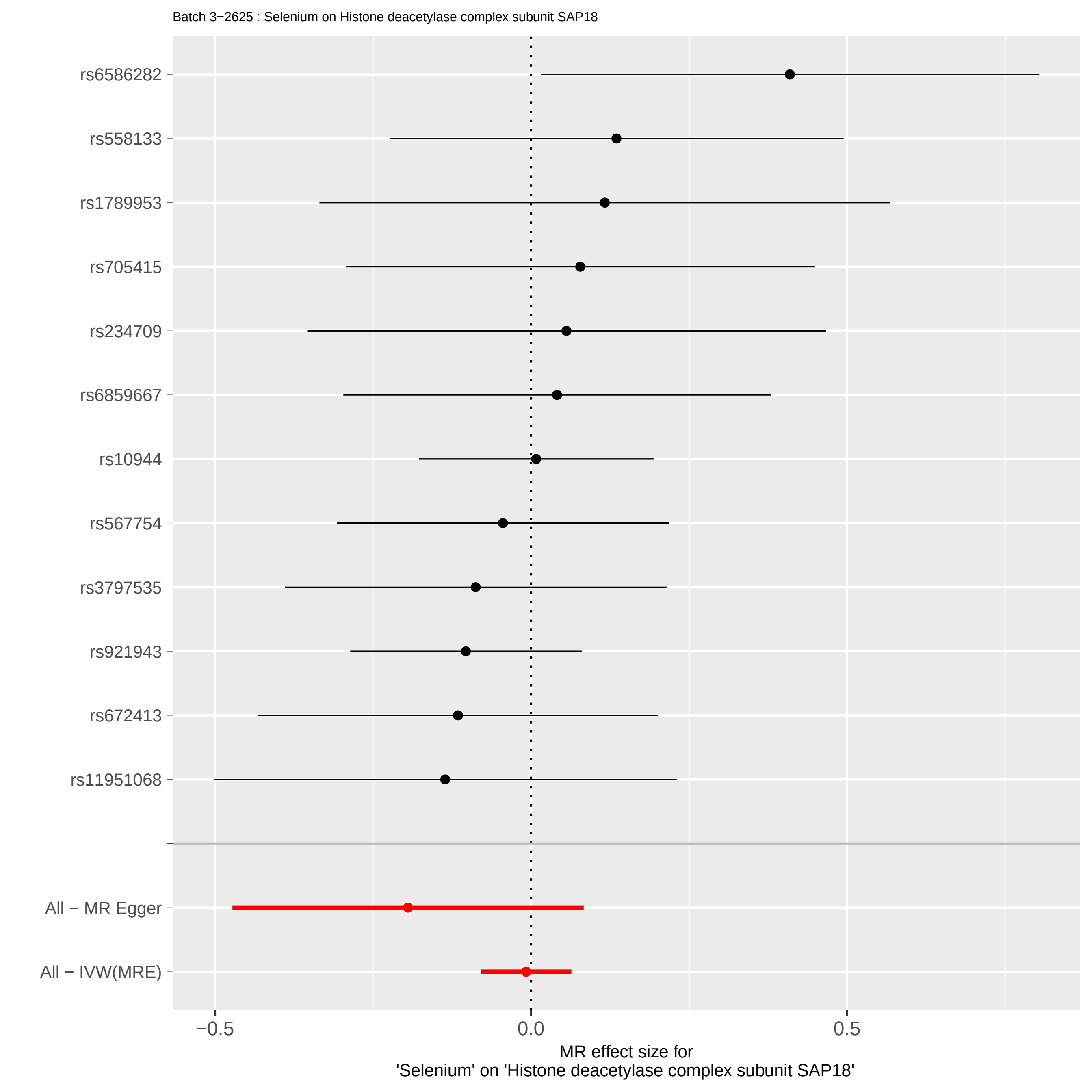

Batch 3−2625 : Selenium on Histone deacetylase complex subunit SAP18
rs6586282
rs558133
rs1789953
rs705415
rs234709
rs6859667
rs10944
rs567754
rs3797535
rs921943
rs672413
rs11951068
All − MR Egger
All − IVW(MRE)
−0.5
0.0
0.5
MR effect size for
'Selenium' on 'Histone deacetylase complex subunit SAP18'

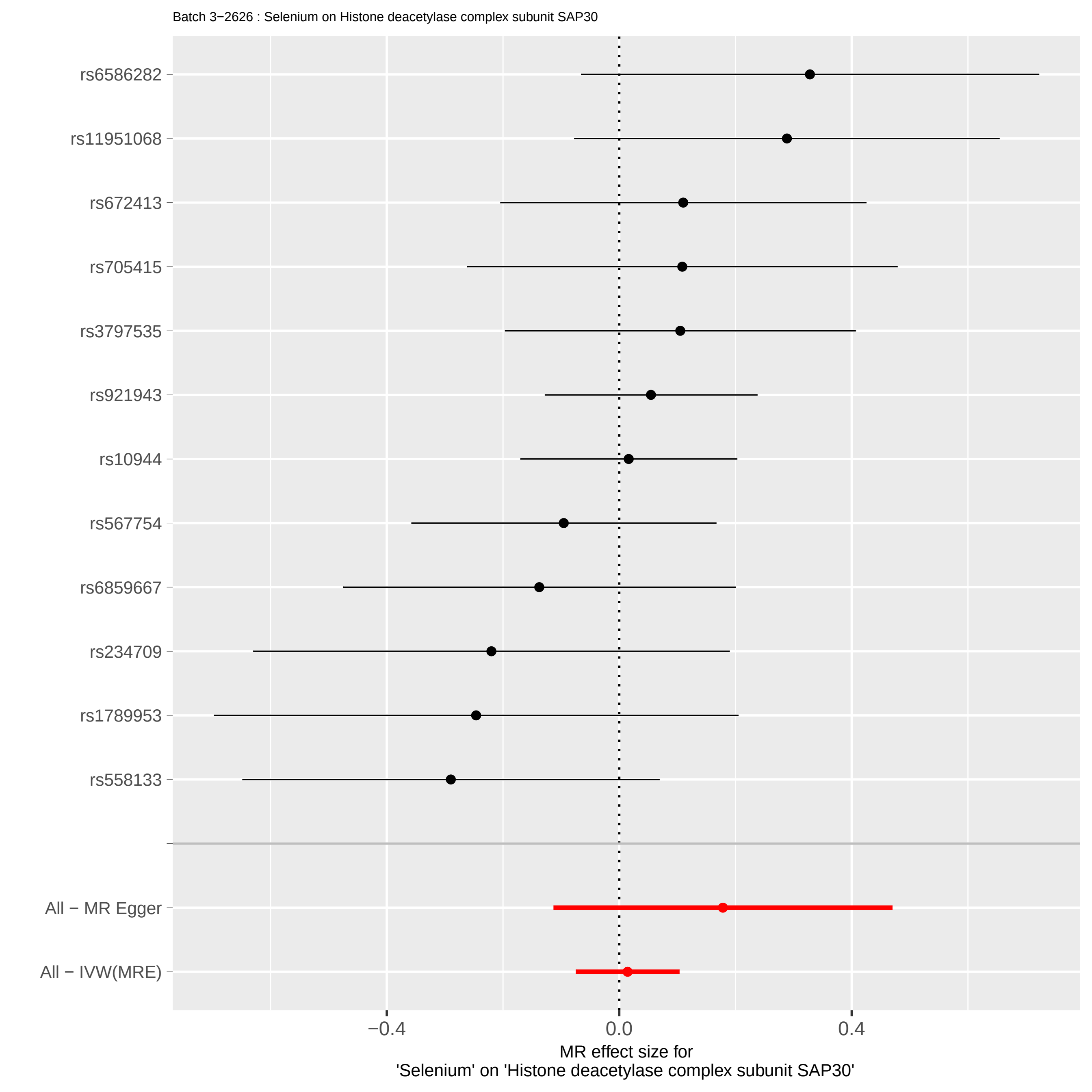

Batch 3−2626 : Selenium on Histone deacetylase complex subunit SAP30
rs6586282
rs11951068
rs672413
rs705415
rs3797535
rs921943
rs10944
rs567754
rs6859667
rs234709
rs1789953
rs558133
All − MR Egger
All − IVW(MRE)
−0.4
0.0
0.4
MR effect size for
'Selenium' on 'Histone deacetylase complex subunit SAP30'

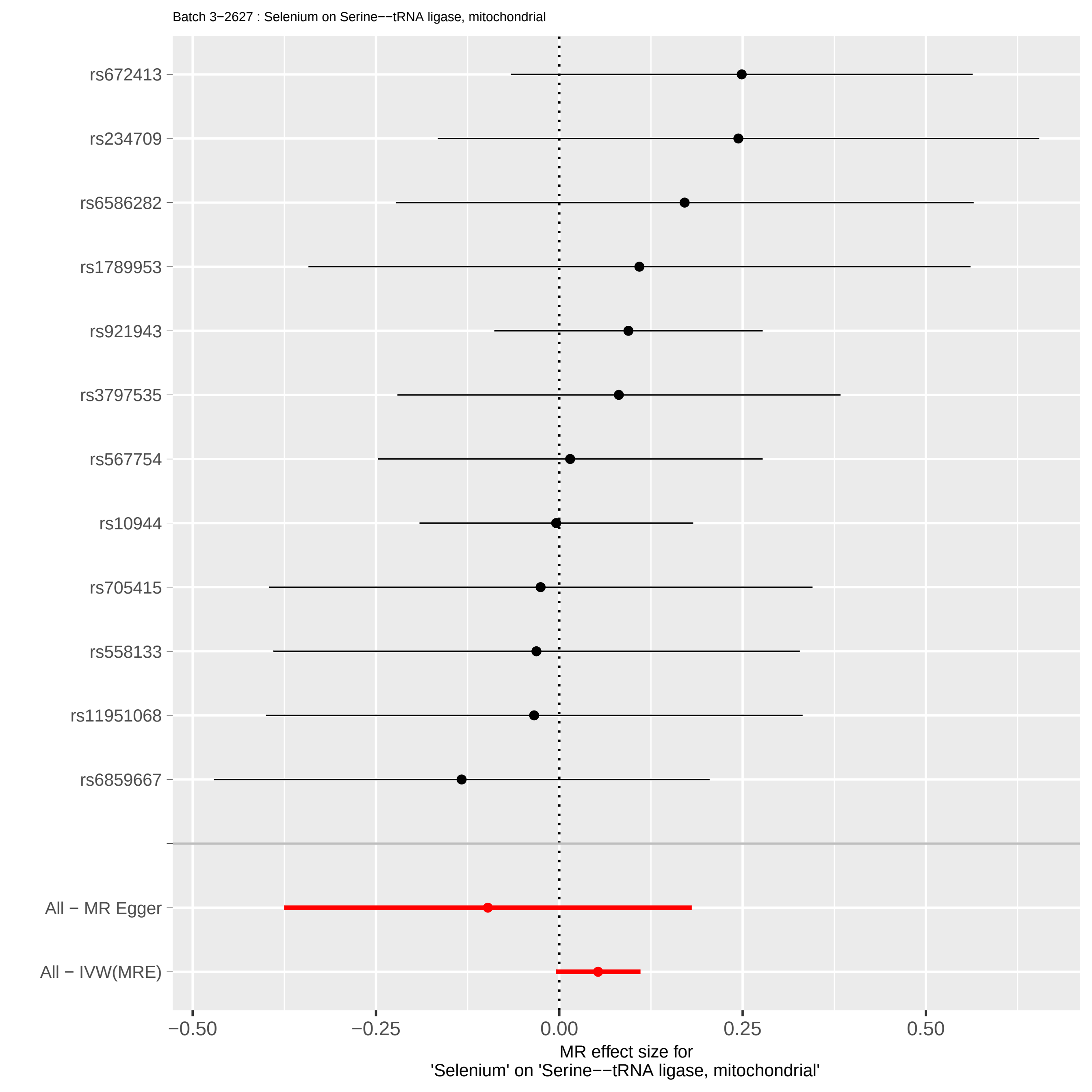

Batch 3−2627 : Selenium on Serine−−tRNA ligase, mitochondrial
rs672413
rs234709
rs6586282
rs1789953
rs921943
rs3797535
rs567754
rs10944
rs705415
rs558133
rs11951068
rs6859667
All − MR Egger
All − IVW(MRE)
−0.50
−0.25
0.00
0.25
0.50
MR effect size for
'Selenium' on 'Serine−−tRNA ligase, mitochondrial'

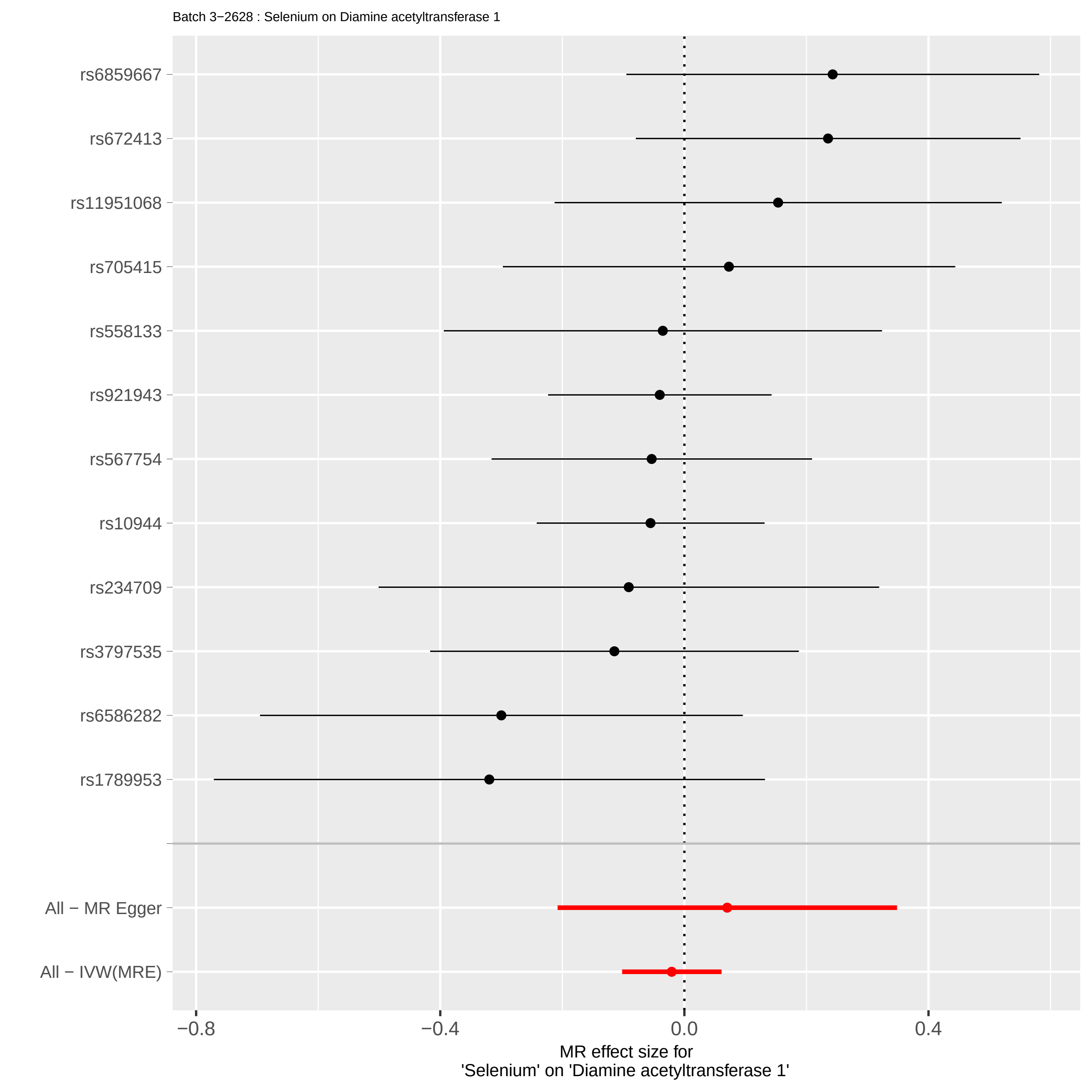

Batch 3−2628 : Selenium on Diamine acetyltransferase 1
rs6859667
rs672413
rs11951068
rs705415
rs558133
rs921943
rs567754
rs10944
rs234709
rs3797535
rs6586282
rs1789953
All − MR Egger
All − IVW(MRE)
−0.8
−0.4
0.0
0.4
MR effect size for
'Selenium' on 'Diamine acetyltransferase 1'

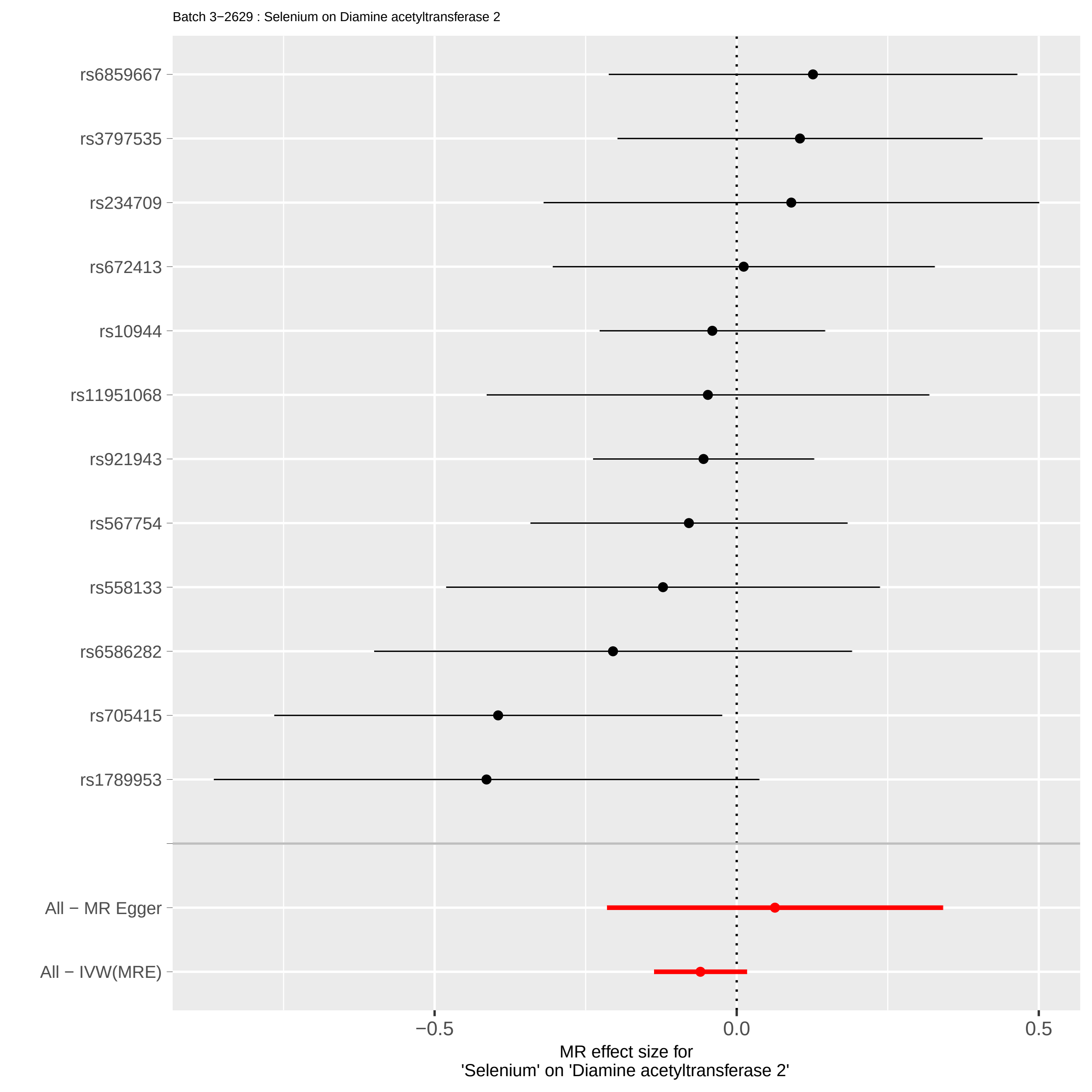

Batch 3−2629 : Selenium on Diamine acetyltransferase 2
rs6859667
rs3797535
rs234709
rs672413
rs10944
rs11951068
rs921943
rs567754
rs558133
rs6586282
rs705415
rs1789953
All − MR Egger
All − IVW(MRE)
−0.5
0.0
0.5
MR effect size for
'Selenium' on 'Diamine acetyltransferase 2'

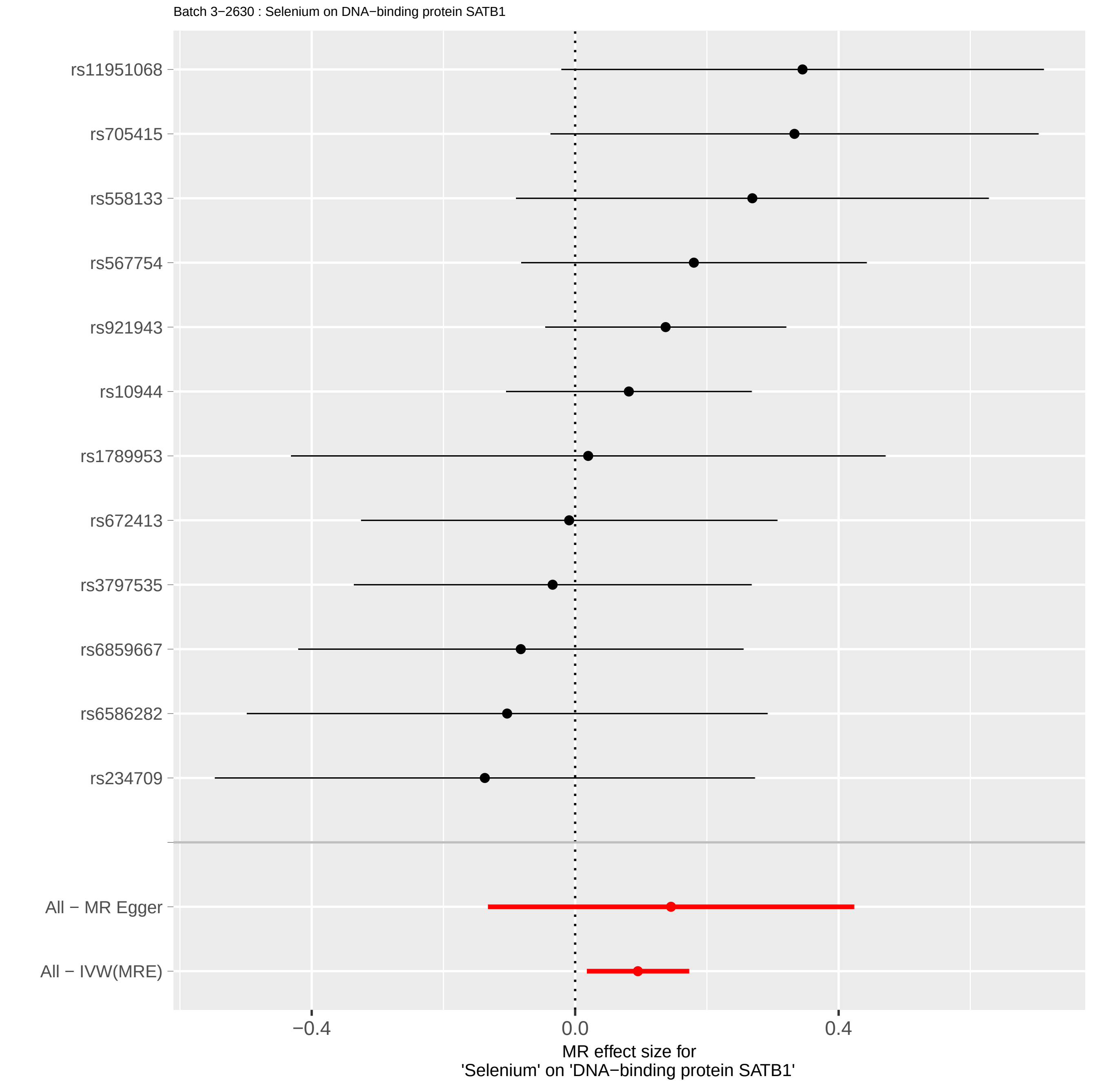

Batch 3−2630 : Selenium on DNA−binding protein SATB1
rs11951068
rs705415
rs558133
rs567754
rs921943
rs10944
rs1789953
rs672413
rs3797535
rs6859667
rs6586282
rs234709
All − MR Egger
All − IVW(MRE)
−0.4
0.0
0.4
MR effect size for
'Selenium' on 'DNA−binding protein SATB1'

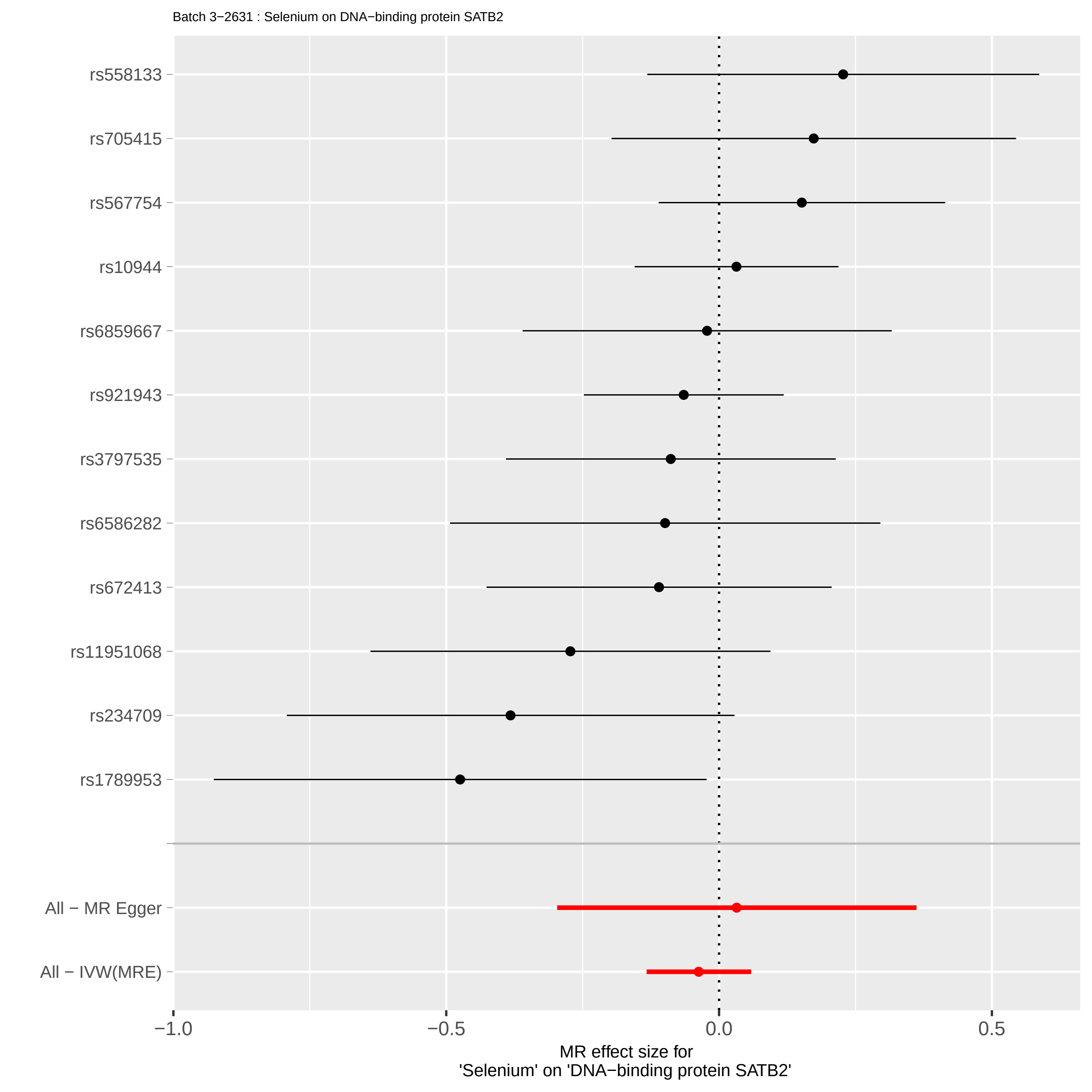

Batch 3−2631 : Selenium on DNA−binding protein SATB2
rs558133
rs705415
rs567754
rs10944
rs6859667
rs921943
rs3797535
rs6586282
rs672413
rs11951068
rs234709
rs1789953
All − MR Egger
All − IVW(MRE)
−1.0
−0.5
0.0
0.5
MR effect size for
'Selenium' on 'DNA−binding protein SATB2'

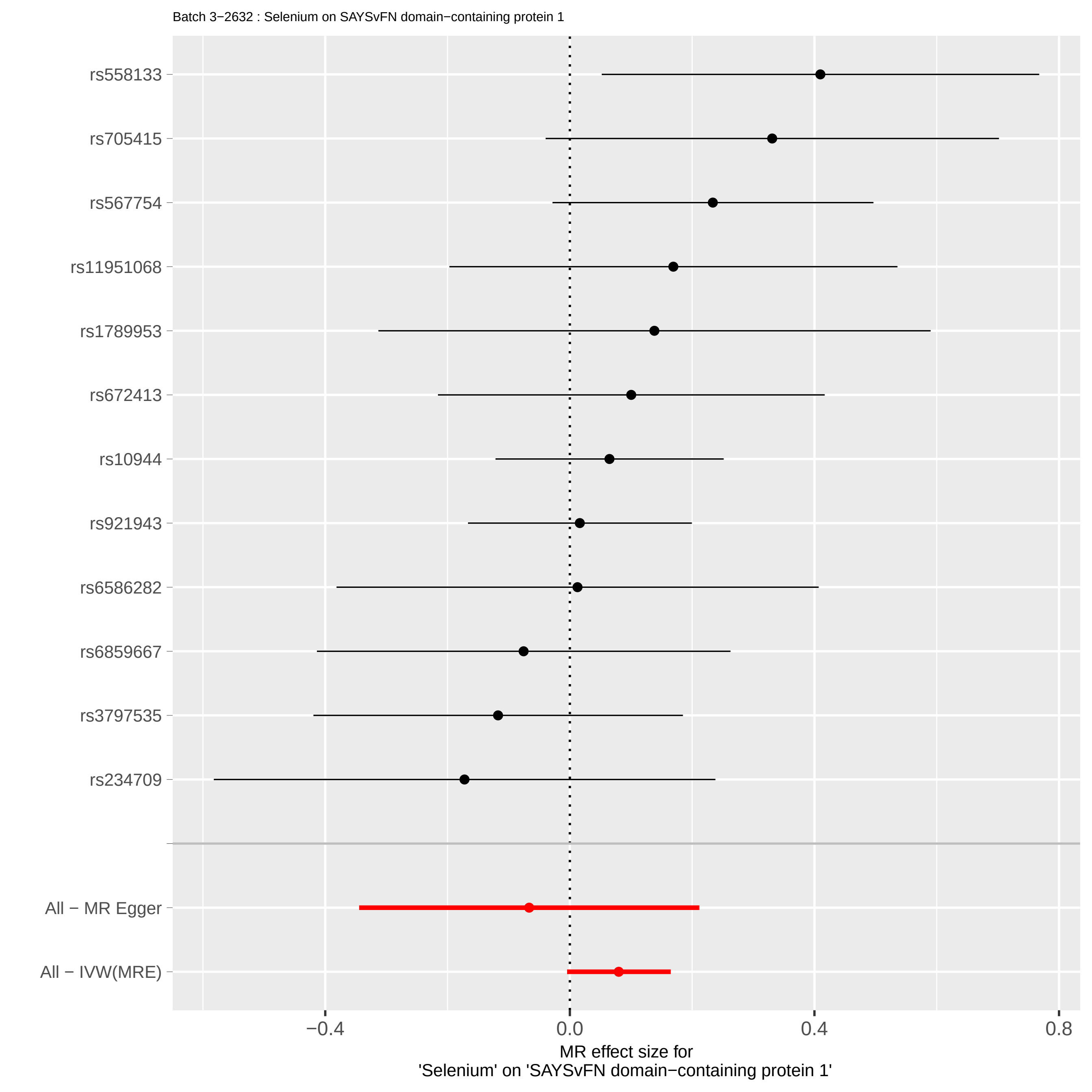

Batch 3−2632 : Selenium on SAYSvFN domain−containing protein 1
rs558133
rs705415
rs567754
rs11951068
rs1789953
rs672413
rs10944
rs921943
rs6586282
rs6859667
rs3797535
rs234709
All − MR Egger
All − IVW(MRE)
−0.4
0.0
0.4
0.8
MR effect size for
'Selenium' on 'SAYSvFN domain−containing protein 1'

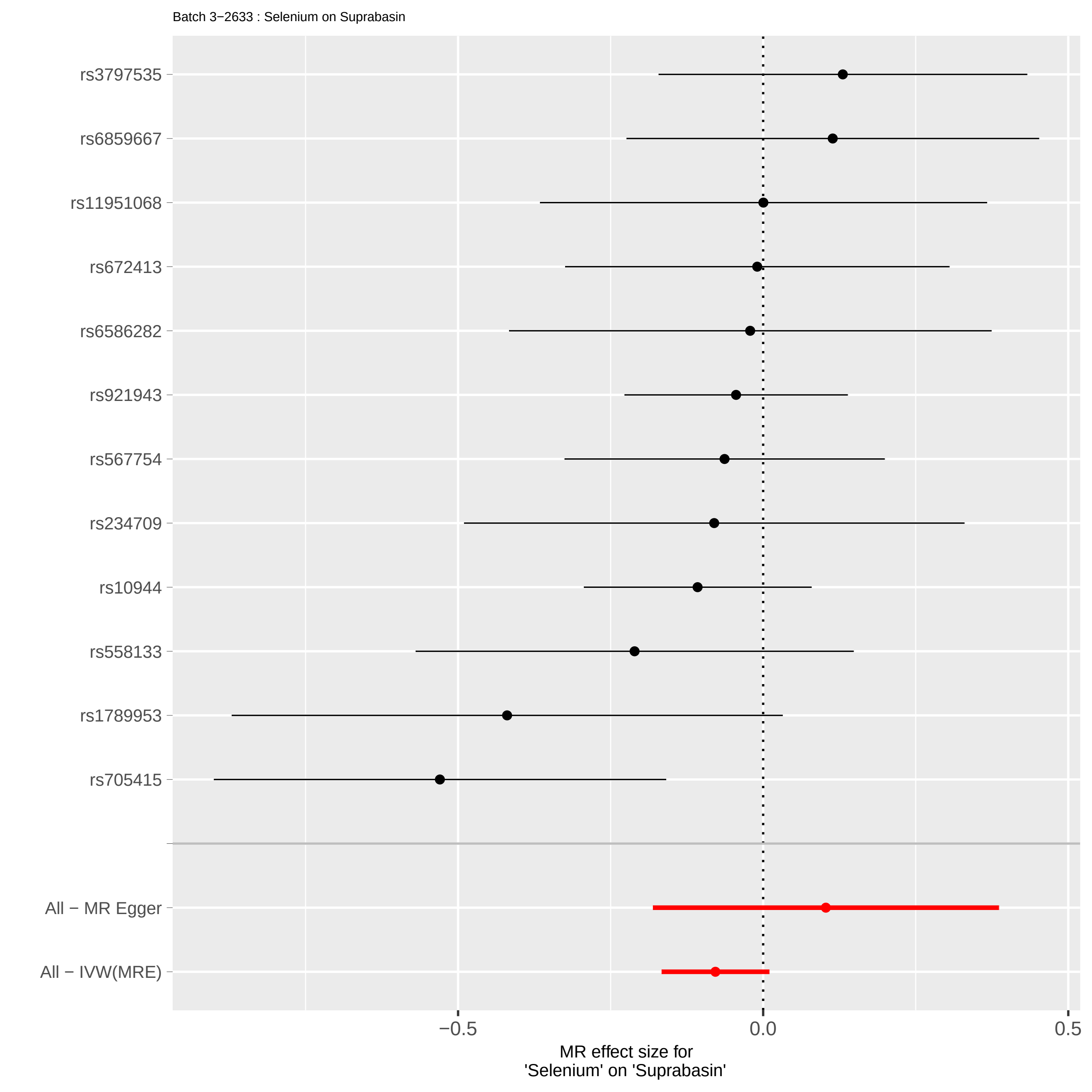
Batch 3−2633 : Selenium on Suprabasin
rs3797535
rs6859667
rs11951068
rs672413
rs6586282
rs921943
rs567754
rs234709
rs10944
rs558133
rs1789953
rs705415
All − MR Egger
All − IVW(MRE)
−0.5
0.0
0.5
MR effect size for
'Selenium' on 'Suprabasin'

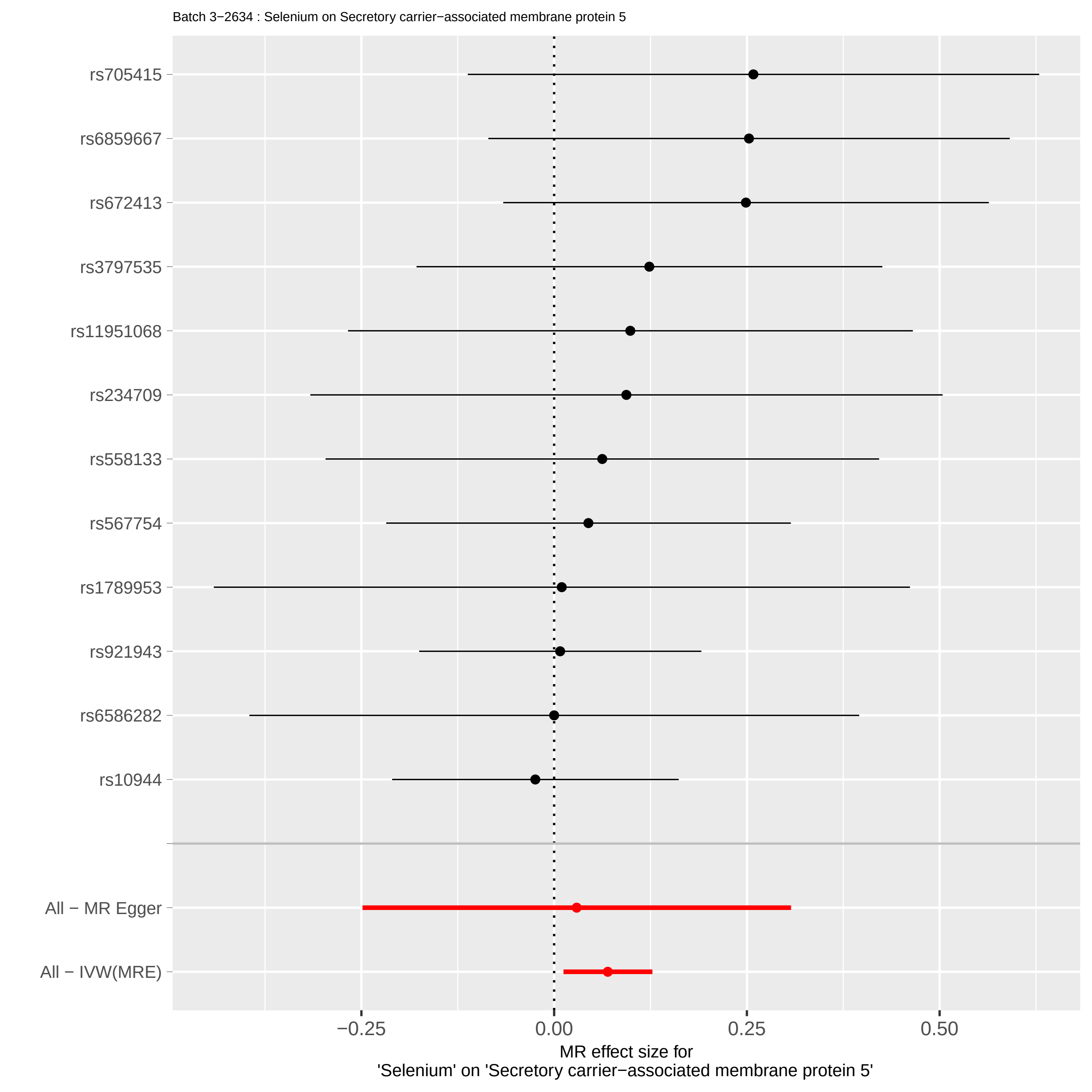

Batch 3−2634 : Selenium on Secretory carrier−associated membrane protein 5
rs705415
rs6859667
rs672413
rs3797535
rs11951068
rs234709
rs558133
rs567754
rs1789953
rs921943
rs6586282
rs10944
All − MR Egger
All − IVW(MRE)
−0.25
0.00
0.25
0.50
MR effect size for
'Selenium' on 'Secretory carrier−associated membrane protein 5'

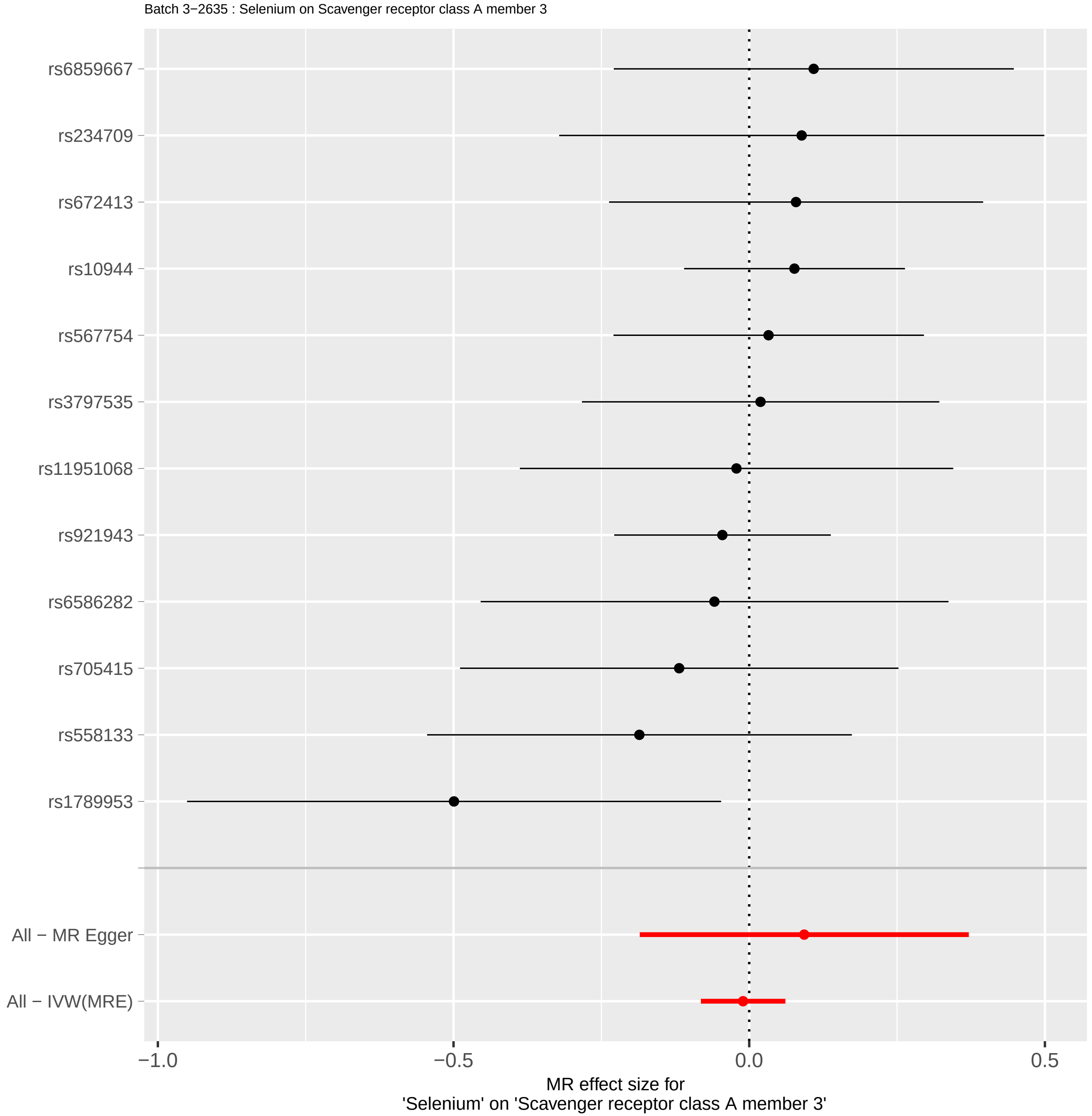

Batch 3−2635 : Selenium on Scavenger receptor class A member 3
rs6859667
rs234709
rs672413
rs10944
rs567754
rs3797535
rs11951068
rs921943
rs6586282
rs705415
rs558133
rs1789953
All − MR Egger
All − IVW(MRE)
−1.0
−0.5
0.0
0.5
MR effect size for
'Selenium' on 'Scavenger receptor class A member 3'

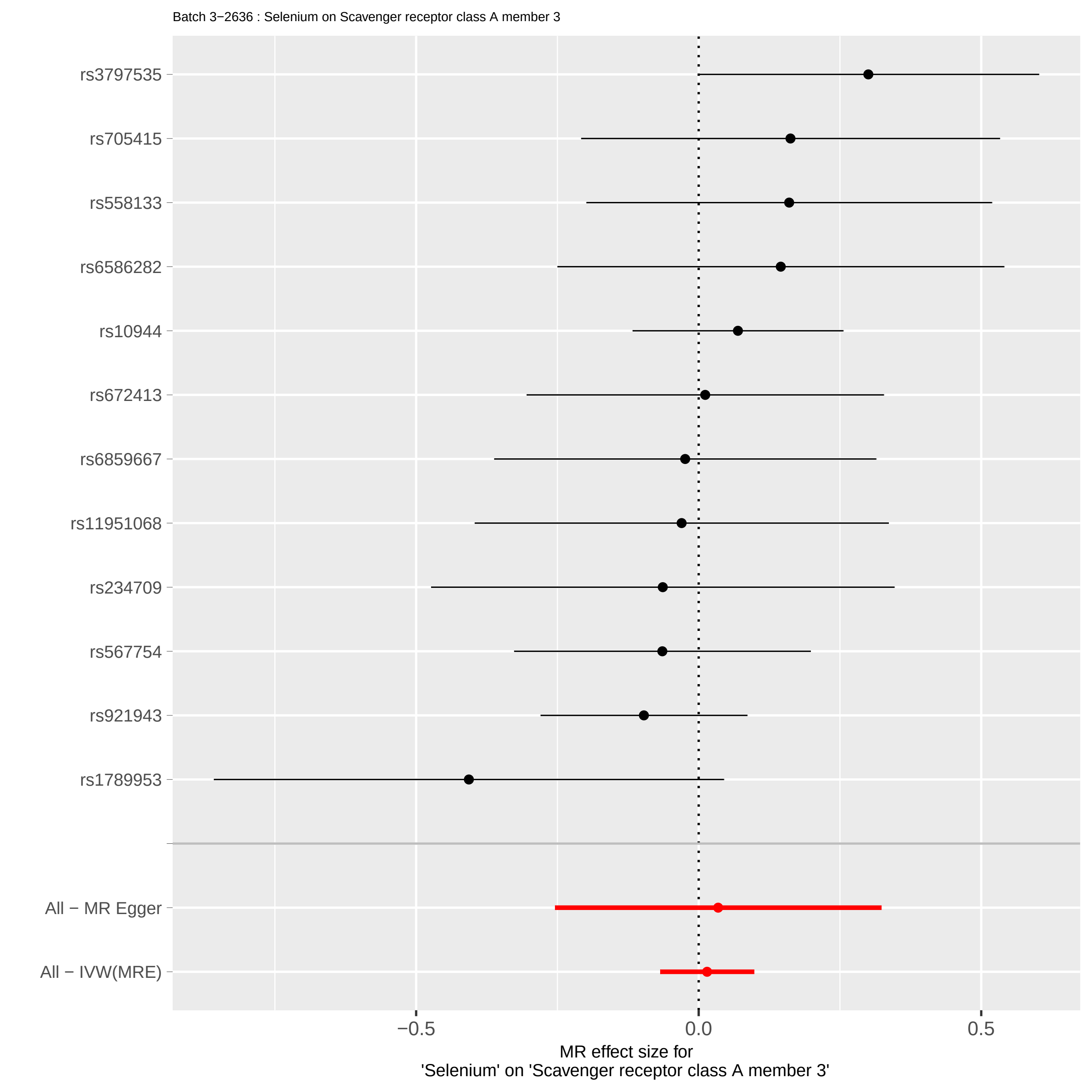
Batch 3−2636 : Selenium on Scavenger receptor class A member 3
rs3797535
rs705415
rs558133
rs6586282
rs10944
rs672413
rs6859667
rs11951068
rs234709
rs567754
rs921943
rs1789953
All − MR Egger
All − IVW(MRE)
−0.5
0.0
0.5
MR effect size for
'Selenium' on 'Scavenger receptor class A member 3'

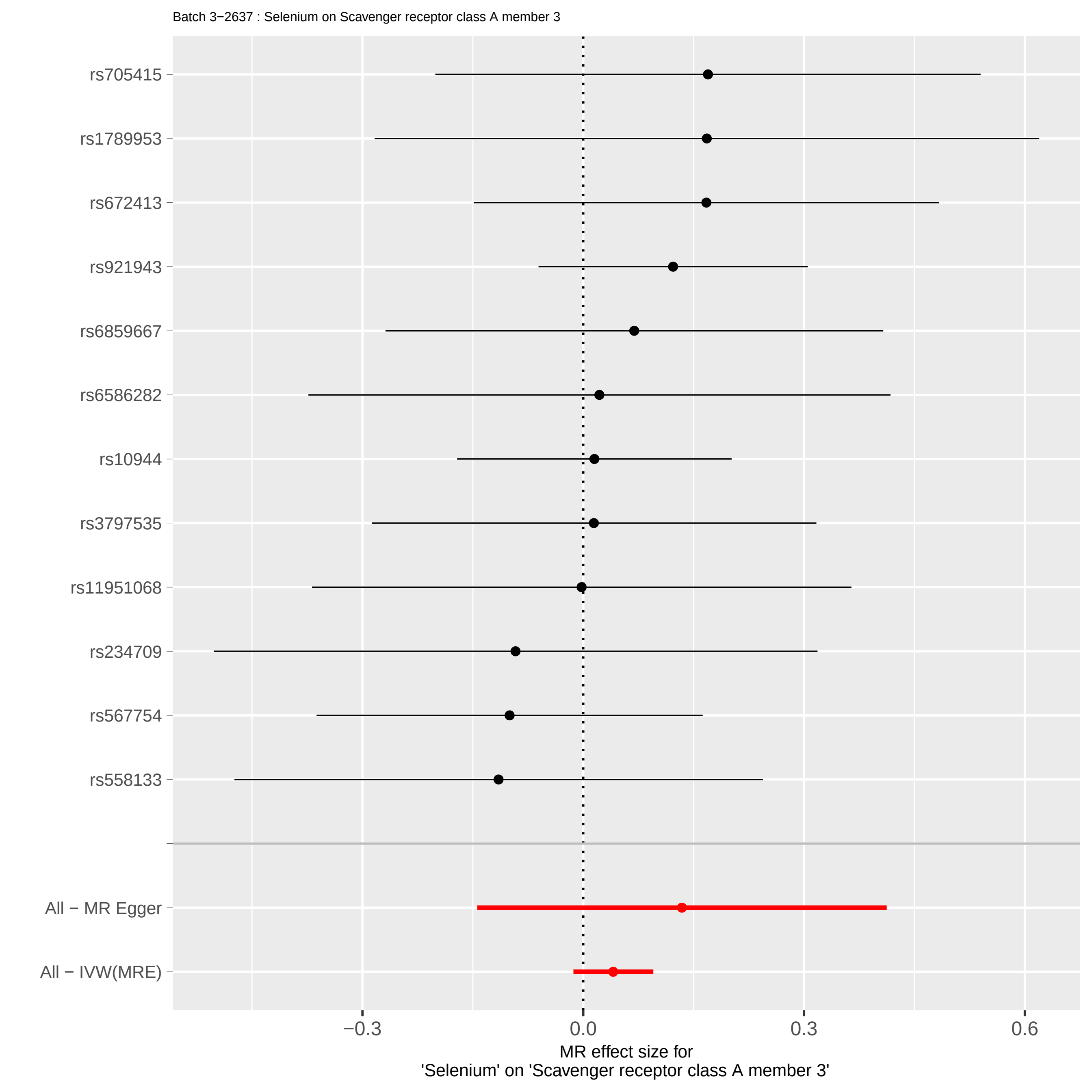
Batch 3−2637 : Selenium on Scavenger receptor class A member 3
rs705415
rs1789953
rs672413
rs921943
rs6859667
rs6586282
rs10944
rs3797535
rs11951068
rs234709
rs567754
rs558133
All − MR Egger
All − IVW(MRE)
−0.3
0.0
0.3
0.6
MR effect size for
'Selenium' on 'Scavenger receptor class A member 3'

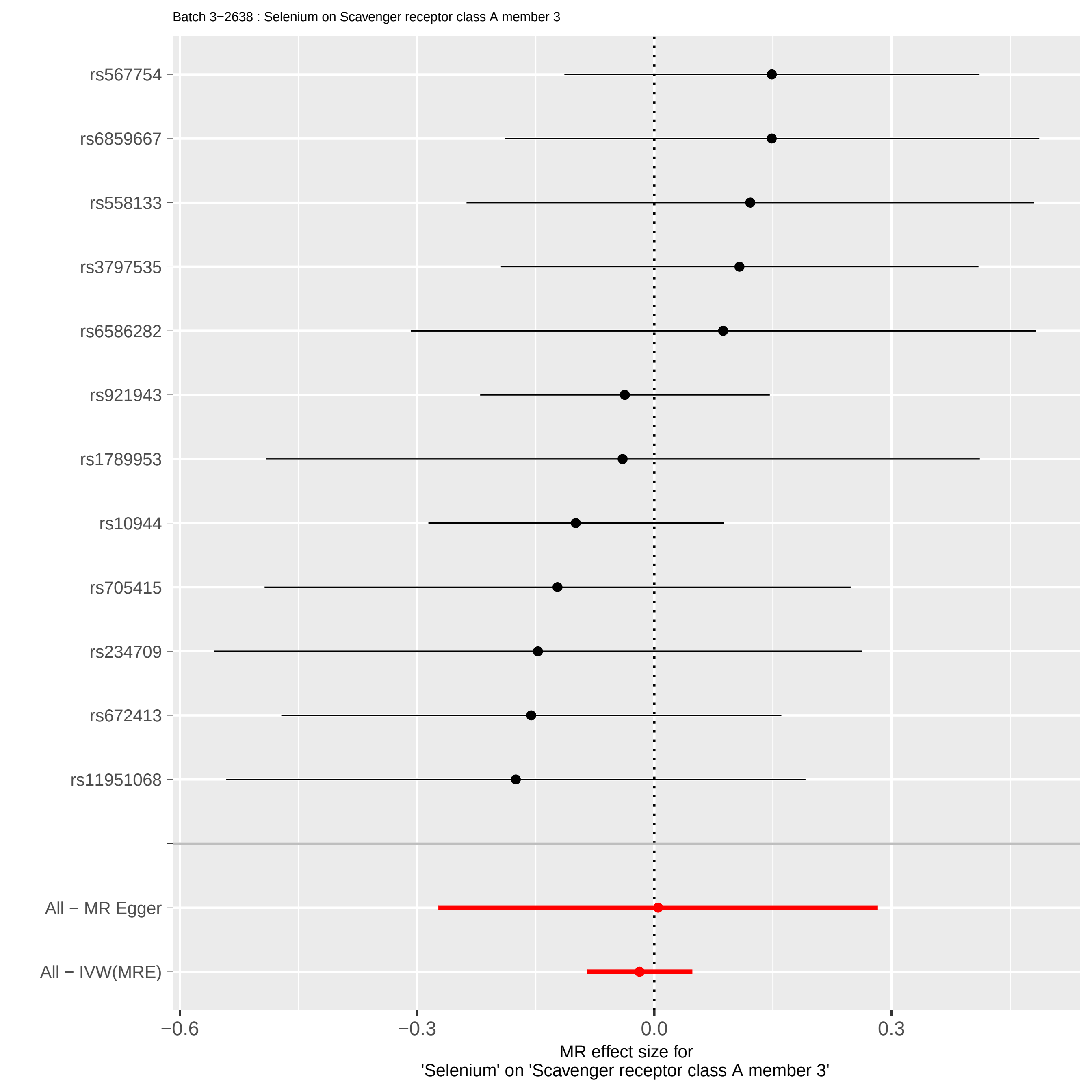

Batch 3−2638 : Selenium on Scavenger receptor class A member 3
rs567754
rs6859667
rs558133
rs3797535
rs6586282
rs921943
rs1789953
rs10944
rs705415
rs234709
rs672413
rs11951068
All − MR Egger
All − IVW(MRE)
−0.6
−0.3
0.0
0.3
MR effect size for
'Selenium' on 'Scavenger receptor class A member 3'

Batch 3−2639 : Selenium on Scavenger receptor class A member 5

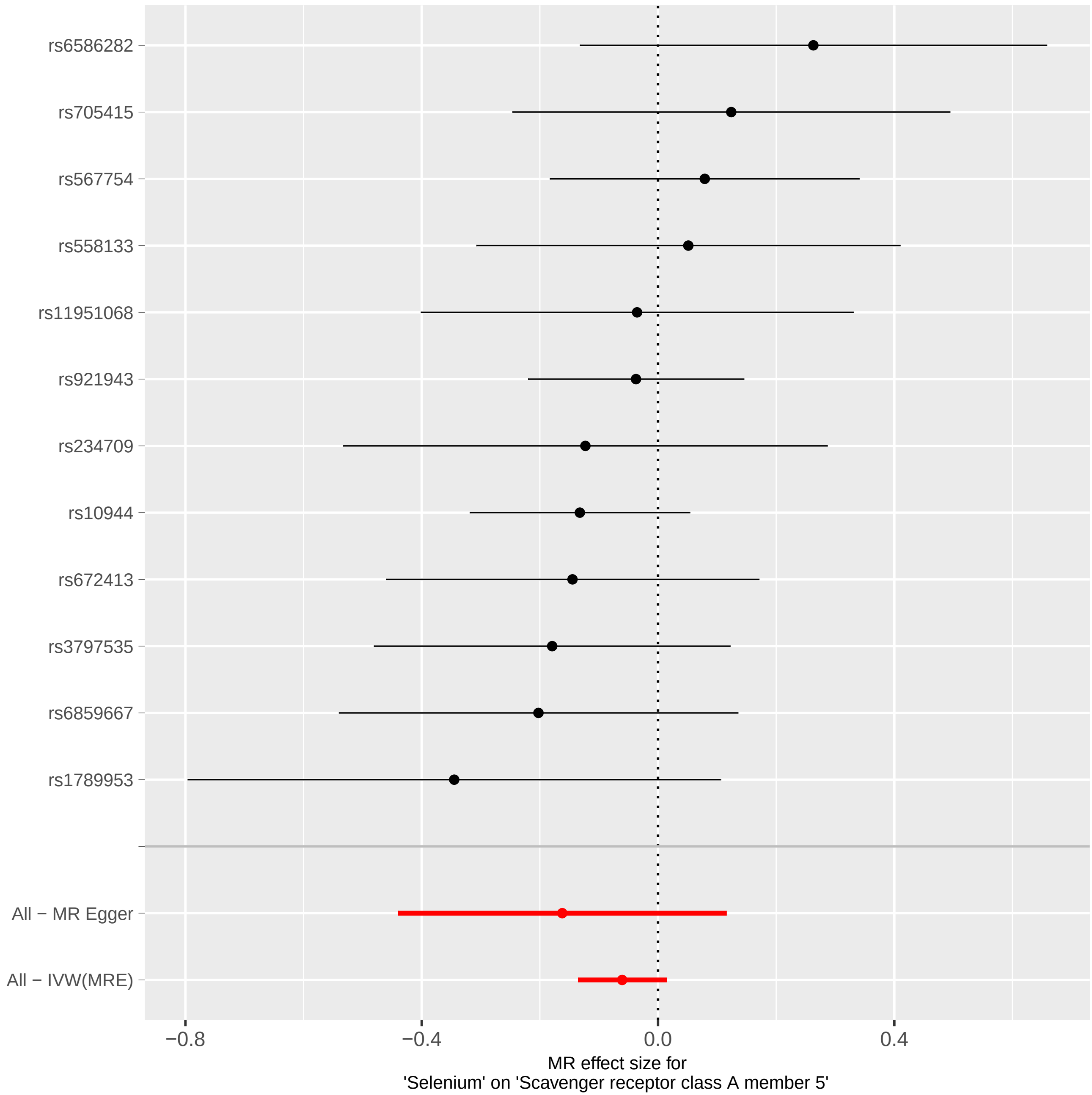

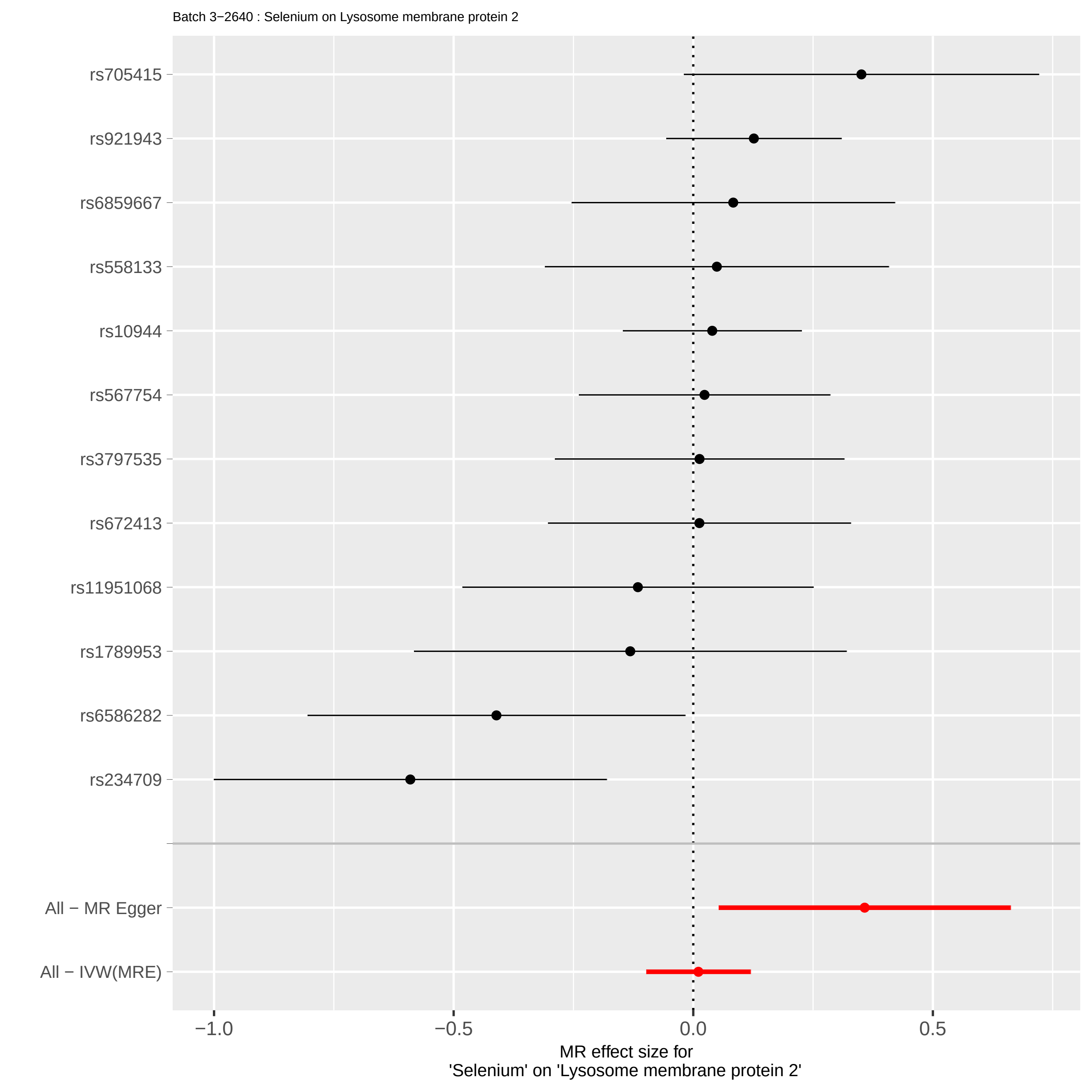

Batch 3−2640 : Selenium on Lysosome membrane protein 2
rs705415
rs921943
rs6859667
rs558133
rs10944
rs567754
rs3797535
rs672413
rs11951068
rs1789953
rs6586282
rs234709
All − MR Egger
All − IVW(MRE)
−1.0
−0.5
0.0
0.5
MR effect size for
'Selenium' on 'Lysosome membrane protein 2'

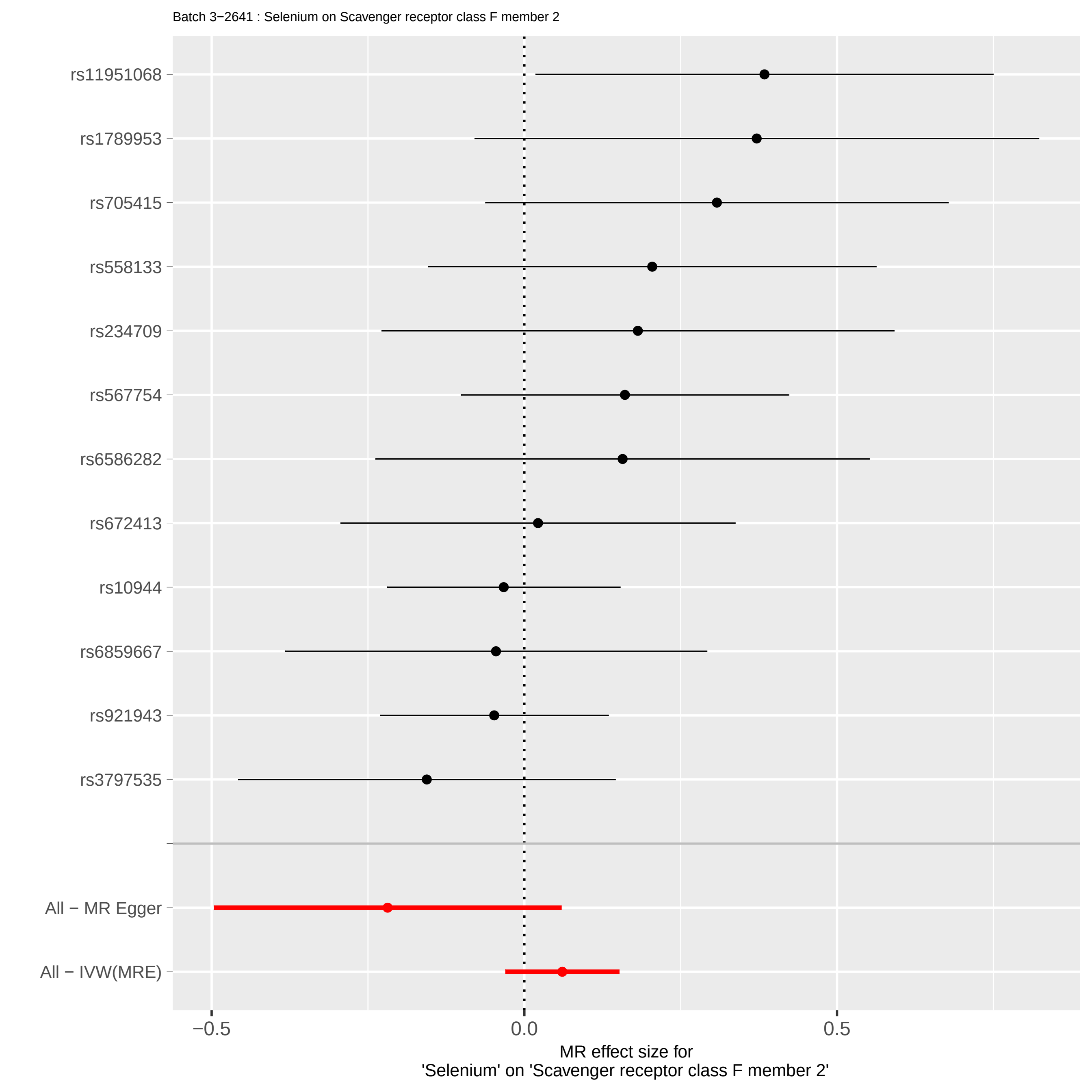

Batch 3−2641 : Selenium on Scavenger receptor class F member 2
rs11951068
rs1789953
rs705415
rs558133
rs234709
rs567754
rs6586282
rs672413
rs10944
rs6859667
rs921943
rs3797535
All − MR Egger
All − IVW(MRE)
−0.5
0.0
0.5
MR effect size for
'Selenium' on 'Scavenger receptor class F member 2'

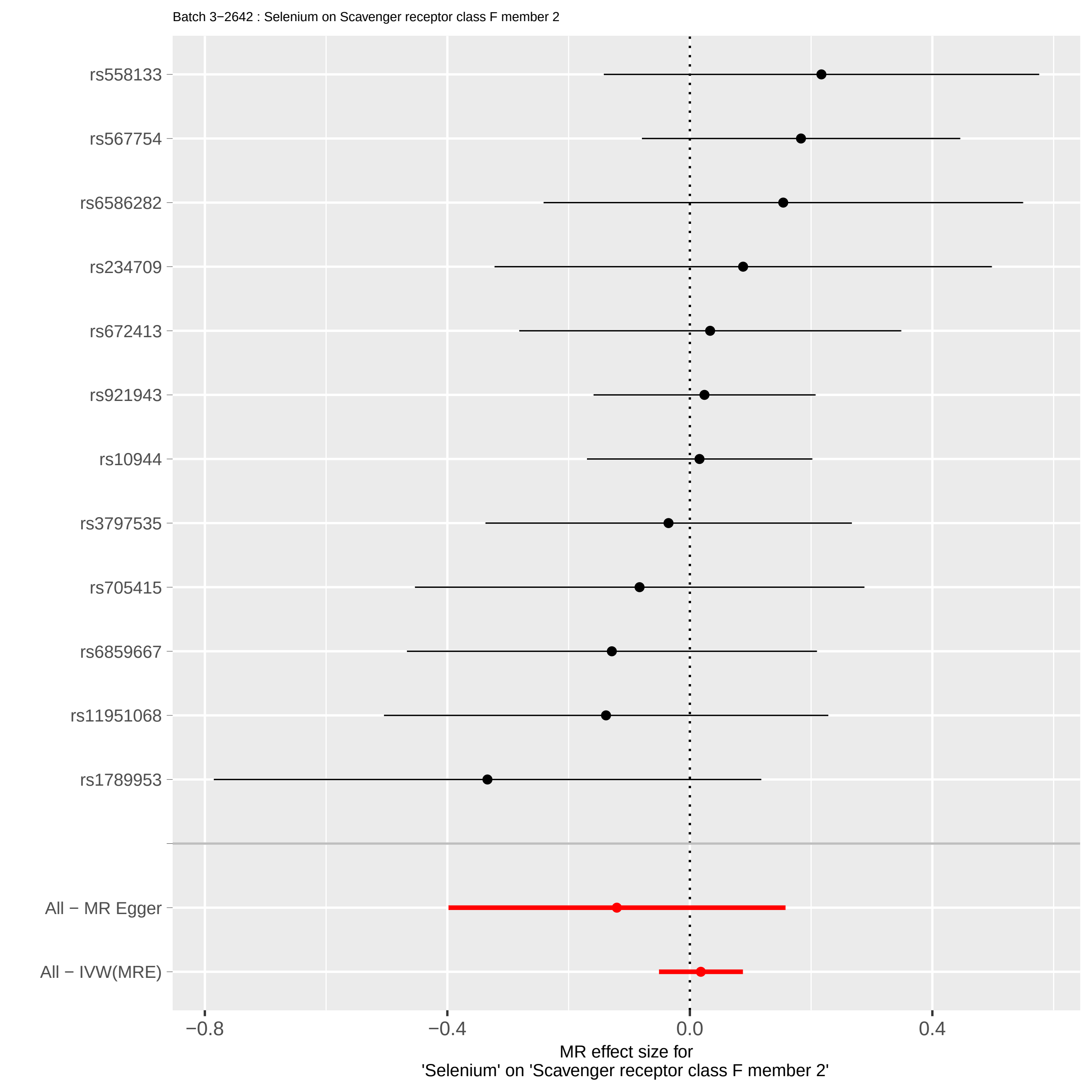
Batch 3−2642 : Selenium on Scavenger receptor class F member 2
rs558133
rs567754
rs6586282
rs234709
rs672413
rs921943
rs10944
rs3797535
rs705415
rs6859667
rs11951068
rs1789953
All − MR Egger
All − IVW(MRE)
−0.8
−0.4
0.0
0.4
MR effect size for
'Selenium' on 'Scavenger receptor class F member 2'

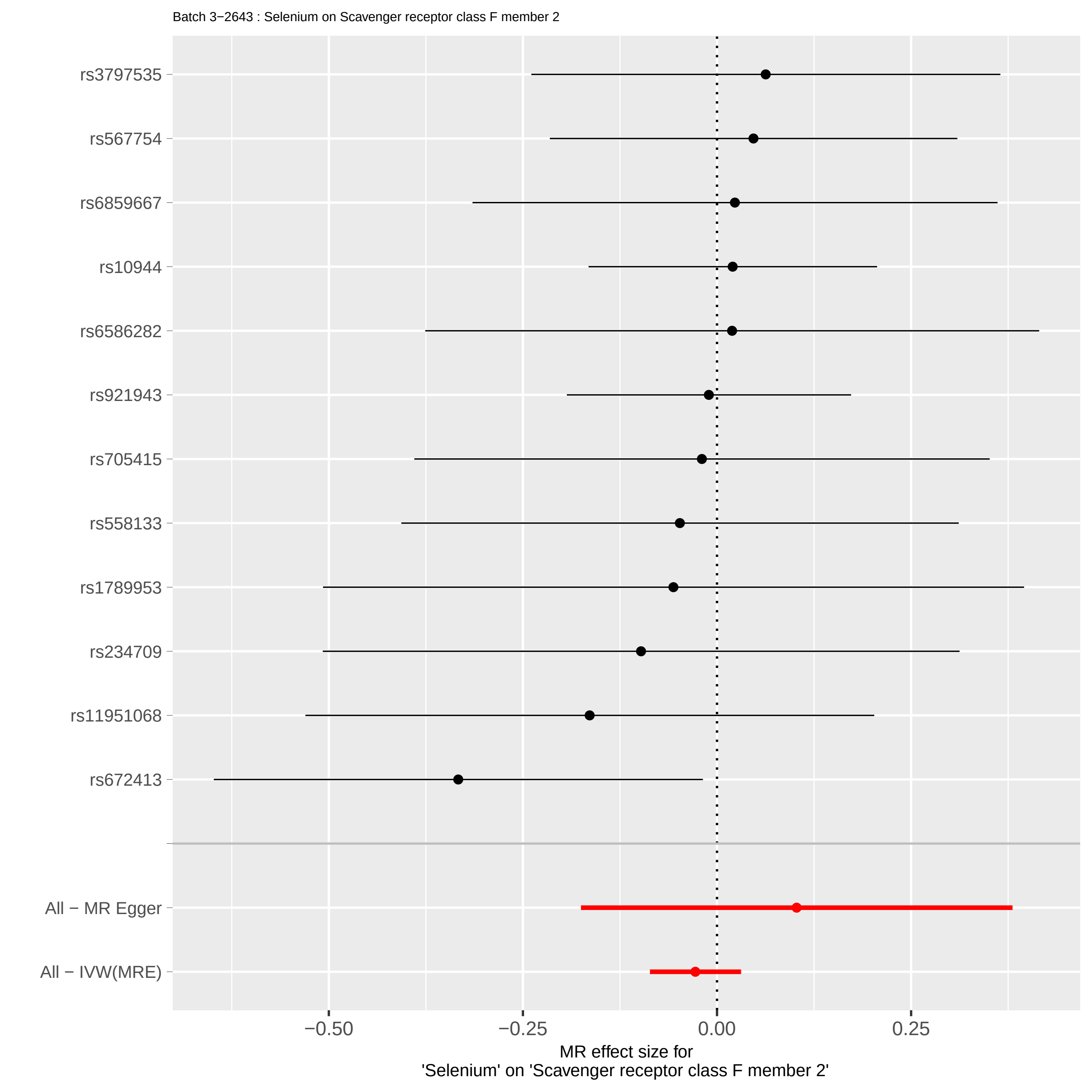

Batch 3−2643 : Selenium on Scavenger receptor class F member 2
rs3797535
rs567754
rs6859667
rs10944
rs6586282
rs921943
rs705415
rs558133
rs1789953
rs234709
rs11951068
rs672413
All − MR Egger
All − IVW(MRE)
−0.50
−0.25
0.00
0.25
MR effect size for
'Selenium' on 'Scavenger receptor class F member 2'

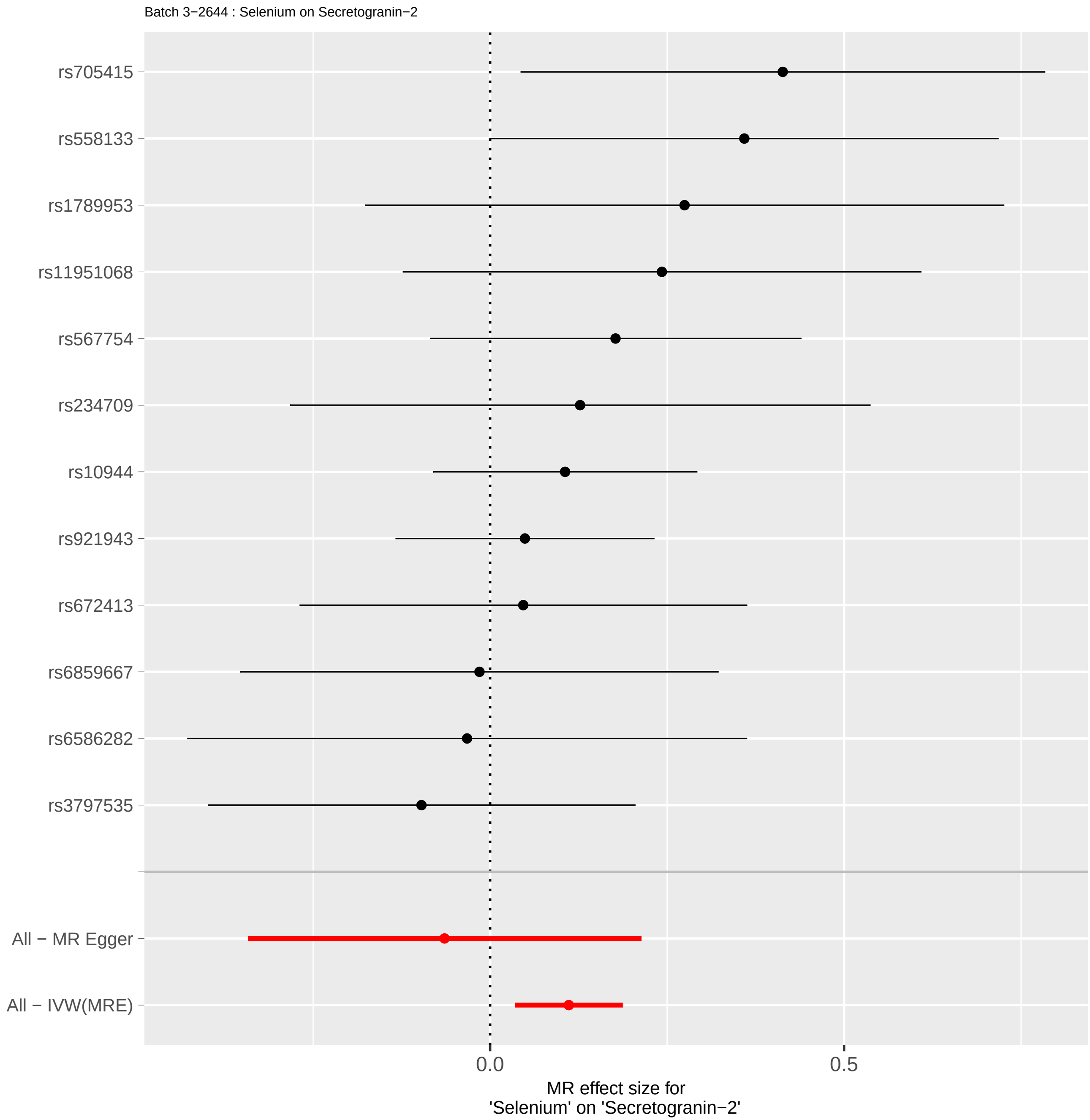
Batch 3−2644 : Selenium on Secretogranin−2
rs705415
rs558133
rs1789953
rs11951068
rs567754
rs234709
rs10944
rs921943
rs672413
rs6859667
rs6586282
rs3797535
All − MR Egger
All − IVW(MRE)
0.0
0.5
MR effect size for
'Selenium' on 'Secretogranin−2'

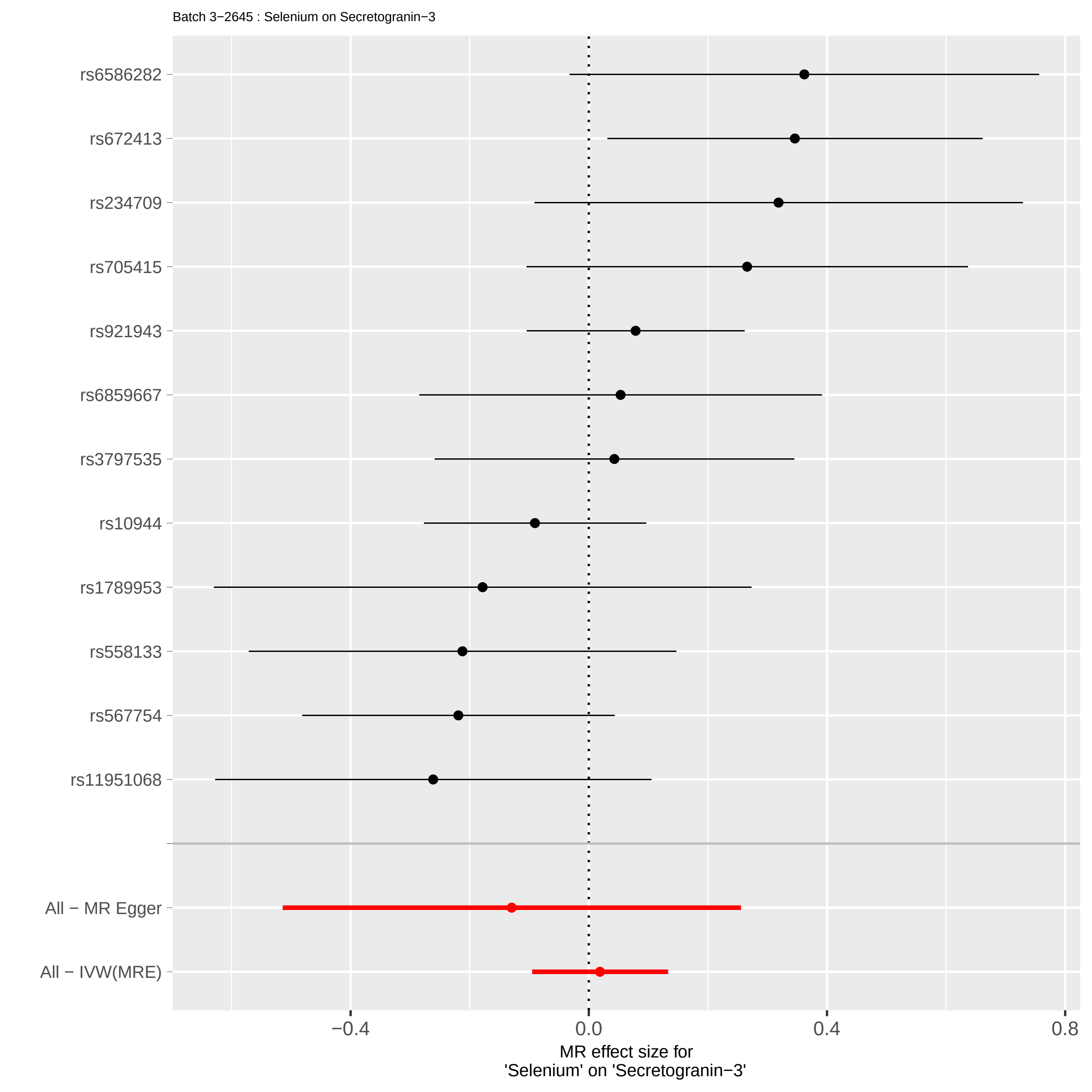

Batch 3−2645 : Selenium on Secretogranin−3
rs6586282
rs672413
rs234709
rs705415
rs921943
rs6859667
rs3797535
rs10944
rs1789953
rs558133
rs567754
rs11951068
All − MR Egger
All − IVW(MRE)
−0.4
0.0
0.4
0.8
MR effect size for
'Selenium' on 'Secretogranin−3'

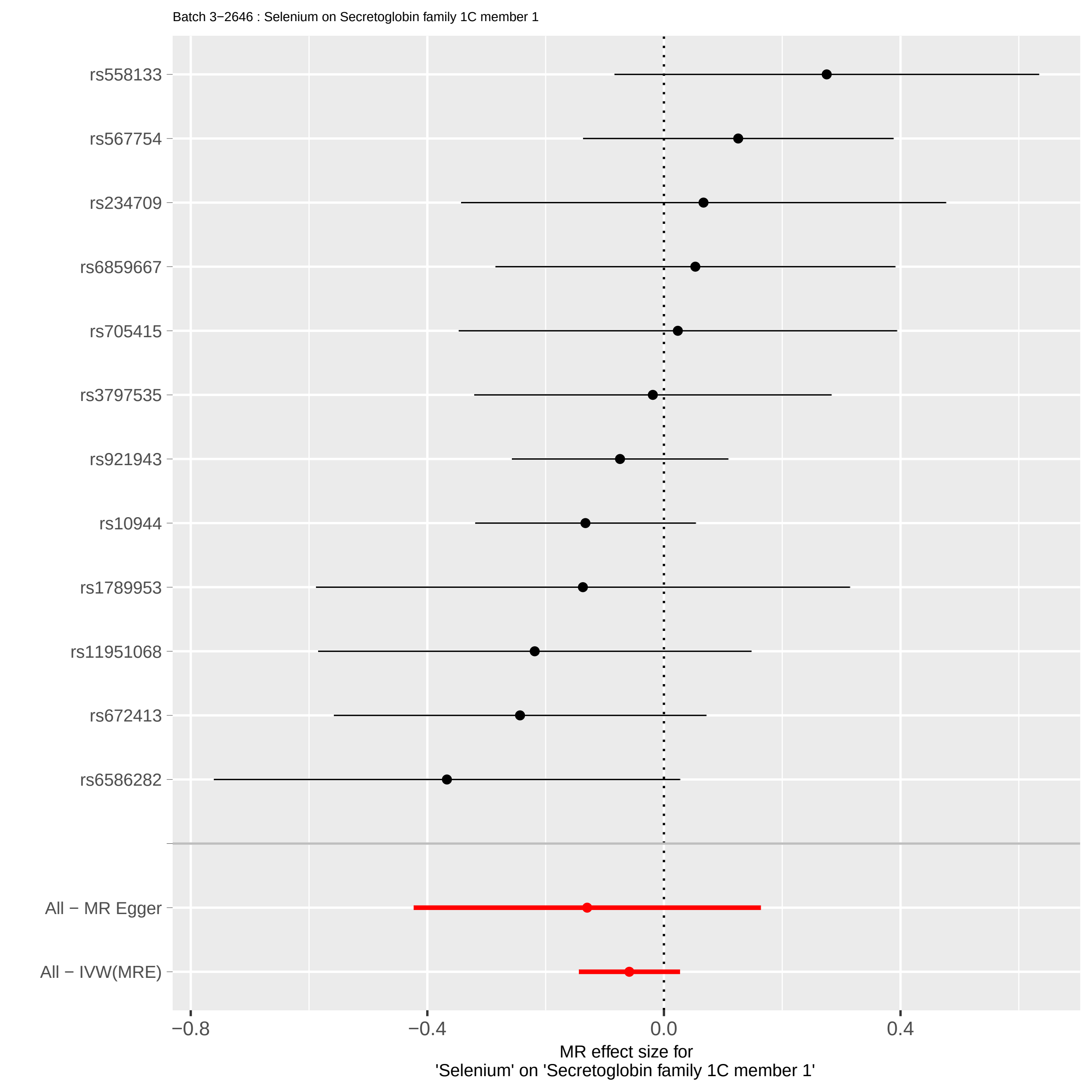

Batch 3−2646 : Selenium on Secretoglobin family 1C member 1
rs558133
rs567754
rs234709
rs6859667
rs705415
rs3797535
rs921943
rs10944
rs1789953
rs11951068
rs672413
rs6586282
All − MR Egger
All − IVW(MRE)
−0.8
−0.4
0.0
0.4
MR effect size for
'Selenium' on 'Secretoglobin family 1C member 1'

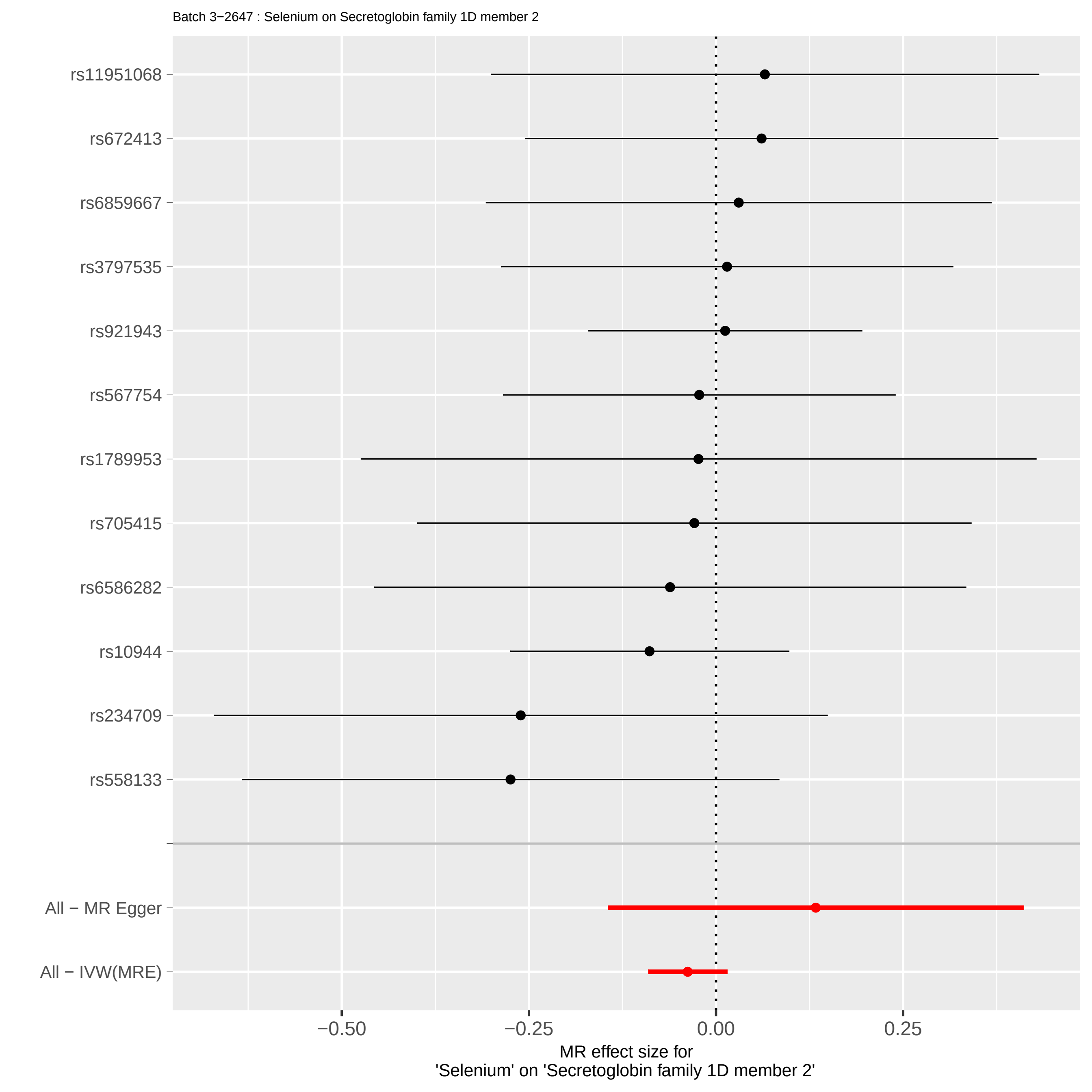

Batch 3−2647 : Selenium on Secretoglobin family 1D member 2
rs11951068
rs672413
rs6859667
rs3797535
rs921943
rs567754
rs1789953
rs705415
rs6586282
rs10944
rs234709
rs558133
All − MR Egger
All − IVW(MRE)
−0.50
−0.25
0.00
0.25
MR effect size for
'Selenium' on 'Secretoglobin family 1D member 2'

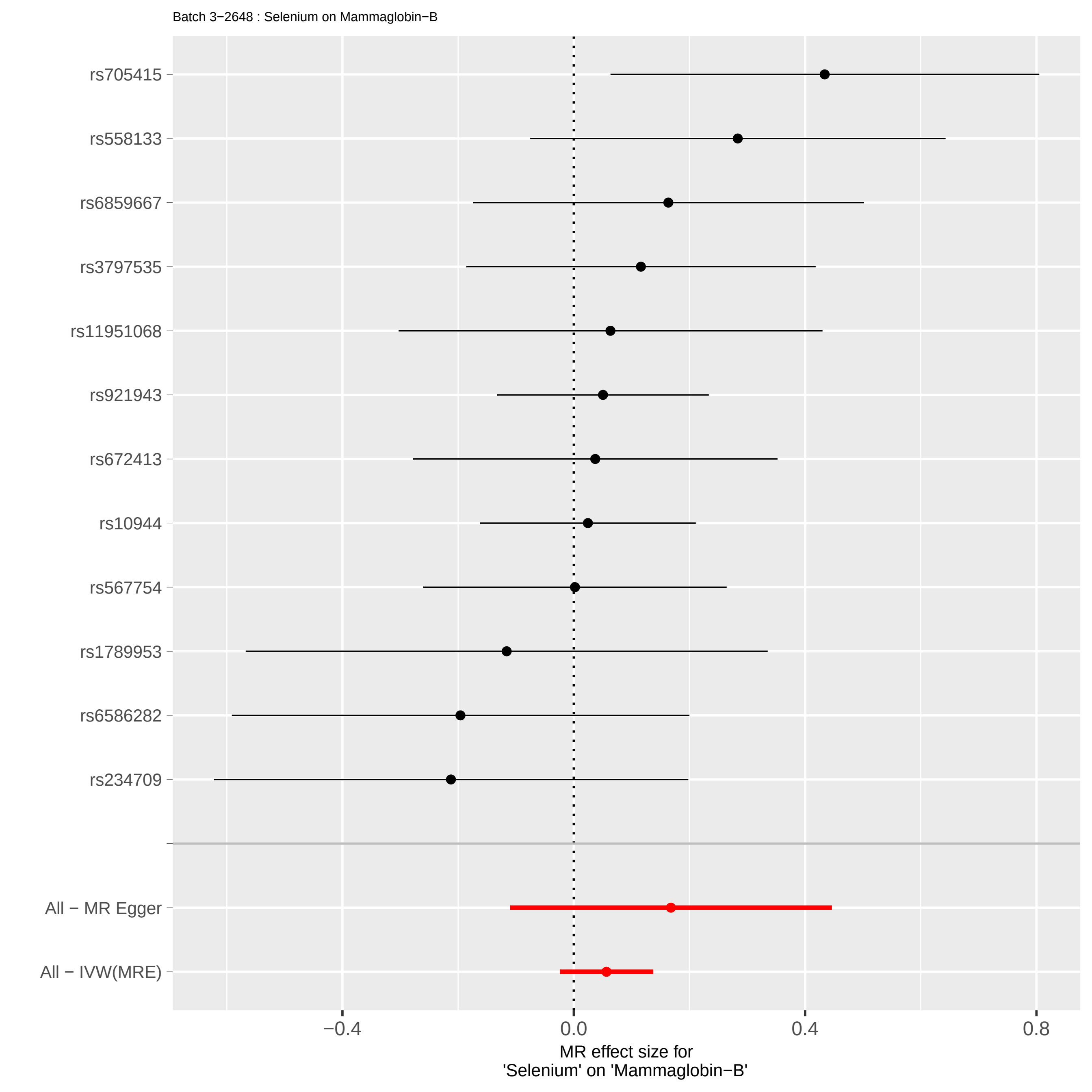
Batch 3−2648 : Selenium on Mammaglobin−B
rs705415
rs558133
rs6859667
rs3797535
rs11951068
rs921943
rs672413
rs10944
rs567754
rs1789953
rs6586282
rs234709
All − MR Egger
All − IVW(MRE)
−0.4
0.0
0.4
0.8
MR effect size for
'Selenium' on 'Mammaglobin−B'

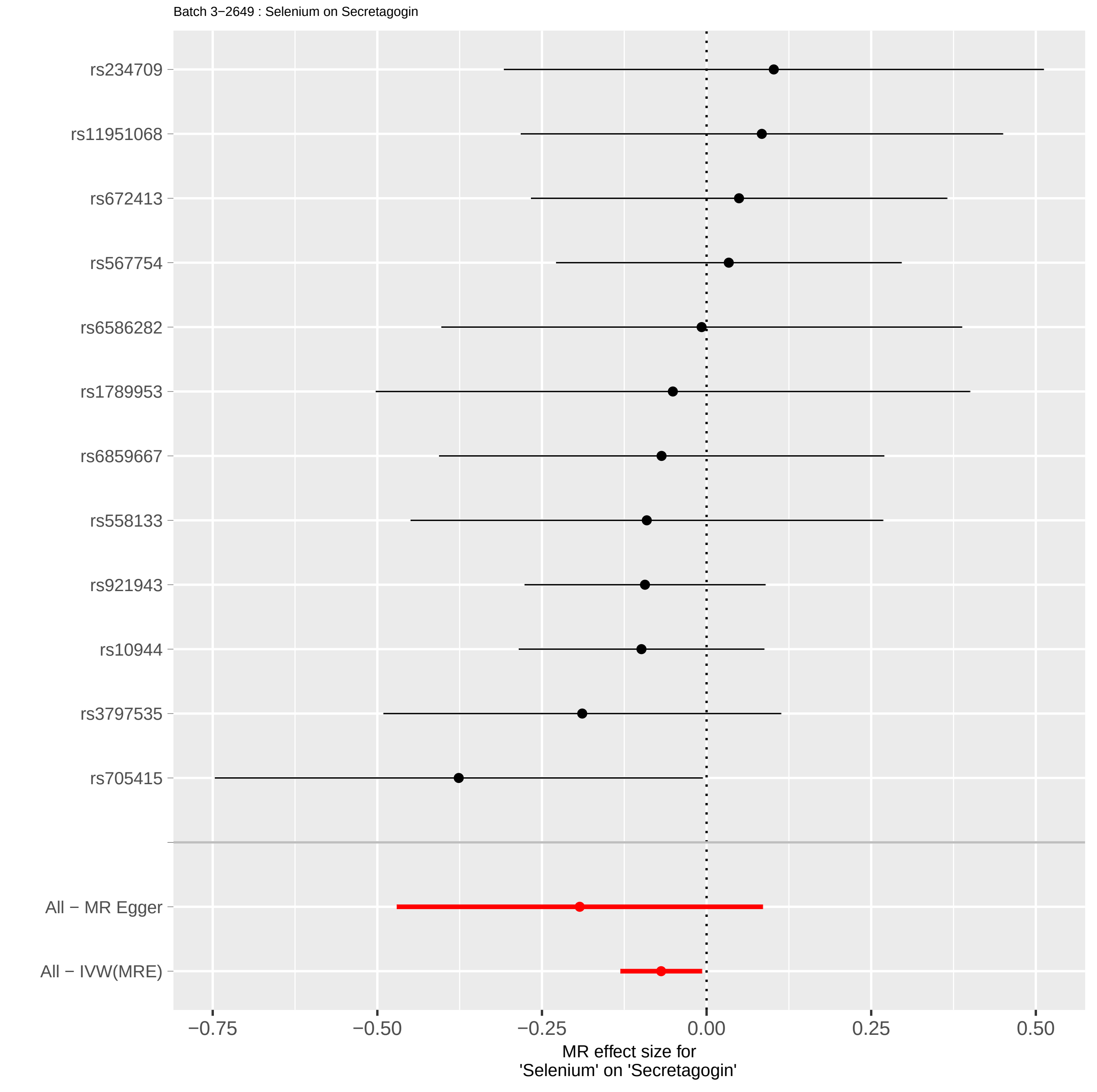

Batch 3−2649 : Selenium on Secretagogin
rs234709
rs11951068
rs672413
rs567754
rs6586282
rs1789953
rs6859667
rs558133
rs921943
rs10944
rs3797535
rs705415
All − MR Egger
All − IVW(MRE)
−0.75
−0.50
−0.25
0.00
0.25
0.50
MR effect size for
'Selenium' on 'Secretagogin'

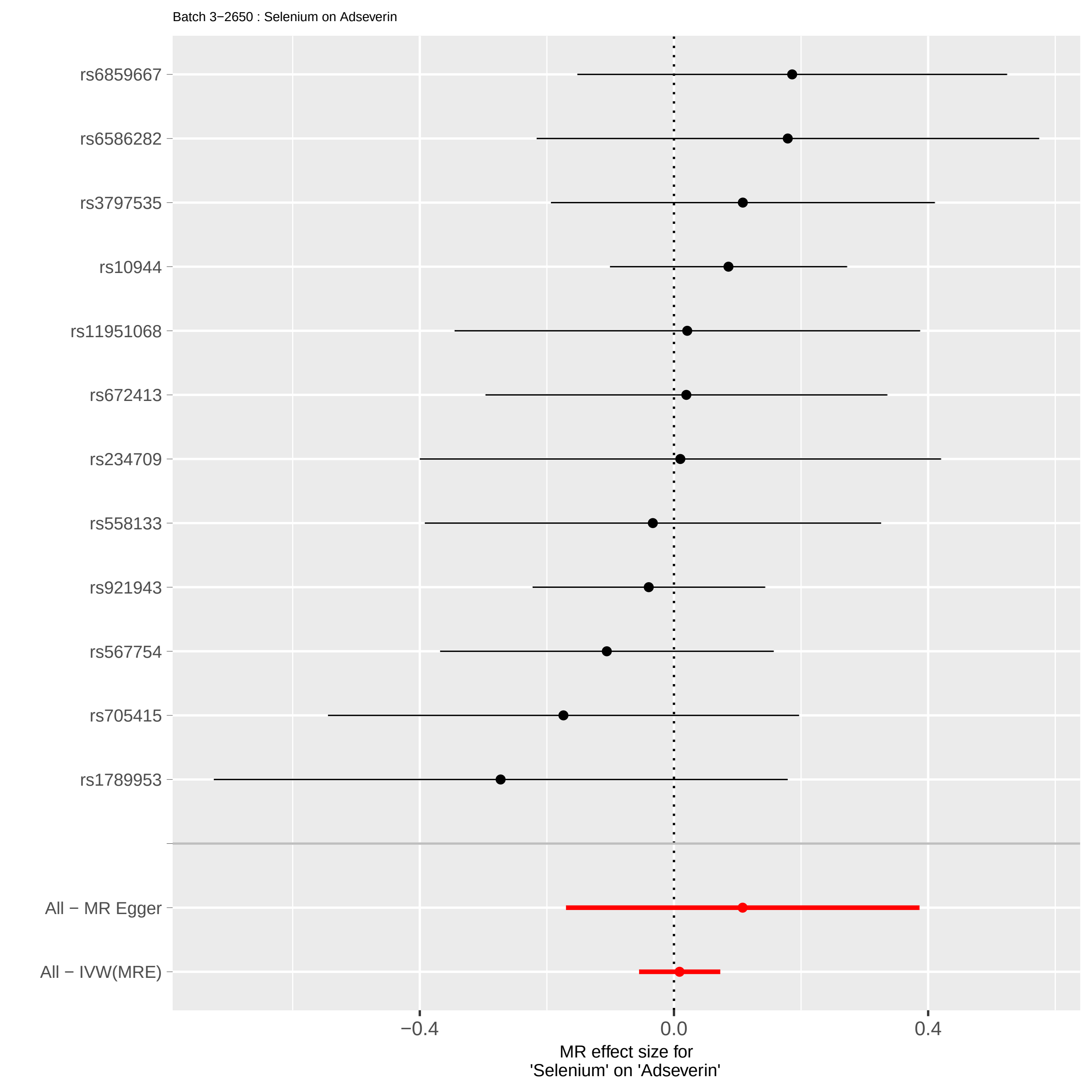

Batch 3−2650 : Selenium on Adseverin
rs6859667
rs6586282
rs3797535
rs10944
rs11951068
rs672413
rs234709
rs558133
rs921943
rs567754
rs705415
rs1789953
All − MR Egger
All − IVW(MRE)
−0.4
0.0
0.4
MR effect size for
'Selenium' on 'Adseverin'

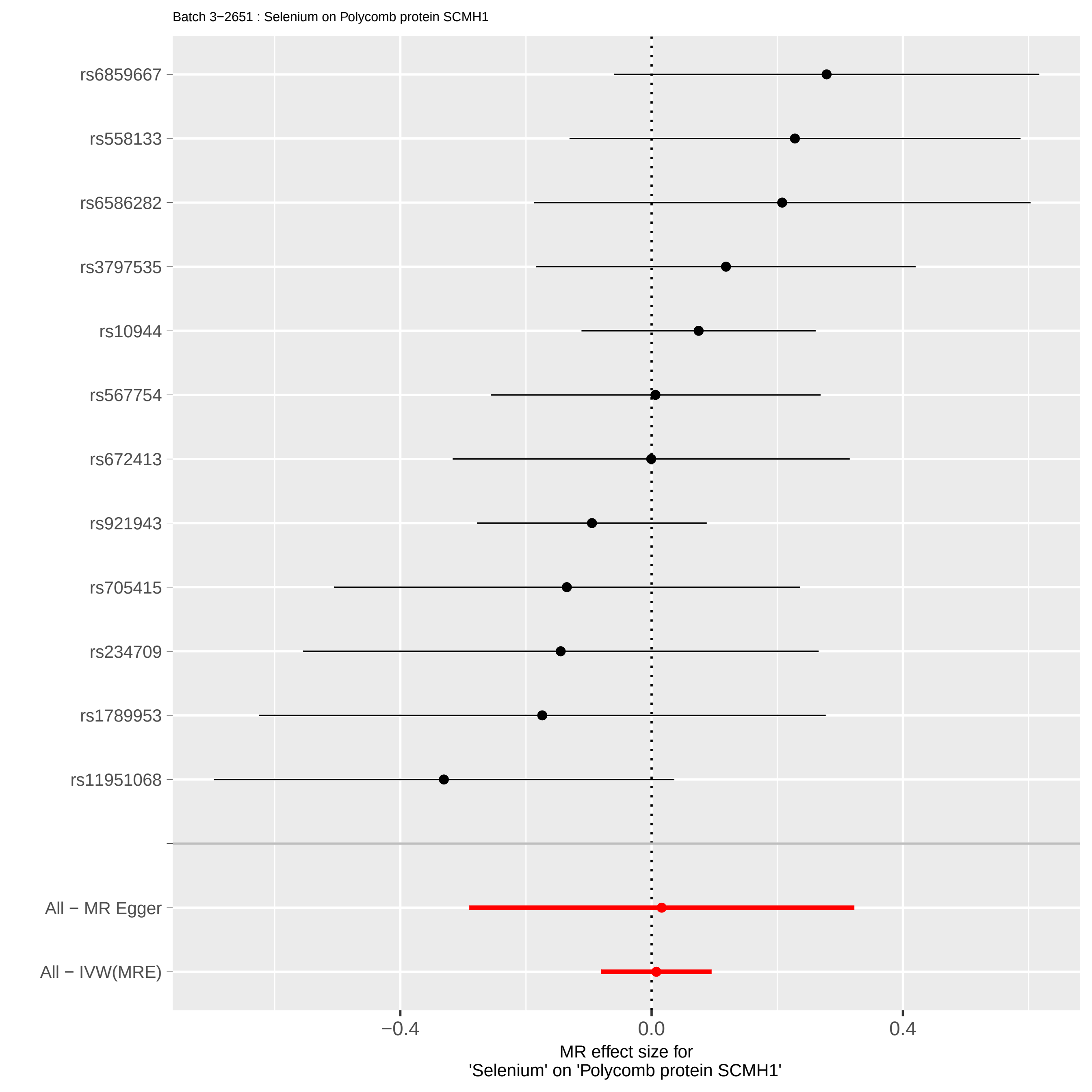
Batch 3−2651 : Selenium on Polycomb protein SCMH1
rs6859667
rs558133
rs6586282
rs3797535
rs10944
rs567754
rs672413
rs921943
rs705415
rs234709
rs1789953
rs11951068
All − MR Egger
All − IVW(MRE)
−0.4
0.0
0.4
MR effect size for
'Selenium' on 'Polycomb protein SCMH1'

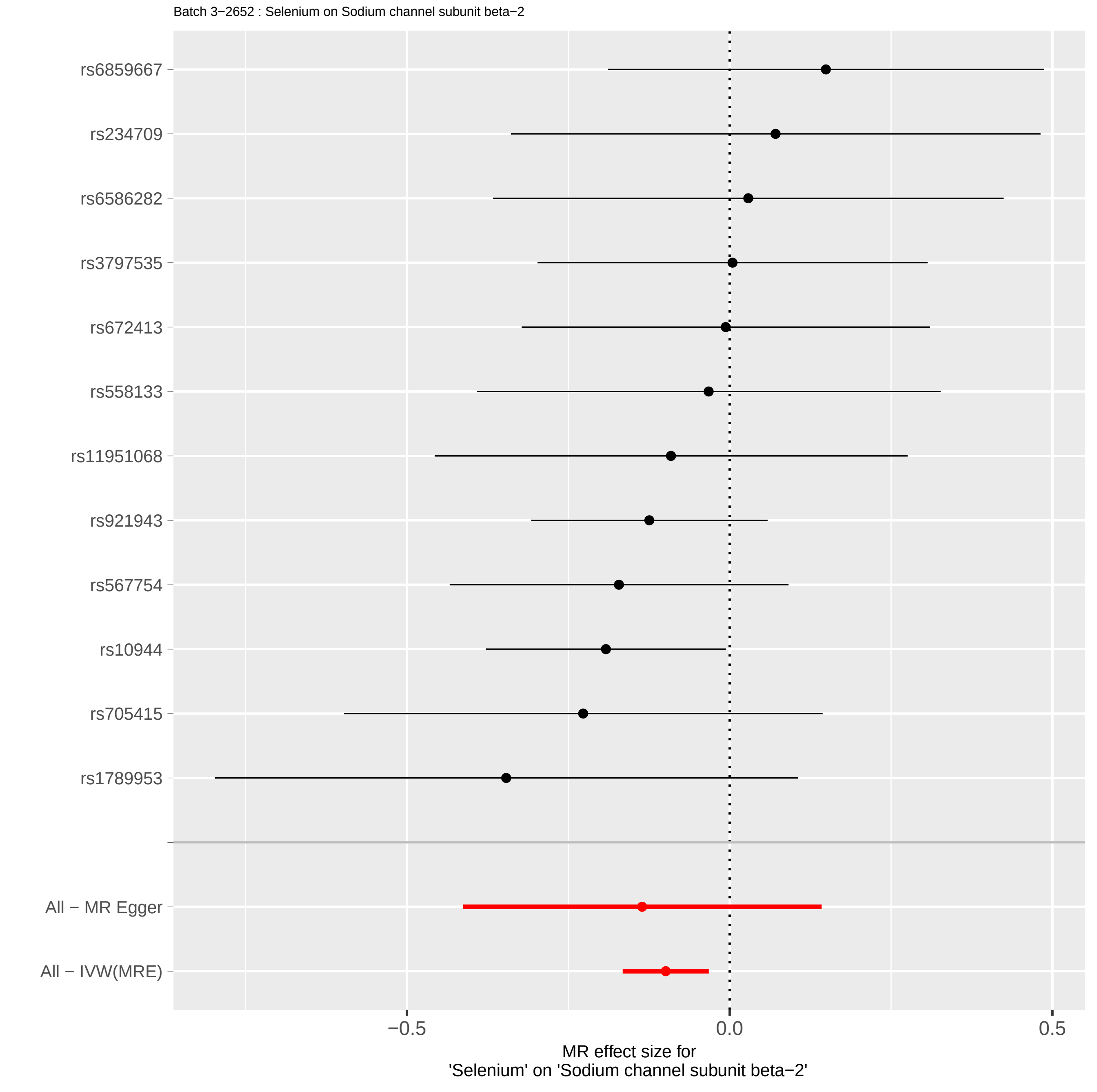

Batch 3−2652 : Selenium on Sodium channel subunit beta−2
rs6859667
rs234709
rs6586282
rs3797535
rs672413
rs558133
rs11951068
rs921943
rs567754
rs10944
rs705415
rs1789953
All − MR Egger
All − IVW(MRE)
−0.5
0.0
0.5
MR effect size for
'Selenium' on 'Sodium channel subunit beta−2'

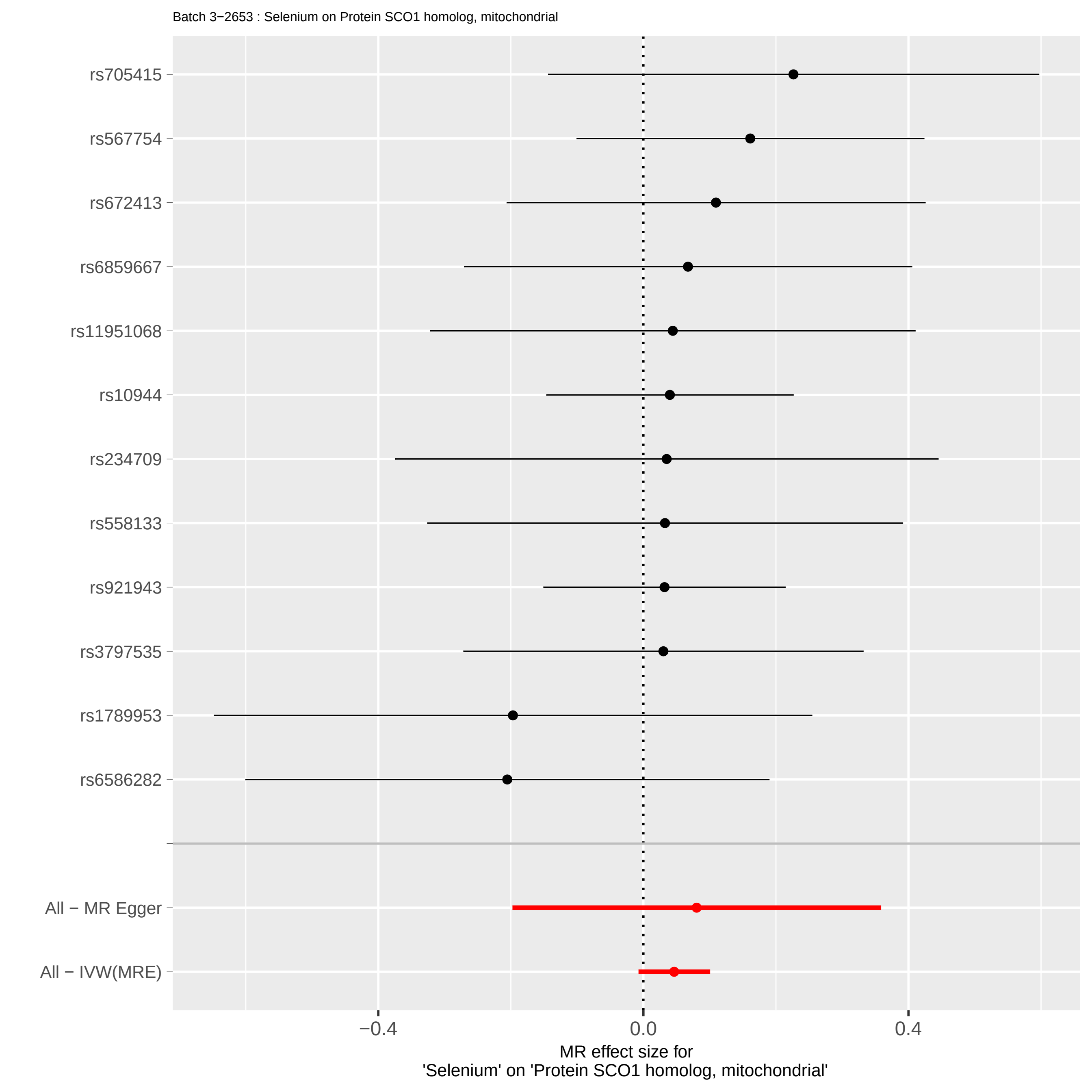

Batch 3−2653 : Selenium on Protein SCO1 homolog, mitochondrial
rs705415
rs567754
rs672413
rs6859667
rs11951068
rs10944
rs234709
rs558133
rs921943
rs3797535
rs1789953
rs6586282
All − MR Egger
All − IVW(MRE)
−0.4
0.0
0.4
MR effect size for
'Selenium' on 'Protein SCO1 homolog, mitochondrial'

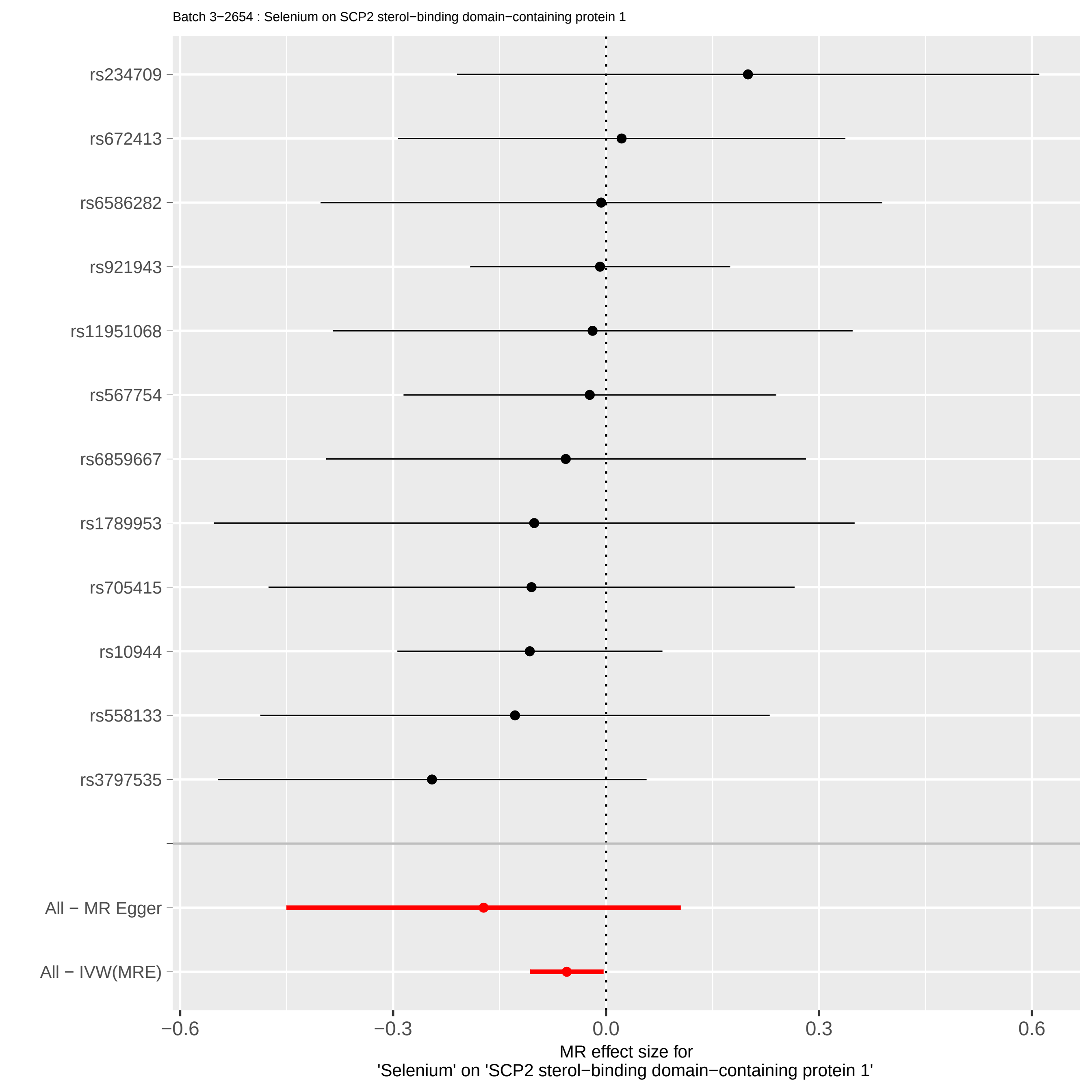

Batch 3−2654 : Selenium on SCP2 sterol−binding domain−containing protein 1
rs234709
rs672413
rs6586282
rs921943
rs11951068
rs567754
rs6859667
rs1789953
rs705415
rs10944
rs558133
rs3797535
All − MR Egger
All − IVW(MRE)
−0.6
−0.3
0.0
0.3
0.6
MR effect size for
'Selenium' on 'SCP2 sterol−binding domain−containing protein 1'

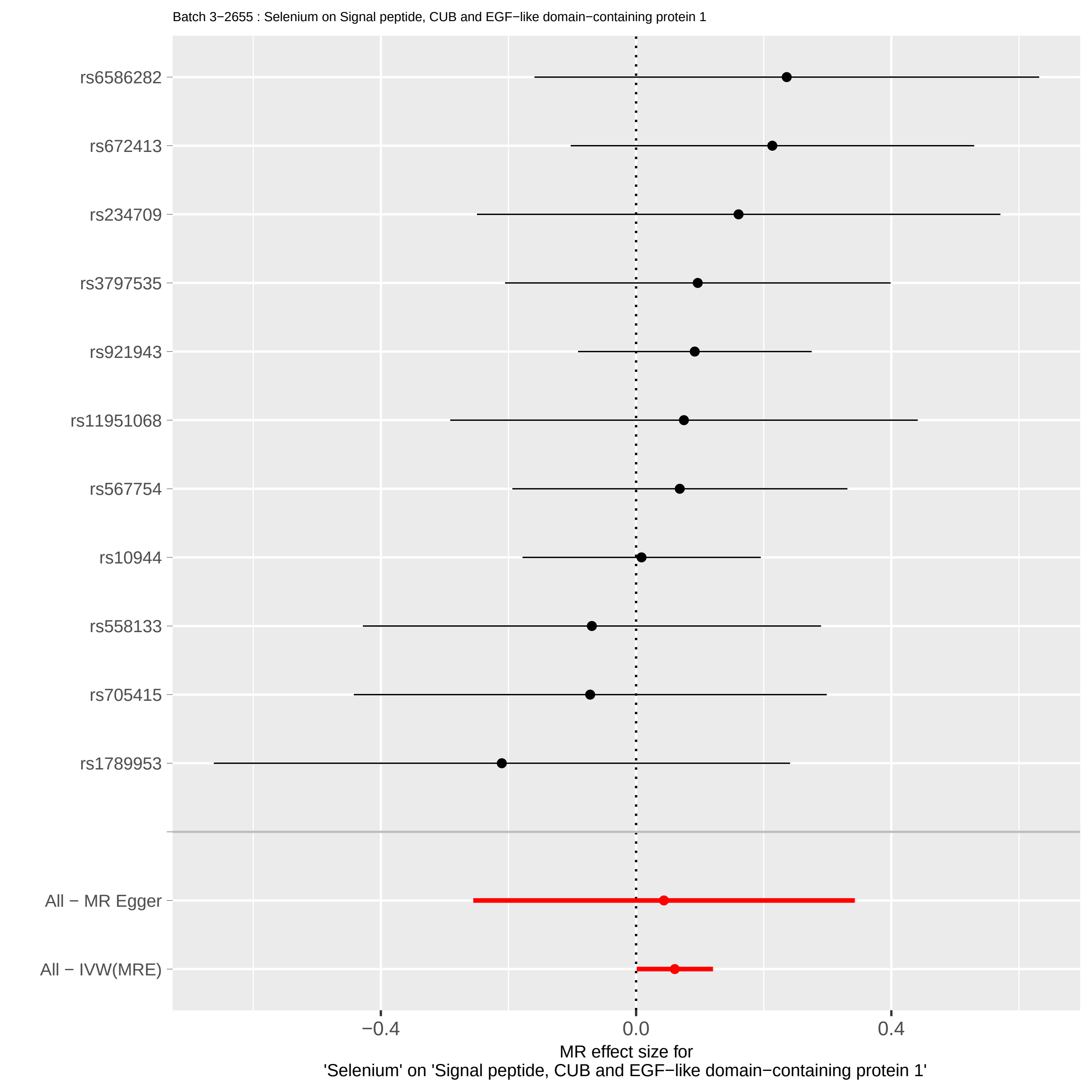

Batch 3−2655 : Selenium on Signal peptide, CUB and EGF−like domain−containing protein 1
rs6586282
rs672413
rs234709
rs3797535
rs921943
rs11951068
rs567754
rs10944
rs558133
rs705415
rs1789953
All − MR Egger
All − IVW(MRE)
−0.4
0.0
0.4
MR effect size for
'Selenium' on 'Signal peptide, CUB and EGF−like domain−containing protein 1'

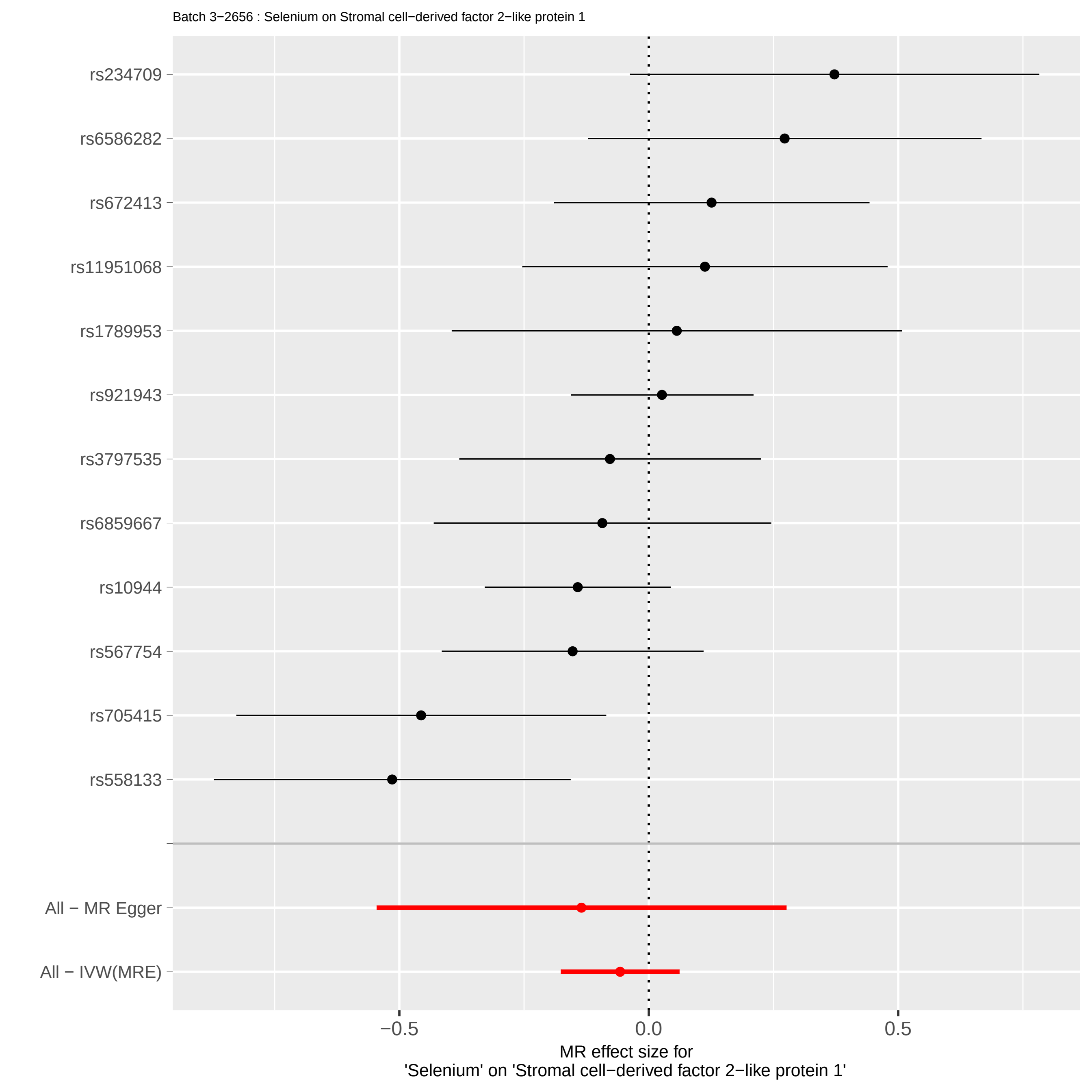

Batch 3−2656 : Selenium on Stromal cell−derived factor 2−like protein 1
rs234709
rs6586282
rs672413
rs11951068
rs1789953
rs921943
rs3797535
rs6859667
rs10944
rs567754
rs705415
rs558133
All − MR Egger
All − IVW(MRE)
−0.5
0.0
0.5
MR effect size for
'Selenium' on 'Stromal cell−derived factor 2−like protein 1'

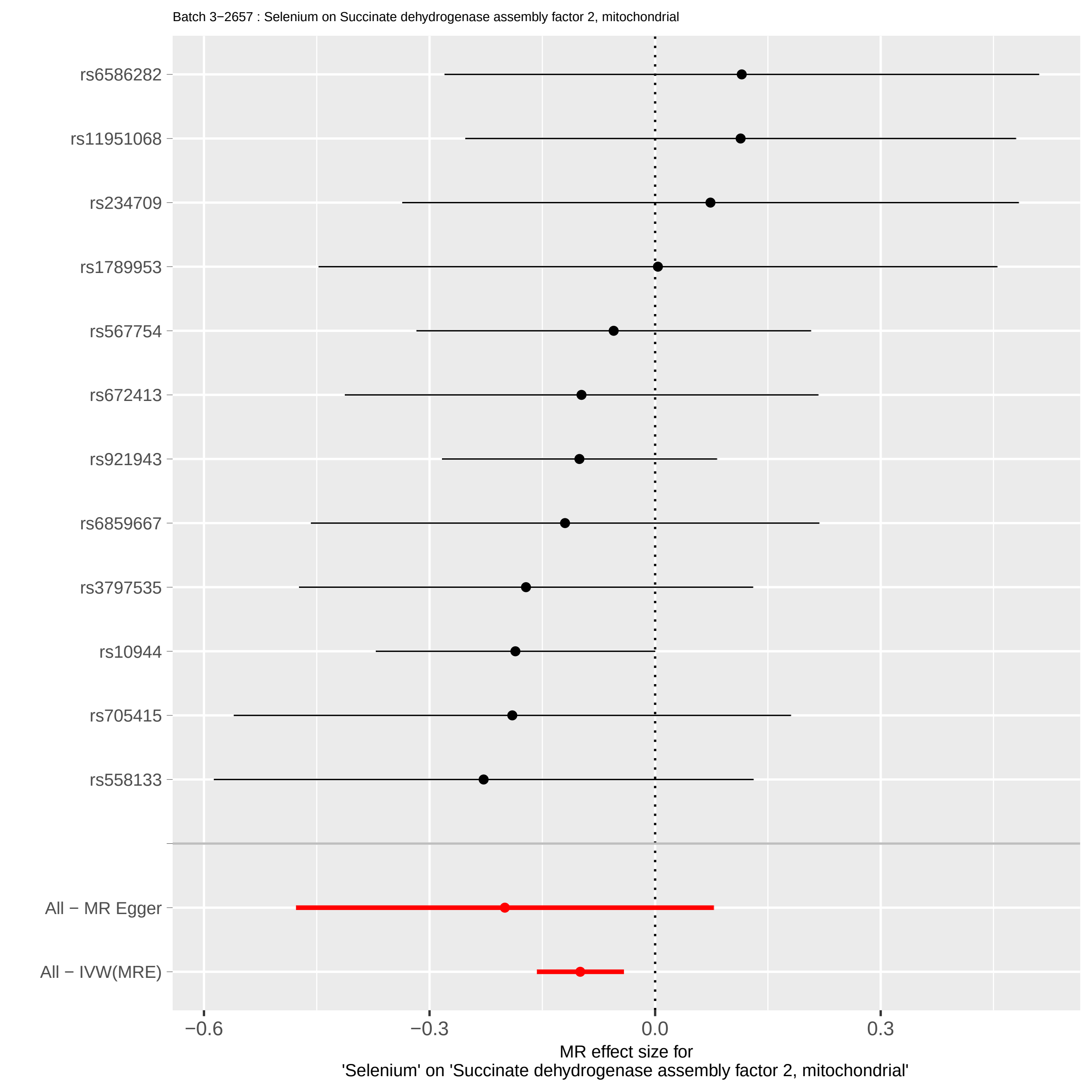

Batch 3−2657 : Selenium on Succinate dehydrogenase assembly factor 2, mitochondrial
rs6586282
rs11951068
rs234709
rs1789953
rs567754
rs672413
rs921943
rs6859667
rs3797535
rs10944
rs705415
rs558133
All − MR Egger
All − IVW(MRE)
−0.6
−0.3
0.0
0.3
MR effect size for
'Selenium' on 'Succinate dehydrogenase assembly factor 2, mitochondrial'

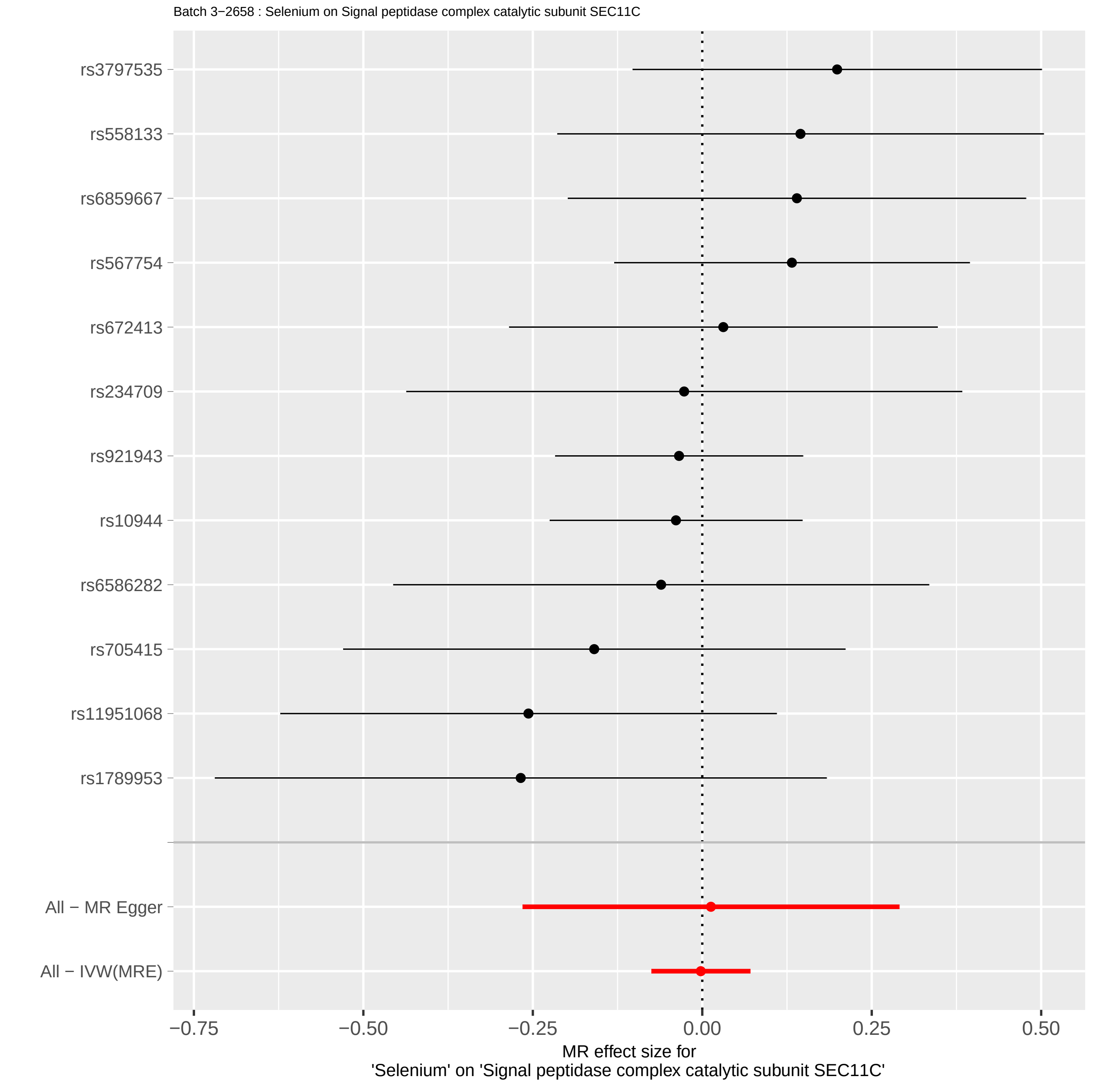

Batch 3−2658 : Selenium on Signal peptidase complex catalytic subunit SEC11C
rs3797535
rs558133
rs6859667
rs567754
rs672413
rs234709
rs921943
rs10944
rs6586282
rs705415
rs11951068
rs1789953
All − MR Egger
All − IVW(MRE)
−0.75
−0.50
−0.25
0.00
0.25
0.50
MR effect size for
'Selenium' on 'Signal peptidase complex catalytic subunit SEC11C'

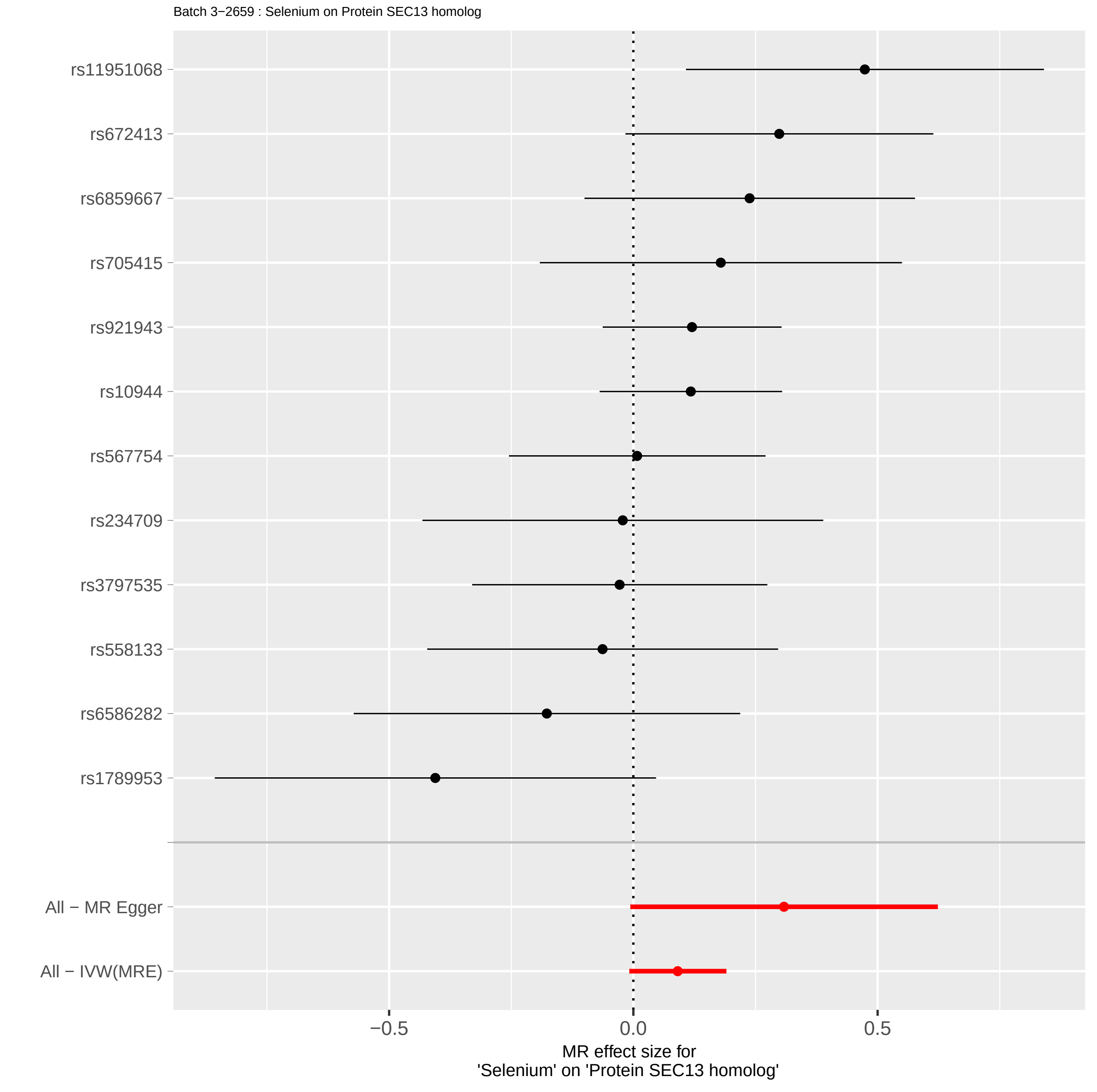

Batch 3−2659 : Selenium on Protein SEC13 homolog
rs11951068
rs672413
rs6859667
rs705415
rs921943
rs10944
rs567754
rs234709
rs3797535
rs558133
rs6586282
rs1789953
All − MR Egger
All − IVW(MRE)
−0.5
0.0
0.5
MR effect size for
'Selenium' on 'Protein SEC13 homolog'

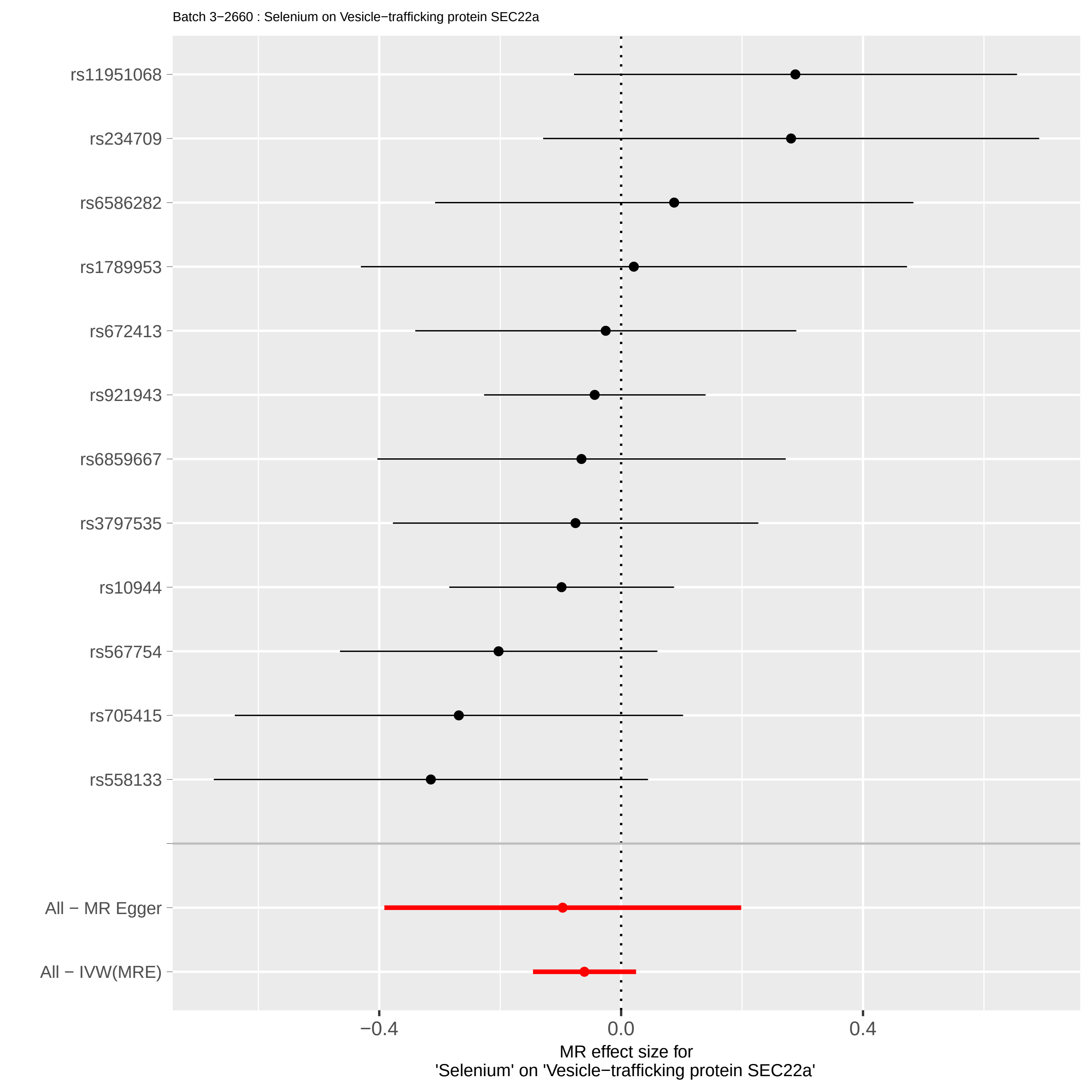

Batch 3−2660 : Selenium on Vesicle−trafficking protein SEC22a
rs11951068
rs234709
rs6586282
rs1789953
rs672413
rs921943
rs6859667
rs3797535
rs10944
rs567754
rs705415
rs558133
All − MR Egger
All − IVW(MRE)
−0.4
0.0
0.4
MR effect size for
'Selenium' on 'Vesicle−trafficking protein SEC22a'

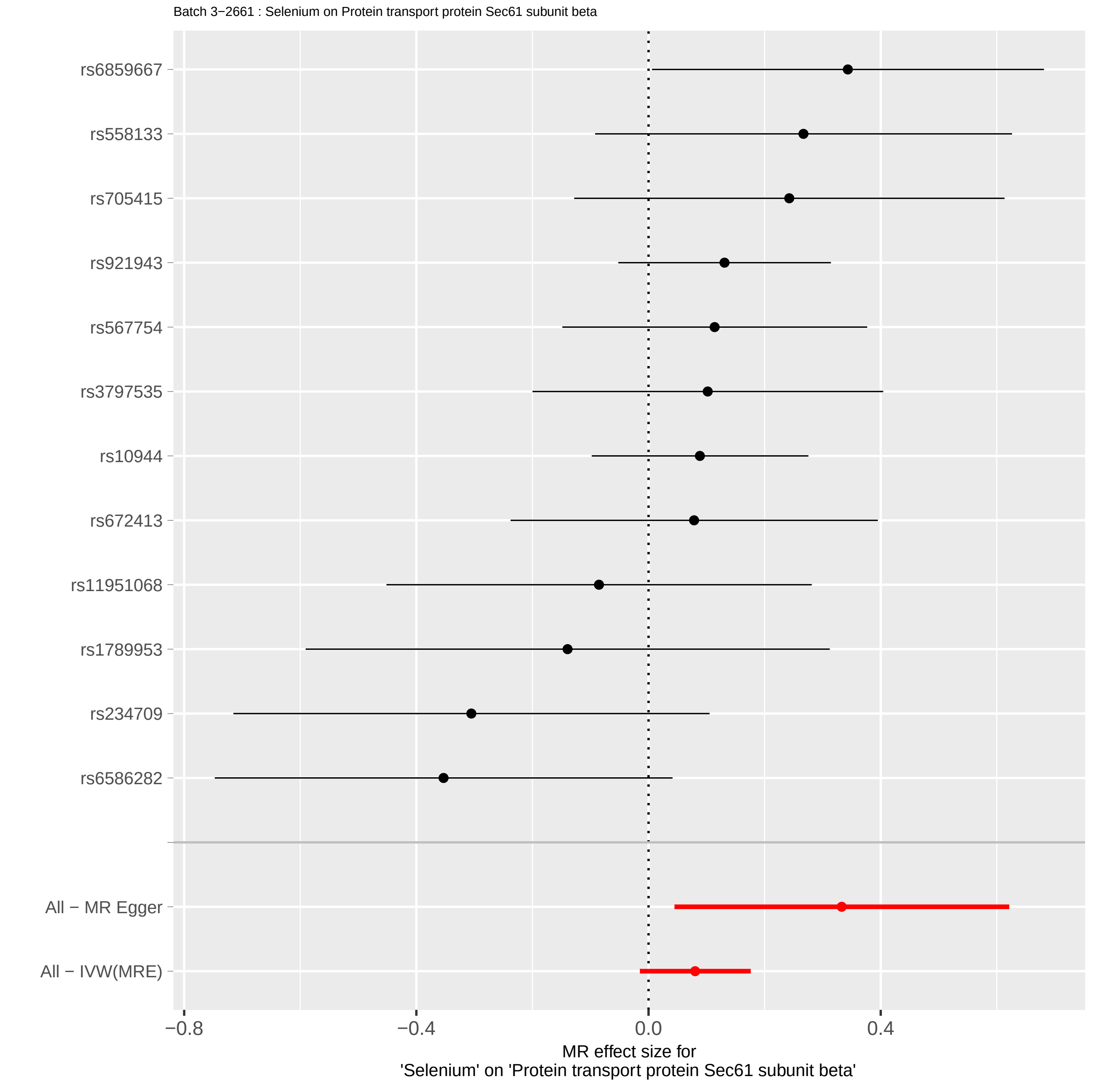

Batch 3−2661 : Selenium on Protein transport protein Sec61 subunit beta
rs6859667
rs558133
rs705415
rs921943
rs567754
rs3797535
rs10944
rs672413
rs11951068
rs1789953
rs234709
rs6586282
All − MR Egger
All − IVW(MRE)
−0.8
−0.4
0.0
0.4
MR effect size for
'Selenium' on 'Protein transport protein Sec61 subunit beta'

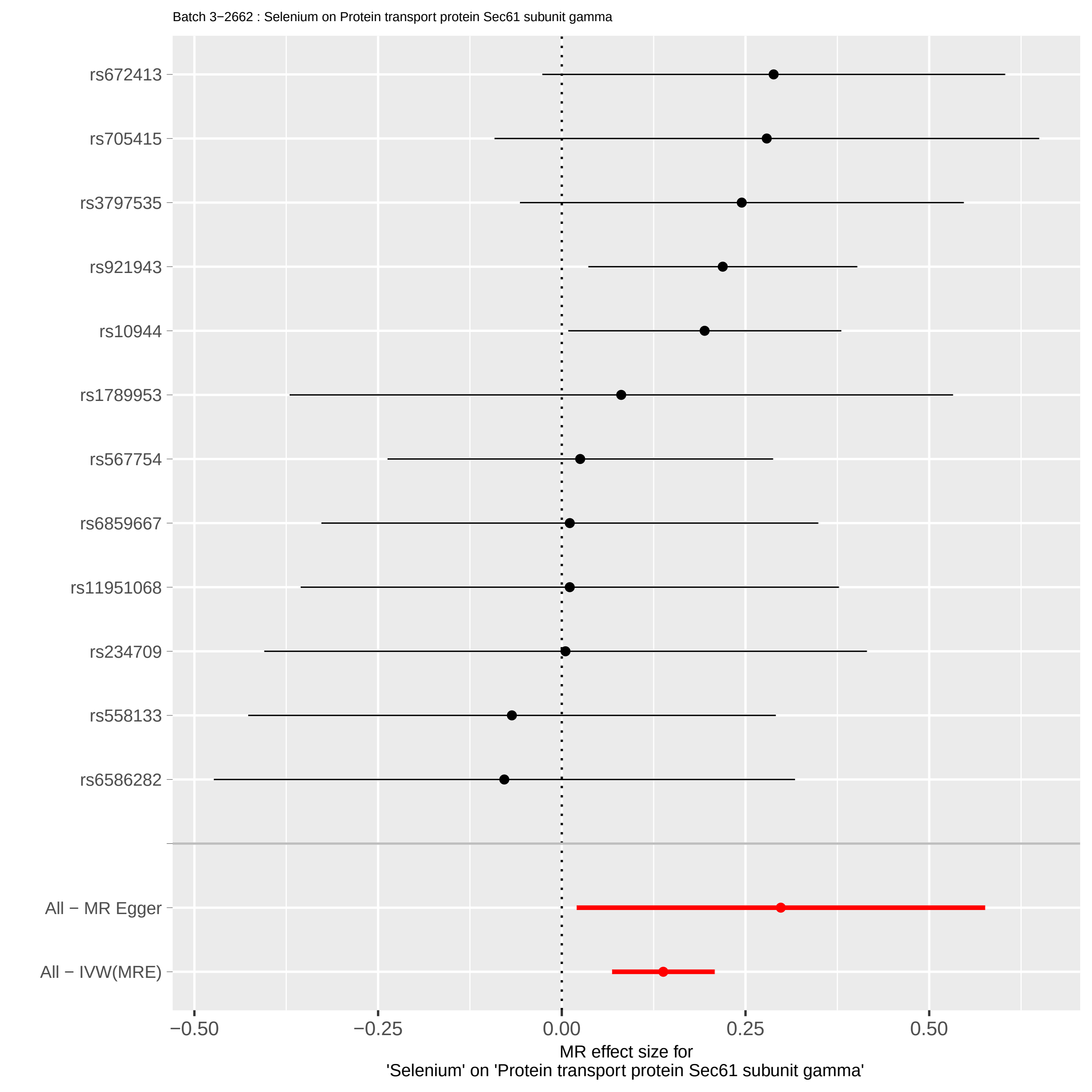

Batch 3−2662 : Selenium on Protein transport protein Sec61 subunit gamma
rs672413
rs705415
rs3797535
rs921943
rs10944
rs1789953
rs567754
rs6859667
rs11951068
rs234709
rs558133
rs6586282
All − MR Egger
All − IVW(MRE)
−0.50
−0.25
0.00
0.25
0.50
MR effect size for
'Selenium' on 'Protein transport protein Sec61 subunit gamma'

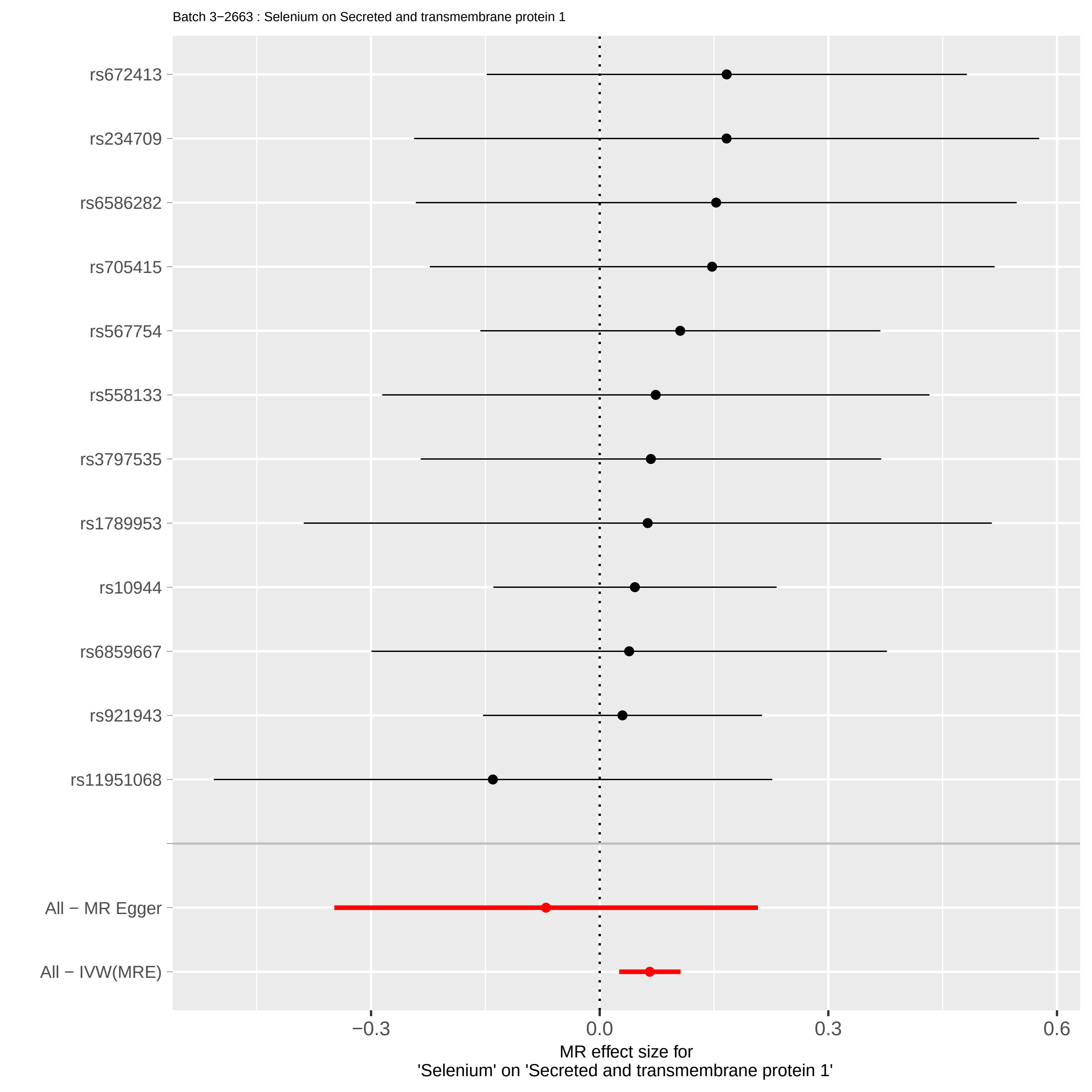

Batch 3−2663 : Selenium on Secreted and transmembrane protein 1
rs672413
rs234709
rs6586282
rs705415
rs567754
rs558133
rs3797535
rs1789953
rs10944
rs6859667
rs921943
rs11951068
All − MR Egger
All − IVW(MRE)
−0.3
0.0
0.3
0.6
MR effect size for
'Selenium' on 'Secreted and transmembrane protein 1'

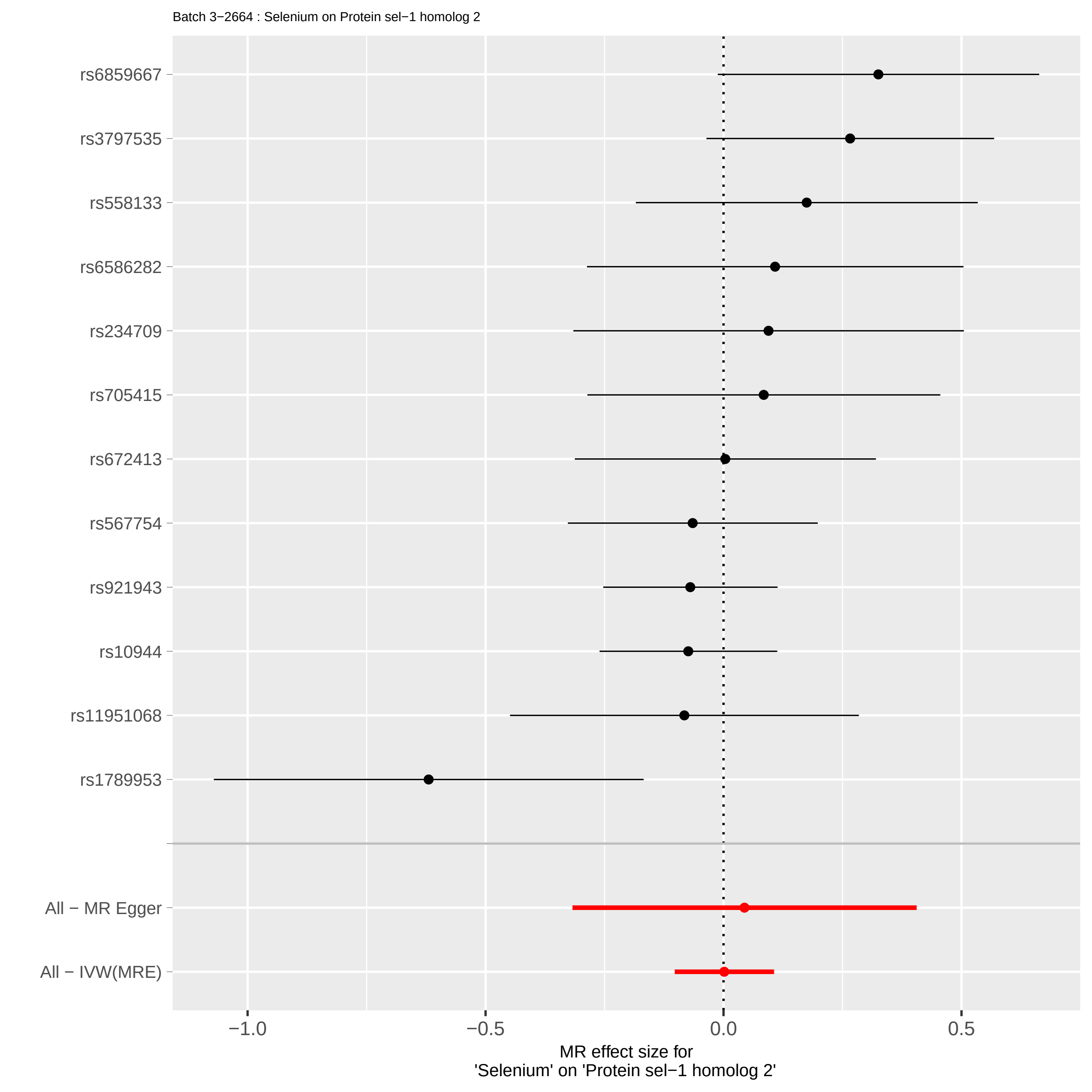
Batch 3−2664 : Selenium on Protein sel−1 homolog 2
rs6859667
rs3797535
rs558133
rs6586282
rs234709
rs705415
rs672413
rs567754
rs921943
rs10944
rs11951068
rs1789953
All − MR Egger
All − IVW(MRE)
−1.0
−0.5
0.0
0.5
MR effect size for
'Selenium' on 'Protein sel−1 homolog 2'

Batch 3−2665 : Selenium on E−selectin

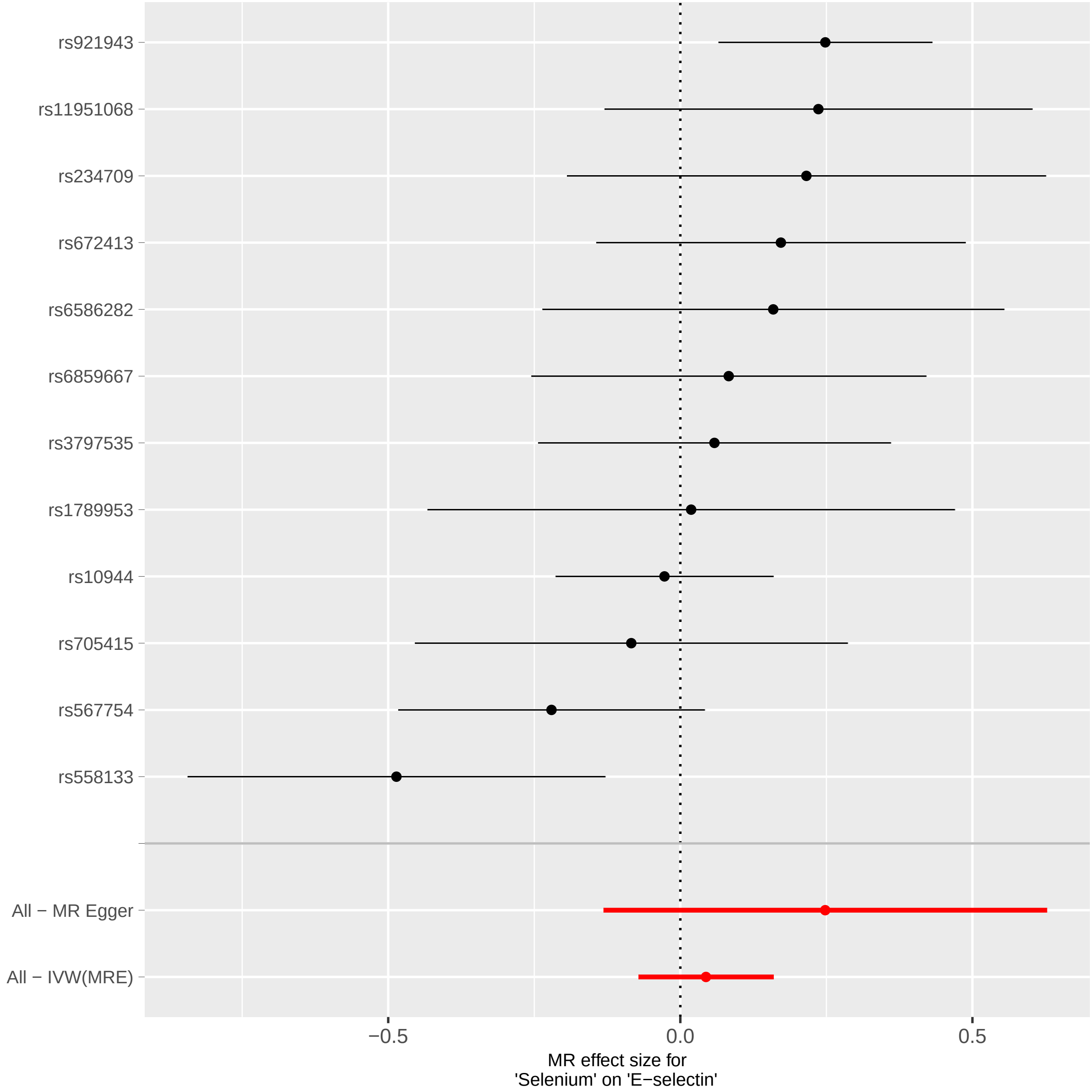

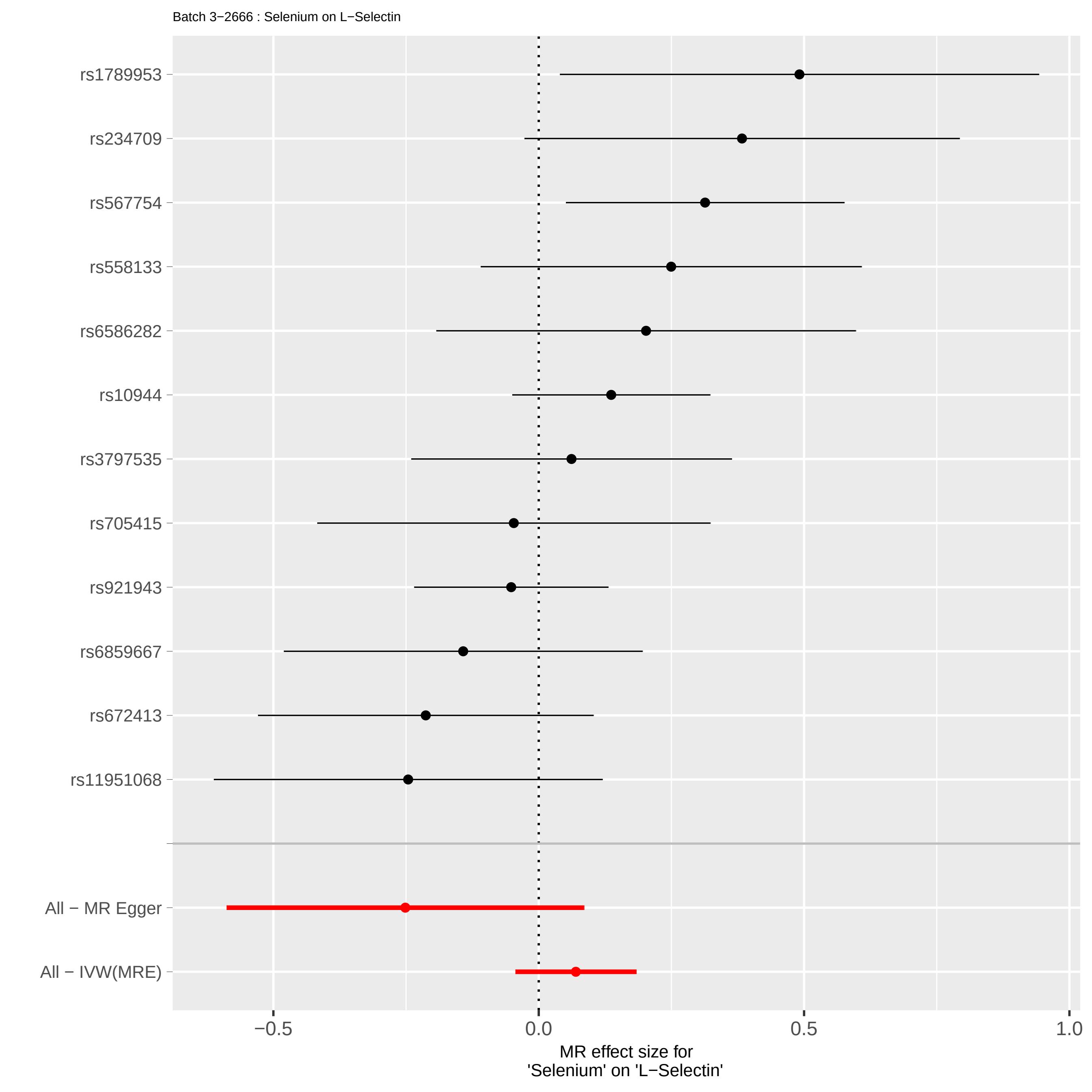

Batch 3−2666 : Selenium on L−Selectin
rs1789953
rs234709
rs567754
rs558133
rs6586282
rs10944
rs3797535
rs705415
rs921943
rs6859667
rs672413
rs11951068
All − MR Egger
All − IVW(MRE)
−0.5
0.0
0.5
1.0
MR effect size for
'Selenium' on 'L−Selectin'

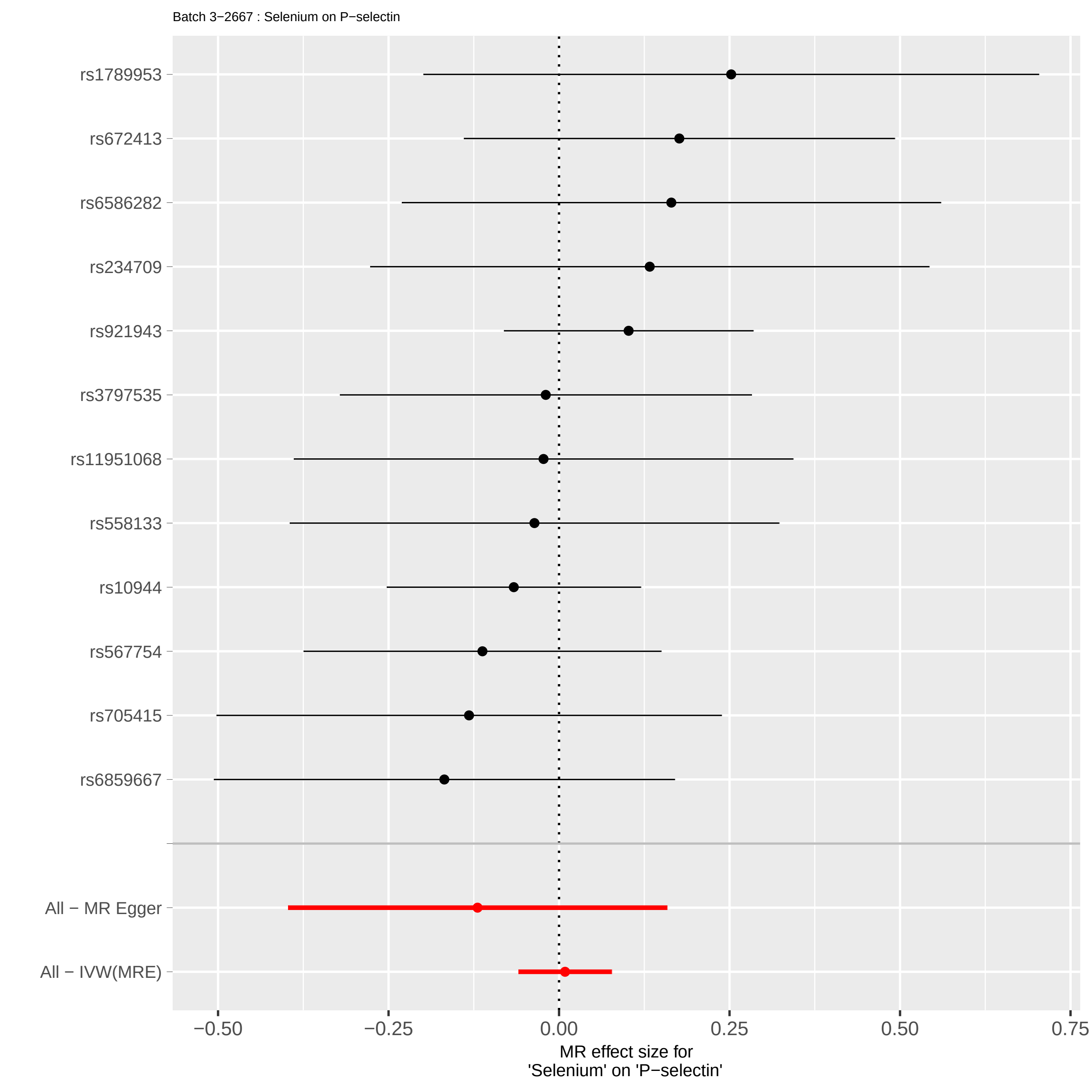

Batch 3−2667 : Selenium on P−selectin
rs1789953
rs672413
rs6586282
rs234709
rs921943
rs3797535
rs11951068
rs558133
rs10944
rs567754
rs705415
rs6859667
All − MR Egger
All − IVW(MRE)
−0.50
−0.25
0.00
0.25
0.50
0.75
MR effect size for
'Selenium' on 'P−selectin'

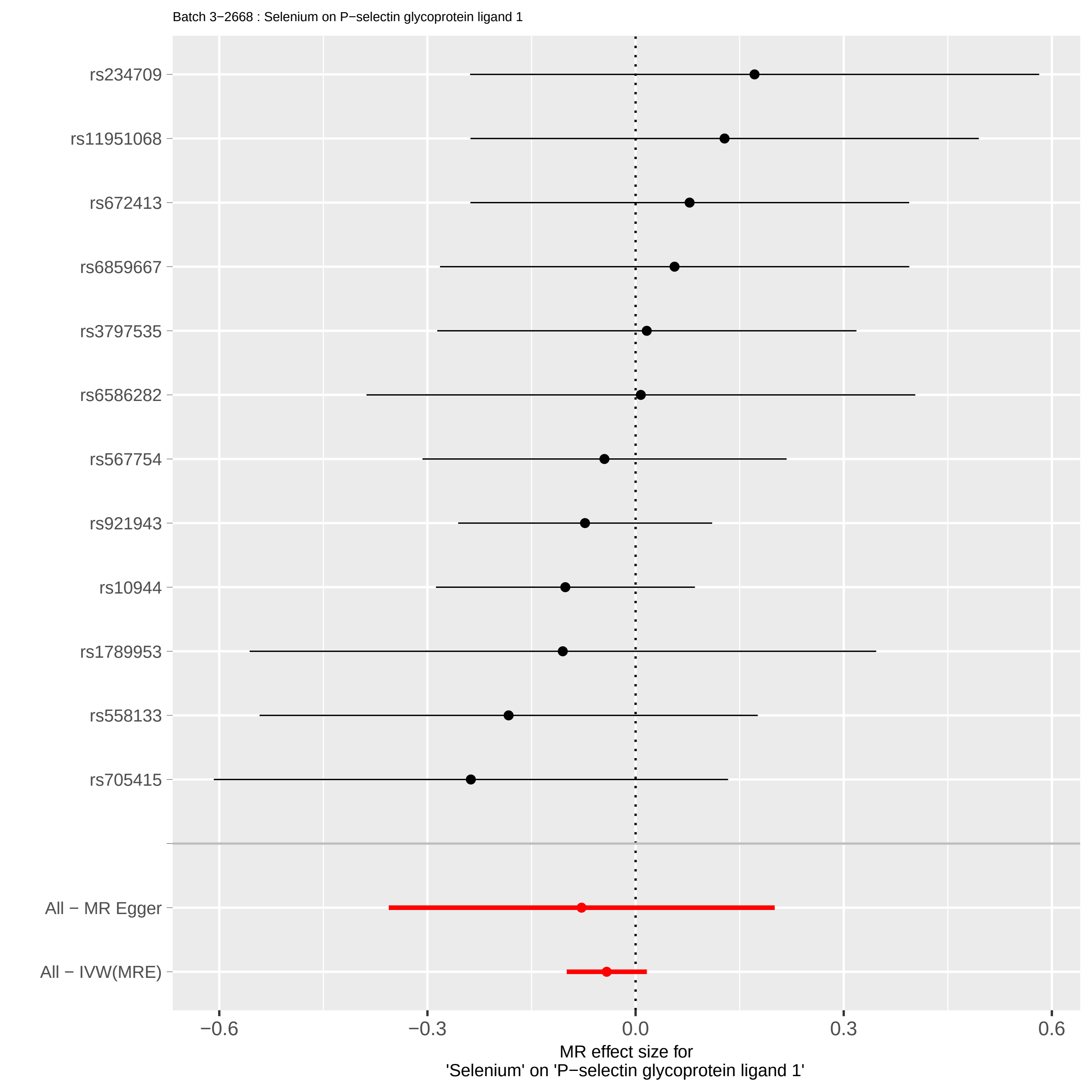
Batch 3−2668 : Selenium on P−selectin glycoprotein ligand 1
rs234709
rs11951068
rs672413
rs6859667
rs3797535
rs6586282
rs567754
rs921943
rs10944
rs1789953
rs558133
rs705415
All − MR Egger
All − IVW(MRE)
−0.6
−0.3
0.0
0.3
0.6
MR effect size for
'Selenium' on 'P−selectin glycoprotein ligand 1'

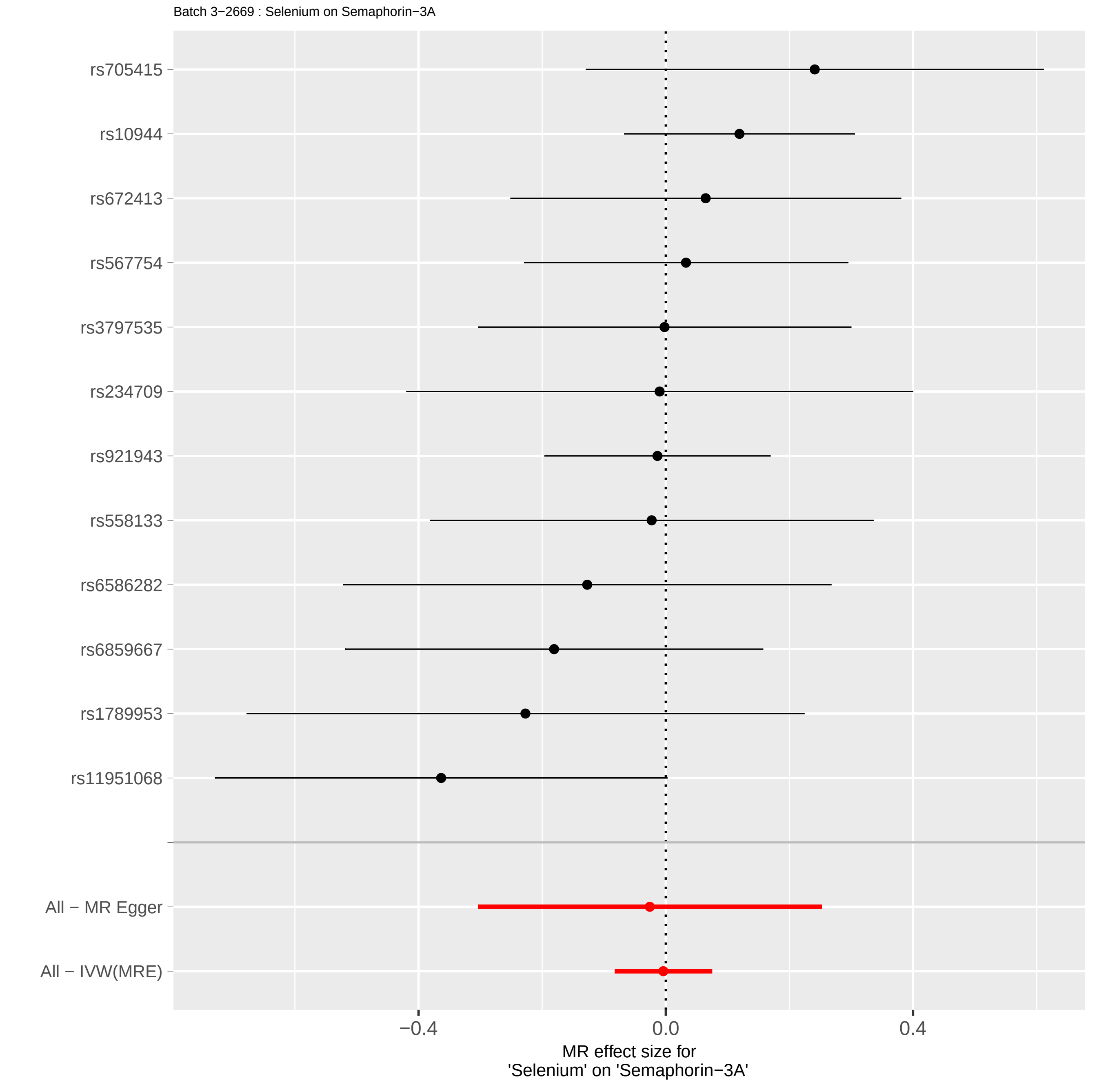

Batch 3−2669 : Selenium on Semaphorin−3A
rs705415
rs10944
rs672413
rs567754
rs3797535
rs234709
rs921943
rs558133
rs6586282
rs6859667
rs1789953
rs11951068
All − MR Egger
All − IVW(MRE)
−0.4
0.0
0.4
MR effect size for
'Selenium' on 'Semaphorin−3A'

Batch 3−2670 : Selenium on Semaphorin−3B

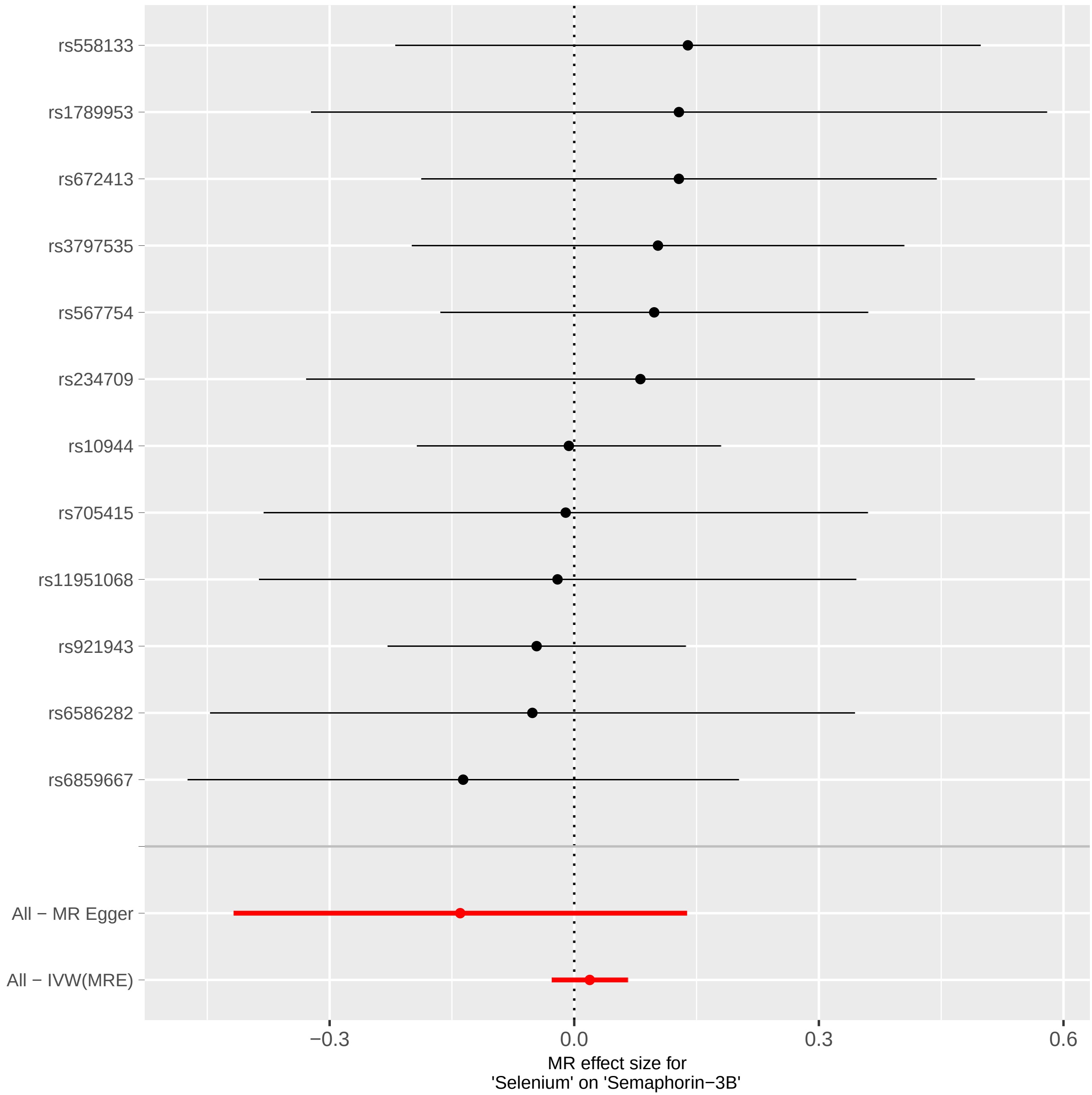

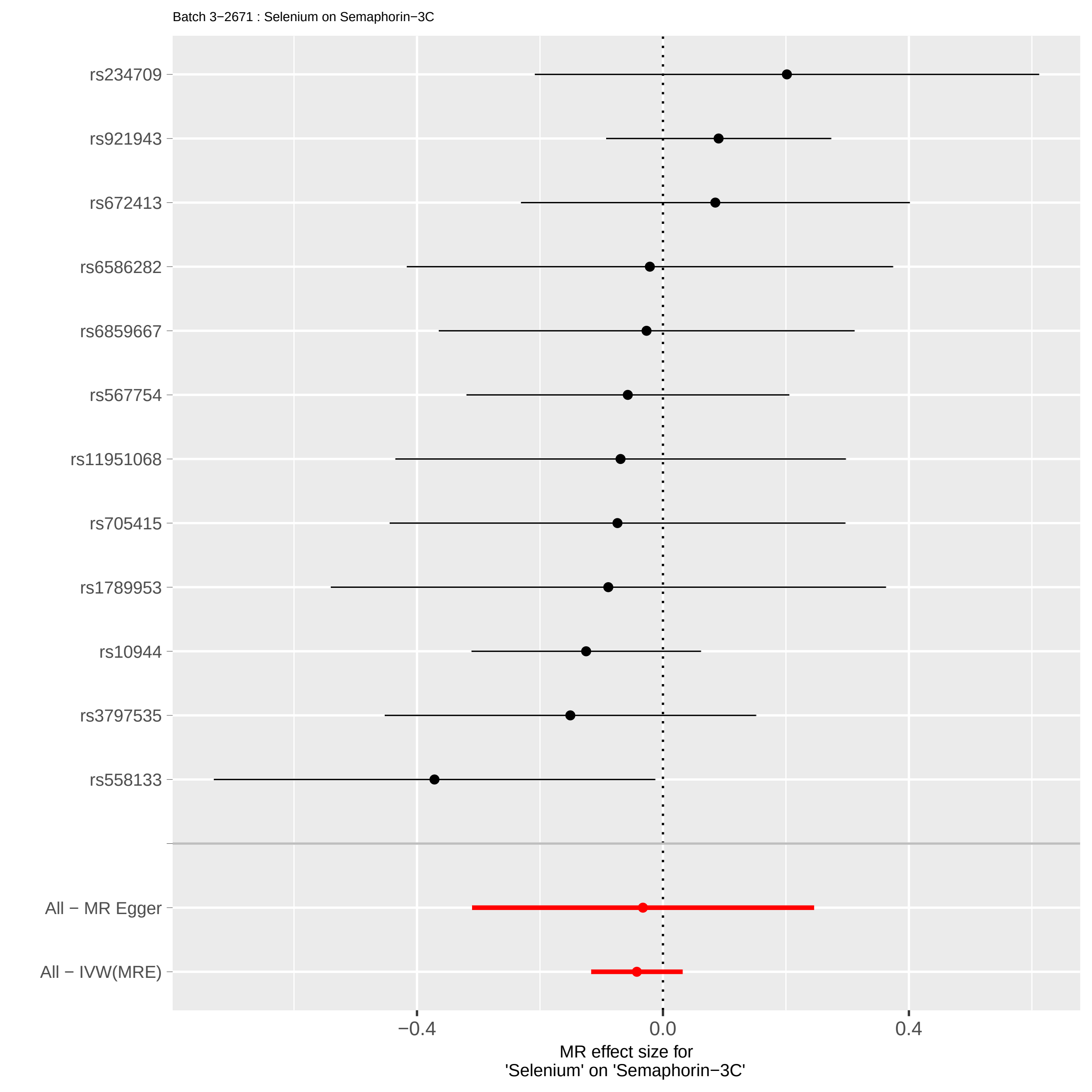
Batch 3−2671 : Selenium on Semaphorin−3C
rs234709
rs921943
rs672413
rs6586282
rs6859667
rs567754
rs11951068
rs705415
rs1789953
rs10944
rs3797535
rs558133
All − MR Egger
All − IVW(MRE)
−0.4
0.0
0.4
MR effect size for
'Selenium' on 'Semaphorin−3C'

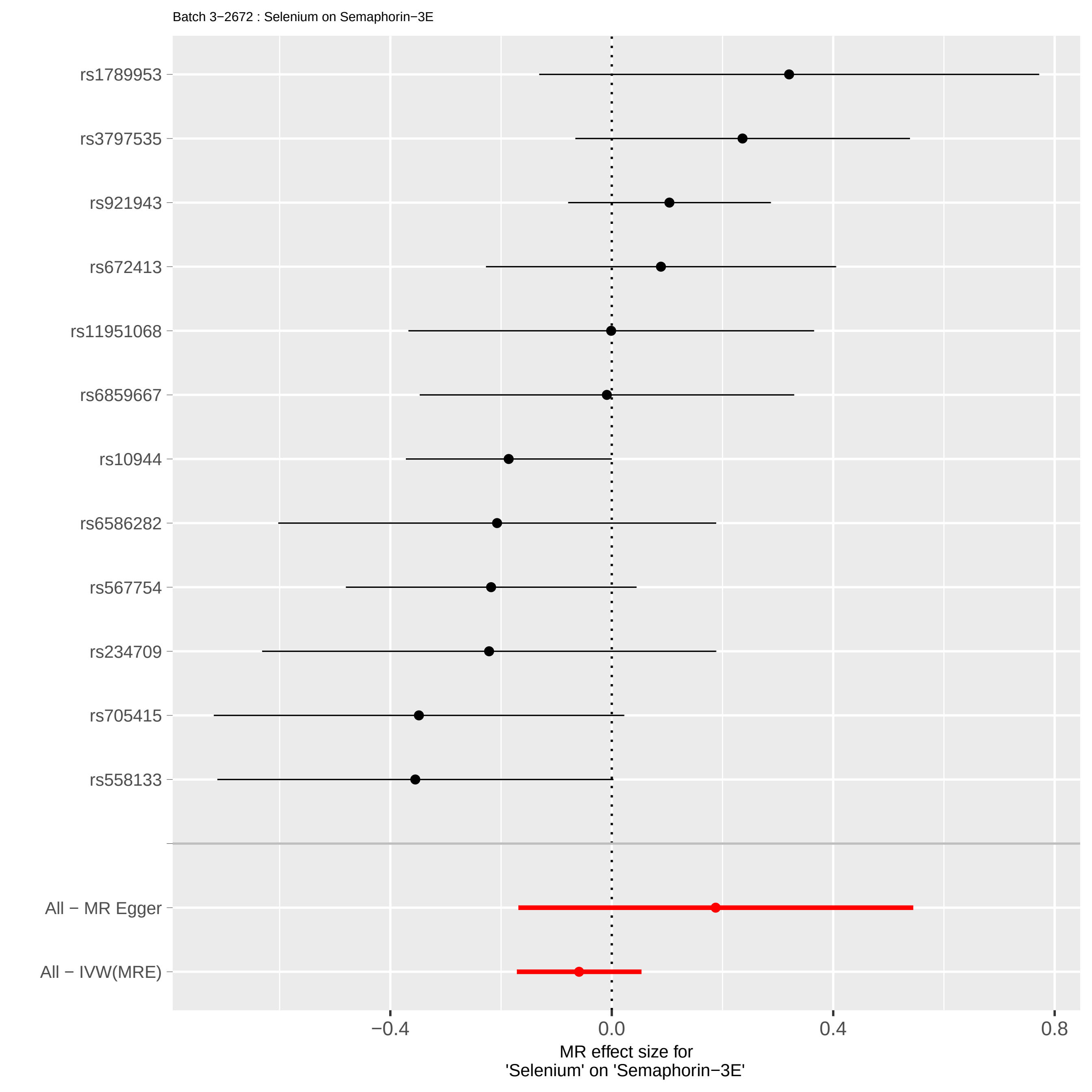

Batch 3−2672 : Selenium on Semaphorin−3E
rs1789953
rs3797535
rs921943
rs672413
rs11951068
rs6859667
rs10944
rs6586282
rs567754
rs234709
rs705415
rs558133
All − MR Egger
All − IVW(MRE)
−0.4
0.0
0.4
0.8
MR effect size for
'Selenium' on 'Semaphorin−3E'

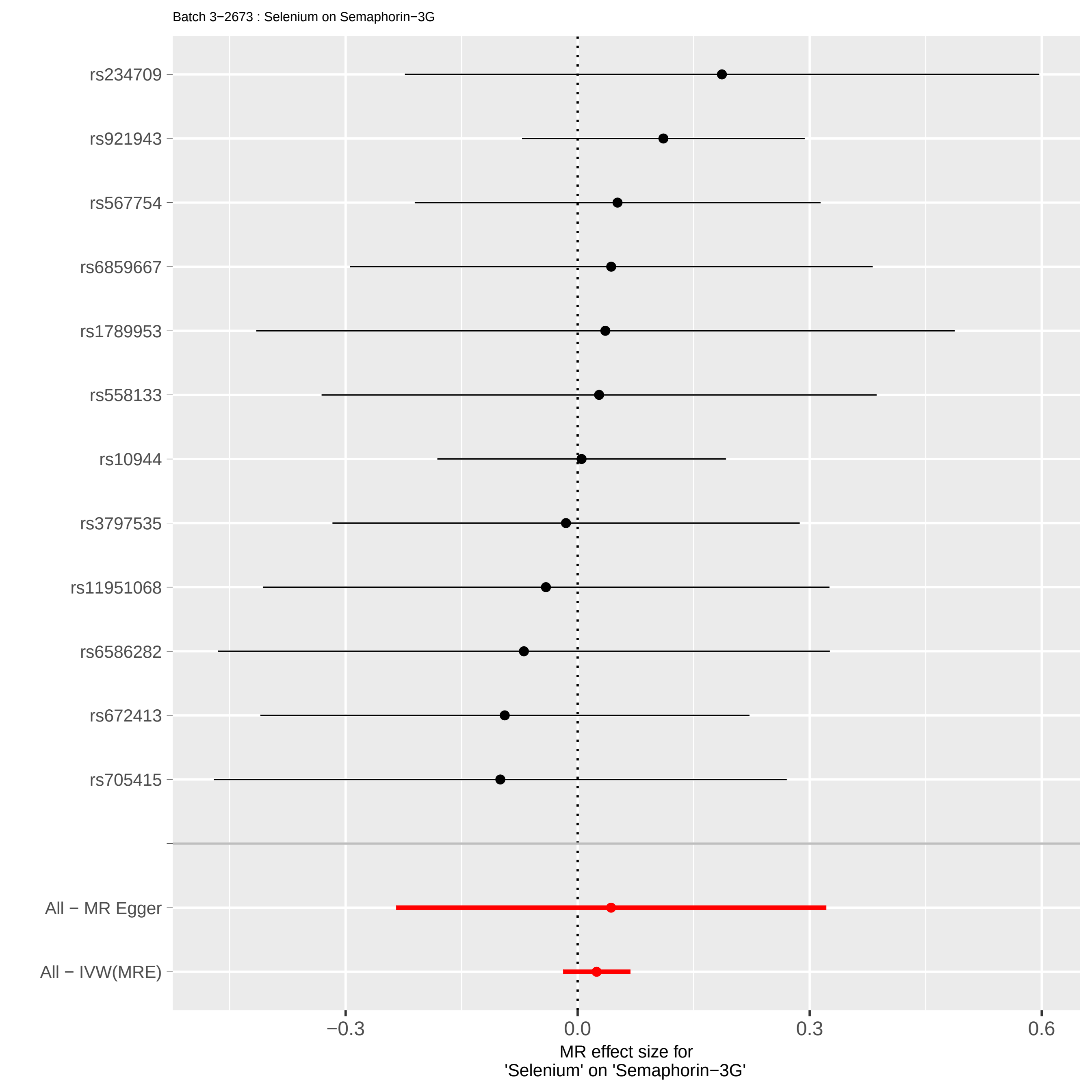
Batch 3−2673 : Selenium on Semaphorin−3G
rs234709
rs921943
rs567754
rs6859667
rs1789953
rs558133
rs10944
rs3797535
rs11951068
rs6586282
rs672413
rs705415
All − MR Egger
All − IVW(MRE)
−0.3
0.0
0.3
0.6
MR effect size for
'Selenium' on 'Semaphorin−3G'

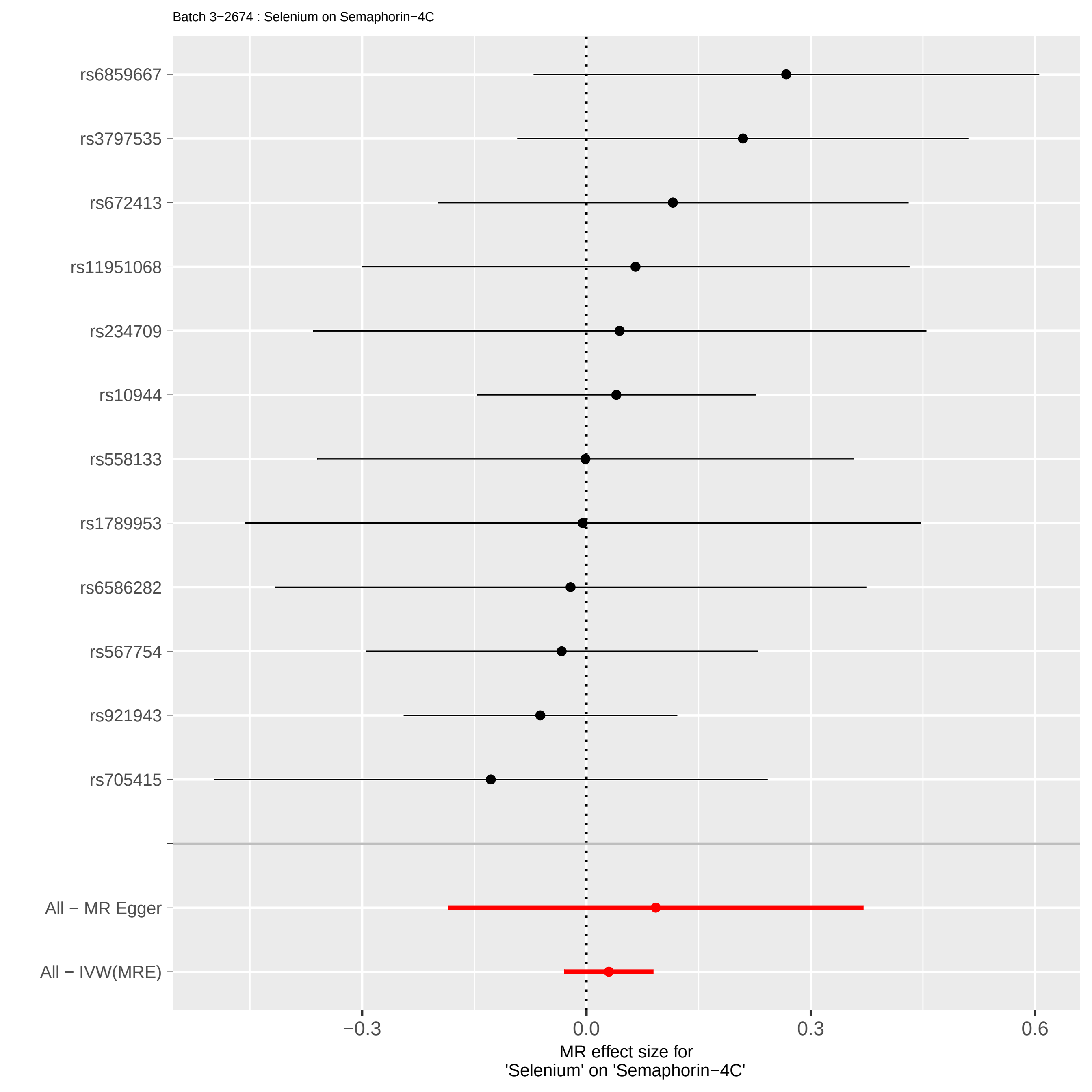

Batch 3−2674 : Selenium on Semaphorin−4C
rs6859667
rs3797535
rs672413
rs11951068
rs234709
rs10944
rs558133
rs1789953
rs6586282
rs567754
rs921943
rs705415
All − MR Egger
All − IVW(MRE)
−0.3
0.0
0.3
0.6
MR effect size for
'Selenium' on 'Semaphorin−4C'

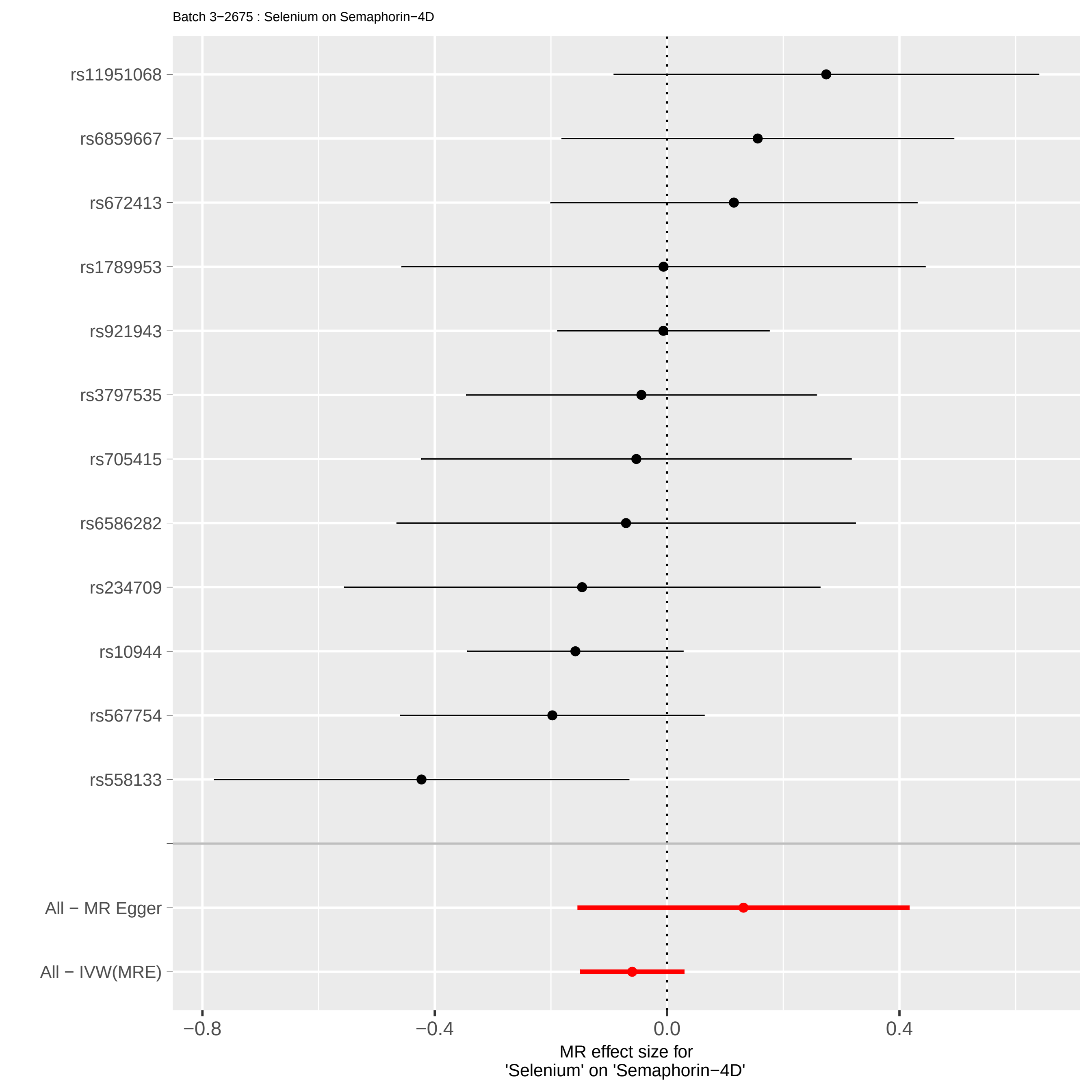

Batch 3−2675 : Selenium on Semaphorin−4D
rs11951068
rs6859667
rs672413
rs1789953
rs921943
rs3797535
rs705415
rs6586282
rs234709
rs10944
rs567754
rs558133
All − MR Egger
All − IVW(MRE)
−0.8
−0.4
0.0
0.4
MR effect size for
'Selenium' on 'Semaphorin−4D'

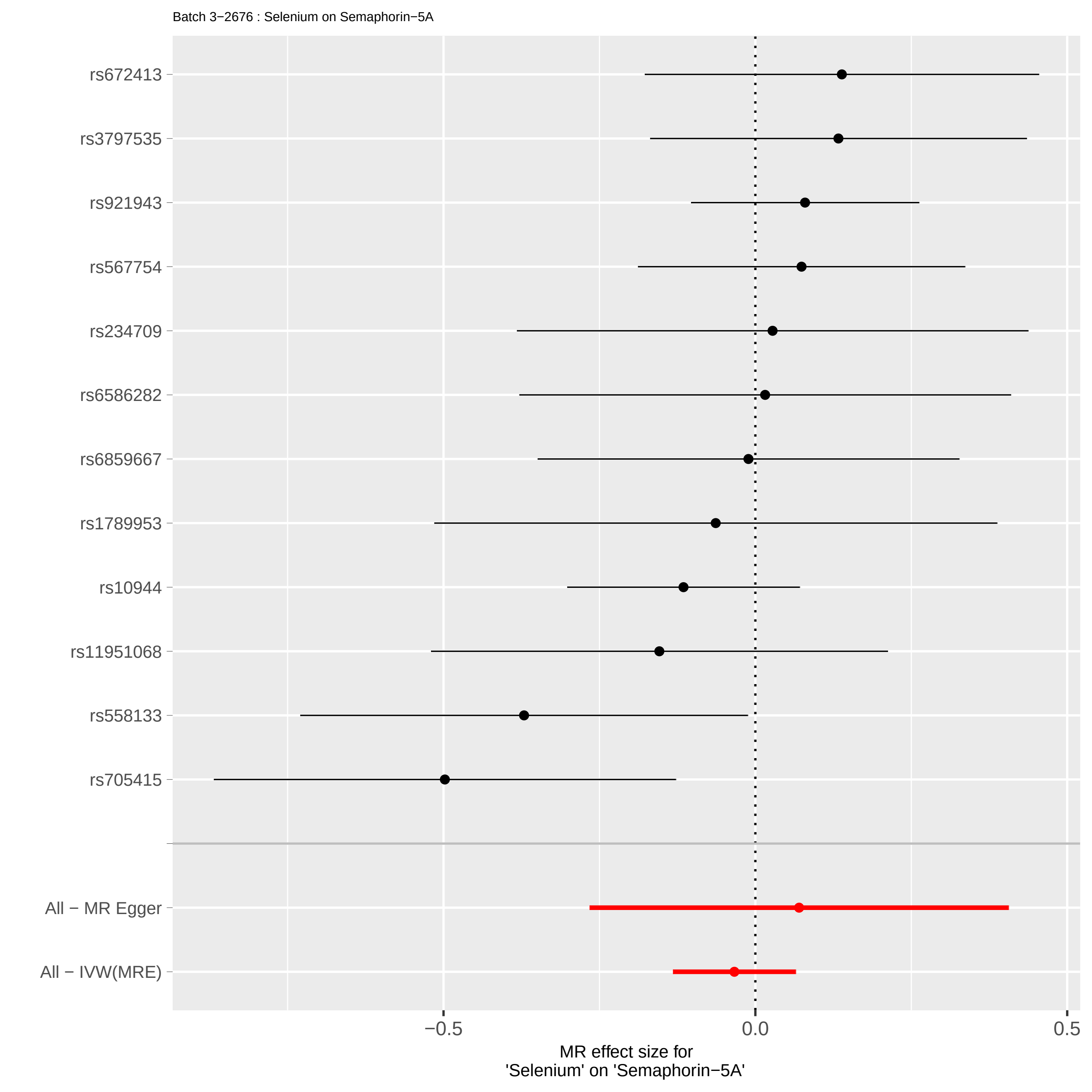

Batch 3−2676 : Selenium on Semaphorin−5A
rs672413
rs3797535
rs921943
rs567754
rs234709
rs6586282
rs6859667
rs1789953
rs10944
rs11951068
rs558133
rs705415
All − MR Egger
All − IVW(MRE)
−0.5
0.0
0.5
MR effect size for
'Selenium' on 'Semaphorin−5A'

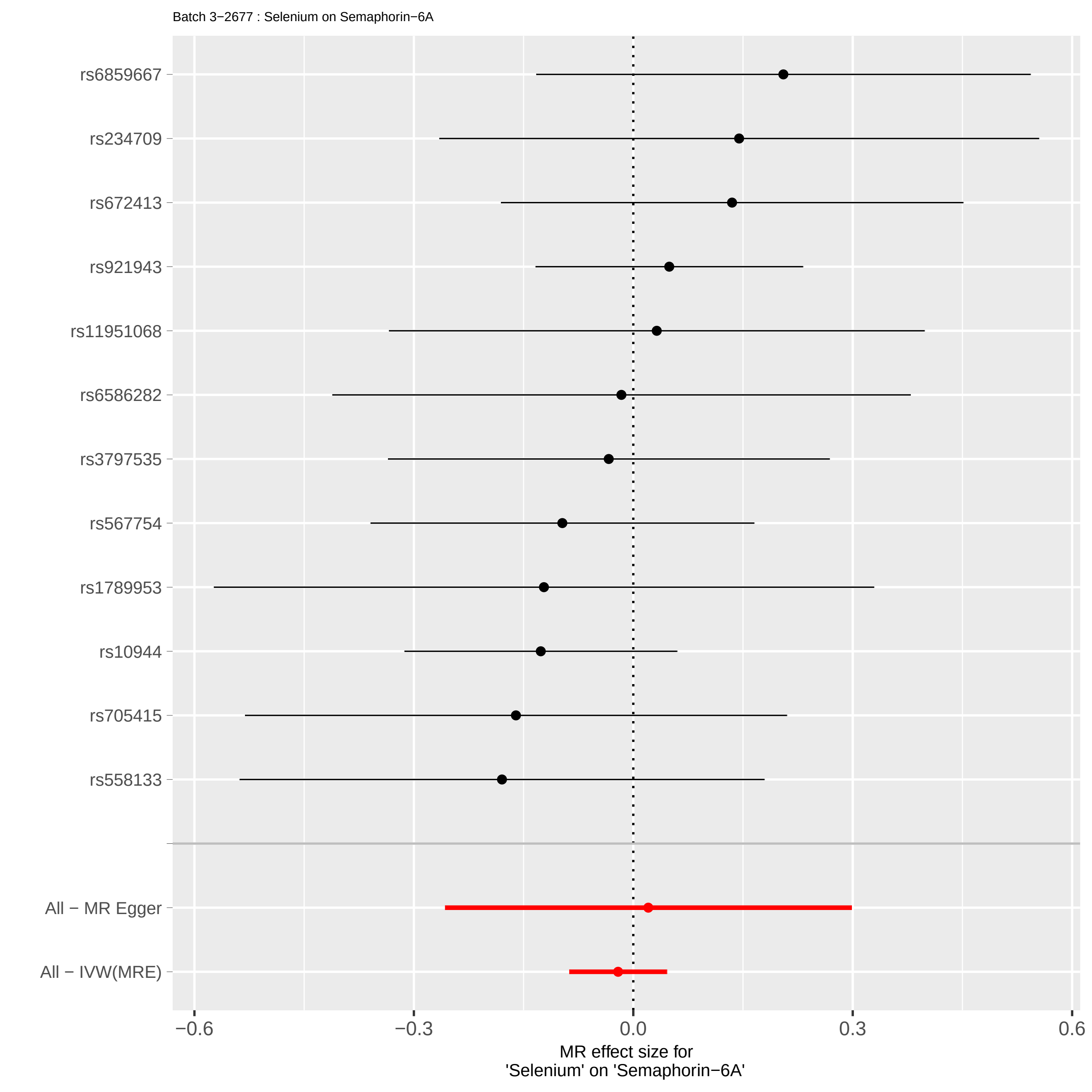

Batch 3−2677 : Selenium on Semaphorin−6A
rs6859667
rs234709
rs672413
rs921943
rs11951068
rs6586282
rs3797535
rs567754
rs1789953
rs10944
rs705415
rs558133
All − MR Egger
All − IVW(MRE)
−0.6
−0.3
0.0
0.3
0.6
MR effect size for
'Selenium' on 'Semaphorin−6A'

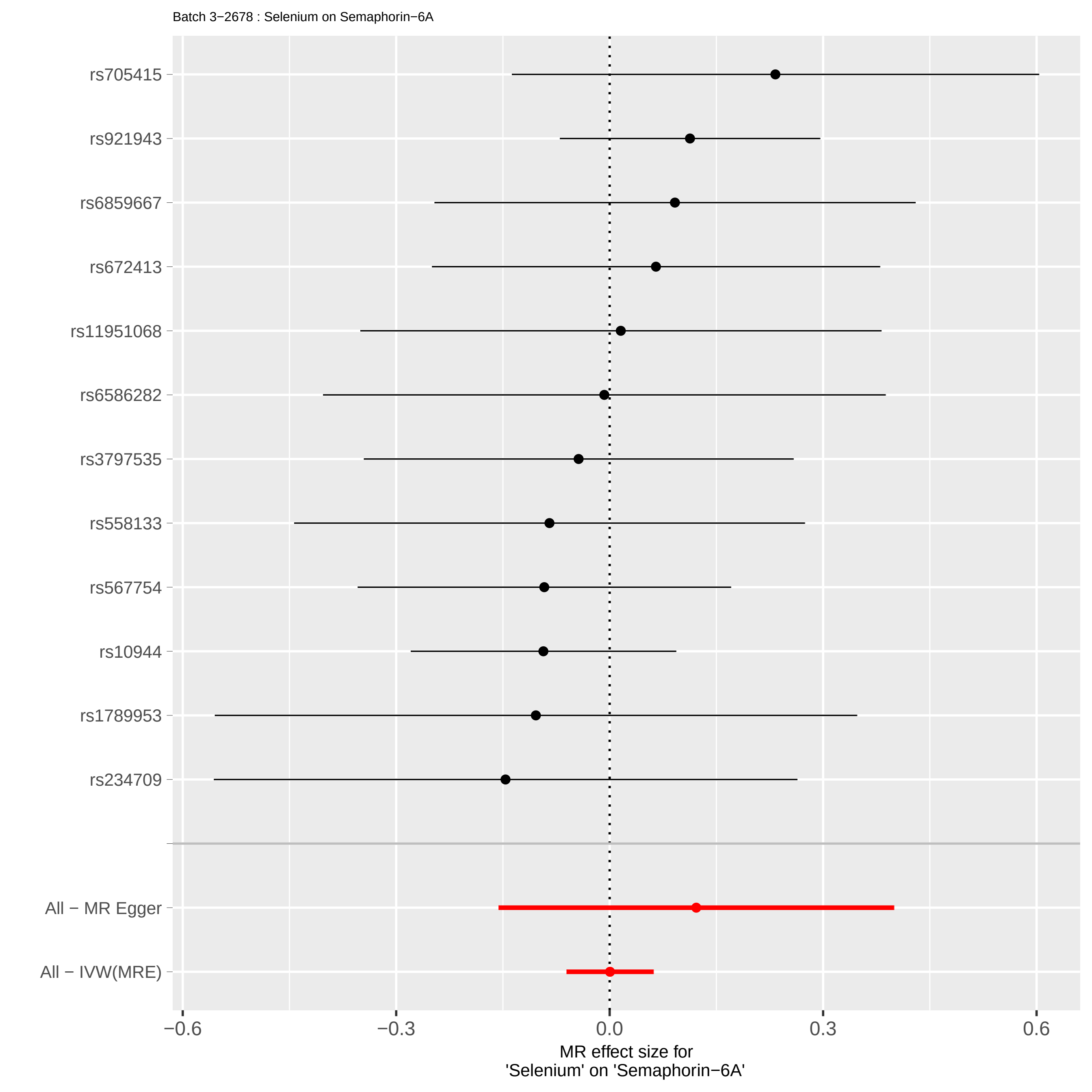

Batch 3−2678 : Selenium on Semaphorin−6A
rs705415
rs921943
rs6859667
rs672413
rs11951068
rs6586282
rs3797535
rs558133
rs567754
rs10944
rs1789953
rs234709
All − MR Egger
All − IVW(MRE)
−0.6
−0.3
0.0
0.3
0.6
MR effect size for
'Selenium' on 'Semaphorin−6A'

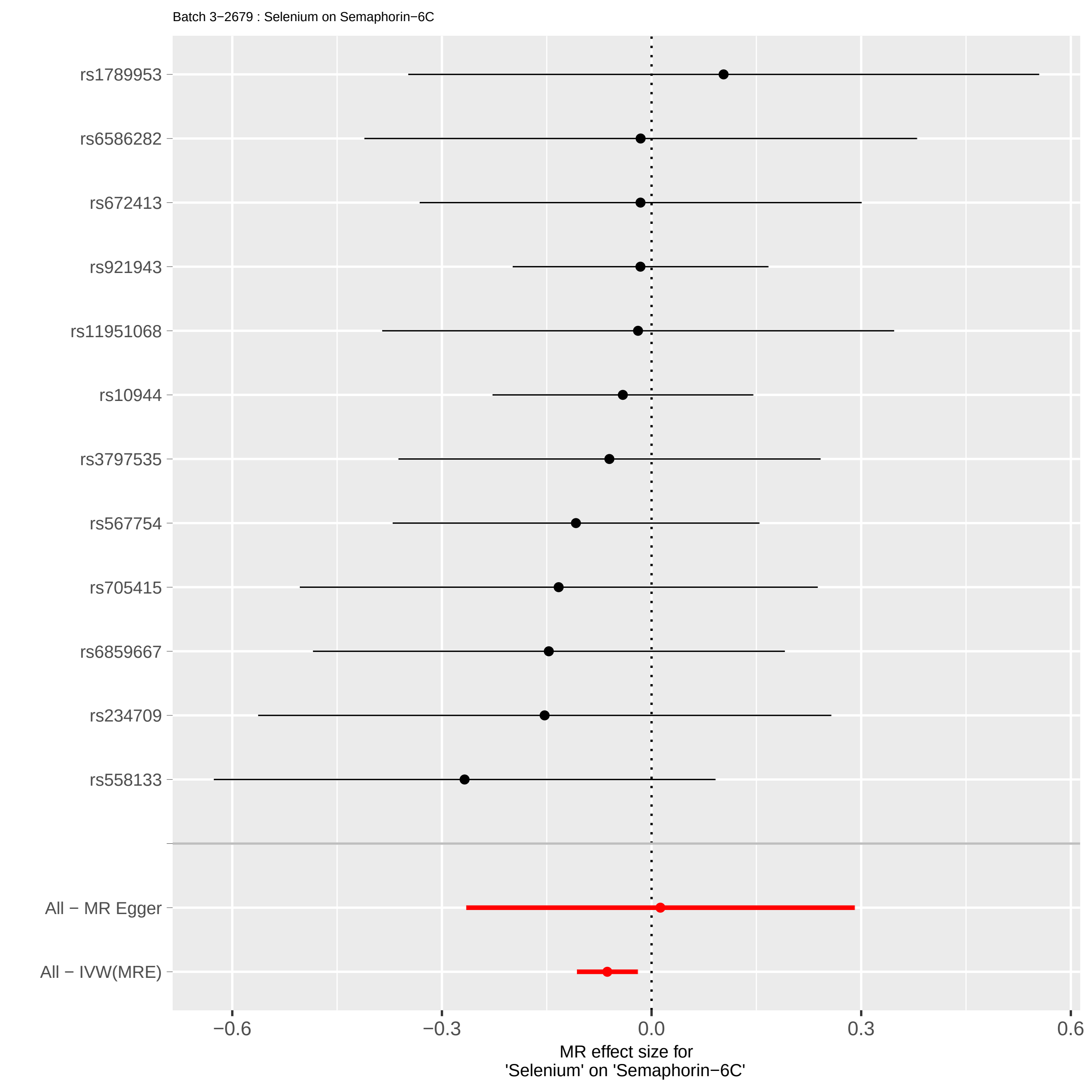
Batch 3−2679 : Selenium on Semaphorin−6C
rs1789953
rs6586282
rs672413
rs921943
rs11951068
rs10944
rs3797535
rs567754
rs705415
rs6859667
rs234709
rs558133
All − MR Egger
All − IVW(MRE)
−0.6
−0.3
0.0
0.3
0.6
MR effect size for
'Selenium' on 'Semaphorin−6C'

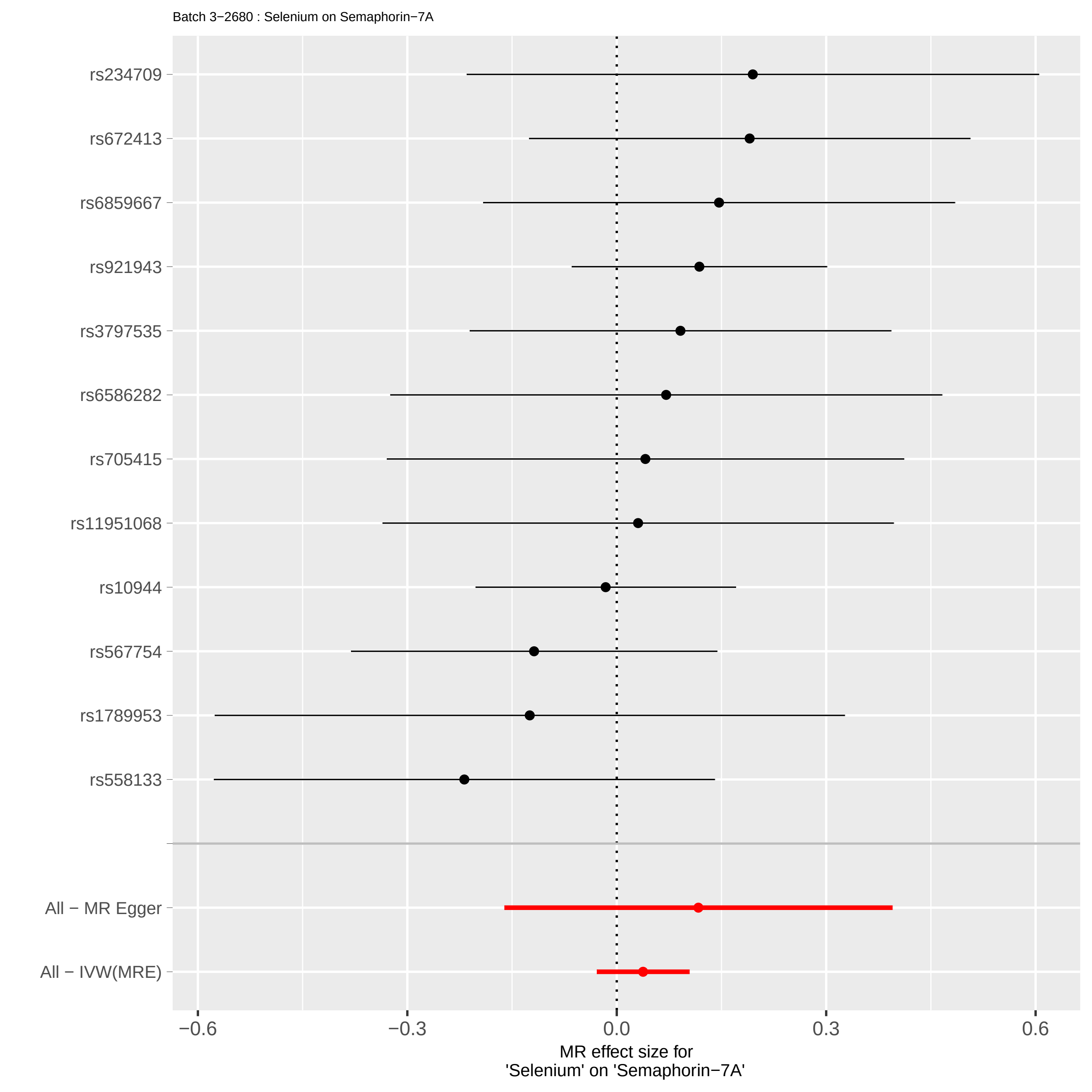

Batch 3−2680 : Selenium on Semaphorin−7A
rs234709
rs672413
rs6859667
rs921943
rs3797535
rs6586282
rs705415
rs11951068
rs10944
rs567754
rs1789953
rs558133
All − MR Egger
All − IVW(MRE)
−0.6
−0.3
0.0
0.3
0.6
MR effect size for
'Selenium' on 'Semaphorin−7A'

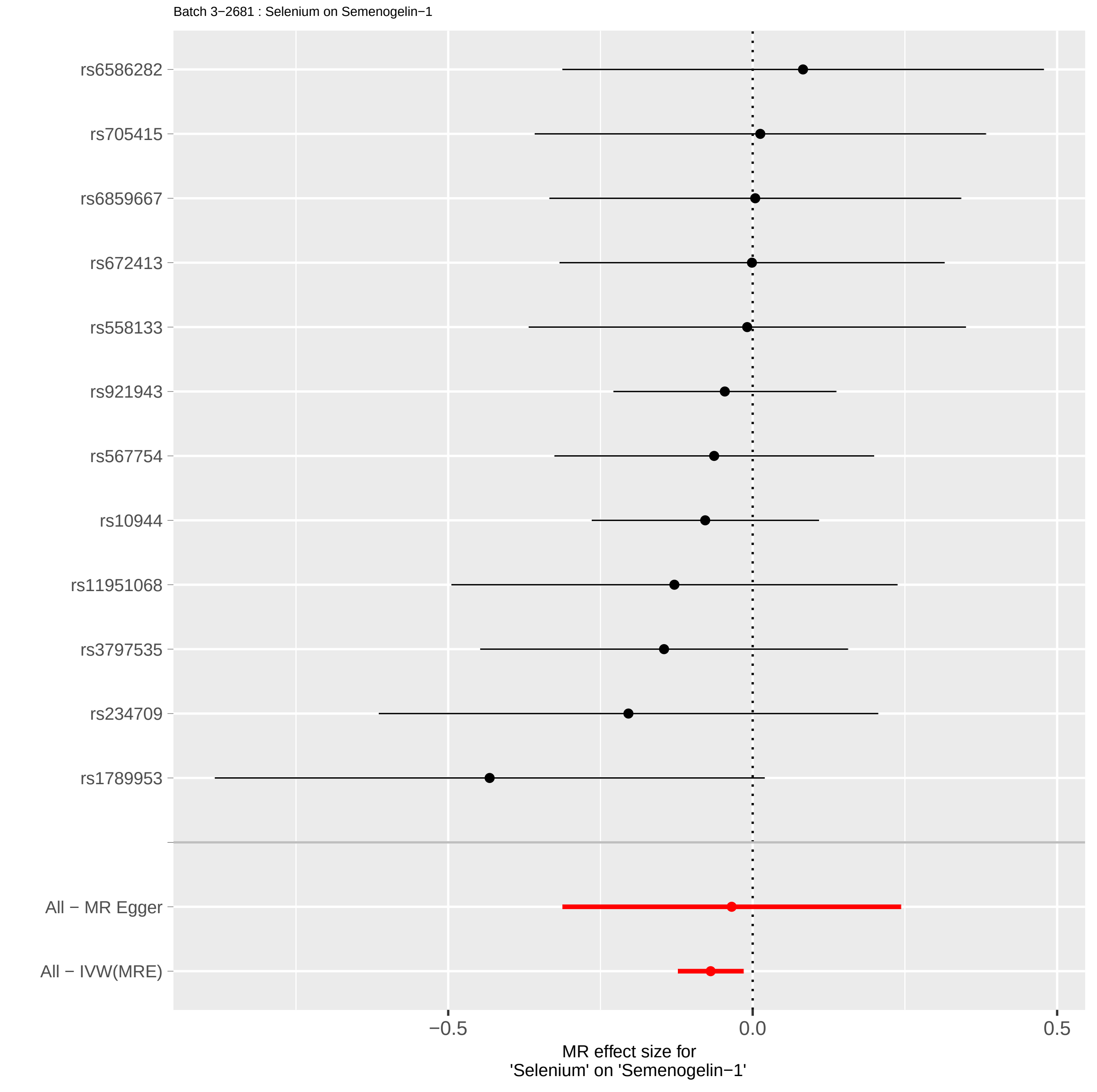

Batch 3−2681 : Selenium on Semenogelin−1
rs6586282
rs705415
rs6859667
rs672413
rs558133
rs921943
rs567754
rs10944
rs11951068
rs3797535
rs234709
rs1789953
All − MR Egger
All − IVW(MRE)
−0.5
0.0
0.5
MR effect size for
'Selenium' on 'Semenogelin−1'

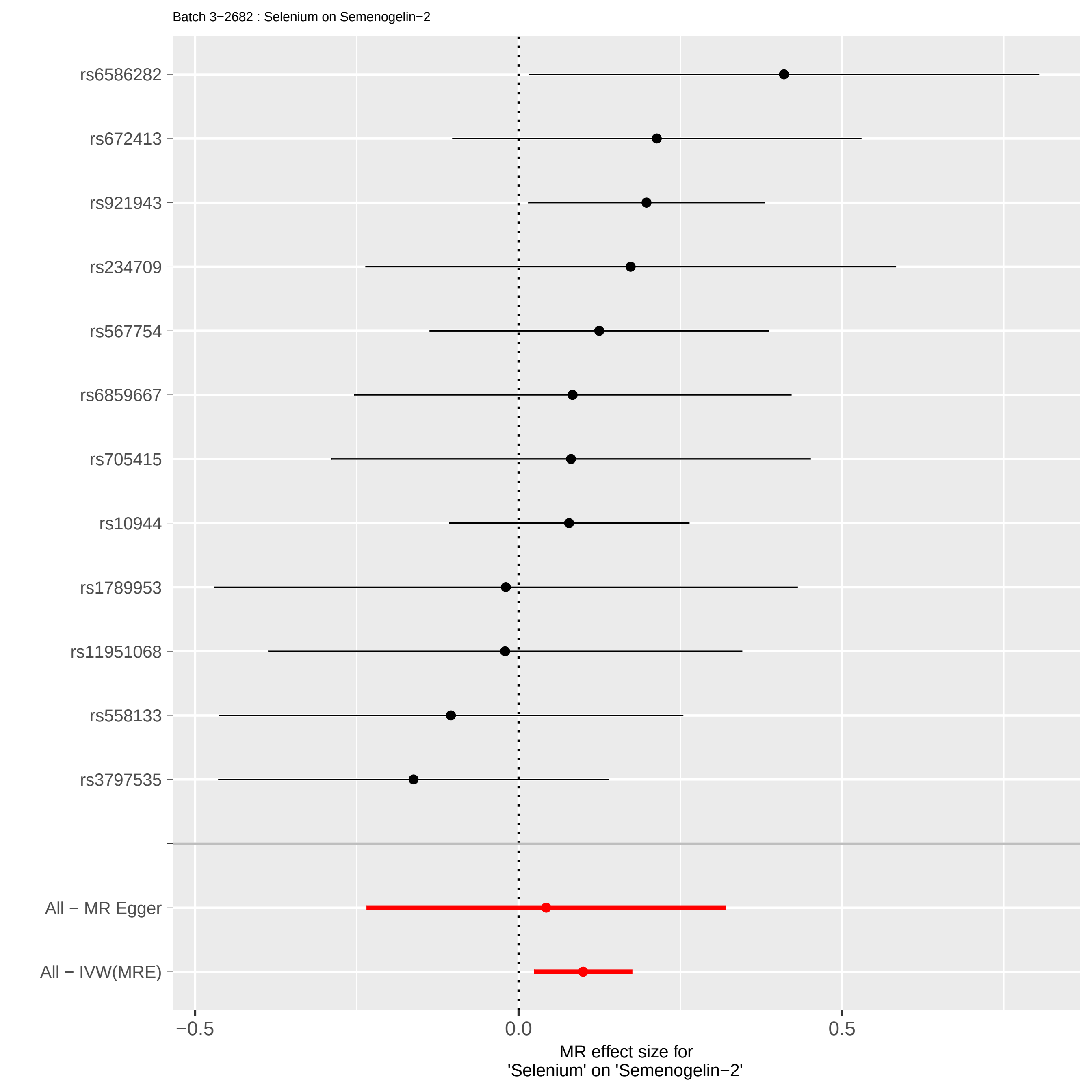
Batch 3−2682 : Selenium on Semenogelin−2
rs6586282
rs672413
rs921943
rs234709
rs567754
rs6859667
rs705415
rs10944
rs1789953
rs11951068
rs558133
rs3797535
All − MR Egger
All − IVW(MRE)
−0.5
0.0
0.5
MR effect size for
'Selenium' on 'Semenogelin−2'

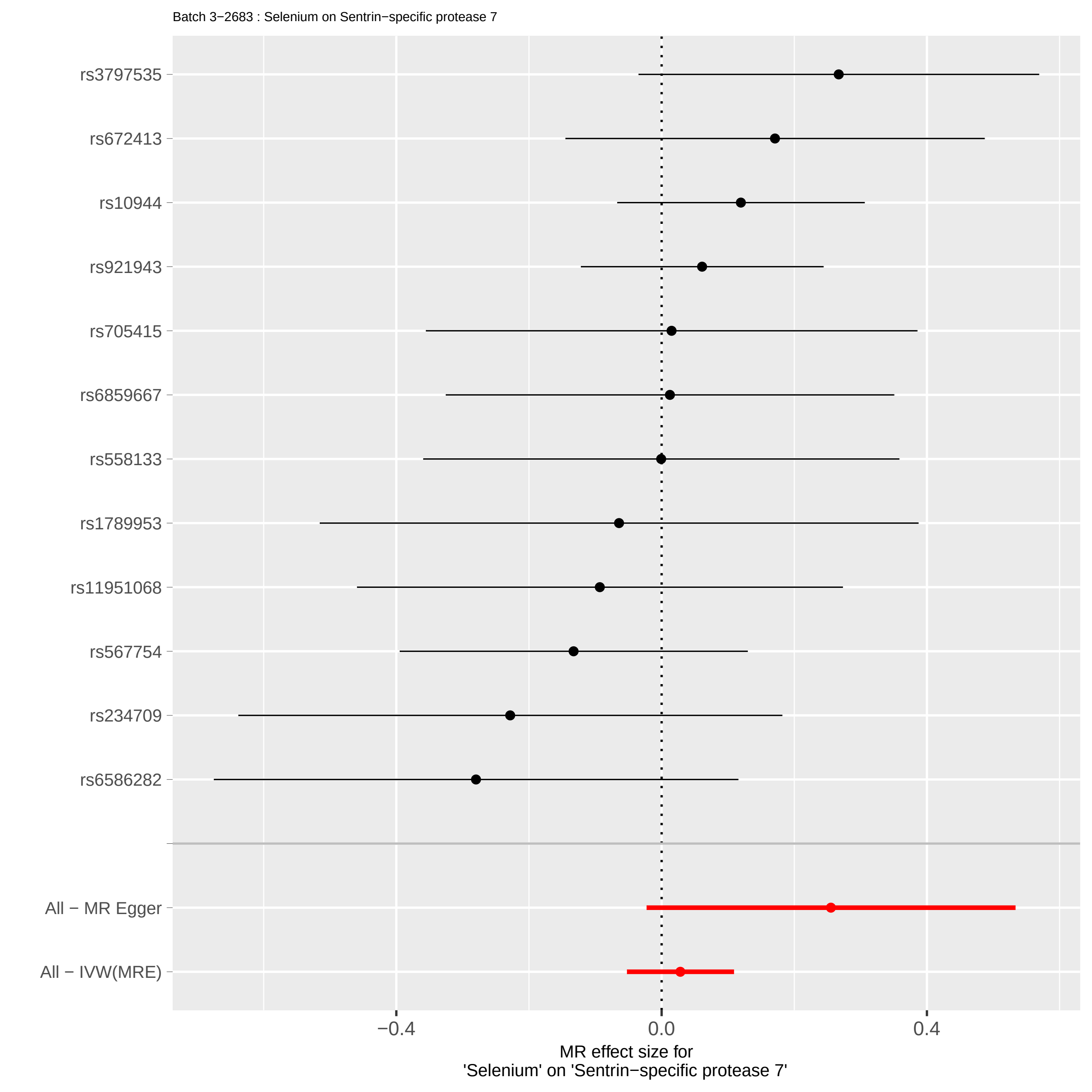

Batch 3−2683 : Selenium on Sentrin−specific protease 7
rs3797535
rs672413
rs10944
rs921943
rs705415
rs6859667
rs558133
rs1789953
rs11951068
rs567754
rs234709
rs6586282
All − MR Egger
All − IVW(MRE)
−0.4
0.0
0.4
MR effect size for
'Selenium' on 'Sentrin−specific protease 7'

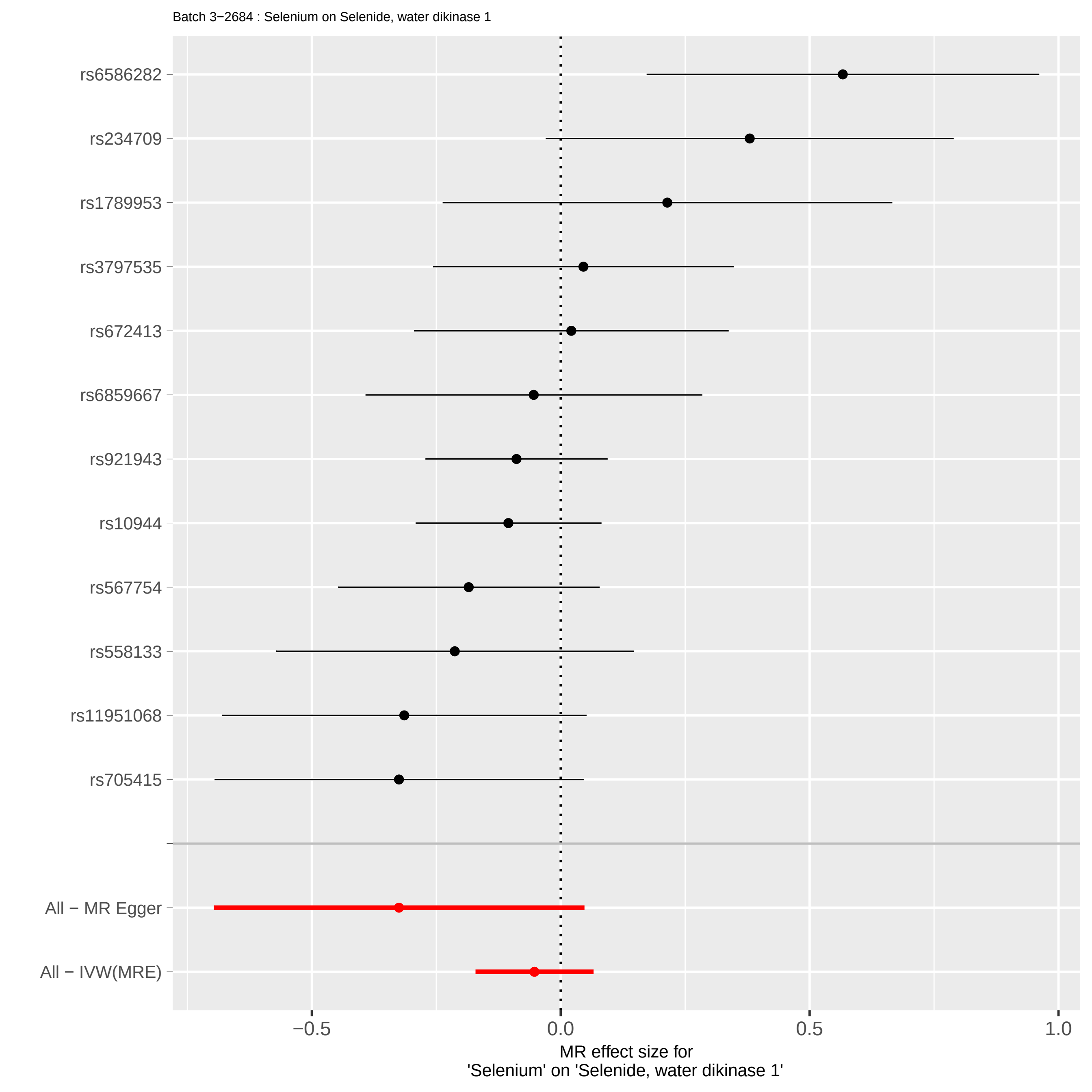
Batch 3−2684 : Selenium on Selenide, water dikinase 1
rs6586282
rs234709
rs1789953
rs3797535
rs672413
rs6859667
rs921943
rs10944
rs567754
rs558133
rs11951068
rs705415
All − MR Egger
All − IVW(MRE)
−0.5
0.0
0.5
1.0
MR effect size for
'Selenium' on 'Selenide, water dikinase 1'

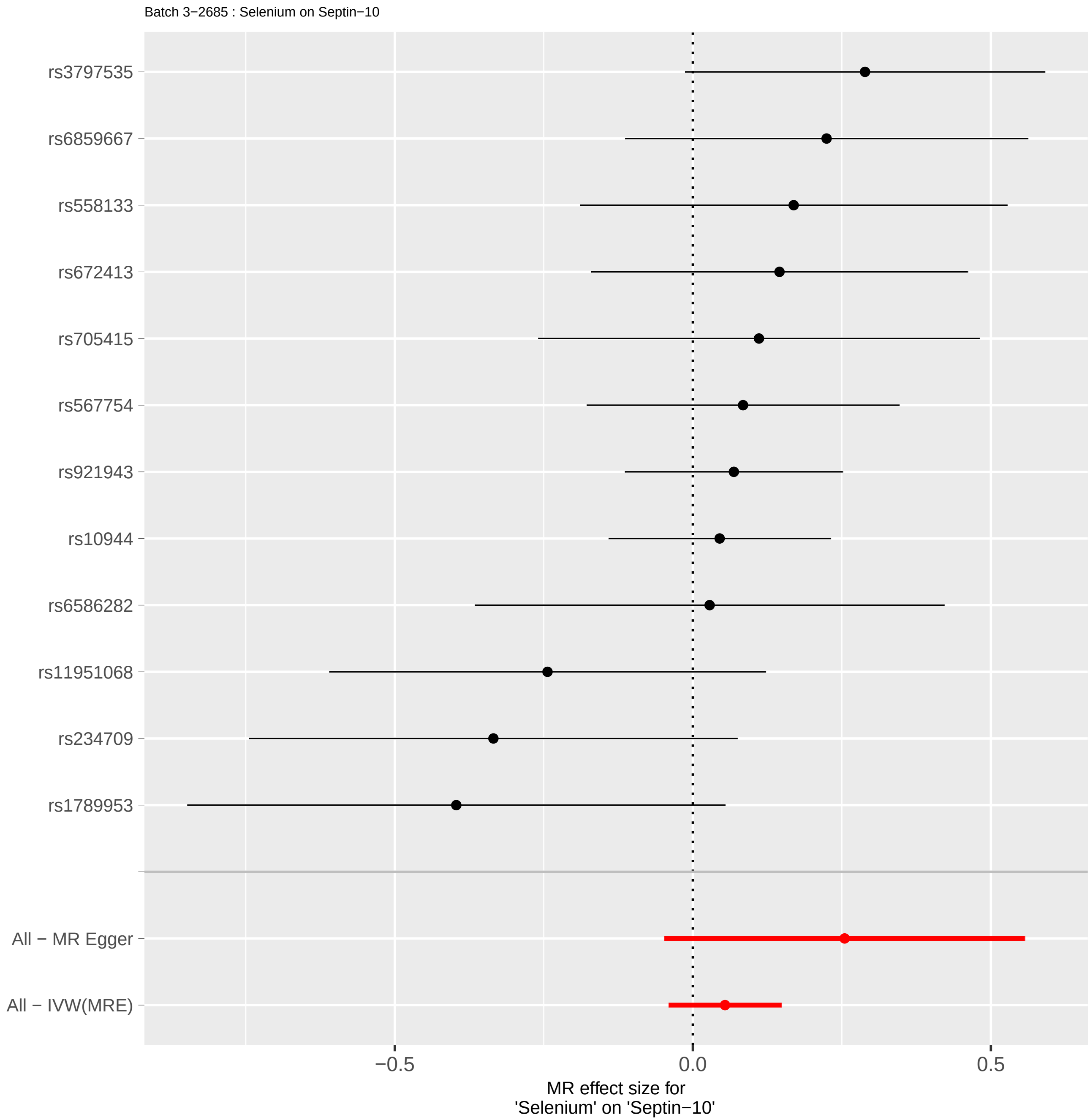

Batch 3−2685 : Selenium on Septin−10
rs3797535
rs6859667
rs558133
rs672413
rs705415
rs567754
rs921943
rs10944
rs6586282
rs11951068
rs234709
rs1789953
All − MR Egger
All − IVW(MRE)
−0.5
0.0
0.5
MR effect size for
'Selenium' on 'Septin−10'

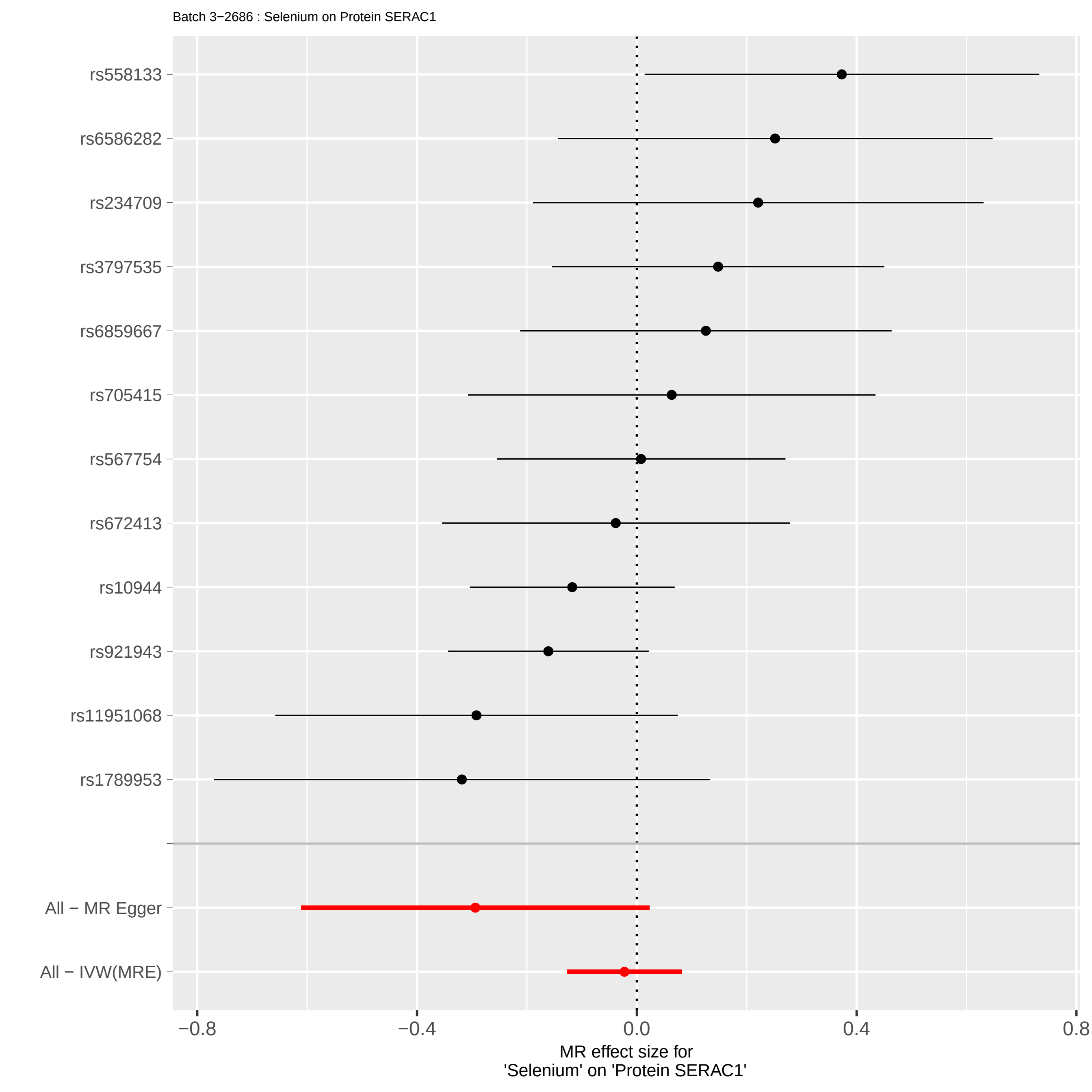

Batch 3−2686 : Selenium on Protein SERAC1
rs558133
rs6586282
rs234709
rs3797535
rs6859667
rs705415
rs567754
rs672413
rs10944
rs921943
rs11951068
rs1789953
All − MR Egger
All − IVW(MRE)
−0.8
−0.4
0.0
0.4
0.8
MR effect size for
'Selenium' on 'Protein SERAC1'

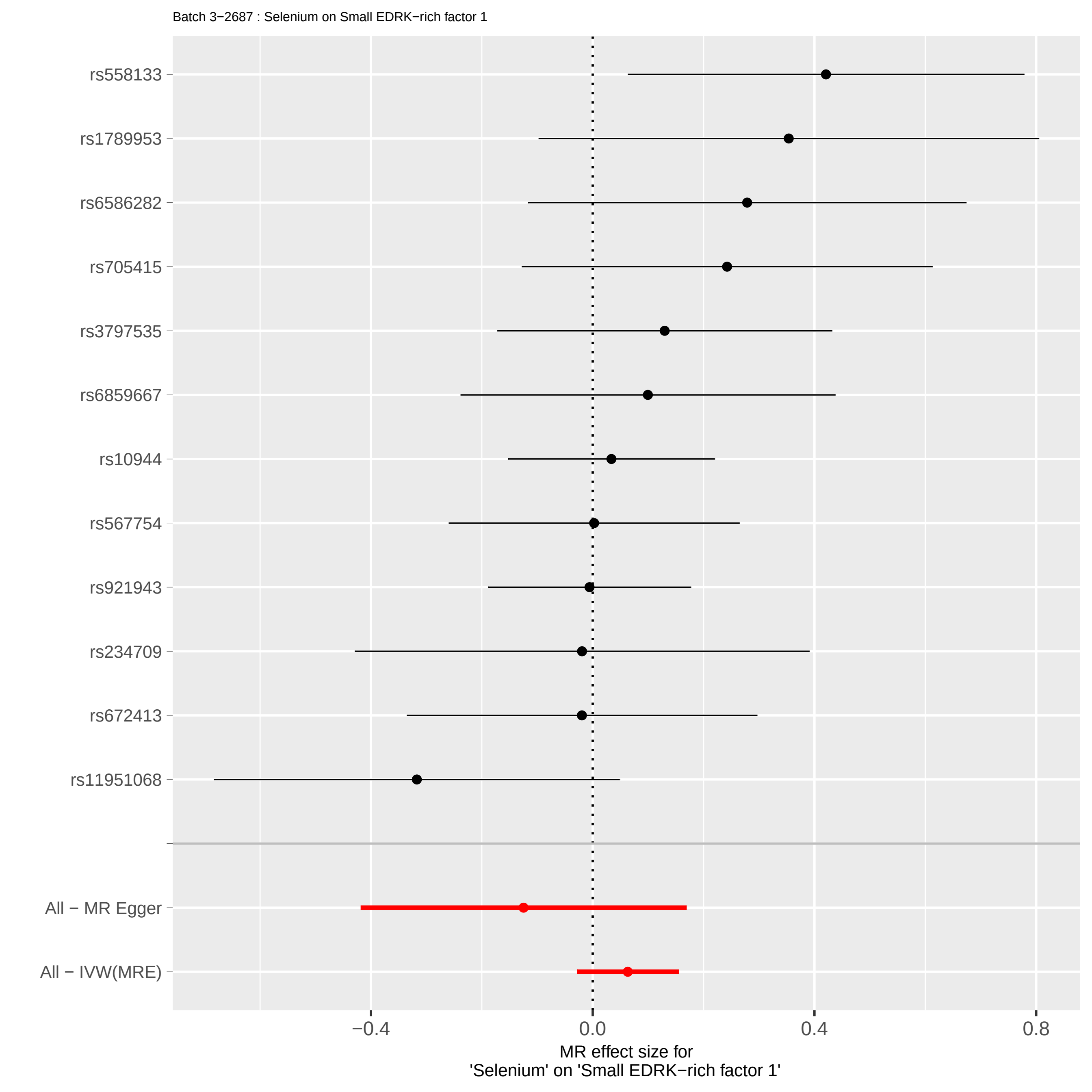
Batch 3−2687 : Selenium on Small EDRK−rich factor 1
rs558133
rs1789953
rs6586282
rs705415
rs3797535
rs6859667
rs10944
rs567754
rs921943
rs234709
rs672413
rs11951068
All − MR Egger
All − IVW(MRE)
−0.4
0.0
0.4
0.8
MR effect size for
'Selenium' on 'Small EDRK−rich factor 1'

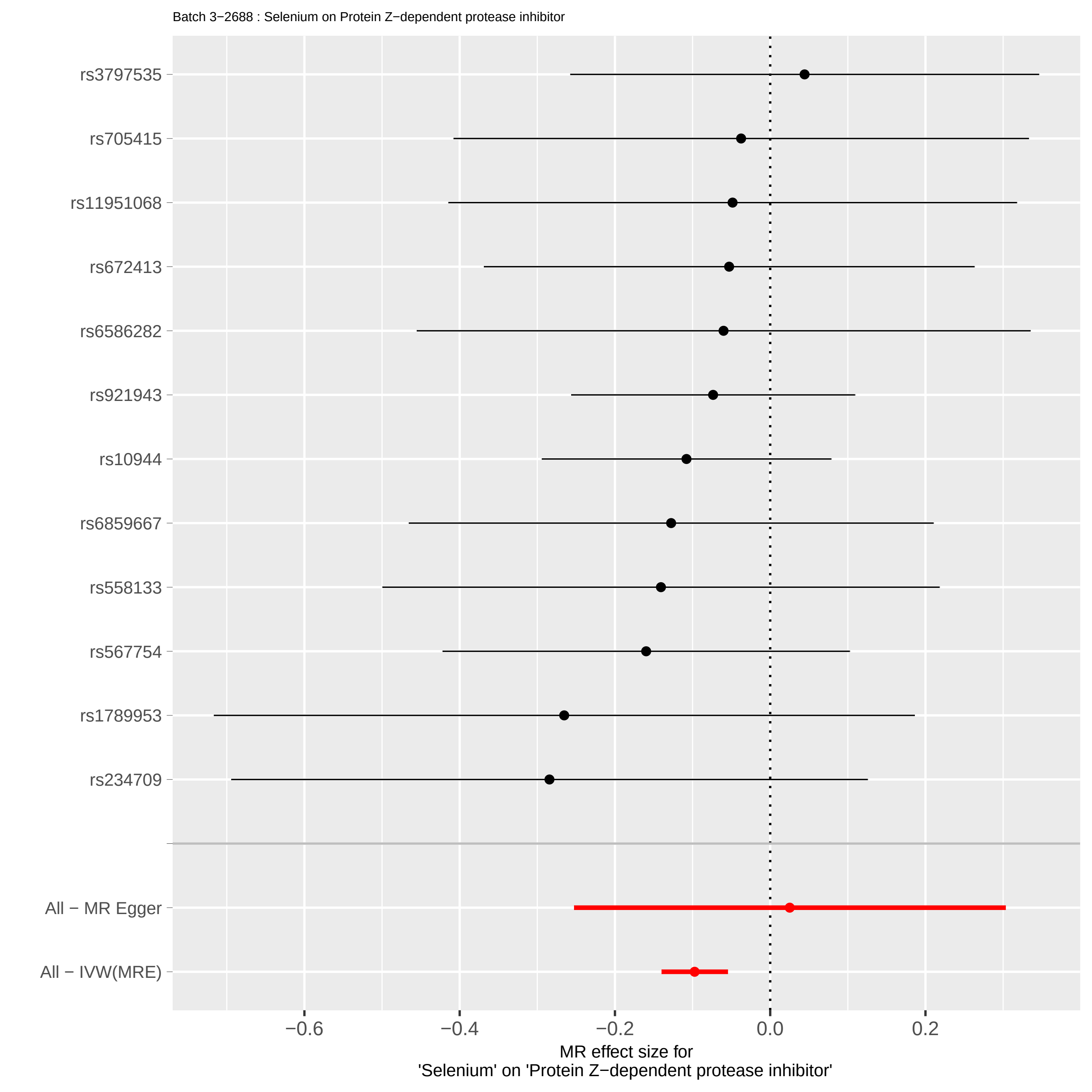
Batch 3−2688 : Selenium on Protein Z−dependent protease inhibitor
rs3797535
rs705415
rs11951068
rs672413
rs6586282
rs921943
rs10944
rs6859667
rs558133
rs567754
rs1789953
rs234709
All − MR Egger
All − IVW(MRE)
−0.6
−0.4
−0.2
0.0
0.2
MR effect size for
'Selenium' on 'Protein Z−dependent protease inhibitor'

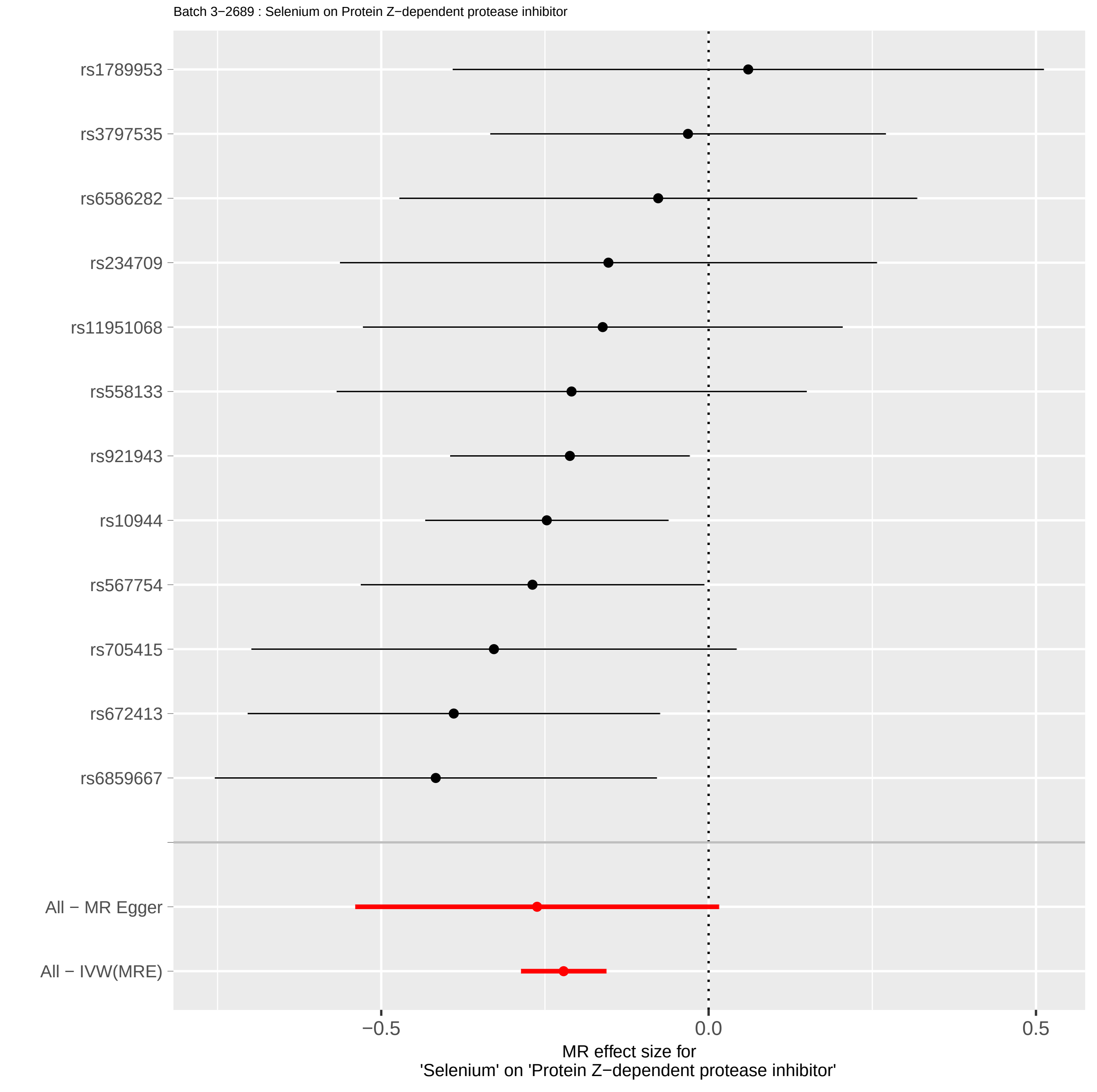
Batch 3−2689 : Selenium on Protein Z−dependent protease inhibitor
rs1789953
rs3797535
rs6586282
rs234709
rs11951068
rs558133
rs921943
rs10944
rs567754
rs705415
rs672413
rs6859667
All − MR Egger
All − IVW(MRE)
−0.5
0.0
0.5
MR effect size for
'Selenium' on 'Protein Z−dependent protease inhibitor'

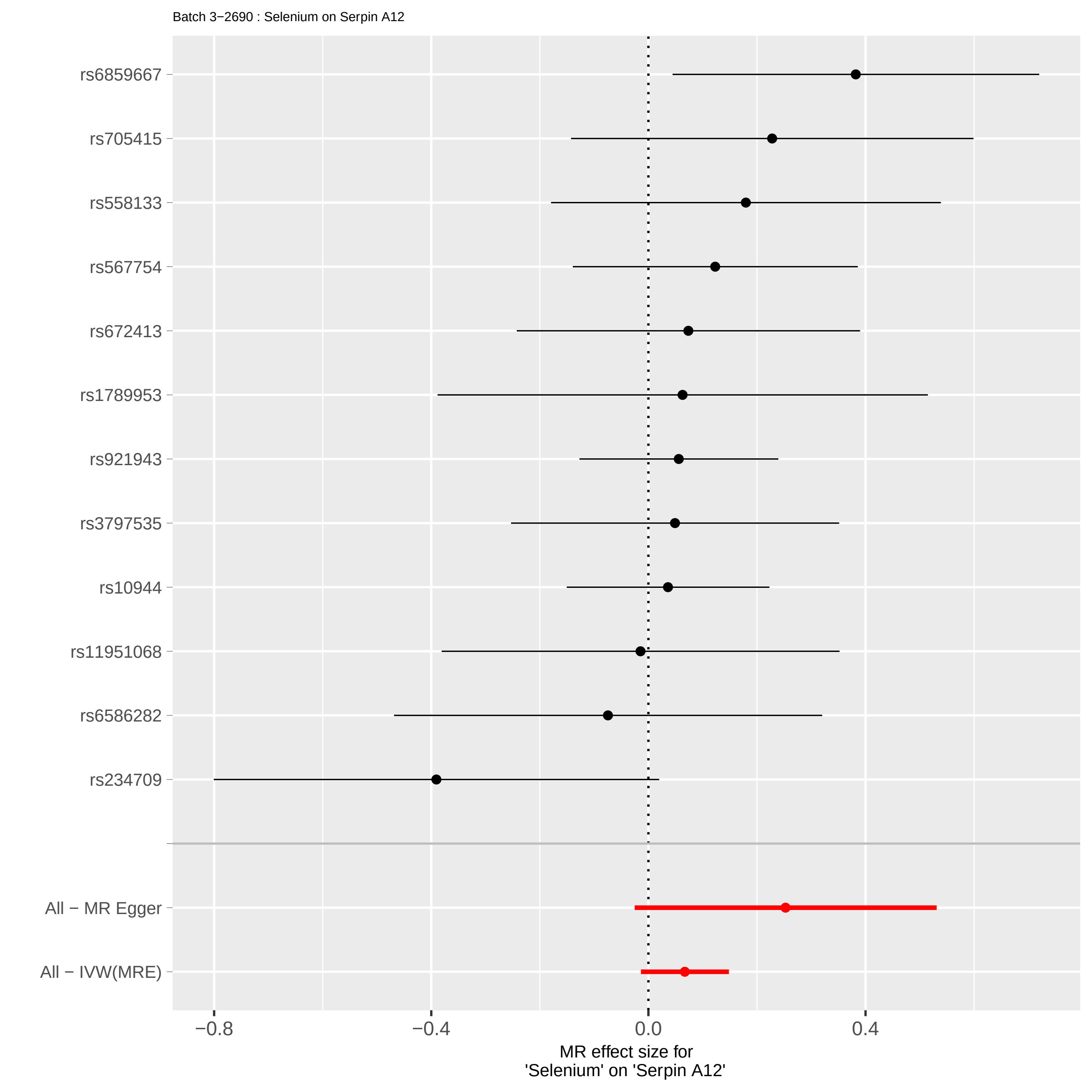

Batch 3−2690 : Selenium on Serpin A12
rs6859667
rs705415
rs558133
rs567754
rs672413
rs1789953
rs921943
rs3797535
rs10944
rs11951068
rs6586282
rs234709
All − MR Egger
All − IVW(MRE)
−0.8
−0.4
0.0
0.4
MR effect size for
'Selenium' on 'Serpin A12'

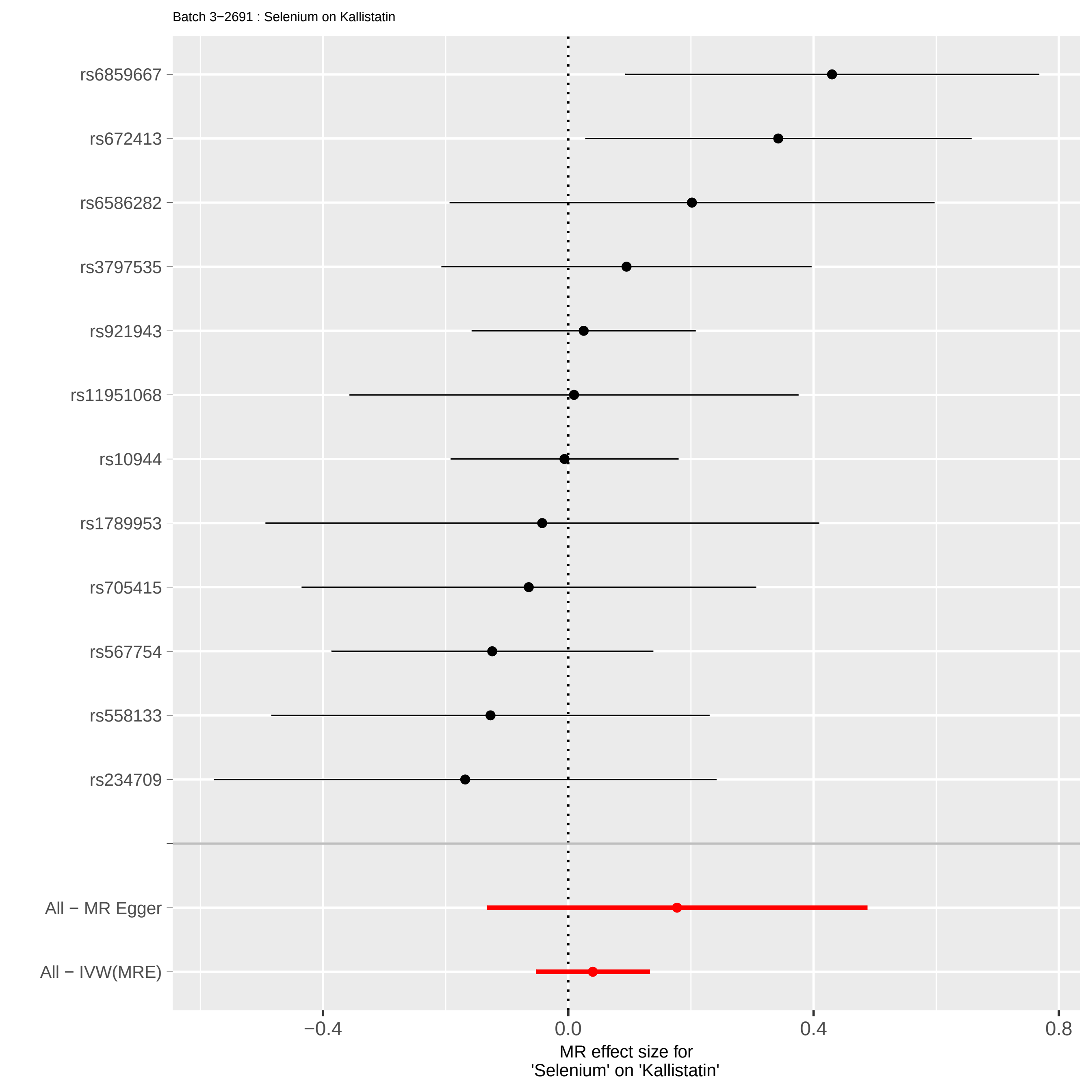

Batch 3−2691 : Selenium on Kallistatin
rs6859667
rs672413
rs6586282
rs3797535
rs921943
rs11951068
rs10944
rs1789953
rs705415
rs567754
rs558133
rs234709
All − MR Egger
All − IVW(MRE)
−0.4
0.0
0.4
0.8
MR effect size for
'Selenium' on 'Kallistatin'

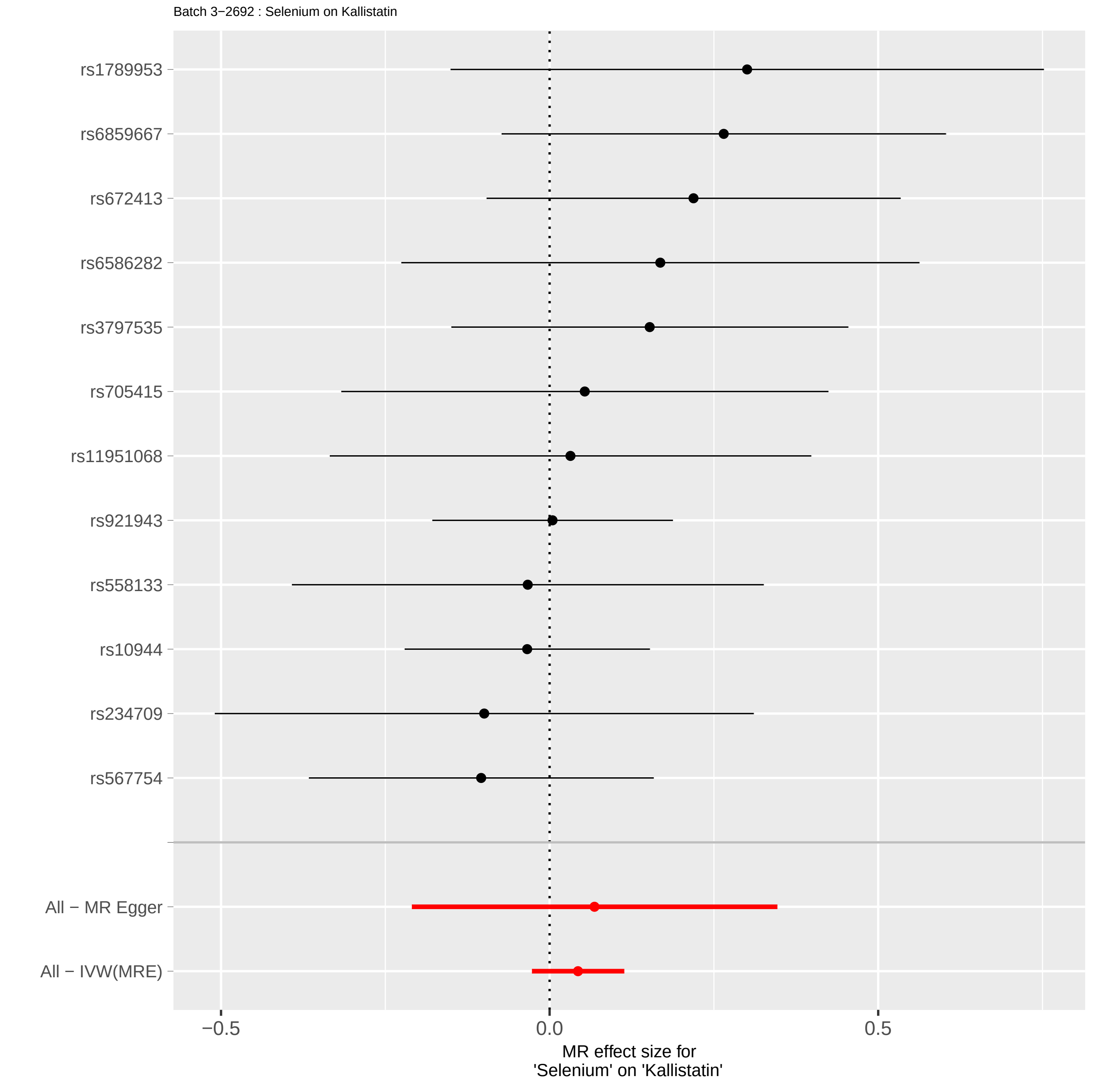

Batch 3−2692 : Selenium on Kallistatin
rs1789953
rs6859667
rs672413
rs6586282
rs3797535
rs705415
rs11951068
rs921943
rs558133
rs10944
rs234709
rs567754
All − MR Egger
All − IVW(MRE)
−0.5
0.0
0.5
MR effect size for
'Selenium' on 'Kallistatin'

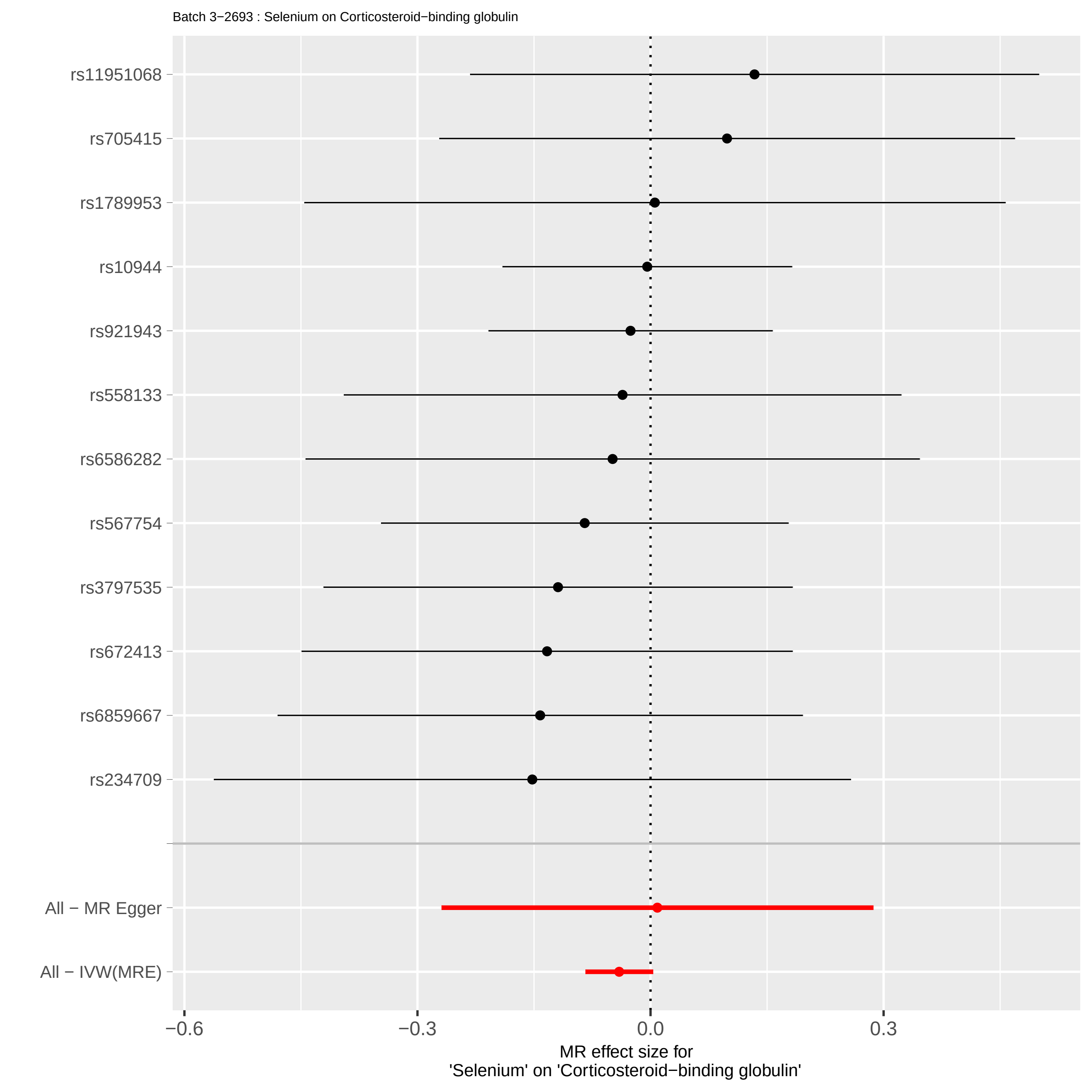

Batch 3−2693 : Selenium on Corticosteroid−binding globulin
rs11951068
rs705415
rs1789953
rs10944
rs921943
rs558133
rs6586282
rs567754
rs3797535
rs672413
rs6859667
rs234709
All − MR Egger
All − IVW(MRE)
−0.6
−0.3
0.0
0.3
MR effect size for
'Selenium' on 'Corticosteroid−binding globulin'

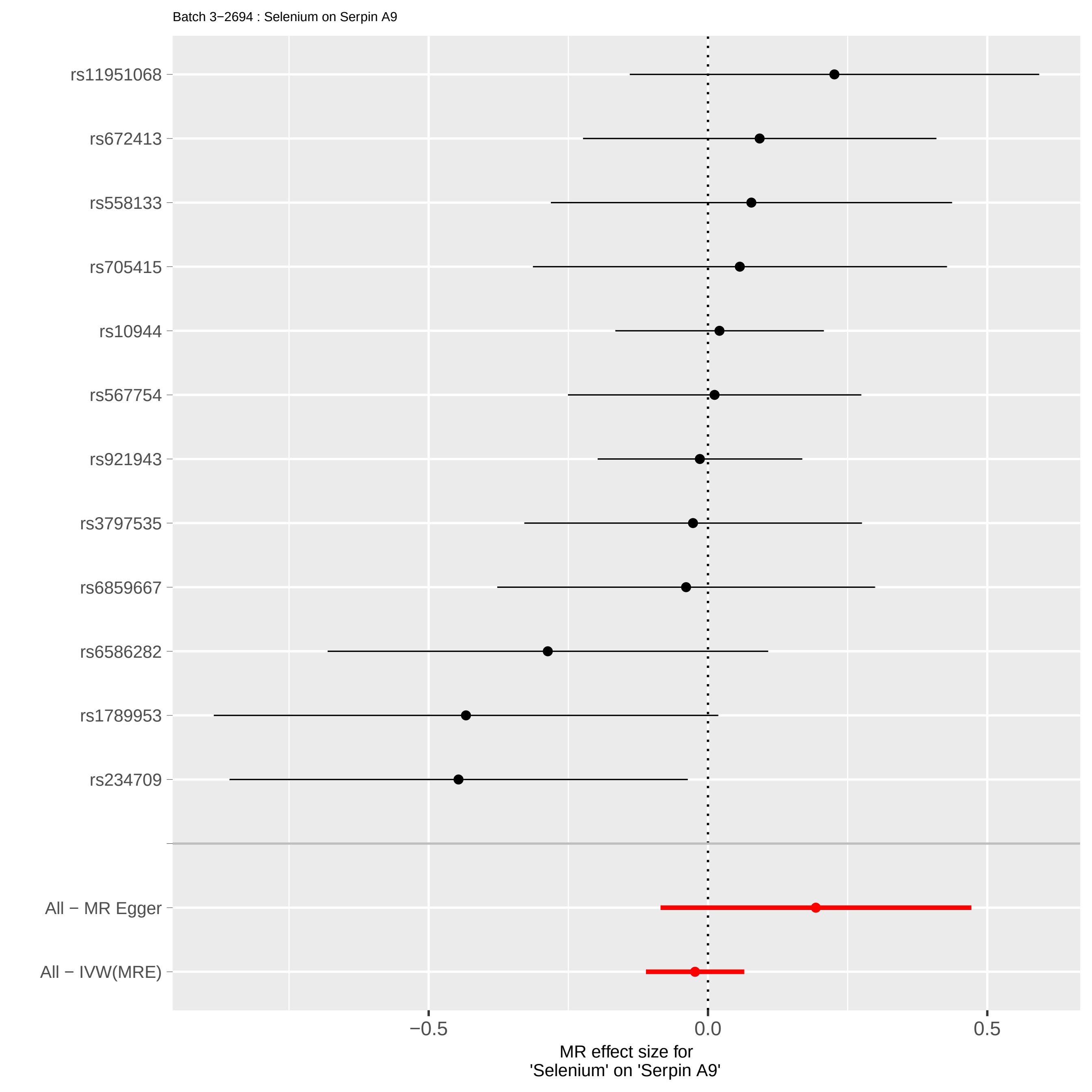
Batch 3−2694 : Selenium on Serpin A9
rs11951068
rs672413
rs558133
rs705415
rs10944
rs567754
rs921943
rs3797535
rs6859667
rs6586282
rs1789953
rs234709
All − MR Egger
All − IVW(MRE)
−0.5
0.0
0.5
MR effect size for
'Selenium' on 'Serpin A9'

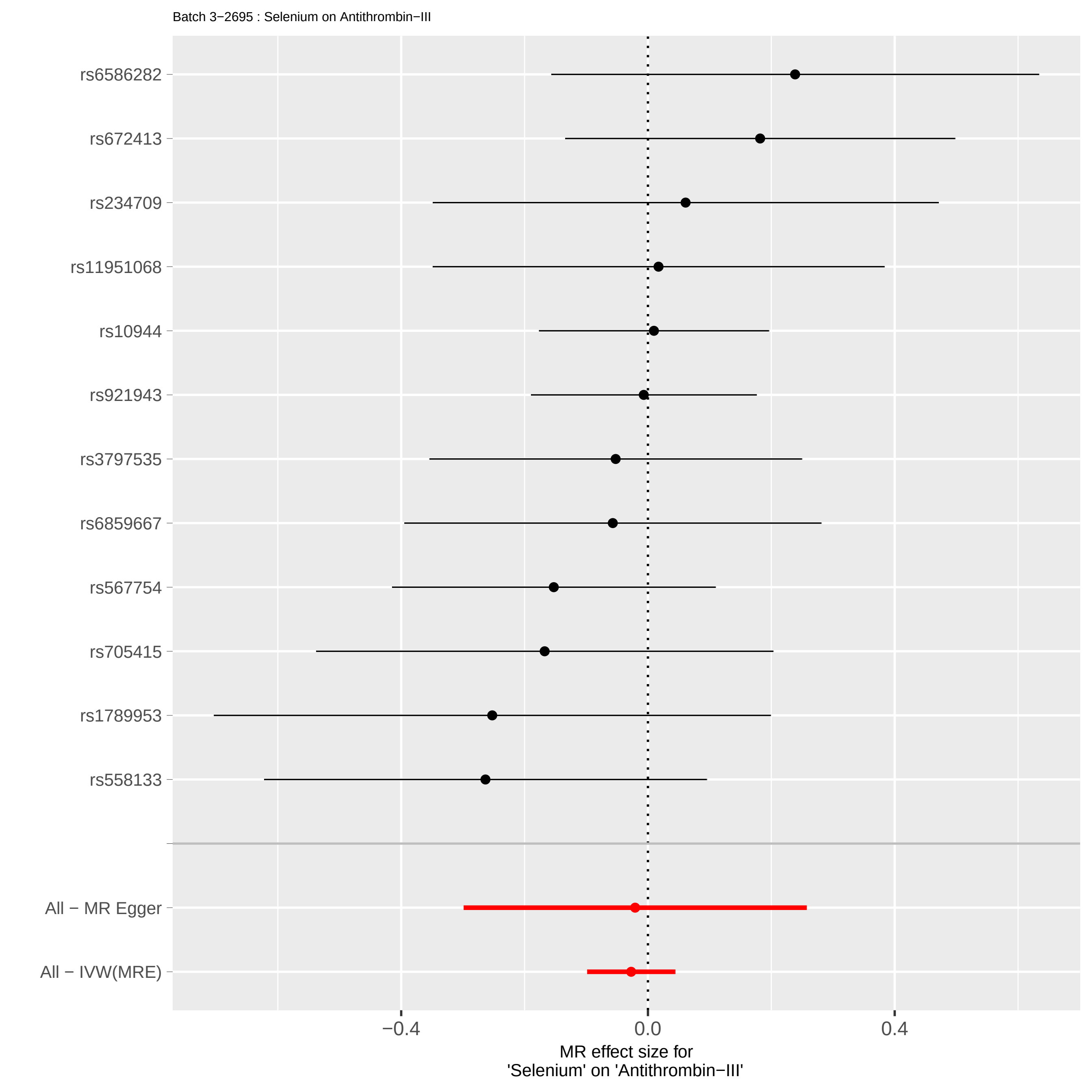

Batch 3−2695 : Selenium on Antithrombin−III
rs6586282
rs672413
rs234709
rs11951068
rs10944
rs921943
rs3797535
rs6859667
rs567754
rs705415
rs1789953
rs558133
All − MR Egger
All − IVW(MRE)
−0.4
0.0
0.4
MR effect size for
'Selenium' on 'Antithrombin−III'

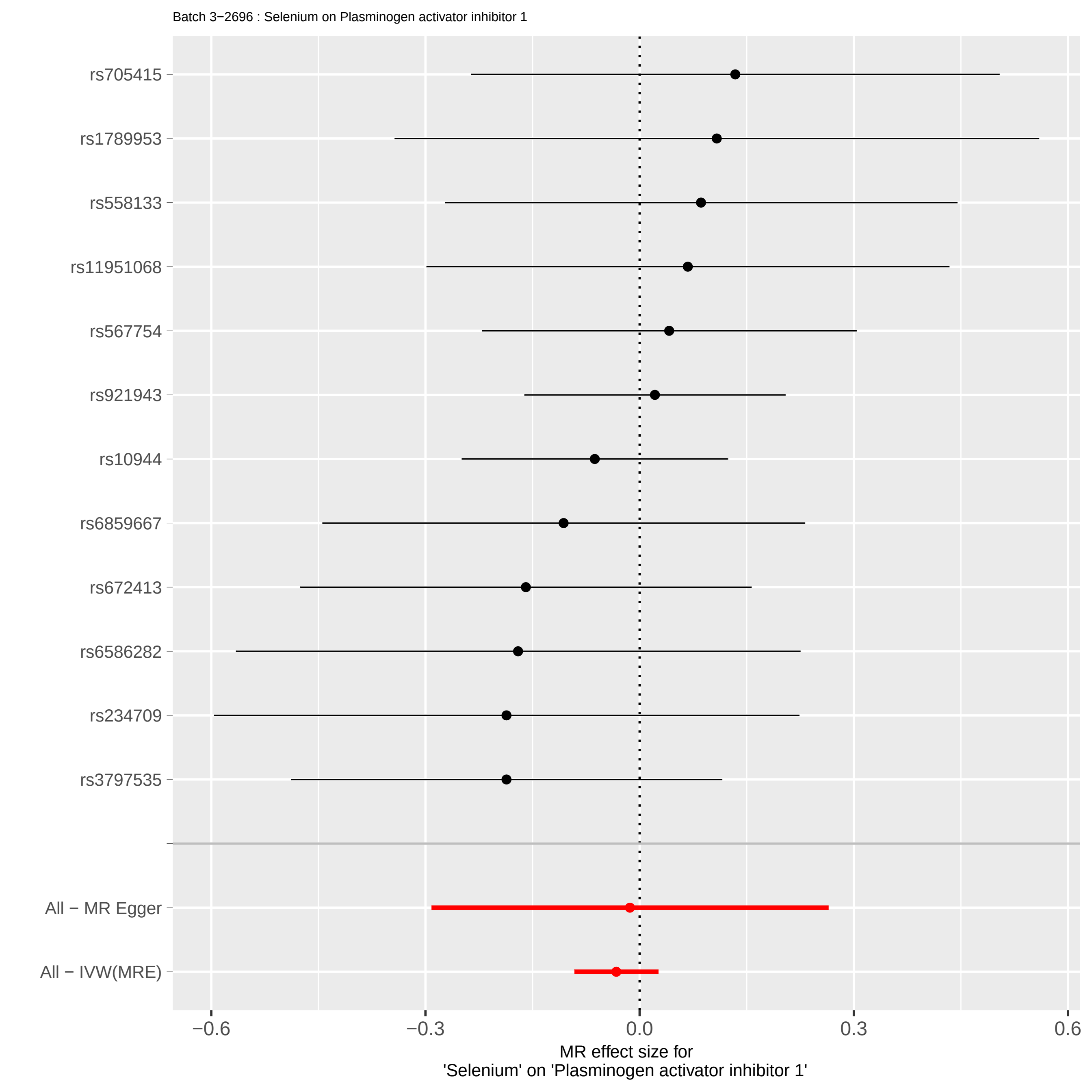
Batch 3−2696 : Selenium on Plasminogen activator inhibitor 1
rs705415
rs1789953
rs558133
rs11951068
rs567754
rs921943
rs10944
rs6859667
rs672413
rs6586282
rs234709
rs3797535
All − MR Egger
All − IVW(MRE)
−0.6
−0.3
0.0
0.3
0.6
MR effect size for
'Selenium' on 'Plasminogen activator inhibitor 1'

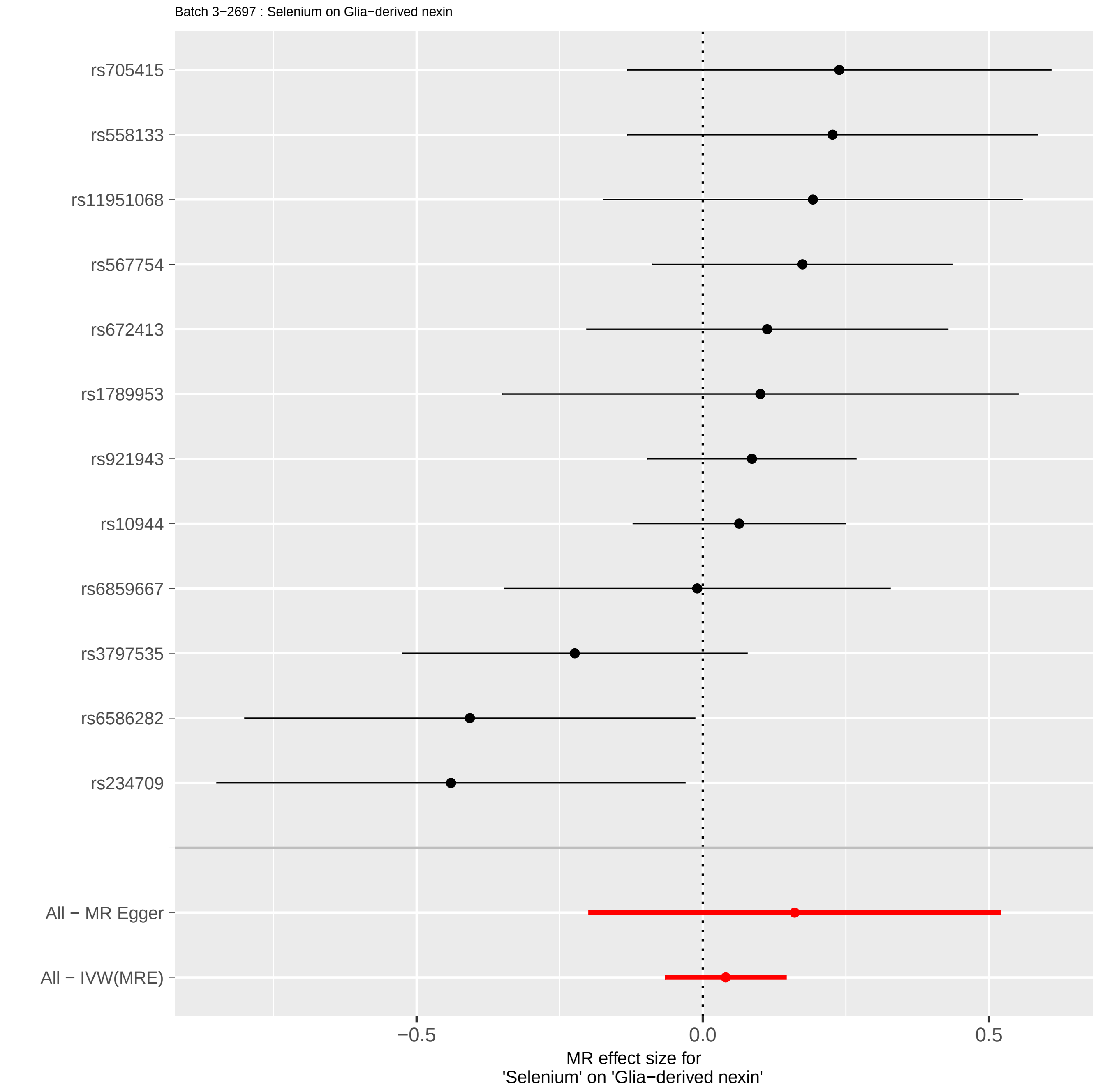

Batch 3−2697 : Selenium on Glia−derived nexin
rs705415
rs558133
rs11951068
rs567754
rs672413
rs1789953
rs921943
rs10944
rs6859667
rs3797535
rs6586282
rs234709
All − MR Egger
All − IVW(MRE)
−0.5
0.0
0.5
MR effect size for
'Selenium' on 'Glia−derived nexin'

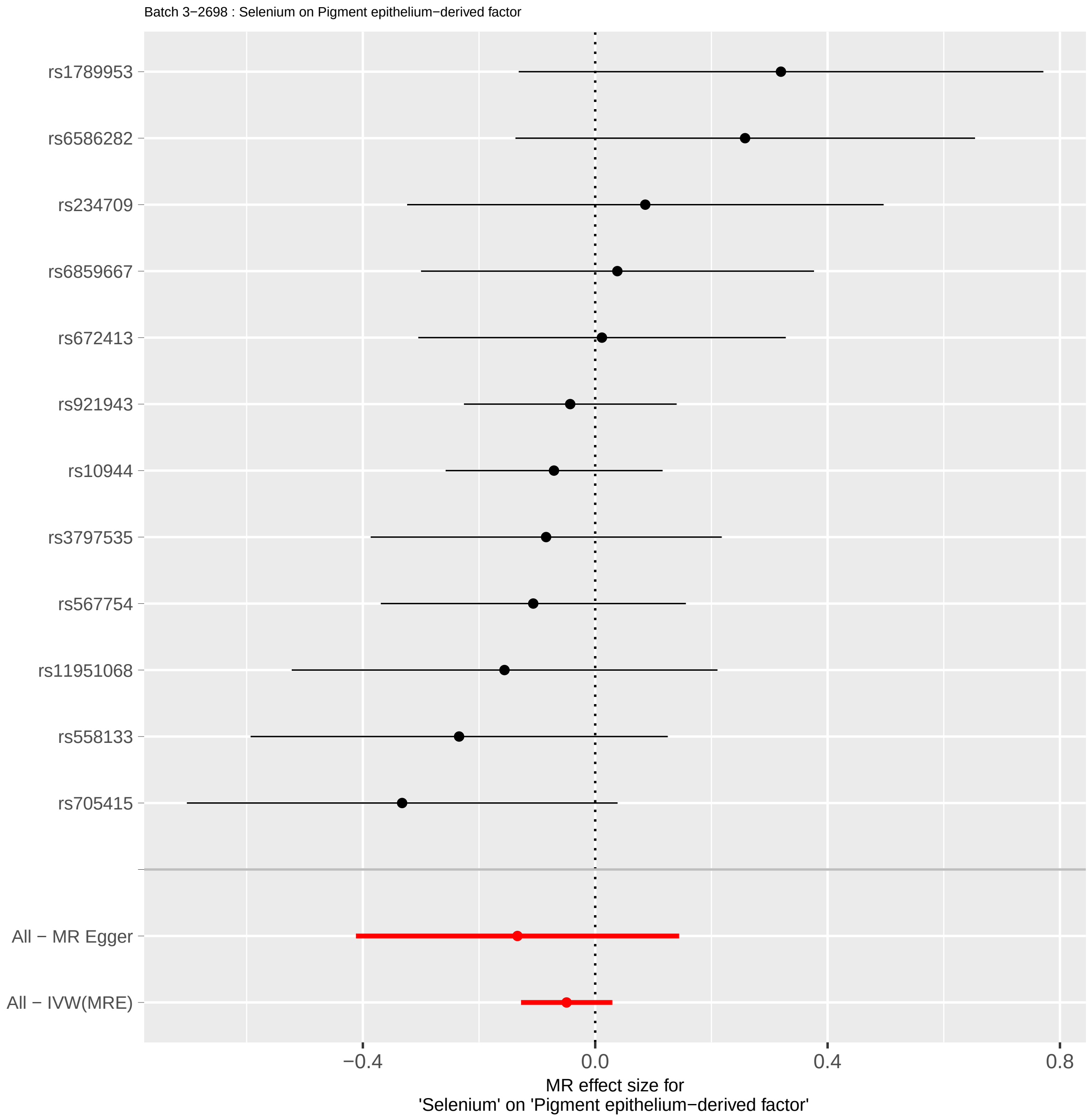

Batch 3−2698 : Selenium on Pigment epithelium−derived factor
rs1789953
rs6586282
rs234709
rs6859667
rs672413
rs921943
rs10944
rs3797535
rs567754
rs11951068
rs558133
rs705415
All − MR Egger
All − IVW(MRE)
−0.4
0.0
0.4
0.8
MR effect size for
'Selenium' on 'Pigment epithelium−derived factor'

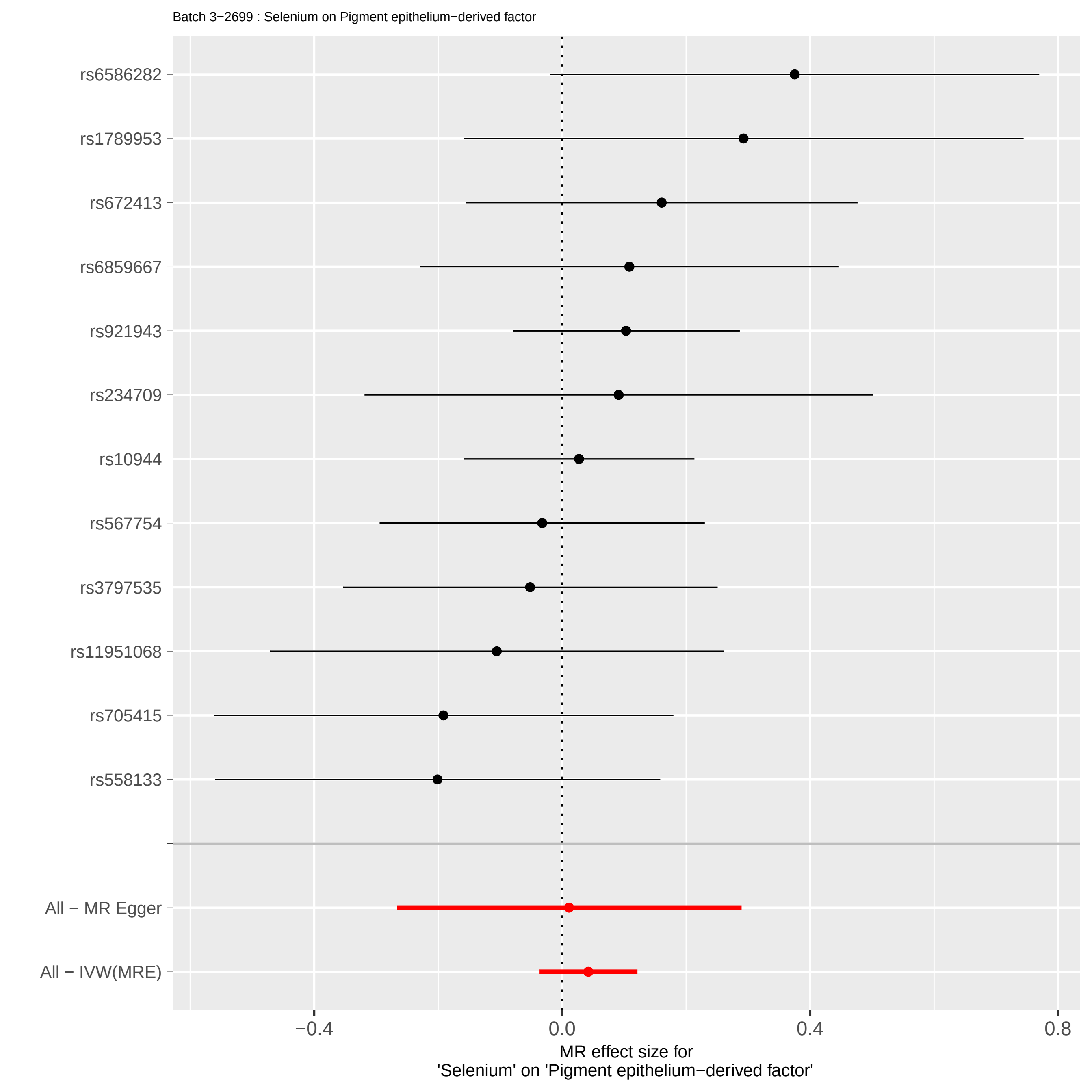
Batch 3−2699 : Selenium on Pigment epithelium−derived factor
rs6586282
rs1789953
rs672413
rs6859667
rs921943
rs234709
rs10944
rs567754
rs3797535
rs11951068
rs705415
rs558133
All − MR Egger
All − IVW(MRE)
−0.4
0.0
0.4
0.8
MR effect size for
'Selenium' on 'Pigment epithelium−derived factor'

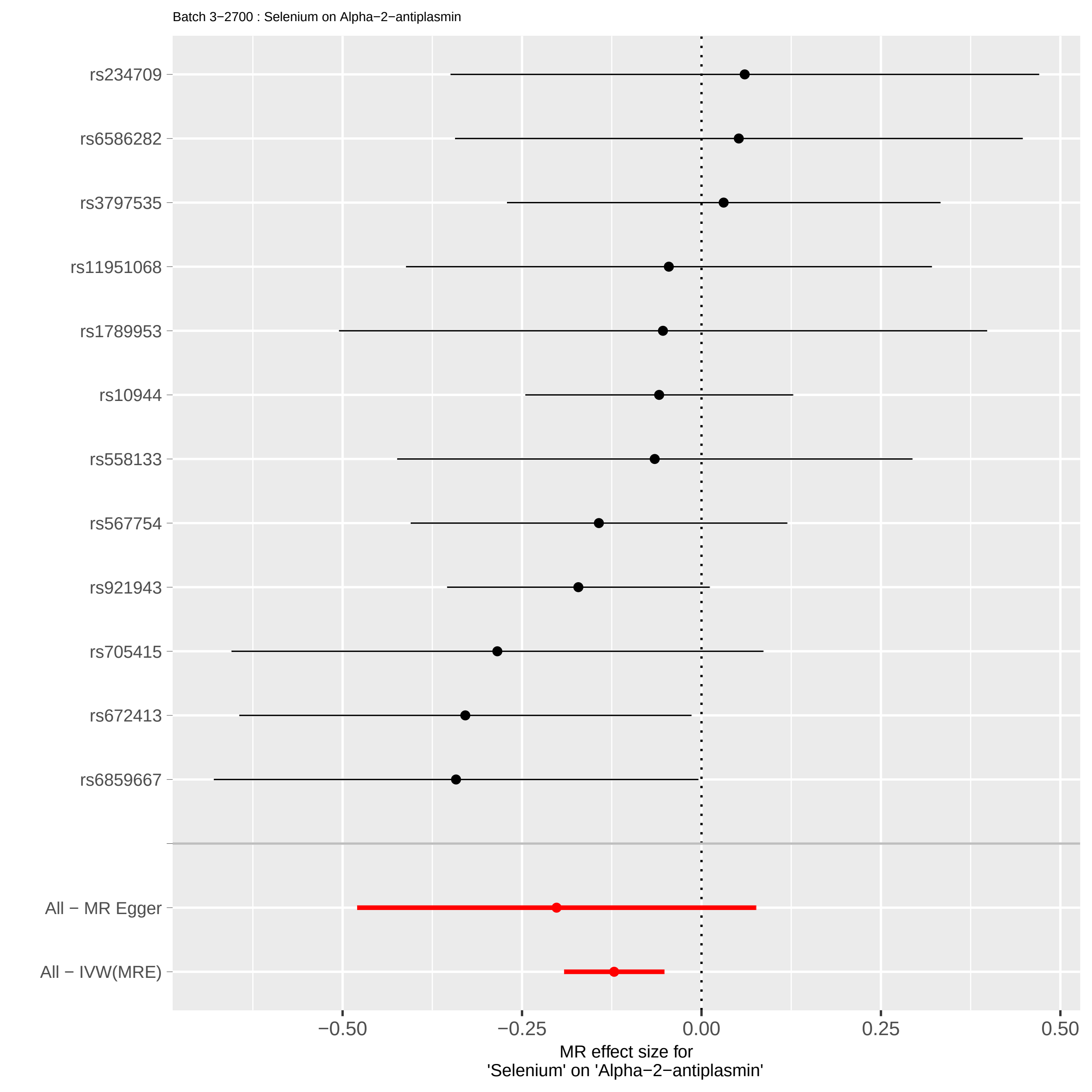
Batch 3−2700 : Selenium on Alpha−2−antiplasmin
rs234709
rs6586282
rs3797535
rs11951068
rs1789953
rs10944
rs558133
rs567754
rs921943
rs705415
rs672413
rs6859667
All − MR Egger
All − IVW(MRE)
−0.50
−0.25
0.00
0.25
0.50
MR effect size for
'Selenium' on 'Alpha−2−antiplasmin'

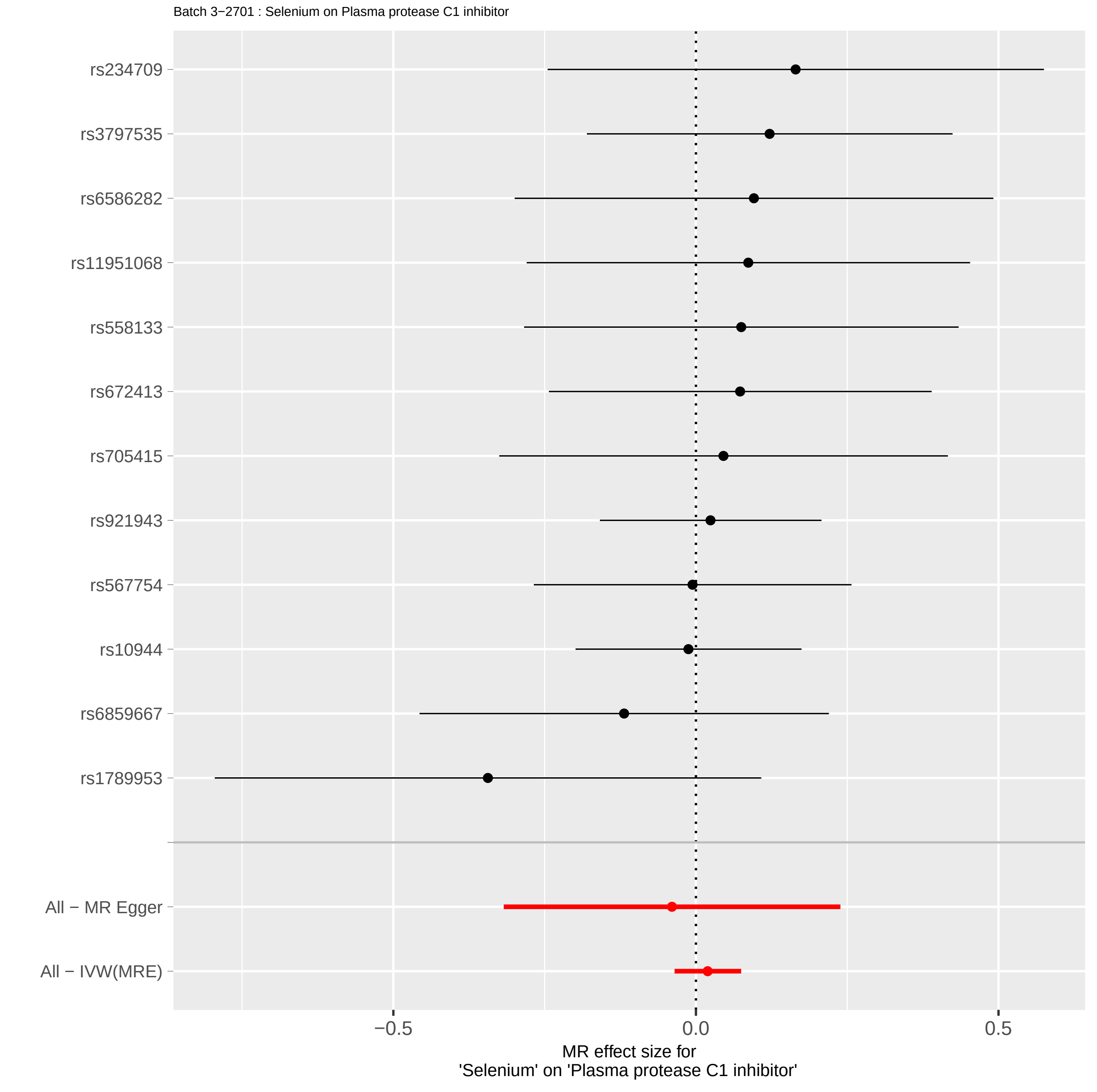

Batch 3−2701 : Selenium on Plasma protease C1 inhibitor
rs234709
rs3797535
rs6586282
rs11951068
rs558133
rs672413
rs705415
rs921943
rs567754
rs10944
rs6859667
rs1789953
All − MR Egger
All − IVW(MRE)
−0.5
0.0
0.5
MR effect size for
'Selenium' on 'Plasma protease C1 inhibitor'

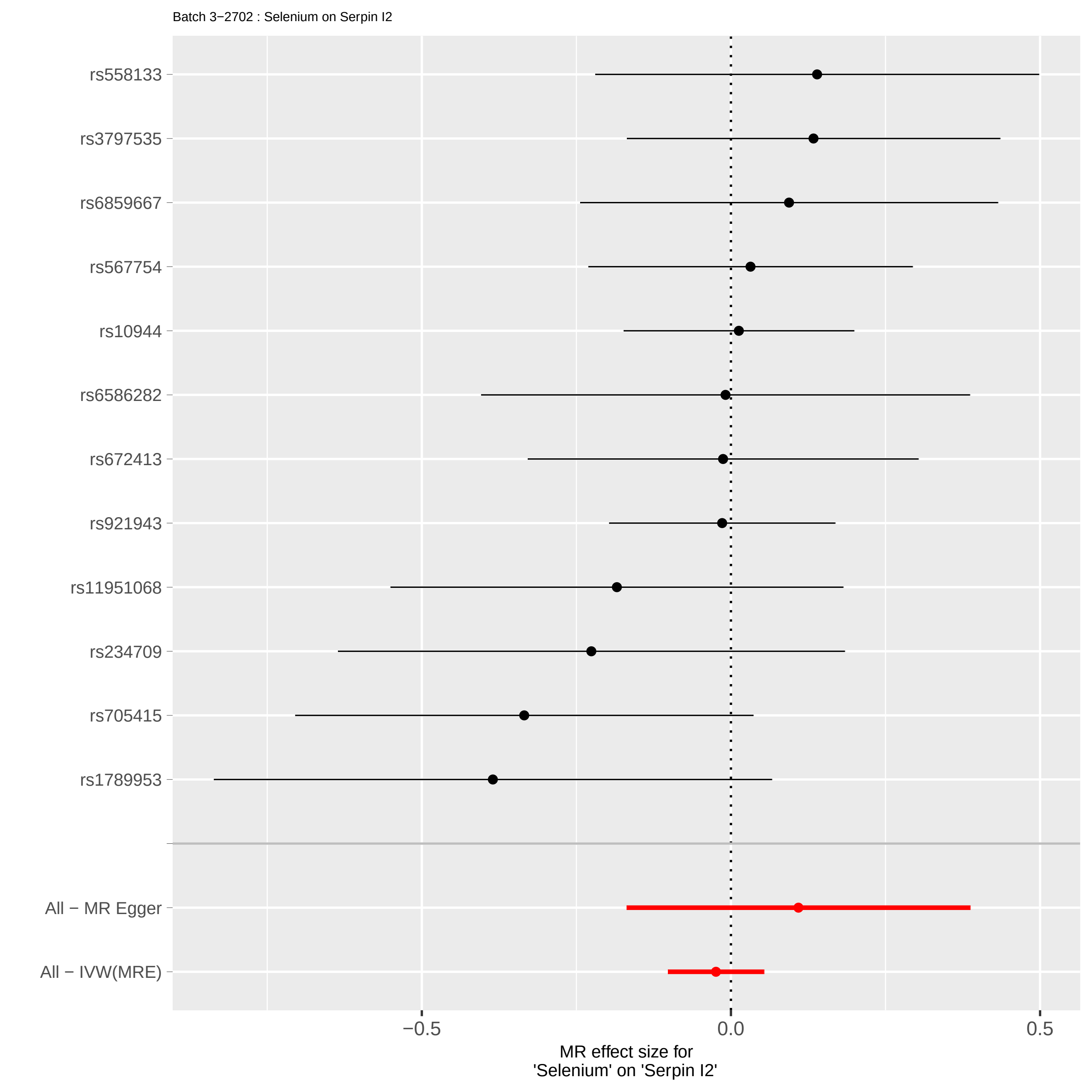

Batch 3−2702 : Selenium on Serpin I2
rs558133
rs3797535
rs6859667
rs567754
rs10944
rs6586282
rs672413
rs921943
rs11951068
rs234709
rs705415
rs1789953
All − MR Egger
All − IVW(MRE)
−0.5
0.0
0.5
MR effect size for
'Selenium' on 'Serpin I2'

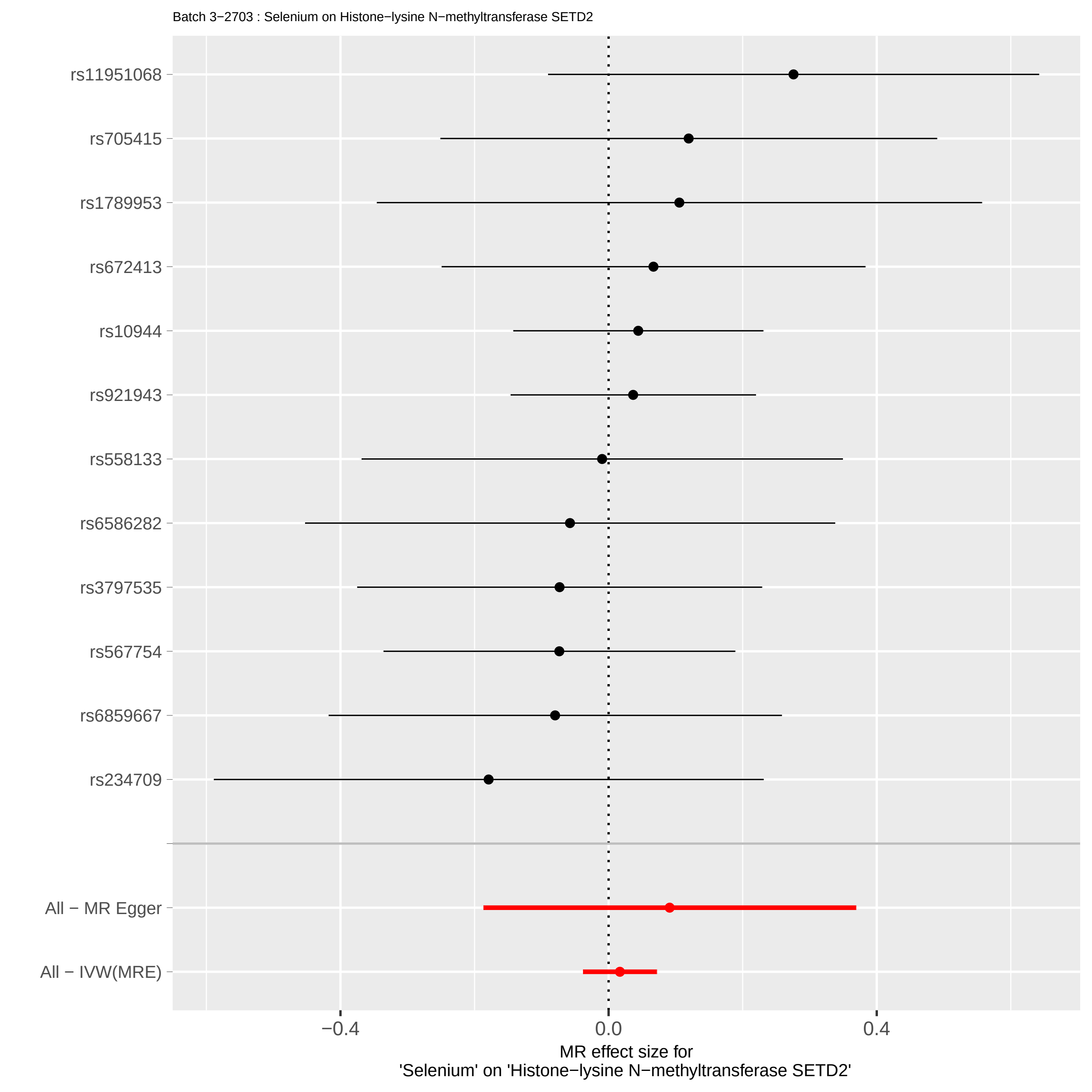
Batch 3−2703 : Selenium on Histone−lysine N−methyltransferase SETD2
rs11951068
rs705415
rs1789953
rs672413
rs10944
rs921943
rs558133
rs6586282
rs3797535
rs567754
rs6859667
rs234709
All − MR Egger
All − IVW(MRE)
−0.4
0.0
0.4
MR effect size for
'Selenium' on 'Histone−lysine N−methyltransferase SETD2'

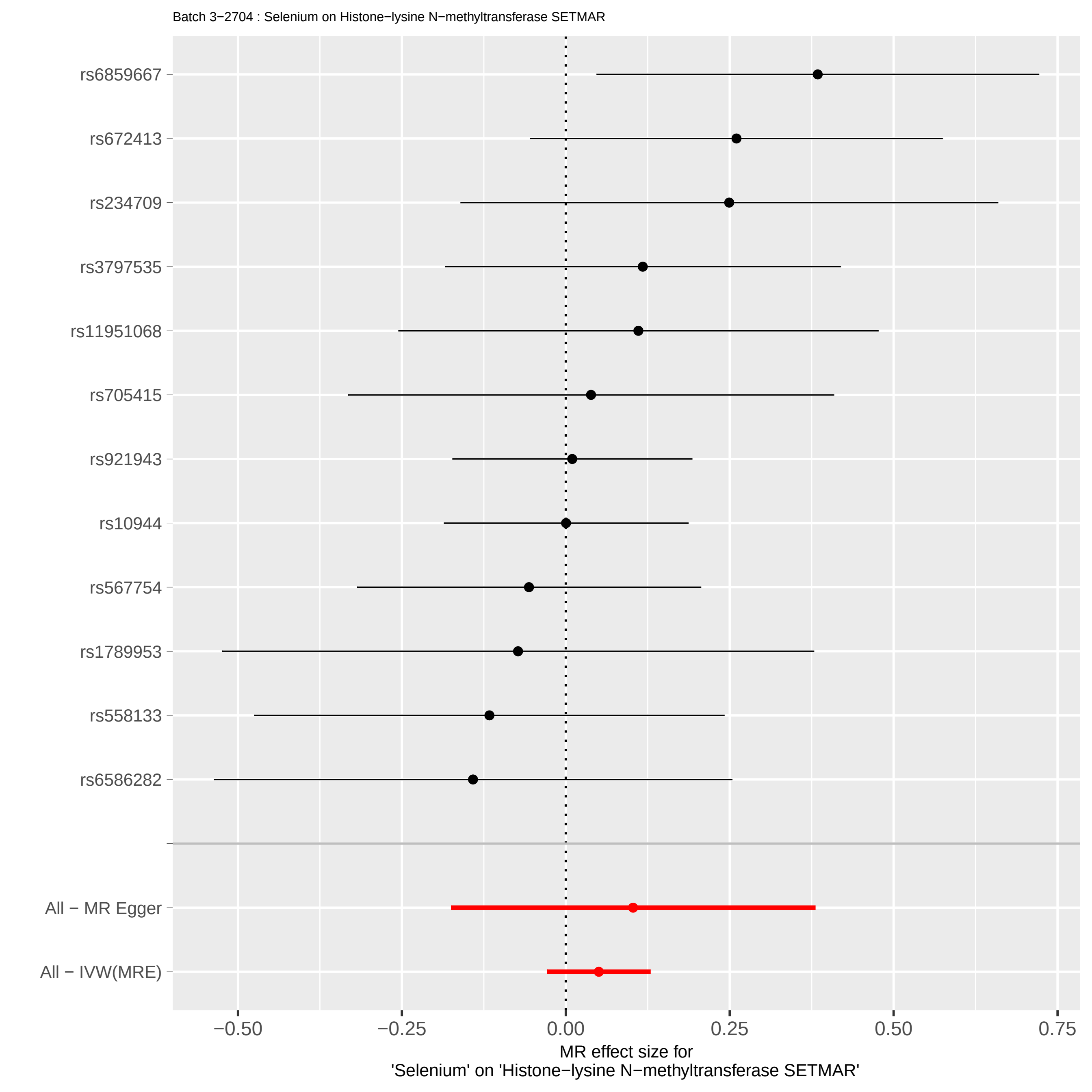

Batch 3−2704 : Selenium on Histone−lysine N−methyltransferase SETMAR
rs6859667
rs672413
rs234709
rs3797535
rs11951068
rs705415
rs921943
rs10944
rs567754
rs1789953
rs558133
rs6586282
All − MR Egger
All − IVW(MRE)
−0.50
−0.25
0.00
0.25
0.50
0.75
MR effect size for
'Selenium' on 'Histone−lysine N−methyltransferase SETMAR'

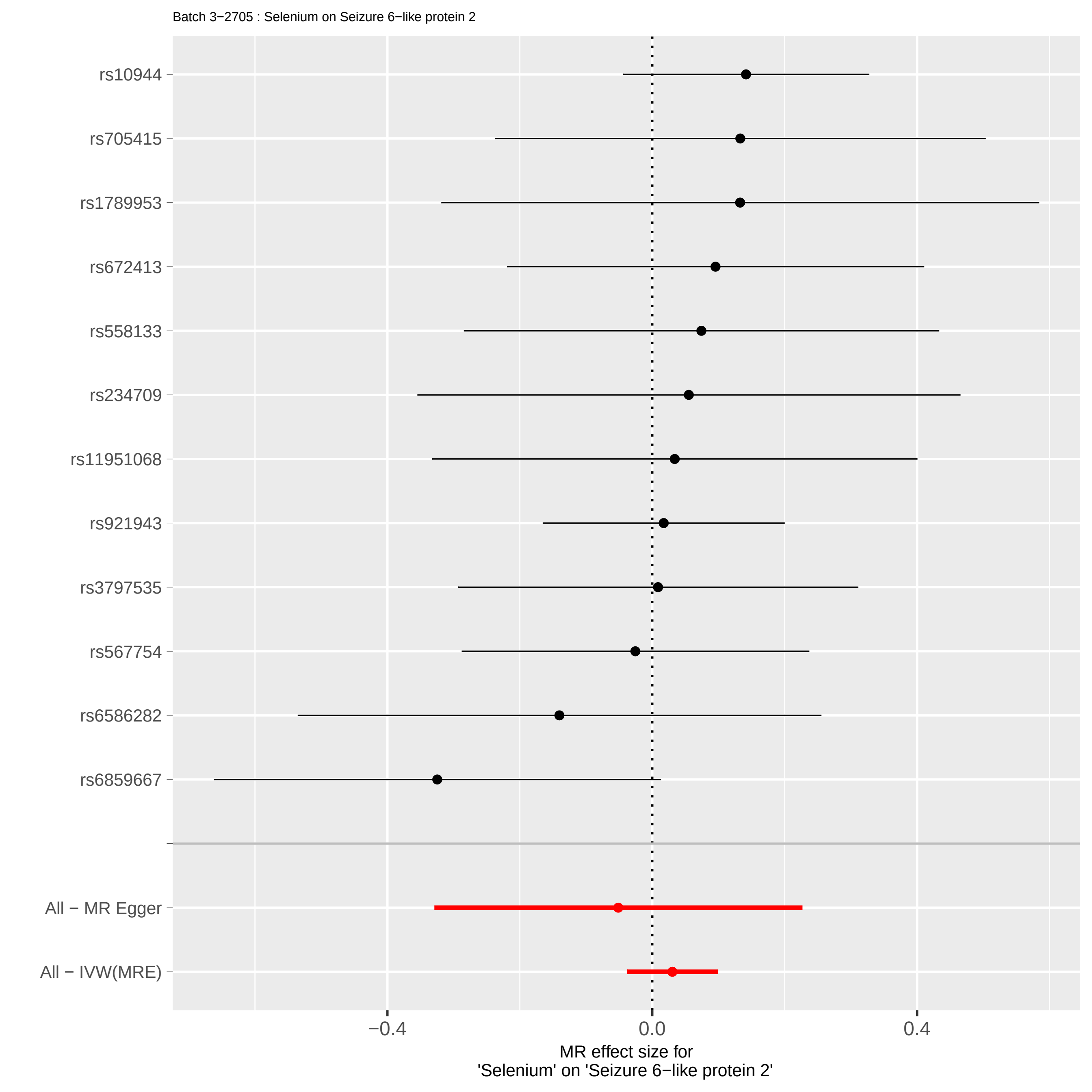
Batch 3−2705 : Selenium on Seizure 6−like protein 2
rs10944
rs705415
rs1789953
rs672413
rs558133
rs234709
rs11951068
rs921943
rs3797535
rs567754
rs6586282
rs6859667
All − MR Egger
All − IVW(MRE)
−0.4
0.0
0.4
MR effect size for
'Selenium' on 'Seizure 6−like protein 2'

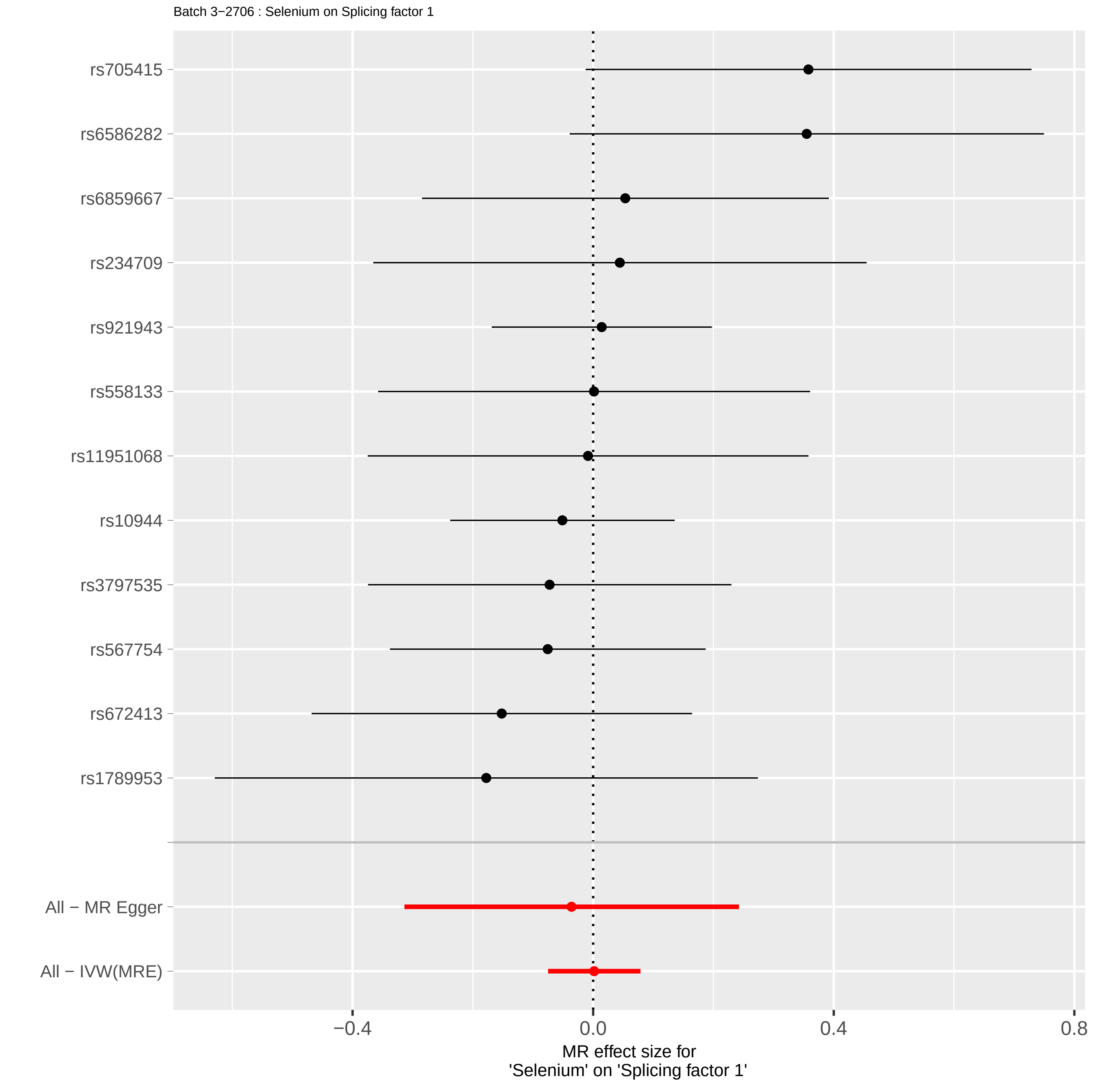

Batch 3−2706 : Selenium on Splicing factor 1
rs705415
rs6586282
rs6859667
rs234709
rs921943
rs558133
rs11951068
rs10944
rs3797535
rs567754
rs672413
rs1789953
All − MR Egger
All − IVW(MRE)
−0.4
0.0
0.4
0.8
MR effect size for
'Selenium' on 'Splicing factor 1'

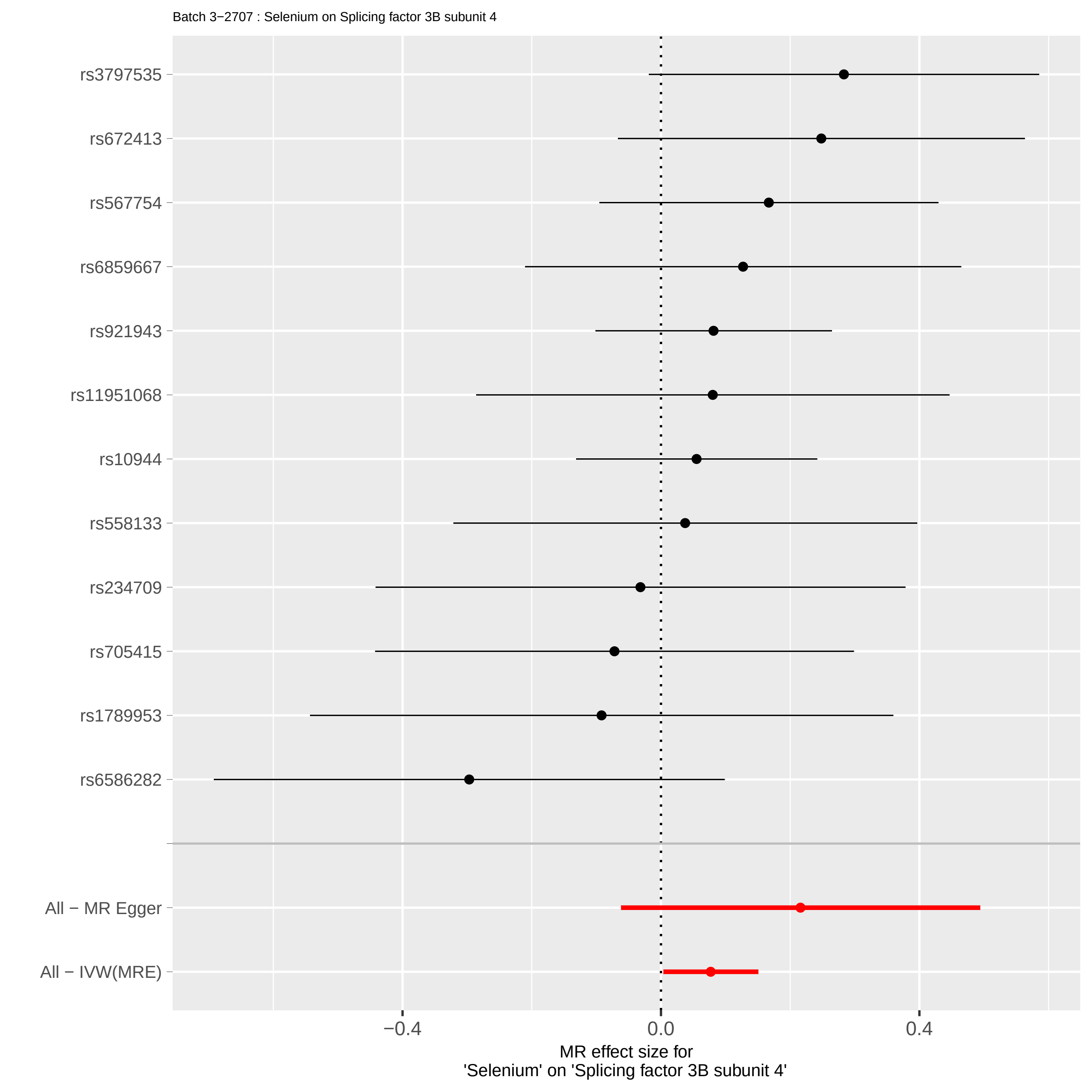

Batch 3−2707 : Selenium on Splicing factor 3B subunit 4
rs3797535
rs672413
rs567754
rs6859667
rs921943
rs11951068
rs10944
rs558133
rs234709
rs705415
rs1789953
rs6586282
All − MR Egger
All − IVW(MRE)
−0.4
0.0
0.4
MR effect size for
'Selenium' on 'Splicing factor 3B subunit 4'

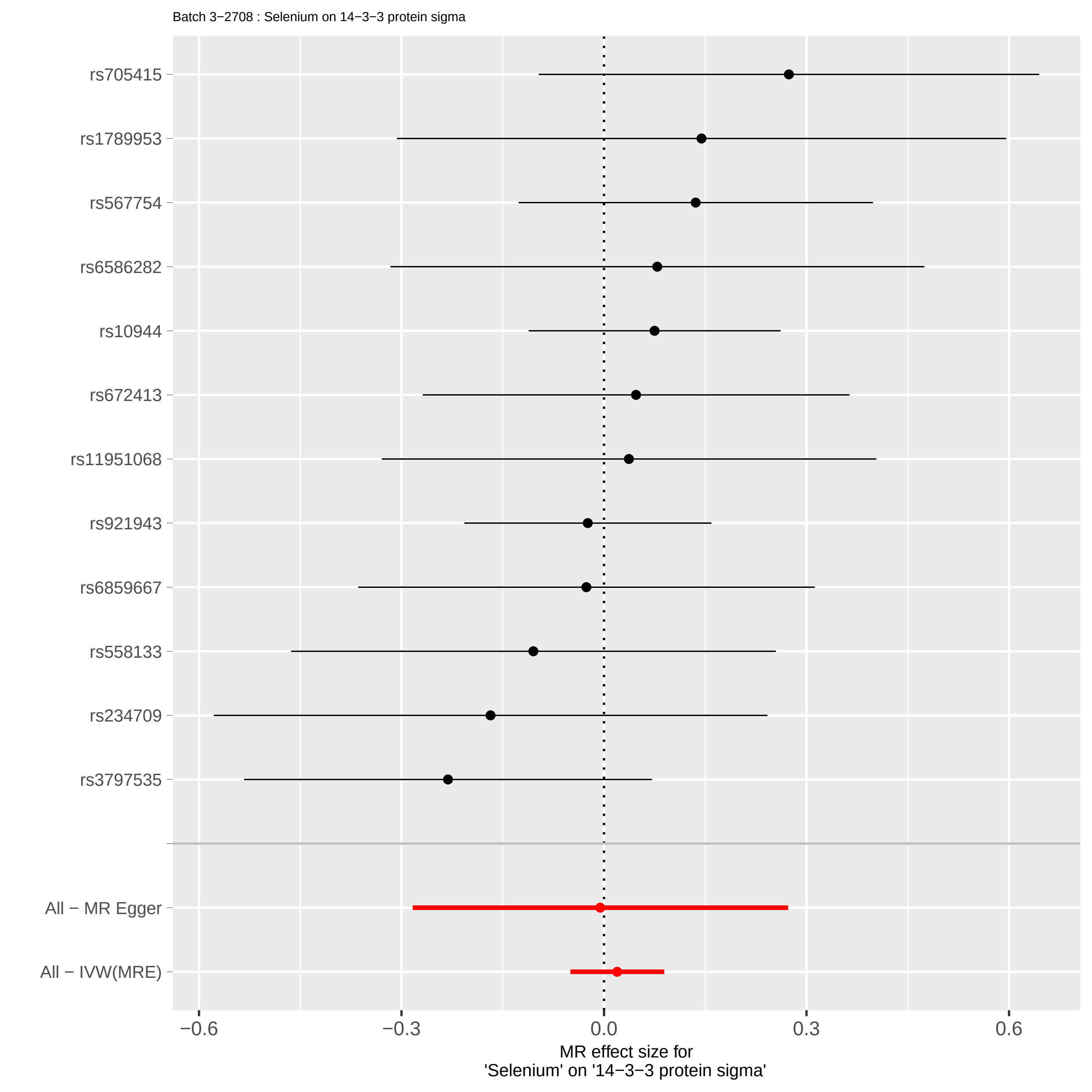
Batch 3−2708 : Selenium on 14−3−3 protein sigma
rs705415
rs1789953
rs567754
rs6586282
rs10944
rs672413
rs11951068
rs921943
rs6859667
rs558133
rs234709
rs3797535
All − MR Egger
All − IVW(MRE)
−0.6
−0.3
0.0
0.3
0.6
MR effect size for
'Selenium' on '14−3−3 protein sigma'

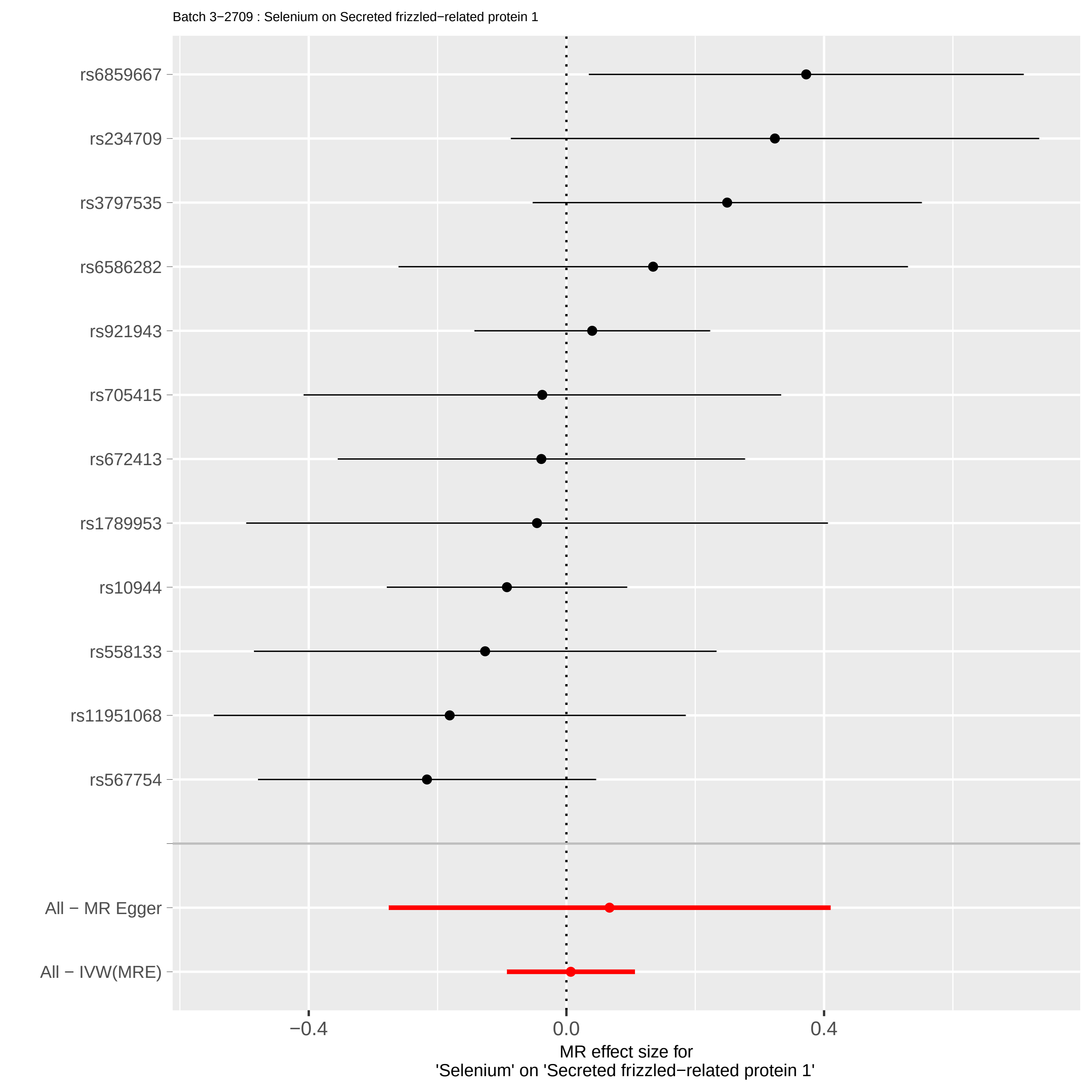
Batch 3−2709 : Selenium on Secreted frizzled−related protein 1
rs6859667
rs234709
rs3797535
rs6586282
rs921943
rs705415
rs672413
rs1789953
rs10944
rs558133
rs11951068
rs567754
All − MR Egger
All − IVW(MRE)
−0.4
0.0
0.4
MR effect size for
'Selenium' on 'Secreted frizzled−related protein 1'

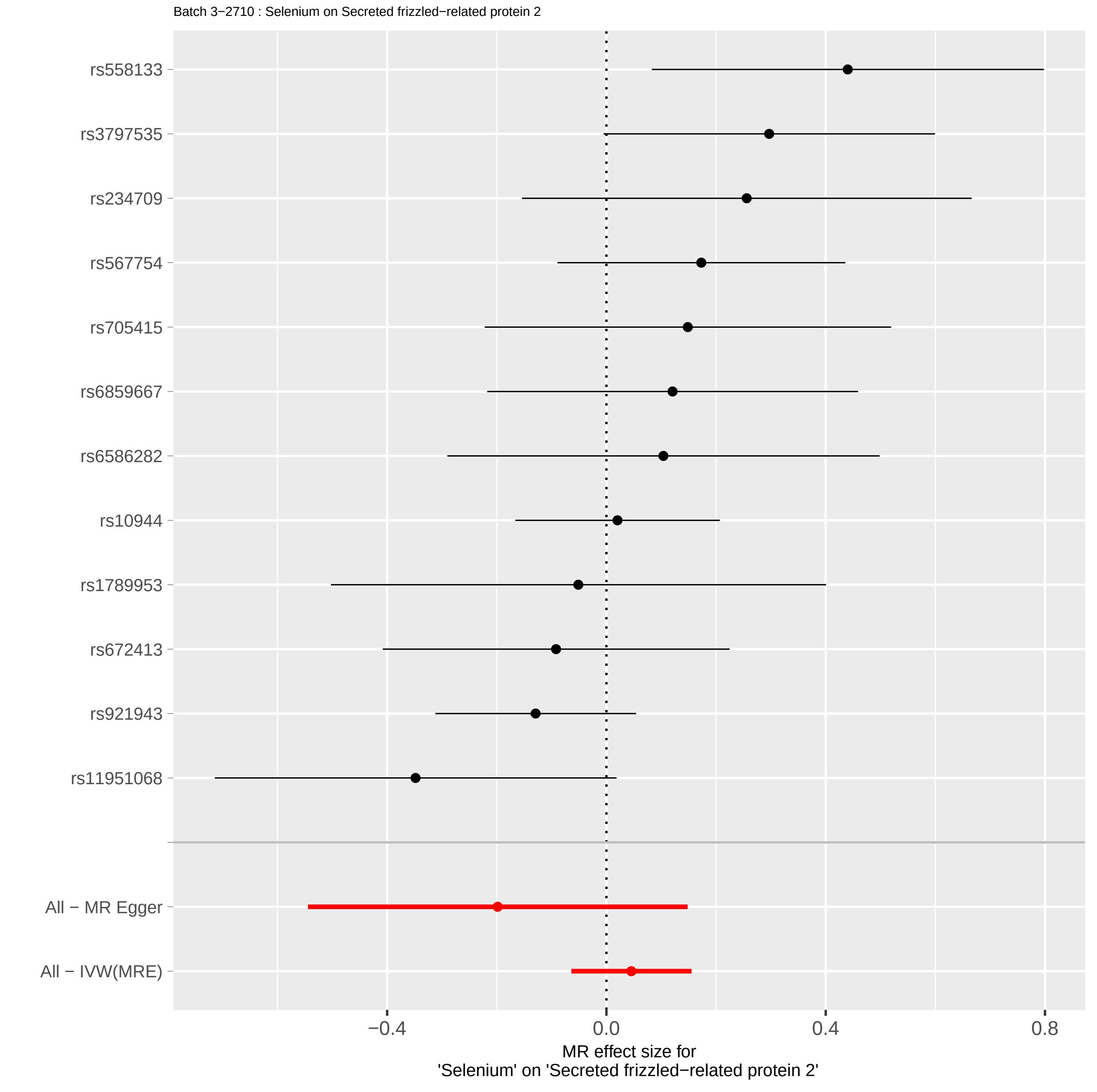

Batch 3−2710 : Selenium on Secreted frizzled−related protein 2
rs558133
rs3797535
rs234709
rs567754
rs705415
rs6859667
rs6586282
rs10944
rs1789953
rs672413
rs921943
rs11951068
All − MR Egger
All − IVW(MRE)
−0.4
0.0
0.4
0.8
MR effect size for
'Selenium' on 'Secreted frizzled−related protein 2'

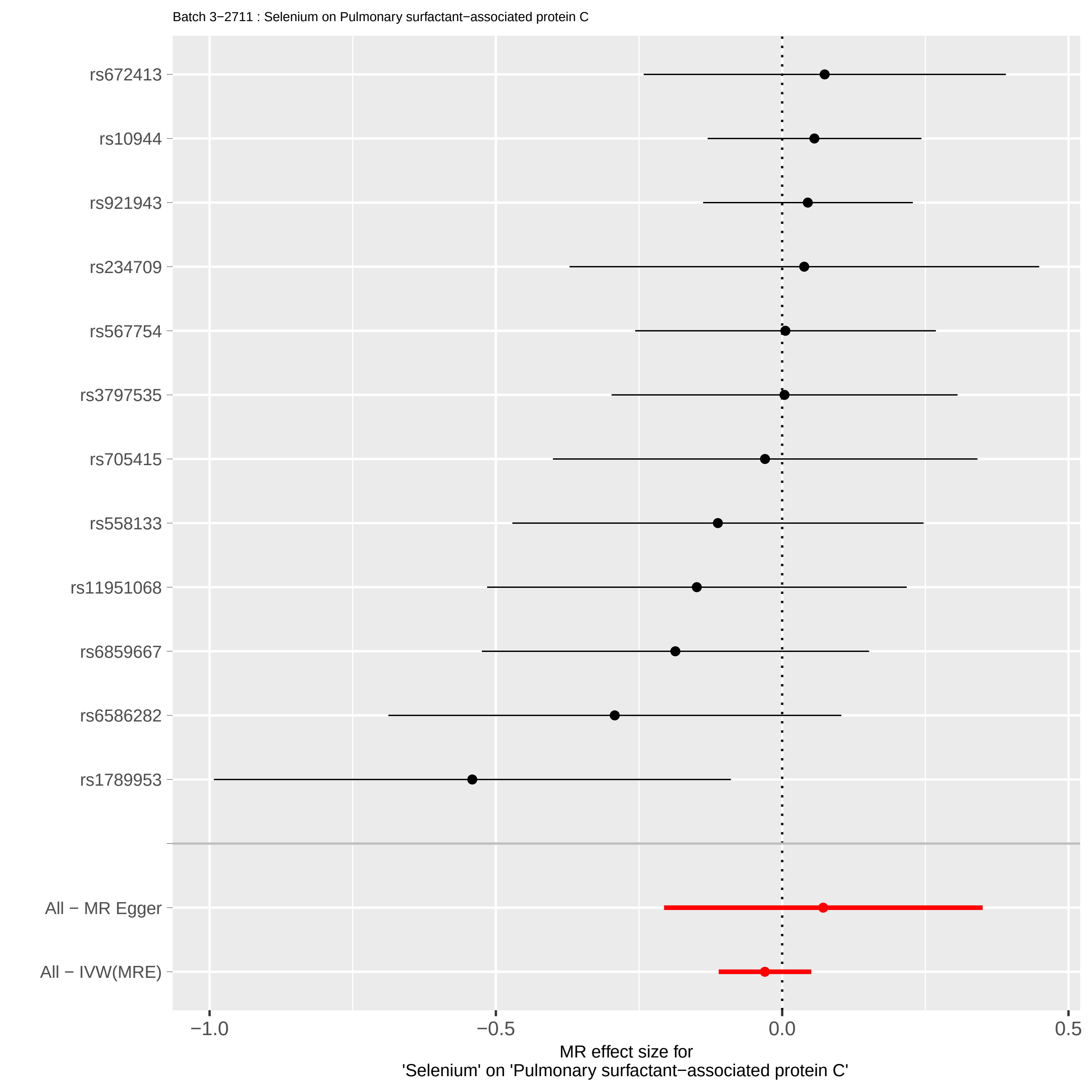

Batch 3−2711 : Selenium on Pulmonary surfactant−associated protein C
rs672413
rs10944
rs921943
rs234709
rs567754
rs3797535
rs705415
rs558133
rs11951068
rs6859667
rs6586282
rs1789953
All − MR Egger
All − IVW(MRE)
−1.0
−0.5
0.0
0.5
MR effect size for
'Selenium' on 'Pulmonary surfactant−associated protein C'

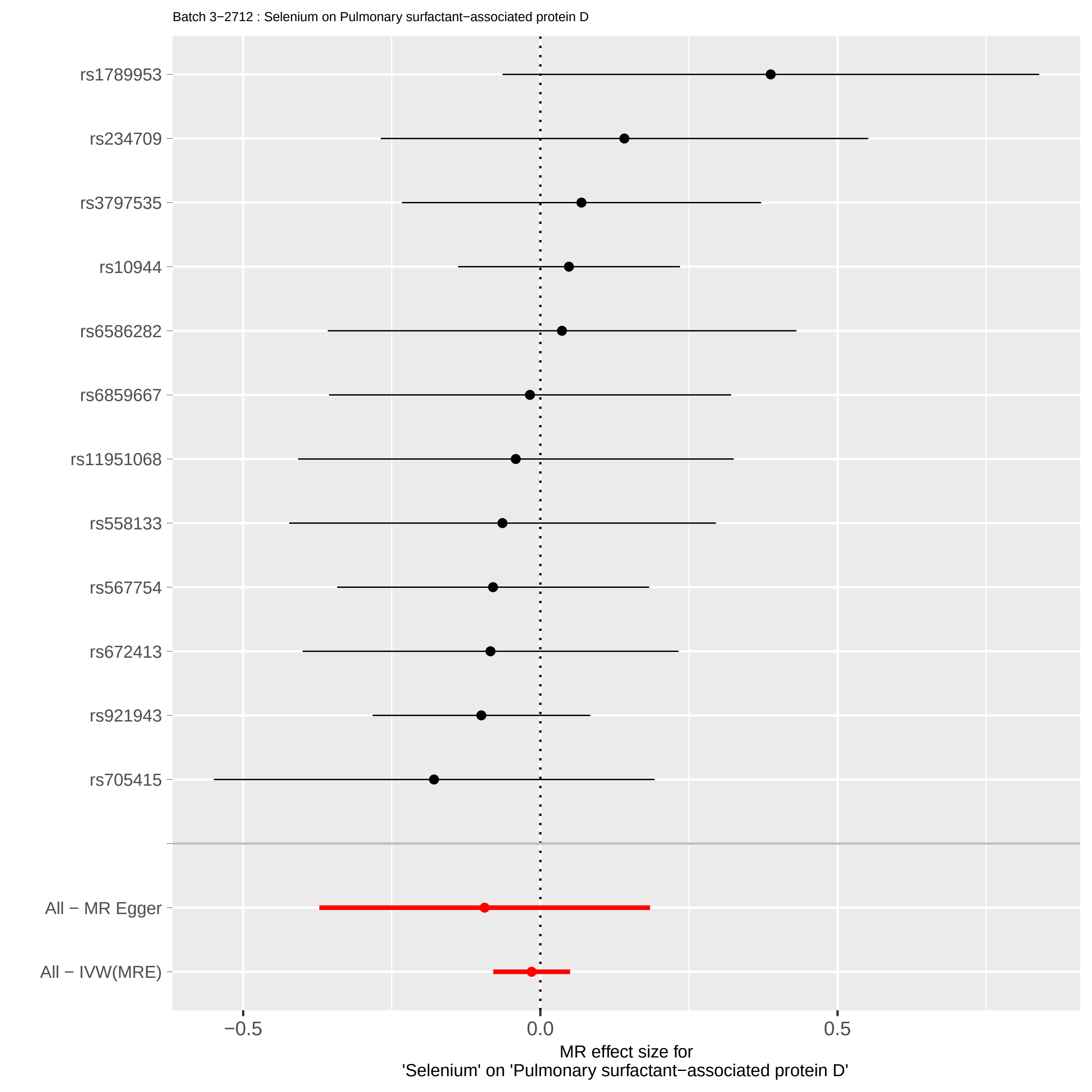

Batch 3−2712 : Selenium on Pulmonary surfactant−associated protein D
rs1789953
rs234709
rs3797535
rs10944
rs6586282
rs6859667
rs11951068
rs558133
rs567754
rs672413
rs921943
rs705415
All − MR Egger
All − IVW(MRE)
−0.5
0.0
0.5
MR effect size for
'Selenium' on 'Pulmonary surfactant−associated protein D'

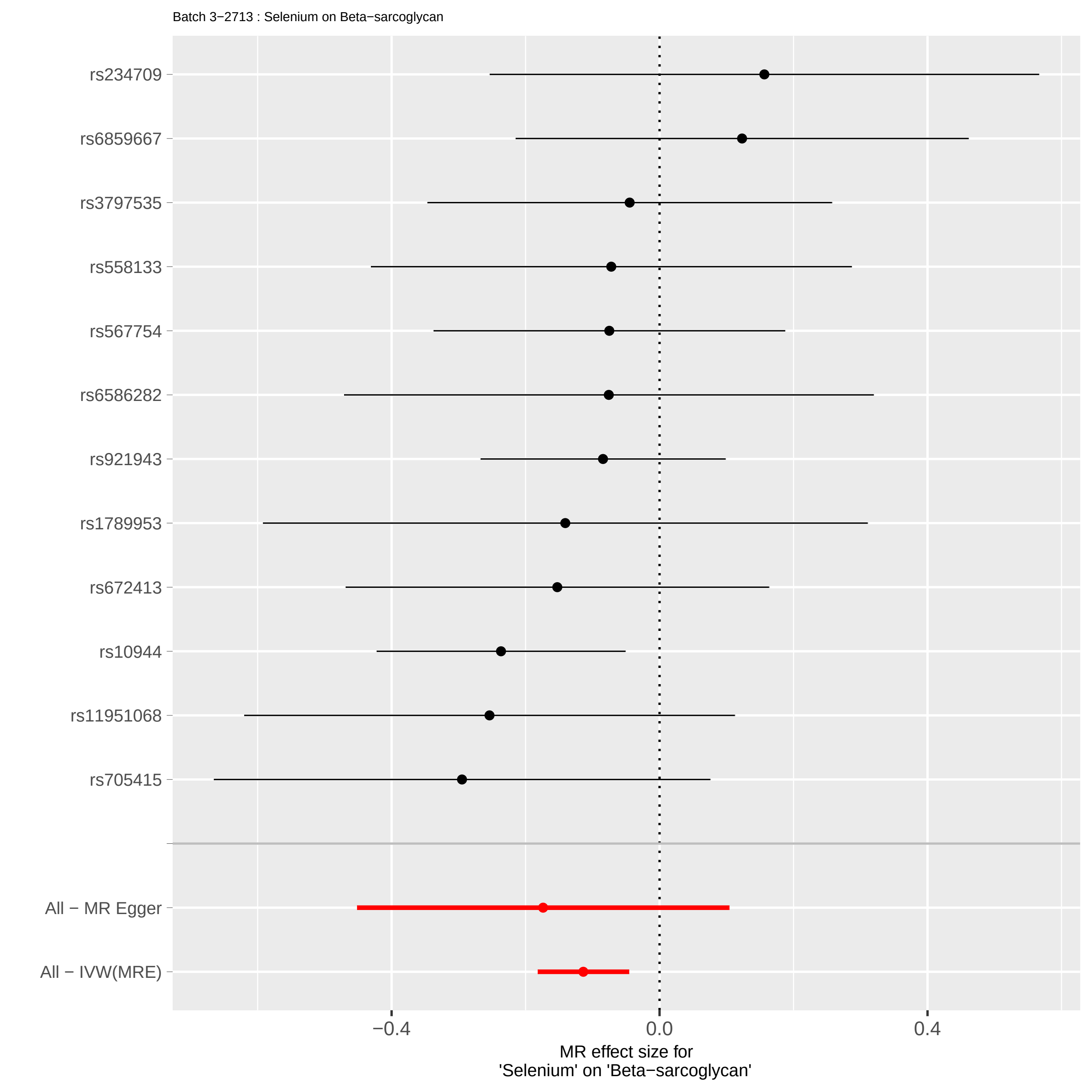

Batch 3−2713 : Selenium on Beta−sarcoglycan
rs234709
rs6859667
rs3797535
rs558133
rs567754
rs6586282
rs921943
rs1789953
rs672413
rs10944
rs11951068
rs705415
All − MR Egger
All − IVW(MRE)
−0.4
0.0
0.4
MR effect size for
'Selenium' on 'Beta−sarcoglycan'

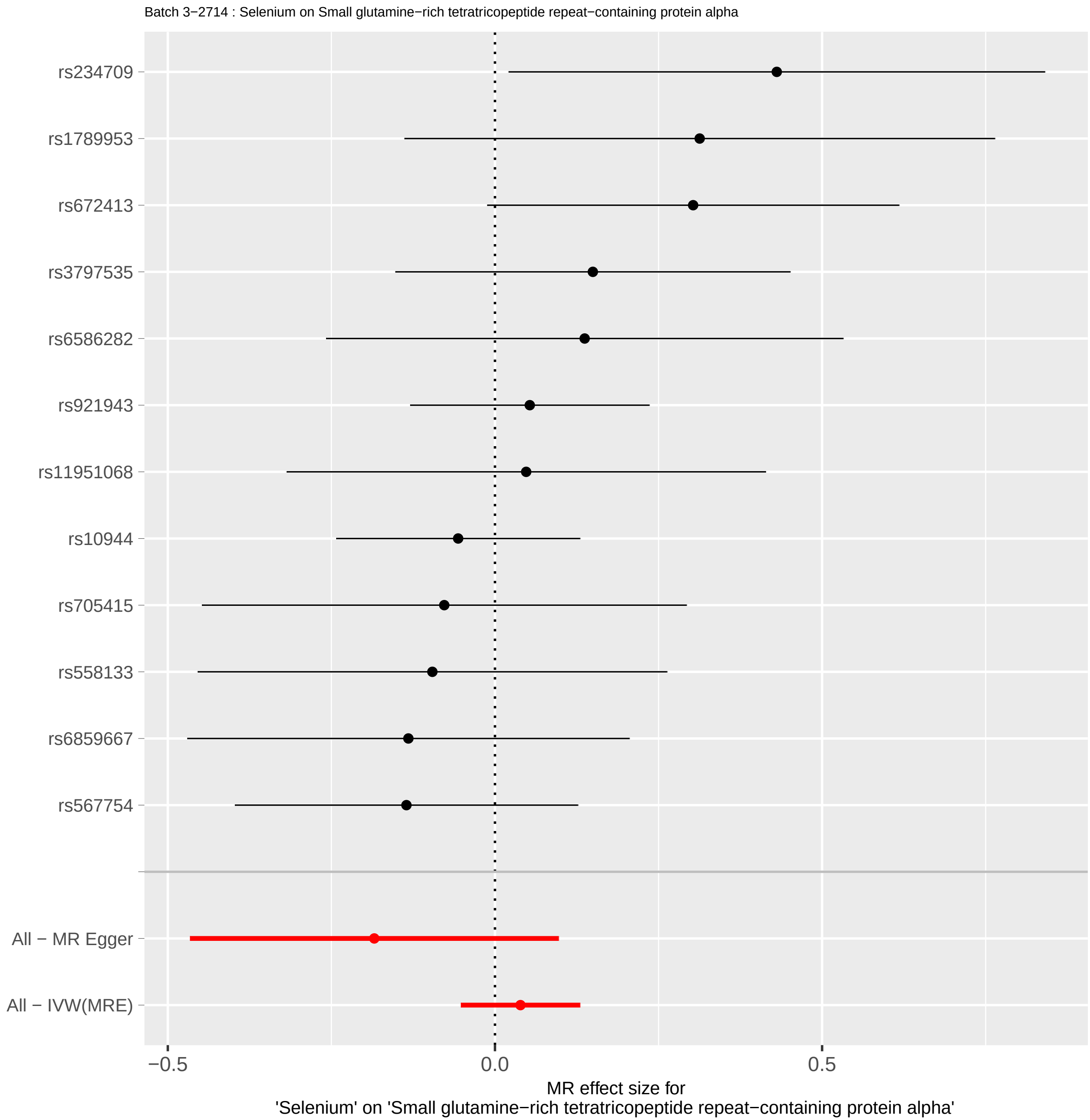
Batch 3−2714 : Selenium on Small glutamine−rich tetratricopeptide repeat−containing protein alpha
rs234709
rs1789953
rs672413
rs3797535
rs6586282
rs921943
rs11951068
rs10944
rs705415
rs558133
rs6859667
rs567754
All − MR Egger
All − IVW(MRE)
−0.5
0.0
0.5
MR effect size for
'Selenium' on 'Small glutamine−rich tetratricopeptide repeat−containing protein alpha'

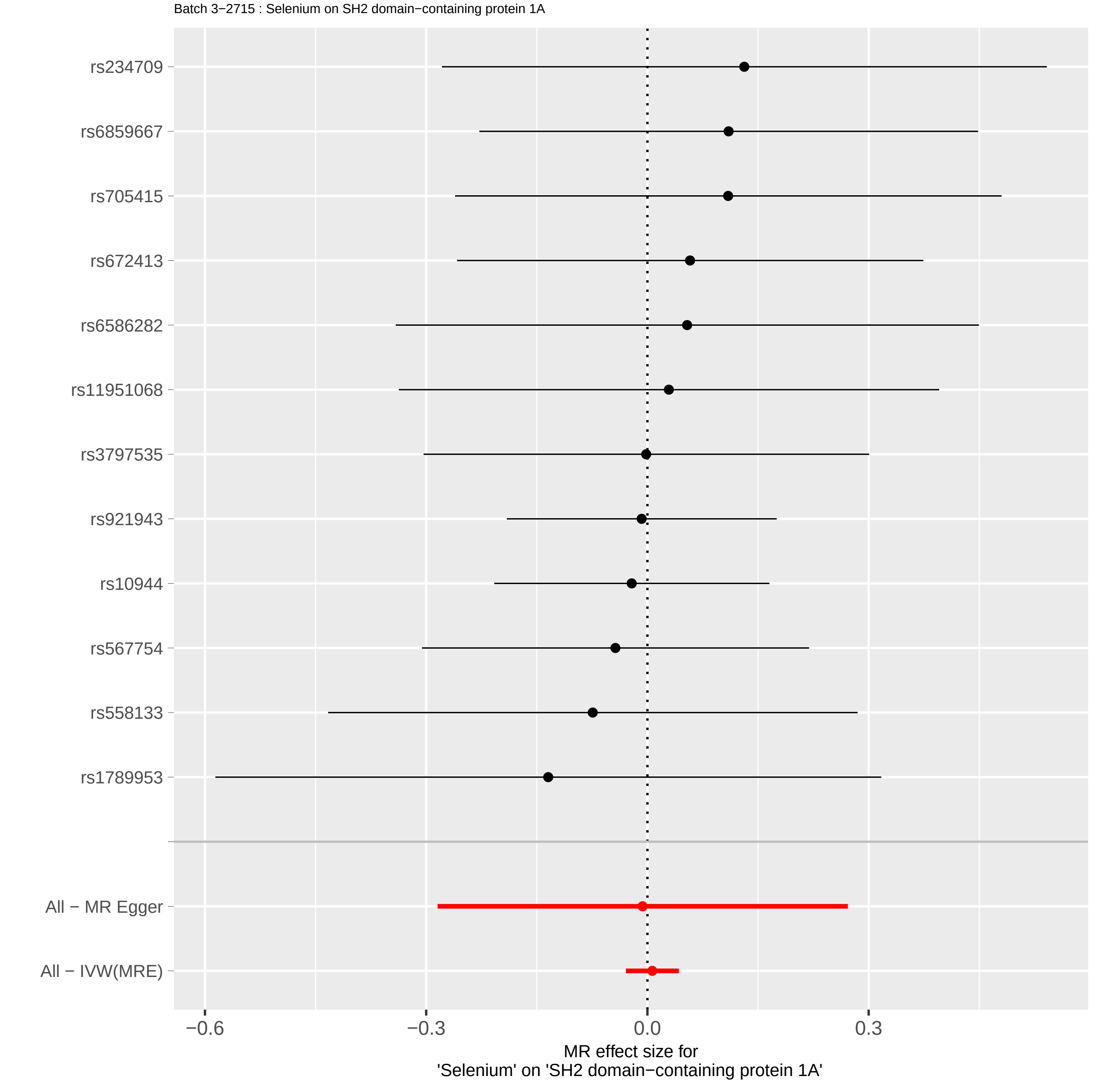
Batch 3−2715 : Selenium on SH2 domain−containing protein 1A
rs234709
rs6859667
rs705415
rs672413
rs6586282
rs11951068
rs3797535
rs921943
rs10944
rs567754
rs558133
rs1789953
All − MR Egger
All − IVW(MRE)
−0.6
−0.3
0.0
0.3
MR effect size for
'Selenium' on 'SH2 domain−containing protein 1A'

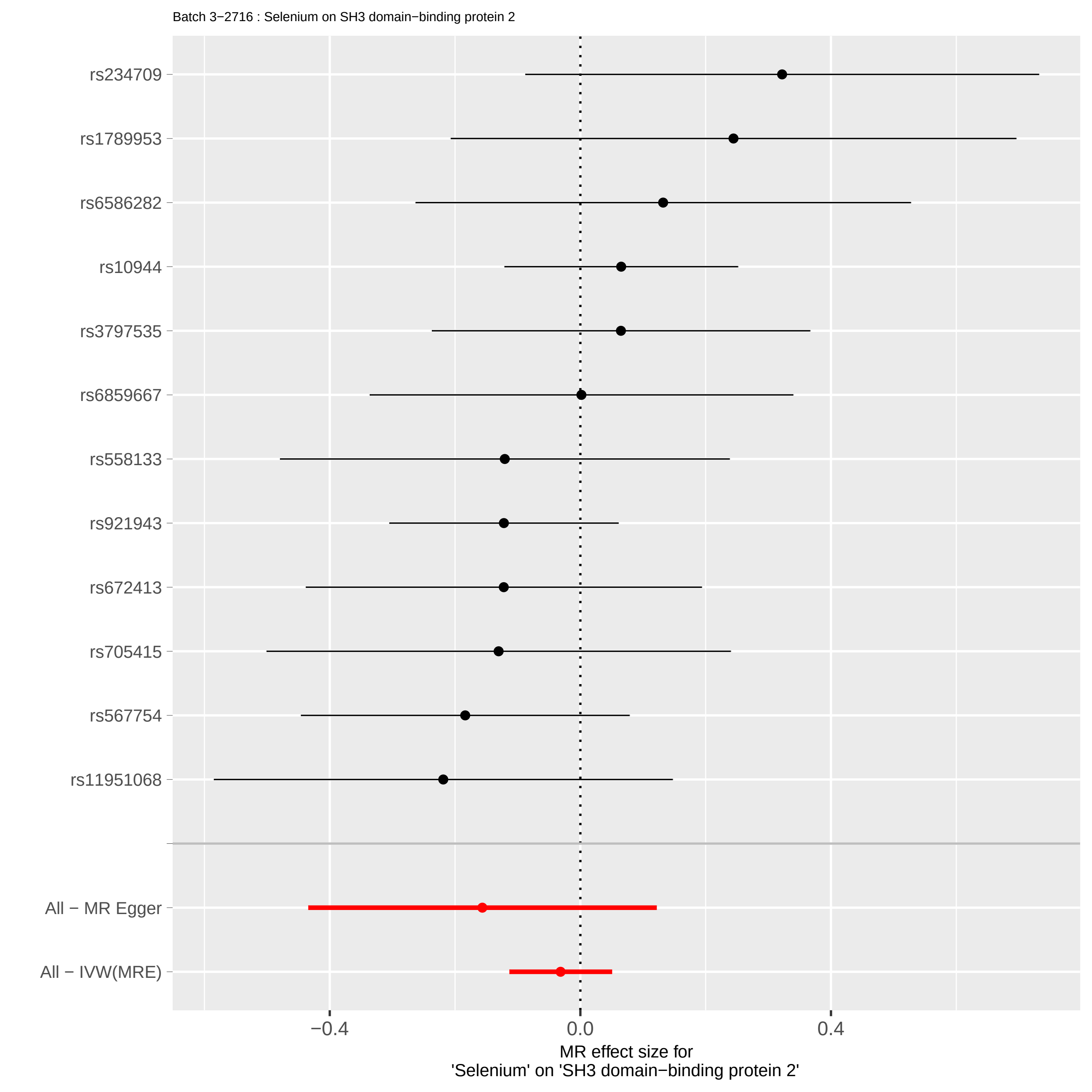

Batch 3−2716 : Selenium on SH3 domain−binding protein 2
rs234709
rs1789953
rs6586282
rs10944
rs3797535
rs6859667
rs558133
rs921943
rs672413
rs705415
rs567754
rs11951068
All − MR Egger
All − IVW(MRE)
−0.4
0.0
0.4
MR effect size for
'Selenium' on 'SH3 domain−binding protein 2'

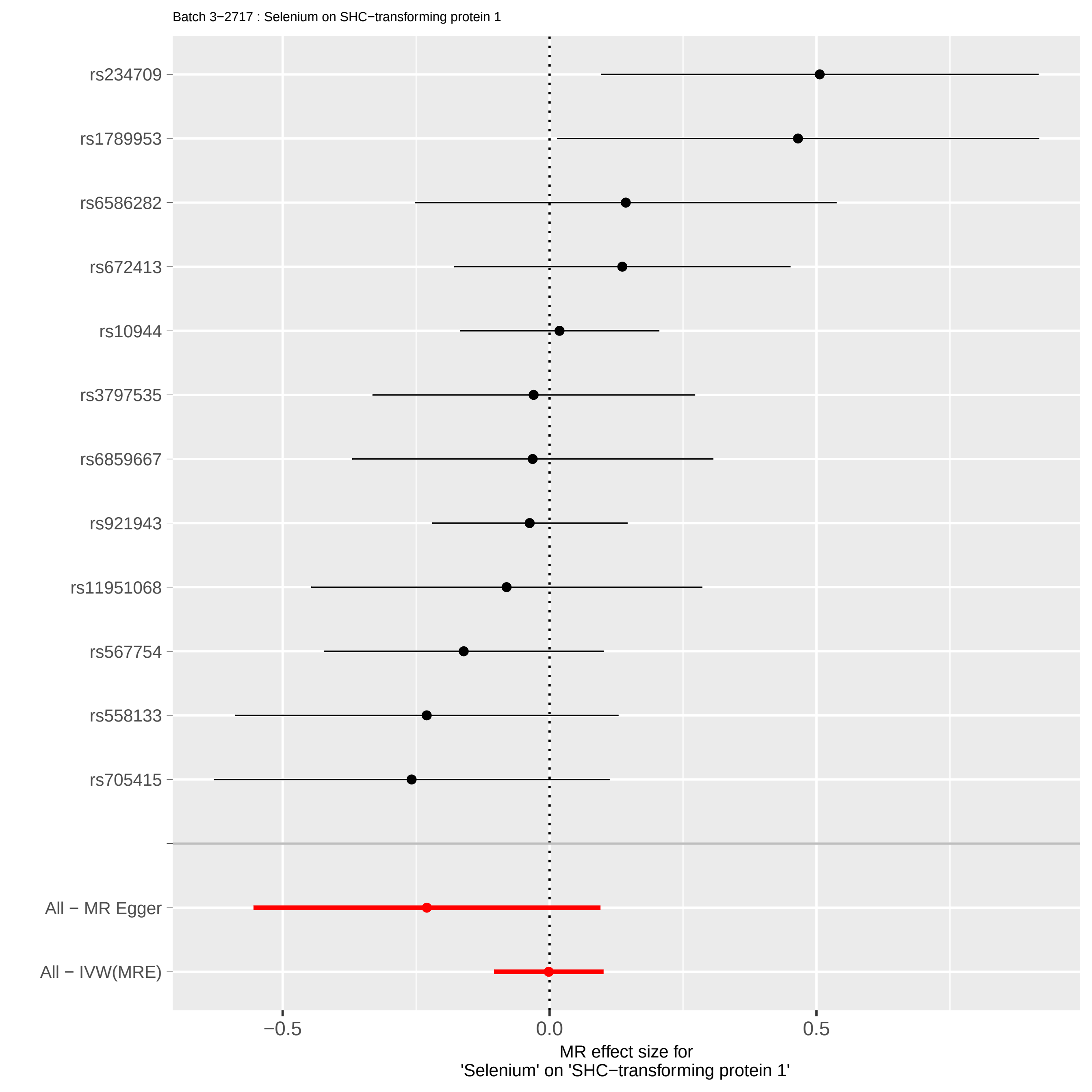
Batch 3−2717 : Selenium on SHC−transforming protein 1
rs234709
rs1789953
rs6586282
rs672413
rs10944
rs3797535
rs6859667
rs921943
rs11951068
rs567754
rs558133
rs705415
All − MR Egger
All − IVW(MRE)
−0.5
0.0
0.5
MR effect size for
'Selenium' on 'SHC−transforming protein 1'

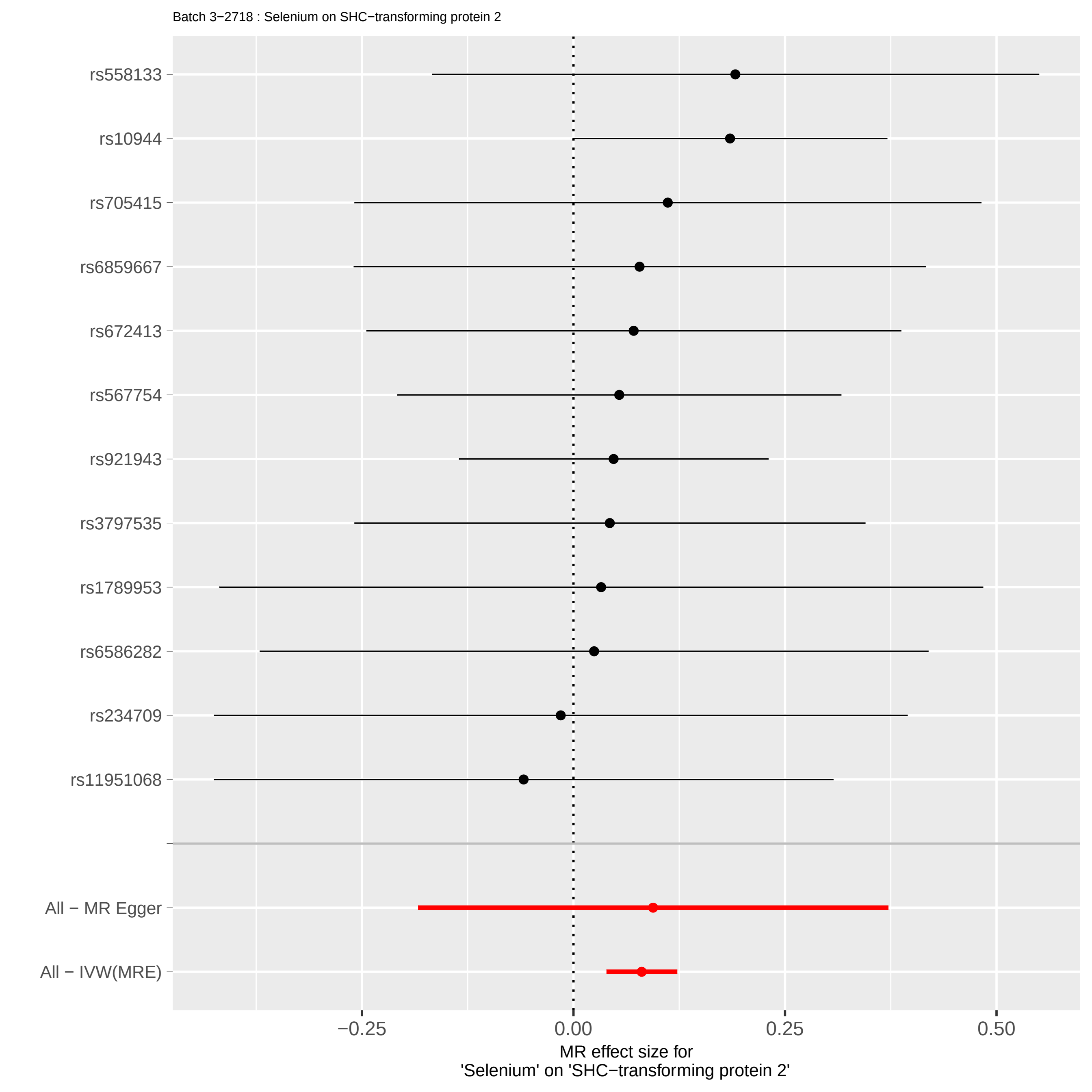

Batch 3−2718 : Selenium on SHC−transforming protein 2
rs558133
rs10944
rs705415
rs6859667
rs672413
rs567754
rs921943
rs3797535
rs1789953
rs6586282
rs234709
rs11951068
All − MR Egger
All − IVW(MRE)
−0.25
0.00
0.25
0.50
MR effect size for
'Selenium' on 'SHC−transforming protein 2'

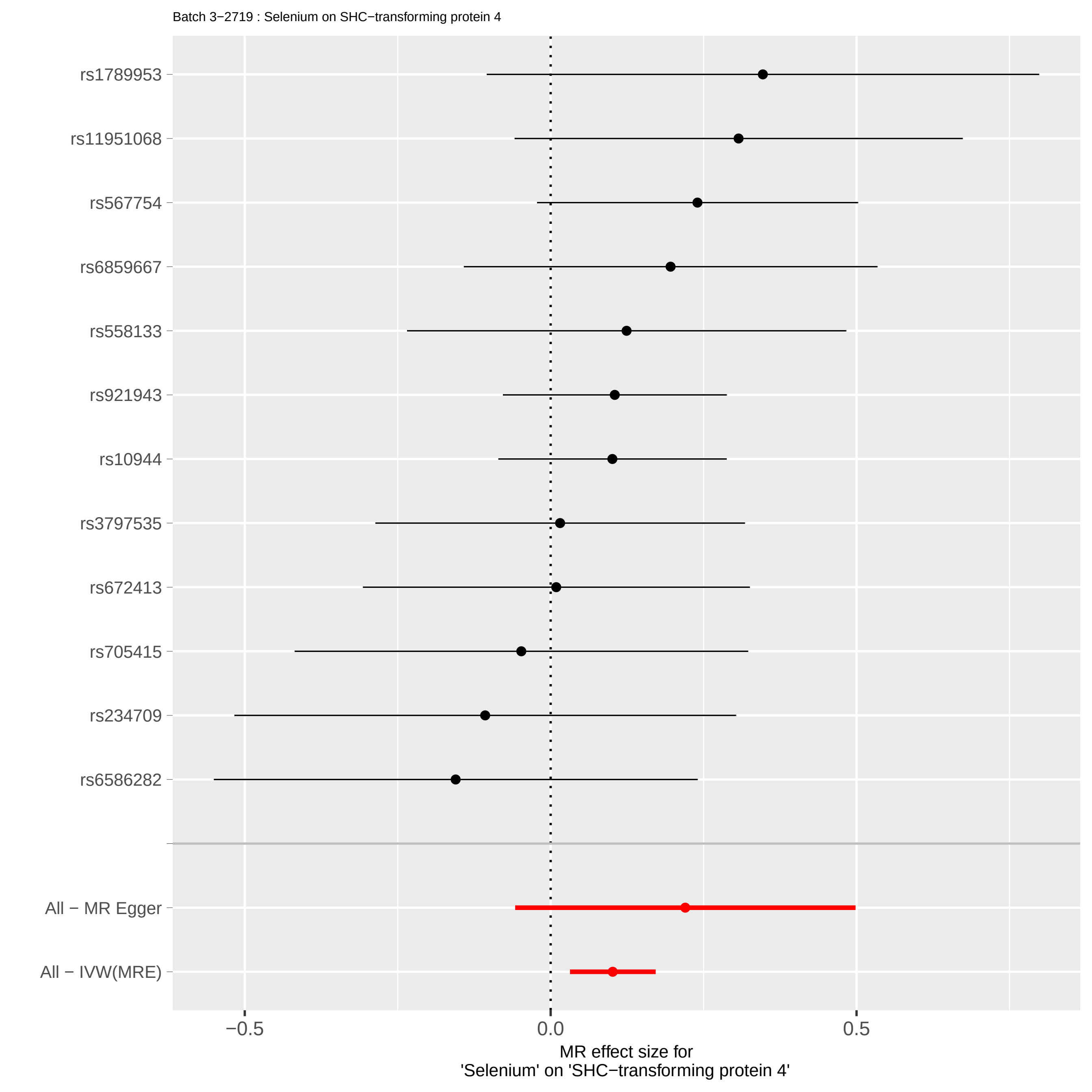

Batch 3−2719 : Selenium on SHC−transforming protein 4
rs1789953
rs11951068
rs567754
rs6859667
rs558133
rs921943
rs10944
rs3797535
rs672413
rs705415
rs234709
rs6586282
All − MR Egger
All − IVW(MRE)
−0.5
0.0
0.5
MR effect size for
'Selenium' on 'SHC−transforming protein 4'

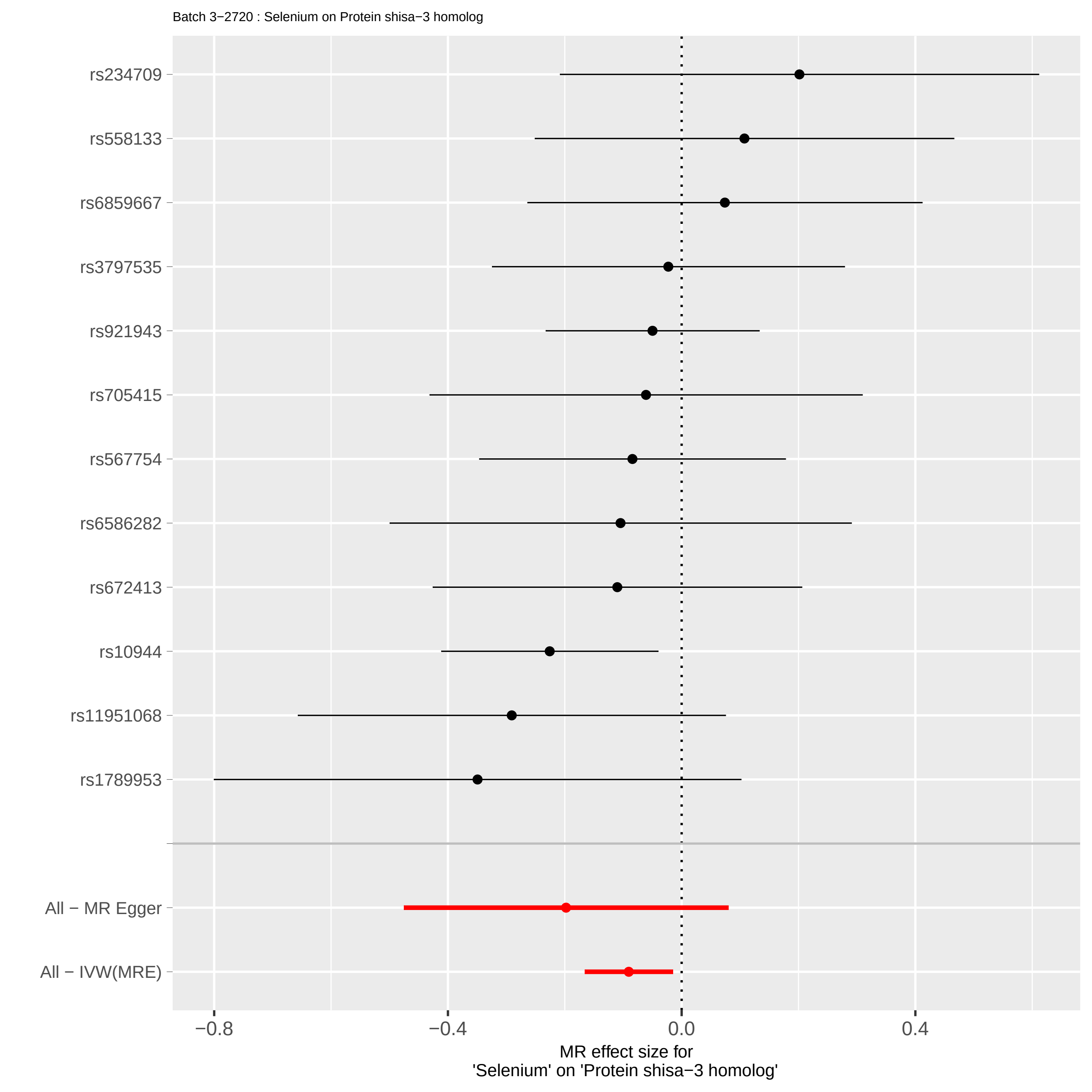
Batch 3−2720 : Selenium on Protein shisa−3 homolog
rs234709
rs558133
rs6859667
rs3797535
rs921943
rs705415
rs567754
rs6586282
rs672413
rs10944
rs11951068
rs1789953
All − MR Egger
All − IVW(MRE)
−0.8
−0.4
0.0
0.4
MR effect size for
'Selenium' on 'Protein shisa−3 homolog'

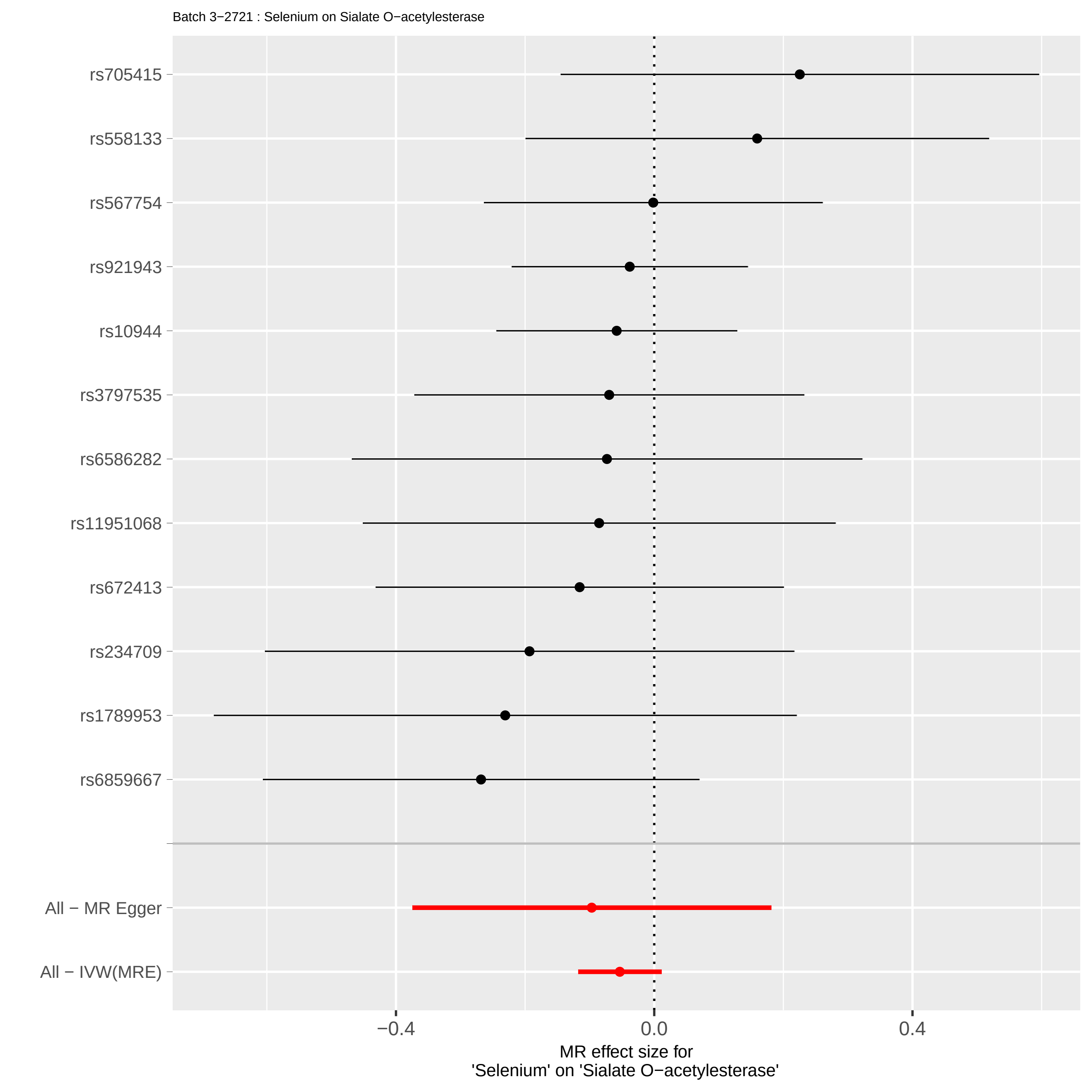

Batch 3−2721 : Selenium on Sialate O−acetylesterase
rs705415
rs558133
rs567754
rs921943
rs10944
rs3797535
rs6586282
rs11951068
rs672413
rs234709
rs1789953
rs6859667
All − MR Egger
All − IVW(MRE)
−0.4
0.0
0.4
MR effect size for
'Selenium' on 'Sialate O−acetylesterase'

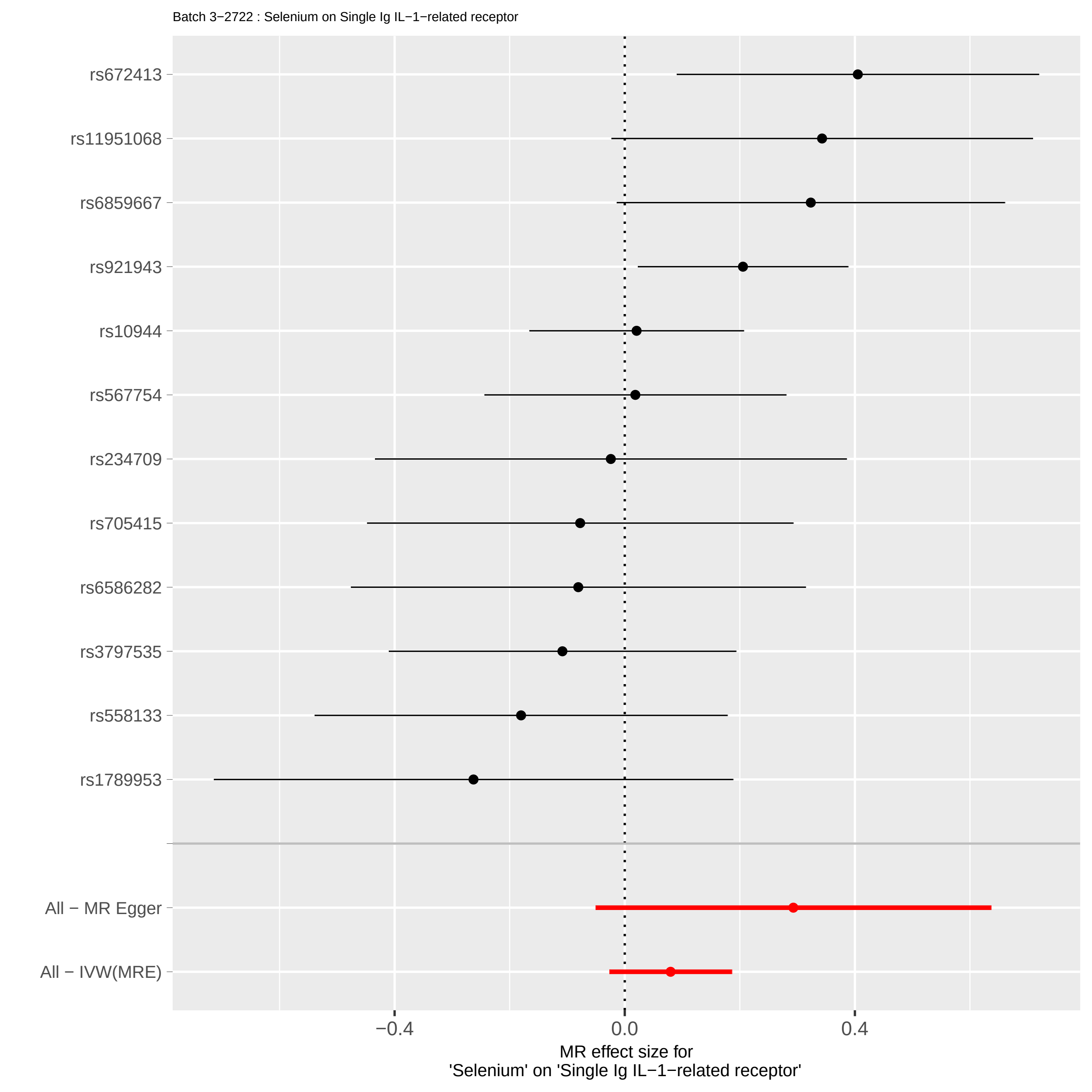
Batch 3−2722 : Selenium on Single Ig IL−1−related receptor
rs672413
rs11951068
rs6859667
rs921943
rs10944
rs567754
rs234709
rs705415
rs6586282
rs3797535
rs558133
rs1789953
All − MR Egger
All − IVW(MRE)
−0.4
0.0
0.4
MR effect size for
'Selenium' on 'Single Ig IL−1−related receptor'

Batch 3−2723 : Selenium on Sialic acid−binding Ig−like lectin 10

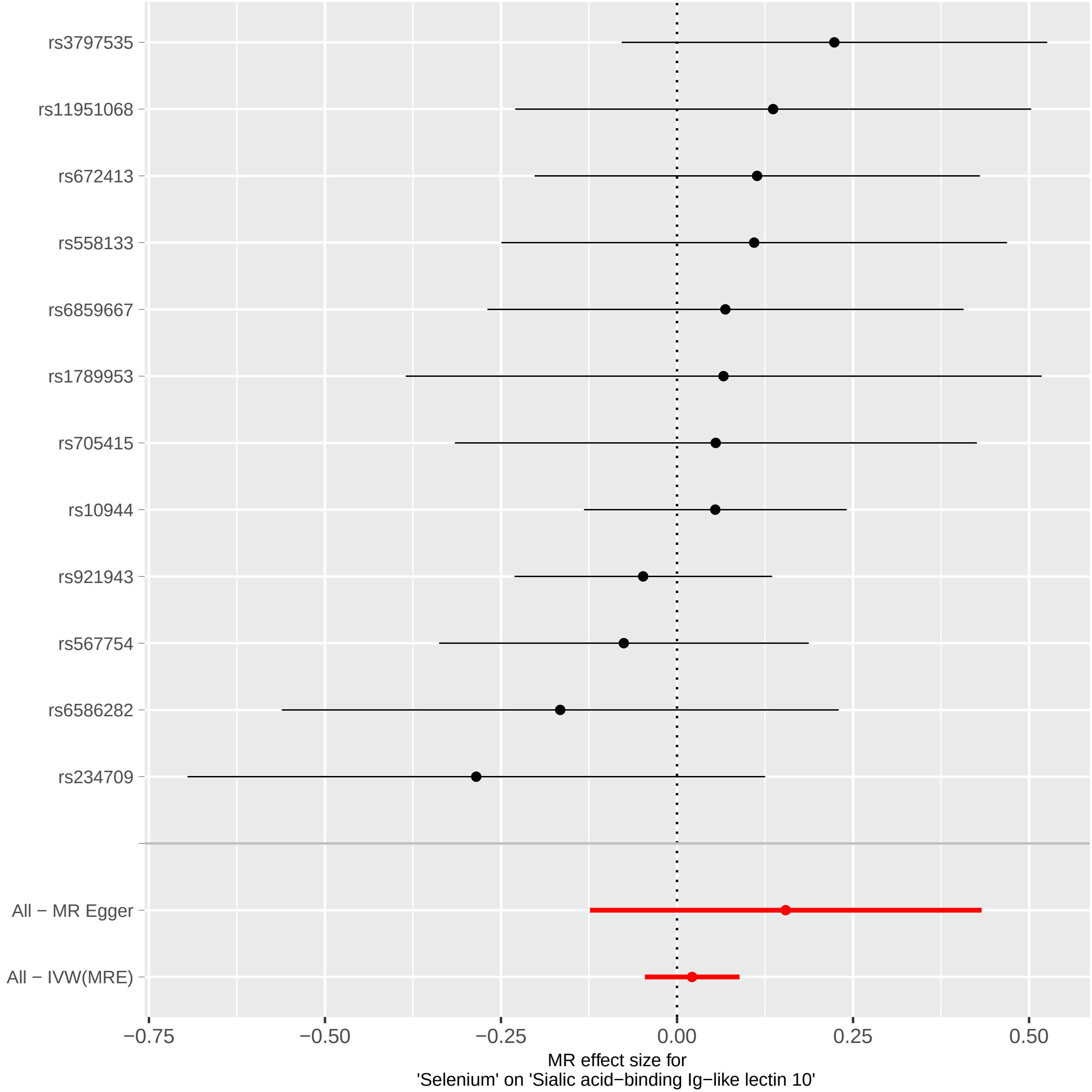

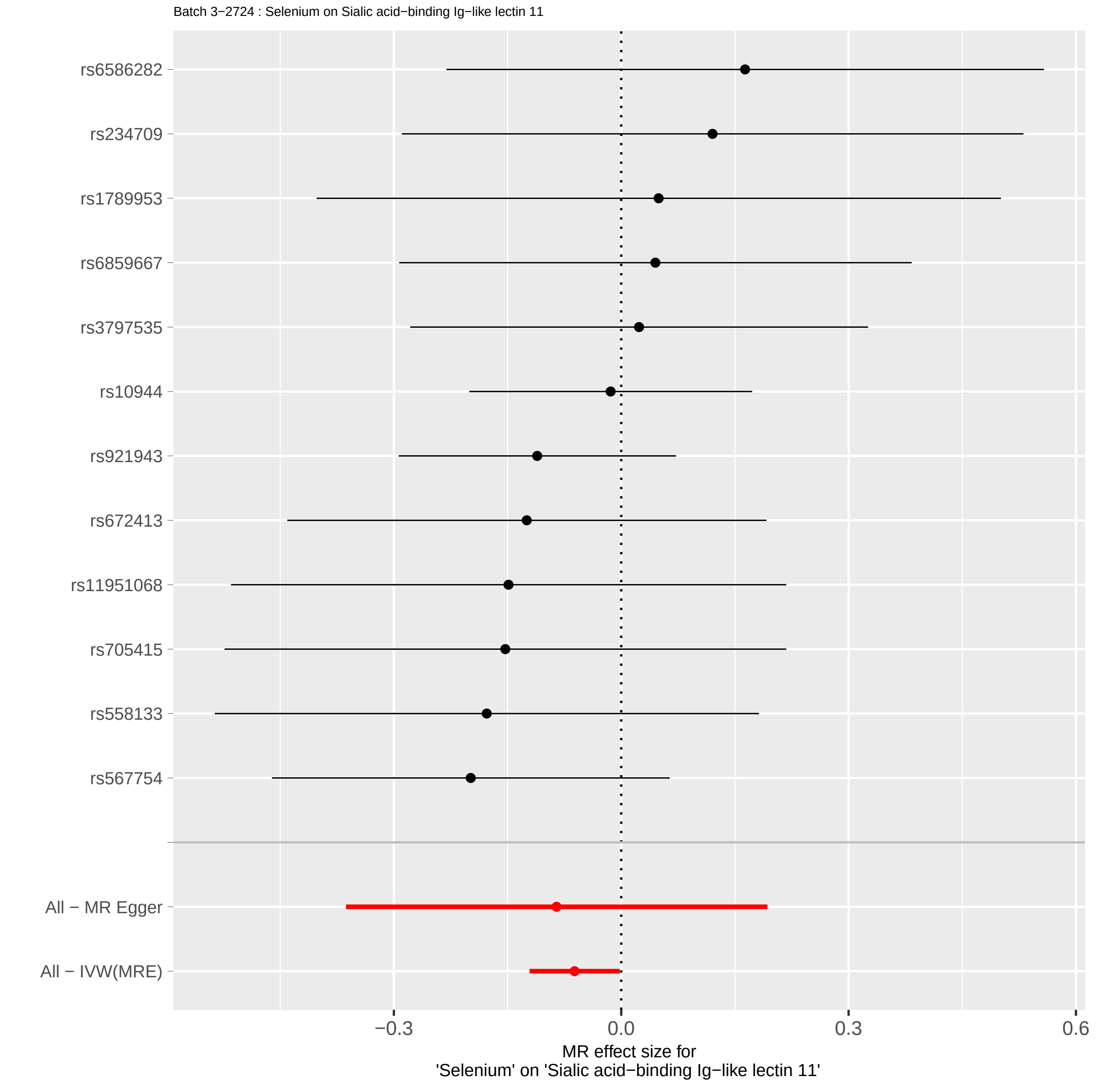

Batch 3−2724 : Selenium on Sialic acid−binding Ig−like lectin 11
rs6586282
rs234709
rs1789953
rs6859667
rs3797535
rs10944
rs921943
rs672413
rs11951068
rs705415
rs558133
rs567754
All − MR Egger
All − IVW(MRE)
−0.3
0.0
0.3
0.6
MR effect size for
'Selenium' on 'Sialic acid−binding Ig−like lectin 11'

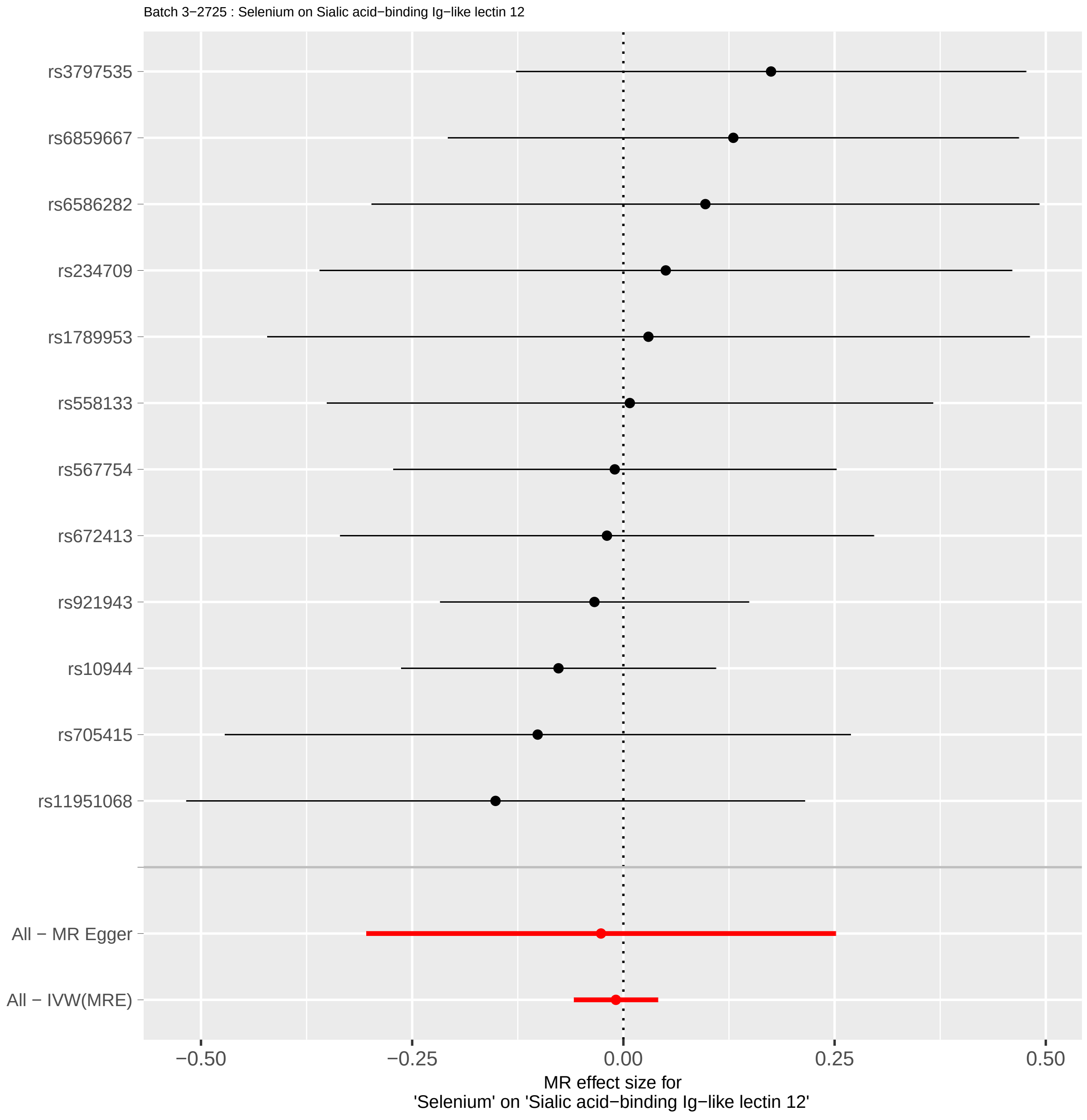

Batch 3−2725 : Selenium on Sialic acid−binding Ig−like lectin 12
rs3797535
rs6859667
rs6586282
rs234709
rs1789953
rs558133
rs567754
rs672413
rs921943
rs10944
rs705415
rs11951068
All − MR Egger
All − IVW(MRE)
−0.50
−0.25
0.00
0.25
0.50
MR effect size for
'Selenium' on 'Sialic acid−binding Ig−like lectin 12'

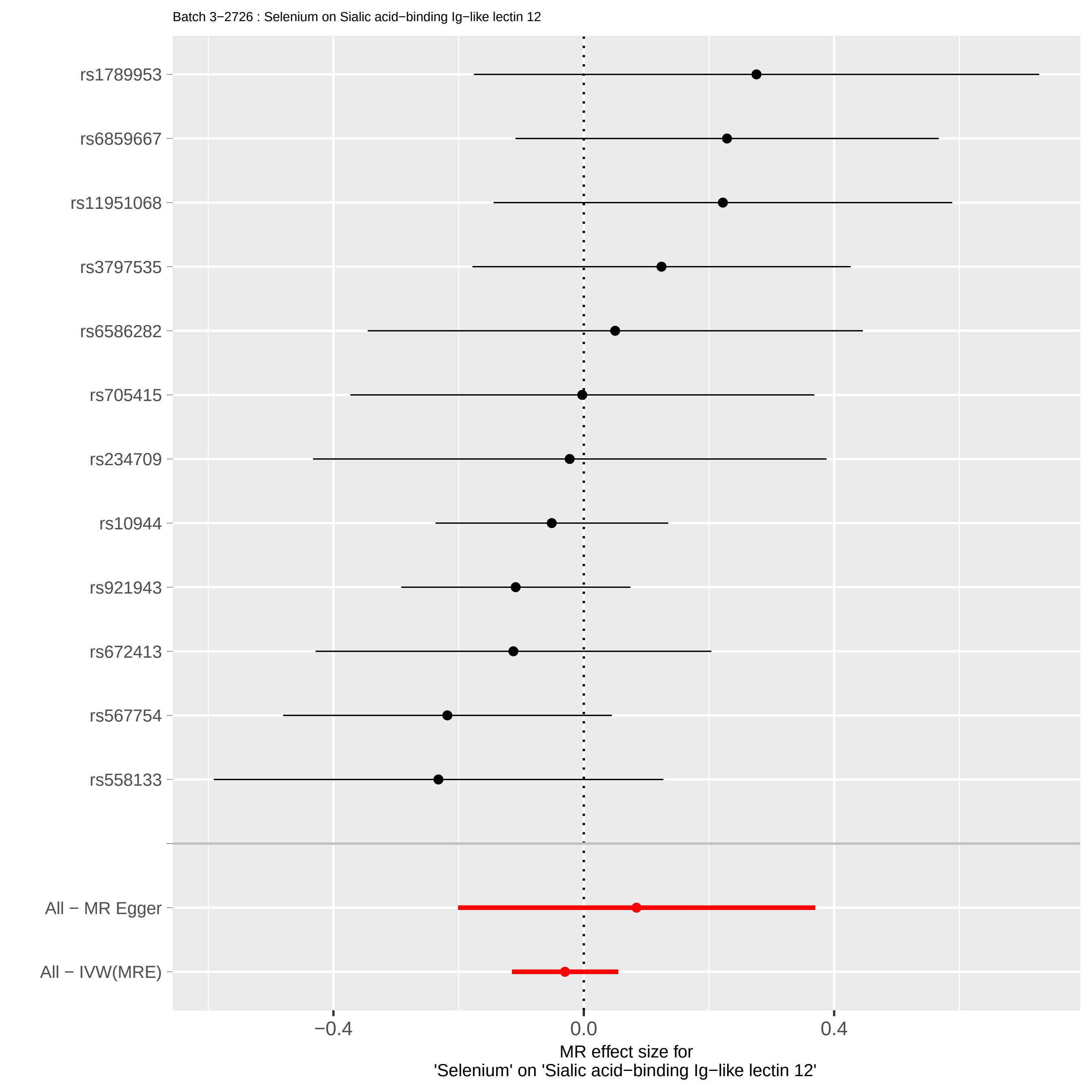
Batch 3−2726 : Selenium on Sialic acid−binding Ig−like lectin 12
rs1789953
rs6859667
rs11951068
rs3797535
rs6586282
rs705415
rs234709
rs10944
rs921943
rs672413
rs567754
rs558133
All − MR Egger
All − IVW(MRE)
−0.4
0.0
0.4
MR effect size for
'Selenium' on 'Sialic acid−binding Ig−like lectin 12'

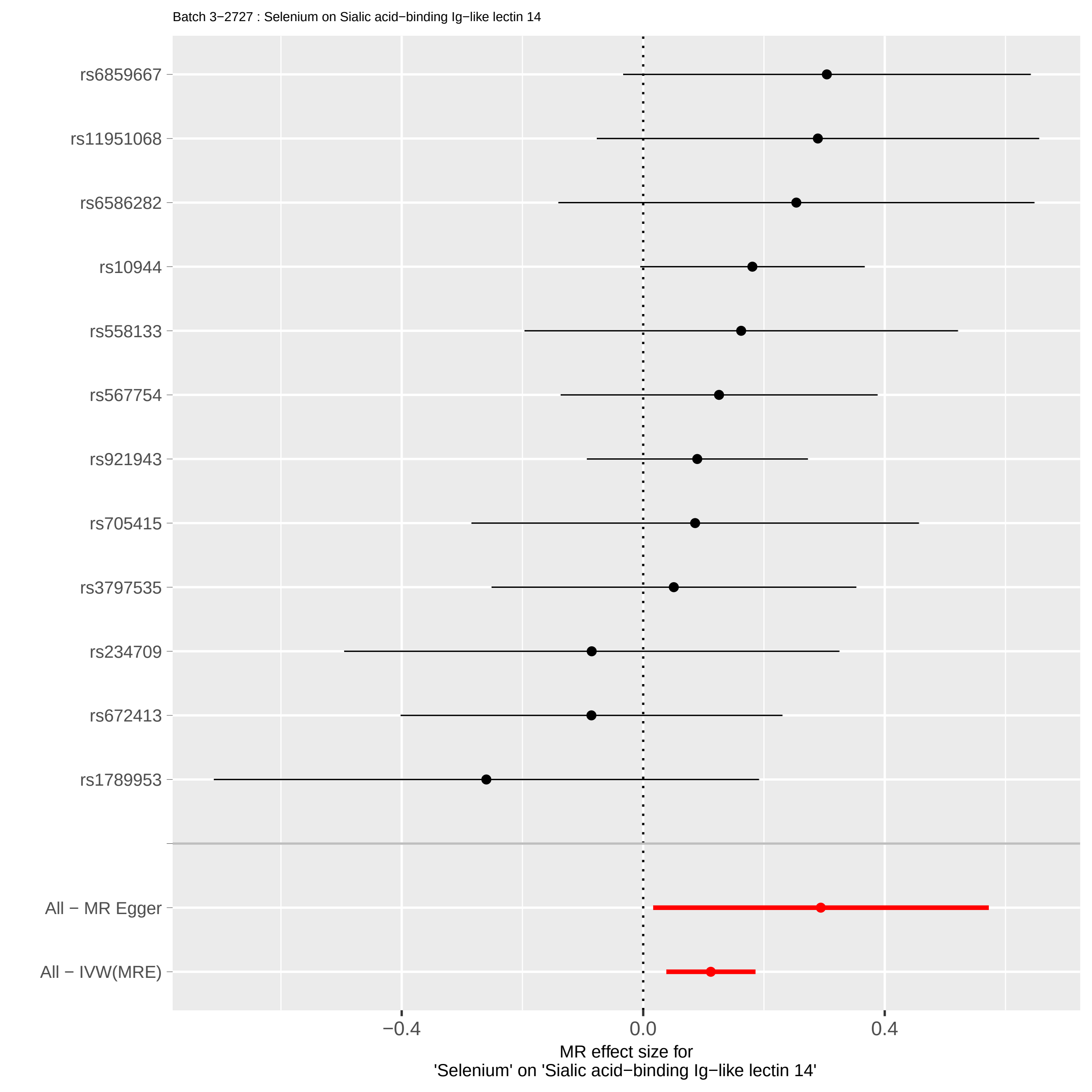

Batch 3−2727 : Selenium on Sialic acid−binding Ig−like lectin 14
rs6859667
rs11951068
rs6586282
rs10944
rs558133
rs567754
rs921943
rs705415
rs3797535
rs234709
rs672413
rs1789953
All − MR Egger
All − IVW(MRE)
−0.4
0.0
0.4
MR effect size for
'Selenium' on 'Sialic acid−binding Ig−like lectin 14'

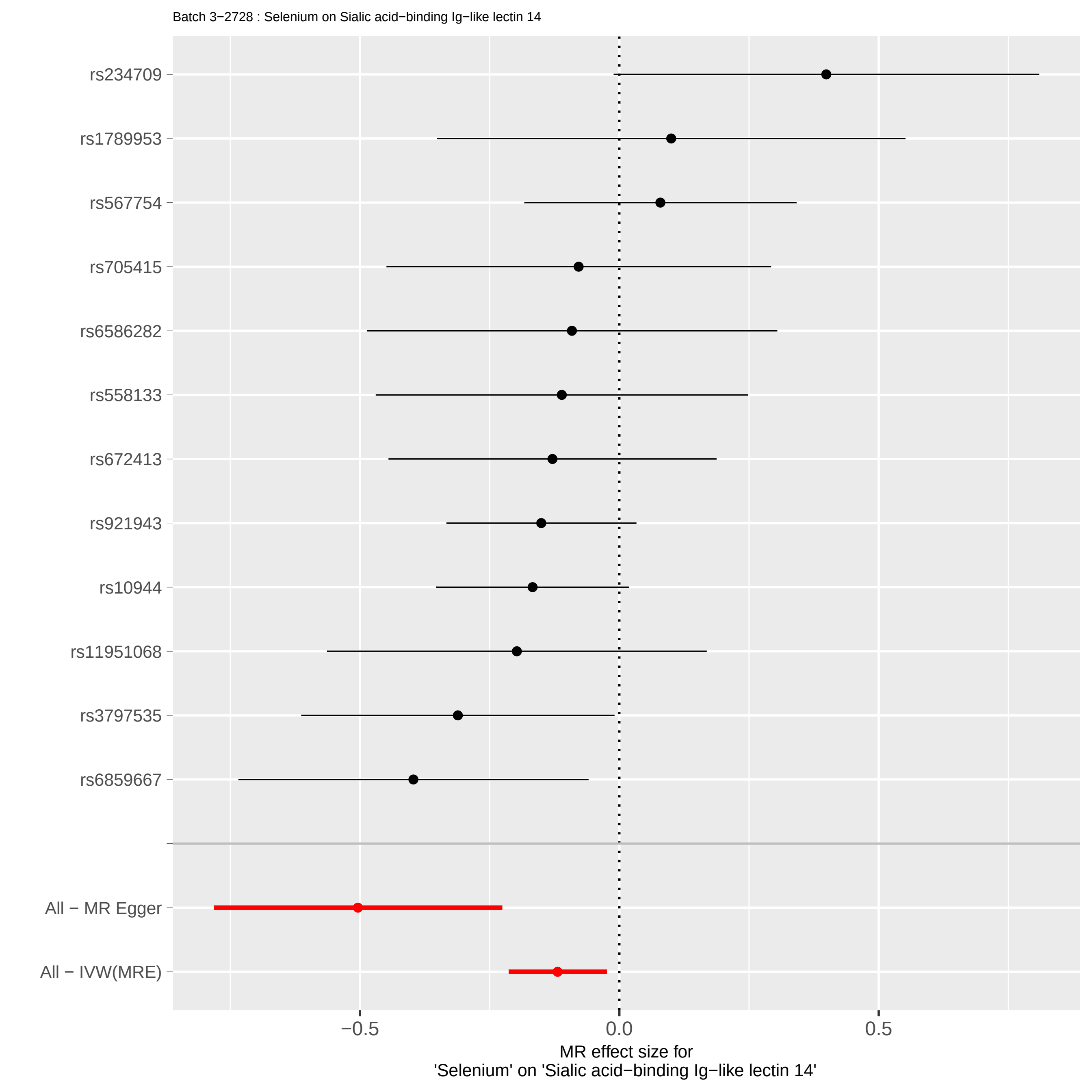

Batch 3−2728 : Selenium on Sialic acid−binding Ig−like lectin 14
rs234709
rs1789953
rs567754
rs705415
rs6586282
rs558133
rs672413
rs921943
rs10944
rs11951068
rs3797535
rs6859667
All − MR Egger
All − IVW(MRE)
−0.5
0.0
0.5
MR effect size for
'Selenium' on 'Sialic acid−binding Ig−like lectin 14'

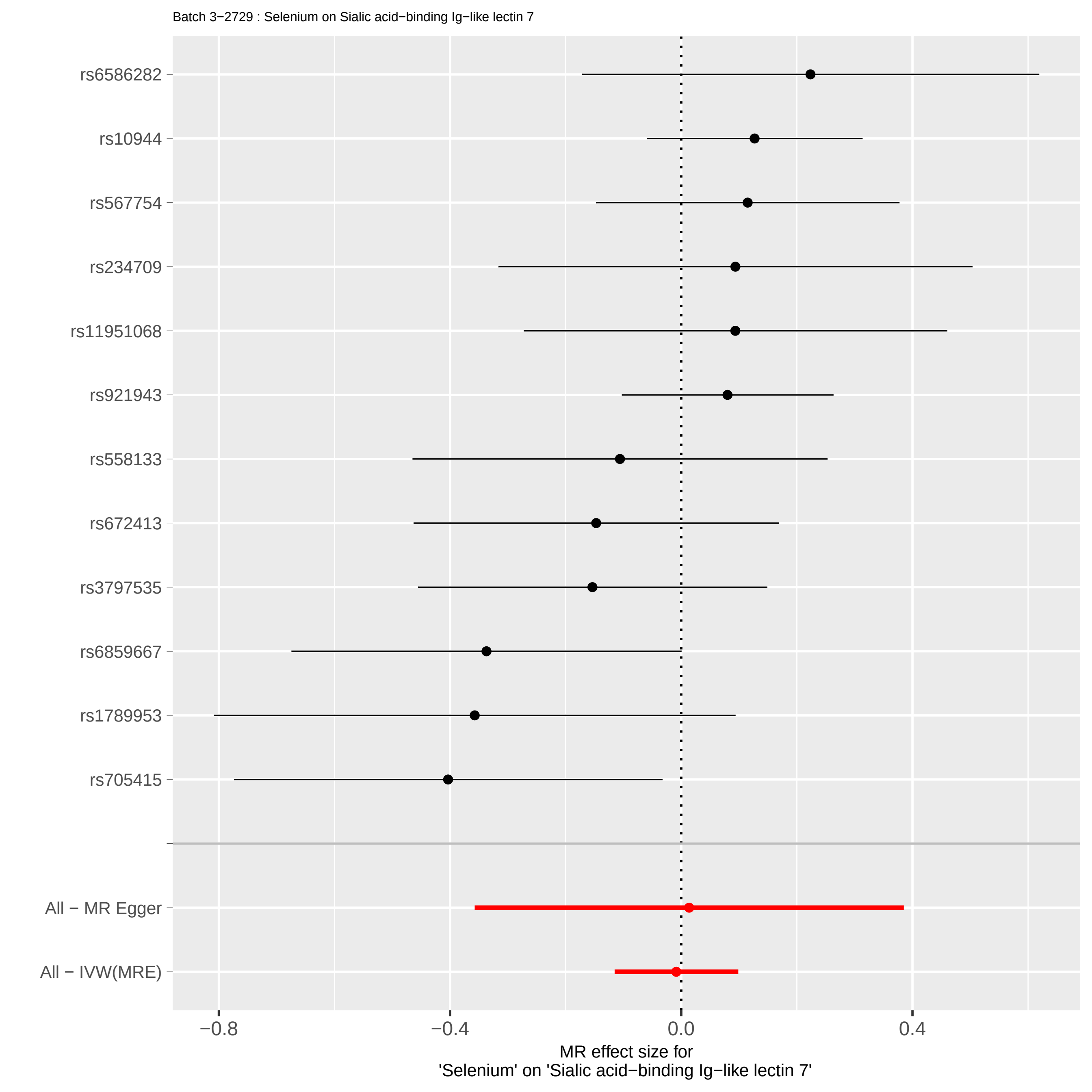

Batch 3−2729 : Selenium on Sialic acid−binding Ig−like lectin 7
rs6586282
rs10944
rs567754
rs234709
rs11951068
rs921943
rs558133
rs672413
rs3797535
rs6859667
rs1789953
rs705415
All − MR Egger
All − IVW(MRE)
−0.8
−0.4
0.0
0.4
MR effect size for
'Selenium' on 'Sialic acid−binding Ig−like lectin 7'

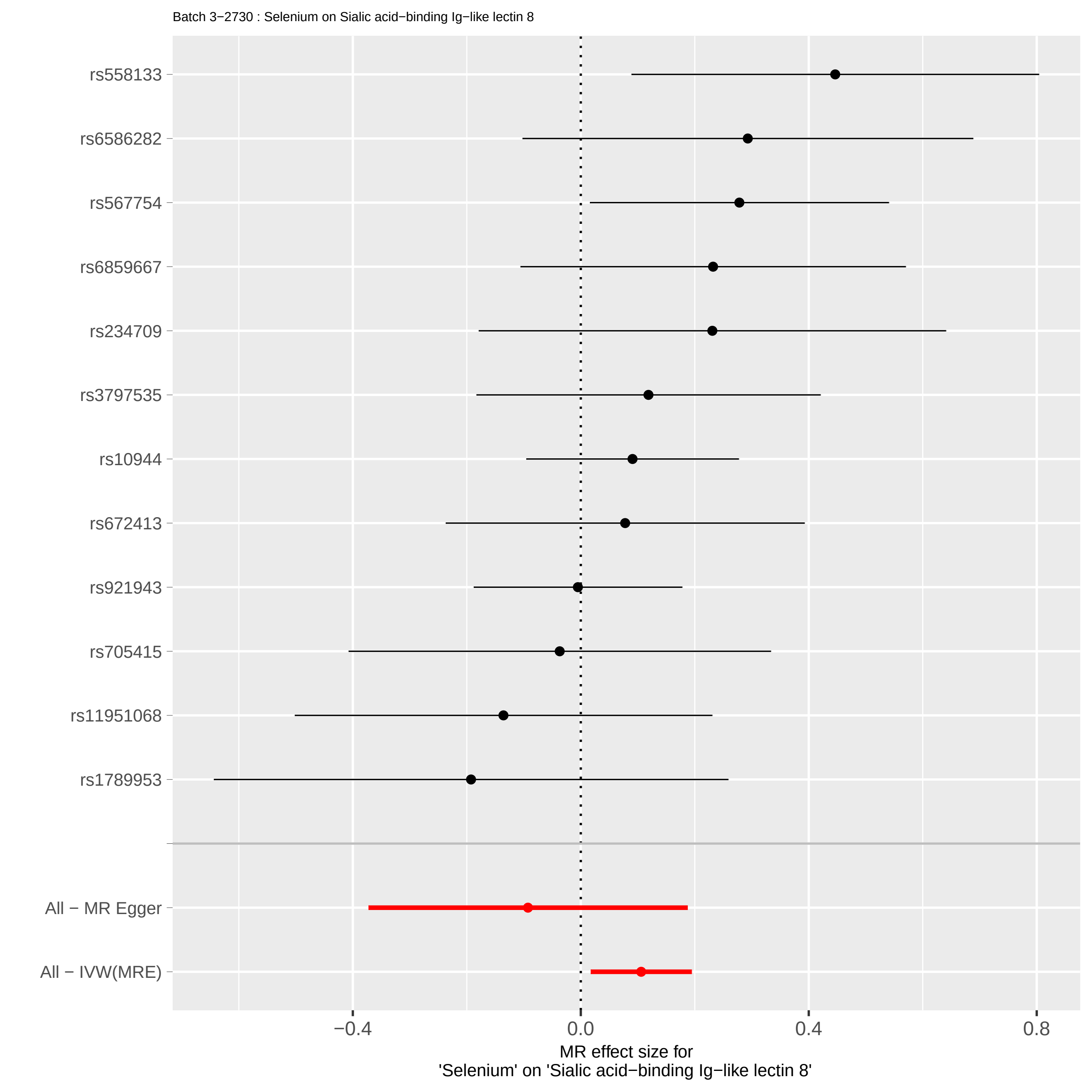

Batch 3−2730 : Selenium on Sialic acid−binding Ig−like lectin 8
rs558133
rs6586282
rs567754
rs6859667
rs234709
rs3797535
rs10944
rs672413
rs921943
rs705415
rs11951068
rs1789953
All − MR Egger
All − IVW(MRE)
−0.4
0.0
0.4
0.8
MR effect size for
'Selenium' on 'Sialic acid−binding Ig−like lectin 8'

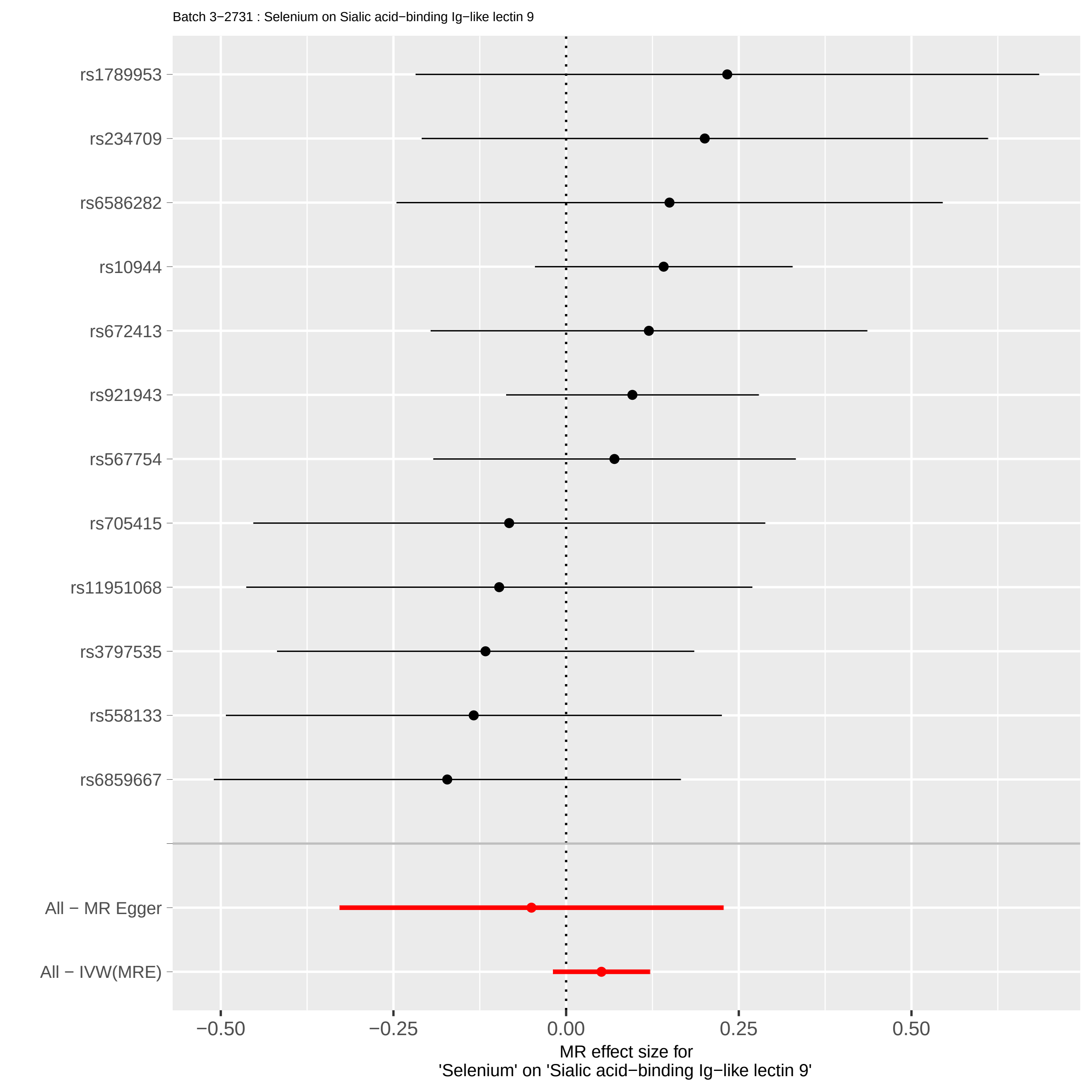

Batch 3−2731 : Selenium on Sialic acid−binding Ig−like lectin 9
rs1789953
rs234709
rs6586282
rs10944
rs672413
rs921943
rs567754
rs705415
rs11951068
rs3797535
rs558133
rs6859667
All − MR Egger
All − IVW(MRE)
−0.50
−0.25
0.00
0.25
0.50
MR effect size for
'Selenium' on 'Sialic acid−binding Ig−like lectin 9'

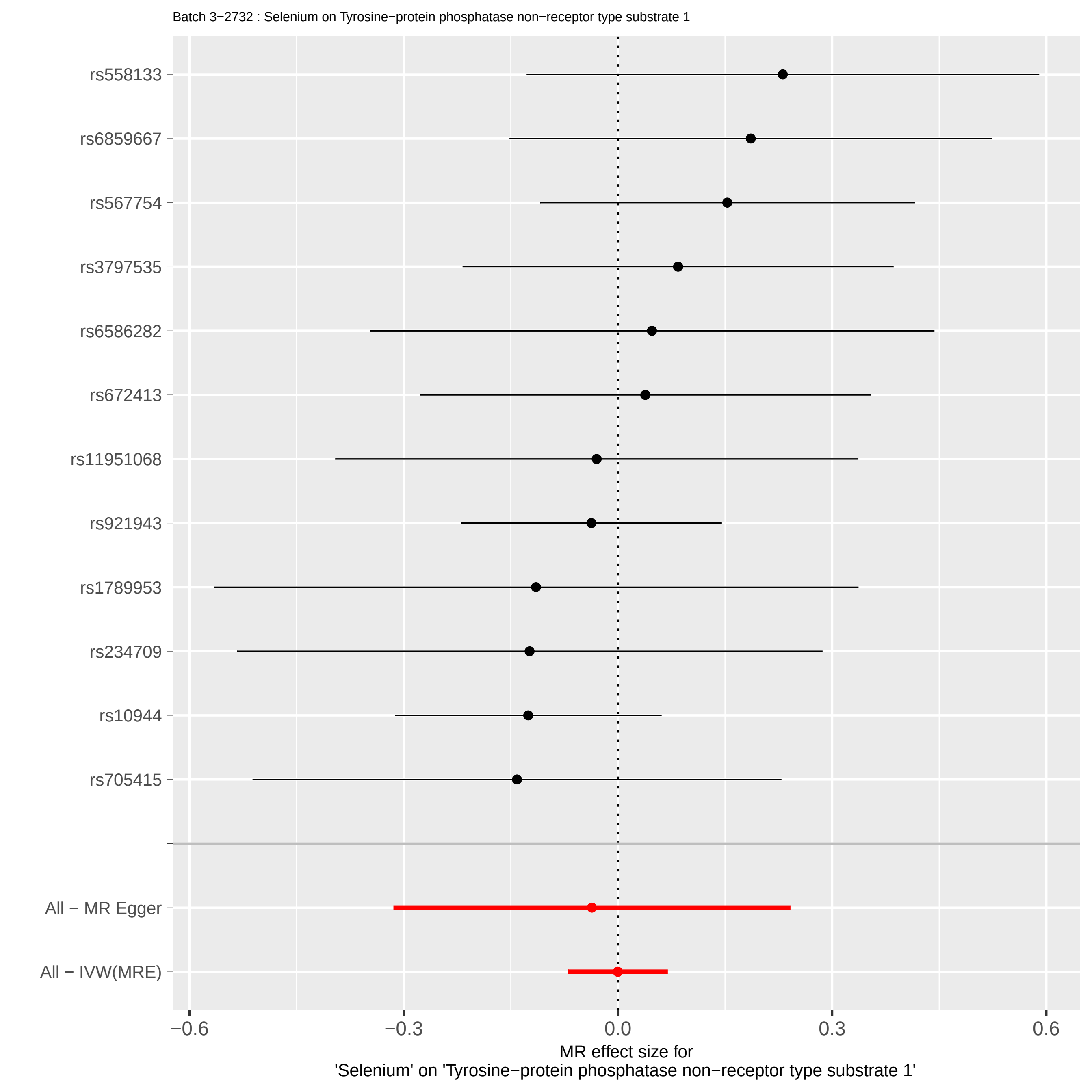
Batch 3−2732 : Selenium on Tyrosine−protein phosphatase non−receptor type substrate 1
rs558133
rs6859667
rs567754
rs3797535
rs6586282
rs672413
rs11951068
rs921943
rs1789953
rs234709
rs10944
rs705415
All − MR Egger
All − IVW(MRE)
−0.6
−0.3
0.0
0.3
0.6
MR effect size for
'Selenium' on 'Tyrosine−protein phosphatase non−receptor type substrate 1'

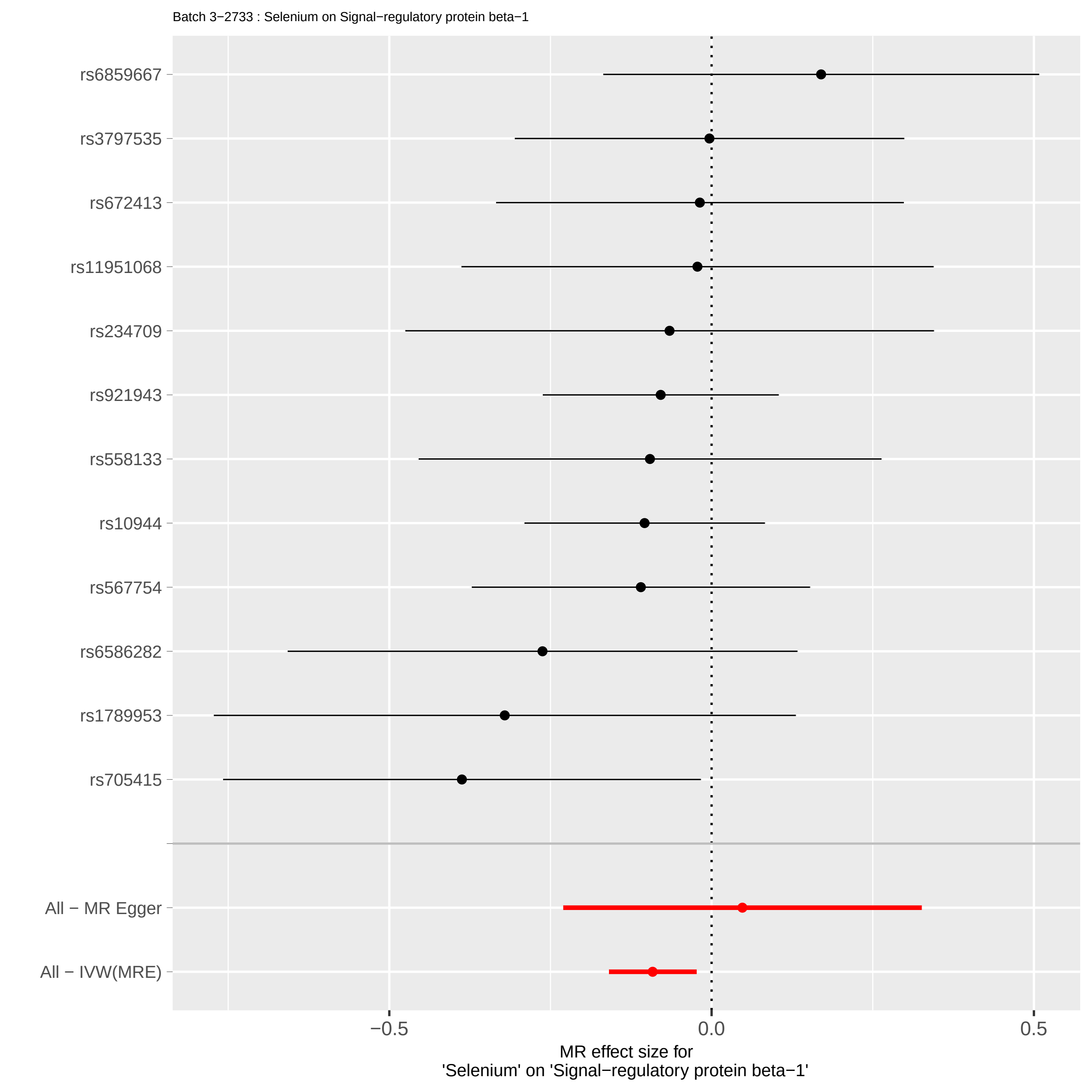
Batch 3−2733 : Selenium on Signal−regulatory protein beta−1
rs6859667
rs3797535
rs672413
rs11951068
rs234709
rs921943
rs558133
rs10944
rs567754
rs6586282
rs1789953
rs705415
All − MR Egger
All − IVW(MRE)
−0.5
0.0
0.5
MR effect size for
'Selenium' on 'Signal−regulatory protein beta−1'

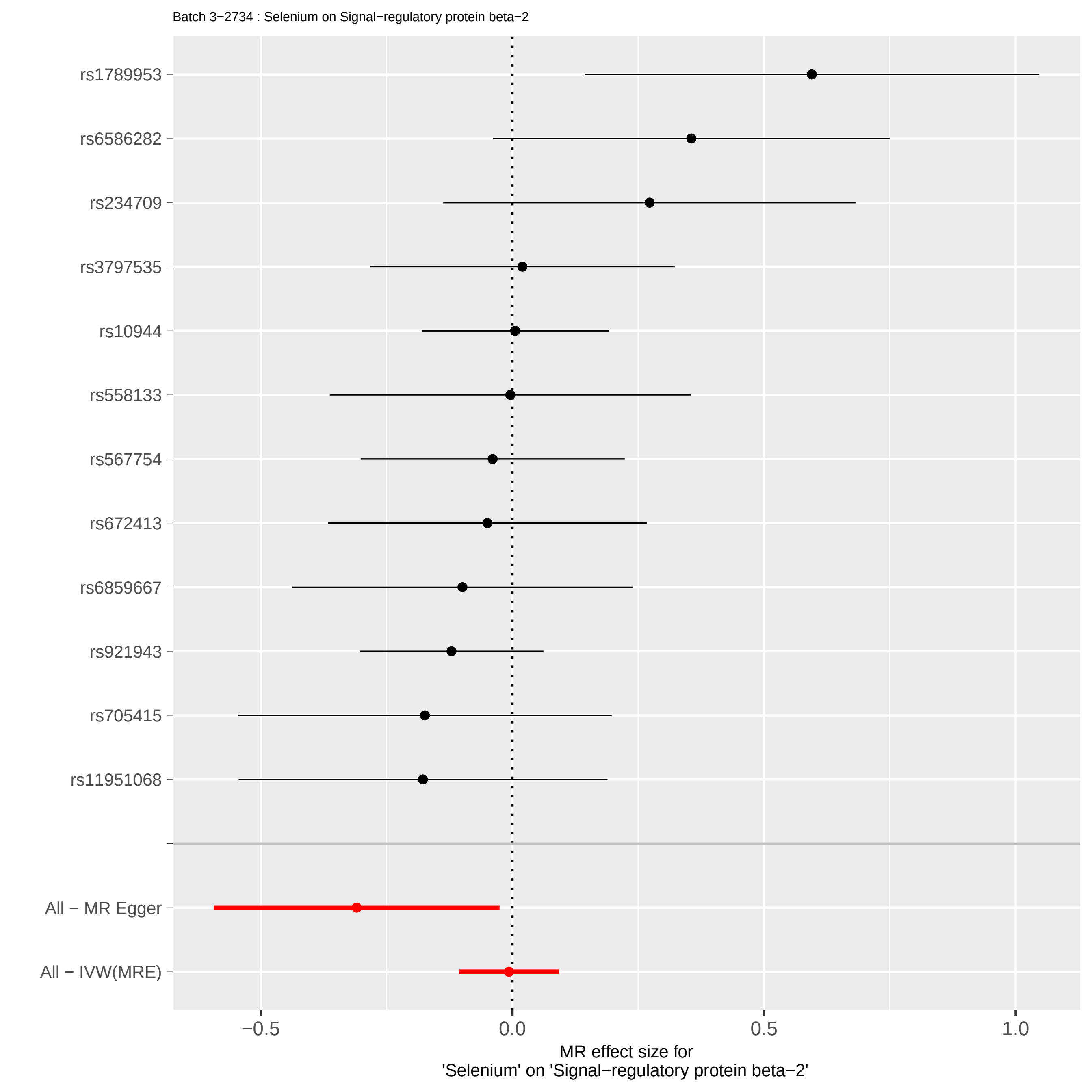

Batch 3−2734 : Selenium on Signal−regulatory protein beta−2
rs1789953
rs6586282
rs234709
rs3797535
rs10944
rs558133
rs567754
rs672413
rs6859667
rs921943
rs705415
rs11951068
All − MR Egger
All − IVW(MRE)
−0.5
0.0
0.5
1.0
MR effect size for
'Selenium' on 'Signal−regulatory protein beta−2'

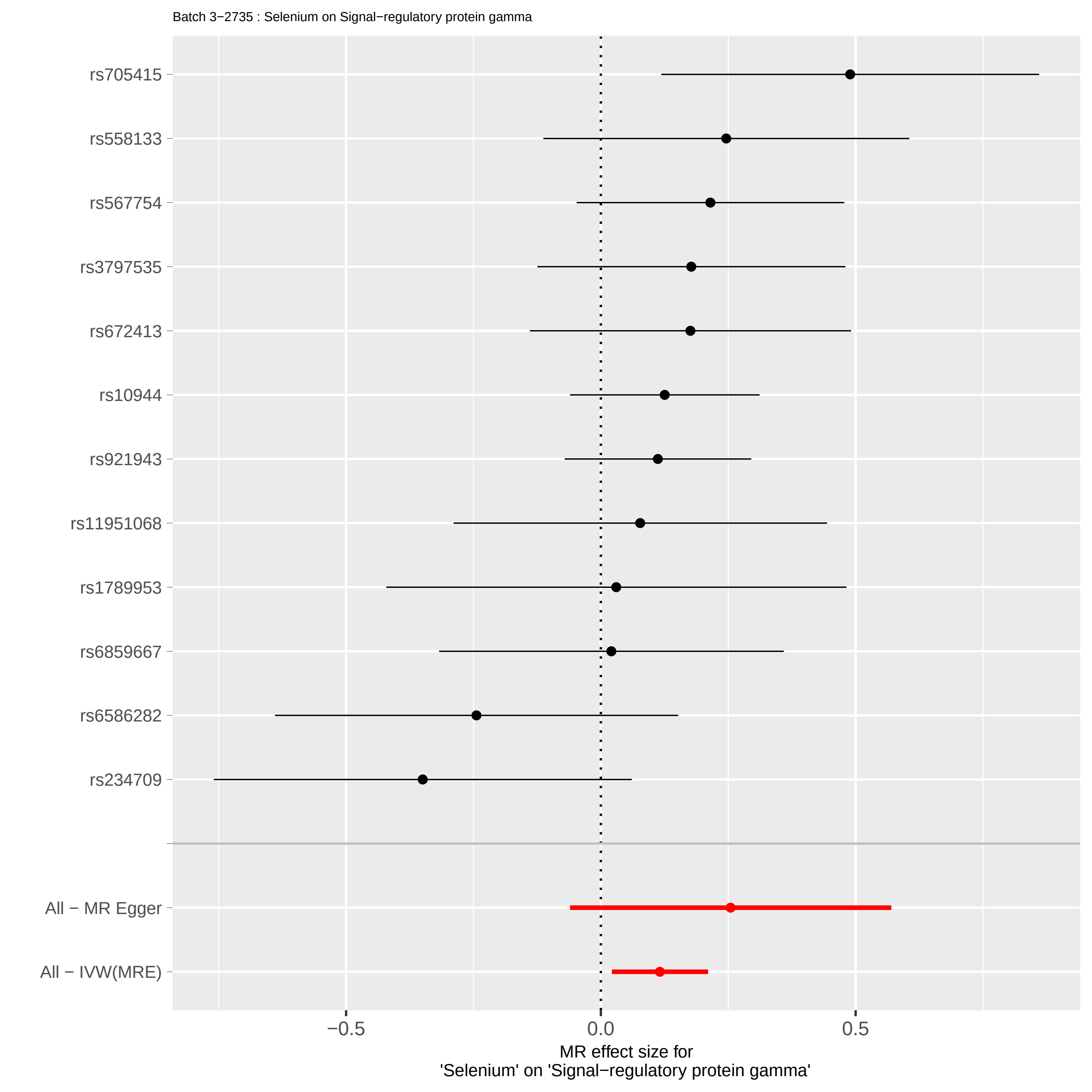

Batch 3−2735 : Selenium on Signal−regulatory protein gamma
rs705415
rs558133
rs567754
rs3797535
rs672413
rs10944
rs921943
rs11951068
rs1789953
rs6859667
rs6586282
rs234709
All − MR Egger
All − IVW(MRE)
−0.5
0.0
0.5
MR effect size for
'Selenium' on 'Signal−regulatory protein gamma'

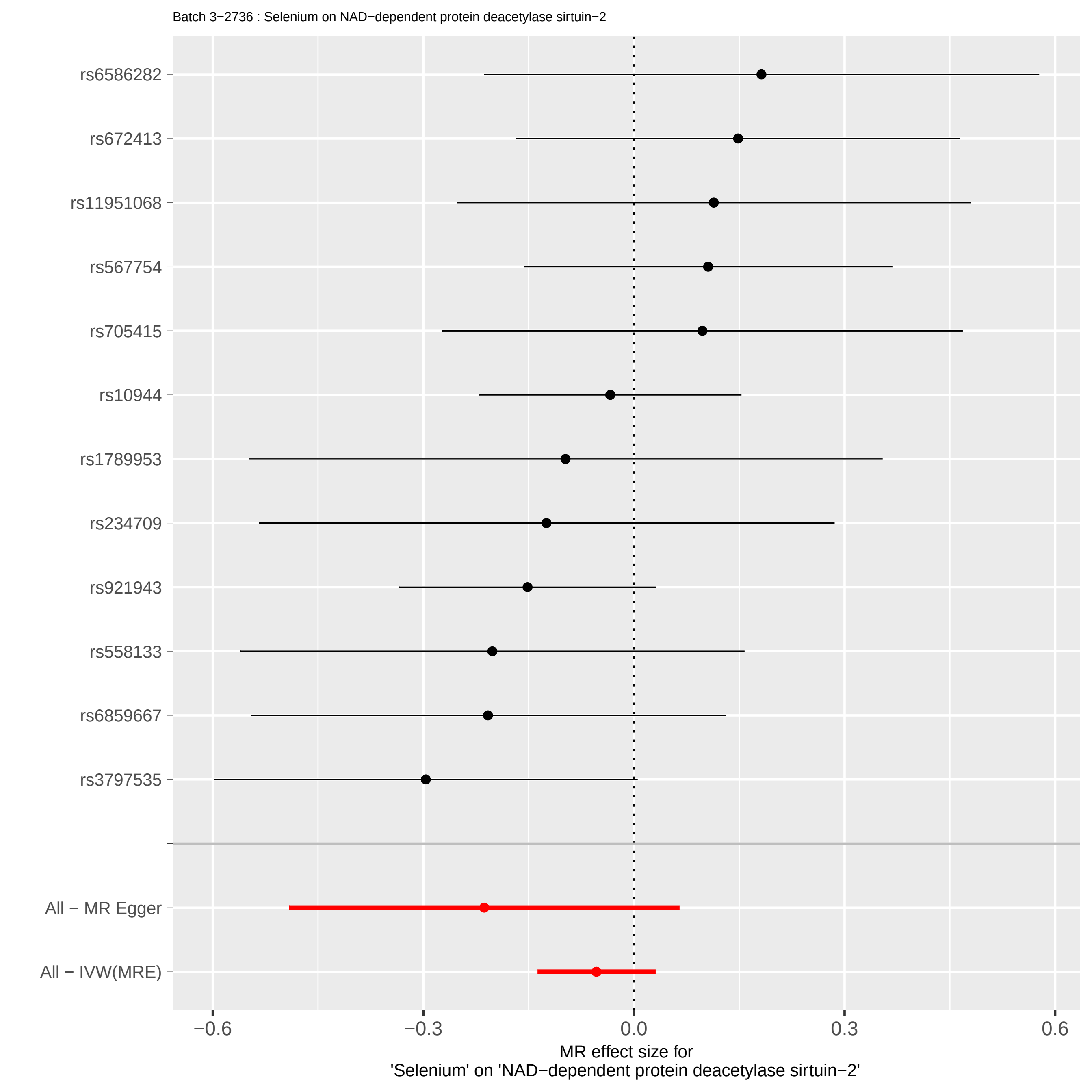

Batch 3−2736 : Selenium on NAD−dependent protein deacetylase sirtuin−2
rs6586282
rs672413
rs11951068
rs567754
rs705415
rs10944
rs1789953
rs234709
rs921943
rs558133
rs6859667
rs3797535
All − MR Egger
All − IVW(MRE)
−0.6
−0.3
0.0
0.3
0.6
MR effect size for
'Selenium' on 'NAD−dependent protein deacetylase sirtuin−2'

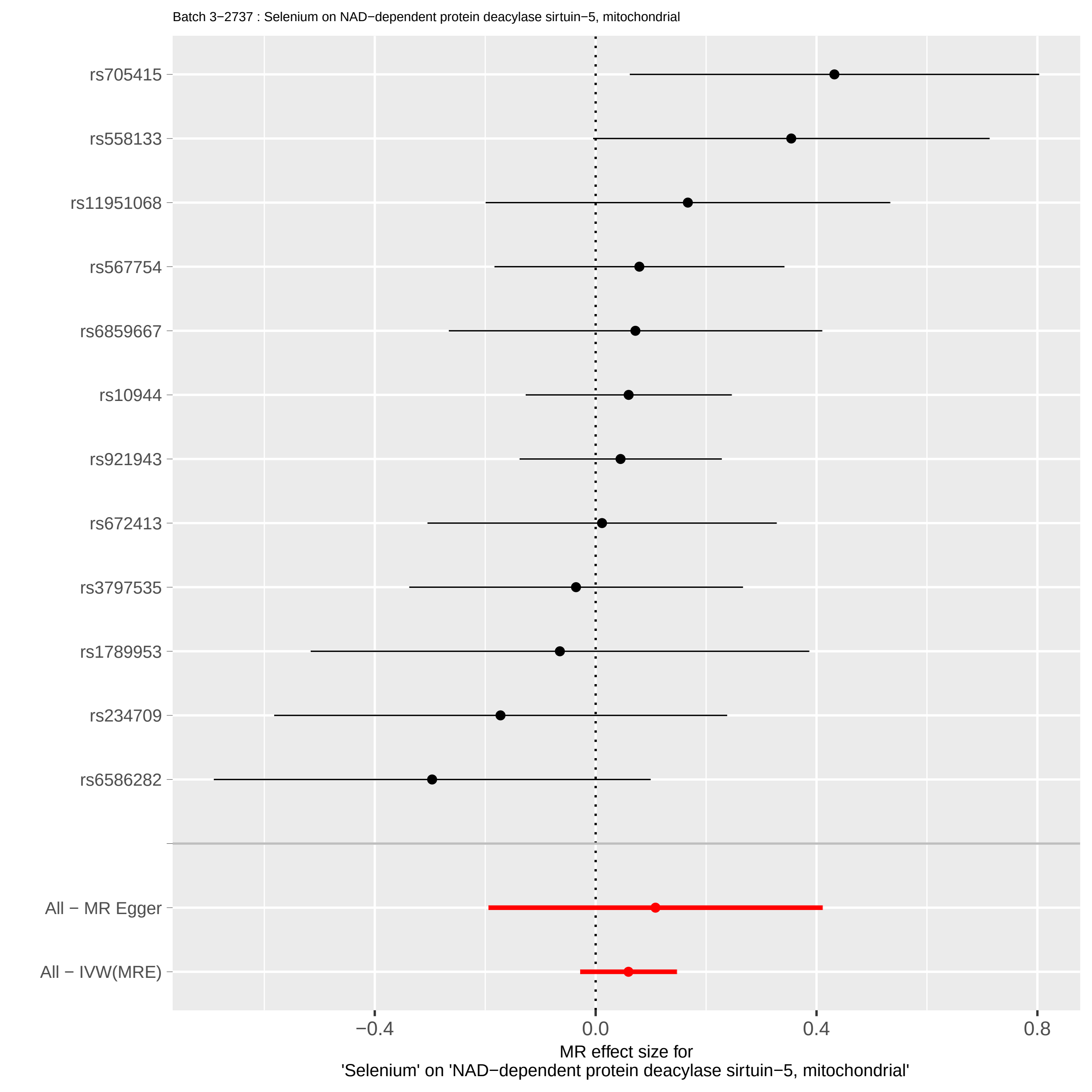
Batch 3−2737 : Selenium on NAD−dependent protein deacylase sirtuin−5, mitochondrial
rs705415
rs558133
rs11951068
rs567754
rs6859667
rs10944
rs921943
rs672413
rs3797535
rs1789953
rs234709
rs6586282
All − MR Egger
All − IVW(MRE)
−0.4
0.0
0.4
0.8
MR effect size for
'Selenium' on 'NAD−dependent protein deacylase sirtuin−5, mitochondrial'

Batch 3−2738 : Selenium on Signaling threshold−regulating transmembrane adapter 1

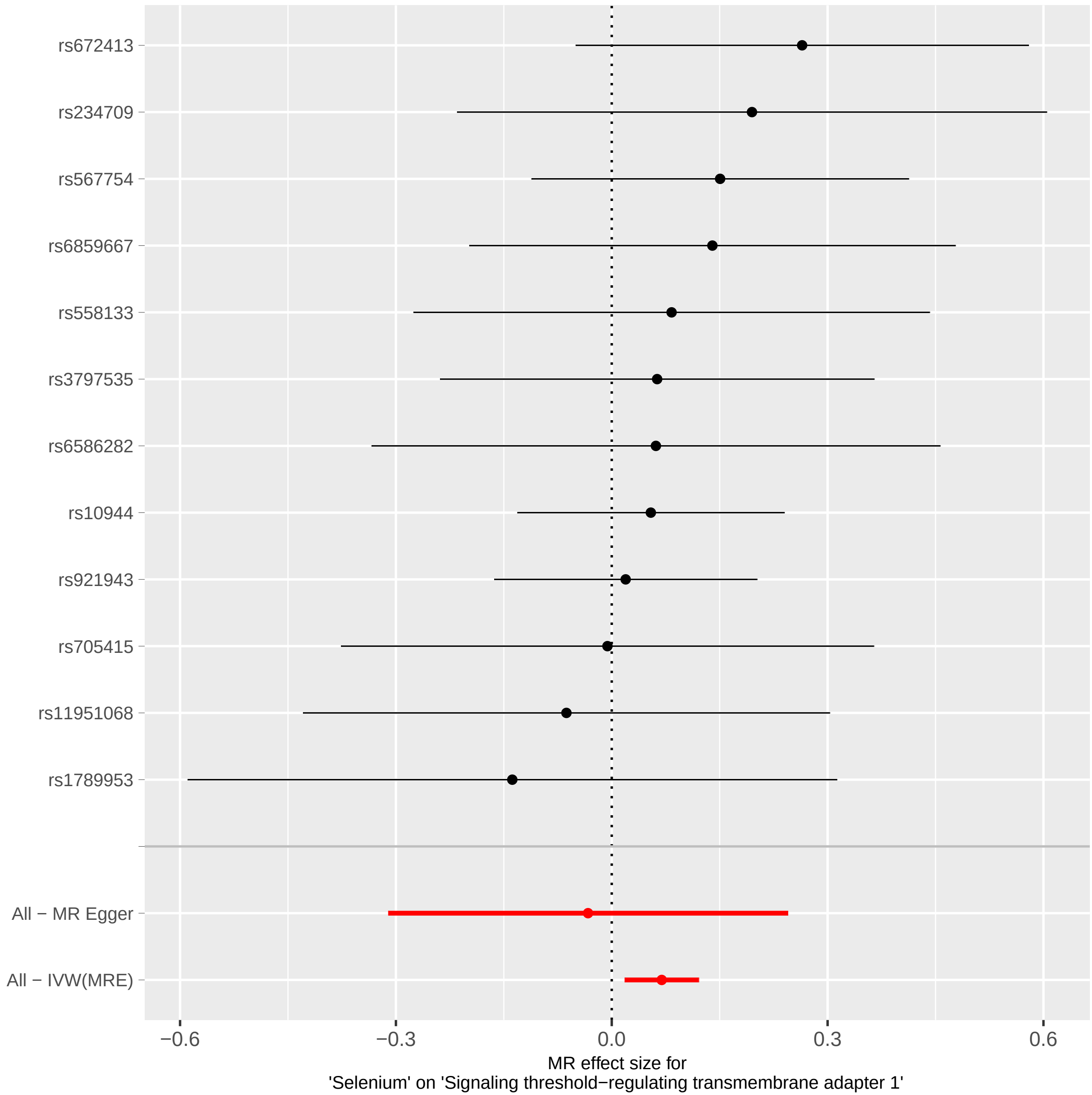

Batch 3−2739 : Selenium on Ski−like protein

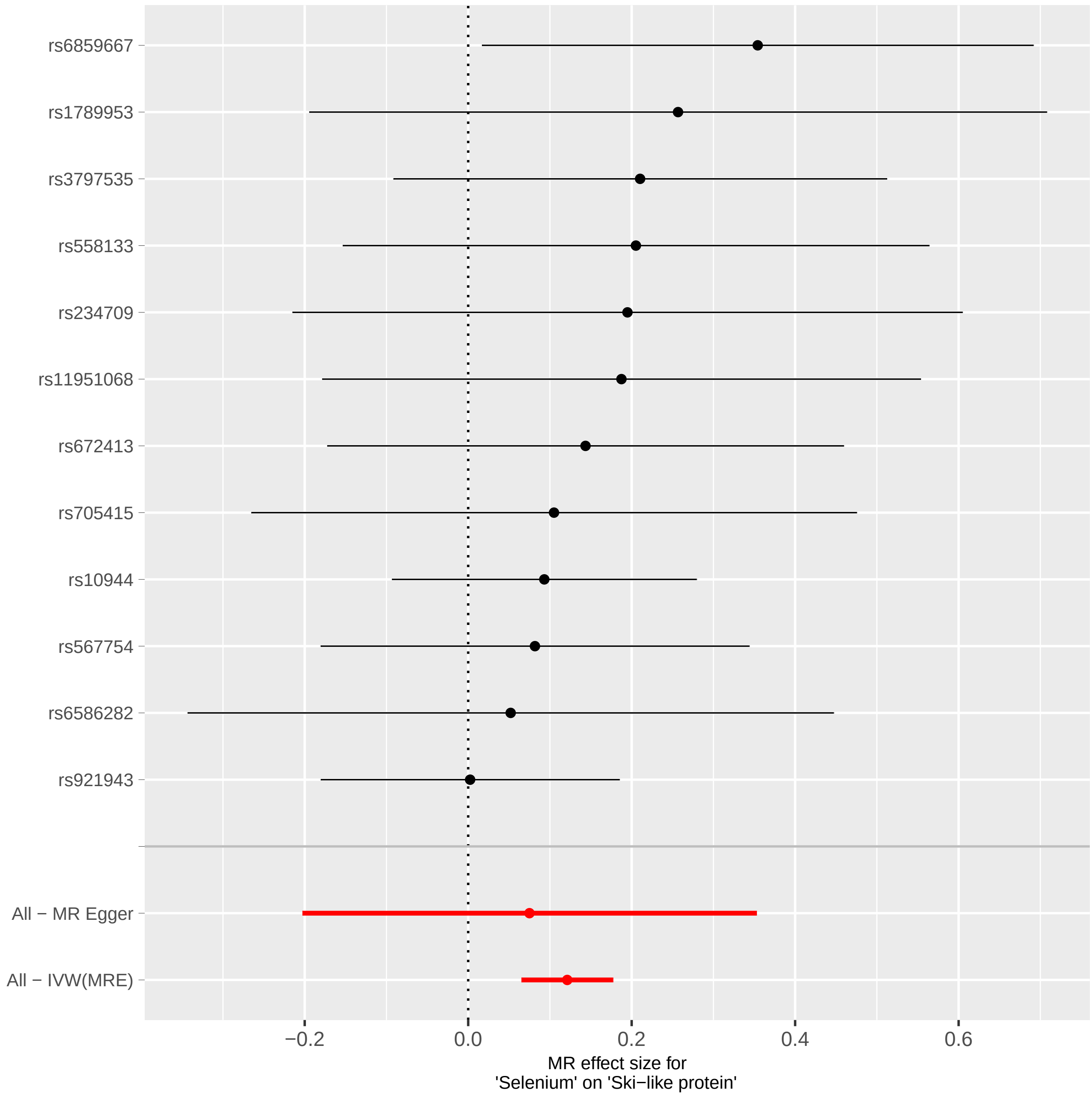

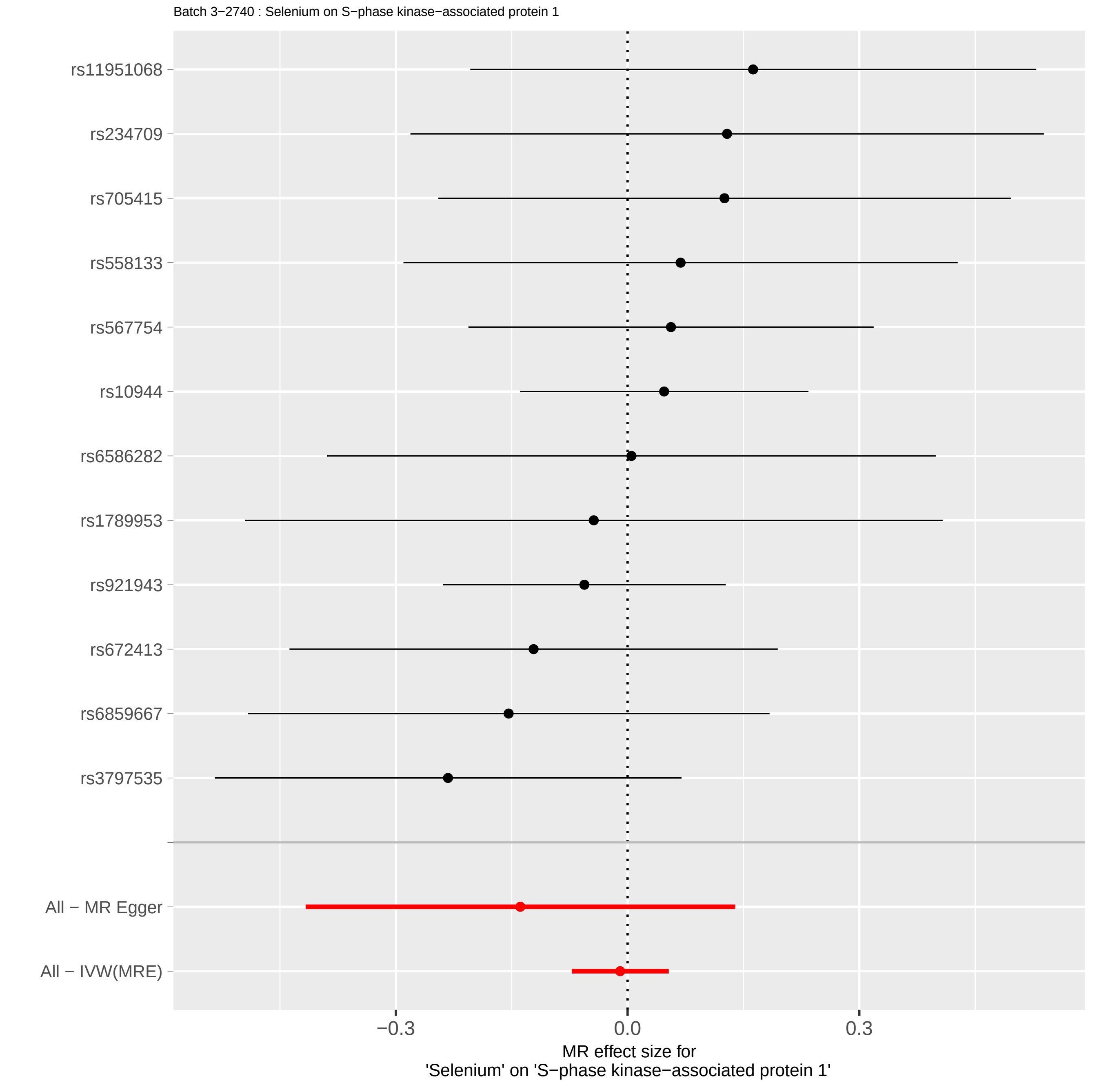
Batch 3−2740 : Selenium on S−phase kinase−associated protein 1
rs11951068
rs234709
rs705415
rs558133
rs567754
rs10944
rs6586282
rs1789953
rs921943
rs672413
rs6859667
rs3797535
All − MR Egger
All − IVW(MRE)
−0.3
0.0
0.3
MR effect size for
'Selenium' on 'S−phase kinase−associated protein 1'

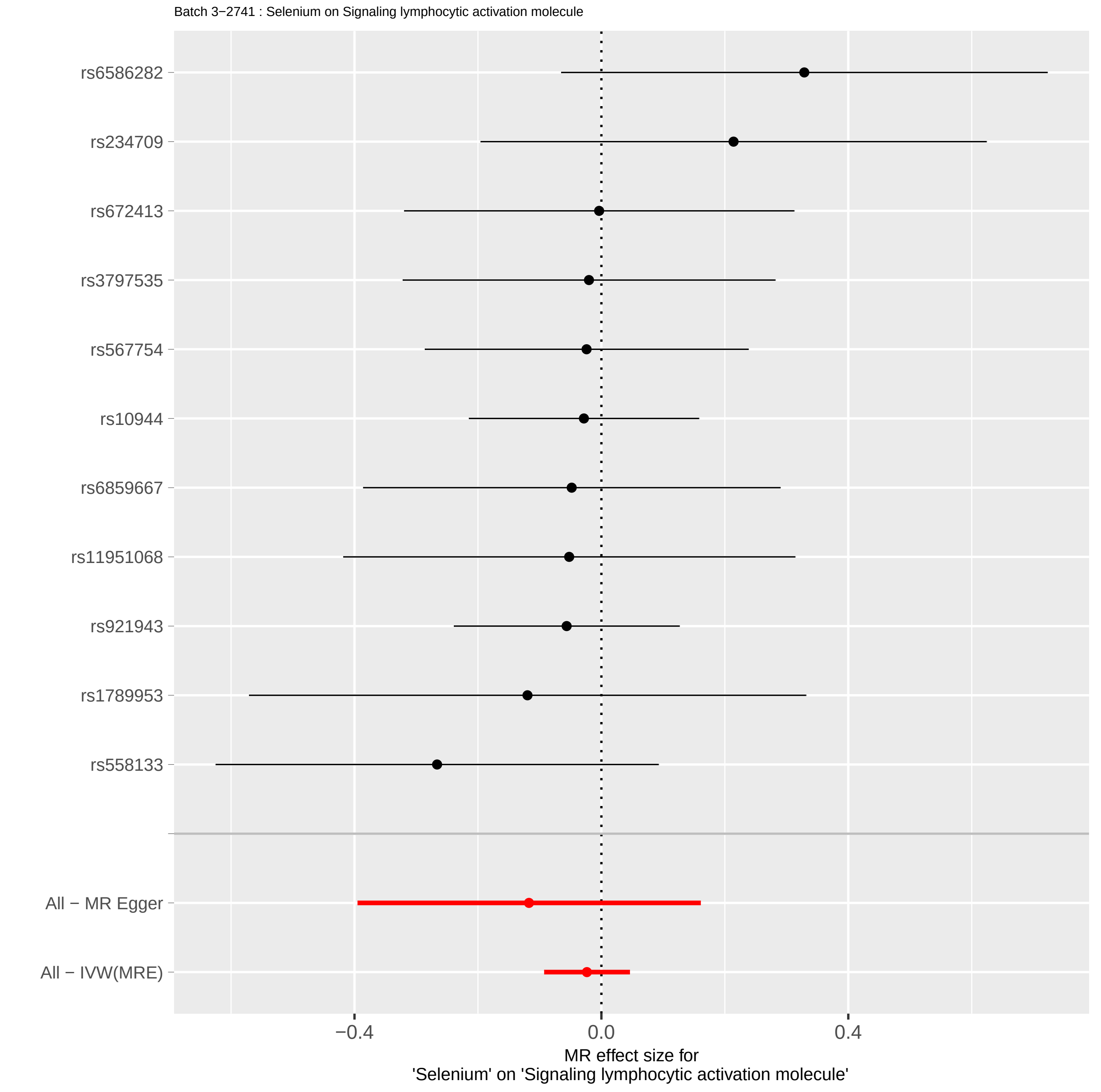
Batch 3−2741 : Selenium on Signaling lymphocytic activation molecule
rs6586282
rs234709
rs672413
rs3797535
rs567754
rs10944
rs6859667
rs11951068
rs921943
rs1789953
rs558133
All − MR Egger
All − IVW(MRE)
−0.4
0.0
0.4
MR effect size for
'Selenium' on 'Signaling lymphocytic activation molecule'

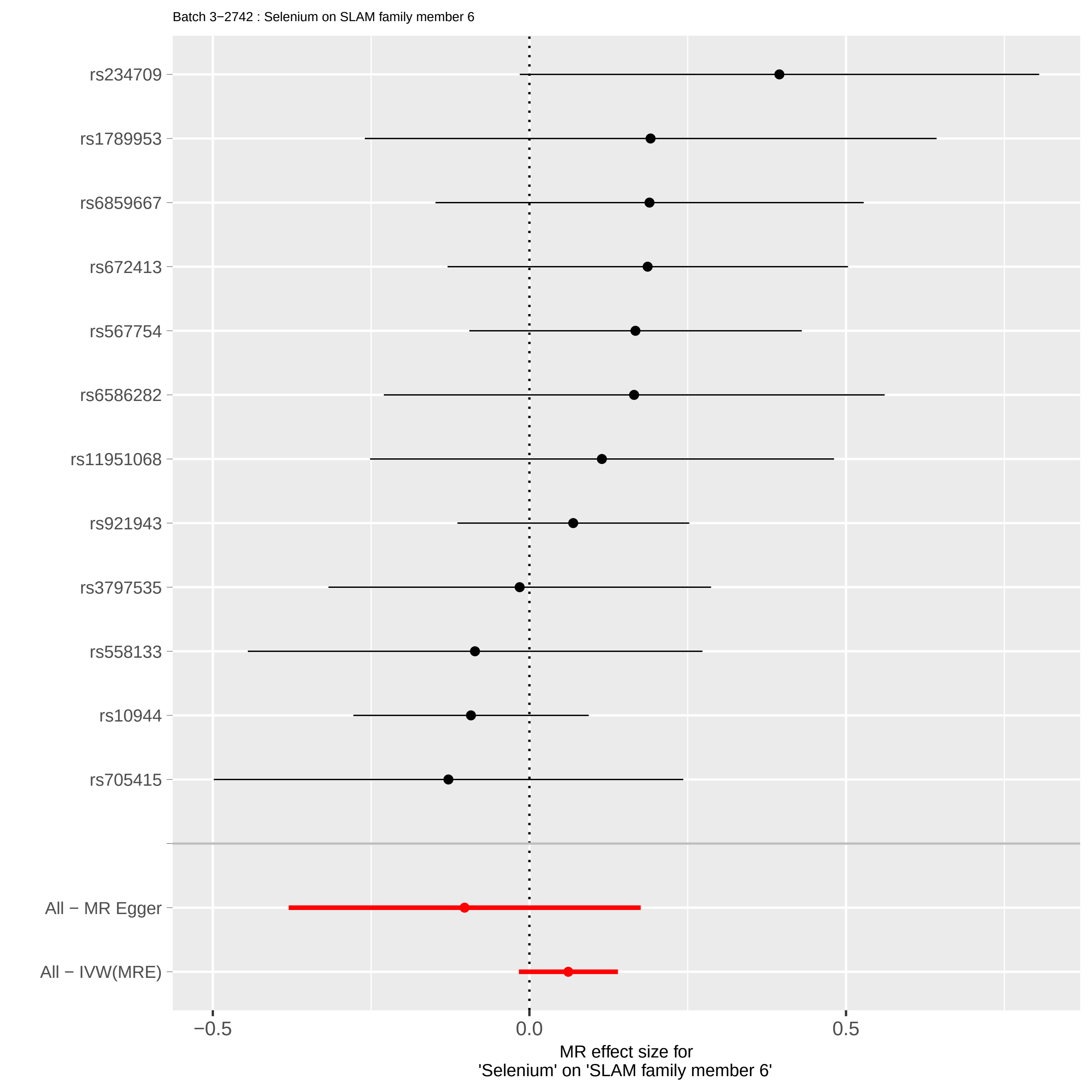
Batch 3−2742 : Selenium on SLAM family member 6
rs234709
rs1789953
rs6859667
rs672413
rs567754
rs6586282
rs11951068
rs921943
rs3797535
rs558133
rs10944
rs705415
All − MR Egger
All − IVW(MRE)
−0.5
0.0
0.5
MR effect size for
'Selenium' on 'SLAM family member 6'

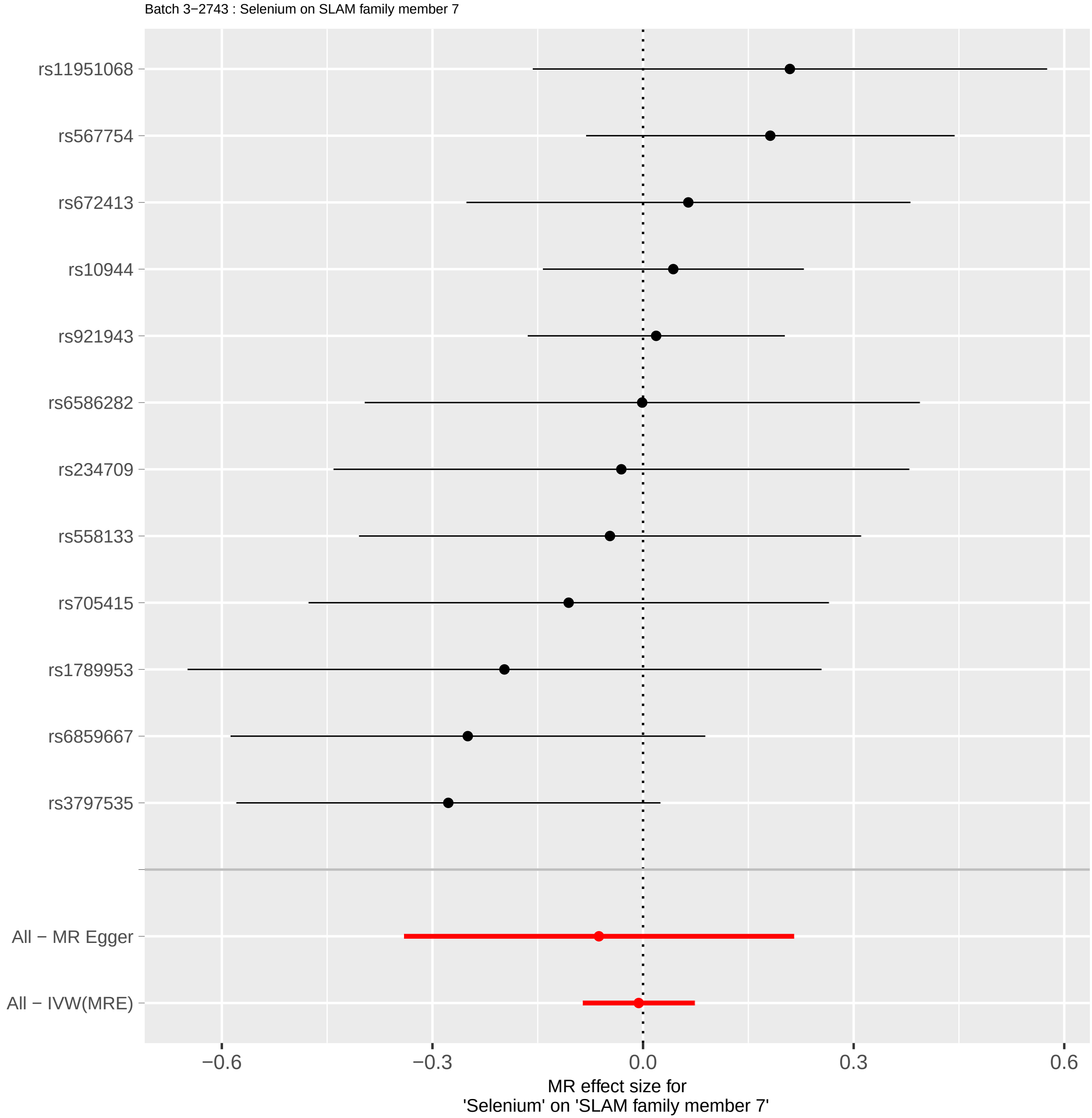

Batch 3−2743 : Selenium on SLAM family member 7
rs11951068
rs567754
rs672413
rs10944
rs921943
rs6586282
rs234709
rs558133
rs705415
rs1789953
rs6859667
rs3797535
All − MR Egger
All − IVW(MRE)
−0.6
−0.3
0.0
0.3
0.6
MR effect size for
'Selenium' on 'SLAM family member 7'

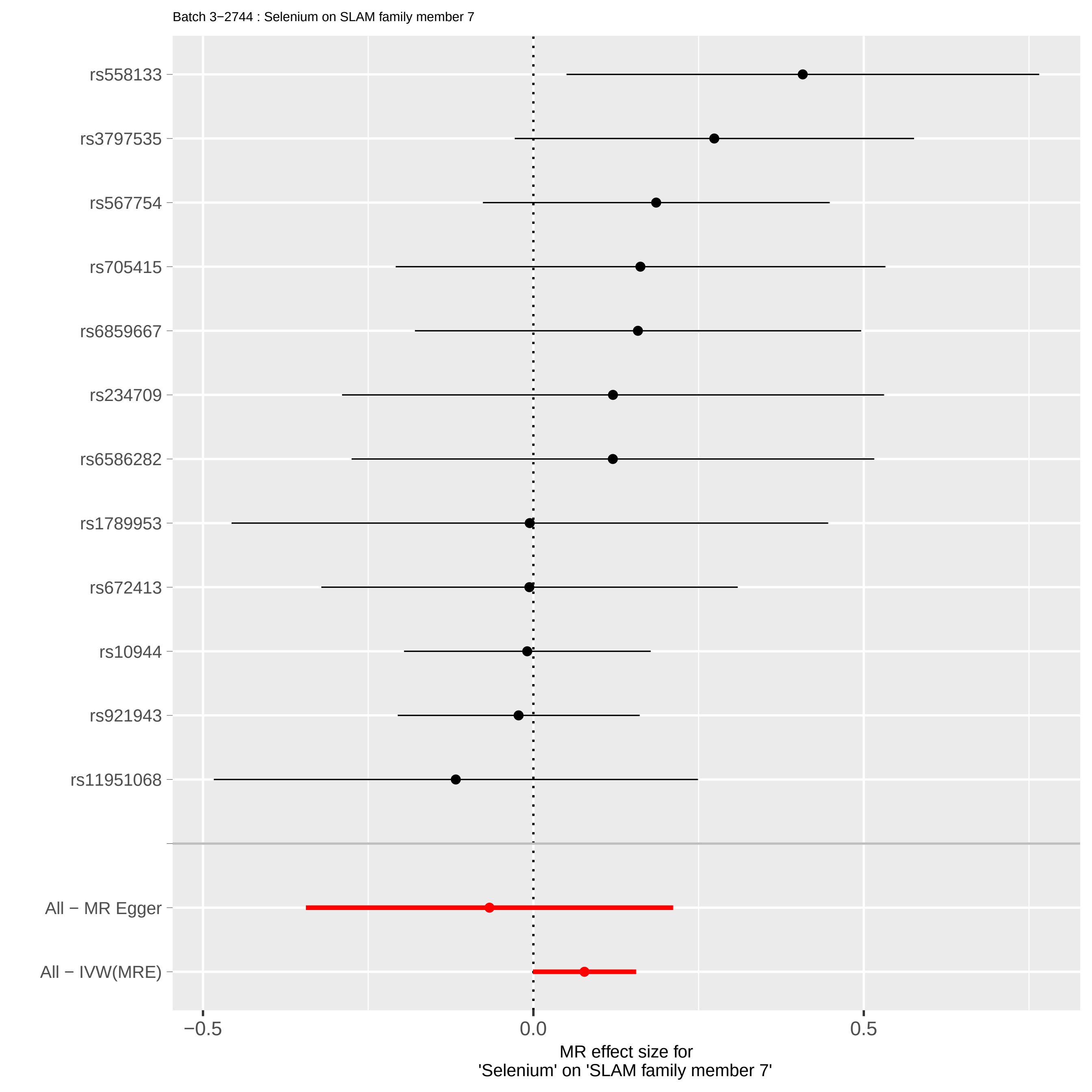

Batch 3−2744 : Selenium on SLAM family member 7
rs558133
rs3797535
rs567754
rs705415
rs6859667
rs234709
rs6586282
rs1789953
rs672413
rs10944
rs921943
rs11951068
All − MR Egger
All − IVW(MRE)
−0.5
0.0
0.5
MR effect size for
'Selenium' on 'SLAM family member 7'

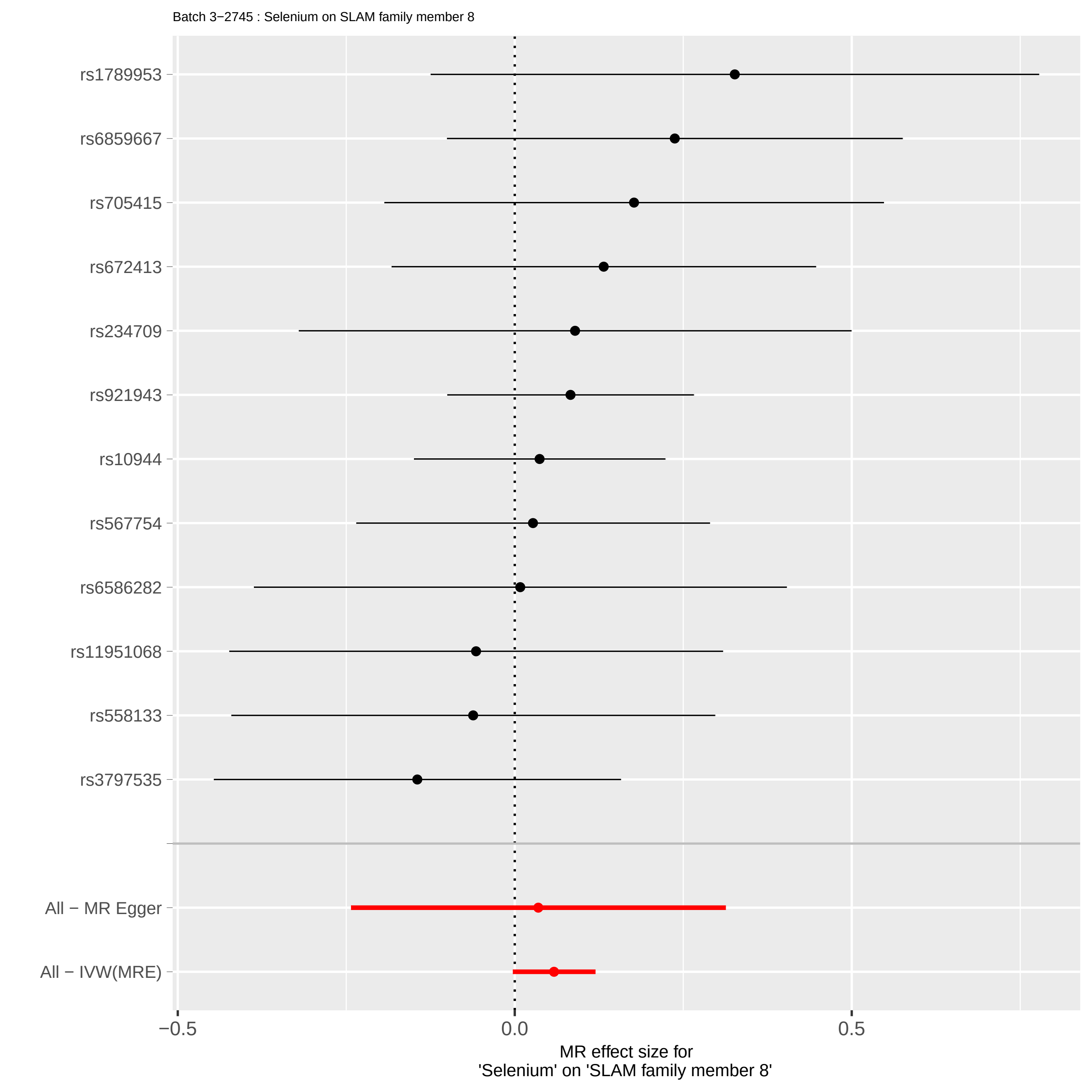

Batch 3−2745 : Selenium on SLAM family member 8
rs1789953
rs6859667
rs705415
rs672413
rs234709
rs921943
rs10944
rs567754
rs6586282
rs11951068
rs558133
rs3797535
All − MR Egger
All − IVW(MRE)
−0.5
0.0
0.5
MR effect size for
'Selenium' on 'SLAM family member 8'

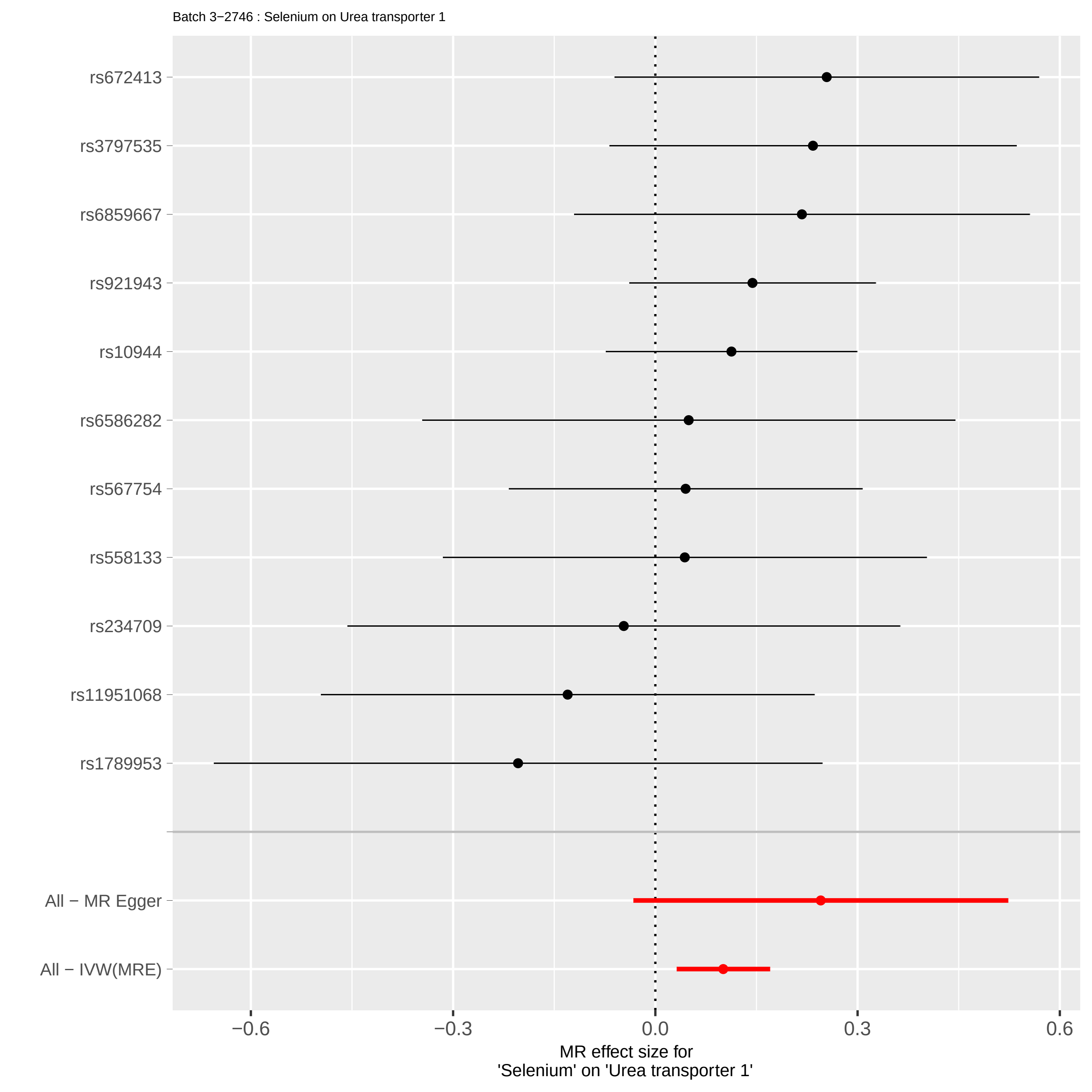

Batch 3−2746 : Selenium on Urea transporter 1
rs672413
rs3797535
rs6859667
rs921943
rs10944
rs6586282
rs567754
rs558133
rs234709
rs11951068
rs1789953
All − MR Egger
All − IVW(MRE)
−0.6
−0.3
0.0
0.3
0.6
MR effect size for
'Selenium' on 'Urea transporter 1'

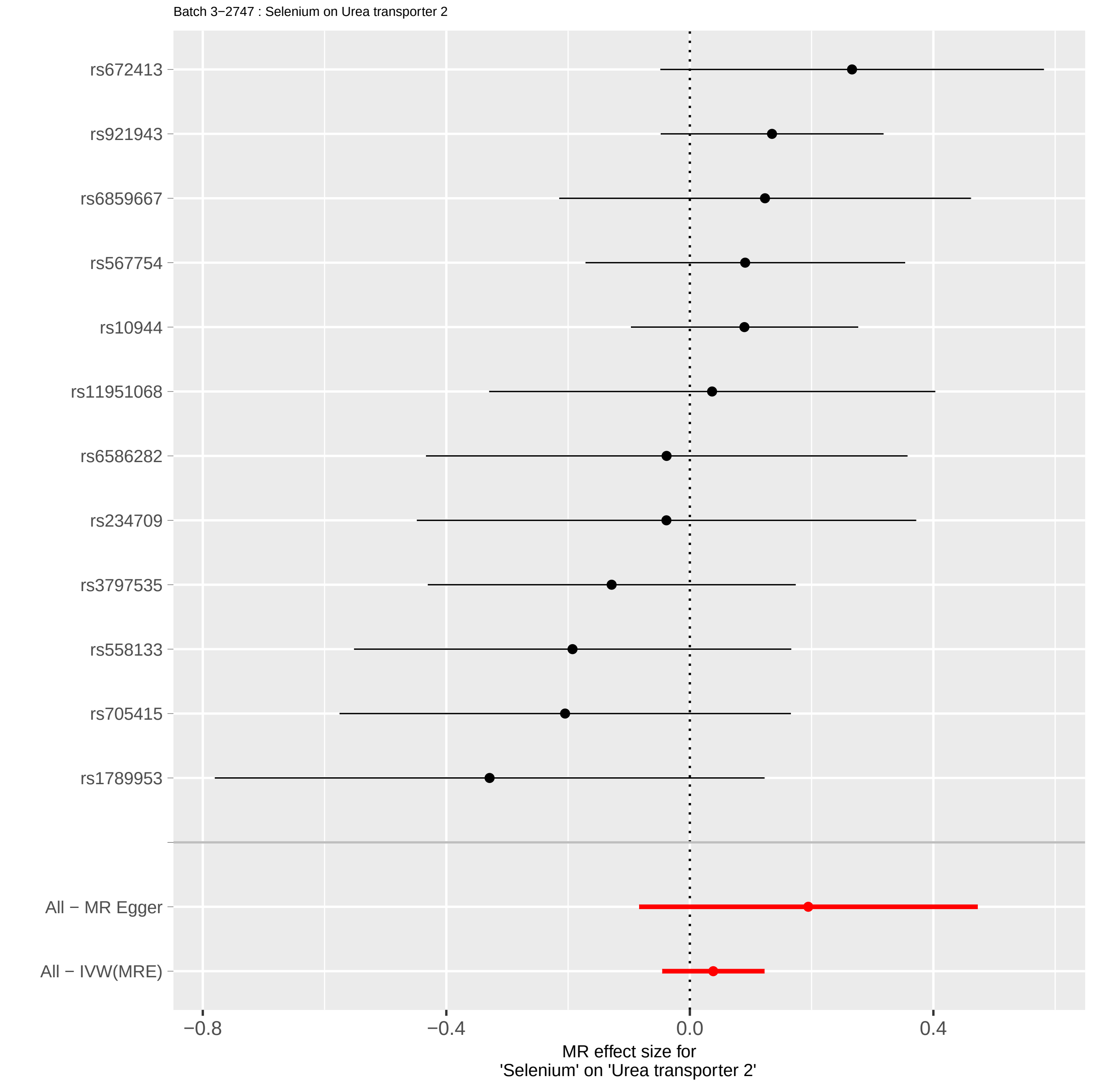

Batch 3−2747 : Selenium on Urea transporter 2
rs672413
rs921943
rs6859667
rs567754
rs10944
rs11951068
rs6586282
rs234709
rs3797535
rs558133
rs705415
rs1789953
All − MR Egger
All − IVW(MRE)
−0.8
−0.4
0.0
0.4
MR effect size for
'Selenium' on 'Urea transporter 2'

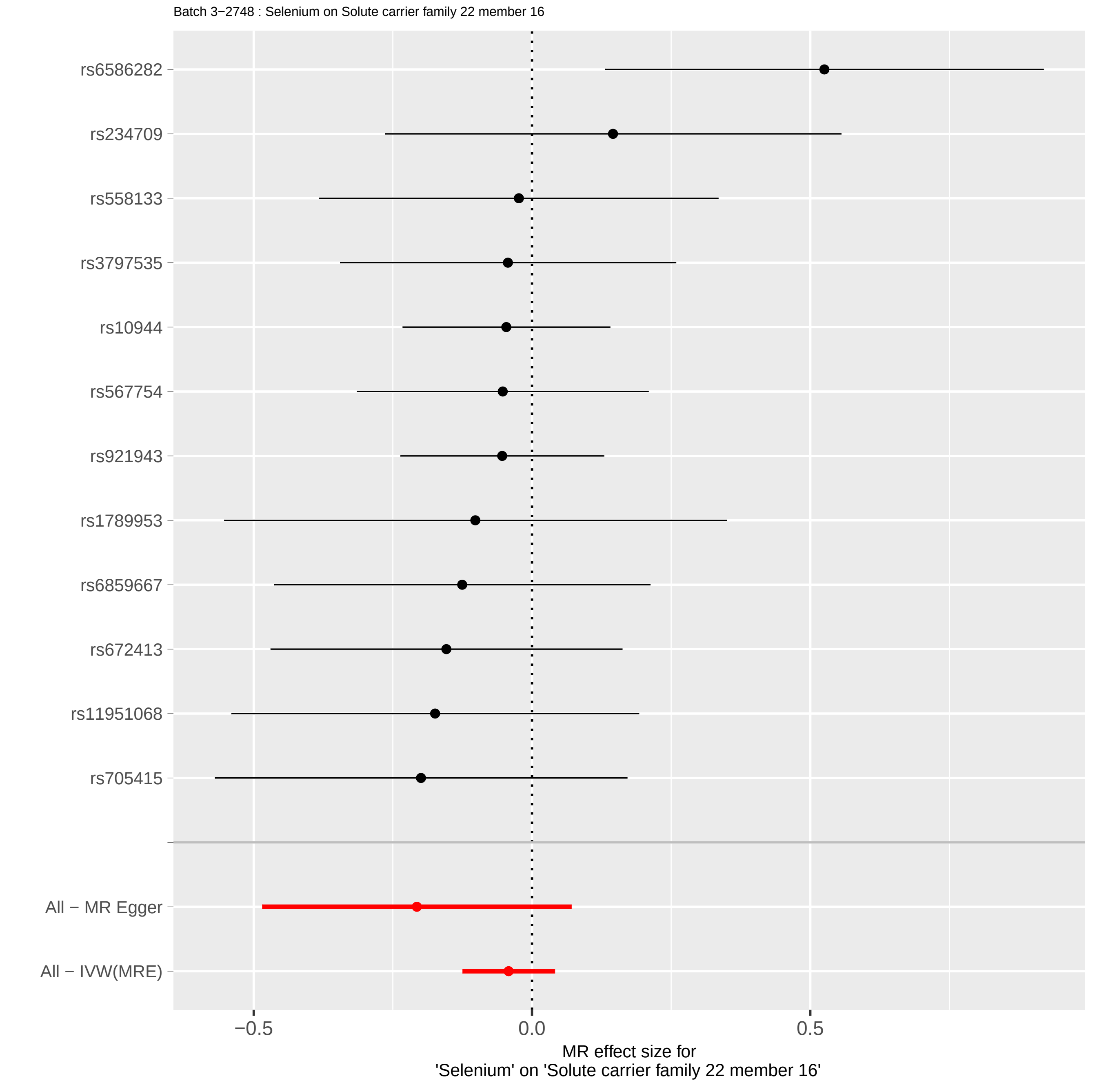

Batch 3−2748 : Selenium on Solute carrier family 22 member 16
rs6586282
rs234709
rs558133
rs3797535
rs10944
rs567754
rs921943
rs1789953
rs6859667
rs672413
rs11951068
rs705415
All − MR Egger
All − IVW(MRE)
−0.5
0.0
0.5
MR effect size for
'Selenium' on 'Solute carrier family 22 member 16'

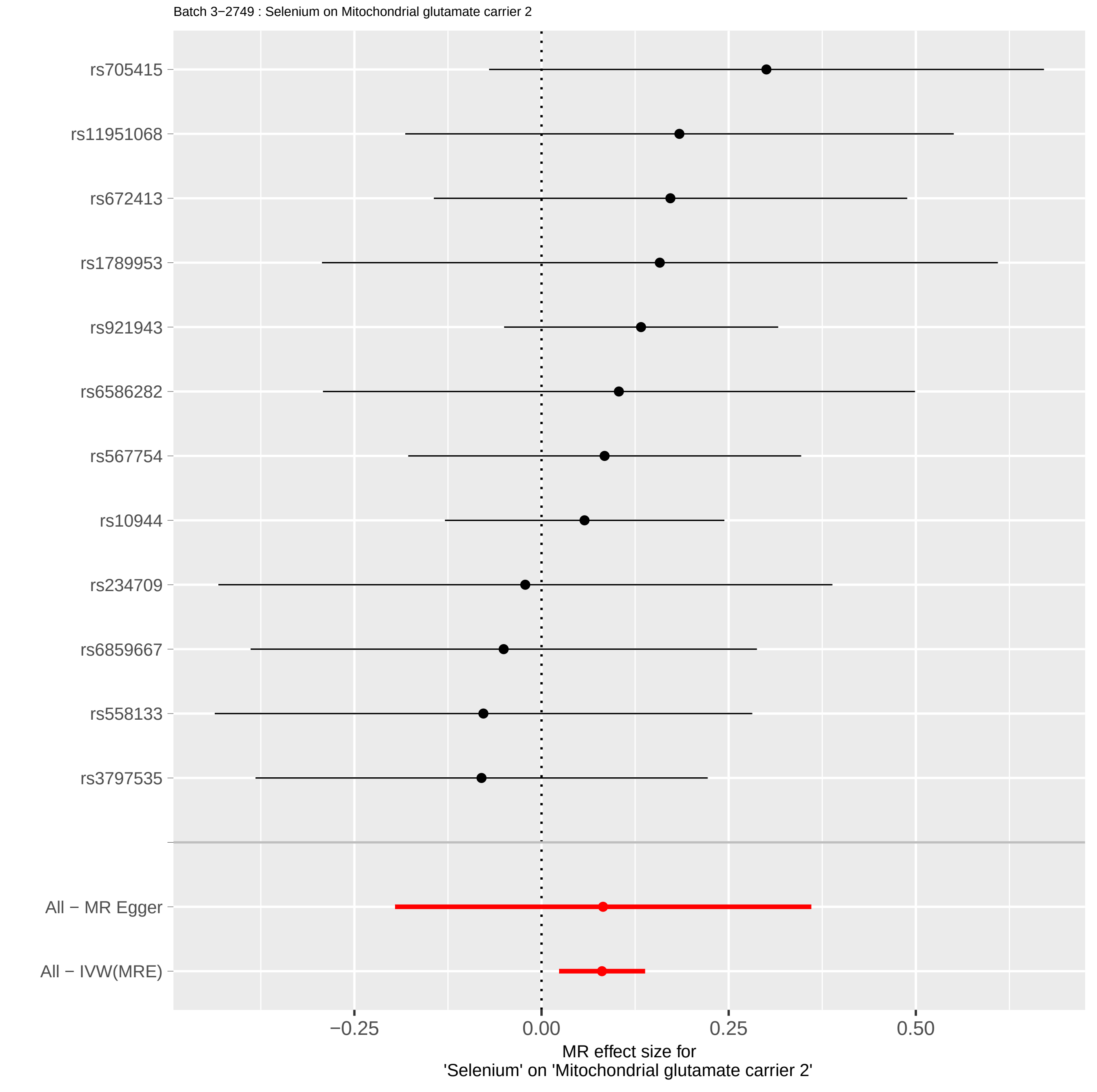

Batch 3−2749 : Selenium on Mitochondrial glutamate carrier 2
rs705415
rs11951068
rs672413
rs1789953
rs921943
rs6586282
rs567754
rs10944
rs234709
rs6859667
rs558133
rs3797535
All − MR Egger
All − IVW(MRE)
−0.25
0.00
0.25
0.50
MR effect size for
'Selenium' on 'Mitochondrial glutamate carrier 2'

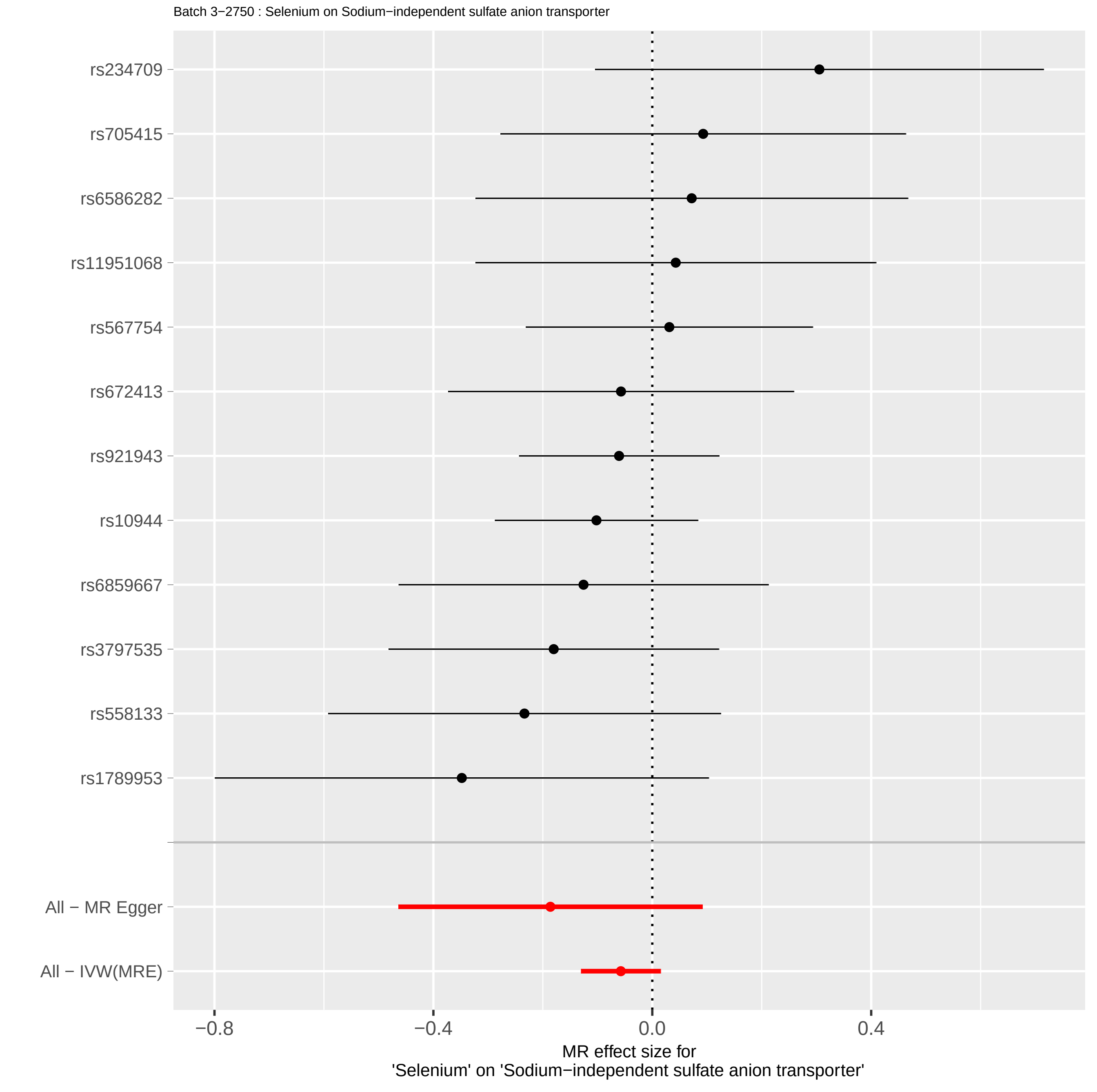

Batch 3−2750 : Selenium on Sodium−independent sulfate anion transporter
rs234709
rs705415
rs6586282
rs11951068
rs567754
rs672413
rs921943
rs10944
rs6859667
rs3797535
rs558133
rs1789953
All − MR Egger
All − IVW(MRE)
−0.8
−0.4
0.0
0.4
MR effect size for
'Selenium' on 'Sodium−independent sulfate anion transporter'

Batch 3−2751 : Selenium on Prestin

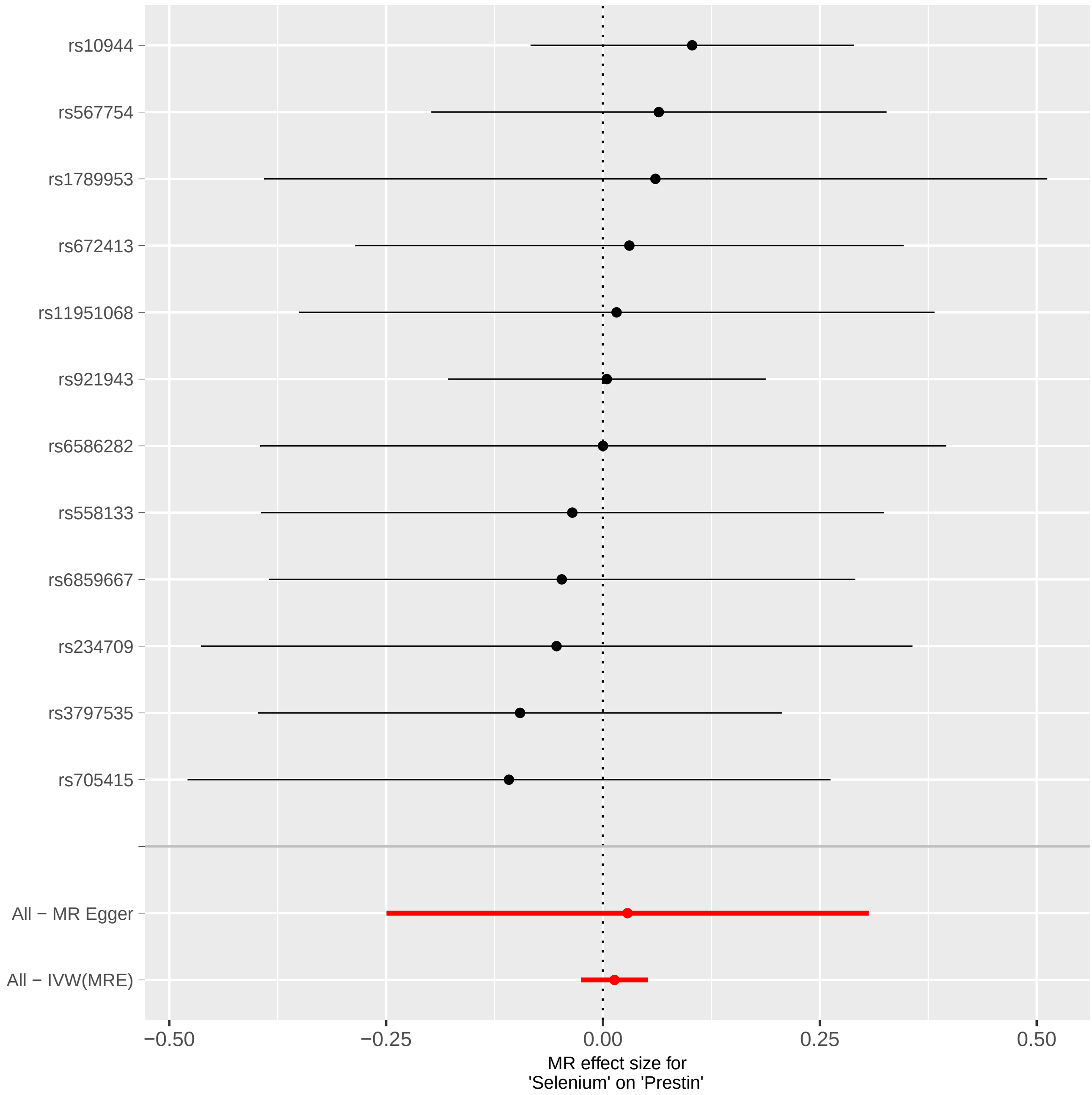

Batch 3−2752 : Selenium on Very long−chain acyl−CoA synthetase

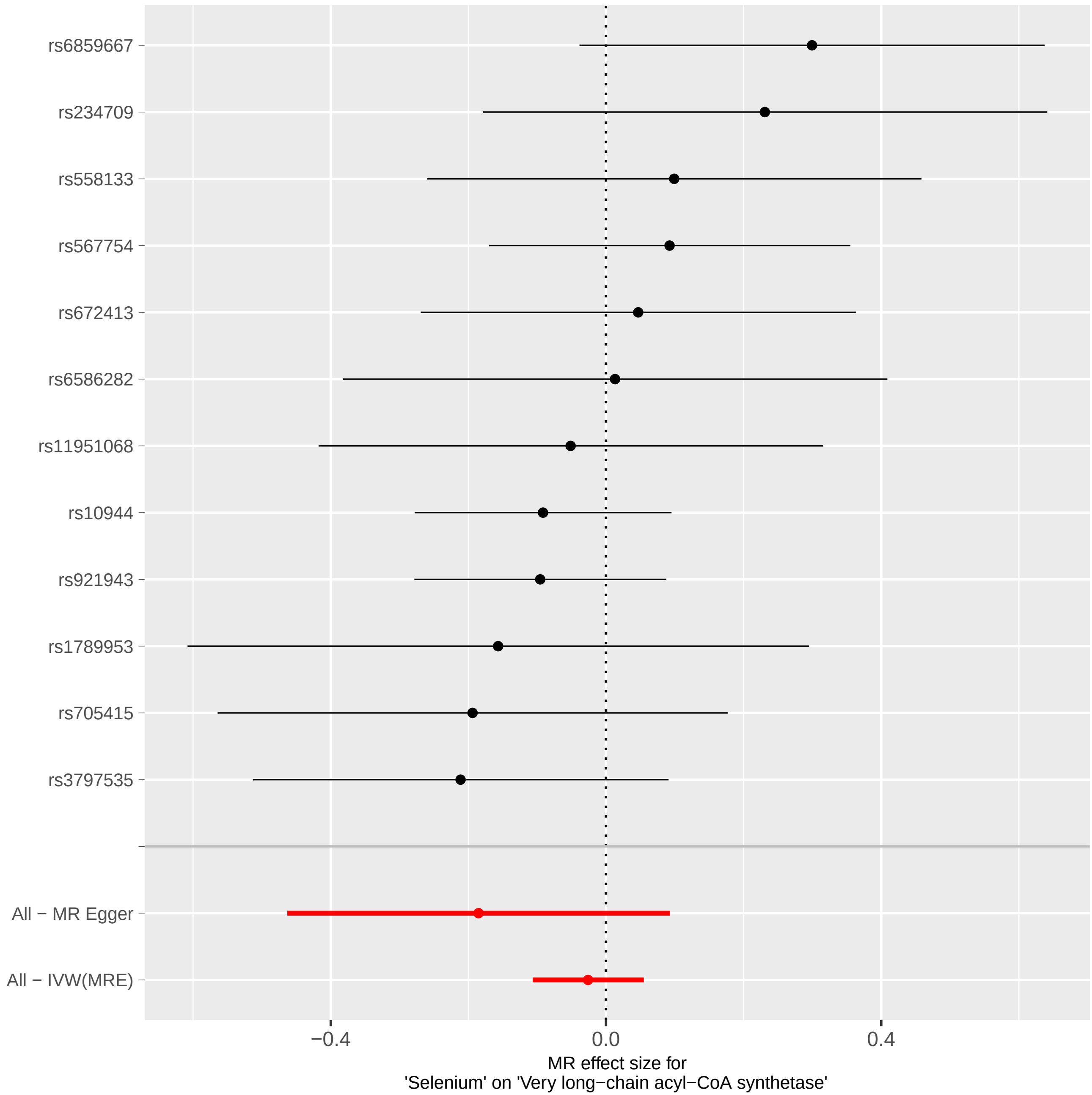

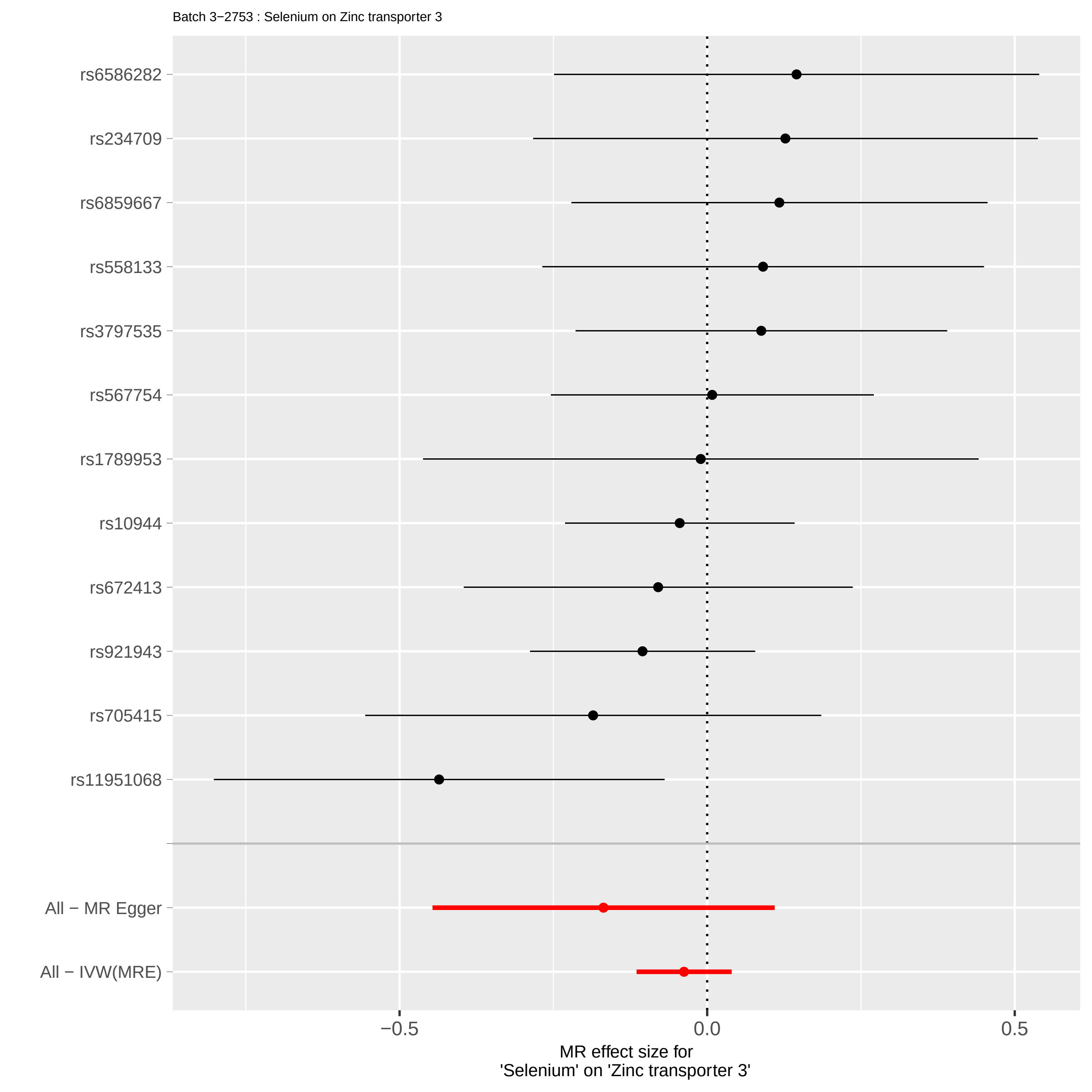
Batch 3−2753 : Selenium on Zinc transporter 3
rs6586282
rs234709
rs6859667
rs558133
rs3797535
rs567754
rs1789953
rs10944
rs672413
rs921943
rs705415
rs11951068
All − MR Egger
All − IVW(MRE)
−0.5
0.0
0.5
MR effect size for
'Selenium' on 'Zinc transporter 3'

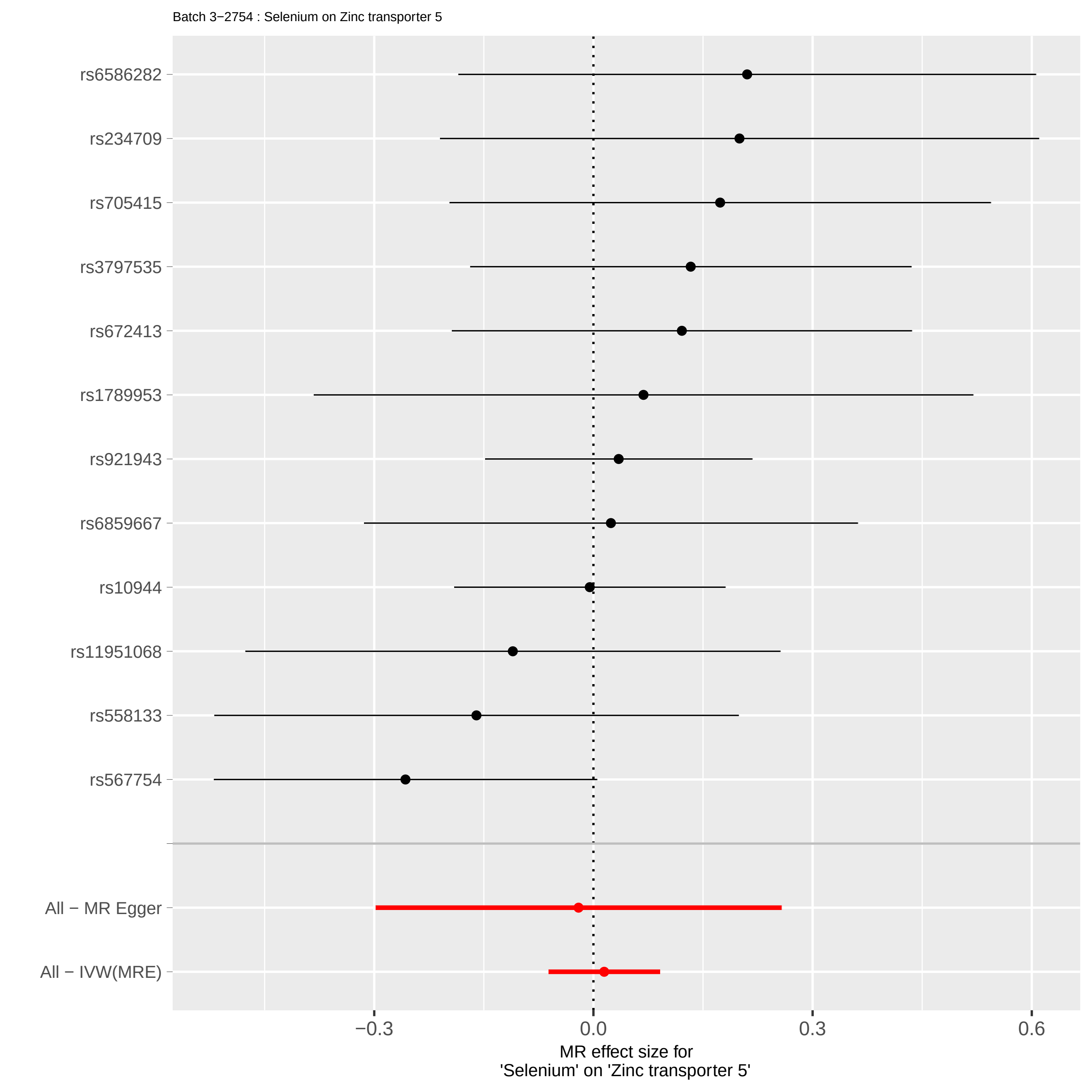

Batch 3−2754 : Selenium on Zinc transporter 5
rs6586282
rs234709
rs705415
rs3797535
rs672413
rs1789953
rs921943
rs6859667
rs10944
rs11951068
rs558133
rs567754
All − MR Egger
All − IVW(MRE)
−0.3
0.0
0.3
0.6
MR effect size for
'Selenium' on 'Zinc transporter 5'

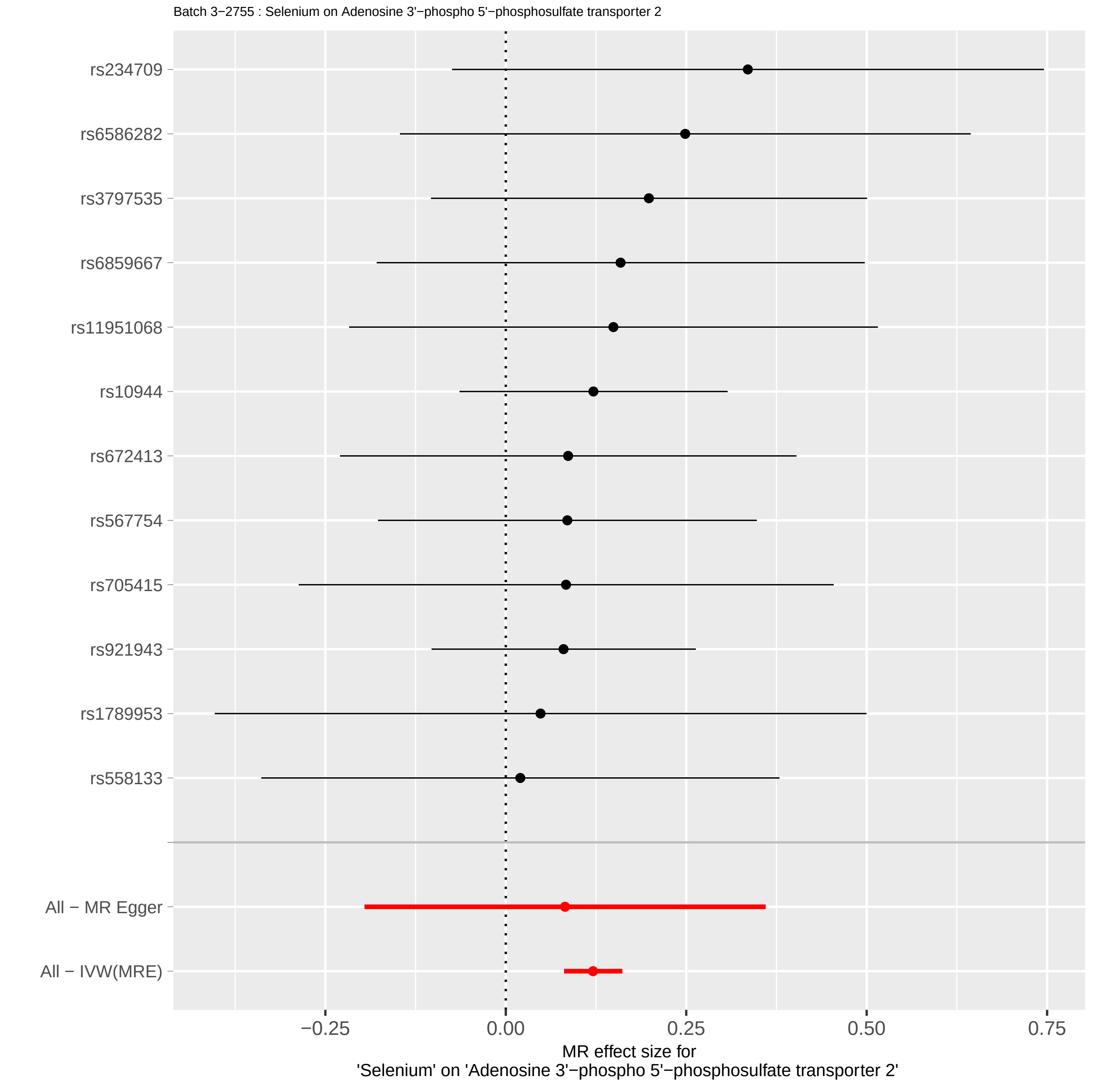
Batch 3−2755 : Selenium on Adenosine 3'−phospho 5'−phosphosulfate transporter 2
rs234709
rs6586282
rs3797535
rs6859667
rs11951068
rs10944
rs672413
rs567754
rs705415
rs921943
rs1789953
rs558133
All − MR Egger
All − IVW(MRE)
−0.25
0.00
0.25
0.50
0.75
MR effect size for
'Selenium' on 'Adenosine 3'−phospho 5'−phosphosulfate transporter 2'

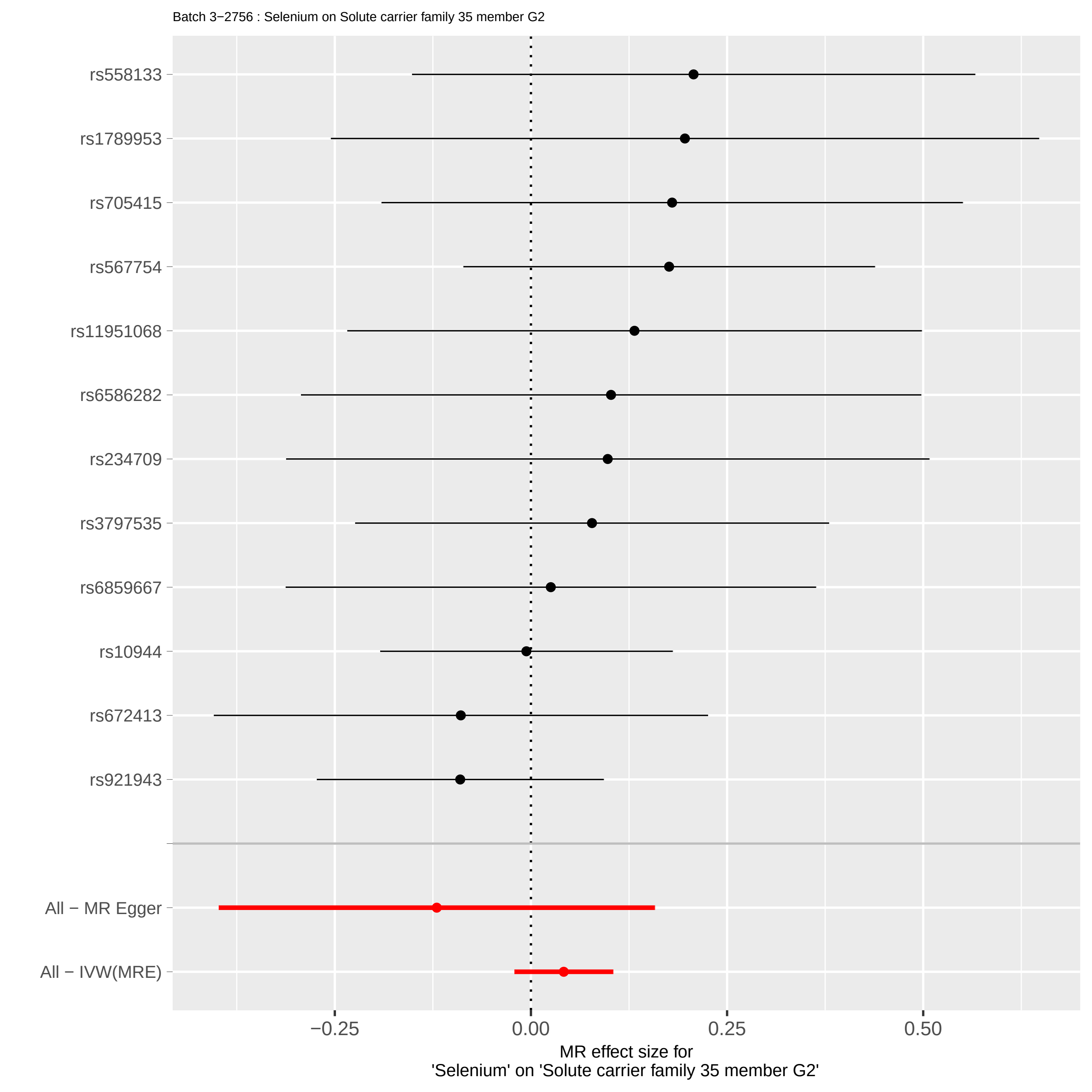
Batch 3−2756 : Selenium on Solute carrier family 35 member G2
rs558133
rs1789953
rs705415
rs567754
rs11951068
rs6586282
rs234709
rs3797535
rs6859667
rs10944
rs672413
rs921943
All − MR Egger
All − IVW(MRE)
−0.25
0.00
0.25
0.50
MR effect size for
'Selenium' on 'Solute carrier family 35 member G2'

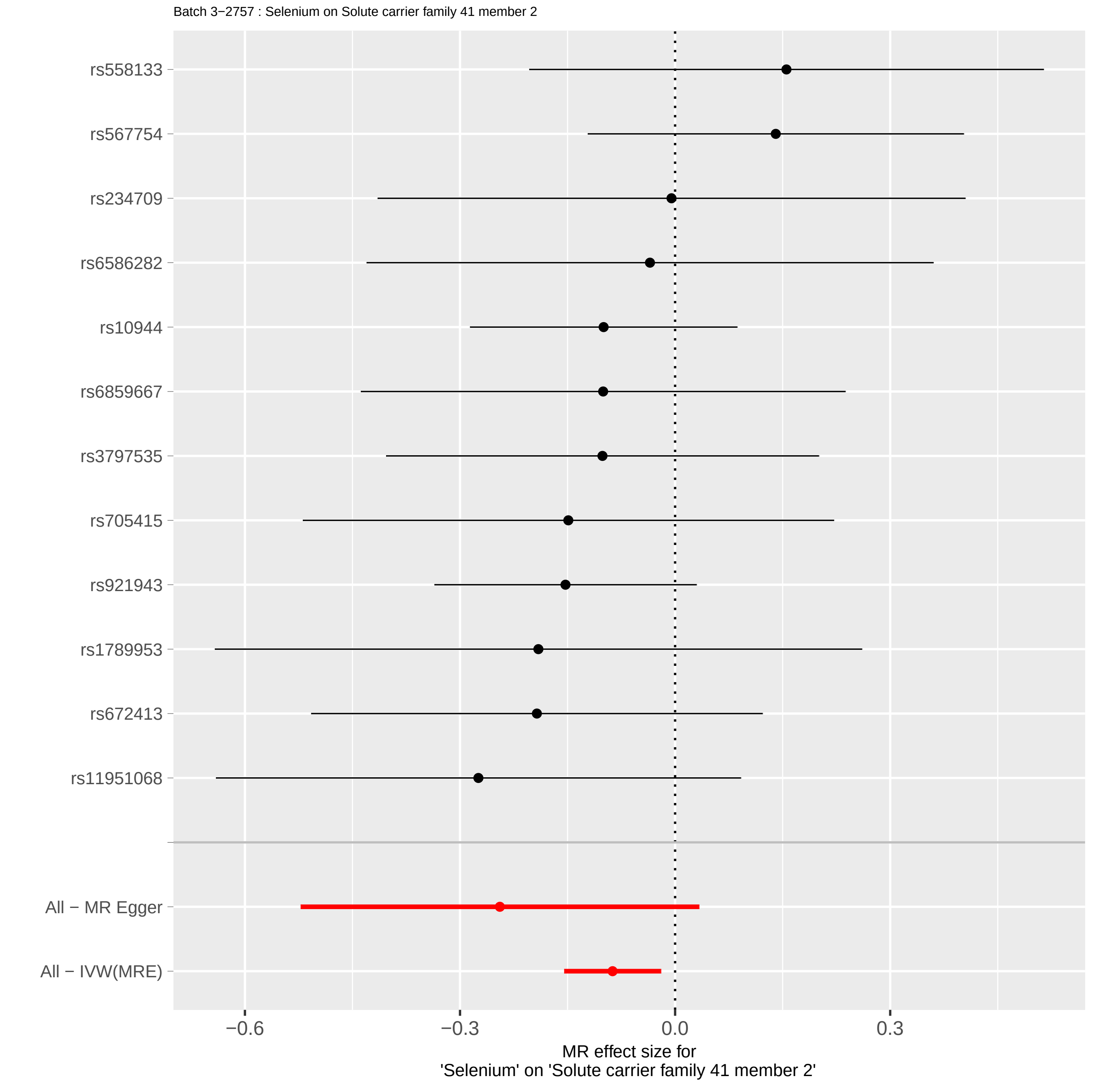

Batch 3−2757 : Selenium on Solute carrier family 41 member 2
rs558133
rs567754
rs234709
rs6586282
rs10944
rs6859667
rs3797535
rs705415
rs921943
rs1789953
rs672413
rs11951068
All − MR Egger
All − IVW(MRE)
−0.6
−0.3
0.0
0.3
MR effect size for
'Selenium' on 'Solute carrier family 41 member 2'

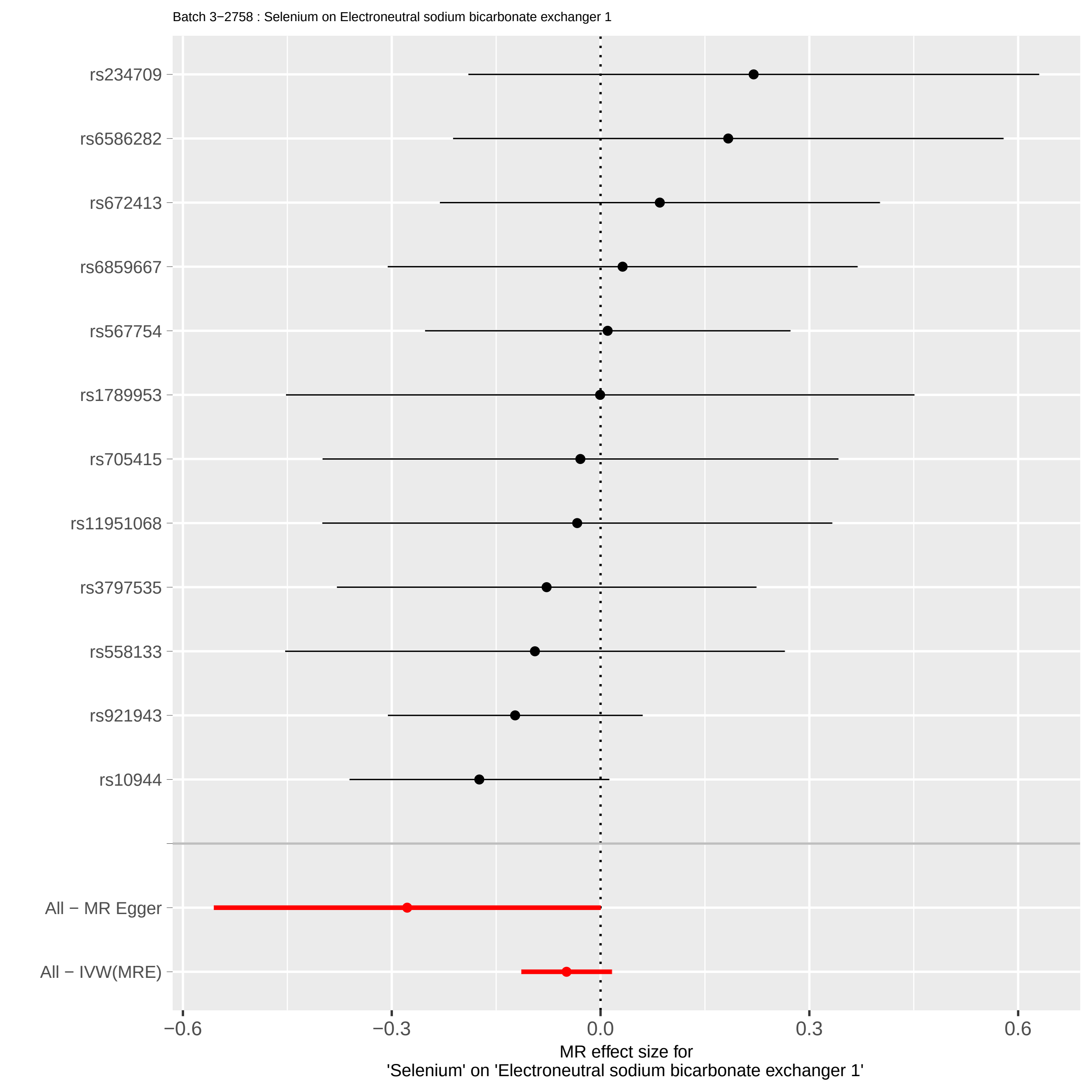

Batch 3−2758 : Selenium on Electroneutral sodium bicarbonate exchanger 1
rs234709
rs6586282
rs672413
rs6859667
rs567754
rs1789953
rs705415
rs11951068
rs3797535
rs558133
rs921943
rs10944
All − MR Egger
All − IVW(MRE)
−0.6
−0.3
0.0
0.3
0.6
MR effect size for
'Selenium' on 'Electroneutral sodium bicarbonate exchanger 1'

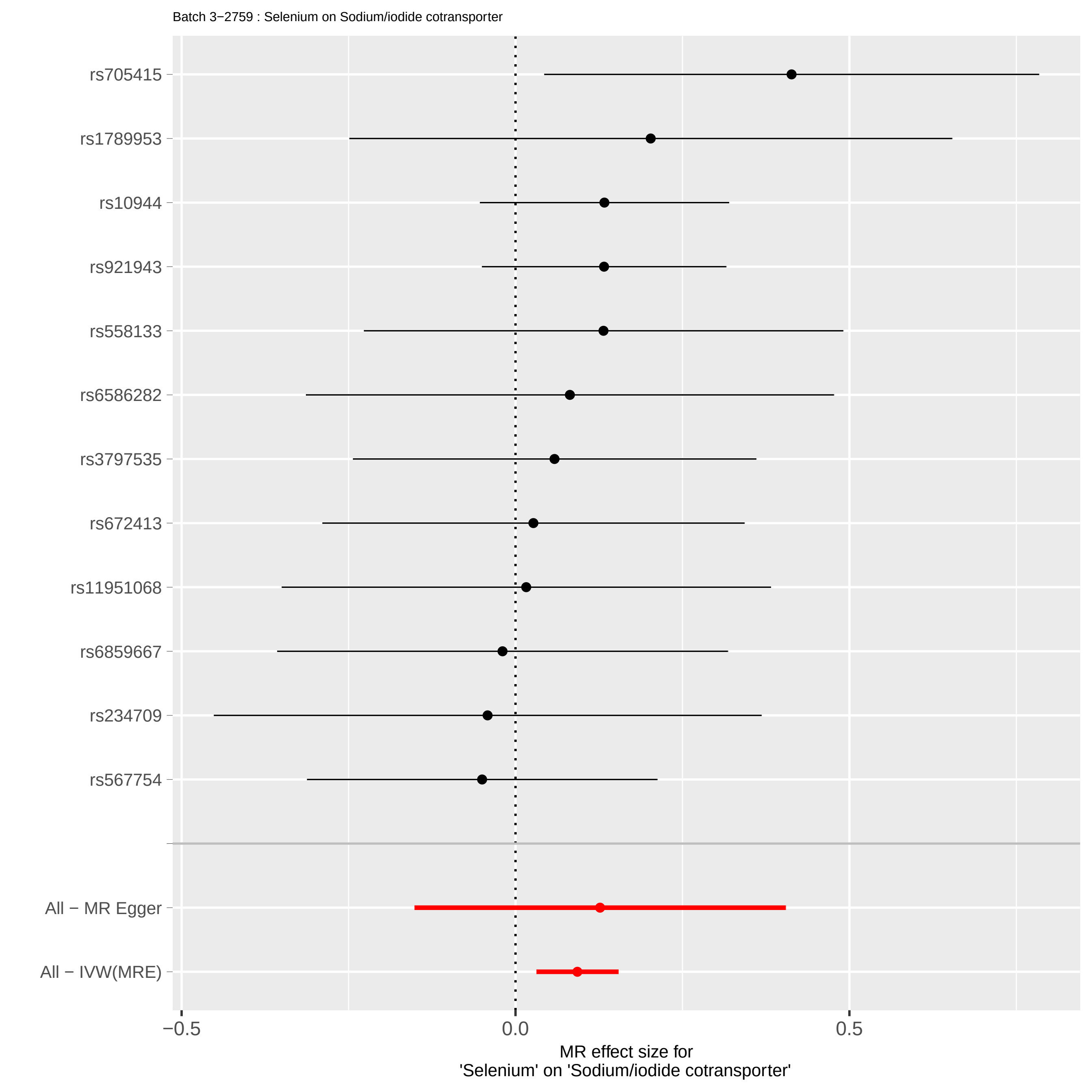

Batch 3−2759 : Selenium on Sodium/iodide cotransporter
rs705415
rs1789953
rs10944
rs921943
rs558133
rs6586282
rs3797535
rs672413
rs11951068
rs6859667
rs234709
rs567754
All − MR Egger
All − IVW(MRE)
−0.5
0.0
0.5
MR effect size for
'Selenium' on 'Sodium/iodide cotransporter'

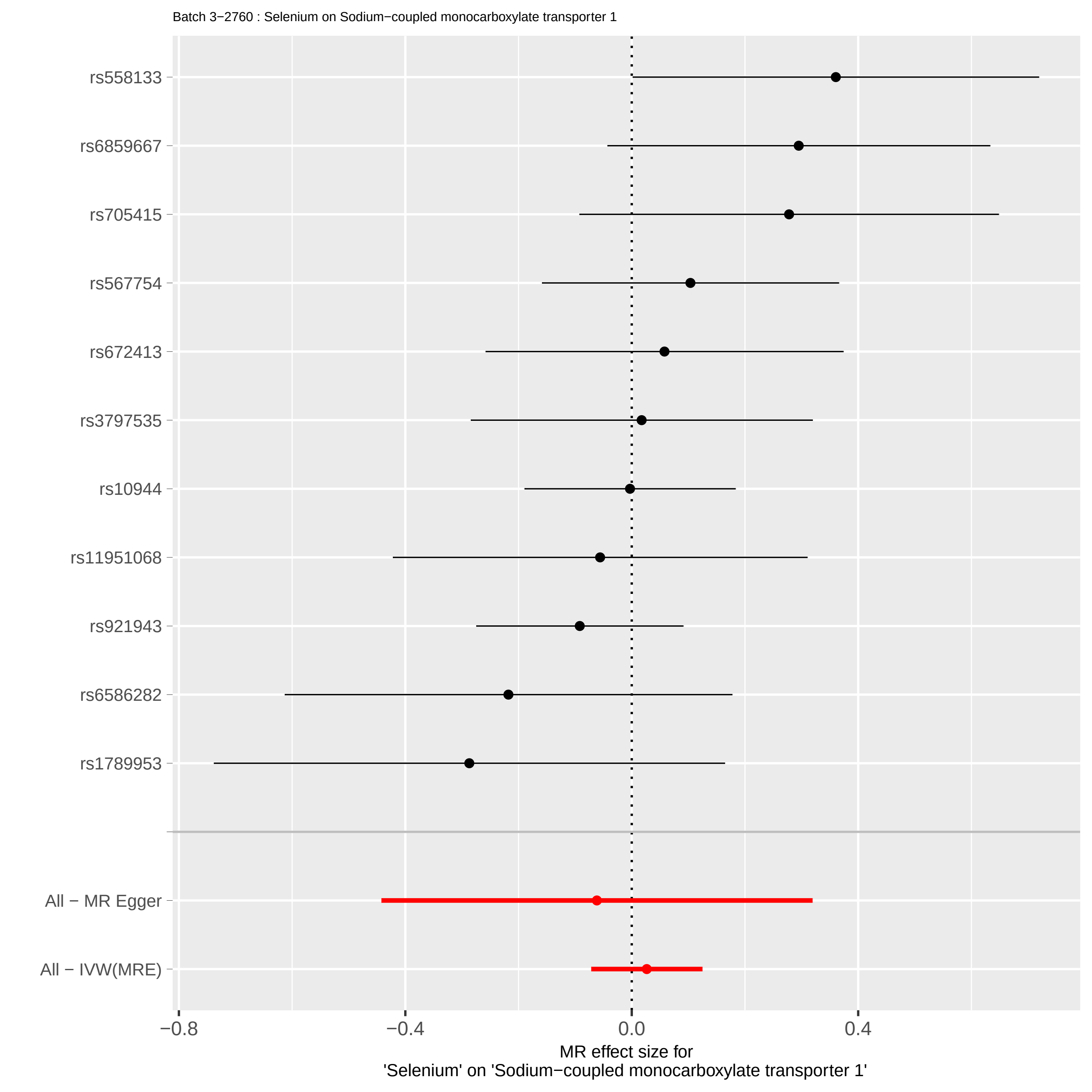

Batch 3−2760 : Selenium on Sodium−coupled monocarboxylate transporter 1
rs558133
rs6859667
rs705415
rs567754
rs672413
rs3797535
rs10944
rs11951068
rs921943
rs6586282
rs1789953
All − MR Egger
All − IVW(MRE)
−0.8
−0.4
0.0
0.4
MR effect size for
'Selenium' on 'Sodium−coupled monocarboxylate transporter 1'

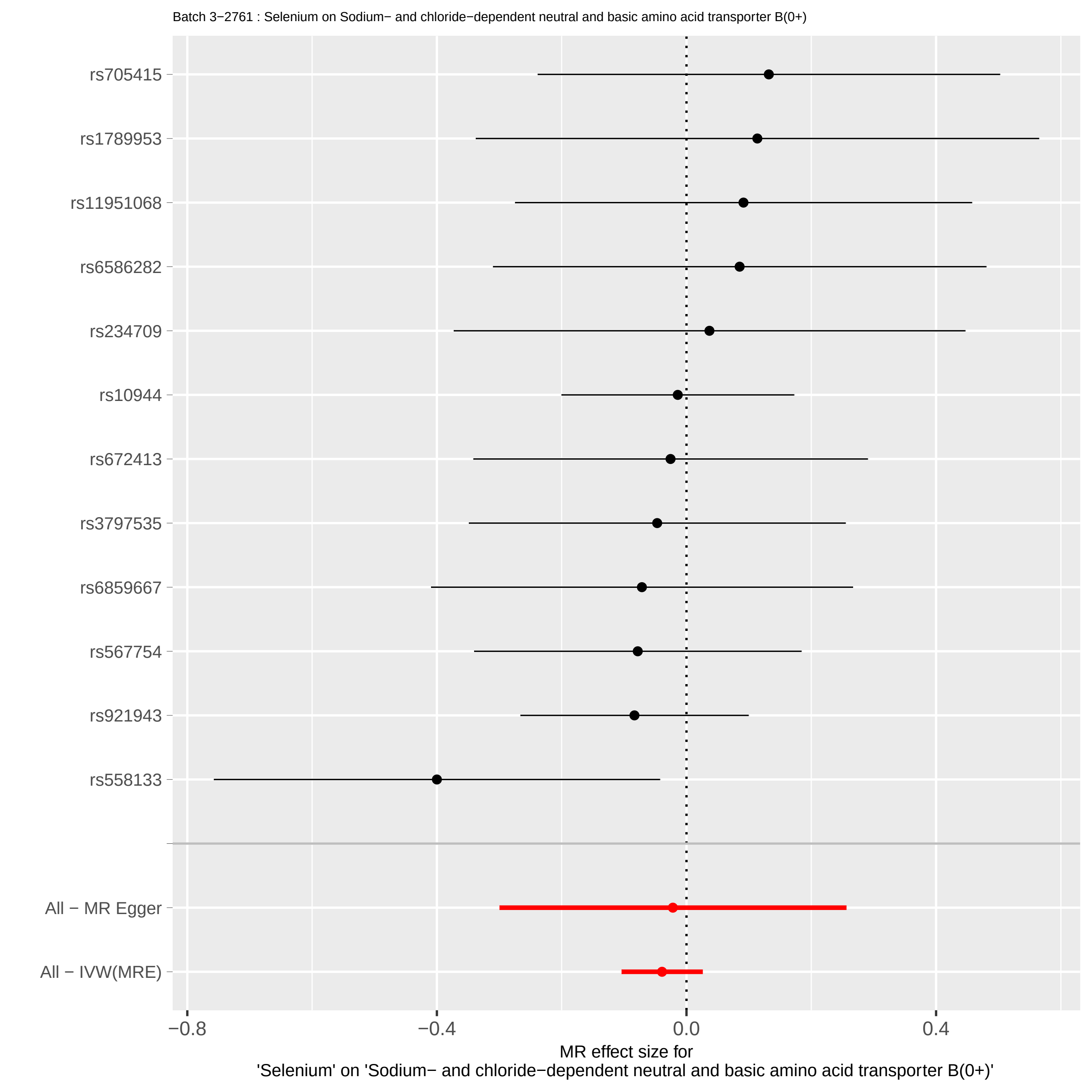
Batch 3−2761 : Selenium on Sodium− and chloride−dependent neutral and basic amino acid transporter B(0+)
rs705415
rs1789953
rs11951068
rs6586282
rs234709
rs10944
rs672413
rs3797535
rs6859667
rs567754
rs921943
rs558133
All − MR Egger
All − IVW(MRE)
−0.8
−0.4
0.0
0.4
MR effect size for
'Selenium' on 'Sodium− and chloride−dependent neutral and basic amino acid transporter B(0+)'

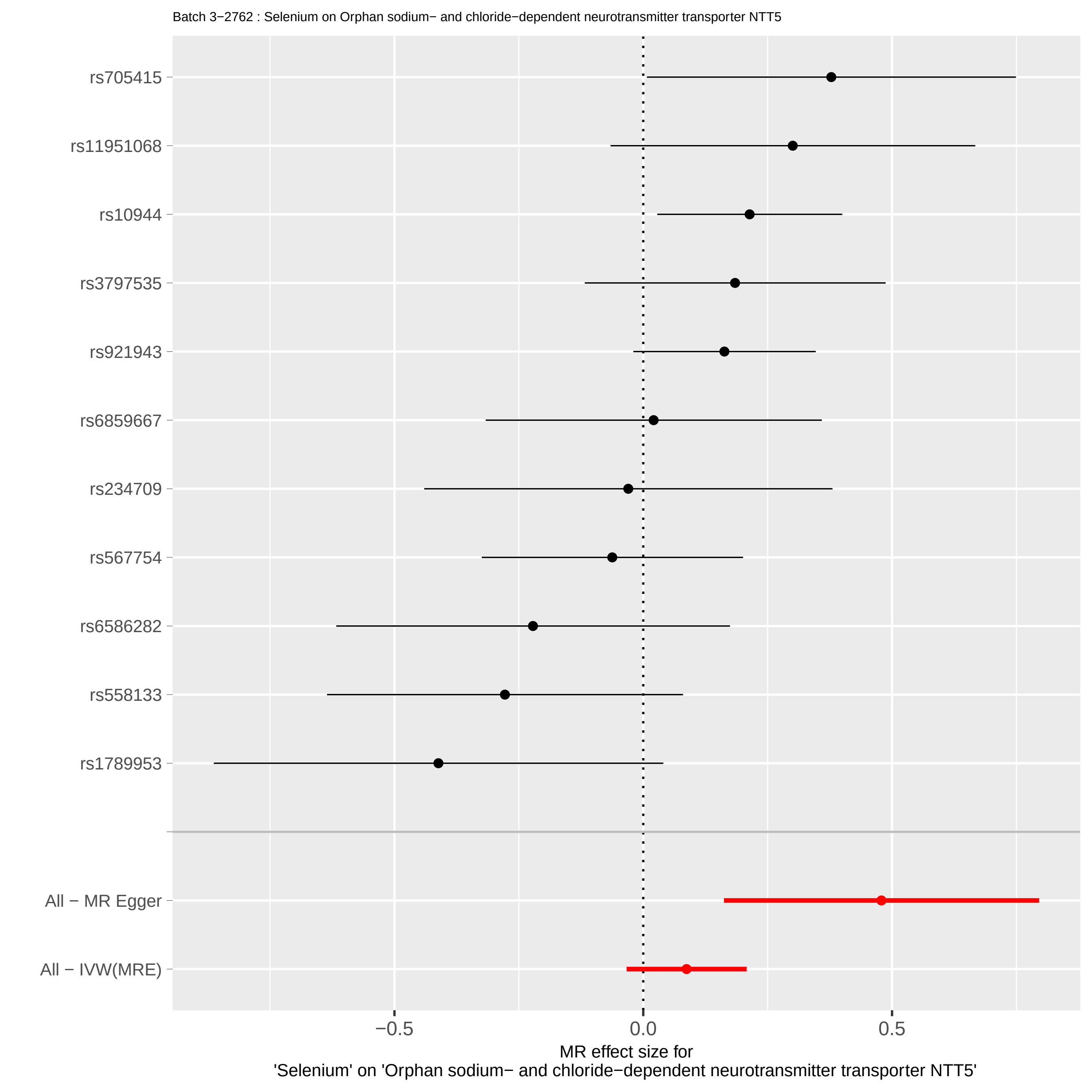
Batch 3−2762 : Selenium on Orphan sodium− and chloride−dependent neurotransmitter transporter NTT5
rs705415
rs11951068
rs10944
rs3797535
rs921943
rs6859667
rs234709
rs567754
rs6586282
rs558133
rs1789953
All − MR Egger
All − IVW(MRE)
−0.5
0.0
0.5
MR effect size for
'Selenium' on 'Orphan sodium− and chloride−dependent neurotransmitter transporter NTT5'

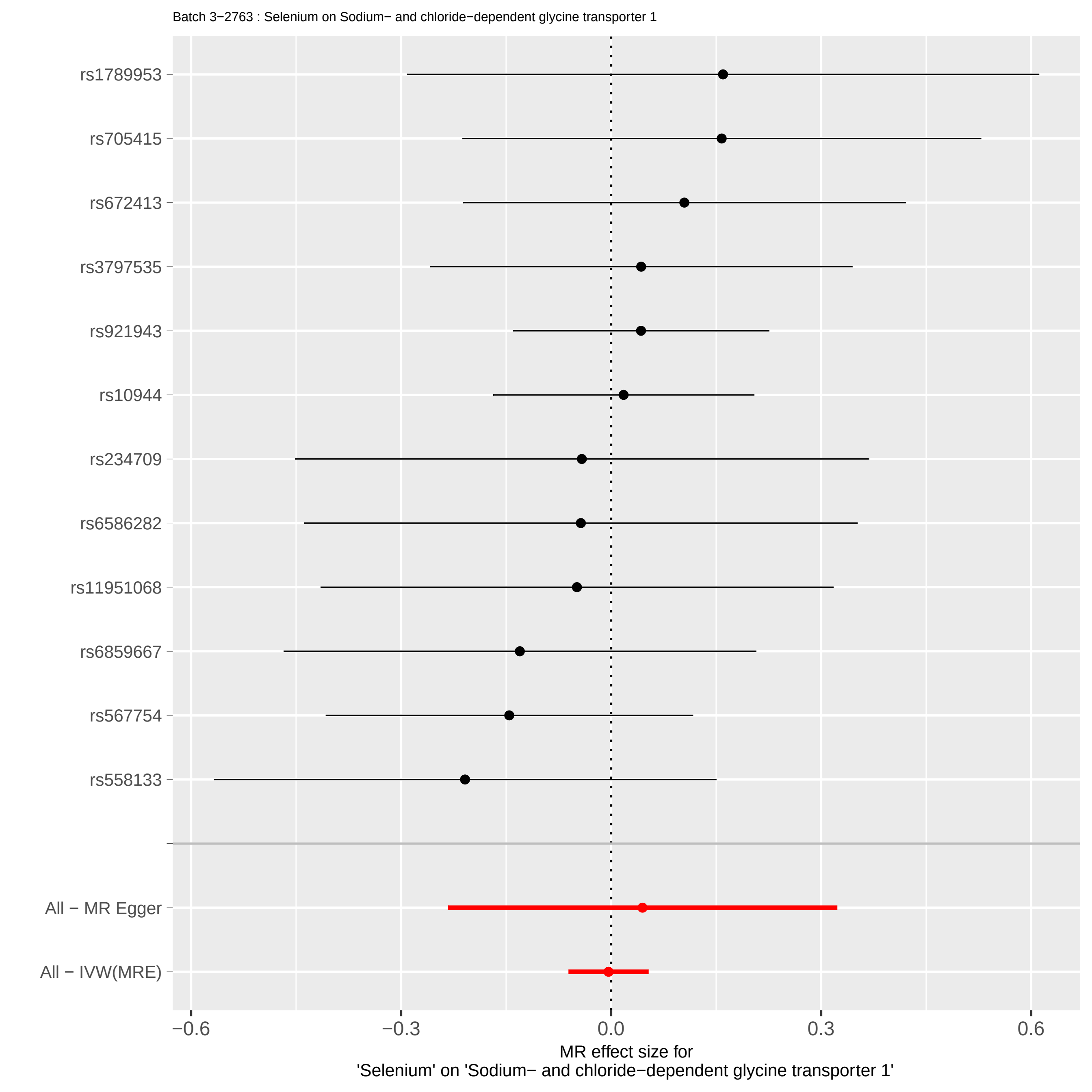

Batch 3−2763 : Selenium on Sodium− and chloride−dependent glycine transporter 1
rs1789953
rs705415
rs672413
rs3797535
rs921943
rs10944
rs234709
rs6586282
rs11951068
rs6859667
rs567754
rs558133
All − MR Egger
All − IVW(MRE)
−0.6
−0.3
0.0
0.3
0.6
MR effect size for
'Selenium' on 'Sodium− and chloride−dependent glycine transporter 1'

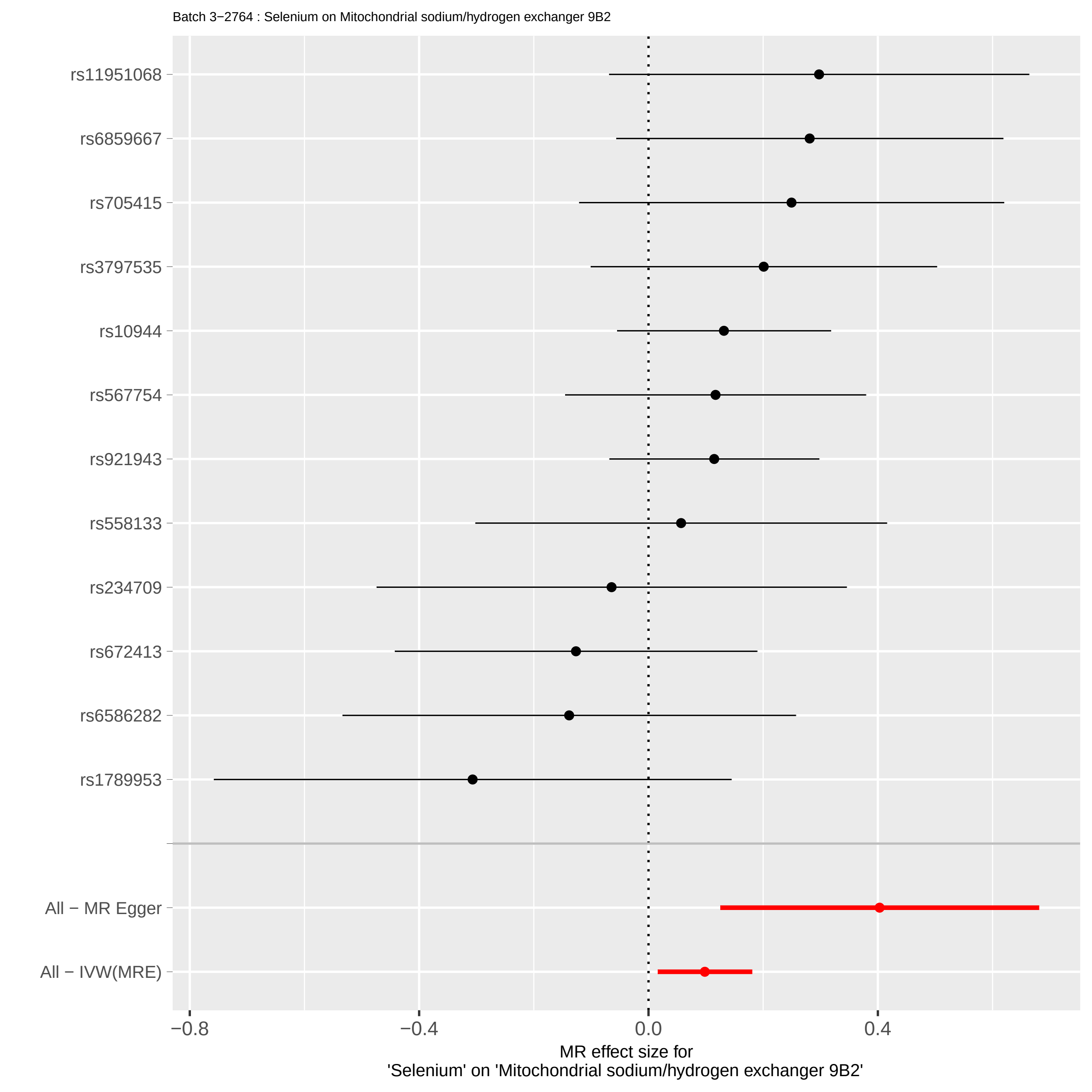

Batch 3−2764 : Selenium on Mitochondrial sodium/hydrogen exchanger 9B2
rs11951068
rs6859667
rs705415
rs3797535
rs10944
rs567754
rs921943
rs558133
rs234709
rs672413
rs6586282
rs1789953
All − MR Egger
All − IVW(MRE)
−0.8
−0.4
0.0
0.4
MR effect size for
'Selenium' on 'Mitochondrial sodium/hydrogen exchanger 9B2'

Batch 3−2765 : Selenium on Solute carrier organic anion transporter family member 5A1

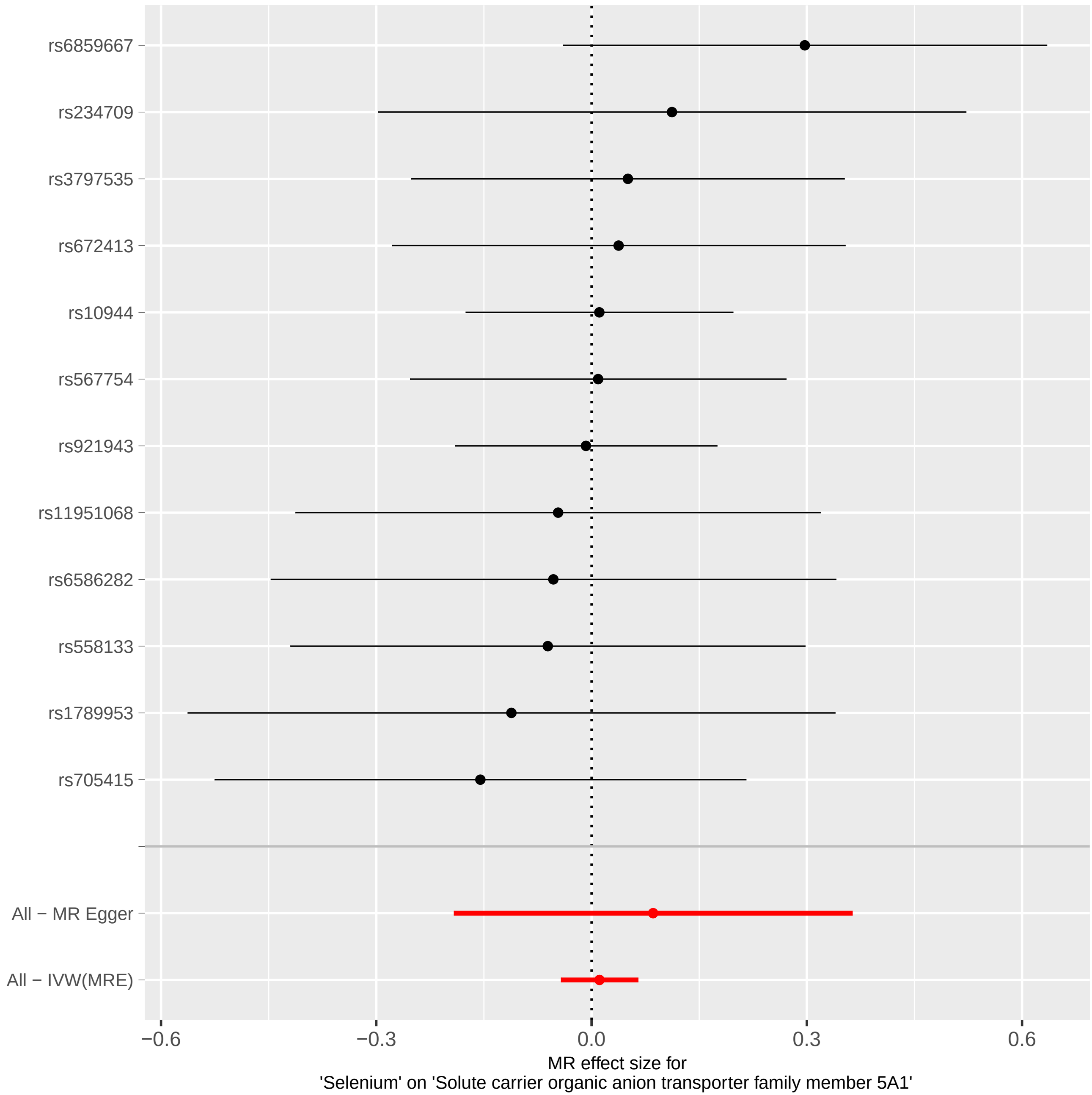

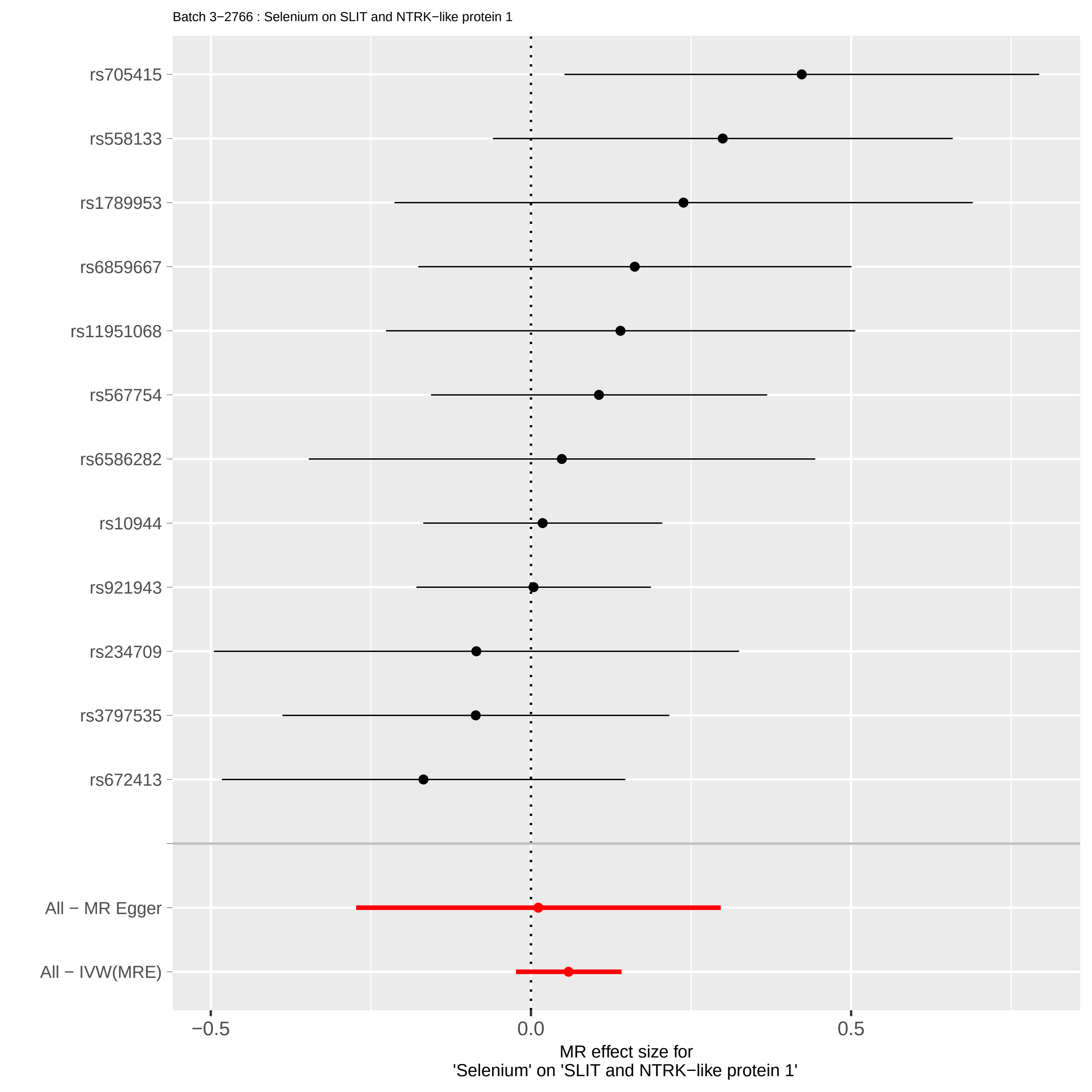

Batch 3−2766 : Selenium on SLIT and NTRK−like protein 1
rs705415
rs558133
rs1789953
rs6859667
rs11951068
rs567754
rs6586282
rs10944
rs921943
rs234709
rs3797535
rs672413
All − MR Egger
All − IVW(MRE)
−0.5
0.0
0.5
MR effect size for
'Selenium' on 'SLIT and NTRK−like protein 1'

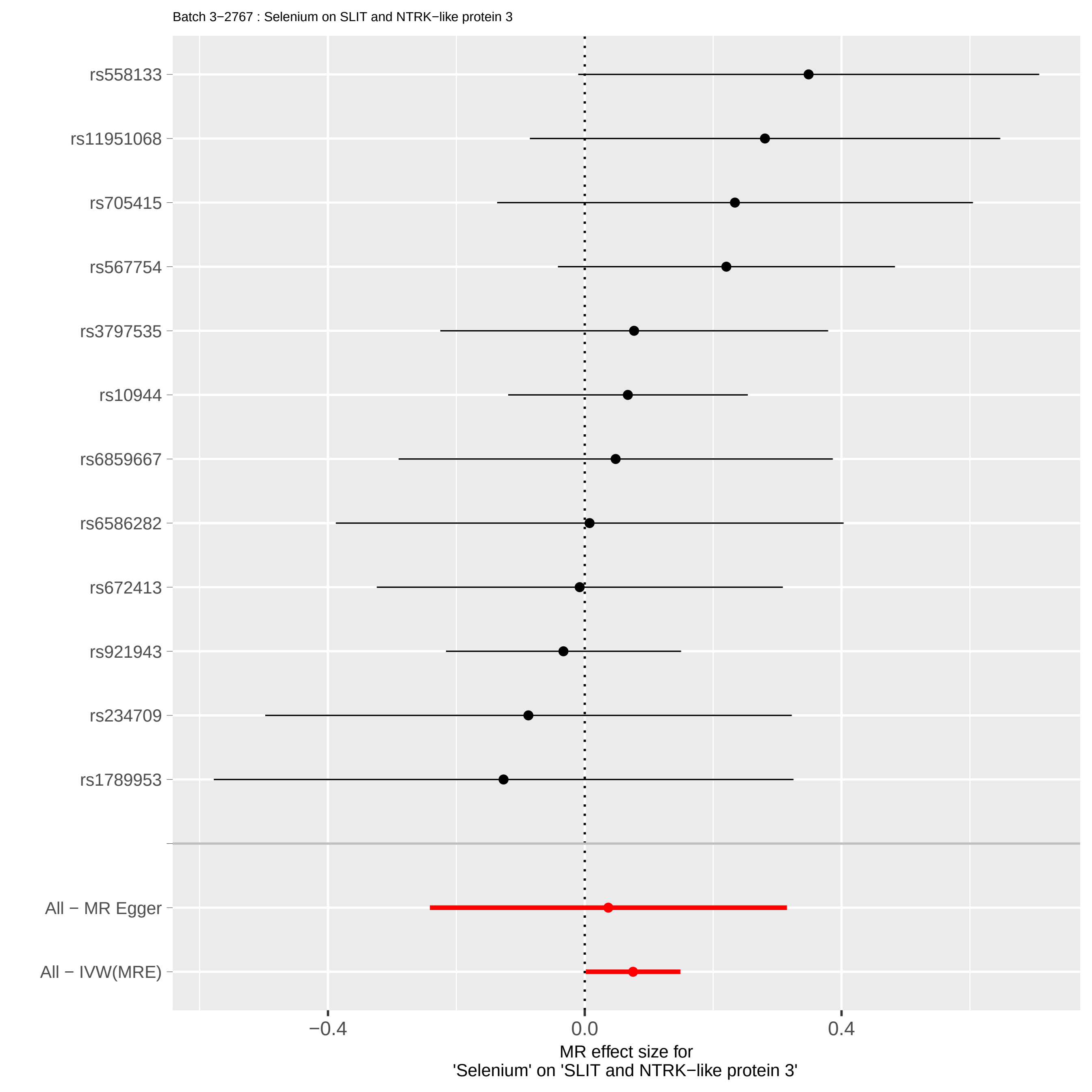
Batch 3−2767 : Selenium on SLIT and NTRK−like protein 3
rs558133
rs11951068
rs705415
rs567754
rs3797535
rs10944
rs6859667
rs6586282
rs672413
rs921943
rs234709
rs1789953
All − MR Egger
All − IVW(MRE)
−0.4
0.0
0.4
MR effect size for
'Selenium' on 'SLIT and NTRK−like protein 3'

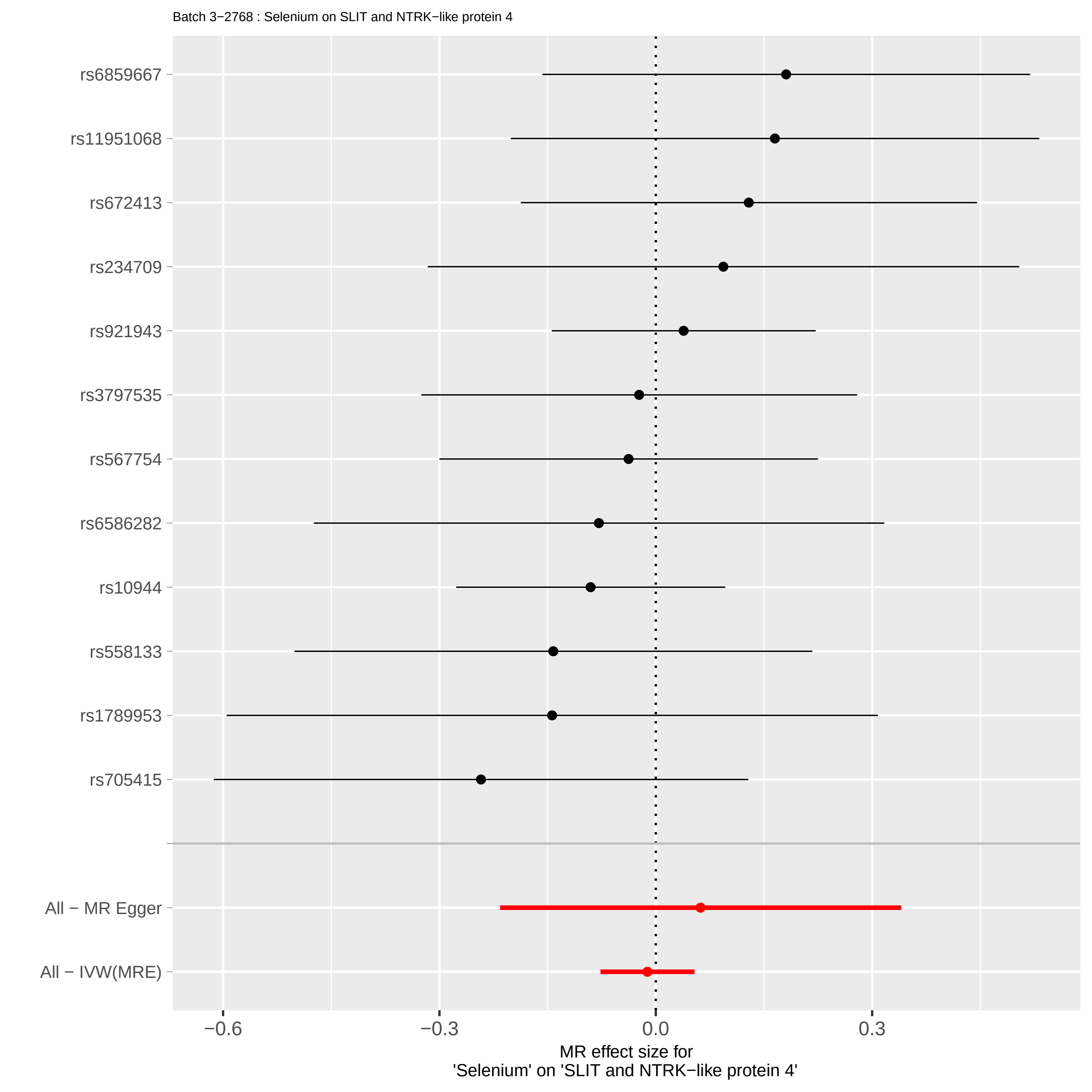

Batch 3−2768 : Selenium on SLIT and NTRK−like protein 4
rs6859667
rs11951068
rs672413
rs234709
rs921943
rs3797535
rs567754
rs6586282
rs10944
rs558133
rs1789953
rs705415
All − MR Egger
All − IVW(MRE)
−0.6
−0.3
0.0
0.3
MR effect size for
'Selenium' on 'SLIT and NTRK−like protein 4'

Batch 3−2769 : Selenium on SLIT and NTRK−like protein 5

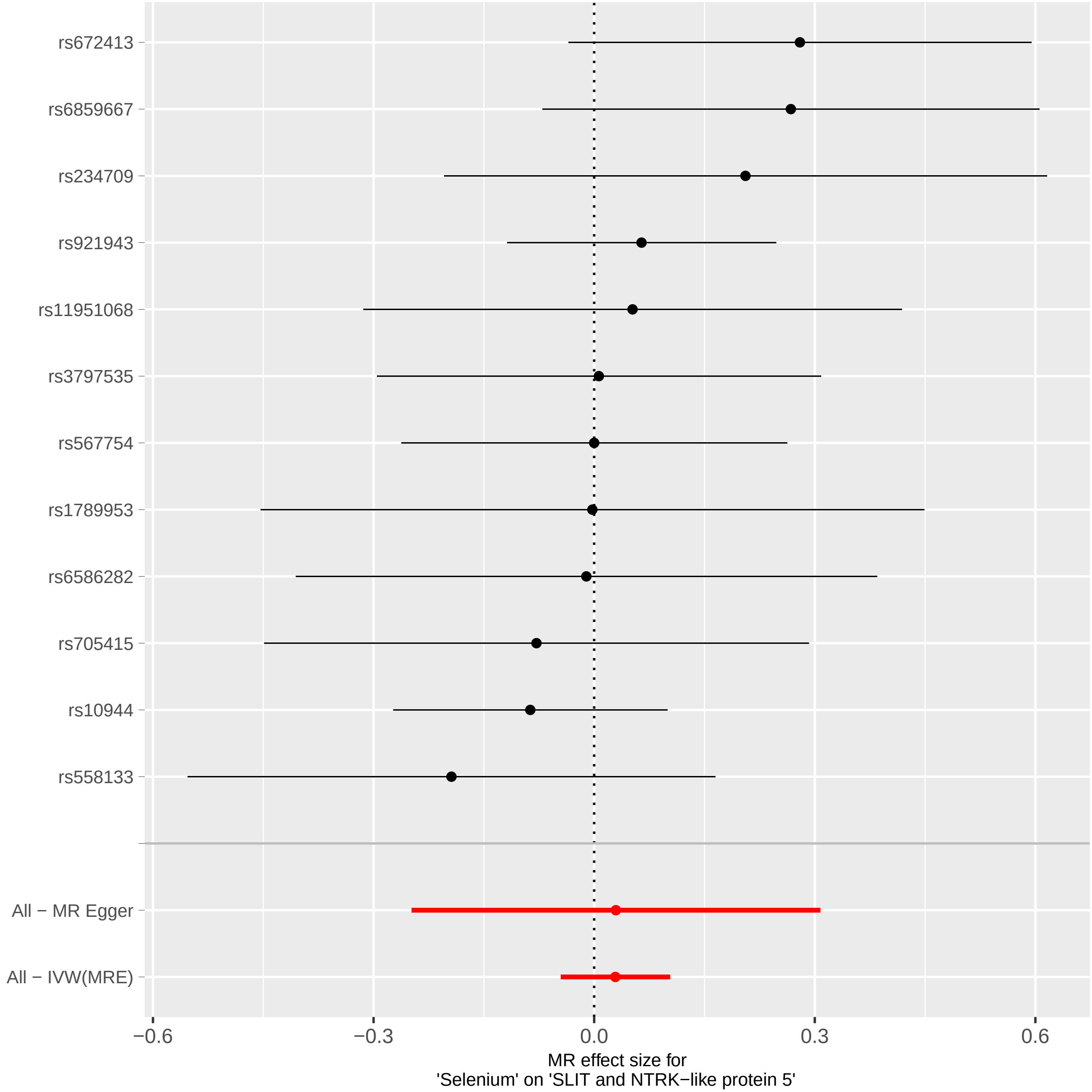

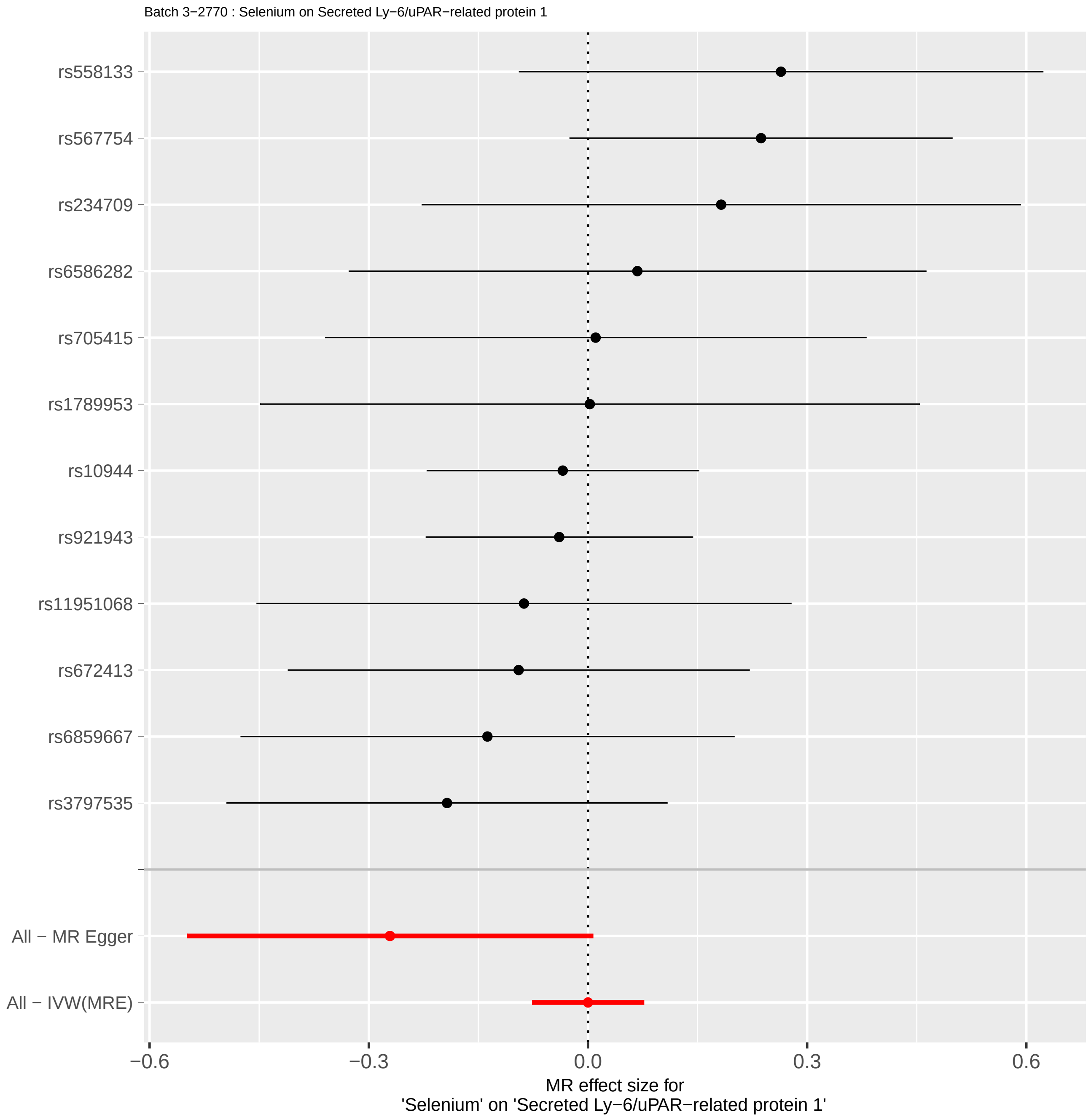

Batch 3−2770 : Selenium on Secreted Ly−6/uPAR−related protein 1
rs558133
rs567754
rs234709
rs6586282
rs705415
rs1789953
rs10944
rs921943
rs11951068
rs672413
rs6859667
rs3797535
All − MR Egger
All − IVW(MRE)
−0.6
−0.3
0.0
0.3
0.6
MR effect size for
'Selenium' on 'Secreted Ly−6/uPAR−related protein 1'

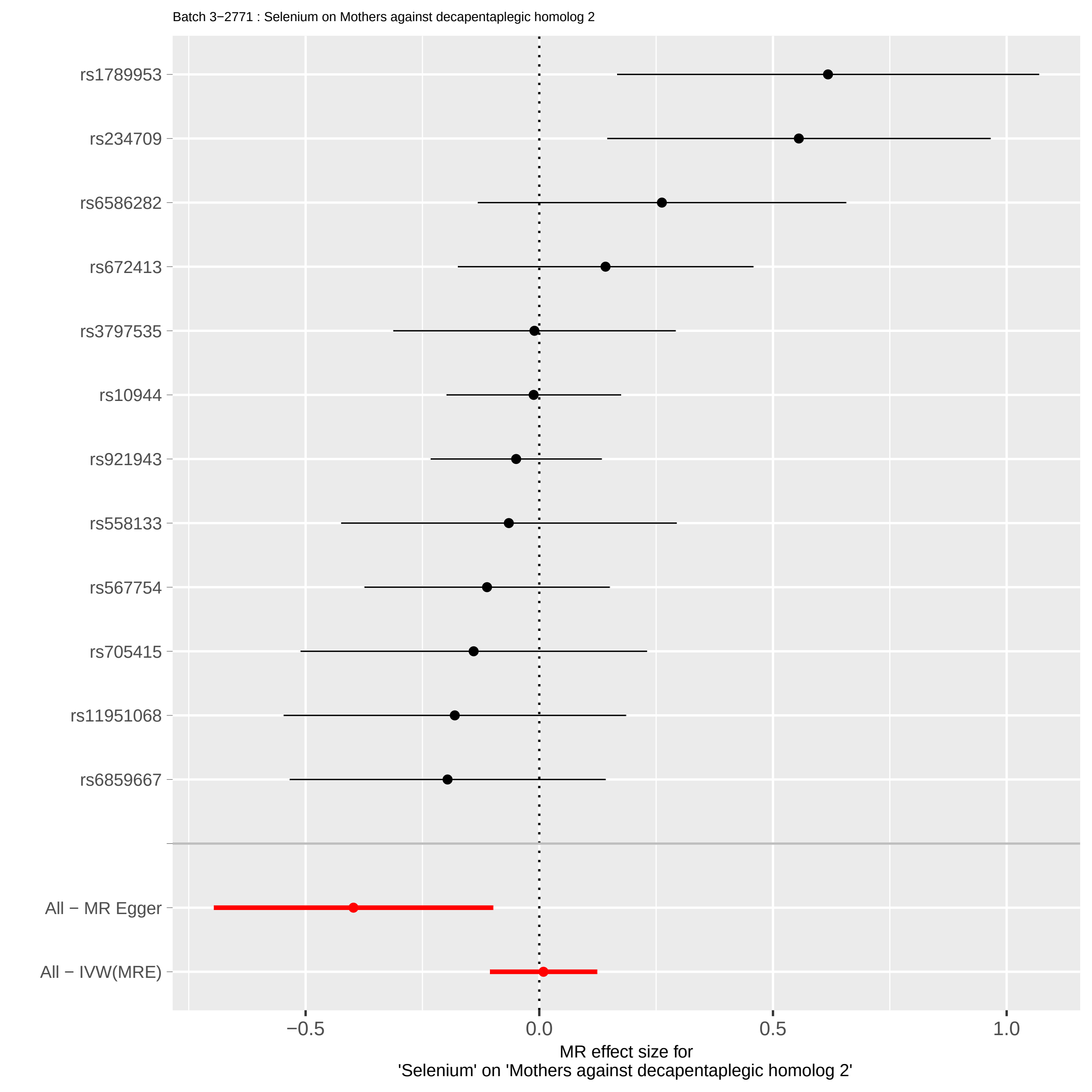

Batch 3−2771 : Selenium on Mothers against decapentaplegic homolog 2
rs1789953
rs234709
rs6586282
rs672413
rs3797535
rs10944
rs921943
rs558133
rs567754
rs705415
rs11951068
rs6859667
All − MR Egger
All − IVW(MRE)
−0.5
0.0
0.5
1.0
MR effect size for
'Selenium' on 'Mothers against decapentaplegic homolog 2'

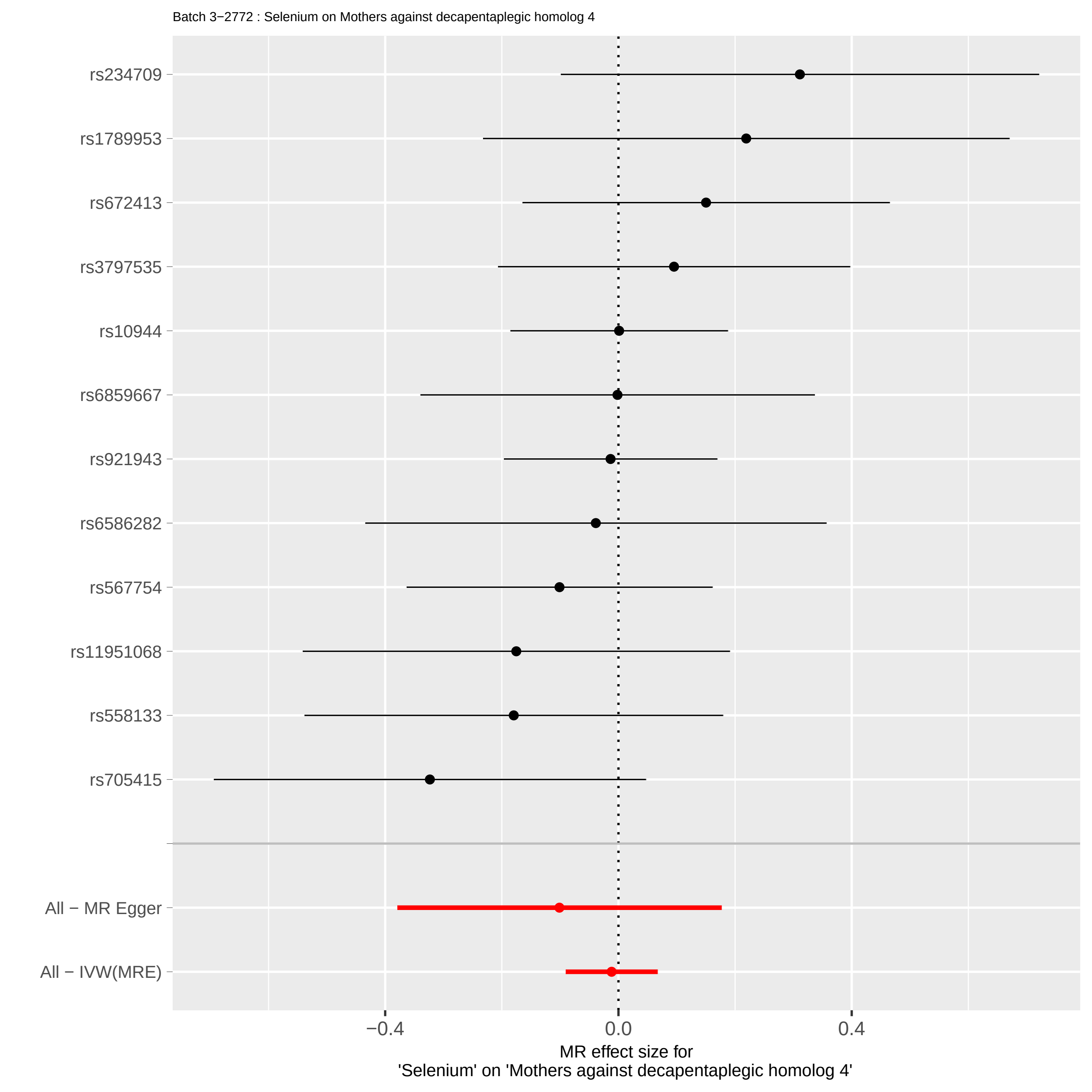

Batch 3−2772 : Selenium on Mothers against decapentaplegic homolog 4
rs234709
rs1789953
rs672413
rs3797535
rs10944
rs6859667
rs921943
rs6586282
rs567754
rs11951068
rs558133
rs705415
All − MR Egger
All − IVW(MRE)
−0.4
0.0
0.4
MR effect size for
'Selenium' on 'Mothers against decapentaplegic homolog 4'

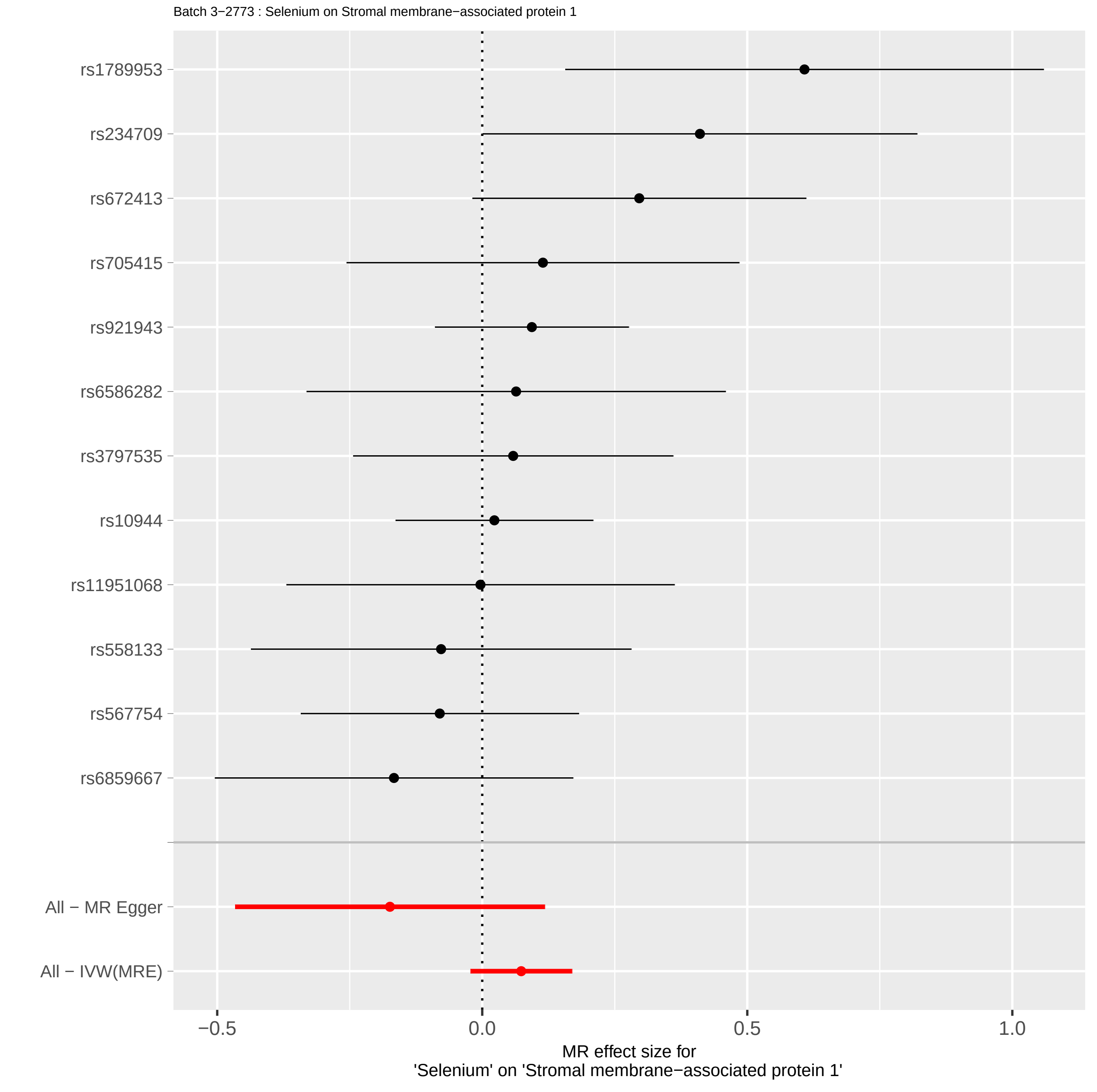

Batch 3−2773 : Selenium on Stromal membrane−associated protein 1
rs1789953
rs234709
rs672413
rs705415
rs921943
rs6586282
rs3797535
rs10944
rs11951068
rs558133
rs567754
rs6859667
All − MR Egger
All − IVW(MRE)
−0.5
0.0
0.5
1.0
MR effect size for
'Selenium' on 'Stromal membrane−associated protein 1'

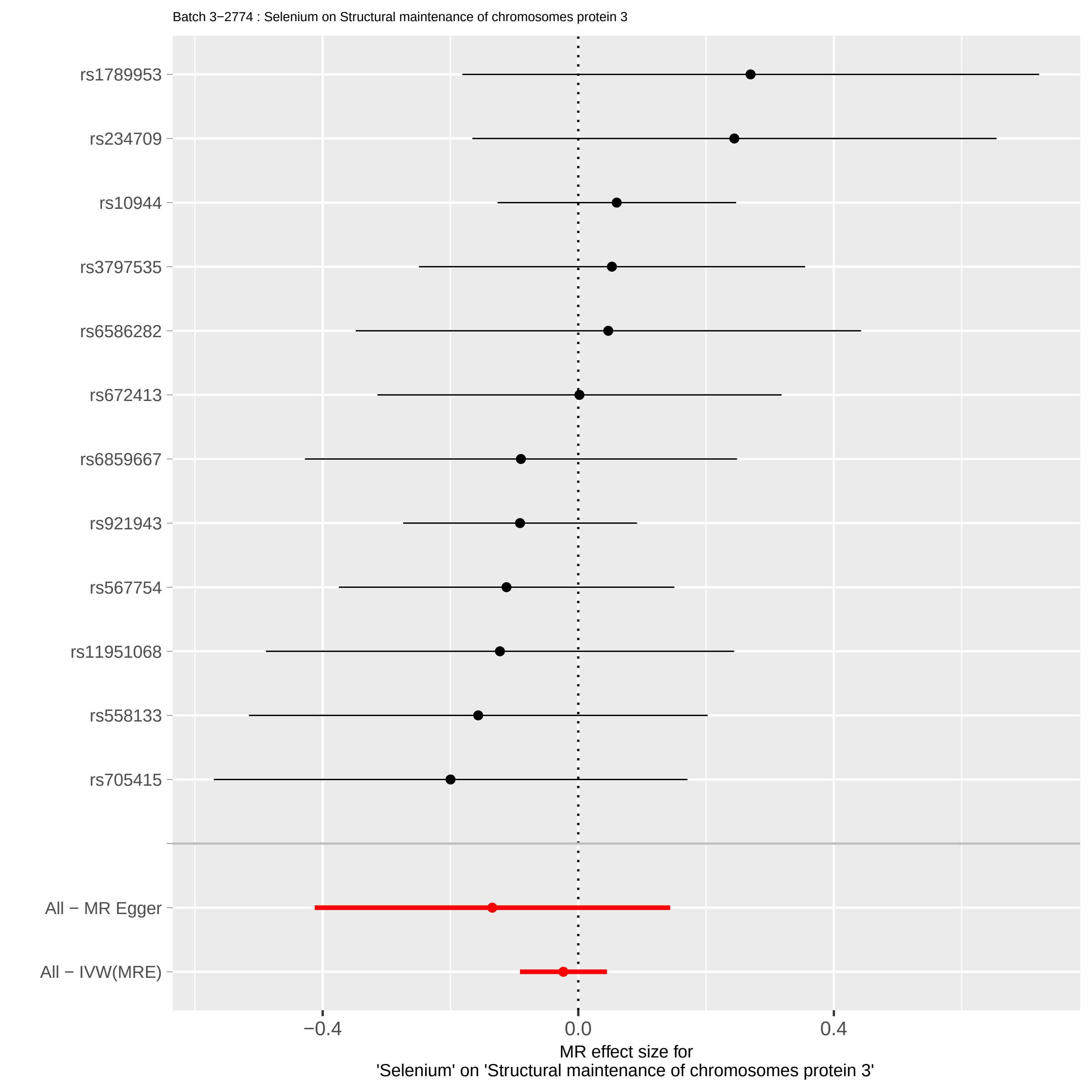

Batch 3−2774 : Selenium on Structural maintenance of chromosomes protein 3
rs1789953
rs234709
rs10944
rs3797535
rs6586282
rs672413
rs6859667
rs921943
rs567754
rs11951068
rs558133
rs705415
All − MR Egger
All − IVW(MRE)
−0.4
0.0
0.4
MR effect size for
'Selenium' on 'Structural maintenance of chromosomes protein 3'

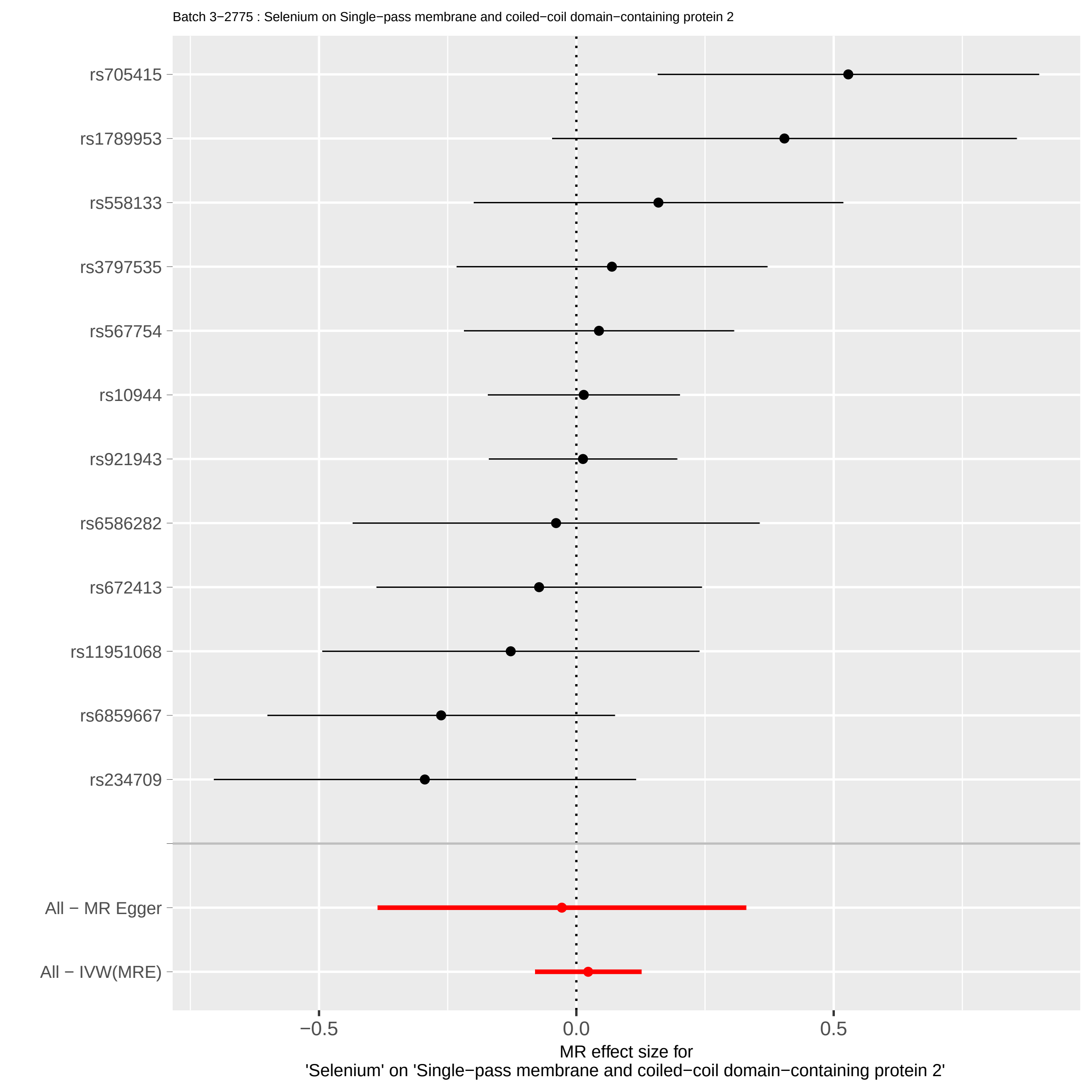

Batch 3−2775 : Selenium on Single−pass membrane and coiled−coil domain−containing protein 2
rs705415
rs1789953
rs558133
rs3797535
rs567754
rs10944
rs921943
rs6586282
rs672413
rs11951068
rs6859667
rs234709
All − MR Egger
All − IVW(MRE)
−0.5
0.0
0.5
MR effect size for
'Selenium' on 'Single−pass membrane and coiled−coil domain−containing protein 2'

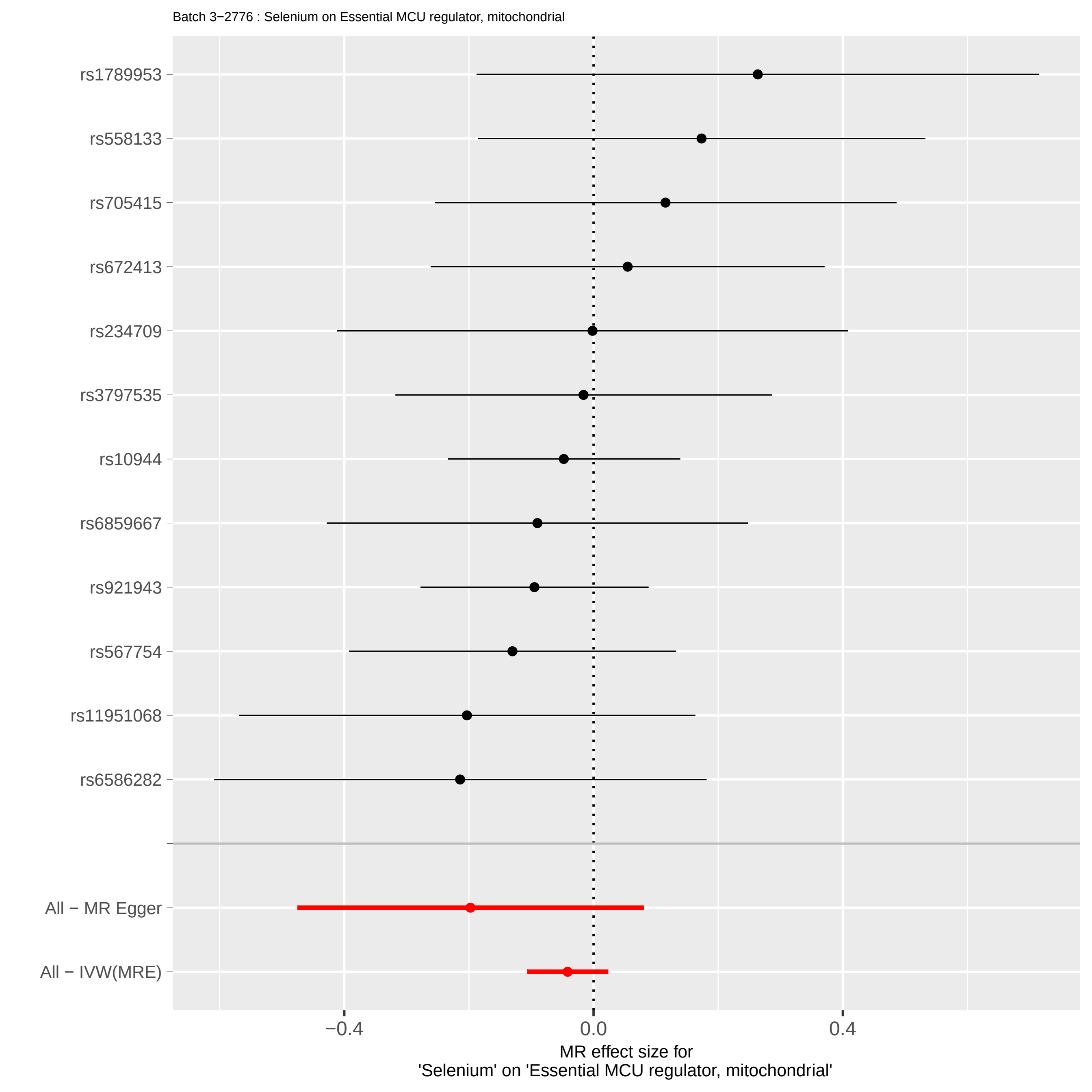
Batch 3−2776 : Selenium on Essential MCU regulator, mitochondrial
rs1789953
rs558133
rs705415
rs672413
rs234709
rs3797535
rs10944
rs6859667
rs921943
rs567754
rs11951068
rs6586282
All − MR Egger
All − IVW(MRE)
−0.4
0.0
0.4
MR effect size for
'Selenium' on 'Essential MCU regulator, mitochondrial'

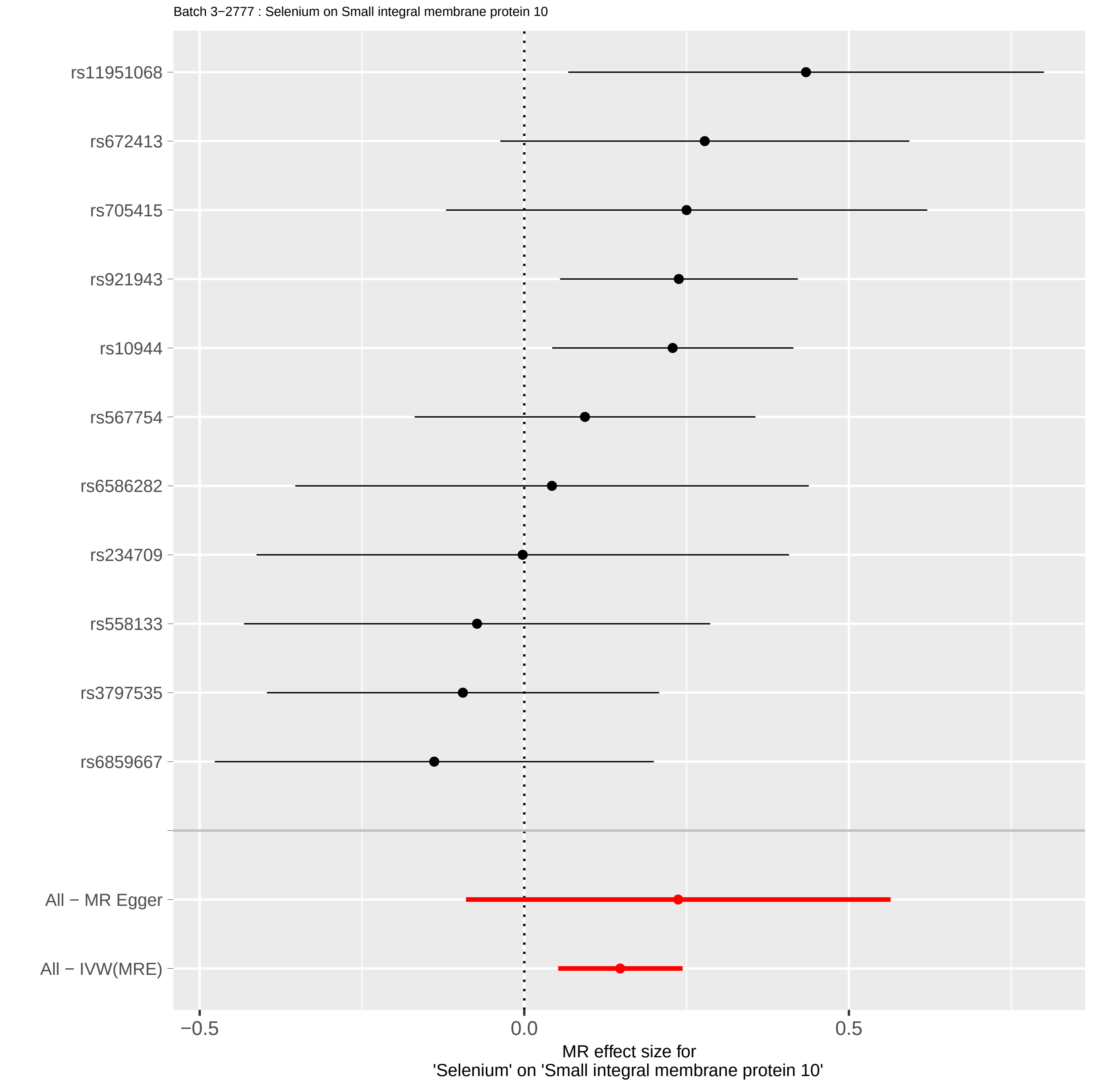

Batch 3−2777 : Selenium on Small integral membrane protein 10
rs11951068
rs672413
rs705415
rs921943
rs10944
rs567754
rs6586282
rs234709
rs558133
rs3797535
rs6859667
All − MR Egger
All − IVW(MRE)
−0.5
0.0
0.5
MR effect size for
'Selenium' on 'Small integral membrane protein 10'

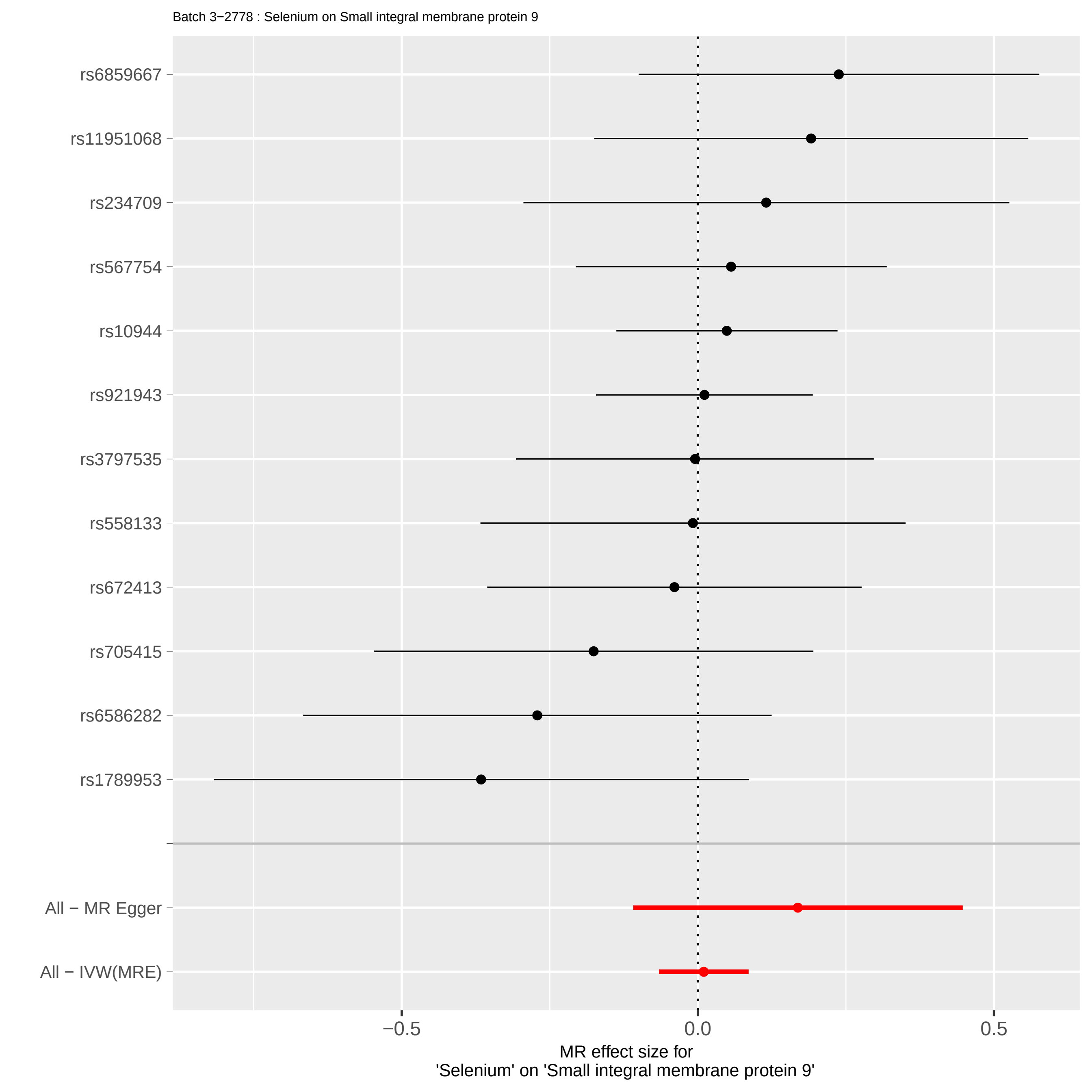

Batch 3−2778 : Selenium on Small integral membrane protein 9
rs6859667
rs11951068
rs234709
rs567754
rs10944
rs921943
rs3797535
rs558133
rs672413
rs705415
rs6586282
rs1789953
All − MR Egger
All − IVW(MRE)
−0.5
0.0
0.5
MR effect size for
'Selenium' on 'Small integral membrane protein 9'

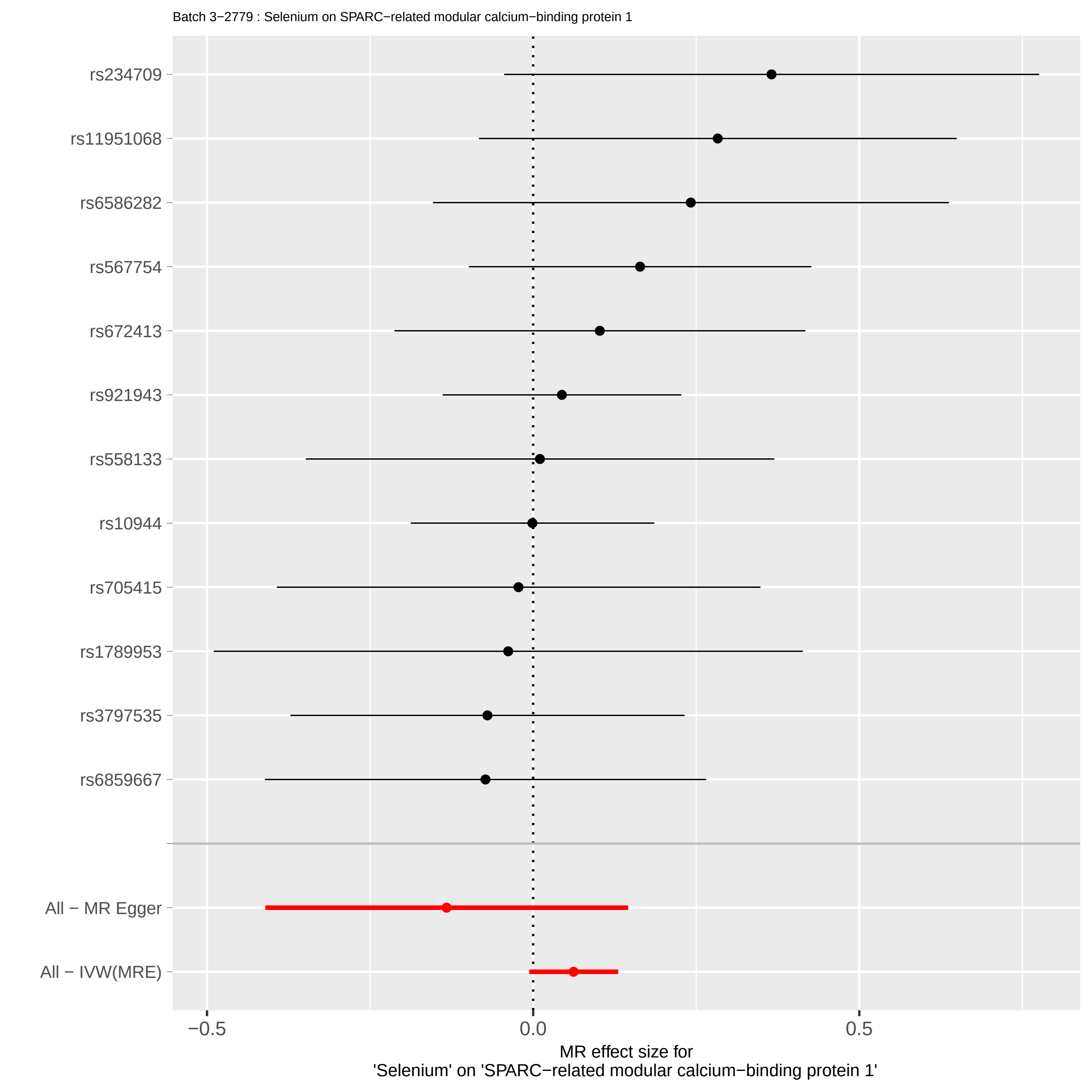

Batch 3−2779 : Selenium on SPARC−related modular calcium−binding protein 1
rs234709
rs11951068
rs6586282
rs567754
rs672413
rs921943
rs558133
rs10944
rs705415
rs1789953
rs3797535
rs6859667
All − MR Egger
All − IVW(MRE)
−0.5
0.0
0.5
MR effect size for
'Selenium' on 'SPARC−related modular calcium−binding protein 1'

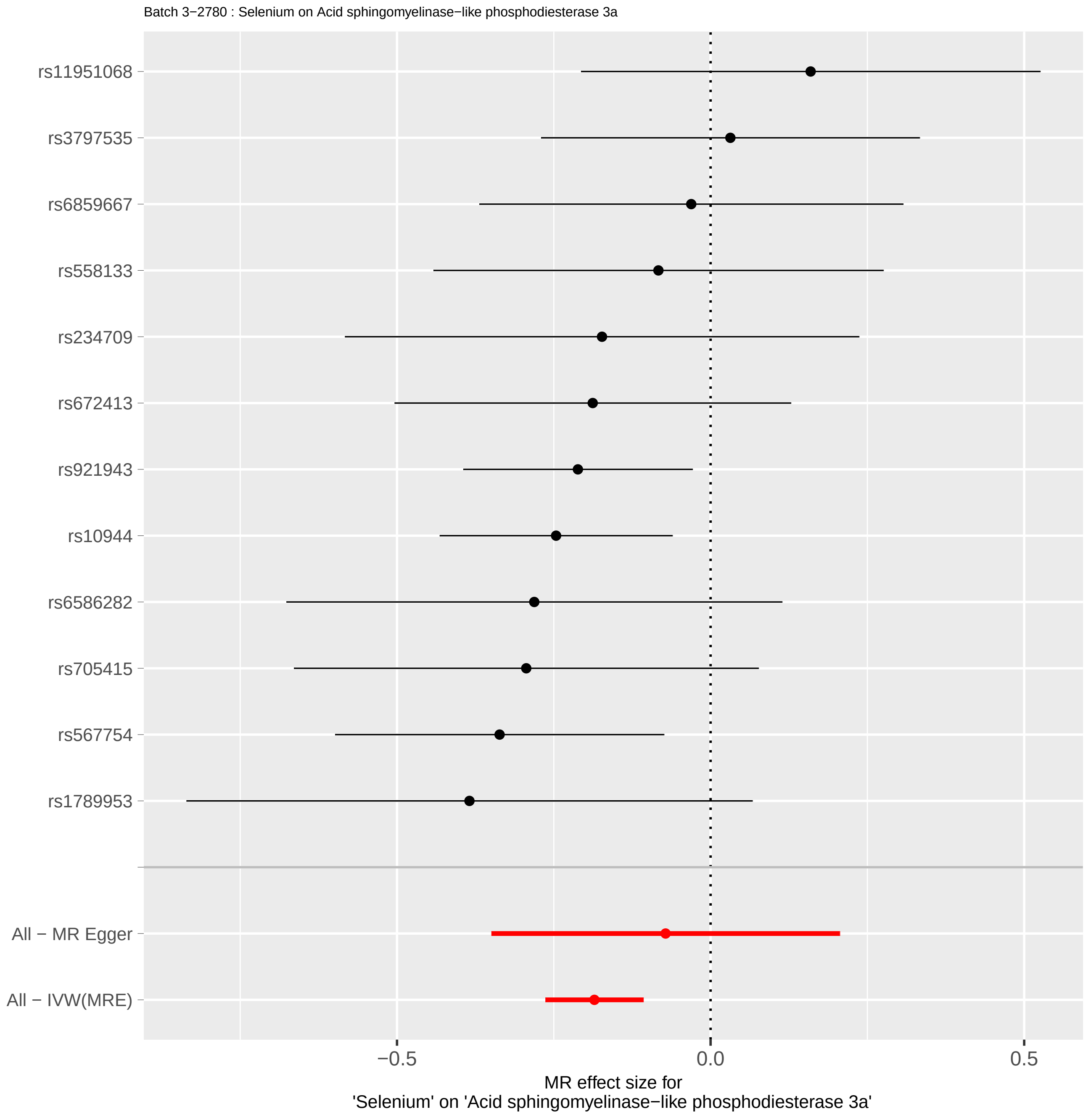

Batch 3−2780 : Selenium on Acid sphingomyelinase−like phosphodiesterase 3a
rs11951068
rs3797535
rs6859667
rs558133
rs234709
rs672413
rs921943
rs10944
rs6586282
rs705415
rs567754
rs1789953
All − MR Egger
All − IVW(MRE)
−0.5
0.0
0.5
MR effect size for
'Selenium' on 'Acid sphingomyelinase−like phosphodiesterase 3a'

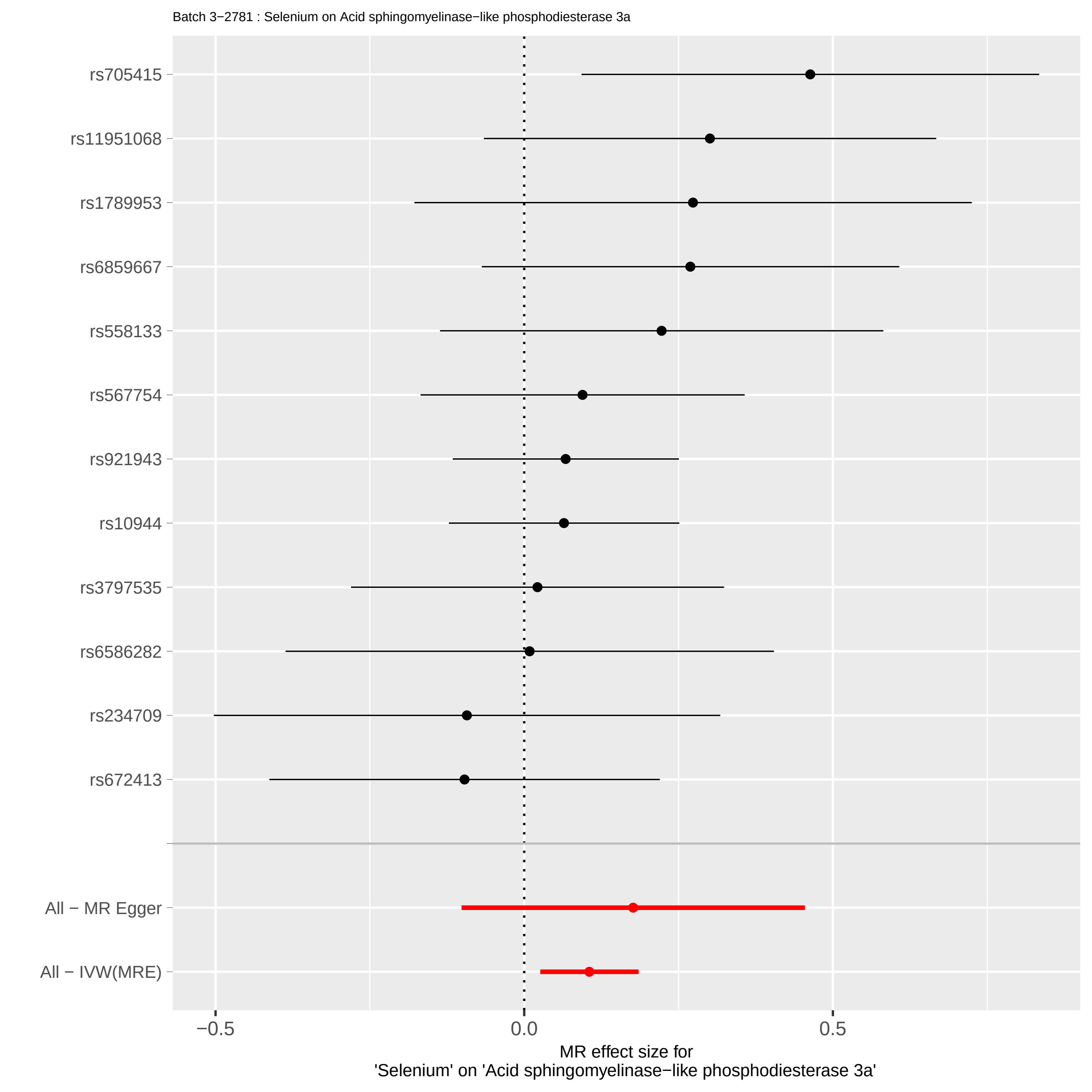

Batch 3−2781 : Selenium on Acid sphingomyelinase−like phosphodiesterase 3a
rs705415
rs11951068
rs1789953
rs6859667
rs558133
rs567754
rs921943
rs10944
rs3797535
rs6586282
rs234709
rs672413
All − MR Egger
All − IVW(MRE)
−0.5
0.0
0.5
MR effect size for
'Selenium' on 'Acid sphingomyelinase−like phosphodiesterase 3a'

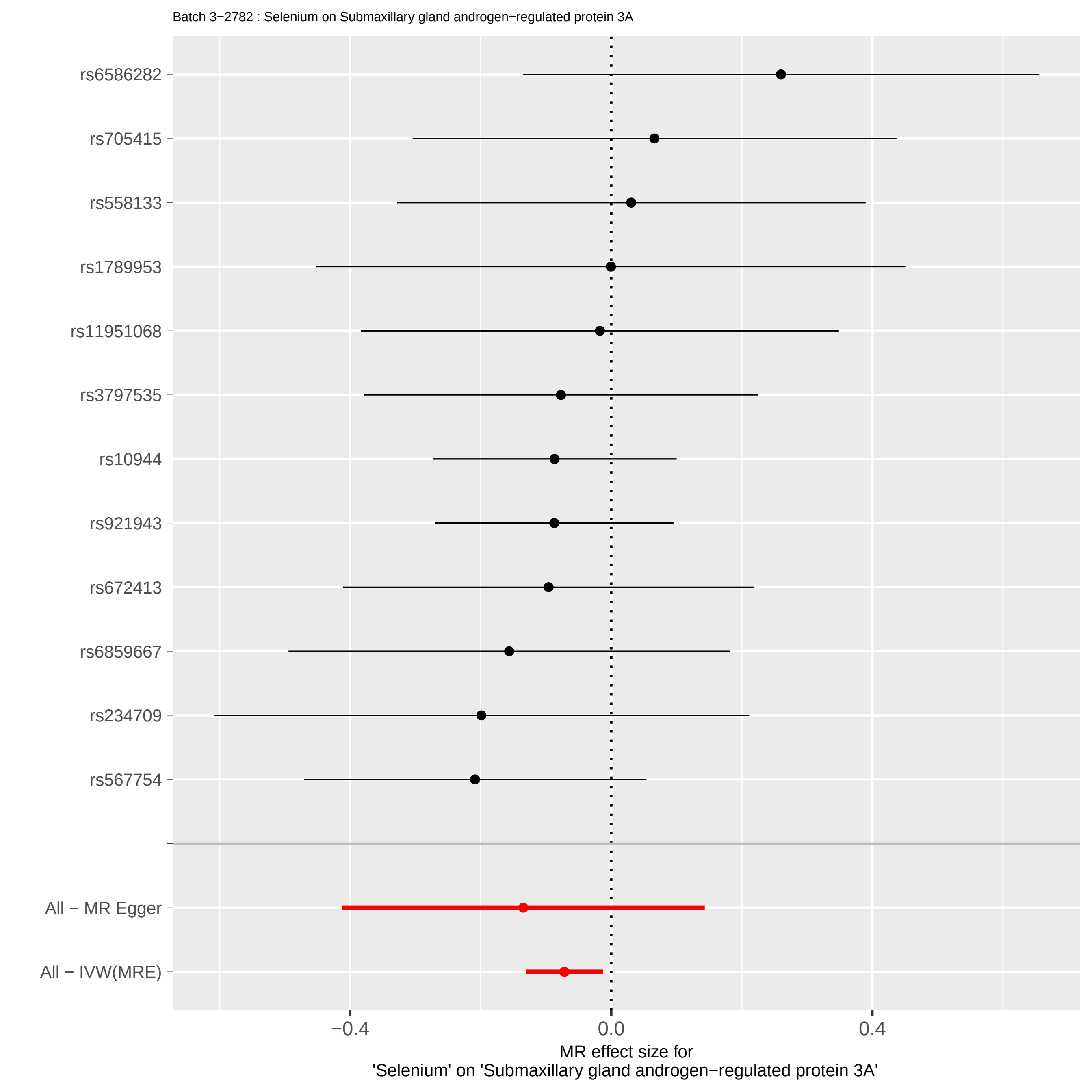

Batch 3−2782 : Selenium on Submaxillary gland androgen−regulated protein 3A
rs6586282
rs705415
rs558133
rs1789953
rs11951068
rs3797535
rs10944
rs921943
rs672413
rs6859667
rs234709
rs567754
All − MR Egger
All − IVW(MRE)
−0.4
0.0
0.4
MR effect size for
'Selenium' on 'Submaxillary gland androgen−regulated protein 3A'

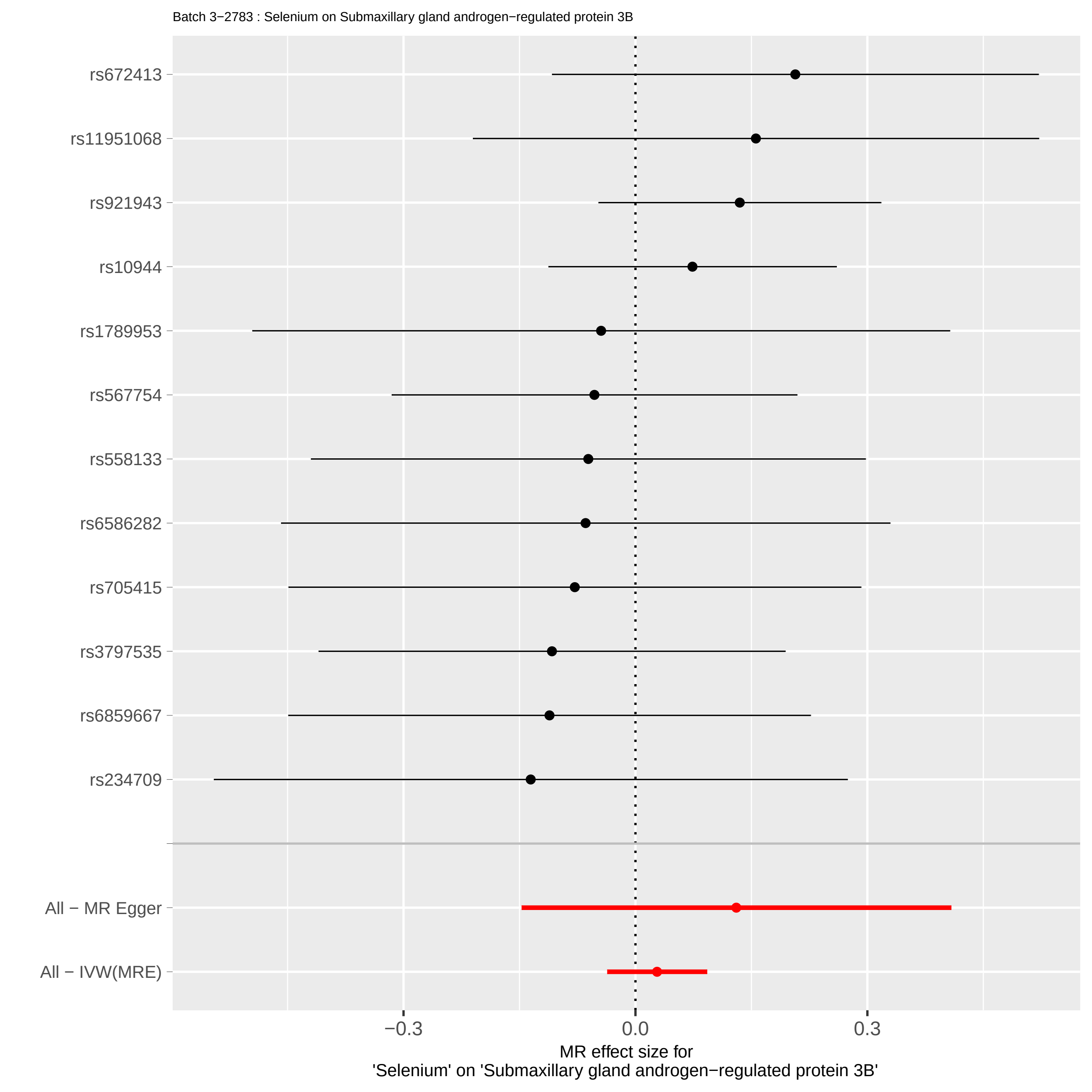

Batch 3−2783 : Selenium on Submaxillary gland androgen−regulated protein 3B
rs672413
rs11951068
rs921943
rs10944
rs1789953
rs567754
rs558133
rs6586282
rs705415
rs3797535
rs6859667
rs234709
All − MR Egger
All − IVW(MRE)
−0.3
0.0
0.3
MR effect size for
'Selenium' on 'Submaxillary gland androgen−regulated protein 3B'

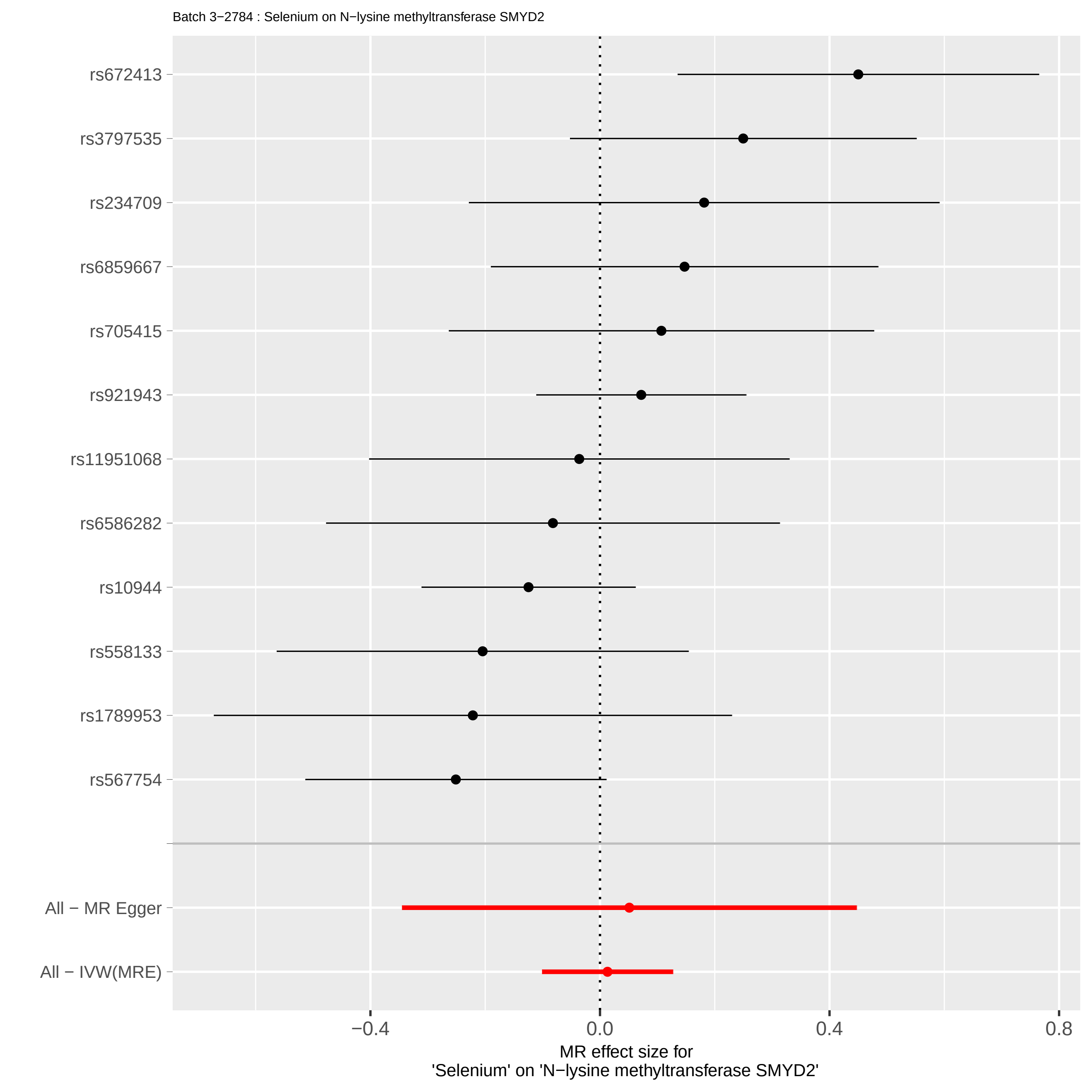

Batch 3−2784 : Selenium on N−lysine methyltransferase SMYD2
rs672413
rs3797535
rs234709
rs6859667
rs705415
rs921943
rs11951068
rs6586282
rs10944
rs558133
rs1789953
rs567754
All − MR Egger
All − IVW(MRE)
−0.4
0.0
0.4
0.8
MR effect size for
'Selenium' on 'N−lysine methyltransferase SMYD2'

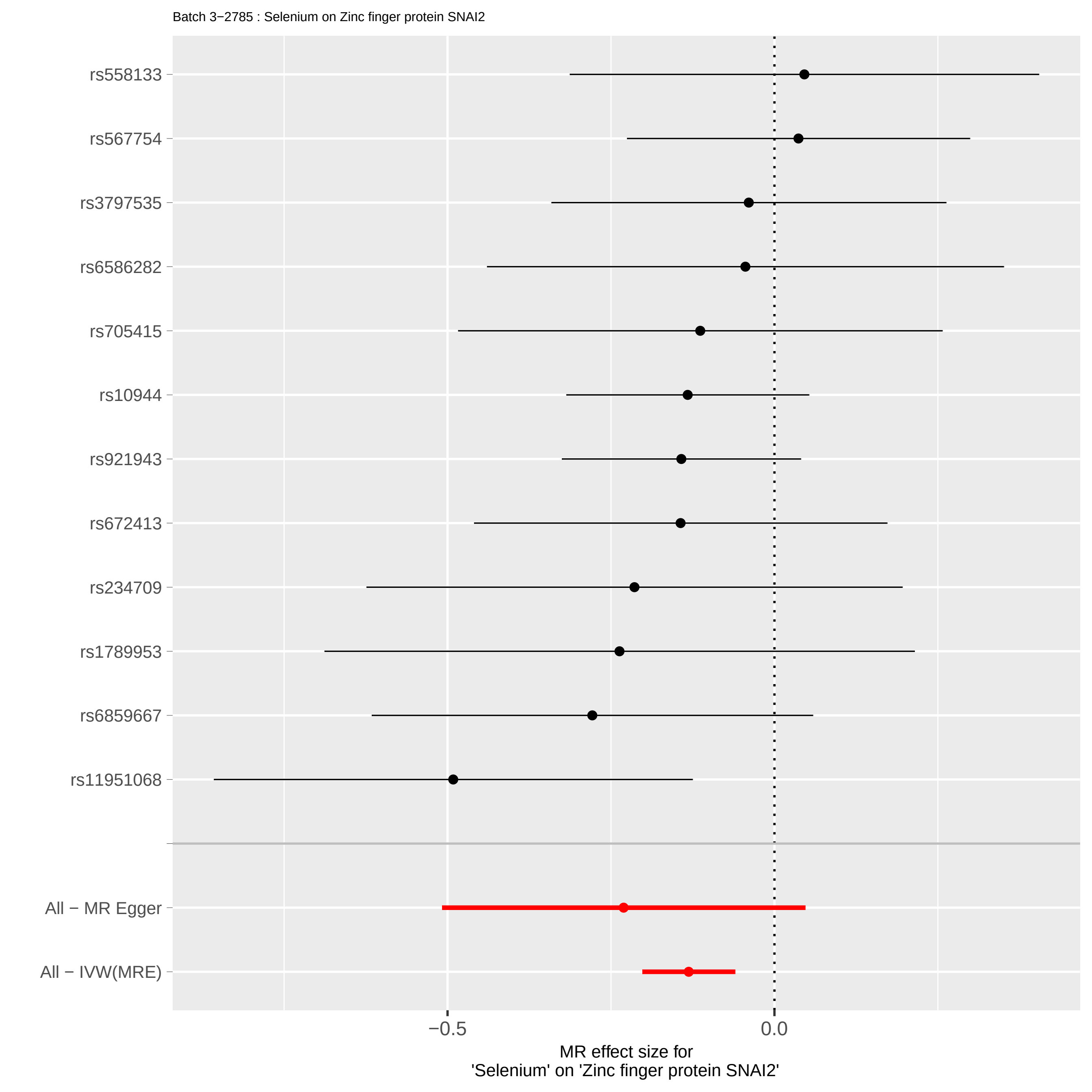

Batch 3−2785 : Selenium on Zinc finger protein SNAI2
rs558133
rs567754
rs3797535
rs6586282
rs705415
rs10944
rs921943
rs672413
rs234709
rs1789953
rs6859667
rs11951068
All − MR Egger
All − IVW(MRE)
−0.5
0.0
MR effect size for
'Selenium' on 'Zinc finger protein SNAI2'

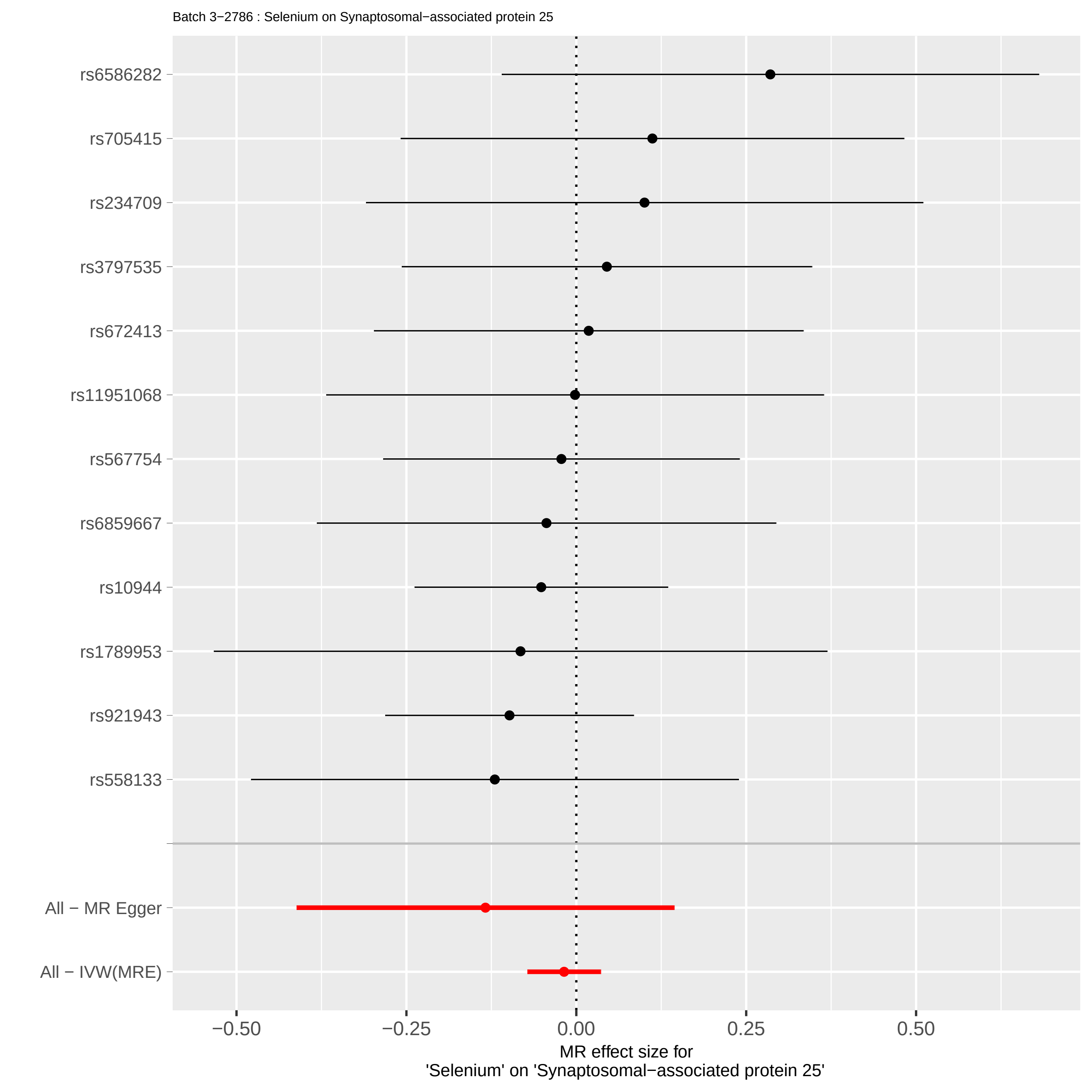

Batch 3−2786 : Selenium on Synaptosomal−associated protein 25
rs6586282
rs705415
rs234709
rs3797535
rs672413
rs11951068
rs567754
rs6859667
rs10944
rs1789953
rs921943
rs558133
All − MR Egger
All − IVW(MRE)
−0.50
−0.25
0.00
0.25
0.50
MR effect size for
'Selenium' on 'Synaptosomal−associated protein 25'

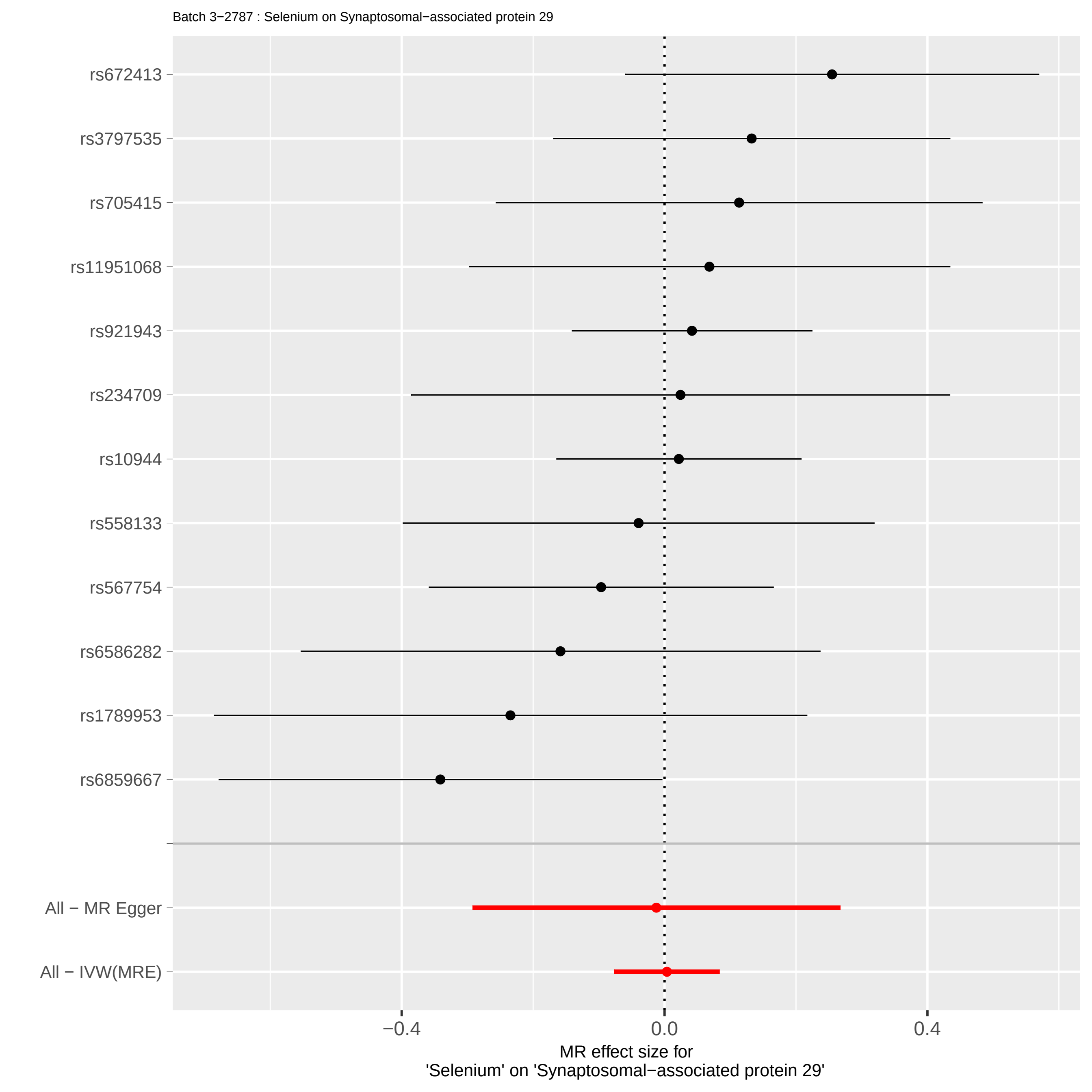

Batch 3−2787 : Selenium on Synaptosomal−associated protein 29
rs672413
rs3797535
rs705415
rs11951068
rs921943
rs234709
rs10944
rs558133
rs567754
rs6586282
rs1789953
rs6859667
All − MR Egger
All − IVW(MRE)
−0.4
0.0
0.4
MR effect size for
'Selenium' on 'Synaptosomal−associated protein 29'

Batch 3−2788 : Selenium on SNARE−associated protein Snapin

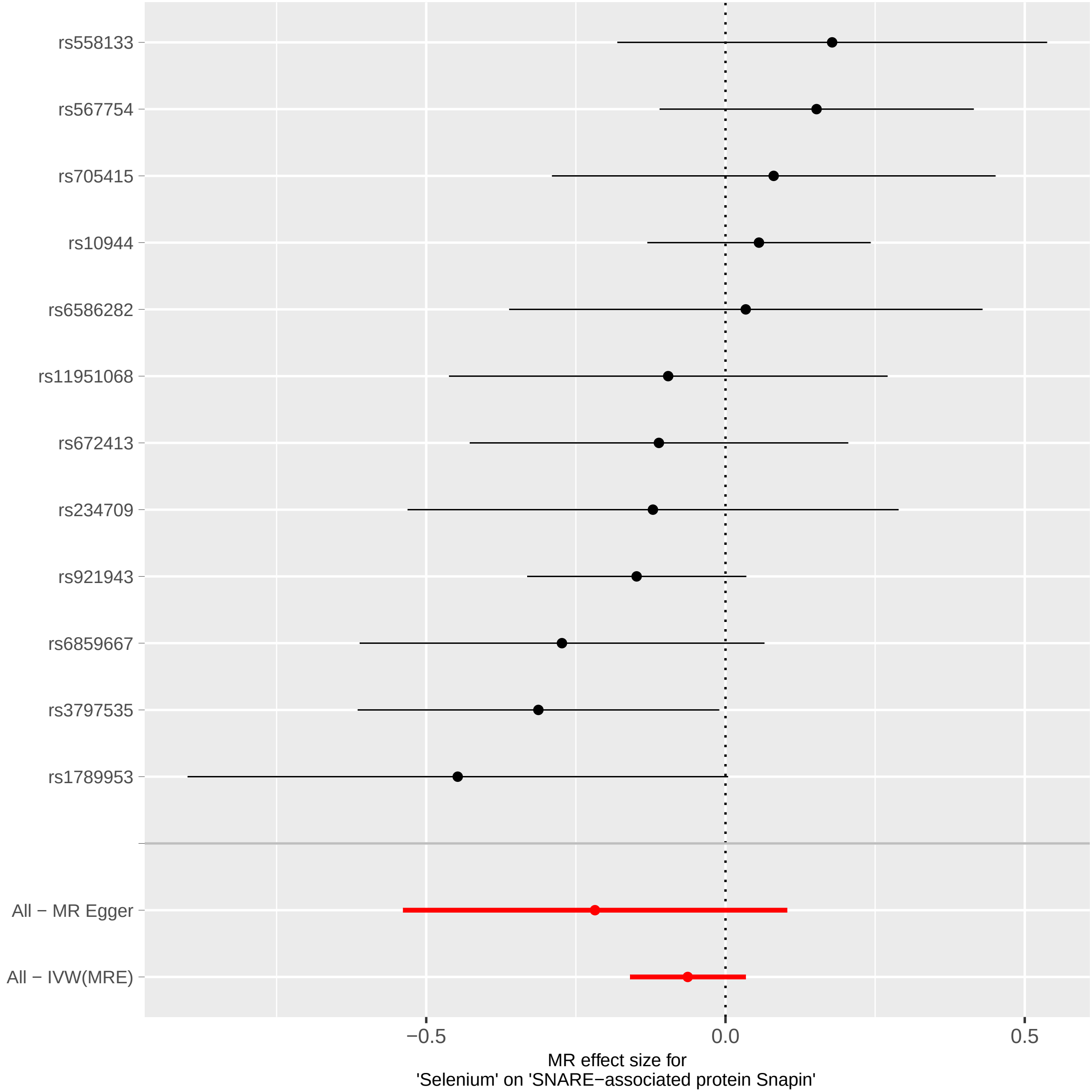

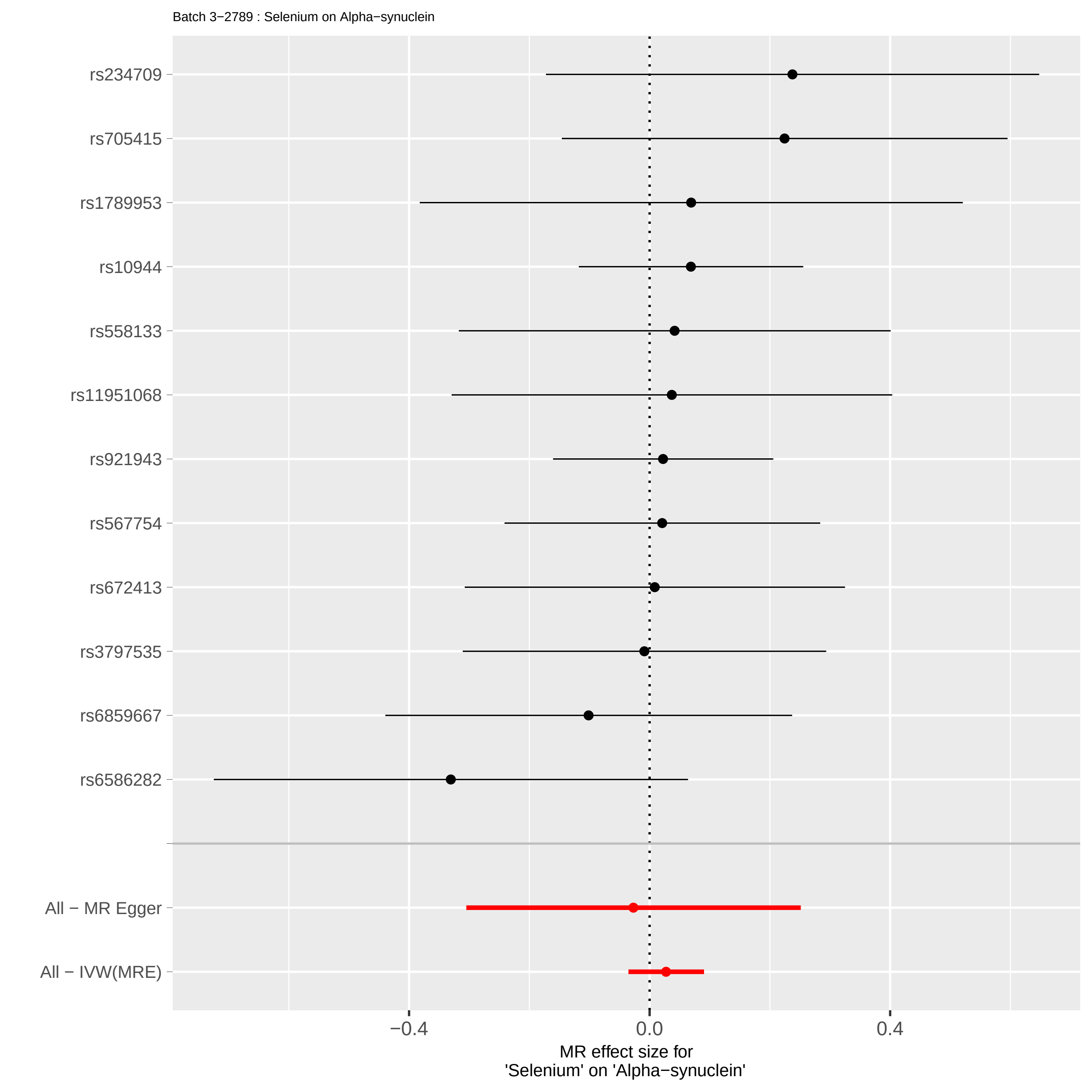
Batch 3−2789 : Selenium on Alpha−synuclein
rs234709
rs705415
rs1789953
rs10944
rs558133
rs11951068
rs921943
rs567754
rs672413
rs3797535
rs6859667
rs6586282
All − MR Egger
All − IVW(MRE)
−0.4
0.0
0.4
MR effect size for
'Selenium' on 'Alpha−synuclein'

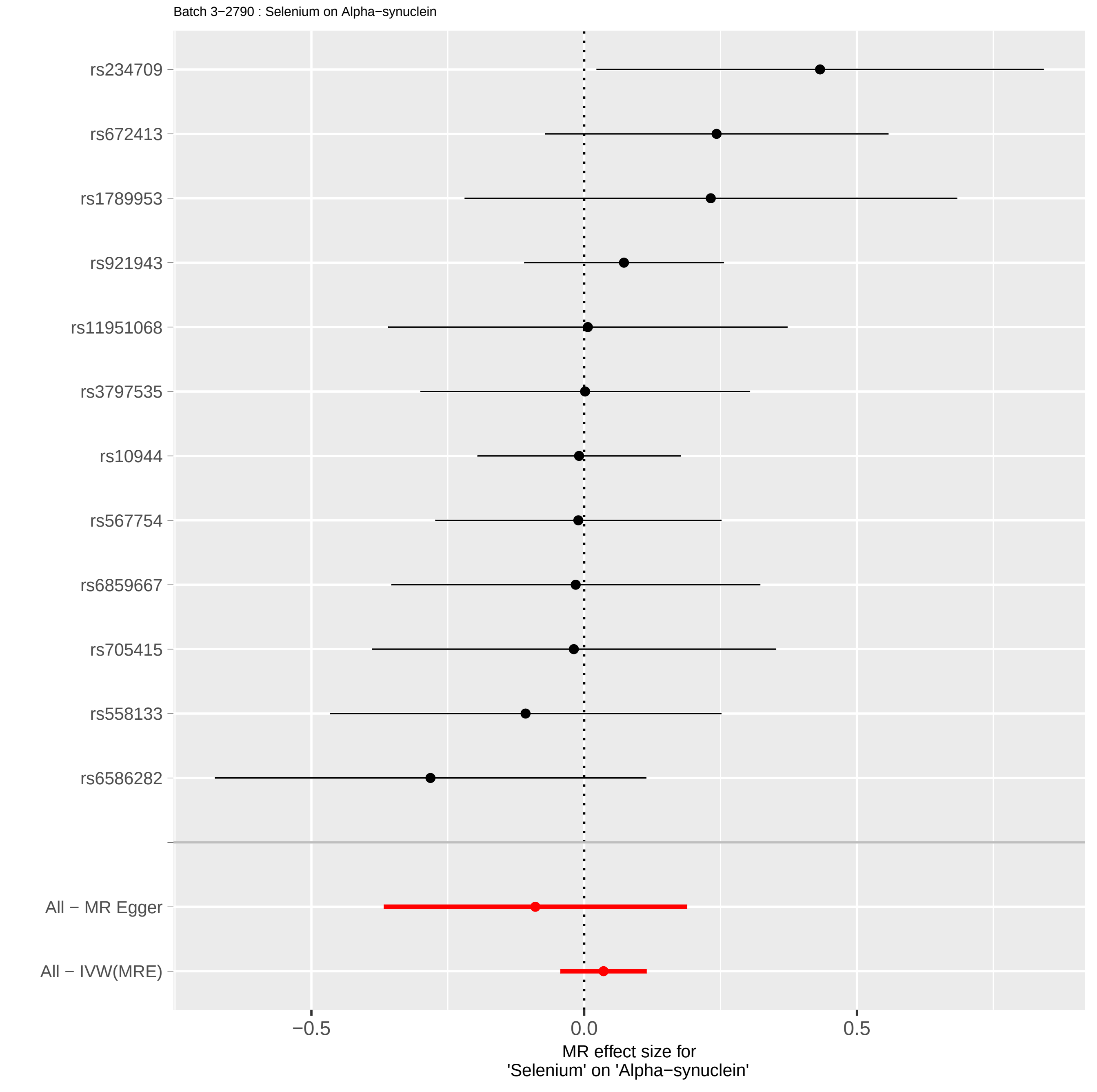

Batch 3−2790 : Selenium on Alpha−synuclein
rs234709
rs672413
rs1789953
rs921943
rs11951068
rs3797535
rs10944
rs567754
rs6859667
rs705415
rs558133
rs6586282
All − MR Egger
All − IVW(MRE)
−0.5
0.0
0.5
MR effect size for
'Selenium' on 'Alpha−synuclein'

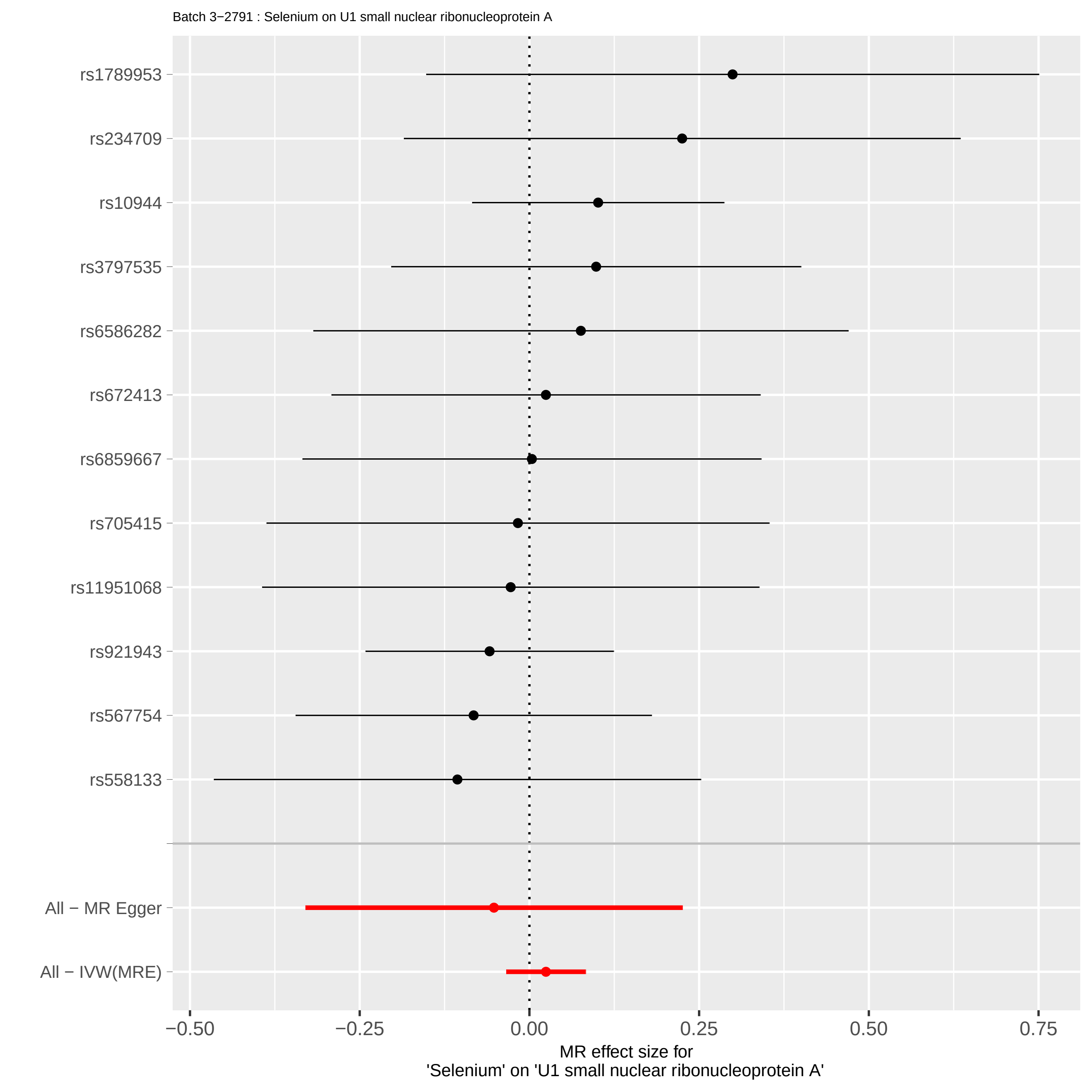

Batch 3−2791 : Selenium on U1 small nuclear ribonucleoprotein A
rs1789953
rs234709
rs10944
rs3797535
rs6586282
rs672413
rs6859667
rs705415
rs11951068
rs921943
rs567754
rs558133
All − MR Egger
All − IVW(MRE)
−0.50
−0.25
0.00
0.25
0.50
0.75
MR effect size for
'Selenium' on 'U1 small nuclear ribonucleoprotein A'

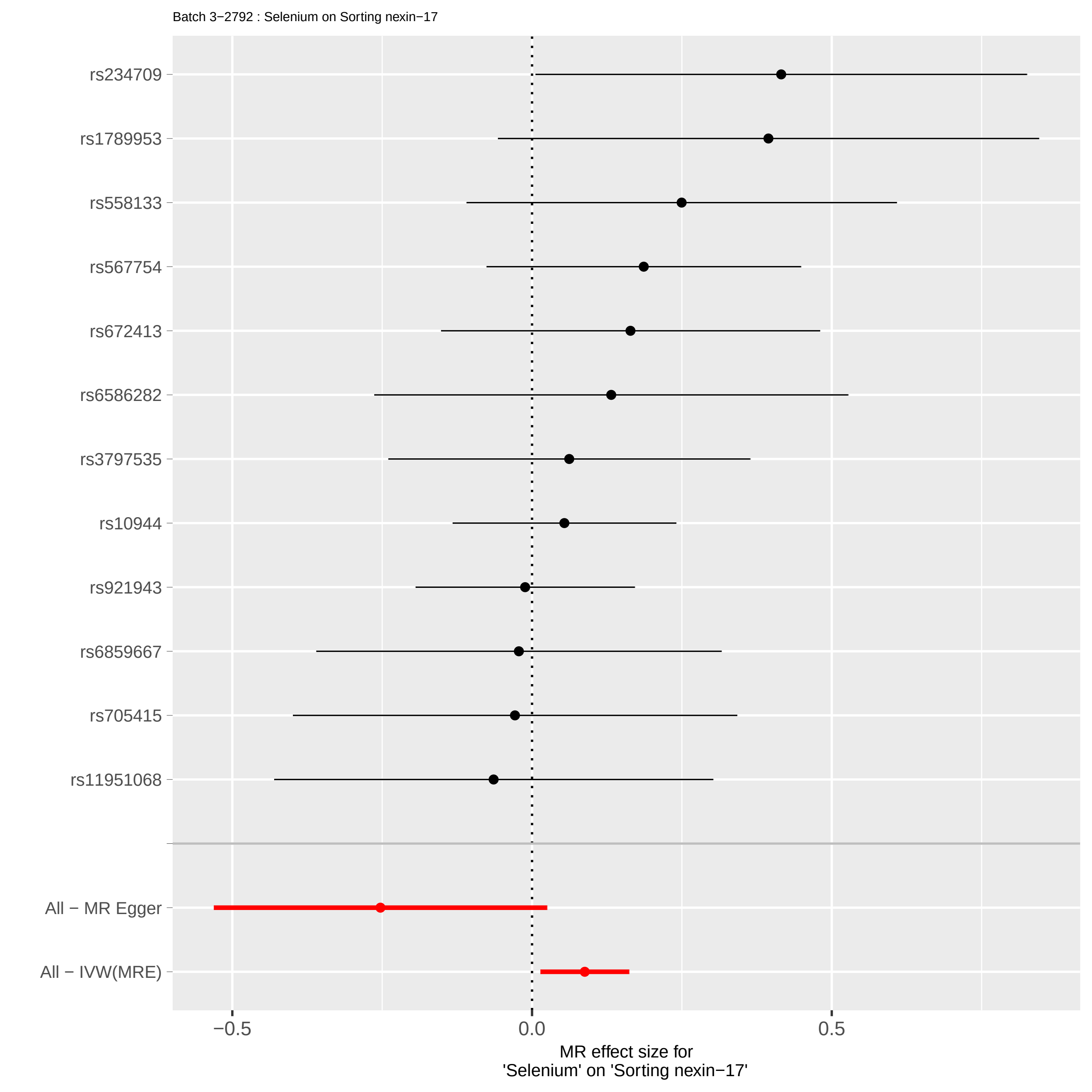

Batch 3−2792 : Selenium on Sorting nexin−17
rs234709
rs1789953
rs558133
rs567754
rs672413
rs6586282
rs3797535
rs10944
rs921943
rs6859667
rs705415
rs11951068
All − MR Egger
All − IVW(MRE)
−0.5
0.0
0.5
MR effect size for
'Selenium' on 'Sorting nexin−17'

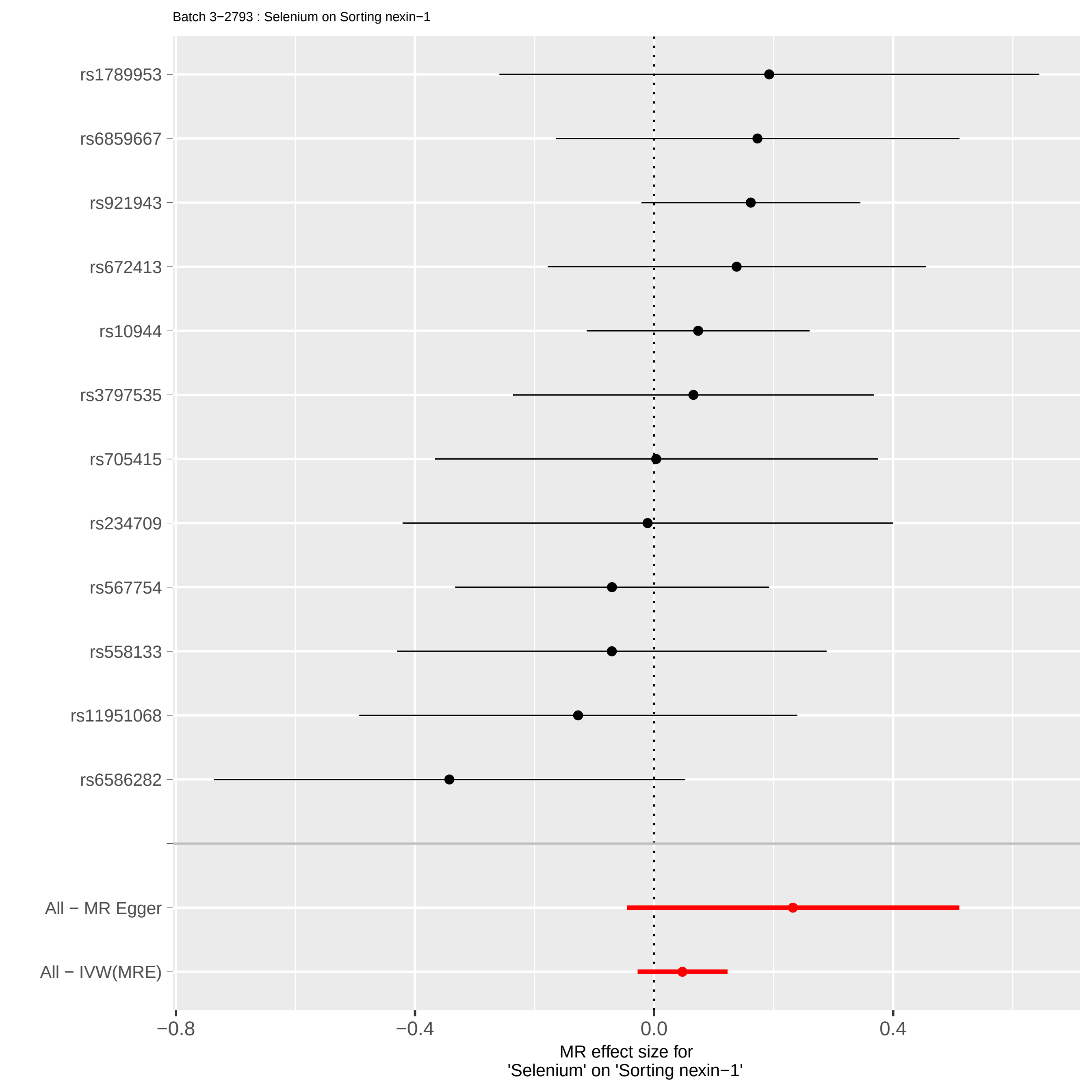

Batch 3−2793 : Selenium on Sorting nexin−1
rs1789953
rs6859667
rs921943
rs672413
rs10944
rs3797535
rs705415
rs234709
rs567754
rs558133
rs11951068
rs6586282
All − MR Egger
All − IVW(MRE)
−0.8
−0.4
0.0
0.4
MR effect size for
'Selenium' on 'Sorting nexin−1'

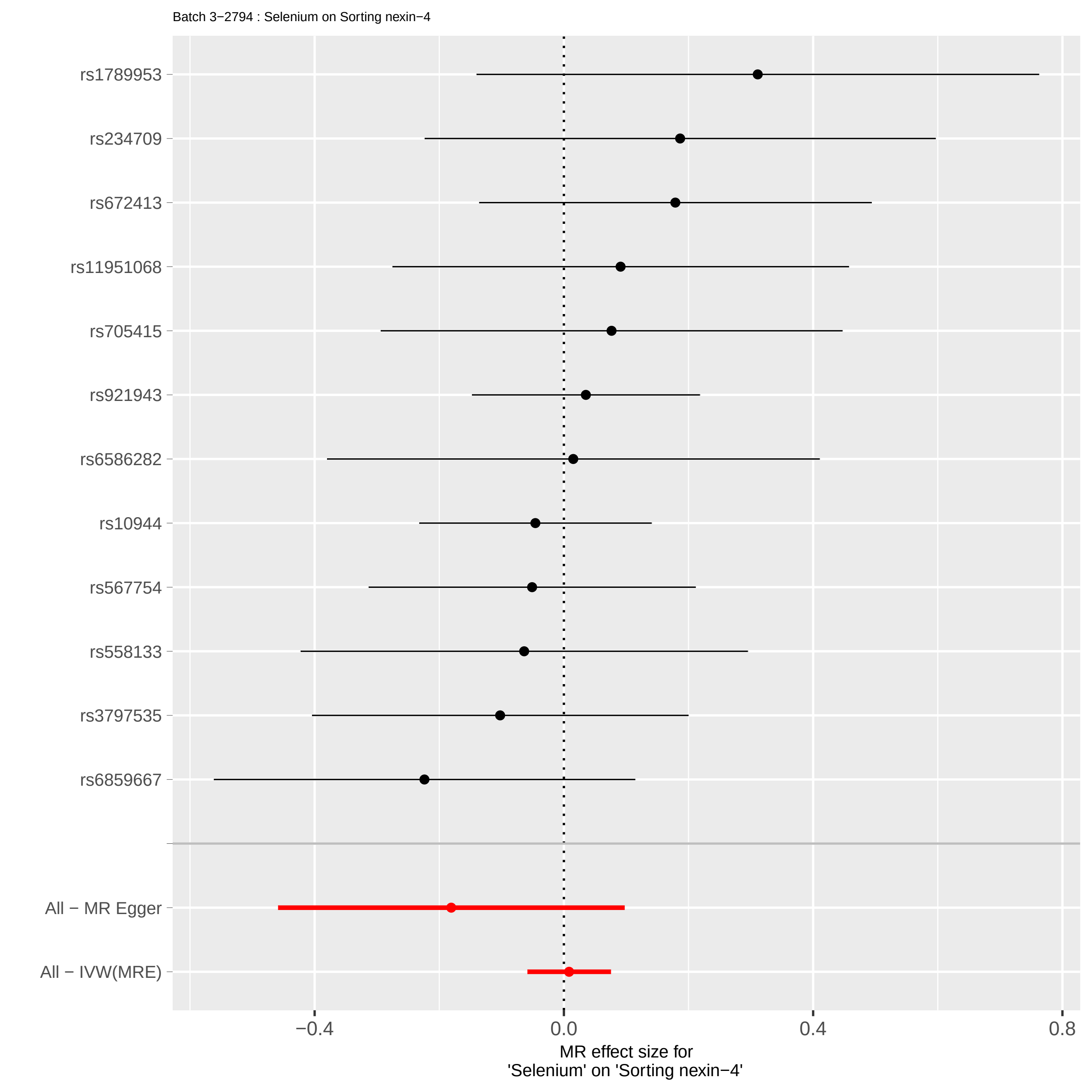
Batch 3−2794 : Selenium on Sorting nexin−4
rs1789953
rs234709
rs672413
rs11951068
rs705415
rs921943
rs6586282
rs10944
rs567754
rs558133
rs3797535
rs6859667
All − MR Egger
All − IVW(MRE)
−0.4
0.0
0.4
0.8
MR effect size for
'Selenium' on 'Sorting nexin−4'

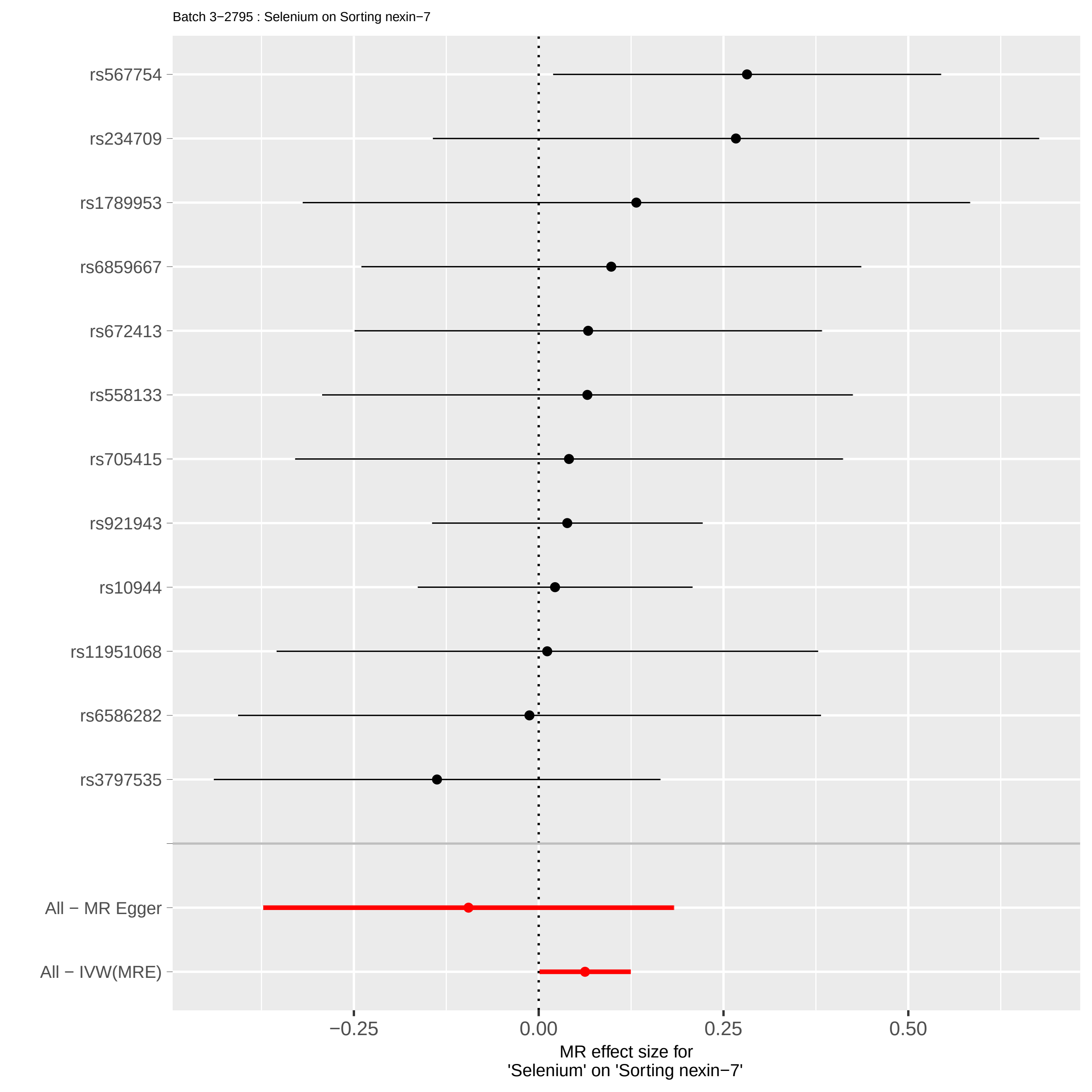

Batch 3−2795 : Selenium on Sorting nexin−7
rs567754
rs234709
rs1789953
rs6859667
rs672413
rs558133
rs705415
rs921943
rs10944
rs11951068
rs6586282
rs3797535
All − MR Egger
All − IVW(MRE)
−0.25
0.00
0.25
0.50
MR effect size for
'Selenium' on 'Sorting nexin−7'

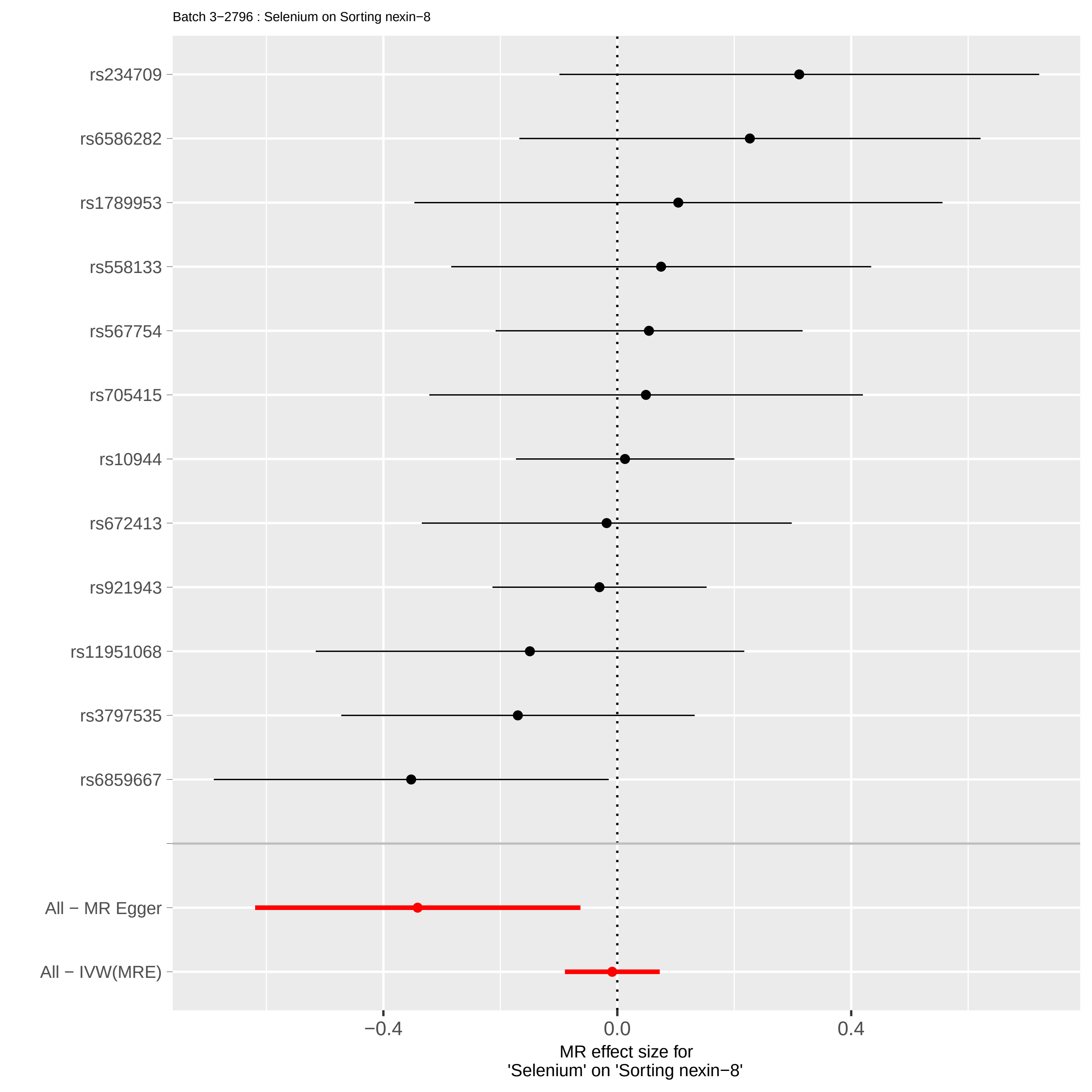
Batch 3−2796 : Selenium on Sorting nexin−8
rs234709
rs6586282
rs1789953
rs558133
rs567754
rs705415
rs10944
rs672413
rs921943
rs11951068
rs3797535
rs6859667
All − MR Egger
All − IVW(MRE)
−0.4
0.0
0.4
MR effect size for
'Selenium' on 'Sorting nexin−8'

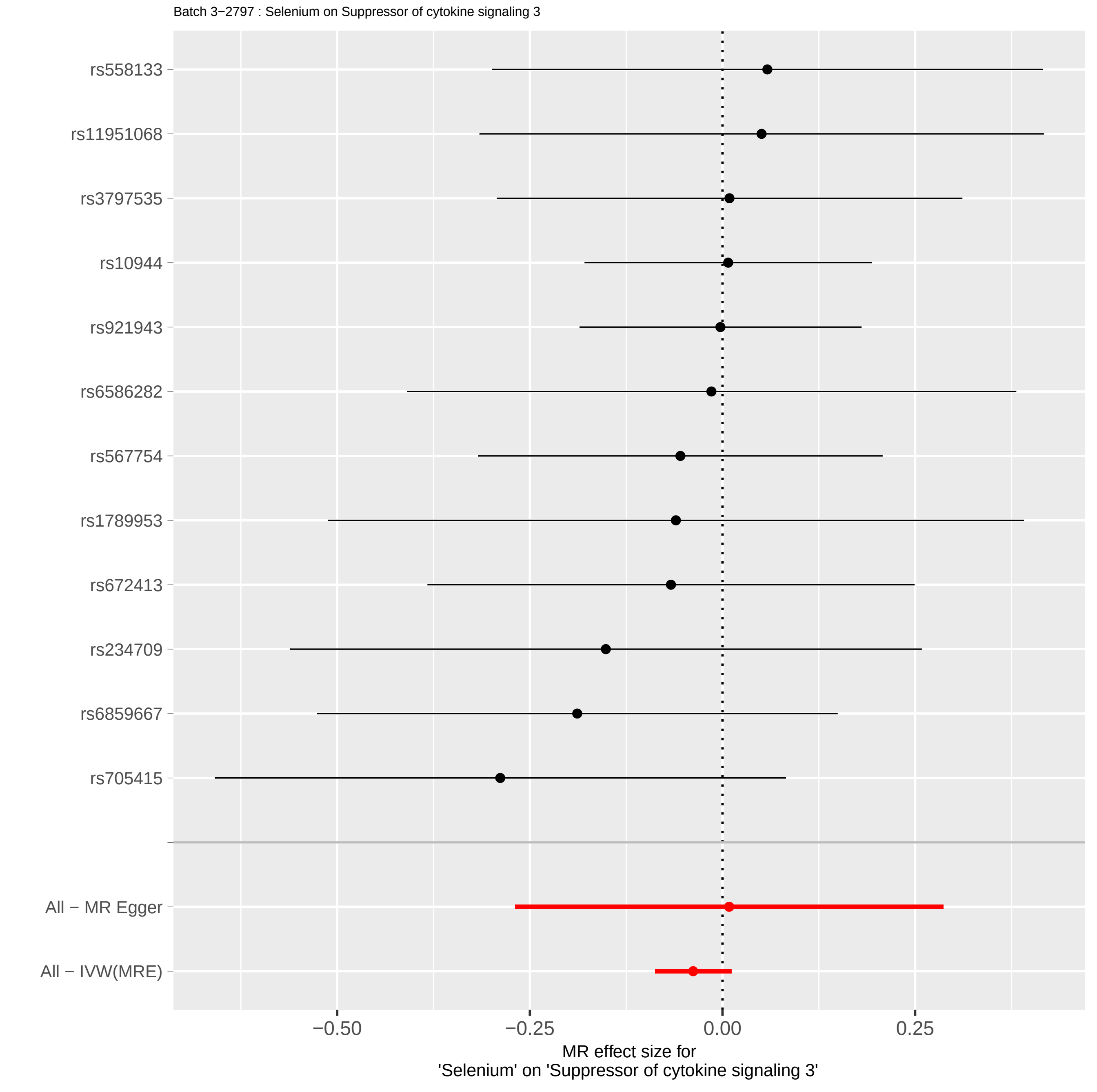

Batch 3−2797 : Selenium on Suppressor of cytokine signaling 3
rs558133
rs11951068
rs3797535
rs10944
rs921943
rs6586282
rs567754
rs1789953
rs672413
rs234709
rs6859667
rs705415
All − MR Egger
All − IVW(MRE)
−0.50
−0.25
0.00
0.25
MR effect size for
'Selenium' on 'Suppressor of cytokine signaling 3'

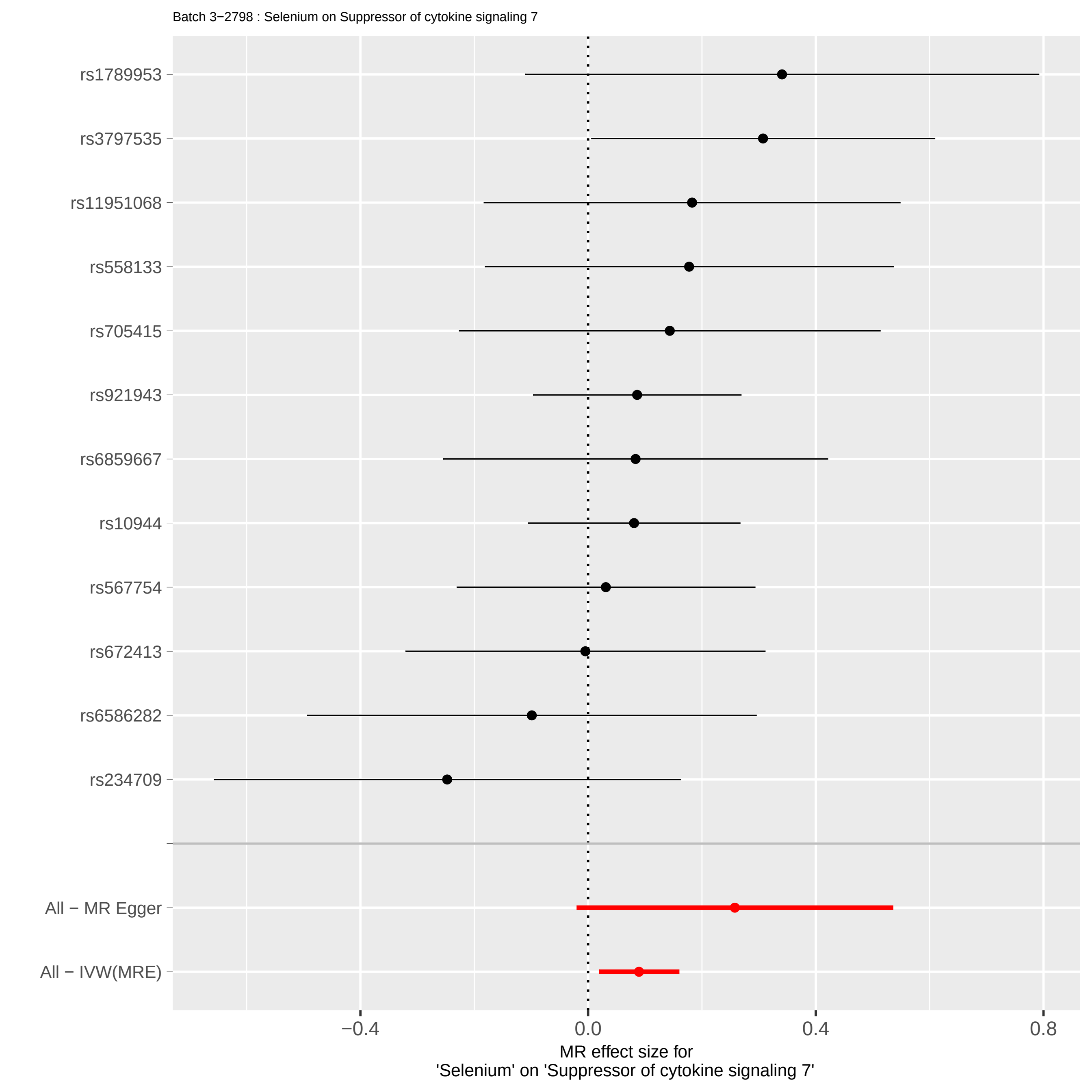

Batch 3−2798 : Selenium on Suppressor of cytokine signaling 7
rs1789953
rs3797535
rs11951068
rs558133
rs705415
rs921943
rs6859667
rs10944
rs567754
rs672413
rs6586282
rs234709
All − MR Egger
All − IVW(MRE)
−0.4
0.0
0.4
0.8
MR effect size for
'Selenium' on 'Suppressor of cytokine signaling 7'

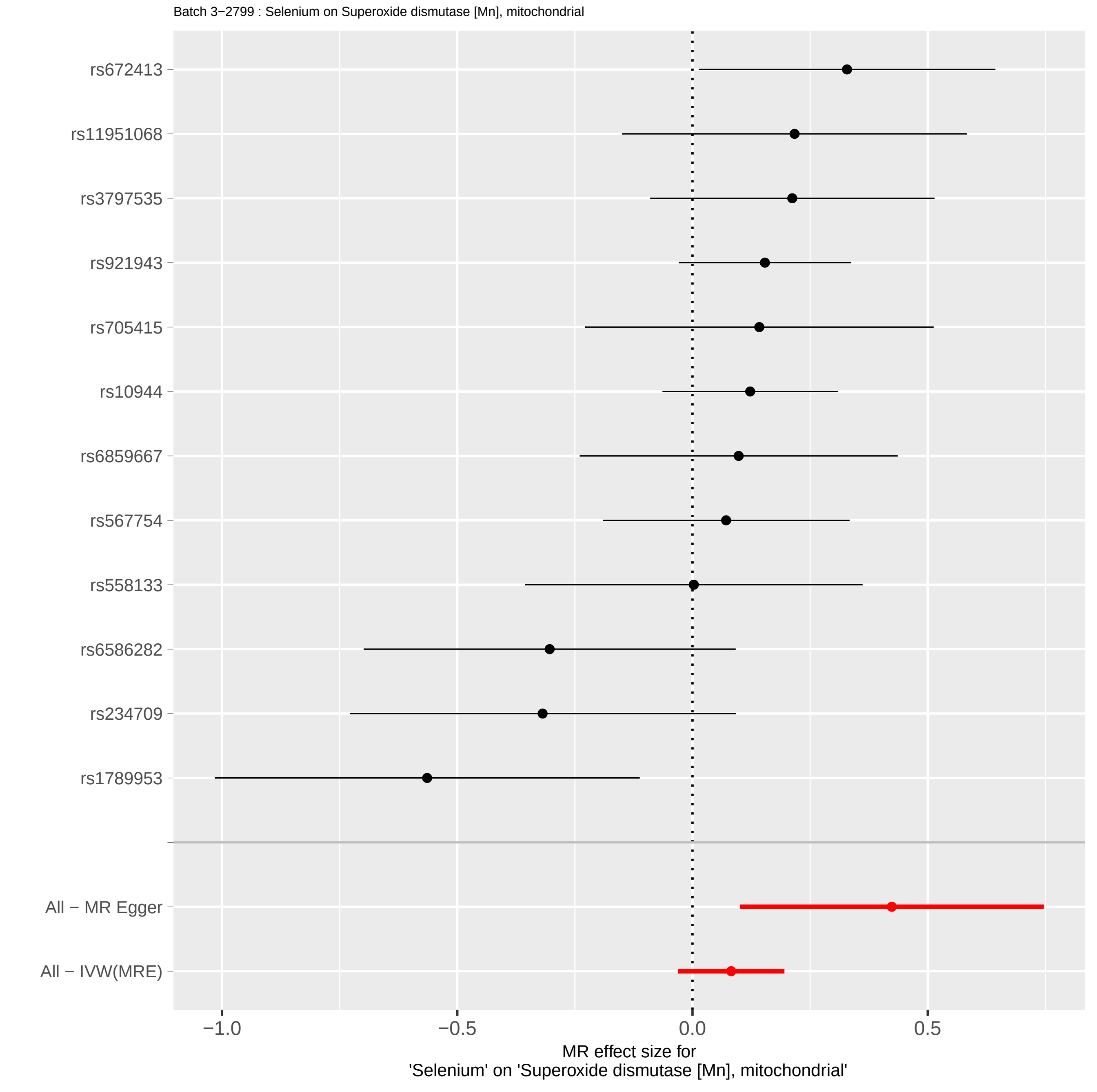

Batch 3−2799 : Selenium on Superoxide dismutase [Mn], mitochondrial
rs672413
rs11951068
rs3797535
rs921943
rs705415
rs10944
rs6859667
rs567754
rs558133
rs6586282
rs234709
rs1789953
All − MR Egger
All − IVW(MRE)
−1.0
−0.5
0.0
0.5
MR effect size for
'Selenium' on 'Superoxide dismutase [Mn], mitochondrial'

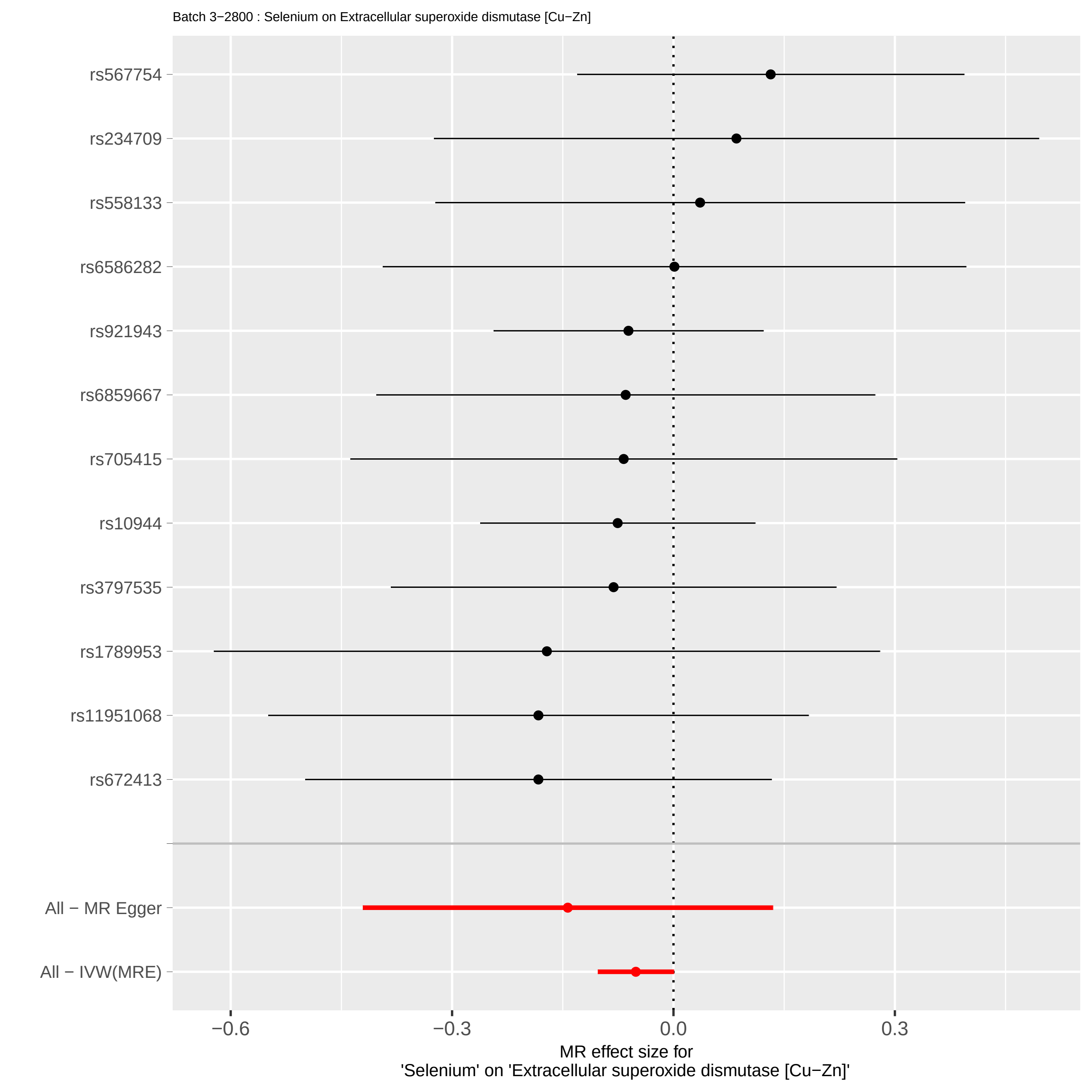

Batch 3−2800 : Selenium on Extracellular superoxide dismutase [Cu−Zn]
rs567754
rs234709
rs558133
rs6586282
rs921943
rs6859667
rs705415
rs10944
rs3797535
rs1789953
rs11951068
rs672413
All − MR Egger
All − IVW(MRE)
−0.6
−0.3
0.0
0.3
MR effect size for
'Selenium' on 'Extracellular superoxide dismutase [Cu−Zn]'

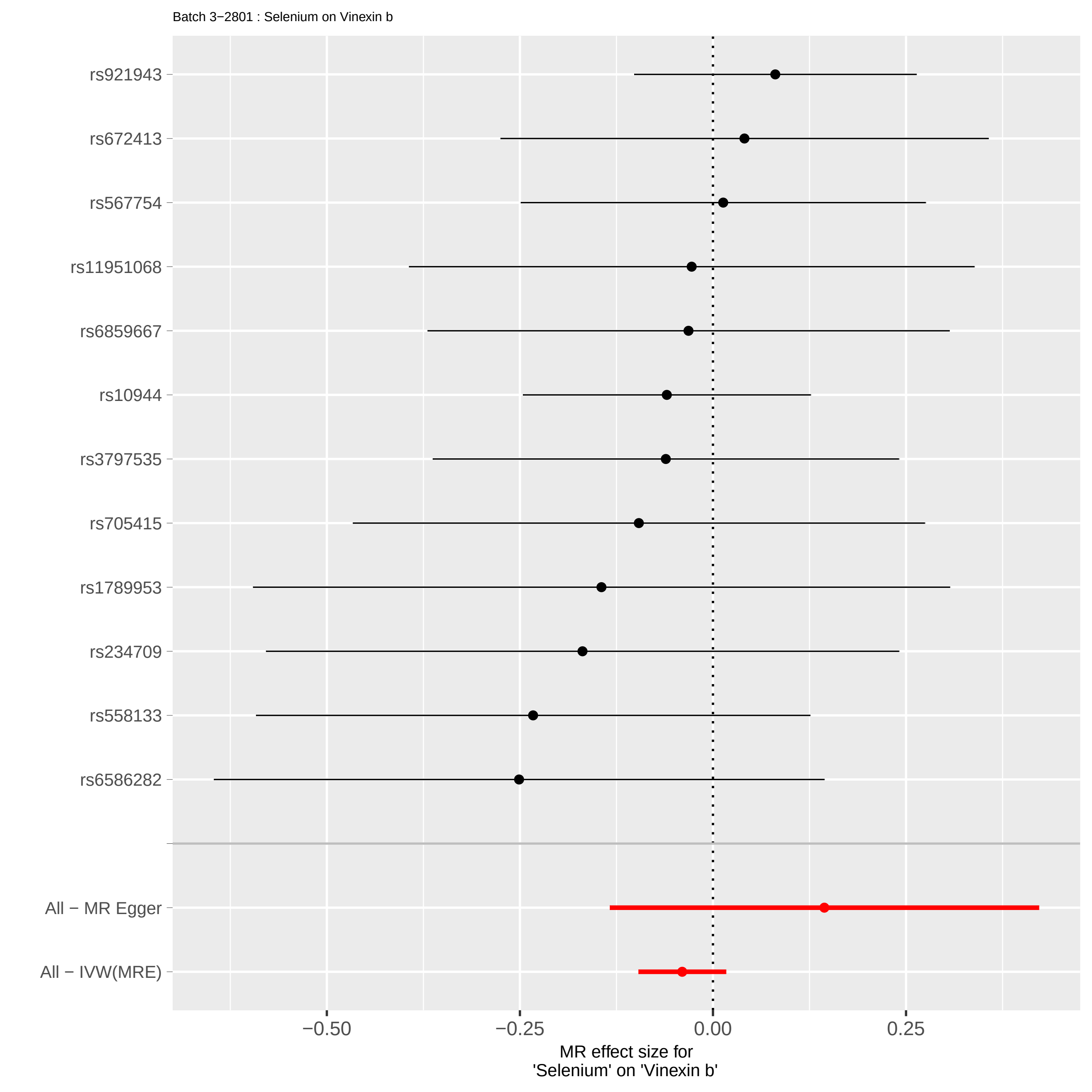

Batch 3−2801 : Selenium on Vinexin b
rs921943
rs672413
rs567754
rs11951068
rs6859667
rs10944
rs3797535
rs705415
rs1789953
rs234709
rs558133
rs6586282
All − MR Egger
All − IVW(MRE)
−0.50
−0.25
0.00
0.25
MR effect size for
'Selenium' on 'Vinexin b'

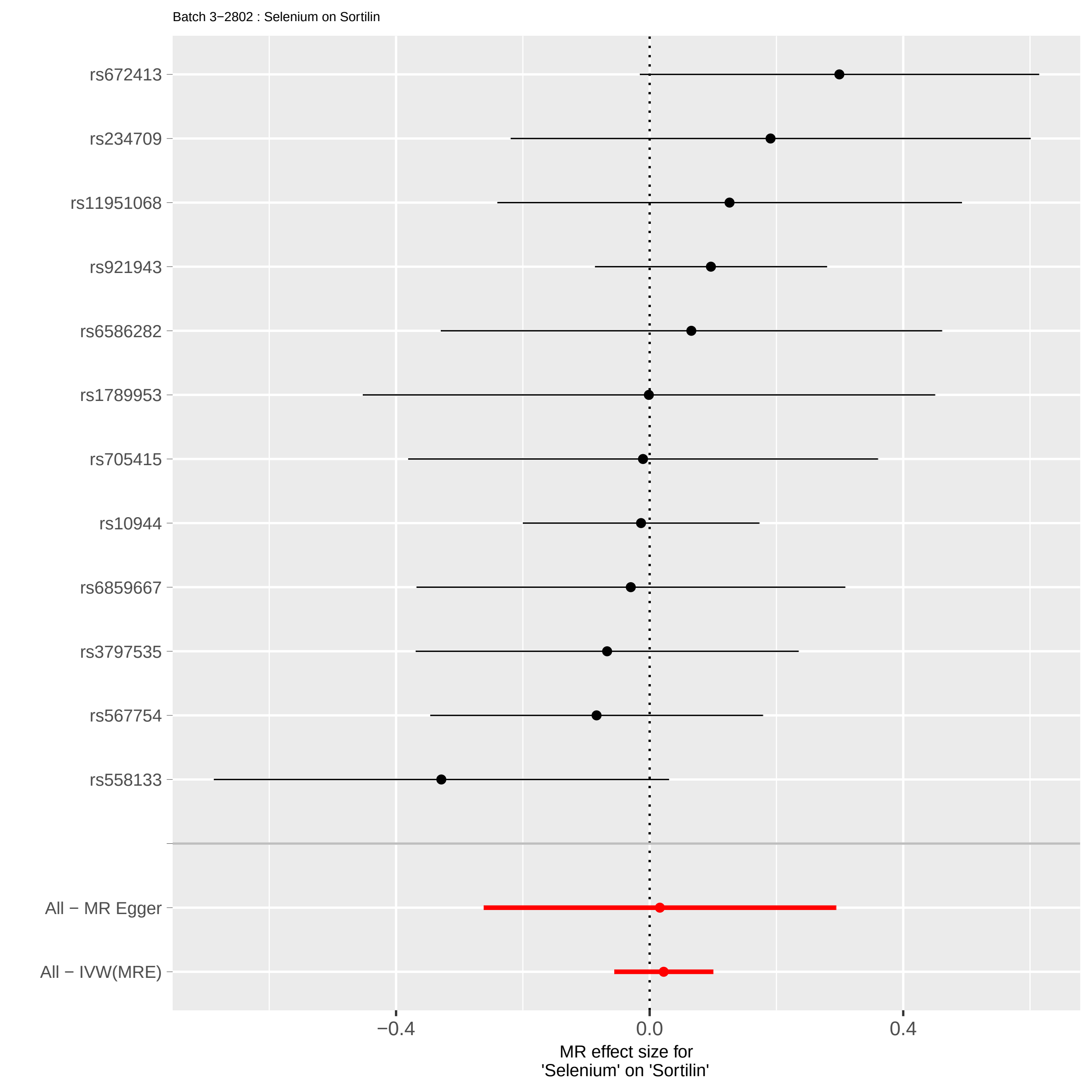

Batch 3−2802 : Selenium on Sortilin
rs672413
rs234709
rs11951068
rs921943
rs6586282
rs1789953
rs705415
rs10944
rs6859667
rs3797535
rs567754
rs558133
All − MR Egger
All − IVW(MRE)
−0.4
0.0
0.4
MR effect size for
'Selenium' on 'Sortilin'

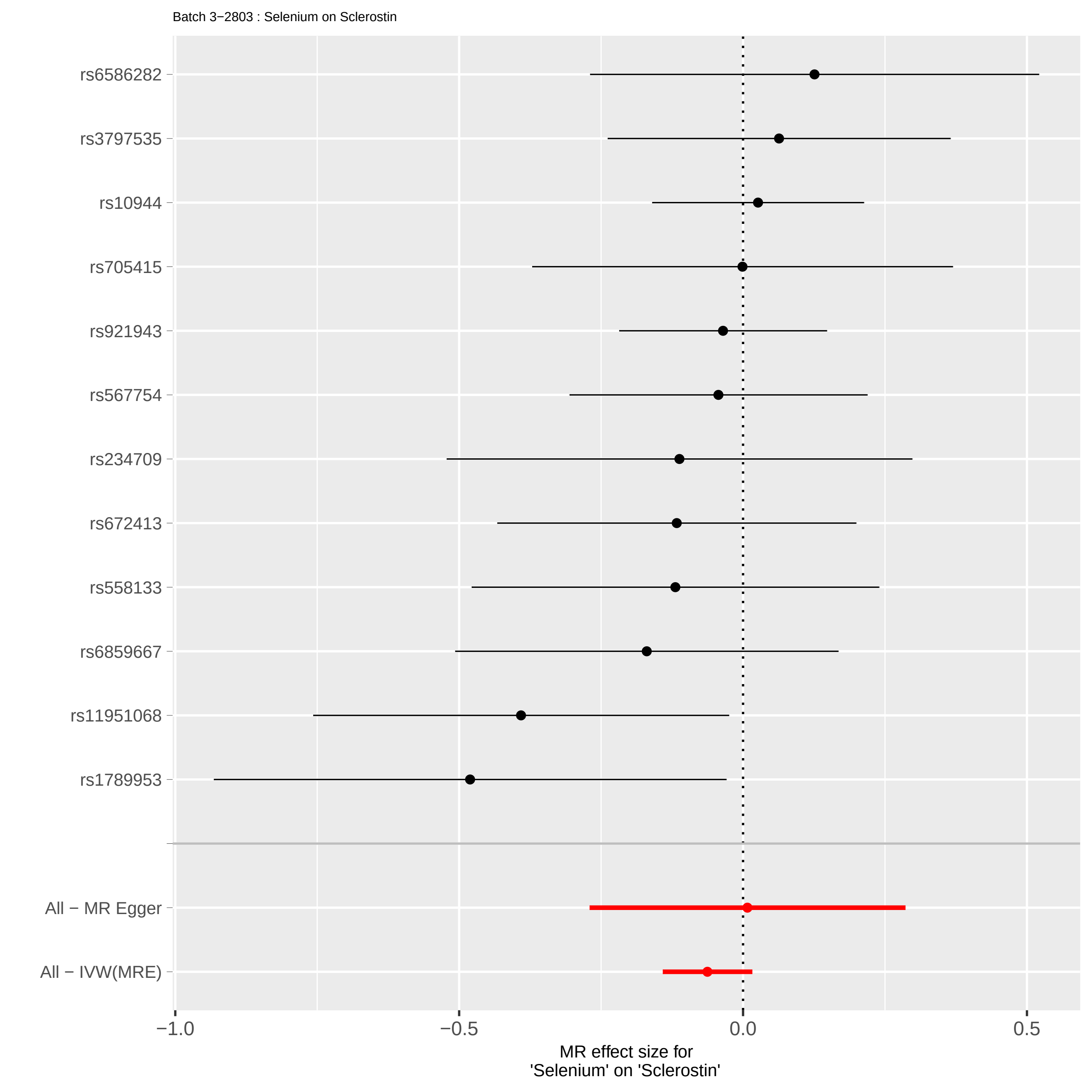
Batch 3−2803 : Selenium on Sclerostin
rs6586282
rs3797535
rs10944
rs705415
rs921943
rs567754
rs234709
rs672413
rs558133
rs6859667
rs11951068
rs1789953
All − MR Egger
All − IVW(MRE)
−1.0
−0.5
0.0
0.5
MR effect size for
'Selenium' on 'Sclerostin'

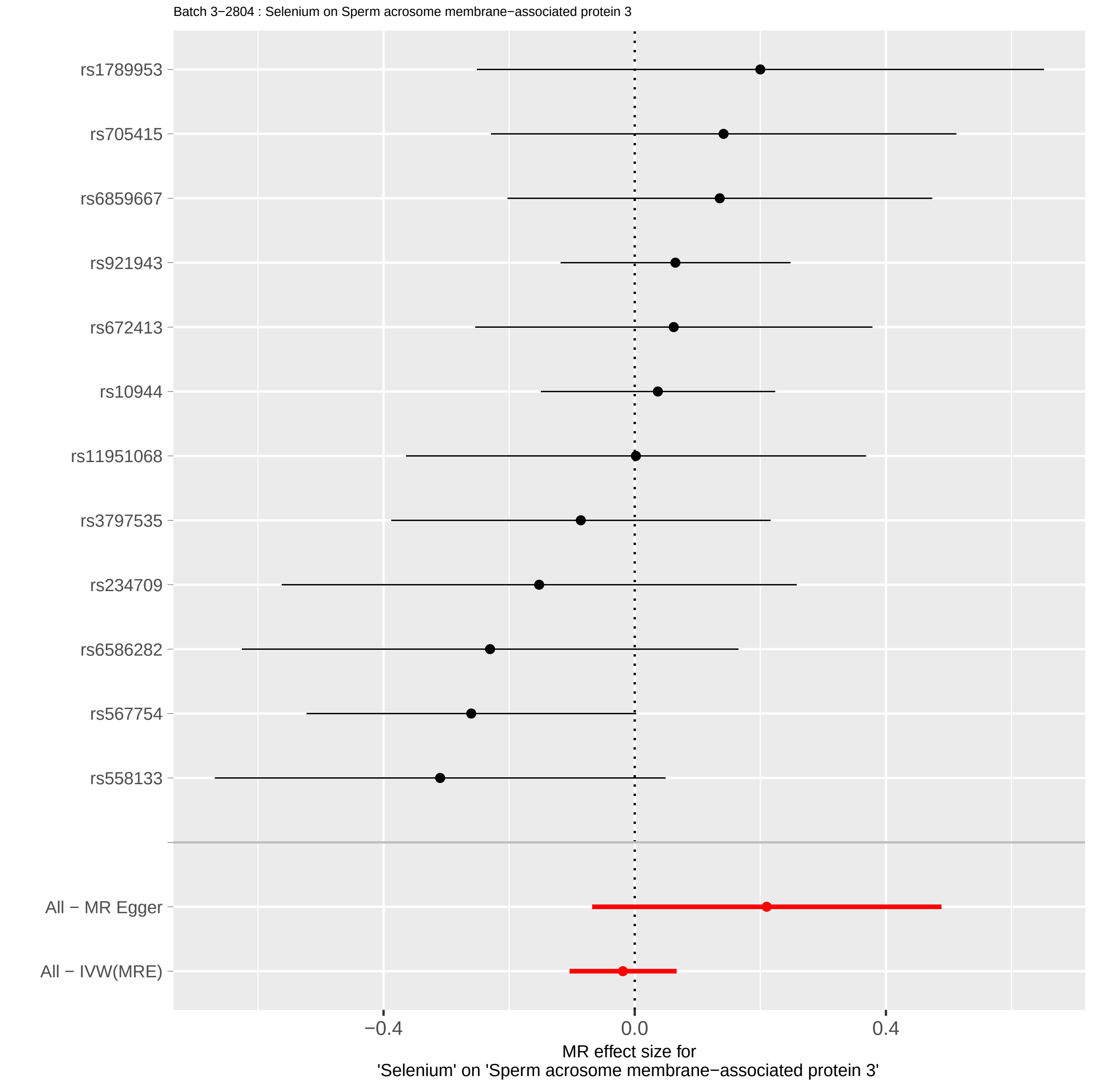
Batch 3−2804 : Selenium on Sperm acrosome membrane−associated protein 3
rs1789953
rs705415
rs6859667
rs921943
rs672413
rs10944
rs11951068
rs3797535
rs234709
rs6586282
rs567754
rs558133
All − MR Egger
All − IVW(MRE)
−0.4
0.0
0.4
MR effect size for
'Selenium' on 'Sperm acrosome membrane−associated protein 3'

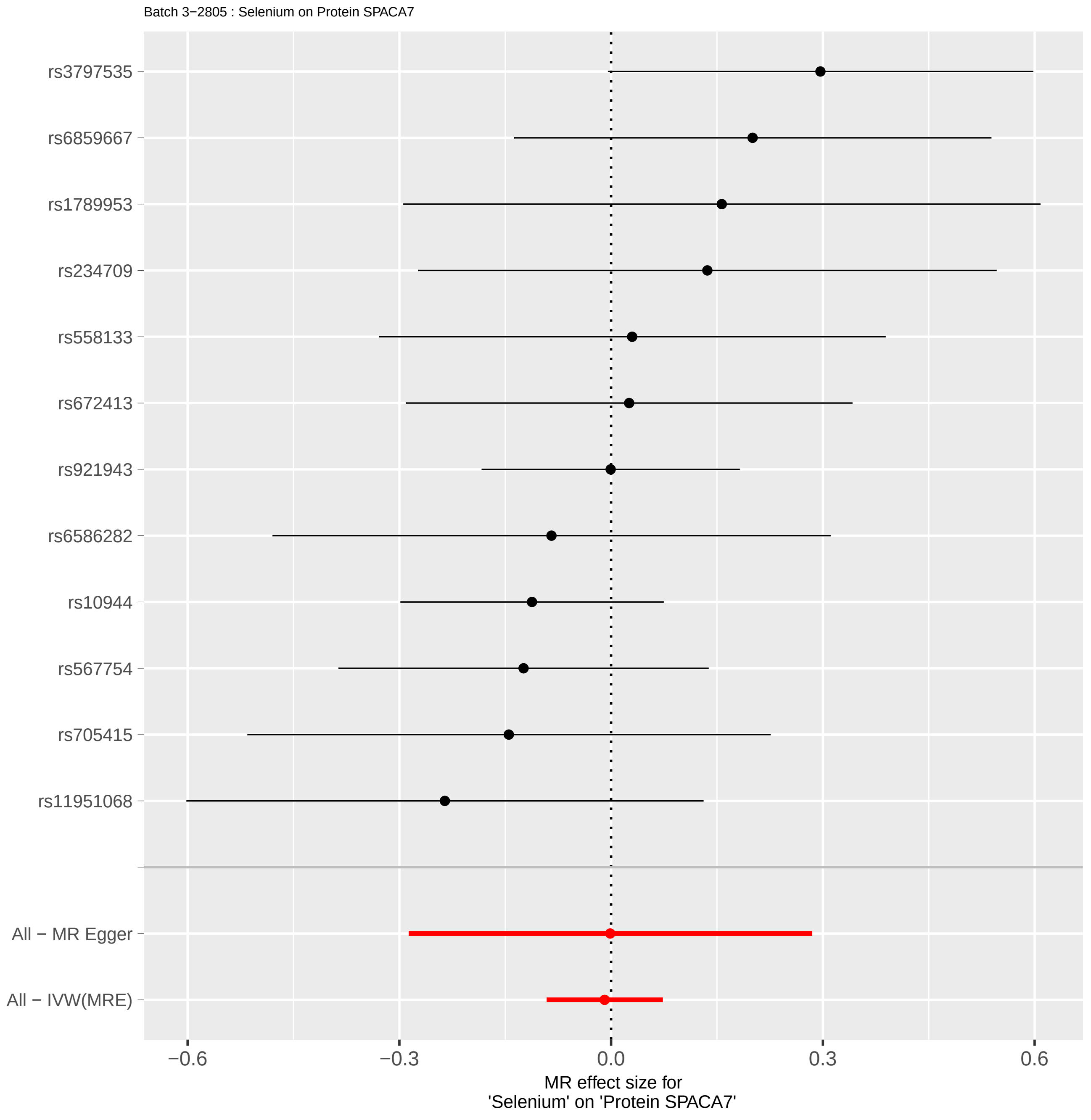
Batch 3−2805 : Selenium on Protein SPACA7
rs3797535
rs6859667
rs1789953
rs234709
rs558133
rs672413
rs921943
rs6586282
rs10944
rs567754
rs705415
rs11951068
All − MR Egger
All − IVW(MRE)
−0.6
−0.3
0.0
0.3
0.6
MR effect size for
'Selenium' on 'Protein SPACA7'

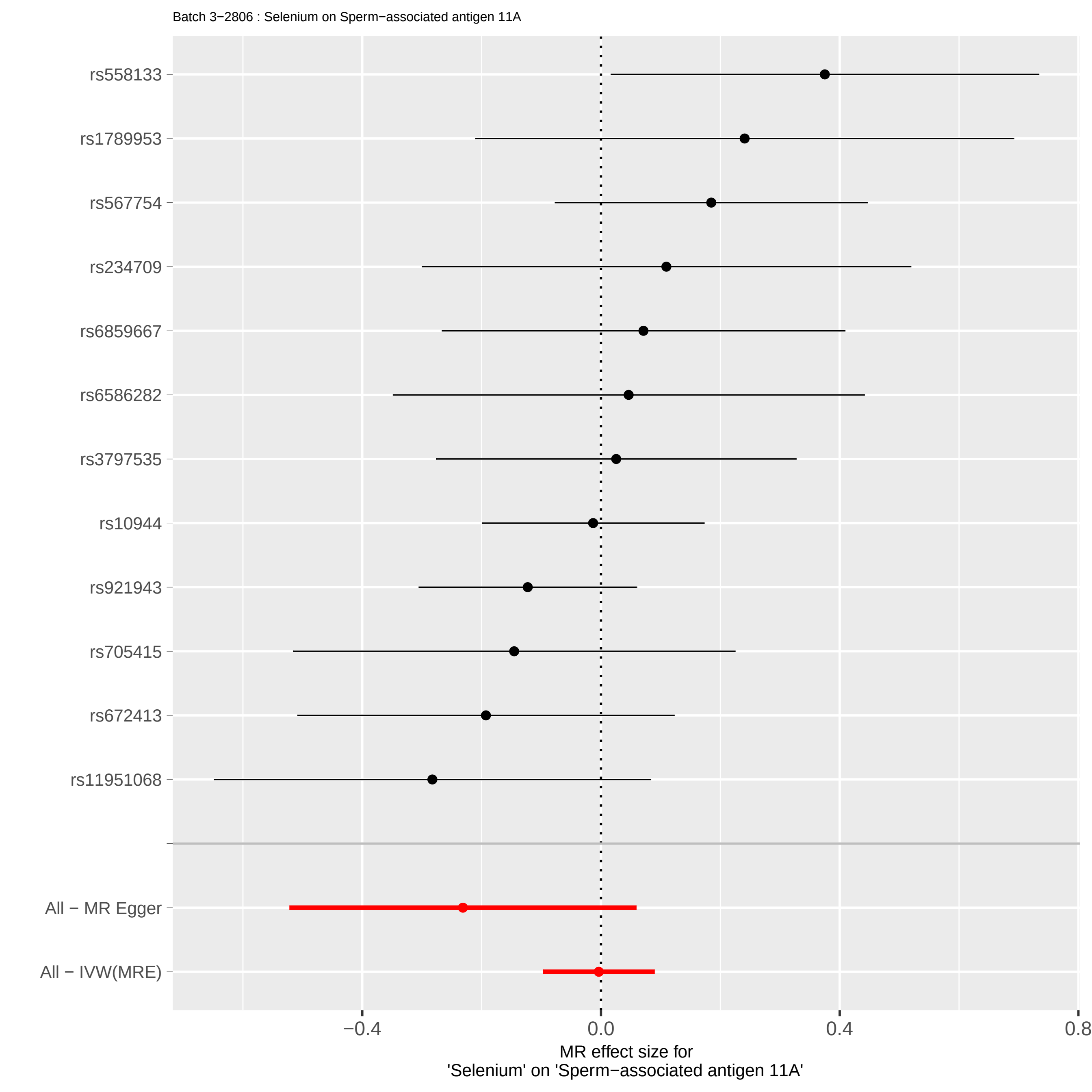

Batch 3−2806 : Selenium on Sperm−associated antigen 11A
rs558133
rs1789953
rs567754
rs234709
rs6859667
rs6586282
rs3797535
rs10944
rs921943
rs705415
rs672413
rs11951068
All − MR Egger
All − IVW(MRE)
−0.4
0.0
0.4
0.8
MR effect size for
'Selenium' on 'Sperm−associated antigen 11A'

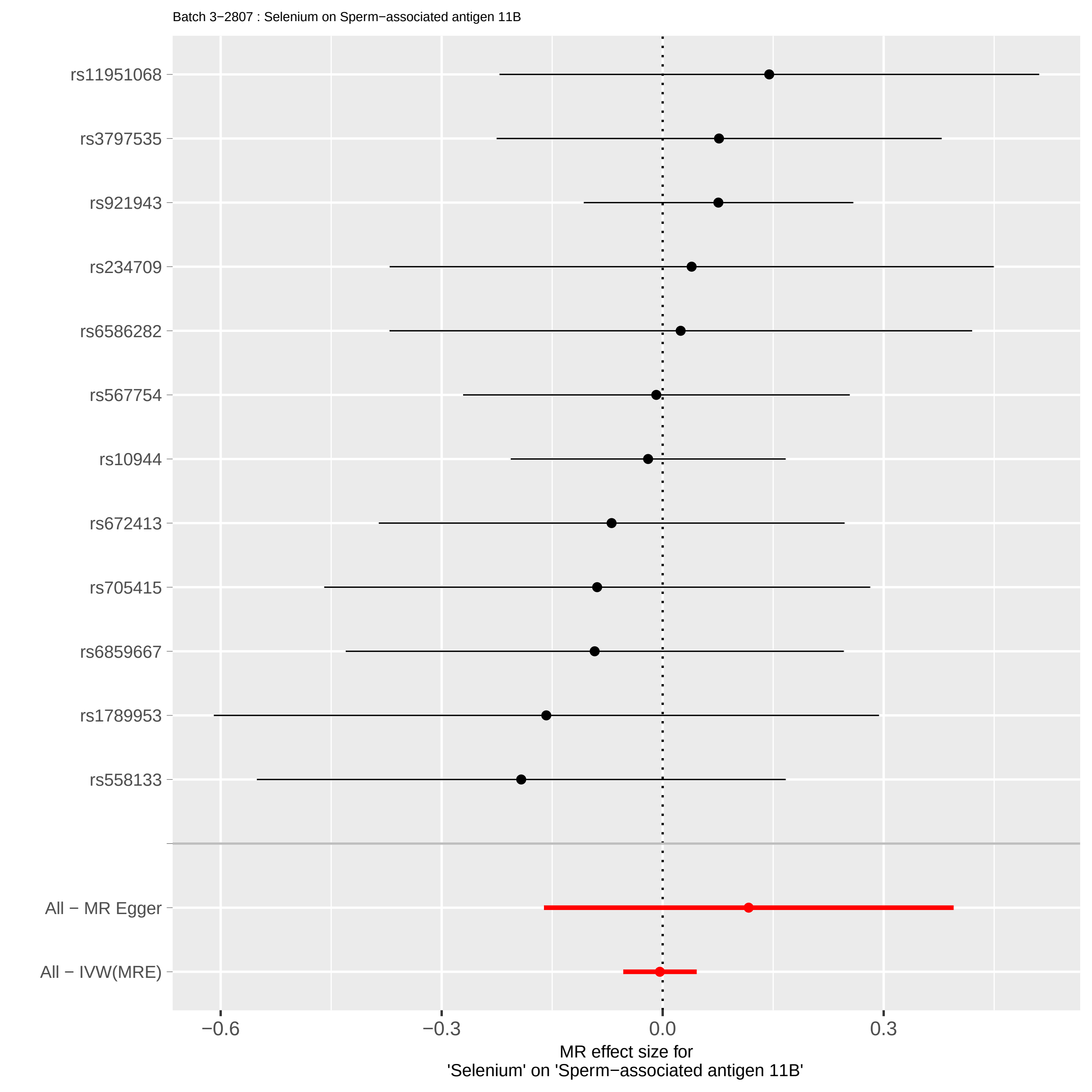
Batch 3−2807 : Selenium on Sperm−associated antigen 11B
rs11951068
rs3797535
rs921943
rs234709
rs6586282
rs567754
rs10944
rs672413
rs705415
rs6859667
rs1789953
rs558133
All − MR Egger
All − IVW(MRE)
−0.6
−0.3
0.0
0.3
MR effect size for
'Selenium' on 'Sperm−associated antigen 11B'

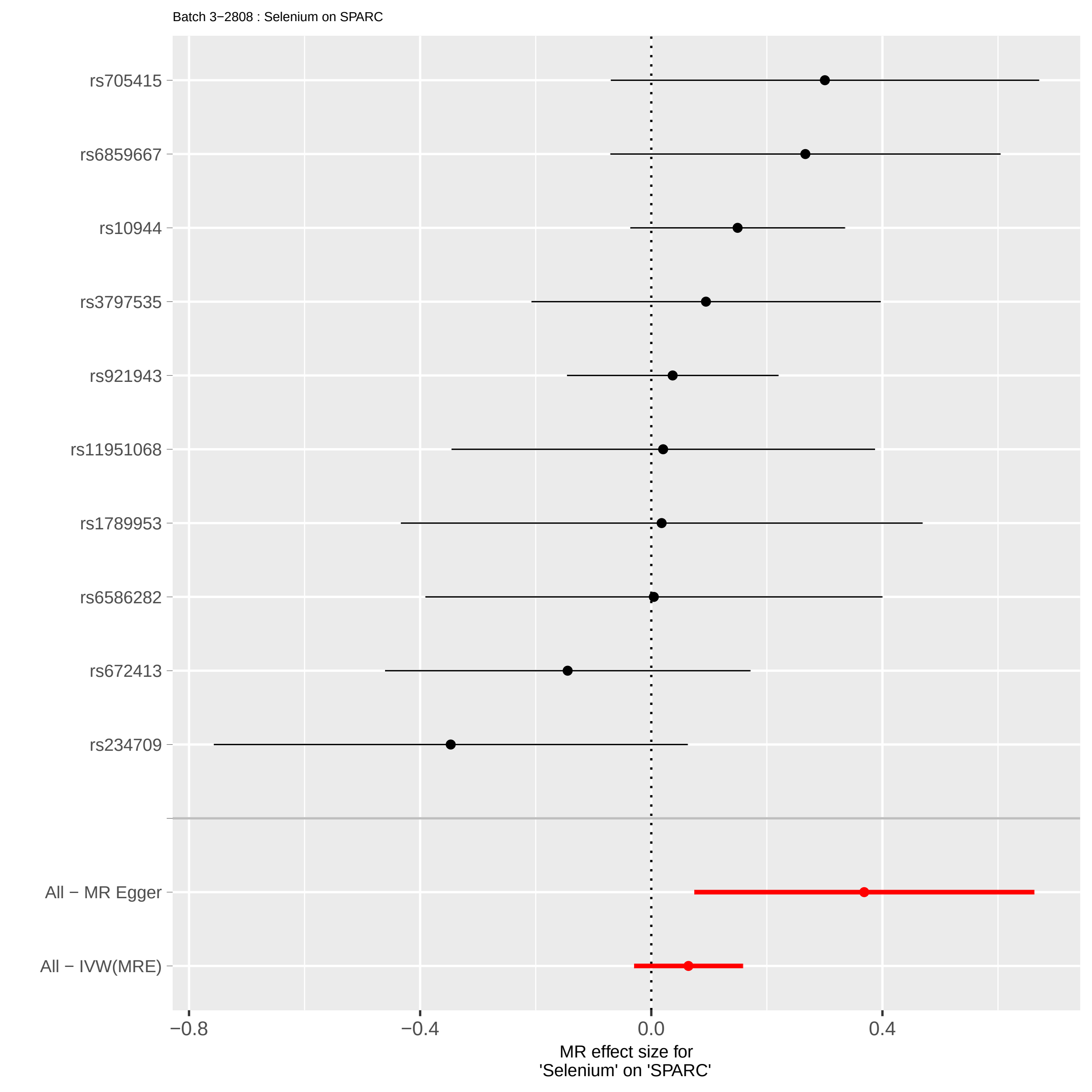
Batch 3−2808 : Selenium on SPARC
rs705415
rs6859667
rs10944
rs3797535
rs921943
rs11951068
rs1789953
rs6586282
rs672413
rs234709
All − MR Egger
All − IVW(MRE)
−0.8
−0.4
0.0
0.4
MR effect size for
'Selenium' on 'SPARC'

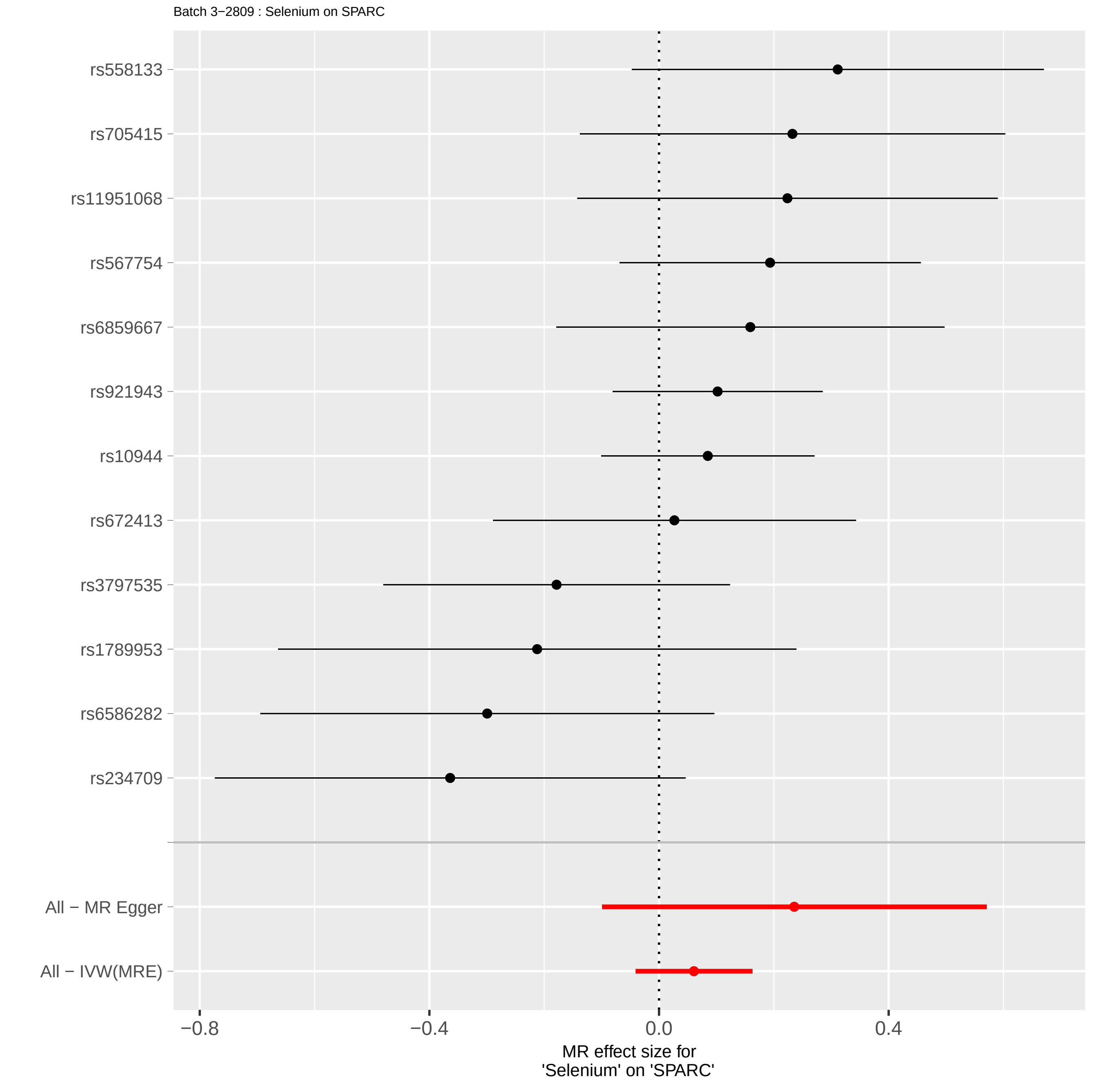

Batch 3−2809 : Selenium on SPARC
rs558133
rs705415
rs11951068
rs567754
rs6859667
rs921943
rs10944
rs672413
rs3797535
rs1789953
rs6586282
rs234709
All − MR Egger
All − IVW(MRE)
−0.8
−0.4
0.0
0.4
MR effect size for
'Selenium' on 'SPARC'

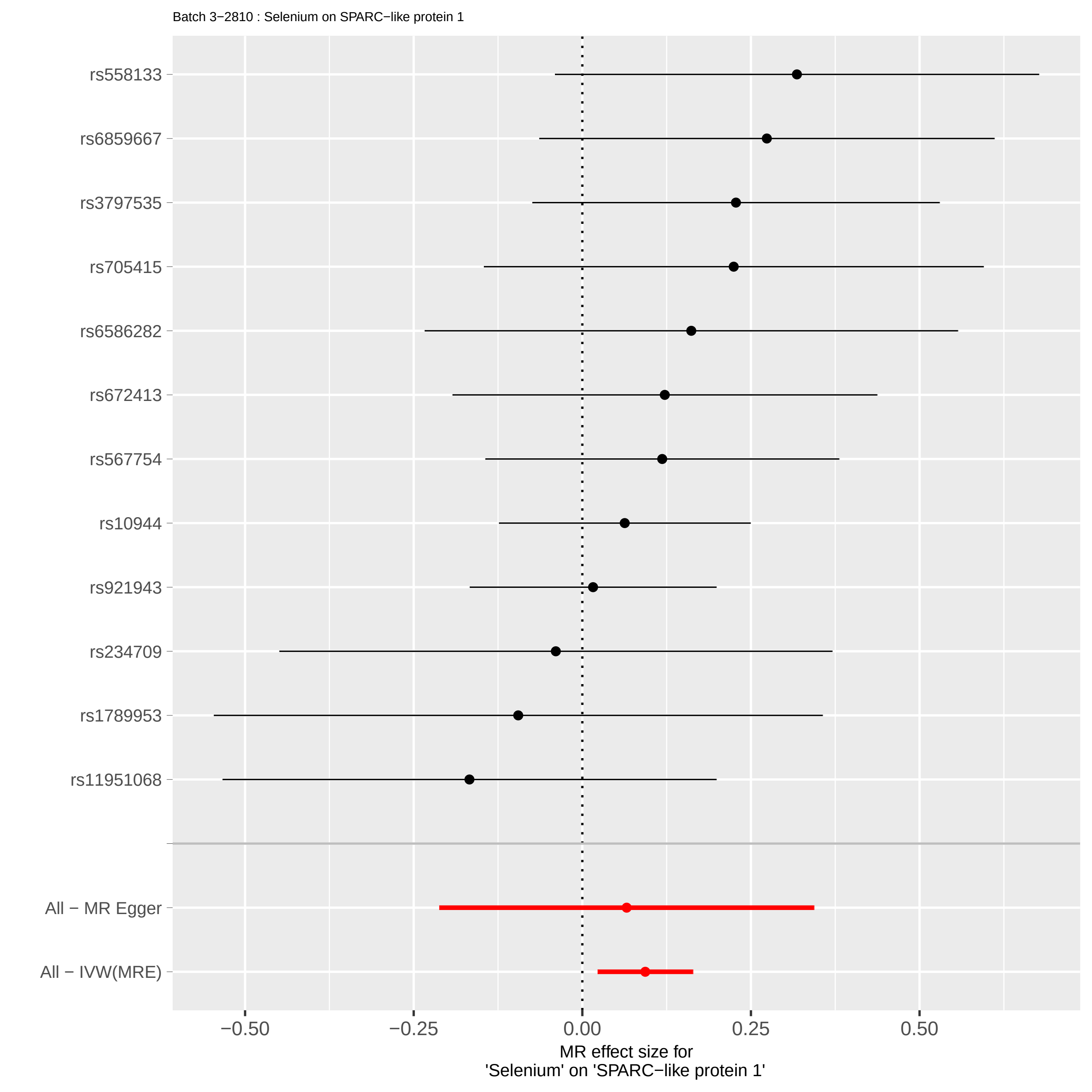

Batch 3−2810 : Selenium on SPARC−like protein 1
rs558133
rs6859667
rs3797535
rs705415
rs6586282
rs672413
rs567754
rs10944
rs921943
rs234709
rs1789953
rs11951068
All − MR Egger
All − IVW(MRE)
−0.50
−0.25
0.00
0.25
0.50
MR effect size for
'Selenium' on 'SPARC−like protein 1'

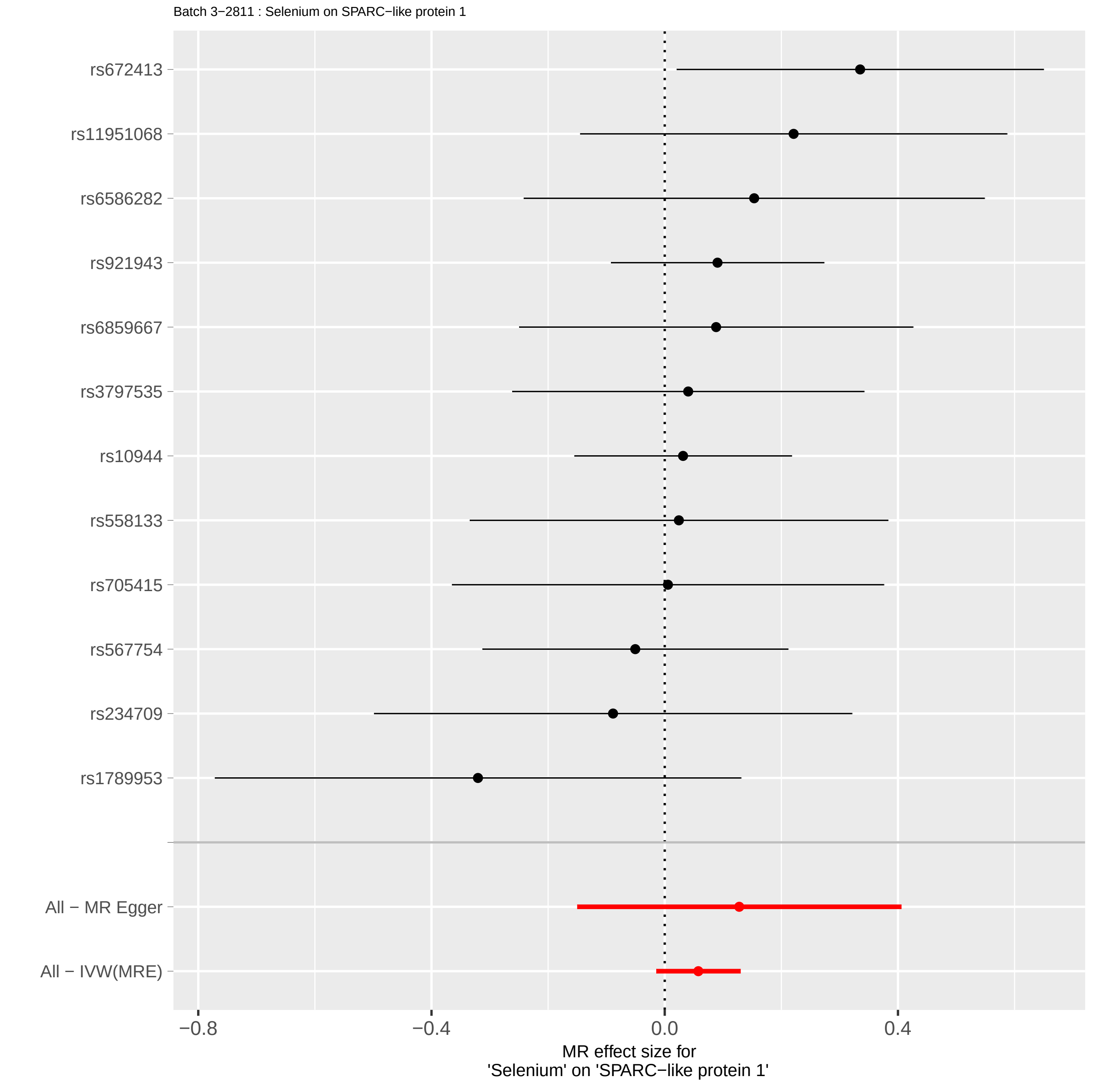

Batch 3−2811 : Selenium on SPARC−like protein 1
rs672413
rs11951068
rs6586282
rs921943
rs6859667
rs3797535
rs10944
rs558133
rs705415
rs567754
rs234709
rs1789953
All − MR Egger
All − IVW(MRE)
−0.8
−0.4
0.0
0.4
MR effect size for
'Selenium' on 'SPARC−like protein 1'

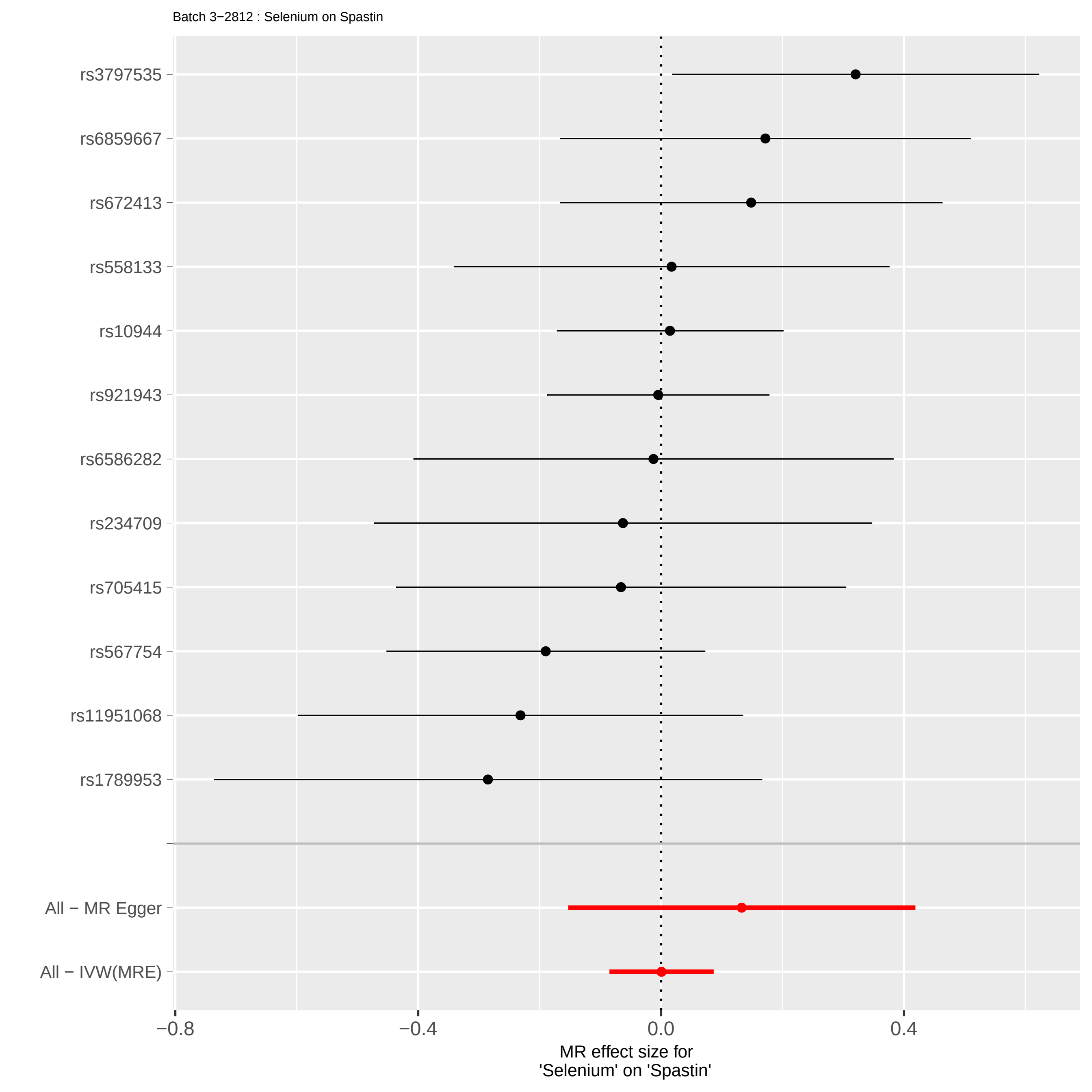

Batch 3−2812 : Selenium on Spastin
rs3797535
rs6859667
rs672413
rs558133
rs10944
rs921943
rs6586282
rs234709
rs705415
rs567754
rs11951068
rs1789953
All − MR Egger
All − IVW(MRE)
−0.8
−0.4
0.0
0.4
MR effect size for
'Selenium' on 'Spastin'

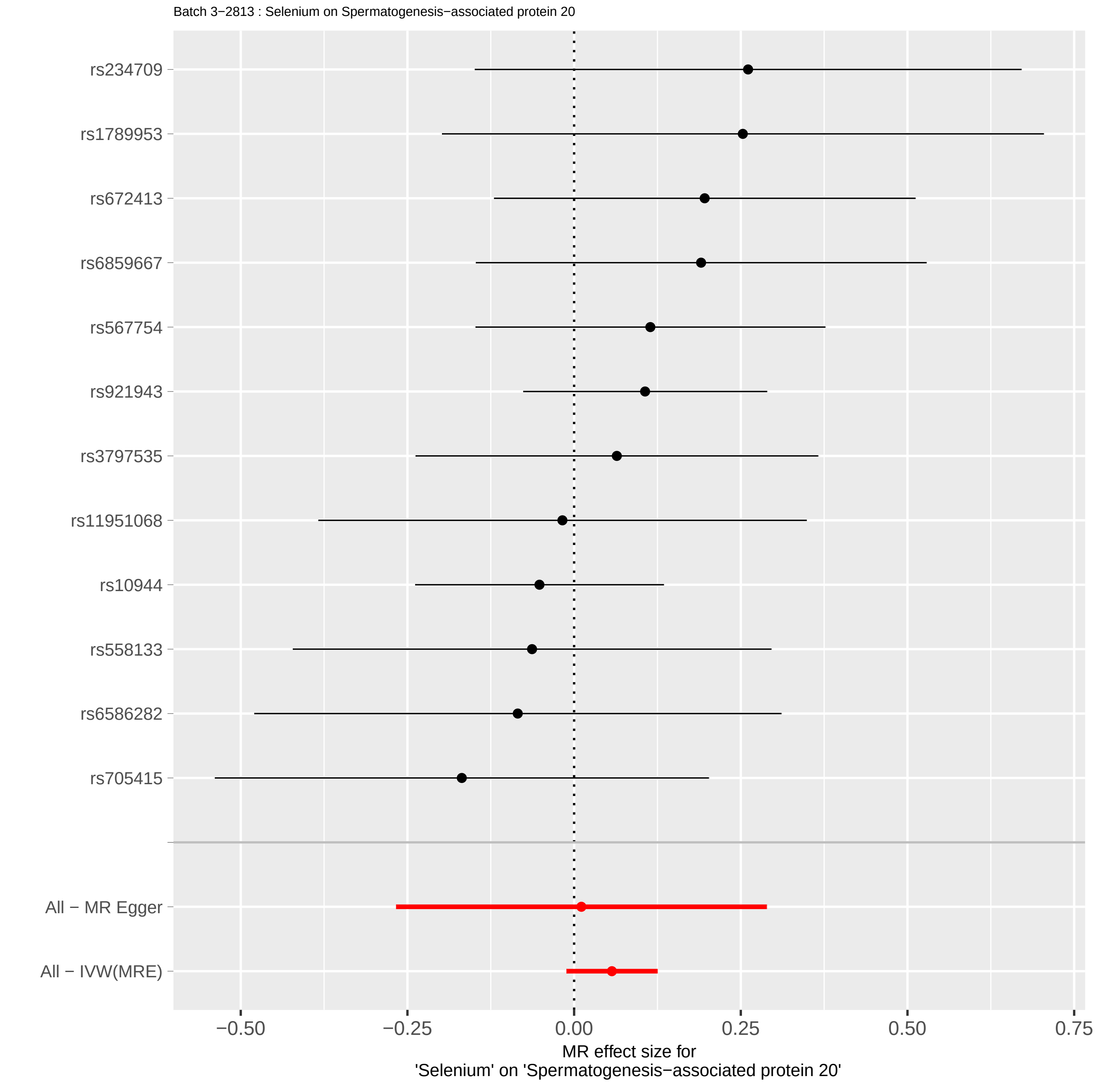

Batch 3−2813 : Selenium on Spermatogenesis−associated protein 20
rs234709
rs1789953
rs672413
rs6859667
rs567754
rs921943
rs3797535
rs11951068
rs10944
rs558133
rs6586282
rs705415
All − MR Egger
All − IVW(MRE)
−0.50
−0.25
0.00
0.25
0.50
0.75
MR effect size for
'Selenium' on 'Spermatogenesis−associated protein 20'

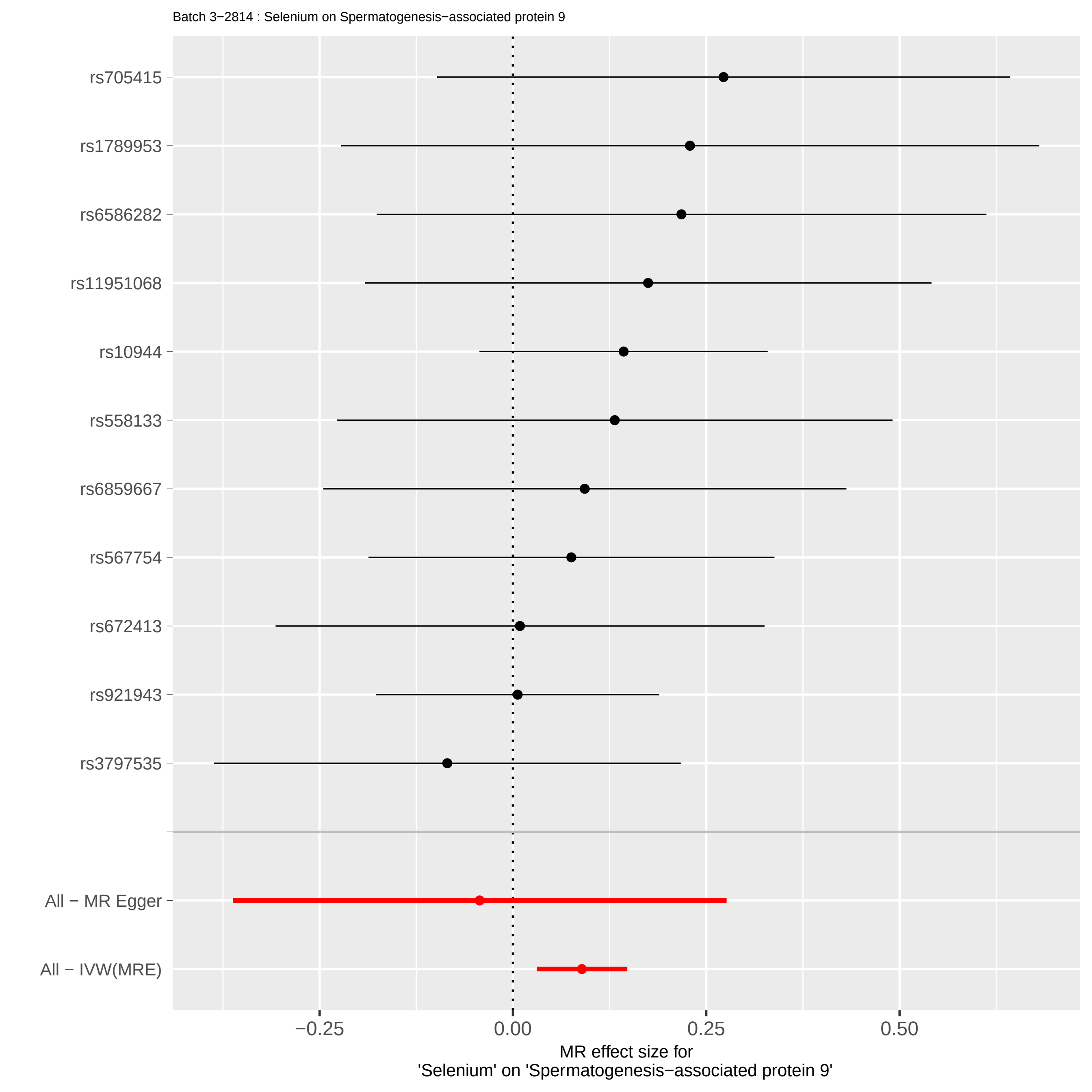
Batch 3−2814 : Selenium on Spermatogenesis−associated protein 9
rs705415
rs1789953
rs6586282
rs11951068
rs10944
rs558133
rs6859667
rs567754
rs672413
rs921943
rs3797535
All − MR Egger
All − IVW(MRE)
−0.25
0.00
0.25
0.50
MR effect size for
'Selenium' on 'Spermatogenesis−associated protein 9'

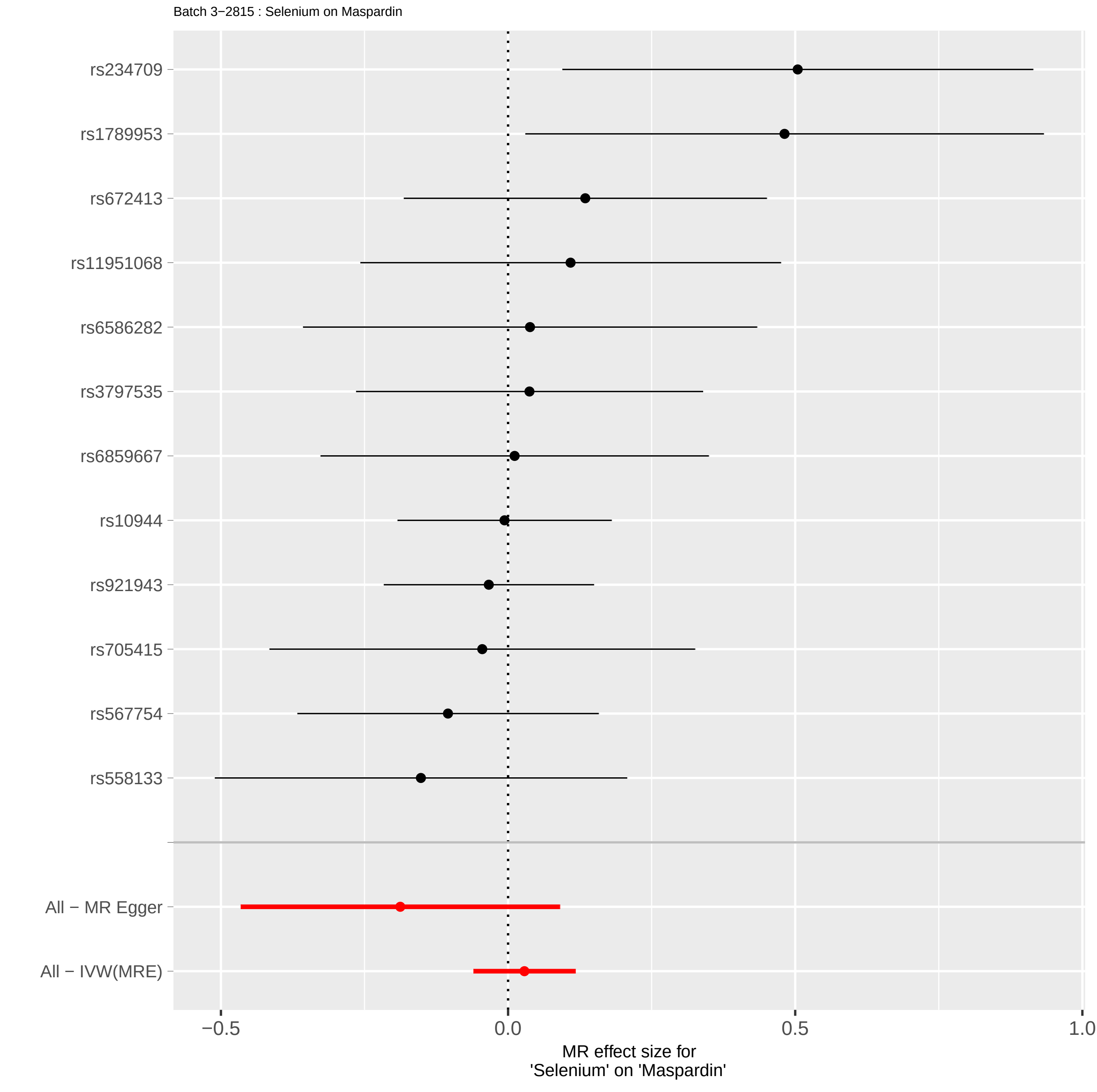

Batch 3−2815 : Selenium on Maspardin
rs234709
rs1789953
rs672413
rs11951068
rs6586282
rs3797535
rs6859667
rs10944
rs921943
rs705415
rs567754
rs558133
All − MR Egger
All − IVW(MRE)
−0.5
0.0
0.5
1.0
MR effect size for
'Selenium' on 'Maspardin'

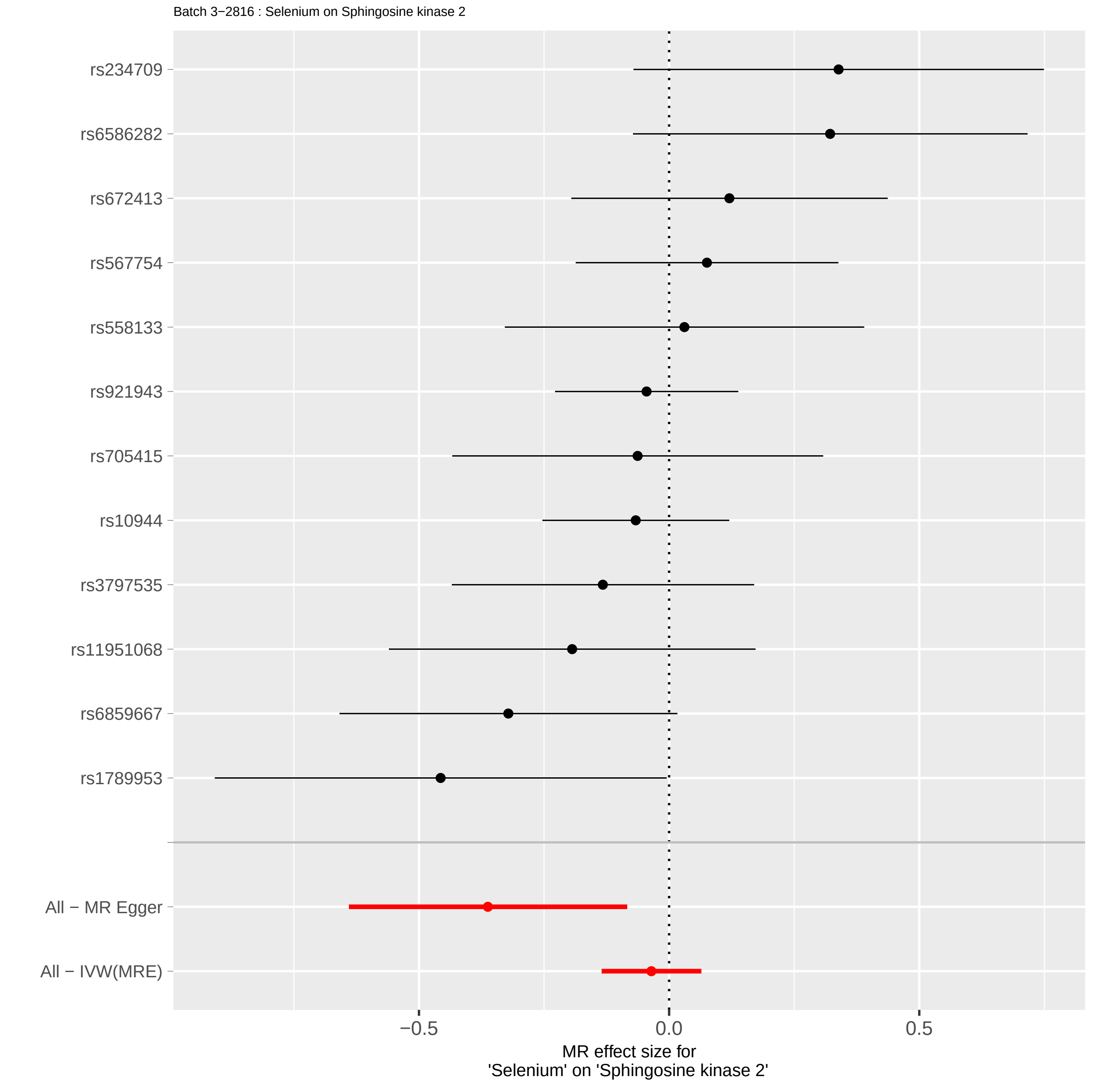
Batch 3−2816 : Selenium on Sphingosine kinase 2
rs234709
rs6586282
rs672413
rs567754
rs558133
rs921943
rs705415
rs10944
rs3797535
rs11951068
rs6859667
rs1789953
All − MR Egger
All − IVW(MRE)
−0.5
0.0
0.5
MR effect size for
'Selenium' on 'Sphingosine kinase 2'

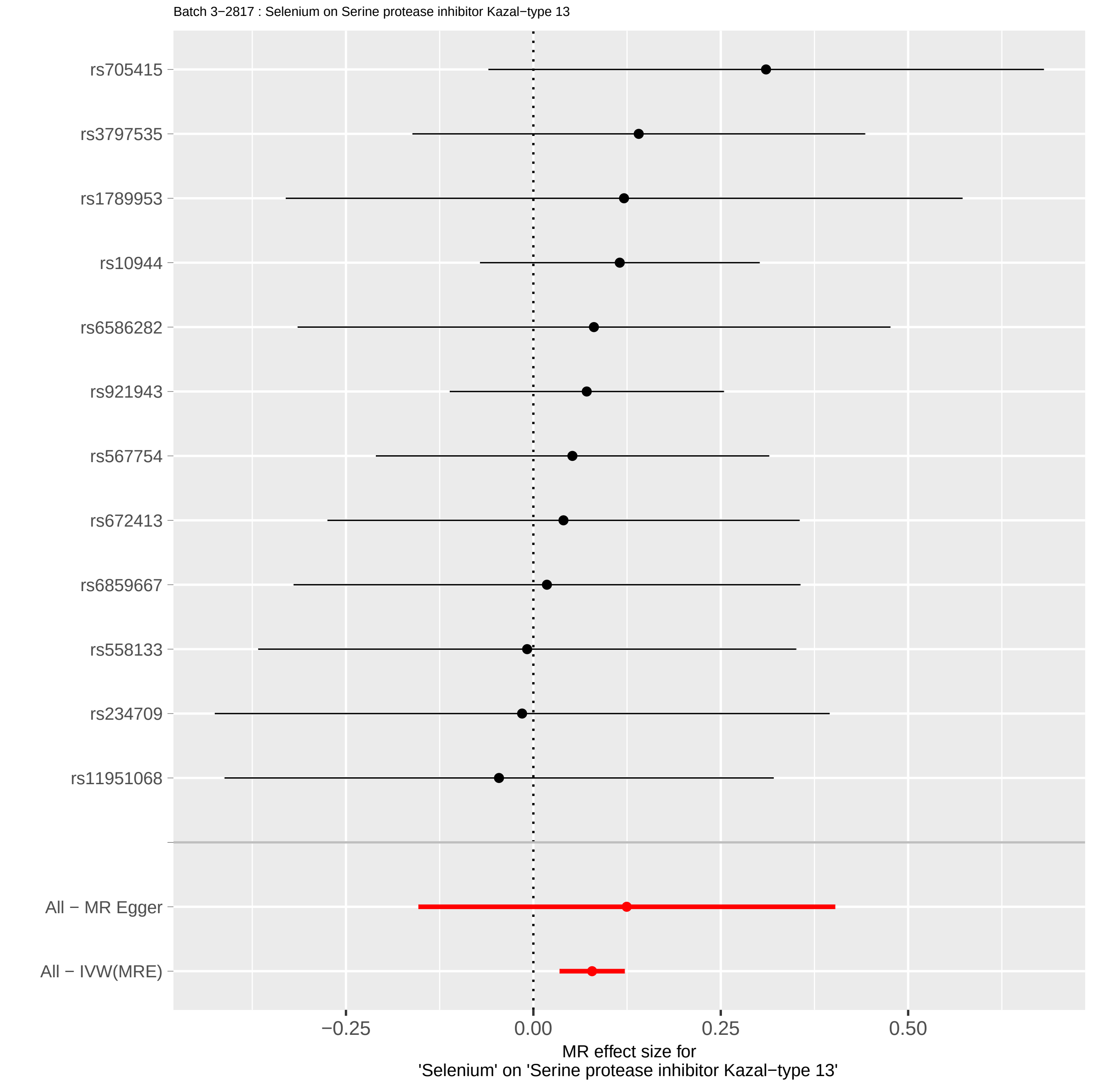
Batch 3−2817 : Selenium on Serine protease inhibitor Kazal−type 13
rs705415
rs3797535
rs1789953
rs10944
rs6586282
rs921943
rs567754
rs672413
rs6859667
rs558133
rs234709
rs11951068
All − MR Egger
All − IVW(MRE)
−0.25
0.00
0.25
0.50
MR effect size for
'Selenium' on 'Serine protease inhibitor Kazal−type 13'

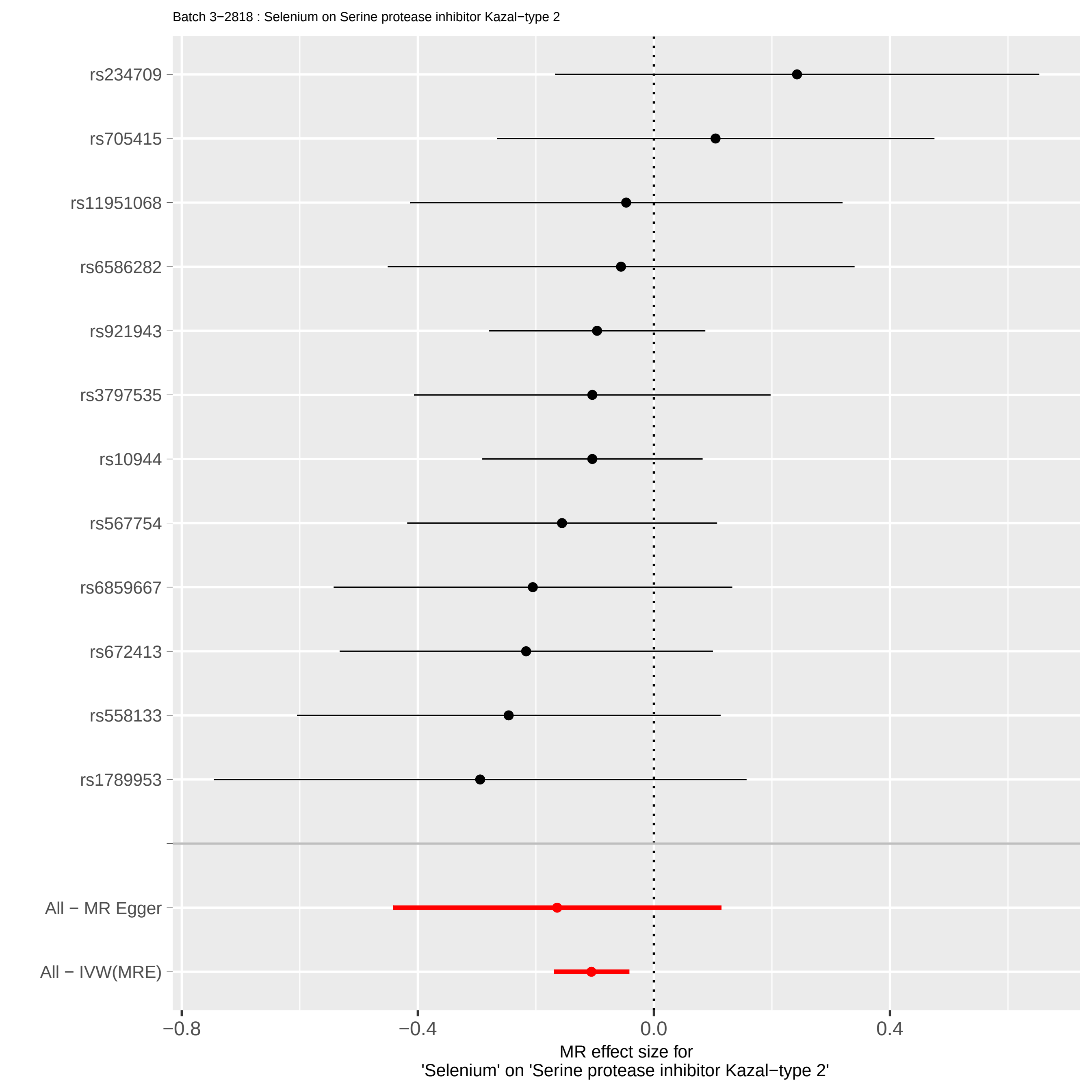

Batch 3−2818 : Selenium on Serine protease inhibitor Kazal−type 2
rs234709
rs705415
rs11951068
rs6586282
rs921943
rs3797535
rs10944
rs567754
rs6859667
rs672413
rs558133
rs1789953
All − MR Egger
All − IVW(MRE)
−0.8
−0.4
0.0
0.4
MR effect size for
'Selenium' on 'Serine protease inhibitor Kazal−type 2'

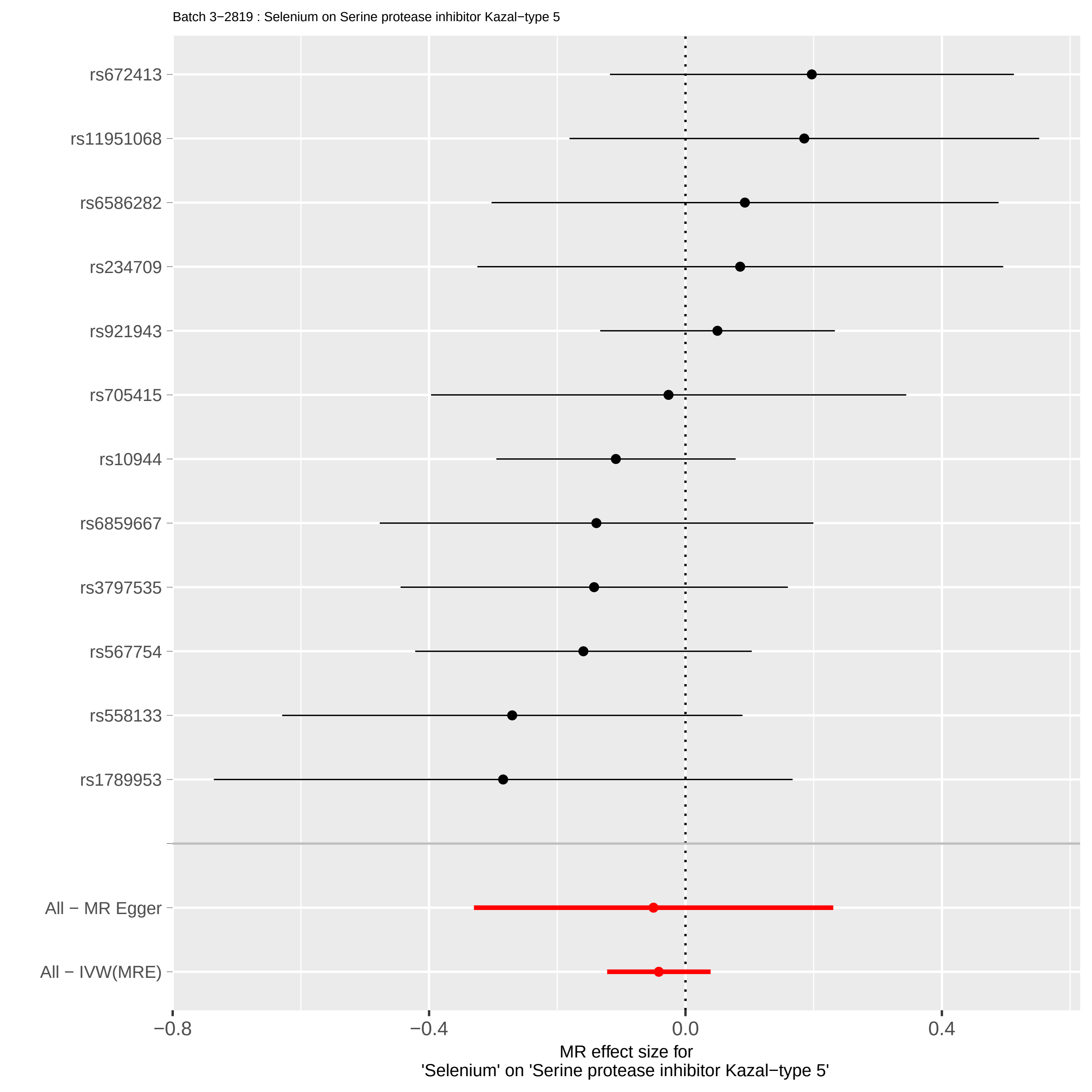

Batch 3−2819 : Selenium on Serine protease inhibitor Kazal−type 5
rs672413
rs11951068
rs6586282
rs234709
rs921943
rs705415
rs10944
rs6859667
rs3797535
rs567754
rs558133
rs1789953
All − MR Egger
All − IVW(MRE)
−0.8
−0.4
0.0
0.4
MR effect size for
'Selenium' on 'Serine protease inhibitor Kazal−type 5'

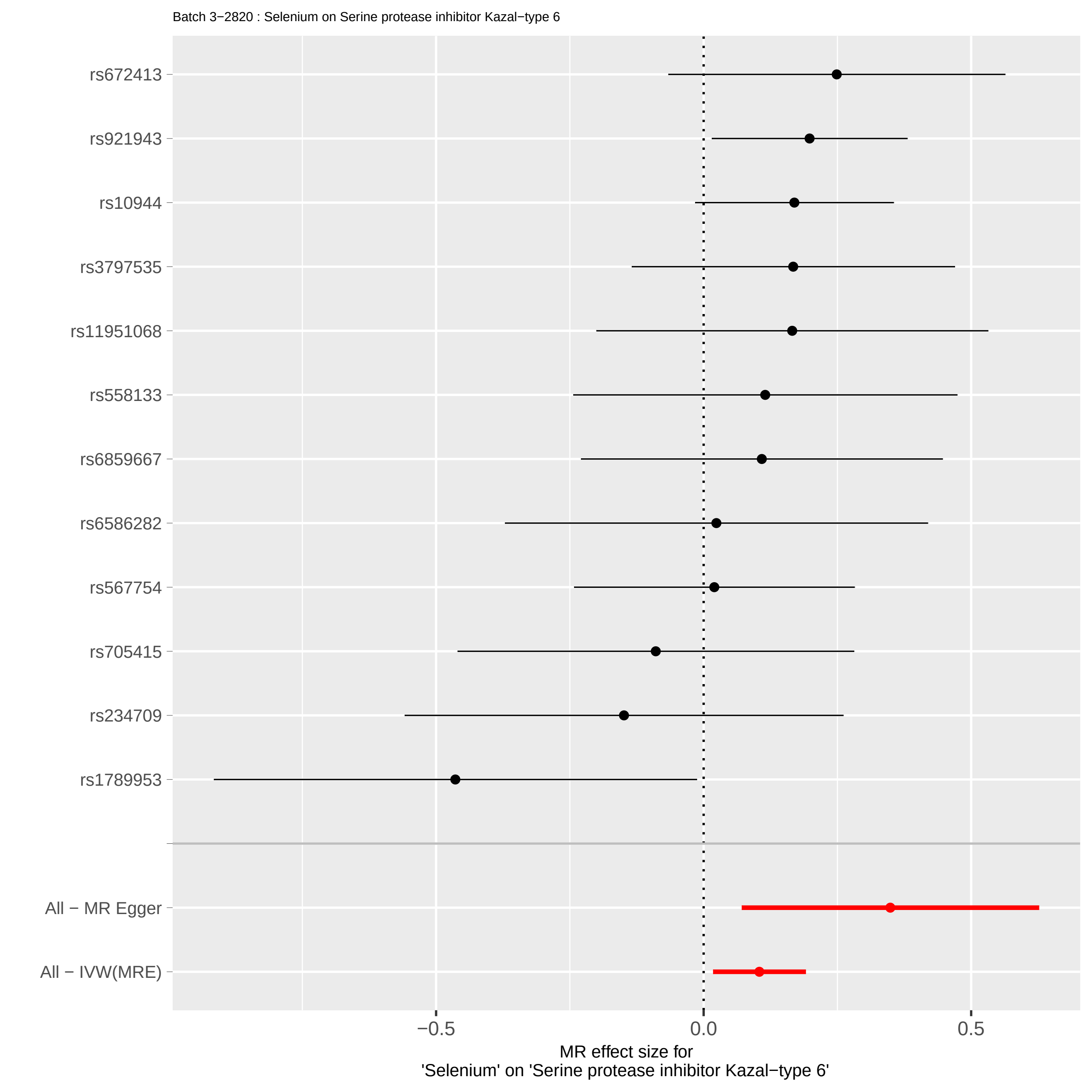
Batch 3−2820 : Selenium on Serine protease inhibitor Kazal−type 6
rs672413
rs921943
rs10944
rs3797535
rs11951068
rs558133
rs6859667
rs6586282
rs567754
rs705415
rs234709
rs1789953
All − MR Egger
All − IVW(MRE)
−0.5
0.0
0.5
MR effect size for
'Selenium' on 'Serine protease inhibitor Kazal−type 6'

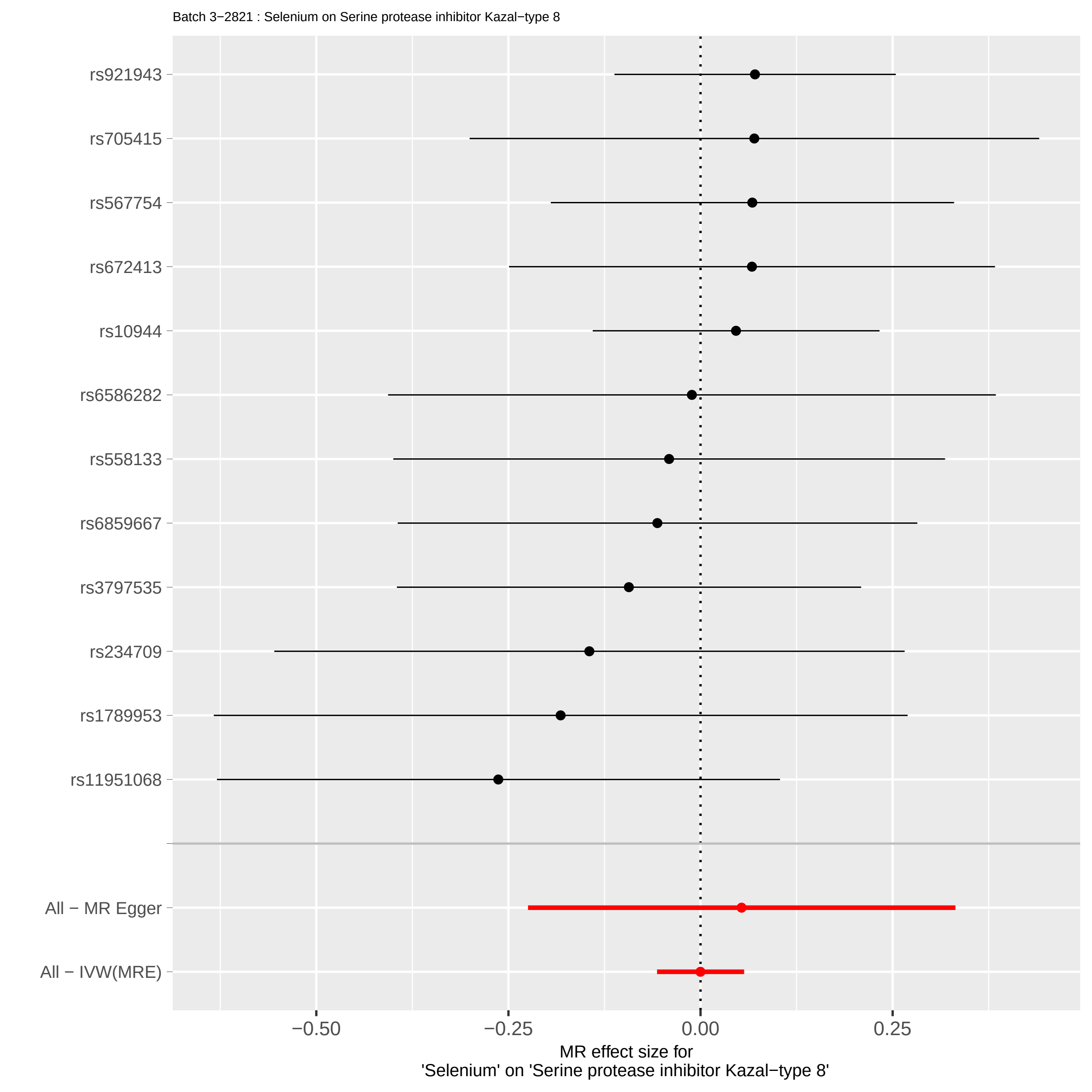
Batch 3−2821 : Selenium on Serine protease inhibitor Kazal−type 8
rs921943
rs705415
rs567754
rs672413
rs10944
rs6586282
rs558133
rs6859667
rs3797535
rs234709
rs1789953
rs11951068
All − MR Egger
All − IVW(MRE)
−0.50
−0.25
0.00
0.25
MR effect size for
'Selenium' on 'Serine protease inhibitor Kazal−type 8'

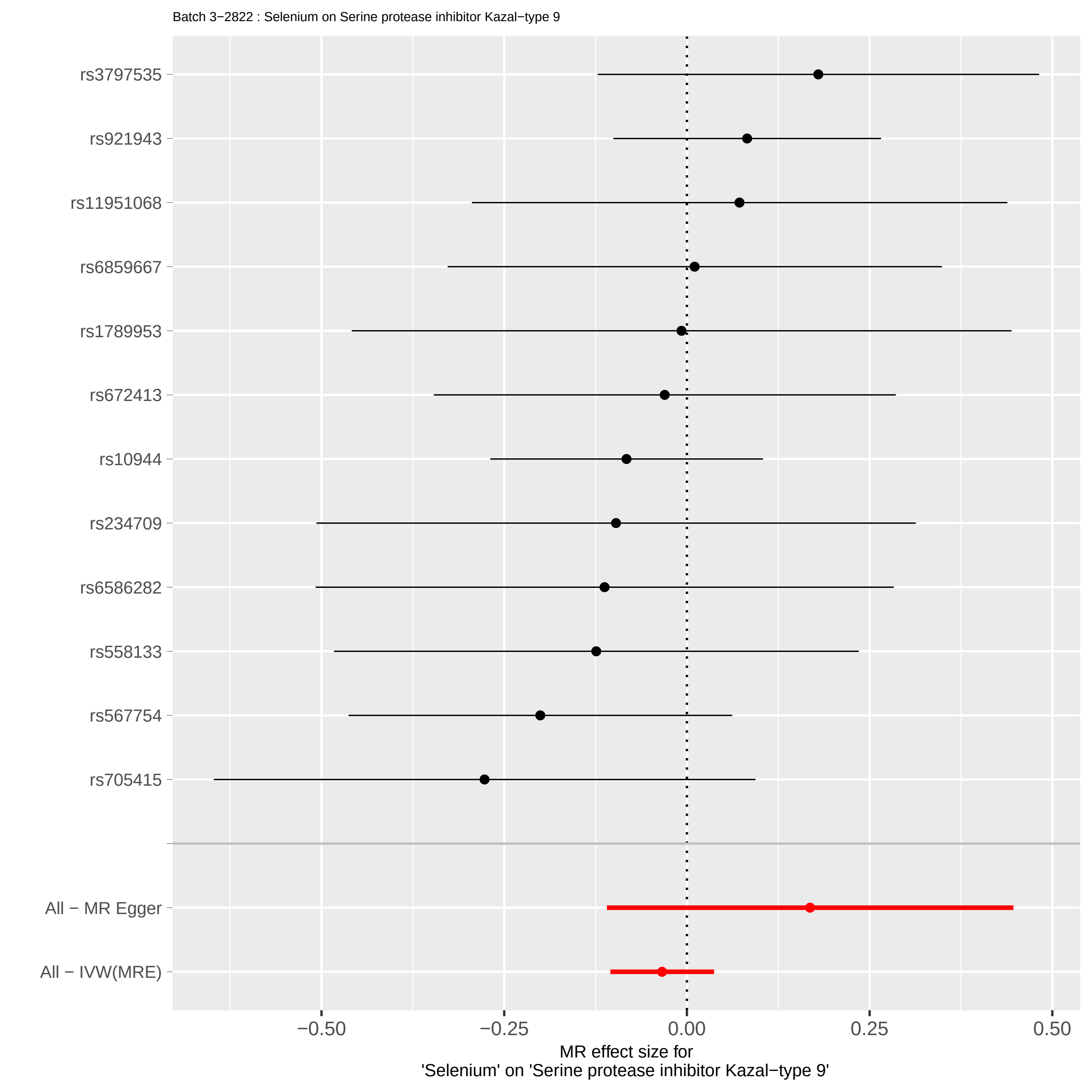

Batch 3−2822 : Selenium on Serine protease inhibitor Kazal−type 9
rs3797535
rs921943
rs11951068
rs6859667
rs1789953
rs672413
rs10944
rs234709
rs6586282
rs558133
rs567754
rs705415
All − MR Egger
All − IVW(MRE)
−0.50
−0.25
0.00
0.25
0.50
MR effect size for
'Selenium' on 'Serine protease inhibitor Kazal−type 9'

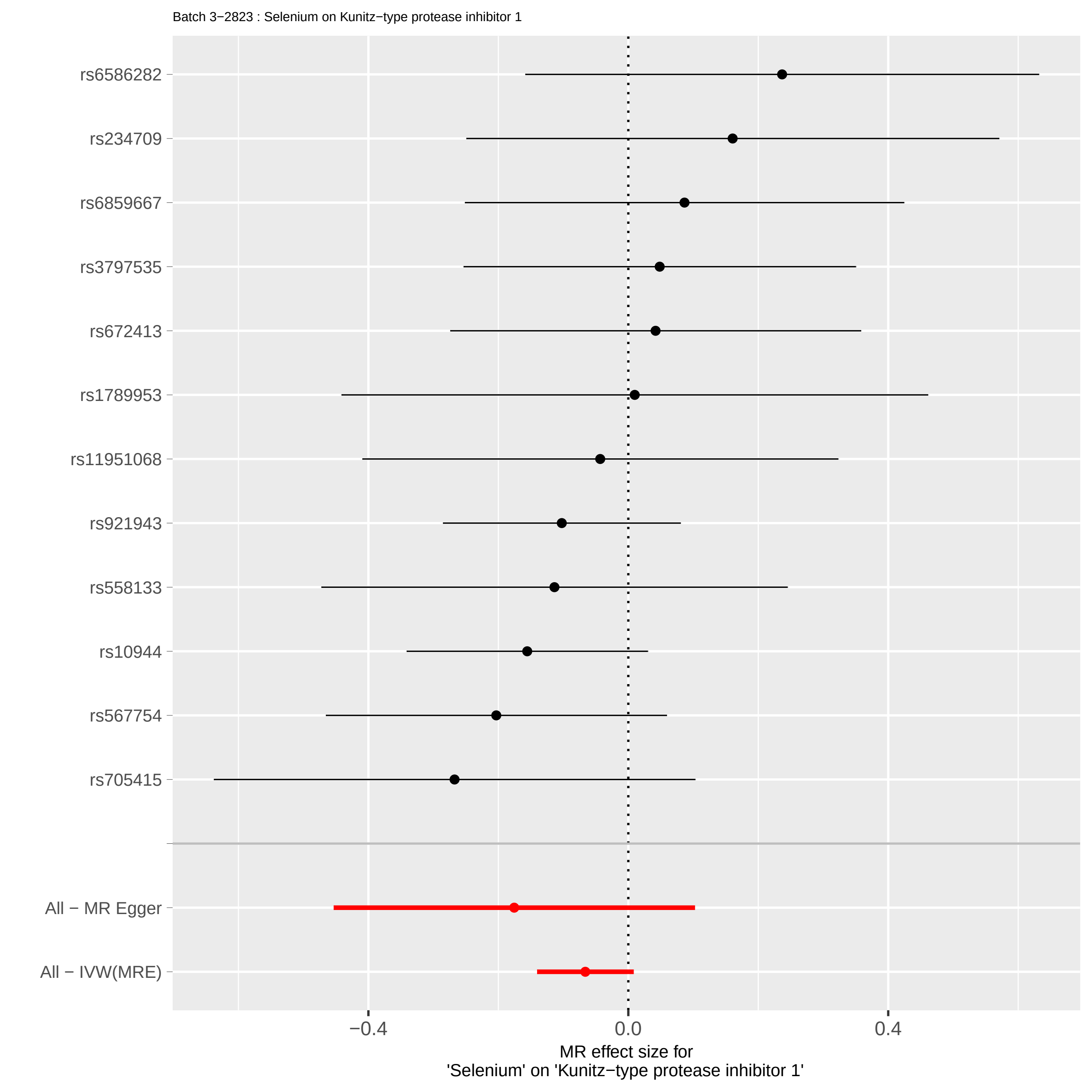
Batch 3−2823 : Selenium on Kunitz−type protease inhibitor 1
rs6586282
rs234709
rs6859667
rs3797535
rs672413
rs1789953
rs11951068
rs921943
rs558133
rs10944
rs567754
rs705415
All − MR Egger
All − IVW(MRE)
−0.4
0.0
0.4
MR effect size for
'Selenium' on 'Kunitz−type protease inhibitor 1'

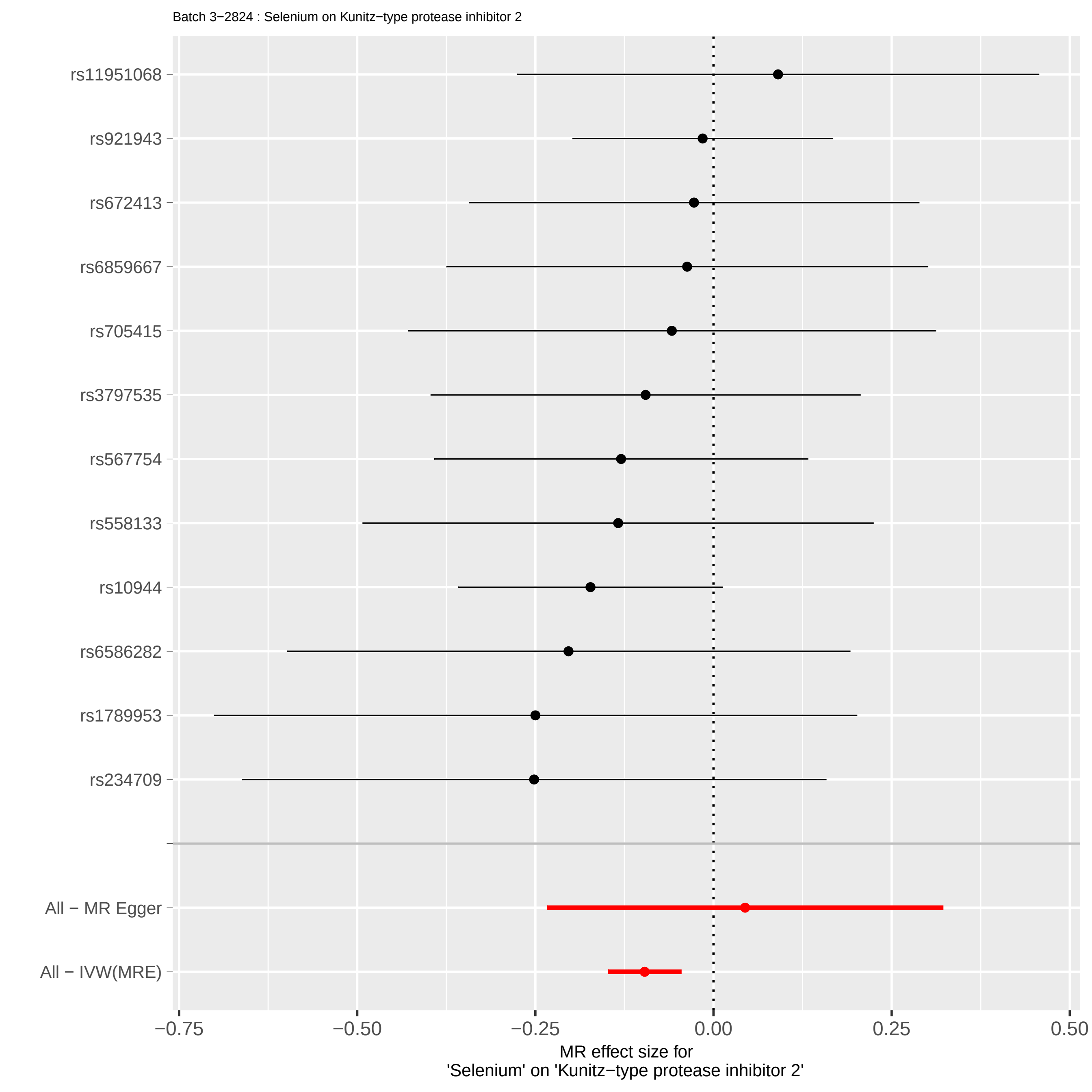
Batch 3−2824 : Selenium on Kunitz−type protease inhibitor 2
rs11951068
rs921943
rs672413
rs6859667
rs705415
rs3797535
rs567754
rs558133
rs10944
rs6586282
rs1789953
rs234709
All − MR Egger
All − IVW(MRE)
−0.75
−0.50
−0.25
0.00
0.25
0.50
MR effect size for
'Selenium' on 'Kunitz−type protease inhibitor 2'

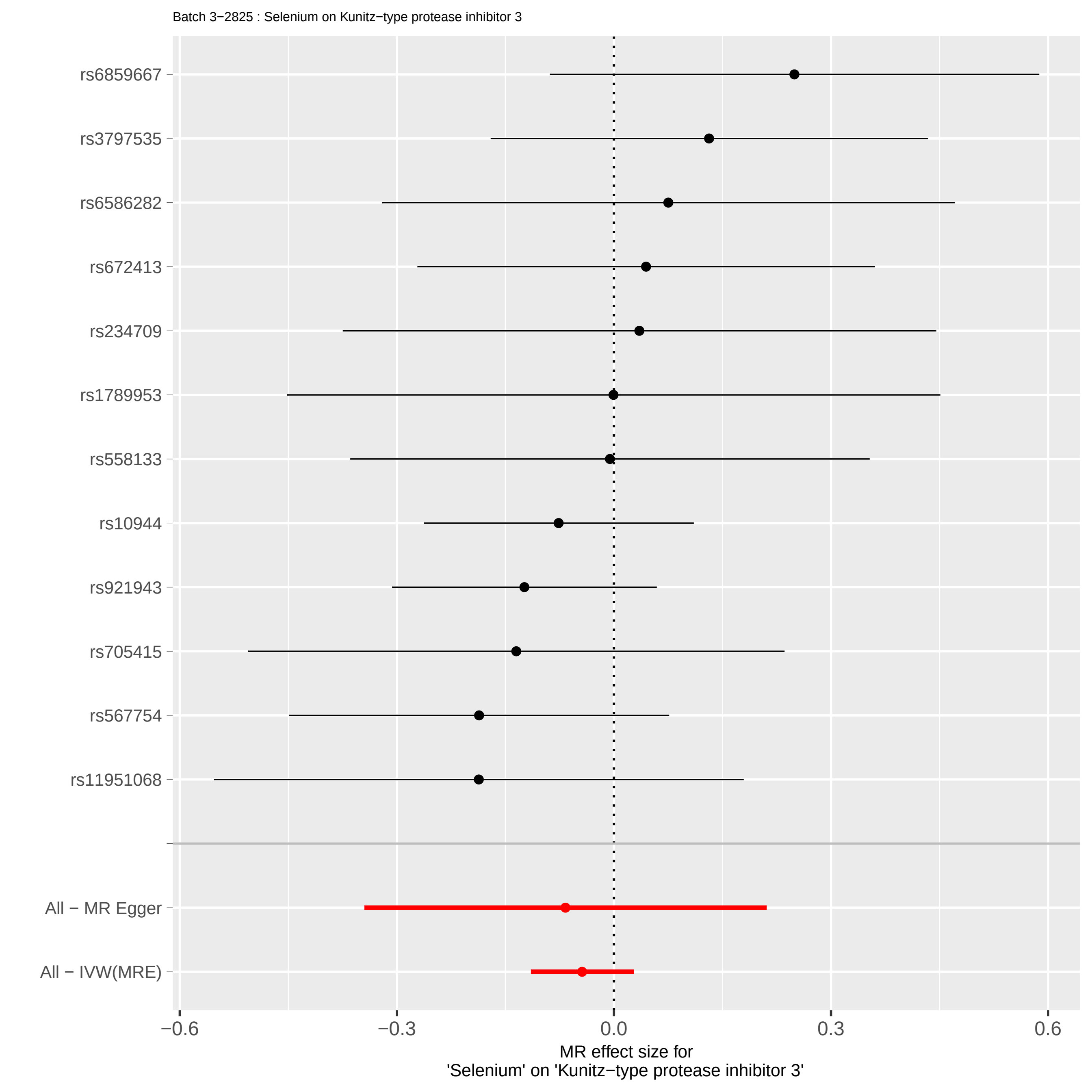
Batch 3−2825 : Selenium on Kunitz−type protease inhibitor 3
rs6859667
rs3797535
rs6586282
rs672413
rs234709
rs1789953
rs558133
rs10944
rs921943
rs705415
rs567754
rs11951068
All − MR Egger
All − IVW(MRE)
−0.6
−0.3
0.0
0.3
0.6
MR effect size for
'Selenium' on 'Kunitz−type protease inhibitor 3'

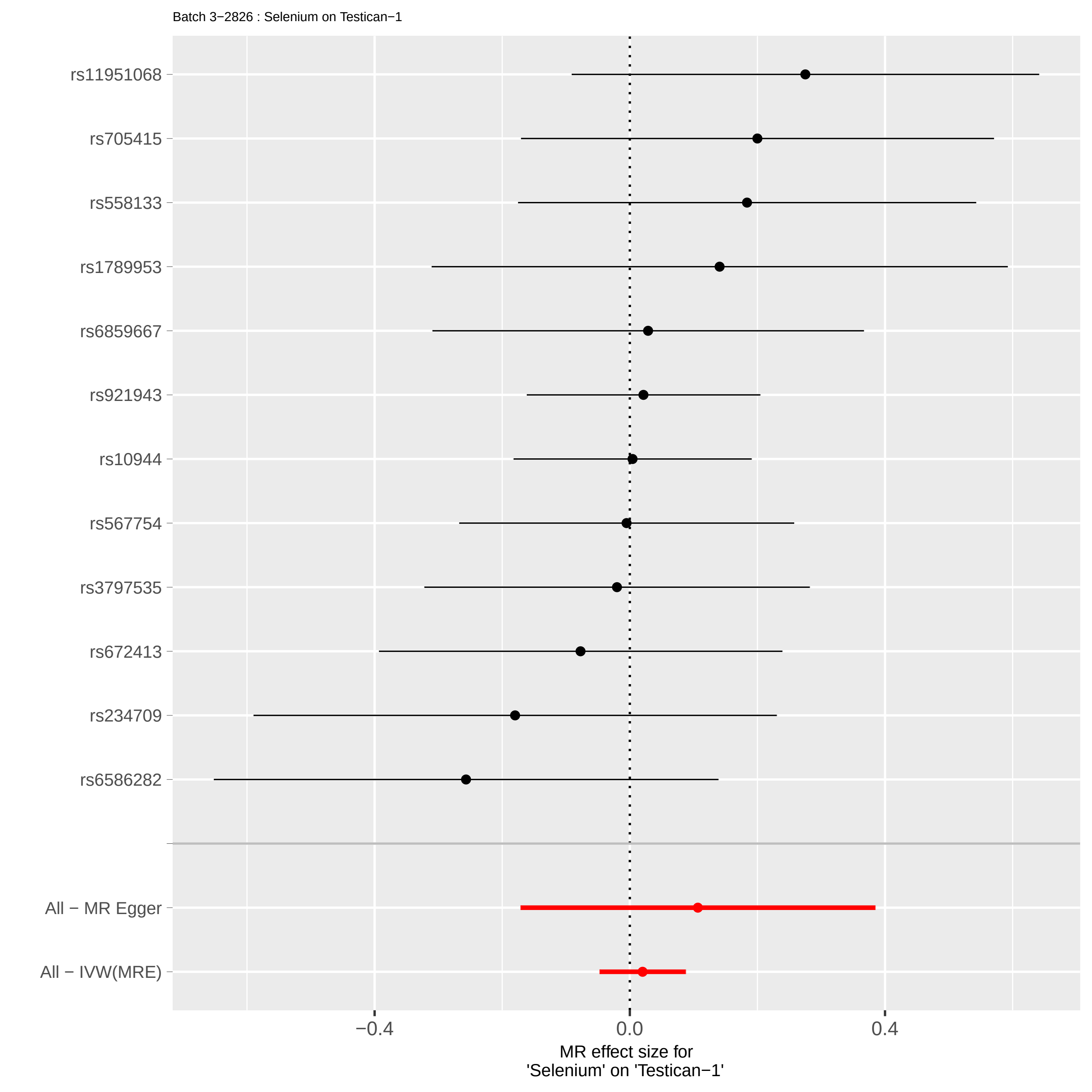

Batch 3−2826 : Selenium on Testican−1
rs11951068
rs705415
rs558133
rs1789953
rs6859667
rs921943
rs10944
rs567754
rs3797535
rs672413
rs234709
rs6586282
All − MR Egger
All − IVW(MRE)
−0.4
0.0
0.4
MR effect size for
'Selenium' on 'Testican−1'

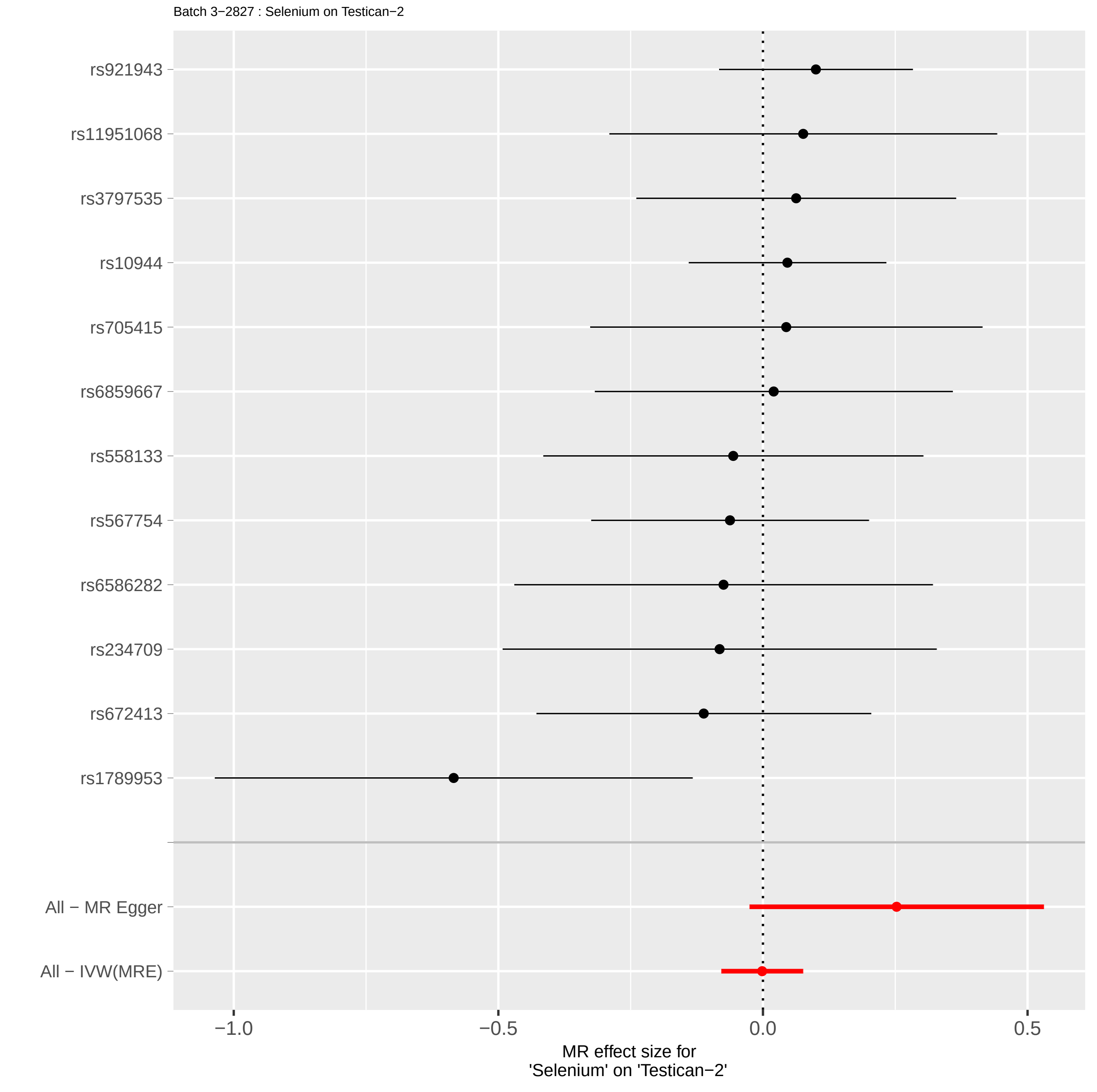

Batch 3−2827 : Selenium on Testican−2
rs921943
rs11951068
rs3797535
rs10944
rs705415
rs6859667
rs558133
rs567754
rs6586282
rs234709
rs672413
rs1789953
All − MR Egger
All − IVW(MRE)
−1.0
−0.5
0.0
0.5
MR effect size for
'Selenium' on 'Testican−2'

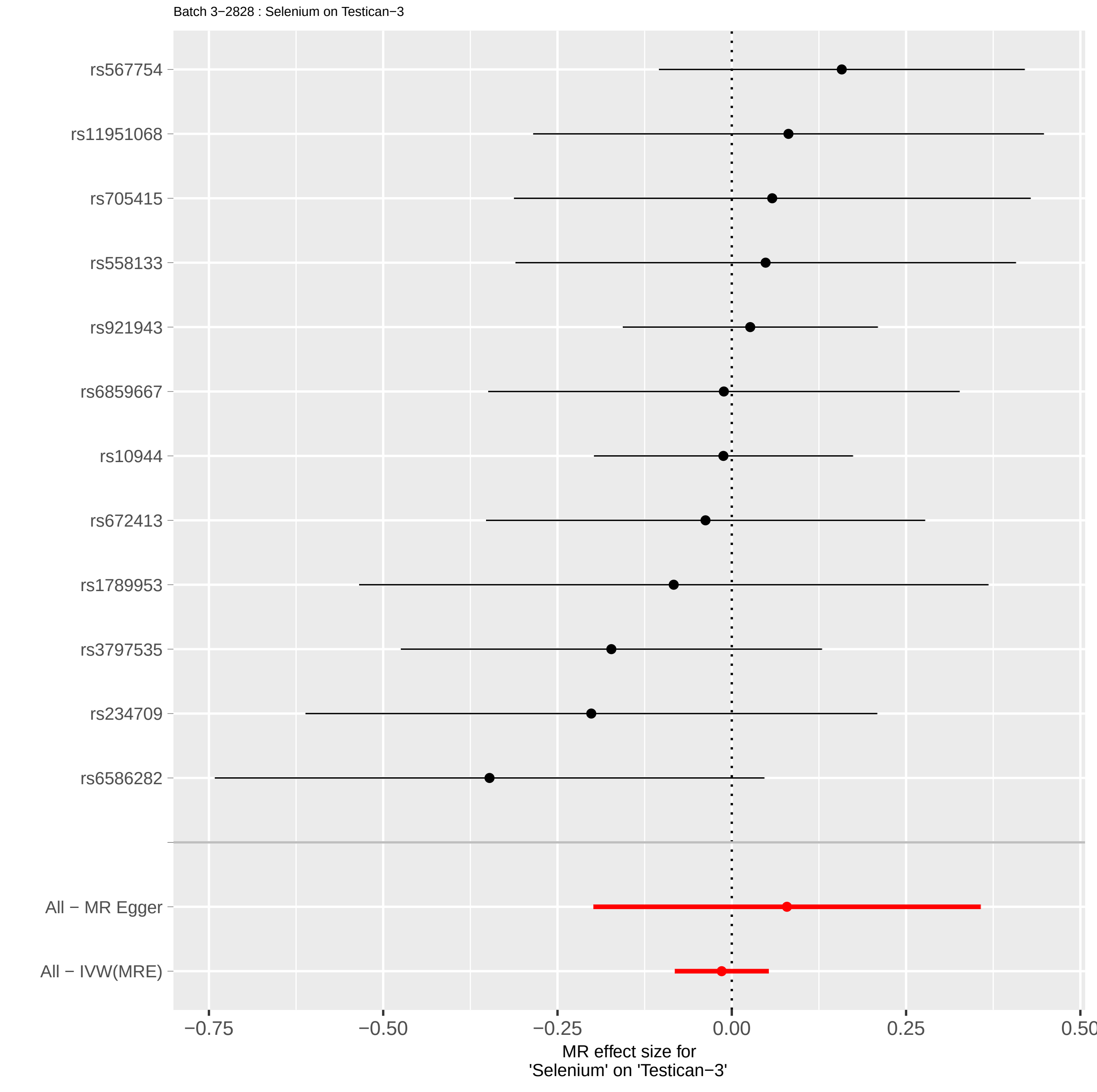

Batch 3−2828 : Selenium on Testican−3
rs567754
rs11951068
rs705415
rs558133
rs921943
rs6859667
rs10944
rs672413
rs1789953
rs3797535
rs234709
rs6586282
All − MR Egger
All − IVW(MRE)
−0.75
−0.50
−0.25
0.00
0.25
0.50
MR effect size for
'Selenium' on 'Testican−3'

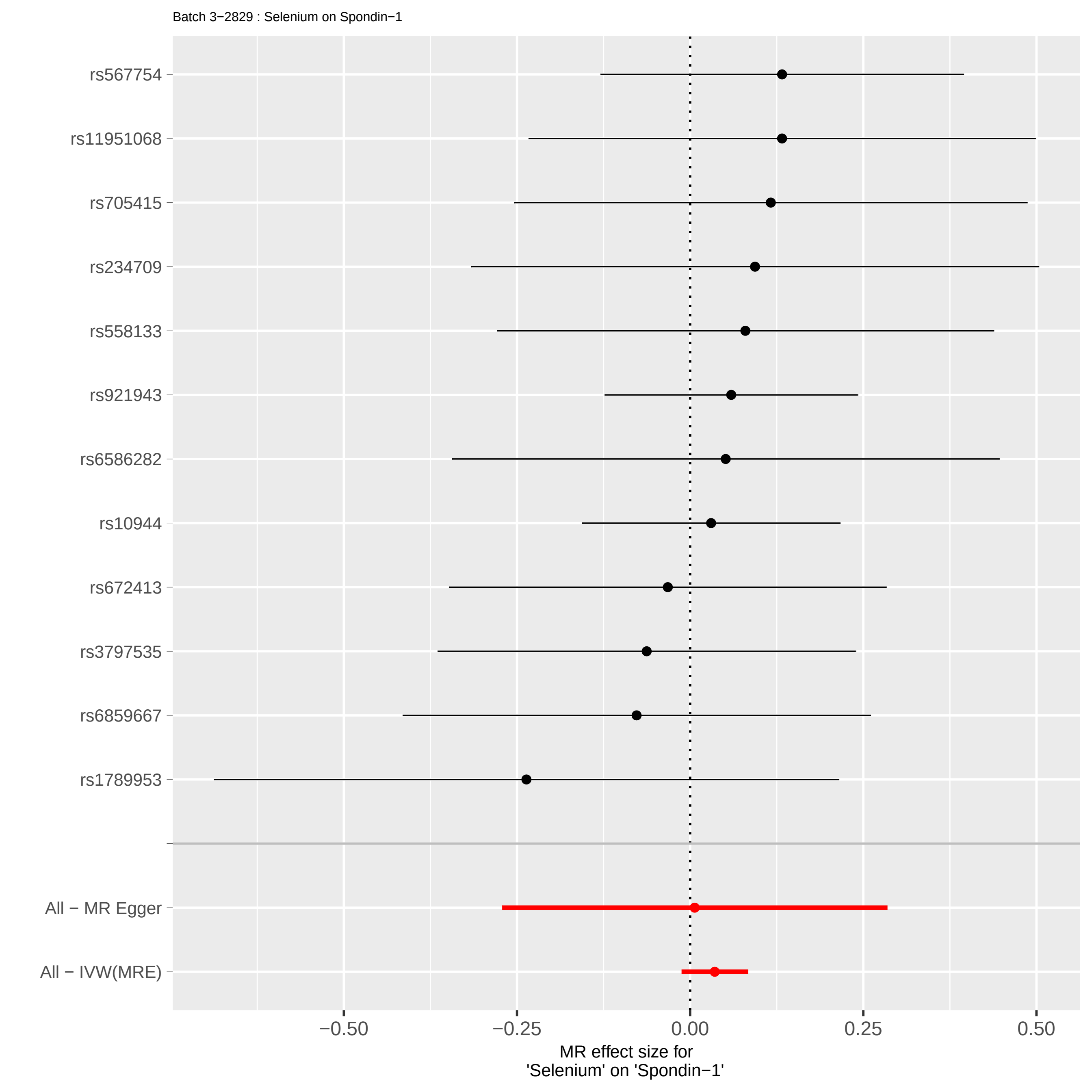
Batch 3−2829 : Selenium on Spondin−1
rs567754
rs11951068
rs705415
rs234709
rs558133
rs921943
rs6586282
rs10944
rs672413
rs3797535
rs6859667
rs1789953
All − MR Egger
All − IVW(MRE)
−0.50
−0.25
0.00
0.25
0.50
MR effect size for
'Selenium' on 'Spondin−1'

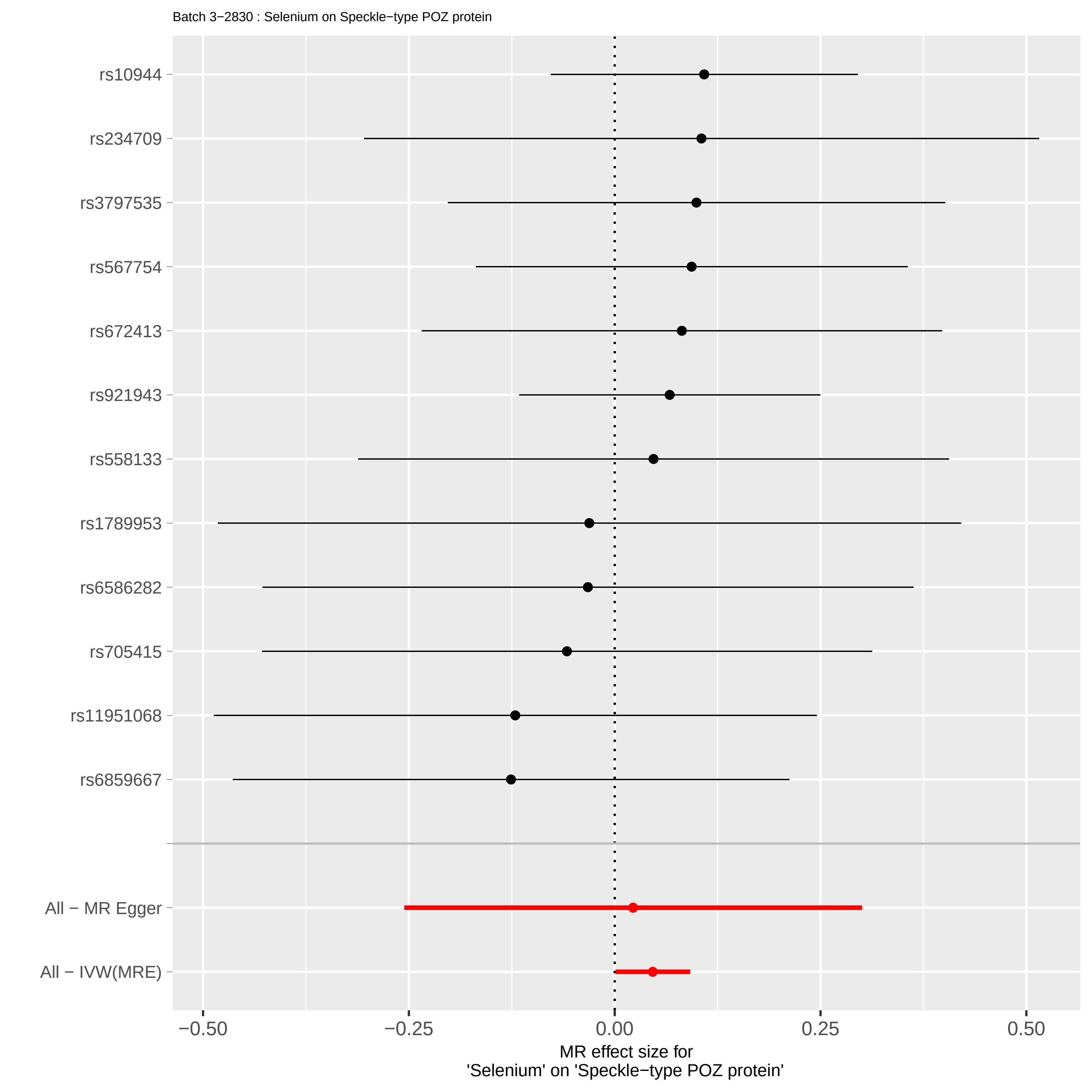

Batch 3−2830 : Selenium on Speckle−type POZ protein
rs10944
rs234709
rs3797535
rs567754
rs672413
rs921943
rs558133
rs1789953
rs6586282
rs705415
rs11951068
rs6859667
All − MR Egger
All − IVW(MRE)
−0.50
−0.25
0.00
0.25
0.50
MR effect size for
'Selenium' on 'Speckle−type POZ protein'

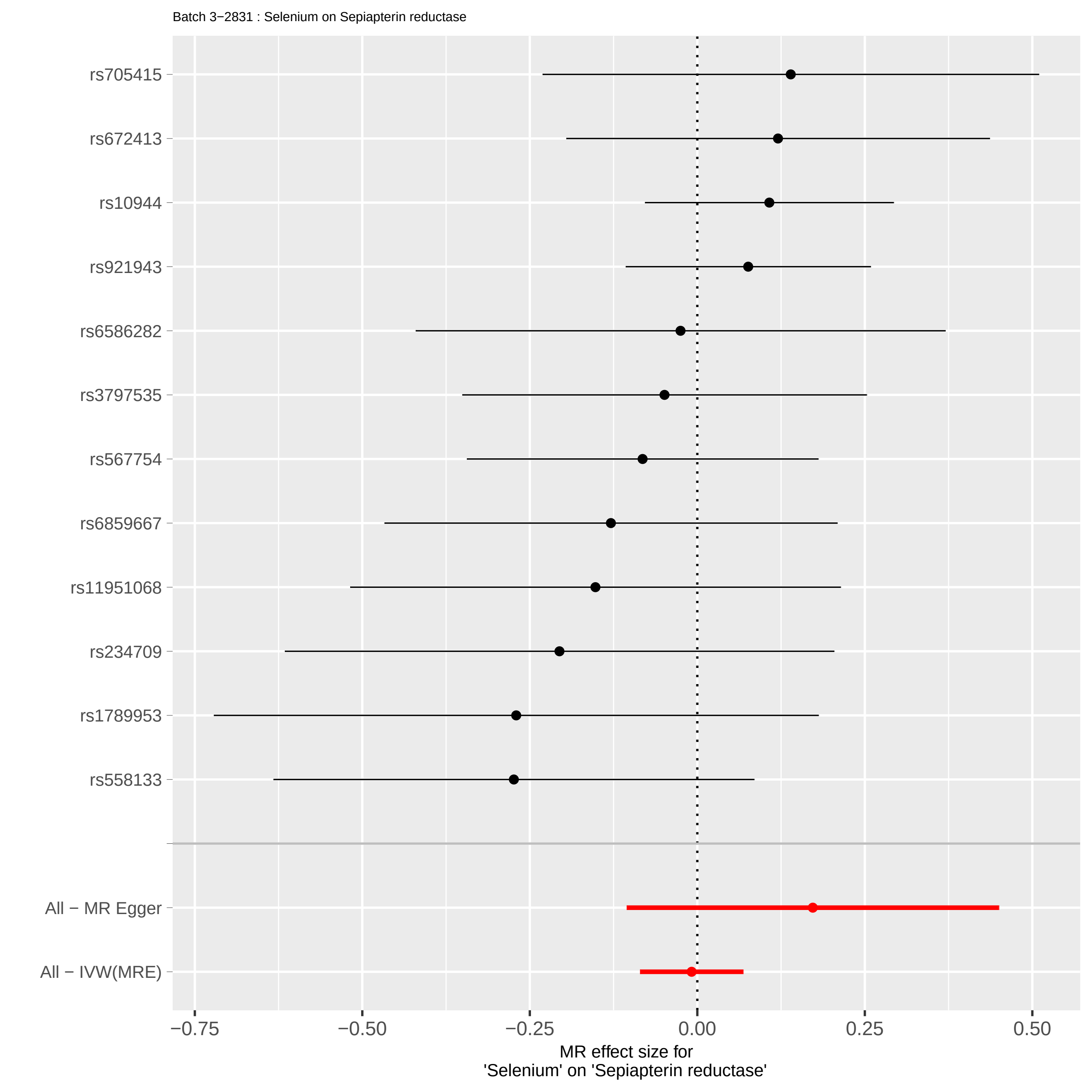

Batch 3−2831 : Selenium on Sepiapterin reductase
rs705415
rs672413
rs10944
rs921943
rs6586282
rs3797535
rs567754
rs6859667
rs11951068
rs234709
rs1789953
rs558133
All − MR Egger
All − IVW(MRE)
−0.75
−0.50
−0.25
0.00
0.25
0.50
MR effect size for
'Selenium' on 'Sepiapterin reductase'

Batch 3−2832 : Selenium on Sprouty−related, EVH1 domain−containing protein 1

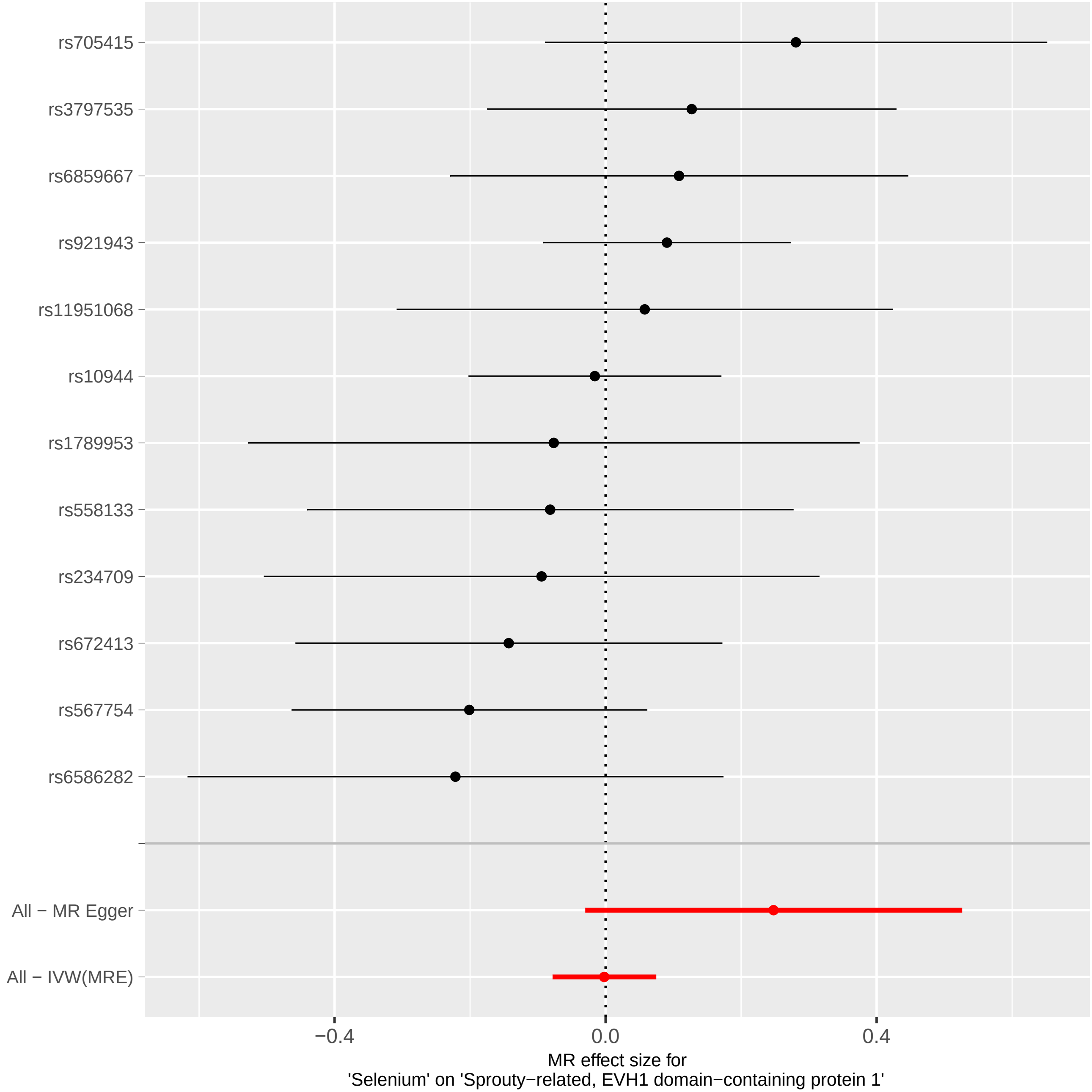

Batch 3−2833 : Selenium on Protein sprouty homolog 1

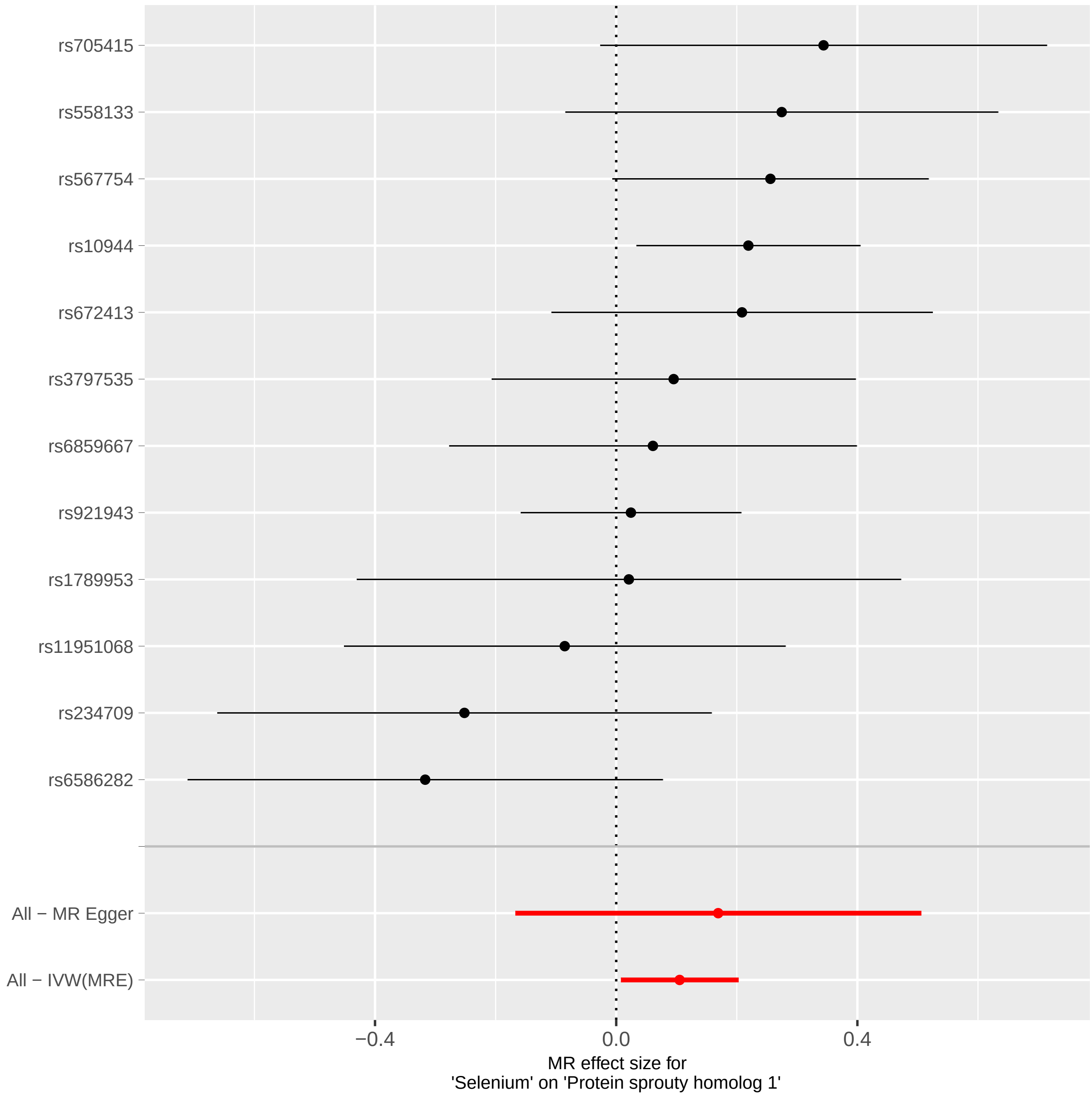

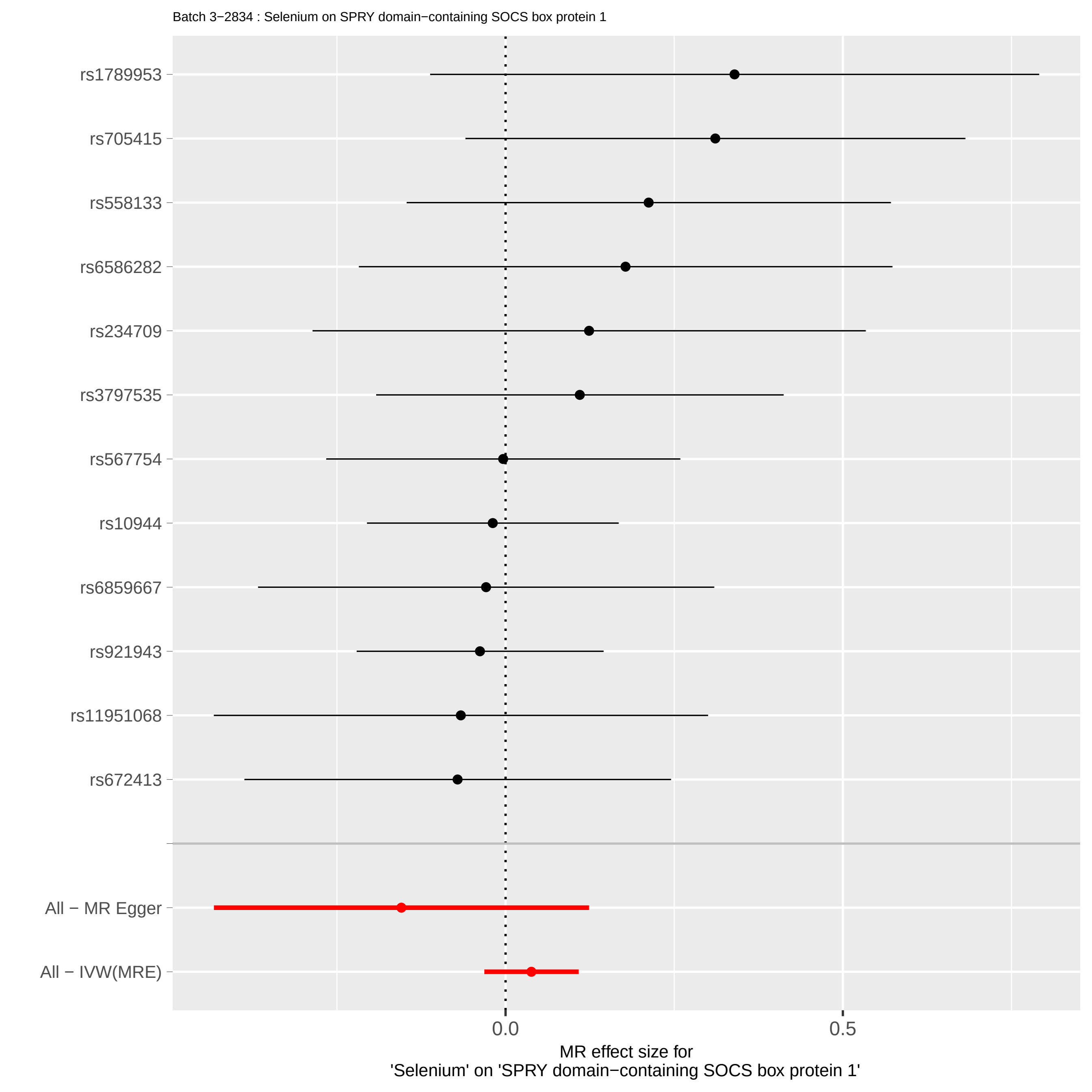

Batch 3−2834 : Selenium on SPRY domain−containing SOCS box protein 1
rs1789953
rs705415
rs558133
rs6586282
rs234709
rs3797535
rs567754
rs10944
rs6859667
rs921943
rs11951068
rs672413
All − MR Egger
All − IVW(MRE)
0.0
0.5
MR effect size for
'Selenium' on 'SPRY domain−containing SOCS box protein 1'

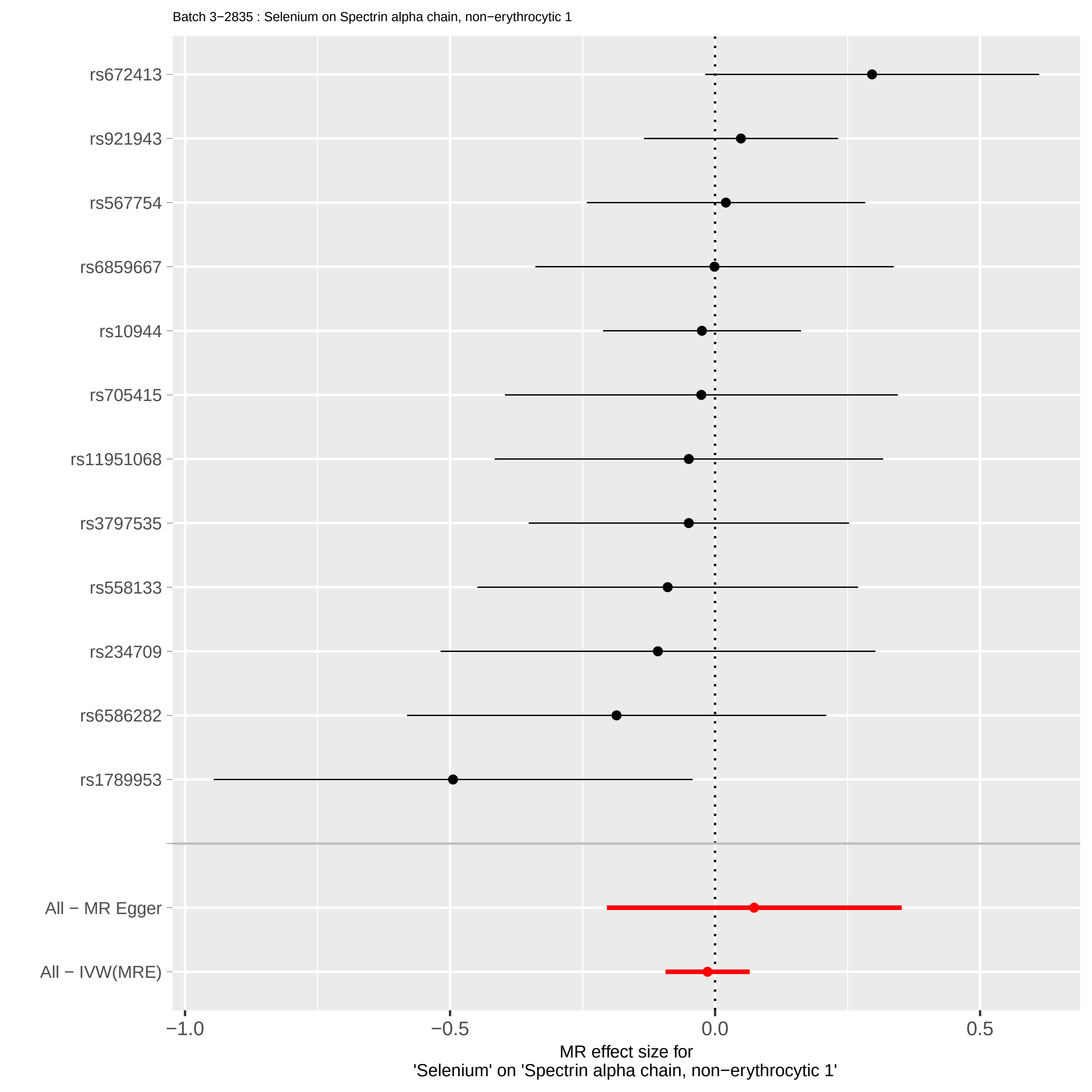

Batch 3−2835 : Selenium on Spectrin alpha chain, non−erythrocytic 1
rs672413
rs921943
rs567754
rs6859667
rs10944
rs705415
rs11951068
rs3797535
rs558133
rs234709
rs6586282
rs1789953
All − MR Egger
All − IVW(MRE)
−1.0
−0.5
0.0
0.5
MR effect size for
'Selenium' on 'Spectrin alpha chain, non−erythrocytic 1'

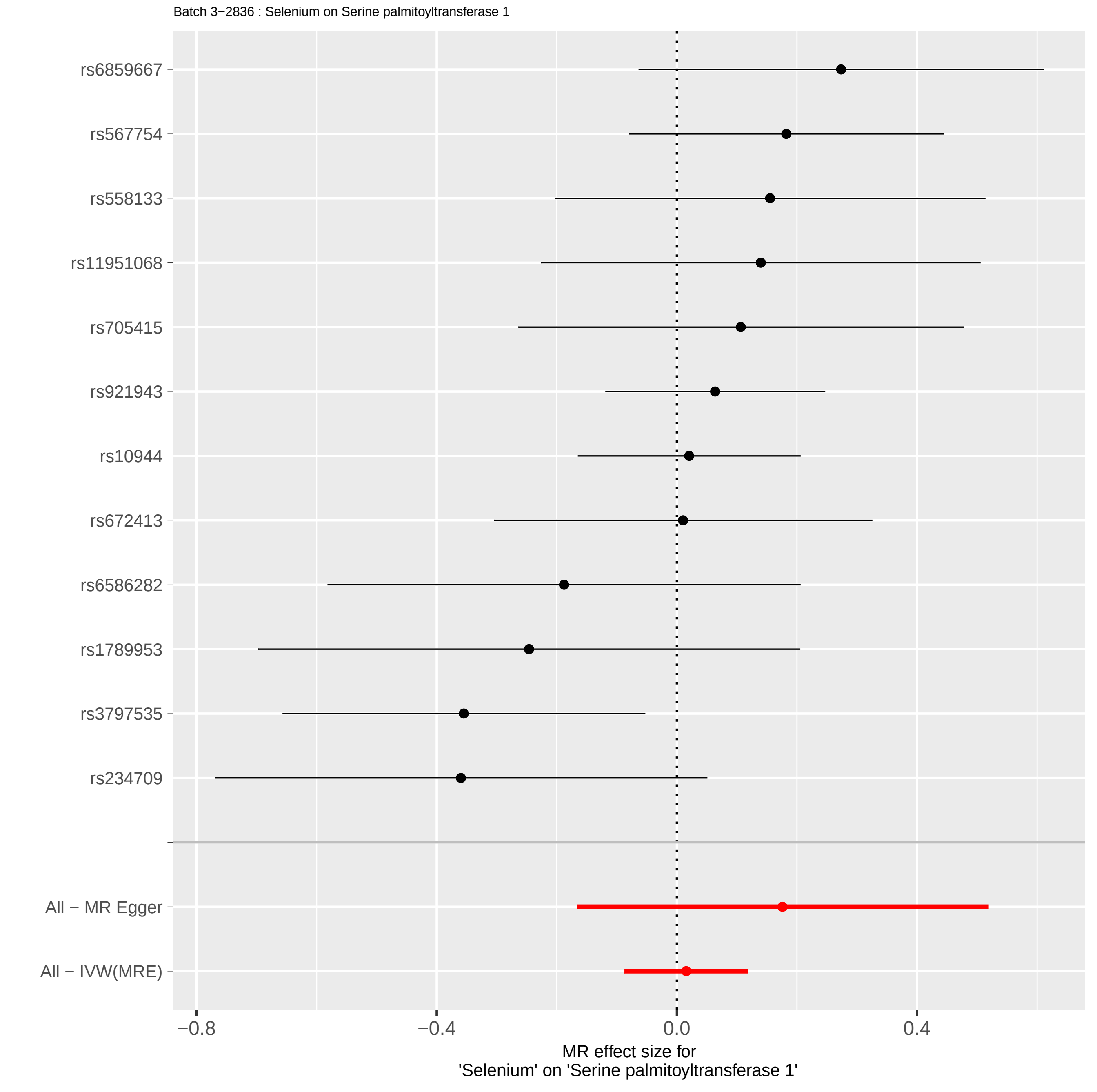

Batch 3−2836 : Selenium on Serine palmitoyltransferase 1
rs6859667
rs567754
rs558133
rs11951068
rs705415
rs921943
rs10944
rs672413
rs6586282
rs1789953
rs3797535
rs234709
All − MR Egger
All − IVW(MRE)
−0.8
−0.4
0.0
0.4
MR effect size for
'Selenium' on 'Serine palmitoyltransferase 1'

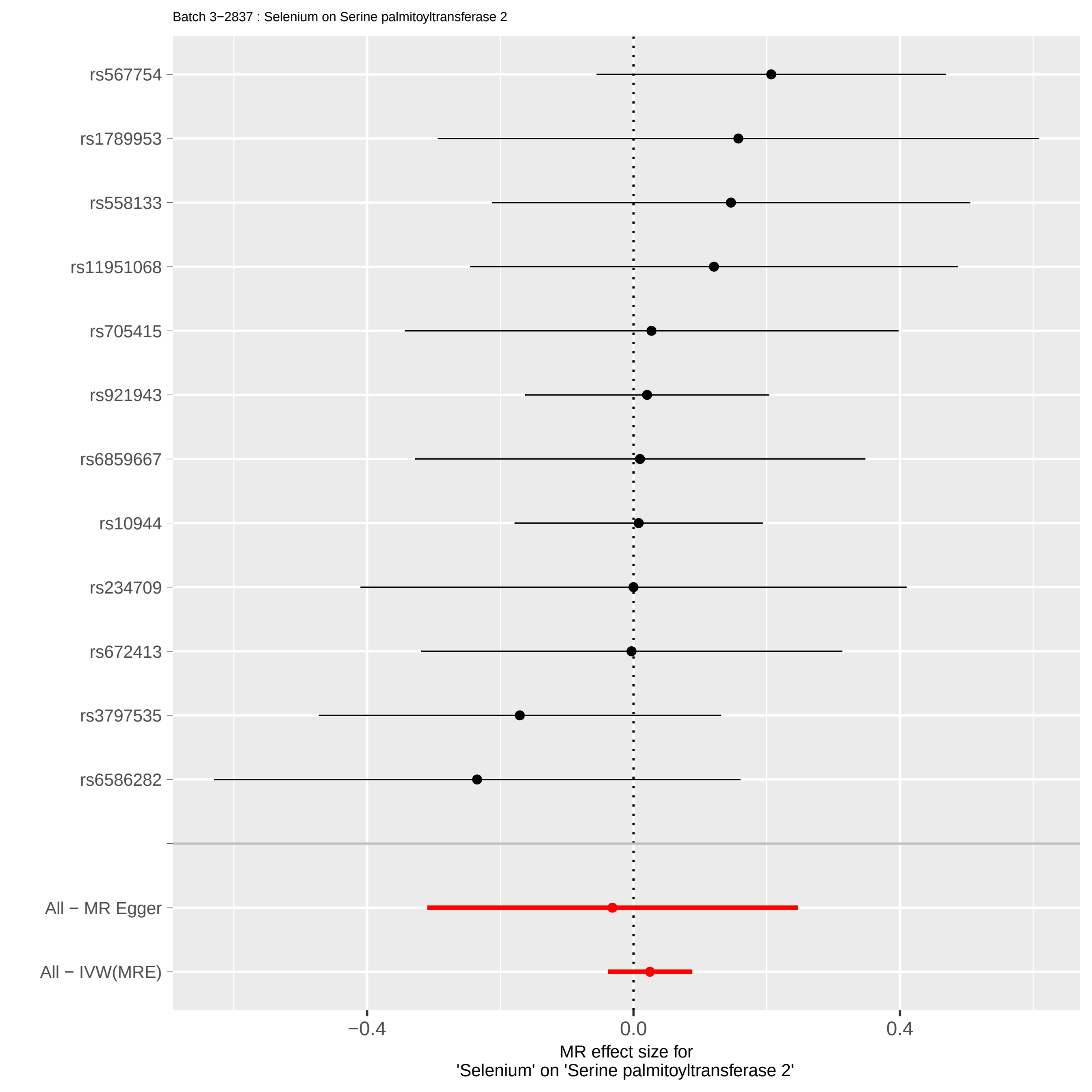
Batch 3−2837 : Selenium on Serine palmitoyltransferase 2
rs567754
rs1789953
rs558133
rs11951068
rs705415
rs921943
rs6859667
rs10944
rs234709
rs672413
rs3797535
rs6586282
All − MR Egger
All − IVW(MRE)
−0.4
0.0
0.4
MR effect size for
'Selenium' on 'Serine palmitoyltransferase 2'

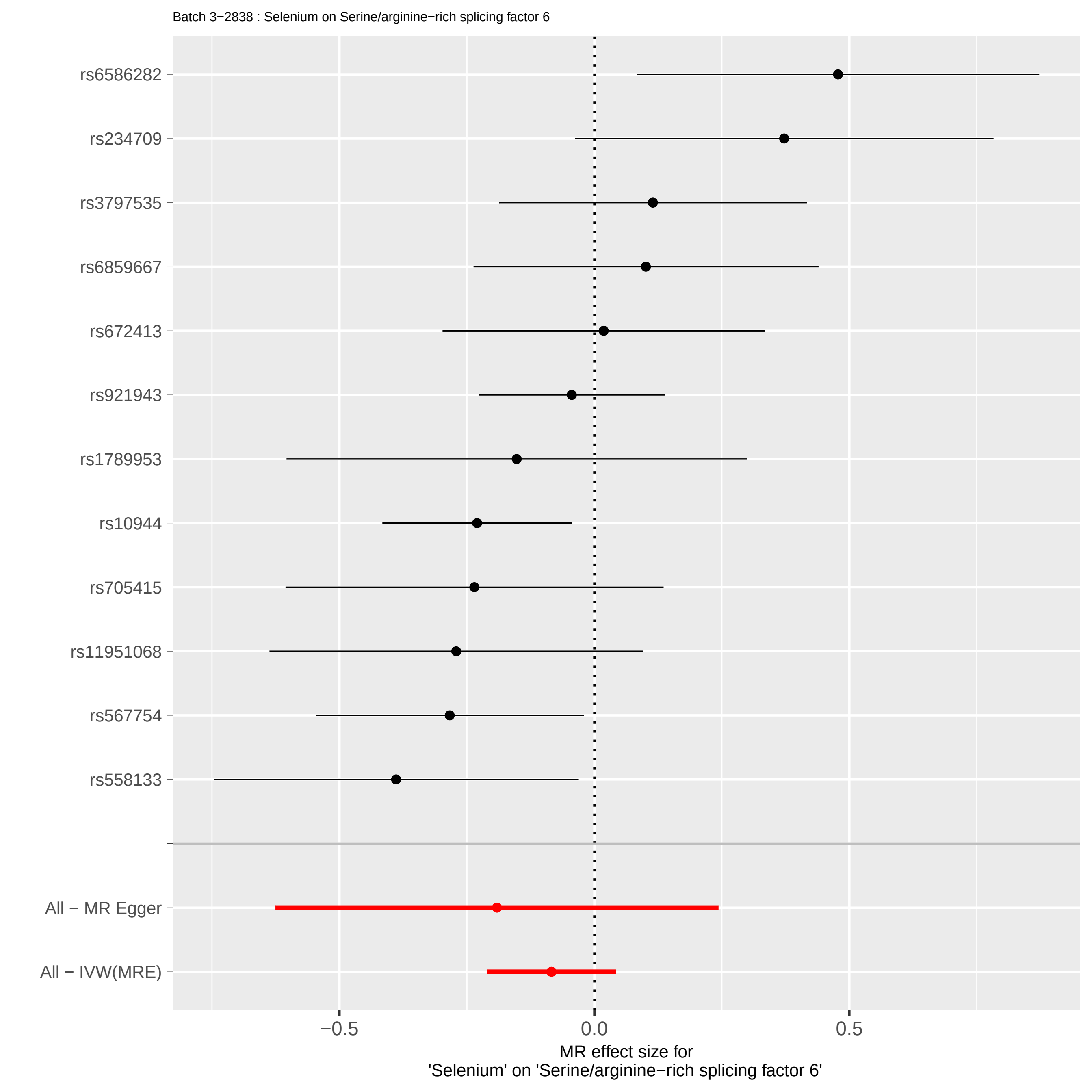
Batch 3−2838 : Selenium on Serine/arginine−rich splicing factor 6
rs6586282
rs234709
rs3797535
rs6859667
rs672413
rs921943
rs1789953
rs10944
rs705415
rs11951068
rs567754
rs558133
All − MR Egger
All − IVW(MRE)
−0.5
0.0
0.5
MR effect size for
'Selenium' on 'Serine/arginine−rich splicing factor 6'

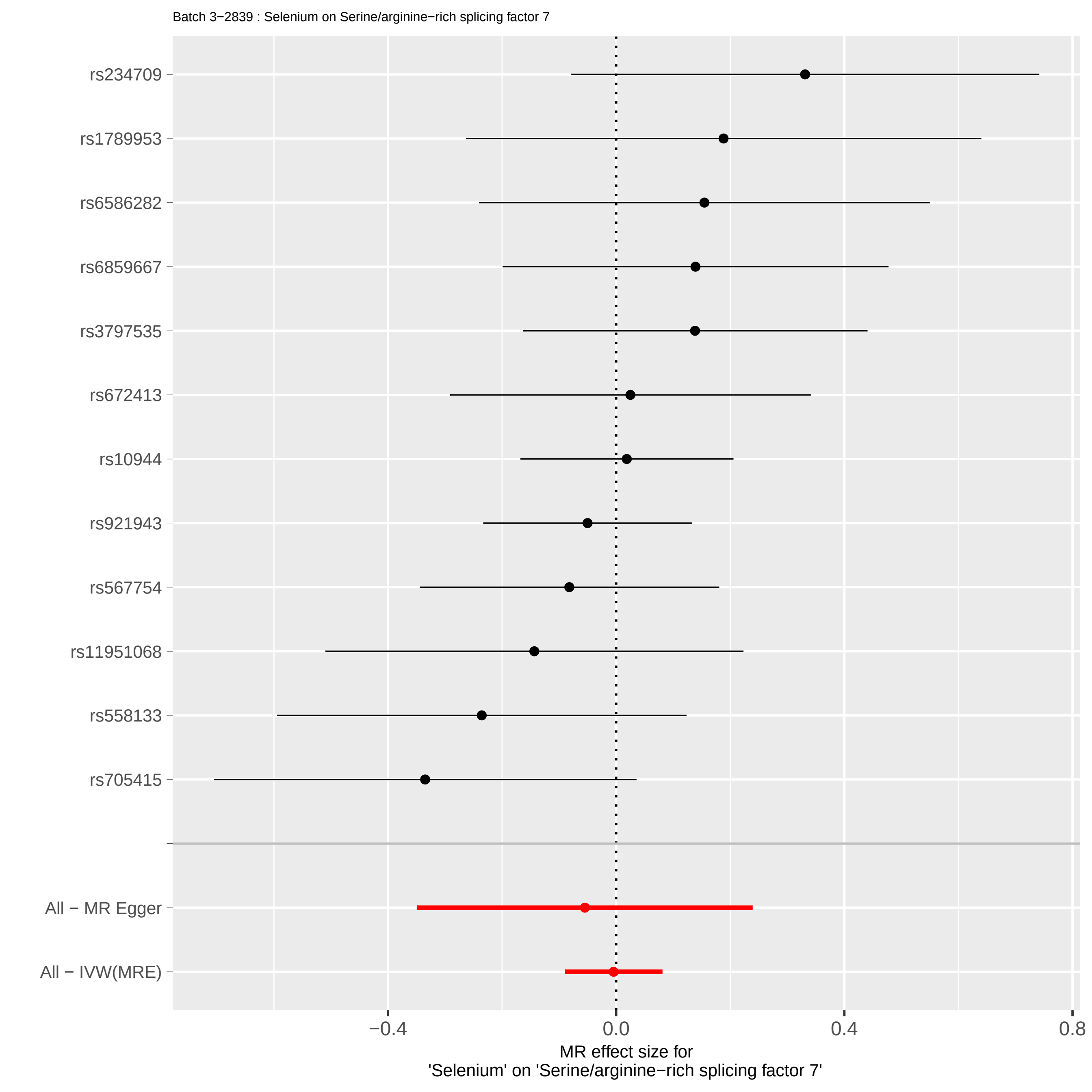

Batch 3−2839 : Selenium on Serine/arginine−rich splicing factor 7
rs234709
rs1789953
rs6586282
rs6859667
rs3797535
rs672413
rs10944
rs921943
rs567754
rs11951068
rs558133
rs705415
All − MR Egger
All − IVW(MRE)
−0.4
0.0
0.4
0.8
MR effect size for
'Selenium' on 'Serine/arginine−rich splicing factor 7'

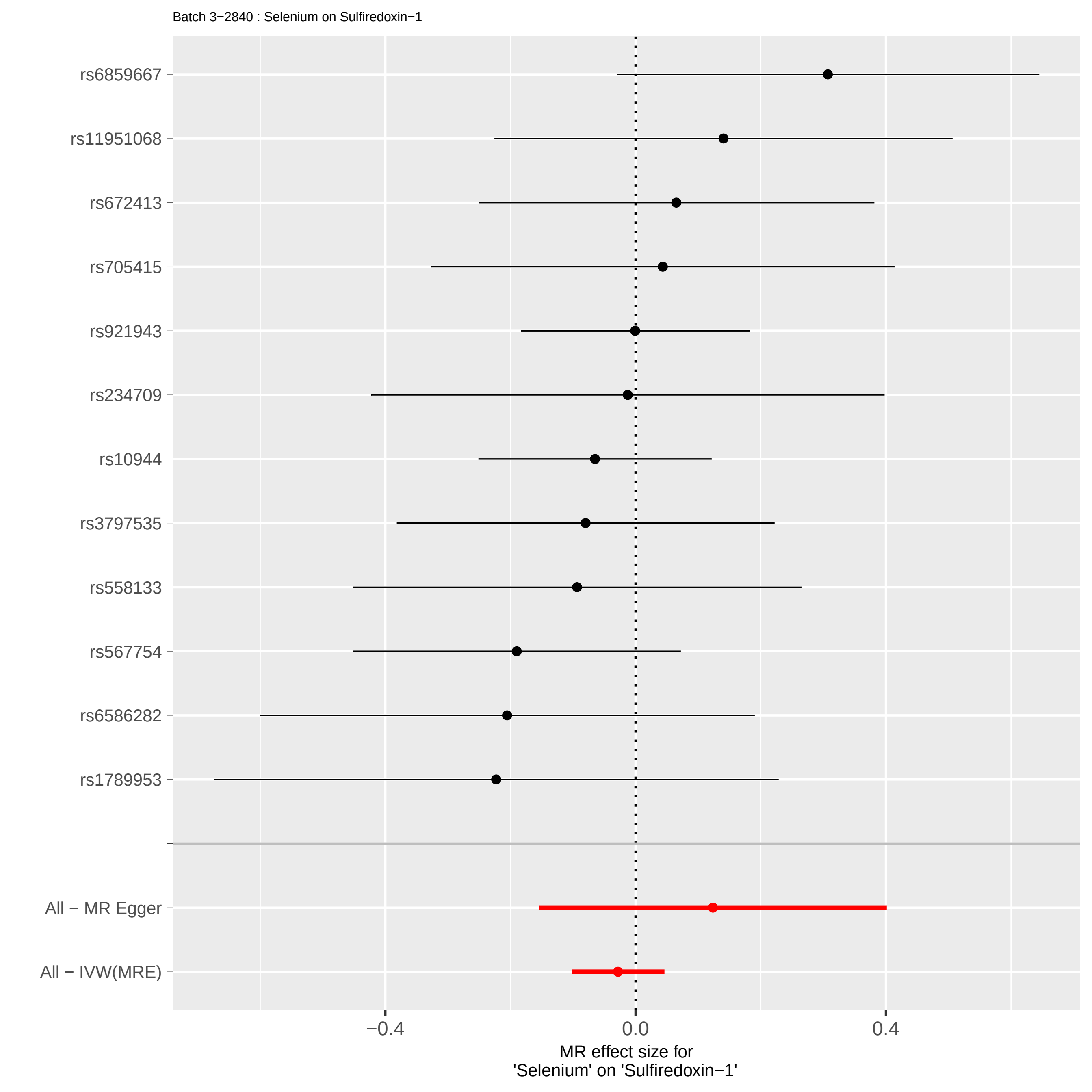
Batch 3−2840 : Selenium on Sulfiredoxin−1
rs6859667
rs11951068
rs672413
rs705415
rs921943
rs234709
rs10944
rs3797535
rs558133
rs567754
rs6586282
rs1789953
All − MR Egger
All − IVW(MRE)
−0.4
0.0
0.4
MR effect size for
'Selenium' on 'Sulfiredoxin−1'

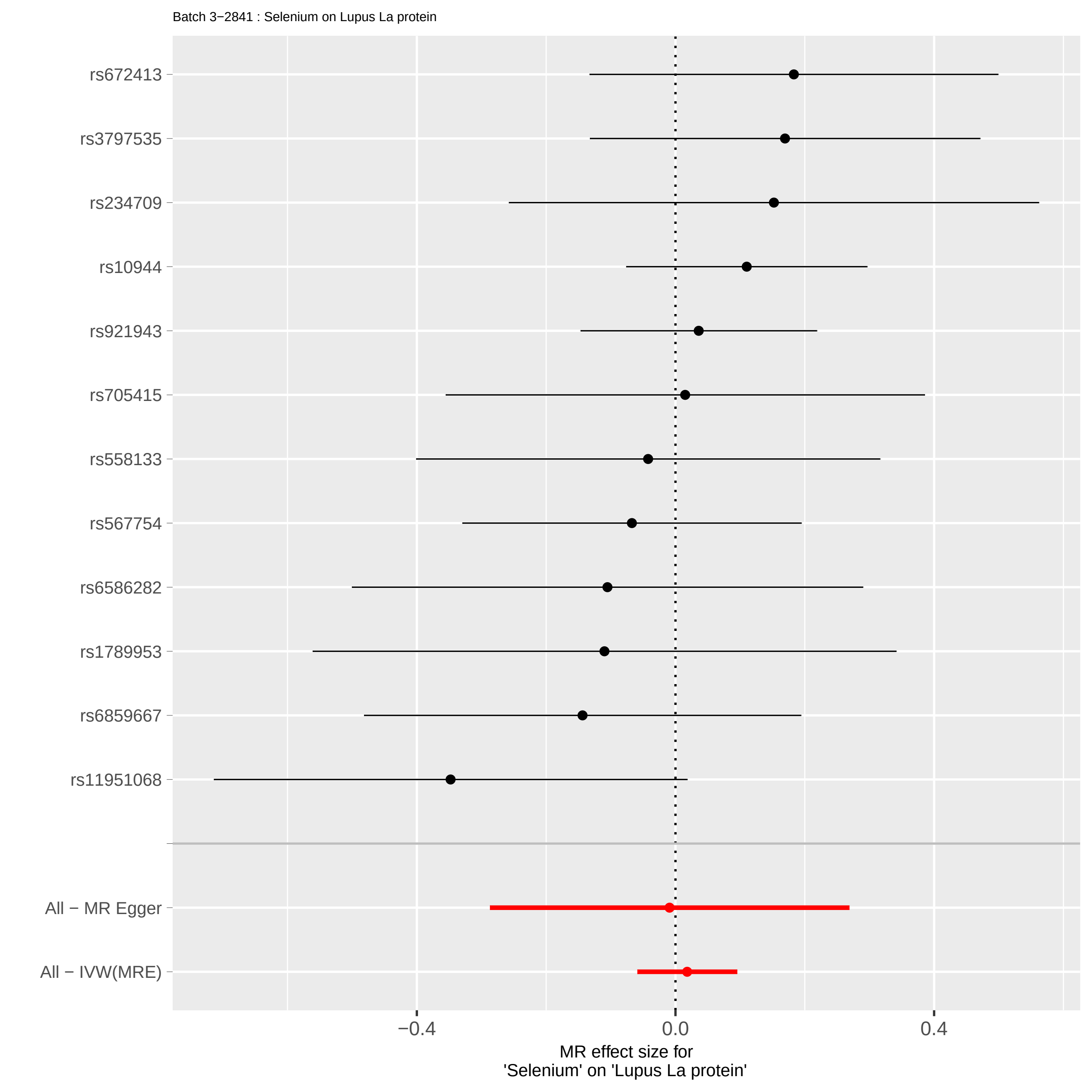

Batch 3−2841 : Selenium on Lupus La protein
rs672413
rs3797535
rs234709
rs10944
rs921943
rs705415
rs558133
rs567754
rs6586282
rs1789953
rs6859667
rs11951068
All − MR Egger
All − IVW(MRE)
−0.4
0.0
0.4
MR effect size for
'Selenium' on 'Lupus La protein'

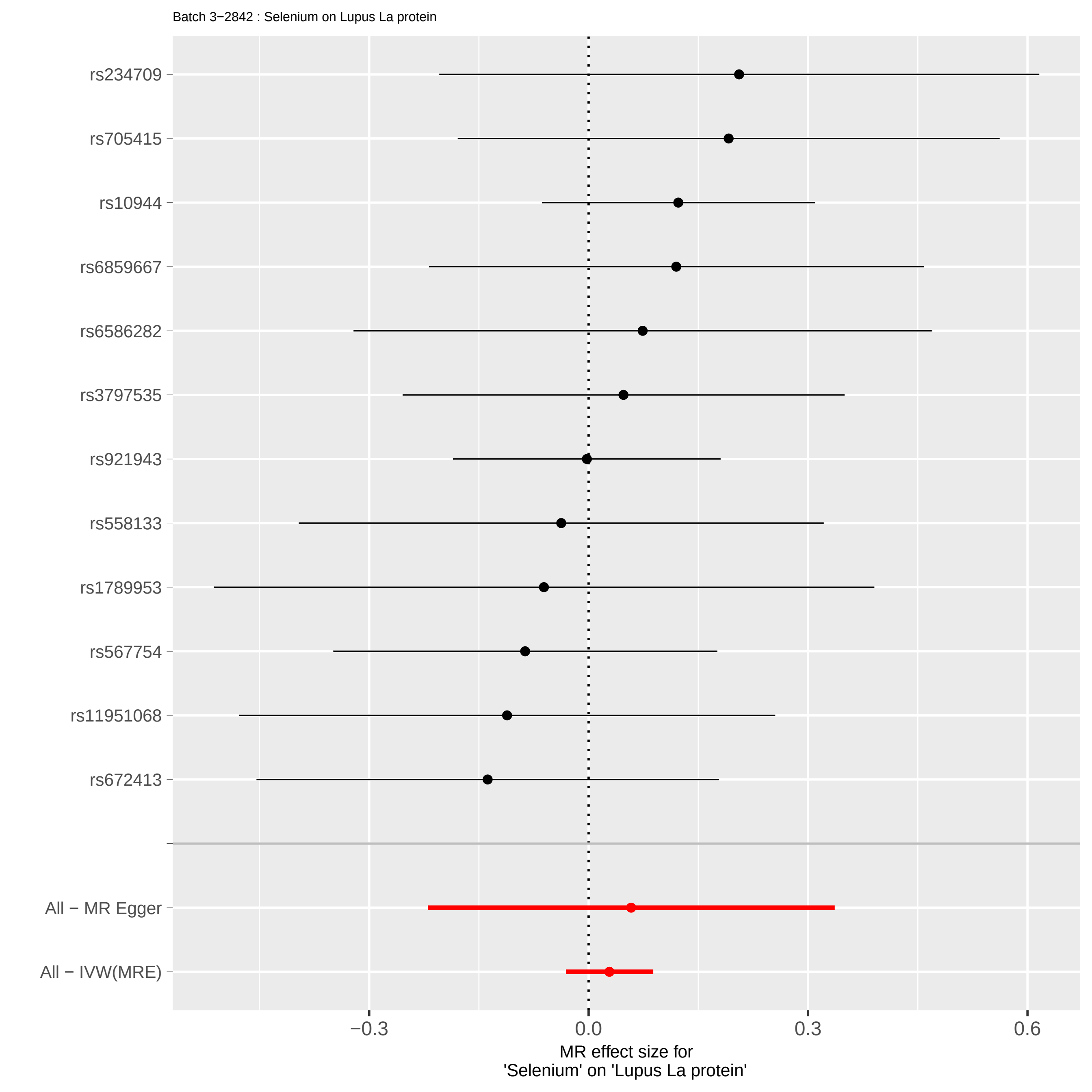
Batch 3−2842 : Selenium on Lupus La protein
rs234709
rs705415
rs10944
rs6859667
rs6586282
rs3797535
rs921943
rs558133
rs1789953
rs567754
rs11951068
rs672413
All − MR Egger
All − IVW(MRE)
−0.3
0.0
0.3
0.6
MR effect size for
'Selenium' on 'Lupus La protein'

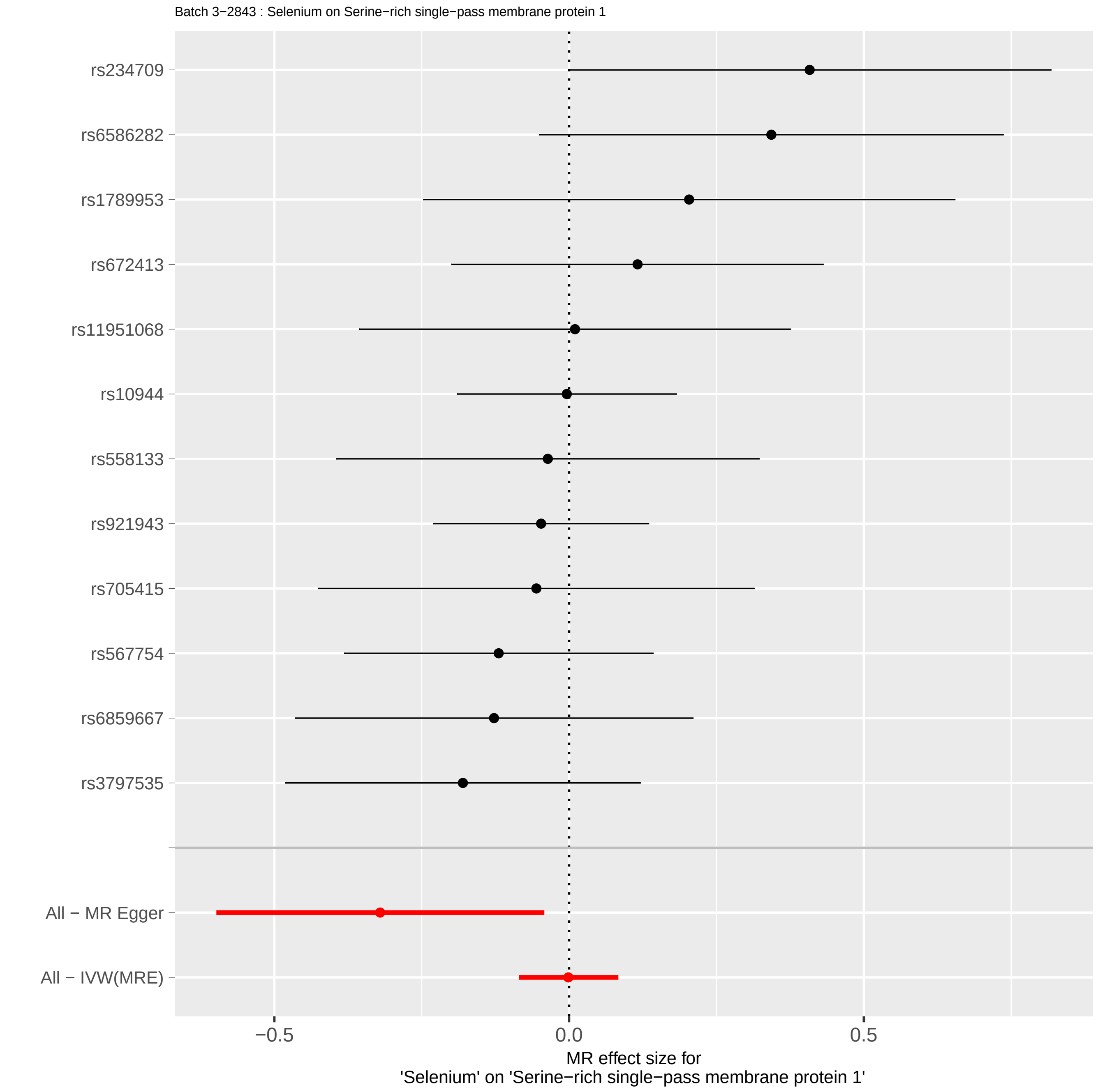

Batch 3−2843 : Selenium on Serine−rich single−pass membrane protein 1
rs234709
rs6586282
rs1789953
rs672413
rs11951068
rs10944
rs558133
rs921943
rs705415
rs567754
rs6859667
rs3797535
All − MR Egger
All − IVW(MRE)
−0.5
0.0
0.5
MR effect size for
'Selenium' on 'Serine−rich single−pass membrane protein 1'

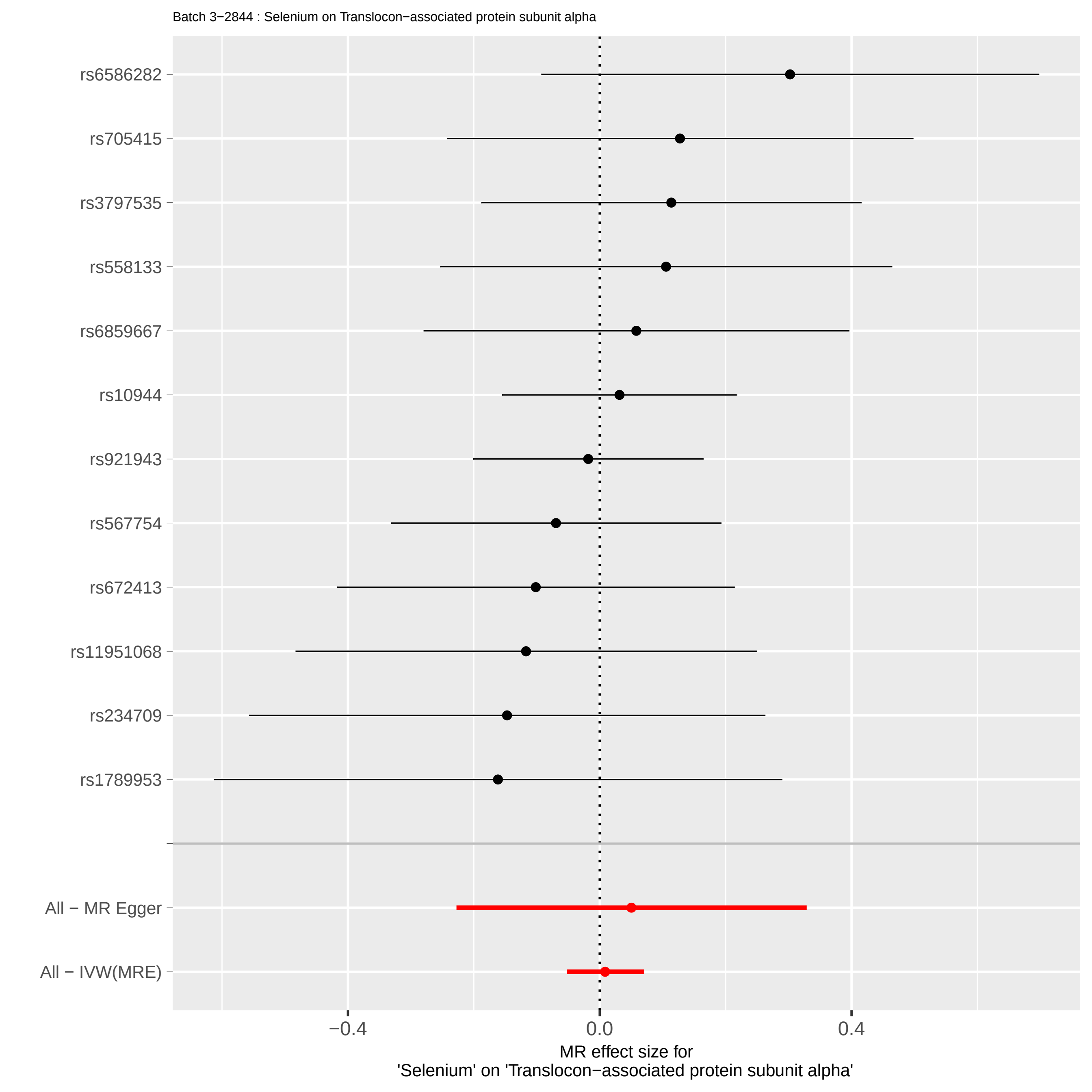
Batch 3−2844 : Selenium on Translocon−associated protein subunit alpha
rs6586282
rs705415
rs3797535
rs558133
rs6859667
rs10944
rs921943
rs567754
rs672413
rs11951068
rs234709
rs1789953
All − MR Egger
All − IVW(MRE)
−0.4
0.0
0.4
MR effect size for
'Selenium' on 'Translocon−associated protein subunit alpha'

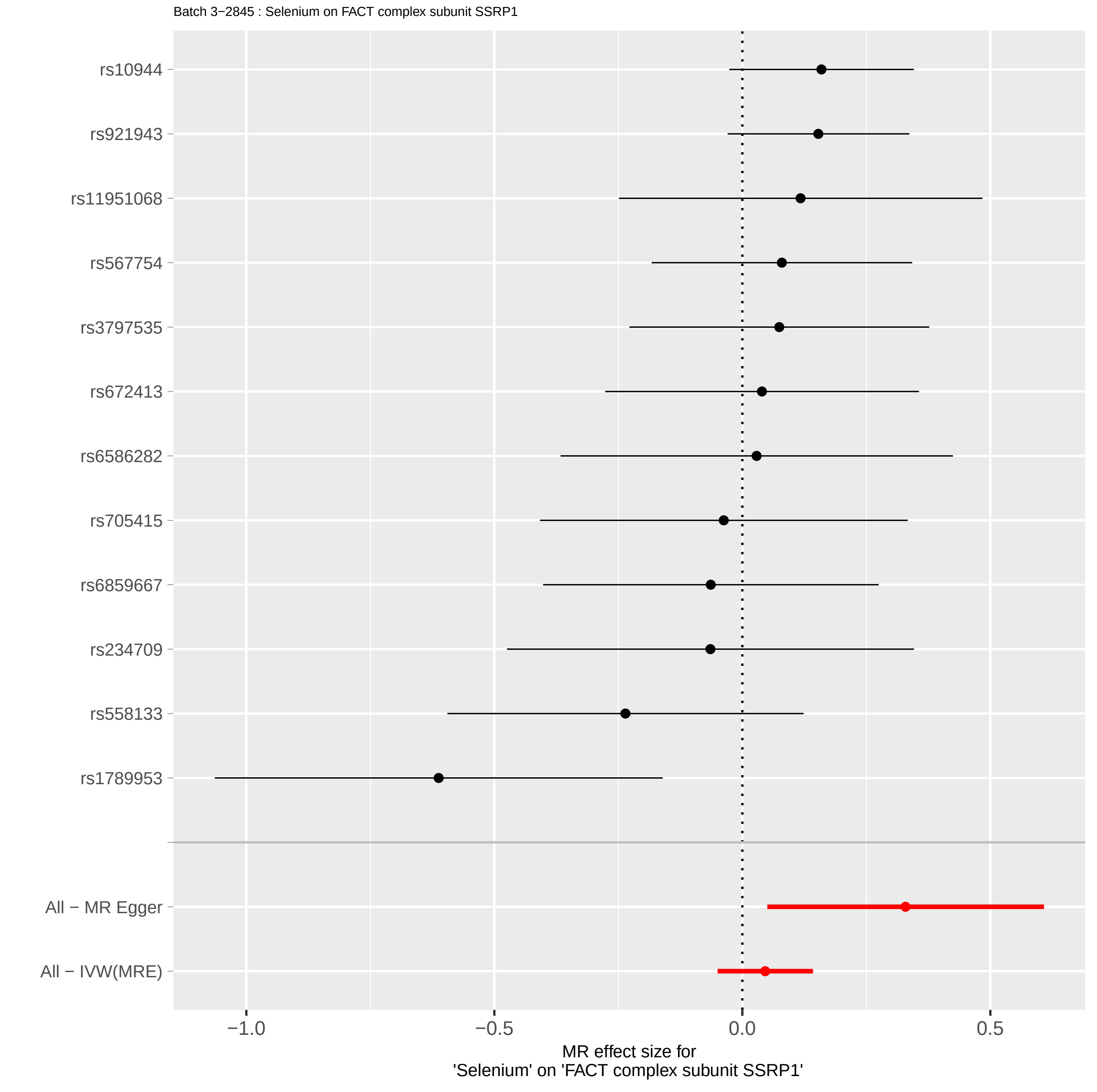

Batch 3−2845 : Selenium on FACT complex subunit SSRP1
rs10944
rs921943
rs11951068
rs567754
rs3797535
rs672413
rs6586282
rs705415
rs6859667
rs234709
rs558133
rs1789953
All − MR Egger
All − IVW(MRE)
−1.0
−0.5
0.0
0.5
MR effect size for
'Selenium' on 'FACT complex subunit SSRP1'

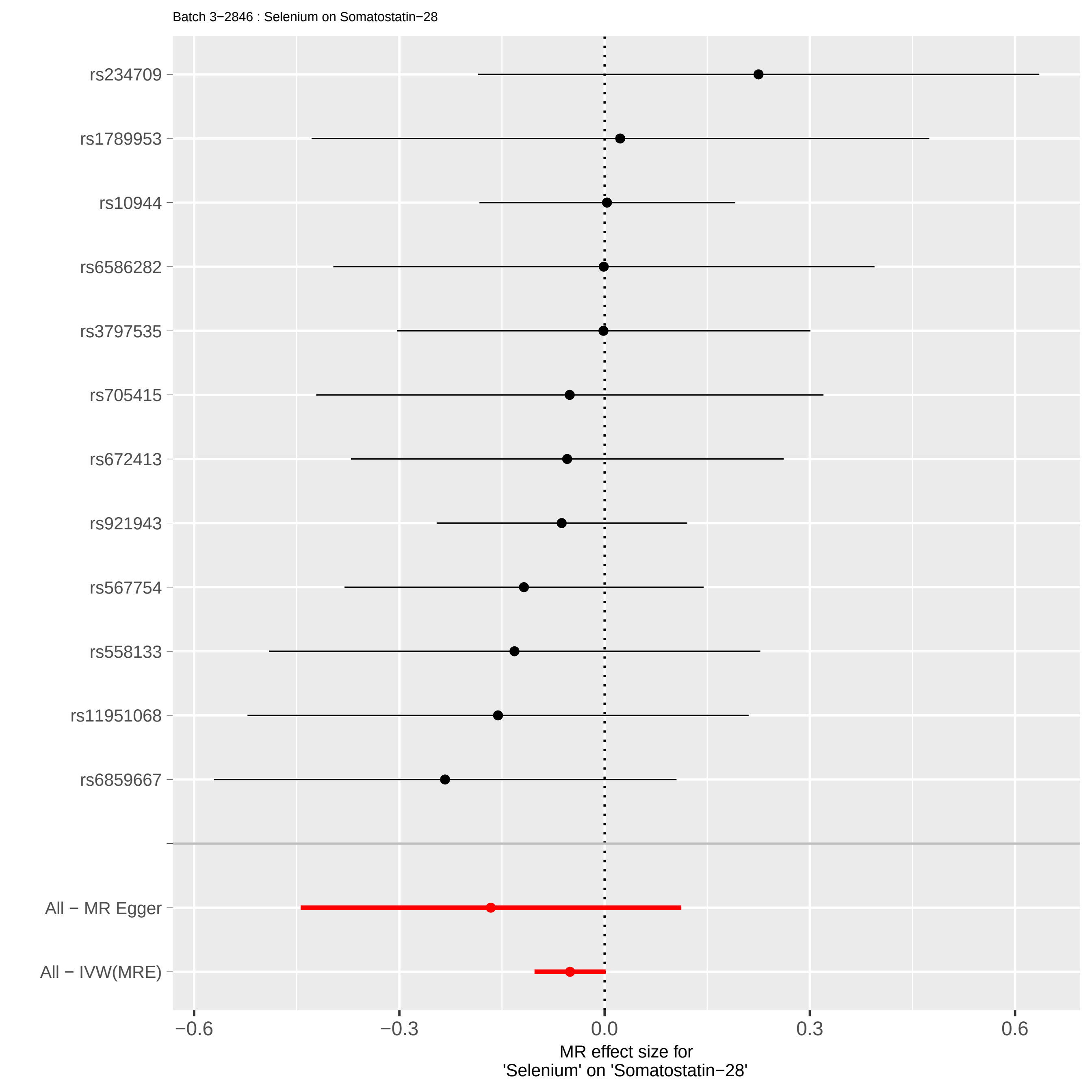

Batch 3−2846 : Selenium on Somatostatin−28
rs234709
rs1789953
rs10944
rs6586282
rs3797535
rs705415
rs672413
rs921943
rs567754
rs558133
rs11951068
rs6859667
All − MR Egger
All − IVW(MRE)
−0.6
−0.3
0.0
0.3
0.6
MR effect size for
'Selenium' on 'Somatostatin−28'

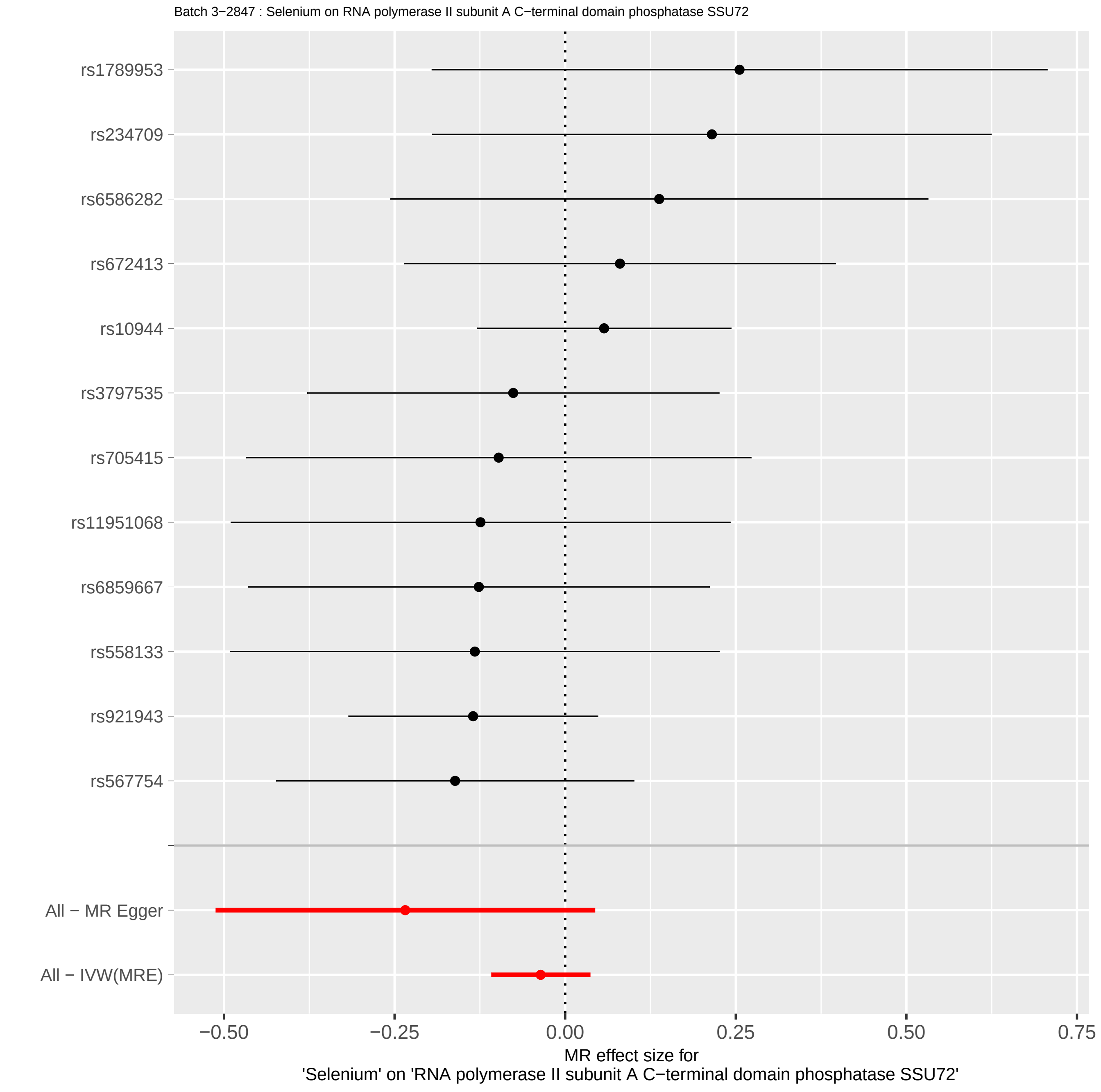
Batch 3−2847 : Selenium on RNA polymerase II subunit A C−terminal domain phosphatase SSU72
rs1789953
rs234709
rs6586282
rs672413
rs10944
rs3797535
rs705415
rs11951068
rs6859667
rs558133
rs921943
rs567754
All − MR Egger
All − IVW(MRE)
−0.50
−0.25
0.00
0.25
0.50
0.75
MR effect size for
'Selenium' on 'RNA polymerase II subunit A C−terminal domain phosphatase SSU72'

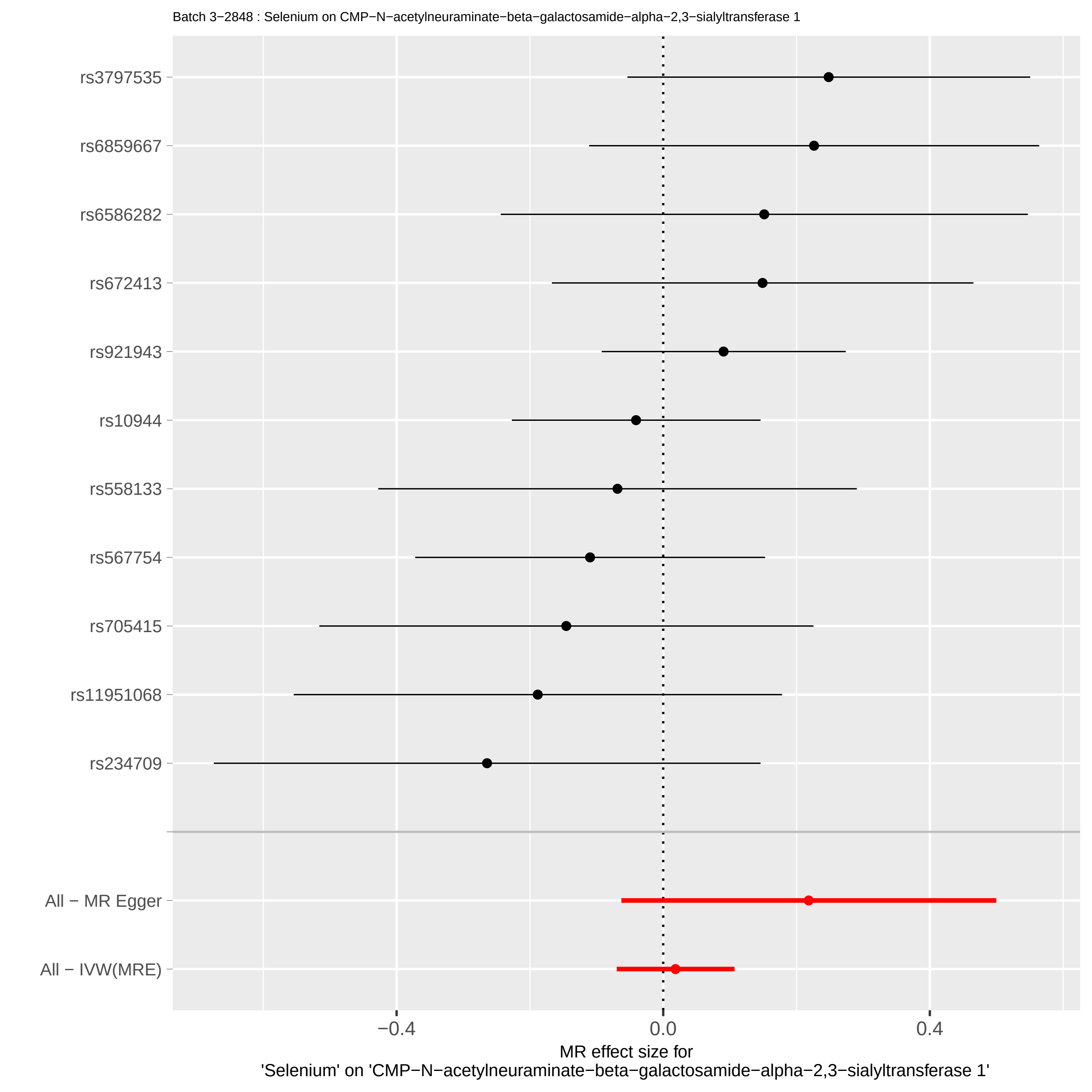

Batch 3−2848 : Selenium on CMP−N−acetylneuraminate−beta−galactosamide−alpha−2,3−sialyltransferase 1
rs3797535
rs6859667
rs6586282
rs672413
rs921943
rs10944
rs558133
rs567754
rs705415
rs11951068
rs234709
All − MR Egger
All − IVW(MRE)
−0.4
0.0
0.4
MR effect size for
'Selenium' on 'CMP−N−acetylneuraminate−beta−galactosamide−alpha−2,3−sialyltransferase 1'

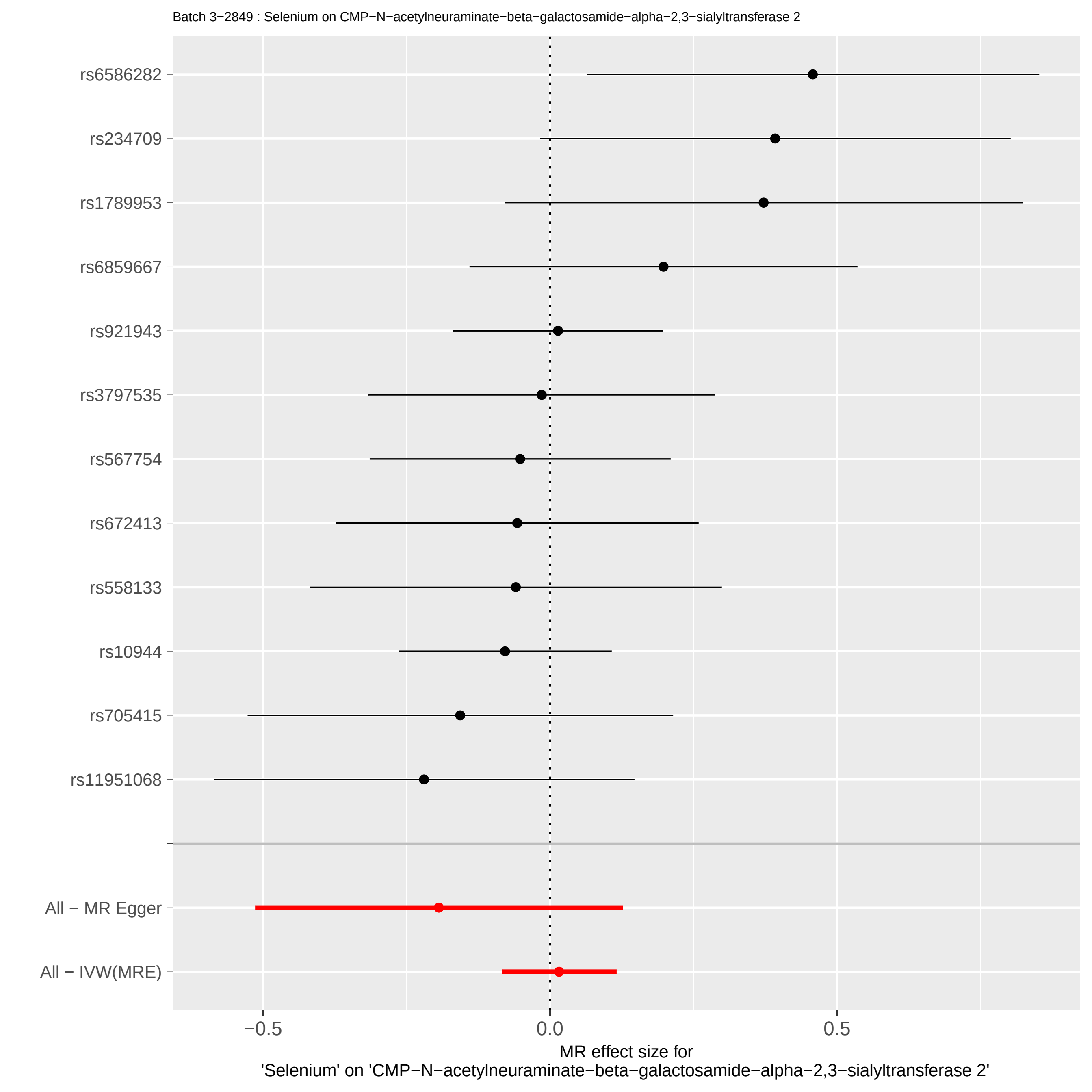

Batch 3−2849 : Selenium on CMP−N−acetylneuraminate−beta−galactosamide−alpha−2,3−sialyltransferase 2
rs6586282
rs234709
rs1789953
rs6859667
rs921943
rs3797535
rs567754
rs672413
rs558133
rs10944
rs705415
rs11951068
All − MR Egger
All − IVW(MRE)
−0.5
0.0
0.5
MR effect size for
'Selenium' on 'CMP−N−acetylneuraminate−beta−galactosamide−alpha−2,3−sialyltransferase 2'

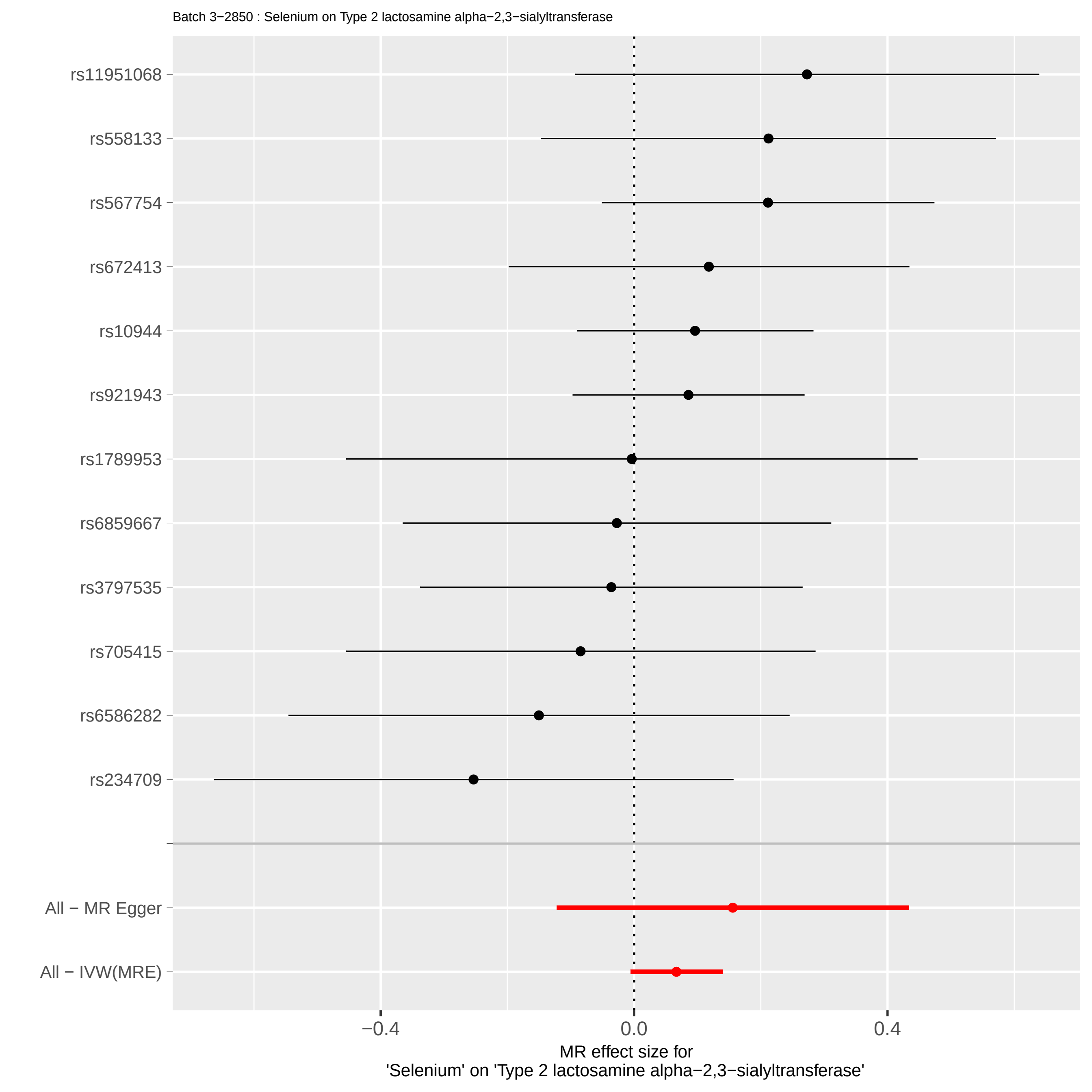
Batch 3−2850 : Selenium on Type 2 lactosamine alpha−2,3−sialyltransferase
rs11951068
rs558133
rs567754
rs672413
rs10944
rs921943
rs1789953
rs6859667
rs3797535
rs705415
rs6586282
rs234709
All − MR Egger
All − IVW(MRE)
−0.4
0.0
0.4
MR effect size for
'Selenium' on 'Type 2 lactosamine alpha−2,3−sialyltransferase'

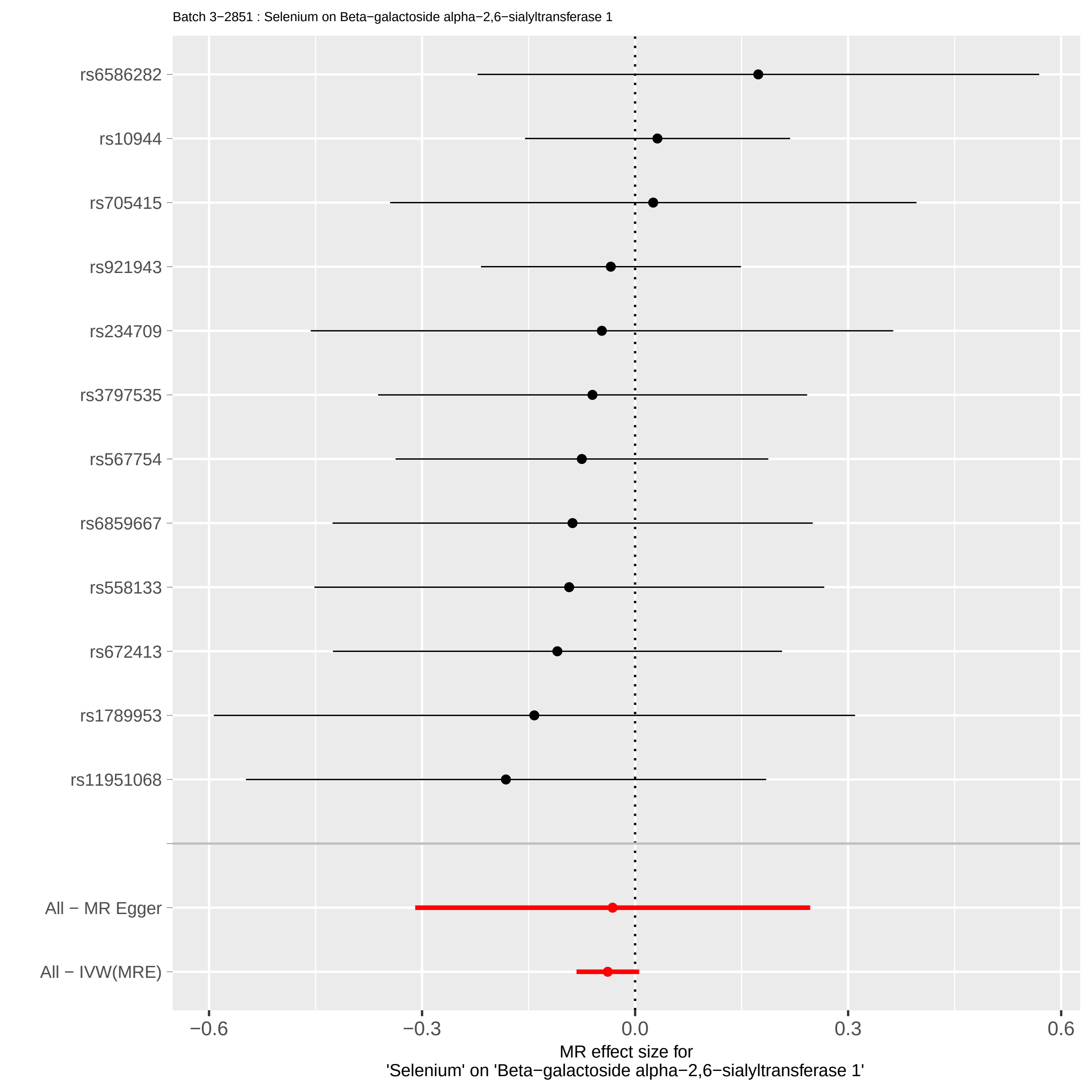
Batch 3−2851 : Selenium on Beta−galactoside alpha−2,6−sialyltransferase 1
rs6586282
rs10944
rs705415
rs921943
rs234709
rs3797535
rs567754
rs6859667
rs558133
rs672413
rs1789953
rs11951068
All − MR Egger
All − IVW(MRE)
−0.6
−0.3
0.0
0.3
0.6
MR effect size for
'Selenium' on 'Beta−galactoside alpha−2,6−sialyltransferase 1'

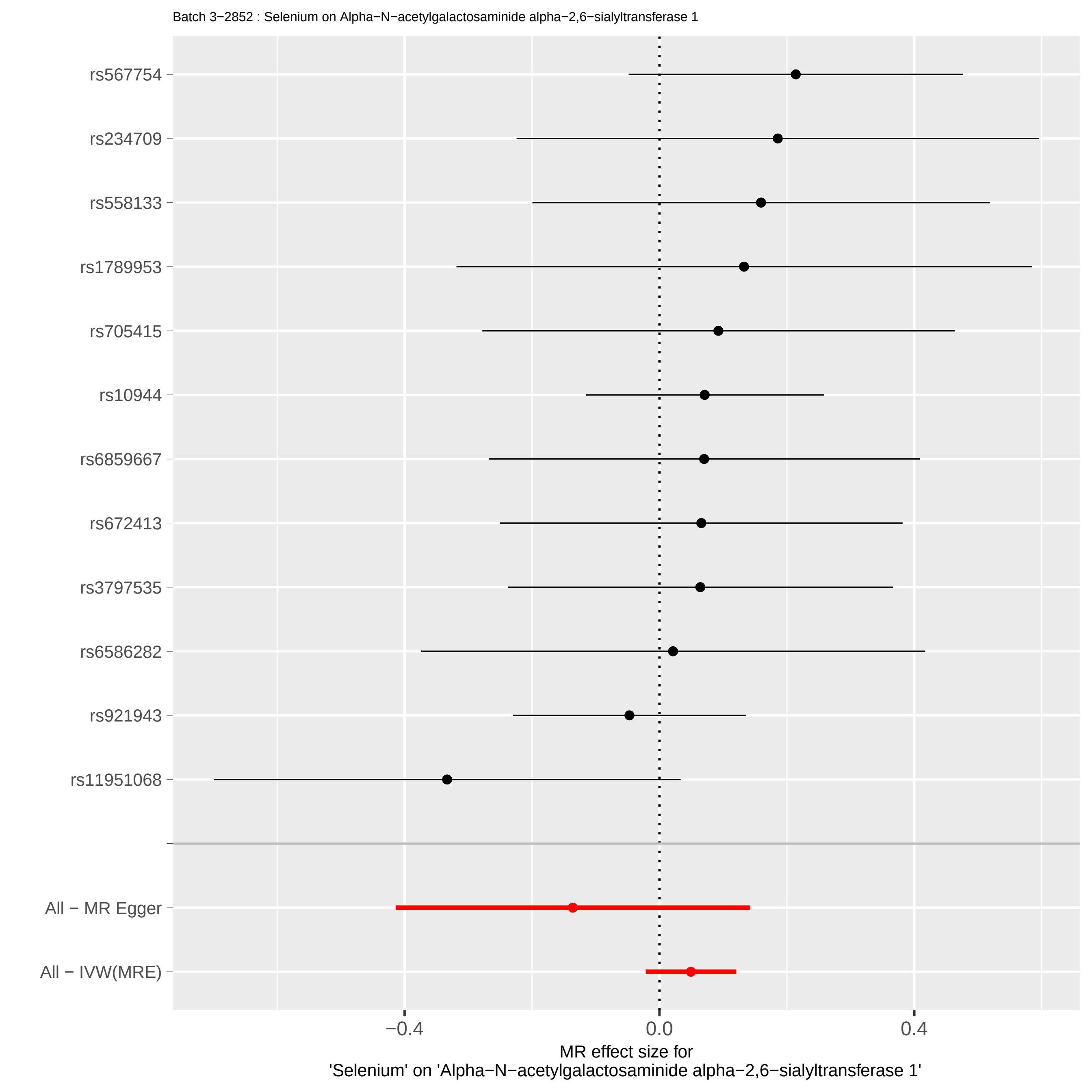

Batch 3−2852 : Selenium on Alpha−N−acetylgalactosaminide alpha−2,6−sialyltransferase 1
rs567754
rs234709
rs558133
rs1789953
rs705415
rs10944
rs6859667
rs672413
rs3797535
rs6586282
rs921943
rs11951068
All − MR Egger
All − IVW(MRE)
−0.4
0.0
0.4
MR effect size for
'Selenium' on 'Alpha−N−acetylgalactosaminide alpha−2,6−sialyltransferase 1'

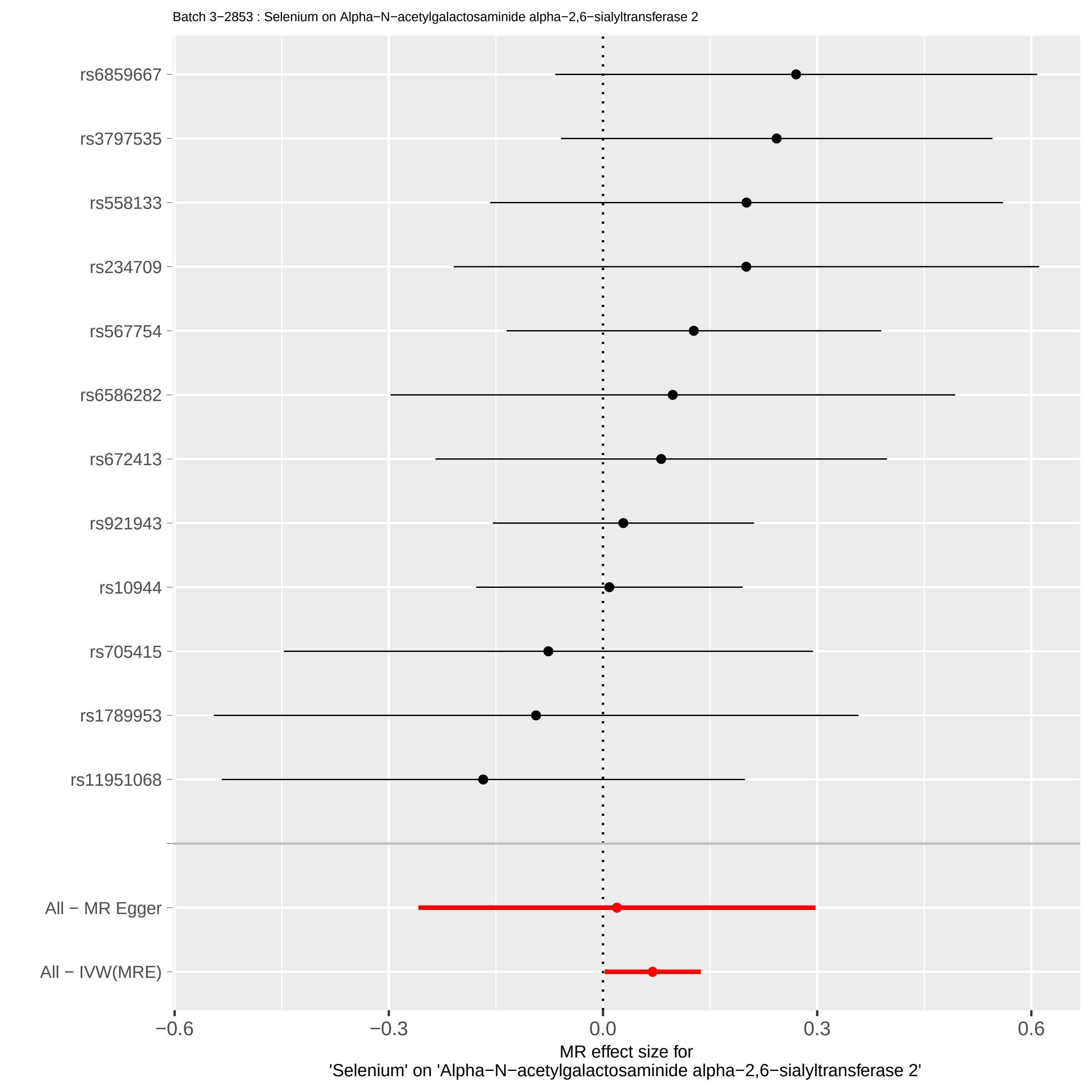

Batch 3−2853 : Selenium on Alpha−N−acetylgalactosaminide alpha−2,6−sialyltransferase 2
rs6859667
rs3797535
rs558133
rs234709
rs567754
rs6586282
rs672413
rs921943
rs10944
rs705415
rs1789953
rs11951068
All − MR Egger
All − IVW(MRE)
−0.6
−0.3
0.0
0.3
0.6
MR effect size for
'Selenium' on 'Alpha−N−acetylgalactosaminide alpha−2,6−sialyltransferase 2'

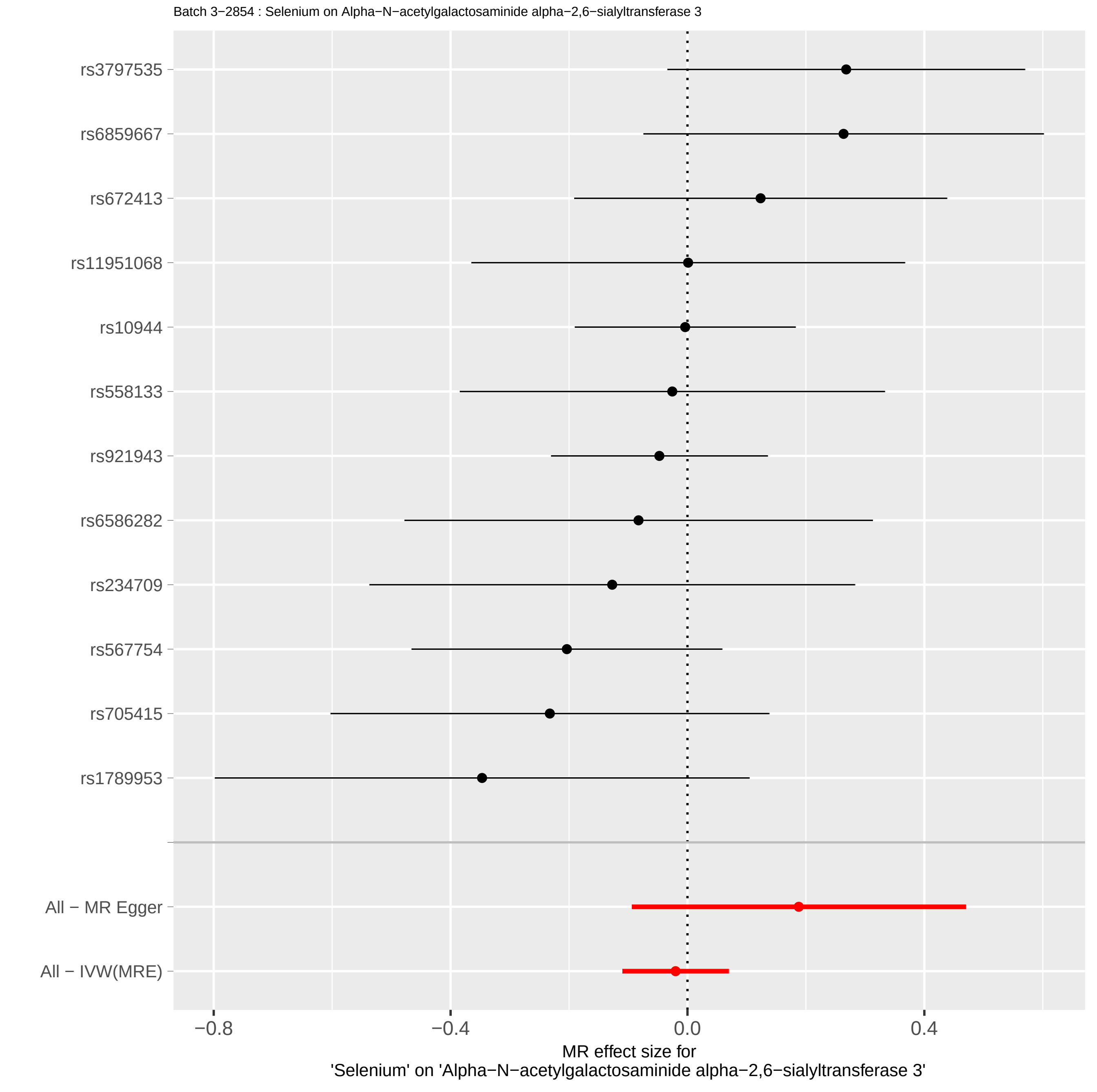
Batch 3−2854 : Selenium on Alpha−N−acetylgalactosaminide alpha−2,6−sialyltransferase 3
rs3797535
rs6859667
rs672413
rs11951068
rs10944
rs558133
rs921943
rs6586282
rs234709
rs567754
rs705415
rs1789953
All − MR Egger
All − IVW(MRE)
−0.8
−0.4
0.0
0.4
MR effect size for
'Selenium' on 'Alpha−N−acetylgalactosaminide alpha−2,6−sialyltransferase 3'

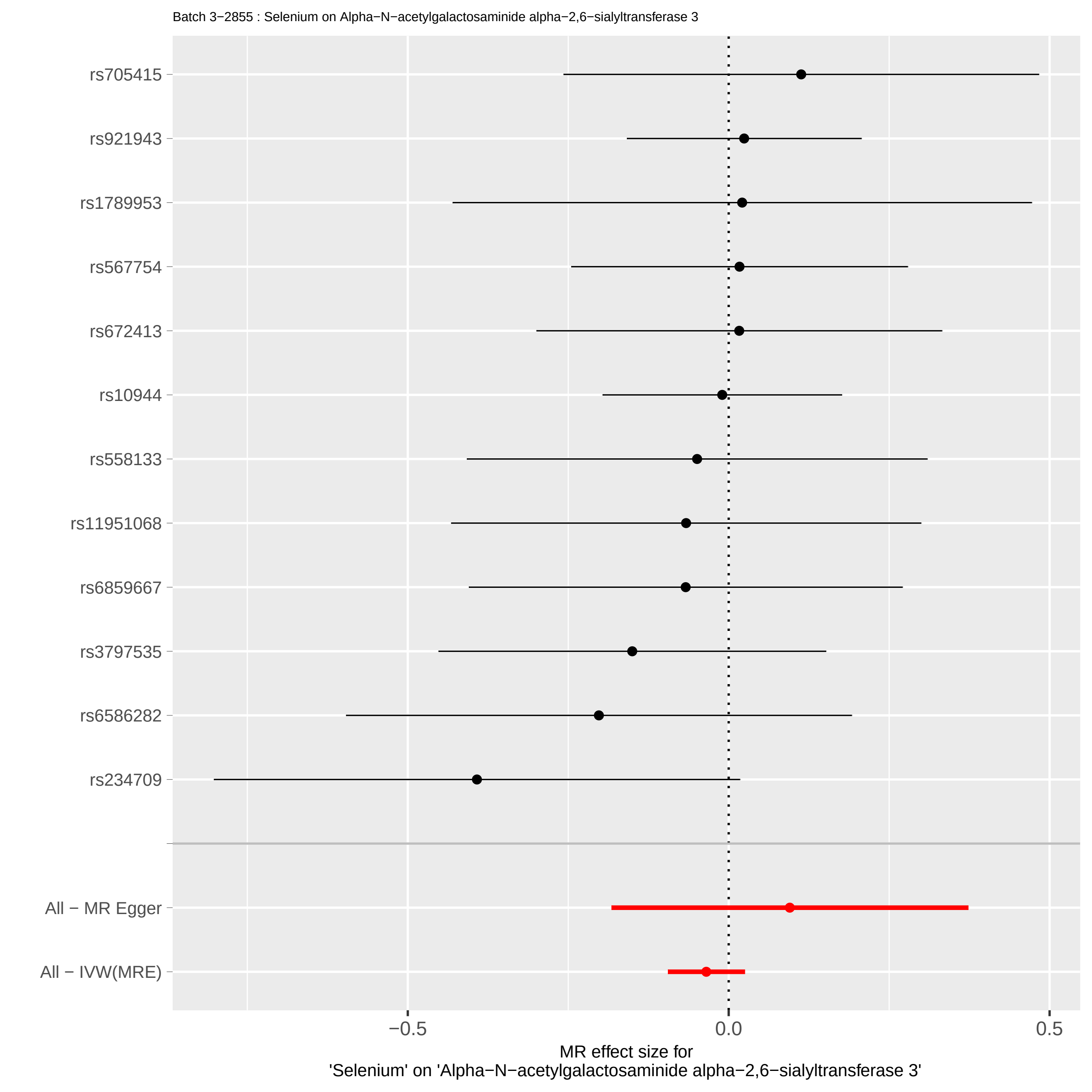

Batch 3−2855 : Selenium on Alpha−N−acetylgalactosaminide alpha−2,6−sialyltransferase 3
rs705415
rs921943
rs1789953
rs567754
rs672413
rs10944
rs558133
rs11951068
rs6859667
rs3797535
rs6586282
rs234709
All − MR Egger
All − IVW(MRE)
−0.5
0.0
0.5
MR effect size for
'Selenium' on 'Alpha−N−acetylgalactosaminide alpha−2,6−sialyltransferase 3'

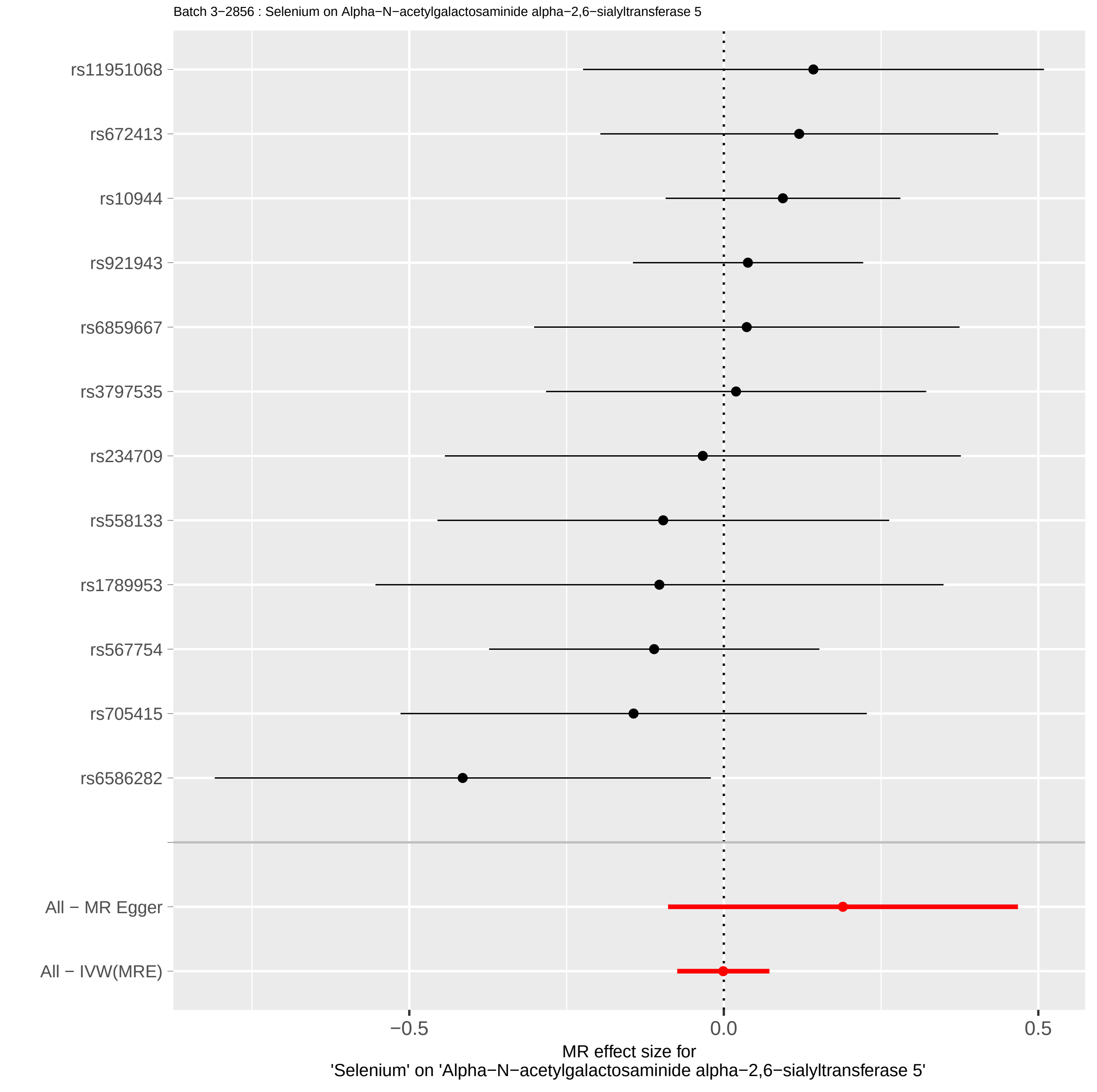
Batch 3−2856 : Selenium on Alpha−N−acetylgalactosaminide alpha−2,6−sialyltransferase 5
rs11951068
rs672413
rs10944
rs921943
rs6859667
rs3797535
rs234709
rs558133
rs1789953
rs567754
rs705415
rs6586282
All − MR Egger
All − IVW(MRE)
−0.5
0.0
0.5
MR effect size for
'Selenium' on 'Alpha−N−acetylgalactosaminide alpha−2,6−sialyltransferase 5'

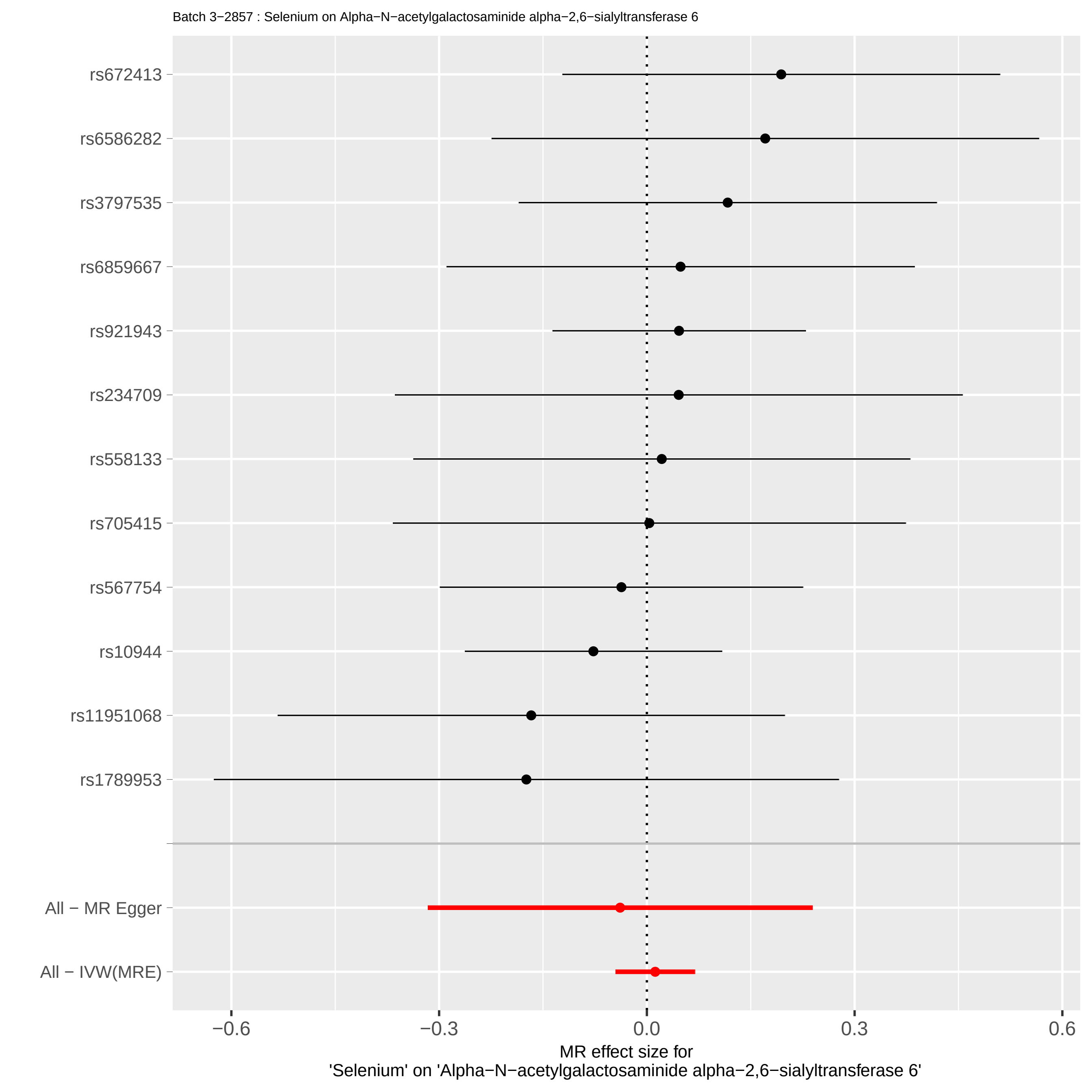

Batch 3−2857 : Selenium on Alpha−N−acetylgalactosaminide alpha−2,6−sialyltransferase 6
rs672413
rs6586282
rs3797535
rs6859667
rs921943
rs234709
rs558133
rs705415
rs567754
rs10944
rs11951068
rs1789953
All − MR Egger
All − IVW(MRE)
−0.6
−0.3
0.0
0.3
0.6
MR effect size for
'Selenium' on 'Alpha−N−acetylgalactosaminide alpha−2,6−sialyltransferase 6'

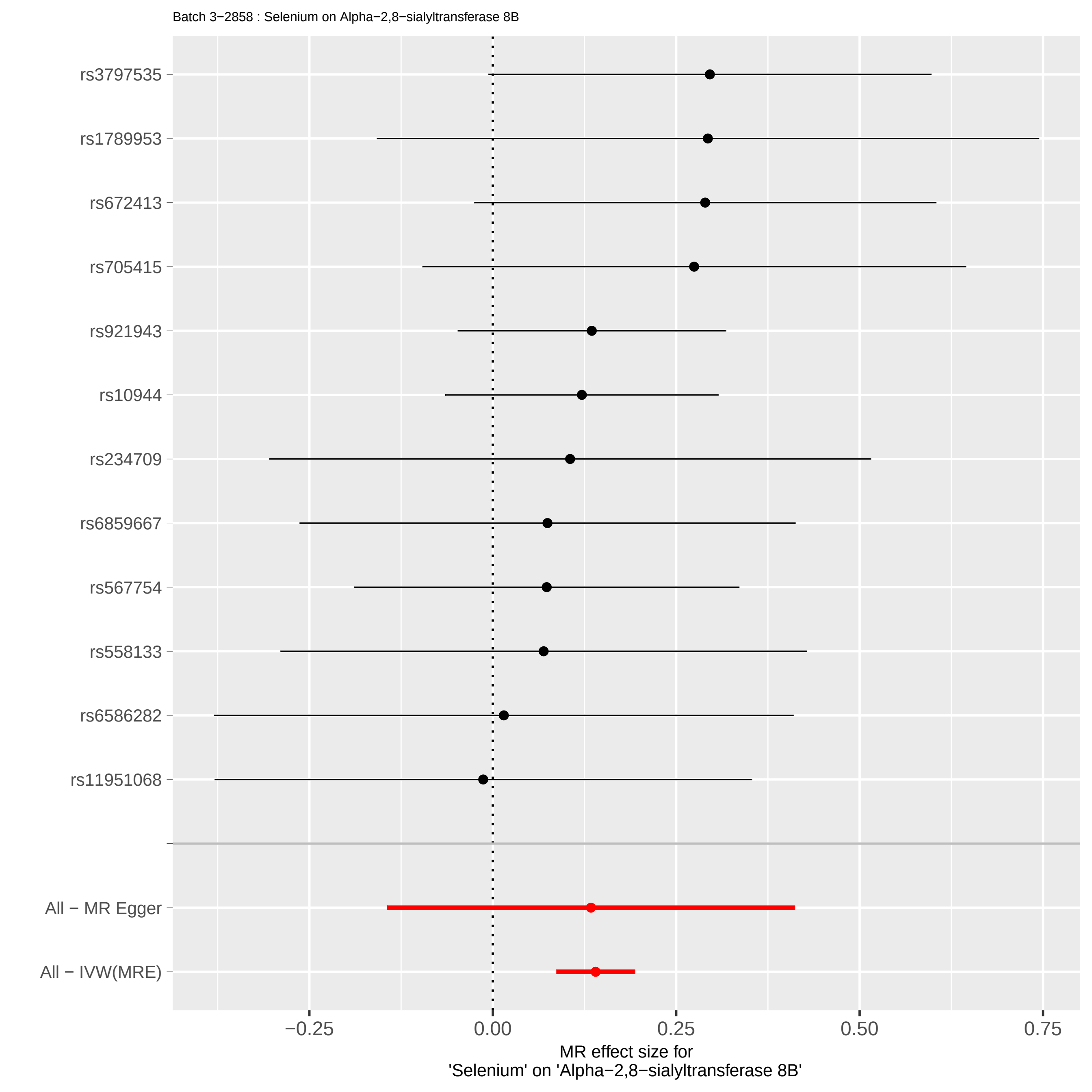
Batch 3−2858 : Selenium on Alpha−2,8−sialyltransferase 8B
rs3797535
rs1789953
rs672413
rs705415
rs921943
rs10944
rs234709
rs6859667
rs567754
rs558133
rs6586282
rs11951068
All − MR Egger
All − IVW(MRE)
−0.25
0.00
0.25
0.50
0.75
MR effect size for
'Selenium' on 'Alpha−2,8−sialyltransferase 8B'

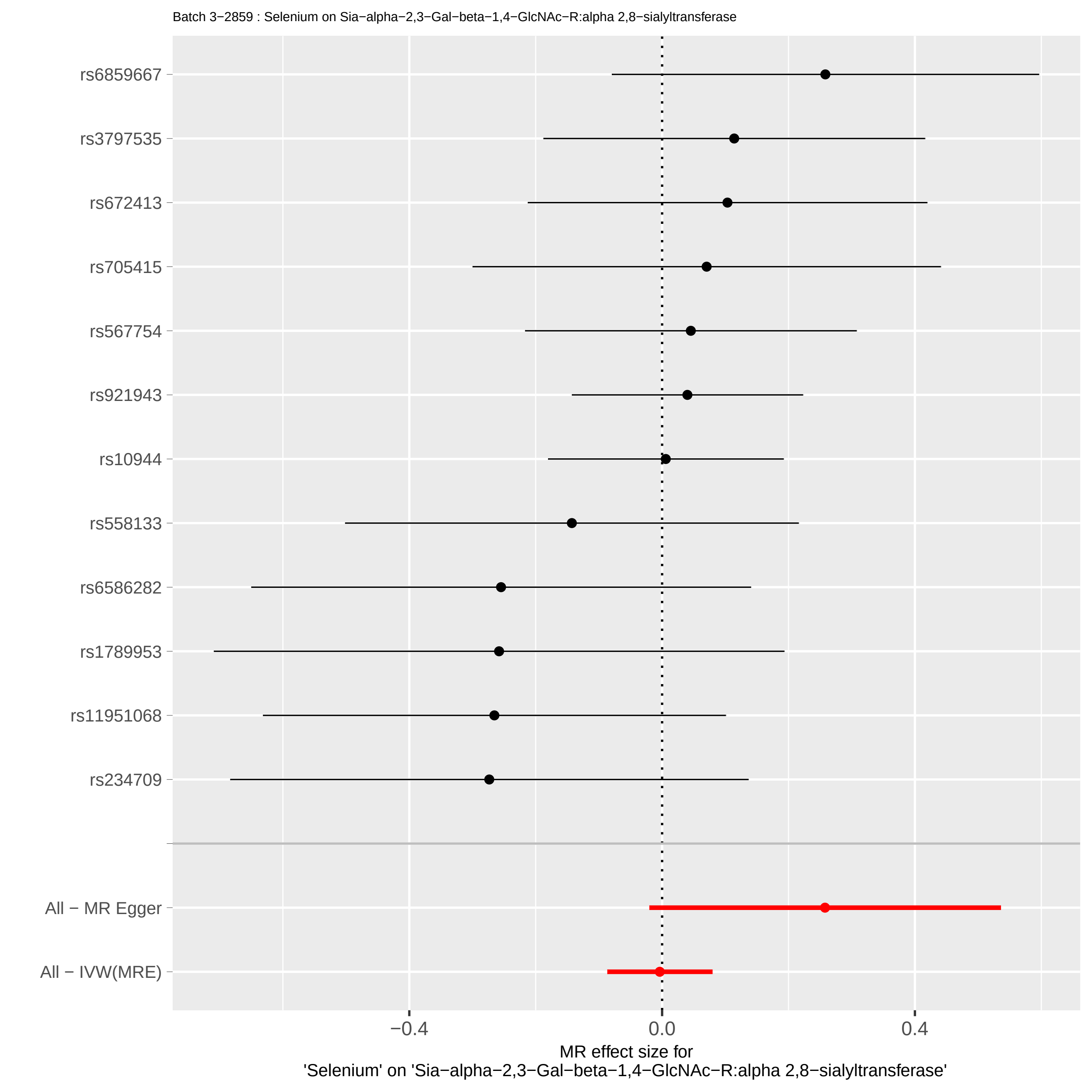
Batch 3−2859 : Selenium on Sia−alpha−2,3−Gal−beta−1,4−GlcNAc−R:alpha 2,8−sialyltransferase
rs6859667
rs3797535
rs672413
rs705415
rs567754
rs921943
rs10944
rs558133
rs6586282
rs1789953
rs11951068
rs234709
All − MR Egger
All − IVW(MRE)
−0.4
0.0
0.4
MR effect size for
'Selenium' on 'Sia−alpha−2,3−Gal−beta−1,4−GlcNAc−R:alpha 2,8−sialyltransferase'

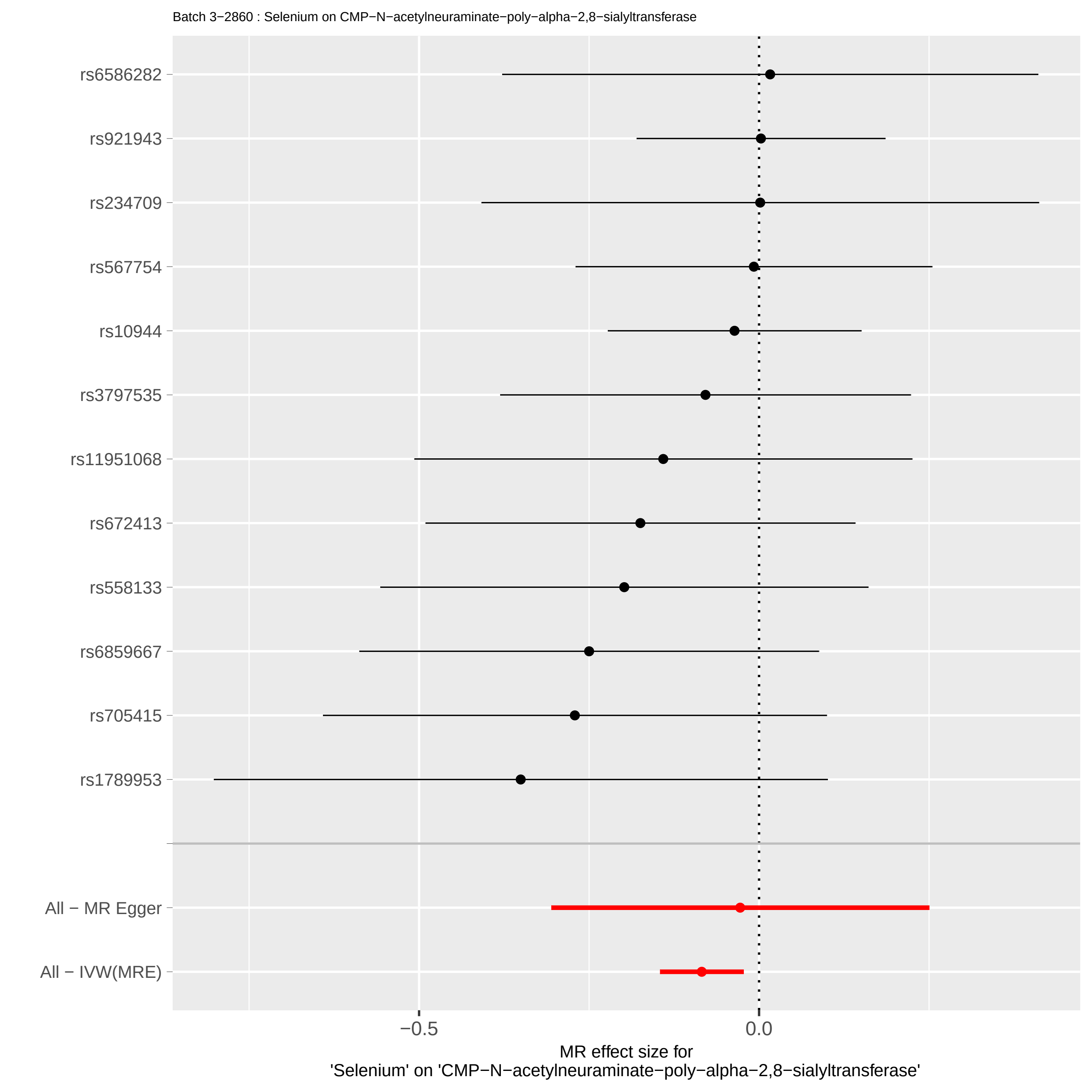

Batch 3−2860 : Selenium on CMP−N−acetylneuraminate−poly−alpha−2,8−sialyltransferase
rs6586282
rs921943
rs234709
rs567754
rs10944
rs3797535
rs11951068
rs672413
rs558133
rs6859667
rs705415
rs1789953
All − MR Egger
All − IVW(MRE)
−0.5
0.0
MR effect size for
'Selenium' on 'CMP−N−acetylneuraminate−poly−alpha−2,8−sialyltransferase'

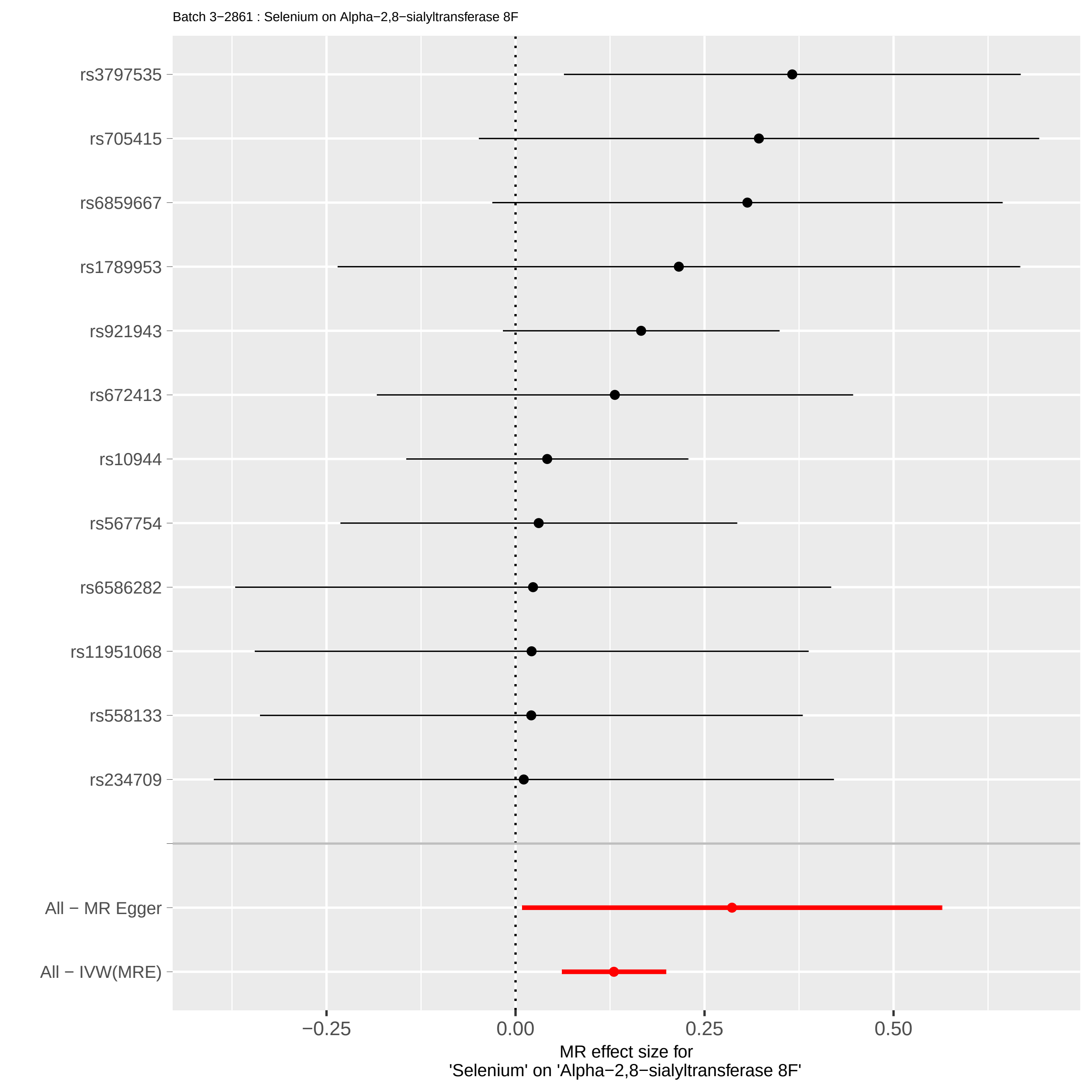
Batch 3−2861 : Selenium on Alpha−2,8−sialyltransferase 8F
rs3797535
rs705415
rs6859667
rs1789953
rs921943
rs672413
rs10944
rs567754
rs6586282
rs11951068
rs558133
rs234709
All − MR Egger
All − IVW(MRE)
−0.25
0.00
0.25
0.50
MR effect size for
'Selenium' on 'Alpha−2,8−sialyltransferase 8F'

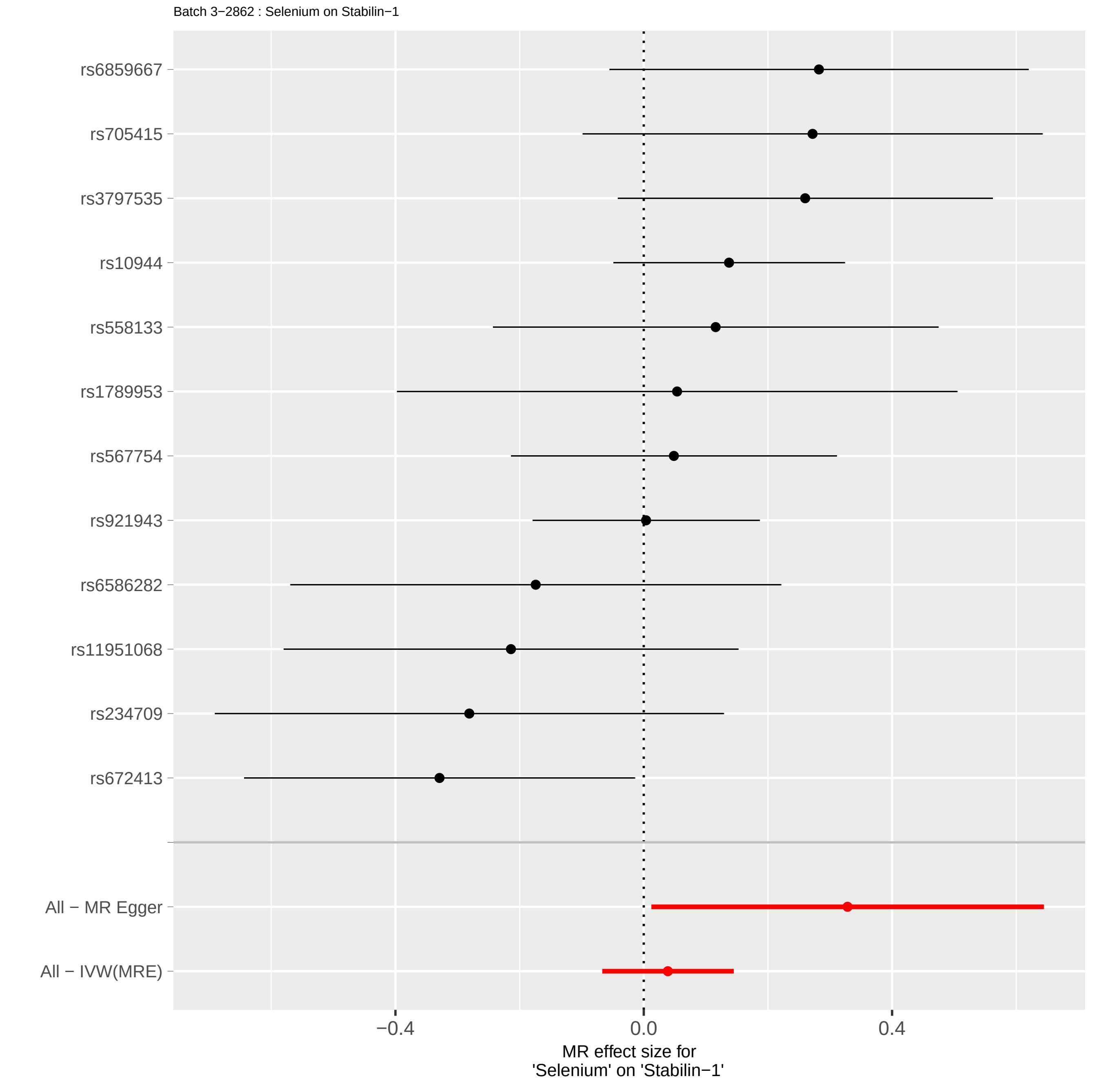

Batch 3−2862 : Selenium on Stabilin−1
rs6859667
rs705415
rs3797535
rs10944
rs558133
rs1789953
rs567754
rs921943
rs6586282
rs11951068
rs234709
rs672413
All − MR Egger
All − IVW(MRE)
−0.4
0.0
0.4
MR effect size for
'Selenium' on 'Stabilin−1'

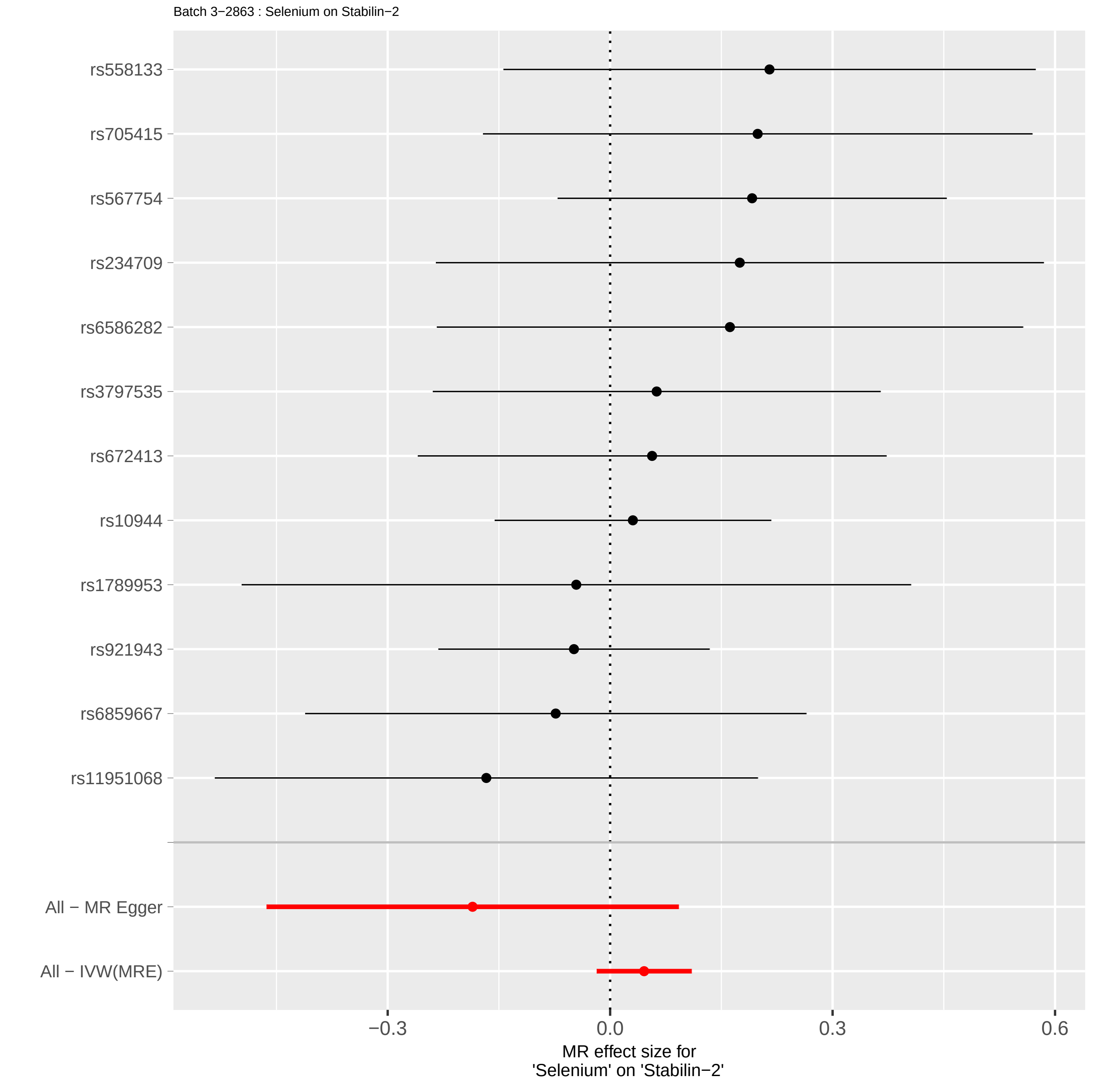
Batch 3−2863 : Selenium on Stabilin−2
rs558133
rs705415
rs567754
rs234709
rs6586282
rs3797535
rs672413
rs10944
rs1789953
rs921943
rs6859667
rs11951068
All − MR Egger
All − IVW(MRE)
−0.3
0.0
0.3
0.6
MR effect size for
'Selenium' on 'Stabilin−2'

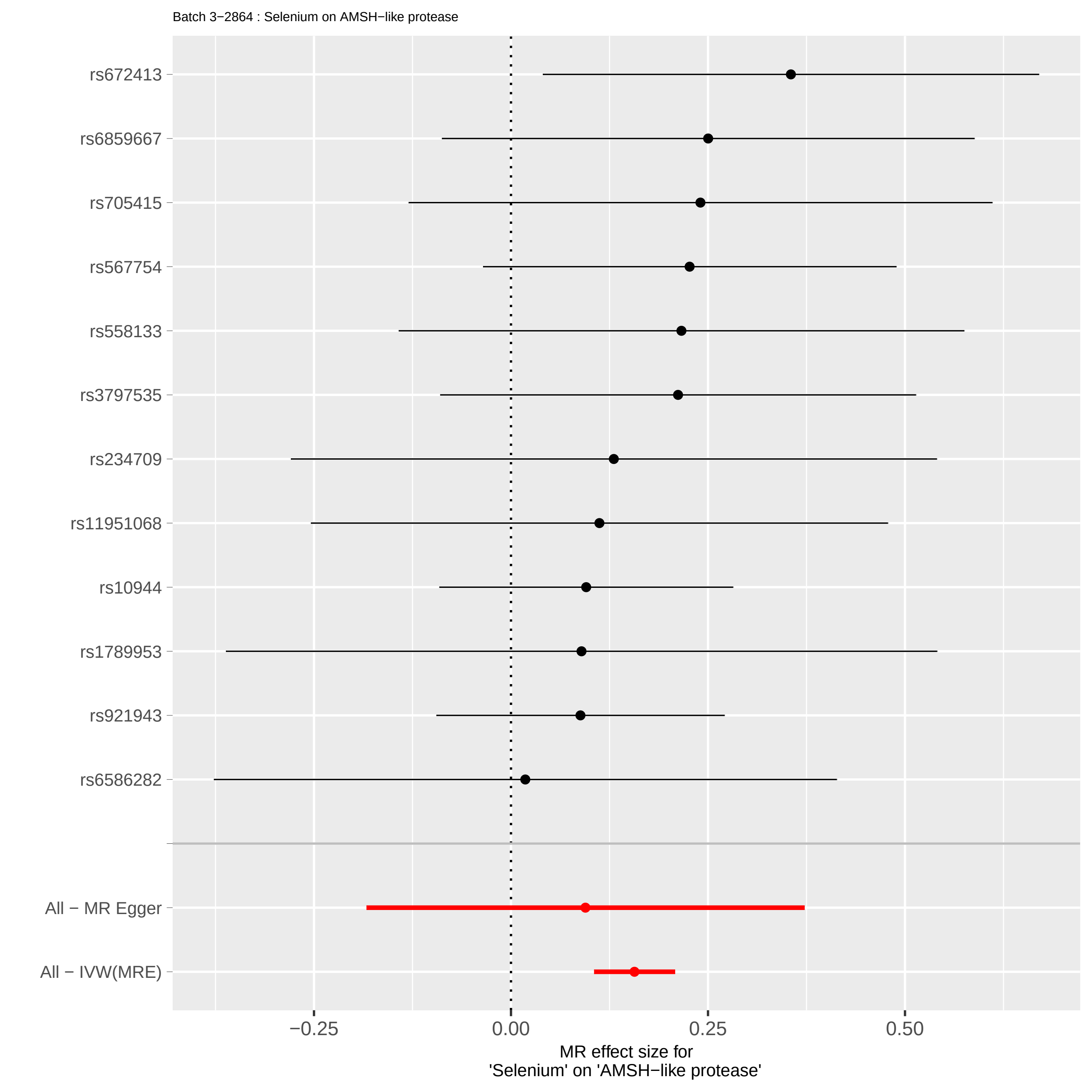
Batch 3−2864 : Selenium on AMSH−like protease
rs672413
rs6859667
rs705415
rs567754
rs558133
rs3797535
rs234709
rs11951068
rs10944
rs1789953
rs921943
rs6586282
All − MR Egger
All − IVW(MRE)
−0.25
0.00
0.25
0.50
MR effect size for
'Selenium' on 'AMSH−like protease'

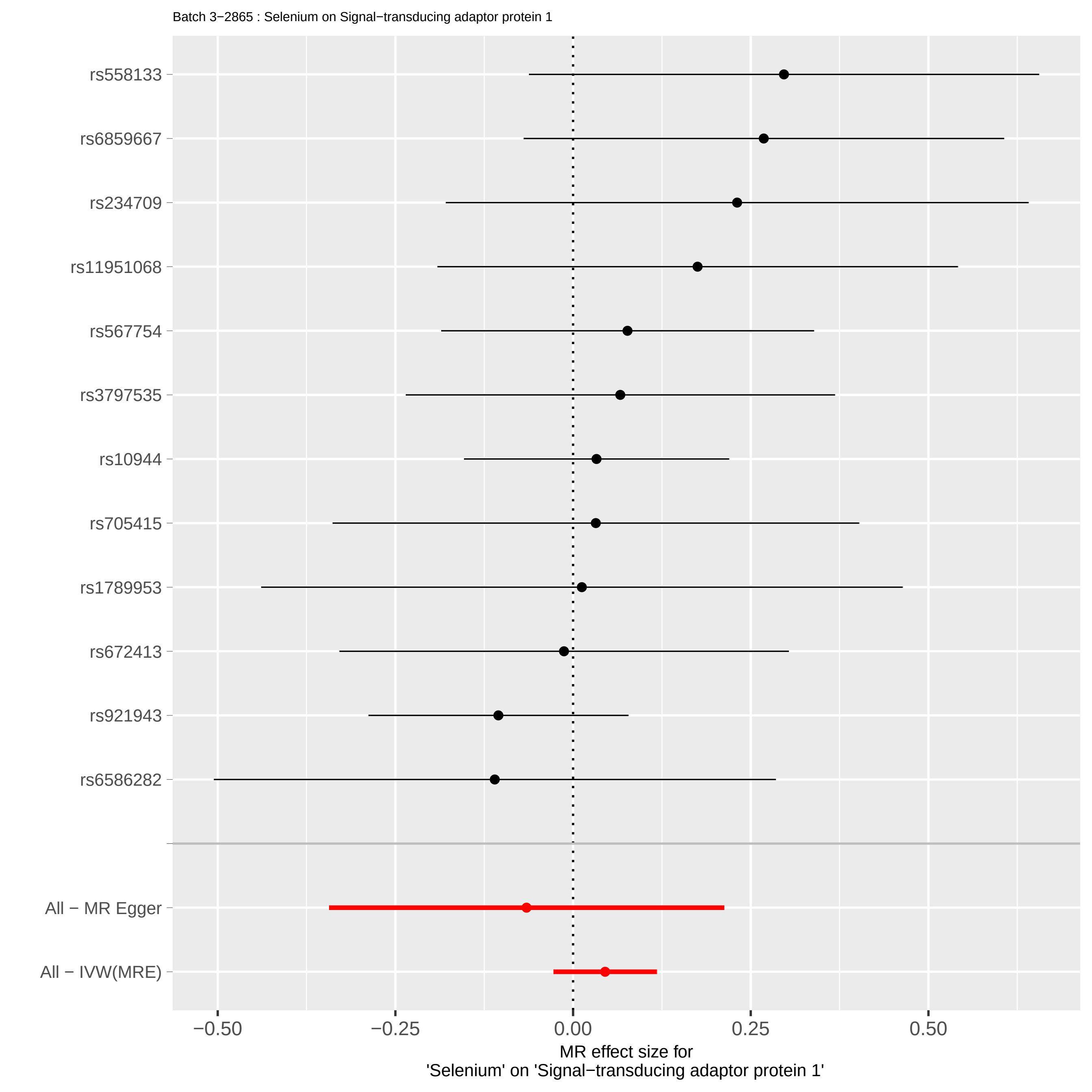

Batch 3−2865 : Selenium on Signal−transducing adaptor protein 1
rs558133
rs6859667
rs234709
rs11951068
rs567754
rs3797535
rs10944
rs705415
rs1789953
rs672413
rs921943
rs6586282
All − MR Egger
All − IVW(MRE)
−0.50
−0.25
0.00
0.25
0.50
MR effect size for
'Selenium' on 'Signal−transducing adaptor protein 1'

Batch 3−2866 : Selenium on Steroidogenic acute regulatory protein, mitochondrial

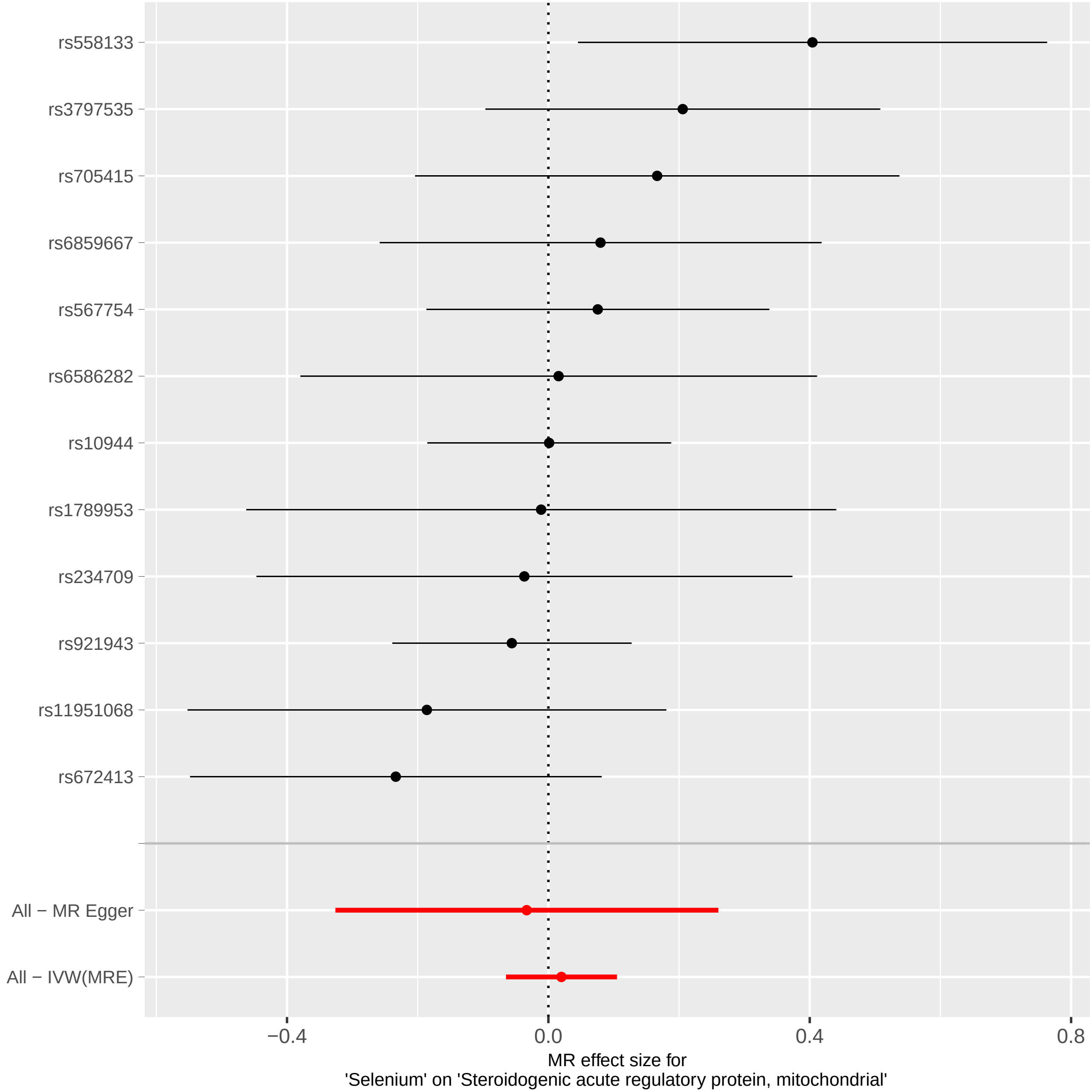

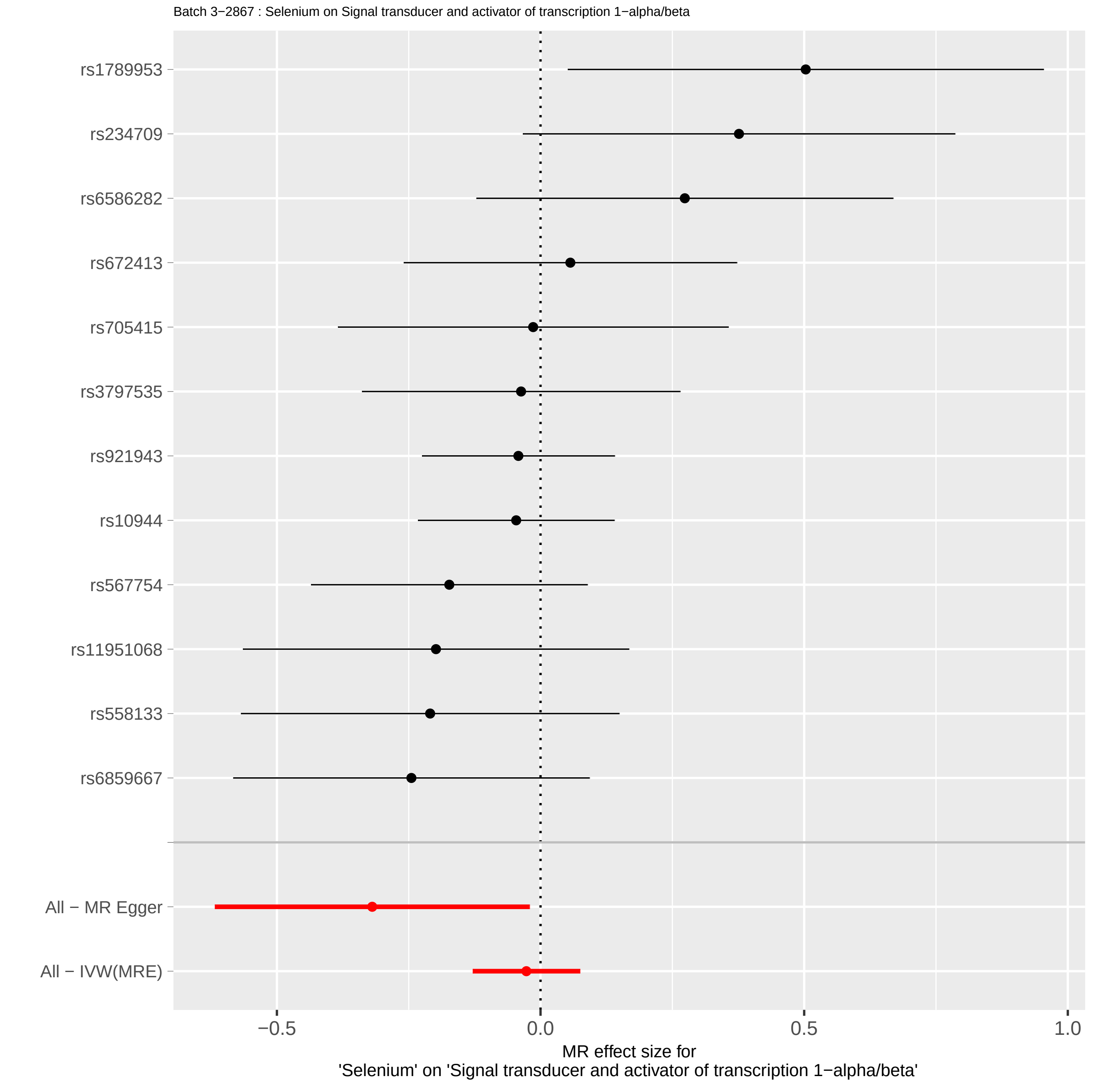

Batch 3−2867 : Selenium on Signal transducer and activator of transcription 1−alpha/beta
rs1789953
rs234709
rs6586282
rs672413
rs705415
rs3797535
rs921943
rs10944
rs567754
rs11951068
rs558133
rs6859667
All − MR Egger
All − IVW(MRE)
−0.5
0.0
0.5
1.0
MR effect size for
'Selenium' on 'Signal transducer and activator of transcription 1−alpha/beta'

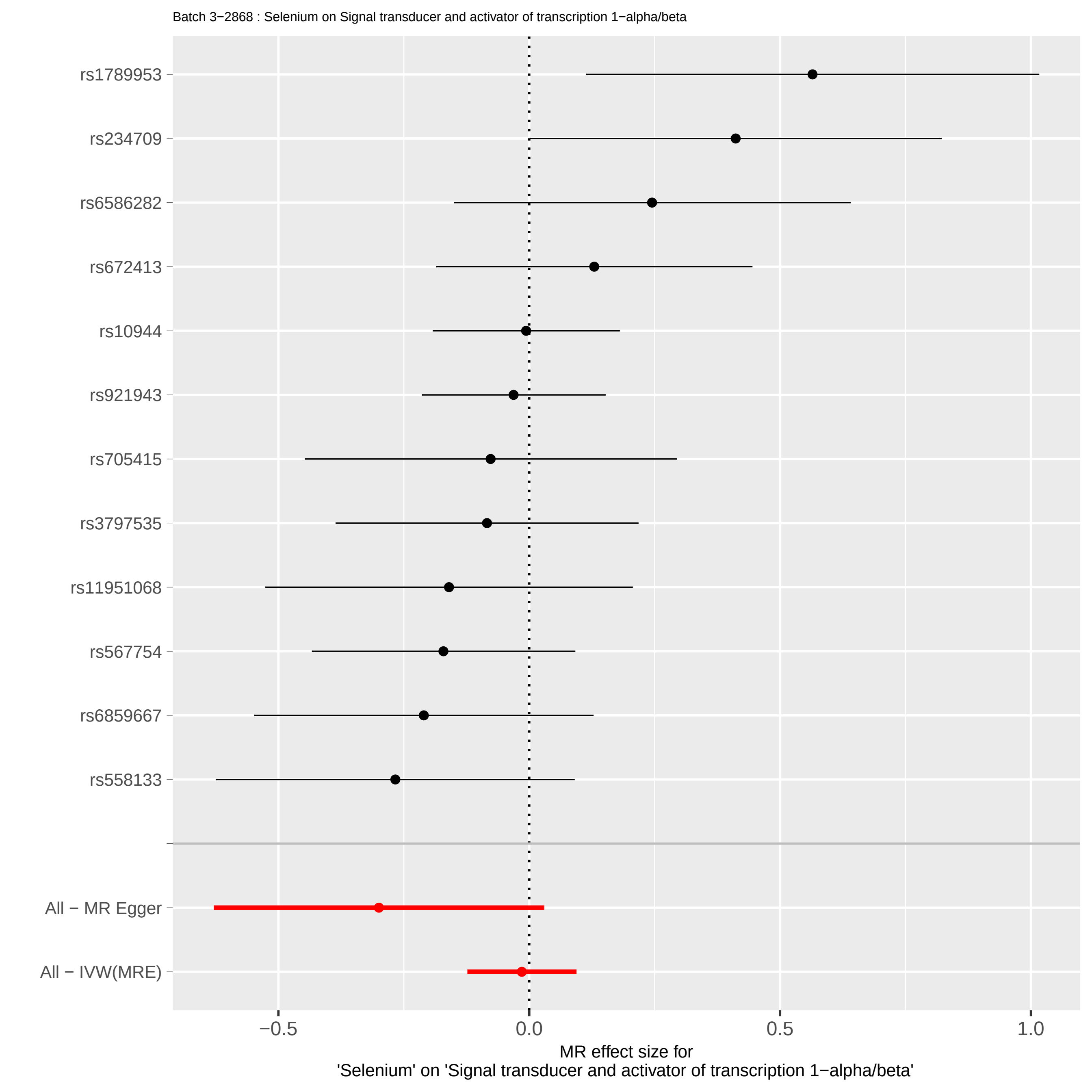
Batch 3−2868 : Selenium on Signal transducer and activator of transcription 1−alpha/beta
rs1789953
rs234709
rs6586282
rs672413
rs10944
rs921943
rs705415
rs3797535
rs11951068
rs567754
rs6859667
rs558133
All − MR Egger
All − IVW(MRE)
−0.5
0.0
0.5
1.0
MR effect size for
'Selenium' on 'Signal transducer and activator of transcription 1−alpha/beta'

Batch 3−2869 : Selenium on Signal transducer and activator of transcription 3

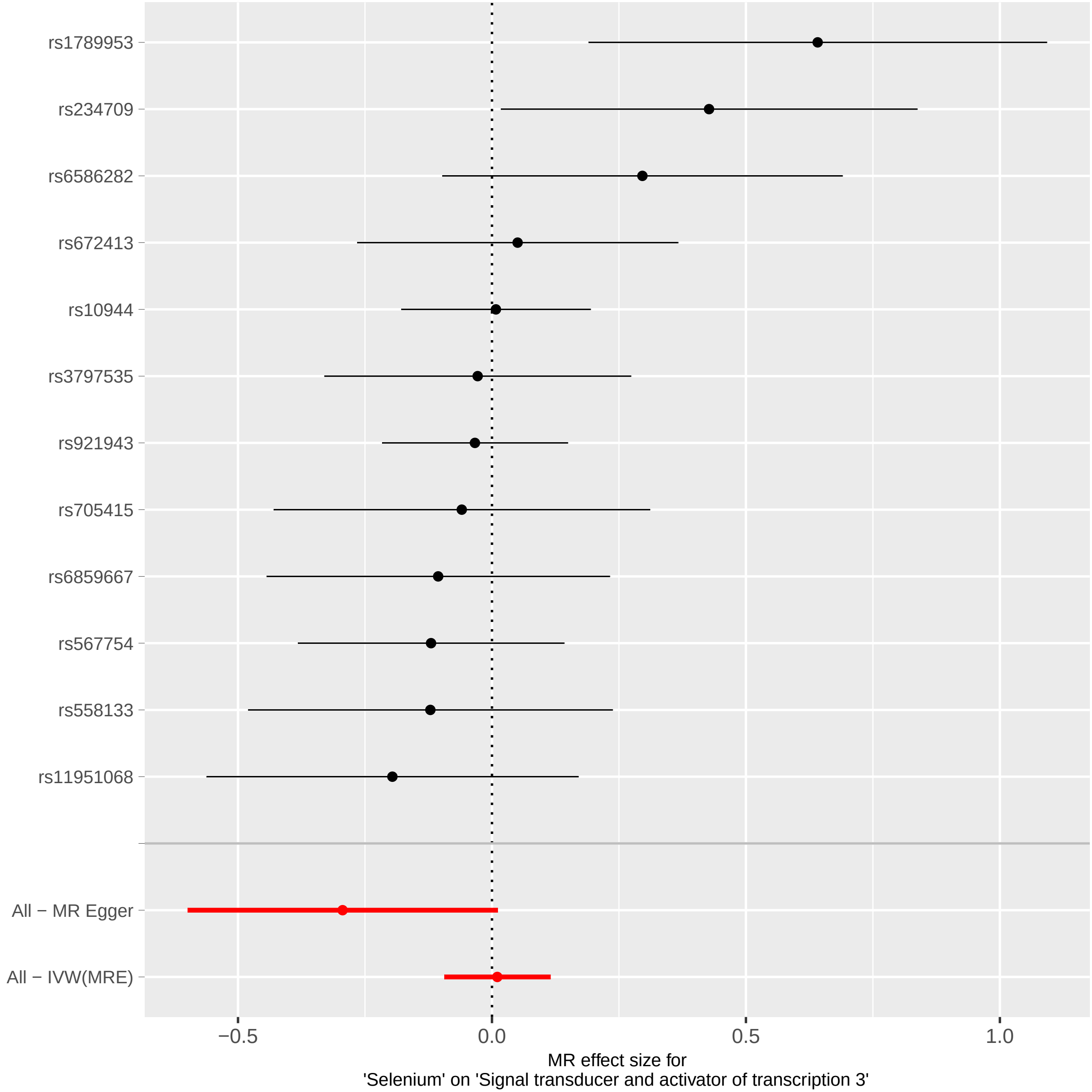

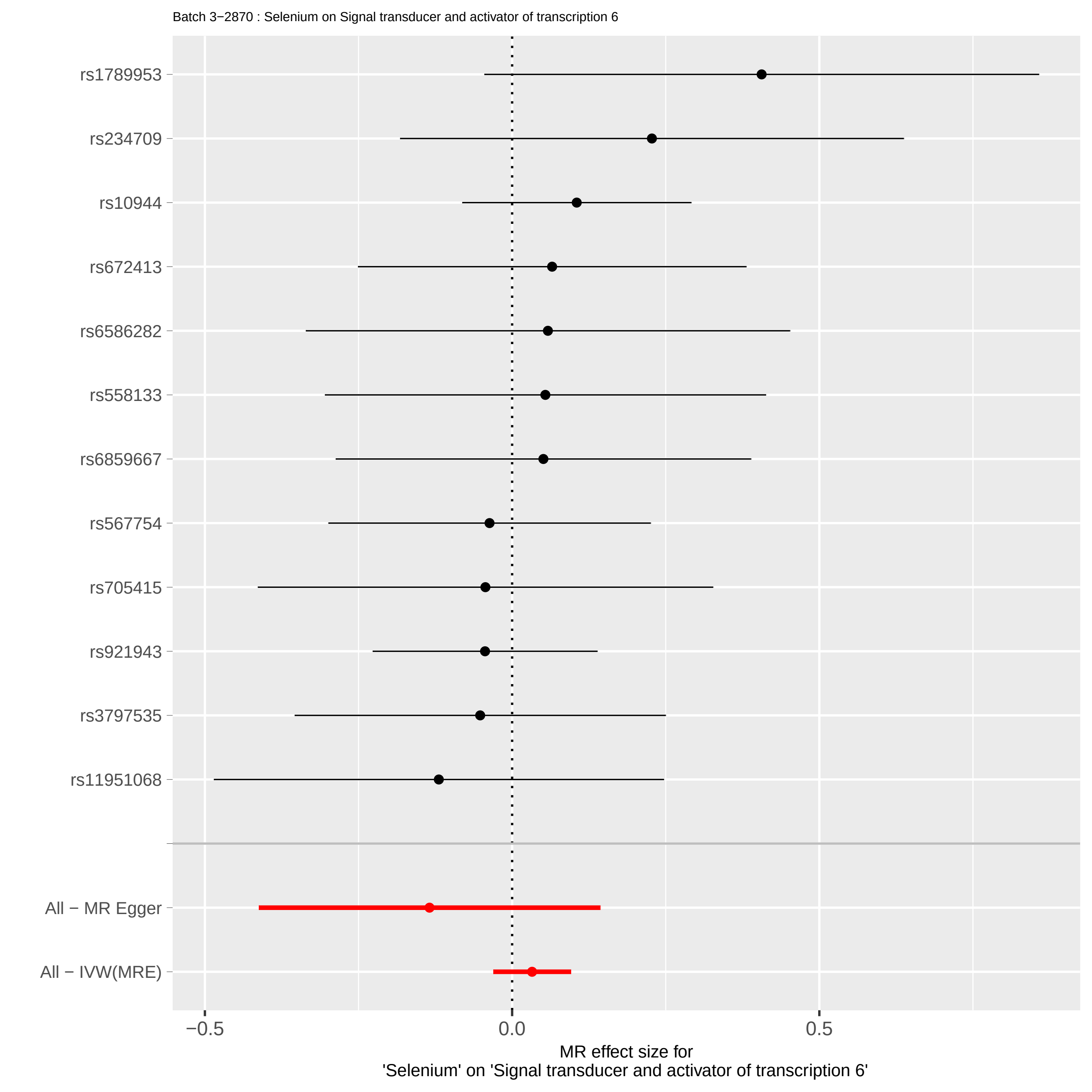

Batch 3−2870 : Selenium on Signal transducer and activator of transcription 6
rs1789953
rs234709
rs10944
rs672413
rs6586282
rs558133
rs6859667
rs567754
rs705415
rs921943
rs3797535
rs11951068
All − MR Egger
All − IVW(MRE)
−0.5
0.0
0.5
MR effect size for
'Selenium' on 'Signal transducer and activator of transcription 6'

Batch 3−2871 : Selenium on Double−stranded RNA−binding protein Staufen homolog 1

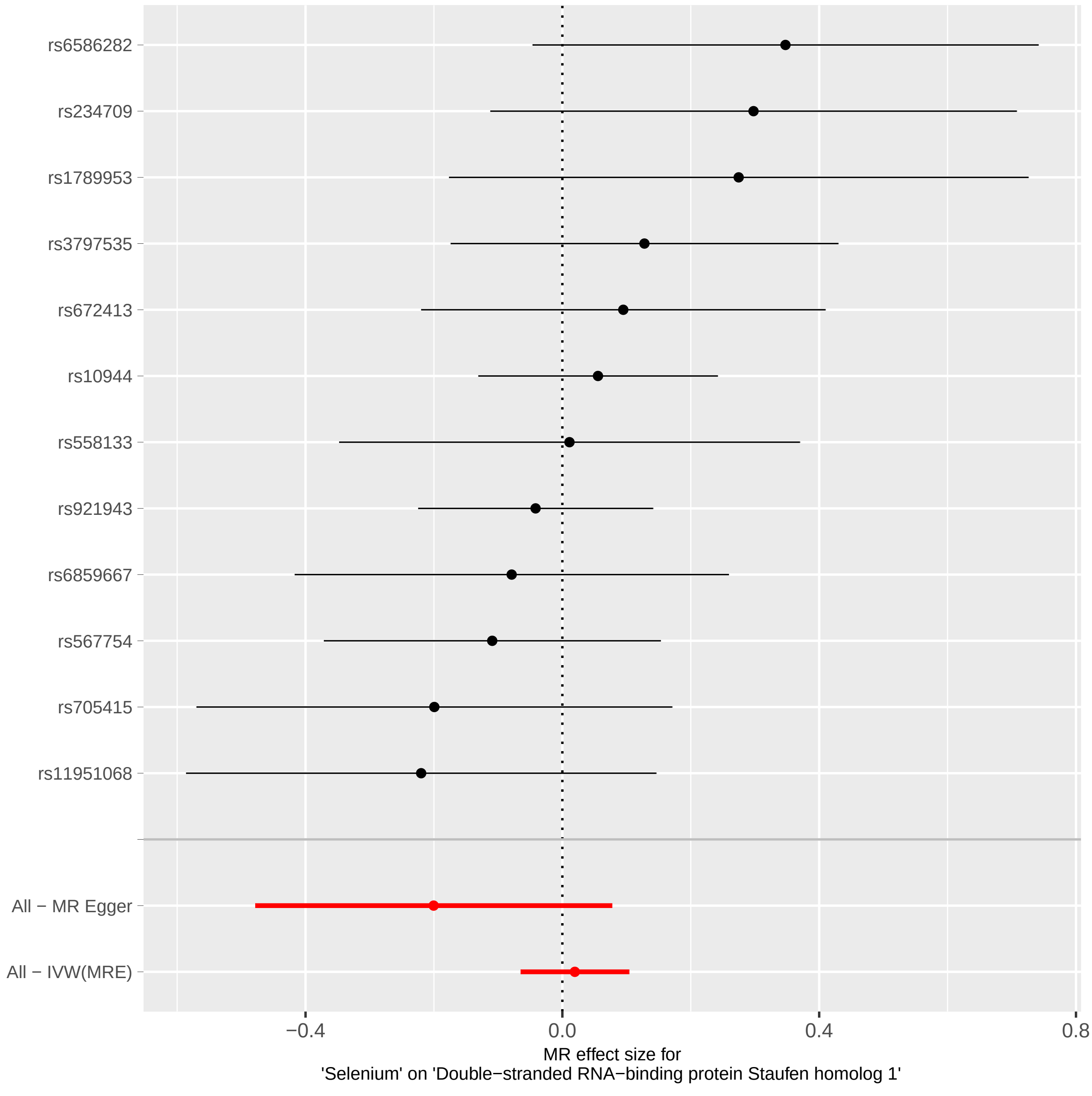

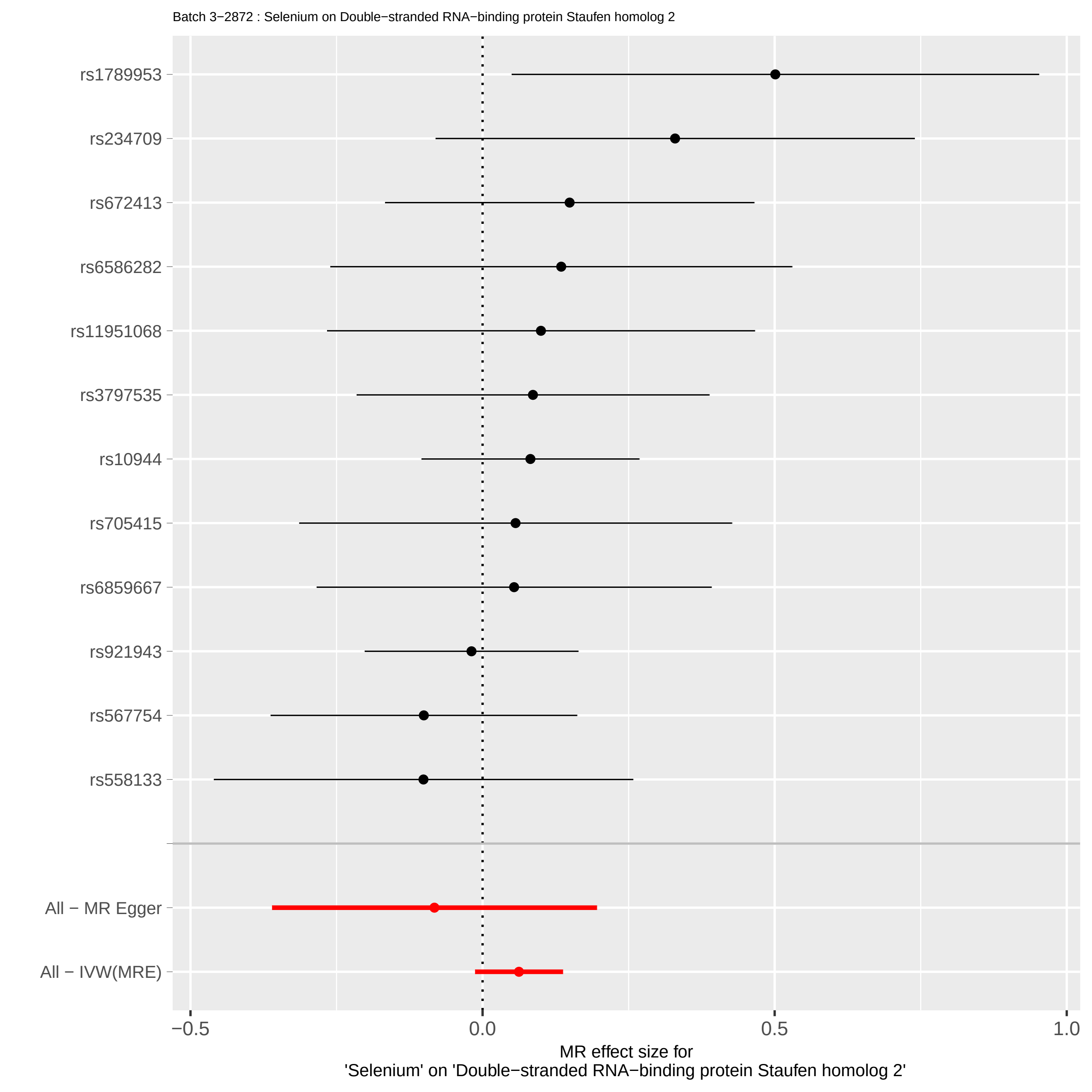

Batch 3−2872 : Selenium on Double−stranded RNA−binding protein Staufen homolog 2
rs1789953
rs234709
rs672413
rs6586282
rs11951068
rs3797535
rs10944
rs705415
rs6859667
rs921943
rs567754
rs558133
All − MR Egger
All − IVW(MRE)
−0.5
0.0
0.5
1.0
MR effect size for
'Selenium' on 'Double−stranded RNA−binding protein Staufen homolog 2'

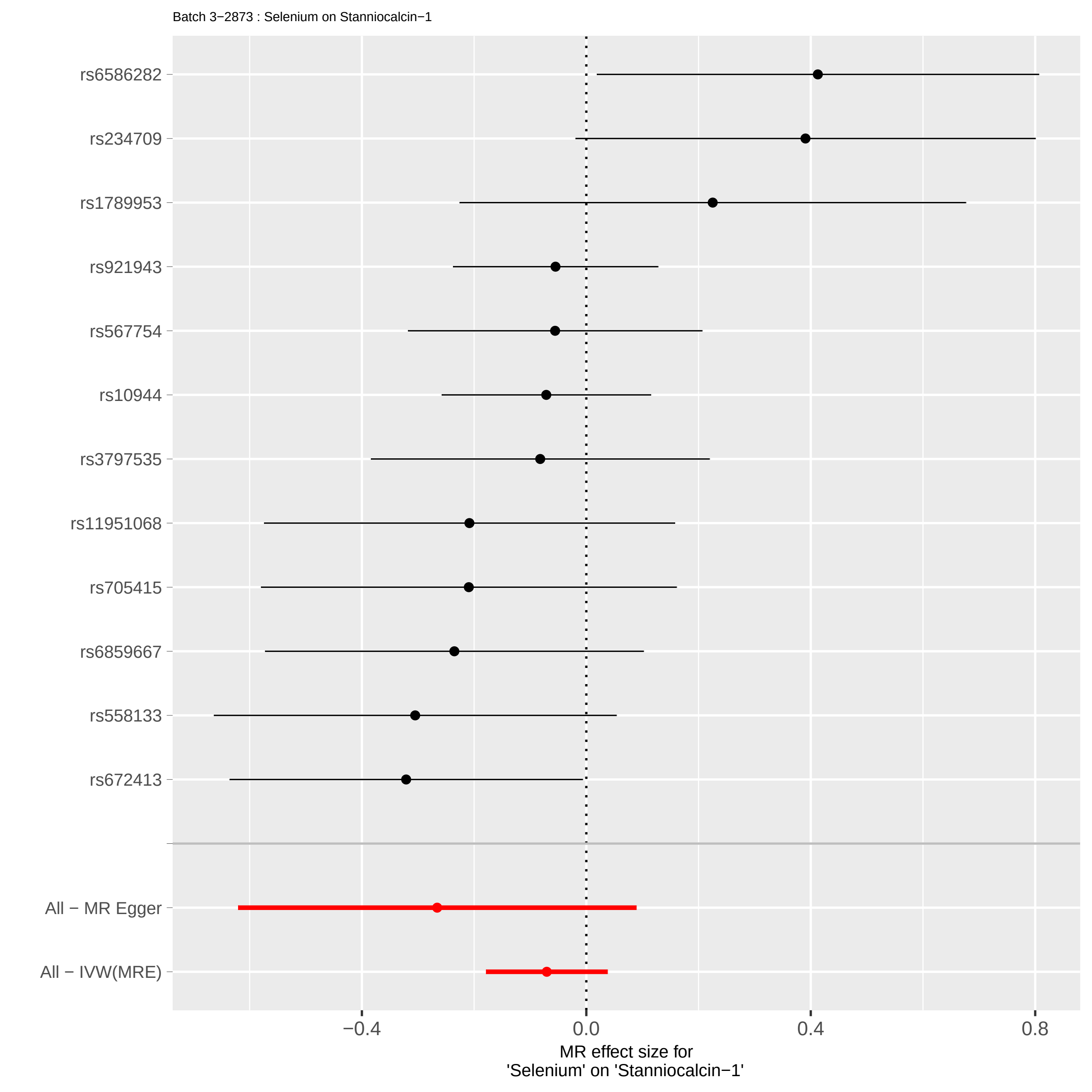

Batch 3−2873 : Selenium on Stanniocalcin−1
rs6586282
rs234709
rs1789953
rs921943
rs567754
rs10944
rs3797535
rs11951068
rs705415
rs6859667
rs558133
rs672413
All − MR Egger
All − IVW(MRE)
−0.4
0.0
0.4
0.8
MR effect size for
'Selenium' on 'Stanniocalcin−1'

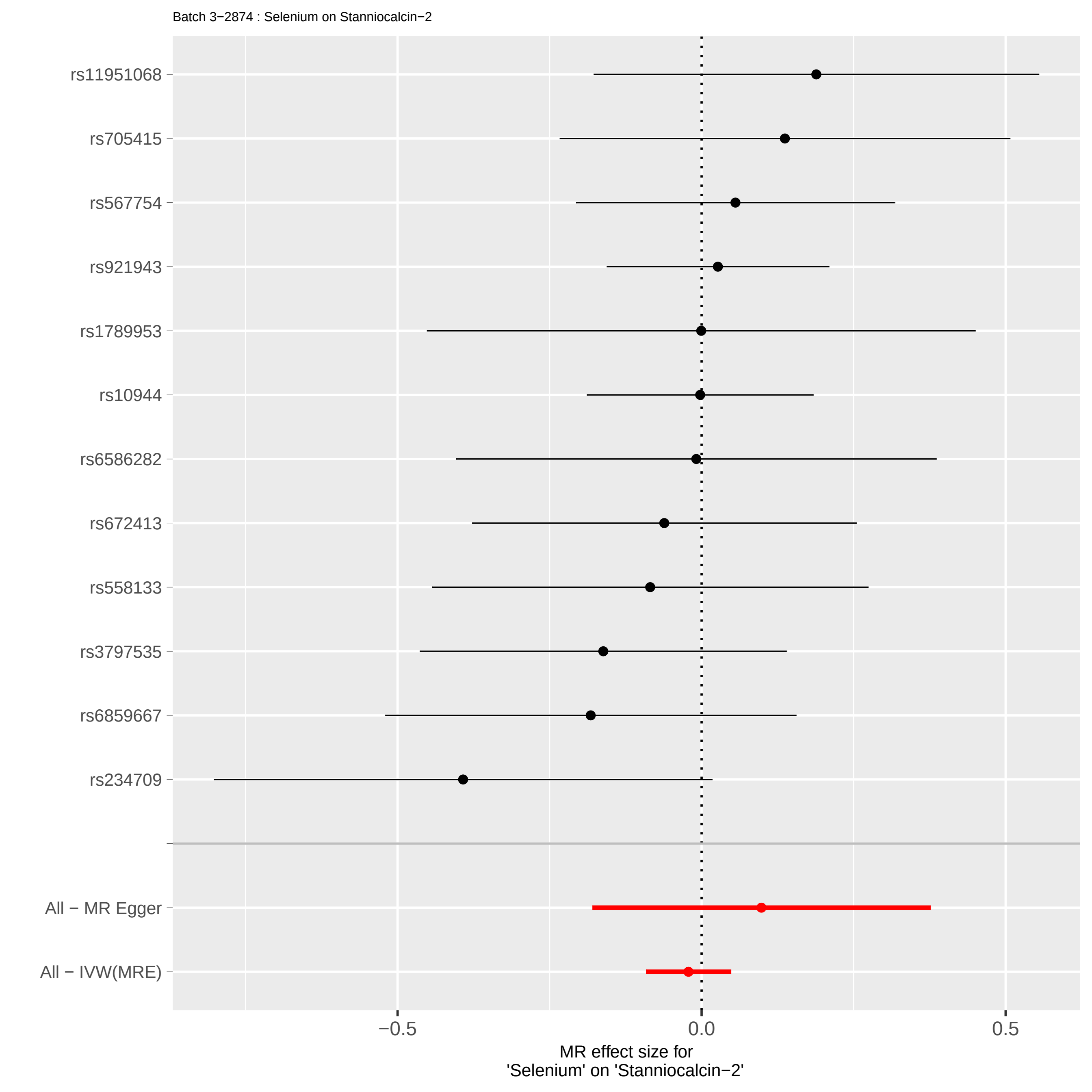

Batch 3−2874 : Selenium on Stanniocalcin−2
rs11951068
rs705415
rs567754
rs921943
rs1789953
rs10944
rs6586282
rs672413
rs558133
rs3797535
rs6859667
rs234709
All − MR Egger
All − IVW(MRE)
−0.5
0.0
0.5
MR effect size for
'Selenium' on 'Stanniocalcin−2'

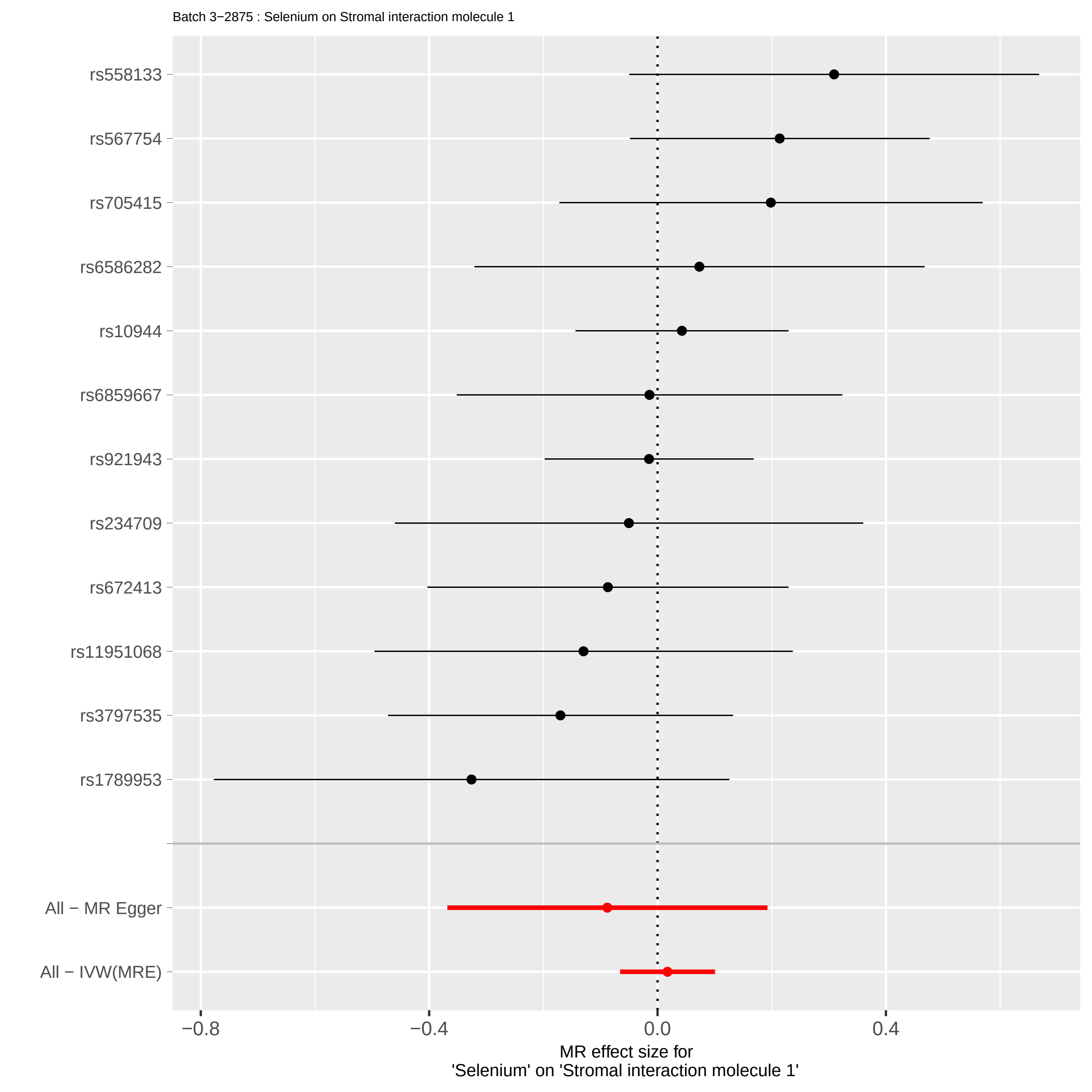

Batch 3−2875 : Selenium on Stromal interaction molecule 1
rs558133
rs567754
rs705415
rs6586282
rs10944
rs6859667
rs921943
rs234709
rs672413
rs11951068
rs3797535
rs1789953
All − MR Egger
All − IVW(MRE)
−0.8
−0.4
0.0
0.4
MR effect size for
'Selenium' on 'Stromal interaction molecule 1'

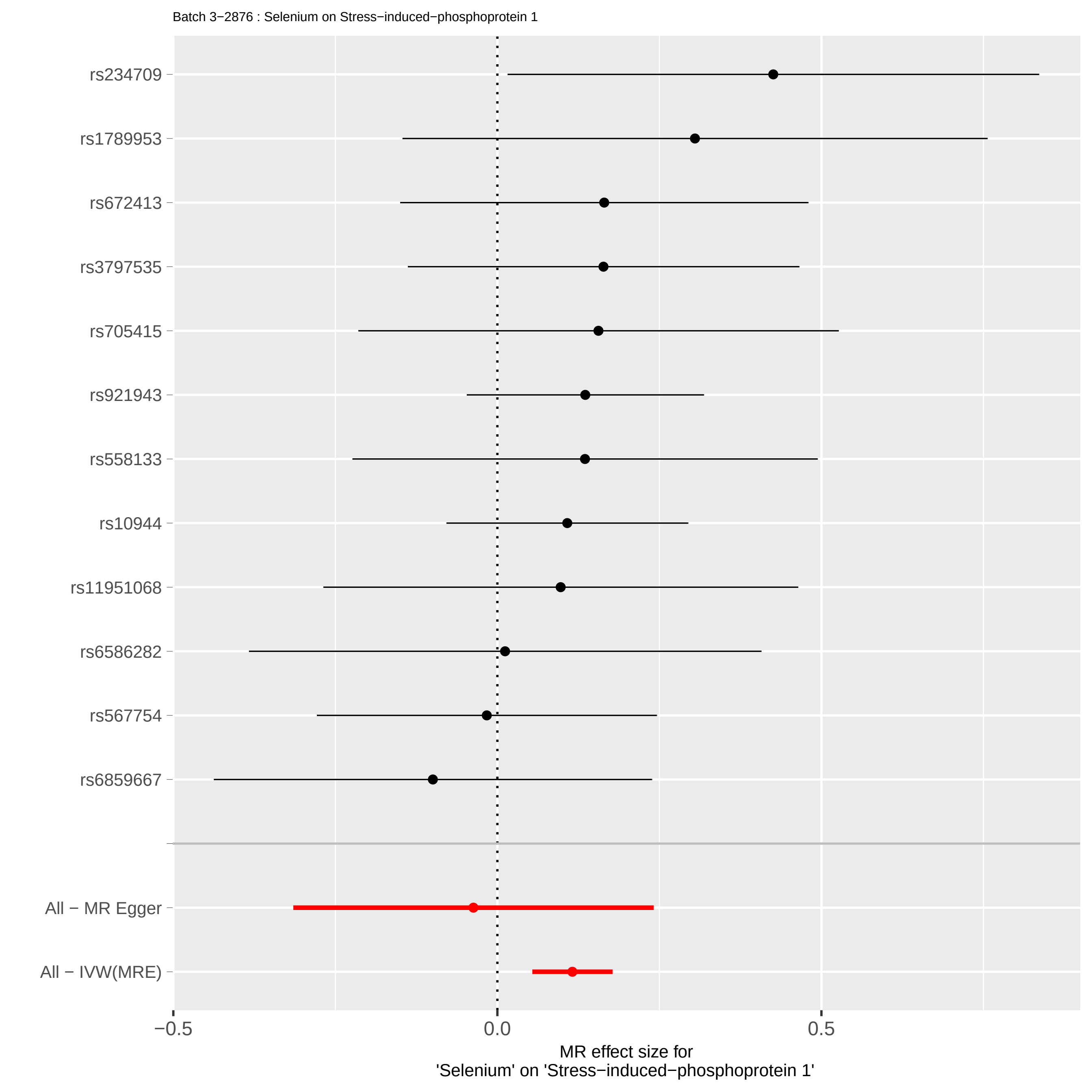
Batch 3−2876 : Selenium on Stress−induced−phosphoprotein 1
rs234709
rs1789953
rs672413
rs3797535
rs705415
rs921943
rs558133
rs10944
rs11951068
rs6586282
rs567754
rs6859667
All − MR Egger
All − IVW(MRE)
−0.5
0.0
0.5
MR effect size for
'Selenium' on 'Stress−induced−phosphoprotein 1'

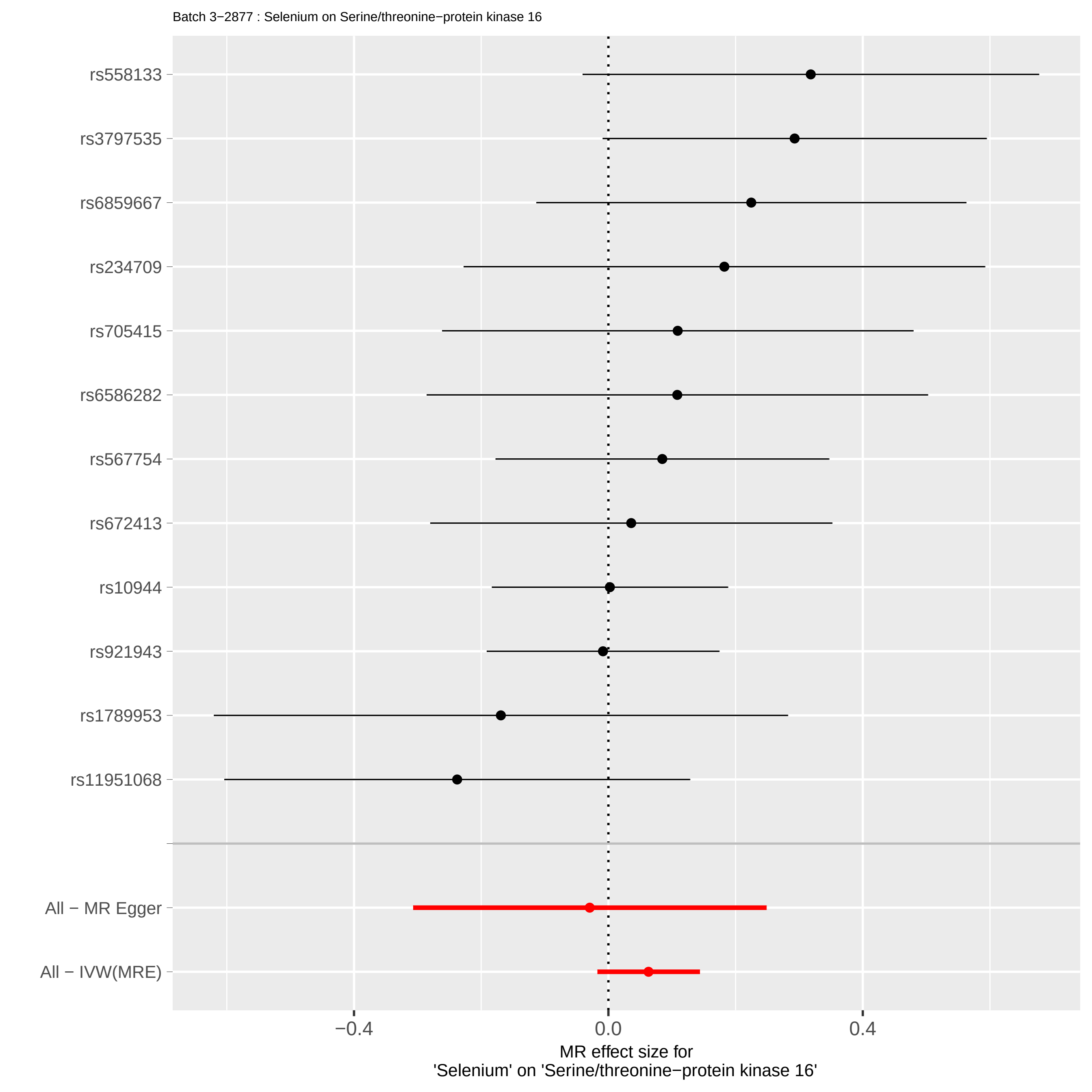
Batch 3−2877 : Selenium on Serine/threonine−protein kinase 16
rs558133
rs3797535
rs6859667
rs234709
rs705415
rs6586282
rs567754
rs672413
rs10944
rs921943
rs1789953
rs11951068
All − MR Egger
All − IVW(MRE)
−0.4
0.0
0.4
MR effect size for
'Selenium' on 'Serine/threonine−protein kinase 16'

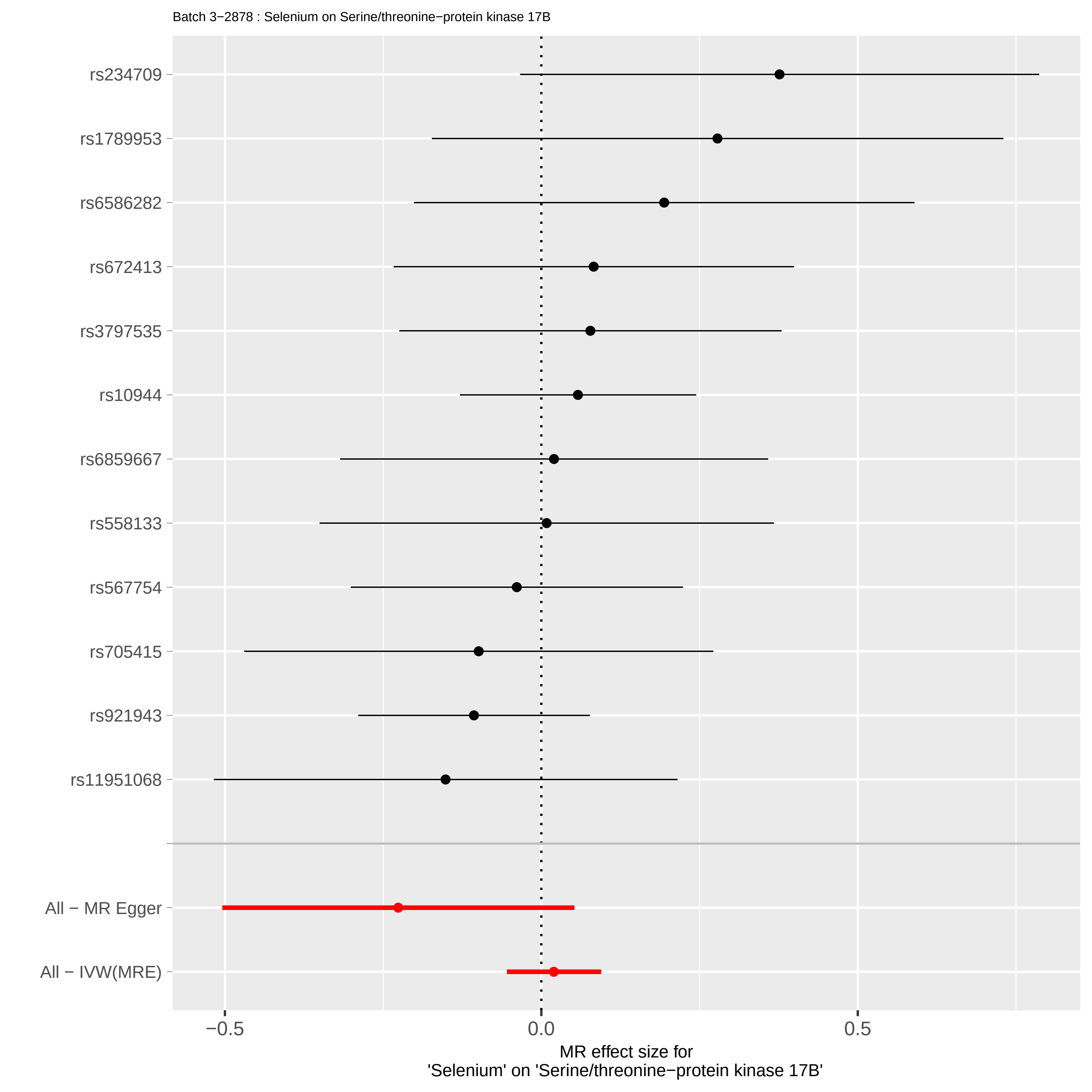
Batch 3−2878 : Selenium on Serine/threonine−protein kinase 17B
rs234709
rs1789953
rs6586282
rs672413
rs3797535
rs10944
rs6859667
rs558133
rs567754
rs705415
rs921943
rs11951068
All − MR Egger
All − IVW(MRE)
−0.5
0.0
0.5
MR effect size for
'Selenium' on 'Serine/threonine−protein kinase 17B'

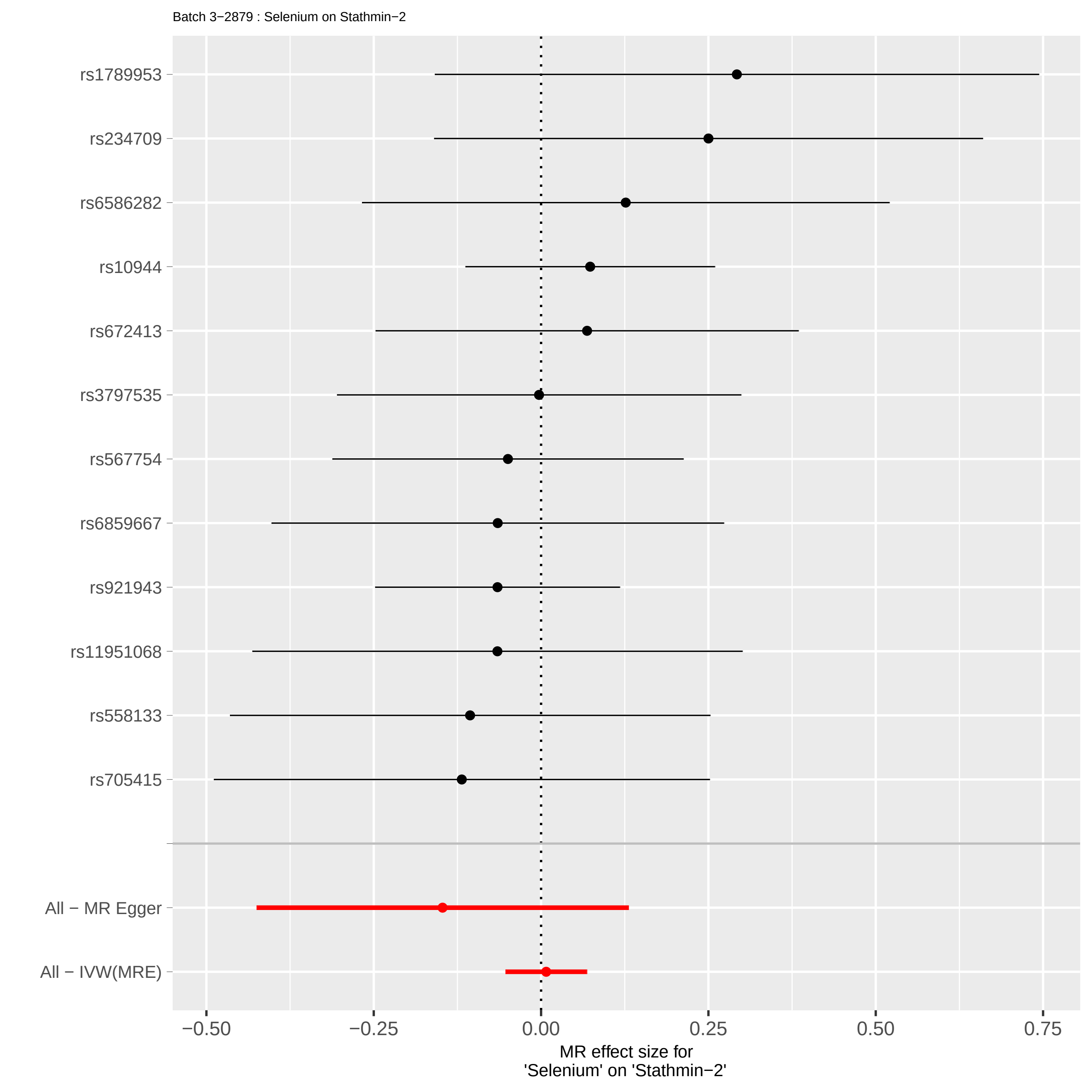

Batch 3−2879 : Selenium on Stathmin−2
rs1789953
rs234709
rs6586282
rs10944
rs672413
rs3797535
rs567754
rs6859667
rs921943
rs11951068
rs558133
rs705415
All − MR Egger
All − IVW(MRE)
−0.50
−0.25
0.00
0.25
0.50
0.75
MR effect size for
'Selenium' on 'Stathmin−2'

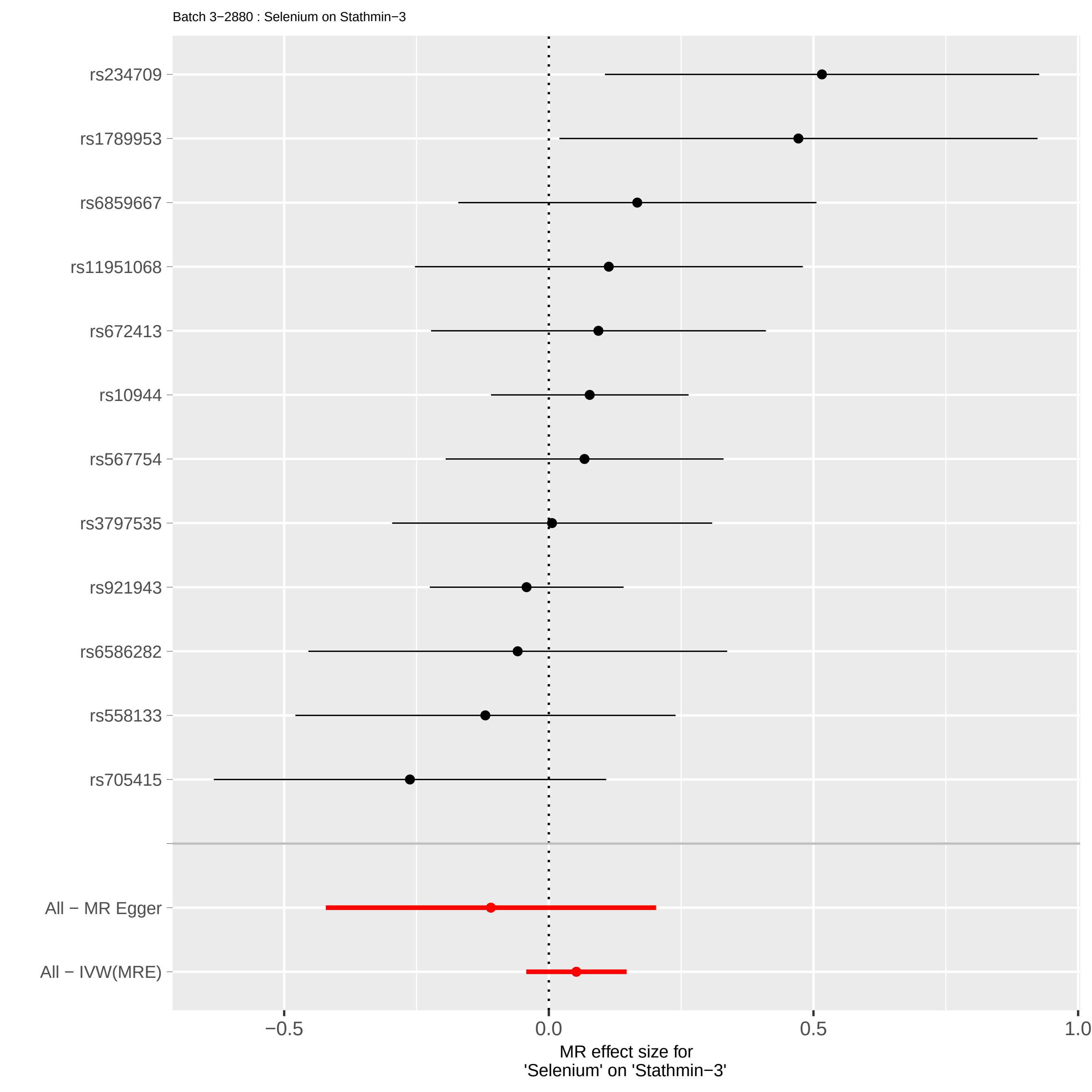
Batch 3−2880 : Selenium on Stathmin−3
rs234709
rs1789953
rs6859667
rs11951068
rs672413
rs10944
rs567754
rs3797535
rs921943
rs6586282
rs558133
rs705415
All − MR Egger
All − IVW(MRE)
−0.5
0.0
0.5
1.0
MR effect size for
'Selenium' on 'Stathmin−3'

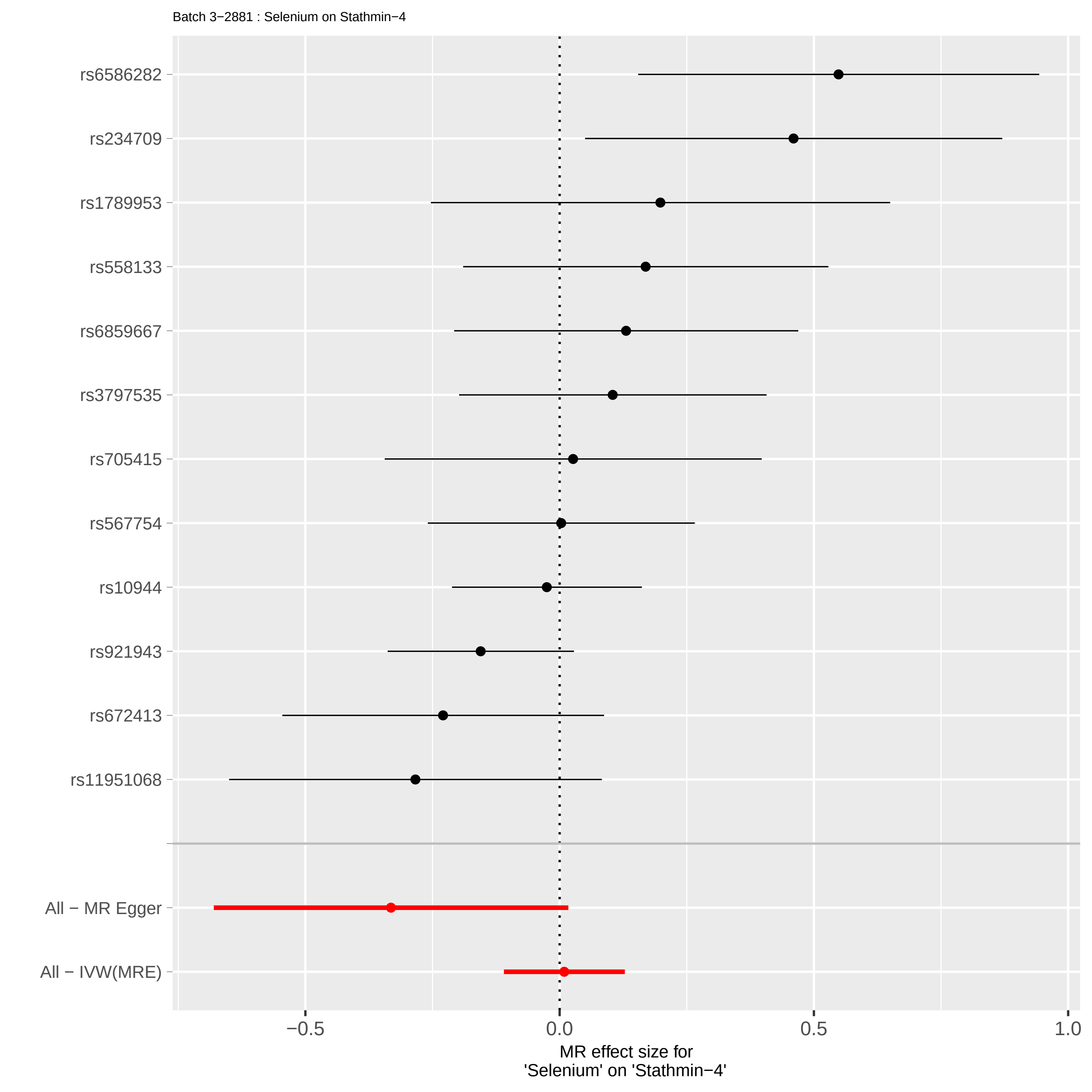
Batch 3−2881 : Selenium on Stathmin−4
rs6586282
rs234709
rs1789953
rs558133
rs6859667
rs3797535
rs705415
rs567754
rs10944
rs921943
rs672413
rs11951068
All − MR Egger
All − IVW(MRE)
−0.5
0.0
0.5
1.0
MR effect size for
'Selenium' on 'Stathmin−4'

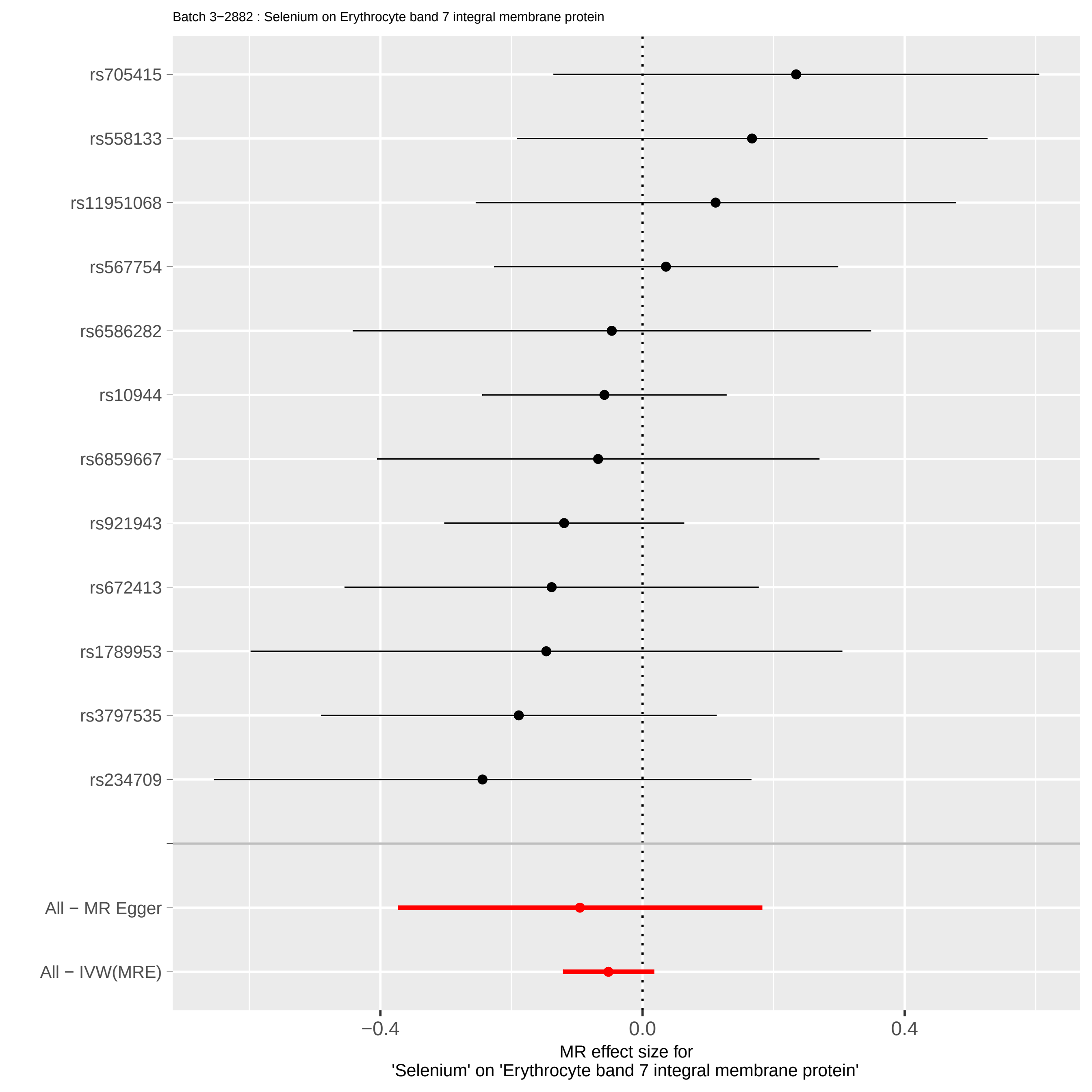

Batch 3−2882 : Selenium on Erythrocyte band 7 integral membrane protein
rs705415
rs558133
rs11951068
rs567754
rs6586282
rs10944
rs6859667
rs921943
rs672413
rs1789953
rs3797535
rs234709
All − MR Egger
All − IVW(MRE)
−0.4
0.0
0.4
MR effect size for
'Selenium' on 'Erythrocyte band 7 integral membrane protein'

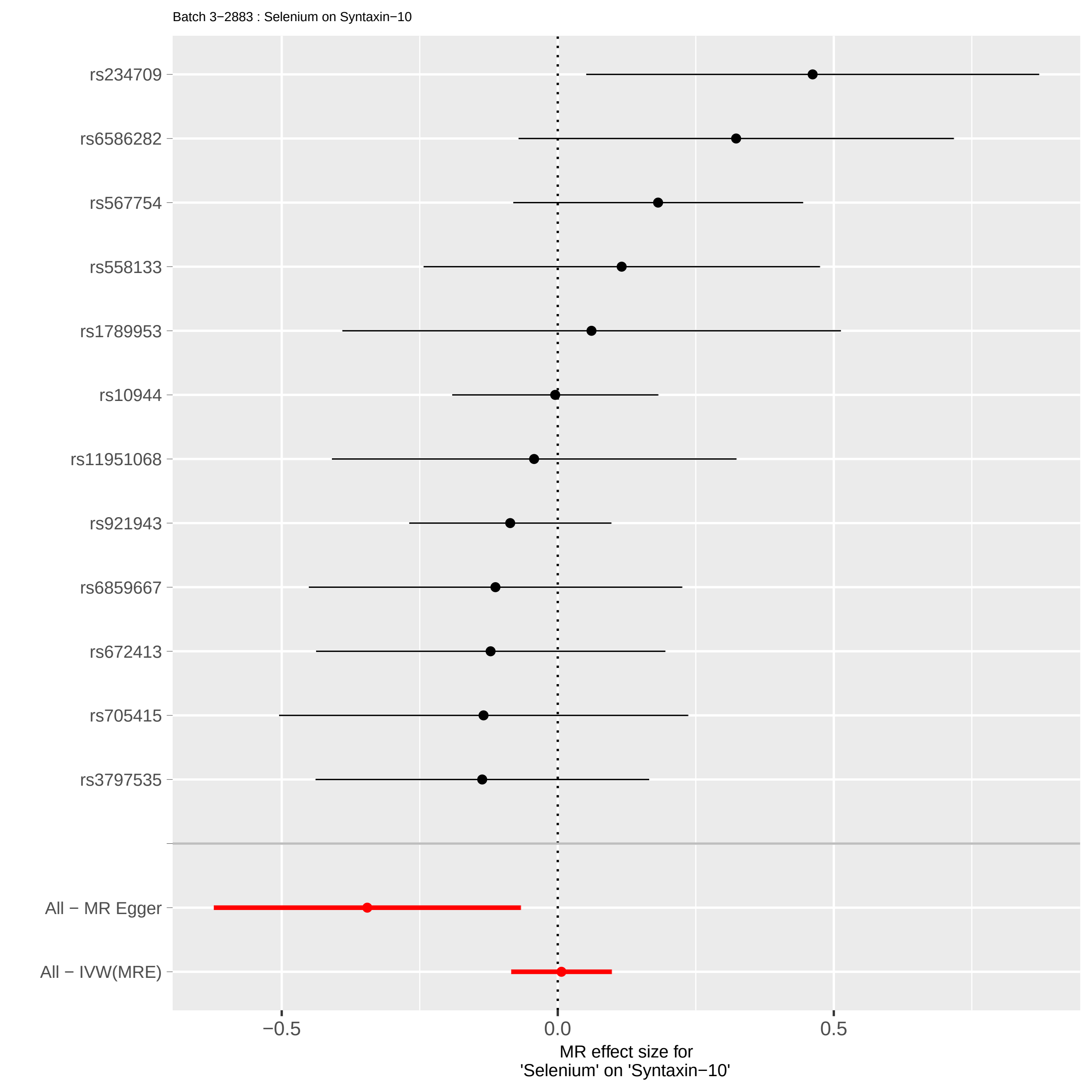
Batch 3−2883 : Selenium on Syntaxin−10
rs234709
rs6586282
rs567754
rs558133
rs1789953
rs10944
rs11951068
rs921943
rs6859667
rs672413
rs705415
rs3797535
All − MR Egger
All − IVW(MRE)
−0.5
0.0
0.5
MR effect size for
'Selenium' on 'Syntaxin−10'

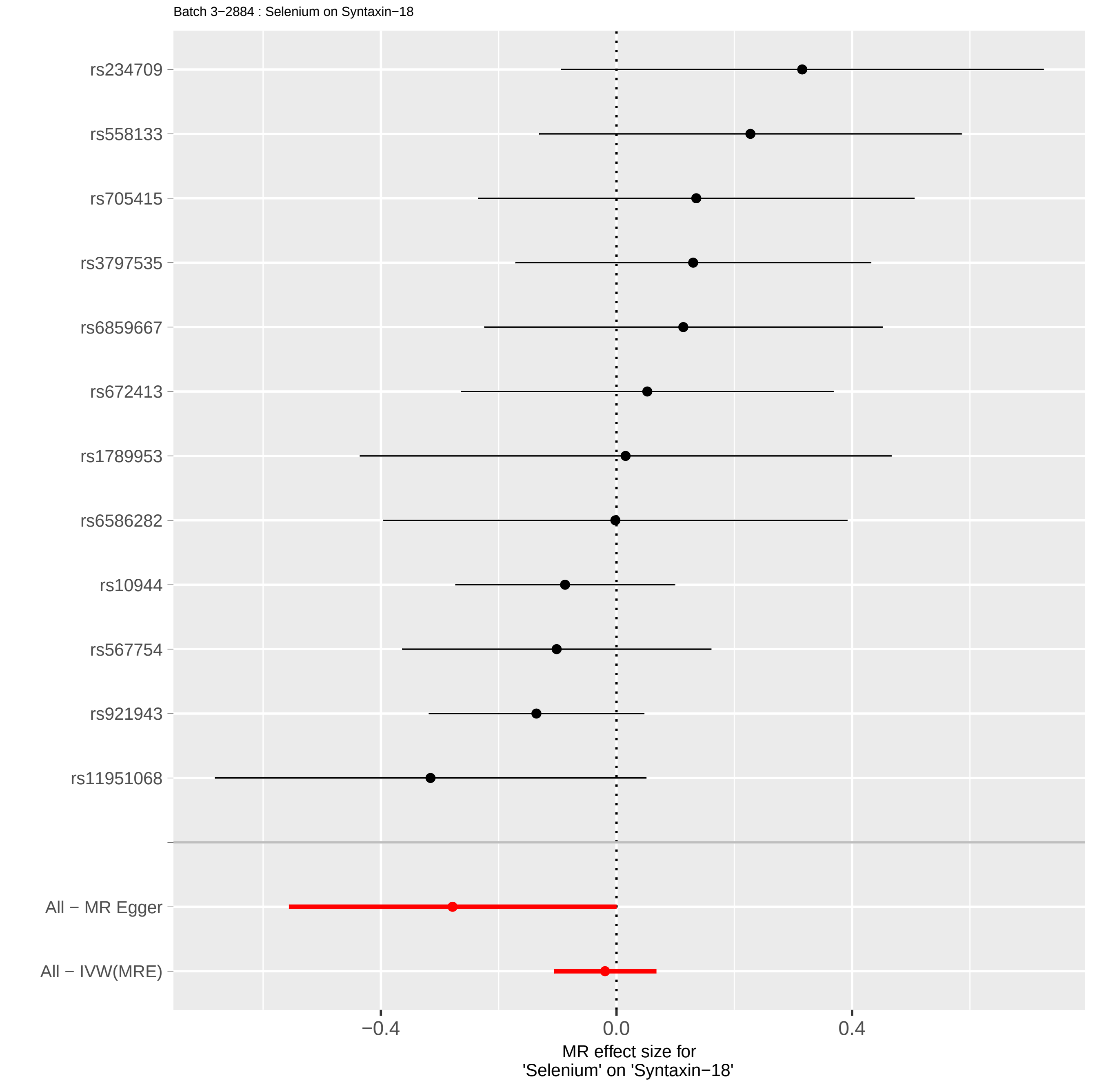

Batch 3−2884 : Selenium on Syntaxin−18
rs234709
rs558133
rs705415
rs3797535
rs6859667
rs672413
rs1789953
rs6586282
rs10944
rs567754
rs921943
rs11951068
All − MR Egger
All − IVW(MRE)
−0.4
0.0
0.4
MR effect size for
'Selenium' on 'Syntaxin−18'

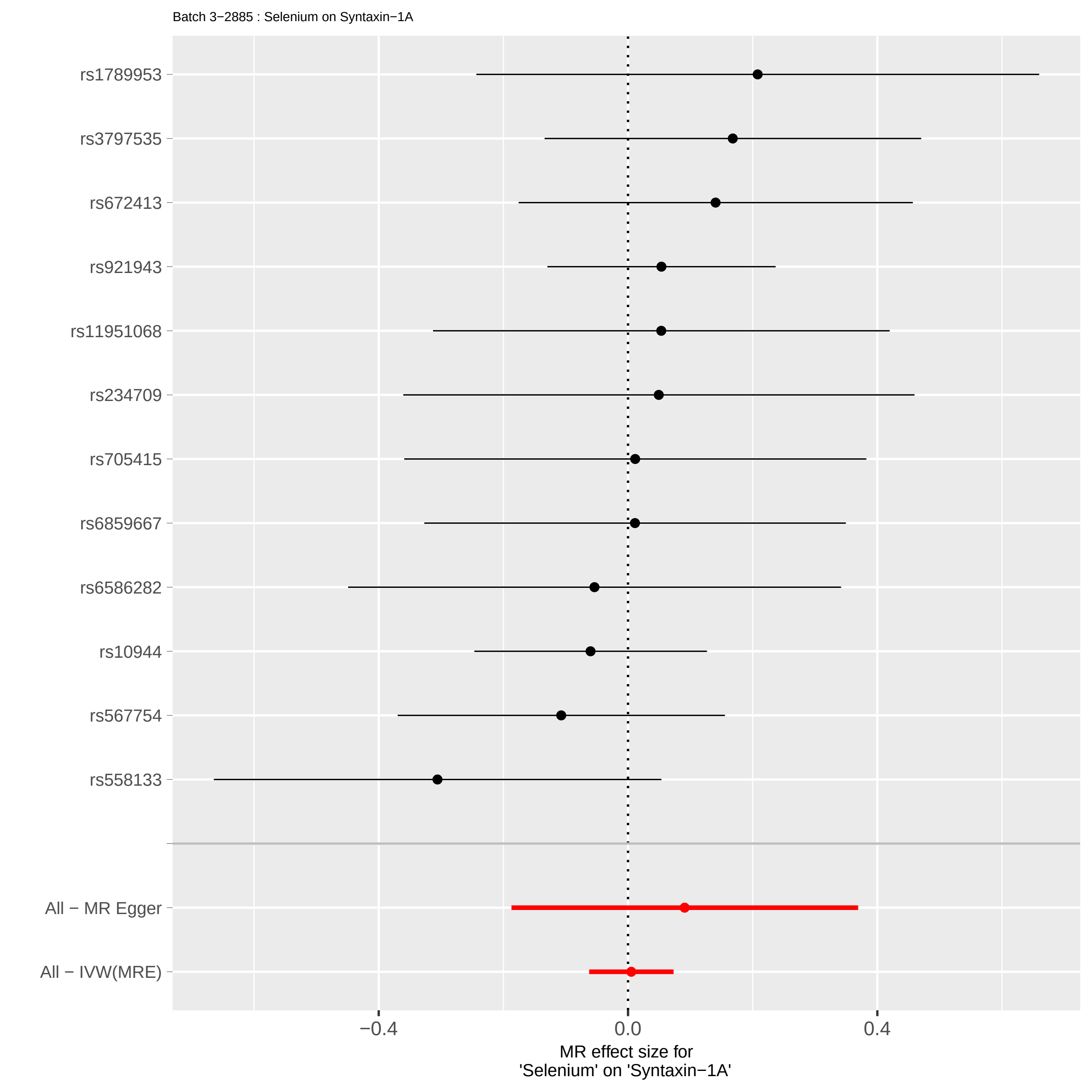

Batch 3−2885 : Selenium on Syntaxin−1A
rs1789953
rs3797535
rs672413
rs921943
rs11951068
rs234709
rs705415
rs6859667
rs6586282
rs10944
rs567754
rs558133
All − MR Egger
All − IVW(MRE)
−0.4
0.0
0.4
MR effect size for
'Selenium' on 'Syntaxin−1A'

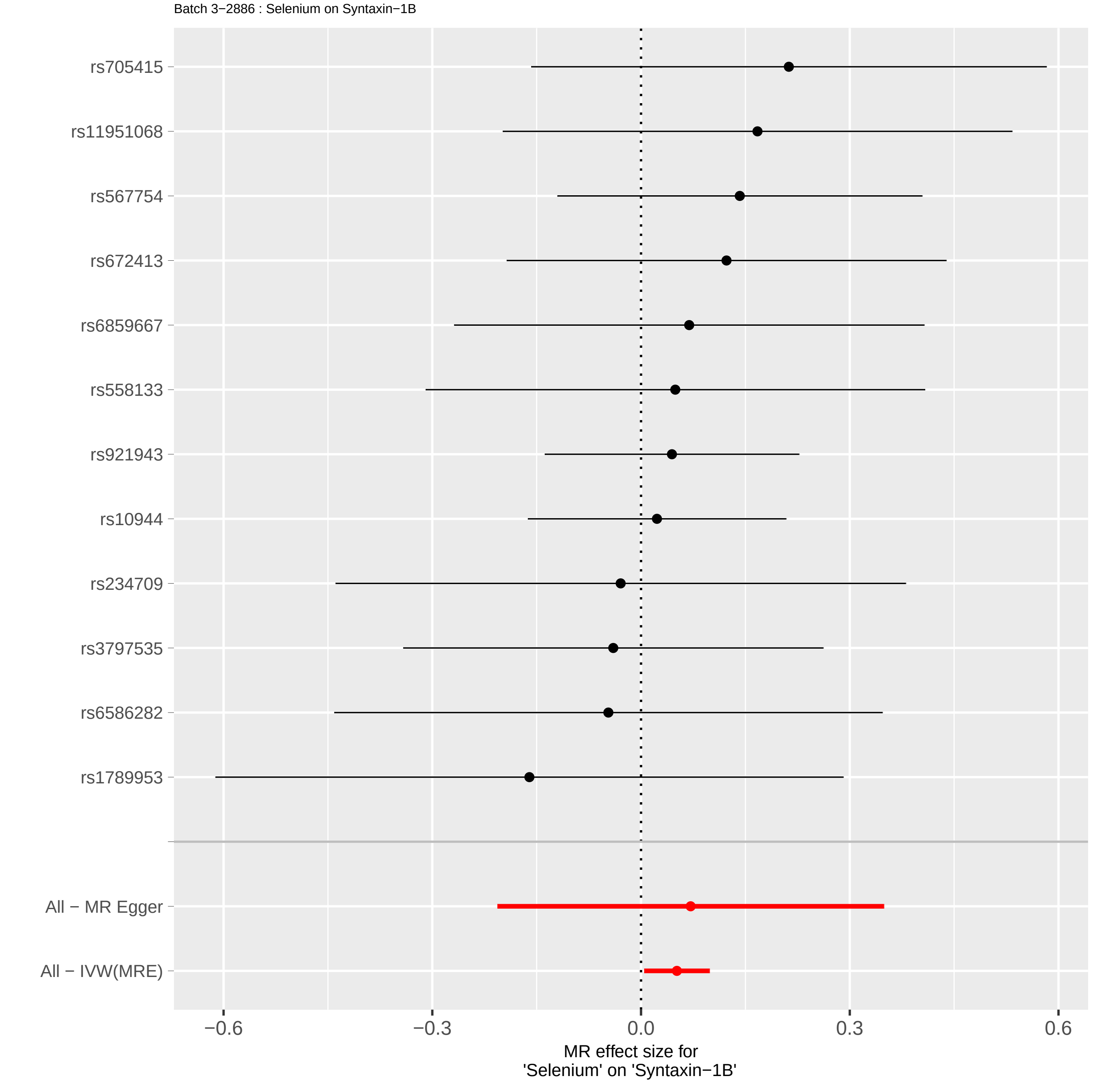

Batch 3−2886 : Selenium on Syntaxin−1B
rs705415
rs11951068
rs567754
rs672413
rs6859667
rs558133
rs921943
rs10944
rs234709
rs3797535
rs6586282
rs1789953
All − MR Egger
All − IVW(MRE)
−0.6
−0.3
0.0
0.3
0.6
MR effect size for
'Selenium' on 'Syntaxin−1B'

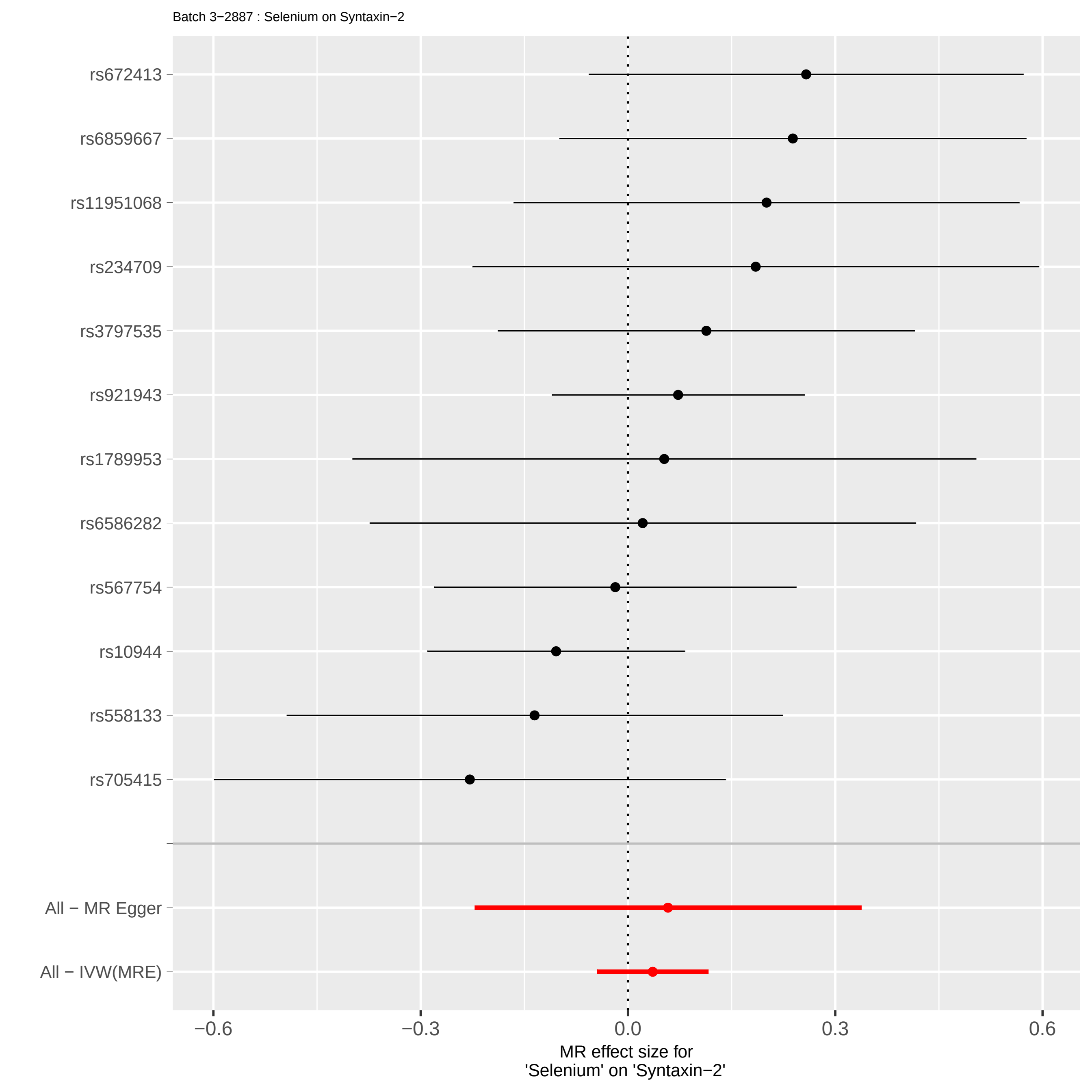

Batch 3−2887 : Selenium on Syntaxin−2
rs672413
rs6859667
rs11951068
rs234709
rs3797535
rs921943
rs1789953
rs6586282
rs567754
rs10944
rs558133
rs705415
All − MR Egger
All − IVW(MRE)
−0.6
−0.3
0.0
0.3
0.6
MR effect size for
'Selenium' on 'Syntaxin−2'

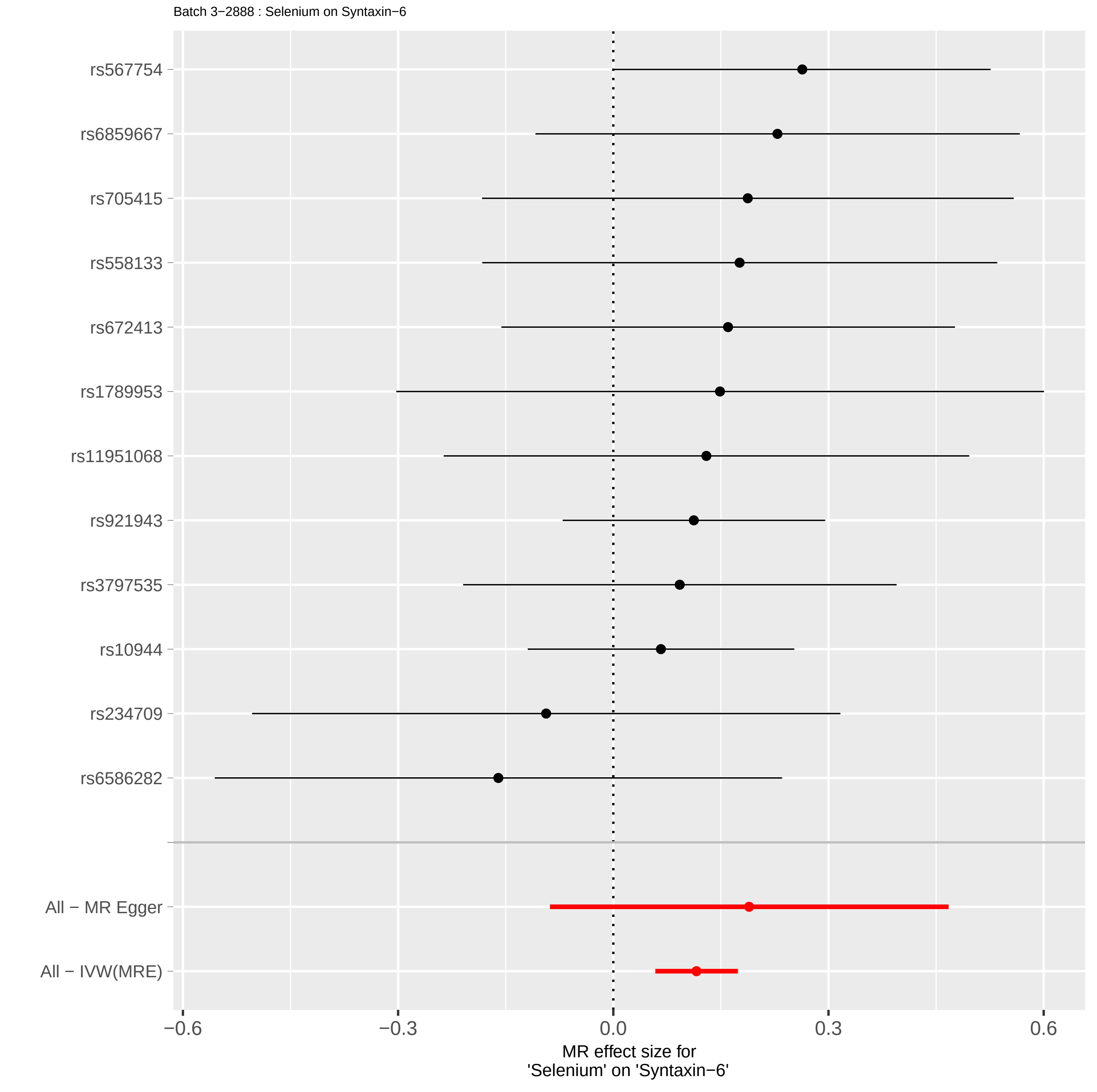

Batch 3−2888 : Selenium on Syntaxin−6
rs567754
rs6859667
rs705415
rs558133
rs672413
rs1789953
rs11951068
rs921943
rs3797535
rs10944
rs234709
rs6586282
All − MR Egger
All − IVW(MRE)
−0.6
−0.3
0.0
0.3
0.6
MR effect size for
'Selenium' on 'Syntaxin−6'

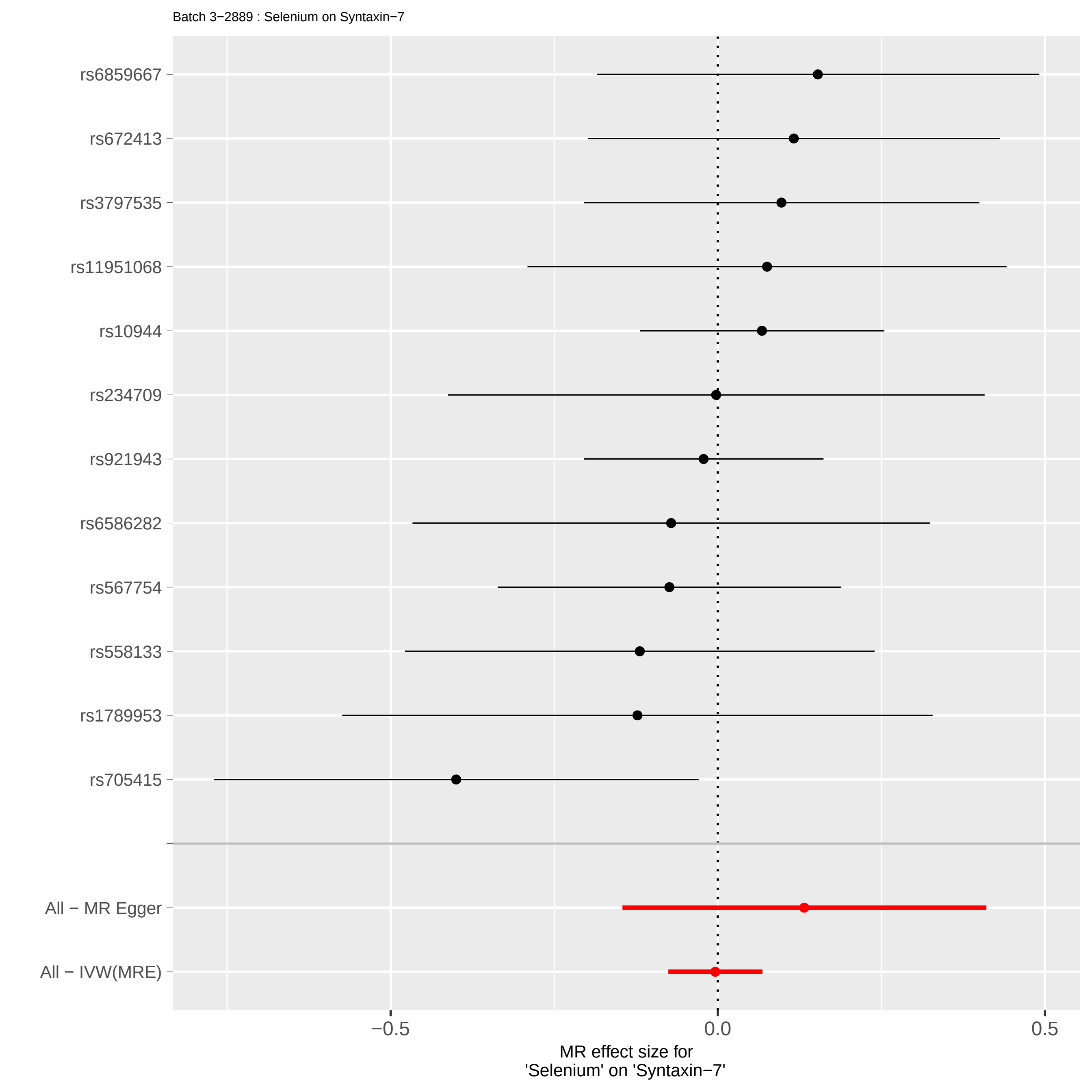

Batch 3−2889 : Selenium on Syntaxin−7
rs6859667
rs672413
rs3797535
rs11951068
rs10944
rs234709
rs921943
rs6586282
rs567754
rs558133
rs1789953
rs705415
All − MR Egger
All − IVW(MRE)
−0.5
0.0
0.5
MR effect size for
'Selenium' on 'Syntaxin−7'

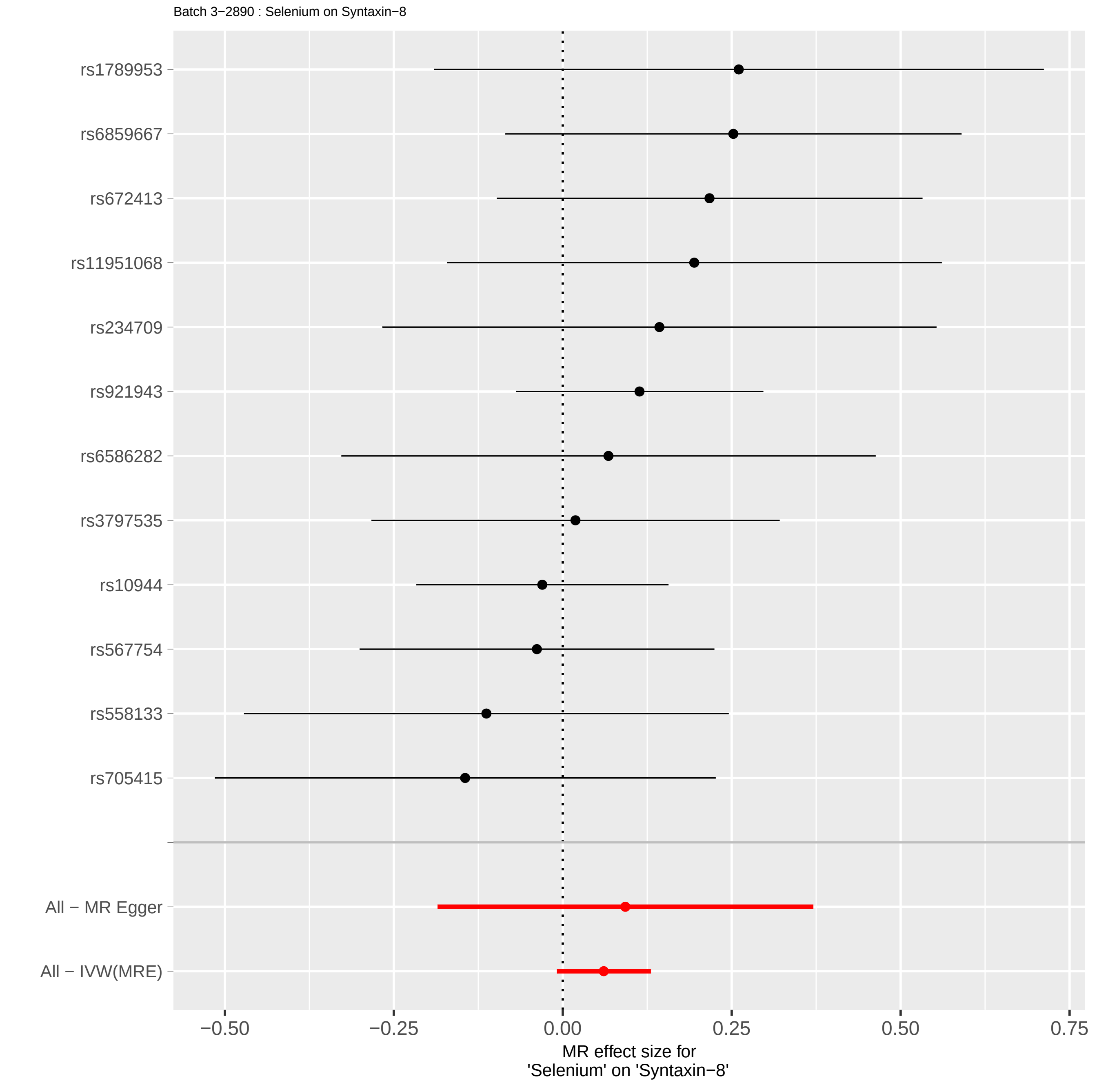

Batch 3−2890 : Selenium on Syntaxin−8
rs1789953
rs6859667
rs672413
rs11951068
rs234709
rs921943
rs6586282
rs3797535
rs10944
rs567754
rs558133
rs705415
All − MR Egger
All − IVW(MRE)
−0.50
−0.25
0.00
0.25
0.50
0.75
MR effect size for
'Selenium' on 'Syntaxin−8'

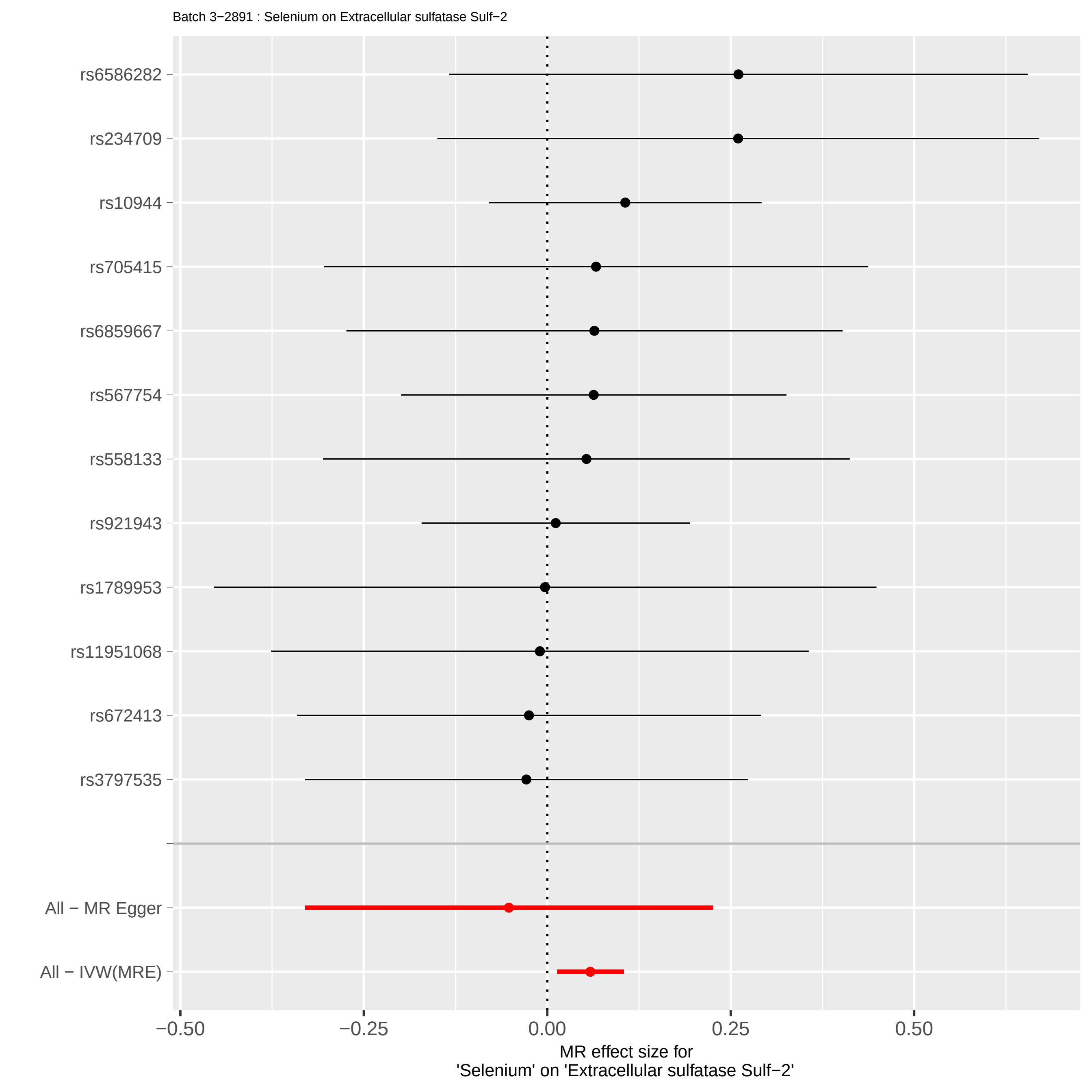

Batch 3−2891 : Selenium on Extracellular sulfatase Sulf−2
rs6586282
rs234709
rs10944
rs705415
rs6859667
rs567754
rs558133
rs921943
rs1789953
rs11951068
rs672413
rs3797535
All − MR Egger
All − IVW(MRE)
−0.50
−0.25
0.00
0.25
0.50
MR effect size for
'Selenium' on 'Extracellular sulfatase Sulf−2'

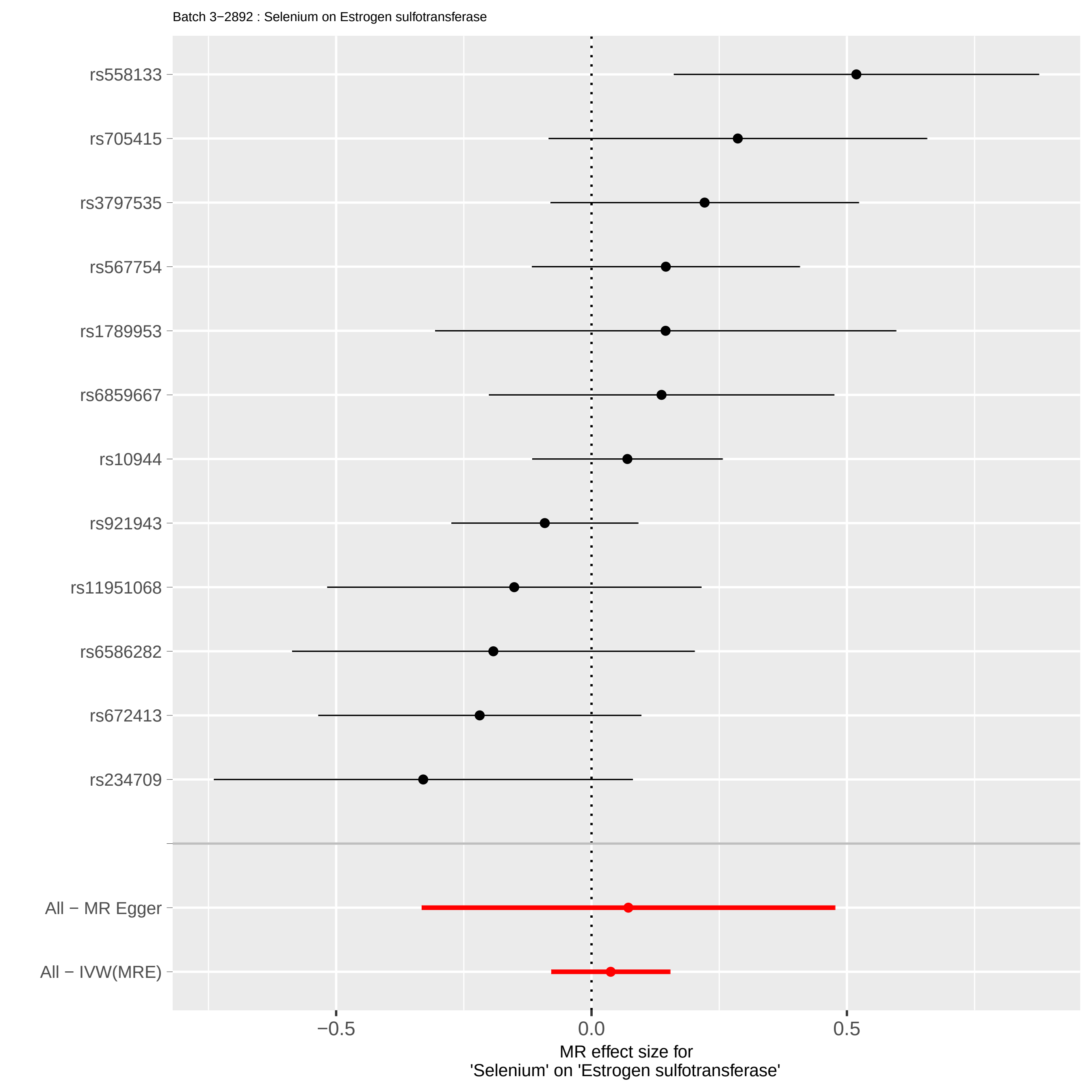
Batch 3−2892 : Selenium on Estrogen sulfotransferase
rs558133
rs705415
rs3797535
rs567754
rs1789953
rs6859667
rs10944
rs921943
rs11951068
rs6586282
rs672413
rs234709
All − MR Egger
All − IVW(MRE)
−0.5
0.0
0.5
MR effect size for
'Selenium' on 'Estrogen sulfotransferase'

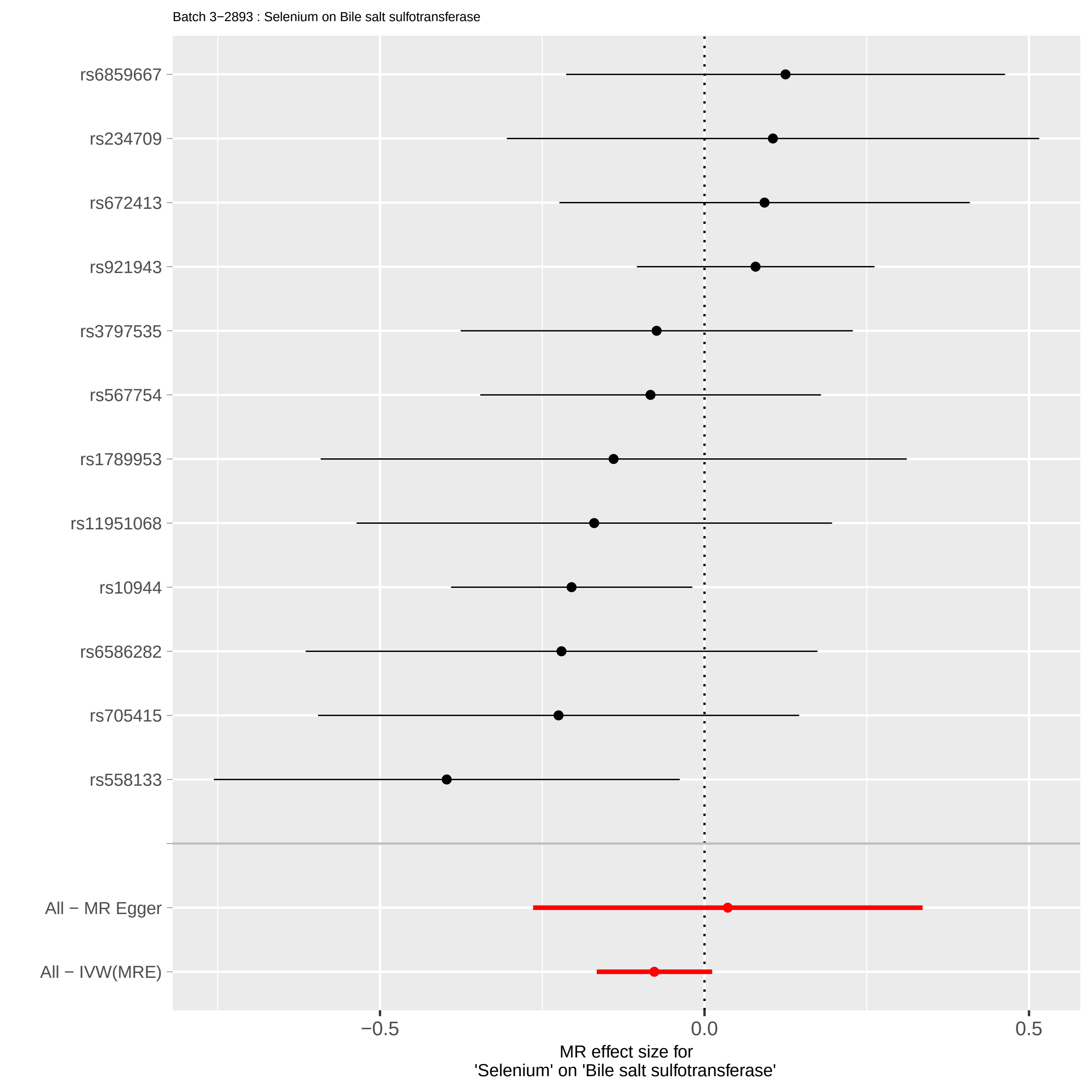

Batch 3−2893 : Selenium on Bile salt sulfotransferase
rs6859667
rs234709
rs672413
rs921943
rs3797535
rs567754
rs1789953
rs11951068
rs10944
rs6586282
rs705415
rs558133
All − MR Egger
All − IVW(MRE)
−0.5
0.0
0.5
MR effect size for
'Selenium' on 'Bile salt sulfotransferase'

Batch 3−2894 : Selenium on Sulfotransferase 4A1

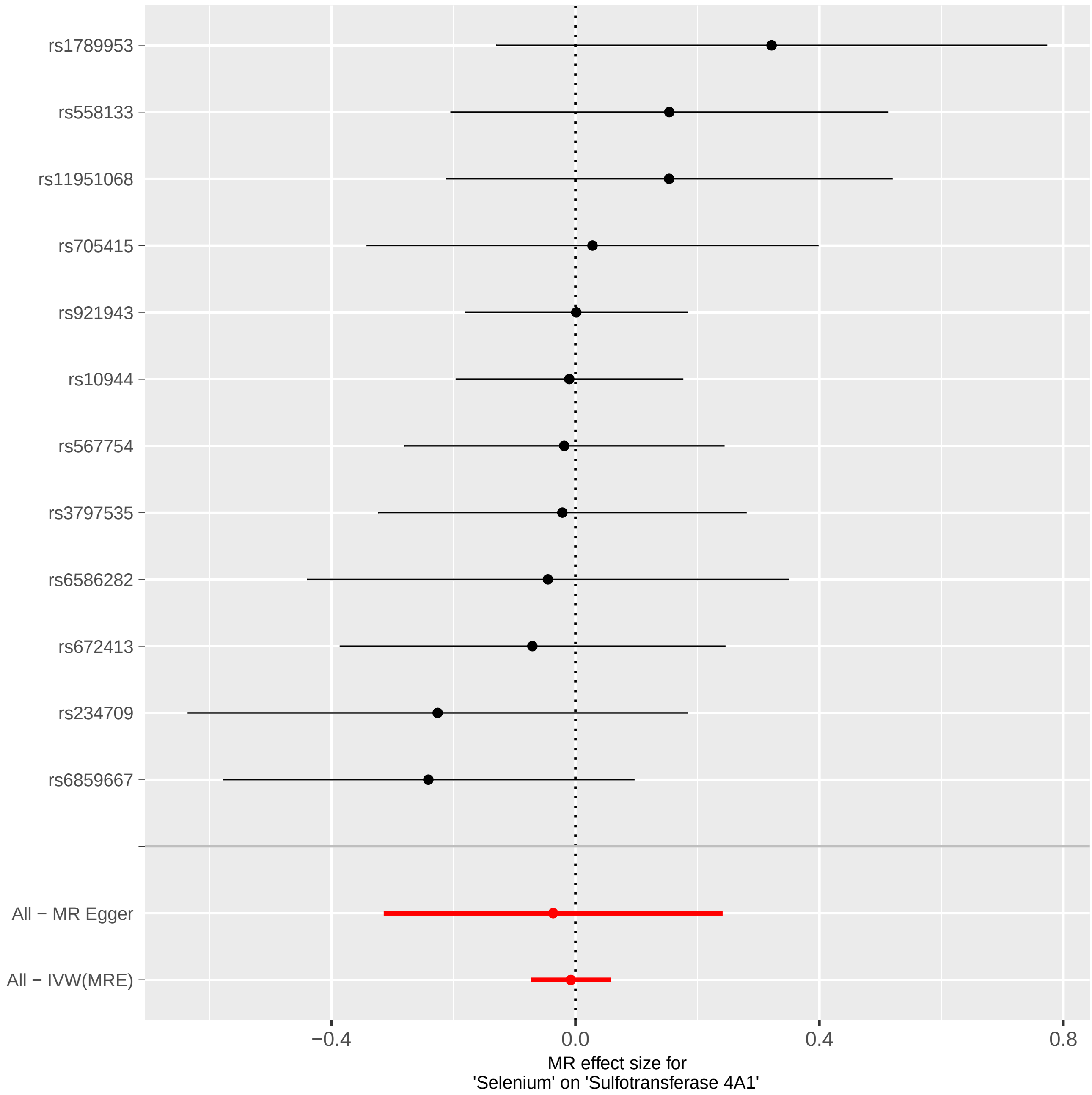

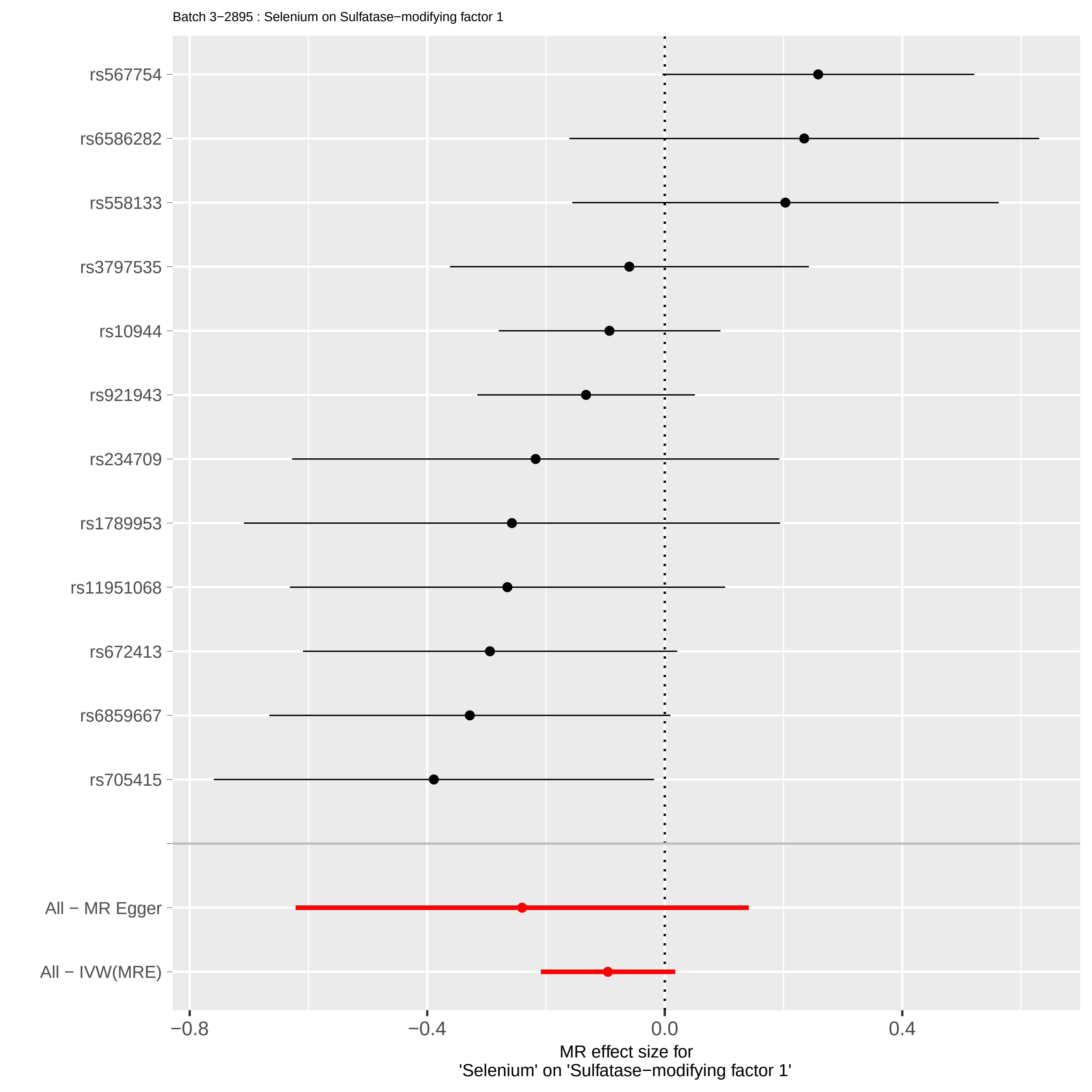
Batch 3−2895 : Selenium on Sulfatase−modifying factor 1
rs567754
rs6586282
rs558133
rs3797535
rs10944
rs921943
rs234709
rs1789953
rs11951068
rs672413
rs6859667
rs705415
All − MR Egger
All − IVW(MRE)
−0.8
−0.4
0.0
0.4
MR effect size for
'Selenium' on 'Sulfatase−modifying factor 1'

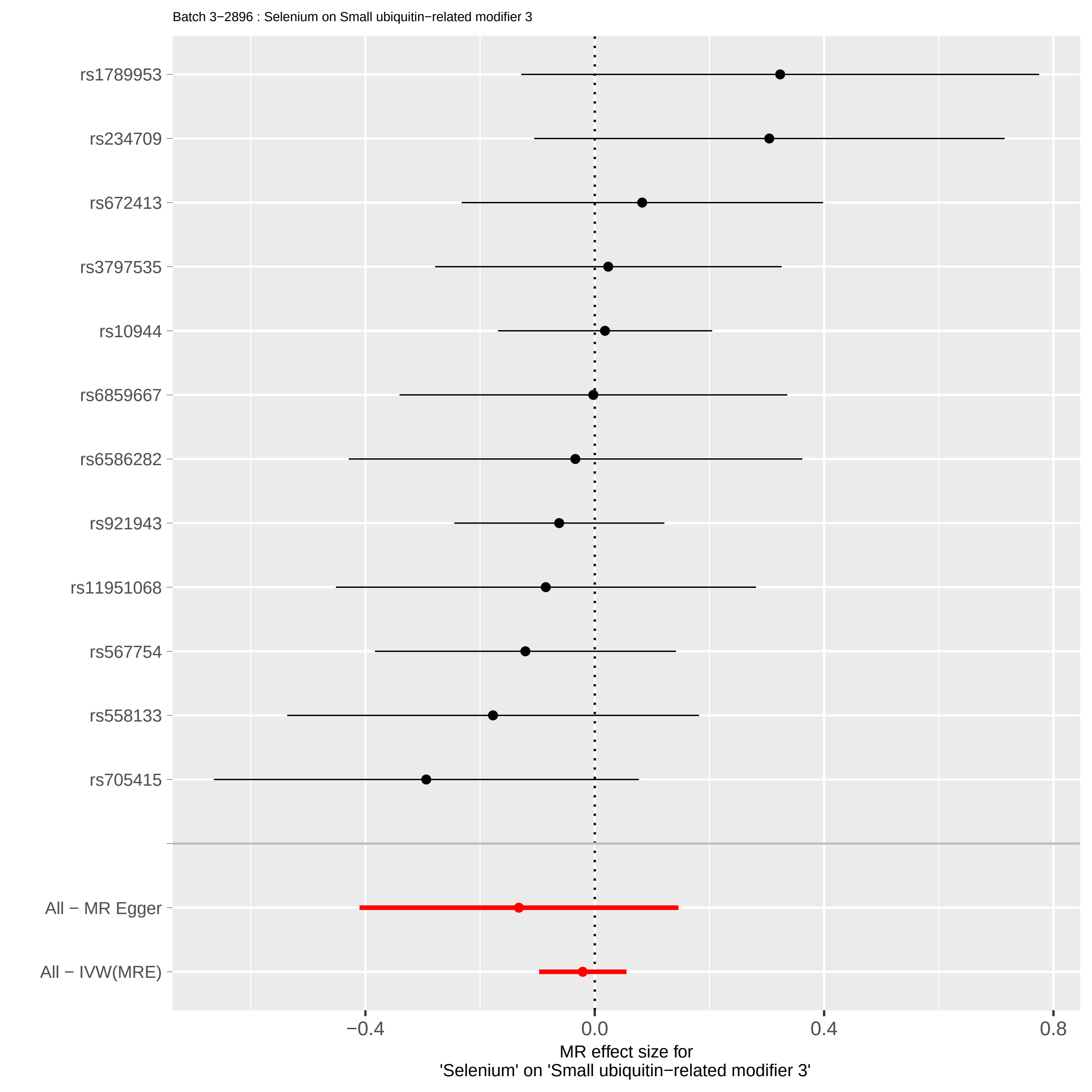
Batch 3−2896 : Selenium on Small ubiquitin−related modifier 3
rs1789953
rs234709
rs672413
rs3797535
rs10944
rs6859667
rs6586282
rs921943
rs11951068
rs567754
rs558133
rs705415
All − MR Egger
All − IVW(MRE)
−0.4
0.0
0.4
0.8
MR effect size for
'Selenium' on 'Small ubiquitin−related modifier 3'

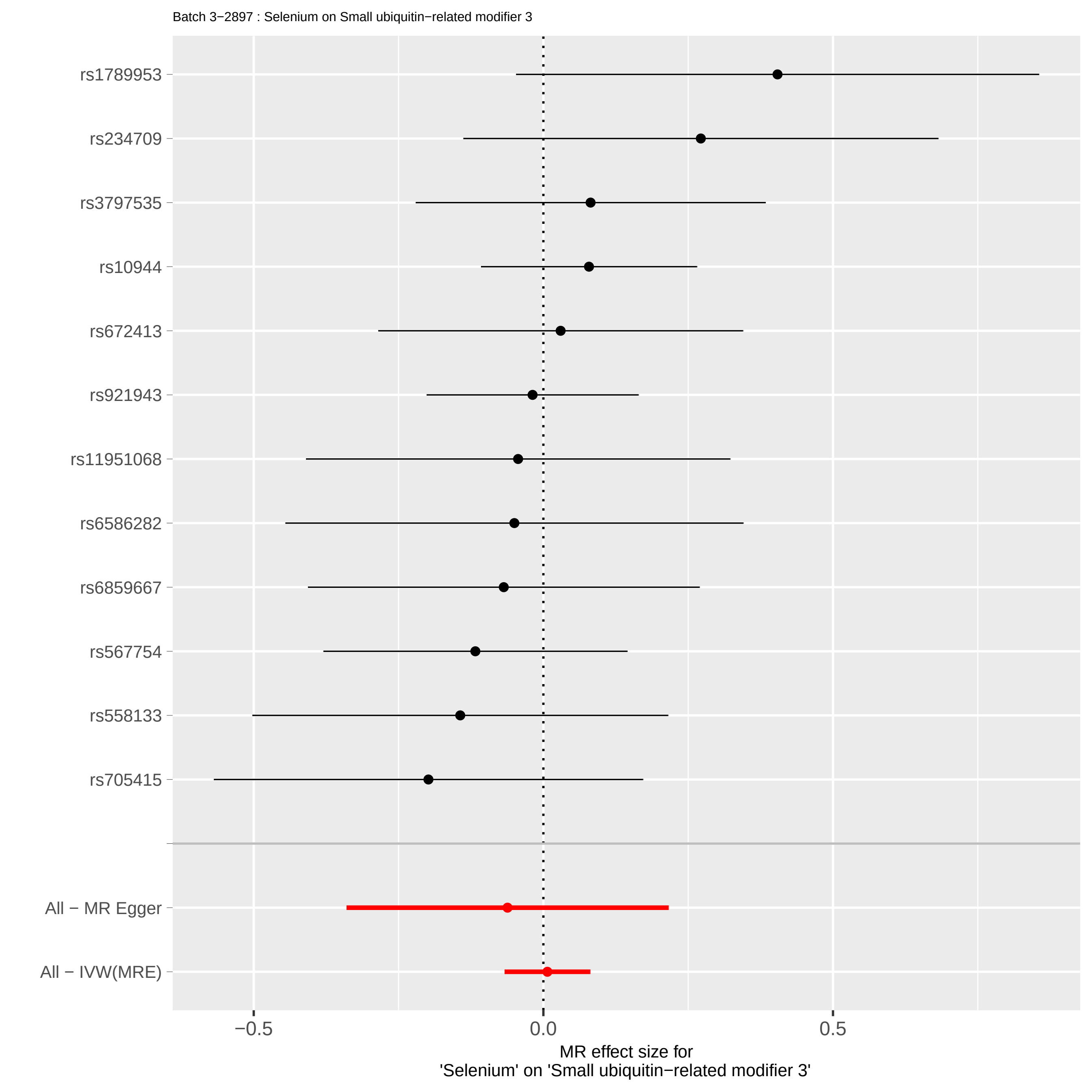
Batch 3−2897 : Selenium on Small ubiquitin−related modifier 3
rs1789953
rs234709
rs3797535
rs10944
rs672413
rs921943
rs11951068
rs6586282
rs6859667
rs567754
rs558133
rs705415
All − MR Egger
All − IVW(MRE)
−0.5
0.0
0.5
MR effect size for
'Selenium' on 'Small ubiquitin−related modifier 3'

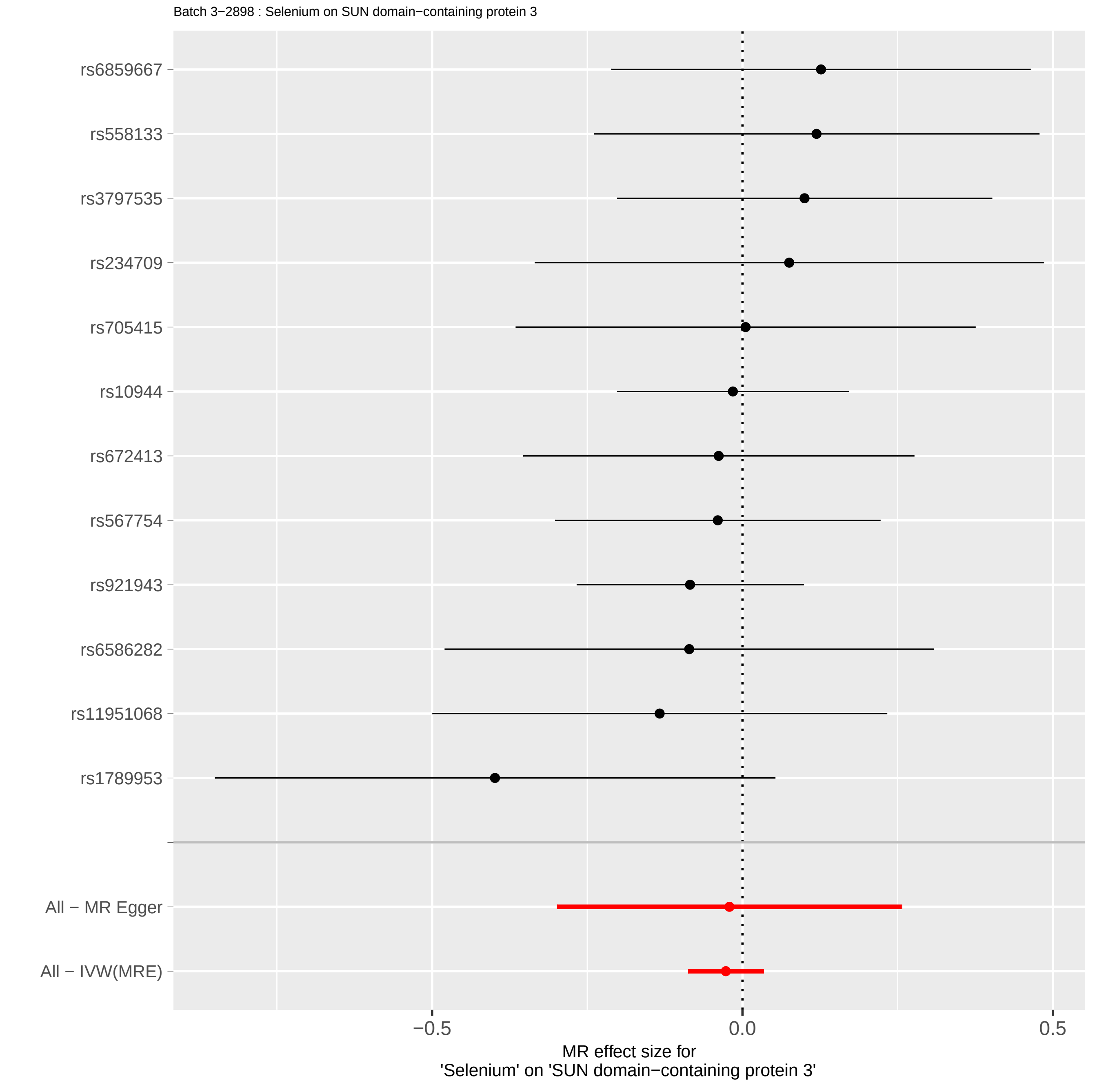

Batch 3−2898 : Selenium on SUN domain−containing protein 3
rs6859667
rs558133
rs3797535
rs234709
rs705415
rs10944
rs672413
rs567754
rs921943
rs6586282
rs11951068
rs1789953
All − MR Egger
All − IVW(MRE)
−0.5
0.0
0.5
MR effect size for
'Selenium' on 'SUN domain−containing protein 3'

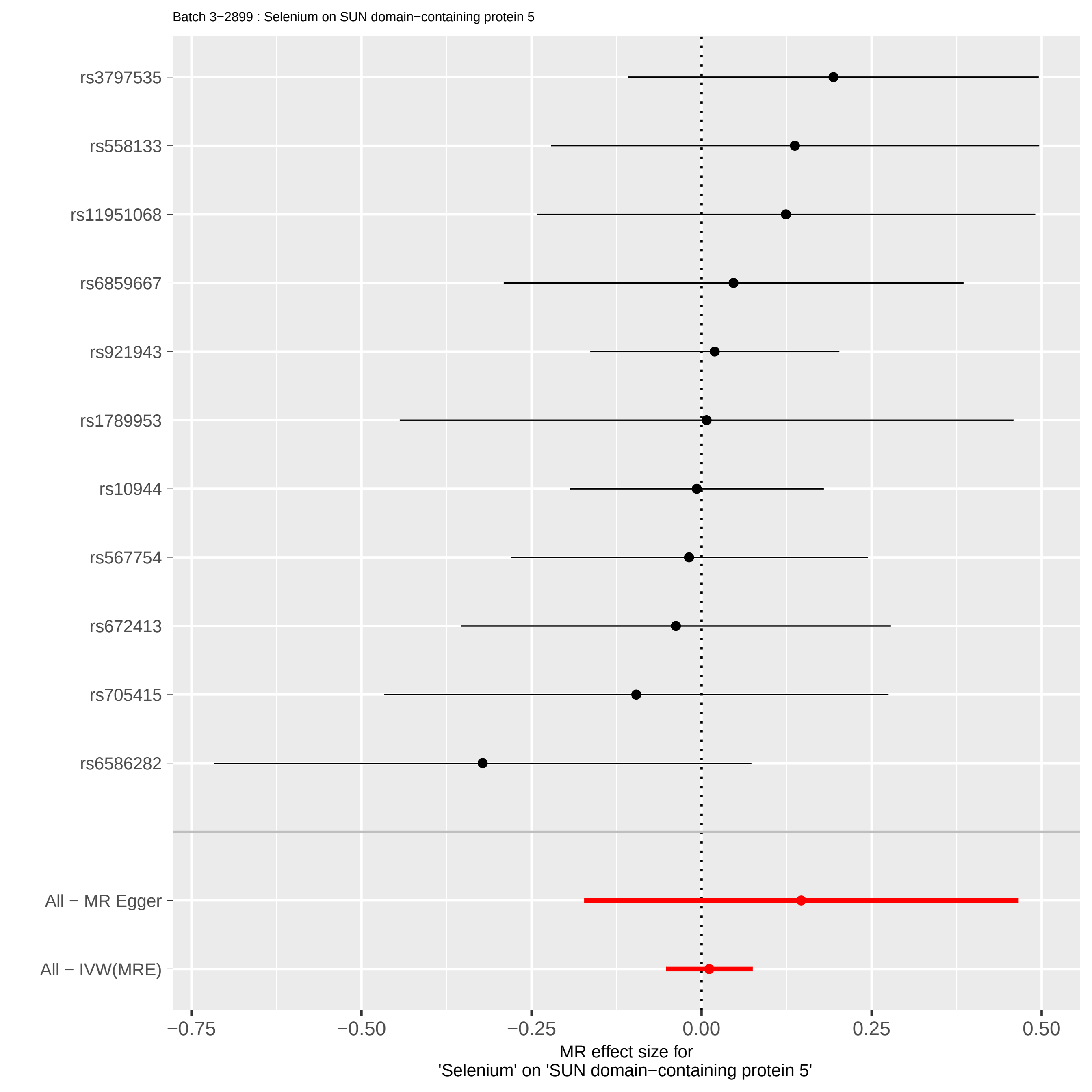

Batch 3−2899 : Selenium on SUN domain−containing protein 5
rs3797535
rs558133
rs11951068
rs6859667
rs921943
rs1789953
rs10944
rs567754
rs672413
rs705415
rs6586282
All − MR Egger
All − IVW(MRE)
−0.75
−0.50
−0.25
0.00
0.25
0.50
MR effect size for
'Selenium' on 'SUN domain−containing protein 5'

Batch 3−2900 : Selenium on Surfeit locus protein 1

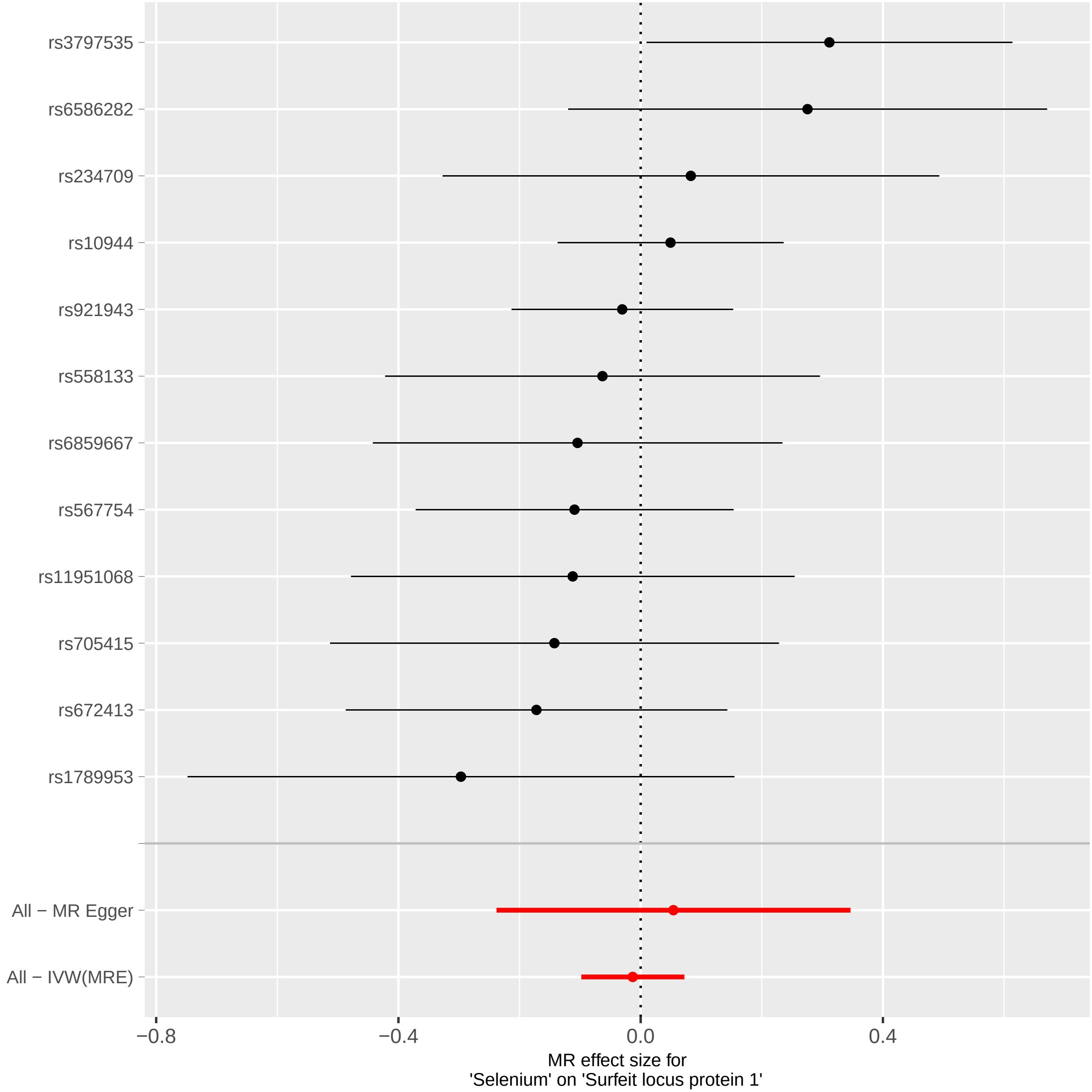

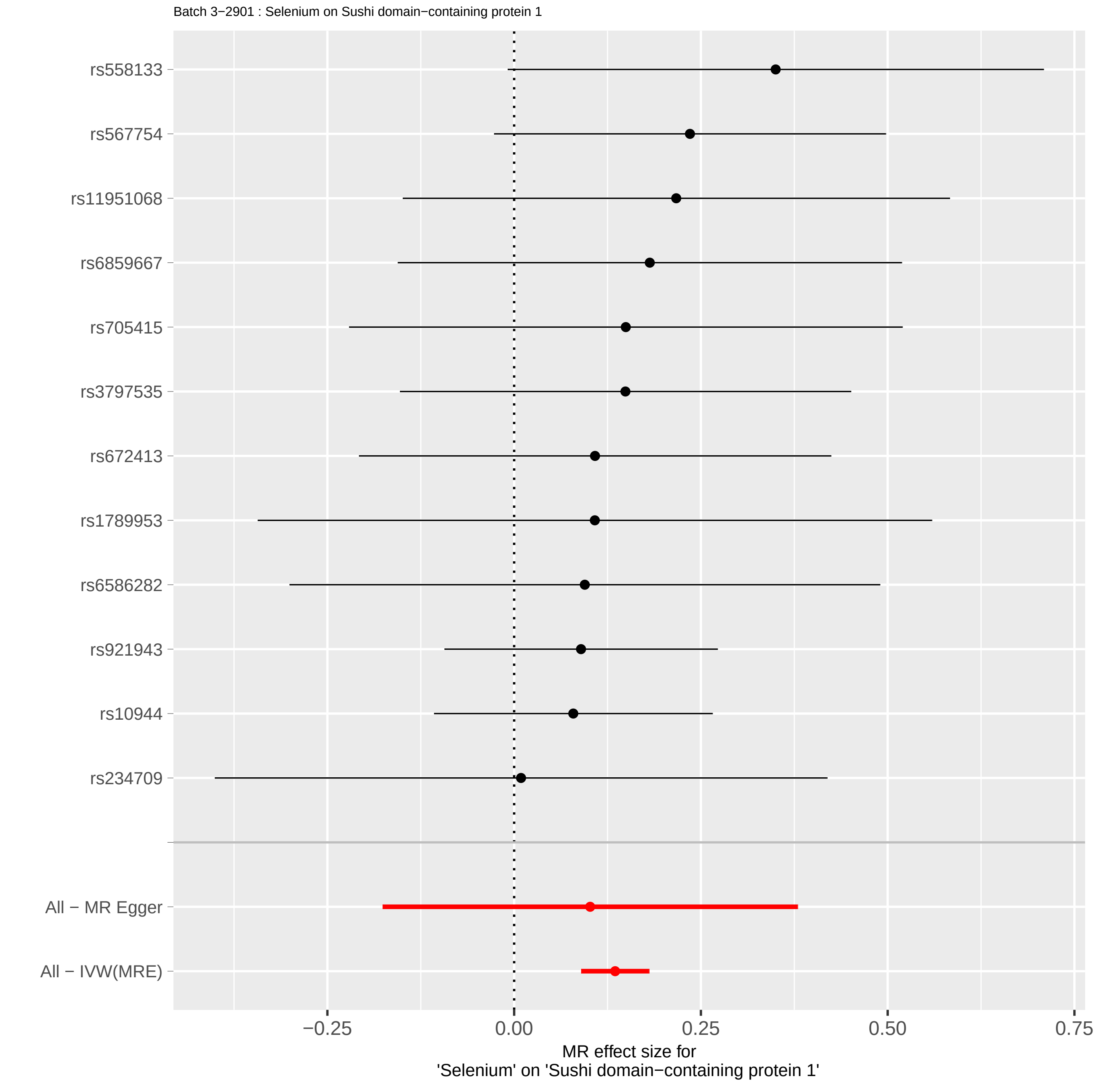

Batch 3−2901 : Selenium on Sushi domain−containing protein 1
rs558133
rs567754
rs11951068
rs6859667
rs705415
rs3797535
rs672413
rs1789953
rs6586282
rs921943
rs10944
rs234709
All − MR Egger
All − IVW(MRE)
−0.25
0.00
0.25
0.50
0.75
MR effect size for
'Selenium' on 'Sushi domain−containing protein 1'

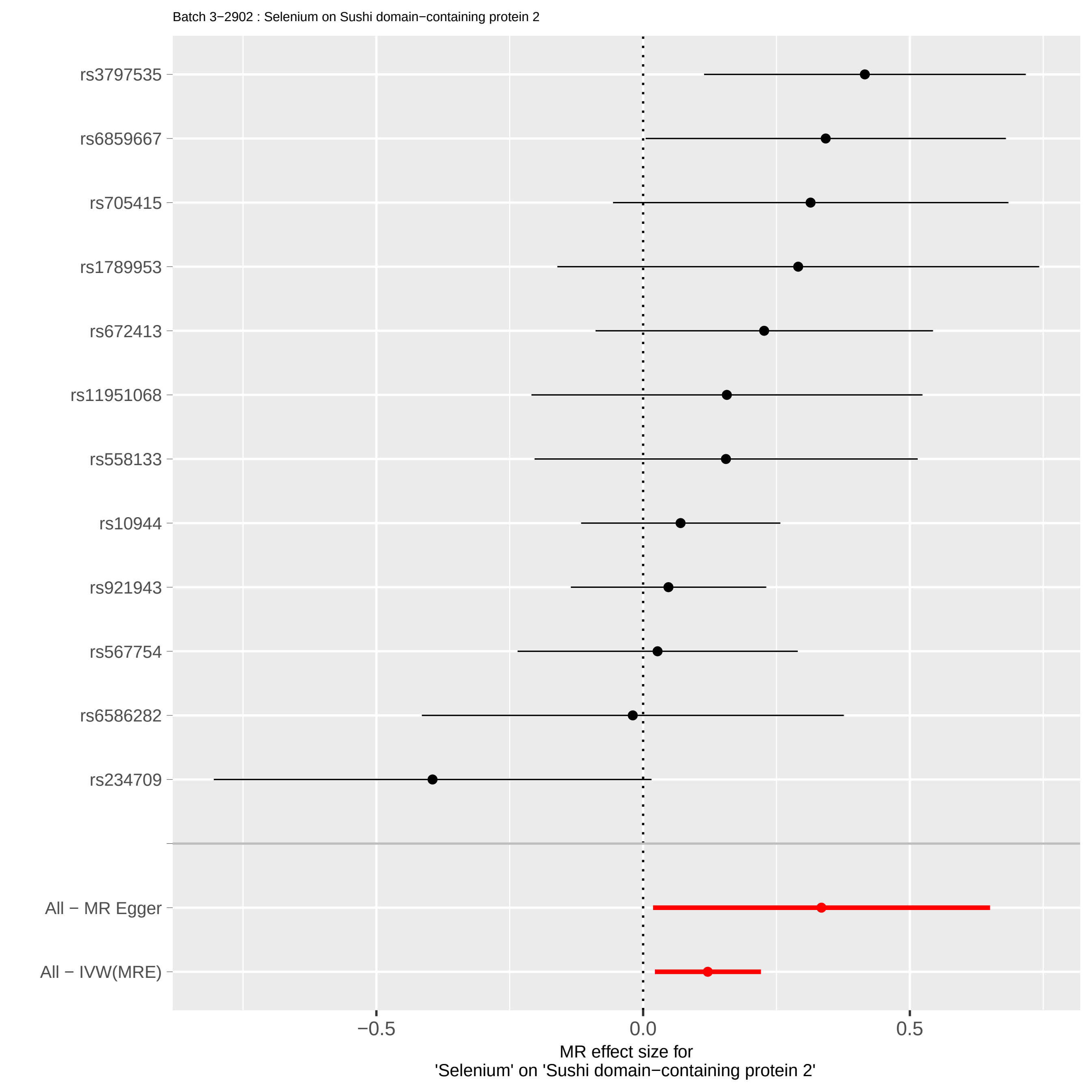

Batch 3−2902 : Selenium on Sushi domain−containing protein 2
rs3797535
rs6859667
rs705415
rs1789953
rs672413
rs11951068
rs558133
rs10944
rs921943
rs567754
rs6586282
rs234709
All − MR Egger
All − IVW(MRE)
−0.5
0.0
0.5
MR effect size for
'Selenium' on 'Sushi domain−containing protein 2'

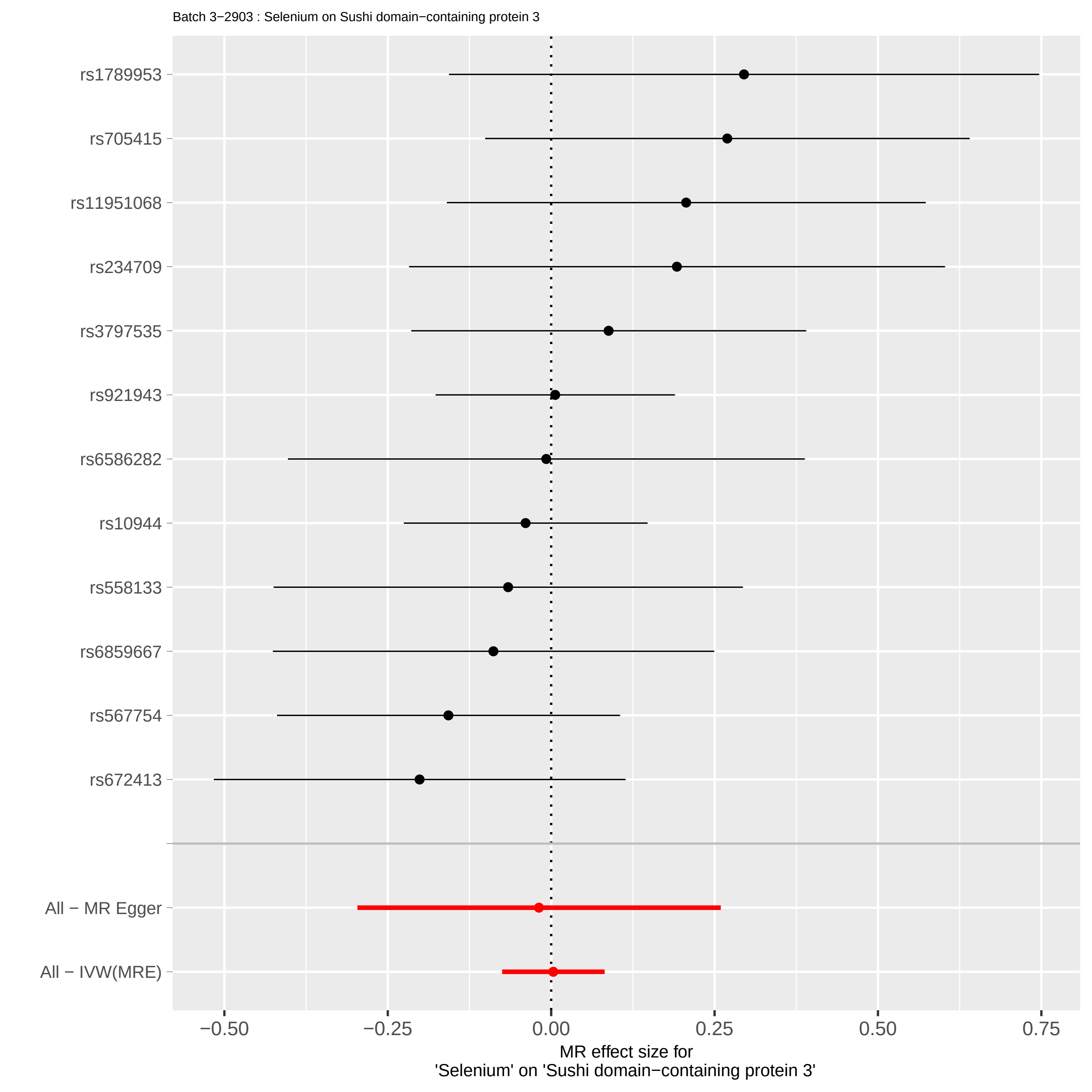

Batch 3−2903 : Selenium on Sushi domain−containing protein 3
rs1789953
rs705415
rs11951068
rs234709
rs3797535
rs921943
rs6586282
rs10944
rs558133
rs6859667
rs567754
rs672413
All − MR Egger
All − IVW(MRE)
−0.50
−0.25
0.00
0.25
0.50
0.75
MR effect size for
'Selenium' on 'Sushi domain−containing protein 3'

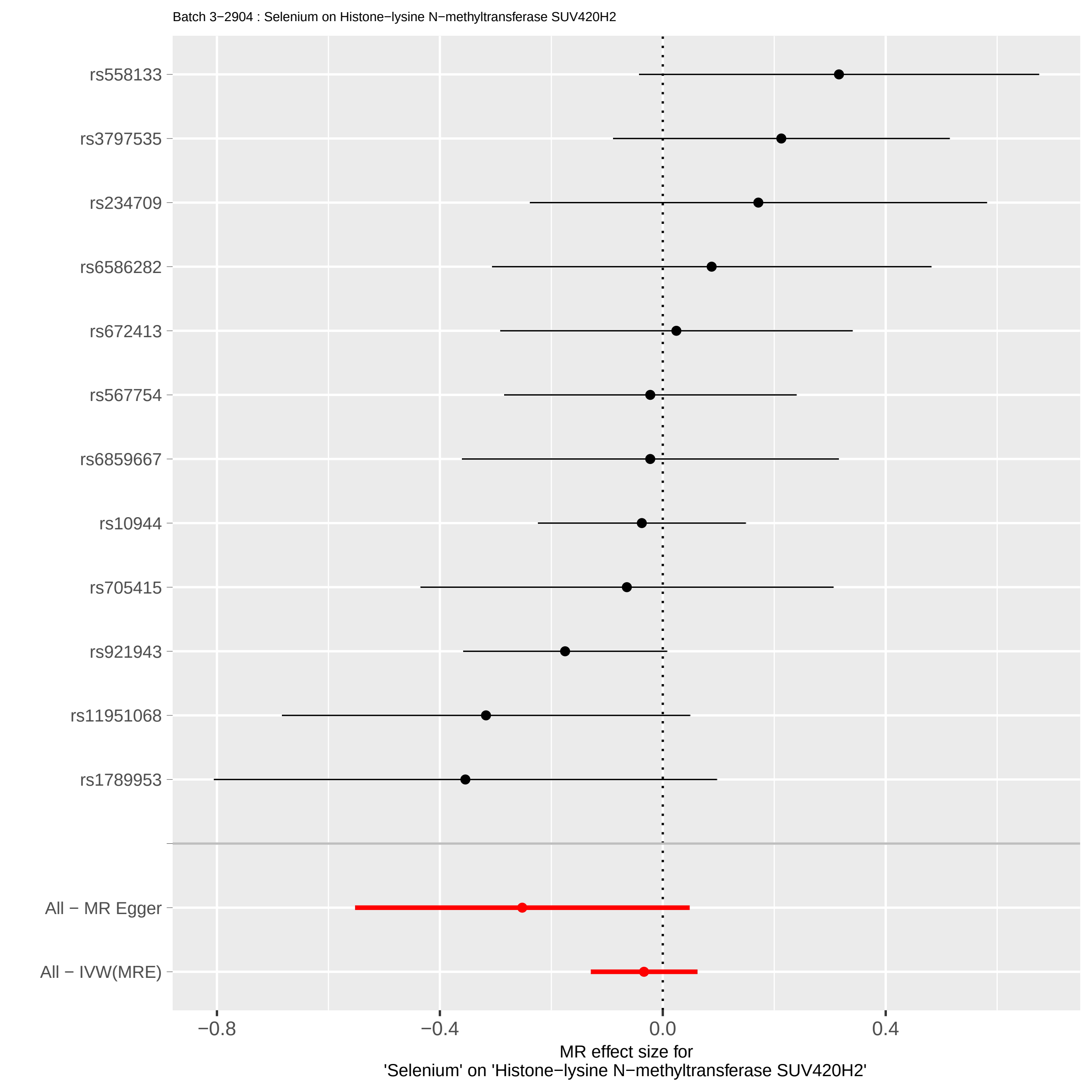

Batch 3−2904 : Selenium on Histone−lysine N−methyltransferase SUV420H2
rs558133
rs3797535
rs234709
rs6586282
rs672413
rs567754
rs6859667
rs10944
rs705415
rs921943
rs11951068
rs1789953
All − MR Egger
All − IVW(MRE)
−0.8
−0.4
0.0
0.4
MR effect size for
'Selenium' on 'Histone−lysine N−methyltransferase SUV420H2'

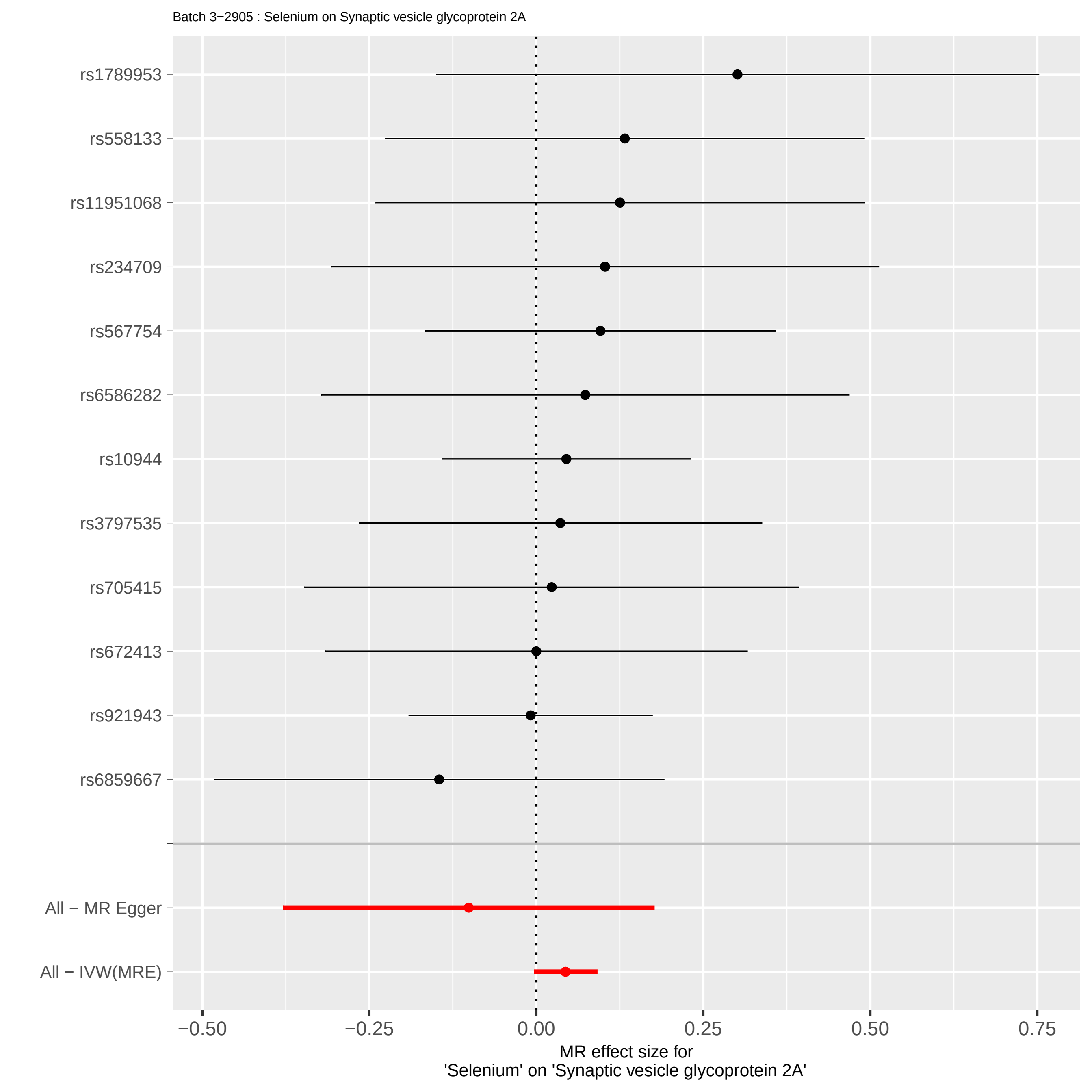

Batch 3−2905 : Selenium on Synaptic vesicle glycoprotein 2A
rs1789953
rs558133
rs11951068
rs234709
rs567754
rs6586282
rs10944
rs3797535
rs705415
rs672413
rs921943
rs6859667
All − MR Egger
All − IVW(MRE)
−0.50
−0.25
0.00
0.25
0.50
0.75
MR effect size for
'Selenium' on 'Synaptic vesicle glycoprotein 2A'

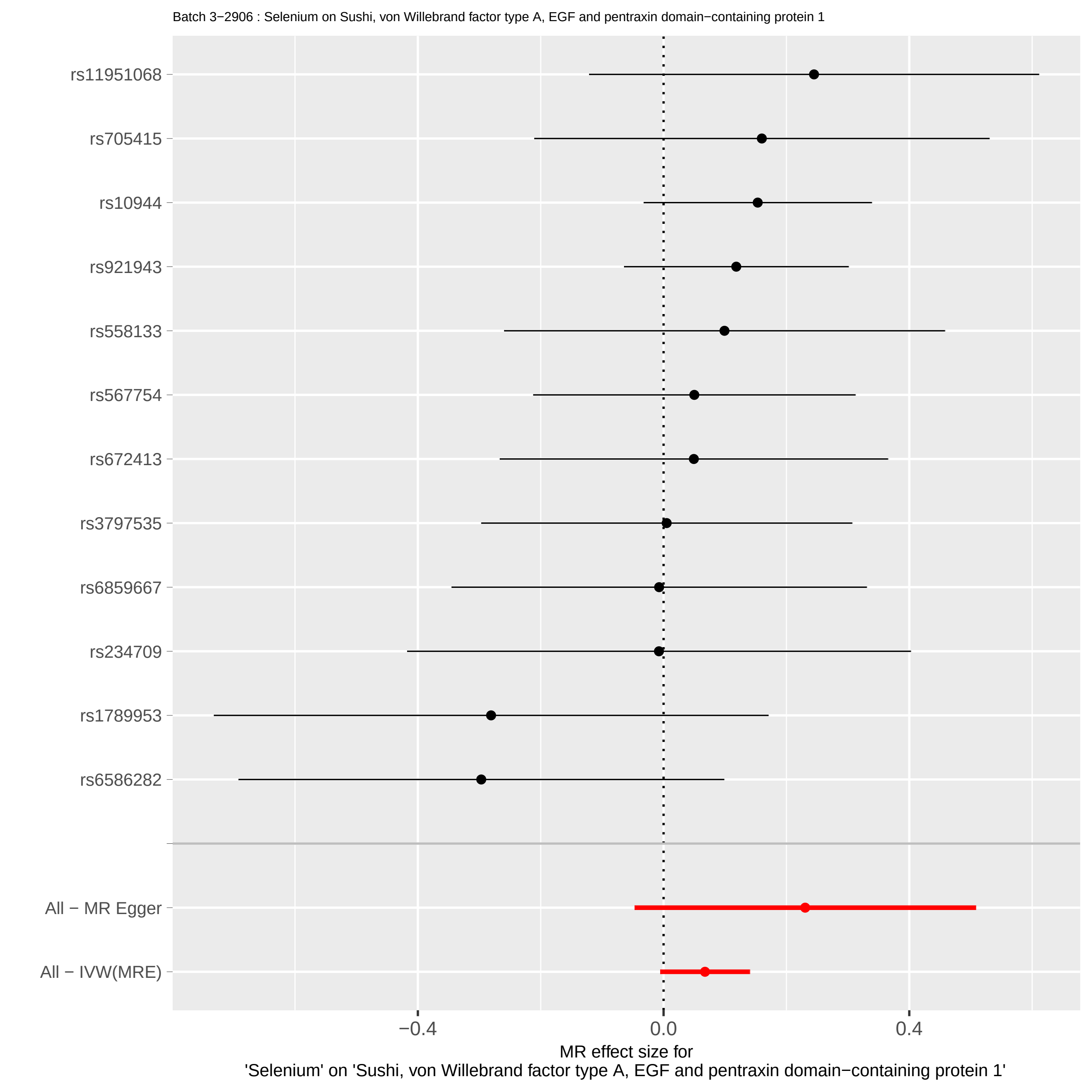

Batch 3−2906 : Selenium on Sushi, von Willebrand factor type A, EGF and pentraxin domain−containing protein 1
rs11951068
rs705415
rs10944
rs921943
rs558133
rs567754
rs672413
rs3797535
rs6859667
rs234709
rs1789953
rs6586282
All − MR Egger
All − IVW(MRE)
−0.4
0.0
0.4
MR effect size for
'Selenium' on 'Sushi, von Willebrand factor type A, EGF and pentraxin domain−containing protein 1'

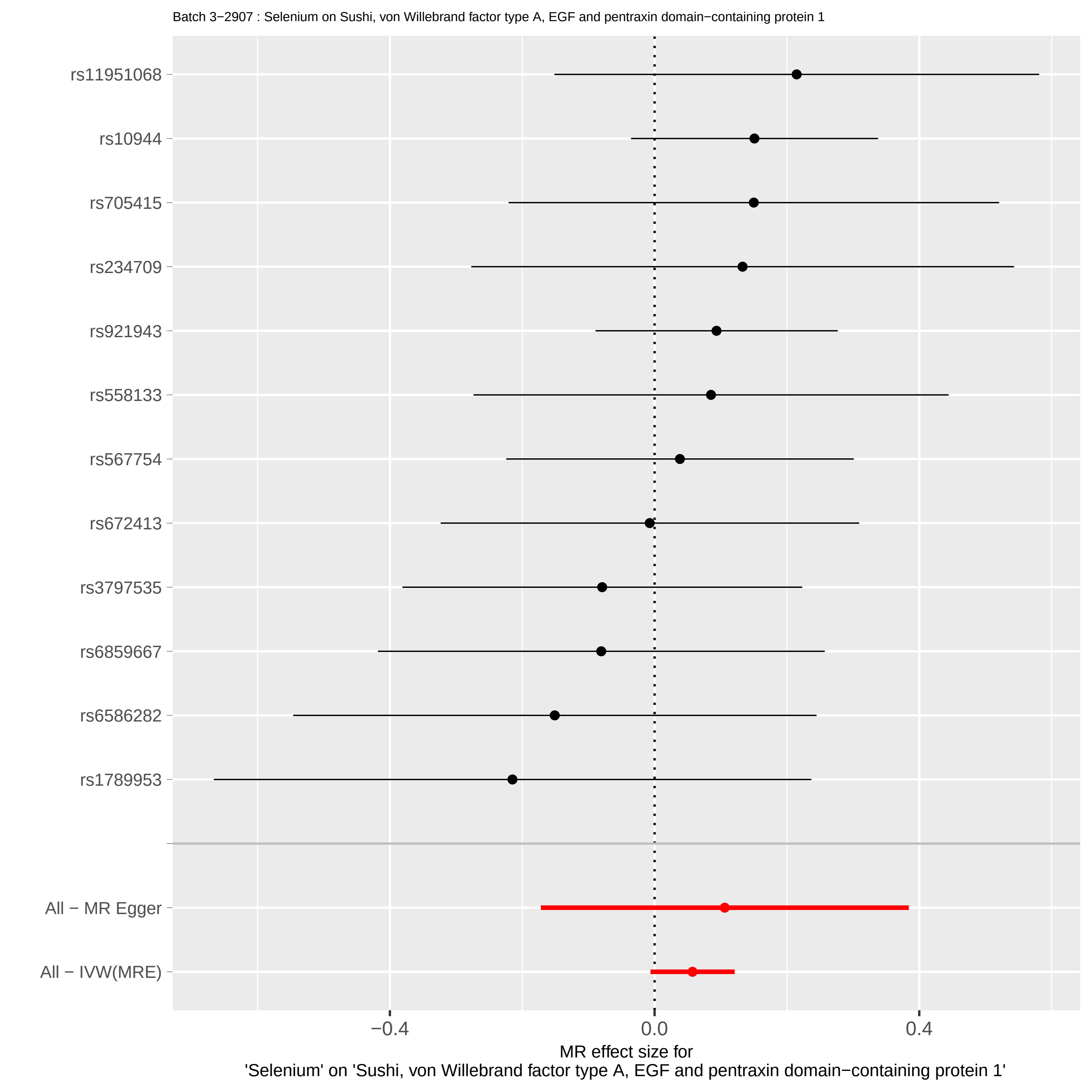
Batch 3−2907 : Selenium on Sushi, von Willebrand factor type A, EGF and pentraxin domain−containing protein 1
rs11951068
rs10944
rs705415
rs234709
rs921943
rs558133
rs567754
rs672413
rs3797535
rs6859667
rs6586282
rs1789953
All − MR Egger
All − IVW(MRE)
−0.4
0.0
0.4
MR effect size for
'Selenium' on 'Sushi, von Willebrand factor type A, EGF and pentraxin domain−containing protein 1'

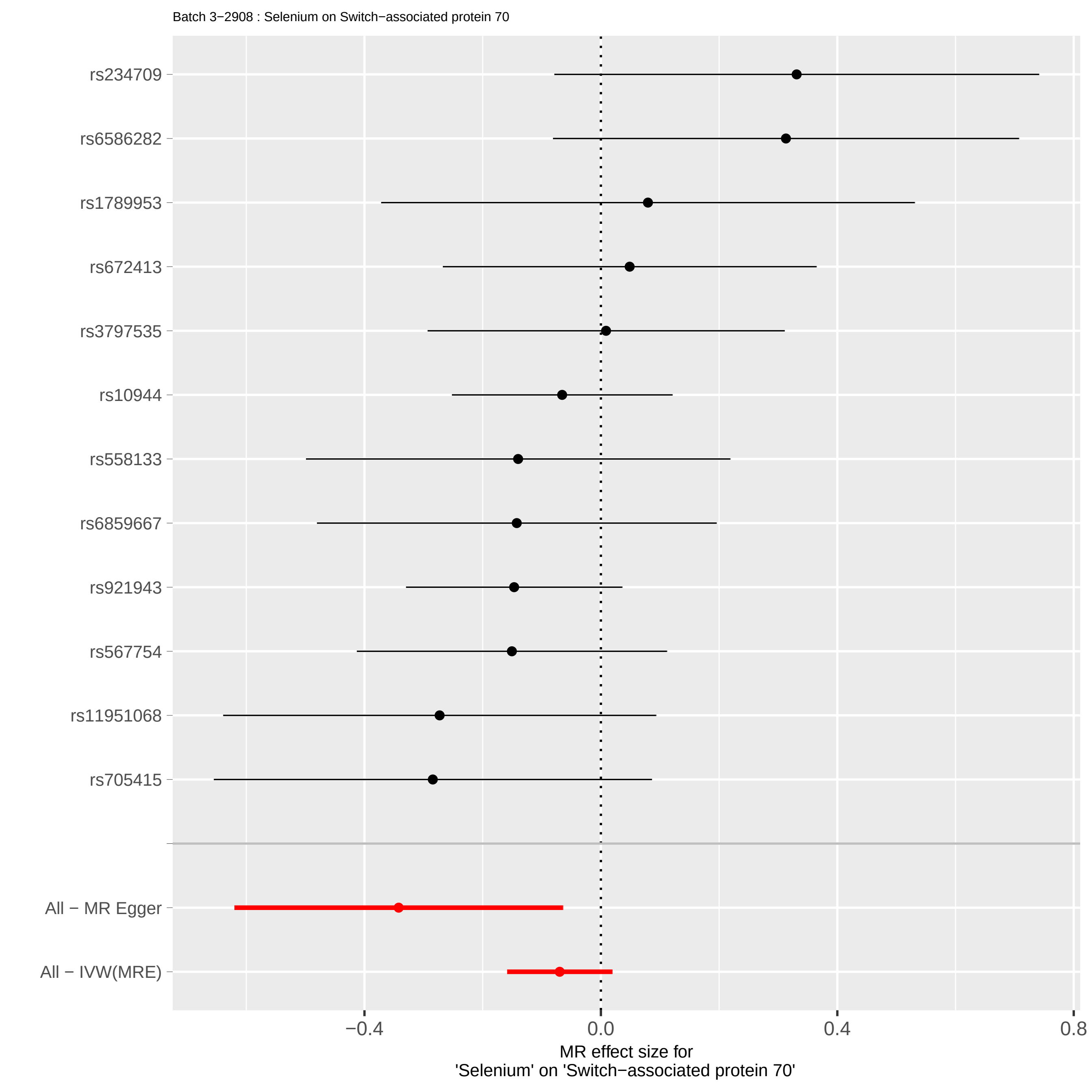

Batch 3−2908 : Selenium on Switch−associated protein 70
rs234709
rs6586282
rs1789953
rs672413
rs3797535
rs10944
rs558133
rs6859667
rs921943
rs567754
rs11951068
rs705415
All − MR Egger
All − IVW(MRE)
−0.4
0.0
0.4
0.8
MR effect size for
'Selenium' on 'Switch−associated protein 70'

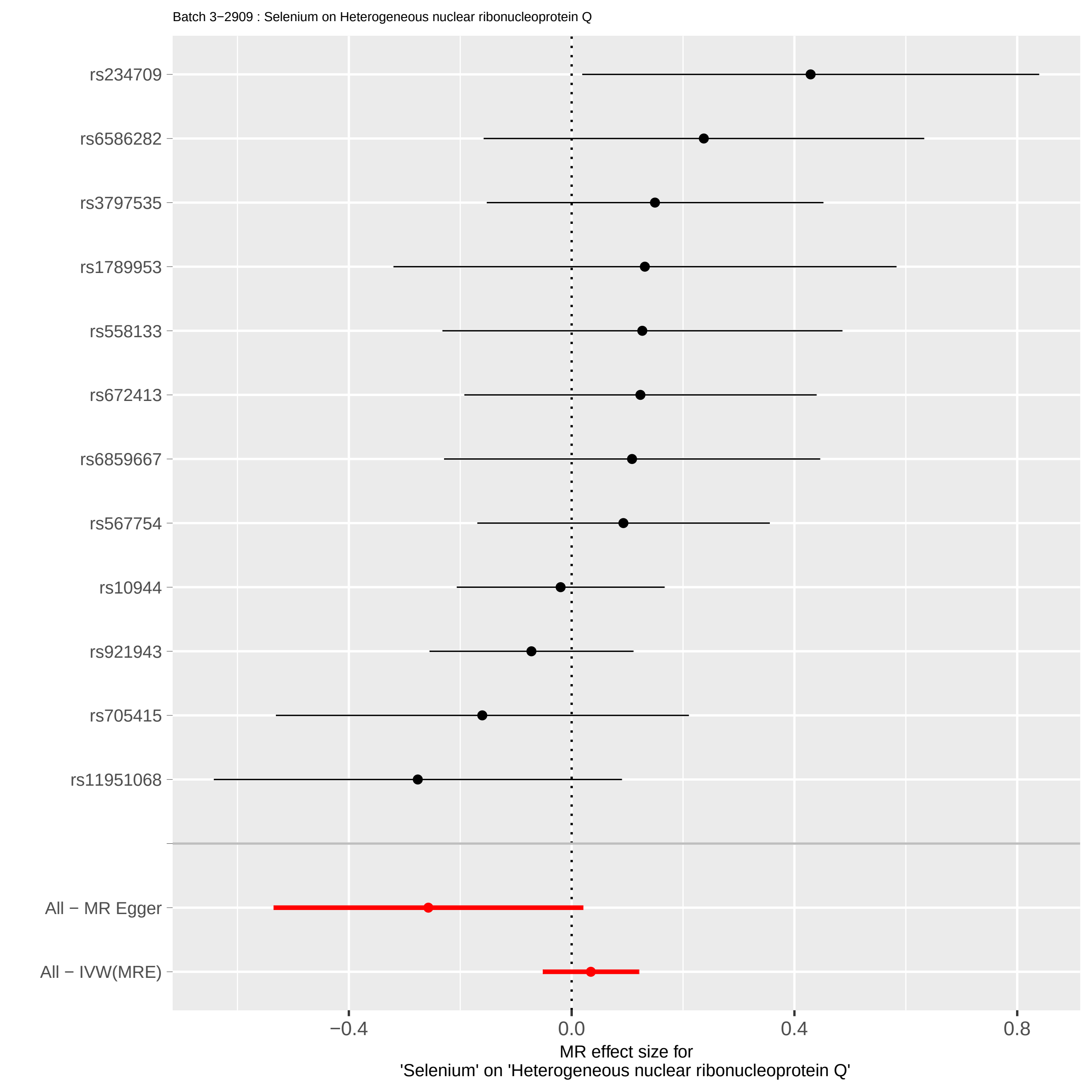

Batch 3−2909 : Selenium on Heterogeneous nuclear ribonucleoprotein Q
rs234709
rs6586282
rs3797535
rs1789953
rs558133
rs672413
rs6859667
rs567754
rs10944
rs921943
rs705415
rs11951068
All − MR Egger
All − IVW(MRE)
−0.4
0.0
0.4
0.8
MR effect size for
'Selenium' on 'Heterogeneous nuclear ribonucleoprotein Q'

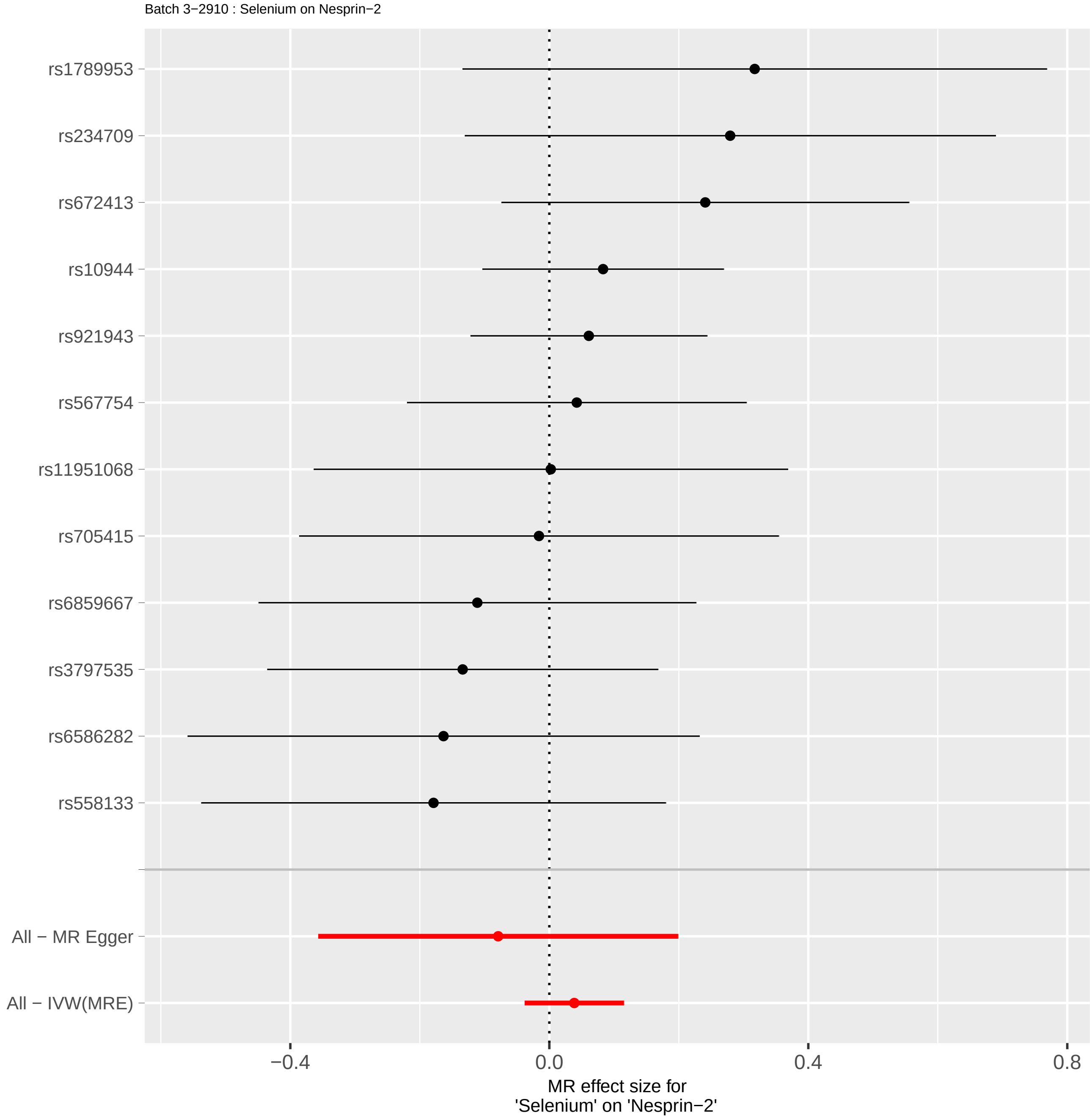

Batch 3−2910 : Selenium on Nesprin−2
rs1789953
rs234709
rs672413
rs10944
rs921943
rs567754
rs11951068
rs705415
rs6859667
rs3797535
rs6586282
rs558133
All − MR Egger
All − IVW(MRE)
−0.4
0.0
0.4
0.8
MR effect size for
'Selenium' on 'Nesprin−2'

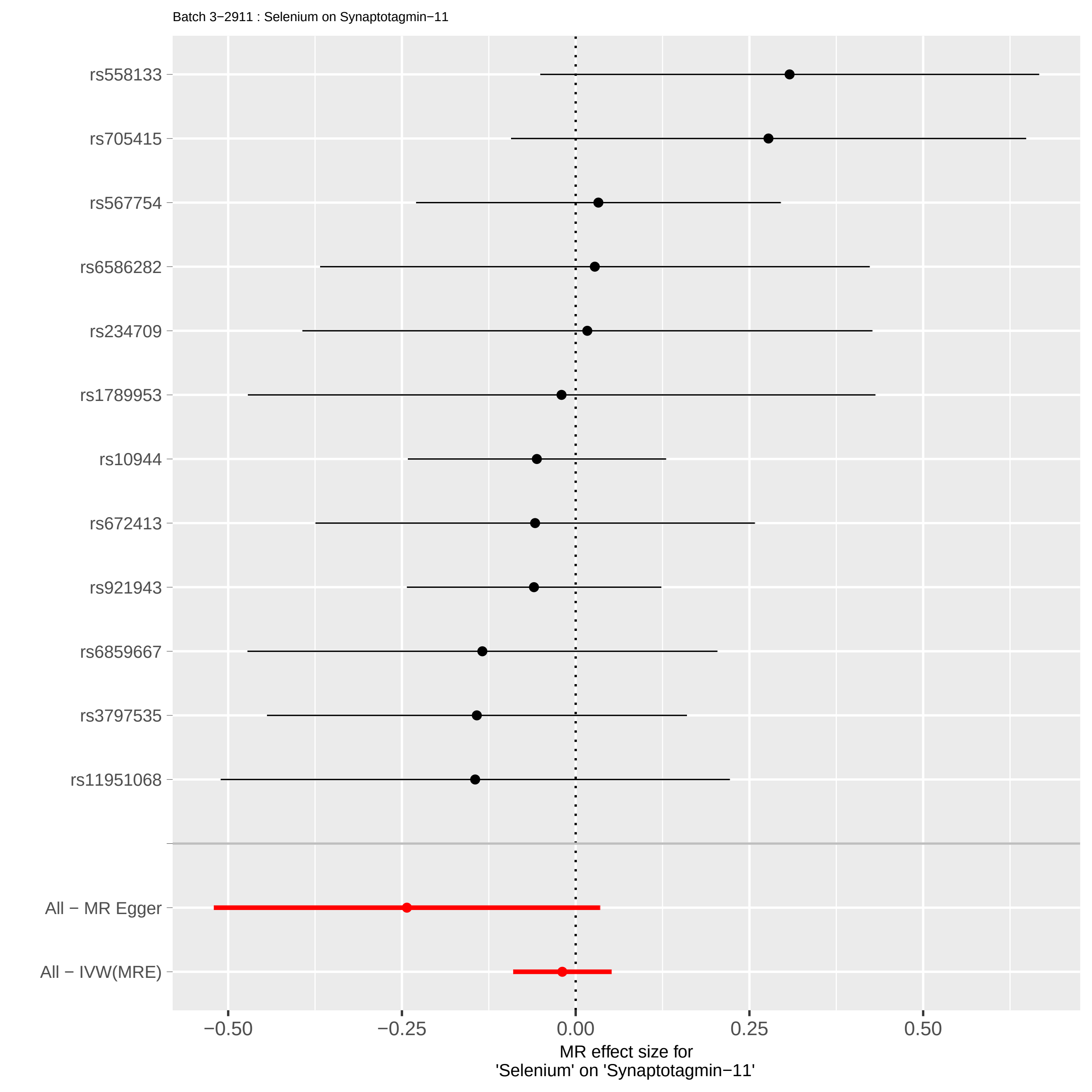

Batch 3−2911 : Selenium on Synaptotagmin−11
rs558133
rs705415
rs567754
rs6586282
rs234709
rs1789953
rs10944
rs672413
rs921943
rs6859667
rs3797535
rs11951068
All − MR Egger
All − IVW(MRE)
−0.50
−0.25
0.00
0.25
0.50
MR effect size for
'Selenium' on 'Synaptotagmin−11'

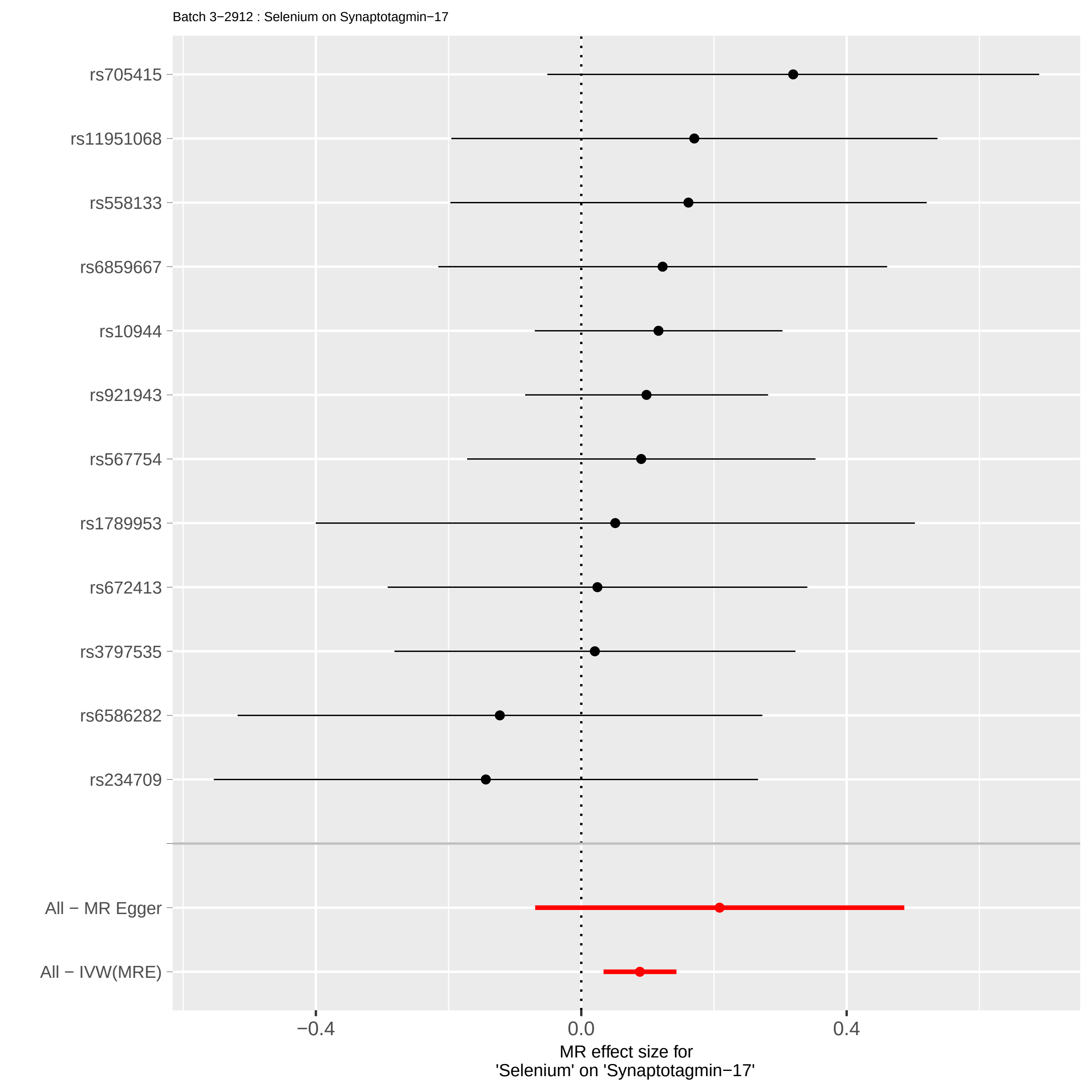

Batch 3−2912 : Selenium on Synaptotagmin−17
rs705415
rs11951068
rs558133
rs6859667
rs10944
rs921943
rs567754
rs1789953
rs672413
rs3797535
rs6586282
rs234709
All − MR Egger
All − IVW(MRE)
−0.4
0.0
0.4
MR effect size for
'Selenium' on 'Synaptotagmin−17'

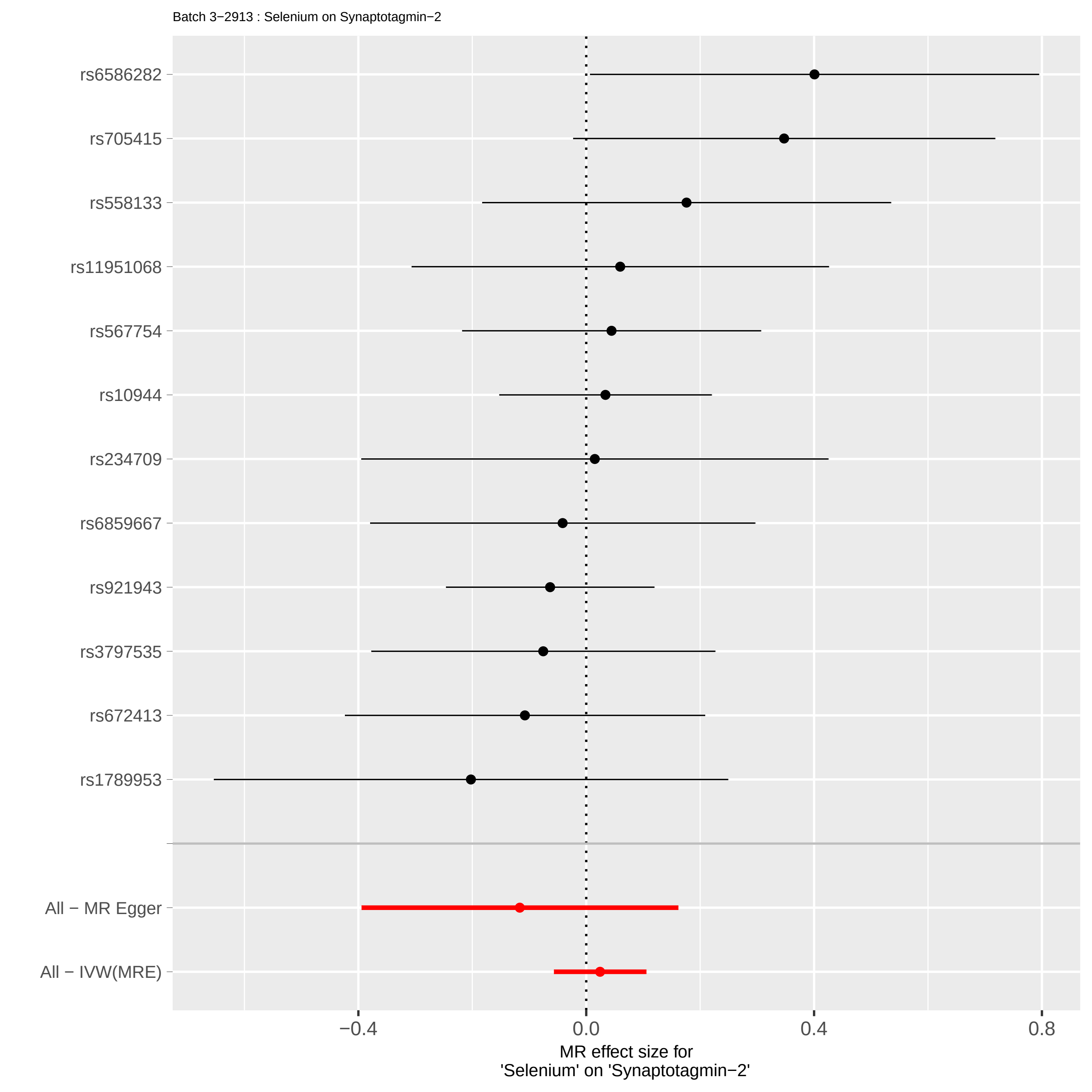

Batch 3−2913 : Selenium on Synaptotagmin−2
rs6586282
rs705415
rs558133
rs11951068
rs567754
rs10944
rs234709
rs6859667
rs921943
rs3797535
rs672413
rs1789953
All − MR Egger
All − IVW(MRE)
−0.4
0.0
0.4
0.8
MR effect size for
'Selenium' on 'Synaptotagmin−2'

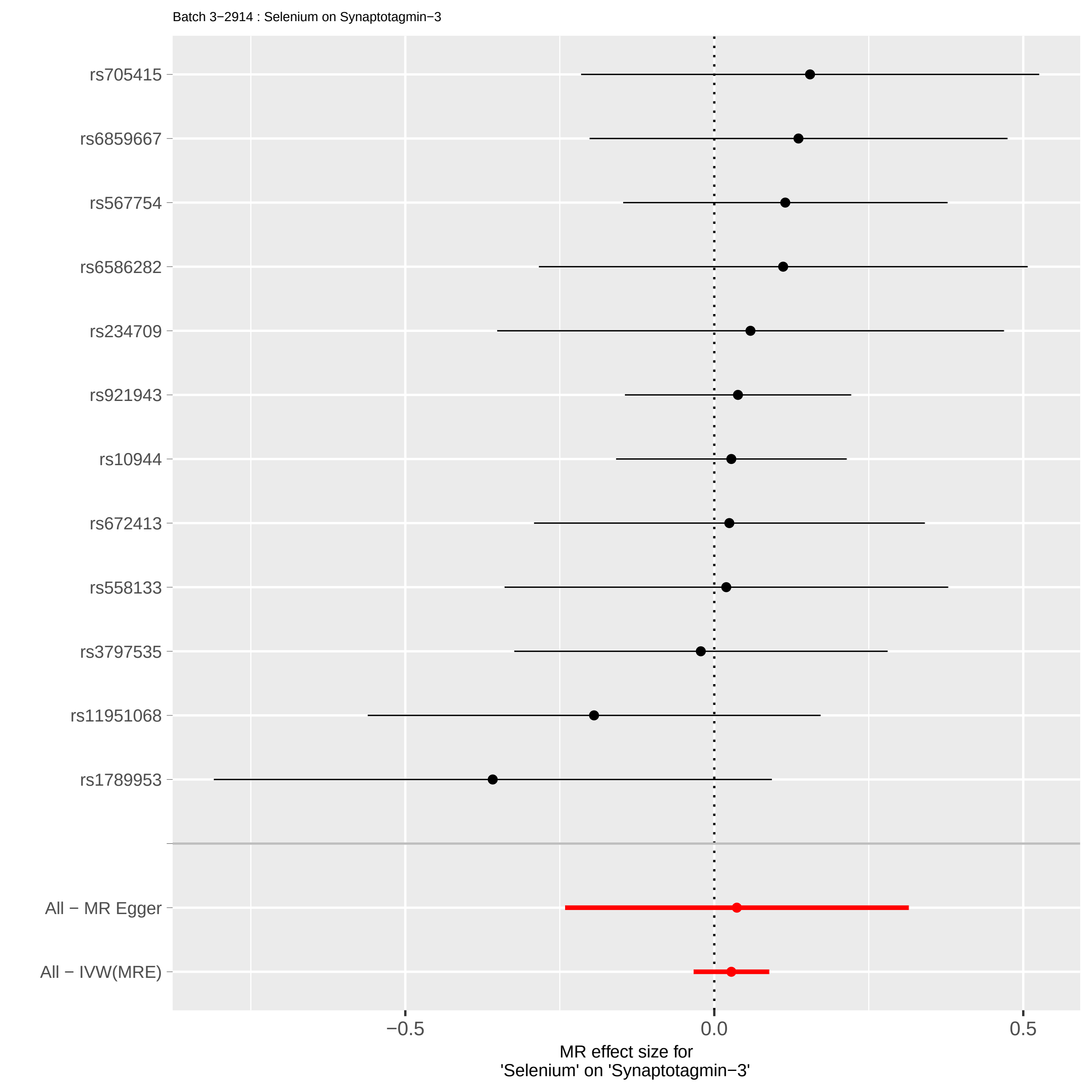

Batch 3−2914 : Selenium on Synaptotagmin−3
rs705415
rs6859667
rs567754
rs6586282
rs234709
rs921943
rs10944
rs672413
rs558133
rs3797535
rs11951068
rs1789953
All − MR Egger
All − IVW(MRE)
−0.5
0.0
0.5
MR effect size for
'Selenium' on 'Synaptotagmin−3'

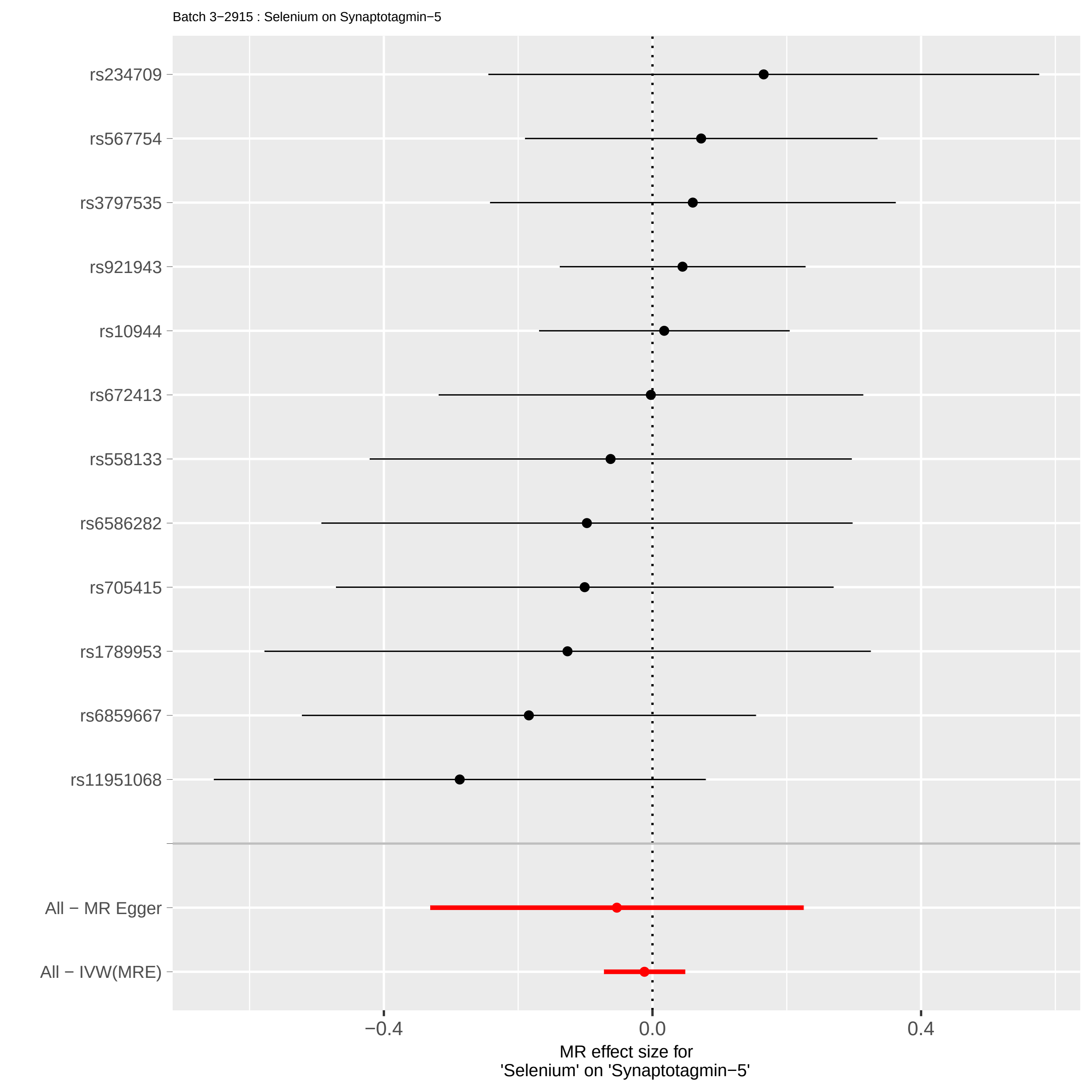

Batch 3−2915 : Selenium on Synaptotagmin−5
rs234709
rs567754
rs3797535
rs921943
rs10944
rs672413
rs558133
rs6586282
rs705415
rs1789953
rs6859667
rs11951068
All − MR Egger
All − IVW(MRE)
−0.4
0.0
0.4
MR effect size for
'Selenium' on 'Synaptotagmin−5'

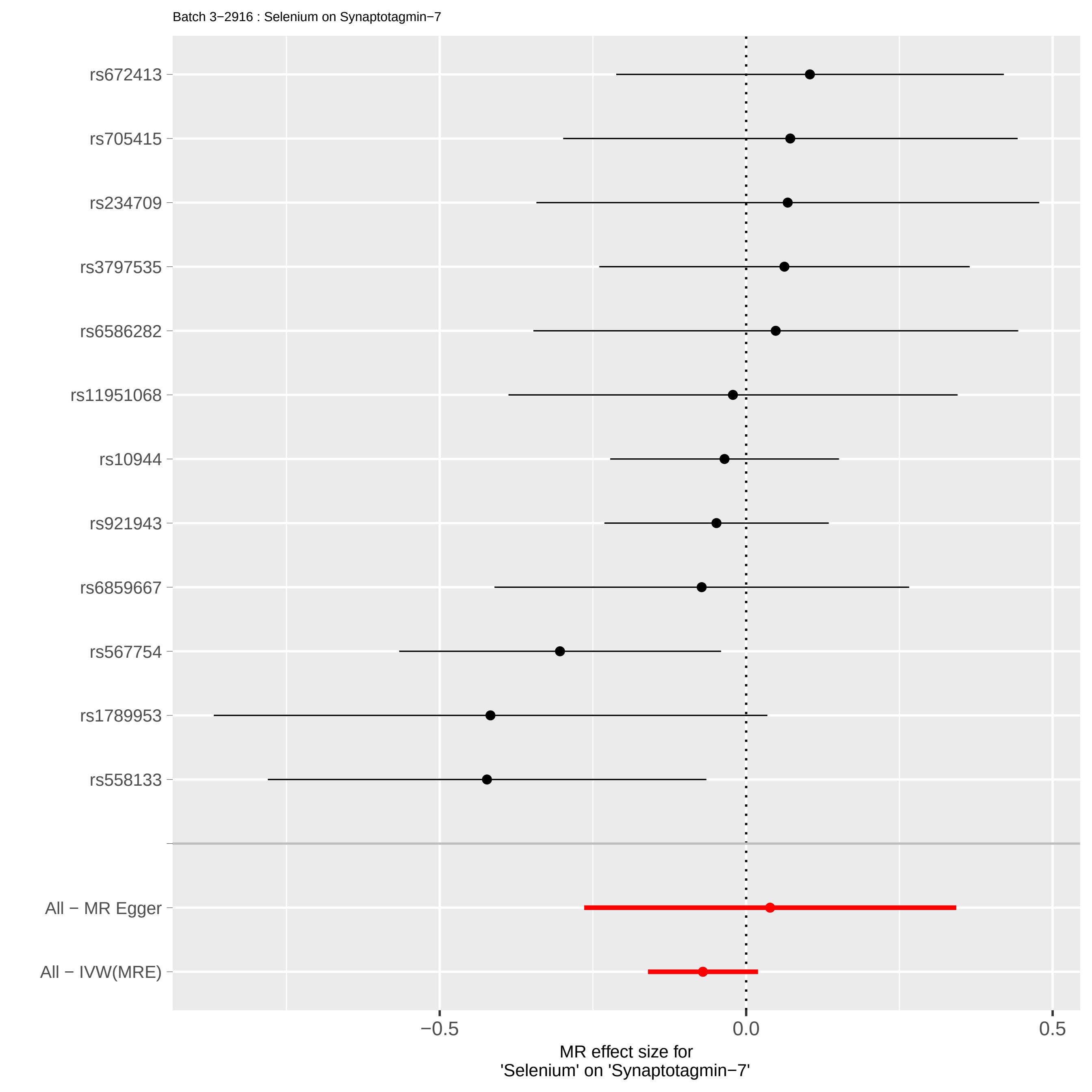

Batch 3−2916 : Selenium on Synaptotagmin−7
rs672413
rs705415
rs234709
rs3797535
rs6586282
rs11951068
rs10944
rs921943
rs6859667
rs567754
rs1789953
rs558133
All − MR Egger
All − IVW(MRE)
−0.5
0.0
0.5
MR effect size for
'Selenium' on 'Synaptotagmin−7'

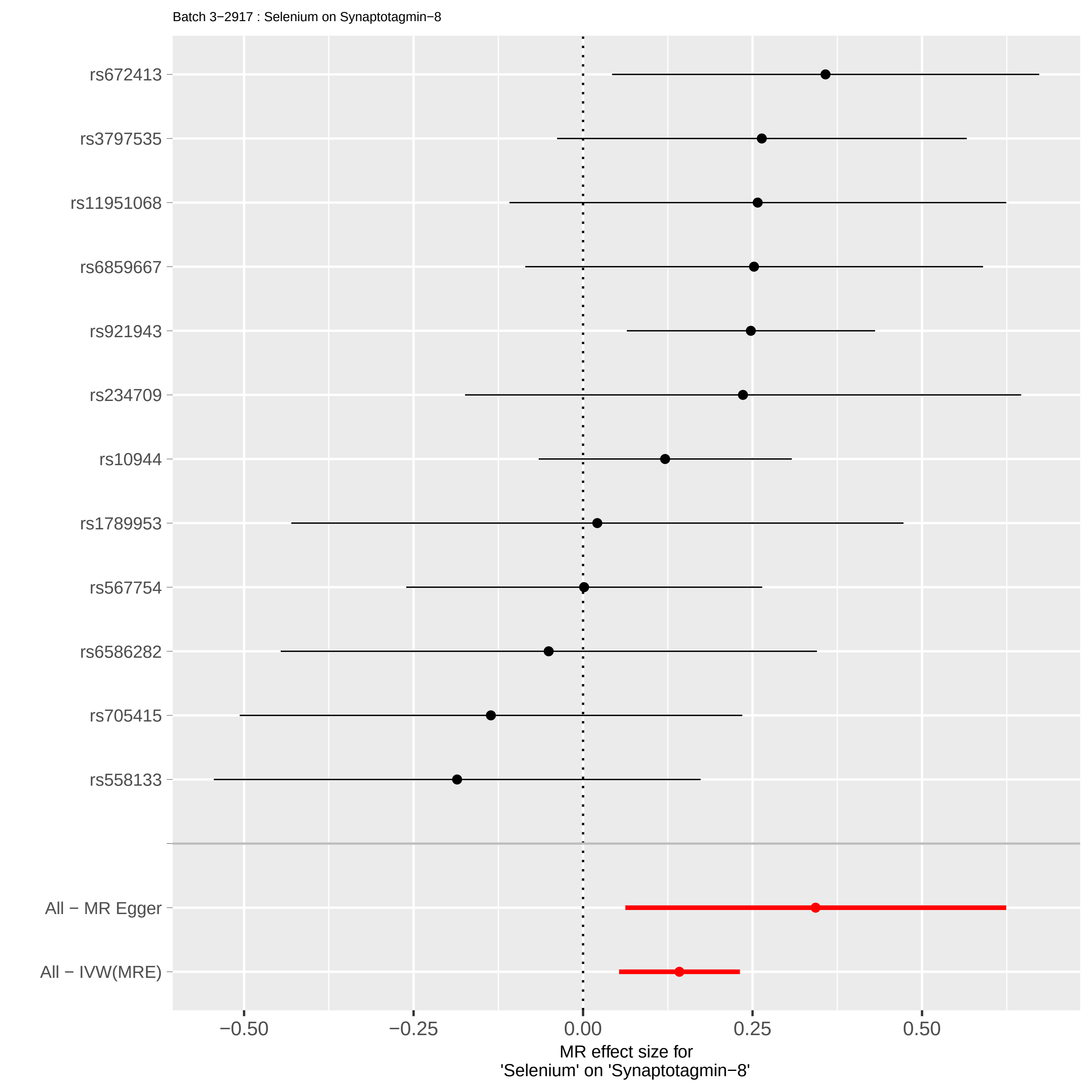
Batch 3−2917 : Selenium on Synaptotagmin−8
rs672413
rs3797535
rs11951068
rs6859667
rs921943
rs234709
rs10944
rs1789953
rs567754
rs6586282
rs705415
rs558133
All − MR Egger
All − IVW(MRE)
−0.50
−0.25
0.00
0.25
0.50
MR effect size for
'Selenium' on 'Synaptotagmin−8'

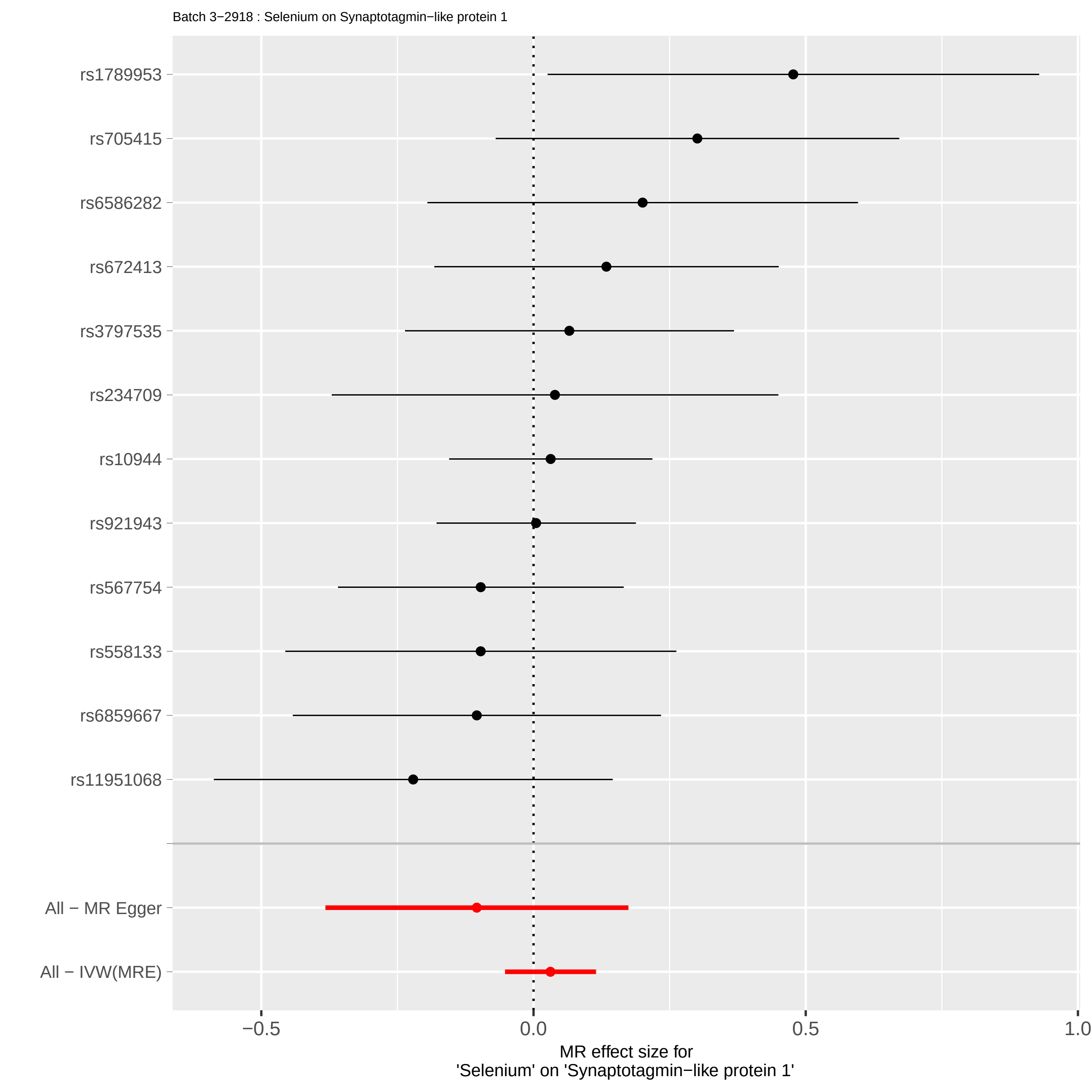
Batch 3−2918 : Selenium on Synaptotagmin−like protein 1
rs1789953
rs705415
rs6586282
rs672413
rs3797535
rs234709
rs10944
rs921943
rs567754
rs558133
rs6859667
rs11951068
All − MR Egger
All − IVW(MRE)
−0.5
0.0
0.5
1.0
MR effect size for
'Selenium' on 'Synaptotagmin−like protein 1'

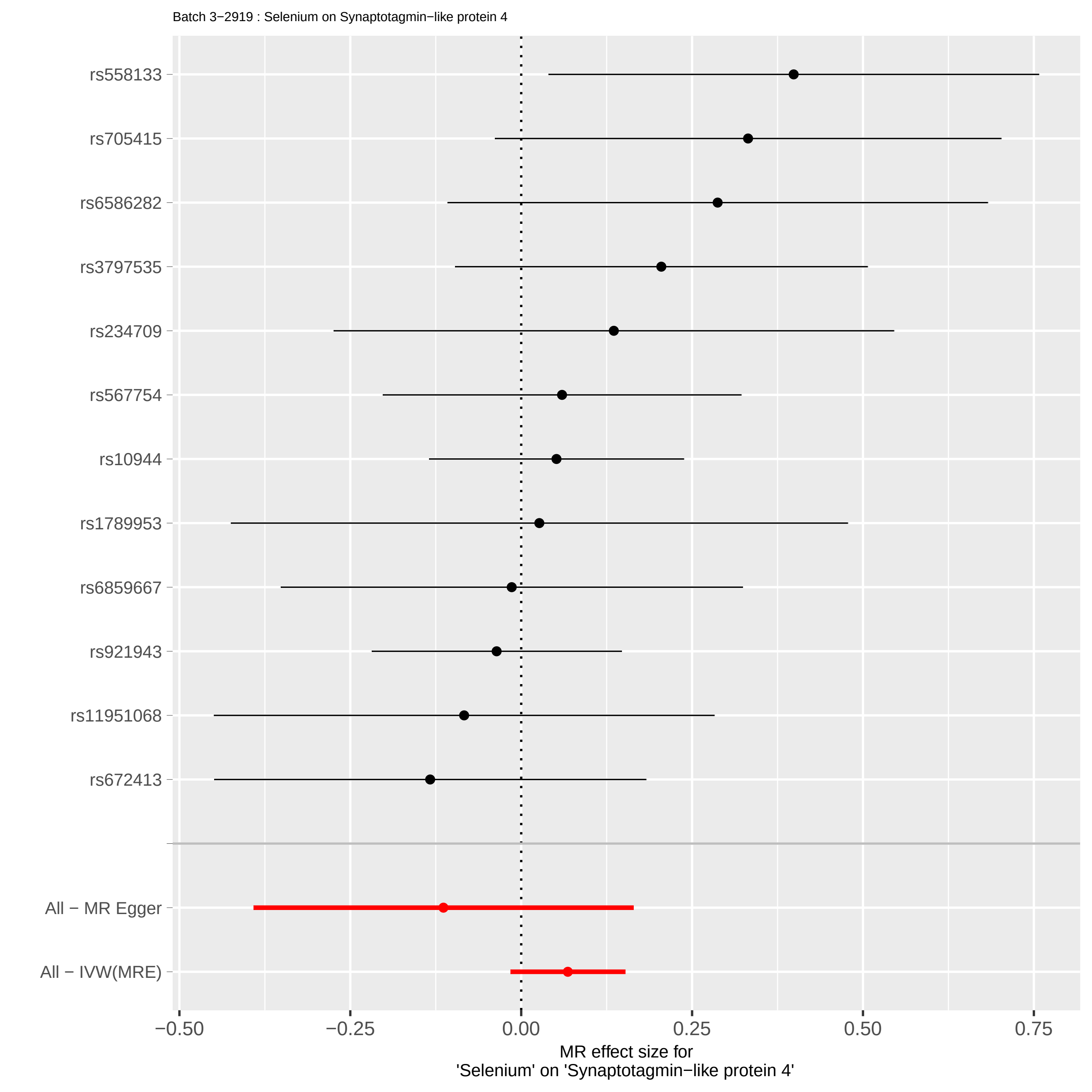

Batch 3−2919 : Selenium on Synaptotagmin−like protein 4
rs558133
rs705415
rs6586282
rs3797535
rs234709
rs567754
rs10944
rs1789953
rs6859667
rs921943
rs11951068
rs672413
All − MR Egger
All − IVW(MRE)
−0.50
−0.25
0.00
0.25
0.50
0.75
MR effect size for
'Selenium' on 'Synaptotagmin−like protein 4'

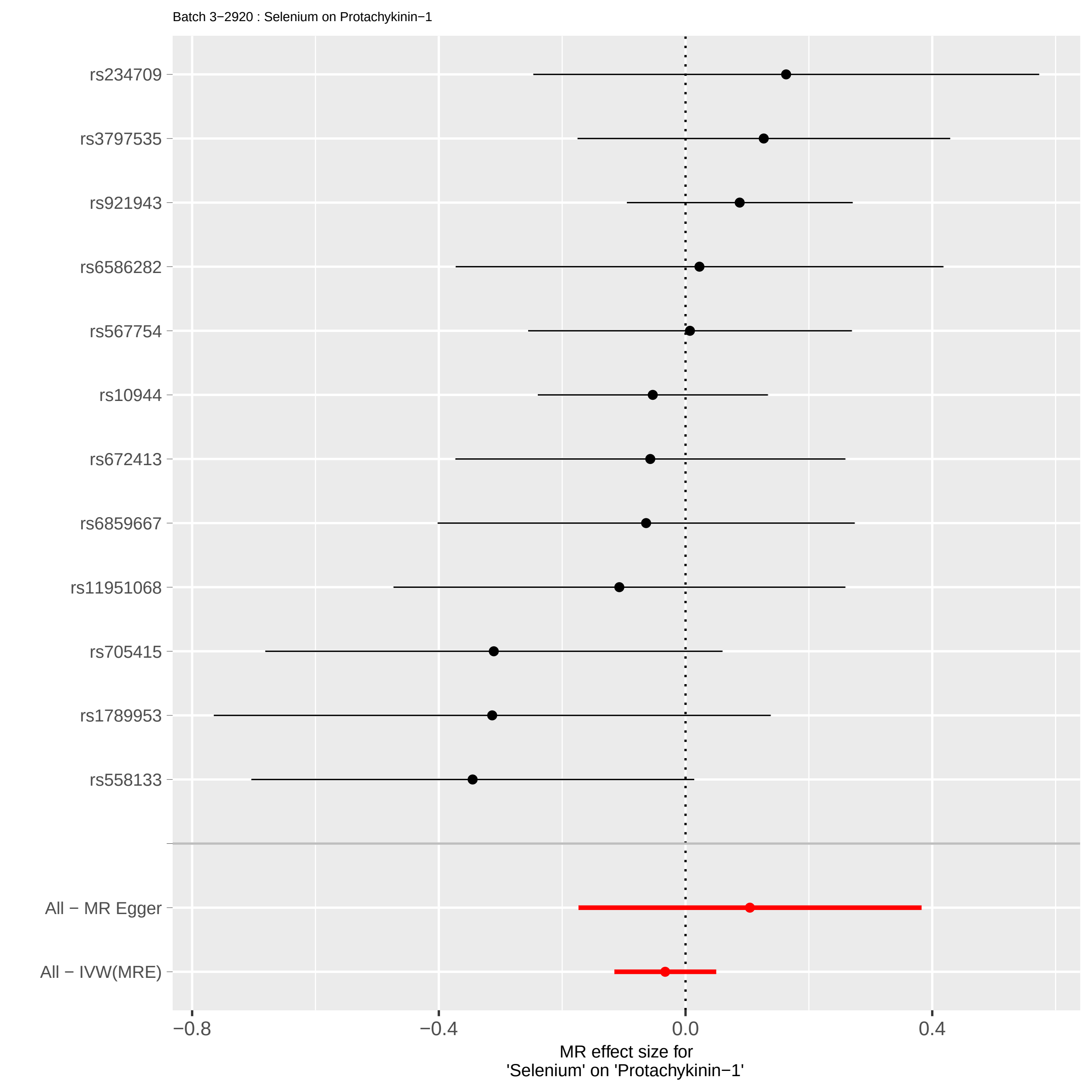
Batch 3−2920 : Selenium on Protachykinin−1
rs234709
rs3797535
rs921943
rs6586282
rs567754
rs10944
rs672413
rs6859667
rs11951068
rs705415
rs1789953
rs558133
All − MR Egger
All − IVW(MRE)
−0.8
−0.4
0.0
0.4
MR effect size for
'Selenium' on 'Protachykinin−1'

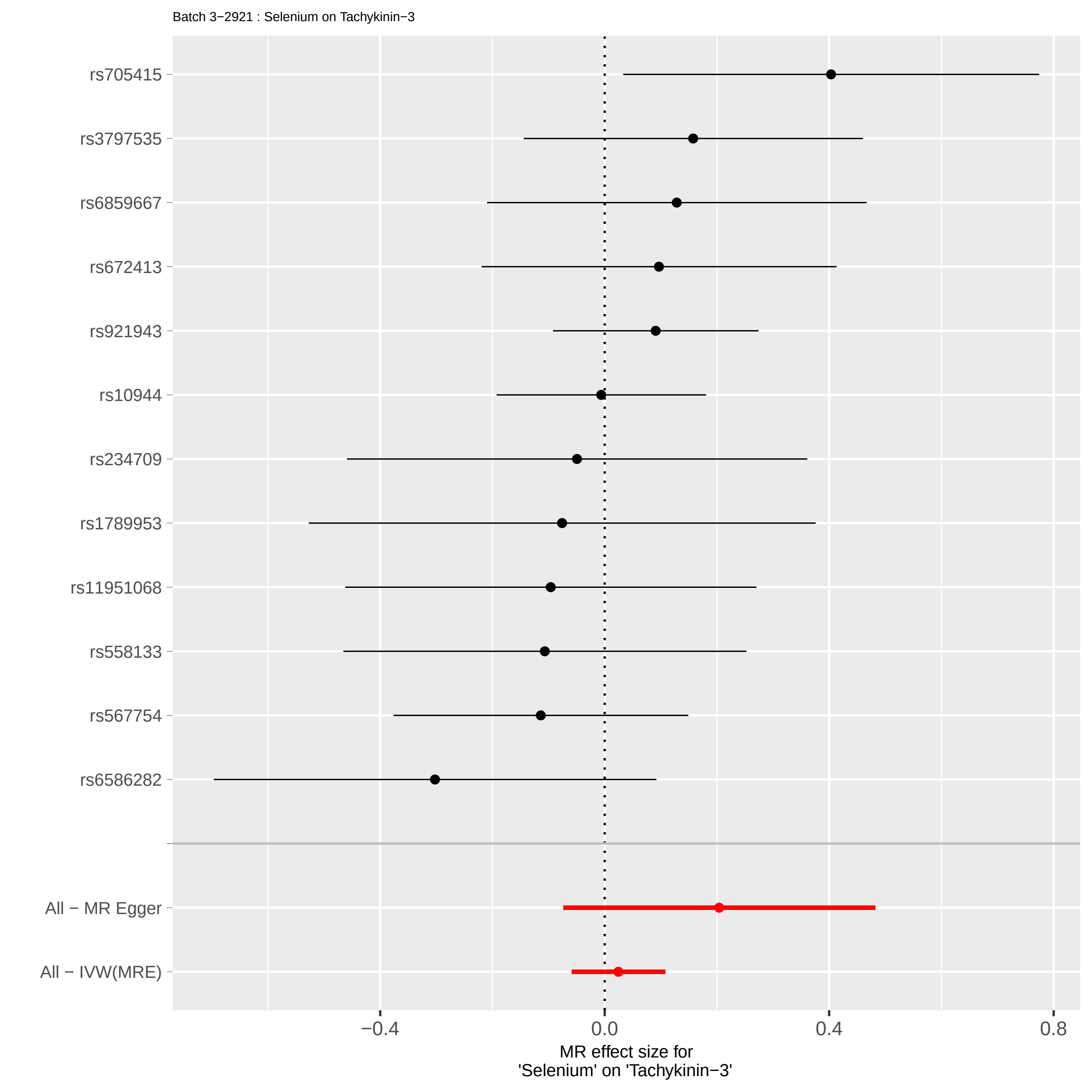
Batch 3−2921 : Selenium on Tachykinin−3
rs705415
rs3797535
rs6859667
rs672413
rs921943
rs10944
rs234709
rs1789953
rs11951068
rs558133
rs567754
rs6586282
All − MR Egger
All − IVW(MRE)
−0.4
0.0
0.4
0.8
MR effect size for
'Selenium' on 'Tachykinin−3'

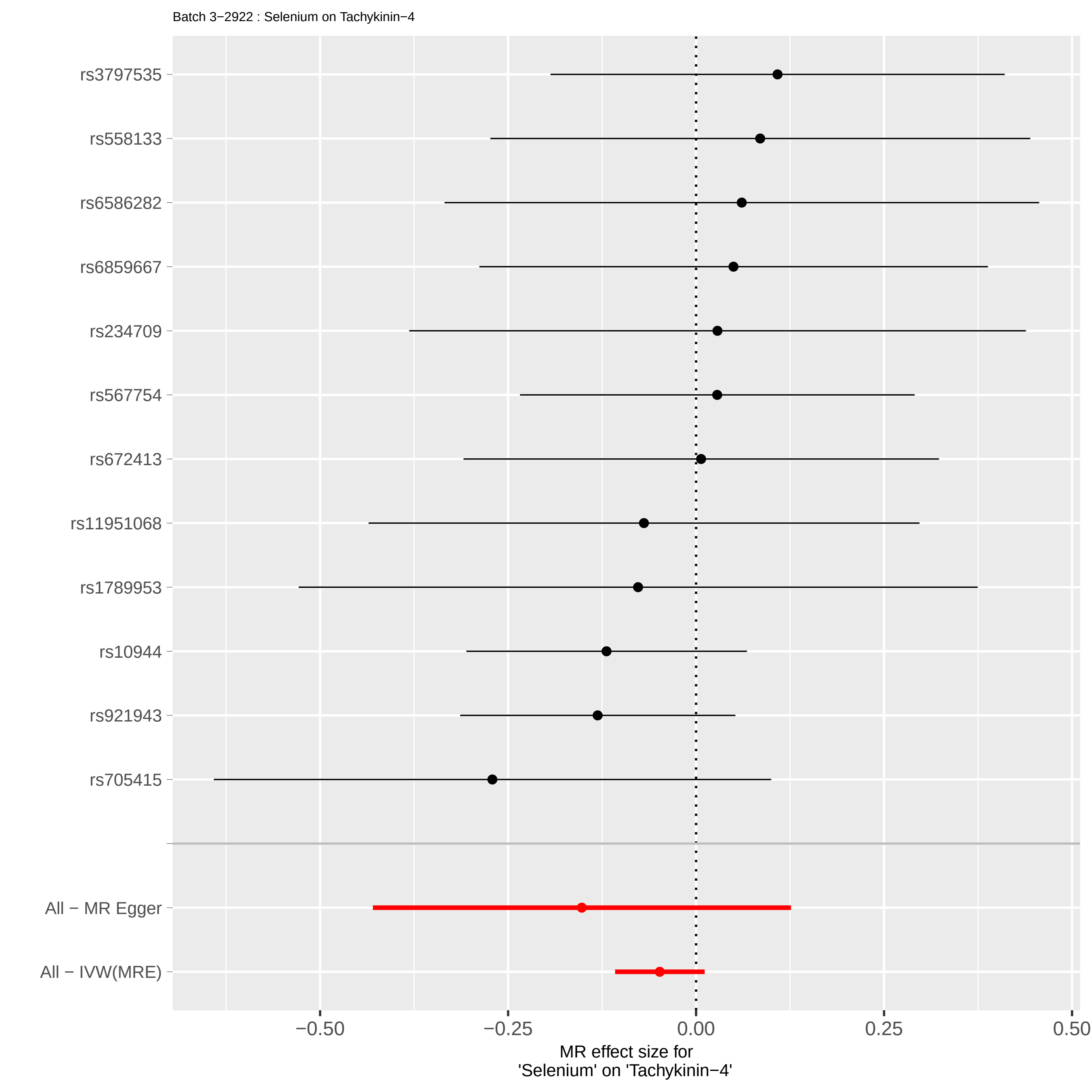

Batch 3−2922 : Selenium on Tachykinin−4
rs3797535
rs558133
rs6586282
rs6859667
rs234709
rs567754
rs672413
rs11951068
rs1789953
rs10944
rs921943
rs705415
All − MR Egger
All − IVW(MRE)
−0.50
−0.25
0.00
0.25
0.50
MR effect size for
'Selenium' on 'Tachykinin−4'

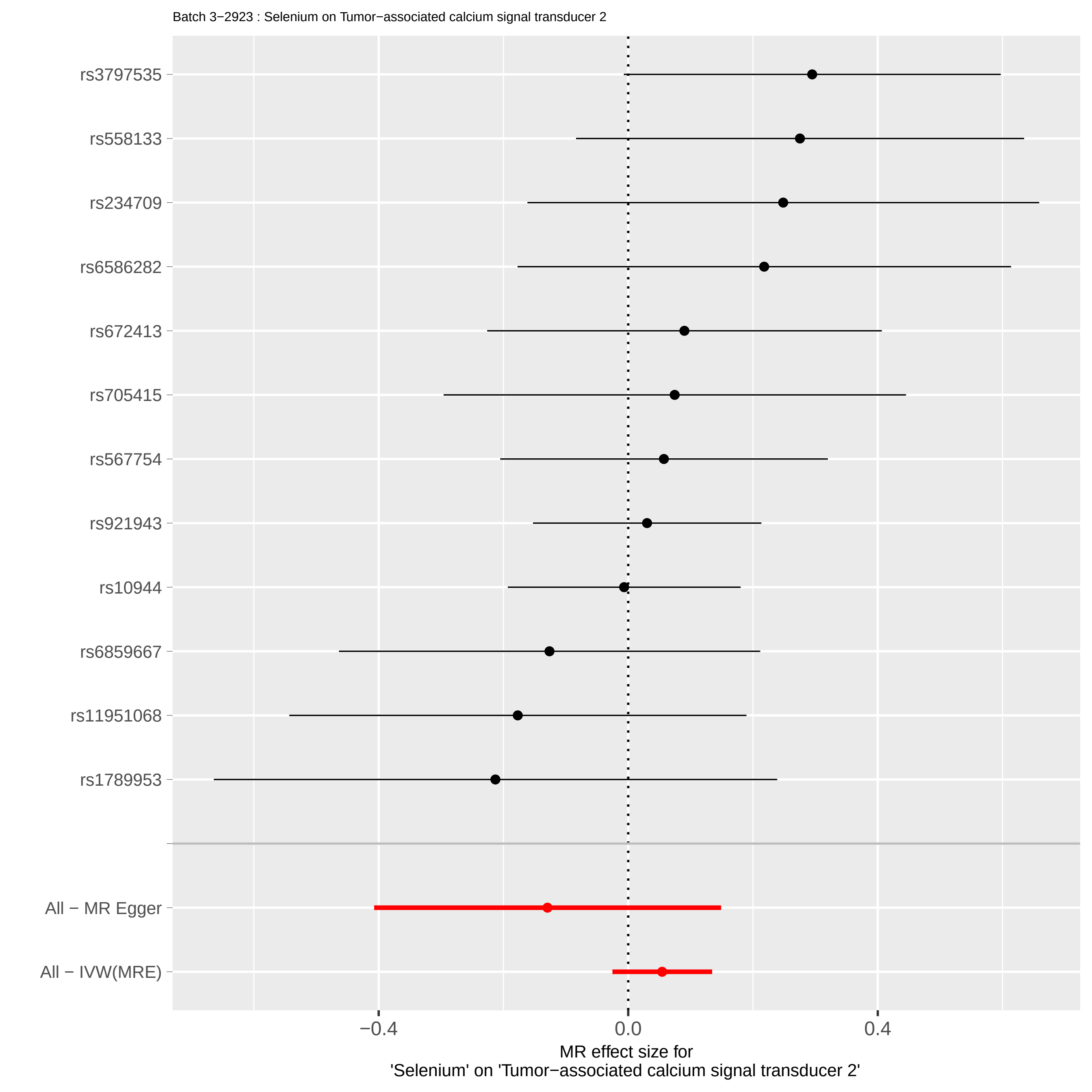
Batch 3−2923 : Selenium on Tumor−associated calcium signal transducer 2
rs3797535
rs558133
rs234709
rs6586282
rs672413
rs705415
rs567754
rs921943
rs10944
rs6859667
rs11951068
rs1789953
All − MR Egger
All − IVW(MRE)
−0.4
0.0
0.4
MR effect size for
'Selenium' on 'Tumor−associated calcium signal transducer 2'

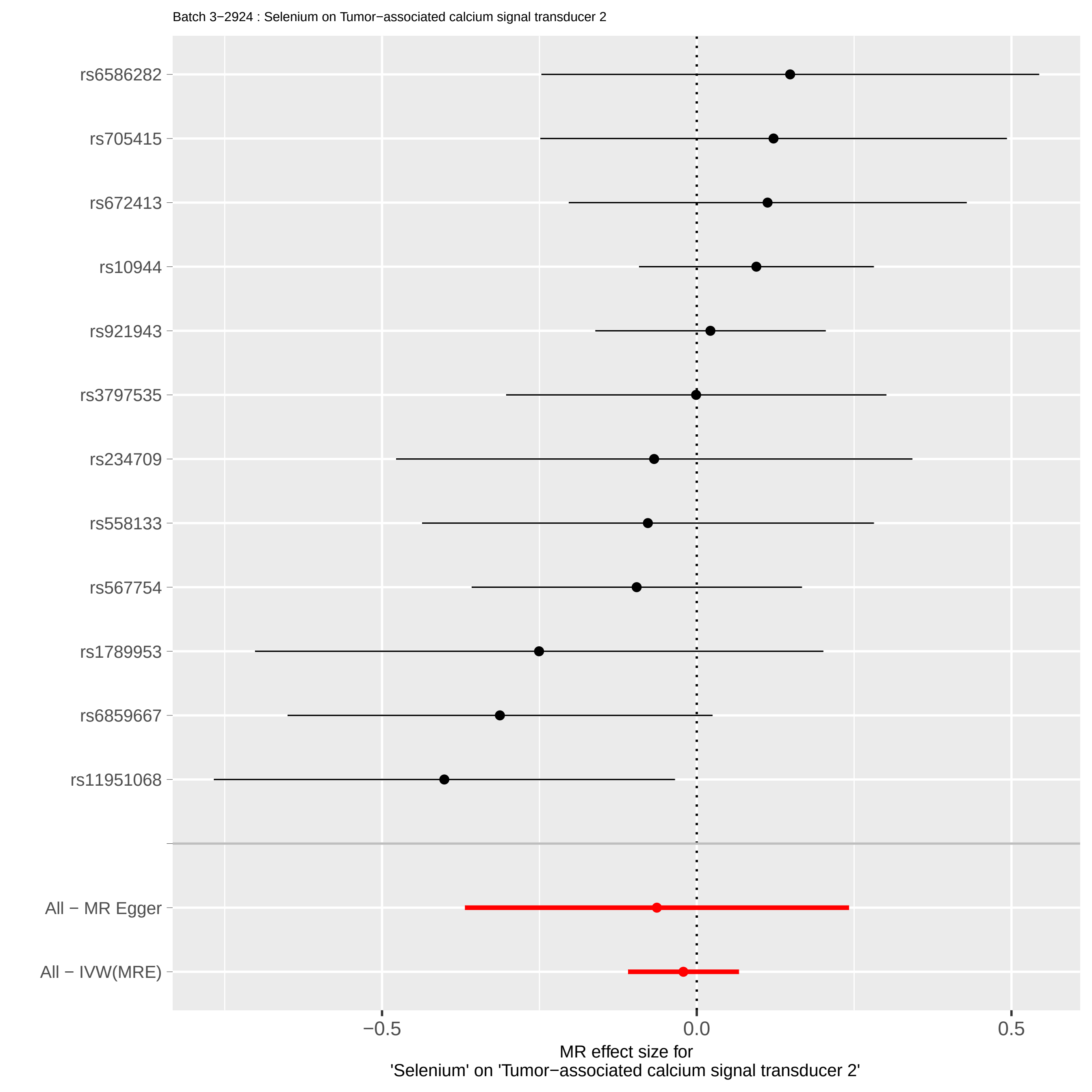
Batch 3−2924 : Selenium on Tumor−associated calcium signal transducer 2
rs6586282
rs705415
rs672413
rs10944
rs921943
rs3797535
rs234709
rs558133
rs567754
rs1789953
rs6859667
rs11951068
All − MR Egger
All − IVW(MRE)
−0.5
0.0
0.5
MR effect size for
'Selenium' on 'Tumor−associated calcium signal transducer 2'

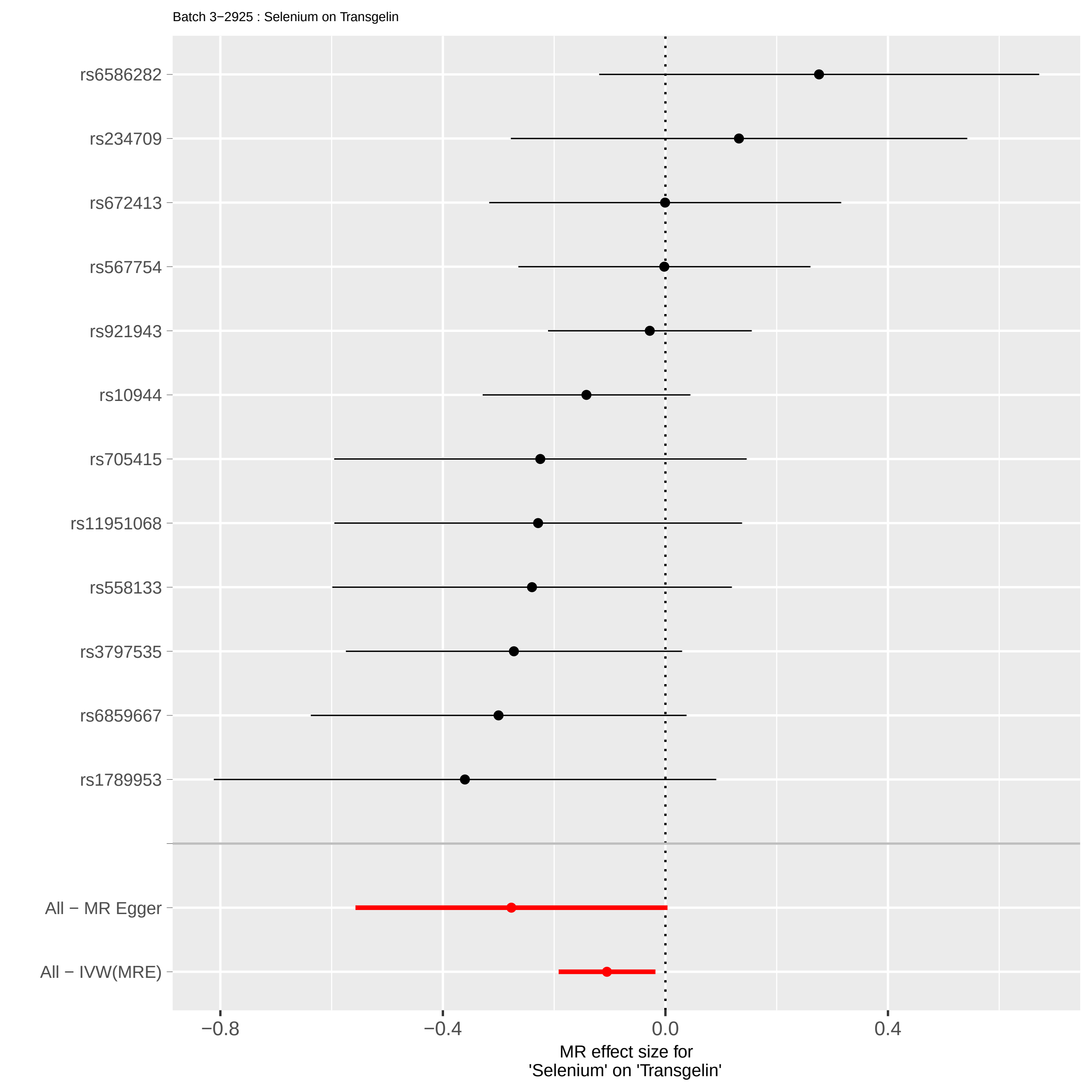

Batch 3−2925 : Selenium on Transgelin
rs6586282
rs234709
rs672413
rs567754
rs921943
rs10944
rs705415
rs11951068
rs558133
rs3797535
rs6859667
rs1789953
All − MR Egger
All − IVW(MRE)
−0.8
−0.4
0.0
0.4
MR effect size for
'Selenium' on 'Transgelin'

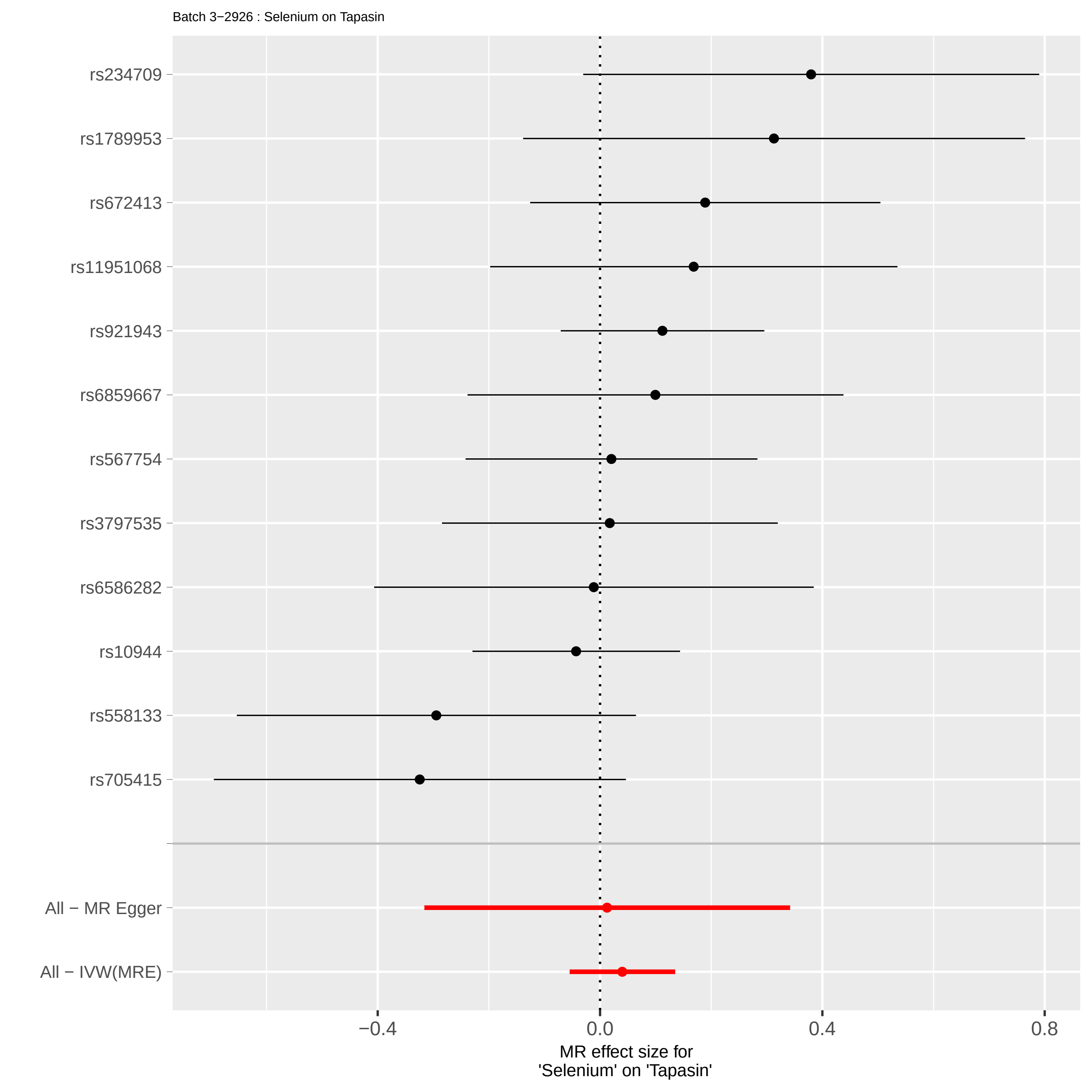

Batch 3−2926 : Selenium on Tapasin
rs234709
rs1789953
rs672413
rs11951068
rs921943
rs6859667
rs567754
rs3797535
rs6586282
rs10944
rs558133
rs705415
All − MR Egger
All − IVW(MRE)
−0.4
0.0
0.4
0.8
MR effect size for
'Selenium' on 'Tapasin'

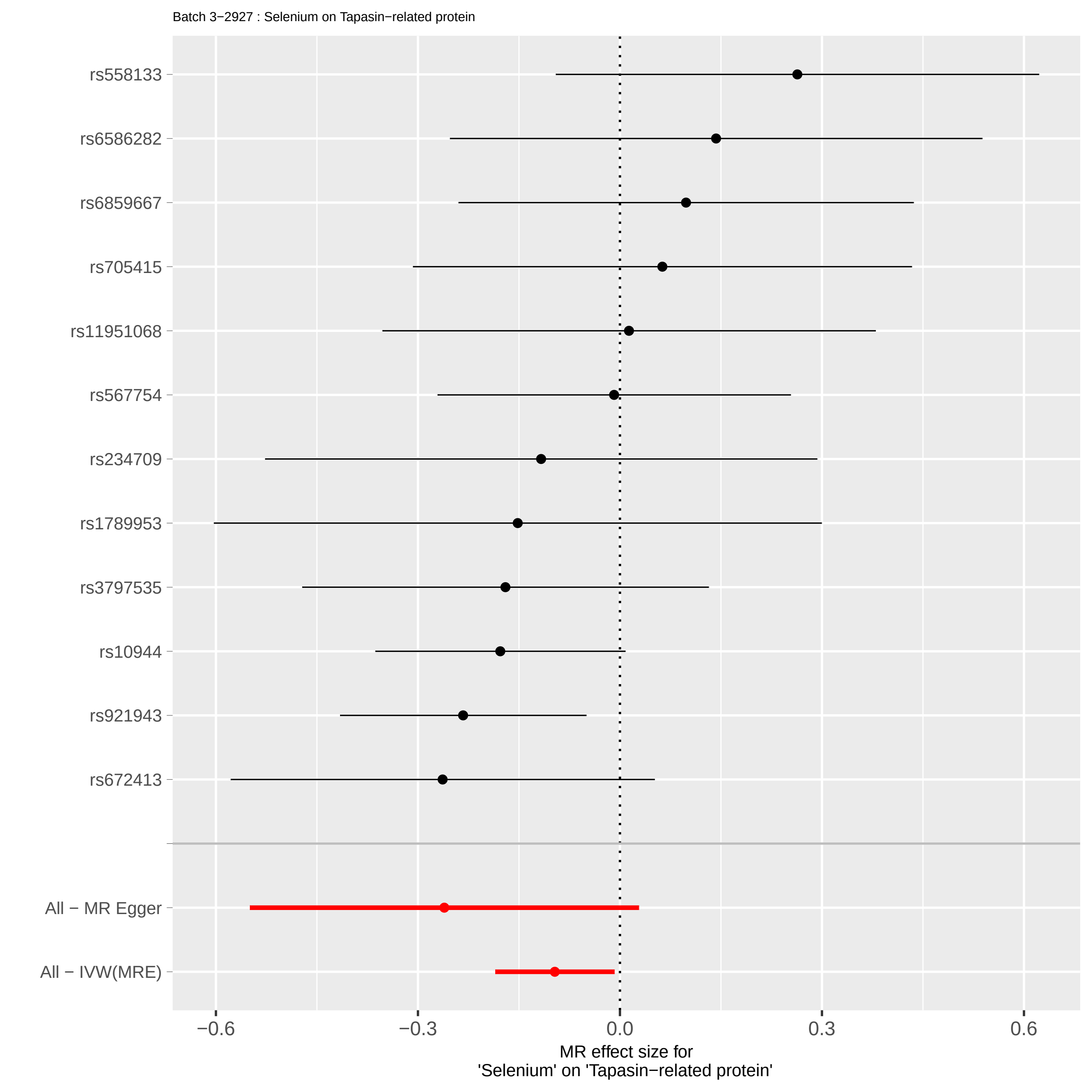

Batch 3−2927 : Selenium on Tapasin−related protein
rs558133
rs6586282
rs6859667
rs705415
rs11951068
rs567754
rs234709
rs1789953
rs3797535
rs10944
rs921943
rs672413
All − MR Egger
All − IVW(MRE)
−0.6
−0.3
0.0
0.3
0.6
MR effect size for
'Selenium' on 'Tapasin−related protein'

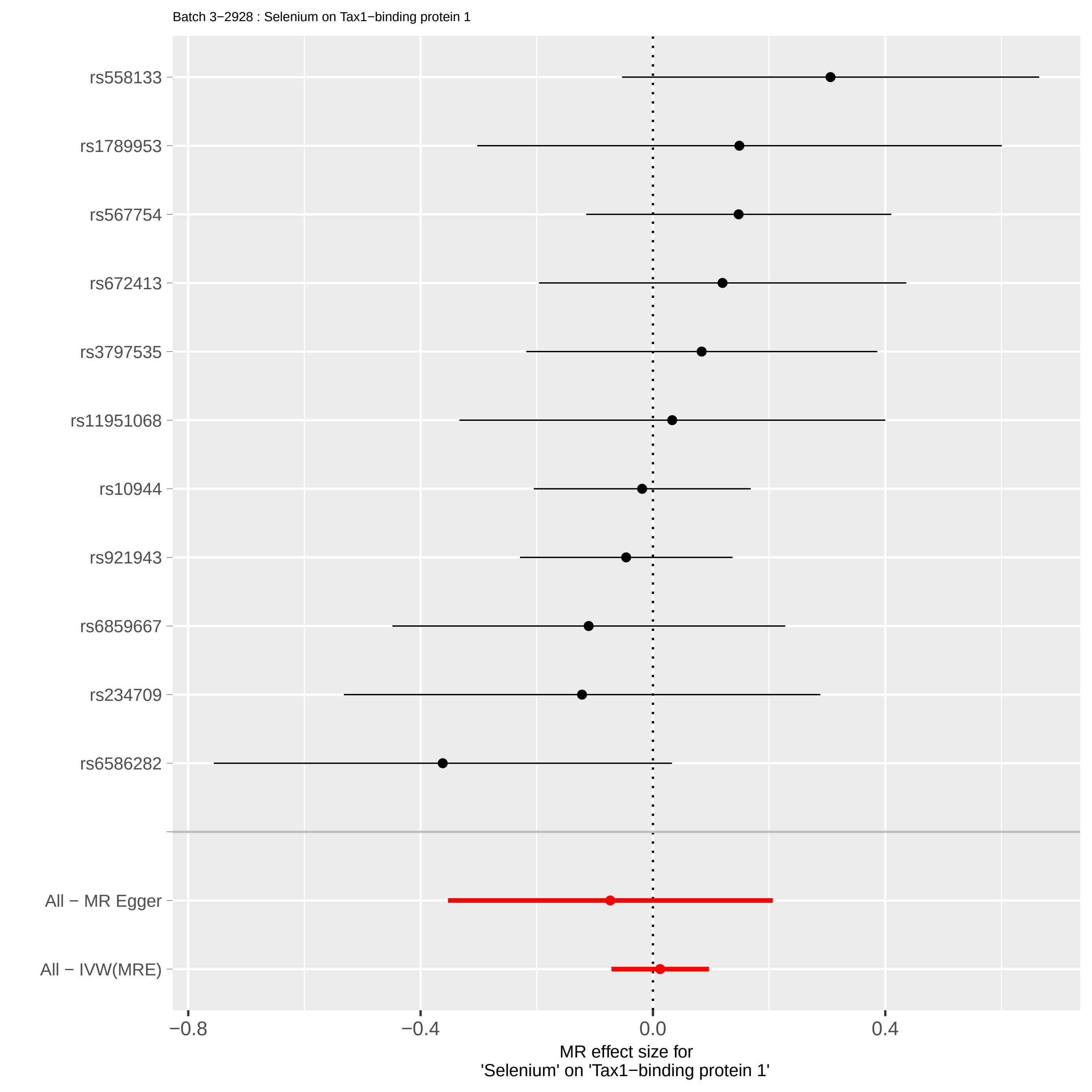

Batch 3−2928 : Selenium on Tax1−binding protein 1
rs558133
rs1789953
rs567754
rs672413
rs3797535
rs11951068
rs10944
rs921943
rs6859667
rs234709
rs6586282
All − MR Egger
All − IVW(MRE)
−0.8
−0.4
0.0
0.4
MR effect size for
'Selenium' on 'Tax1−binding protein 1'

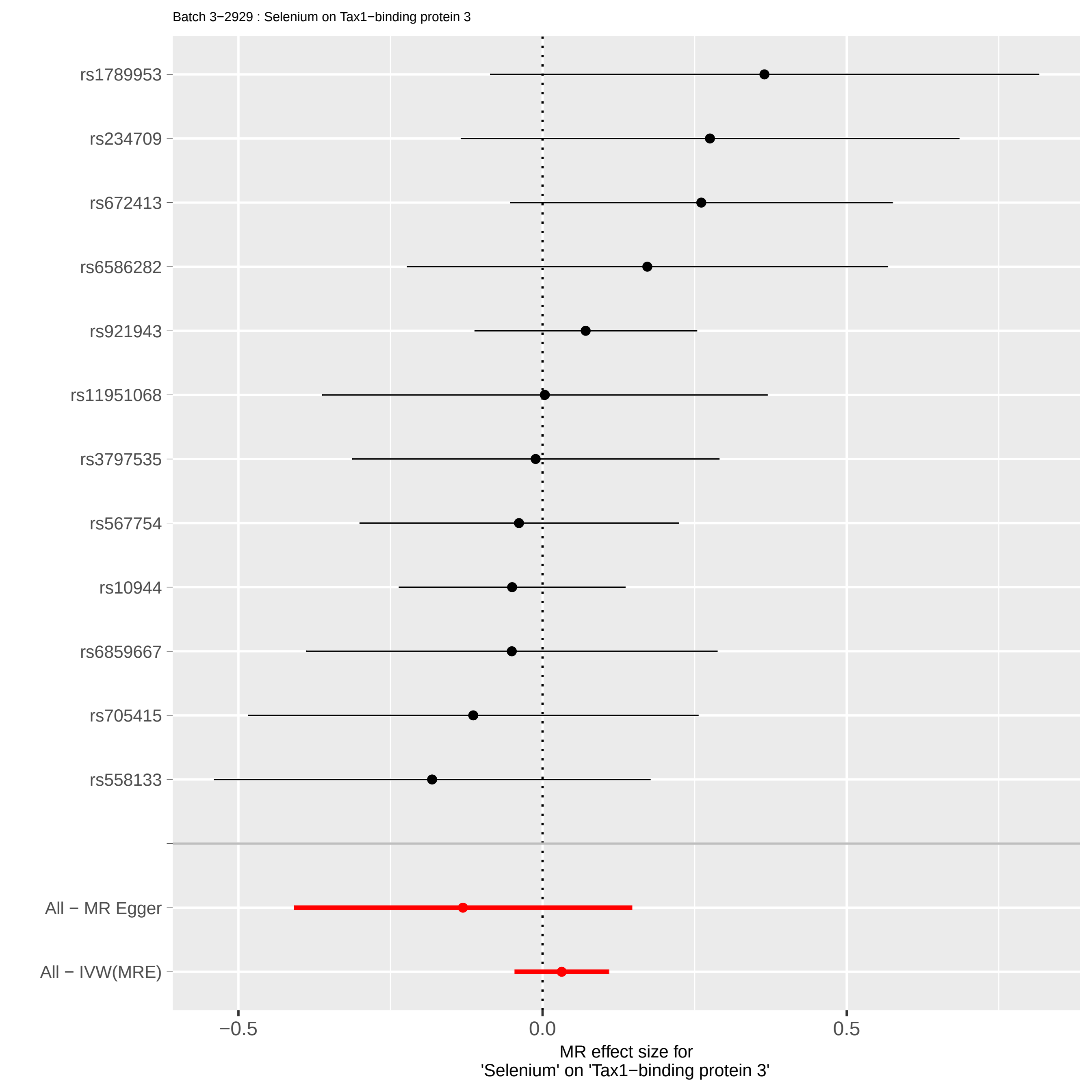

Batch 3−2929 : Selenium on Tax1−binding protein 3
rs1789953
rs234709
rs672413
rs6586282
rs921943
rs11951068
rs3797535
rs567754
rs10944
rs6859667
rs705415
rs558133
All − MR Egger
All − IVW(MRE)
−0.5
0.0
0.5
MR effect size for
'Selenium' on 'Tax1−binding protein 3'

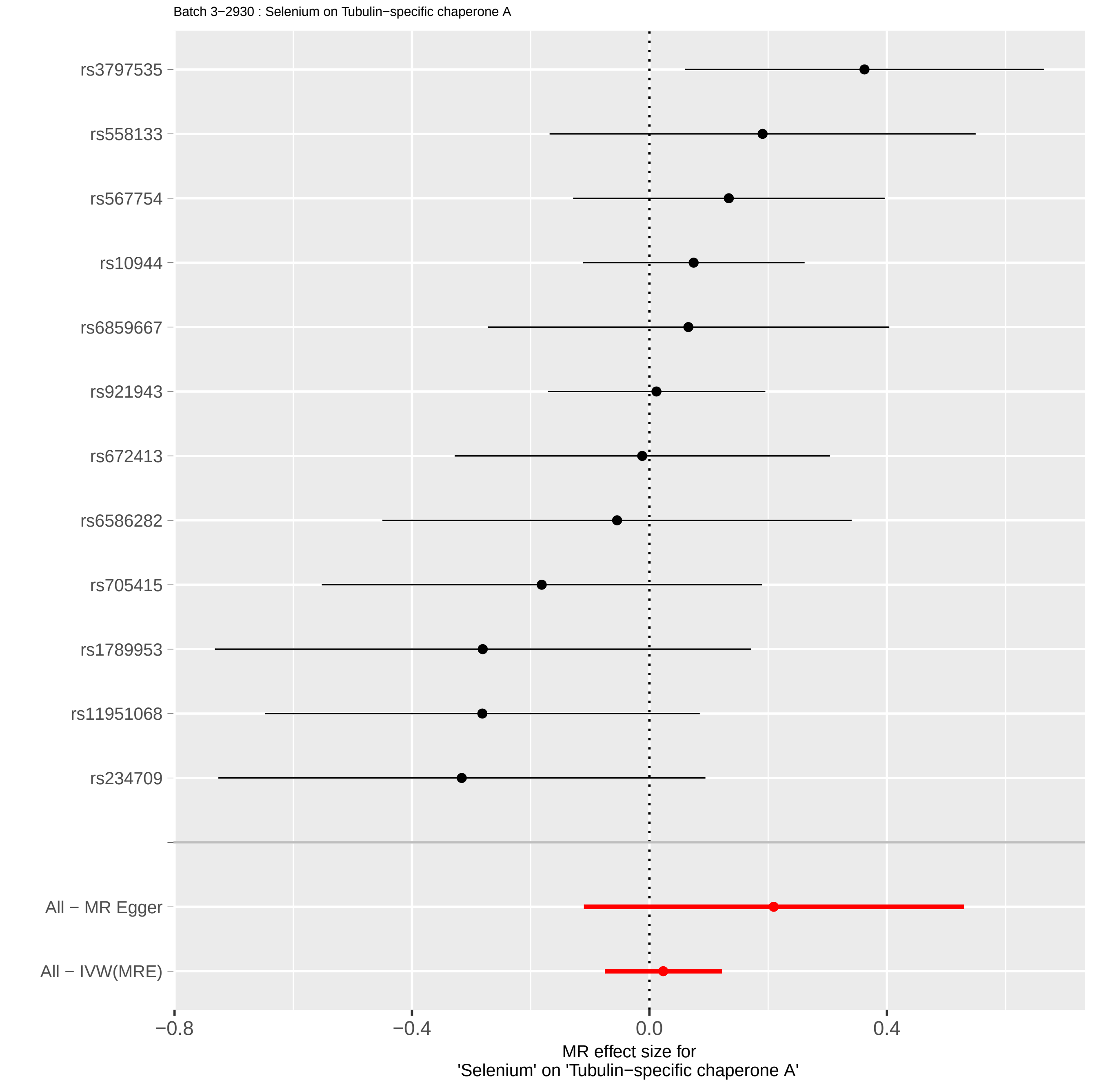

Batch 3−2930 : Selenium on Tubulin−specific chaperone A
rs3797535
rs558133
rs567754
rs10944
rs6859667
rs921943
rs672413
rs6586282
rs705415
rs1789953
rs11951068
rs234709
All − MR Egger
All − IVW(MRE)
−0.8
−0.4
0.0
0.4
MR effect size for
'Selenium' on 'Tubulin−specific chaperone A'

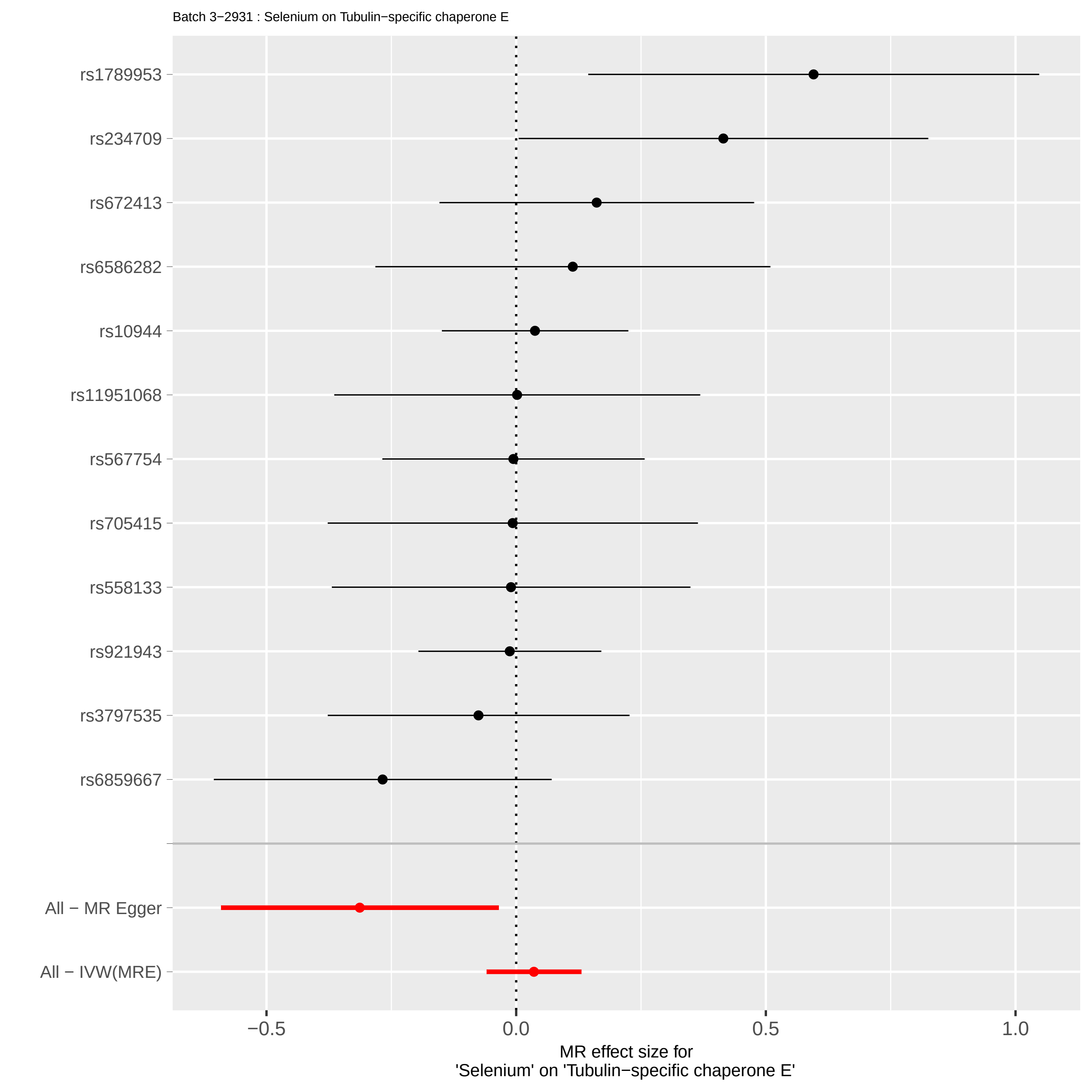

Batch 3−2931 : Selenium on Tubulin−specific chaperone E
rs1789953
rs234709
rs672413
rs6586282
rs10944
rs11951068
rs567754
rs705415
rs558133
rs921943
rs3797535
rs6859667
All − MR Egger
All − IVW(MRE)
−0.5
0.0
0.5
1.0
MR effect size for
'Selenium' on 'Tubulin−specific chaperone E'

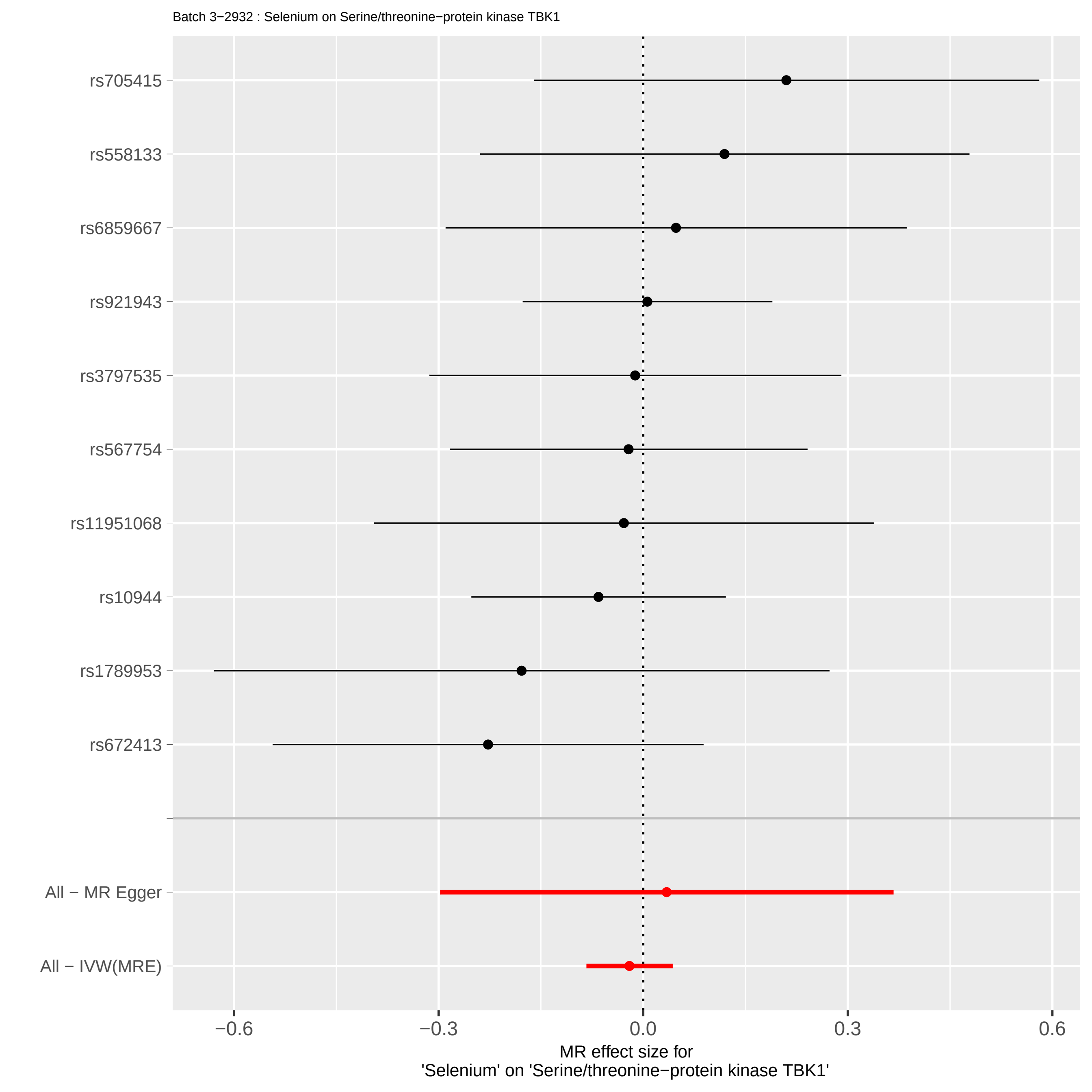

Batch 3−2932 : Selenium on Serine/threonine−protein kinase TBK1
rs705415
rs558133
rs6859667
rs921943
rs3797535
rs567754
rs11951068
rs10944
rs1789953
rs672413
All − MR Egger
All − IVW(MRE)
−0.6
−0.3
0.0
0.3
0.6
MR effect size for
'Selenium' on 'Serine/threonine−protein kinase TBK1'

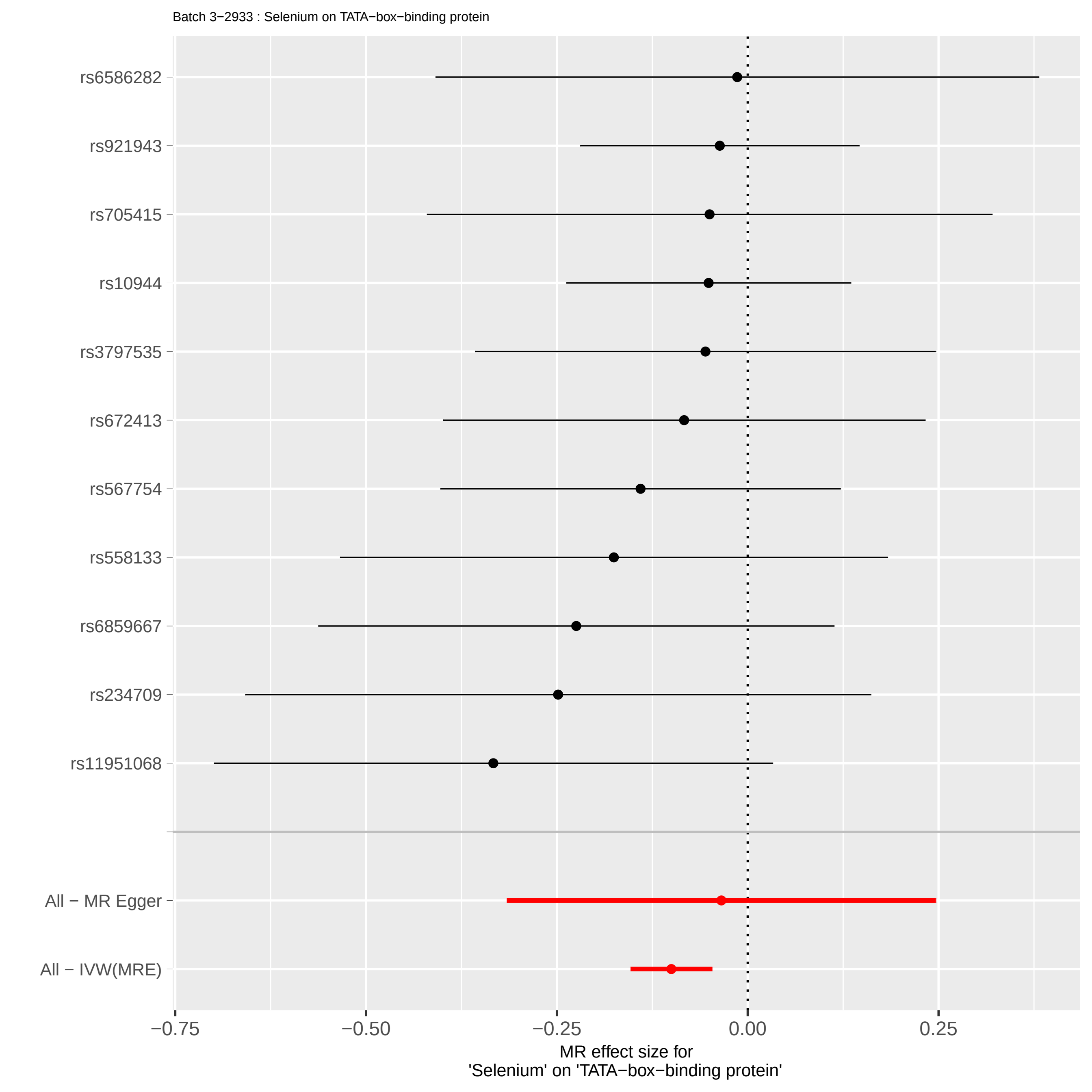

Batch 3−2933 : Selenium on TATA−box−binding protein
rs6586282
rs921943
rs705415
rs10944
rs3797535
rs672413
rs567754
rs558133
rs6859667
rs234709
rs11951068
All − MR Egger
All − IVW(MRE)
−0.75
−0.50
−0.25
0.00
0.25
MR effect size for
'Selenium' on 'TATA−box−binding protein'

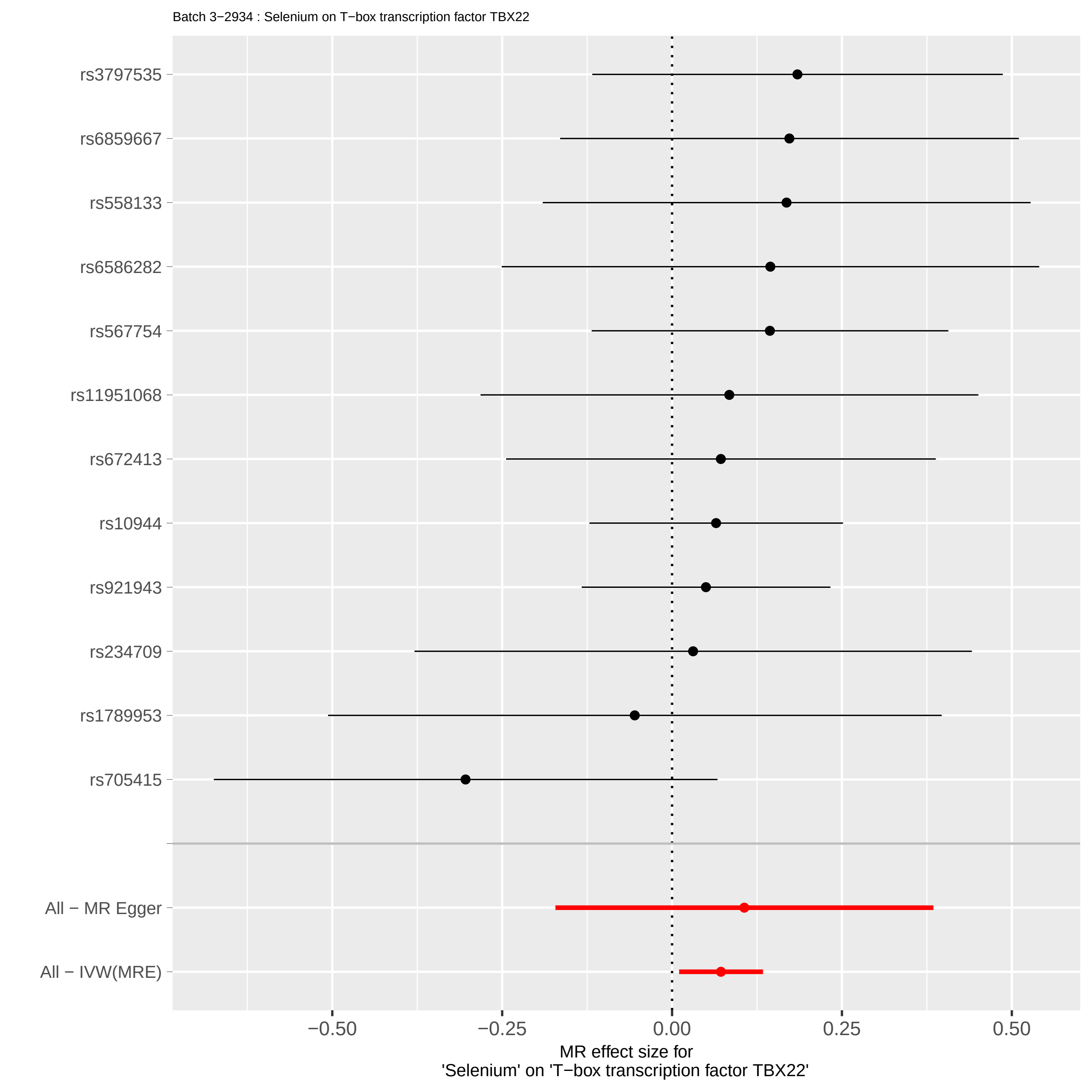

Batch 3−2934 : Selenium on T−box transcription factor TBX22
rs3797535
rs6859667
rs558133
rs6586282
rs567754
rs11951068
rs672413
rs10944
rs921943
rs234709
rs1789953
rs705415
All − MR Egger
All − IVW(MRE)
−0.50
−0.25
0.00
0.25
0.50
MR effect size for
'Selenium' on 'T−box transcription factor TBX22'

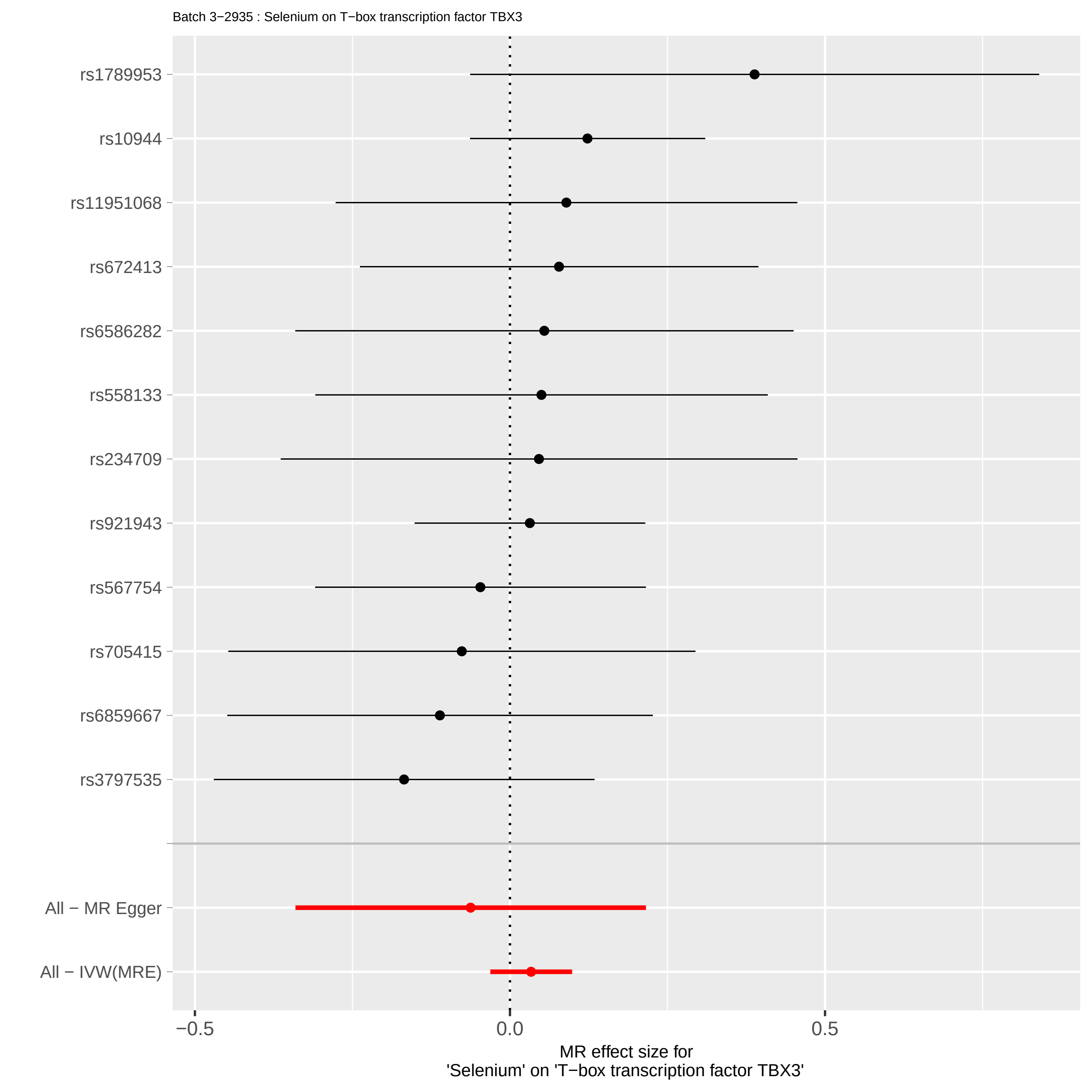

Batch 3−2935 : Selenium on T−box transcription factor TBX3
rs1789953
rs10944
rs11951068
rs672413
rs6586282
rs558133
rs234709
rs921943
rs567754
rs705415
rs6859667
rs3797535
All − MR Egger
All − IVW(MRE)
−0.5
0.0
0.5
MR effect size for
'Selenium' on 'T−box transcription factor TBX3'

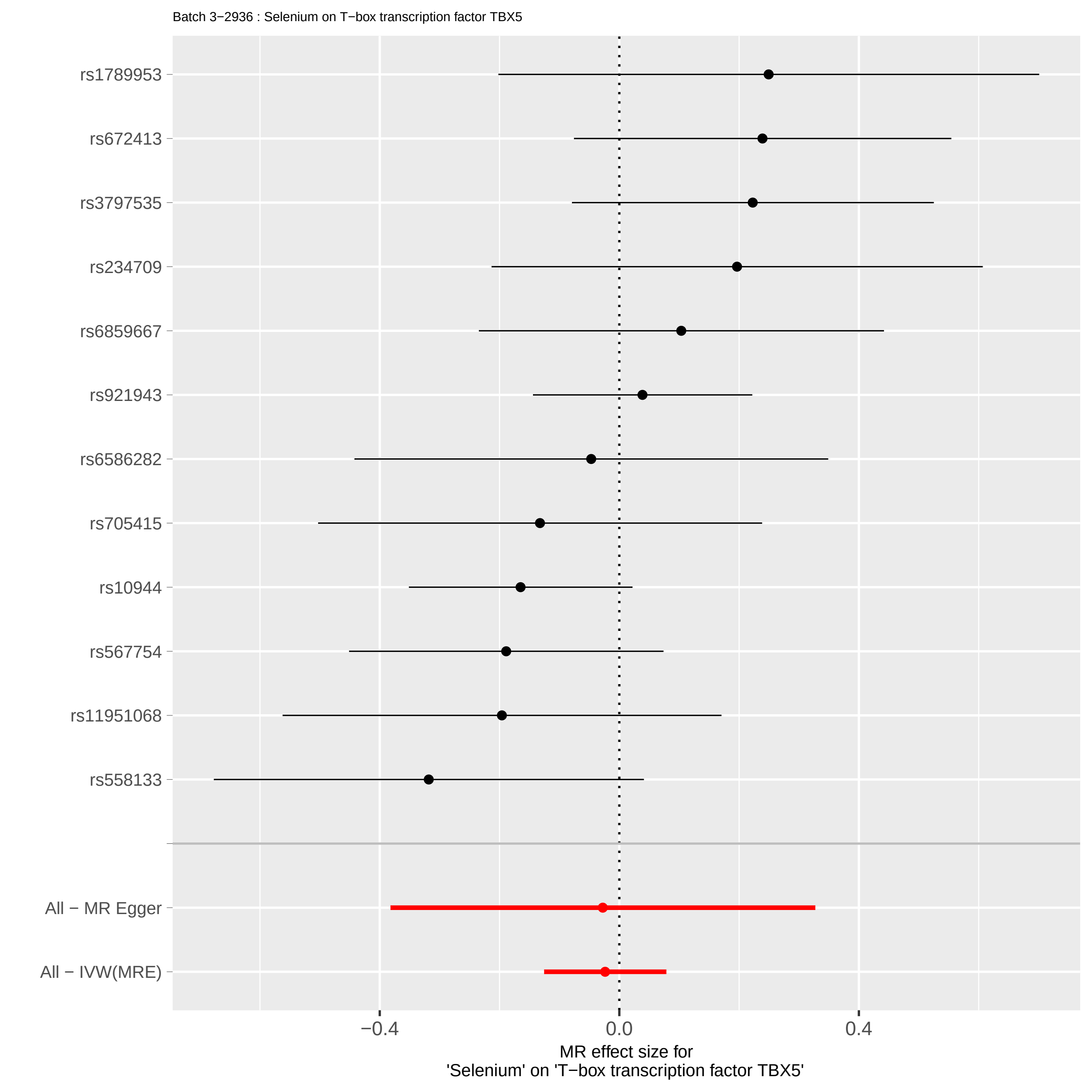

Batch 3−2936 : Selenium on T−box transcription factor TBX5
rs1789953
rs672413
rs3797535
rs234709
rs6859667
rs921943
rs6586282
rs705415
rs10944
rs567754
rs11951068
rs558133
All − MR Egger
All − IVW(MRE)
−0.4
0.0
0.4
MR effect size for
'Selenium' on 'T−box transcription factor TBX5'

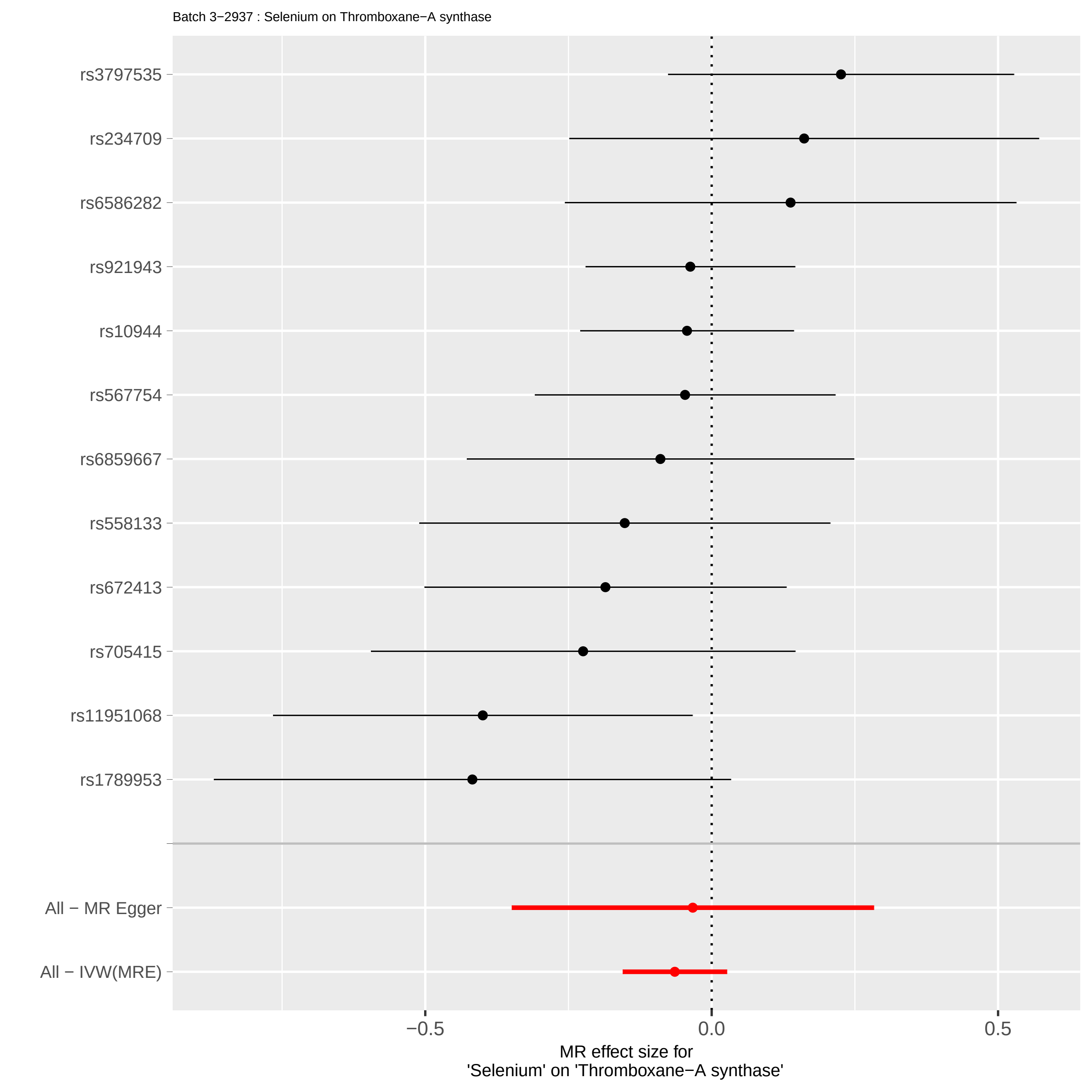

Batch 3−2937 : Selenium on Thromboxane−A synthase
rs3797535
rs234709
rs6586282
rs921943
rs10944
rs567754
rs6859667
rs558133
rs672413
rs705415
rs11951068
rs1789953
All − MR Egger
All − IVW(MRE)
−0.5
0.0
0.5
MR effect size for
'Selenium' on 'Thromboxane−A synthase'

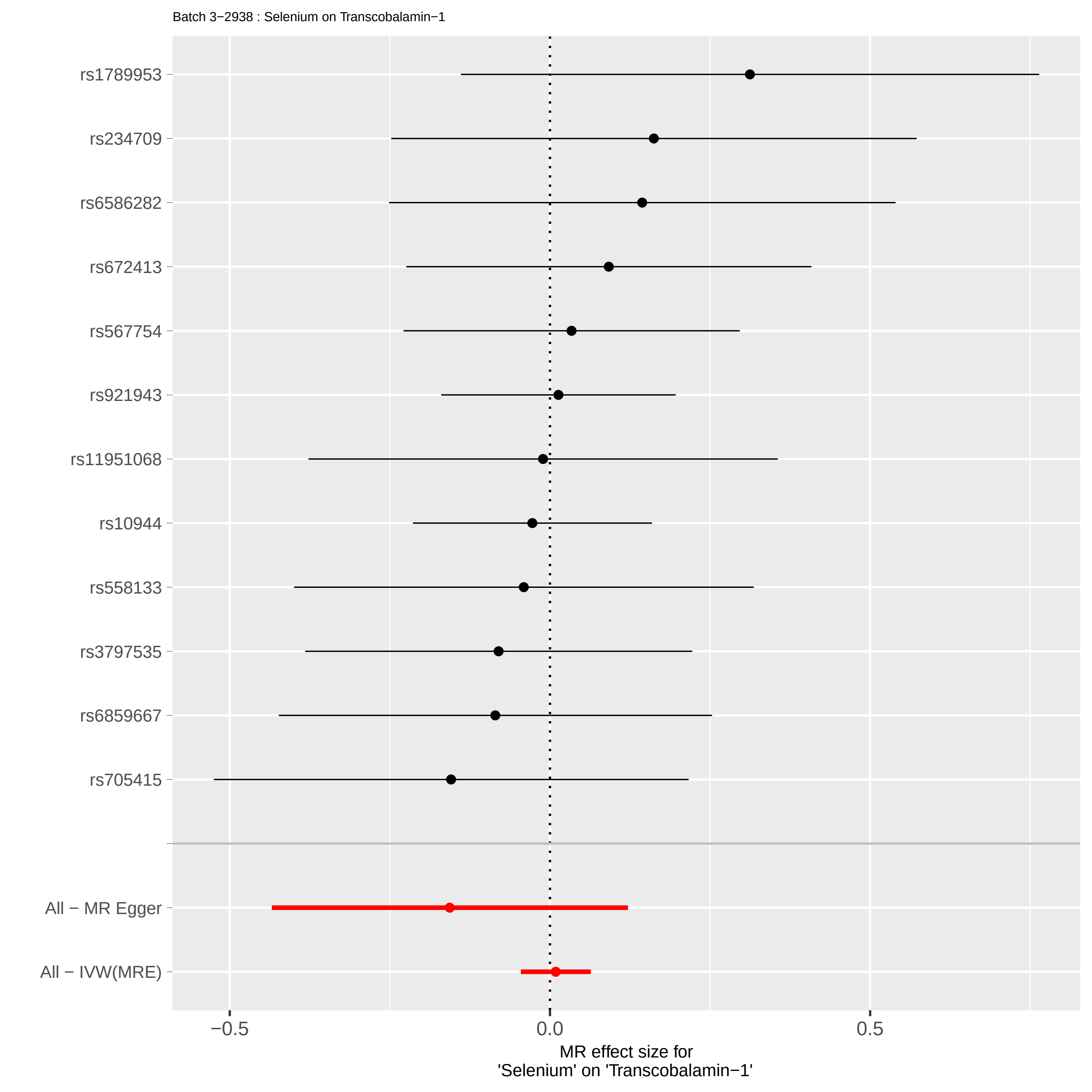
Batch 3−2938 : Selenium on Transcobalamin−1
rs1789953
rs234709
rs6586282
rs672413
rs567754
rs921943
rs11951068
rs10944
rs558133
rs3797535
rs6859667
rs705415
All − MR Egger
All − IVW(MRE)
−0.5
0.0
0.5
MR effect size for
'Selenium' on 'Transcobalamin−1'

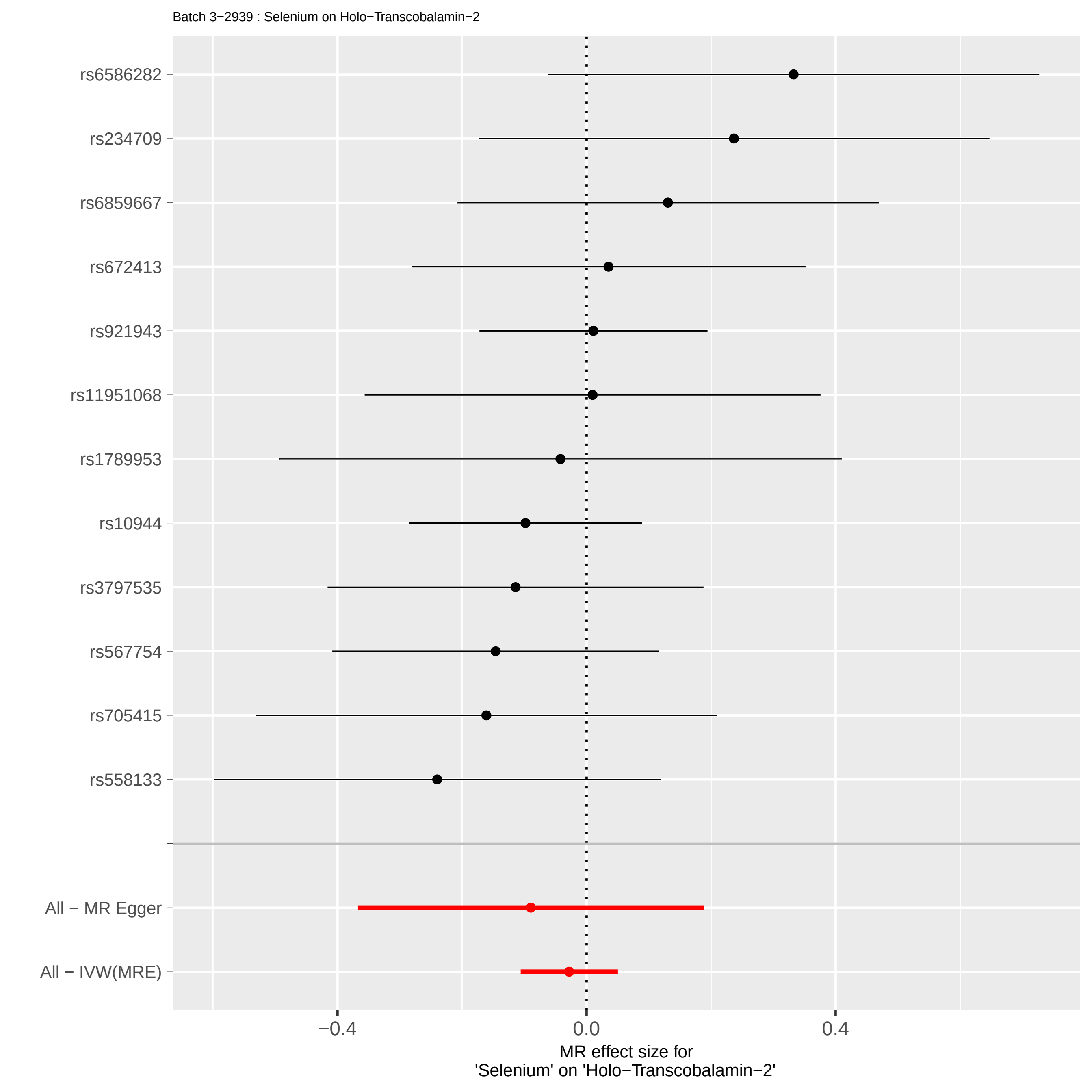

Batch 3−2939 : Selenium on Holo−Transcobalamin−2
rs6586282
rs234709
rs6859667
rs672413
rs921943
rs11951068
rs1789953
rs10944
rs3797535
rs567754
rs705415
rs558133
All − MR Egger
All − IVW(MRE)
−0.4
0.0
0.4
MR effect size for
'Selenium' on 'Holo−Transcobalamin−2'

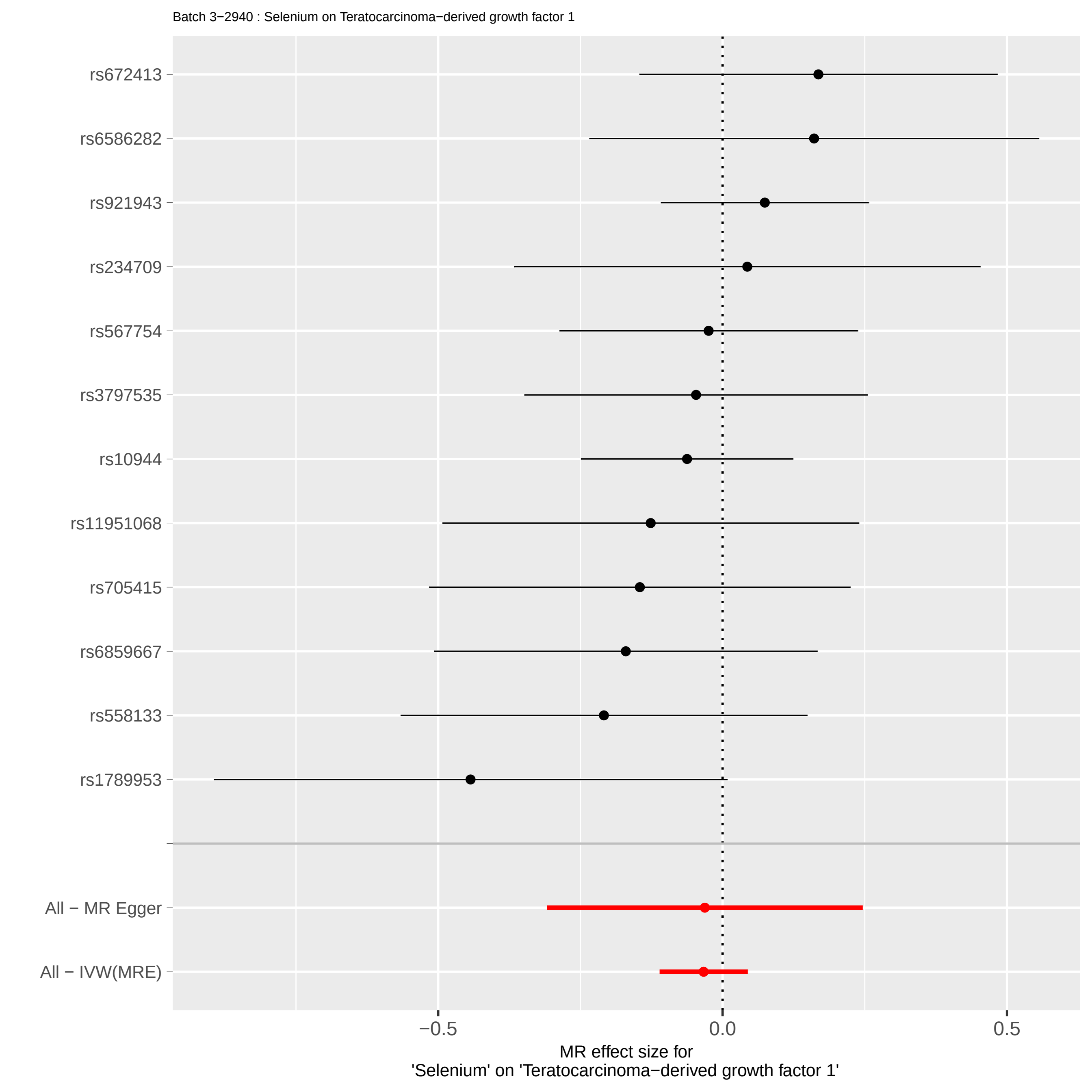

Batch 3−2940 : Selenium on Teratocarcinoma−derived growth factor 1
rs672413
rs6586282
rs921943
rs234709
rs567754
rs3797535
rs10944
rs11951068
rs705415
rs6859667
rs558133
rs1789953
All − MR Egger
All − IVW(MRE)
−0.5
0.0
0.5
MR effect size for
'Selenium' on 'Teratocarcinoma−derived growth factor 1'

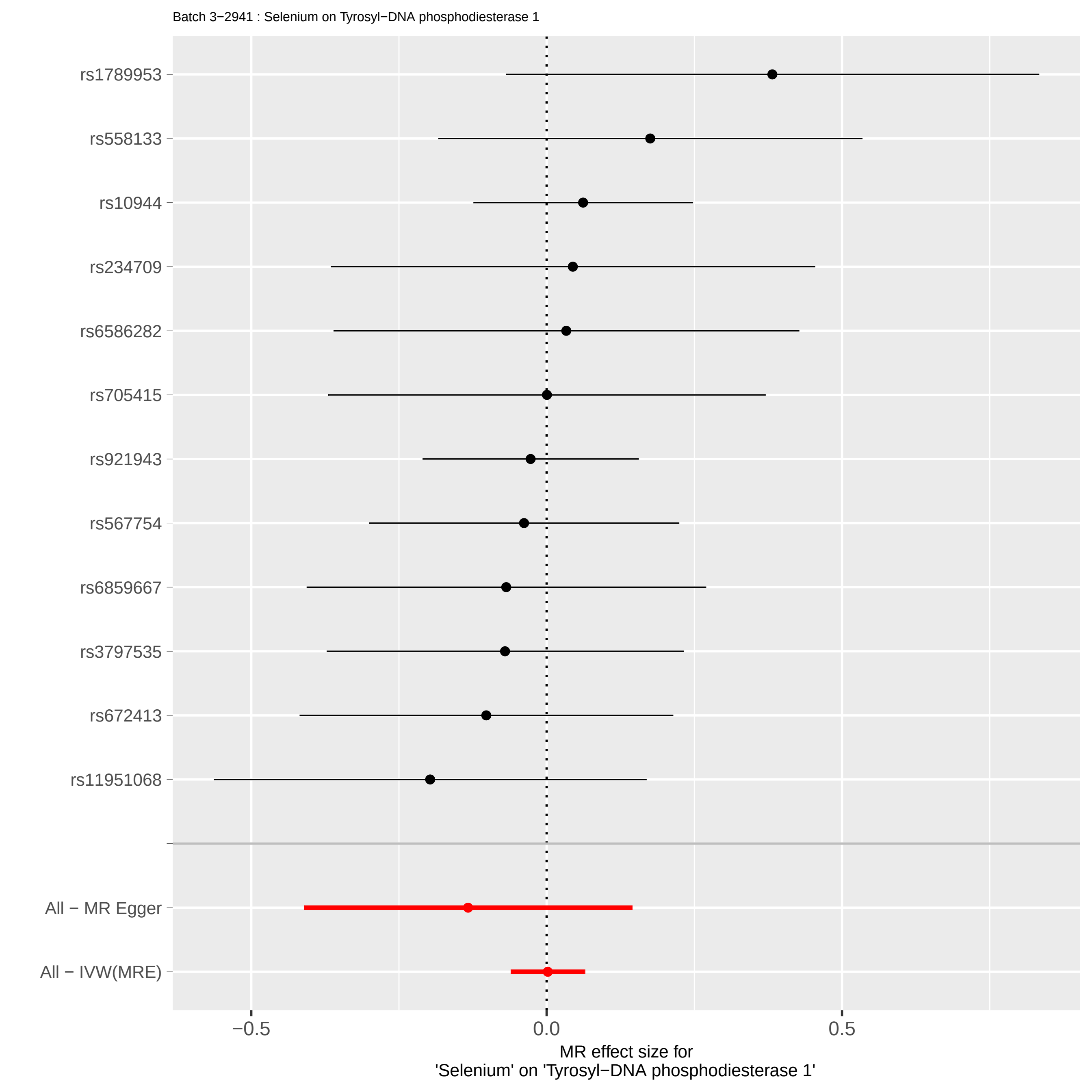
Batch 3−2941 : Selenium on Tyrosyl−DNA phosphodiesterase 1
rs1789953
rs558133
rs10944
rs234709
rs6586282
rs705415
rs921943
rs567754
rs6859667
rs3797535
rs672413
rs11951068
All − MR Egger
All − IVW(MRE)
−0.5
0.0
0.5
MR effect size for
'Selenium' on 'Tyrosyl−DNA phosphodiesterase 1'

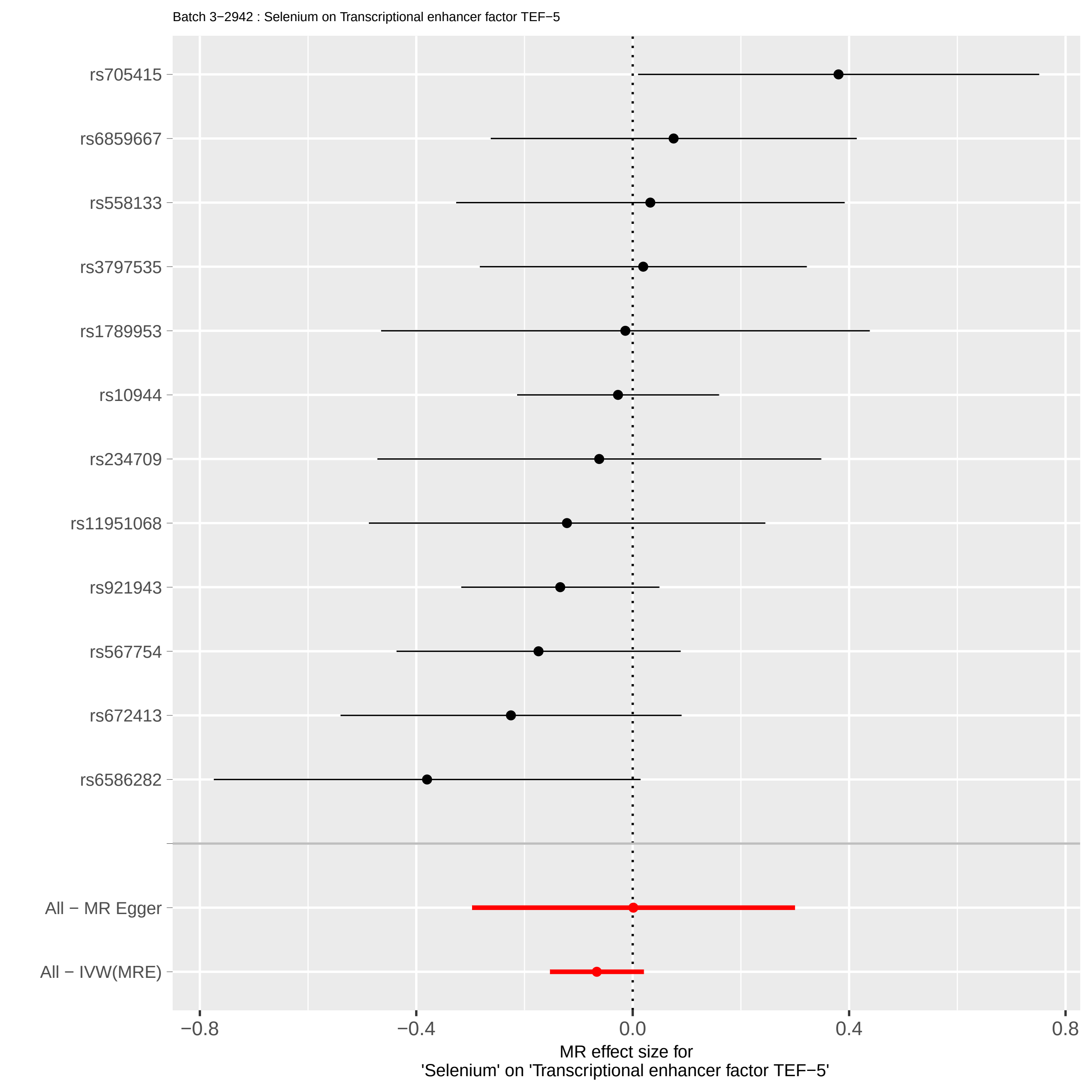
Batch 3−2942 : Selenium on Transcriptional enhancer factor TEF−5
rs705415
rs6859667
rs558133
rs3797535
rs1789953
rs10944
rs234709
rs11951068
rs921943
rs567754
rs672413
rs6586282
All − MR Egger
All − IVW(MRE)
−0.8
−0.4
0.0
0.4
0.8
MR effect size for
'Selenium' on 'Transcriptional enhancer factor TEF−5'

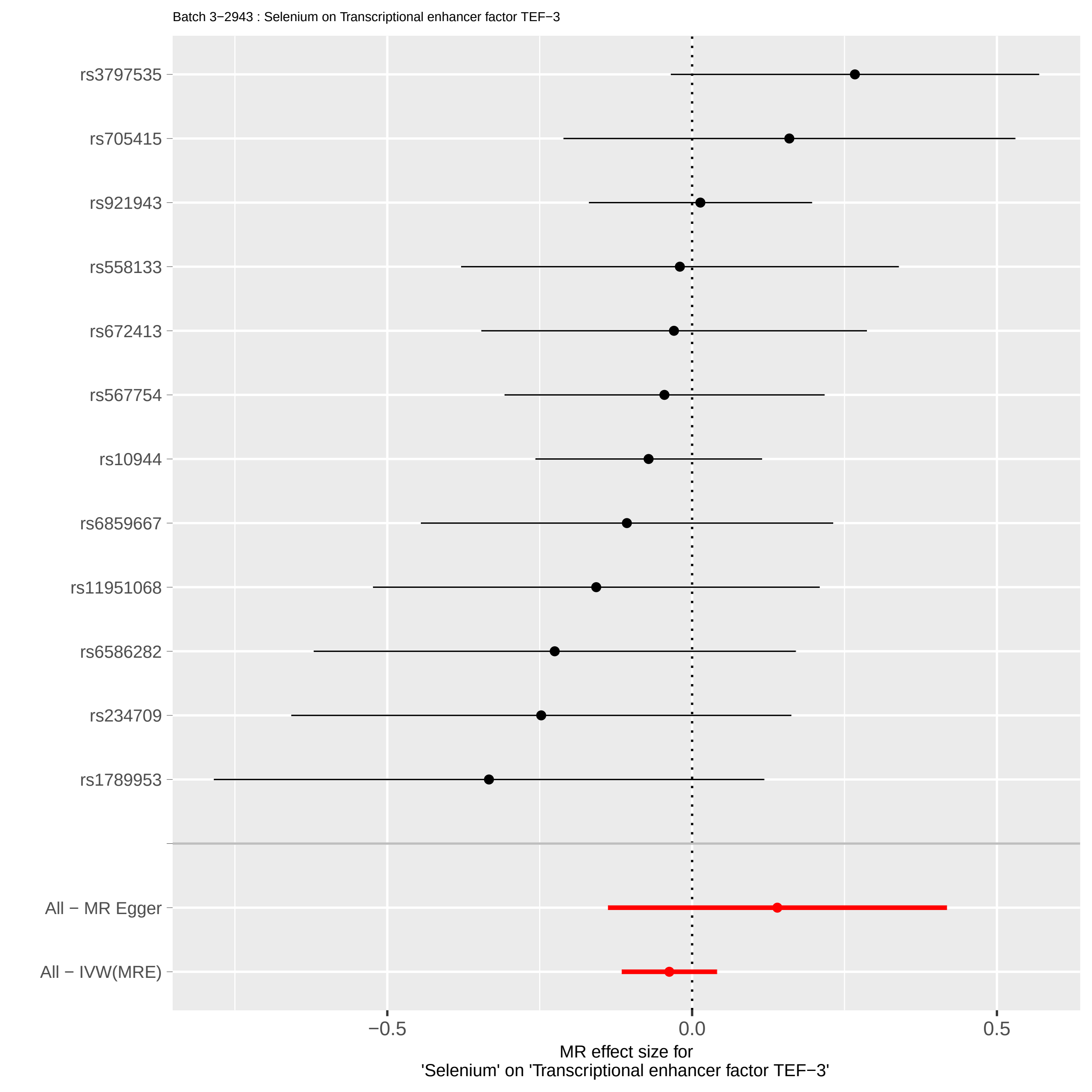

Batch 3−2943 : Selenium on Transcriptional enhancer factor TEF−3
rs3797535
rs705415
rs921943
rs558133
rs672413
rs567754
rs10944
rs6859667
rs11951068
rs6586282
rs234709
rs1789953
All − MR Egger
All − IVW(MRE)
−0.5
0.0
0.5
MR effect size for
'Selenium' on 'Transcriptional enhancer factor TEF−3'

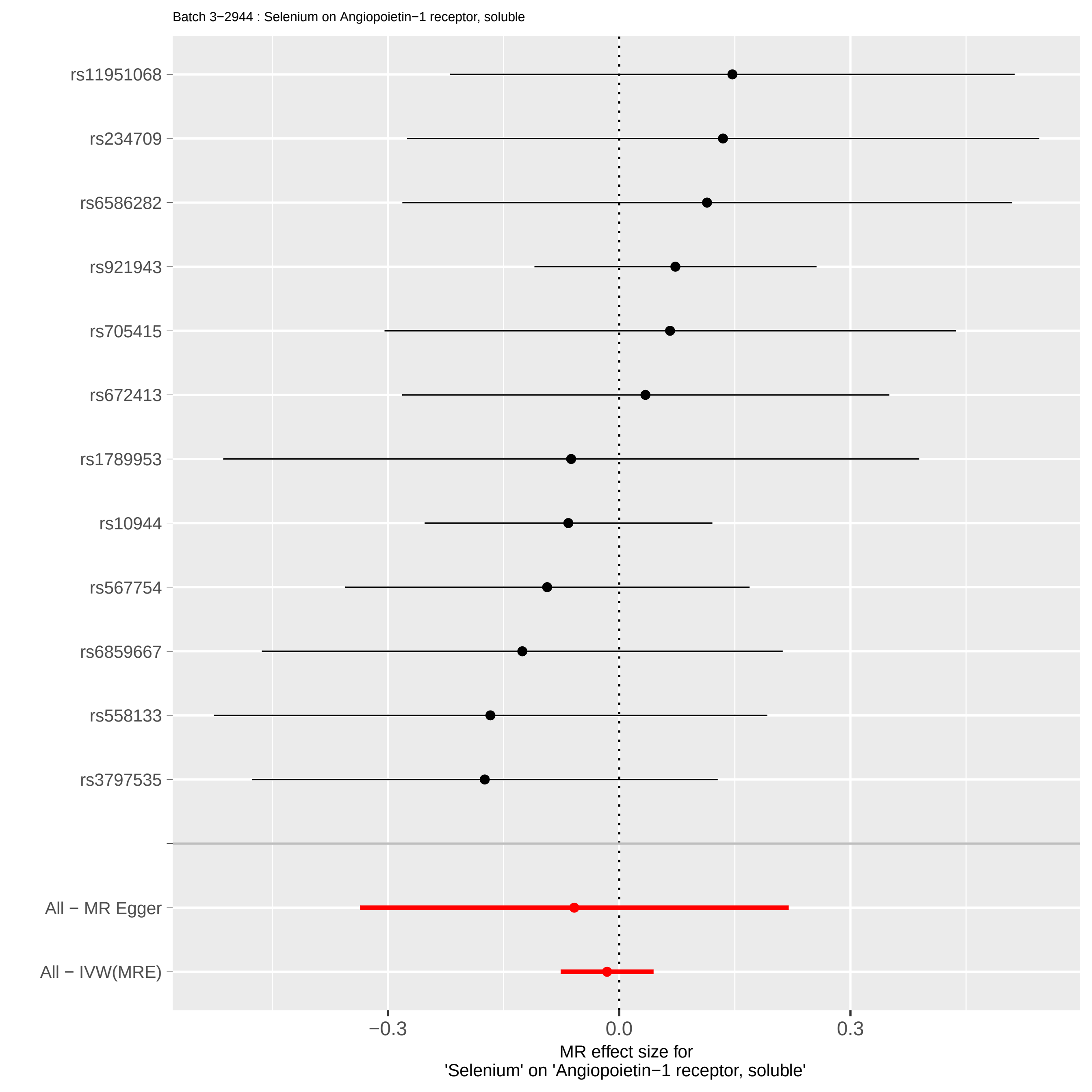

Batch 3−2944 : Selenium on Angiopoietin−1 receptor, soluble
rs11951068
rs234709
rs6586282
rs921943
rs705415
rs672413
rs1789953
rs10944
rs567754
rs6859667
rs558133
rs3797535
All − MR Egger
All − IVW(MRE)
−0.3
0.0
0.3
MR effect size for
'Selenium' on 'Angiopoietin−1 receptor, soluble'

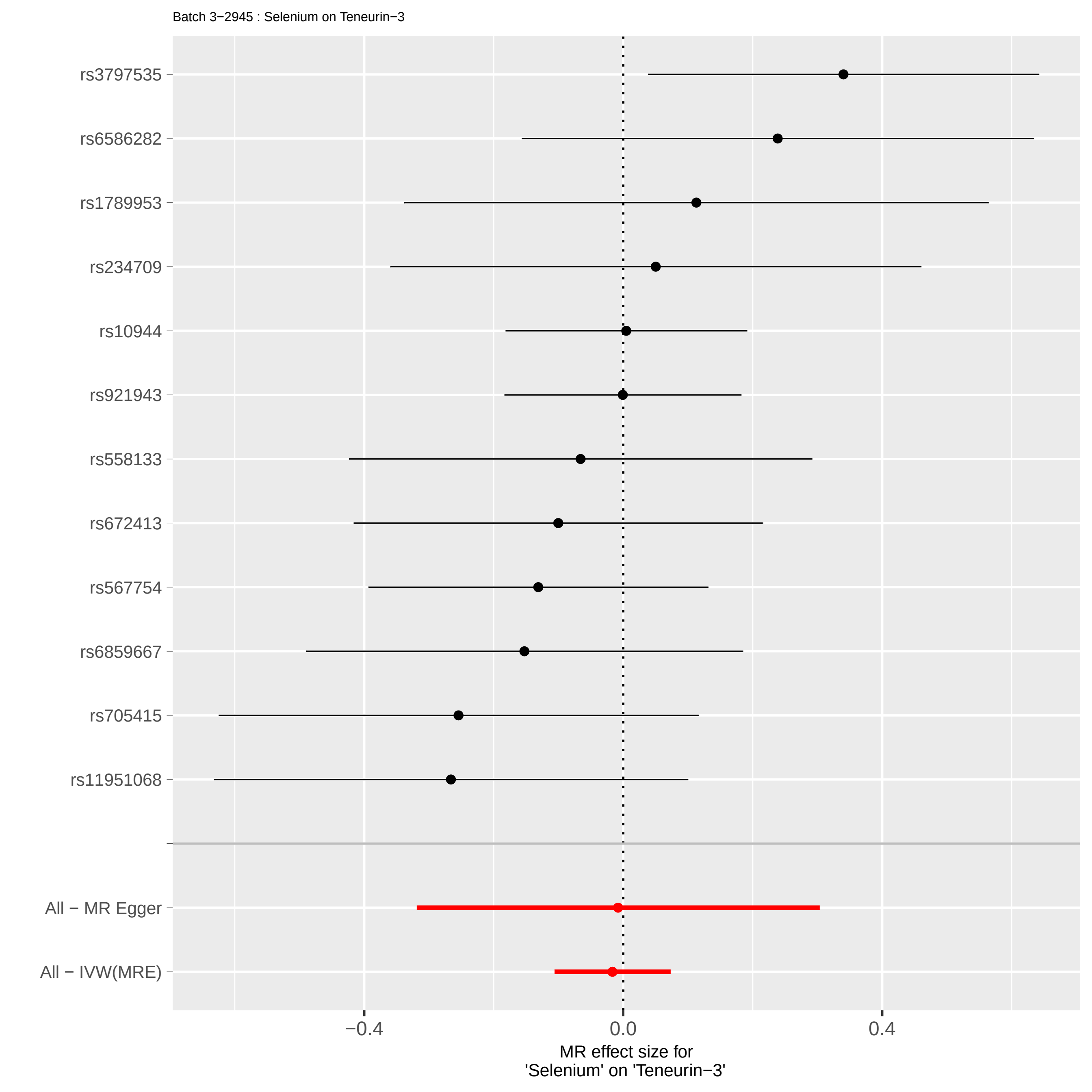

Batch 3−2945 : Selenium on Teneurin−3
rs3797535
rs6586282
rs1789953
rs234709
rs10944
rs921943
rs558133
rs672413
rs567754
rs6859667
rs705415
rs11951068
All − MR Egger
All − IVW(MRE)
−0.4
0.0
0.4
MR effect size for
'Selenium' on 'Teneurin−3'

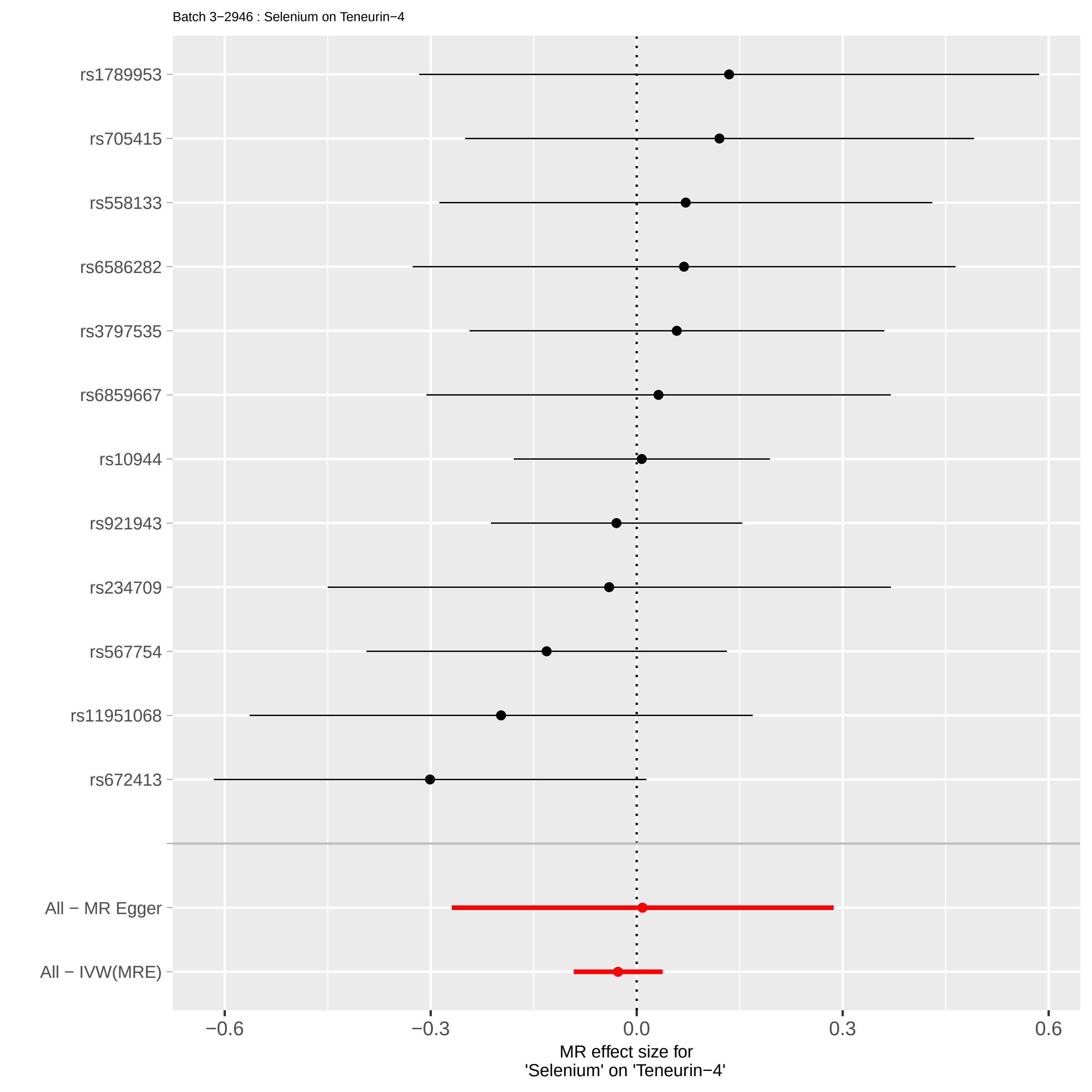

Batch 3−2946 : Selenium on Teneurin−4
rs1789953
rs705415
rs558133
rs6586282
rs3797535
rs6859667
rs10944
rs921943
rs234709
rs567754
rs11951068
rs672413
All − MR Egger
All − IVW(MRE)
−0.6
−0.3
0.0
0.3
0.6
MR effect size for
'Selenium' on 'Teneurin−4'

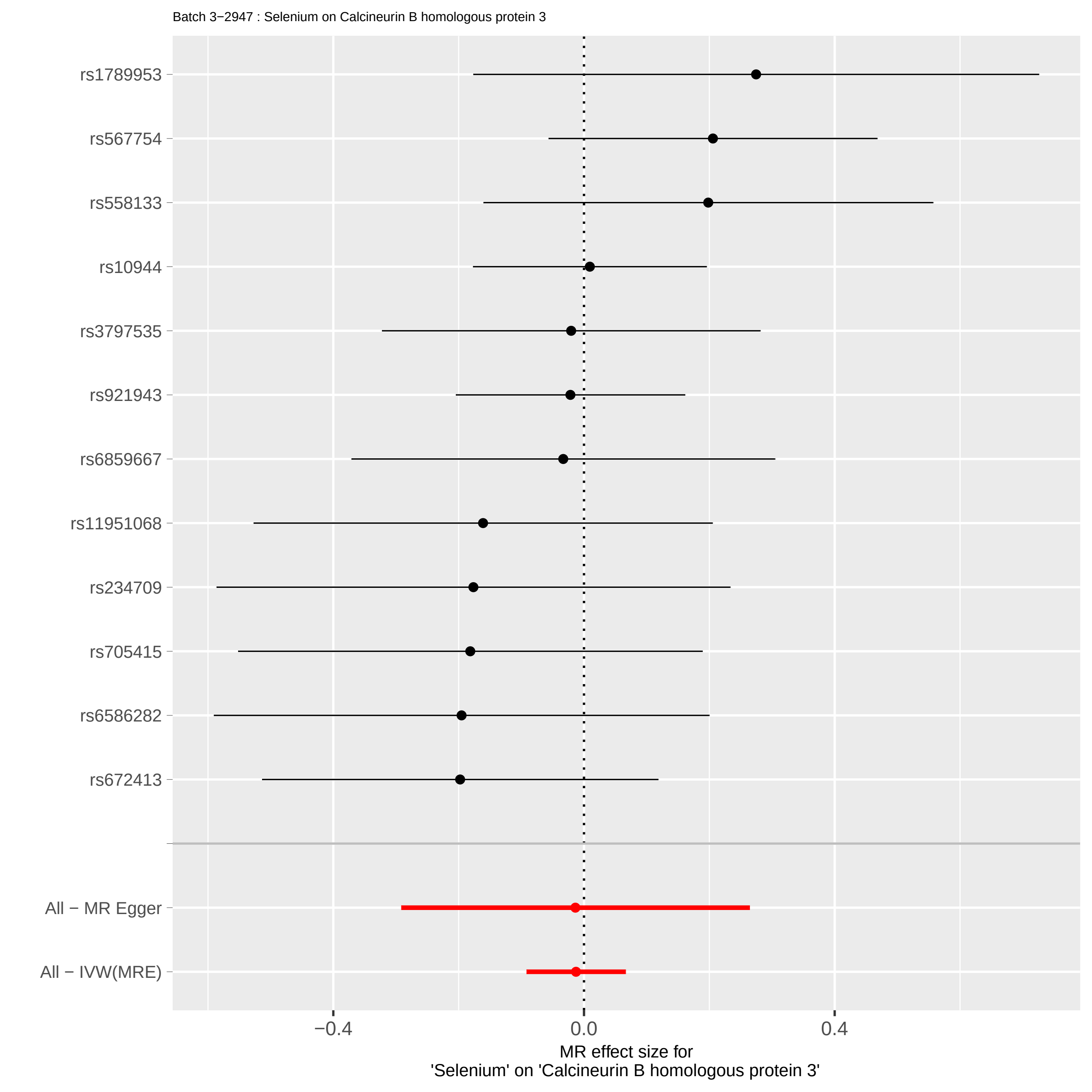

Batch 3−2947 : Selenium on Calcineurin B homologous protein 3
rs1789953
rs567754
rs558133
rs10944
rs3797535
rs921943
rs6859667
rs11951068
rs234709
rs705415
rs6586282
rs672413
All − MR Egger
All − IVW(MRE)
−0.4
0.0
0.4
MR effect size for
'Selenium' on 'Calcineurin B homologous protein 3'

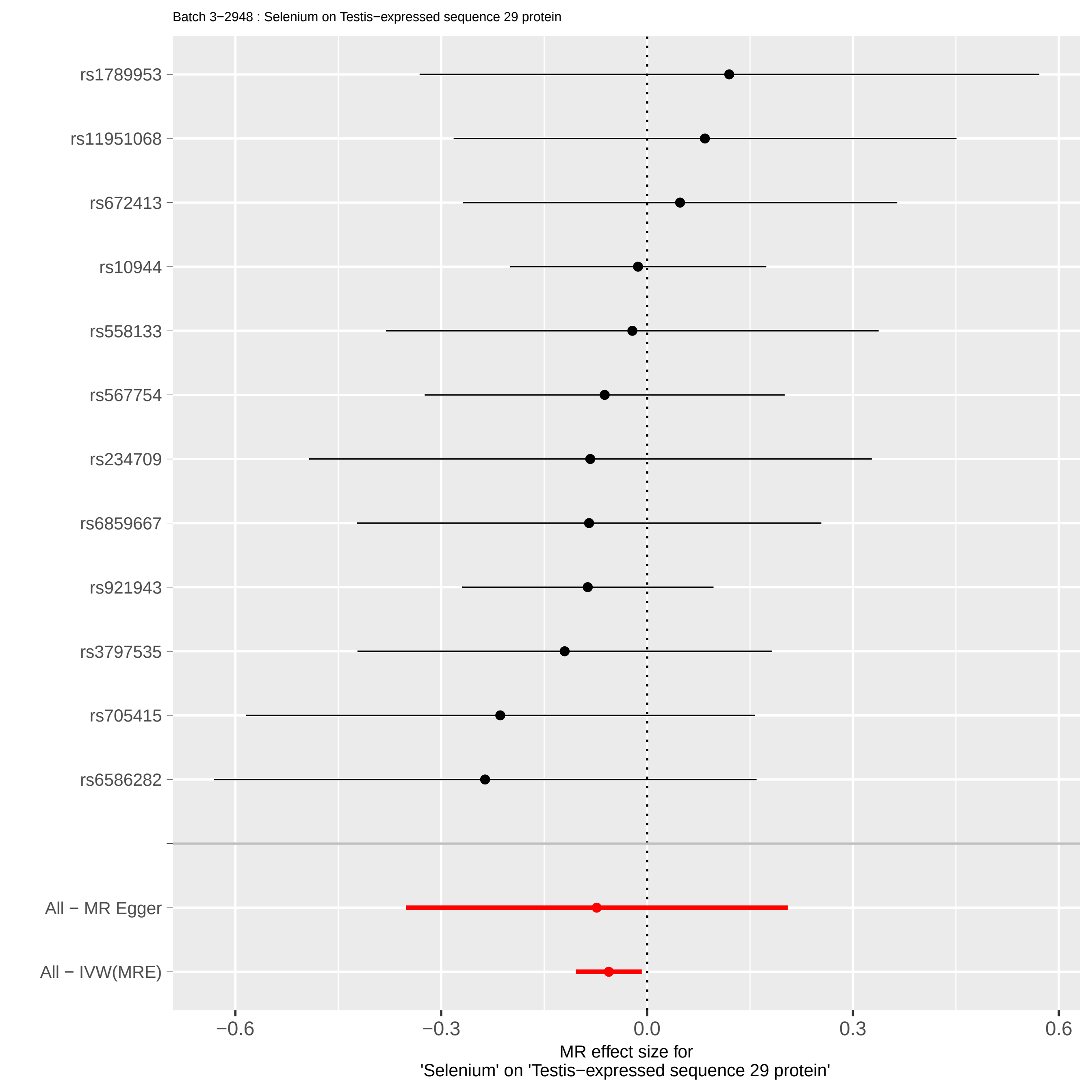

Batch 3−2948 : Selenium on Testis−expressed sequence 29 protein
rs1789953
rs11951068
rs672413
rs10944
rs558133
rs567754
rs234709
rs6859667
rs921943
rs3797535
rs705415
rs6586282
All − MR Egger
All − IVW(MRE)
−0.6
−0.3
0.0
0.3
0.6
MR effect size for
'Selenium' on 'Testis−expressed sequence 29 protein'

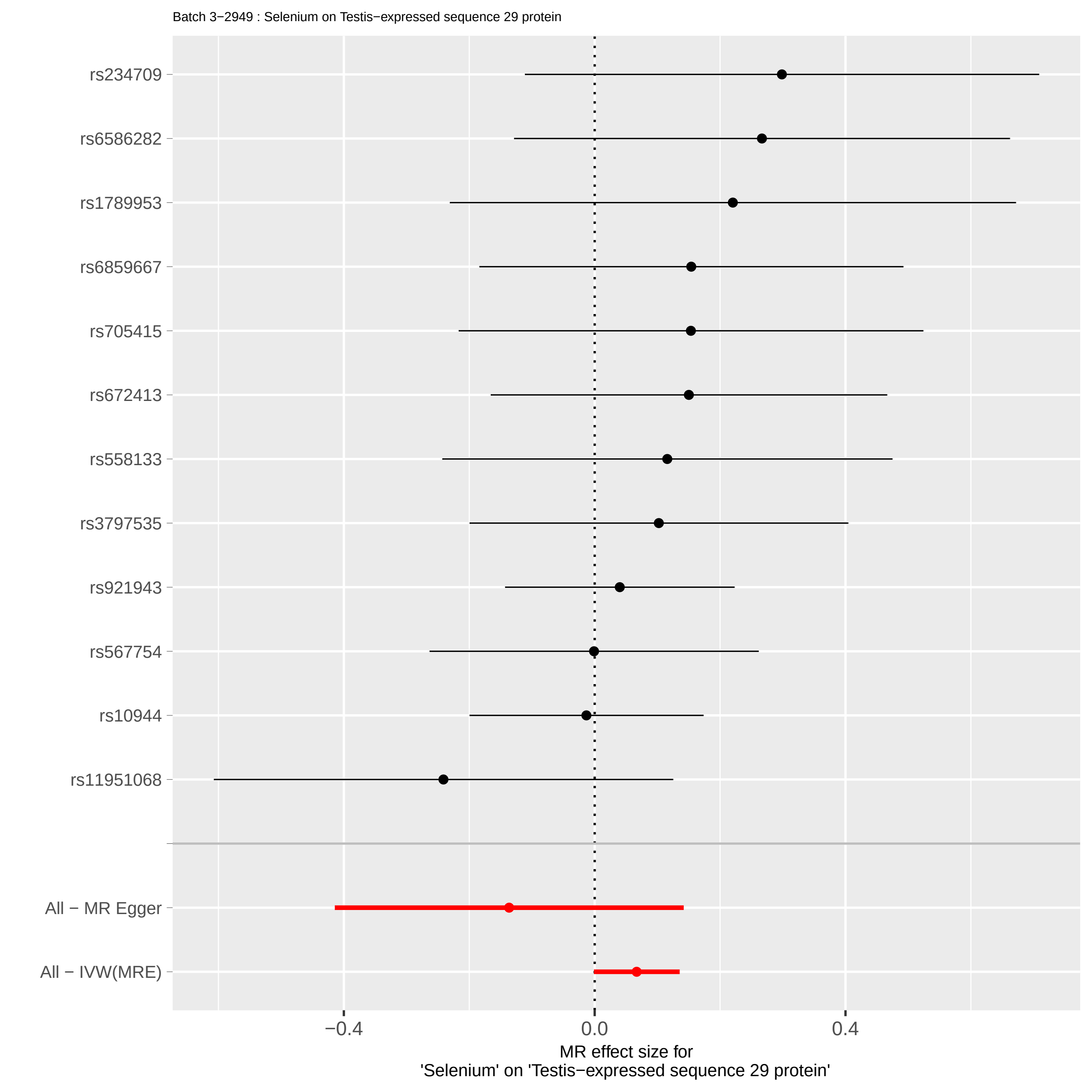
Batch 3−2949 : Selenium on Testis−expressed sequence 29 protein
rs234709
rs6586282
rs1789953
rs6859667
rs705415
rs672413
rs558133
rs3797535
rs921943
rs567754
rs10944
rs11951068
All − MR Egger
All − IVW(MRE)
−0.4
0.0
0.4
MR effect size for
'Selenium' on 'Testis−expressed sequence 29 protein'

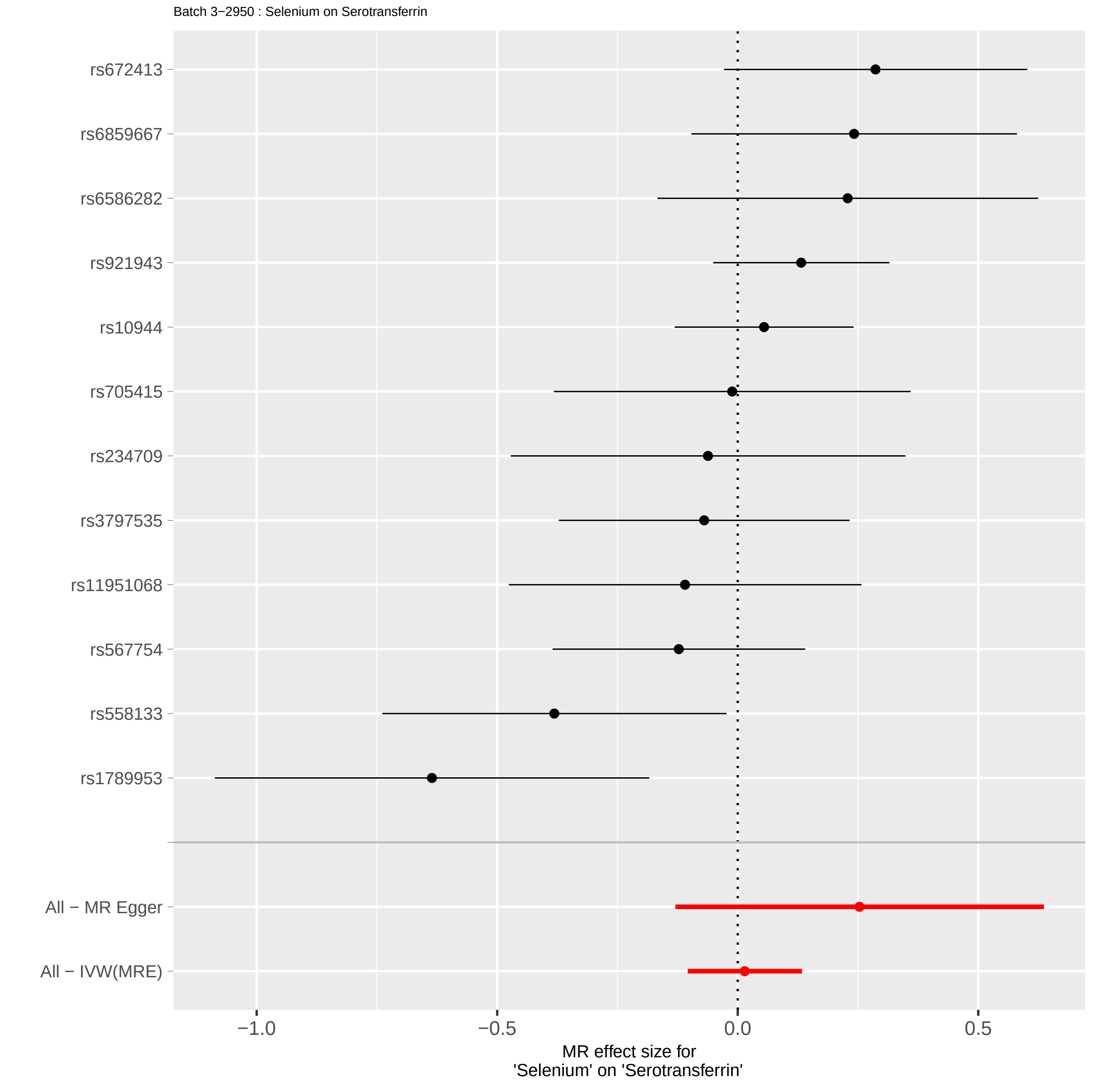
Batch 3−2950 : Selenium on Serotransferrin
rs672413
rs6859667
rs6586282
rs921943
rs10944
rs705415
rs234709
rs3797535
rs11951068
rs567754
rs558133
rs1789953
All − MR Egger
All − IVW(MRE)
−1.0
−0.5
0.0
0.5
MR effect size for
'Selenium' on 'Serotransferrin'

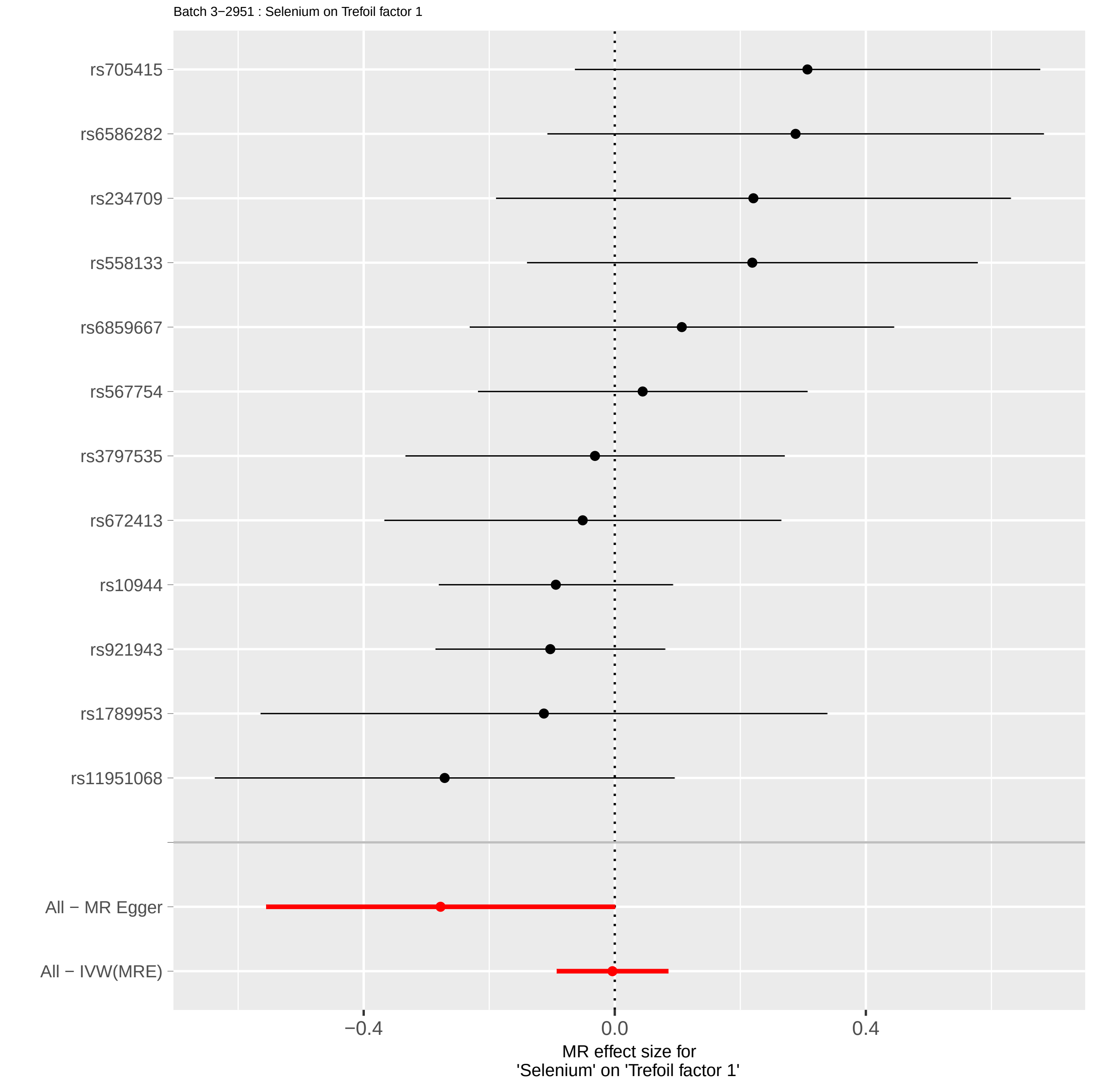
Batch 3−2951 : Selenium on Trefoil factor 1
rs705415
rs6586282
rs234709
rs558133
rs6859667
rs567754
rs3797535
rs672413
rs10944
rs921943
rs1789953
rs11951068
All − MR Egger
All − IVW(MRE)
−0.4
0.0
0.4
MR effect size for
'Selenium' on 'Trefoil factor 1'

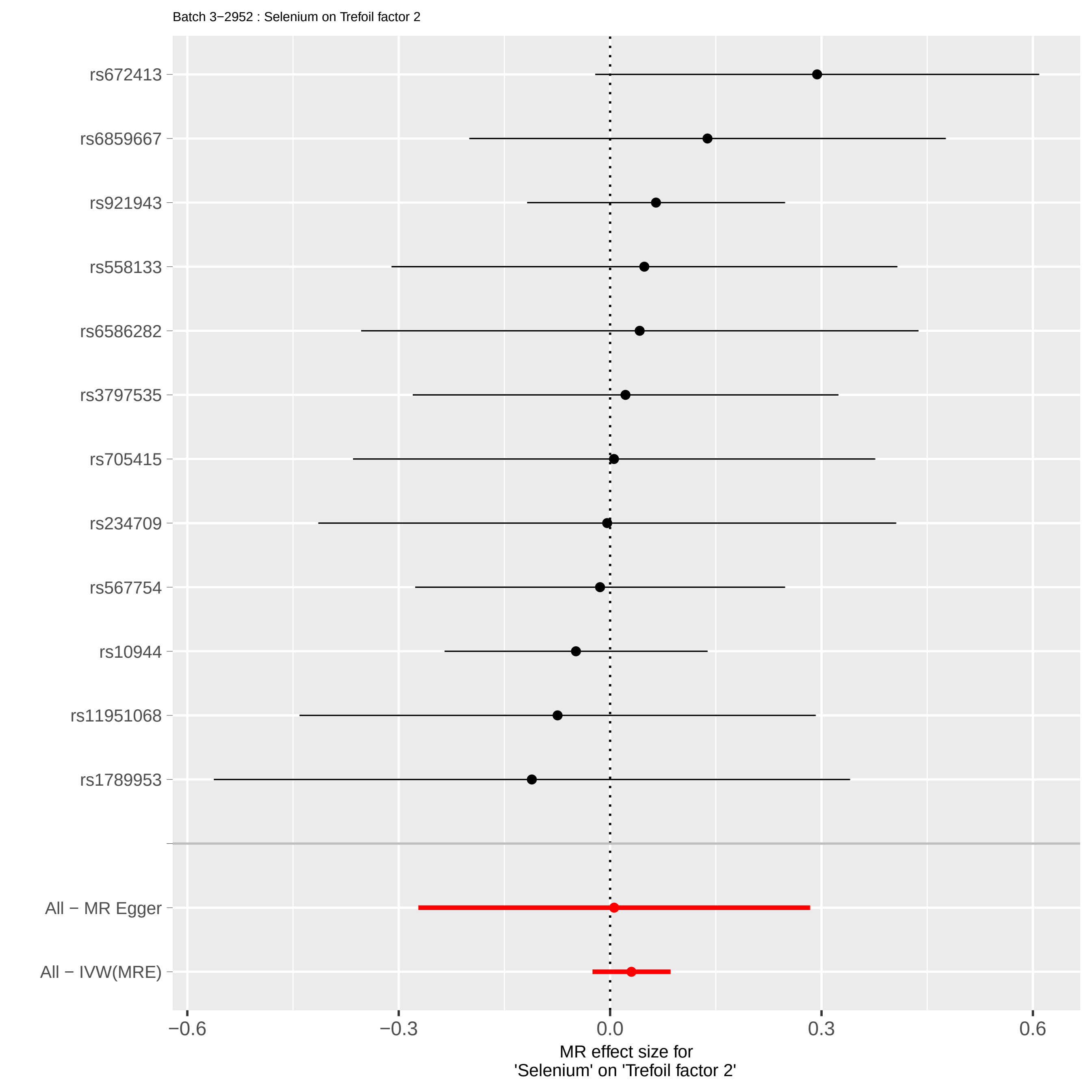

Batch 3−2952 : Selenium on Trefoil factor 2
rs672413
rs6859667
rs921943
rs558133
rs6586282
rs3797535
rs705415
rs234709
rs567754
rs10944
rs11951068
rs1789953
All − MR Egger
All − IVW(MRE)
−0.6
−0.3
0.0
0.3
0.6
MR effect size for
'Selenium' on 'Trefoil factor 2'

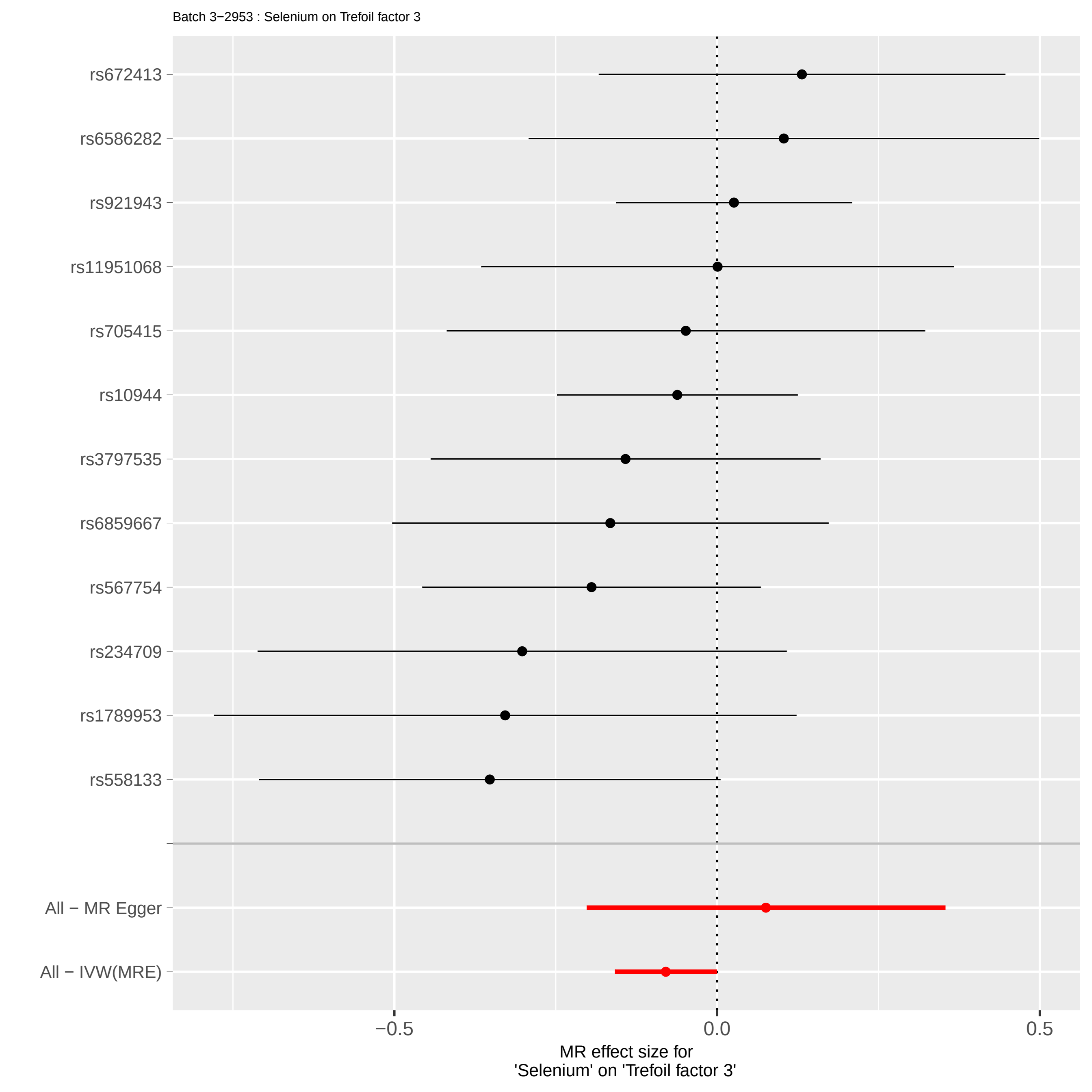

Batch 3−2953 : Selenium on Trefoil factor 3
rs672413
rs6586282
rs921943
rs11951068
rs705415
rs10944
rs3797535
rs6859667
rs567754
rs234709
rs1789953
rs558133
All − MR Egger
All − IVW(MRE)
−0.5
0.0
0.5
MR effect size for
'Selenium' on 'Trefoil factor 3'

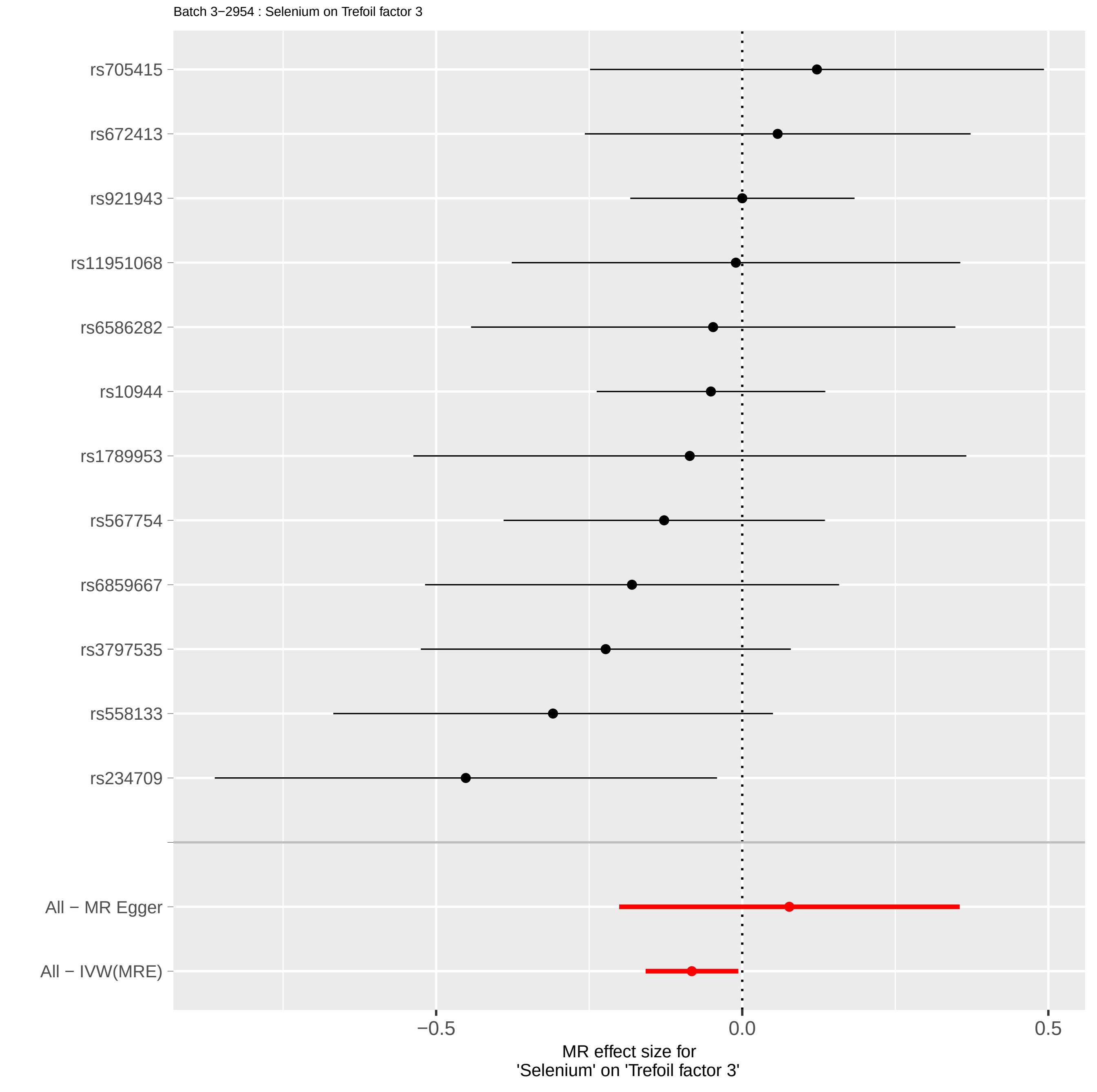

Batch 3−2954 : Selenium on Trefoil factor 3
rs705415
rs672413
rs921943
rs11951068
rs6586282
rs10944
rs1789953
rs567754
rs6859667
rs3797535
rs558133
rs234709
All − MR Egger
All − IVW(MRE)
−0.5
0.0
0.5
MR effect size for
'Selenium' on 'Trefoil factor 3'

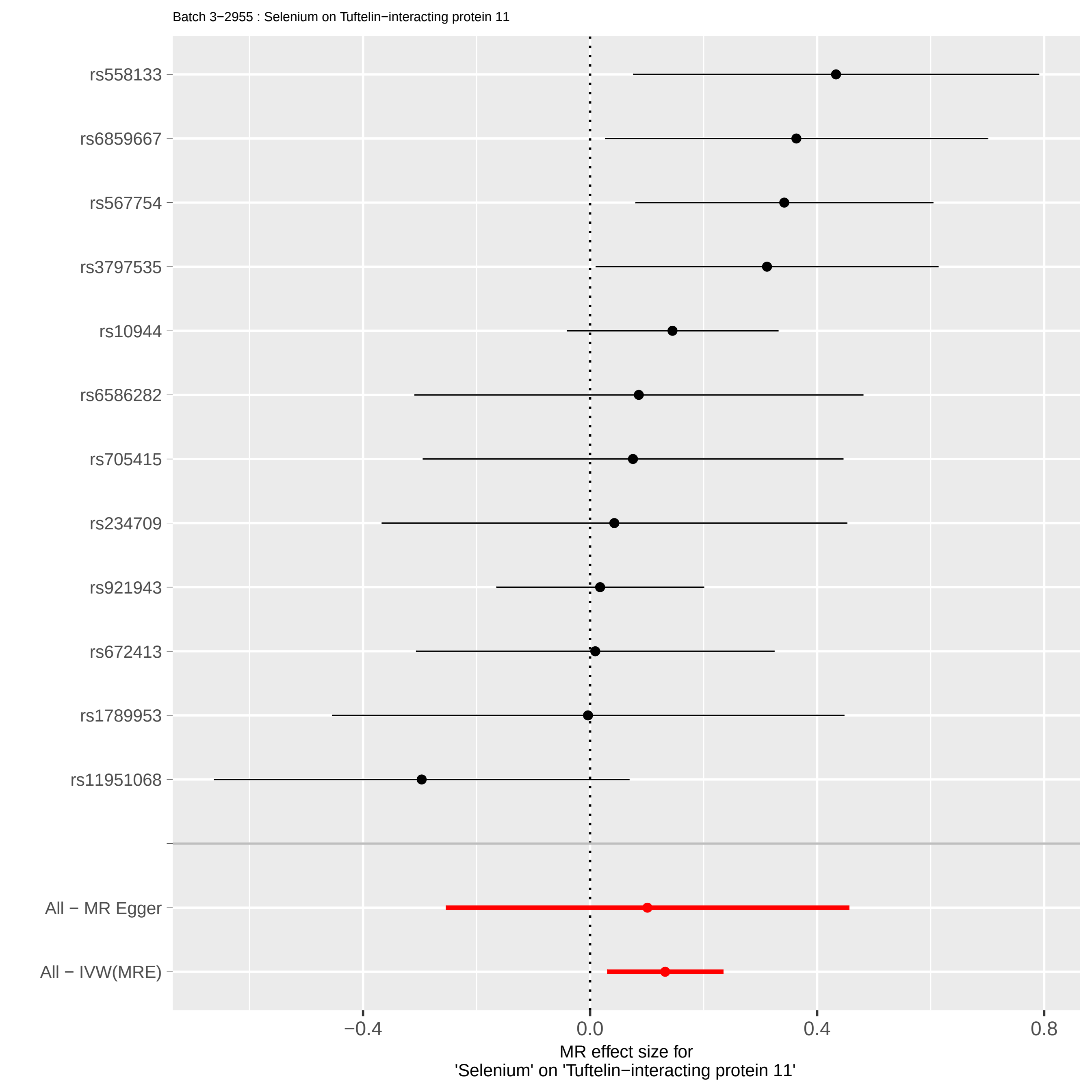

Batch 3−2955 : Selenium on Tuftelin−interacting protein 11
rs558133
rs6859667
rs567754
rs3797535
rs10944
rs6586282
rs705415
rs234709
rs921943
rs672413
rs1789953
rs11951068
All − MR Egger
All − IVW(MRE)
−0.4
0.0
0.4
0.8
MR effect size for
'Selenium' on 'Tuftelin−interacting protein 11'

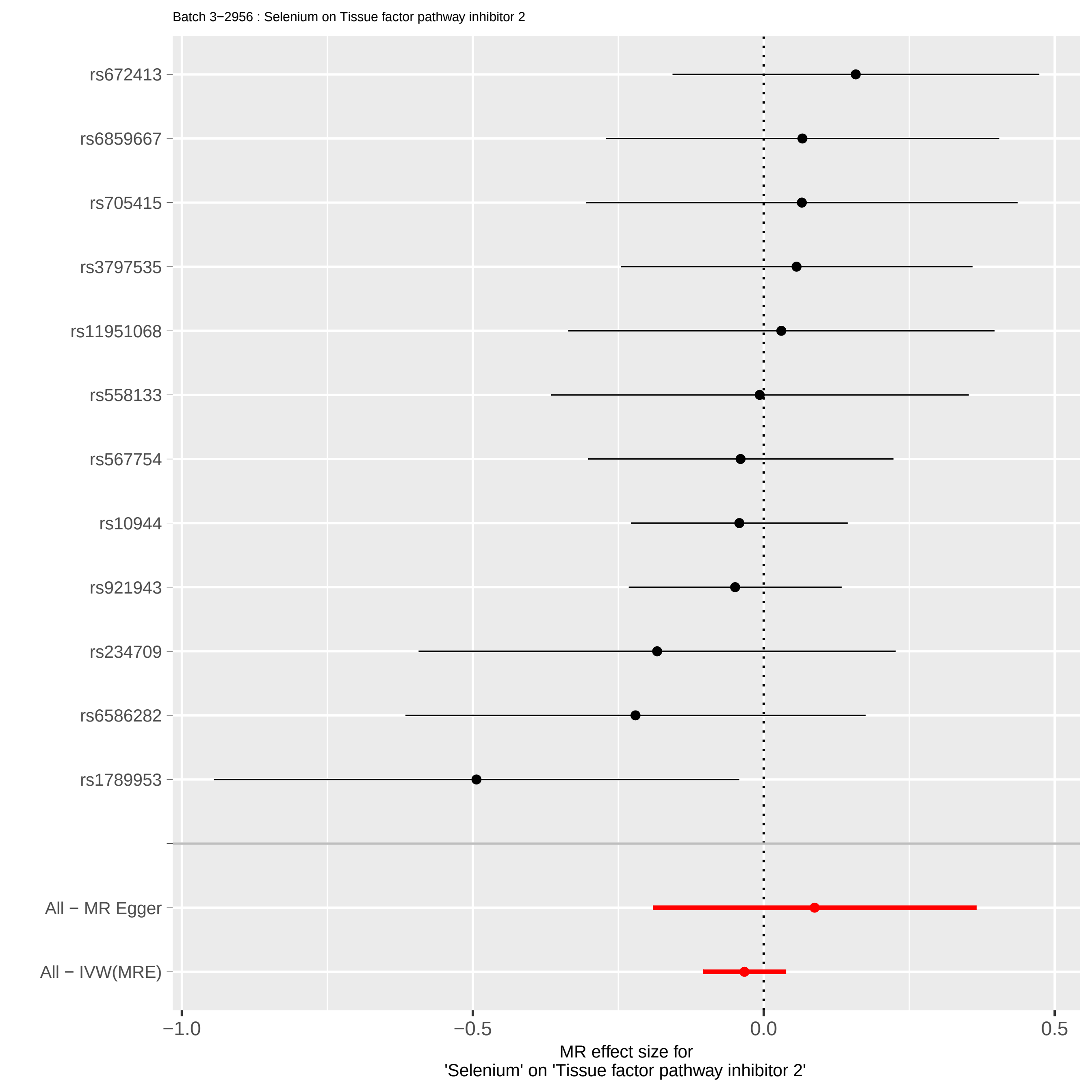
Batch 3−2956 : Selenium on Tissue factor pathway inhibitor 2
rs672413
rs6859667
rs705415
rs3797535
rs11951068
rs558133
rs567754
rs10944
rs921943
rs234709
rs6586282
rs1789953
All − MR Egger
All − IVW(MRE)
−1.0
−0.5
0.0
0.5
MR effect size for
'Selenium' on 'Tissue factor pathway inhibitor 2'

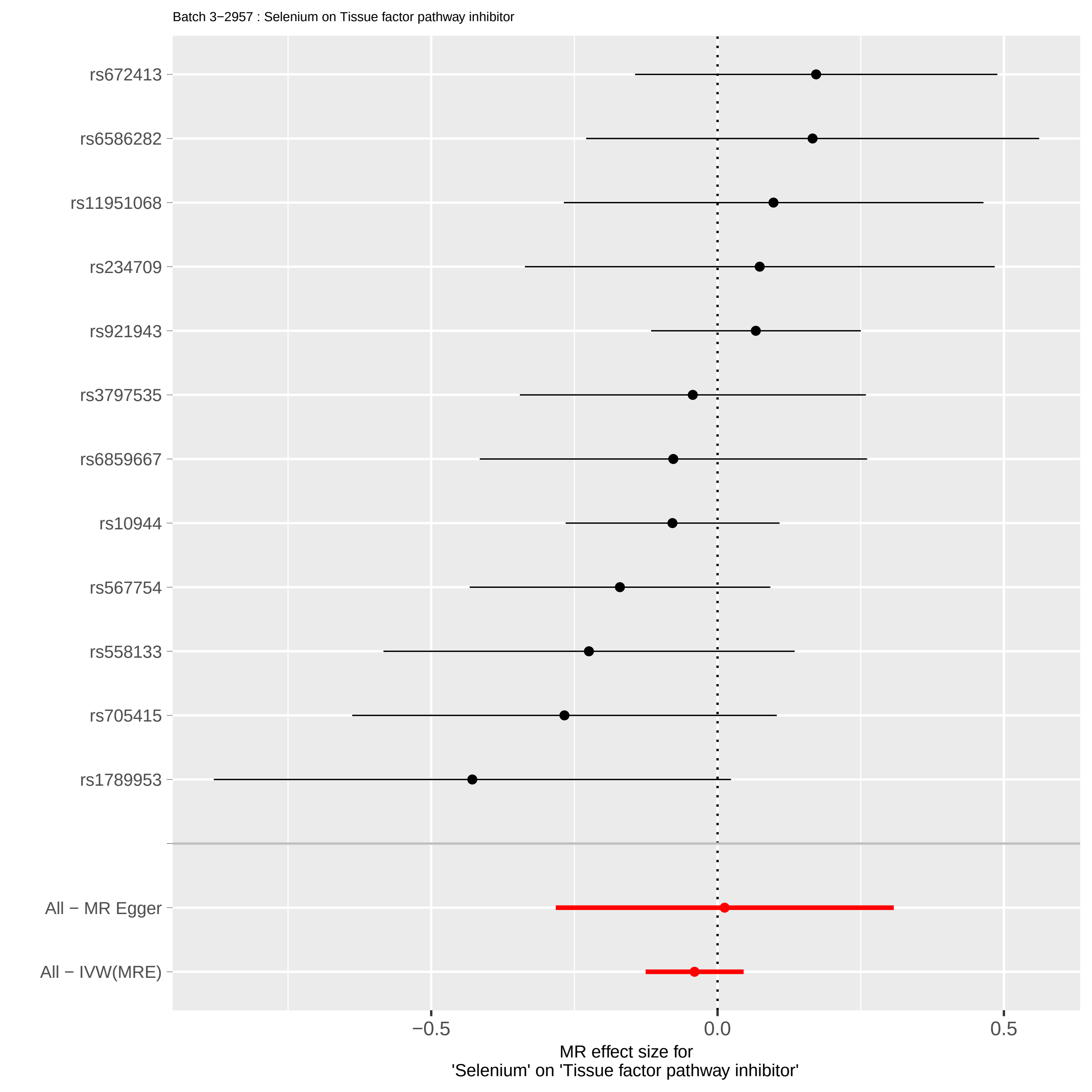
Batch 3−2957 : Selenium on Tissue factor pathway inhibitor
rs672413
rs6586282
rs11951068
rs234709
rs921943
rs3797535
rs6859667
rs10944
rs567754
rs558133
rs705415
rs1789953
All − MR Egger
All − IVW(MRE)
−0.5
0.0
0.5
MR effect size for
'Selenium' on 'Tissue factor pathway inhibitor'

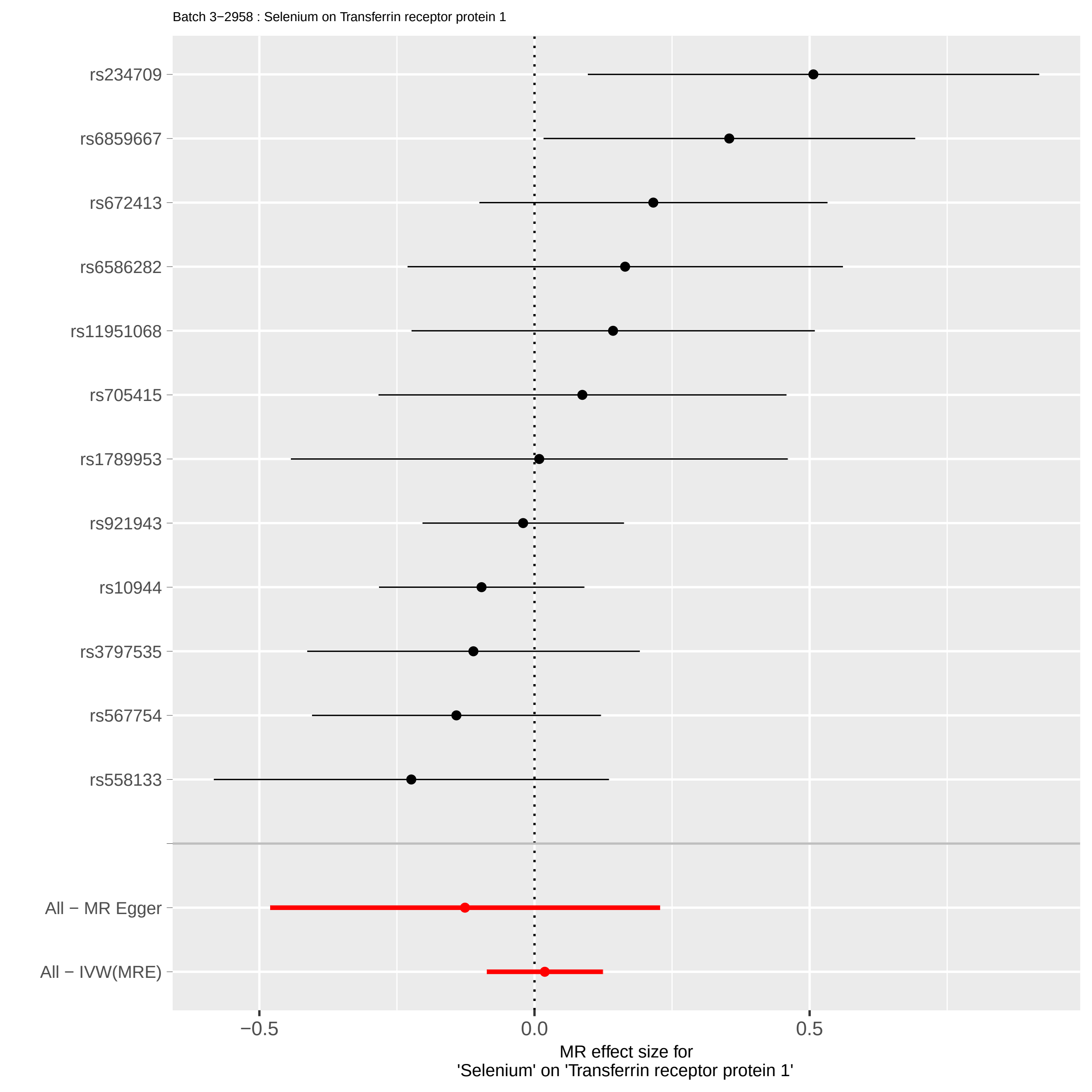
Batch 3−2958 : Selenium on Transferrin receptor protein 1
rs234709
rs6859667
rs672413
rs6586282
rs11951068
rs705415
rs1789953
rs921943
rs10944
rs3797535
rs567754
rs558133
All − MR Egger
All − IVW(MRE)
−0.5
0.0
0.5
MR effect size for
'Selenium' on 'Transferrin receptor protein 1'

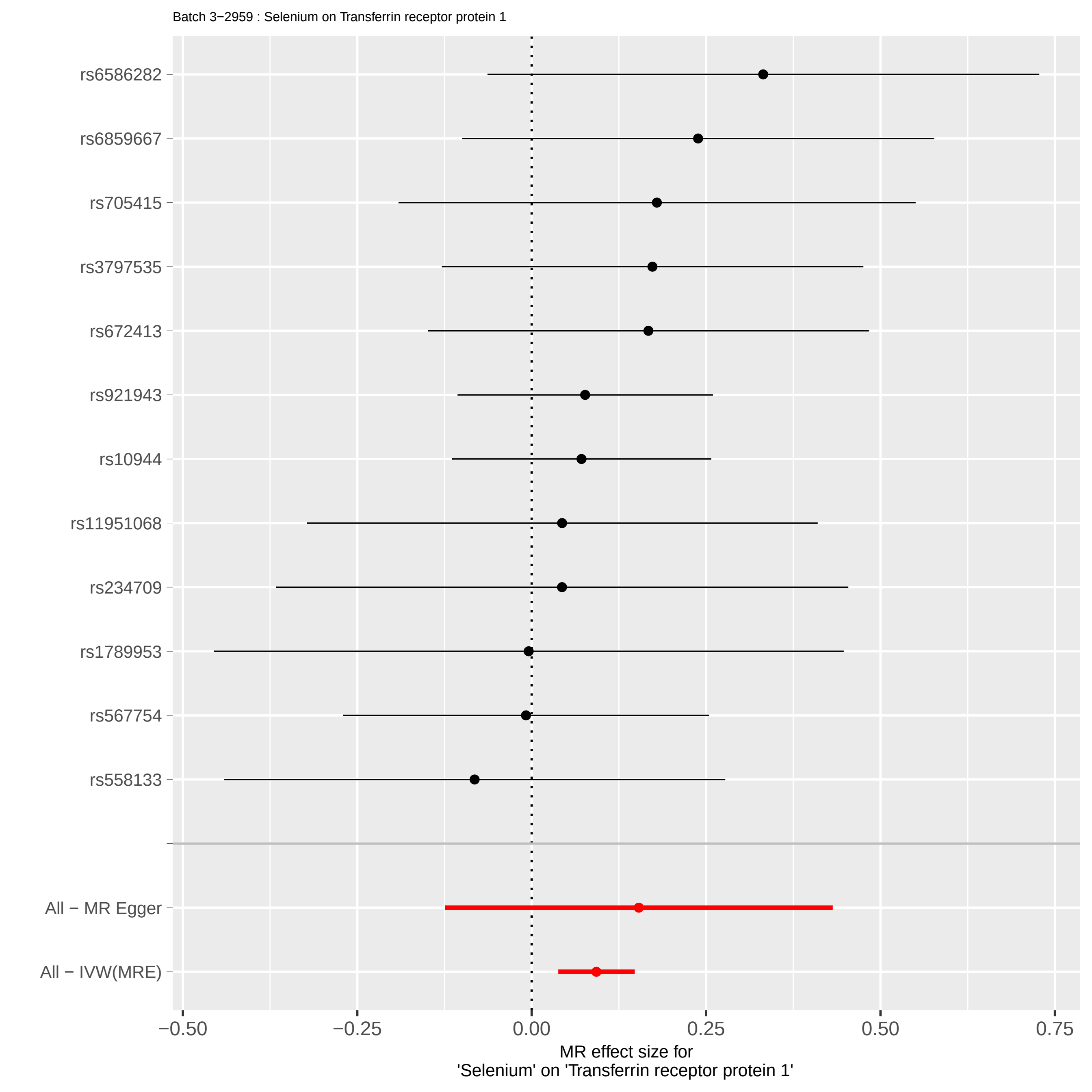

Batch 3−2959 : Selenium on Transferrin receptor protein 1
rs6586282
rs6859667
rs705415
rs3797535
rs672413
rs921943
rs10944
rs11951068
rs234709
rs1789953
rs567754
rs558133
All − MR Egger
All − IVW(MRE)
−0.50
−0.25
0.00
0.25
0.50
0.75
MR effect size for
'Selenium' on 'Transferrin receptor protein 1'

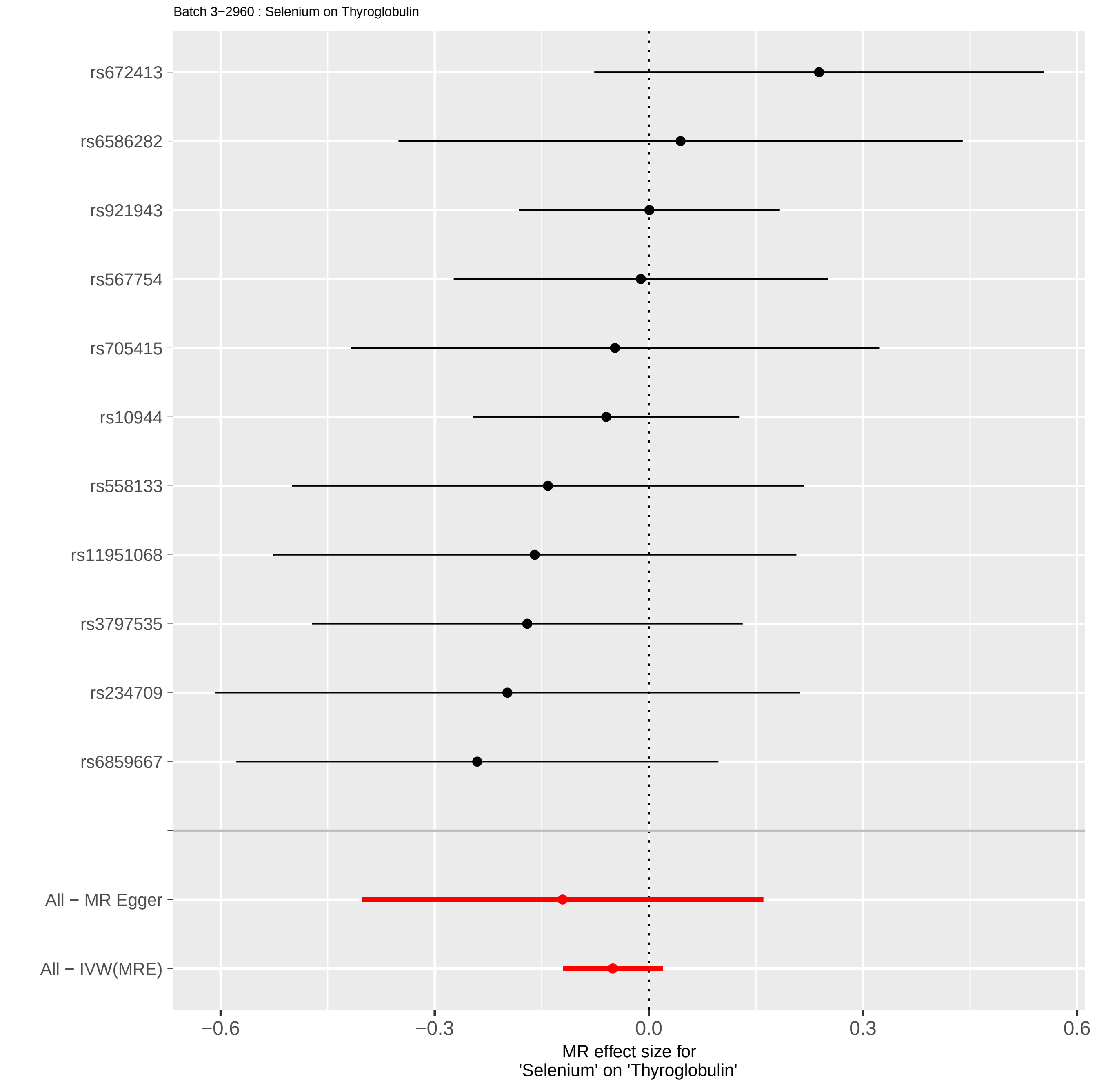

Batch 3−2960 : Selenium on Thyroglobulin
rs672413
rs6586282
rs921943
rs567754
rs705415
rs10944
rs558133
rs11951068
rs3797535
rs234709
rs6859667
All − MR Egger
All − IVW(MRE)
−0.6
−0.3
0.0
0.3
0.6
MR effect size for
'Selenium' on 'Thyroglobulin'

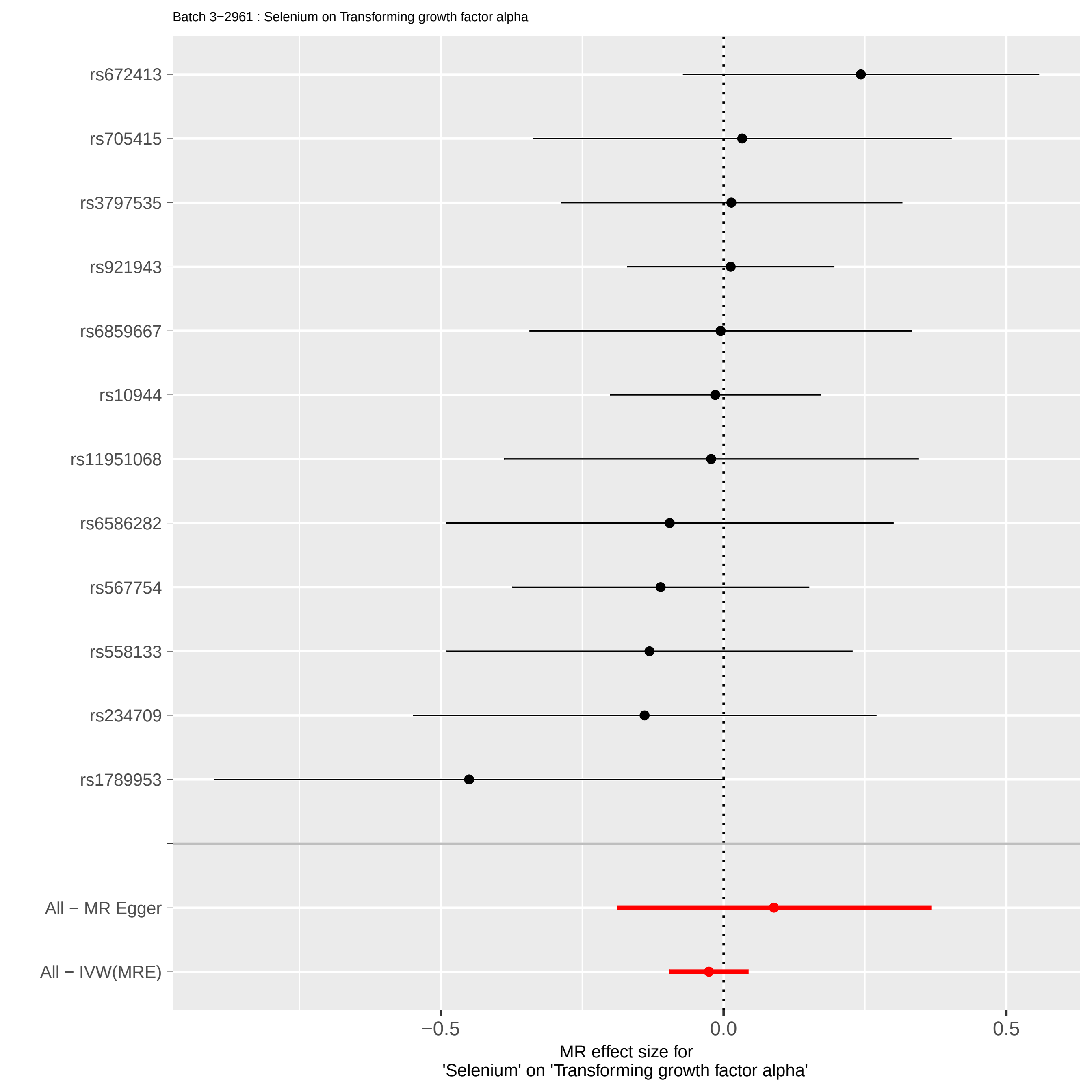

Batch 3−2961 : Selenium on Transforming growth factor alpha
rs672413
rs705415
rs3797535
rs921943
rs6859667
rs10944
rs11951068
rs6586282
rs567754
rs558133
rs234709
rs1789953
All − MR Egger
All − IVW(MRE)
−0.5
0.0
0.5
MR effect size for
'Selenium' on 'Transforming growth factor alpha'

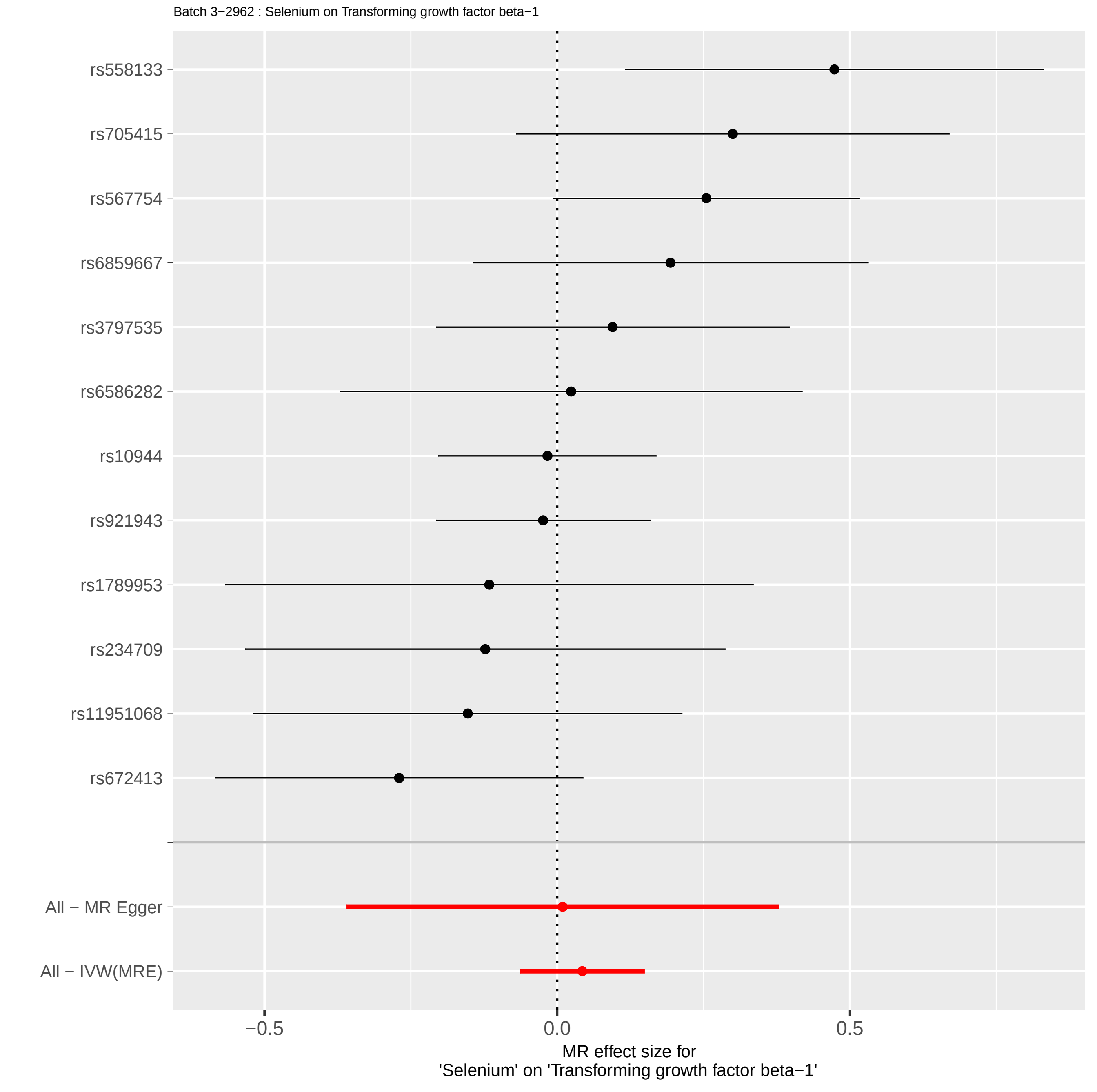

Batch 3−2962 : Selenium on Transforming growth factor beta−1
rs558133
rs705415
rs567754
rs6859667
rs3797535
rs6586282
rs10944
rs921943
rs1789953
rs234709
rs11951068
rs672413
All − MR Egger
All − IVW(MRE)
−0.5
0.0
0.5
MR effect size for
'Selenium' on 'Transforming growth factor beta−1'

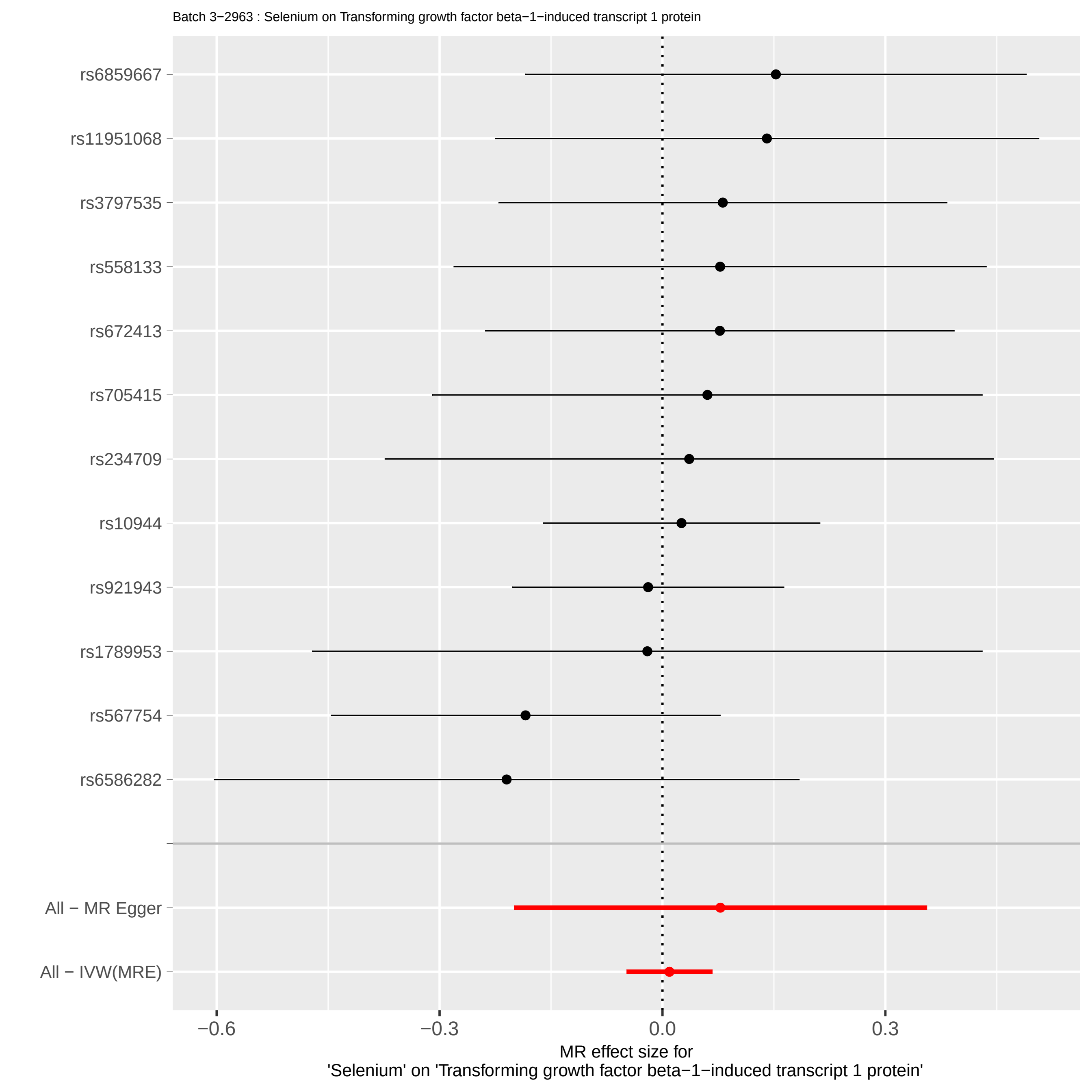
Batch 3−2963 : Selenium on Transforming growth factor beta−1−induced transcript 1 protein
rs6859667
rs11951068
rs3797535
rs558133
rs672413
rs705415
rs234709
rs10944
rs921943
rs1789953
rs567754
rs6586282
All − MR Egger
All − IVW(MRE)
−0.6
−0.3
0.0
0.3
MR effect size for
'Selenium' on 'Transforming growth factor beta−1−induced transcript 1 protein'

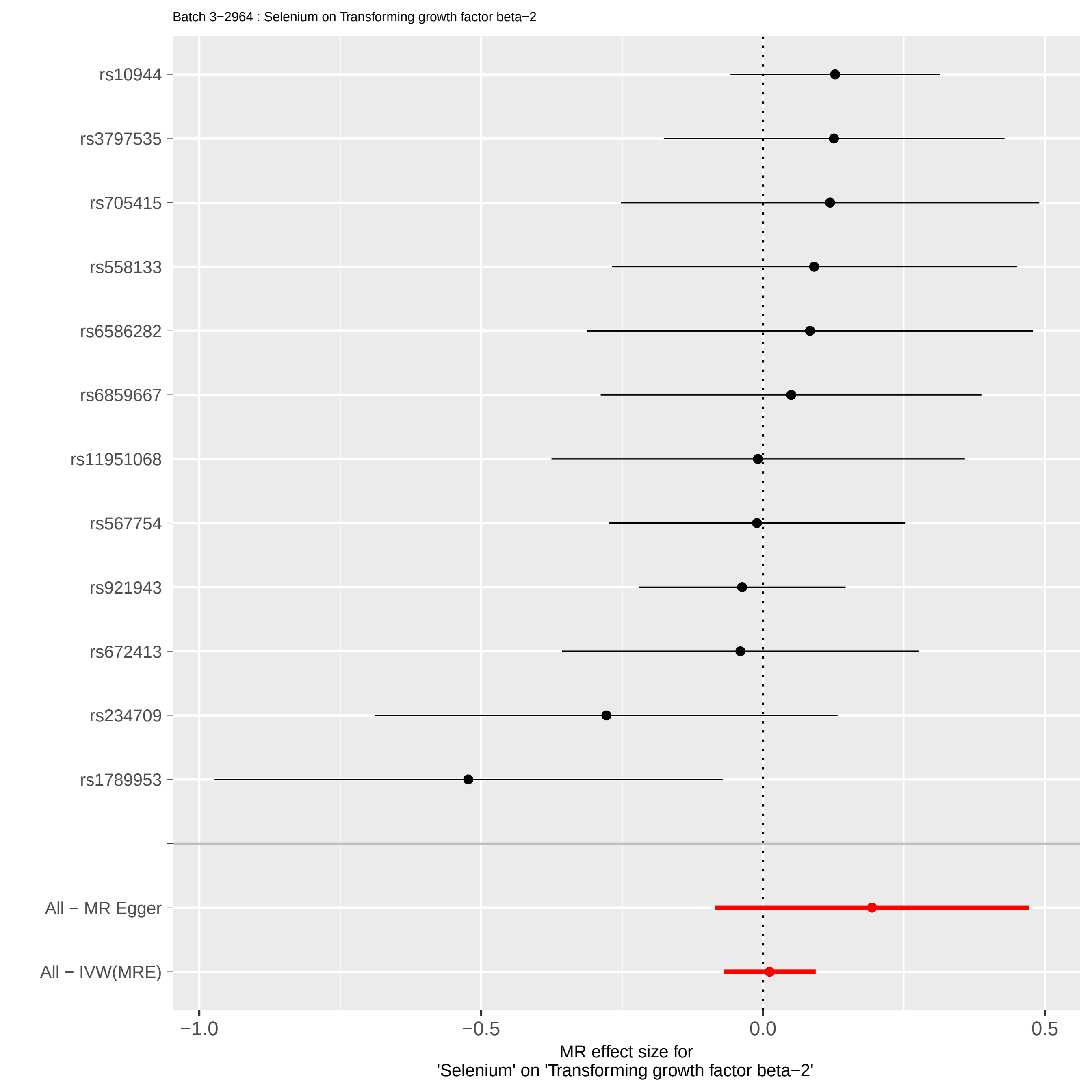

Batch 3−2964 : Selenium on Transforming growth factor beta−2
rs10944
rs3797535
rs705415
rs558133
rs6586282
rs6859667
rs11951068
rs567754
rs921943
rs672413
rs234709
rs1789953
All − MR Egger
All − IVW(MRE)
−1.0
−0.5
0.0
0.5
MR effect size for
'Selenium' on 'Transforming growth factor beta−2'

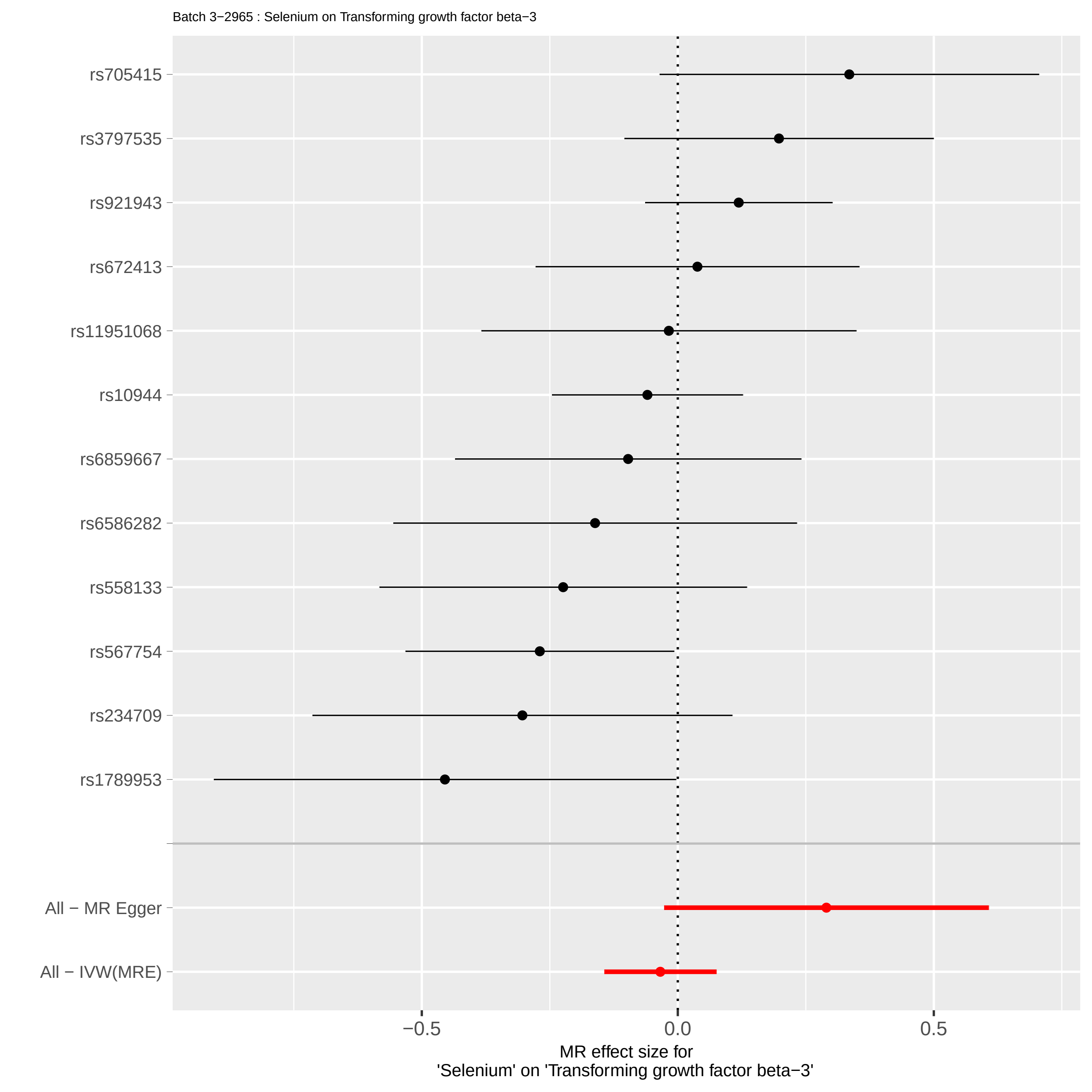

Batch 3−2965 : Selenium on Transforming growth factor beta−3
rs705415
rs3797535
rs921943
rs672413
rs11951068
rs10944
rs6859667
rs6586282
rs558133
rs567754
rs234709
rs1789953
All − MR Egger
All − IVW(MRE)
−0.5
0.0
0.5
MR effect size for
'Selenium' on 'Transforming growth factor beta−3'

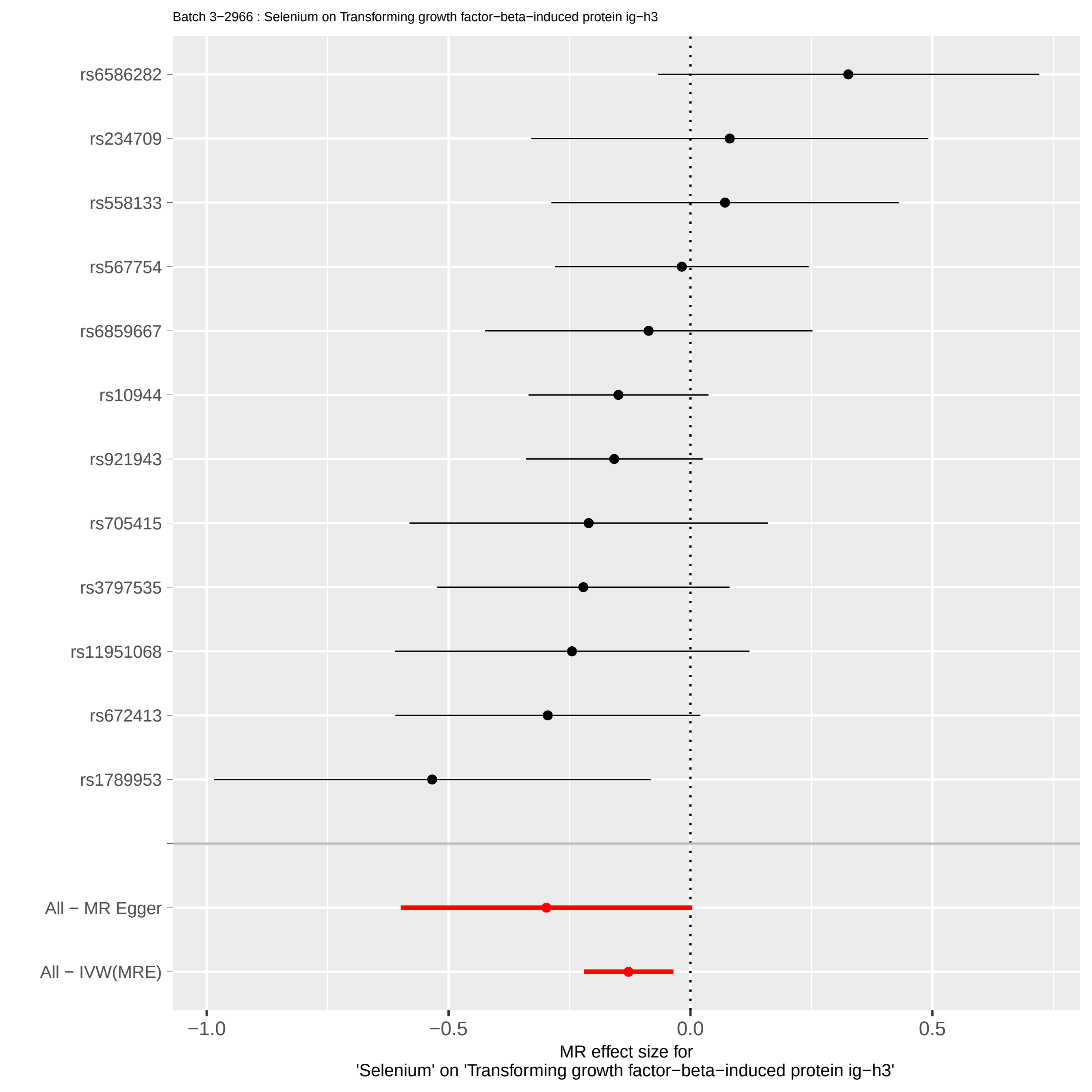

Batch 3−2966 : Selenium on Transforming growth factor−beta−induced protein ig−h3
rs6586282
rs234709
rs558133
rs567754
rs6859667
rs10944
rs921943
rs705415
rs3797535
rs11951068
rs672413
rs1789953
All − MR Egger
All − IVW(MRE)
−1.0
−0.5
0.0
0.5
MR effect size for
'Selenium' on 'Transforming growth factor−beta−induced protein ig−h3'

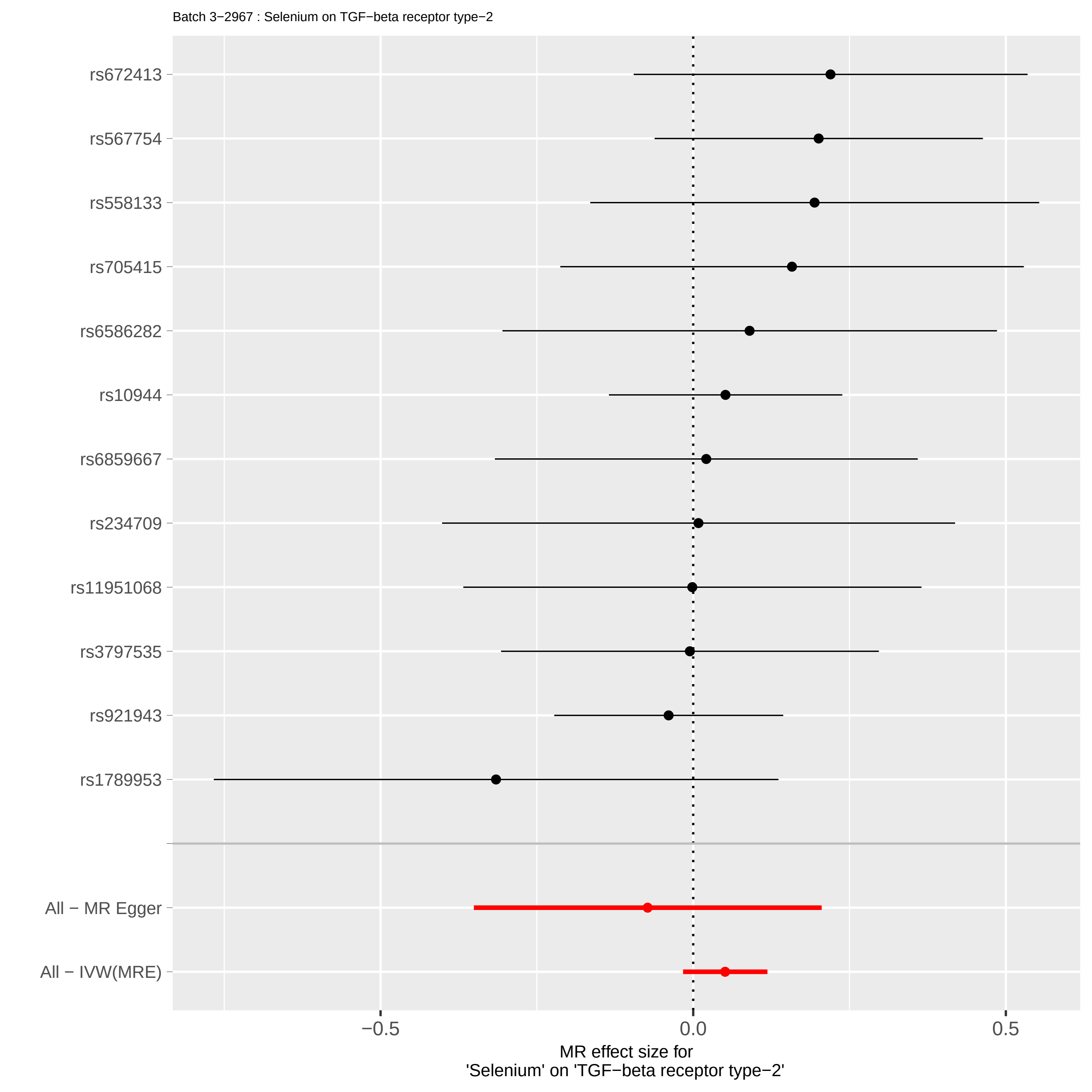

Batch 3−2967 : Selenium on TGF−beta receptor type−2
rs672413
rs567754
rs558133
rs705415
rs6586282
rs10944
rs6859667
rs234709
rs11951068
rs3797535
rs921943
rs1789953
All − MR Egger
All − IVW(MRE)
−0.5
0.0
0.5
MR effect size for
'Selenium' on 'TGF−beta receptor type−2'

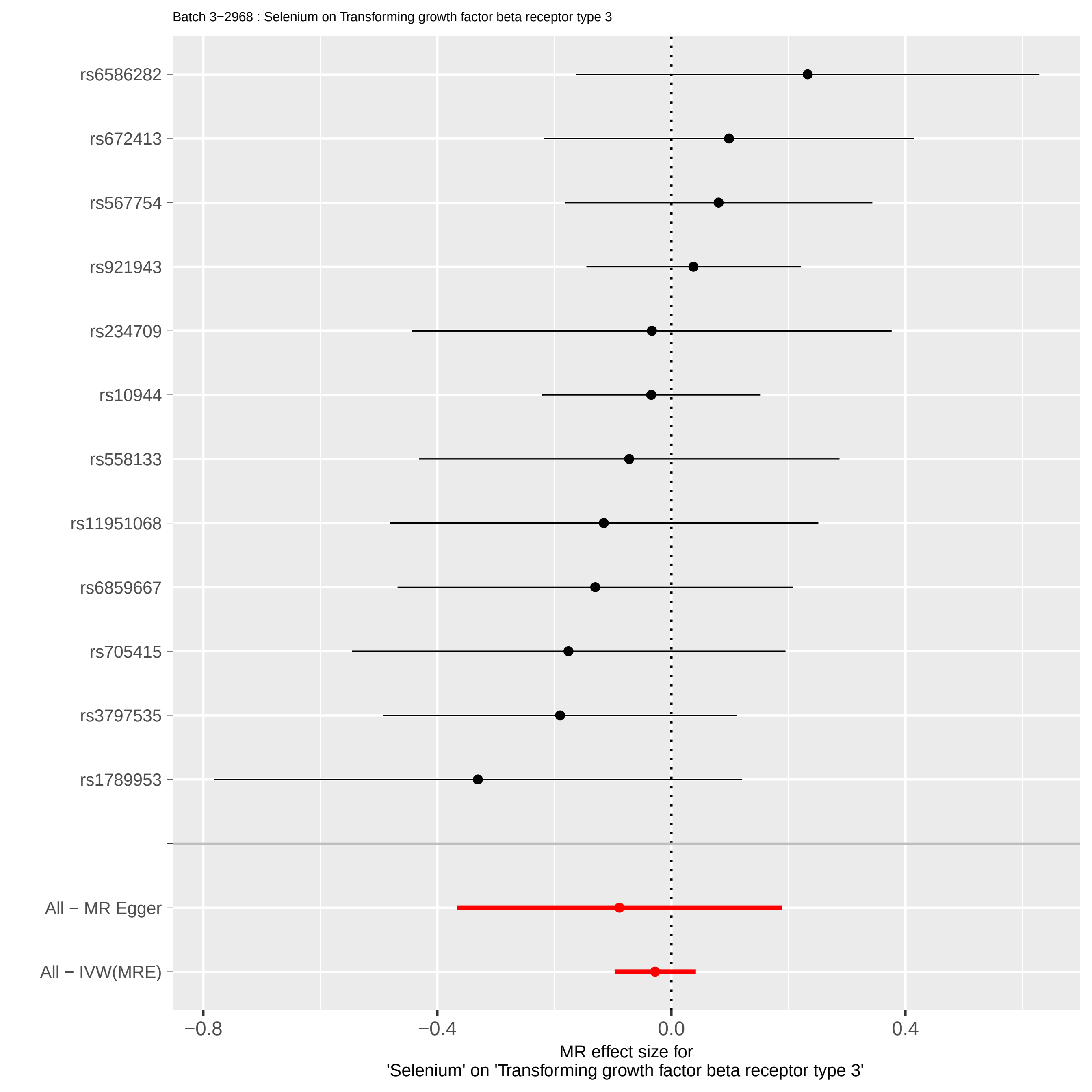

Batch 3−2968 : Selenium on Transforming growth factor beta receptor type 3
rs6586282
rs672413
rs567754
rs921943
rs234709
rs10944
rs558133
rs11951068
rs6859667
rs705415
rs3797535
rs1789953
All − MR Egger
All − IVW(MRE)
−0.8
−0.4
0.0
0.4
MR effect size for
'Selenium' on 'Transforming growth factor beta receptor type 3'

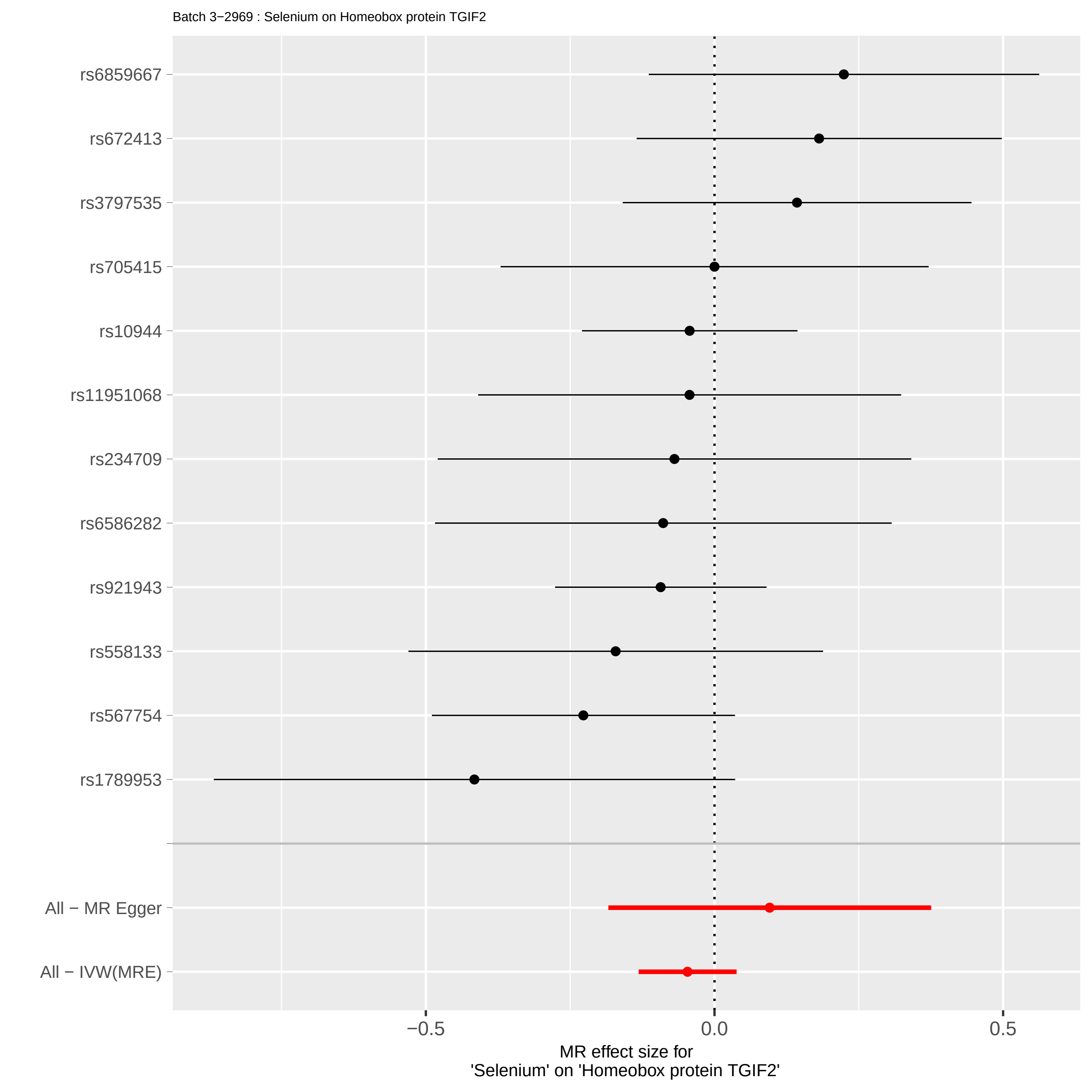
Batch 3−2969 : Selenium on Homeobox protein TGIF2
rs6859667
rs672413
rs3797535
rs705415
rs10944
rs11951068
rs234709
rs6586282
rs921943
rs558133
rs567754
rs1789953
All − MR Egger
All − IVW(MRE)
−0.5
0.0
0.5
MR effect size for
'Selenium' on 'Homeobox protein TGIF2'

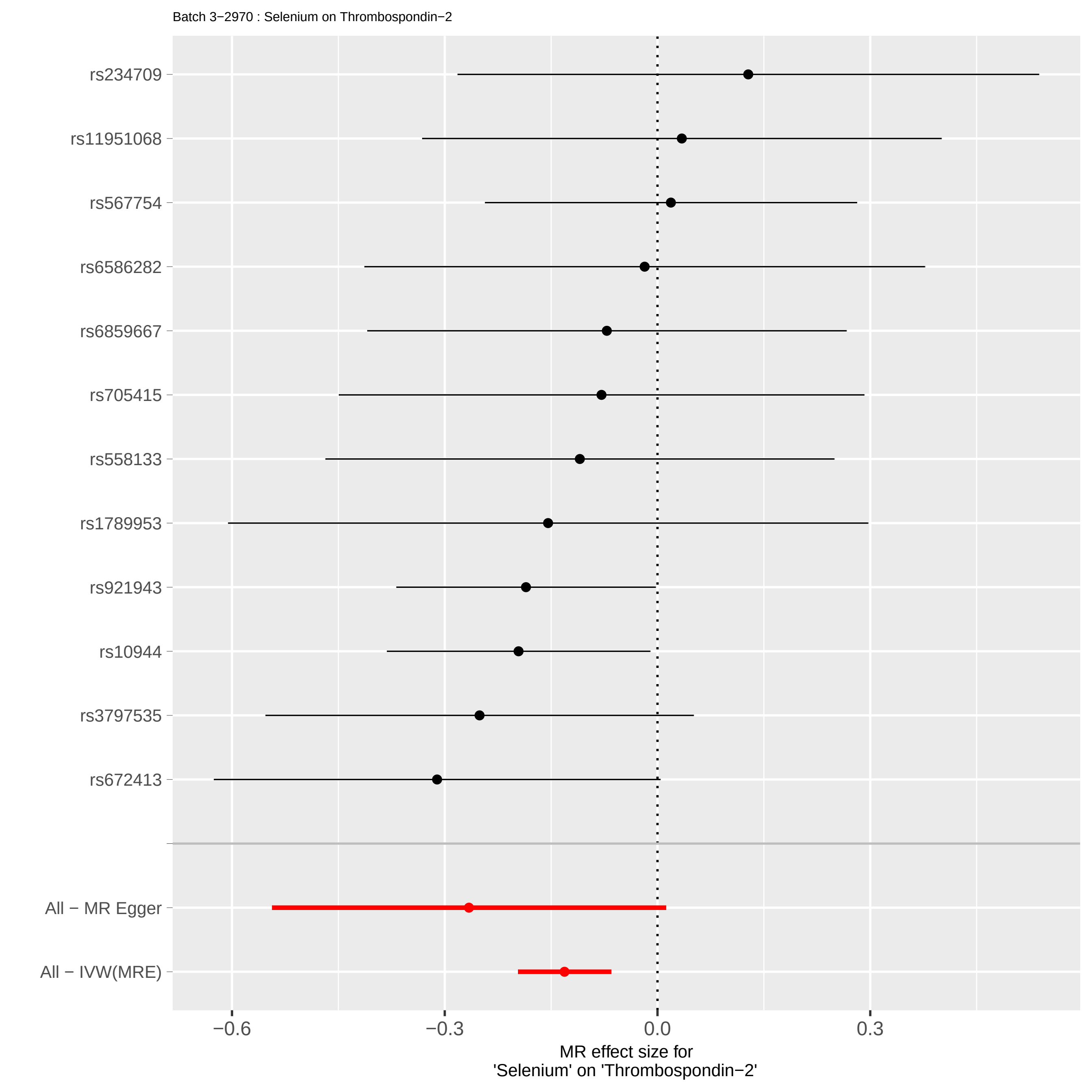

Batch 3−2970 : Selenium on Thrombospondin−2
rs234709
rs11951068
rs567754
rs6586282
rs6859667
rs705415
rs558133
rs1789953
rs921943
rs10944
rs3797535
rs672413
All − MR Egger
All − IVW(MRE)
−0.6
−0.3
0.0
0.3
MR effect size for
'Selenium' on 'Thrombospondin−2'

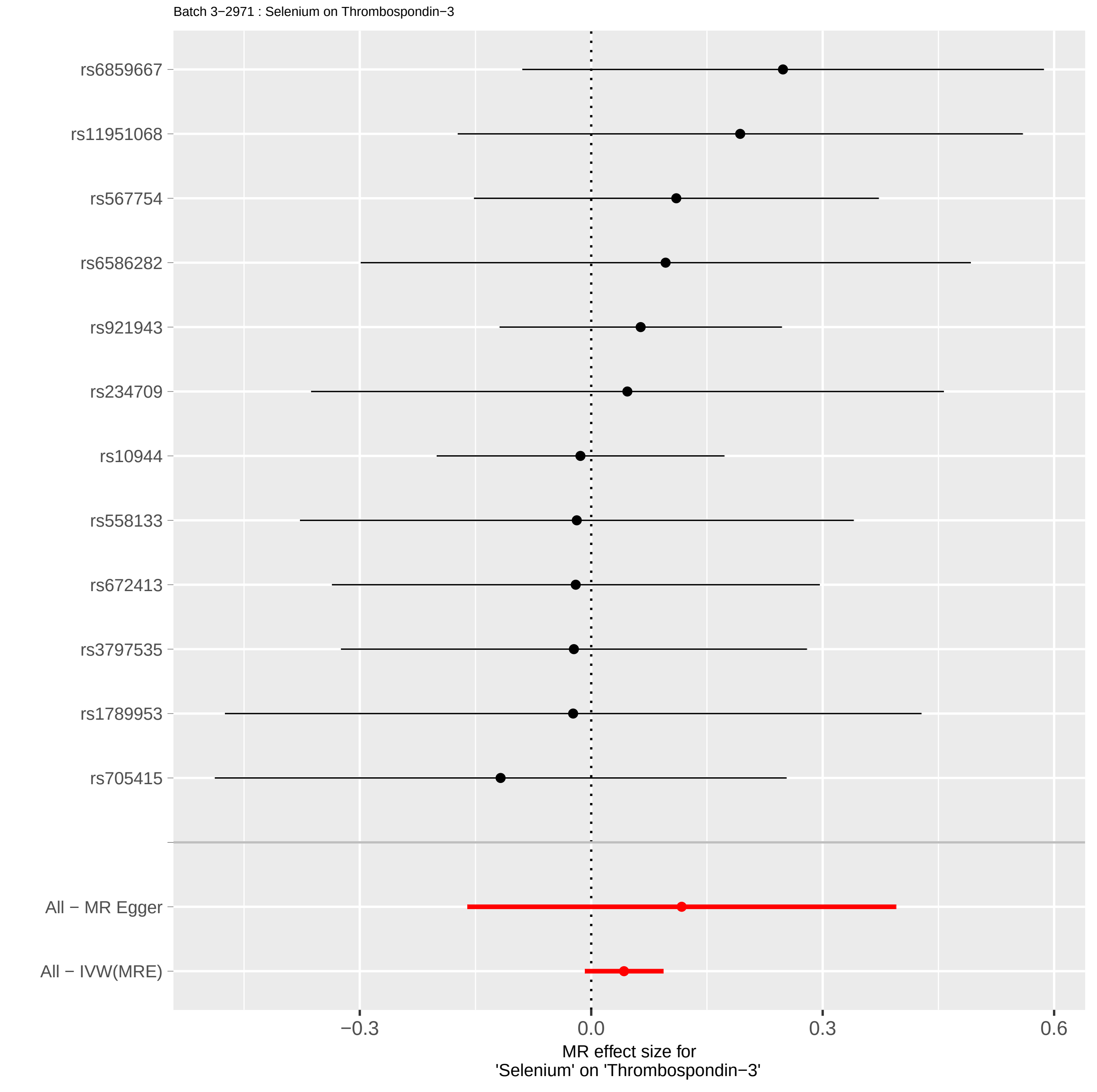

Batch 3−2971 : Selenium on Thrombospondin−3
rs6859667
rs11951068
rs567754
rs6586282
rs921943
rs234709
rs10944
rs558133
rs672413
rs3797535
rs1789953
rs705415
All − MR Egger
All − IVW(MRE)
−0.3
0.0
0.3
0.6
MR effect size for
'Selenium' on 'Thrombospondin−3'

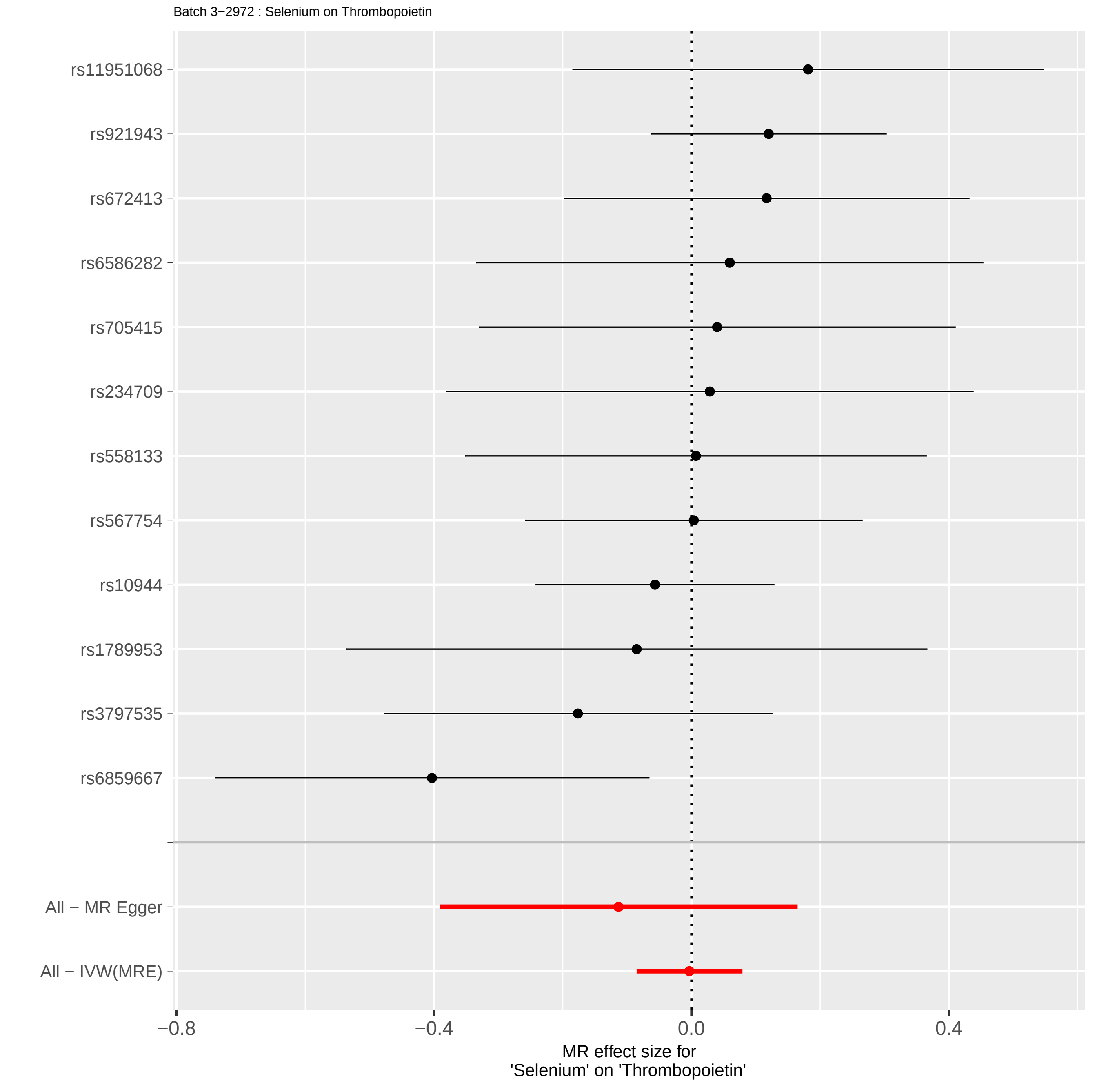

Batch 3−2972 : Selenium on Thrombopoietin
rs11951068
rs921943
rs672413
rs6586282
rs705415
rs234709
rs558133
rs567754
rs10944
rs1789953
rs3797535
rs6859667
All − MR Egger
All − IVW(MRE)
−0.8
−0.4
0.0
0.4
MR effect size for
'Selenium' on 'Thrombopoietin'

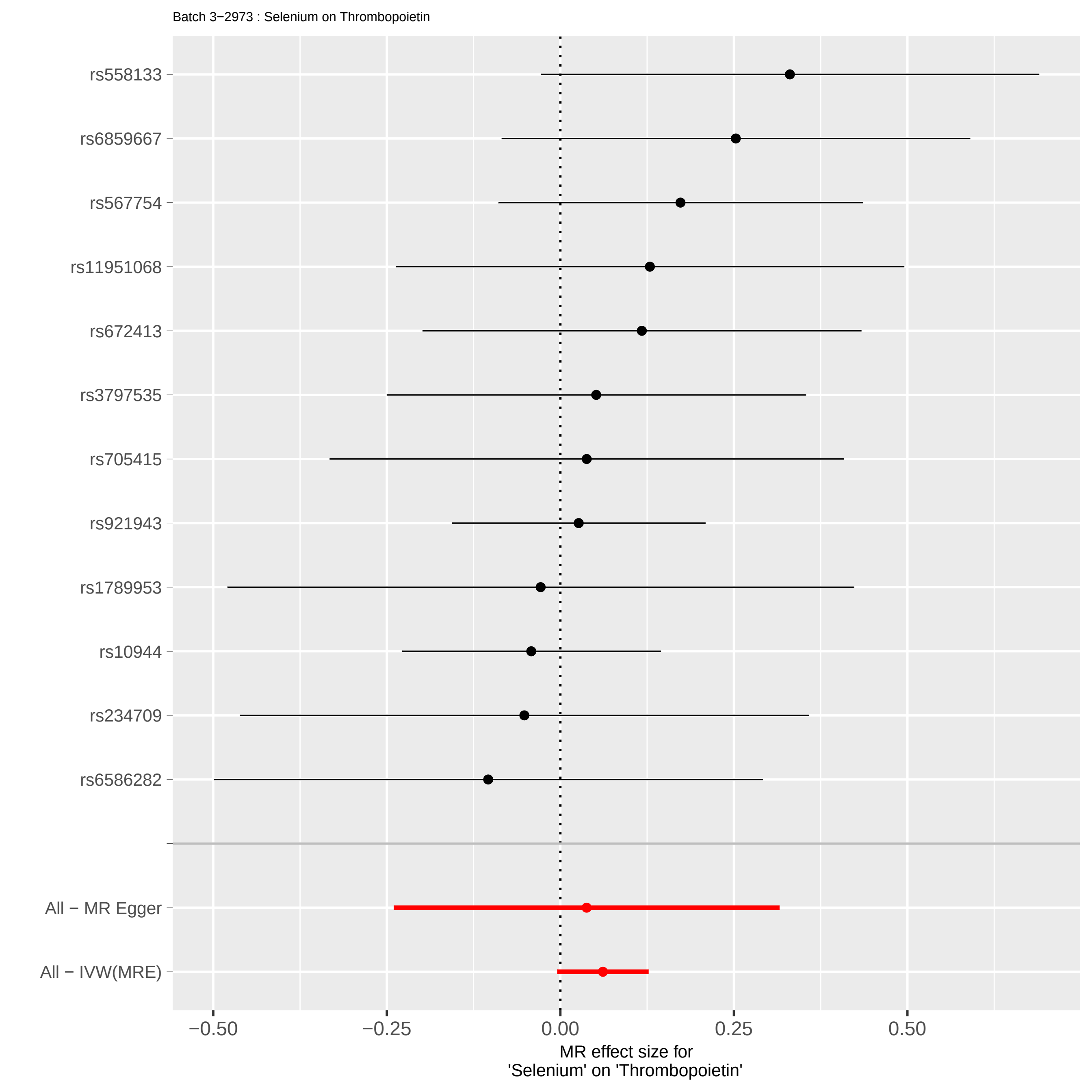

Batch 3−2973 : Selenium on Thrombopoietin
rs558133
rs6859667
rs567754
rs11951068
rs672413
rs3797535
rs705415
rs921943
rs1789953
rs10944
rs234709
rs6586282
All − MR Egger
All − IVW(MRE)
−0.50
−0.25
0.00
0.25
0.50
MR effect size for
'Selenium' on 'Thrombopoietin'

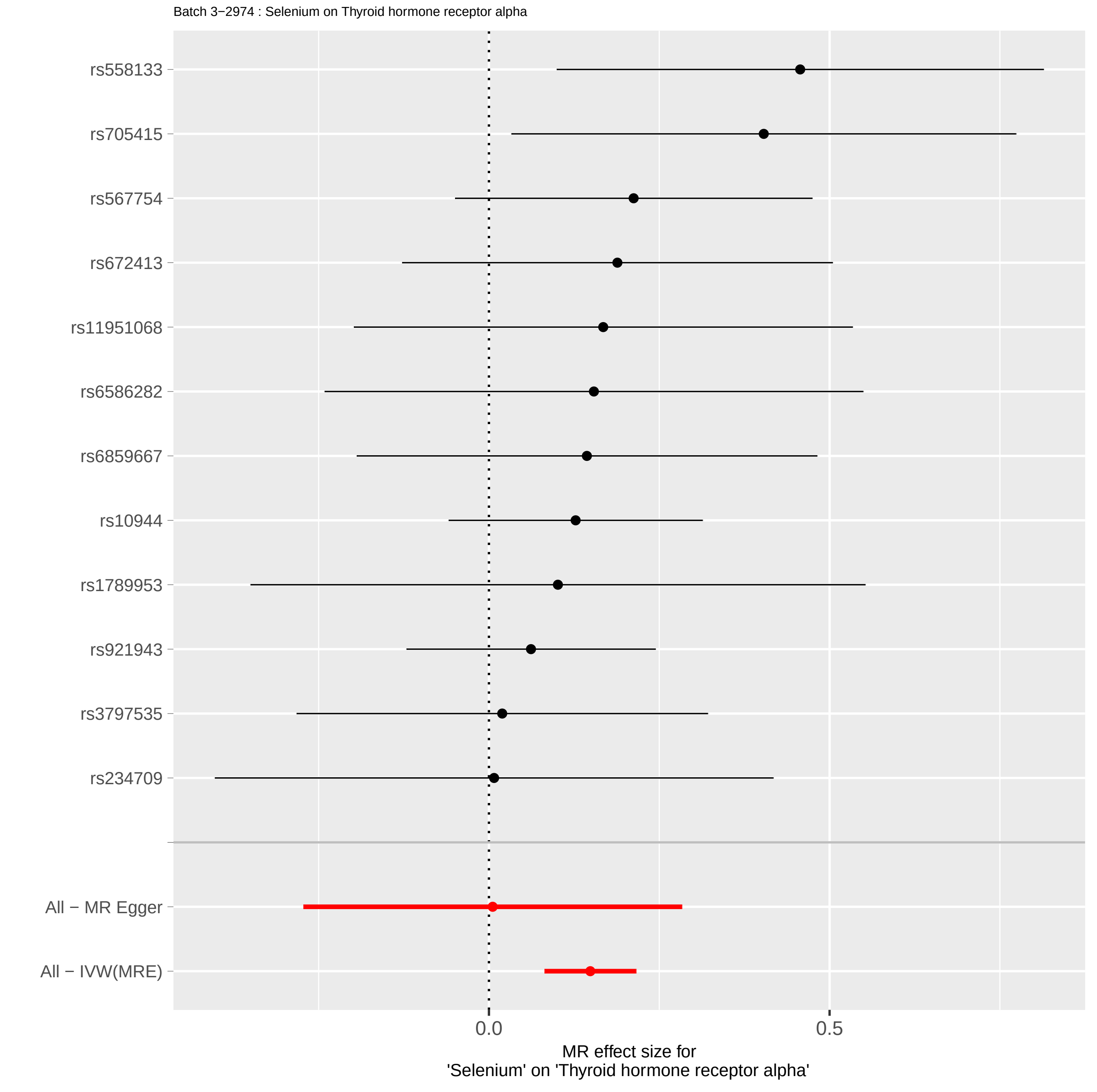
Batch 3−2974 : Selenium on Thyroid hormone receptor alpha
rs558133
rs705415
rs567754
rs672413
rs11951068
rs6586282
rs6859667
rs10944
rs1789953
rs921943
rs3797535
rs234709
All − MR Egger
All − IVW(MRE)
0.0
0.5
MR effect size for
'Selenium' on 'Thyroid hormone receptor alpha'

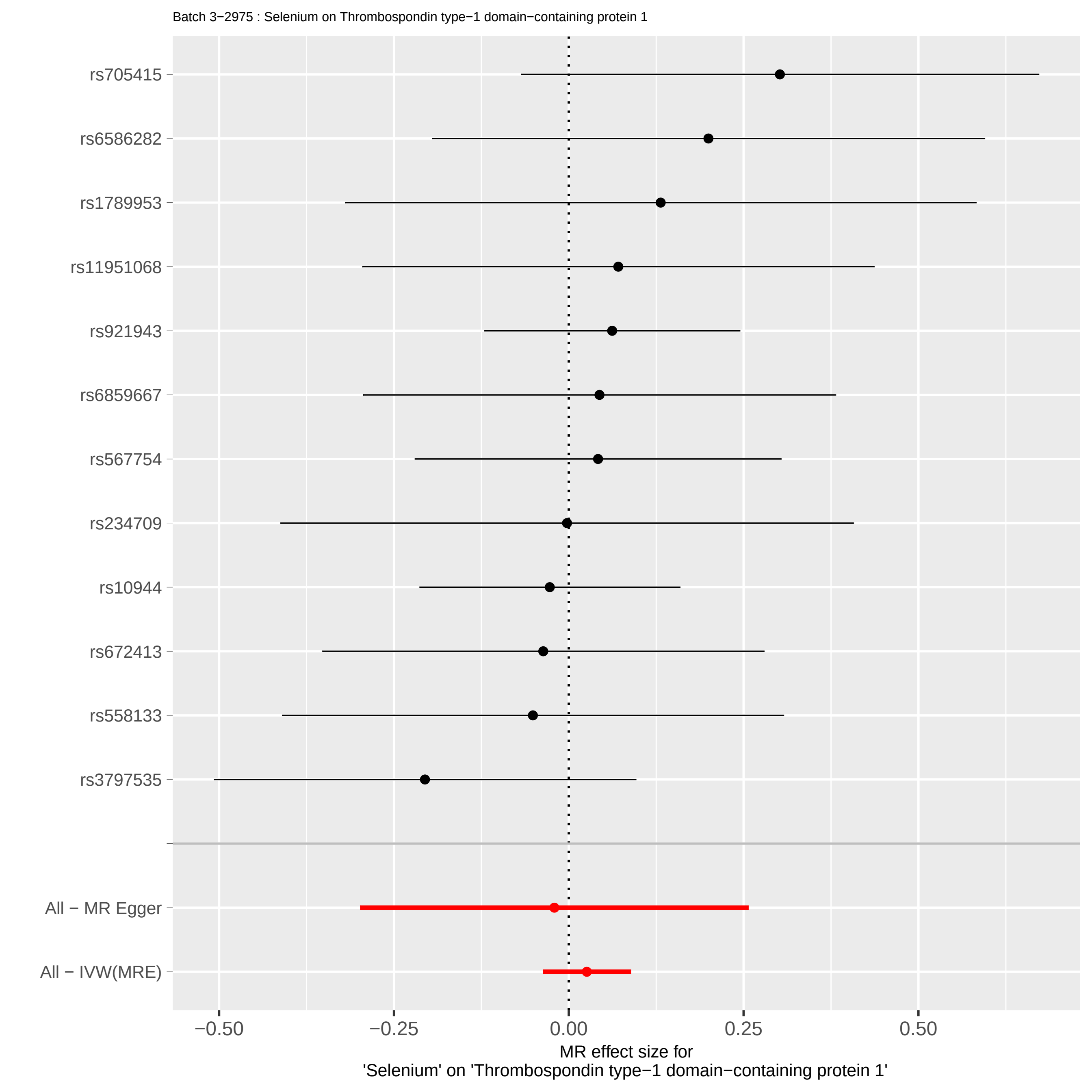

Batch 3−2975 : Selenium on Thrombospondin type−1 domain−containing protein 1
rs705415
rs6586282
rs1789953
rs11951068
rs921943
rs6859667
rs567754
rs234709
rs10944
rs672413
rs558133
rs3797535
All − MR Egger
All − IVW(MRE)
−0.50
−0.25
0.00
0.25
0.50
MR effect size for
'Selenium' on 'Thrombospondin type−1 domain−containing protein 1'

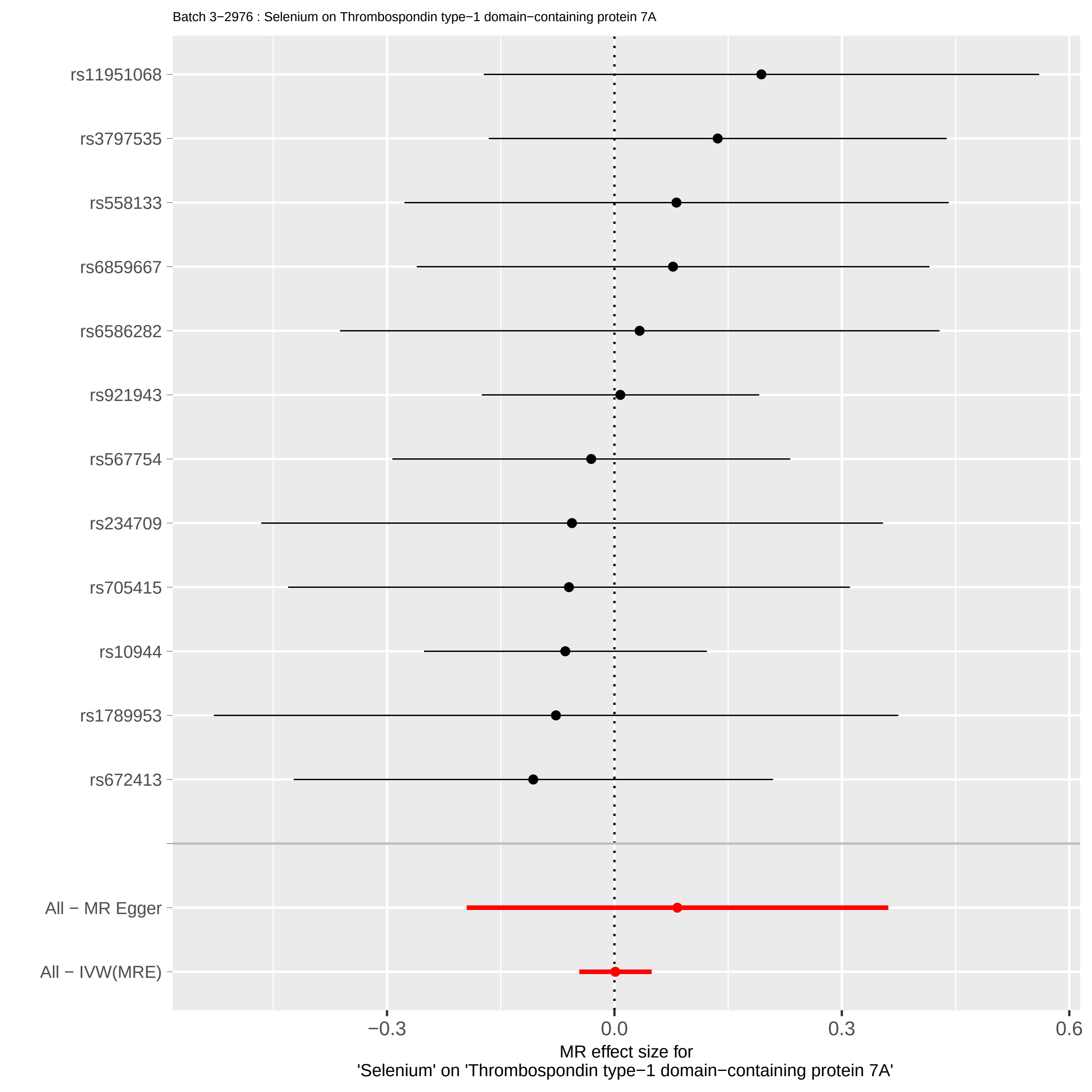

Batch 3−2976 : Selenium on Thrombospondin type−1 domain−containing protein 7A
rs11951068
rs3797535
rs558133
rs6859667
rs6586282
rs921943
rs567754
rs234709
rs705415
rs10944
rs1789953
rs672413
All − MR Egger
All − IVW(MRE)
−0.3
0.0
0.3
0.6
MR effect size for
'Selenium' on 'Thrombospondin type−1 domain−containing protein 7A'

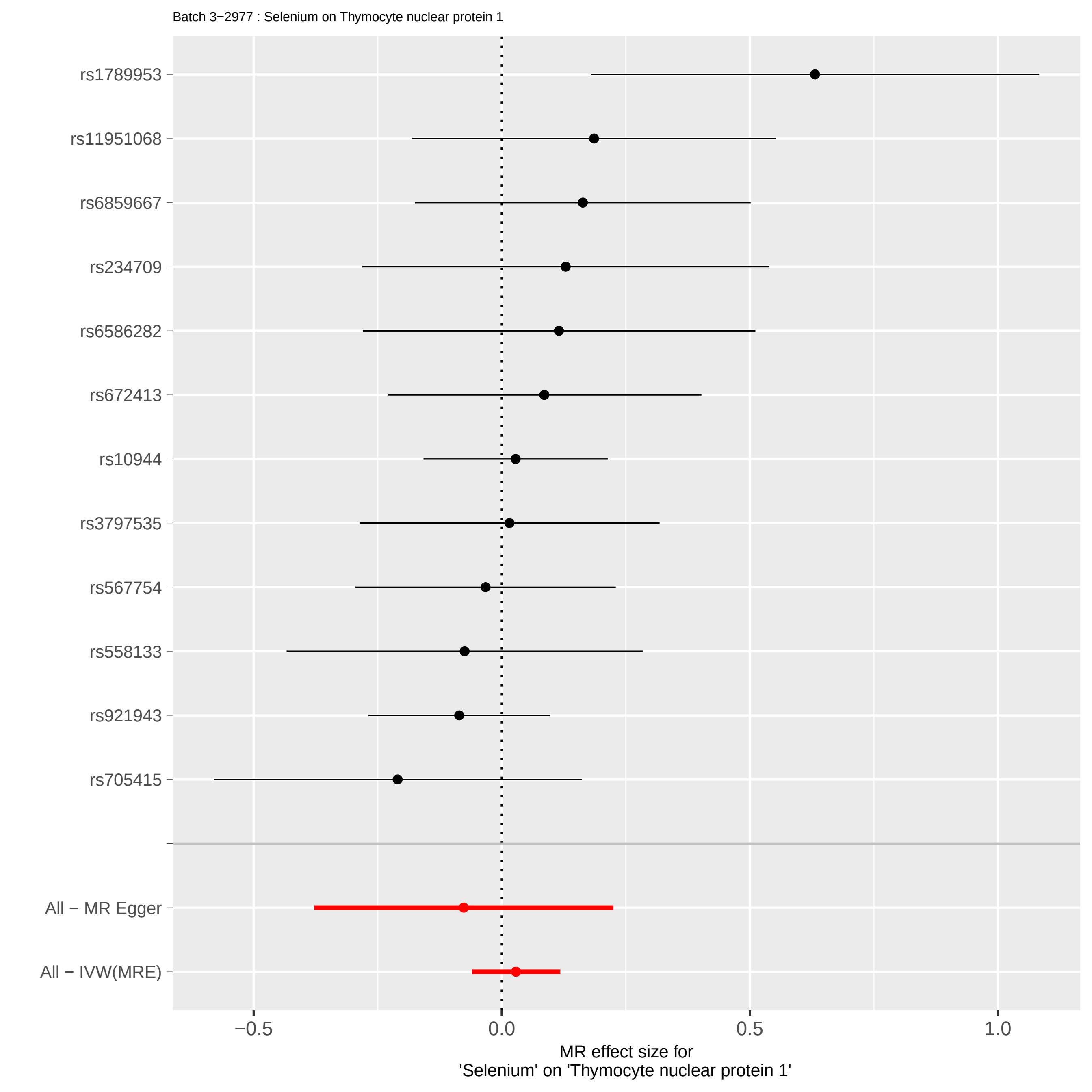
Batch 3−2977 : Selenium on Thymocyte nuclear protein 1
rs1789953
rs11951068
rs6859667
rs234709
rs6586282
rs672413
rs10944
rs3797535
rs567754
rs558133
rs921943
rs705415
All − MR Egger
All − IVW(MRE)
−0.5
0.0
0.5
1.0
MR effect size for
'Selenium' on 'Thymocyte nuclear protein 1'

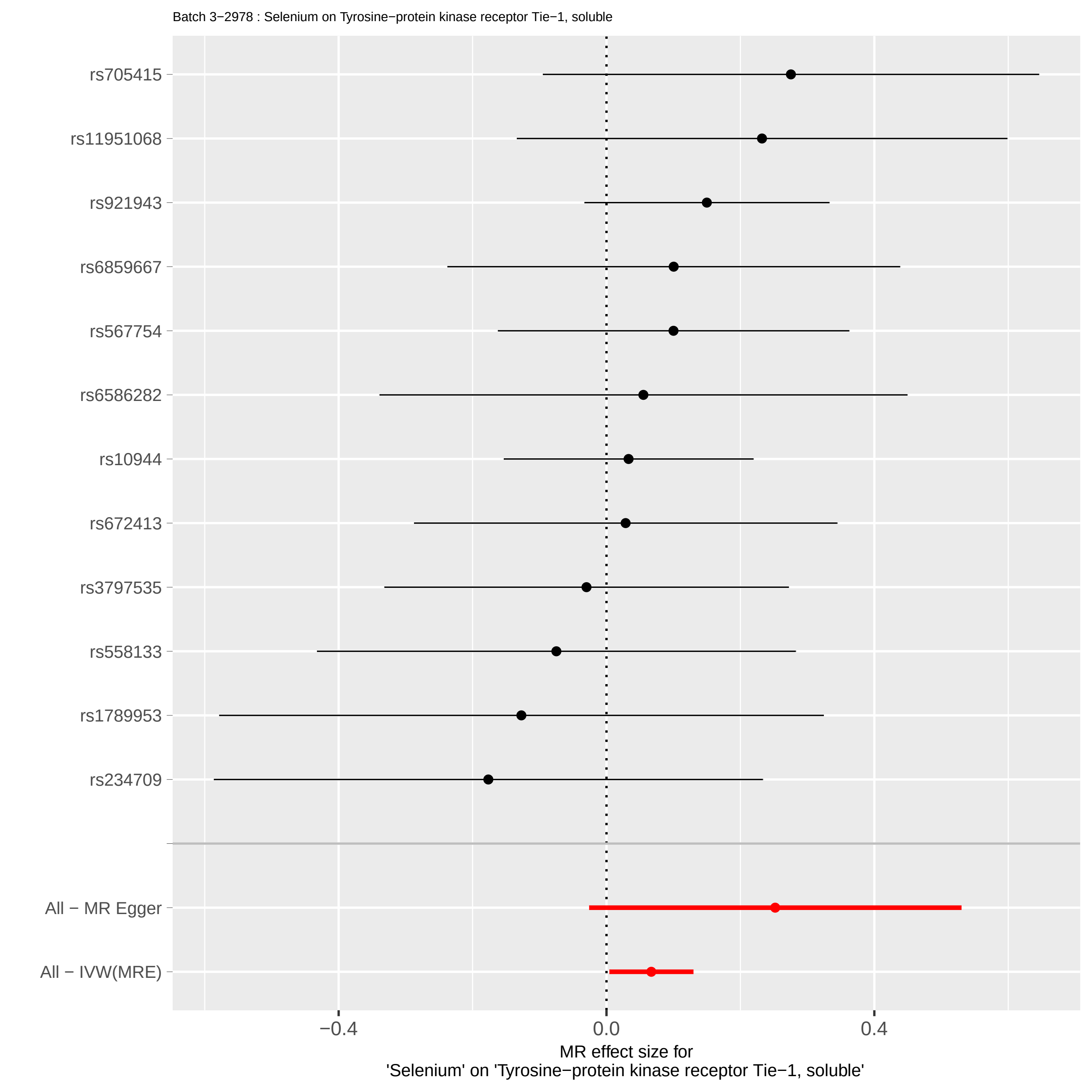
Batch 3−2978 : Selenium on Tyrosine−protein kinase receptor Tie−1, soluble
rs705415
rs11951068
rs921943
rs6859667
rs567754
rs6586282
rs10944
rs672413
rs3797535
rs558133
rs1789953
rs234709
All − MR Egger
All − IVW(MRE)
−0.4
0.0
0.4
MR effect size for
'Selenium' on 'Tyrosine−protein kinase receptor Tie−1, soluble'

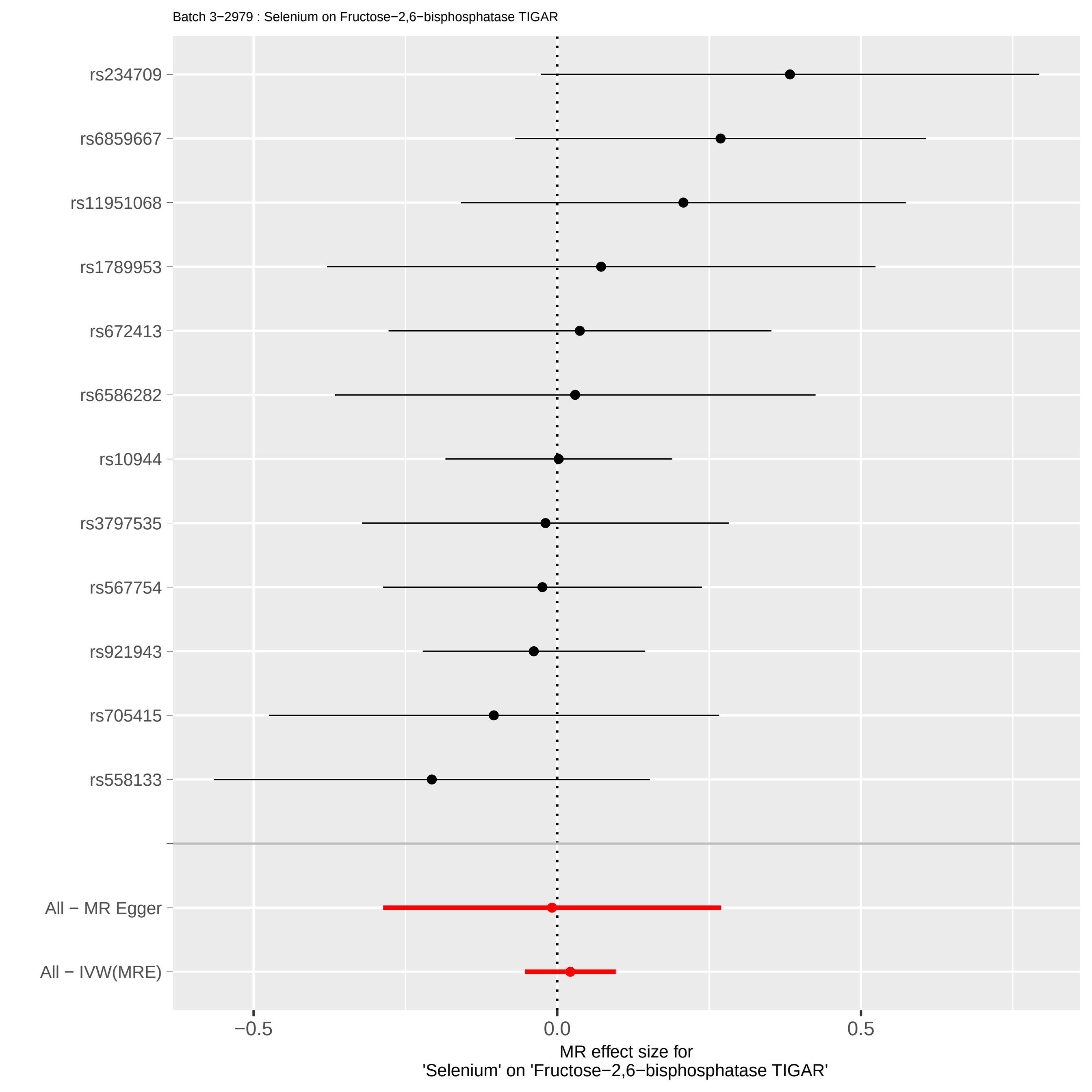
Batch 3−2979 : Selenium on Fructose−2,6−bisphosphatase TIGAR
rs234709
rs6859667
rs11951068
rs1789953
rs672413
rs6586282
rs10944
rs3797535
rs567754
rs921943
rs705415
rs558133
All − MR Egger
All − IVW(MRE)
−0.5
0.0
0.5
MR effect size for
'Selenium' on 'Fructose−2,6−bisphosphatase TIGAR'

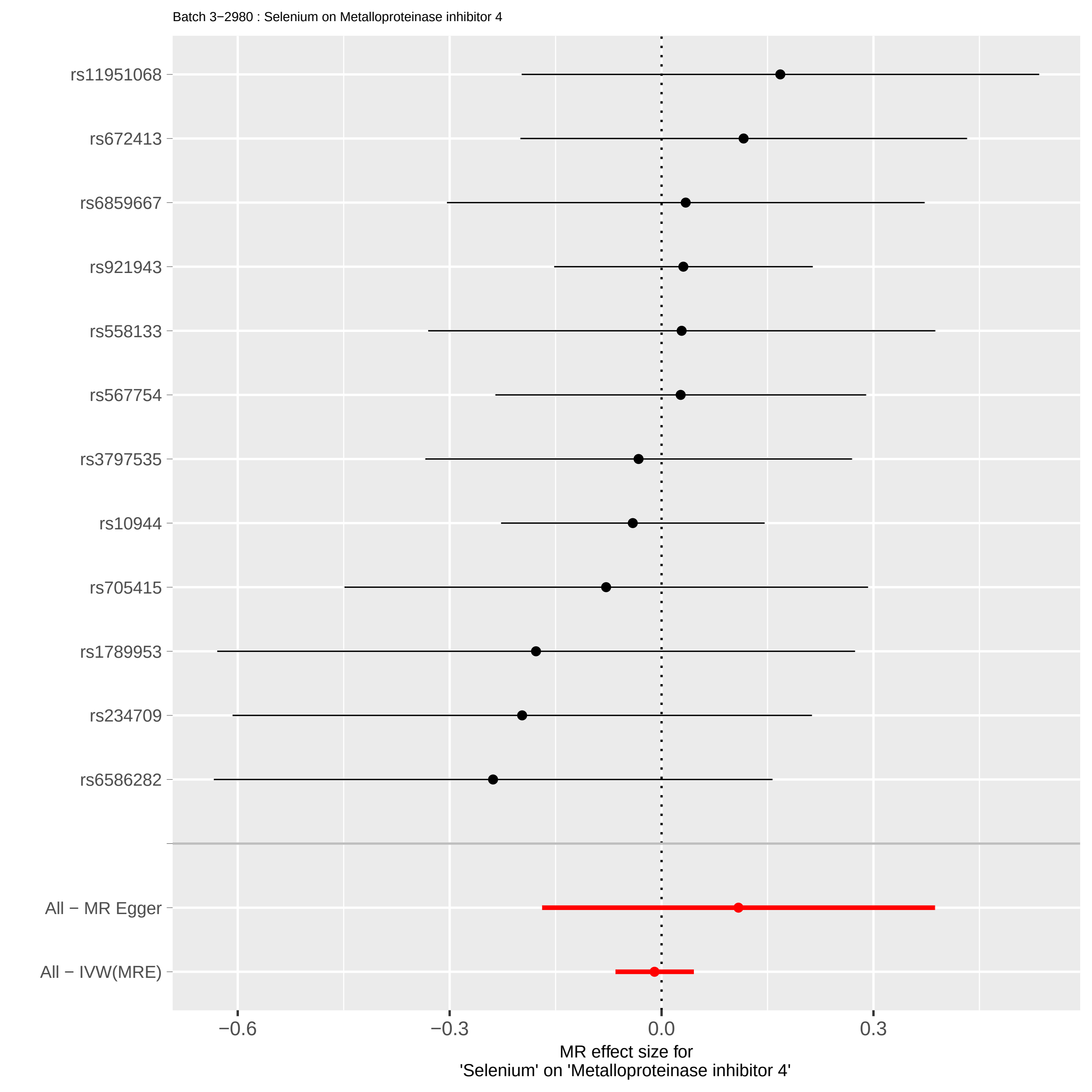

Batch 3−2980 : Selenium on Metalloproteinase inhibitor 4
rs11951068
rs672413
rs6859667
rs921943
rs558133
rs567754
rs3797535
rs10944
rs705415
rs1789953
rs234709
rs6586282
All − MR Egger
All − IVW(MRE)
−0.6
−0.3
0.0
0.3
MR effect size for
'Selenium' on 'Metalloproteinase inhibitor 4'

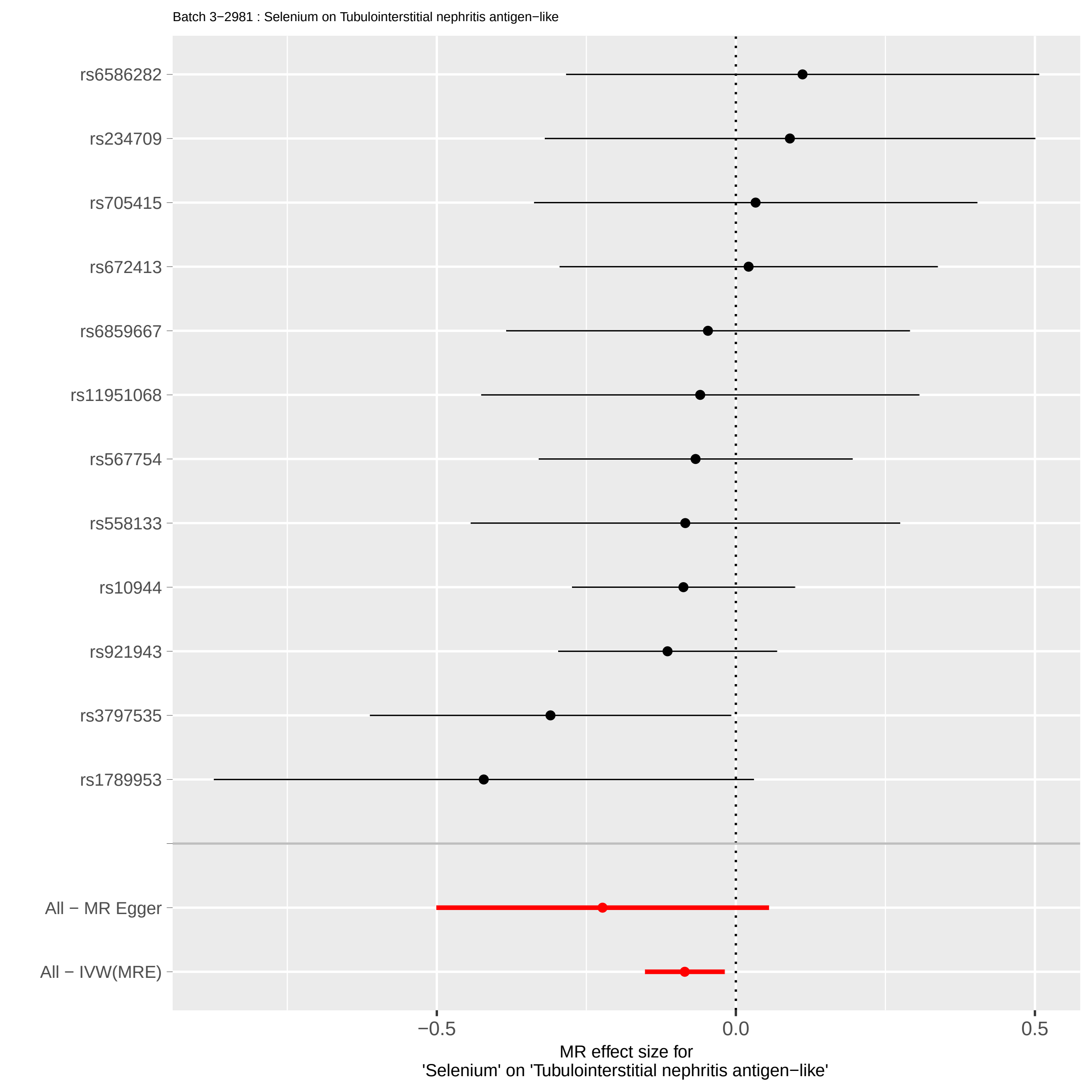

Batch 3−2981 : Selenium on Tubulointerstitial nephritis antigen−like
rs6586282
rs234709
rs705415
rs672413
rs6859667
rs11951068
rs567754
rs558133
rs10944
rs921943
rs3797535
rs1789953
All − MR Egger
All − IVW(MRE)
−0.5
0.0
0.5
MR effect size for
'Selenium' on 'Tubulointerstitial nephritis antigen−like'

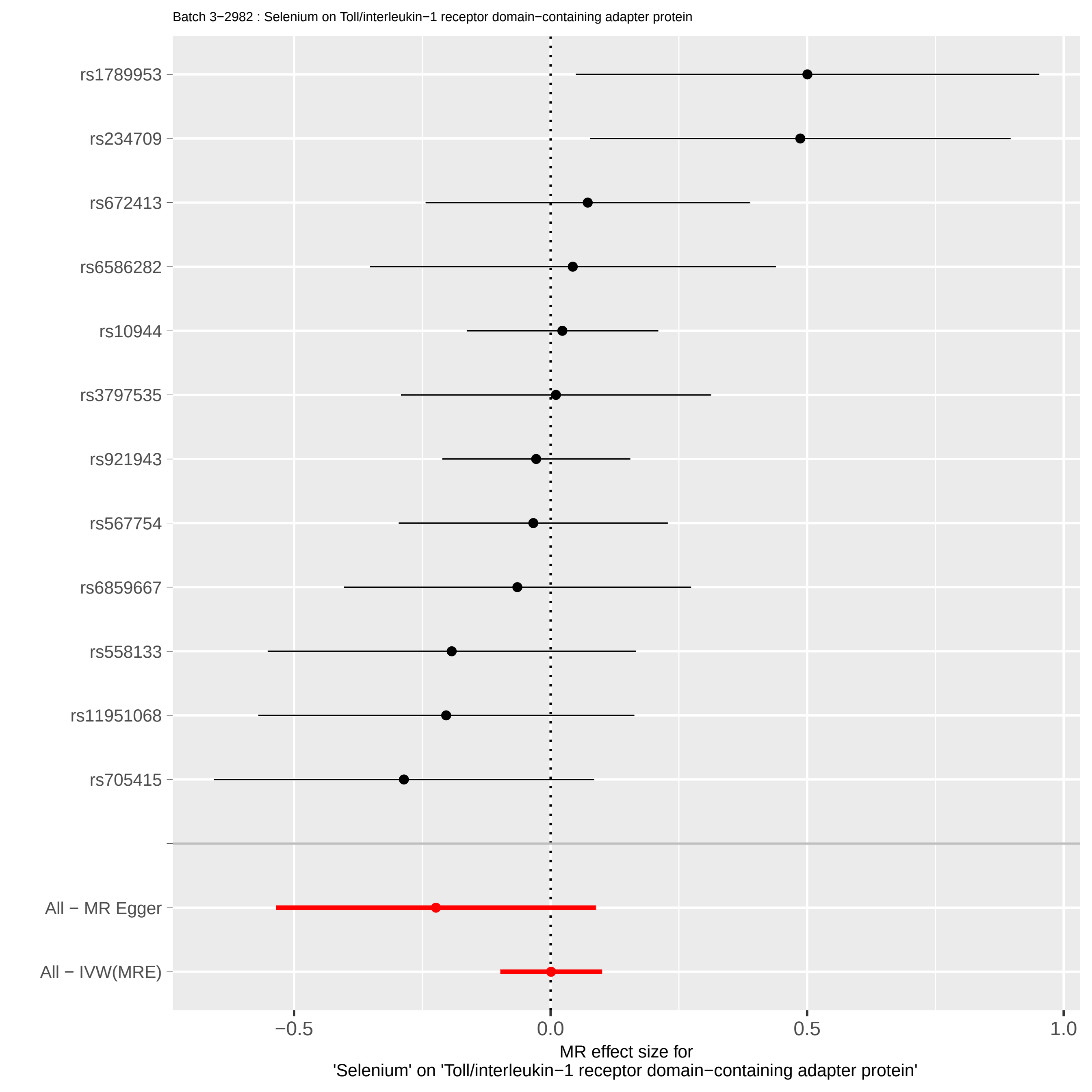
Batch 3−2982 : Selenium on Toll/interleukin−1 receptor domain−containing adapter protein
rs1789953
rs234709
rs672413
rs6586282
rs10944
rs3797535
rs921943
rs567754
rs6859667
rs558133
rs11951068
rs705415
All − MR Egger
All − IVW(MRE)
−0.5
0.0
0.5
1.0
MR effect size for
'Selenium' on 'Toll/interleukin−1 receptor domain−containing adapter protein'

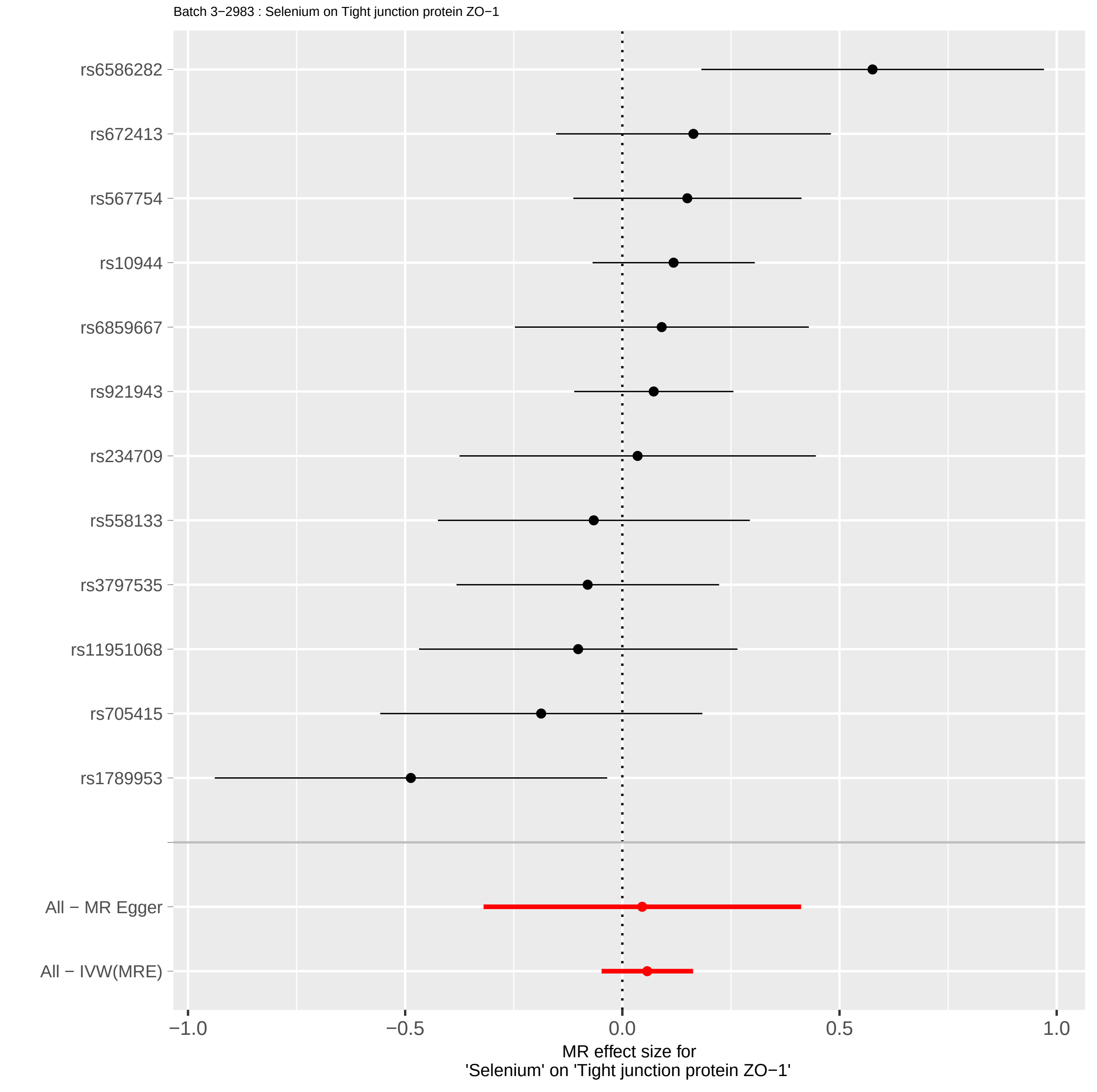

Batch 3−2983 : Selenium on Tight junction protein ZO−1
rs6586282
rs672413
rs567754
rs10944
rs6859667
rs921943
rs234709
rs558133
rs3797535
rs11951068
rs705415
rs1789953
All − MR Egger
All − IVW(MRE)
−1.0
−0.5
0.0
0.5
1.0
MR effect size for
'Selenium' on 'Tight junction protein ZO−1'

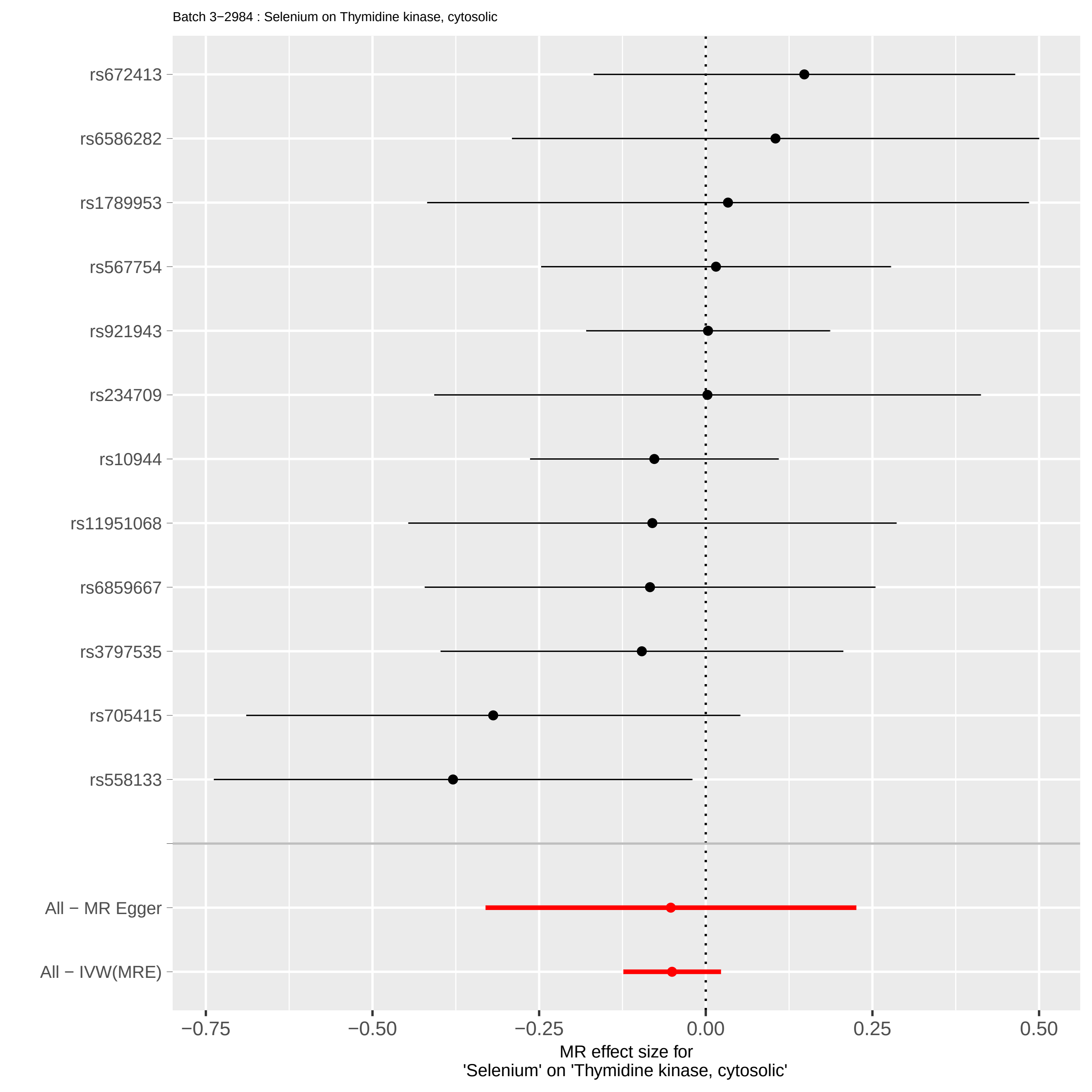

Batch 3−2984 : Selenium on Thymidine kinase, cytosolic
rs672413
rs6586282
rs1789953
rs567754
rs921943
rs234709
rs10944
rs11951068
rs6859667
rs3797535
rs705415
rs558133
All − MR Egger
All − IVW(MRE)
−0.75
−0.50
−0.25
0.00
0.25
0.50
MR effect size for
'Selenium' on 'Thymidine kinase, cytosolic'

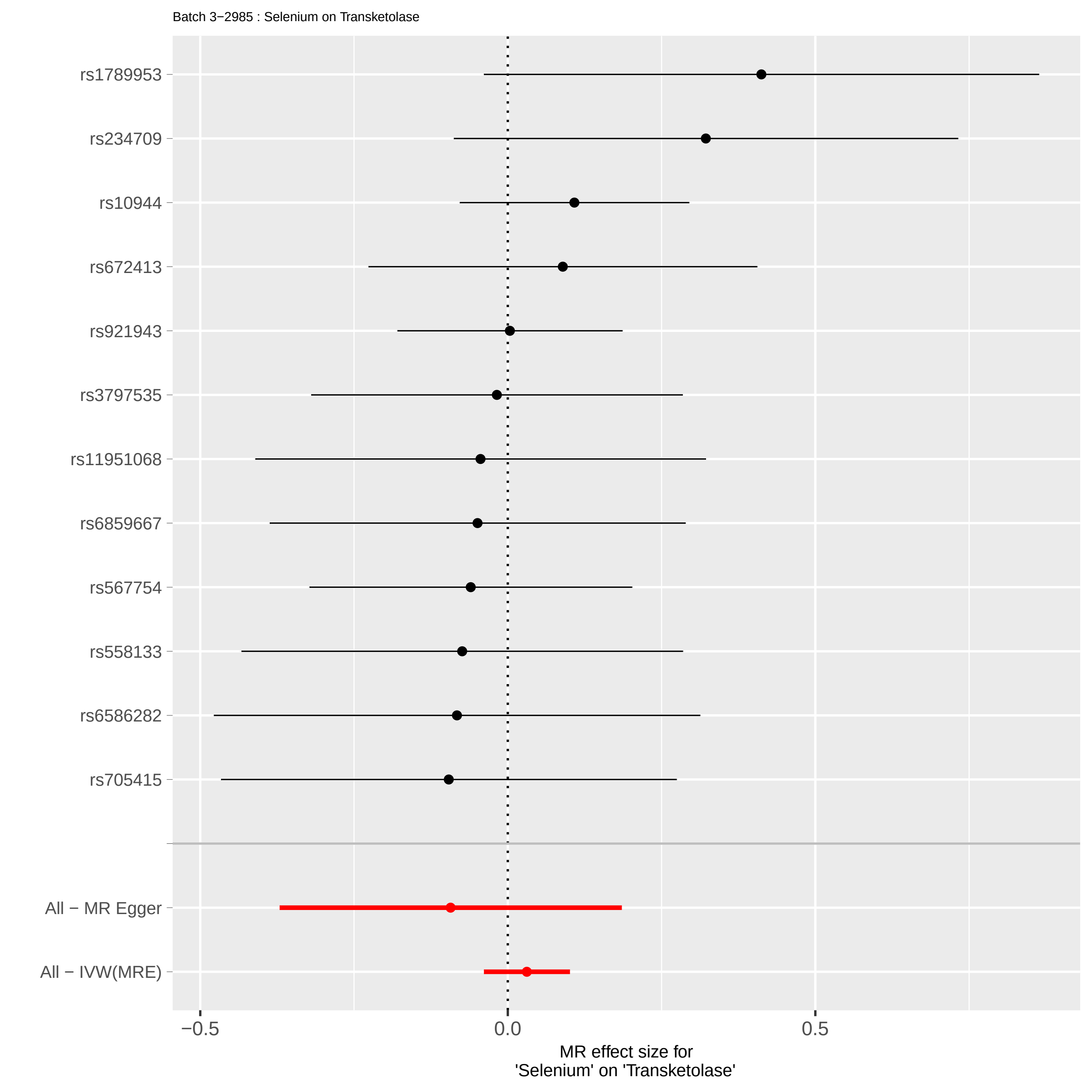

Batch 3−2985 : Selenium on Transketolase
rs1789953
rs234709
rs10944
rs672413
rs921943
rs3797535
rs11951068
rs6859667
rs567754
rs558133
rs6586282
rs705415
All − MR Egger
All − IVW(MRE)
−0.5
0.0
0.5
MR effect size for
'Selenium' on 'Transketolase'

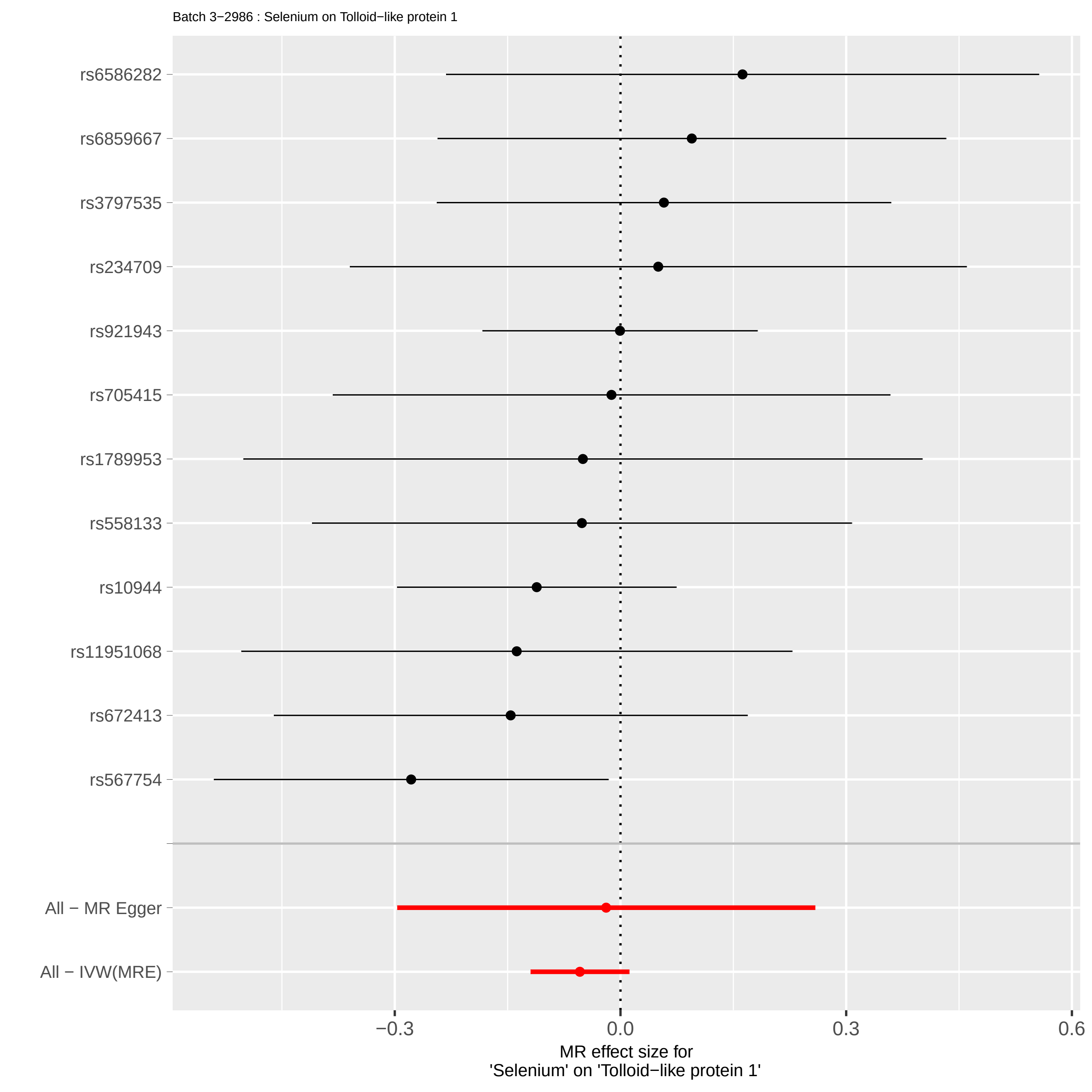

Batch 3−2986 : Selenium on Tolloid−like protein 1
rs6586282
rs6859667
rs3797535
rs234709
rs921943
rs705415
rs1789953
rs558133
rs10944
rs11951068
rs672413
rs567754
All − MR Egger
All − IVW(MRE)
−0.3
0.0
0.3
0.6
MR effect size for
'Selenium' on 'Tolloid−like protein 1'

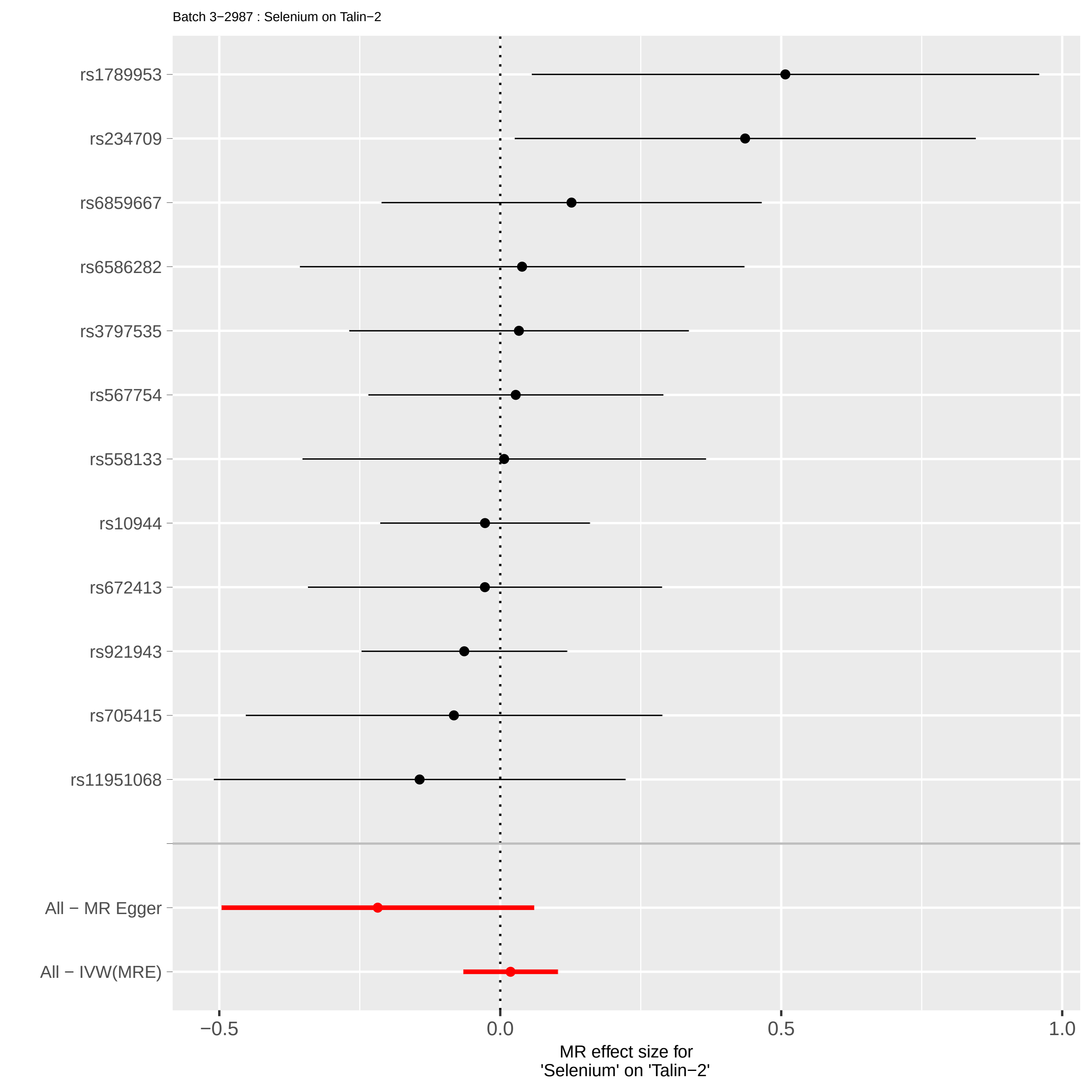

Batch 3−2987 : Selenium on Talin−2
rs1789953
rs234709
rs6859667
rs6586282
rs3797535
rs567754
rs558133
rs10944
rs672413
rs921943
rs705415
rs11951068
All − MR Egger
All − IVW(MRE)
−0.5
0.0
0.5
1.0
MR effect size for
'Selenium' on 'Talin−2'

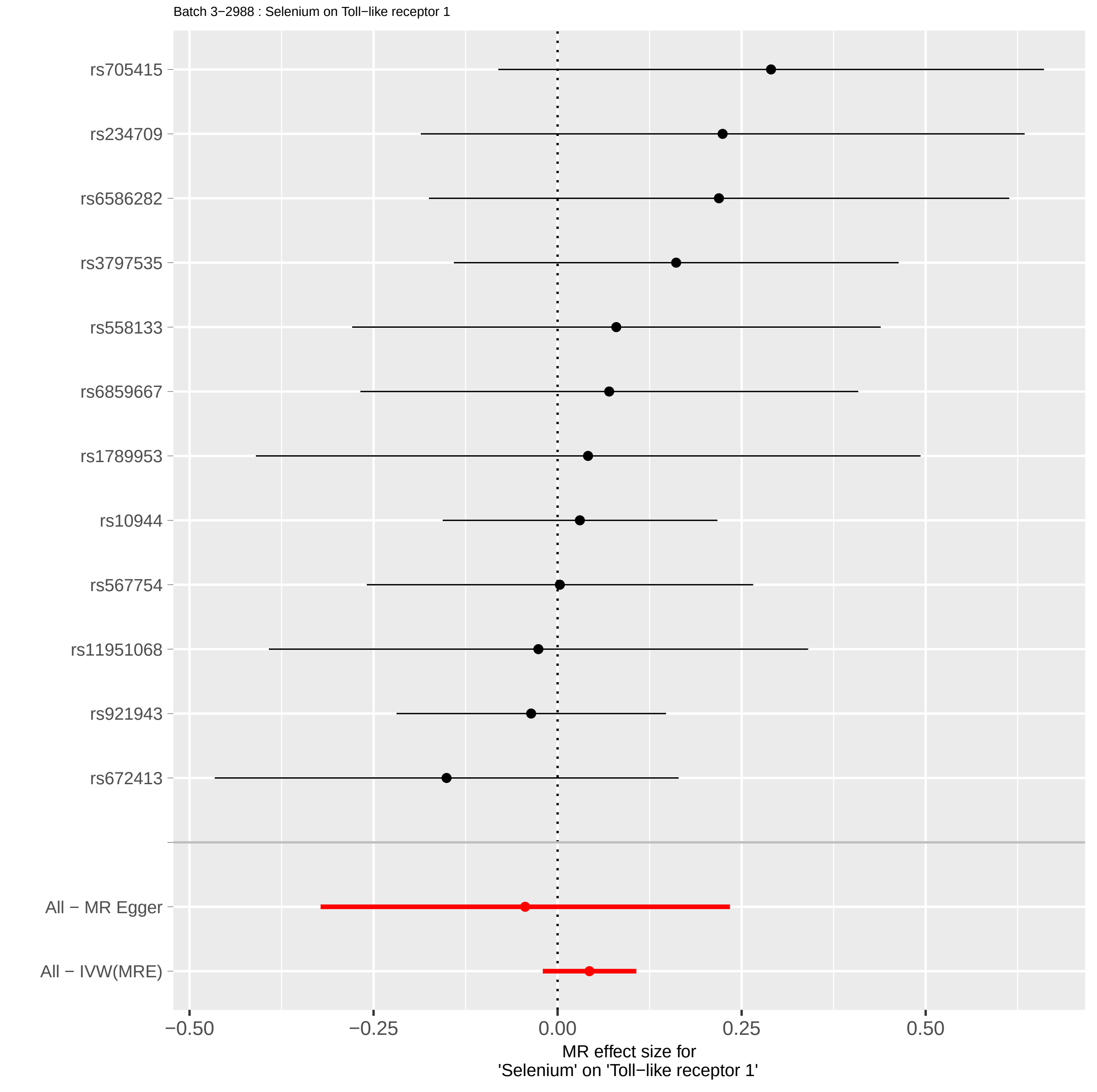

Batch 3−2988 : Selenium on Toll−like receptor 1
rs705415
rs234709
rs6586282
rs3797535
rs558133
rs6859667
rs1789953
rs10944
rs567754
rs11951068
rs921943
rs672413
All − MR Egger
All − IVW(MRE)
−0.50
−0.25
0.00
0.25
0.50
MR effect size for
'Selenium' on 'Toll−like receptor 1'

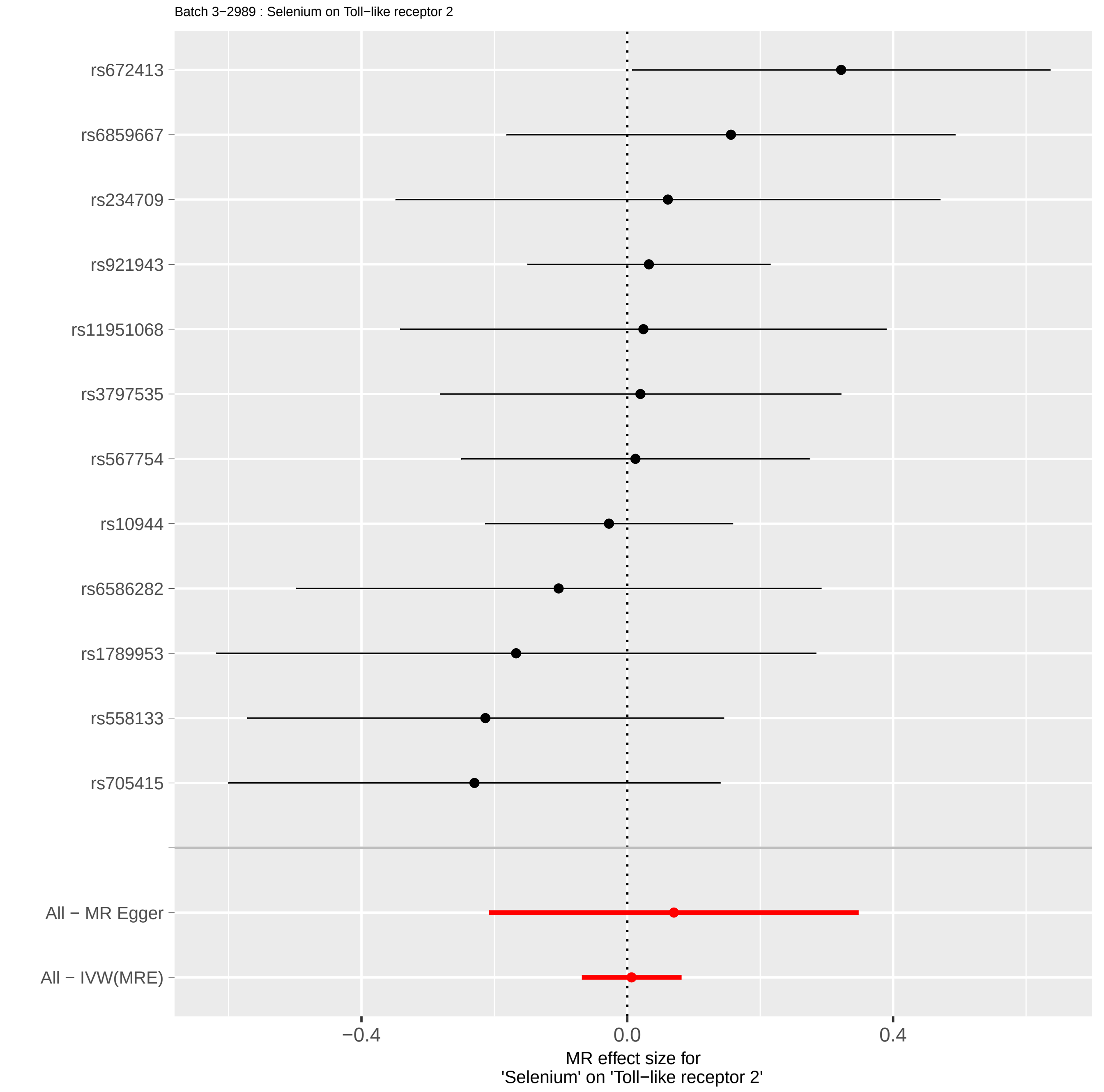

Batch 3−2989 : Selenium on Toll−like receptor 2
rs672413
rs6859667
rs234709
rs921943
rs11951068
rs3797535
rs567754
rs10944
rs6586282
rs1789953
rs558133
rs705415
All − MR Egger
All − IVW(MRE)
−0.4
0.0
0.4
MR effect size for
'Selenium' on 'Toll−like receptor 2'

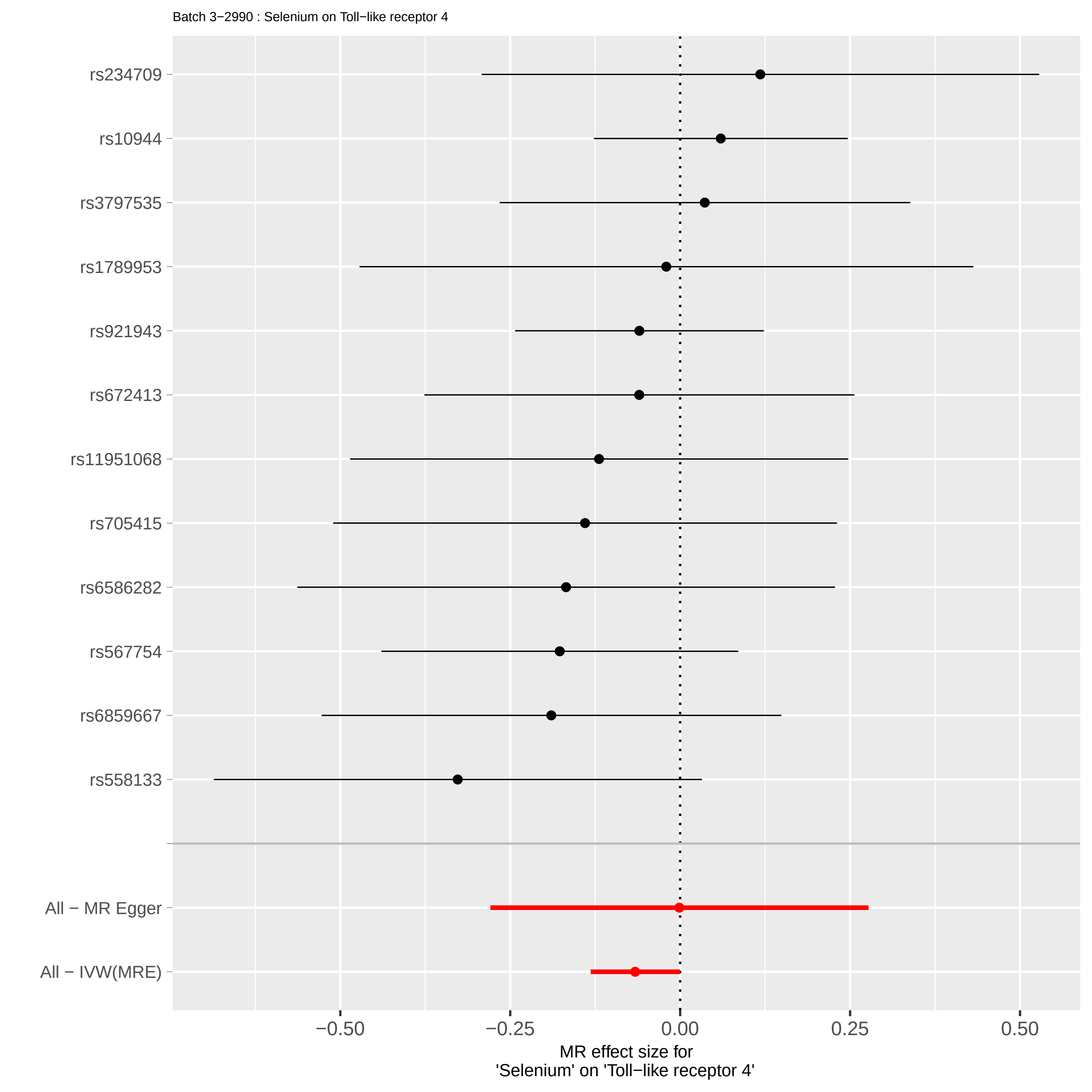

Batch 3−2990 : Selenium on Toll−like receptor 4
rs234709
rs10944
rs3797535
rs1789953
rs921943
rs672413
rs11951068
rs705415
rs6586282
rs567754
rs6859667
rs558133
All − MR Egger
All − IVW(MRE)
−0.50
−0.25
0.00
0.25
0.50
MR effect size for
'Selenium' on 'Toll−like receptor 4'

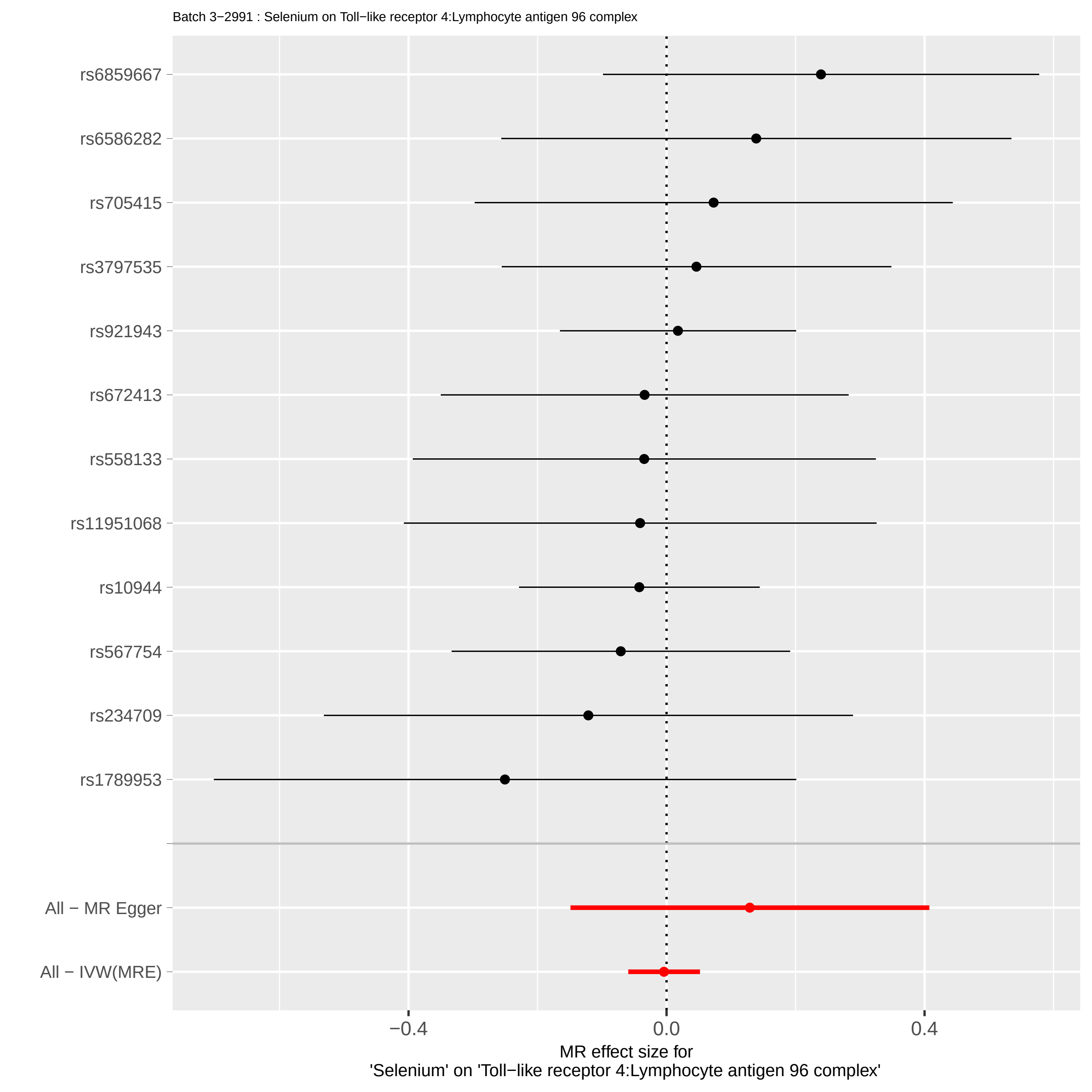

Batch 3−2991 : Selenium on Toll−like receptor 4:Lymphocyte antigen 96 complex
rs6859667
rs6586282
rs705415
rs3797535
rs921943
rs672413
rs558133
rs11951068
rs10944
rs567754
rs234709
rs1789953
All − MR Egger
All − IVW(MRE)
−0.4
0.0
0.4
MR effect size for
'Selenium' on 'Toll−like receptor 4:Lymphocyte antigen 96 complex'

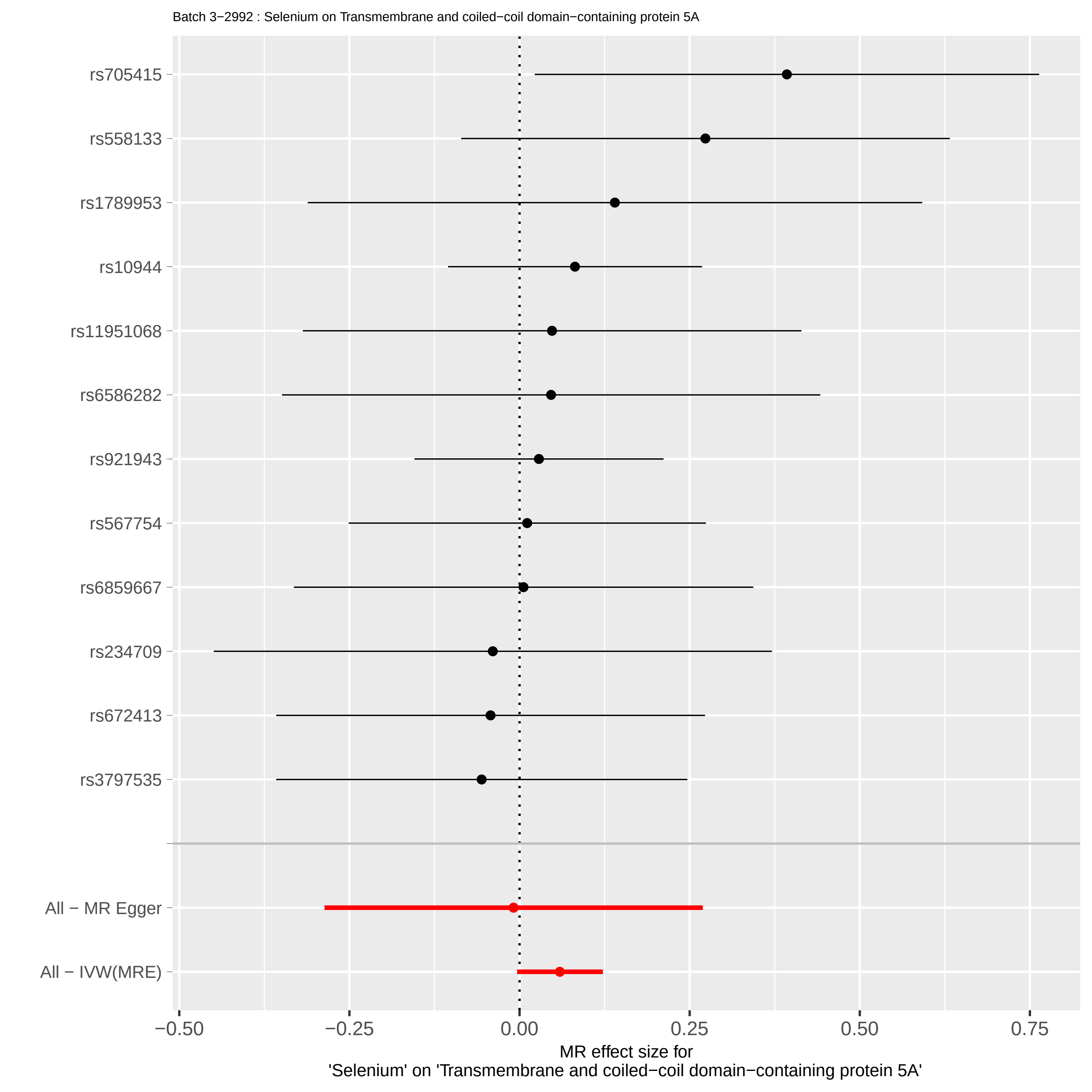

Batch 3−2992 : Selenium on Transmembrane and coiled−coil domain−containing protein 5A
rs705415
rs558133
rs1789953
rs10944
rs11951068
rs6586282
rs921943
rs567754
rs6859667
rs234709
rs672413
rs3797535
All − MR Egger
All − IVW(MRE)
−0.50
−0.25
0.00
0.25
0.50
0.75
MR effect size for
'Selenium' on 'Transmembrane and coiled−coil domain−containing protein 5A'

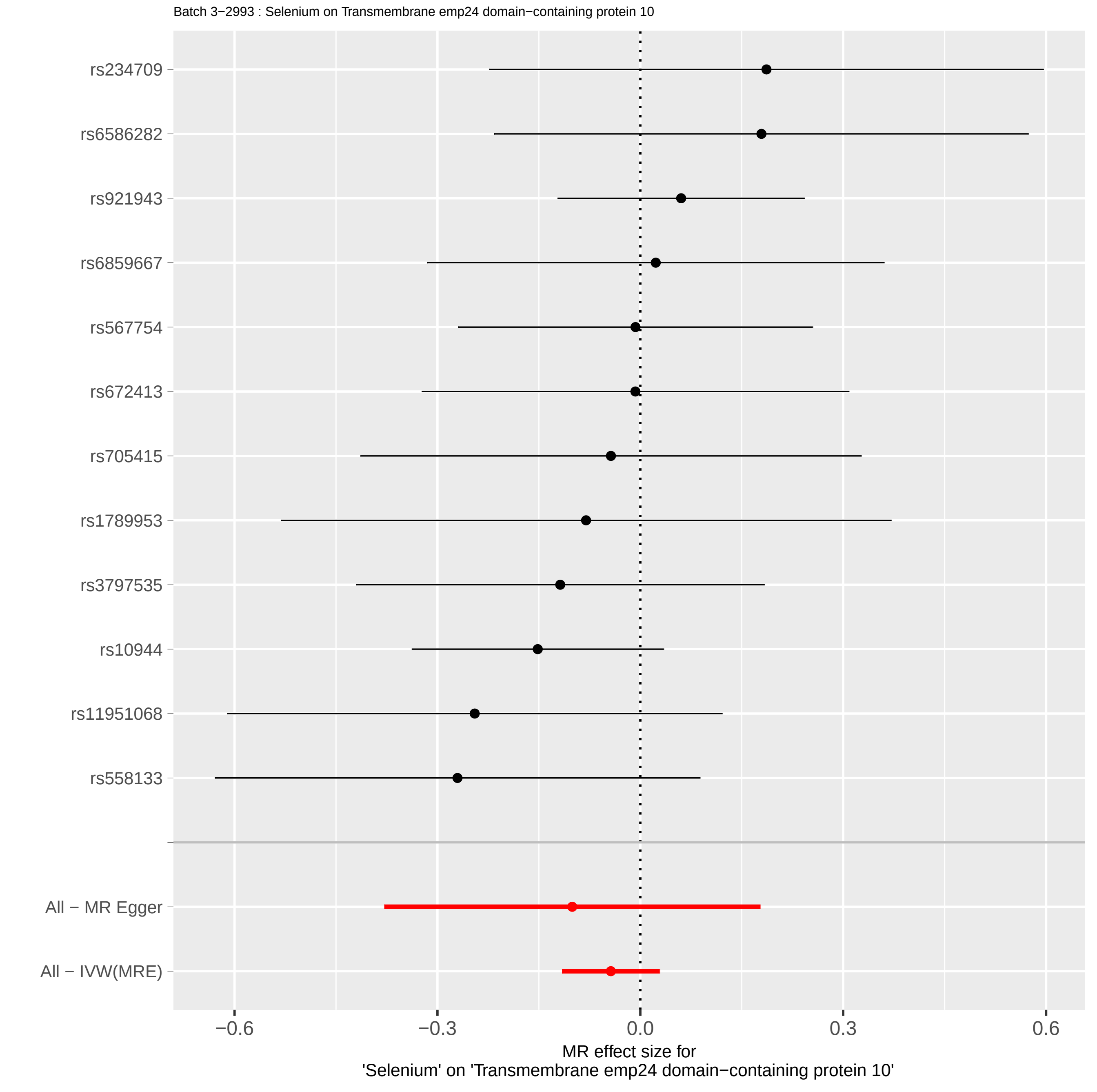

Batch 3−2993 : Selenium on Transmembrane emp24 domain−containing protein 10
rs234709
rs6586282
rs921943
rs6859667
rs567754
rs672413
rs705415
rs1789953
rs3797535
rs10944
rs11951068
rs558133
All − MR Egger
All − IVW(MRE)
−0.6
−0.3
0.0
0.3
0.6
MR effect size for
'Selenium' on 'Transmembrane emp24 domain−containing protein 10'

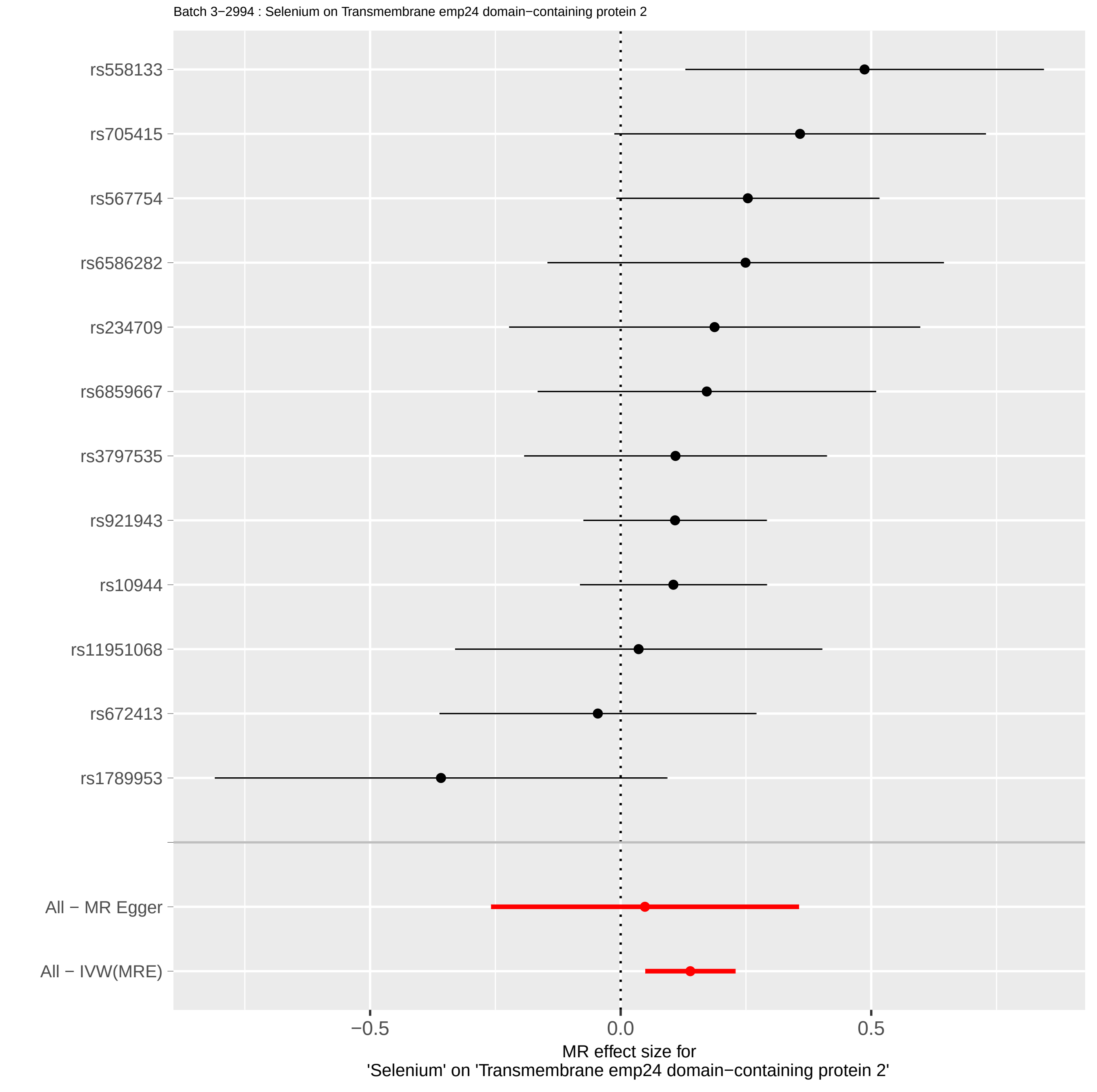

Batch 3−2994 : Selenium on Transmembrane emp24 domain−containing protein 2
rs558133
rs705415
rs567754
rs6586282
rs234709
rs6859667
rs3797535
rs921943
rs10944
rs11951068
rs672413
rs1789953
All − MR Egger
All − IVW(MRE)
−0.5
0.0
0.5
MR effect size for
'Selenium' on 'Transmembrane emp24 domain−containing protein 2'

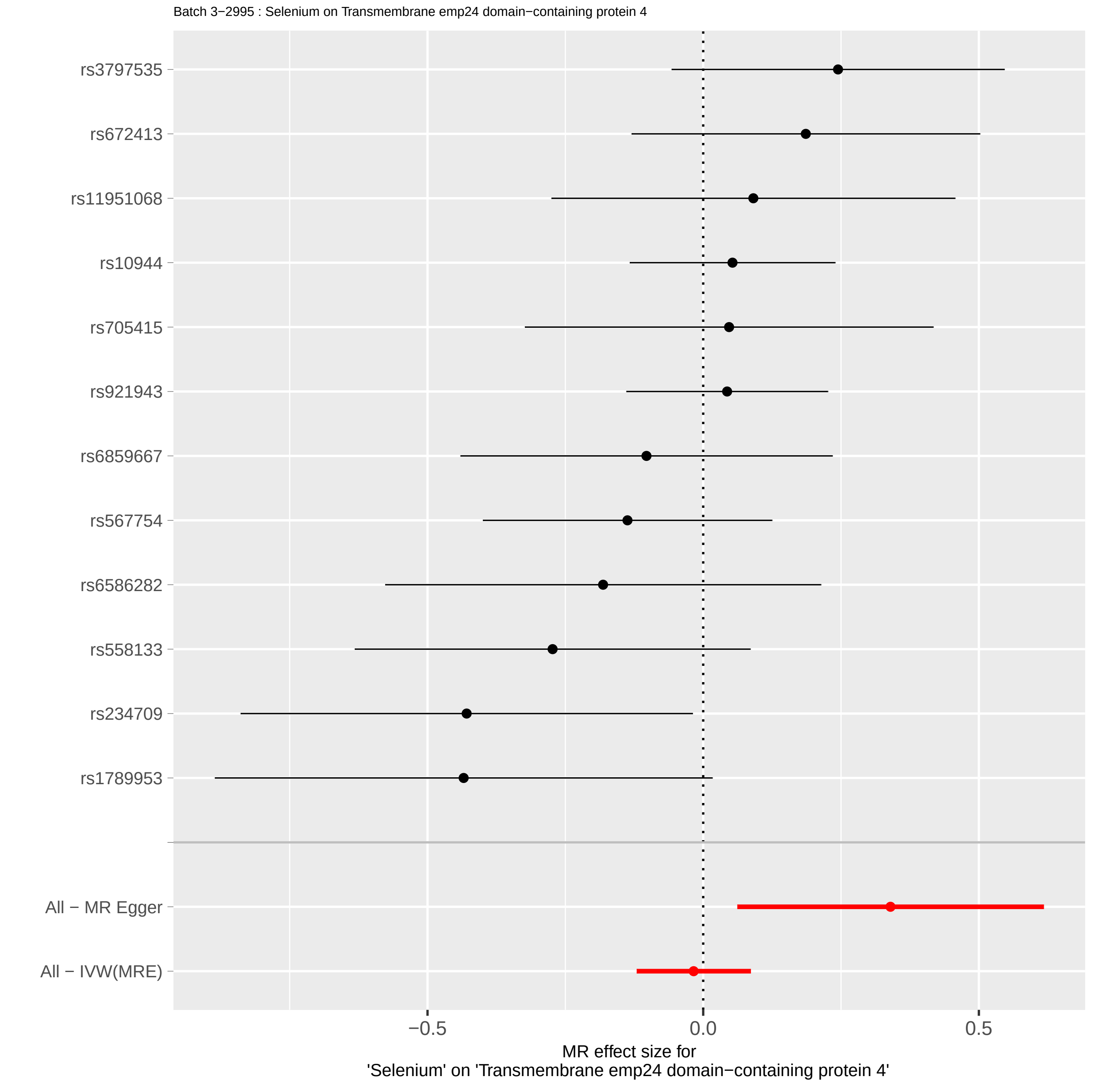
Batch 3−2995 : Selenium on Transmembrane emp24 domain−containing protein 4
rs3797535
rs672413
rs11951068
rs10944
rs705415
rs921943
rs6859667
rs567754
rs6586282
rs558133
rs234709
rs1789953
All − MR Egger
All − IVW(MRE)
−0.5
0.0
0.5
MR effect size for
'Selenium' on 'Transmembrane emp24 domain−containing protein 4'

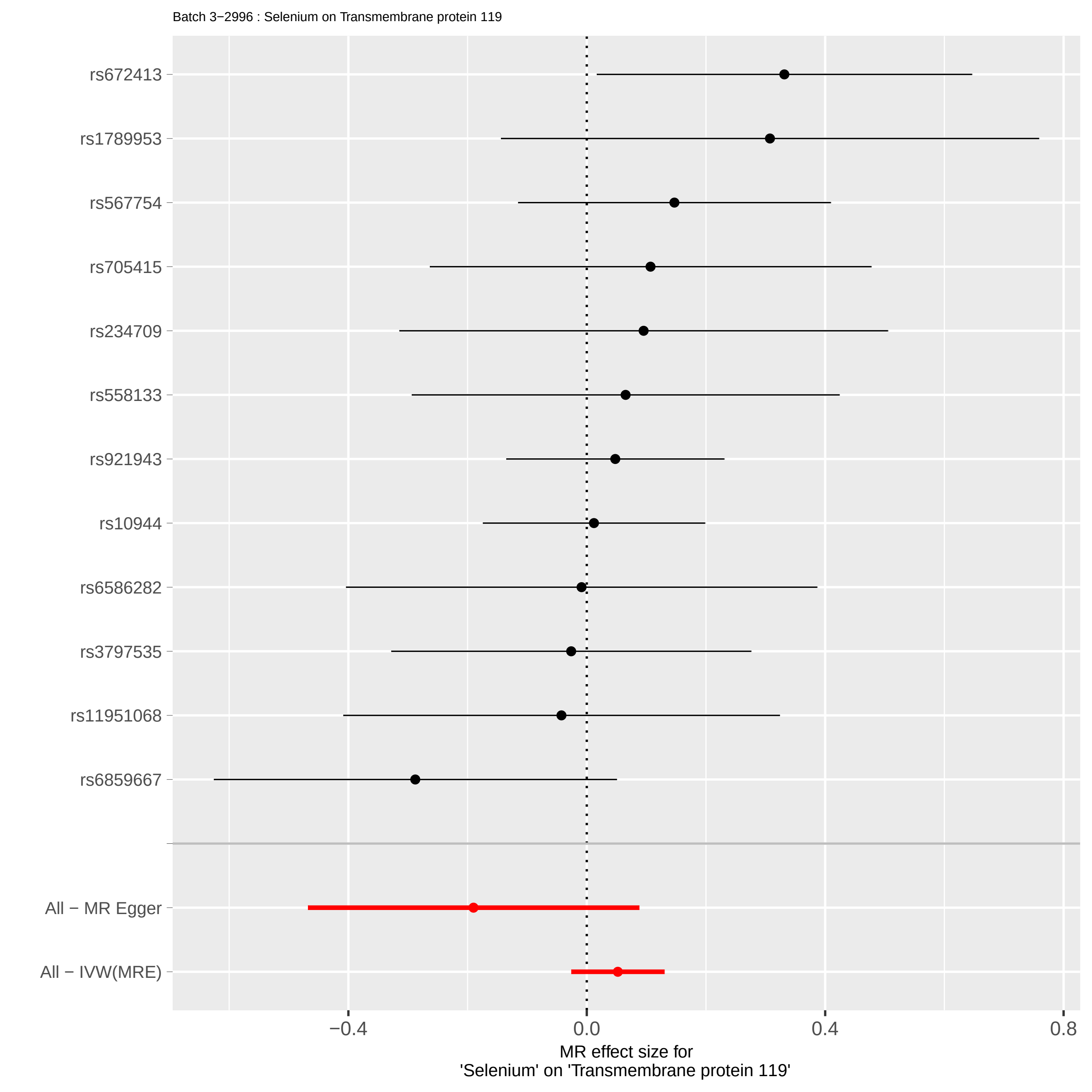

Batch 3−2996 : Selenium on Transmembrane protein 119
rs672413
rs1789953
rs567754
rs705415
rs234709
rs558133
rs921943
rs10944
rs6586282
rs3797535
rs11951068
rs6859667
All − MR Egger
All − IVW(MRE)
−0.4
0.0
0.4
0.8
MR effect size for
'Selenium' on 'Transmembrane protein 119'

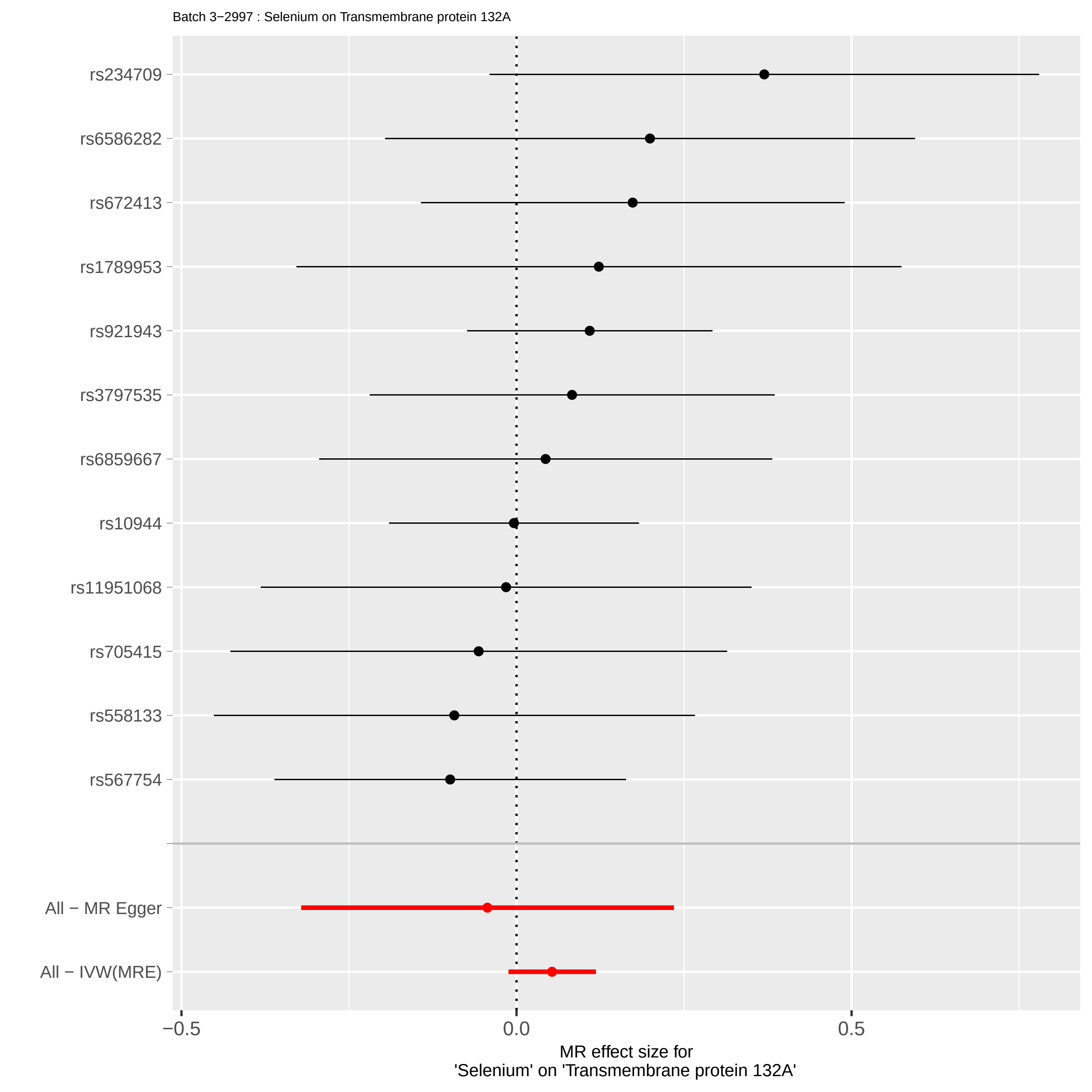
Batch 3−2997 : Selenium on Transmembrane protein 132A
rs234709
rs6586282
rs672413
rs1789953
rs921943
rs3797535
rs6859667
rs10944
rs11951068
rs705415
rs558133
rs567754
All − MR Egger
All − IVW(MRE)
−0.5
0.0
0.5
MR effect size for
'Selenium' on 'Transmembrane protein 132A'

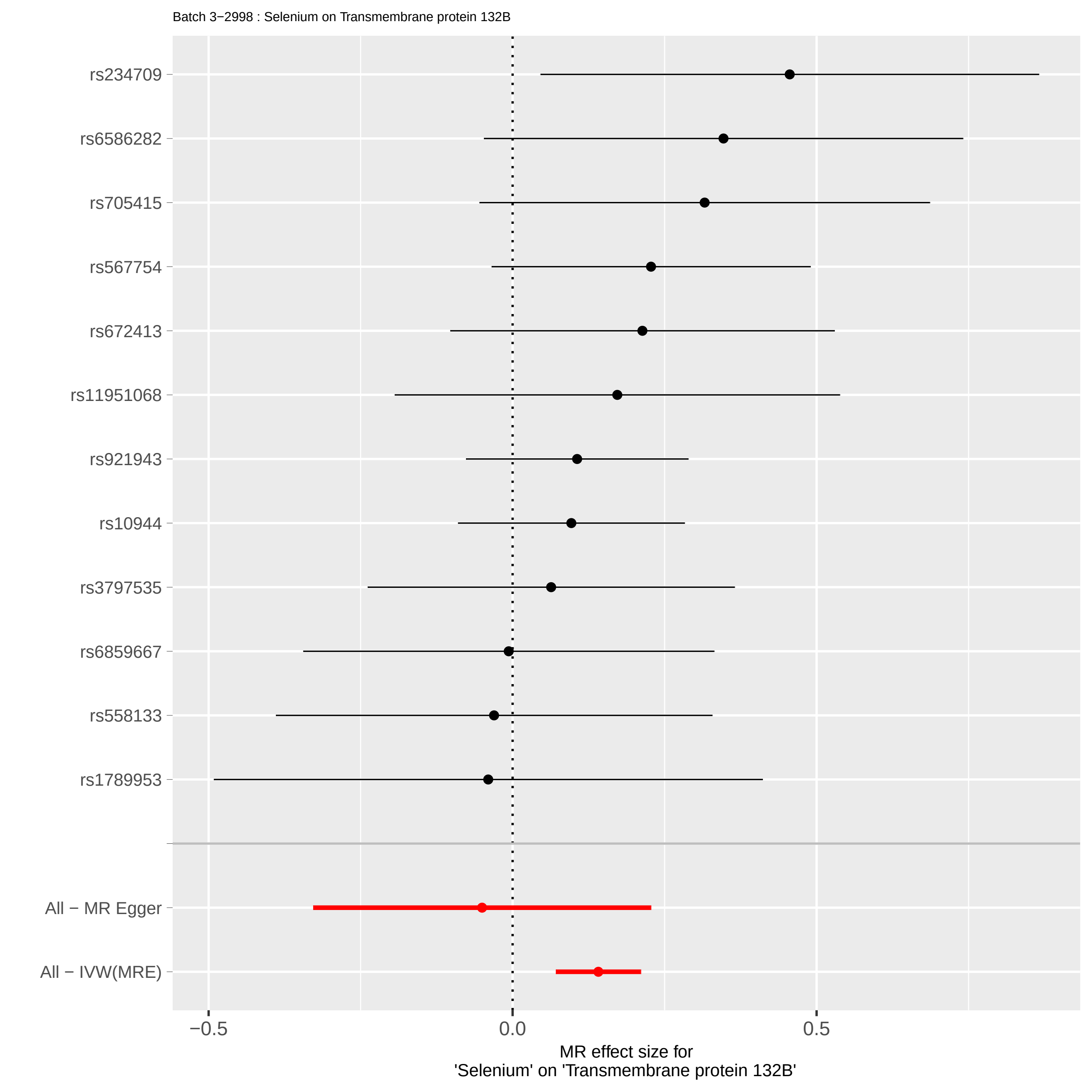

Batch 3−2998 : Selenium on Transmembrane protein 132B
rs234709
rs6586282
rs705415
rs567754
rs672413
rs11951068
rs921943
rs10944
rs3797535
rs6859667
rs558133
rs1789953
All − MR Egger
All − IVW(MRE)
−0.5
0.0
0.5
MR effect size for
'Selenium' on 'Transmembrane protein 132B'

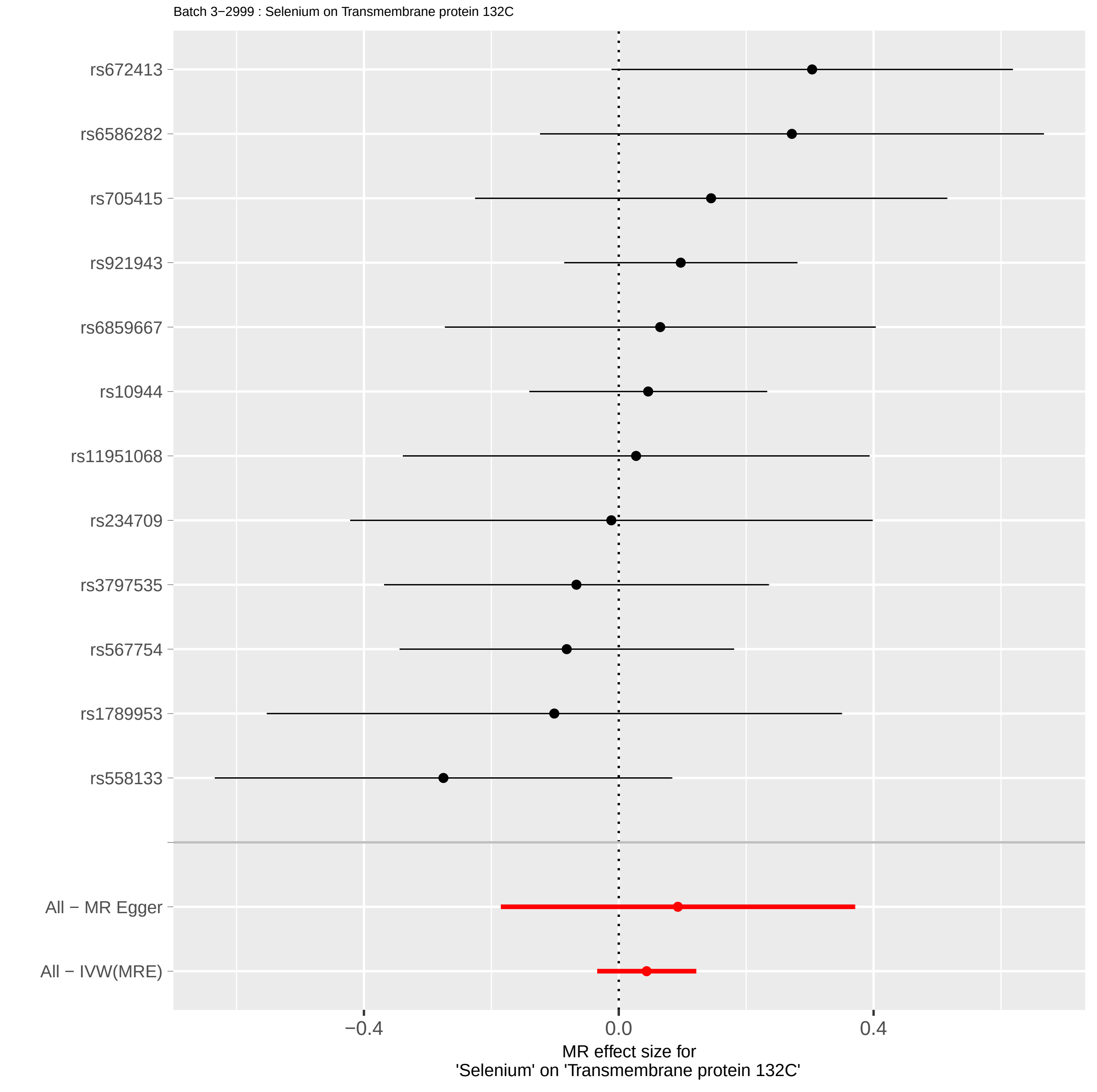

Batch 3−2999 : Selenium on Transmembrane protein 132C
rs672413
rs6586282
rs705415
rs921943
rs6859667
rs10944
rs11951068
rs234709
rs3797535
rs567754
rs1789953
rs558133
All − MR Egger
All − IVW(MRE)
−0.4
0.0
0.4
MR effect size for
'Selenium' on 'Transmembrane protein 132C'

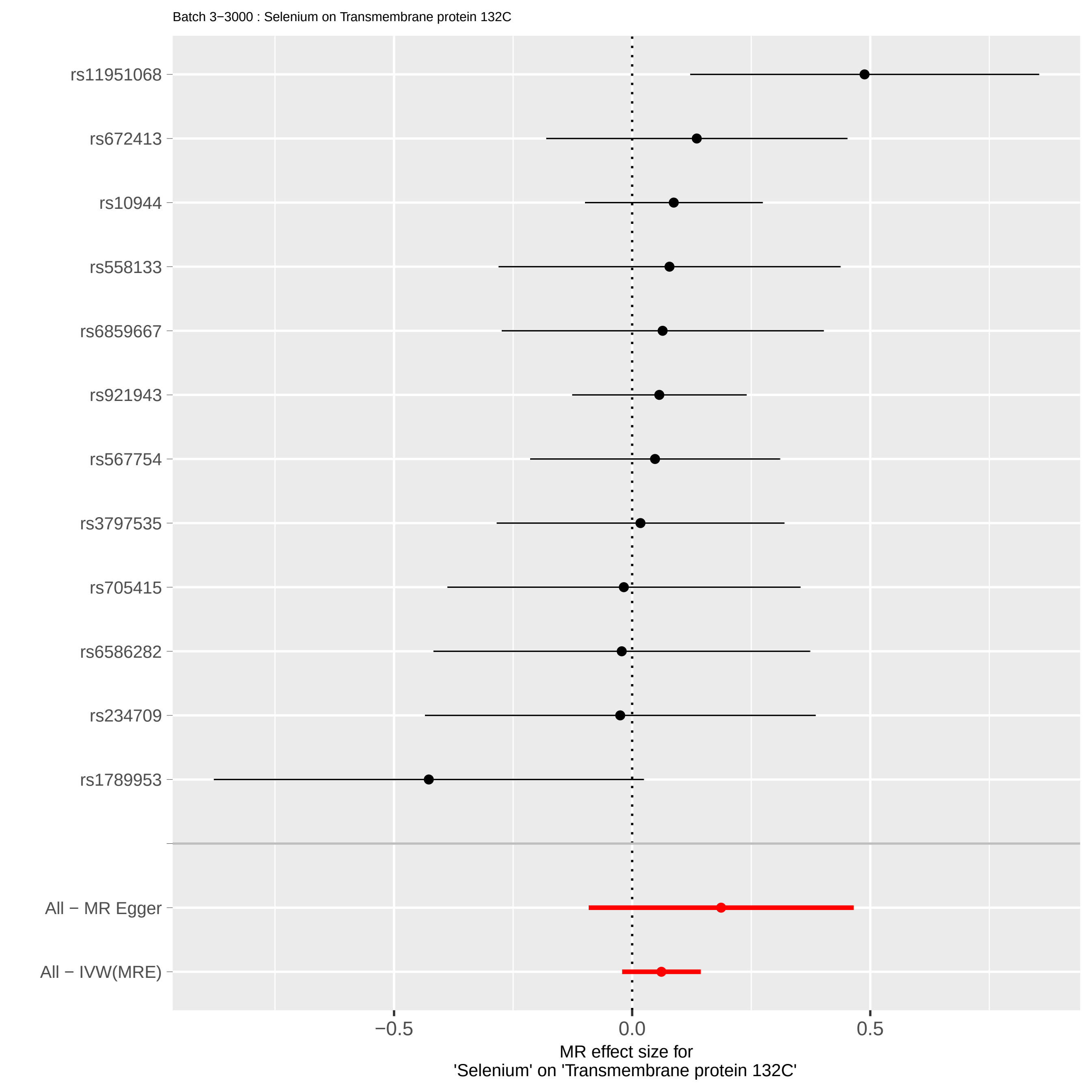

Batch 3−3000 : Selenium on Transmembrane protein 132C
rs11951068
rs672413
rs10944
rs558133
rs6859667
rs921943
rs567754
rs3797535
rs705415
rs6586282
rs234709
rs1789953
All − MR Egger
All − IVW(MRE)
−0.5
0.0
0.5
MR effect size for
'Selenium' on 'Transmembrane protein 132C'

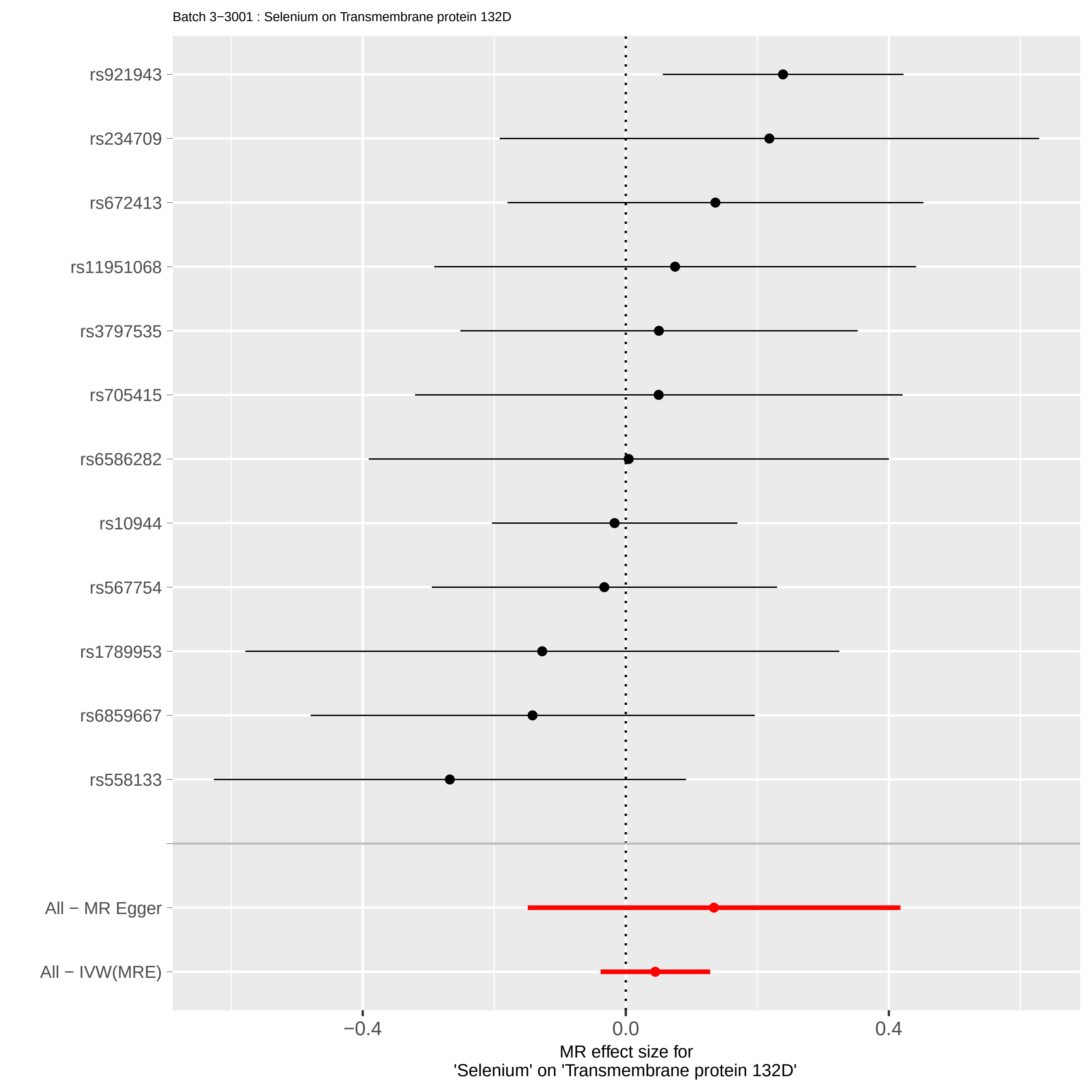

Batch 3−3001 : Selenium on Transmembrane protein 132D
rs921943
rs234709
rs672413
rs11951068
rs3797535
rs705415
rs6586282
rs10944
rs567754
rs1789953
rs6859667
rs558133
All − MR Egger
All − IVW(MRE)
−0.4
0.0
0.4
MR effect size for
'Selenium' on 'Transmembrane protein 132D'

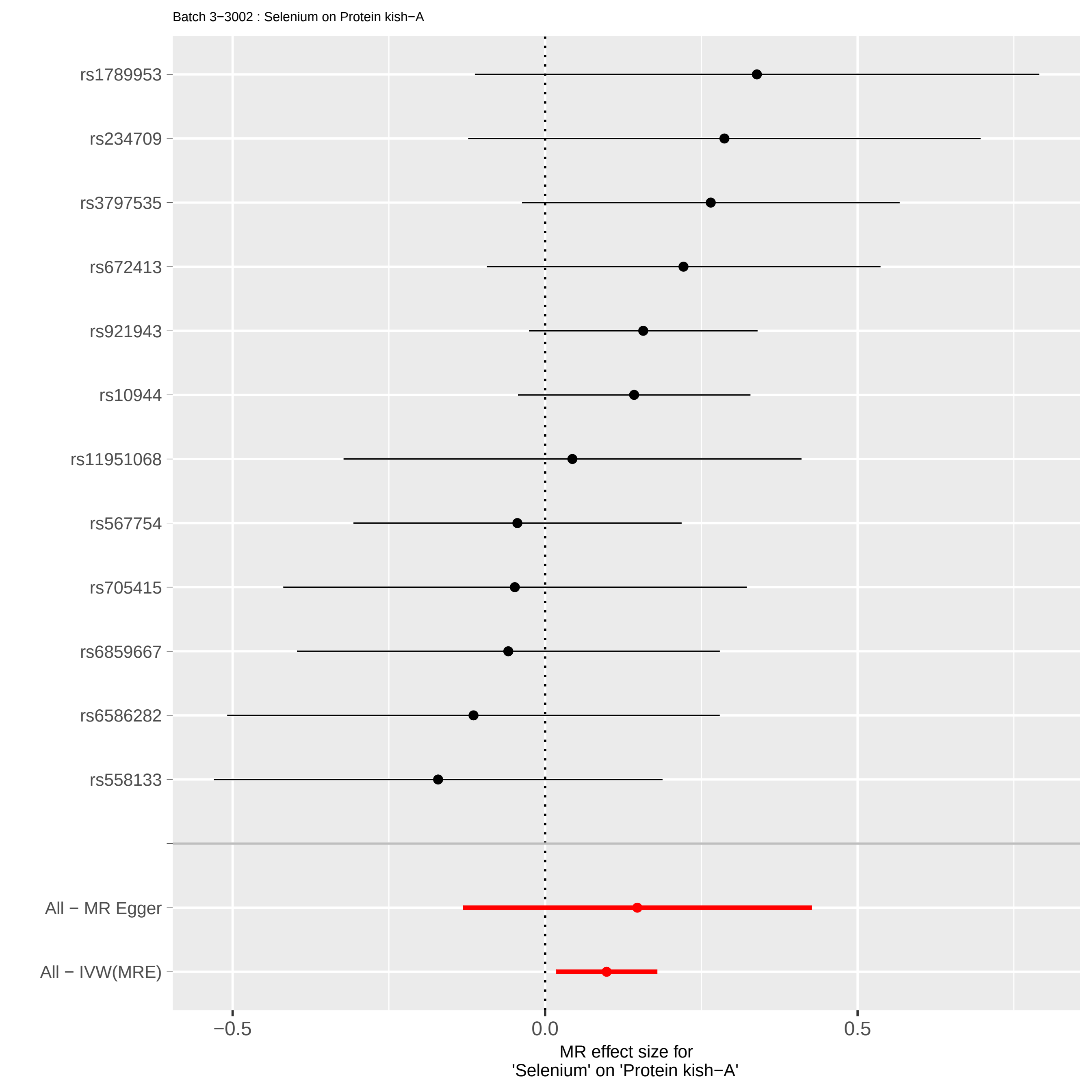
Batch 3−3002 : Selenium on Protein kish−A
rs1789953
rs234709
rs3797535
rs672413
rs921943
rs10944
rs11951068
rs567754
rs705415
rs6859667
rs6586282
rs558133
All − MR Egger
All − IVW(MRE)
−0.5
0.0
0.5
MR effect size for
'Selenium' on 'Protein kish−A'

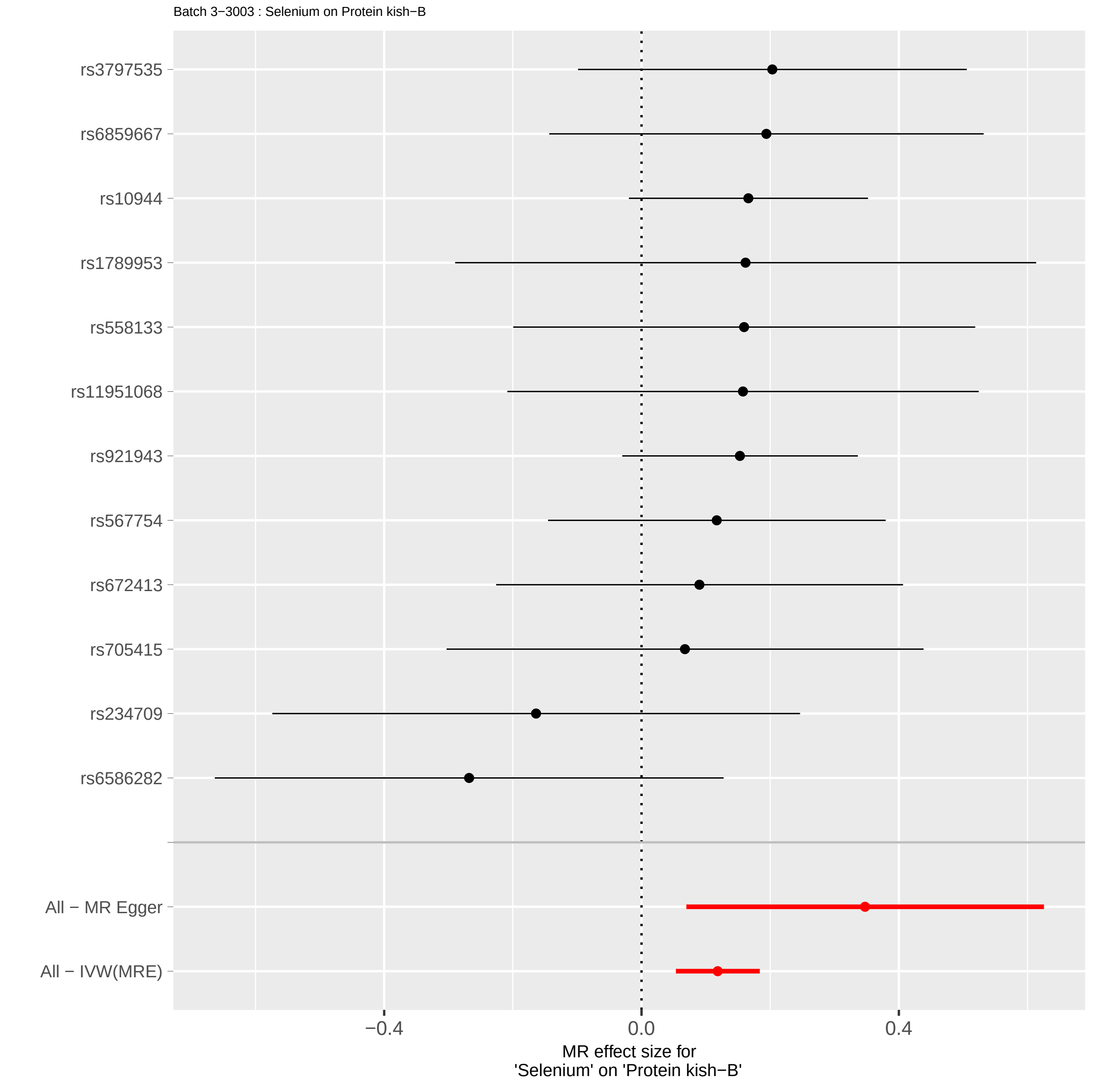

Batch 3−3003 : Selenium on Protein kish−B
rs3797535
rs6859667
rs10944
rs1789953
rs558133
rs11951068
rs921943
rs567754
rs672413
rs705415
rs234709
rs6586282
All − MR Egger
All − IVW(MRE)
−0.4
0.0
0.4
MR effect size for
'Selenium' on 'Protein kish−B'

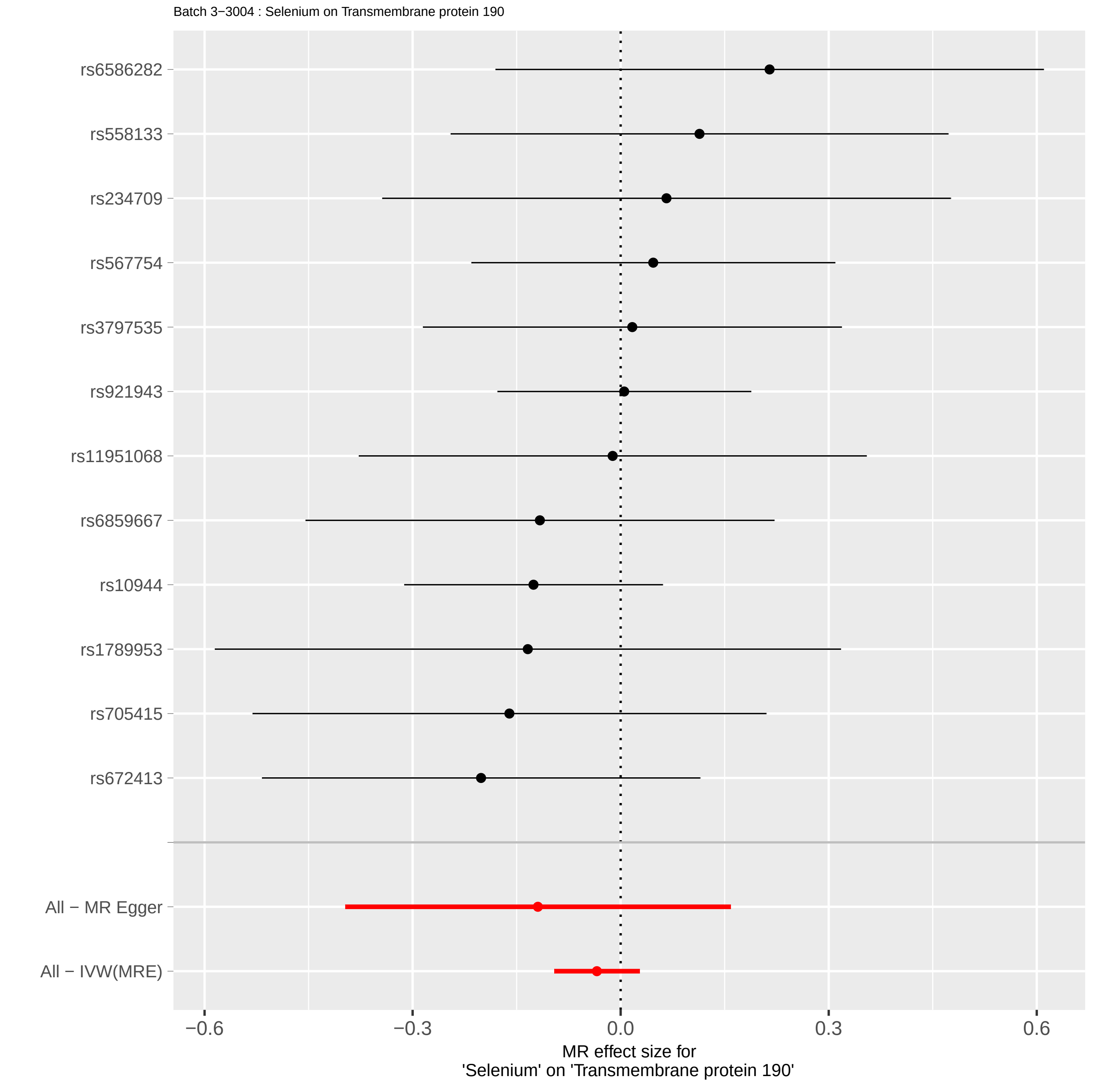

Batch 3−3004 : Selenium on Transmembrane protein 190
rs6586282
rs558133
rs234709
rs567754
rs3797535
rs921943
rs11951068
rs6859667
rs10944
rs1789953
rs705415
rs672413
All − MR Egger
All − IVW(MRE)
−0.6
−0.3
0.0
0.3
0.6
MR effect size for
'Selenium' on 'Transmembrane protein 190'

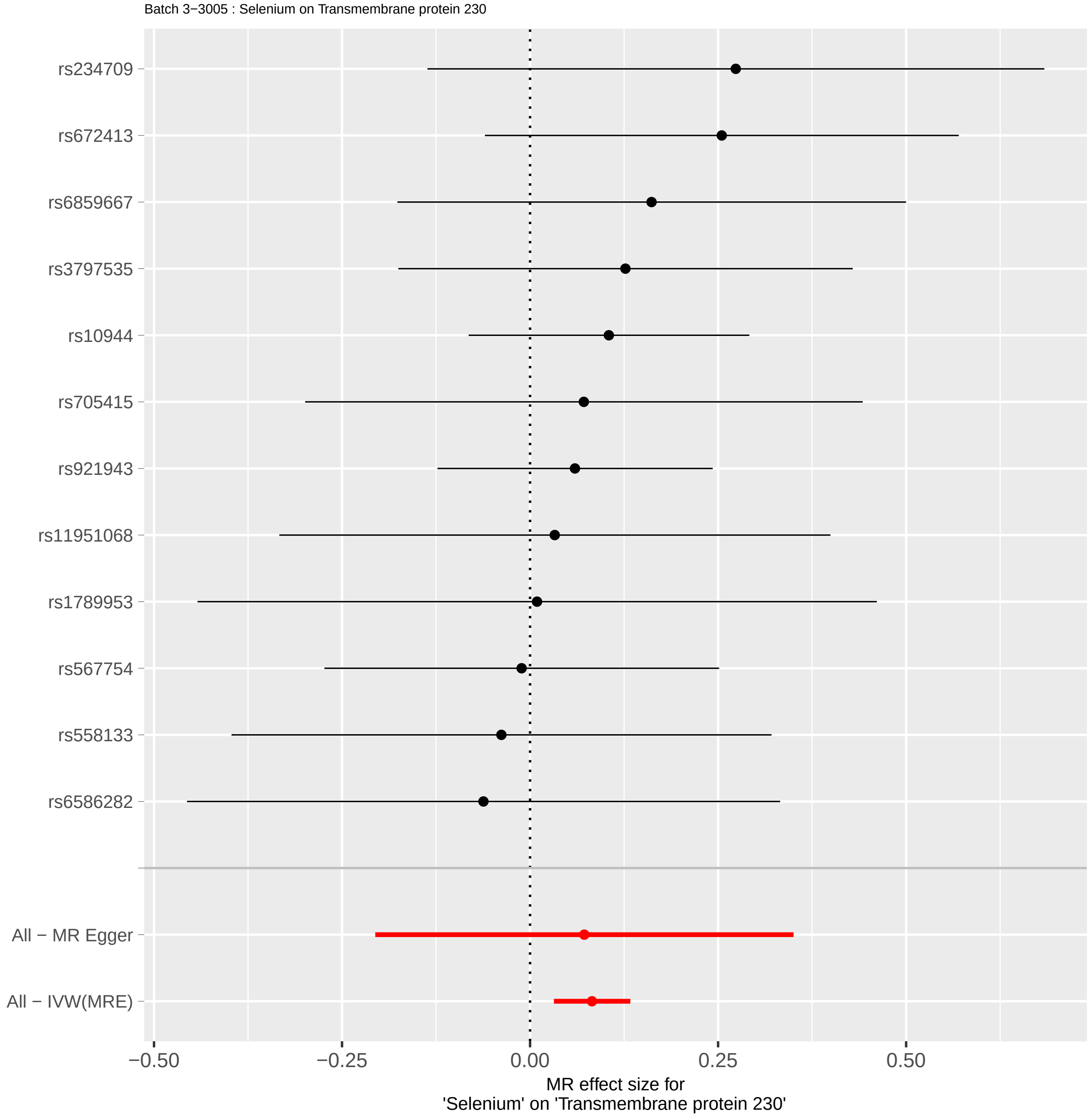

Batch 3−3005 : Selenium on Transmembrane protein 230
rs234709
rs672413
rs6859667
rs3797535
rs10944
rs705415
rs921943
rs11951068
rs1789953
rs567754
rs558133
rs6586282
All − MR Egger
All − IVW(MRE)
−0.50
−0.25
0.00
0.25
0.50
MR effect size for
'Selenium' on 'Transmembrane protein 230'

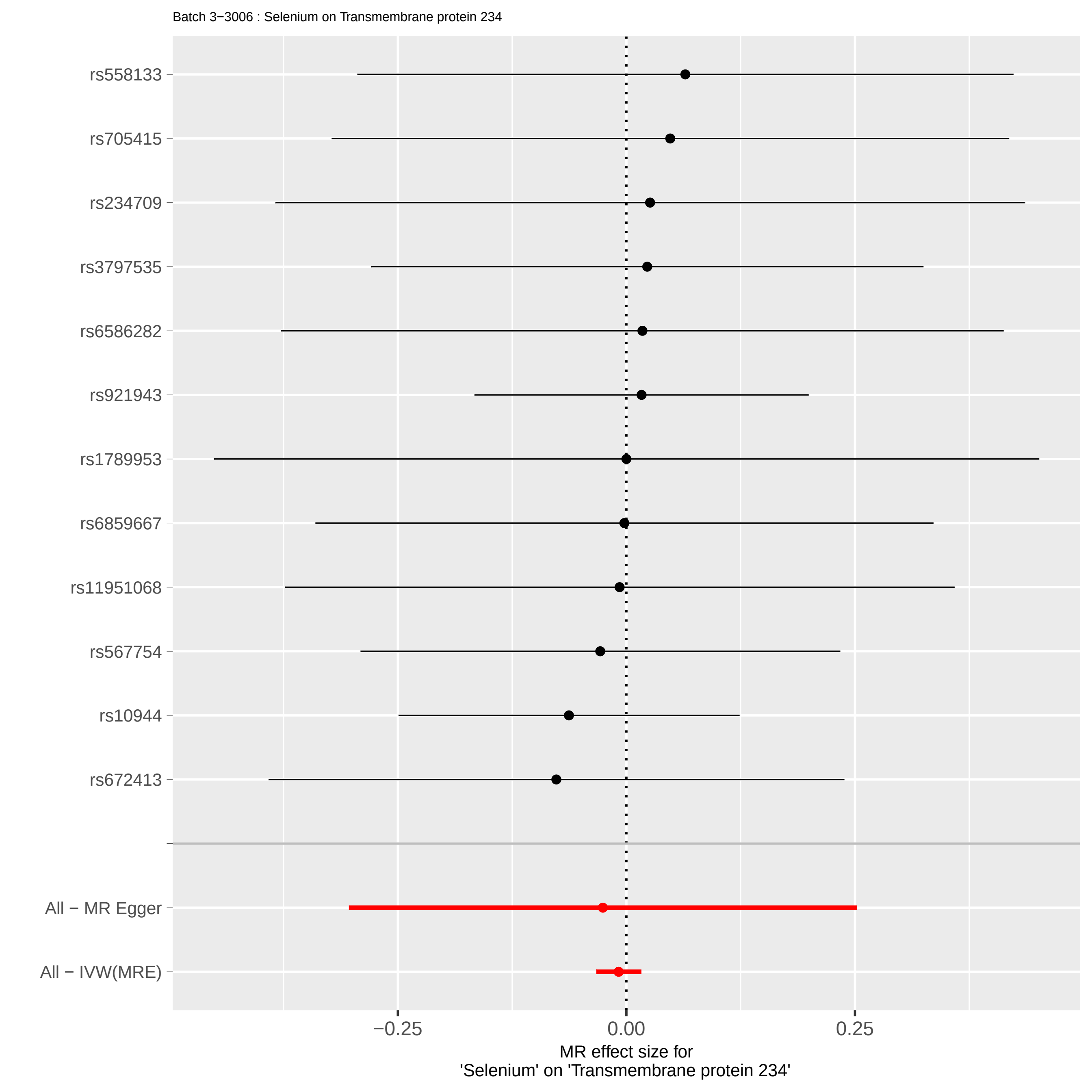

Batch 3−3006 : Selenium on Transmembrane protein 234
rs558133
rs705415
rs234709
rs3797535
rs6586282
rs921943
rs1789953
rs6859667
rs11951068
rs567754
rs10944
rs672413
All − MR Egger
All − IVW(MRE)
−0.25
0.00
0.25
MR effect size for
'Selenium' on 'Transmembrane protein 234'

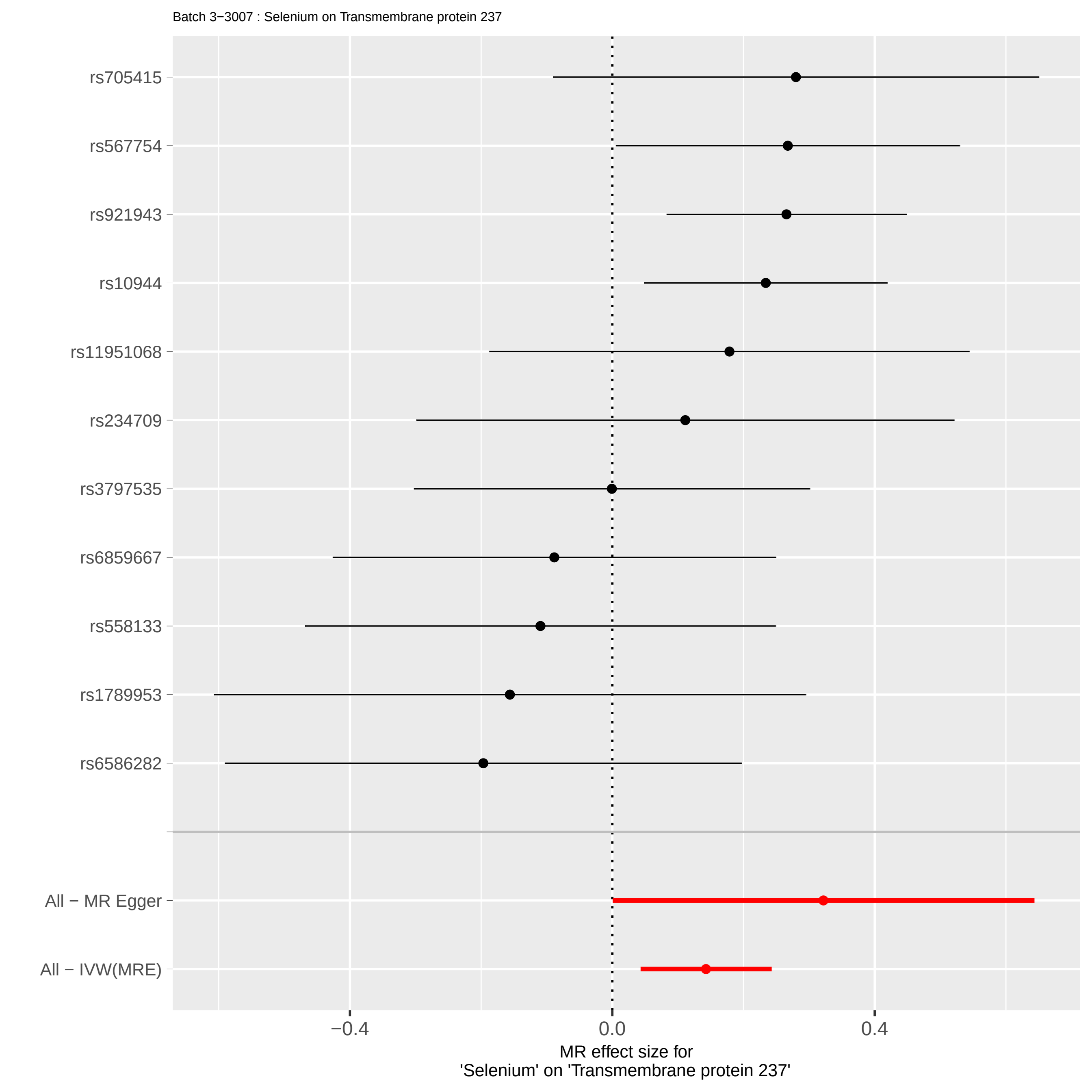

Batch 3−3007 : Selenium on Transmembrane protein 237
rs705415
rs567754
rs921943
rs10944
rs11951068
rs234709
rs3797535
rs6859667
rs558133
rs1789953
rs6586282
All − MR Egger
All − IVW(MRE)
−0.4
0.0
0.4
MR effect size for
'Selenium' on 'Transmembrane protein 237'

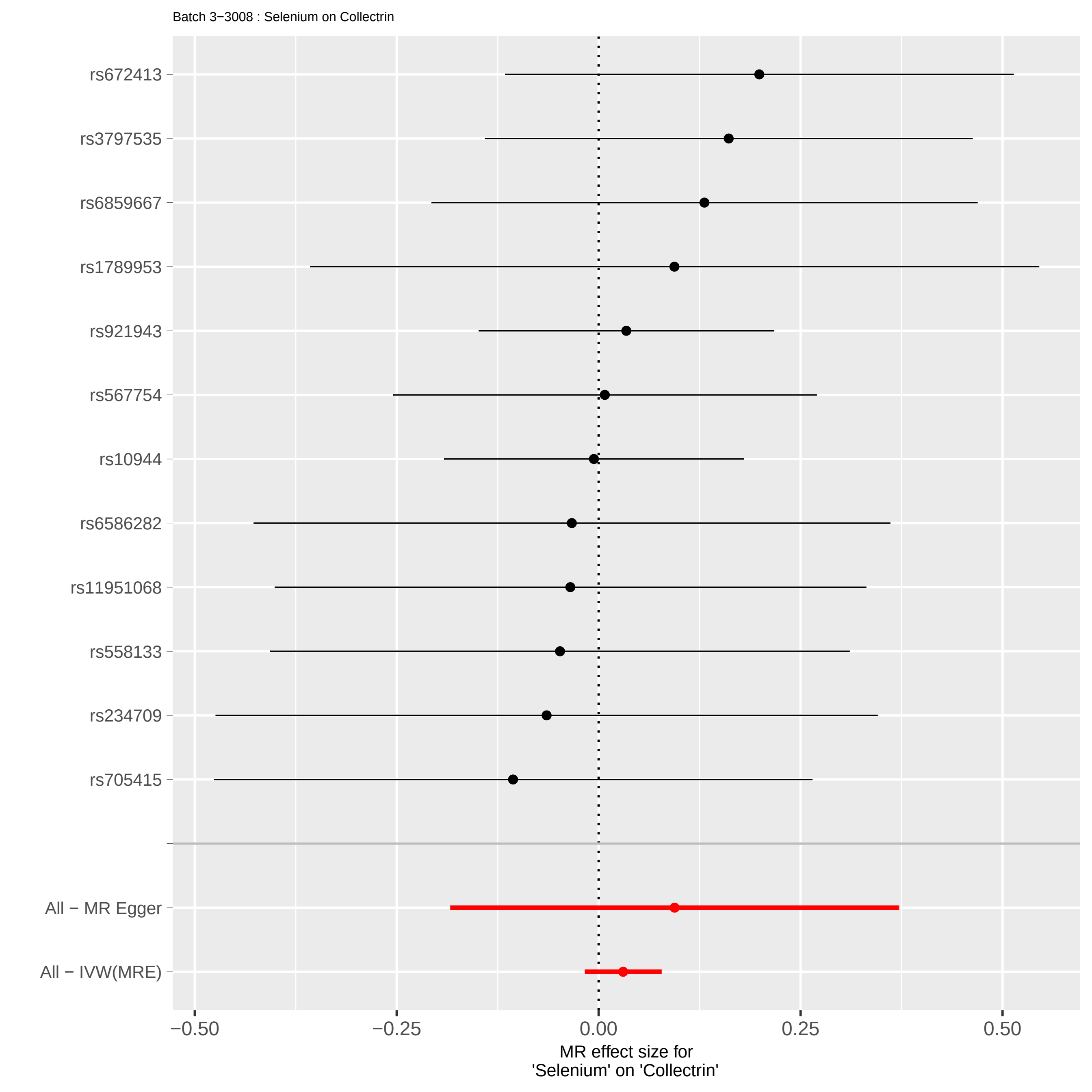
Batch 3−3008 : Selenium on Collectrin
rs672413
rs3797535
rs6859667
rs1789953
rs921943
rs567754
rs10944
rs6586282
rs11951068
rs558133
rs234709
rs705415
All − MR Egger
All − IVW(MRE)
−0.50
−0.25
0.00
0.25
0.50
MR effect size for
'Selenium' on 'Collectrin'

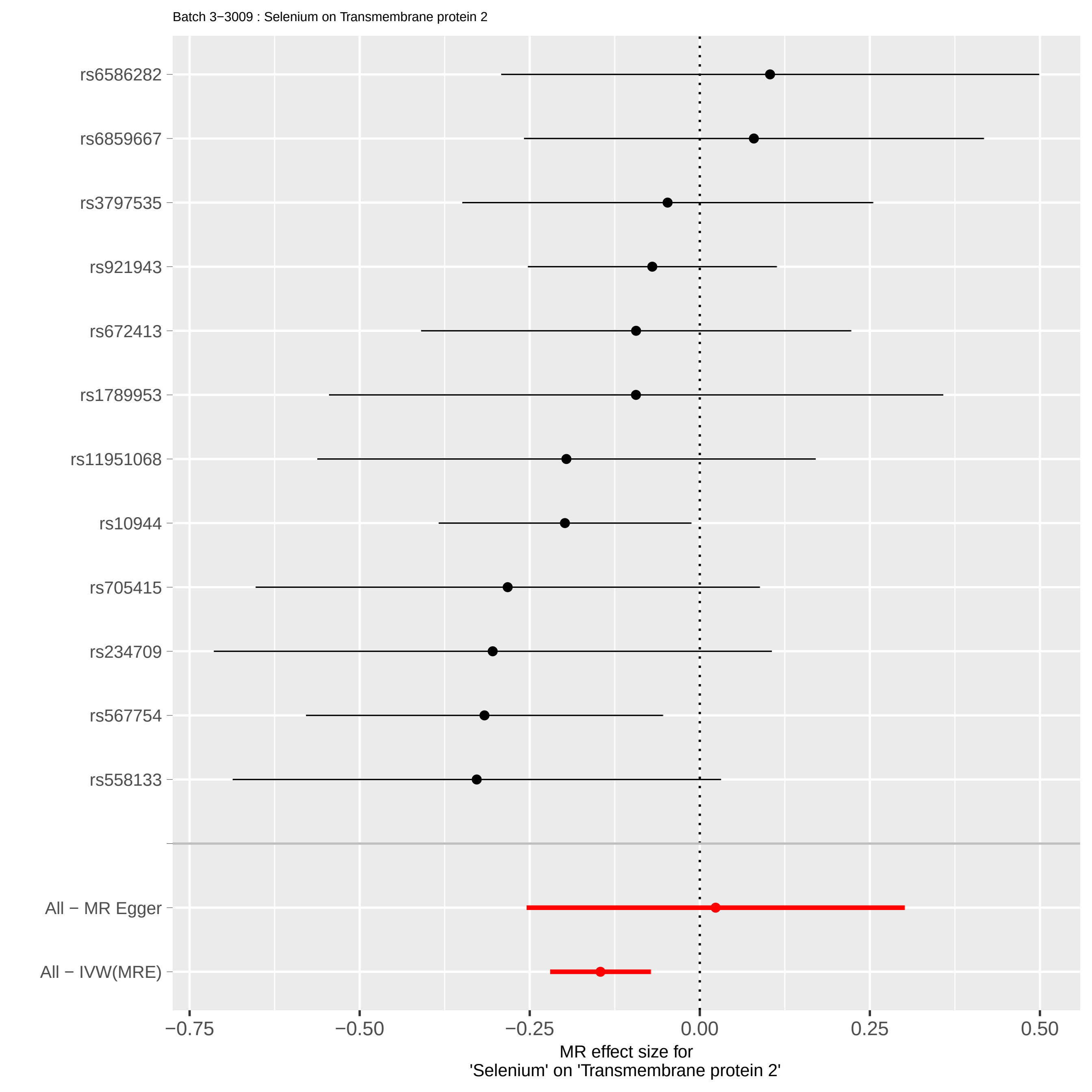

Batch 3−3009 : Selenium on Transmembrane protein 2
rs6586282
rs6859667
rs3797535
rs921943
rs672413
rs1789953
rs11951068
rs10944
rs705415
rs234709
rs567754
rs558133
All − MR Egger
All − IVW(MRE)
−0.75
−0.50
−0.25
0.00
0.25
0.50
MR effect size for
'Selenium' on 'Transmembrane protein 2'

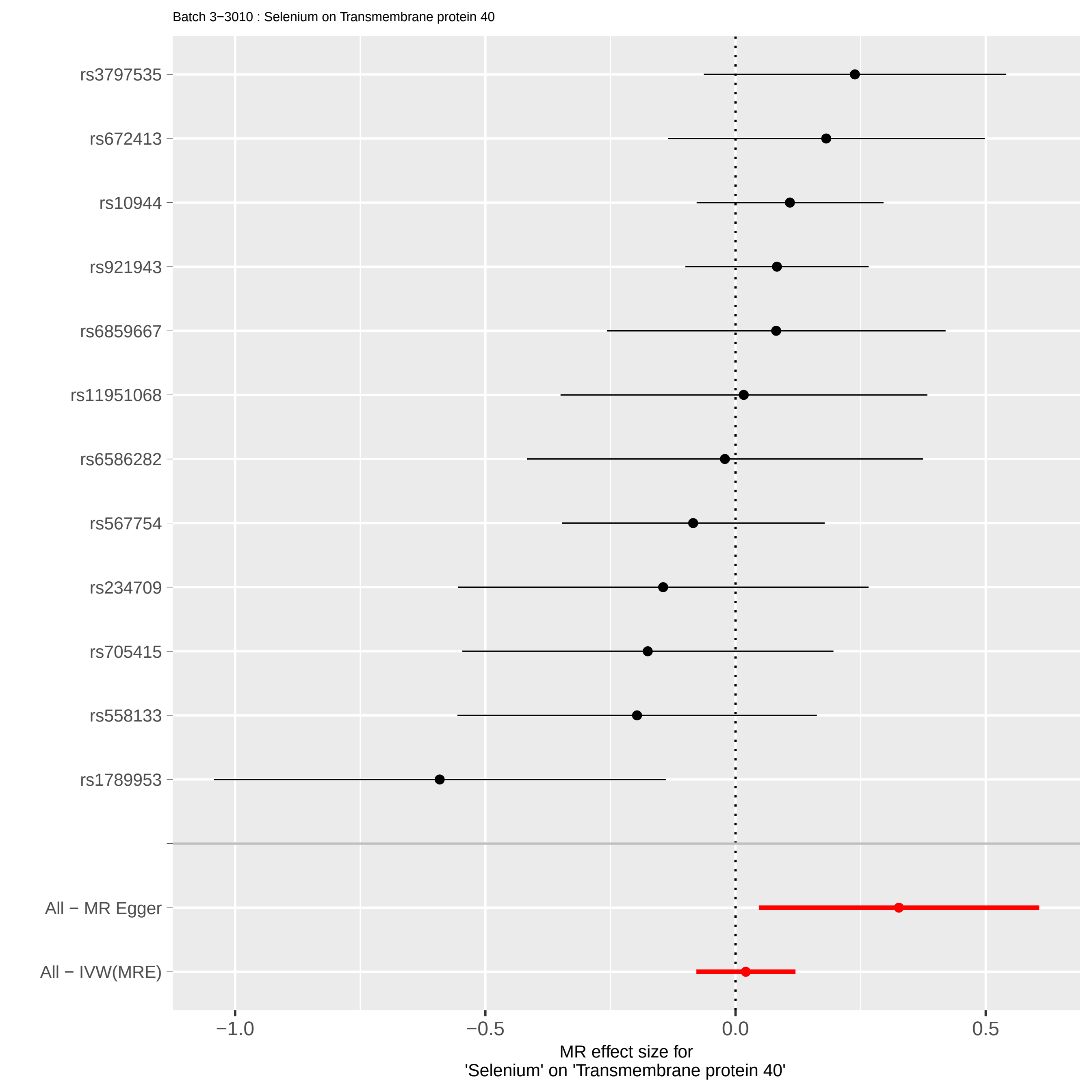

Batch 3−3010 : Selenium on Transmembrane protein 40
rs3797535
rs672413
rs10944
rs921943
rs6859667
rs11951068
rs6586282
rs567754
rs234709
rs705415
rs558133
rs1789953
All − MR Egger
All − IVW(MRE)
−1.0
−0.5
0.0
0.5
MR effect size for
'Selenium' on 'Transmembrane protein 40'

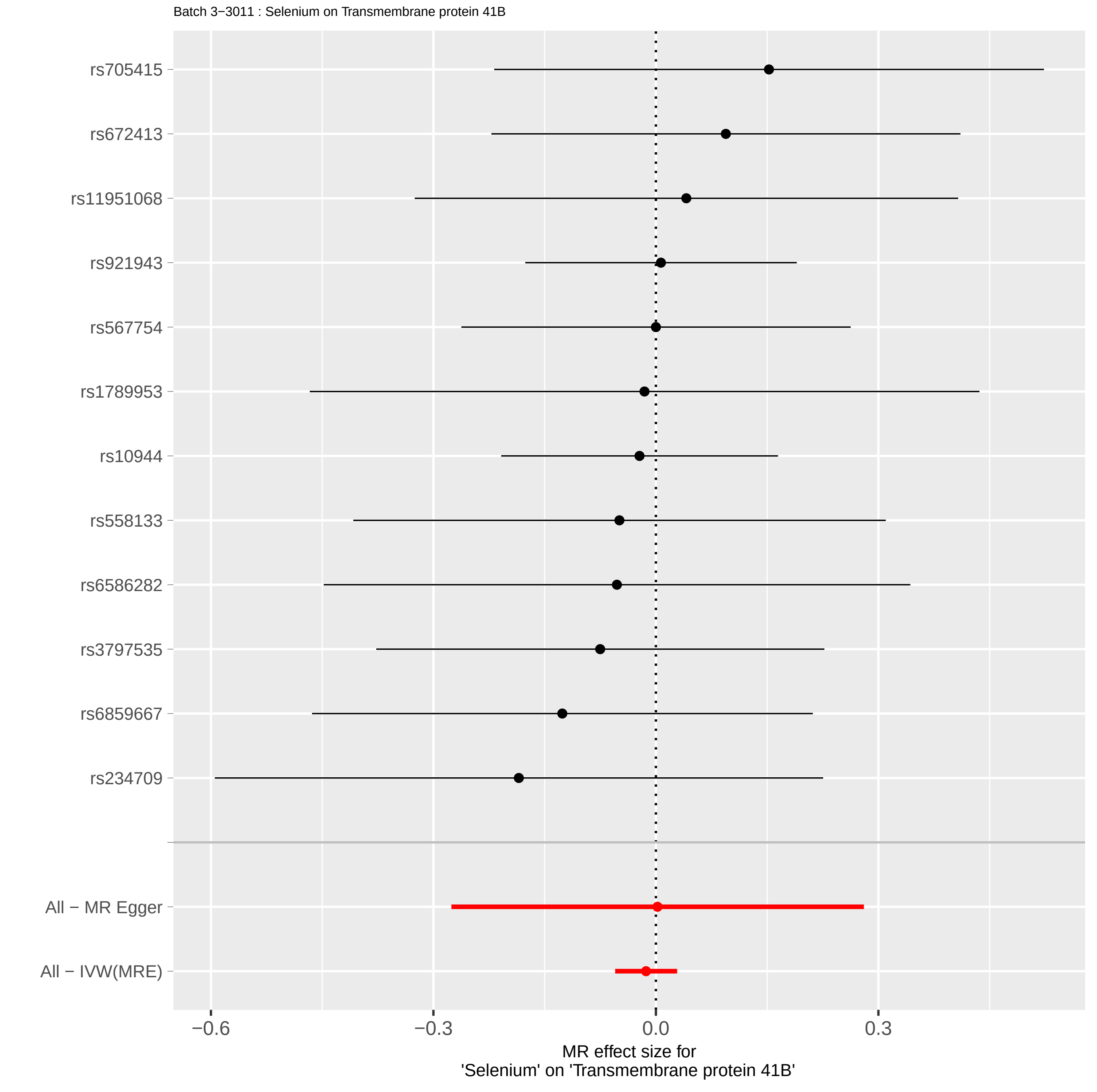

Batch 3−3011 : Selenium on Transmembrane protein 41B
rs705415
rs672413
rs11951068
rs921943
rs567754
rs1789953
rs10944
rs558133
rs6586282
rs3797535
rs6859667
rs234709
All − MR Egger
All − IVW(MRE)
−0.6
−0.3
0.0
0.3
MR effect size for
'Selenium' on 'Transmembrane protein 41B'

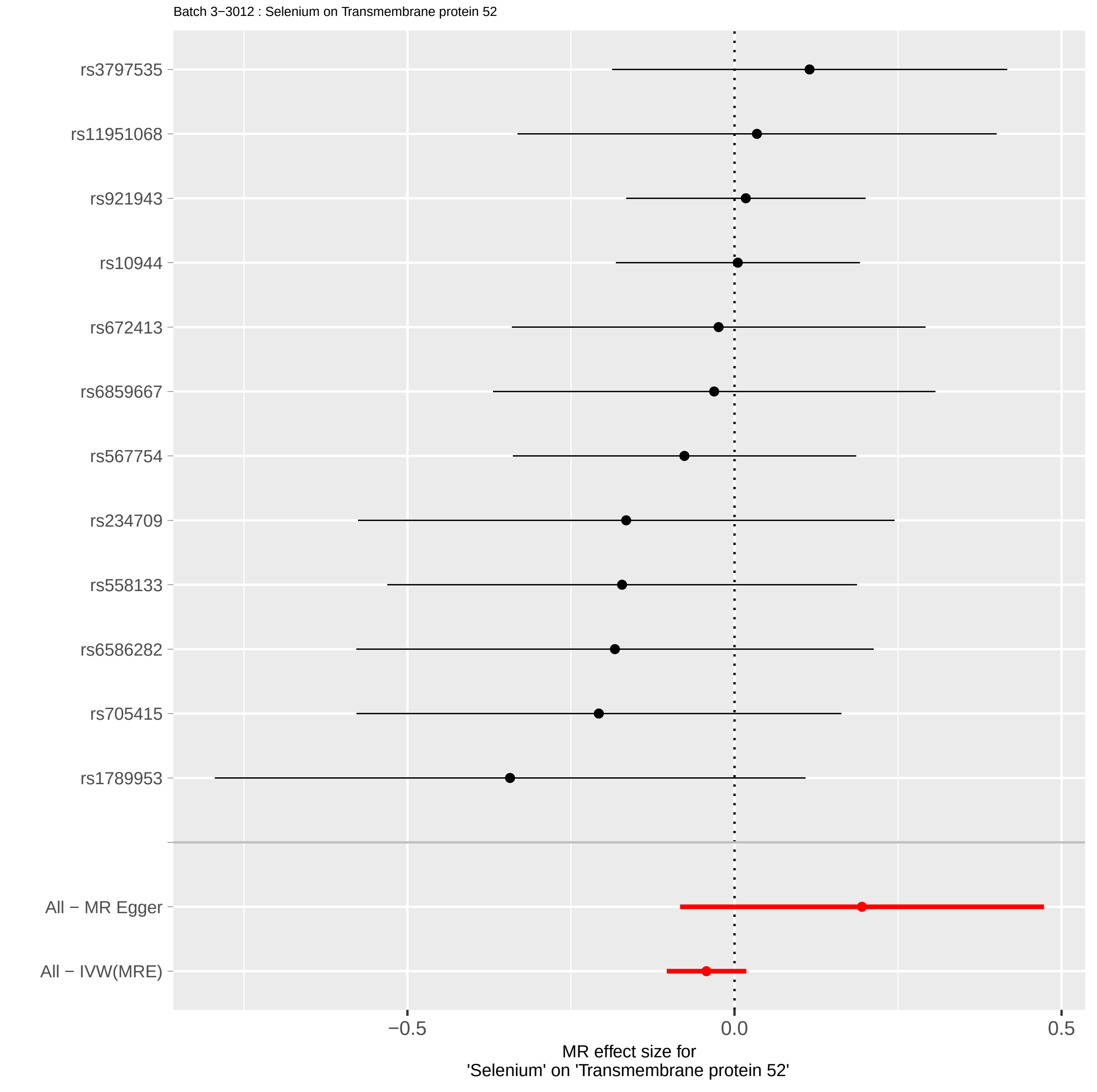

Batch 3−3012 : Selenium on Transmembrane protein 52
rs3797535
rs11951068
rs921943
rs10944
rs672413
rs6859667
rs567754
rs234709
rs558133
rs6586282
rs705415
rs1789953
All − MR Egger
All − IVW(MRE)
−0.5
0.0
0.5
MR effect size for
'Selenium' on 'Transmembrane protein 52'

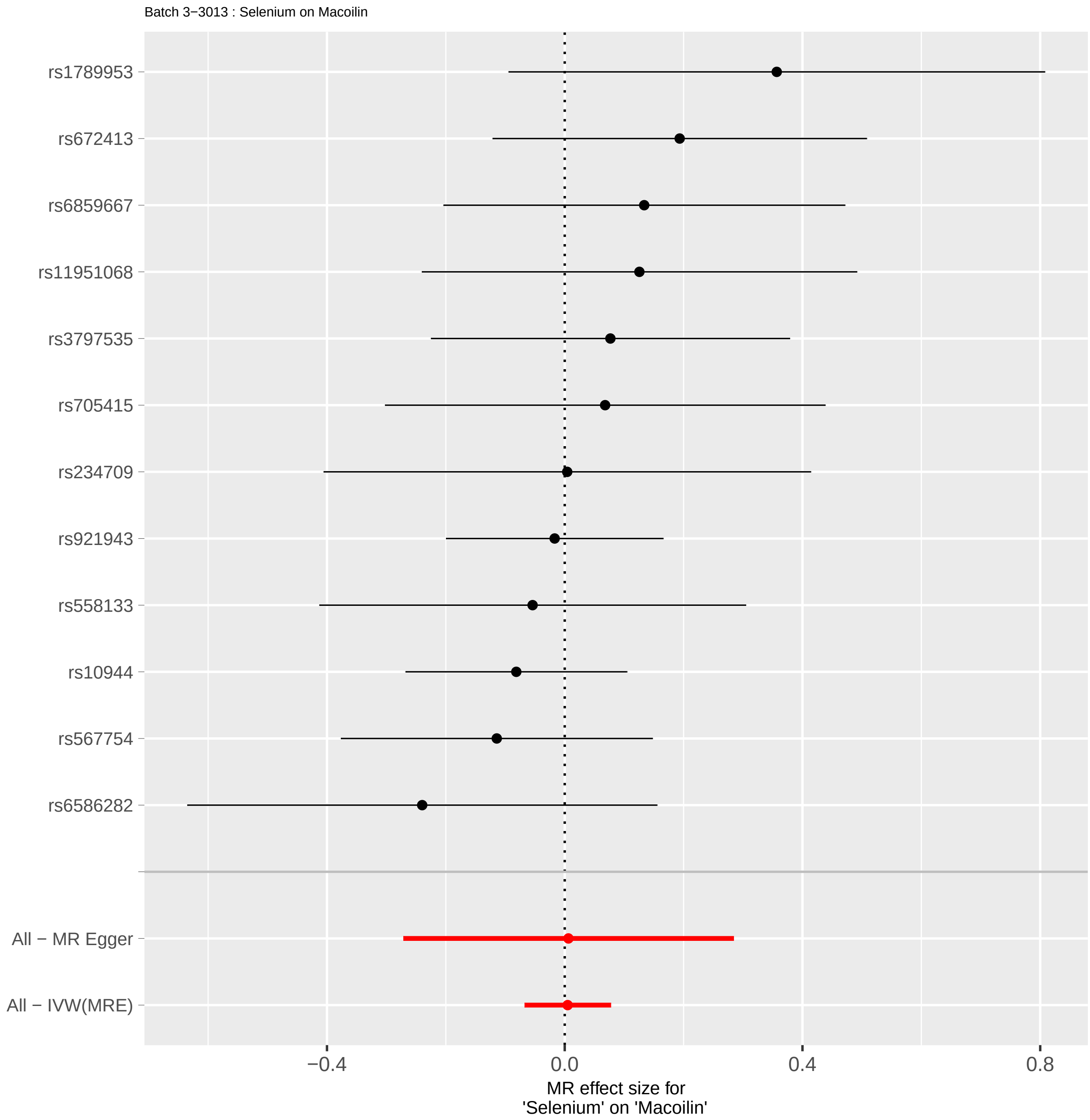
Batch 3−3013 : Selenium on Macoilin
rs1789953
rs672413
rs6859667
rs11951068
rs3797535
rs705415
rs234709
rs921943
rs558133
rs10944
rs567754
rs6586282
All − MR Egger
All − IVW(MRE)
−0.4
0.0
0.4
0.8
MR effect size for
'Selenium' on 'Macoilin'

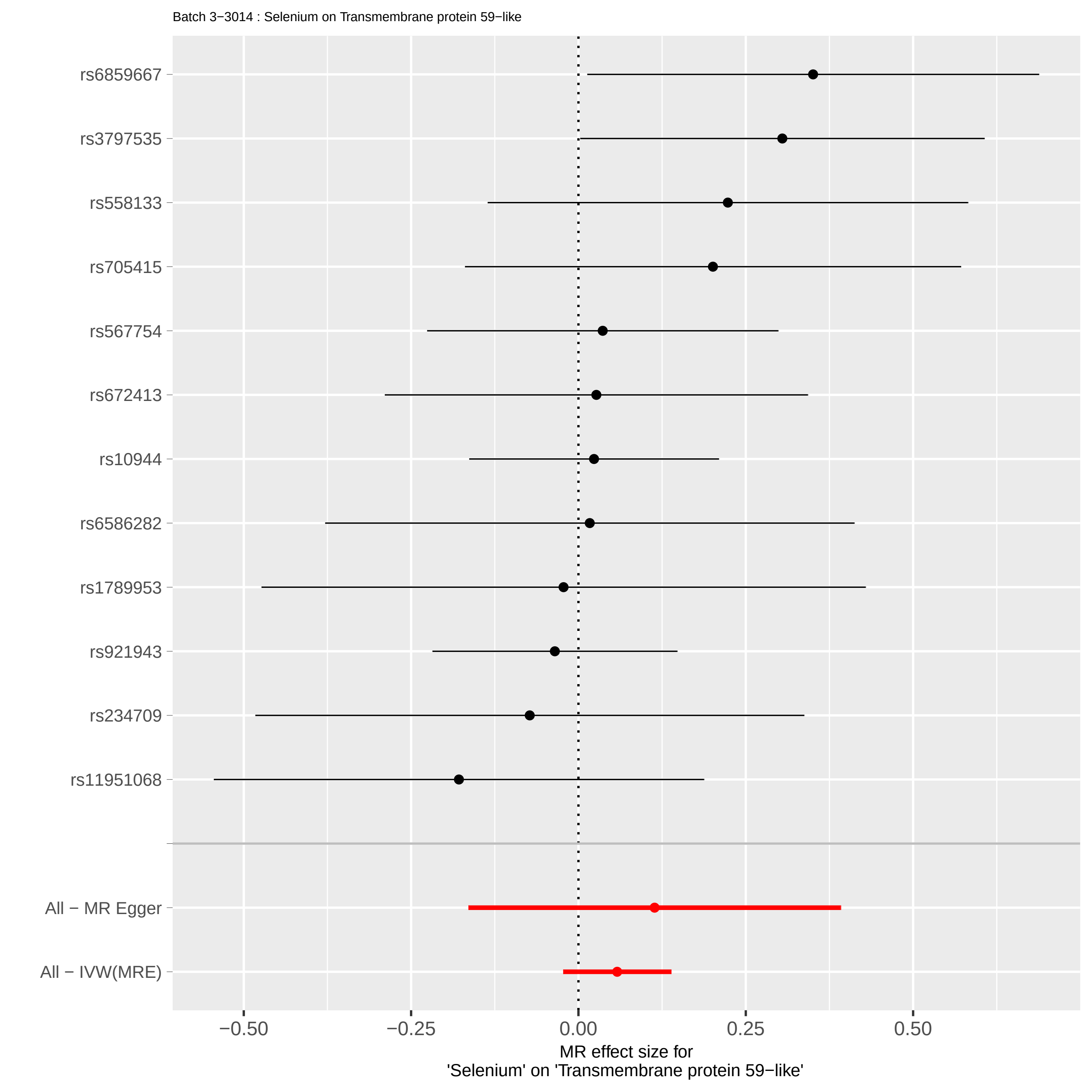

Batch 3−3014 : Selenium on Transmembrane protein 59−like
rs6859667
rs3797535
rs558133
rs705415
rs567754
rs672413
rs10944
rs6586282
rs1789953
rs921943
rs234709
rs11951068
All − MR Egger
All − IVW(MRE)
−0.50
−0.25
0.00
0.25
0.50
MR effect size for
'Selenium' on 'Transmembrane protein 59−like'

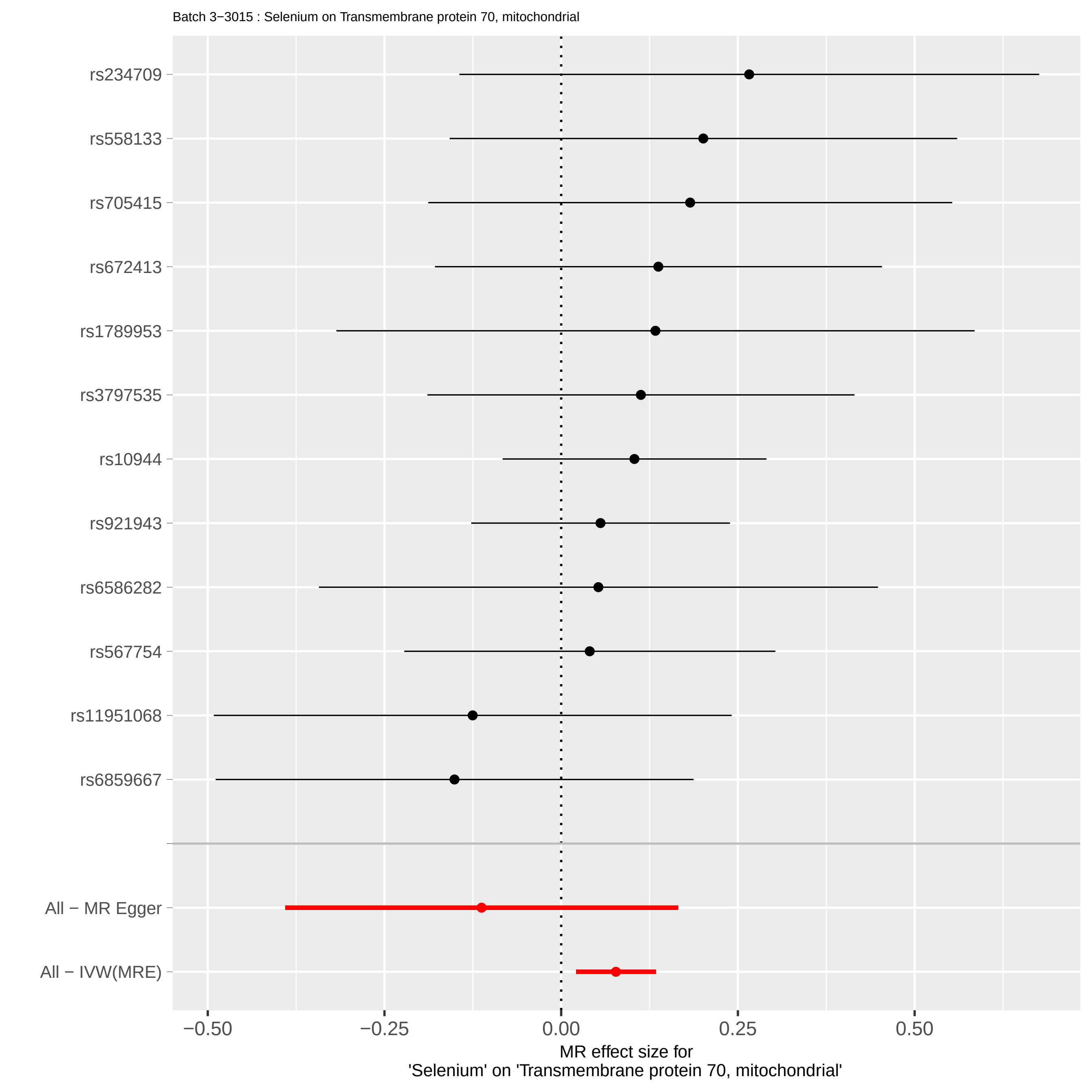
Batch 3−3015 : Selenium on Transmembrane protein 70, mitochondrial
rs234709
rs558133
rs705415
rs672413
rs1789953
rs3797535
rs10944
rs921943
rs6586282
rs567754
rs11951068
rs6859667
All − MR Egger
All − IVW(MRE)
−0.50
−0.25
0.00
0.25
0.50
MR effect size for
'Selenium' on 'Transmembrane protein 70, mitochondrial'

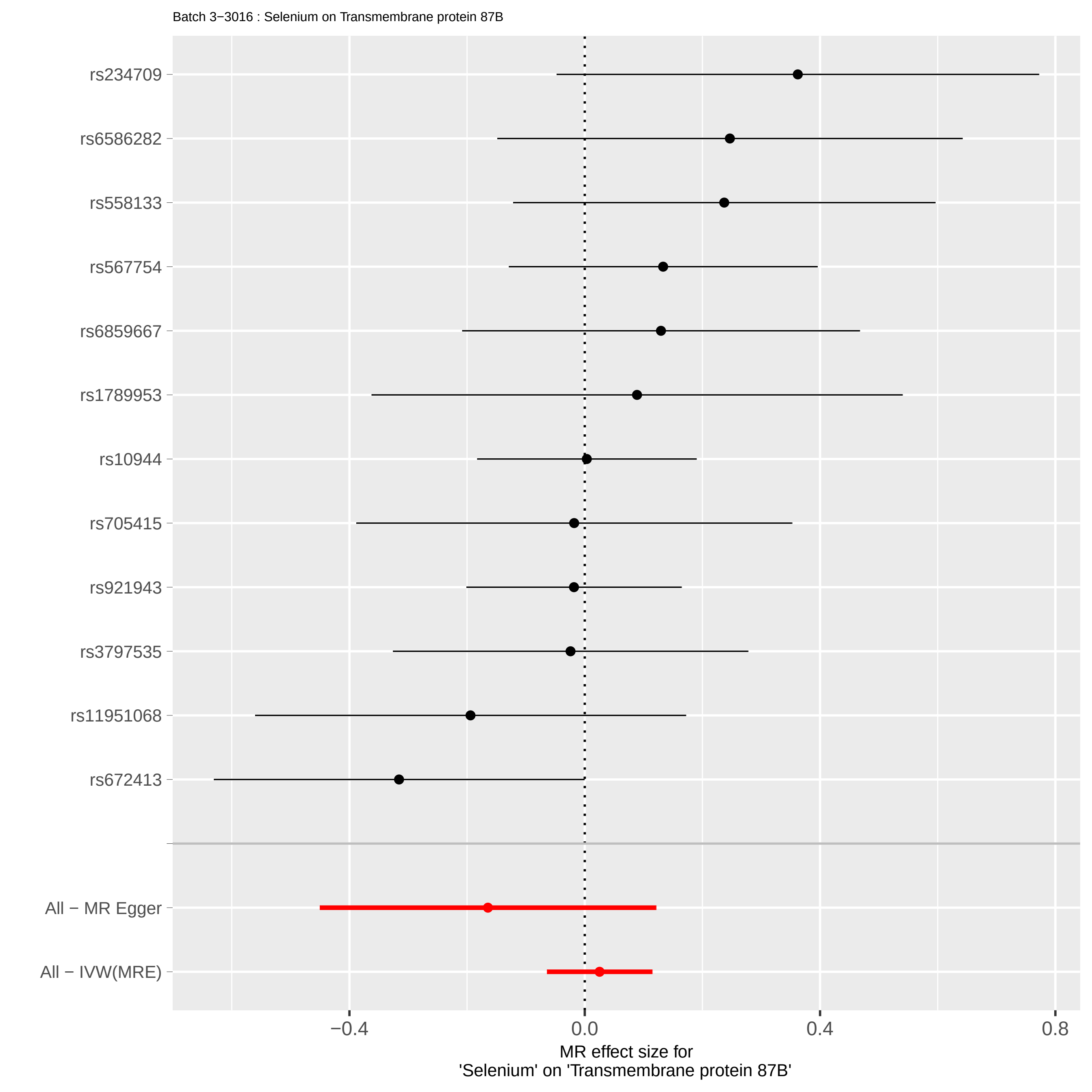

Batch 3−3016 : Selenium on Transmembrane protein 87B
rs234709
rs6586282
rs558133
rs567754
rs6859667
rs1789953
rs10944
rs705415
rs921943
rs3797535
rs11951068
rs672413
All − MR Egger
All − IVW(MRE)
−0.4
0.0
0.4
0.8
MR effect size for
'Selenium' on 'Transmembrane protein 87B'

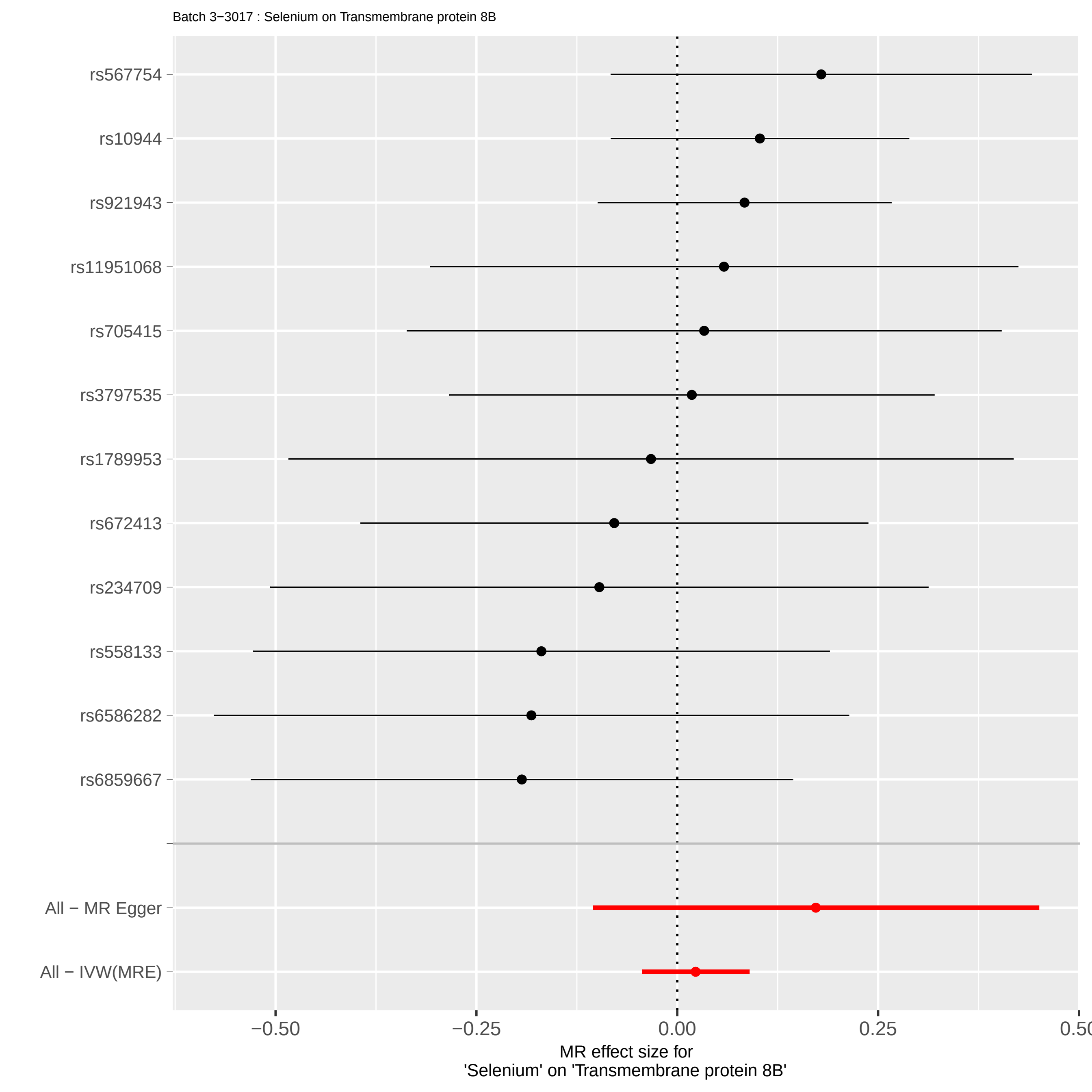
Batch 3−3017 : Selenium on Transmembrane protein 8B
rs567754
rs10944
rs921943
rs11951068
rs705415
rs3797535
rs1789953
rs672413
rs234709
rs558133
rs6586282
rs6859667
All − MR Egger
All − IVW(MRE)
−0.50
−0.25
0.00
0.25
0.50
MR effect size for
'Selenium' on 'Transmembrane protein 8B'

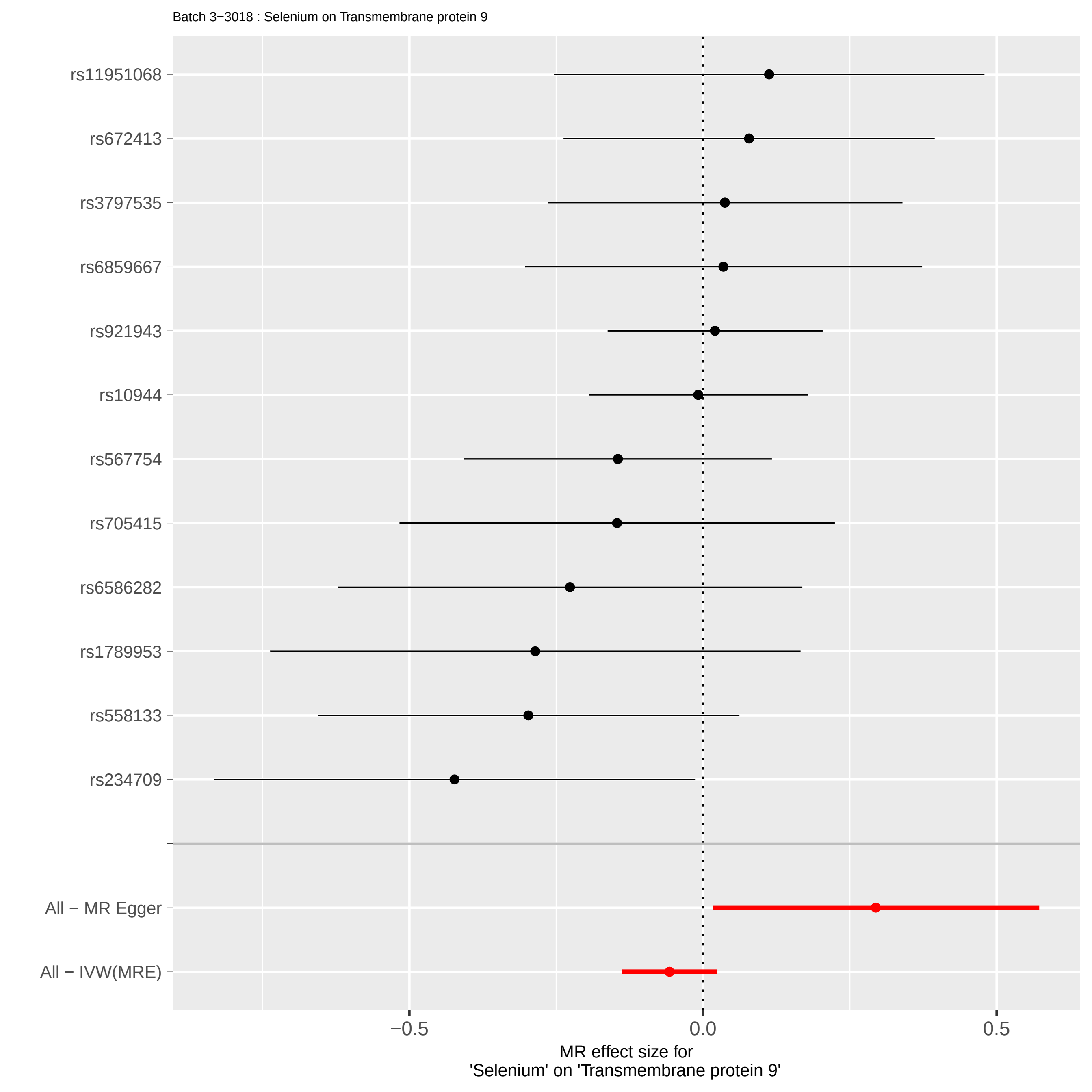
Batch 3−3018 : Selenium on Transmembrane protein 9
rs11951068
rs672413
rs3797535
rs6859667
rs921943
rs10944
rs567754
rs705415
rs6586282
rs1789953
rs558133
rs234709
All − MR Egger
All − IVW(MRE)
−0.5
0.0
0.5
MR effect size for
'Selenium' on 'Transmembrane protein 9'

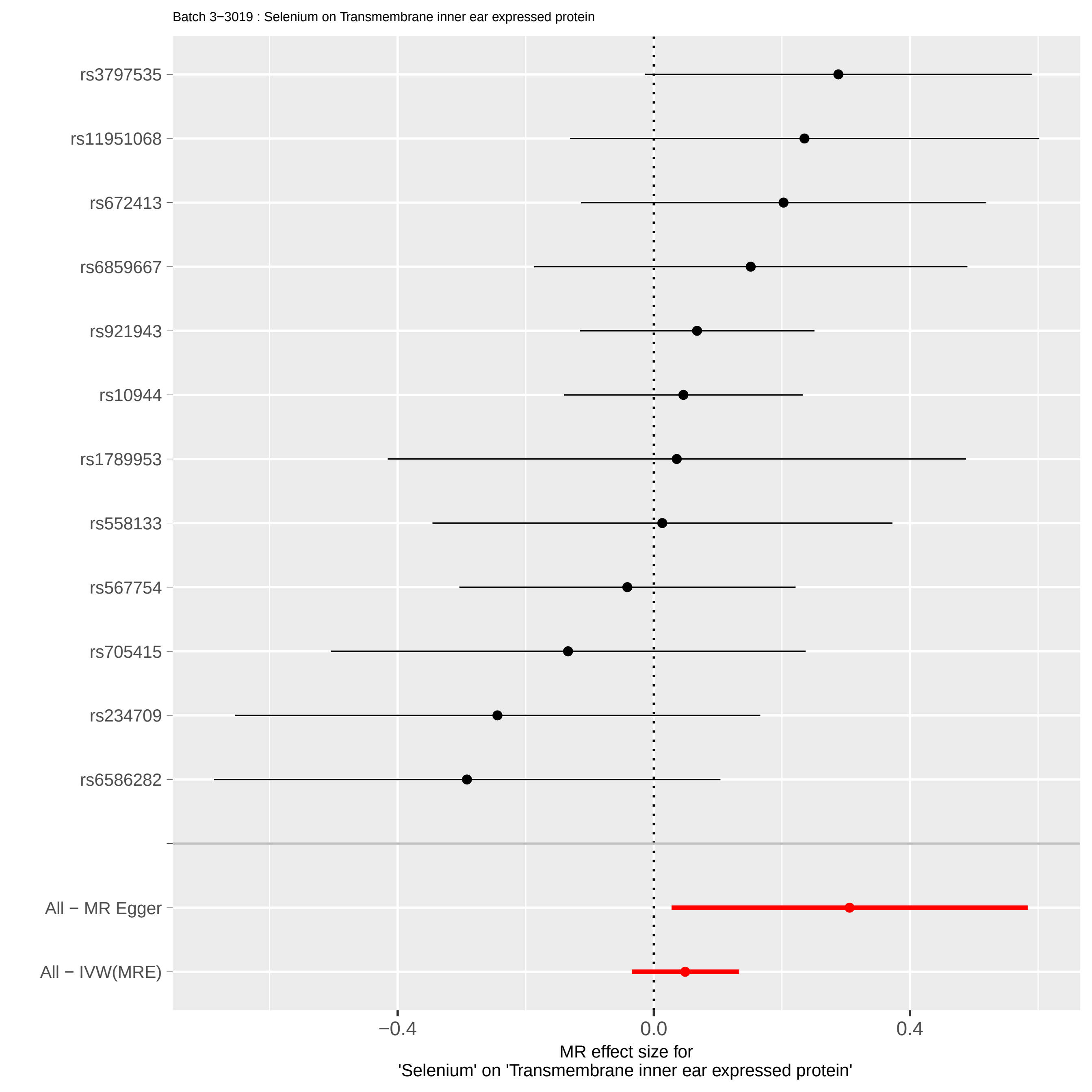

Batch 3−3019 : Selenium on Transmembrane inner ear expressed protein
rs3797535
rs11951068
rs672413
rs6859667
rs921943
rs10944
rs1789953
rs558133
rs567754
rs705415
rs234709
rs6586282
All − MR Egger
All − IVW(MRE)
−0.4
0.0
0.4
MR effect size for
'Selenium' on 'Transmembrane inner ear expressed protein'

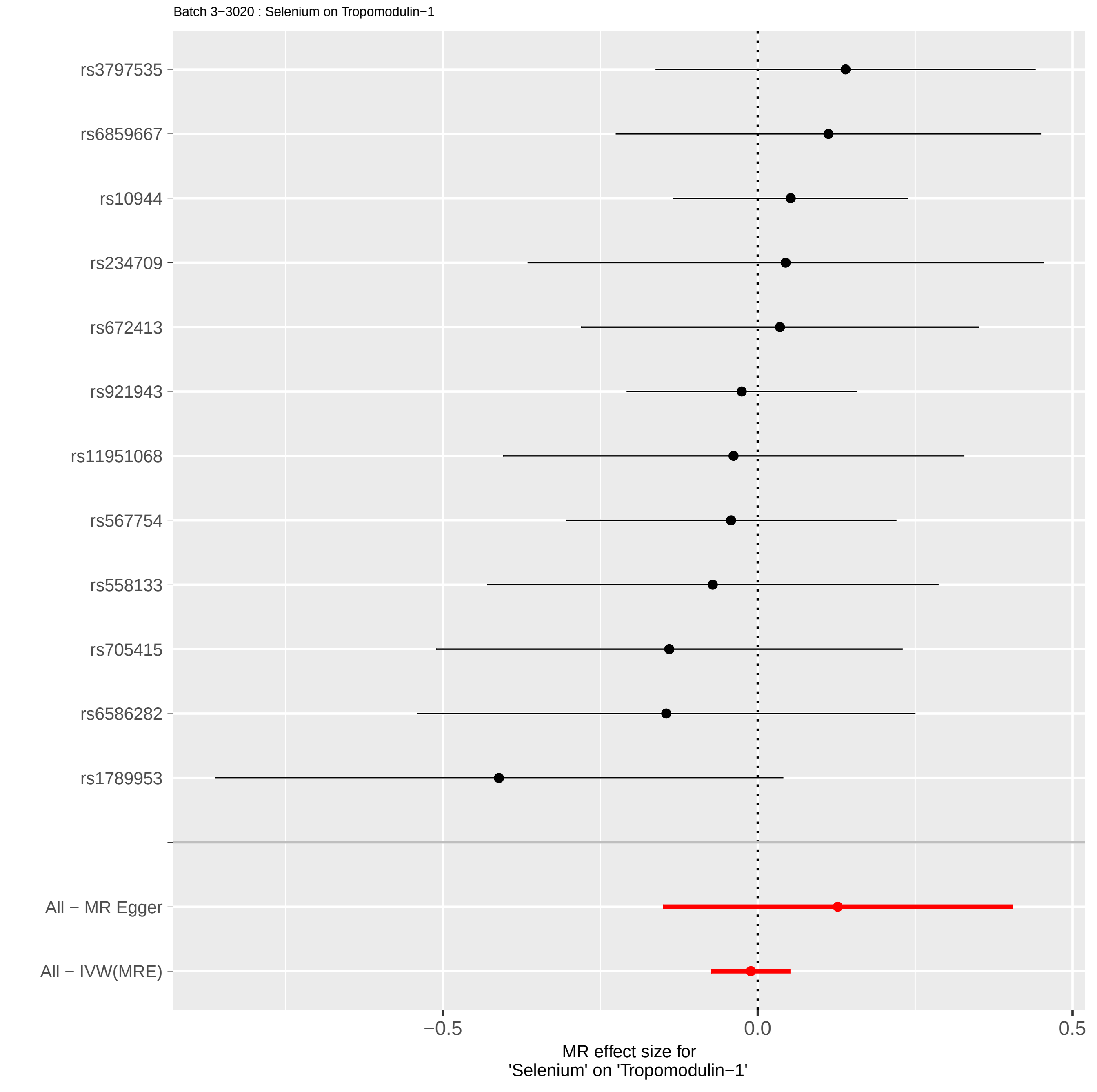
Batch 3−3020 : Selenium on Tropomodulin−1
rs3797535
rs6859667
rs10944
rs234709
rs672413
rs921943
rs11951068
rs567754
rs558133
rs705415
rs6586282
rs1789953
All − MR Egger
All − IVW(MRE)
−0.5
0.0
0.5
MR effect size for
'Selenium' on 'Tropomodulin−1'

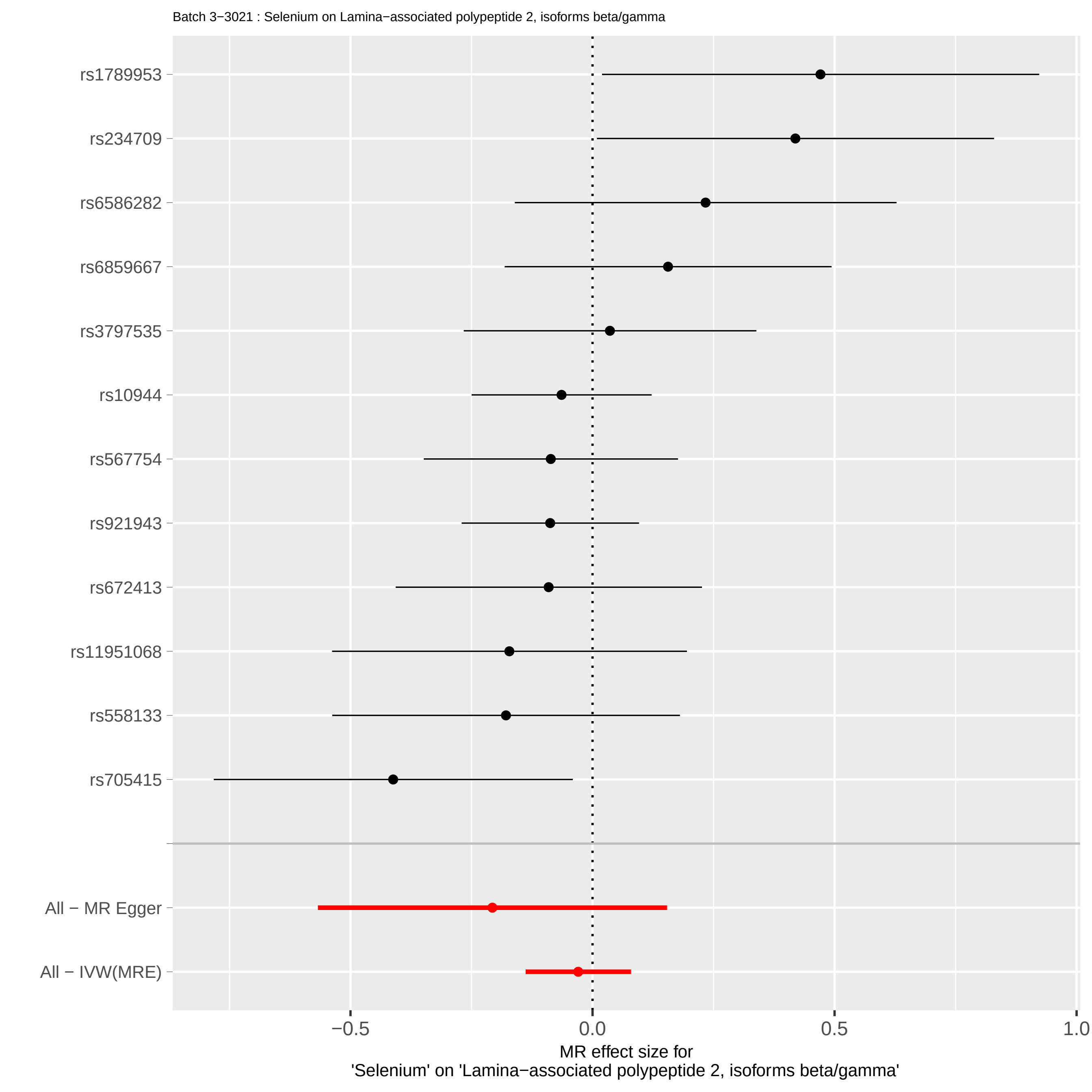
Batch 3−3021 : Selenium on Lamina−associated polypeptide 2, isoforms beta/gamma
rs1789953
rs234709
rs6586282
rs6859667
rs3797535
rs10944
rs567754
rs921943
rs672413
rs11951068
rs558133
rs705415
All − MR Egger
All − IVW(MRE)
−0.5
0.0
0.5
1.0
MR effect size for
'Selenium' on 'Lamina−associated polypeptide 2, isoforms beta/gamma'

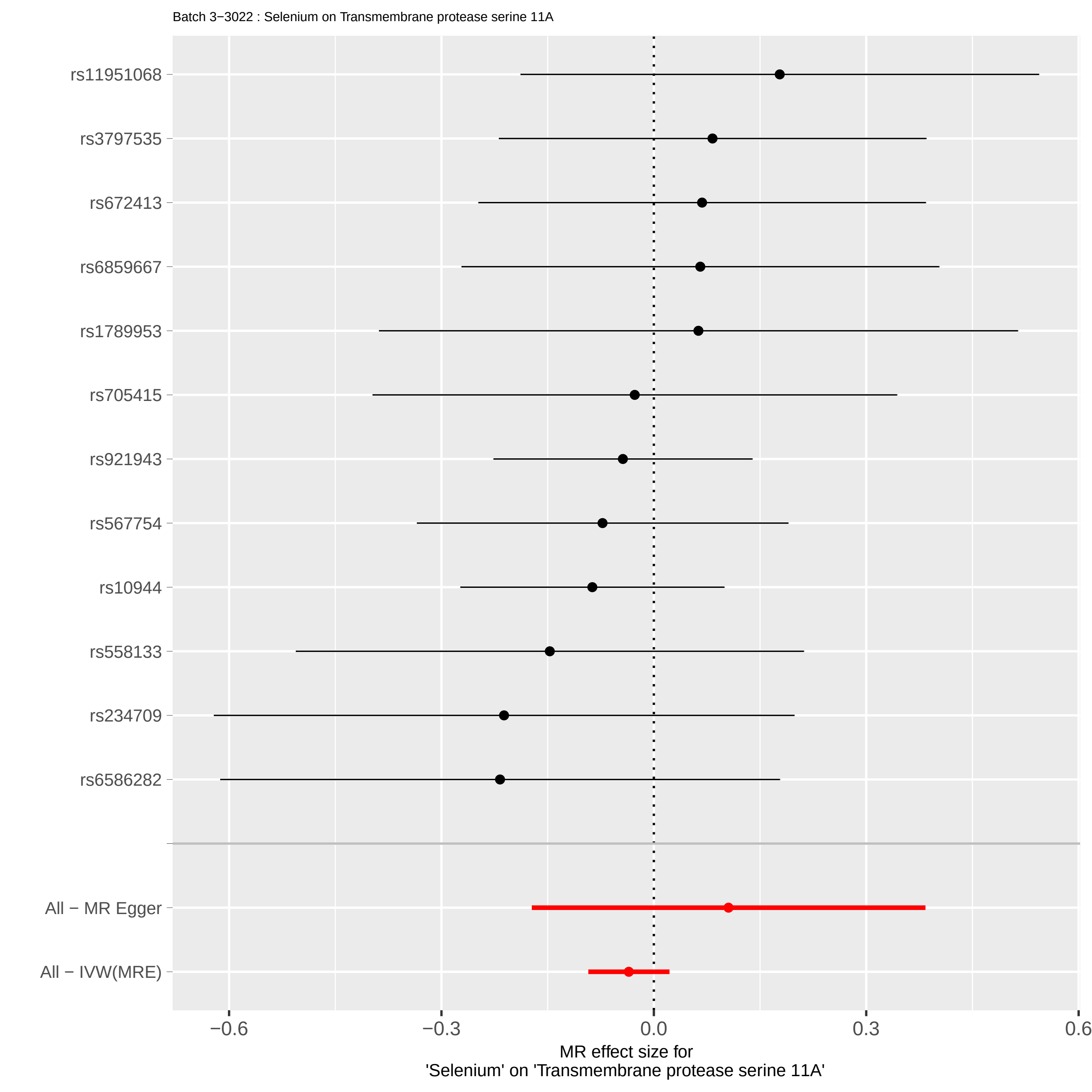

Batch 3−3022 : Selenium on Transmembrane protease serine 11A
rs11951068
rs3797535
rs672413
rs6859667
rs1789953
rs705415
rs921943
rs567754
rs10944
rs558133
rs234709
rs6586282
All − MR Egger
All − IVW(MRE)
−0.6
−0.3
0.0
0.3
0.6
MR effect size for
'Selenium' on 'Transmembrane protease serine 11A'

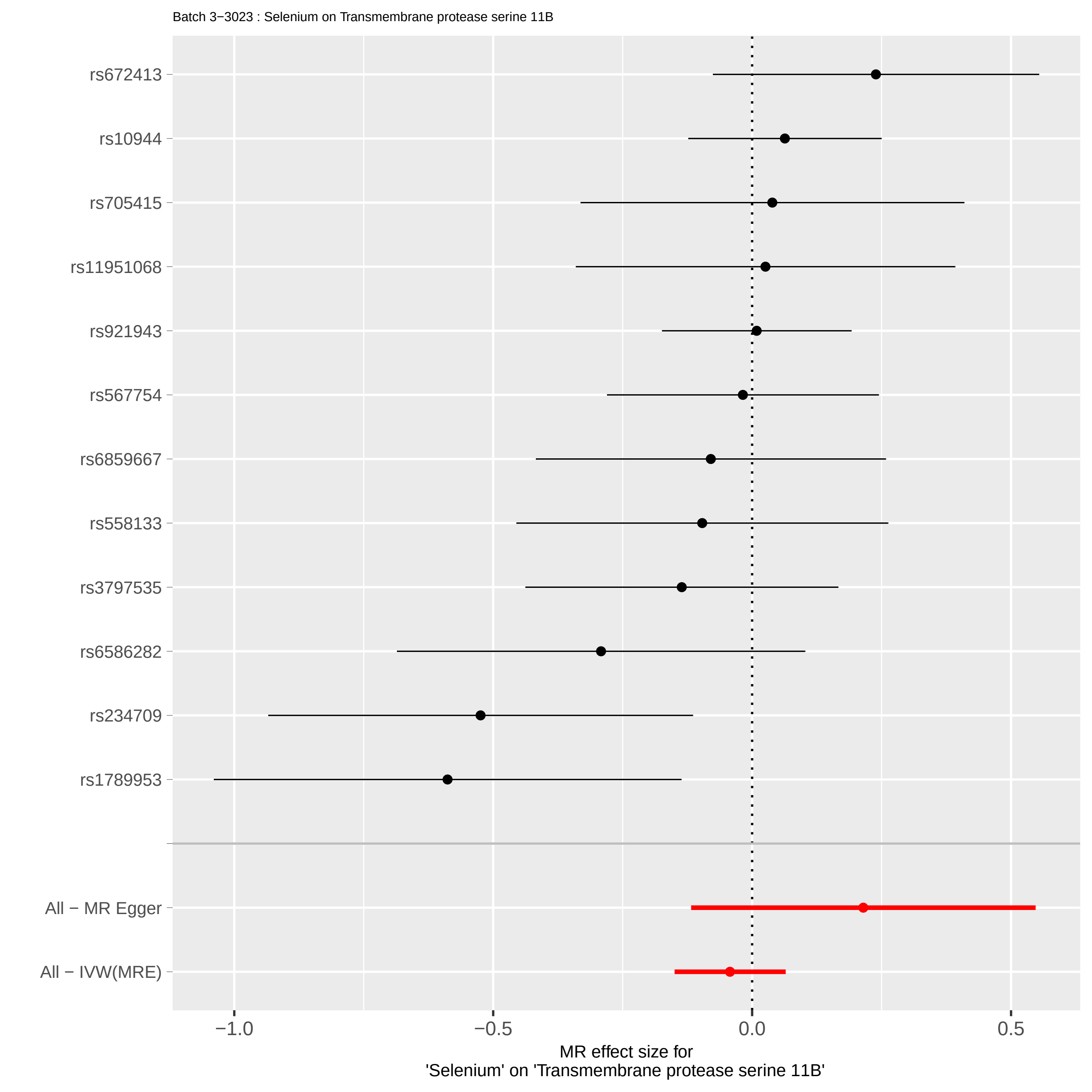

Batch 3−3023 : Selenium on Transmembrane protease serine 11B
rs672413
rs10944
rs705415
rs11951068
rs921943
rs567754
rs6859667
rs558133
rs3797535
rs6586282
rs234709
rs1789953
All − MR Egger
All − IVW(MRE)
−1.0
−0.5
0.0
0.5
MR effect size for
'Selenium' on 'Transmembrane protease serine 11B'

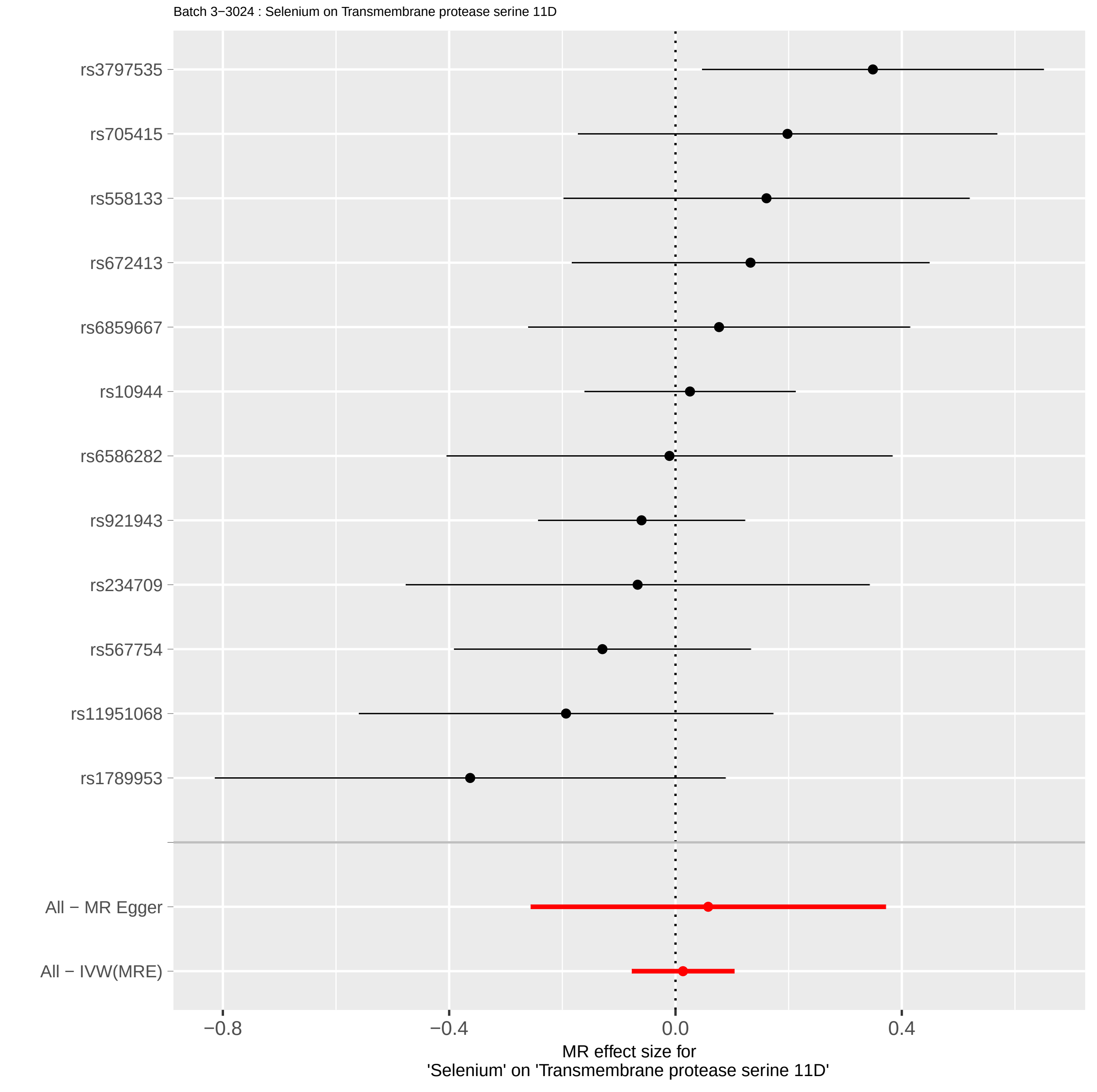

Batch 3−3024 : Selenium on Transmembrane protease serine 11D
rs3797535
rs705415
rs558133
rs672413
rs6859667
rs10944
rs6586282
rs921943
rs234709
rs567754
rs11951068
rs1789953
All − MR Egger
All − IVW(MRE)
−0.8
−0.4
0.0
0.4
MR effect size for
'Selenium' on 'Transmembrane protease serine 11D'

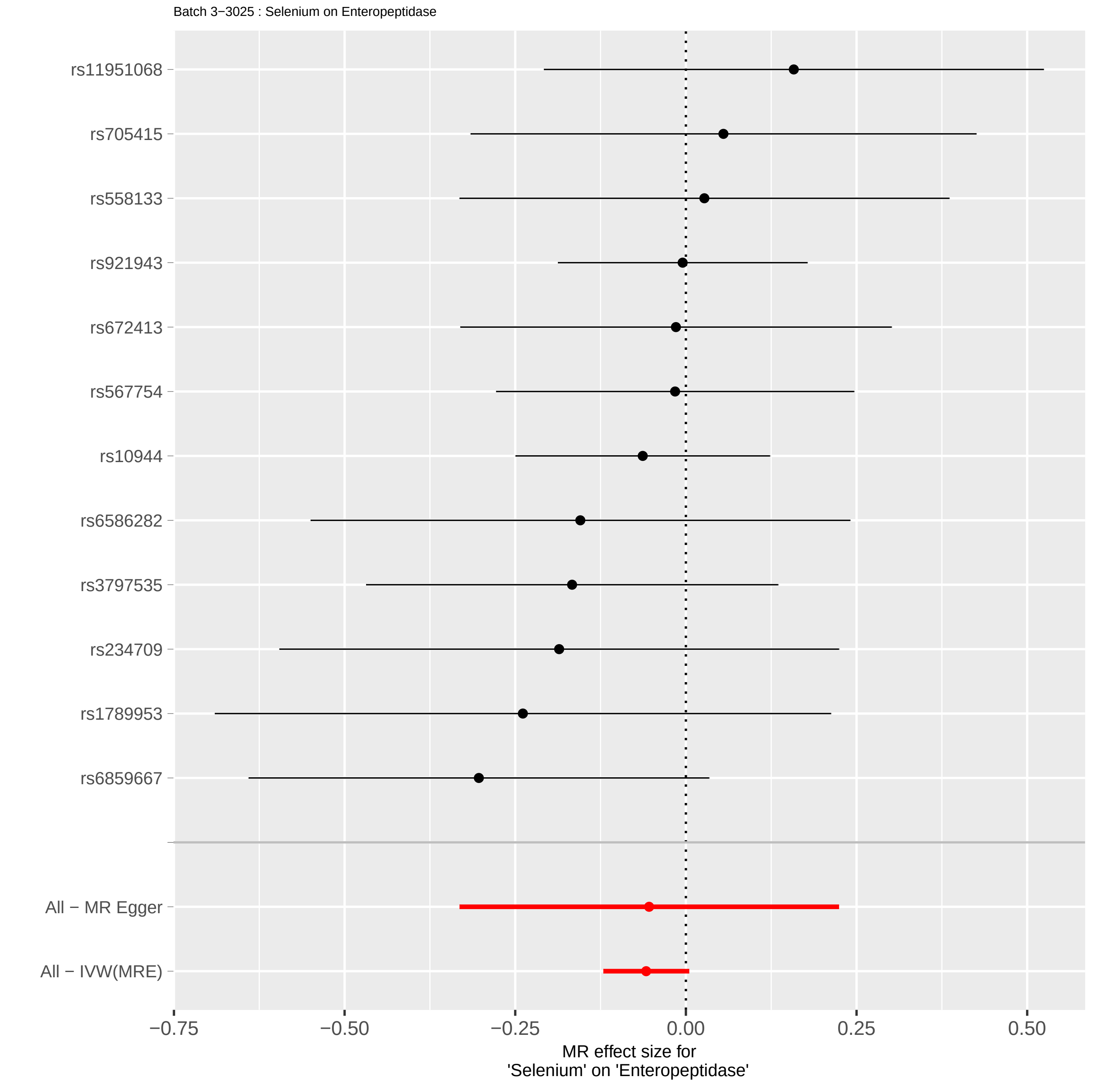
Batch 3−3025 : Selenium on Enteropeptidase
rs11951068
rs705415
rs558133
rs921943
rs672413
rs567754
rs10944
rs6586282
rs3797535
rs234709
rs1789953
rs6859667
All − MR Egger
All − IVW(MRE)
−0.75
−0.50
−0.25
0.00
0.25
0.50
MR effect size for
'Selenium' on 'Enteropeptidase'

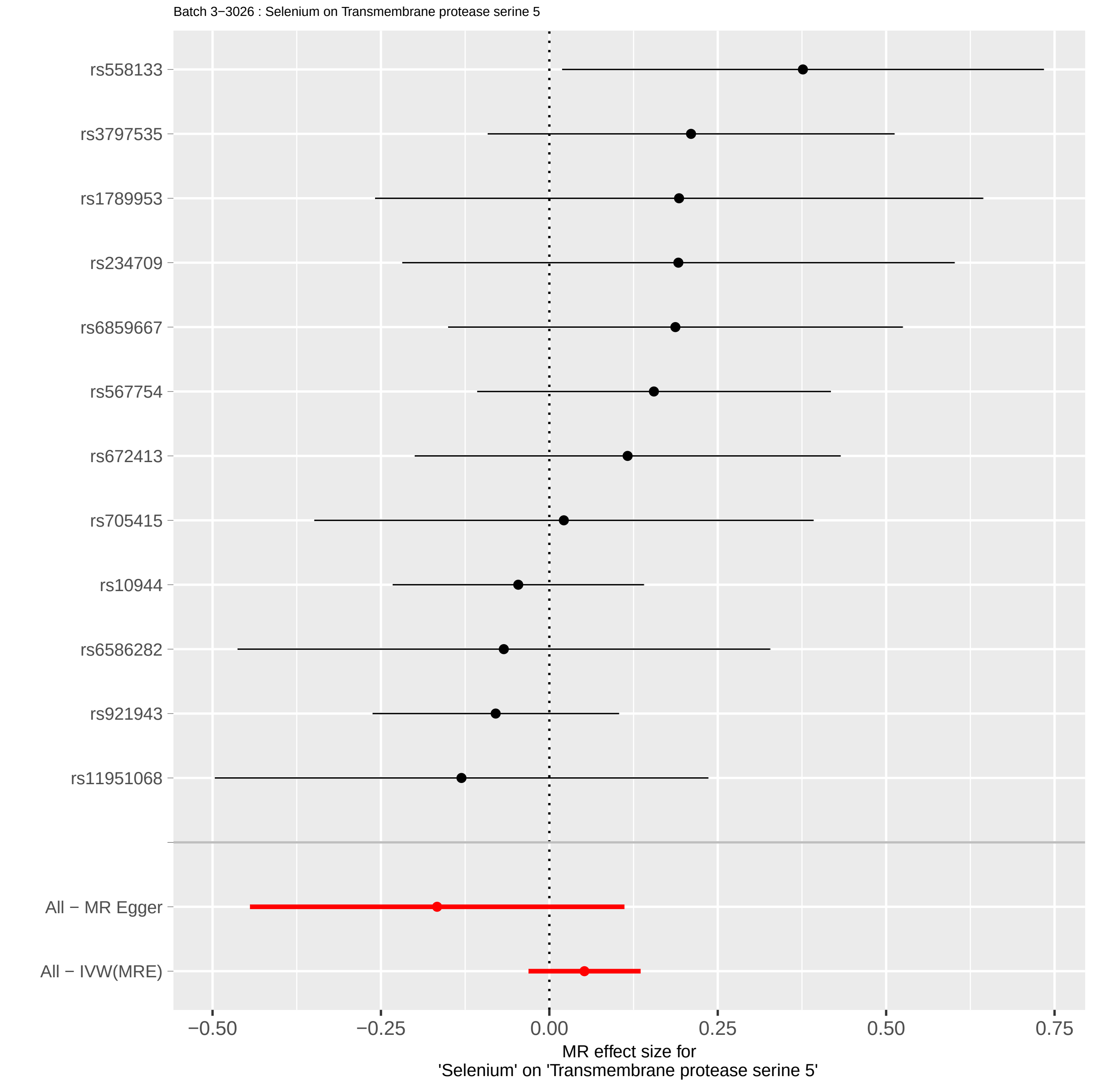

Batch 3−3026 : Selenium on Transmembrane protease serine 5
rs558133
rs3797535
rs1789953
rs234709
rs6859667
rs567754
rs672413
rs705415
rs10944
rs6586282
rs921943
rs11951068
All − MR Egger
All − IVW(MRE)
−0.50
−0.25
0.00
0.25
0.50
0.75
MR effect size for
'Selenium' on 'Transmembrane protease serine 5'

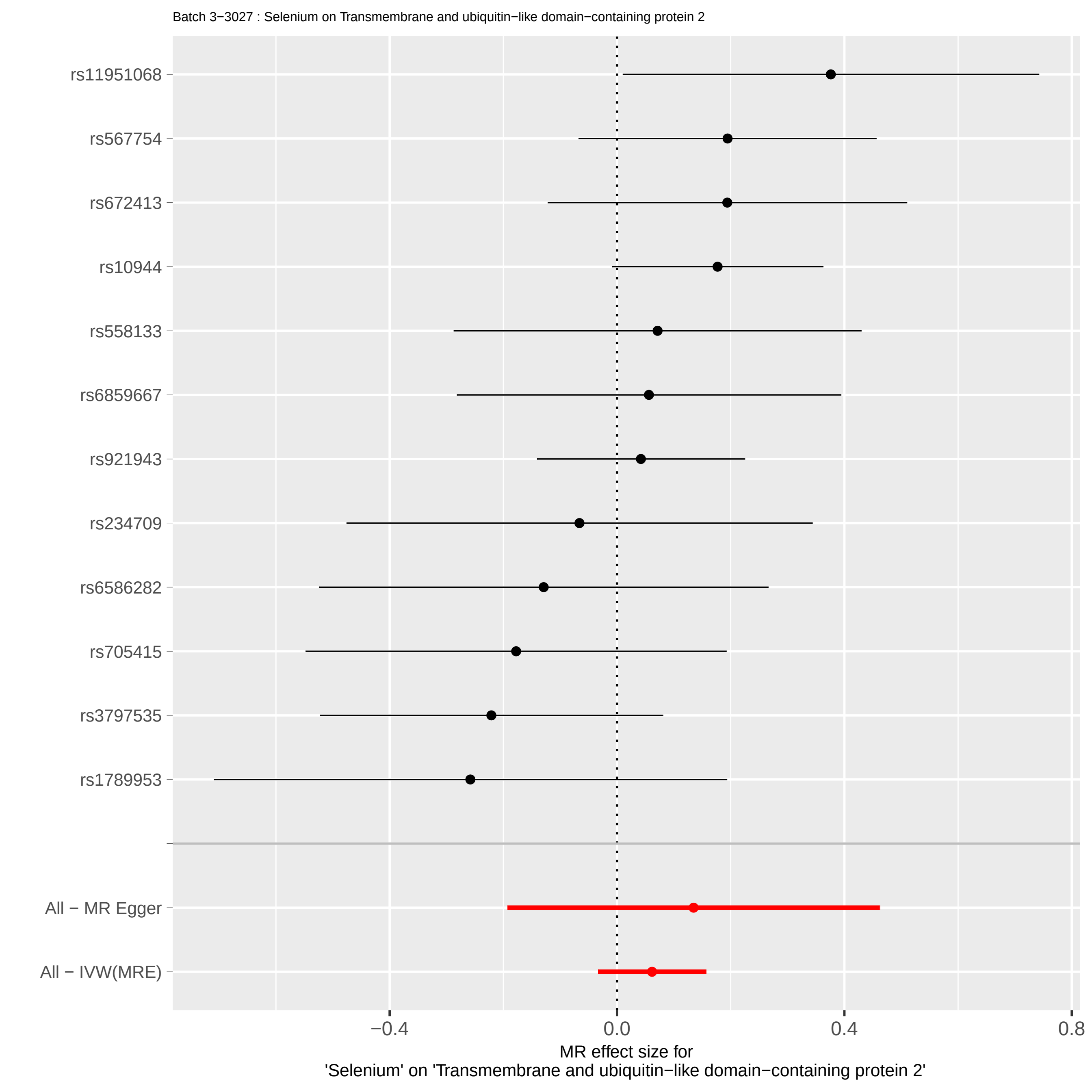

Batch 3−3027 : Selenium on Transmembrane and ubiquitin−like domain−containing protein 2
rs11951068
rs567754
rs672413
rs10944
rs558133
rs6859667
rs921943
rs234709
rs6586282
rs705415
rs3797535
rs1789953
All − MR Egger
All − IVW(MRE)
−0.4
0.0
0.4
0.8
MR effect size for
'Selenium' on 'Transmembrane and ubiquitin−like domain−containing protein 2'

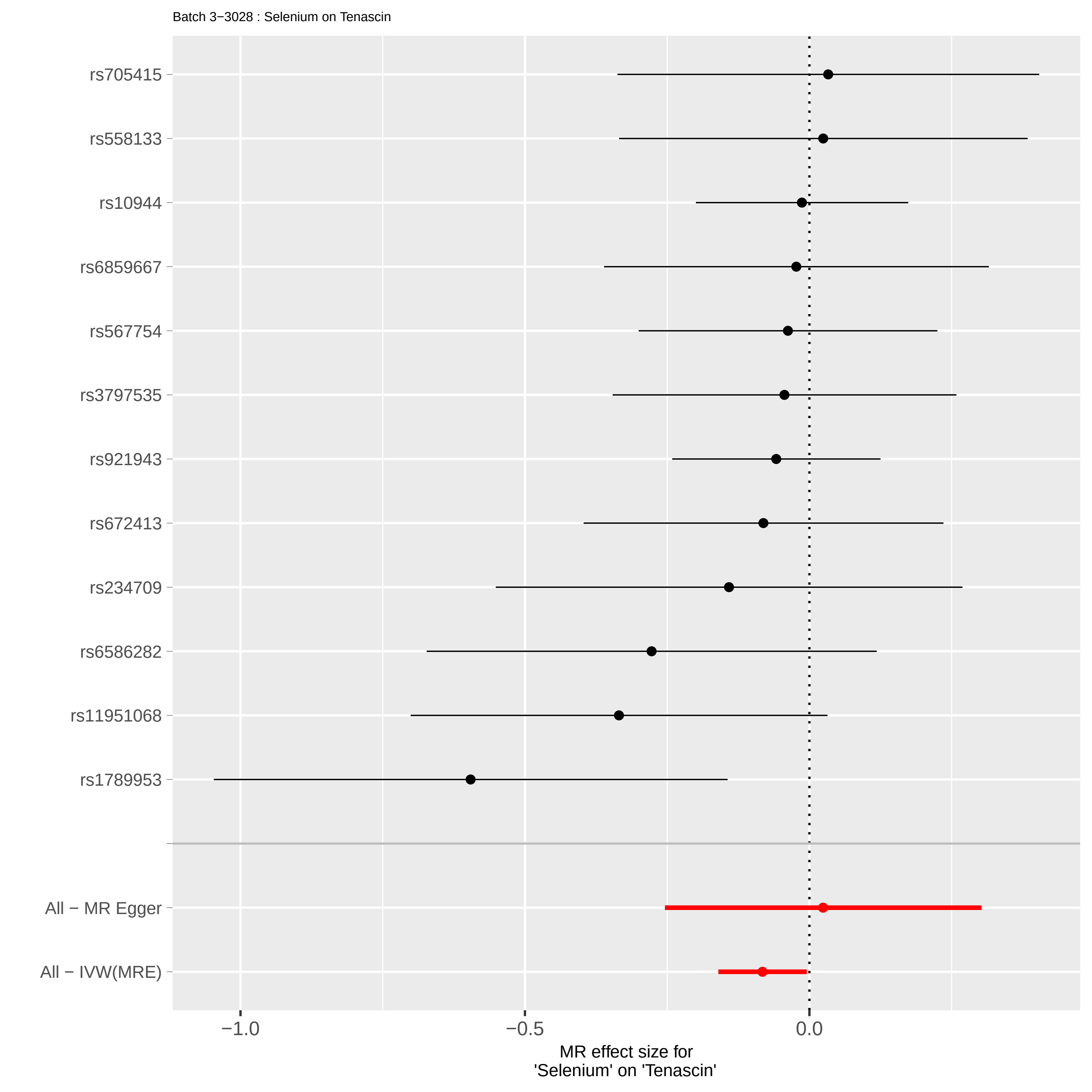

Batch 3−3028 : Selenium on Tenascin
rs705415
rs558133
rs10944
rs6859667
rs567754
rs3797535
rs921943
rs672413
rs234709
rs6586282
rs11951068
rs1789953
All − MR Egger
All − IVW(MRE)
−1.0
−0.5
0.0
MR effect size for
'Selenium' on 'Tenascin'

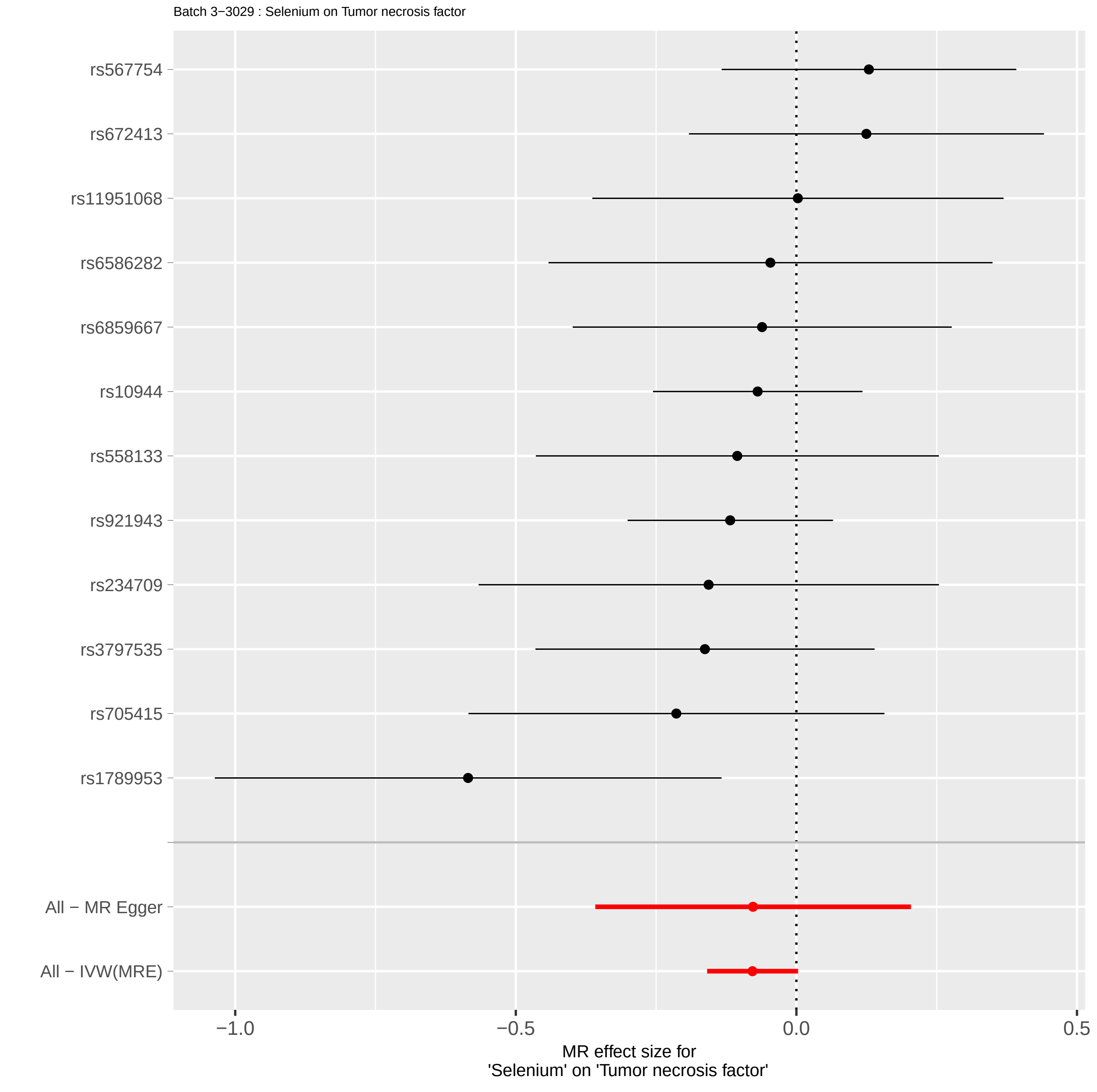

Batch 3−3029 : Selenium on Tumor necrosis factor
rs567754
rs672413
rs11951068
rs6586282
rs6859667
rs10944
rs558133
rs921943
rs234709
rs3797535
rs705415
rs1789953
All − MR Egger
All − IVW(MRE)
−1.0
−0.5
0.0
0.5
MR effect size for
'Selenium' on 'Tumor necrosis factor'

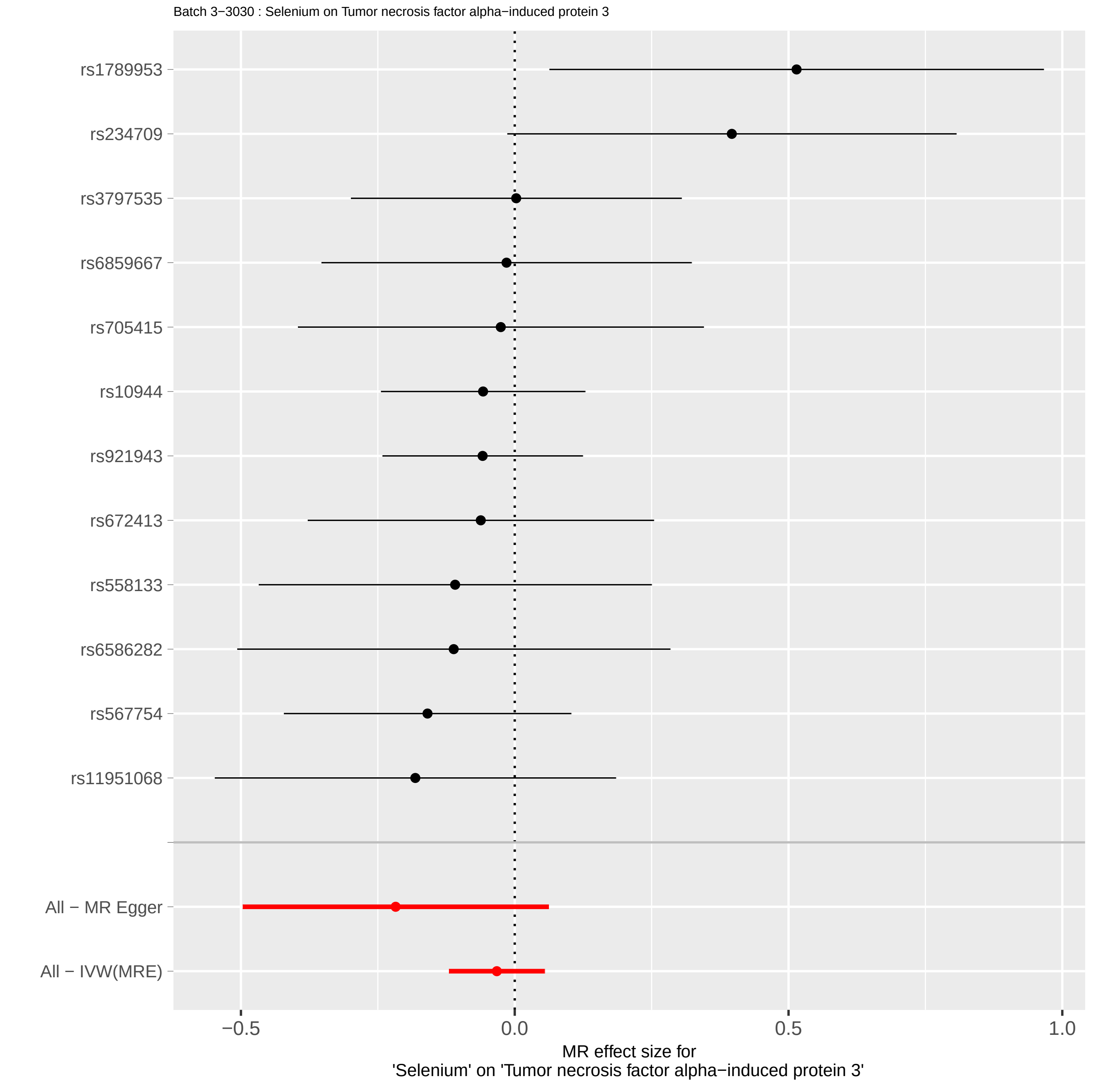

Batch 3−3030 : Selenium on Tumor necrosis factor alpha−induced protein 3
rs1789953
rs234709
rs3797535
rs6859667
rs705415
rs10944
rs921943
rs672413
rs558133
rs6586282
rs567754
rs11951068
All − MR Egger
All − IVW(MRE)
−0.5
0.0
0.5
1.0
MR effect size for
'Selenium' on 'Tumor necrosis factor alpha−induced protein 3'

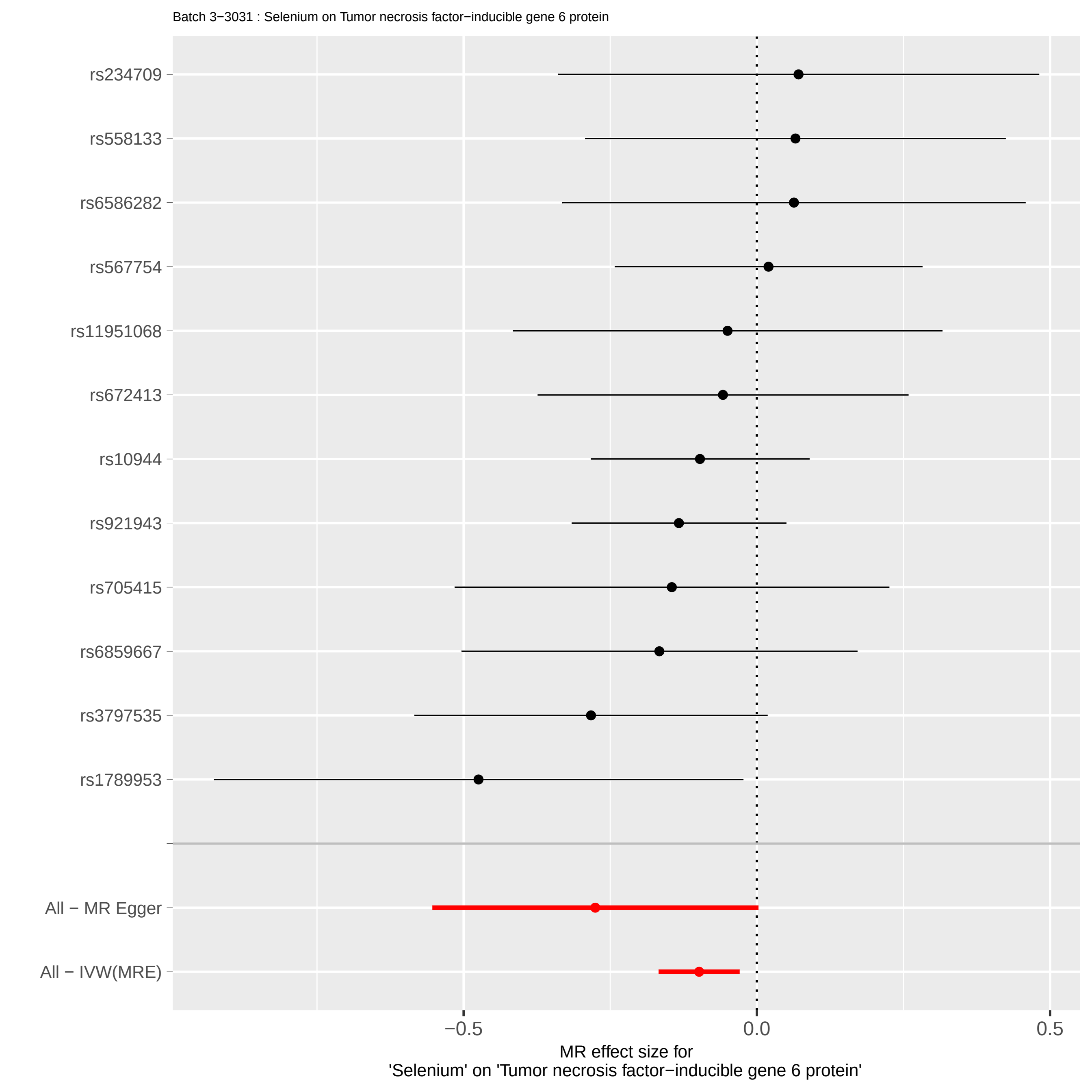
Batch 3−3031 : Selenium on Tumor necrosis factor−inducible gene 6 protein
rs234709
rs558133
rs6586282
rs567754
rs11951068
rs672413
rs10944
rs921943
rs705415
rs6859667
rs3797535
rs1789953
All − MR Egger
All − IVW(MRE)
−0.5
0.0
0.5
MR effect size for
'Selenium' on 'Tumor necrosis factor−inducible gene 6 protein'

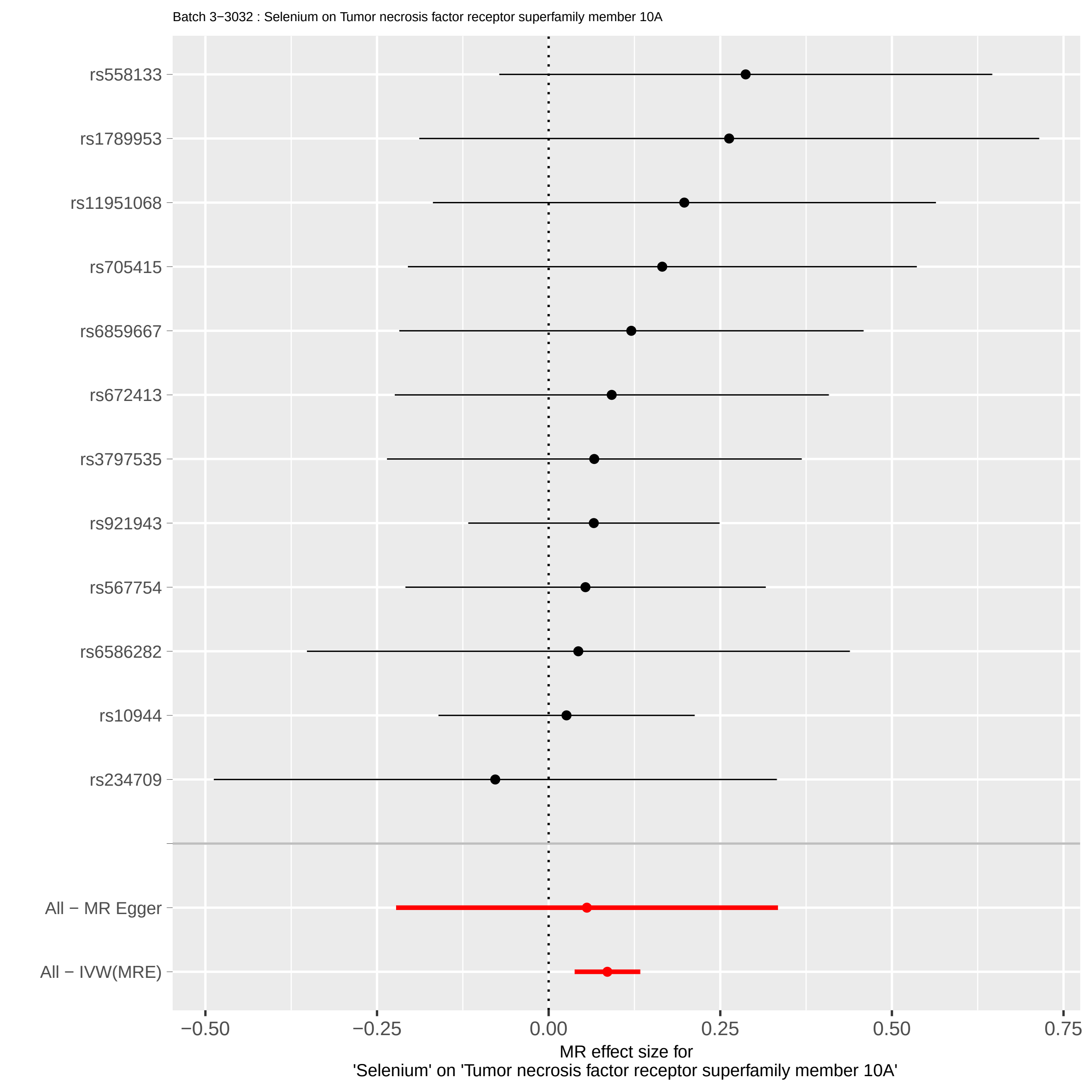

Batch 3−3032 : Selenium on Tumor necrosis factor receptor superfamily member 10A
rs558133
rs1789953
rs11951068
rs705415
rs6859667
rs672413
rs3797535
rs921943
rs567754
rs6586282
rs10944
rs234709
All − MR Egger
All − IVW(MRE)
−0.50
−0.25
0.00
0.25
0.50
0.75
MR effect size for
'Selenium' on 'Tumor necrosis factor receptor superfamily member 10A'

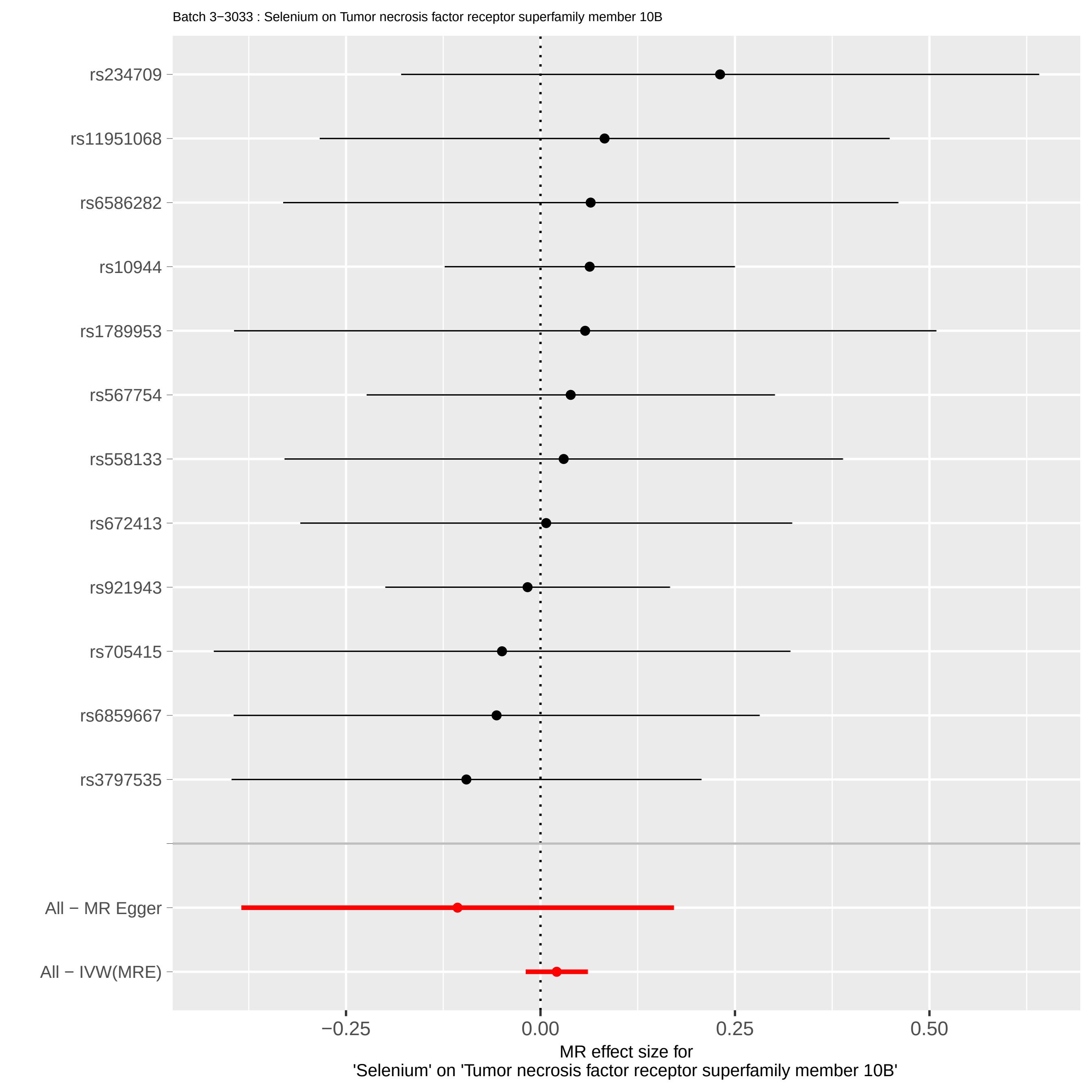

Batch 3−3033 : Selenium on Tumor necrosis factor receptor superfamily member 10B
rs234709
rs11951068
rs6586282
rs10944
rs1789953
rs567754
rs558133
rs672413
rs921943
rs705415
rs6859667
rs3797535
All − MR Egger
All − IVW(MRE)
−0.25
0.00
0.25
0.50
MR effect size for
'Selenium' on 'Tumor necrosis factor receptor superfamily member 10B'

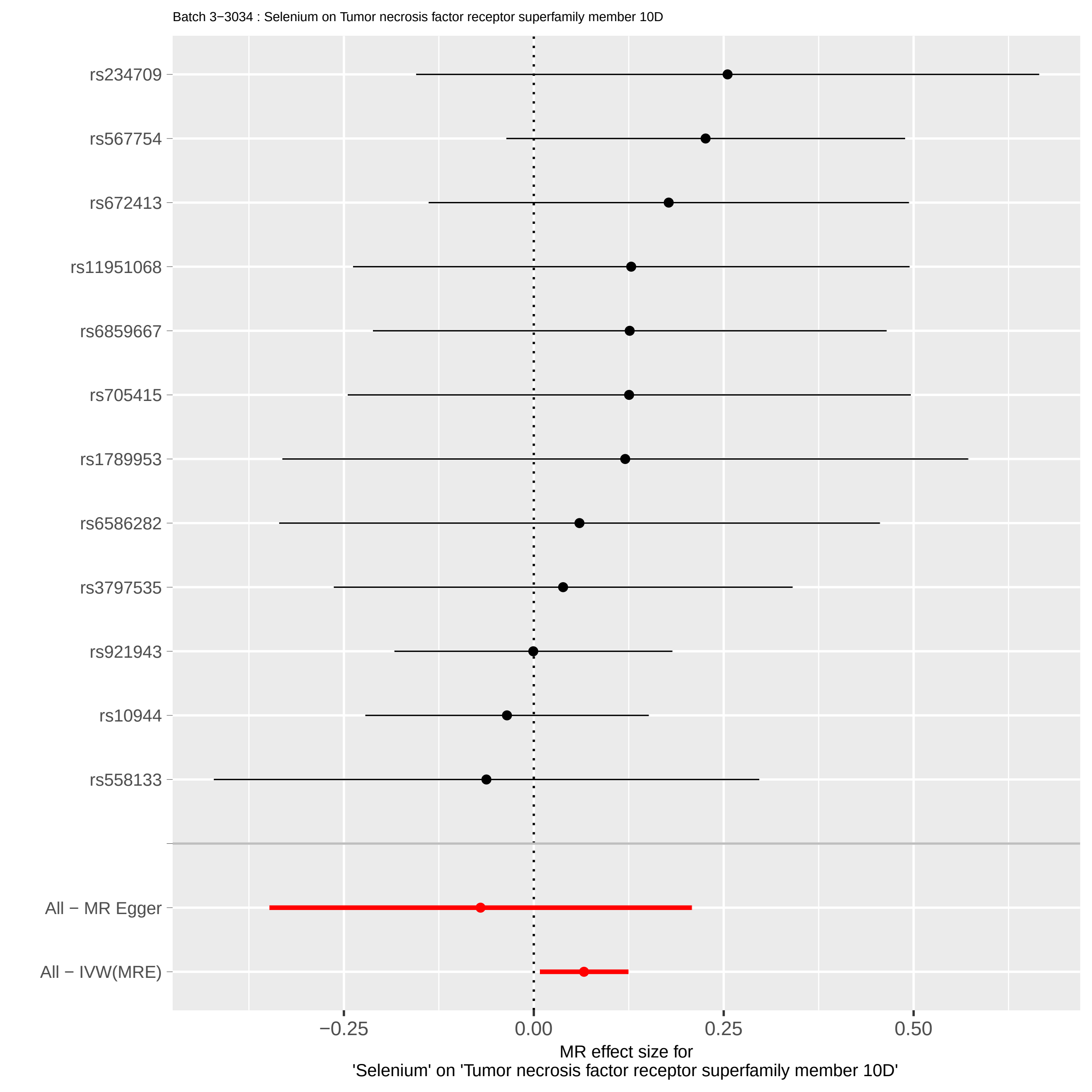
Batch 3−3034 : Selenium on Tumor necrosis factor receptor superfamily member 10D
rs234709
rs567754
rs672413
rs11951068
rs6859667
rs705415
rs1789953
rs6586282
rs3797535
rs921943
rs10944
rs558133
All − MR Egger
All − IVW(MRE)
−0.25
0.00
0.25
0.50
MR effect size for
'Selenium' on 'Tumor necrosis factor receptor superfamily member 10D'

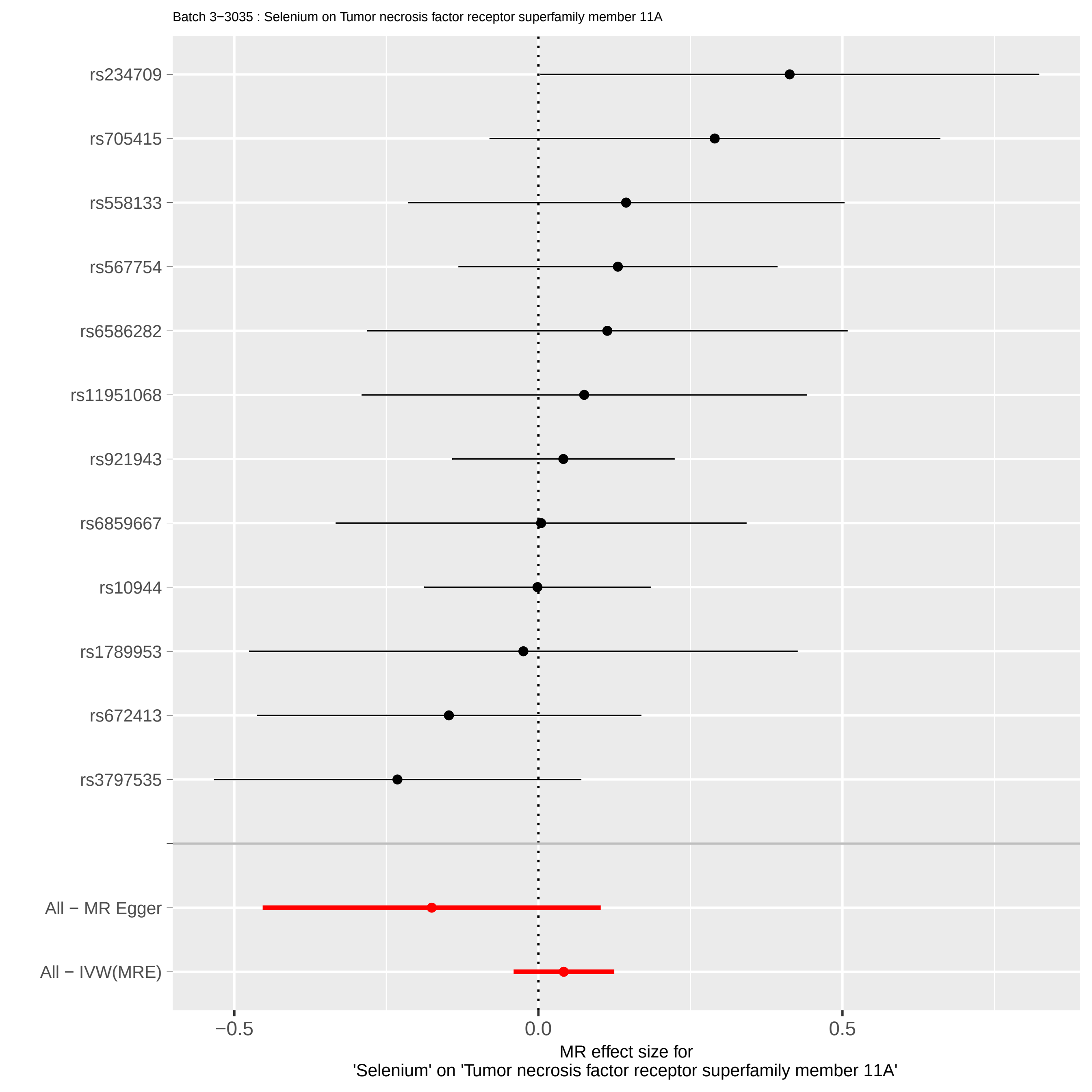
Batch 3−3035 : Selenium on Tumor necrosis factor receptor superfamily member 11A
rs234709
rs705415
rs558133
rs567754
rs6586282
rs11951068
rs921943
rs6859667
rs10944
rs1789953
rs672413
rs3797535
All − MR Egger
All − IVW(MRE)
−0.5
0.0
0.5
MR effect size for
'Selenium' on 'Tumor necrosis factor receptor superfamily member 11A'

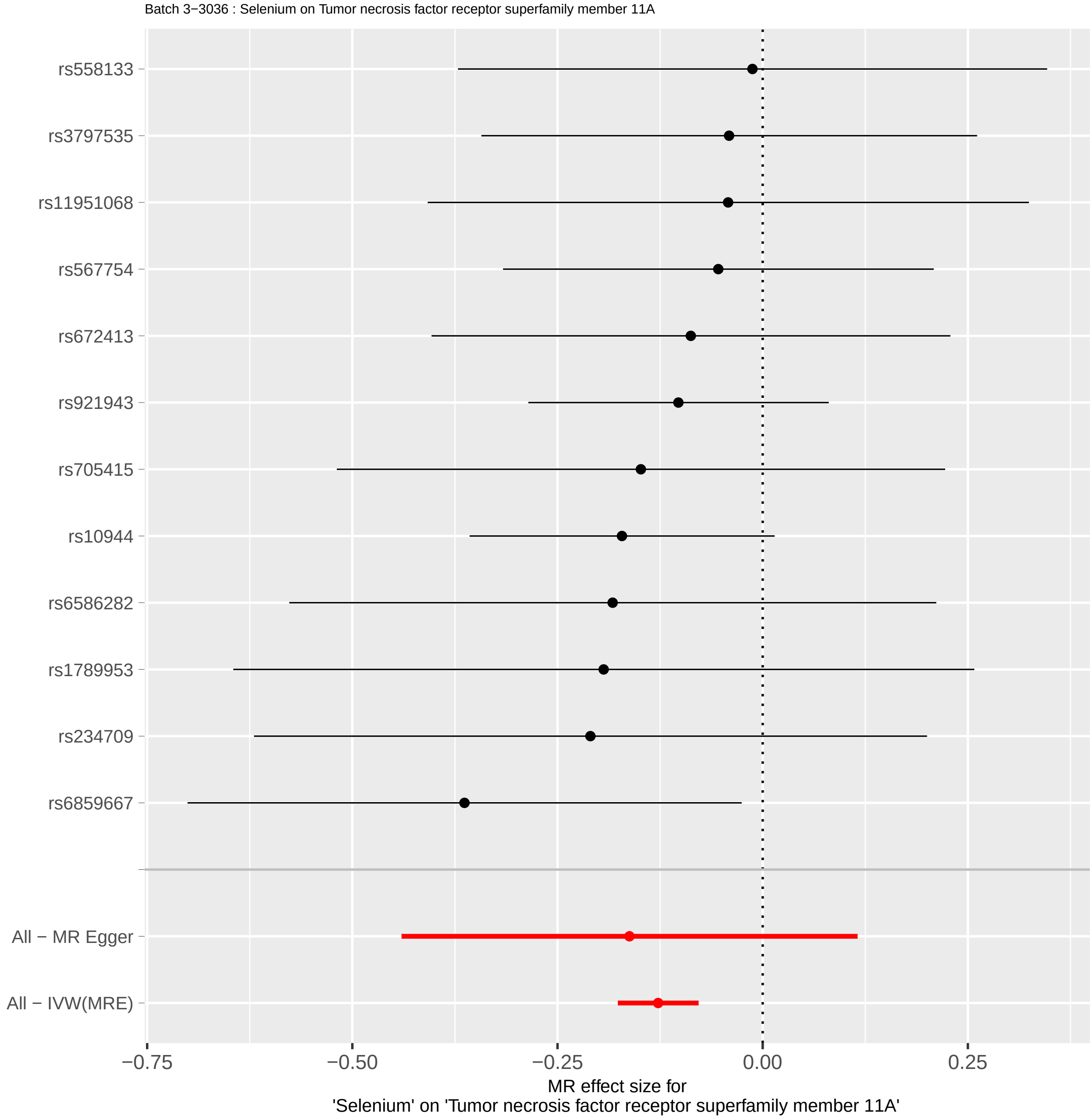

Batch 3−3036 : Selenium on Tumor necrosis factor receptor superfamily member 11A
rs558133
rs3797535
rs11951068
rs567754
rs672413
rs921943
rs705415
rs10944
rs6586282
rs1789953
rs234709
rs6859667
All − MR Egger
All − IVW(MRE)
−0.75
−0.50
−0.25
0.00
0.25
MR effect size for
'Selenium' on 'Tumor necrosis factor receptor superfamily member 11A'

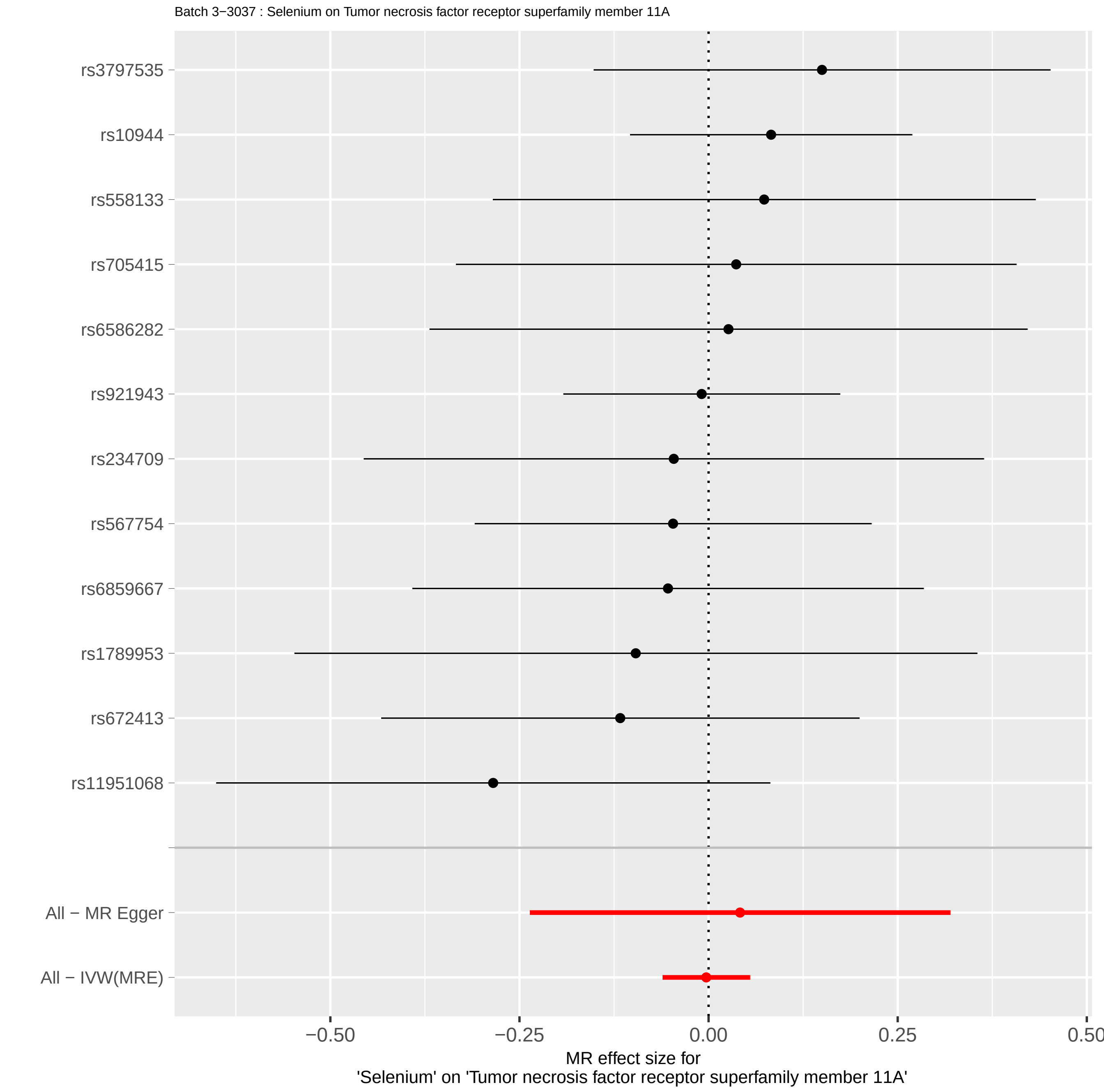
Batch 3−3037 : Selenium on Tumor necrosis factor receptor superfamily member 11A
rs3797535
rs10944
rs558133
rs705415
rs6586282
rs921943
rs234709
rs567754
rs6859667
rs1789953
rs672413
rs11951068
All − MR Egger
All − IVW(MRE)
−0.50
−0.25
0.00
0.25
0.50
MR effect size for
'Selenium' on 'Tumor necrosis factor receptor superfamily member 11A'

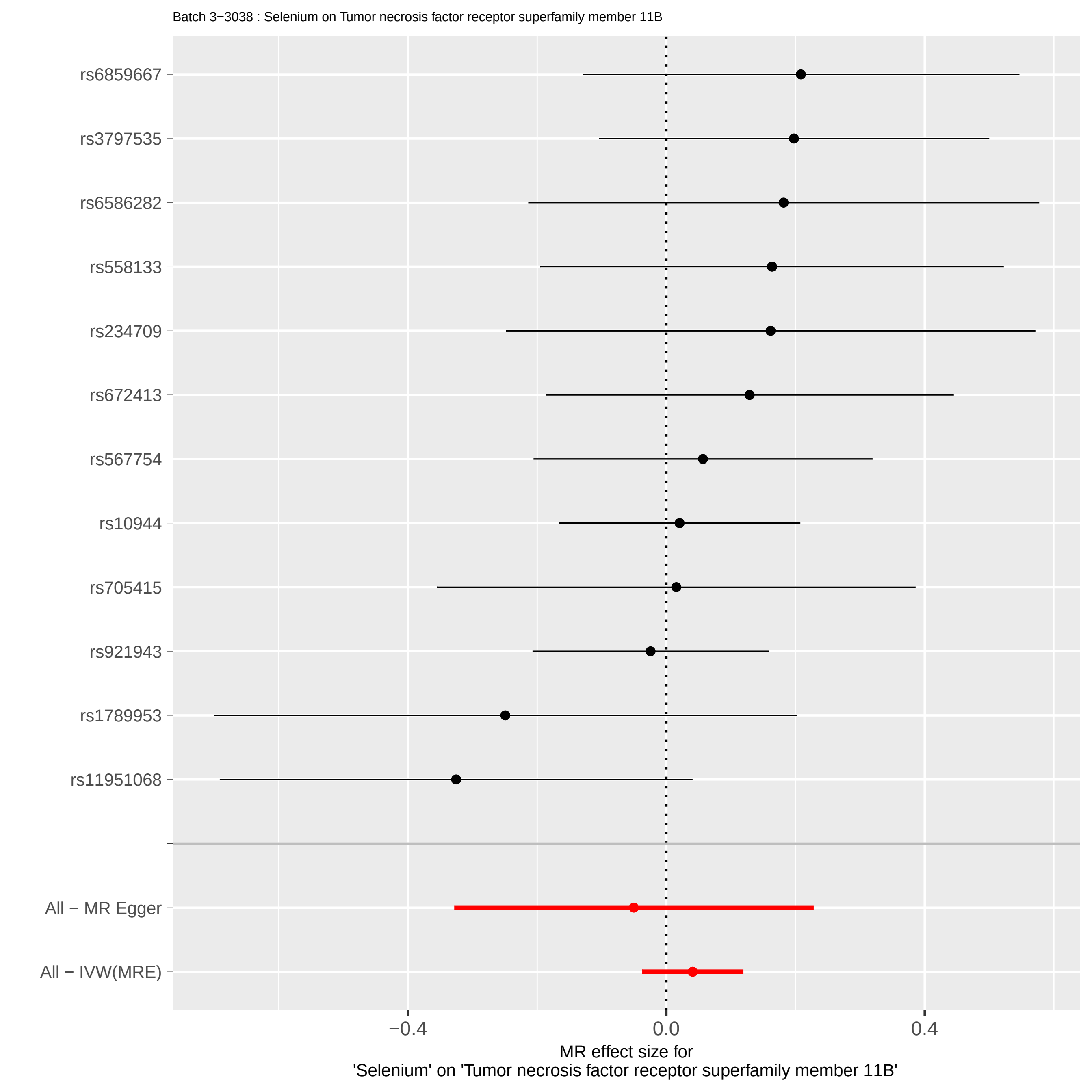
Batch 3−3038 : Selenium on Tumor necrosis factor receptor superfamily member 11B
rs6859667
rs3797535
rs6586282
rs558133
rs234709
rs672413
rs567754
rs10944
rs705415
rs921943
rs1789953
rs11951068
All − MR Egger
All − IVW(MRE)
−0.4
0.0
0.4
MR effect size for
'Selenium' on 'Tumor necrosis factor receptor superfamily member 11B'

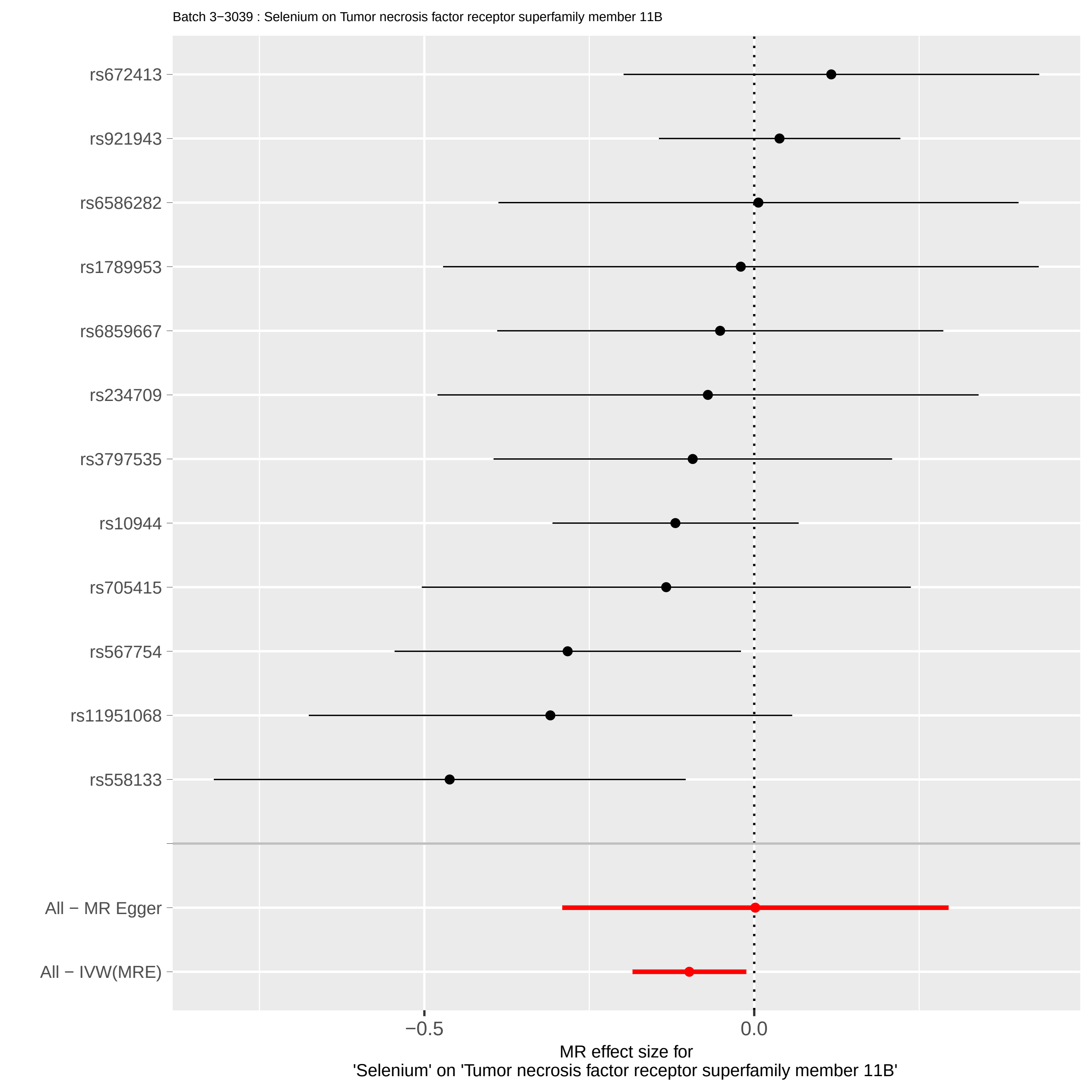

Batch 3−3039 : Selenium on Tumor necrosis factor receptor superfamily member 11B
rs672413
rs921943
rs6586282
rs1789953
rs6859667
rs234709
rs3797535
rs10944
rs705415
rs567754
rs11951068
rs558133
All − MR Egger
All − IVW(MRE)
−0.5
0.0
MR effect size for
'Selenium' on 'Tumor necrosis factor receptor superfamily member 11B'

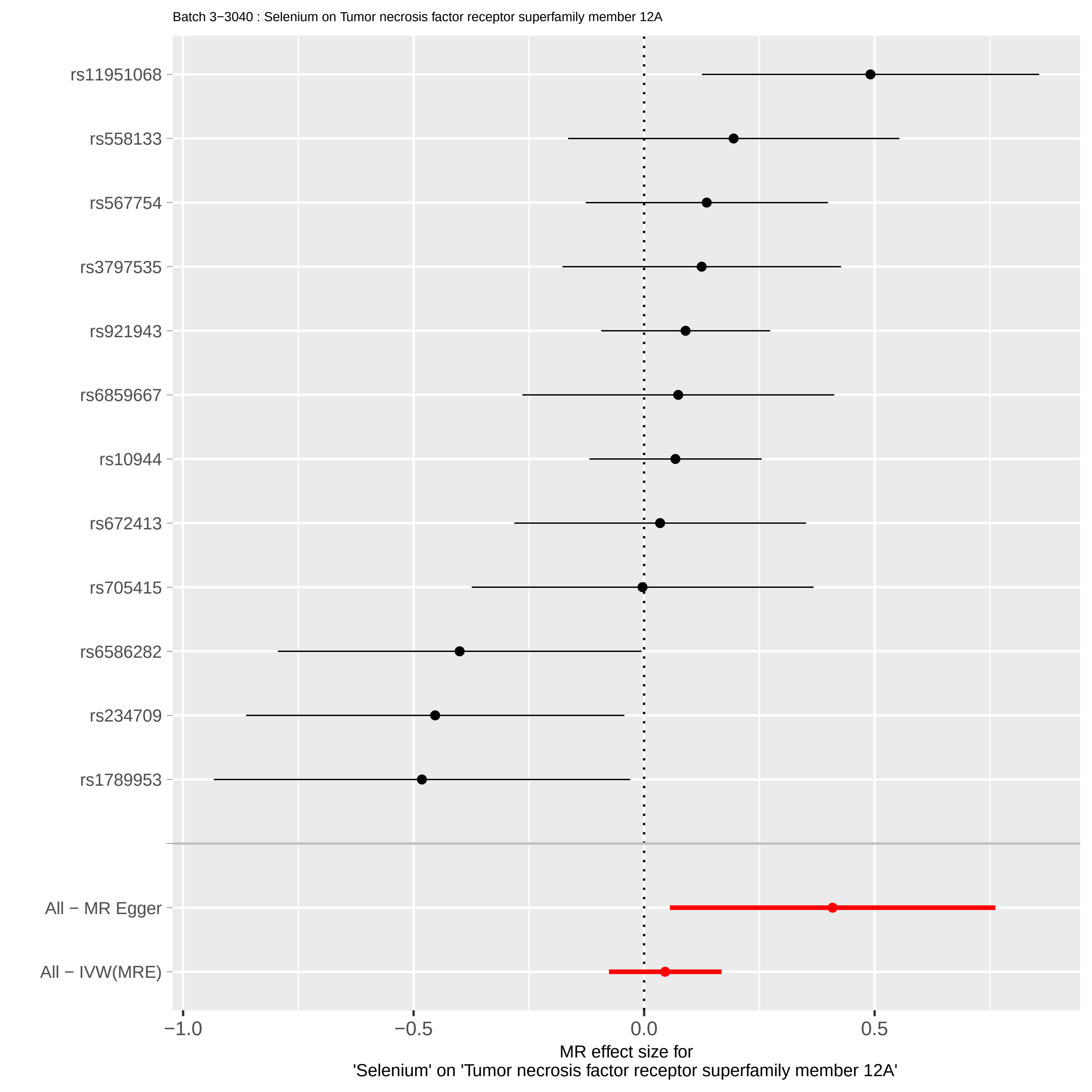

Batch 3−3040 : Selenium on Tumor necrosis factor receptor superfamily member 12A
rs11951068
rs558133
rs567754
rs3797535
rs921943
rs6859667
rs10944
rs672413
rs705415
rs6586282
rs234709
rs1789953
All − MR Egger
All − IVW(MRE)
−1.0
−0.5
0.0
0.5
MR effect size for
'Selenium' on 'Tumor necrosis factor receptor superfamily member 12A'

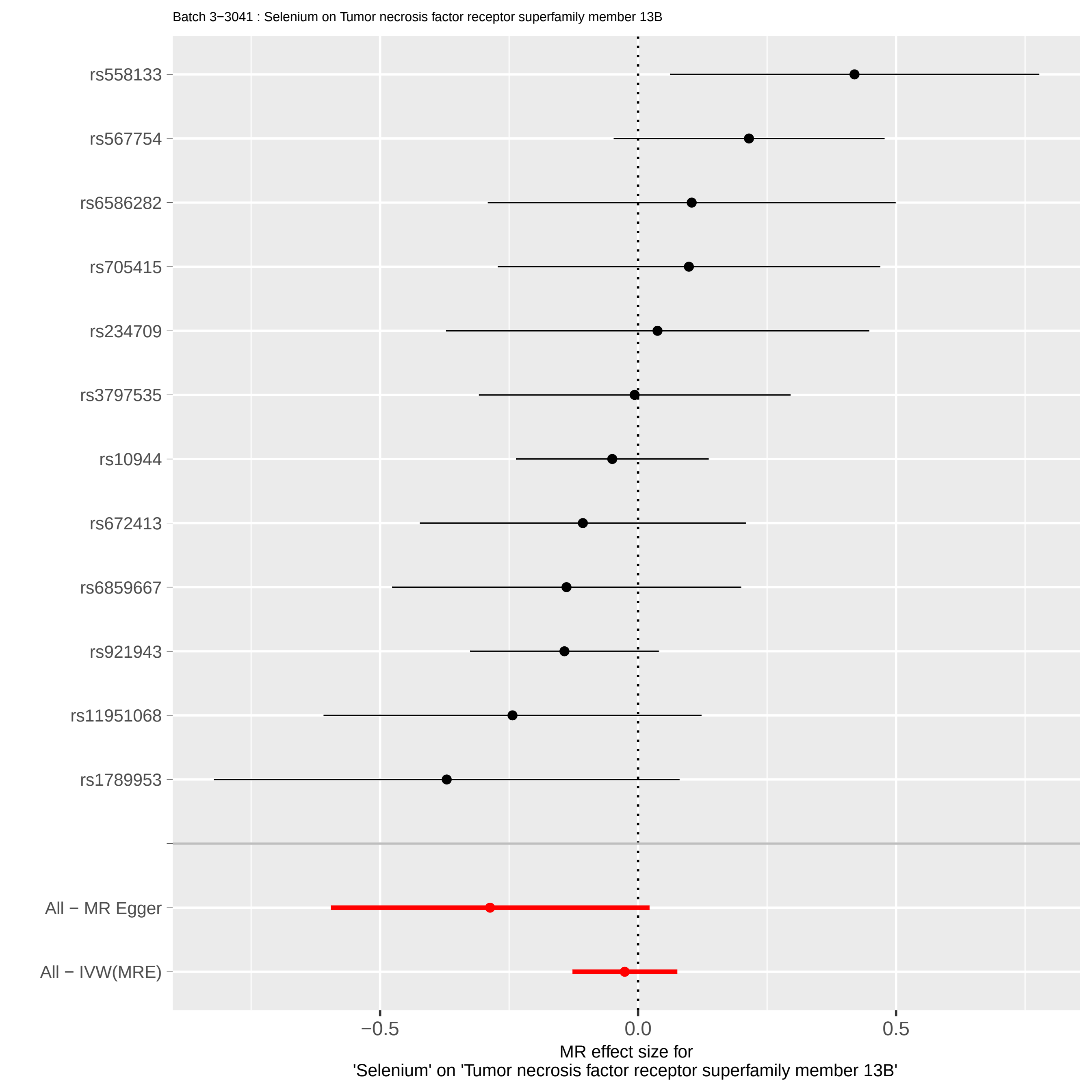

Batch 3−3041 : Selenium on Tumor necrosis factor receptor superfamily member 13B
rs558133
rs567754
rs6586282
rs705415
rs234709
rs3797535
rs10944
rs672413
rs6859667
rs921943
rs11951068
rs1789953
All − MR Egger
All − IVW(MRE)
−0.5
0.0
0.5
MR effect size for
'Selenium' on 'Tumor necrosis factor receptor superfamily member 13B'

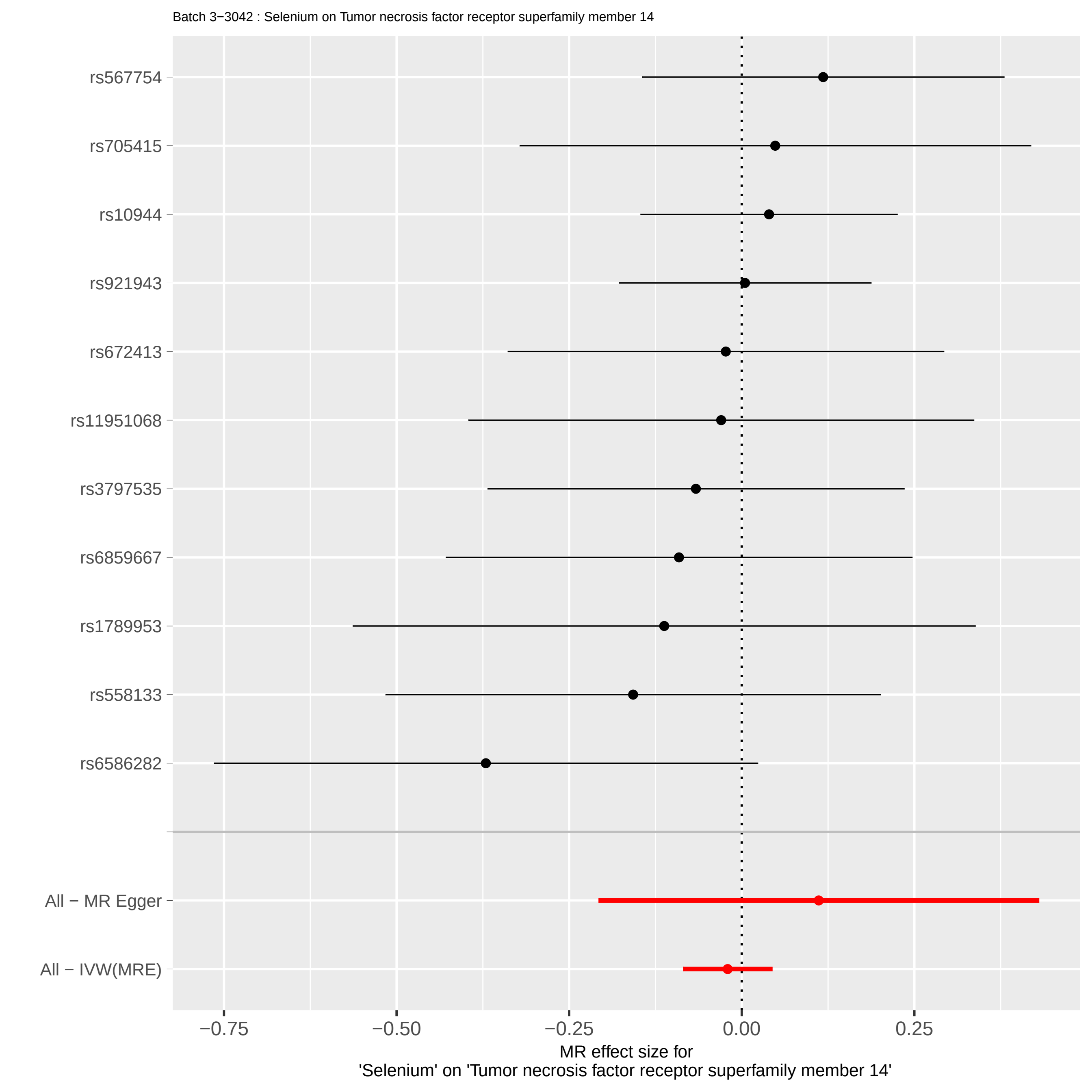

Batch 3−3042 : Selenium on Tumor necrosis factor receptor superfamily member 14
rs567754
rs705415
rs10944
rs921943
rs672413
rs11951068
rs3797535
rs6859667
rs1789953
rs558133
rs6586282
All − MR Egger
All − IVW(MRE)
−0.75
−0.50
−0.25
0.00
0.25
MR effect size for
'Selenium' on 'Tumor necrosis factor receptor superfamily member 14'

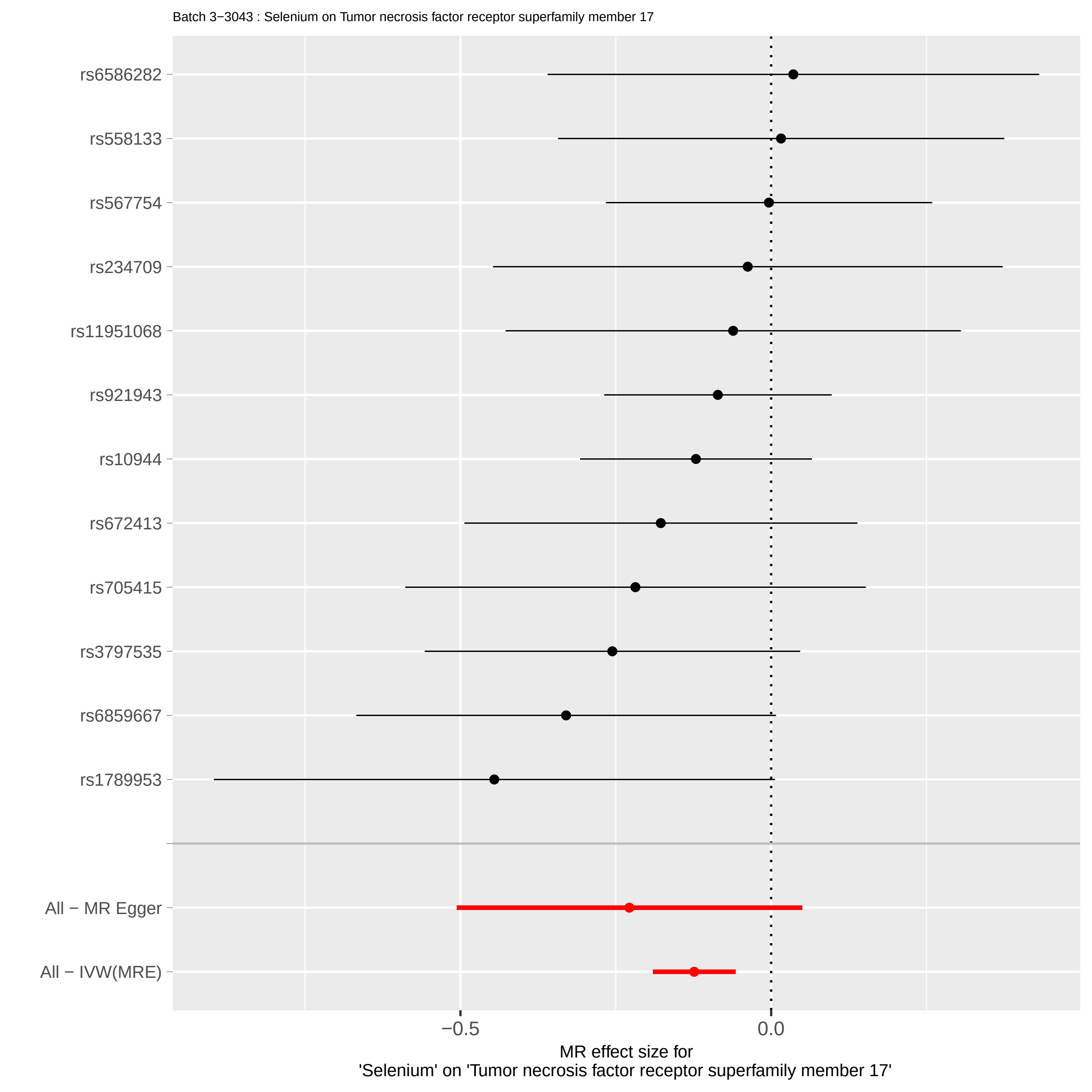

Batch 3−3043 : Selenium on Tumor necrosis factor receptor superfamily member 17
rs6586282
rs558133
rs567754
rs234709
rs11951068
rs921943
rs10944
rs672413
rs705415
rs3797535
rs6859667
rs1789953
All − MR Egger
All − IVW(MRE)
−0.5
0.0
MR effect size for
'Selenium' on 'Tumor necrosis factor receptor superfamily member 17'

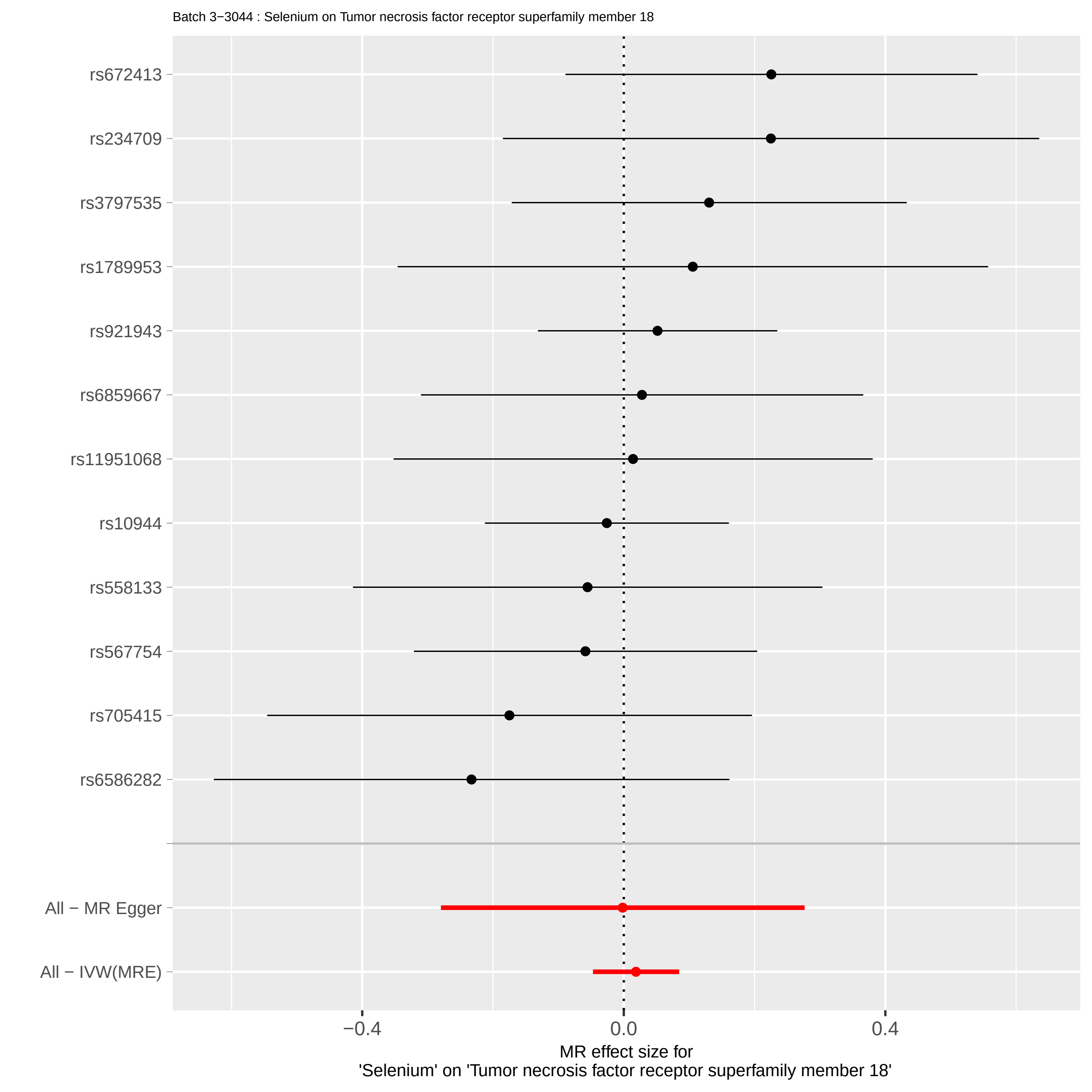

Batch 3−3044 : Selenium on Tumor necrosis factor receptor superfamily member 18
rs672413
rs234709
rs3797535
rs1789953
rs921943
rs6859667
rs11951068
rs10944
rs558133
rs567754
rs705415
rs6586282
All − MR Egger
All − IVW(MRE)
−0.4
0.0
0.4
MR effect size for
'Selenium' on 'Tumor necrosis factor receptor superfamily member 18'

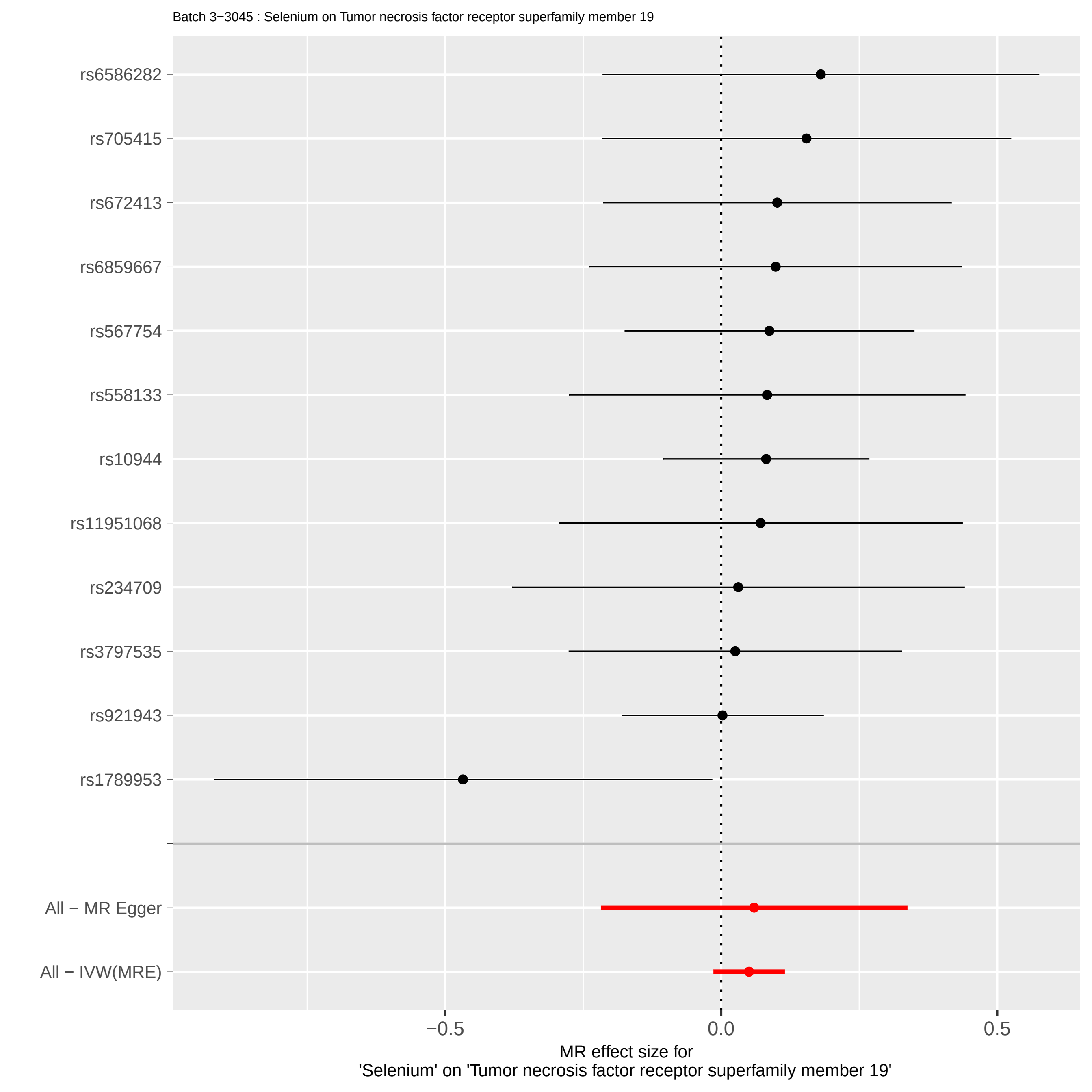

Batch 3−3045 : Selenium on Tumor necrosis factor receptor superfamily member 19
rs6586282
rs705415
rs672413
rs6859667
rs567754
rs558133
rs10944
rs11951068
rs234709
rs3797535
rs921943
rs1789953
All − MR Egger
All − IVW(MRE)
−0.5
0.0
0.5
MR effect size for
'Selenium' on 'Tumor necrosis factor receptor superfamily member 19'

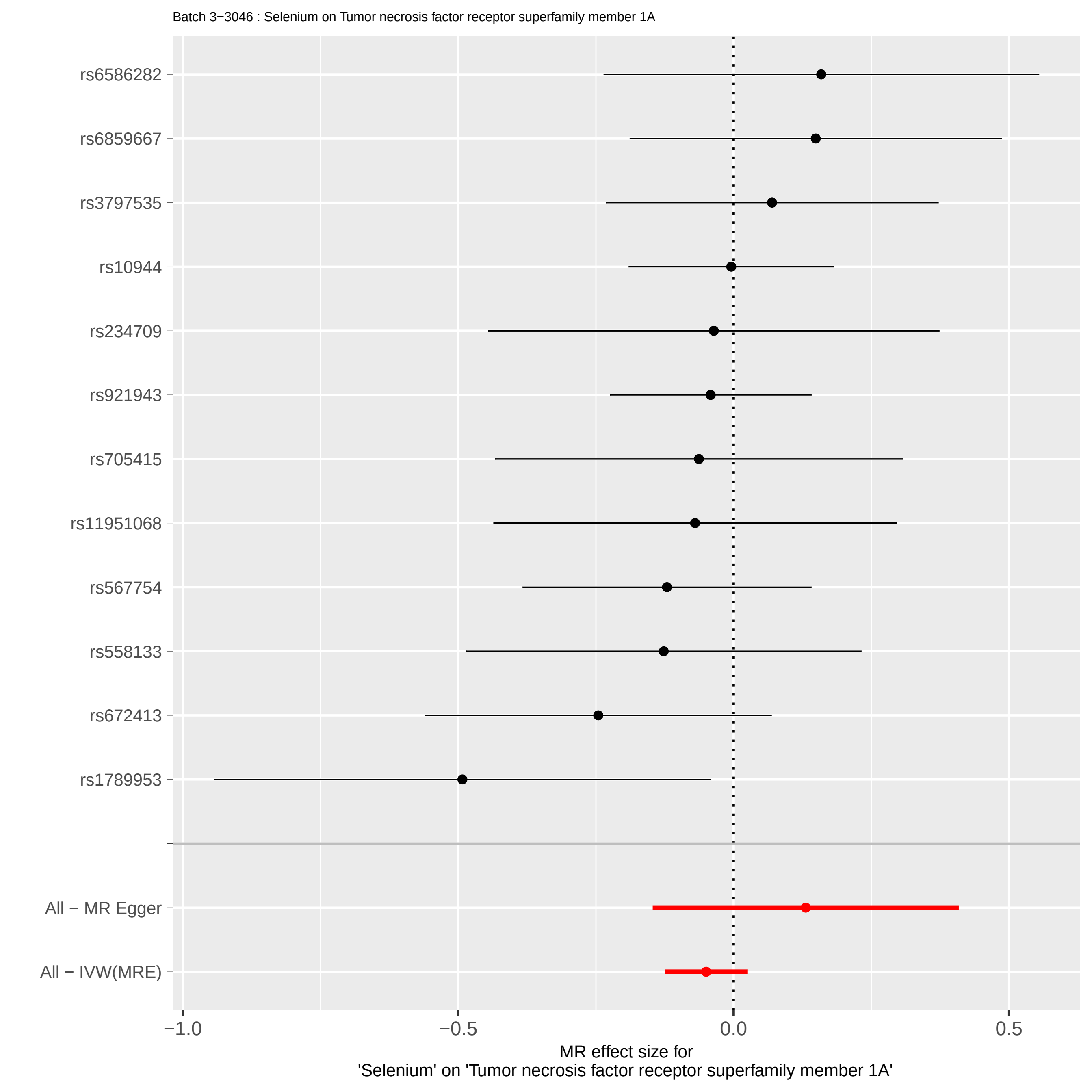

Batch 3−3046 : Selenium on Tumor necrosis factor receptor superfamily member 1A
rs6586282
rs6859667
rs3797535
rs10944
rs234709
rs921943
rs705415
rs11951068
rs567754
rs558133
rs672413
rs1789953
All − MR Egger
All − IVW(MRE)
−1.0
−0.5
0.0
0.5
MR effect size for
'Selenium' on 'Tumor necrosis factor receptor superfamily member 1A'

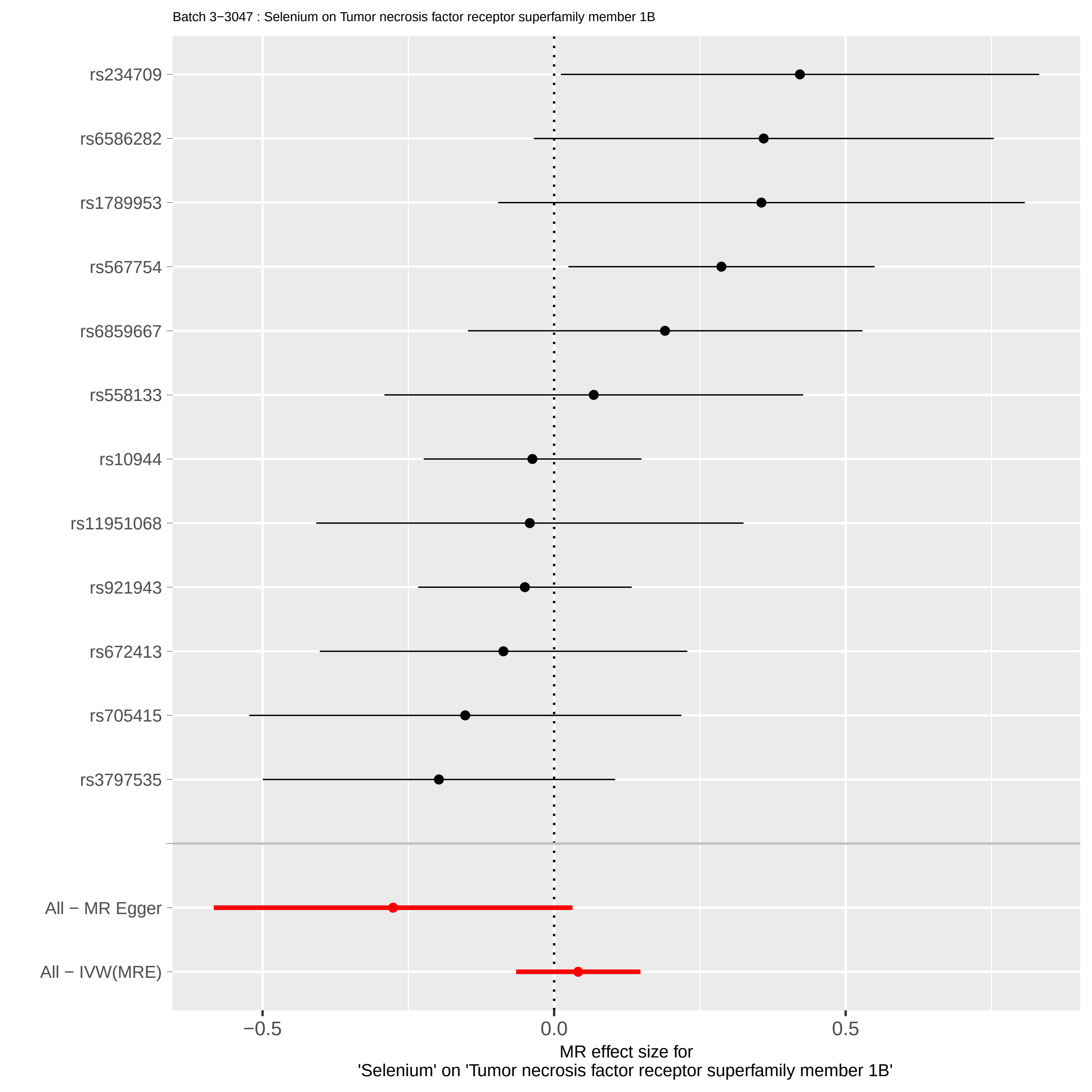

Batch 3−3047 : Selenium on Tumor necrosis factor receptor superfamily member 1B
rs234709
rs6586282
rs1789953
rs567754
rs6859667
rs558133
rs10944
rs11951068
rs921943
rs672413
rs705415
rs3797535
All − MR Egger
All − IVW(MRE)
−0.5
0.0
0.5
MR effect size for
'Selenium' on 'Tumor necrosis factor receptor superfamily member 1B'

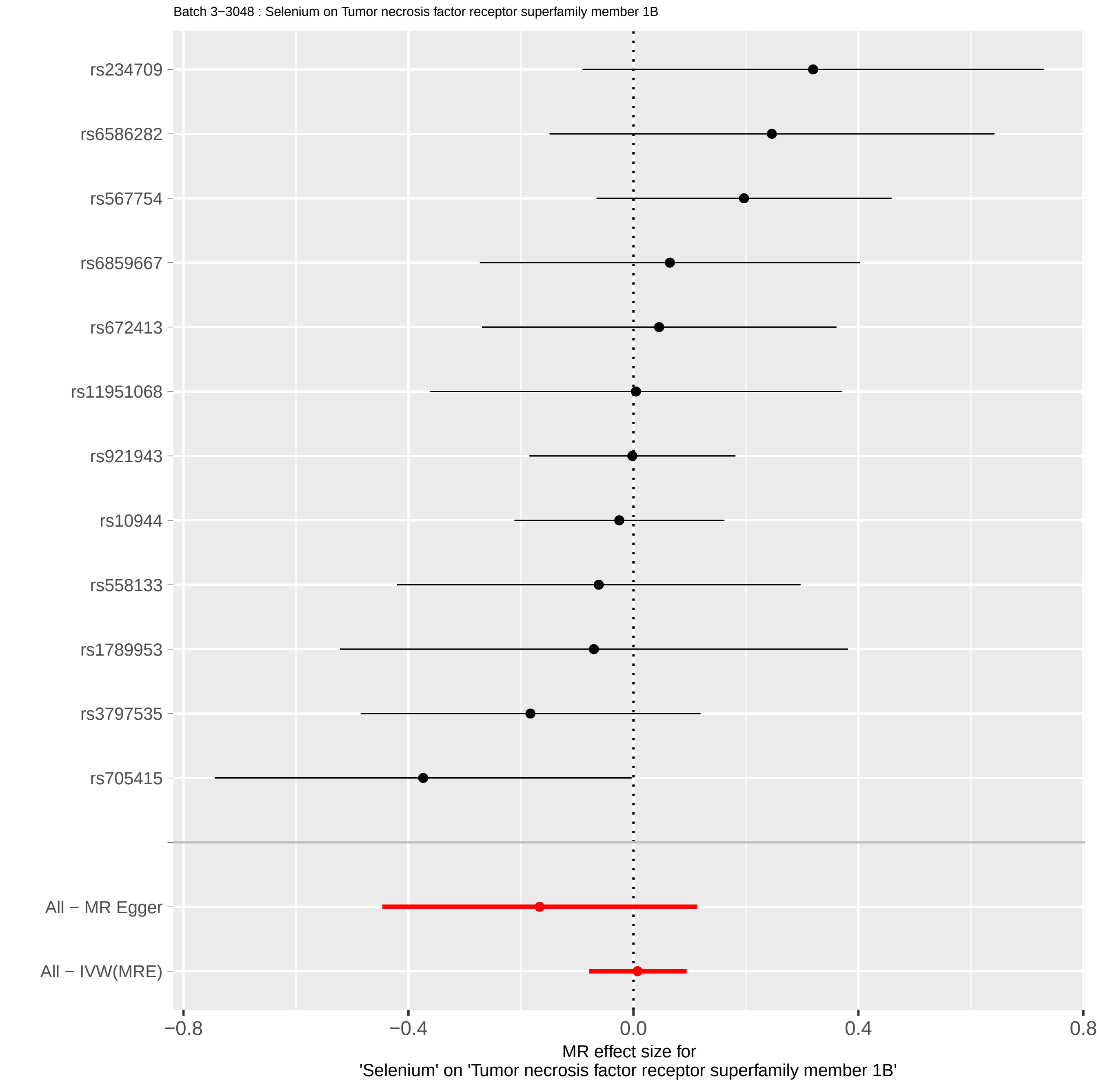

Batch 3−3048 : Selenium on Tumor necrosis factor receptor superfamily member 1B
rs234709
rs6586282
rs567754
rs6859667
rs672413
rs11951068
rs921943
rs10944
rs558133
rs1789953
rs3797535
rs705415
All − MR Egger
All − IVW(MRE)
−0.8
−0.4
0.0
0.4
0.8
MR effect size for
'Selenium' on 'Tumor necrosis factor receptor superfamily member 1B'

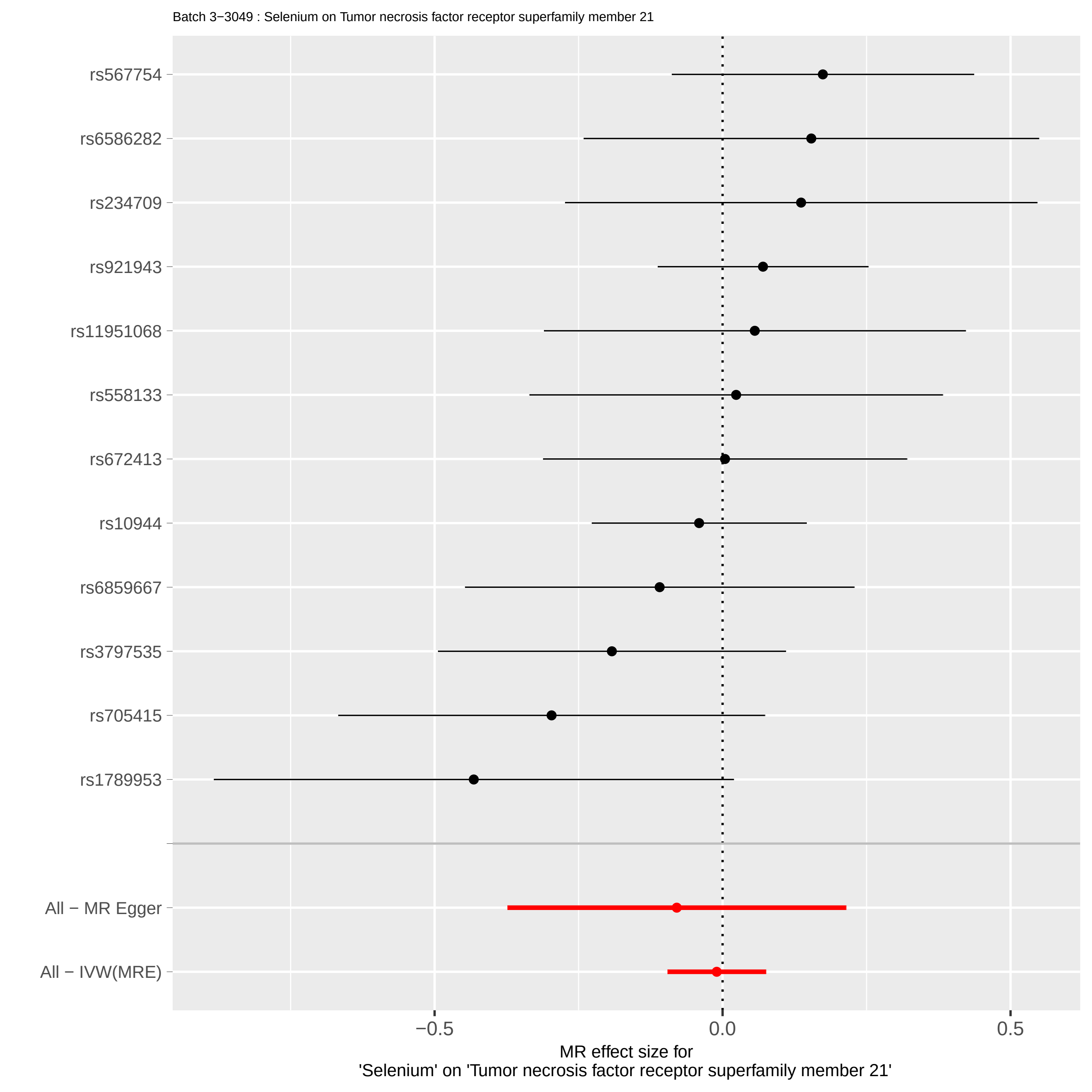

Batch 3−3049 : Selenium on Tumor necrosis factor receptor superfamily member 21
rs567754
rs6586282
rs234709
rs921943
rs11951068
rs558133
rs672413
rs10944
rs6859667
rs3797535
rs705415
rs1789953
All − MR Egger
All − IVW(MRE)
−0.5
0.0
0.5
MR effect size for
'Selenium' on 'Tumor necrosis factor receptor superfamily member 21'

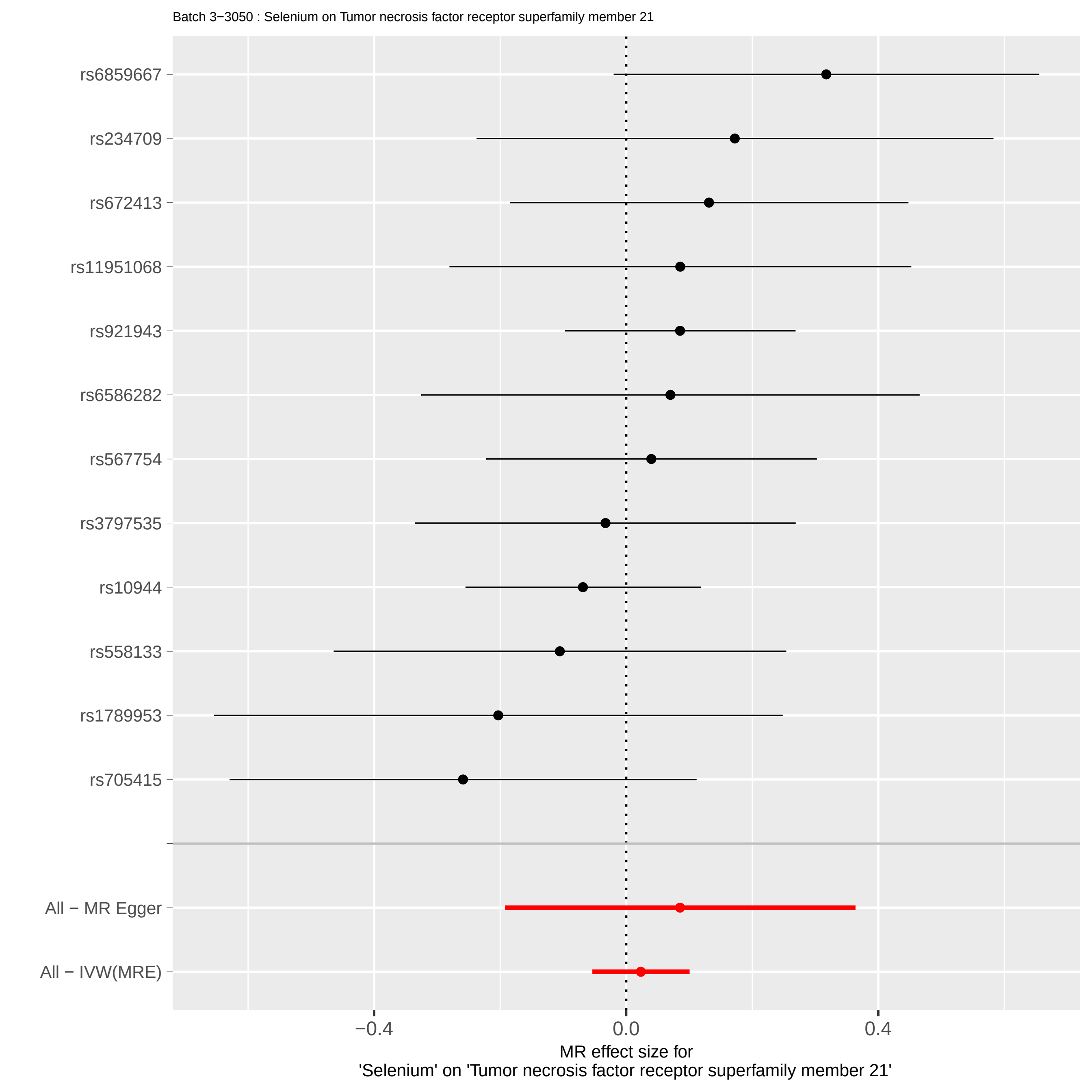

Batch 3−3050 : Selenium on Tumor necrosis factor receptor superfamily member 21
rs6859667
rs234709
rs672413
rs11951068
rs921943
rs6586282
rs567754
rs3797535
rs10944
rs558133
rs1789953
rs705415
All − MR Egger
All − IVW(MRE)
−0.4
0.0
0.4
MR effect size for
'Selenium' on 'Tumor necrosis factor receptor superfamily member 21'

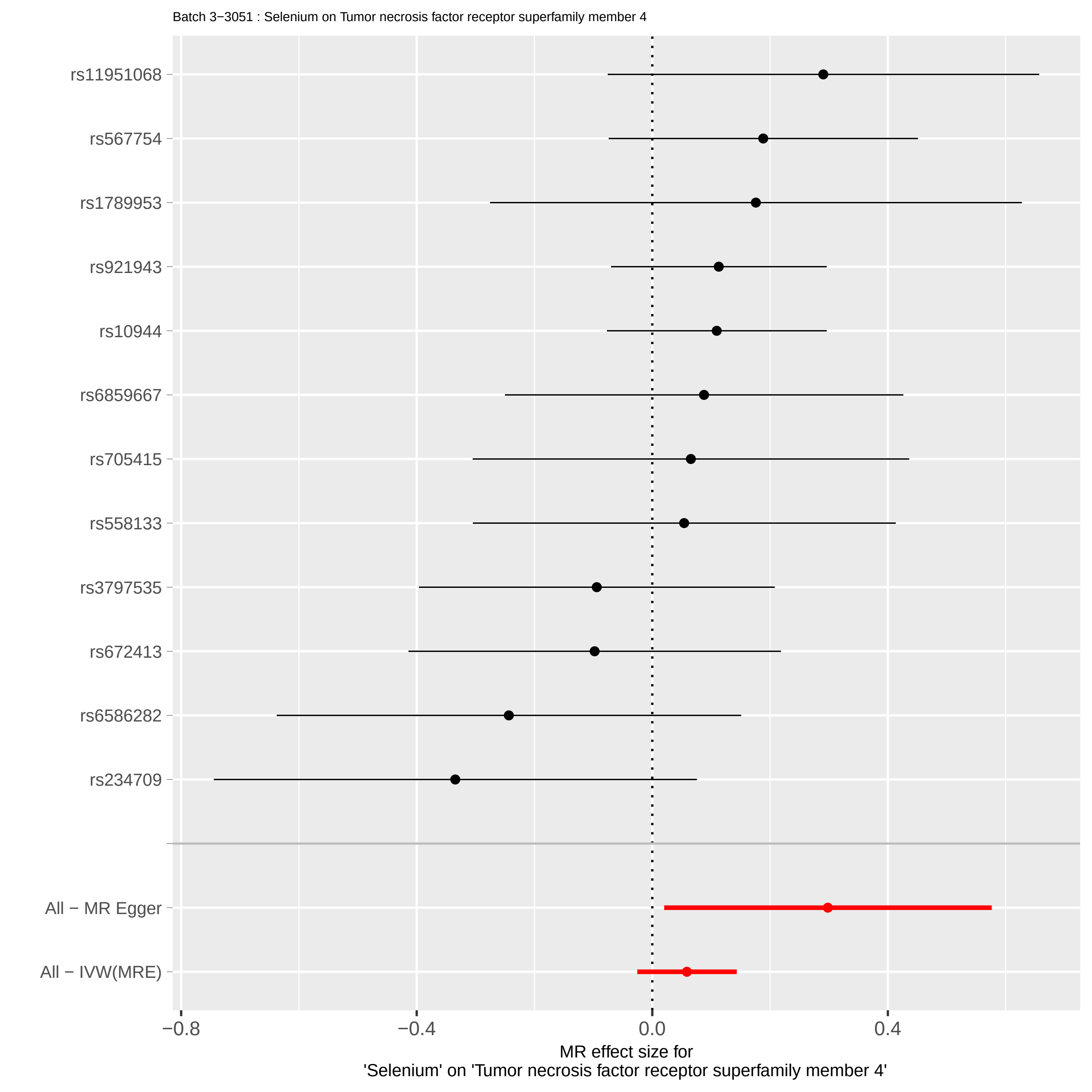

Batch 3−3051 : Selenium on Tumor necrosis factor receptor superfamily member 4
rs11951068
rs567754
rs1789953
rs921943
rs10944
rs6859667
rs705415
rs558133
rs3797535
rs672413
rs6586282
rs234709
All − MR Egger
All − IVW(MRE)
−0.8
−0.4
0.0
0.4
MR effect size for
'Selenium' on 'Tumor necrosis factor receptor superfamily member 4'

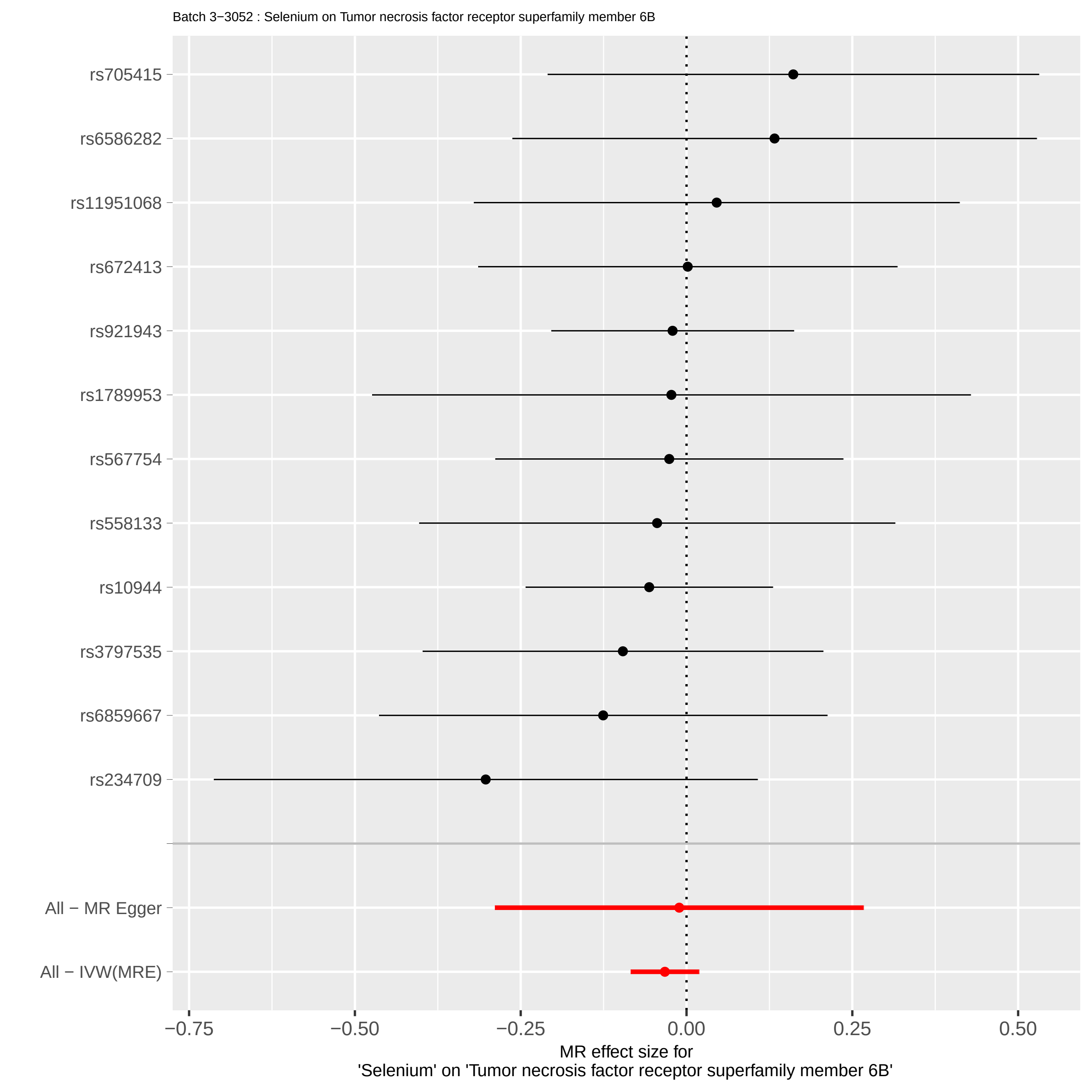
Batch 3−3052 : Selenium on Tumor necrosis factor receptor superfamily member 6B
rs705415
rs6586282
rs11951068
rs672413
rs921943
rs1789953
rs567754
rs558133
rs10944
rs3797535
rs6859667
rs234709
All − MR Egger
All − IVW(MRE)
−0.75
−0.50
−0.25
0.00
0.25
0.50
MR effect size for
'Selenium' on 'Tumor necrosis factor receptor superfamily member 6B'

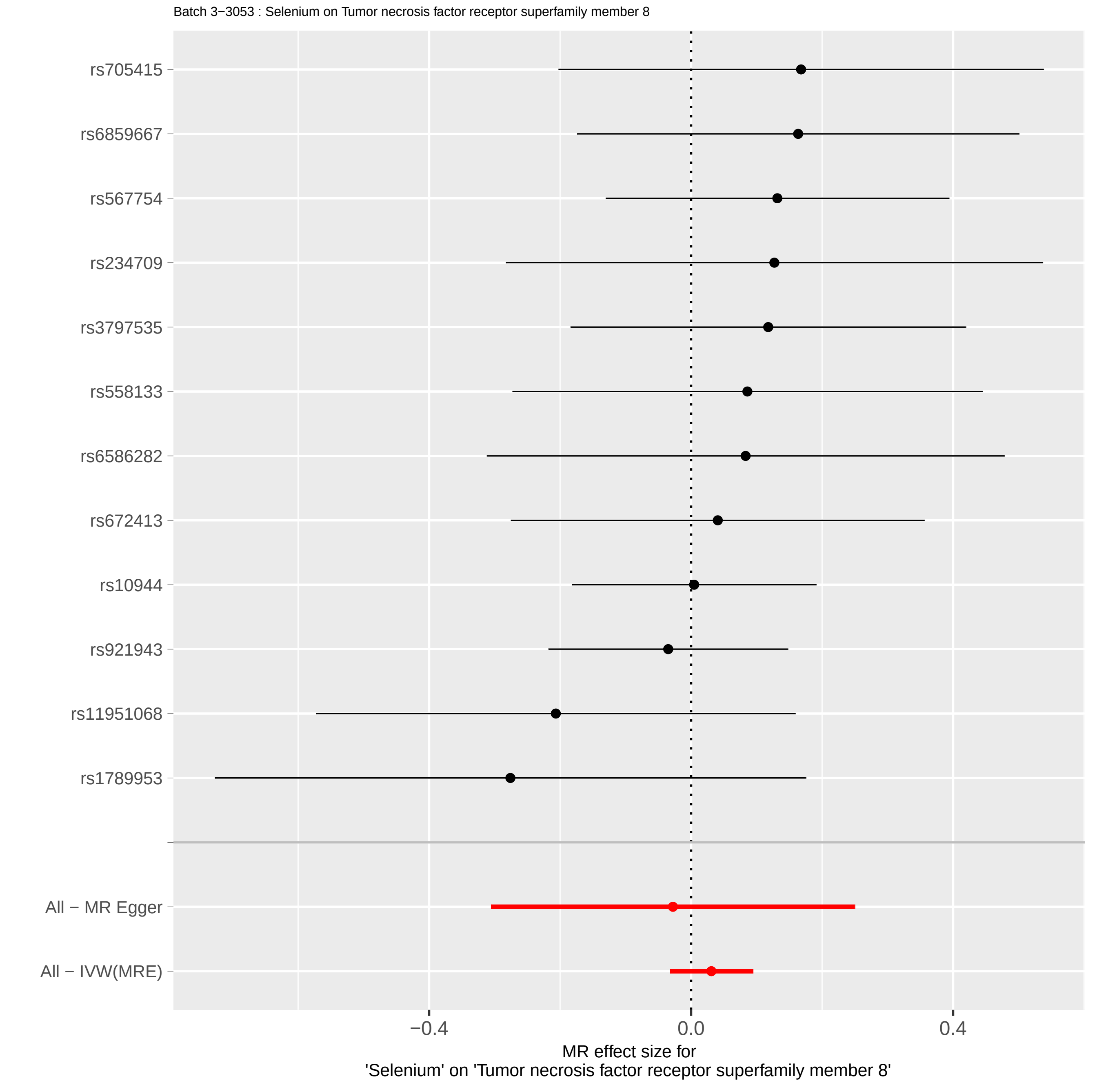

Batch 3−3053 : Selenium on Tumor necrosis factor receptor superfamily member 8
rs705415
rs6859667
rs567754
rs234709
rs3797535
rs558133
rs6586282
rs672413
rs10944
rs921943
rs11951068
rs1789953
All − MR Egger
All − IVW(MRE)
−0.4
0.0
0.4
MR effect size for
'Selenium' on 'Tumor necrosis factor receptor superfamily member 8'

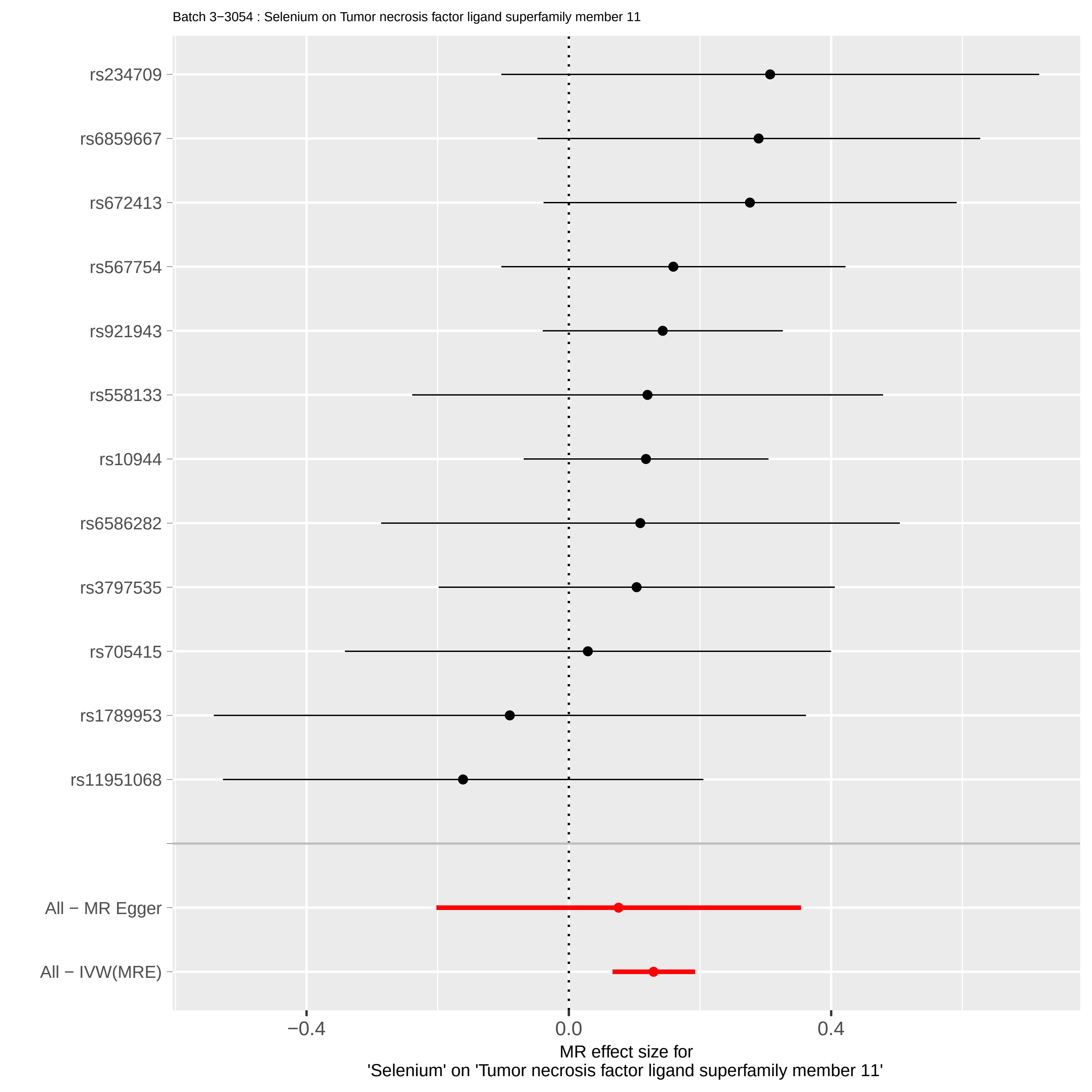

Batch 3−3054 : Selenium on Tumor necrosis factor ligand superfamily member 11
rs234709
rs6859667
rs672413
rs567754
rs921943
rs558133
rs10944
rs6586282
rs3797535
rs705415
rs1789953
rs11951068
All − MR Egger
All − IVW(MRE)
−0.4
0.0
0.4
MR effect size for
'Selenium' on 'Tumor necrosis factor ligand superfamily member 11'

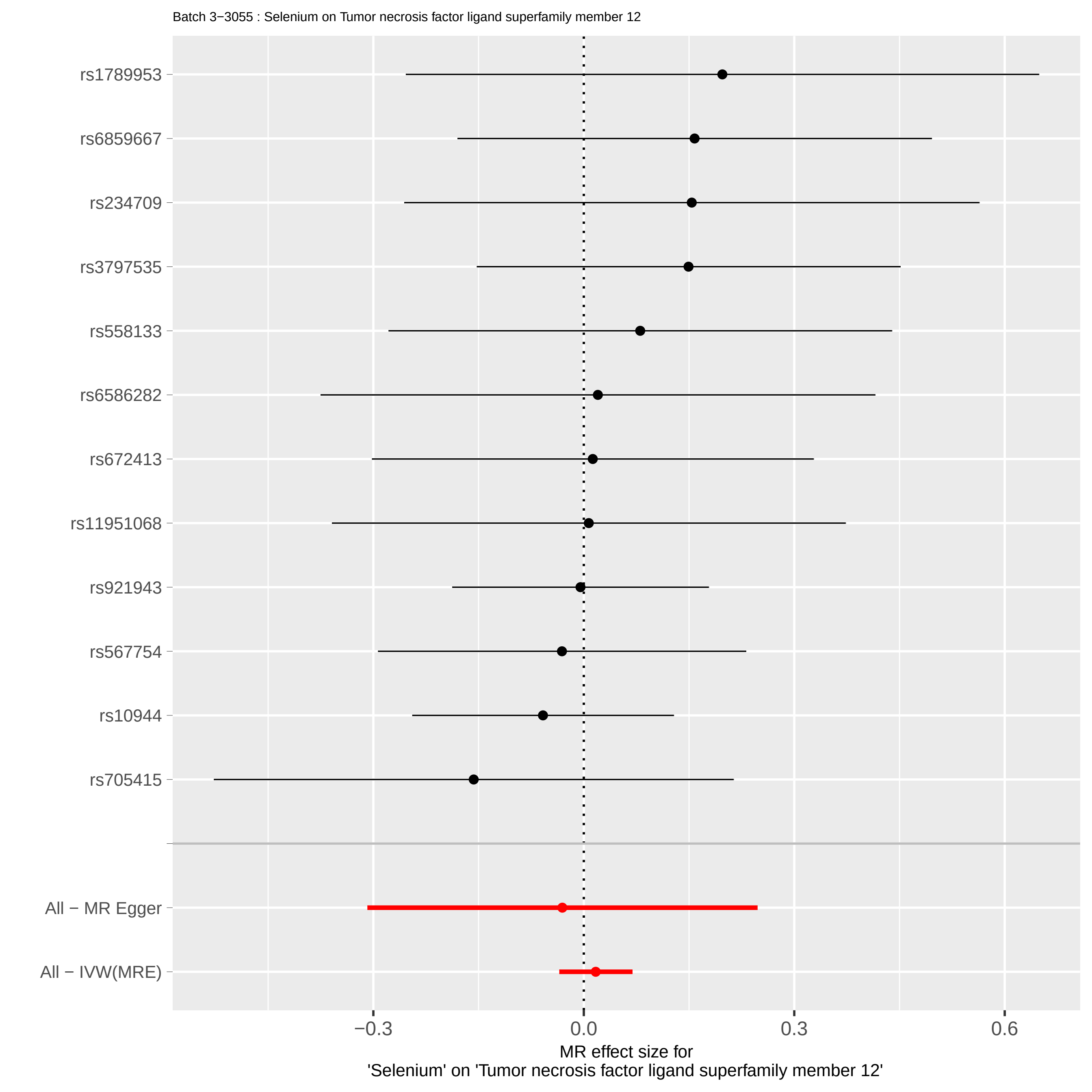

Batch 3−3055 : Selenium on Tumor necrosis factor ligand superfamily member 12
rs1789953
rs6859667
rs234709
rs3797535
rs558133
rs6586282
rs672413
rs11951068
rs921943
rs567754
rs10944
rs705415
All − MR Egger
All − IVW(MRE)
−0.3
0.0
0.3
0.6
MR effect size for
'Selenium' on 'Tumor necrosis factor ligand superfamily member 12'

Batch 3−3056 : Selenium on Tumor necrosis factor ligand superfamily member 13B

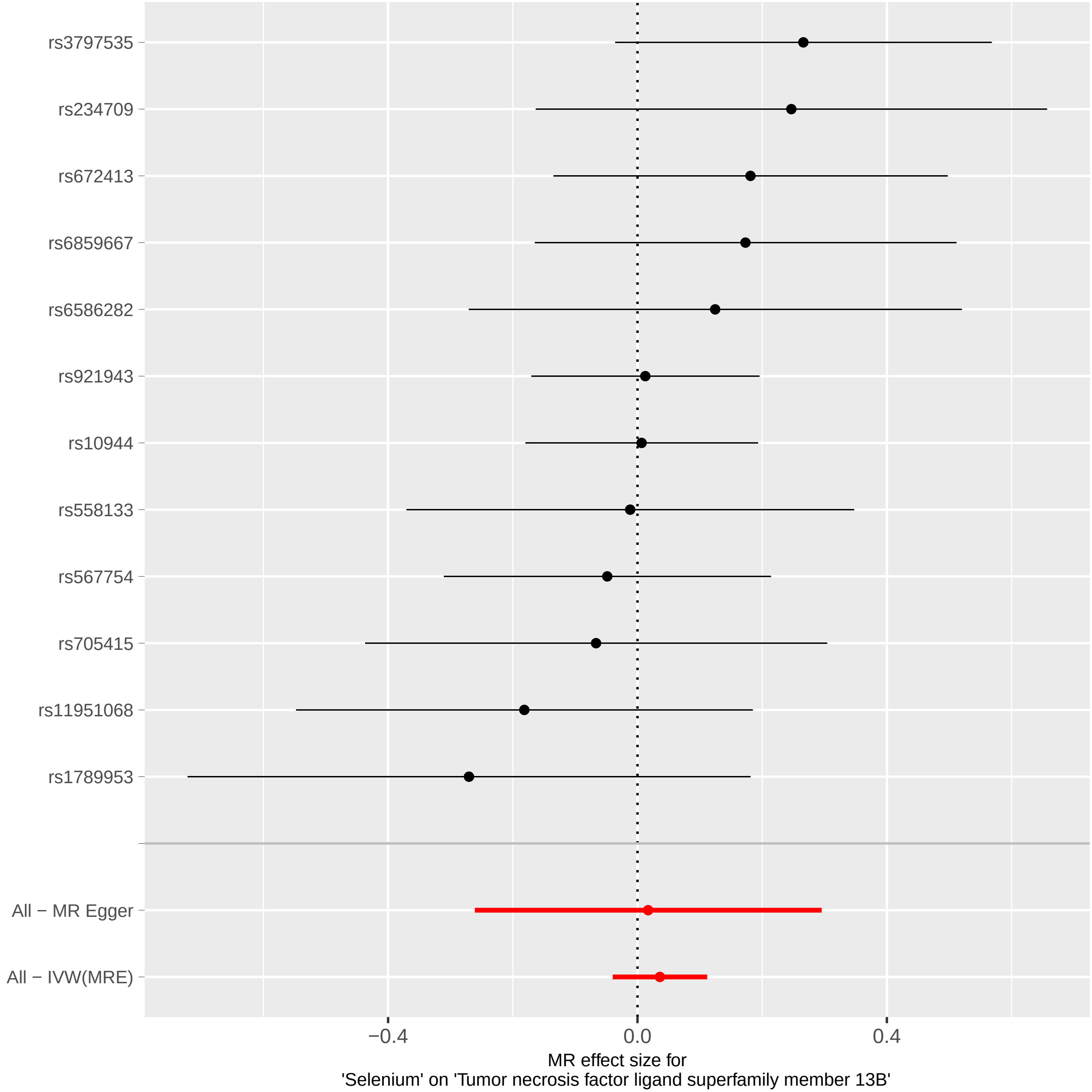

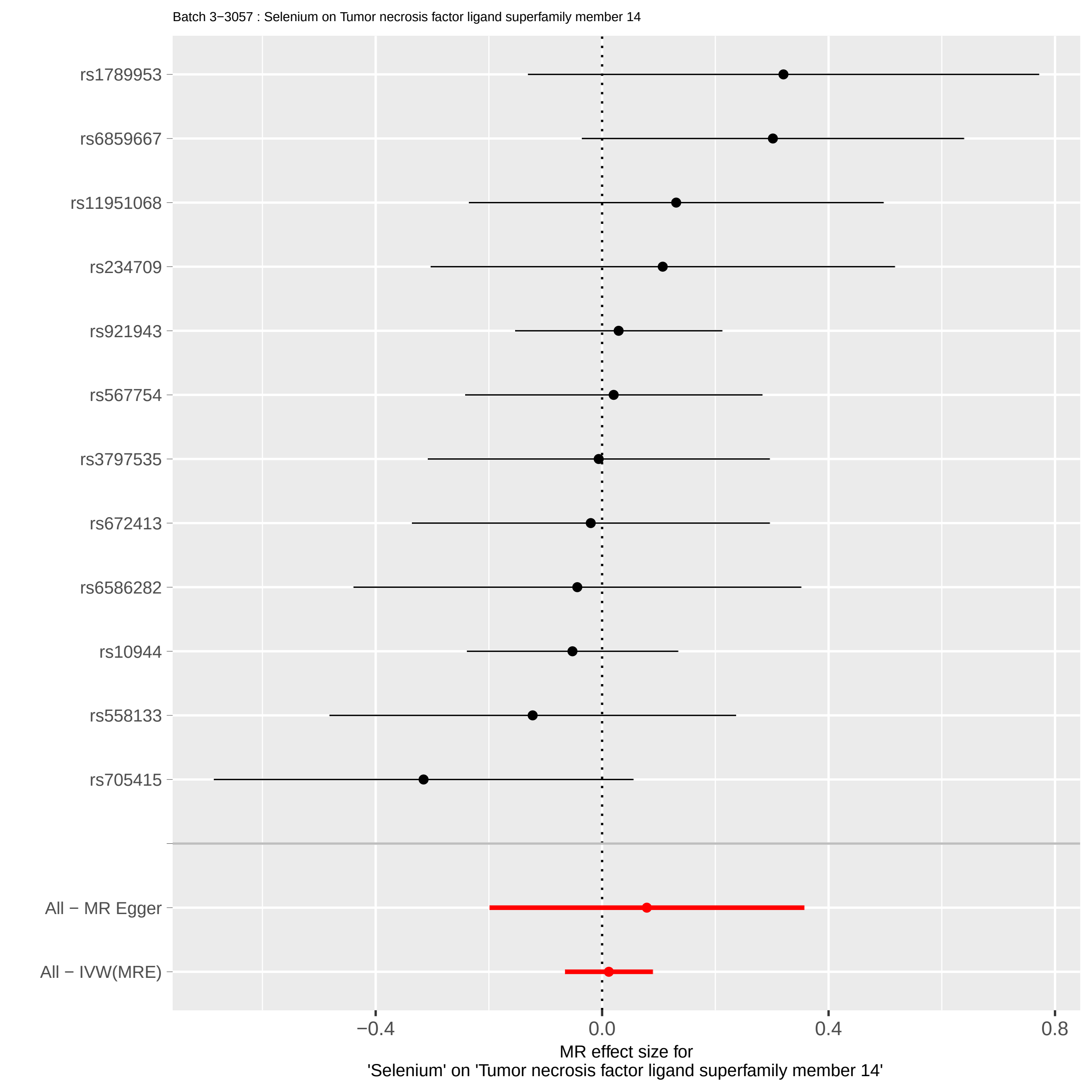

Batch 3−3057 : Selenium on Tumor necrosis factor ligand superfamily member 14
rs1789953
rs6859667
rs11951068
rs234709
rs921943
rs567754
rs3797535
rs672413
rs6586282
rs10944
rs558133
rs705415
All − MR Egger
All − IVW(MRE)
−0.4
0.0
0.4
0.8
MR effect size for
'Selenium' on 'Tumor necrosis factor ligand superfamily member 14'

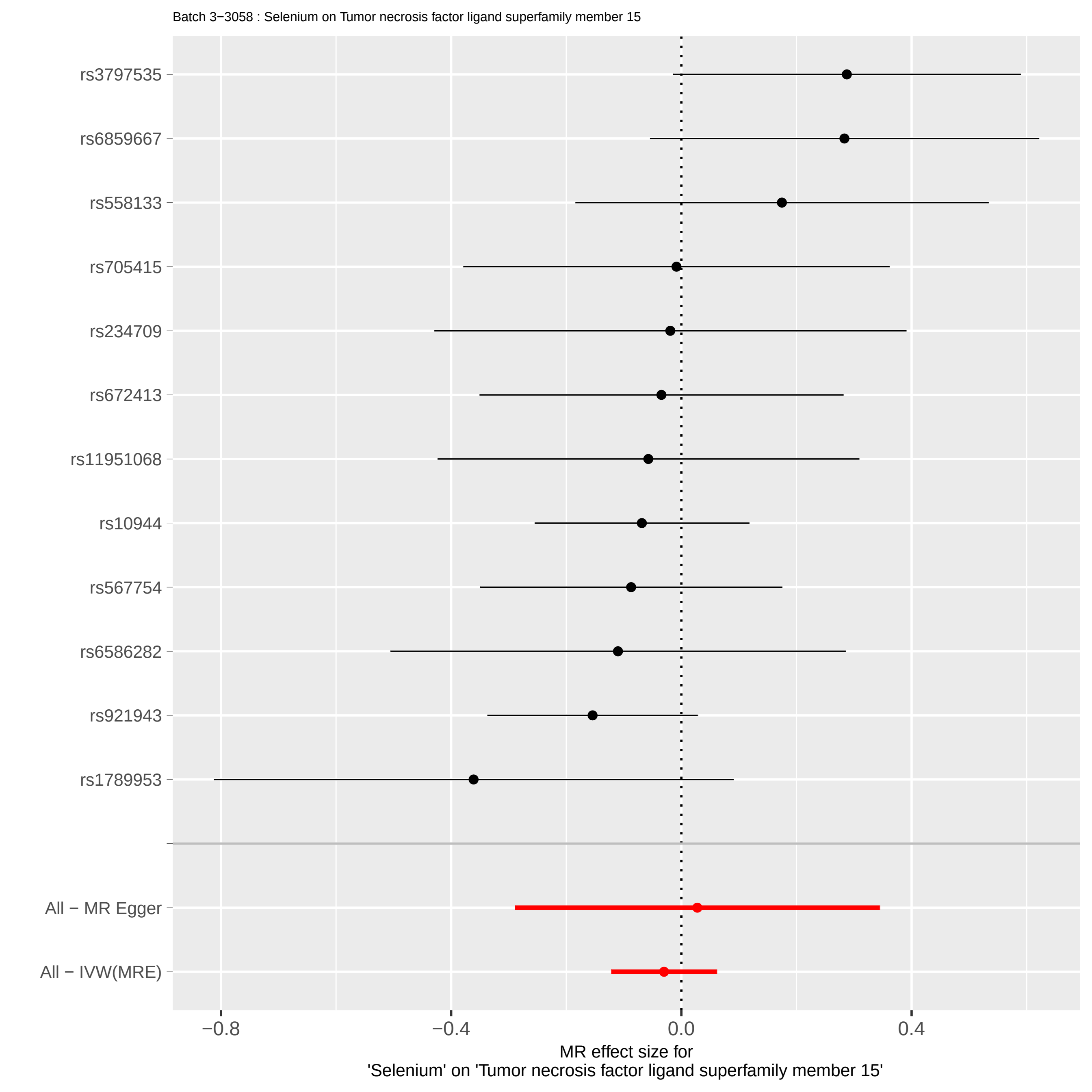

Batch 3−3058 : Selenium on Tumor necrosis factor ligand superfamily member 15
rs3797535
rs6859667
rs558133
rs705415
rs234709
rs672413
rs11951068
rs10944
rs567754
rs6586282
rs921943
rs1789953
All − MR Egger
All − IVW(MRE)
−0.8
−0.4
0.0
0.4
MR effect size for
'Selenium' on 'Tumor necrosis factor ligand superfamily member 15'

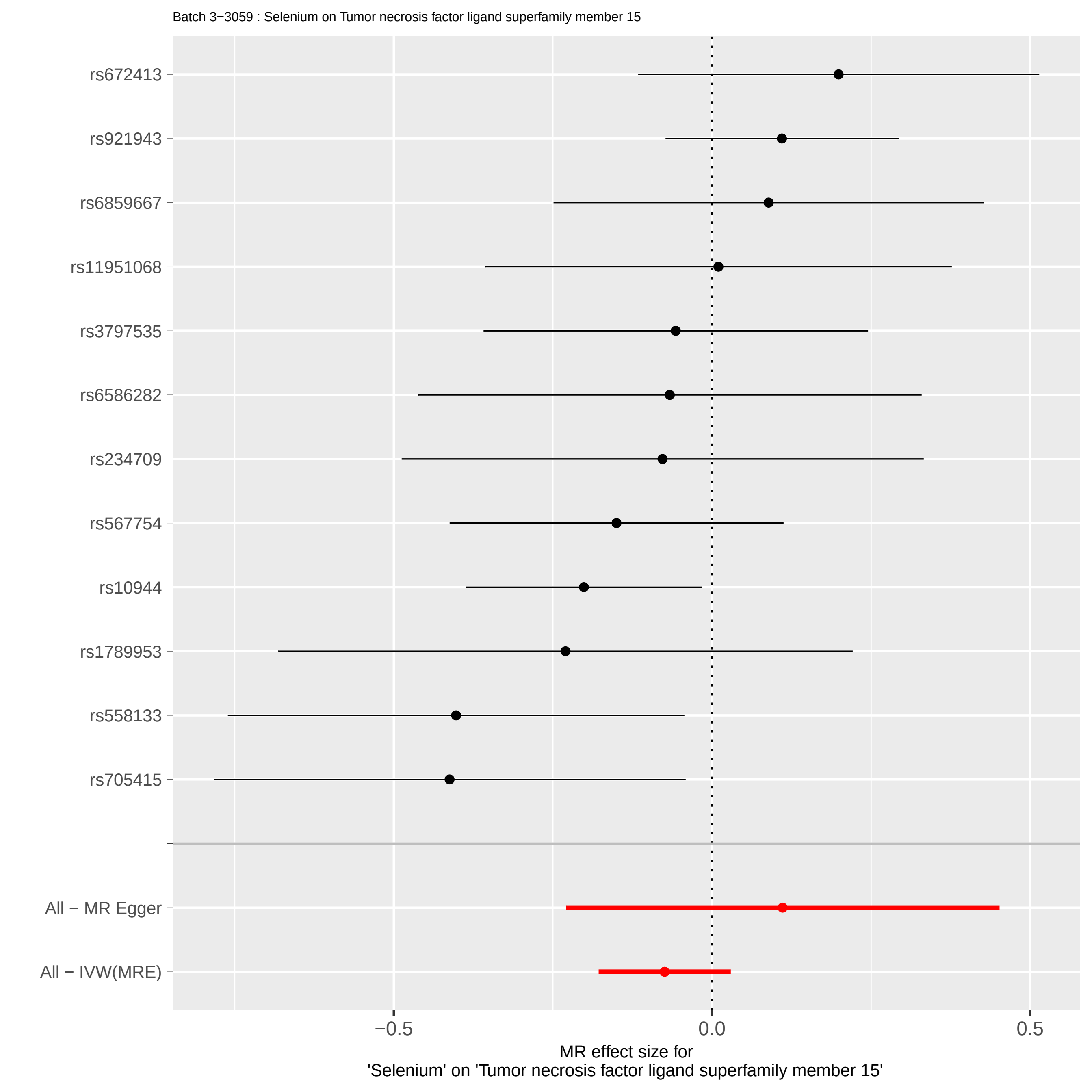
Batch 3−3059 : Selenium on Tumor necrosis factor ligand superfamily member 15
rs672413
rs921943
rs6859667
rs11951068
rs3797535
rs6586282
rs234709
rs567754
rs10944
rs1789953
rs558133
rs705415
All − MR Egger
All − IVW(MRE)
−0.5
0.0
0.5
MR effect size for
'Selenium' on 'Tumor necrosis factor ligand superfamily member 15'

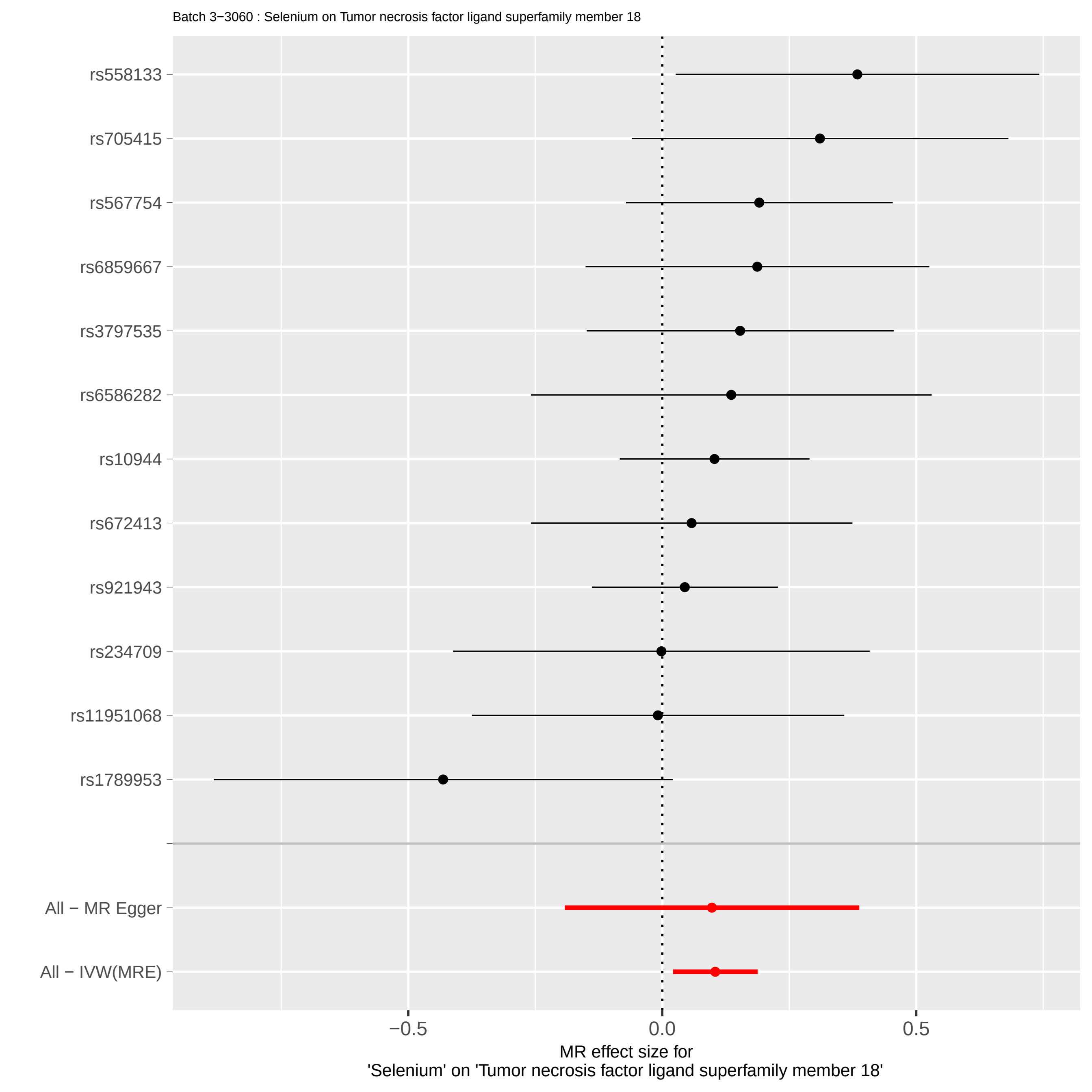

Batch 3−3060 : Selenium on Tumor necrosis factor ligand superfamily member 18
rs558133
rs705415
rs567754
rs6859667
rs3797535
rs6586282
rs10944
rs672413
rs921943
rs234709
rs11951068
rs1789953
All − MR Egger
All − IVW(MRE)
−0.5
0.0
0.5
MR effect size for
'Selenium' on 'Tumor necrosis factor ligand superfamily member 18'

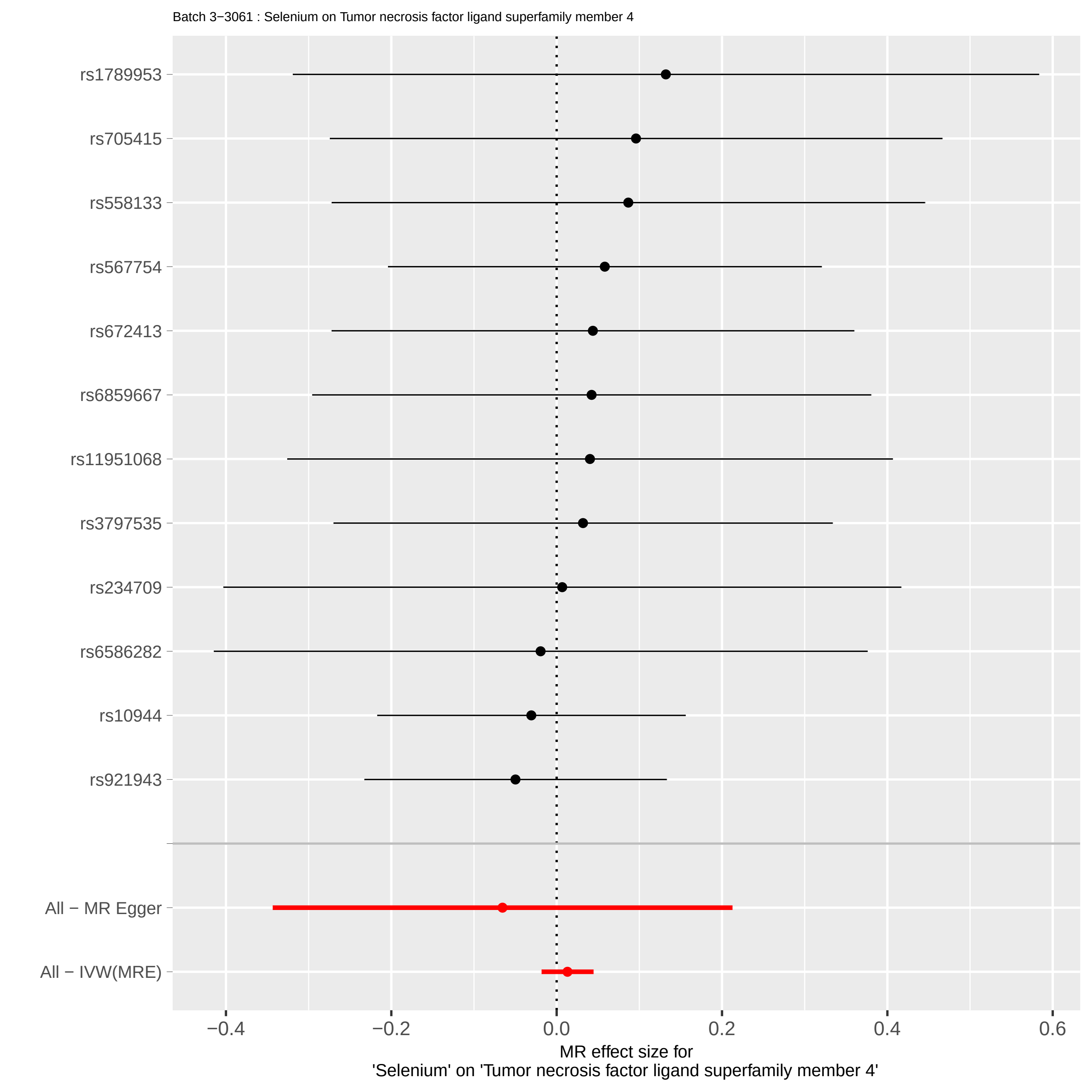

Batch 3−3061 : Selenium on Tumor necrosis factor ligand superfamily member 4
rs1789953
rs705415
rs558133
rs567754
rs672413
rs6859667
rs11951068
rs3797535
rs234709
rs6586282
rs10944
rs921943
All − MR Egger
All − IVW(MRE)
−0.4
−0.2
0.0
0.2
0.4
0.6
MR effect size for
'Selenium' on 'Tumor necrosis factor ligand superfamily member 4'

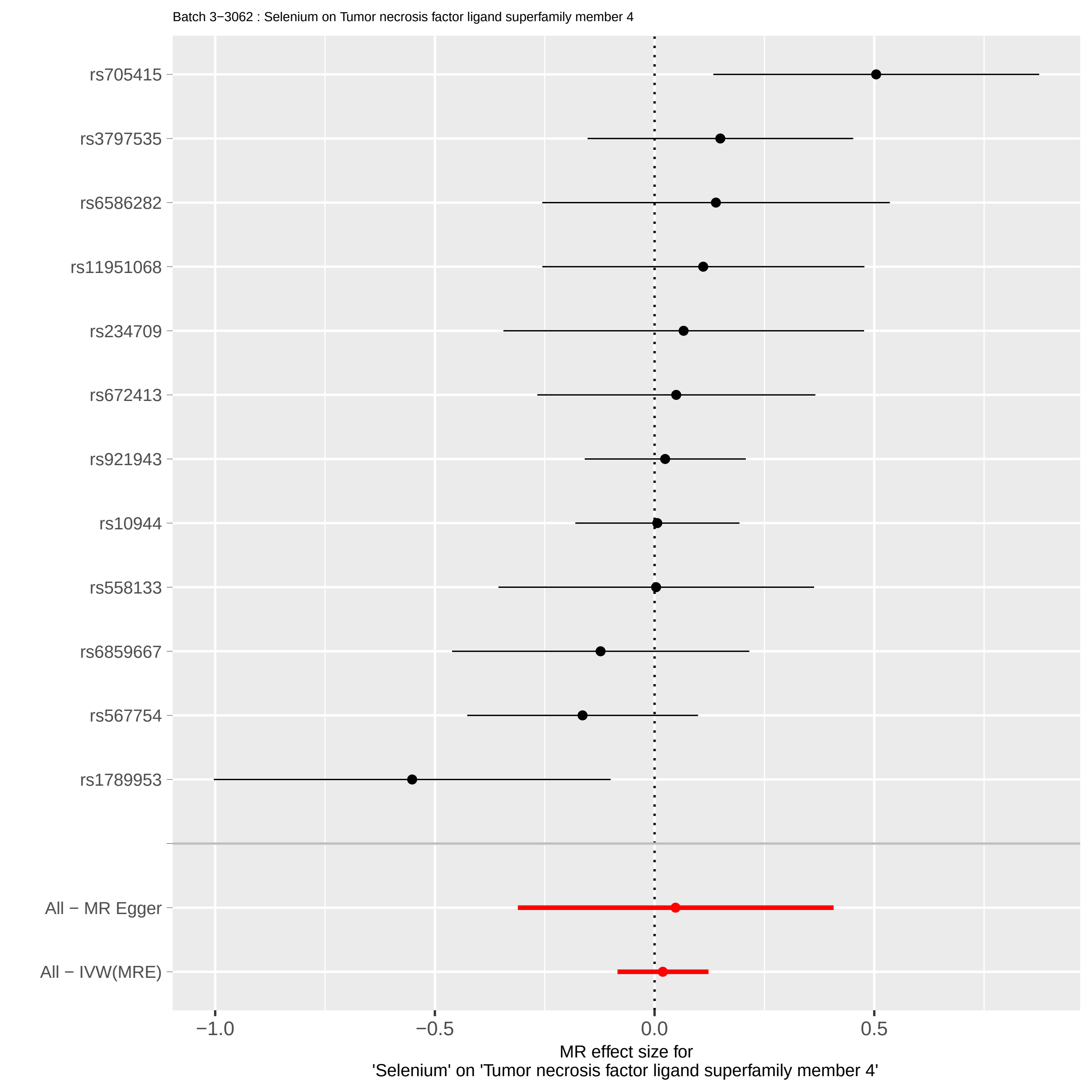
Batch 3−3062 : Selenium on Tumor necrosis factor ligand superfamily member 4
rs705415
rs3797535
rs6586282
rs11951068
rs234709
rs672413
rs921943
rs10944
rs558133
rs6859667
rs567754
rs1789953
All − MR Egger
All − IVW(MRE)
−1.0
−0.5
0.0
0.5
MR effect size for
'Selenium' on 'Tumor necrosis factor ligand superfamily member 4'

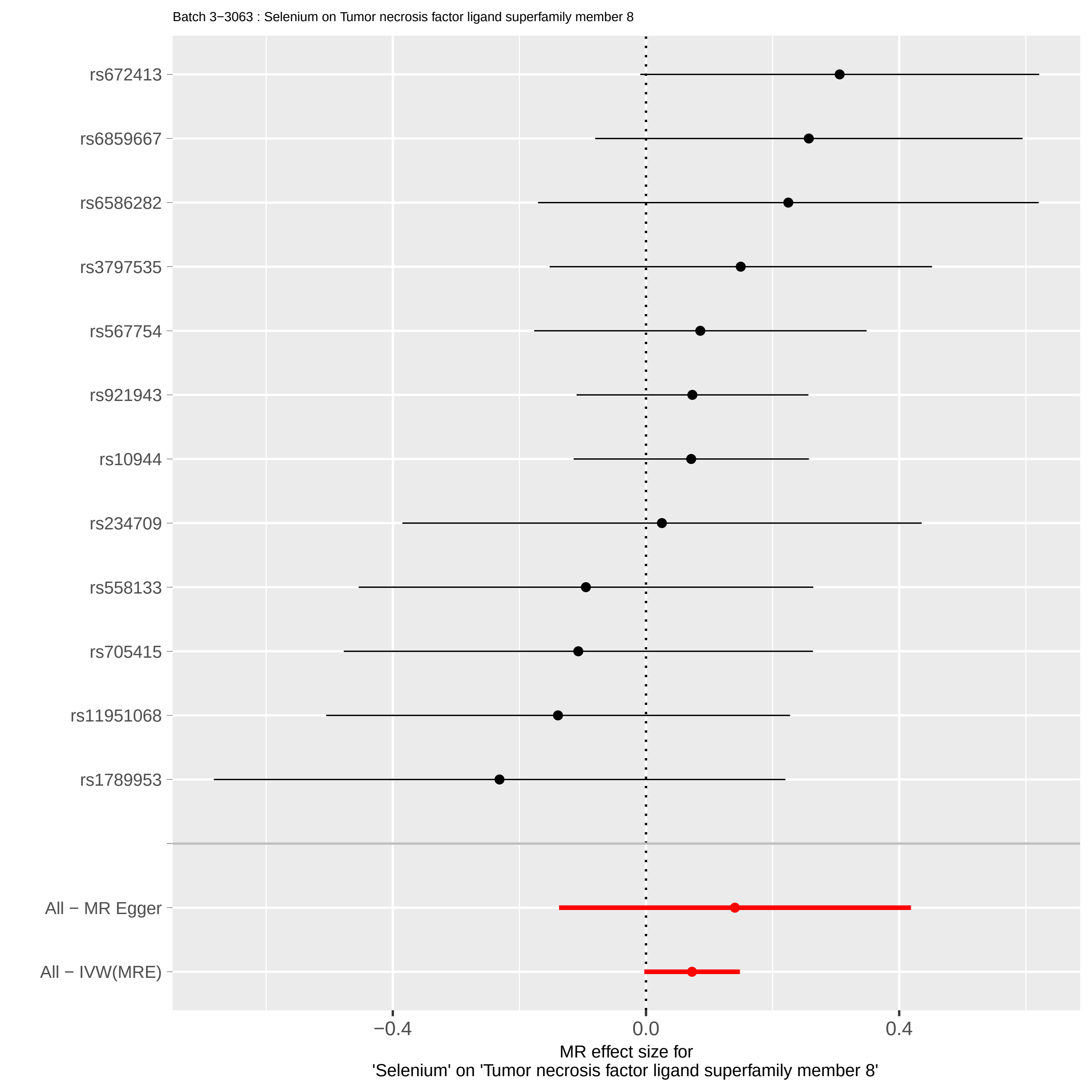

Batch 3−3063 : Selenium on Tumor necrosis factor ligand superfamily member 8
rs672413
rs6859667
rs6586282
rs3797535
rs567754
rs921943
rs10944
rs234709
rs558133
rs705415
rs11951068
rs1789953
All − MR Egger
All − IVW(MRE)
−0.4
0.0
0.4
MR effect size for
'Selenium' on 'Tumor necrosis factor ligand superfamily member 8'

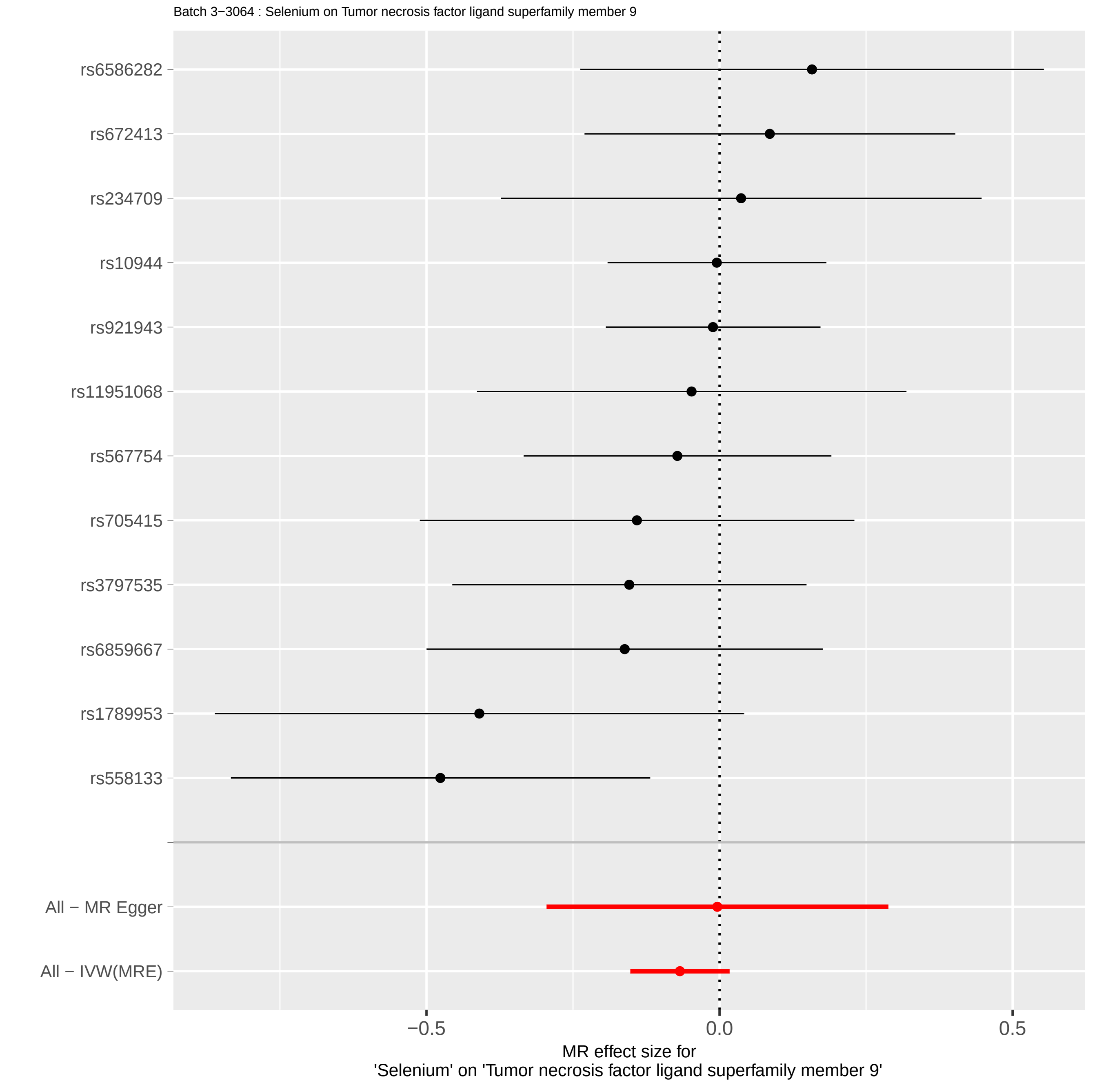

Batch 3−3064 : Selenium on Tumor necrosis factor ligand superfamily member 9
rs6586282
rs672413
rs234709
rs10944
rs921943
rs11951068
rs567754
rs705415
rs3797535
rs6859667
rs1789953
rs558133
All − MR Egger
All − IVW(MRE)
−0.5
0.0
0.5
MR effect size for
'Selenium' on 'Tumor necrosis factor ligand superfamily member 9'

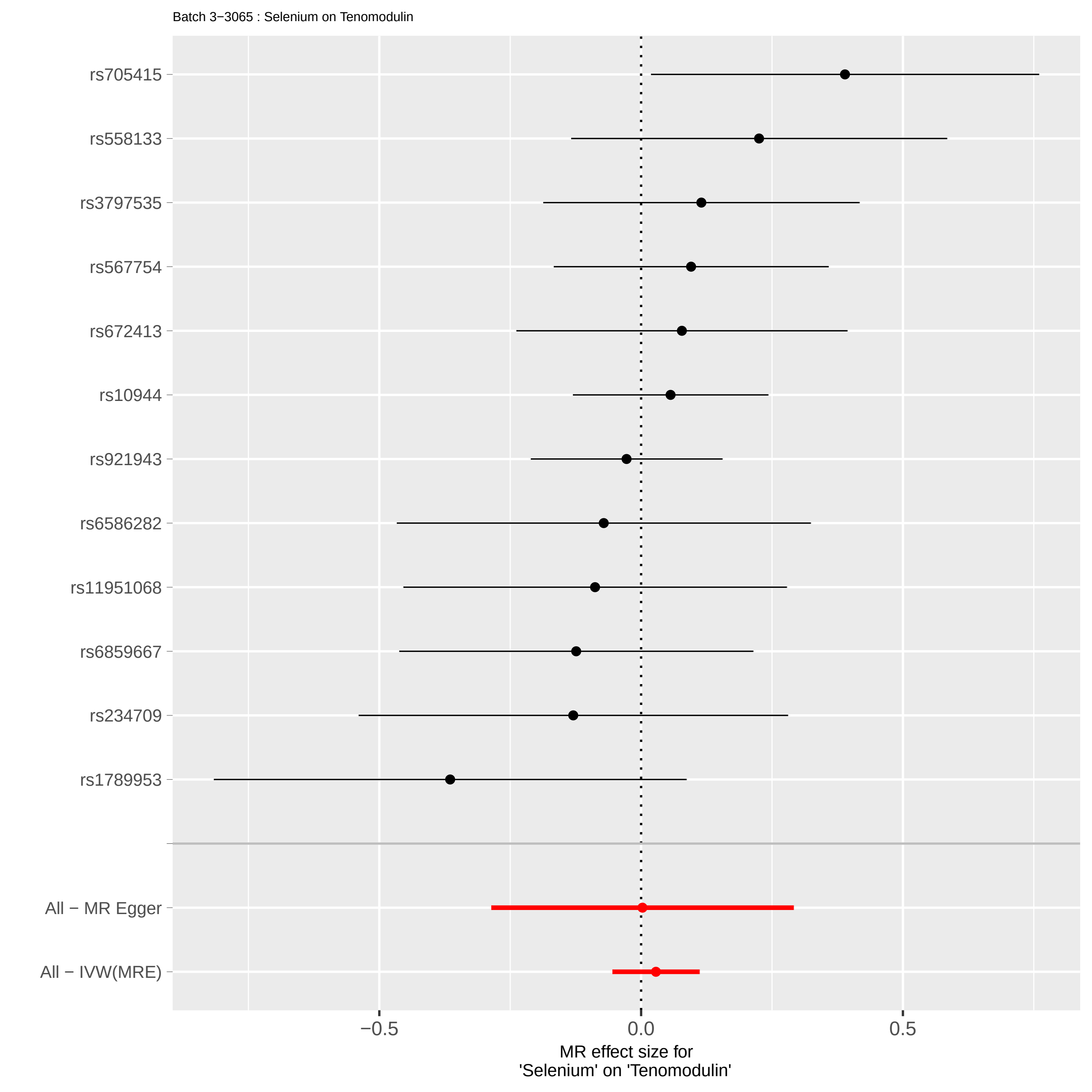

Batch 3−3065 : Selenium on Tenomodulin
rs705415
rs558133
rs3797535
rs567754
rs672413
rs10944
rs921943
rs6586282
rs11951068
rs6859667
rs234709
rs1789953
All − MR Egger
All − IVW(MRE)
−0.5
0.0
0.5
MR effect size for
'Selenium' on 'Tenomodulin'

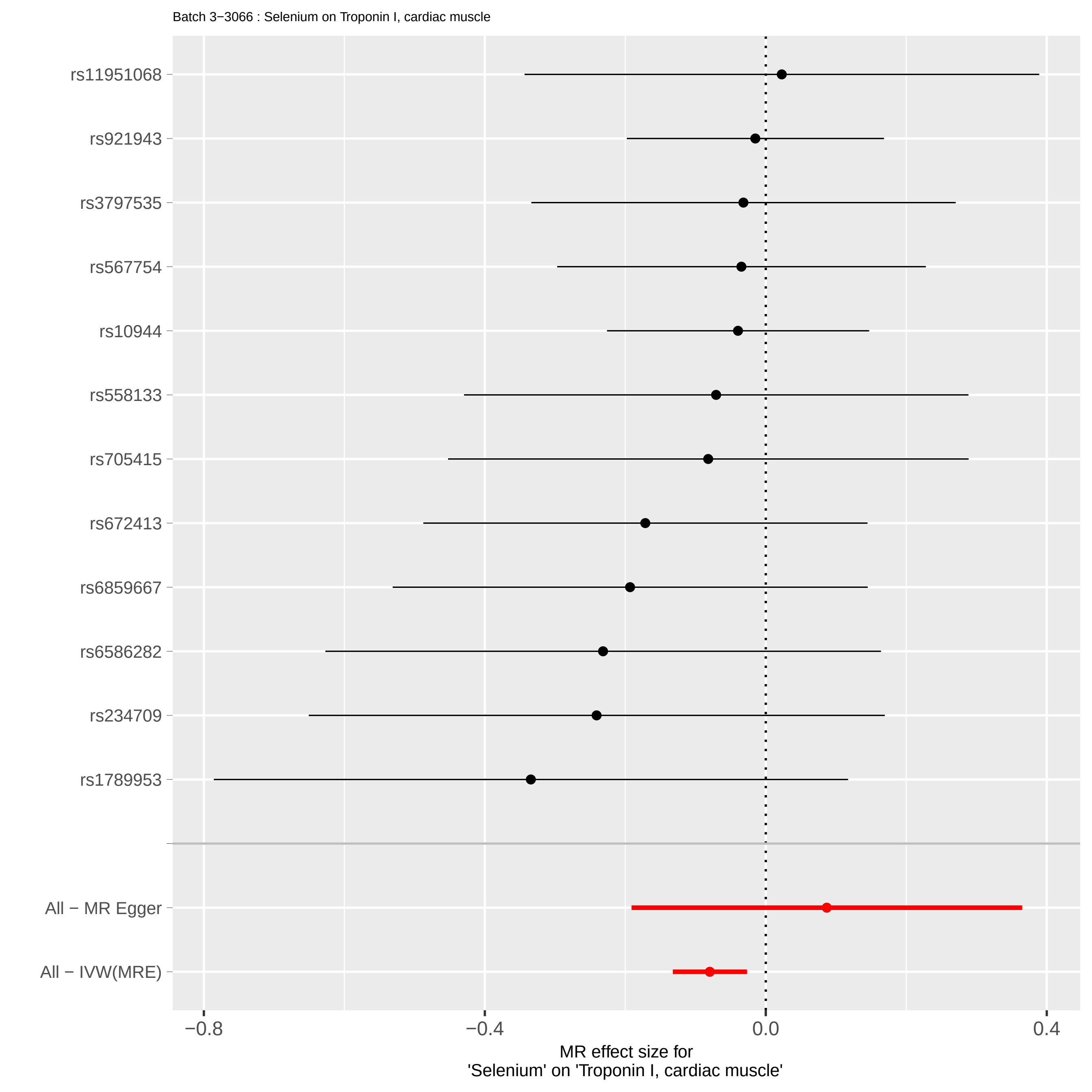

Batch 3−3066 : Selenium on Troponin I, cardiac muscle
rs11951068
rs921943
rs3797535
rs567754
rs10944
rs558133
rs705415
rs672413
rs6859667
rs6586282
rs234709
rs1789953
All − MR Egger
All − IVW(MRE)
−0.8
−0.4
0.0
0.4
MR effect size for
'Selenium' on 'Troponin I, cardiac muscle'

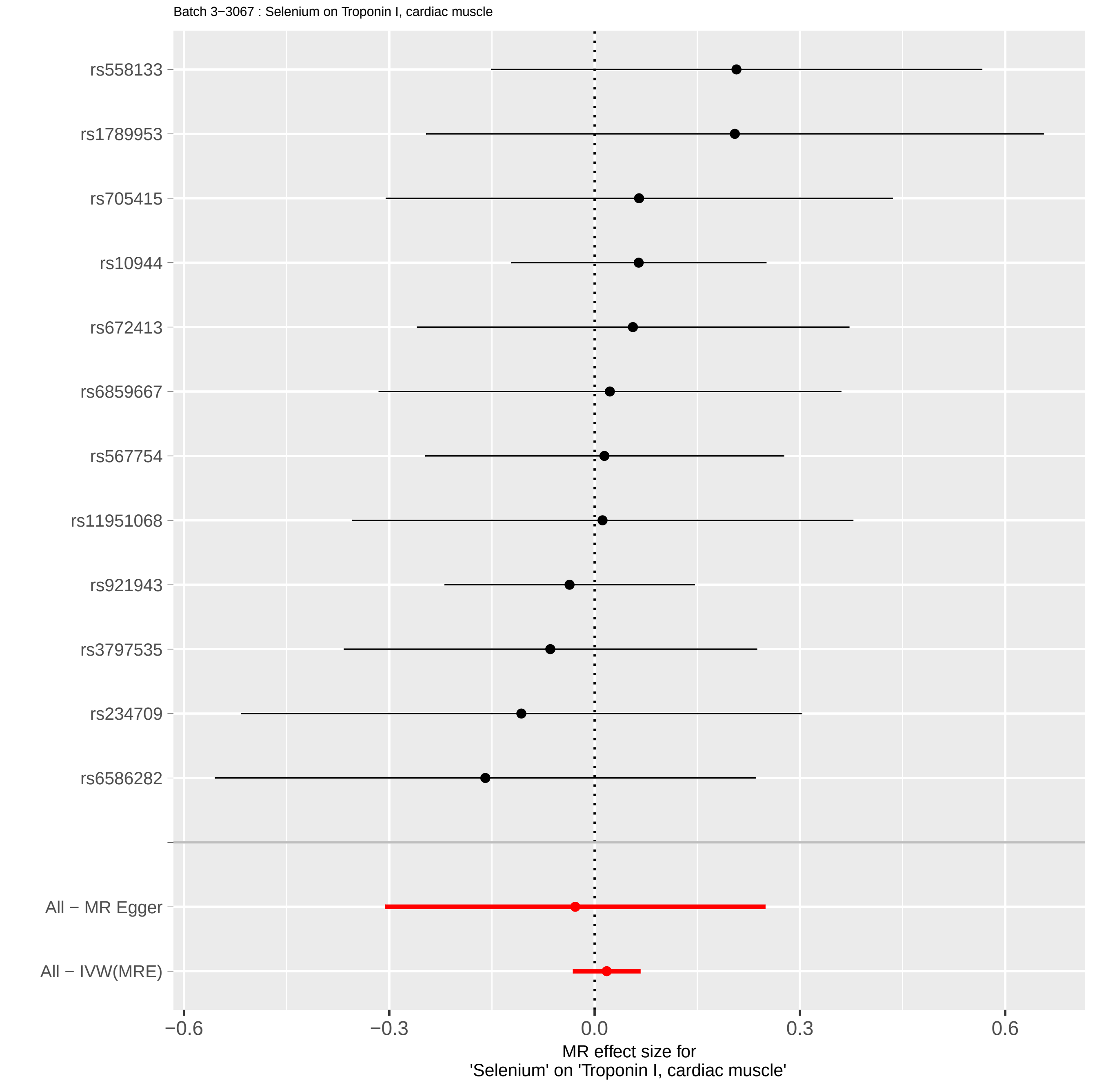

Batch 3−3067 : Selenium on Troponin I, cardiac muscle
rs558133
rs1789953
rs705415
rs10944
rs672413
rs6859667
rs567754
rs11951068
rs921943
rs3797535
rs234709
rs6586282
All − MR Egger
All − IVW(MRE)
−0.6
−0.3
0.0
0.3
0.6
MR effect size for
'Selenium' on 'Troponin I, cardiac muscle'

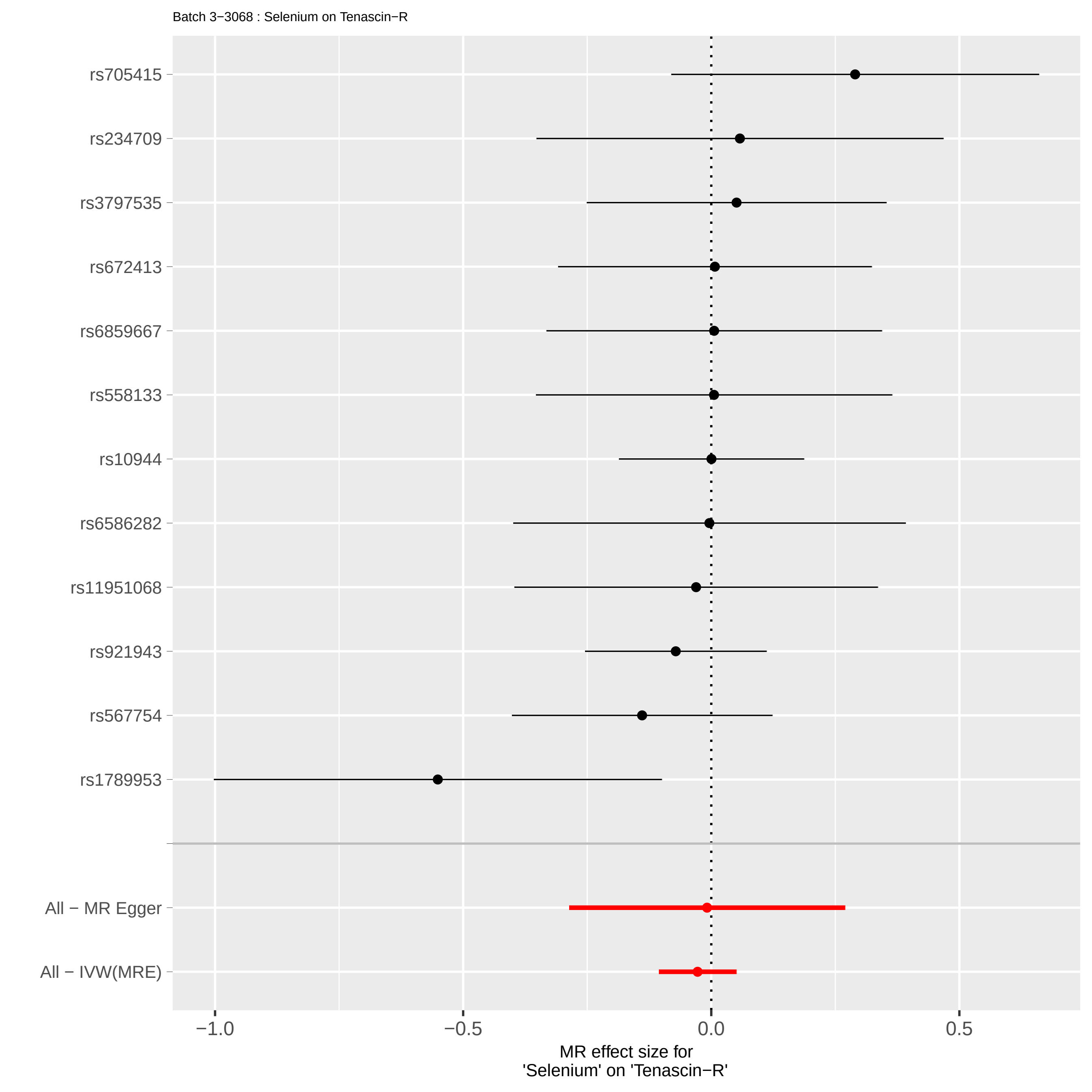

Batch 3−3068 : Selenium on Tenascin−R
rs705415
rs234709
rs3797535
rs672413
rs6859667
rs558133
rs10944
rs6586282
rs11951068
rs921943
rs567754
rs1789953
All − MR Egger
All − IVW(MRE)
−1.0
−0.5
0.0
0.5
MR effect size for
'Selenium' on 'Tenascin−R'

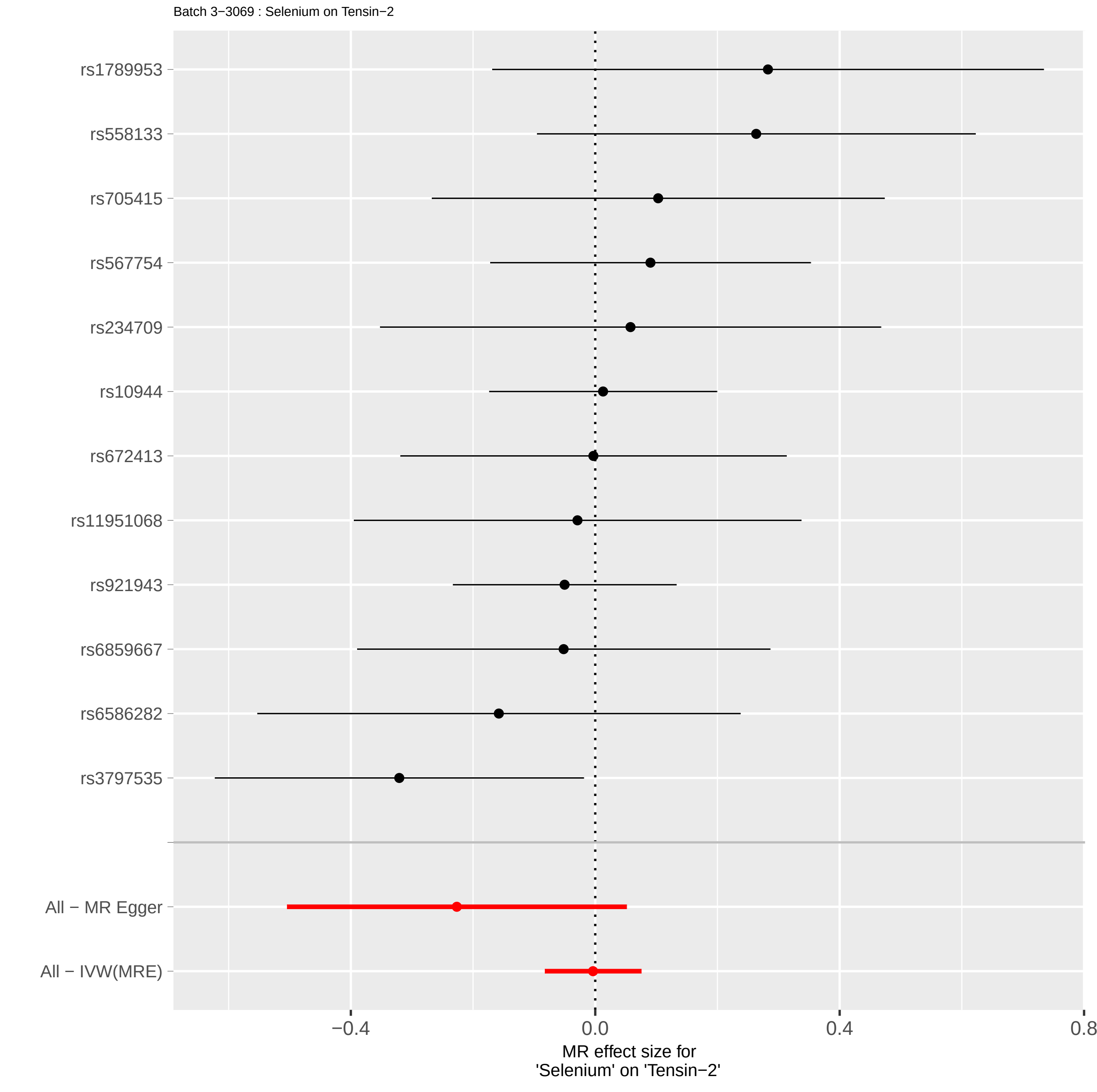

Batch 3−3069 : Selenium on Tensin−2
rs1789953
rs558133
rs705415
rs567754
rs234709
rs10944
rs672413
rs11951068
rs921943
rs6859667
rs6586282
rs3797535
All − MR Egger
All − IVW(MRE)
−0.4
0.0
0.4
0.8
MR effect size for
'Selenium' on 'Tensin−2'

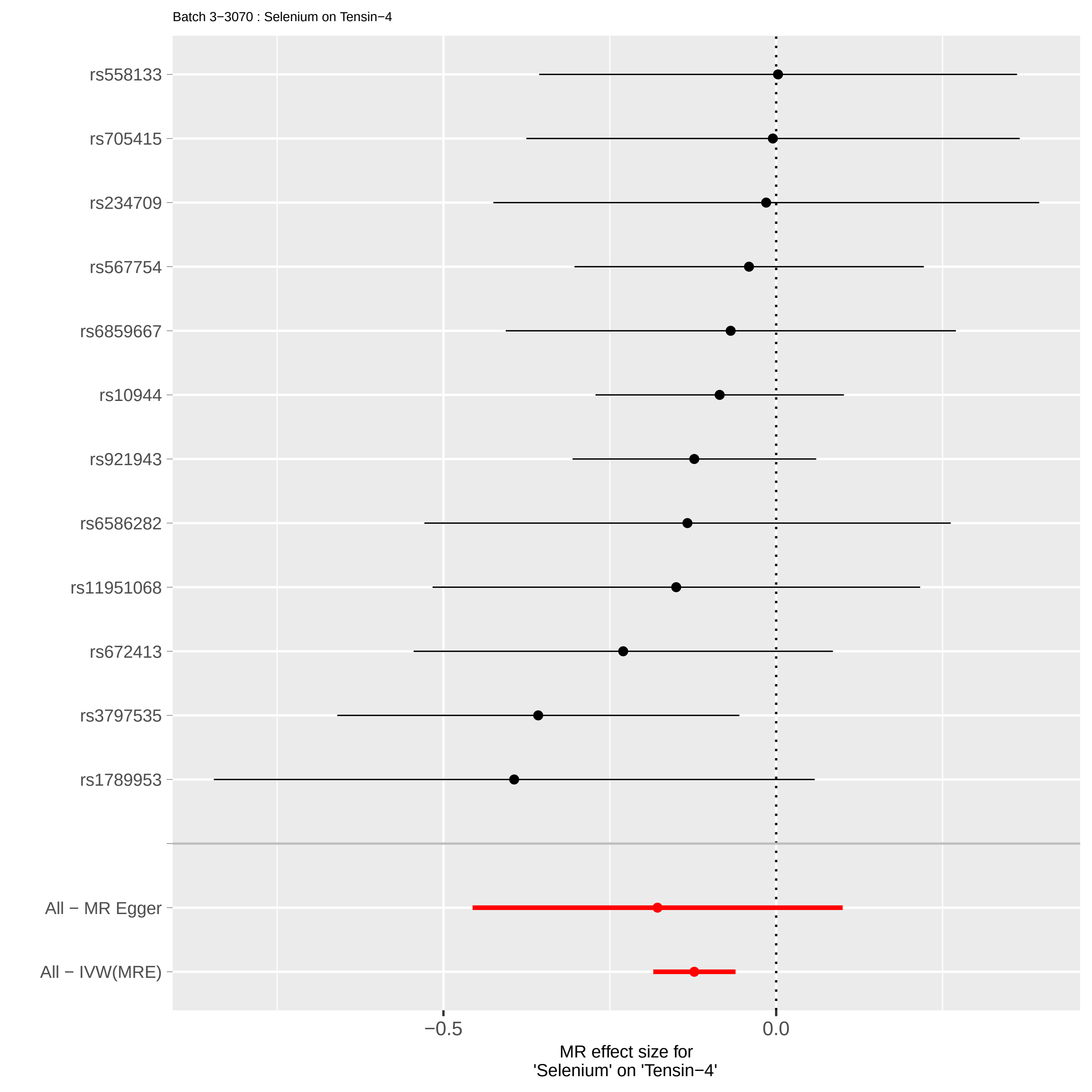
Batch 3−3070 : Selenium on Tensin−4
rs558133
rs705415
rs234709
rs567754
rs6859667
rs10944
rs921943
rs6586282
rs11951068
rs672413
rs3797535
rs1789953
All − MR Egger
All − IVW(MRE)
−0.5
0.0
MR effect size for
'Selenium' on 'Tensin−4'

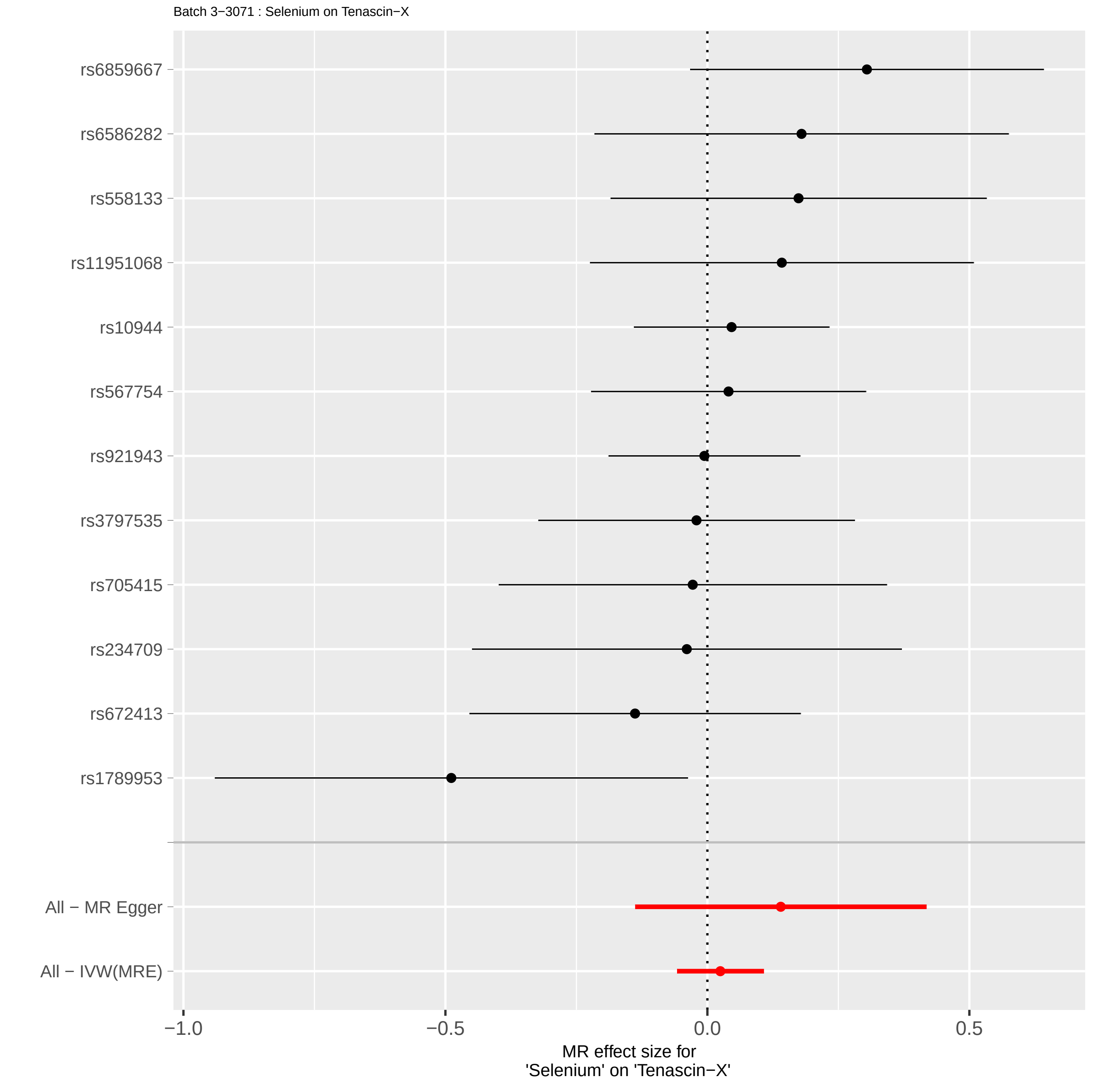
Batch 3−3071 : Selenium on Tenascin−X
rs6859667
rs6586282
rs558133
rs11951068
rs10944
rs567754
rs921943
rs3797535
rs705415
rs234709
rs672413
rs1789953
All − MR Egger
All − IVW(MRE)
−1.0
−0.5
0.0
0.5
MR effect size for
'Selenium' on 'Tenascin−X'

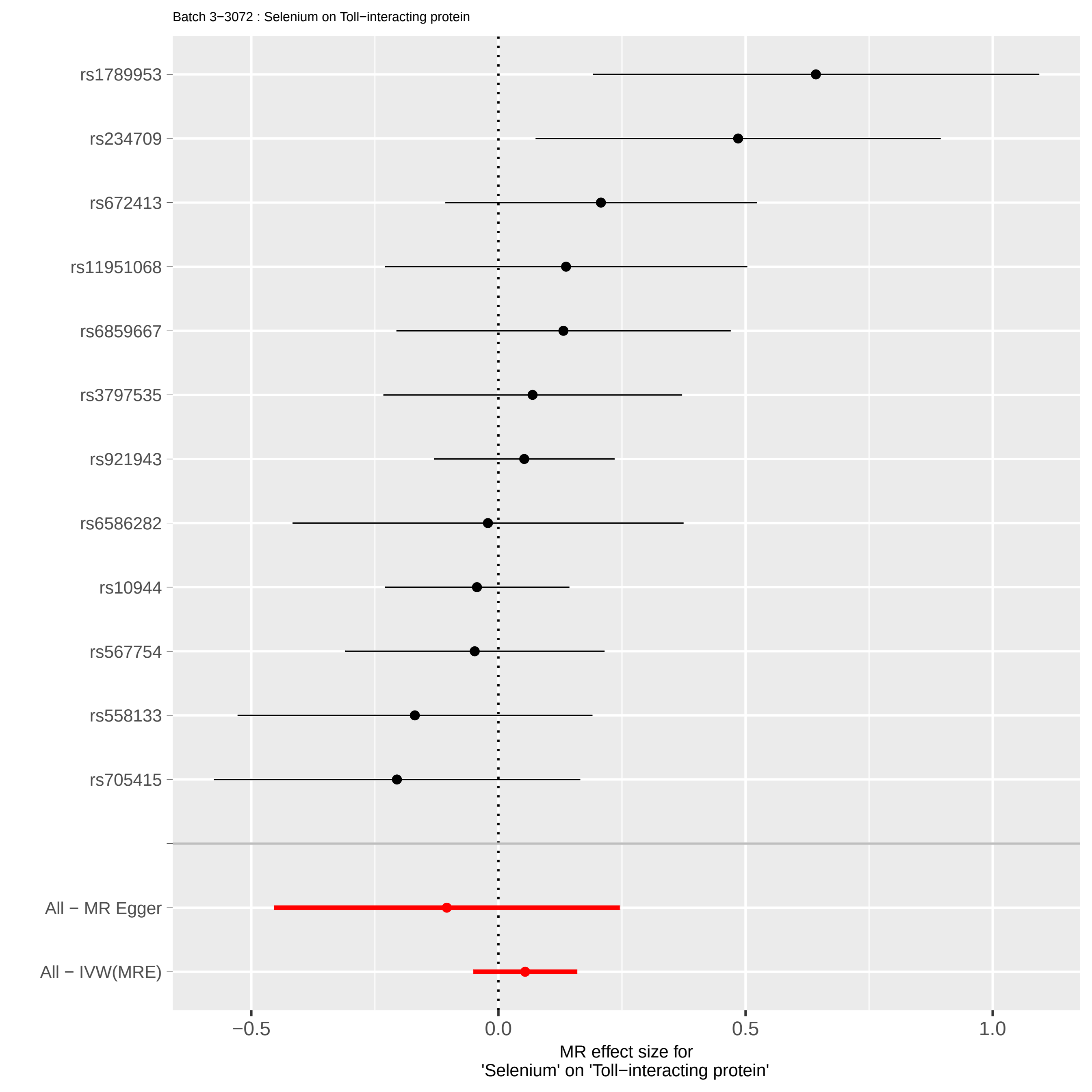
Batch 3−3072 : Selenium on Toll−interacting protein
rs1789953
rs234709
rs672413
rs11951068
rs6859667
rs3797535
rs921943
rs6586282
rs10944
rs567754
rs558133
rs705415
All − MR Egger
All − IVW(MRE)
−0.5
0.0
0.5
1.0
MR effect size for
'Selenium' on 'Toll−interacting protein'

Batch 3−3073 : Selenium on TOM1−like protein 1

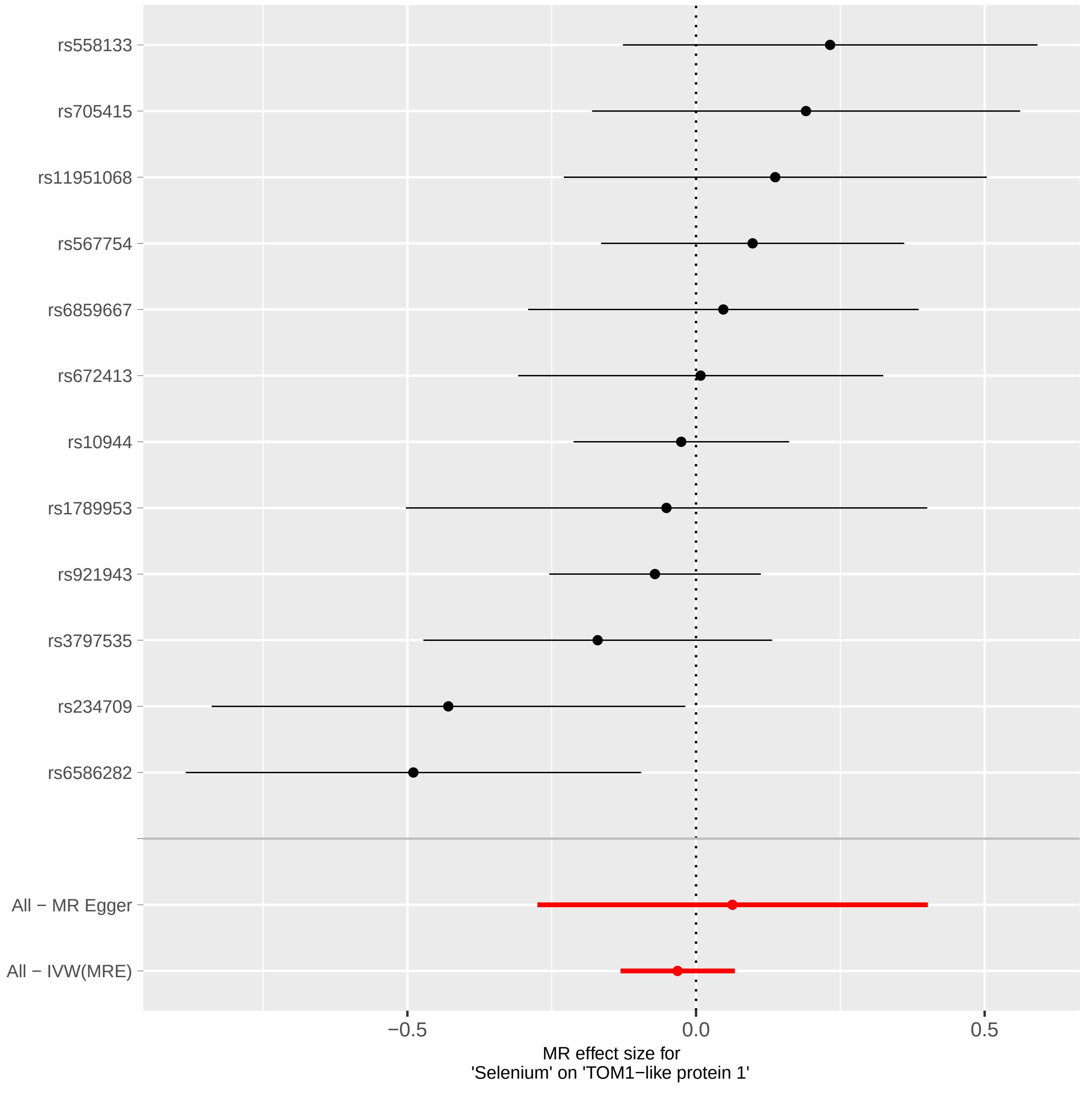

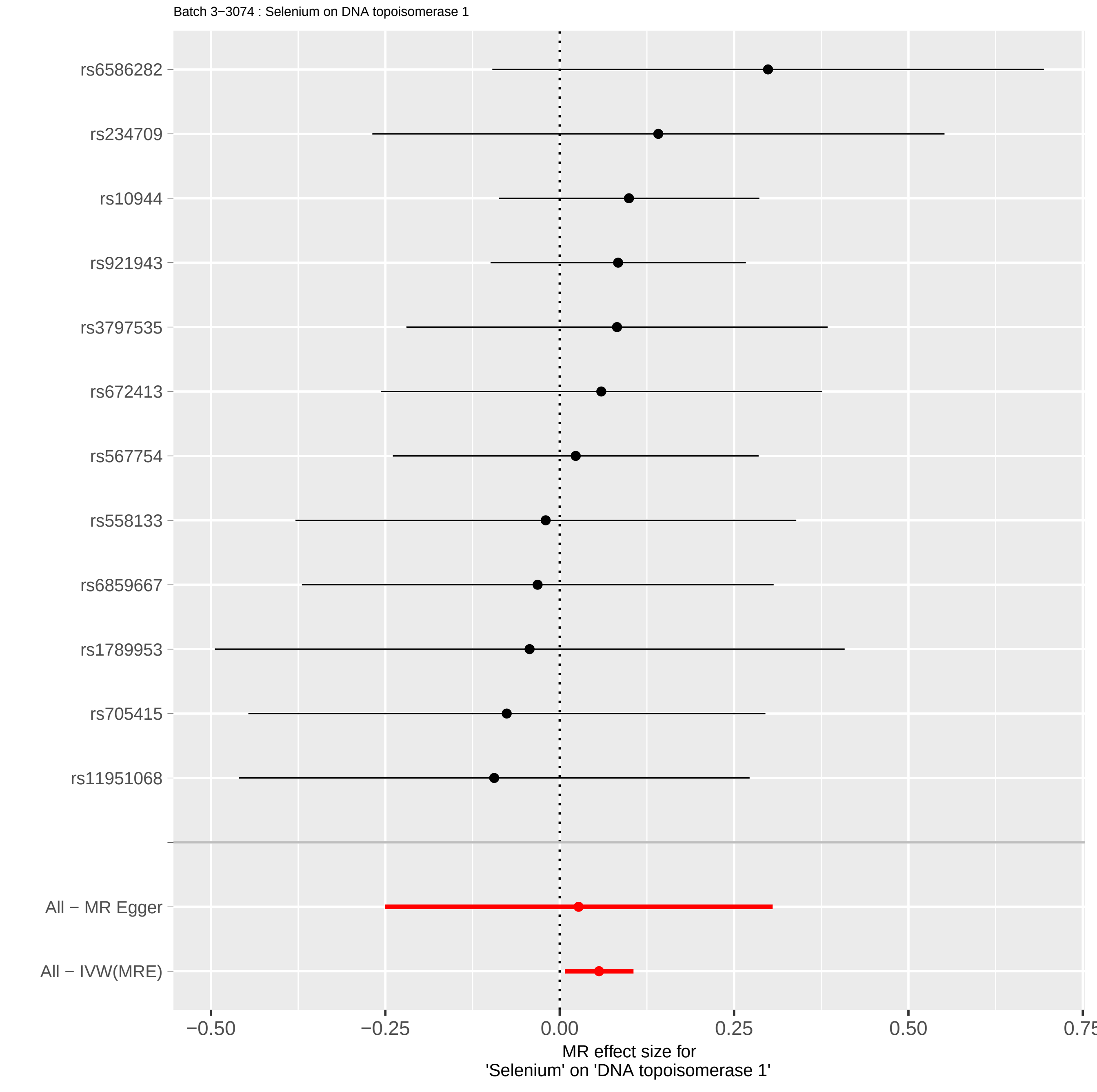
Batch 3−3074 : Selenium on DNA topoisomerase 1
rs6586282
rs234709
rs10944
rs921943
rs3797535
rs672413
rs567754
rs558133
rs6859667
rs1789953
rs705415
rs11951068
All − MR Egger
All − IVW(MRE)
−0.50
−0.25
0.00
0.25
0.50
0.75
MR effect size for
'Selenium' on 'DNA topoisomerase 1'

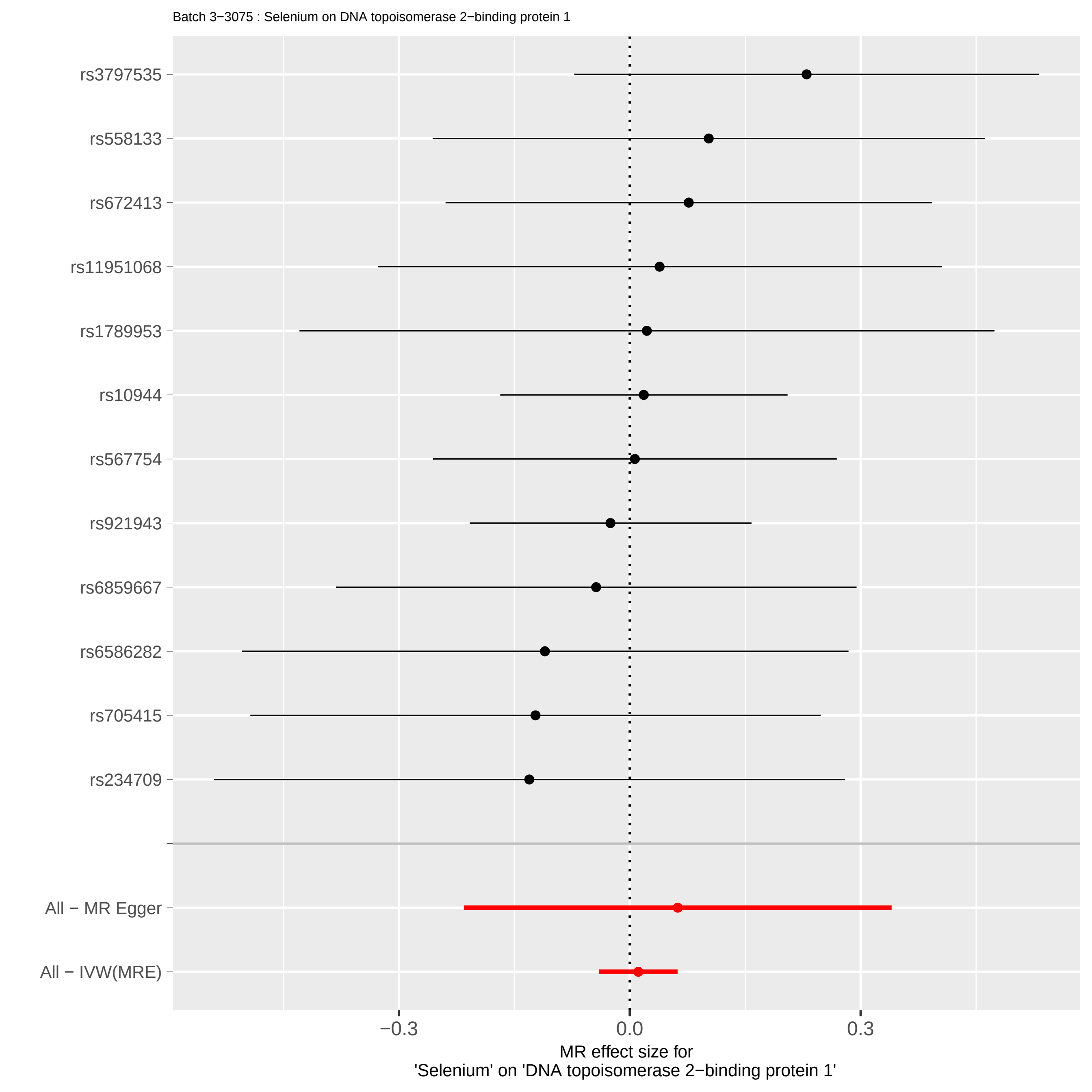

Batch 3−3075 : Selenium on DNA topoisomerase 2−binding protein 1
rs3797535
rs558133
rs672413
rs11951068
rs1789953
rs10944
rs567754
rs921943
rs6859667
rs6586282
rs705415
rs234709
All − MR Egger
All − IVW(MRE)
−0.3
0.0
0.3
MR effect size for
'Selenium' on 'DNA topoisomerase 2−binding protein 1'

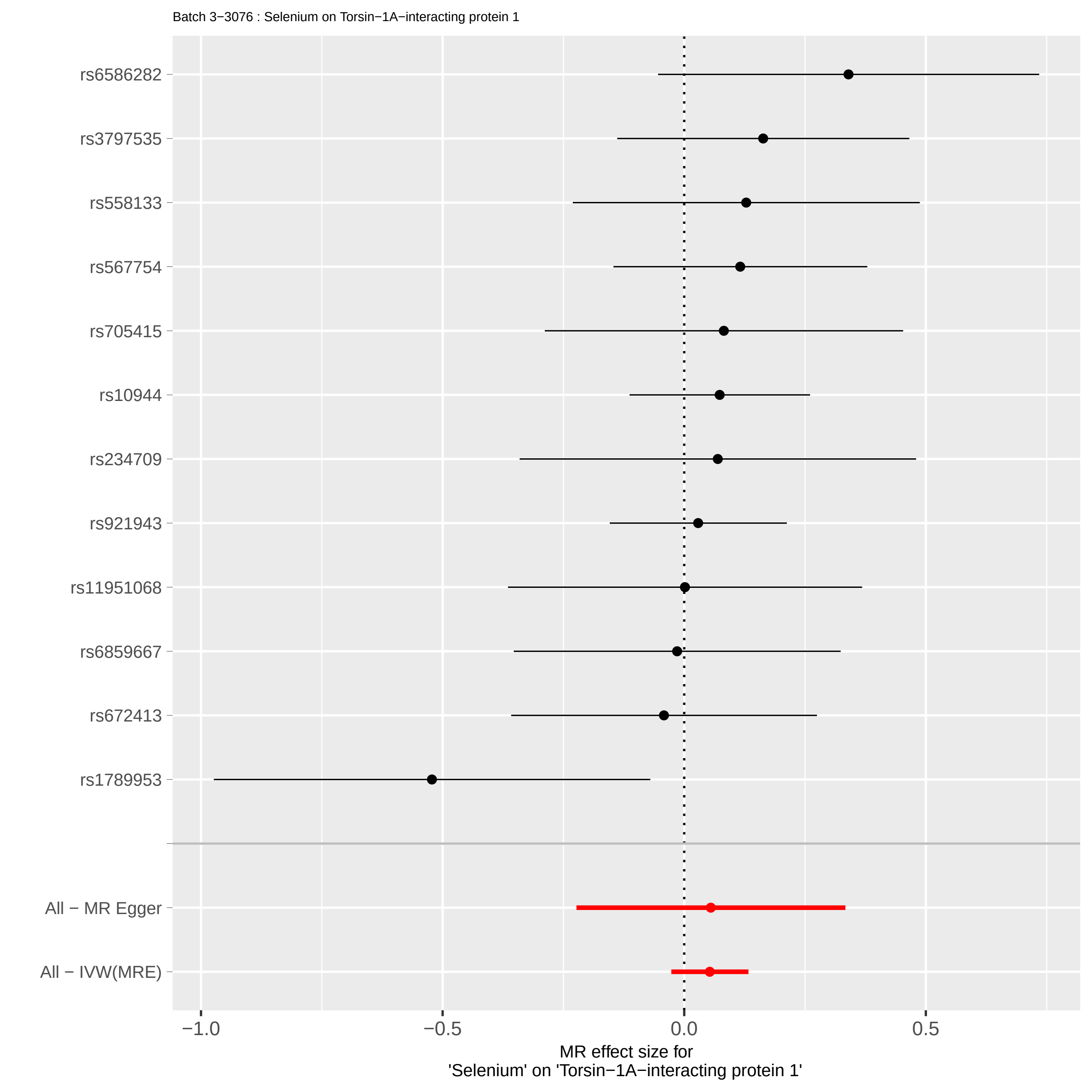

Batch 3−3076 : Selenium on Torsin−1A−interacting protein 1
rs6586282
rs3797535
rs558133
rs567754
rs705415
rs10944
rs234709
rs921943
rs11951068
rs6859667
rs672413
rs1789953
All − MR Egger
All − IVW(MRE)
−1.0
−0.5
0.0
0.5
MR effect size for
'Selenium' on 'Torsin−1A−interacting protein 1'

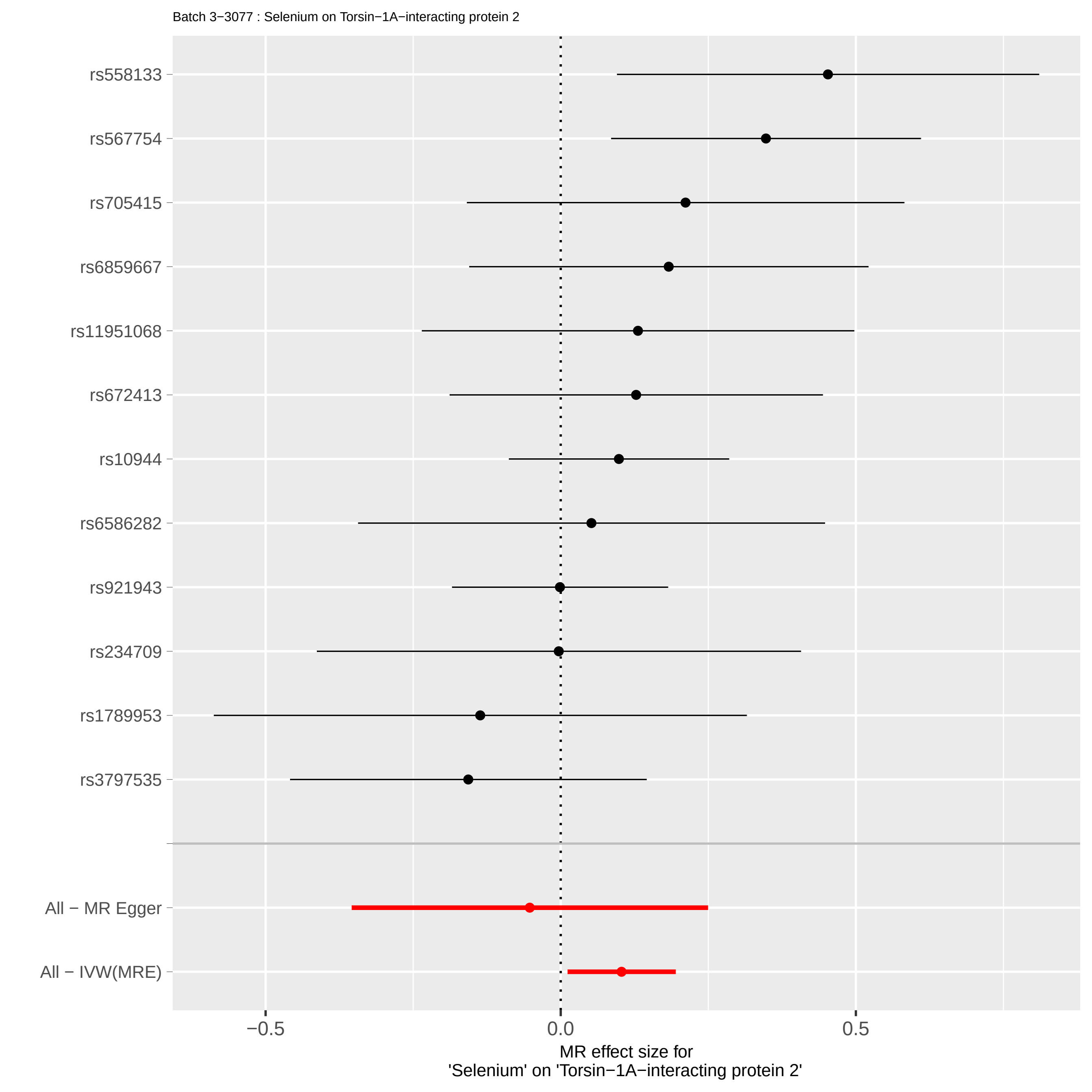
Batch 3−3077 : Selenium on Torsin−1A−interacting protein 2
rs558133
rs567754
rs705415
rs6859667
rs11951068
rs672413
rs10944
rs6586282
rs921943
rs234709
rs1789953
rs3797535
All − MR Egger
All − IVW(MRE)
−0.5
0.0
0.5
MR effect size for
'Selenium' on 'Torsin−1A−interacting protein 2'

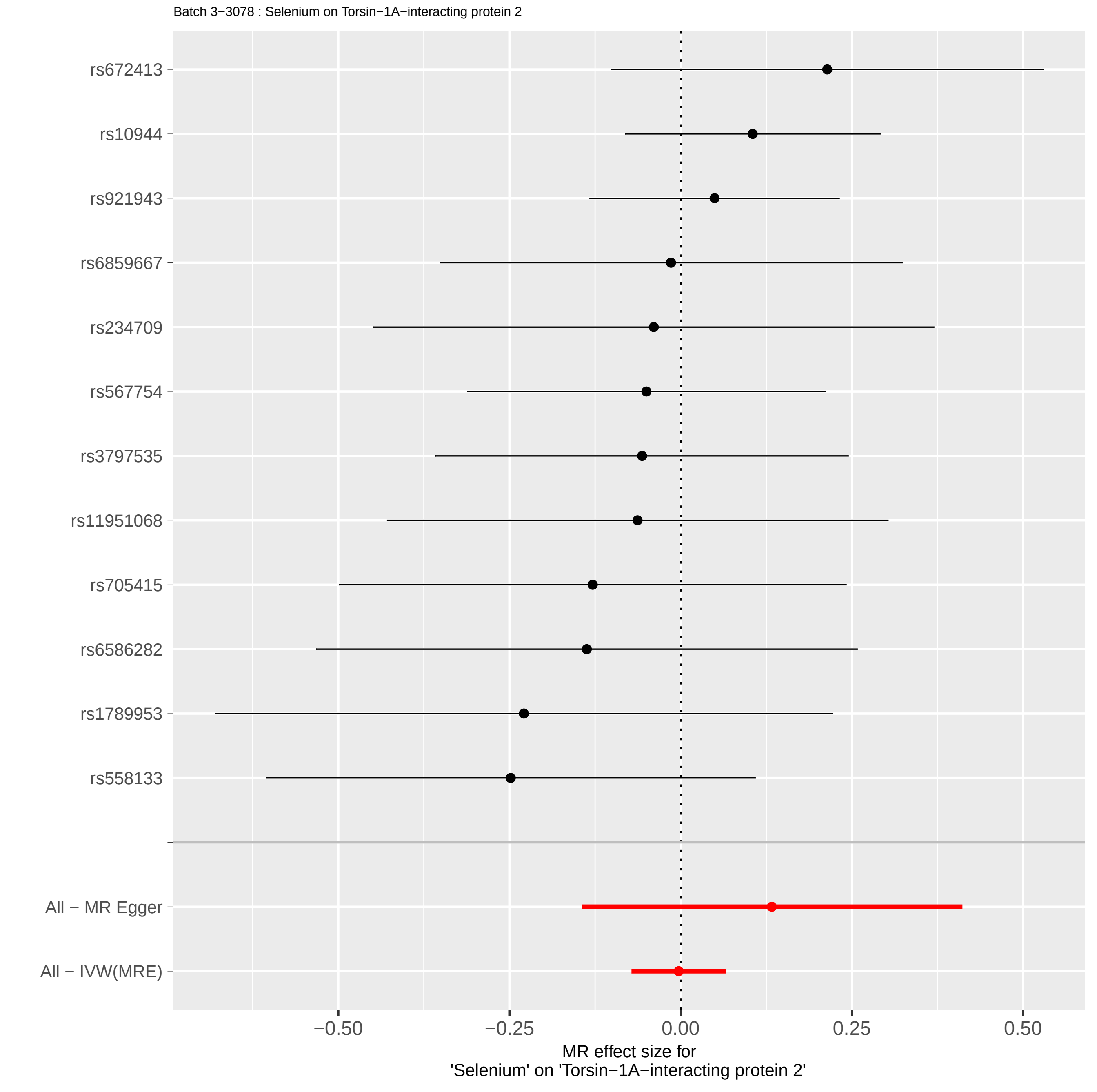

Batch 3−3078 : Selenium on Torsin−1A−interacting protein 2
rs672413
rs10944
rs921943
rs6859667
rs234709
rs567754
rs3797535
rs11951068
rs705415
rs6586282
rs1789953
rs558133
All − MR Egger
All − IVW(MRE)
−0.50
−0.25
0.00
0.25
0.50
MR effect size for
'Selenium' on 'Torsin−1A−interacting protein 2'

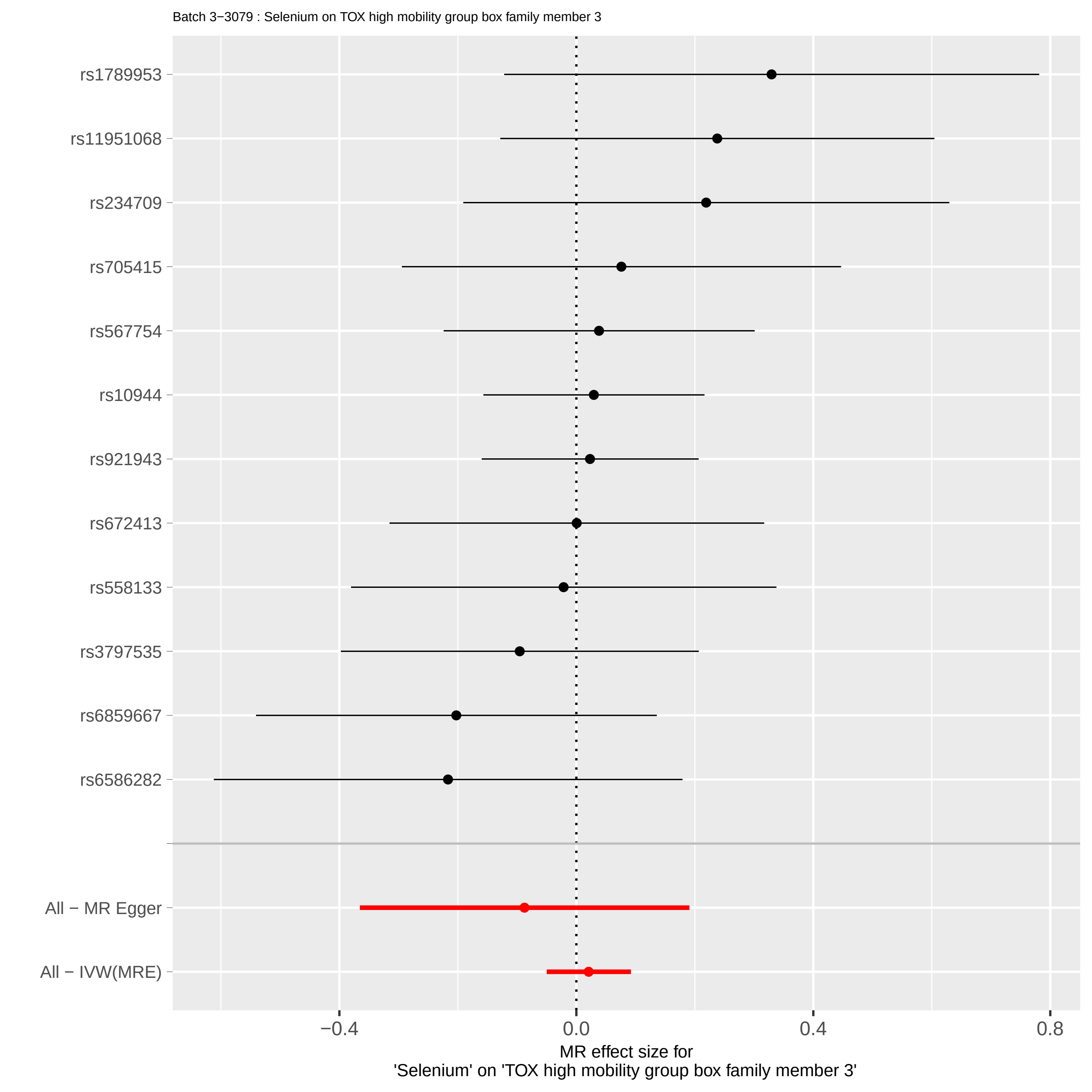

Batch 3−3079 : Selenium on TOX high mobility group box family member 3
rs1789953
rs11951068
rs234709
rs705415
rs567754
rs10944
rs921943
rs672413
rs558133
rs3797535
rs6859667
rs6586282
All − MR Egger
All − IVW(MRE)
−0.4
0.0
0.4
0.8
MR effect size for
'Selenium' on 'TOX high mobility group box family member 3'

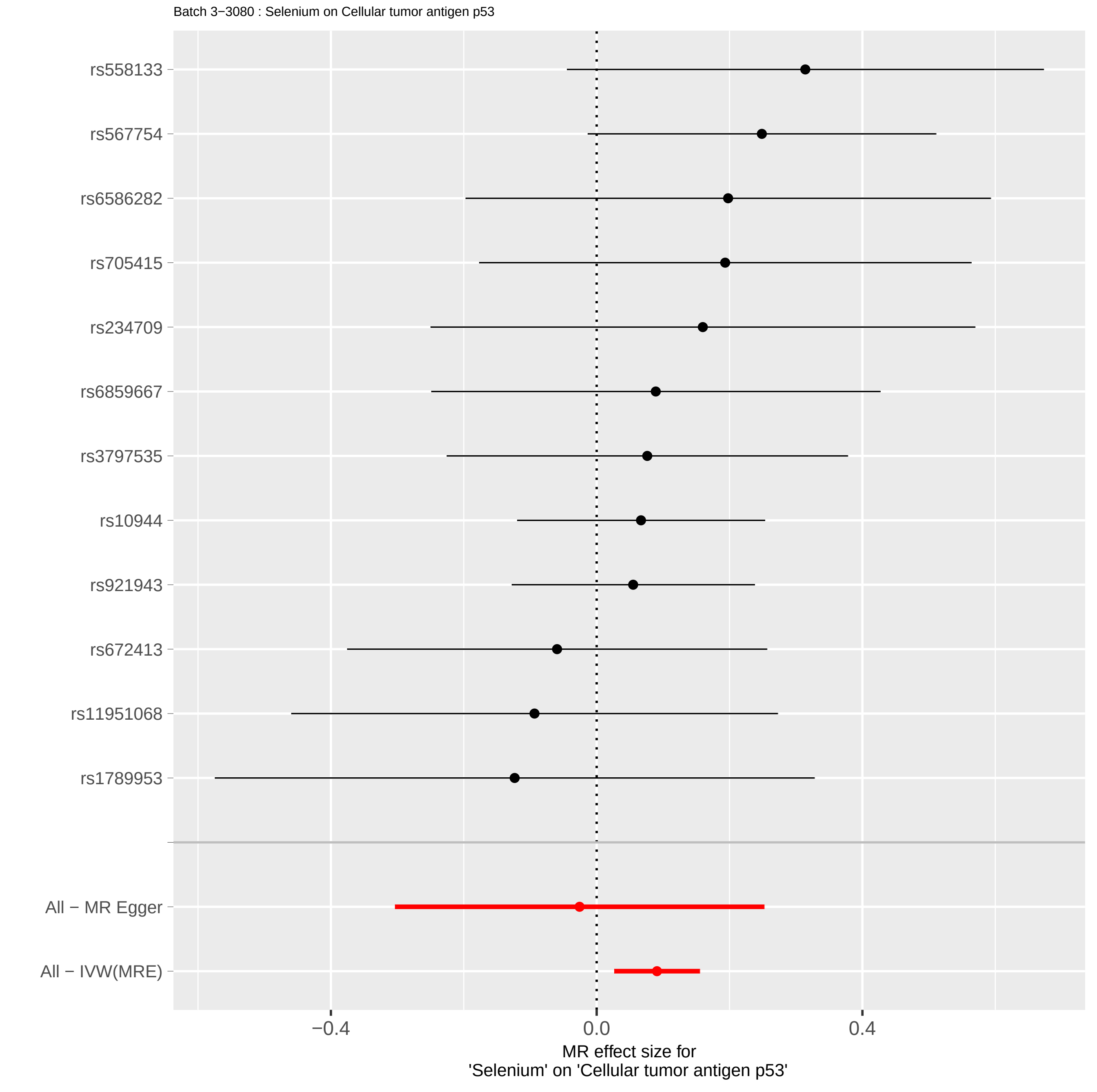

Batch 3−3080 : Selenium on Cellular tumor antigen p53
rs558133
rs567754
rs6586282
rs705415
rs234709
rs6859667
rs3797535
rs10944
rs921943
rs672413
rs11951068
rs1789953
All − MR Egger
All − IVW(MRE)
−0.4
0.0
0.4
MR effect size for
'Selenium' on 'Cellular tumor antigen p53'

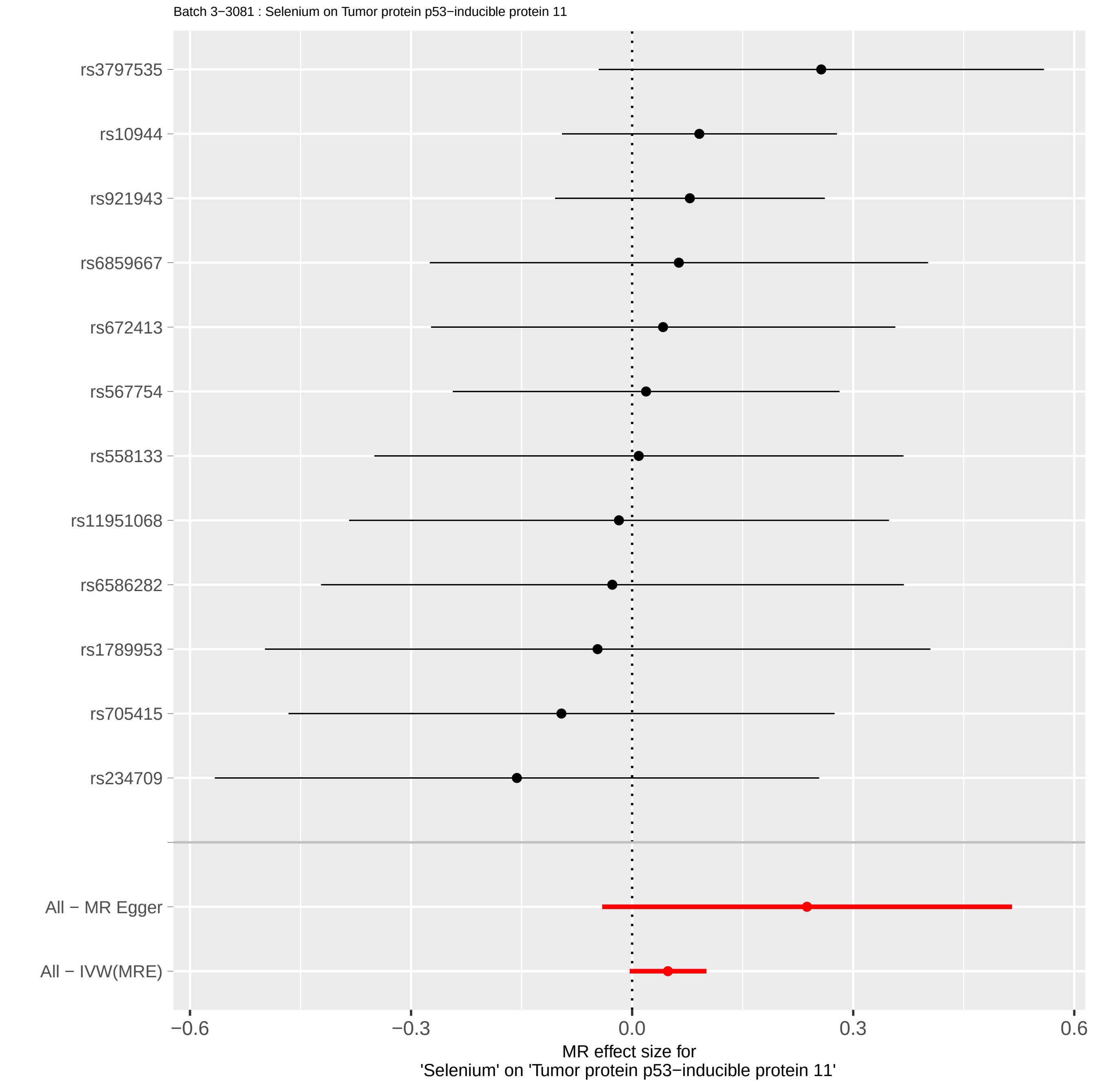

Batch 3−3081 : Selenium on Tumor protein p53−inducible protein 11
rs3797535
rs10944
rs921943
rs6859667
rs672413
rs567754
rs558133
rs11951068
rs6586282
rs1789953
rs705415
rs234709
All − MR Egger
All − IVW(MRE)
−0.6
−0.3
0.0
0.3
0.6
MR effect size for
'Selenium' on 'Tumor protein p53−inducible protein 11'

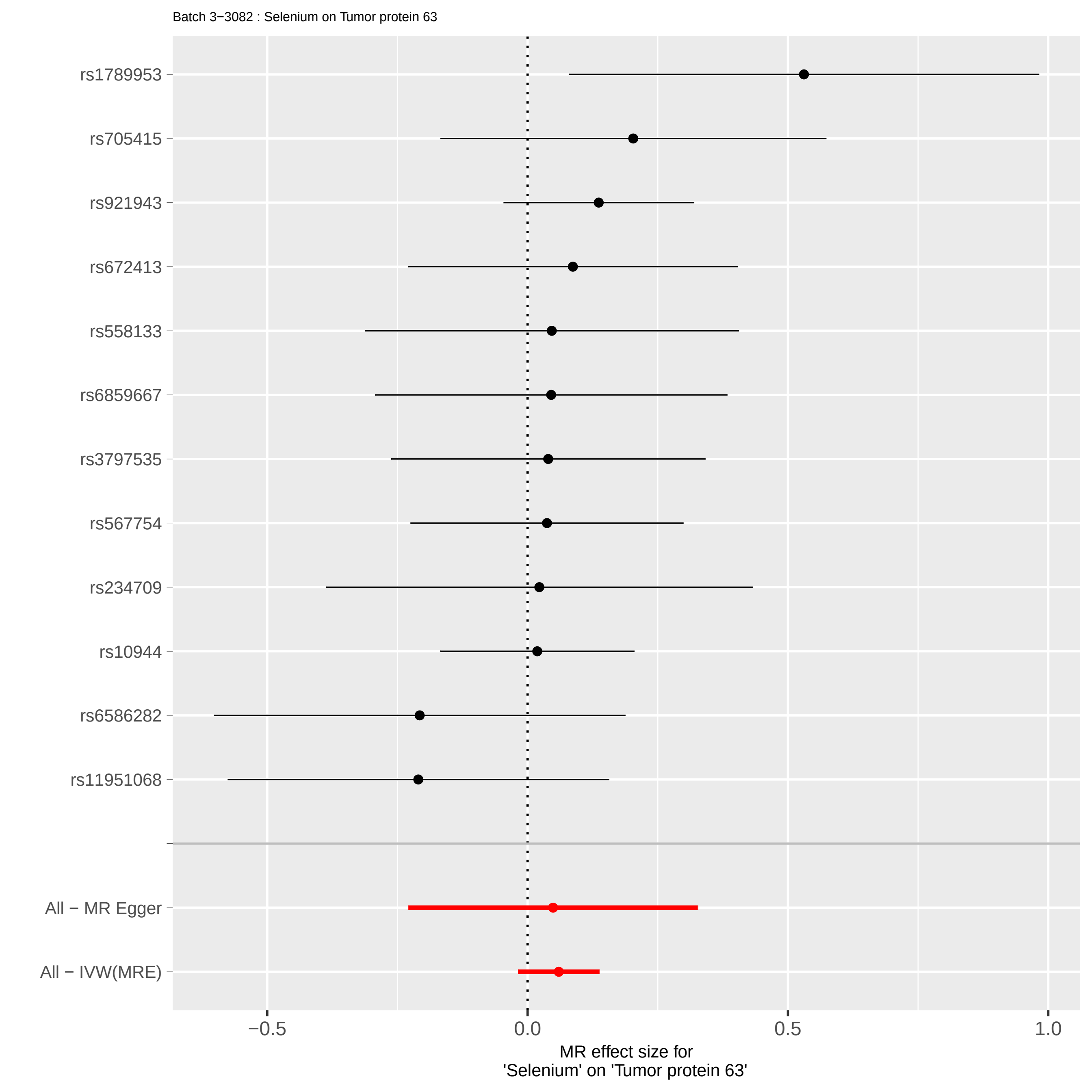

Batch 3−3082 : Selenium on Tumor protein 63
rs1789953
rs705415
rs921943
rs672413
rs558133
rs6859667
rs3797535
rs567754
rs234709
rs10944
rs6586282
rs11951068
All − MR Egger
All − IVW(MRE)
−0.5
0.0
0.5
1.0
MR effect size for
'Selenium' on 'Tumor protein 63'

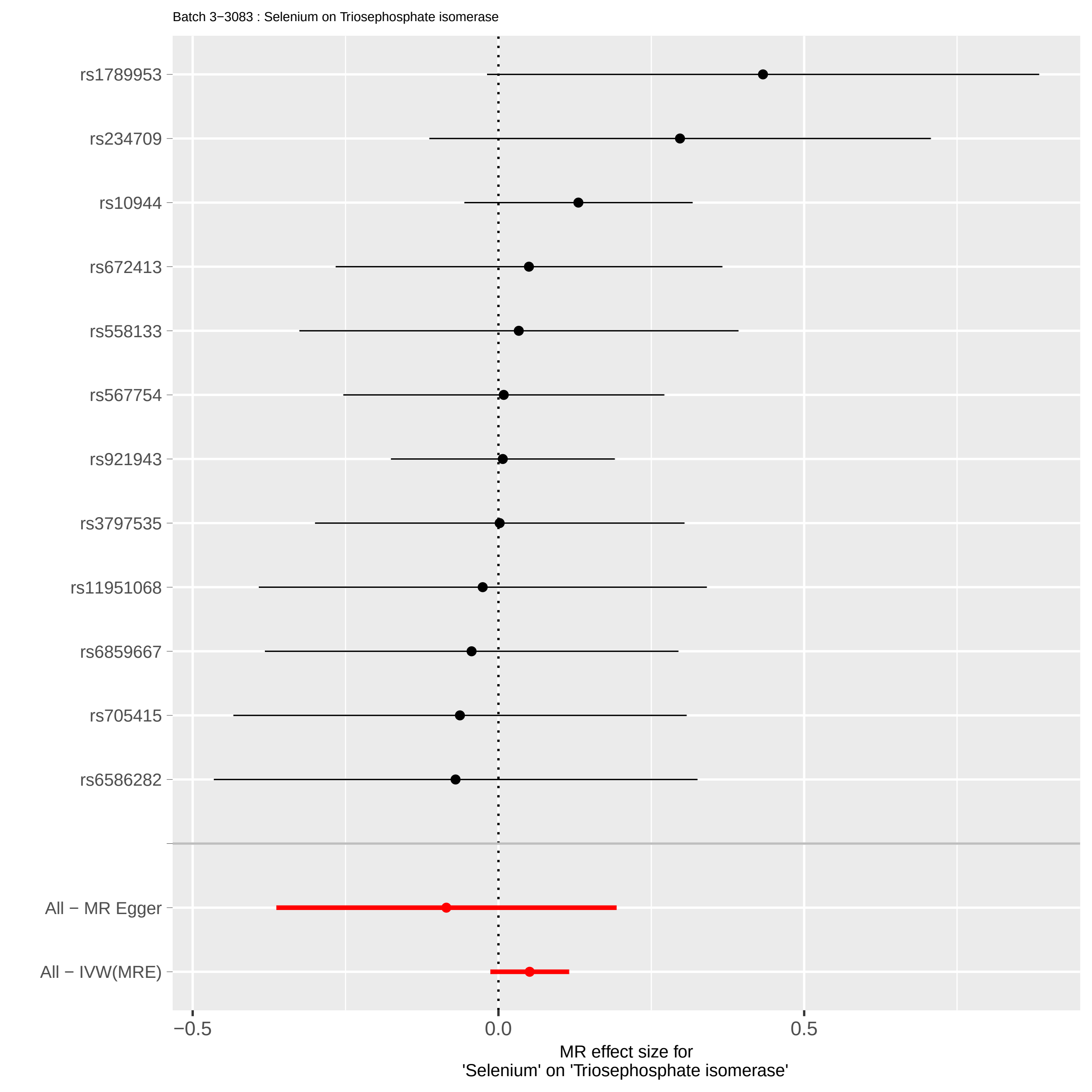

Batch 3−3083 : Selenium on Triosephosphate isomerase
rs1789953
rs234709
rs10944
rs672413
rs558133
rs567754
rs921943
rs3797535
rs11951068
rs6859667
rs705415
rs6586282
All − MR Egger
All − IVW(MRE)
−0.5
0.0
0.5
MR effect size for
'Selenium' on 'Triosephosphate isomerase'

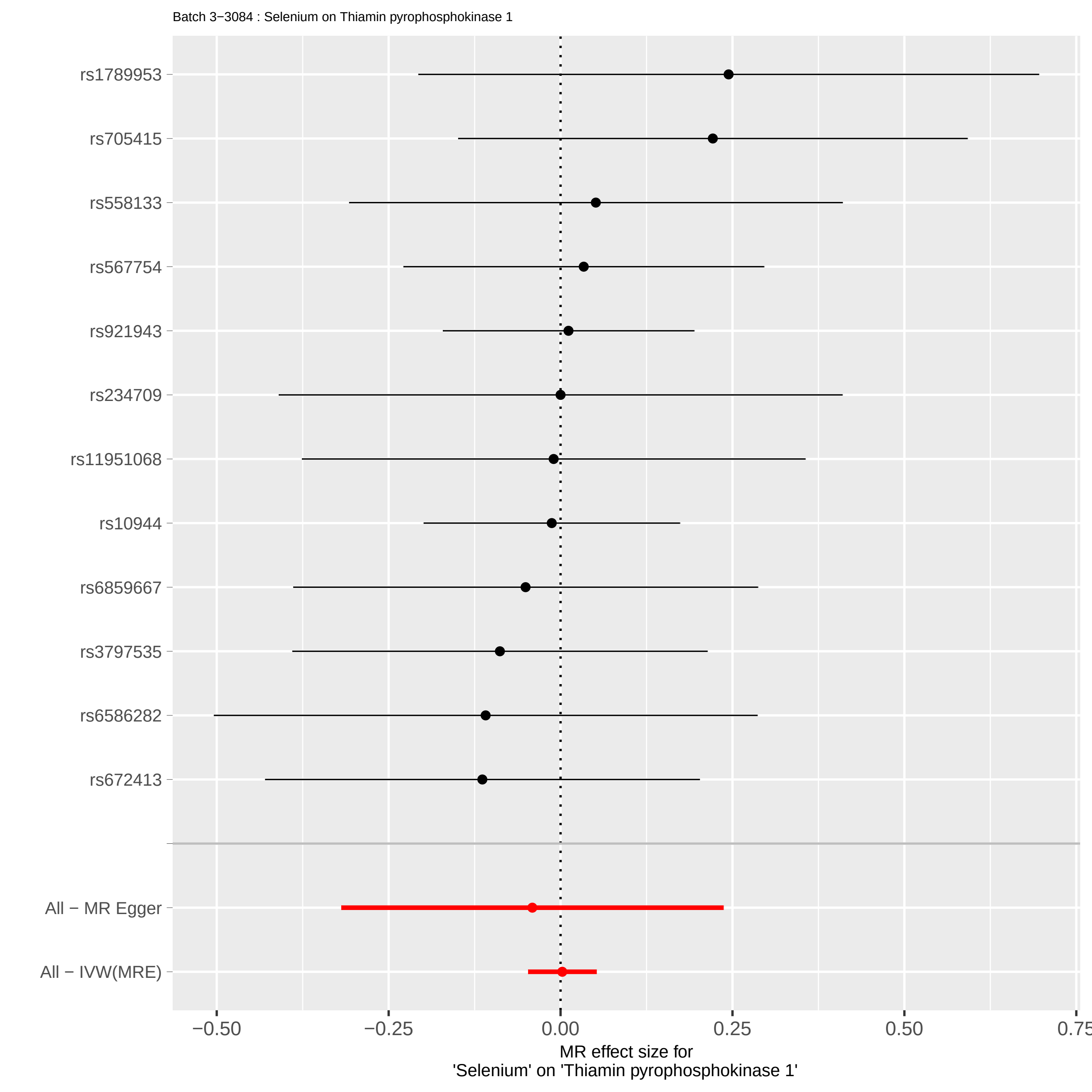

Batch 3−3084 : Selenium on Thiamin pyrophosphokinase 1
rs1789953
rs705415
rs558133
rs567754
rs921943
rs234709
rs11951068
rs10944
rs6859667
rs3797535
rs6586282
rs672413
All − MR Egger
All − IVW(MRE)
−0.50
−0.25
0.00
0.25
0.50
0.75
MR effect size for
'Selenium' on 'Thiamin pyrophosphokinase 1'

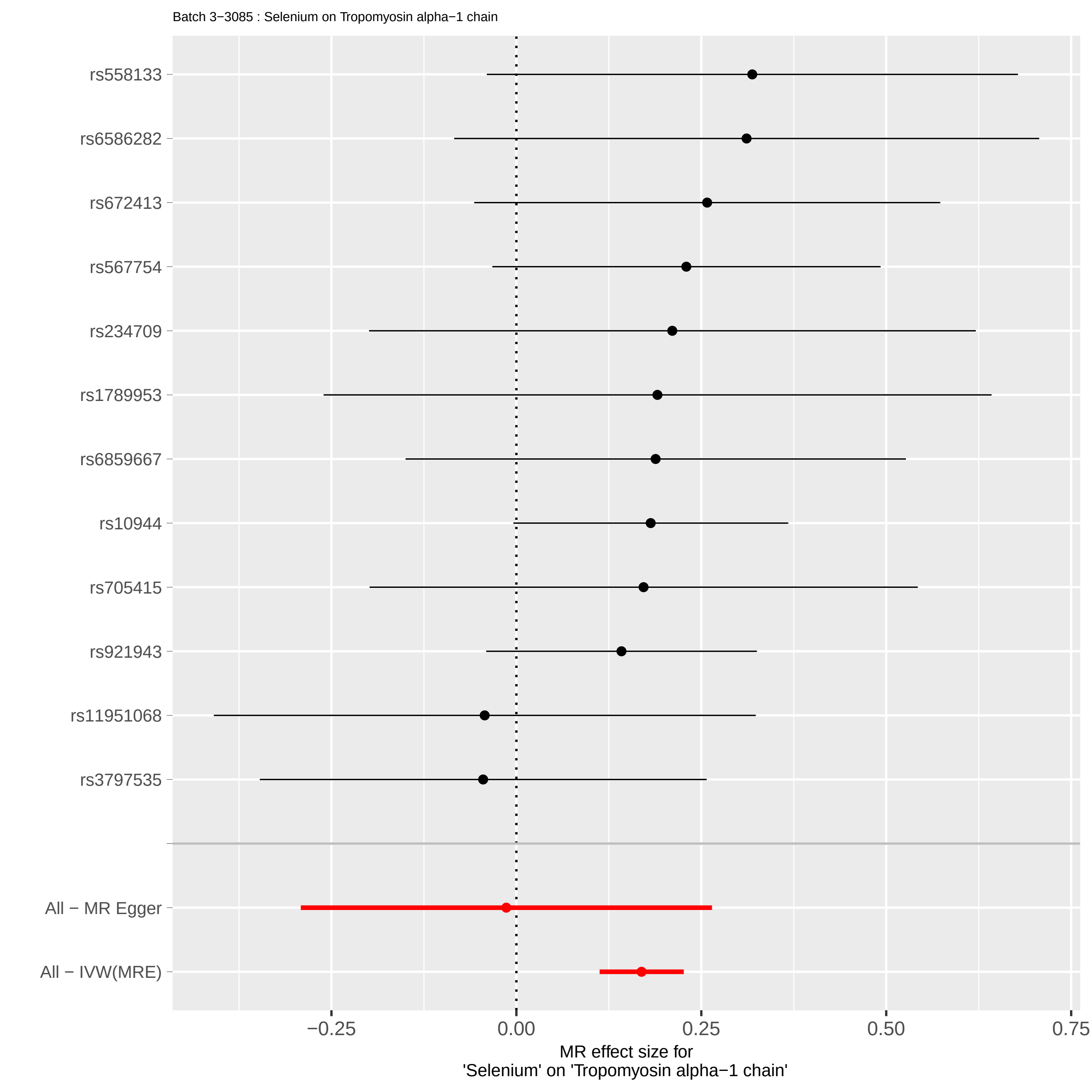

Batch 3−3085 : Selenium on Tropomyosin alpha−1 chain
rs558133
rs6586282
rs672413
rs567754
rs234709
rs1789953
rs6859667
rs10944
rs705415
rs921943
rs11951068
rs3797535
All − MR Egger
All − IVW(MRE)
−0.25
0.00
0.25
0.50
0.75
MR effect size for
'Selenium' on 'Tropomyosin alpha−1 chain'

Batch 3−3086 : Selenium on Tropomyosin beta chain

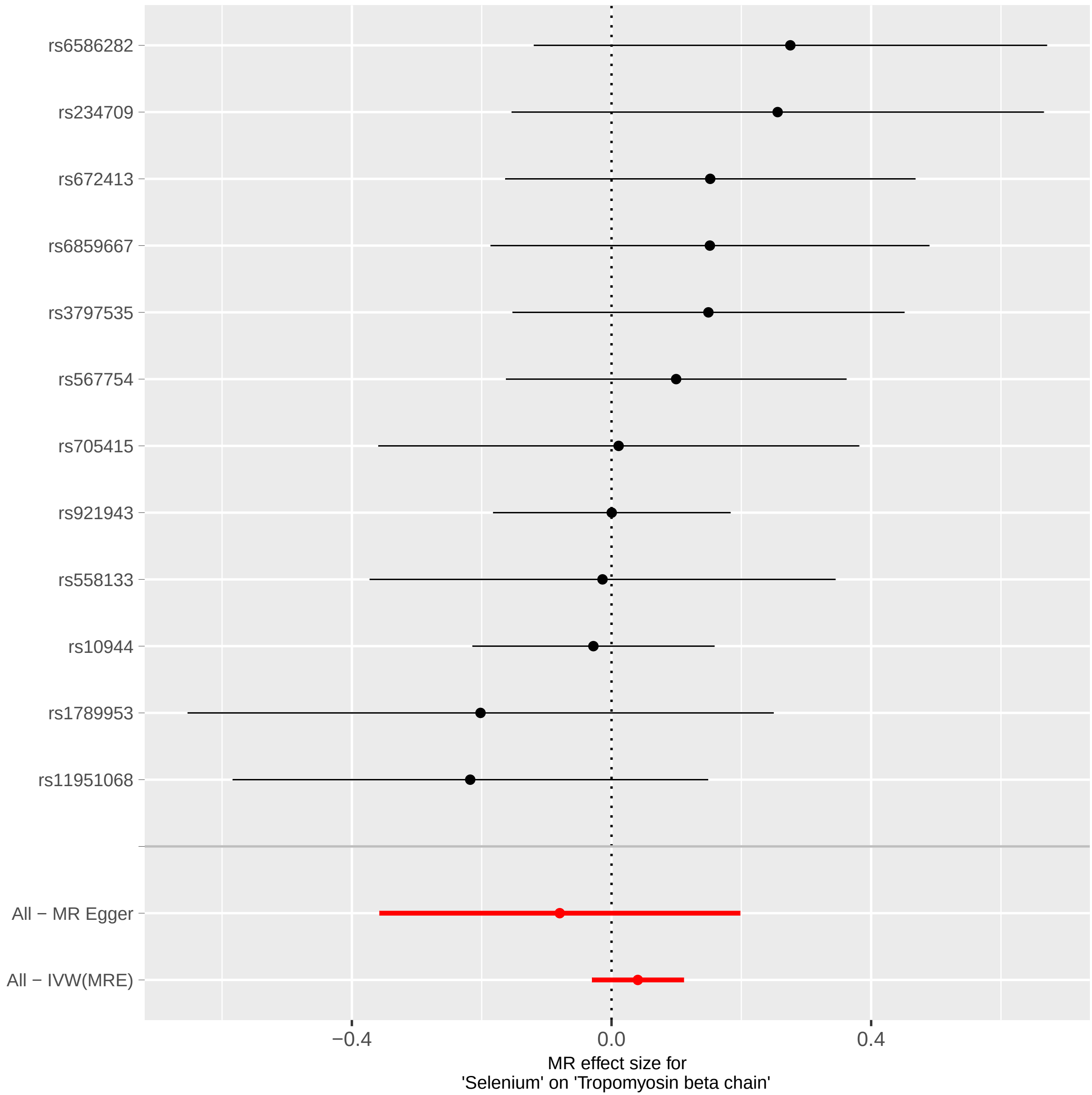

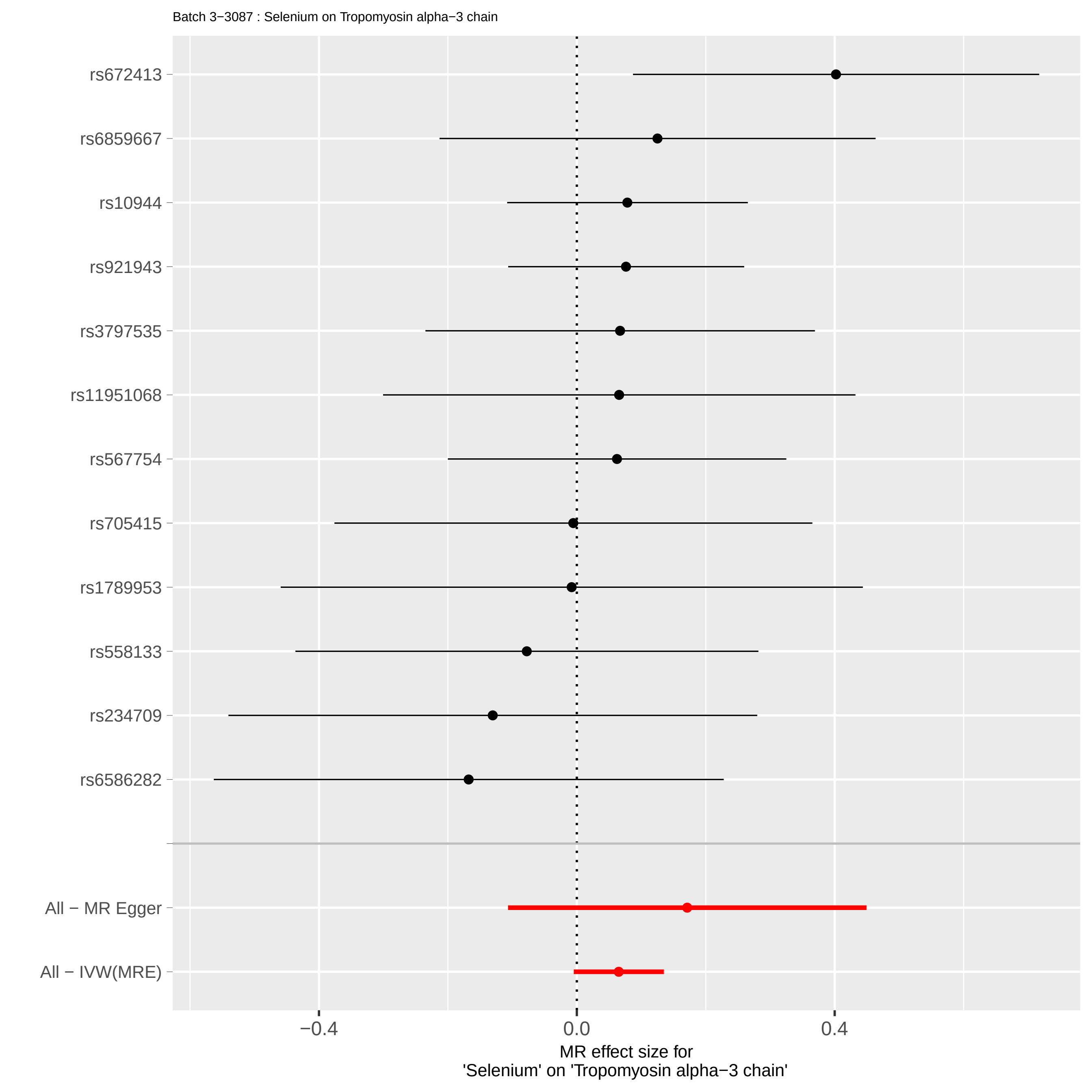

Batch 3−3087 : Selenium on Tropomyosin alpha−3 chain
rs672413
rs6859667
rs10944
rs921943
rs3797535
rs11951068
rs567754
rs705415
rs1789953
rs558133
rs234709
rs6586282
All − MR Egger
All − IVW(MRE)
−0.4
0.0
0.4
MR effect size for
'Selenium' on 'Tropomyosin alpha−3 chain'

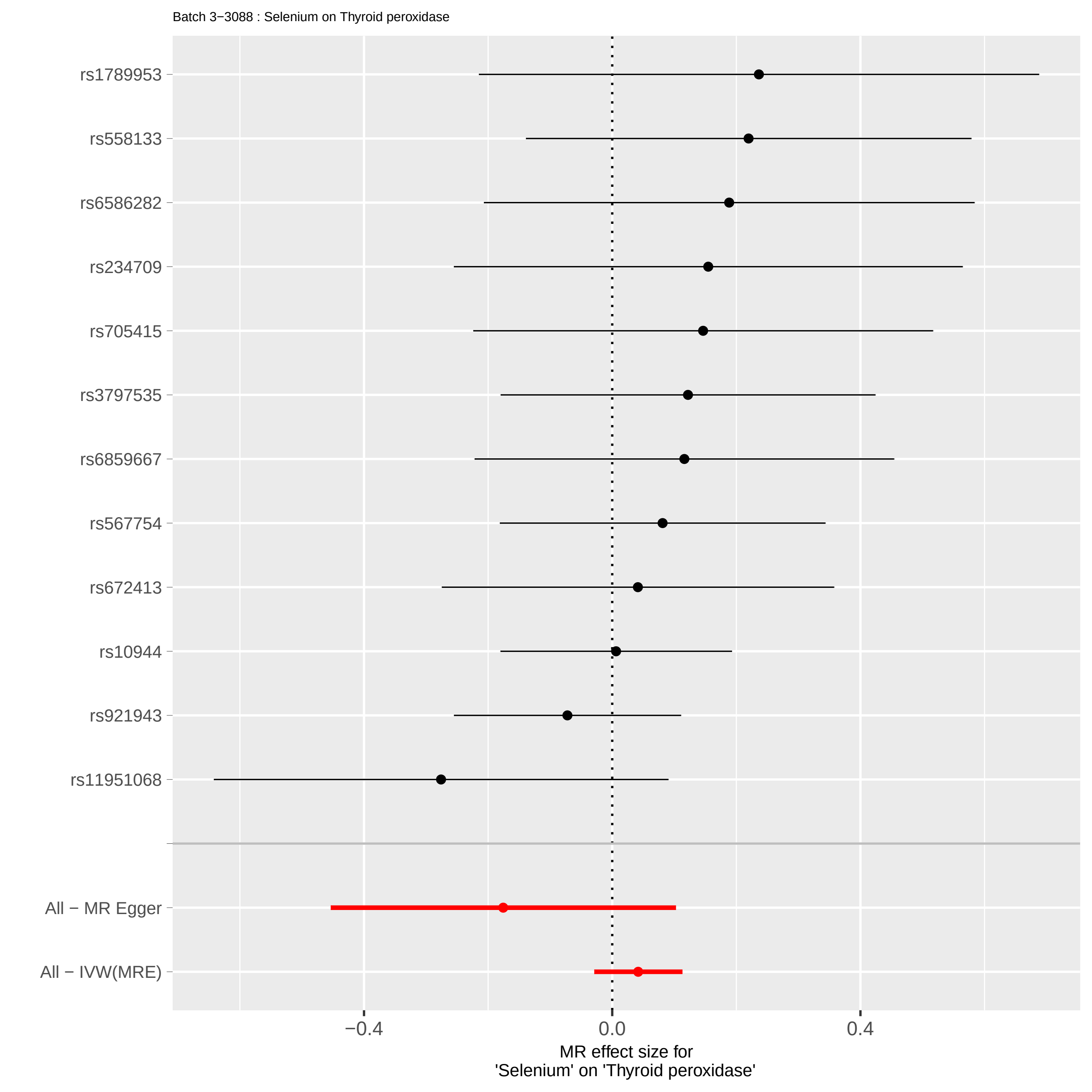

Batch 3−3088 : Selenium on Thyroid peroxidase
rs1789953
rs558133
rs6586282
rs234709
rs705415
rs3797535
rs6859667
rs567754
rs672413
rs10944
rs921943
rs11951068
All − MR Egger
All − IVW(MRE)
−0.4
0.0
0.4
MR effect size for
'Selenium' on 'Thyroid peroxidase'

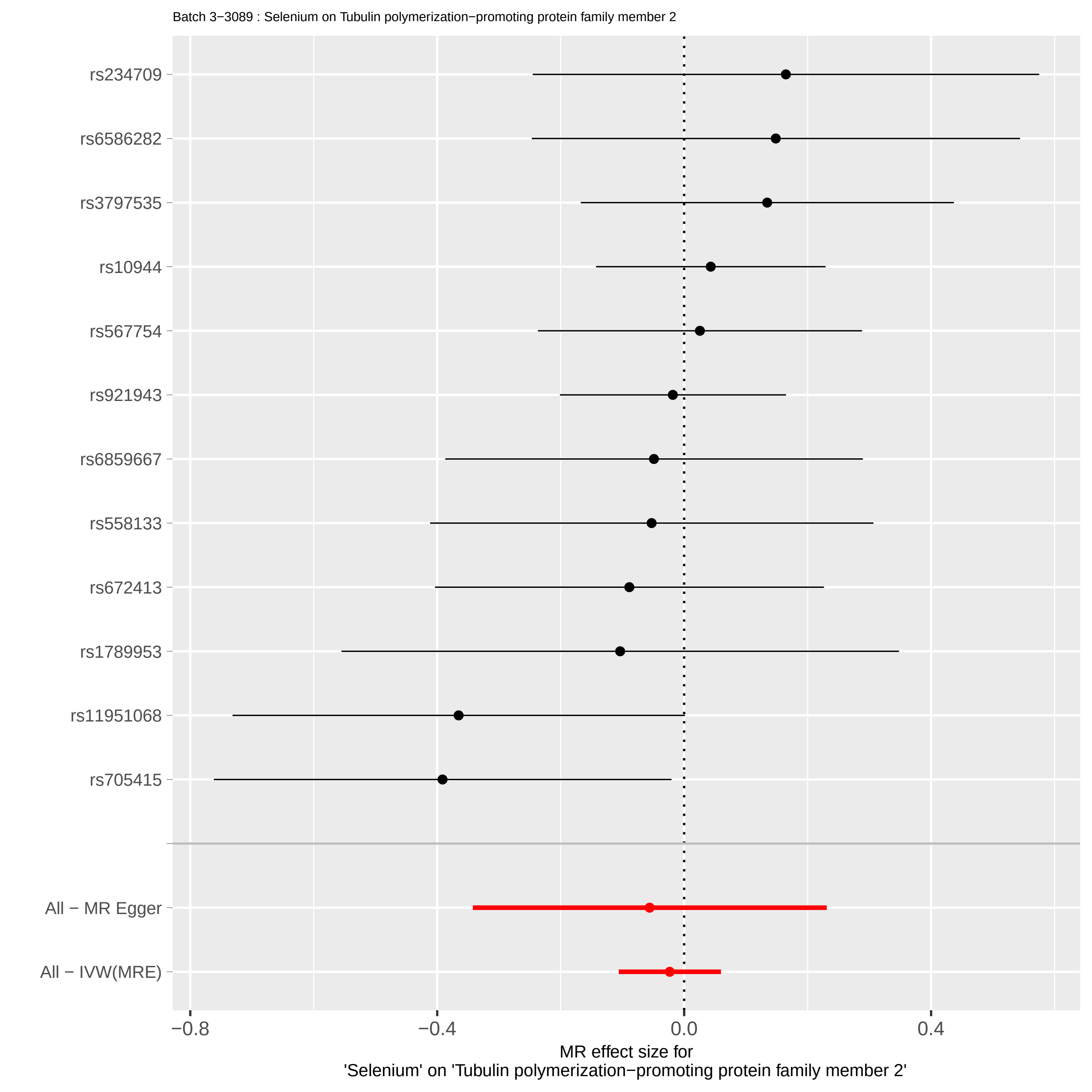

Batch 3−3089 : Selenium on Tubulin polymerization−promoting protein family member 2
rs234709
rs6586282
rs3797535
rs10944
rs567754
rs921943
rs6859667
rs558133
rs672413
rs1789953
rs11951068
rs705415
All − MR Egger
All − IVW(MRE)
−0.8
−0.4
0.0
0.4
MR effect size for
'Selenium' on 'Tubulin polymerization−promoting protein family member 2'

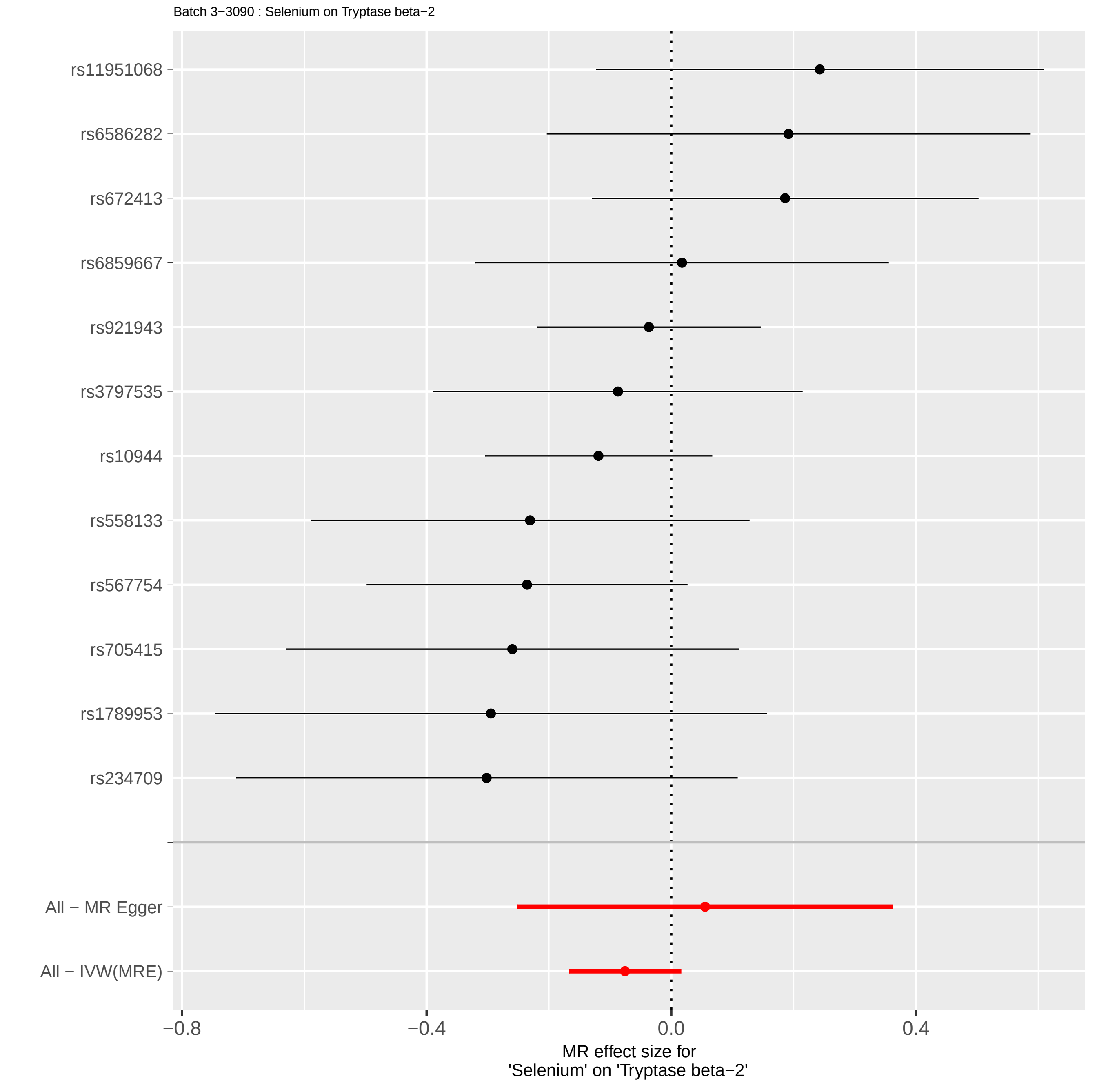

Batch 3−3090 : Selenium on Tryptase beta−2
rs11951068
rs6586282
rs672413
rs6859667
rs921943
rs3797535
rs10944
rs558133
rs567754
rs705415
rs1789953
rs234709
All − MR Egger
All − IVW(MRE)
−0.8
−0.4
0.0
0.4
MR effect size for
'Selenium' on 'Tryptase beta−2'

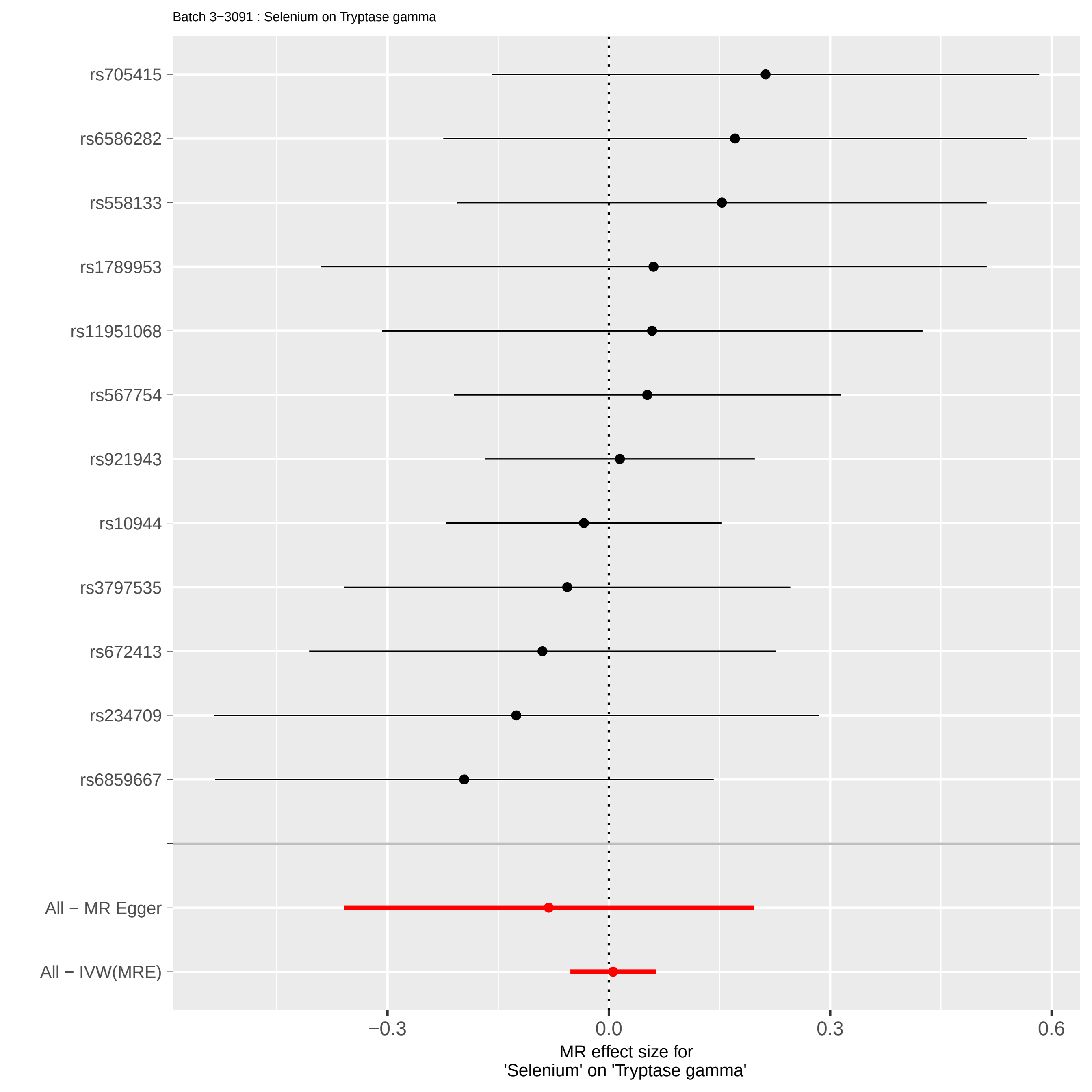
Batch 3−3091 : Selenium on Tryptase gamma
rs705415
rs6586282
rs558133
rs1789953
rs11951068
rs567754
rs921943
rs10944
rs3797535
rs672413
rs234709
rs6859667
All − MR Egger
All − IVW(MRE)
−0.3
0.0
0.3
0.6
MR effect size for
'Selenium' on 'Tryptase gamma'

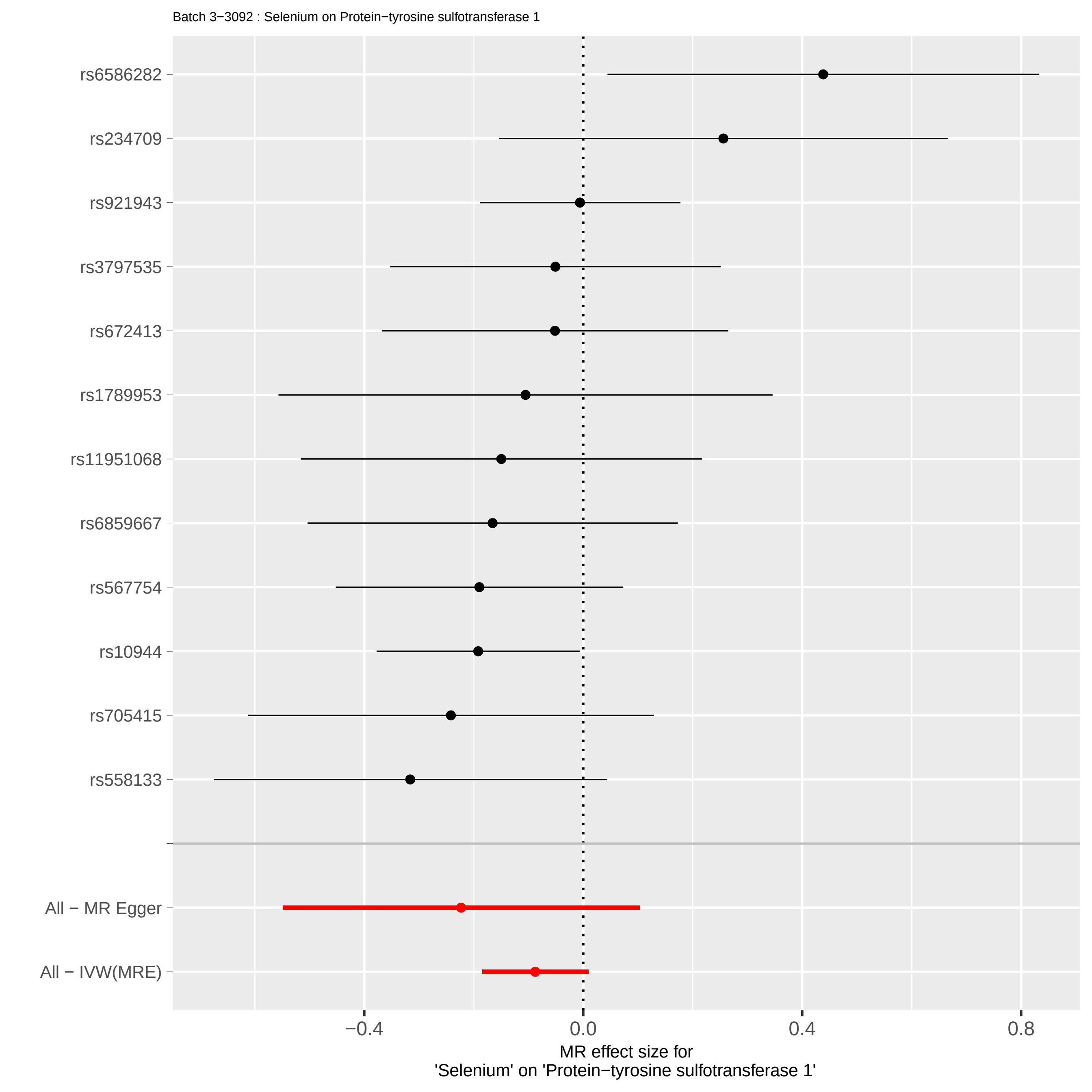

Batch 3−3092 : Selenium on Protein−tyrosine sulfotransferase 1
rs6586282
rs234709
rs921943
rs3797535
rs672413
rs1789953
rs11951068
rs6859667
rs567754
rs10944
rs705415
rs558133
All − MR Egger
All − IVW(MRE)
−0.4
0.0
0.4
0.8
MR effect size for
'Selenium' on 'Protein−tyrosine sulfotransferase 1'

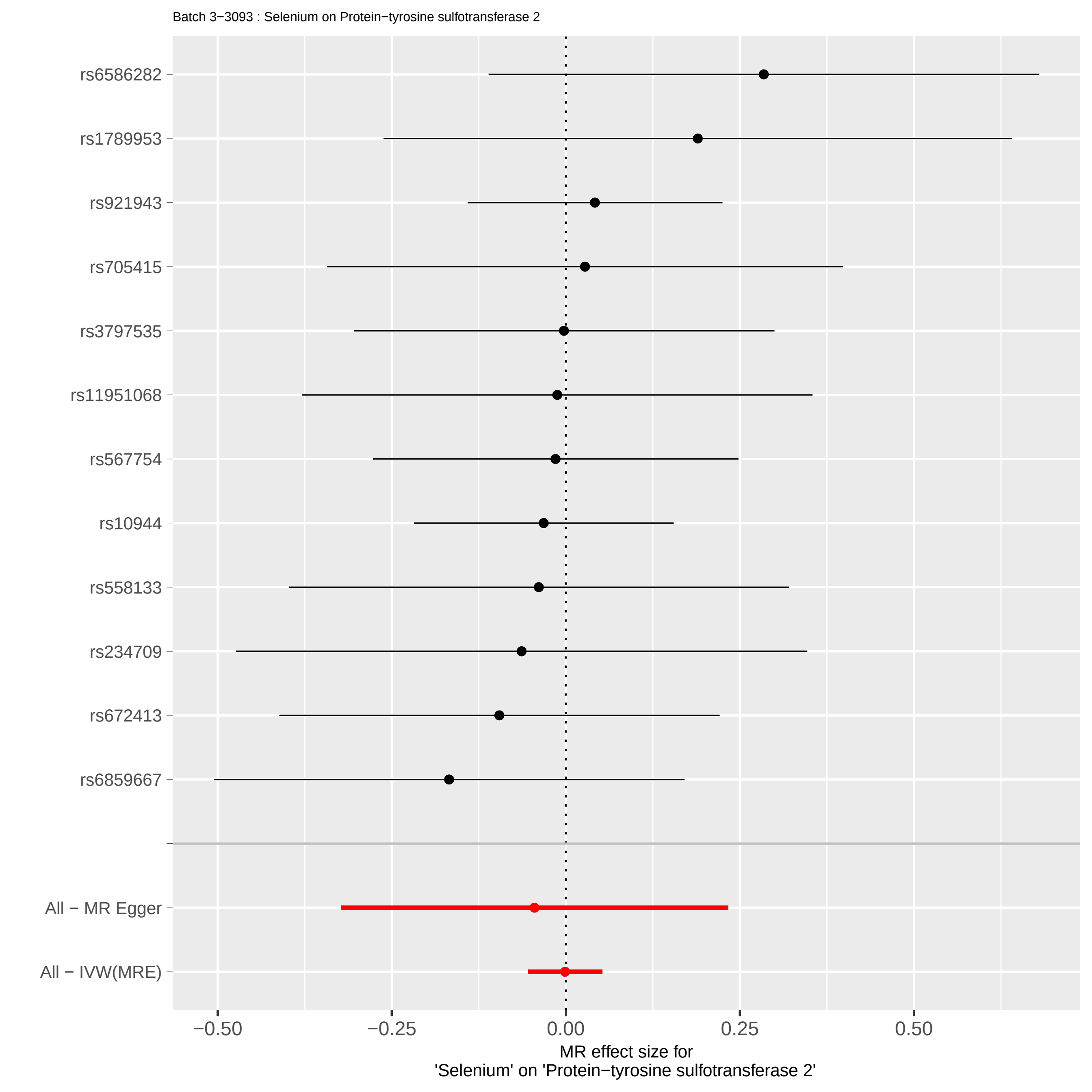

Batch 3−3093 : Selenium on Protein−tyrosine sulfotransferase 2
rs6586282
rs1789953
rs921943
rs705415
rs3797535
rs11951068
rs567754
rs10944
rs558133
rs234709
rs672413
rs6859667
All − MR Egger
All − IVW(MRE)
−0.50
−0.25
0.00
0.25
0.50
MR effect size for
'Selenium' on 'Protein−tyrosine sulfotransferase 2'

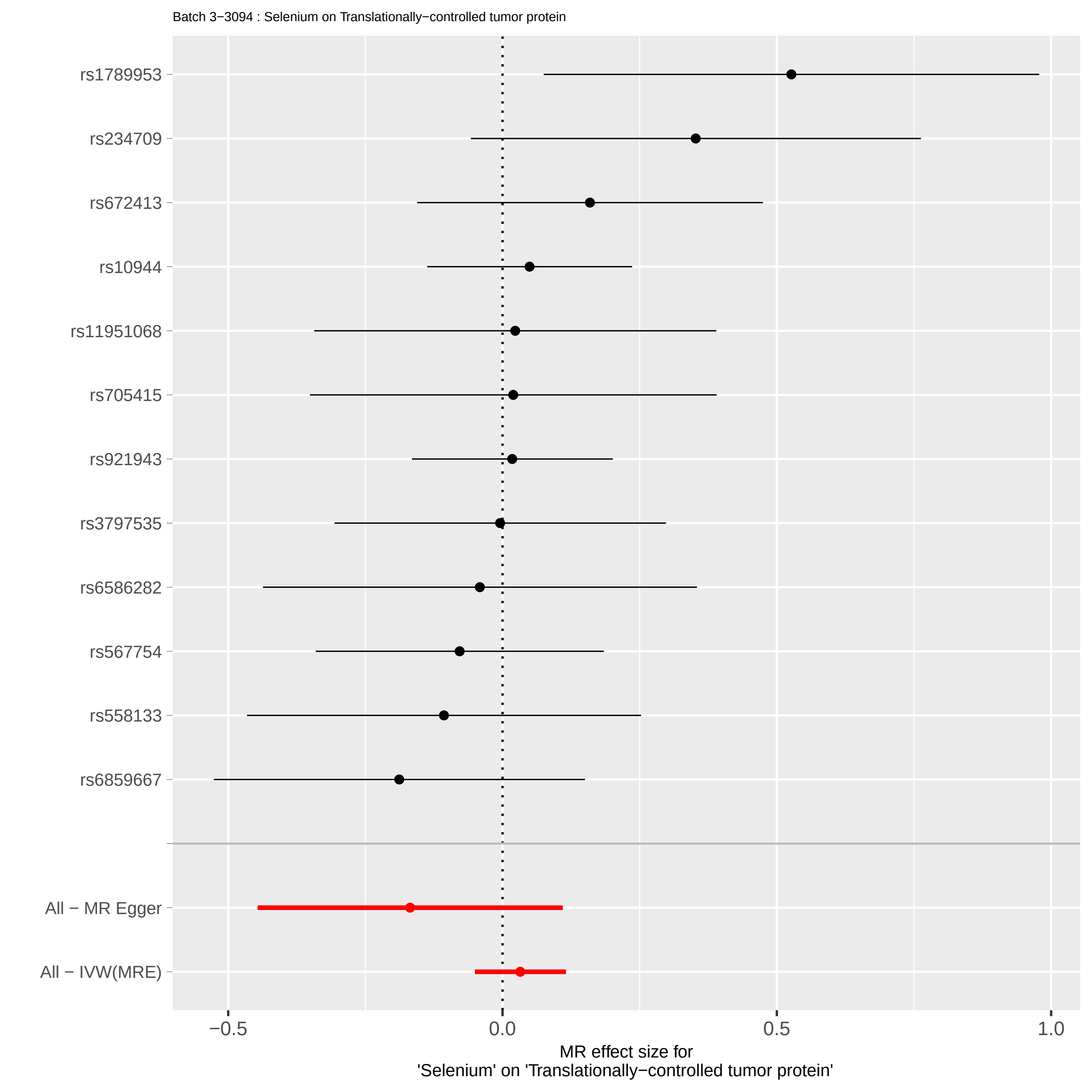

Batch 3−3094 : Selenium on Translationally−controlled tumor protein
rs1789953
rs234709
rs672413
rs10944
rs11951068
rs705415
rs921943
rs3797535
rs6586282
rs567754
rs558133
rs6859667
All − MR Egger
All − IVW(MRE)
−0.5
0.0
0.5
1.0
MR effect size for
'Selenium' on 'Translationally−controlled tumor protein'

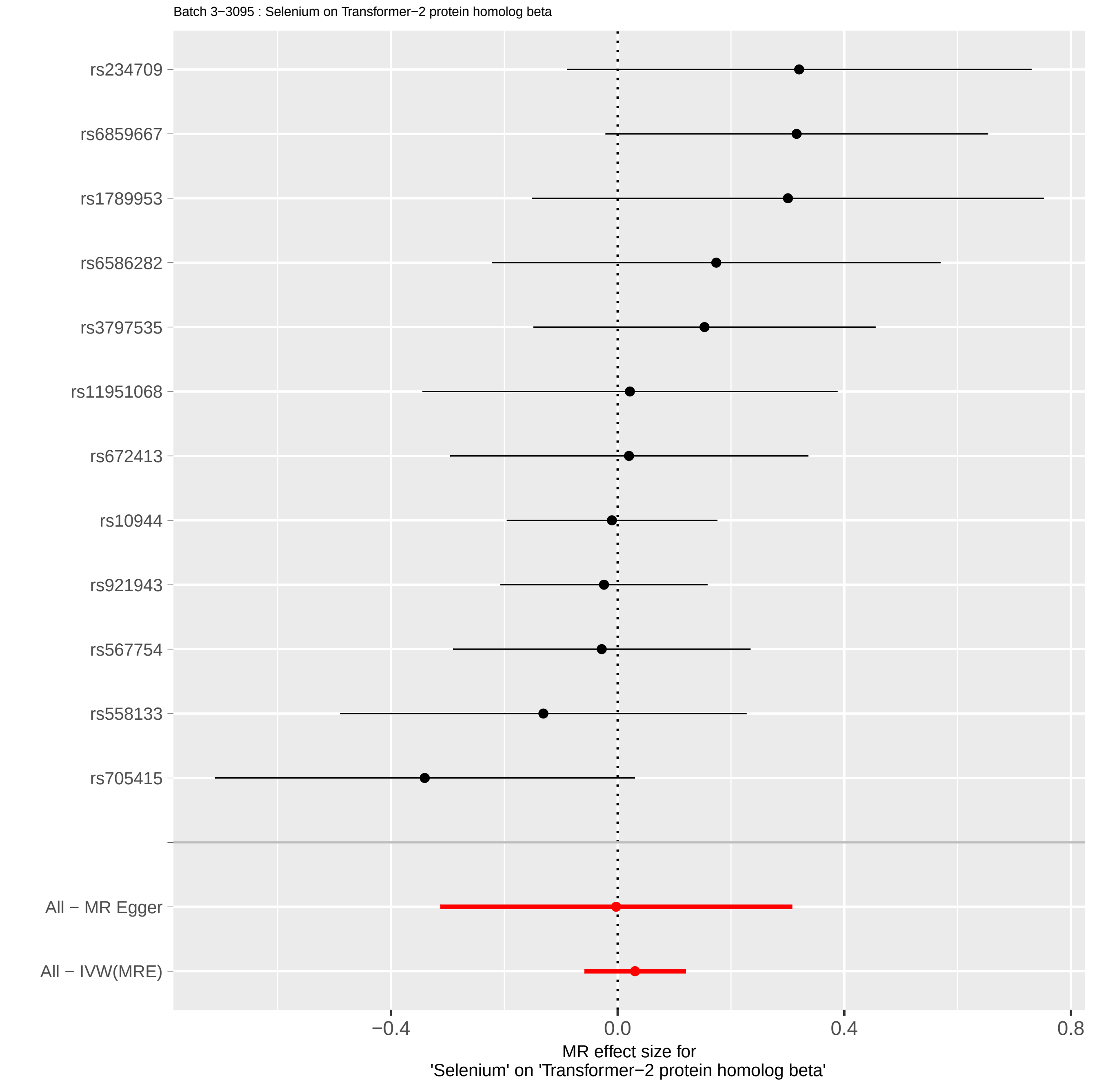

Batch 3−3095 : Selenium on Transformer−2 protein homolog beta
rs234709
rs6859667
rs1789953
rs6586282
rs3797535
rs11951068
rs672413
rs10944
rs921943
rs567754
rs558133
rs705415
All − MR Egger
All − IVW(MRE)
−0.4
0.0
0.4
0.8
MR effect size for
'Selenium' on 'Transformer−2 protein homolog beta'

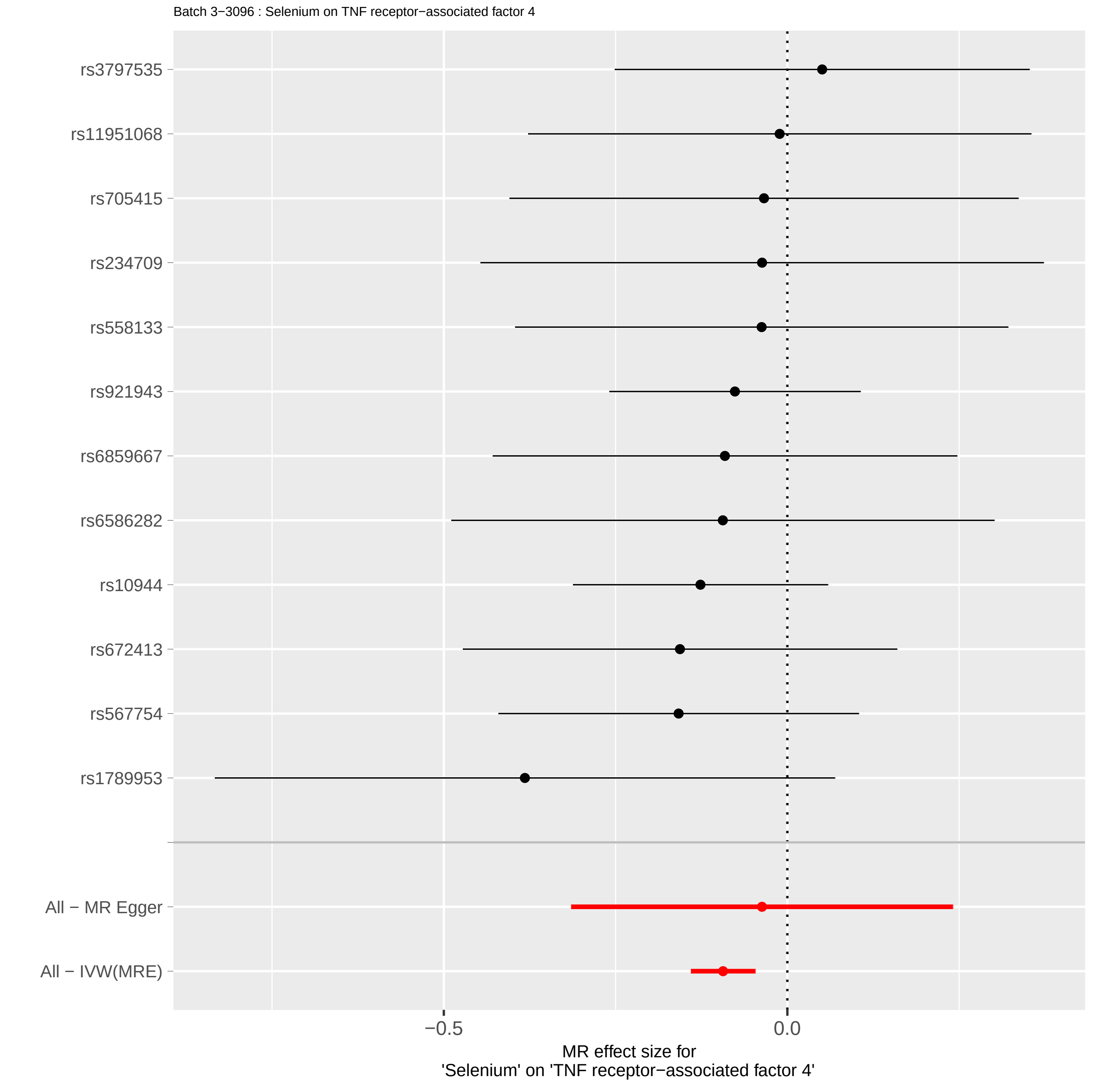
Batch 3−3096 : Selenium on TNF receptor−associated factor 4
rs3797535
rs11951068
rs705415
rs234709
rs558133
rs921943
rs6859667
rs6586282
rs10944
rs672413
rs567754
rs1789953
All − MR Egger
All − IVW(MRE)
−0.5
0.0
MR effect size for
'Selenium' on 'TNF receptor−associated factor 4'

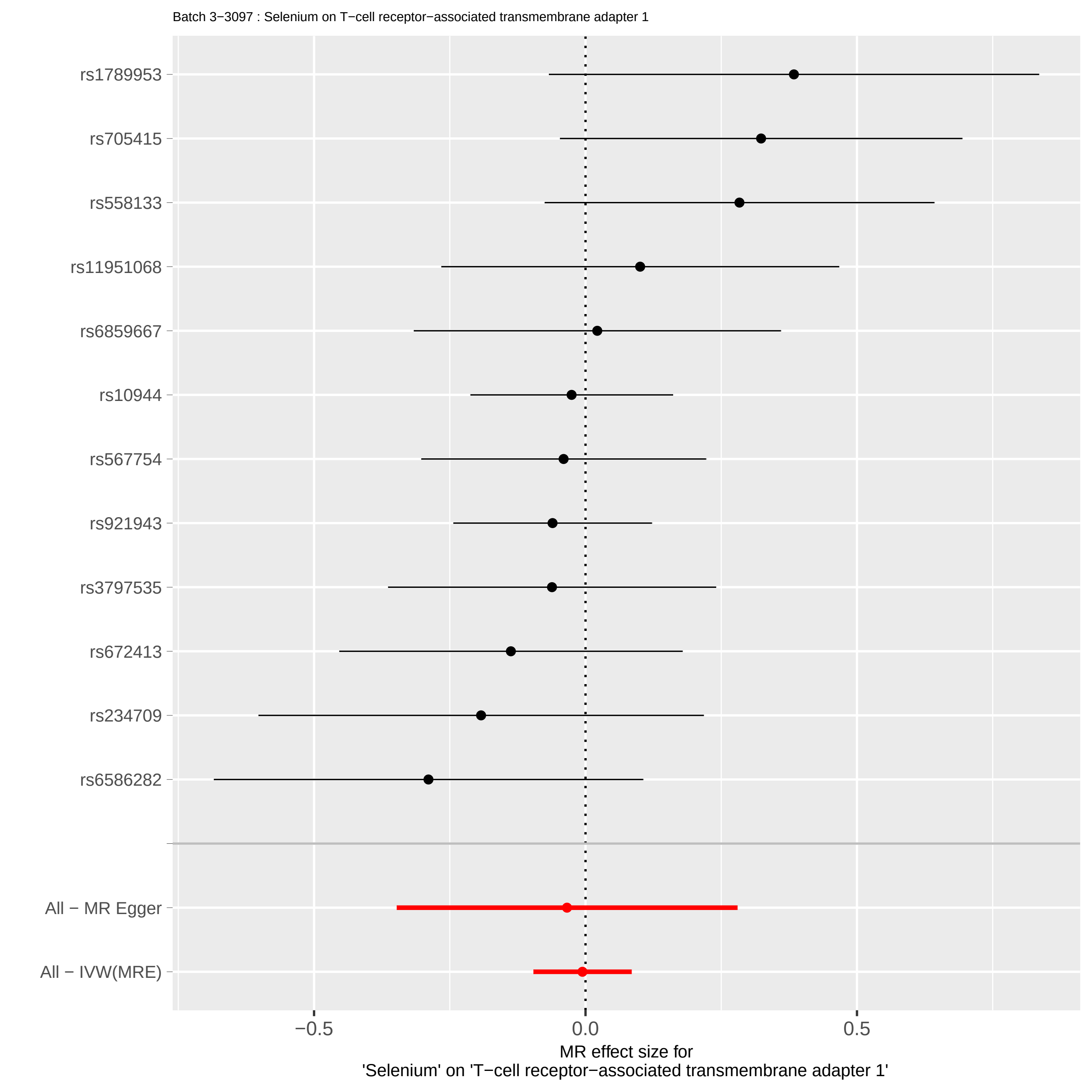

Batch 3−3097 : Selenium on T−cell receptor−associated transmembrane adapter 1
rs1789953
rs705415
rs558133
rs11951068
rs6859667
rs10944
rs567754
rs921943
rs3797535
rs672413
rs234709
rs6586282
All − MR Egger
All − IVW(MRE)
−0.5
0.0
0.5
MR effect size for
'Selenium' on 'T−cell receptor−associated transmembrane adapter 1'

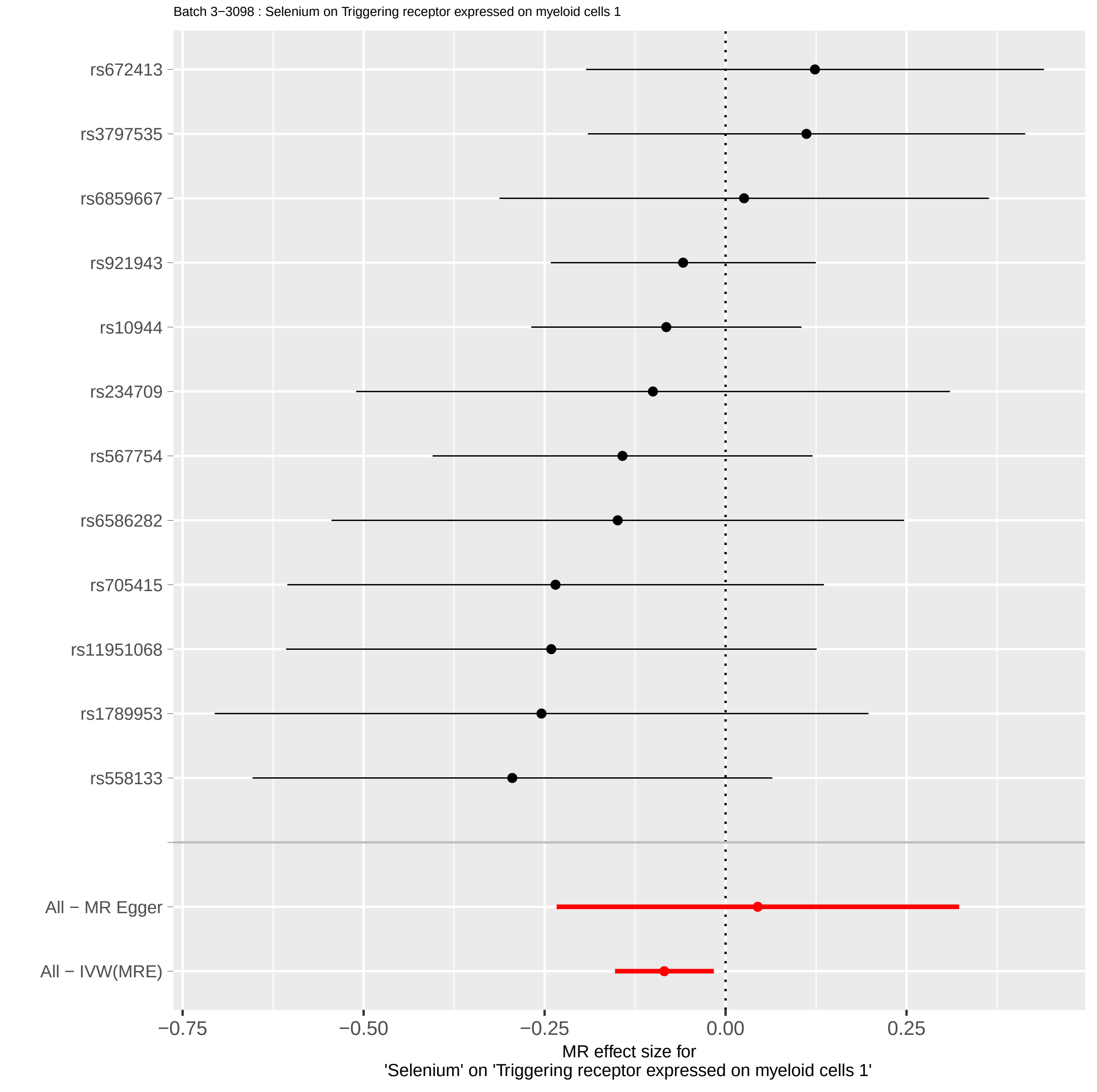

Batch 3−3098 : Selenium on Triggering receptor expressed on myeloid cells 1
rs672413
rs3797535
rs6859667
rs921943
rs10944
rs234709
rs567754
rs6586282
rs705415
rs11951068
rs1789953
rs558133
All − MR Egger
All − IVW(MRE)
−0.75
−0.50
−0.25
0.00
0.25
MR effect size for
'Selenium' on 'Triggering receptor expressed on myeloid cells 1'

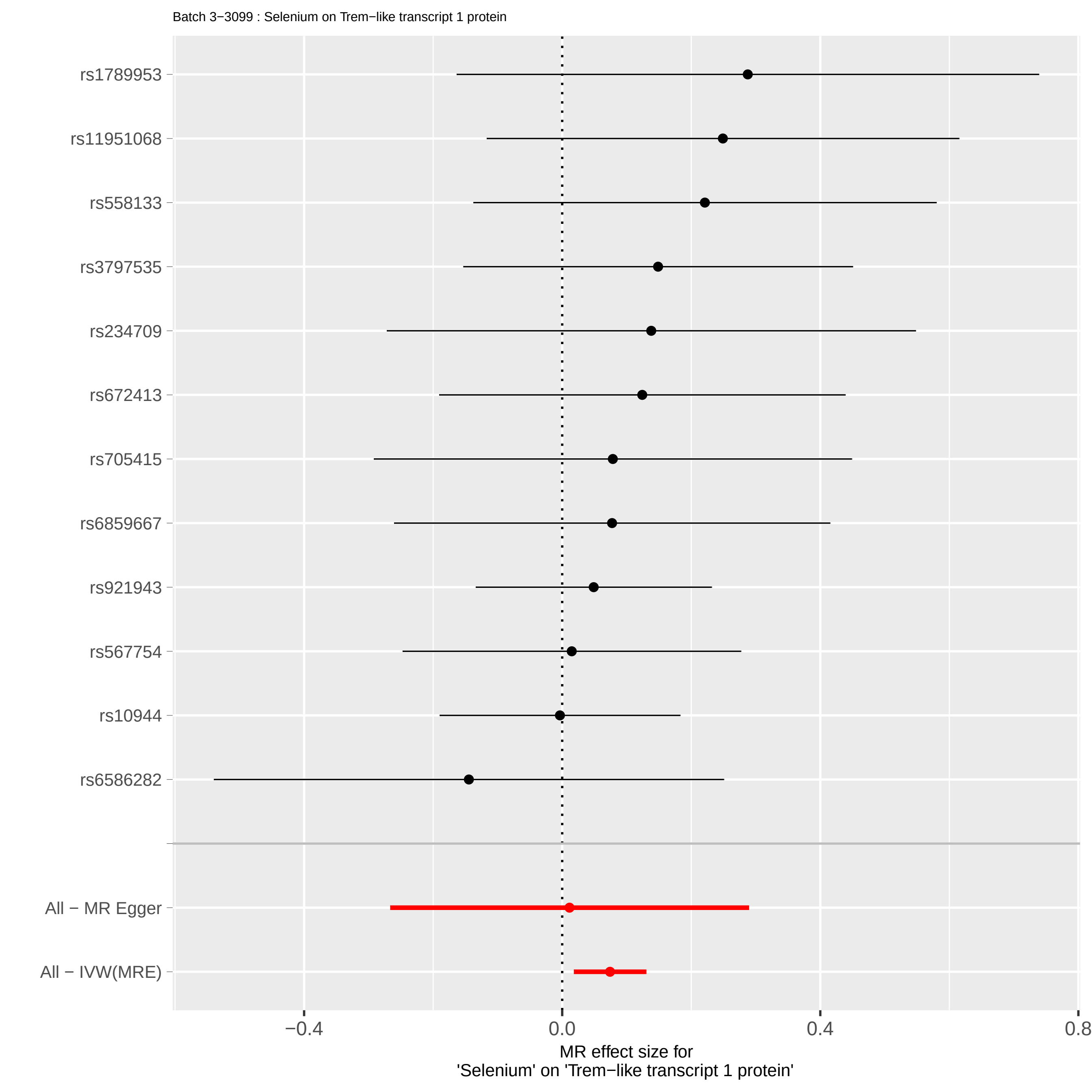

Batch 3−3099 : Selenium on Trem−like transcript 1 protein
rs1789953
rs11951068
rs558133
rs3797535
rs234709
rs672413
rs705415
rs6859667
rs921943
rs567754
rs10944
rs6586282
All − MR Egger
All − IVW(MRE)
−0.4
0.0
0.4
0.8
MR effect size for
'Selenium' on 'Trem−like transcript 1 protein'

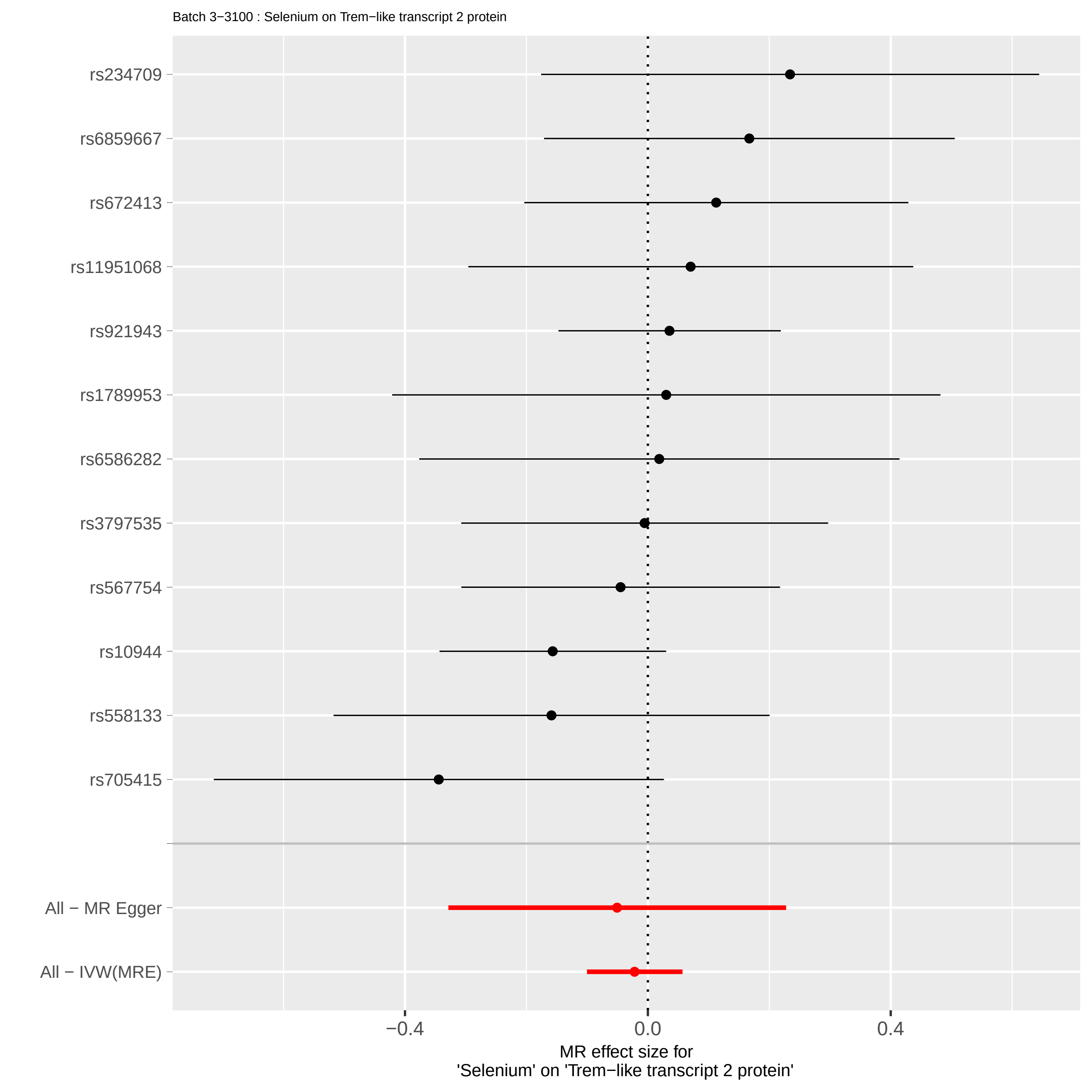
Batch 3−3100 : Selenium on Trem−like transcript 2 protein
rs234709
rs6859667
rs672413
rs11951068
rs921943
rs1789953
rs6586282
rs3797535
rs567754
rs10944
rs558133
rs705415
All − MR Egger
All − IVW(MRE)
−0.4
0.0
0.4
MR effect size for
'Selenium' on 'Trem−like transcript 2 protein'

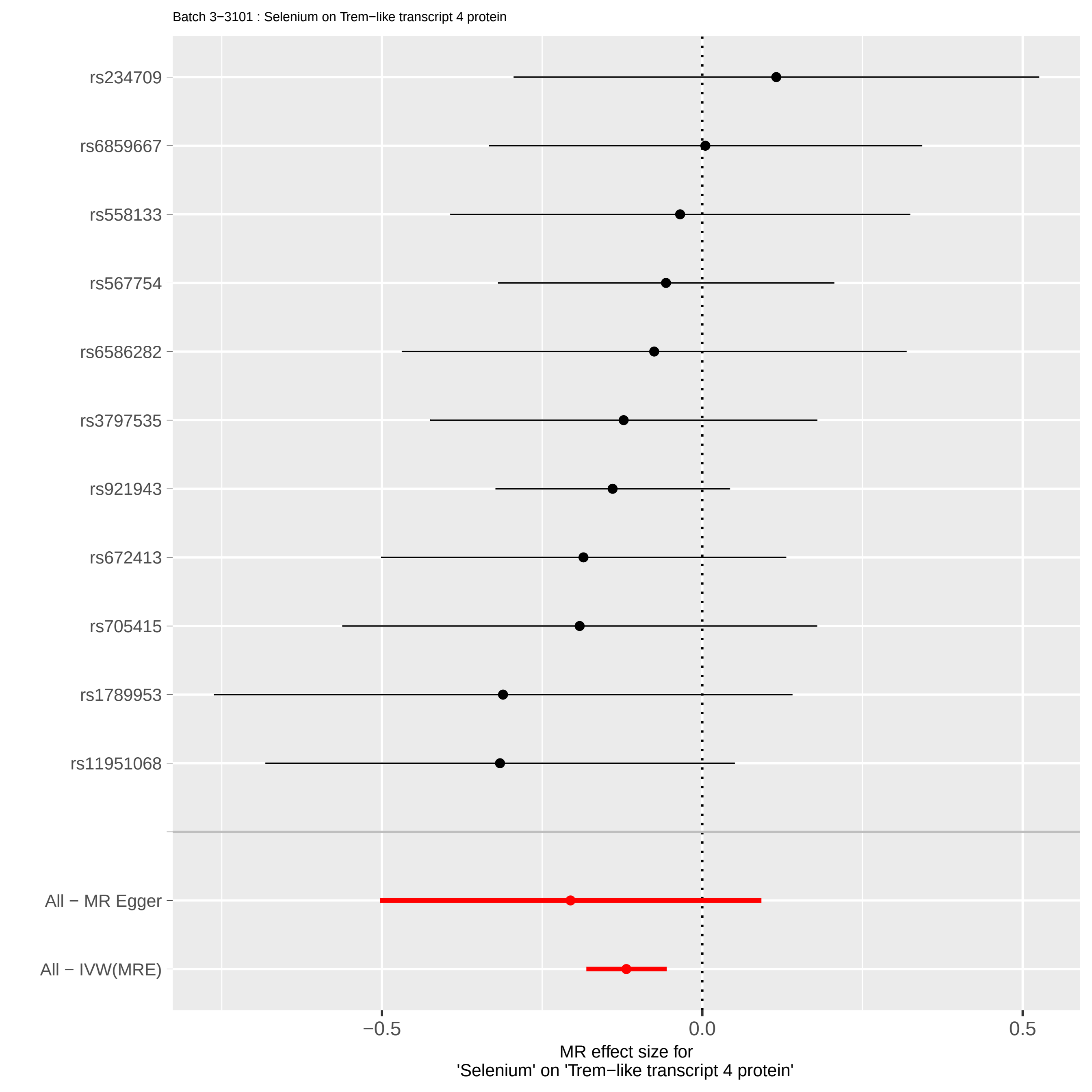

Batch 3−3101 : Selenium on Trem−like transcript 4 protein
rs234709
rs6859667
rs558133
rs567754
rs6586282
rs3797535
rs921943
rs672413
rs705415
rs1789953
rs11951068
All − MR Egger
All − IVW(MRE)
−0.5
0.0
0.5
MR effect size for
'Selenium' on 'Trem−like transcript 4 protein'

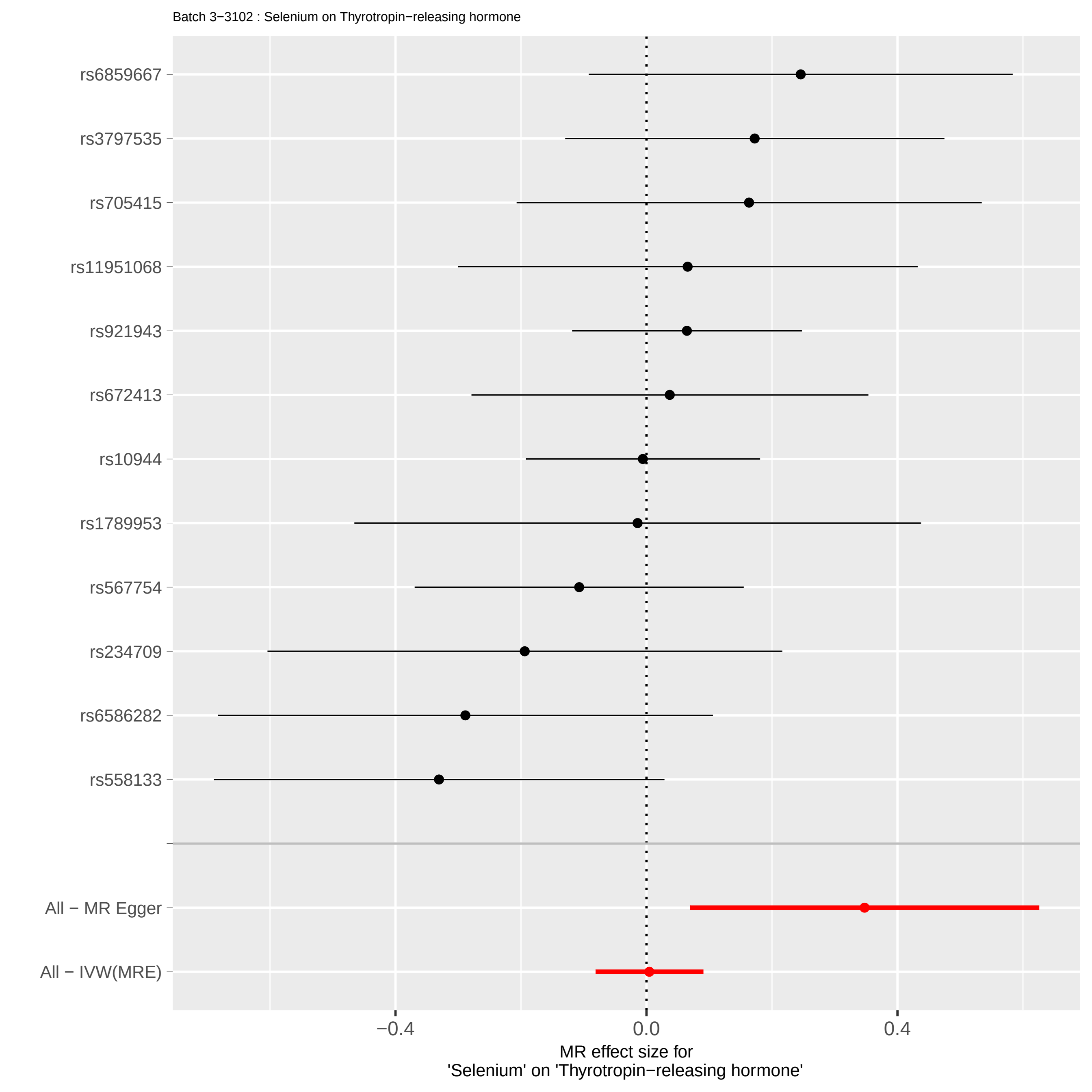

Batch 3−3102 : Selenium on Thyrotropin−releasing hormone
rs6859667
rs3797535
rs705415
rs11951068
rs921943
rs672413
rs10944
rs1789953
rs567754
rs234709
rs6586282
rs558133
All − MR Egger
All − IVW(MRE)
−0.4
0.0
0.4
MR effect size for
'Selenium' on 'Thyrotropin−releasing hormone'

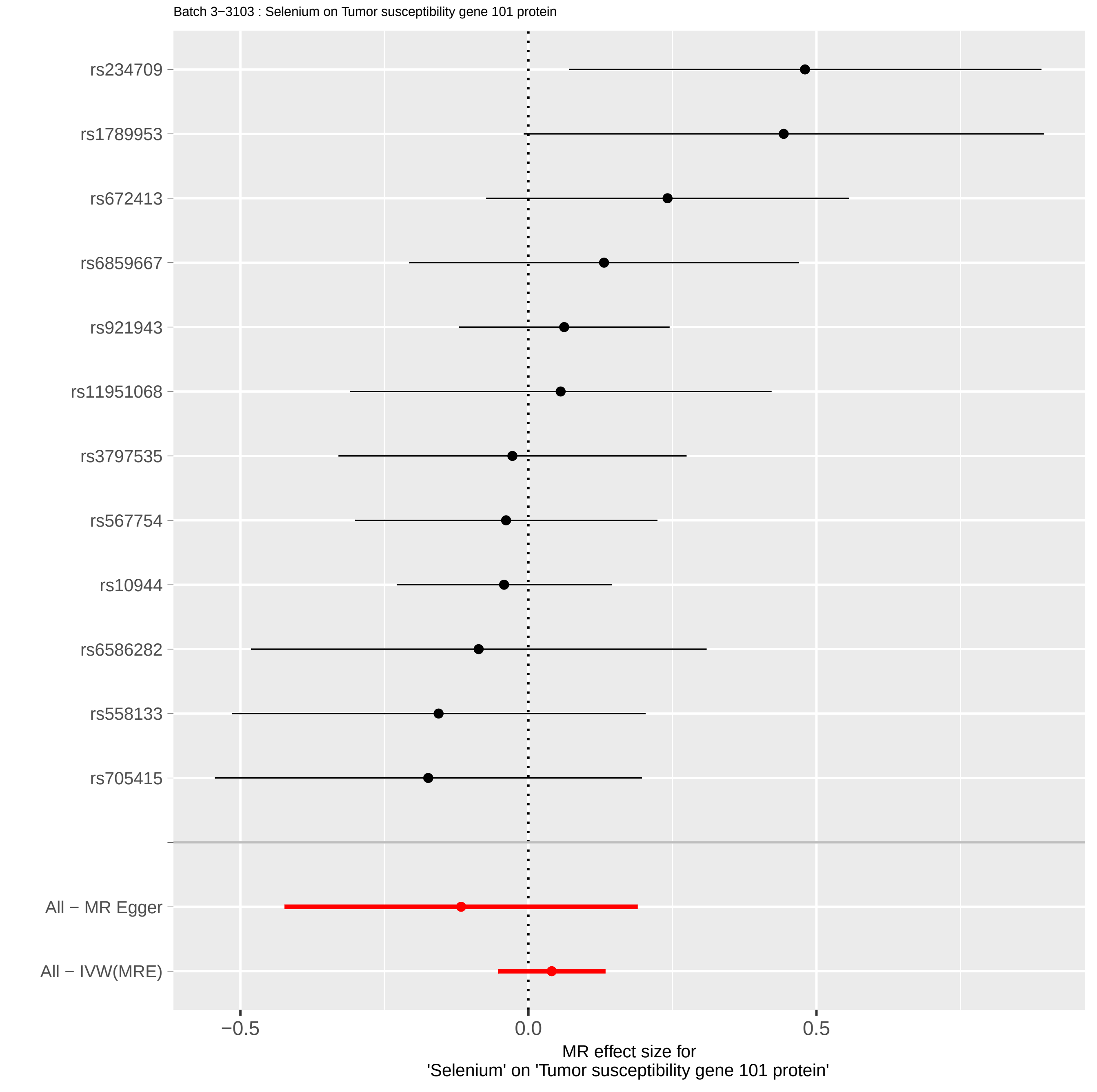

Batch 3−3103 : Selenium on Tumor susceptibility gene 101 protein
rs234709
rs1789953
rs672413
rs6859667
rs921943
rs11951068
rs3797535
rs567754
rs10944
rs6586282
rs558133
rs705415
All − MR Egger
All − IVW(MRE)
−0.5
0.0
0.5
MR effect size for
'Selenium' on 'Tumor susceptibility gene 101 protein'

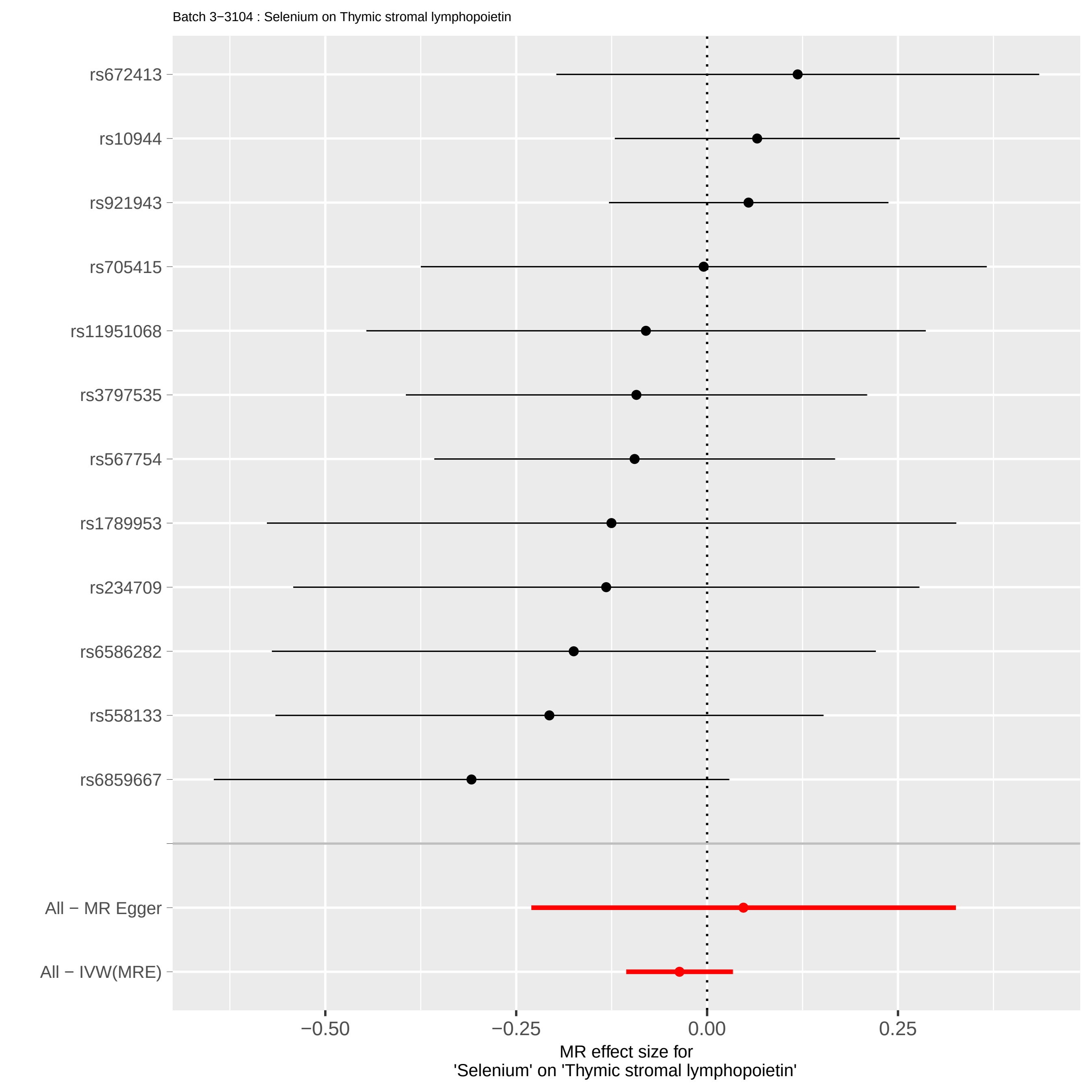

Batch 3−3104 : Selenium on Thymic stromal lymphopoietin
rs672413
rs10944
rs921943
rs705415
rs11951068
rs3797535
rs567754
rs1789953
rs234709
rs6586282
rs558133
rs6859667
All − MR Egger
All − IVW(MRE)
−0.50
−0.25
0.00
0.25
MR effect size for
'Selenium' on 'Thymic stromal lymphopoietin'

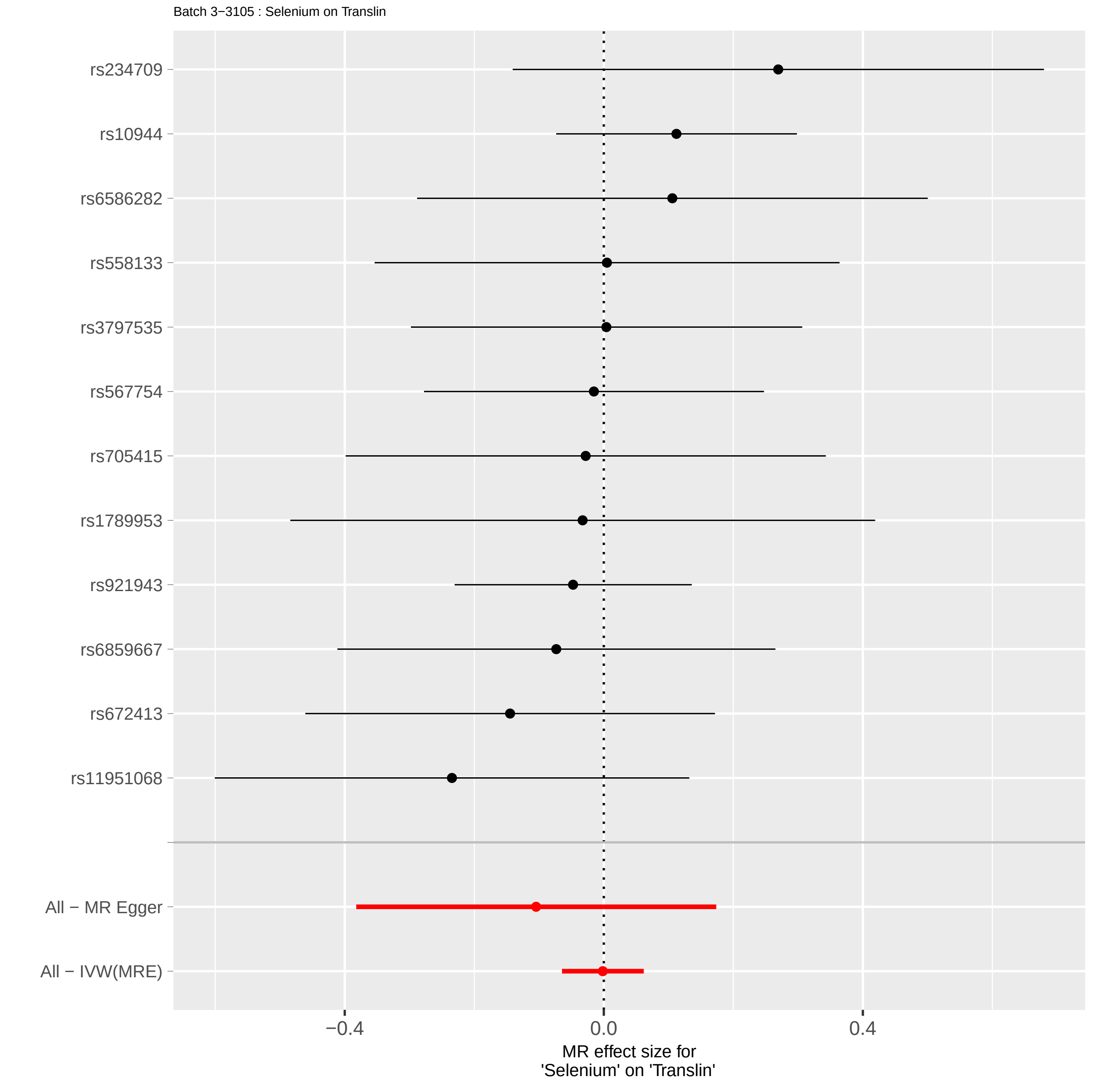

Batch 3−3105 : Selenium on Translin
rs234709
rs10944
rs6586282
rs558133
rs3797535
rs567754
rs705415
rs1789953
rs921943
rs6859667
rs672413
rs11951068
All − MR Egger
All − IVW(MRE)
−0.4
0.0
0.4
MR effect size for
'Selenium' on 'Translin'

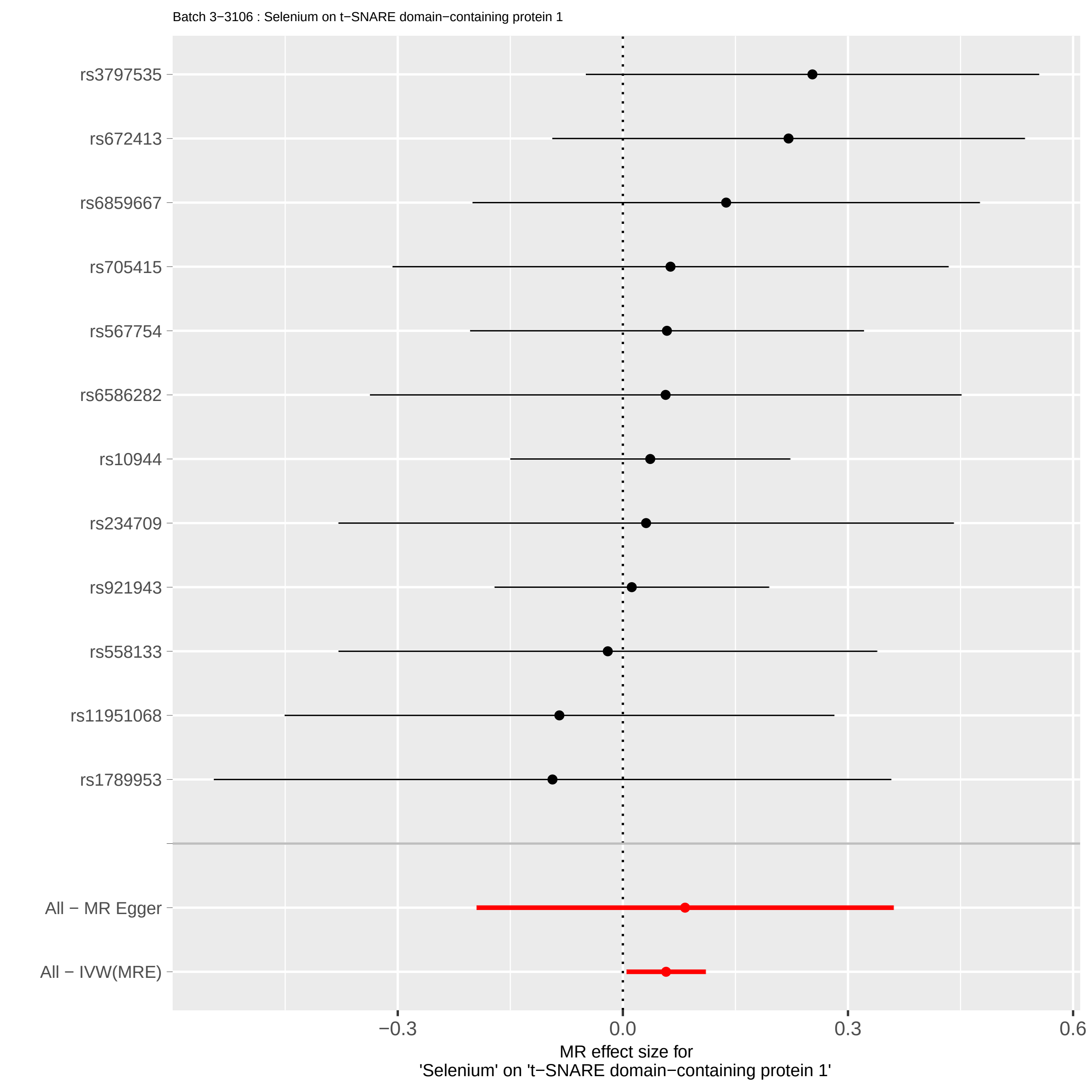

Batch 3−3106 : Selenium on t−SNARE domain−containing protein 1
rs3797535
rs672413
rs6859667
rs705415
rs567754
rs6586282
rs10944
rs234709
rs921943
rs558133
rs11951068
rs1789953
All − MR Egger
All − IVW(MRE)
−0.3
0.0
0.3
0.6
MR effect size for
'Selenium' on 't−SNARE domain−containing protein 1'

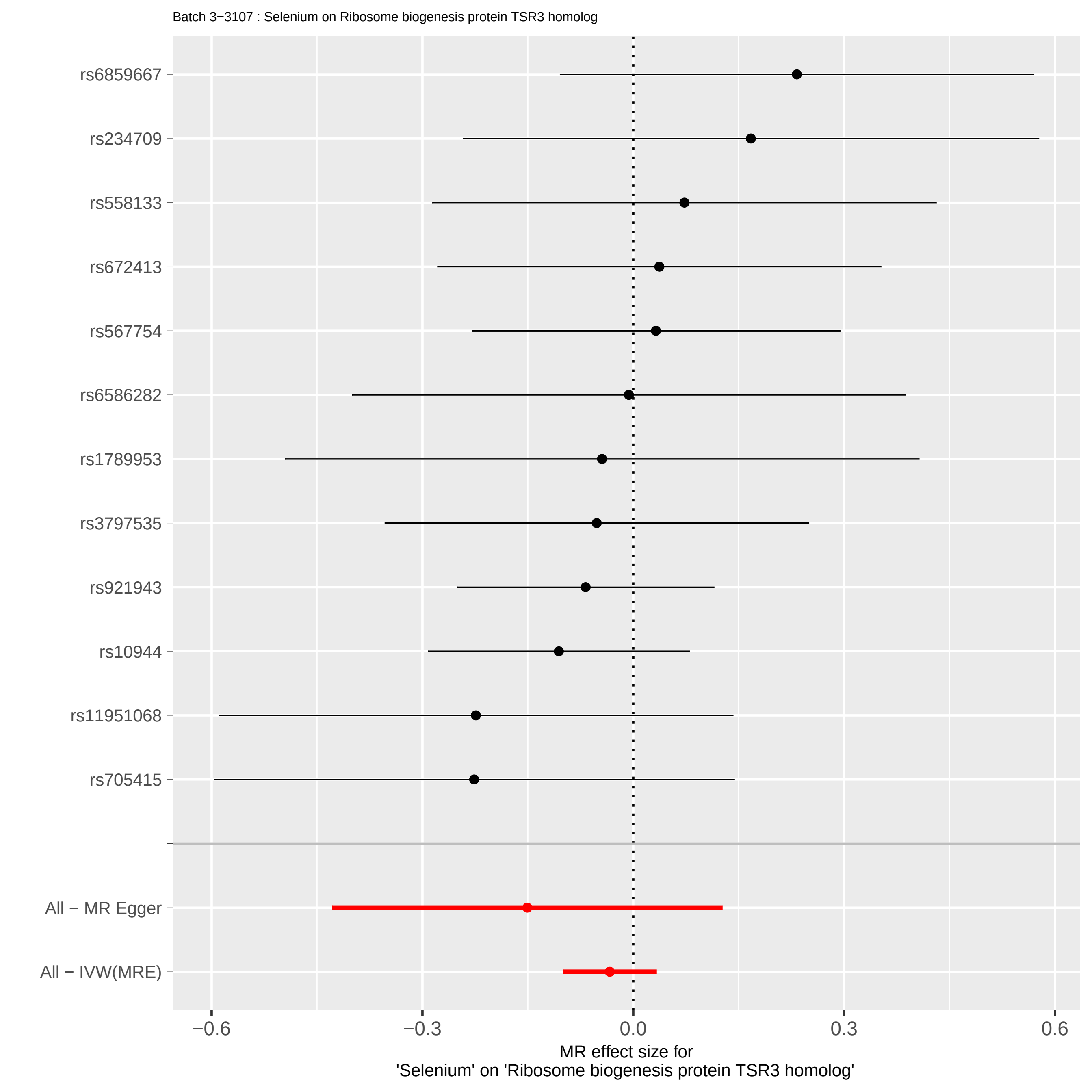

Batch 3−3107 : Selenium on Ribosome biogenesis protein TSR3 homolog
rs6859667
rs234709
rs558133
rs672413
rs567754
rs6586282
rs1789953
rs3797535
rs921943
rs10944
rs11951068
rs705415
All − MR Egger
All − IVW(MRE)
−0.6
−0.3
0.0
0.3
0.6
MR effect size for
'Selenium' on 'Ribosome biogenesis protein TSR3 homolog'

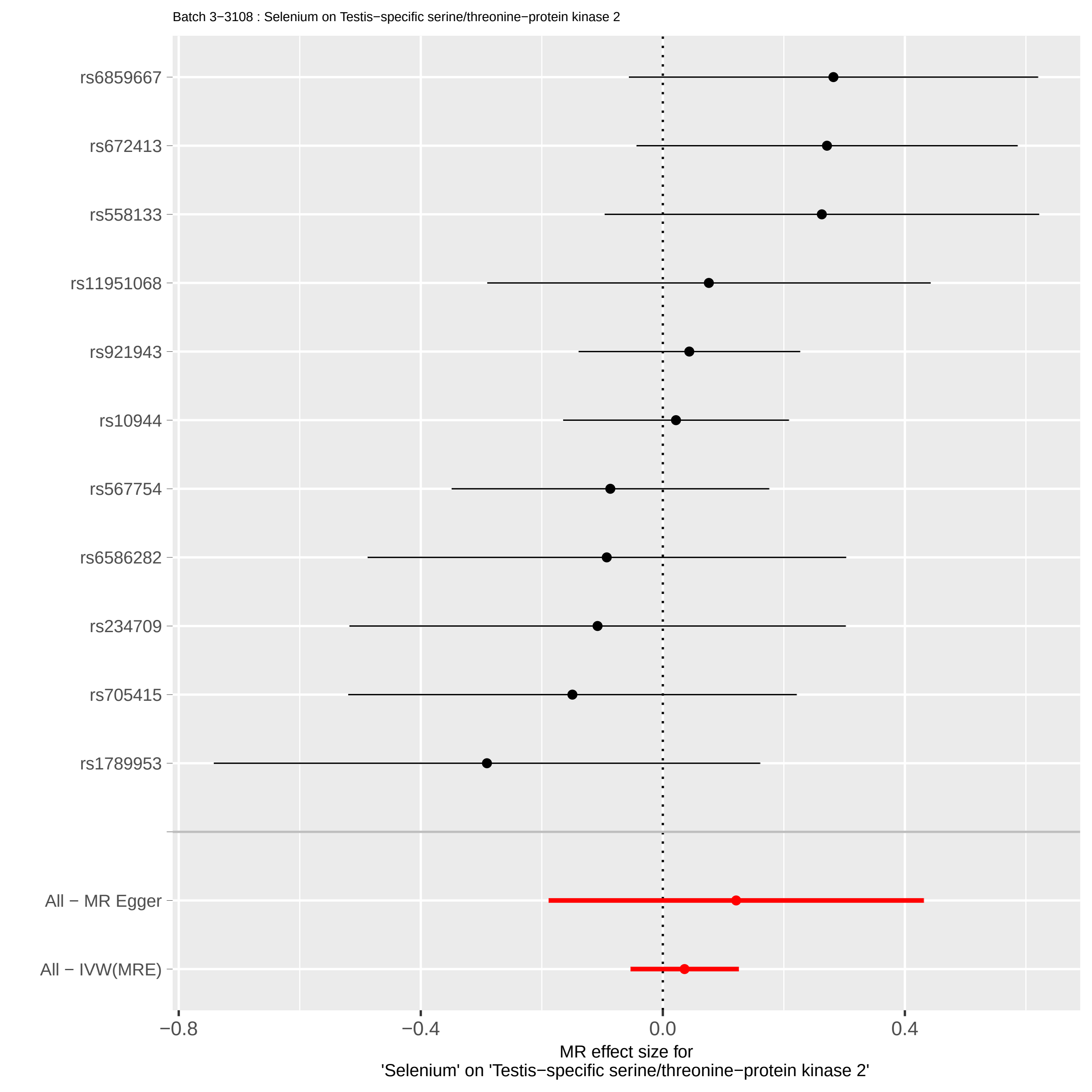

Batch 3−3108 : Selenium on Testis−specific serine/threonine−protein kinase 2
rs6859667
rs672413
rs558133
rs11951068
rs921943
rs10944
rs567754
rs6586282
rs234709
rs705415
rs1789953
All − MR Egger
All − IVW(MRE)
−0.8
−0.4
0.0
0.4
MR effect size for
'Selenium' on 'Testis−specific serine/threonine−protein kinase 2'

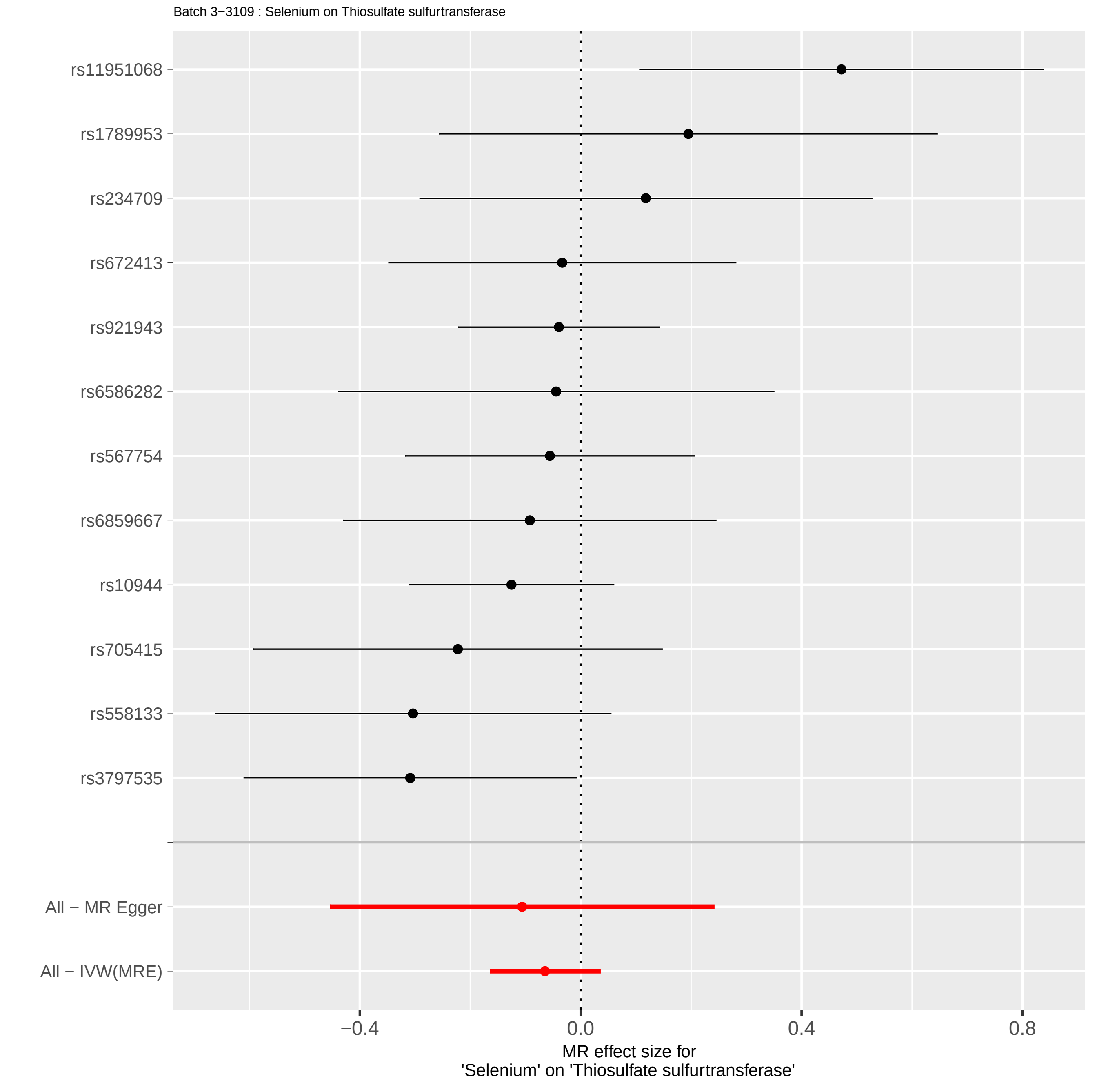
Batch 3−3109 : Selenium on Thiosulfate sulfurtransferase
rs11951068
rs1789953
rs234709
rs672413
rs921943
rs6586282
rs567754
rs6859667
rs10944
rs705415
rs558133
rs3797535
All − MR Egger
All − IVW(MRE)
−0.4
0.0
0.4
0.8
MR effect size for
'Selenium' on 'Thiosulfate sulfurtransferase'

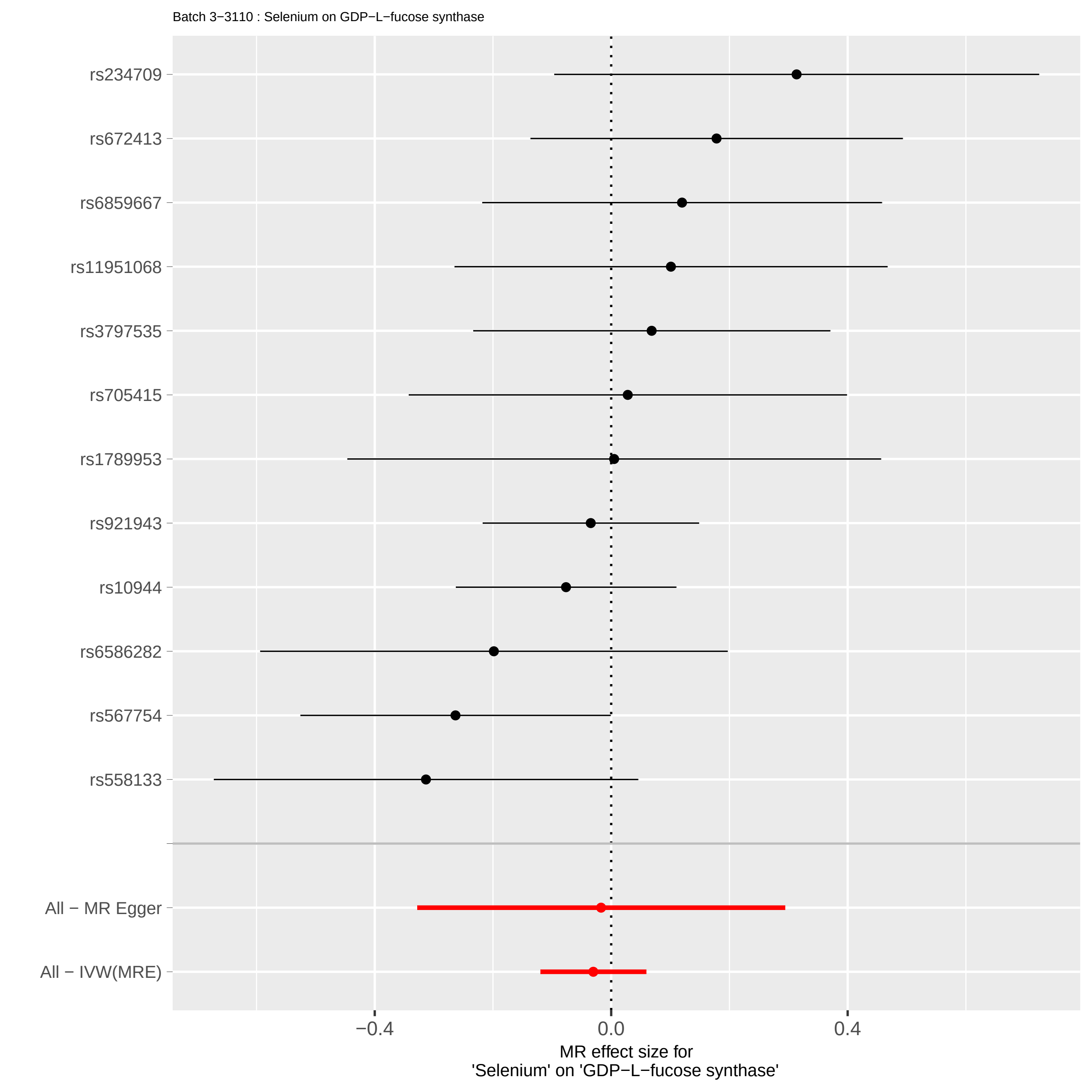

Batch 3−3110 : Selenium on GDP−L−fucose synthase
rs234709
rs672413
rs6859667
rs11951068
rs3797535
rs705415
rs1789953
rs921943
rs10944
rs6586282
rs567754
rs558133
All − MR Egger
All − IVW(MRE)
−0.4
0.0
0.4
MR effect size for
'Selenium' on 'GDP−L−fucose synthase'

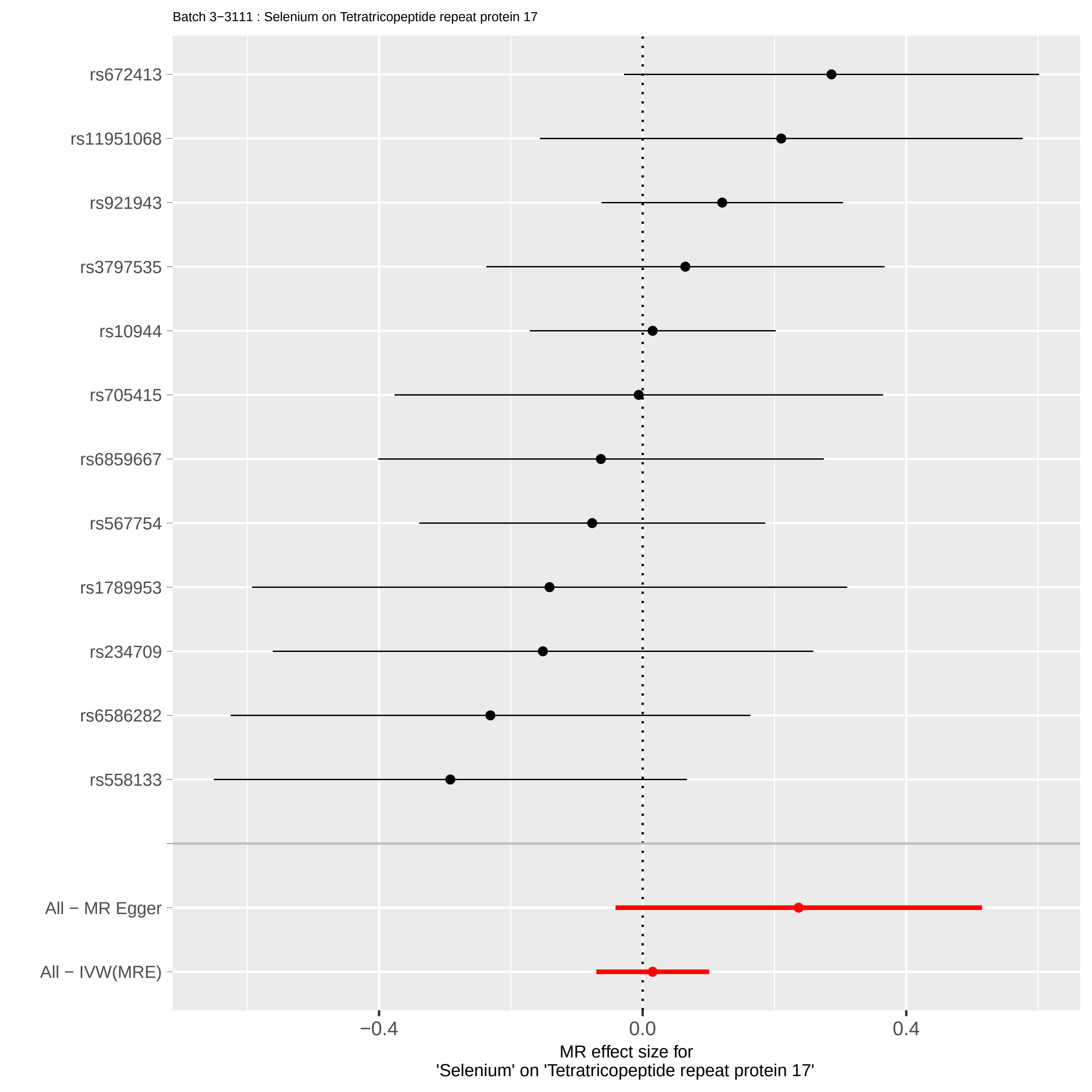

Batch 3−3111 : Selenium on Tetratricopeptide repeat protein 17
rs672413
rs11951068
rs921943
rs3797535
rs10944
rs705415
rs6859667
rs567754
rs1789953
rs234709
rs6586282
rs558133
All − MR Egger
All − IVW(MRE)
−0.4
0.0
0.4
MR effect size for
'Selenium' on 'Tetratricopeptide repeat protein 17'

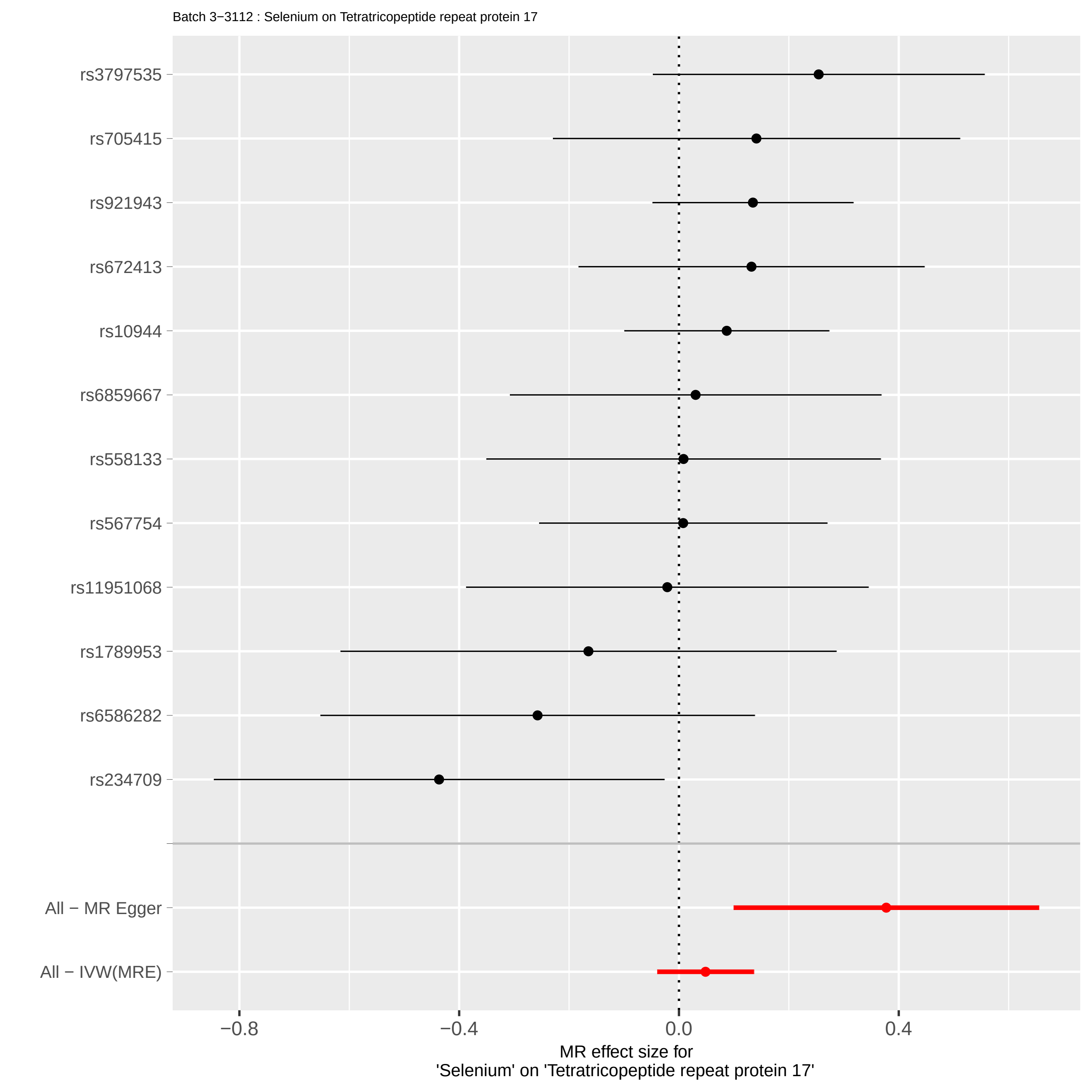

Batch 3−3112 : Selenium on Tetratricopeptide repeat protein 17
rs3797535
rs705415
rs921943
rs672413
rs10944
rs6859667
rs558133
rs567754
rs11951068
rs1789953
rs6586282
rs234709
All − MR Egger
All − IVW(MRE)
−0.8
−0.4
0.0
0.4
MR effect size for
'Selenium' on 'Tetratricopeptide repeat protein 17'

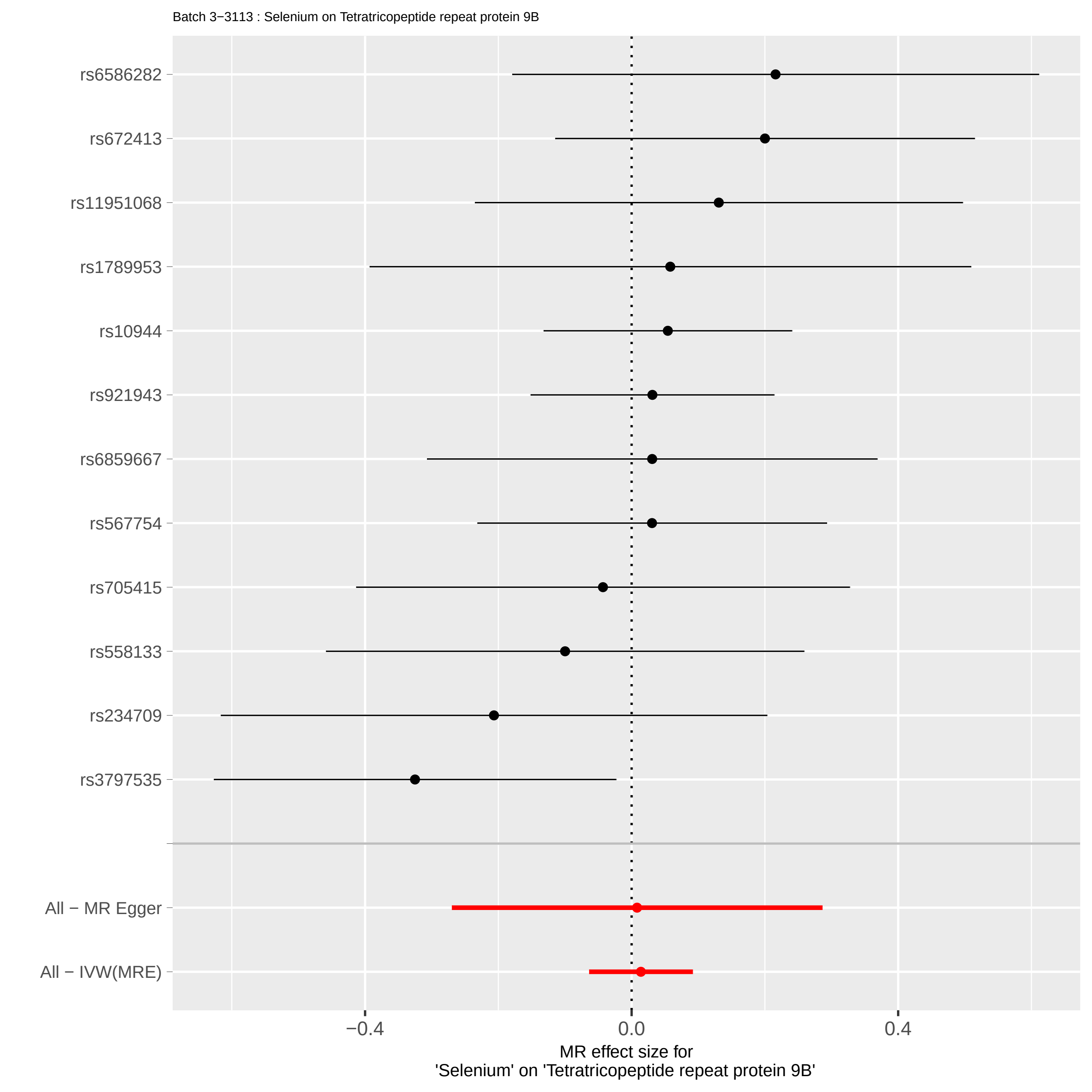

Batch 3−3113 : Selenium on Tetratricopeptide repeat protein 9B
rs6586282
rs672413
rs11951068
rs1789953
rs10944
rs921943
rs6859667
rs567754
rs705415
rs558133
rs234709
rs3797535
All − MR Egger
All − IVW(MRE)
−0.4
0.0
0.4
MR effect size for
'Selenium' on 'Tetratricopeptide repeat protein 9B'

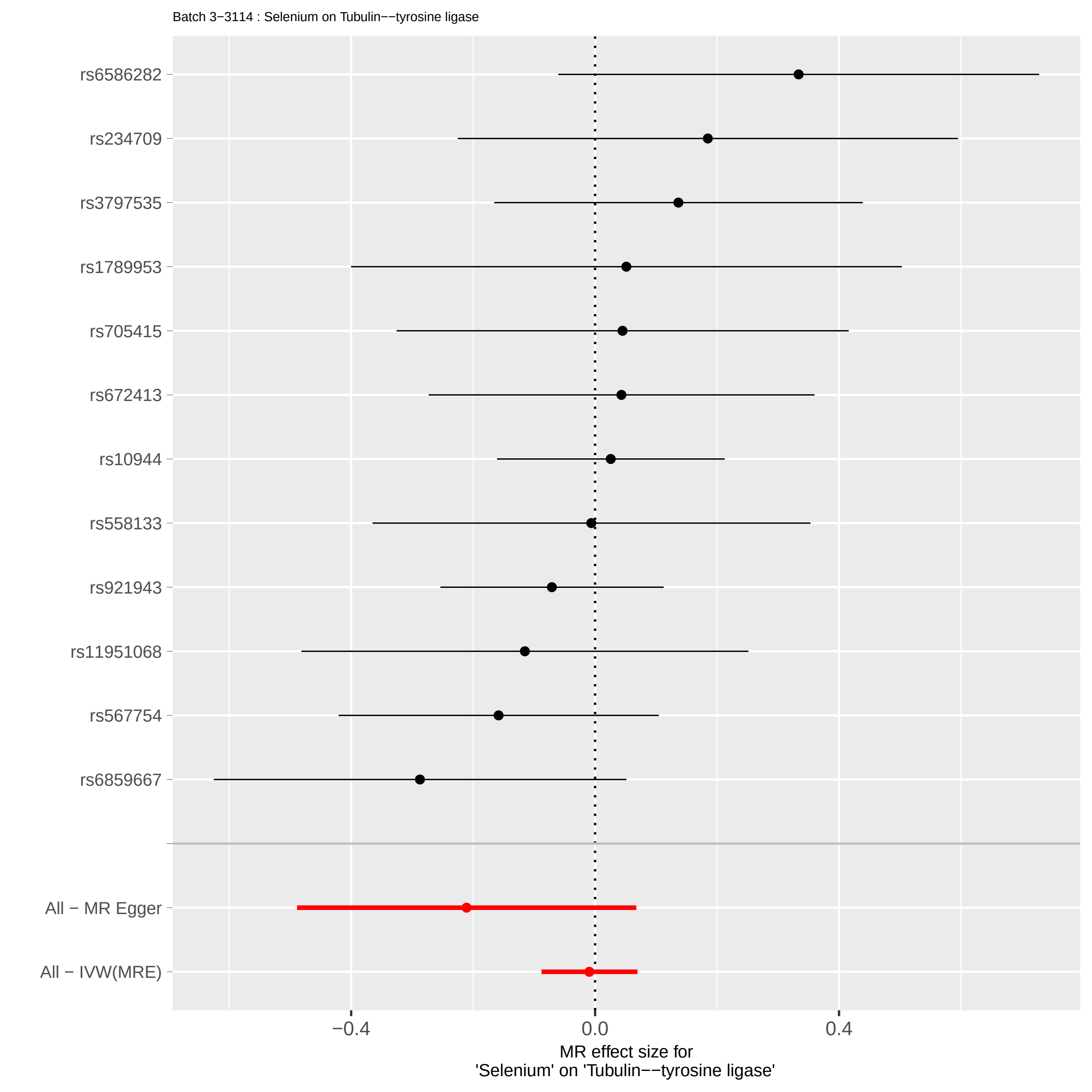

Batch 3−3114 : Selenium on Tubulin−−tyrosine ligase
rs6586282
rs234709
rs3797535
rs1789953
rs705415
rs672413
rs10944
rs558133
rs921943
rs11951068
rs567754
rs6859667
All − MR Egger
All − IVW(MRE)
−0.4
0.0
0.4
MR effect size for
'Selenium' on 'Tubulin−−tyrosine ligase'

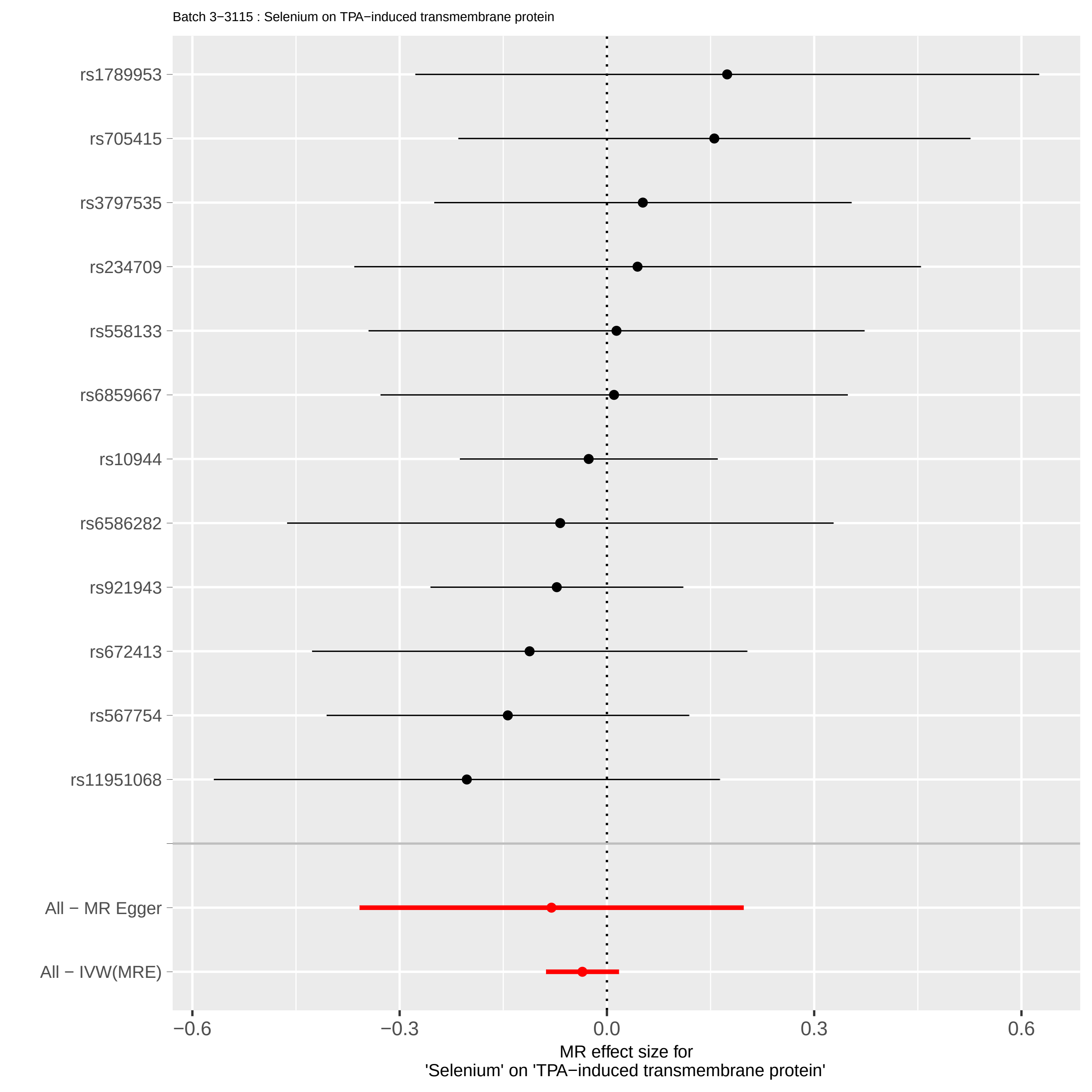

Batch 3−3115 : Selenium on TPA−induced transmembrane protein
rs1789953
rs705415
rs3797535
rs234709
rs558133
rs6859667
rs10944
rs6586282
rs921943
rs672413
rs567754
rs11951068
All − MR Egger
All − IVW(MRE)
−0.6
−0.3
0.0
0.3
0.6
MR effect size for
'Selenium' on 'TPA−induced transmembrane protein'

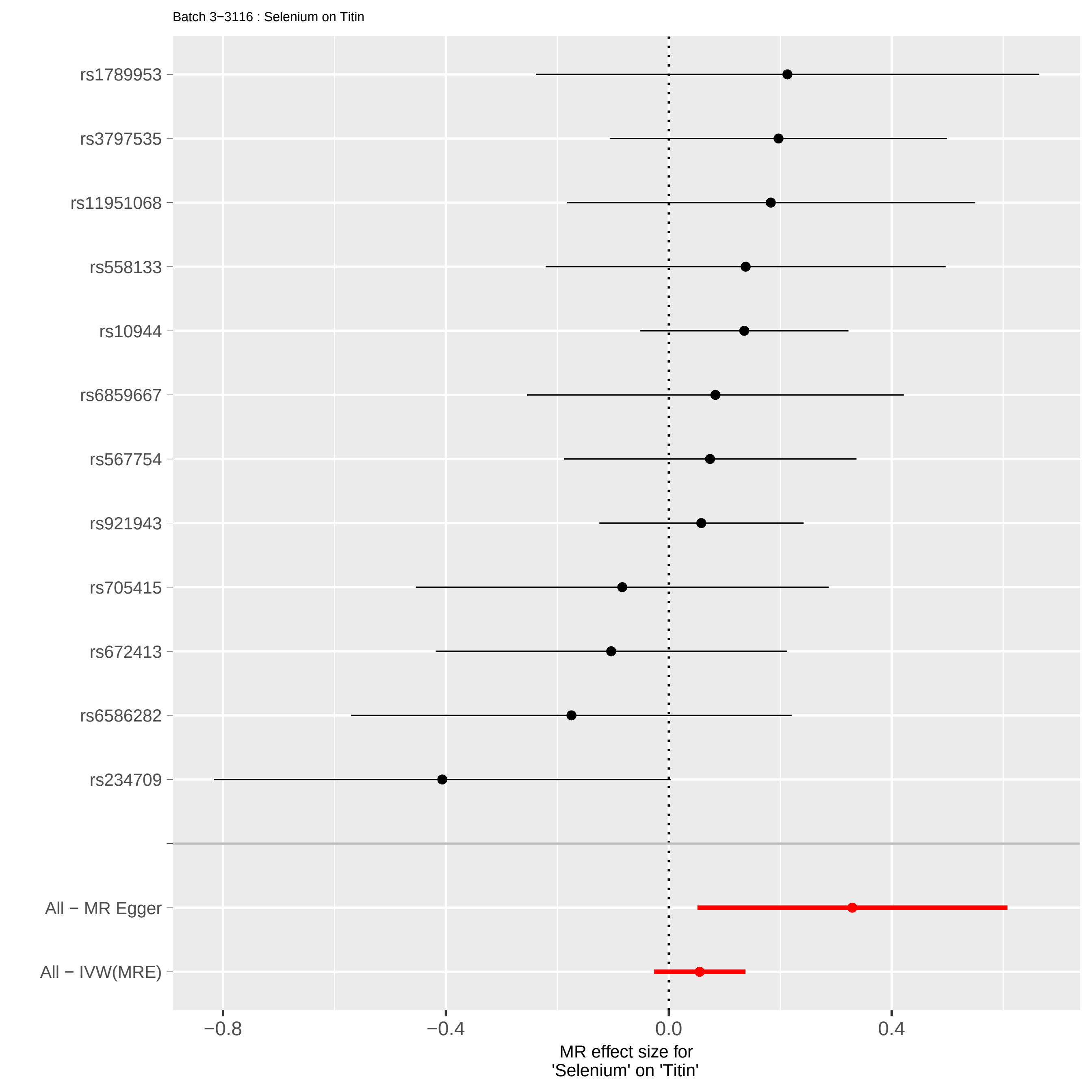

Batch 3−3116 : Selenium on Titin
rs1789953
rs3797535
rs11951068
rs558133
rs10944
rs6859667
rs567754
rs921943
rs705415
rs672413
rs6586282
rs234709
All − MR Egger
All − IVW(MRE)
−0.8
−0.4
0.0
0.4
MR effect size for
'Selenium' on 'Titin'

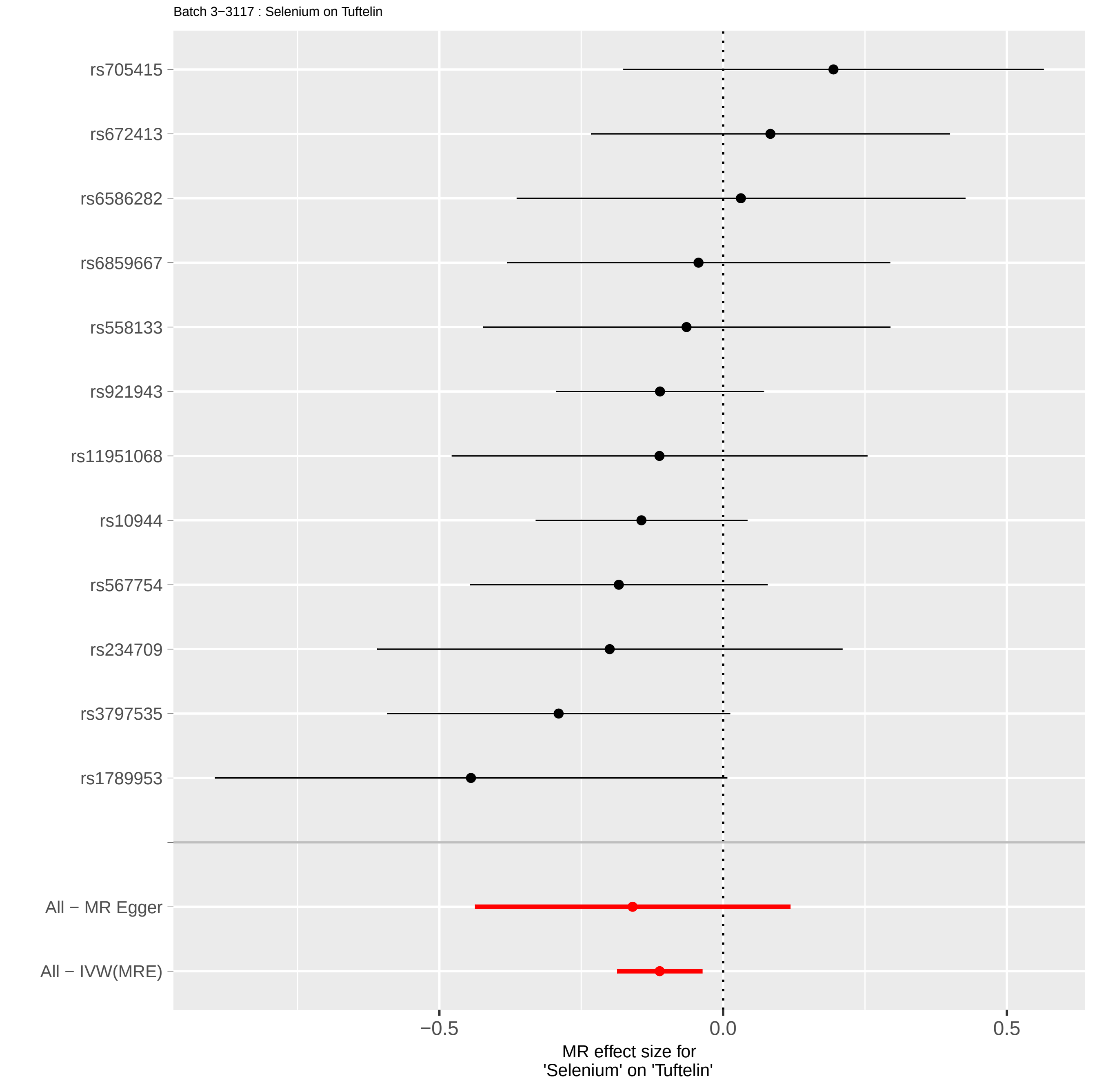

Batch 3−3117 : Selenium on Tuftelin
rs705415
rs672413
rs6586282
rs6859667
rs558133
rs921943
rs11951068
rs10944
rs567754
rs234709
rs3797535
rs1789953
All − MR Egger
All − IVW(MRE)
−0.5
0.0
0.5
MR effect size for
'Selenium' on 'Tuftelin'

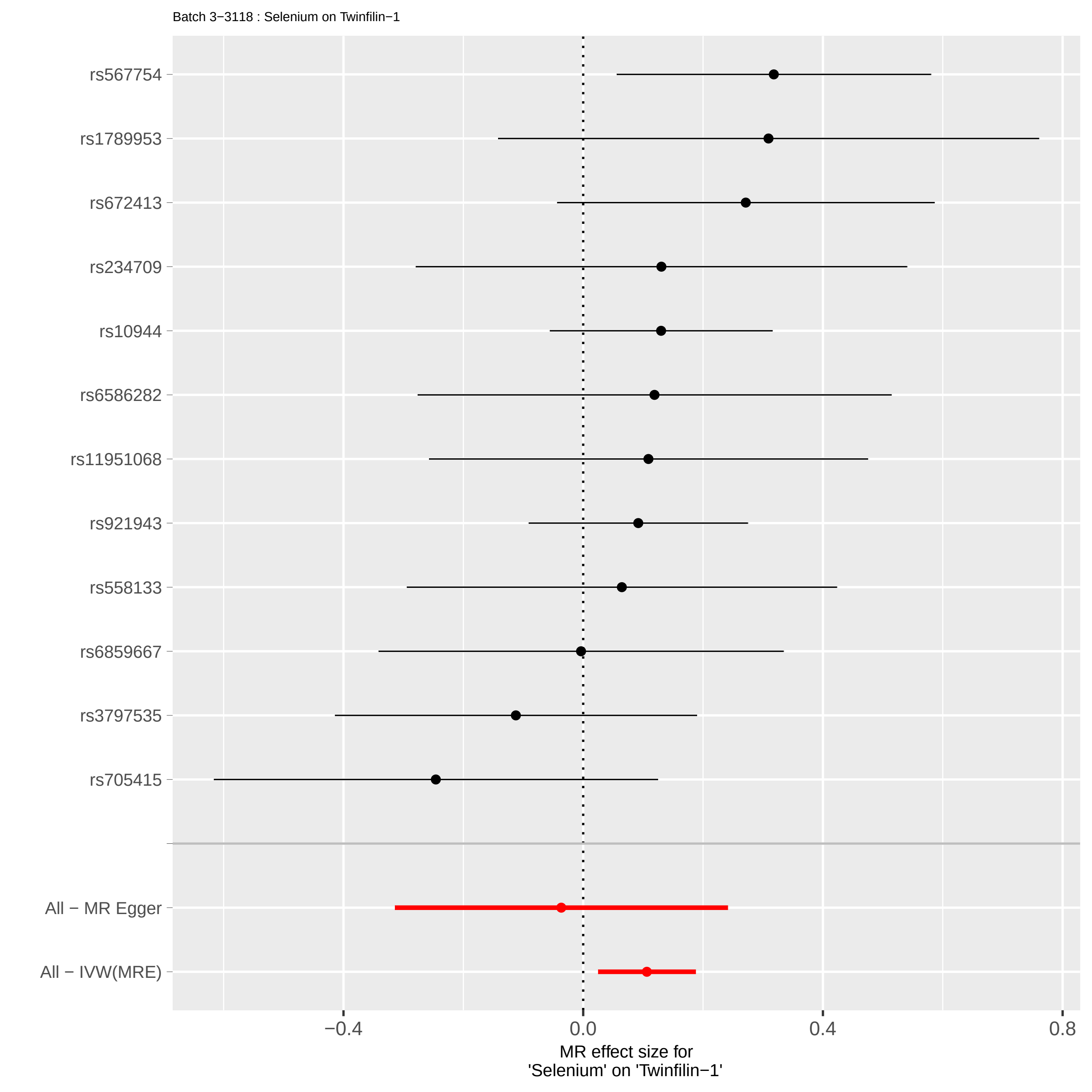

Batch 3−3118 : Selenium on Twinfilin−1
rs567754
rs1789953
rs672413
rs234709
rs10944
rs6586282
rs11951068
rs921943
rs558133
rs6859667
rs3797535
rs705415
All − MR Egger
All − IVW(MRE)
−0.4
0.0
0.4
0.8
MR effect size for
'Selenium' on 'Twinfilin−1'

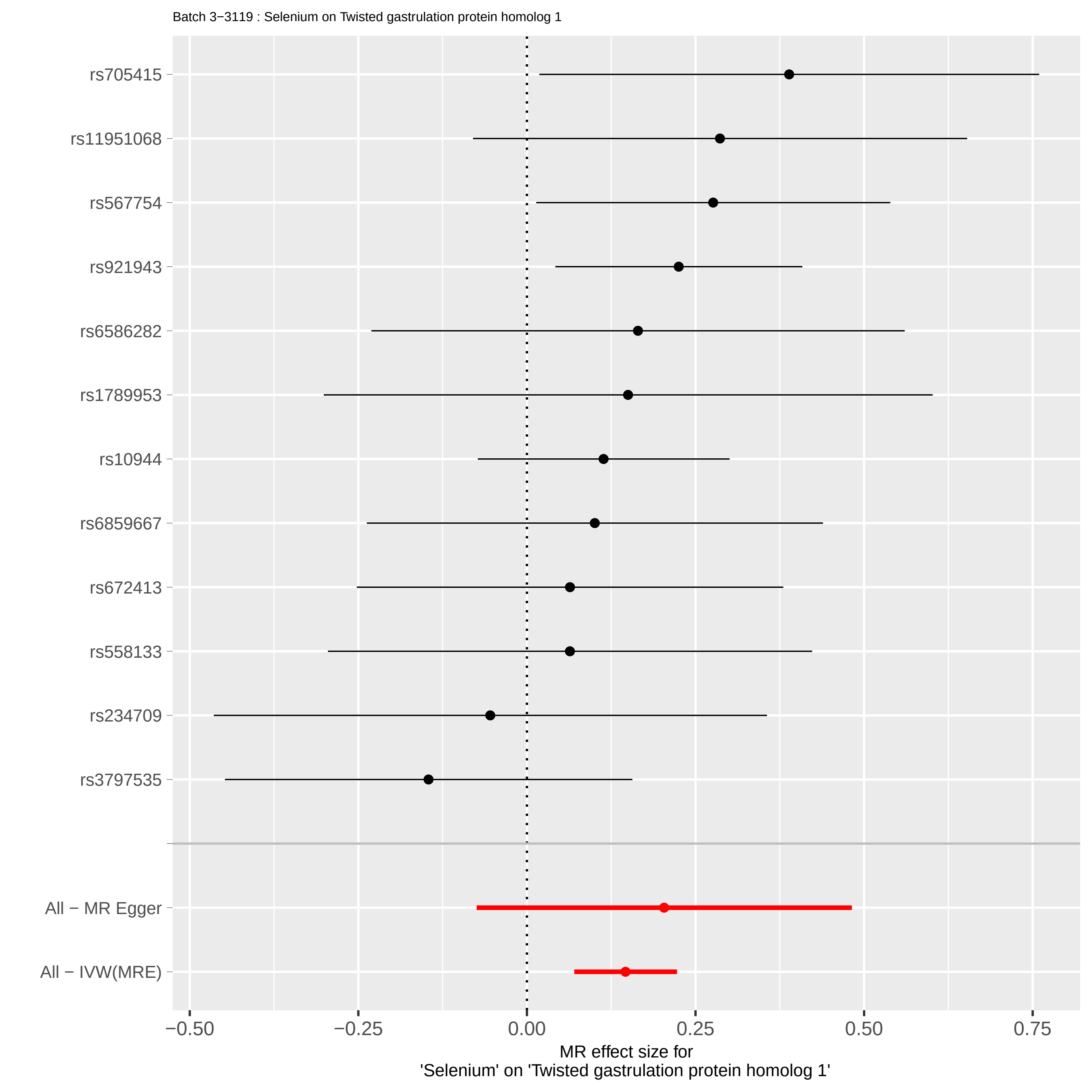

Batch 3−3119 : Selenium on Twisted gastrulation protein homolog 1
rs705415
rs11951068
rs567754
rs921943
rs6586282
rs1789953
rs10944
rs6859667
rs672413
rs558133
rs234709
rs3797535
All − MR Egger
All − IVW(MRE)
−0.50
−0.25
0.00
0.25
0.50
0.75
MR effect size for
'Selenium' on 'Twisted gastrulation protein homolog 1'

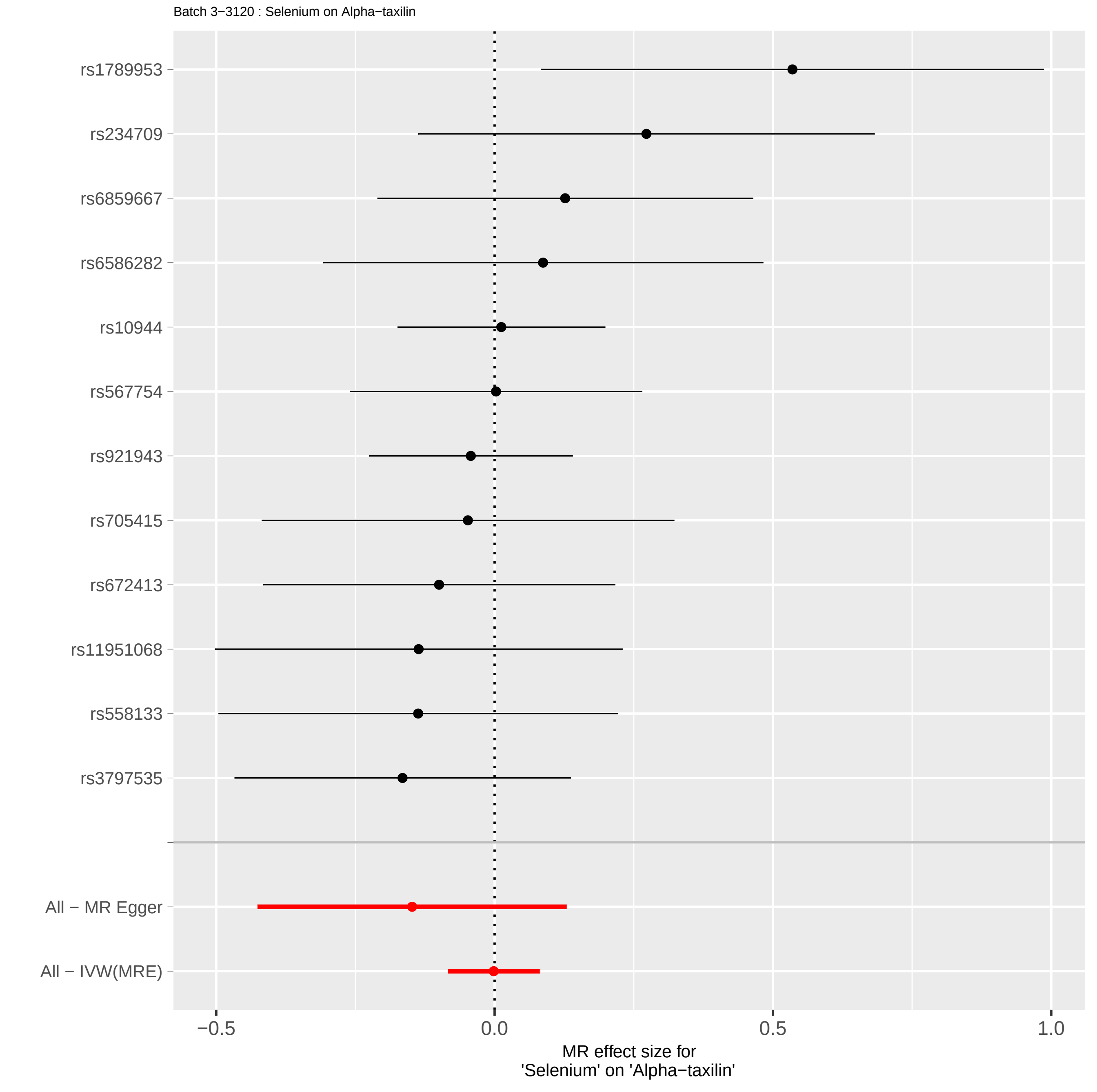

Batch 3−3120 : Selenium on Alpha−taxilin
rs1789953
rs234709
rs6859667
rs6586282
rs10944
rs567754
rs921943
rs705415
rs672413
rs11951068
rs558133
rs3797535
All − MR Egger
All − IVW(MRE)
−0.5
0.0
0.5
1.0
MR effect size for
'Selenium' on 'Alpha−taxilin'

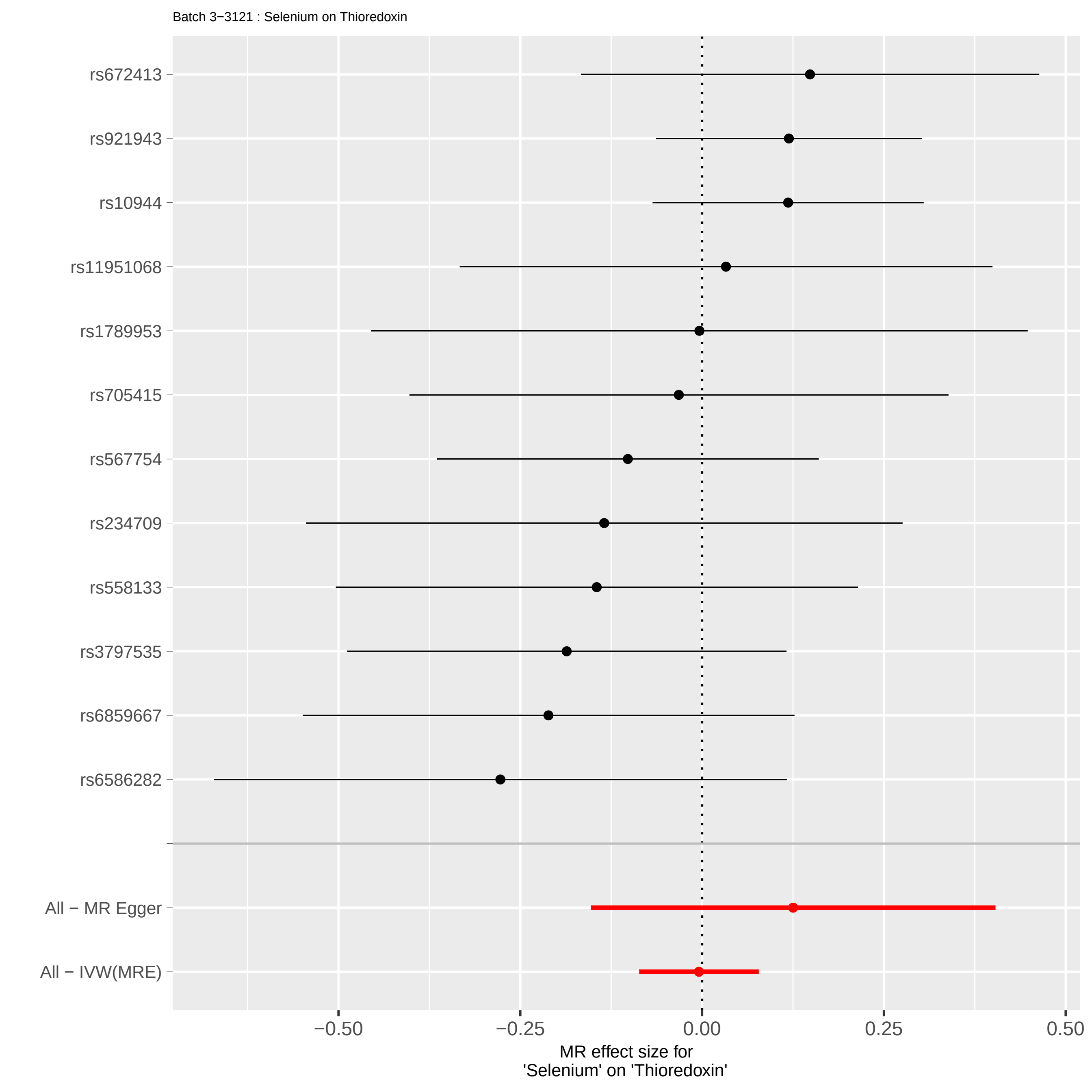

Batch 3−3121 : Selenium on Thioredoxin
rs672413
rs921943
rs10944
rs11951068
rs1789953
rs705415
rs567754
rs234709
rs558133
rs3797535
rs6859667
rs6586282
All − MR Egger
All − IVW(MRE)
−0.50
−0.25
0.00
0.25
0.50
MR effect size for
'Selenium' on 'Thioredoxin'

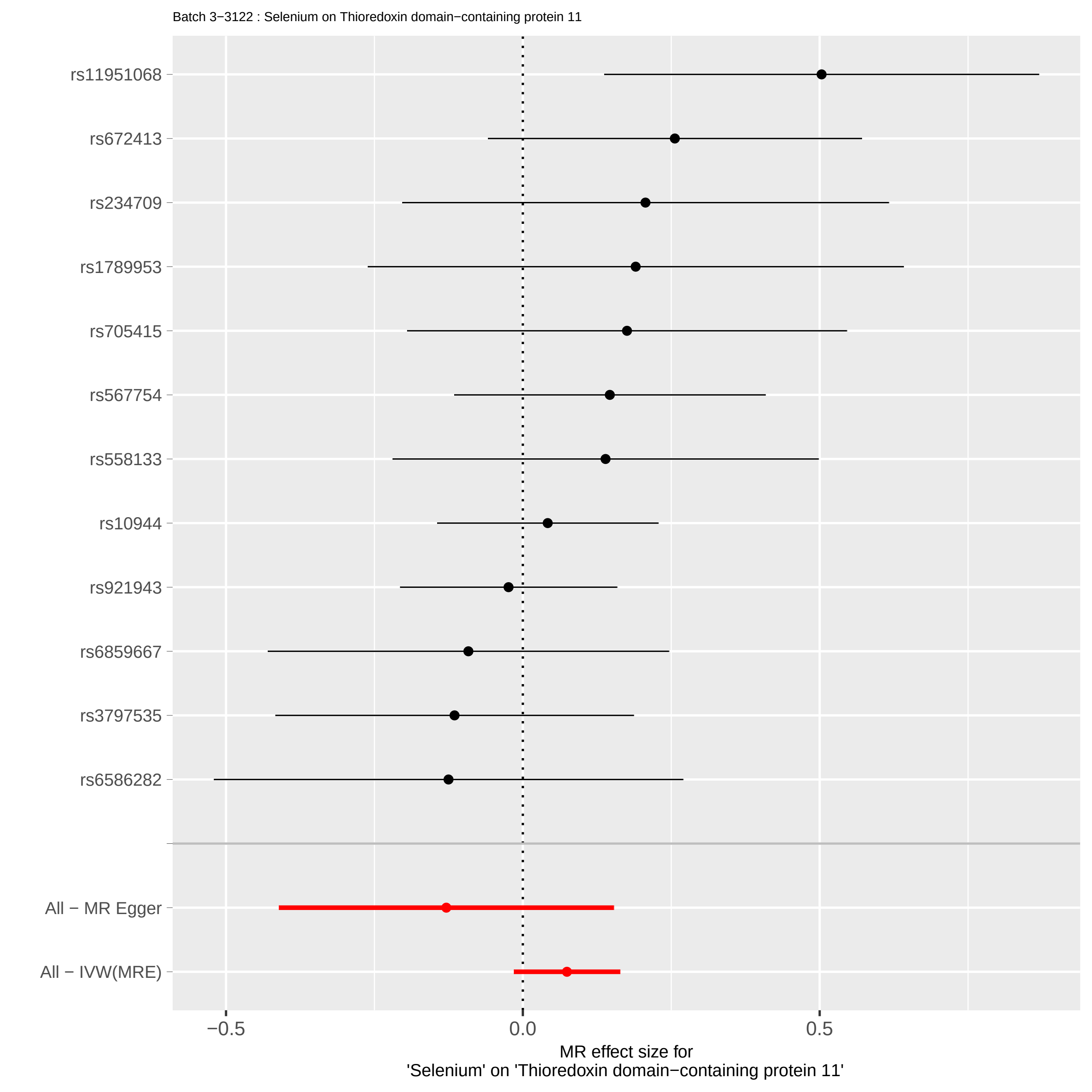

Batch 3−3122 : Selenium on Thioredoxin domain−containing protein 11
rs11951068
rs672413
rs234709
rs1789953
rs705415
rs567754
rs558133
rs10944
rs921943
rs6859667
rs3797535
rs6586282
All − MR Egger
All − IVW(MRE)
−0.5
0.0
0.5
MR effect size for
'Selenium' on 'Thioredoxin domain−containing protein 11'

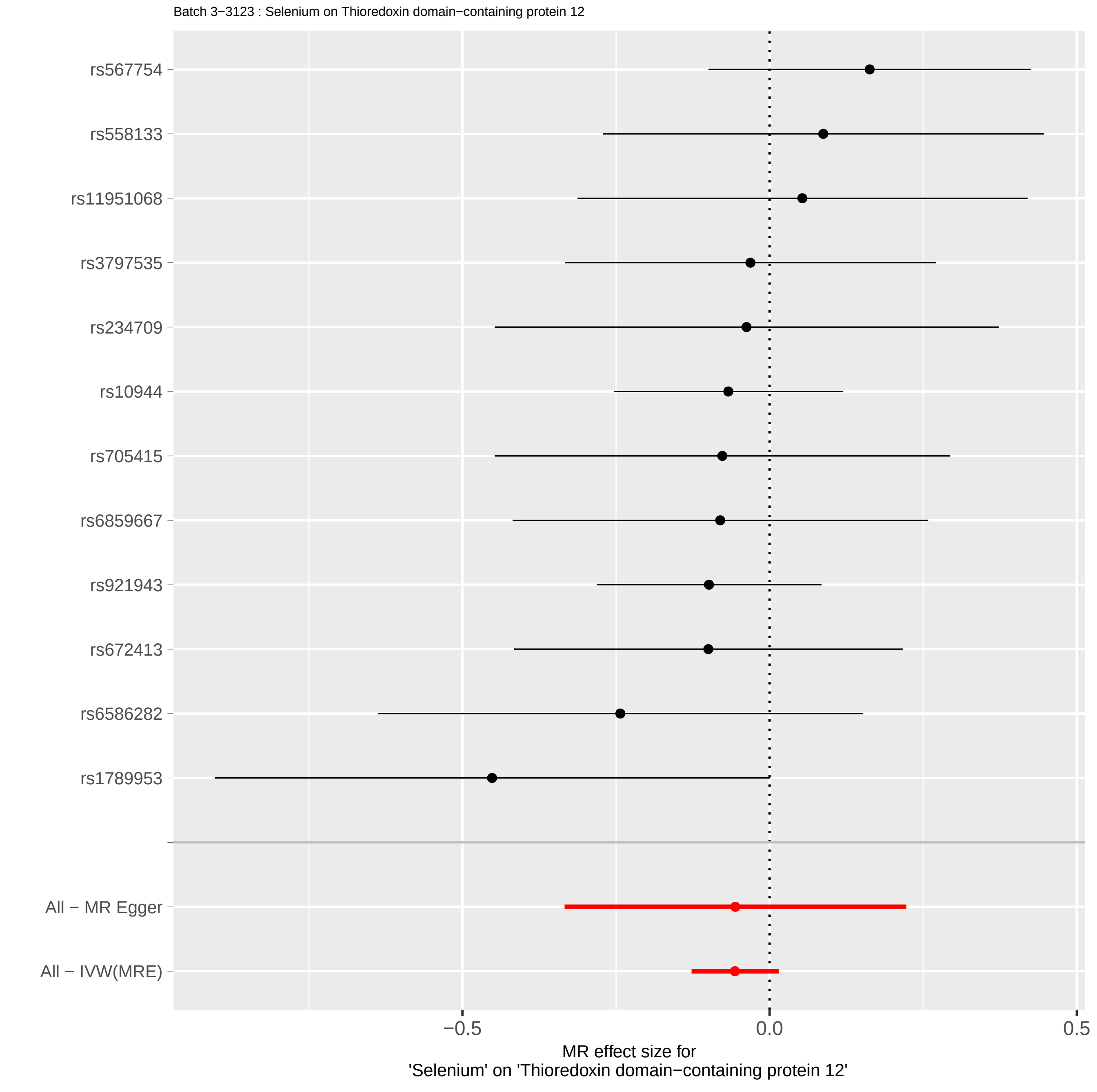

Batch 3−3123 : Selenium on Thioredoxin domain−containing protein 12
rs567754
rs558133
rs11951068
rs3797535
rs234709
rs10944
rs705415
rs6859667
rs921943
rs672413
rs6586282
rs1789953
All − MR Egger
All − IVW(MRE)
−0.5
0.0
0.5
MR effect size for
'Selenium' on 'Thioredoxin domain−containing protein 12'

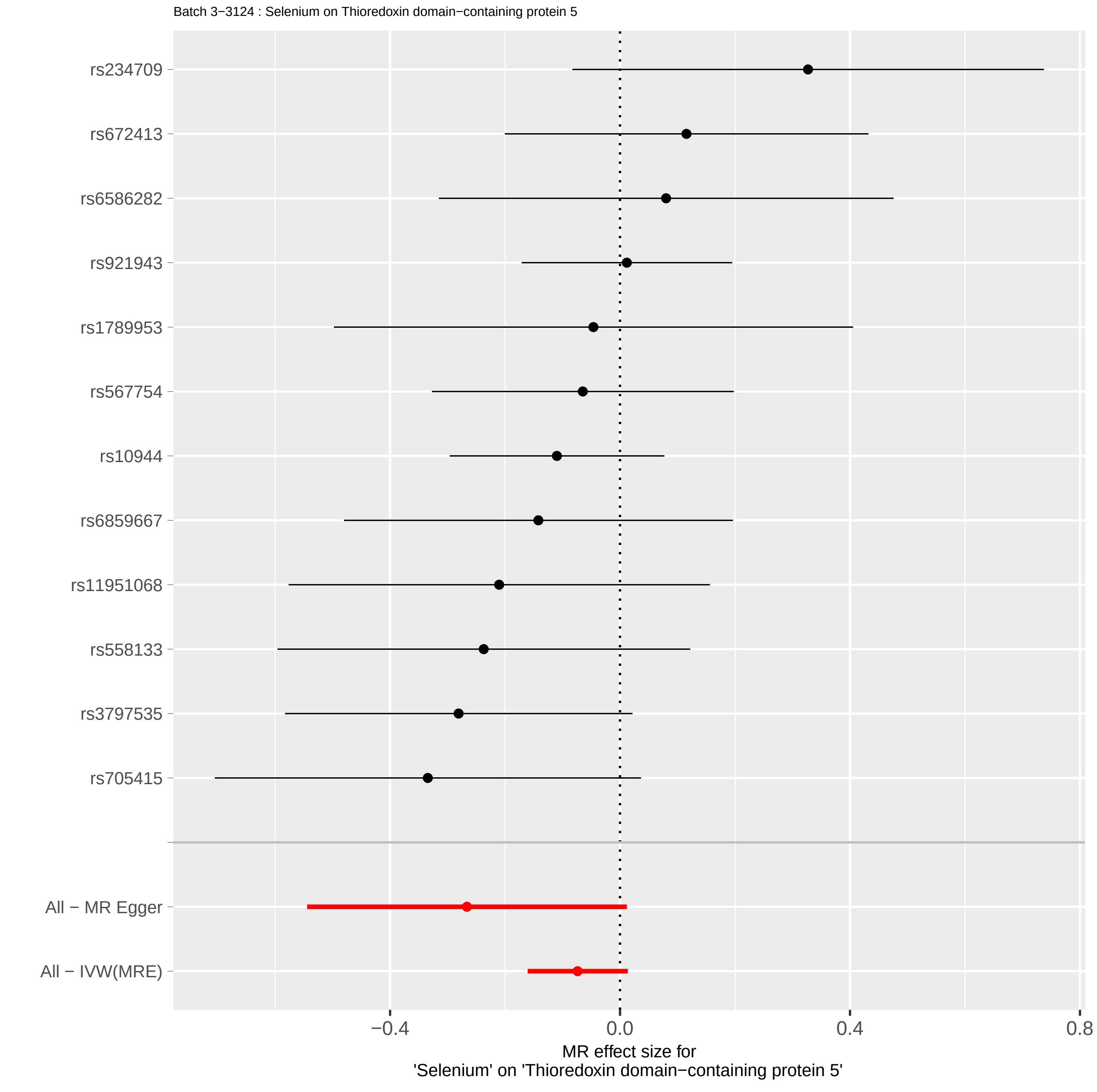

Batch 3−3124 : Selenium on Thioredoxin domain−containing protein 5
rs234709
rs672413
rs6586282
rs921943
rs1789953
rs567754
rs10944
rs6859667
rs11951068
rs558133
rs3797535
rs705415
All − MR Egger
All − IVW(MRE)
−0.4
0.0
0.4
0.8
MR effect size for
'Selenium' on 'Thioredoxin domain−containing protein 5'

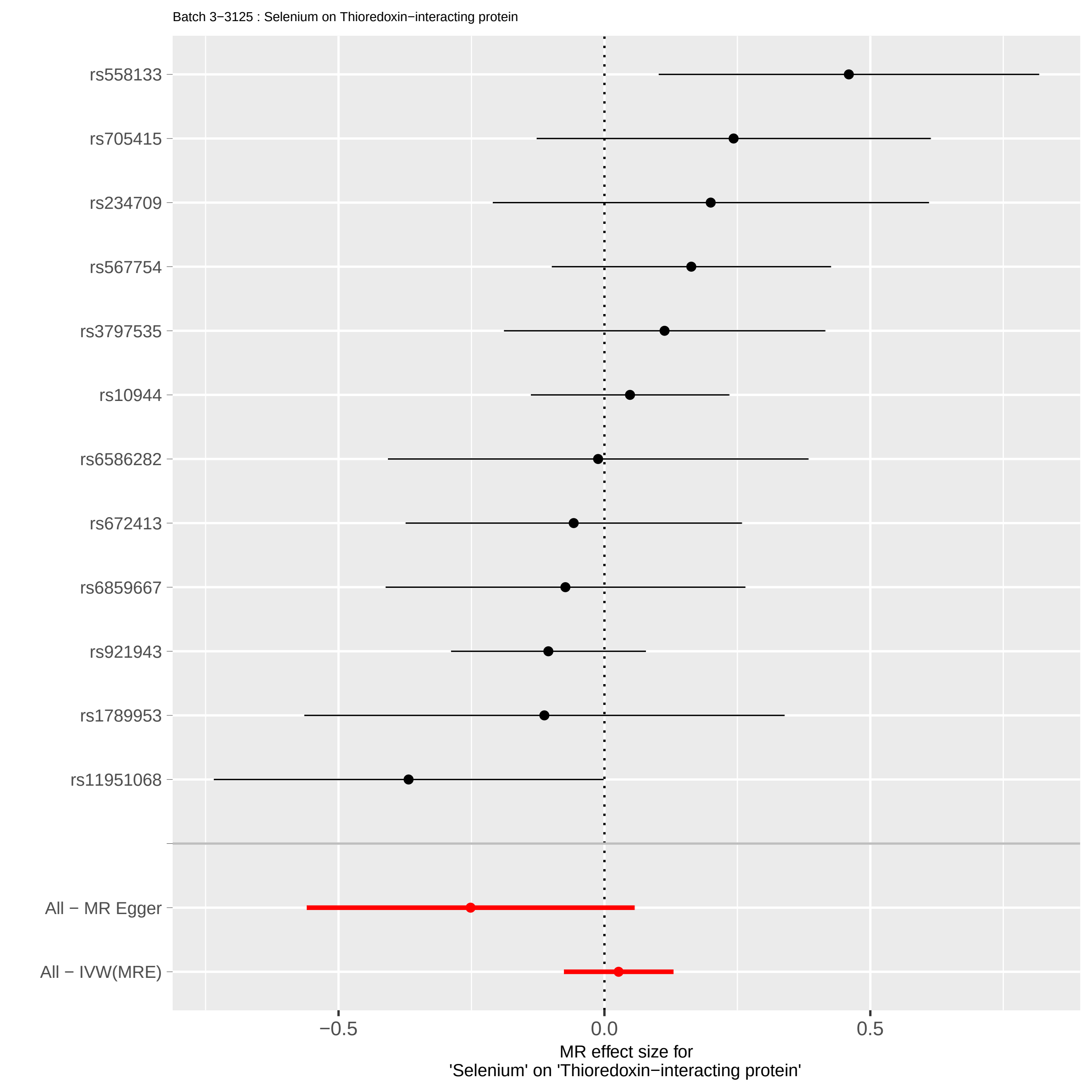

Batch 3−3125 : Selenium on Thioredoxin−interacting protein
rs558133
rs705415
rs234709
rs567754
rs3797535
rs10944
rs6586282
rs672413
rs6859667
rs921943
rs1789953
rs11951068
All − MR Egger
All − IVW(MRE)
−0.5
0.0
0.5
MR effect size for
'Selenium' on 'Thioredoxin−interacting protein'

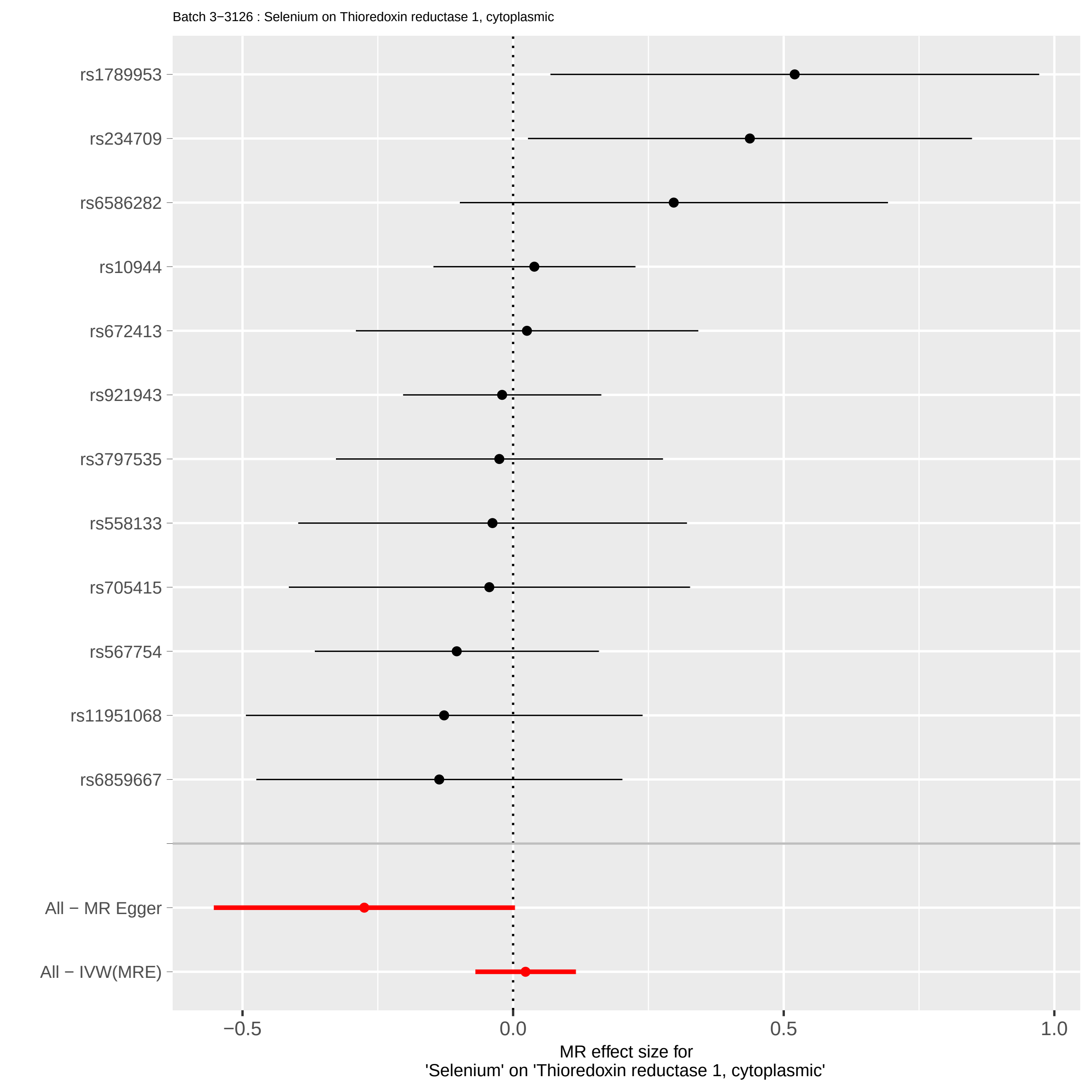
Batch 3−3126 : Selenium on Thioredoxin reductase 1, cytoplasmic
rs1789953
rs234709
rs6586282
rs10944
rs672413
rs921943
rs3797535
rs558133
rs705415
rs567754
rs11951068
rs6859667
All − MR Egger
All − IVW(MRE)
−0.5
0.0
0.5
1.0
MR effect size for
'Selenium' on 'Thioredoxin reductase 1, cytoplasmic'

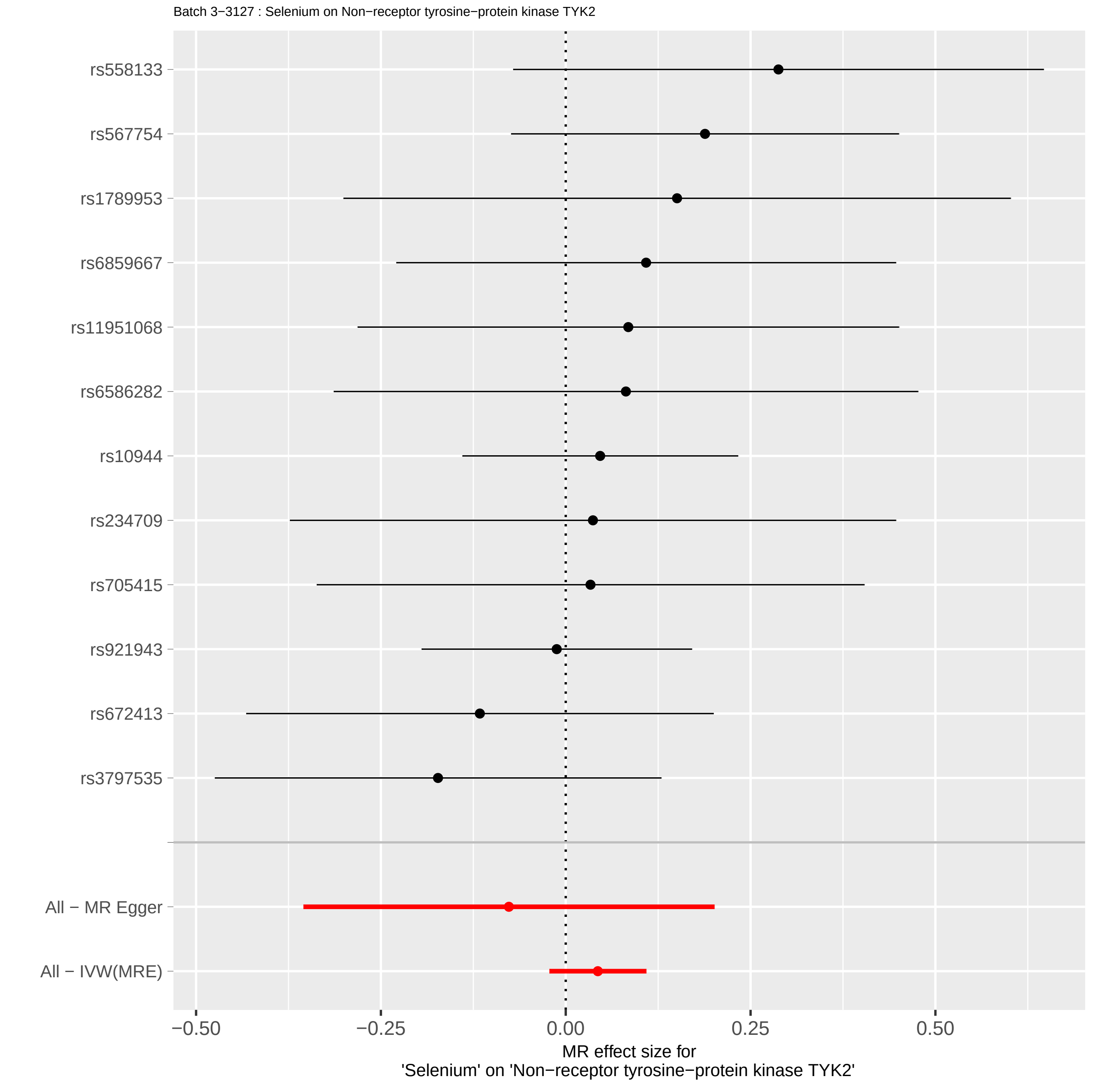

Batch 3−3127 : Selenium on Non−receptor tyrosine−protein kinase TYK2
rs558133
rs567754
rs1789953
rs6859667
rs11951068
rs6586282
rs10944
rs234709
rs705415
rs921943
rs672413
rs3797535
All − MR Egger
All − IVW(MRE)
−0.50
−0.25
0.00
0.25
0.50
MR effect size for
'Selenium' on 'Non−receptor tyrosine−protein kinase TYK2'

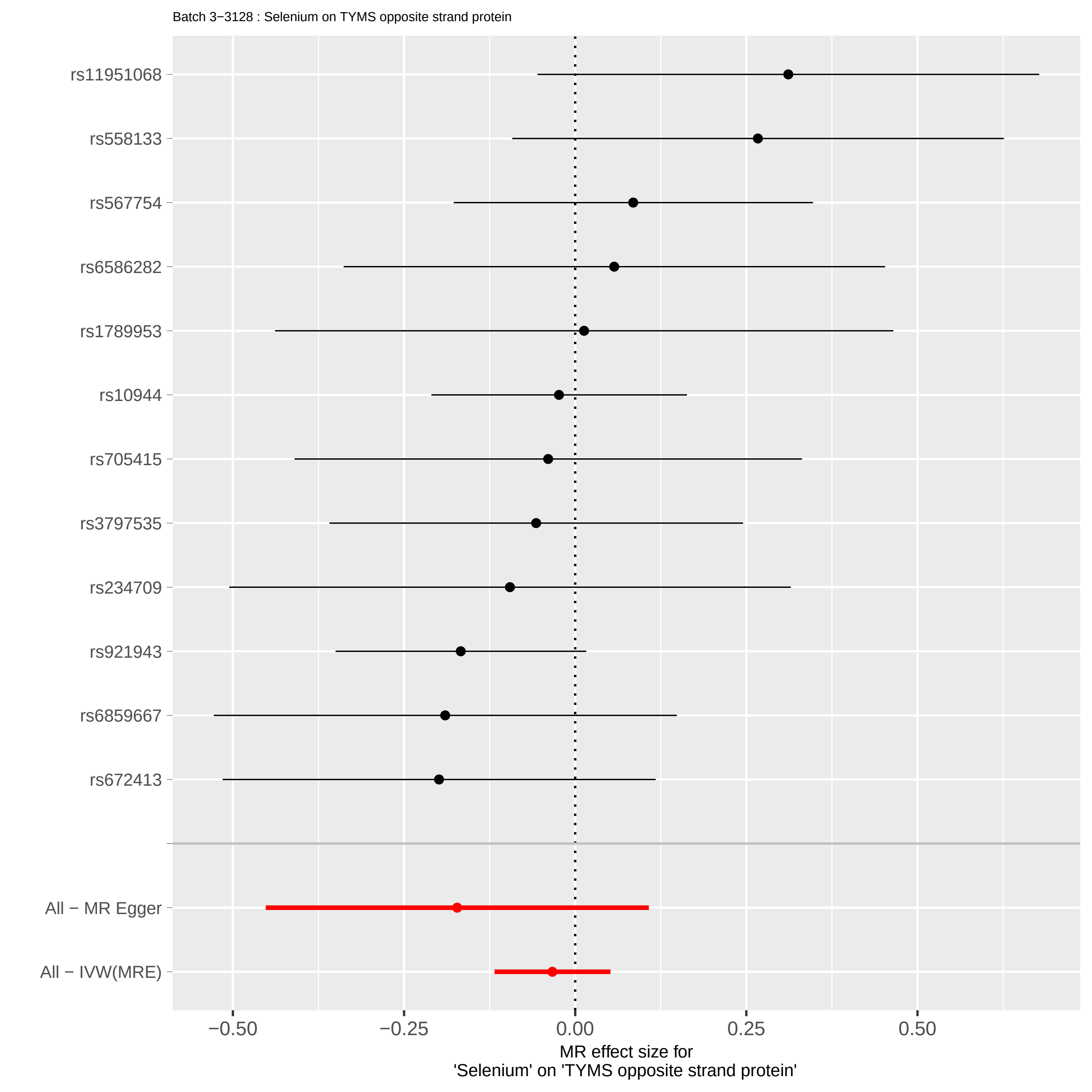

Batch 3−3128 : Selenium on TYMS opposite strand protein
rs11951068
rs558133
rs567754
rs6586282
rs1789953
rs10944
rs705415
rs3797535
rs234709
rs921943
rs6859667
rs672413
All − MR Egger
All − IVW(MRE)
−0.50
−0.25
0.00
0.25
0.50
MR effect size for
'Selenium' on 'TYMS opposite strand protein'

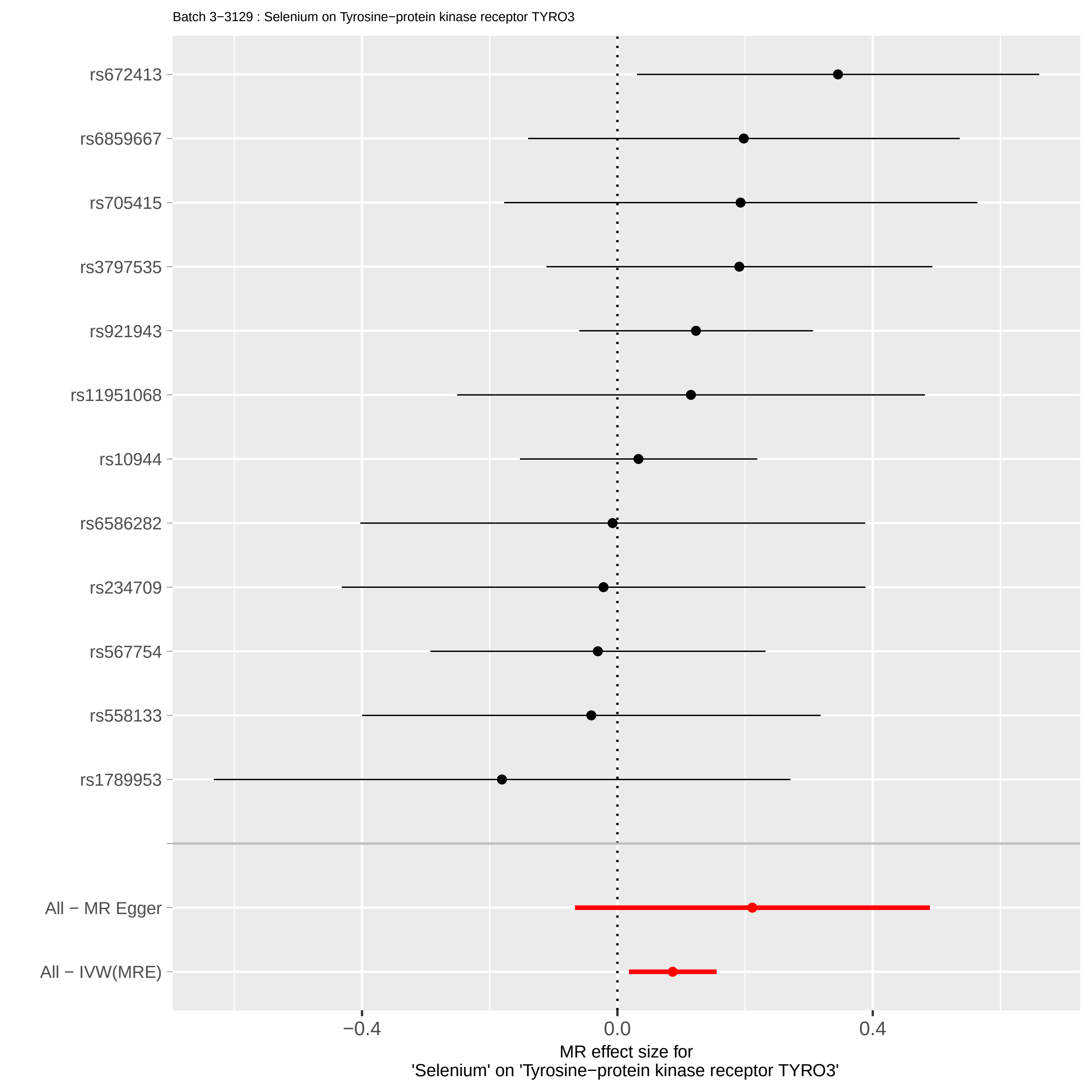

Batch 3−3129 : Selenium on Tyrosine−protein kinase receptor TYRO3
rs672413
rs6859667
rs705415
rs3797535
rs921943
rs11951068
rs10944
rs6586282
rs234709
rs567754
rs558133
rs1789953
All − MR Egger
All − IVW(MRE)
−0.4
0.0
0.4
MR effect size for
'Selenium' on 'Tyrosine−protein kinase receptor TYRO3'

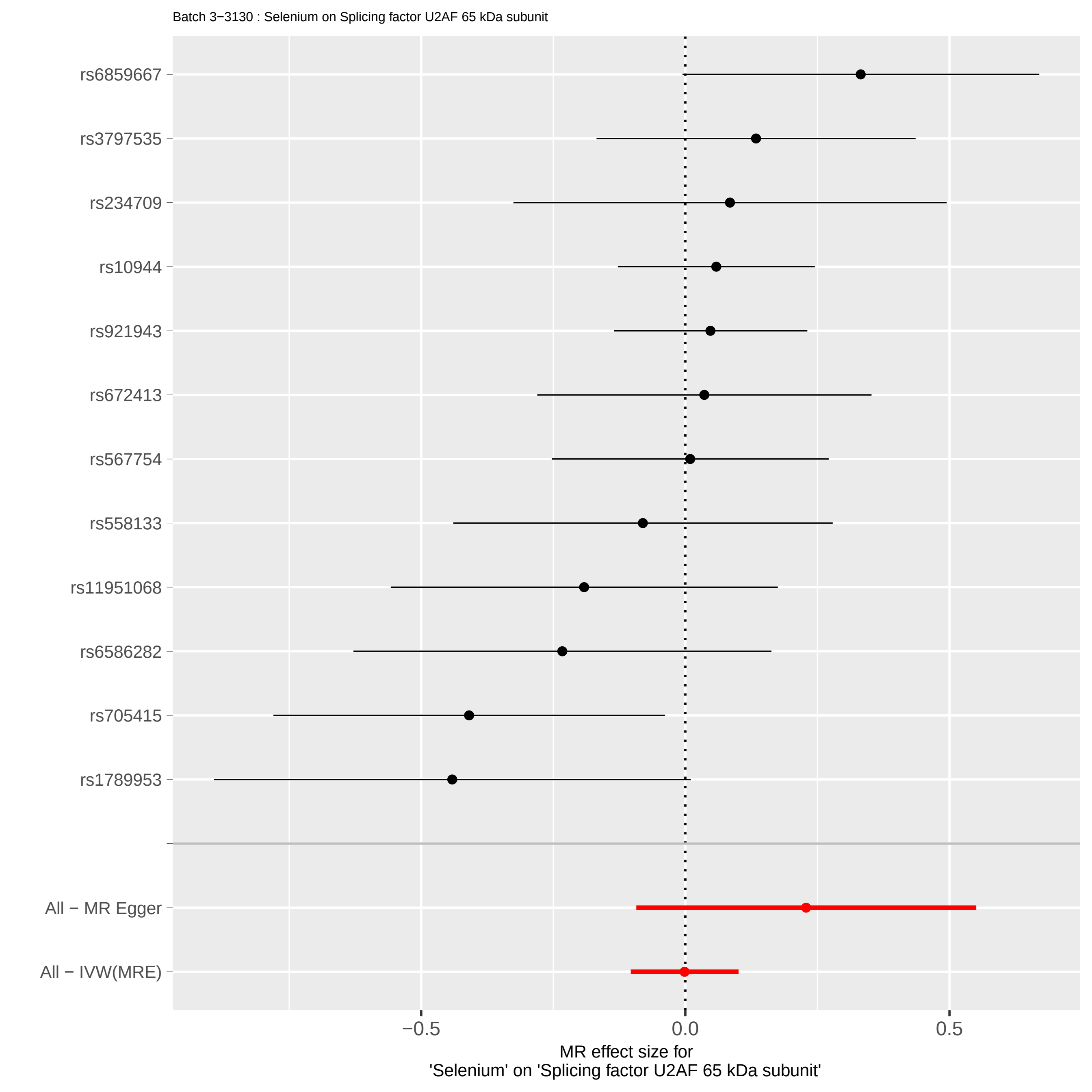
Batch 3−3130 : Selenium on Splicing factor U2AF 65 kDa subunit
rs6859667
rs3797535
rs234709
rs10944
rs921943
rs672413
rs567754
rs558133
rs11951068
rs6586282
rs705415
rs1789953
All − MR Egger
All − IVW(MRE)
−0.5
0.0
0.5
MR effect size for
'Selenium' on 'Splicing factor U2AF 65 kDa subunit'

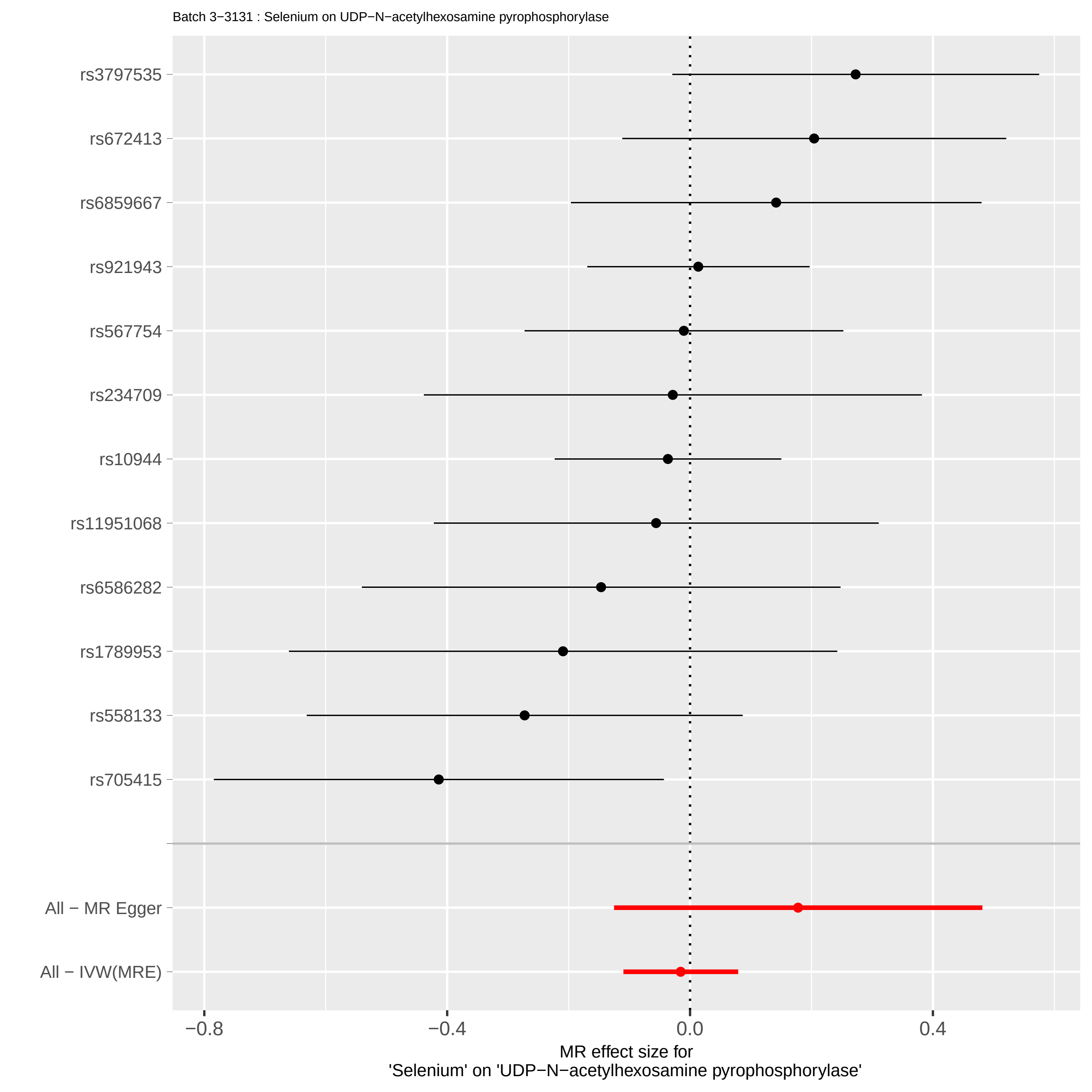

Batch 3−3131 : Selenium on UDP−N−acetylhexosamine pyrophosphorylase
rs3797535
rs672413
rs6859667
rs921943
rs567754
rs234709
rs10944
rs11951068
rs6586282
rs1789953
rs558133
rs705415
All − MR Egger
All − IVW(MRE)
−0.8
−0.4
0.0
0.4
MR effect size for
'Selenium' on 'UDP−N−acetylhexosamine pyrophosphorylase'

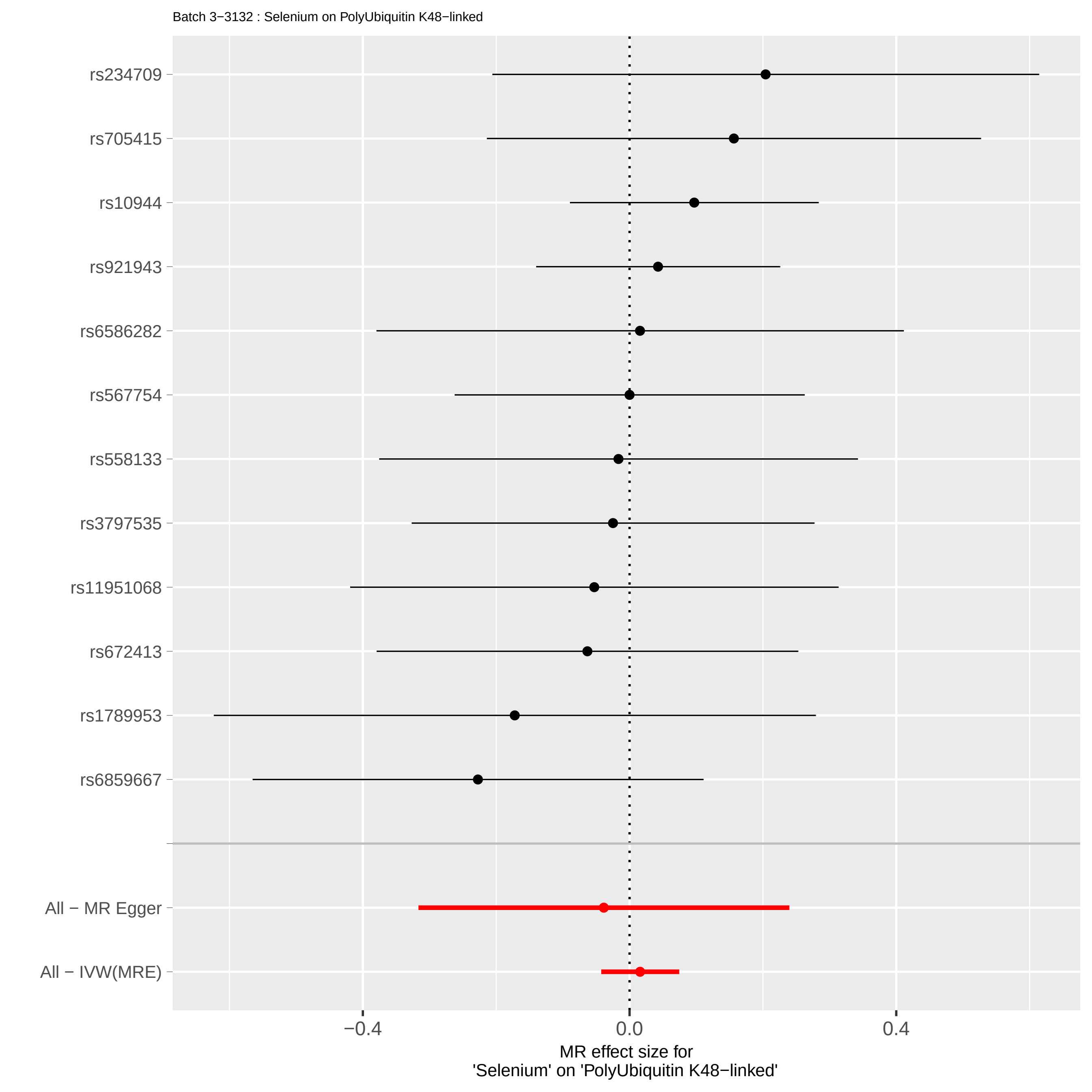

Batch 3−3132 : Selenium on PolyUbiquitin K48−linked
rs234709
rs705415
rs10944
rs921943
rs6586282
rs567754
rs558133
rs3797535
rs11951068
rs672413
rs1789953
rs6859667
All − MR Egger
All − IVW(MRE)
−0.4
0.0
0.4
MR effect size for
'Selenium' on 'PolyUbiquitin K48−linked'

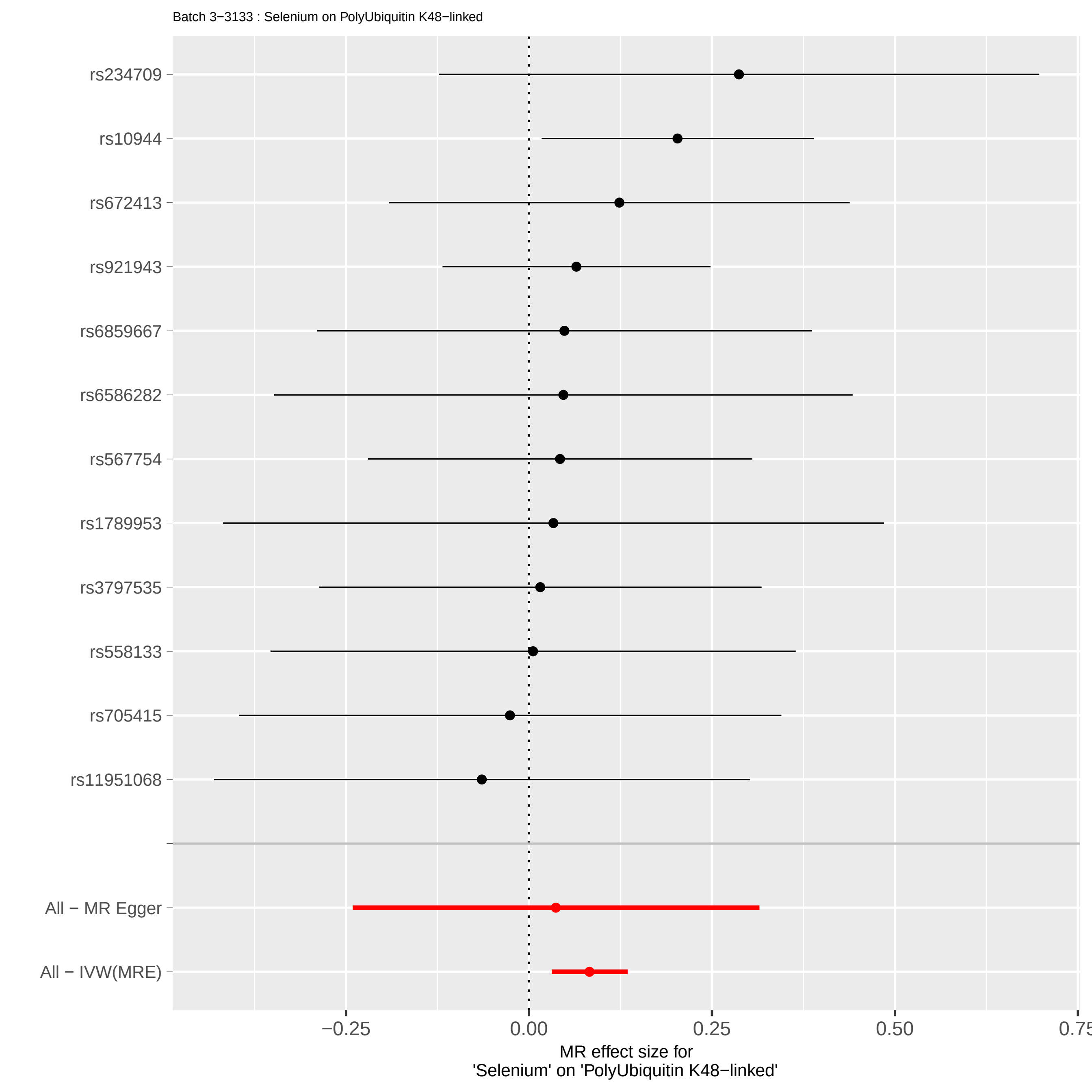

Batch 3−3133 : Selenium on PolyUbiquitin K48−linked
rs234709
rs10944
rs672413
rs921943
rs6859667
rs6586282
rs567754
rs1789953
rs3797535
rs558133
rs705415
rs11951068
All − MR Egger
All − IVW(MRE)
−0.25
0.00
0.25
0.50
0.75
MR effect size for
'Selenium' on 'PolyUbiquitin K48−linked'

Batch 3−3134 : Selenium on PolyUbiquitin K63−linked

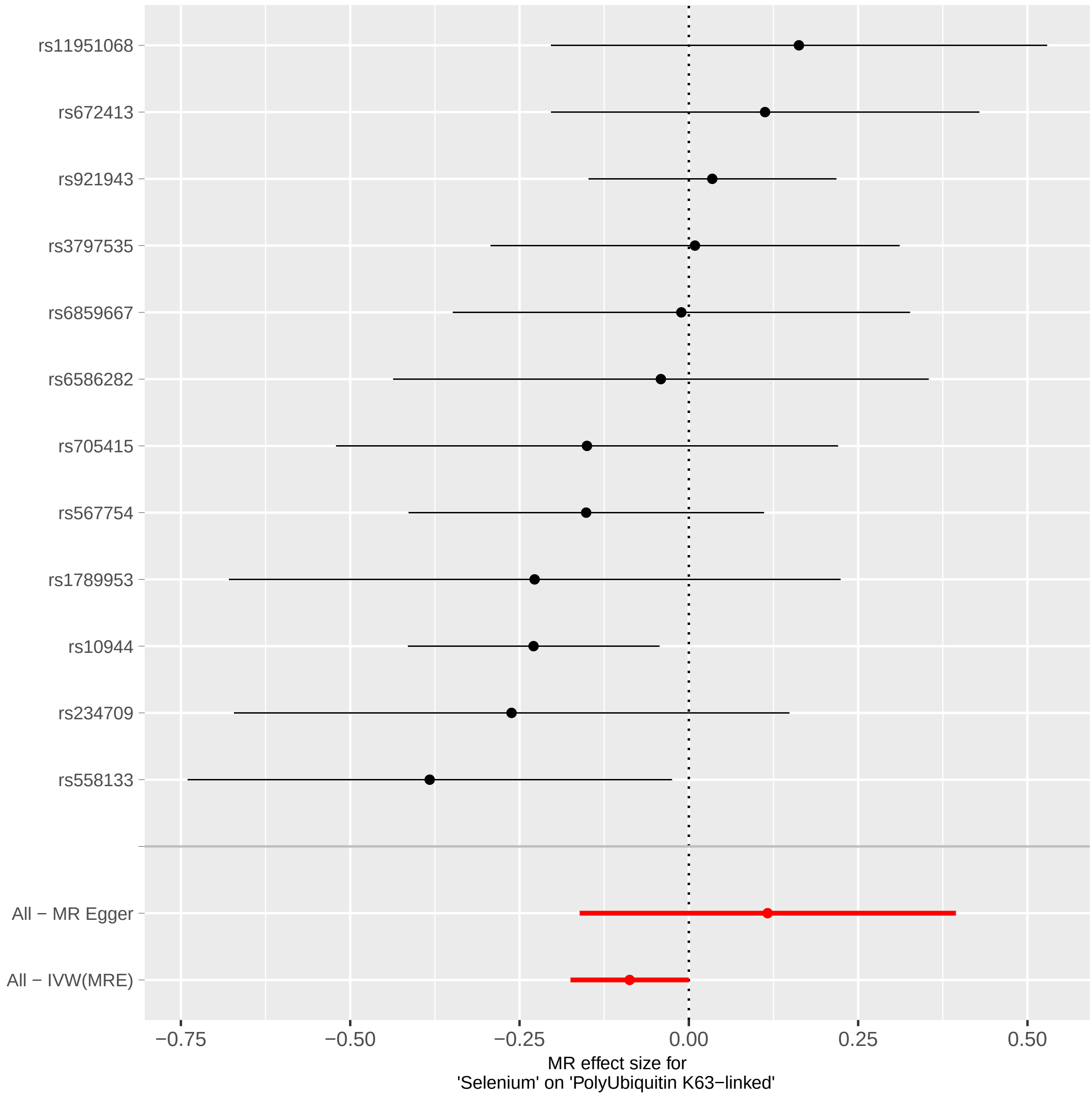

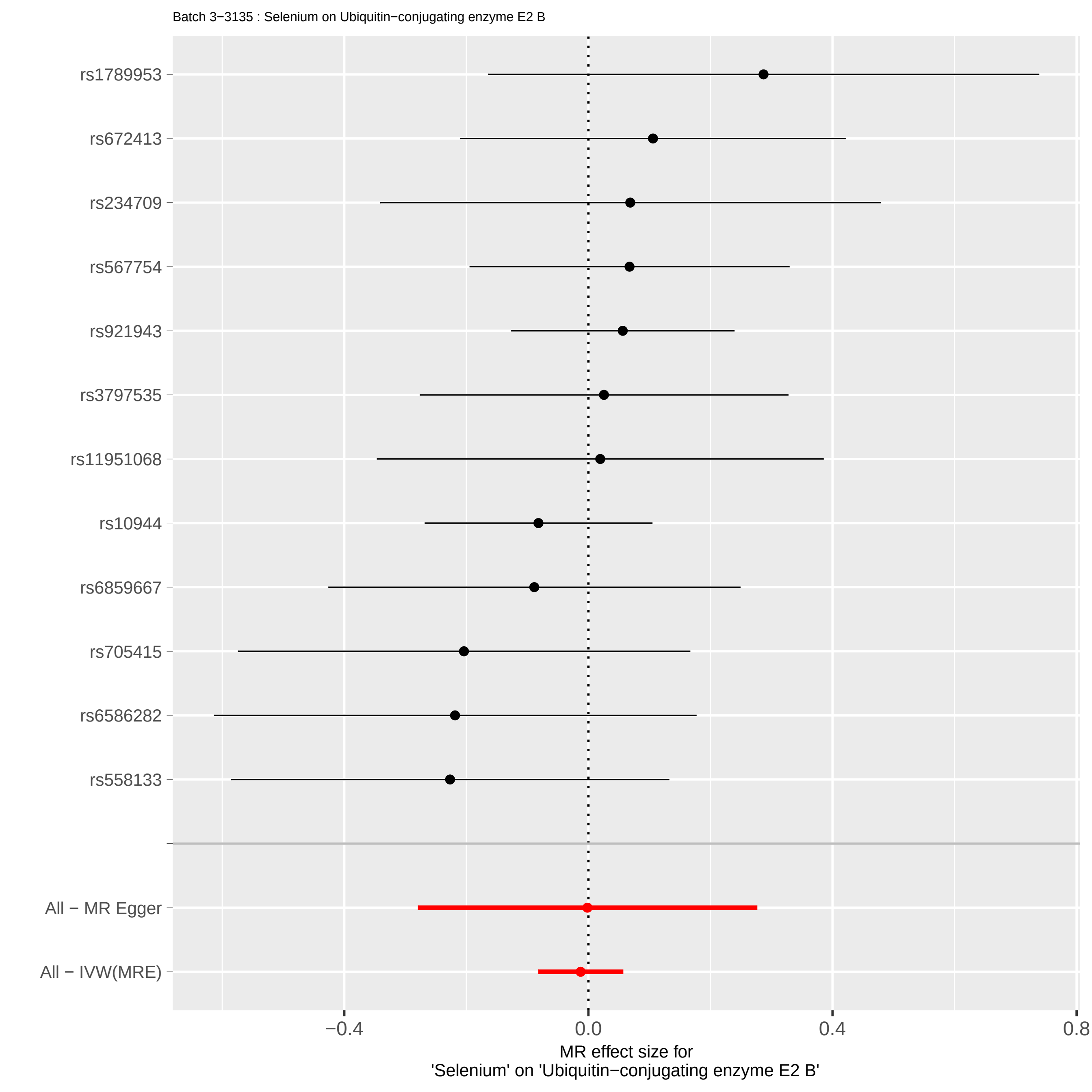

Batch 3−3135 : Selenium on Ubiquitin−conjugating enzyme E2 B
rs1789953
rs672413
rs234709
rs567754
rs921943
rs3797535
rs11951068
rs10944
rs6859667
rs705415
rs6586282
rs558133
All − MR Egger
All − IVW(MRE)
−0.4
0.0
0.4
0.8
MR effect size for
'Selenium' on 'Ubiquitin−conjugating enzyme E2 B'

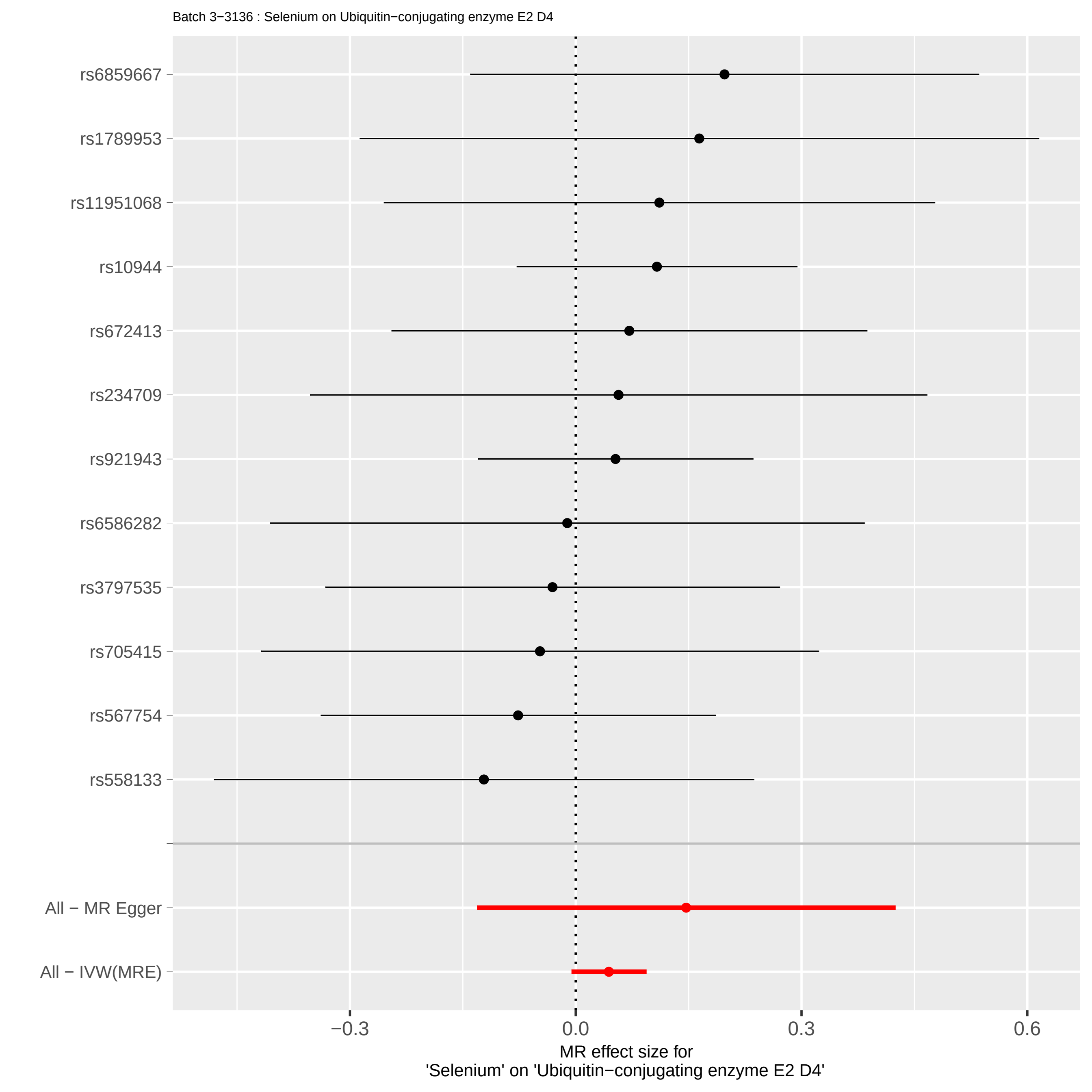

Batch 3−3136 : Selenium on Ubiquitin−conjugating enzyme E2 D4
rs6859667
rs1789953
rs11951068
rs10944
rs672413
rs234709
rs921943
rs6586282
rs3797535
rs705415
rs567754
rs558133
All − MR Egger
All − IVW(MRE)
−0.3
0.0
0.3
0.6
MR effect size for
'Selenium' on 'Ubiquitin−conjugating enzyme E2 D4'

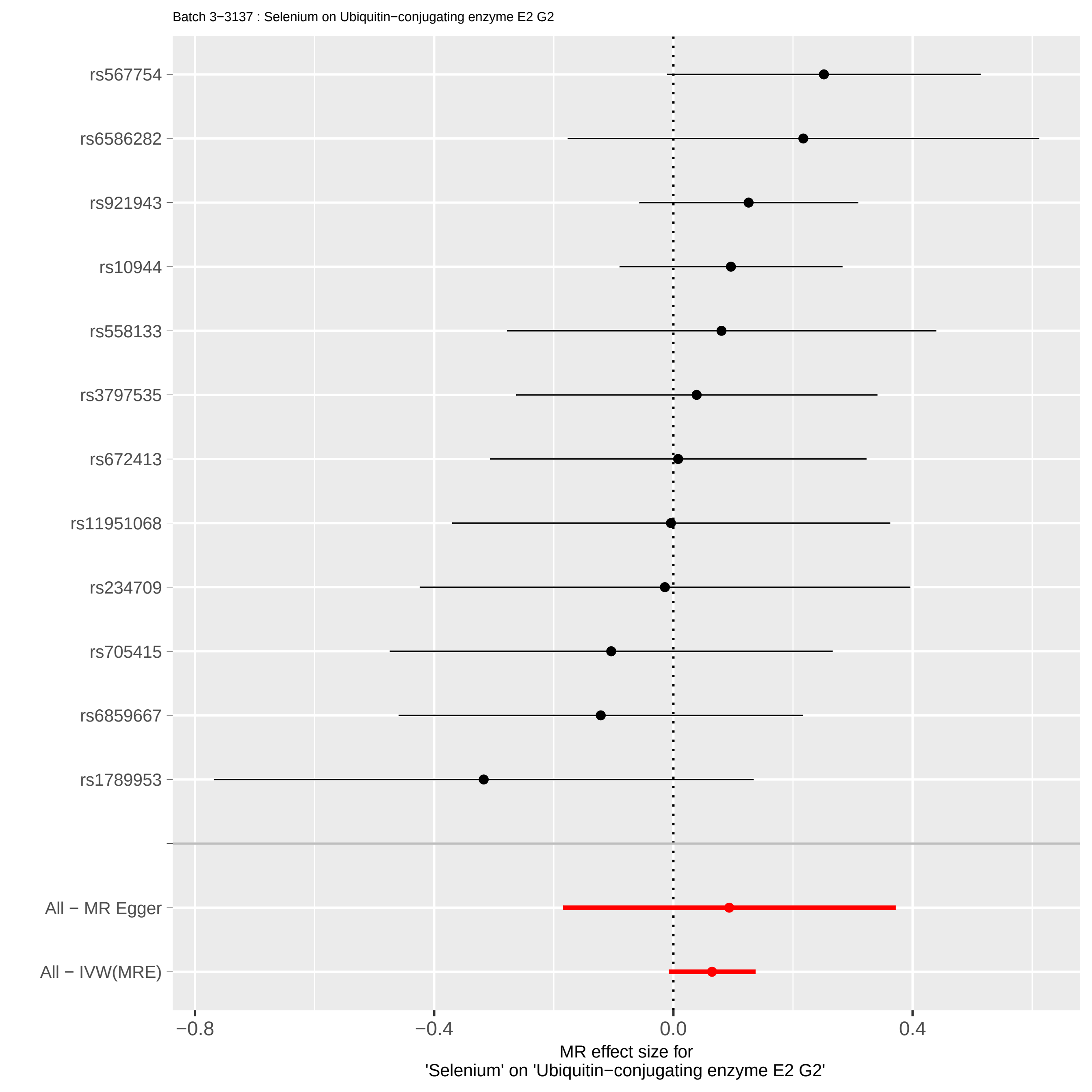
Batch 3−3137 : Selenium on Ubiquitin−conjugating enzyme E2 G2
rs567754
rs6586282
rs921943
rs10944
rs558133
rs3797535
rs672413
rs11951068
rs234709
rs705415
rs6859667
rs1789953
All − MR Egger
All − IVW(MRE)
−0.8
−0.4
0.0
0.4
MR effect size for
'Selenium' on 'Ubiquitin−conjugating enzyme E2 G2'

Batch 3−3138 : Selenium on SUMO−conjugating enzyme UBC9

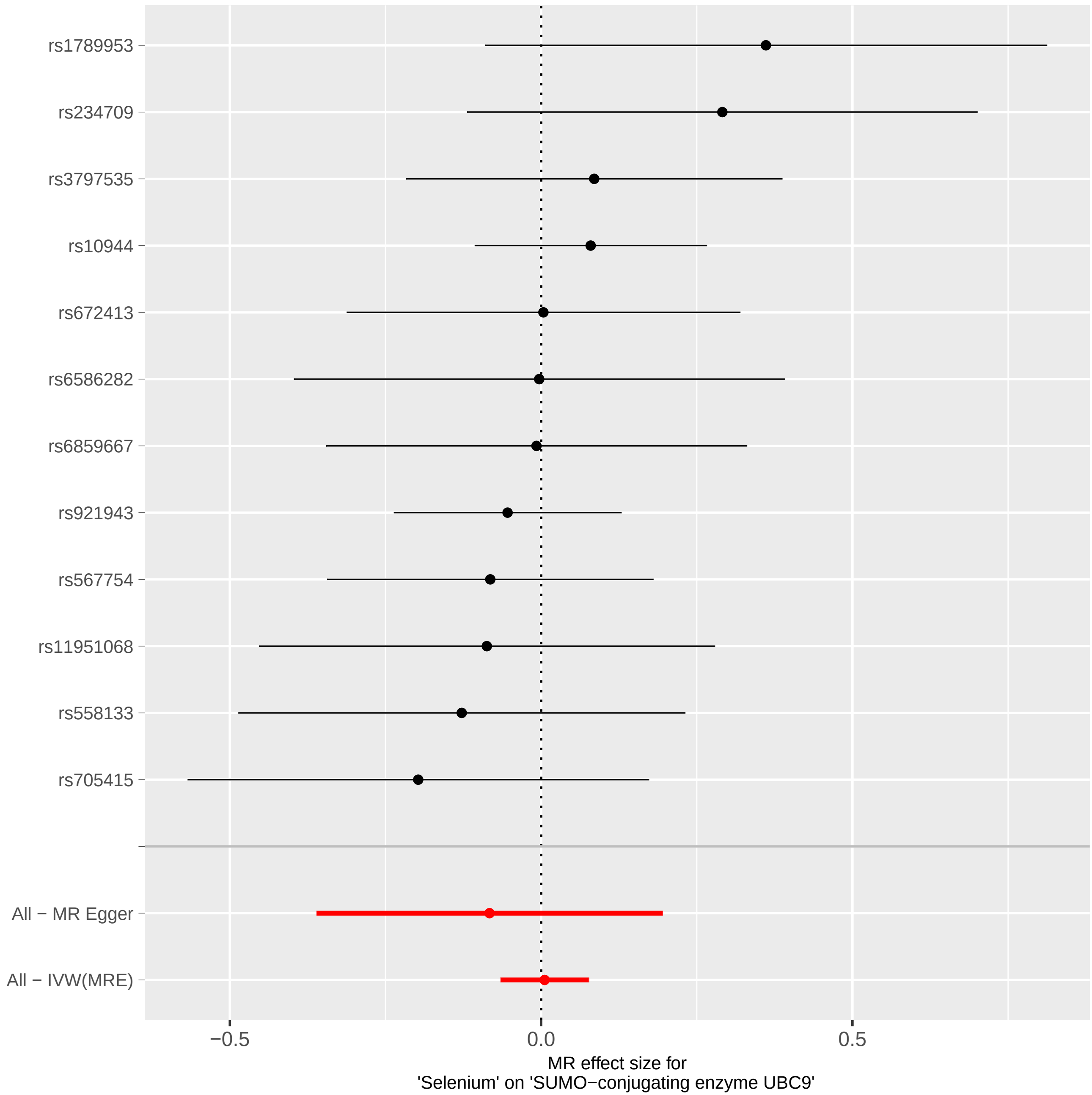

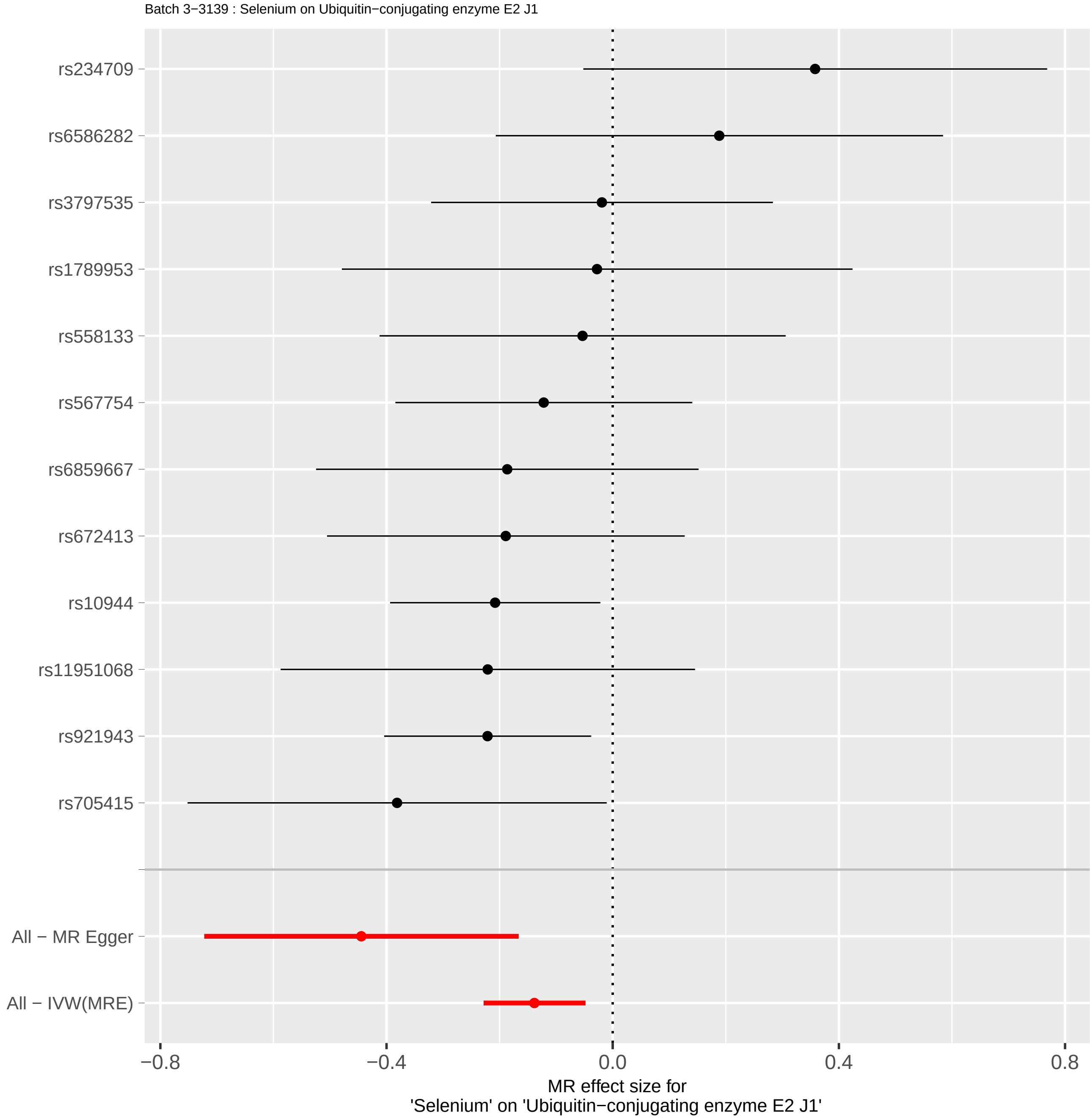
Batch 3−3139 : Selenium on Ubiquitin−conjugating enzyme E2 J1
rs234709
rs6586282
rs3797535
rs1789953
rs558133
rs567754
rs6859667
rs672413
rs10944
rs11951068
rs921943
rs705415
All − MR Egger
All − IVW(MRE)
−0.8
−0.4
0.0
0.4
0.8
MR effect size for
'Selenium' on 'Ubiquitin−conjugating enzyme E2 J1'

Batch 3−3140 : Selenium on Ubiquitin−conjugating enzyme E2 J2

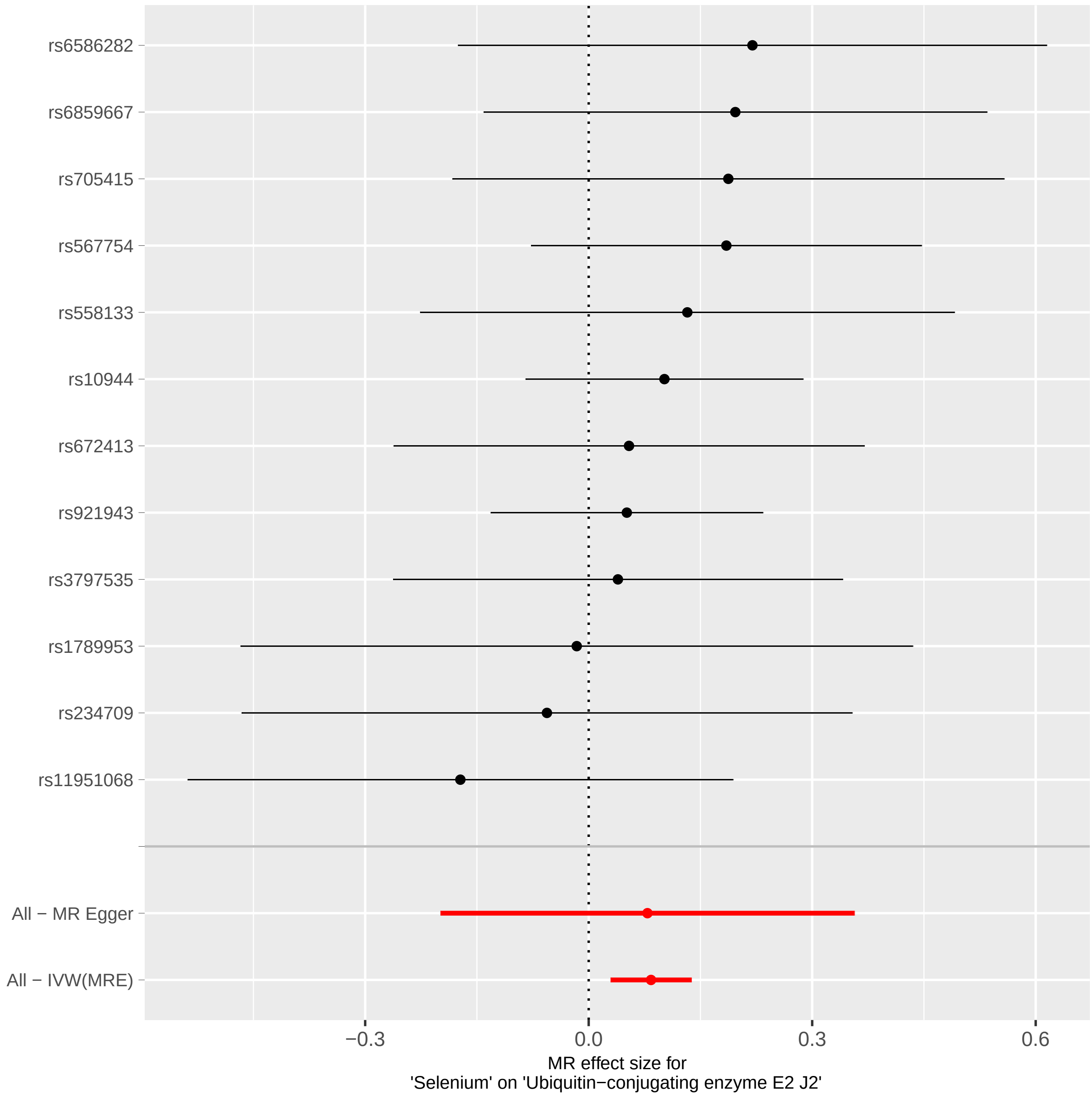

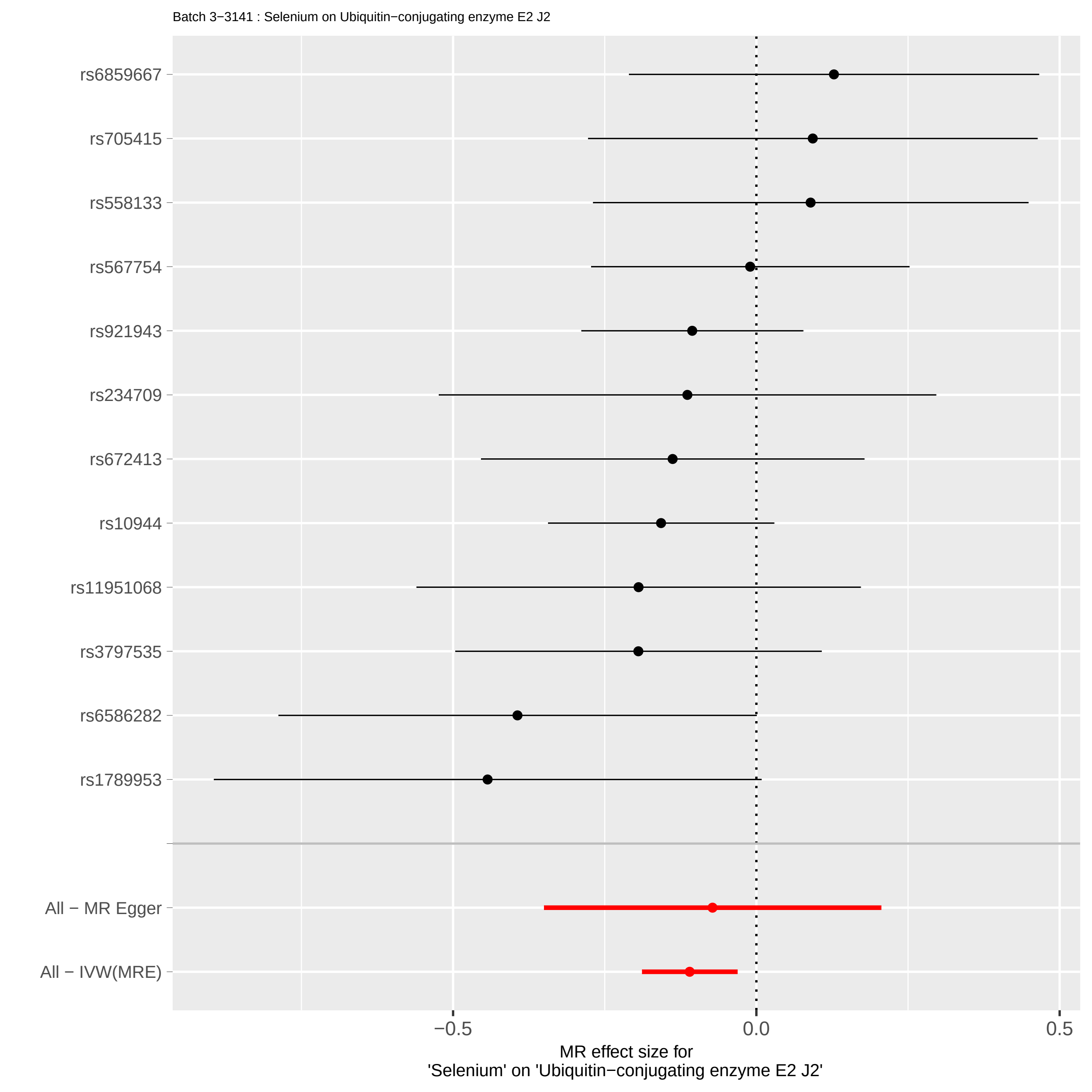

Batch 3−3141 : Selenium on Ubiquitin−conjugating enzyme E2 J2
rs6859667
rs705415
rs558133
rs567754
rs921943
rs234709
rs672413
rs10944
rs11951068
rs3797535
rs6586282
rs1789953
All − MR Egger
All − IVW(MRE)
−0.5
0.0
0.5
MR effect size for
'Selenium' on 'Ubiquitin−conjugating enzyme E2 J2'

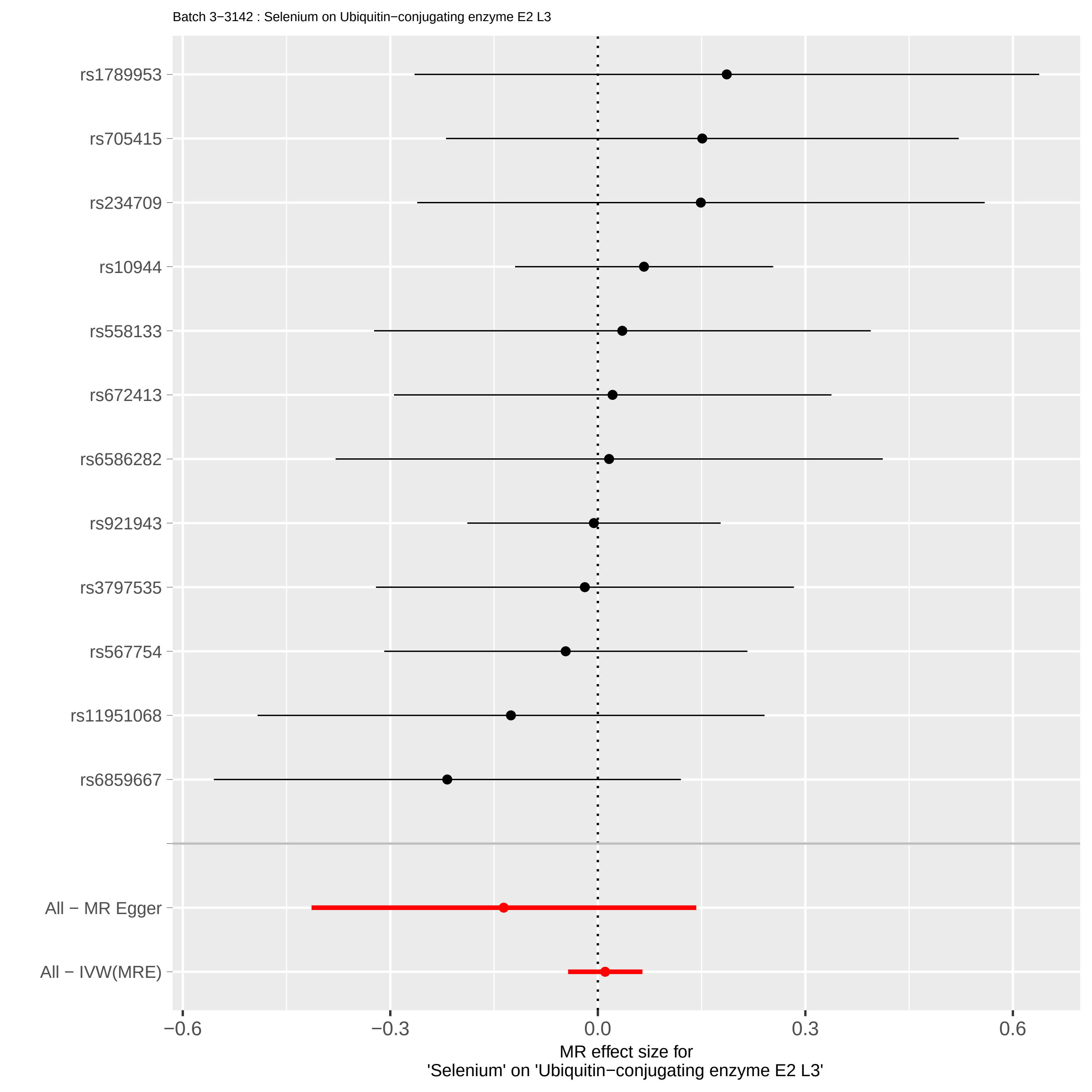

Batch 3−3142 : Selenium on Ubiquitin−conjugating enzyme E2 L3
rs1789953
rs705415
rs234709
rs10944
rs558133
rs672413
rs6586282
rs921943
rs3797535
rs567754
rs11951068
rs6859667
All − MR Egger
All − IVW(MRE)
−0.6
−0.3
0.0
0.3
0.6
MR effect size for
'Selenium' on 'Ubiquitin−conjugating enzyme E2 L3'

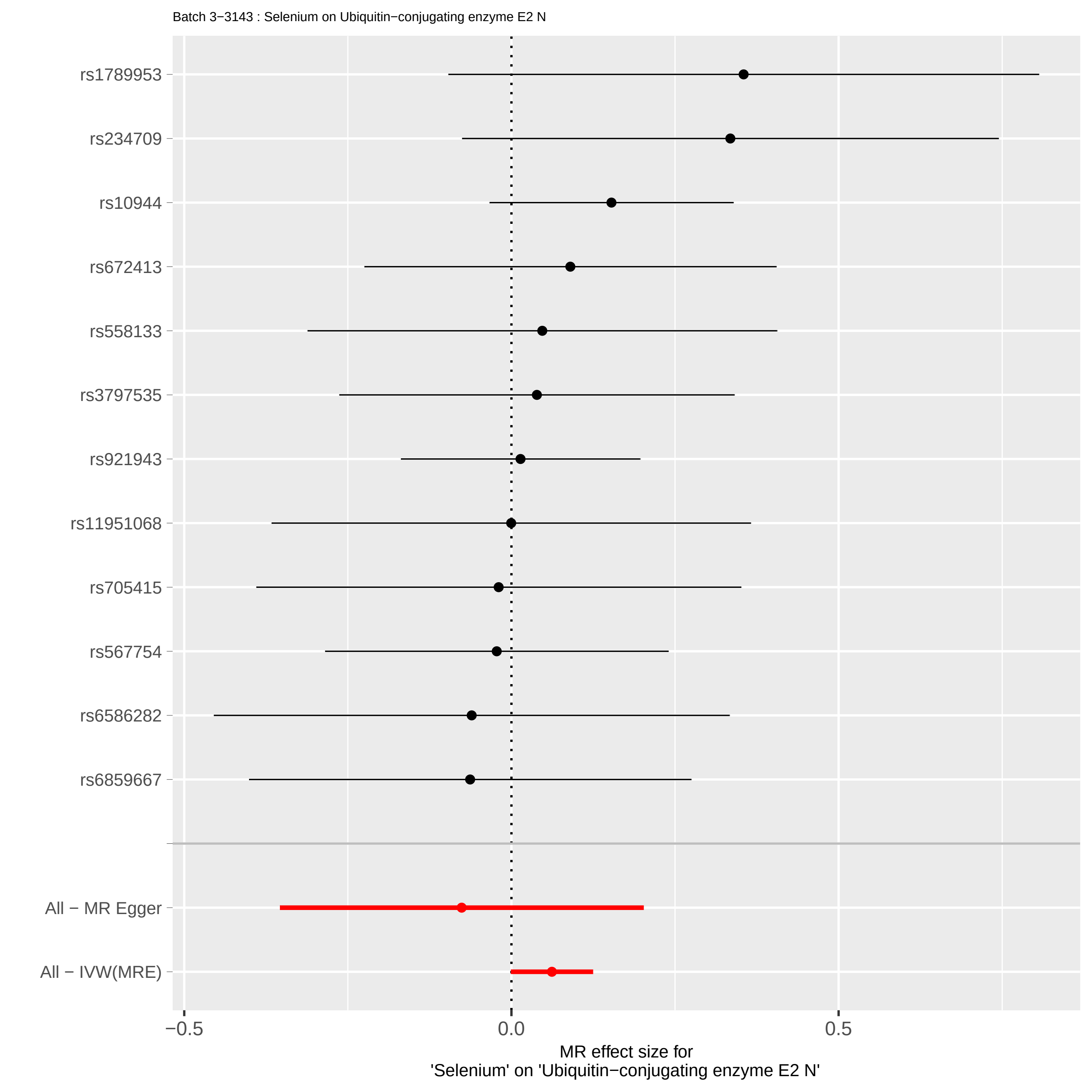
Batch 3−3143 : Selenium on Ubiquitin−conjugating enzyme E2 N
rs1789953
rs234709
rs10944
rs672413
rs558133
rs3797535
rs921943
rs11951068
rs705415
rs567754
rs6586282
rs6859667
All − MR Egger
All − IVW(MRE)
−0.5
0.0
0.5
MR effect size for
'Selenium' on 'Ubiquitin−conjugating enzyme E2 N'

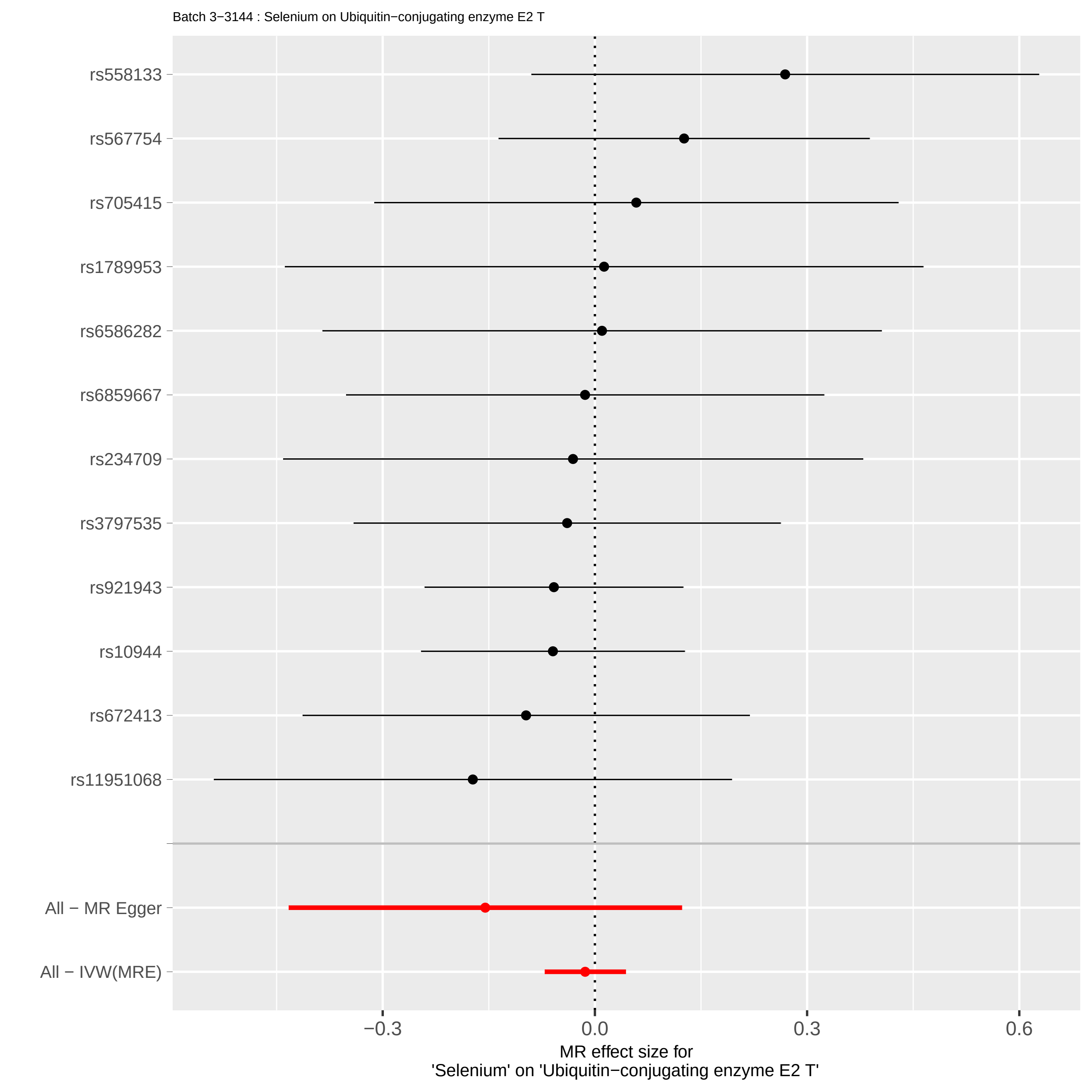

Batch 3−3144 : Selenium on Ubiquitin−conjugating enzyme E2 T
rs558133
rs567754
rs705415
rs1789953
rs6586282
rs6859667
rs234709
rs3797535
rs921943
rs10944
rs672413
rs11951068
All − MR Egger
All − IVW(MRE)
−0.3
0.0
0.3
0.6
MR effect size for
'Selenium' on 'Ubiquitin−conjugating enzyme E2 T'

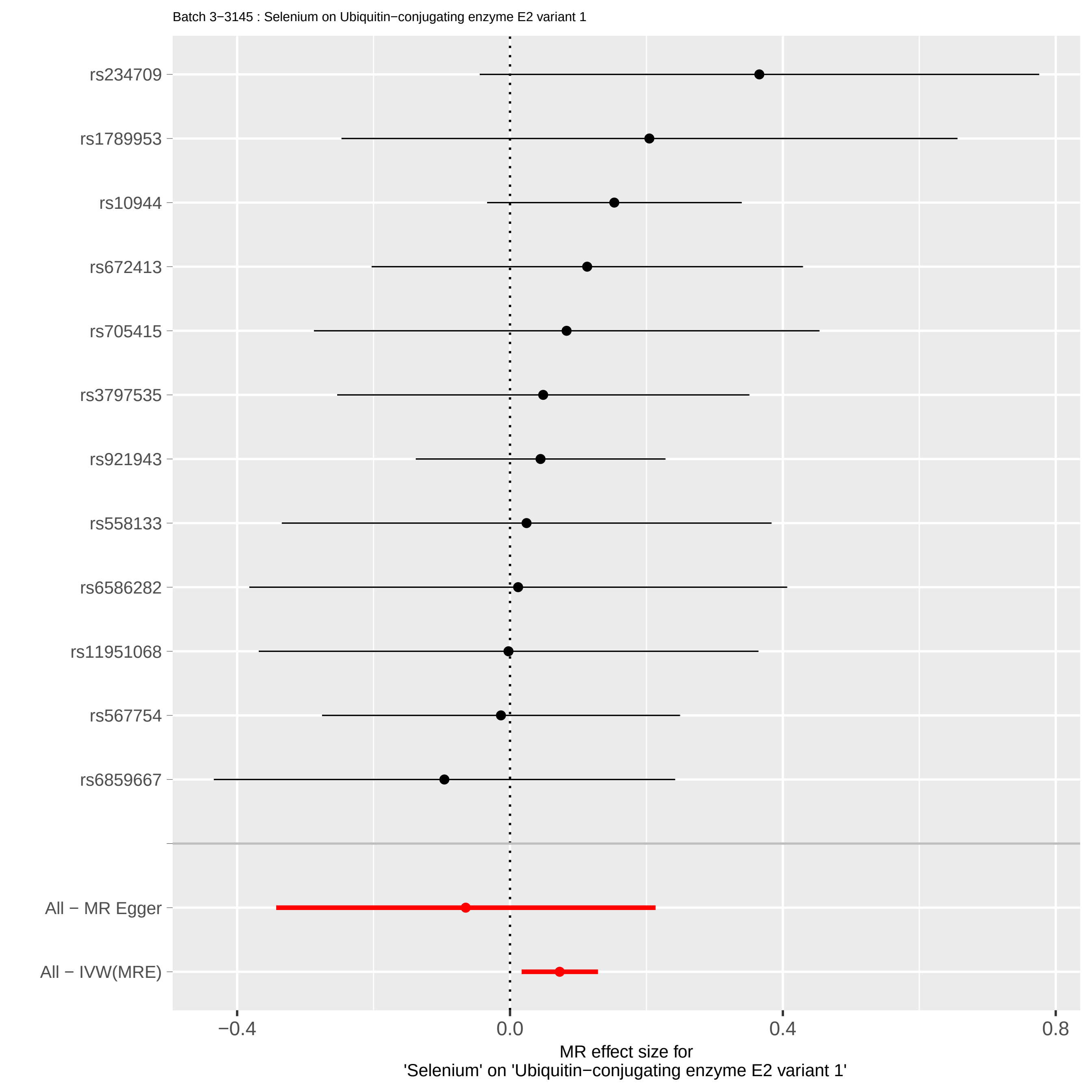

Batch 3−3145 : Selenium on Ubiquitin−conjugating enzyme E2 variant 1
rs234709
rs1789953
rs10944
rs672413
rs705415
rs3797535
rs921943
rs558133
rs6586282
rs11951068
rs567754
rs6859667
All − MR Egger
All − IVW(MRE)
−0.4
0.0
0.4
0.8
MR effect size for
'Selenium' on 'Ubiquitin−conjugating enzyme E2 variant 1'

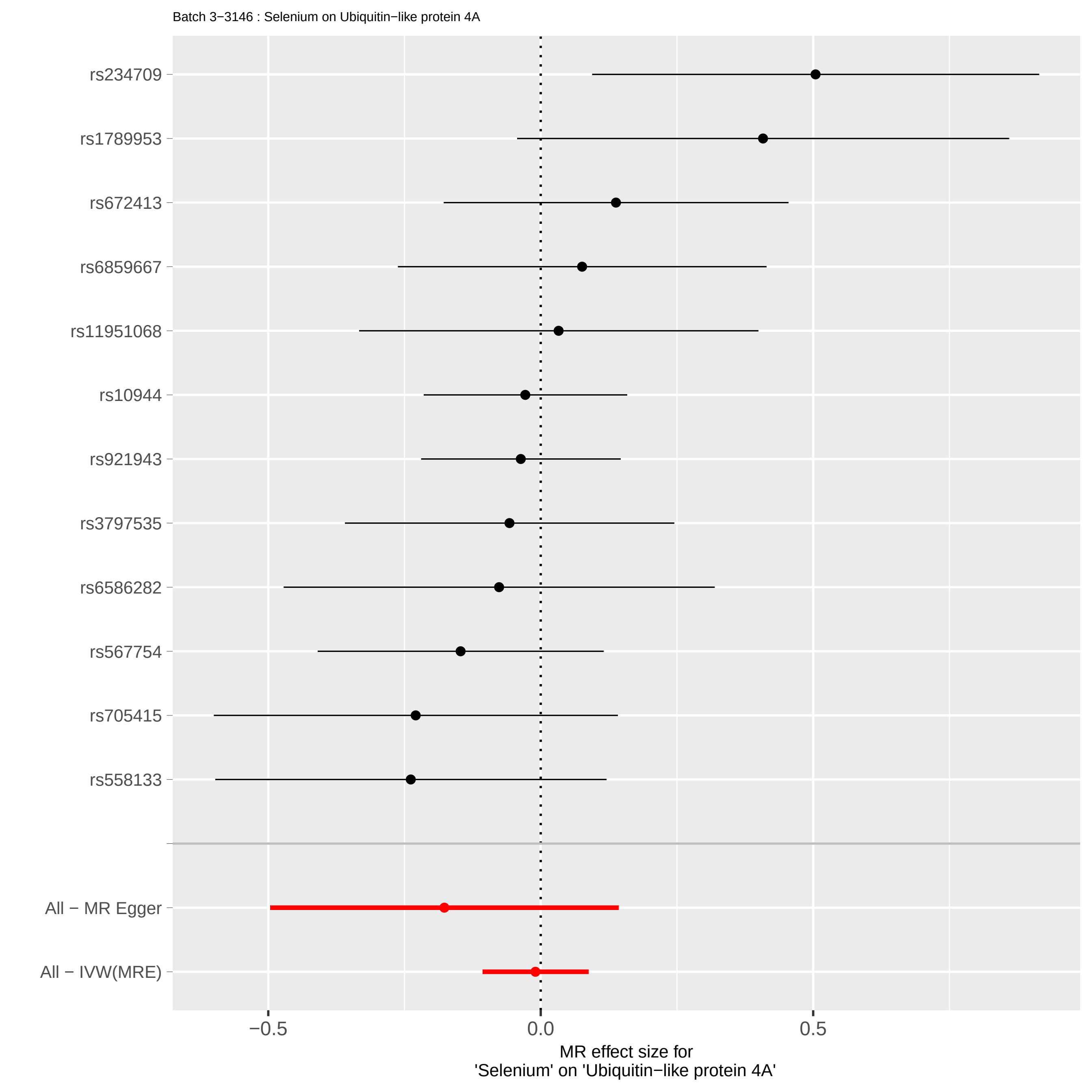

Batch 3−3146 : Selenium on Ubiquitin−like protein 4A
rs234709
rs1789953
rs672413
rs6859667
rs11951068
rs10944
rs921943
rs3797535
rs6586282
rs567754
rs705415
rs558133
All − MR Egger
All − IVW(MRE)
−0.5
0.0
0.5
MR effect size for
'Selenium' on 'Ubiquitin−like protein 4A'

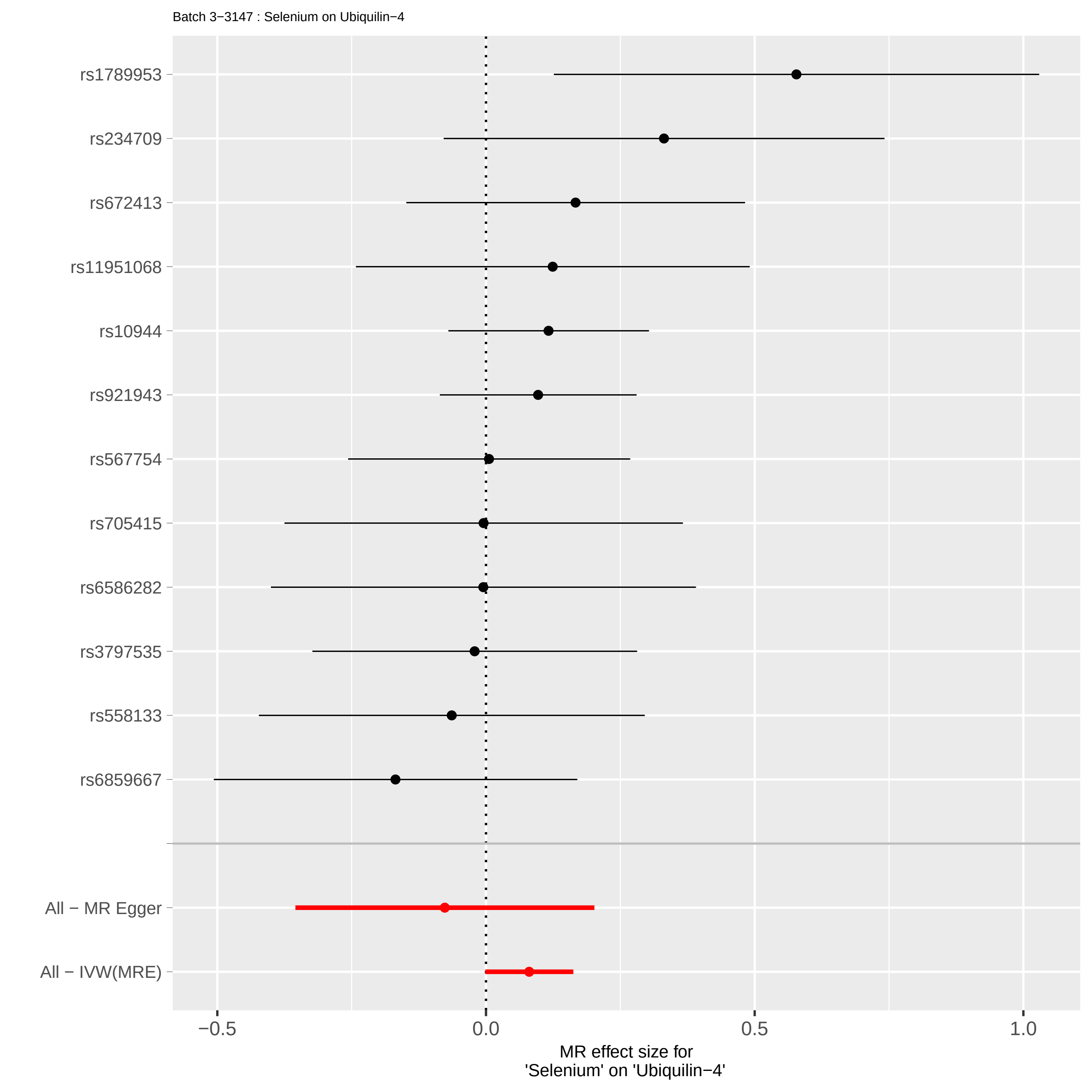

Batch 3−3147 : Selenium on Ubiquilin−4
rs1789953
rs234709
rs672413
rs11951068
rs10944
rs921943
rs567754
rs705415
rs6586282
rs3797535
rs558133
rs6859667
All − MR Egger
All − IVW(MRE)
−0.5
0.0
0.5
1.0
MR effect size for
'Selenium' on 'Ubiquilin−4'

Batch 3−3148 : Selenium on Ubiquitin domain−containing protein 2

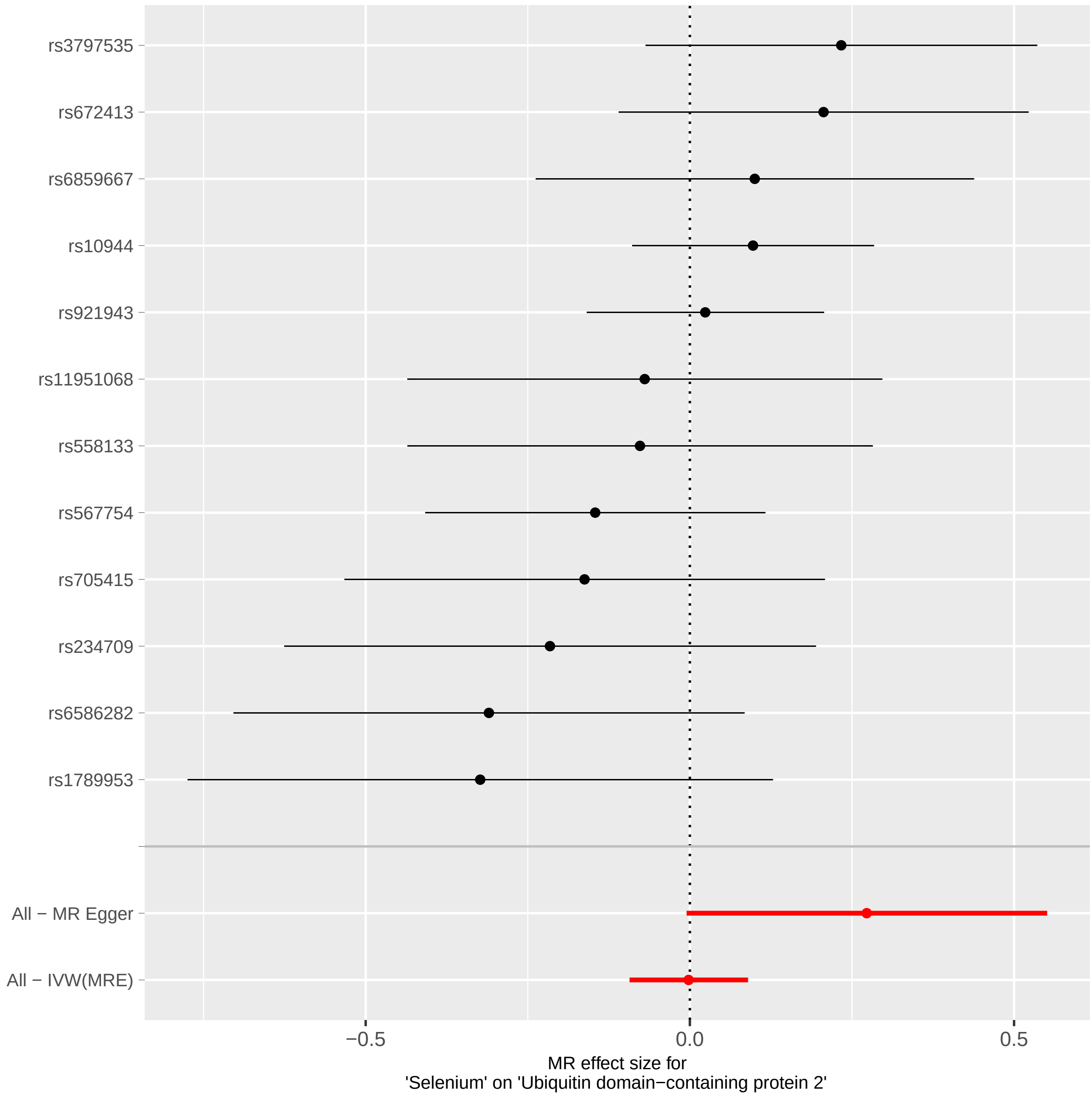

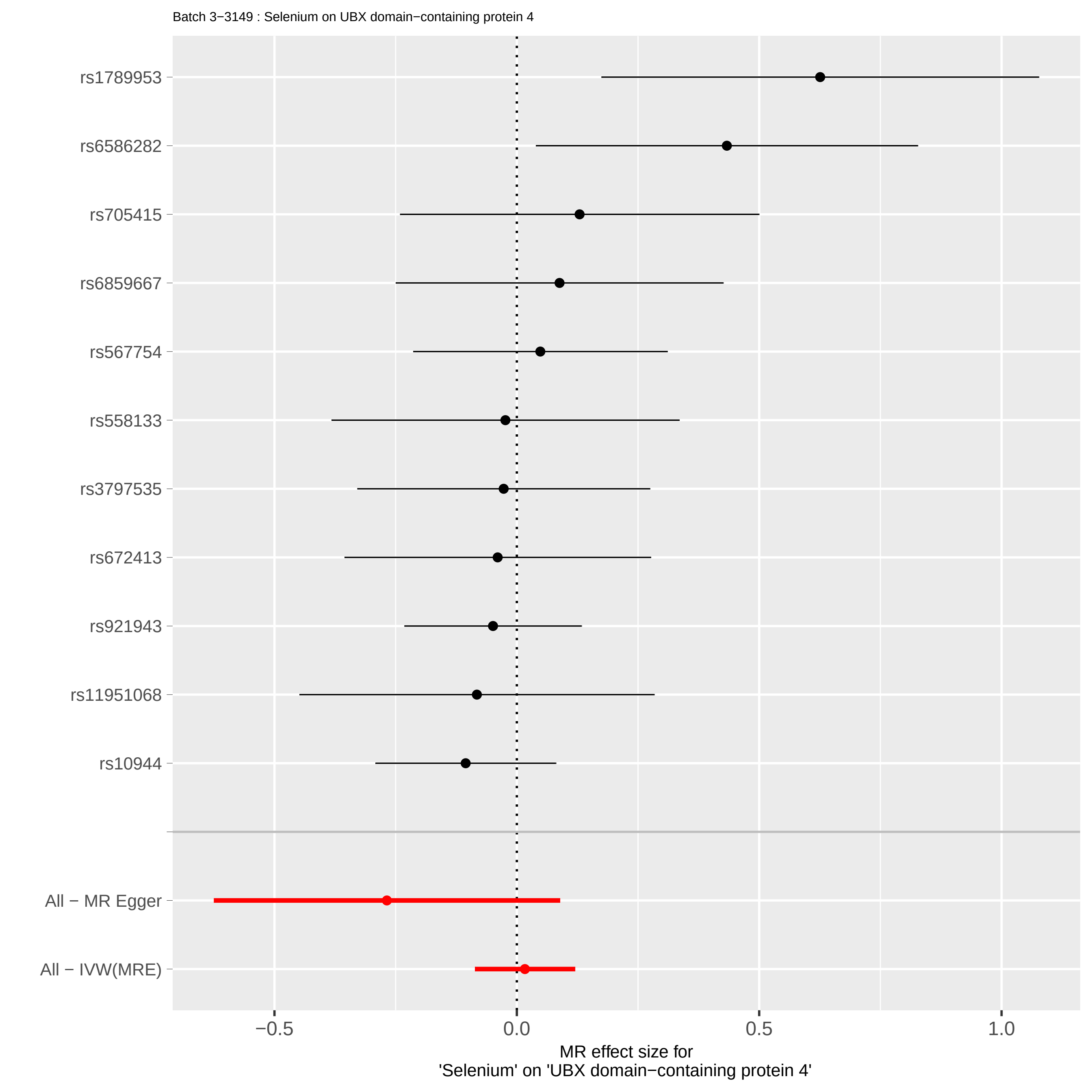

Batch 3−3149 : Selenium on UBX domain−containing protein 4
rs1789953
rs6586282
rs705415
rs6859667
rs567754
rs558133
rs3797535
rs672413
rs921943
rs11951068
rs10944
All − MR Egger
All − IVW(MRE)
−0.5
0.0
0.5
1.0
MR effect size for
'Selenium' on 'UBX domain−containing protein 4'

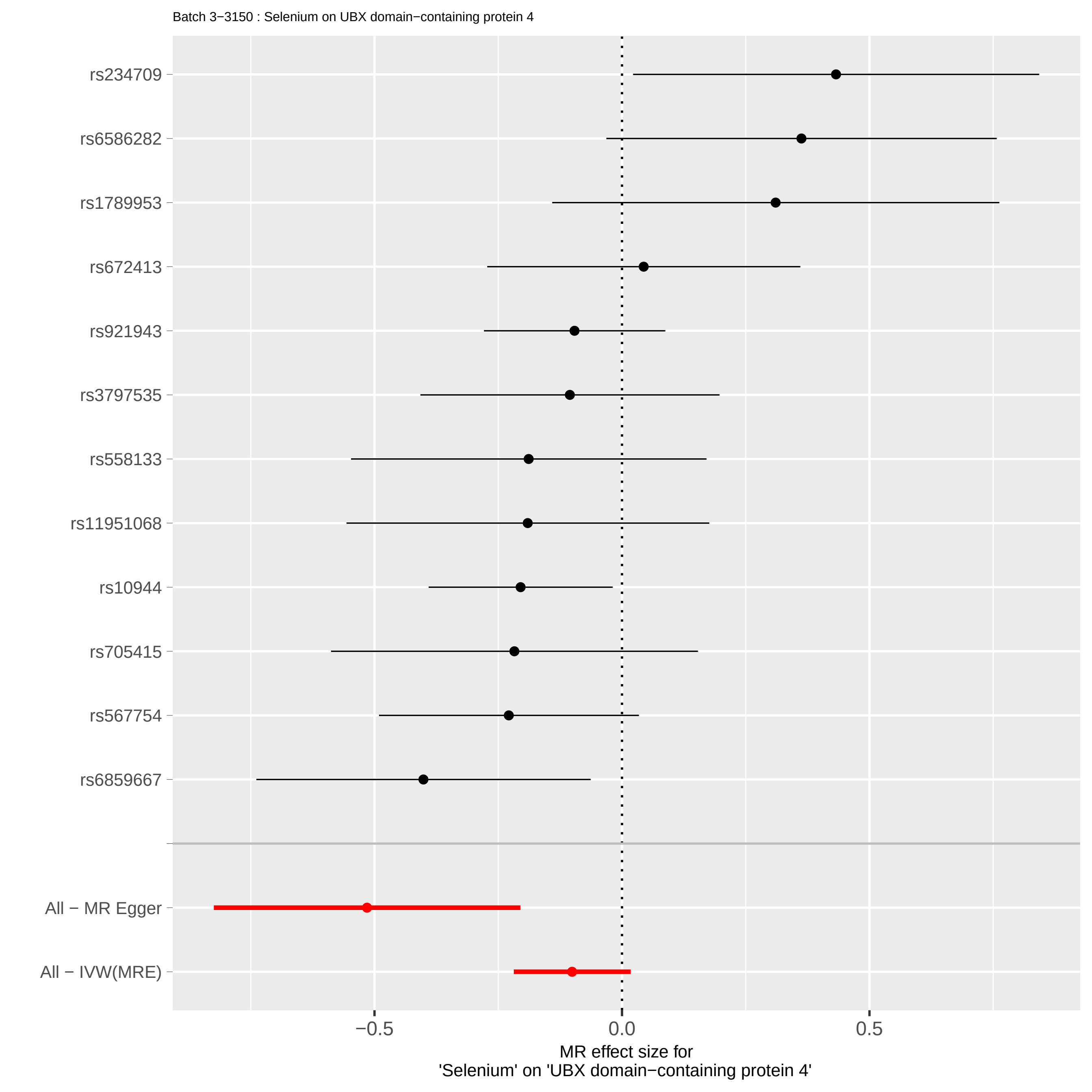

Batch 3−3150 : Selenium on UBX domain−containing protein 4
rs234709
rs6586282
rs1789953
rs672413
rs921943
rs3797535
rs558133
rs11951068
rs10944
rs705415
rs567754
rs6859667
All − MR Egger
All − IVW(MRE)
−0.5
0.0
0.5
MR effect size for
'Selenium' on 'UBX domain−containing protein 4'

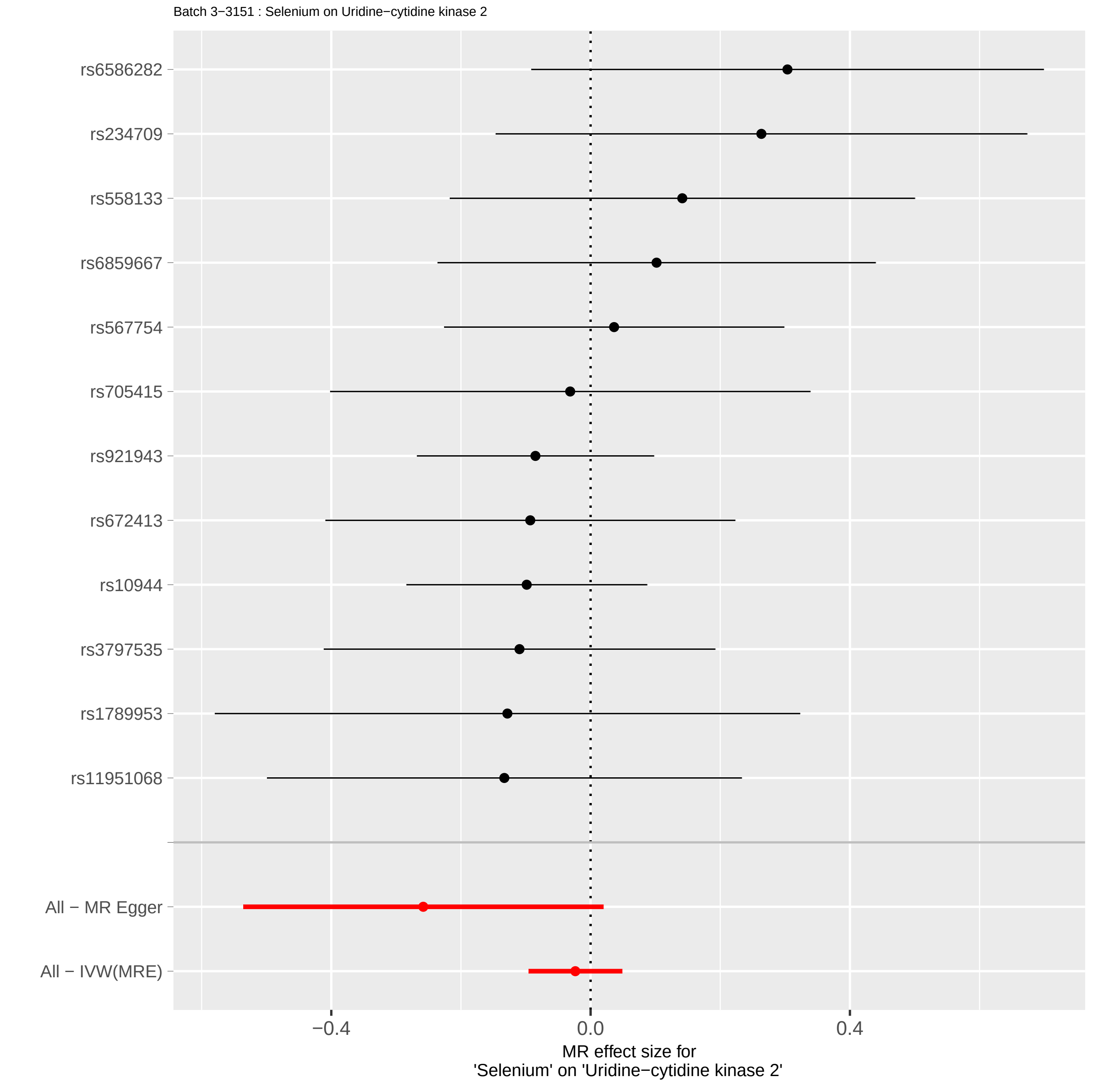
Batch 3−3151 : Selenium on Uridine−cytidine kinase 2
rs6586282
rs234709
rs558133
rs6859667
rs567754
rs705415
rs921943
rs672413
rs10944
rs3797535
rs1789953
rs11951068
All − MR Egger
All − IVW(MRE)
−0.4
0.0
0.4
MR effect size for
'Selenium' on 'Uridine−cytidine kinase 2'

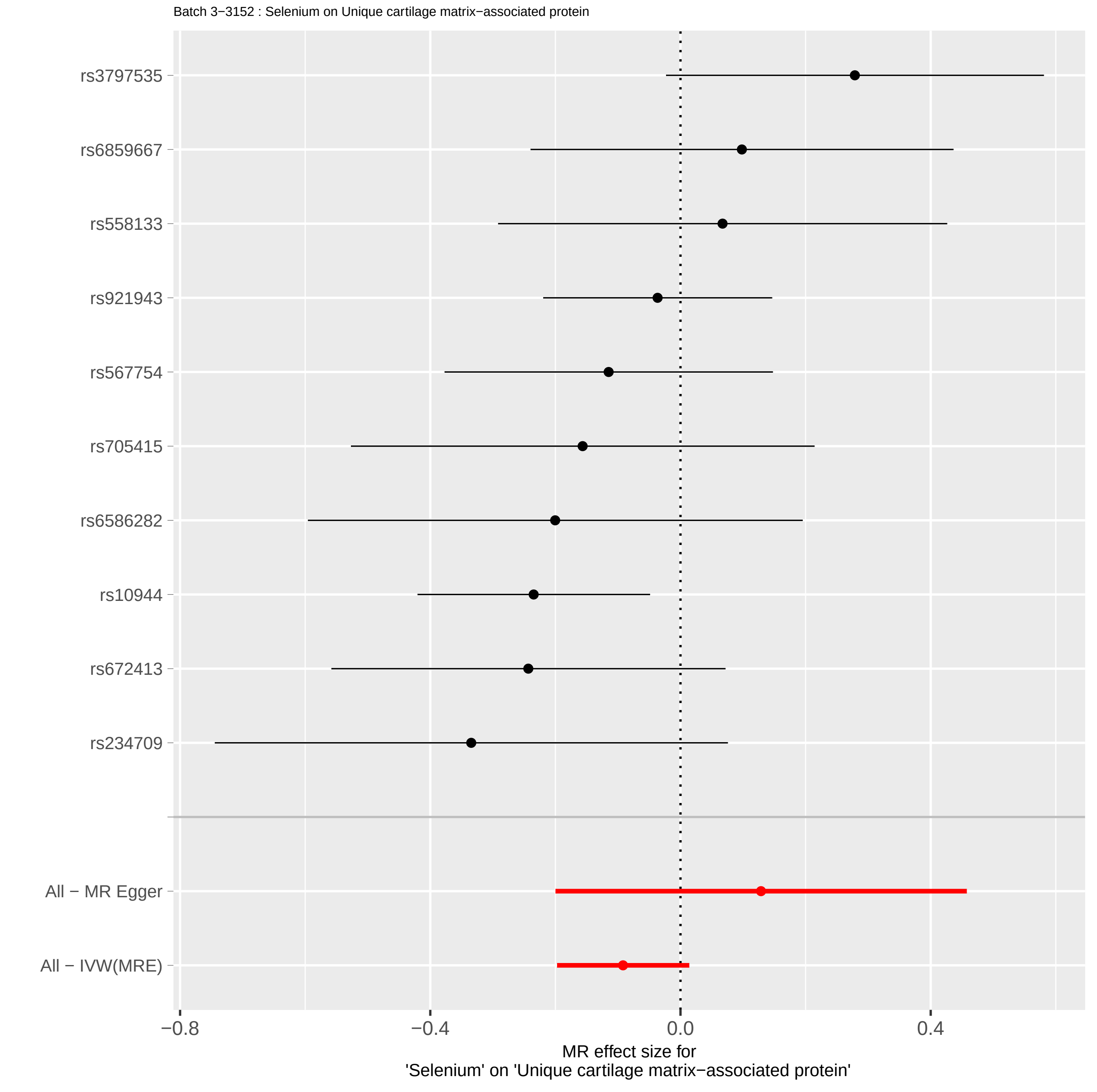

Batch 3−3152 : Selenium on Unique cartilage matrix−associated protein
rs3797535
rs6859667
rs558133
rs921943
rs567754
rs705415
rs6586282
rs10944
rs672413
rs234709
All − MR Egger
All − IVW(MRE)
−0.8
−0.4
0.0
0.4
MR effect size for
'Selenium' on 'Unique cartilage matrix−associated protein'

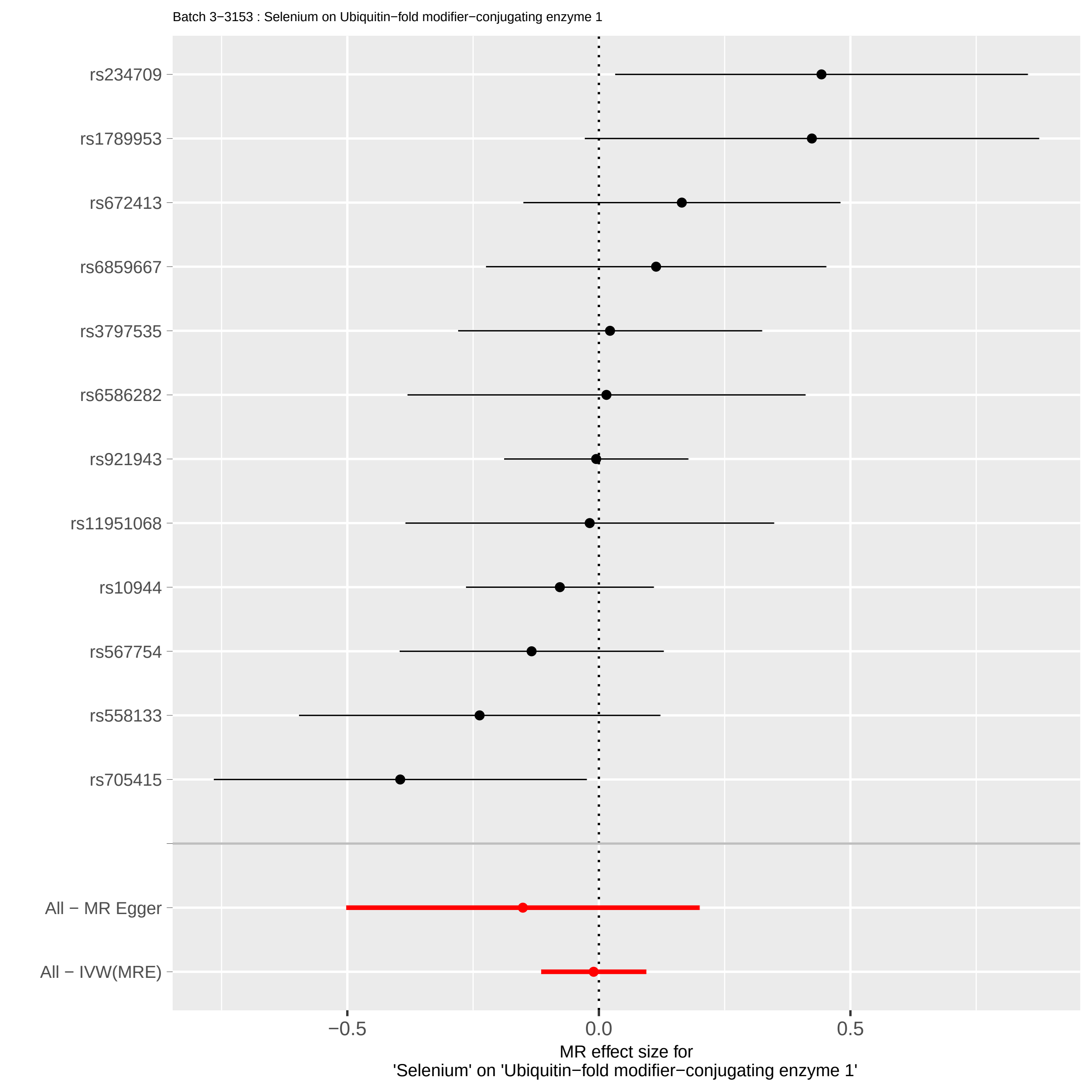

Batch 3−3153 : Selenium on Ubiquitin−fold modifier−conjugating enzyme 1
rs234709
rs1789953
rs672413
rs6859667
rs3797535
rs6586282
rs921943
rs11951068
rs10944
rs567754
rs558133
rs705415
All − MR Egger
All − IVW(MRE)
−0.5
0.0
0.5
MR effect size for
'Selenium' on 'Ubiquitin−fold modifier−conjugating enzyme 1'

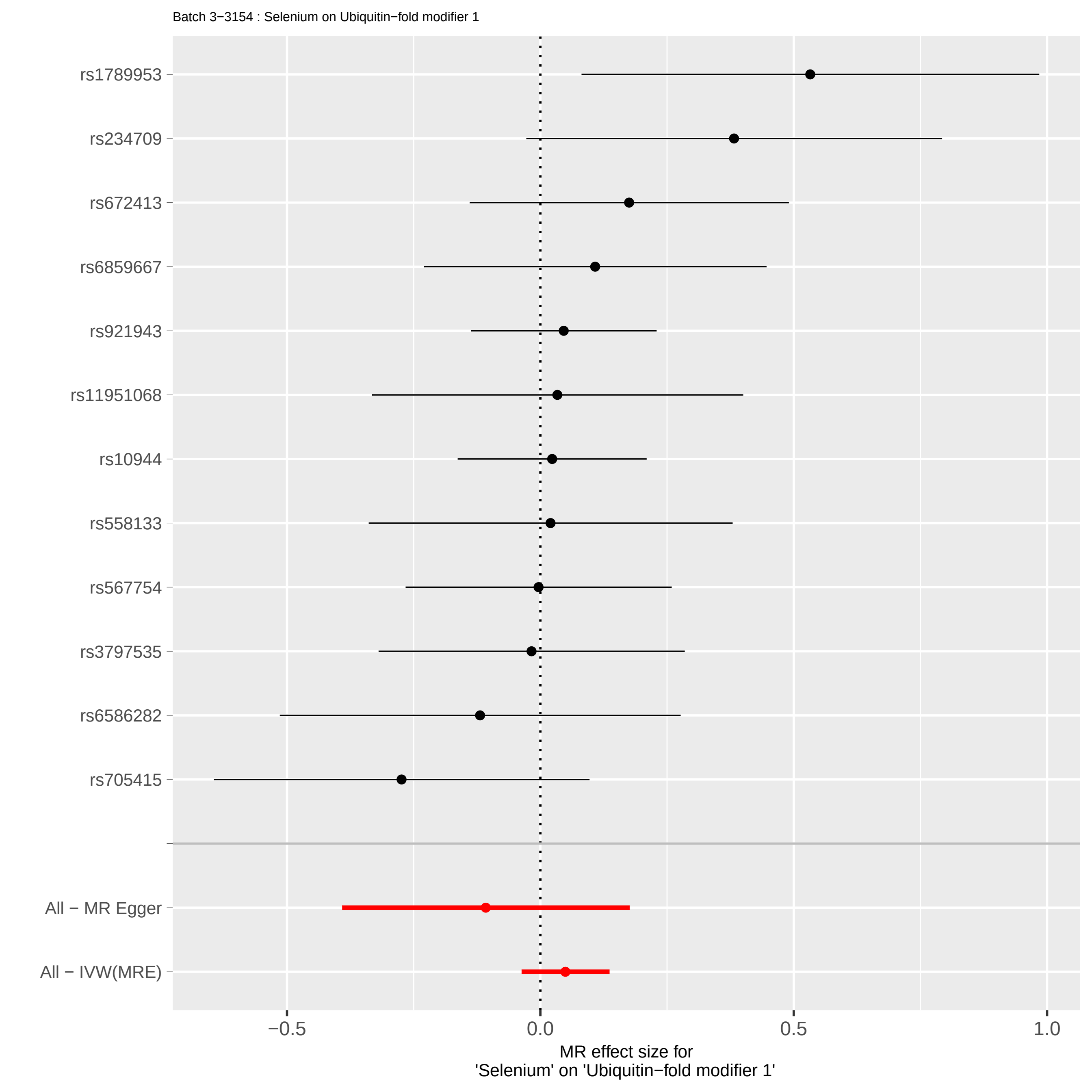
Batch 3−3154 : Selenium on Ubiquitin−fold modifier 1
rs1789953
rs234709
rs672413
rs6859667
rs921943
rs11951068
rs10944
rs558133
rs567754
rs3797535
rs6586282
rs705415
All − MR Egger
All − IVW(MRE)
−0.5
0.0
0.5
1.0
MR effect size for
'Selenium' on 'Ubiquitin−fold modifier 1'

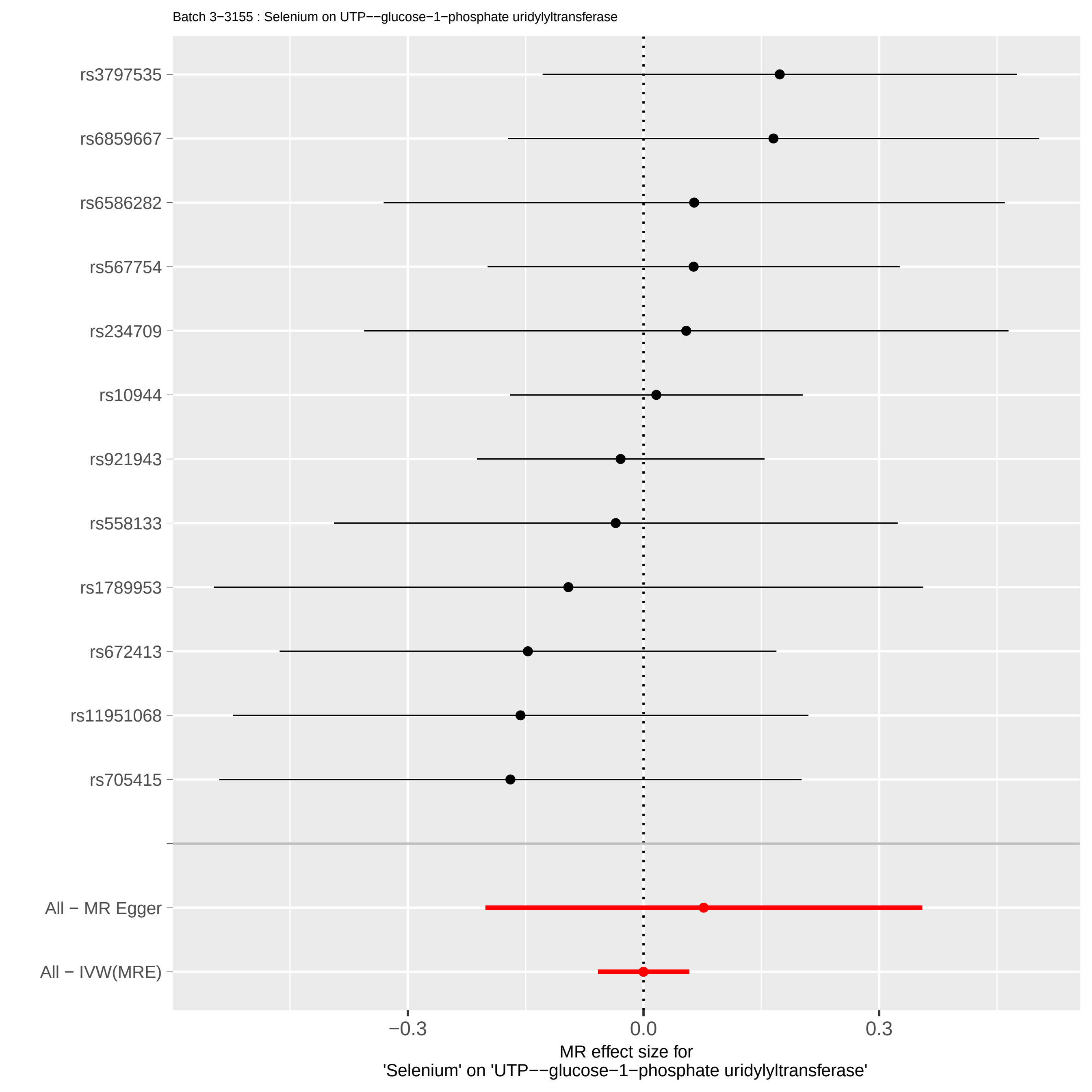
Batch 3−3155 : Selenium on UTP−−glucose−1−phosphate uridylyltransferase
rs3797535
rs6859667
rs6586282
rs567754
rs234709
rs10944
rs921943
rs558133
rs1789953
rs672413
rs11951068
rs705415
All − MR Egger
All − IVW(MRE)
−0.3
0.0
0.3
MR effect size for
'Selenium' on 'UTP−−glucose−1−phosphate uridylyltransferase'

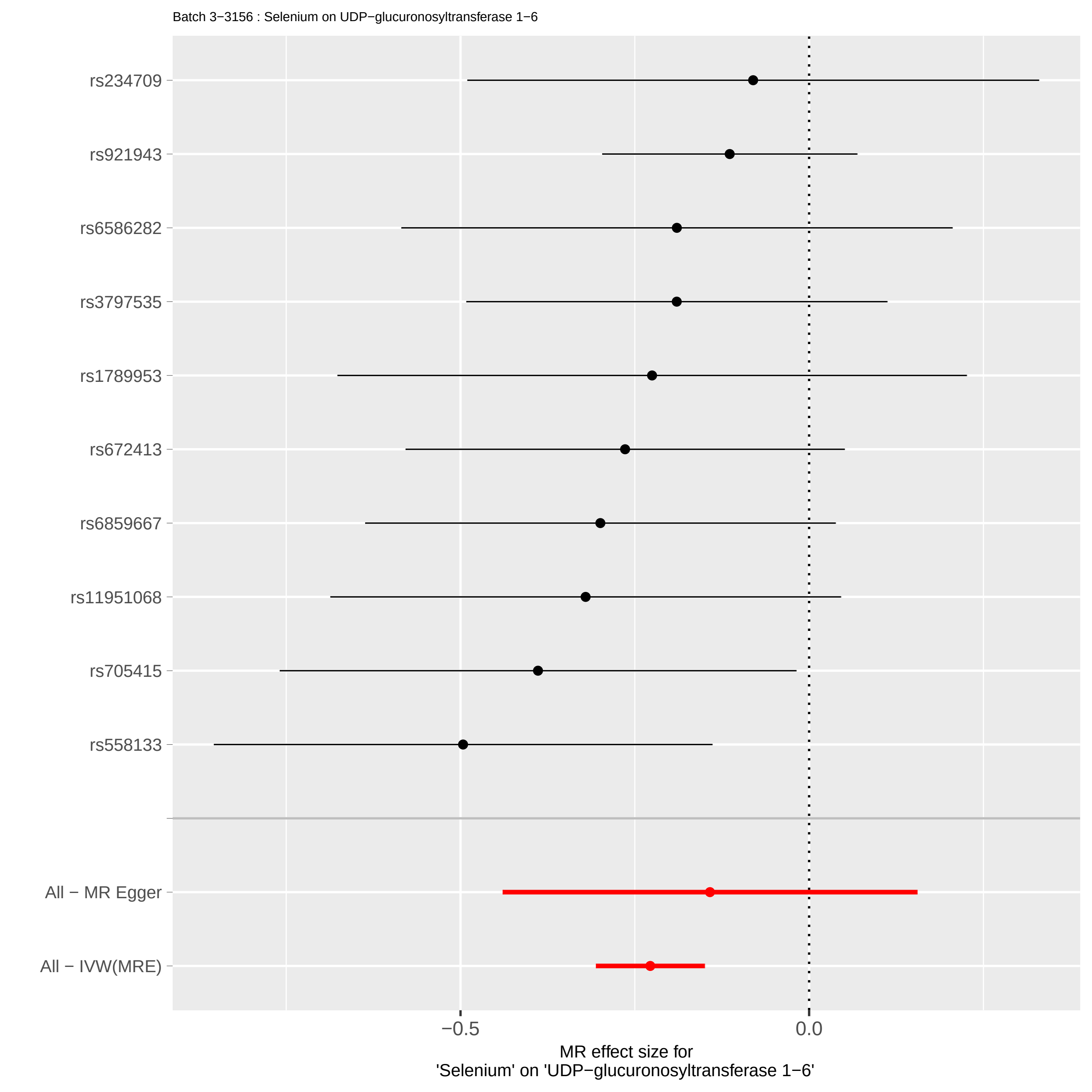
Batch 3−3156 : Selenium on UDP−glucuronosyltransferase 1−6
rs234709
rs921943
rs6586282
rs3797535
rs1789953
rs672413
rs6859667
rs11951068
rs705415
rs558133
All − MR Egger
All − IVW(MRE)
−0.5
0.0
MR effect size for
'Selenium' on 'UDP−glucuronosyltransferase 1−6'

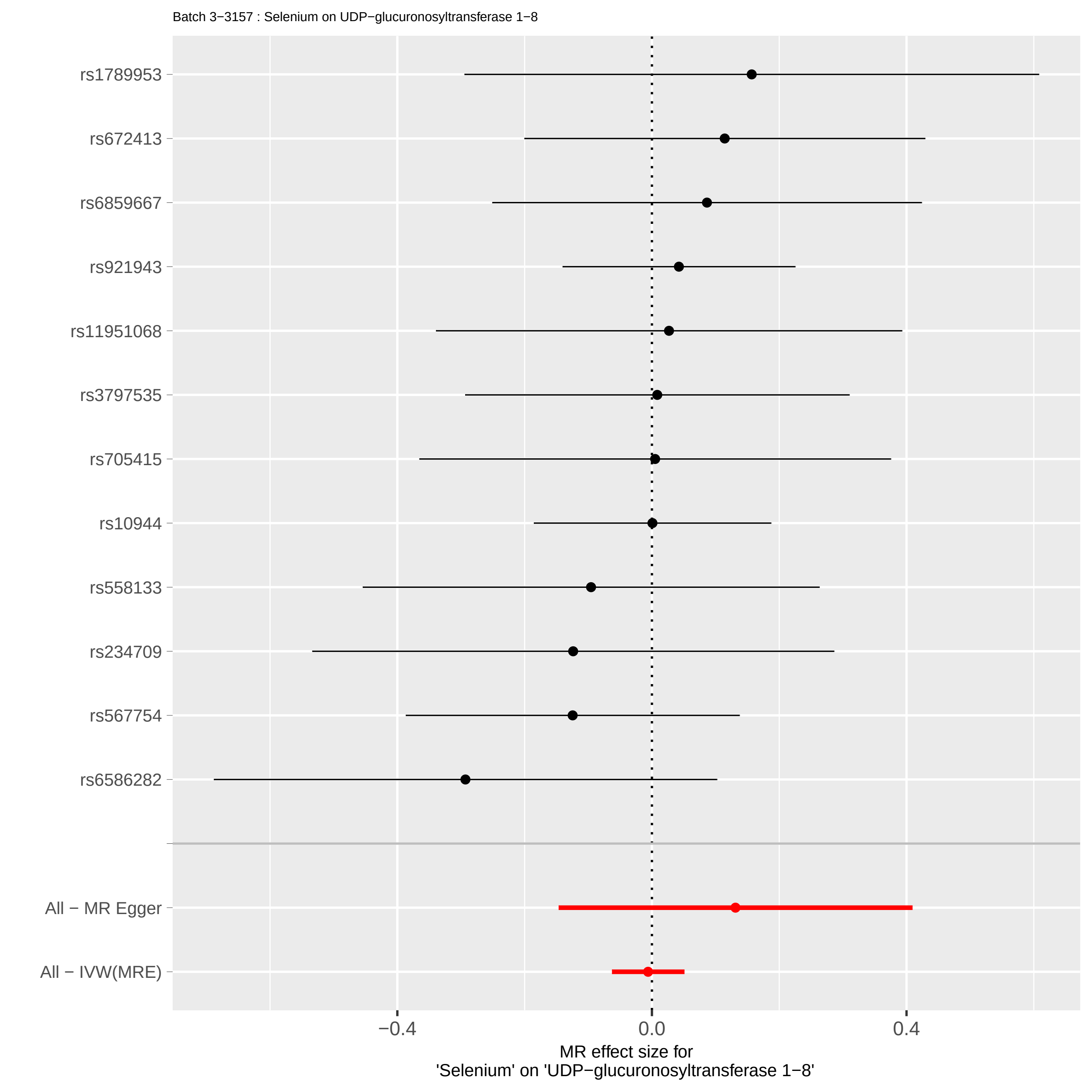

Batch 3−3157 : Selenium on UDP−glucuronosyltransferase 1−8
rs1789953
rs672413
rs6859667
rs921943
rs11951068
rs3797535
rs705415
rs10944
rs558133
rs234709
rs567754
rs6586282
All − MR Egger
All − IVW(MRE)
−0.4
0.0
0.4
MR effect size for
'Selenium' on 'UDP−glucuronosyltransferase 1−8'

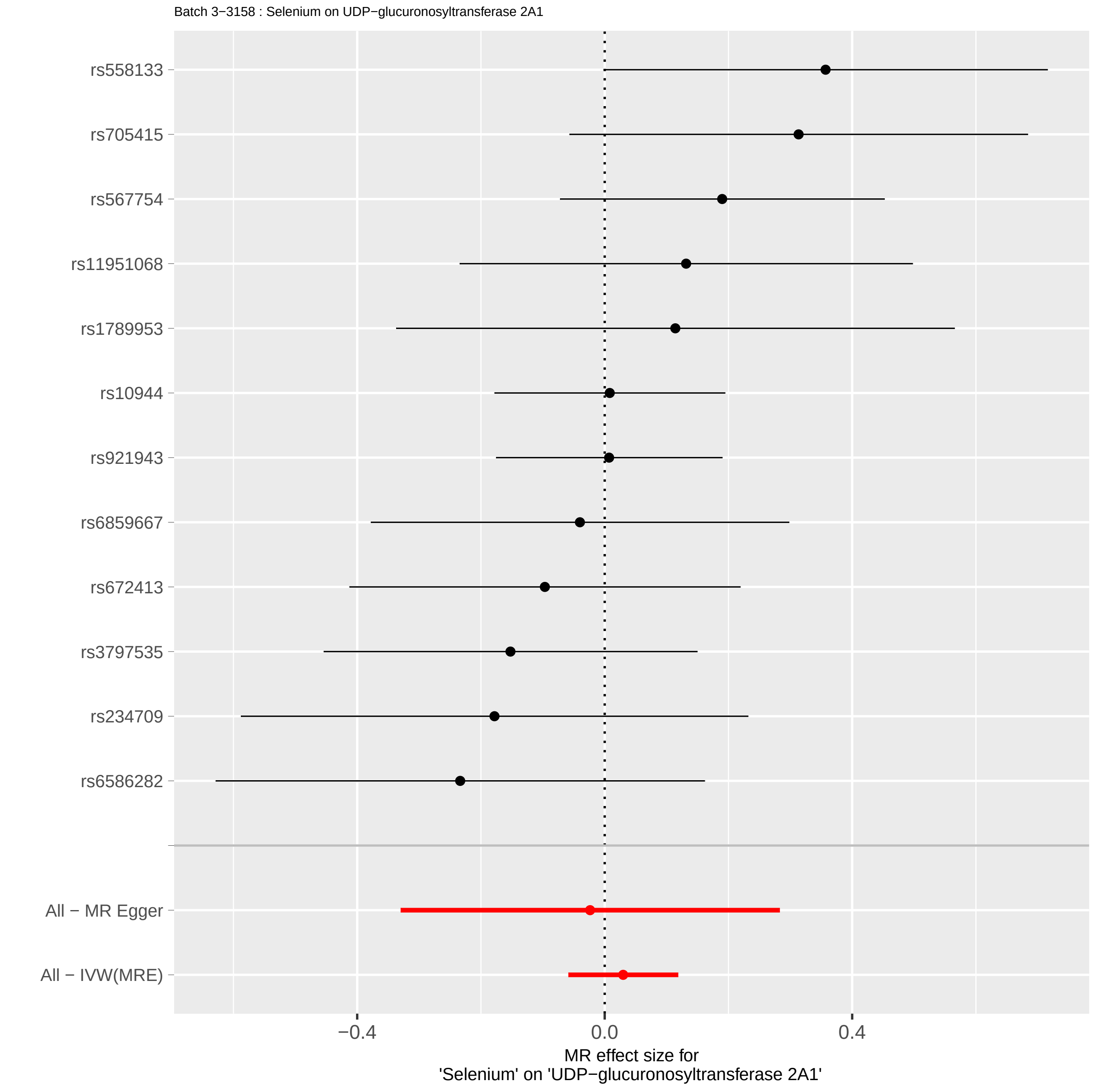

Batch 3−3158 : Selenium on UDP−glucuronosyltransferase 2A1
rs558133
rs705415
rs567754
rs11951068
rs1789953
rs10944
rs921943
rs6859667
rs672413
rs3797535
rs234709
rs6586282
All − MR Egger
All − IVW(MRE)
−0.4
0.0
0.4
MR effect size for
'Selenium' on 'UDP−glucuronosyltransferase 2A1'

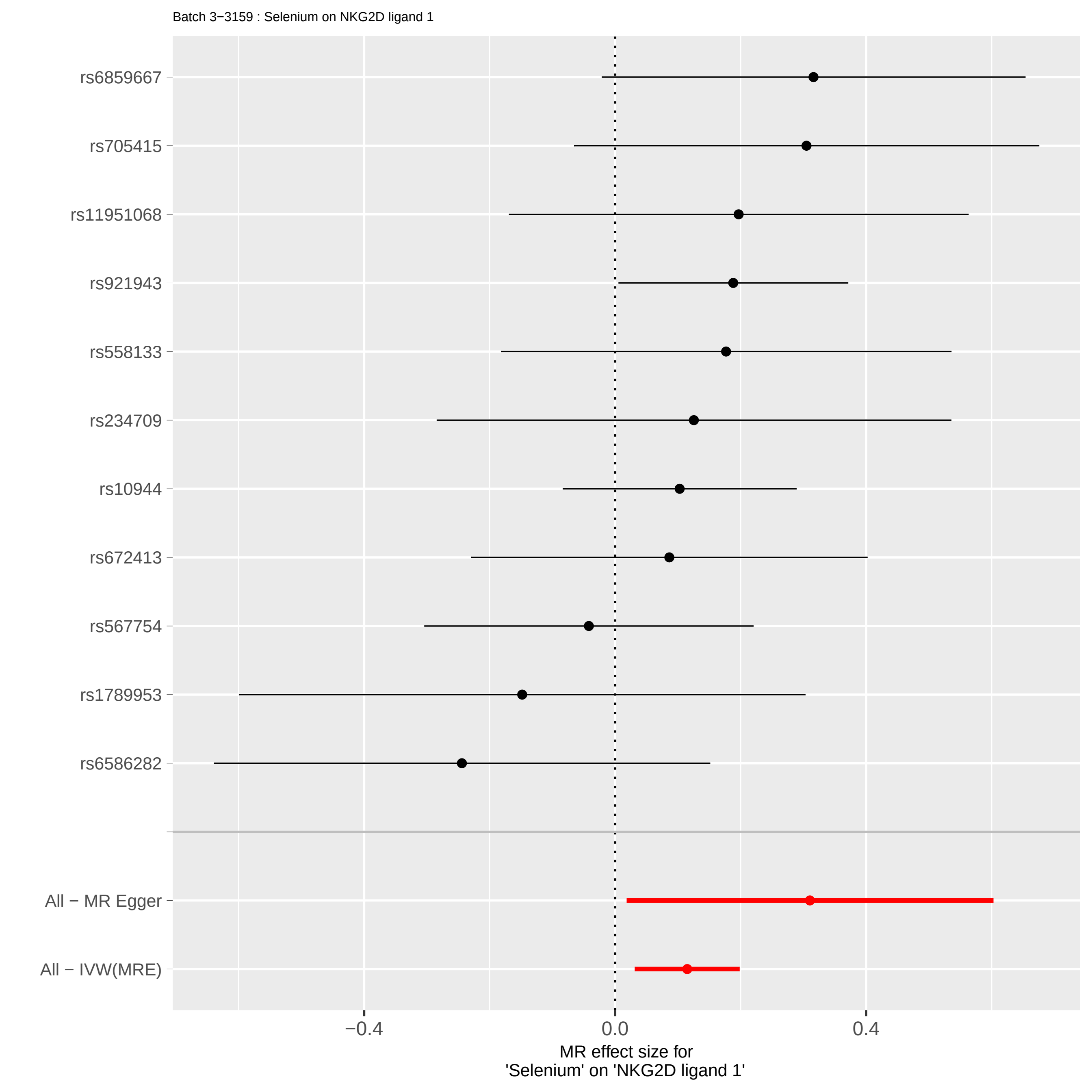
Batch 3−3159 : Selenium on NKG2D ligand 1
rs6859667
rs705415
rs11951068
rs921943
rs558133
rs234709
rs10944
rs672413
rs567754
rs1789953
rs6586282
All − MR Egger
All − IVW(MRE)
−0.4
0.0
0.4
MR effect size for
'Selenium' on 'NKG2D ligand 1'

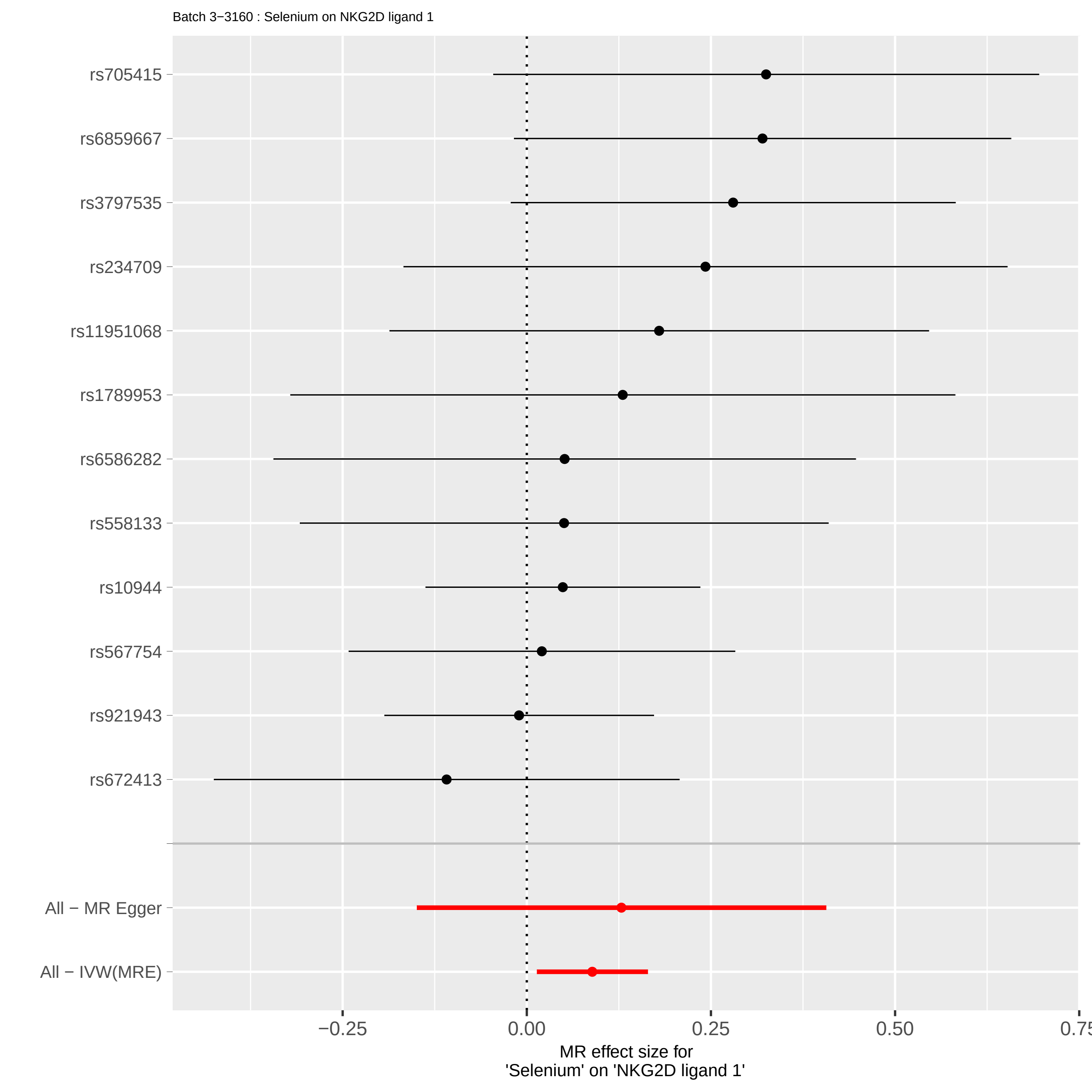

Batch 3−3160 : Selenium on NKG2D ligand 1
rs705415
rs6859667
rs3797535
rs234709
rs11951068
rs1789953
rs6586282
rs558133
rs10944
rs567754
rs921943
rs672413
All − MR Egger
All − IVW(MRE)
−0.25
0.00
0.25
0.50
0.75
MR effect size for
'Selenium' on 'NKG2D ligand 1'

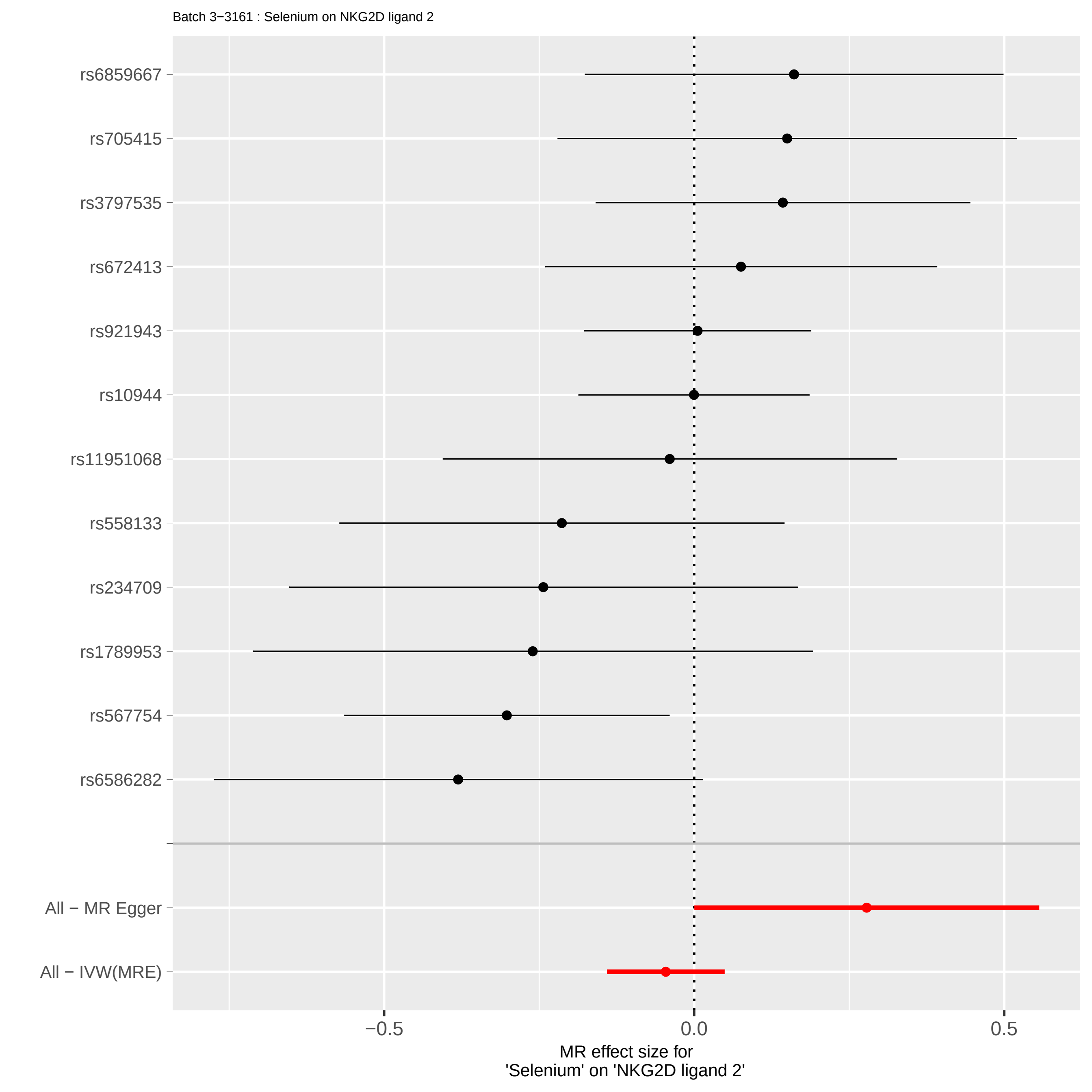

Batch 3−3161 : Selenium on NKG2D ligand 2
rs6859667
rs705415
rs3797535
rs672413
rs921943
rs10944
rs11951068
rs558133
rs234709
rs1789953
rs567754
rs6586282
All − MR Egger
All − IVW(MRE)
−0.5
0.0
0.5
MR effect size for
'Selenium' on 'NKG2D ligand 2'

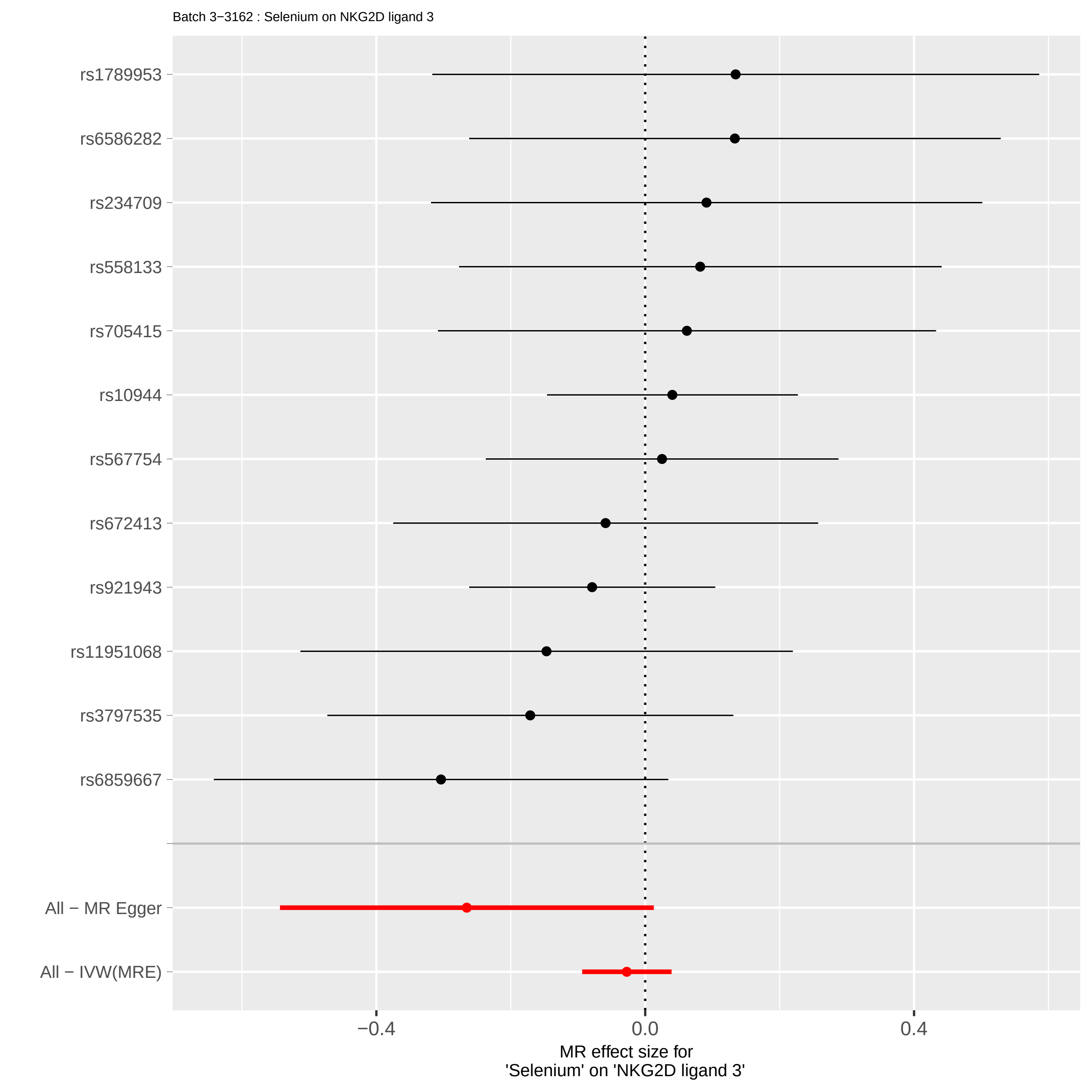
Batch 3−3162 : Selenium on NKG2D ligand 3
rs1789953
rs6586282
rs234709
rs558133
rs705415
rs10944
rs567754
rs672413
rs921943
rs11951068
rs3797535
rs6859667
All − MR Egger
All − IVW(MRE)
−0.4
0.0
0.4
MR effect size for
'Selenium' on 'NKG2D ligand 3'

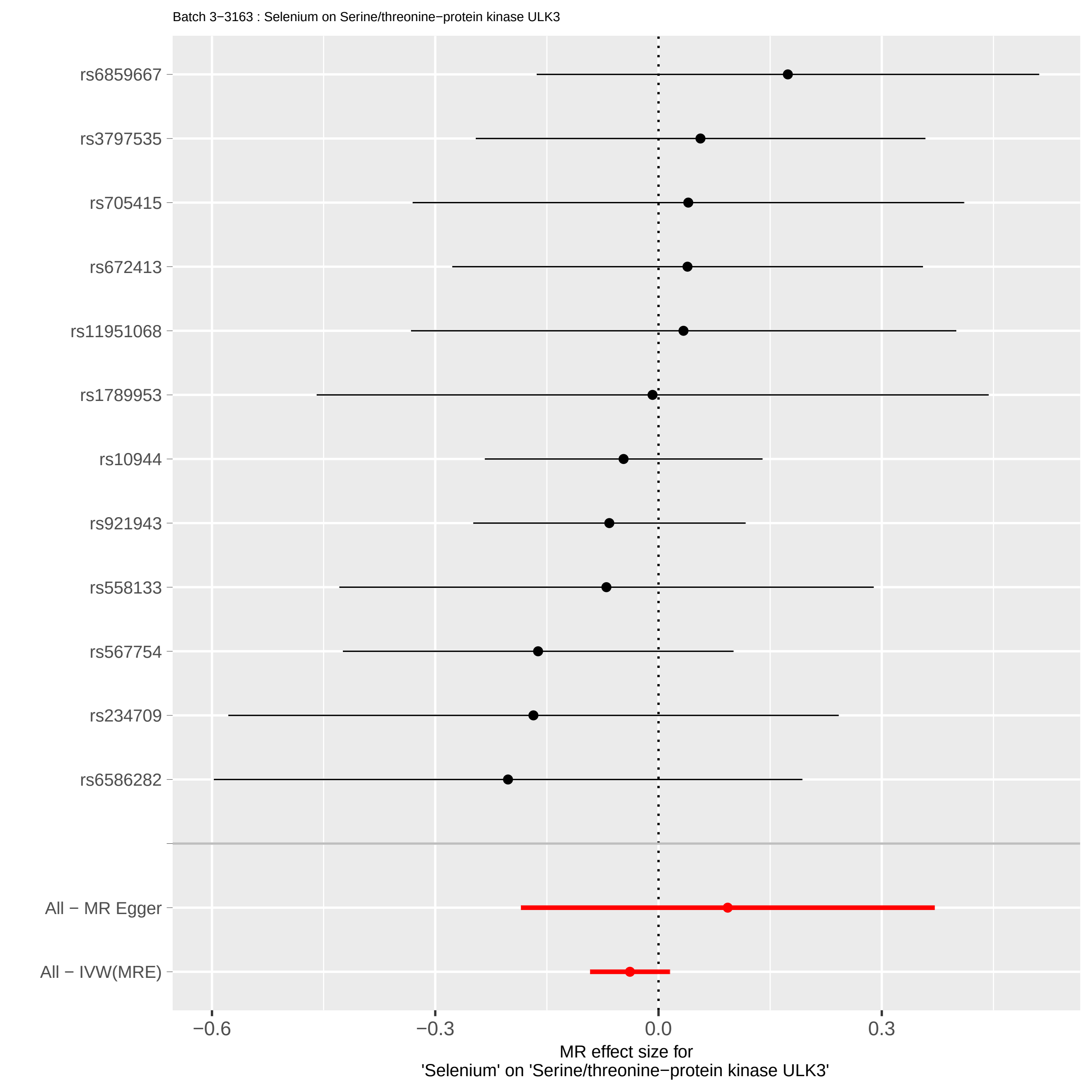

Batch 3−3163 : Selenium on Serine/threonine−protein kinase ULK3
rs6859667
rs3797535
rs705415
rs672413
rs11951068
rs1789953
rs10944
rs921943
rs558133
rs567754
rs234709
rs6586282
All − MR Egger
All − IVW(MRE)
−0.6
−0.3
0.0
0.3
MR effect size for
'Selenium' on 'Serine/threonine−protein kinase ULK3'

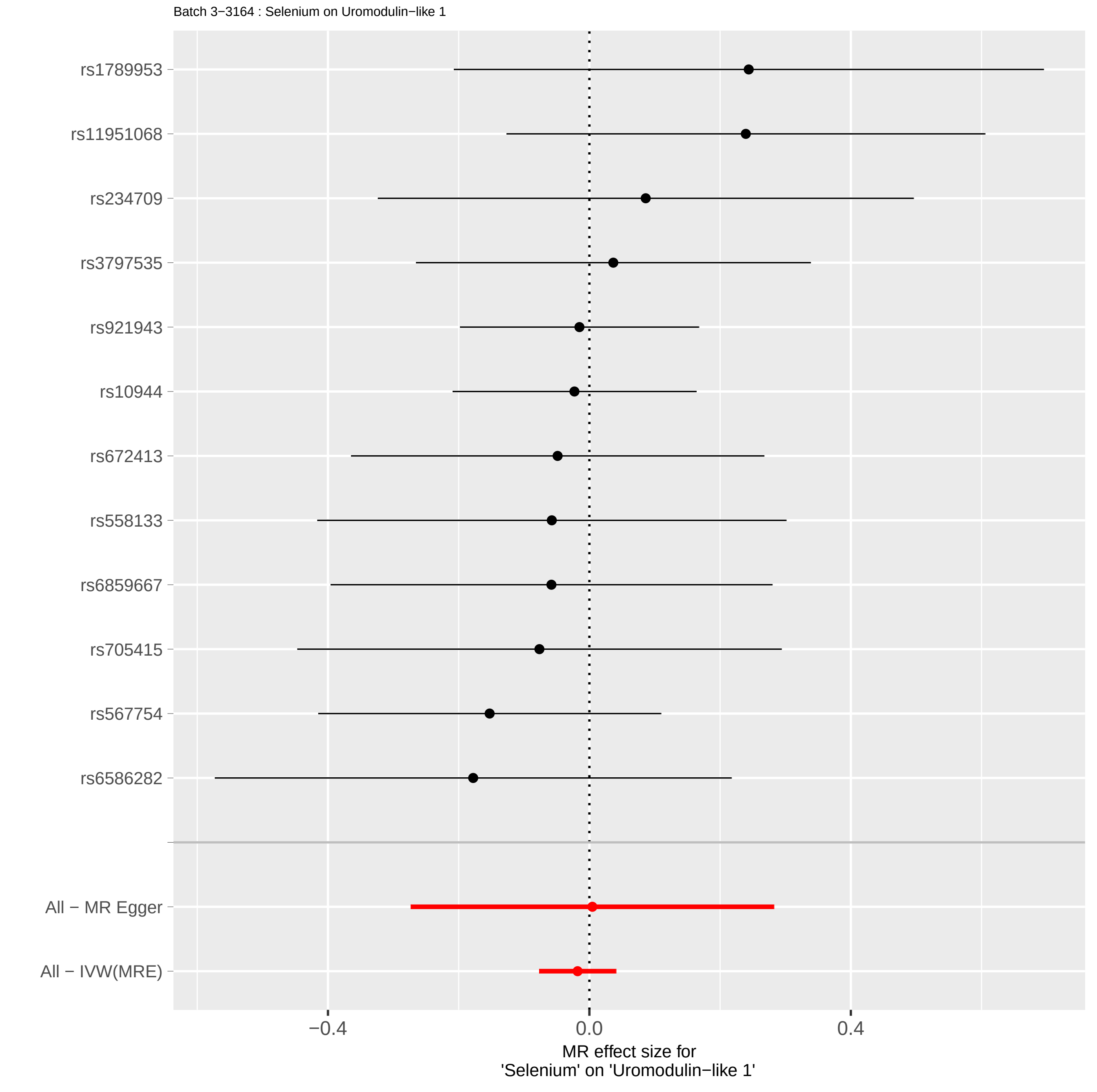

Batch 3−3164 : Selenium on Uromodulin−like 1
rs1789953
rs11951068
rs234709
rs3797535
rs921943
rs10944
rs672413
rs558133
rs6859667
rs705415
rs567754
rs6586282
All − MR Egger
All − IVW(MRE)
−0.4
0.0
0.4
MR effect size for
'Selenium' on 'Uromodulin−like 1'

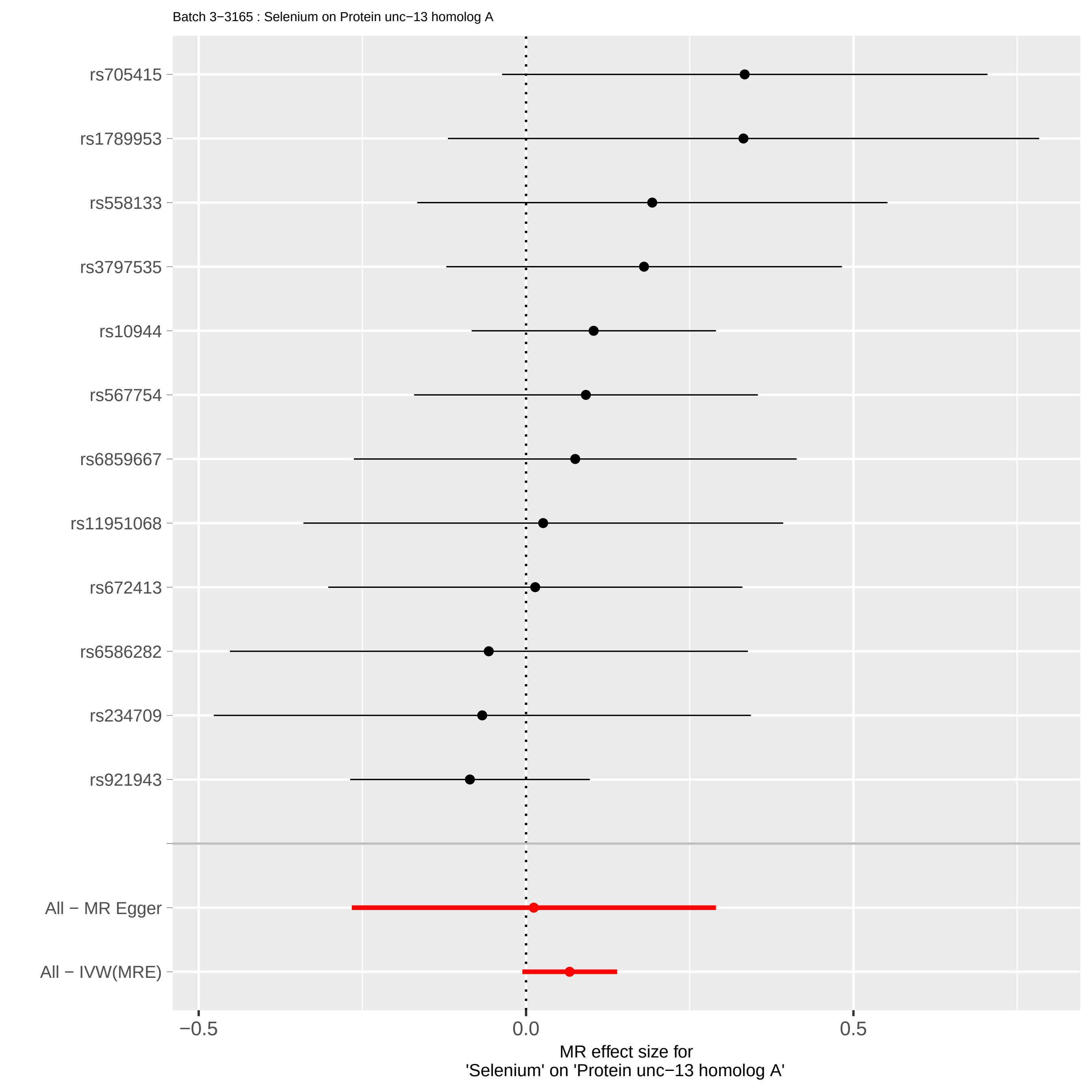
Batch 3−3165 : Selenium on Protein unc−13 homolog A
rs705415
rs1789953
rs558133
rs3797535
rs10944
rs567754
rs6859667
rs11951068
rs672413
rs6586282
rs234709
rs921943
All − MR Egger
All − IVW(MRE)
−0.5
0.0
0.5
MR effect size for
'Selenium' on 'Protein unc−13 homolog A'

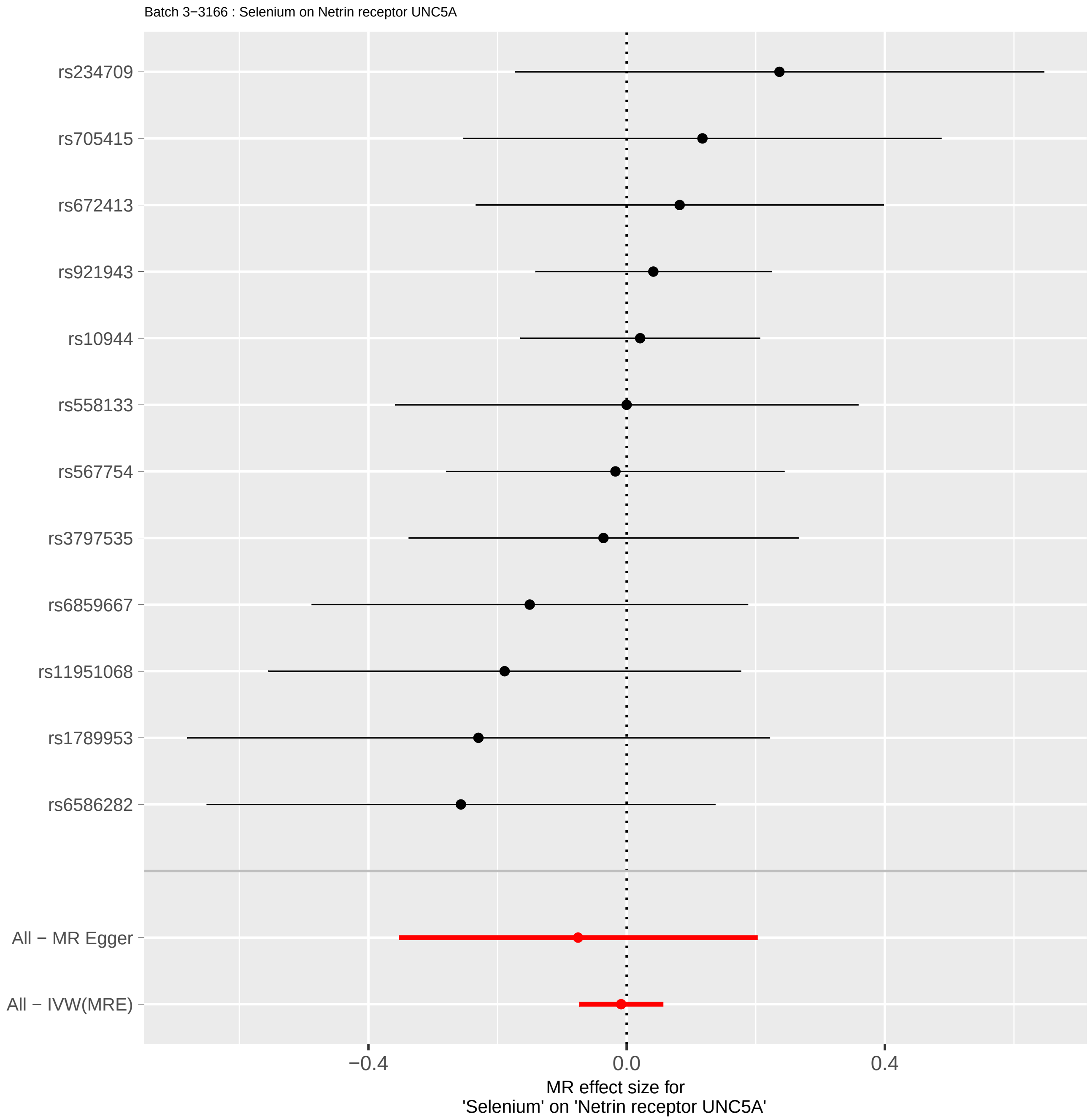

Batch 3−3166 : Selenium on Netrin receptor UNC5A
rs234709
rs705415
rs672413
rs921943
rs10944
rs558133
rs567754
rs3797535
rs6859667
rs11951068
rs1789953
rs6586282
All − MR Egger
All − IVW(MRE)
−0.4
0.0
0.4
MR effect size for
'Selenium' on 'Netrin receptor UNC5A'

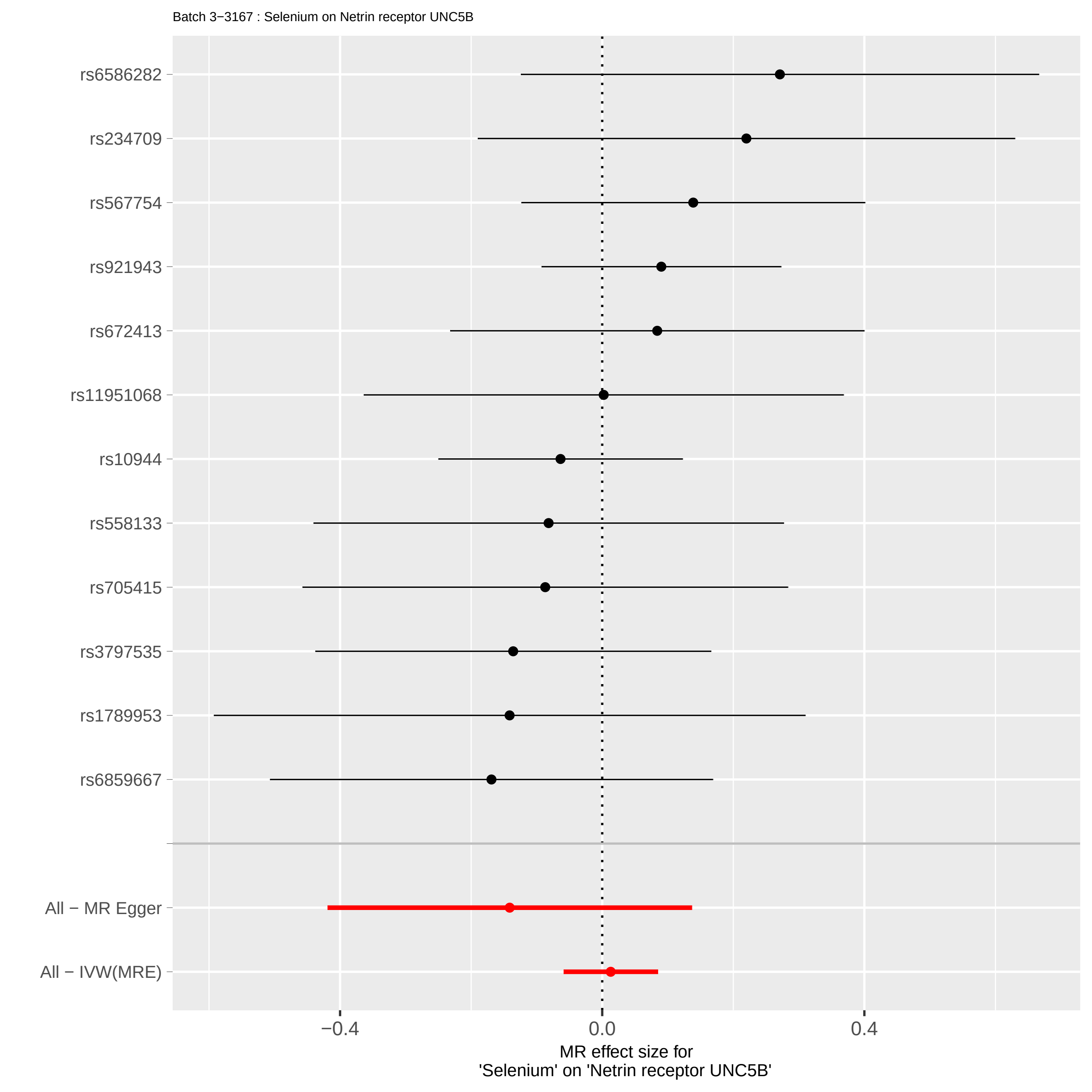
Batch 3−3167 : Selenium on Netrin receptor UNC5B
rs6586282
rs234709
rs567754
rs921943
rs672413
rs11951068
rs10944
rs558133
rs705415
rs3797535
rs1789953
rs6859667
All − MR Egger
All − IVW(MRE)
−0.4
0.0
0.4
MR effect size for
'Selenium' on 'Netrin receptor UNC5B'

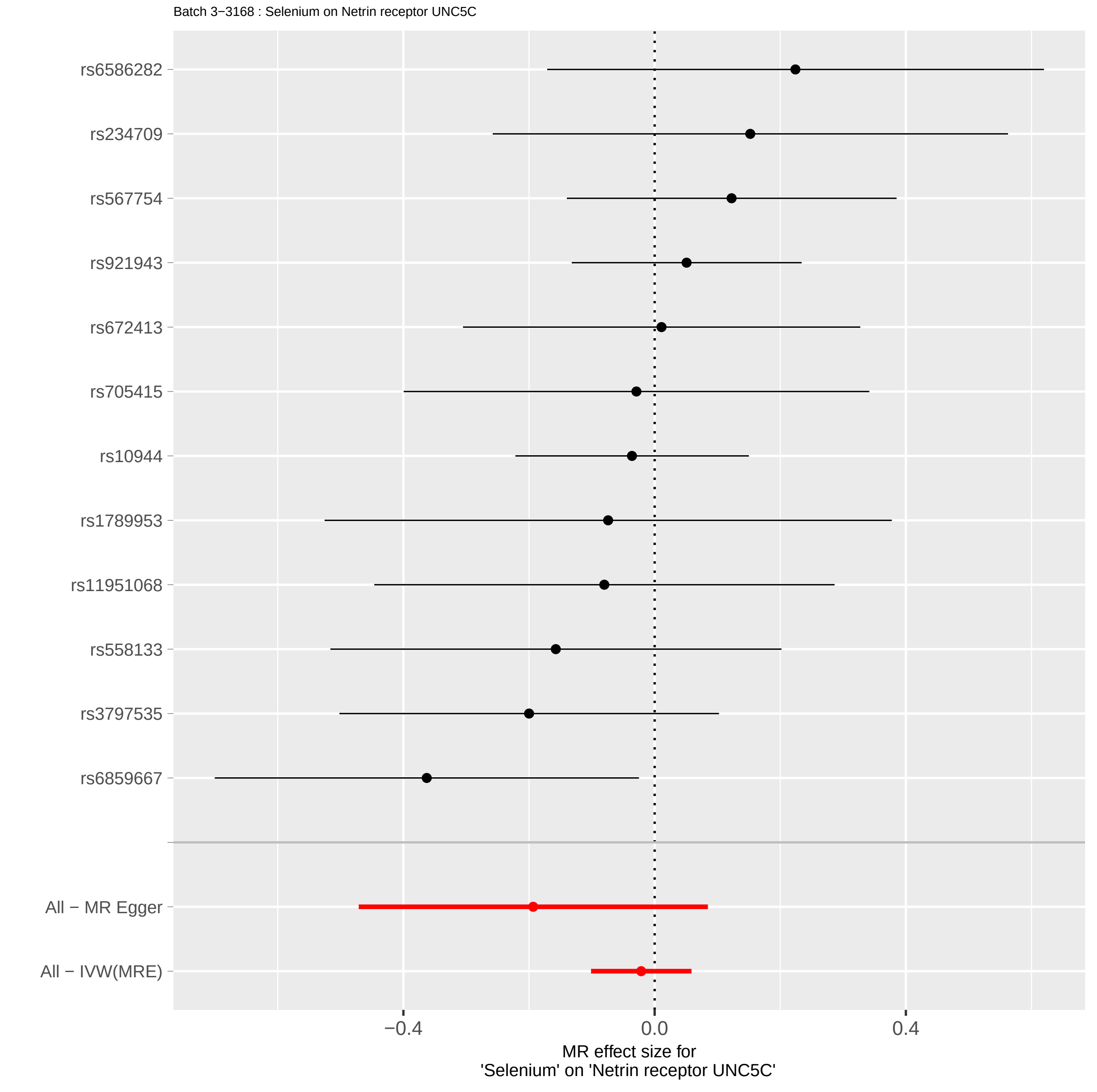

Batch 3−3168 : Selenium on Netrin receptor UNC5C
rs6586282
rs234709
rs567754
rs921943
rs672413
rs705415
rs10944
rs1789953
rs11951068
rs558133
rs3797535
rs6859667
All − MR Egger
All − IVW(MRE)
−0.4
0.0
0.4
MR effect size for
'Selenium' on 'Netrin receptor UNC5C'

Batch 3−3169 : Selenium on Netrin receptor UNC5D

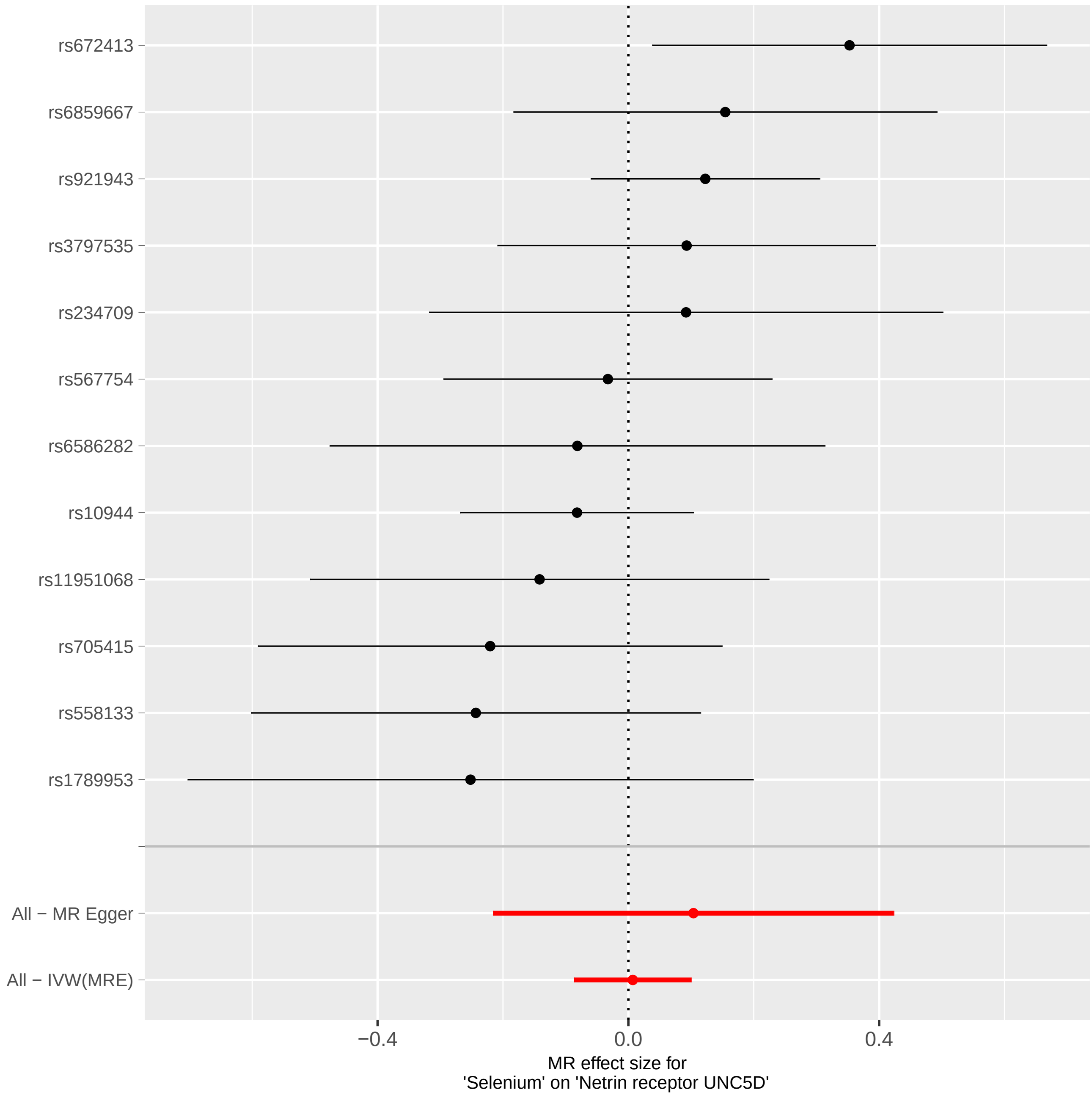

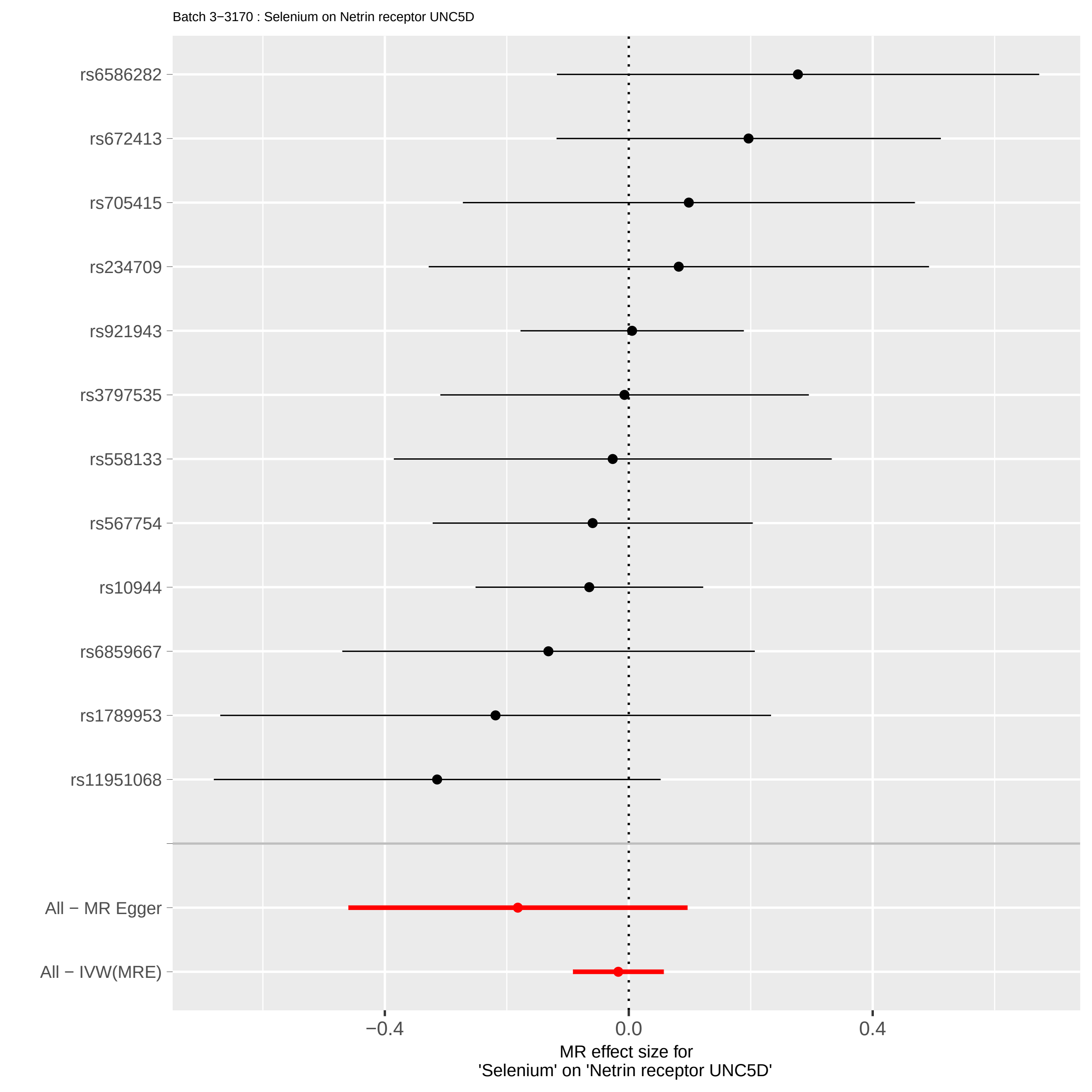

Batch 3−3170 : Selenium on Netrin receptor UNC5D
rs6586282
rs672413
rs705415
rs234709
rs921943
rs3797535
rs558133
rs567754
rs10944
rs6859667
rs1789953
rs11951068
All − MR Egger
All − IVW(MRE)
−0.4
0.0
0.4
MR effect size for
'Selenium' on 'Netrin receptor UNC5D'

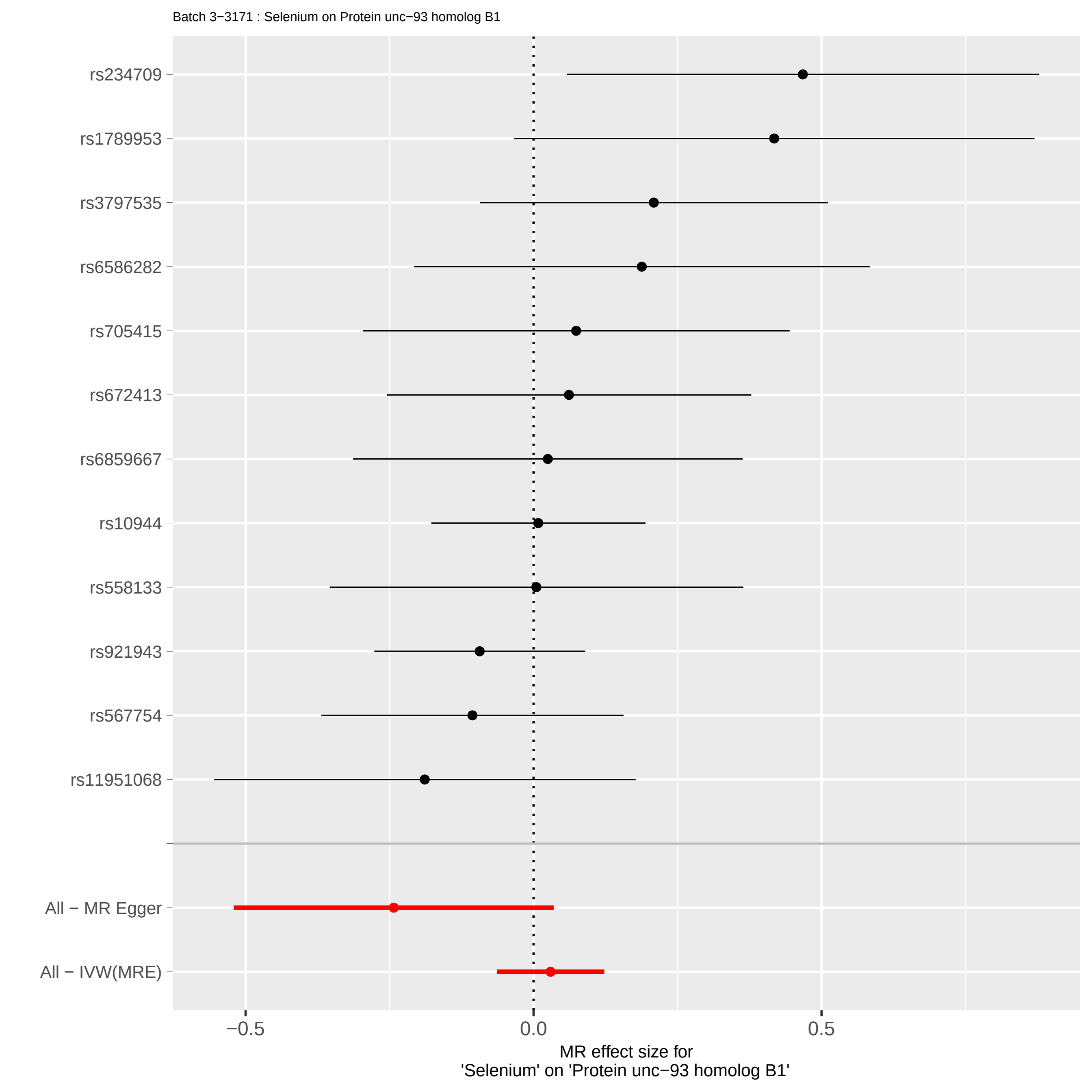

Batch 3−3171 : Selenium on Protein unc−93 homolog B1
rs234709
rs1789953
rs3797535
rs6586282
rs705415
rs672413
rs6859667
rs10944
rs558133
rs921943
rs567754
rs11951068
All − MR Egger
All − IVW(MRE)
−0.5
0.0
0.5
MR effect size for
'Selenium' on 'Protein unc−93 homolog B1'

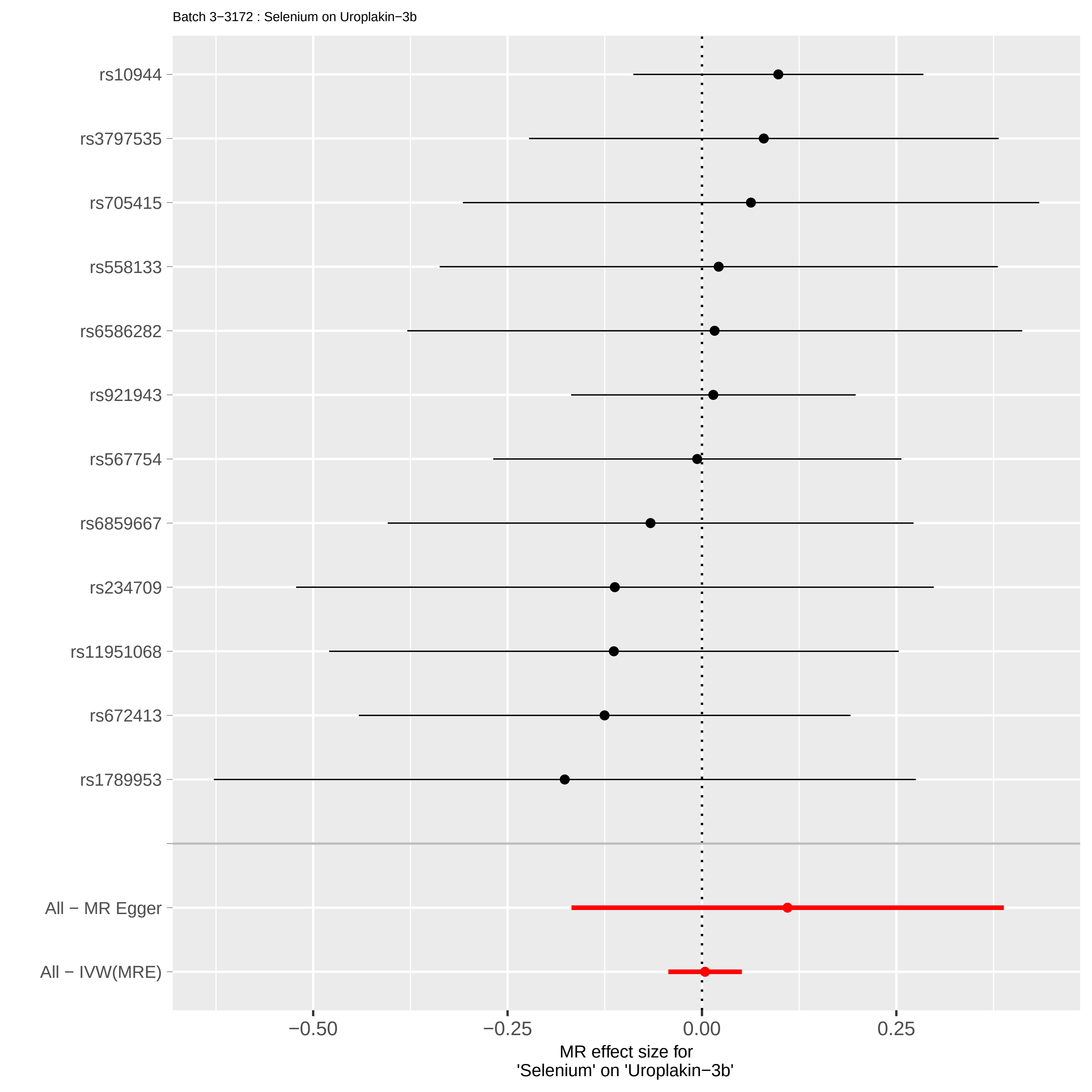

Batch 3−3172 : Selenium on Uroplakin−3b
rs10944
rs3797535
rs705415
rs558133
rs6586282
rs921943
rs567754
rs6859667
rs234709
rs11951068
rs672413
rs1789953
All − MR Egger
All − IVW(MRE)
−0.50
−0.25
0.00
0.25
MR effect size for
'Selenium' on 'Uroplakin−3b'

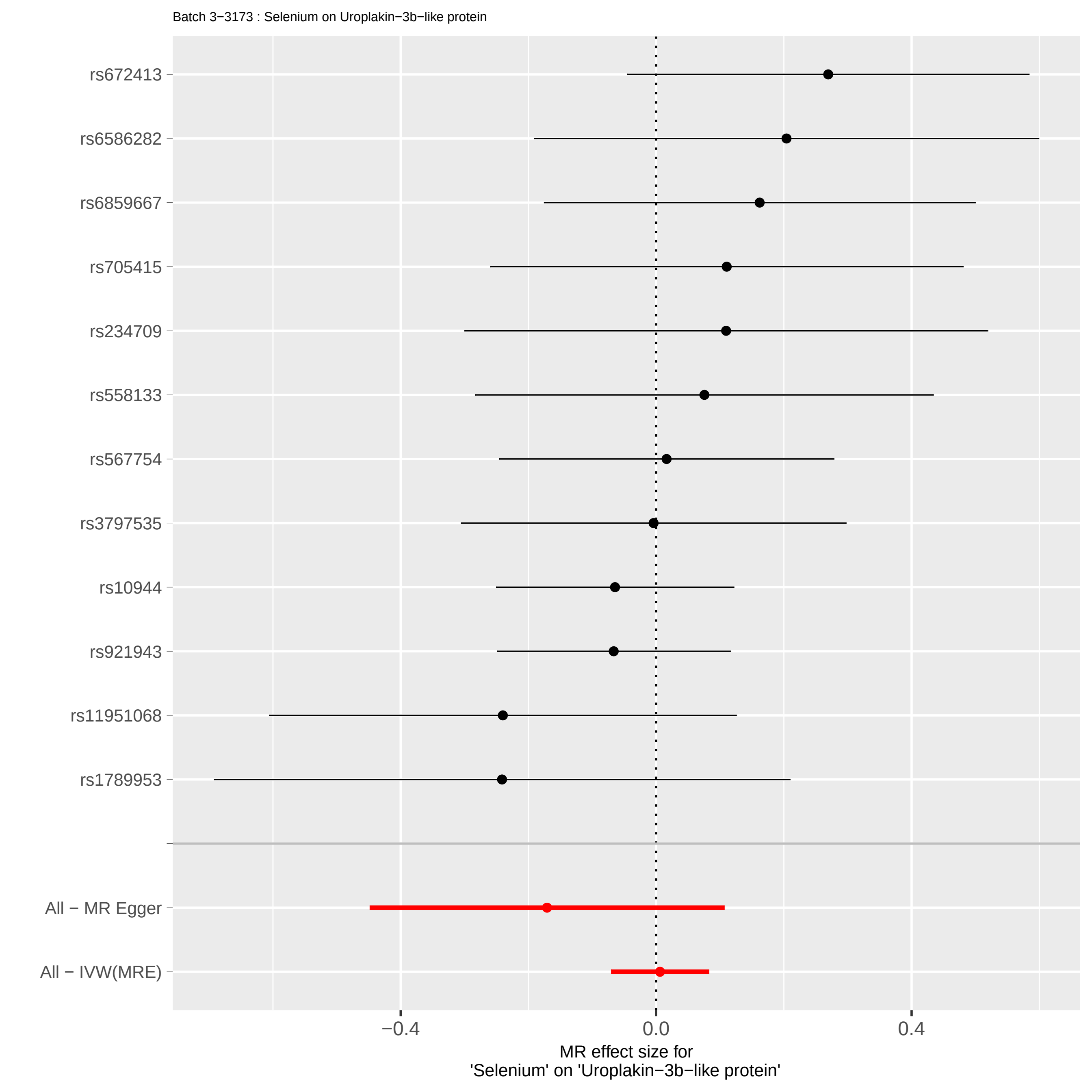

Batch 3−3173 : Selenium on Uroplakin−3b−like protein
rs672413
rs6586282
rs6859667
rs705415
rs234709
rs558133
rs567754
rs3797535
rs10944
rs921943
rs11951068
rs1789953
All − MR Egger
All − IVW(MRE)
−0.4
0.0
0.4
MR effect size for
'Selenium' on 'Uroplakin−3b−like protein'

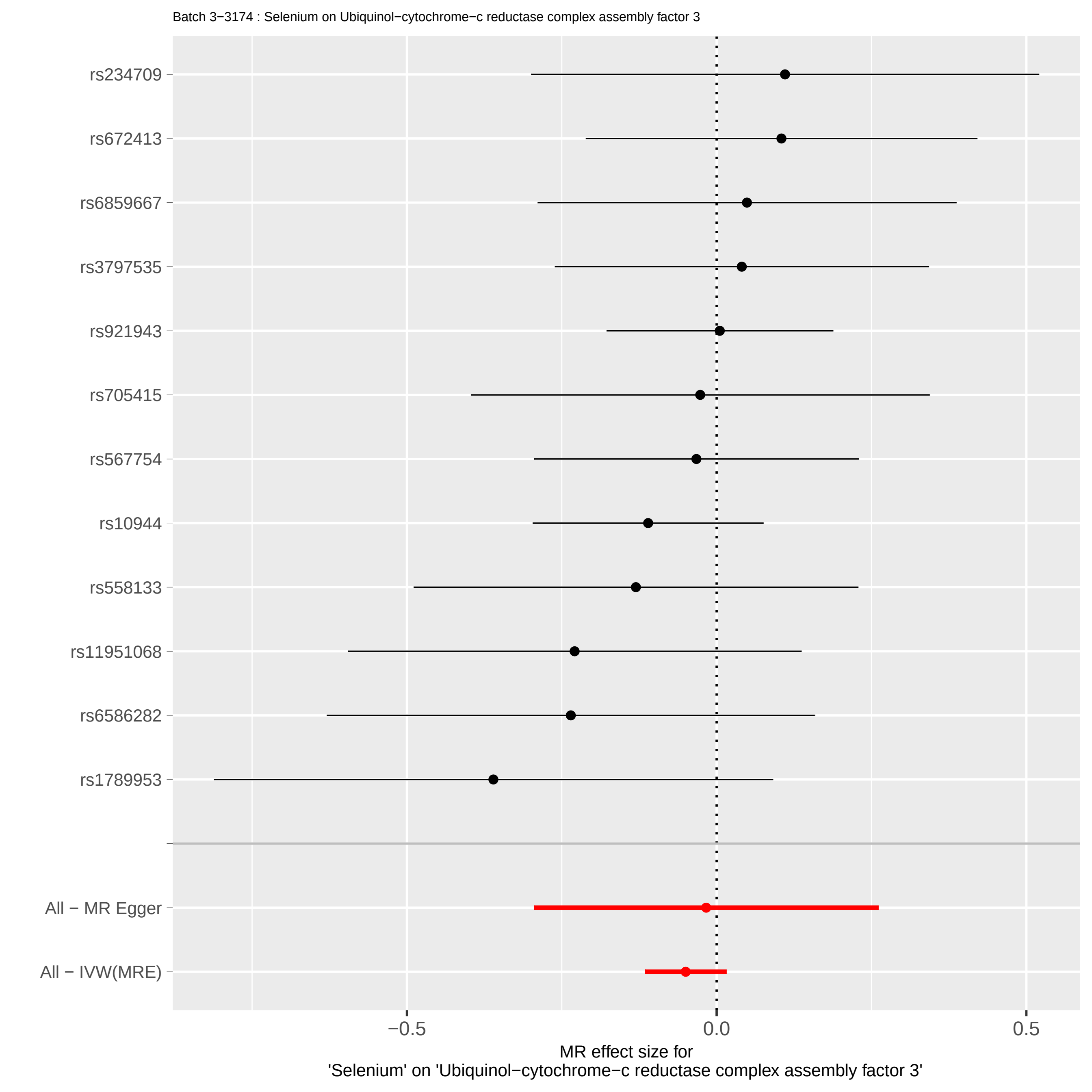

Batch 3−3174 : Selenium on Ubiquinol−cytochrome−c reductase complex assembly factor 3
rs234709
rs672413
rs6859667
rs3797535
rs921943
rs705415
rs567754
rs10944
rs558133
rs11951068
rs6586282
rs1789953
All − MR Egger
All − IVW(MRE)
−0.5
0.0
0.5
MR effect size for
'Selenium' on 'Ubiquinol−cytochrome−c reductase complex assembly factor 3'

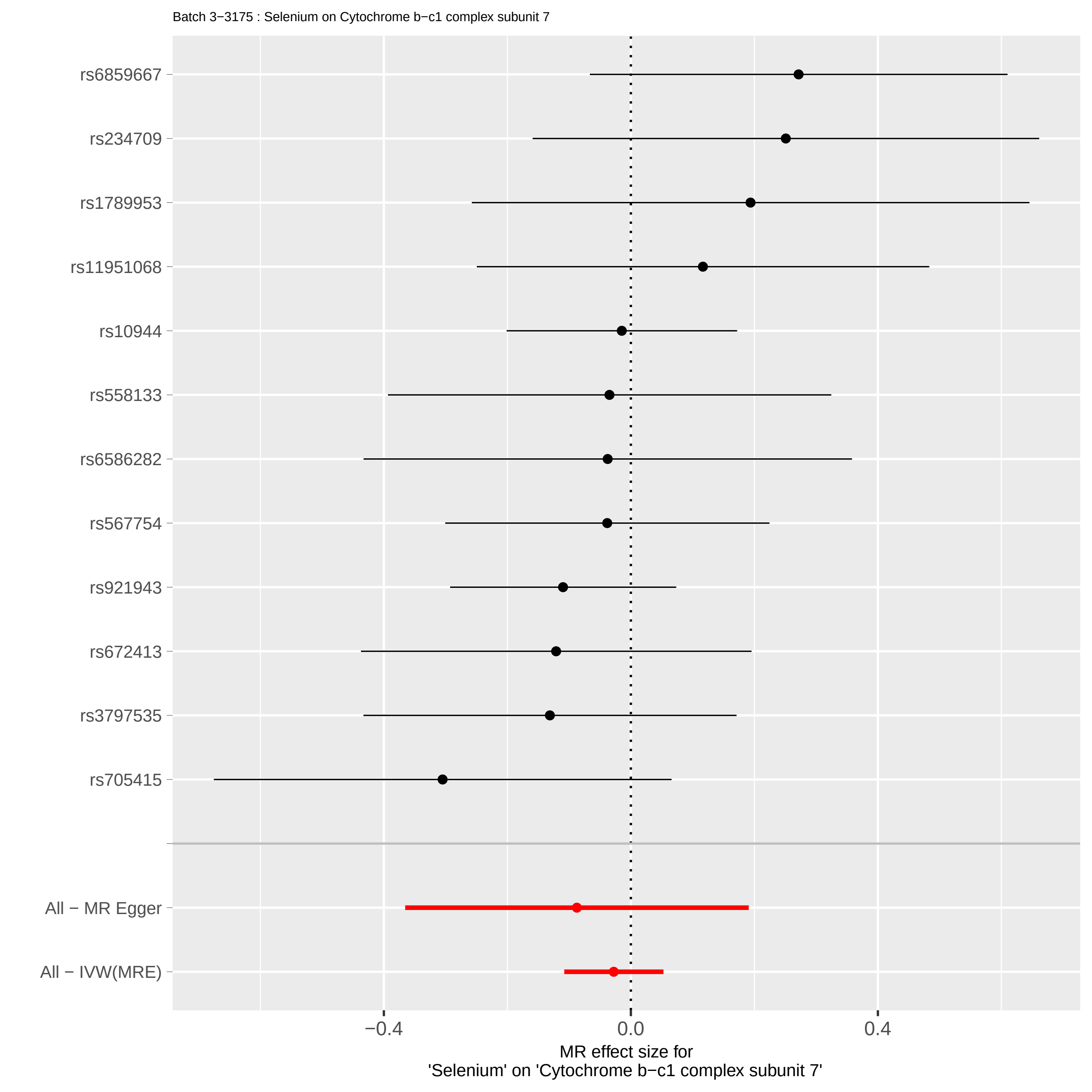

Batch 3−3175 : Selenium on Cytochrome b−c1 complex subunit 7
rs6859667
rs234709
rs1789953
rs11951068
rs10944
rs558133
rs6586282
rs567754
rs921943
rs672413
rs3797535
rs705415
All − MR Egger
All − IVW(MRE)
−0.4
0.0
0.4
MR effect size for
'Selenium' on 'Cytochrome b−c1 complex subunit 7'

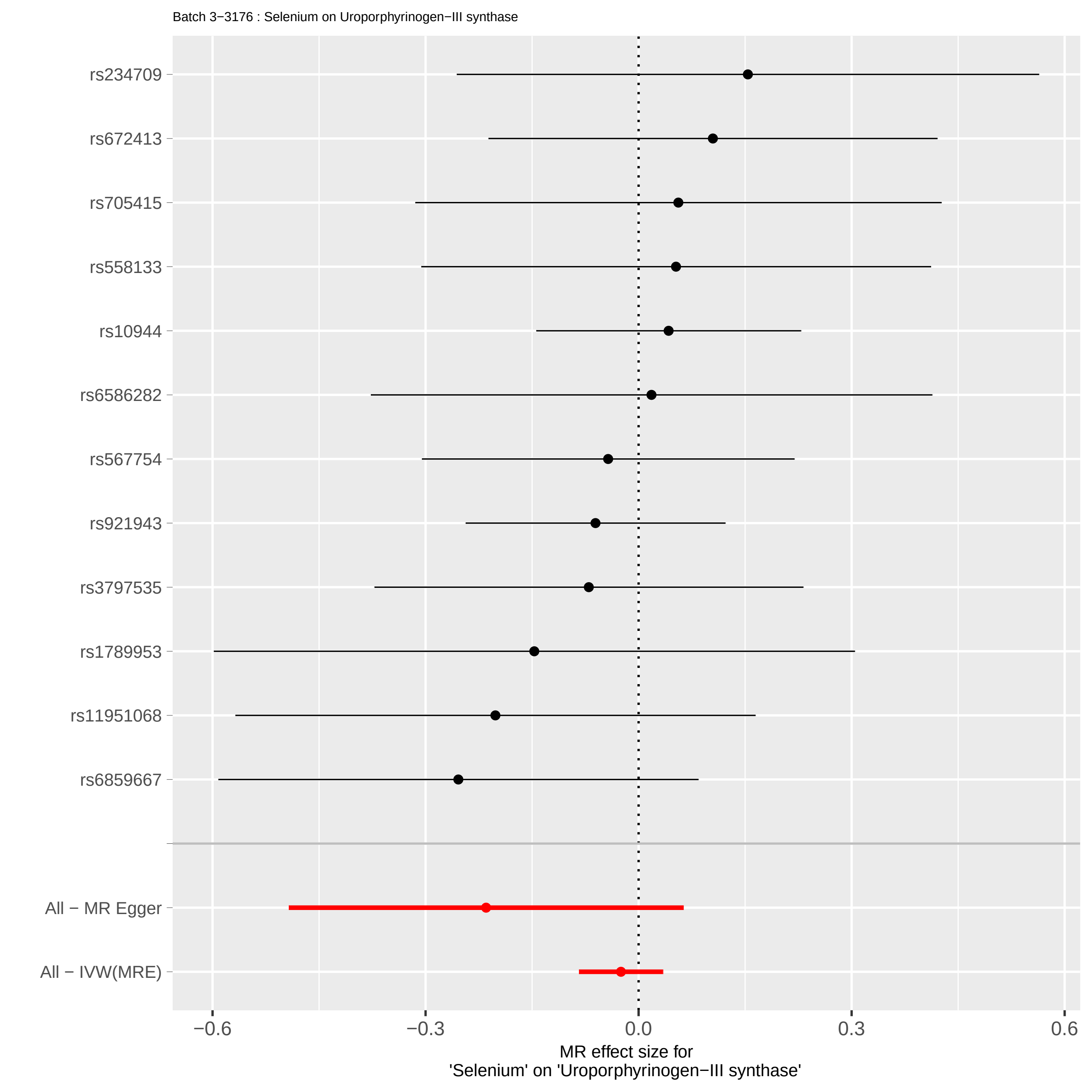

Batch 3−3176 : Selenium on Uroporphyrinogen−III synthase
rs234709
rs672413
rs705415
rs558133
rs10944
rs6586282
rs567754
rs921943
rs3797535
rs1789953
rs11951068
rs6859667
All − MR Egger
All − IVW(MRE)
−0.6
−0.3
0.0
0.3
0.6
MR effect size for
'Selenium' on 'Uroporphyrinogen−III synthase'

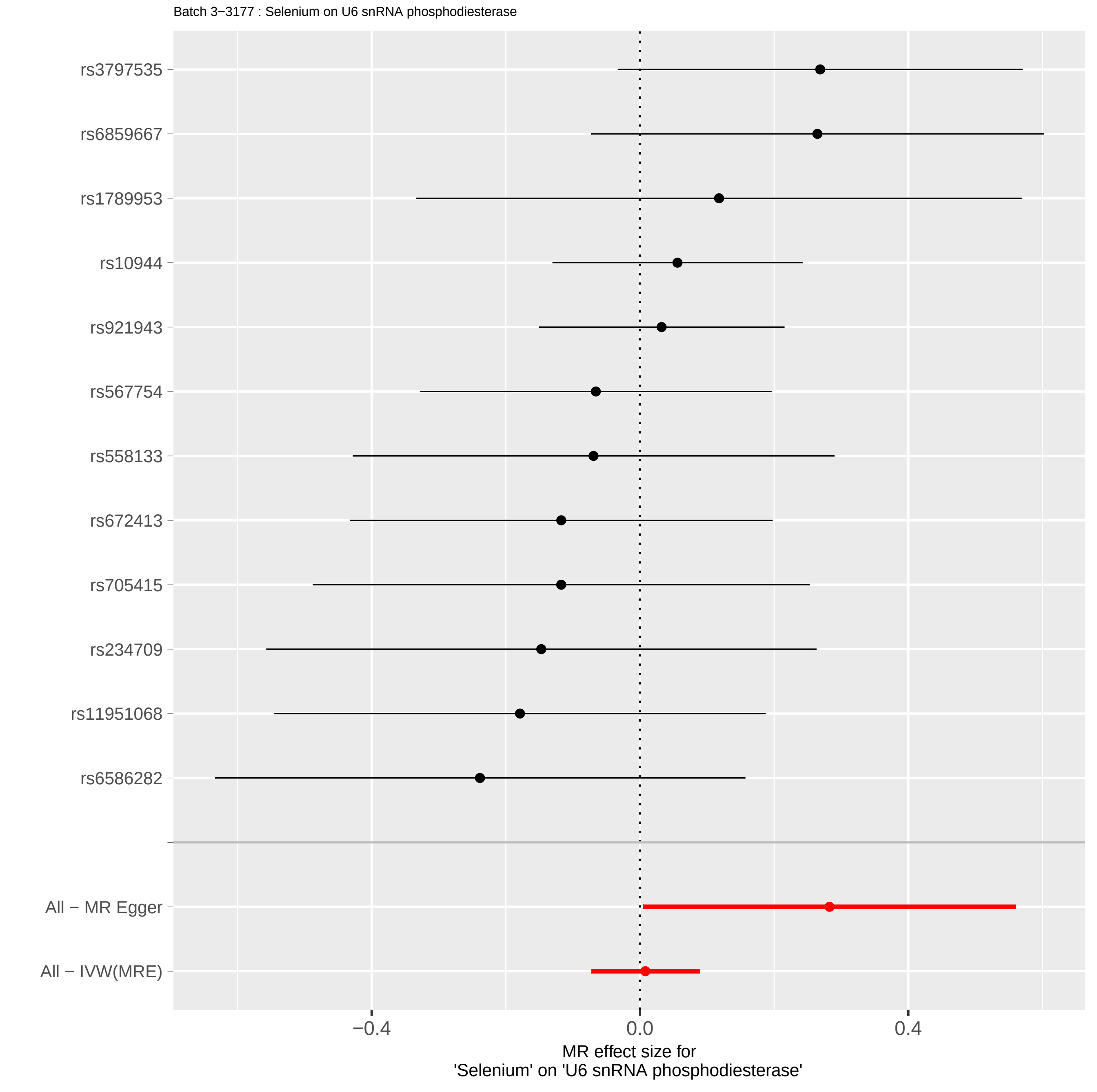

Batch 3−3177 : Selenium on U6 snRNA phosphodiesterase
rs3797535
rs6859667
rs1789953
rs10944
rs921943
rs567754
rs558133
rs672413
rs705415
rs234709
rs11951068
rs6586282
All − MR Egger
All − IVW(MRE)
−0.4
0.0
0.4
MR effect size for
'Selenium' on 'U6 snRNA phosphodiesterase'

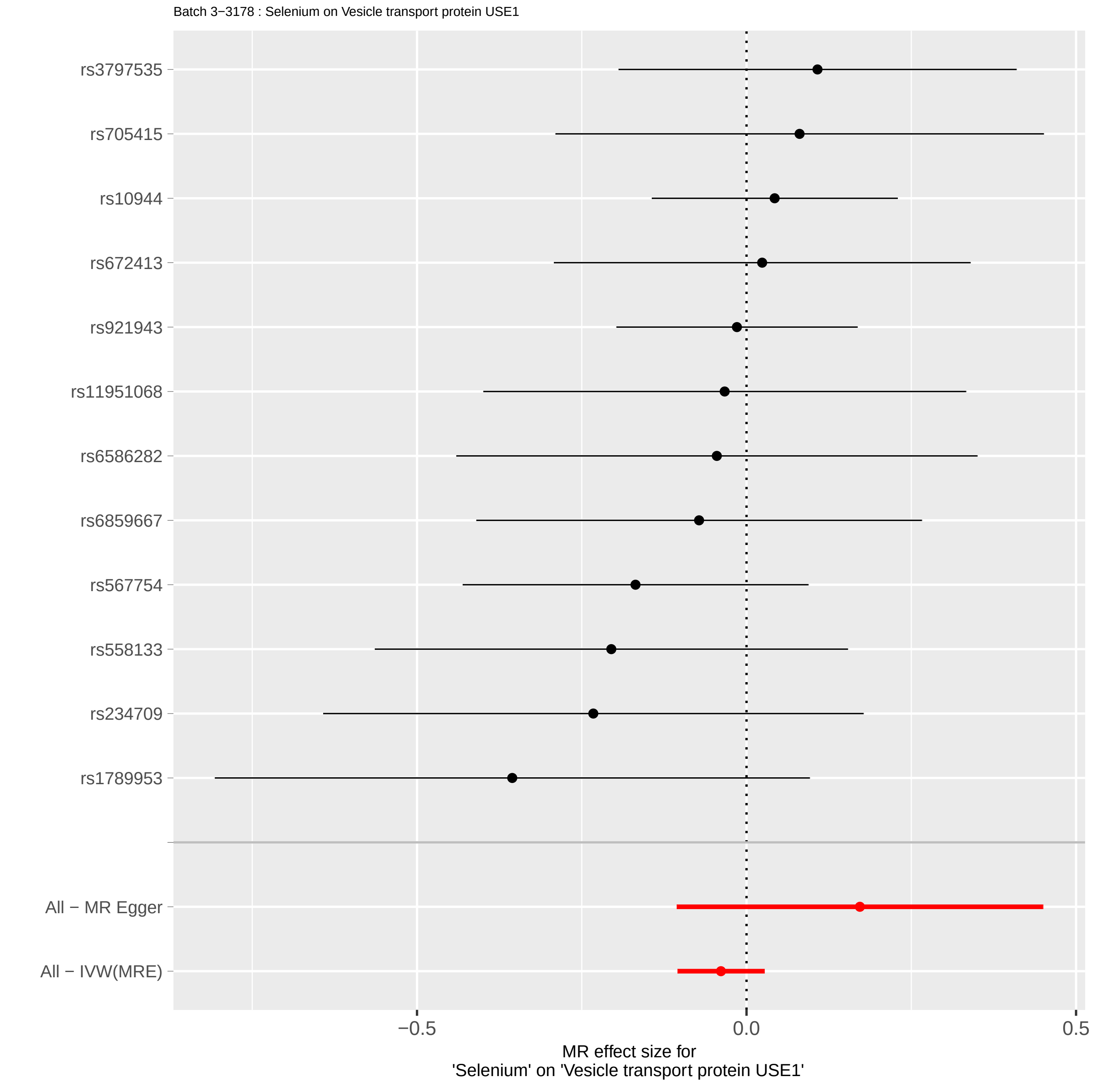

Batch 3−3178 : Selenium on Vesicle transport protein USE1
rs3797535
rs705415
rs10944
rs672413
rs921943
rs11951068
rs6586282
rs6859667
rs567754
rs558133
rs234709
rs1789953
All − MR Egger
All − IVW(MRE)
−0.5
0.0
0.5
MR effect size for
'Selenium' on 'Vesicle transport protein USE1'

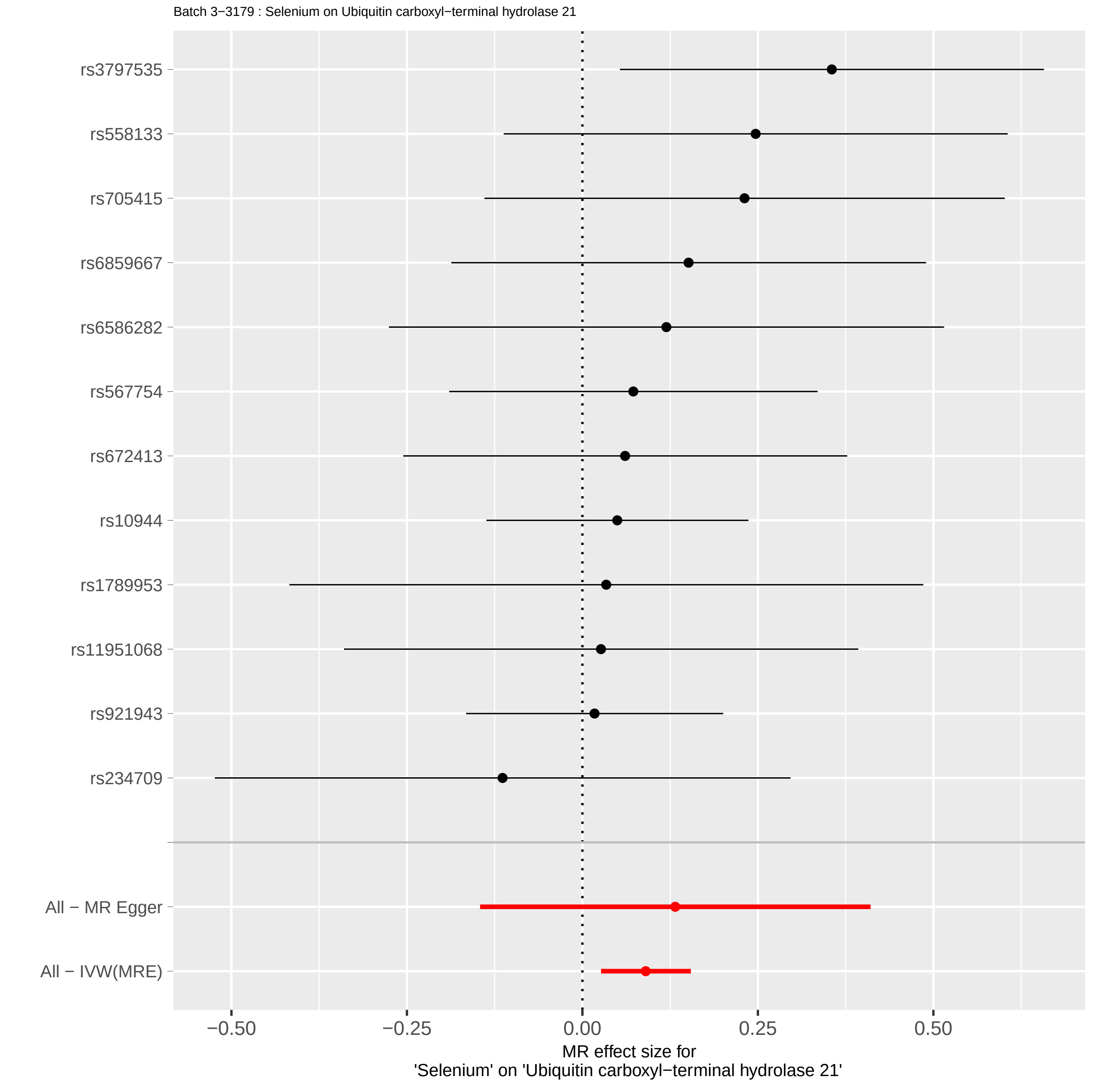
Batch 3−3179 : Selenium on Ubiquitin carboxyl−terminal hydrolase 21
rs3797535
rs558133
rs705415
rs6859667
rs6586282
rs567754
rs672413
rs10944
rs1789953
rs11951068
rs921943
rs234709
All − MR Egger
All − IVW(MRE)
−0.50
−0.25
0.00
0.25
0.50
MR effect size for
'Selenium' on 'Ubiquitin carboxyl−terminal hydrolase 21'

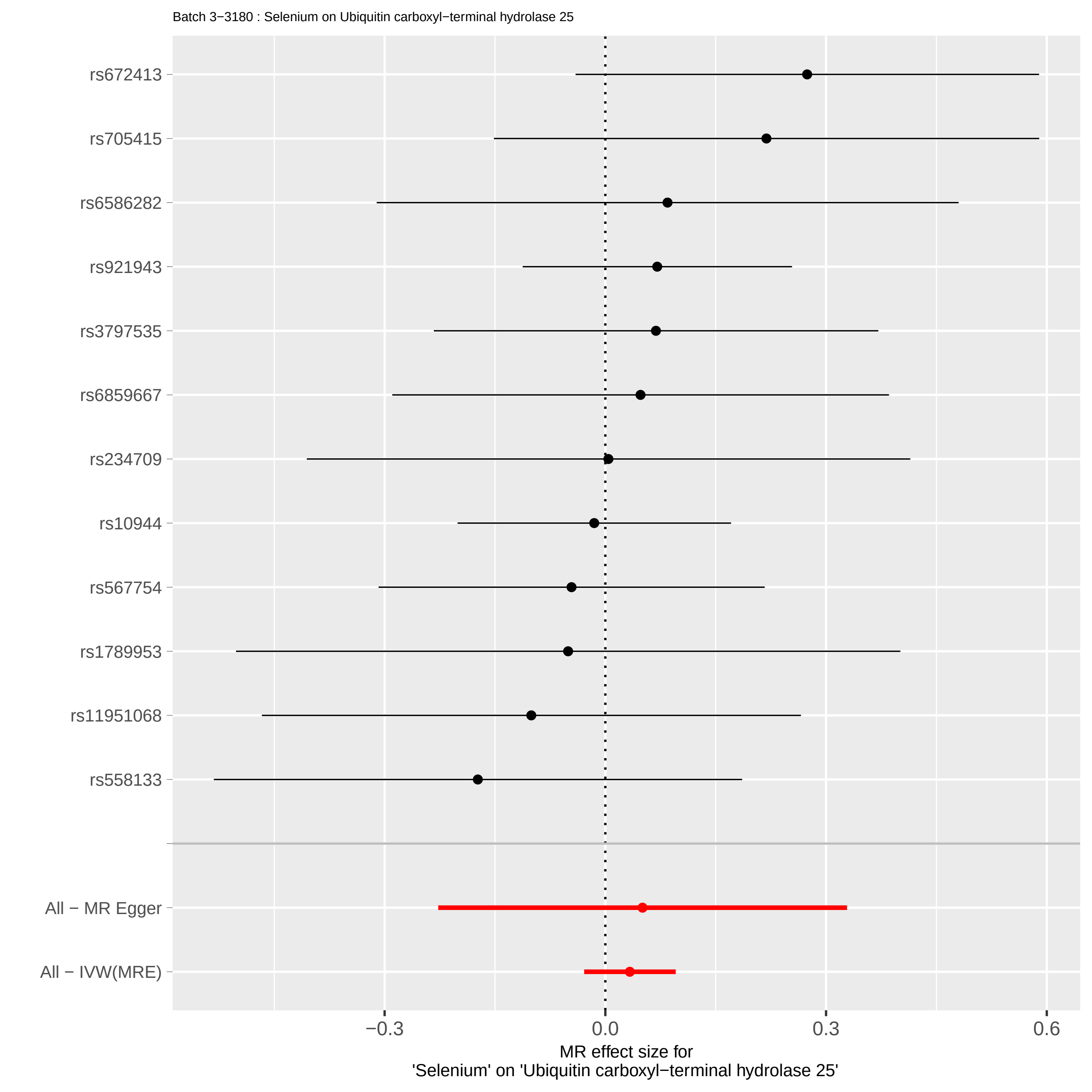

Batch 3−3180 : Selenium on Ubiquitin carboxyl−terminal hydrolase 25
rs672413
rs705415
rs6586282
rs921943
rs3797535
rs6859667
rs234709
rs10944
rs567754
rs1789953
rs11951068
rs558133
All − MR Egger
All − IVW(MRE)
−0.3
0.0
0.3
0.6
MR effect size for
'Selenium' on 'Ubiquitin carboxyl−terminal hydrolase 25'

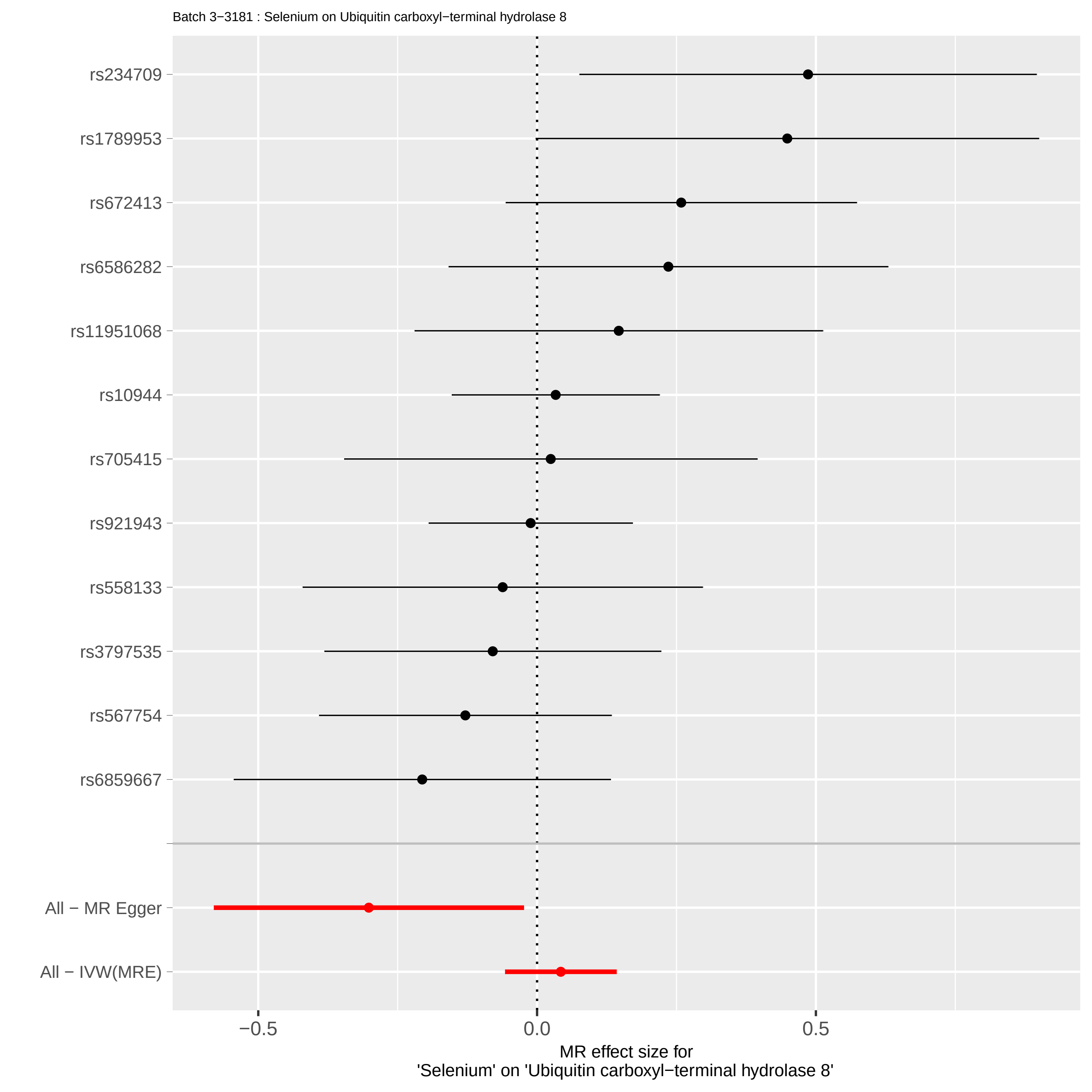

Batch 3−3181 : Selenium on Ubiquitin carboxyl−terminal hydrolase 8
rs234709
rs1789953
rs672413
rs6586282
rs11951068
rs10944
rs705415
rs921943
rs558133
rs3797535
rs567754
rs6859667
All − MR Egger
All − IVW(MRE)
−0.5
0.0
0.5
MR effect size for
'Selenium' on 'Ubiquitin carboxyl−terminal hydrolase 8'

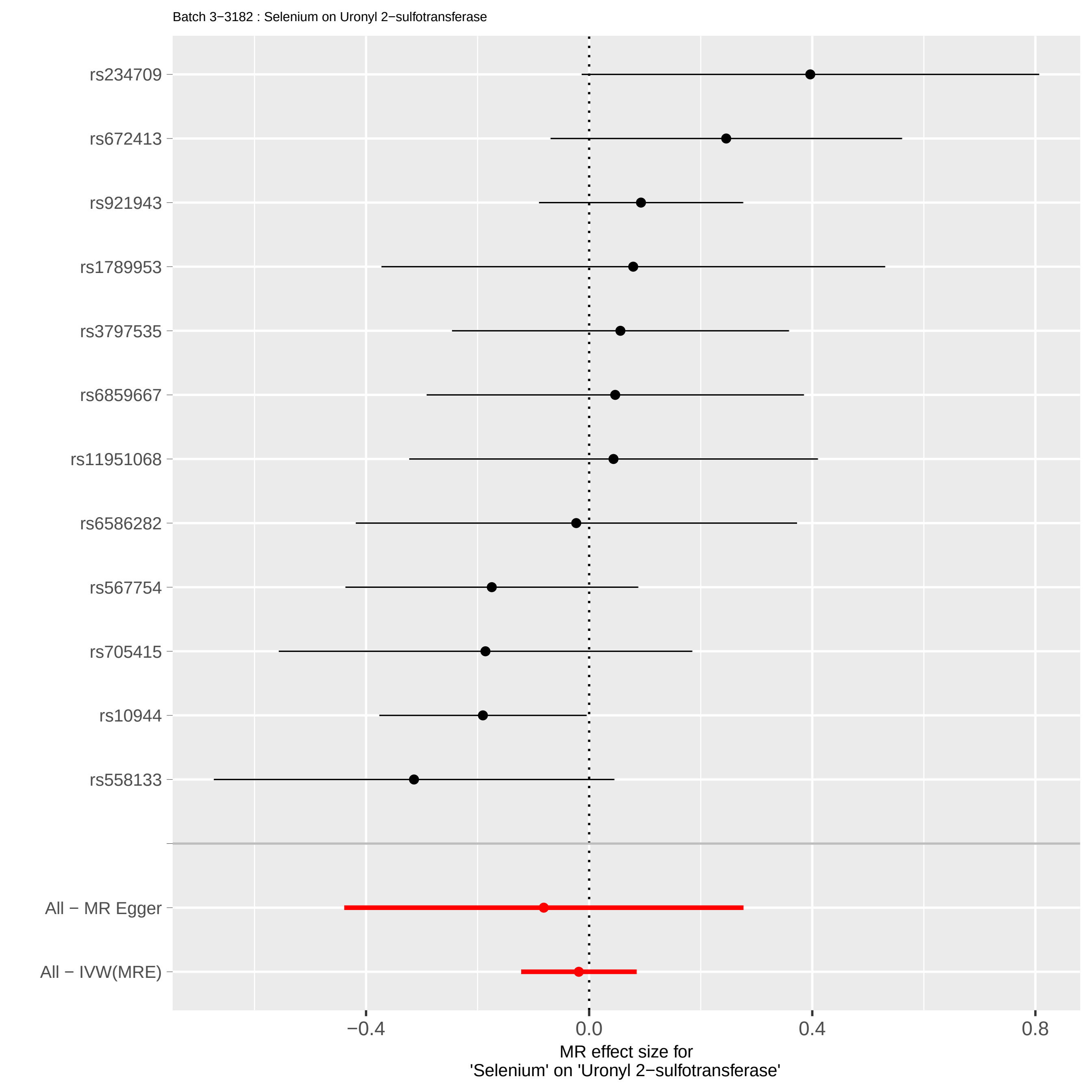

Batch 3−3182 : Selenium on Uronyl 2−sulfotransferase
rs234709
rs672413
rs921943
rs1789953
rs3797535
rs6859667
rs11951068
rs6586282
rs567754
rs705415
rs10944
rs558133
All − MR Egger
All − IVW(MRE)
−0.4
0.0
0.4
0.8
MR effect size for
'Selenium' on 'Uronyl 2−sulfotransferase'

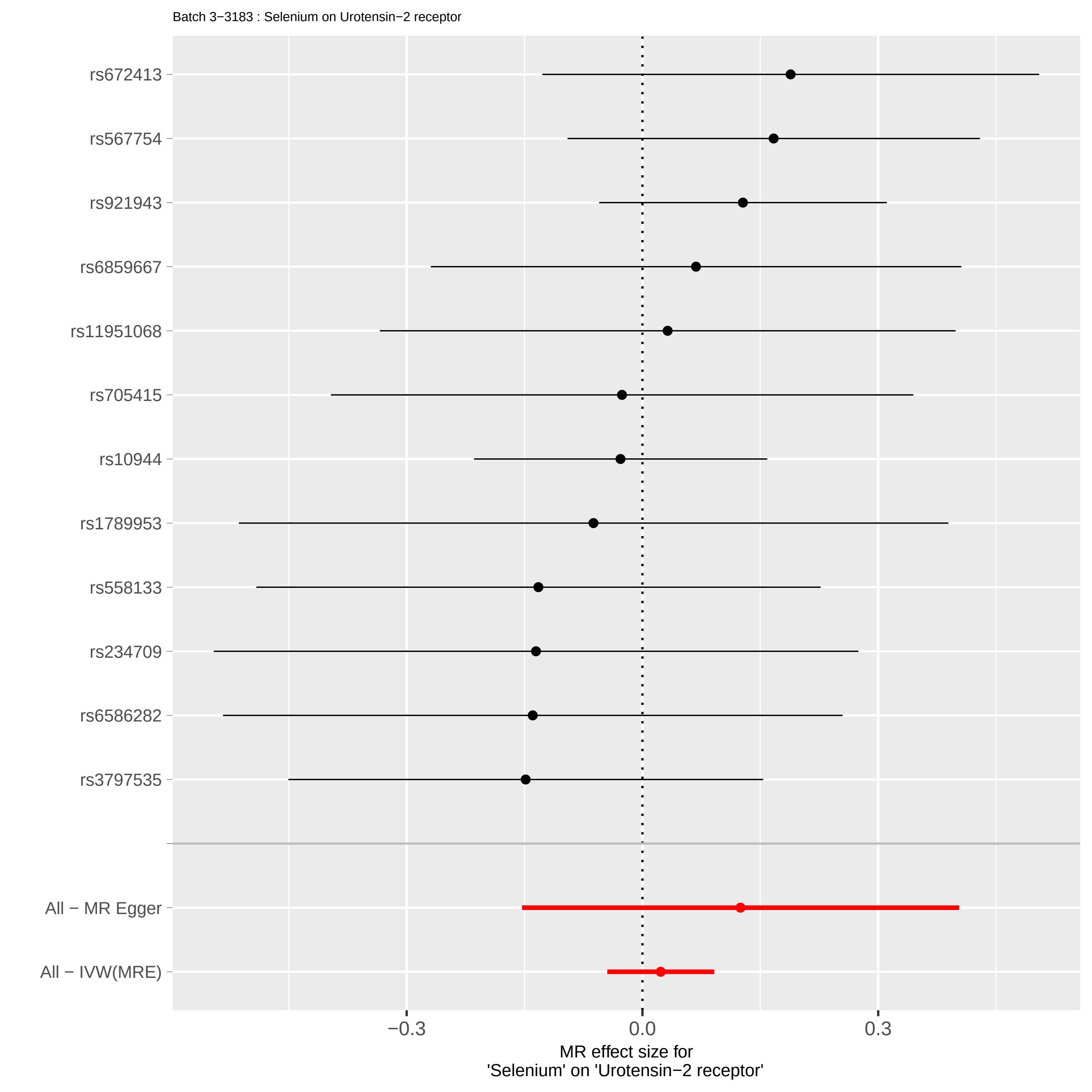

Batch 3−3183 : Selenium on Urotensin−2 receptor
rs672413
rs567754
rs921943
rs6859667
rs11951068
rs705415
rs10944
rs1789953
rs558133
rs234709
rs6586282
rs3797535
All − MR Egger
All − IVW(MRE)
−0.3
0.0
0.3
MR effect size for
'Selenium' on 'Urotensin−2 receptor'

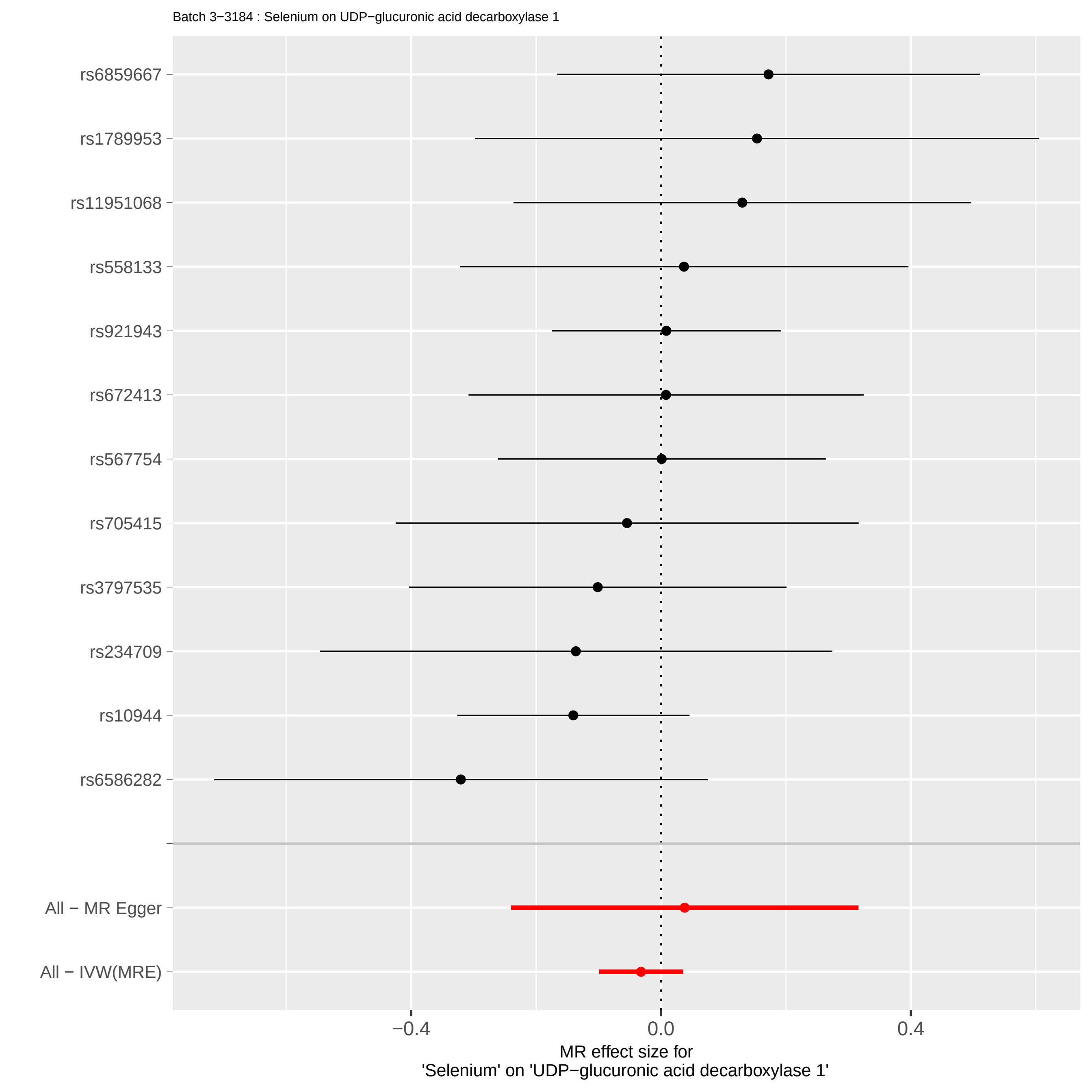
Batch 3−3184 : Selenium on UDP−glucuronic acid decarboxylase 1
rs6859667
rs1789953
rs11951068
rs558133
rs921943
rs672413
rs567754
rs705415
rs3797535
rs234709
rs10944
rs6586282
All − MR Egger
All − IVW(MRE)
−0.4
0.0
0.4
MR effect size for
'Selenium' on 'UDP−glucuronic acid decarboxylase 1'

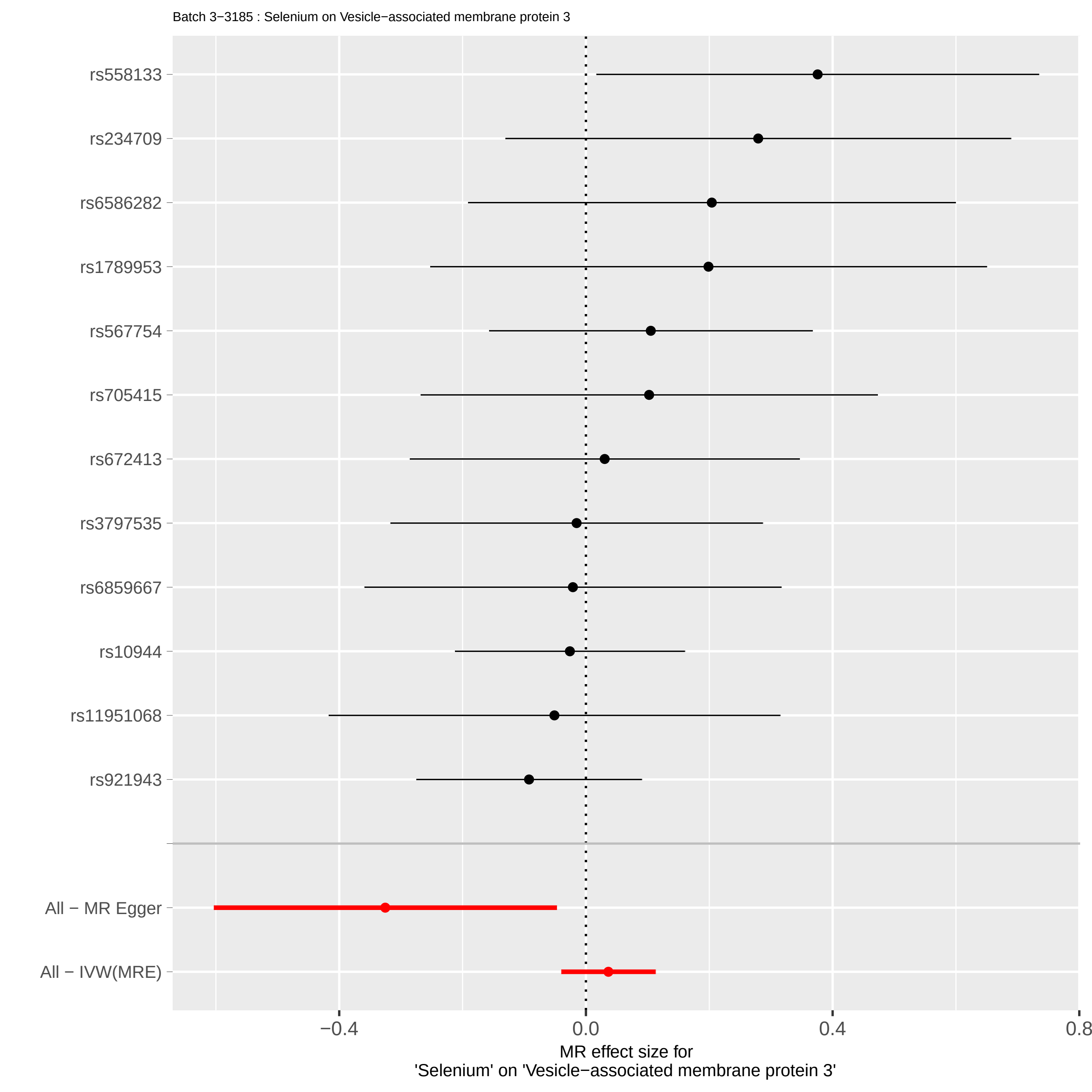

Batch 3−3185 : Selenium on Vesicle−associated membrane protein 3
rs558133
rs234709
rs6586282
rs1789953
rs567754
rs705415
rs672413
rs3797535
rs6859667
rs10944
rs11951068
rs921943
All − MR Egger
All − IVW(MRE)
−0.4
0.0
0.4
0.8
MR effect size for
'Selenium' on 'Vesicle−associated membrane protein 3'

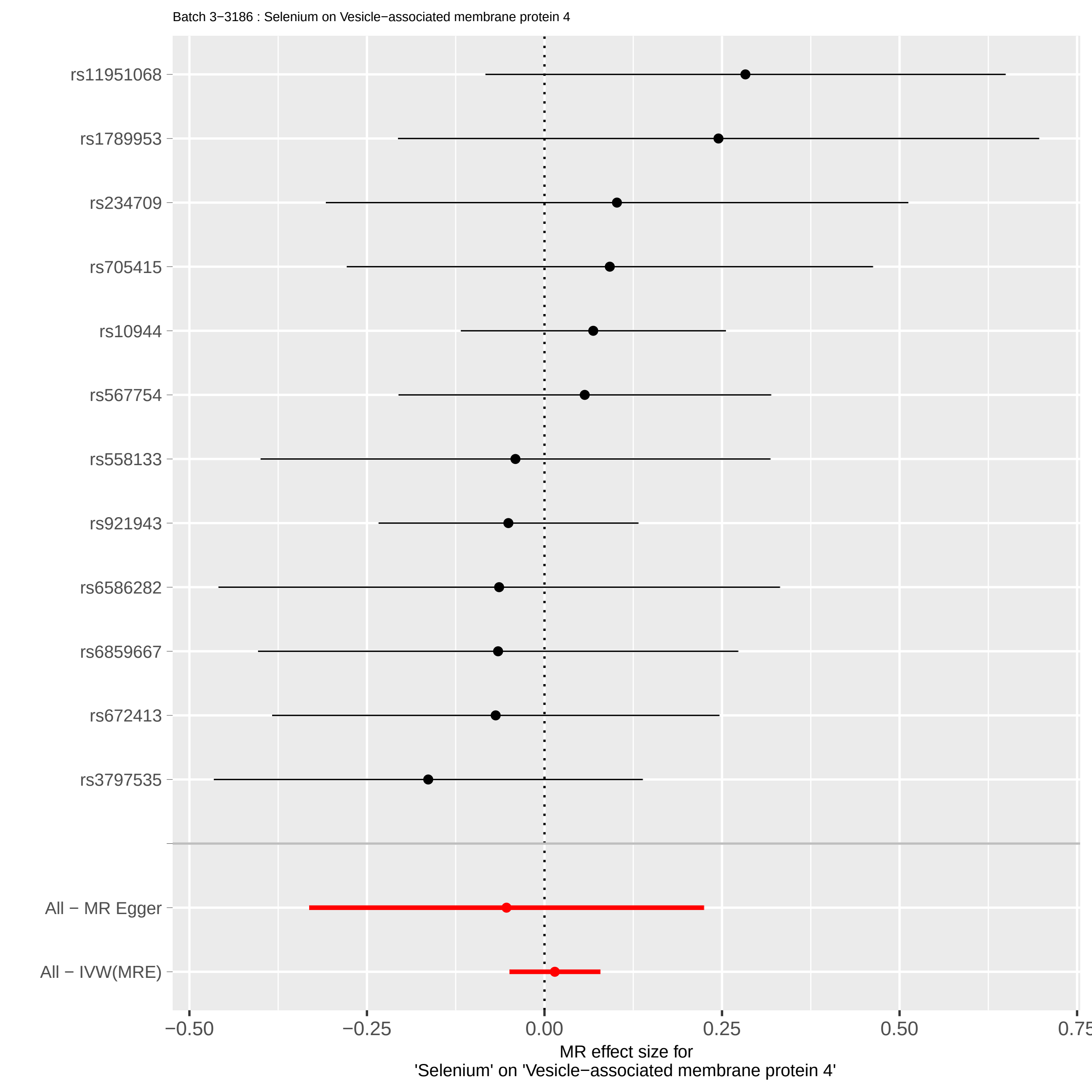

Batch 3−3186 : Selenium on Vesicle−associated membrane protein 4
rs11951068
rs1789953
rs234709
rs705415
rs10944
rs567754
rs558133
rs921943
rs6586282
rs6859667
rs672413
rs3797535
All − MR Egger
All − IVW(MRE)
−0.50
−0.25
0.00
0.25
0.50
0.75
MR effect size for
'Selenium' on 'Vesicle−associated membrane protein 4'

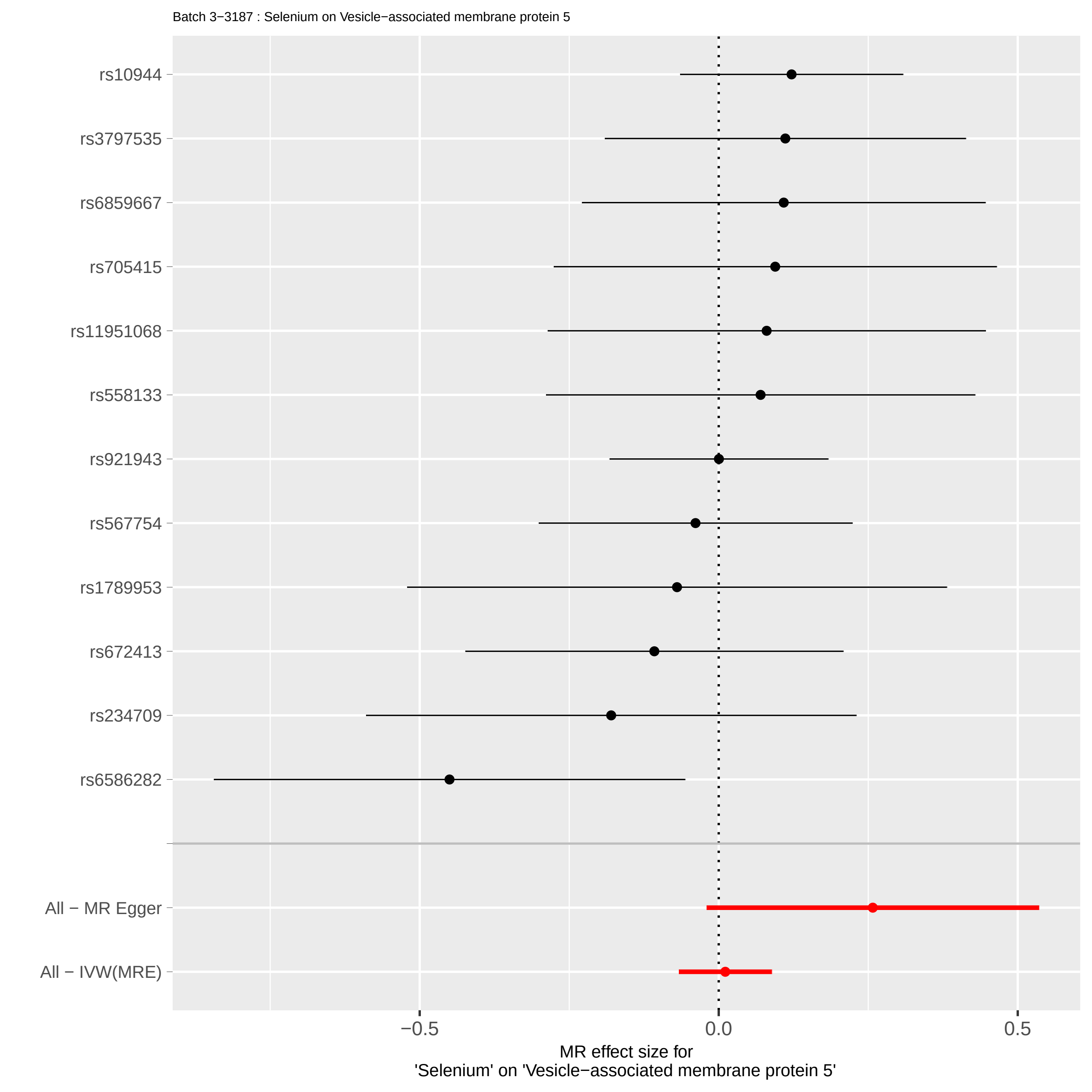

Batch 3−3187 : Selenium on Vesicle−associated membrane protein 5
rs10944
rs3797535
rs6859667
rs705415
rs11951068
rs558133
rs921943
rs567754
rs1789953
rs672413
rs234709
rs6586282
All − MR Egger
All − IVW(MRE)
−0.5
0.0
0.5
MR effect size for
'Selenium' on 'Vesicle−associated membrane protein 5'

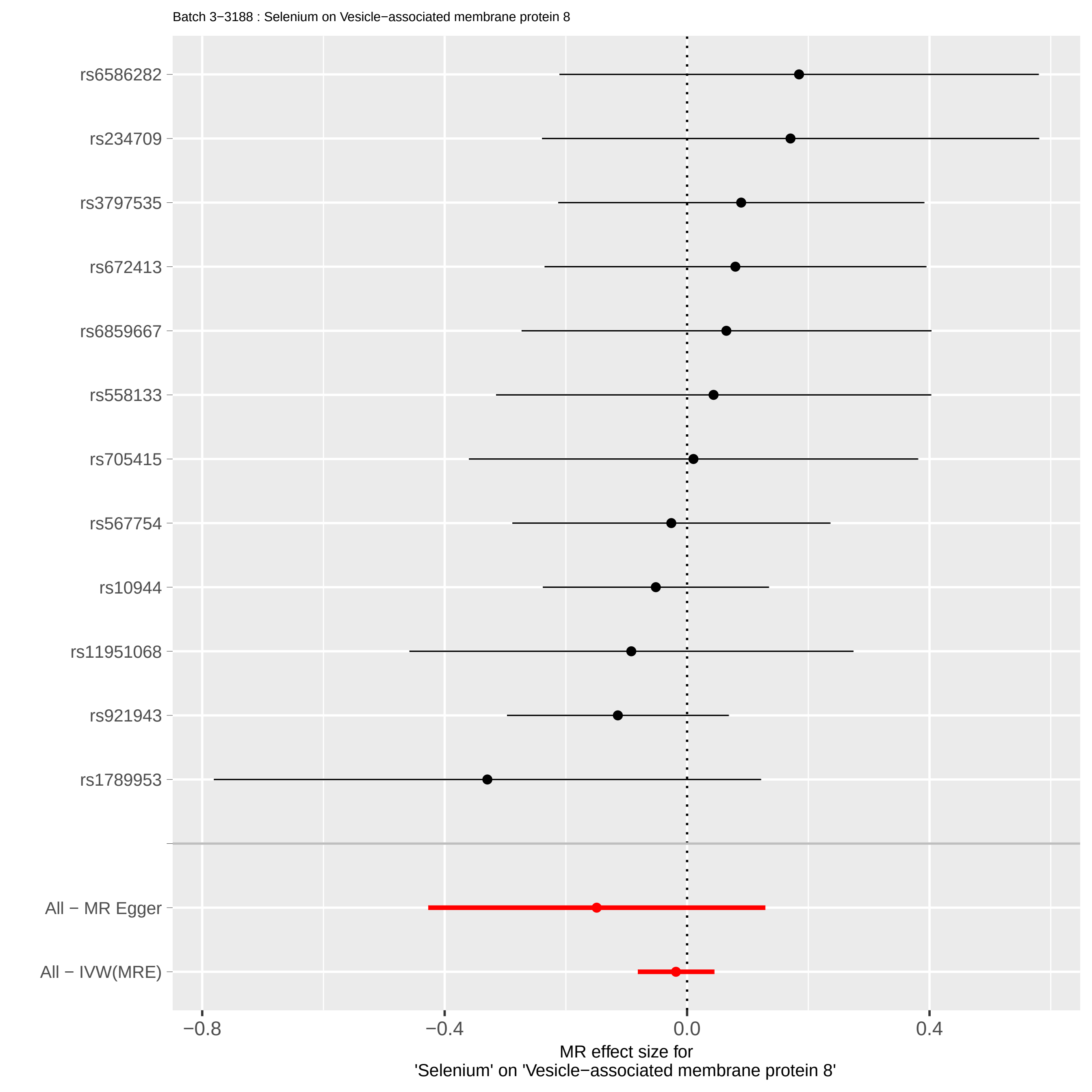

Batch 3−3188 : Selenium on Vesicle−associated membrane protein 8
rs6586282
rs234709
rs3797535
rs672413
rs6859667
rs558133
rs705415
rs567754
rs10944
rs11951068
rs921943
rs1789953
All − MR Egger
All − IVW(MRE)
−0.8
−0.4
0.0
0.4
MR effect size for
'Selenium' on 'Vesicle−associated membrane protein 8'

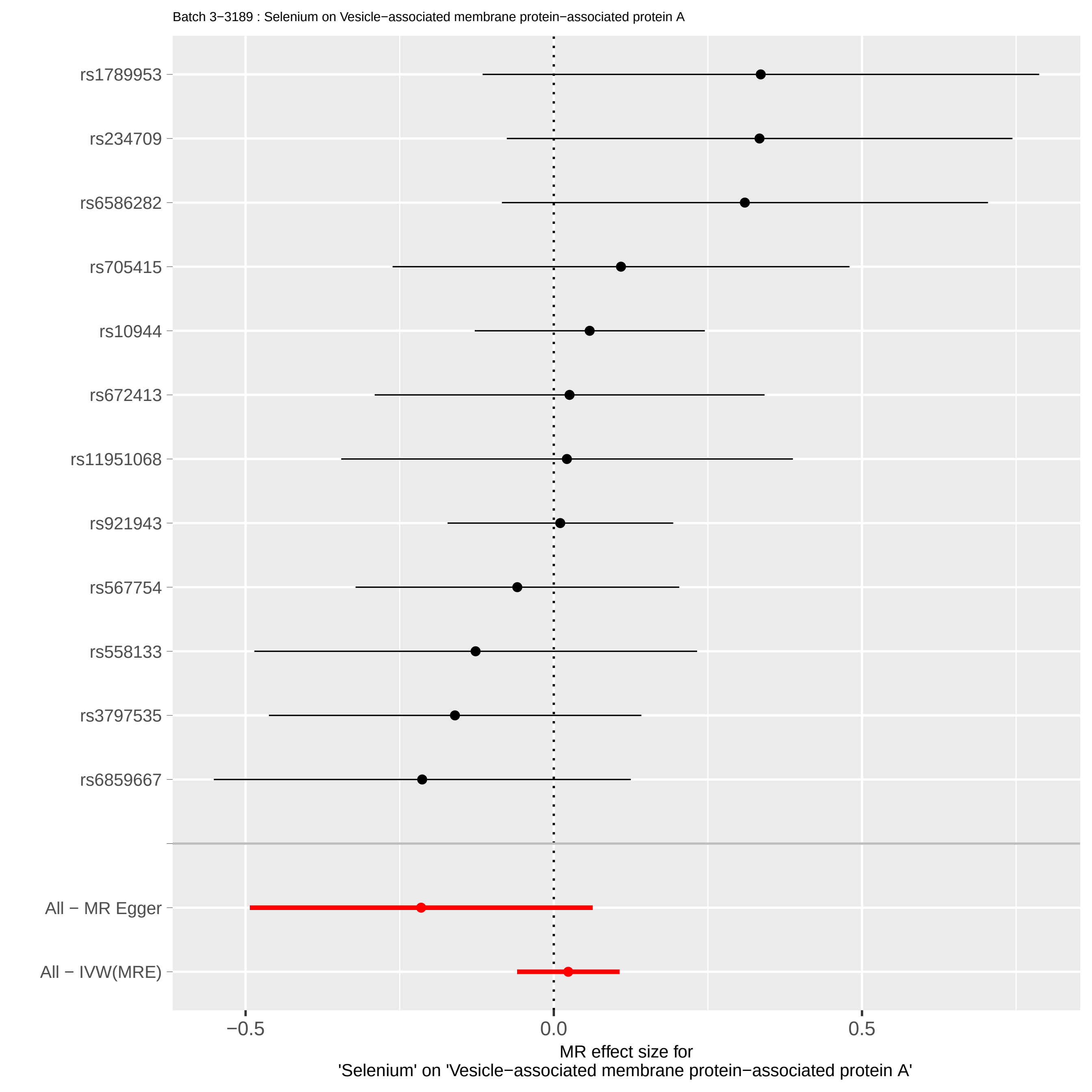

Batch 3−3189 : Selenium on Vesicle−associated membrane protein−associated protein A
rs1789953
rs234709
rs6586282
rs705415
rs10944
rs672413
rs11951068
rs921943
rs567754
rs558133
rs3797535
rs6859667
All − MR Egger
All − IVW(MRE)
−0.5
0.0
0.5
MR effect size for
'Selenium' on 'Vesicle−associated membrane protein−associated protein A'

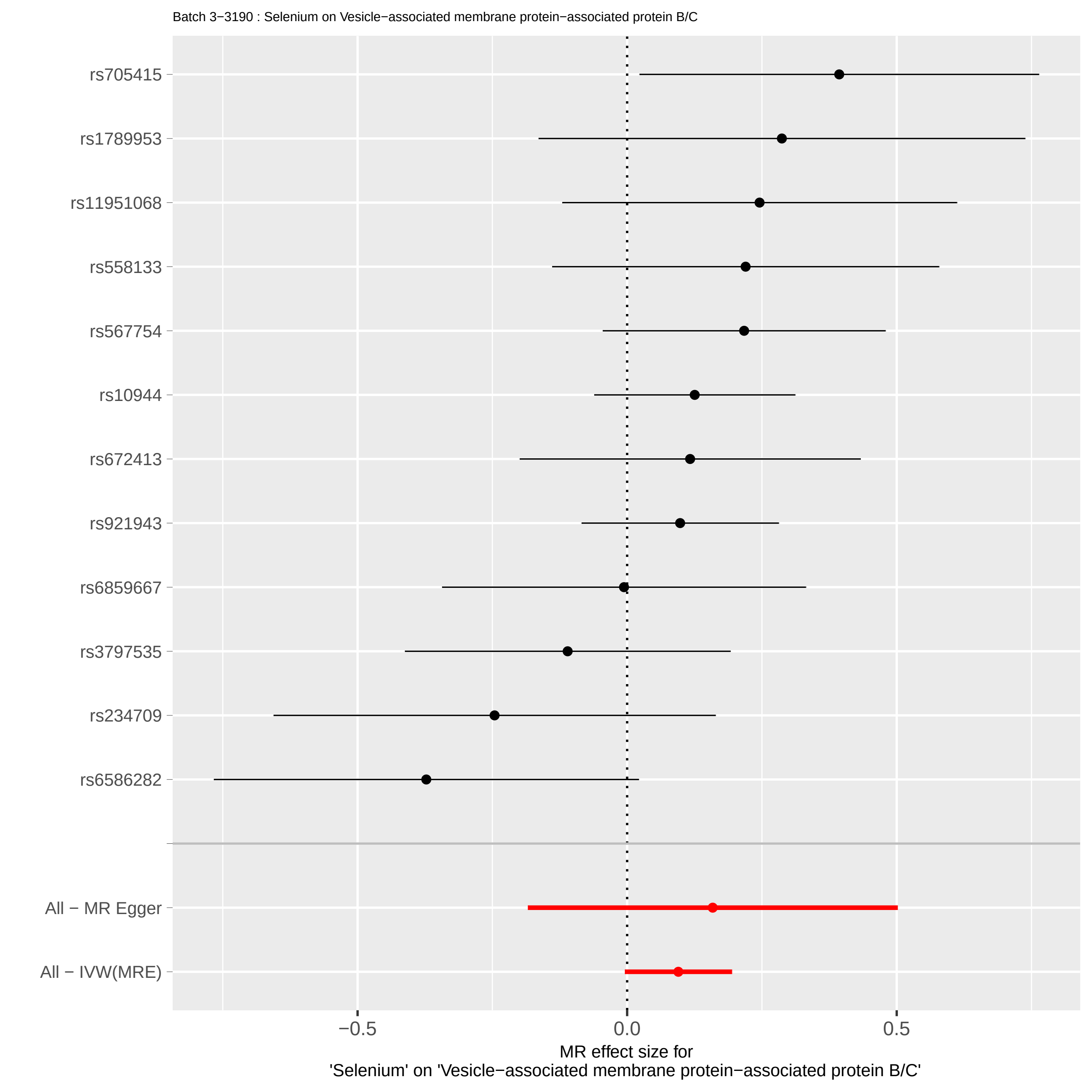

Batch 3−3190 : Selenium on Vesicle−associated membrane protein−associated protein B/C
rs705415
rs1789953
rs11951068
rs558133
rs567754
rs10944
rs672413
rs921943
rs6859667
rs3797535
rs234709
rs6586282
All − MR Egger
All − IVW(MRE)
−0.5
0.0
0.5
MR effect size for
'Selenium' on 'Vesicle−associated membrane protein−associated protein B/C'

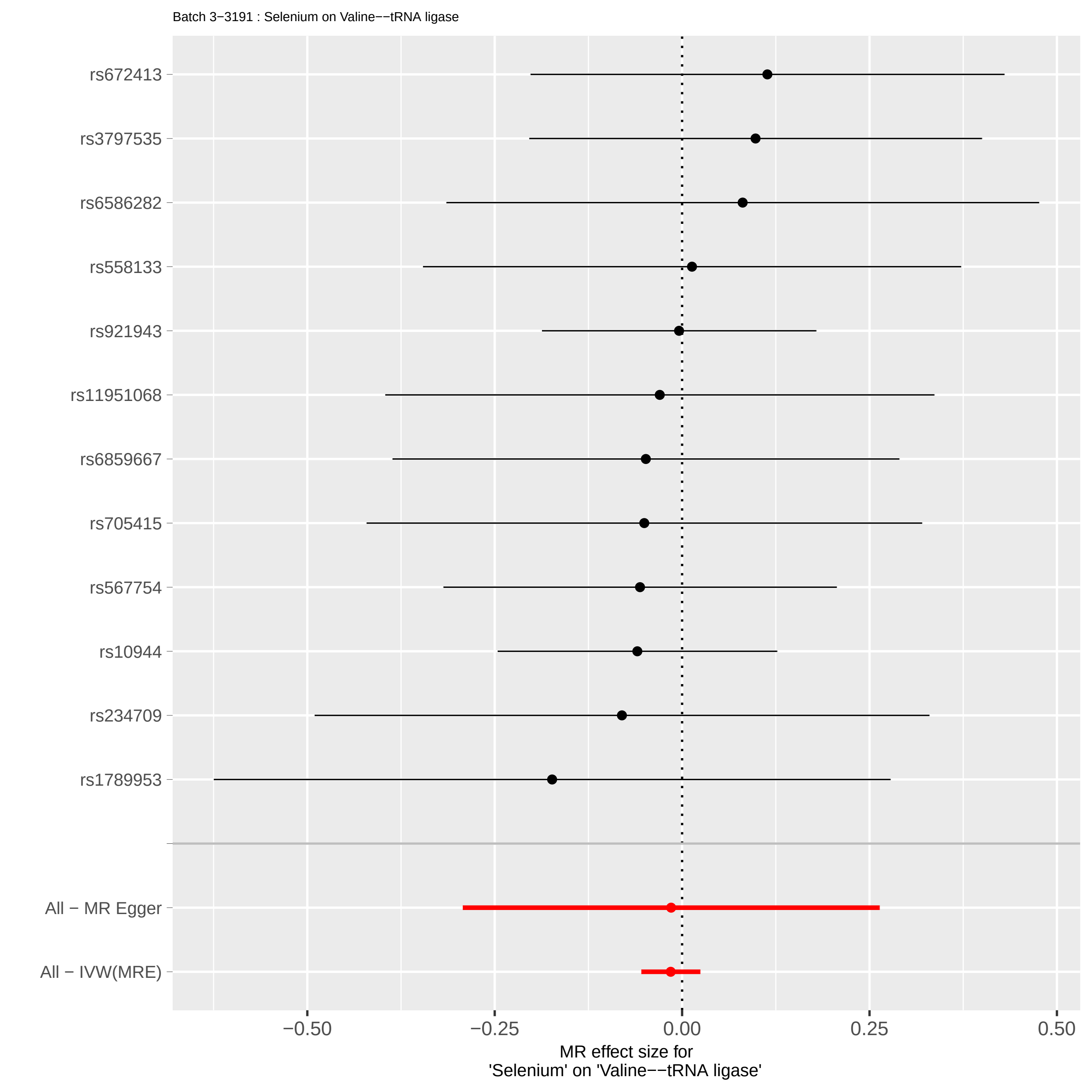
Batch 3−3191 : Selenium on Valine−−tRNA ligase
rs672413
rs3797535
rs6586282
rs558133
rs921943
rs11951068
rs6859667
rs705415
rs567754
rs10944
rs234709
rs1789953
All − MR Egger
All − IVW(MRE)
−0.50
−0.25
0.00
0.25
0.50
MR effect size for
'Selenium' on 'Valine−−tRNA ligase'

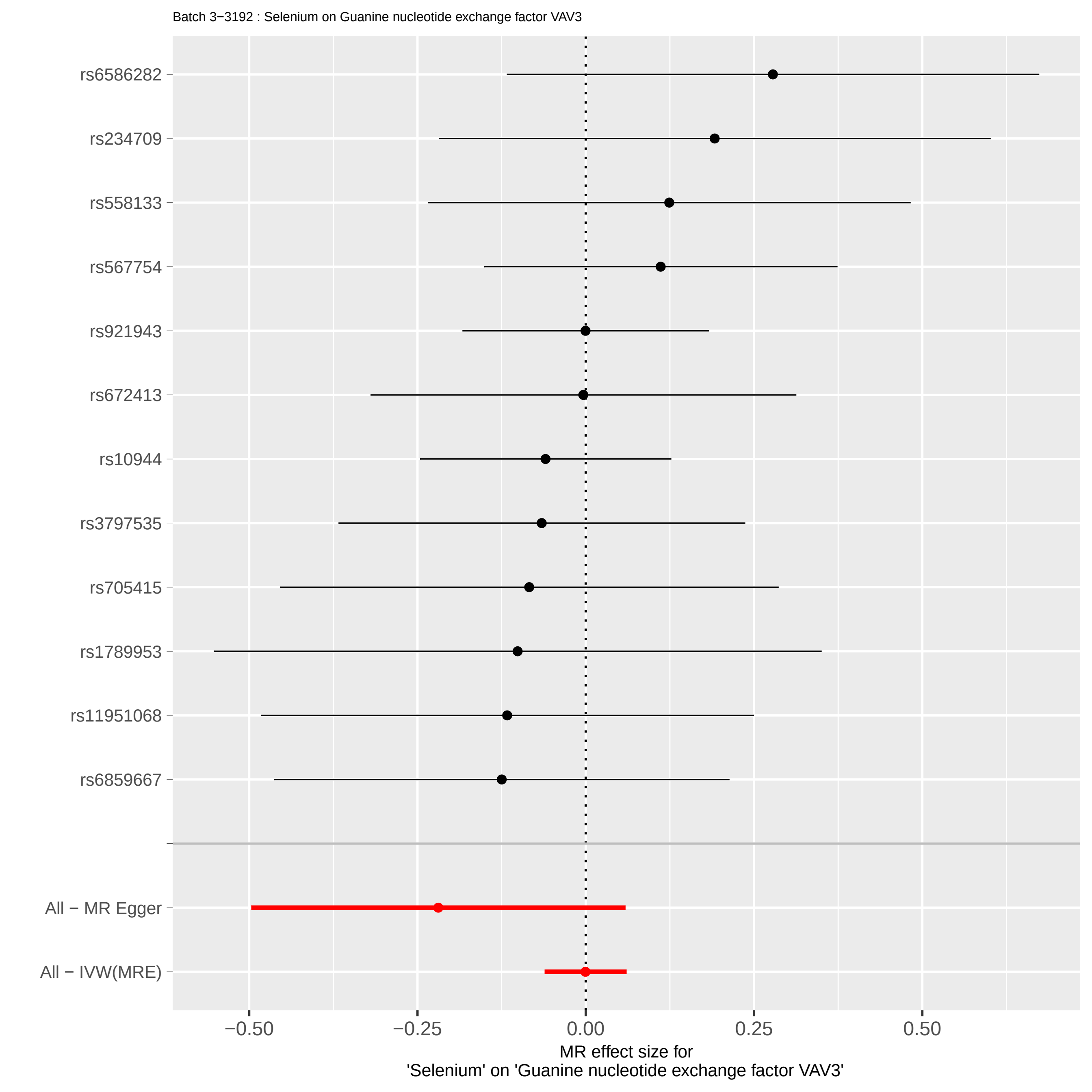

Batch 3−3192 : Selenium on Guanine nucleotide exchange factor VAV3
rs6586282
rs234709
rs558133
rs567754
rs921943
rs672413
rs10944
rs3797535
rs705415
rs1789953
rs11951068
rs6859667
All − MR Egger
All − IVW(MRE)
−0.50
−0.25
0.00
0.25
0.50
MR effect size for
'Selenium' on 'Guanine nucleotide exchange factor VAV3'

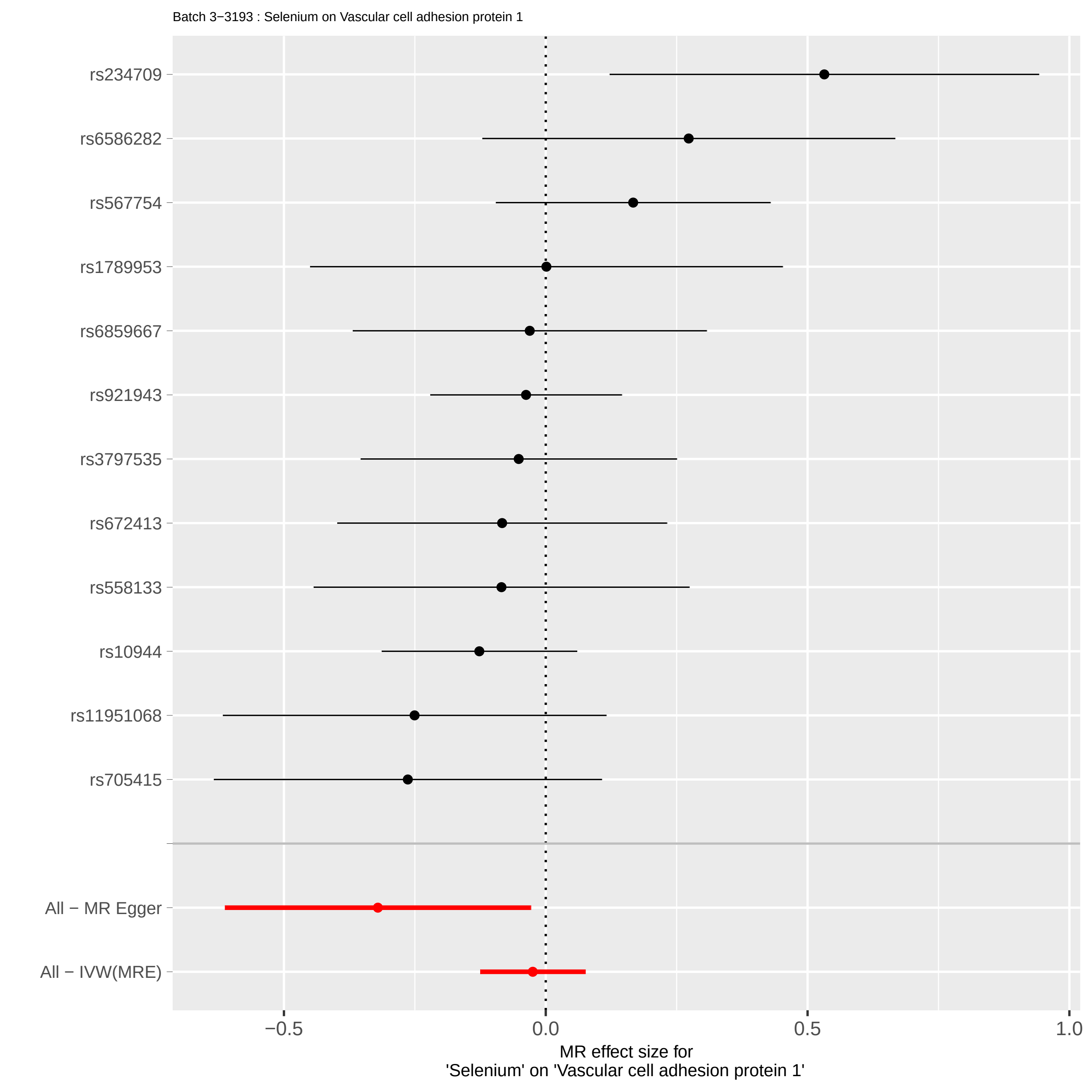

Batch 3−3193 : Selenium on Vascular cell adhesion protein 1
rs234709
rs6586282
rs567754
rs1789953
rs6859667
rs921943
rs3797535
rs672413
rs558133
rs10944
rs11951068
rs705415
All − MR Egger
All − IVW(MRE)
−0.5
0.0
0.5
1.0
MR effect size for
'Selenium' on 'Vascular cell adhesion protein 1'

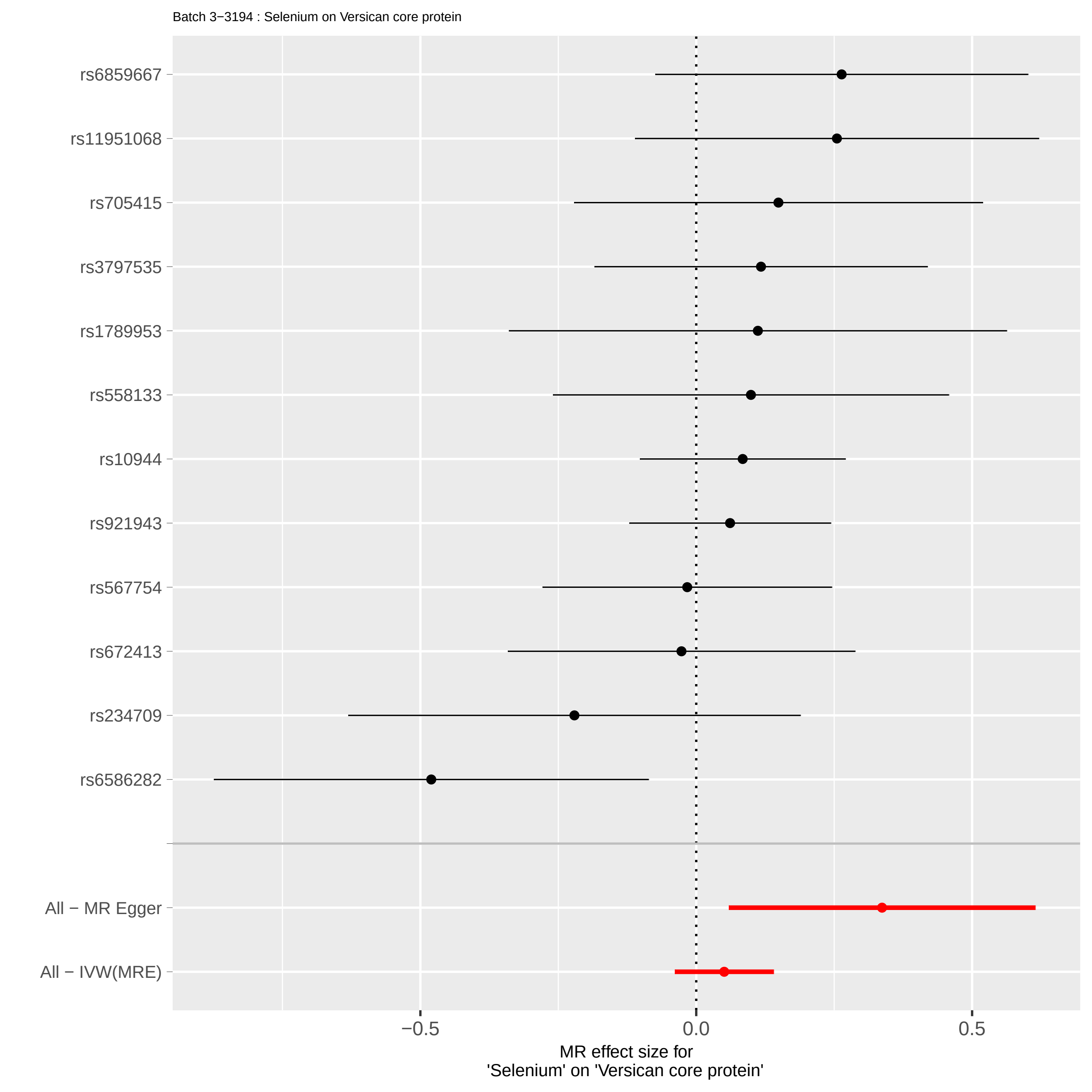

Batch 3−3194 : Selenium on Versican core protein
rs6859667
rs11951068
rs705415
rs3797535
rs1789953
rs558133
rs10944
rs921943
rs567754
rs672413
rs234709
rs6586282
All − MR Egger
All − IVW(MRE)
−0.5
0.0
0.5
MR effect size for
'Selenium' on 'Versican core protein'

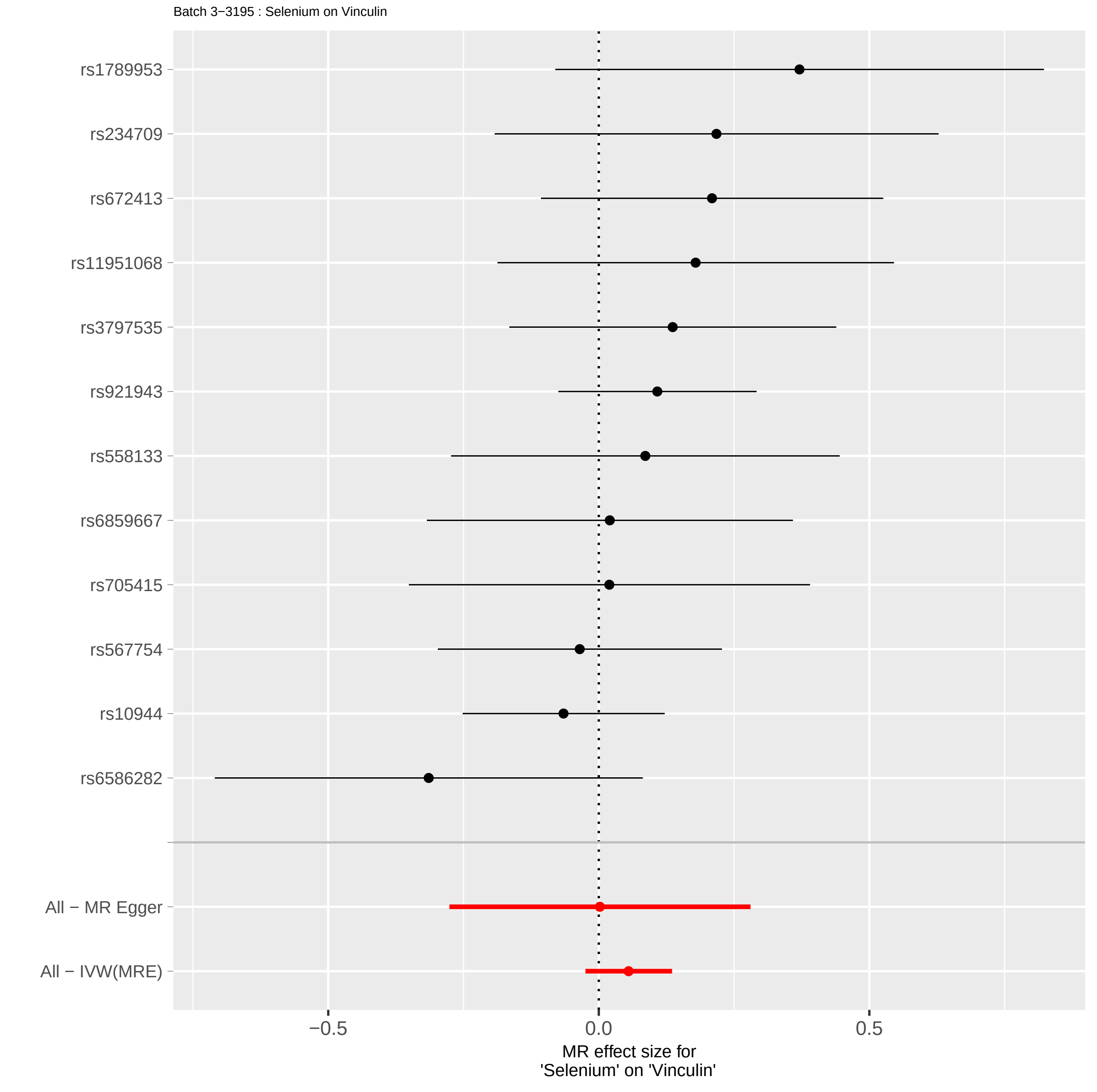

Batch 3−3195 : Selenium on Vinculin
rs1789953
rs234709
rs672413
rs11951068
rs3797535
rs921943
rs558133
rs6859667
rs705415
rs567754
rs10944
rs6586282
All − MR Egger
All − IVW(MRE)
−0.5
0.0
0.5
MR effect size for
'Selenium' on 'Vinculin'

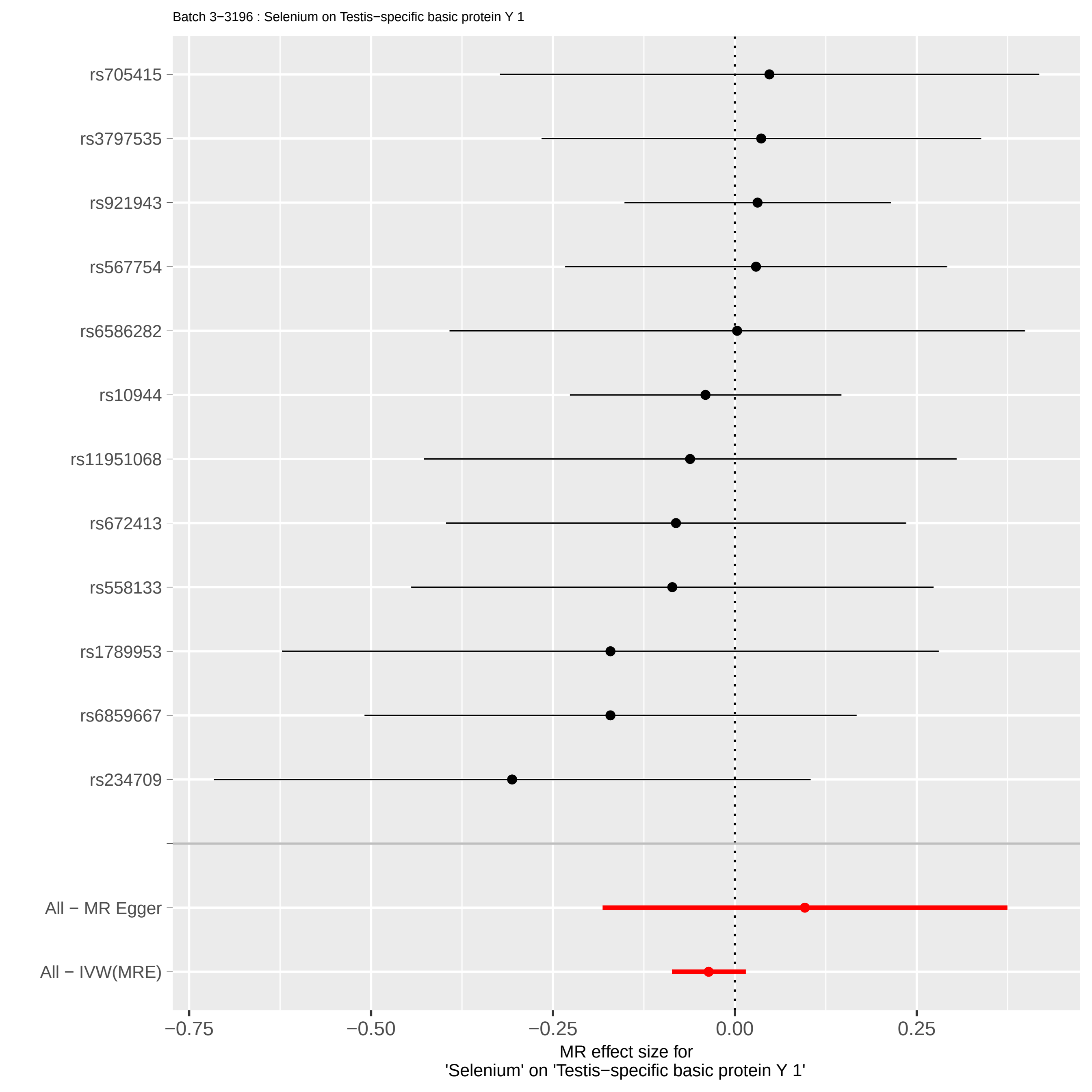

Batch 3−3196 : Selenium on Testis−specific basic protein Y 1
rs705415
rs3797535
rs921943
rs567754
rs6586282
rs10944
rs11951068
rs672413
rs558133
rs1789953
rs6859667
rs234709
All − MR Egger
All − IVW(MRE)
−0.75
−0.50
−0.25
0.00
0.25
MR effect size for
'Selenium' on 'Testis−specific basic protein Y 1'

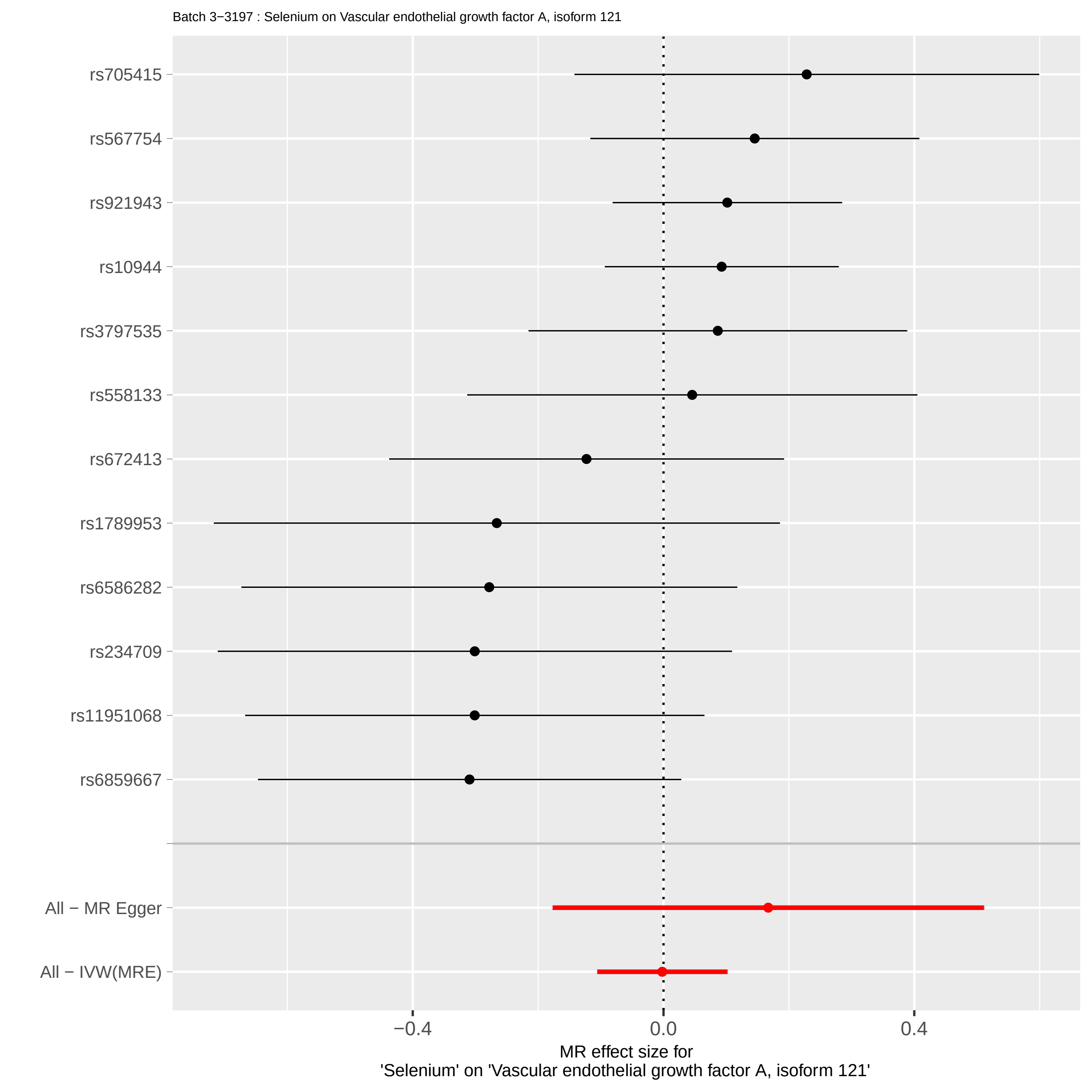
Batch 3−3197 : Selenium on Vascular endothelial growth factor A, isoform 121
rs705415
rs567754
rs921943
rs10944
rs3797535
rs558133
rs672413
rs1789953
rs6586282
rs234709
rs11951068
rs6859667
All − MR Egger
All − IVW(MRE)
−0.4
0.0
0.4
MR effect size for
'Selenium' on 'Vascular endothelial growth factor A, isoform 121'

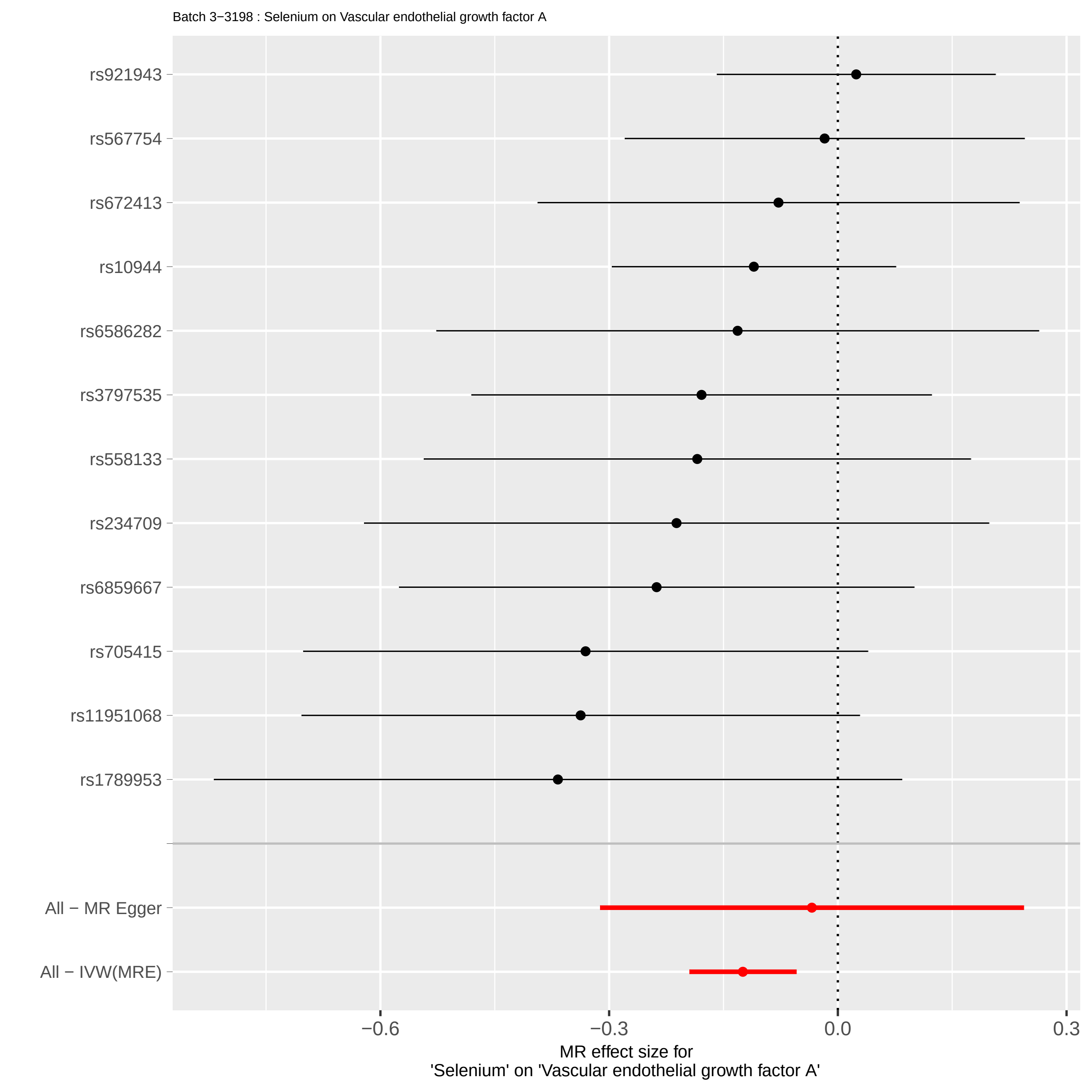
Batch 3−3198 : Selenium on Vascular endothelial growth factor A
rs921943
rs567754
rs672413
rs10944
rs6586282
rs3797535
rs558133
rs234709
rs6859667
rs705415
rs11951068
rs1789953
All − MR Egger
All − IVW(MRE)
−0.6
−0.3
0.0
0.3
MR effect size for
'Selenium' on 'Vascular endothelial growth factor A'

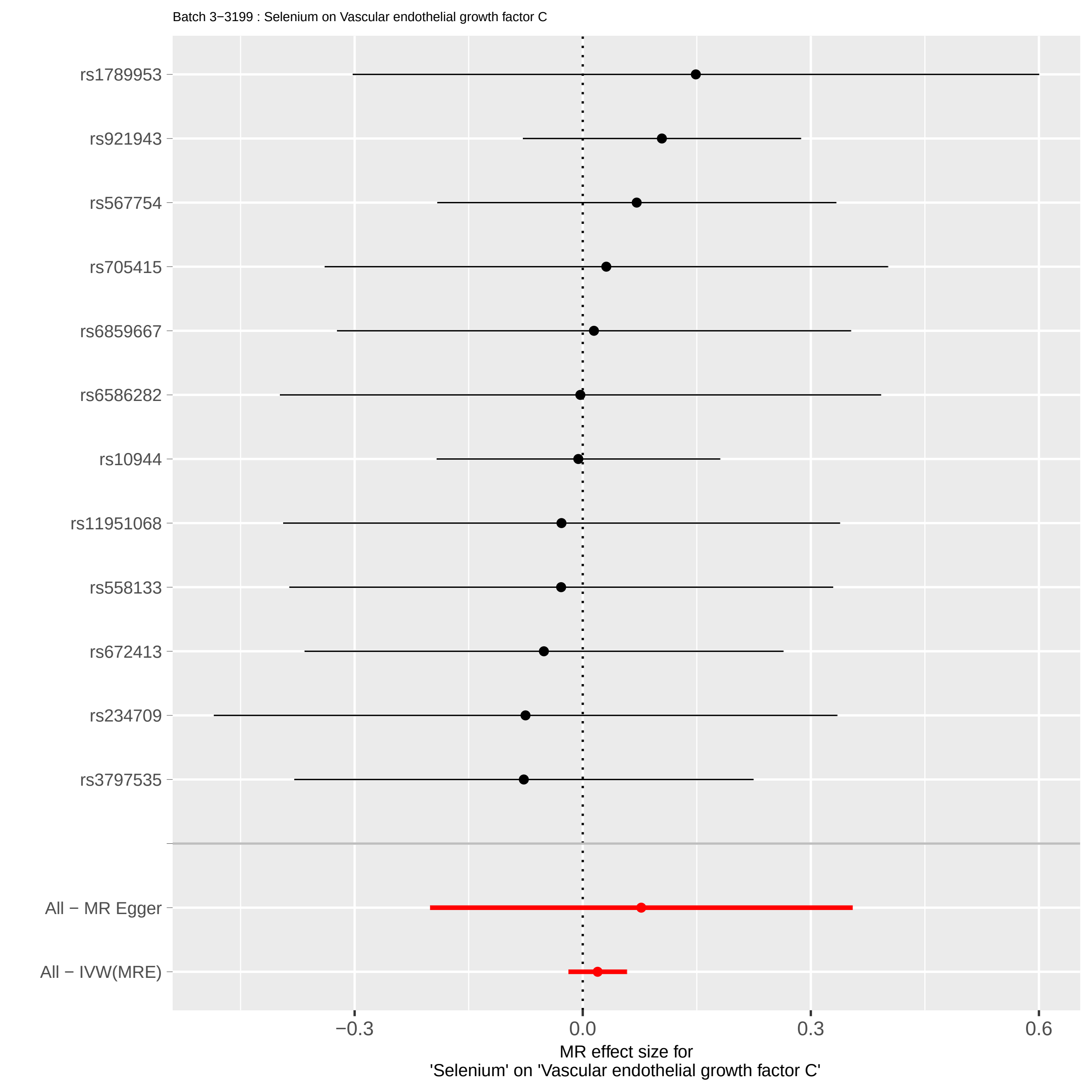

Batch 3−3199 : Selenium on Vascular endothelial growth factor C
rs1789953
rs921943
rs567754
rs705415
rs6859667
rs6586282
rs10944
rs11951068
rs558133
rs672413
rs234709
rs3797535
All − MR Egger
All − IVW(MRE)
−0.3
0.0
0.3
0.6
MR effect size for
'Selenium' on 'Vascular endothelial growth factor C'

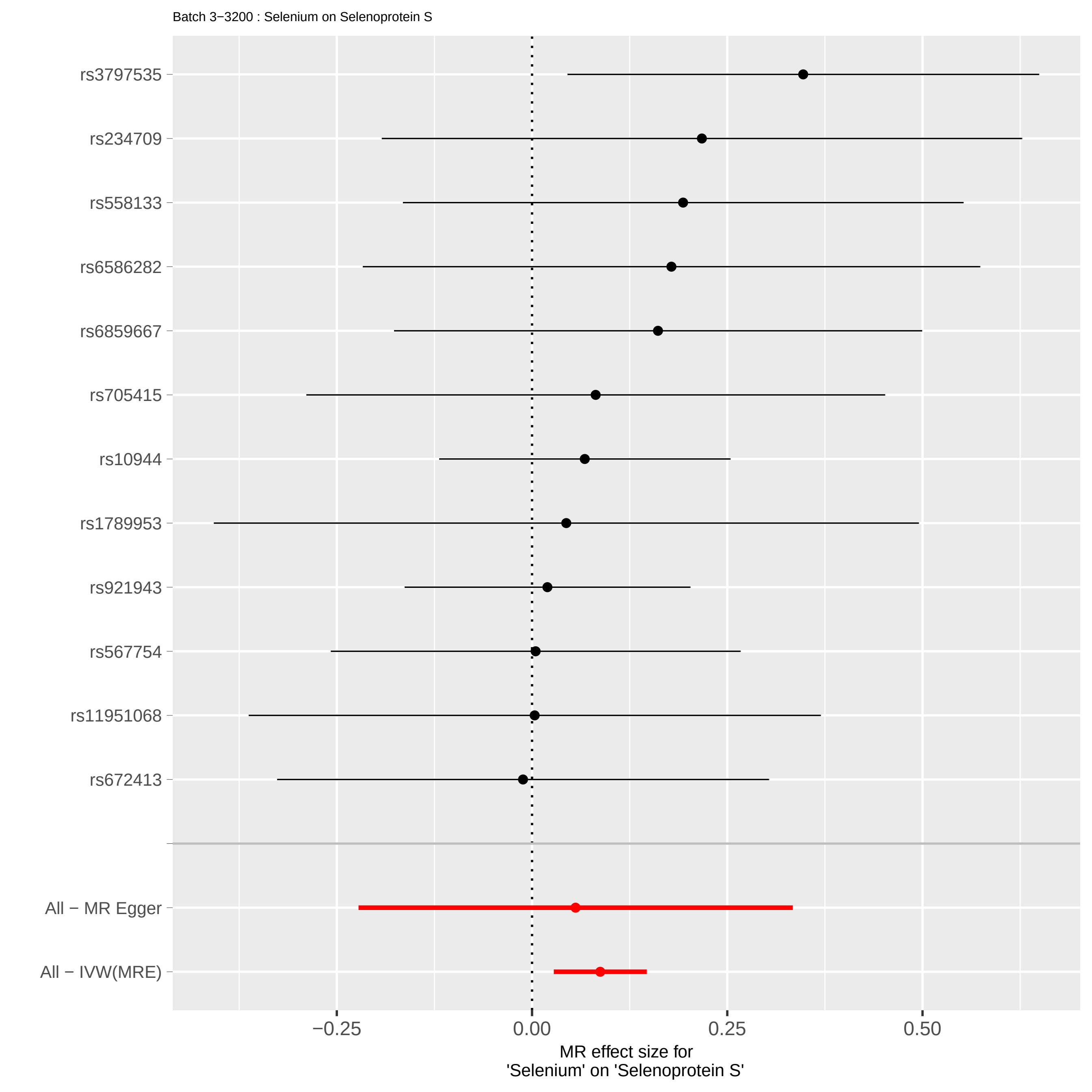

Batch 3−3200 : Selenium on Selenoprotein S
rs3797535
rs234709
rs558133
rs6586282
rs6859667
rs705415
rs10944
rs1789953
rs921943
rs567754
rs11951068
rs672413
All − MR Egger
All − IVW(MRE)
−0.25
0.00
0.25
0.50
MR effect size for
'Selenium' on 'Selenoprotein S'

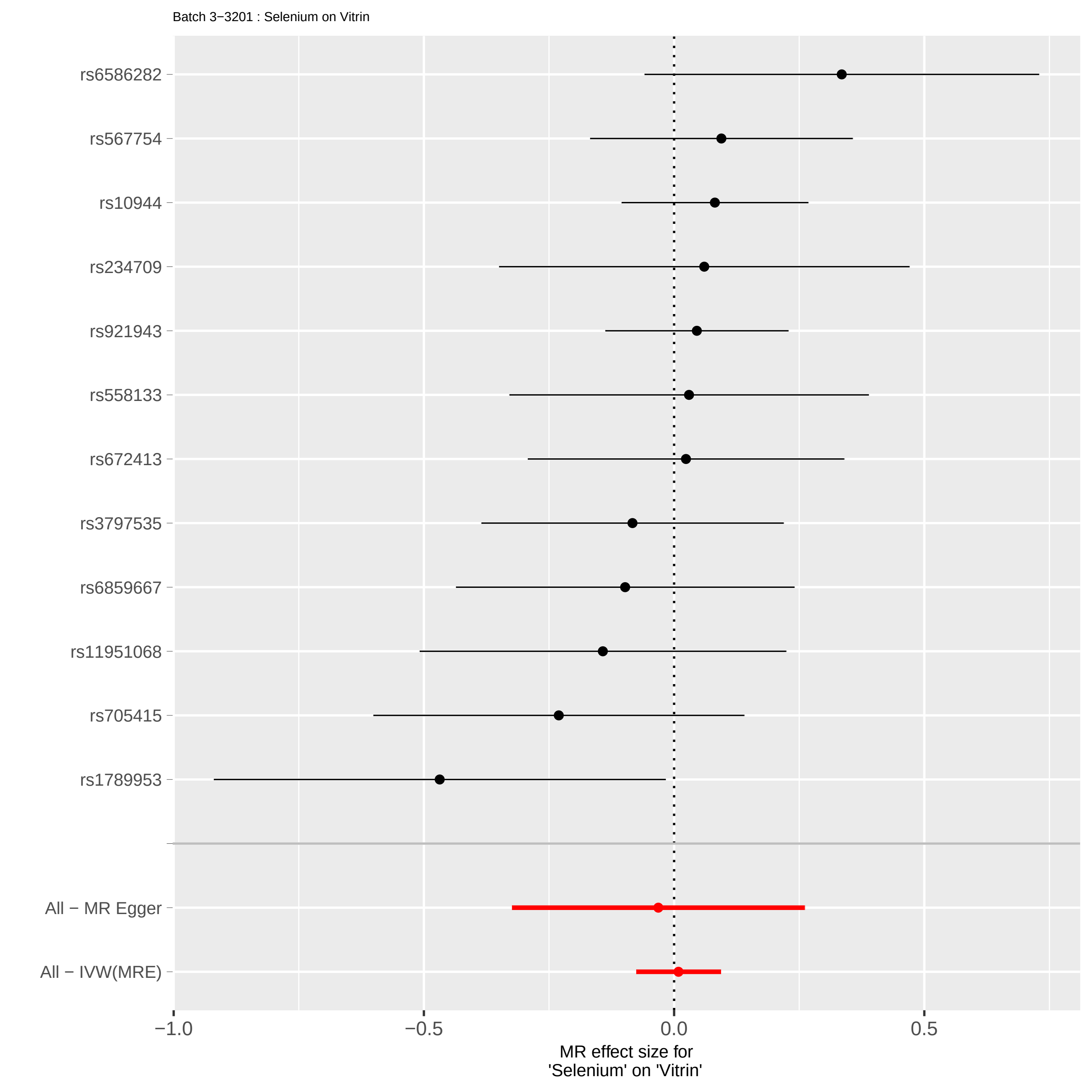

Batch 3−3201 : Selenium on Vitrin
rs6586282
rs567754
rs10944
rs234709
rs921943
rs558133
rs672413
rs3797535
rs6859667
rs11951068
rs705415
rs1789953
All − MR Egger
All − IVW(MRE)
−1.0
−0.5
0.0
0.5
MR effect size for
'Selenium' on 'Vitrin'

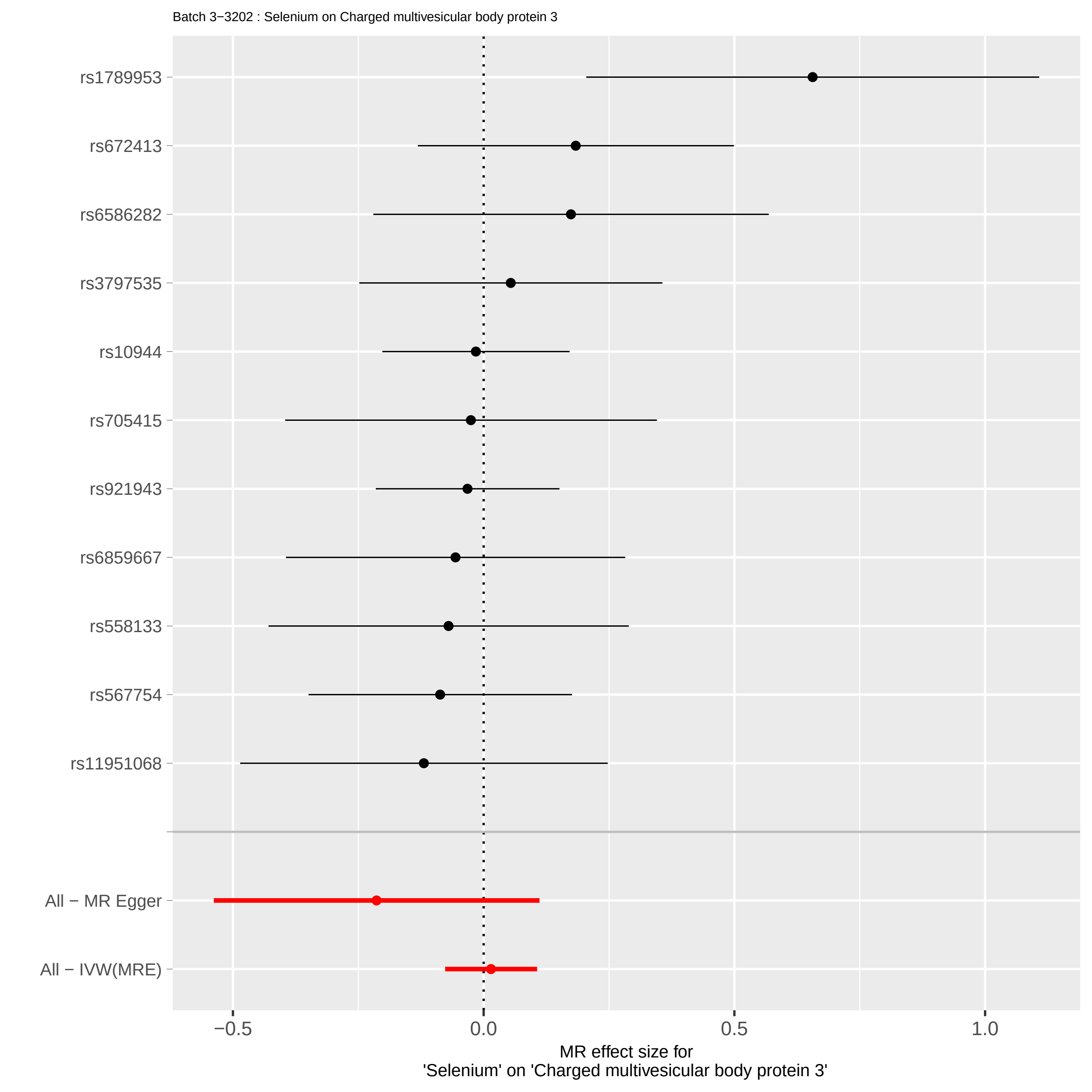
Batch 3−3202 : Selenium on Charged multivesicular body protein 3
rs1789953
rs672413
rs6586282
rs3797535
rs10944
rs705415
rs921943
rs6859667
rs558133
rs567754
rs11951068
All − MR Egger
All − IVW(MRE)
−0.5
0.0
0.5
1.0
MR effect size for
'Selenium' on 'Charged multivesicular body protein 3'

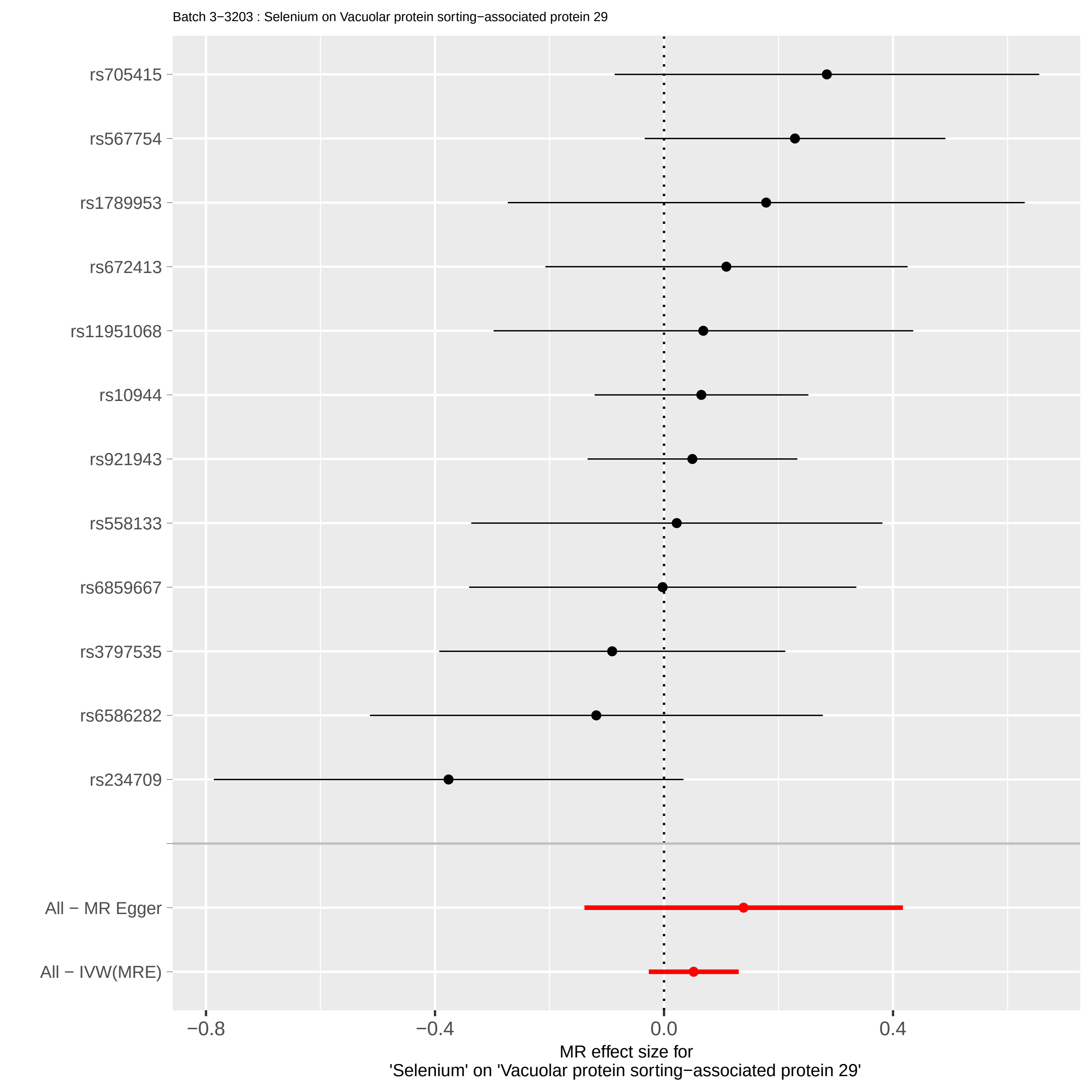

Batch 3−3203 : Selenium on Vacuolar protein sorting−associated protein 29
rs705415
rs567754
rs1789953
rs672413
rs11951068
rs10944
rs921943
rs558133
rs6859667
rs3797535
rs6586282
rs234709
All − MR Egger
All − IVW(MRE)
−0.8
−0.4
0.0
0.4
MR effect size for
'Selenium' on 'Vacuolar protein sorting−associated protein 29'

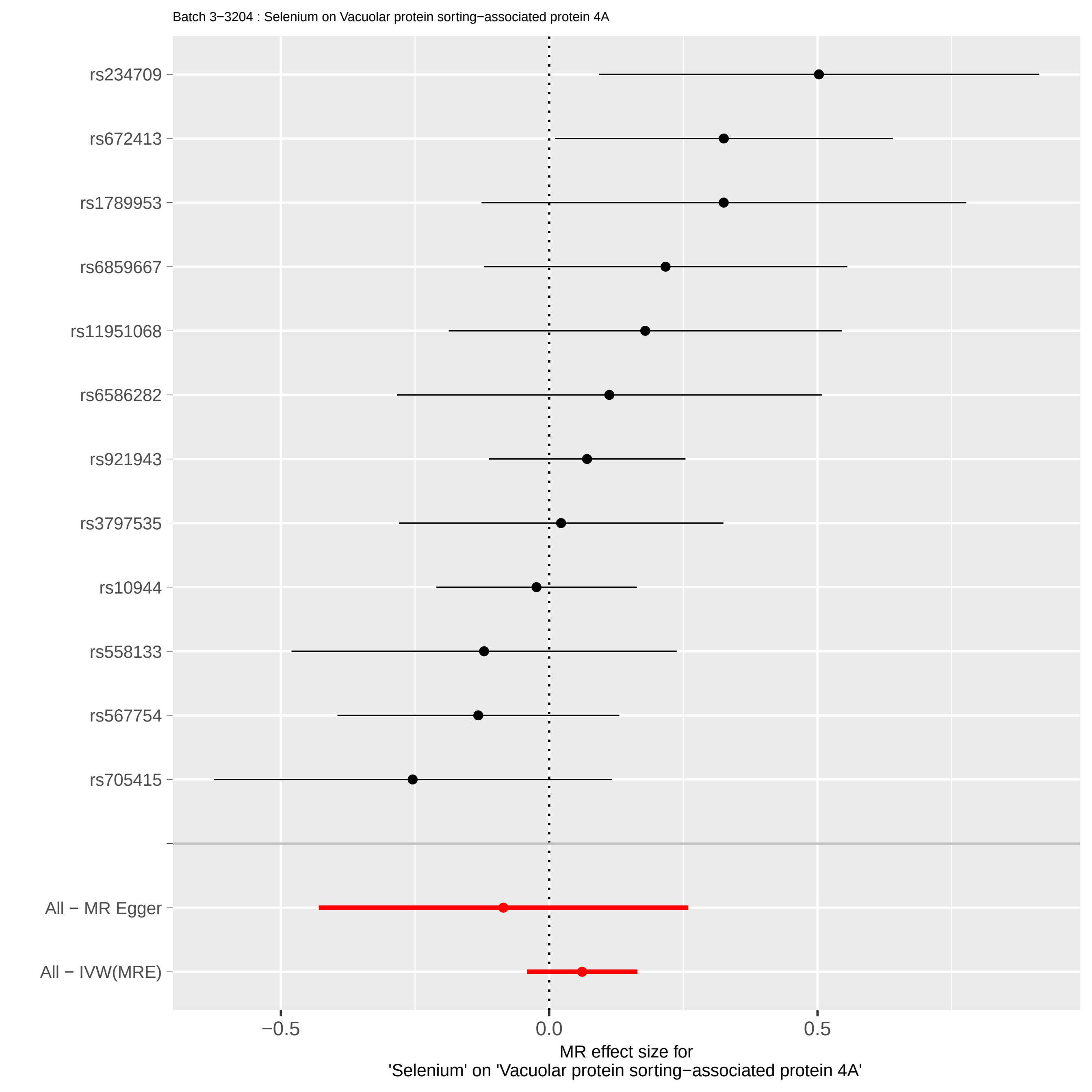

Batch 3−3204 : Selenium on Vacuolar protein sorting−associated protein 4A
rs234709
rs672413
rs1789953
rs6859667
rs11951068
rs6586282
rs921943
rs3797535
rs10944
rs558133
rs567754
rs705415
All − MR Egger
All − IVW(MRE)
−0.5
0.0
0.5
MR effect size for
'Selenium' on 'Vacuolar protein sorting−associated protein 4A'

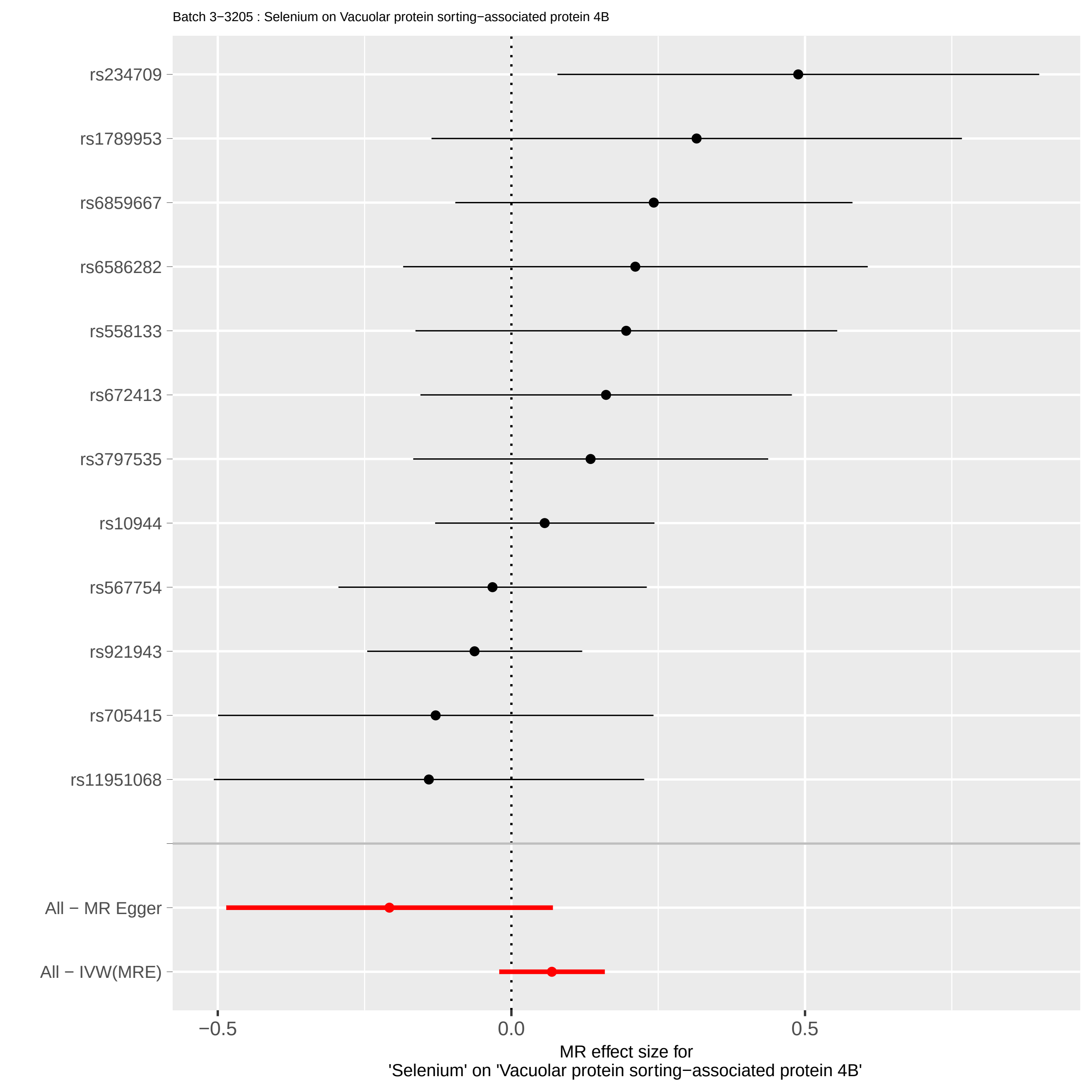
Batch 3−3205 : Selenium on Vacuolar protein sorting−associated protein 4B
rs234709
rs1789953
rs6859667
rs6586282
rs558133
rs672413
rs3797535
rs10944
rs567754
rs921943
rs705415
rs11951068
All − MR Egger
All − IVW(MRE)
−0.5
0.0
0.5
MR effect size for
'Selenium' on 'Vacuolar protein sorting−associated protein 4B'

Batch 3−3206 : Selenium on Serine/threonine−protein kinase VRK1

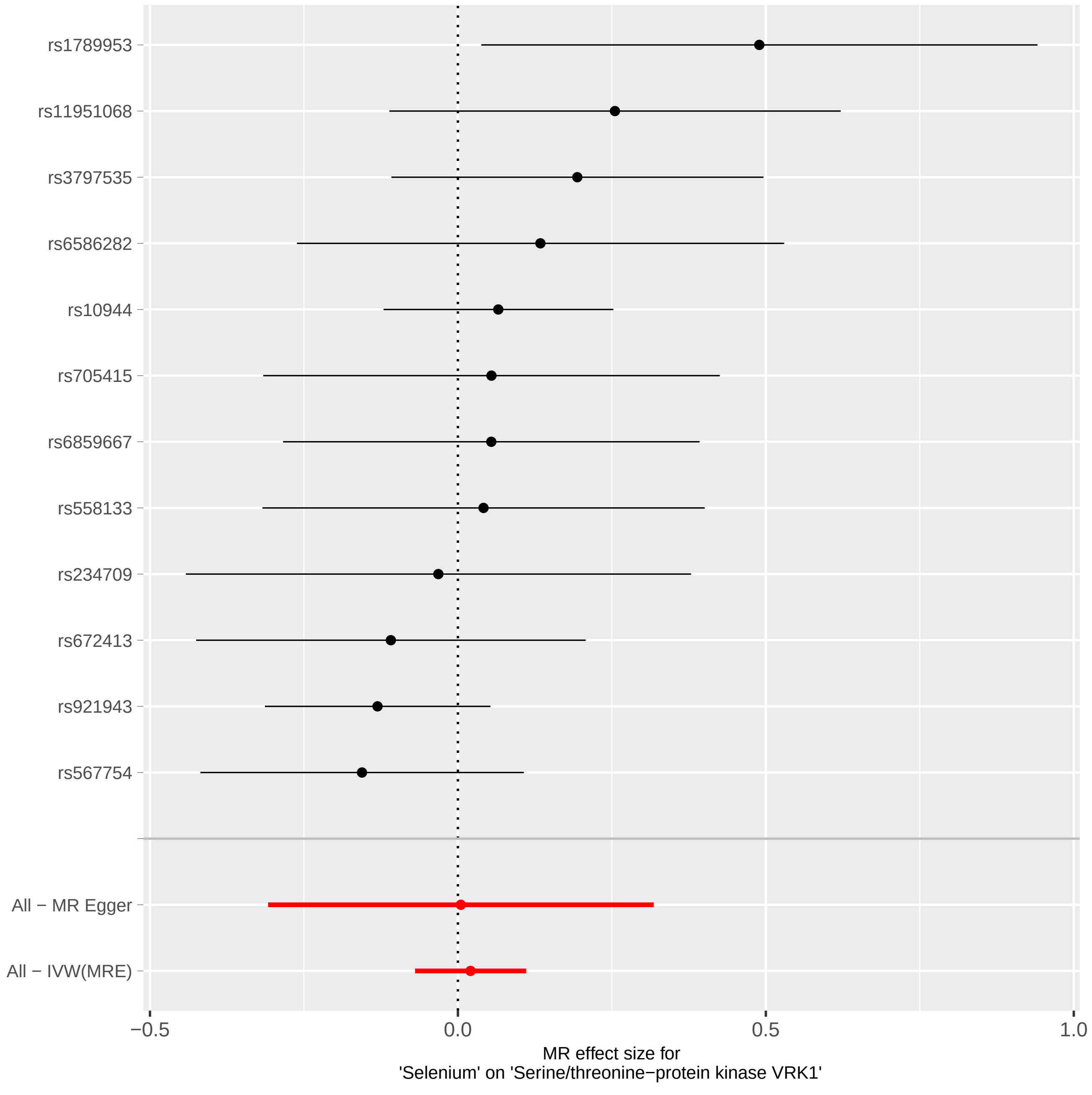

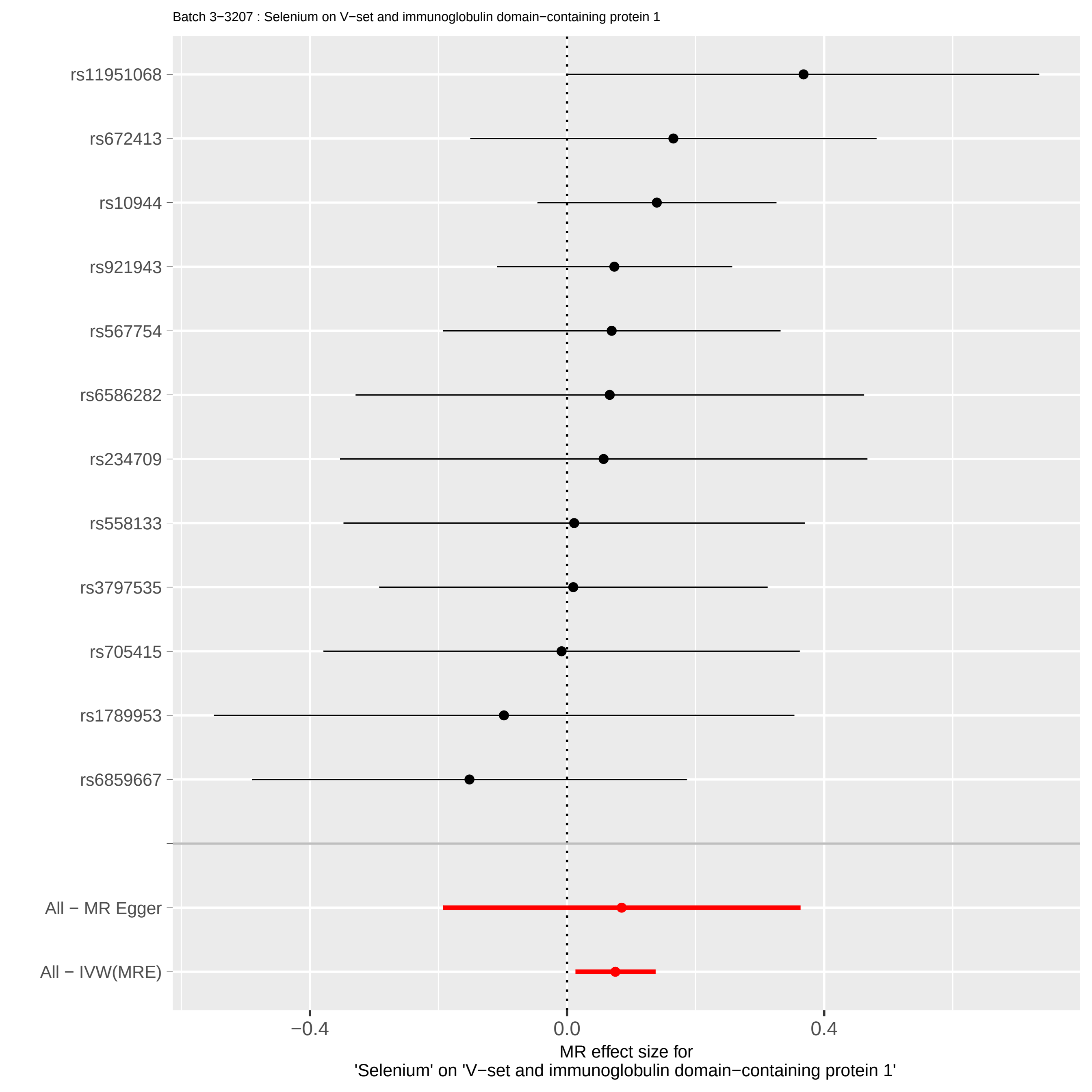

Batch 3−3207 : Selenium on V−set and immunoglobulin domain−containing protein 1
rs11951068
rs672413
rs10944
rs921943
rs567754
rs6586282
rs234709
rs558133
rs3797535
rs705415
rs1789953
rs6859667
All − MR Egger
All − IVW(MRE)
−0.4
0.0
0.4
MR effect size for
'Selenium' on 'V−set and immunoglobulin domain−containing protein 1'

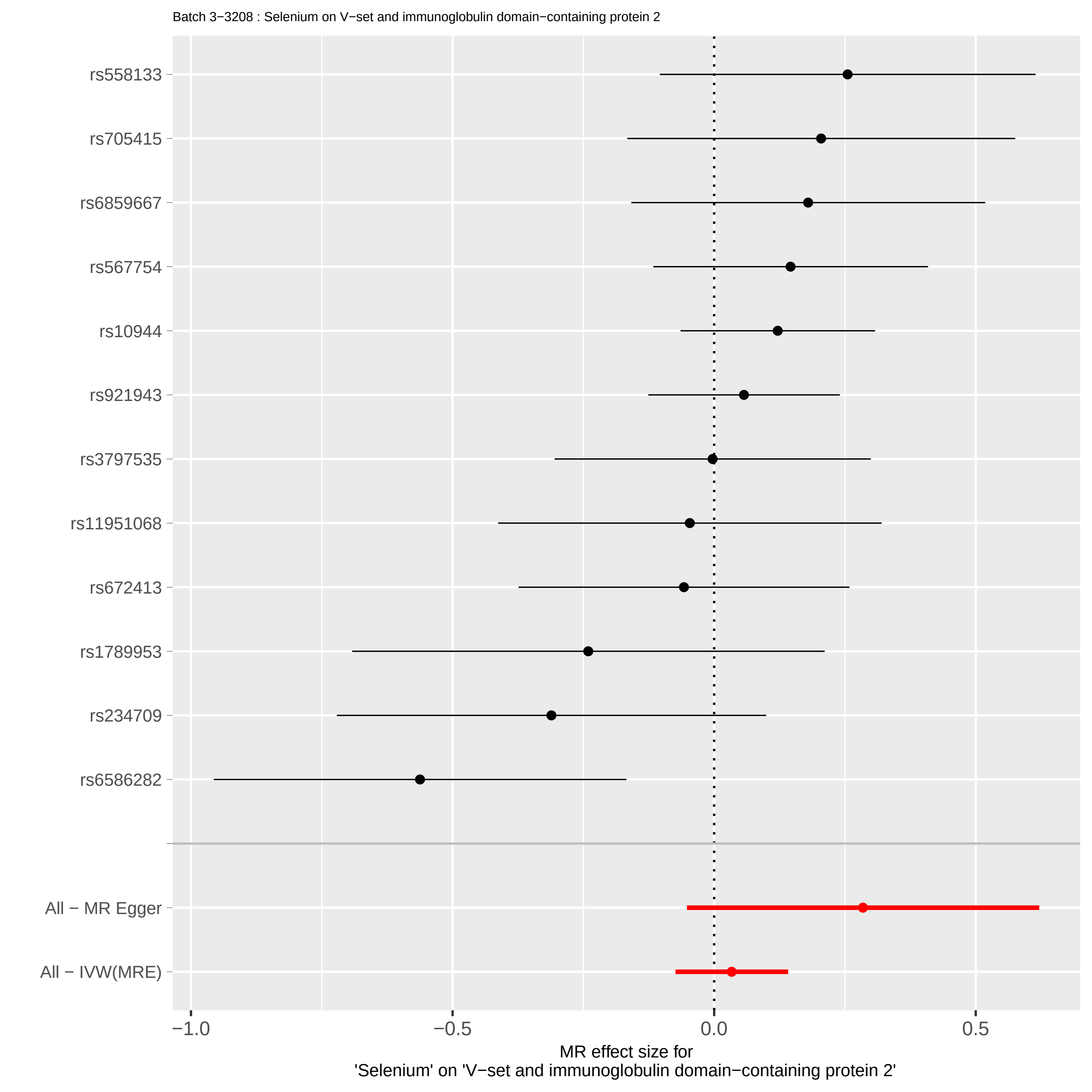

Batch 3−3208 : Selenium on V−set and immunoglobulin domain−containing protein 2
rs558133
rs705415
rs6859667
rs567754
rs10944
rs921943
rs3797535
rs11951068
rs672413
rs1789953
rs234709
rs6586282
All − MR Egger
All − IVW(MRE)
−1.0
−0.5
0.0
0.5
MR effect size for
'Selenium' on 'V−set and immunoglobulin domain−containing protein 2'

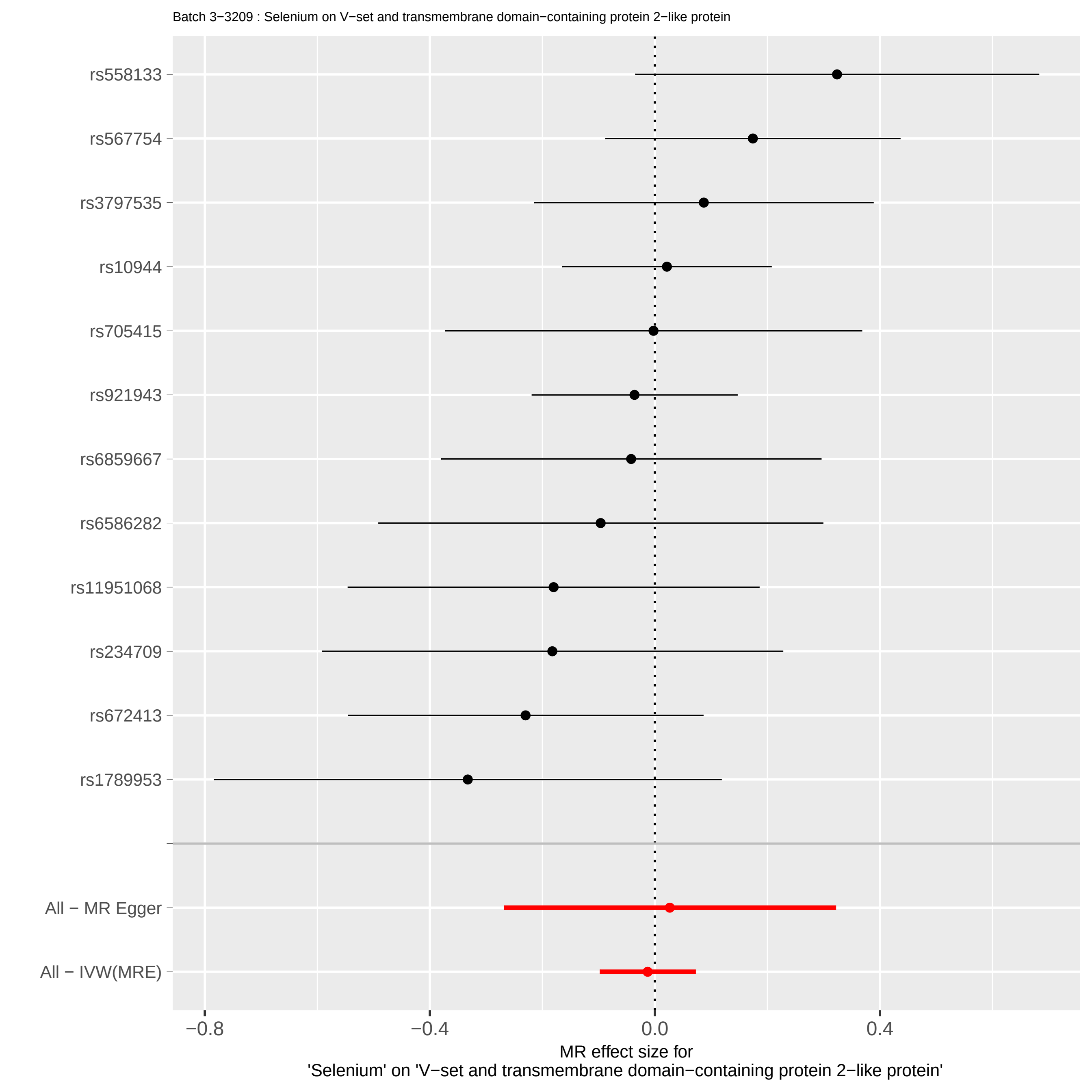
Batch 3−3209 : Selenium on V−set and transmembrane domain−containing protein 2−like protein
rs558133
rs567754
rs3797535
rs10944
rs705415
rs921943
rs6859667
rs6586282
rs11951068
rs234709
rs672413
rs1789953
All − MR Egger
All − IVW(MRE)
−0.8
−0.4
0.0
0.4
MR effect size for
'Selenium' on 'V−set and transmembrane domain−containing protein 2−like protein'

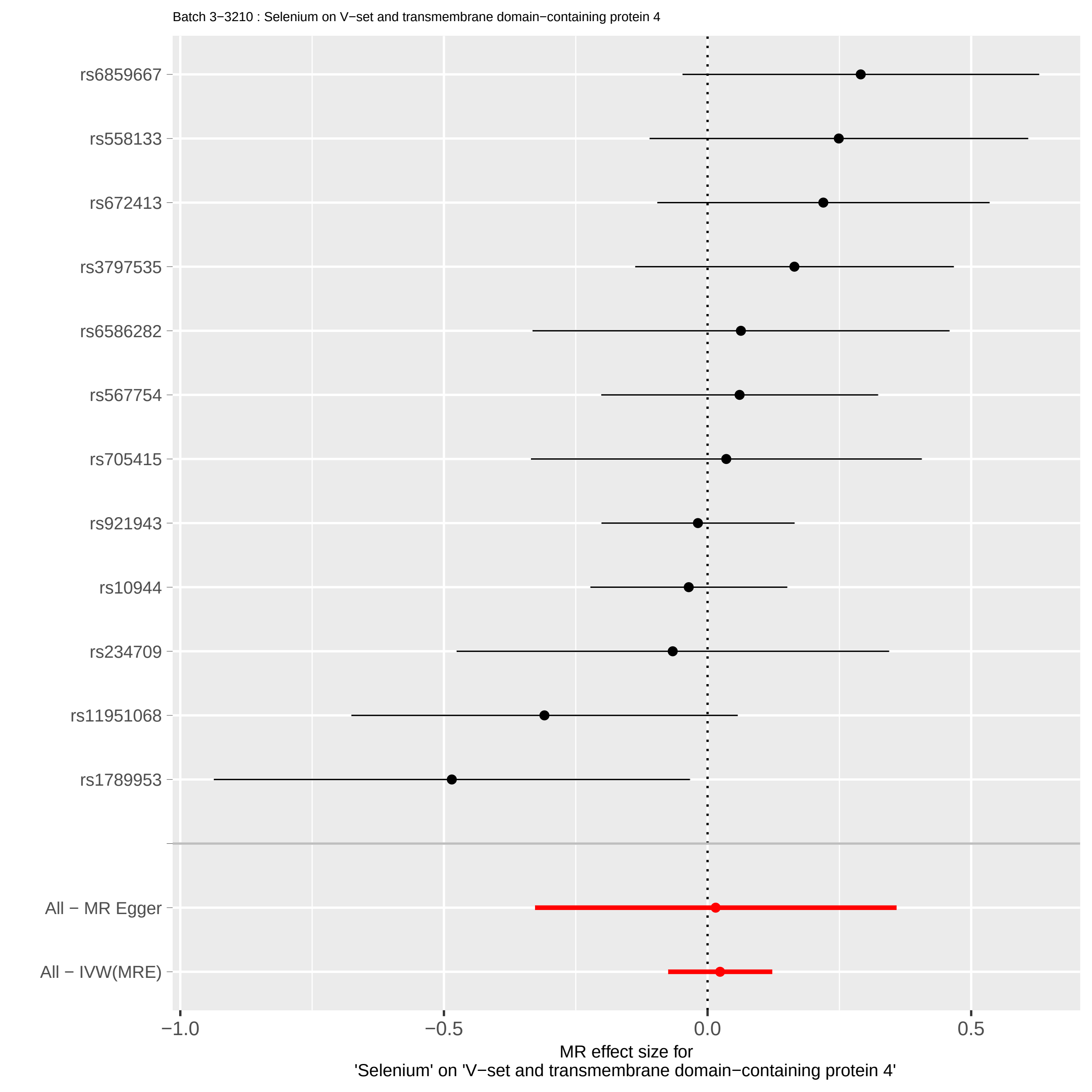
Batch 3−3210 : Selenium on V−set and transmembrane domain−containing protein 4
rs6859667
rs558133
rs672413
rs3797535
rs6586282
rs567754
rs705415
rs921943
rs10944
rs234709
rs11951068
rs1789953
All − MR Egger
All − IVW(MRE)
−1.0
−0.5
0.0
0.5
MR effect size for
'Selenium' on 'V−set and transmembrane domain−containing protein 4'

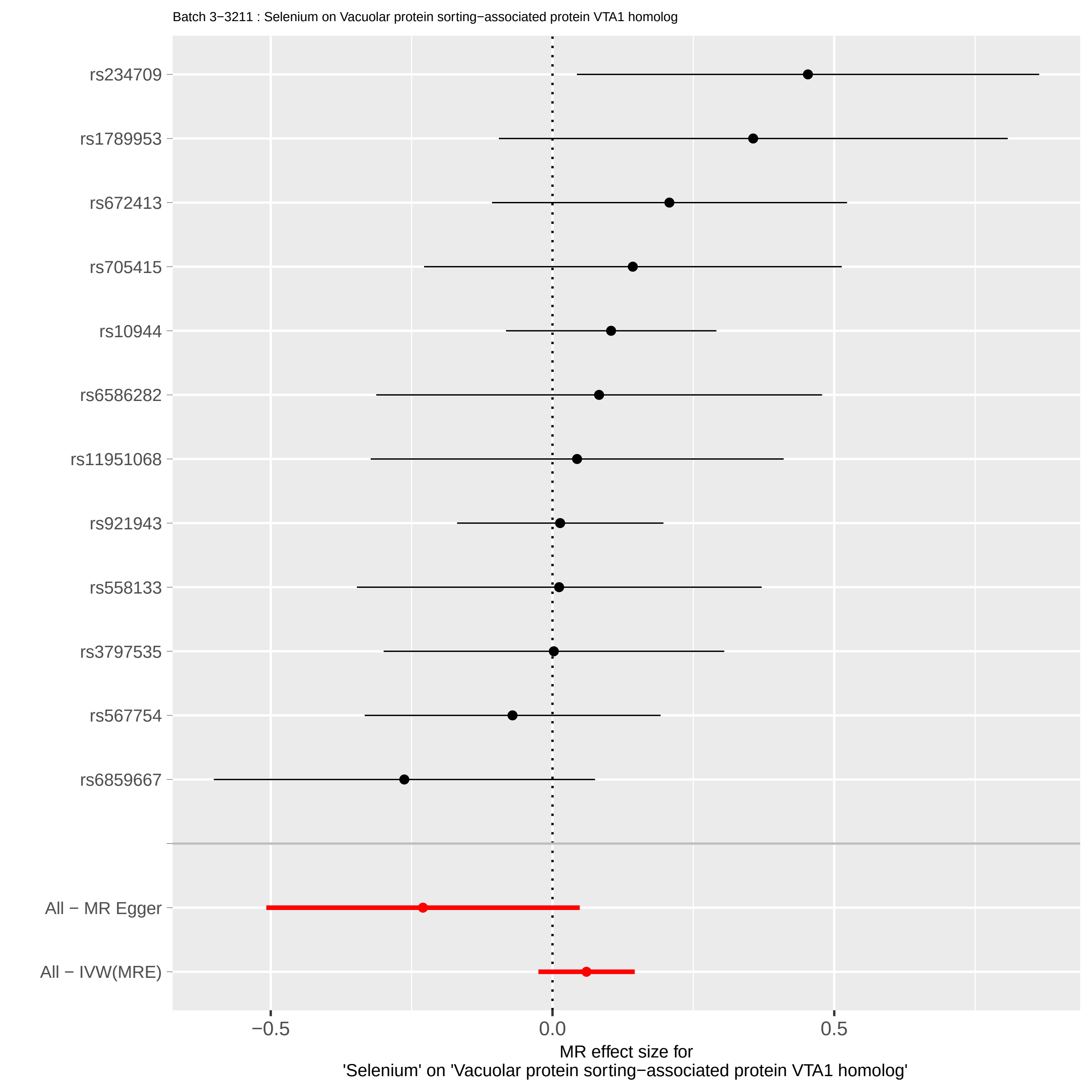
Batch 3−3211 : Selenium on Vacuolar protein sorting−associated protein VTA1 homolog
rs234709
rs1789953
rs672413
rs705415
rs10944
rs6586282
rs11951068
rs921943
rs558133
rs3797535
rs567754
rs6859667
All − MR Egger
All − IVW(MRE)
−0.5
0.0
0.5
MR effect size for
'Selenium' on 'Vacuolar protein sorting−associated protein VTA1 homolog'

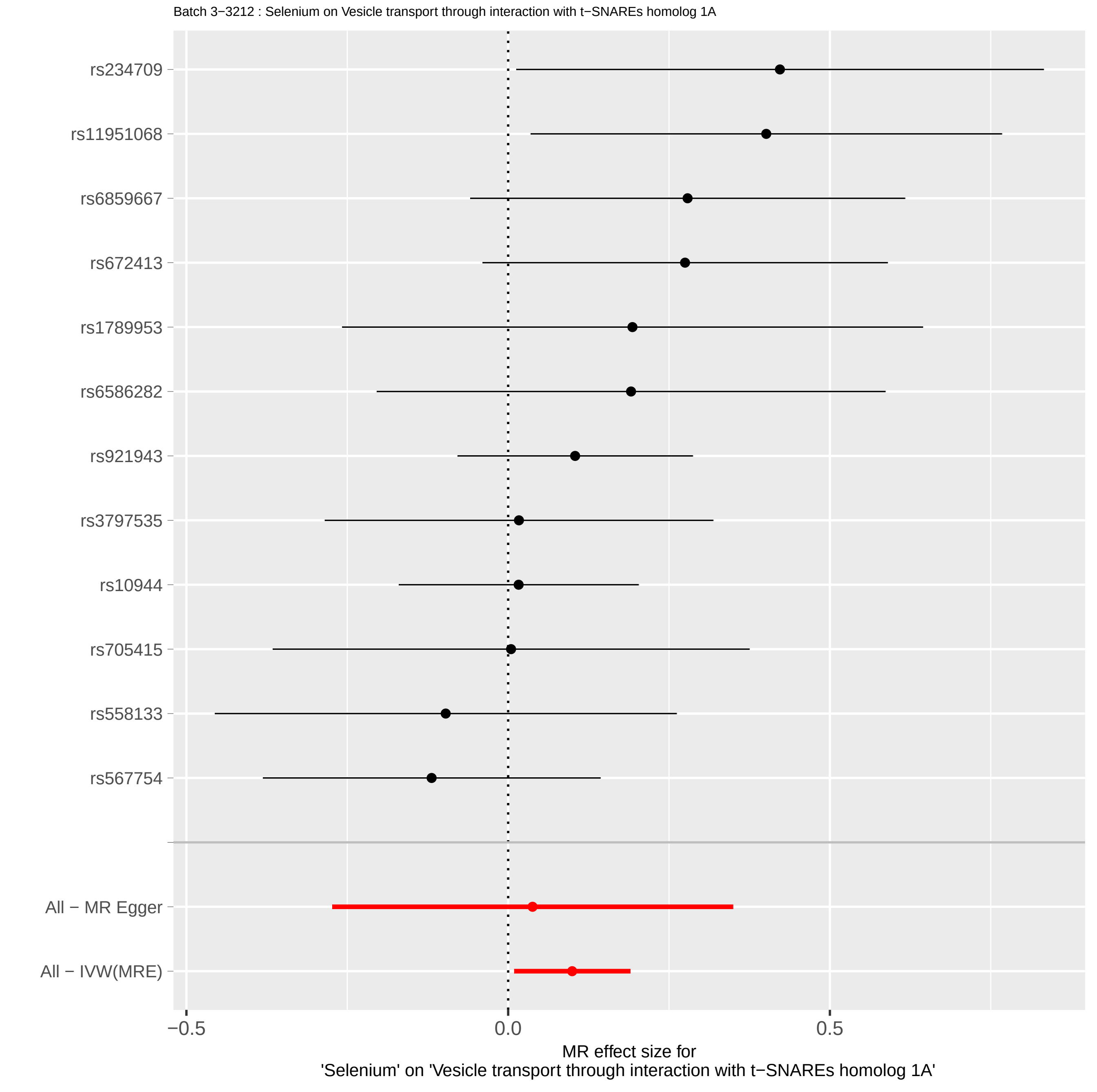

Batch 3−3212 : Selenium on Vesicle transport through interaction with t−SNAREs homolog 1A
rs234709
rs11951068
rs6859667
rs672413
rs1789953
rs6586282
rs921943
rs3797535
rs10944
rs705415
rs558133
rs567754
All − MR Egger
All − IVW(MRE)
−0.5
0.0
0.5
MR effect size for
'Selenium' on 'Vesicle transport through interaction with t−SNAREs homolog 1A'

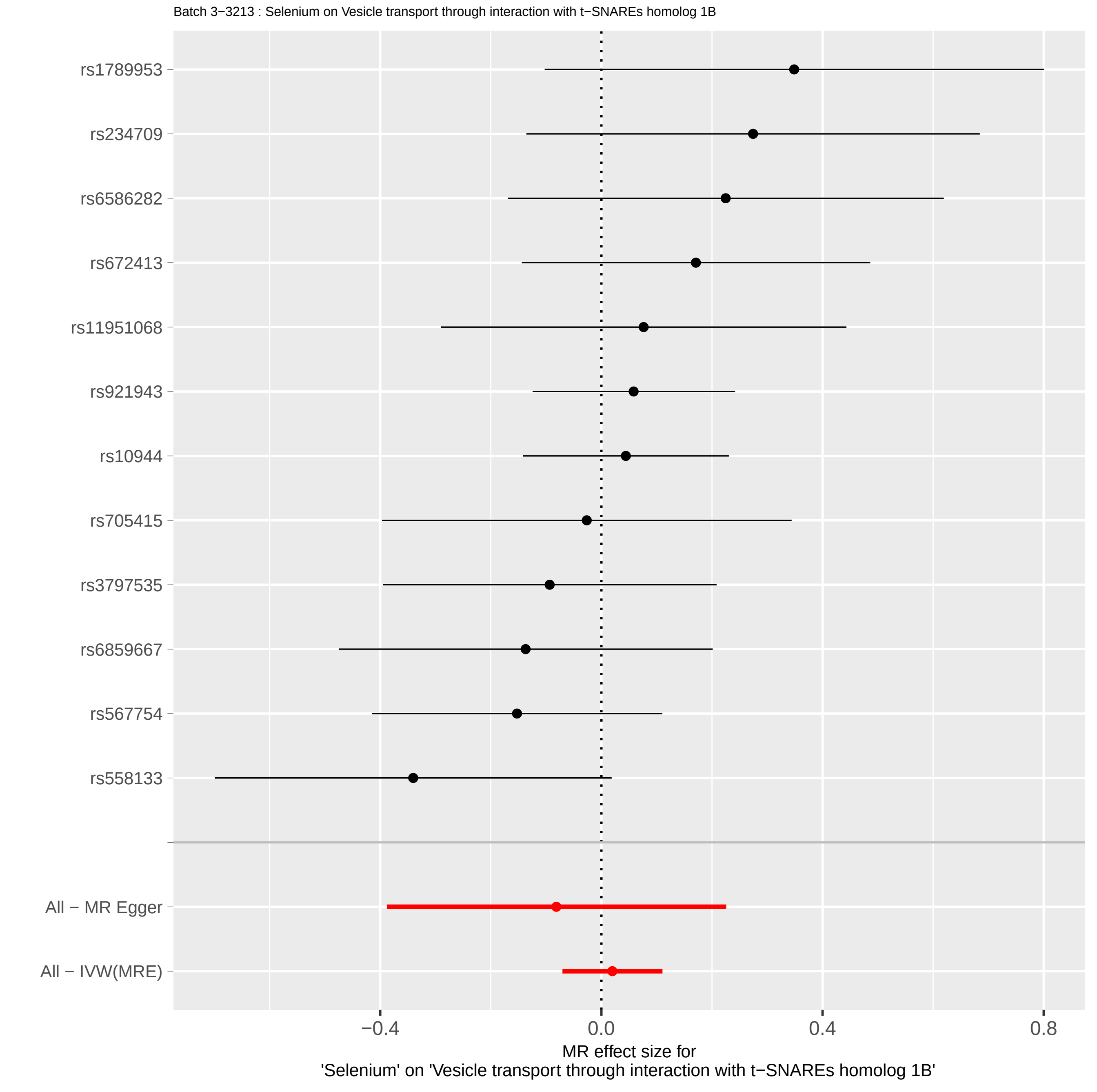
Batch 3−3213 : Selenium on Vesicle transport through interaction with t−SNAREs homolog 1B
rs1789953
rs234709
rs6586282
rs672413
rs11951068
rs921943
rs10944
rs705415
rs3797535
rs6859667
rs567754
rs558133
All − MR Egger
All − IVW(MRE)
−0.4
0.0
0.4
0.8
MR effect size for
'Selenium' on 'Vesicle transport through interaction with t−SNAREs homolog 1B'

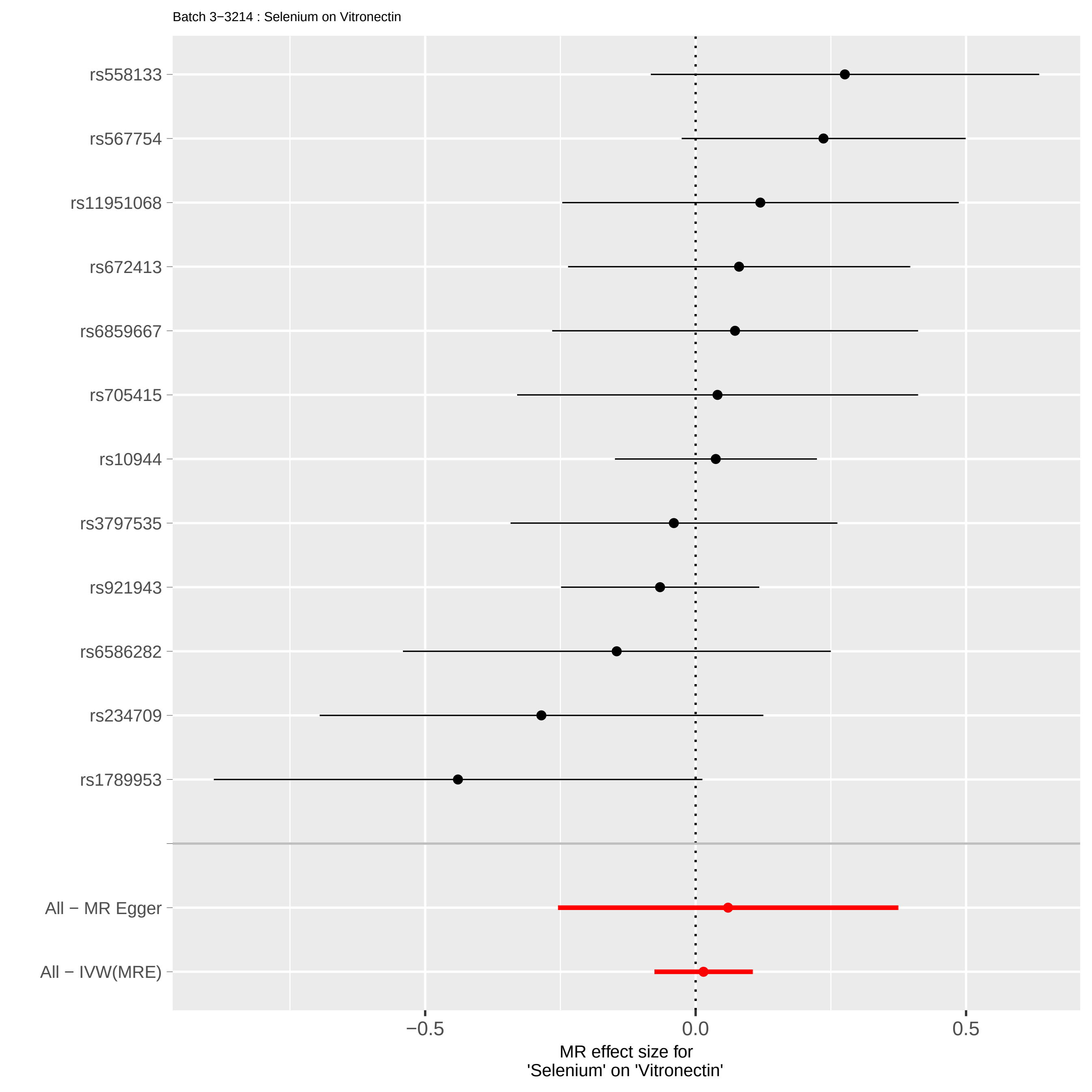

Batch 3−3214 : Selenium on Vitronectin
rs558133
rs567754
rs11951068
rs672413
rs6859667
rs705415
rs10944
rs3797535
rs921943
rs6586282
rs234709
rs1789953
All − MR Egger
All − IVW(MRE)
−0.5
0.0
0.5
MR effect size for
'Selenium' on 'Vitronectin'

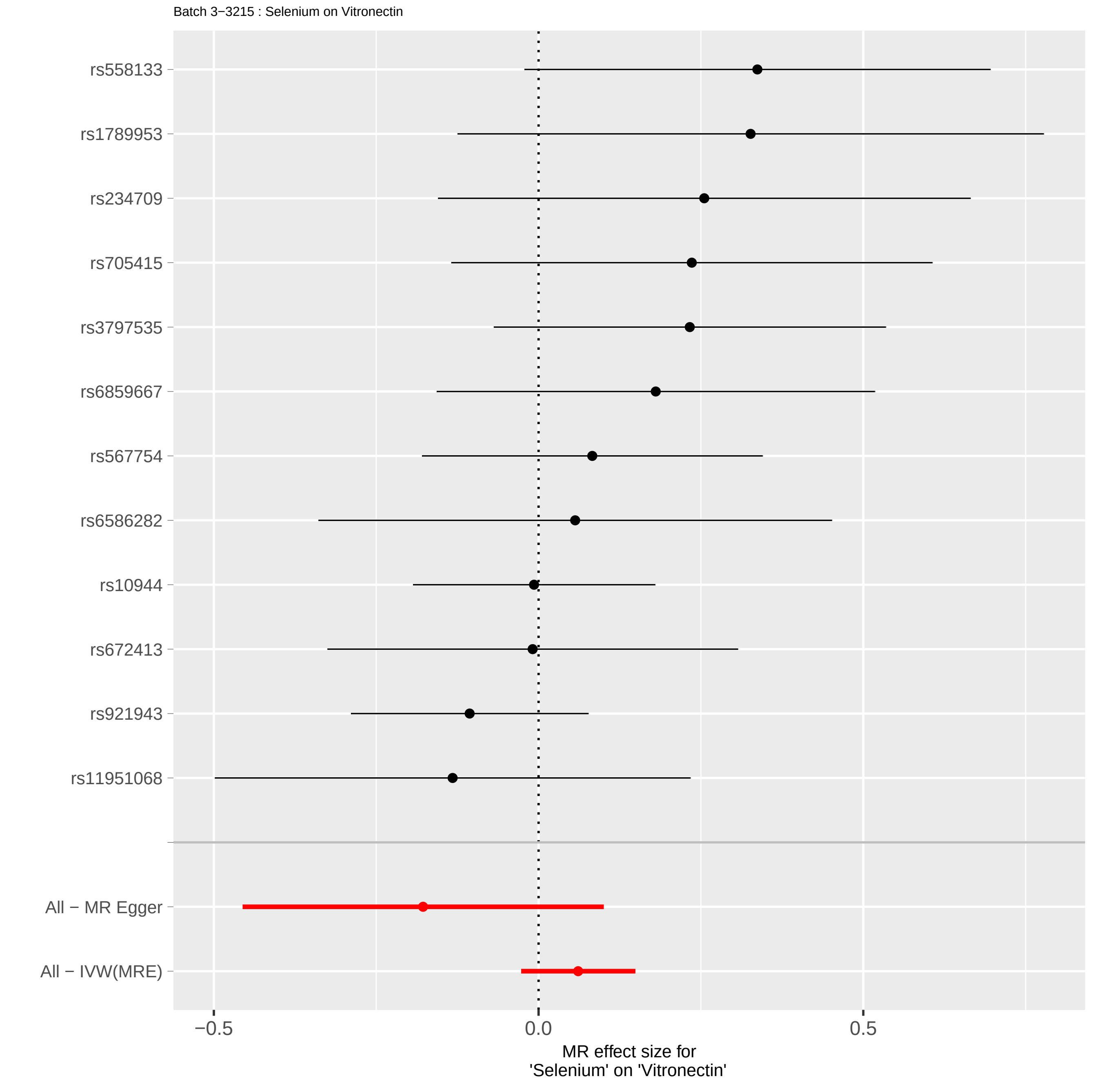
Batch 3−3215 : Selenium on Vitronectin
rs558133
rs1789953
rs234709
rs705415
rs3797535
rs6859667
rs567754
rs6586282
rs10944
rs672413
rs921943
rs11951068
All − MR Egger
All − IVW(MRE)
−0.5
0.0
0.5
MR effect size for
'Selenium' on 'Vitronectin'

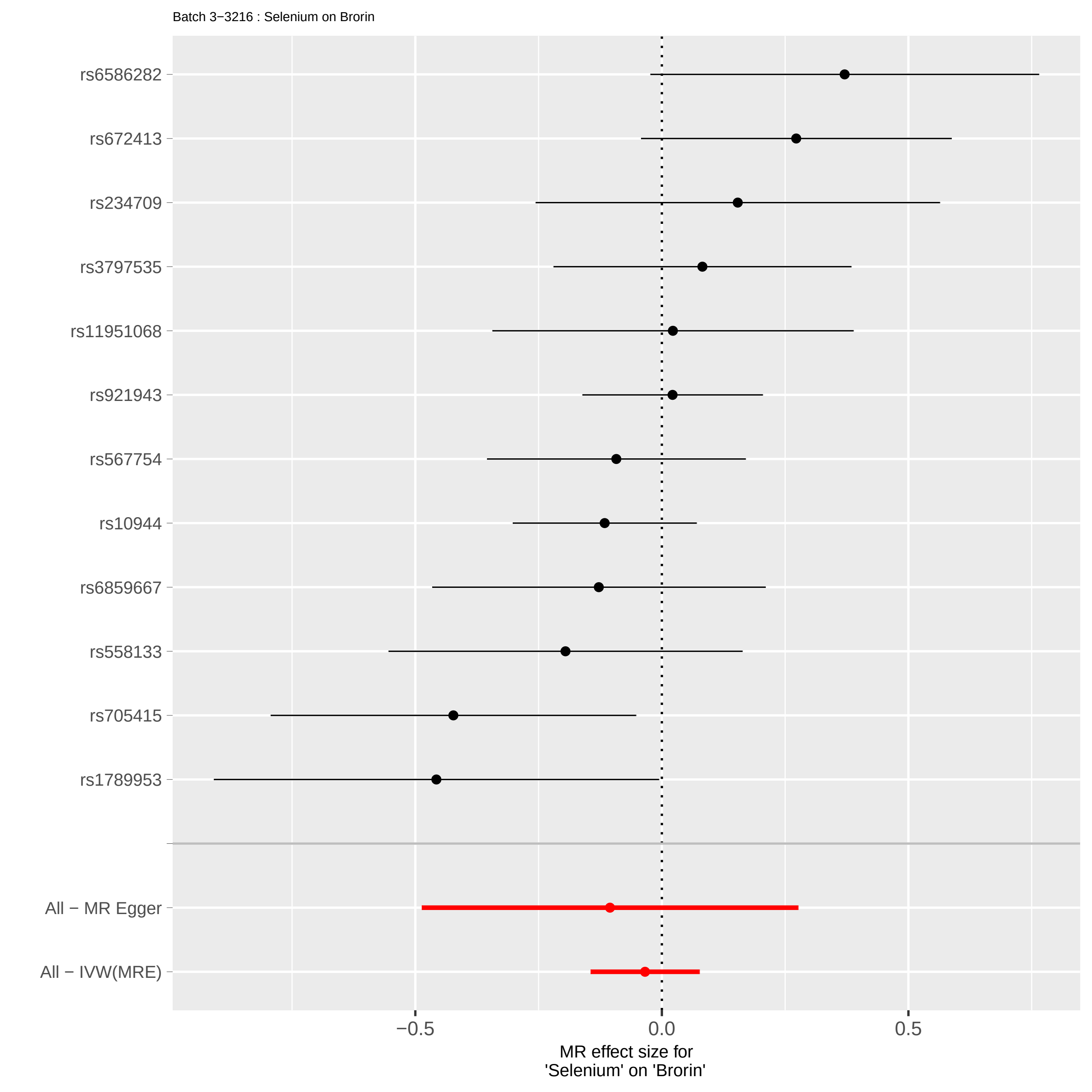
Batch 3−3216 : Selenium on Brorin
rs6586282
rs672413
rs234709
rs3797535
rs11951068
rs921943
rs567754
rs10944
rs6859667
rs558133
rs705415
rs1789953
All − MR Egger
All − IVW(MRE)
−0.5
0.0
0.5
MR effect size for
'Selenium' on 'Brorin'

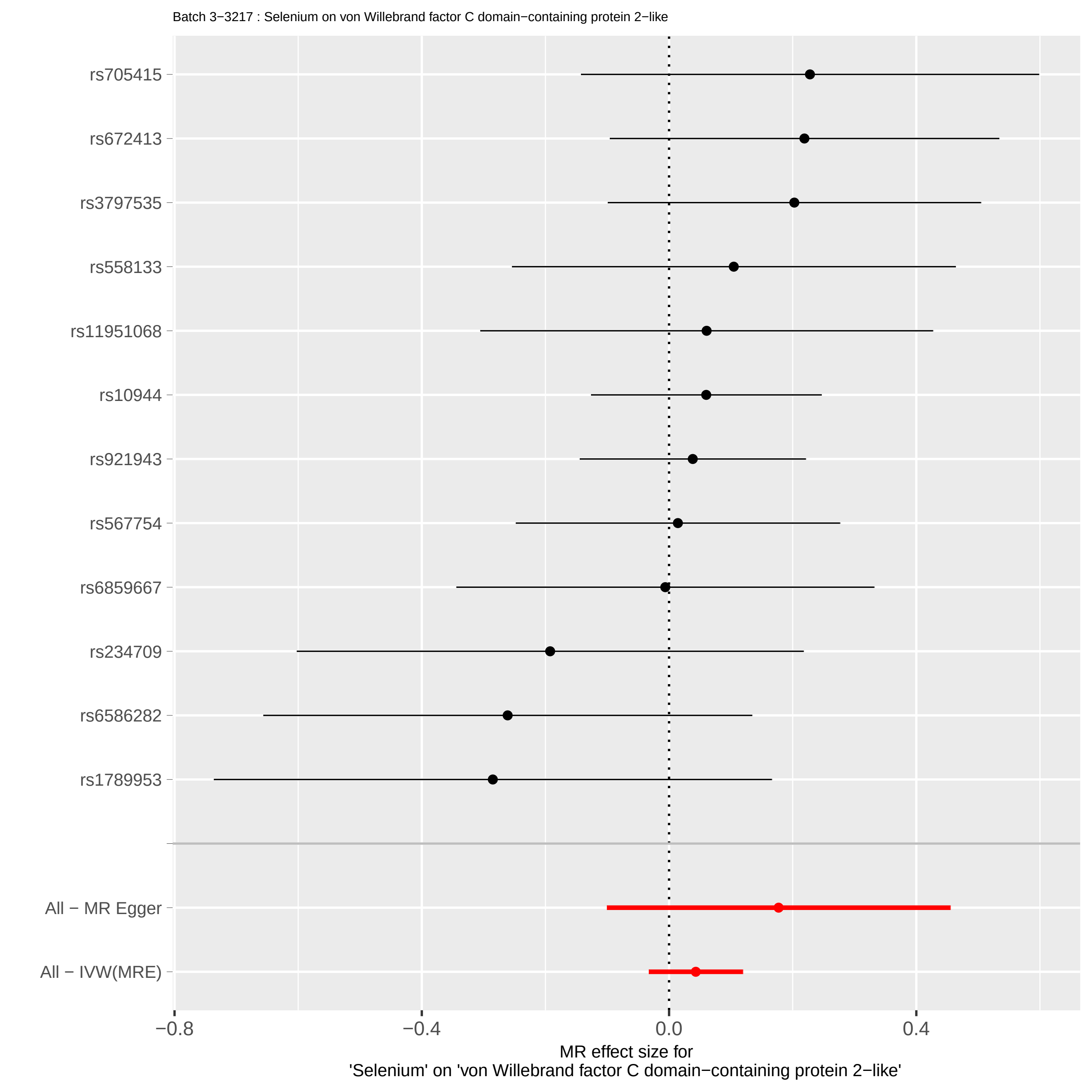

Batch 3−3217 : Selenium on von Willebrand factor C domain−containing protein 2−like
rs705415
rs672413
rs3797535
rs558133
rs11951068
rs10944
rs921943
rs567754
rs6859667
rs234709
rs6586282
rs1789953
All − MR Egger
All − IVW(MRE)
−0.8
−0.4
0.0
0.4
MR effect size for
'Selenium' on 'von Willebrand factor C domain−containing protein 2−like'

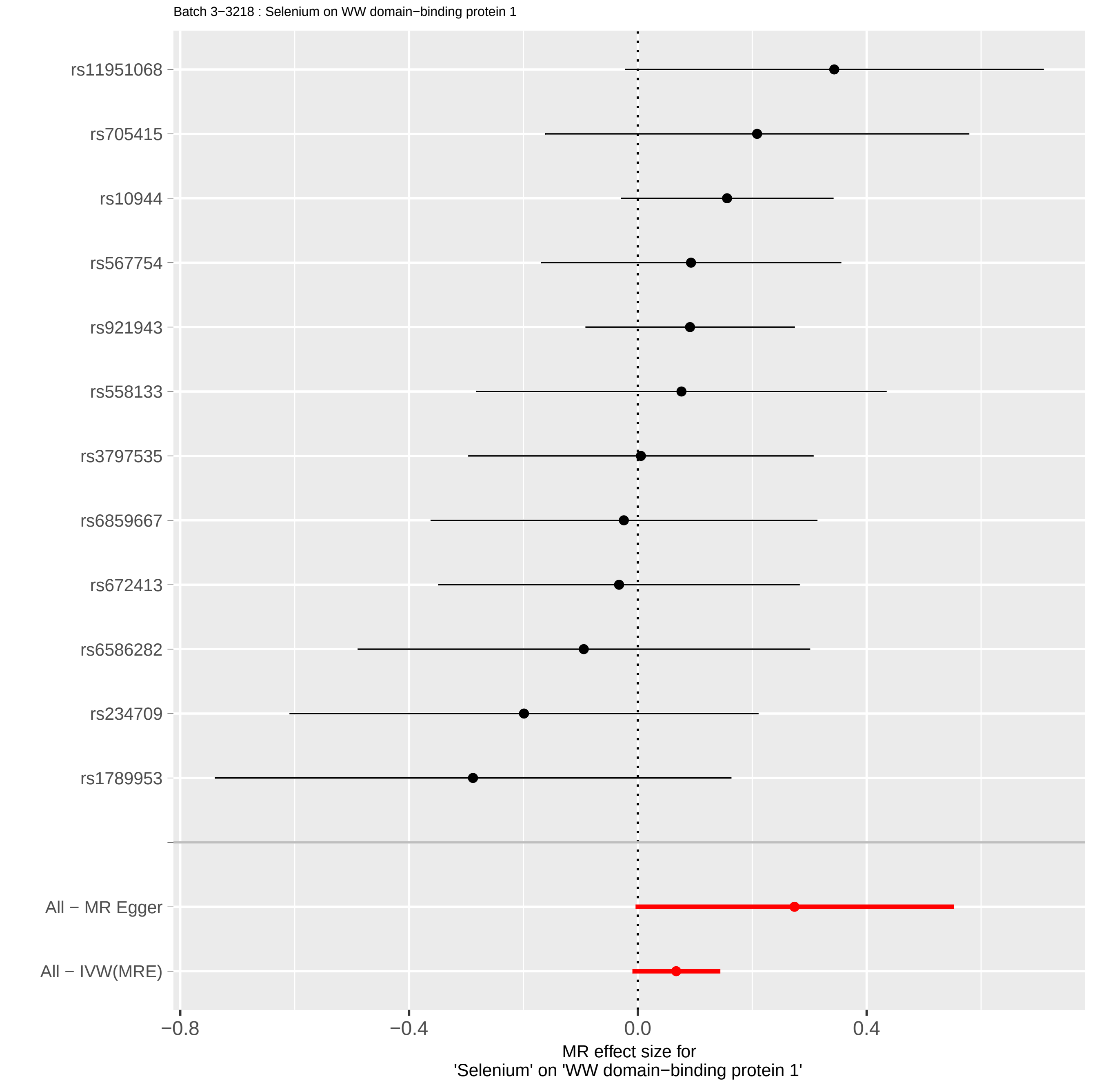

Batch 3−3218 : Selenium on WW domain−binding protein 1
rs11951068
rs705415
rs10944
rs567754
rs921943
rs558133
rs3797535
rs6859667
rs672413
rs6586282
rs234709
rs1789953
All − MR Egger
All − IVW(MRE)
−0.8
−0.4
0.0
0.4
MR effect size for
'Selenium' on 'WW domain−binding protein 1'

Batch 3−3219 : Selenium on WW domain binding protein 1−like

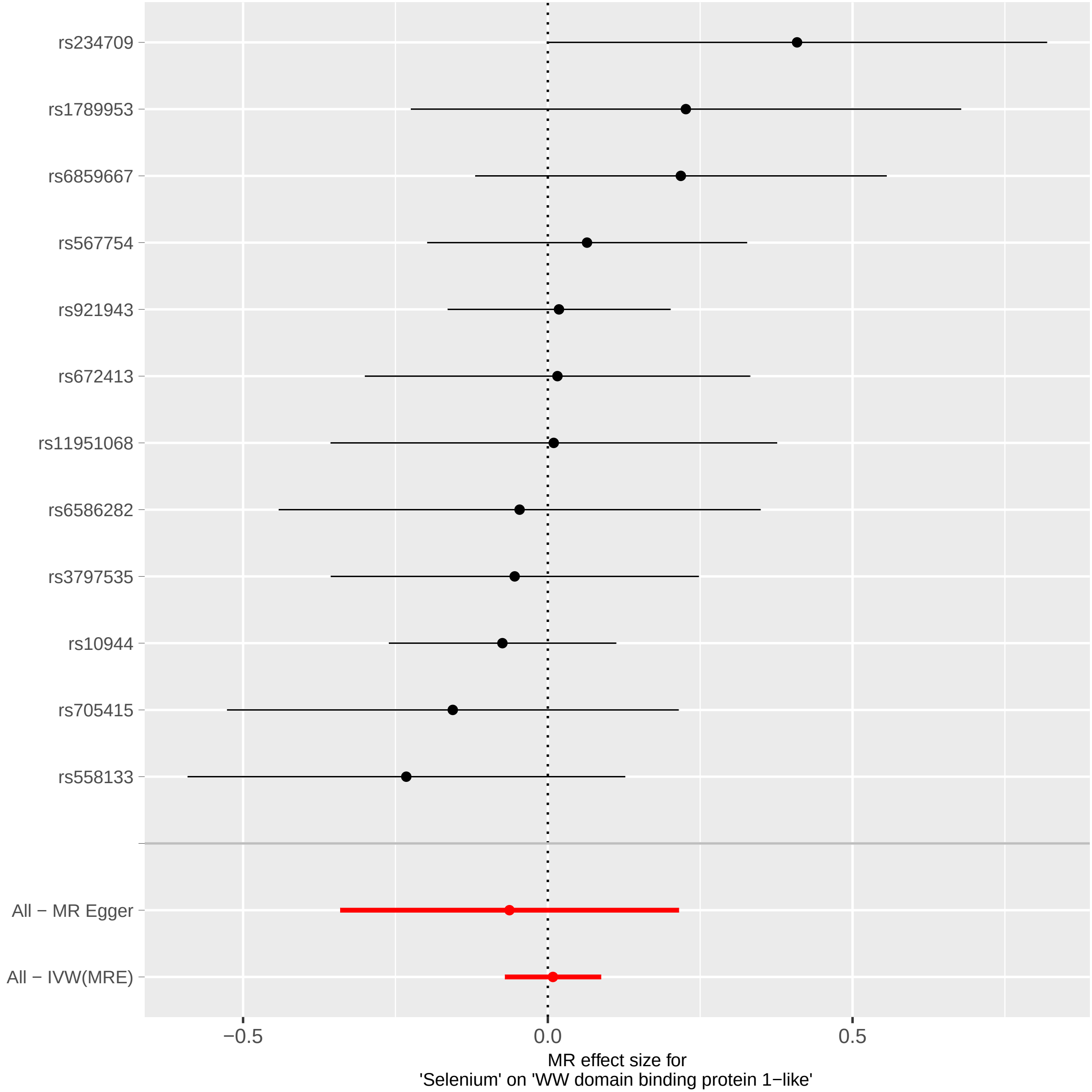

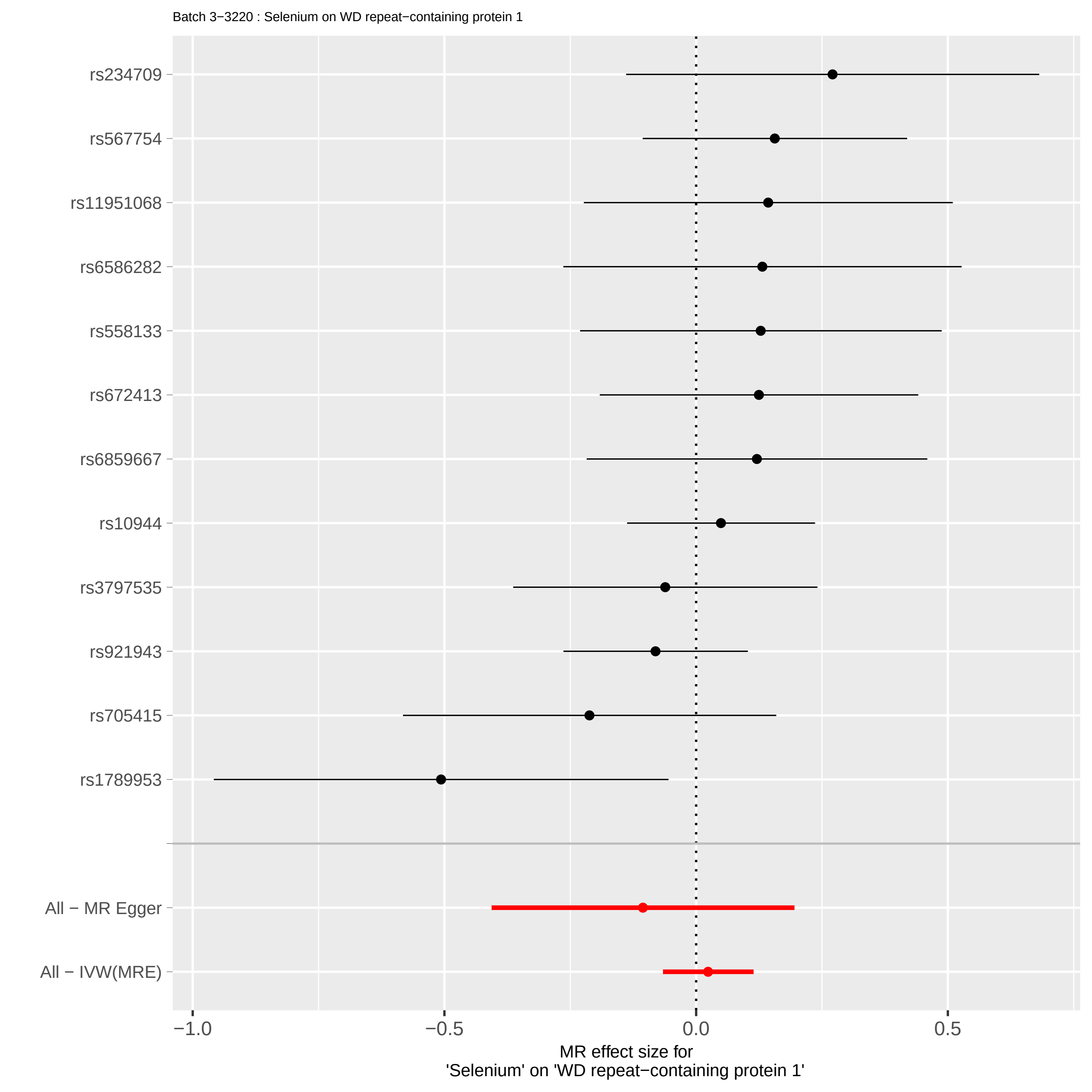

Batch 3−3220 : Selenium on WD repeat−containing protein 1
rs234709
rs567754
rs11951068
rs6586282
rs558133
rs672413
rs6859667
rs10944
rs3797535
rs921943
rs705415
rs1789953
All − MR Egger
All − IVW(MRE)
−1.0
−0.5
0.0
0.5
MR effect size for
'Selenium' on 'WD repeat−containing protein 1'

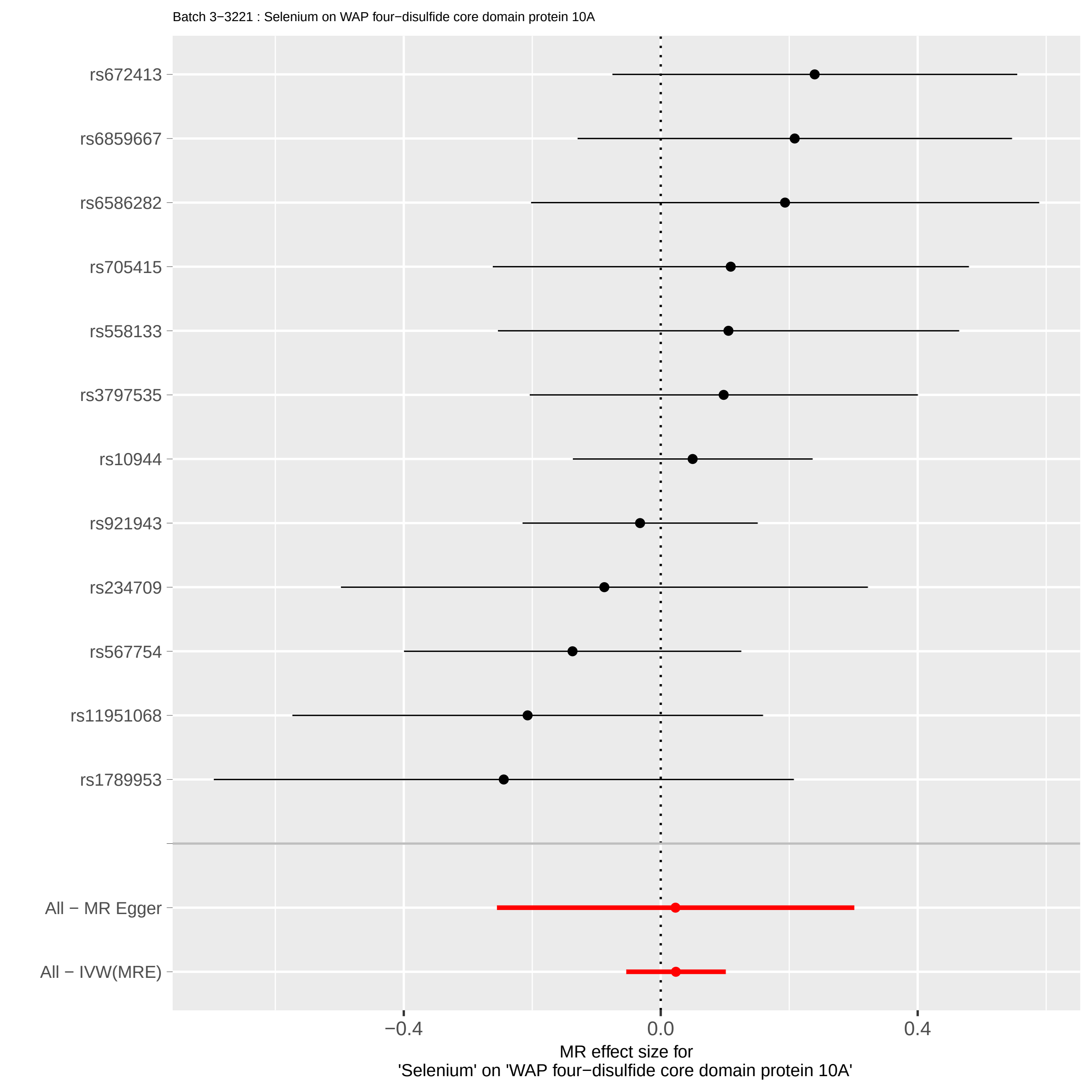

Batch 3−3221 : Selenium on WAP four−disulfide core domain protein 10A
rs672413
rs6859667
rs6586282
rs705415
rs558133
rs3797535
rs10944
rs921943
rs234709
rs567754
rs11951068
rs1789953
All − MR Egger
All − IVW(MRE)
−0.4
0.0
0.4
MR effect size for
'Selenium' on 'WAP four−disulfide core domain protein 10A'

Batch 3−3222 : Selenium on WAP four−disulfide core domain protein 12

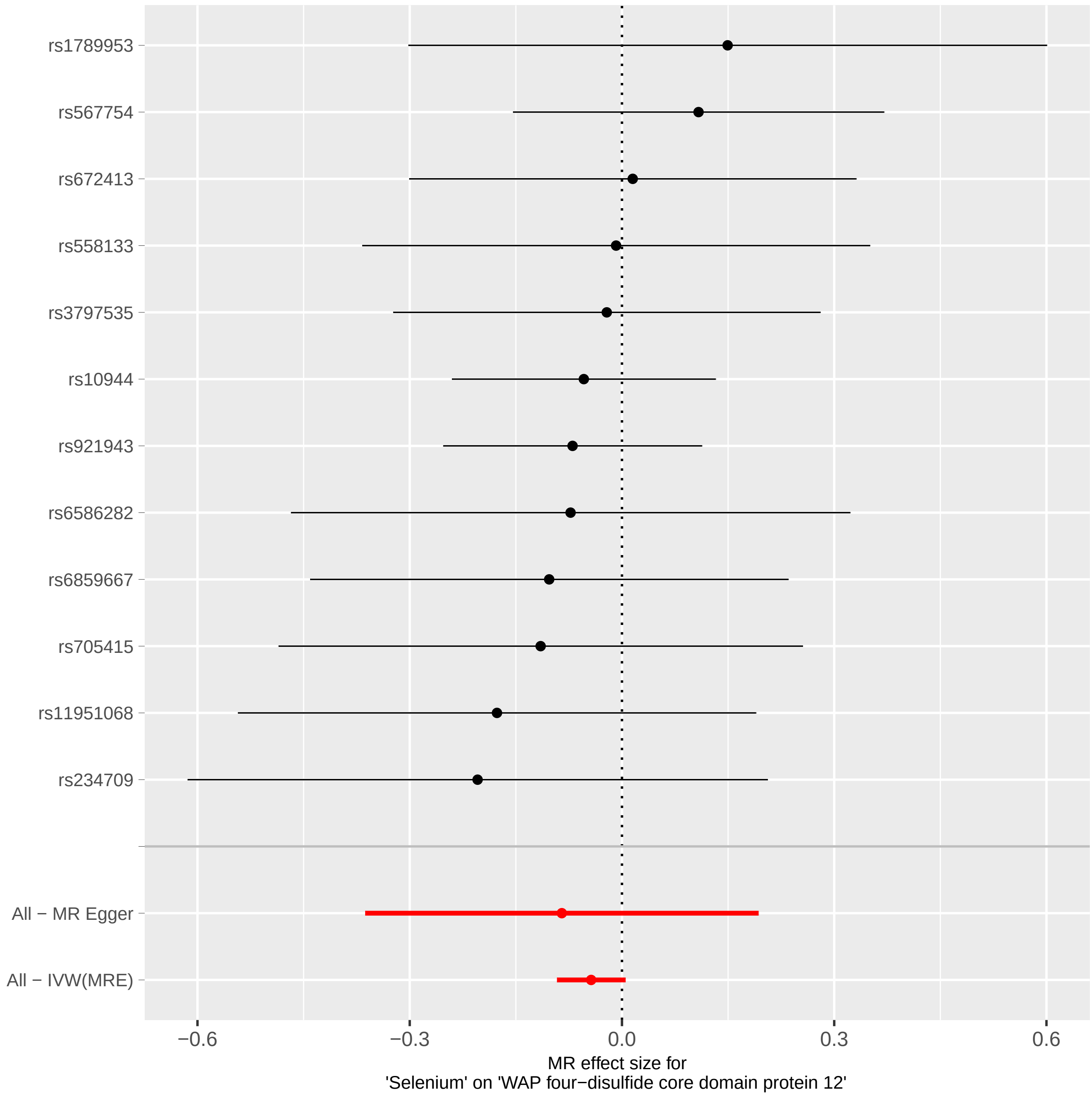

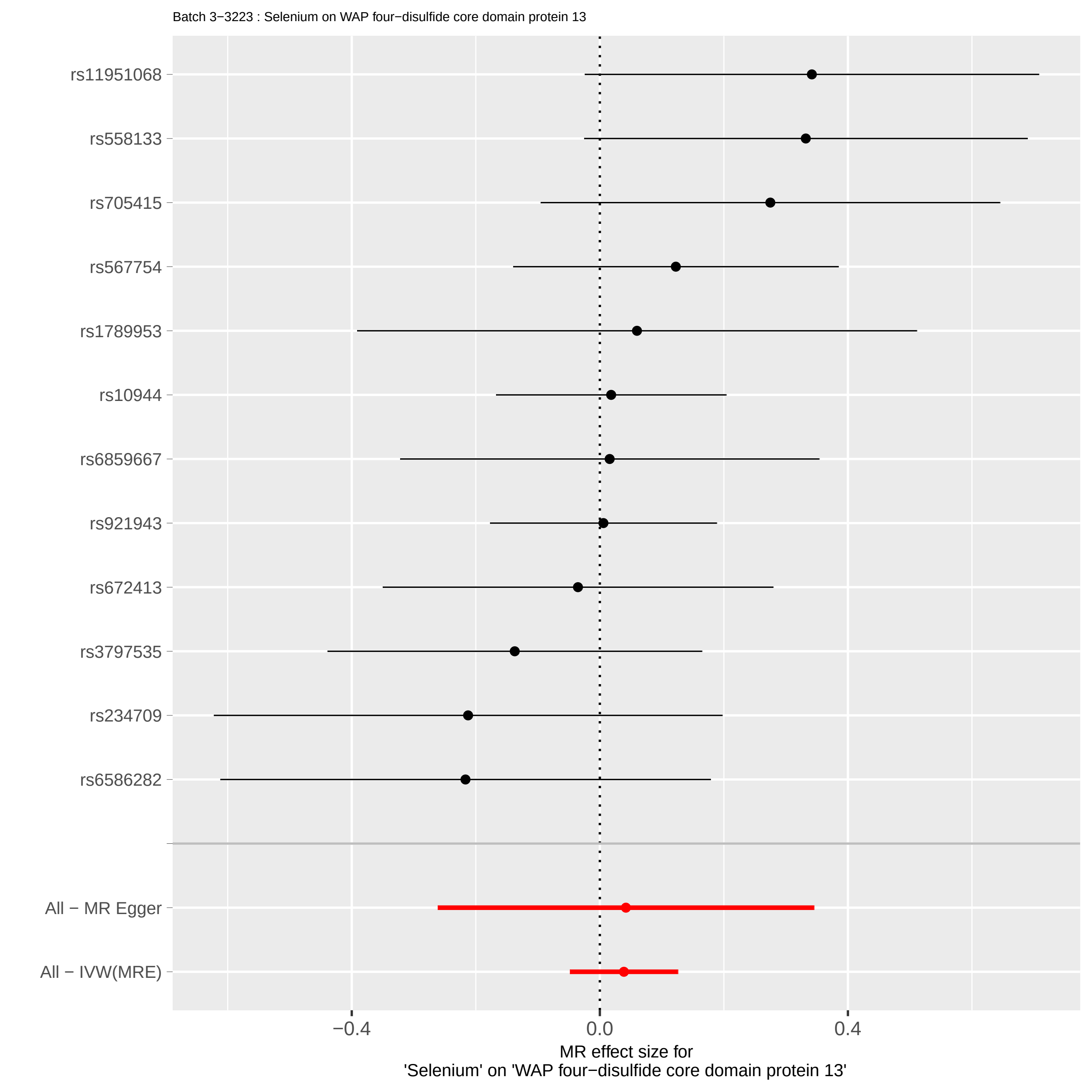
Batch 3−3223 : Selenium on WAP four−disulfide core domain protein 13
rs11951068
rs558133
rs705415
rs567754
rs1789953
rs10944
rs6859667
rs921943
rs672413
rs3797535
rs234709
rs6586282
All − MR Egger
All − IVW(MRE)
−0.4
0.0
0.4
MR effect size for
'Selenium' on 'WAP four−disulfide core domain protein 13'

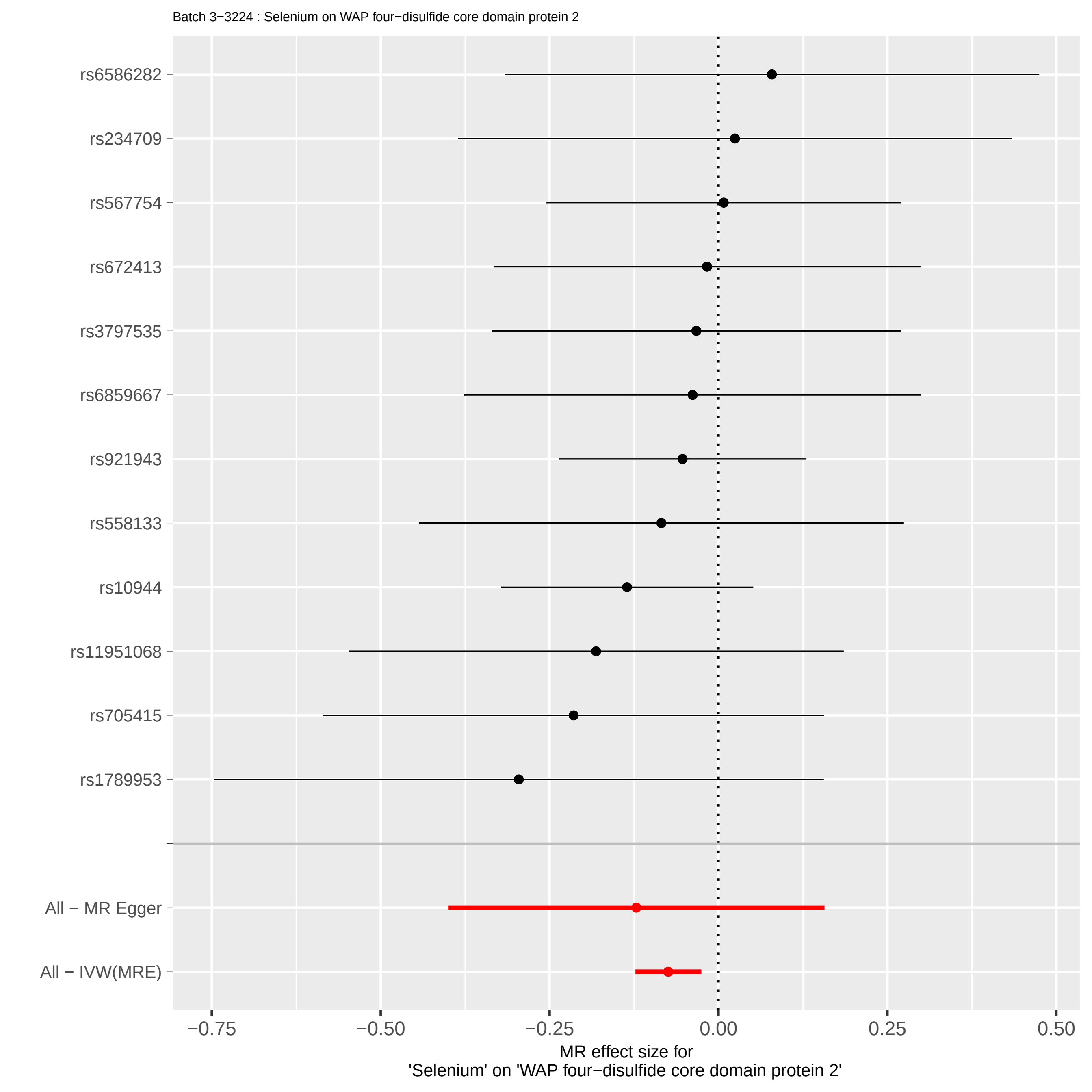
Batch 3−3224 : Selenium on WAP four−disulfide core domain protein 2
rs6586282
rs234709
rs567754
rs672413
rs3797535
rs6859667
rs921943
rs558133
rs10944
rs11951068
rs705415
rs1789953
All − MR Egger
All − IVW(MRE)
−0.75
−0.50
−0.25
0.00
0.25
0.50
MR effect size for
'Selenium' on 'WAP four−disulfide core domain protein 2'

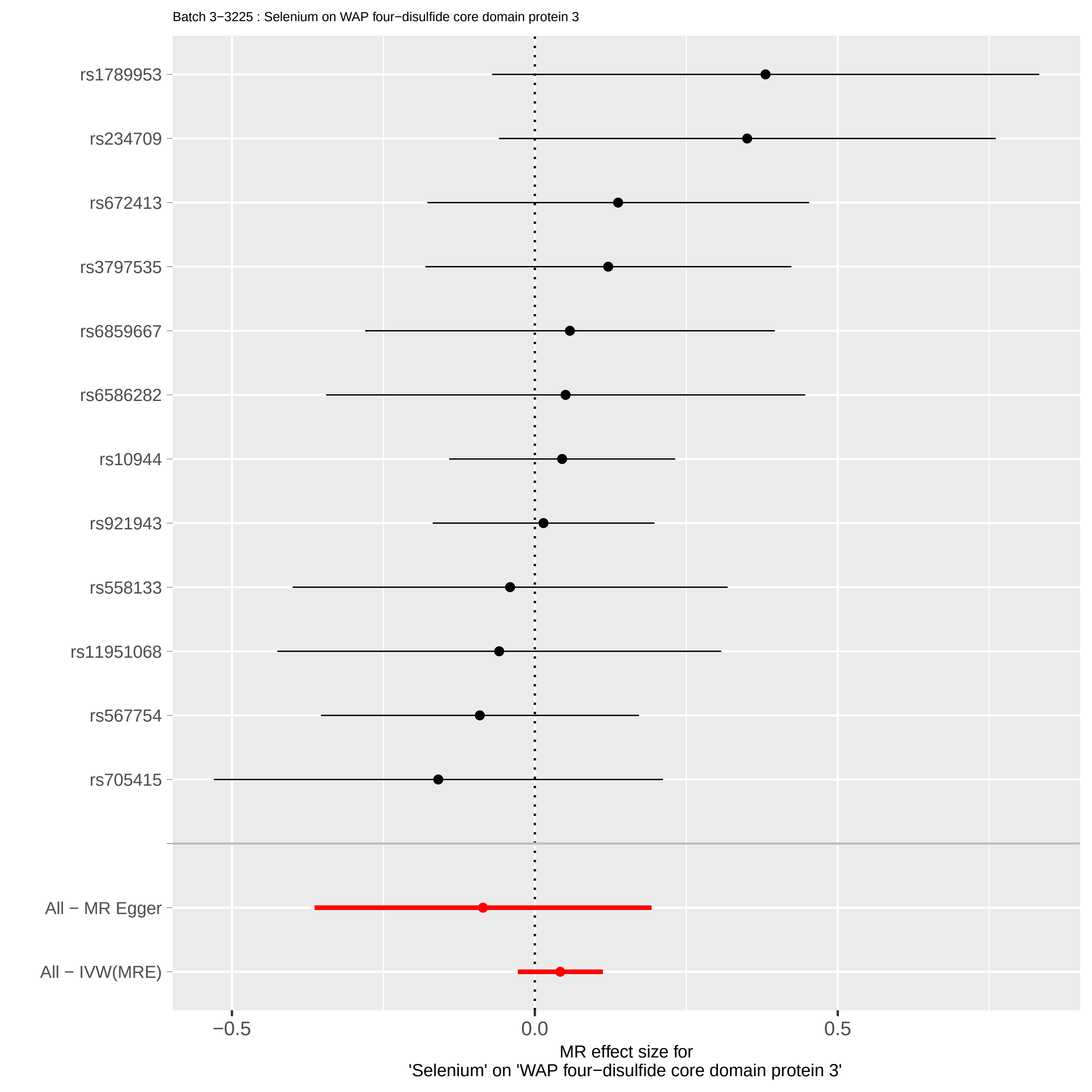

Batch 3−3225 : Selenium on WAP four−disulfide core domain protein 3
rs1789953
rs234709
rs672413
rs3797535
rs6859667
rs6586282
rs10944
rs921943
rs558133
rs11951068
rs567754
rs705415
All − MR Egger
All − IVW(MRE)
−0.5
0.0
0.5
MR effect size for
'Selenium' on 'WAP four−disulfide core domain protein 3'

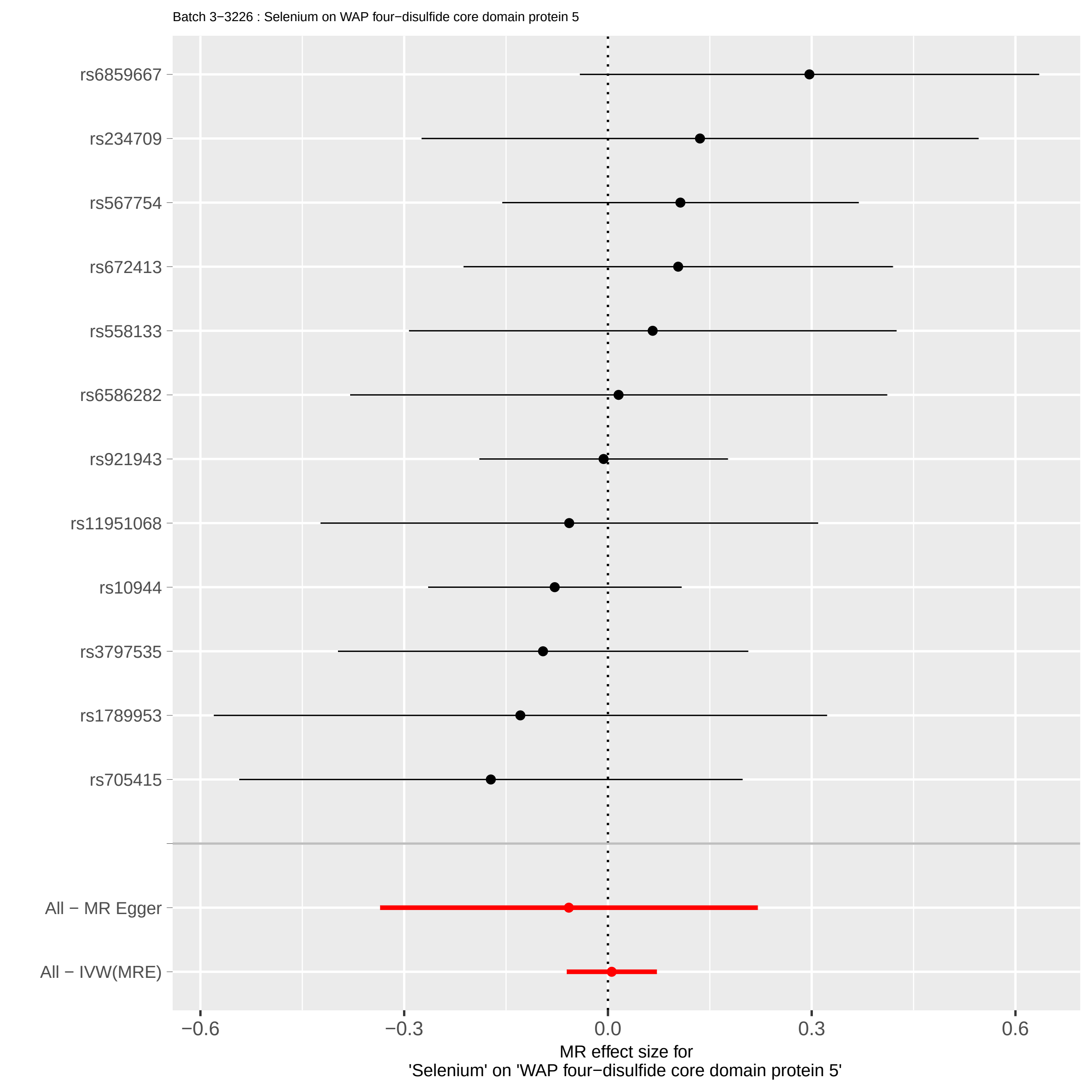

Batch 3−3226 : Selenium on WAP four−disulfide core domain protein 5
rs6859667
rs234709
rs567754
rs672413
rs558133
rs6586282
rs921943
rs11951068
rs10944
rs3797535
rs1789953
rs705415
All − MR Egger
All − IVW(MRE)
−0.6
−0.3
0.0
0.3
0.6
MR effect size for
'Selenium' on 'WAP four−disulfide core domain protein 5'

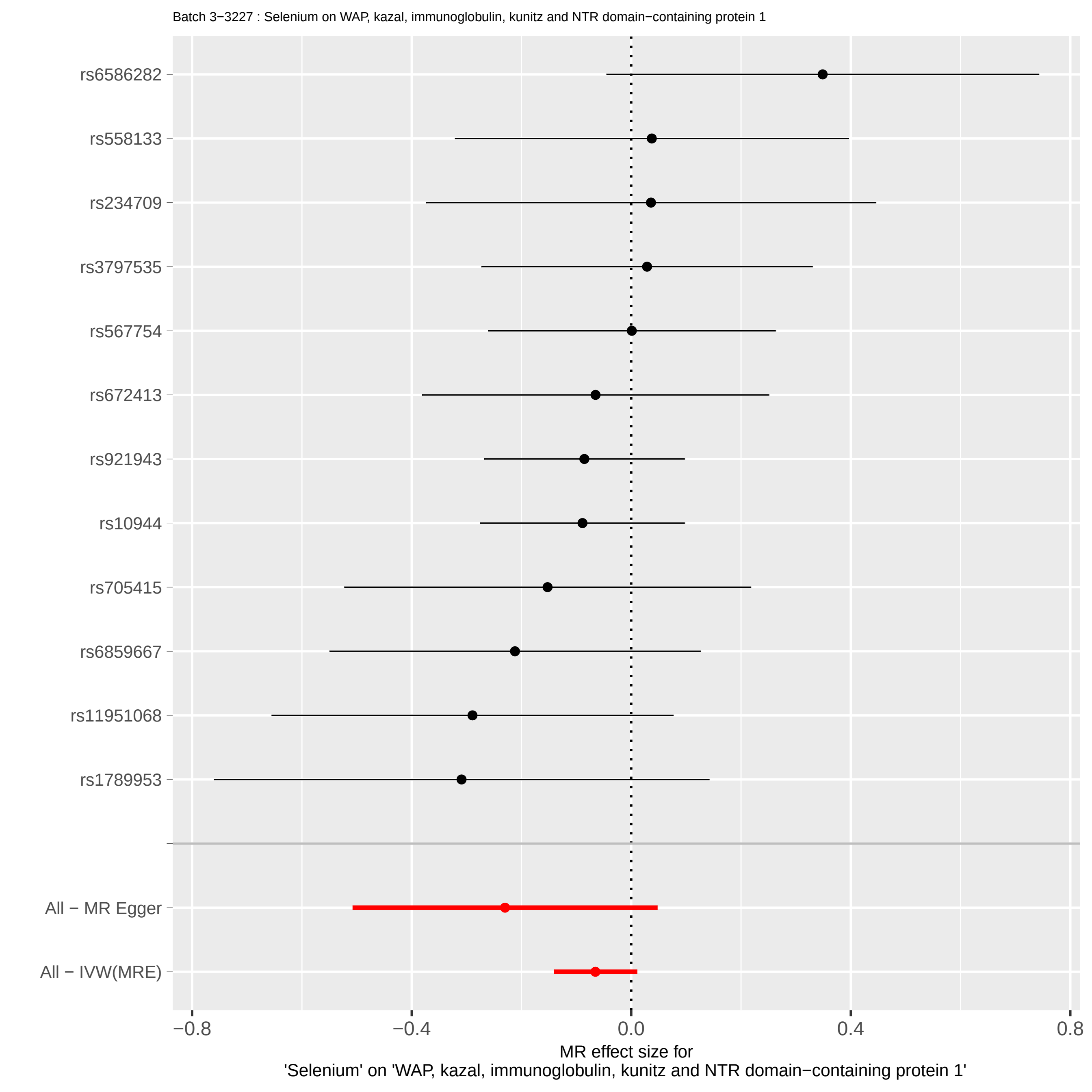

Batch 3−3227 : Selenium on WAP, kazal, immunoglobulin, kunitz and NTR domain−containing protein 1
rs6586282
rs558133
rs234709
rs3797535
rs567754
rs672413
rs921943
rs10944
rs705415
rs6859667
rs11951068
rs1789953
All − MR Egger
All − IVW(MRE)
−0.8
−0.4
0.0
0.4
0.8
MR effect size for
'Selenium' on 'WAP, kazal, immunoglobulin, kunitz and NTR domain−containing protein 1'

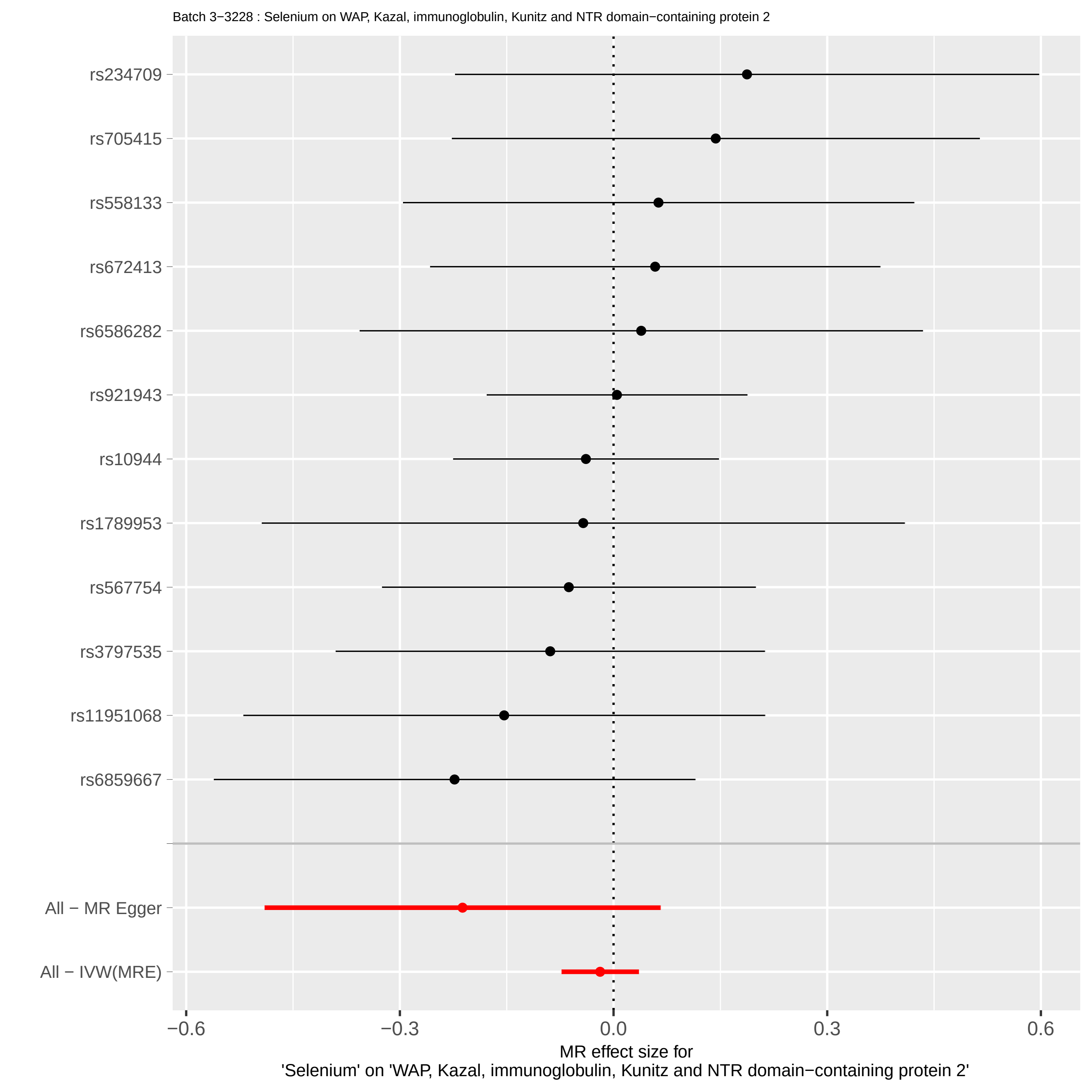
Batch 3−3228 : Selenium on WAP, Kazal, immunoglobulin, Kunitz and NTR domain−containing protein 2
rs234709
rs705415
rs558133
rs672413
rs6586282
rs921943
rs10944
rs1789953
rs567754
rs3797535
rs11951068
rs6859667
All − MR Egger
All − IVW(MRE)
−0.6
−0.3
0.0
0.3
0.6
MR effect size for
'Selenium' on 'WAP, Kazal, immunoglobulin, Kunitz and NTR domain−containing protein 2'

Batch 3−3229 : Selenium on WAP, Kazal, immunoglobulin, Kunitz and NTR domain−containing protein 2

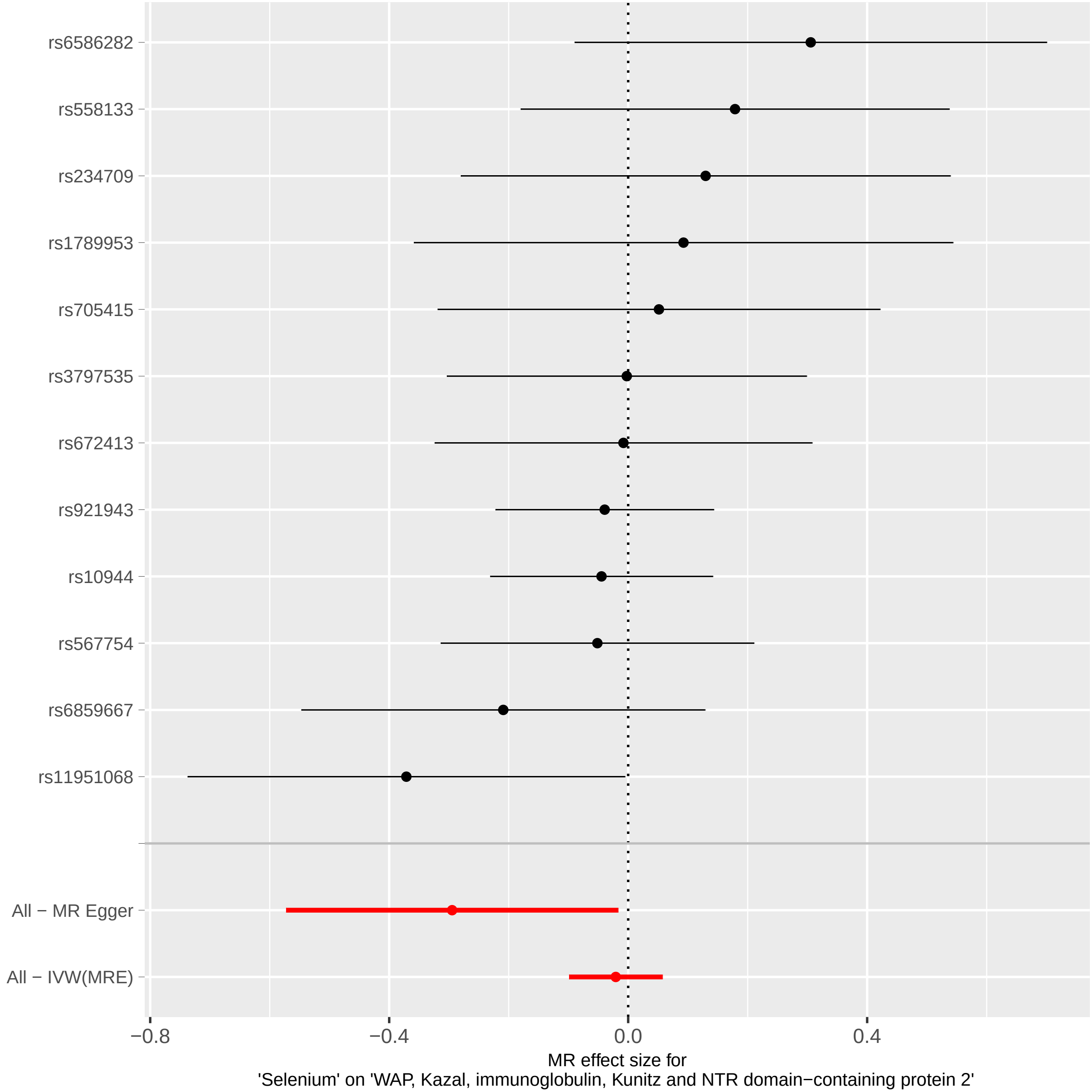

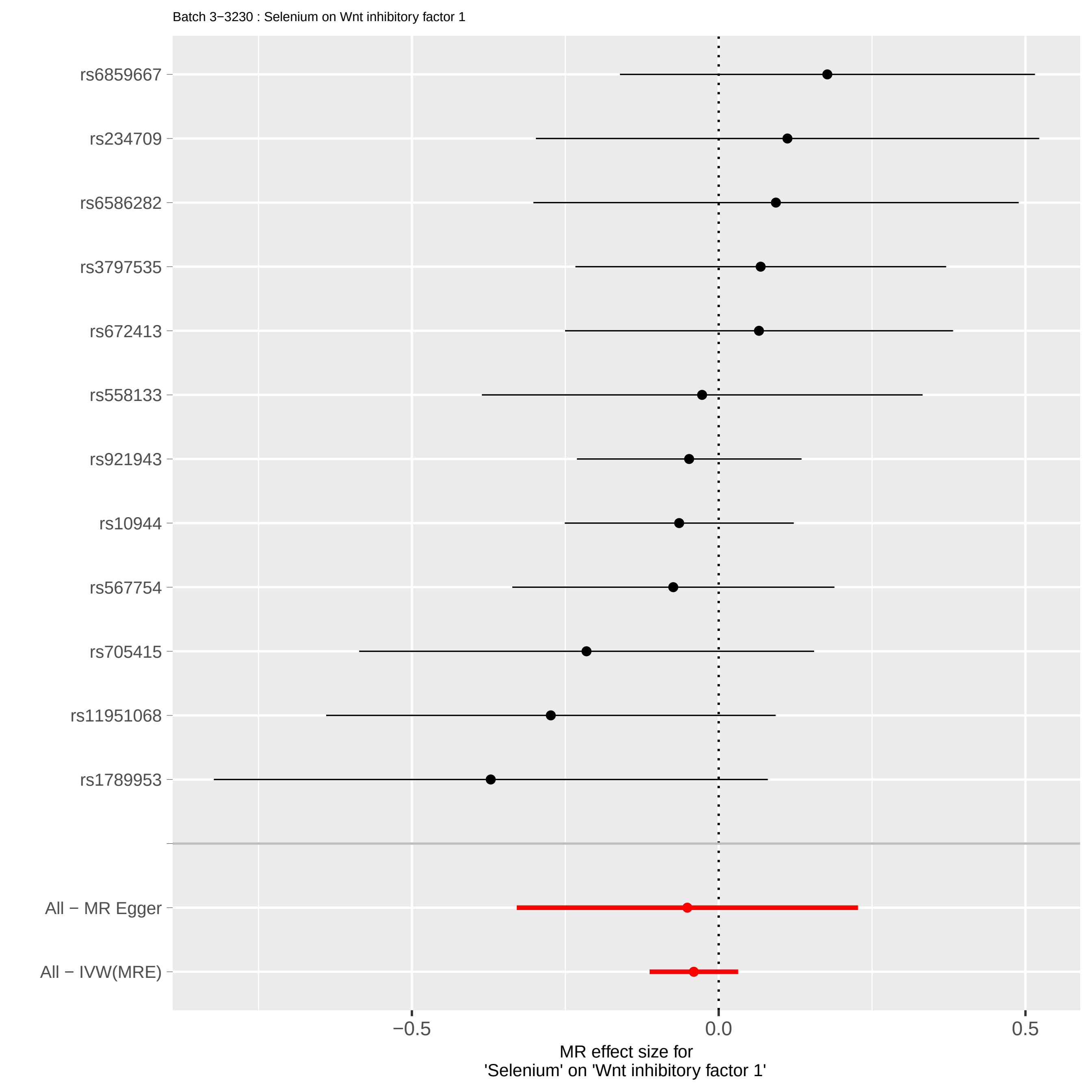
Batch 3−3230 : Selenium on Wnt inhibitory factor 1
rs6859667
rs234709
rs6586282
rs3797535
rs672413
rs558133
rs921943
rs10944
rs567754
rs705415
rs11951068
rs1789953
All − MR Egger
All − IVW(MRE)
−0.5
0.0
0.5
MR effect size for
'Selenium' on 'Wnt inhibitory factor 1'

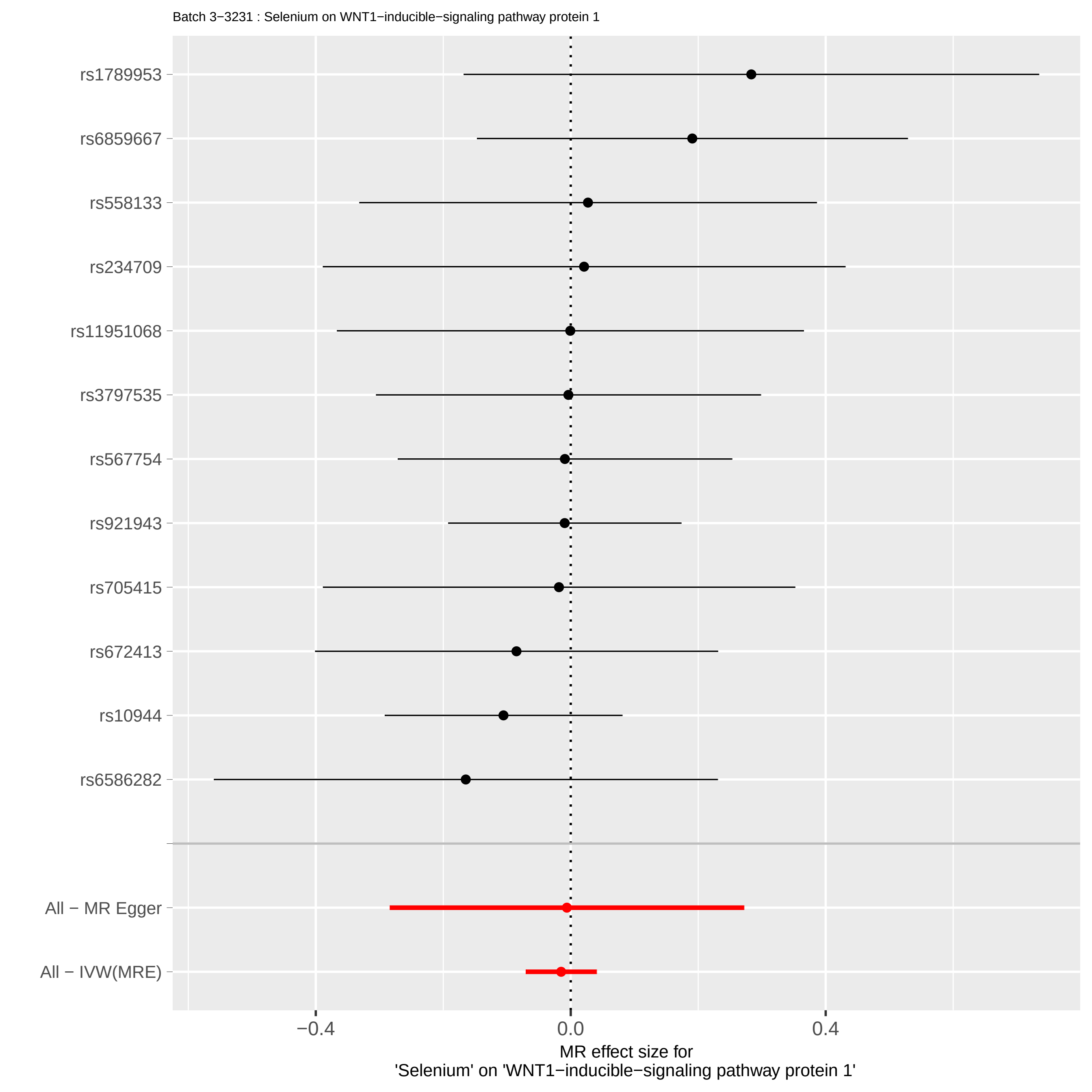

Batch 3−3231 : Selenium on WNT1−inducible−signaling pathway protein 1
rs1789953
rs6859667
rs558133
rs234709
rs11951068
rs3797535
rs567754
rs921943
rs705415
rs672413
rs10944
rs6586282
All − MR Egger
All − IVW(MRE)
−0.4
0.0
0.4
MR effect size for
'Selenium' on 'WNT1−inducible−signaling pathway protein 1'

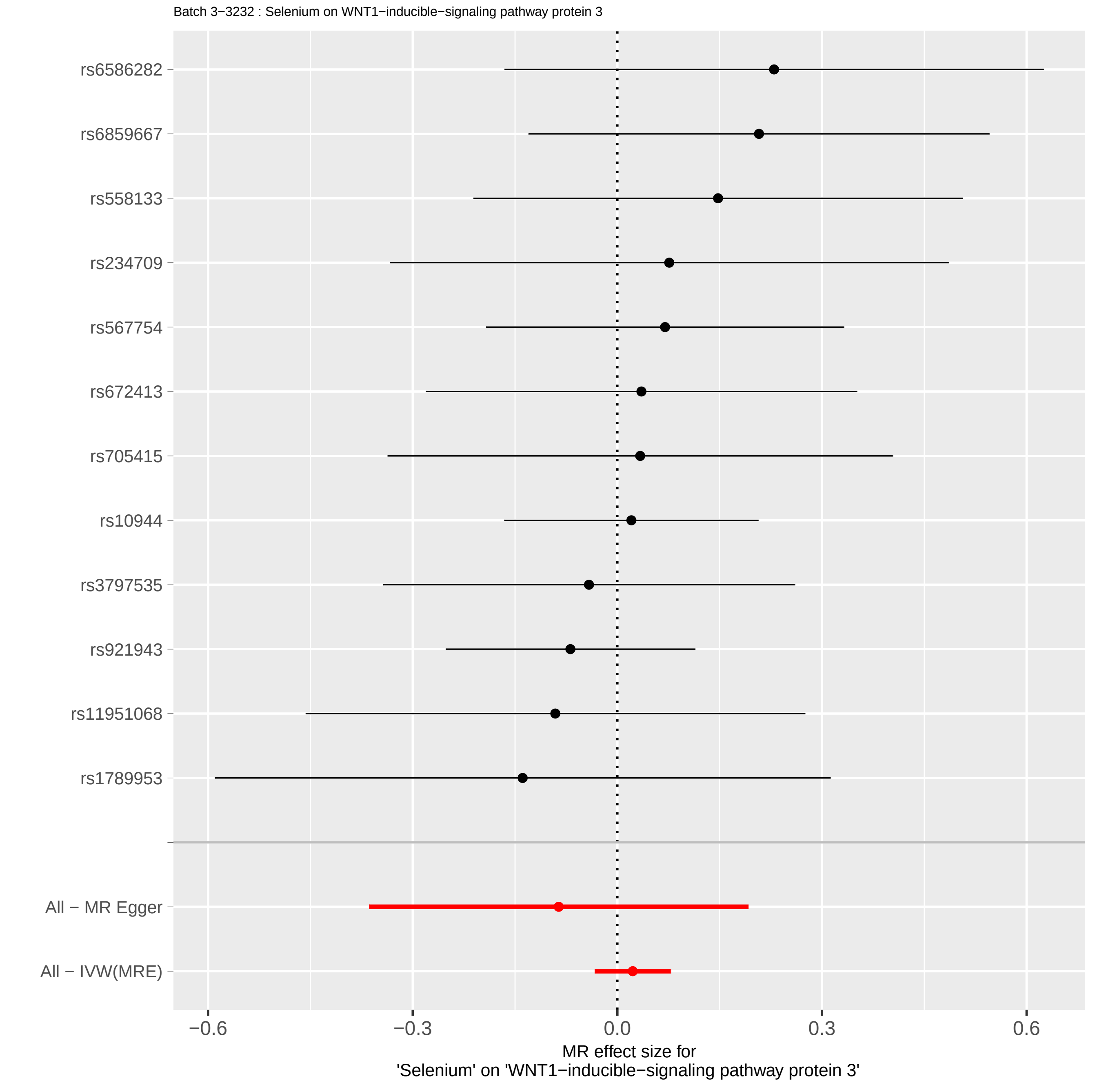

Batch 3−3232 : Selenium on WNT1−inducible−signaling pathway protein 3
rs6586282
rs6859667
rs558133
rs234709
rs567754
rs672413
rs705415
rs10944
rs3797535
rs921943
rs11951068
rs1789953
All − MR Egger
All − IVW(MRE)
−0.6
−0.3
0.0
0.3
0.6
MR effect size for
'Selenium' on 'WNT1−inducible−signaling pathway protein 3'

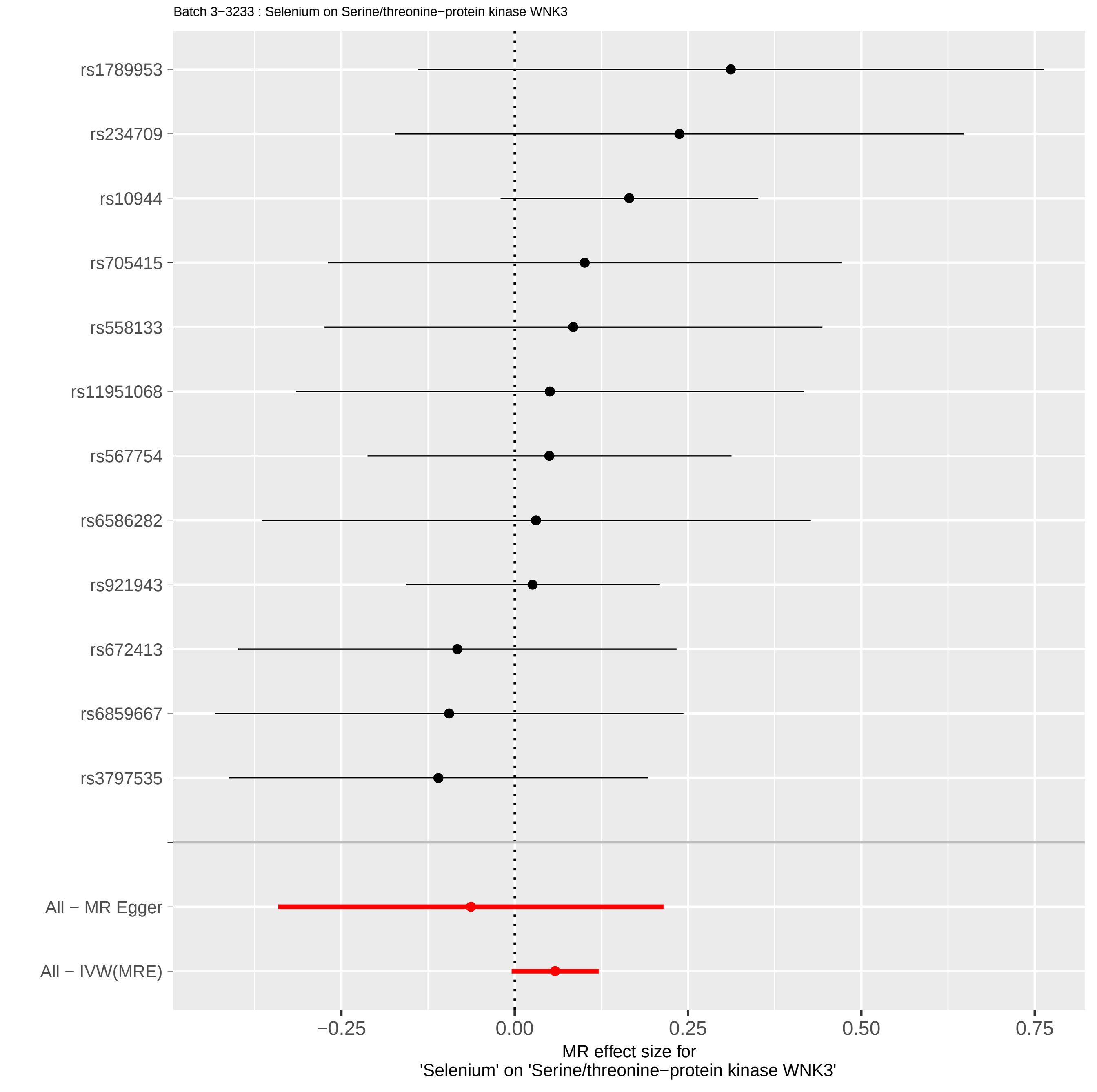
Batch 3−3233 : Selenium on Serine/threonine−protein kinase WNK3
rs1789953
rs234709
rs10944
rs705415
rs558133
rs11951068
rs567754
rs6586282
rs921943
rs672413
rs6859667
rs3797535
All − MR Egger
All − IVW(MRE)
−0.25
0.00
0.25
0.50
0.75
MR effect size for
'Selenium' on 'Serine/threonine−protein kinase WNK3'

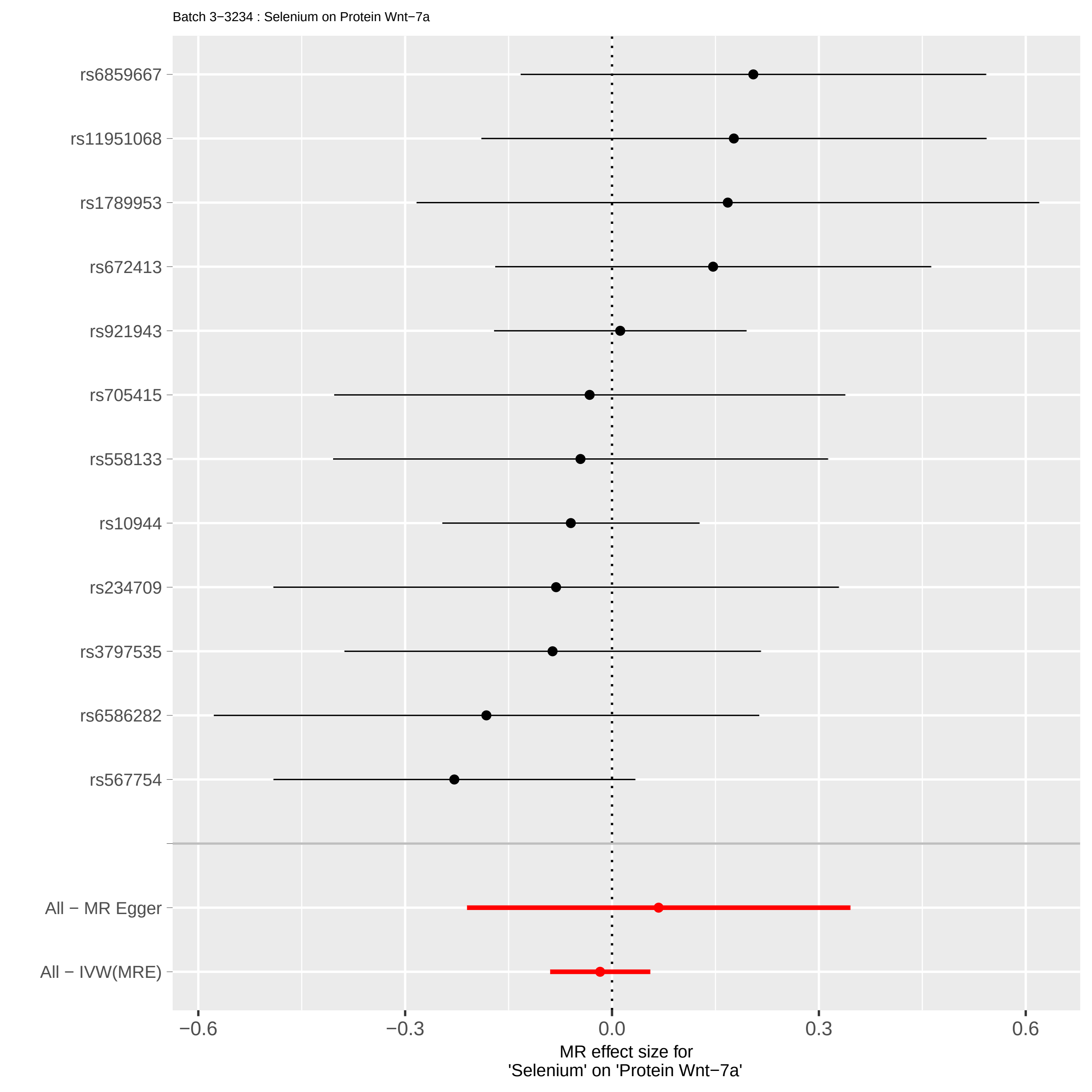

Batch 3−3234 : Selenium on Protein Wnt−7a
rs6859667
rs11951068
rs1789953
rs672413
rs921943
rs705415
rs558133
rs10944
rs234709
rs3797535
rs6586282
rs567754
All − MR Egger
All − IVW(MRE)
−0.6
−0.3
0.0
0.3
0.6
MR effect size for
'Selenium' on 'Protein Wnt−7a'

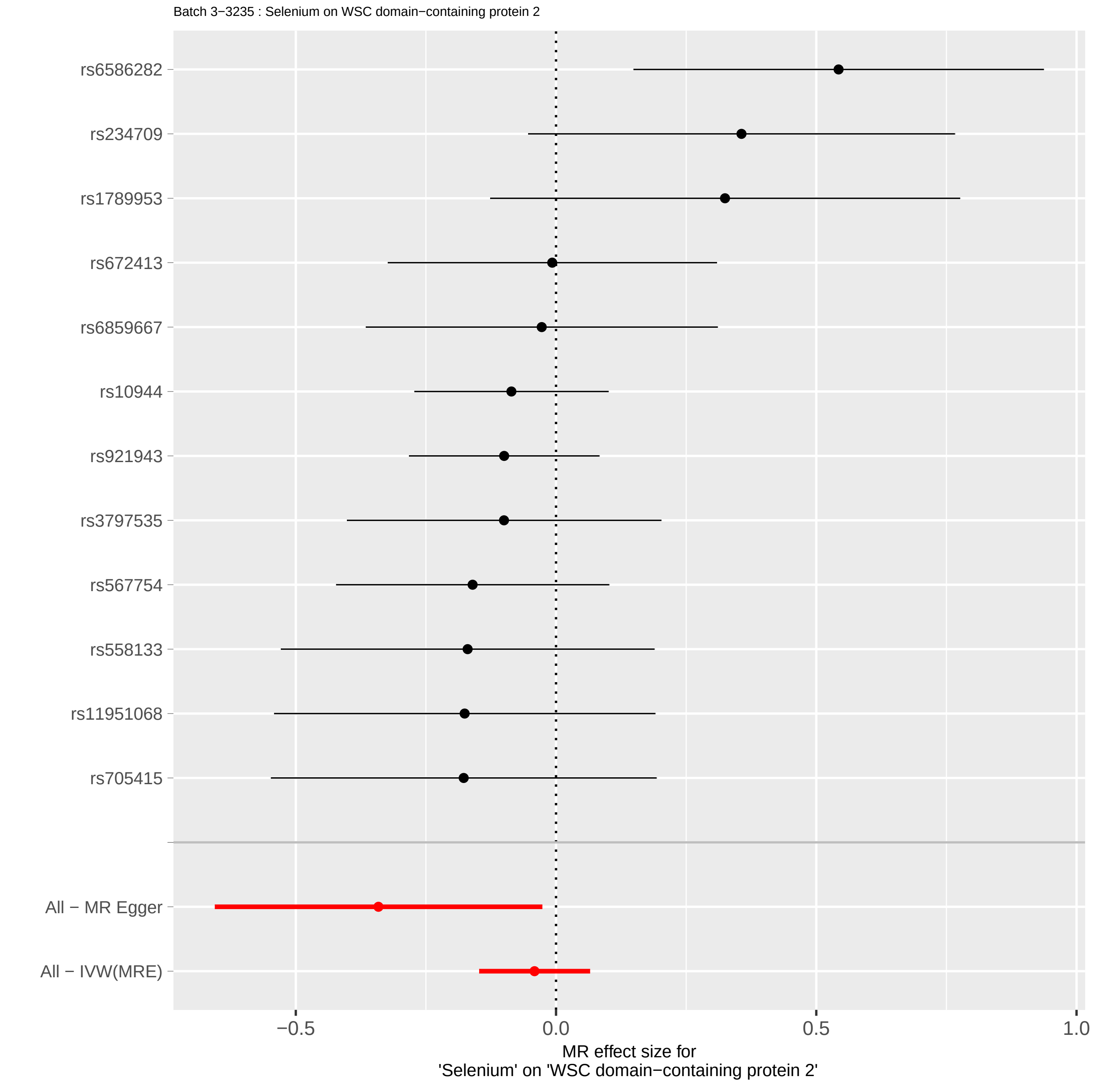

Batch 3−3235 : Selenium on WSC domain−containing protein 2
rs6586282
rs234709
rs1789953
rs672413
rs6859667
rs10944
rs921943
rs3797535
rs567754
rs558133
rs11951068
rs705415
All − MR Egger
All − IVW(MRE)
−0.5
0.0
0.5
1.0
MR effect size for
'Selenium' on 'WSC domain−containing protein 2'

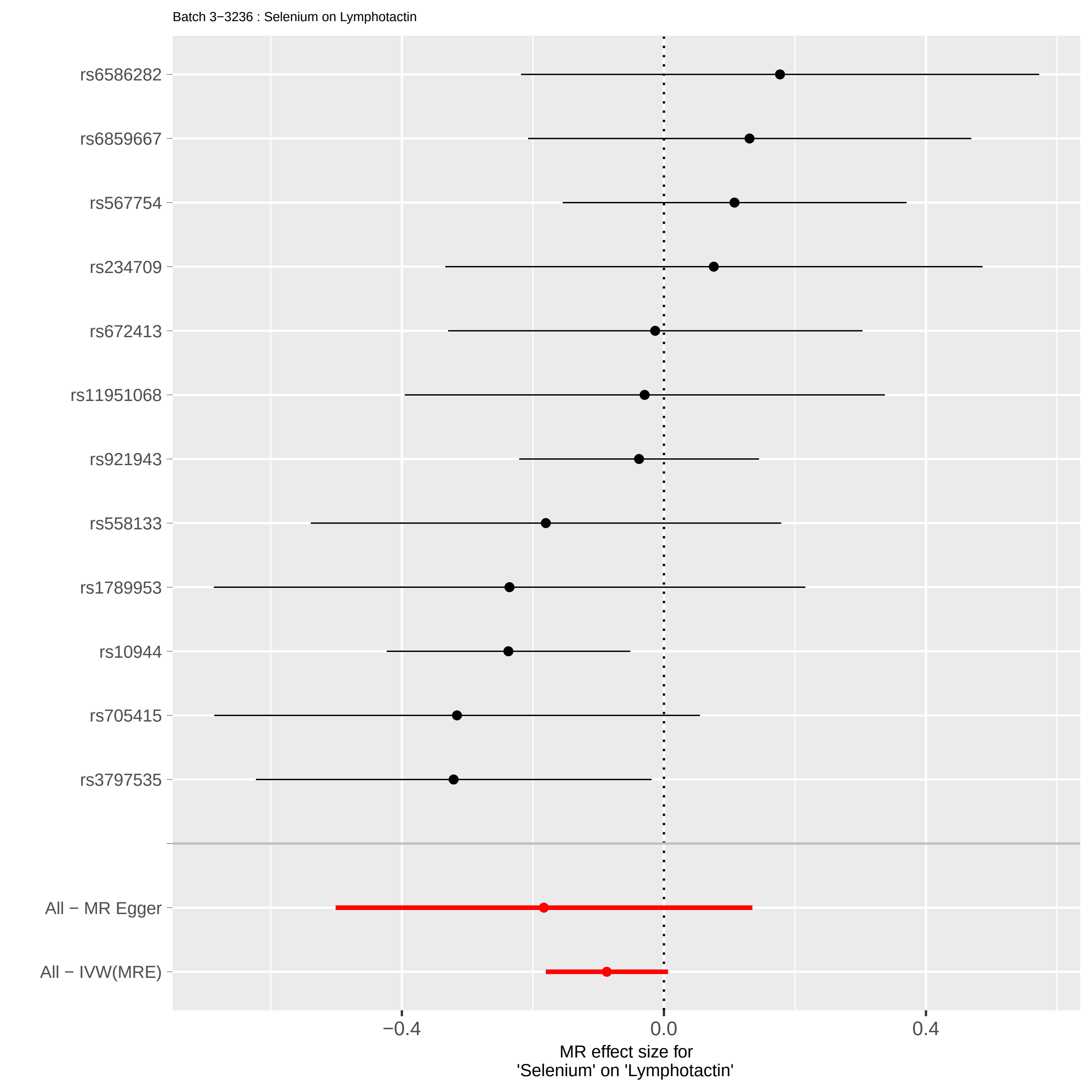
Batch 3−3236 : Selenium on Lymphotactin
rs6586282
rs6859667
rs567754
rs234709
rs672413
rs11951068
rs921943
rs558133
rs1789953
rs10944
rs705415
rs3797535
All − MR Egger
All − IVW(MRE)
−0.4
0.0
0.4
MR effect size for
'Selenium' on 'Lymphotactin'

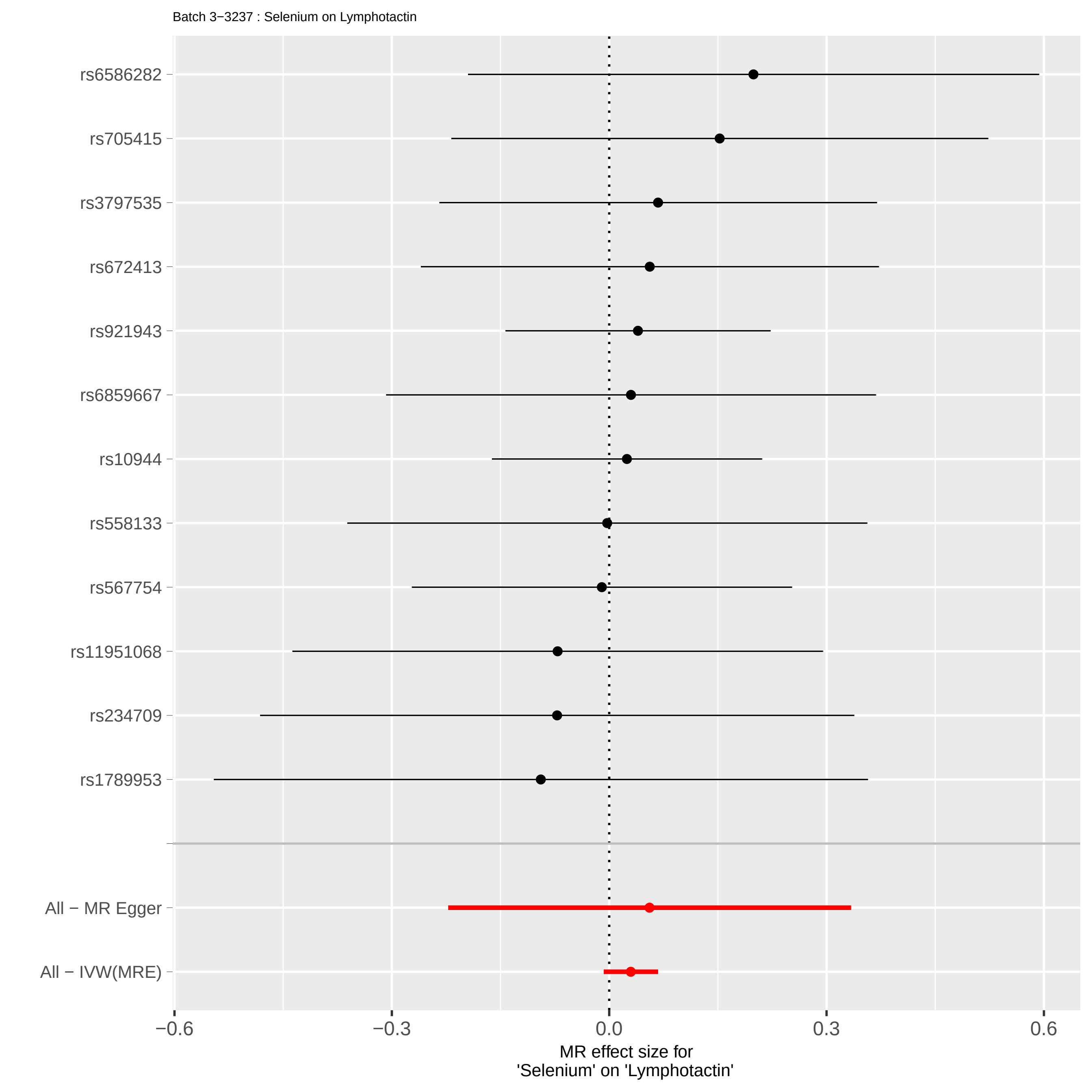

Batch 3−3237 : Selenium on Lymphotactin
rs6586282
rs705415
rs3797535
rs672413
rs921943
rs6859667
rs10944
rs558133
rs567754
rs11951068
rs234709
rs1789953
All − MR Egger
All − IVW(MRE)
−0.6
−0.3
0.0
0.3
0.6
MR effect size for
'Selenium' on 'Lymphotactin'

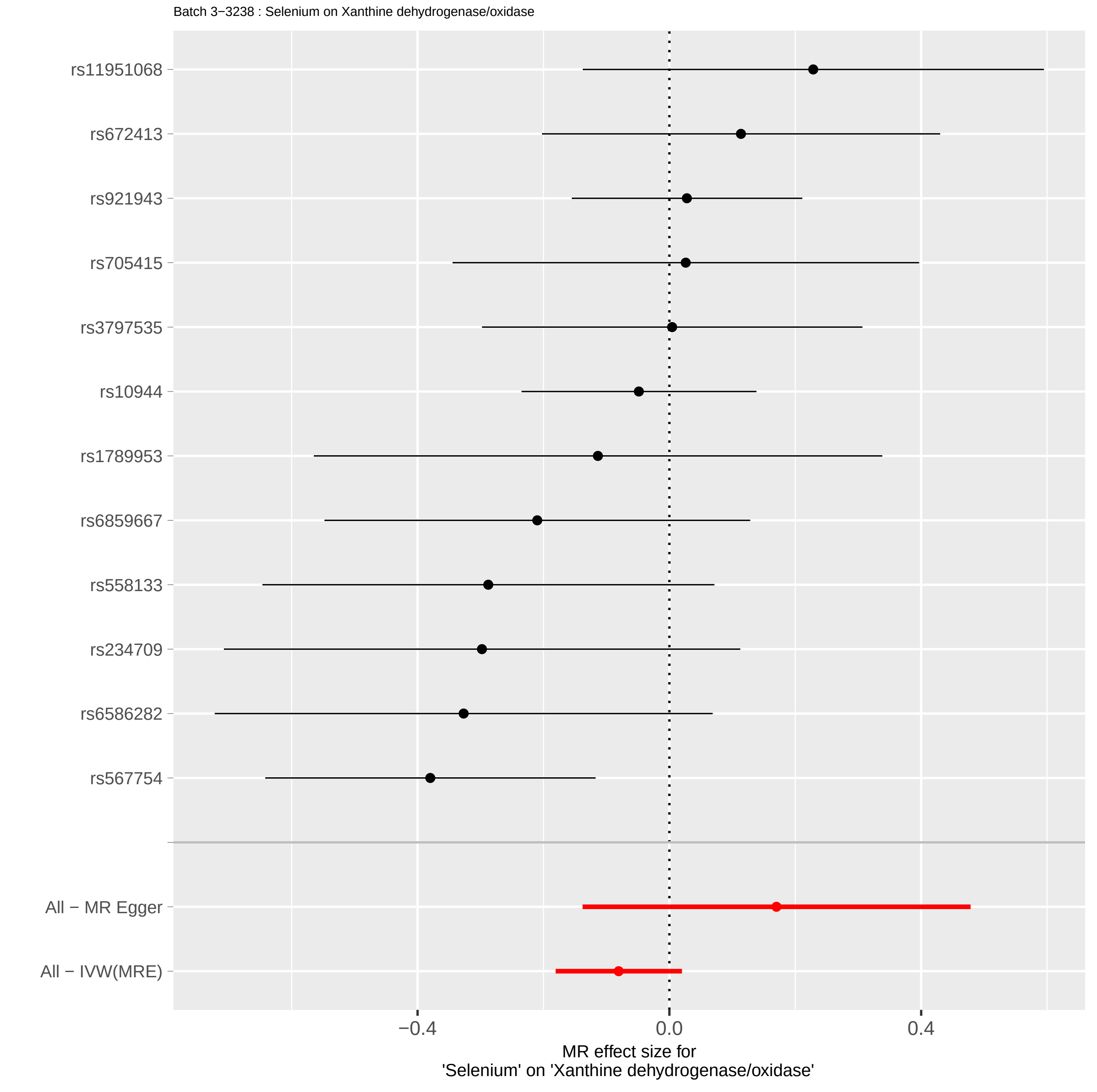
Batch 3−3238 : Selenium on Xanthine dehydrogenase/oxidase
rs11951068
rs672413
rs921943
rs705415
rs3797535
rs10944
rs1789953
rs6859667
rs558133
rs234709
rs6586282
rs567754
All − MR Egger
All − IVW(MRE)
−0.4
0.0
0.4
MR effect size for
'Selenium' on 'Xanthine dehydrogenase/oxidase'

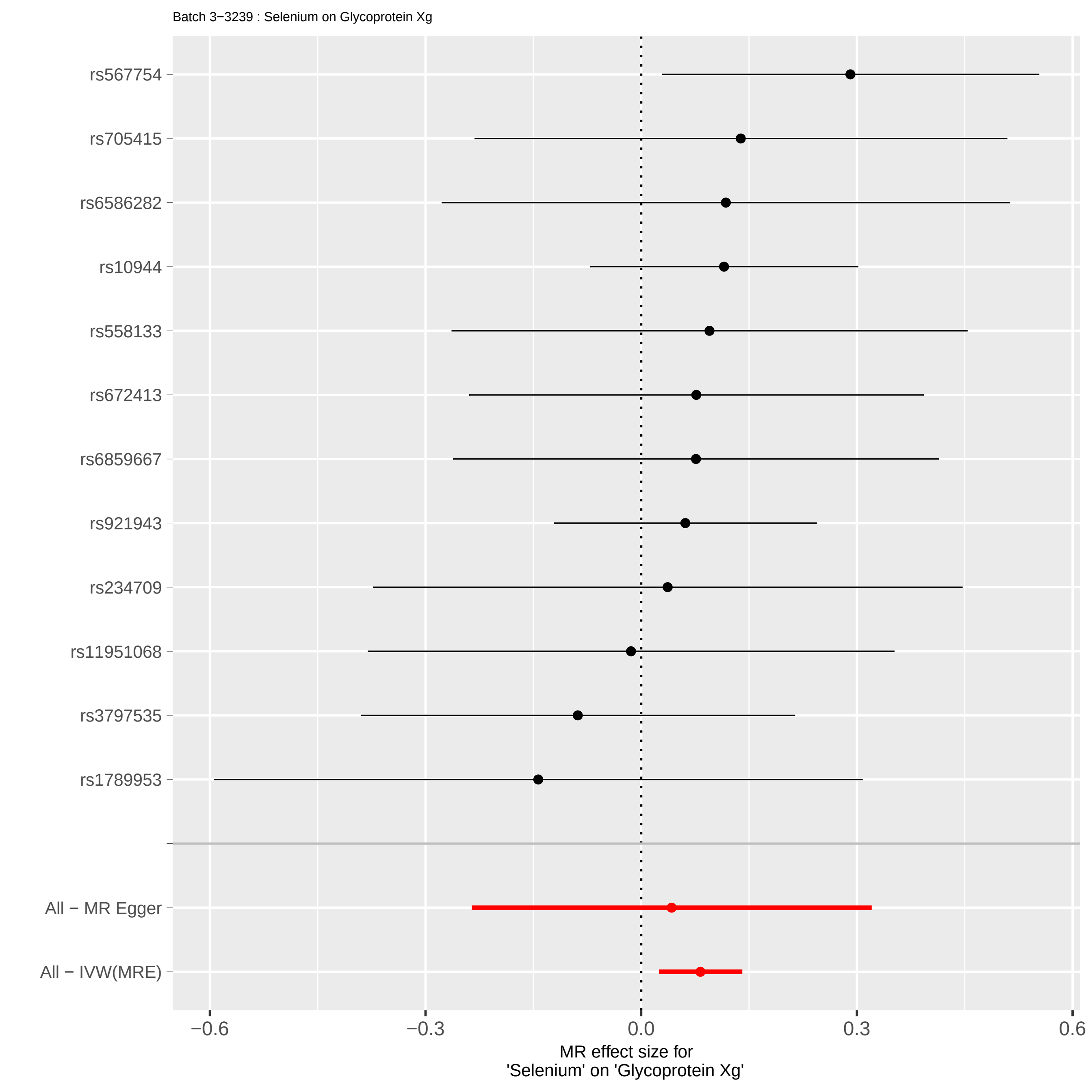

Batch 3−3239 : Selenium on Glycoprotein Xg
rs567754
rs705415
rs6586282
rs10944
rs558133
rs672413
rs6859667
rs921943
rs234709
rs11951068
rs3797535
rs1789953
All − MR Egger
All − IVW(MRE)
−0.6
−0.3
0.0
0.3
0.6
MR effect size for
'Selenium' on 'Glycoprotein Xg'

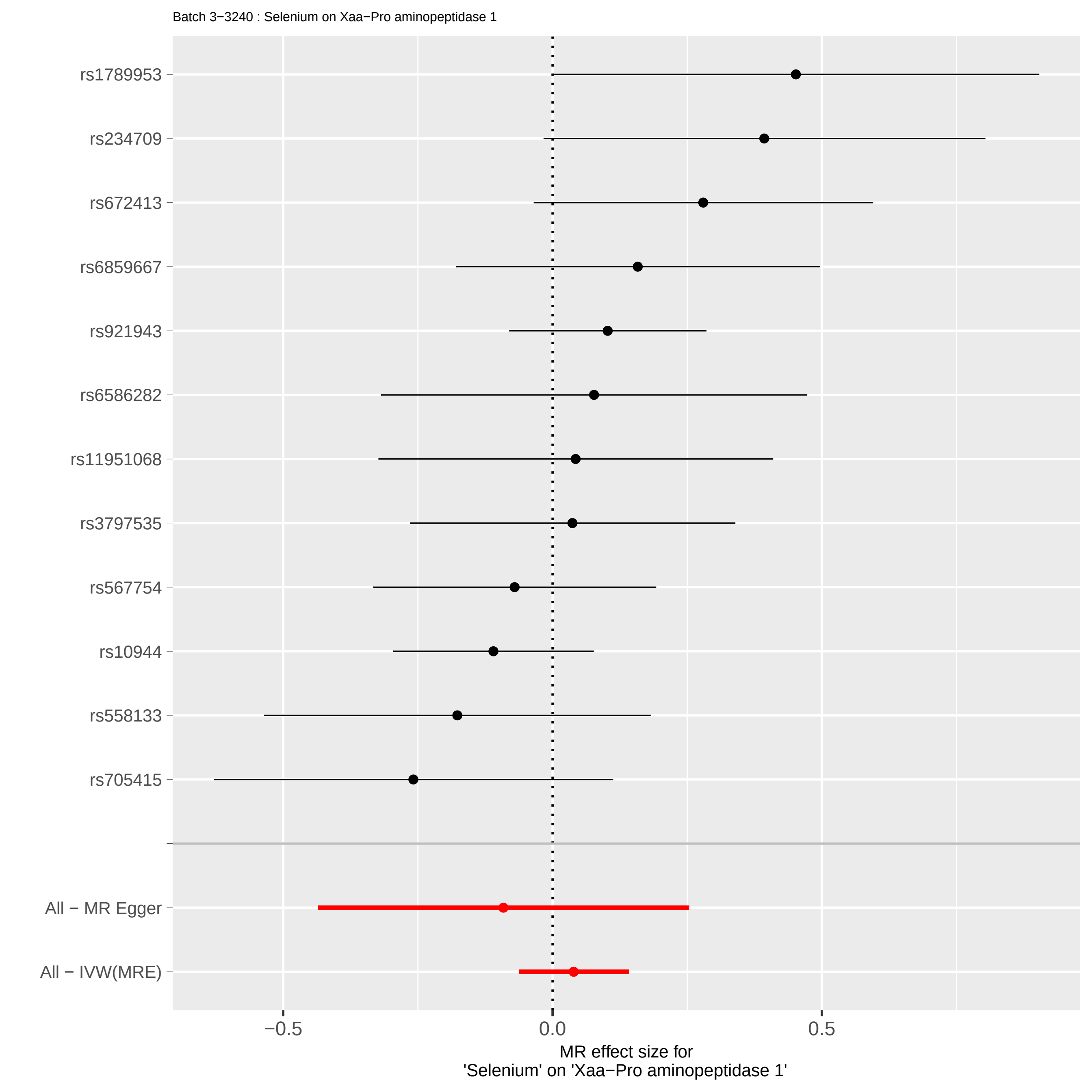
Batch 3−3240 : Selenium on Xaa−Pro aminopeptidase 1
rs1789953
rs234709
rs672413
rs6859667
rs921943
rs6586282
rs11951068
rs3797535
rs567754
rs10944
rs558133
rs705415
All − MR Egger
All − IVW(MRE)
−0.5
0.0
0.5
MR effect size for
'Selenium' on 'Xaa−Pro aminopeptidase 1'

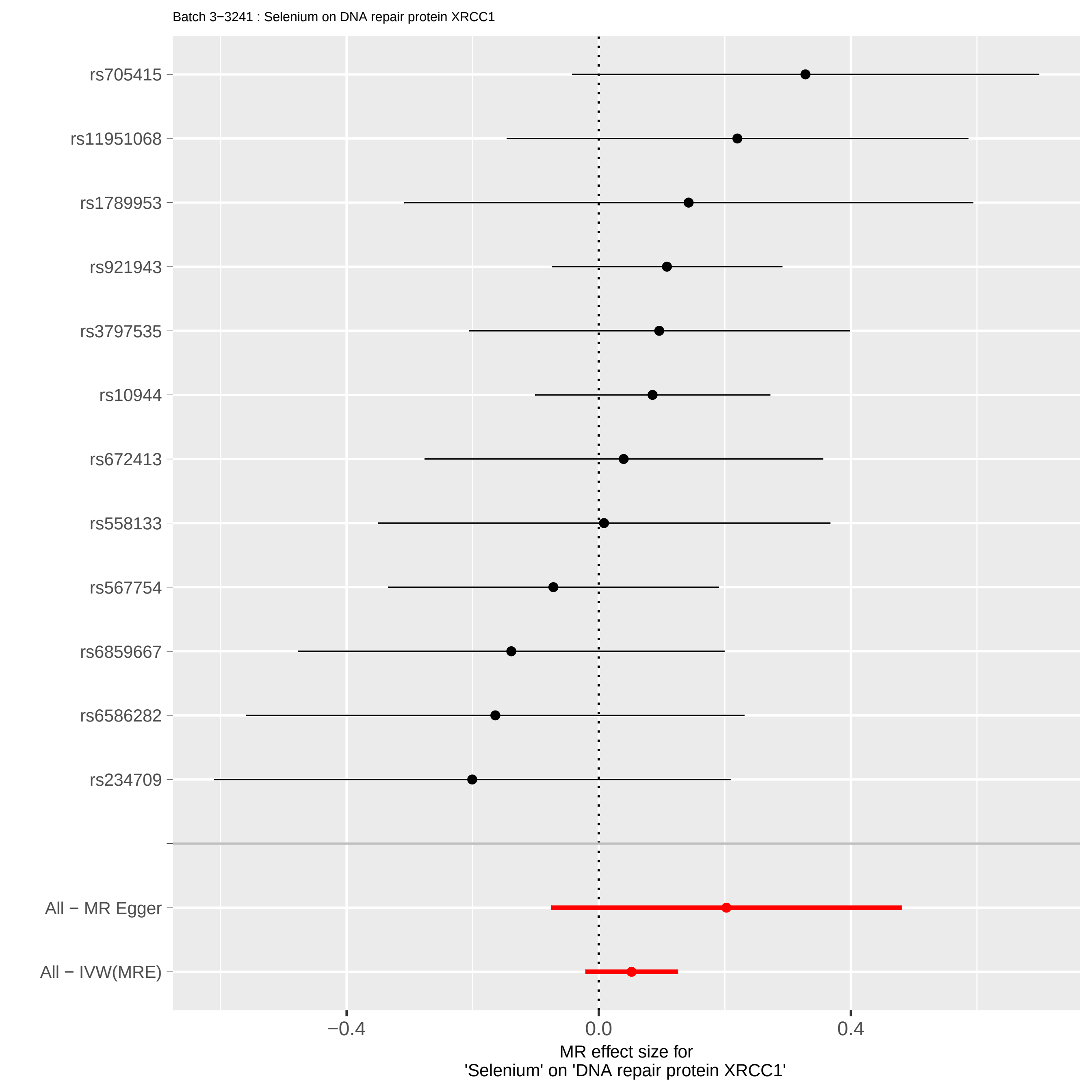

Batch 3−3241 : Selenium on DNA repair protein XRCC1
rs705415
rs11951068
rs1789953
rs921943
rs3797535
rs10944
rs672413
rs558133
rs567754
rs6859667
rs6586282
rs234709
All − MR Egger
All − IVW(MRE)
−0.4
0.0
0.4
MR effect size for
'Selenium' on 'DNA repair protein XRCC1'

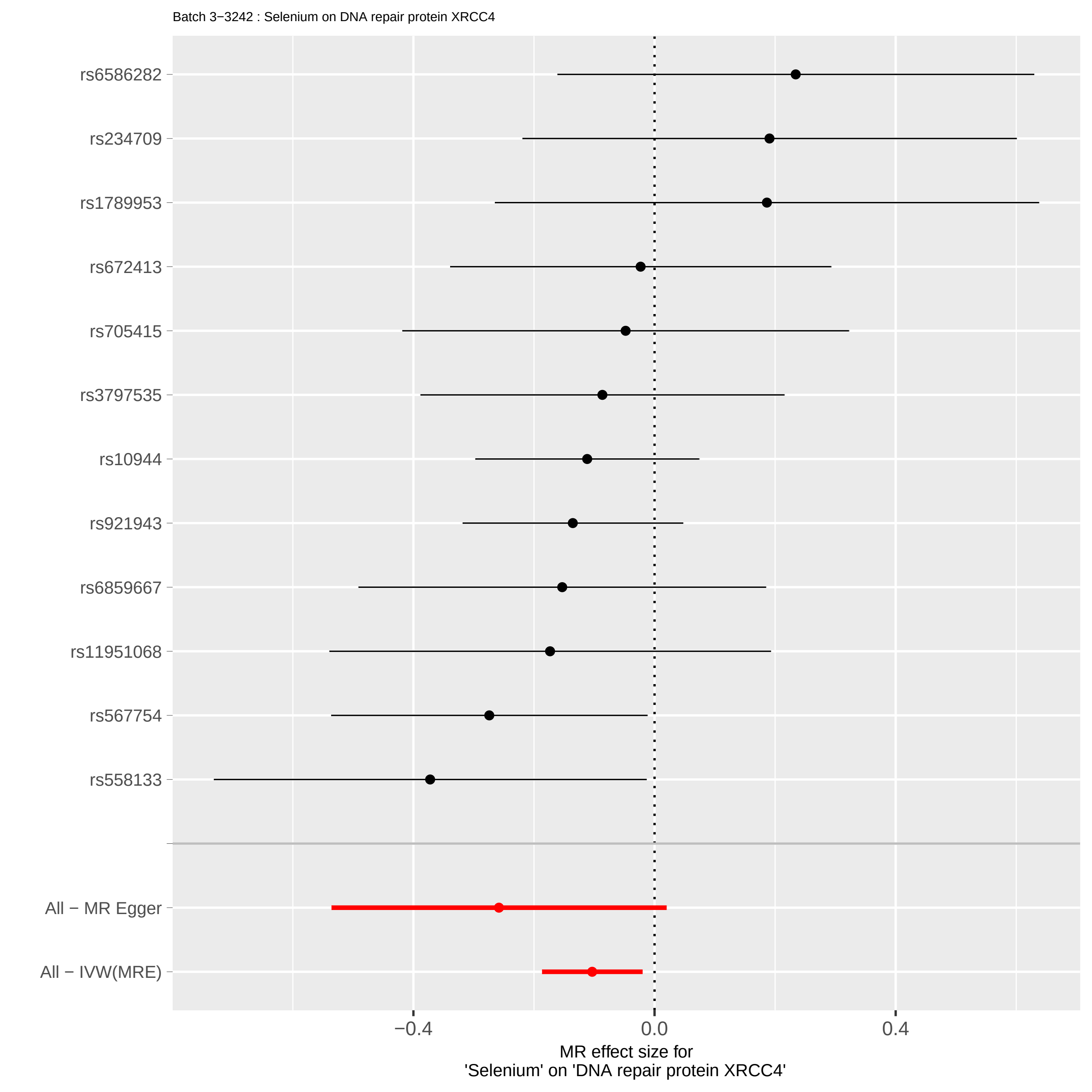

Batch 3−3242 : Selenium on DNA repair protein XRCC4
rs6586282
rs234709
rs1789953
rs672413
rs705415
rs3797535
rs10944
rs921943
rs6859667
rs11951068
rs567754
rs558133
All − MR Egger
All − IVW(MRE)
−0.4
0.0
0.4
MR effect size for
'Selenium' on 'DNA repair protein XRCC4'

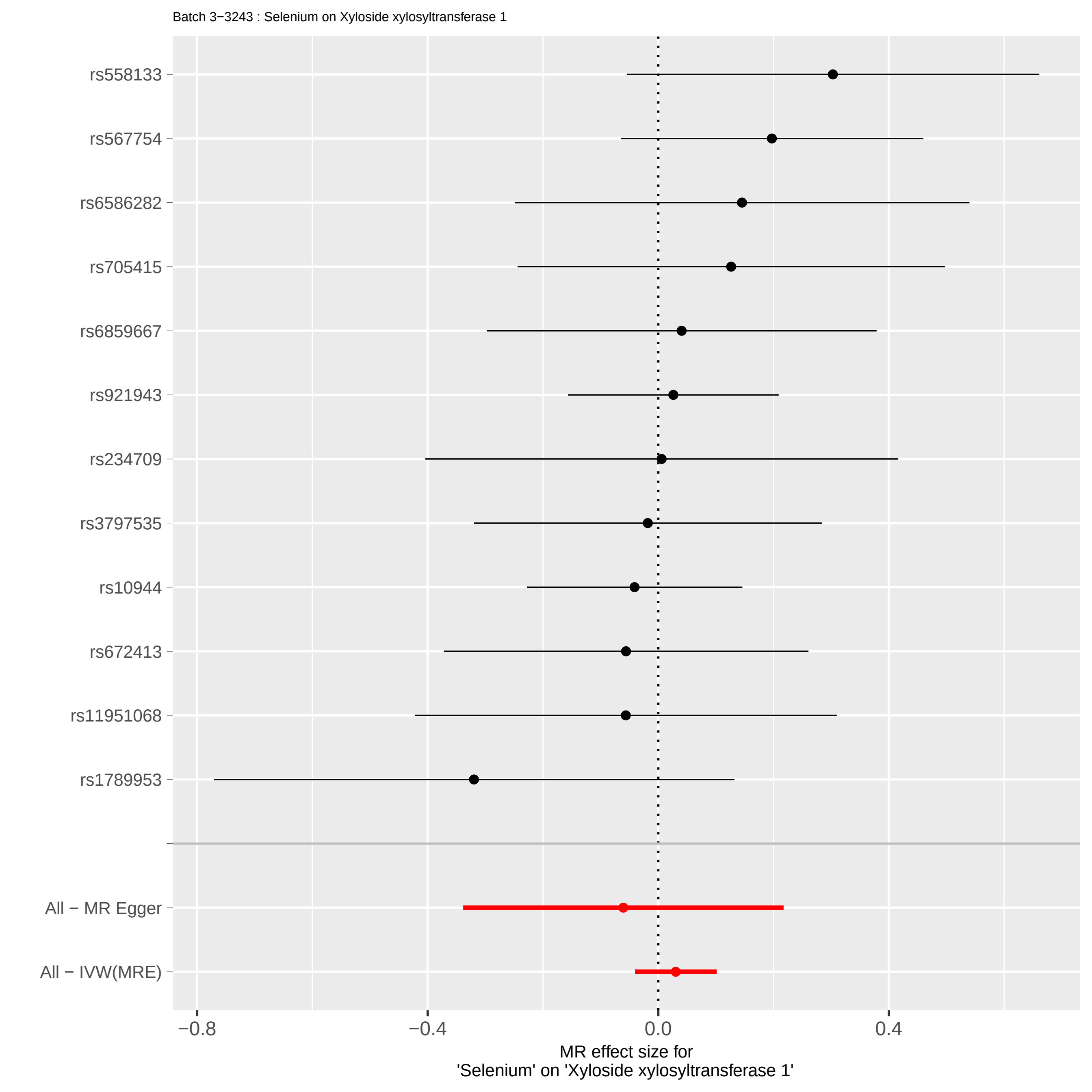

Batch 3−3243 : Selenium on Xyloside xylosyltransferase 1
rs558133
rs567754
rs6586282
rs705415
rs6859667
rs921943
rs234709
rs3797535
rs10944
rs672413
rs11951068
rs1789953
All − MR Egger
All − IVW(MRE)
−0.8
−0.4
0.0
0.4
MR effect size for
'Selenium' on 'Xyloside xylosyltransferase 1'

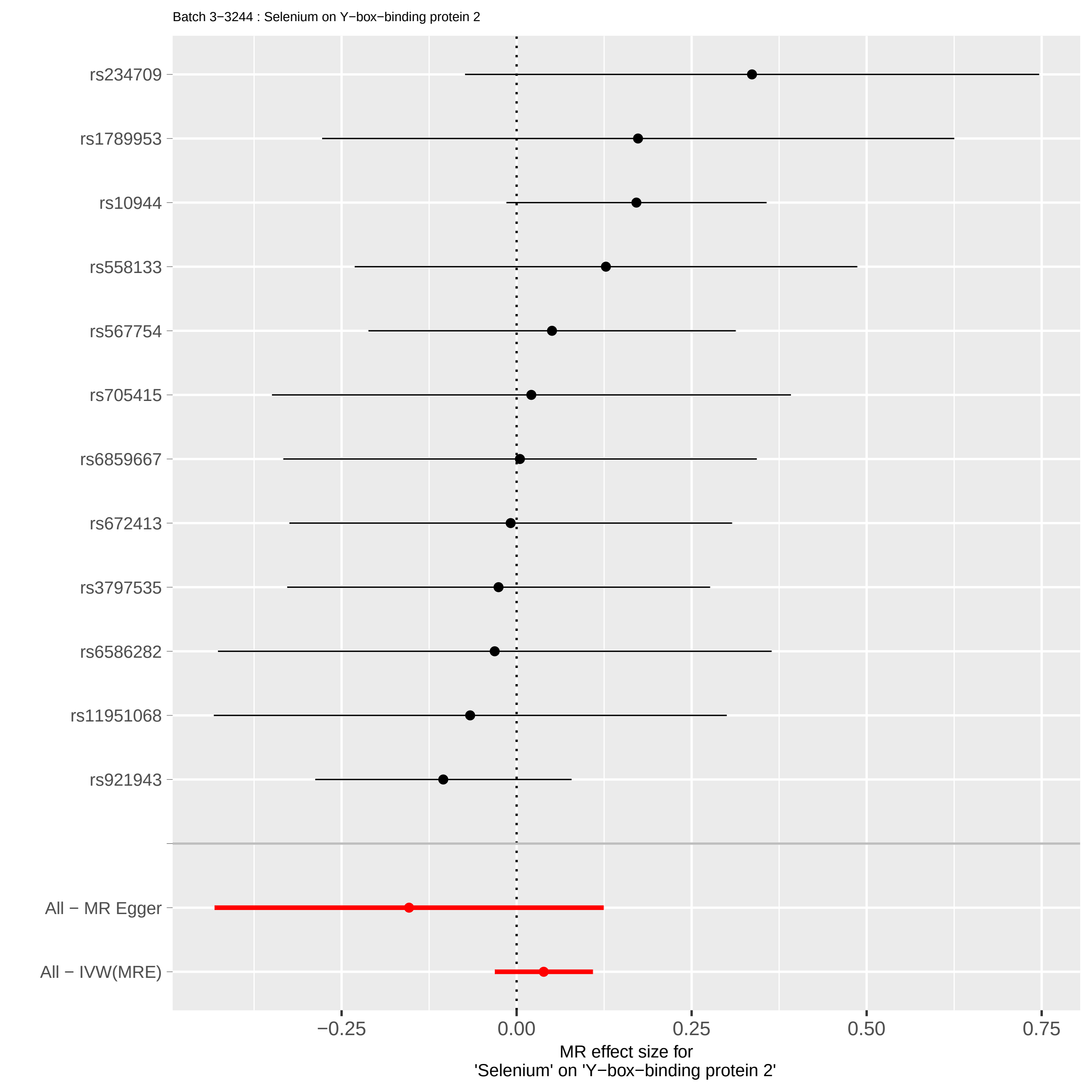

Batch 3−3244 : Selenium on Y−box−binding protein 2
rs234709
rs1789953
rs10944
rs558133
rs567754
rs705415
rs6859667
rs672413
rs3797535
rs6586282
rs11951068
rs921943
All − MR Egger
All − IVW(MRE)
−0.25
0.00
0.25
0.50
0.75
MR effect size for
'Selenium' on 'Y−box−binding protein 2'

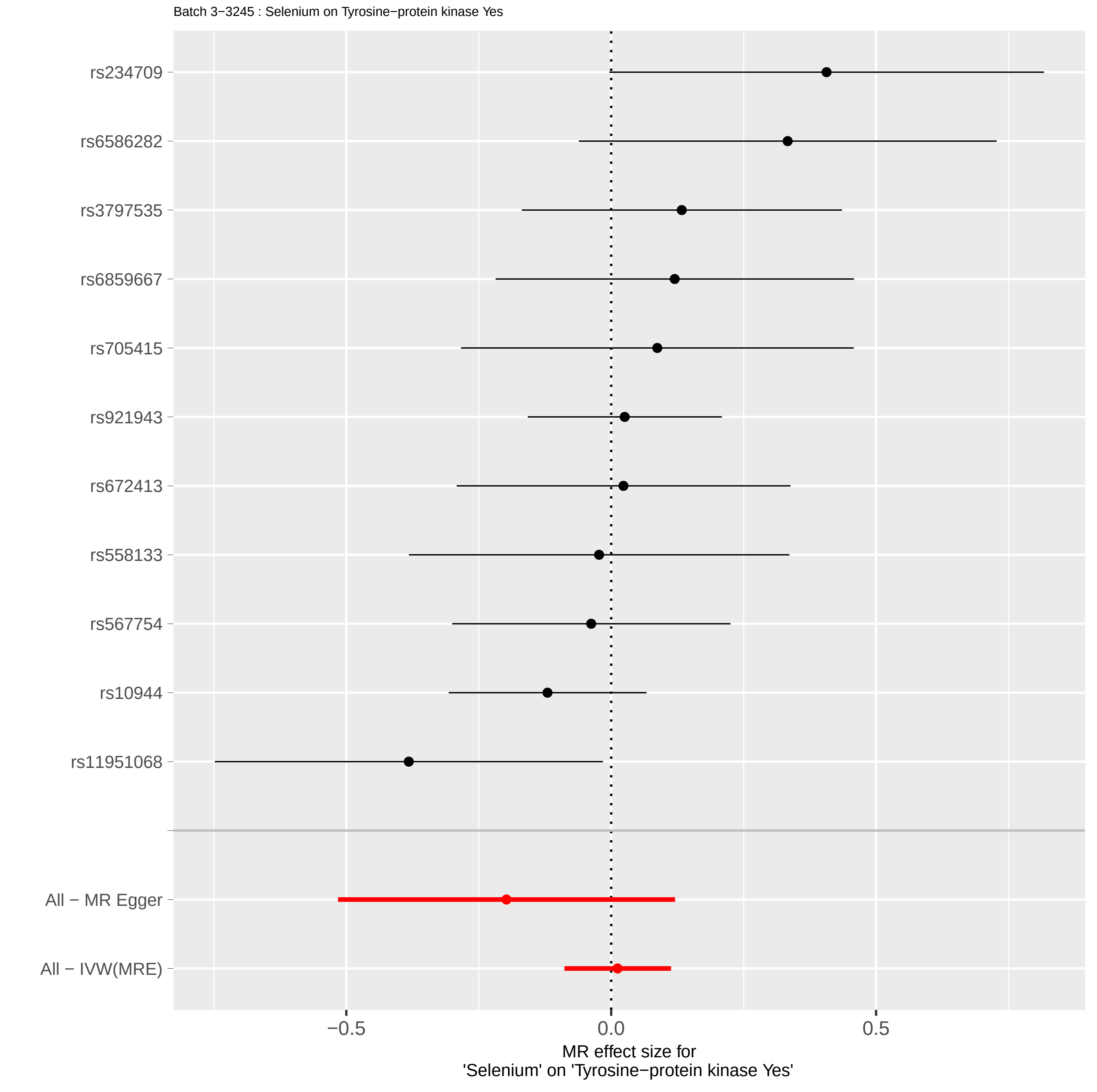
Batch 3−3245 : Selenium on Tyrosine−protein kinase Yes
rs234709
rs6586282
rs3797535
rs6859667
rs705415
rs921943
rs672413
rs558133
rs567754
rs10944
rs11951068
All − MR Egger
All − IVW(MRE)
−0.5
0.0
0.5
MR effect size for
'Selenium' on 'Tyrosine−protein kinase Yes'

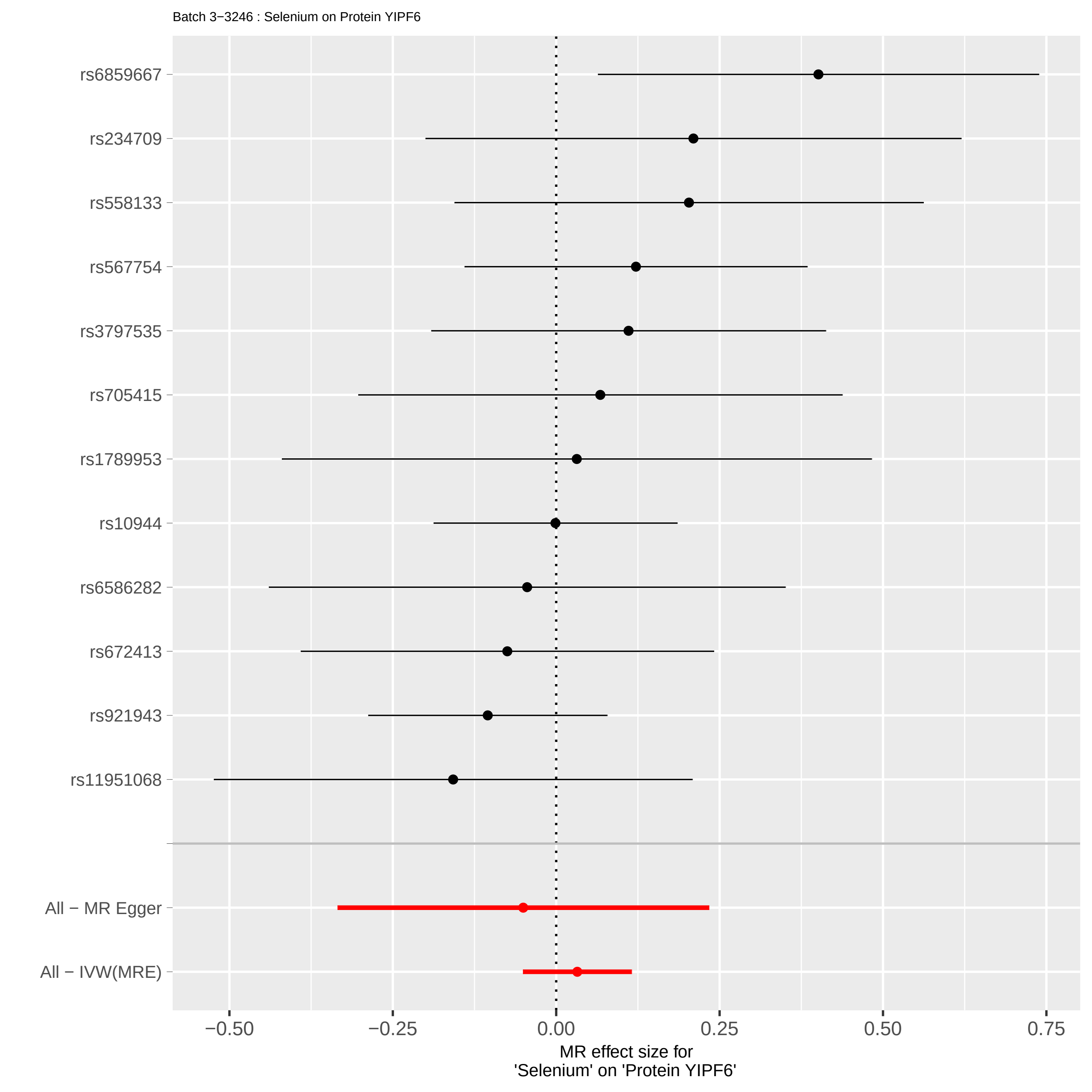

Batch 3−3246 : Selenium on Protein YIPF6
rs6859667
rs234709
rs558133
rs567754
rs3797535
rs705415
rs1789953
rs10944
rs6586282
rs672413
rs921943
rs11951068
All − MR Egger
All − IVW(MRE)
−0.50
−0.25
0.00
0.25
0.50
0.75
MR effect size for
'Selenium' on 'Protein YIPF6'

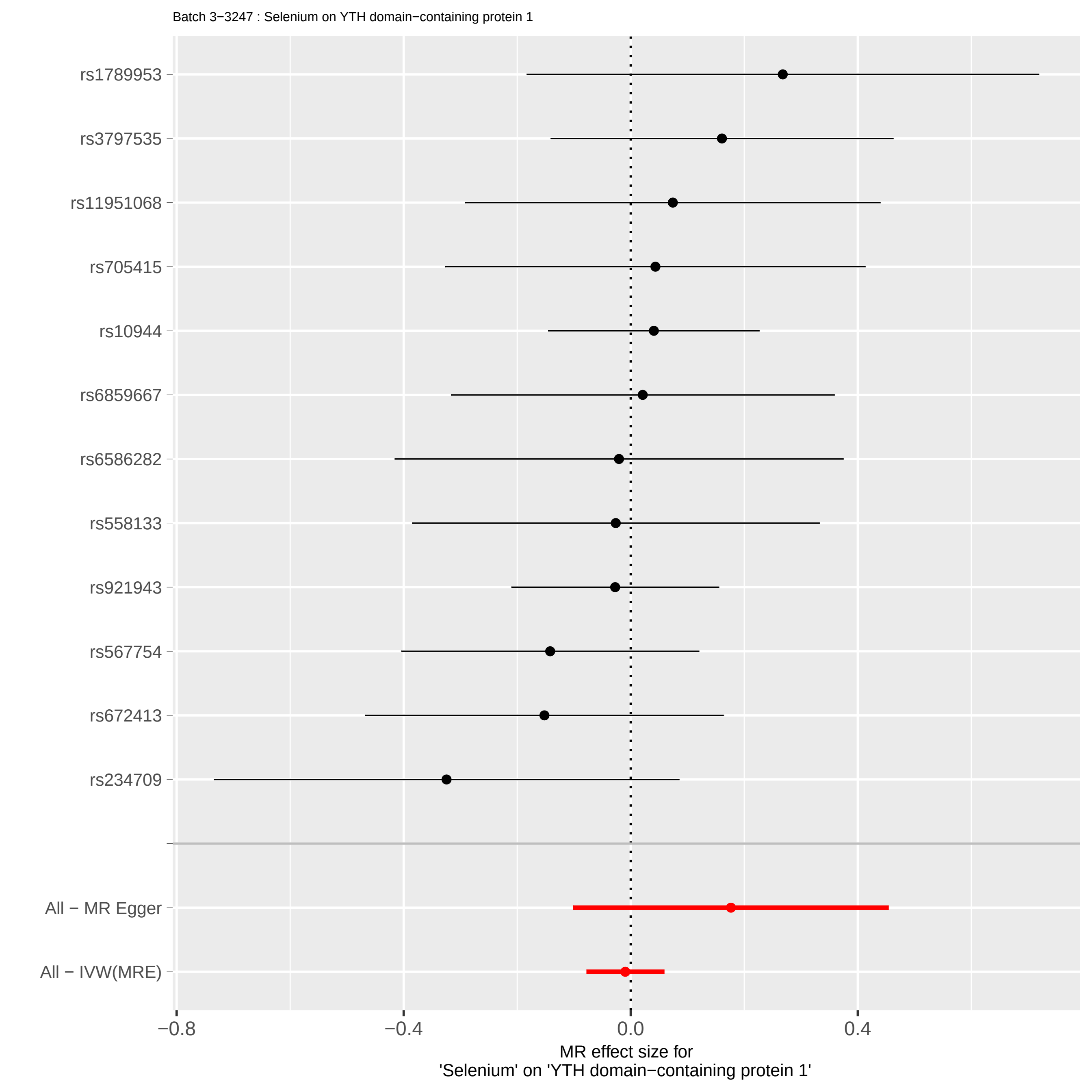

Batch 3−3247 : Selenium on YTH domain−containing protein 1
rs1789953
rs3797535
rs11951068
rs705415
rs10944
rs6859667
rs6586282
rs558133
rs921943
rs567754
rs672413
rs234709
All − MR Egger
All − IVW(MRE)
−0.8
−0.4
0.0
0.4
MR effect size for
'Selenium' on 'YTH domain−containing protein 1'

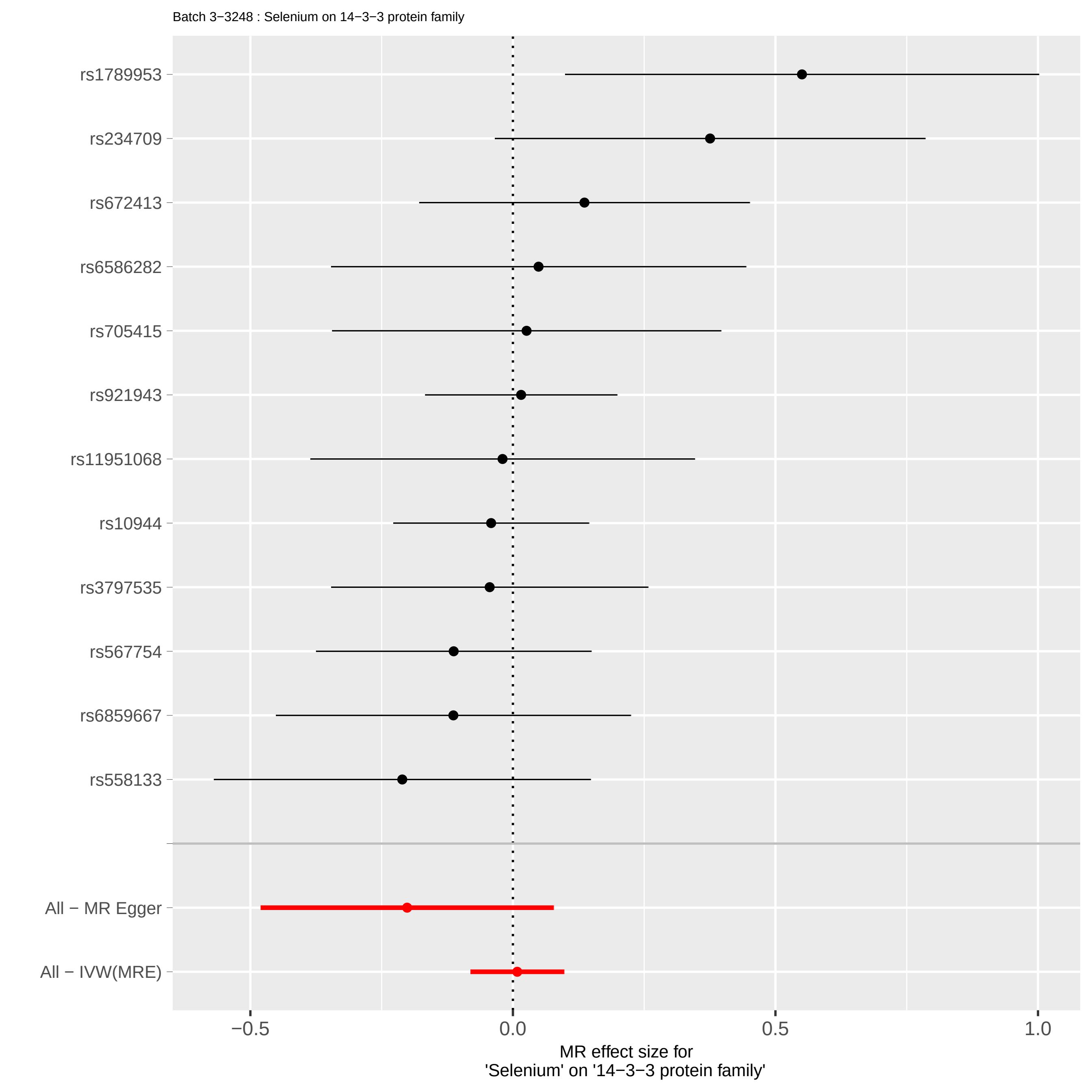

Batch 3−3248 : Selenium on 14−3−3 protein family
rs1789953
rs234709
rs672413
rs6586282
rs705415
rs921943
rs11951068
rs10944
rs3797535
rs567754
rs6859667
rs558133
All − MR Egger
All − IVW(MRE)
−0.5
0.0
0.5
1.0
MR effect size for
'Selenium' on '14−3−3 protein family'

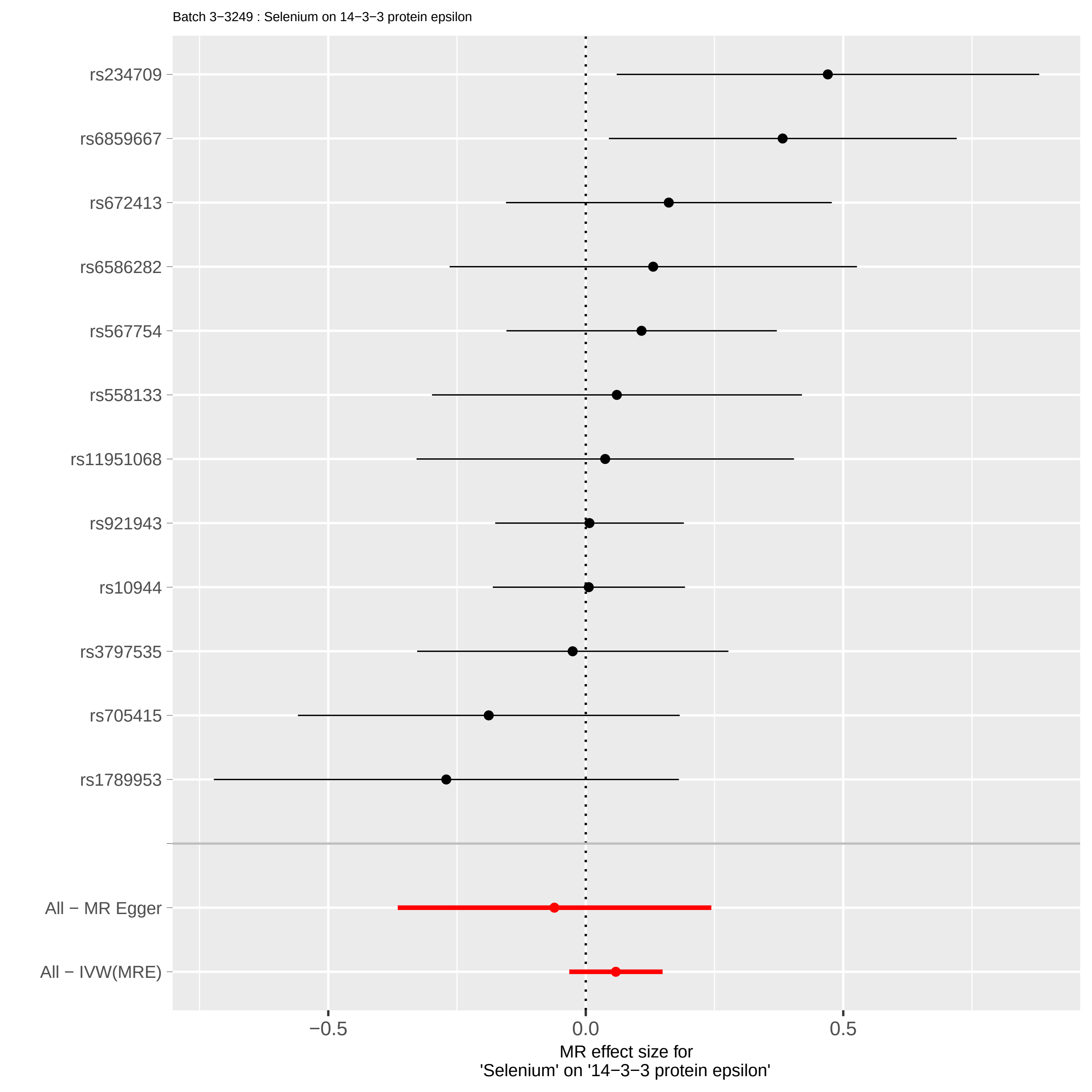

Batch 3−3249 : Selenium on 14−3−3 protein epsilon
rs234709
rs6859667
rs672413
rs6586282
rs567754
rs558133
rs11951068
rs921943
rs10944
rs3797535
rs705415
rs1789953
All − MR Egger
All − IVW(MRE)
−0.5
0.0
0.5
MR effect size for
'Selenium' on '14−3−3 protein epsilon'

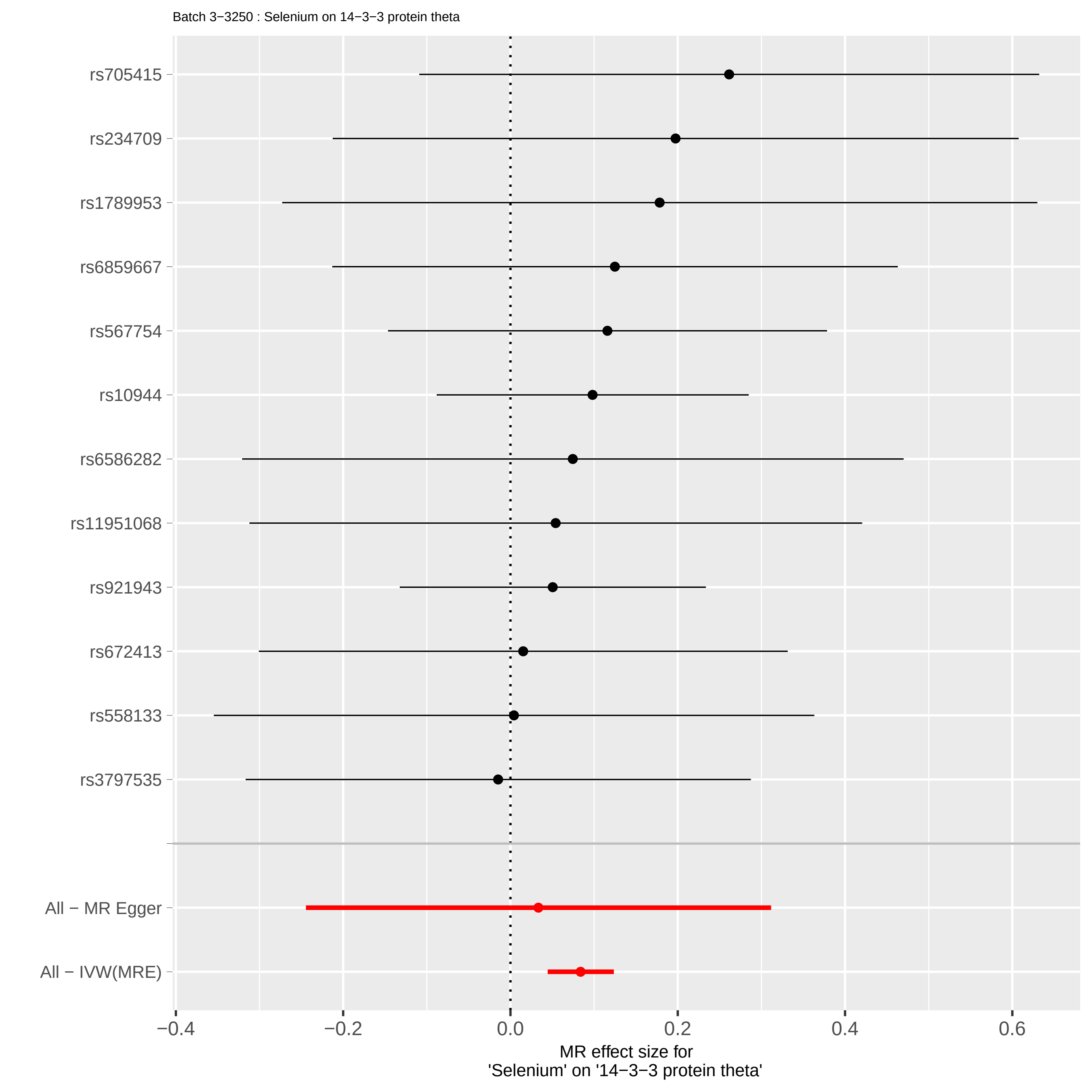

Batch 3−3250 : Selenium on 14−3−3 protein theta
rs705415
rs234709
rs1789953
rs6859667
rs567754
rs10944
rs6586282
rs11951068
rs921943
rs672413
rs558133
rs3797535
All − MR Egger
All − IVW(MRE)
−0.4
−0.2
0.0
0.2
0.4
0.6
MR effect size for
'Selenium' on '14−3−3 protein theta'

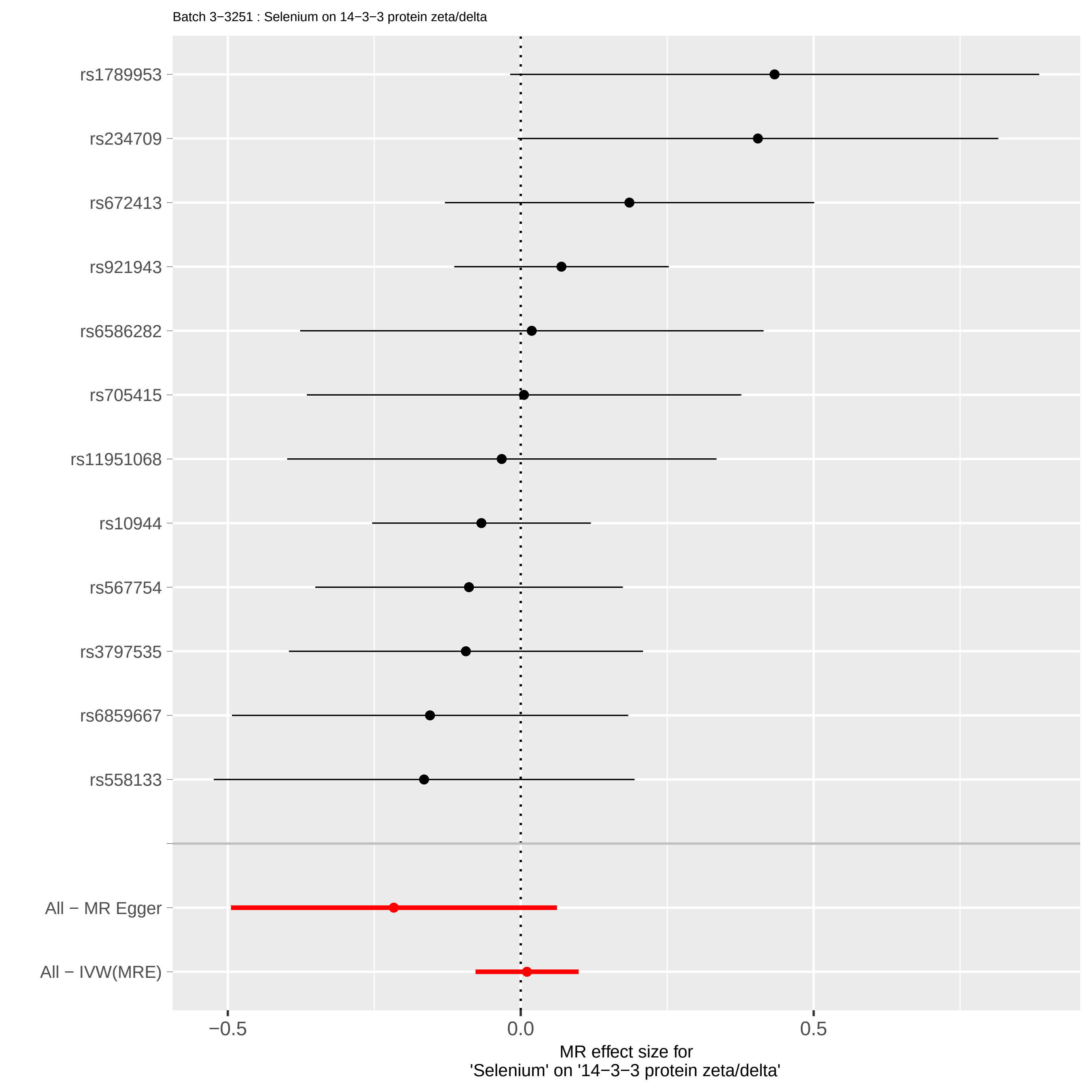

Batch 3−3251 : Selenium on 14−3−3 protein zeta/delta
rs1789953
rs234709
rs672413
rs921943
rs6586282
rs705415
rs11951068
rs10944
rs567754
rs3797535
rs6859667
rs558133
All − MR Egger
All − IVW(MRE)
−0.5
0.0
0.5
MR effect size for
'Selenium' on '14−3−3 protein zeta/delta'

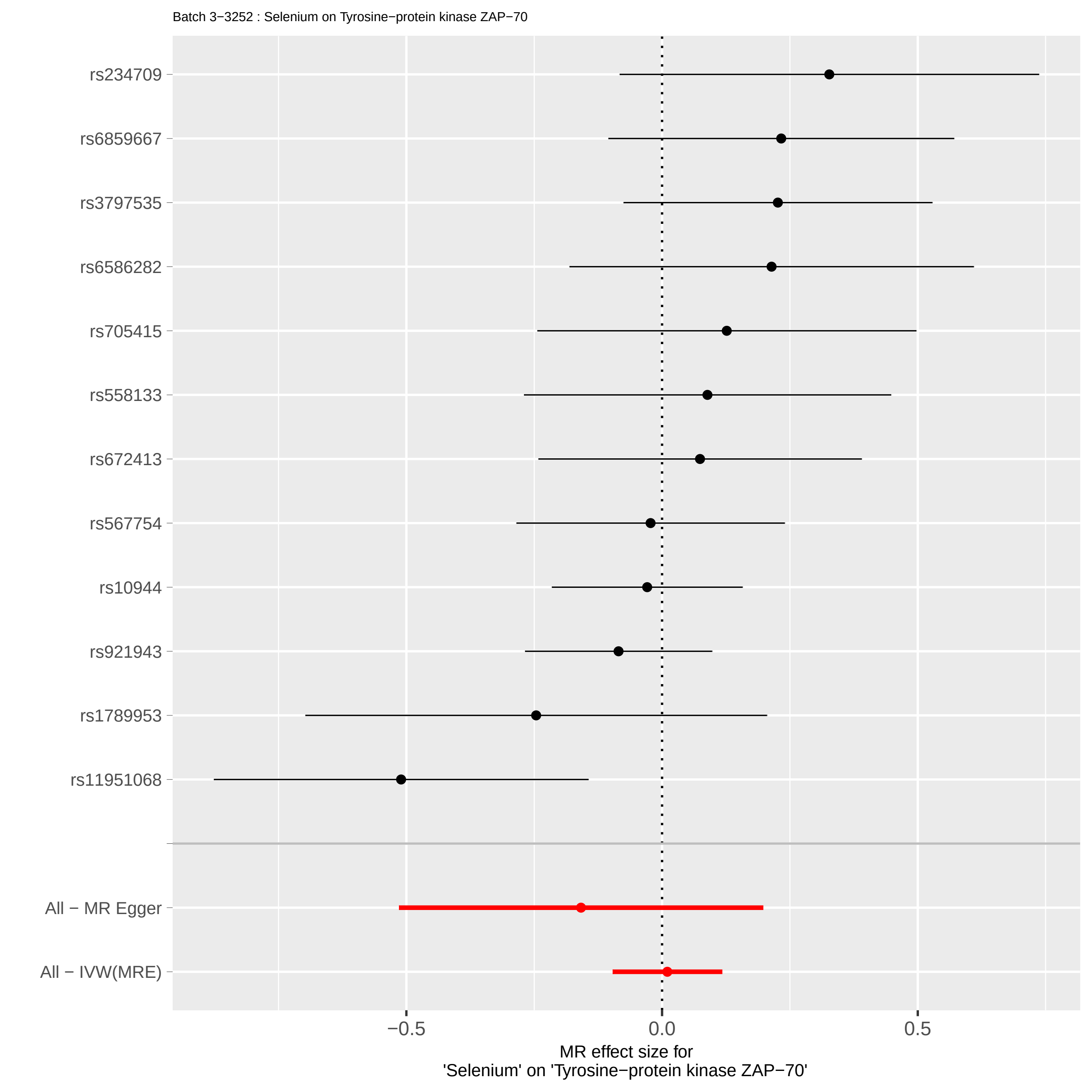

Batch 3−3252 : Selenium on Tyrosine−protein kinase ZAP−70
rs234709
rs6859667
rs3797535
rs6586282
rs705415
rs558133
rs672413
rs567754
rs10944
rs921943
rs1789953
rs11951068
All − MR Egger
All − IVW(MRE)
−0.5
0.0
0.5
MR effect size for
'Selenium' on 'Tyrosine−protein kinase ZAP−70'

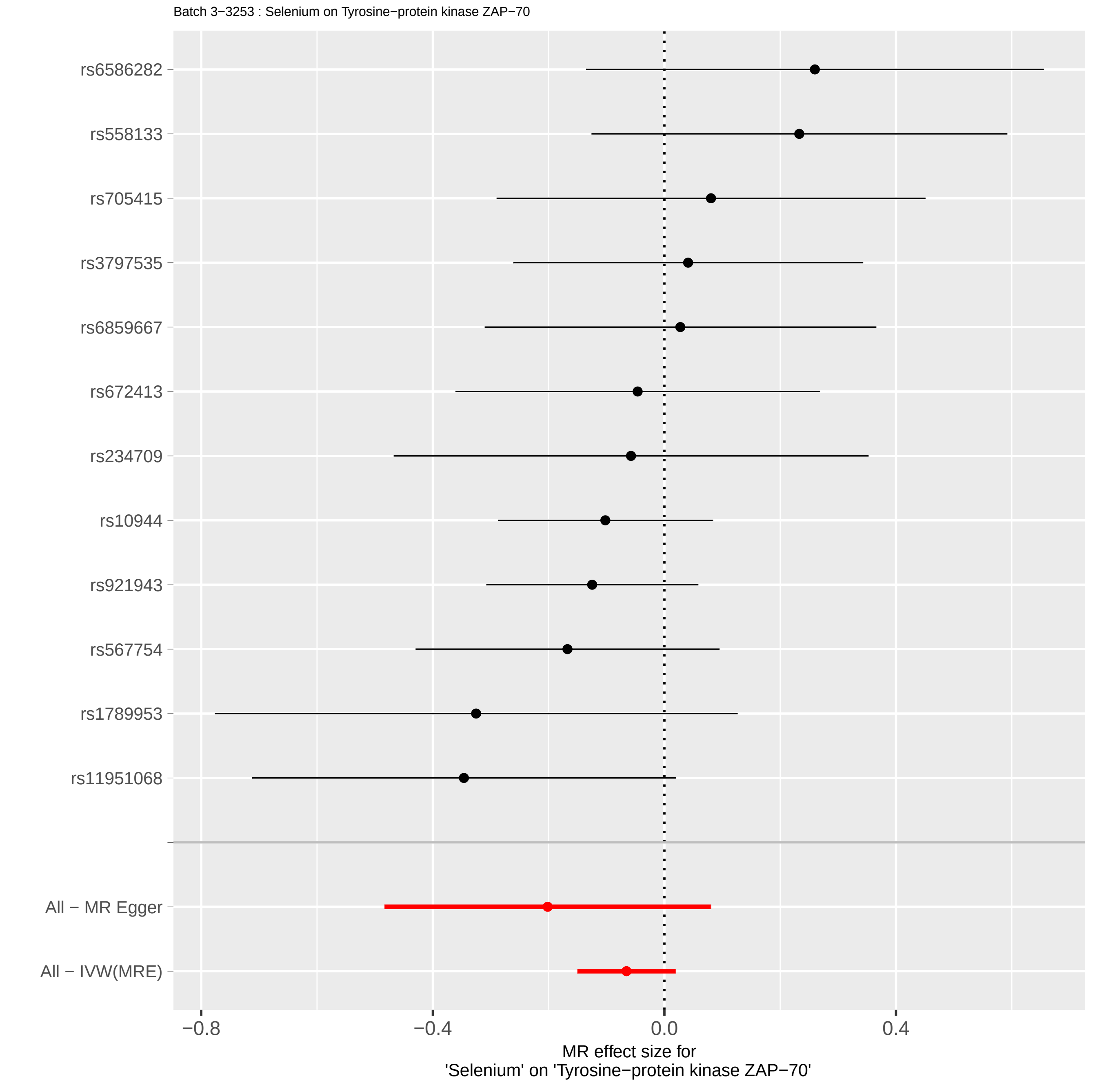

Batch 3−3253 : Selenium on Tyrosine−protein kinase ZAP−70
rs6586282
rs558133
rs705415
rs3797535
rs6859667
rs672413
rs234709
rs10944
rs921943
rs567754
rs1789953
rs11951068
All − MR Egger
All − IVW(MRE)
−0.8
−0.4
0.0
0.4
MR effect size for
'Selenium' on 'Tyrosine−protein kinase ZAP−70'

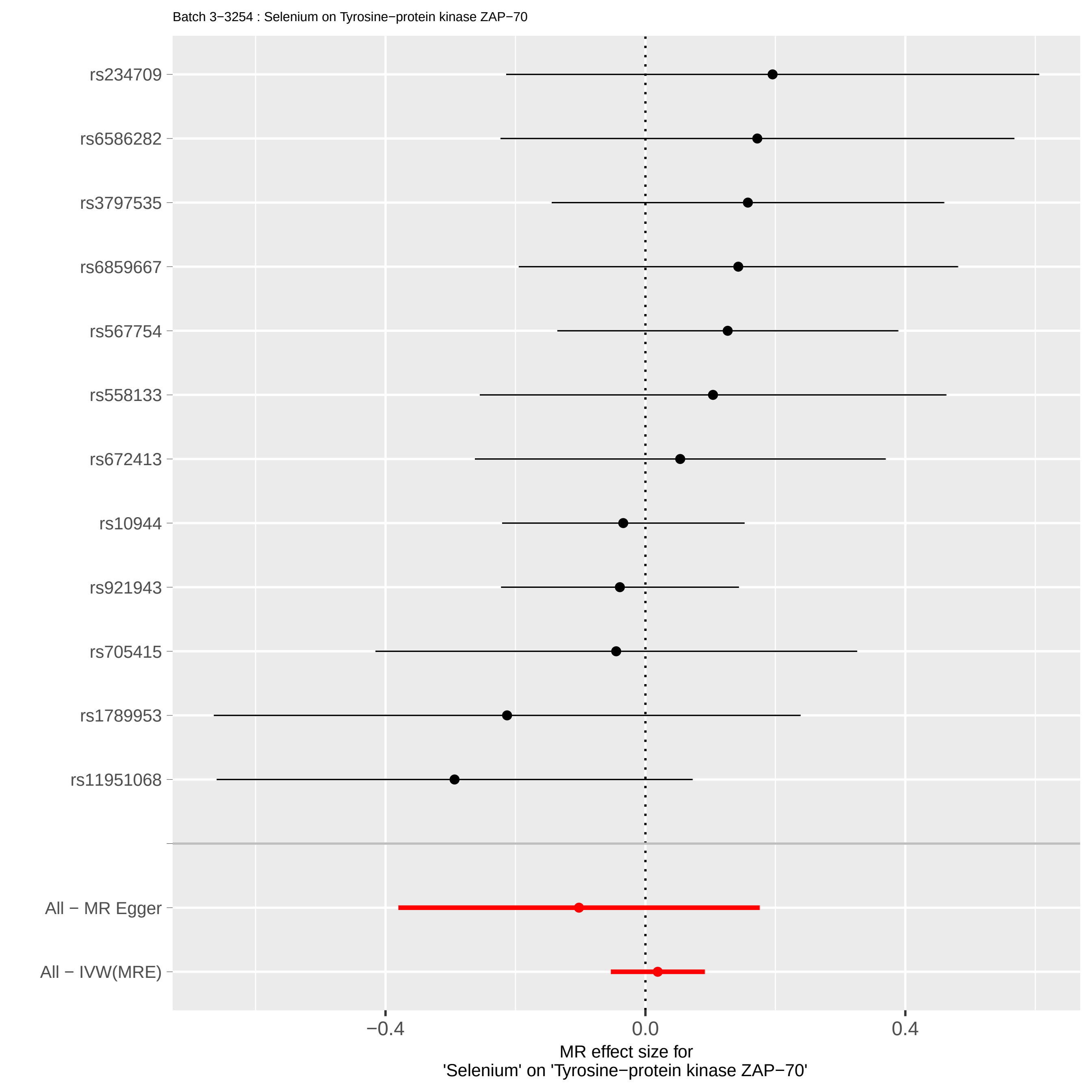

Batch 3−3254 : Selenium on Tyrosine−protein kinase ZAP−70
rs234709
rs6586282
rs3797535
rs6859667
rs567754
rs558133
rs672413
rs10944
rs921943
rs705415
rs1789953
rs11951068
All − MR Egger
All − IVW(MRE)
−0.4
0.0
0.4
MR effect size for
'Selenium' on 'Tyrosine−protein kinase ZAP−70'

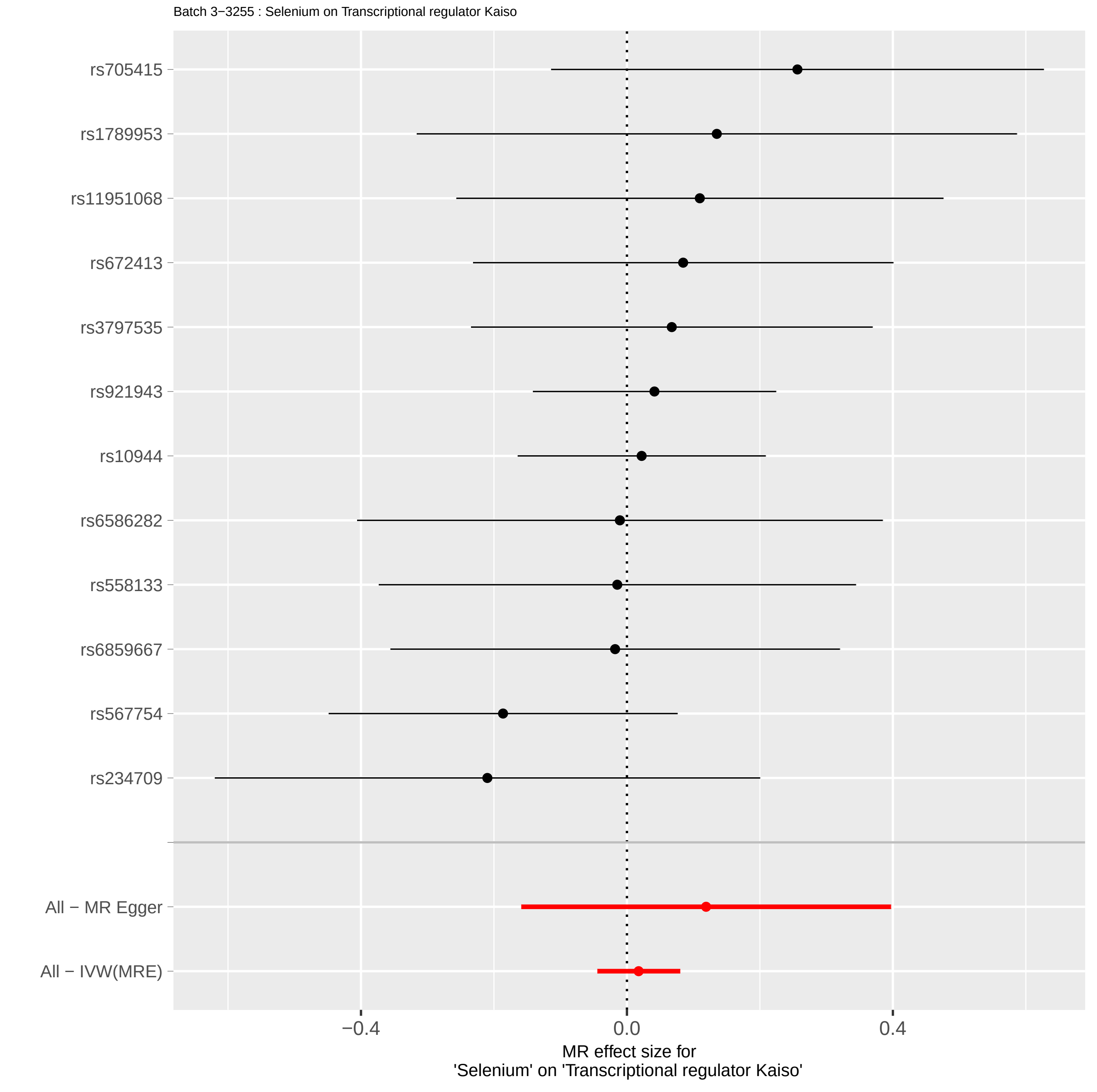
Batch 3−3255 : Selenium on Transcriptional regulator Kaiso
rs705415
rs1789953
rs11951068
rs672413
rs3797535
rs921943
rs10944
rs6586282
rs558133
rs6859667
rs567754
rs234709
All − MR Egger
All − IVW(MRE)
−0.4
0.0
0.4
MR effect size for
'Selenium' on 'Transcriptional regulator Kaiso'

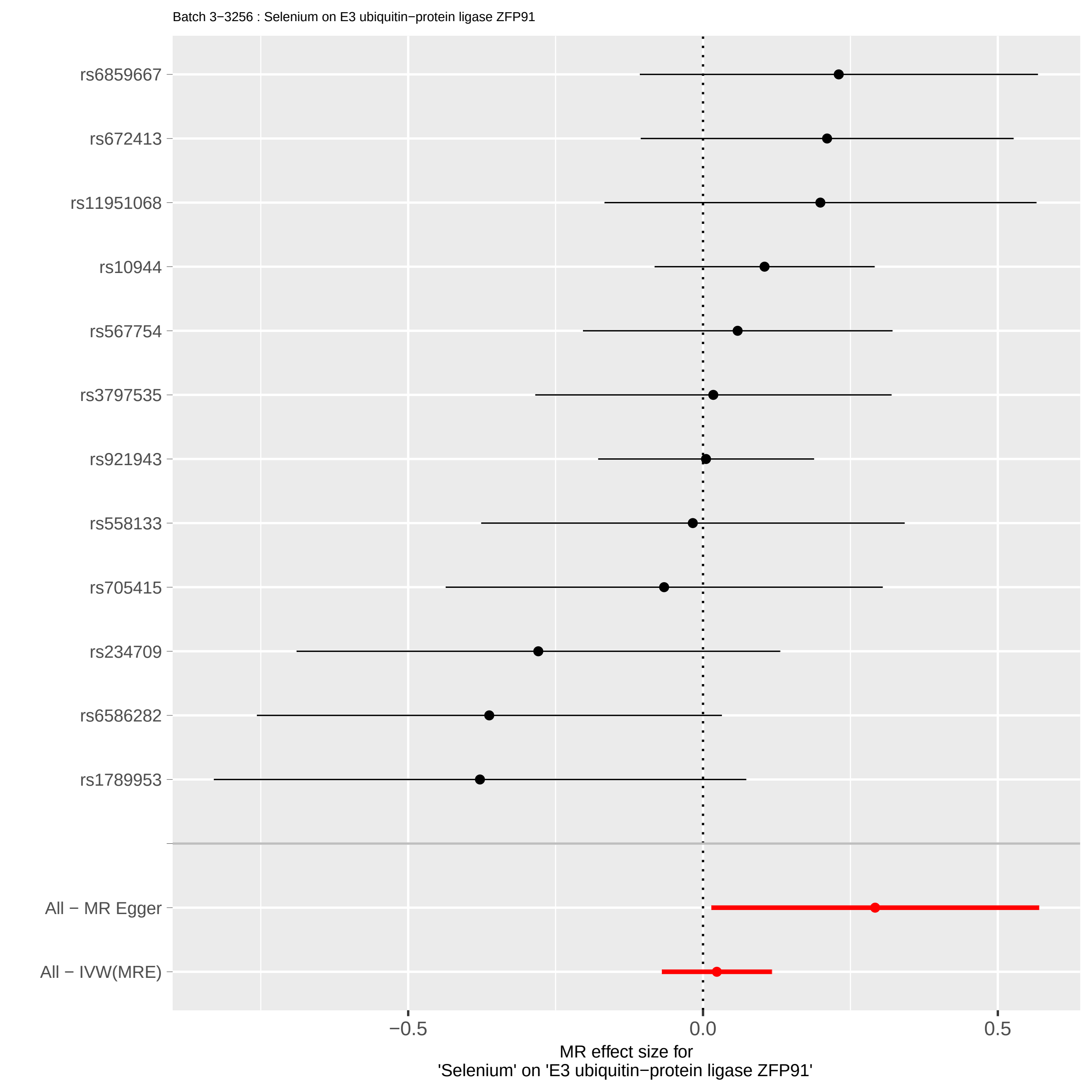
Batch 3−3256 : Selenium on E3 ubiquitin−protein ligase ZFP91
rs6859667
rs672413
rs11951068
rs10944
rs567754
rs3797535
rs921943
rs558133
rs705415
rs234709
rs6586282
rs1789953
All − MR Egger
All − IVW(MRE)
−0.5
0.0
0.5
MR effect size for
'Selenium' on 'E3 ubiquitin−protein ligase ZFP91'

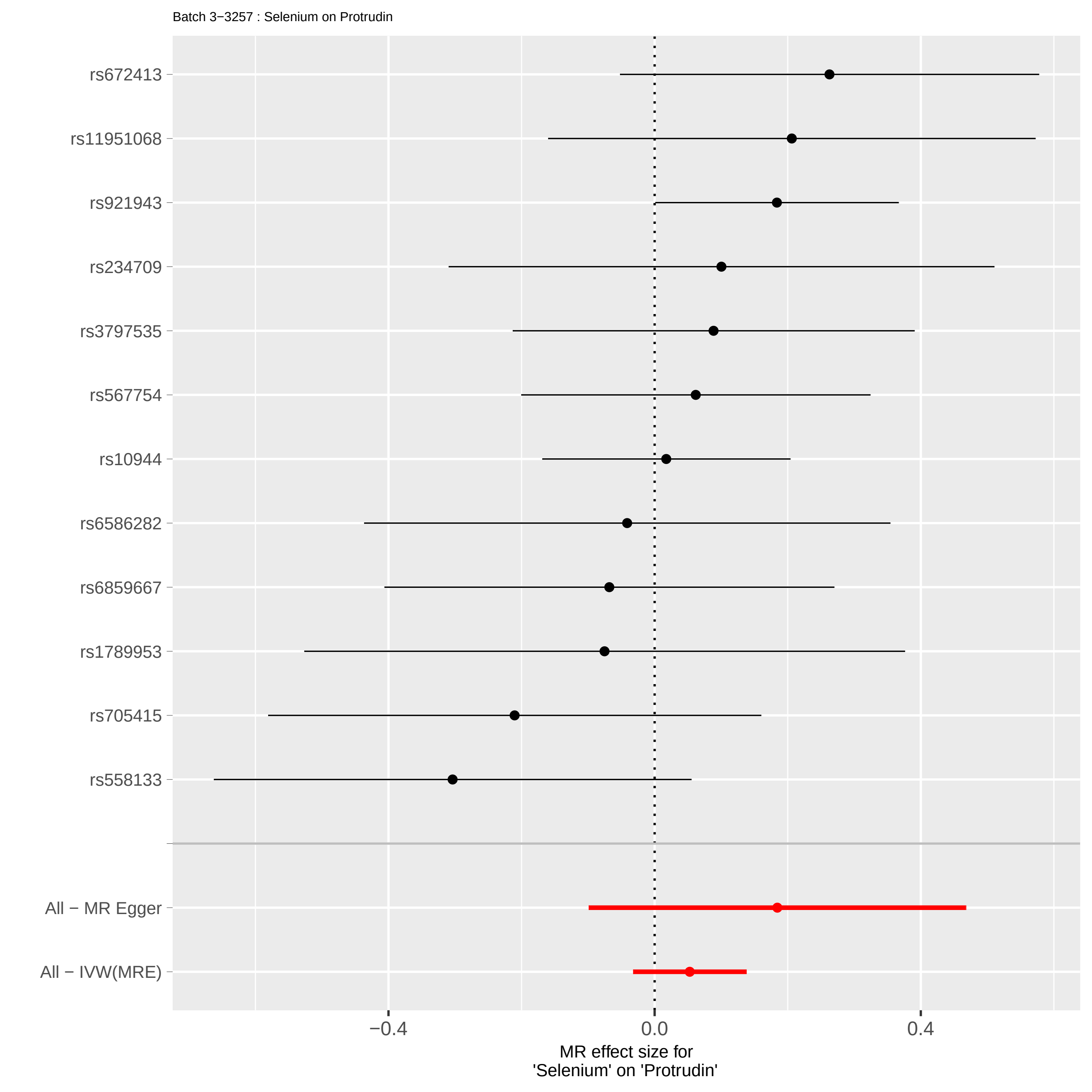
Batch 3−3257 : Selenium on Protrudin
rs672413
rs11951068
rs921943
rs234709
rs3797535
rs567754
rs10944
rs6586282
rs6859667
rs1789953
rs705415
rs558133
All − MR Egger
All − IVW(MRE)
−0.4
0.0
0.4
MR effect size for
'Selenium' on 'Protrudin'

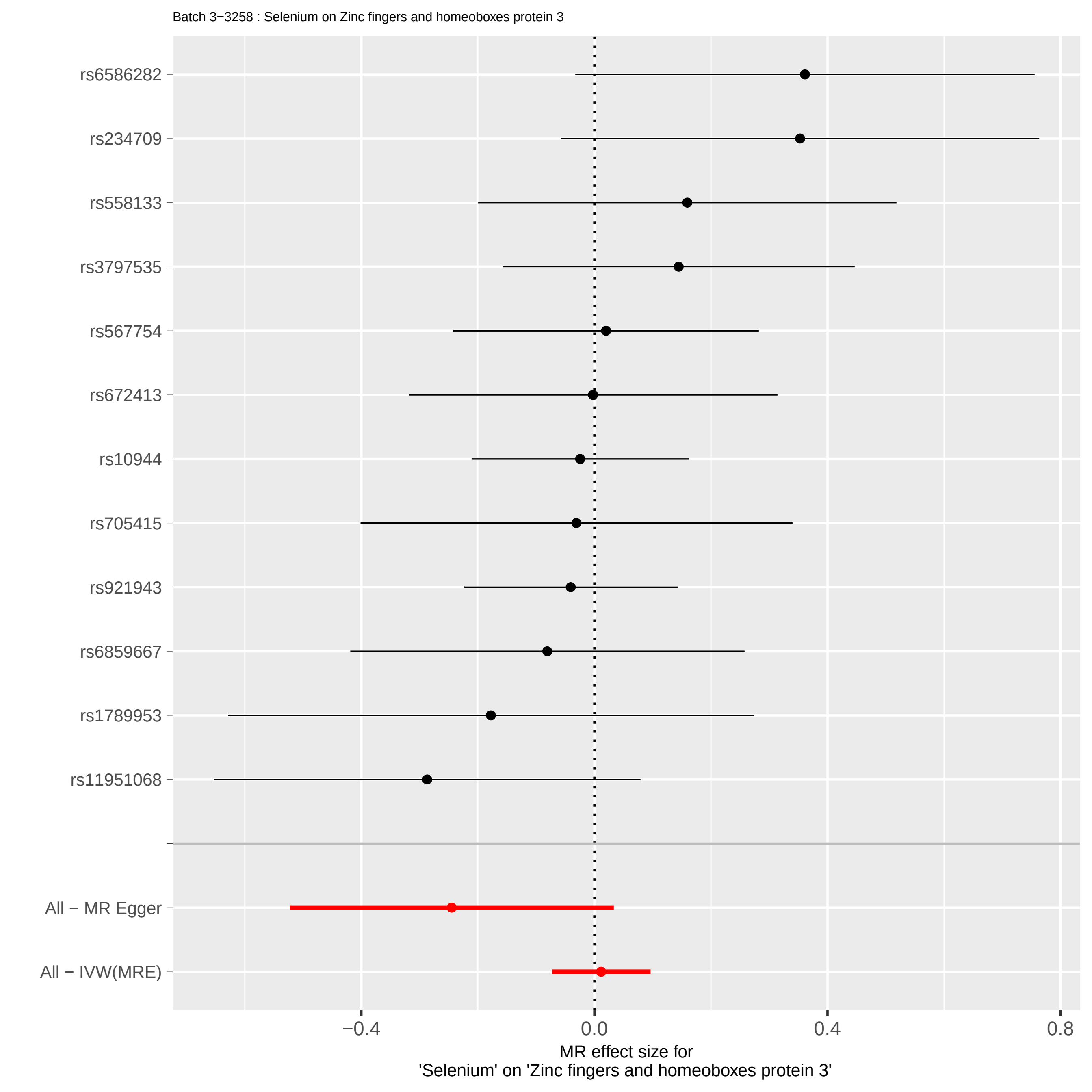

Batch 3−3258 : Selenium on Zinc fingers and homeoboxes protein 3
rs6586282
rs234709
rs558133
rs3797535
rs567754
rs672413
rs10944
rs705415
rs921943
rs6859667
rs1789953
rs11951068
All − MR Egger
All − IVW(MRE)
−0.4
0.0
0.4
0.8
MR effect size for
'Selenium' on 'Zinc fingers and homeoboxes protein 3'

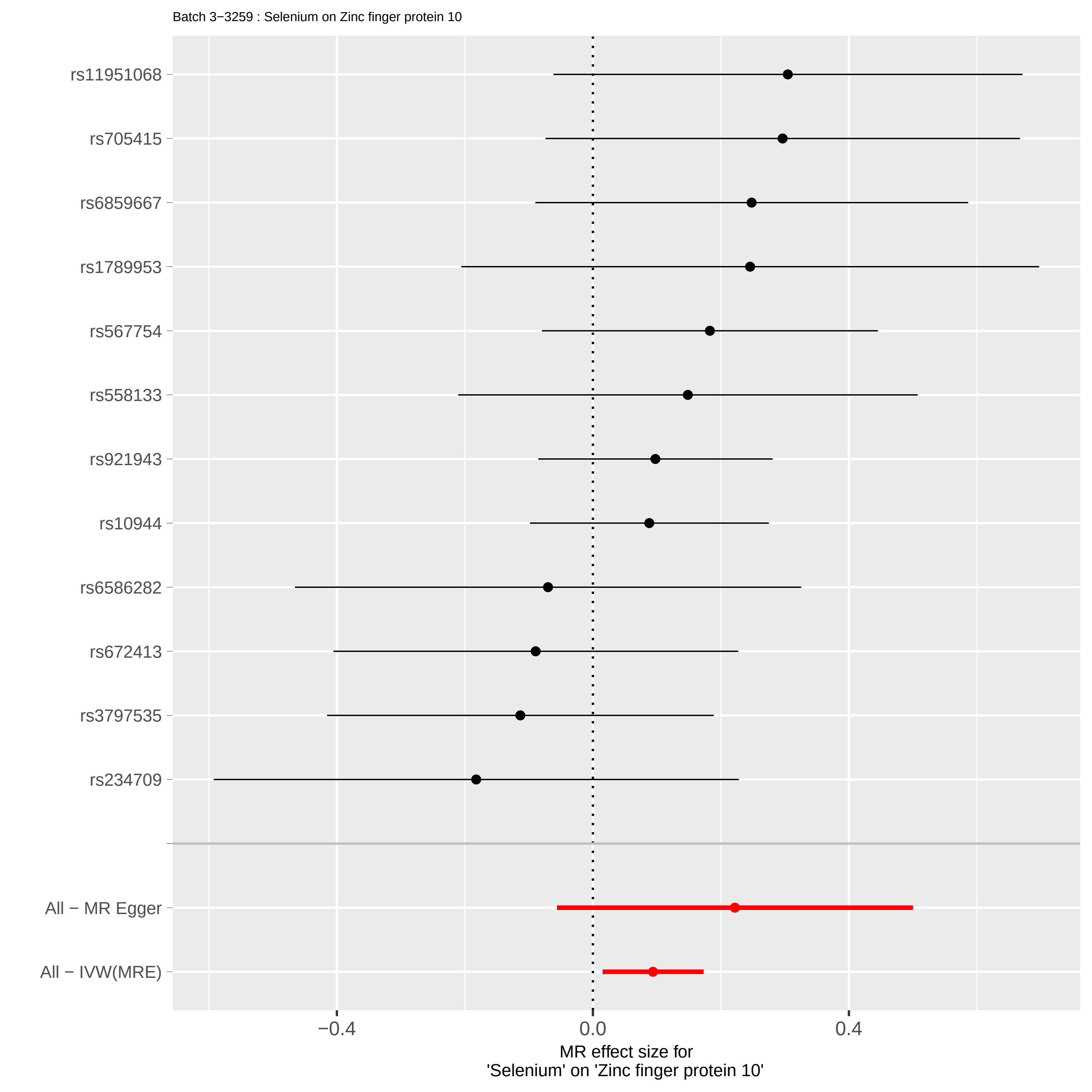

Batch 3−3259 : Selenium on Zinc finger protein 10
rs11951068
rs705415
rs6859667
rs1789953
rs567754
rs558133
rs921943
rs10944
rs6586282
rs672413
rs3797535
rs234709
All − MR Egger
All − IVW(MRE)
−0.4
0.0
0.4
MR effect size for
'Selenium' on 'Zinc finger protein 10'

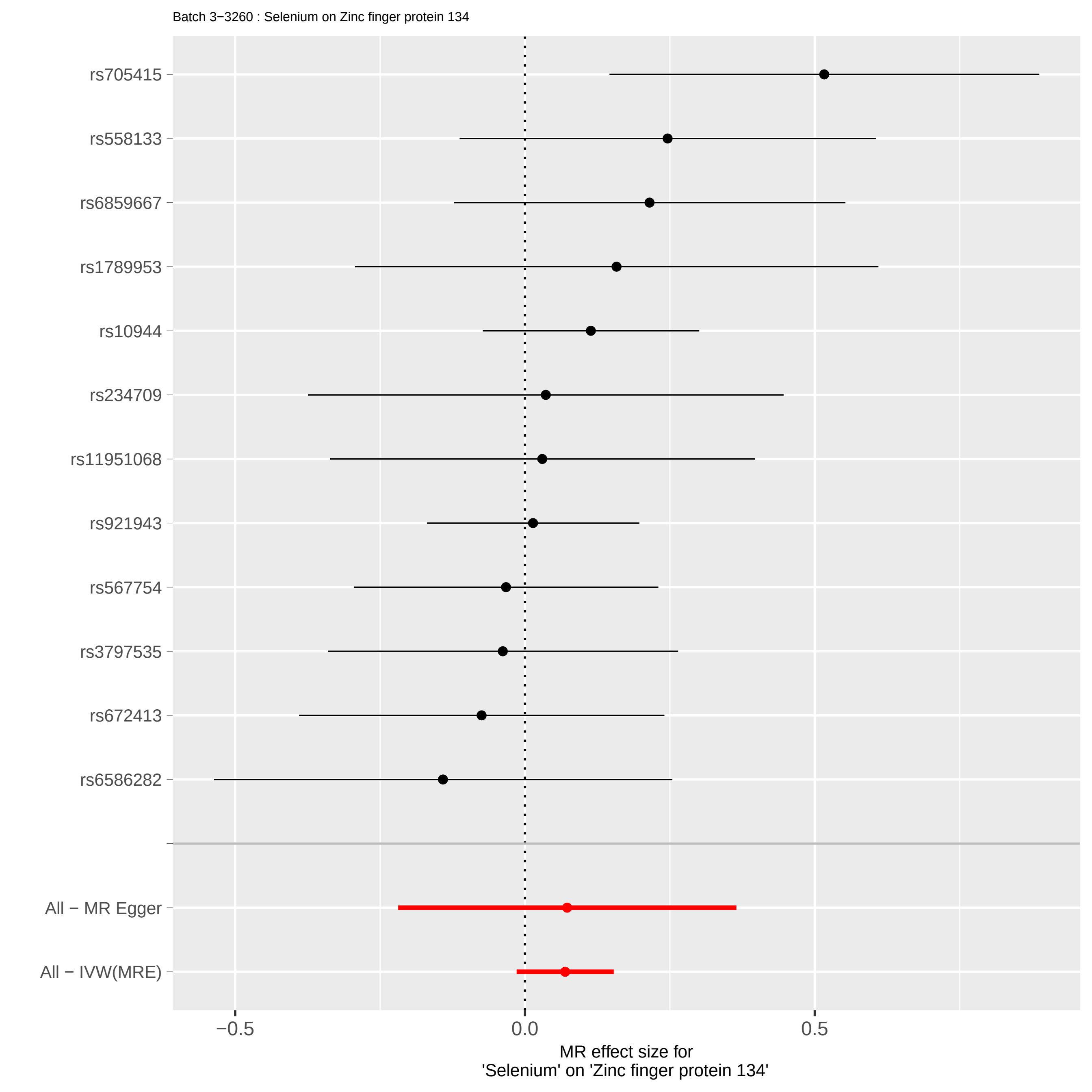

Batch 3−3260 : Selenium on Zinc finger protein 134
rs705415
rs558133
rs6859667
rs1789953
rs10944
rs234709
rs11951068
rs921943
rs567754
rs3797535
rs672413
rs6586282
All − MR Egger
All − IVW(MRE)
−0.5
0.0
0.5
MR effect size for
'Selenium' on 'Zinc finger protein 134'

Batch 3−3261 : Selenium on Zinc finger protein 174

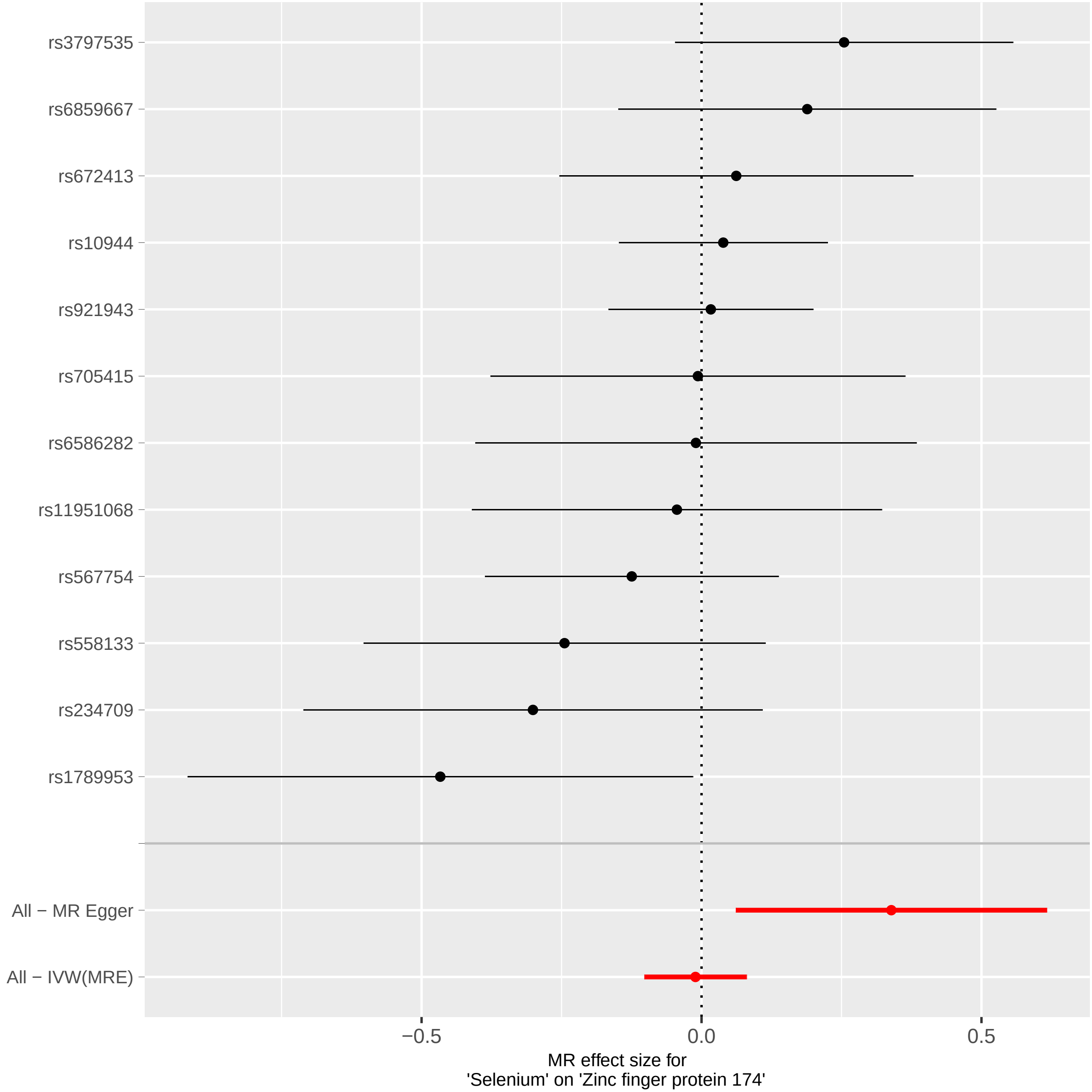

Batch 3−3262 : Selenium on Zinc finger protein 175

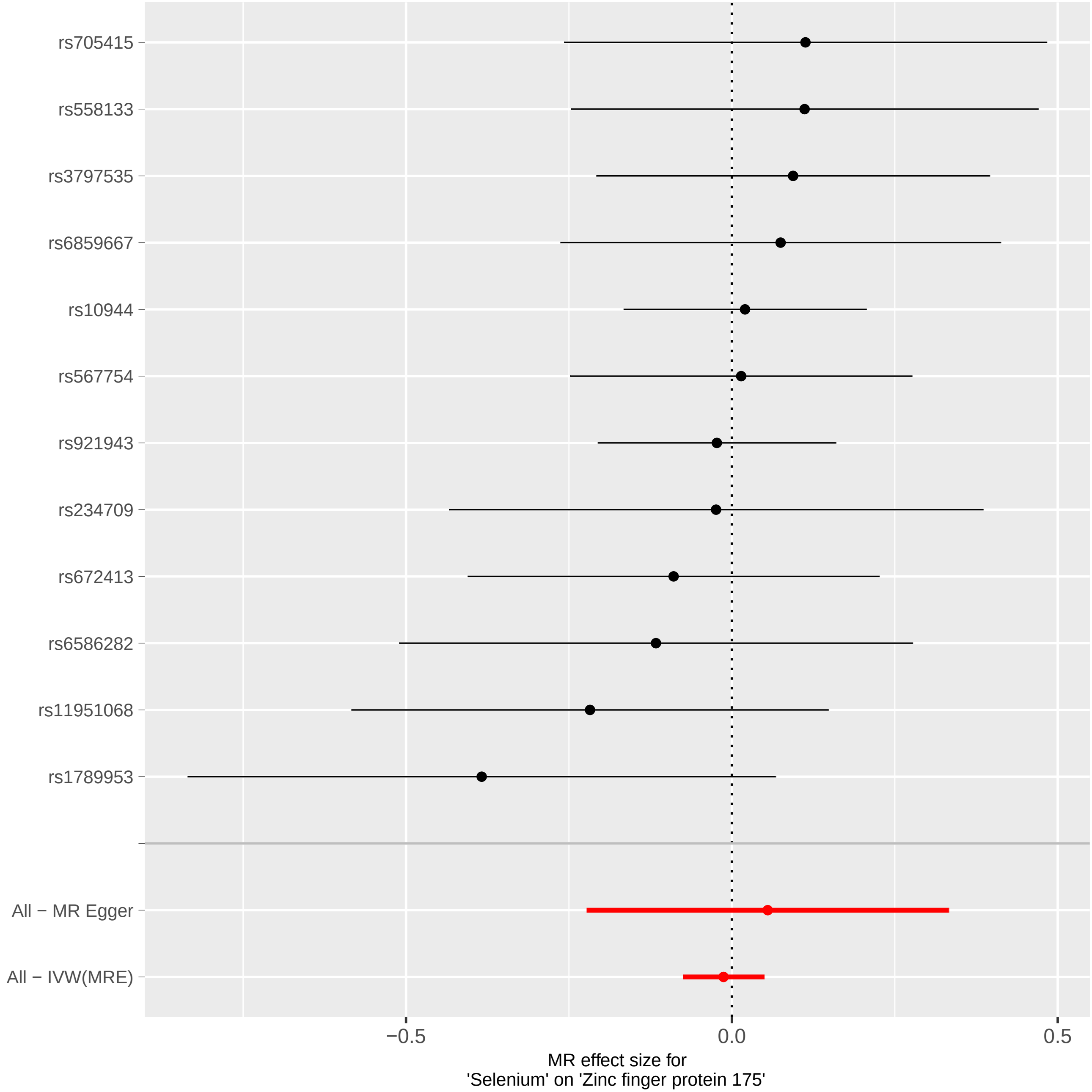

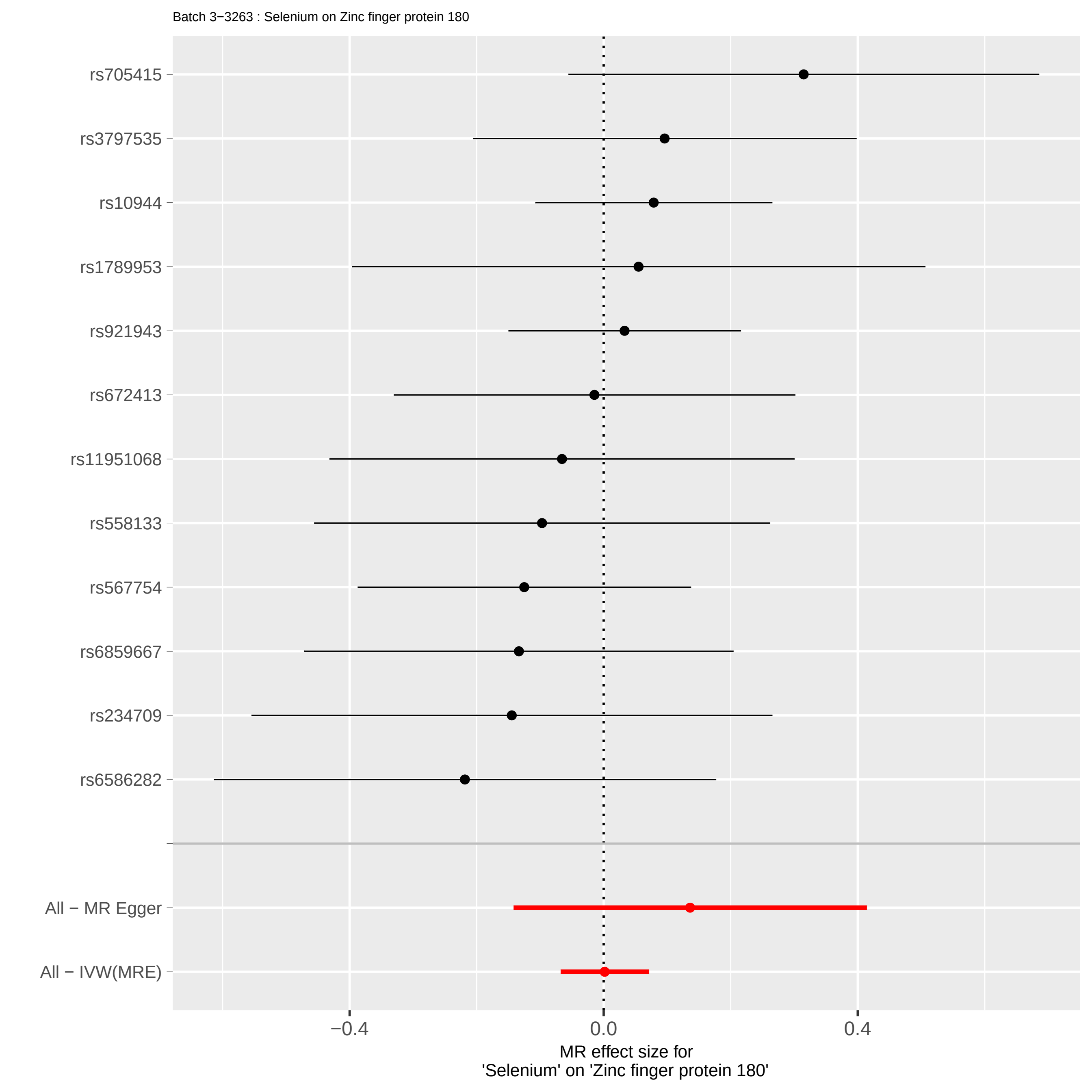

Batch 3−3263 : Selenium on Zinc finger protein 180
rs705415
rs3797535
rs10944
rs1789953
rs921943
rs672413
rs11951068
rs558133
rs567754
rs6859667
rs234709
rs6586282
All − MR Egger
All − IVW(MRE)
−0.4
0.0
0.4
MR effect size for
'Selenium' on 'Zinc finger protein 180'

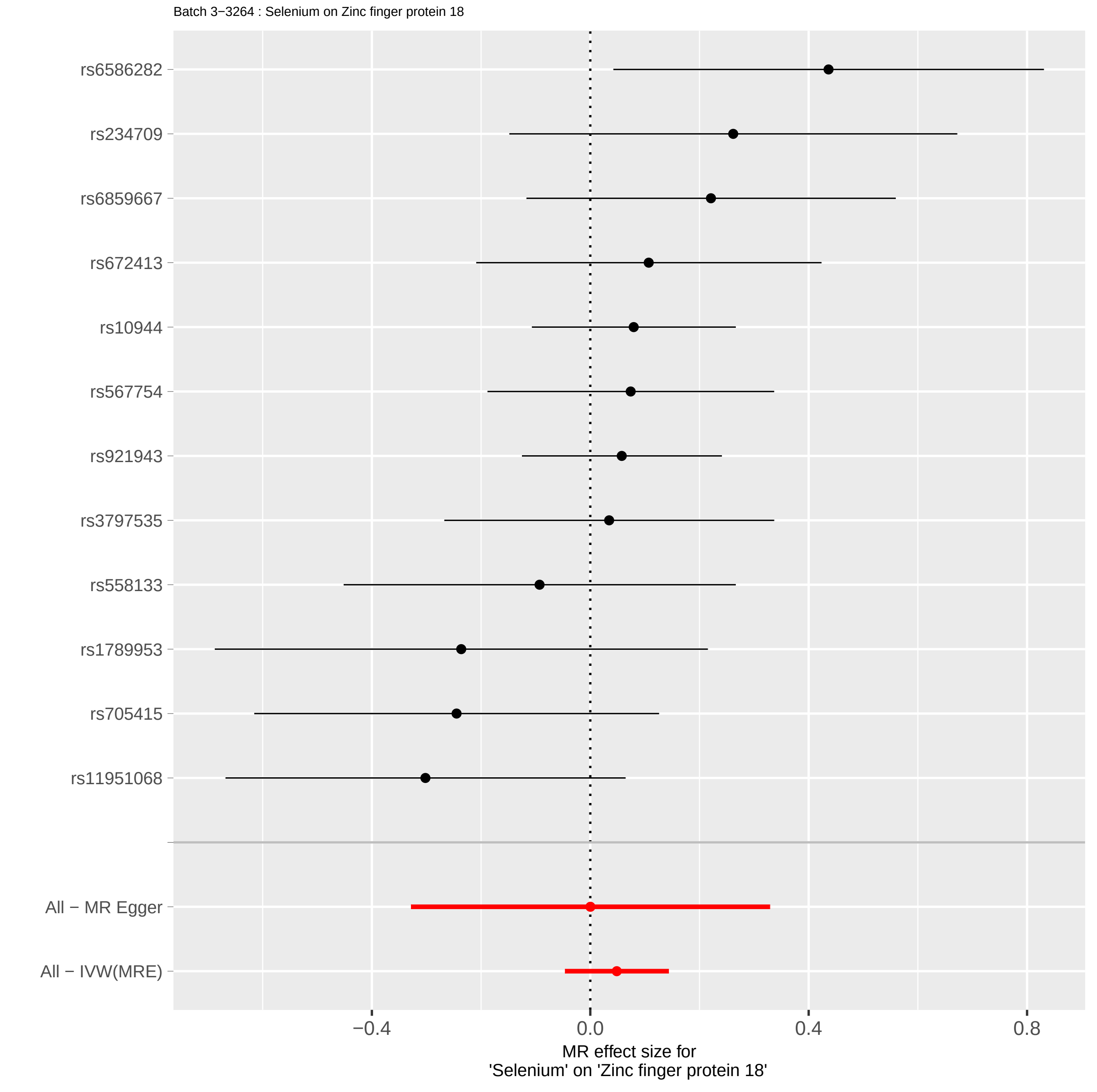

Batch 3−3264 : Selenium on Zinc finger protein 18
rs6586282
rs234709
rs6859667
rs672413
rs10944
rs567754
rs921943
rs3797535
rs558133
rs1789953
rs705415
rs11951068
All − MR Egger
All − IVW(MRE)
−0.4
0.0
0.4
0.8
MR effect size for
'Selenium' on 'Zinc finger protein 18'

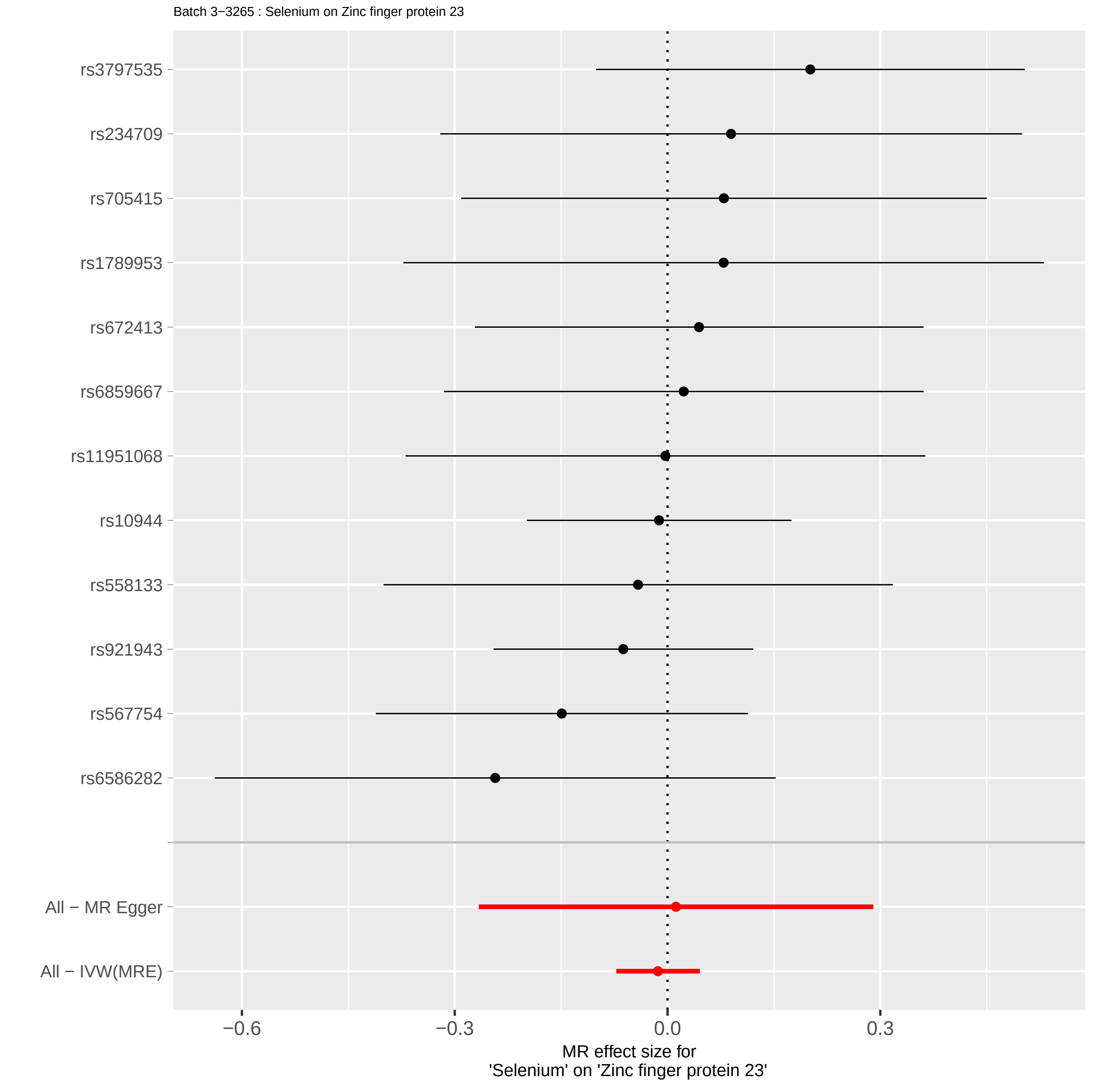
Batch 3−3265 : Selenium on Zinc finger protein 23
rs3797535
rs234709
rs705415
rs1789953
rs672413
rs6859667
rs11951068
rs10944
rs558133
rs921943
rs567754
rs6586282
All − MR Egger
All − IVW(MRE)
−0.6
−0.3
0.0
0.3
MR effect size for
'Selenium' on 'Zinc finger protein 23'

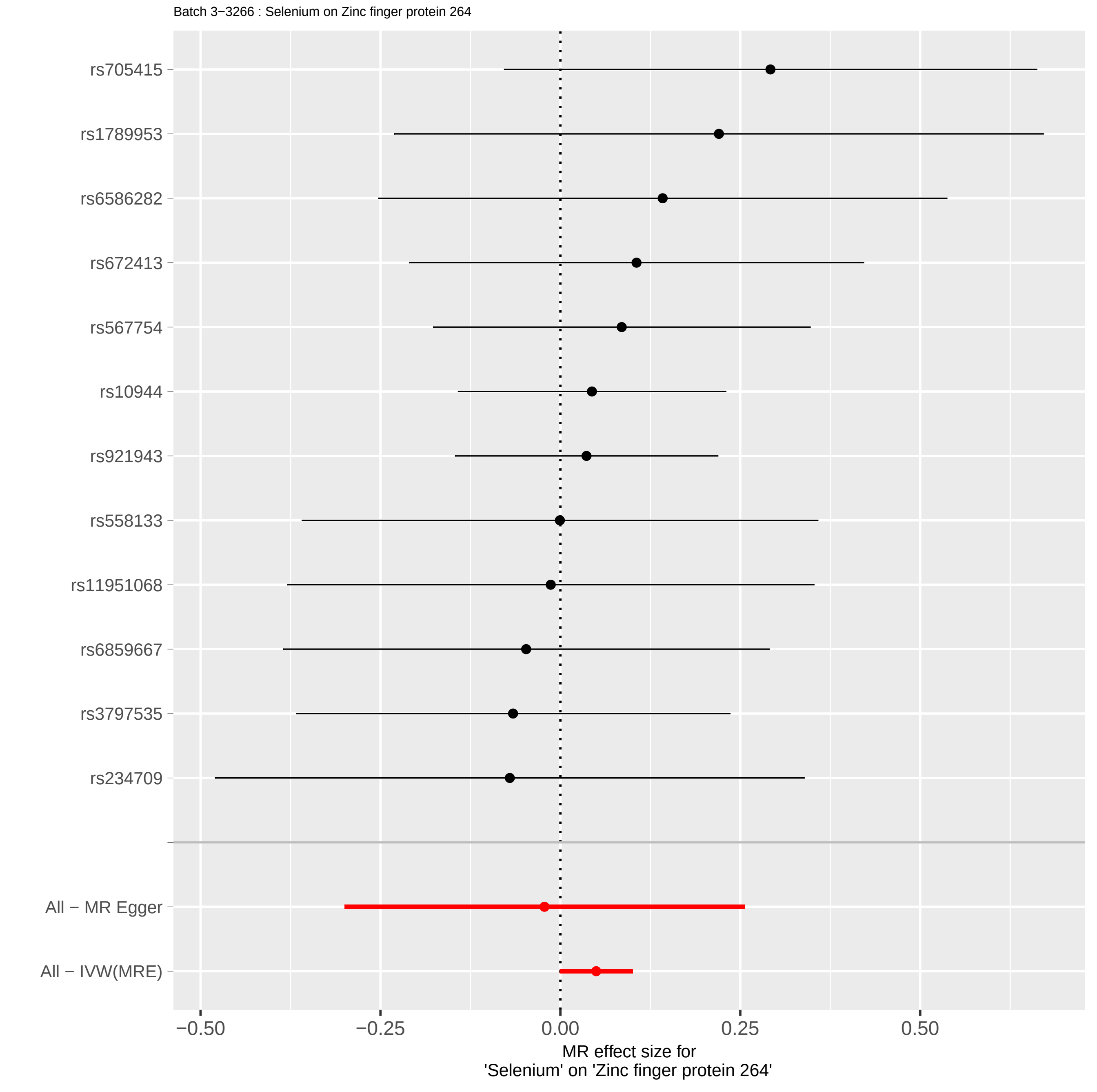
Batch 3−3266 : Selenium on Zinc finger protein 264
rs705415
rs1789953
rs6586282
rs672413
rs567754
rs10944
rs921943
rs558133
rs11951068
rs6859667
rs3797535
rs234709
All − MR Egger
All − IVW(MRE)
−0.50
−0.25
0.00
0.25
0.50
MR effect size for
'Selenium' on 'Zinc finger protein 264'

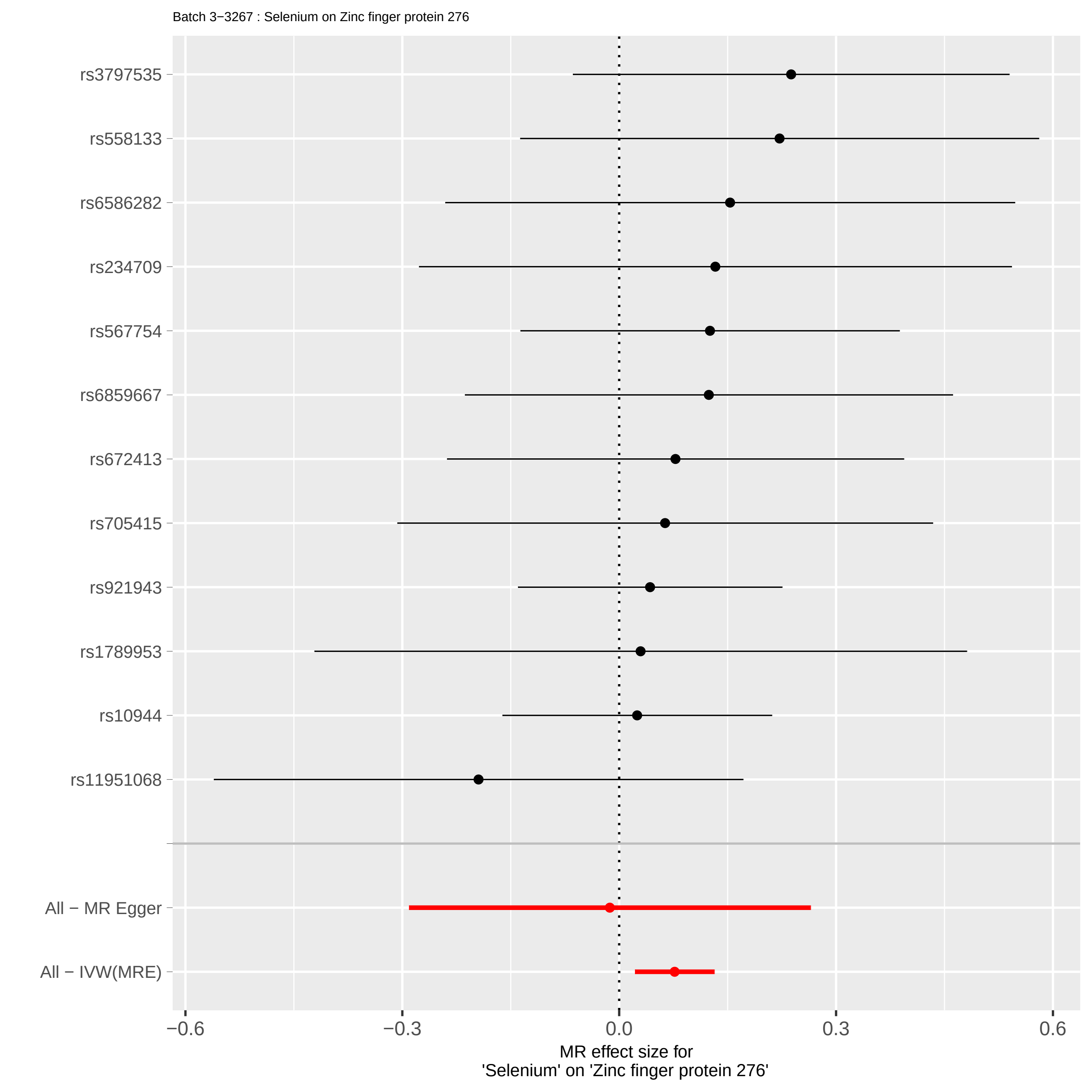
Batch 3−3267 : Selenium on Zinc finger protein 276
rs3797535
rs558133
rs6586282
rs234709
rs567754
rs6859667
rs672413
rs705415
rs921943
rs1789953
rs10944
rs11951068
All − MR Egger
All − IVW(MRE)
−0.6
−0.3
0.0
0.3
0.6
MR effect size for
'Selenium' on 'Zinc finger protein 276'

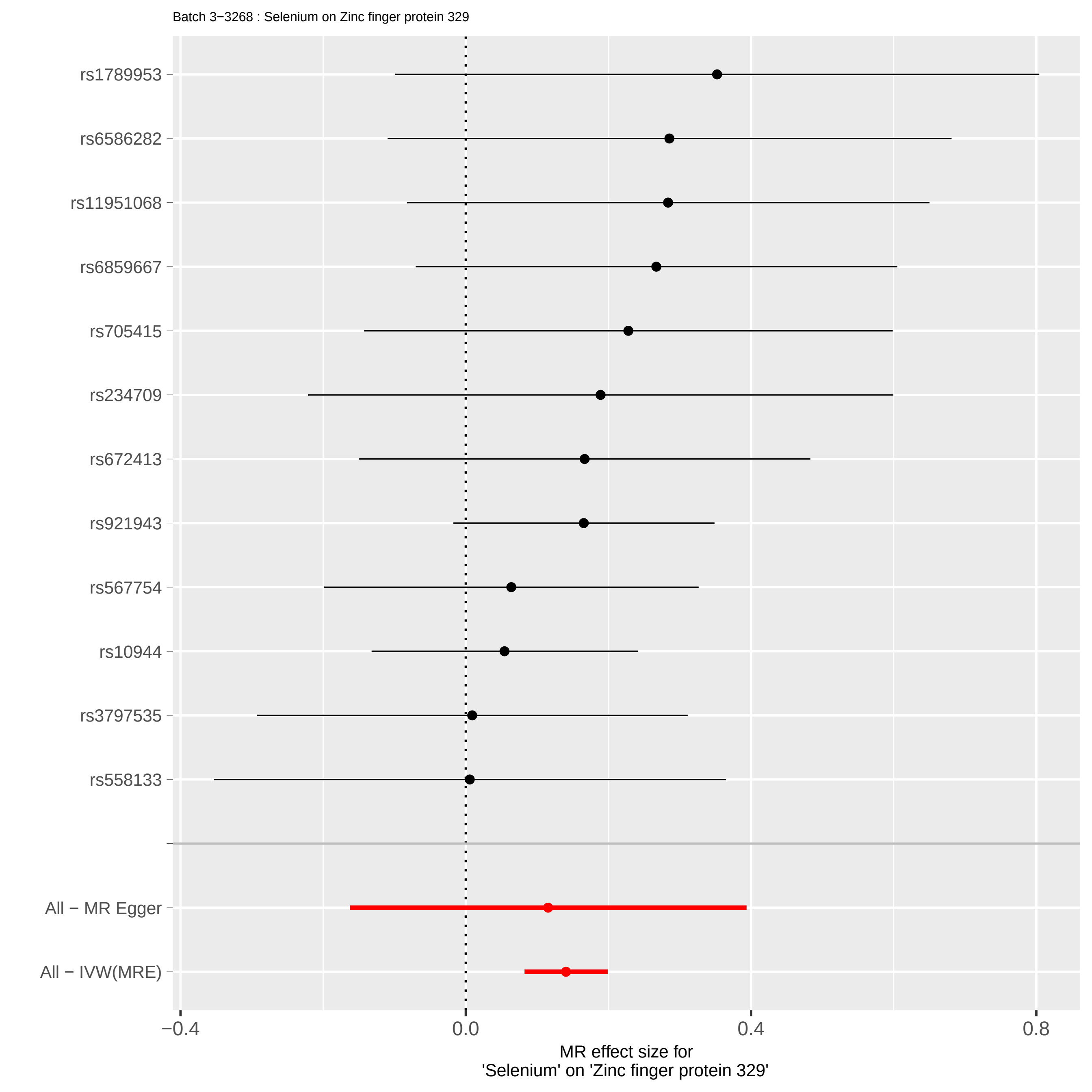
Batch 3−3268 : Selenium on Zinc finger protein 329
rs1789953
rs6586282
rs11951068
rs6859667
rs705415
rs234709
rs672413
rs921943
rs567754
rs10944
rs3797535
rs558133
All − MR Egger
All − IVW(MRE)
−0.4
0.0
0.4
0.8
MR effect size for
'Selenium' on 'Zinc finger protein 329'

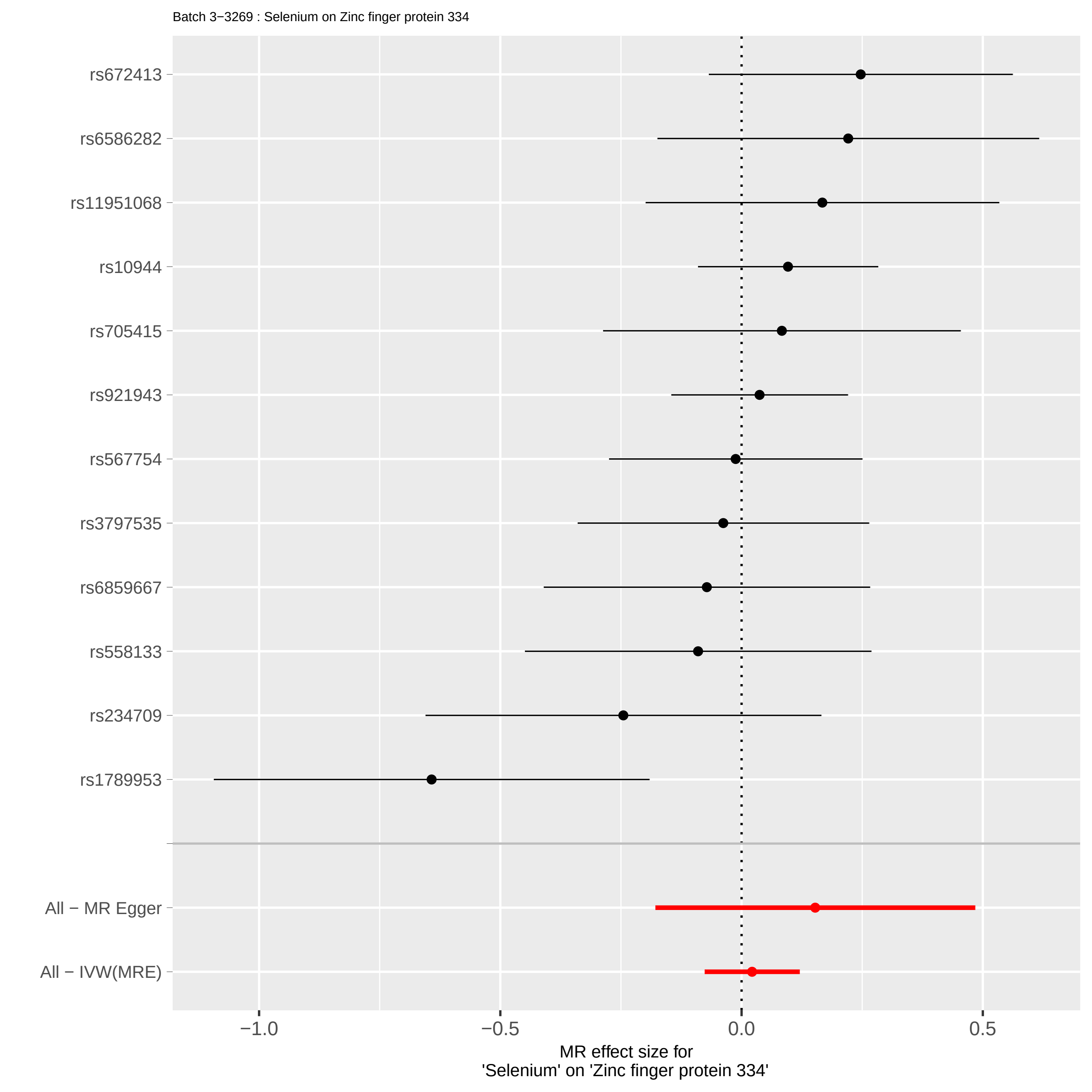

Batch 3−3269 : Selenium on Zinc finger protein 334
rs672413
rs6586282
rs11951068
rs10944
rs705415
rs921943
rs567754
rs3797535
rs6859667
rs558133
rs234709
rs1789953
All − MR Egger
All − IVW(MRE)
−1.0
−0.5
0.0
0.5
MR effect size for
'Selenium' on 'Zinc finger protein 334'

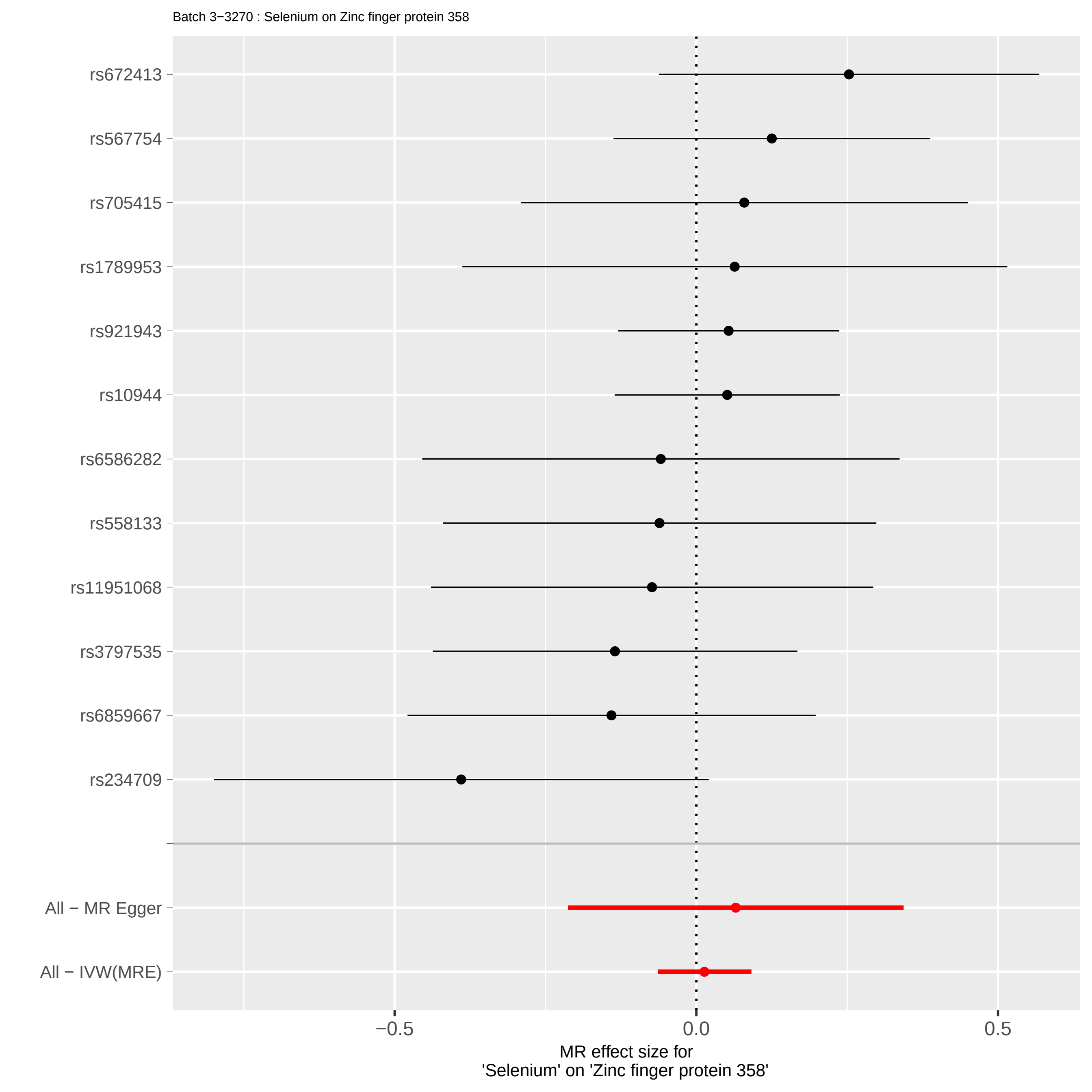

Batch 3−3270 : Selenium on Zinc finger protein 358
rs672413
rs567754
rs705415
rs1789953
rs921943
rs10944
rs6586282
rs558133
rs11951068
rs3797535
rs6859667
rs234709
All − MR Egger
All − IVW(MRE)
−0.5
0.0
0.5
MR effect size for
'Selenium' on 'Zinc finger protein 358'

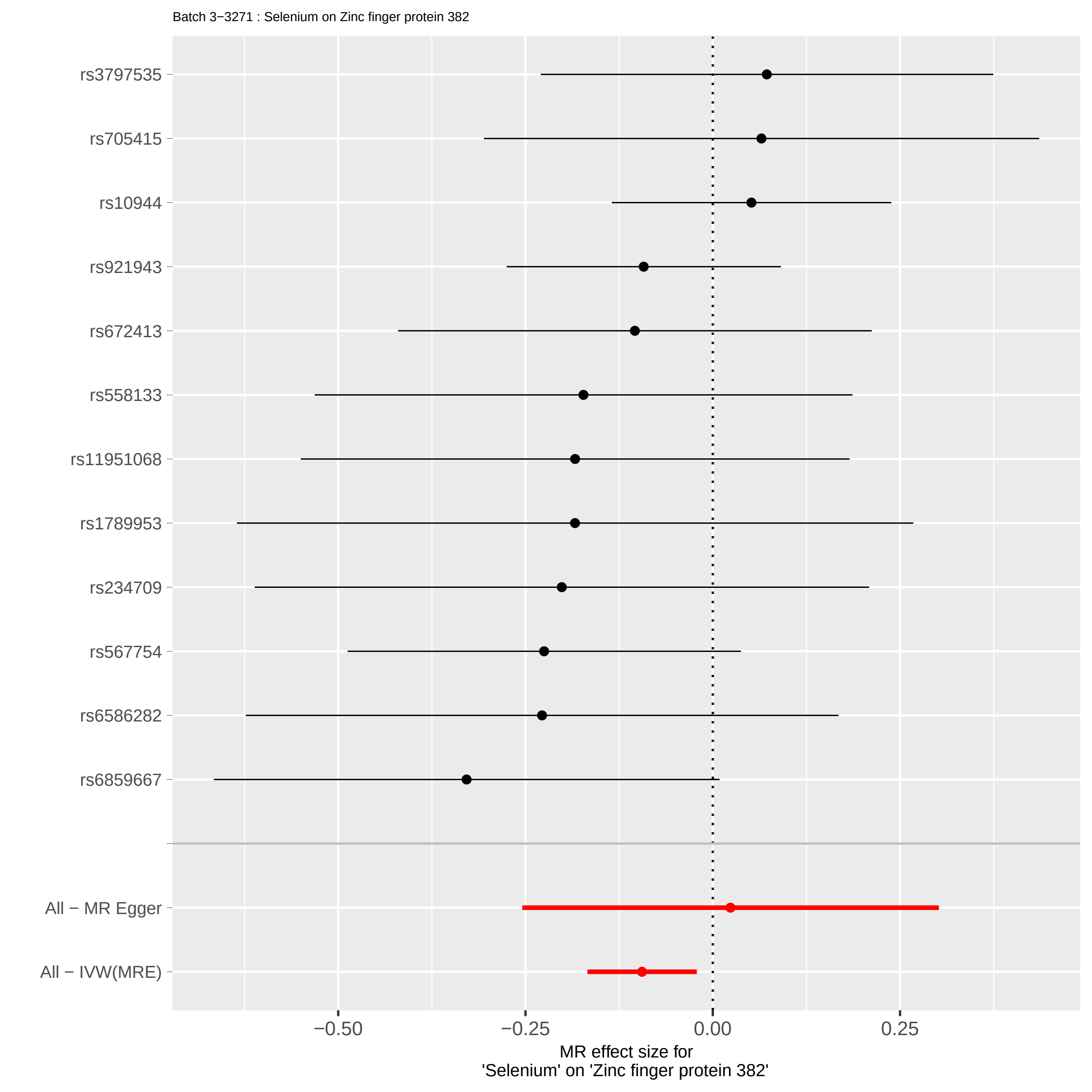

Batch 3−3271 : Selenium on Zinc finger protein 382
rs3797535
rs705415
rs10944
rs921943
rs672413
rs558133
rs11951068
rs1789953
rs234709
rs567754
rs6586282
rs6859667
All − MR Egger
All − IVW(MRE)
−0.50
−0.25
0.00
0.25
MR effect size for
'Selenium' on 'Zinc finger protein 382'

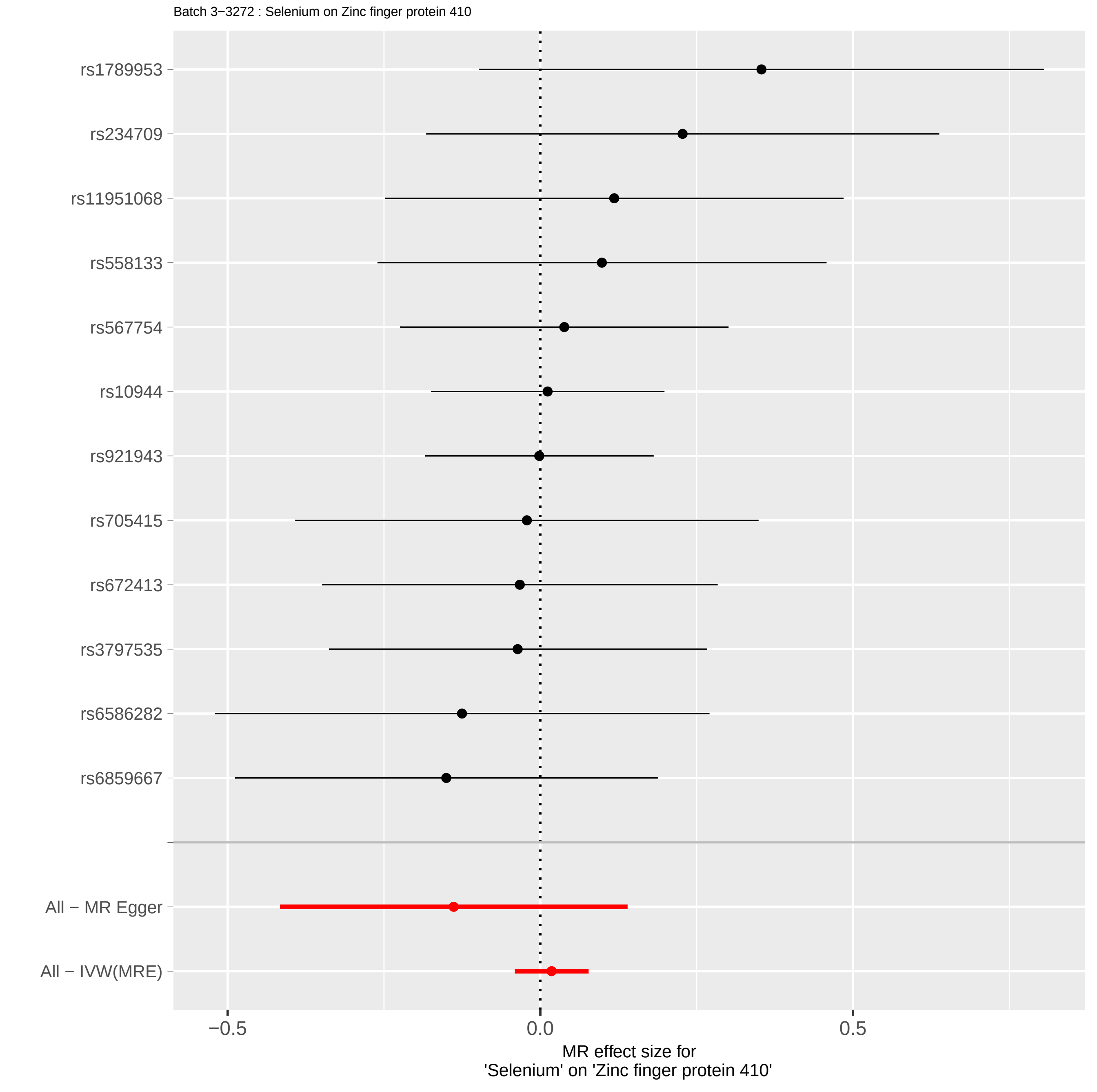

Batch 3−3272 : Selenium on Zinc finger protein 410
rs1789953
rs234709
rs11951068
rs558133
rs567754
rs10944
rs921943
rs705415
rs672413
rs3797535
rs6586282
rs6859667
All − MR Egger
All − IVW(MRE)
−0.5
0.0
0.5
MR effect size for
'Selenium' on 'Zinc finger protein 410'

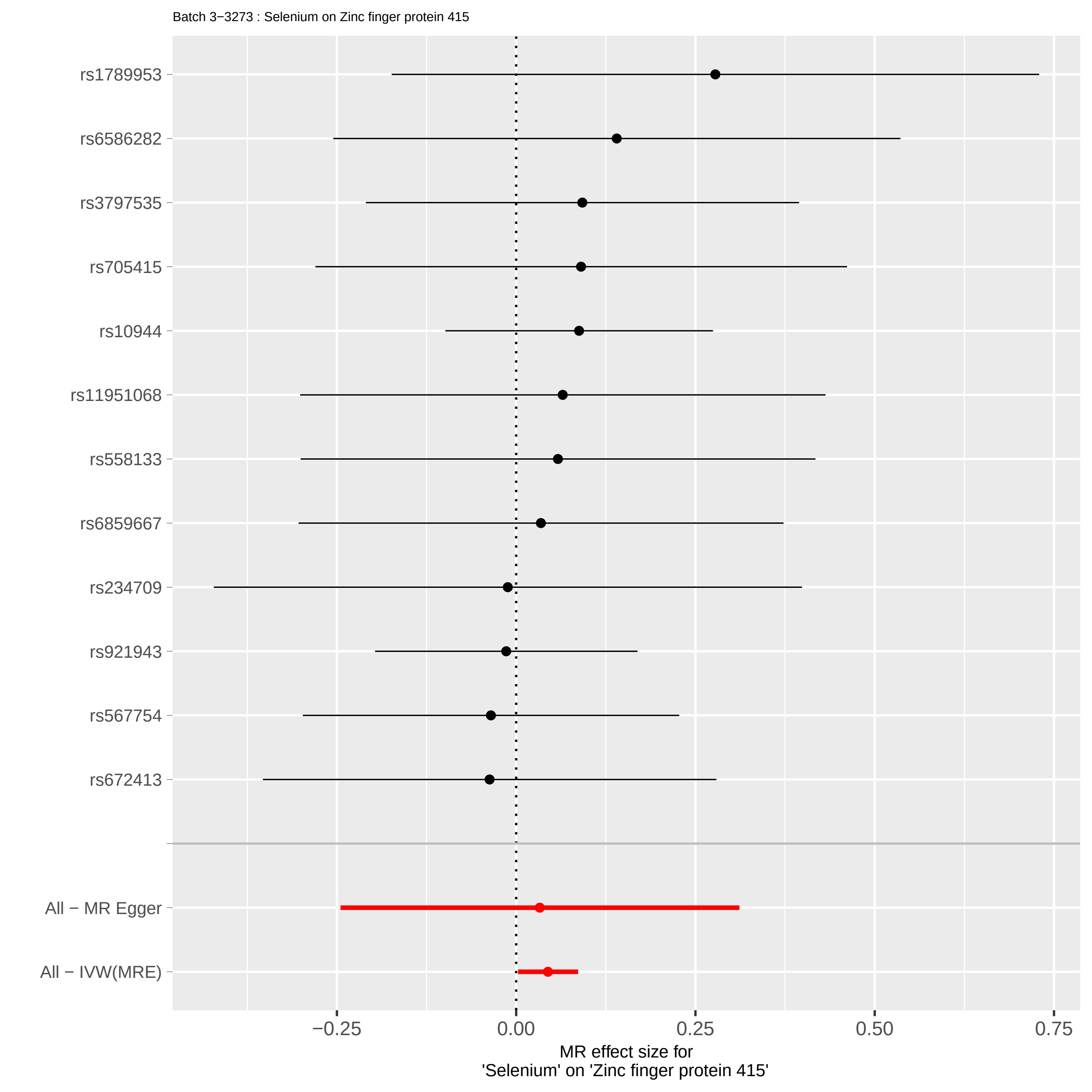

Batch 3−3273 : Selenium on Zinc finger protein 415
rs1789953
rs6586282
rs3797535
rs705415
rs10944
rs11951068
rs558133
rs6859667
rs234709
rs921943
rs567754
rs672413
All − MR Egger
All − IVW(MRE)
−0.25
0.00
0.25
0.50
0.75
MR effect size for
'Selenium' on 'Zinc finger protein 415'

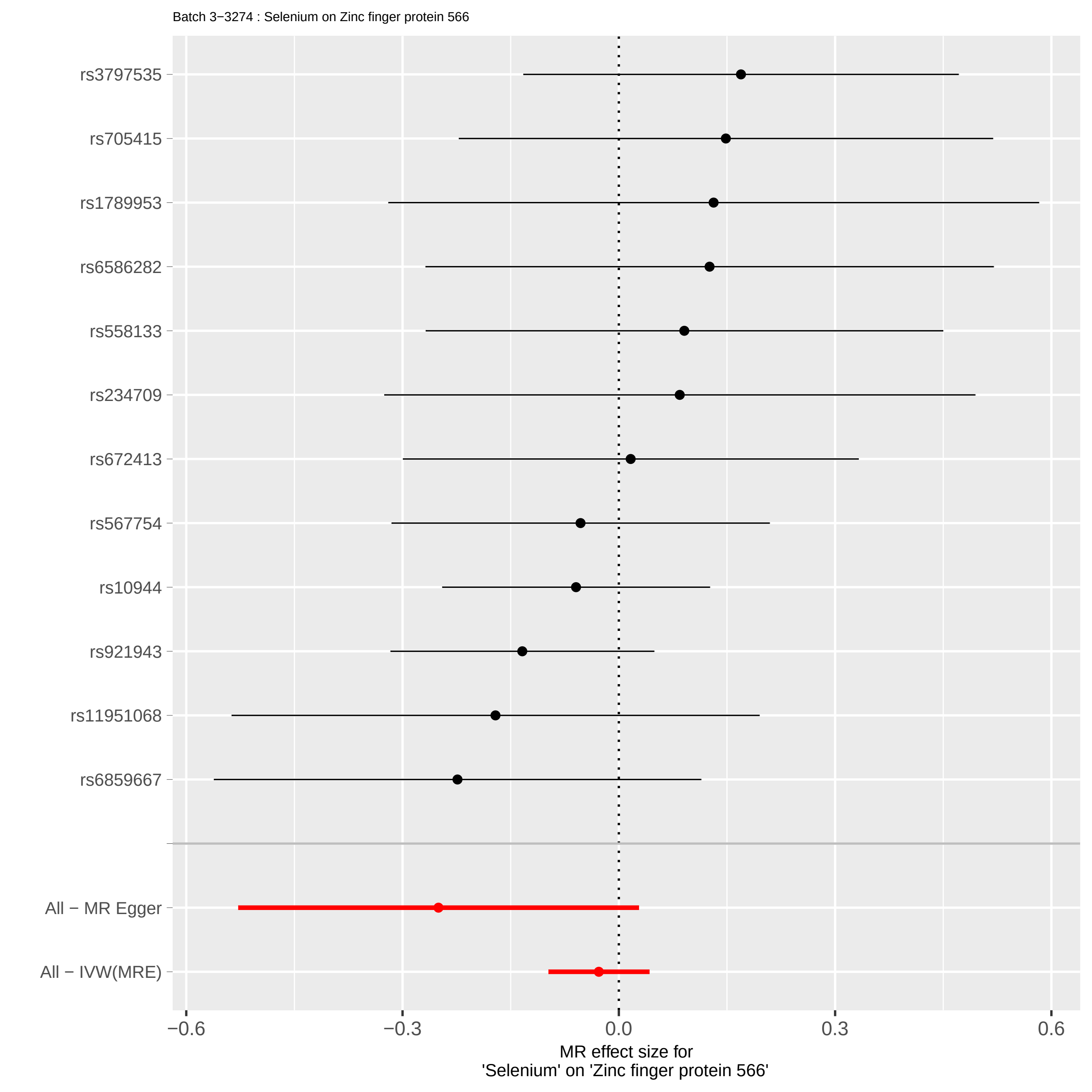

Batch 3−3274 : Selenium on Zinc finger protein 566
rs3797535
rs705415
rs1789953
rs6586282
rs558133
rs234709
rs672413
rs567754
rs10944
rs921943
rs11951068
rs6859667
All − MR Egger
All − IVW(MRE)
−0.6
−0.3
0.0
0.3
0.6
MR effect size for
'Selenium' on 'Zinc finger protein 566'

Batch 3−3275 : Selenium on Zinc finger protein 75D

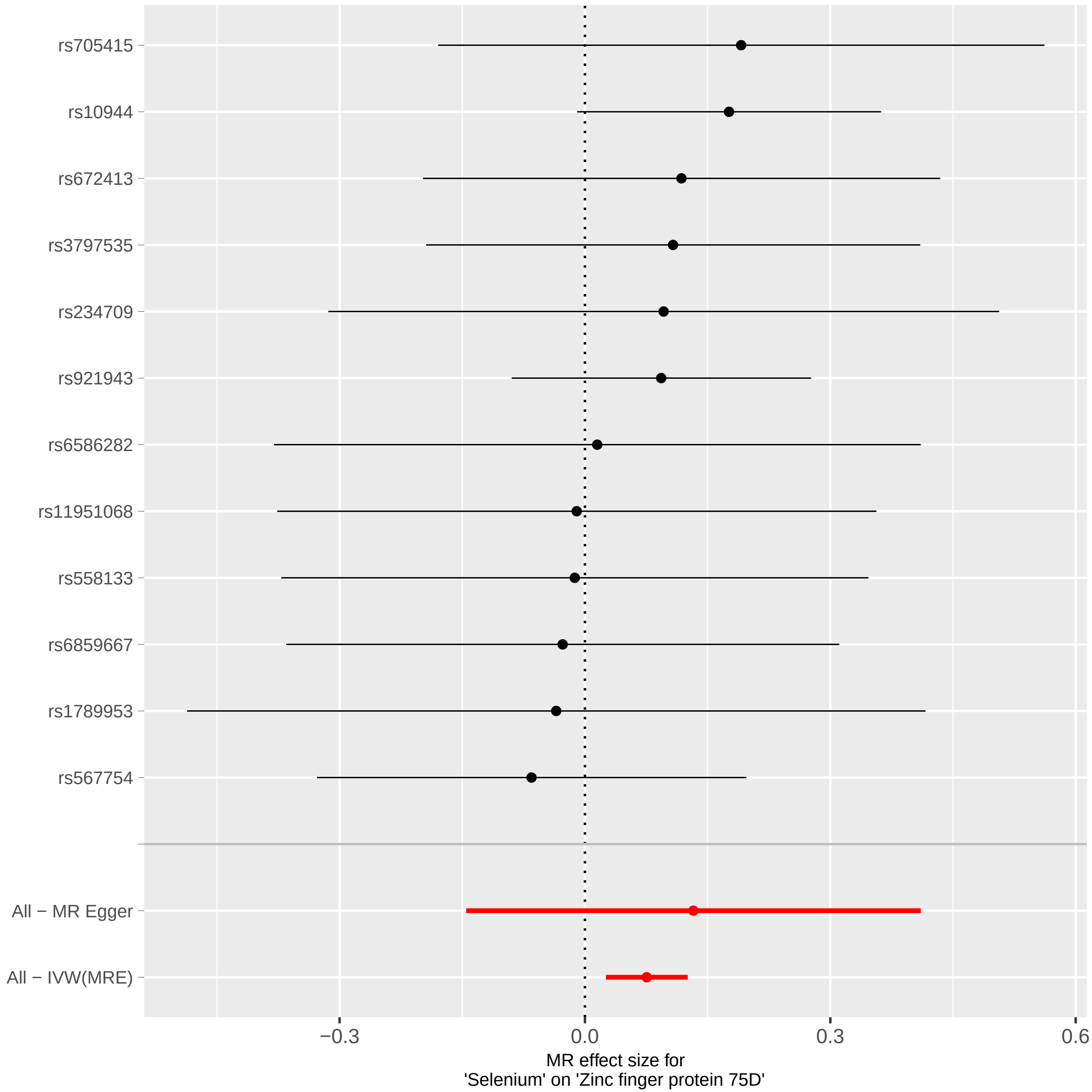

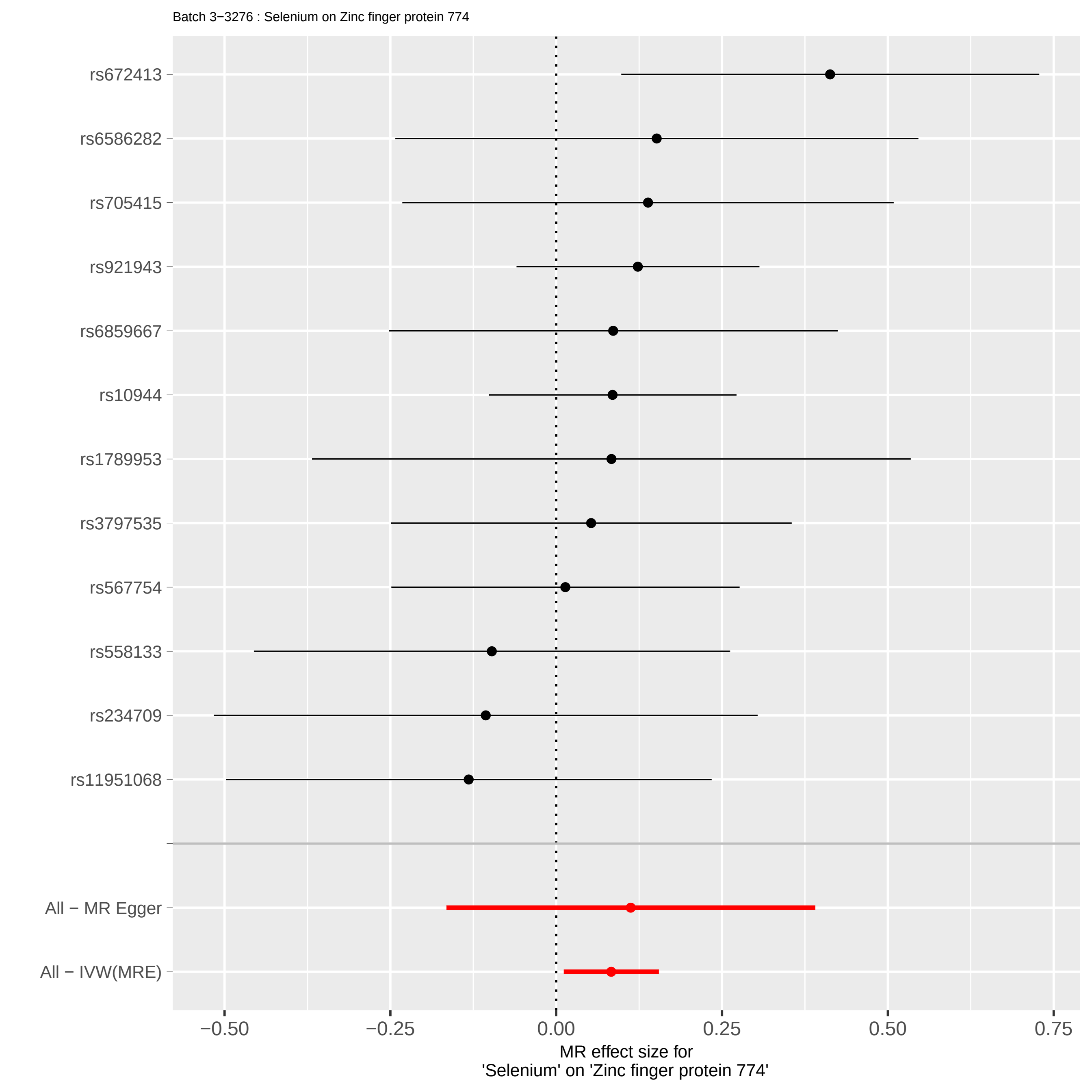
Batch 3−3276 : Selenium on Zinc finger protein 774
rs672413
rs6586282
rs705415
rs921943
rs6859667
rs10944
rs1789953
rs3797535
rs567754
rs558133
rs234709
rs11951068
All − MR Egger
All − IVW(MRE)
−0.50
−0.25
0.00
0.25
0.50
0.75
MR effect size for
'Selenium' on 'Zinc finger protein 774'

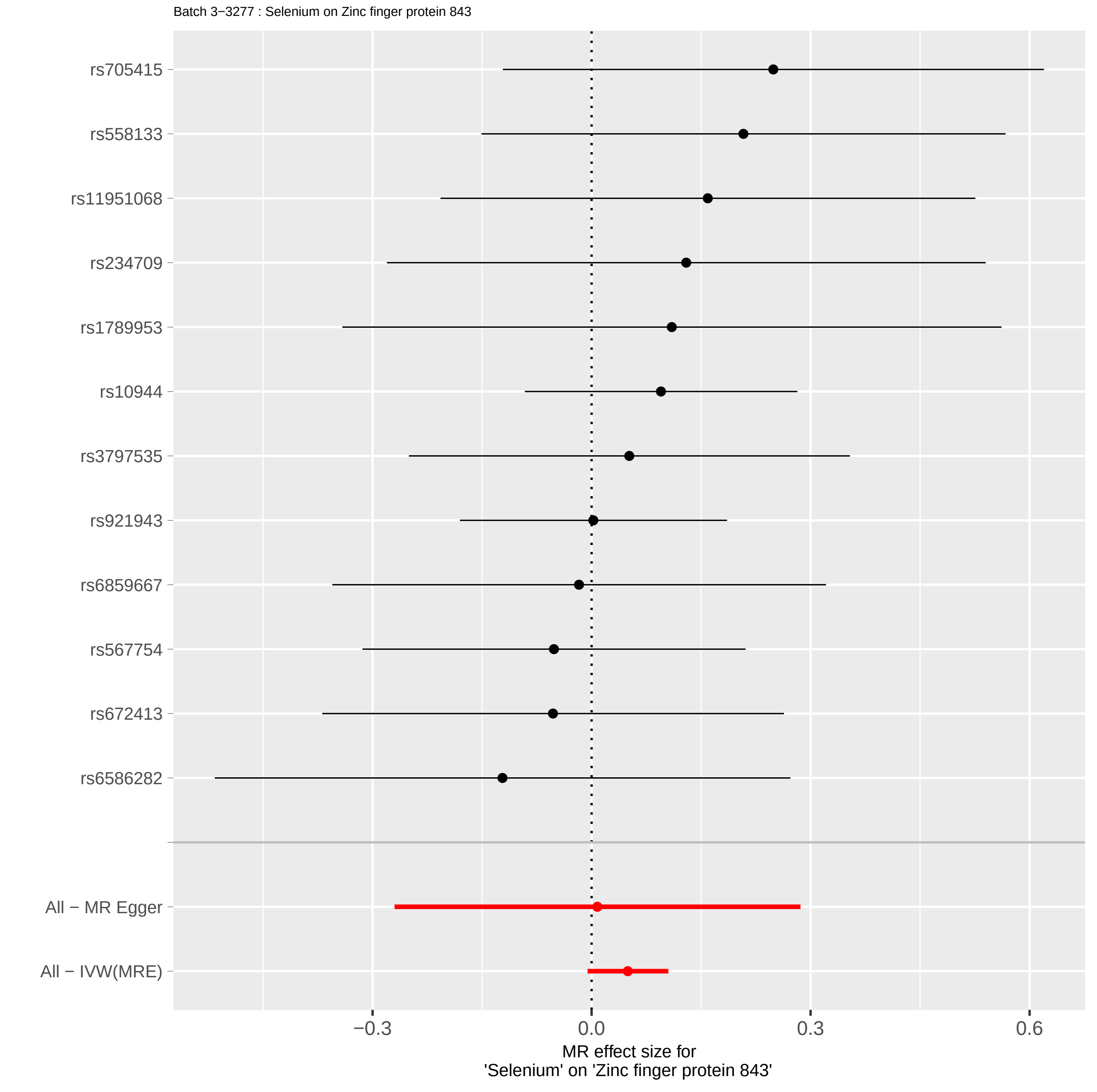

Batch 3−3277 : Selenium on Zinc finger protein 843
rs705415
rs558133
rs11951068
rs234709
rs1789953
rs10944
rs3797535
rs921943
rs6859667
rs567754
rs672413
rs6586282
All − MR Egger
All − IVW(MRE)
−0.3
0.0
0.3
0.6
MR effect size for
'Selenium' on 'Zinc finger protein 843'

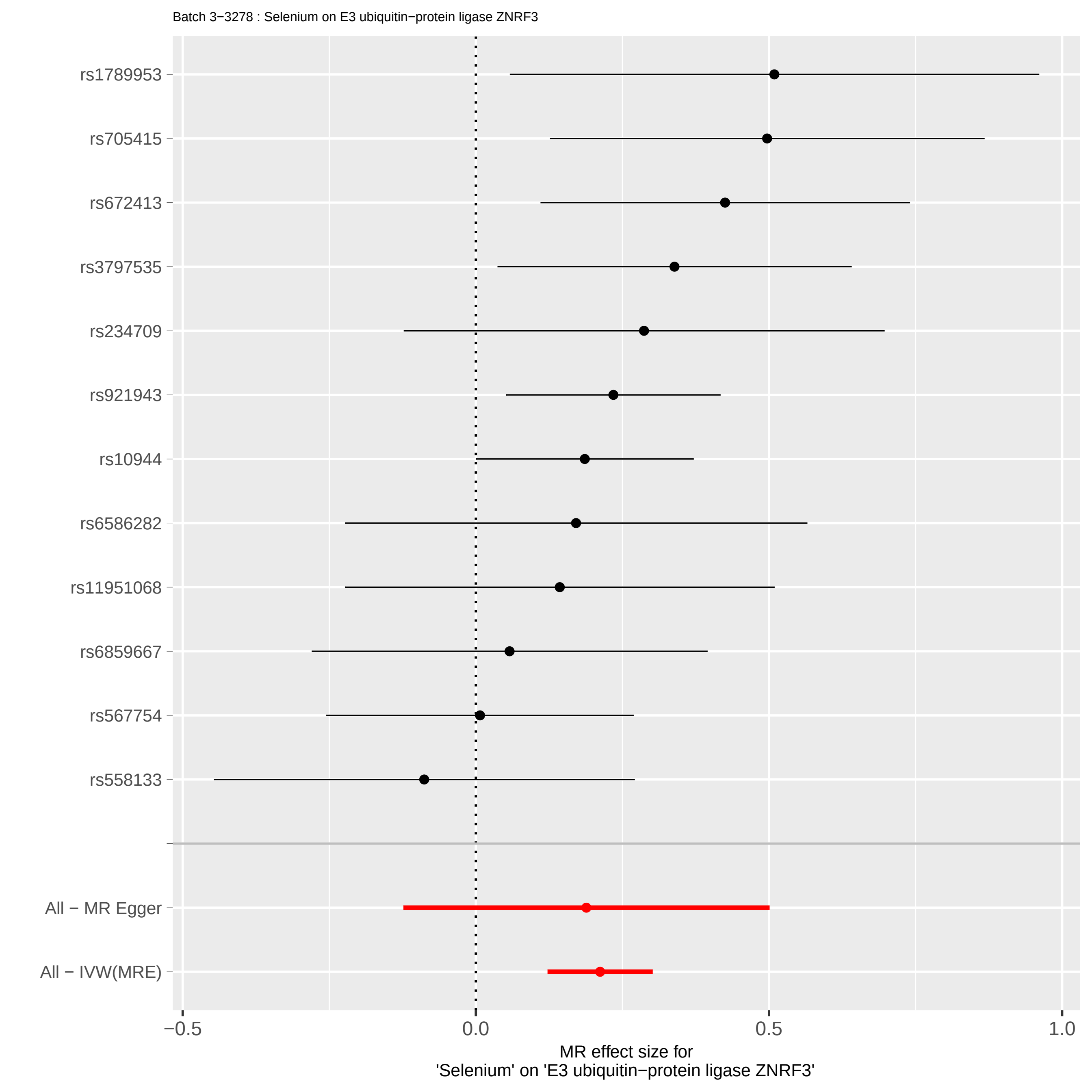

Batch 3−3278 : Selenium on E3 ubiquitin−protein ligase ZNRF3
rs1789953
rs705415
rs672413
rs3797535
rs234709
rs921943
rs10944
rs6586282
rs11951068
rs6859667
rs567754
rs558133
All − MR Egger
All − IVW(MRE)
−0.5
0.0
0.5
1.0
MR effect size for
'Selenium' on 'E3 ubiquitin−protein ligase ZNRF3'

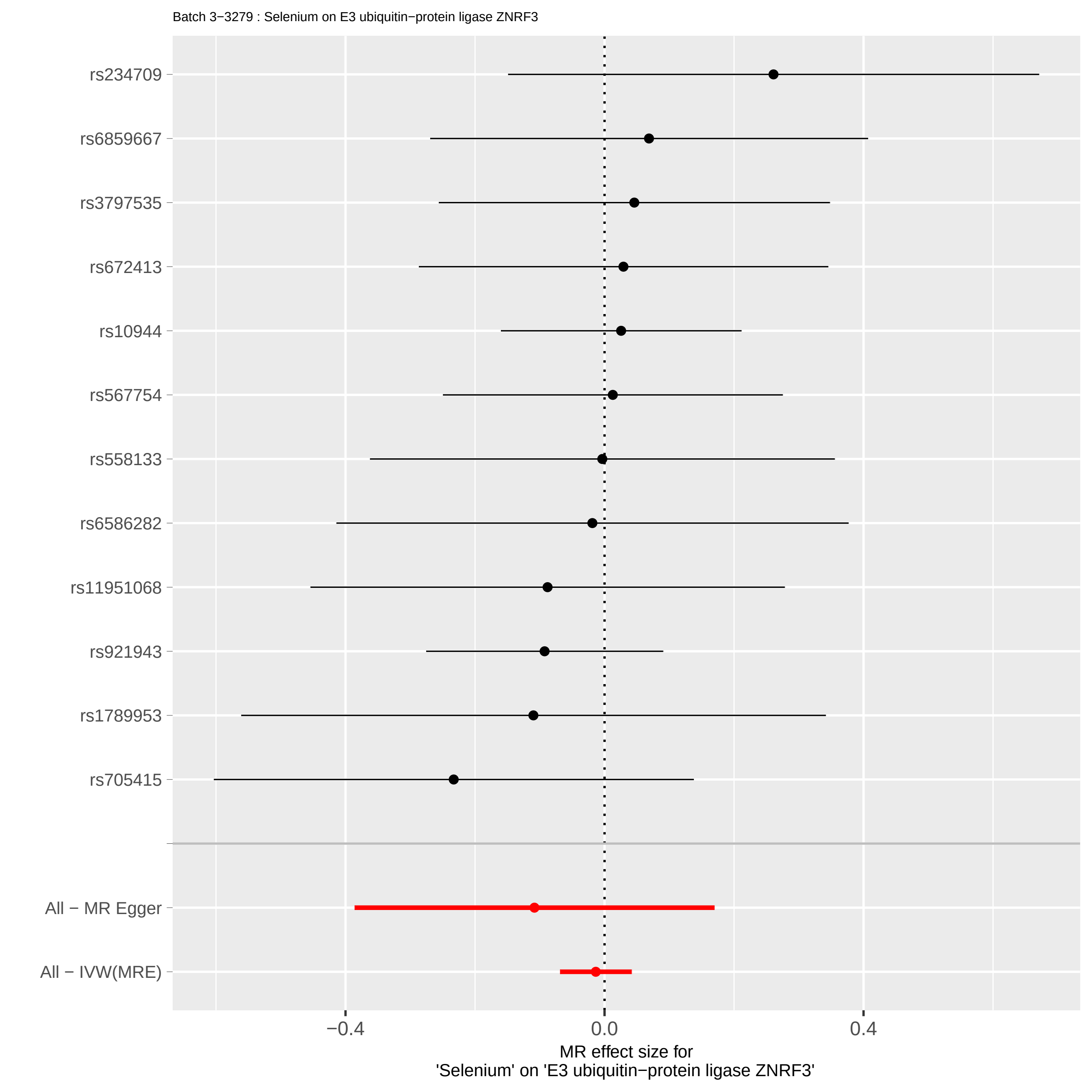
Batch 3−3279 : Selenium on E3 ubiquitin−protein ligase ZNRF3
rs234709
rs6859667
rs3797535
rs672413
rs10944
rs567754
rs558133
rs6586282
rs11951068
rs921943
rs1789953
rs705415
All − MR Egger
All − IVW(MRE)
−0.4
0.0
0.4
MR effect size for
'Selenium' on 'E3 ubiquitin−protein ligase ZNRF3'

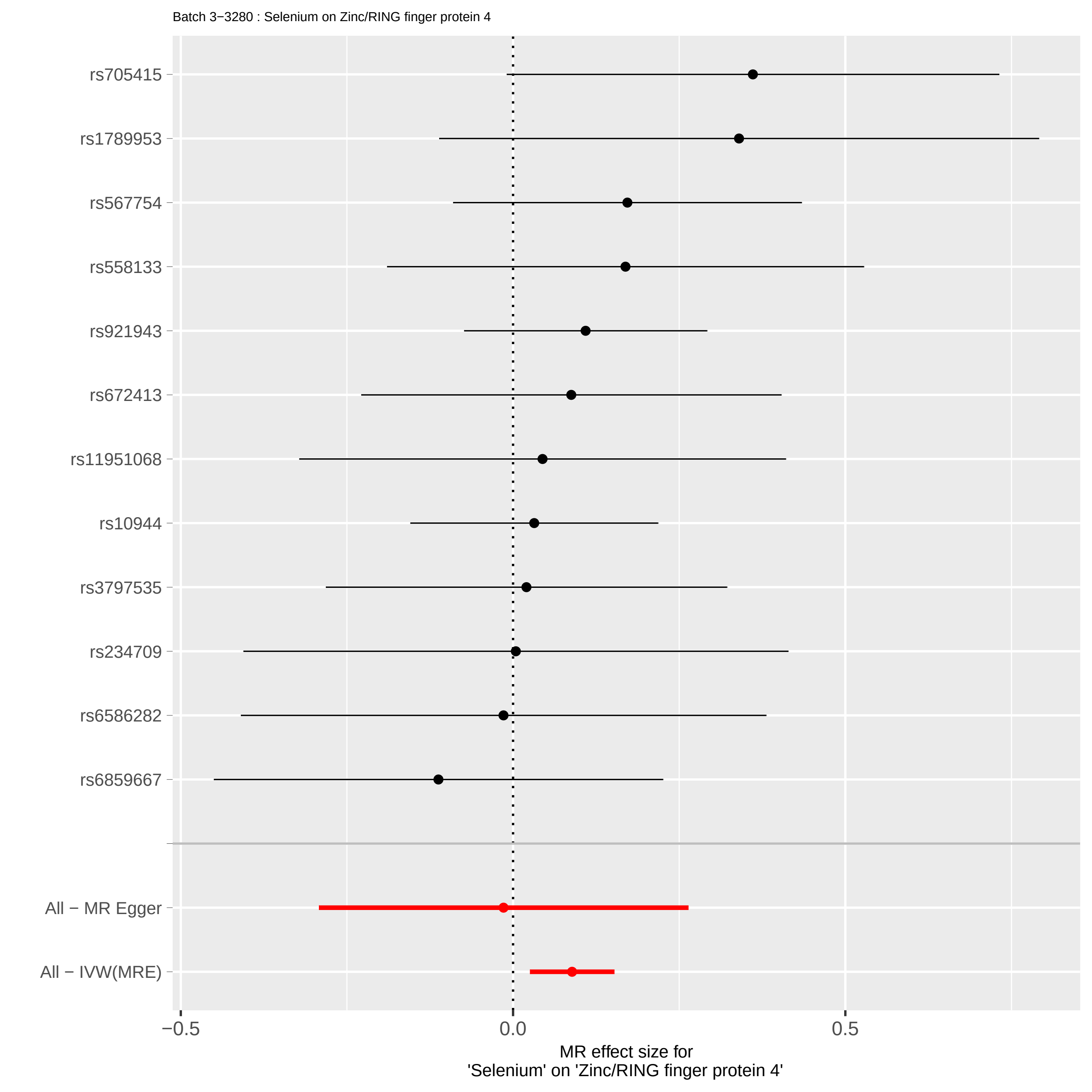

Batch 3−3280 : Selenium on Zinc/RING finger protein 4
rs705415
rs1789953
rs567754
rs558133
rs921943
rs672413
rs11951068
rs10944
rs3797535
rs234709
rs6586282
rs6859667
All − MR Egger
All − IVW(MRE)
−0.5
0.0
0.5
MR effect size for
'Selenium' on 'Zinc/RING finger protein 4'

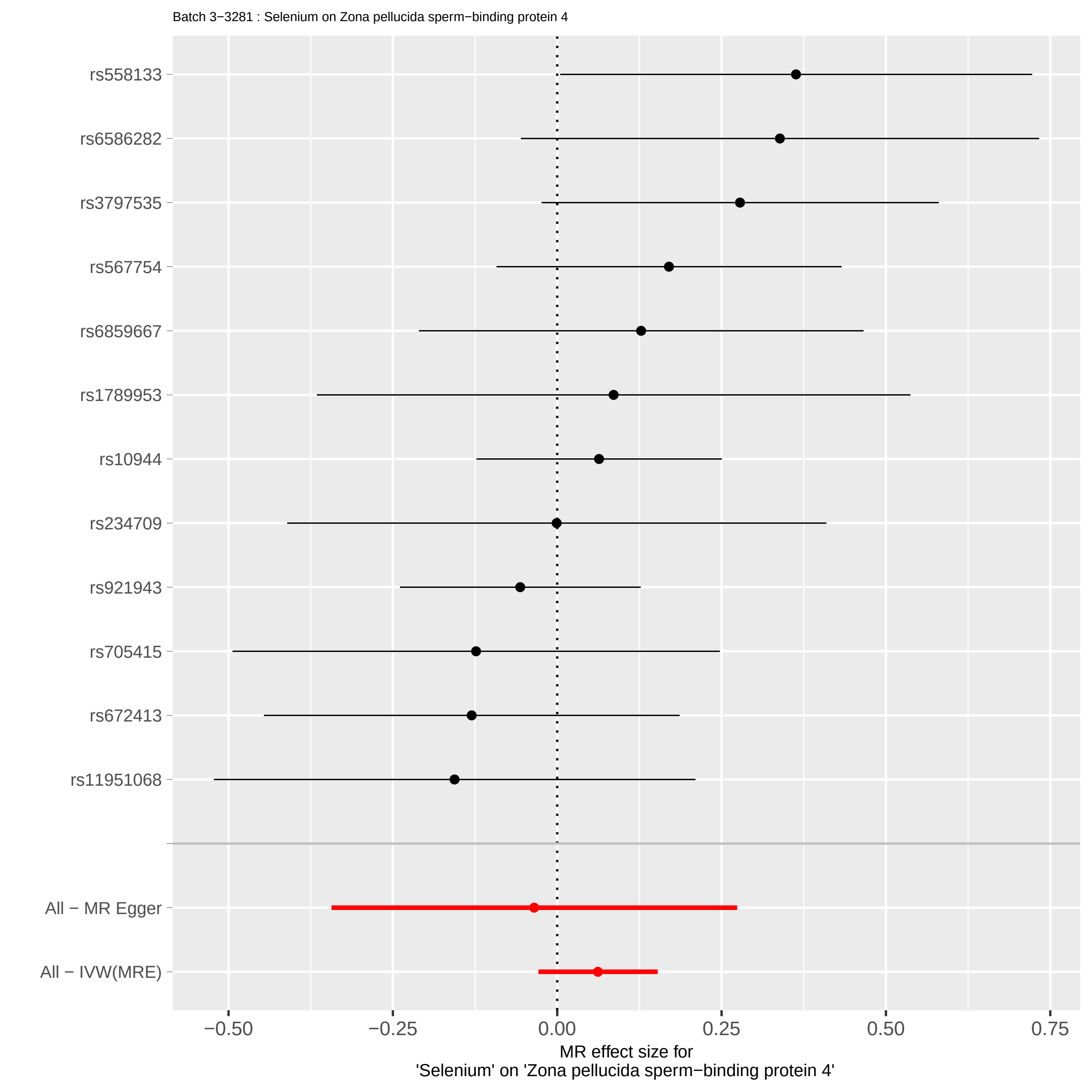

Batch 3−3281 : Selenium on Zona pellucida sperm−binding protein 4
rs558133
rs6586282
rs3797535
rs567754
rs6859667
rs1789953
rs10944
rs234709
rs921943
rs705415
rs672413
rs11951068
All − MR Egger
All − IVW(MRE)
−0.50
−0.25
0.00
0.25
0.50
0.75
MR effect size for
'Selenium' on 'Zona pellucida sperm−binding protein 4'

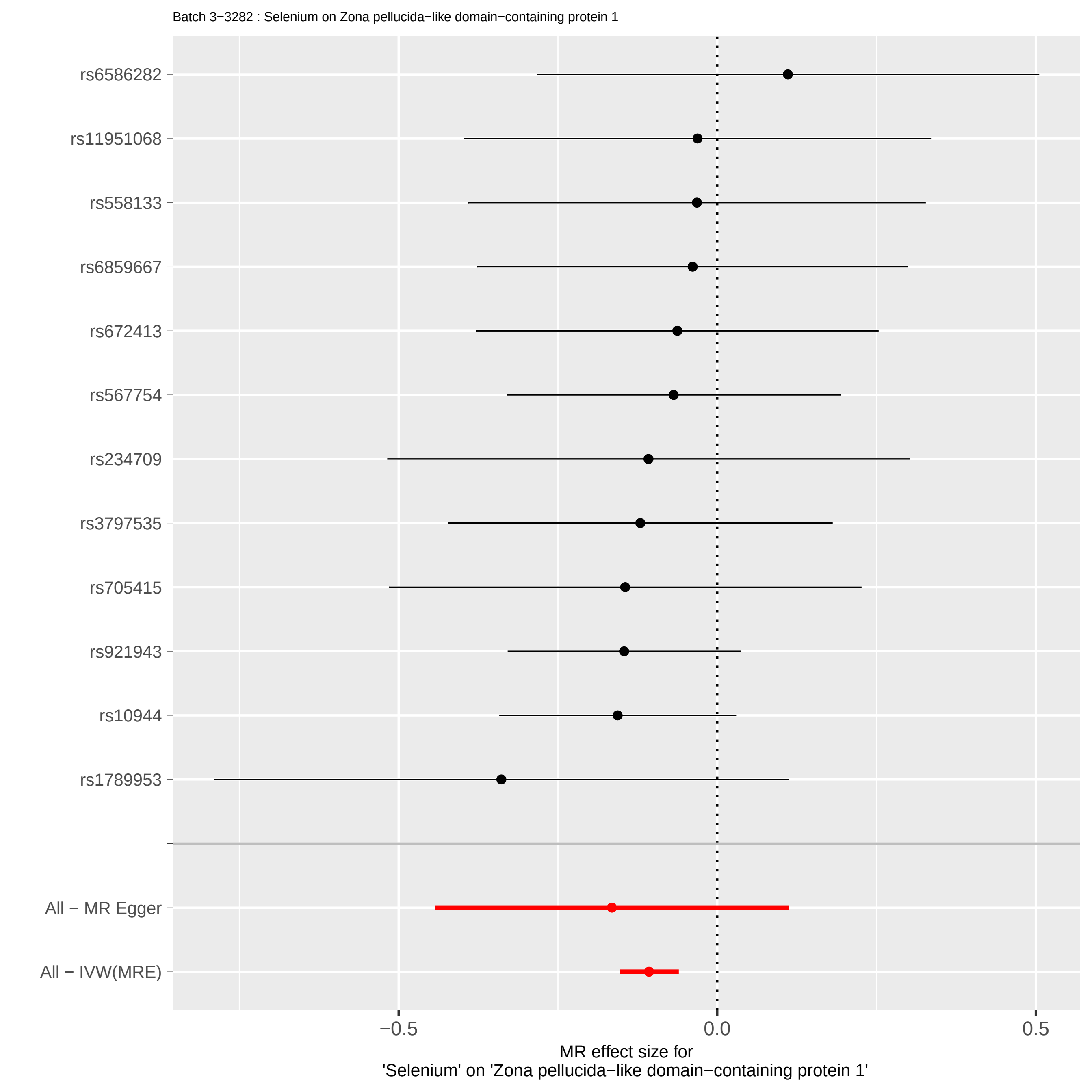

Batch 3−3282 : Selenium on Zona pellucida−like domain−containing protein 1
rs6586282
rs11951068
rs558133
rs6859667
rs672413
rs567754
rs234709
rs3797535
rs705415
rs921943
rs10944
rs1789953
All − MR Egger
All − IVW(MRE)
−0.5
0.0
0.5
MR effect size for
'Selenium' on 'Zona pellucida−like domain−containing protein 1'

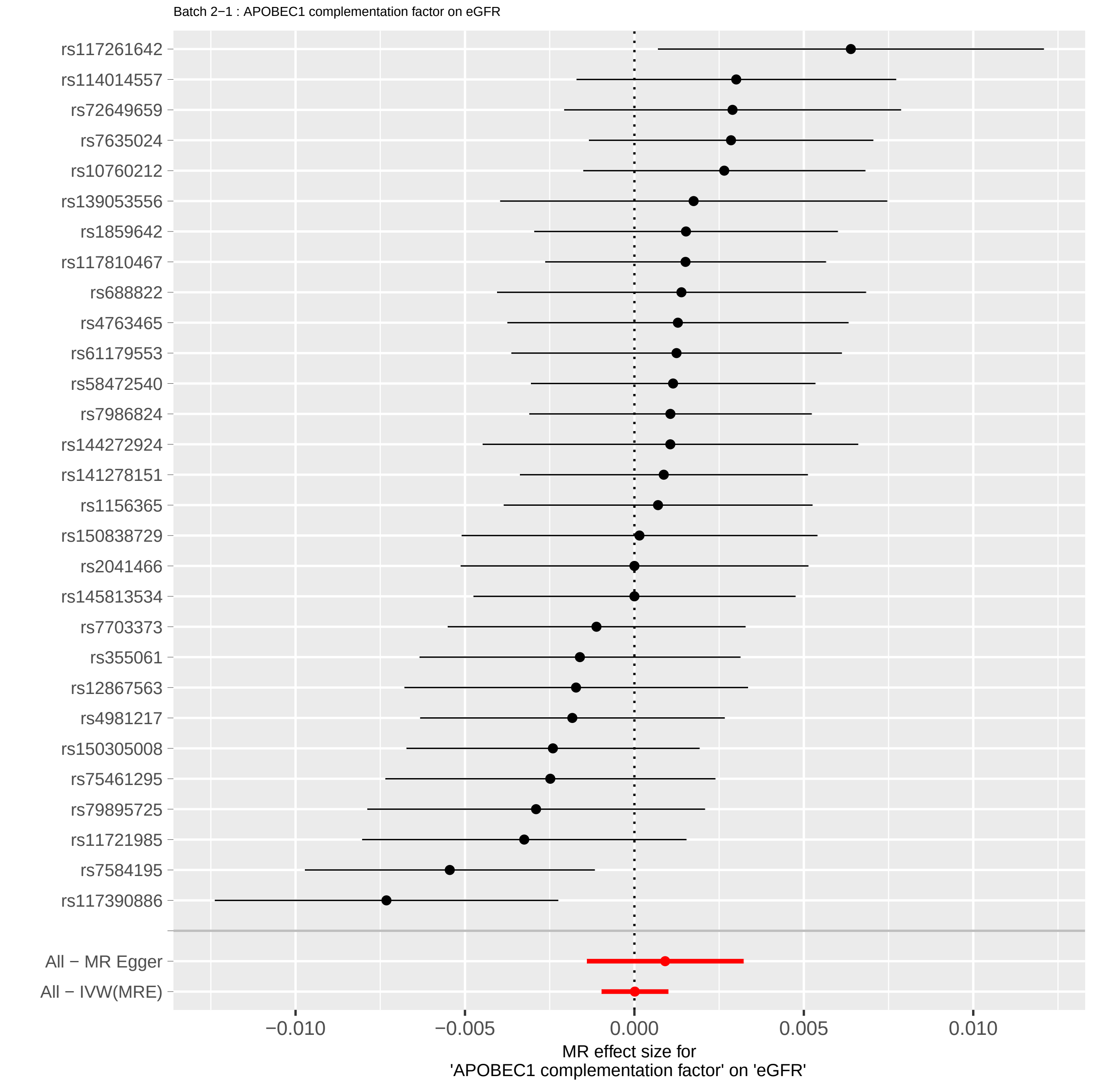

Batch 2−1 : APOBEC1 complementation factor on eGFR
rs117261642
rs114014557
rs72649659
rs7635024
rs10760212
rs139053556
rs1859642
rs117810467
rs688822
rs4763465
rs61179553
rs58472540
rs7986824
rs144272924
rs141278151
rs1156365
rs150838729
rs2041466
rs145813534
rs7703373
rs355061
rs12867563
rs4981217
rs150305008
rs75461295
rs79895725
rs11721985
rs7584195
rs117390886
All − MR Egger
All − IVW(MRE)
−0.010
−0.005
0.000
0.005
0.010
MR effect size for
'APOBEC1 complementation factor' on 'eGFR'

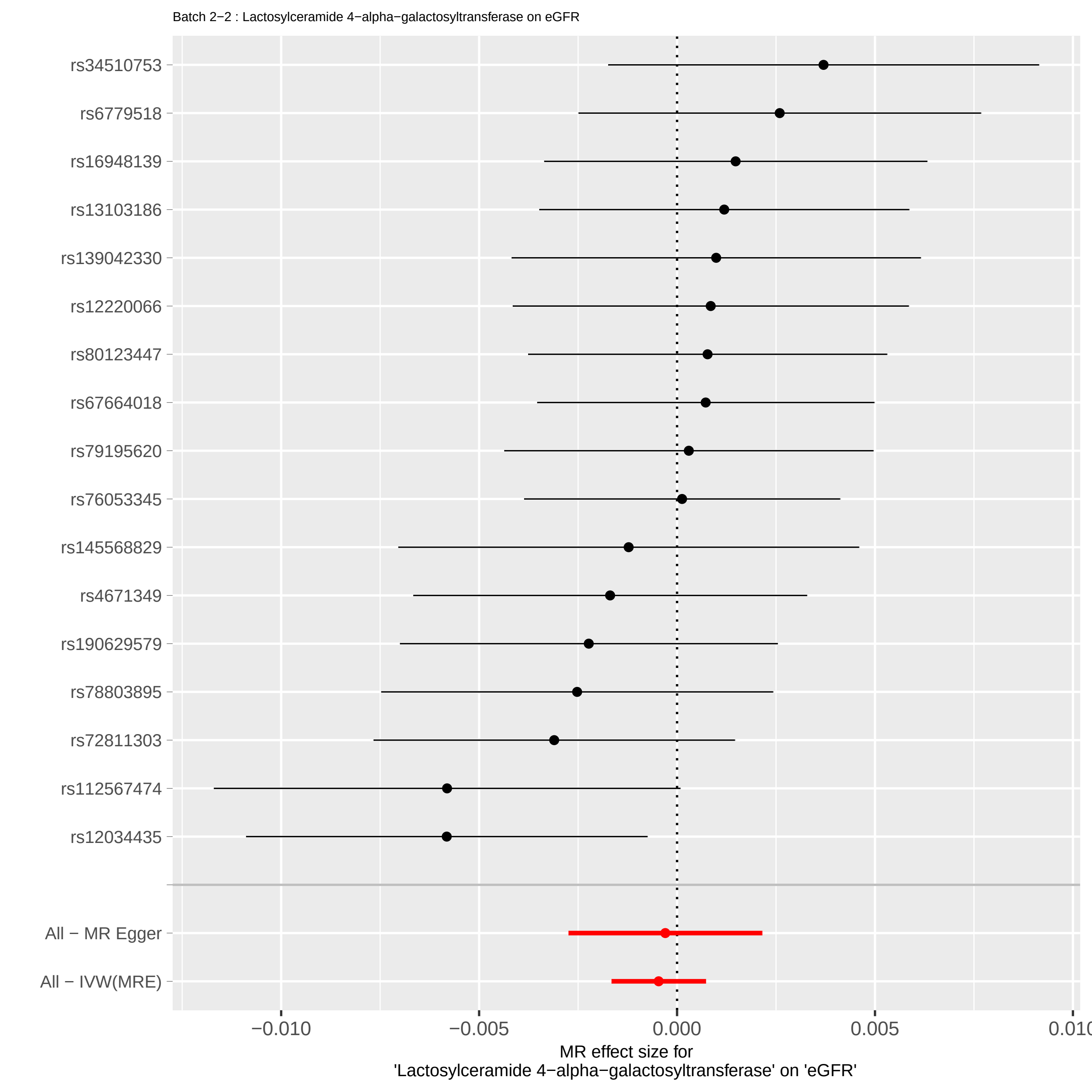

Batch 2−2 : Lactosylceramide 4−alpha−galactosyltransferase on eGFR
rs34510753
rs6779518
rs16948139
rs13103186
rs139042330
rs12220066
rs80123447
rs67664018
rs79195620
rs76053345
rs145568829
rs4671349
rs190629579
rs78803895
rs72811303
rs112567474
rs12034435
All − MR Egger
All − IVW(MRE)
−0.010
−0.005
0.000
0.005
0.010
MR effect size for
'Lactosylceramide 4−alpha−galactosyltransferase' on 'eGFR'

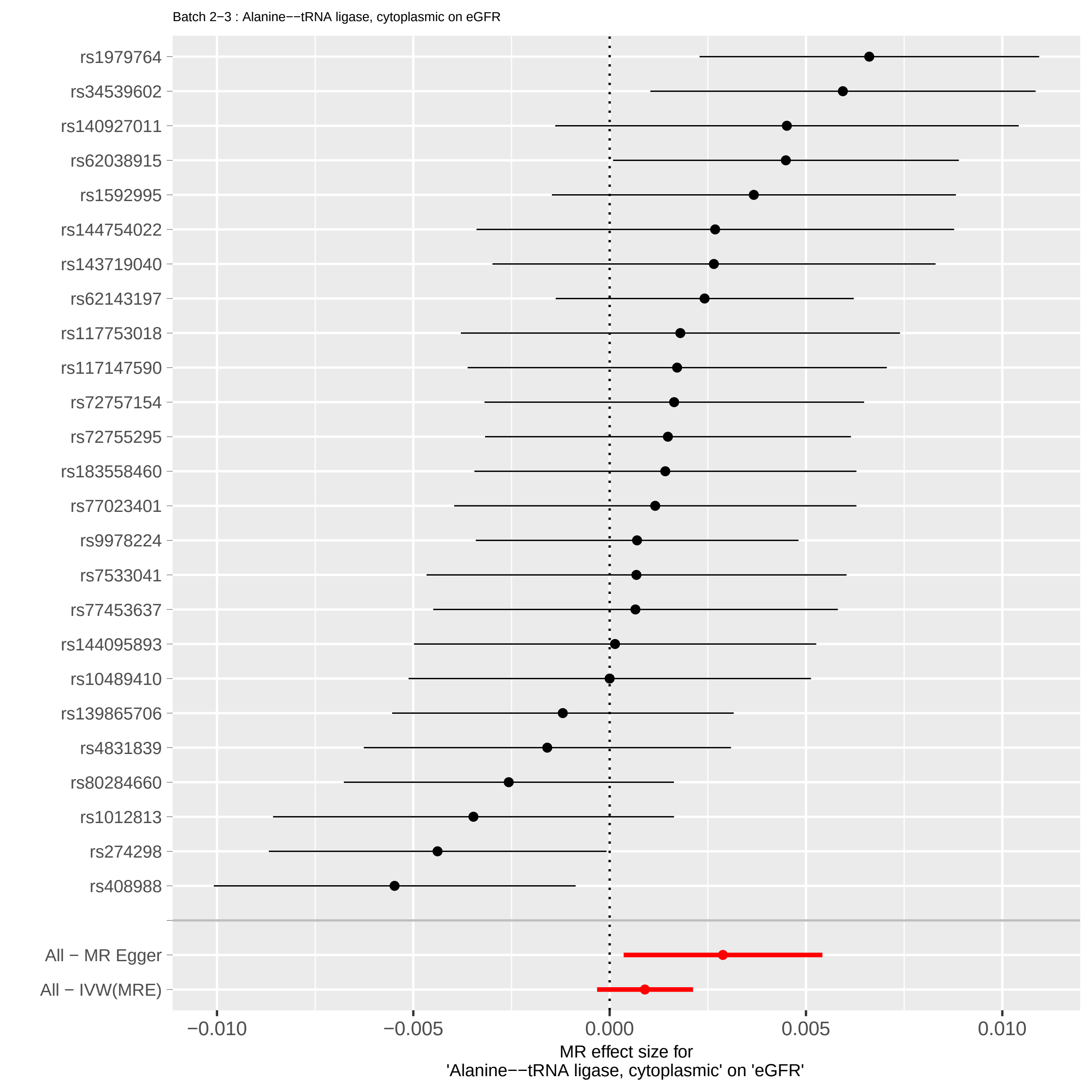

Batch 2−3 : Alanine−−tRNA ligase, cytoplasmic on eGFR
rs1979764
rs34539602
rs140927011
rs62038915
rs1592995
rs144754022
rs143719040
rs62143197
rs117753018
rs117147590
rs72757154
rs72755295
rs183558460
rs77023401
rs9978224
rs7533041
rs77453637
rs144095893
rs10489410
rs139865706
rs4831839
rs80284660
rs1012813
rs274298
rs408988
All − MR Egger
All − IVW(MRE)
−0.010
−0.005
0.000
0.005
0.010
MR effect size for
'Alanine−−tRNA ligase, cytoplasmic' on 'eGFR'

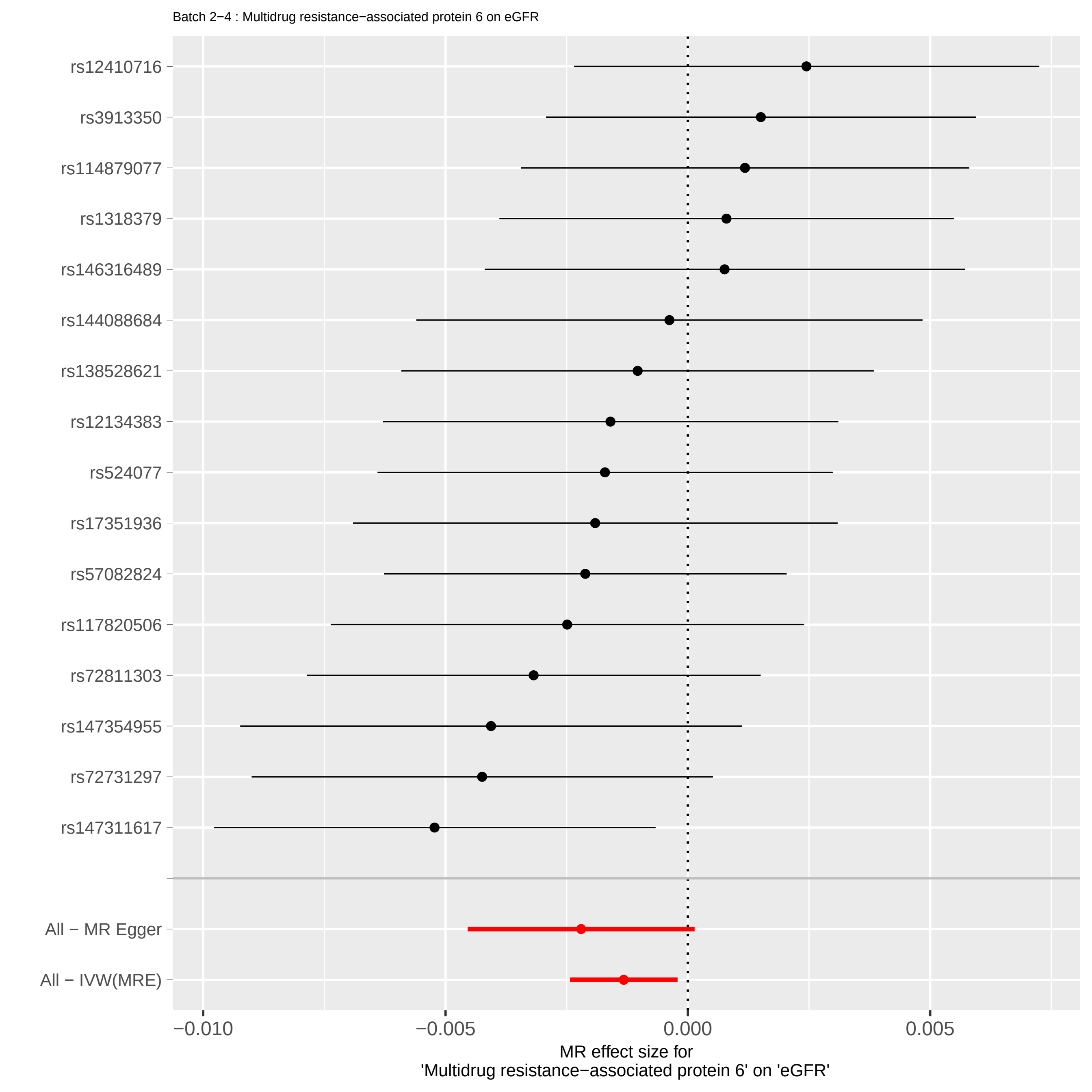

Batch 2−4 : Multidrug resistance−associated protein 6 on eGFR
rs12410716
rs3913350
rs114879077
rs1318379
rs146316489
rs144088684
rs138528621
rs12134383
rs524077
rs17351936
rs57082824
rs117820506
rs72811303
rs147354955
rs72731297
rs147311617
All − MR Egger
All − IVW(MRE)
−0.010
−0.005
0.000
0.005
MR effect size for
'Multidrug resistance−associated protein 6' on 'eGFR'

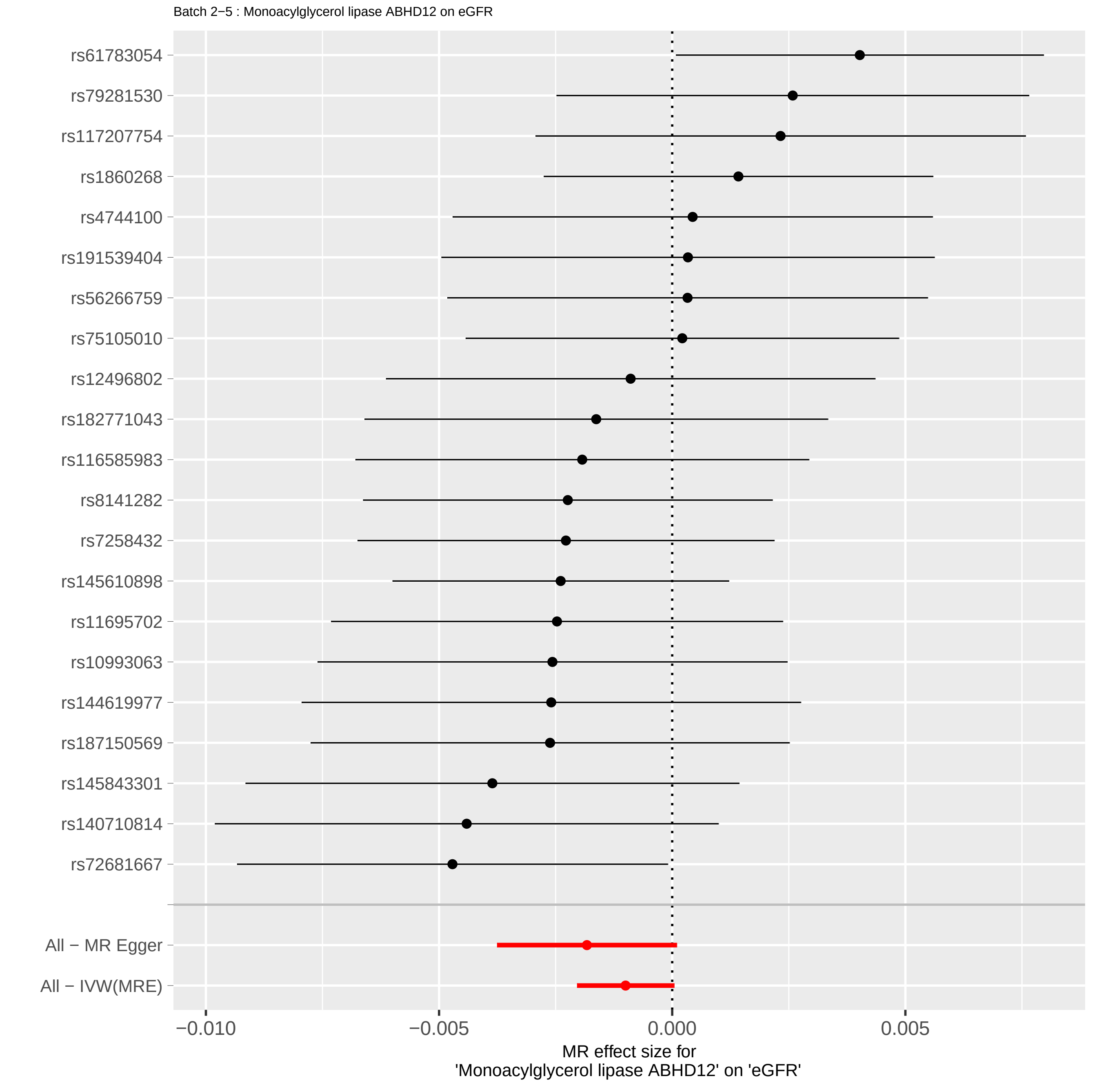
Batch 2−5 : Monoacylglycerol lipase ABHD12 on eGFR
rs61783054
rs79281530
rs117207754
rs1860268
rs4744100
rs191539404
rs56266759
rs75105010
rs12496802
rs182771043
rs116585983
rs8141282
rs7258432
rs145610898
rs11695702
rs10993063
rs144619977
rs187150569
rs145843301
rs140710814
rs72681667
All − MR Egger
All − IVW(MRE)
−0.010
−0.005
0.000
0.005
MR effect size for
'Monoacylglycerol lipase ABHD12' on 'eGFR'

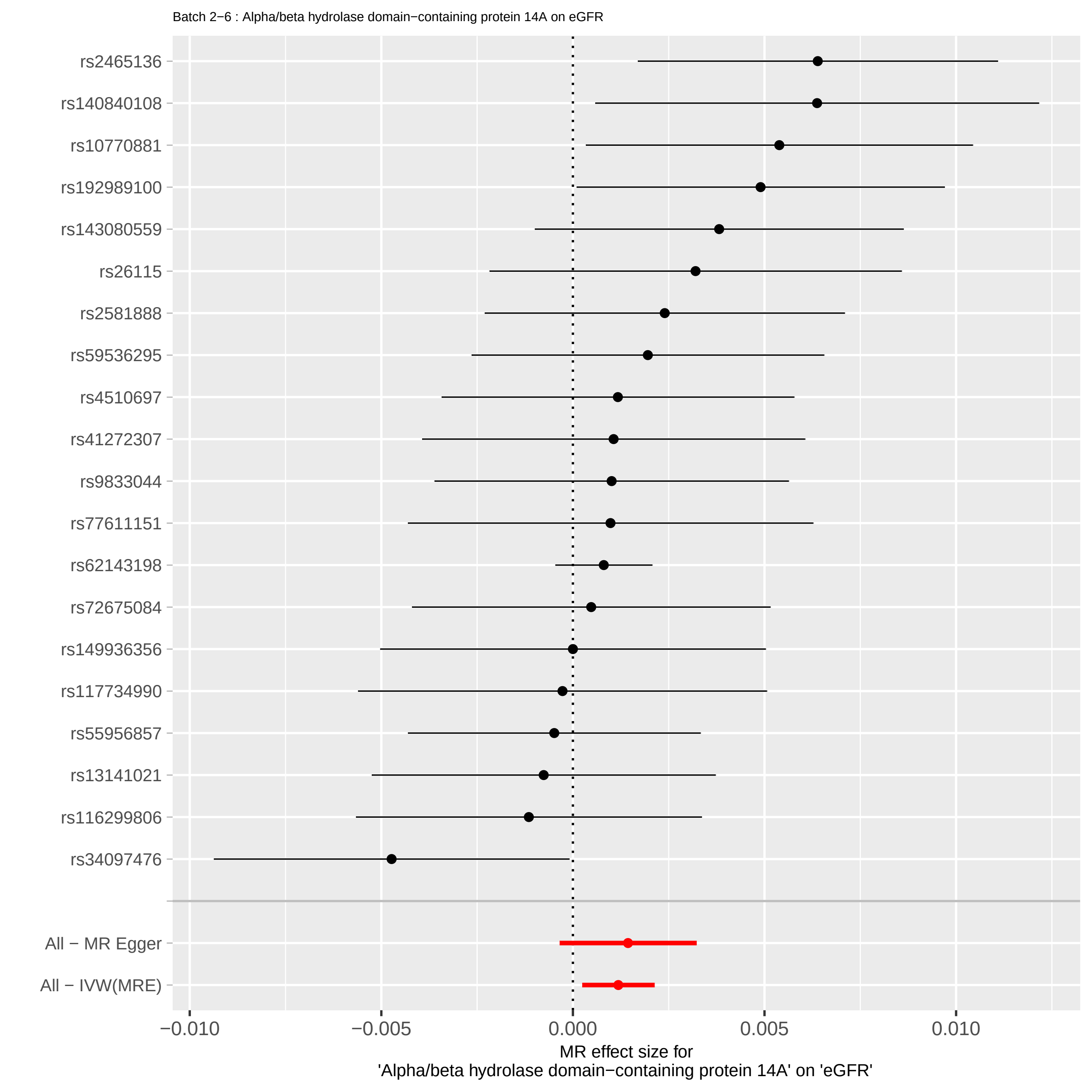
Batch 2−6 : Alpha/beta hydrolase domain−containing protein 14A on eGFR
rs2465136
rs140840108
rs10770881
rs192989100
rs143080559
rs26115
rs2581888
rs59536295
rs4510697
rs41272307
rs9833044
rs77611151
rs62143198
rs72675084
rs149936356
rs117734990
rs55956857
rs13141021
rs116299806
rs34097476
All − MR Egger
All − IVW(MRE)
−0.010
−0.005
0.000
0.005
0.010
MR effect size for
'Alpha/beta hydrolase domain−containing protein 14A' on 'eGFR'

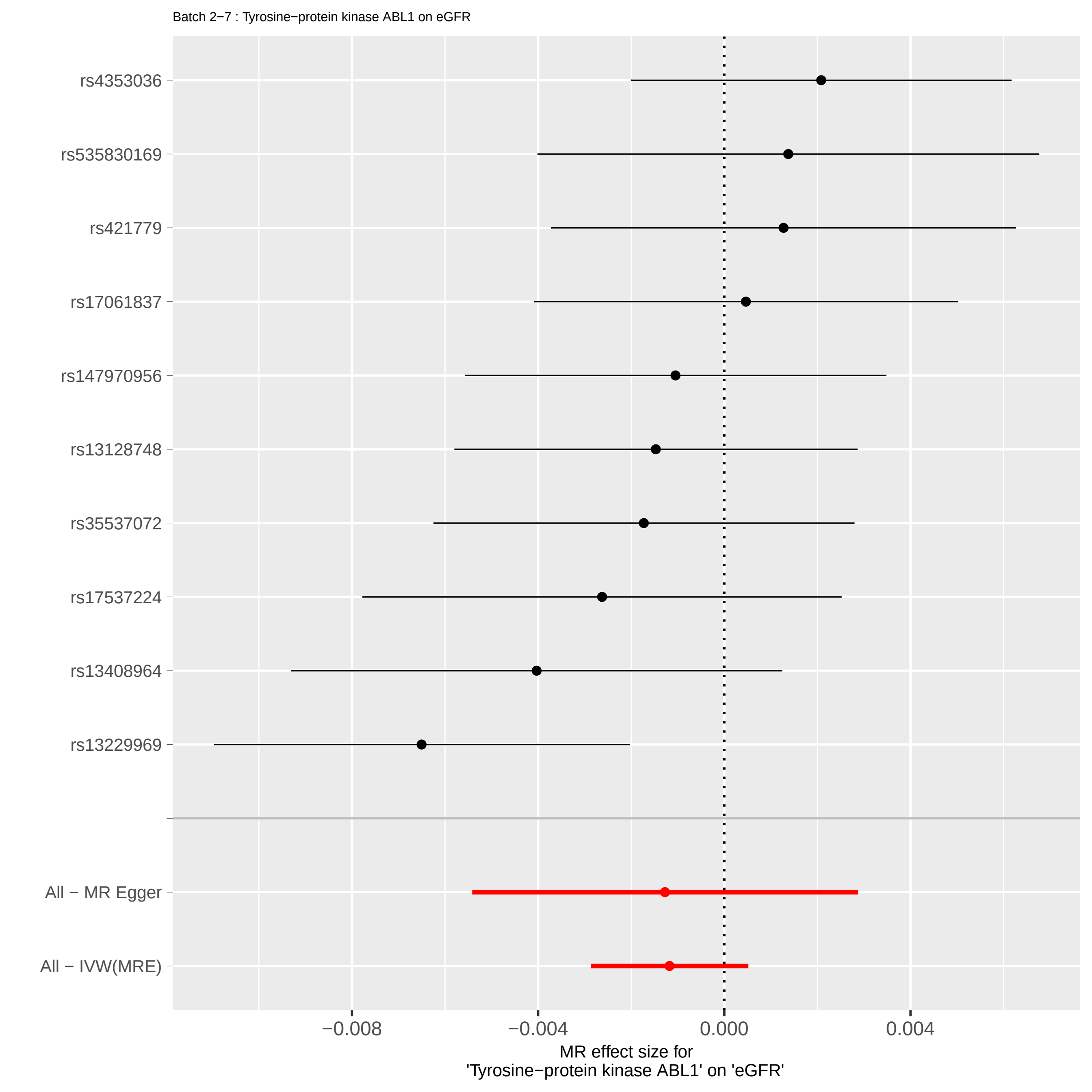
Batch 2−7 : Tyrosine−protein kinase ABL1 on eGFR
rs4353036
rs535830169
rs421779
rs17061837
rs147970956
rs13128748
rs35537072
rs17537224
rs13408964
rs13229969
All − MR Egger
All − IVW(MRE)
−0.008
−0.004
0.000
0.004
MR effect size for
'Tyrosine−protein kinase ABL1' on 'eGFR'

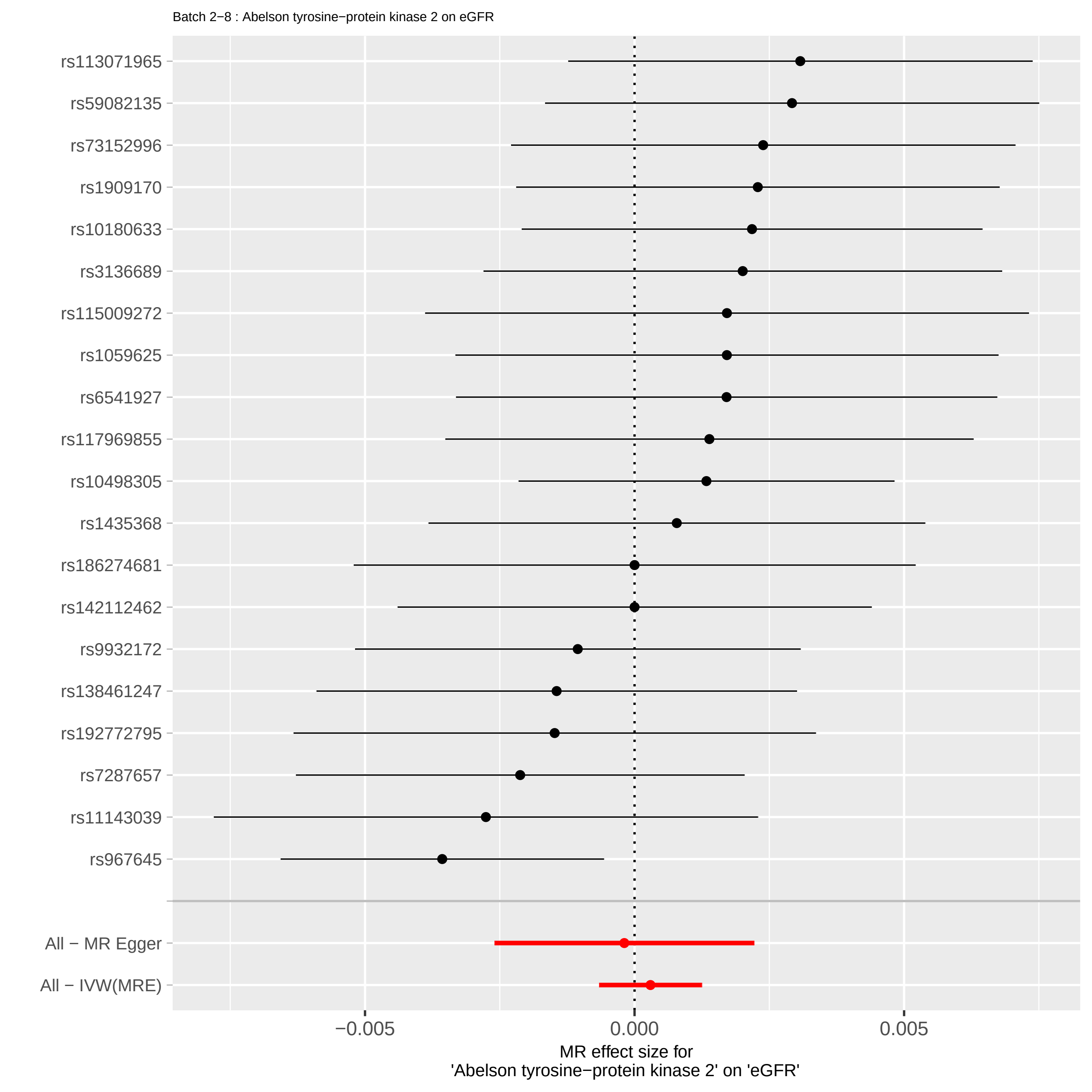
Batch 2−8 : Abelson tyrosine−protein kinase 2 on eGFR
rs113071965
rs59082135
rs73152996
rs1909170
rs10180633
rs3136689
rs115009272
rs1059625
rs6541927
rs117969855
rs10498305
rs1435368
rs186274681
rs142112462
rs9932172
rs138461247
rs192772795
rs7287657
rs11143039
rs967645
All − MR Egger
All − IVW(MRE)
−0.005
0.000
0.005
MR effect size for
'Abelson tyrosine−protein kinase 2' on 'eGFR'

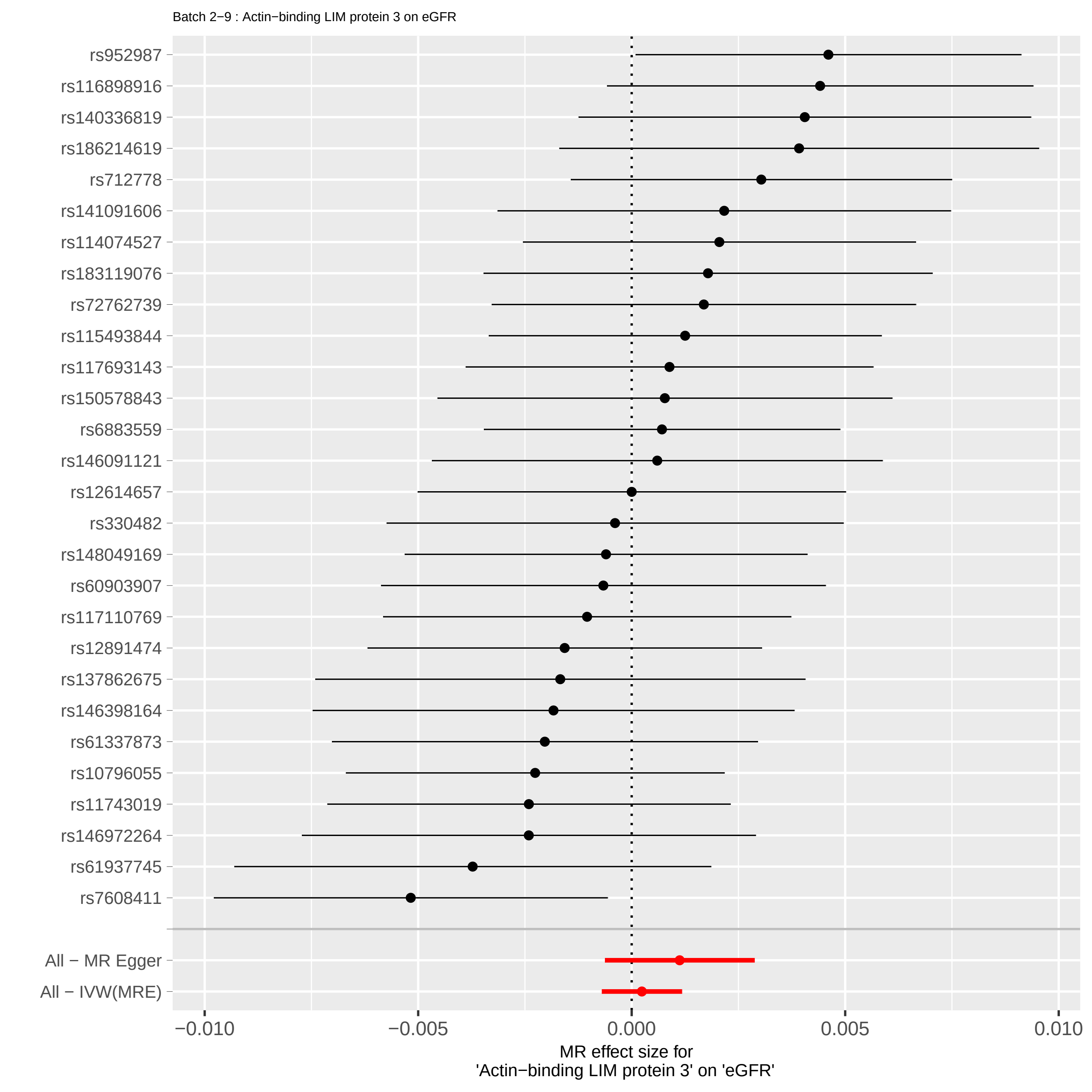

Batch 2−9 : Actin−binding LIM protein 3 on eGFR
rs952987
rs116898916
rs140336819
rs186214619
rs712778
rs141091606
rs114074527
rs183119076
rs72762739
rs115493844
rs117693143
rs150578843
rs6883559
rs146091121
rs12614657
rs330482
rs148049169
rs60903907
rs117110769
rs12891474
rs137862675
rs146398164
rs61337873
rs10796055
rs11743019
rs146972264
rs61937745
rs7608411
All − MR Egger
All − IVW(MRE)
−0.010
−0.005
0.000
0.005
0.010
MR effect size for
'Actin−binding LIM protein 3' on 'eGFR'

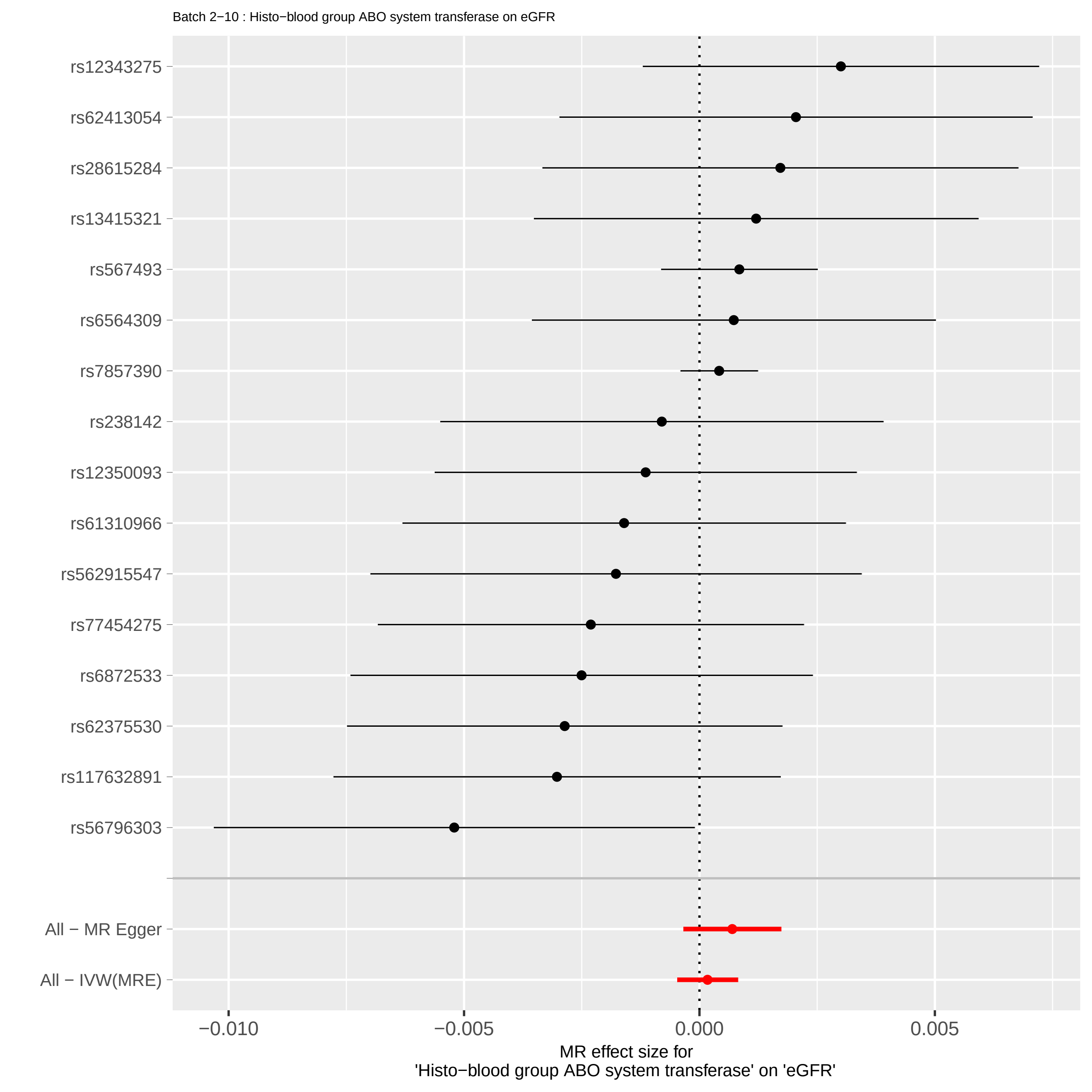
Batch 2−10 : Histo−blood group ABO system transferase on eGFR
rs12343275
rs62413054
rs28615284
rs13415321
rs567493
rs6564309
rs7857390
rs238142
rs12350093
rs61310966
rs562915547
rs77454275
rs6872533
rs62375530
rs117632891
rs56796303
All − MR Egger
All − IVW(MRE)
−0.010
−0.005
0.000
0.005
MR effect size for
'Histo−blood group ABO system transferase' on 'eGFR'

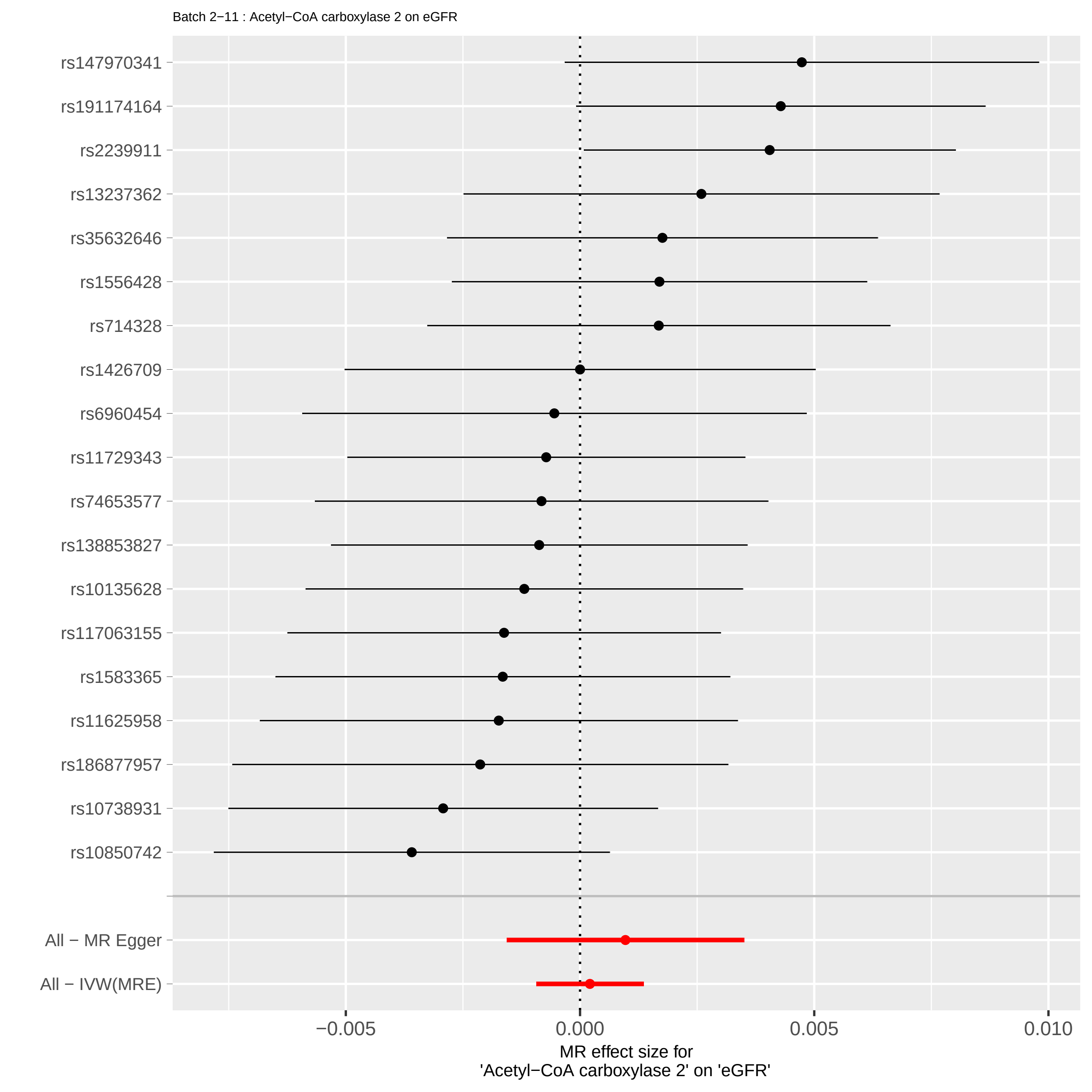

Batch 2−11 : Acetyl−CoA carboxylase 2 on eGFR
rs147970341
rs191174164
rs2239911
rs13237362
rs35632646
rs1556428
rs714328
rs1426709
rs6960454
rs11729343
rs74653577
rs138853827
rs10135628
rs117063155
rs1583365
rs11625958
rs186877957
rs10738931
rs10850742
All − MR Egger
All − IVW(MRE)
−0.005
0.000
0.005
0.010
MR effect size for
'Acetyl−CoA carboxylase 2' on 'eGFR'

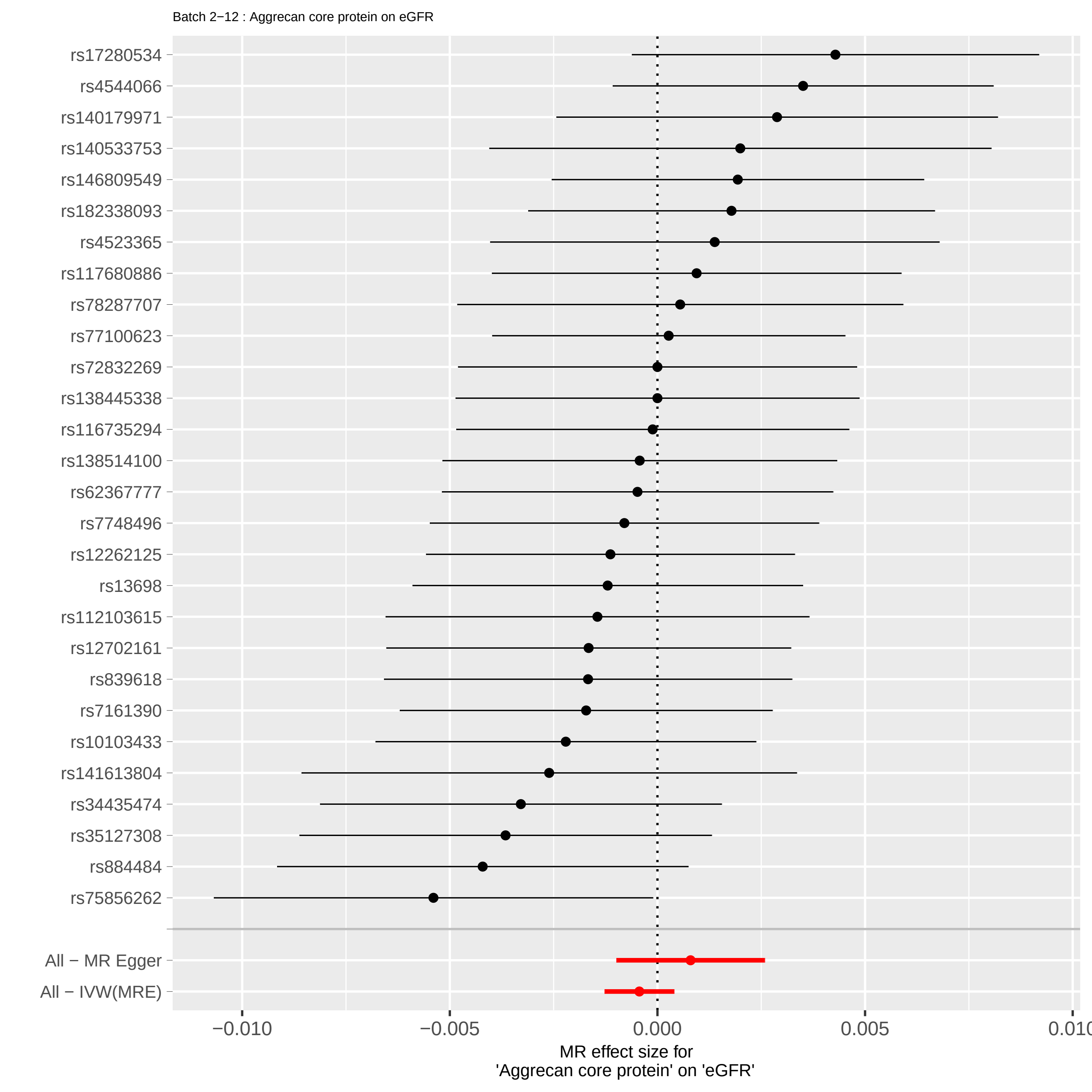

Batch 2−12 : Aggrecan core protein on eGFR
rs17280534
rs4544066
rs140179971
rs140533753
rs146809549
rs182338093
rs4523365
rs117680886
rs78287707
rs77100623
rs72832269
rs138445338
rs116735294
rs138514100
rs62367777
rs7748496
rs12262125
rs13698
rs112103615
rs12702161
rs839618
rs7161390
rs10103433
rs141613804
rs34435474
rs35127308
rs884484
rs75856262
All − MR Egger
All − IVW(MRE)
−0.010
−0.005
0.000
0.005
0.010
MR effect size for
'Aggrecan core protein' on 'eGFR'

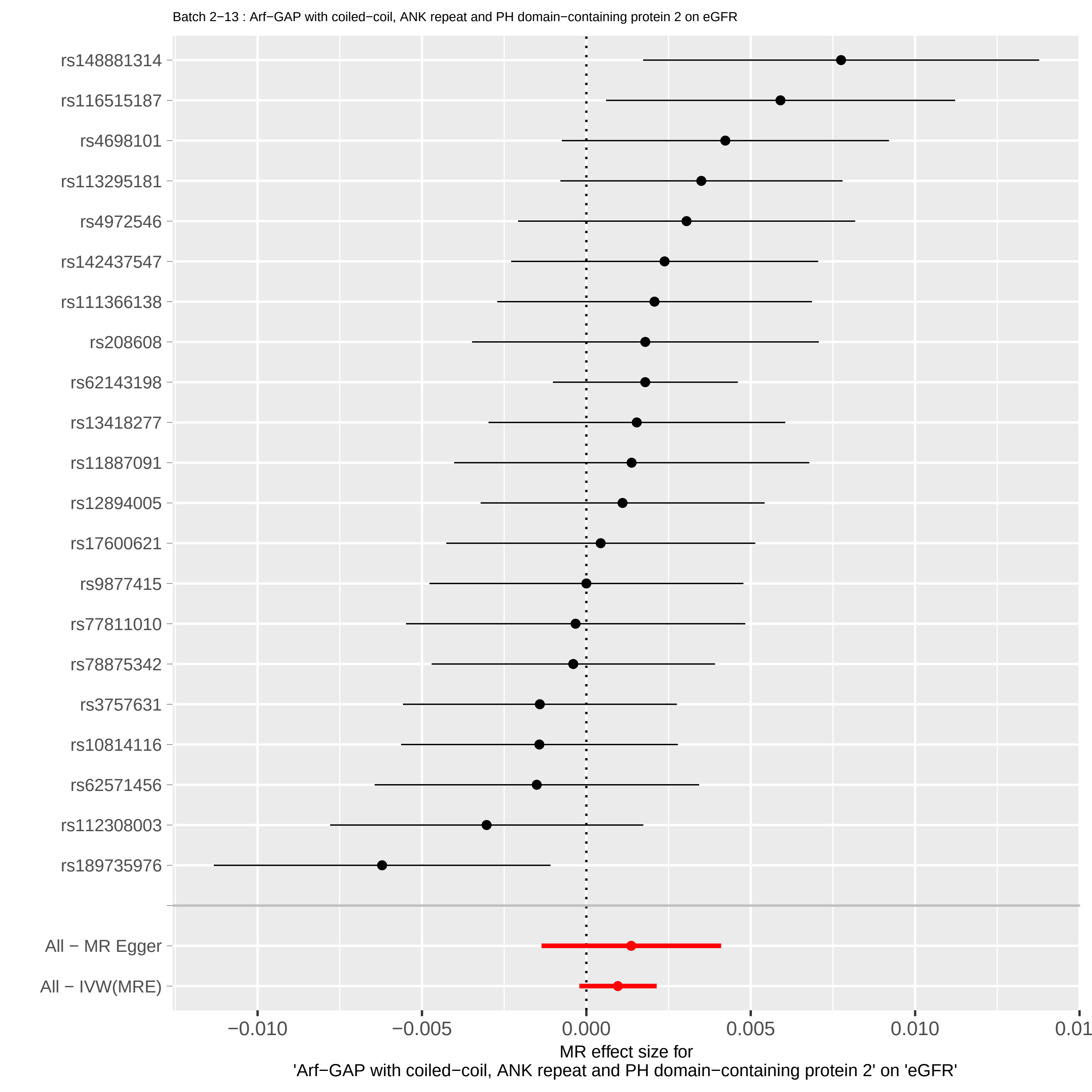

Batch 2−13 : Arf−GAP with coiled−coil, ANK repeat and PH domain−containing protein 2 on eGFR
rs148881314
rs116515187
rs4698101
rs113295181
rs4972546
rs142437547
rs111366138
rs208608
rs62143198
rs13418277
rs11887091
rs12894005
rs17600621
rs9877415
rs77811010
rs78875342
rs3757631
rs10814116
rs62571456
rs112308003
rs189735976
All − MR Egger
All − IVW(MRE)
−0.010
−0.005
0.000
0.005
0.010
0.01
MR effect size for
'Arf−GAP with coiled−coil, ANK repeat and PH domain−containing protein 2' on 'eGFR'

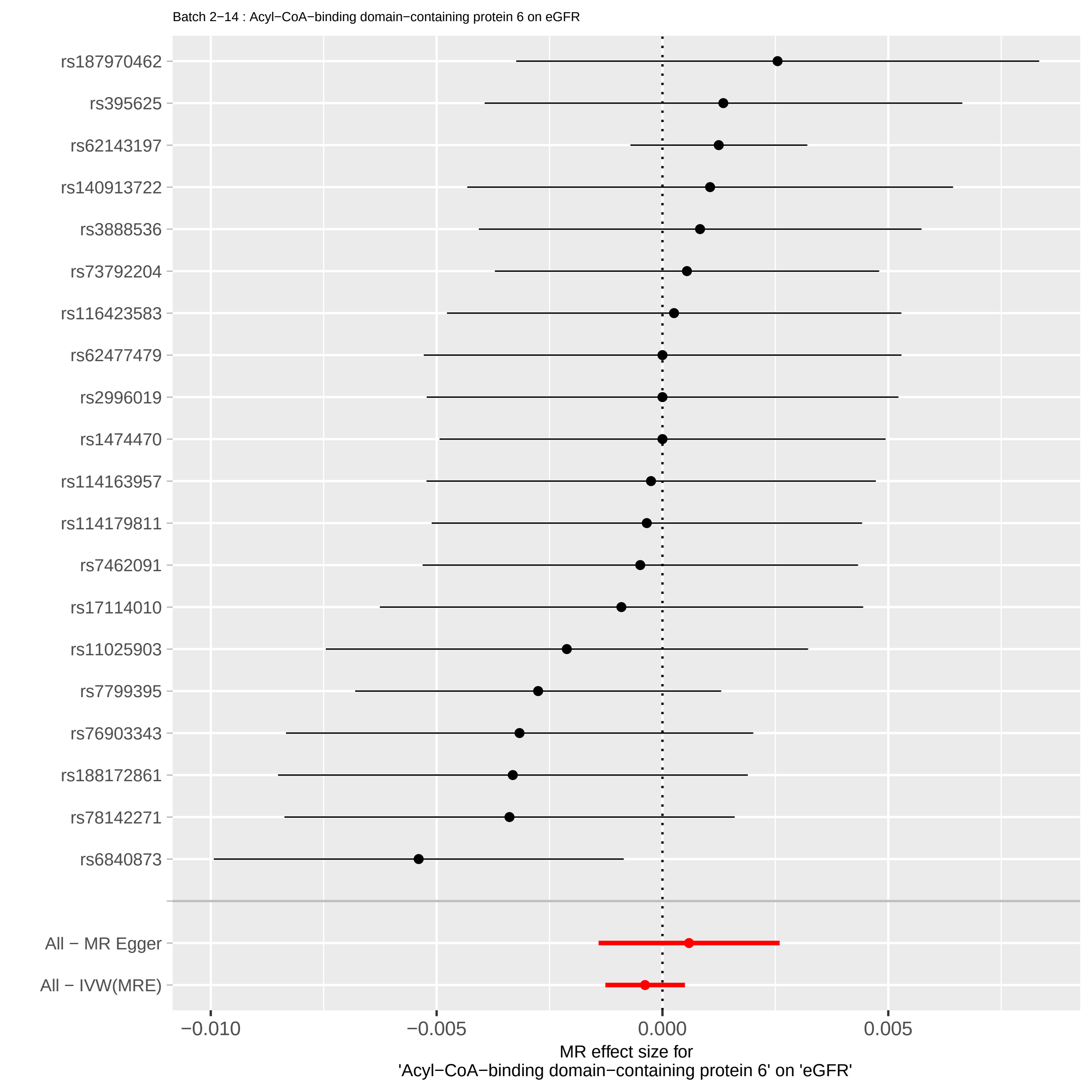

Batch 2−14 : Acyl−CoA−binding domain−containing protein 6 on eGFR
rs187970462
rs395625
rs62143197
rs140913722
rs3888536
rs73792204
rs116423583
rs62477479
rs2996019
rs1474470
rs114163957
rs114179811
rs7462091
rs17114010
rs11025903
rs7799395
rs76903343
rs188172861
rs78142271
rs6840873
All − MR Egger
All − IVW(MRE)
−0.010
−0.005
0.000
0.005
MR effect size for
'Acyl−CoA−binding domain−containing protein 6' on 'eGFR'

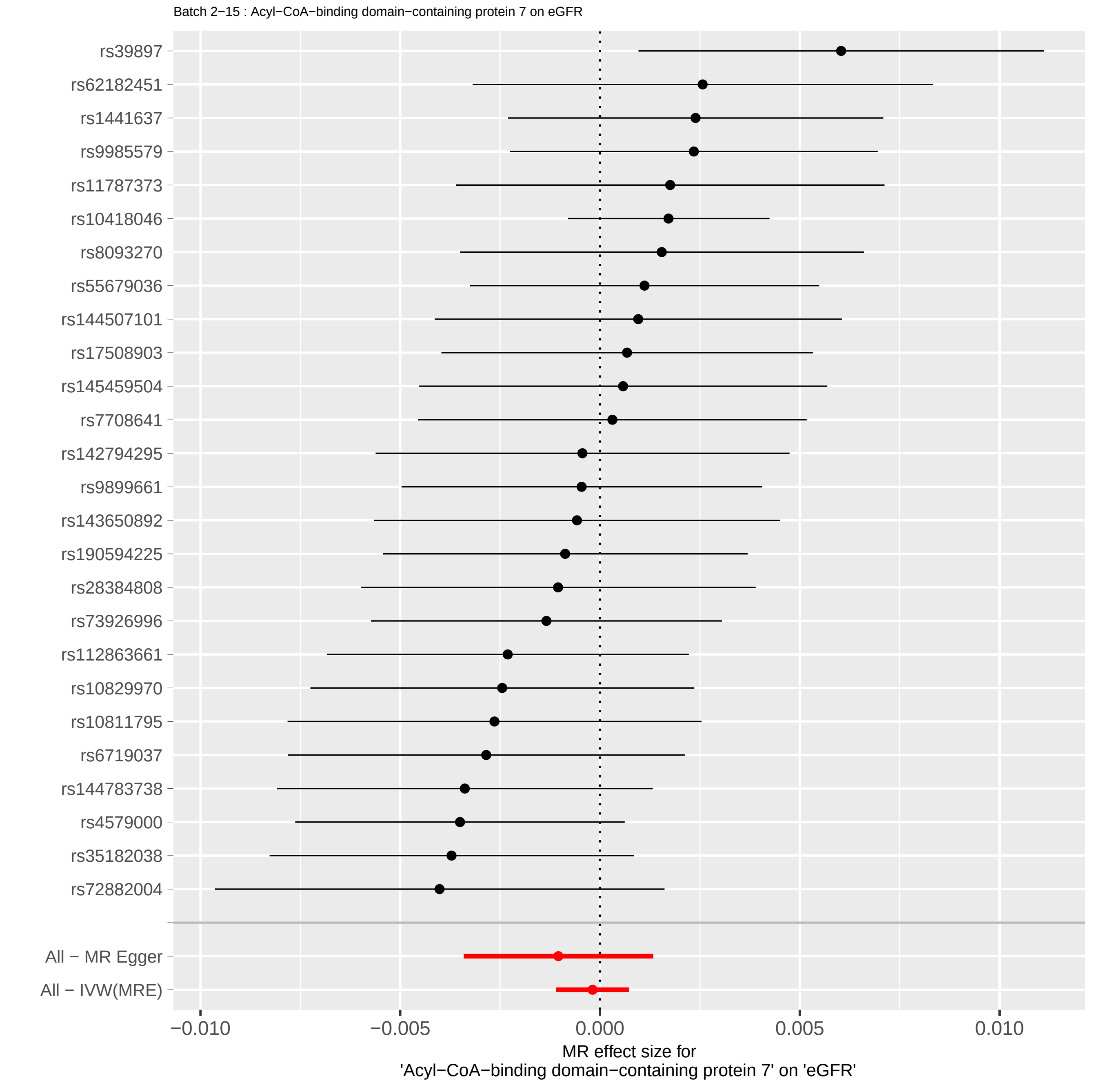

Batch 2−15 : Acyl−CoA−binding domain−containing protein 7 on eGFR
rs39897
rs62182451
rs1441637
rs9985579
rs11787373
rs10418046
rs8093270
rs55679036
rs144507101
rs17508903
rs145459504
rs7708641
rs142794295
rs9899661
rs143650892
rs190594225
rs28384808
rs73926996
rs112863661
rs10829970
rs10811795
rs6719037
rs144783738
rs4579000
rs35182038
rs72882004
All − MR Egger
All − IVW(MRE)
−0.010
−0.005
0.000
0.005
0.010
MR effect size for
'Acyl−CoA−binding domain−containing protein 7' on 'eGFR'

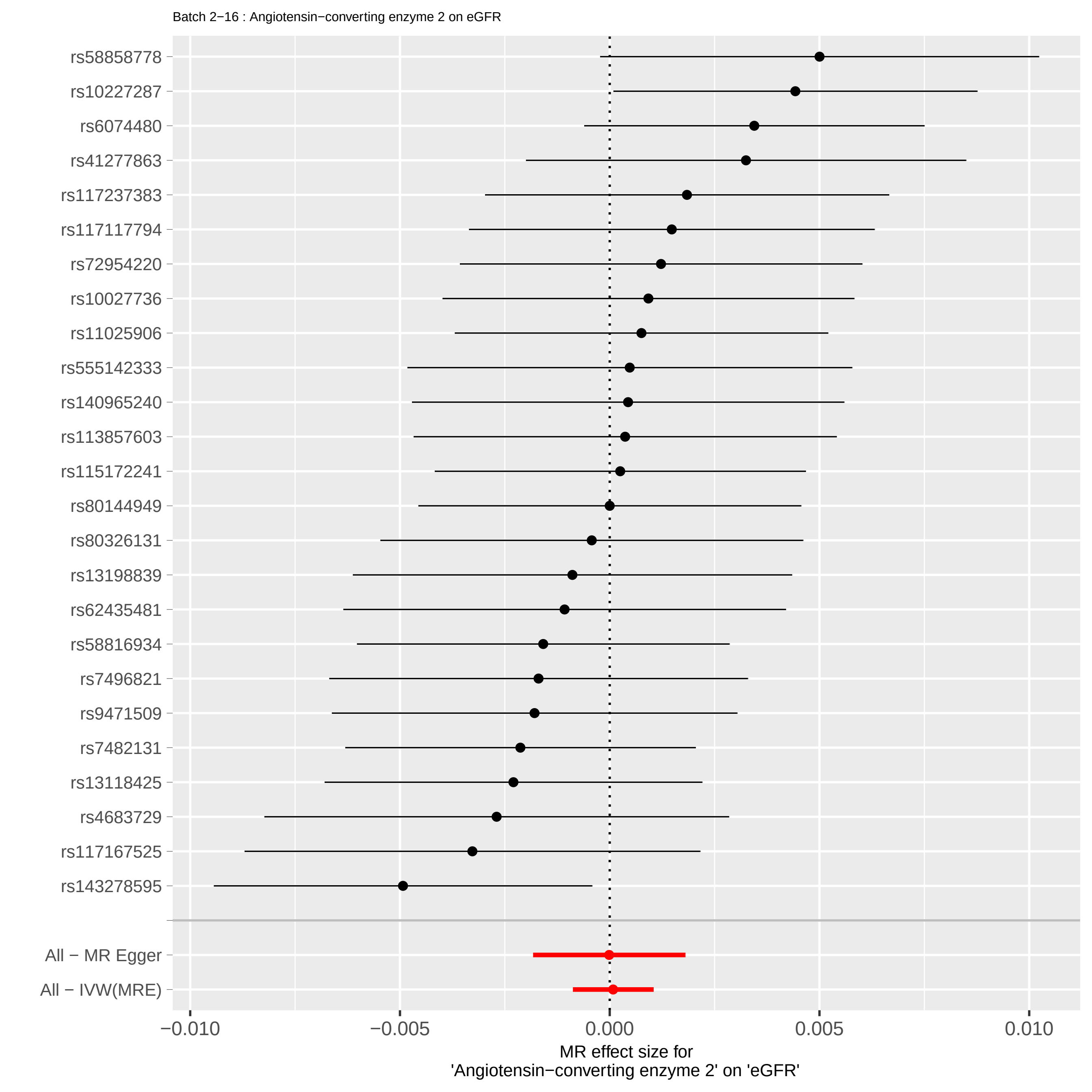

Batch 2−16 : Angiotensin−converting enzyme 2 on eGFR
rs58858778
rs10227287
rs6074480
rs41277863
rs117237383
rs117117794
rs72954220
rs10027736
rs11025906
rs555142333
rs140965240
rs113857603
rs115172241
rs80144949
rs80326131
rs13198839
rs62435481
rs58816934
rs7496821
rs9471509
rs7482131
rs13118425
rs4683729
rs117167525
rs143278595
All − MR Egger
All − IVW(MRE)
−0.010
−0.005
0.000
0.005
0.010
MR effect size for
'Angiotensin−converting enzyme 2' on 'eGFR'

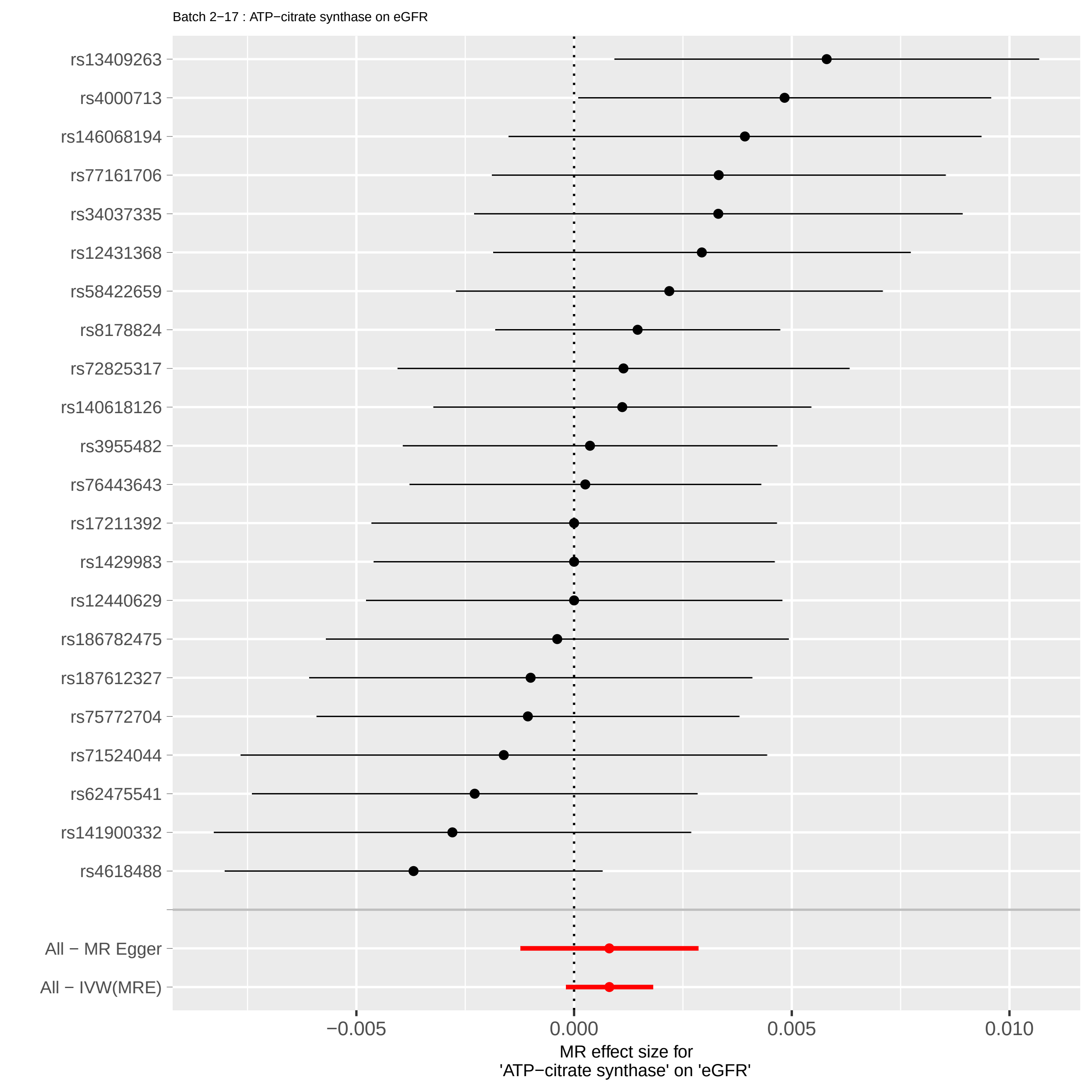

Batch 2−17 : ATP−citrate synthase on eGFR
rs13409263
rs4000713
rs146068194
rs77161706
rs34037335
rs12431368
rs58422659
rs8178824
rs72825317
rs140618126
rs3955482
rs76443643
rs17211392
rs1429983
rs12440629
rs186782475
rs187612327
rs75772704
rs71524044
rs62475541
rs141900332
rs4618488
All − MR Egger
All − IVW(MRE)
−0.005
0.000
0.005
0.010
MR effect size for
'ATP−citrate synthase' on 'eGFR'

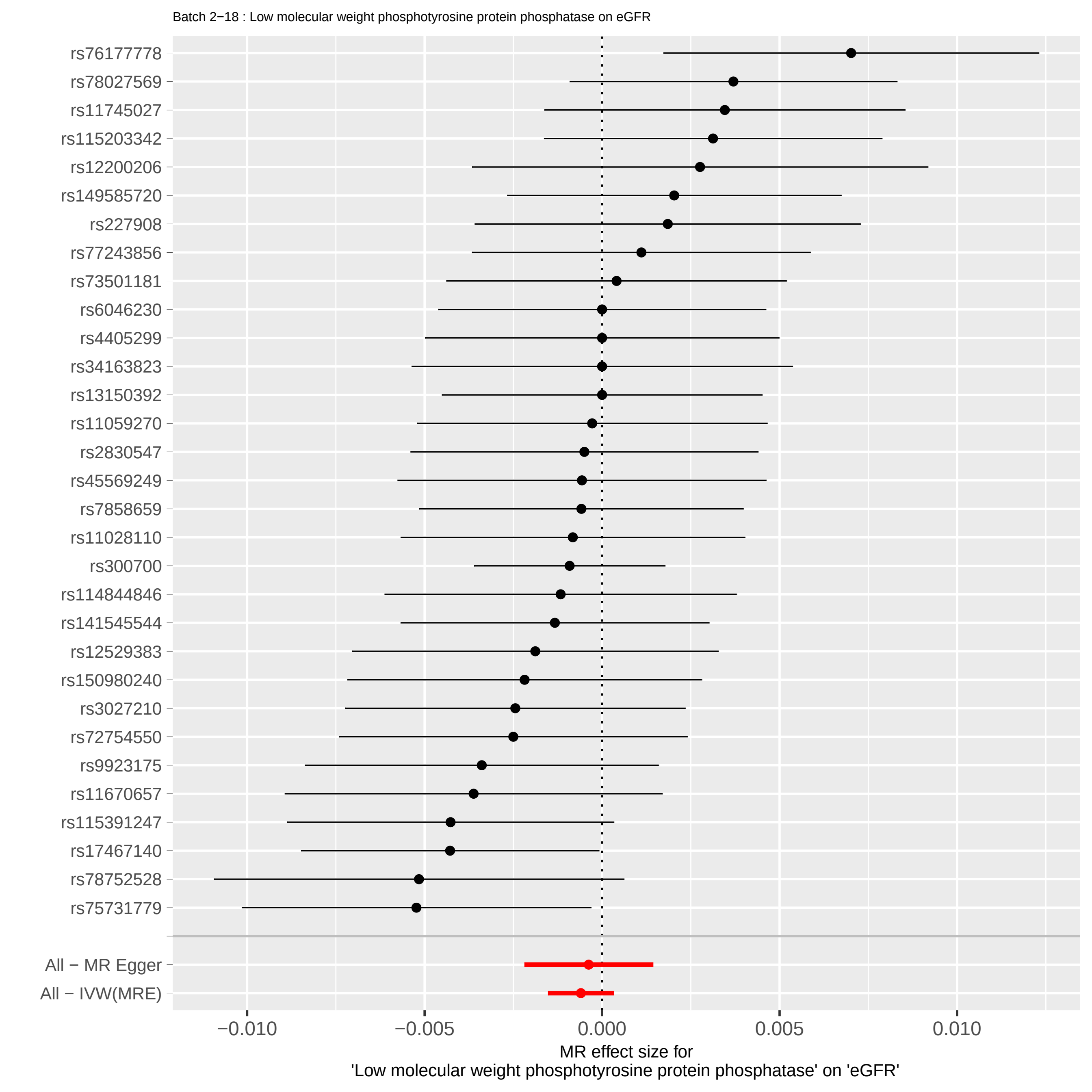
Batch 2−18 : Low molecular weight phosphotyrosine protein phosphatase on eGFR
rs76177778
rs78027569
rs11745027
rs115203342
rs12200206
rs149585720
rs227908
rs77243856
rs73501181
rs6046230
rs4405299
rs34163823
rs13150392
rs11059270
rs2830547
rs45569249
rs7858659
rs11028110
rs300700
rs114844846
rs141545544
rs12529383
rs150980240
rs3027210
rs72754550
rs9923175
rs11670657
rs115391247
rs17467140
rs78752528
rs75731779
All − MR Egger
All − IVW(MRE)
−0.010
−0.005
0.000
0.005
0.010
MR effect size for
'Low molecular weight phosphotyrosine protein phosphatase' on 'eGFR'

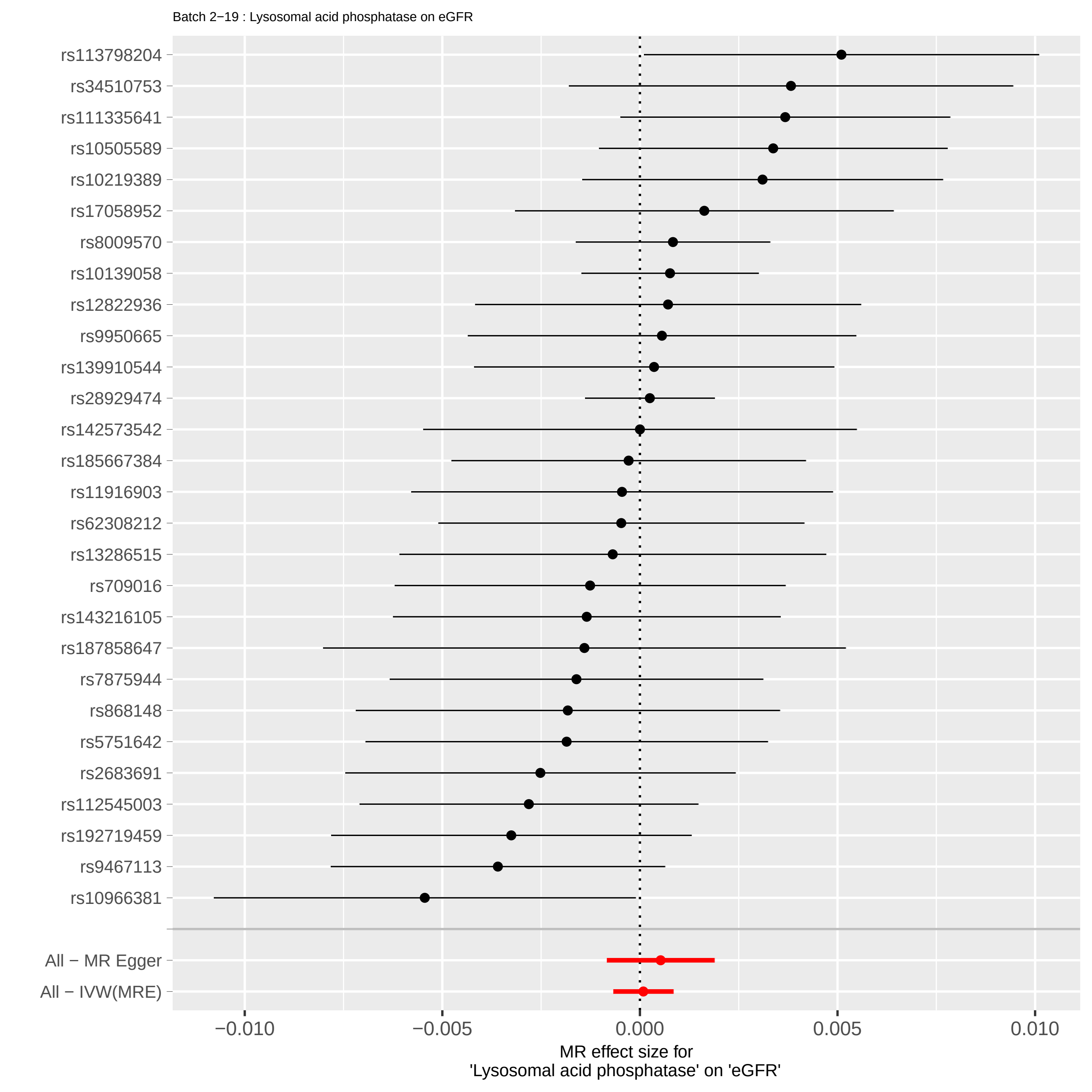

Batch 2−19 : Lysosomal acid phosphatase on eGFR
rs113798204
rs34510753
rs111335641
rs10505589
rs10219389
rs17058952
rs8009570
rs10139058
rs12822936
rs9950665
rs139910544
rs28929474
rs142573542
rs185667384
rs11916903
rs62308212
rs13286515
rs709016
rs143216105
rs187858647
rs7875944
rs868148
rs5751642
rs2683691
rs112545003
rs192719459
rs9467113
rs10966381
All − MR Egger
All − IVW(MRE)
−0.010
−0.005
0.000
0.005
0.010
MR effect size for
'Lysosomal acid phosphatase' on 'eGFR'

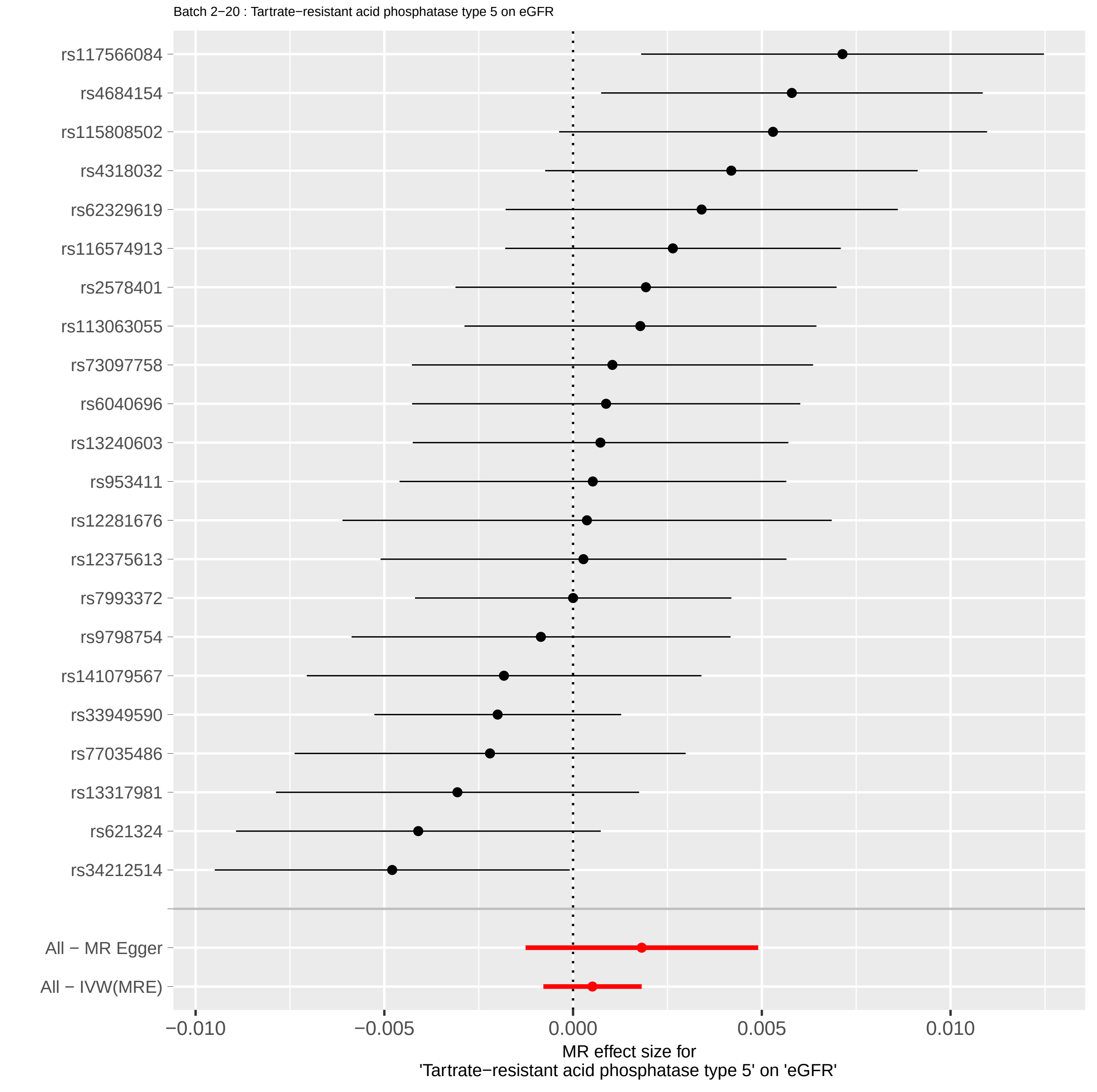

Batch 2−20 : Tartrate−resistant acid phosphatase type 5 on eGFR
rs117566084
rs4684154
rs115808502
rs4318032
rs62329619
rs116574913
rs2578401
rs113063055
rs73097758
rs6040696
rs13240603
rs953411
rs12281676
rs12375613
rs7993372
rs9798754
rs141079567
rs33949590
rs77035486
rs13317981
rs621324
rs34212514
All − MR Egger
All − IVW(MRE)
−0.010
−0.005
0.000
0.005
0.010
MR effect size for
'Tartrate−resistant acid phosphatase type 5' on 'eGFR'

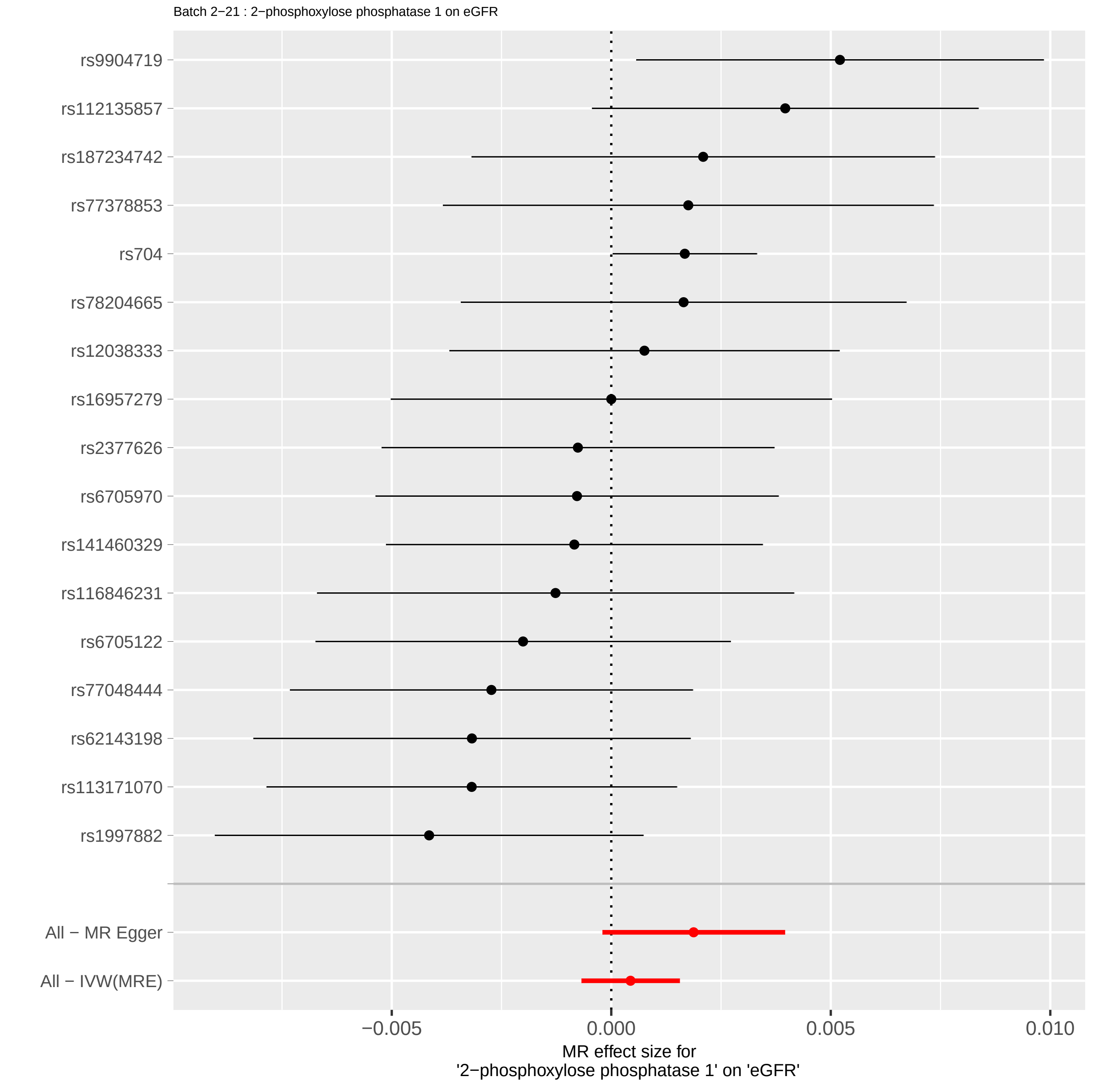

Batch 2−21 : 2−phosphoxylose phosphatase 1 on eGFR
rs9904719
rs112135857
rs187234742
rs77378853
rs704
rs78204665
rs12038333
rs16957279
rs2377626
rs6705970
rs141460329
rs116846231
rs6705122
rs77048444
rs62143198
rs113171070
rs1997882
All − MR Egger
All − IVW(MRE)
−0.005
0.000
0.005
0.010
MR effect size for
'2−phosphoxylose phosphatase 1' on 'eGFR'

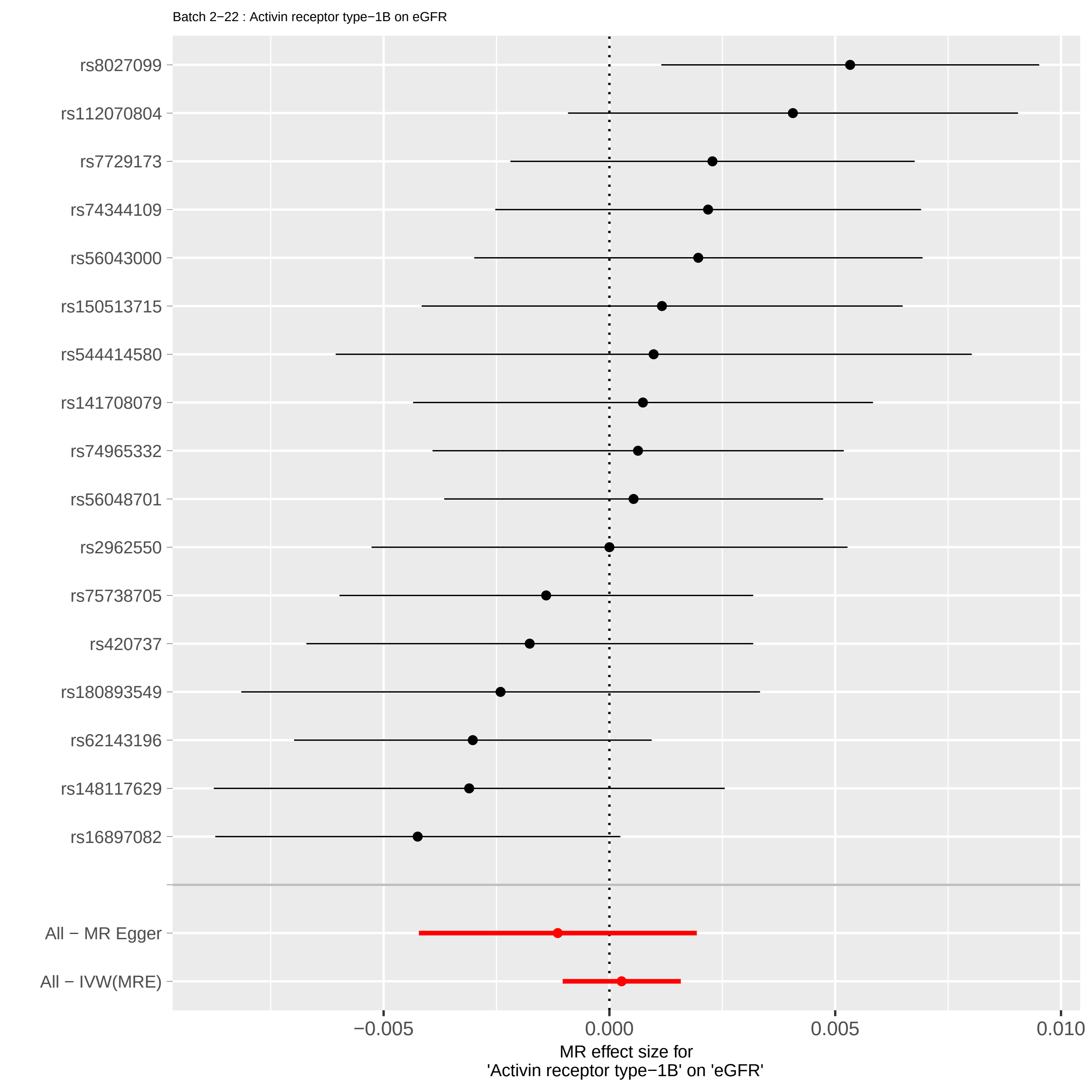

Batch 2−22 : Activin receptor type−1B on eGFR
rs8027099
rs112070804
rs7729173
rs74344109
rs56043000
rs150513715
rs544414580
rs141708079
rs74965332
rs56048701
rs2962550
rs75738705
rs420737
rs180893549
rs62143196
rs148117629
rs16897082
All − MR Egger
All − IVW(MRE)
−0.005
0.000
0.005
0.010
MR effect size for
'Activin receptor type−1B' on 'eGFR'

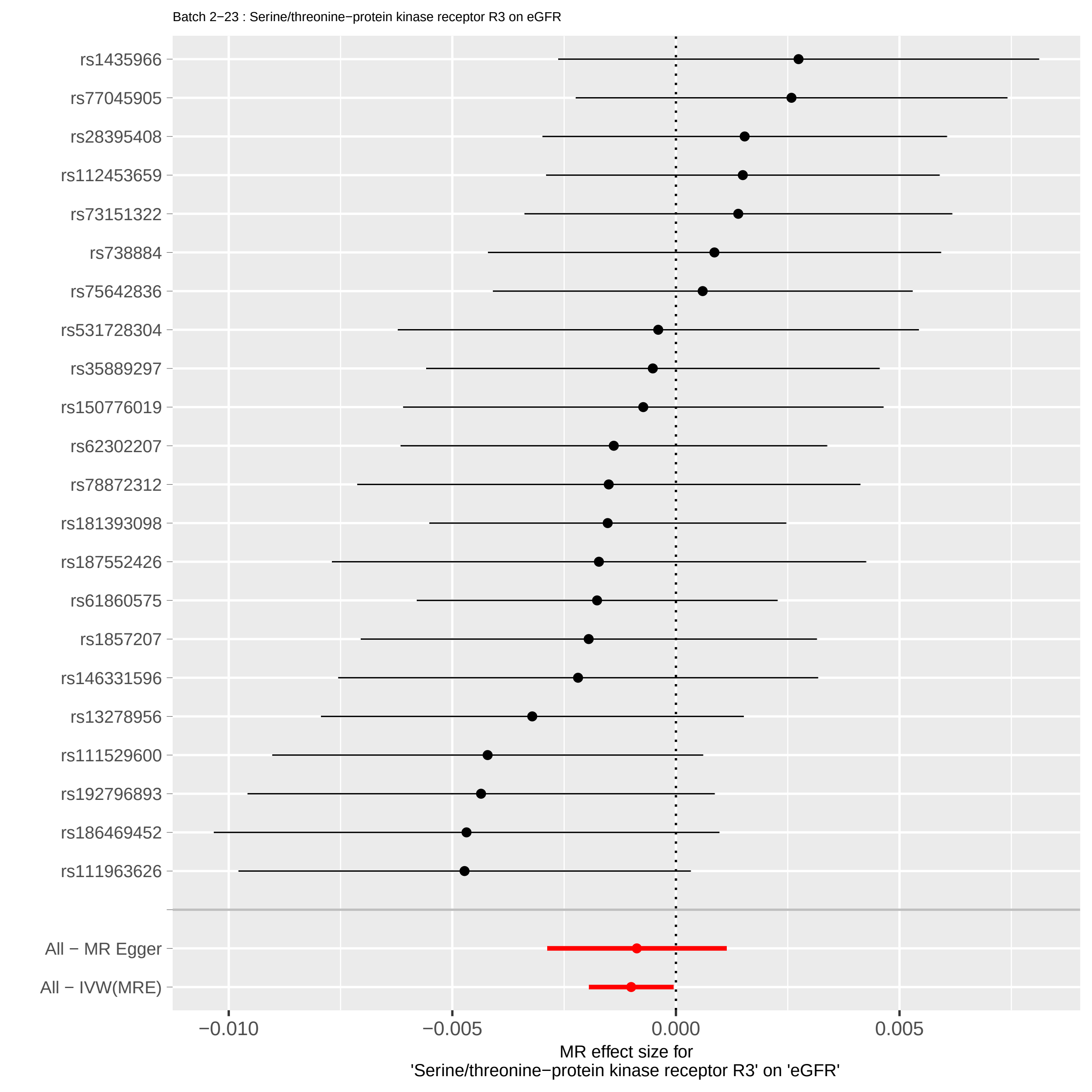

Batch 2−23 : Serine/threonine−protein kinase receptor R3 on eGFR
rs1435966
rs77045905
rs28395408
rs112453659
rs73151322
rs738884
rs75642836
rs531728304
rs35889297
rs150776019
rs62302207
rs78872312
rs181393098
rs187552426
rs61860575
rs1857207
rs146331596
rs13278956
rs111529600
rs192796893
rs186469452
rs111963626
All − MR Egger
All − IVW(MRE)
−0.010
−0.005
0.000
0.005
MR effect size for
'Serine/threonine−protein kinase receptor R3' on 'eGFR'

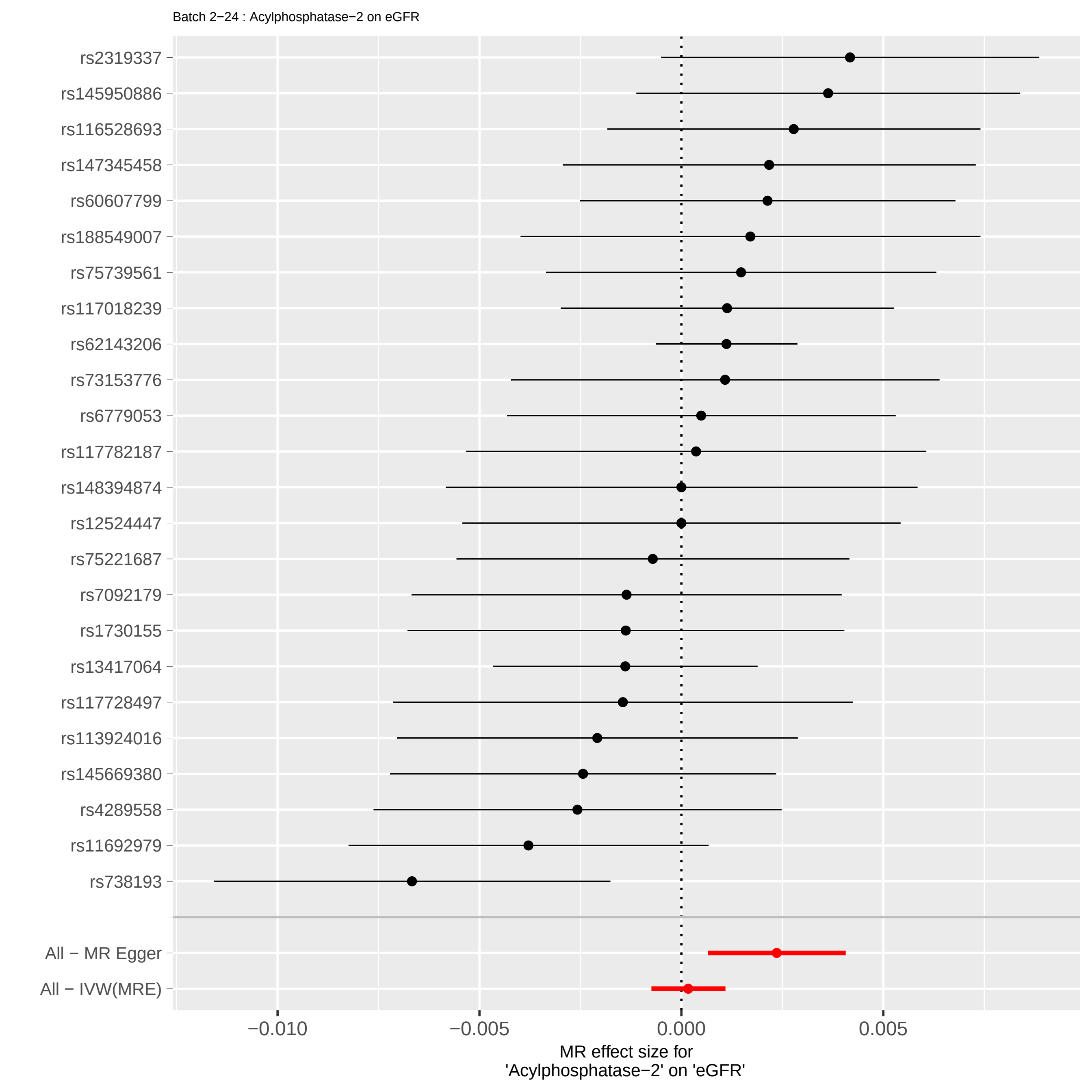

Batch 2−24 : Acylphosphatase−2 on eGFR
rs2319337
rs145950886
rs116528693
rs147345458
rs60607799
rs188549007
rs75739561
rs117018239
rs62143206
rs73153776
rs6779053
rs117782187
rs148394874
rs12524447
rs75221687
rs7092179
rs1730155
rs13417064
rs117728497
rs113924016
rs145669380
rs4289558
rs11692979
rs738193
All − MR Egger
All − IVW(MRE)
−0.010
−0.005
0.000
0.005
MR effect size for
'Acylphosphatase−2' on 'eGFR'

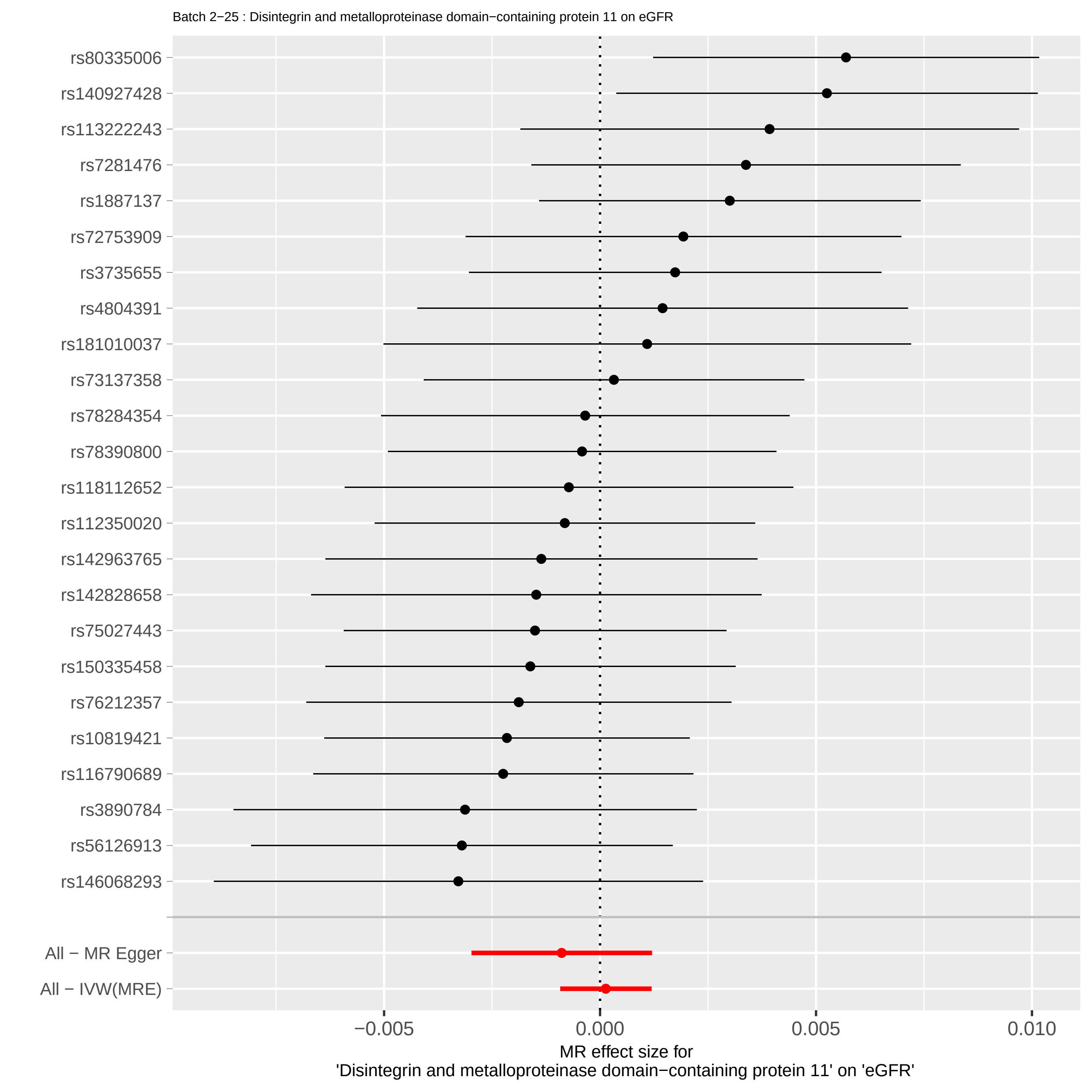
Batch 2−25 : Disintegrin and metalloproteinase domain−containing protein 11 on eGFR
rs80335006
rs140927428
rs113222243
rs7281476
rs1887137
rs72753909
rs3735655
rs4804391
rs181010037
rs73137358
rs78284354
rs78390800
rs118112652
rs112350020
rs142963765
rs142828658
rs75027443
rs150335458
rs76212357
rs10819421
rs116790689
rs3890784
rs56126913
rs146068293
All − MR Egger
All − IVW(MRE)
−0.005
0.000
0.005
0.010
MR effect size for
'Disintegrin and metalloproteinase domain−containing protein 11' on 'eGFR'

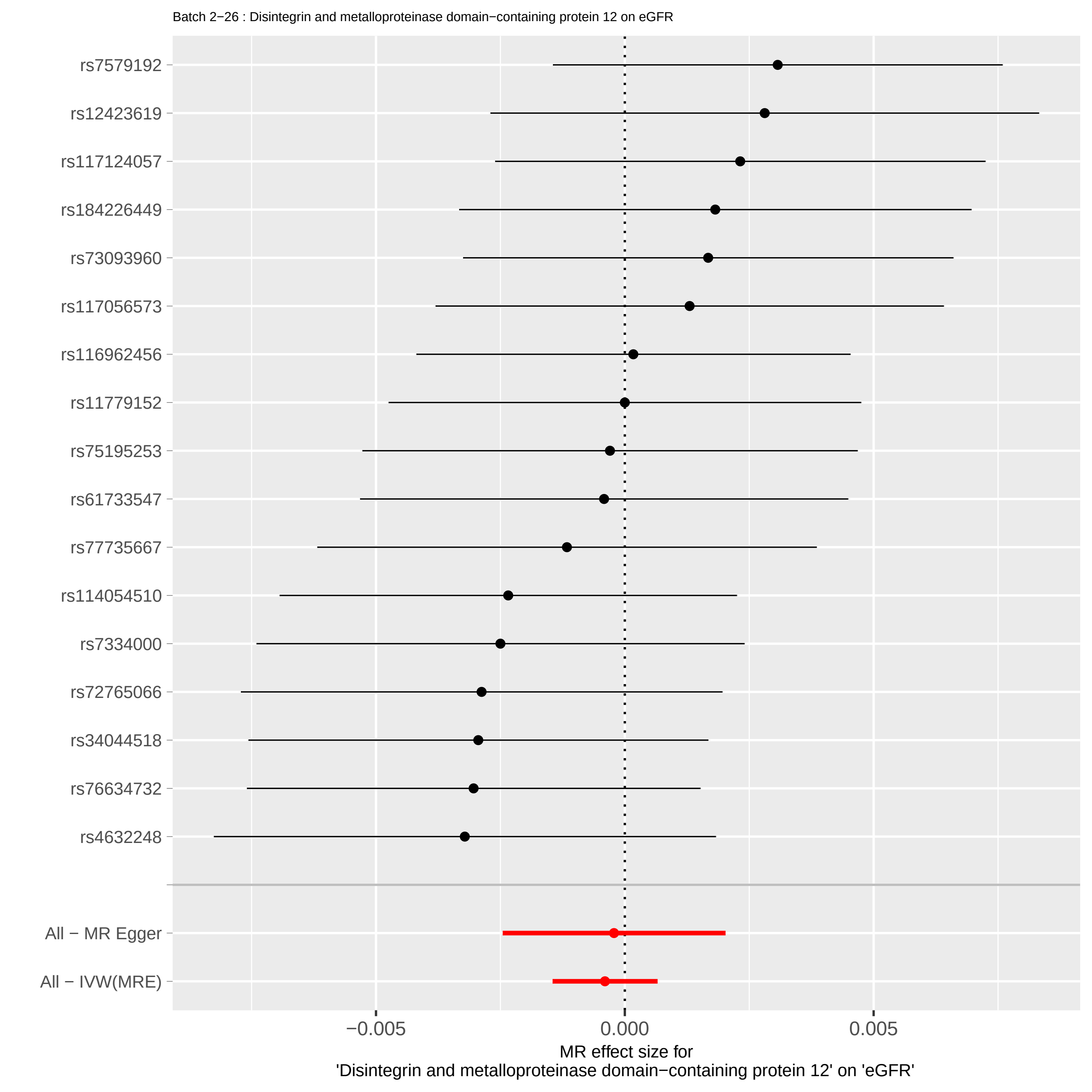

Batch 2−26 : Disintegrin and metalloproteinase domain−containing protein 12 on eGFR
rs7579192
rs12423619
rs117124057
rs184226449
rs73093960
rs117056573
rs116962456
rs11779152
rs75195253
rs61733547
rs77735667
rs114054510
rs7334000
rs72765066
rs34044518
rs76634732
rs4632248
All − MR Egger
All − IVW(MRE)
−0.005
0.000
0.005
MR effect size for
'Disintegrin and metalloproteinase domain−containing protein 12' on 'eGFR'

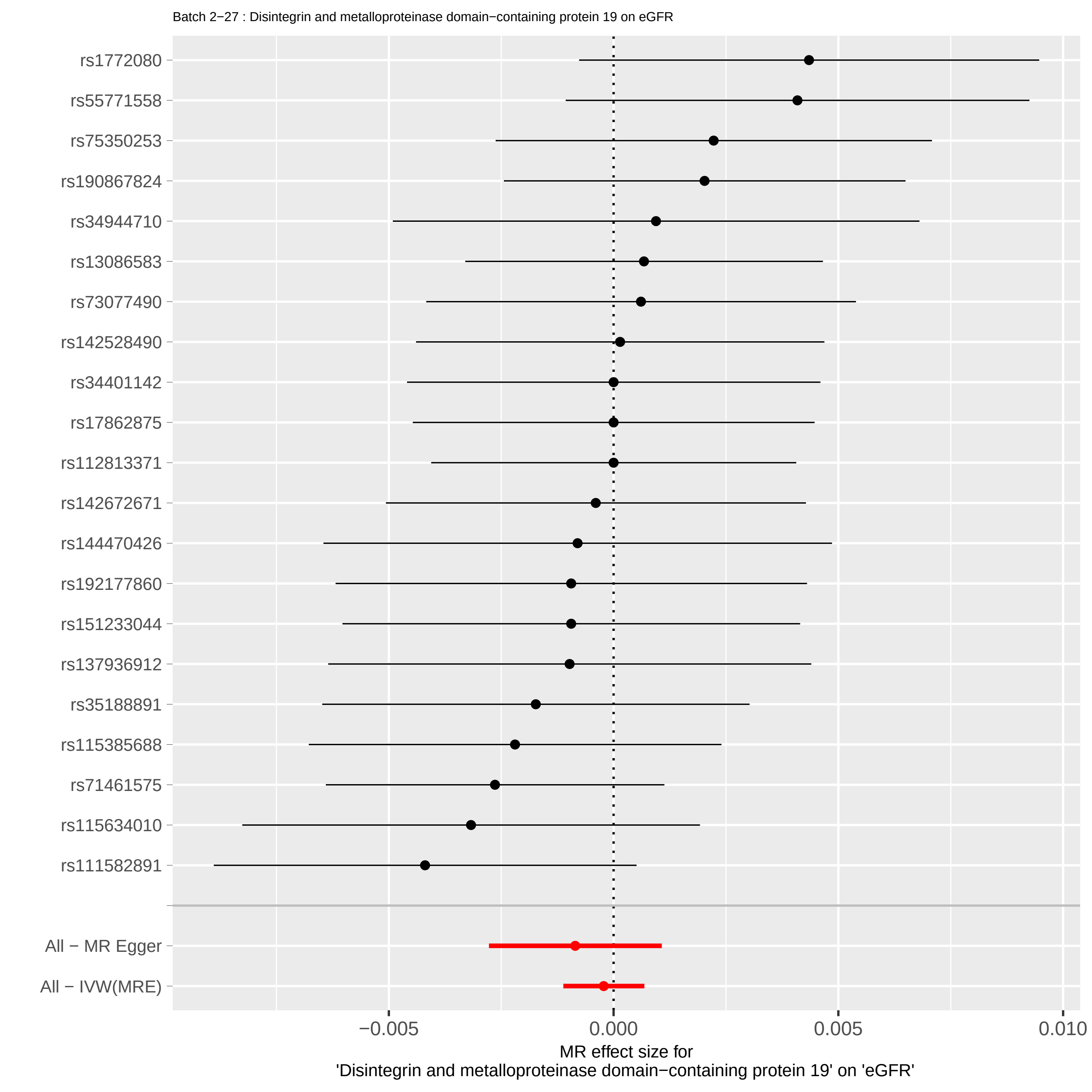

Batch 2−27 : Disintegrin and metalloproteinase domain−containing protein 19 on eGFR
rs1772080
rs55771558
rs75350253
rs190867824
rs34944710
rs13086583
rs73077490
rs142528490
rs34401142
rs17862875
rs112813371
rs142672671
rs144470426
rs192177860
rs151233044
rs137936912
rs35188891
rs115385688
rs71461575
rs115634010
rs111582891
All − MR Egger
All − IVW(MRE)
−0.005
0.000
0.005
0.010
MR effect size for
'Disintegrin and metalloproteinase domain−containing protein 19' on 'eGFR'

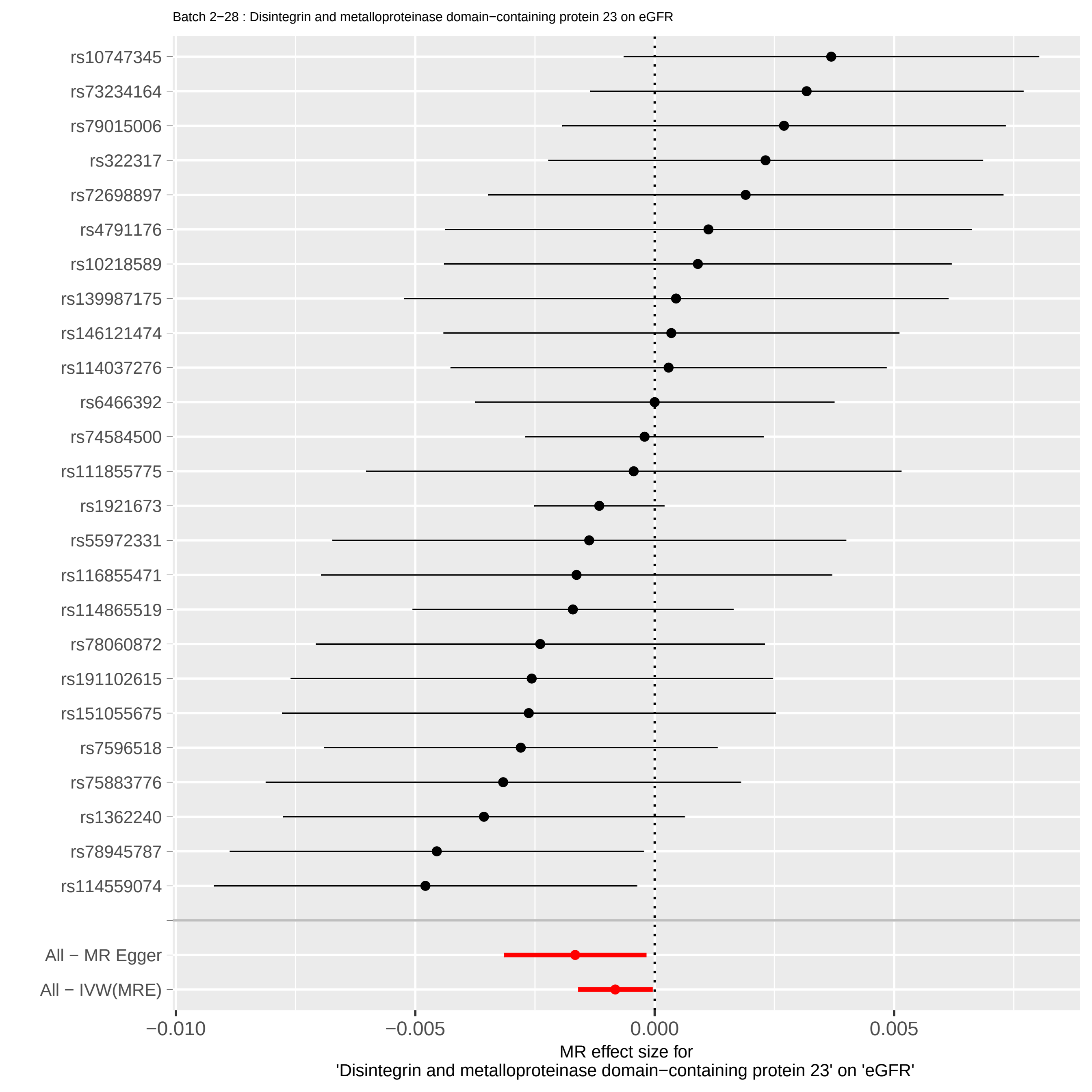
Batch 2−28 : Disintegrin and metalloproteinase domain−containing protein 23 on eGFR
rs10747345
rs73234164
rs79015006
rs322317
rs72698897
rs4791176
rs10218589
rs139987175
rs146121474
rs114037276
rs6466392
rs74584500
rs111855775
rs1921673
rs55972331
rs116855471
rs114865519
rs78060872
rs191102615
rs151055675
rs7596518
rs75883776
rs1362240
rs78945787
rs114559074
All − MR Egger
All − IVW(MRE)
−0.010
−0.005
0.000
0.005
MR effect size for
'Disintegrin and metalloproteinase domain−containing protein 23' on 'eGFR'

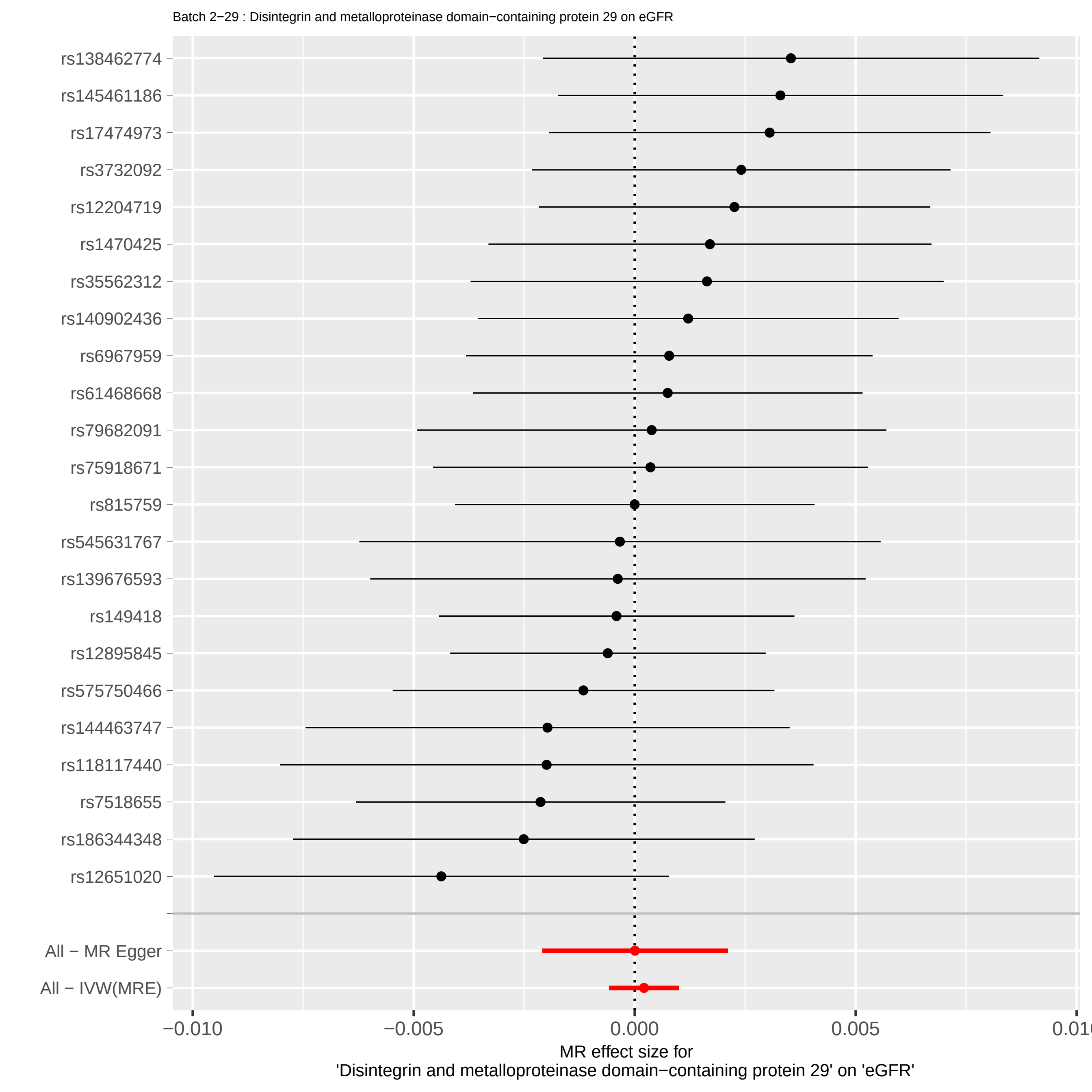

Batch 2−29 : Disintegrin and metalloproteinase domain−containing protein 29 on eGFR
rs138462774
rs145461186
rs17474973
rs3732092
rs12204719
rs1470425
rs35562312
rs140902436
rs6967959
rs61468668
rs79682091
rs75918671
rs815759
rs545631767
rs139676593
rs149418
rs12895845
rs575750466
rs144463747
rs118117440
rs7518655
rs186344348
rs12651020
All − MR Egger
All − IVW(MRE)
−0.010
−0.005
0.000
0.005
MR effect size for
'Disintegrin and metalloproteinase domain−containing protein 29' on 'eGFR'

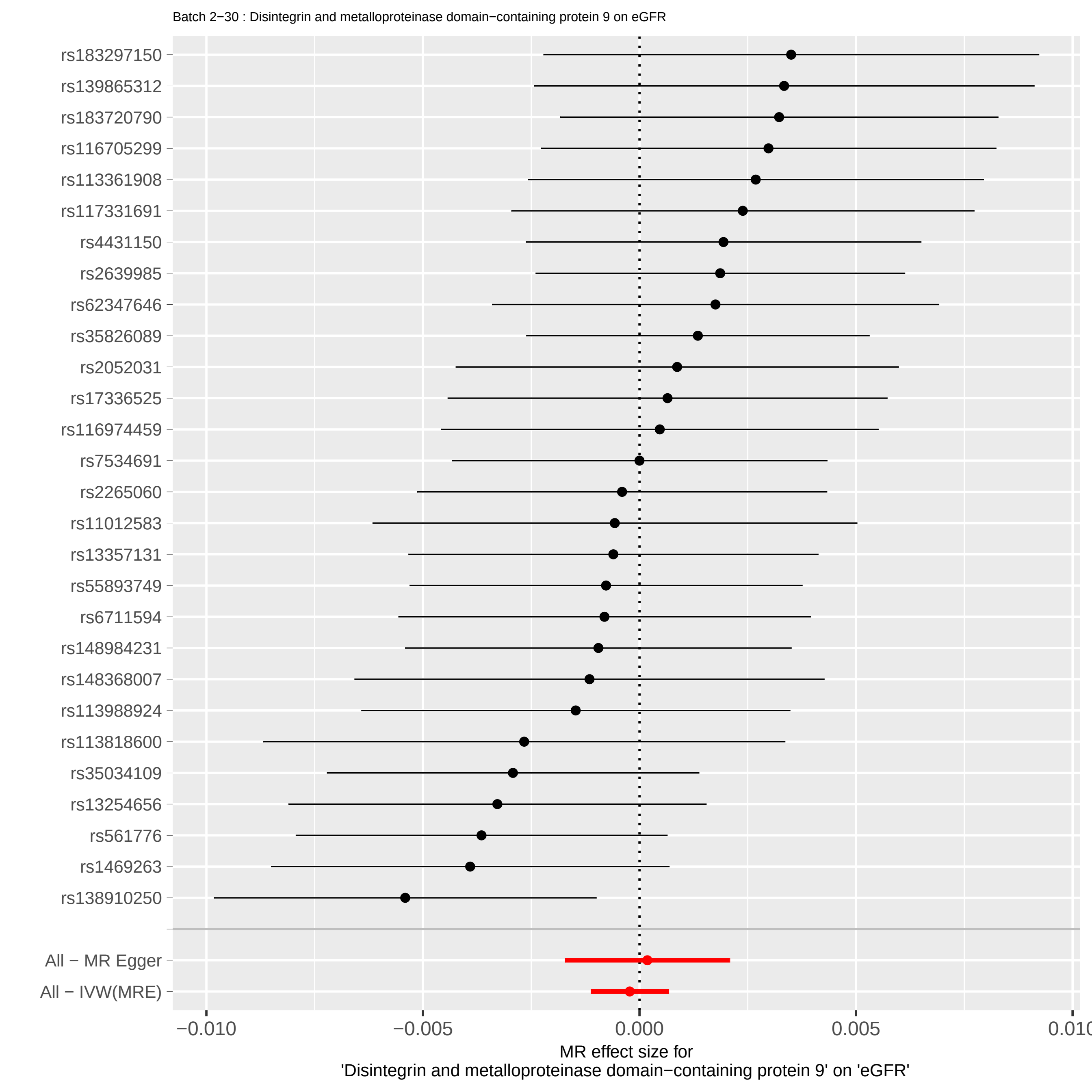

Batch 2−30 : Disintegrin and metalloproteinase domain−containing protein 9 on eGFR
rs183297150
rs139865312
rs183720790
rs116705299
rs113361908
rs117331691
rs4431150
rs2639985
rs62347646
rs35826089
rs2052031
rs17336525
rs116974459
rs7534691
rs2265060
rs11012583
rs13357131
rs55893749
rs6711594
rs148984231
rs148368007
rs113988924
rs113818600
rs35034109
rs13254656
rs561776
rs1469263
rs138910250
All − MR Egger
All − IVW(MRE)
−0.010
−0.005
0.000
0.005
0.010
MR effect size for
'Disintegrin and metalloproteinase domain−containing protein 9' on 'eGFR'

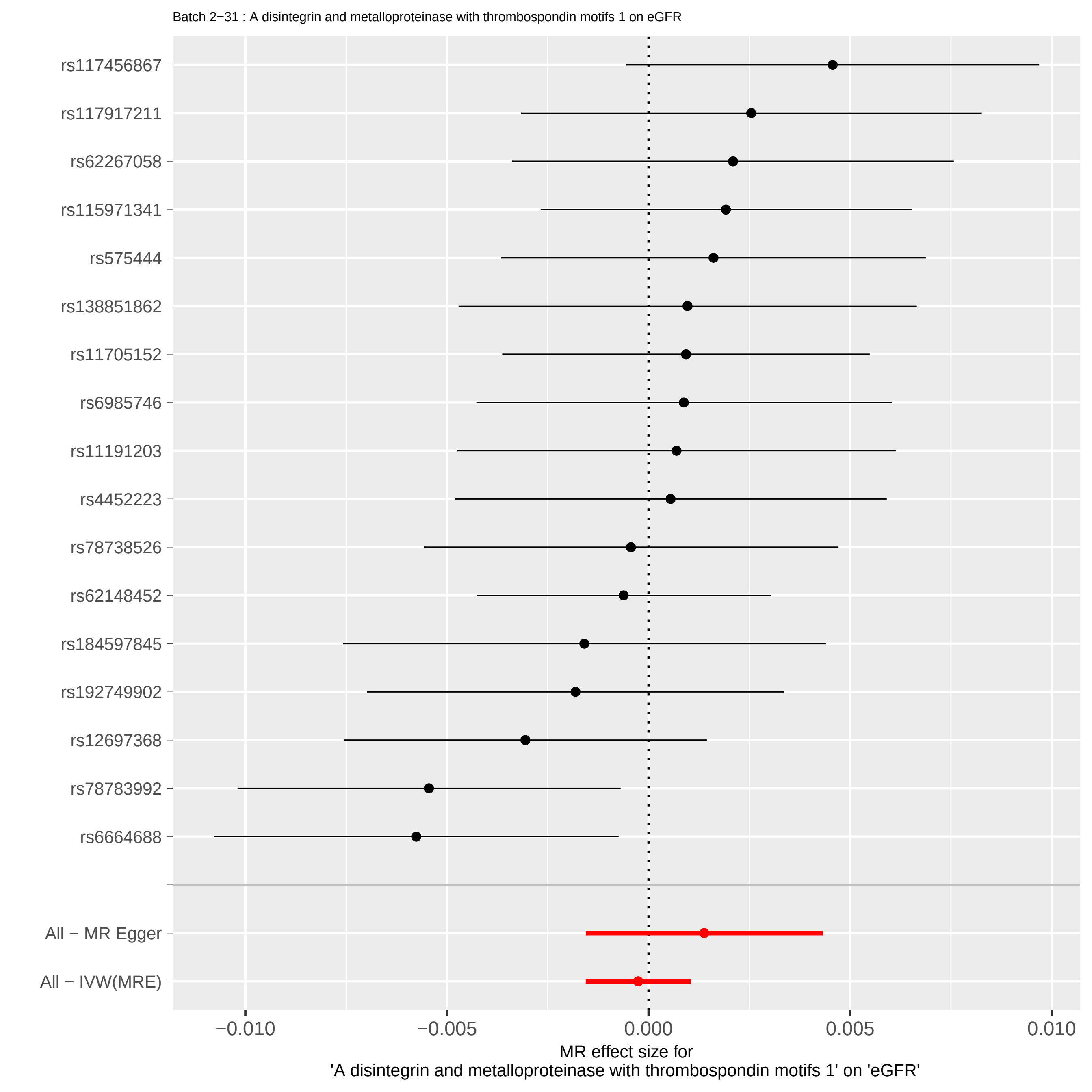

Batch 2−31 : A disintegrin and metalloproteinase with thrombospondin motifs 1 on eGFR
rs117456867
rs117917211
rs62267058
rs115971341
rs575444
rs138851862
rs11705152
rs6985746
rs11191203
rs4452223
rs78738526
rs62148452
rs184597845
rs192749902
rs12697368
rs78783992
rs6664688
All − MR Egger
All − IVW(MRE)
−0.010
−0.005
0.000
0.005
0.010
MR effect size for
'A disintegrin and metalloproteinase with thrombospondin motifs 1' on 'eGFR'

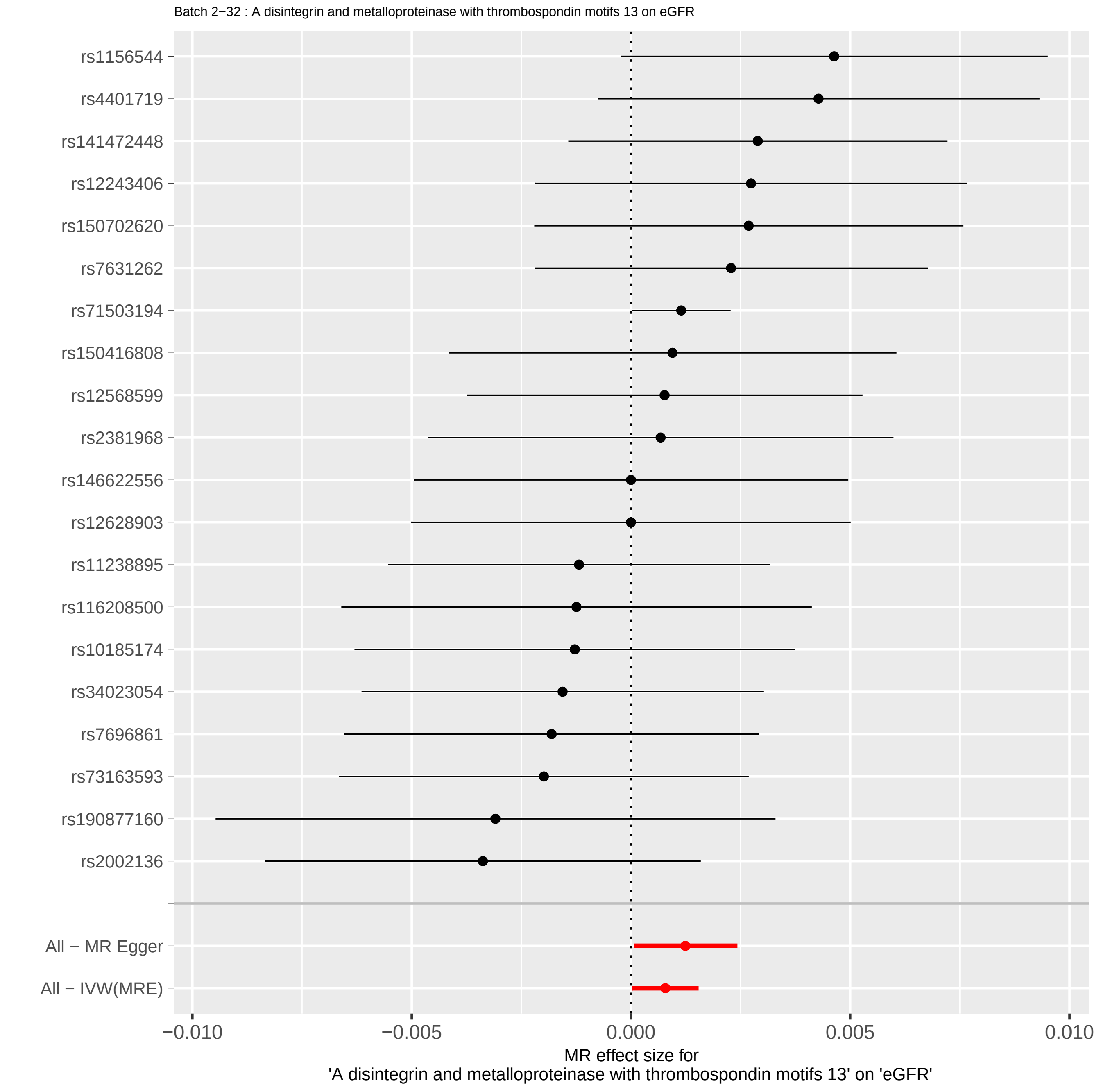

Batch 2−32 : A disintegrin and metalloproteinase with thrombospondin motifs 13 on eGFR
rs1156544
rs4401719
rs141472448
rs12243406
rs150702620
rs7631262
rs71503194
rs150416808
rs12568599
rs2381968
rs146622556
rs12628903
rs11238895
rs116208500
rs10185174
rs34023054
rs7696861
rs73163593
rs190877160
rs2002136
All − MR Egger
All − IVW(MRE)
−0.010
−0.005
0.000
0.005
0.010
MR effect size for
'A disintegrin and metalloproteinase with thrombospondin motifs 13' on 'eGFR'

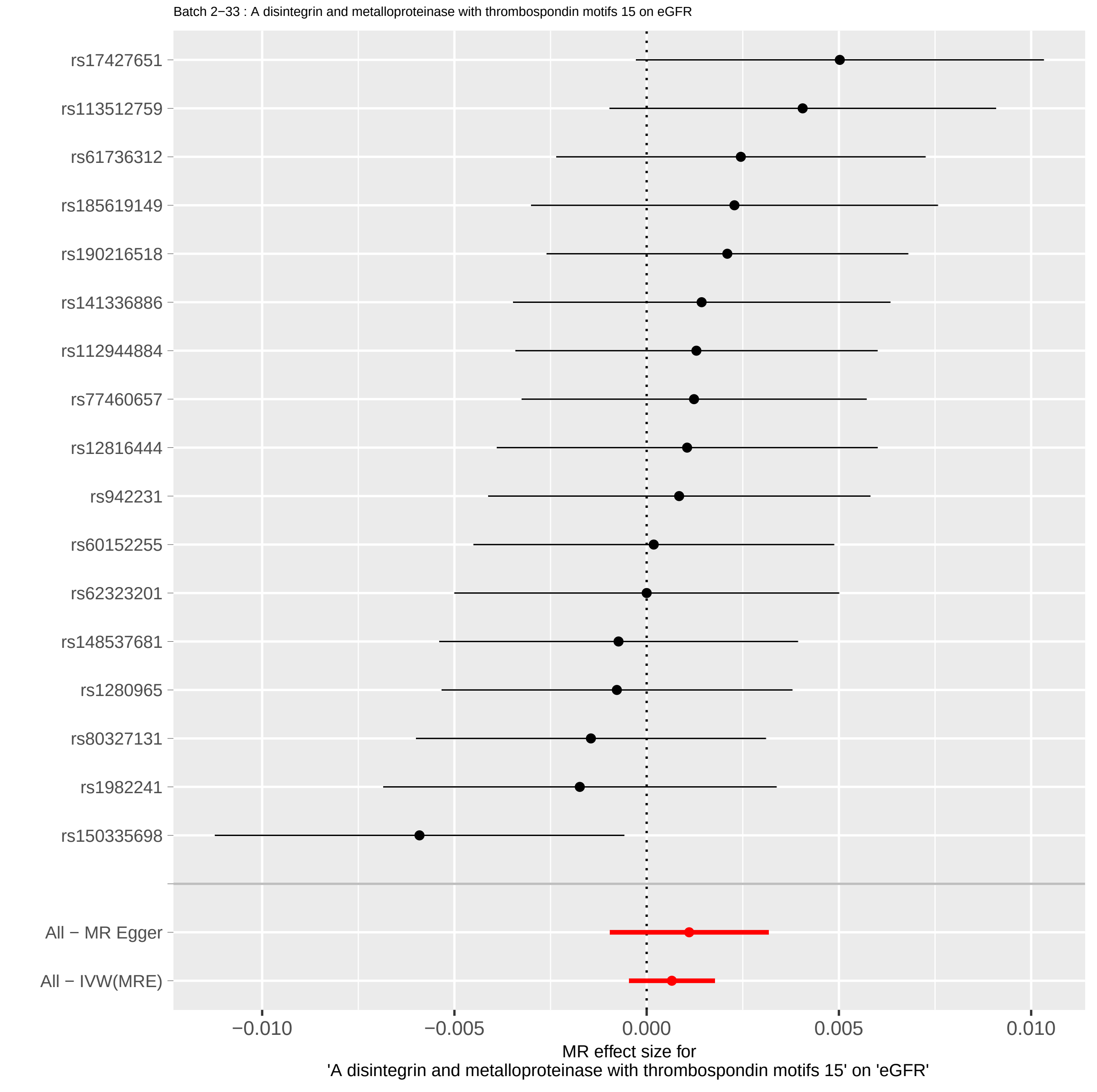

Batch 2−33 : A disintegrin and metalloproteinase with thrombospondin motifs 15 on eGFR
rs17427651
rs113512759
rs61736312
rs185619149
rs190216518
rs141336886
rs112944884
rs77460657
rs12816444
rs942231
rs60152255
rs62323201
rs148537681
rs1280965
rs80327131
rs1982241
rs150335698
All − MR Egger
All − IVW(MRE)
−0.010
−0.005
0.000
0.005
0.010
MR effect size for
'A disintegrin and metalloproteinase with thrombospondin motifs 15' on 'eGFR'

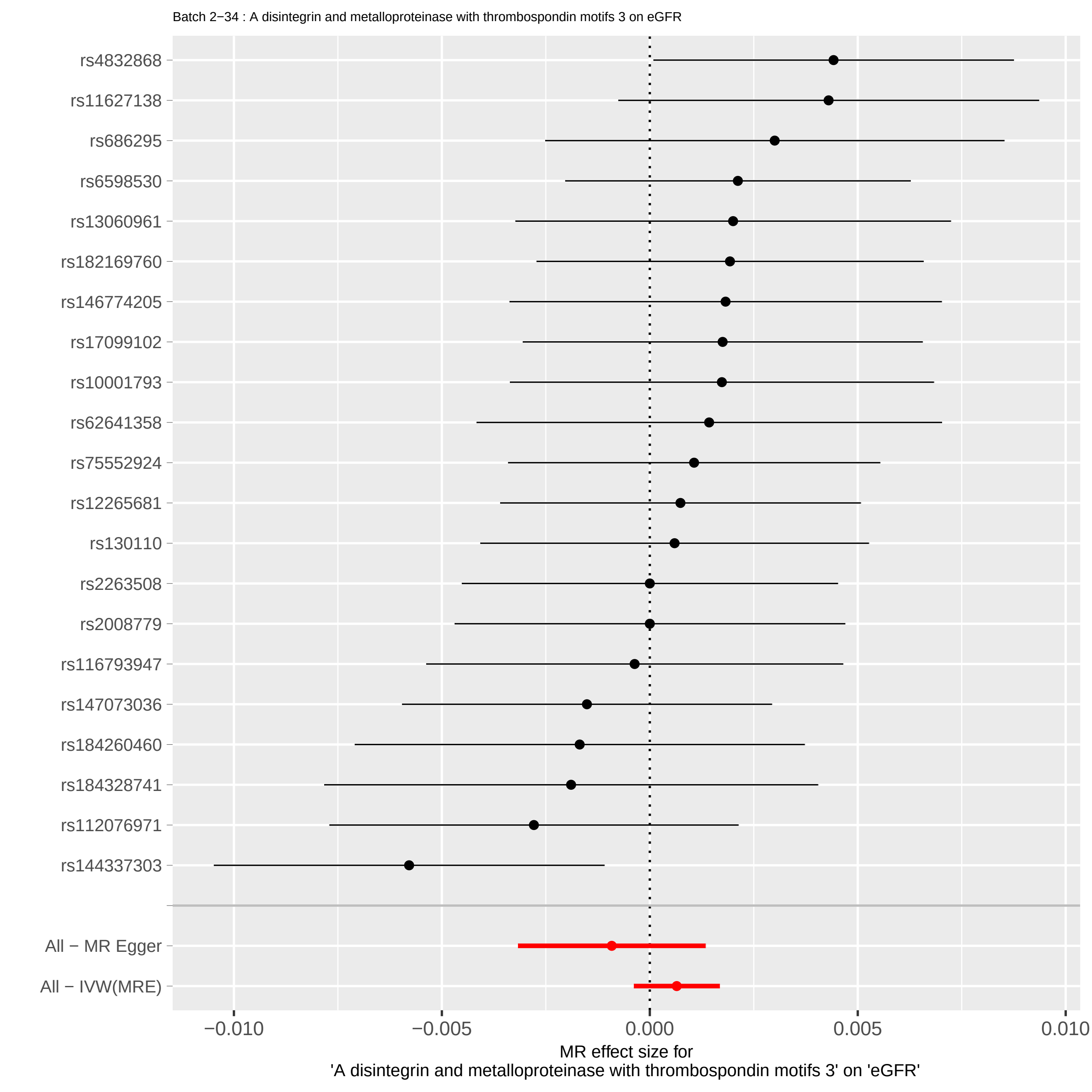
Batch 2−34 : A disintegrin and metalloproteinase with thrombospondin motifs 3 on eGFR
rs4832868
rs11627138
rs686295
rs6598530
rs13060961
rs182169760
rs146774205
rs17099102
rs10001793
rs62641358
rs75552924
rs12265681
rs130110
rs2263508
rs2008779
rs116793947
rs147073036
rs184260460
rs184328741
rs112076971
rs144337303
All − MR Egger
All − IVW(MRE)
−0.010
−0.005
0.000
0.005
0.010
MR effect size for
'A disintegrin and metalloproteinase with thrombospondin motifs 3' on 'eGFR'

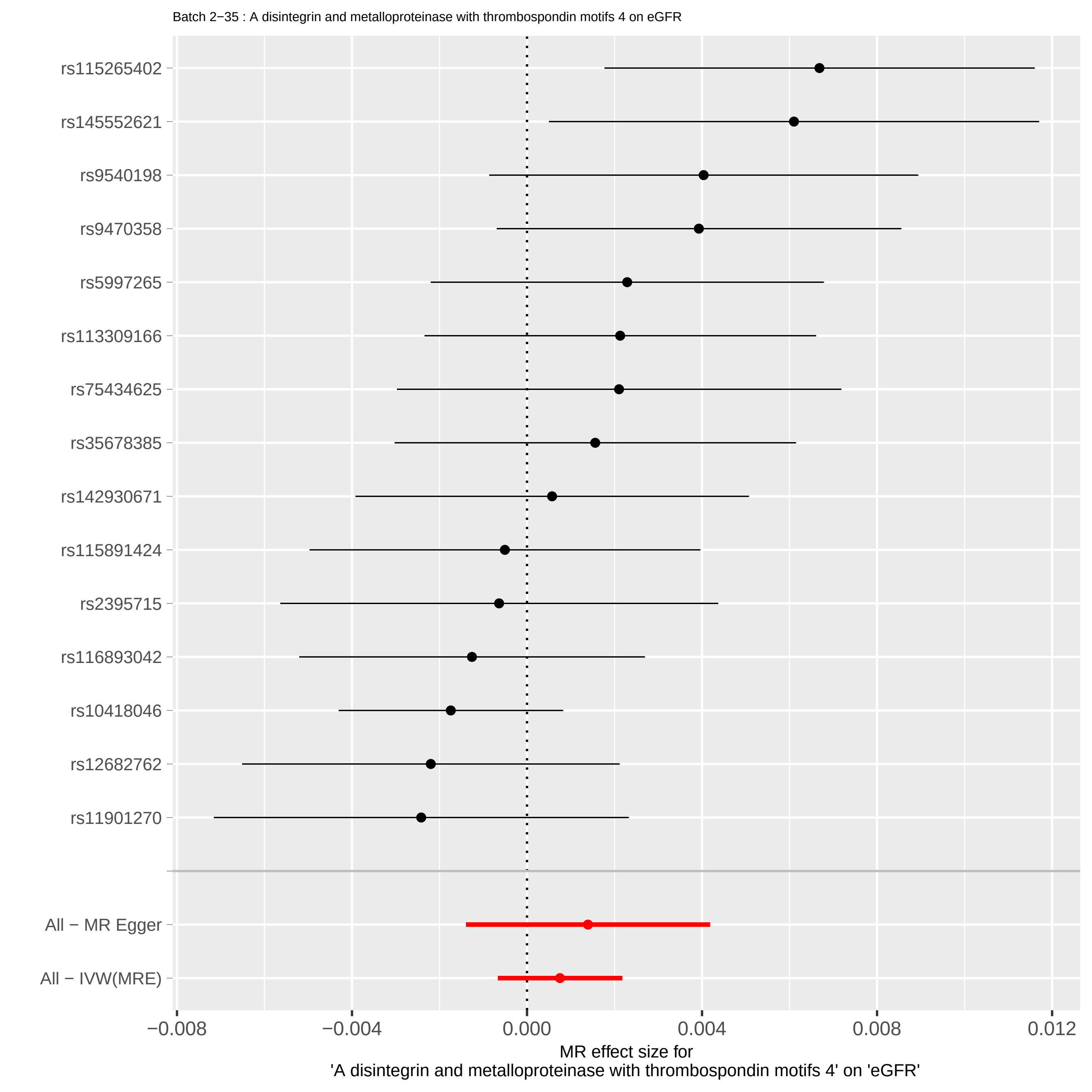

Batch 2−35 : A disintegrin and metalloproteinase with thrombospondin motifs 4 on eGFR
rs115265402
rs145552621
rs9540198
rs9470358
rs5997265
rs113309166
rs75434625
rs35678385
rs142930671
rs115891424
rs2395715
rs116893042
rs10418046
rs12682762
rs11901270
All − MR Egger
All − IVW(MRE)
−0.008
−0.004
0.000
0.004
0.008
0.012
MR effect size for
'A disintegrin and metalloproteinase with thrombospondin motifs 4' on 'eGFR'

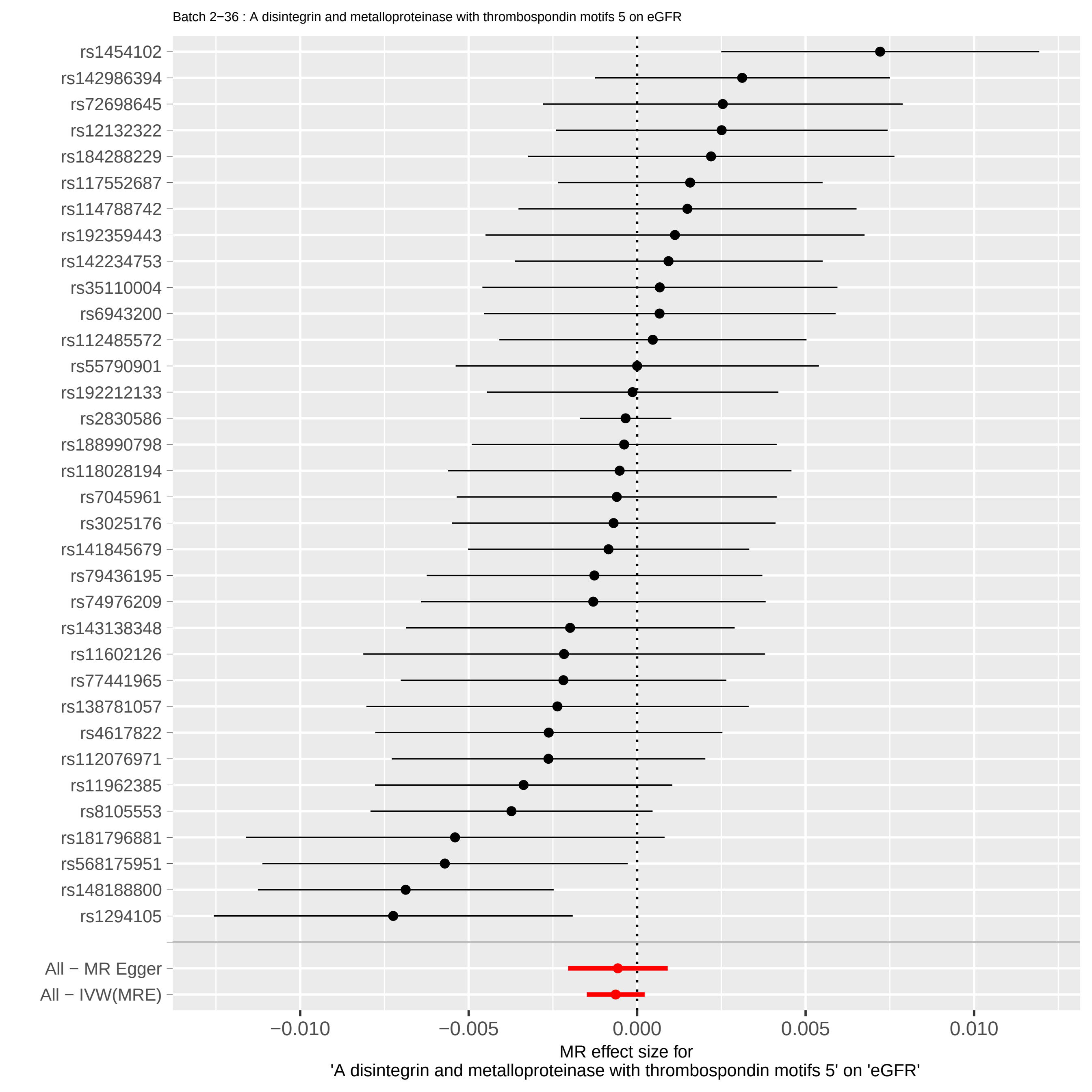

Batch 2−36 : A disintegrin and metalloproteinase with thrombospondin motifs 5 on eGFR
rs1454102
rs142986394
rs72698645
rs12132322
rs184288229
rs117552687
rs114788742
rs192359443
rs142234753
rs35110004
rs6943200
rs112485572
rs55790901
rs192212133
rs2830586
rs188990798
rs118028194
rs7045961
rs3025176
rs141845679
rs79436195
rs74976209
rs143138348
rs11602126
rs77441965
rs138781057
rs4617822
rs112076971
rs11962385
rs8105553
rs181796881
rs568175951
rs148188800
rs1294105
All − MR Egger
All − IVW(MRE)
−0.010
−0.005
0.000
0.005
0.010
MR effect size for
'A disintegrin and metalloproteinase with thrombospondin motifs 5' on 'eGFR'

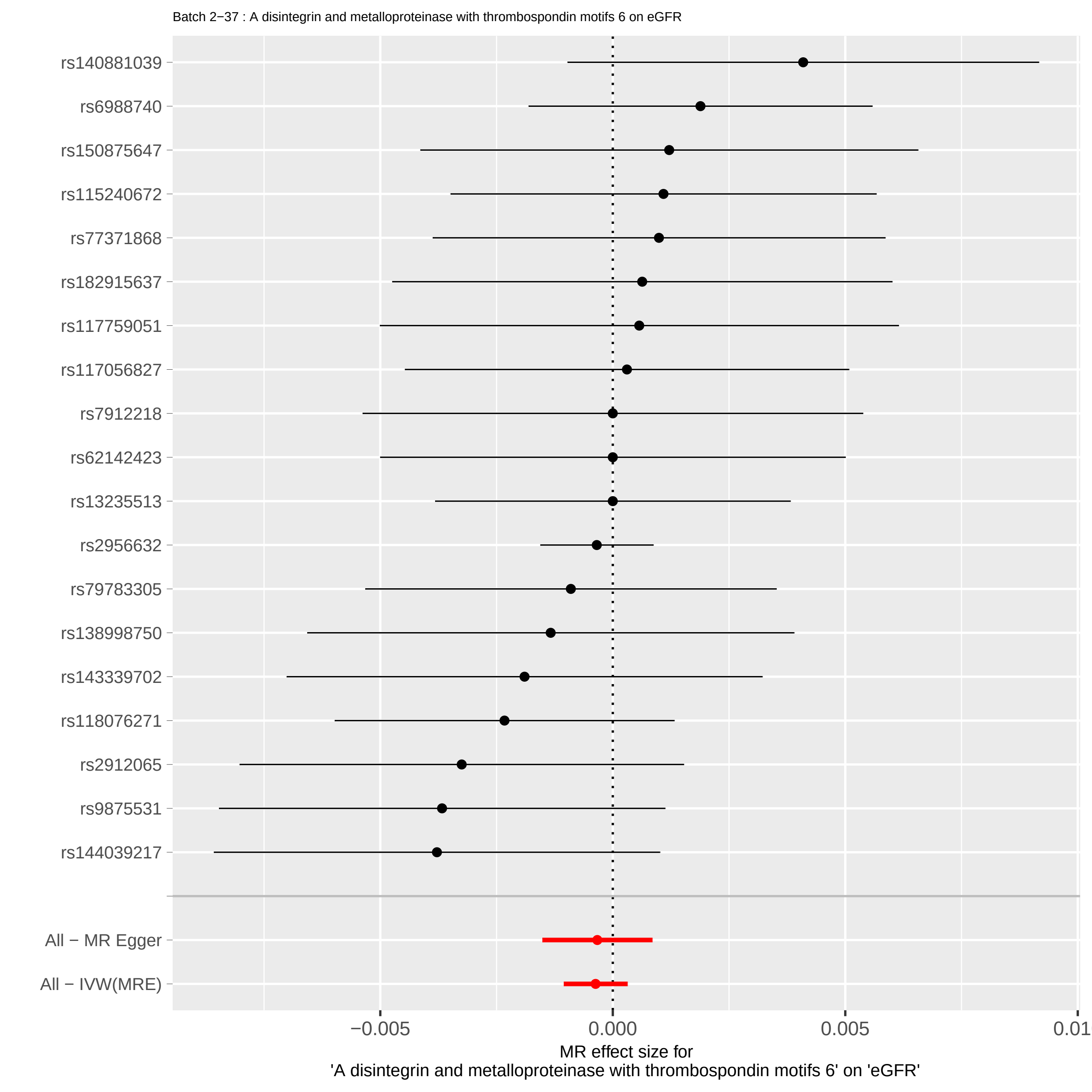

Batch 2−37 : A disintegrin and metalloproteinase with thrombospondin motifs 6 on eGFR
rs140881039
rs6988740
rs150875647
rs115240672
rs77371868
rs182915637
rs117759051
rs117056827
rs7912218
rs62142423
rs13235513
rs2956632
rs79783305
rs138998750
rs143339702
rs118076271
rs2912065
rs9875531
rs144039217
All − MR Egger
All − IVW(MRE)
−0.005
0.000
0.005
0.01
MR effect size for
'A disintegrin and metalloproteinase with thrombospondin motifs 6' on 'eGFR'

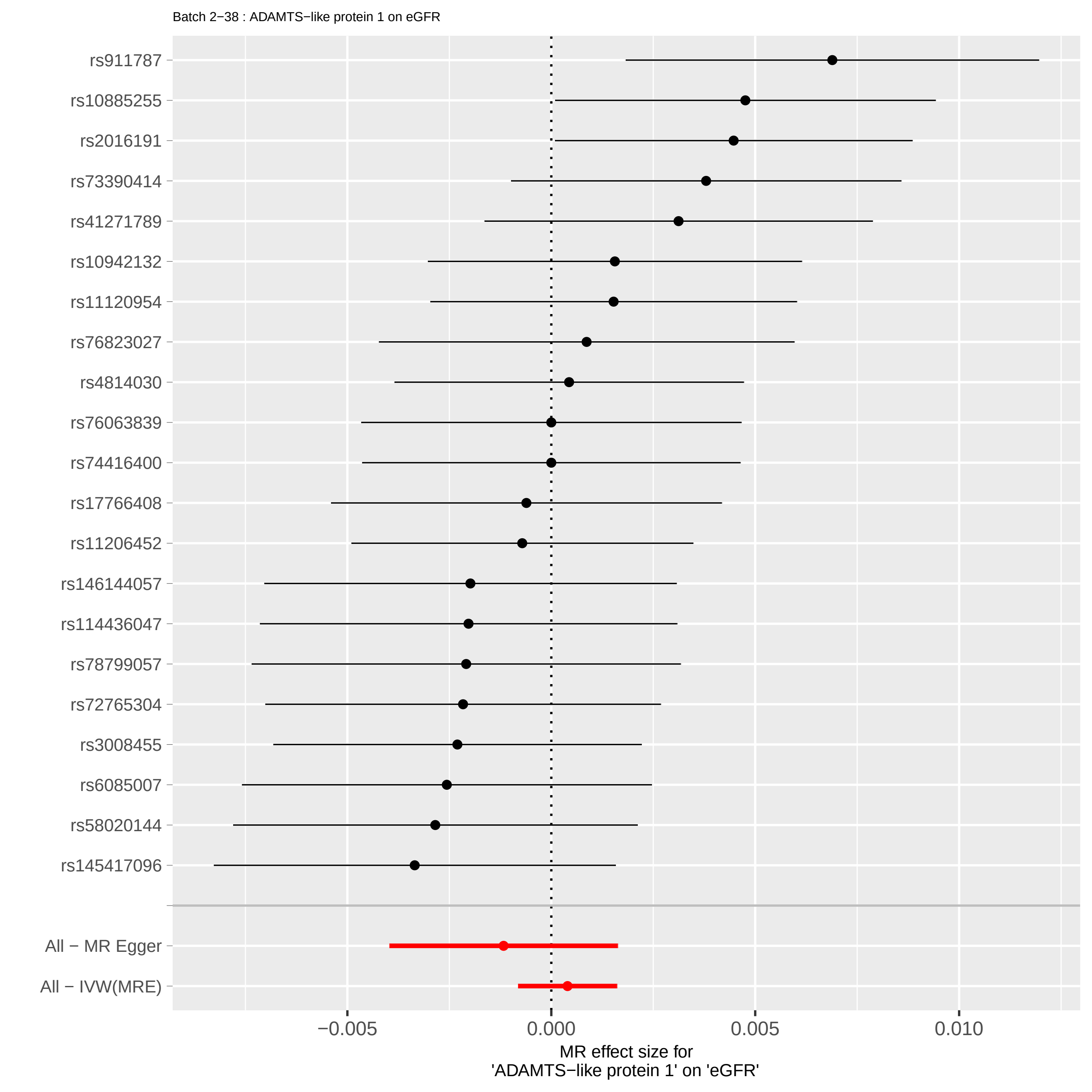
Batch 2−38 : ADAMTS−like protein 1 on eGFR
rs911787
rs10885255
rs2016191
rs73390414
rs41271789
rs10942132
rs11120954
rs76823027
rs4814030
rs76063839
rs74416400
rs17766408
rs11206452
rs146144057
rs114436047
rs78799057
rs72765304
rs3008455
rs6085007
rs58020144
rs145417096
All − MR Egger
All − IVW(MRE)
−0.005
0.000
0.005
0.010
MR effect size for
'ADAMTS−like protein 1' on 'eGFR'

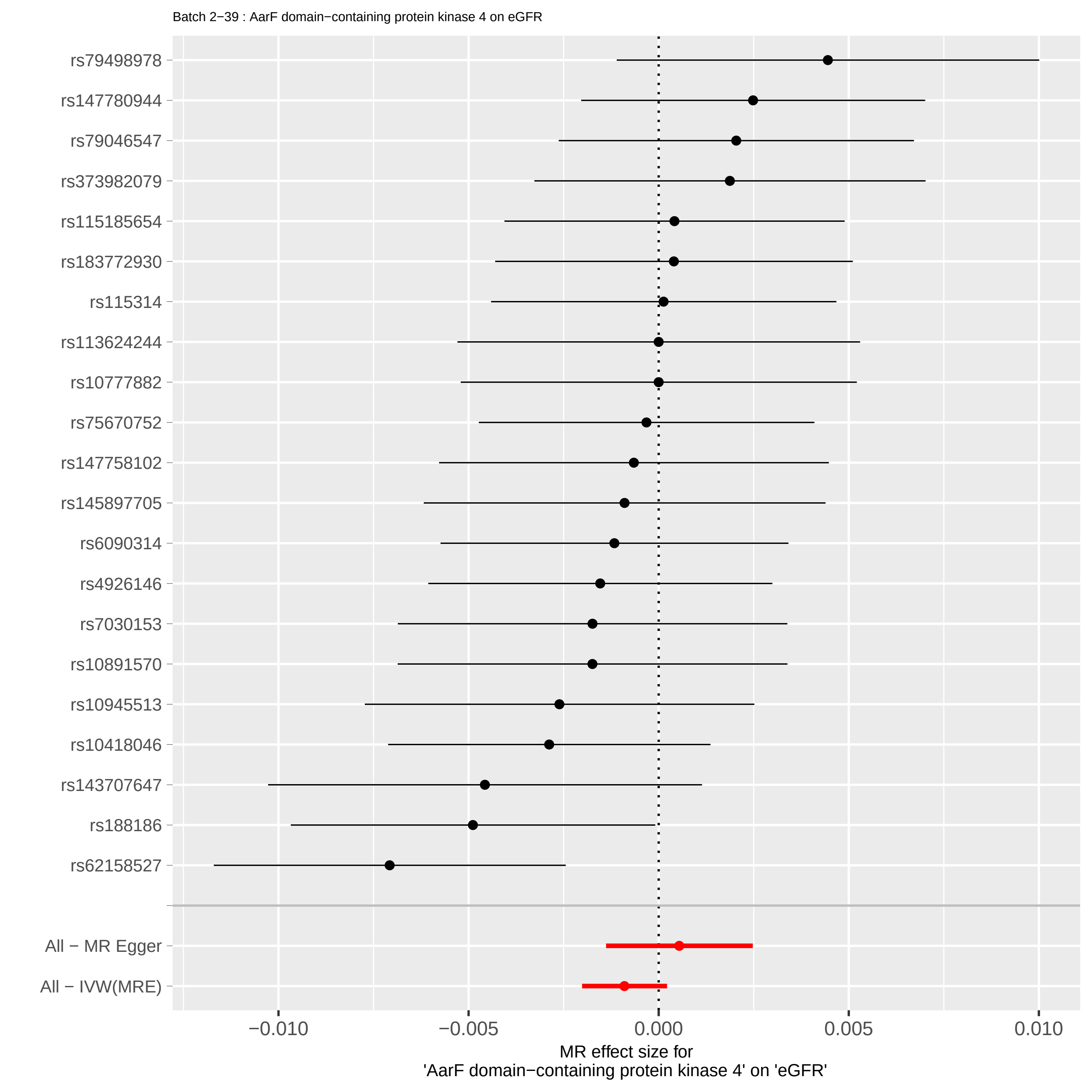
Batch 2−39 : AarF domain−containing protein kinase 4 on eGFR
rs79498978
rs147780944
rs79046547
rs373982079
rs115185654
rs183772930
rs115314
rs113624244
rs10777882
rs75670752
rs147758102
rs145897705
rs6090314
rs4926146
rs7030153
rs10891570
rs10945513
rs10418046
rs143707647
rs188186
rs62158527
All − MR Egger
All − IVW(MRE)
−0.010
−0.005
0.000
0.005
0.010
MR effect size for
'AarF domain−containing protein kinase 4' on 'eGFR'

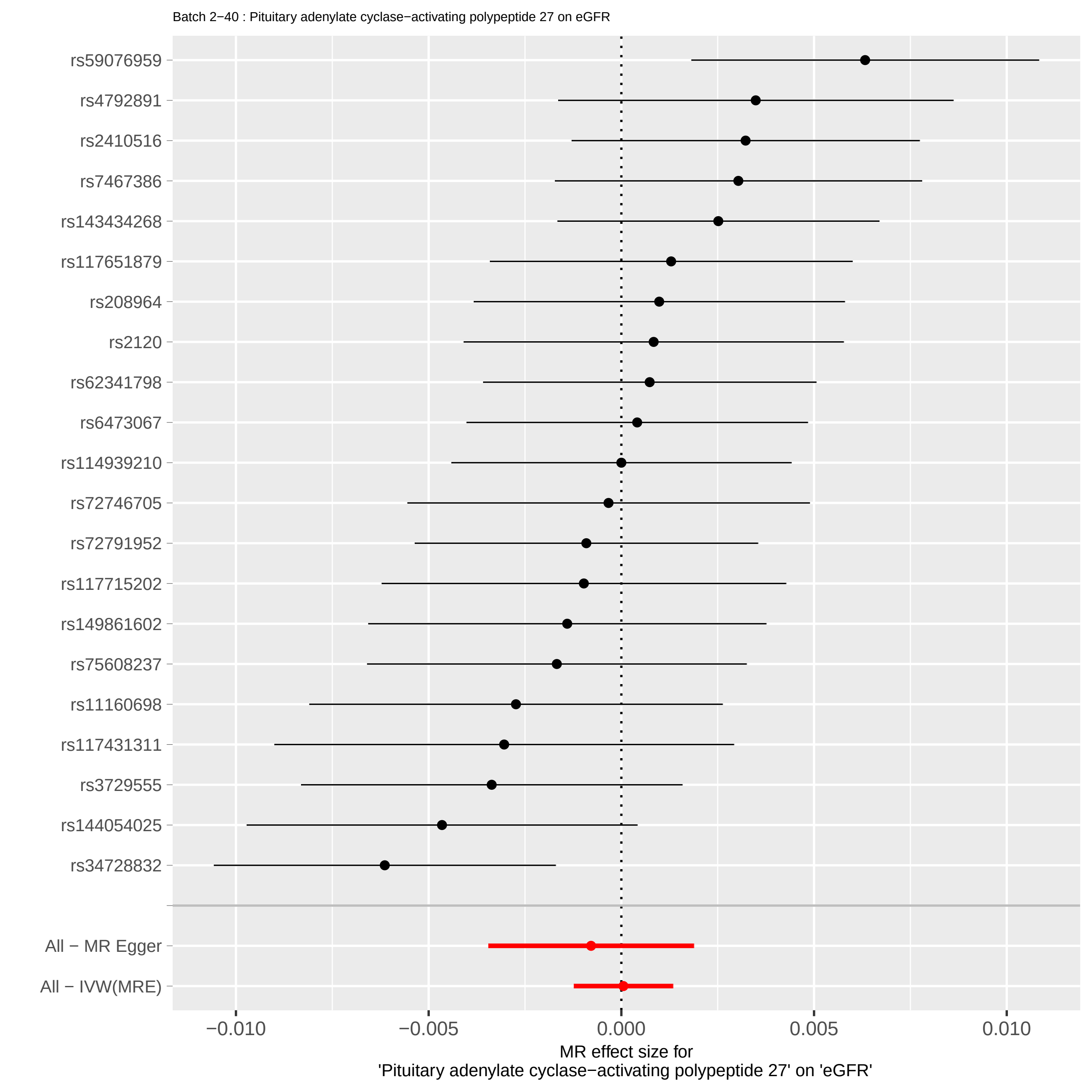

Batch 2−40 : Pituitary adenylate cyclase−activating polypeptide 27 on eGFR
rs59076959
rs4792891
rs2410516
rs7467386
rs143434268
rs117651879
rs208964
rs2120
rs62341798
rs6473067
rs114939210
rs72746705
rs72791952
rs117715202
rs149861602
rs75608237
rs11160698
rs117431311
rs3729555
rs144054025
rs34728832
All − MR Egger
All − IVW(MRE)
−0.010
−0.005
0.000
0.005
0.010
MR effect size for
'Pituitary adenylate cyclase−activating polypeptide 27' on 'eGFR'

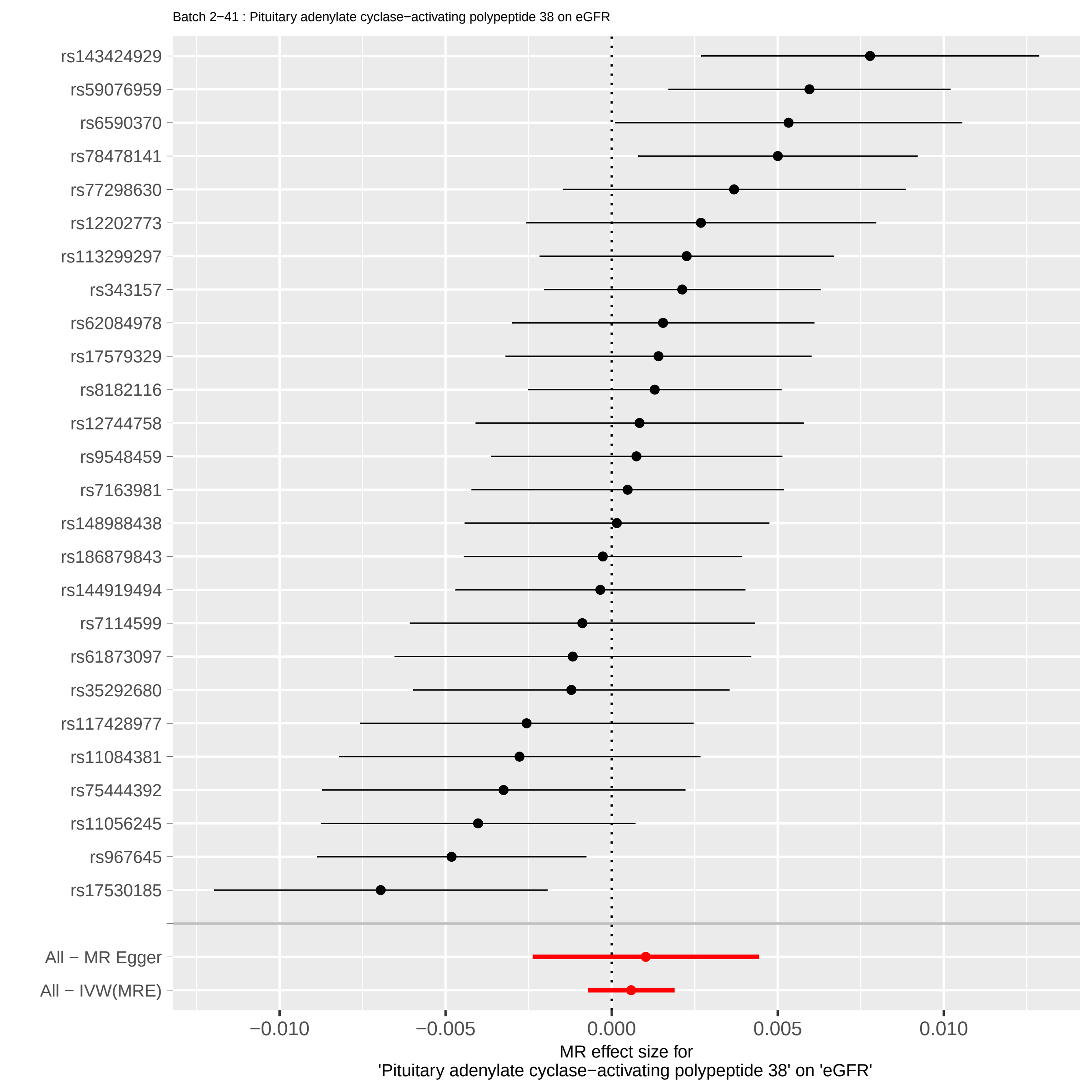

Batch 2−41 : Pituitary adenylate cyclase−activating polypeptide 38 on eGFR
rs143424929
rs59076959
rs6590370
rs78478141
rs77298630
rs12202773
rs113299297
rs343157
rs62084978
rs17579329
rs8182116
rs12744758
rs9548459
rs7163981
rs148988438
rs186879843
rs144919494
rs7114599
rs61873097
rs35292680
rs117428977
rs11084381
rs75444392
rs11056245
rs967645
rs17530185
All − MR Egger
All − IVW(MRE)
−0.010
−0.005
0.000
0.005
0.010
MR effect size for
'Pituitary adenylate cyclase−activating polypeptide 38' on 'eGFR'

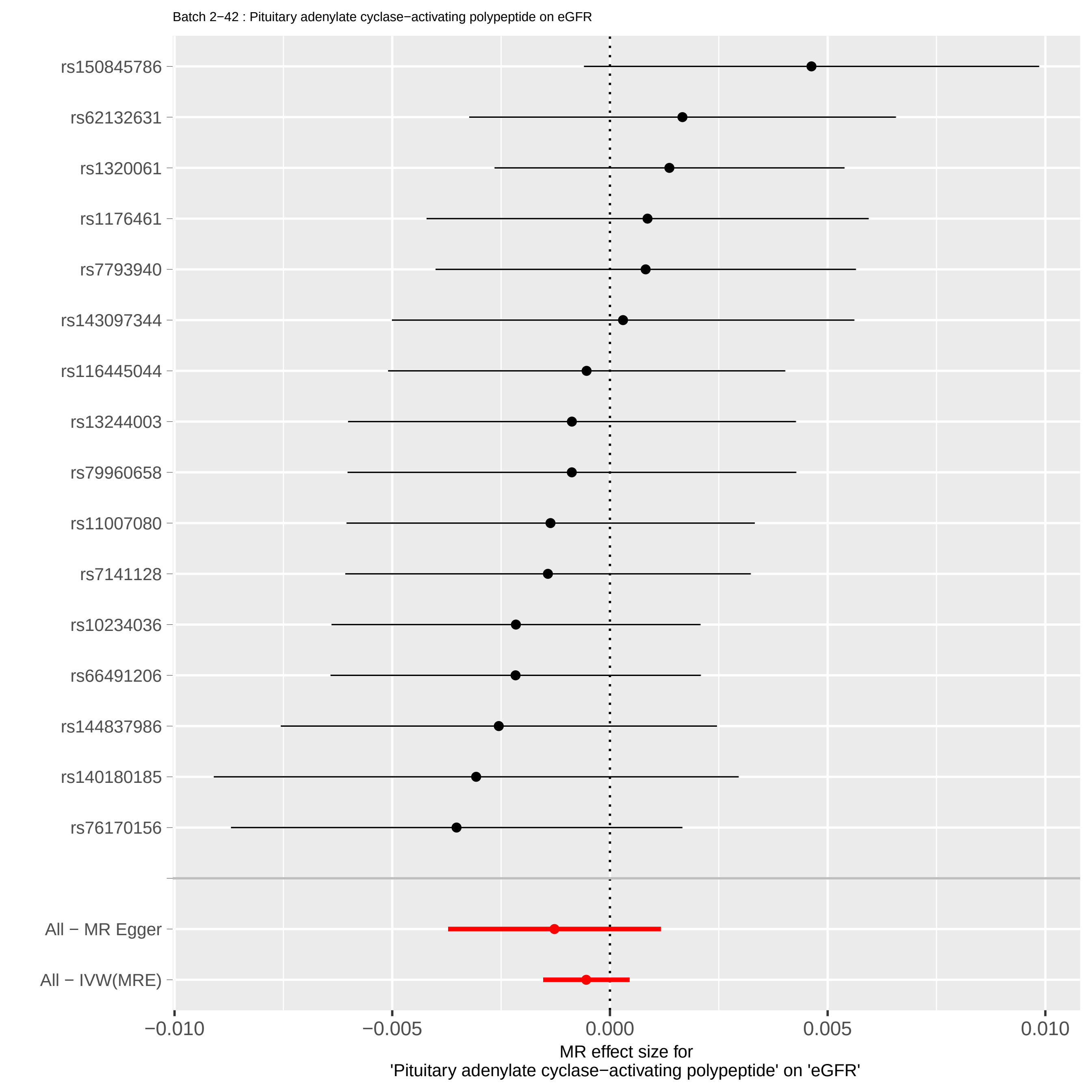

Batch 2−42 : Pituitary adenylate cyclase−activating polypeptide on eGFR
rs150845786
rs62132631
rs1320061
rs1176461
rs7793940
rs143097344
rs116445044
rs13244003
rs79960658
rs11007080
rs7141128
rs10234036
rs66491206
rs144837986
rs140180185
rs76170156
All − MR Egger
All − IVW(MRE)
−0.010
−0.005
0.000
0.005
0.010
MR effect size for
'Pituitary adenylate cyclase−activating polypeptide' on 'eGFR'

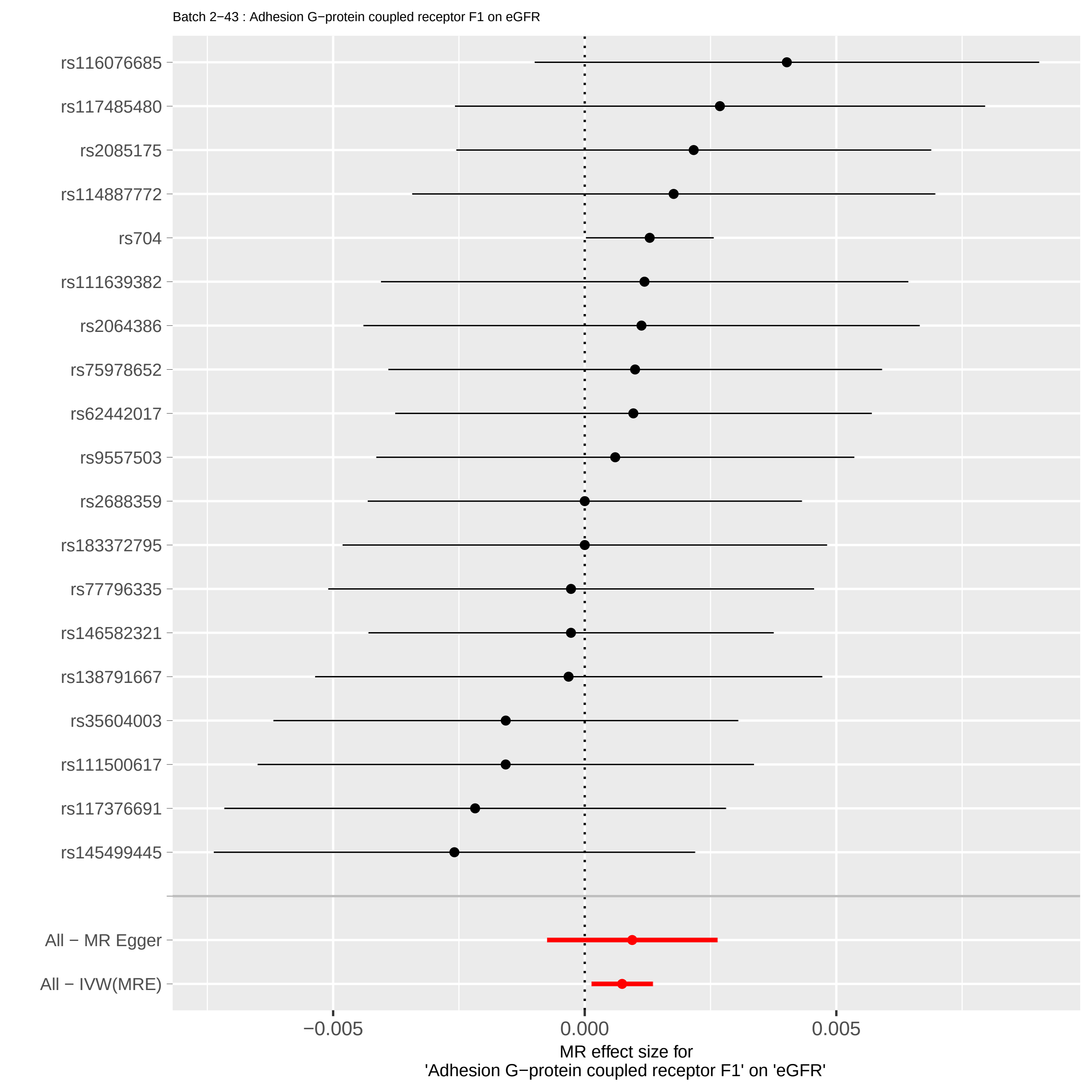
Batch 2−43 : Adhesion G−protein coupled receptor F1 on eGFR
rs116076685
rs117485480
rs2085175
rs114887772
rs704
rs111639382
rs2064386
rs75978652
rs62442017
rs9557503
rs2688359
rs183372795
rs77796335
rs146582321
rs138791667
rs35604003
rs111500617
rs117376691
rs145499445
All − MR Egger
All − IVW(MRE)
−0.005
0.000
0.005
MR effect size for
'Adhesion G−protein coupled receptor F1' on 'eGFR'

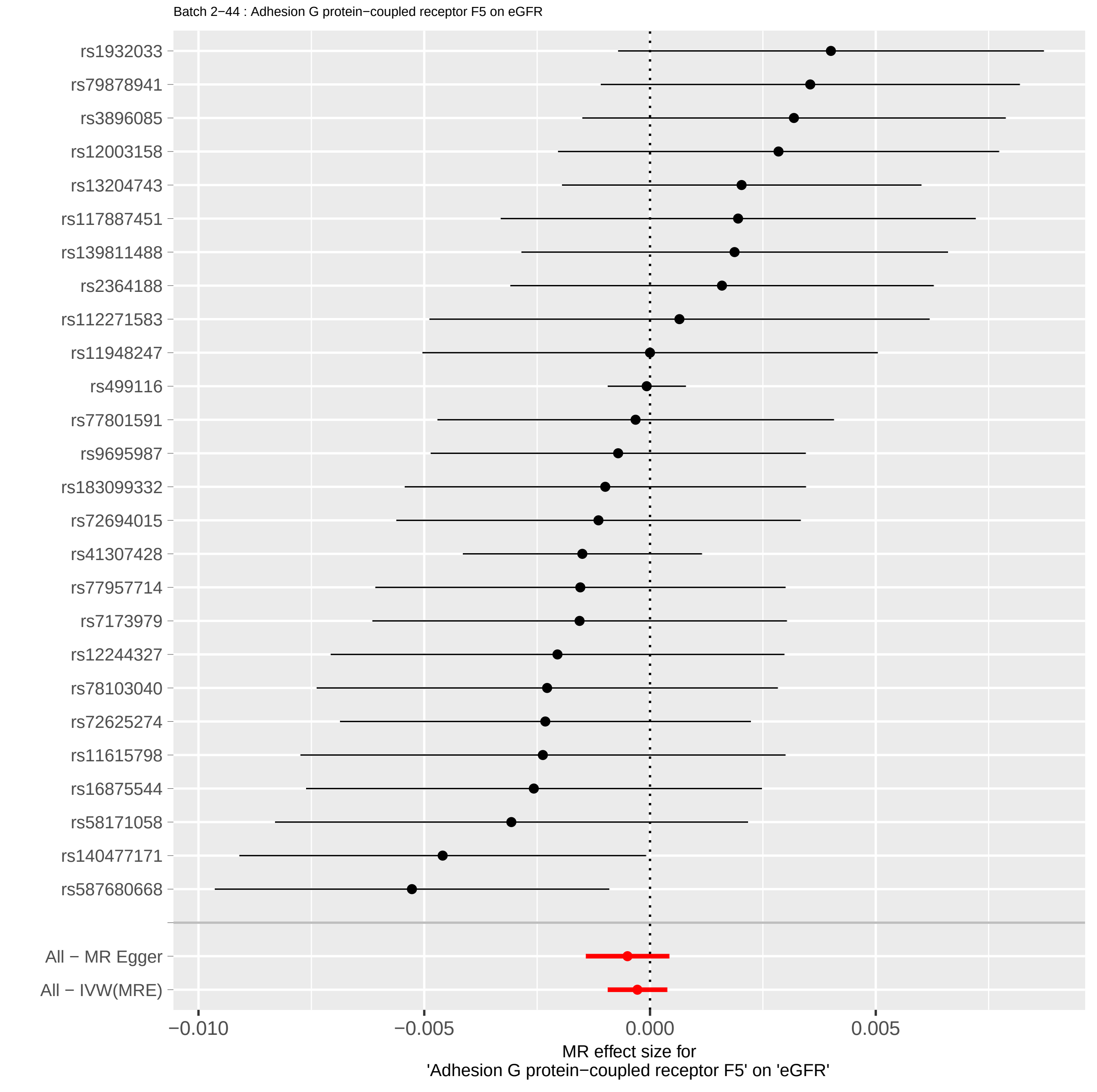

Batch 2−44 : Adhesion G protein−coupled receptor F5 on eGFR
rs1932033
rs79878941
rs3896085
rs12003158
rs13204743
rs117887451
rs139811488
rs2364188
rs112271583
rs11948247
rs499116
rs77801591
rs9695987
rs183099332
rs72694015
rs41307428
rs77957714
rs7173979
rs12244327
rs78103040
rs72625274
rs11615798
rs16875544
rs58171058
rs140477171
rs587680668
All − MR Egger
All − IVW(MRE)
−0.010
−0.005
0.000
0.005
MR effect size for
'Adhesion G protein−coupled receptor F5' on 'eGFR'

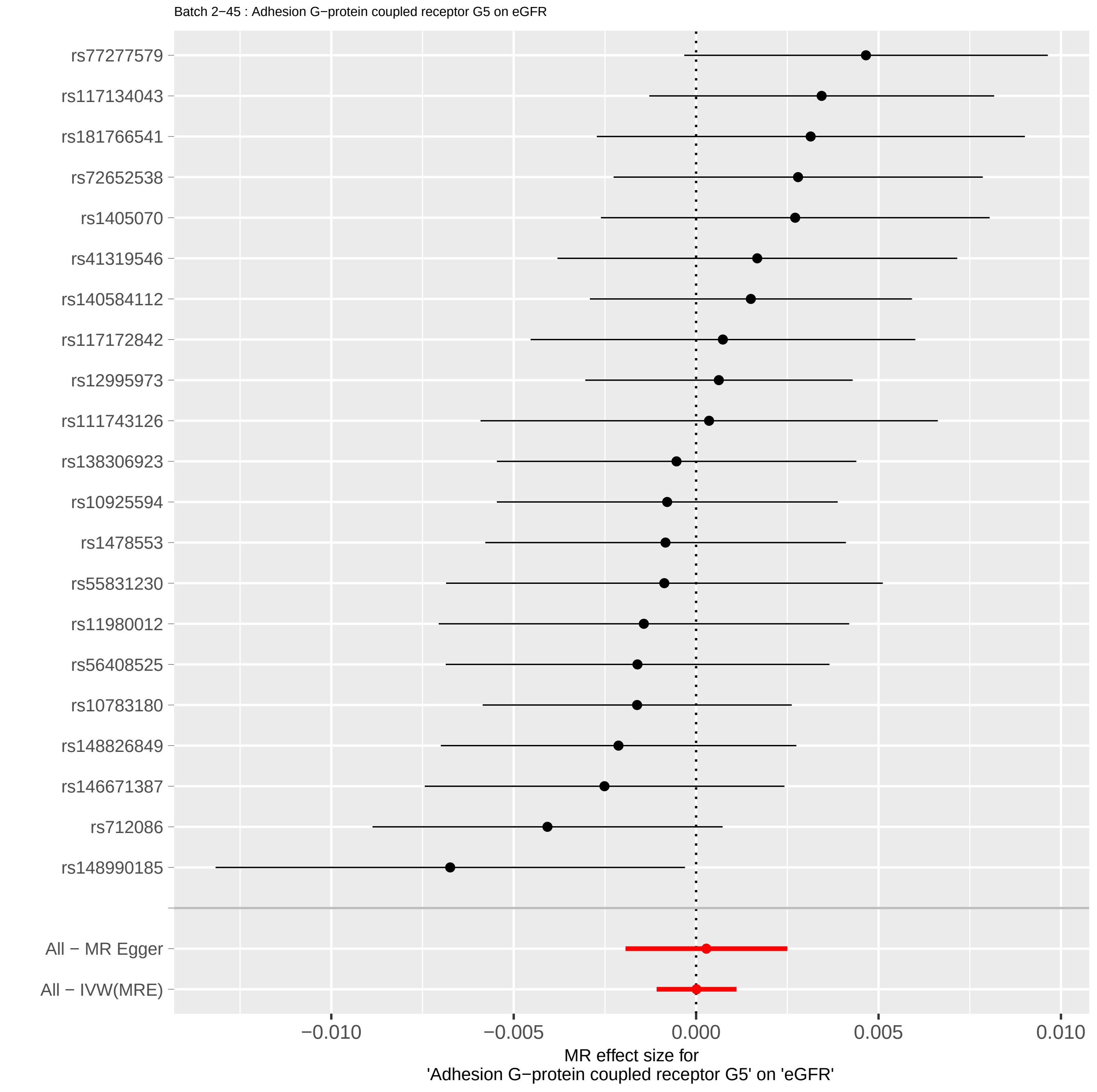

Batch 2−45 : Adhesion G−protein coupled receptor G5 on eGFR
rs77277579
rs117134043
rs181766541
rs72652538
rs1405070
rs41319546
rs140584112
rs117172842
rs12995973
rs111743126
rs138306923
rs10925594
rs1478553
rs55831230
rs11980012
rs56408525
rs10783180
rs148826849
rs146671387
rs712086
rs148990185
All − MR Egger
All − IVW(MRE)
−0.010
−0.005
0.000
0.005
0.010
MR effect size for
'Adhesion G−protein coupled receptor G5' on 'eGFR'

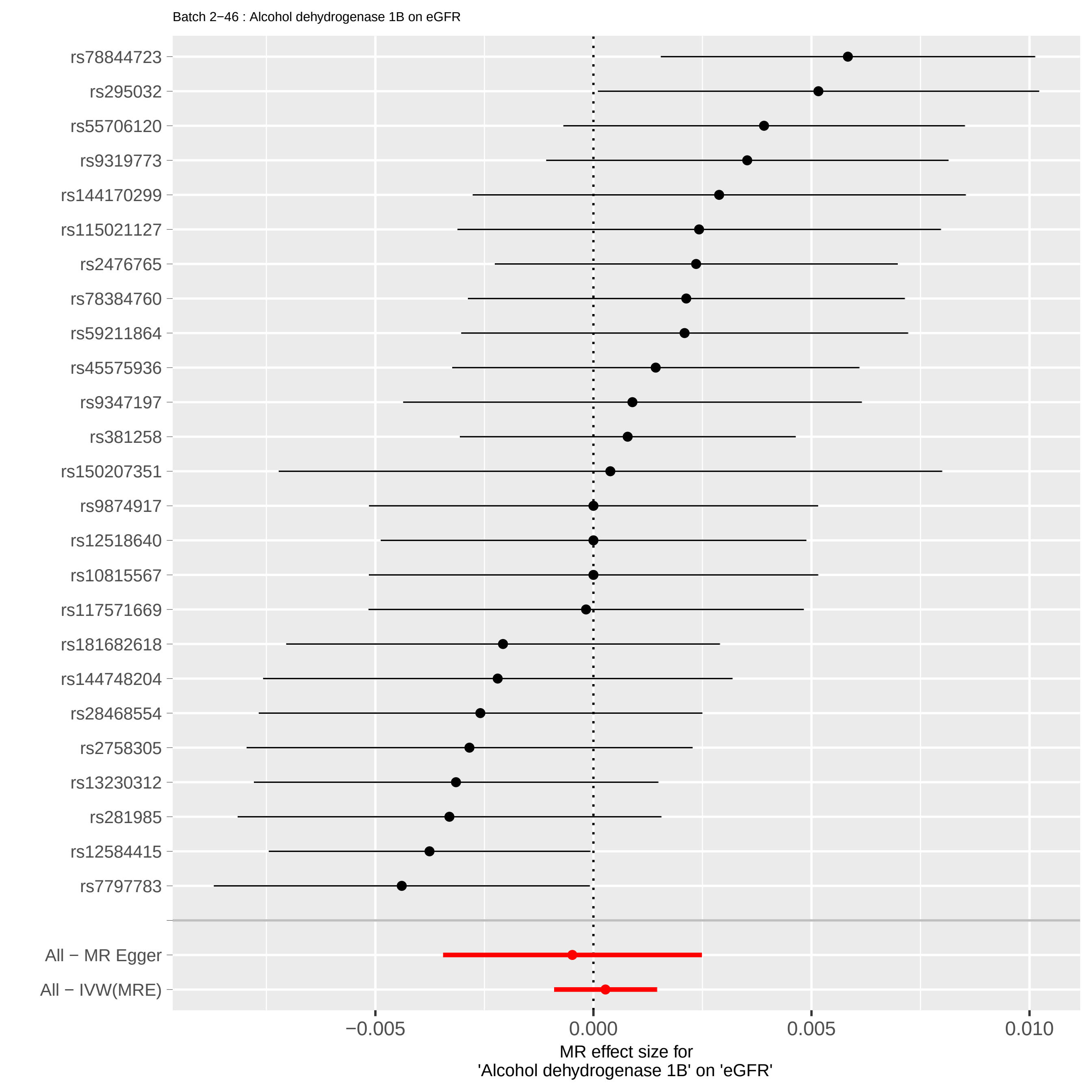
Batch 2−46 : Alcohol dehydrogenase 1B on eGFR
rs78844723
rs295032
rs55706120
rs9319773
rs144170299
rs115021127
rs2476765
rs78384760
rs59211864
rs45575936
rs9347197
rs381258
rs150207351
rs9874917
rs12518640
rs10815567
rs117571669
rs181682618
rs144748204
rs28468554
rs2758305
rs13230312
rs281985
rs12584415
rs7797783
All − MR Egger
All − IVW(MRE)
−0.005
0.000
0.005
0.010
MR effect size for
'Alcohol dehydrogenase 1B' on 'eGFR'

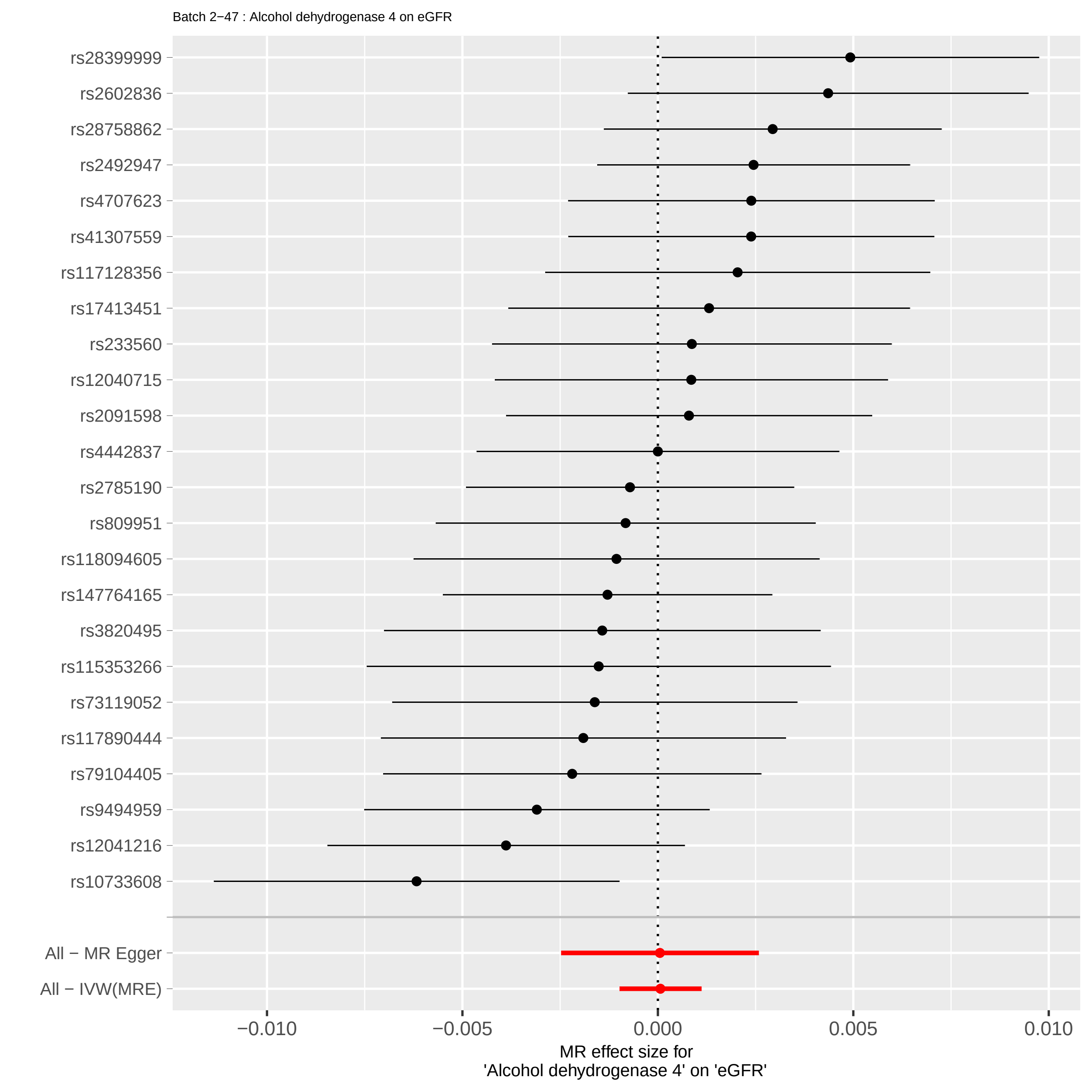
Batch 2−47 : Alcohol dehydrogenase 4 on eGFR
rs28399999
rs2602836
rs28758862
rs2492947
rs4707623
rs41307559
rs117128356
rs17413451
rs233560
rs12040715
rs2091598
rs4442837
rs2785190
rs809951
rs118094605
rs147764165
rs3820495
rs115353266
rs73119052
rs117890444
rs79104405
rs9494959
rs12041216
rs10733608
All − MR Egger
All − IVW(MRE)
−0.010
−0.005
0.000
0.005
0.010
MR effect size for
'Alcohol dehydrogenase 4' on 'eGFR'

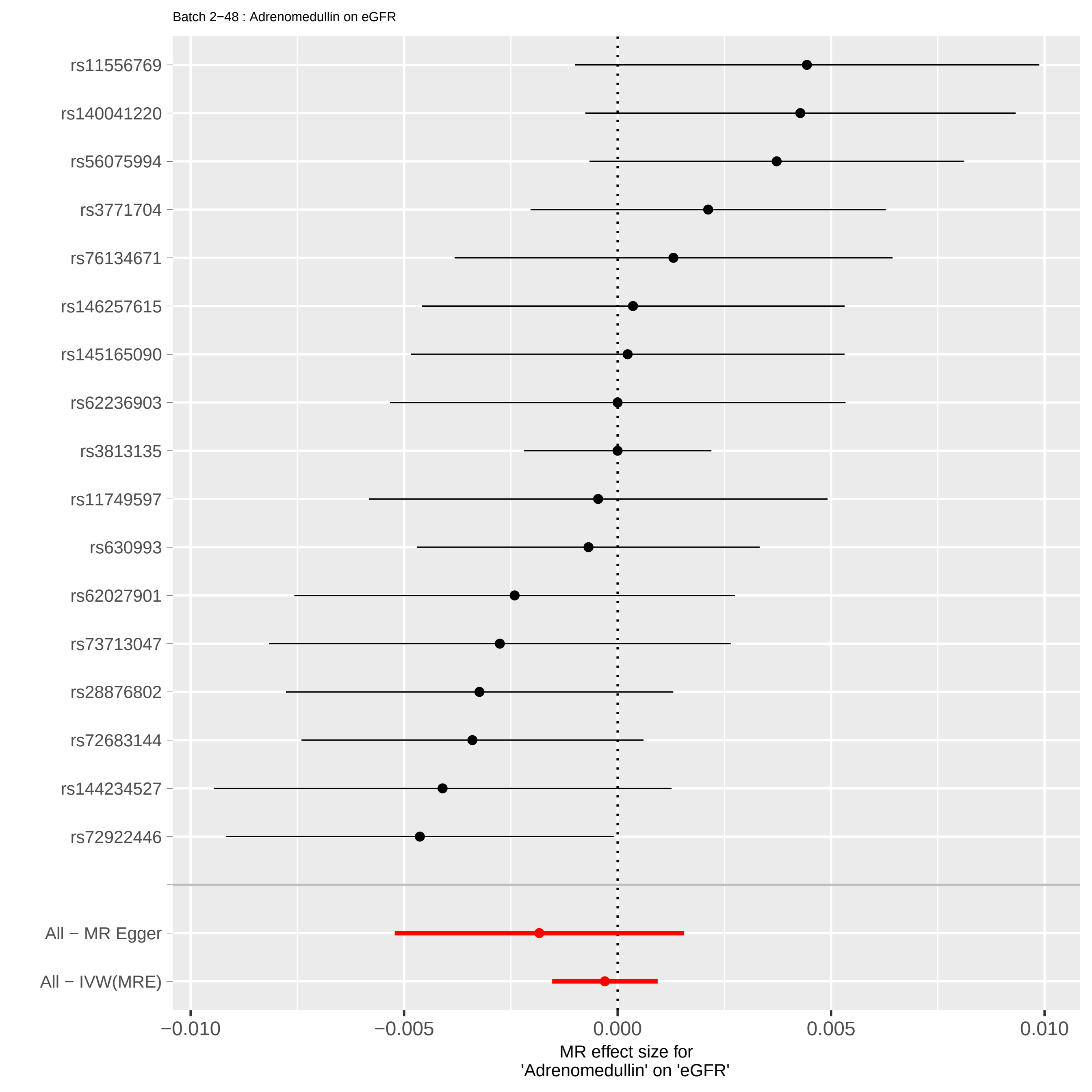

Batch 2−48 : Adrenomedullin on eGFR
rs11556769
rs140041220
rs56075994
rs3771704
rs76134671
rs146257615
rs145165090
rs62236903
rs3813135
rs11749597
rs630993
rs62027901
rs73713047
rs28876802
rs72683144
rs144234527
rs72922446
All − MR Egger
All − IVW(MRE)
−0.010
−0.005
0.000
0.005
0.010
MR effect size for
'Adrenomedullin' on 'eGFR'

Batch 2−49 : ADP−dependent glucokinase on eGFR

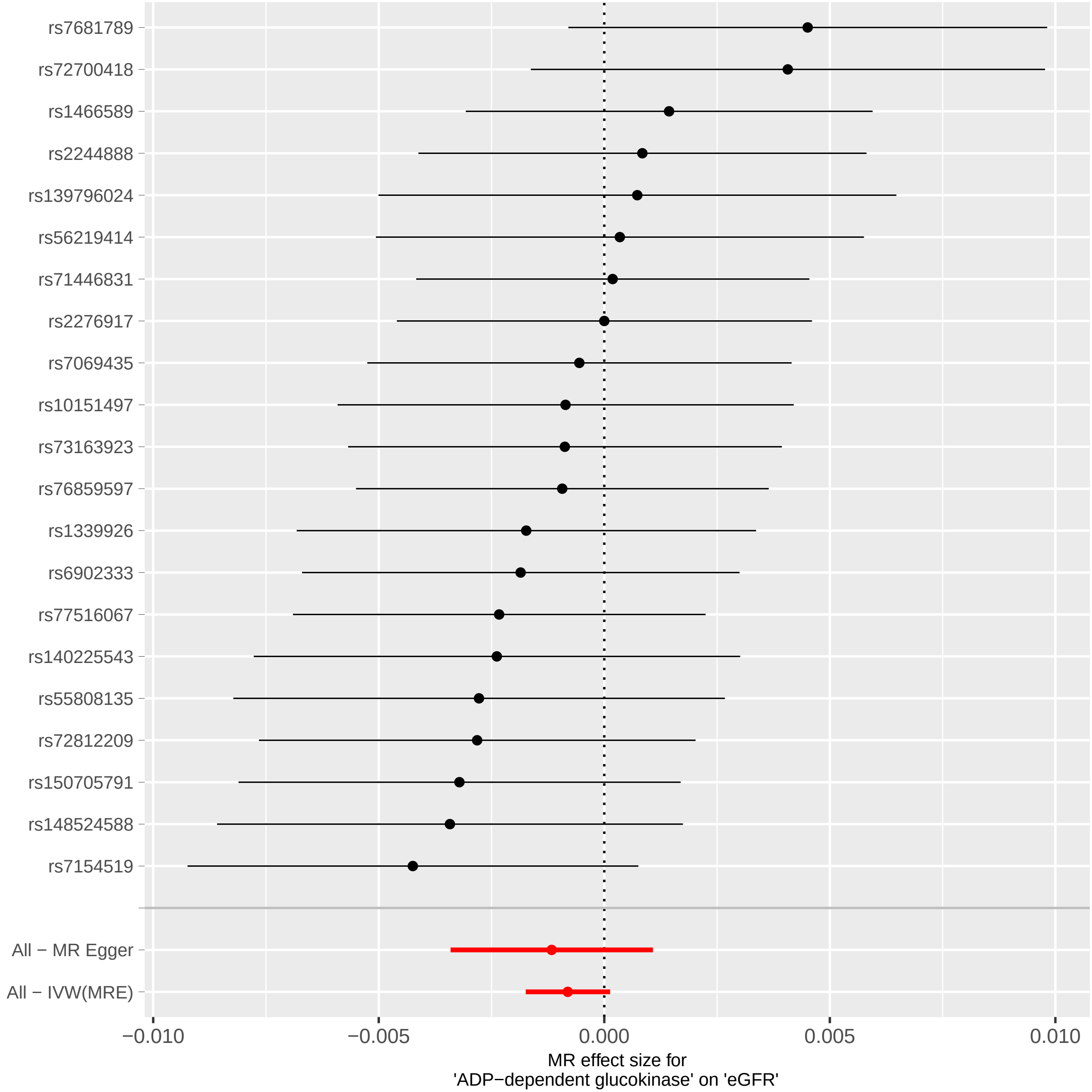

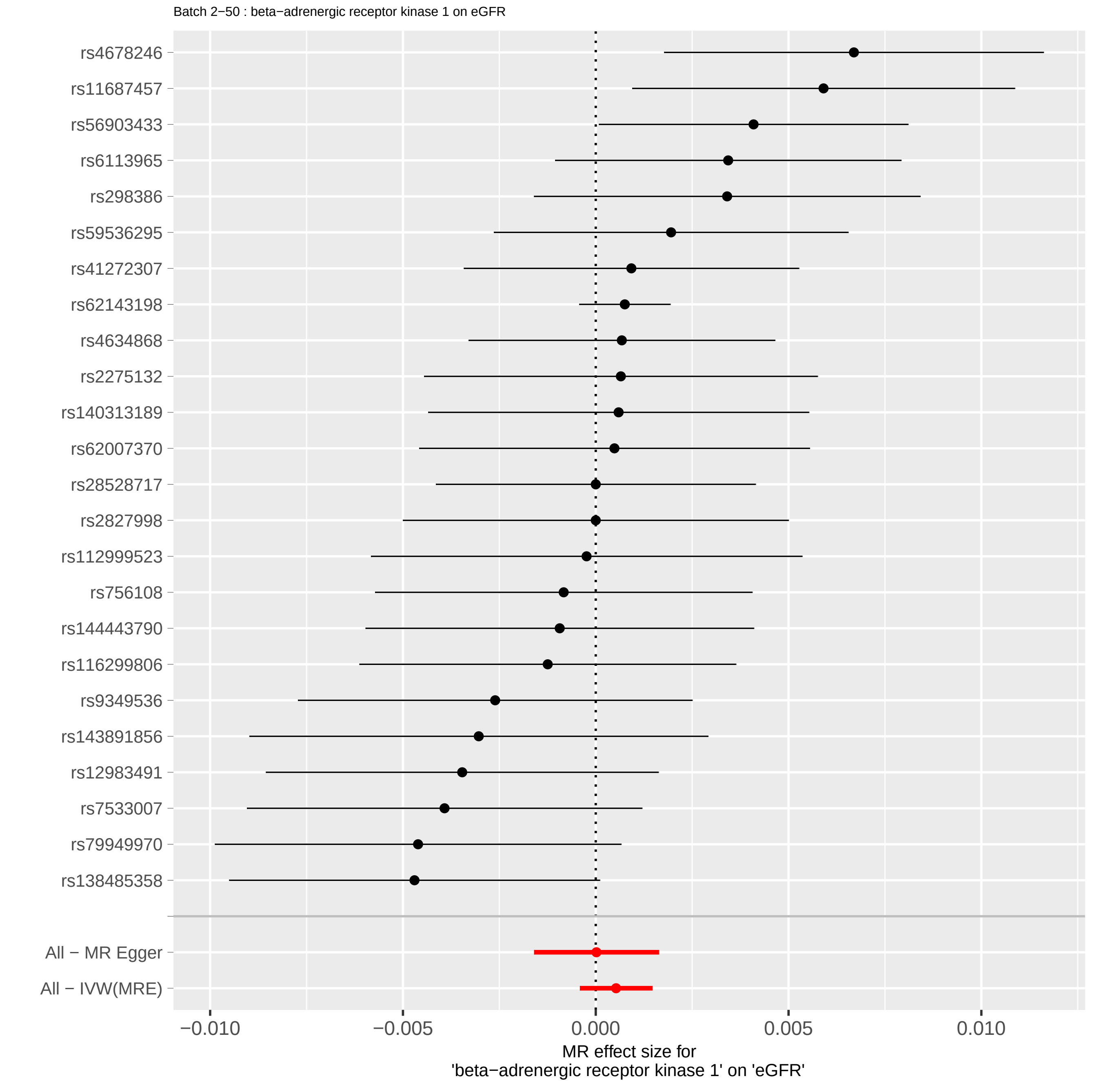

Batch 2−50 : beta−adrenergic receptor kinase 1 on eGFR
rs4678246
rs11687457
rs56903433
rs6113965
rs298386
rs59536295
rs41272307
rs62143198
rs4634868
rs2275132
rs140313189
rs62007370
rs28528717
rs2827998
rs112999523
rs756108
rs144443790
rs116299806
rs9349536
rs143891856
rs12983491
rs7533007
rs79949970
rs138485358
All − MR Egger
All − IVW(MRE)
−0.010
−0.005
0.000
0.005
0.010
MR effect size for
'beta−adrenergic receptor kinase 1' on 'eGFR'

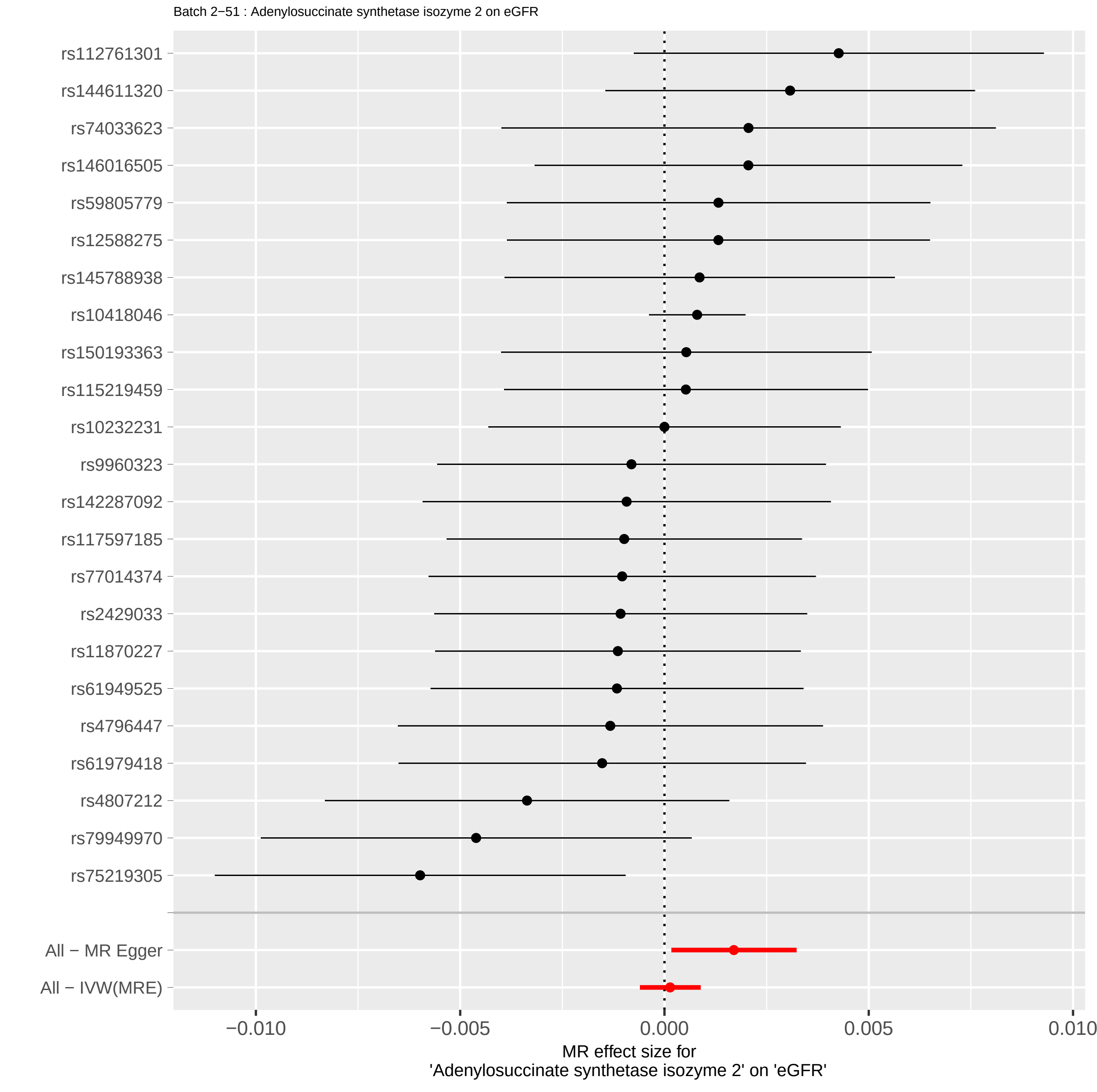

Batch 2−51 : Adenylosuccinate synthetase isozyme 2 on eGFR
rs112761301
rs144611320
rs74033623
rs146016505
rs59805779
rs12588275
rs145788938
rs10418046
rs150193363
rs115219459
rs10232231
rs9960323
rs142287092
rs117597185
rs77014374
rs2429033
rs11870227
rs61949525
rs4796447
rs61979418
rs4807212
rs79949970
rs75219305
All − MR Egger
All − IVW(MRE)
−0.010
−0.005
0.000
0.005
0.010
MR effect size for
'Adenylosuccinate synthetase isozyme 2' on 'eGFR'

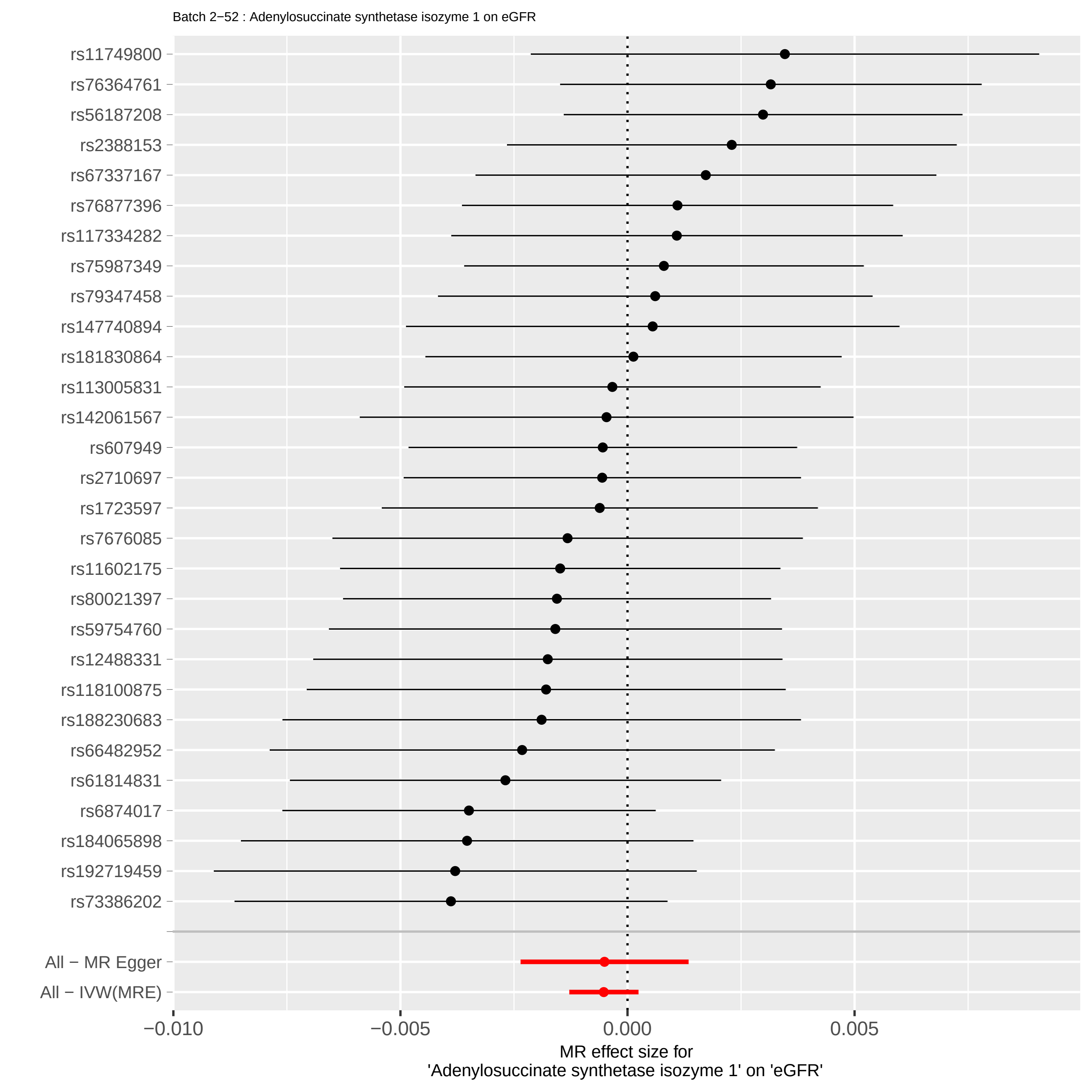

Batch 2−52 : Adenylosuccinate synthetase isozyme 1 on eGFR
rs11749800
rs76364761
rs56187208
rs2388153
rs67337167
rs76877396
rs117334282
rs75987349
rs79347458
rs147740894
rs181830864
rs113005831
rs142061567
rs607949
rs2710697
rs1723597
rs7676085
rs11602175
rs80021397
rs59754760
rs12488331
rs118100875
rs188230683
rs66482952
rs61814831
rs6874017
rs184065898
rs192719459
rs73386202
All − MR Egger
All − IVW(MRE)
−0.010
−0.005
0.000
0.005
MR effect size for
'Adenylosuccinate synthetase isozyme 1' on 'eGFR'

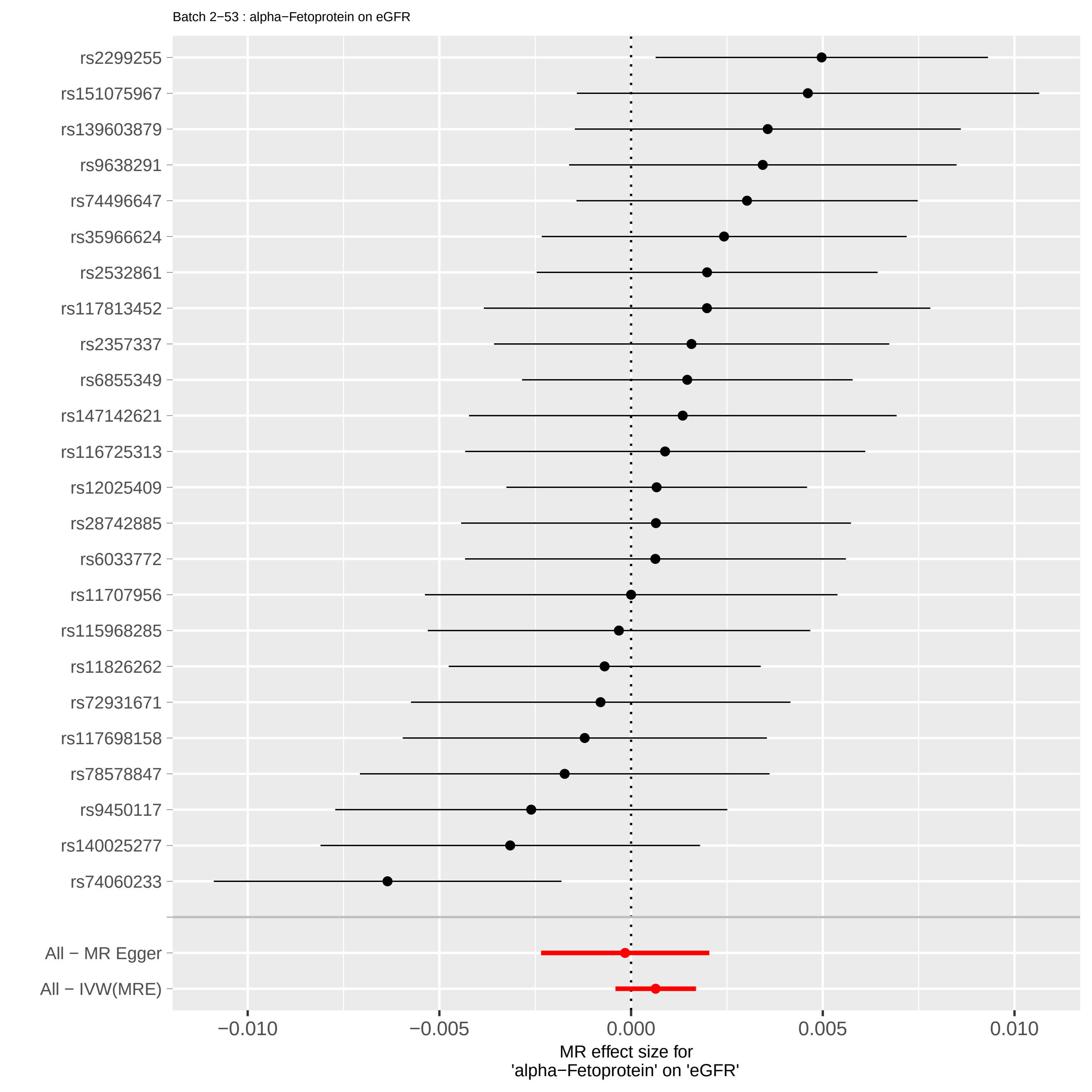
Batch 2−53 : alpha−Fetoprotein on eGFR
rs2299255
rs151075967
rs139603879
rs9638291
rs74496647
rs35966624
rs2532861
rs117813452
rs2357337
rs6855349
rs147142621
rs116725313
rs12025409
rs28742885
rs6033772
rs11707956
rs115968285
rs11826262
rs72931671
rs117698158
rs78578847
rs9450117
rs140025277
rs74060233
All − MR Egger
All − IVW(MRE)
−0.010
−0.005
0.000
0.005
0.010
MR effect size for
'alpha−Fetoprotein' on 'eGFR'

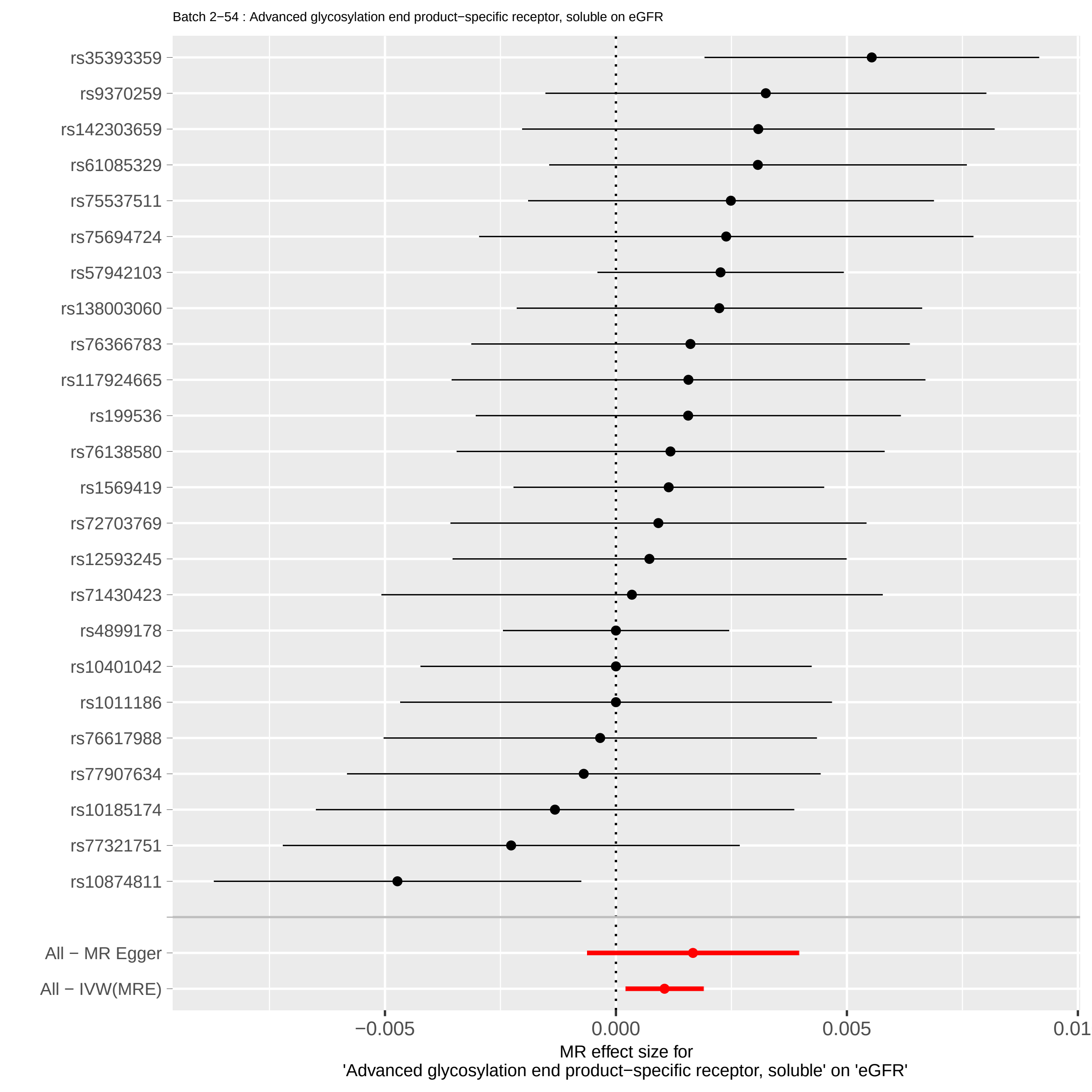

Batch 2−54 : Advanced glycosylation end product−specific receptor, soluble on eGFR
rs35393359
rs9370259
rs142303659
rs61085329
rs75537511
rs75694724
rs57942103
rs138003060
rs76366783
rs117924665
rs199536
rs76138580
rs1569419
rs72703769
rs12593245
rs71430423
rs4899178
rs10401042
rs1011186
rs76617988
rs77907634
rs10185174
rs77321751
rs10874811
All − MR Egger
All − IVW(MRE)
−0.005
0.000
0.005
0.01
MR effect size for
'Advanced glycosylation end product−specific receptor, soluble' on 'eGFR'

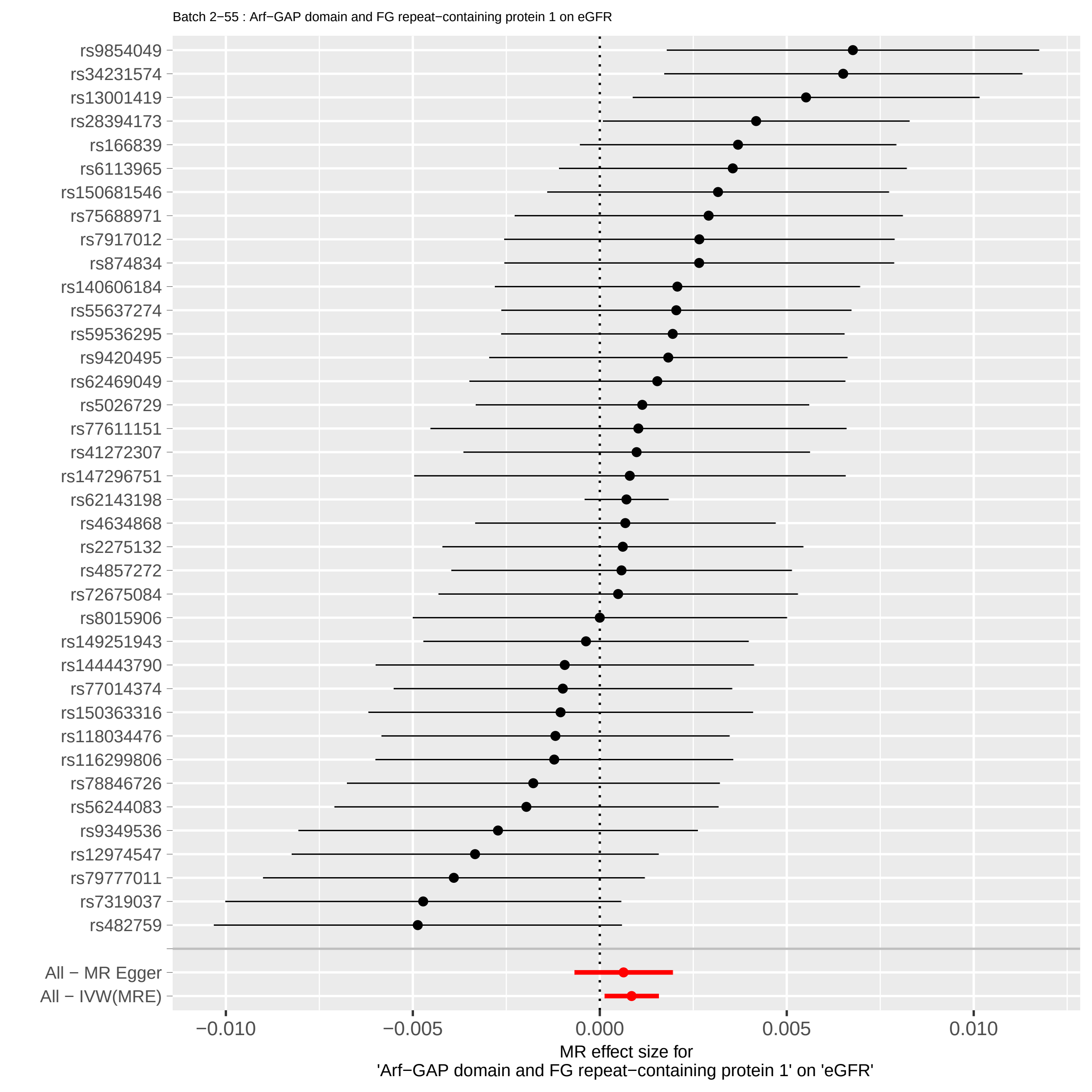
Batch 2−55 : Arf−GAP domain and FG repeat−containing protein 1 on eGFR
rs9854049
rs34231574
rs13001419
rs28394173
rs166839
rs6113965
rs150681546
rs75688971
rs7917012
rs874834
rs140606184
rs55637274
rs59536295
rs9420495
rs62469049
rs5026729
rs77611151
rs41272307
rs147296751
rs62143198
rs4634868
rs2275132
rs4857272
rs72675084
rs8015906
rs149251943
rs144443790
rs77014374
rs150363316
rs118034476
rs116299806
rs78846726
rs56244083
rs9349536
rs12974547
rs79777011
rs7319037
rs482759
All − MR Egger
All − IVW(MRE)
−0.010
−0.005
0.000
0.005
0.010
MR effect size for
'Arf−GAP domain and FG repeat−containing protein 1' on 'eGFR'

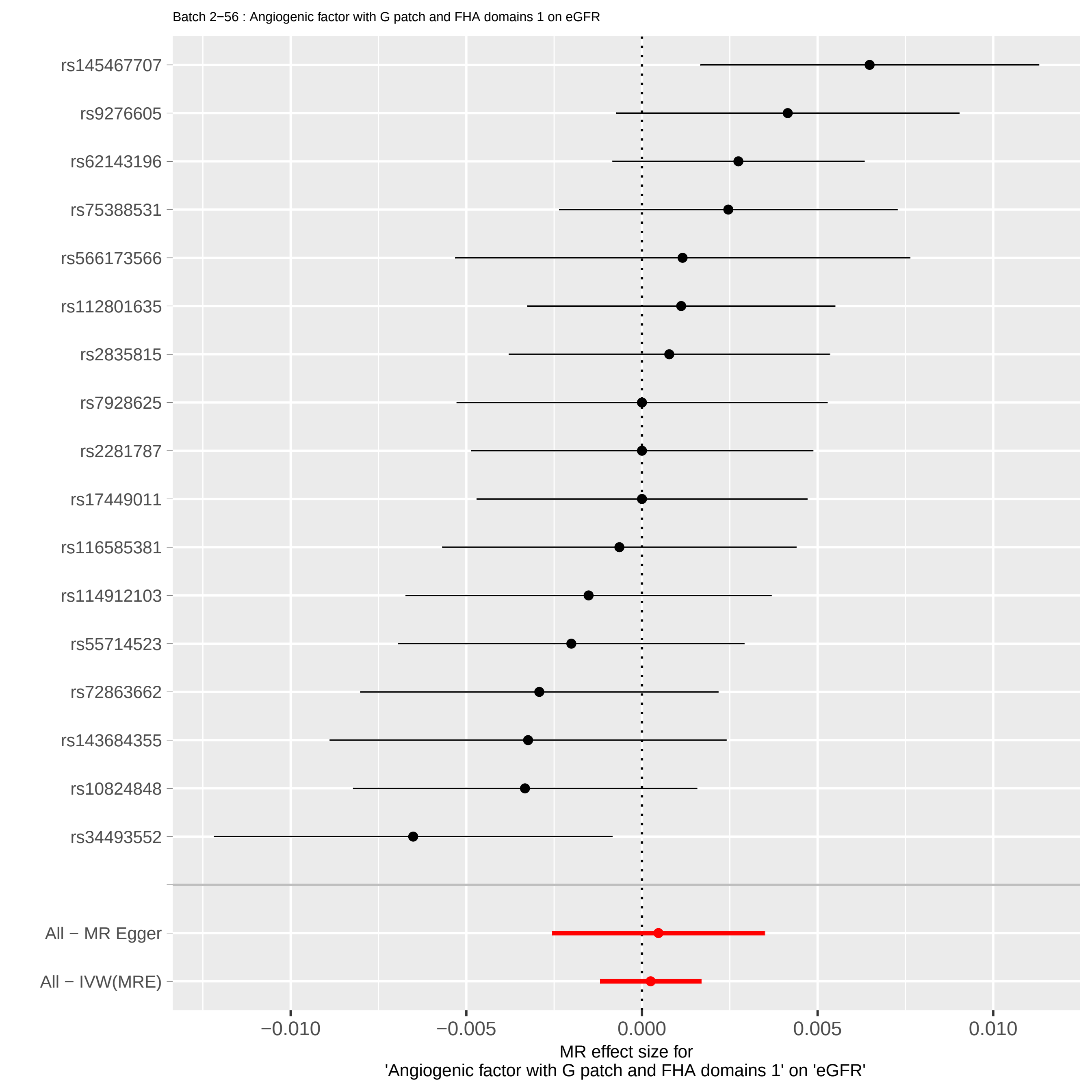

Batch 2−56 : Angiogenic factor with G patch and FHA domains 1 on eGFR
rs145467707
rs9276605
rs62143196
rs75388531
rs566173566
rs112801635
rs2835815
rs7928625
rs2281787
rs17449011
rs116585381
rs114912103
rs55714523
rs72863662
rs143684355
rs10824848
rs34493552
All − MR Egger
All − IVW(MRE)
−0.010
−0.005
0.000
0.005
0.010
MR effect size for
'Angiogenic factor with G patch and FHA domains 1' on 'eGFR'

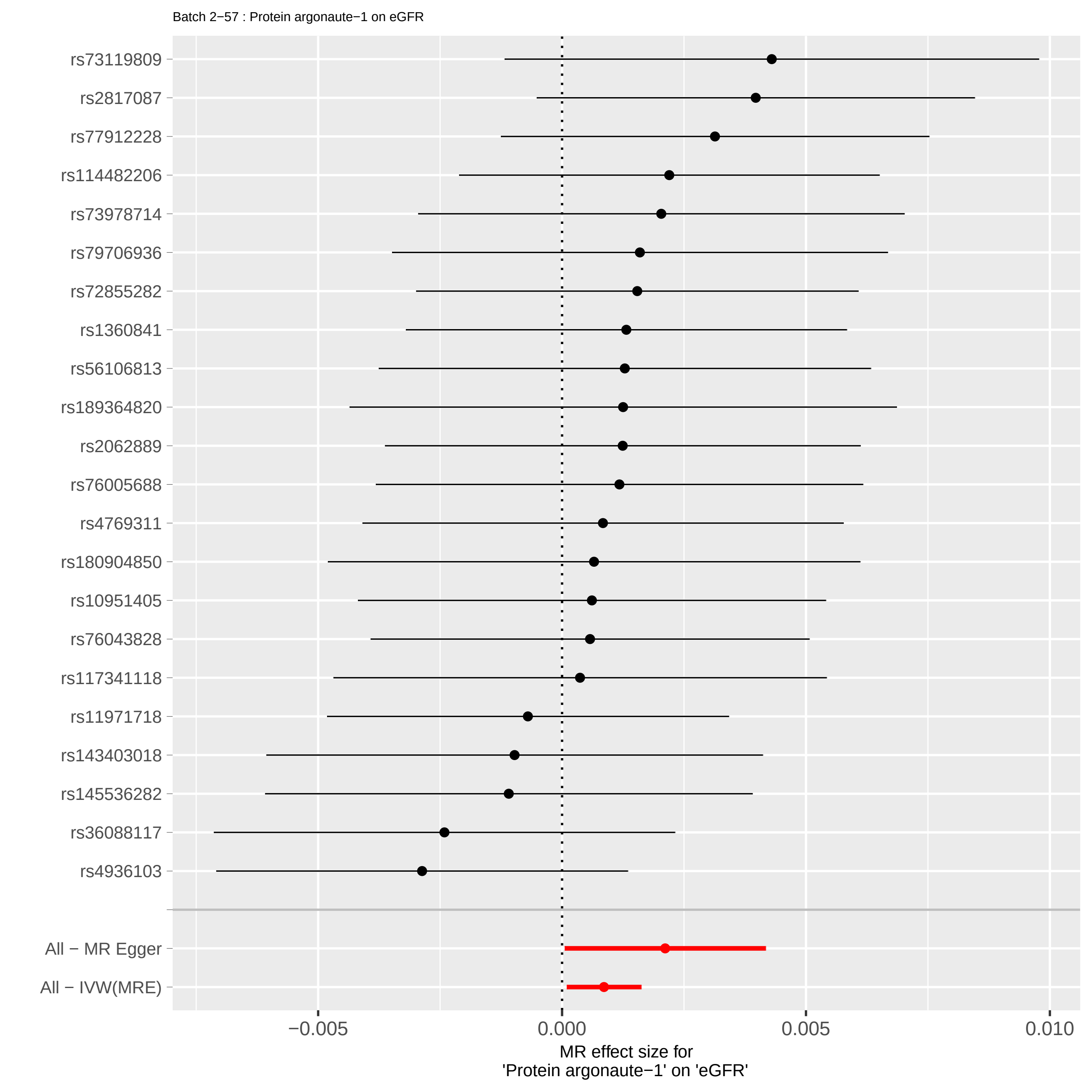

Batch 2−57 : Protein argonaute−1 on eGFR
rs73119809
rs2817087
rs77912228
rs114482206
rs73978714
rs79706936
rs72855282
rs1360841
rs56106813
rs189364820
rs2062889
rs76005688
rs4769311
rs180904850
rs10951405
rs76043828
rs117341118
rs11971718
rs143403018
rs145536282
rs36088117
rs4936103
All − MR Egger
All − IVW(MRE)
−0.005
0.000
0.005
0.010
MR effect size for
'Protein argonaute−1' on 'eGFR'

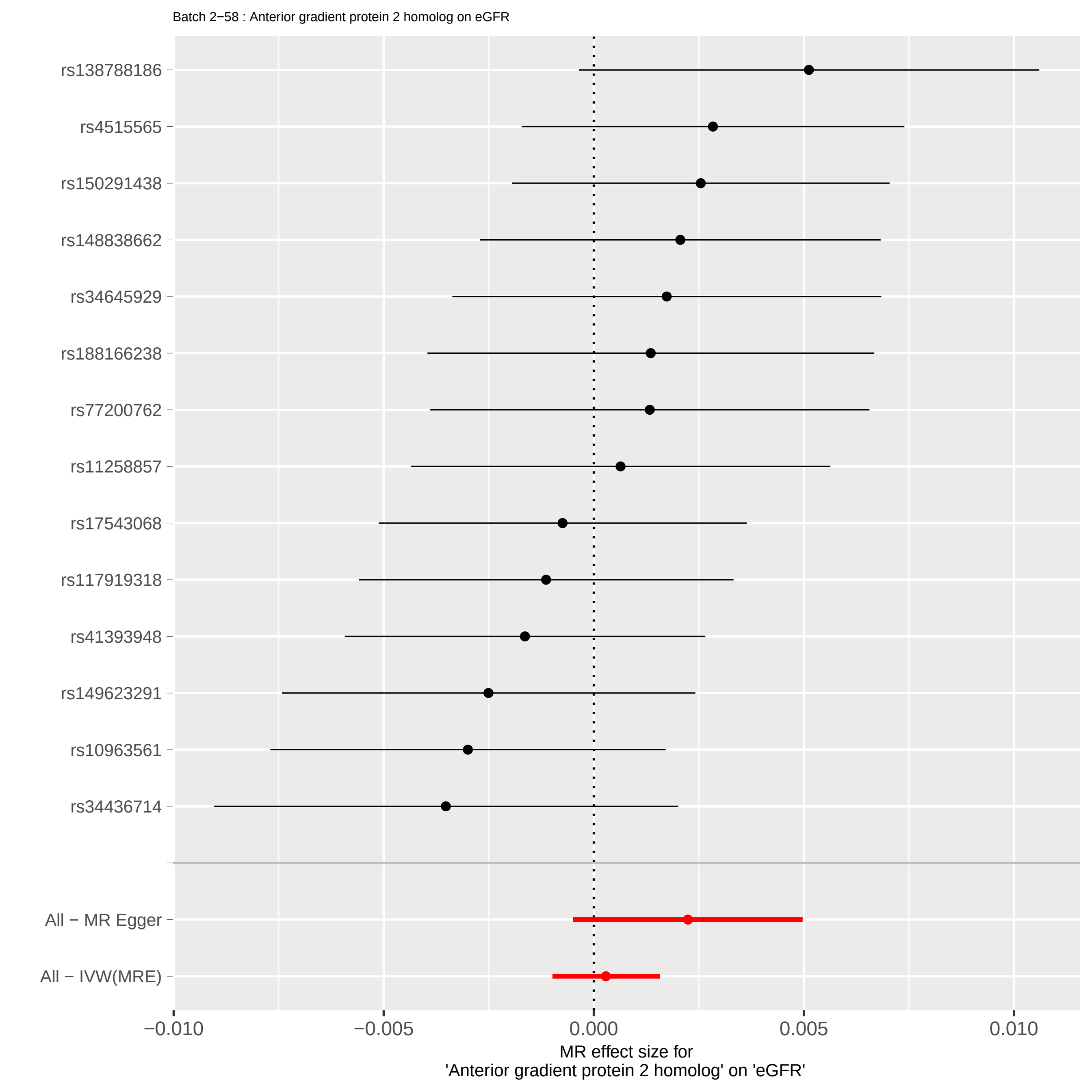

Batch 2−58 : Anterior gradient protein 2 homolog on eGFR
rs138788186
rs4515565
rs150291438
rs148838662
rs34645929
rs188166238
rs77200762
rs11258857
rs17543068
rs117919318
rs41393948
rs149623291
rs10963561
rs34436714
All − MR Egger
All − IVW(MRE)
−0.010
−0.005
0.000
0.005
0.010
MR effect size for
'Anterior gradient protein 2 homolog' on 'eGFR'

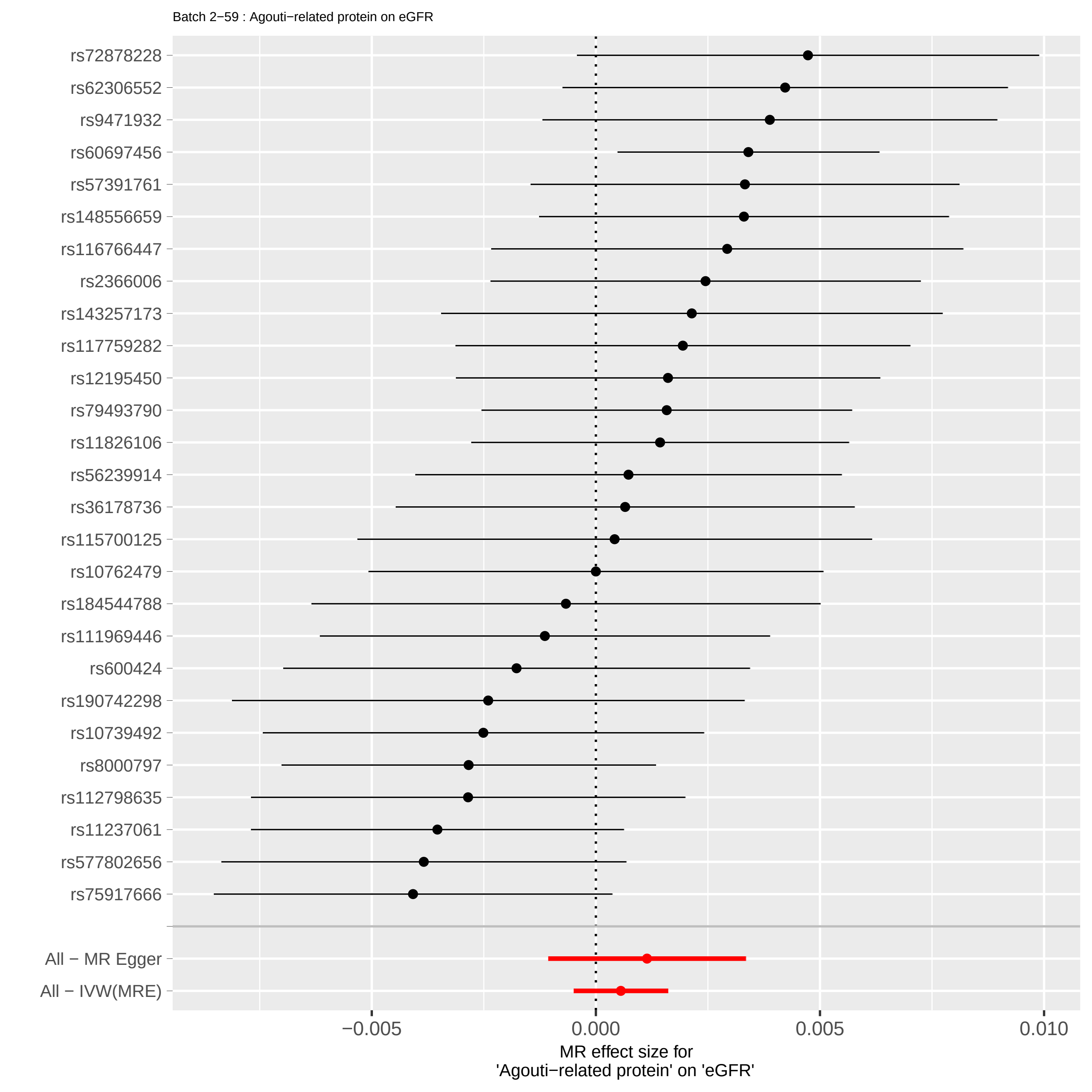

Batch 2−59 : Agouti−related protein on eGFR
rs72878228
rs62306552
rs9471932
rs60697456
rs57391761
rs148556659
rs116766447
rs2366006
rs143257173
rs117759282
rs12195450
rs79493790
rs11826106
rs56239914
rs36178736
rs115700125
rs10762479
rs184544788
rs111969446
rs600424
rs190742298
rs10739492
rs8000797
rs112798635
rs11237061
rs577802656
rs75917666
All − MR Egger
All − IVW(MRE)
−0.005
0.000
0.005
0.010
MR effect size for
'Agouti−related protein' on 'eGFR'

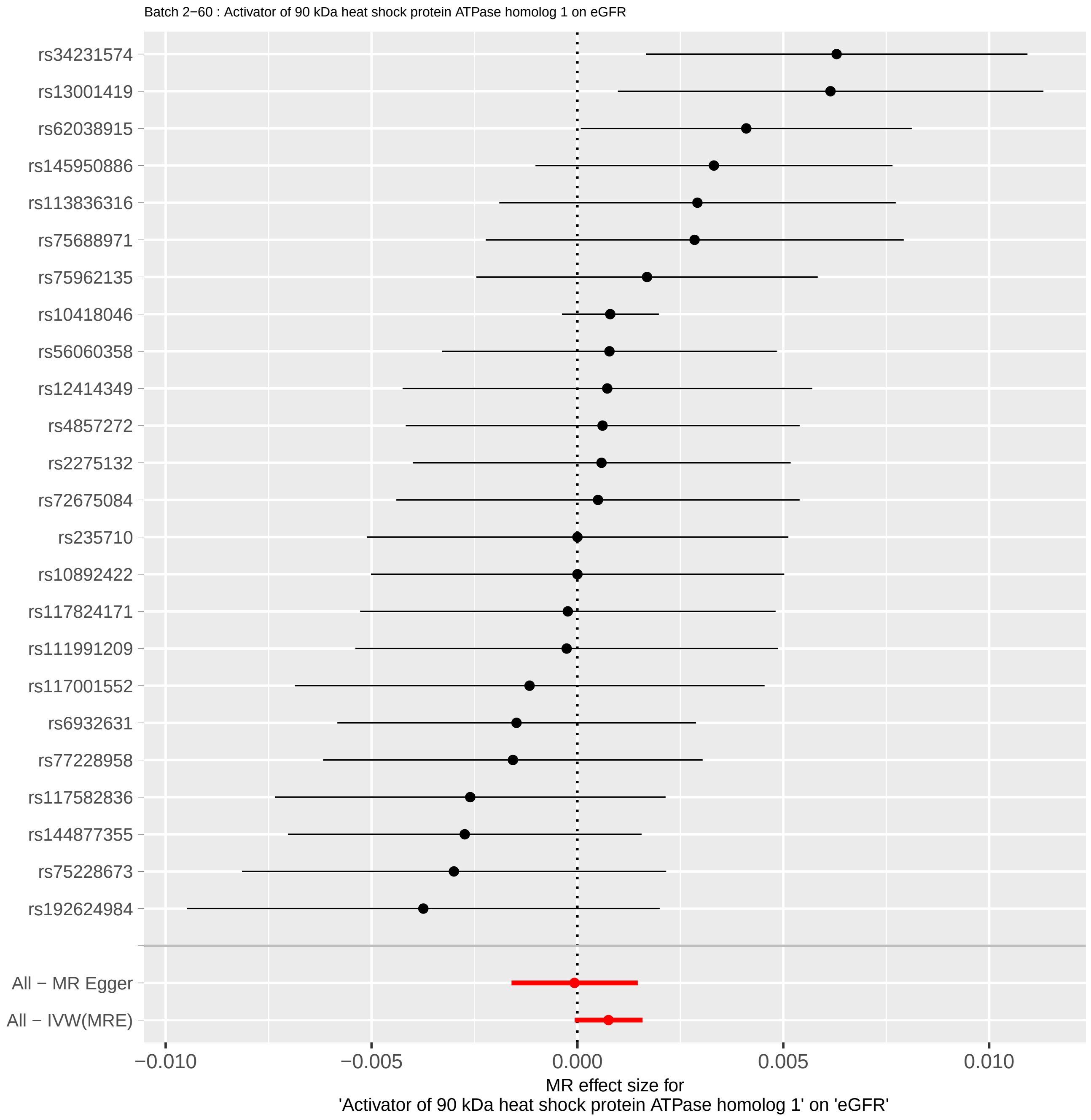
Batch 2−60 : Activator of 90 kDa heat shock protein ATPase homolog 1 on eGFR
rs34231574
rs13001419
rs62038915
rs145950886
rs113836316
rs75688971
rs75962135
rs10418046
rs56060358
rs12414349
rs4857272
rs2275132
rs72675084
rs235710
rs10892422
rs117824171
rs111991209
rs117001552
rs6932631
rs77228958
rs117582836
rs144877355
rs75228673
rs192624984
All − MR Egger
All − IVW(MRE)
−0.010
−0.005
0.000
0.005
0.010
MR effect size for
'Activator of 90 kDa heat shock protein ATPase homolog 1' on 'eGFR'

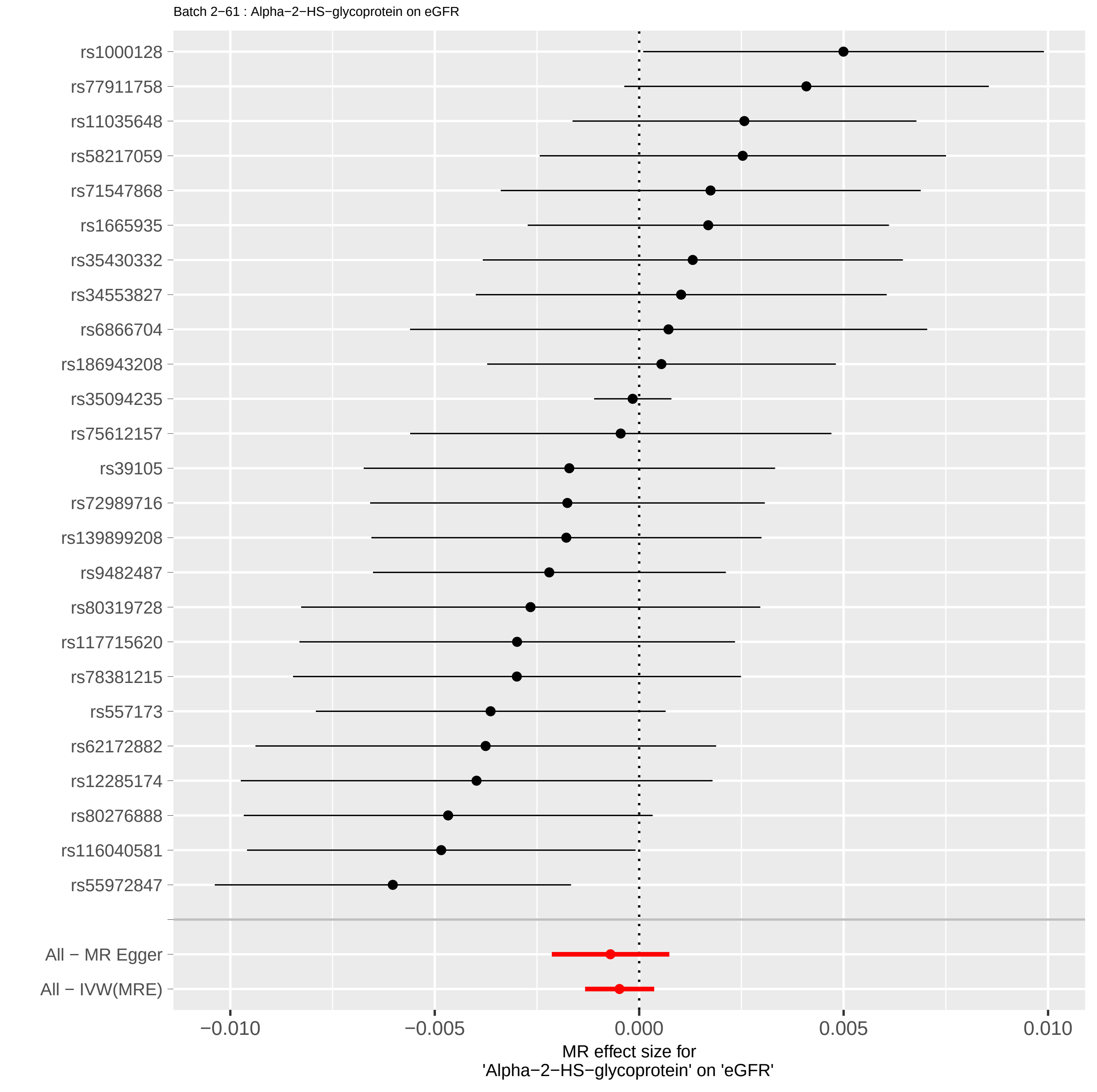

Batch 2−61 : Alpha−2−HS−glycoprotein on eGFR
rs1000128
rs77911758
rs11035648
rs58217059
rs71547868
rs1665935
rs35430332
rs34553827
rs6866704
rs186943208
rs35094235
rs75612157
rs39105
rs72989716
rs139899208
rs9482487
rs80319728
rs117715620
rs78381215
rs557173
rs62172882
rs12285174
rs80276888
rs116040581
rs55972847
All − MR Egger
All − IVW(MRE)
−0.010
−0.005
0.000
0.005
0.010
MR effect size for
'Alpha−2−HS−glycoprotein' on 'eGFR'

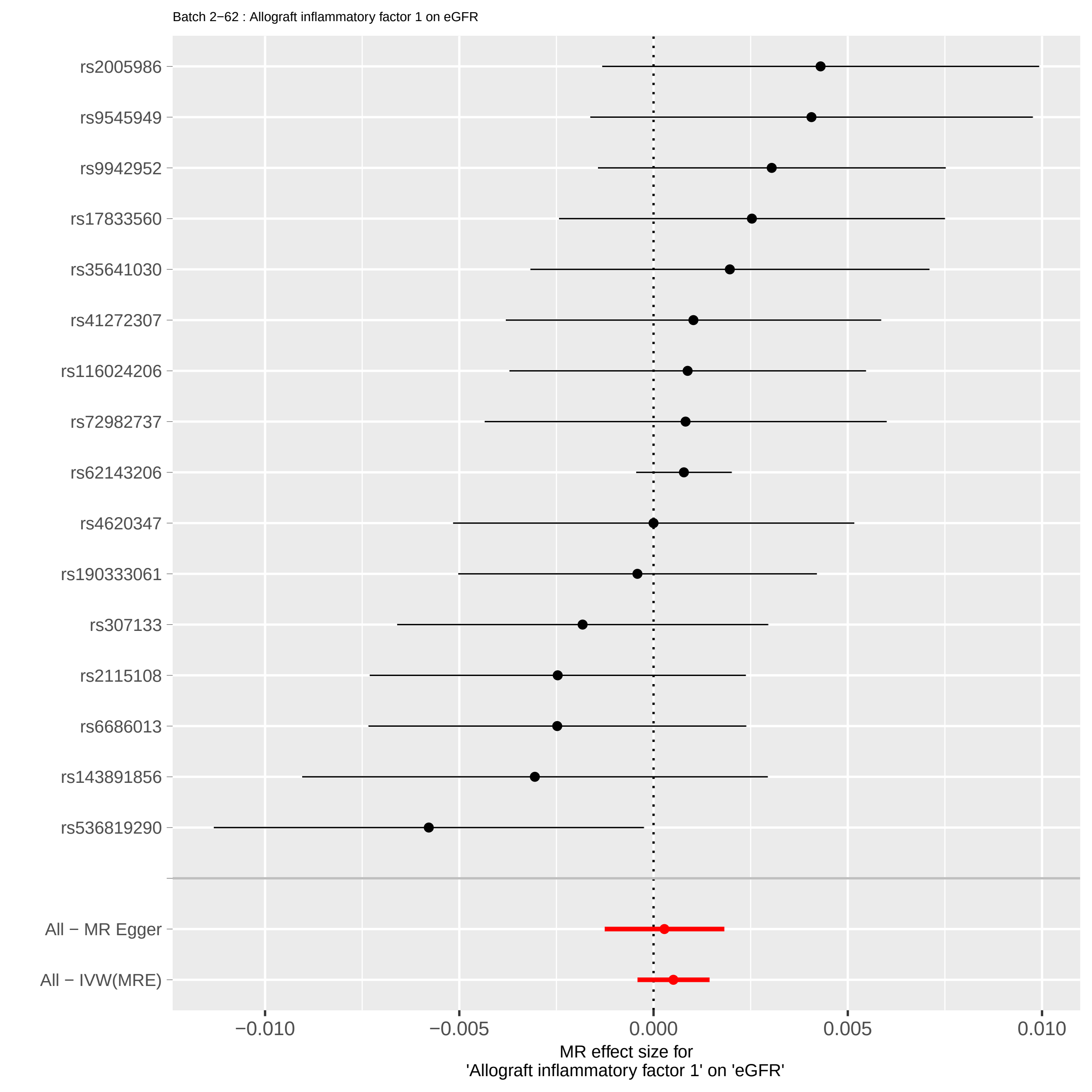
Batch 2−62 : Allograft inflammatory factor 1 on eGFR
rs2005986
rs9545949
rs9942952
rs17833560
rs35641030
rs41272307
rs116024206
rs72982737
rs62143206
rs4620347
rs190333061
rs307133
rs2115108
rs6686013
rs143891856
rs536819290
All − MR Egger
All − IVW(MRE)
−0.010
−0.005
0.000
0.005
0.010
MR effect size for
'Allograft inflammatory factor 1' on 'eGFR'

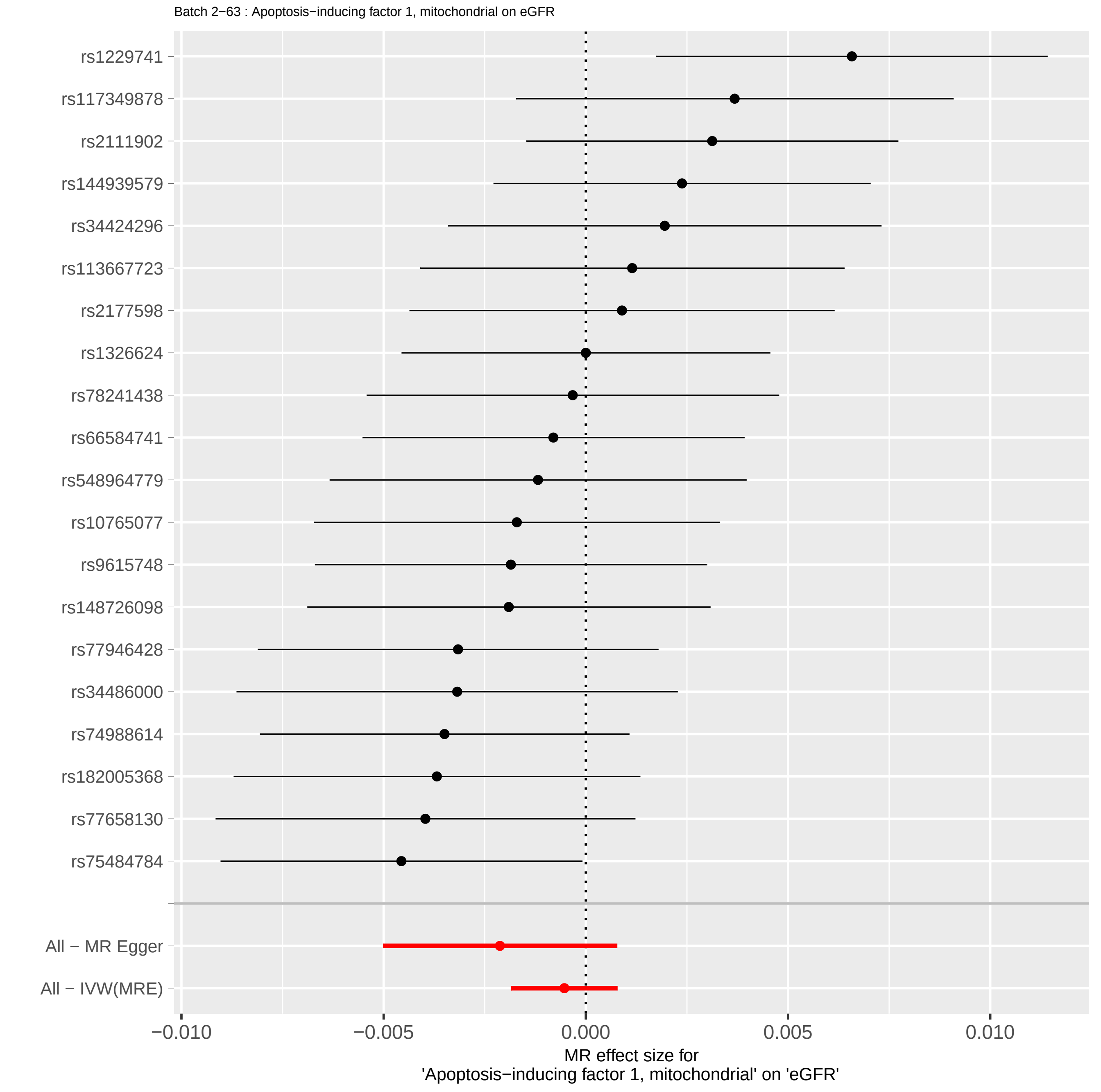

Batch 2−63 : Apoptosis−inducing factor 1, mitochondrial on eGFR
rs1229741
rs117349878
rs2111902
rs144939579
rs34424296
rs113667723
rs2177598
rs1326624
rs78241438
rs66584741
rs548964779
rs10765077
rs9615748
rs148726098
rs77946428
rs34486000
rs74988614
rs182005368
rs77658130
rs75484784
All − MR Egger
All − IVW(MRE)
−0.010
−0.005
0.000
0.005
0.010
MR effect size for
'Apoptosis−inducing factor 1, mitochondrial' on 'eGFR'

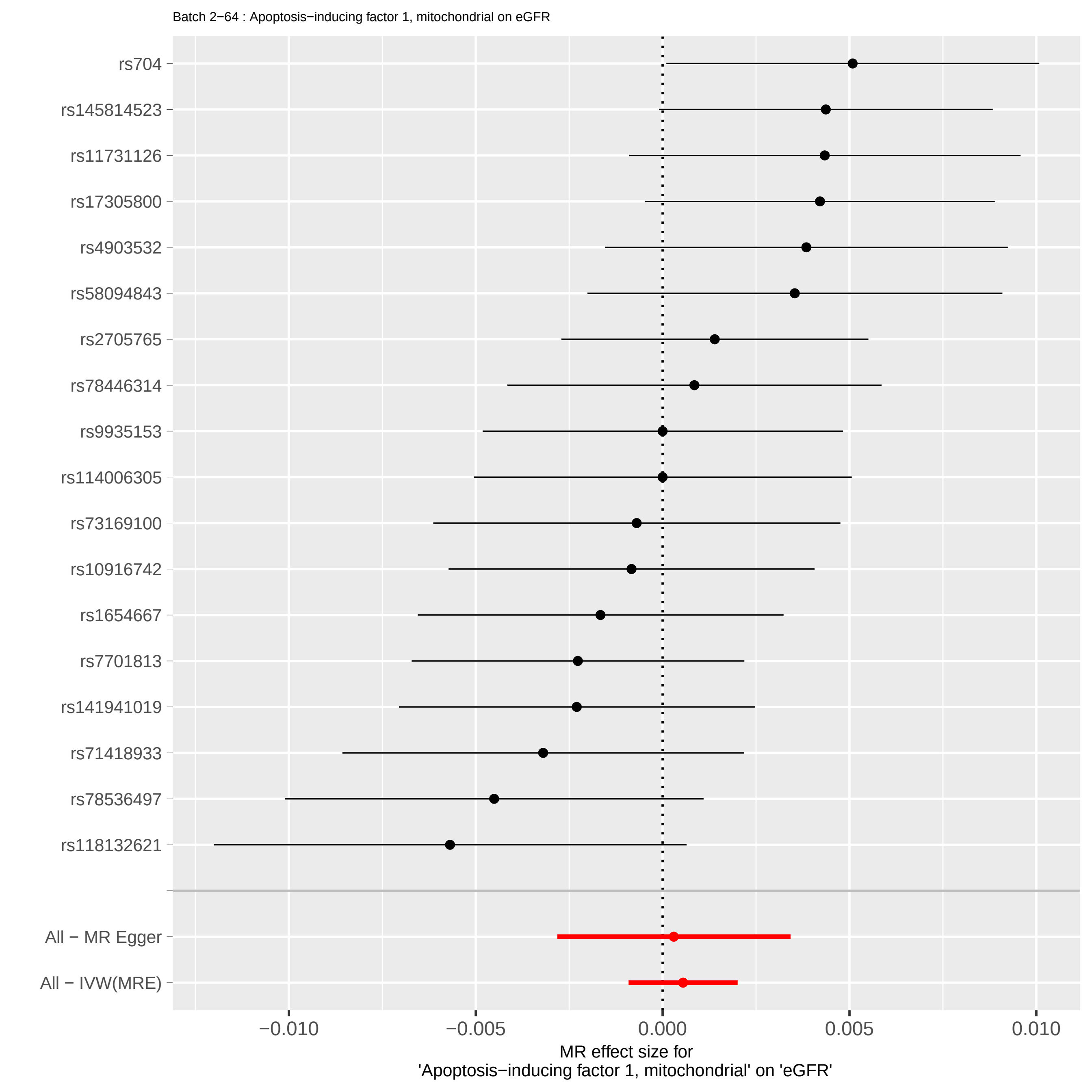

Batch 2−64 : Apoptosis−inducing factor 1, mitochondrial on eGFR
rs704
rs145814523
rs11731126
rs17305800
rs4903532
rs58094843
rs2705765
rs78446314
rs9935153
rs114006305
rs73169100
rs10916742
rs1654667
rs7701813
rs141941019
rs71418933
rs78536497
rs118132621
All − MR Egger
All − IVW(MRE)
−0.010
−0.005
0.000
0.005
0.010
MR effect size for
'Apoptosis−inducing factor 1, mitochondrial' on 'eGFR'

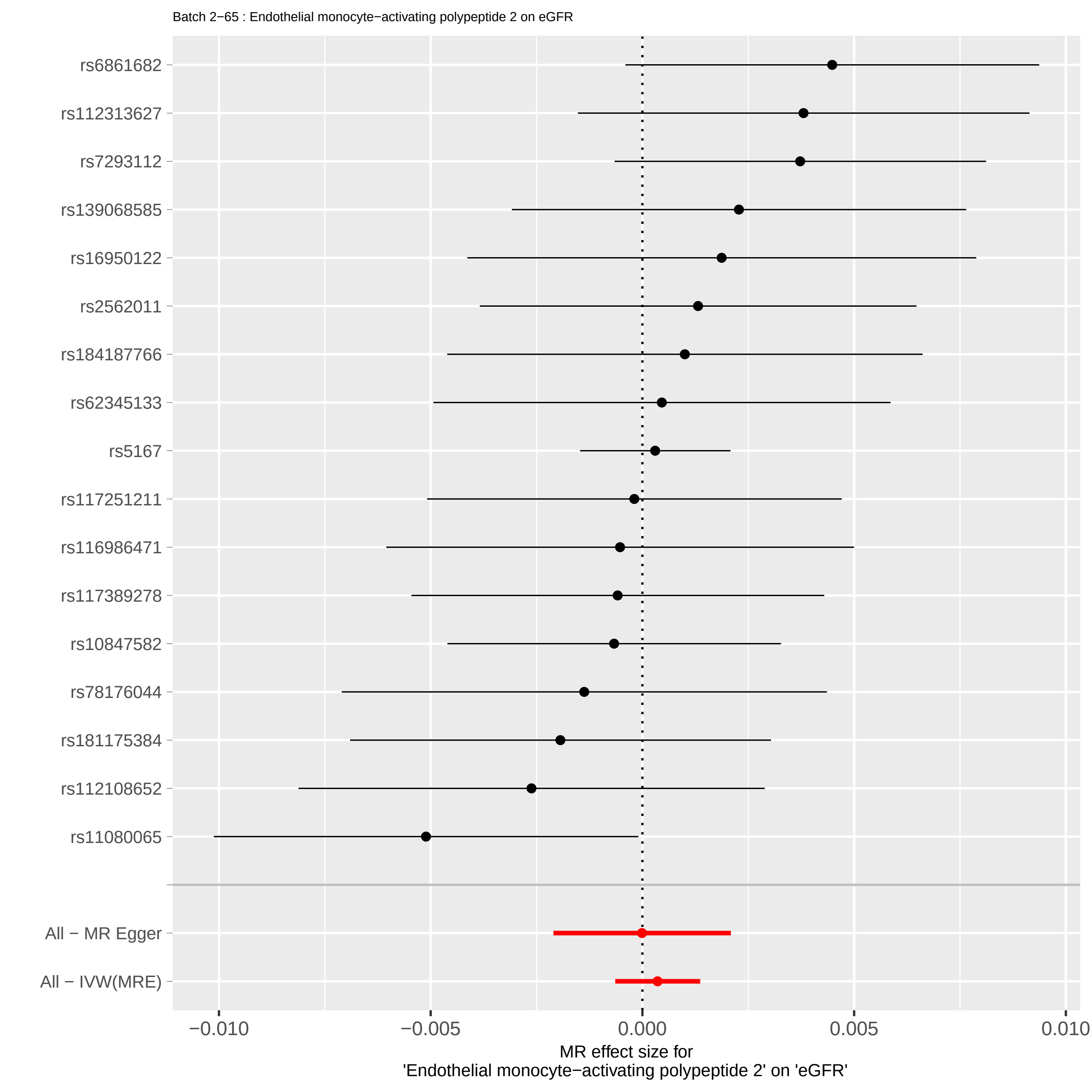
Batch 2−65 : Endothelial monocyte−activating polypeptide 2 on eGFR
rs6861682
rs112313627
rs7293112
rs139068585
rs16950122
rs2562011
rs184187766
rs62345133
rs5167
rs117251211
rs116986471
rs117389278
rs10847582
rs78176044
rs181175384
rs112108652
rs11080065
All − MR Egger
All − IVW(MRE)
−0.010
−0.005
0.000
0.005
0.010
MR effect size for
'Endothelial monocyte−activating polypeptide 2' on 'eGFR'

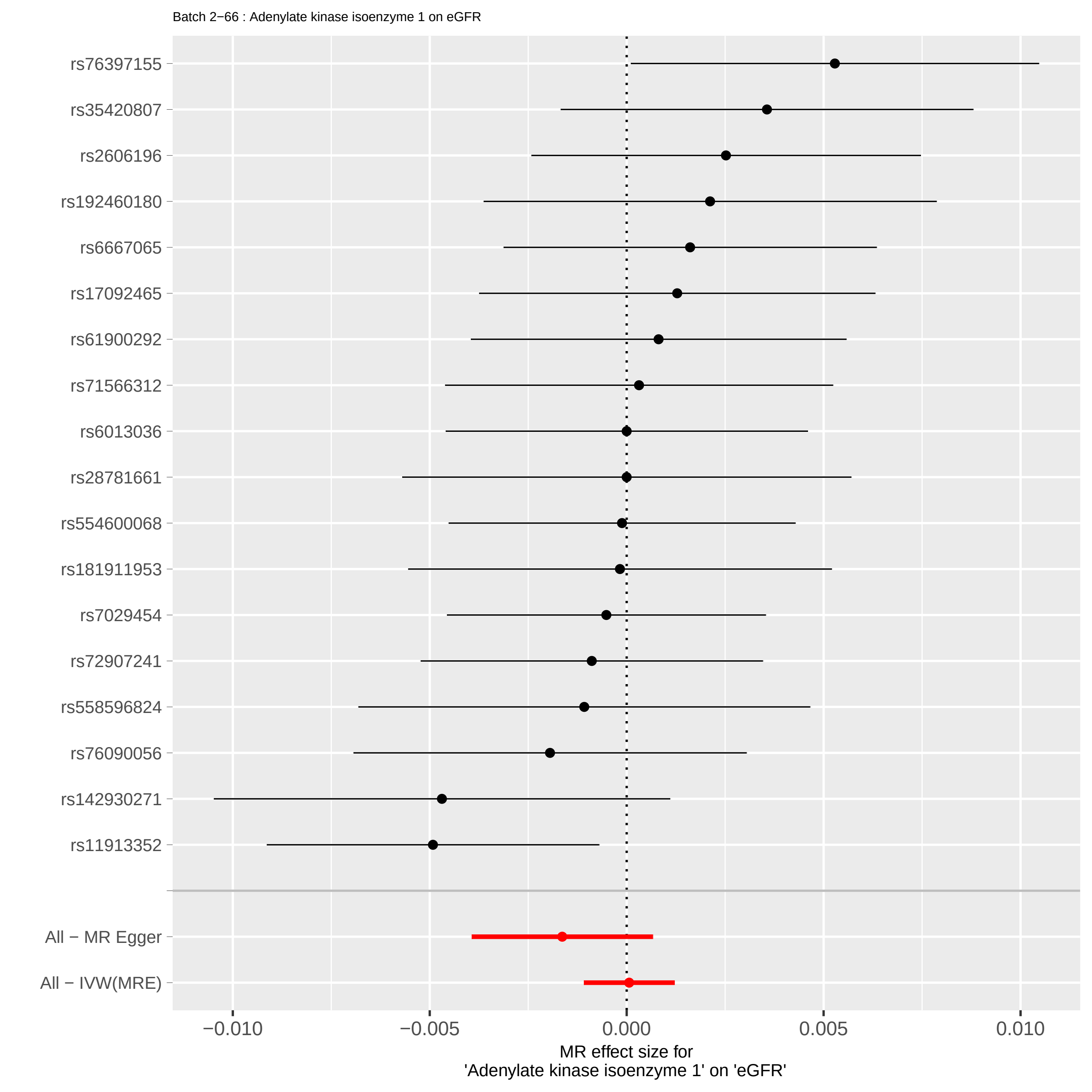
Batch 2−66 : Adenylate kinase isoenzyme 1 on eGFR
rs76397155
rs35420807
rs2606196
rs192460180
rs6667065
rs17092465
rs61900292
rs71566312
rs6013036
rs28781661
rs554600068
rs181911953
rs7029454
rs72907241
rs558596824
rs76090056
rs142930271
rs11913352
All − MR Egger
All − IVW(MRE)
−0.010
−0.005
0.000
0.005
0.010
MR effect size for
'Adenylate kinase isoenzyme 1' on 'eGFR'

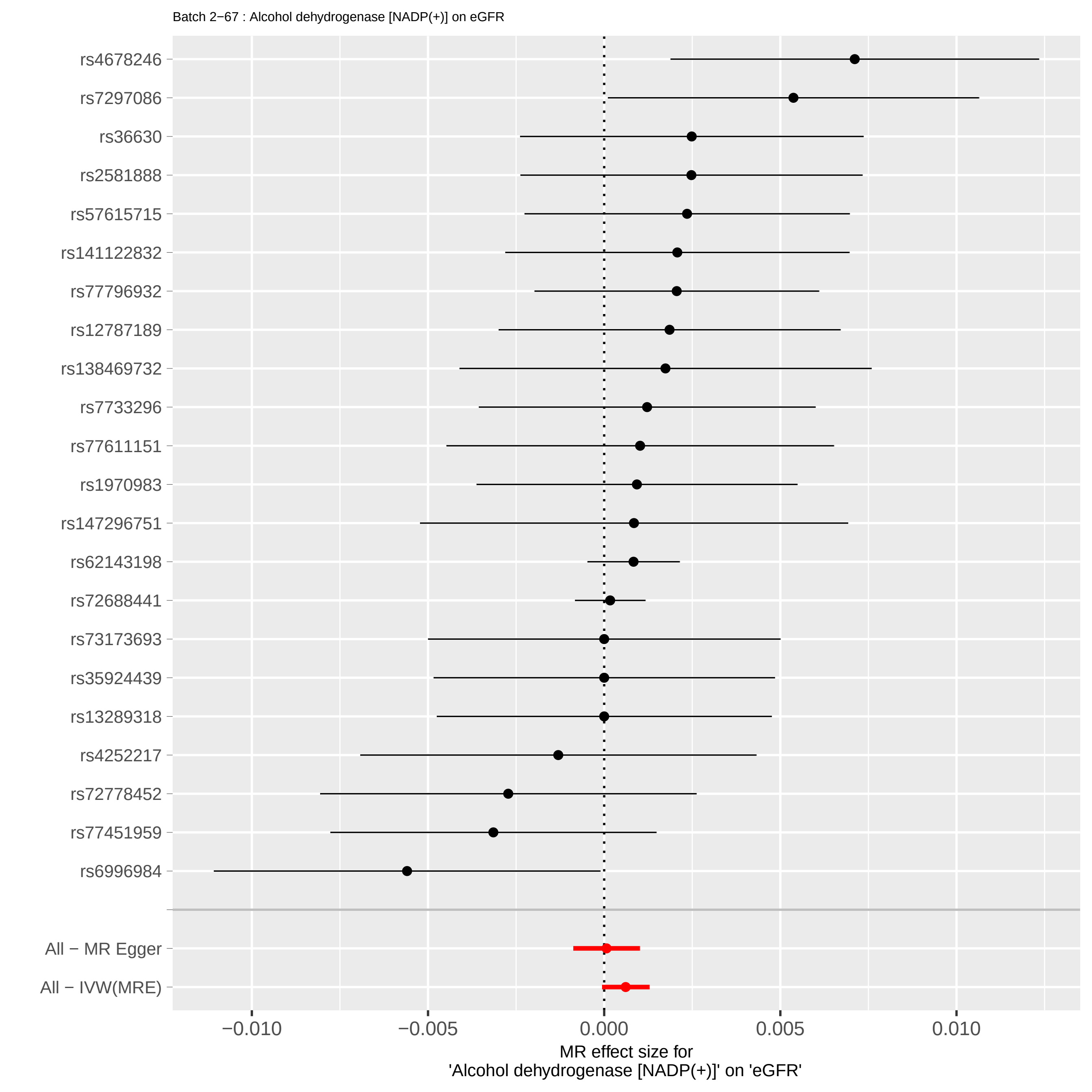
Batch 2−67 : Alcohol dehydrogenase [NADP(+)] on eGFR
rs4678246
rs7297086
rs36630
rs2581888
rs57615715
rs141122832
rs77796932
rs12787189
rs138469732
rs7733296
rs77611151
rs1970983
rs147296751
rs62143198
rs72688441
rs73173693
rs35924439
rs13289318
rs4252217
rs72778452
rs77451959
rs6996984
All − MR Egger
All − IVW(MRE)
−0.010
−0.005
0.000
0.005
0.010
MR effect size for
'Alcohol dehydrogenase [NADP(+)]' on 'eGFR'

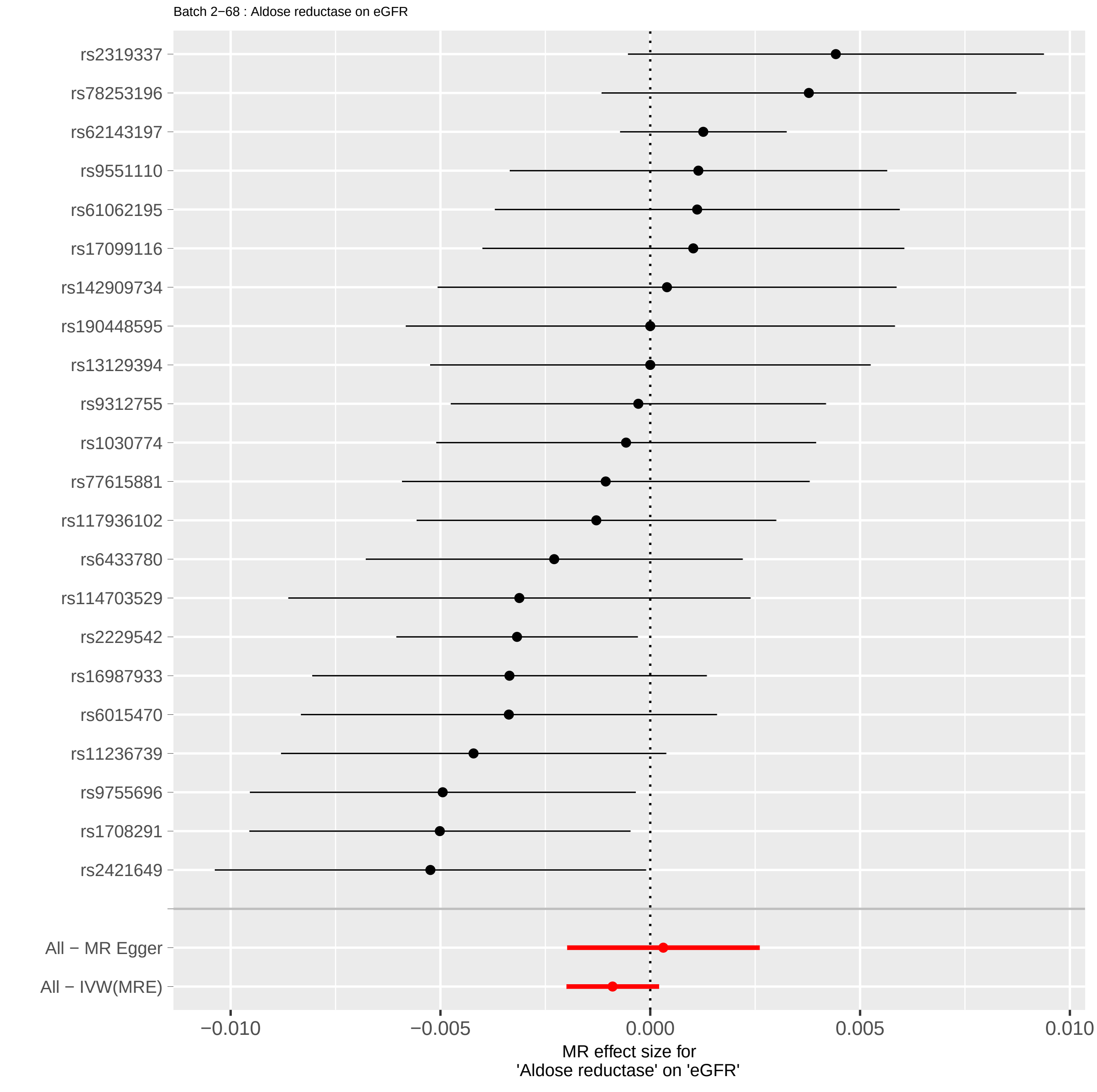
Batch 2−68 : Aldose reductase on eGFR
rs2319337
rs78253196
rs62143197
rs9551110
rs61062195
rs17099116
rs142909734
rs190448595
rs13129394
rs9312755
rs1030774
rs77615881
rs117936102
rs6433780
rs114703529
rs2229542
rs16987933
rs6015470
rs11236739
rs9755696
rs1708291
rs2421649
All − MR Egger
All − IVW(MRE)
−0.010
−0.005
0.000
0.005
0.010
MR effect size for
'Aldose reductase' on 'eGFR'

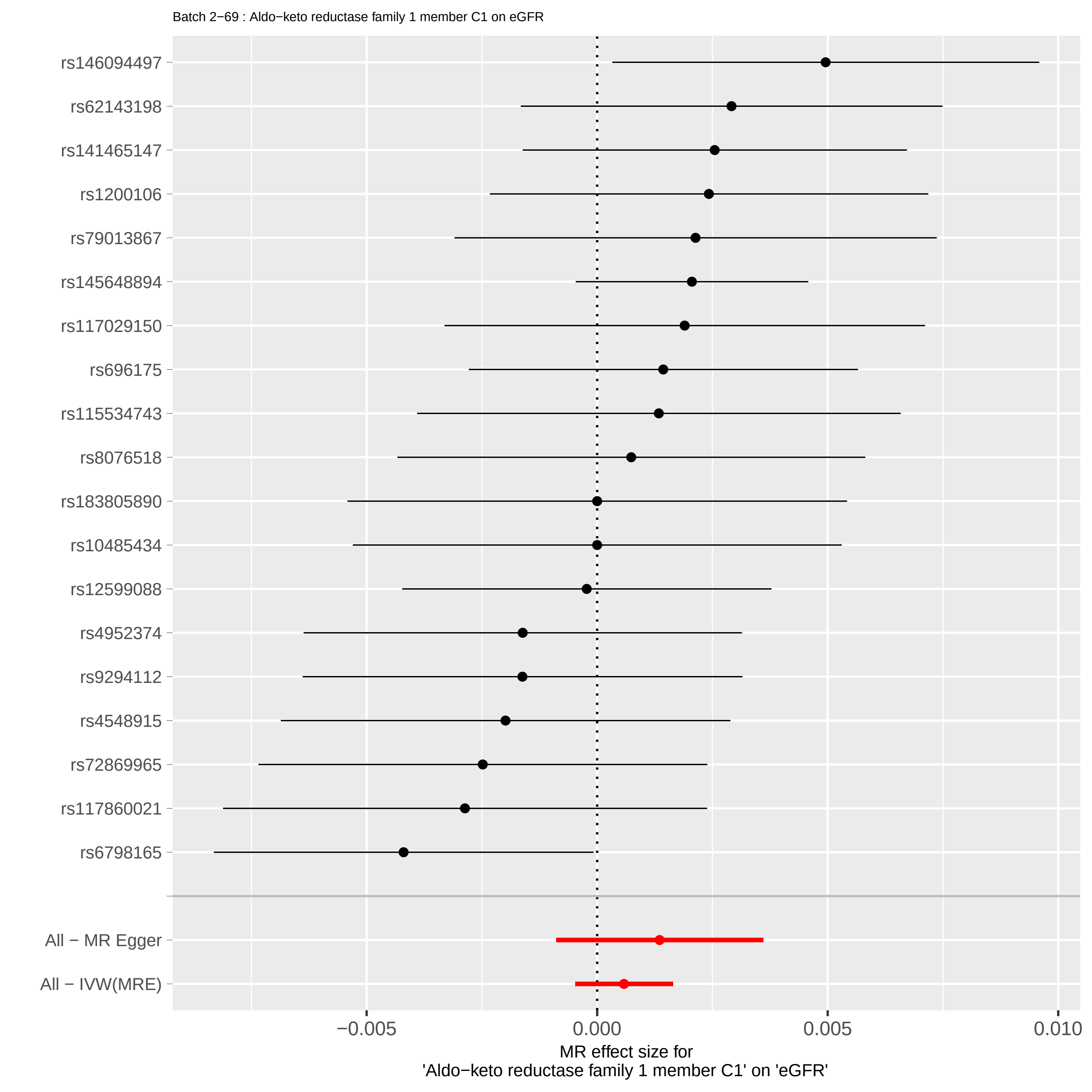

Batch 2−69 : Aldo−keto reductase family 1 member C1 on eGFR
rs146094497
rs62143198
rs141465147
rs1200106
rs79013867
rs145648894
rs117029150
rs696175
rs115534743
rs8076518
rs183805890
rs10485434
rs12599088
rs4952374
rs9294112
rs4548915
rs72869965
rs117860021
rs6798165
All − MR Egger
All − IVW(MRE)
−0.005
0.000
0.005
0.010
MR effect size for
'Aldo−keto reductase family 1 member C1' on 'eGFR'

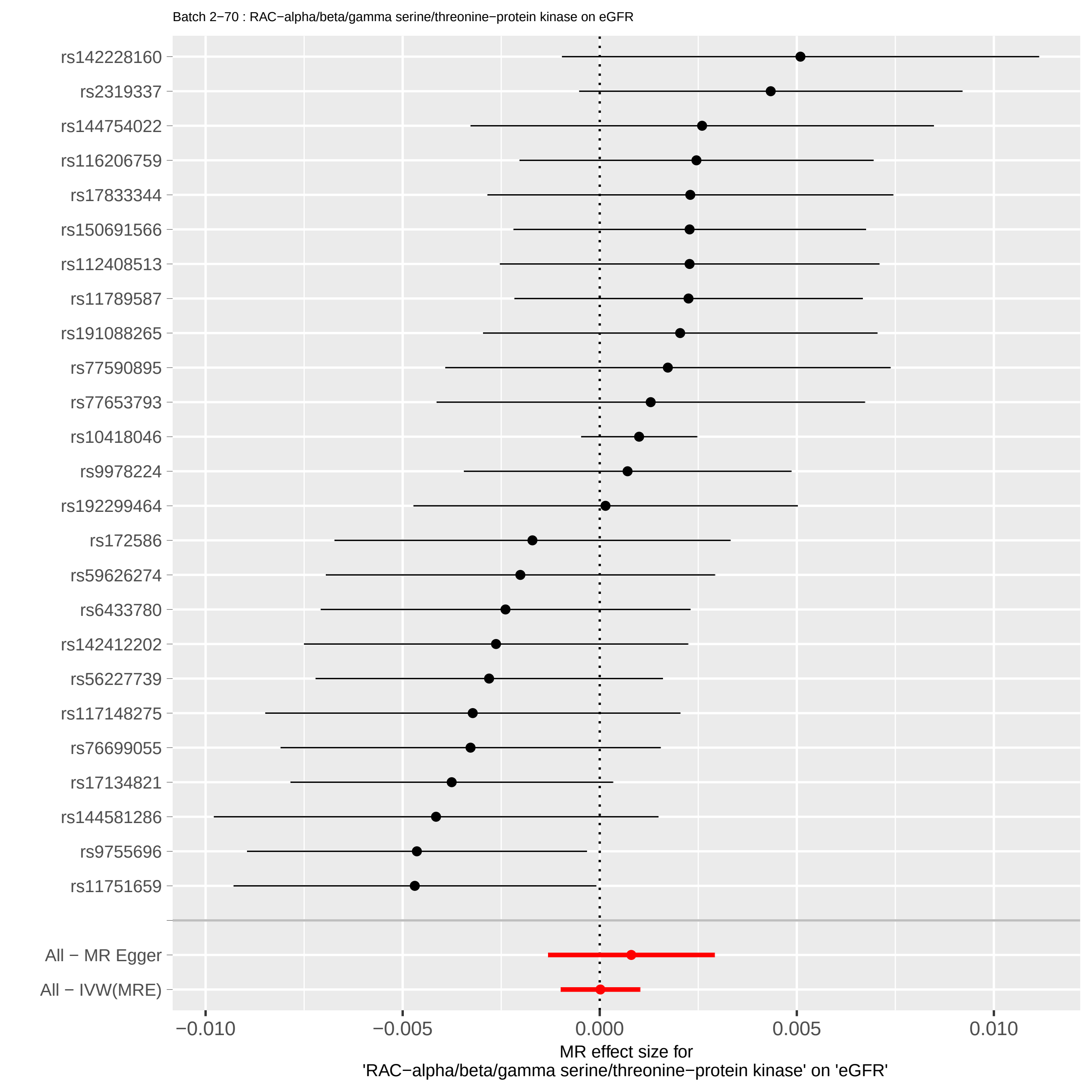

Batch 2−70 : RAC−alpha/beta/gamma serine/threonine−protein kinase on eGFR
rs142228160
rs2319337
rs144754022
rs116206759
rs17833344
rs150691566
rs112408513
rs11789587
rs191088265
rs77590895
rs77653793
rs10418046
rs9978224
rs192299464
rs172586
rs59626274
rs6433780
rs142412202
rs56227739
rs117148275
rs76699055
rs17134821
rs144581286
rs9755696
rs11751659
All − MR Egger
All − IVW(MRE)
−0.010
−0.005
0.000
0.005
0.010
MR effect size for
'RAC−alpha/beta/gamma serine/threonine−protein kinase' on 'eGFR'

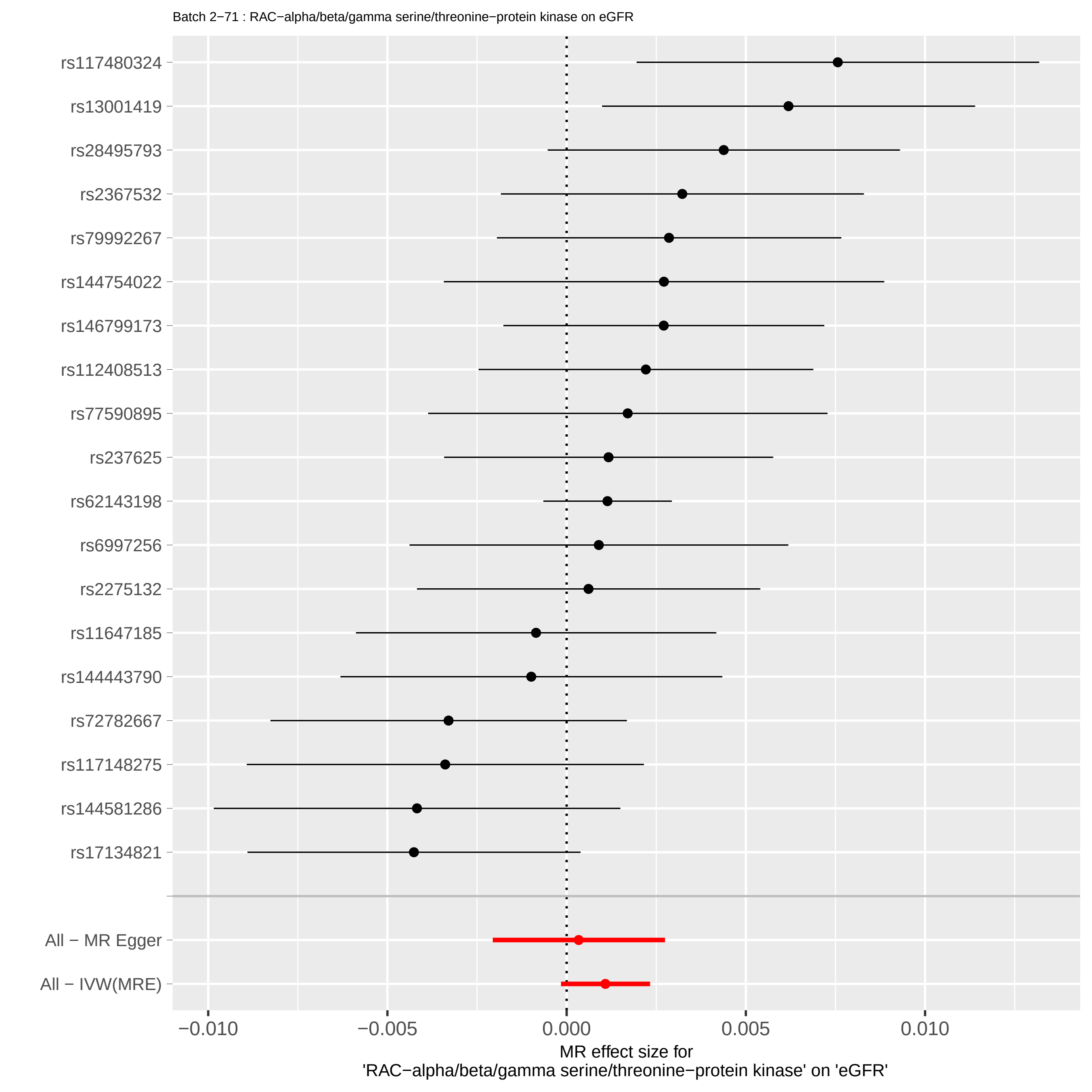
Batch 2−71 : RAC−alpha/beta/gamma serine/threonine−protein kinase on eGFR
rs117480324
rs13001419
rs28495793
rs2367532
rs79992267
rs144754022
rs146799173
rs112408513
rs77590895
rs237625
rs62143198
rs6997256
rs2275132
rs11647185
rs144443790
rs72782667
rs117148275
rs144581286
rs17134821
All − MR Egger
All − IVW(MRE)
−0.010
−0.005
0.000
0.005
0.010
MR effect size for
'RAC−alpha/beta/gamma serine/threonine−protein kinase' on 'eGFR'

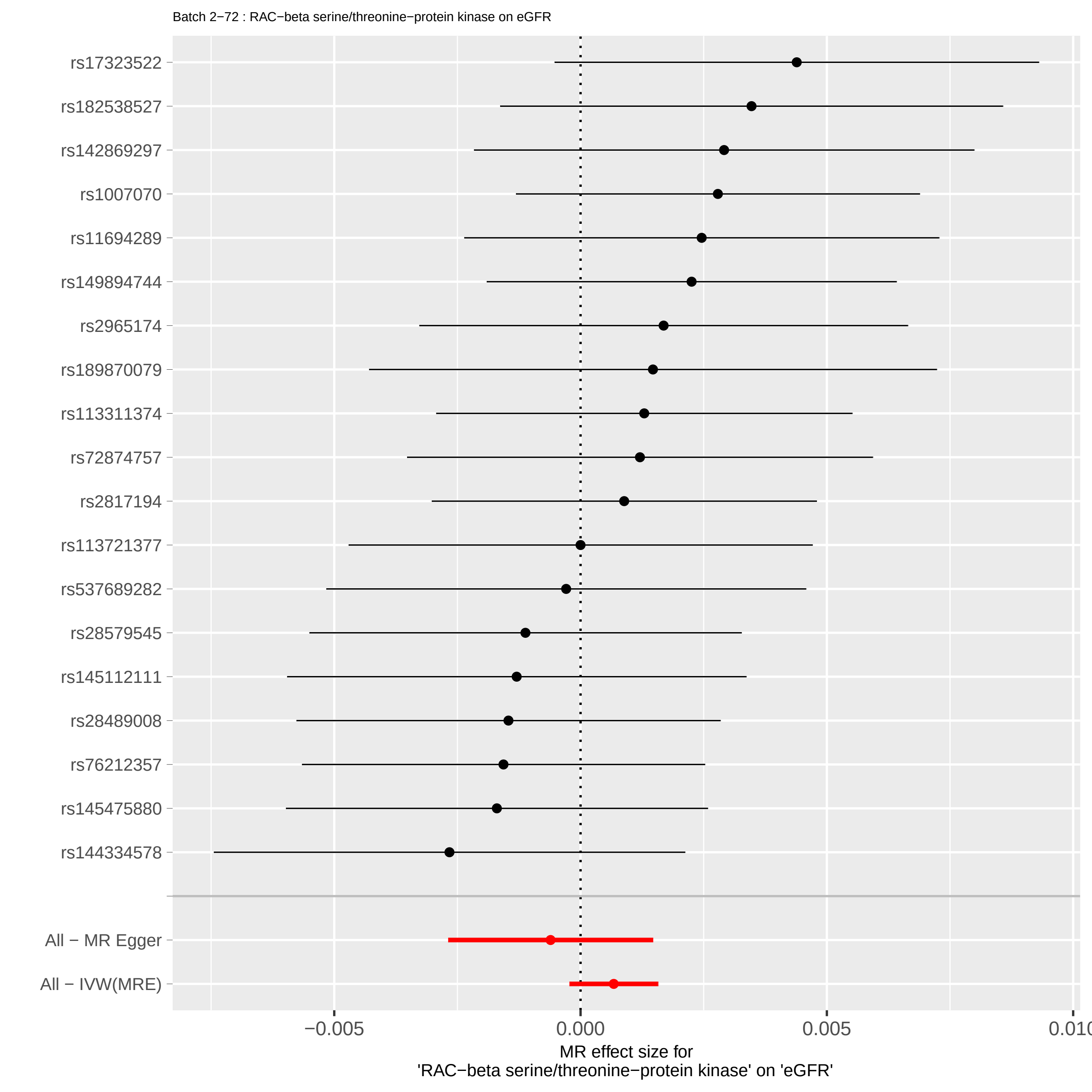

Batch 2−72 : RAC−beta serine/threonine−protein kinase on eGFR
rs17323522
rs182538527
rs142869297
rs1007070
rs11694289
rs149894744
rs2965174
rs189870079
rs113311374
rs72874757
rs2817194
rs113721377
rs537689282
rs28579545
rs145112111
rs28489008
rs76212357
rs145475880
rs144334578
All − MR Egger
All − IVW(MRE)
−0.005
0.000
0.005
0.010
MR effect size for
'RAC−beta serine/threonine−protein kinase' on 'eGFR'

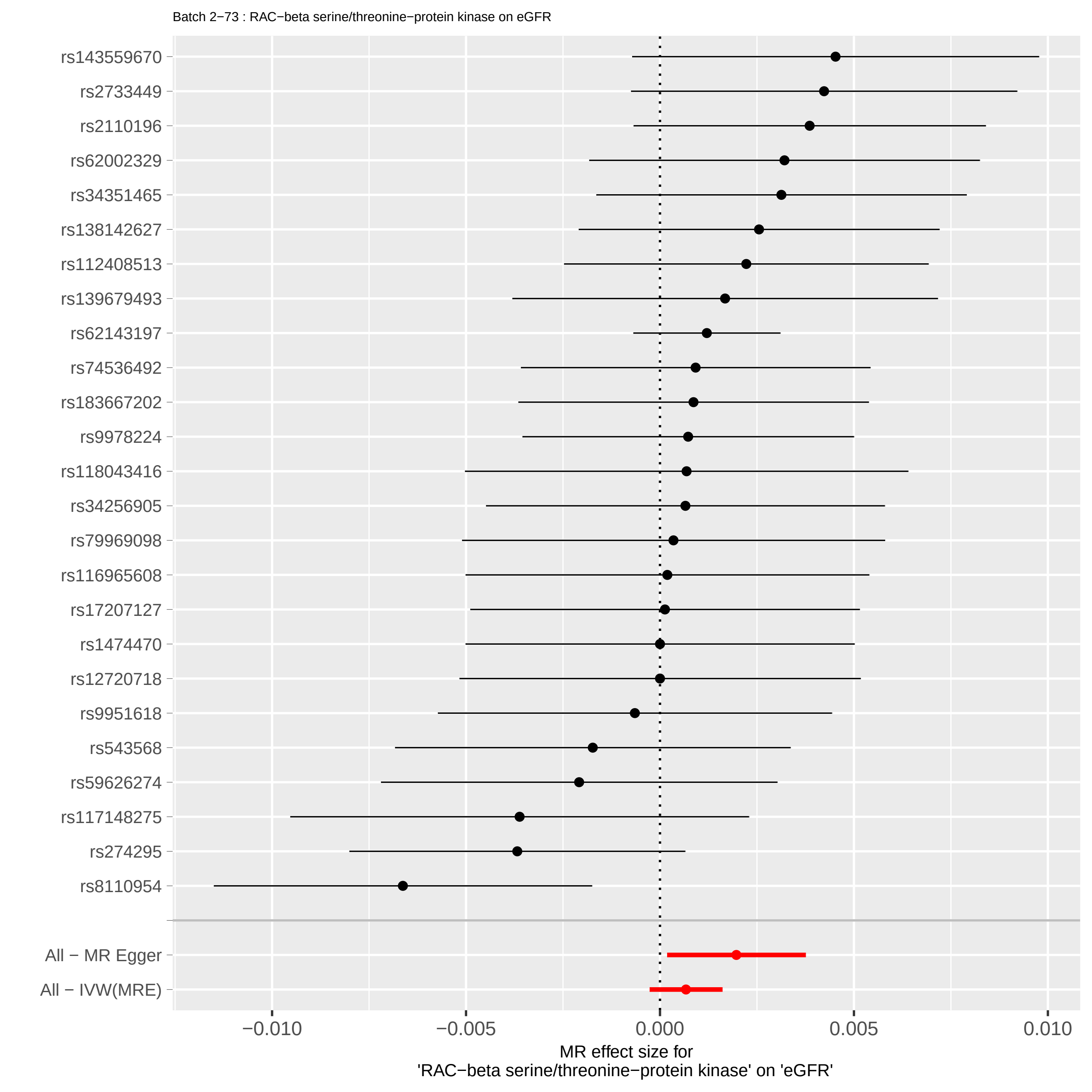

Batch 2−73 : RAC−beta serine/threonine−protein kinase on eGFR
rs143559670
rs2733449
rs2110196
rs62002329
rs34351465
rs138142627
rs112408513
rs139679493
rs62143197
rs74536492
rs183667202
rs9978224
rs118043416
rs34256905
rs79969098
rs116965608
rs17207127
rs1474470
rs12720718
rs9951618
rs543568
rs59626274
rs117148275
rs274295
rs8110954
All − MR Egger
All − IVW(MRE)
−0.010
−0.005
0.000
0.005
0.010
MR effect size for
'RAC−beta serine/threonine−protein kinase' on 'eGFR'

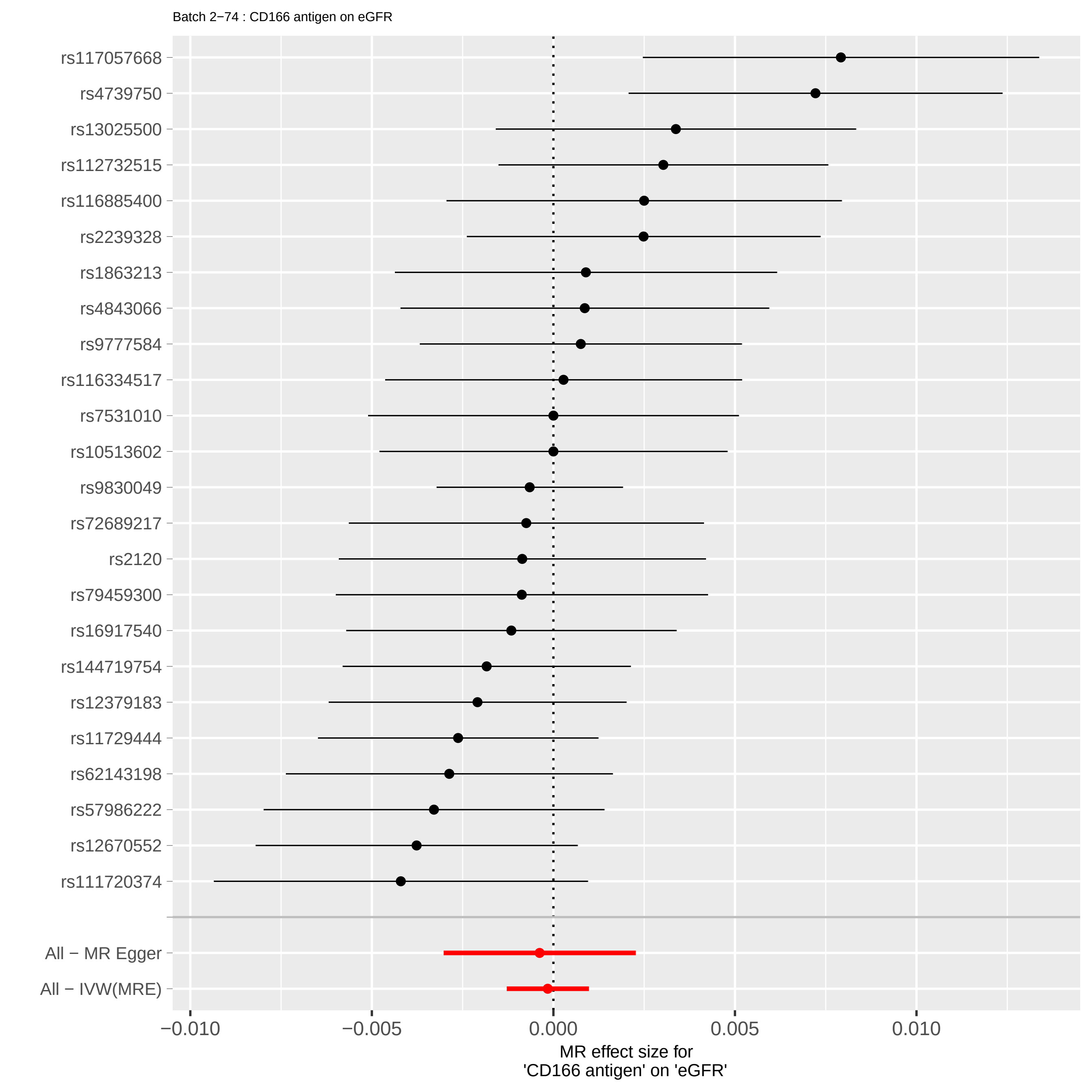
Batch 2−74 : CD166 antigen on eGFR
rs117057668
rs4739750
rs13025500
rs112732515
rs116885400
rs2239328
rs1863213
rs4843066
rs9777584
rs116334517
rs7531010
rs10513602
rs9830049
rs72689217
rs2120
rs79459300
rs16917540
rs144719754
rs12379183
rs11729444
rs62143198
rs57986222
rs12670552
rs111720374
All − MR Egger
All − IVW(MRE)
−0.010
−0.005
0.000
0.005
0.010
MR effect size for
'CD166 antigen' on 'eGFR'

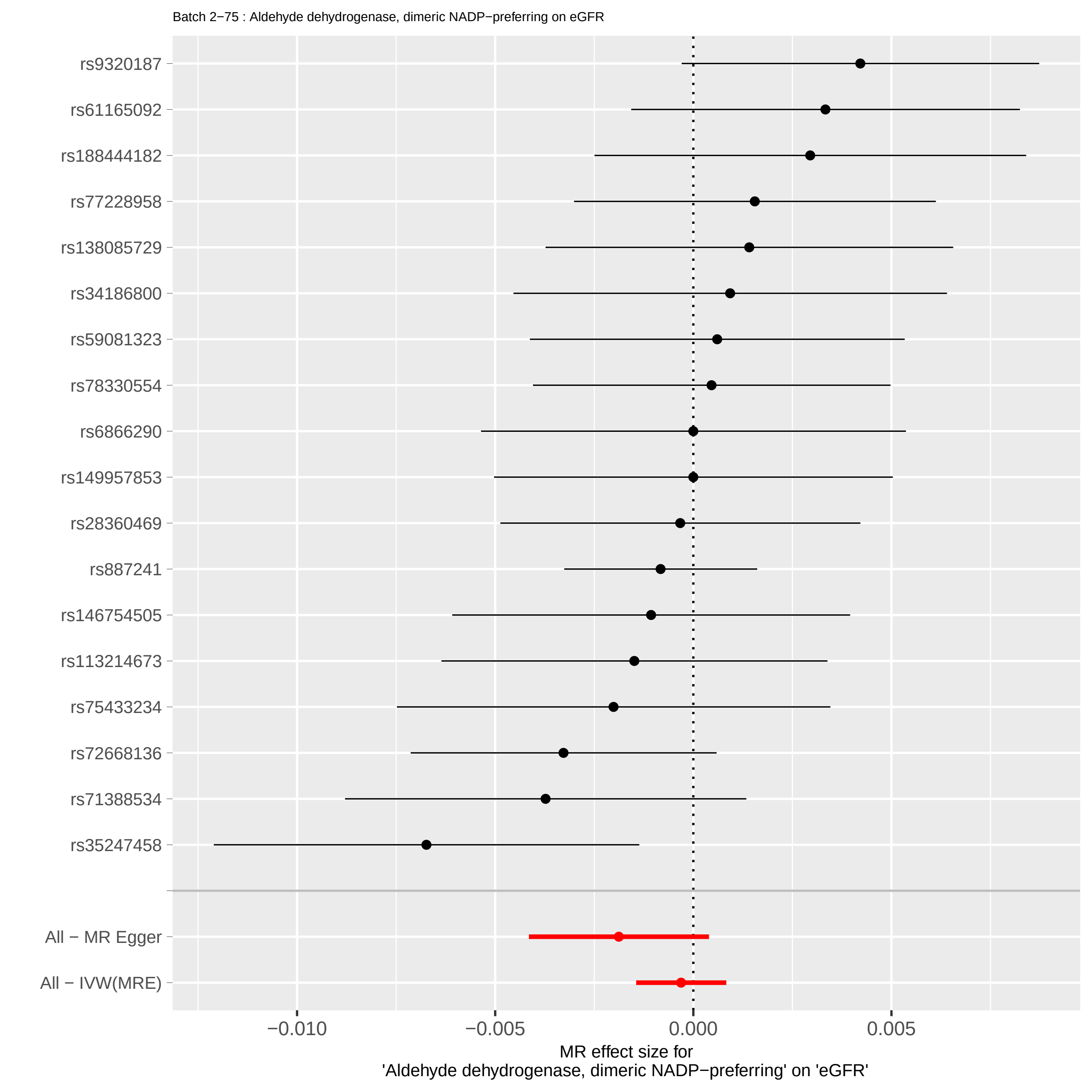

Batch 2−75 : Aldehyde dehydrogenase, dimeric NADP−preferring on eGFR
rs9320187
rs61165092
rs188444182
rs77228958
rs138085729
rs34186800
rs59081323
rs78330554
rs6866290
rs149957853
rs28360469
rs887241
rs146754505
rs113214673
rs75433234
rs72668136
rs71388534
rs35247458
All − MR Egger
All − IVW(MRE)
−0.010
−0.005
0.000
0.005
MR effect size for
'Aldehyde dehydrogenase, dimeric NADP−preferring' on 'eGFR'

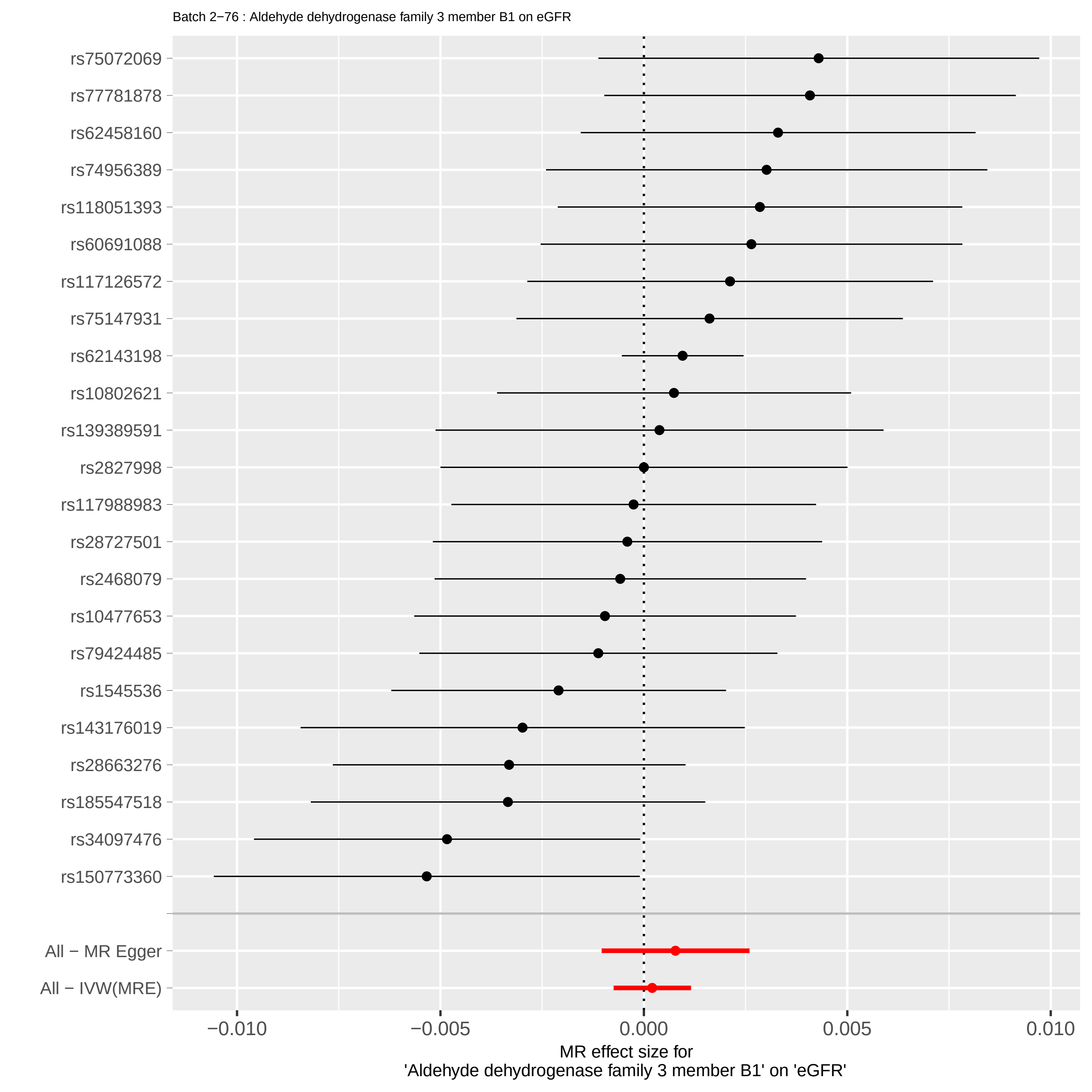

Batch 2−76 : Aldehyde dehydrogenase family 3 member B1 on eGFR
rs75072069
rs77781878
rs62458160
rs74956389
rs118051393
rs60691088
rs117126572
rs75147931
rs62143198
rs10802621
rs139389591
rs2827998
rs117988983
rs28727501
rs2468079
rs10477653
rs79424485
rs1545536
rs143176019
rs28663276
rs185547518
rs34097476
rs150773360
All − MR Egger
All − IVW(MRE)
−0.010
−0.005
0.000
0.005
0.010
MR effect size for
'Aldehyde dehydrogenase family 3 member B1' on 'eGFR'

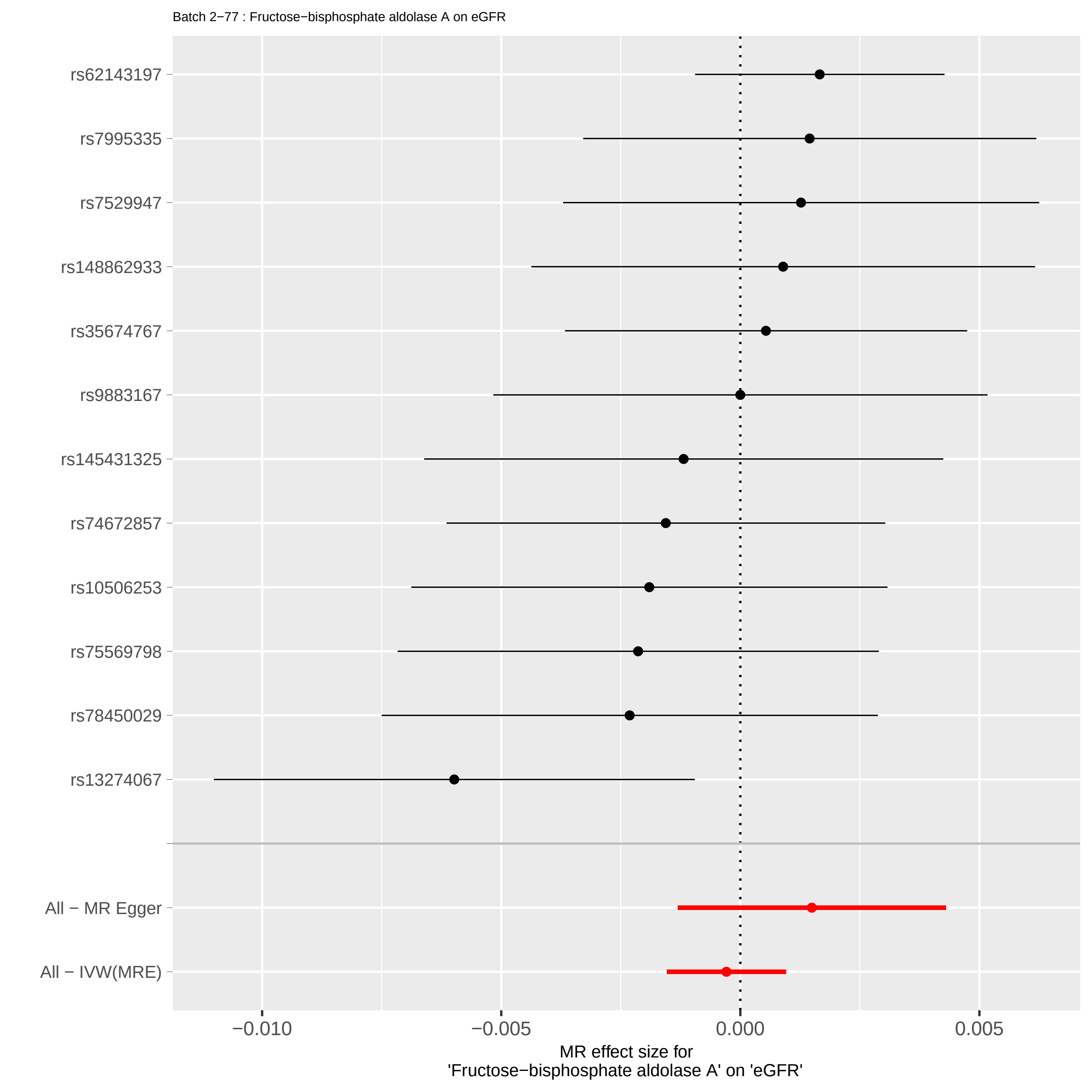

Batch 2−77 : Fructose−bisphosphate aldolase A on eGFR
rs62143197
rs7995335
rs7529947
rs148862933
rs35674767
rs9883167
rs145431325
rs74672857
rs10506253
rs75569798
rs78450029
rs13274067
All − MR Egger
All − IVW(MRE)
−0.010
−0.005
0.000
0.005
MR effect size for
'Fructose−bisphosphate aldolase A' on 'eGFR'

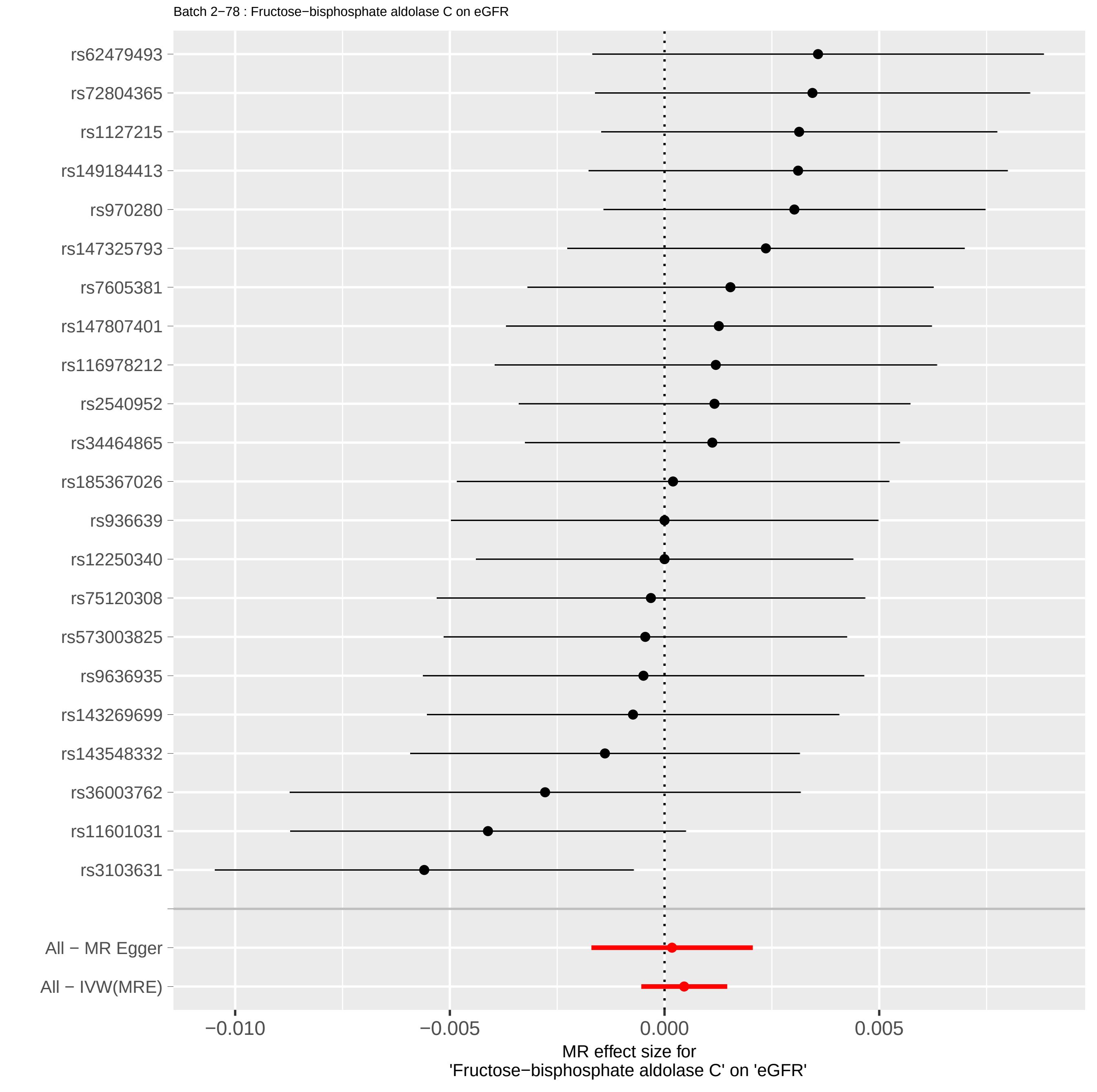

Batch 2−78 : Fructose−bisphosphate aldolase C on eGFR
rs62479493
rs72804365
rs1127215
rs149184413
rs970280
rs147325793
rs7605381
rs147807401
rs116978212
rs2540952
rs34464865
rs185367026
rs936639
rs12250340
rs75120308
rs573003825
rs9636935
rs143269699
rs143548332
rs36003762
rs11601031
rs3103631
All − MR Egger
All − IVW(MRE)
−0.010
−0.005
0.000
0.005
MR effect size for
'Fructose−bisphosphate aldolase C' on 'eGFR'

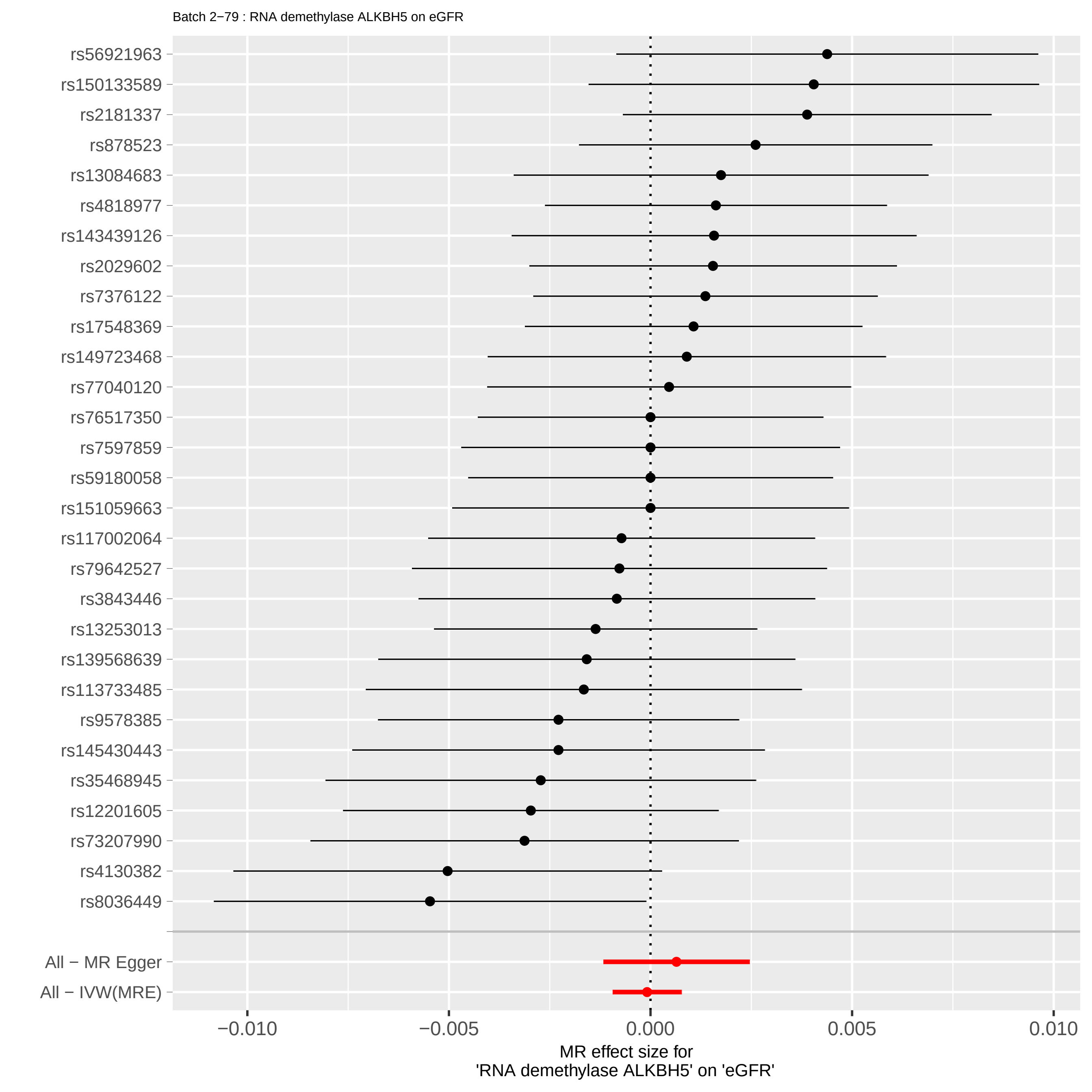

Batch 2−79 : RNA demethylase ALKBH5 on eGFR
rs56921963
rs150133589
rs2181337
rs878523
rs13084683
rs4818977
rs143439126
rs2029602
rs7376122
rs17548369
rs149723468
rs77040120
rs76517350
rs7597859
rs59180058
rs151059663
rs117002064
rs79642527
rs3843446
rs13253013
rs139568639
rs113733485
rs9578385
rs145430443
rs35468945
rs12201605
rs73207990
rs4130382
rs8036449
All − MR Egger
All − IVW(MRE)
−0.010
−0.005
0.000
0.005
0.010
MR effect size for
'RNA demethylase ALKBH5' on 'eGFR'

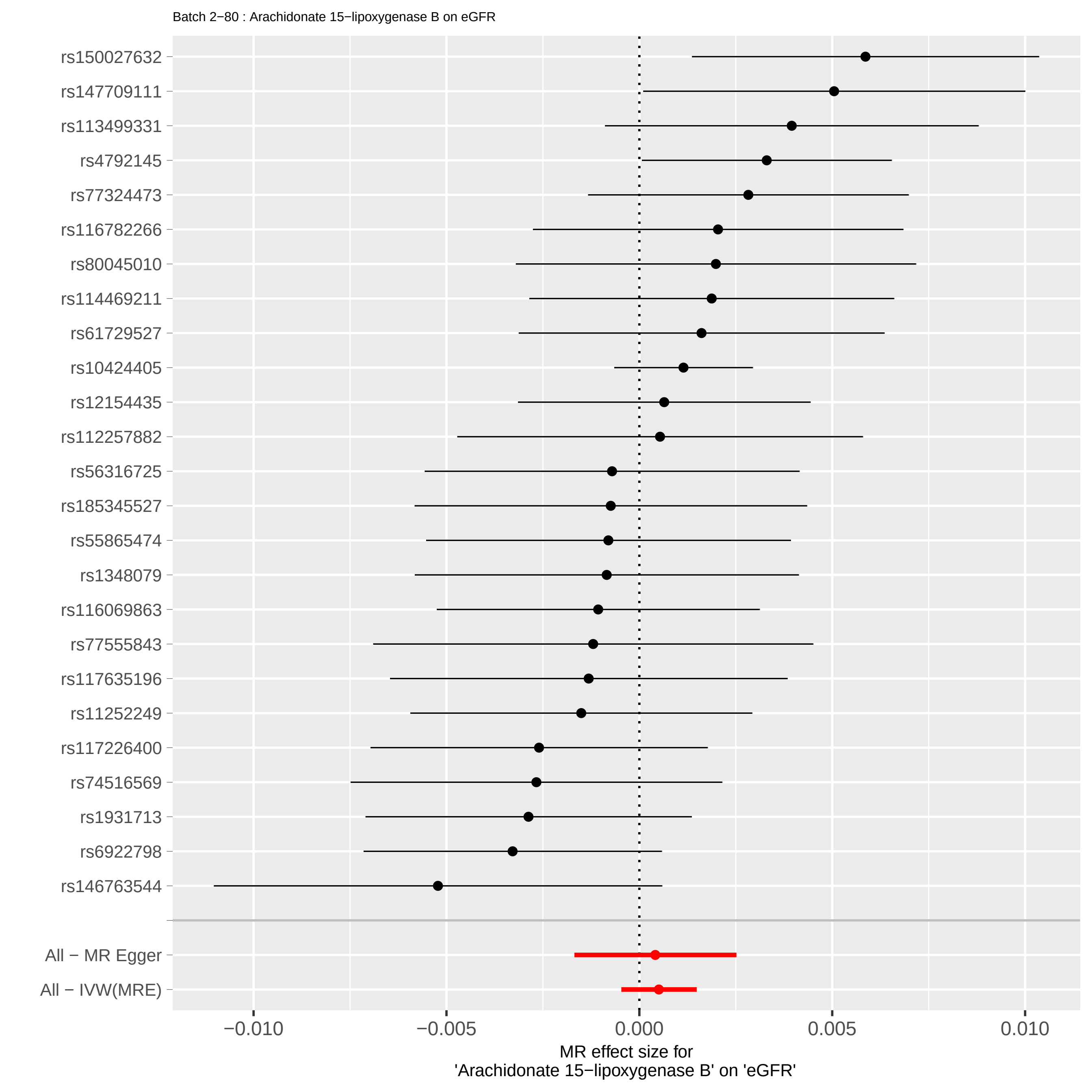

Batch 2−80 : Arachidonate 15−lipoxygenase B on eGFR
rs150027632
rs147709111
rs113499331
rs4792145
rs77324473
rs116782266
rs80045010
rs114469211
rs61729527
rs10424405
rs12154435
rs112257882
rs56316725
rs185345527
rs55865474
rs1348079
rs116069863
rs77555843
rs117635196
rs11252249
rs117226400
rs74516569
rs1931713
rs6922798
rs146763544
All − MR Egger
All − IVW(MRE)
−0.010
−0.005
0.000
0.005
0.010
MR effect size for
'Arachidonate 15−lipoxygenase B' on 'eGFR'

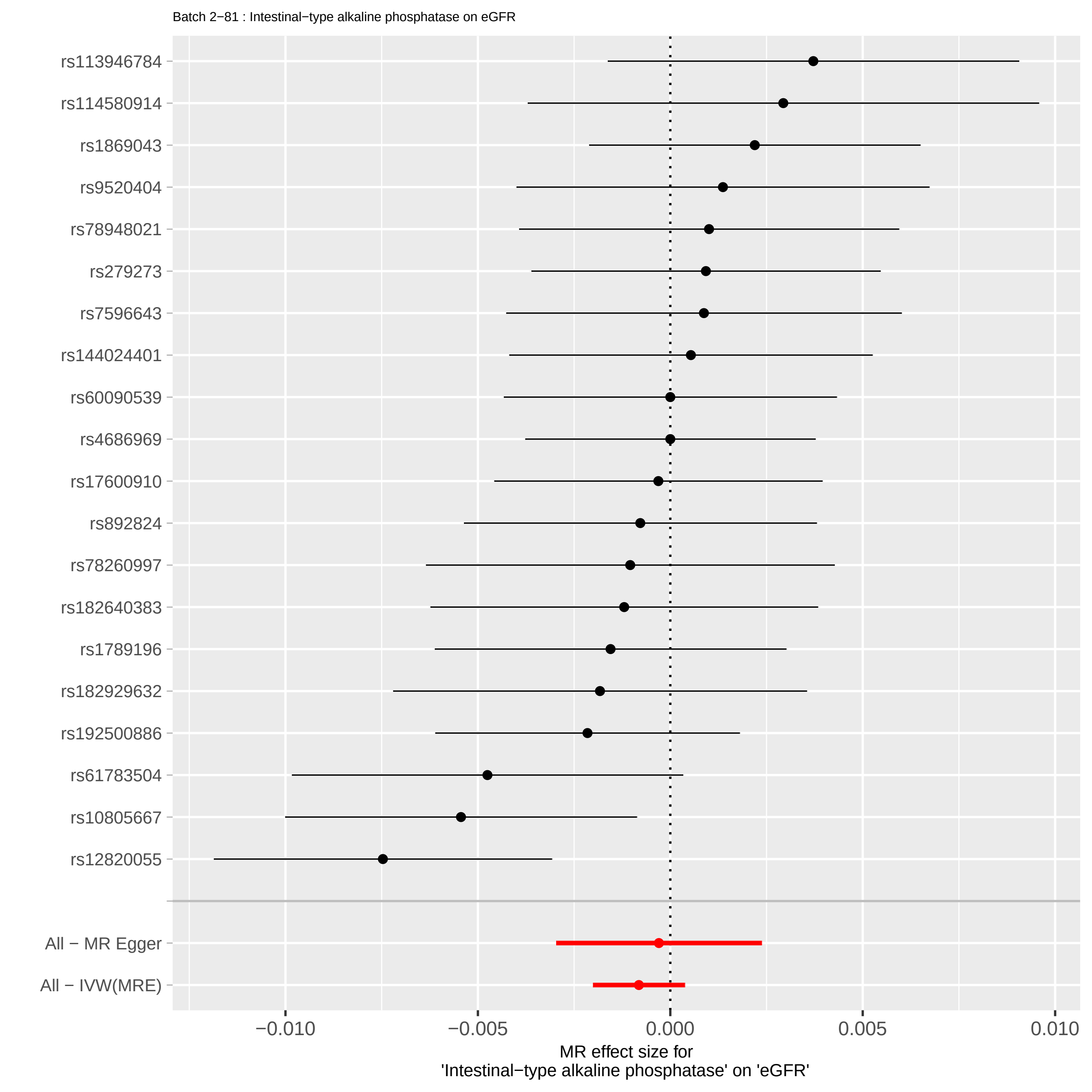

Batch 2−81 : Intestinal−type alkaline phosphatase on eGFR
rs113946784
rs114580914
rs1869043
rs9520404
rs78948021
rs279273
rs7596643
rs144024401
rs60090539
rs4686969
rs17600910
rs892824
rs78260997
rs182640383
rs1789196
rs182929632
rs192500886
rs61783504
rs10805667
rs12820055
All − MR Egger
All − IVW(MRE)
−0.010
−0.005
0.000
0.005
0.010
MR effect size for
'Intestinal−type alkaline phosphatase' on 'eGFR'

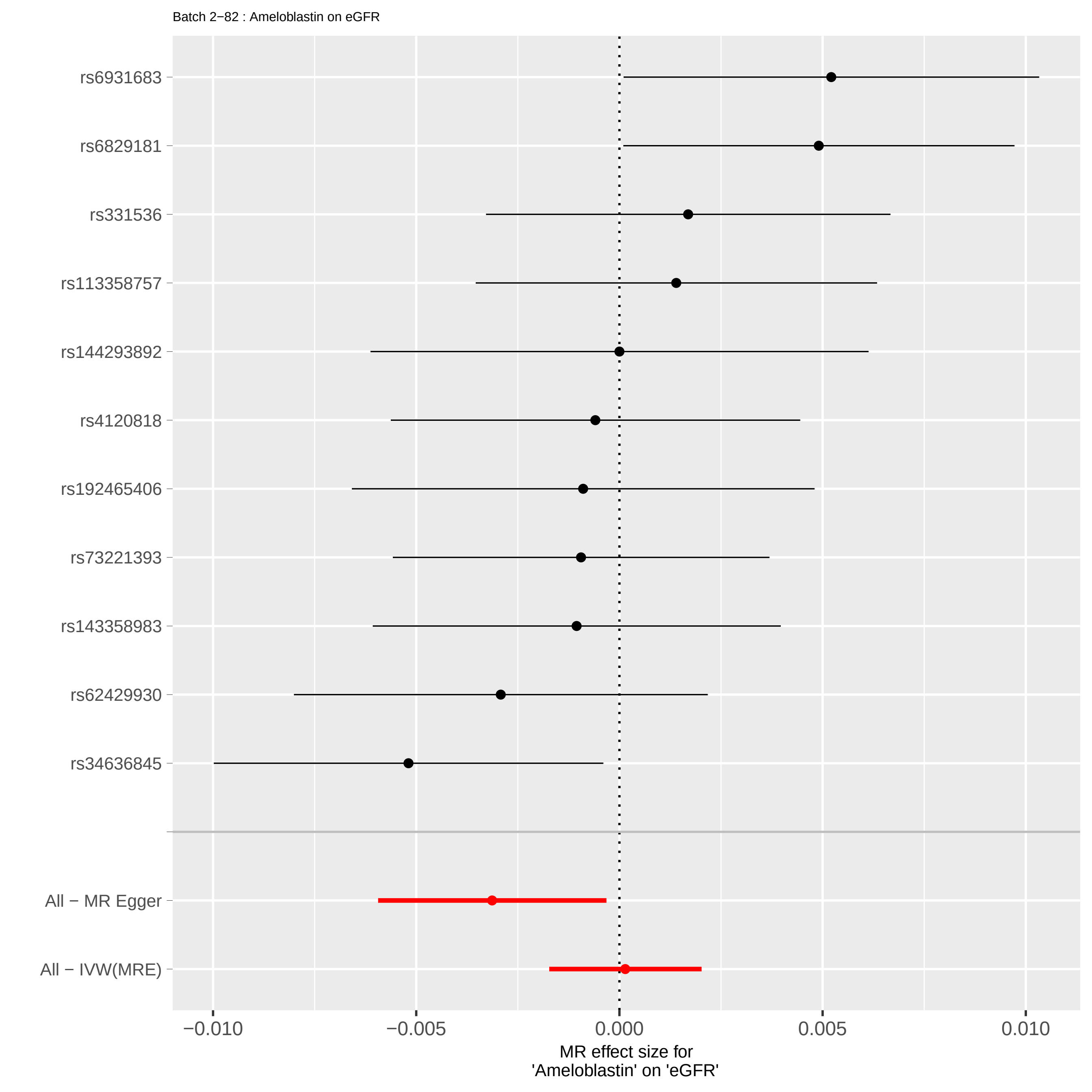
Batch 2−82 : Ameloblastin on eGFR
rs6931683
rs6829181
rs331536
rs113358757
rs144293892
rs4120818
rs192465406
rs73221393
rs143358983
rs62429930
rs34636845
All − MR Egger
All − IVW(MRE)
−0.010
−0.005
0.000
0.005
0.010
MR effect size for
'Ameloblastin' on 'eGFR'

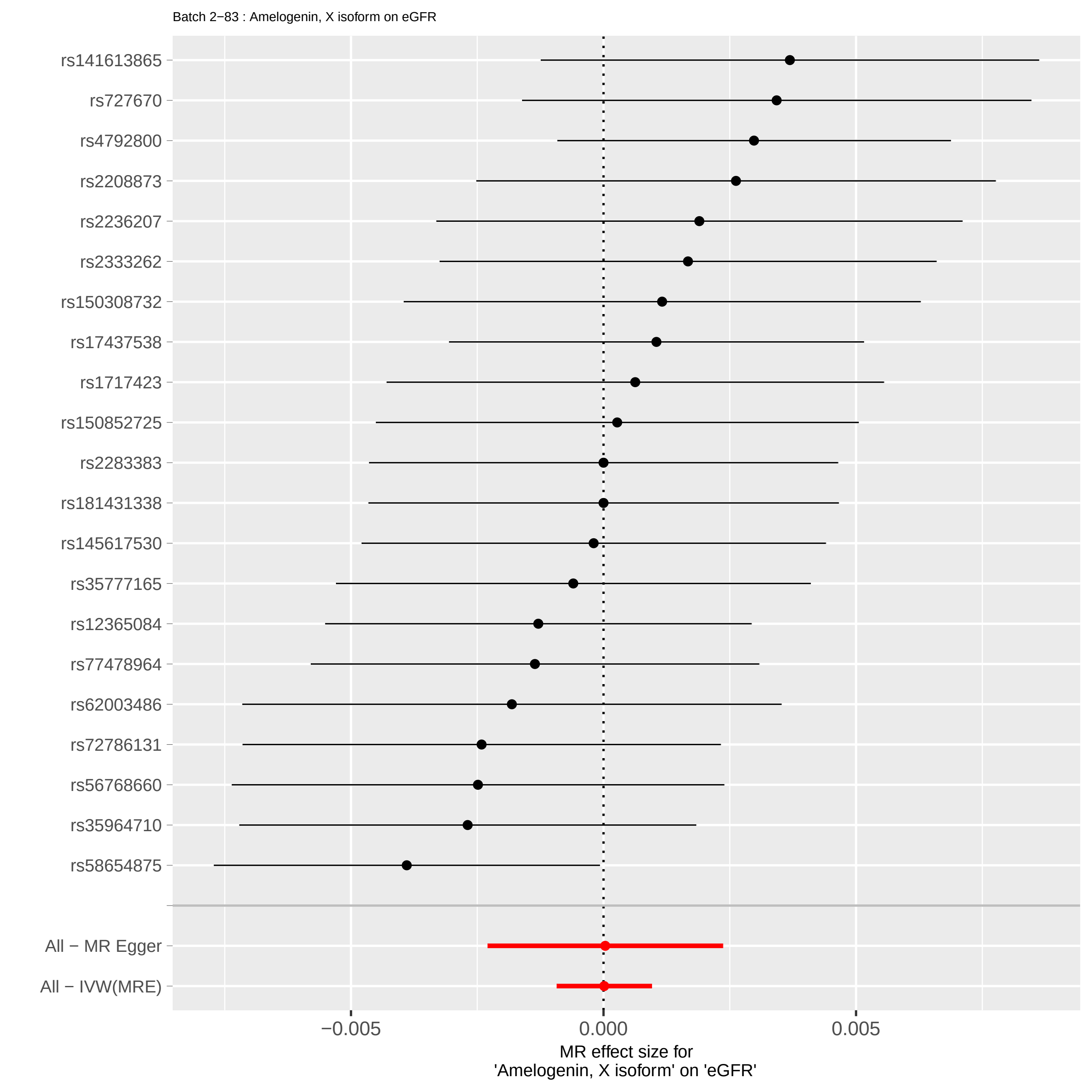

Batch 2−83 : Amelogenin, X isoform on eGFR
rs141613865
rs727670
rs4792800
rs2208873
rs2236207
rs2333262
rs150308732
rs17437538
rs1717423
rs150852725
rs2283383
rs181431338
rs145617530
rs35777165
rs12365084
rs77478964
rs62003486
rs72786131
rs56768660
rs35964710
rs58654875
All − MR Egger
All − IVW(MRE)
−0.005
0.000
0.005
MR effect size for
'Amelogenin, X isoform' on 'eGFR'

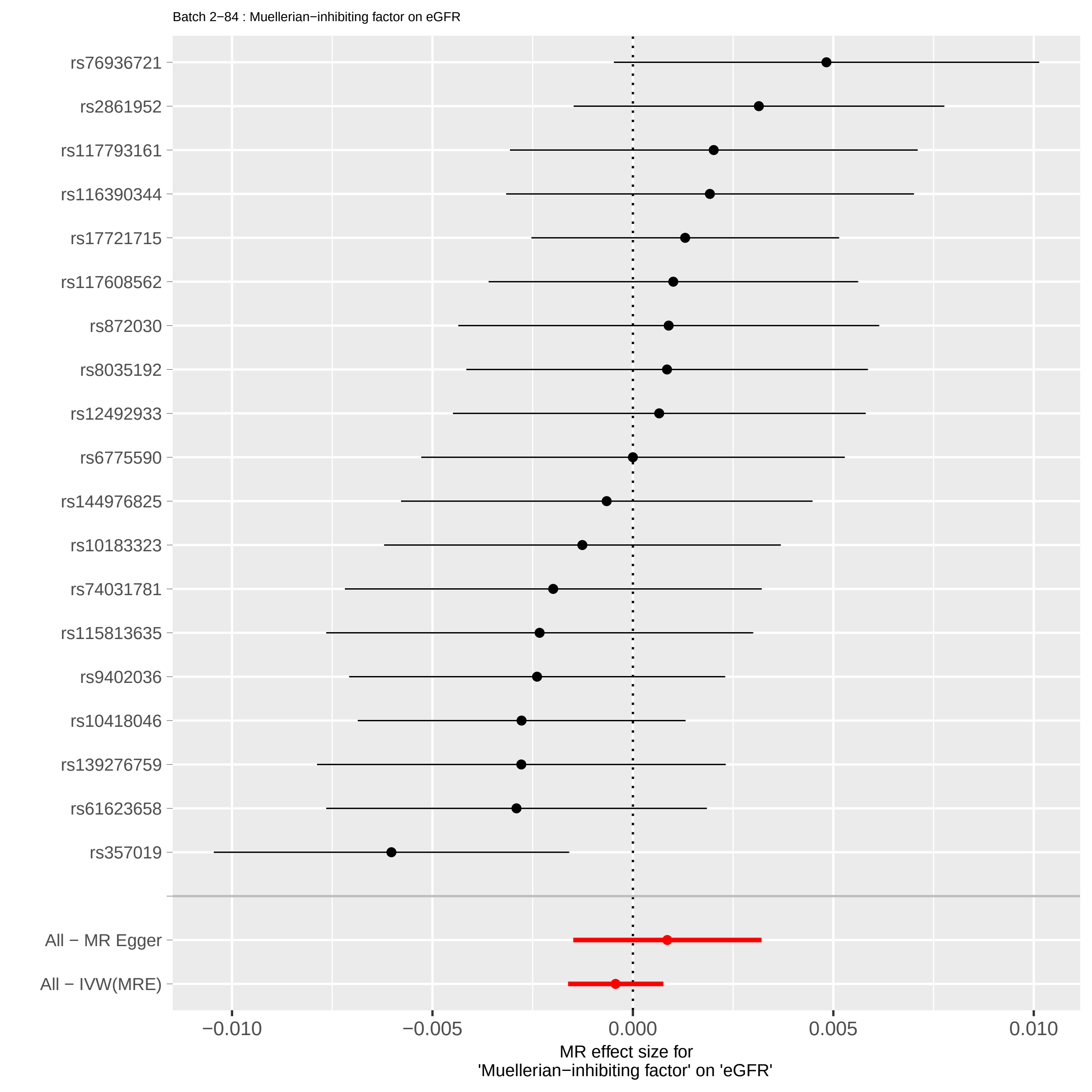
Batch 2−84 : Muellerian−inhibiting factor on eGFR
rs76936721
rs2861952
rs117793161
rs116390344
rs17721715
rs117608562
rs872030
rs8035192
rs12492933
rs6775590
rs144976825
rs10183323
rs74031781
rs115813635
rs9402036
rs10418046
rs139276759
rs61623658
rs357019
All − MR Egger
All − IVW(MRE)
−0.010
−0.005
0.000
0.005
0.010
MR effect size for
'Muellerian−inhibiting factor' on 'eGFR'

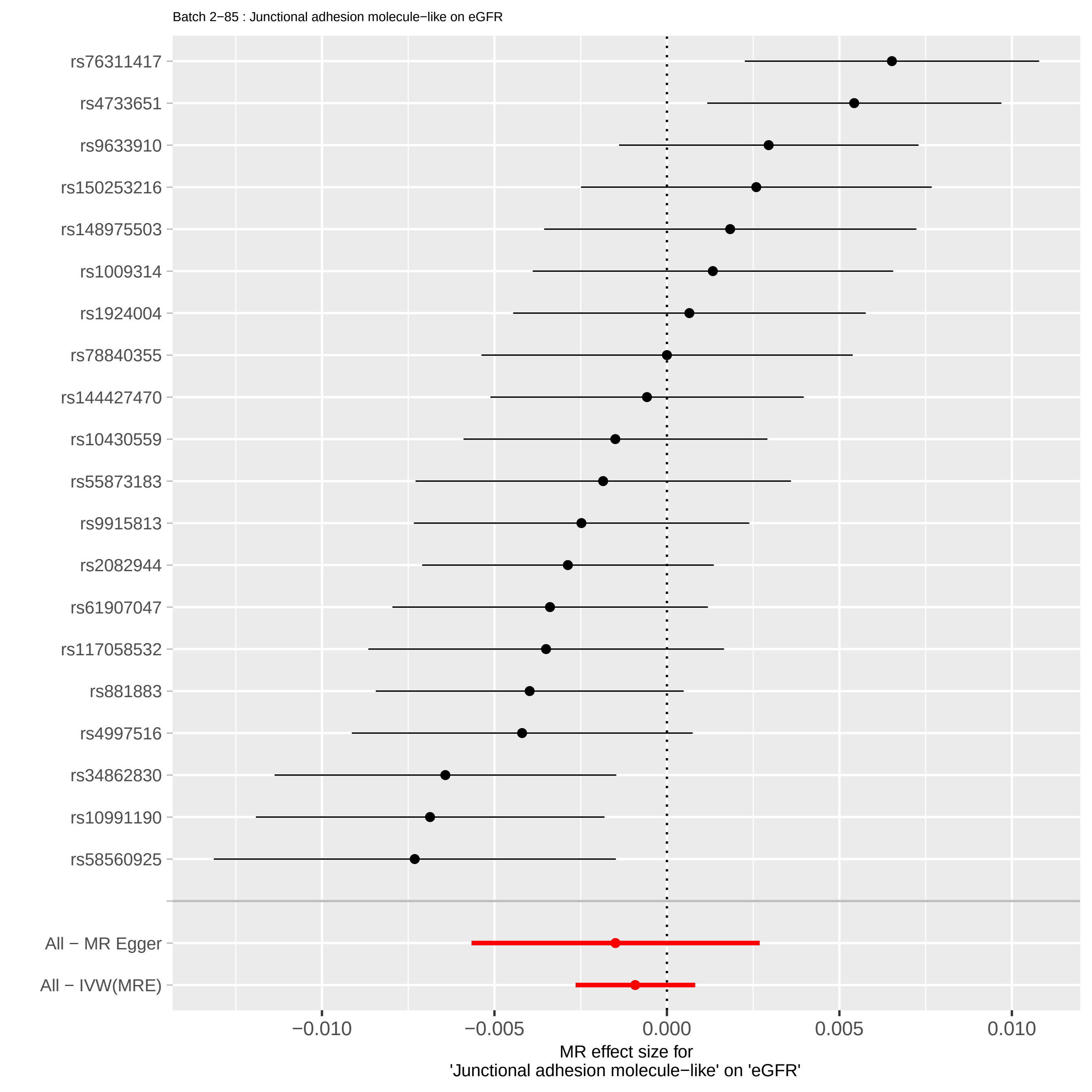

Batch 2−85 : Junctional adhesion molecule−like on eGFR
rs76311417
rs4733651
rs9633910
rs150253216
rs148975503
rs1009314
rs1924004
rs78840355
rs144427470
rs10430559
rs55873183
rs9915813
rs2082944
rs61907047
rs117058532
rs881883
rs4997516
rs34862830
rs10991190
rs58560925
All − MR Egger
All − IVW(MRE)
−0.010
−0.005
0.000
0.005
0.010
MR effect size for
'Junctional adhesion molecule−like' on 'eGFR'

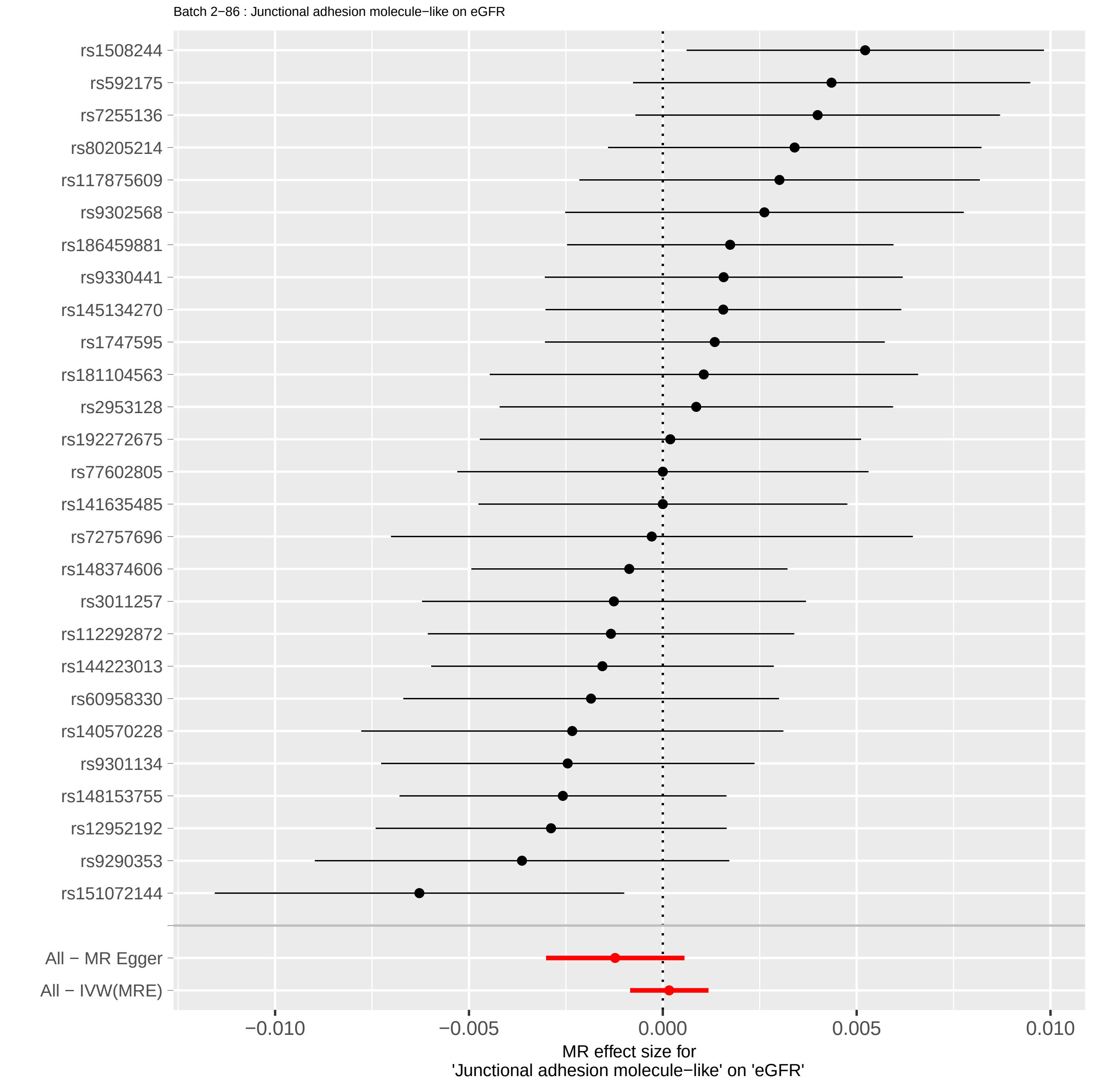

Batch 2−86 : Junctional adhesion molecule−like on eGFR
rs1508244
rs592175
rs7255136
rs80205214
rs117875609
rs9302568
rs186459881
rs9330441
rs145134270
rs1747595
rs181104563
rs2953128
rs192272675
rs77602805
rs141635485
rs72757696
rs148374606
rs3011257
rs112292872
rs144223013
rs60958330
rs140570228
rs9301134
rs148153755
rs12952192
rs9290353
rs151072144
All − MR Egger
All − IVW(MRE)
−0.010
−0.005
0.000
0.005
0.010
MR effect size for
'Junctional adhesion molecule−like' on 'eGFR'

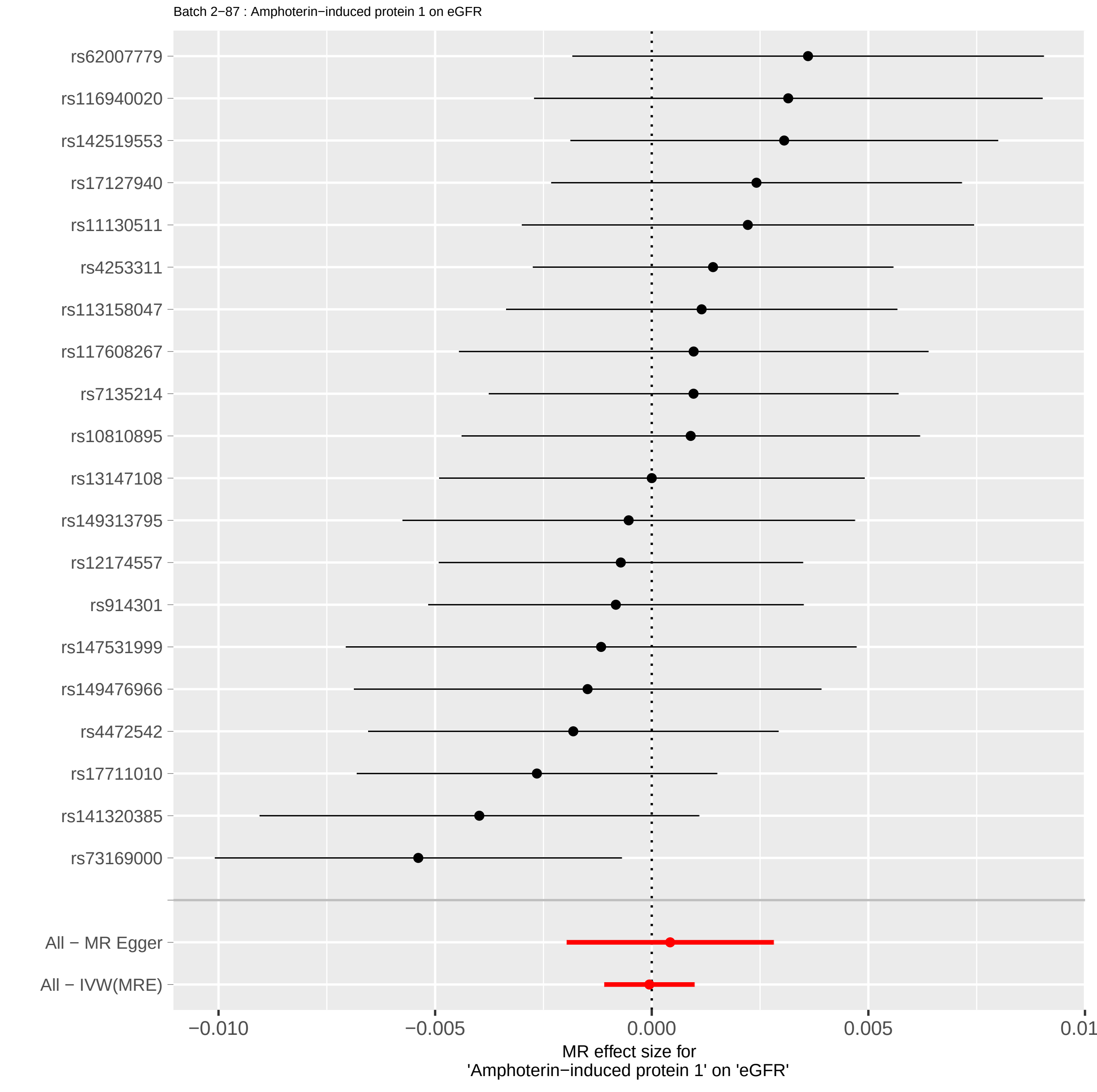

Batch 2−87 : Amphoterin−induced protein 1 on eGFR
rs62007779
rs116940020
rs142519553
rs17127940
rs11130511
rs4253311
rs113158047
rs117608267
rs7135214
rs10810895
rs13147108
rs149313795
rs12174557
rs914301
rs147531999
rs149476966
rs4472542
rs17711010
rs141320385
rs73169000
All − MR Egger
All − IVW(MRE)
−0.010
−0.005
0.000
0.005
0.01
MR effect size for
'Amphoterin−induced protein 1' on 'eGFR'

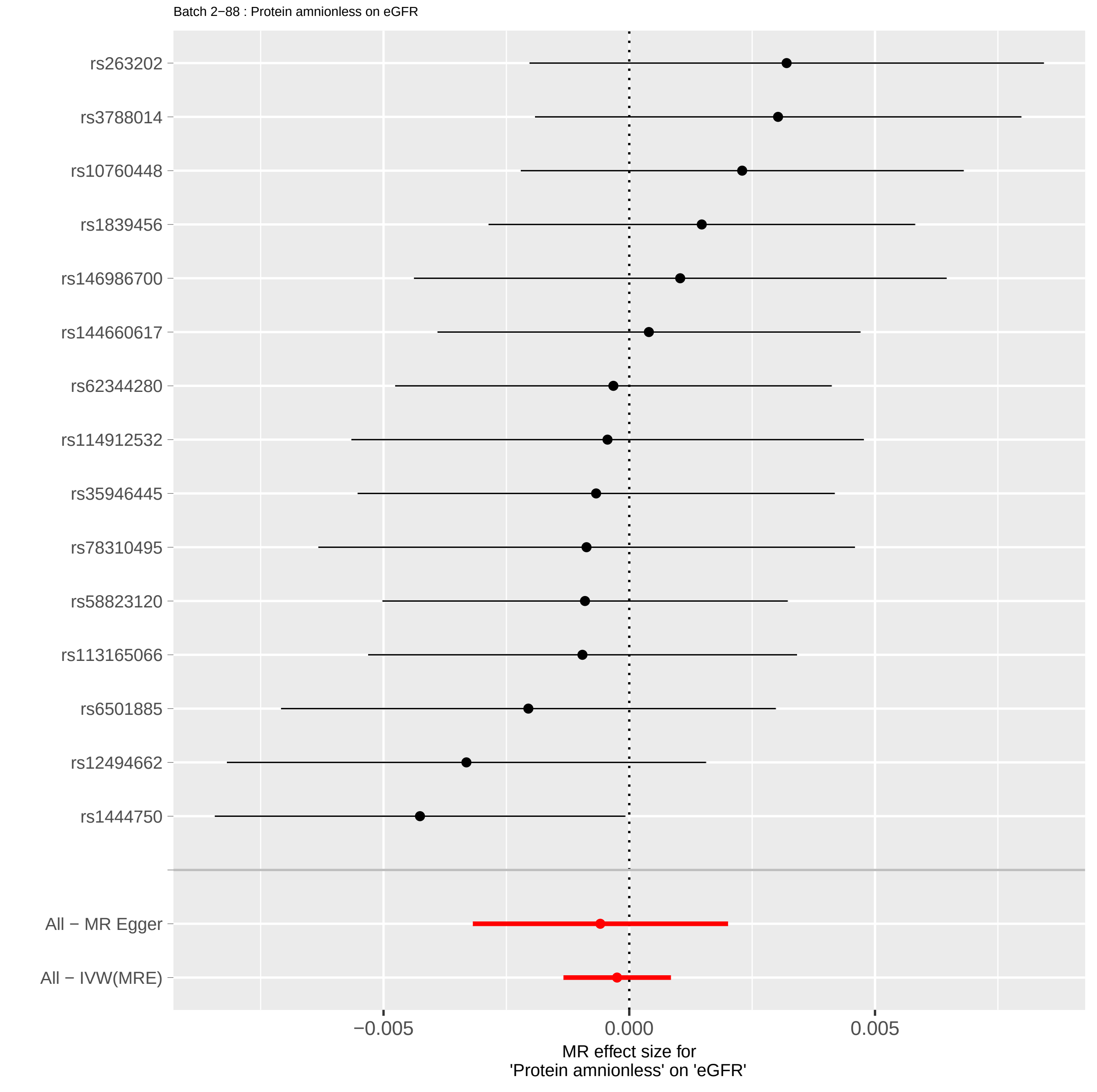

Batch 2−88 : Protein amnionless on eGFR
rs263202
rs3788014
rs10760448
rs1839456
rs146986700
rs144660617
rs62344280
rs114912532
rs35946445
rs78310495
rs58823120
rs113165066
rs6501885
rs12494662
rs1444750
All − MR Egger
All − IVW(MRE)
−0.005
0.000
0.005
MR effect size for
'Protein amnionless' on 'eGFR'

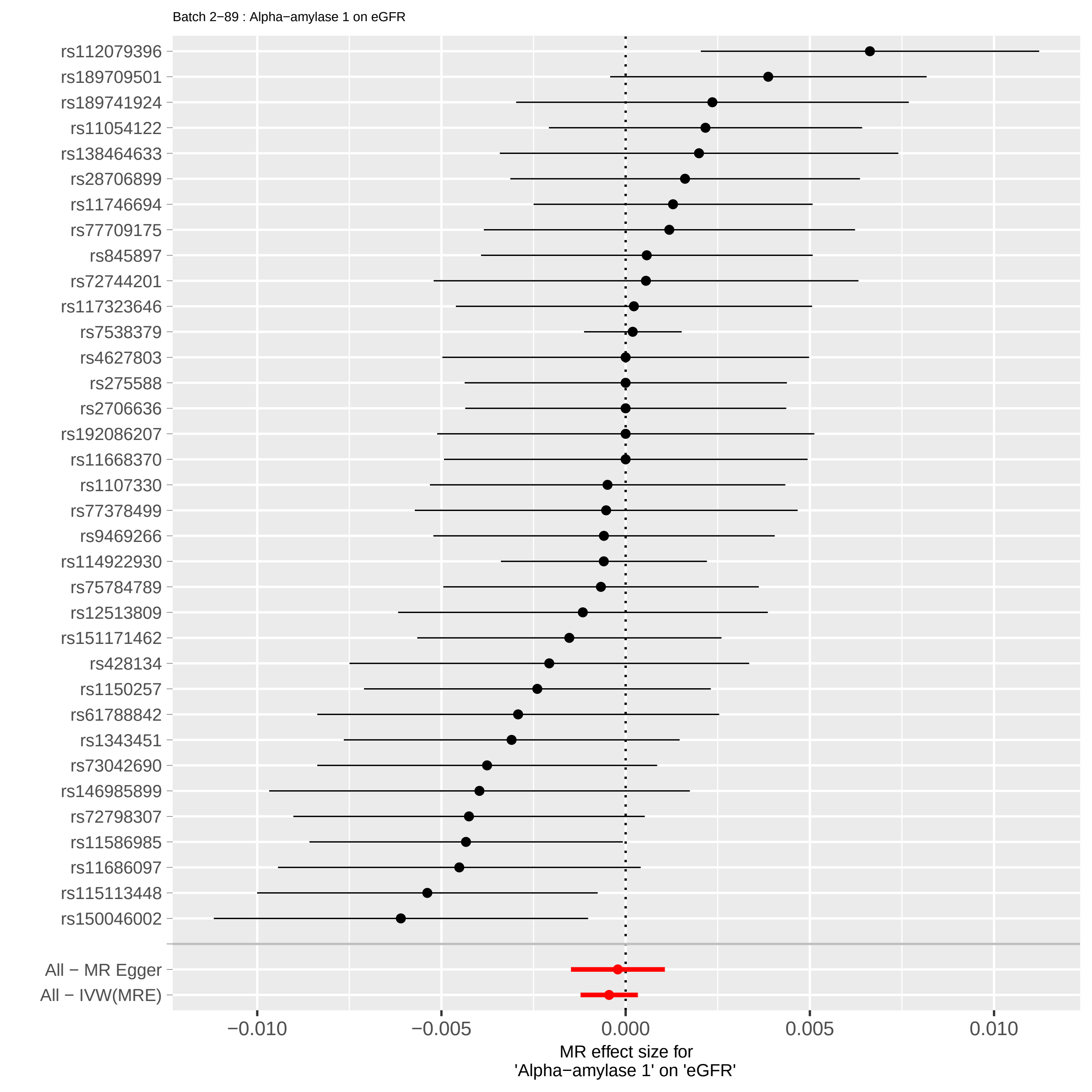
Batch 2−89 : Alpha−amylase 1 on eGFR
rs112079396
rs189709501
rs189741924
rs11054122
rs138464633
rs28706899
rs11746694
rs77709175
rs845897
rs72744201
rs117323646
rs7538379
rs4627803
rs275588
rs2706636
rs192086207
rs11668370
rs1107330
rs77378499
rs9469266
rs114922930
rs75784789
rs12513809
rs151171462
rs428134
rs1150257
rs61788842
rs1343451
rs73042690
rs146985899
rs72798307
rs11586985
rs11686097
rs115113448
rs150046002
All − MR Egger
All − IVW(MRE)
−0.010
−0.005
0.000
0.005
0.010
MR effect size for
'Alpha−amylase 1' on 'eGFR'

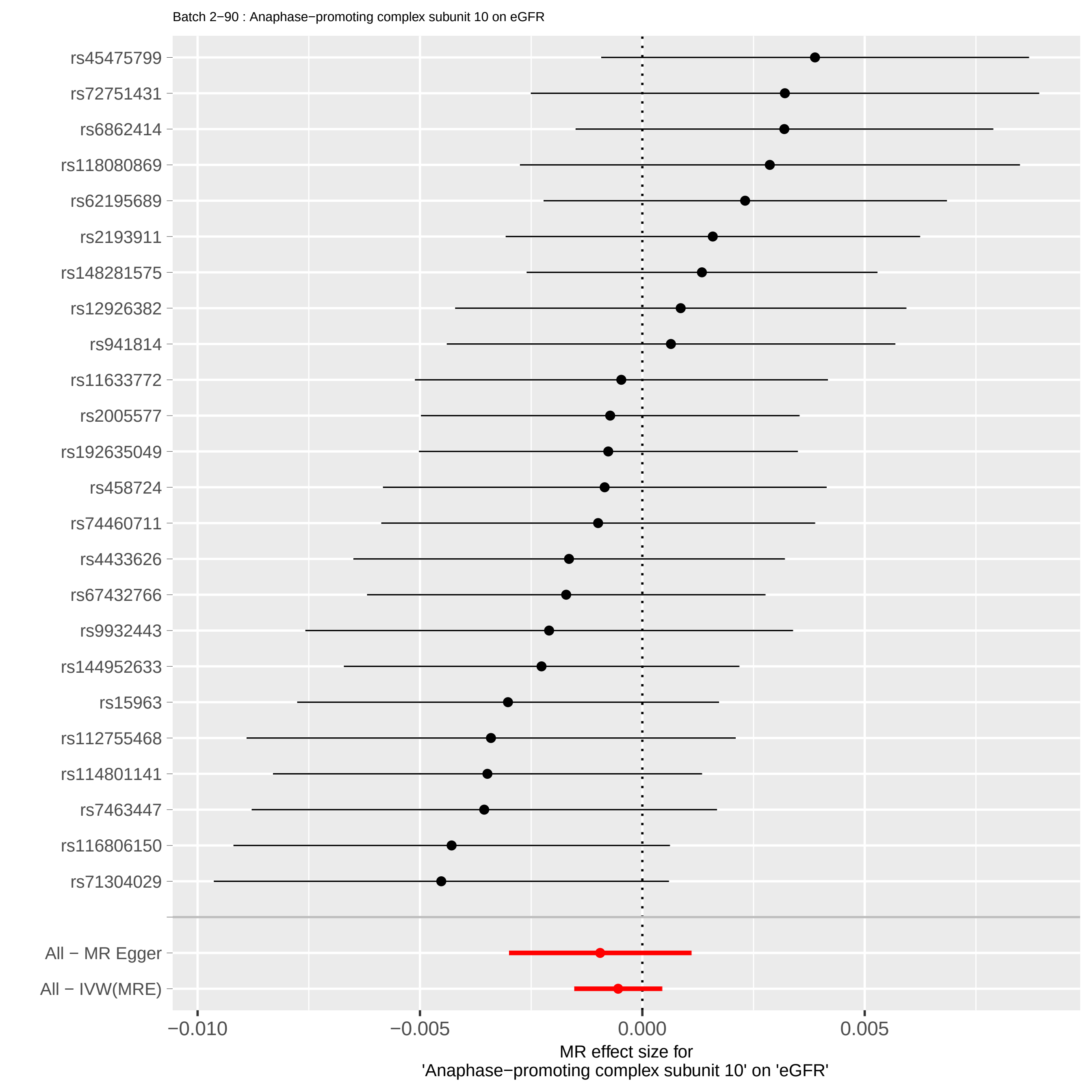

Batch 2−90 : Anaphase−promoting complex subunit 10 on eGFR
rs45475799
rs72751431
rs6862414
rs118080869
rs62195689
rs2193911
rs148281575
rs12926382
rs941814
rs11633772
rs2005577
rs192635049
rs458724
rs74460711
rs4433626
rs67432766
rs9932443
rs144952633
rs15963
rs112755468
rs114801141
rs7463447
rs116806150
rs71304029
All − MR Egger
All − IVW(MRE)
−0.010
−0.005
0.000
0.005
MR effect size for
'Anaphase−promoting complex subunit 10' on 'eGFR'

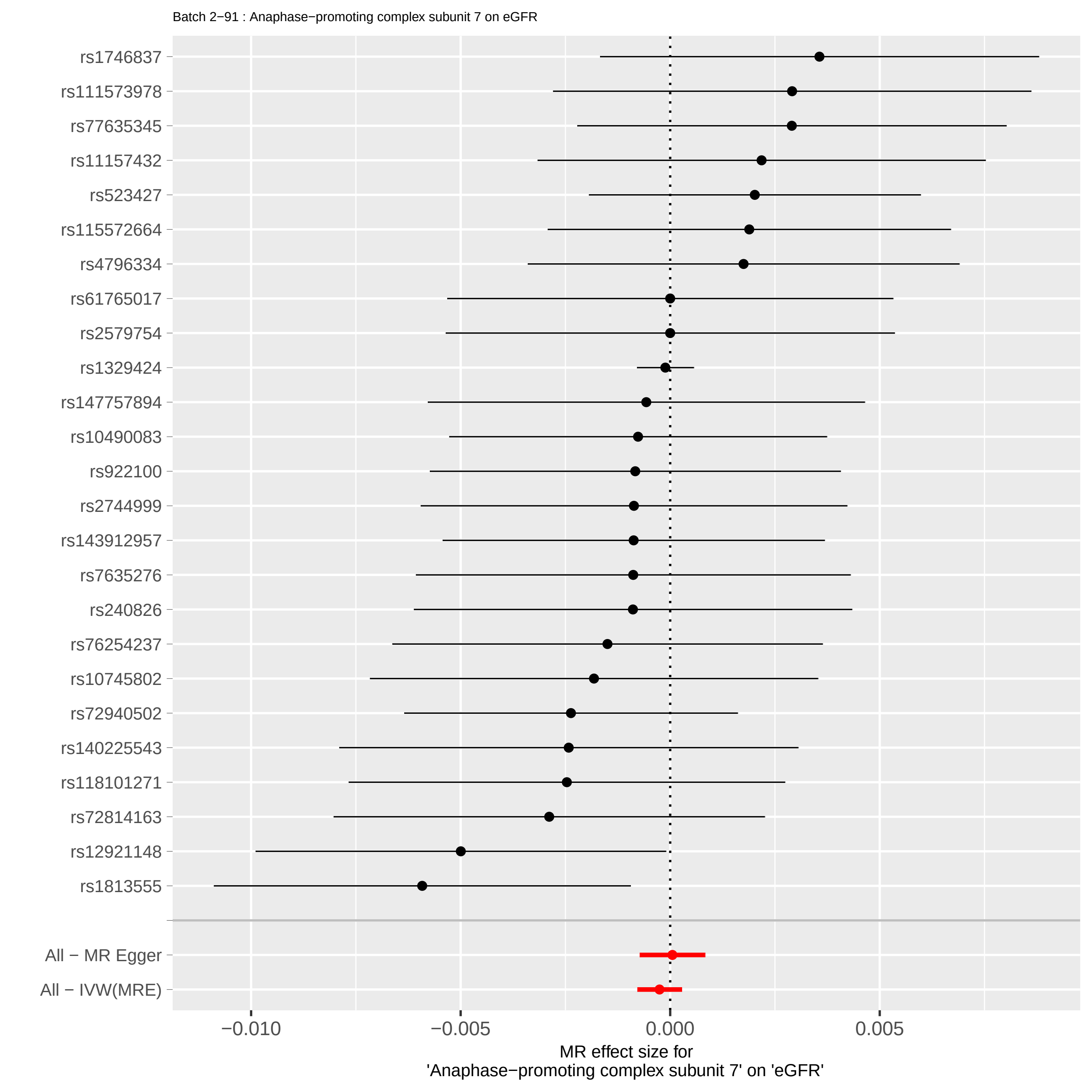

Batch 2−91 : Anaphase−promoting complex subunit 7 on eGFR
rs1746837
rs111573978
rs77635345
rs11157432
rs523427
rs115572664
rs4796334
rs61765017
rs2579754
rs1329424
rs147757894
rs10490083
rs922100
rs2744999
rs143912957
rs7635276
rs240826
rs76254237
rs10745802
rs72940502
rs140225543
rs118101271
rs72814163
rs12921148
rs1813555
All − MR Egger
All − IVW(MRE)
−0.010
−0.005
0.000
0.005
MR effect size for
'Anaphase−promoting complex subunit 7' on 'eGFR'

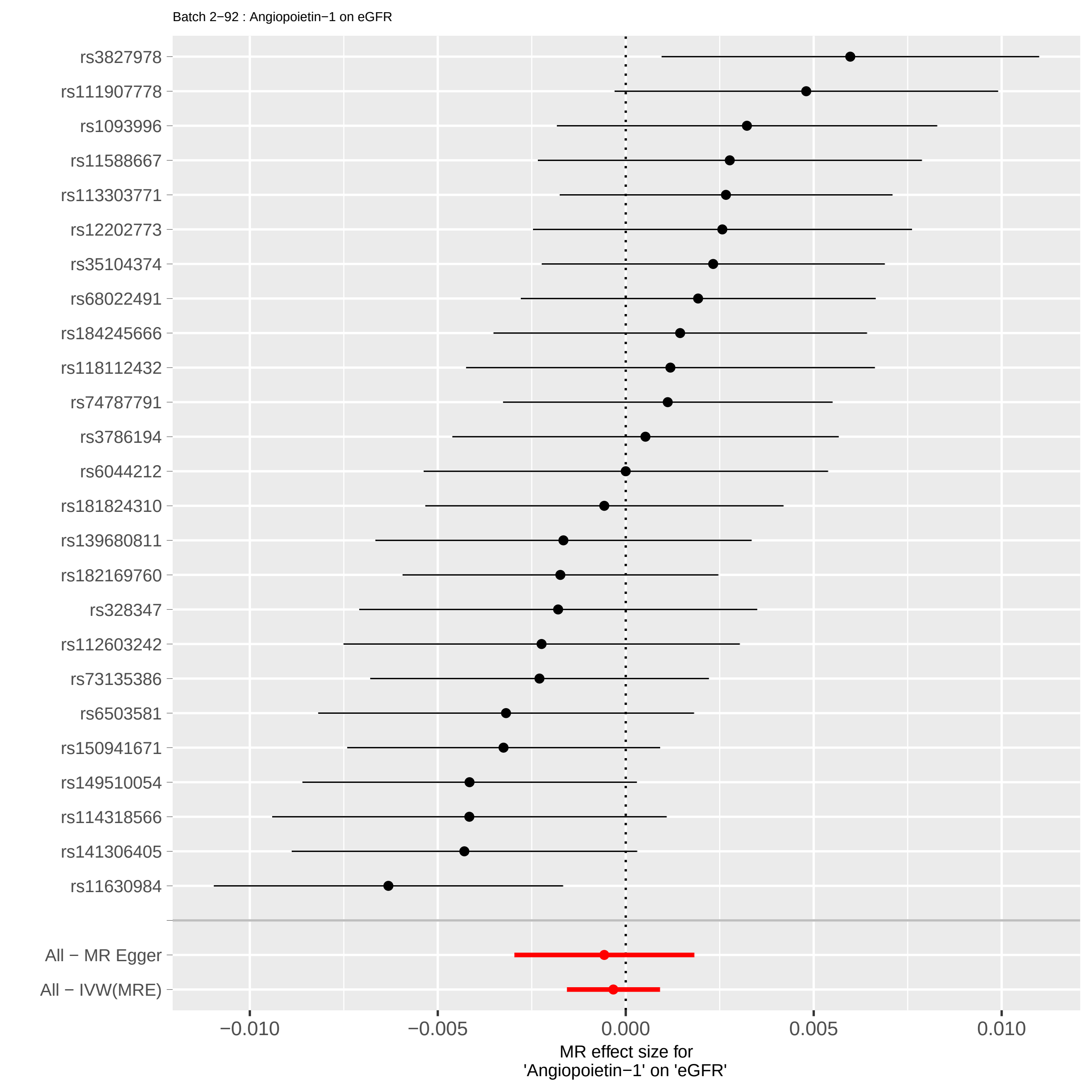
Batch 2−92 : Angiopoietin−1 on eGFR
rs3827978
rs111907778
rs1093996
rs11588667
rs113303771
rs12202773
rs35104374
rs68022491
rs184245666
rs118112432
rs74787791
rs3786194
rs6044212
rs181824310
rs139680811
rs182169760
rs328347
rs112603242
rs73135386
rs6503581
rs150941671
rs149510054
rs114318566
rs141306405
rs11630984
All − MR Egger
All − IVW(MRE)
−0.010
−0.005
0.000
0.005
0.010
MR effect size for
'Angiopoietin−1' on 'eGFR'

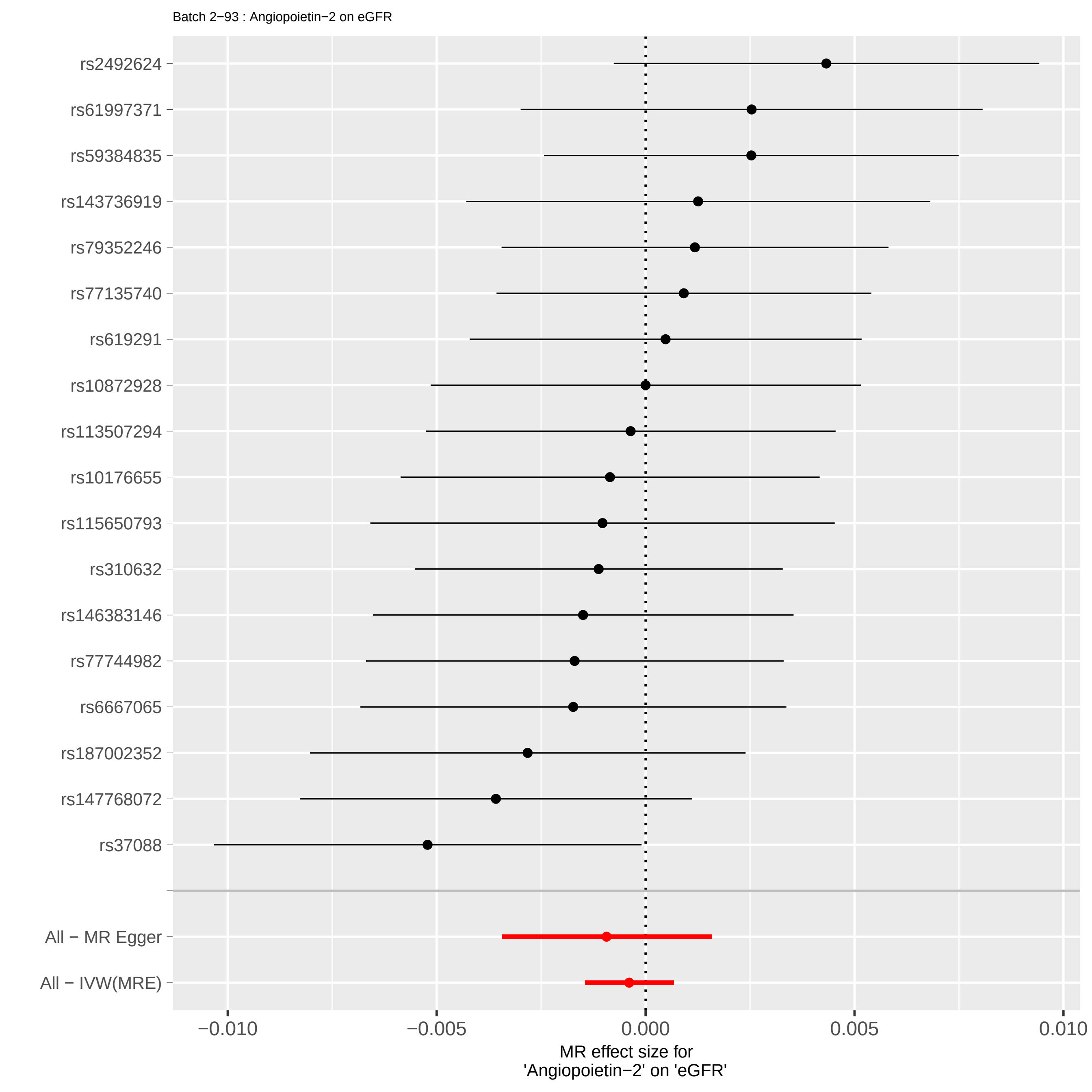

Batch 2−93 : Angiopoietin−2 on eGFR
rs2492624
rs61997371
rs59384835
rs143736919
rs79352246
rs77135740
rs619291
rs10872928
rs113507294
rs10176655
rs115650793
rs310632
rs146383146
rs77744982
rs6667065
rs187002352
rs147768072
rs37088
All − MR Egger
All − IVW(MRE)
−0.010
−0.005
0.000
0.005
0.010
MR effect size for
'Angiopoietin−2' on 'eGFR'

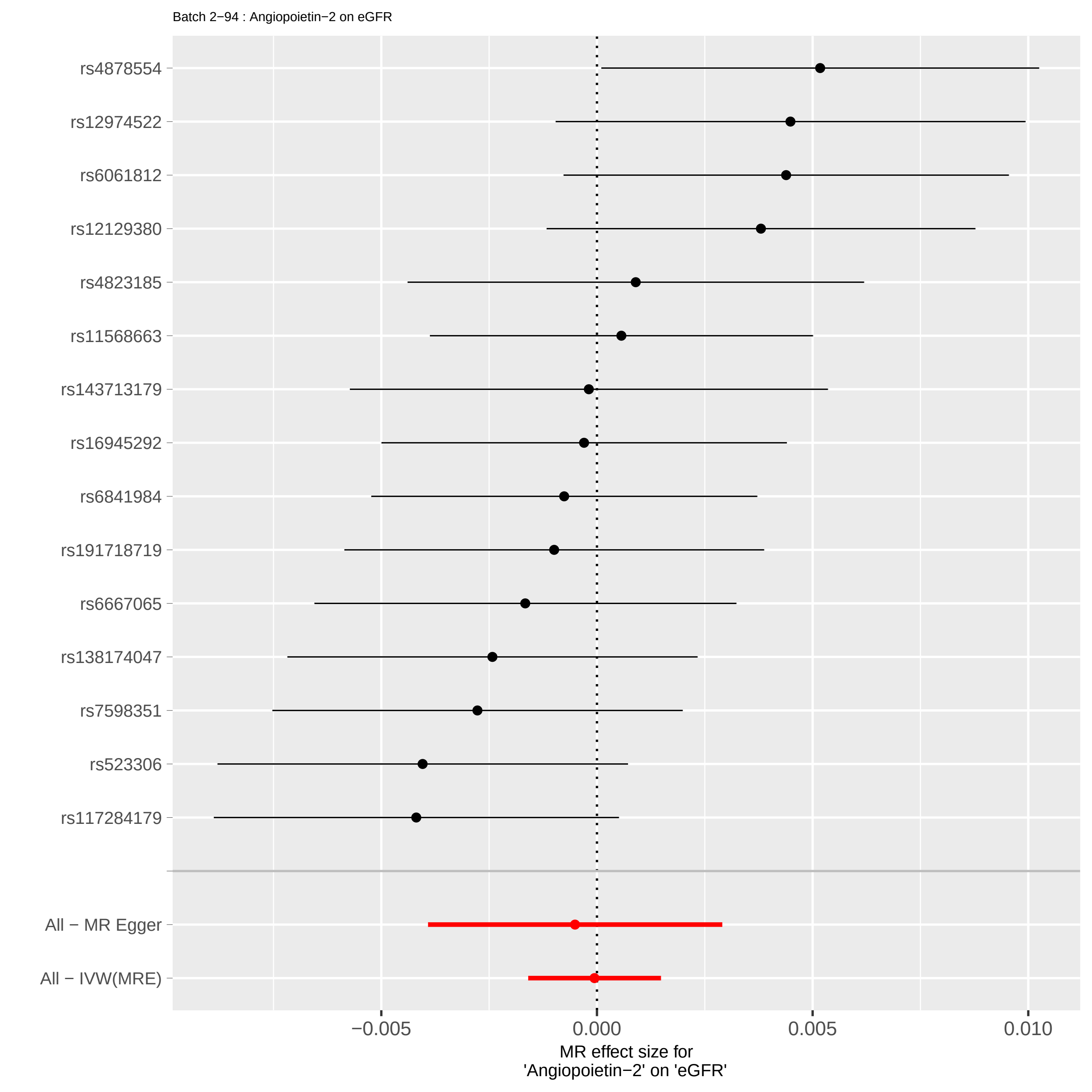
Batch 2−94 : Angiopoietin−2 on eGFR
rs4878554
rs12974522
rs6061812
rs12129380
rs4823185
rs11568663
rs143713179
rs16945292
rs6841984
rs191718719
rs6667065
rs138174047
rs7598351
rs523306
rs117284179
All − MR Egger
All − IVW(MRE)
−0.005
0.000
0.005
0.010
MR effect size for
'Angiopoietin−2' on 'eGFR'

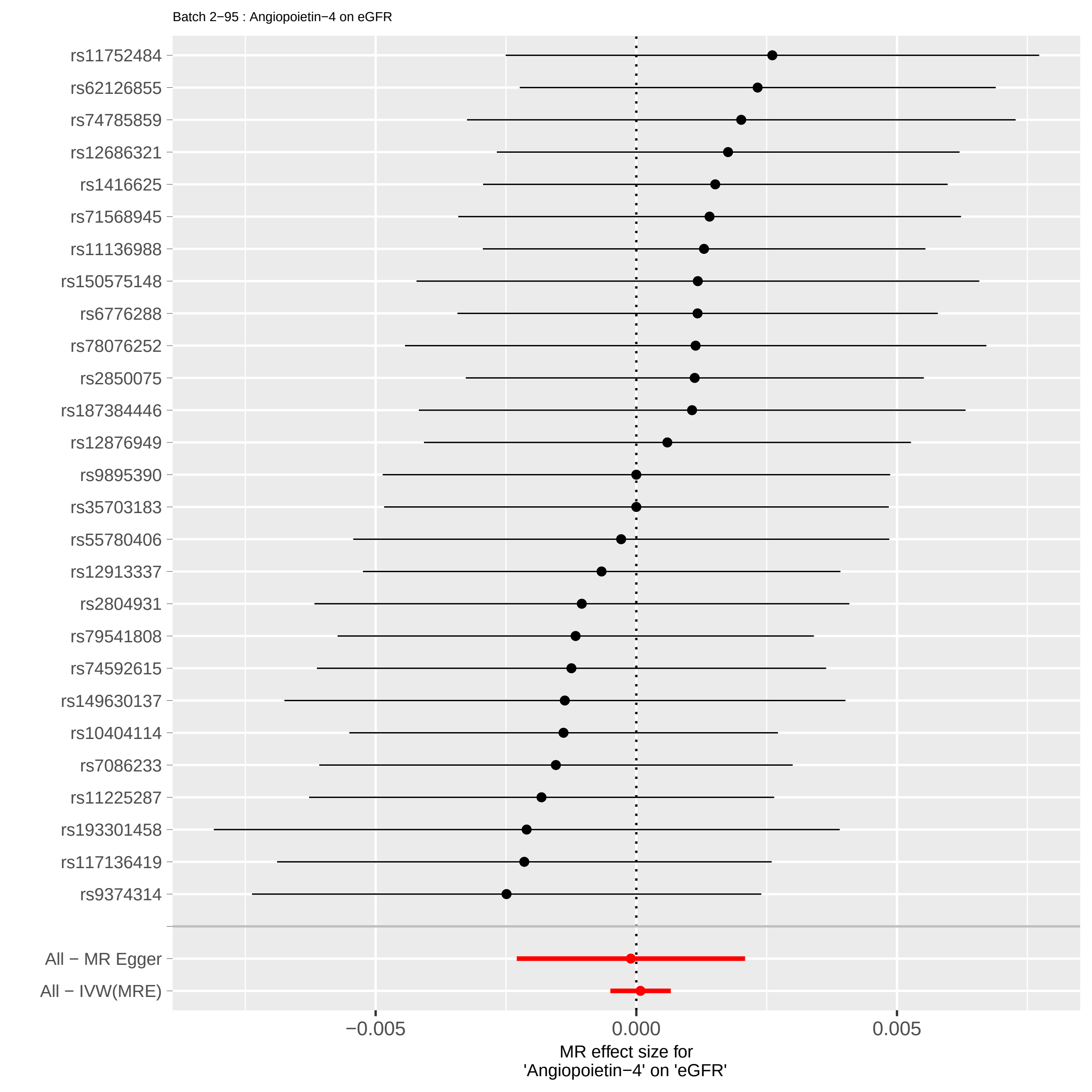

Batch 2−95 : Angiopoietin−4 on eGFR
rs11752484
rs62126855
rs74785859
rs12686321
rs1416625
rs71568945
rs11136988
rs150575148
rs6776288
rs78076252
rs2850075
rs187384446
rs12876949
rs9895390
rs35703183
rs55780406
rs12913337
rs2804931
rs79541808
rs74592615
rs149630137
rs10404114
rs7086233
rs11225287
rs193301458
rs117136419
rs9374314
All − MR Egger
All − IVW(MRE)
−0.005
0.000
0.005
MR effect size for
'Angiopoietin−4' on 'eGFR'

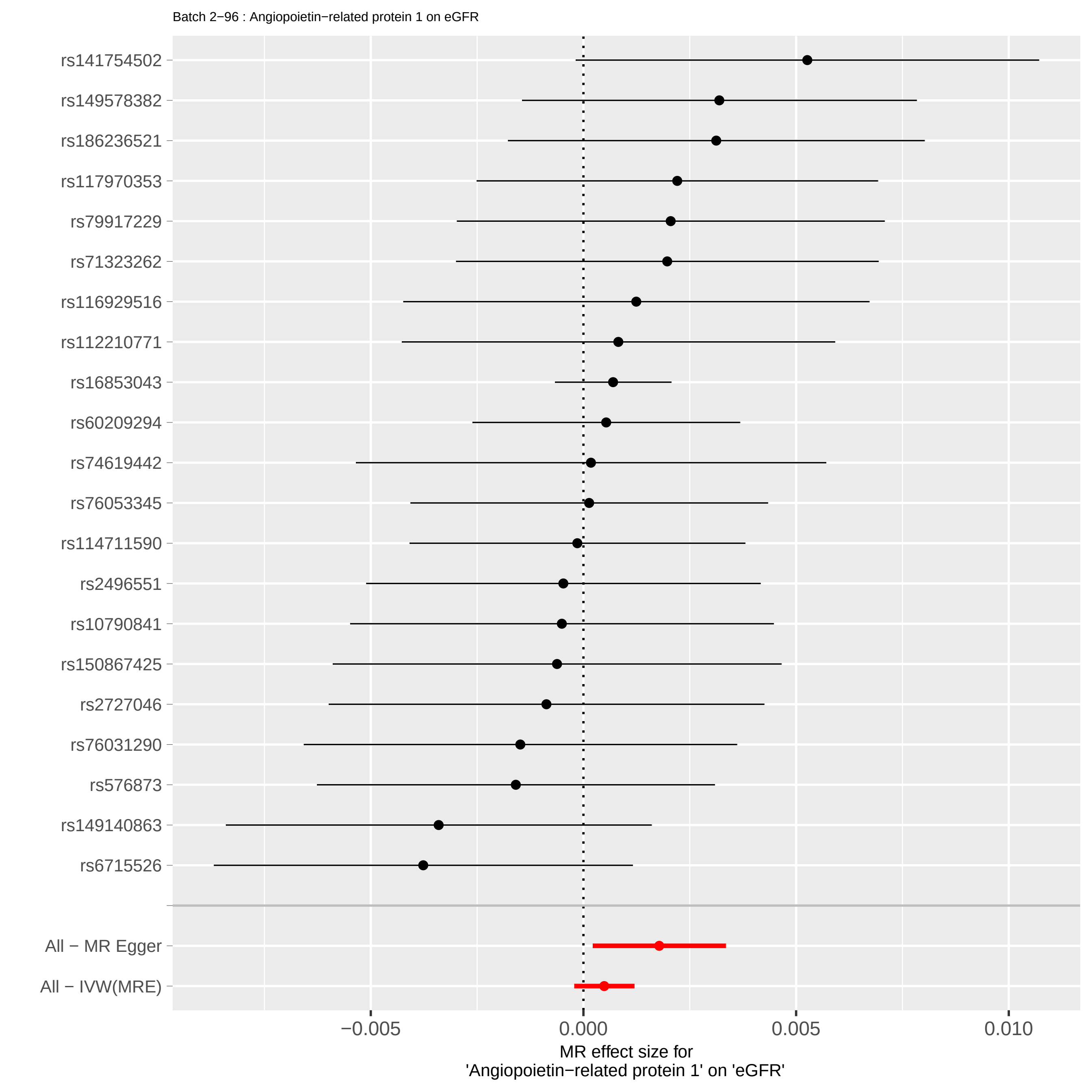

Batch 2−96 : Angiopoietin−related protein 1 on eGFR
rs141754502
rs149578382
rs186236521
rs117970353
rs79917229
rs71323262
rs116929516
rs112210771
rs16853043
rs60209294
rs74619442
rs76053345
rs114711590
rs2496551
rs10790841
rs150867425
rs2727046
rs76031290
rs576873
rs149140863
rs6715526
All − MR Egger
All − IVW(MRE)
−0.005
0.000
0.005
0.010
MR effect size for
'Angiopoietin−related protein 1' on 'eGFR'

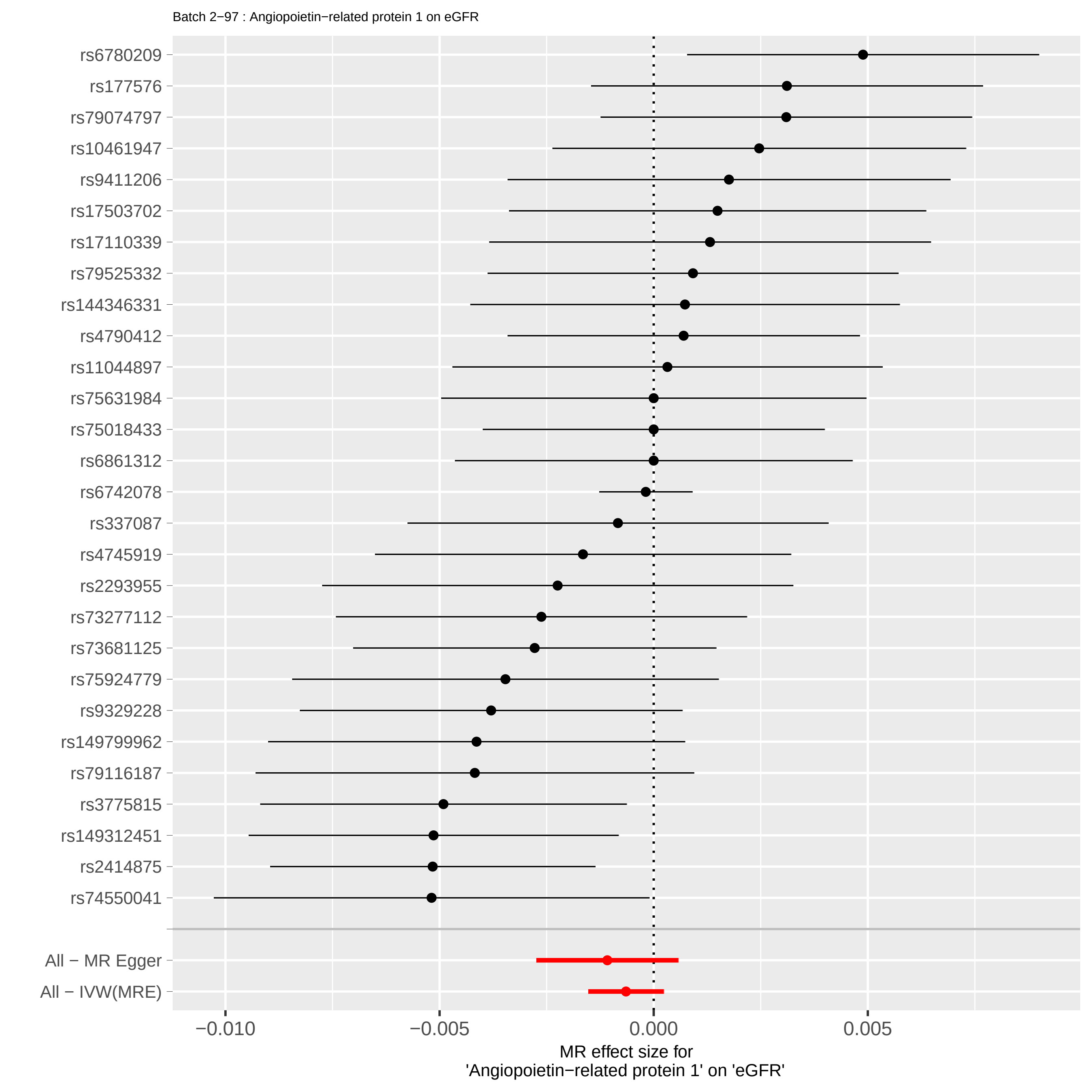

Batch 2−97 : Angiopoietin−related protein 1 on eGFR
rs6780209
rs177576
rs79074797
rs10461947
rs9411206
rs17503702
rs17110339
rs79525332
rs144346331
rs4790412
rs11044897
rs75631984
rs75018433
rs6861312
rs6742078
rs337087
rs4745919
rs2293955
rs73277112
rs73681125
rs75924779
rs9329228
rs149799962
rs79116187
rs3775815
rs149312451
rs2414875
rs74550041
All − MR Egger
All − IVW(MRE)
−0.010
−0.005
0.000
0.005
MR effect size for
'Angiopoietin−related protein 1' on 'eGFR'

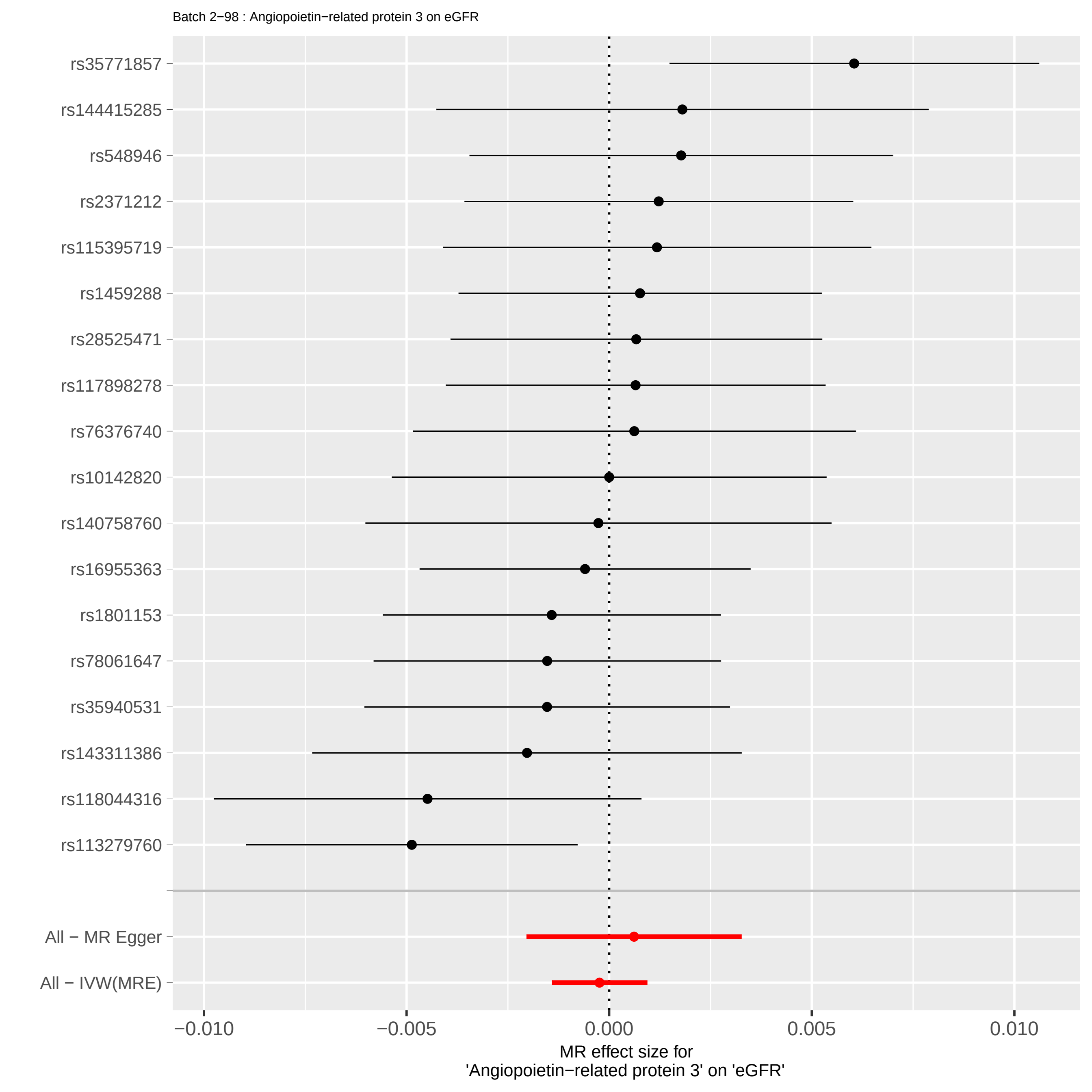

Batch 2−98 : Angiopoietin−related protein 3 on eGFR
rs35771857
rs144415285
rs548946
rs2371212
rs115395719
rs1459288
rs28525471
rs117898278
rs76376740
rs10142820
rs140758760
rs16955363
rs1801153
rs78061647
rs35940531
rs143311386
rs118044316
rs113279760
All − MR Egger
All − IVW(MRE)
−0.010
−0.005
0.000
0.005
0.010
MR effect size for
'Angiopoietin−related protein 3' on 'eGFR'

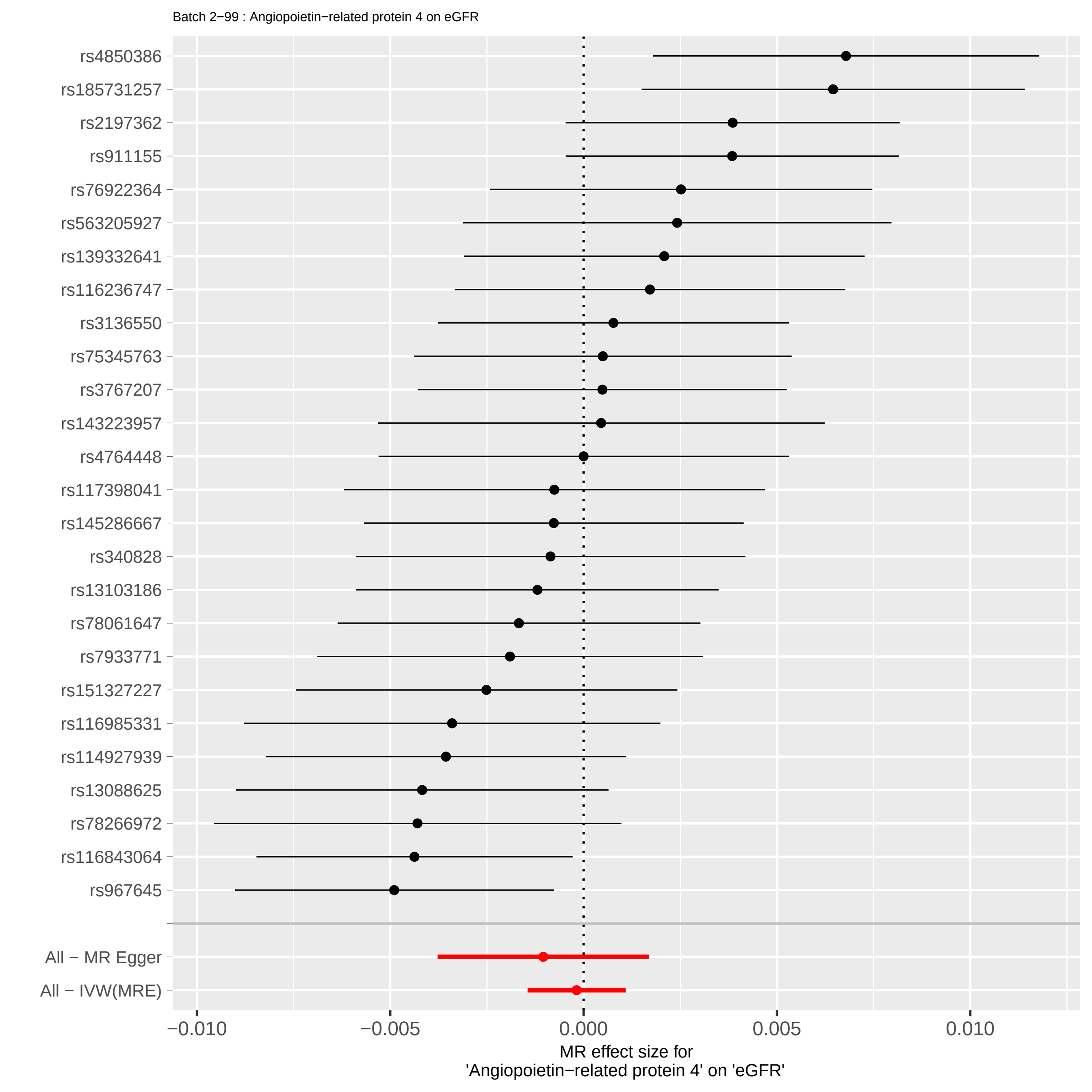

Batch 2−99 : Angiopoietin−related protein 4 on eGFR
rs4850386
rs185731257
rs2197362
rs911155
rs76922364
rs563205927
rs139332641
rs116236747
rs3136550
rs75345763
rs3767207
rs143223957
rs4764448
rs117398041
rs145286667
rs340828
rs13103186
rs78061647
rs7933771
rs151327227
rs116985331
rs114927939
rs13088625
rs78266972
rs116843064
rs967645
All − MR Egger
All − IVW(MRE)
−0.010
−0.005
0.000
0.005
0.010
MR effect size for
'Angiopoietin−related protein 4' on 'eGFR'

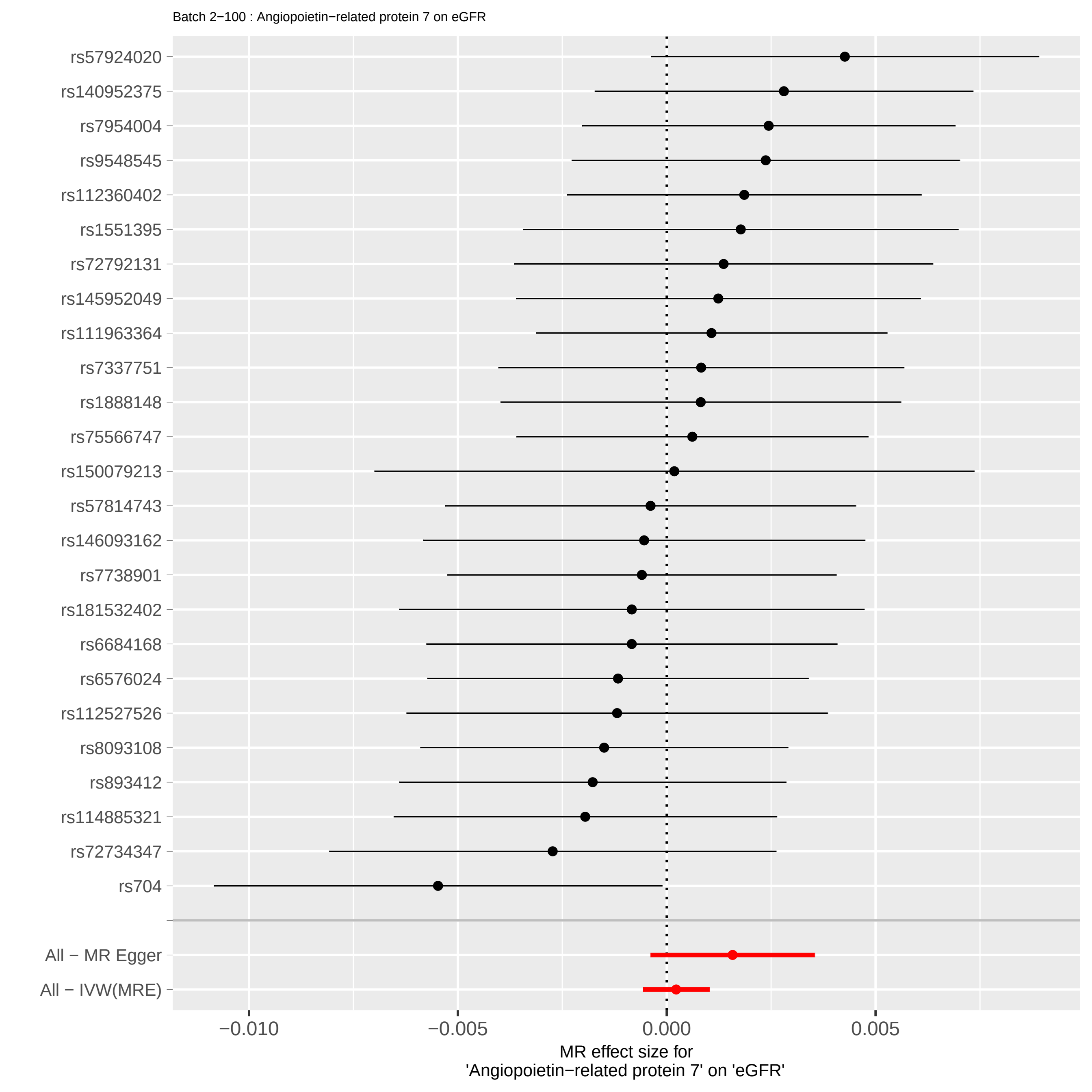

Batch 2−100 : Angiopoietin−related protein 7 on eGFR
rs57924020
rs140952375
rs7954004
rs9548545
rs112360402
rs1551395
rs72792131
rs145952049
rs111963364
rs7337751
rs1888148
rs75566747
rs150079213
rs57814743
rs146093162
rs7738901
rs181532402
rs6684168
rs6576024
rs112527526
rs8093108
rs893412
rs114885321
rs72734347
rs704
All − MR Egger
All − IVW(MRE)
−0.010
−0.005
0.000
0.005
MR effect size for
'Angiopoietin−related protein 7' on 'eGFR'

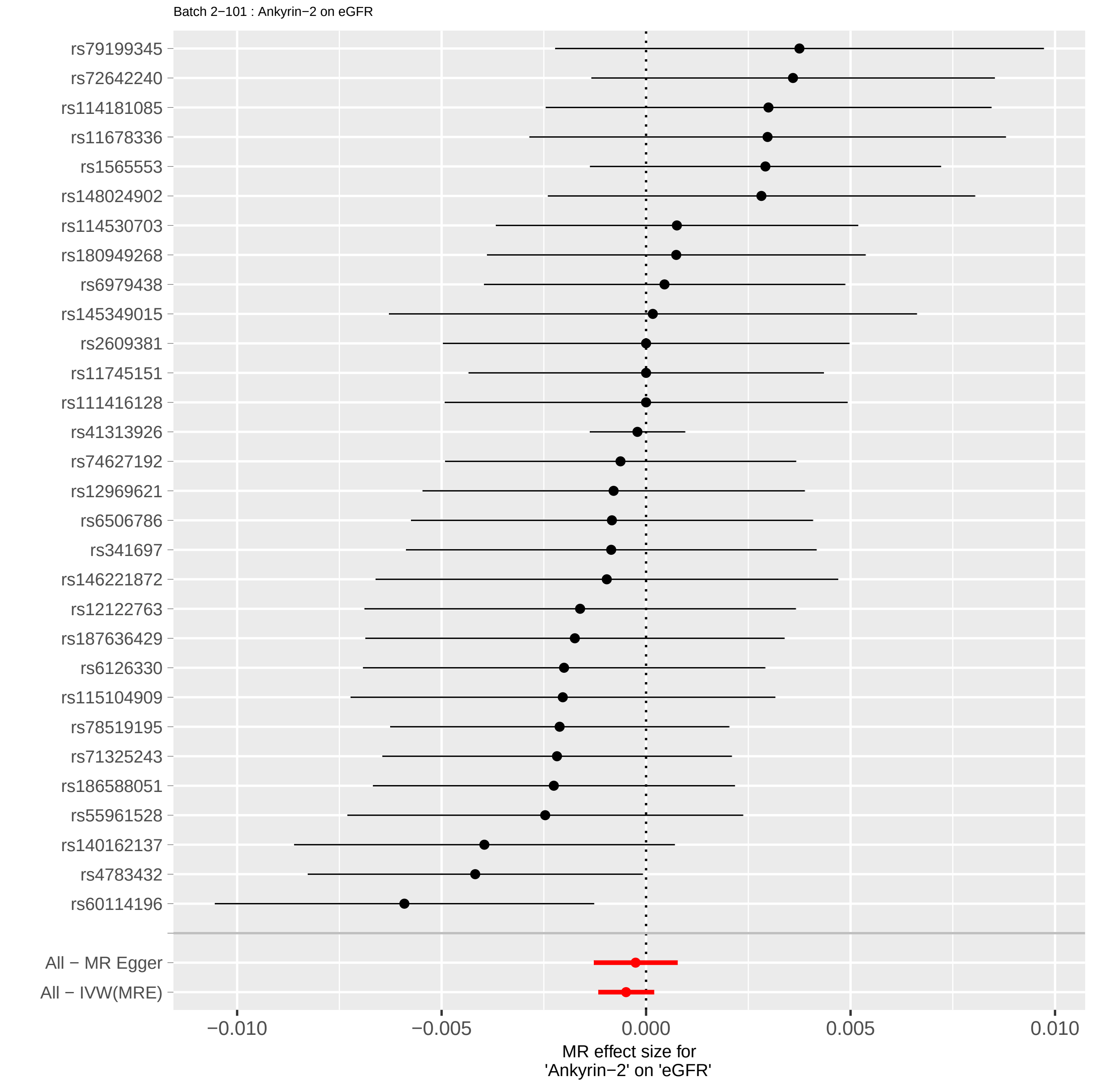

Batch 2−101 : Ankyrin−2 on eGFR
rs79199345
rs72642240
rs114181085
rs11678336
rs1565553
rs148024902
rs114530703
rs180949268
rs6979438
rs145349015
rs2609381
rs11745151
rs111416128
rs41313926
rs74627192
rs12969621
rs6506786
rs341697
rs146221872
rs12122763
rs187636429
rs6126330
rs115104909
rs78519195
rs71325243
rs186588051
rs55961528
rs140162137
rs4783432
rs60114196
All − MR Egger
All − IVW(MRE)
−0.010
−0.005
0.000
0.005
0.010
MR effect size for
'Ankyrin−2' on 'eGFR'

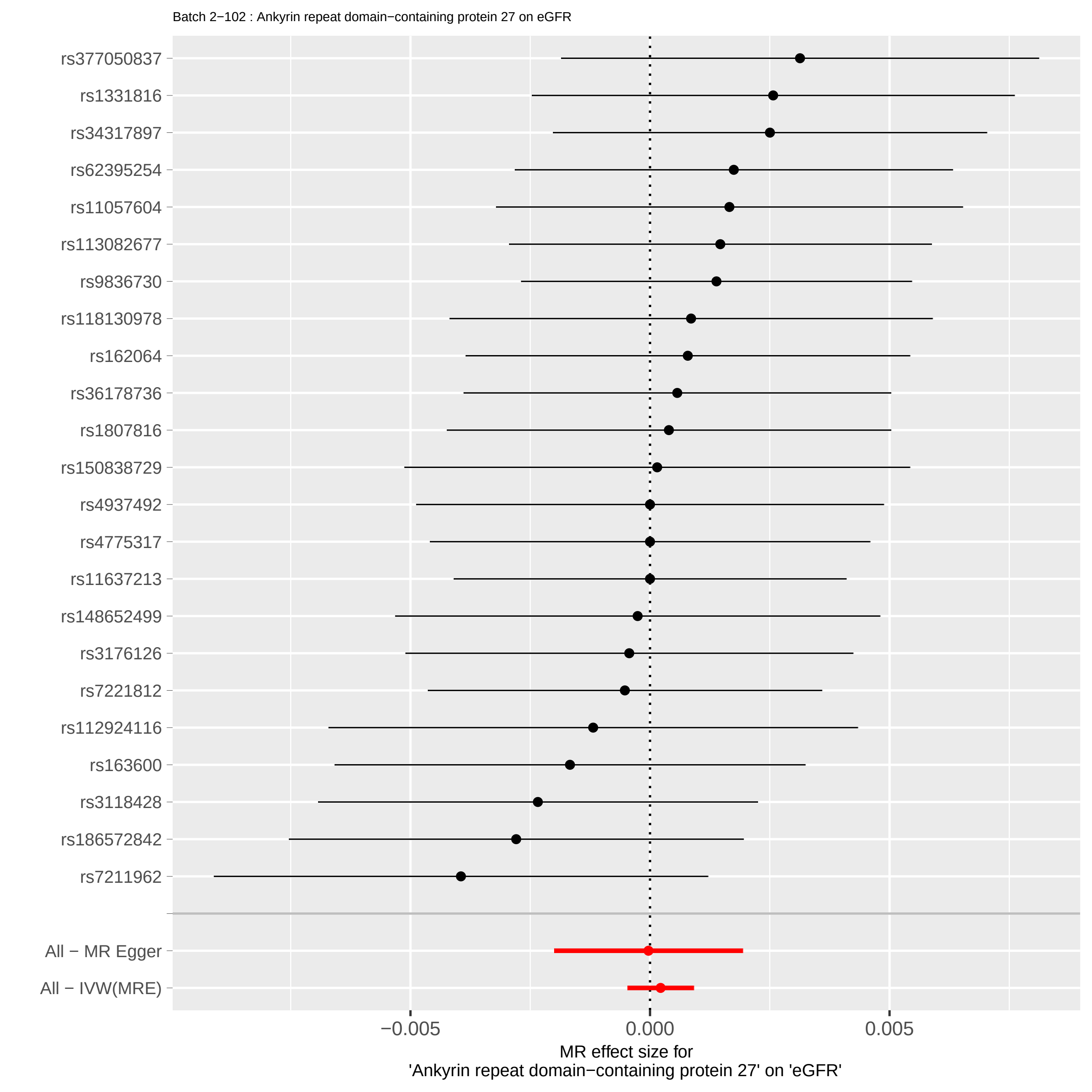

Batch 2−102 : Ankyrin repeat domain−containing protein 27 on eGFR
rs377050837
rs1331816
rs34317897
rs62395254
rs11057604
rs113082677
rs9836730
rs118130978
rs162064
rs36178736
rs1807816
rs150838729
rs4937492
rs4775317
rs11637213
rs148652499
rs3176126
rs7221812
rs112924116
rs163600
rs3118428
rs186572842
rs7211962
All − MR Egger
All − IVW(MRE)
−0.005
0.000
0.005
MR effect size for
'Ankyrin repeat domain−containing protein 27' on 'eGFR'

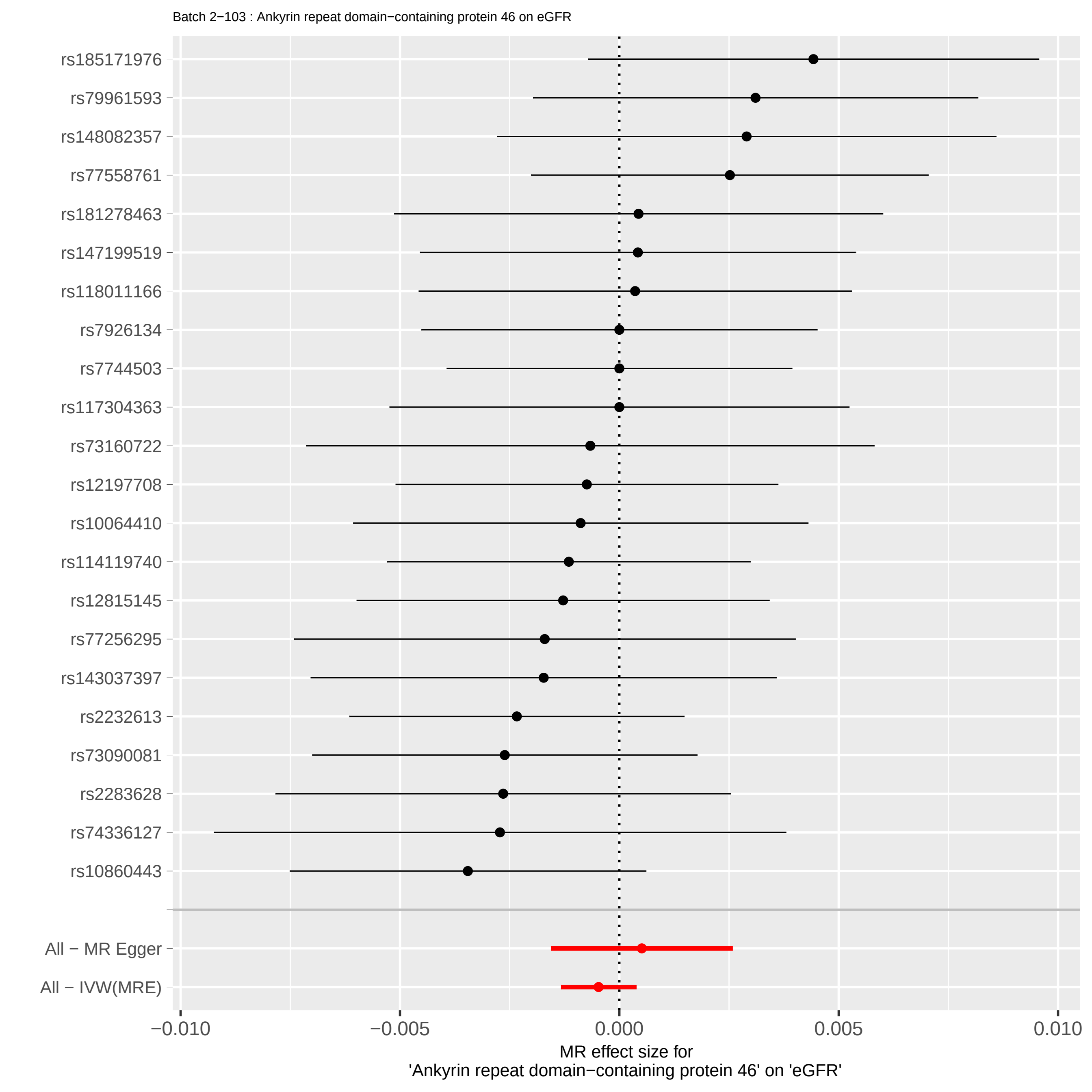

Batch 2−103 : Ankyrin repeat domain−containing protein 46 on eGFR
rs185171976
rs79961593
rs148082357
rs77558761
rs181278463
rs147199519
rs118011166
rs7926134
rs7744503
rs117304363
rs73160722
rs12197708
rs10064410
rs114119740
rs12815145
rs77256295
rs143037397
rs2232613
rs73090081
rs2283628
rs74336127
rs10860443
All − MR Egger
All − IVW(MRE)
−0.010
−0.005
0.000
0.005
0.010
MR effect size for
'Ankyrin repeat domain−containing protein 46' on 'eGFR'

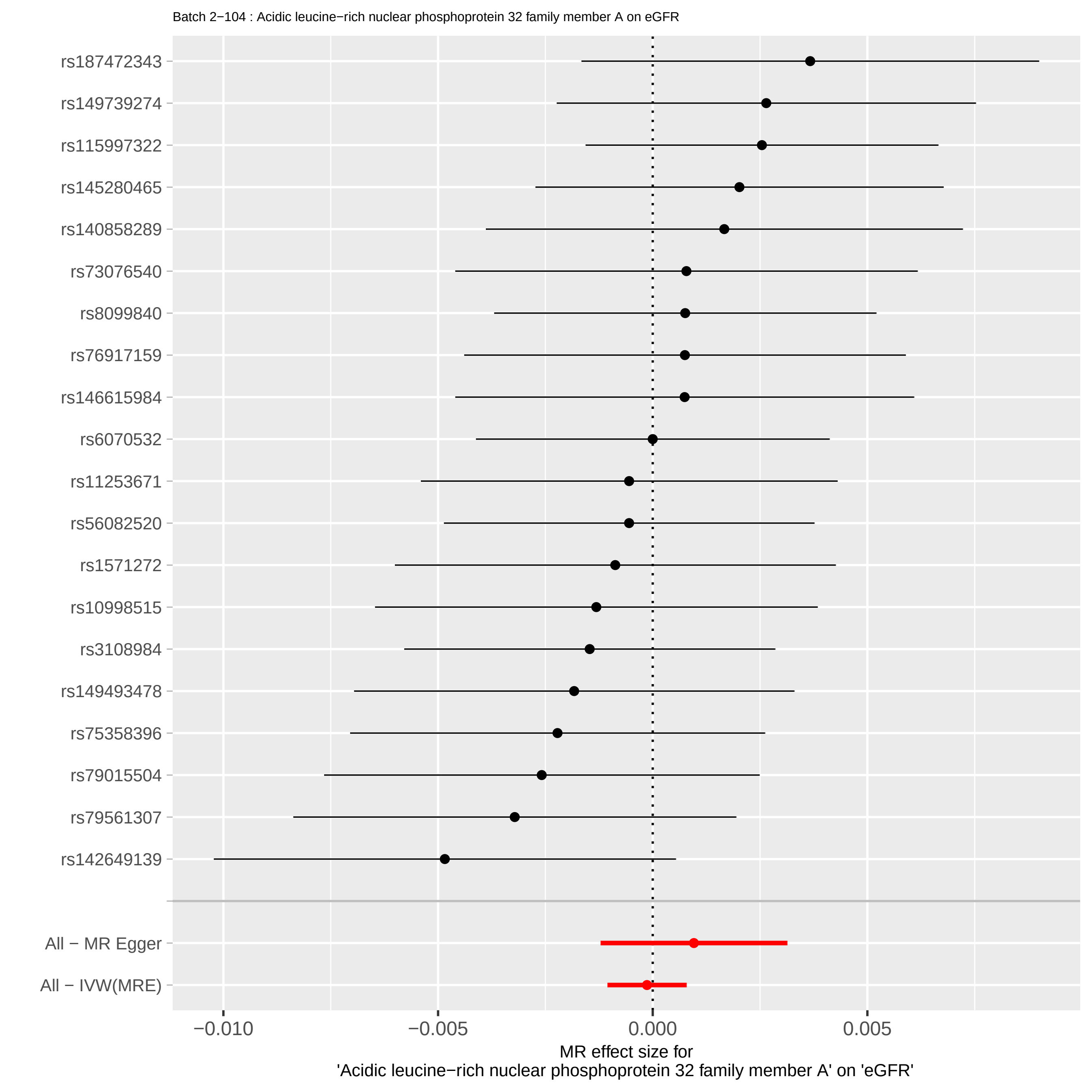

Batch 2−104 : Acidic leucine−rich nuclear phosphoprotein 32 family member A on eGFR
rs187472343
rs149739274
rs115997322
rs145280465
rs140858289
rs73076540
rs8099840
rs76917159
rs146615984
rs6070532
rs11253671
rs56082520
rs1571272
rs10998515
rs3108984
rs149493478
rs75358396
rs79015504
rs79561307
rs142649139
All − MR Egger
All − IVW(MRE)
−0.010
−0.005
0.000
0.005
MR effect size for
'Acidic leucine−rich nuclear phosphoprotein 32 family member A' on 'eGFR'

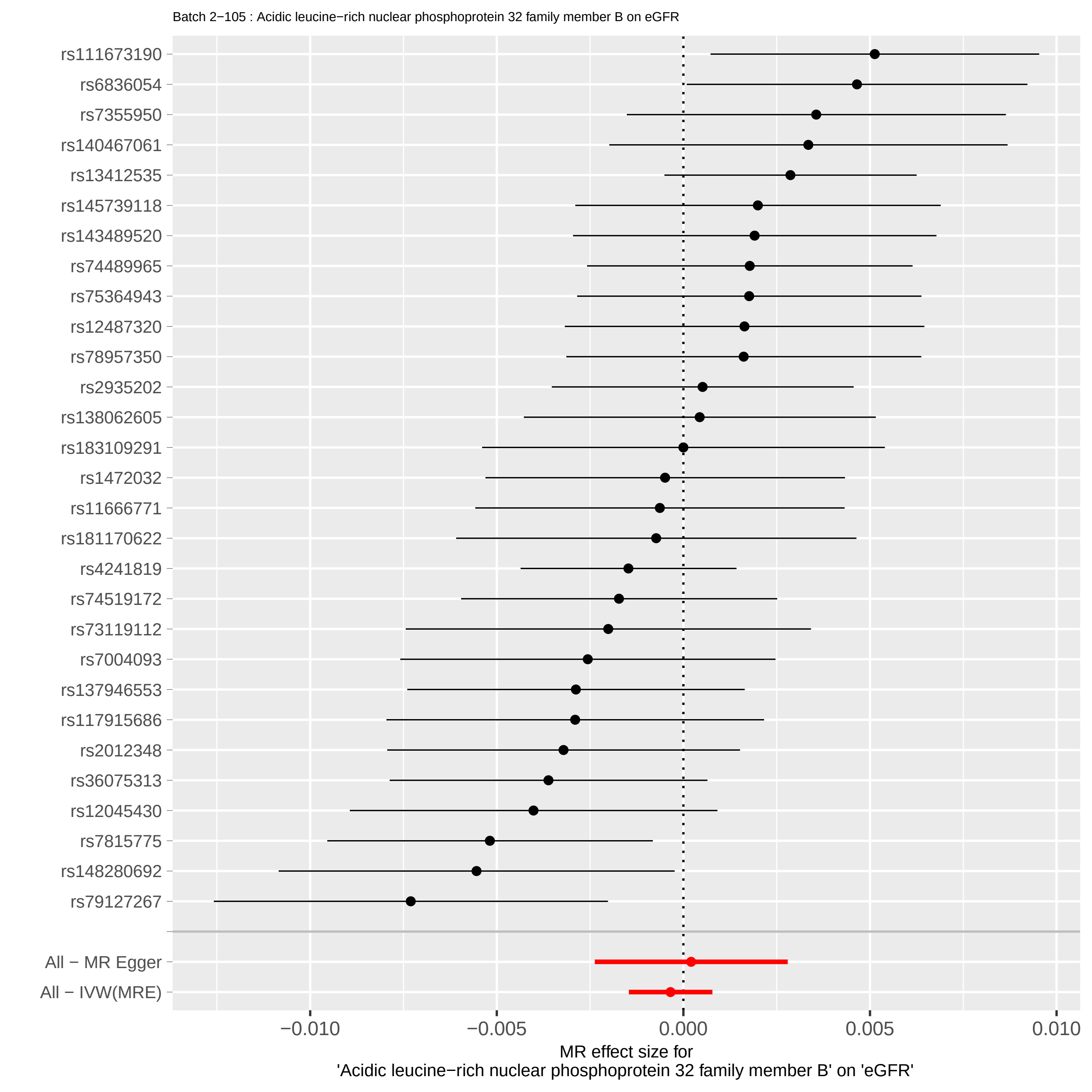
Batch 2−105 : Acidic leucine−rich nuclear phosphoprotein 32 family member B on eGFR
rs111673190
rs6836054
rs7355950
rs140467061
rs13412535
rs145739118
rs143489520
rs74489965
rs75364943
rs12487320
rs78957350
rs2935202
rs138062605
rs183109291
rs1472032
rs11666771
rs181170622
rs4241819
rs74519172
rs73119112
rs7004093
rs137946553
rs117915686
rs2012348
rs36075313
rs12045430
rs7815775
rs148280692
rs79127267
All − MR Egger
All − IVW(MRE)
−0.010
−0.005
0.000
0.005
0.010
MR effect size for
'Acidic leucine−rich nuclear phosphoprotein 32 family member B' on 'eGFR'

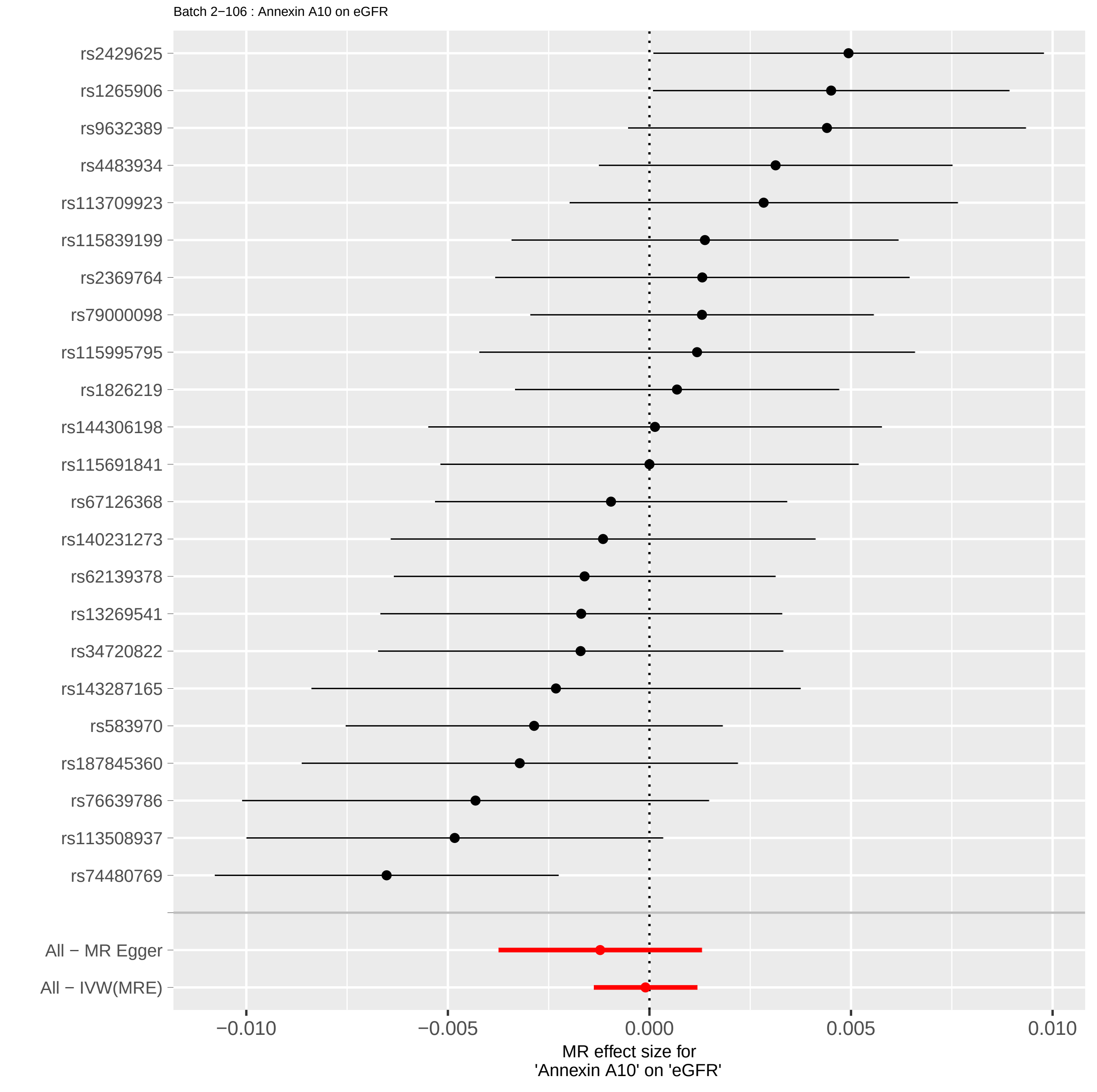
Batch 2−106 : Annexin A10 on eGFR
rs2429625
rs1265906
rs9632389
rs4483934
rs113709923
rs115839199
rs2369764
rs79000098
rs115995795
rs1826219
rs144306198
rs115691841
rs67126368
rs140231273
rs62139378
rs13269541
rs34720822
rs143287165
rs583970
rs187845360
rs76639786
rs113508937
rs74480769
All − MR Egger
All − IVW(MRE)
−0.010
−0.005
0.000
0.005
0.010
MR effect size for
'Annexin A10' on 'eGFR'

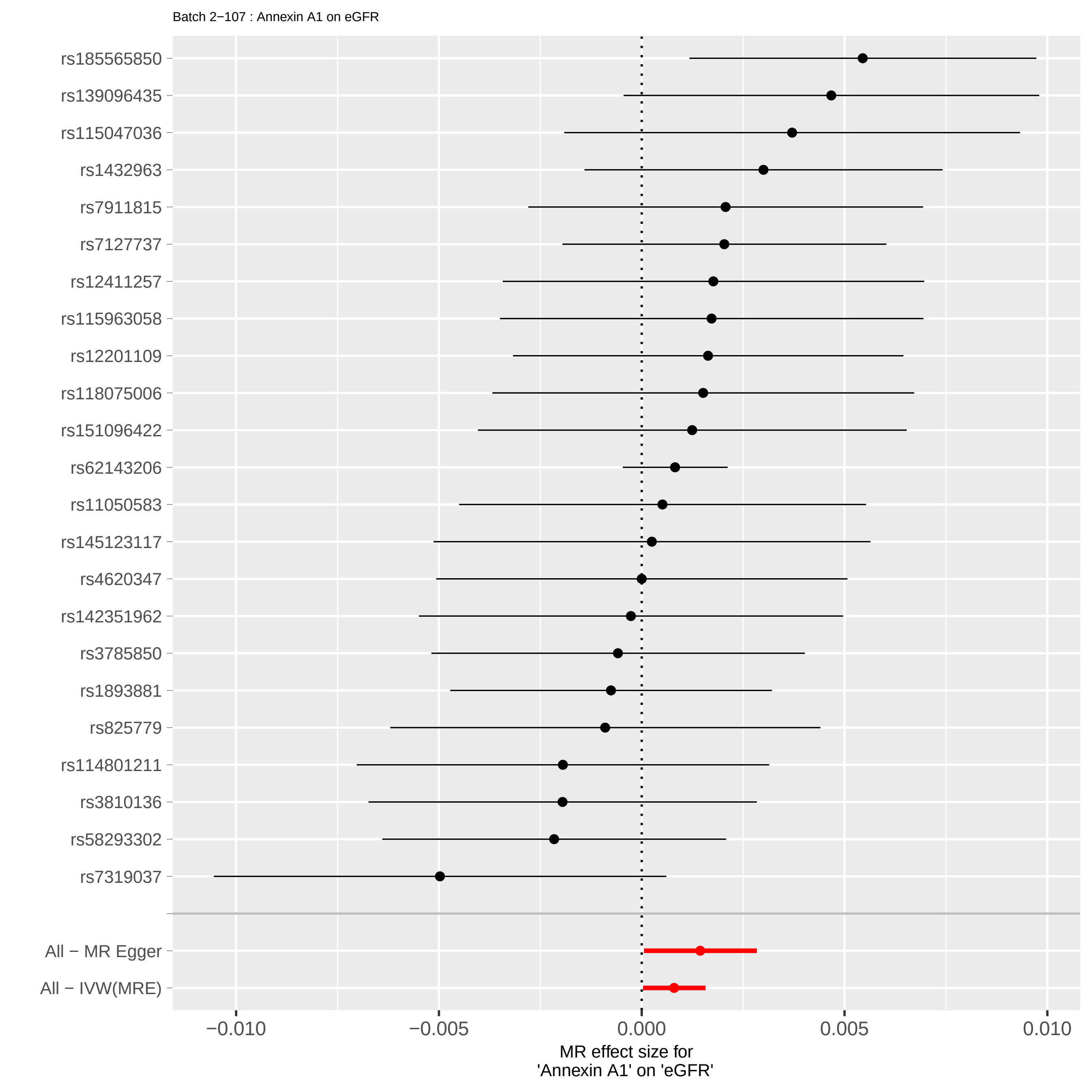

Batch 2−107 : Annexin A1 on eGFR
rs185565850
rs139096435
rs115047036
rs1432963
rs7911815
rs7127737
rs12411257
rs115963058
rs12201109
rs118075006
rs151096422
rs62143206
rs11050583
rs145123117
rs4620347
rs142351962
rs3785850
rs1893881
rs825779
rs114801211
rs3810136
rs58293302
rs7319037
All − MR Egger
All − IVW(MRE)
−0.010
−0.005
0.000
0.005
0.010
MR effect size for
'Annexin A1' on 'eGFR'

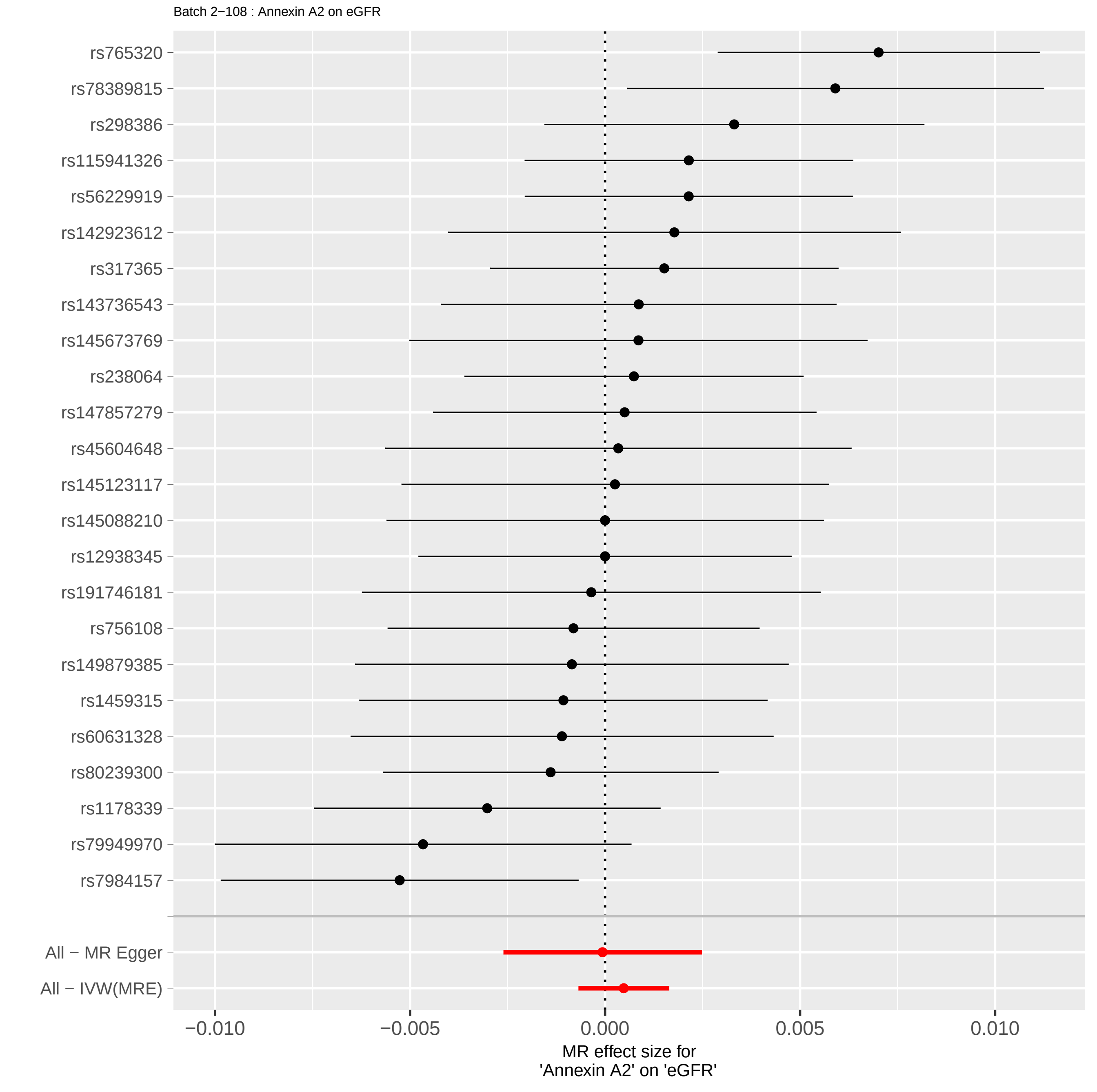

Batch 2−108 : Annexin A2 on eGFR
rs765320
rs78389815
rs298386
rs115941326
rs56229919
rs142923612
rs317365
rs143736543
rs145673769
rs238064
rs147857279
rs45604648
rs145123117
rs145088210
rs12938345
rs191746181
rs756108
rs149879385
rs1459315
rs60631328
rs80239300
rs1178339
rs79949970
rs7984157
All − MR Egger
All − IVW(MRE)
−0.010
−0.005
0.000
0.005
0.010
MR effect size for
'Annexin A2' on 'eGFR'

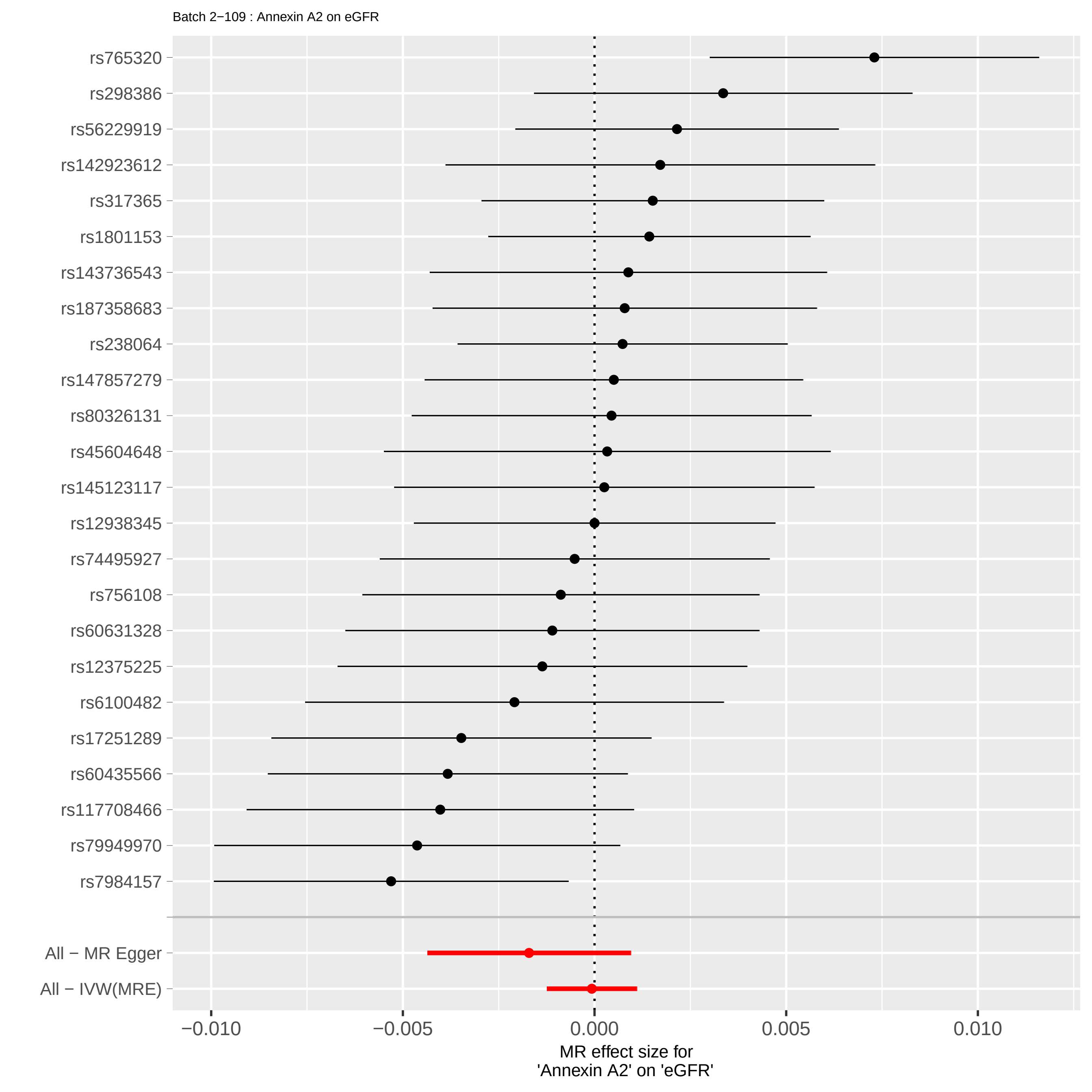
Batch 2−109 : Annexin A2 on eGFR
rs765320
rs298386
rs56229919
rs142923612
rs317365
rs1801153
rs143736543
rs187358683
rs238064
rs147857279
rs80326131
rs45604648
rs145123117
rs12938345
rs74495927
rs756108
rs60631328
rs12375225
rs6100482
rs17251289
rs60435566
rs117708466
rs79949970
rs7984157
All − MR Egger
All − IVW(MRE)
−0.010
−0.005
0.000
0.005
0.010
MR effect size for
'Annexin A2' on 'eGFR'

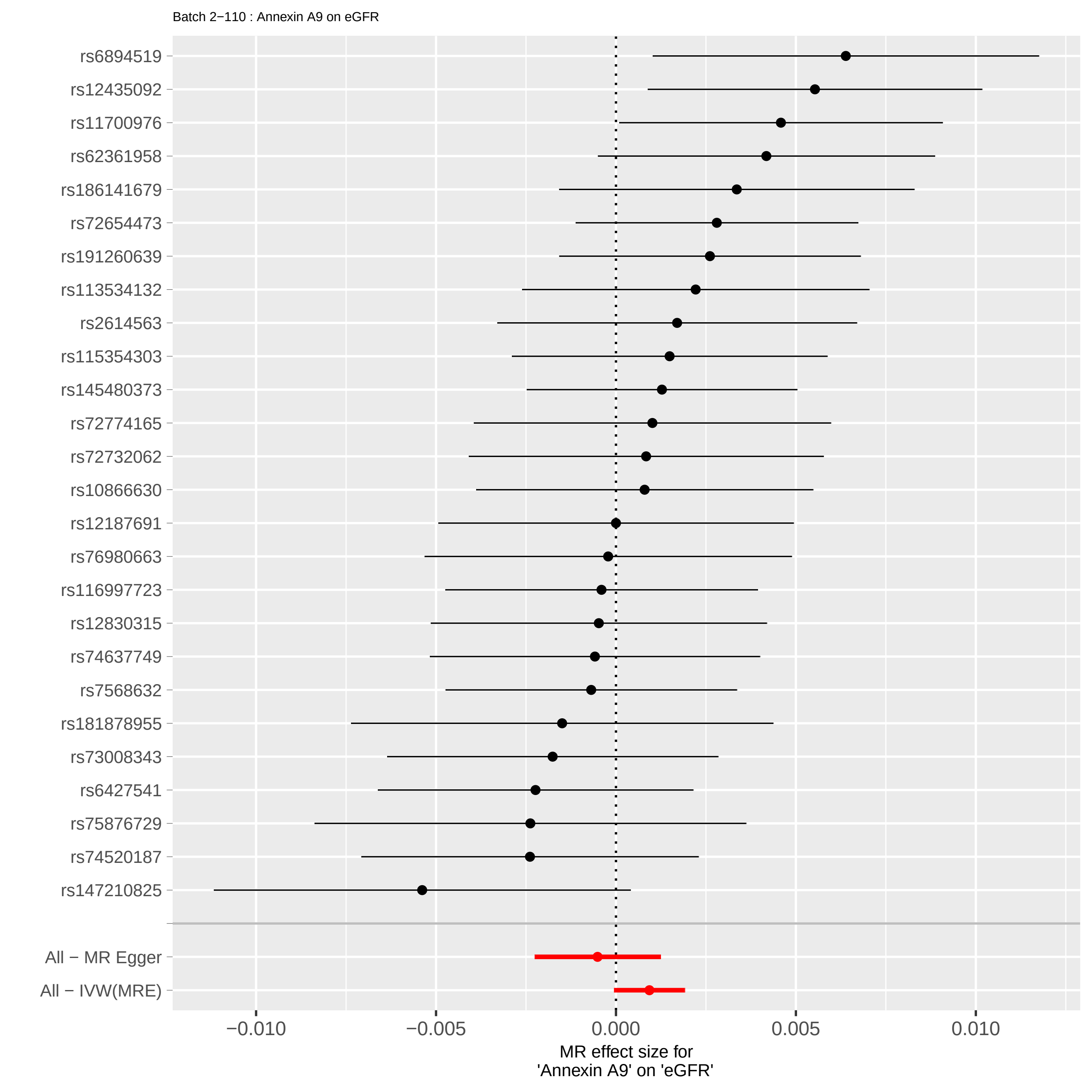
Batch 2−110 : Annexin A9 on eGFR
rs6894519
rs12435092
rs11700976
rs62361958
rs186141679
rs72654473
rs191260639
rs113534132
rs2614563
rs115354303
rs145480373
rs72774165
rs72732062
rs10866630
rs12187691
rs76980663
rs116997723
rs12830315
rs74637749
rs7568632
rs181878955
rs73008343
rs6427541
rs75876729
rs74520187
rs147210825
All − MR Egger
All − IVW(MRE)
−0.010
−0.005
0.000
0.005
0.010
MR effect size for
'Annexin A9' on 'eGFR'

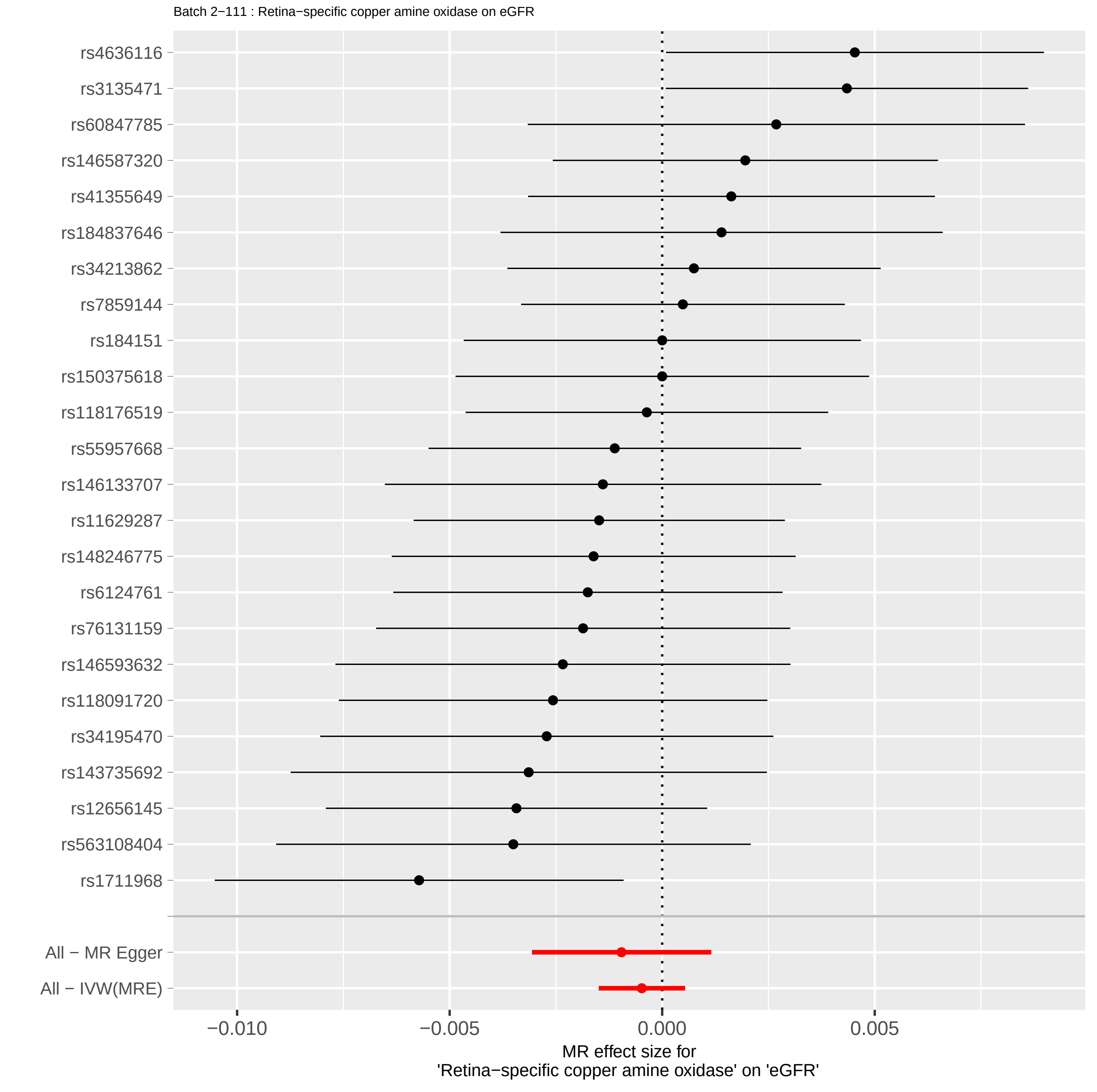

Batch 2−111 : Retina−specific copper amine oxidase on eGFR
rs4636116
rs3135471
rs60847785
rs146587320
rs41355649
rs184837646
rs34213862
rs7859144
rs184151
rs150375618
rs118176519
rs55957668
rs146133707
rs11629287
rs148246775
rs6124761
rs76131159
rs146593632
rs118091720
rs34195470
rs143735692
rs12656145
rs563108404
rs1711968
All − MR Egger
All − IVW(MRE)
−0.010
−0.005
0.000
0.005
MR effect size for
'Retina−specific copper amine oxidase' on 'eGFR'

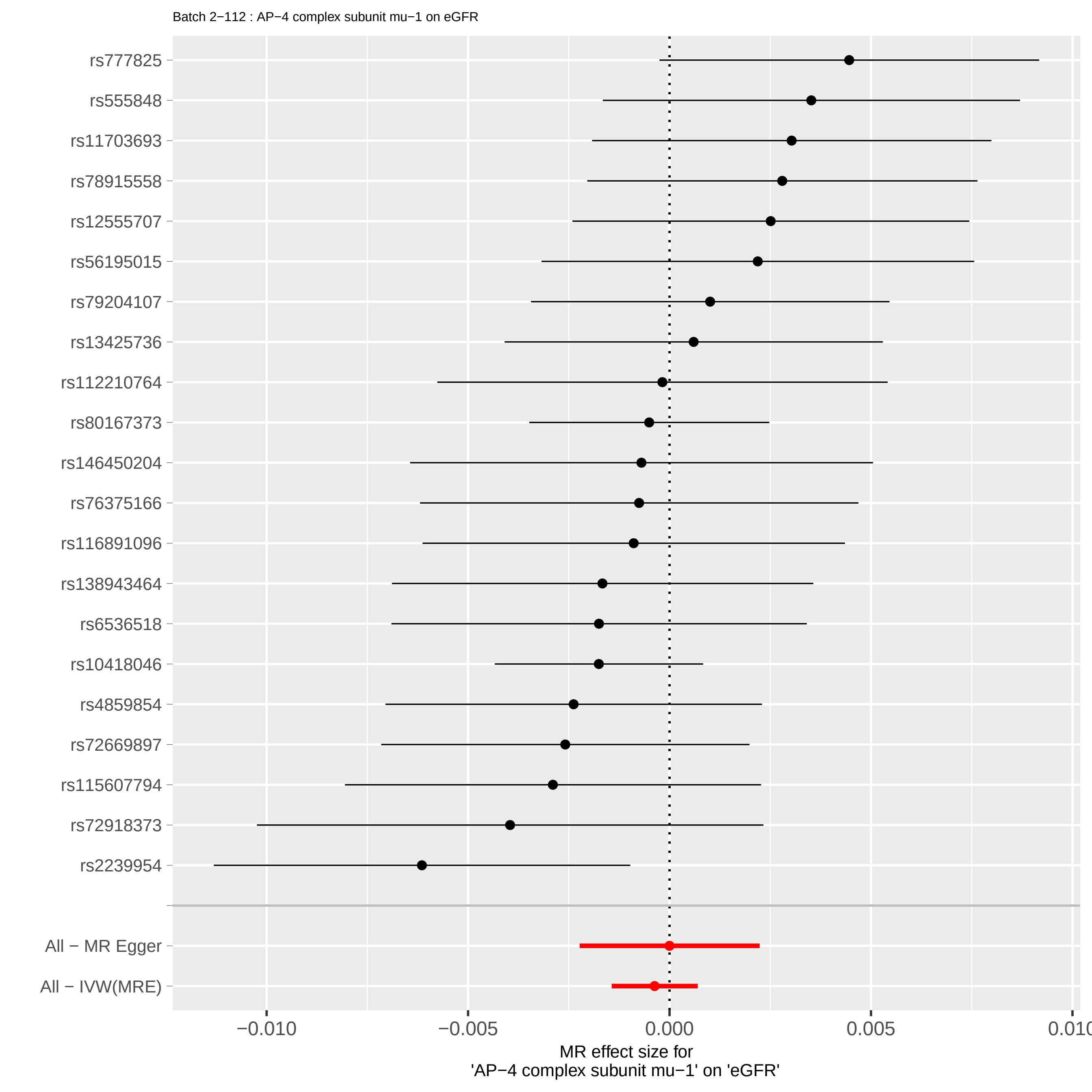
Batch 2−112 : AP−4 complex subunit mu−1 on eGFR
rs777825
rs555848
rs11703693
rs78915558
rs12555707
rs56195015
rs79204107
rs13425736
rs112210764
rs80167373
rs146450204
rs76375166
rs116891096
rs138943464
rs6536518
rs10418046
rs4859854
rs72669897
rs115607794
rs72918373
rs2239954
All − MR Egger
All − IVW(MRE)
−0.010
−0.005
0.000
0.005
0.010
MR effect size for
'AP−4 complex subunit mu−1' on 'eGFR'

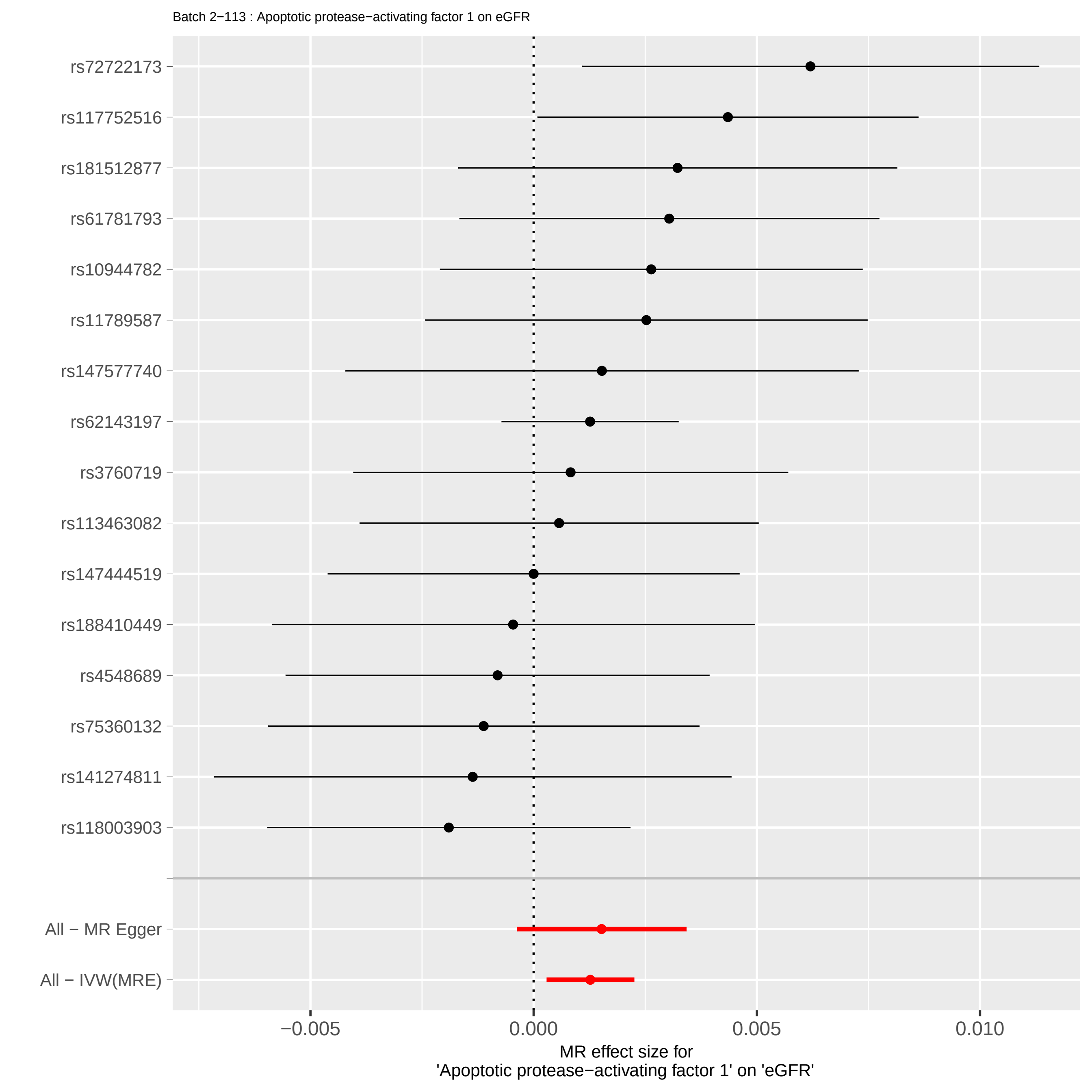

Batch 2−113 : Apoptotic protease−activating factor 1 on eGFR
rs72722173
rs117752516
rs181512877
rs61781793
rs10944782
rs11789587
rs147577740
rs62143197
rs3760719
rs113463082
rs147444519
rs188410449
rs4548689
rs75360132
rs141274811
rs118003903
All − MR Egger
All − IVW(MRE)
−0.005
0.000
0.005
0.010
MR effect size for
'Apoptotic protease−activating factor 1' on 'eGFR'

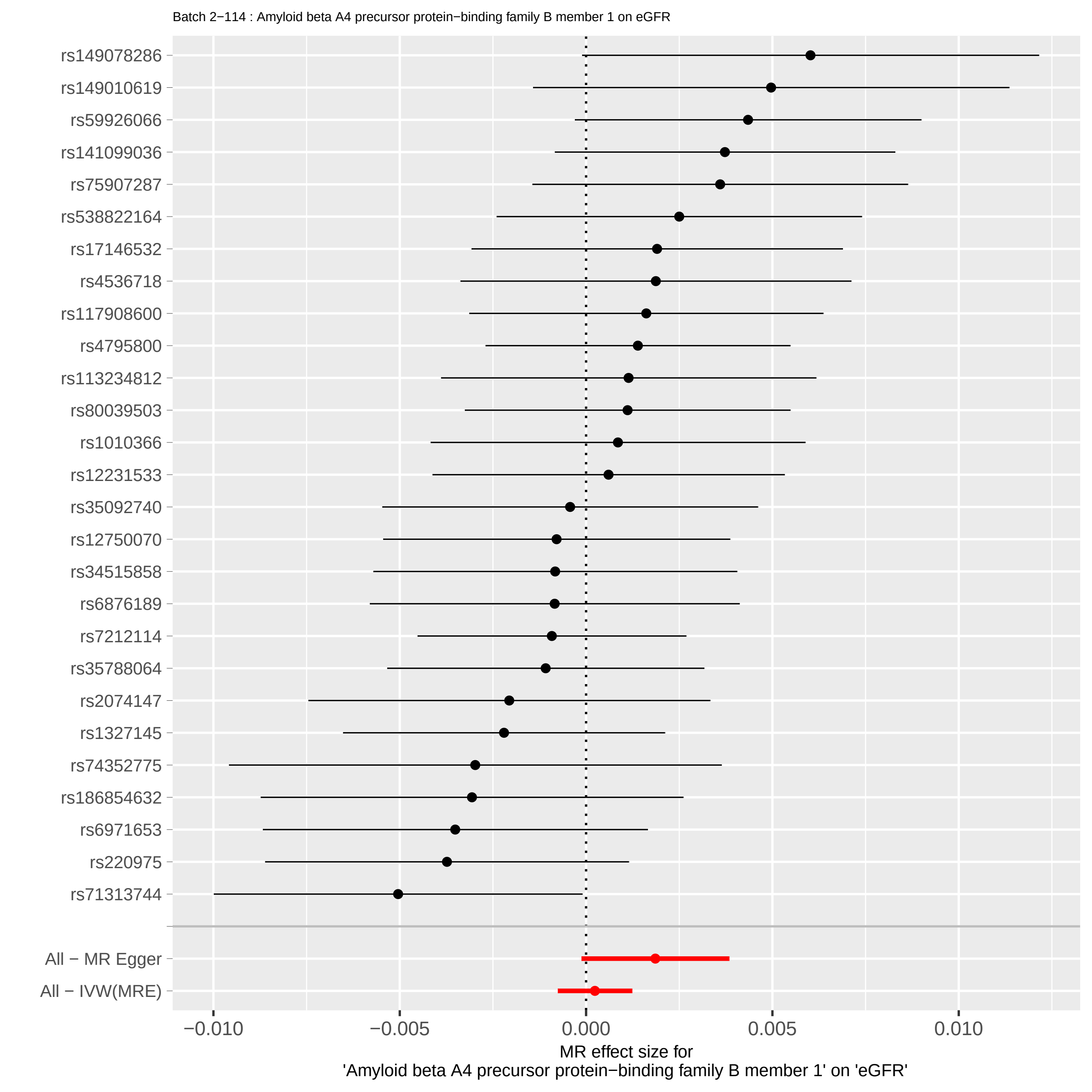

Batch 2−114 : Amyloid beta A4 precursor protein−binding family B member 1 on eGFR
rs149078286
rs149010619
rs59926066
rs141099036
rs75907287
rs538822164
rs17146532
rs4536718
rs117908600
rs4795800
rs113234812
rs80039503
rs1010366
rs12231533
rs35092740
rs12750070
rs34515858
rs6876189
rs7212114
rs35788064
rs2074147
rs1327145
rs74352775
rs186854632
rs6971653
rs220975
rs71313744
All − MR Egger
All − IVW(MRE)
−0.010
−0.005
0.000
0.005
0.010
MR effect size for
'Amyloid beta A4 precursor protein−binding family B member 1' on 'eGFR'

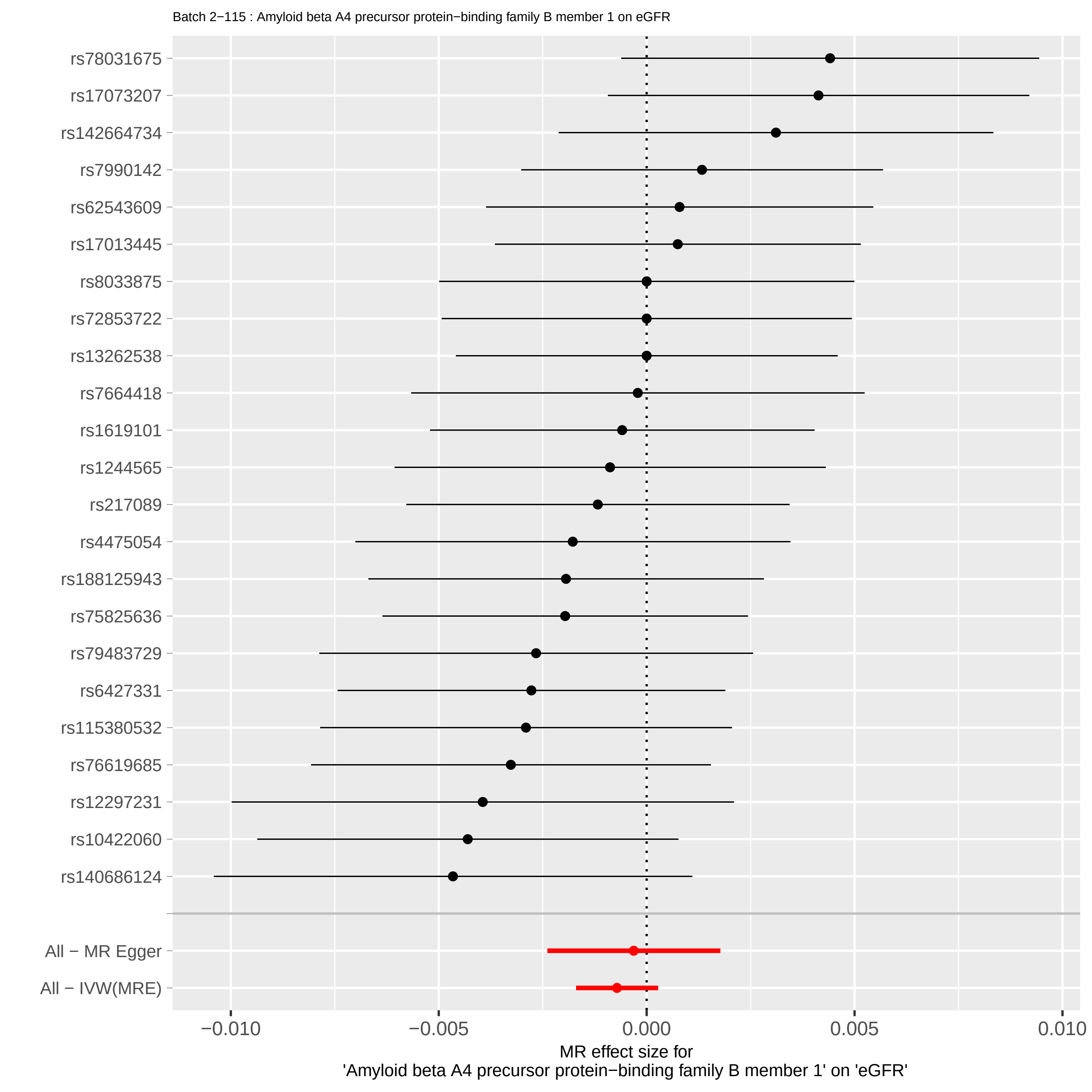

Batch 2−115 : Amyloid beta A4 precursor protein−binding family B member 1 on eGFR
rs78031675
rs17073207
rs142664734
rs7990142
rs62543609
rs17013445
rs8033875
rs72853722
rs13262538
rs7664418
rs1619101
rs1244565
rs217089
rs4475054
rs188125943
rs75825636
rs79483729
rs6427331
rs115380532
rs76619685
rs12297231
rs10422060
rs140686124
All − MR Egger
All − IVW(MRE)
−0.010
−0.005
0.000
0.005
0.010
MR effect size for
'Amyloid beta A4 precursor protein−binding family B member 1' on 'eGFR'

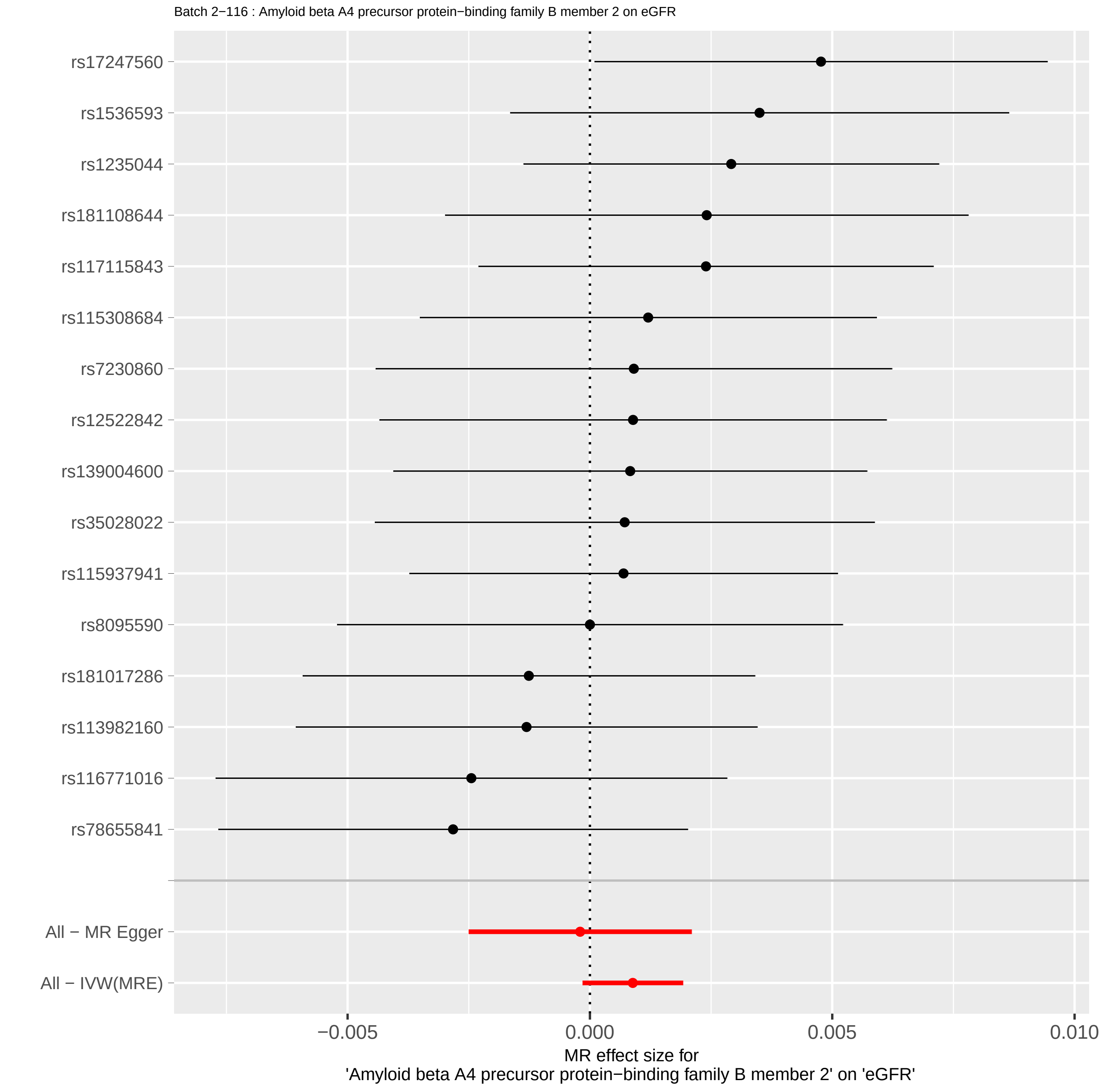

Batch 2−116 : Amyloid beta A4 precursor protein−binding family B member 2 on eGFR
rs17247560
rs1536593
rs1235044
rs181108644
rs117115843
rs115308684
rs7230860
rs12522842
rs139004600
rs35028022
rs115937941
rs8095590
rs181017286
rs113982160
rs116771016
rs78655841
All − MR Egger
All − IVW(MRE)
−0.005
0.000
0.005
0.010
MR effect size for
'Amyloid beta A4 precursor protein−binding family B member 2' on 'eGFR'

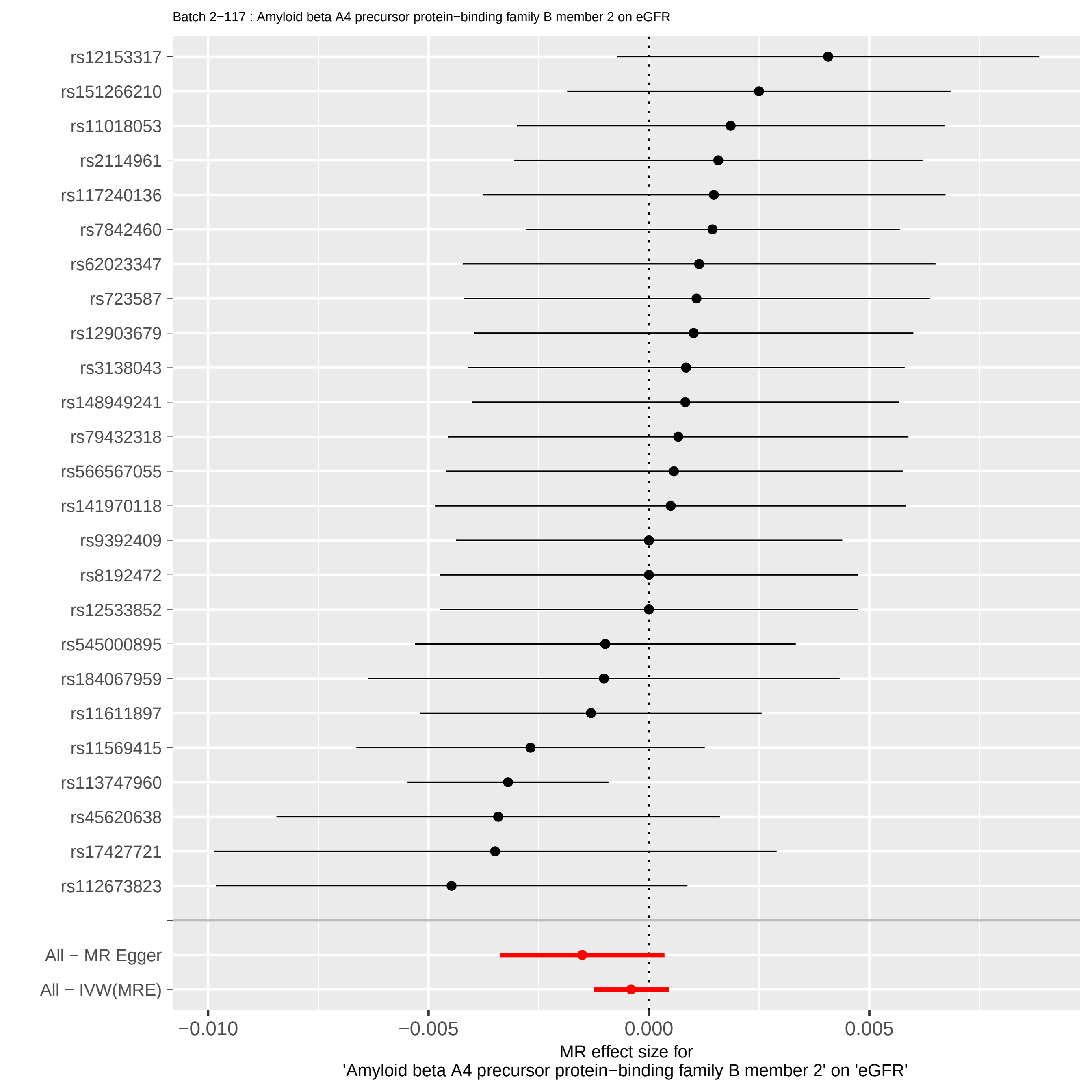
Batch 2−117 : Amyloid beta A4 precursor protein−binding family B member 2 on eGFR
rs12153317
rs151266210
rs11018053
rs2114961
rs117240136
rs7842460
rs62023347
rs723587
rs12903679
rs3138043
rs148949241
rs79432318
rs566567055
rs141970118
rs9392409
rs8192472
rs12533852
rs545000895
rs184067959
rs11611897
rs11569415
rs113747960
rs45620638
rs17427721
rs112673823
All − MR Egger
All − IVW(MRE)
−0.010
−0.005
0.000
0.005
MR effect size for
'Amyloid beta A4 precursor protein−binding family B member 2' on 'eGFR'

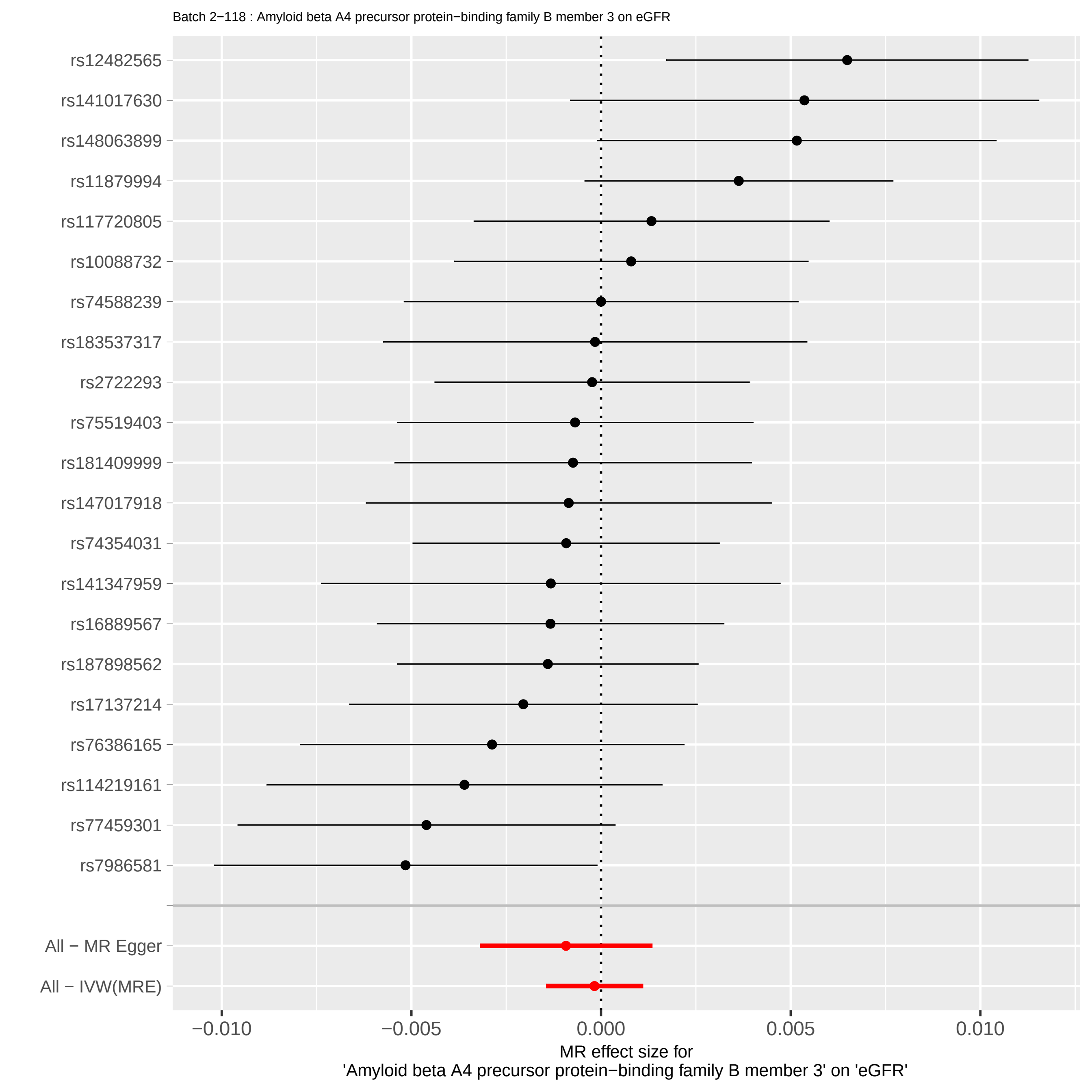
Batch 2−118 : Amyloid beta A4 precursor protein−binding family B member 3 on eGFR
rs12482565
rs141017630
rs148063899
rs11879994
rs117720805
rs10088732
rs74588239
rs183537317
rs2722293
rs75519403
rs181409999
rs147017918
rs74354031
rs141347959
rs16889567
rs187898562
rs17137214
rs76386165
rs114219161
rs77459301
rs7986581
All − MR Egger
All − IVW(MRE)
−0.010
−0.005
0.000
0.005
0.010
MR effect size for
'Amyloid beta A4 precursor protein−binding family B member 3' on 'eGFR'

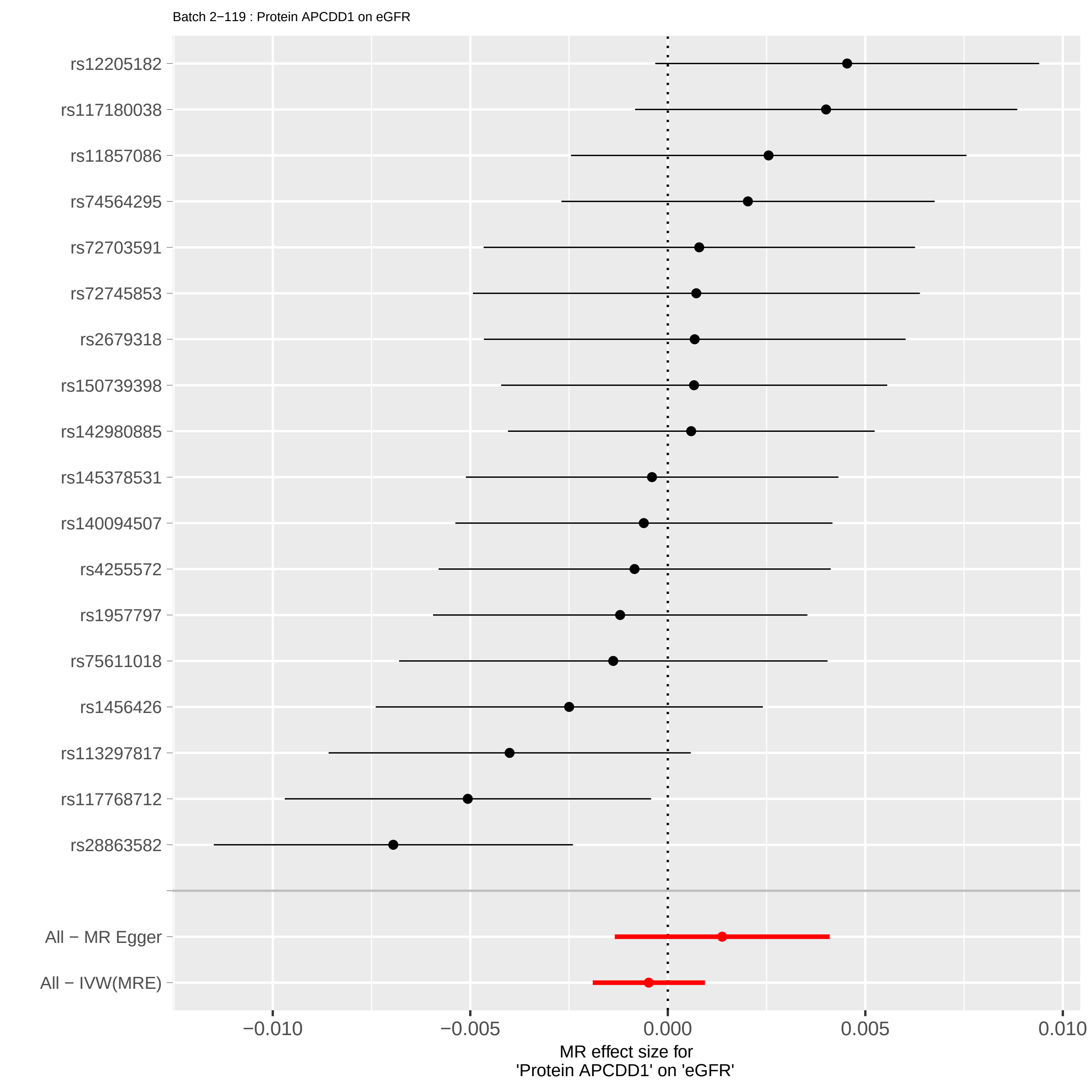
Batch 2−119 : Protein APCDD1 on eGFR
rs12205182
rs117180038
rs11857086
rs74564295
rs72703591
rs72745853
rs2679318
rs150739398
rs142980885
rs145378531
rs140094507
rs4255572
rs1957797
rs75611018
rs1456426
rs113297817
rs117768712
rs28863582
All − MR Egger
All − IVW(MRE)
−0.010
−0.005
0.000
0.005
0.010
MR effect size for
'Protein APCDD1' on 'eGFR'

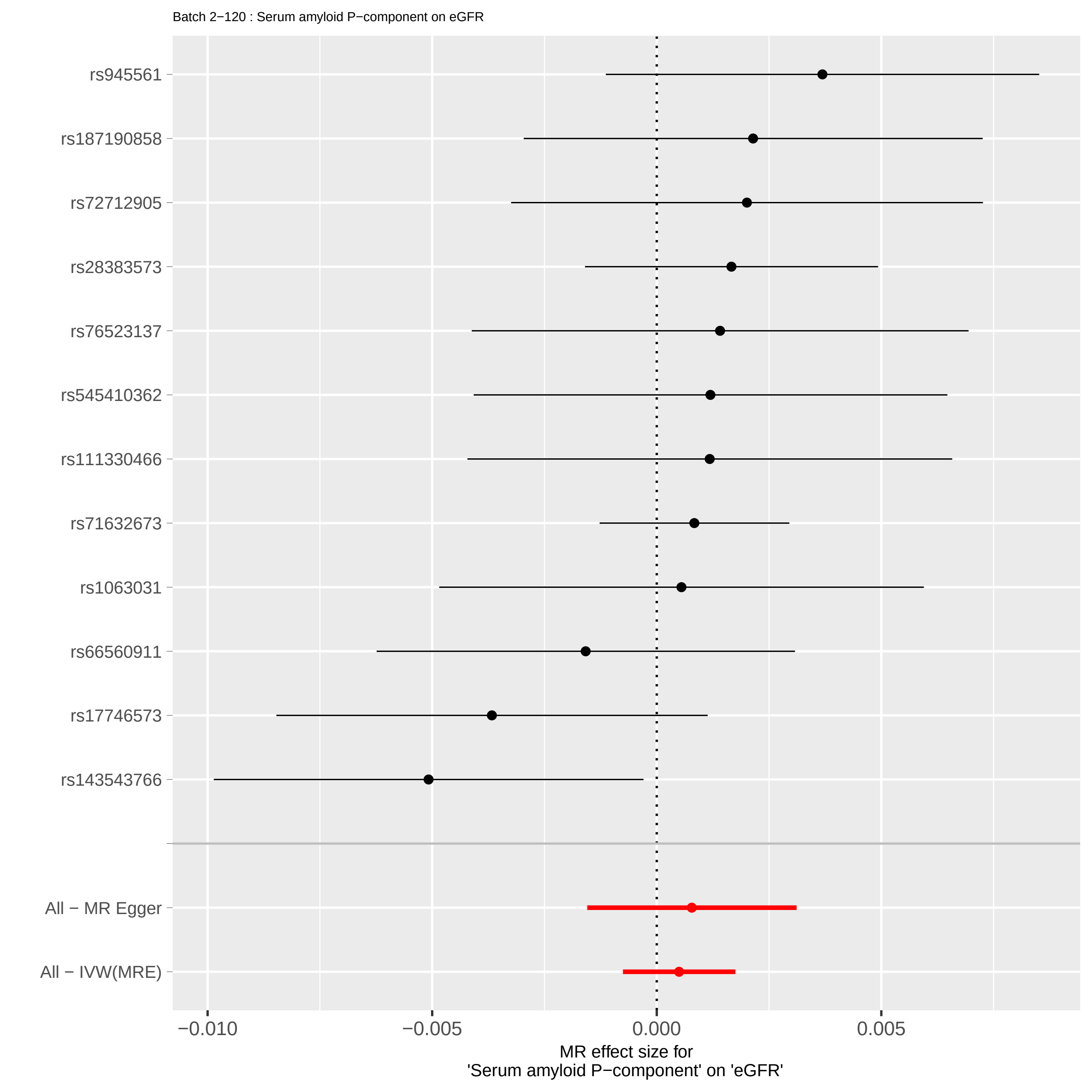
Batch 2−120 : Serum amyloid P−component on eGFR
rs945561
rs187190858
rs72712905
rs28383573
rs76523137
rs545410362
rs111330466
rs71632673
rs1063031
rs66560911
rs17746573
rs143543766
All − MR Egger
All − IVW(MRE)
−0.010
−0.005
0.000
0.005
MR effect size for
'Serum amyloid P−component' on 'eGFR'

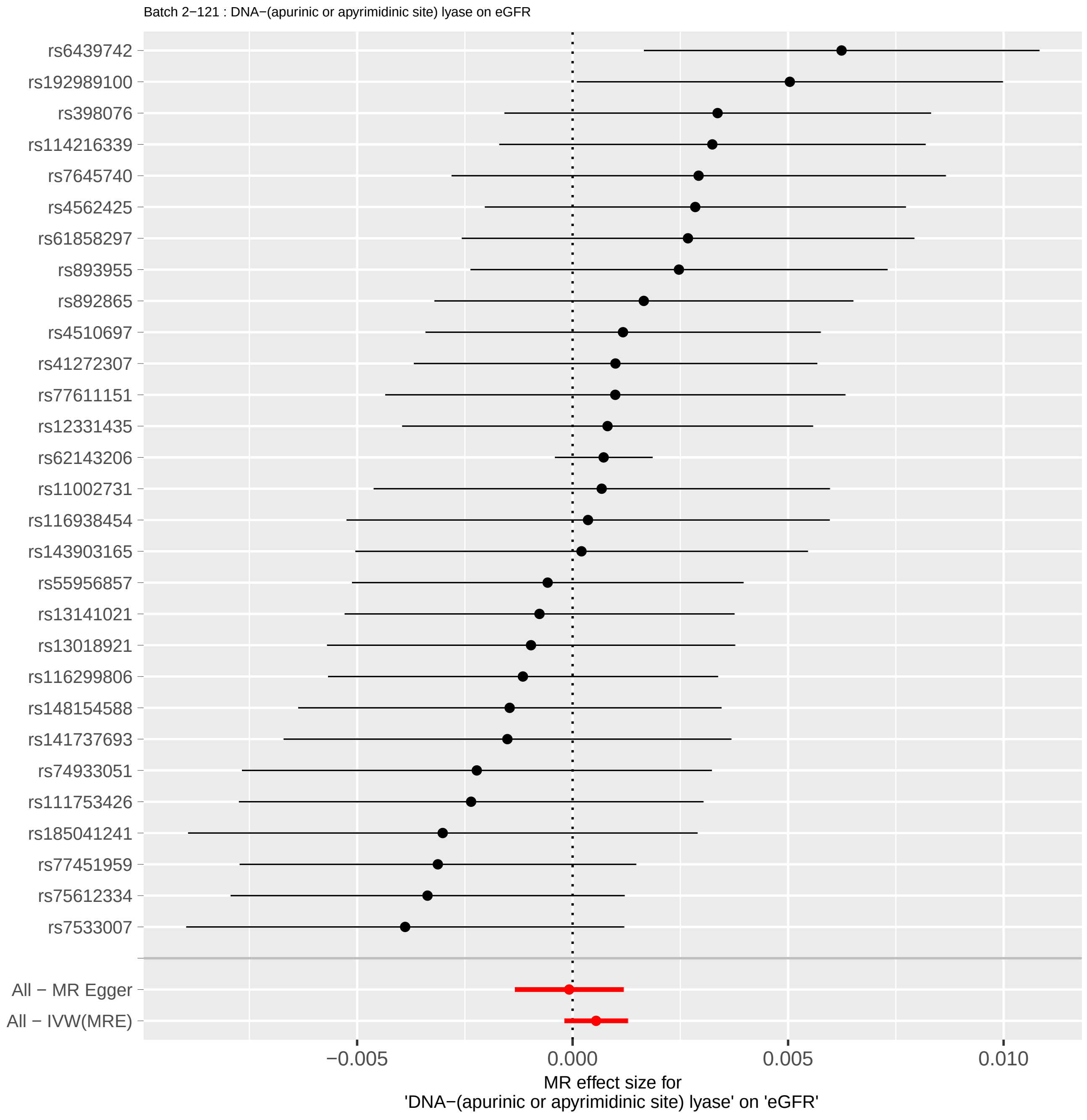
Batch 2−121 : DNA−(apurinic or apyrimidinic site) lyase on eGFR
rs6439742
rs192989100
rs398076
rs114216339
rs7645740
rs4562425
rs61858297
rs893955
rs892865
rs4510697
rs41272307
rs77611151
rs12331435
rs62143206
rs11002731
rs116938454
rs143903165
rs55956857
rs13141021
rs13018921
rs116299806
rs148154588
rs141737693
rs74933051
rs111753426
rs185041241
rs77451959
rs75612334
rs7533007
All − MR Egger
All − IVW(MRE)
−0.005
0.000
0.005
0.010
MR effect size for
'DNA−(apurinic or apyrimidinic site) lyase' on 'eGFR'

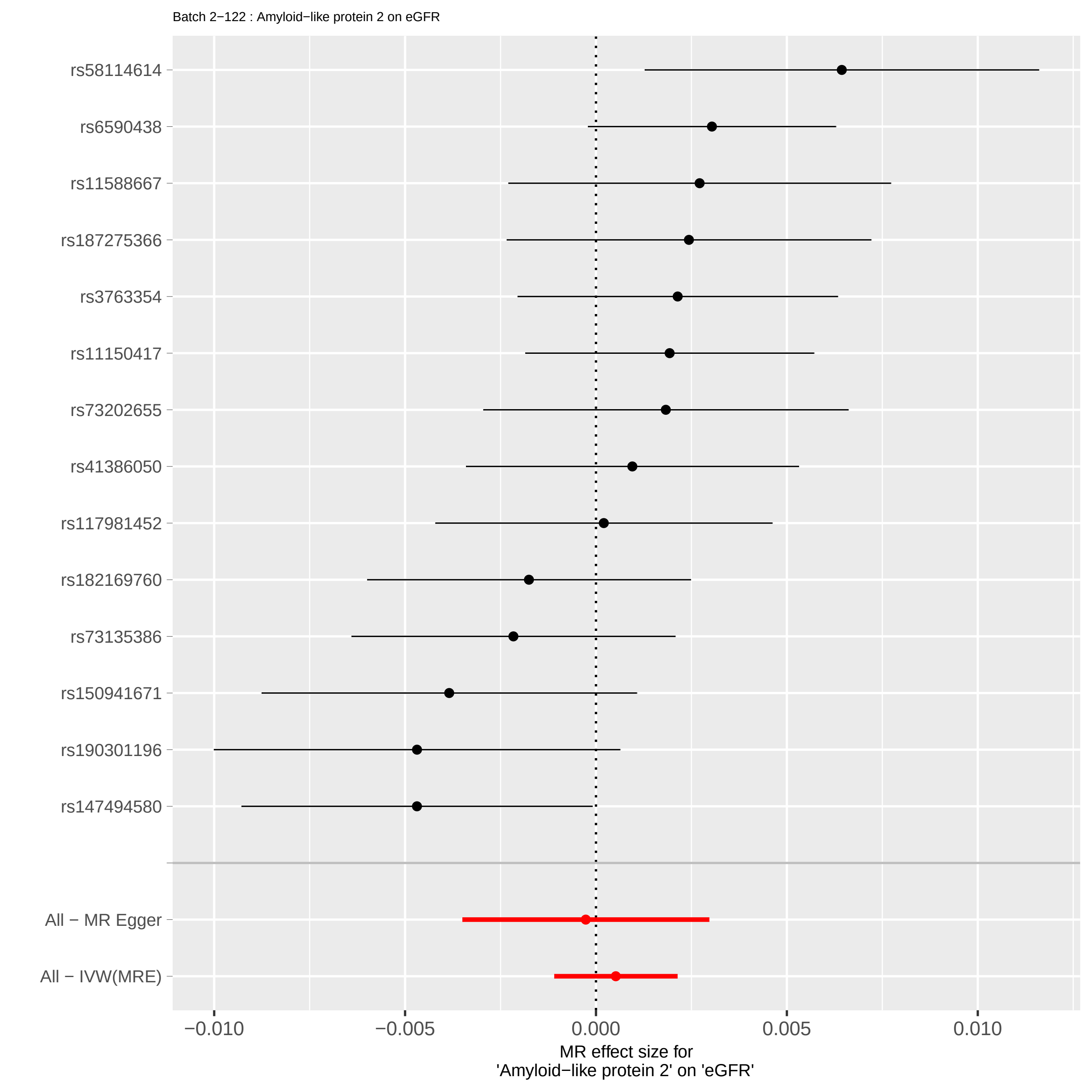
Batch 2−122 : Amyloid−like protein 2 on eGFR
rs58114614
rs6590438
rs11588667
rs187275366
rs3763354
rs11150417
rs73202655
rs41386050
rs117981452
rs182169760
rs73135386
rs150941671
rs190301196
rs147494580
All − MR Egger
All − IVW(MRE)
−0.010
−0.005
0.000
0.005
0.010
MR effect size for
'Amyloid−like protein 2' on 'eGFR'

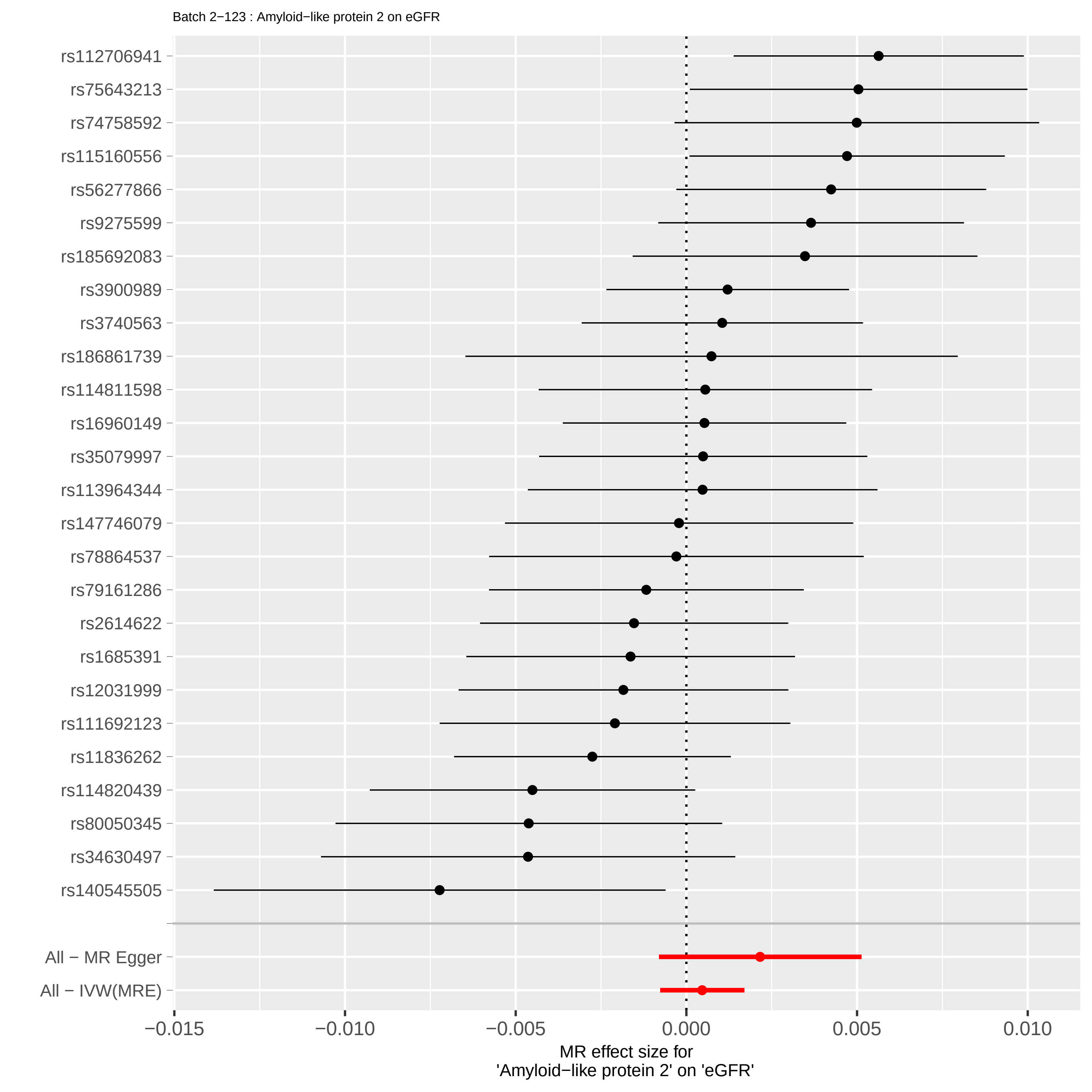

Batch 2−123 : Amyloid−like protein 2 on eGFR
rs112706941
rs75643213
rs74758592
rs115160556
rs56277866
rs9275599
rs185692083
rs3900989
rs3740563
rs186861739
rs114811598
rs16960149
rs35079997
rs113964344
rs147746079
rs78864537
rs79161286
rs2614622
rs1685391
rs12031999
rs111692123
rs11836262
rs114820439
rs80050345
rs34630497
rs140545505
All − MR Egger
All − IVW(MRE)
−0.015
−0.010
−0.005
0.000
0.005
0.010
MR effect size for
'Amyloid−like protein 2' on 'eGFR'

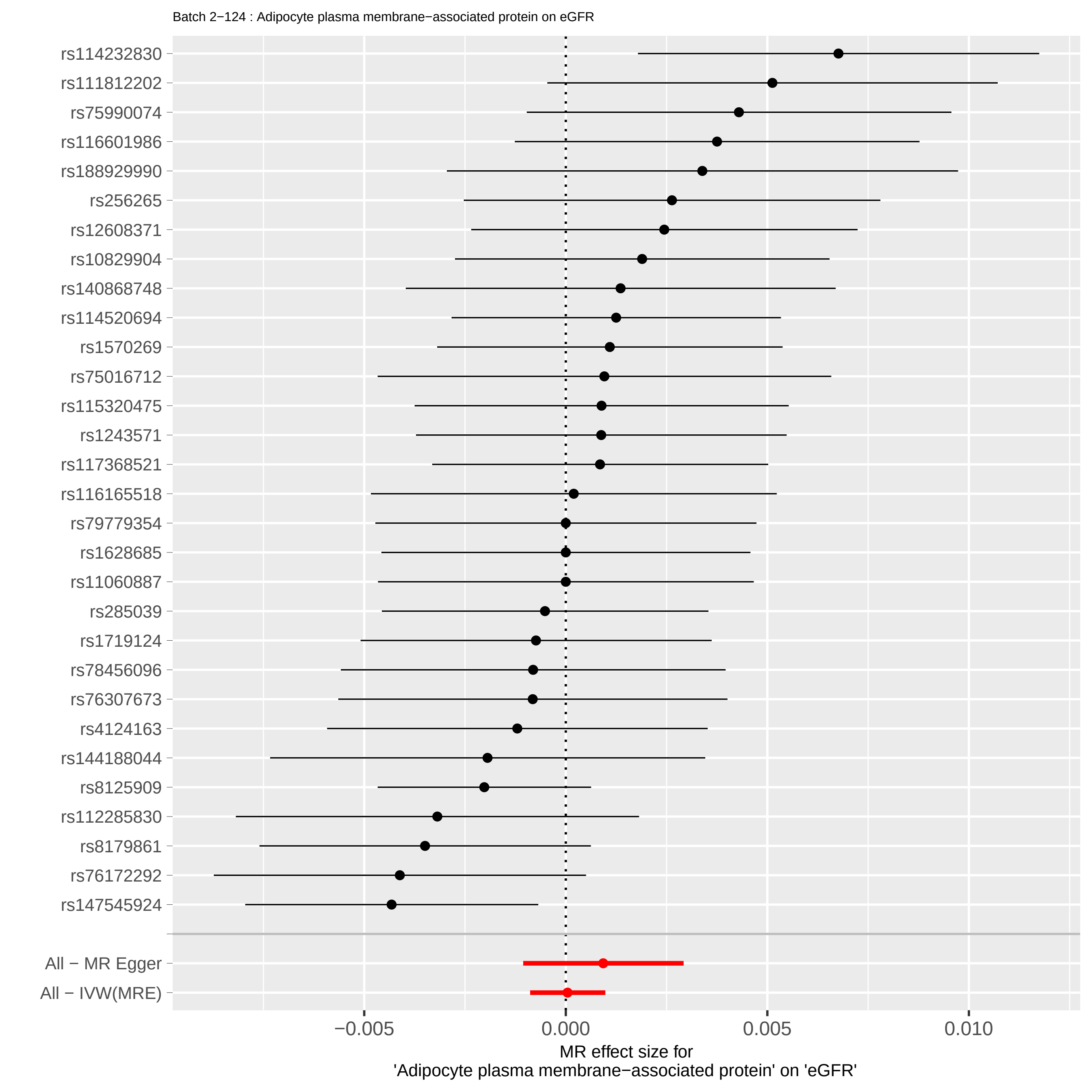

Batch 2−124 : Adipocyte plasma membrane−associated protein on eGFR
rs114232830
rs111812202
rs75990074
rs116601986
rs188929990
rs256265
rs12608371
rs10829904
rs140868748
rs114520694
rs1570269
rs75016712
rs115320475
rs1243571
rs117368521
rs116165518
rs79779354
rs1628685
rs11060887
rs285039
rs1719124
rs78456096
rs76307673
rs4124163
rs144188044
rs8125909
rs112285830
rs8179861
rs76172292
rs147545924
All − MR Egger
All − IVW(MRE)
−0.005
0.000
0.005
0.010
MR effect size for
'Adipocyte plasma membrane−associated protein' on 'eGFR'

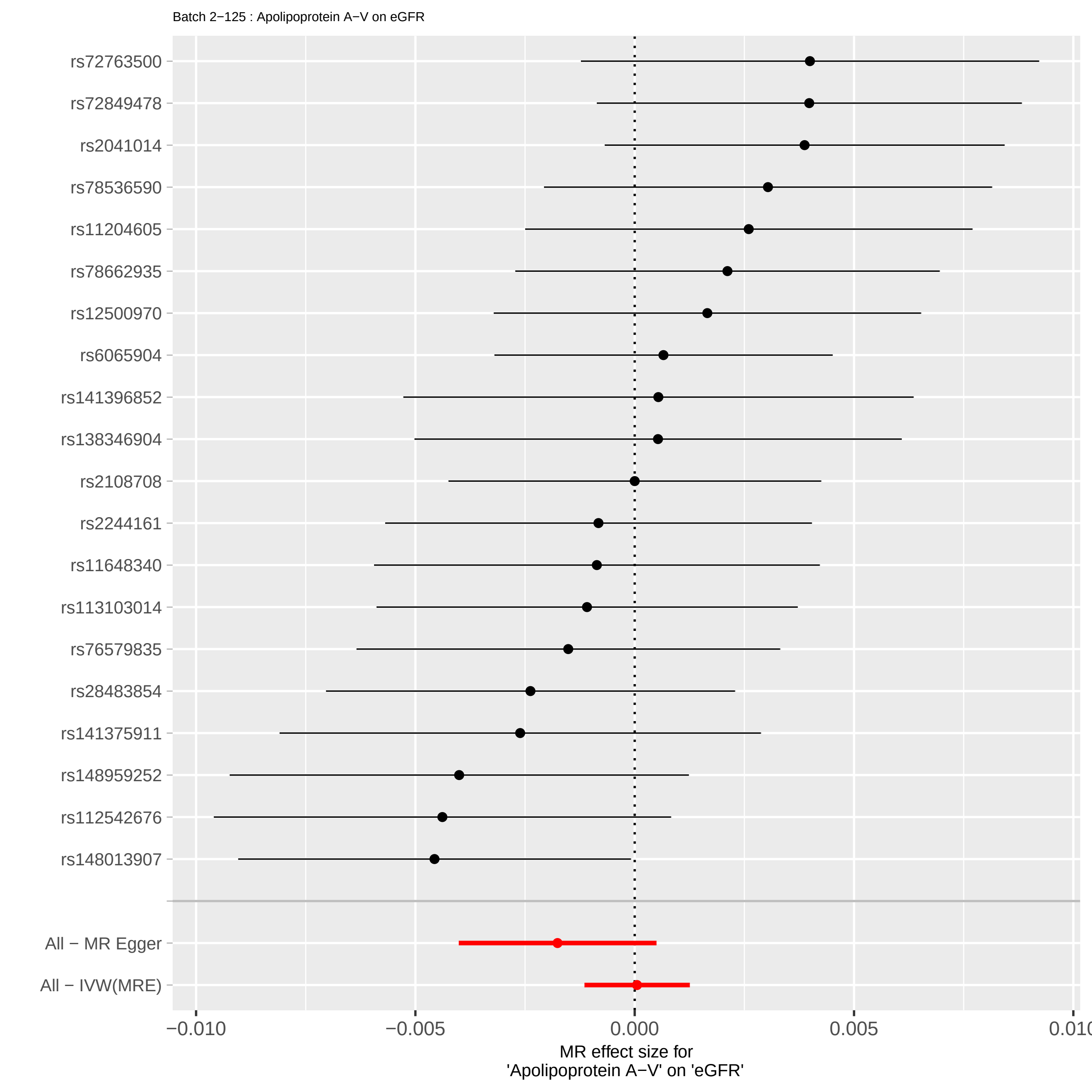
Batch 2−125 : Apolipoprotein A−V on eGFR
rs72763500
rs72849478
rs2041014
rs78536590
rs11204605
rs78662935
rs12500970
rs6065904
rs141396852
rs138346904
rs2108708
rs2244161
rs11648340
rs113103014
rs76579835
rs28483854
rs141375911
rs148959252
rs112542676
rs148013907
All − MR Egger
All − IVW(MRE)
−0.010
−0.005
0.000
0.005
MR effect size for
'Apolipoprotein A−V' on 'eGFR'

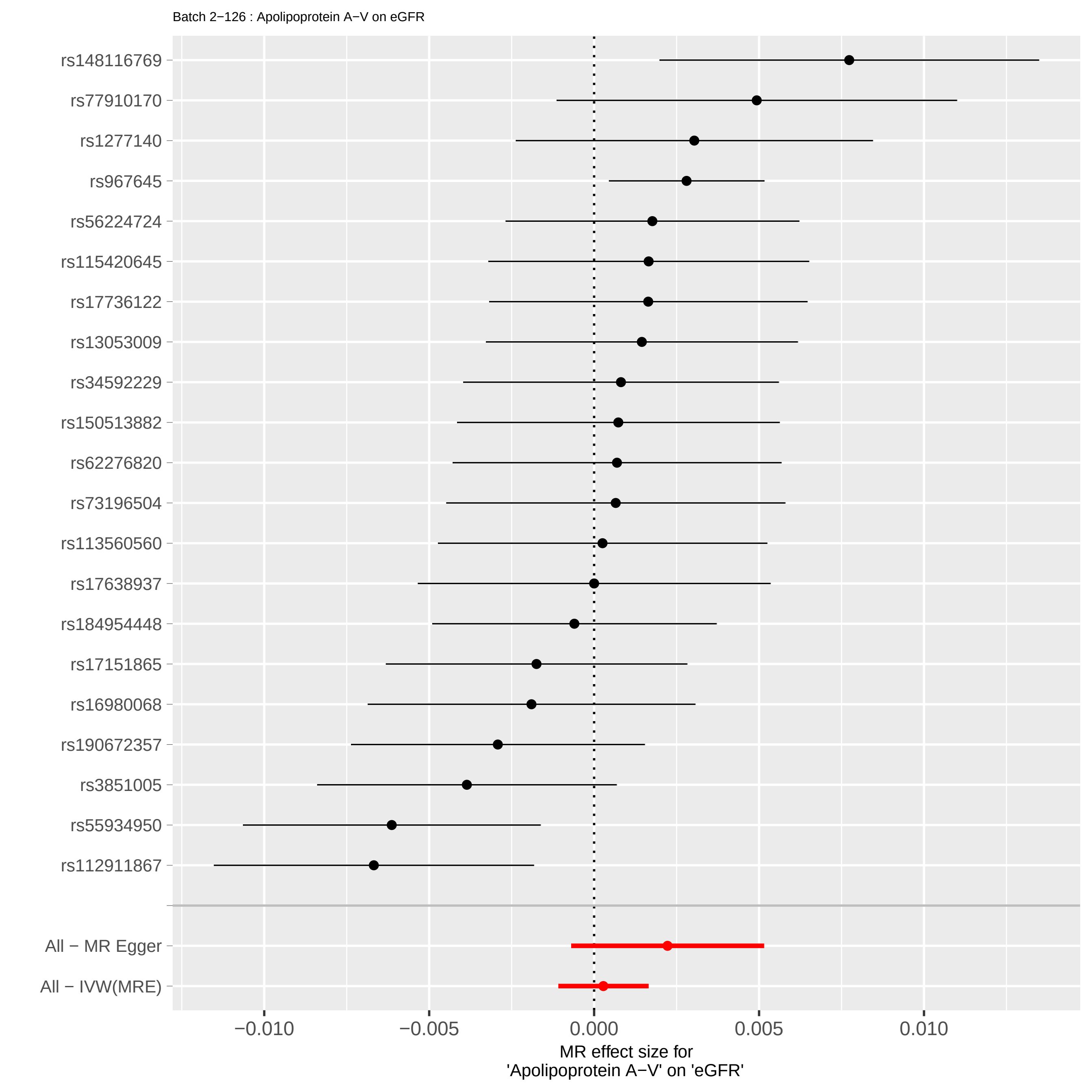

Batch 2−126 : Apolipoprotein A−V on eGFR
rs148116769
rs77910170
rs1277140
rs967645
rs56224724
rs115420645
rs17736122
rs13053009
rs34592229
rs150513882
rs62276820
rs73196504
rs113560560
rs17638937
rs184954448
rs17151865
rs16980068
rs190672357
rs3851005
rs55934950
rs112911867
All − MR Egger
All − IVW(MRE)
−0.010
−0.005
0.000
0.005
0.010
MR effect size for
'Apolipoprotein A−V' on 'eGFR'

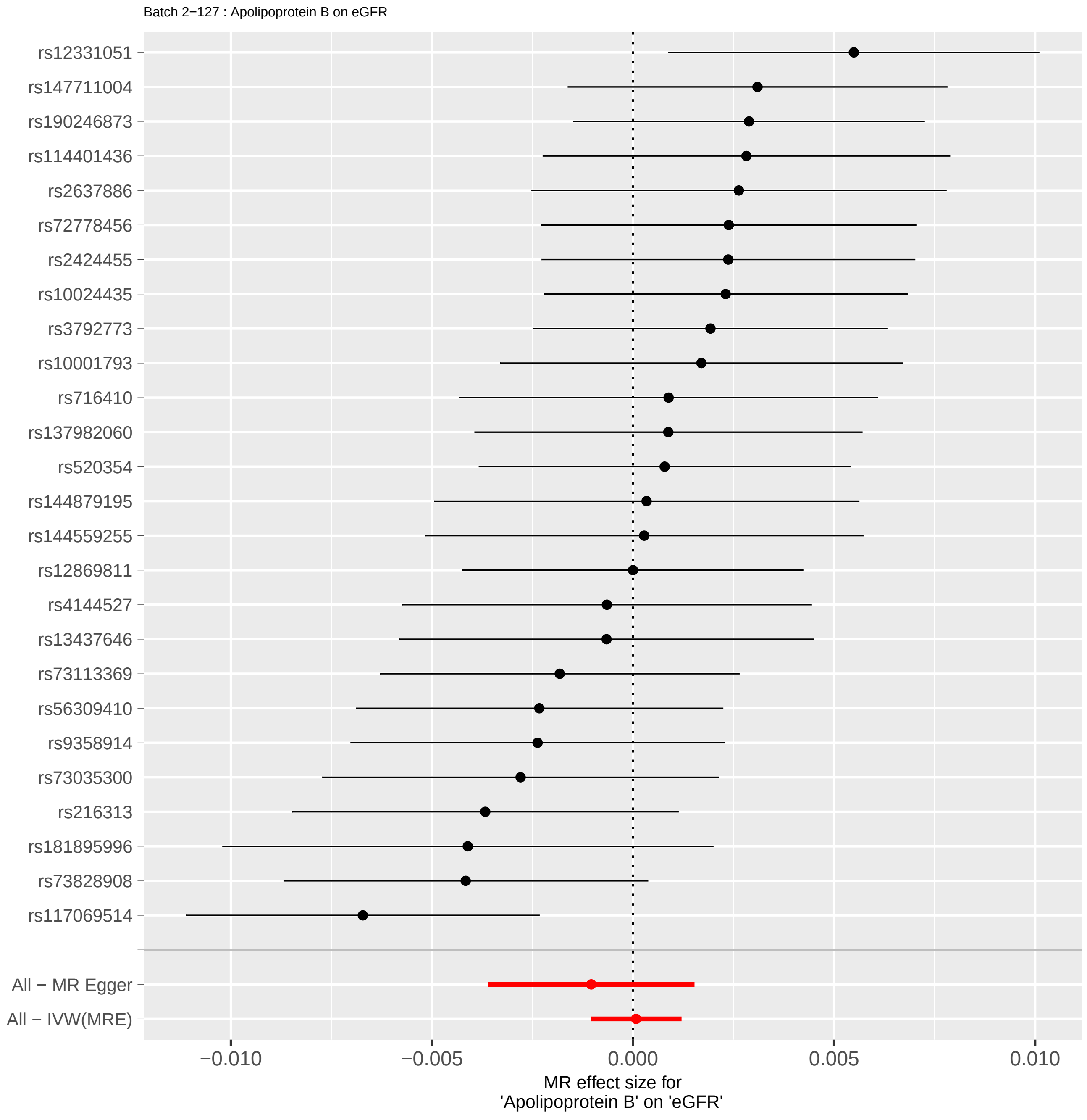

Batch 2−127 : Apolipoprotein B on eGFR
rs12331051
rs147711004
rs190246873
rs114401436
rs2637886
rs72778456
rs2424455
rs10024435
rs3792773
rs10001793
rs716410
rs137982060
rs520354
rs144879195
rs144559255
rs12869811
rs4144527
rs13437646
rs73113369
rs56309410
rs9358914
rs73035300
rs216313
rs181895996
rs73828908
rs117069514
All − MR Egger
All − IVW(MRE)
−0.010
−0.005
0.000
0.005
0.010
MR effect size for
'Apolipoprotein B' on 'eGFR'

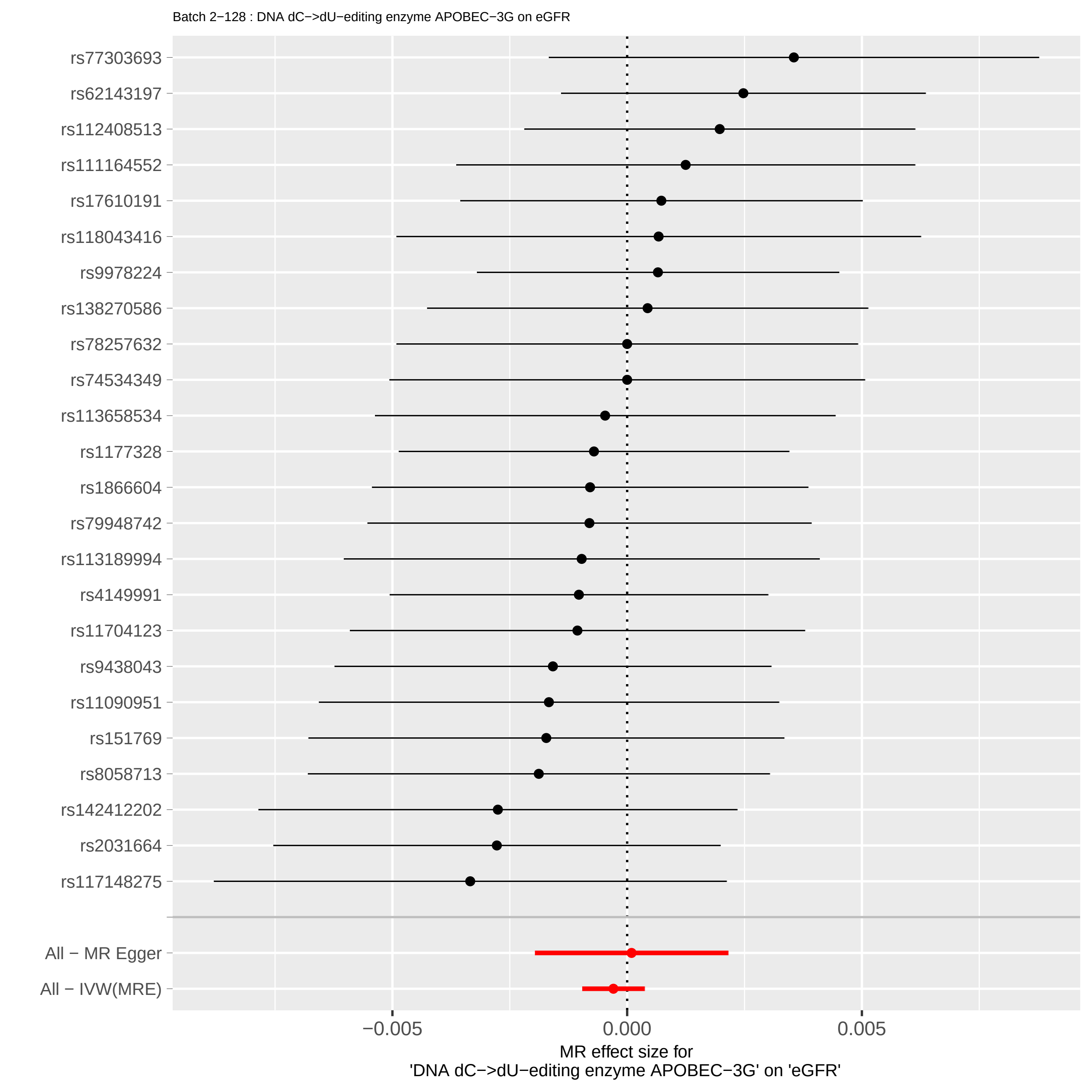

Batch 2−128 : DNA dC−>dU−editing enzyme APOBEC−3G on eGFR
rs77303693
rs62143197
rs112408513
rs111164552
rs17610191
rs118043416
rs9978224
rs138270586
rs78257632
rs74534349
rs113658534
rs1177328
rs1866604
rs79948742
rs113189994
rs4149991
rs11704123
rs9438043
rs11090951
rs151769
rs8058713
rs142412202
rs2031664
rs117148275
All − MR Egger
All − IVW(MRE)
−0.005
0.000
0.005
MR effect size for
'DNA dC−>dU−editing enzyme APOBEC−3G' on 'eGFR'

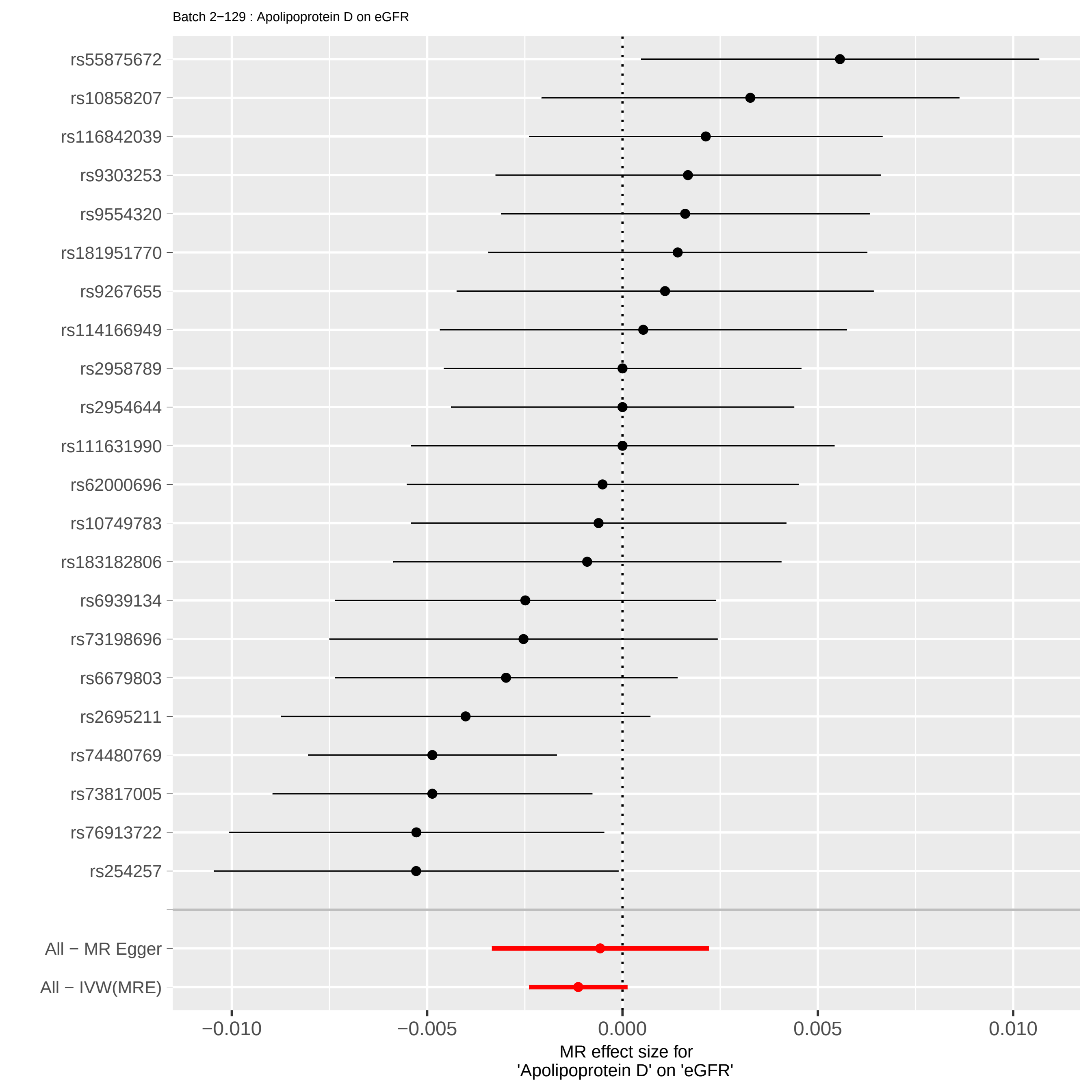
Batch 2−129 : Apolipoprotein D on eGFR
rs55875672
rs10858207
rs116842039
rs9303253
rs9554320
rs181951770
rs9267655
rs114166949
rs2958789
rs2954644
rs111631990
rs62000696
rs10749783
rs183182806
rs6939134
rs73198696
rs6679803
rs2695211
rs74480769
rs73817005
rs76913722
rs254257
All − MR Egger
All − IVW(MRE)
−0.010
−0.005
0.000
0.005
0.010
MR effect size for
'Apolipoprotein D' on 'eGFR'

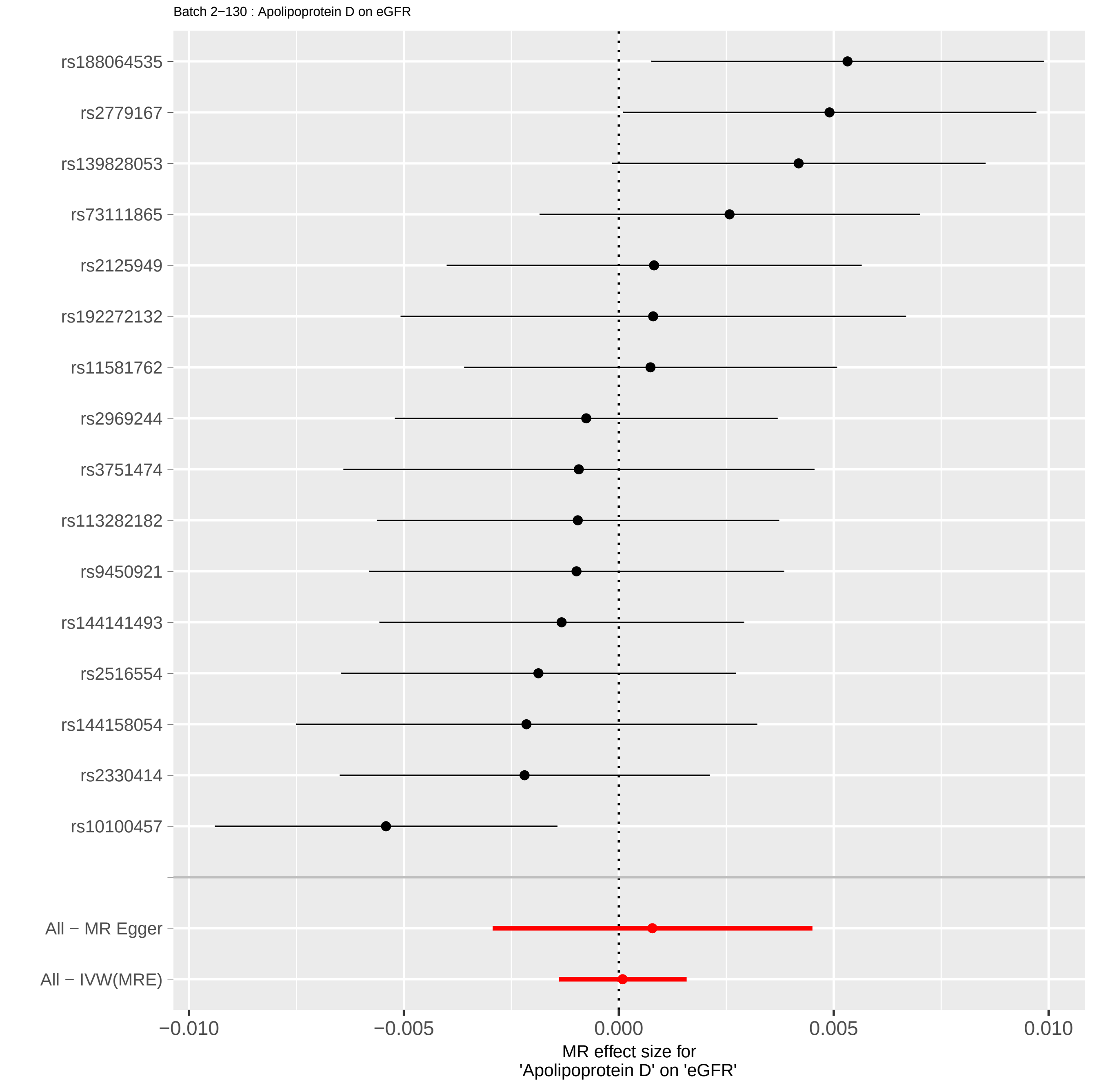
Batch 2−130 : Apolipoprotein D on eGFR
rs188064535
rs2779167
rs139828053
rs73111865
rs2125949
rs192272132
rs11581762
rs2969244
rs3751474
rs113282182
rs9450921
rs144141493
rs2516554
rs144158054
rs2330414
rs10100457
All − MR Egger
All − IVW(MRE)
−0.010
−0.005
0.000
0.005
0.010
MR effect size for
'Apolipoprotein D' on 'eGFR'

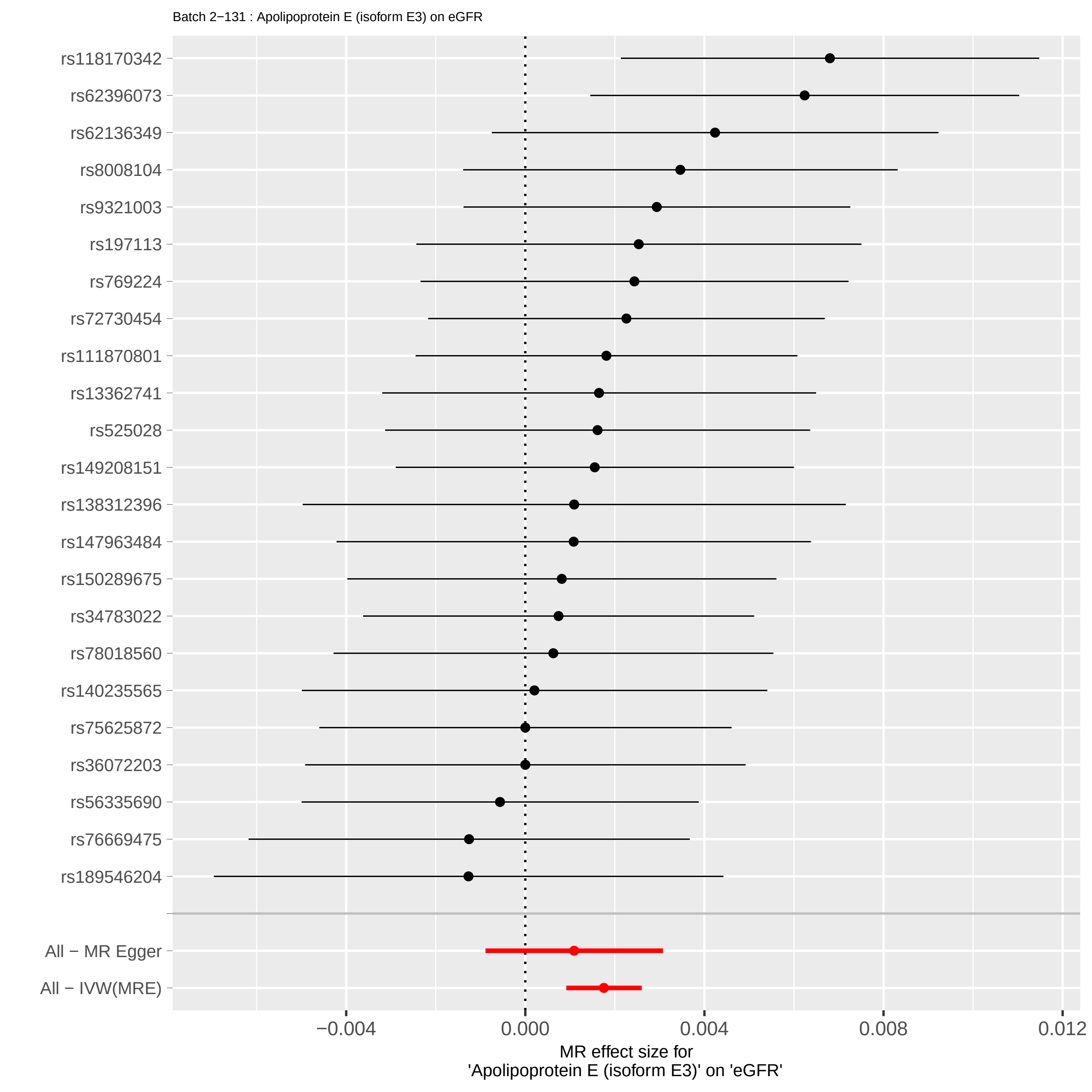

Batch 2−131 : Apolipoprotein E (isoform E3) on eGFR
rs118170342
rs62396073
rs62136349
rs8008104
rs9321003
rs197113
rs769224
rs72730454
rs111870801
rs13362741
rs525028
rs149208151
rs138312396
rs147963484
rs150289675
rs34783022
rs78018560
rs140235565
rs75625872
rs36072203
rs56335690
rs76669475
rs189546204
All − MR Egger
All − IVW(MRE)
−0.004
0.000
0.004
0.008
0.012
MR effect size for
'Apolipoprotein E (isoform E3)' on 'eGFR'

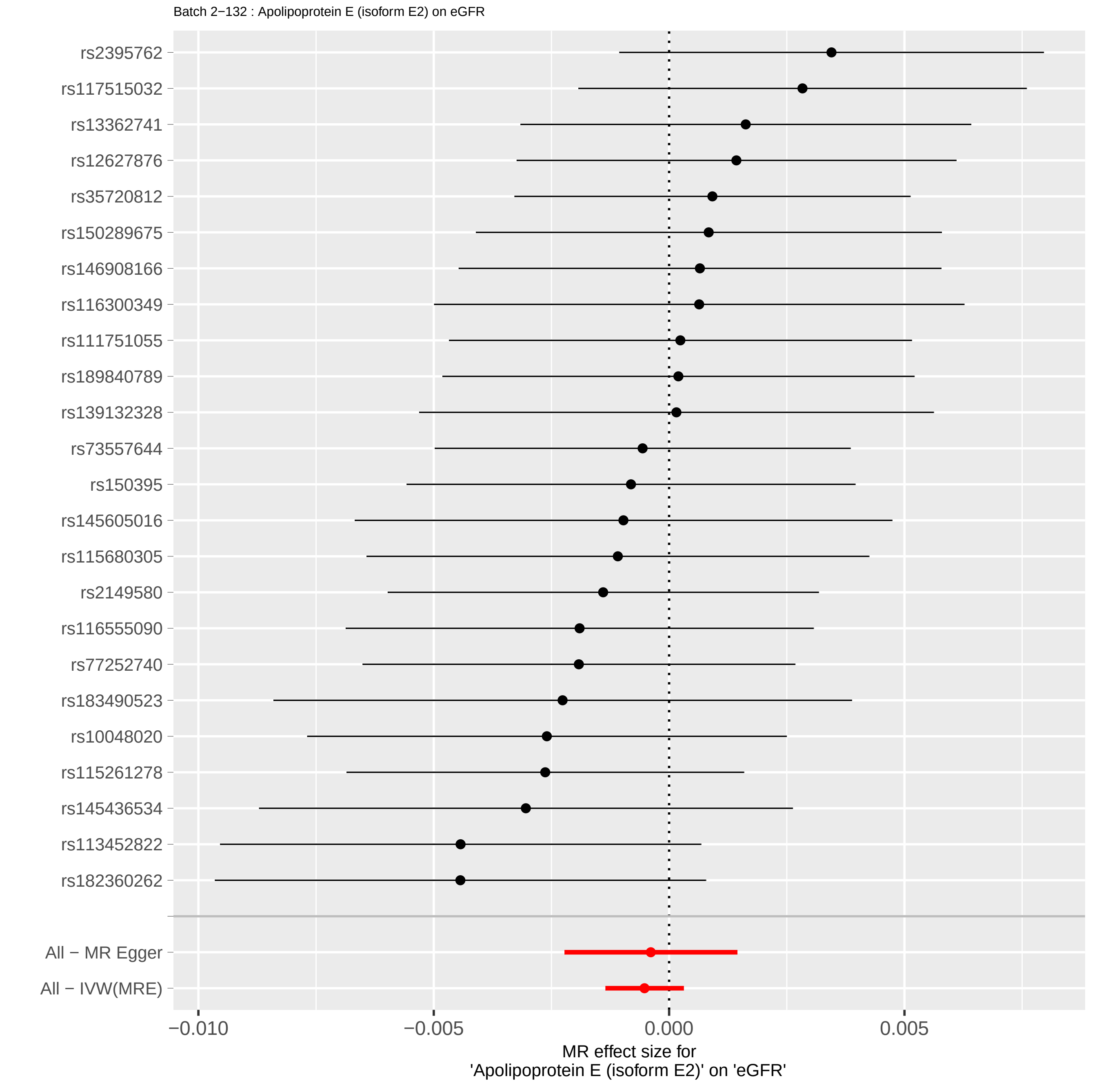
Batch 2−132 : Apolipoprotein E (isoform E2) on eGFR
rs2395762
rs117515032
rs13362741
rs12627876
rs35720812
rs150289675
rs146908166
rs116300349
rs111751055
rs189840789
rs139132328
rs73557644
rs150395
rs145605016
rs115680305
rs2149580
rs116555090
rs77252740
rs183490523
rs10048020
rs115261278
rs145436534
rs113452822
rs182360262
All − MR Egger
All − IVW(MRE)
−0.010
−0.005
0.000
0.005
MR effect size for
'Apolipoprotein E (isoform E2)' on 'eGFR'

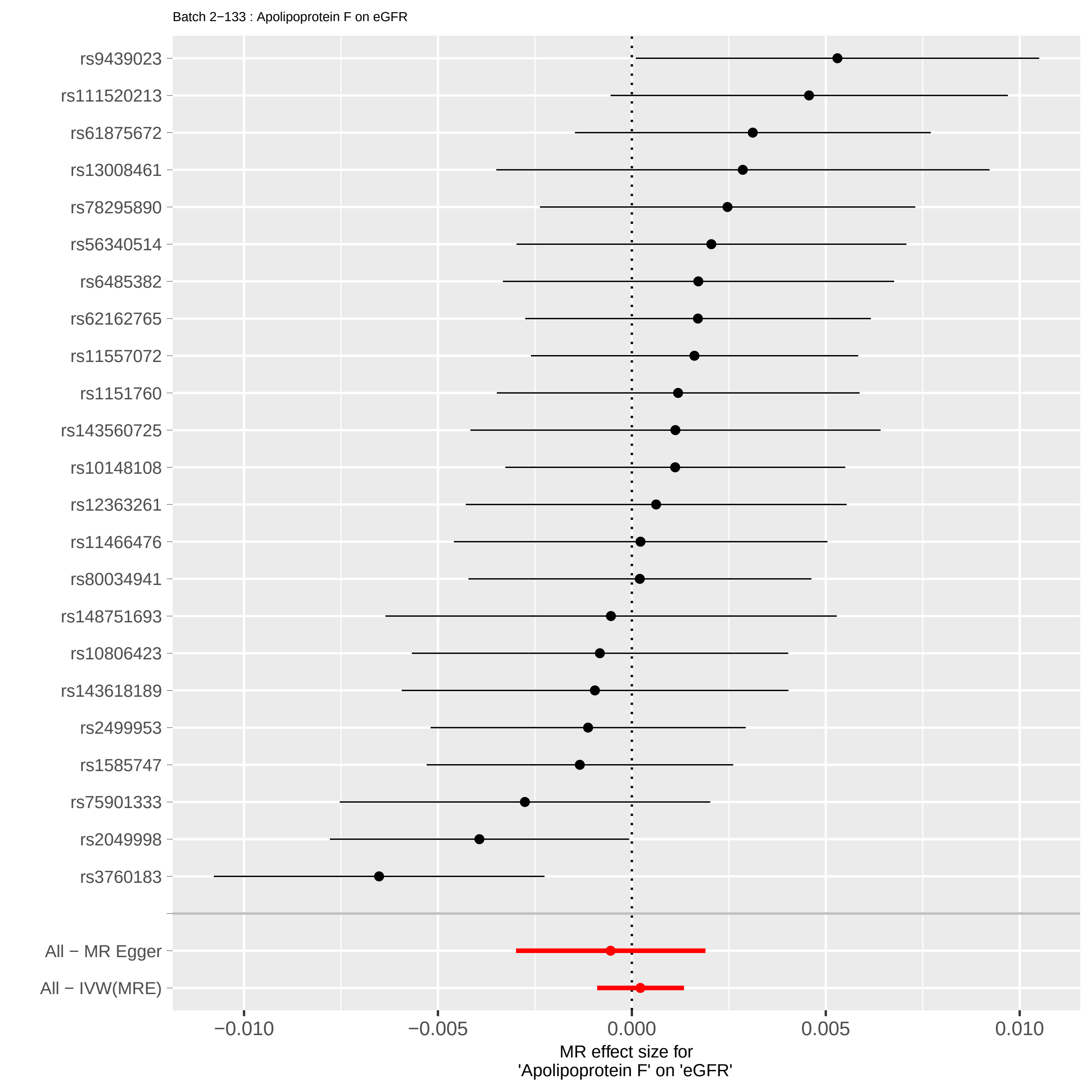

Batch 2−133 : Apolipoprotein F on eGFR
rs9439023
rs111520213
rs61875672
rs13008461
rs78295890
rs56340514
rs6485382
rs62162765
rs11557072
rs1151760
rs143560725
rs10148108
rs12363261
rs11466476
rs80034941
rs148751693
rs10806423
rs143618189
rs2499953
rs1585747
rs75901333
rs2049998
rs3760183
All − MR Egger
All − IVW(MRE)
−0.010
−0.005
0.000
0.005
0.010
MR effect size for
'Apolipoprotein F' on 'eGFR'

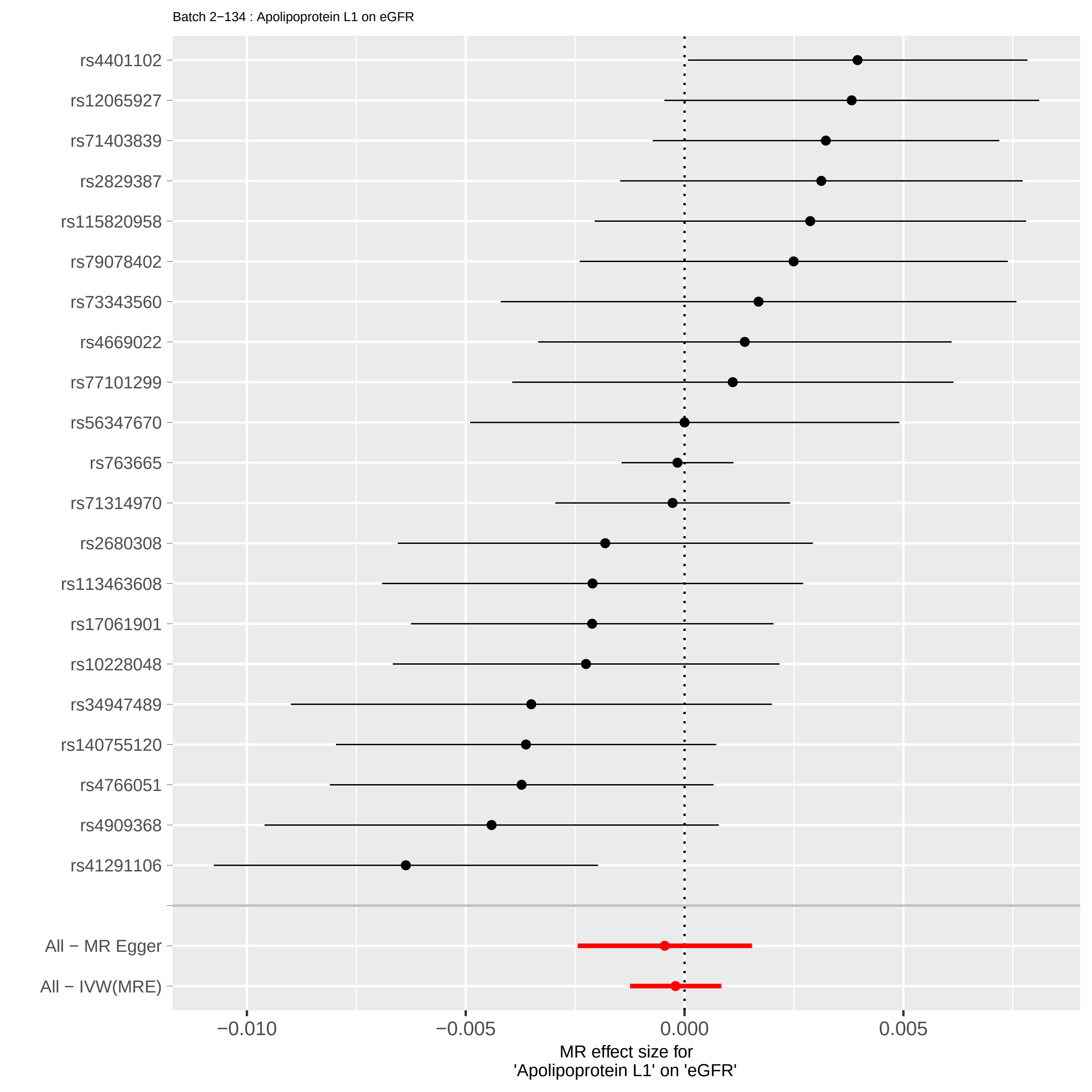

Batch 2−134 : Apolipoprotein L1 on eGFR
rs4401102
rs12065927
rs71403839
rs2829387
rs115820958
rs79078402
rs73343560
rs4669022
rs77101299
rs56347670
rs763665
rs71314970
rs2680308
rs113463608
rs17061901
rs10228048
rs34947489
rs140755120
rs4766051
rs4909368
rs41291106
All − MR Egger
All − IVW(MRE)
−0.010
−0.005
0.000
0.005
MR effect size for
'Apolipoprotein L1' on 'eGFR'

Batch 2−135 : Apolipoprotein L1 on eGFR

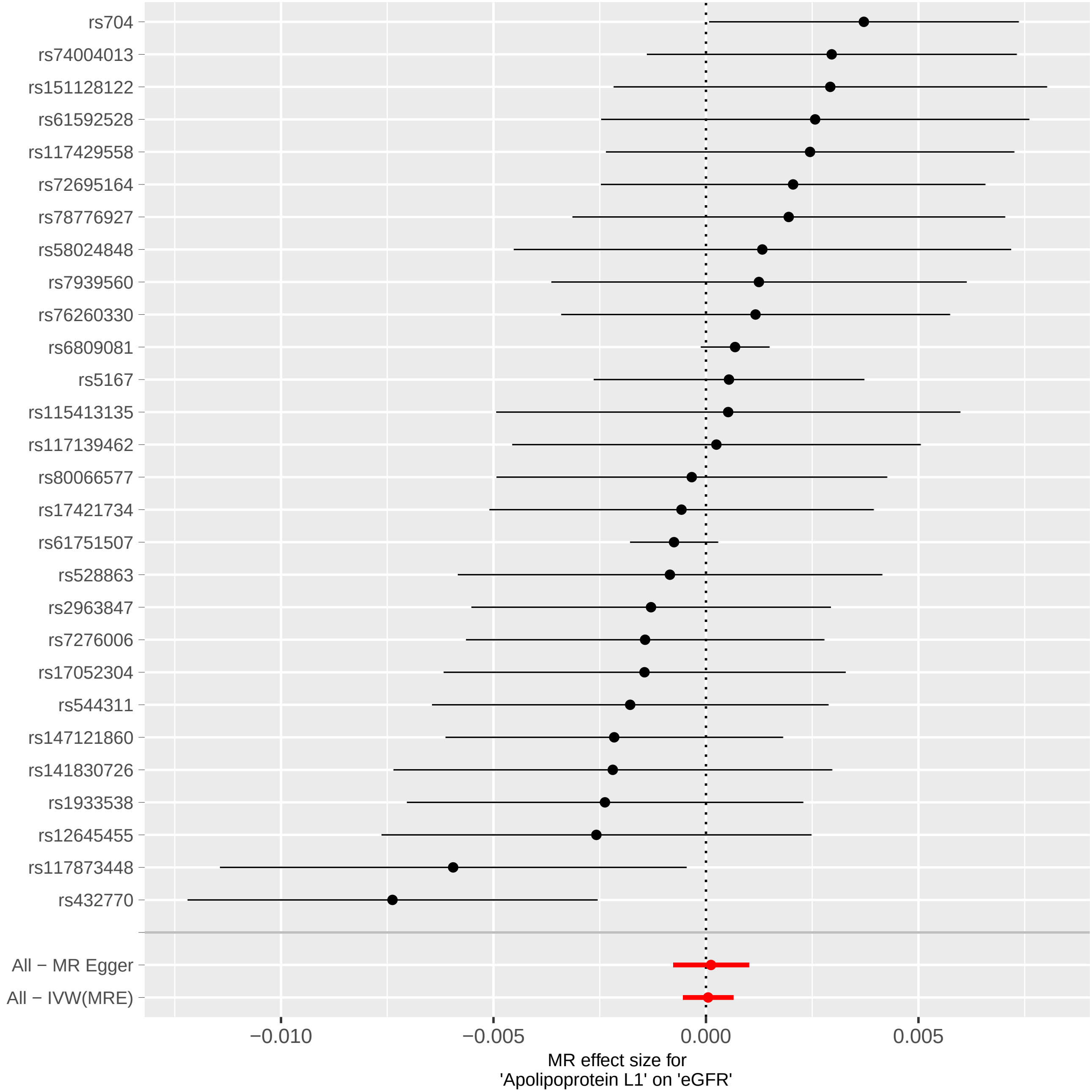

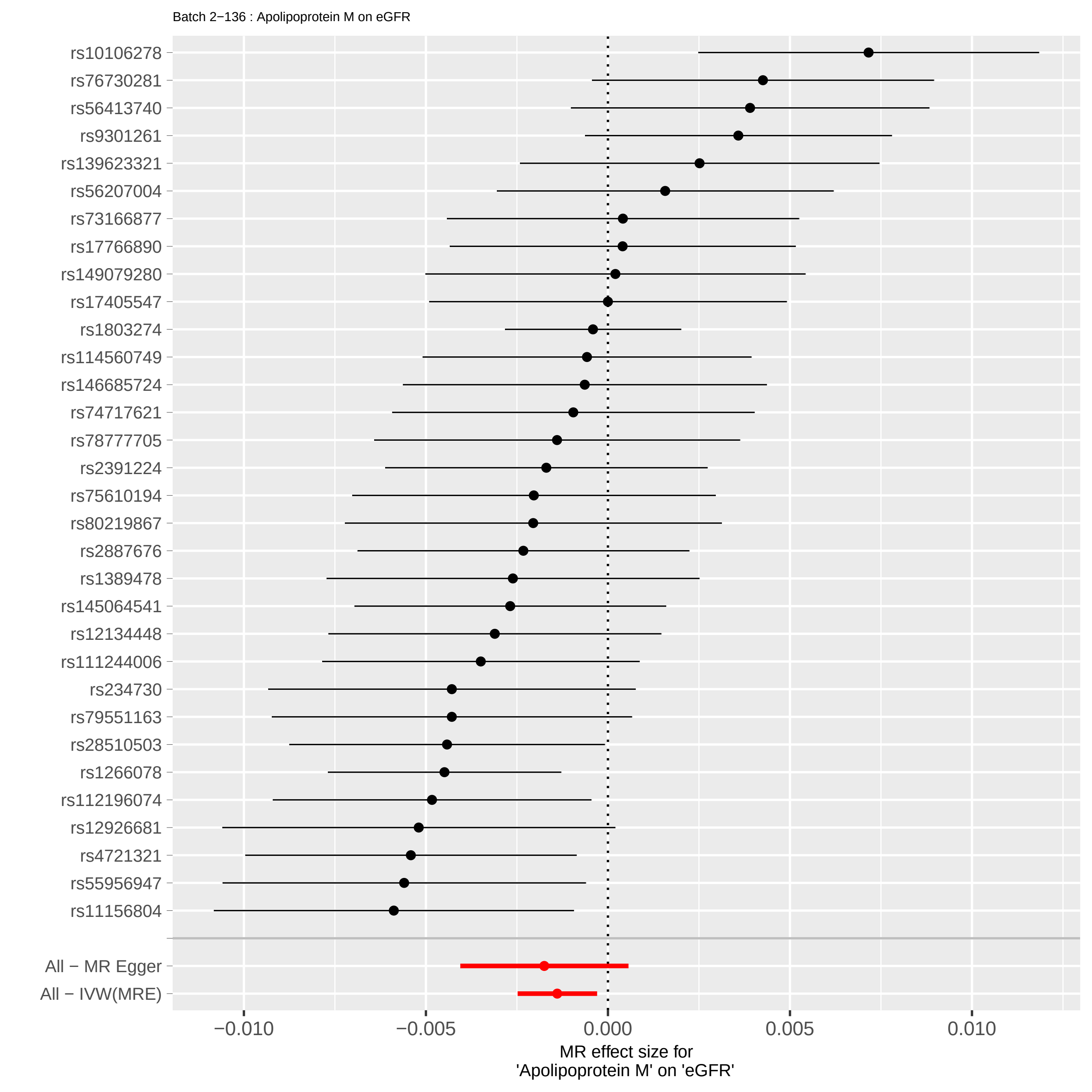

Batch 2−136 : Apolipoprotein M on eGFR
rs10106278
rs76730281
rs56413740
rs9301261
rs139623321
rs56207004
rs73166877
rs17766890
rs149079280
rs17405547
rs1803274
rs114560749
rs146685724
rs74717621
rs78777705
rs2391224
rs75610194
rs80219867
rs2887676
rs1389478
rs145064541
rs12134448
rs111244006
rs234730
rs79551163
rs28510503
rs1266078
rs112196074
rs12926681
rs4721321
rs55956947
rs11156804
All − MR Egger
All − IVW(MRE)
−0.010
−0.005
0.000
0.005
0.010
MR effect size for
'Apolipoprotein M' on 'eGFR'

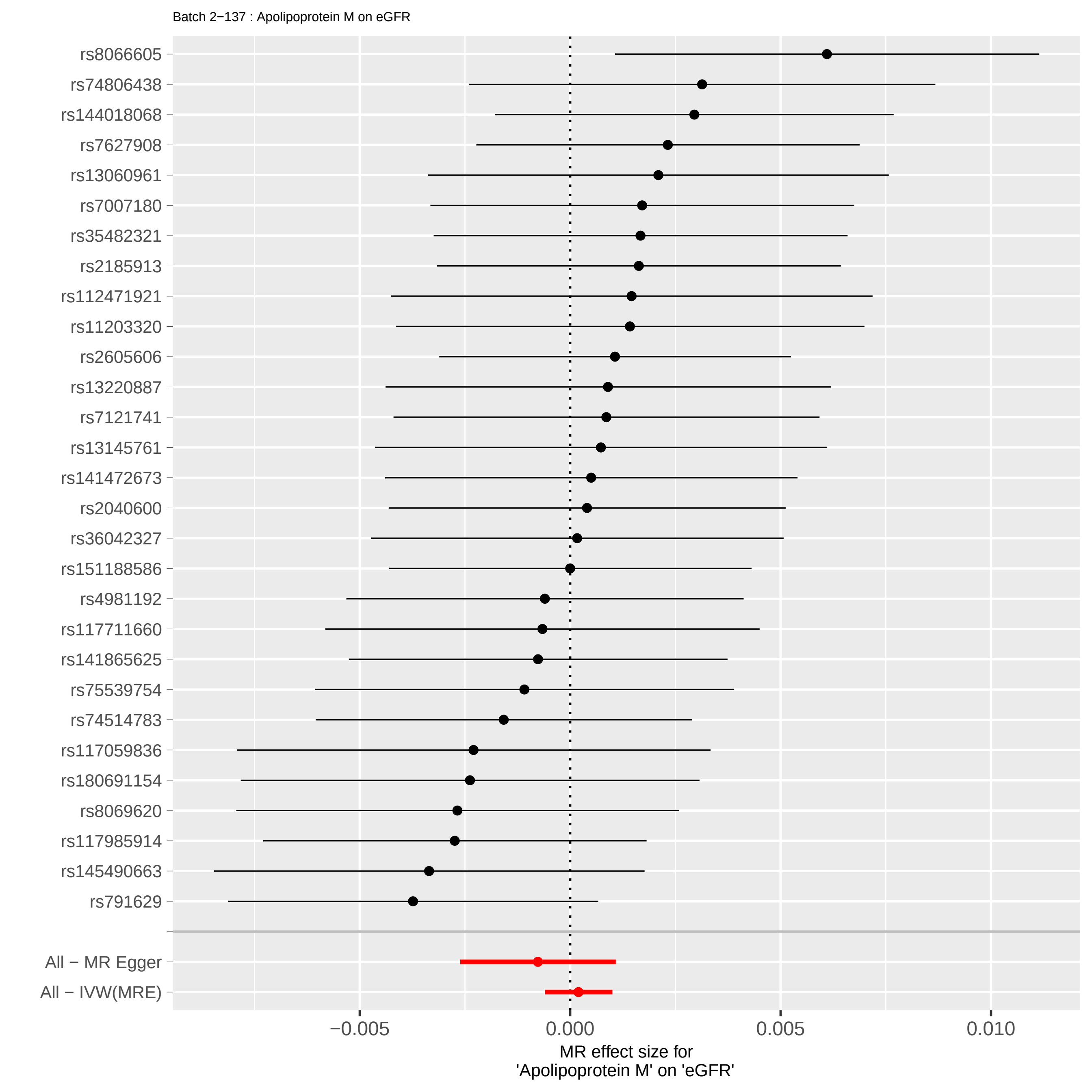

Batch 2−137 : Apolipoprotein M on eGFR
rs8066605
rs74806438
rs144018068
rs7627908
rs13060961
rs7007180
rs35482321
rs2185913
rs112471921
rs11203320
rs2605606
rs13220887
rs7121741
rs13145761
rs141472673
rs2040600
rs36042327
rs151188586
rs4981192
rs117711660
rs141865625
rs75539754
rs74514783
rs117059836
rs180691154
rs8069620
rs117985914
rs145490663
rs791629
All − MR Egger
All − IVW(MRE)
−0.005
0.000
0.005
0.010
MR effect size for
'Apolipoprotein M' on 'eGFR'

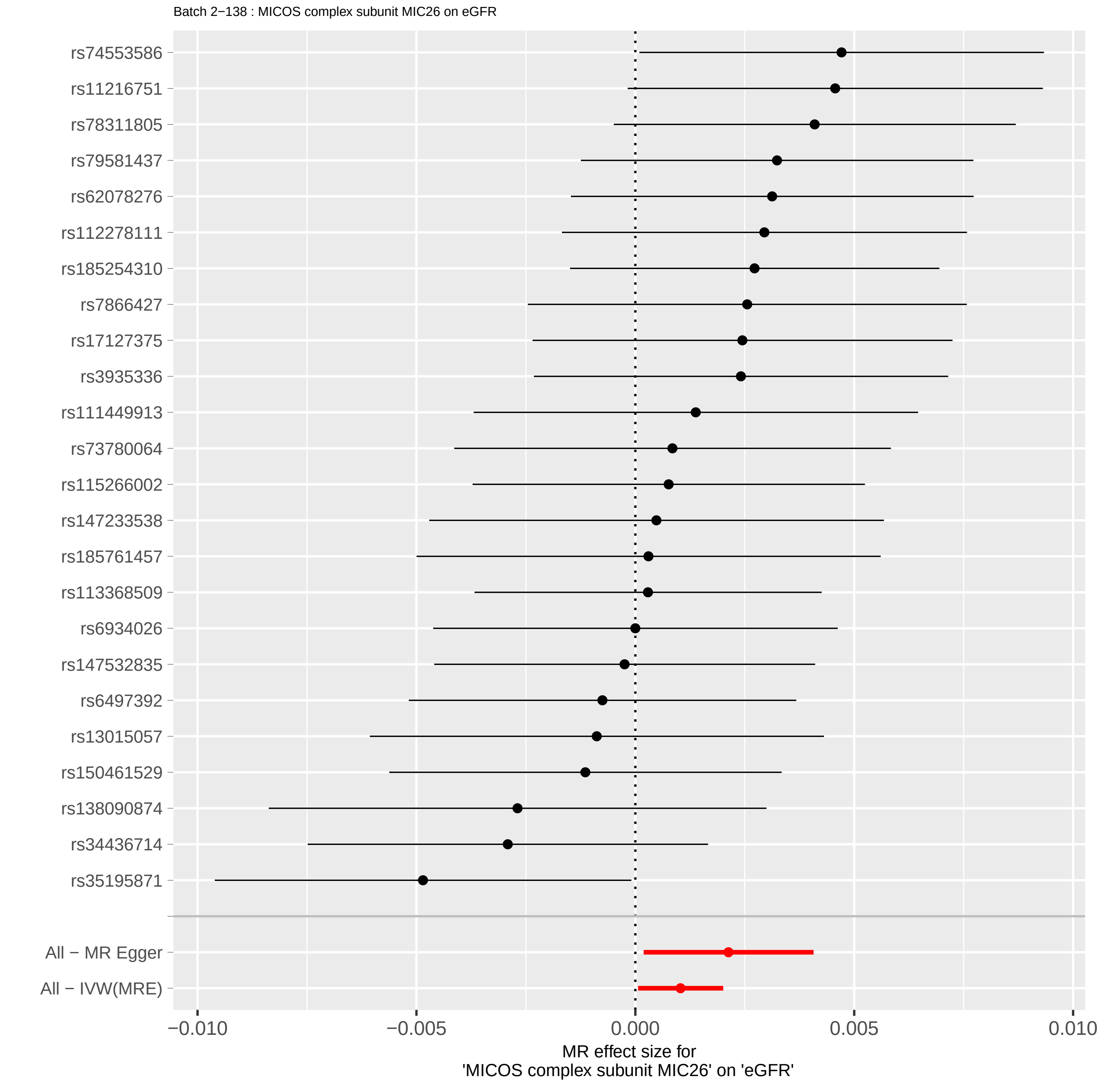

Batch 2−138 : MICOS complex subunit MIC26 on eGFR
rs74553586
rs11216751
rs78311805
rs79581437
rs62078276
rs112278111
rs185254310
rs7866427
rs17127375
rs3935336
rs111449913
rs73780064
rs115266002
rs147233538
rs185761457
rs113368509
rs6934026
rs147532835
rs6497392
rs13015057
rs150461529
rs138090874
rs34436714
rs35195871
All − MR Egger
All − IVW(MRE)
−0.010
−0.005
0.000
0.005
0.010
MR effect size for
'MICOS complex subunit MIC26' on 'eGFR'

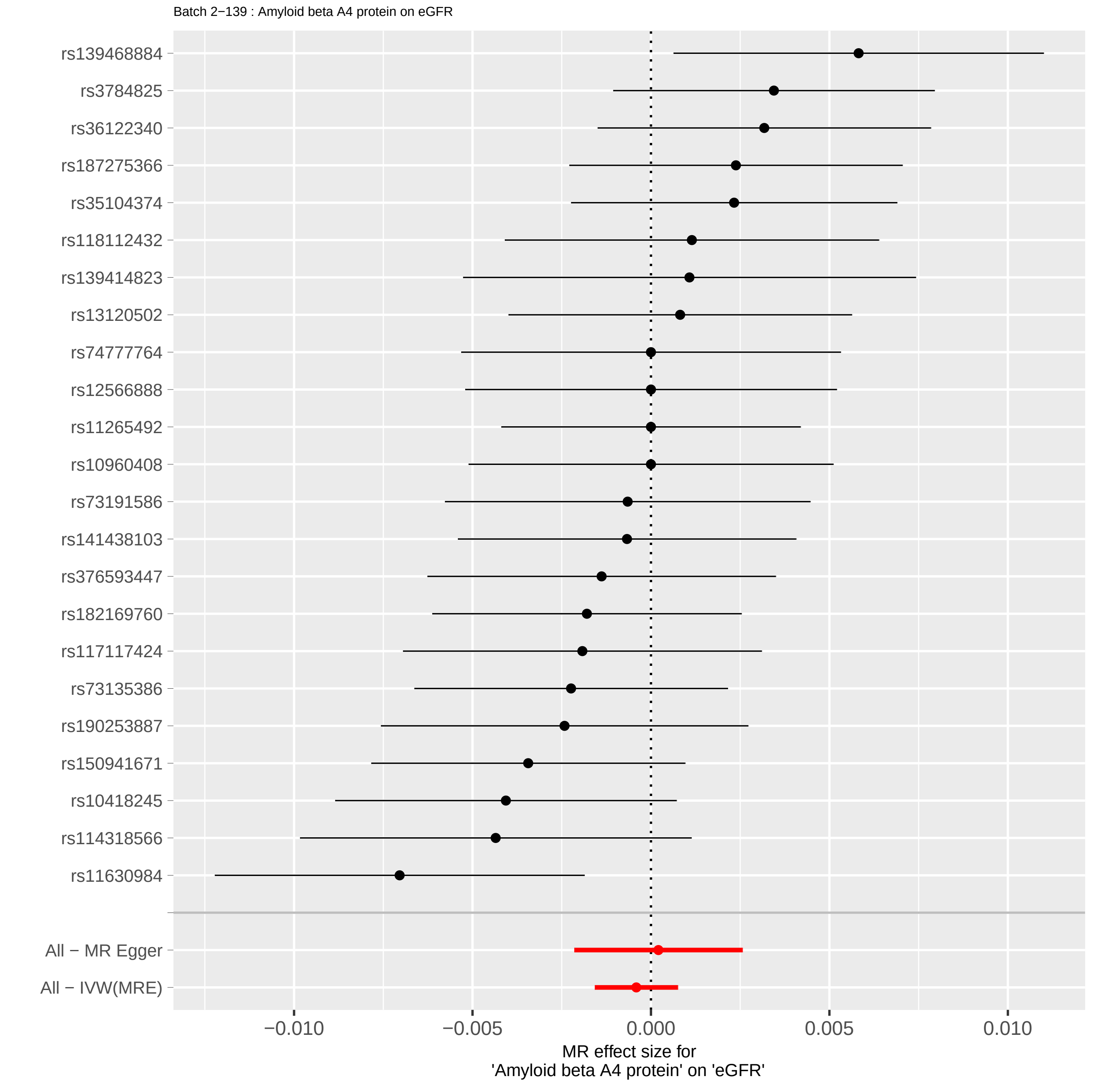
Batch 2−139 : Amyloid beta A4 protein on eGFR
rs139468884
rs3784825
rs36122340
rs187275366
rs35104374
rs118112432
rs139414823
rs13120502
rs74777764
rs12566888
rs11265492
rs10960408
rs73191586
rs141438103
rs376593447
rs182169760
rs117117424
rs73135386
rs190253887
rs150941671
rs10418245
rs114318566
rs11630984
All − MR Egger
All − IVW(MRE)
−0.010
−0.005
0.000
0.005
0.010
MR effect size for
'Amyloid beta A4 protein' on 'eGFR'

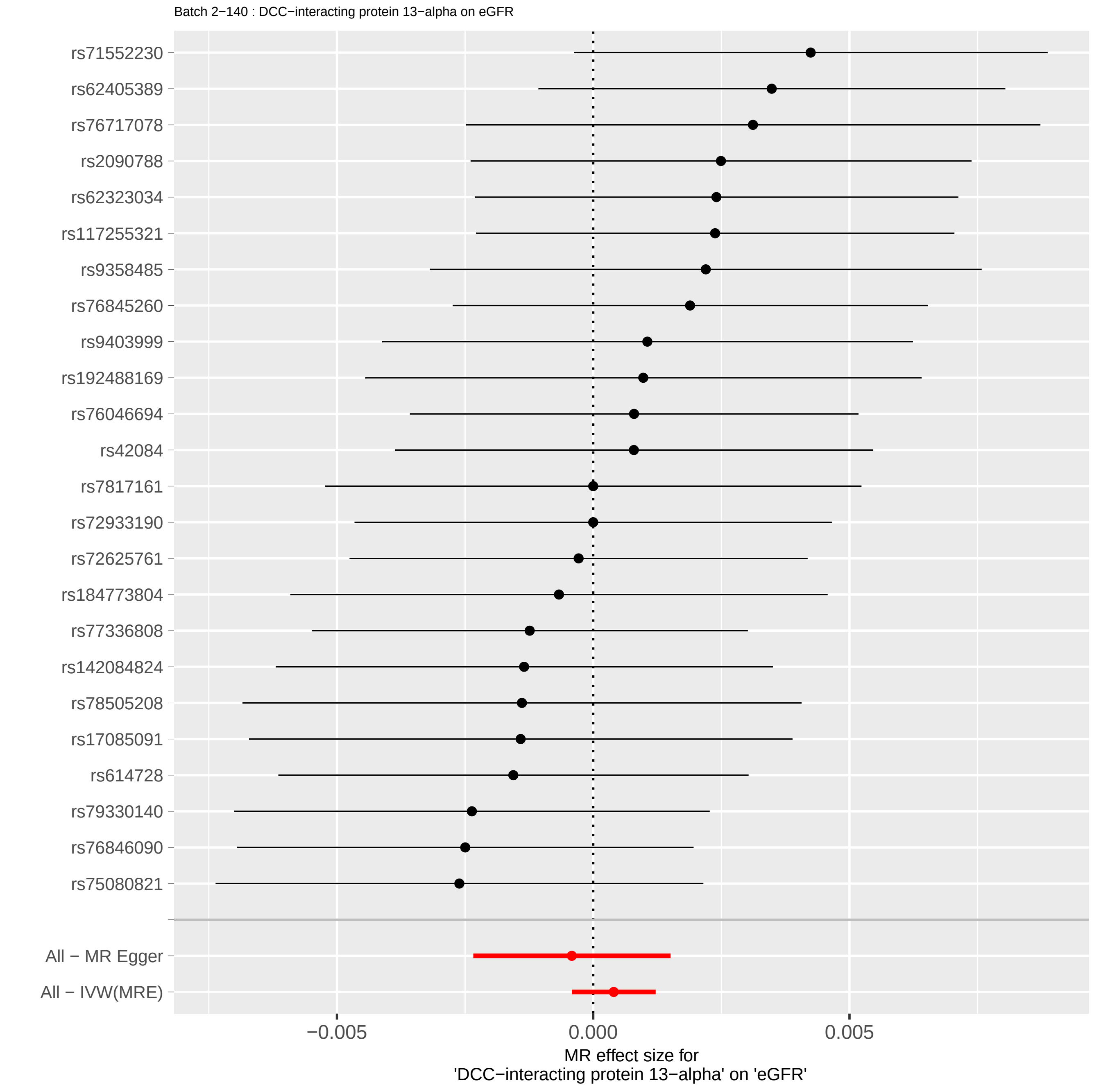
Batch 2−140 : DCC−interacting protein 13−alpha on eGFR
rs71552230
rs62405389
rs76717078
rs2090788
rs62323034
rs117255321
rs9358485
rs76845260
rs9403999
rs192488169
rs76046694
rs42084
rs7817161
rs72933190
rs72625761
rs184773804
rs77336808
rs142084824
rs78505208
rs17085091
rs614728
rs79330140
rs76846090
rs75080821
All − MR Egger
All − IVW(MRE)
−0.005
0.000
0.005
MR effect size for
'DCC−interacting protein 13−alpha' on 'eGFR'

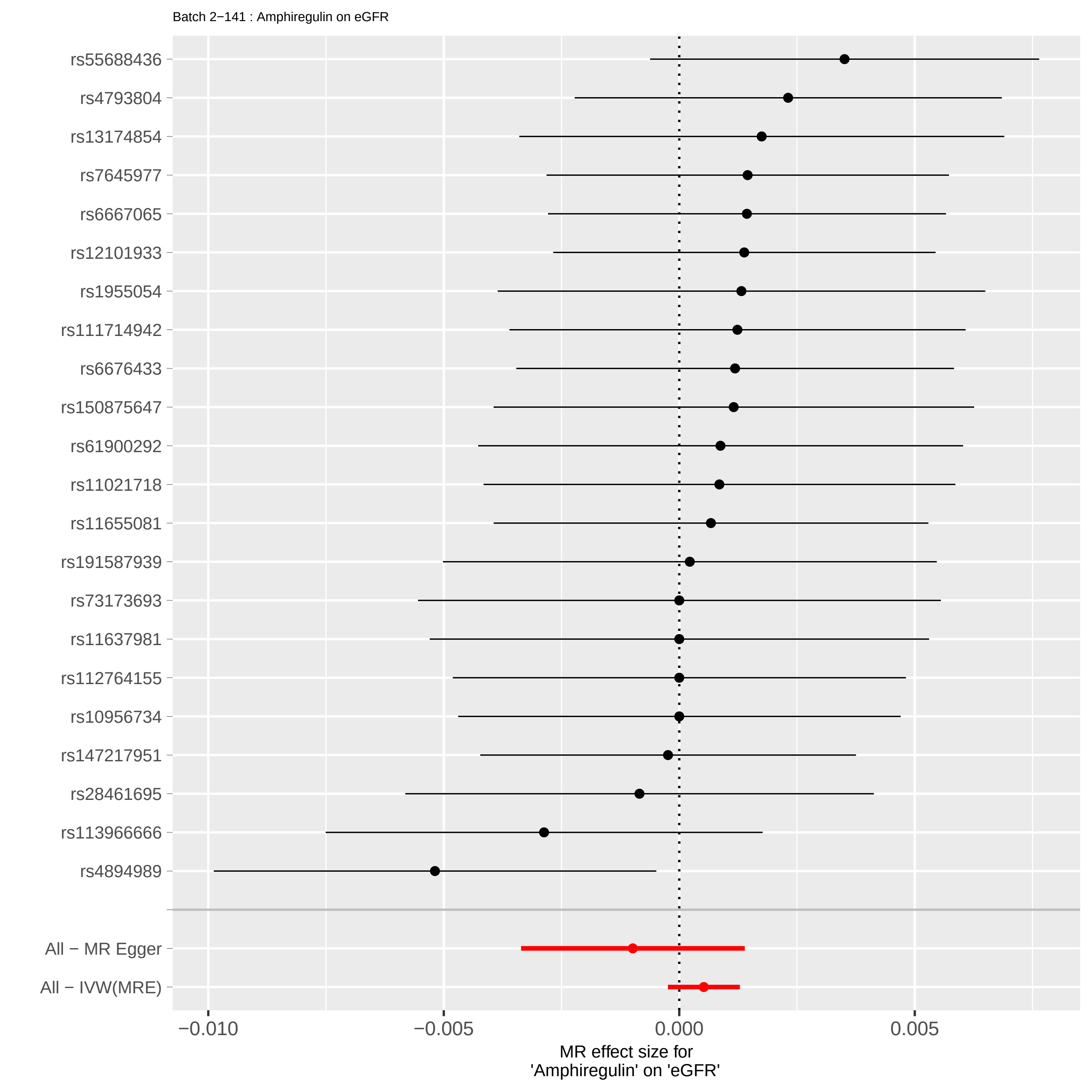

Batch 2−141 : Amphiregulin on eGFR
rs55688436
rs4793804
rs13174854
rs7645977
rs6667065
rs12101933
rs1955054
rs111714942
rs6676433
rs150875647
rs61900292
rs11021718
rs11655081
rs191587939
rs73173693
rs11637981
rs112764155
rs10956734
rs147217951
rs28461695
rs113966666
rs4894989
All − MR Egger
All − IVW(MRE)
−0.010
−0.005
0.000
0.005
MR effect size for
'Amphiregulin' on 'eGFR'

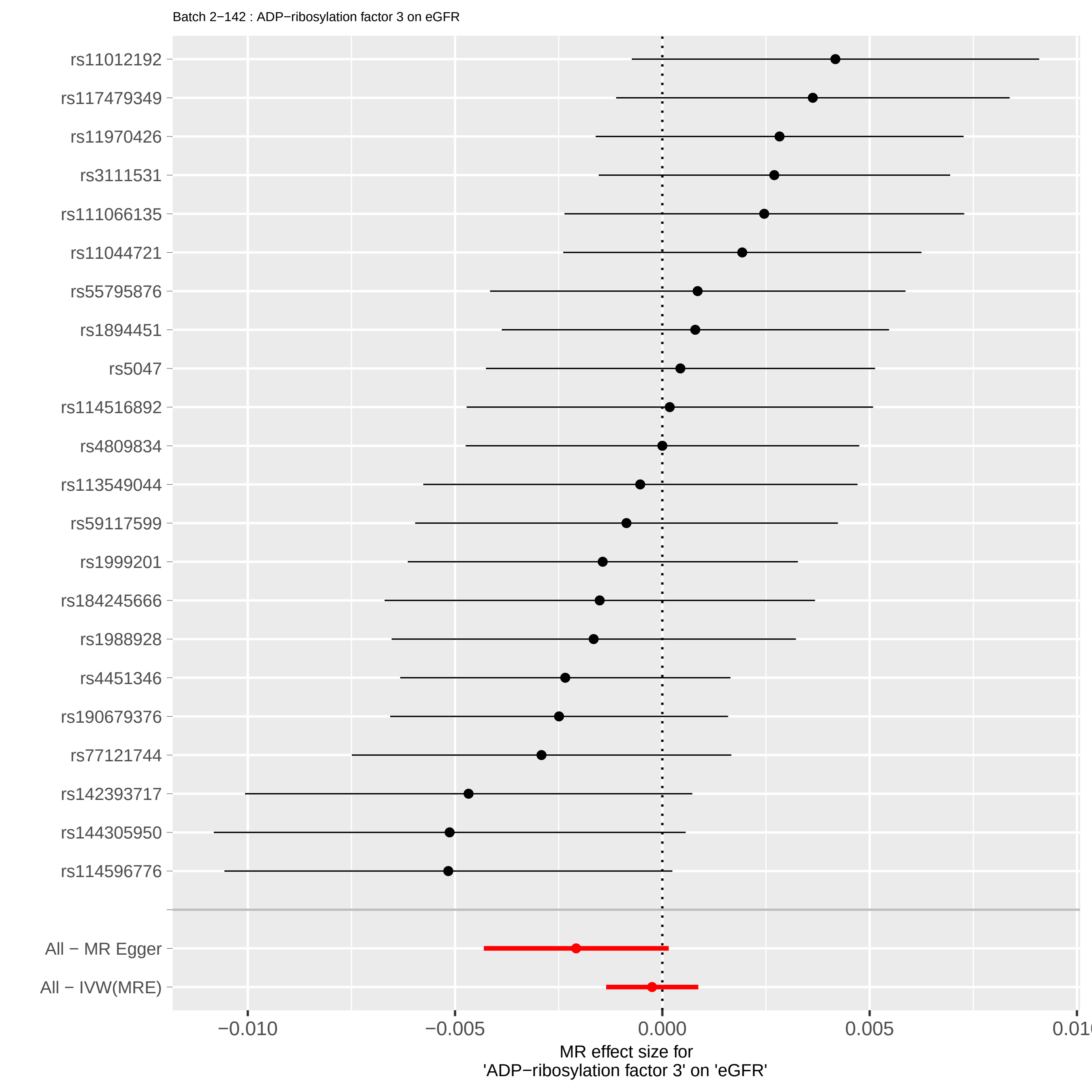

Batch 2−142 : ADP−ribosylation factor 3 on eGFR
rs11012192
rs117479349
rs11970426
rs3111531
rs111066135
rs11044721
rs55795876
rs1894451
rs5047
rs114516892
rs4809834
rs113549044
rs59117599
rs1999201
rs184245666
rs1988928
rs4451346
rs190679376
rs77121744
rs142393717
rs144305950
rs114596776
All − MR Egger
All − IVW(MRE)
−0.010
−0.005
0.000
0.005
0.01
MR effect size for
'ADP−ribosylation factor 3' on 'eGFR'

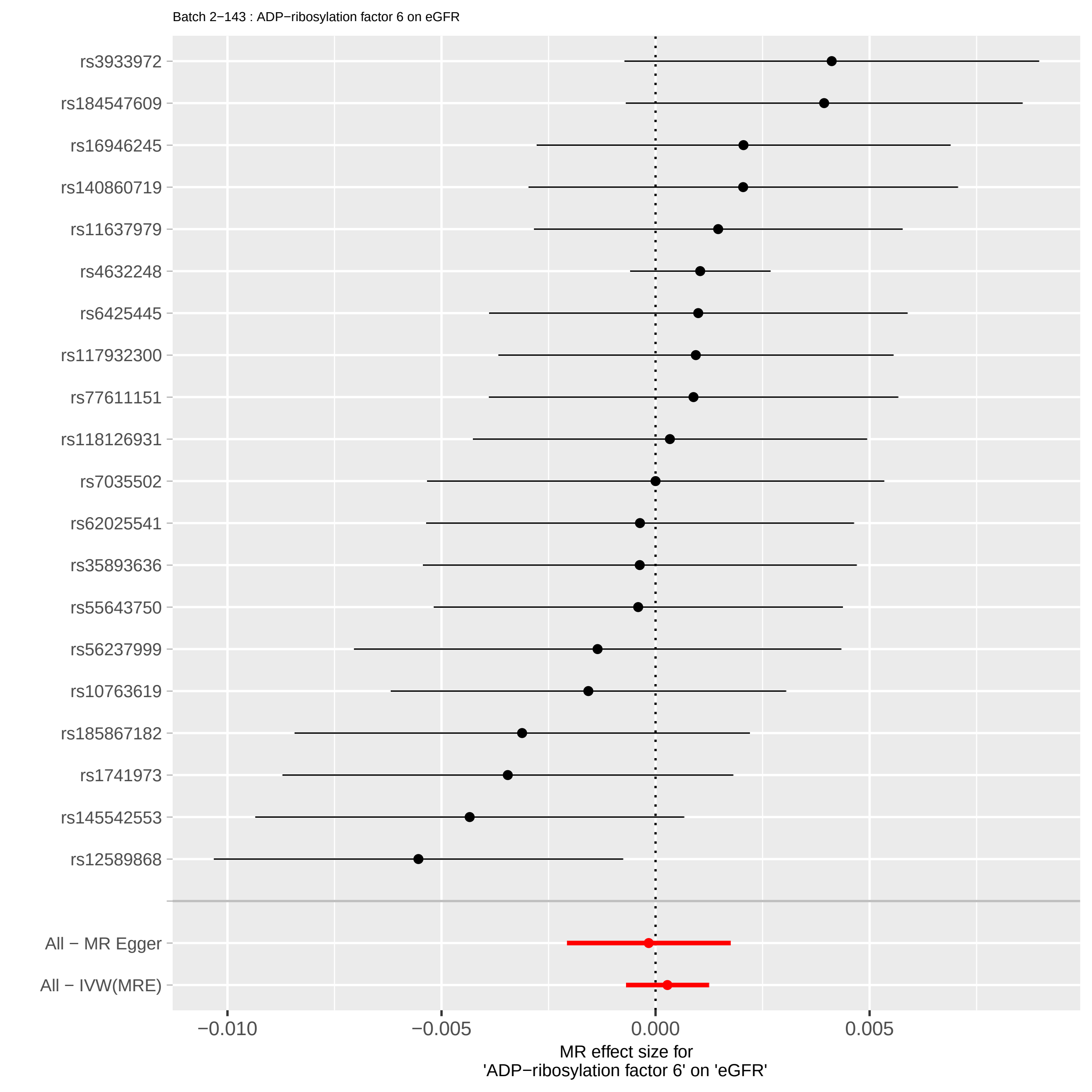

Batch 2−143 : ADP−ribosylation factor 6 on eGFR
rs3933972
rs184547609
rs16946245
rs140860719
rs11637979
rs4632248
rs6425445
rs117932300
rs77611151
rs118126931
rs7035502
rs62025541
rs35893636
rs55643750
rs56237999
rs10763619
rs185867182
rs1741973
rs145542553
rs12589868
All − MR Egger
All − IVW(MRE)
−0.010
−0.005
0.000
0.005
MR effect size for
'ADP−ribosylation factor 6' on 'eGFR'

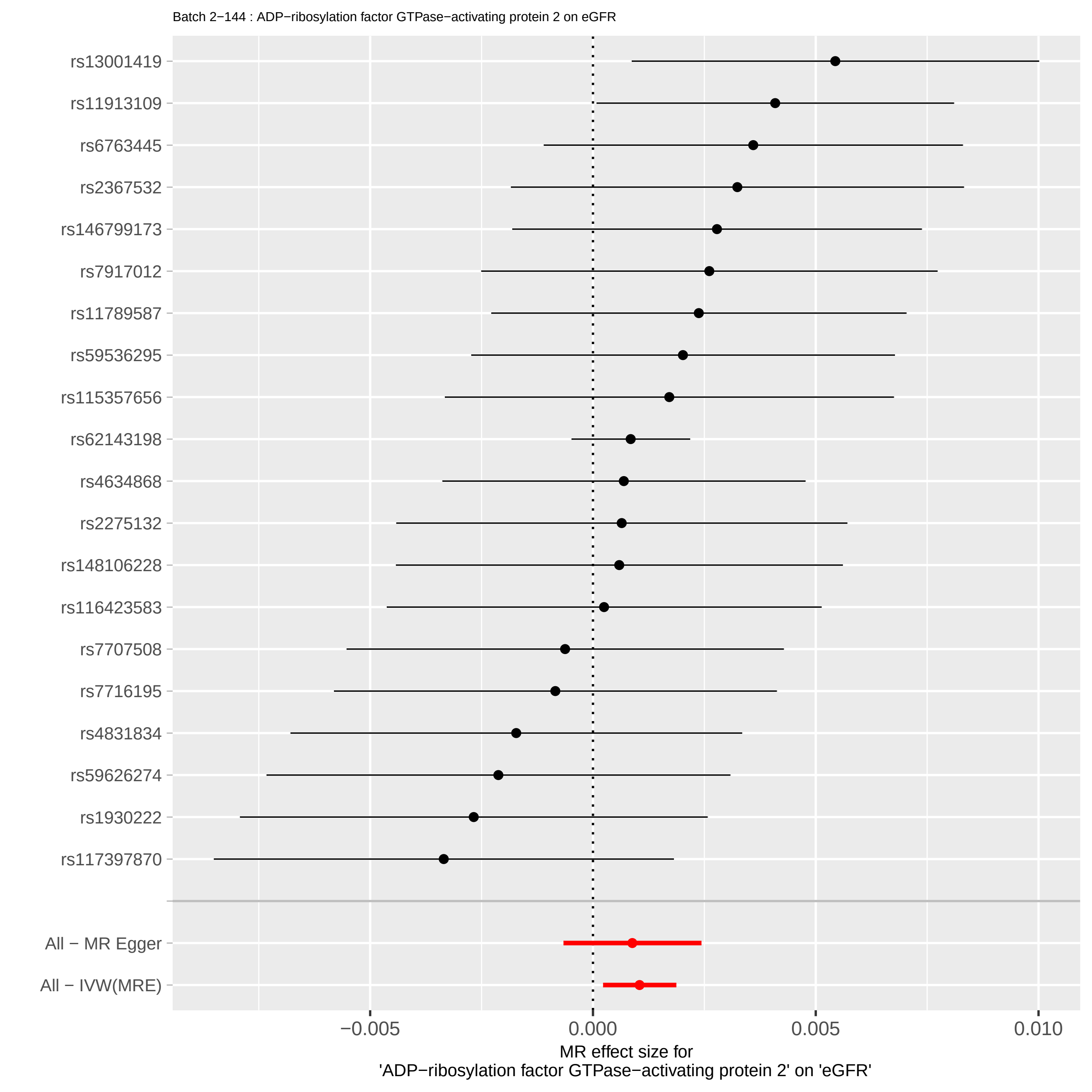
Batch 2−144 : ADP−ribosylation factor GTPase−activating protein 2 on eGFR
rs13001419
rs11913109
rs6763445
rs2367532
rs146799173
rs7917012
rs11789587
rs59536295
rs115357656
rs62143198
rs4634868
rs2275132
rs148106228
rs116423583
rs7707508
rs7716195
rs4831834
rs59626274
rs1930222
rs117397870
All − MR Egger
All − IVW(MRE)
−0.005
0.000
0.005
0.010
MR effect size for
'ADP−ribosylation factor GTPase−activating protein 2' on 'eGFR'

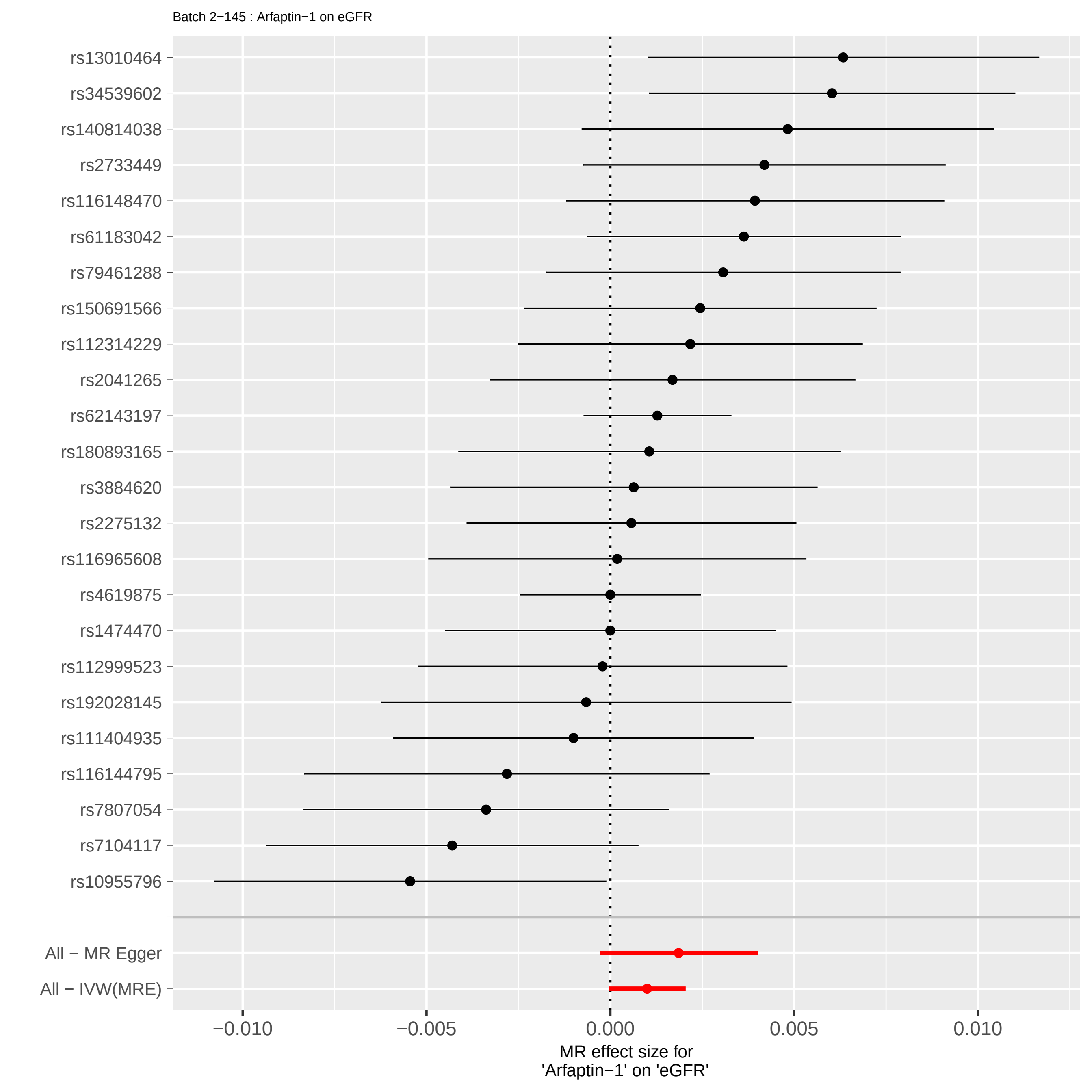

Batch 2−145 : Arfaptin−1 on eGFR
rs13010464
rs34539602
rs140814038
rs2733449
rs116148470
rs61183042
rs79461288
rs150691566
rs112314229
rs2041265
rs62143197
rs180893165
rs3884620
rs2275132
rs116965608
rs4619875
rs1474470
rs112999523
rs192028145
rs111404935
rs116144795
rs7807054
rs7104117
rs10955796
All − MR Egger
All − IVW(MRE)
−0.010
−0.005
0.000
0.005
0.010
MR effect size for
'Arfaptin−1' on 'eGFR'

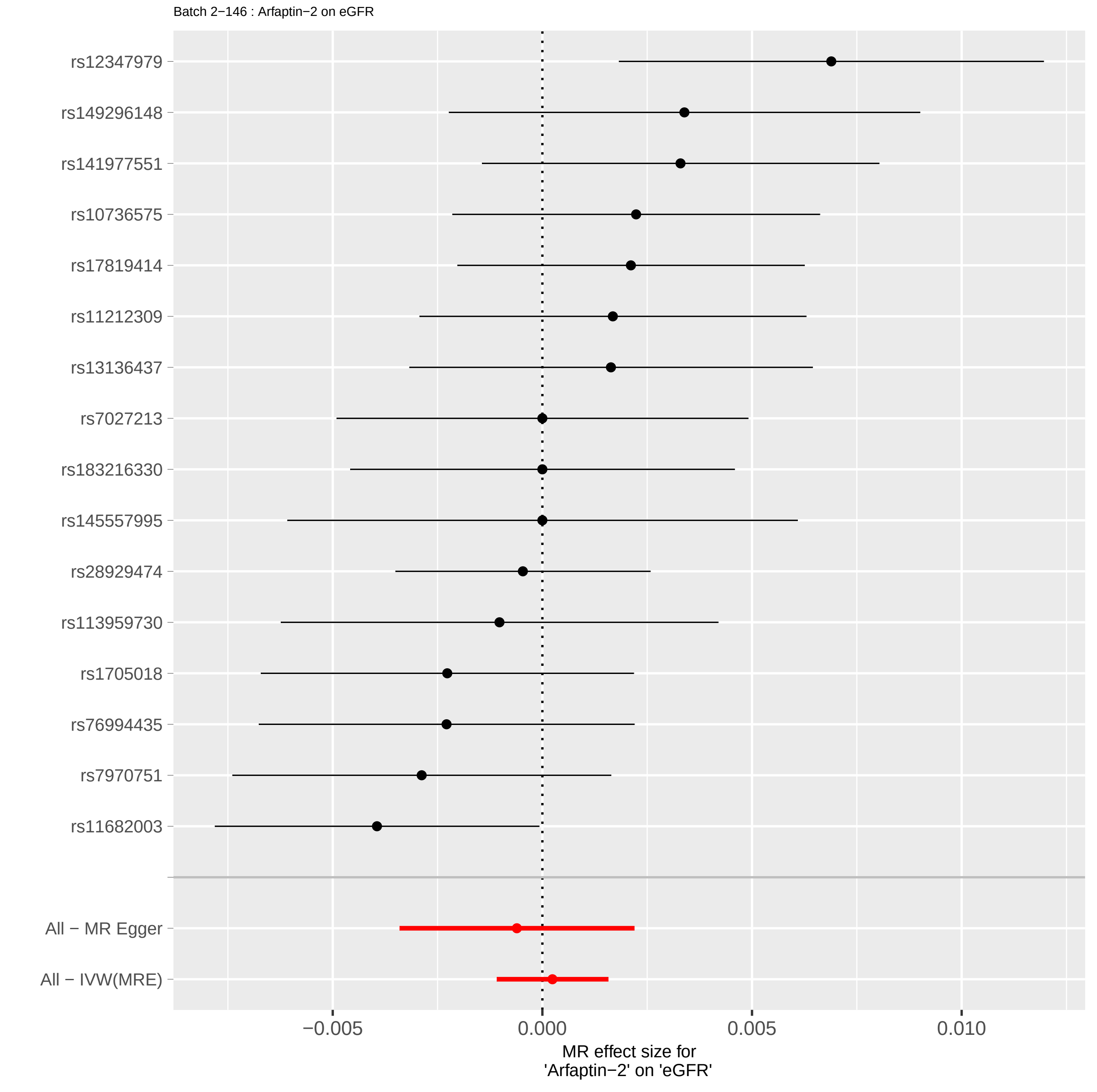
Batch 2−146 : Arfaptin−2 on eGFR
rs12347979
rs149296148
rs141977551
rs10736575
rs17819414
rs11212309
rs13136437
rs7027213
rs183216330
rs145557995
rs28929474
rs113959730
rs1705018
rs76994435
rs7970751
rs11682003
All − MR Egger
All − IVW(MRE)
−0.005
0.000
0.005
0.010
MR effect size for
'Arfaptin−2' on 'eGFR'

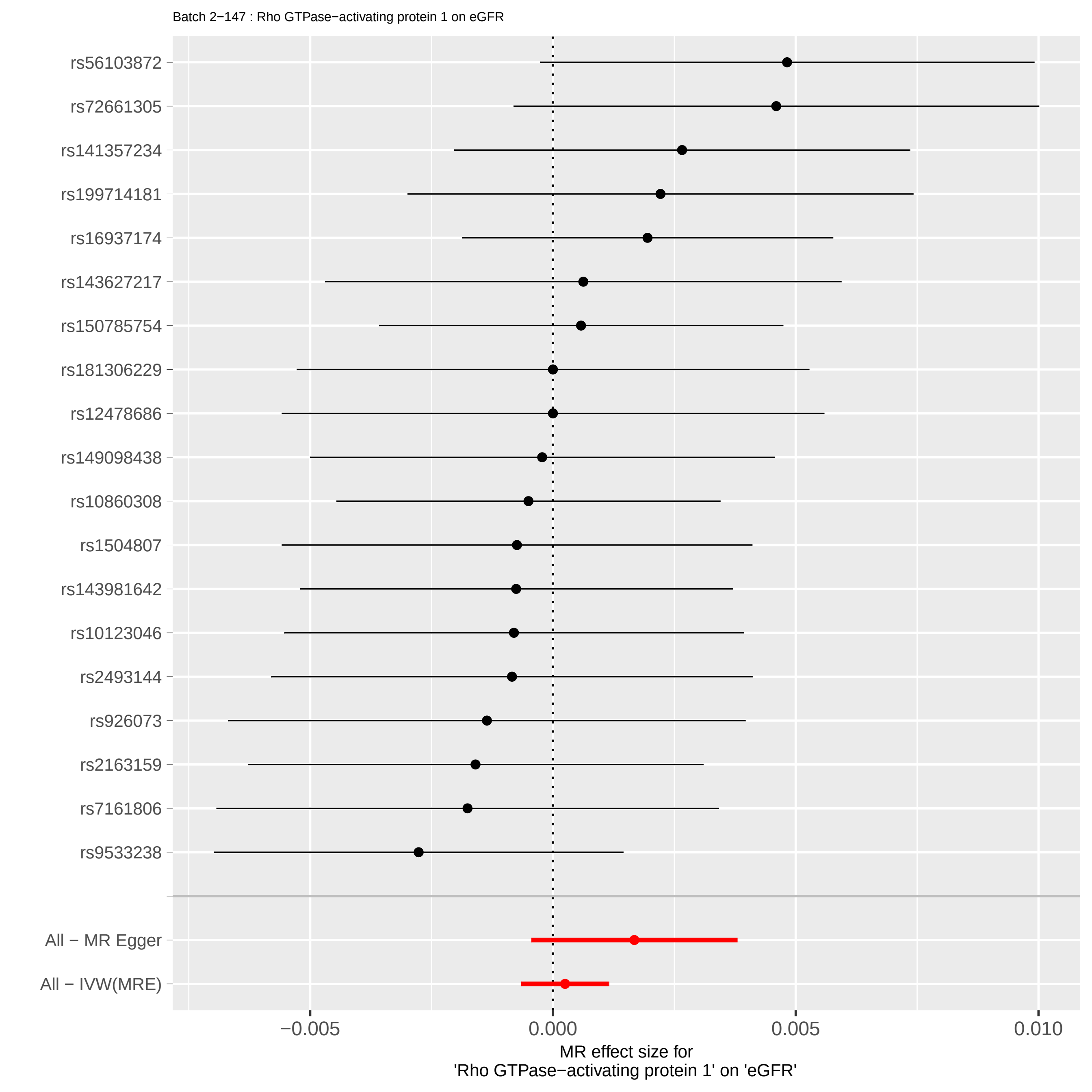
Batch 2−147 : Rho GTPase−activating protein 1 on eGFR
rs56103872
rs72661305
rs141357234
rs199714181
rs16937174
rs143627217
rs150785754
rs181306229
rs12478686
rs149098438
rs10860308
rs1504807
rs143981642
rs10123046
rs2493144
rs926073
rs2163159
rs7161806
rs9533238
All − MR Egger
All − IVW(MRE)
−0.005
0.000
0.005
0.010
MR effect size for
'Rho GTPase−activating protein 1' on 'eGFR'

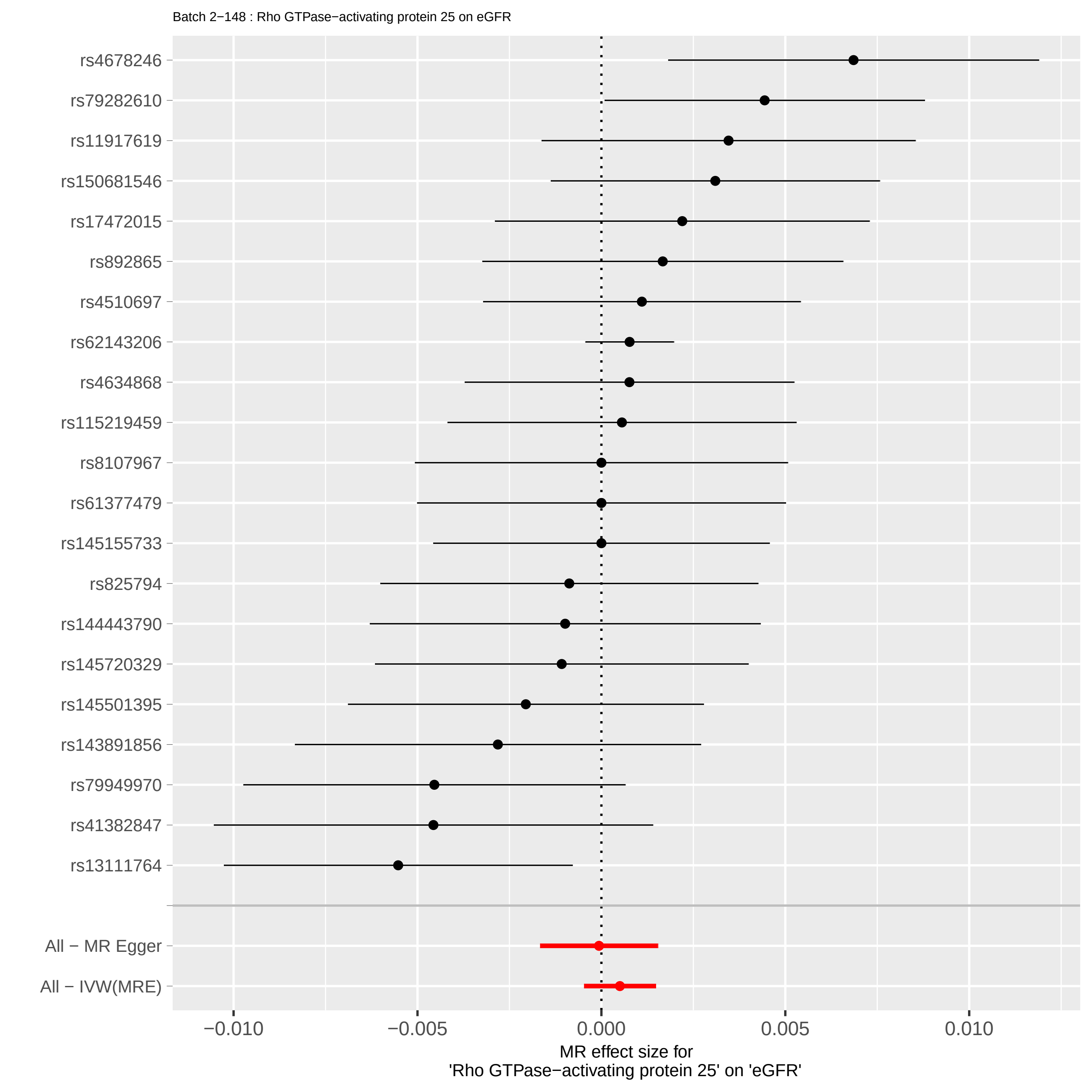

Batch 2−148 : Rho GTPase−activating protein 25 on eGFR
rs4678246
rs79282610
rs11917619
rs150681546
rs17472015
rs892865
rs4510697
rs62143206
rs4634868
rs115219459
rs8107967
rs61377479
rs145155733
rs825794
rs144443790
rs145720329
rs145501395
rs143891856
rs79949970
rs41382847
rs13111764
All − MR Egger
All − IVW(MRE)
−0.010
−0.005
0.000
0.005
0.010
MR effect size for
'Rho GTPase−activating protein 25' on 'eGFR'

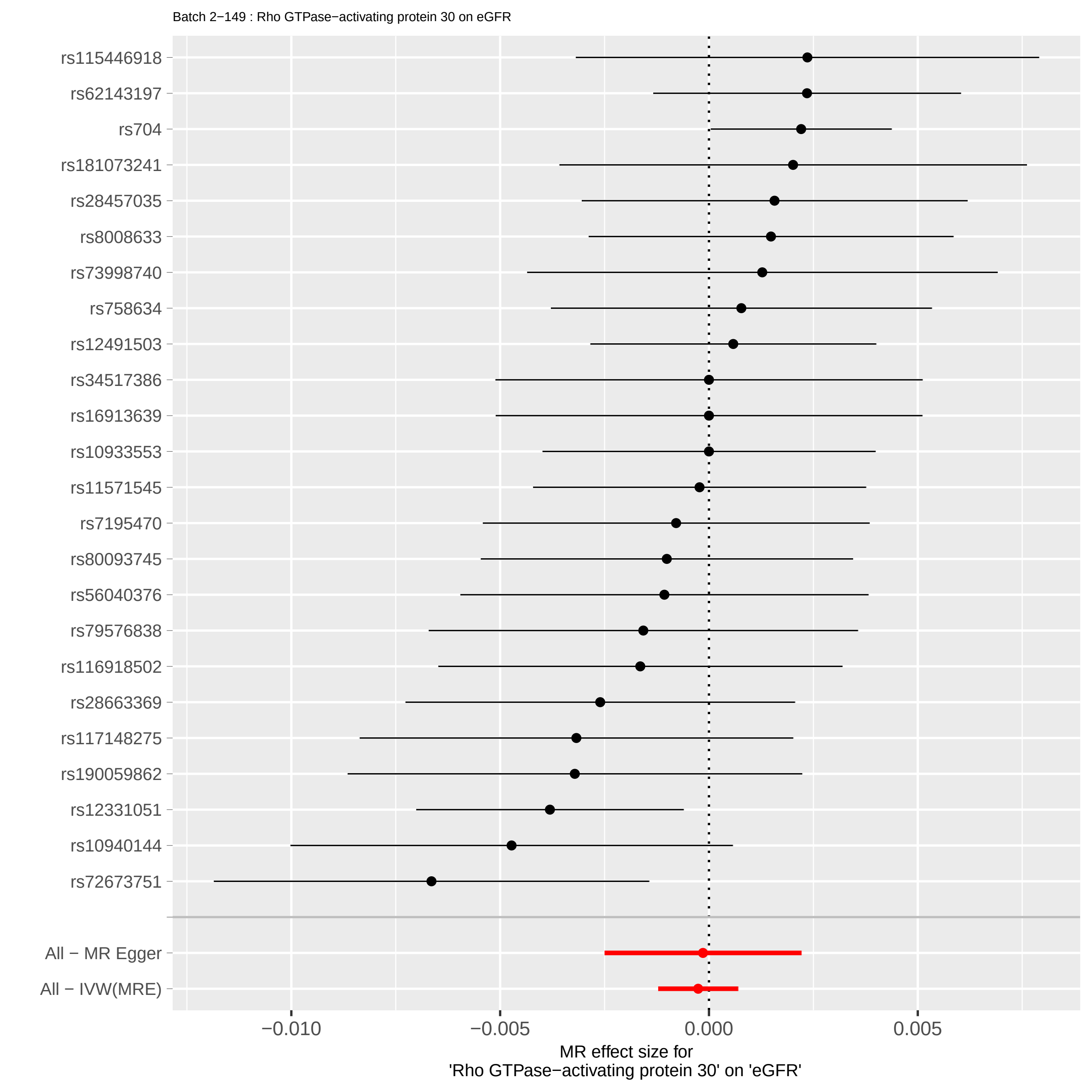

Batch 2−149 : Rho GTPase−activating protein 30 on eGFR
rs115446918
rs62143197
rs704
rs181073241
rs28457035
rs8008633
rs73998740
rs758634
rs12491503
rs34517386
rs16913639
rs10933553
rs11571545
rs7195470
rs80093745
rs56040376
rs79576838
rs116918502
rs28663369
rs117148275
rs190059862
rs12331051
rs10940144
rs72673751
All − MR Egger
All − IVW(MRE)
−0.010
−0.005
0.000
0.005
MR effect size for
'Rho GTPase−activating protein 30' on 'eGFR'

Batch 2−150 : Rho GTPase−activating protein 36 on eGFR

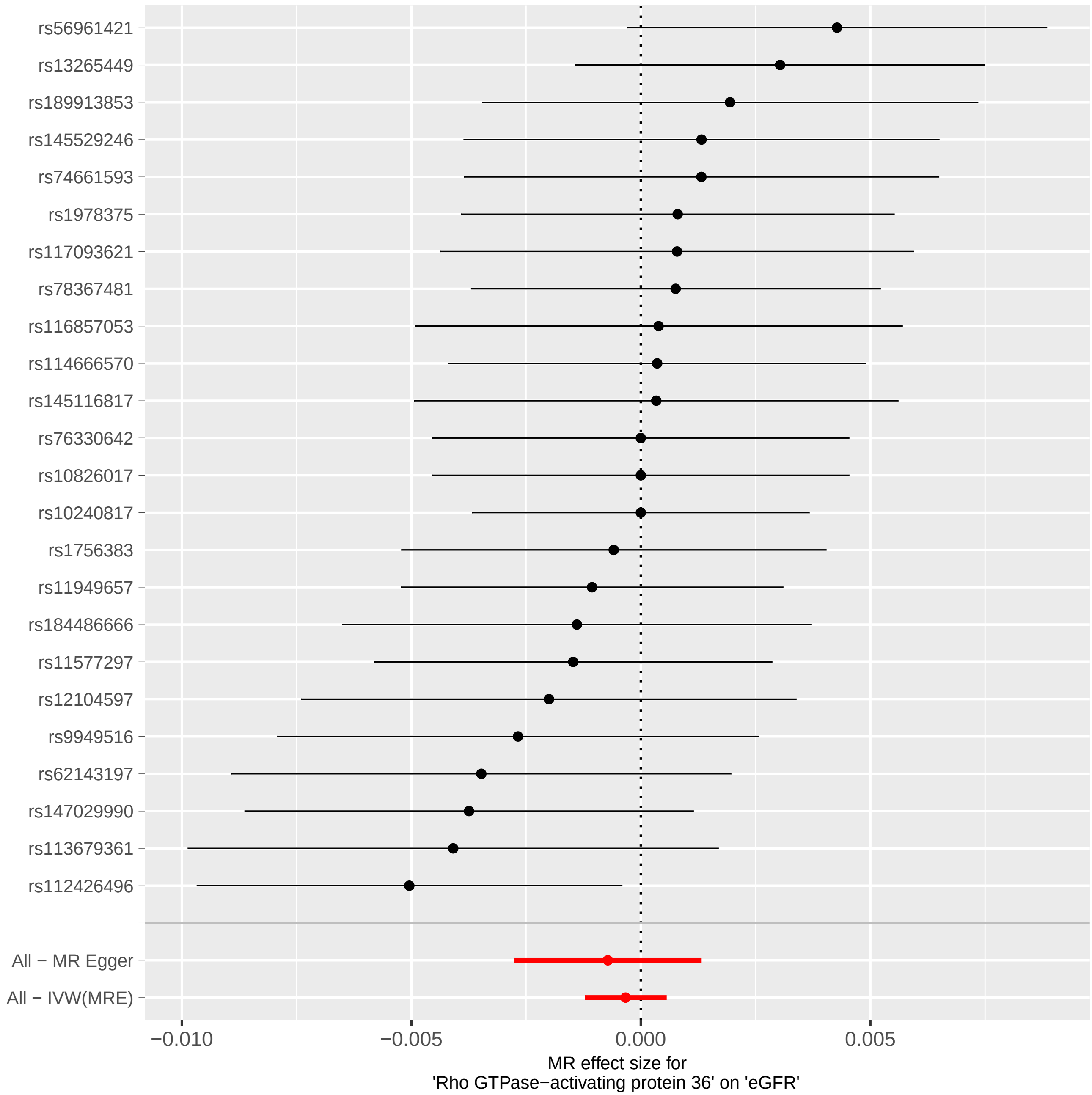

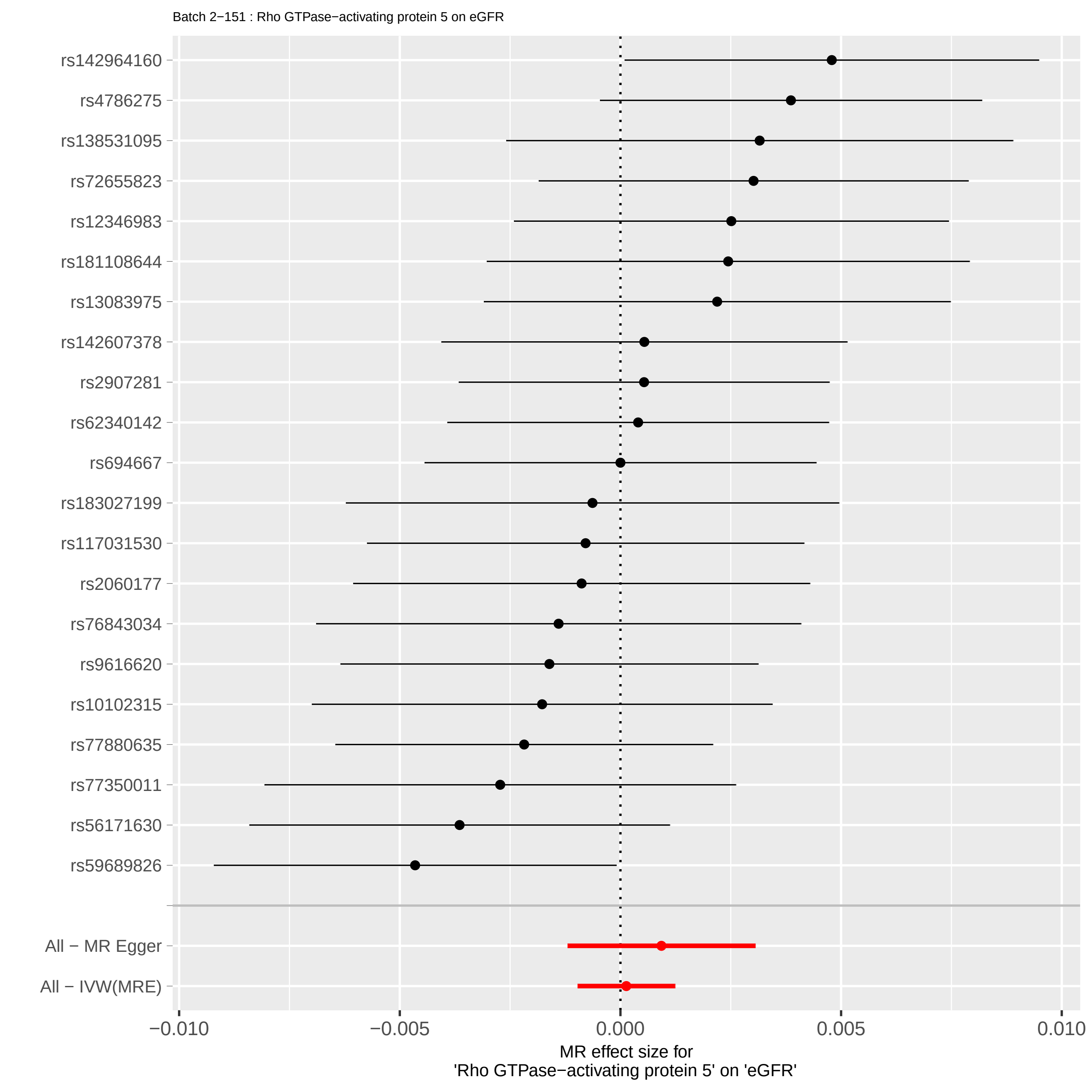

Batch 2−151 : Rho GTPase−activating protein 5 on eGFR
rs142964160
rs4786275
rs138531095
rs72655823
rs12346983
rs181108644
rs13083975
rs142607378
rs2907281
rs62340142
rs694667
rs183027199
rs117031530
rs2060177
rs76843034
rs9616620
rs10102315
rs77880635
rs77350011
rs56171630
rs59689826
All − MR Egger
All − IVW(MRE)
−0.010
−0.005
0.000
0.005
0.010
MR effect size for
'Rho GTPase−activating protein 5' on 'eGFR'

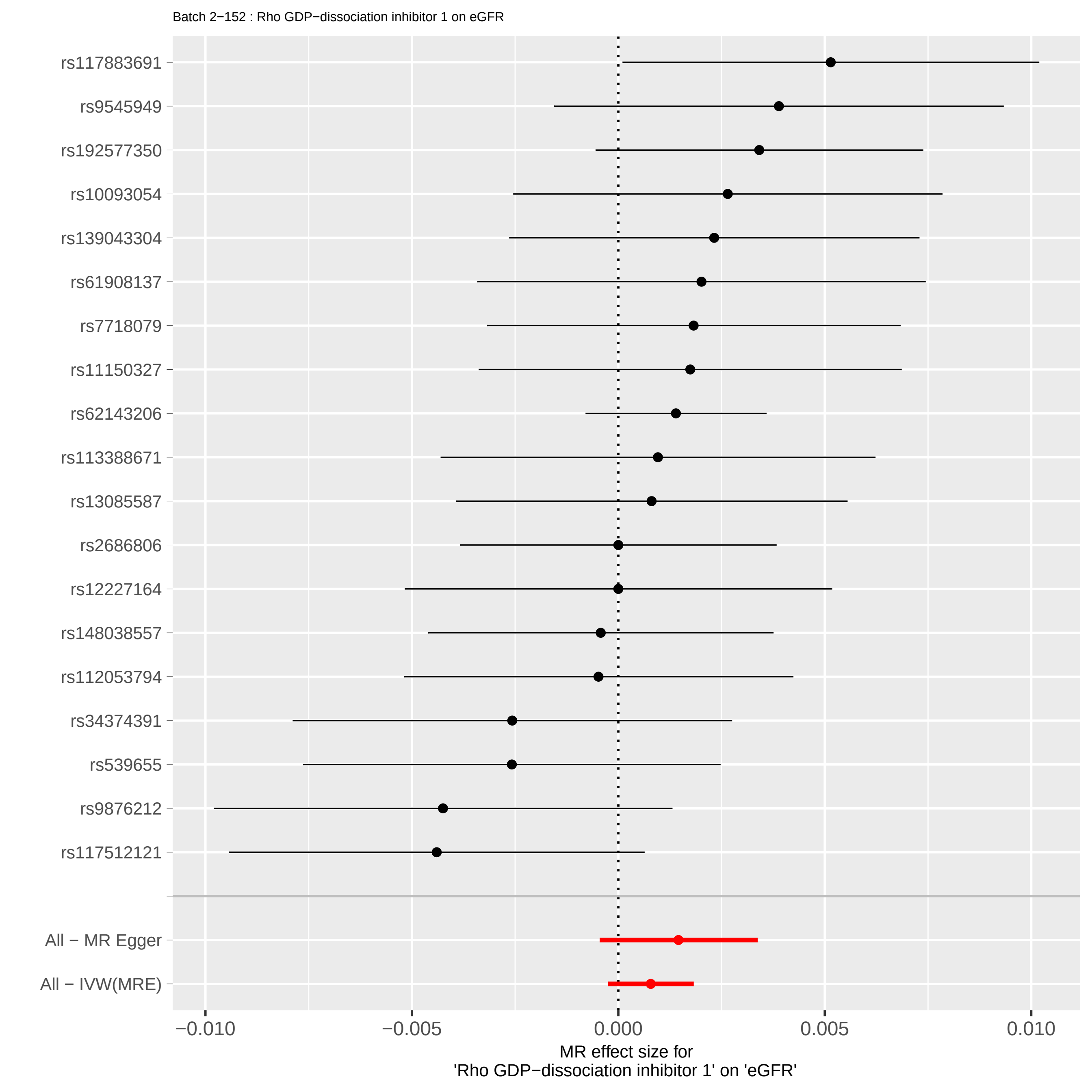
Batch 2−152 : Rho GDP−dissociation inhibitor 1 on eGFR
rs117883691
rs9545949
rs192577350
rs10093054
rs139043304
rs61908137
rs7718079
rs11150327
rs62143206
rs113388671
rs13085587
rs2686806
rs12227164
rs148038557
rs112053794
rs34374391
rs539655
rs9876212
rs117512121
All − MR Egger
All − IVW(MRE)
−0.010
−0.005
0.000
0.005
0.010
MR effect size for
'Rho GDP−dissociation inhibitor 1' on 'eGFR'

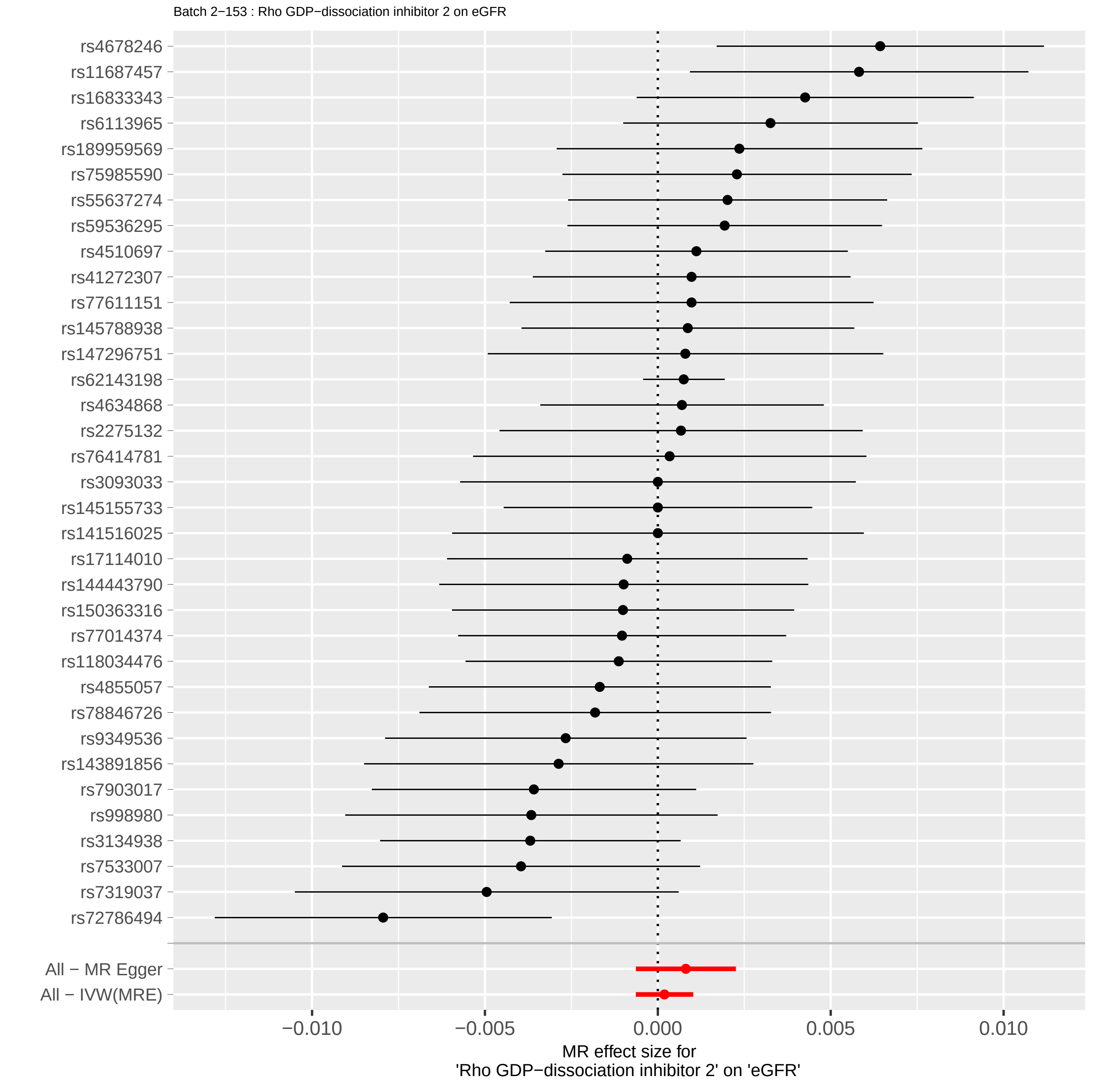

Batch 2−153 : Rho GDP−dissociation inhibitor 2 on eGFR
rs4678246
rs11687457
rs16833343
rs6113965
rs189959569
rs75985590
rs55637274
rs59536295
rs4510697
rs41272307
rs77611151
rs145788938
rs147296751
rs62143198
rs4634868
rs2275132
rs76414781
rs3093033
rs145155733
rs141516025
rs17114010
rs144443790
rs150363316
rs77014374
rs118034476
rs4855057
rs78846726
rs9349536
rs143891856
rs7903017
rs998980
rs3134938
rs7533007
rs7319037
rs72786494
All − MR Egger
All − IVW(MRE)
−0.010
−0.005
0.000
0.005
0.010
MR effect size for
'Rho GDP−dissociation inhibitor 2' on 'eGFR'

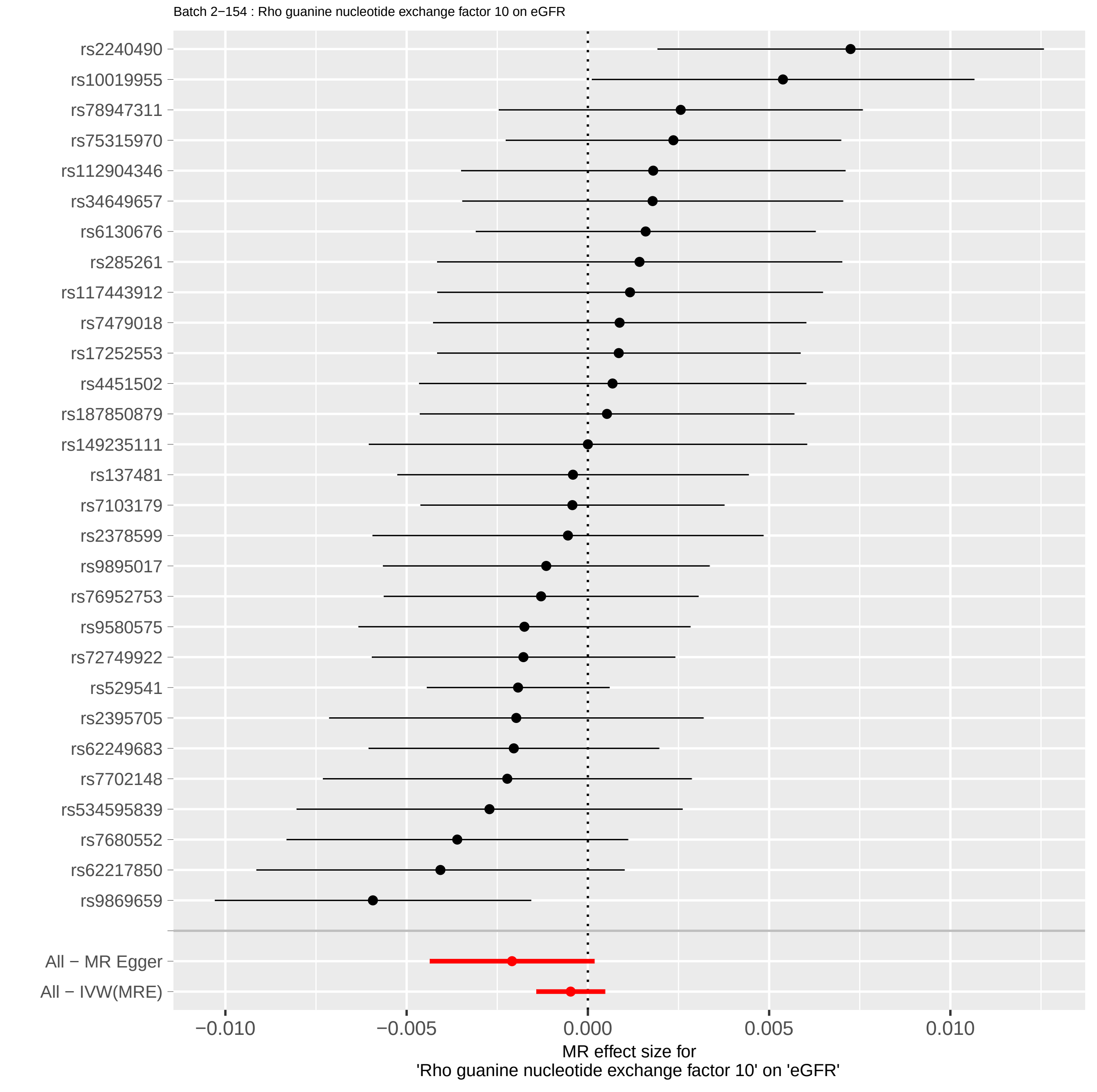
Batch 2−154 : Rho guanine nucleotide exchange factor 10 on eGFR
rs2240490
rs10019955
rs78947311
rs75315970
rs112904346
rs34649657
rs6130676
rs285261
rs117443912
rs7479018
rs17252553
rs4451502
rs187850879
rs149235111
rs137481
rs7103179
rs2378599
rs9895017
rs76952753
rs9580575
rs72749922
rs529541
rs2395705
rs62249683
rs7702148
rs534595839
rs7680552
rs62217850
rs9869659
All − MR Egger
All − IVW(MRE)
−0.010
−0.005
0.000
0.005
0.010
MR effect size for
'Rho guanine nucleotide exchange factor 10' on 'eGFR'

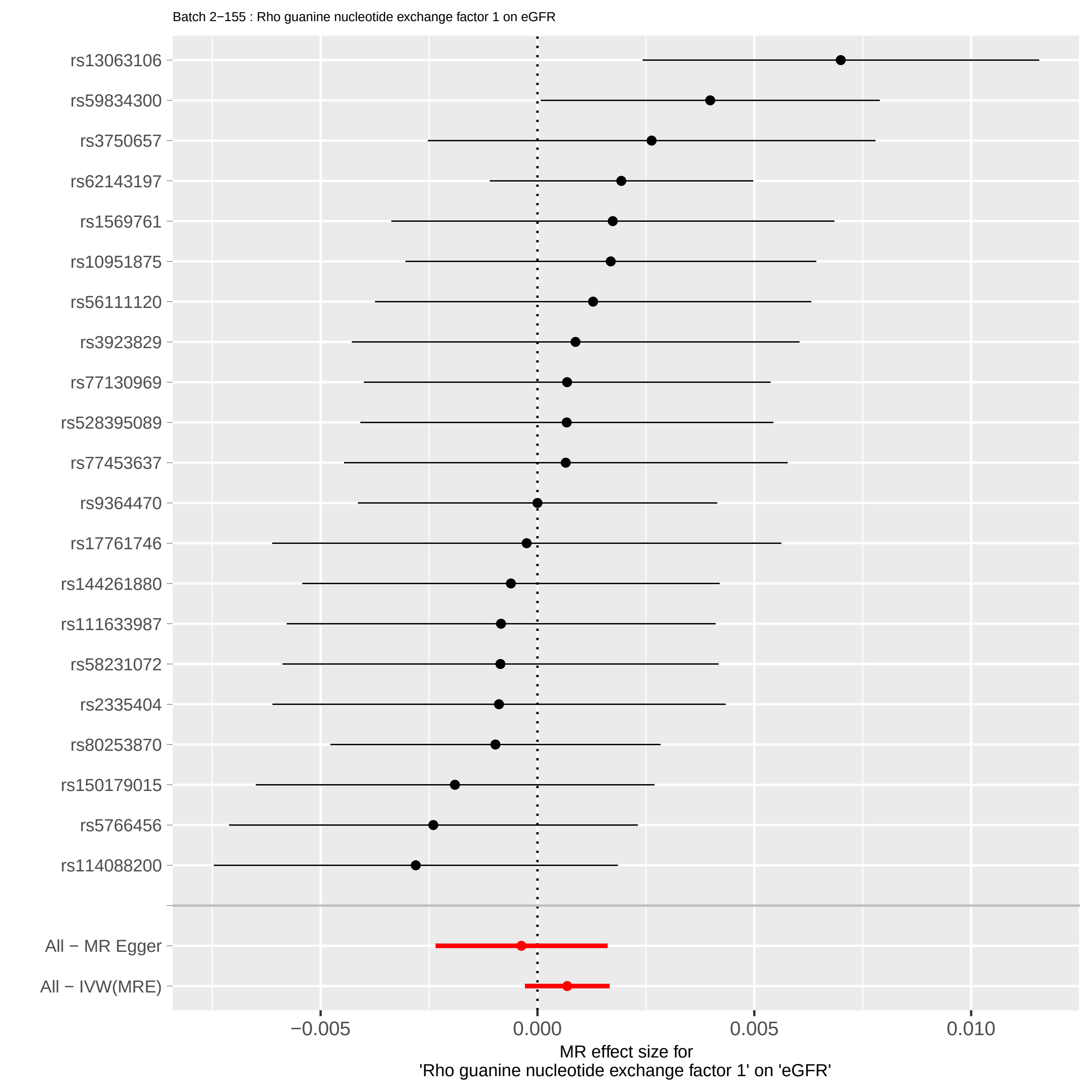

Batch 2−155 : Rho guanine nucleotide exchange factor 1 on eGFR
rs13063106
rs59834300
rs3750657
rs62143197
rs1569761
rs10951875
rs56111120
rs3923829
rs77130969
rs528395089
rs77453637
rs9364470
rs17761746
rs144261880
rs111633987
rs58231072
rs2335404
rs80253870
rs150179015
rs5766456
rs114088200
All − MR Egger
All − IVW(MRE)
−0.005
0.000
0.005
0.010
MR effect size for
'Rho guanine nucleotide exchange factor 1' on 'eGFR'

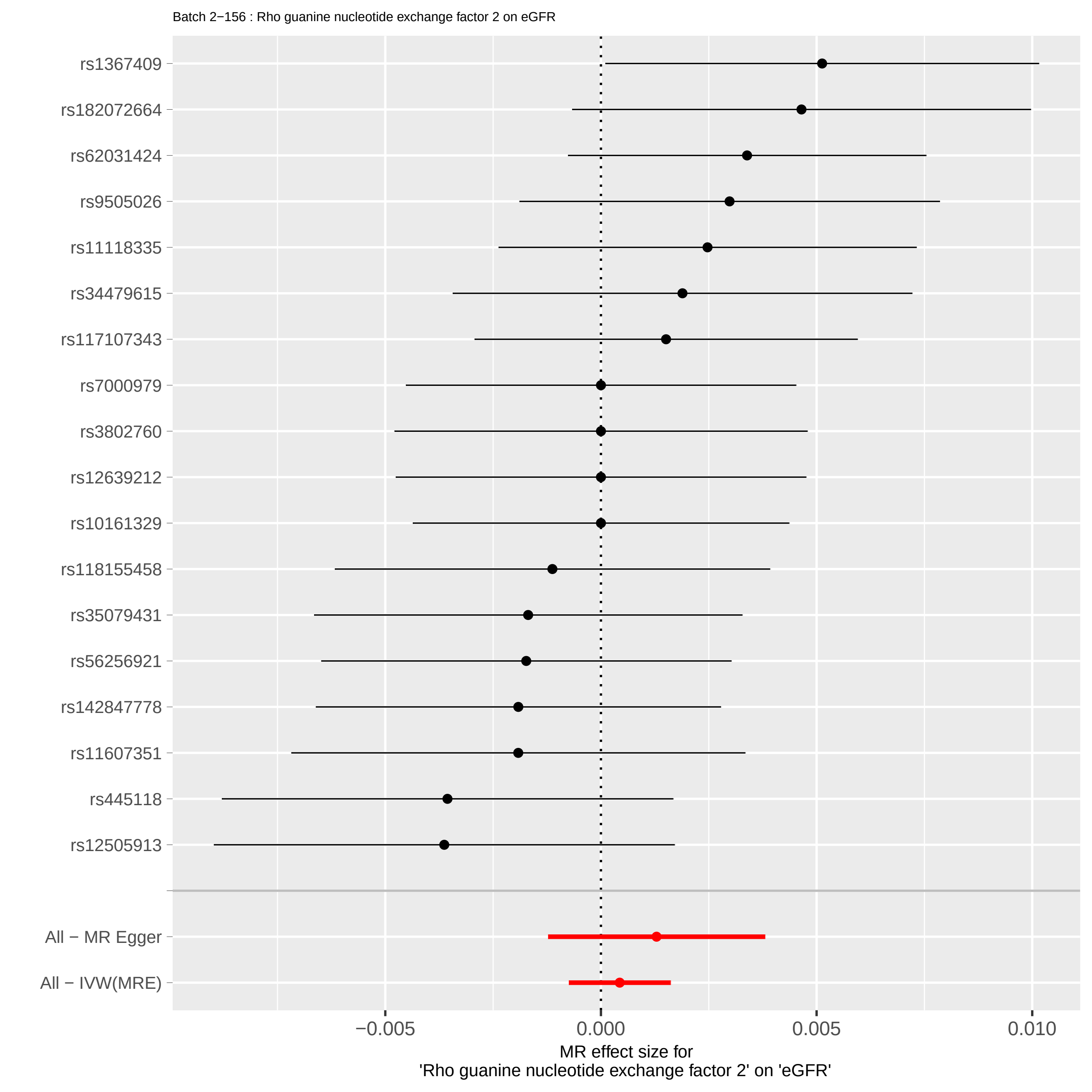
Batch 2−156 : Rho guanine nucleotide exchange factor 2 on eGFR
rs1367409
rs182072664
rs62031424
rs9505026
rs11118335
rs34479615
rs117107343
rs7000979
rs3802760
rs12639212
rs10161329
rs118155458
rs35079431
rs56256921
rs142847778
rs11607351
rs445118
rs12505913
All − MR Egger
All − IVW(MRE)
−0.005
0.000
0.005
0.010
MR effect size for
'Rho guanine nucleotide exchange factor 2' on 'eGFR'

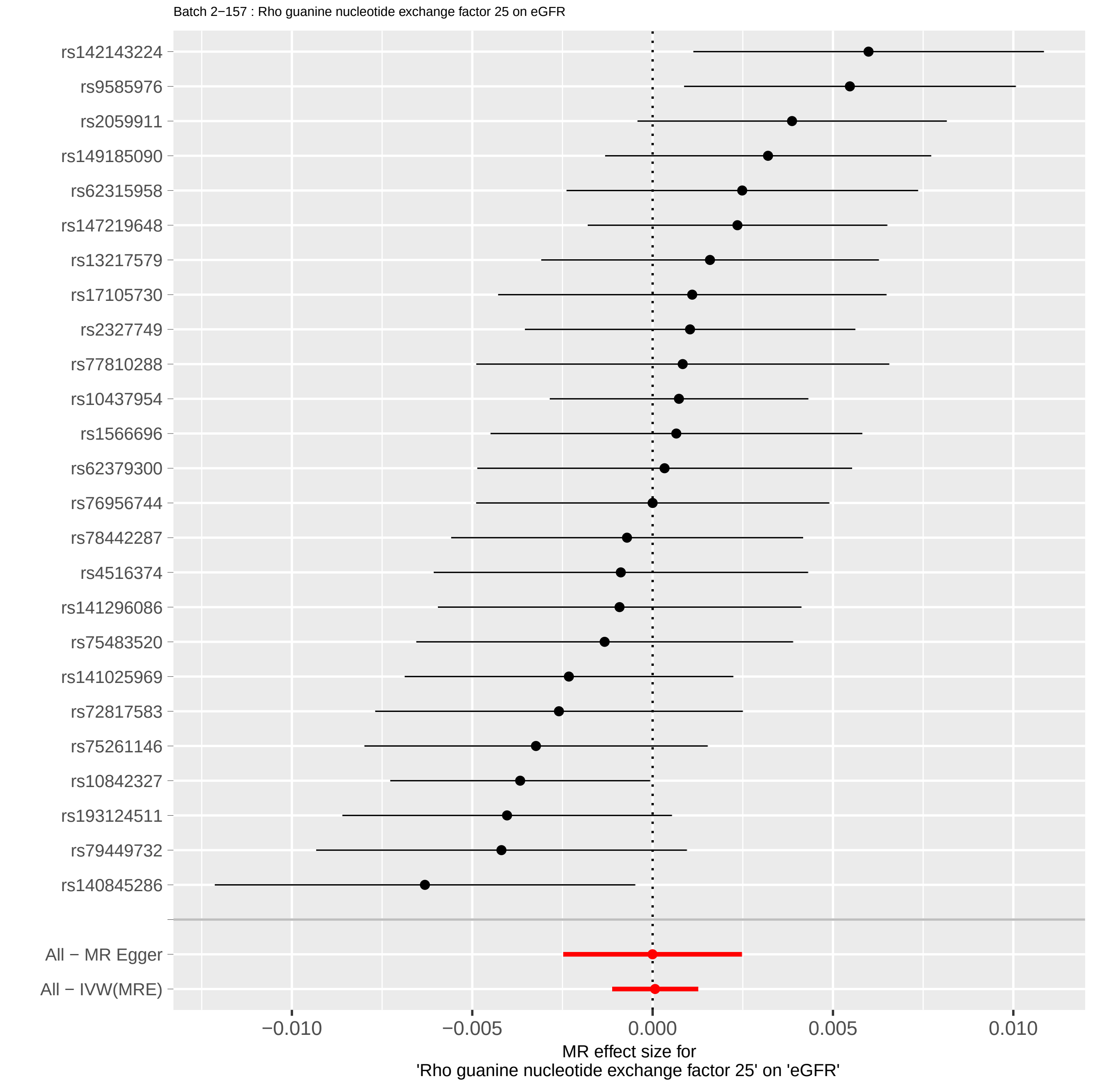

Batch 2−157 : Rho guanine nucleotide exchange factor 25 on eGFR
rs142143224
rs9585976
rs2059911
rs149185090
rs62315958
rs147219648
rs13217579
rs17105730
rs2327749
rs77810288
rs10437954
rs1566696
rs62379300
rs76956744
rs78442287
rs4516374
rs141296086
rs75483520
rs141025969
rs72817583
rs75261146
rs10842327
rs193124511
rs79449732
rs140845286
All − MR Egger
All − IVW(MRE)
−0.010
−0.005
0.000
0.005
0.010
MR effect size for
'Rho guanine nucleotide exchange factor 25' on 'eGFR'

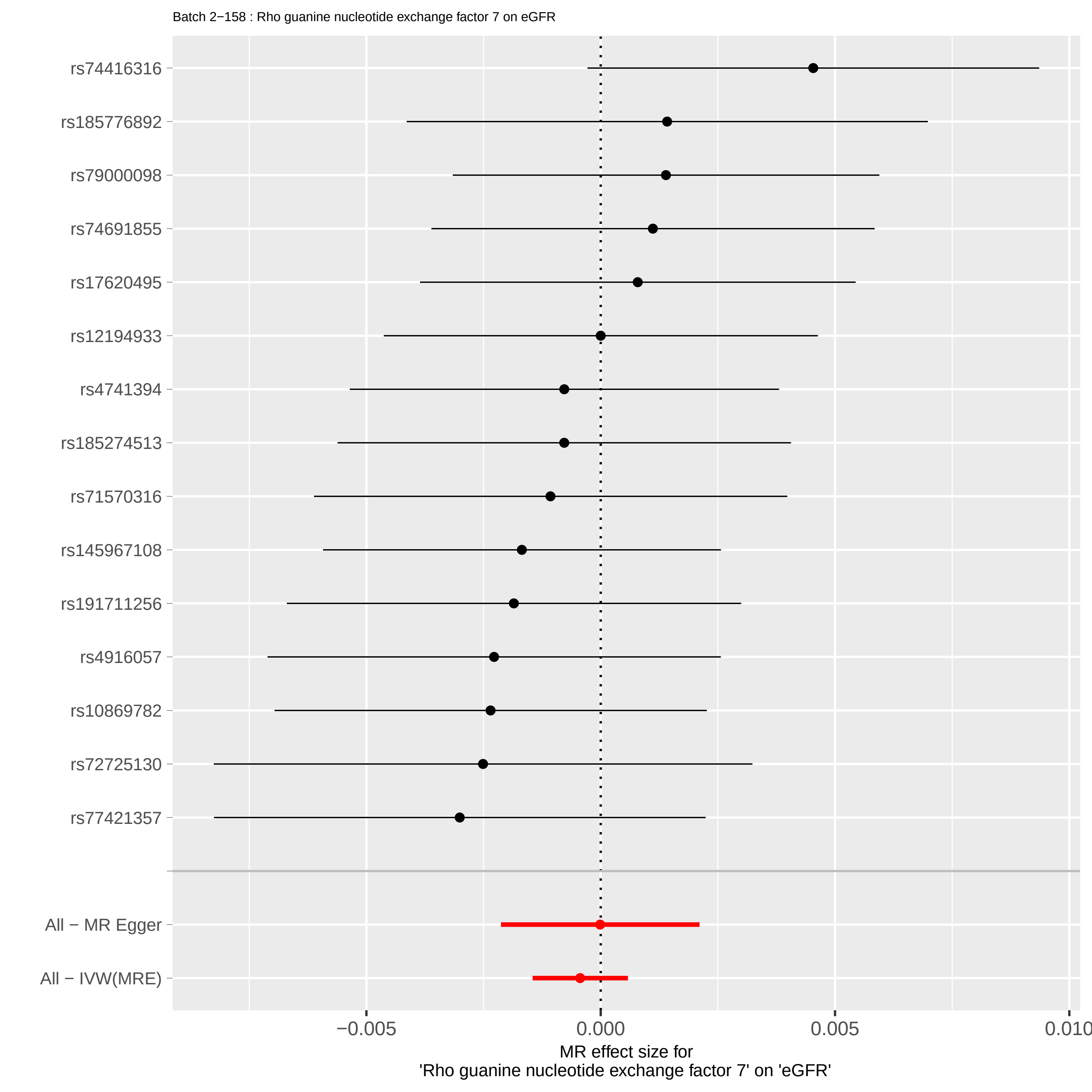

Batch 2−158 : Rho guanine nucleotide exchange factor 7 on eGFR
rs74416316
rs185776892
rs79000098
rs74691855
rs17620495
rs12194933
rs4741394
rs185274513
rs71570316
rs145967108
rs191711256
rs4916057
rs10869782
rs72725130
rs77421357
All − MR Egger
All − IVW(MRE)
−0.005
0.000
0.005
0.010
MR effect size for
'Rho guanine nucleotide exchange factor 7' on 'eGFR'

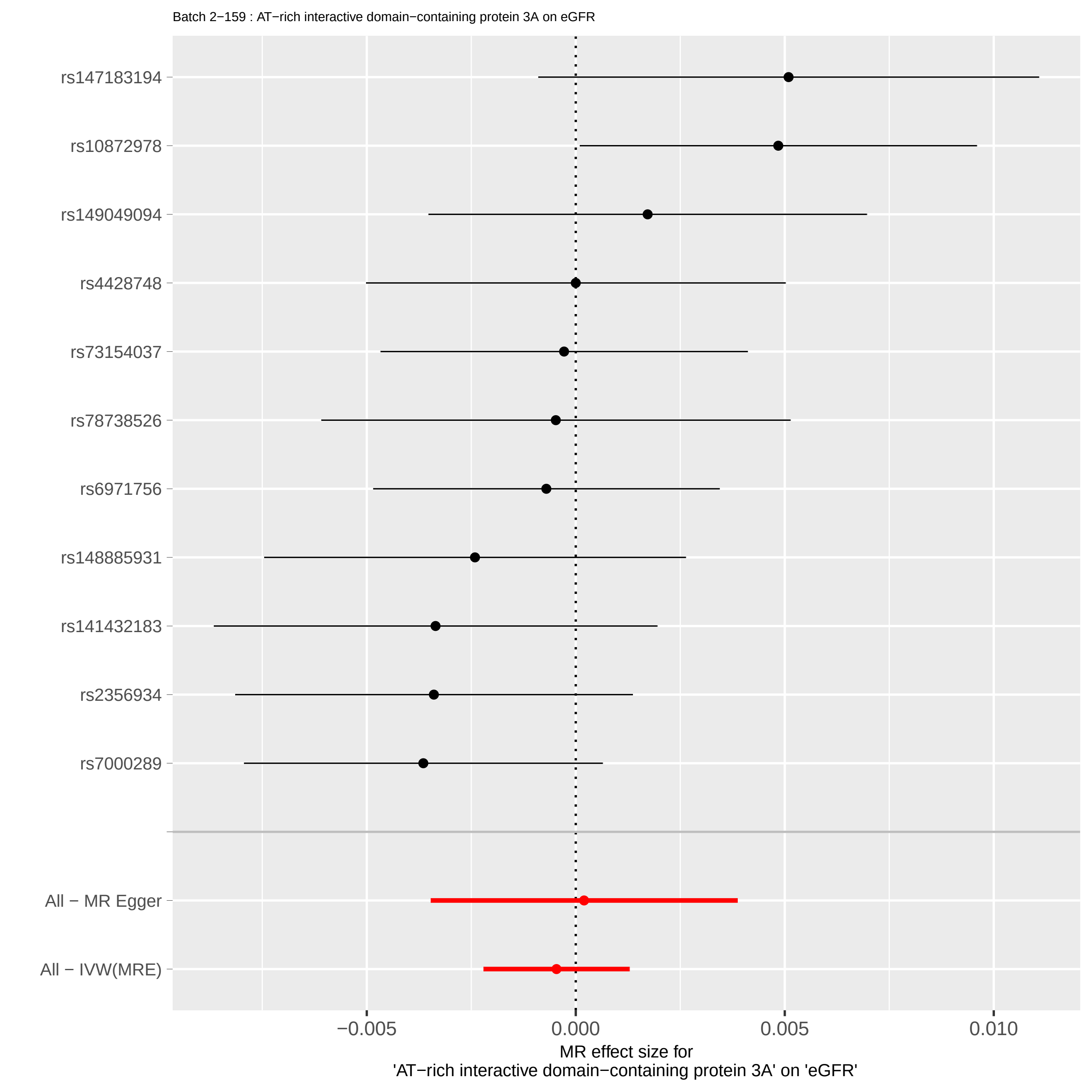

Batch 2−159 : AT−rich interactive domain−containing protein 3A on eGFR
rs147183194
rs10872978
rs149049094
rs4428748
rs73154037
rs78738526
rs6971756
rs148885931
rs141432183
rs2356934
rs7000289
All − MR Egger
All − IVW(MRE)
−0.005
0.000
0.005
0.010
MR effect size for
'AT−rich interactive domain−containing protein 3A' on 'eGFR'

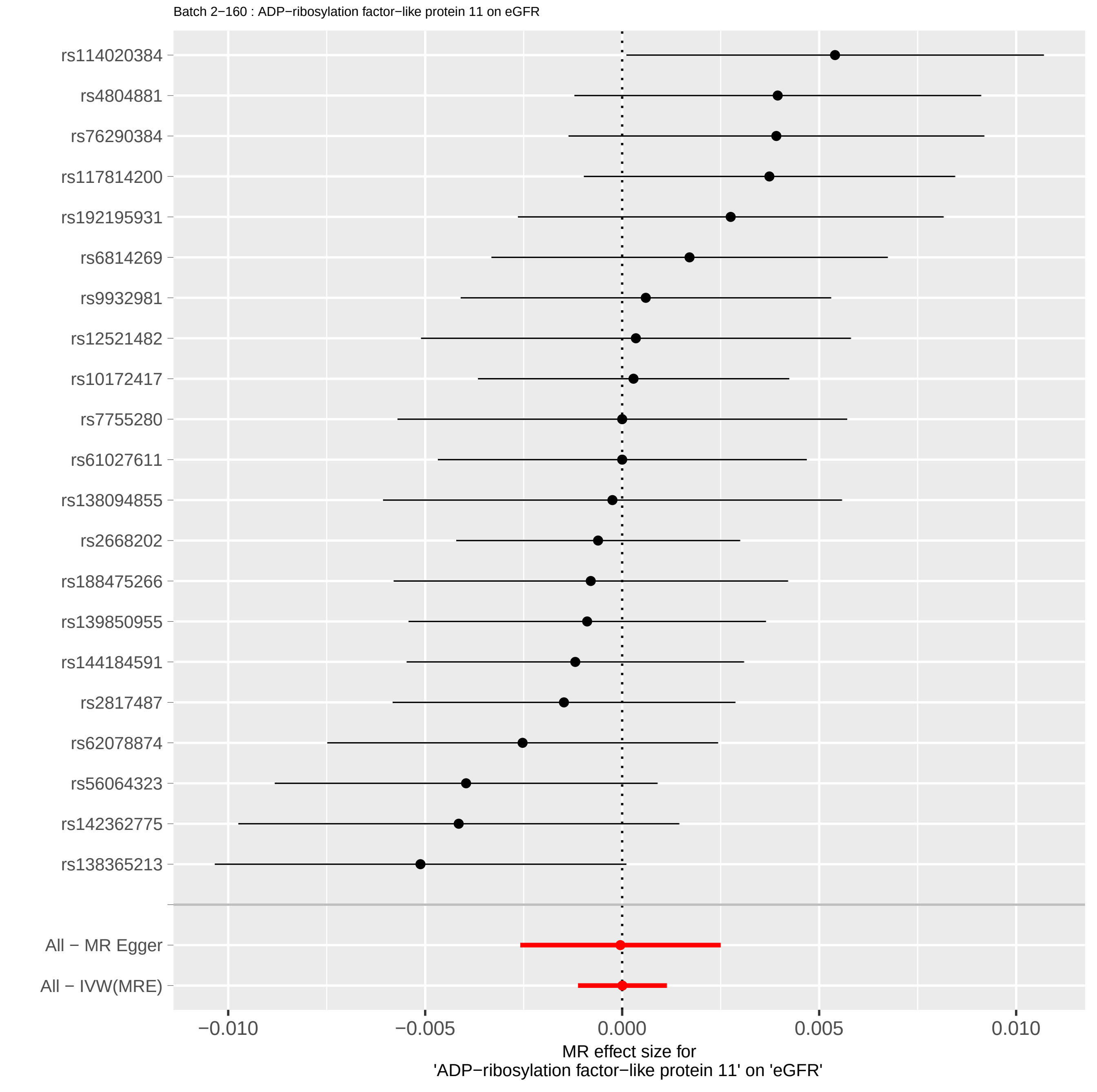

Batch 2−160 : ADP−ribosylation factor−like protein 11 on eGFR
rs114020384
rs4804881
rs76290384
rs117814200
rs192195931
rs6814269
rs9932981
rs12521482
rs10172417
rs7755280
rs61027611
rs138094855
rs2668202
rs188475266
rs139850955
rs144184591
rs2817487
rs62078874
rs56064323
rs142362775
rs138365213
All − MR Egger
All − IVW(MRE)
−0.010
−0.005
0.000
0.005
0.010
MR effect size for
'ADP−ribosylation factor−like protein 11' on 'eGFR'

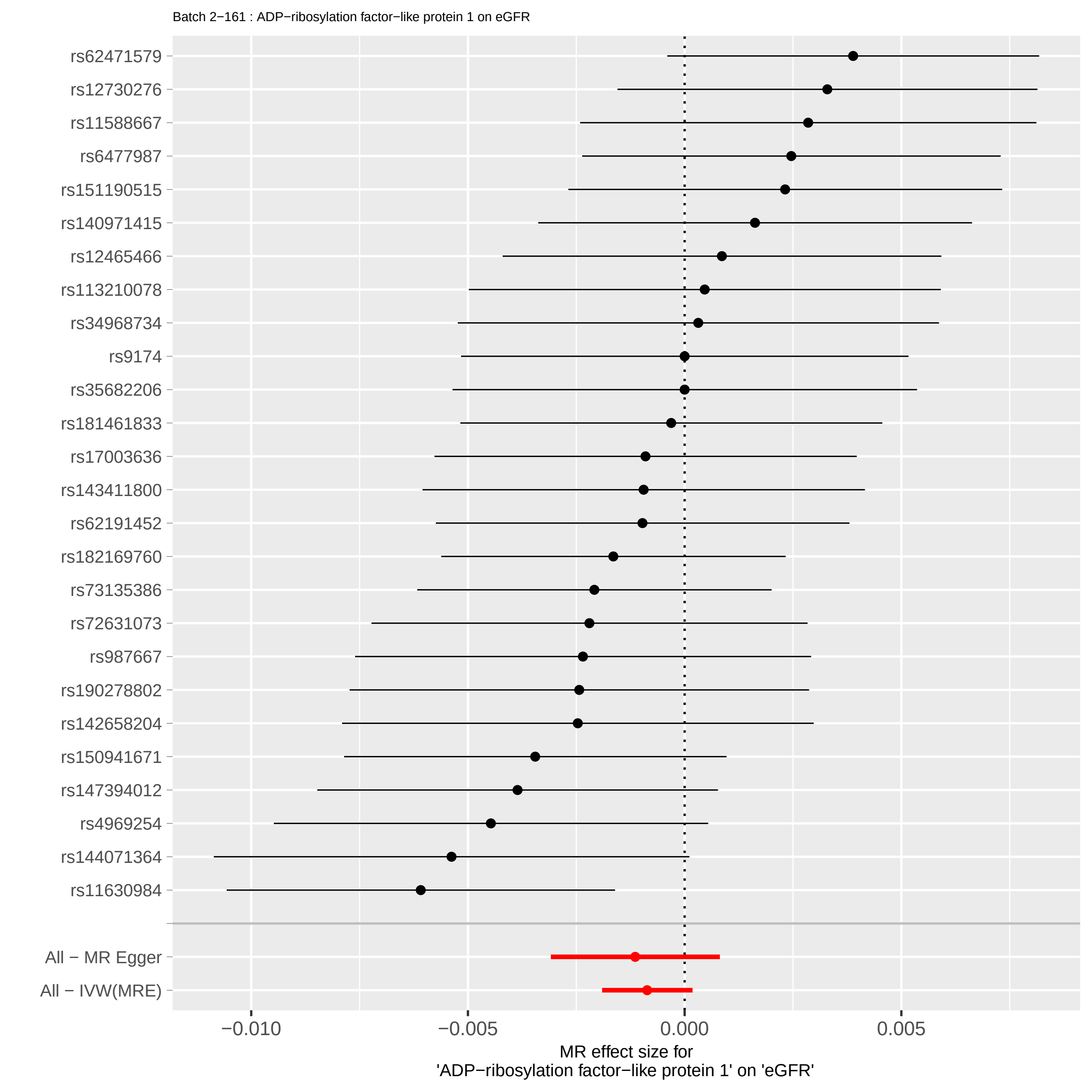

Batch 2−161 : ADP−ribosylation factor−like protein 1 on eGFR
rs62471579
rs12730276
rs11588667
rs6477987
rs151190515
rs140971415
rs12465466
rs113210078
rs34968734
rs9174
rs35682206
rs181461833
rs17003636
rs143411800
rs62191452
rs182169760
rs73135386
rs72631073
rs987667
rs190278802
rs142658204
rs150941671
rs147394012
rs4969254
rs144071364
rs11630984
All − MR Egger
All − IVW(MRE)
−0.010
−0.005
0.000
0.005
MR effect size for
'ADP−ribosylation factor−like protein 1' on 'eGFR'

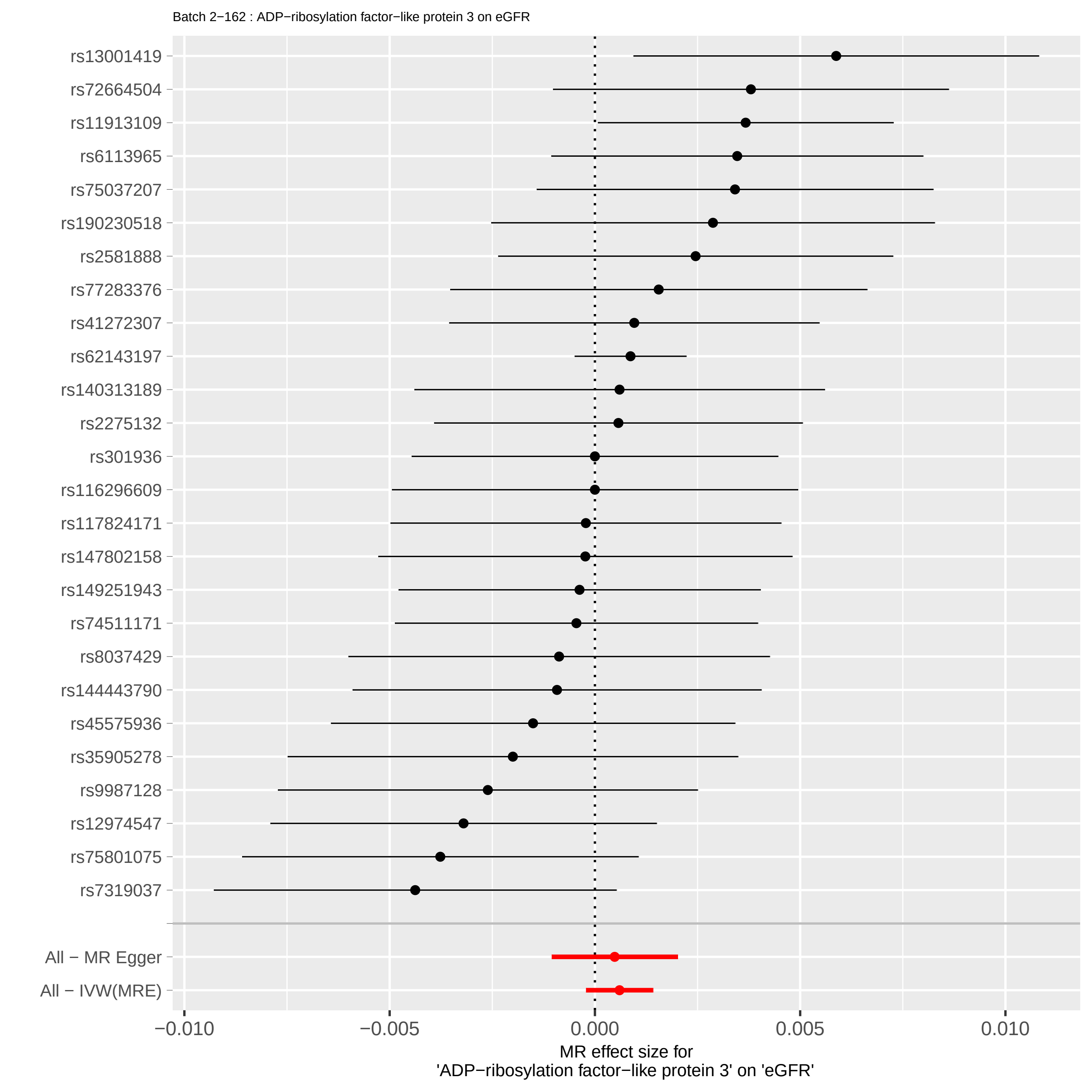

Batch 2−162 : ADP−ribosylation factor−like protein 3 on eGFR
rs13001419
rs72664504
rs11913109
rs6113965
rs75037207
rs190230518
rs2581888
rs77283376
rs41272307
rs62143197
rs140313189
rs2275132
rs301936
rs116296609
rs117824171
rs147802158
rs149251943
rs74511171
rs8037429
rs144443790
rs45575936
rs35905278
rs9987128
rs12974547
rs75801075
rs7319037
All − MR Egger
All − IVW(MRE)
−0.010
−0.005
0.000
0.005
0.010
MR effect size for
'ADP−ribosylation factor−like protein 3' on 'eGFR'

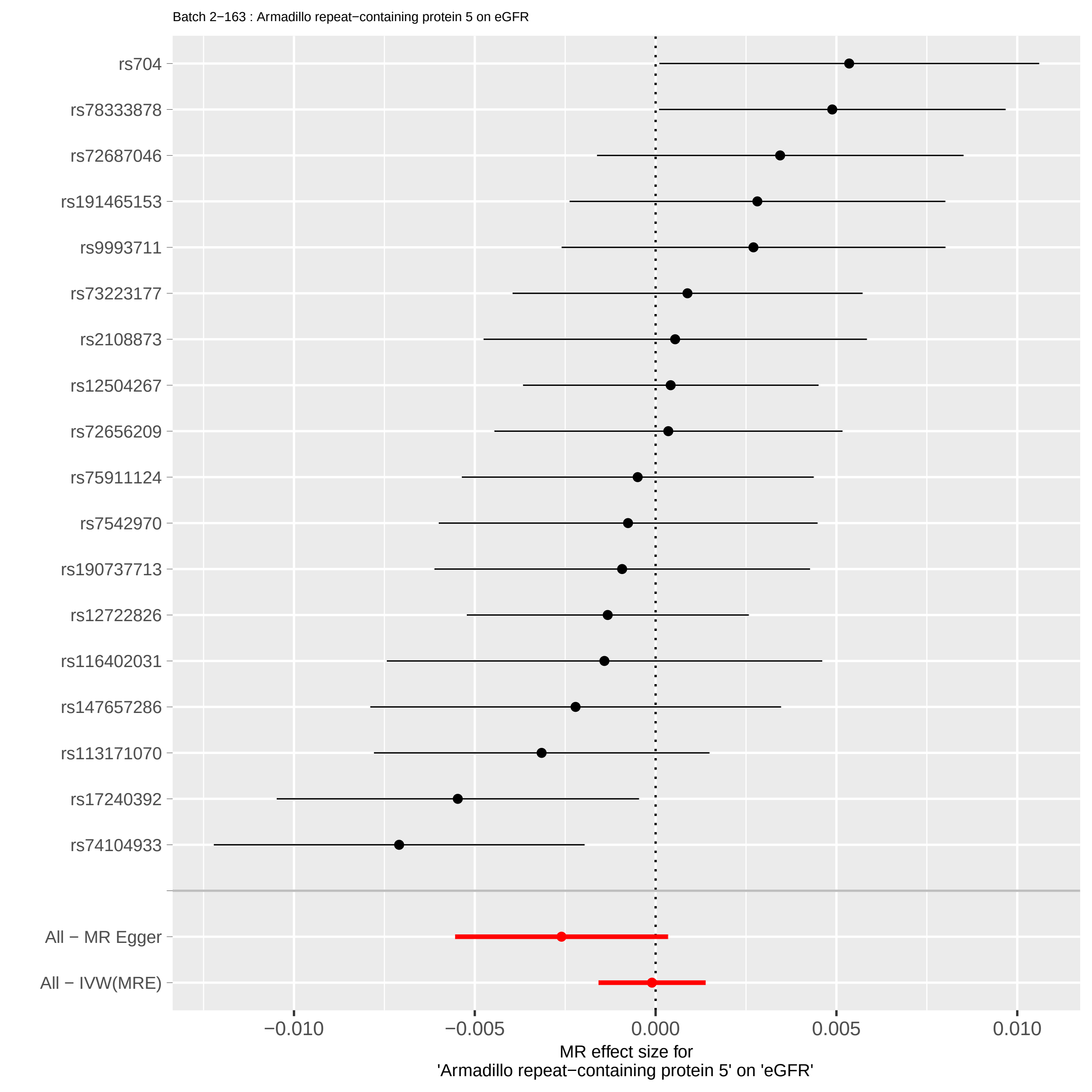

Batch 2−163 : Armadillo repeat−containing protein 5 on eGFR
rs704
rs78333878
rs72687046
rs191465153
rs9993711
rs73223177
rs2108873
rs12504267
rs72656209
rs75911124
rs7542970
rs190737713
rs12722826
rs116402031
rs147657286
rs113171070
rs17240392
rs74104933
All − MR Egger
All − IVW(MRE)
−0.010
−0.005
0.000
0.005
0.010
MR effect size for
'Armadillo repeat−containing protein 5' on 'eGFR'

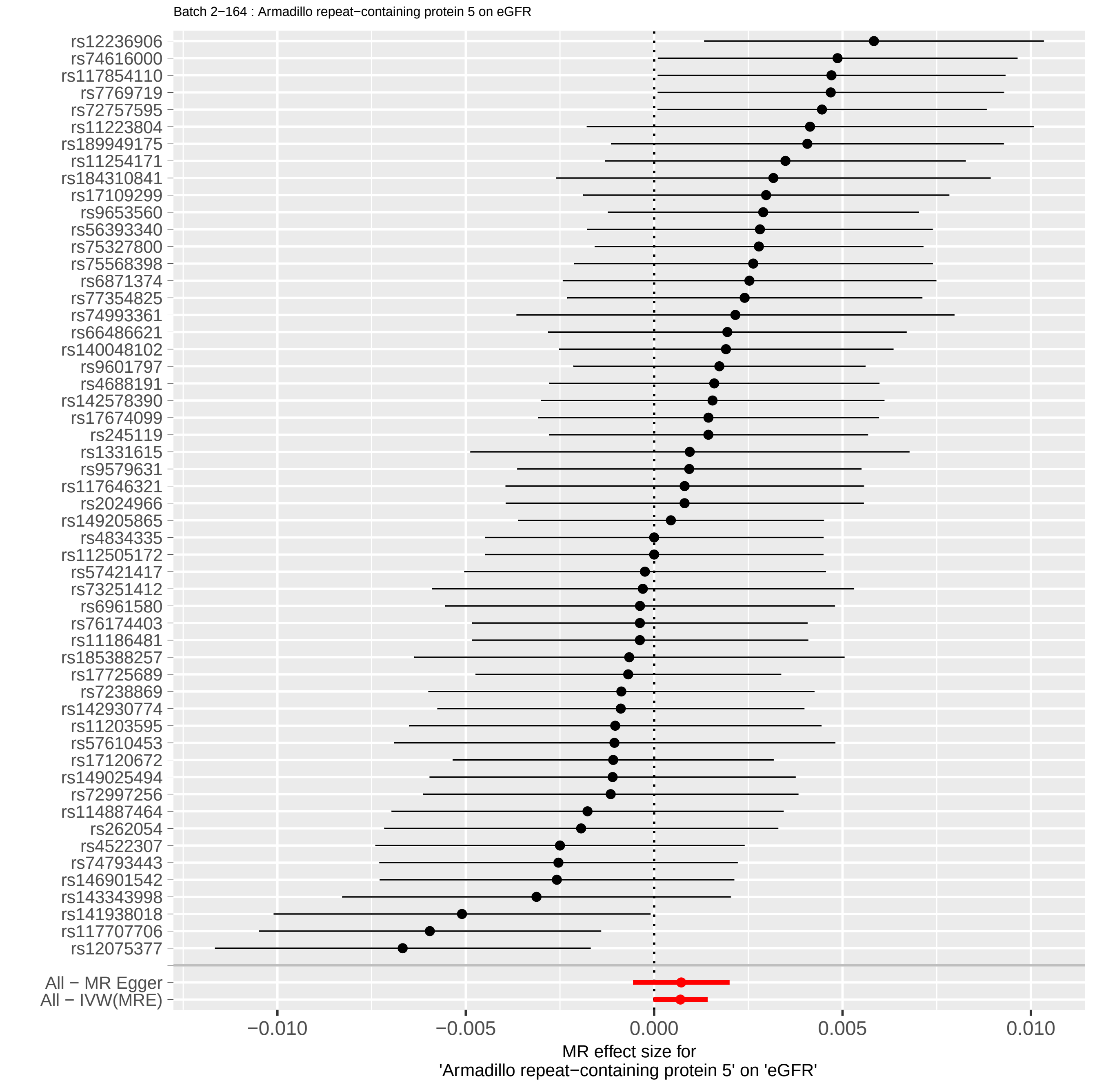

Batch 2−164 : Armadillo repeat−containing protein 5 on eGFR
rs12236906
rs74616000
rs117854110
rs7769719
rs72757595
rs11223804
rs189949175
rs11254171
rs184310841
rs17109299
rs9653560
rs56393340
rs75327800
rs75568398
rs6871374
rs77354825
rs74993361
rs66486621
rs140048102
rs9601797
rs4688191
rs142578390
rs17674099
rs245119
rs1331615
rs9579631
rs117646321
rs2024966
rs149205865
rs4834335
rs112505172
rs57421417
rs73251412
rs6961580
rs76174403
rs11186481
rs185388257
rs17725689
rs7238869
rs142930774
rs11203595
rs57610453
rs17120672
rs149025494
rs72997256
rs114887464
rs262054
rs4522307
rs74793443
rs146901542
rs143343998
rs141938018
rs117707706
rs12075377
All − MR Egger
All − IVW(MRE)
−0.010
−0.005
0.000
0.005
0.010
MR effect size for
'Armadillo repeat−containing protein 5' on 'eGFR'

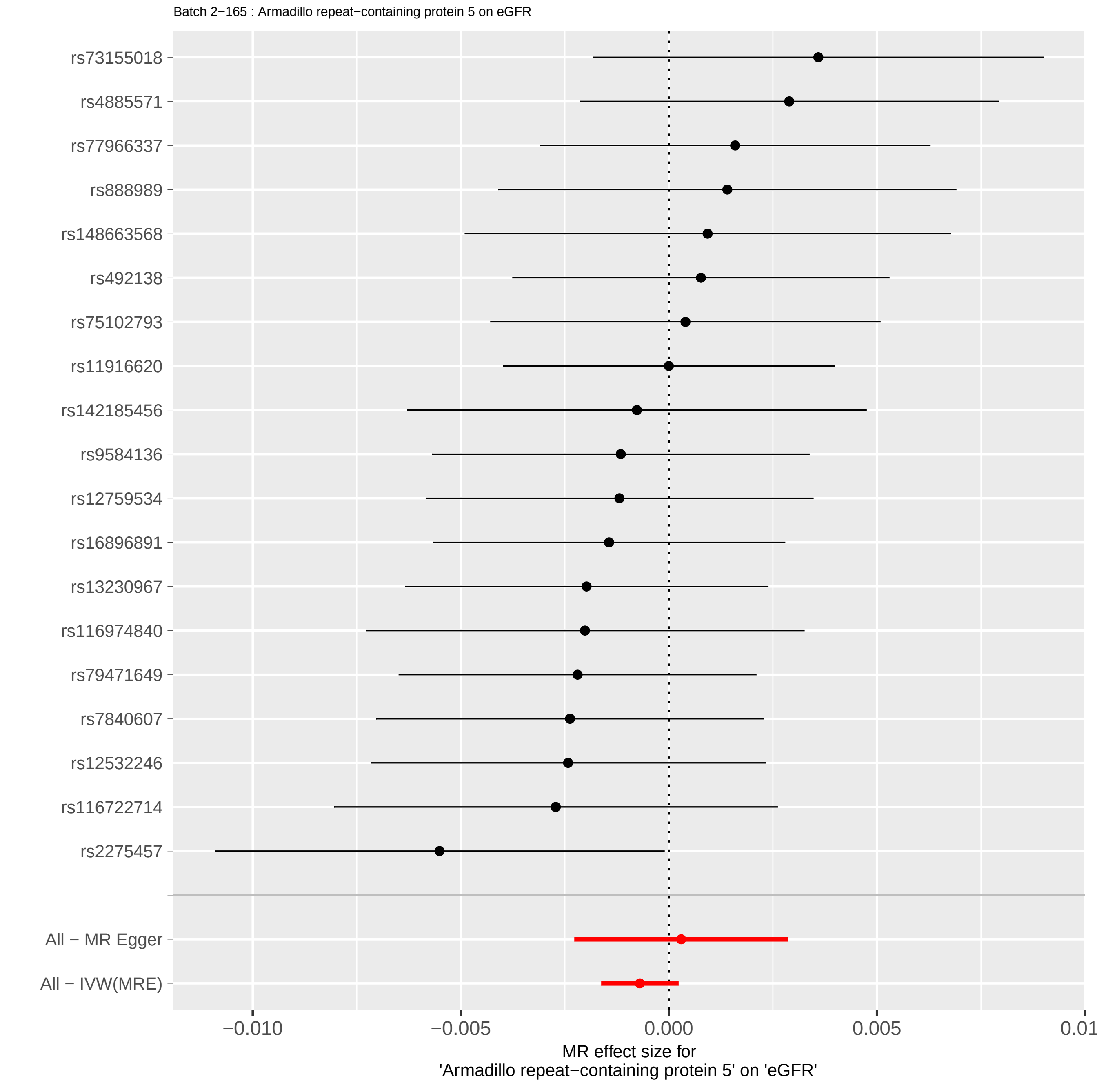

Batch 2−165 : Armadillo repeat−containing protein 5 on eGFR
rs73155018
rs4885571
rs77966337
rs888989
rs148663568
rs492138
rs75102793
rs11916620
rs142185456
rs9584136
rs12759534
rs16896891
rs13230967
rs116974840
rs79471649
rs7840607
rs12532246
rs116722714
rs2275457
All − MR Egger
All − IVW(MRE)
−0.010
−0.005
0.000
0.005
0.01
MR effect size for
'Armadillo repeat−containing protein 5' on 'eGFR'

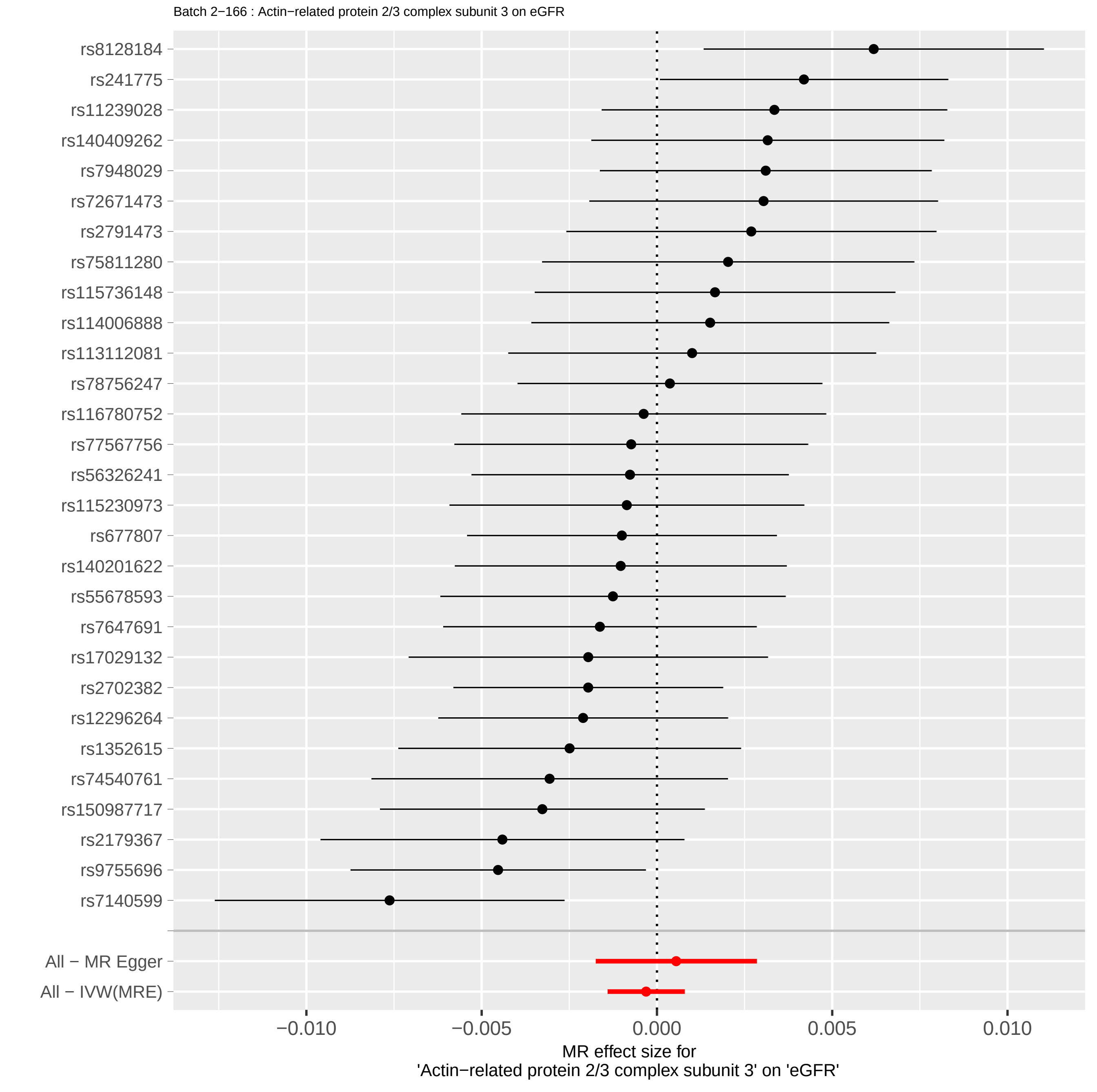
Batch 2−166 : Actin−related protein 2/3 complex subunit 3 on eGFR
rs8128184
rs241775
rs11239028
rs140409262
rs7948029
rs72671473
rs2791473
rs75811280
rs115736148
rs114006888
rs113112081
rs78756247
rs116780752
rs77567756
rs56326241
rs115230973
rs677807
rs140201622
rs55678593
rs7647691
rs17029132
rs2702382
rs12296264
rs1352615
rs74540761
rs150987717
rs2179367
rs9755696
rs7140599
All − MR Egger
All − IVW(MRE)
−0.010
−0.005
0.000
0.005
0.010
MR effect size for
'Actin−related protein 2/3 complex subunit 3' on 'eGFR'

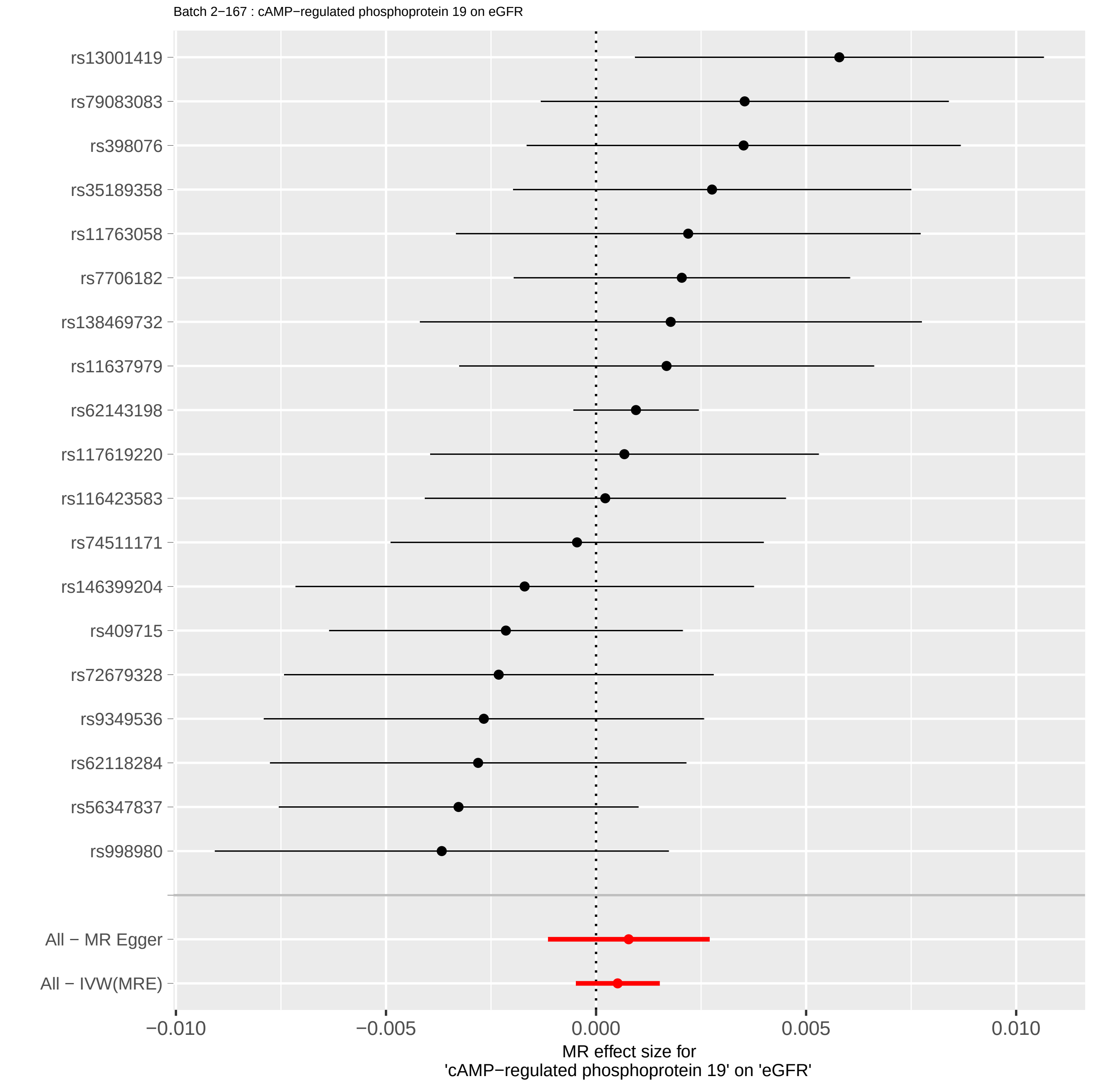

Batch 2−167 : cAMP−regulated phosphoprotein 19 on eGFR
rs13001419
rs79083083
rs398076
rs35189358
rs11763058
rs7706182
rs138469732
rs11637979
rs62143198
rs117619220
rs116423583
rs74511171
rs146399204
rs409715
rs72679328
rs9349536
rs62118284
rs56347837
rs998980
All − MR Egger
All − IVW(MRE)
−0.010
−0.005
0.000
0.005
0.010
MR effect size for
'cAMP−regulated phosphoprotein 19' on 'eGFR'

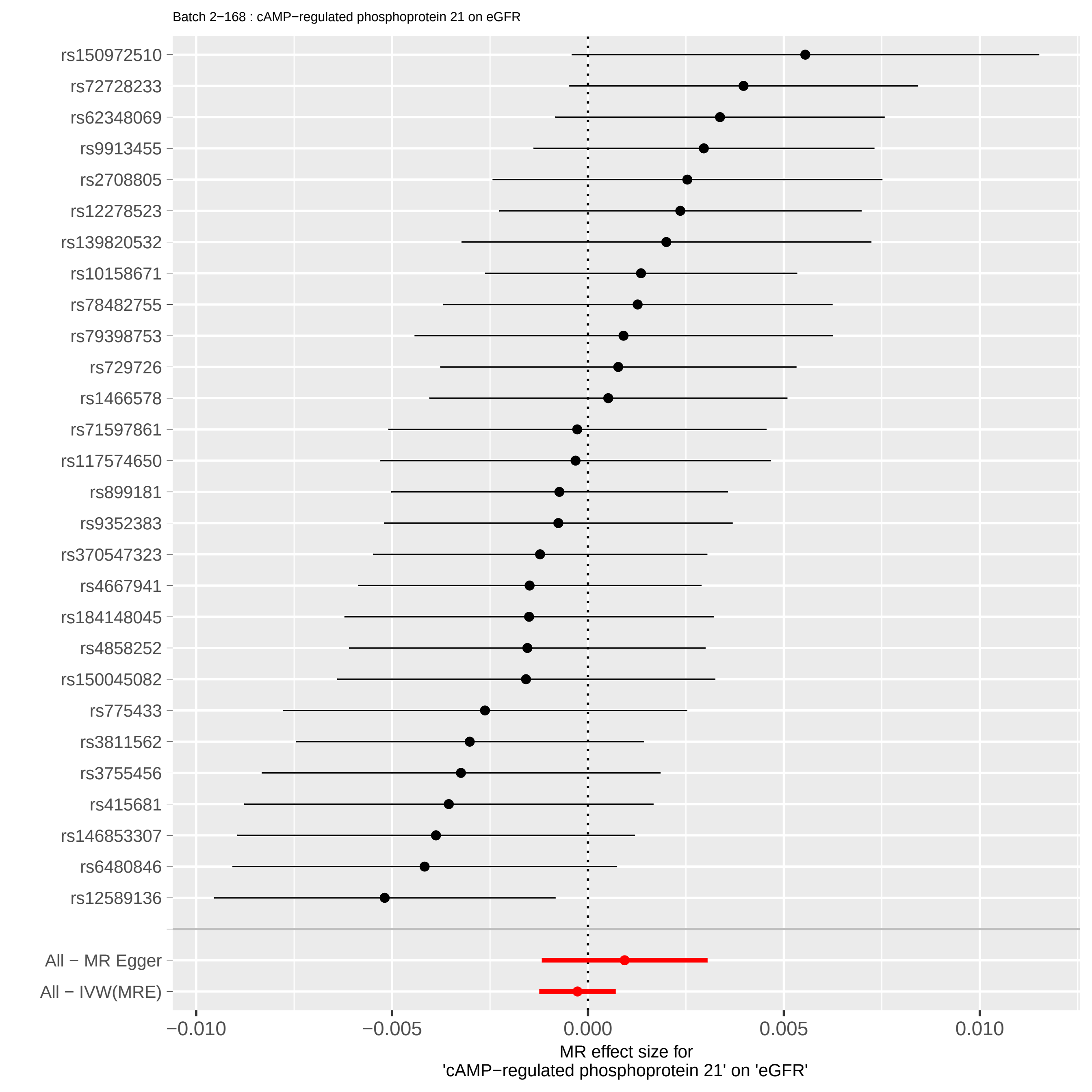

Batch 2−168 : cAMP−regulated phosphoprotein 21 on eGFR
rs150972510
rs72728233
rs62348069
rs9913455
rs2708805
rs12278523
rs139820532
rs10158671
rs78482755
rs79398753
rs729726
rs1466578
rs71597861
rs117574650
rs899181
rs9352383
rs370547323
rs4667941
rs184148045
rs4858252
rs150045082
rs775433
rs3811562
rs3755456
rs415681
rs146853307
rs6480846
rs12589136
All − MR Egger
All − IVW(MRE)
−0.010
−0.005
0.000
0.005
0.010
MR effect size for
'cAMP−regulated phosphoprotein 21' on 'eGFR'

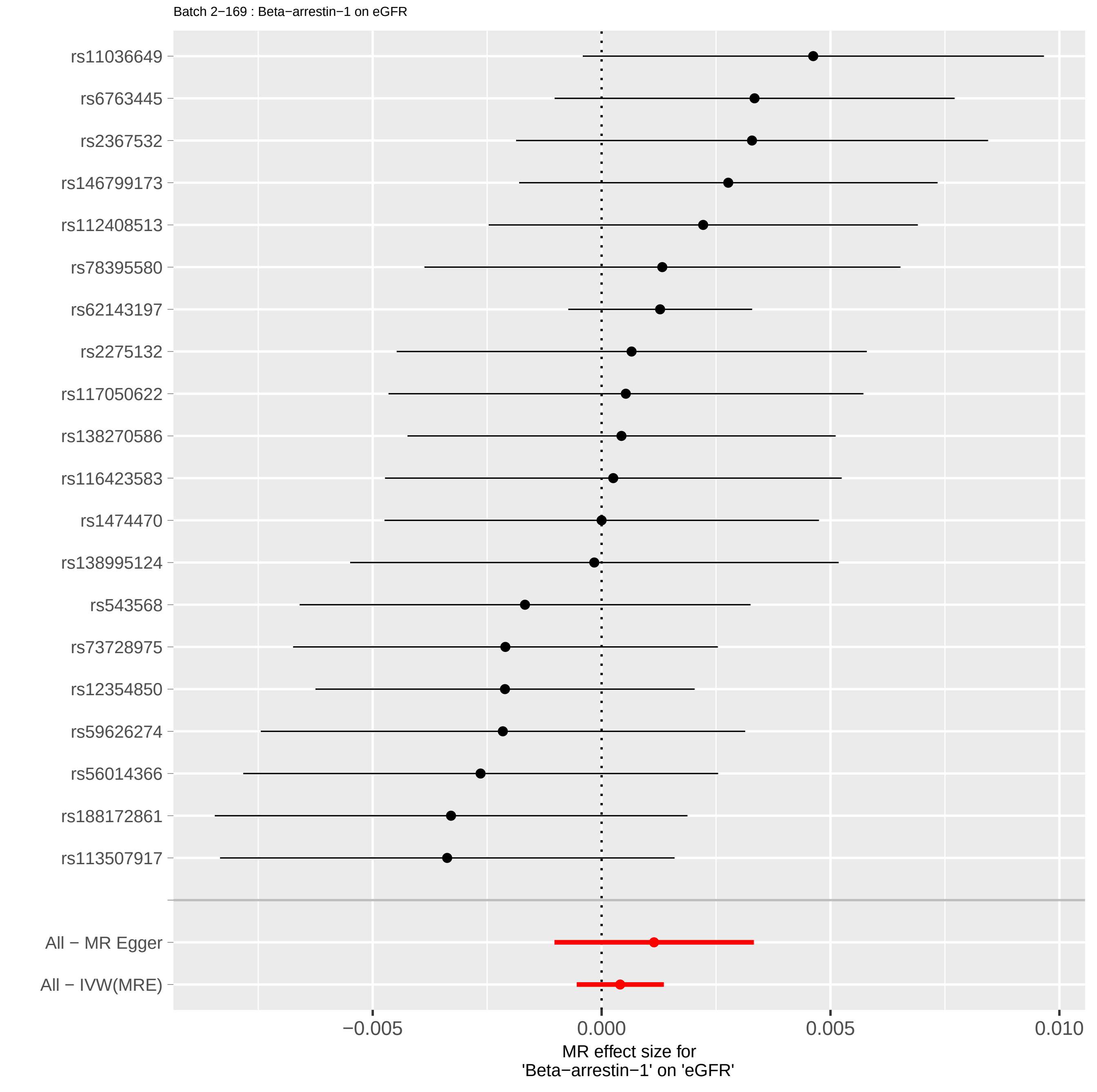

Batch 2−169 : Beta−arrestin−1 on eGFR
rs11036649
rs6763445
rs2367532
rs146799173
rs112408513
rs78395580
rs62143197
rs2275132
rs117050622
rs138270586
rs116423583
rs1474470
rs138995124
rs543568
rs73728975
rs12354850
rs59626274
rs56014366
rs188172861
rs113507917
All − MR Egger
All − IVW(MRE)
−0.005
0.000
0.005
0.010
MR effect size for
'Beta−arrestin−1' on 'eGFR'

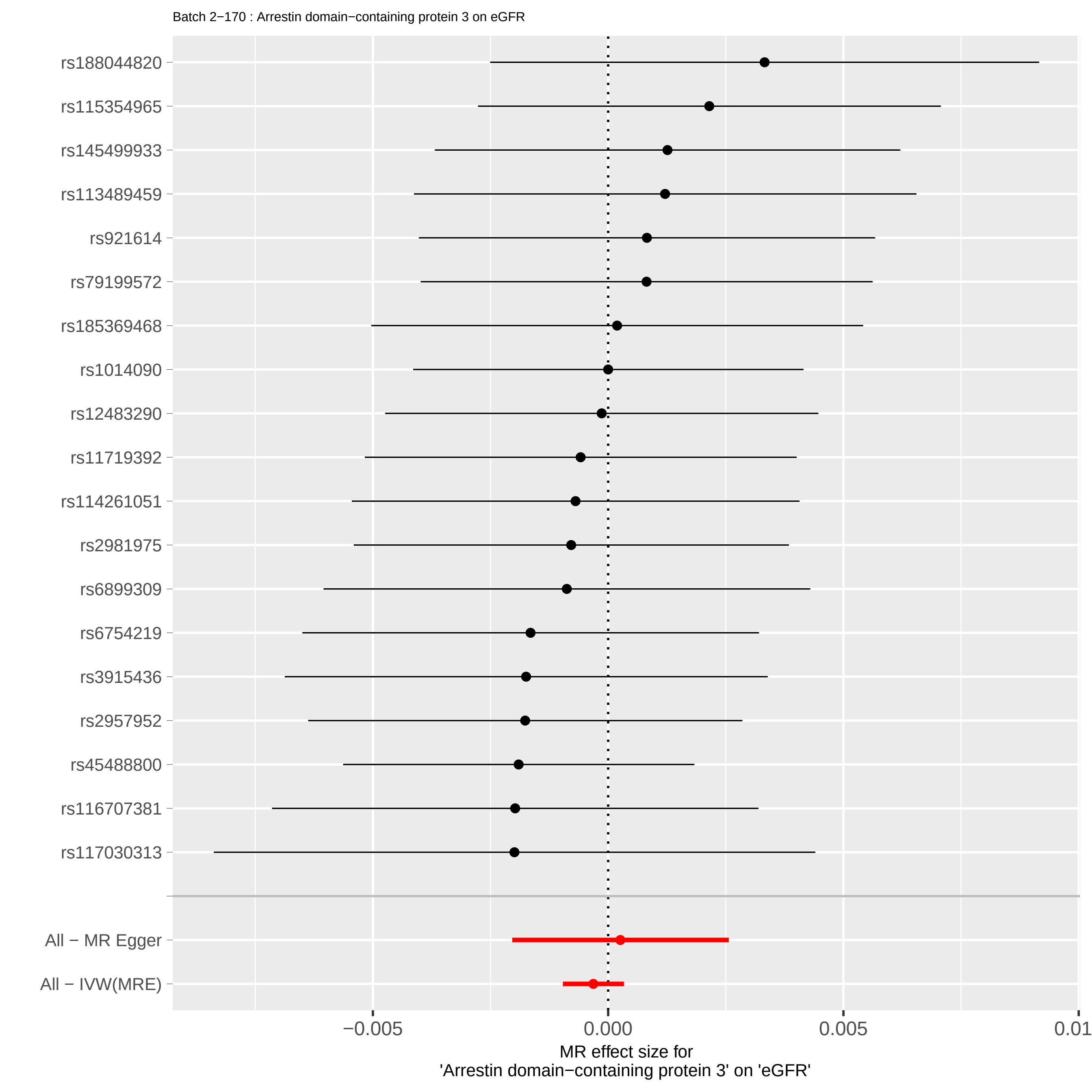

Batch 2−170 : Arrestin domain−containing protein 3 on eGFR
rs188044820
rs115354965
rs145499933
rs113489459
rs921614
rs79199572
rs185369468
rs1014090
rs12483290
rs11719392
rs114261051
rs2981975
rs6899309
rs6754219
rs3915436
rs2957952
rs45488800
rs116707381
rs117030313
All − MR Egger
All − IVW(MRE)
−0.005
0.000
0.005
0.01
MR effect size for
'Arrestin domain−containing protein 3' on 'eGFR'

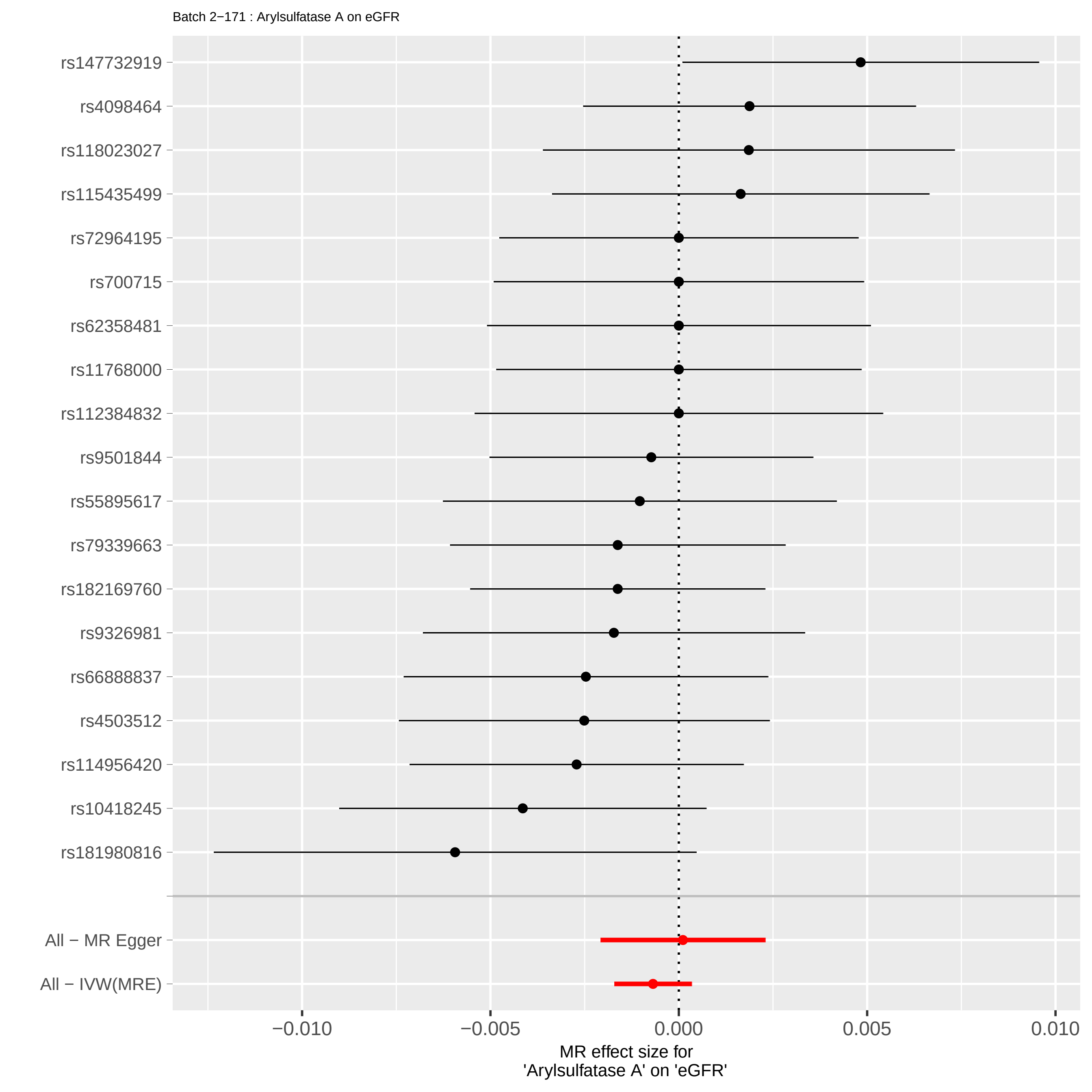

Batch 2−171 : Arylsulfatase A on eGFR
rs147732919
rs4098464
rs118023027
rs115435499
rs72964195
rs700715
rs62358481
rs11768000
rs112384832
rs9501844
rs55895617
rs79339663
rs182169760
rs9326981
rs66888837
rs4503512
rs114956420
rs10418245
rs181980816
All − MR Egger
All − IVW(MRE)
−0.010
−0.005
0.000
0.005
0.010
MR effect size for
'Arylsulfatase A' on 'eGFR'

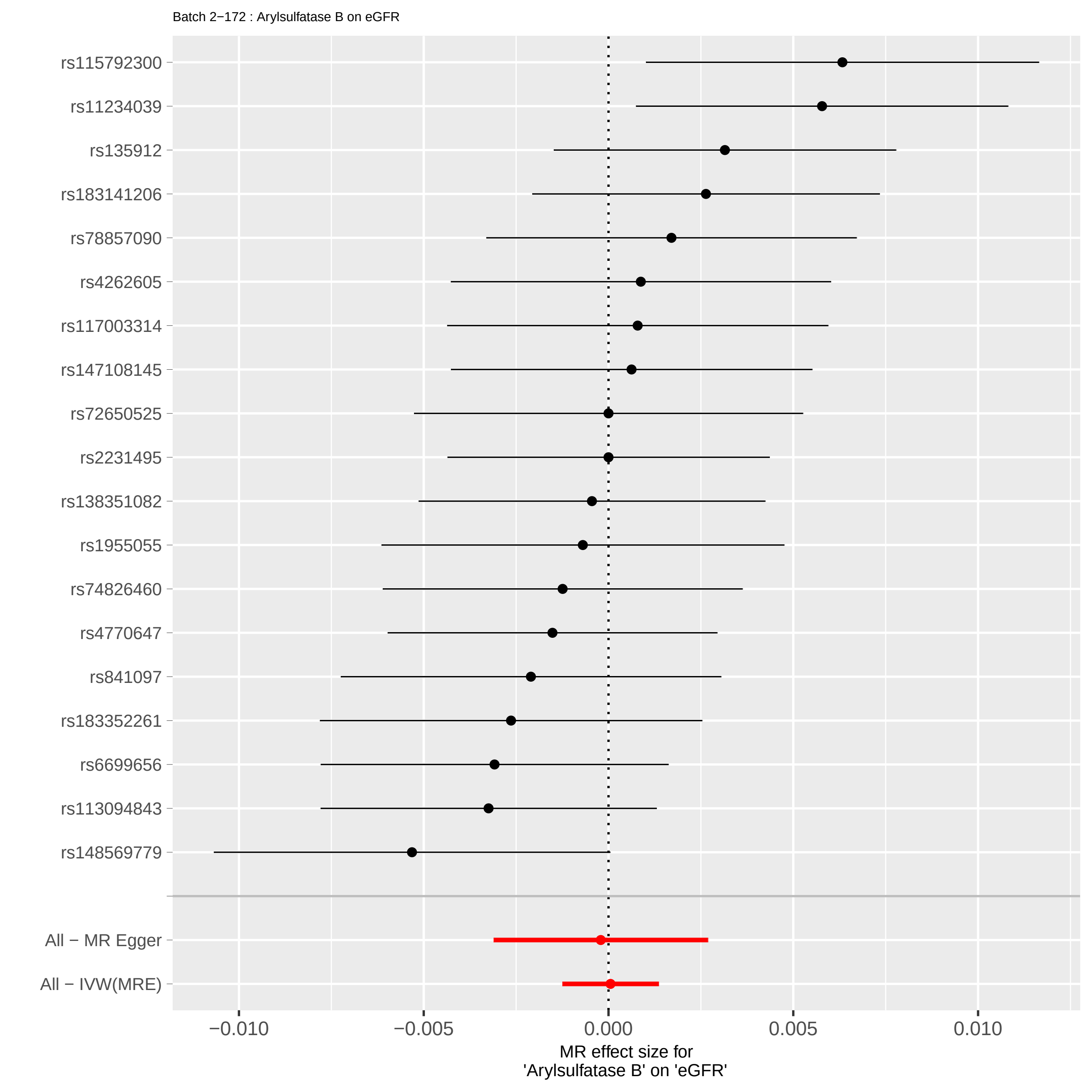

Batch 2−172 : Arylsulfatase B on eGFR
rs115792300
rs11234039
rs135912
rs183141206
rs78857090
rs4262605
rs117003314
rs147108145
rs72650525
rs2231495
rs138351082
rs1955055
rs74826460
rs4770647
rs841097
rs183352261
rs6699656
rs113094843
rs148569779
All − MR Egger
All − IVW(MRE)
−0.010
−0.005
0.000
0.005
0.010
MR effect size for
'Arylsulfatase B' on 'eGFR'

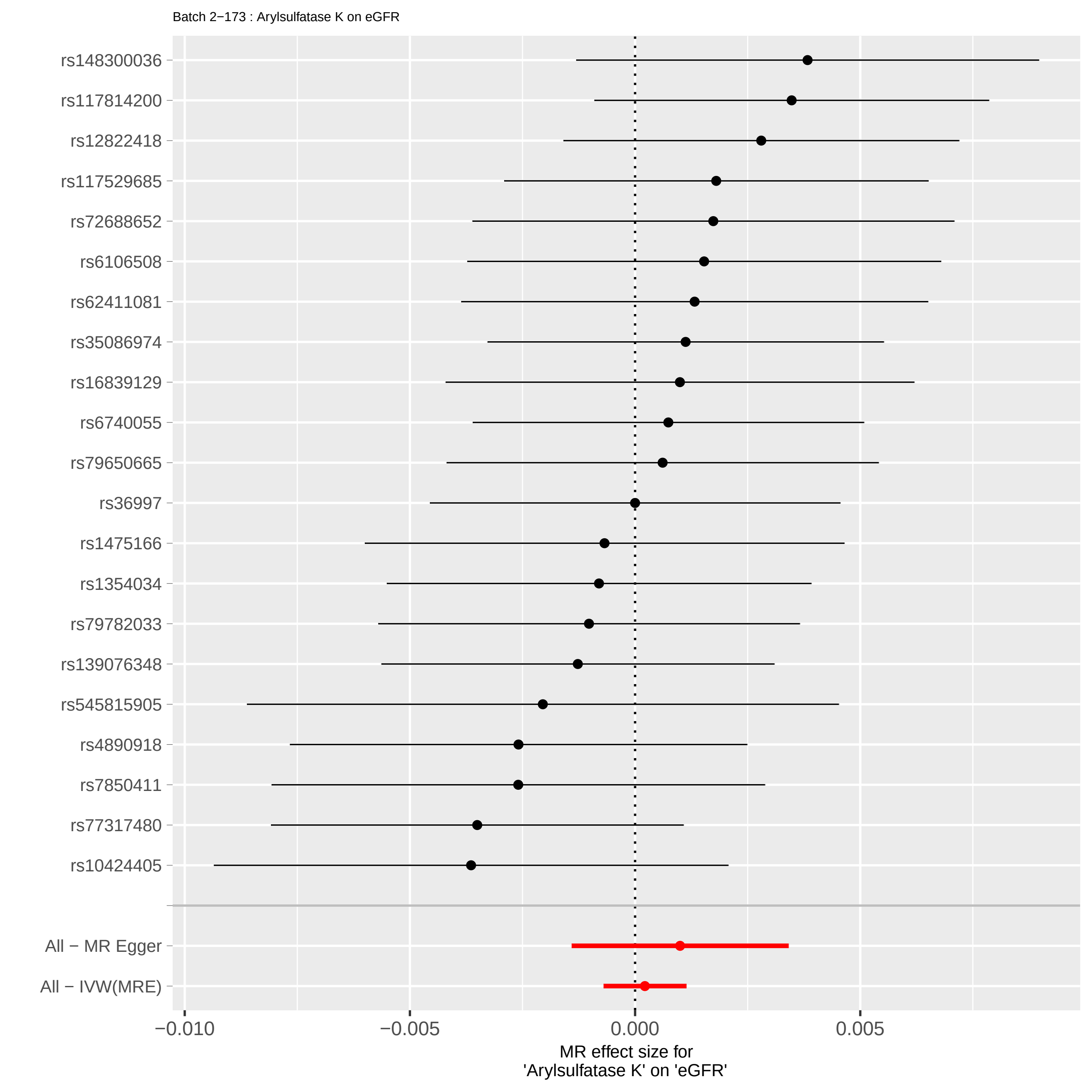

Batch 2−173 : Arylsulfatase K on eGFR
rs148300036
rs117814200
rs12822418
rs117529685
rs72688652
rs6106508
rs62411081
rs35086974
rs16839129
rs6740055
rs79650665
rs36997
rs1475166
rs1354034
rs79782033
rs139076348
rs545815905
rs4890918
rs7850411
rs77317480
rs10424405
All − MR Egger
All − IVW(MRE)
−0.010
−0.005
0.000
0.005
MR effect size for
'Arylsulfatase K' on 'eGFR'

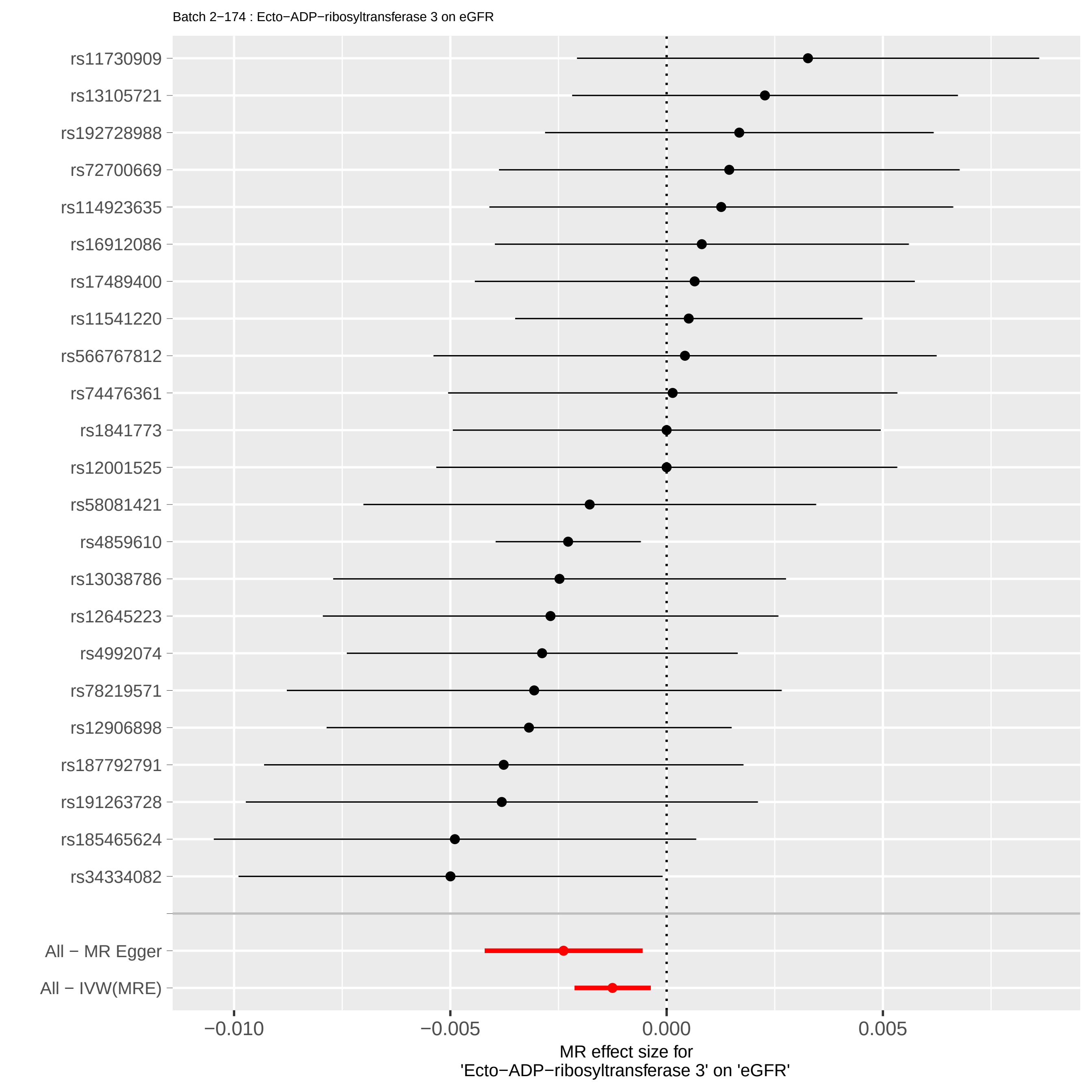
Batch 2−174 : Ecto−ADP−ribosyltransferase 3 on eGFR
rs11730909
rs13105721
rs192728988
rs72700669
rs114923635
rs16912086
rs17489400
rs11541220
rs566767812
rs74476361
rs1841773
rs12001525
rs58081421
rs4859610
rs13038786
rs12645223
rs4992074
rs78219571
rs12906898
rs187792791
rs191263728
rs185465624
rs34334082
All − MR Egger
All − IVW(MRE)
−0.010
−0.005
0.000
0.005
MR effect size for
'Ecto−ADP−ribosyltransferase 3' on 'eGFR'

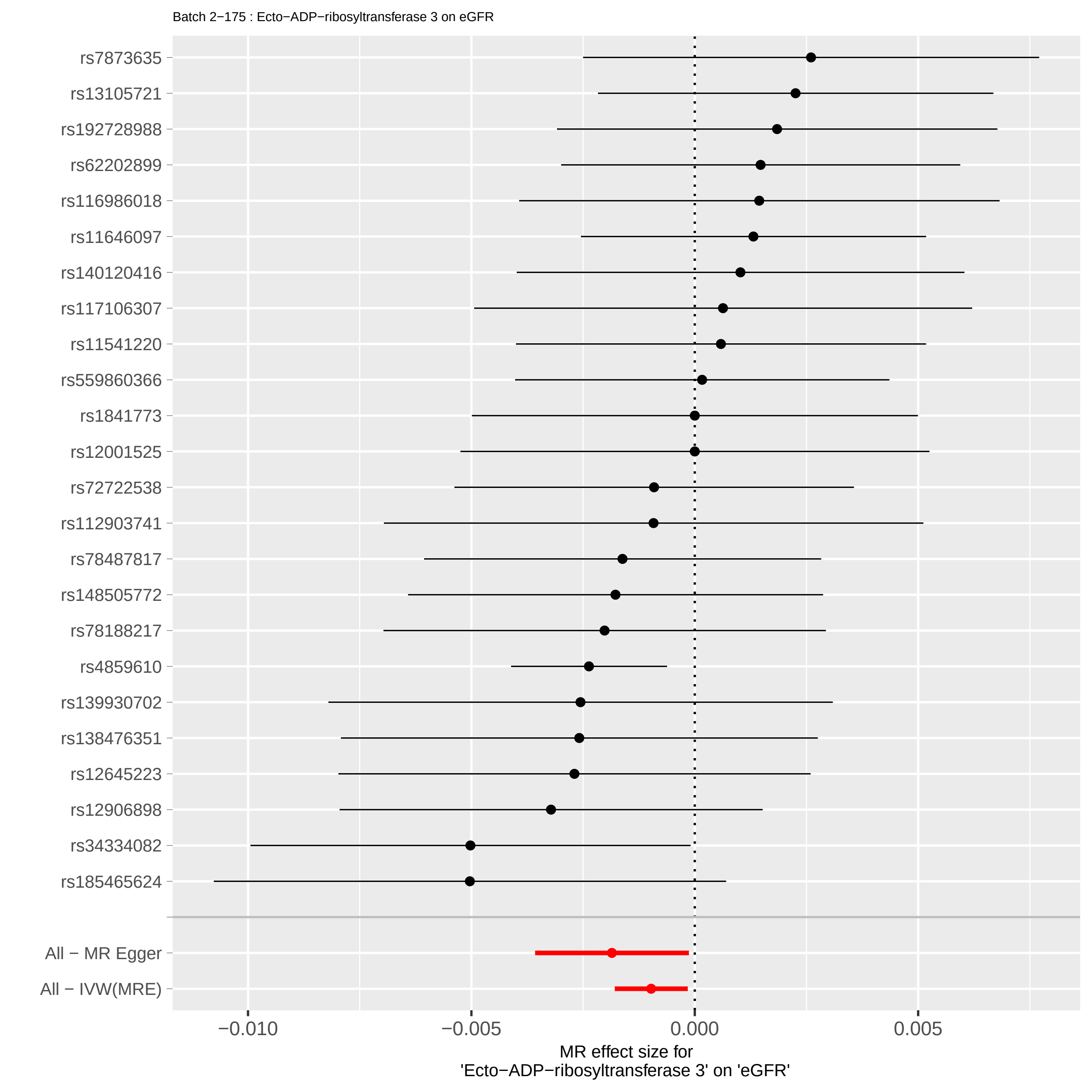
Batch 2−175 : Ecto−ADP−ribosyltransferase 3 on eGFR
rs7873635
rs13105721
rs192728988
rs62202899
rs116986018
rs11646097
rs140120416
rs117106307
rs11541220
rs559860366
rs1841773
rs12001525
rs72722538
rs112903741
rs78487817
rs148505772
rs78188217
rs4859610
rs139930702
rs138476351
rs12645223
rs12906898
rs34334082
rs185465624
All − MR Egger
All − IVW(MRE)
−0.010
−0.005
0.000
0.005
MR effect size for
'Ecto−ADP−ribosyltransferase 3' on 'eGFR'

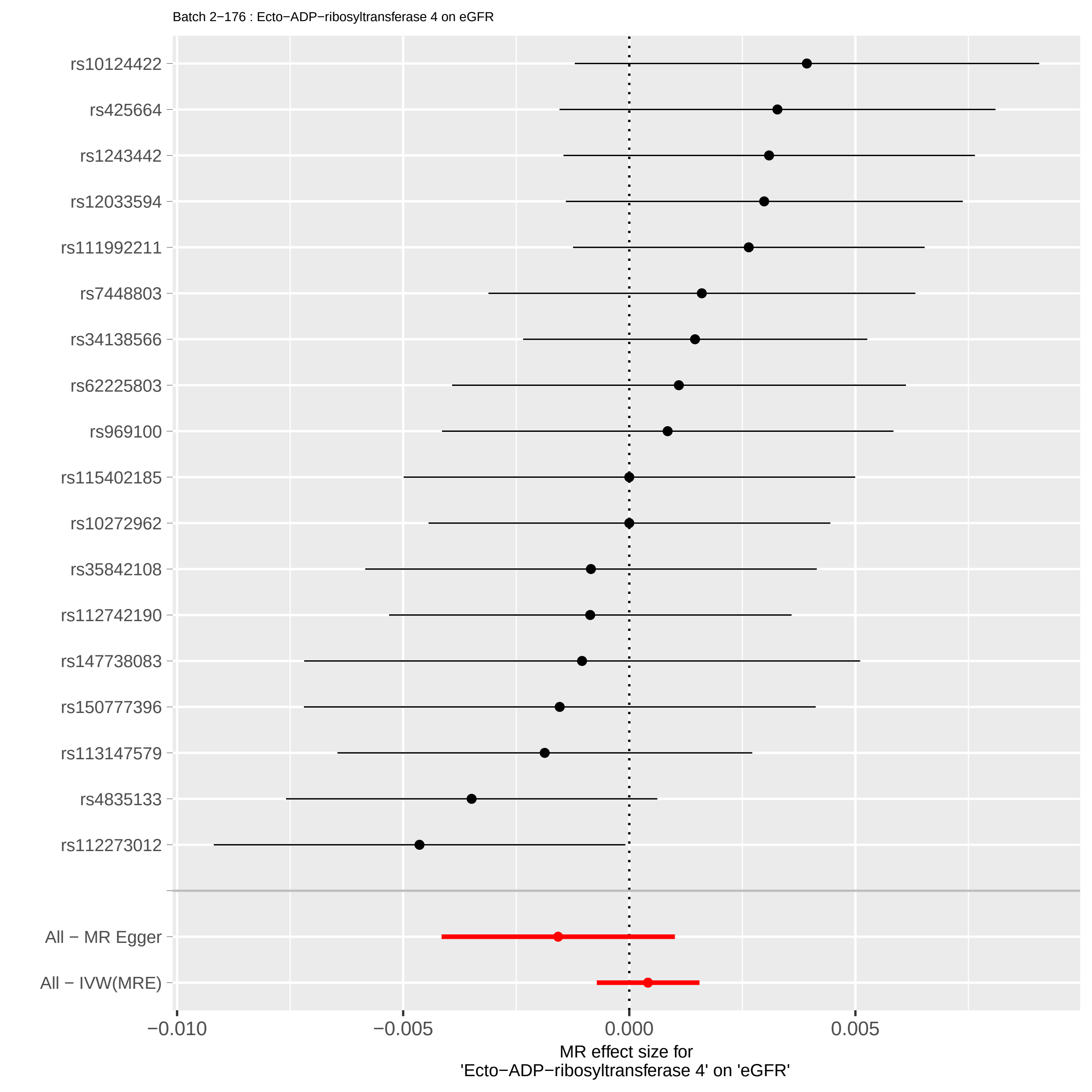

Batch 2−176 : Ecto−ADP−ribosyltransferase 4 on eGFR
rs10124422
rs425664
rs1243442
rs12033594
rs111992211
rs7448803
rs34138566
rs62225803
rs969100
rs115402185
rs10272962
rs35842108
rs112742190
rs147738083
rs150777396
rs113147579
rs4835133
rs112273012
All − MR Egger
All − IVW(MRE)
−0.010
−0.005
0.000
0.005
MR effect size for
'Ecto−ADP−ribosyltransferase 4' on 'eGFR'

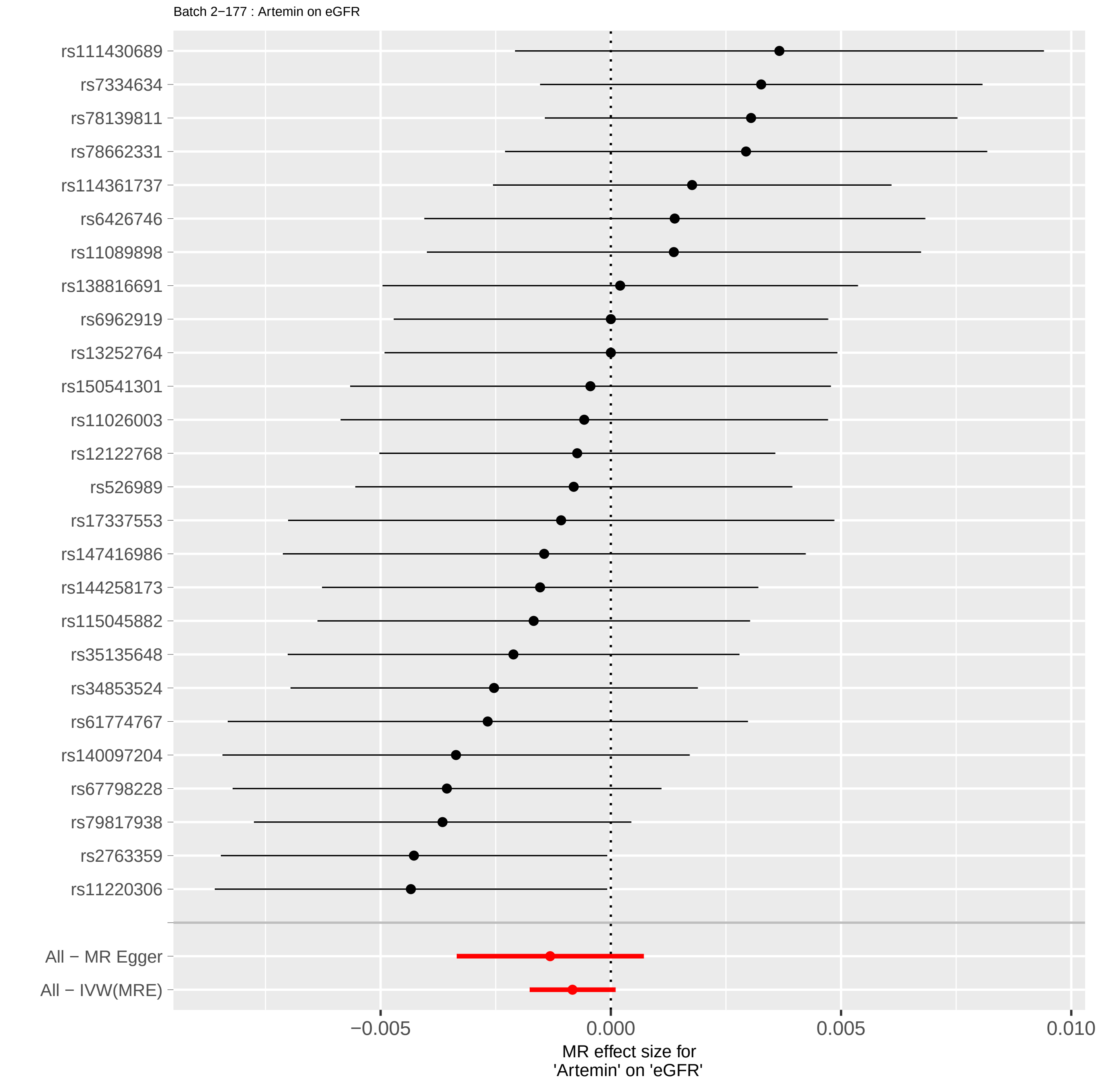
Batch 2−177 : Artemin on eGFR
rs111430689
rs7334634
rs78139811
rs78662331
rs114361737
rs6426746
rs11089898
rs138816691
rs6962919
rs13252764
rs150541301
rs11026003
rs12122768
rs526989
rs17337553
rs147416986
rs144258173
rs115045882
rs35135648
rs34853524
rs61774767
rs140097204
rs67798228
rs79817938
rs2763359
rs11220306
All − MR Egger
All − IVW(MRE)
−0.005
0.000
0.005
0.010
MR effect size for
'Artemin' on 'eGFR'

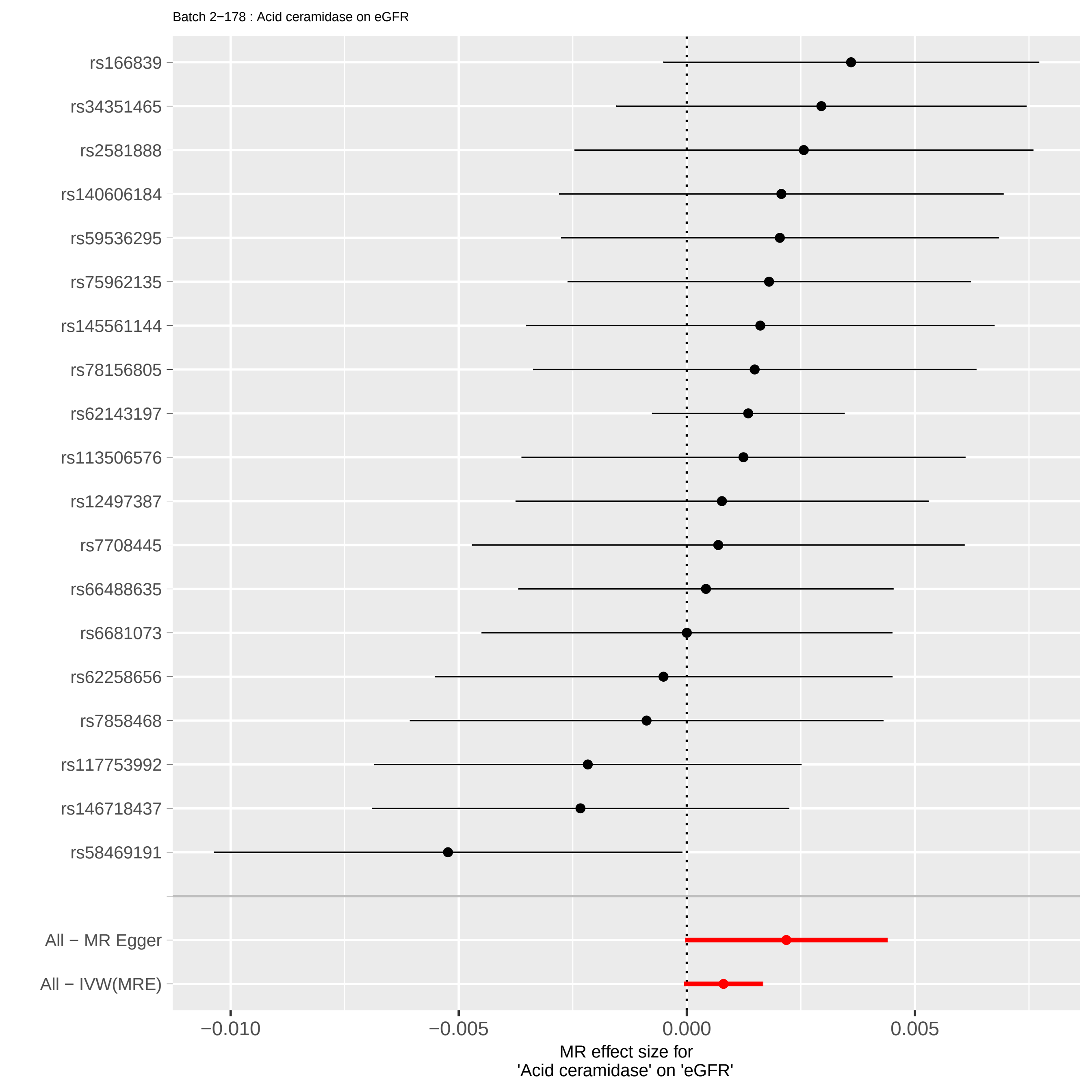

Batch 2−178 : Acid ceramidase on eGFR
rs166839
rs34351465
rs2581888
rs140606184
rs59536295
rs75962135
rs145561144
rs78156805
rs62143197
rs113506576
rs12497387
rs7708445
rs66488635
rs6681073
rs62258656
rs7858468
rs117753992
rs146718437
rs58469191
All − MR Egger
All − IVW(MRE)
−0.010
−0.005
0.000
0.005
MR effect size for
'Acid ceramidase' on 'eGFR'

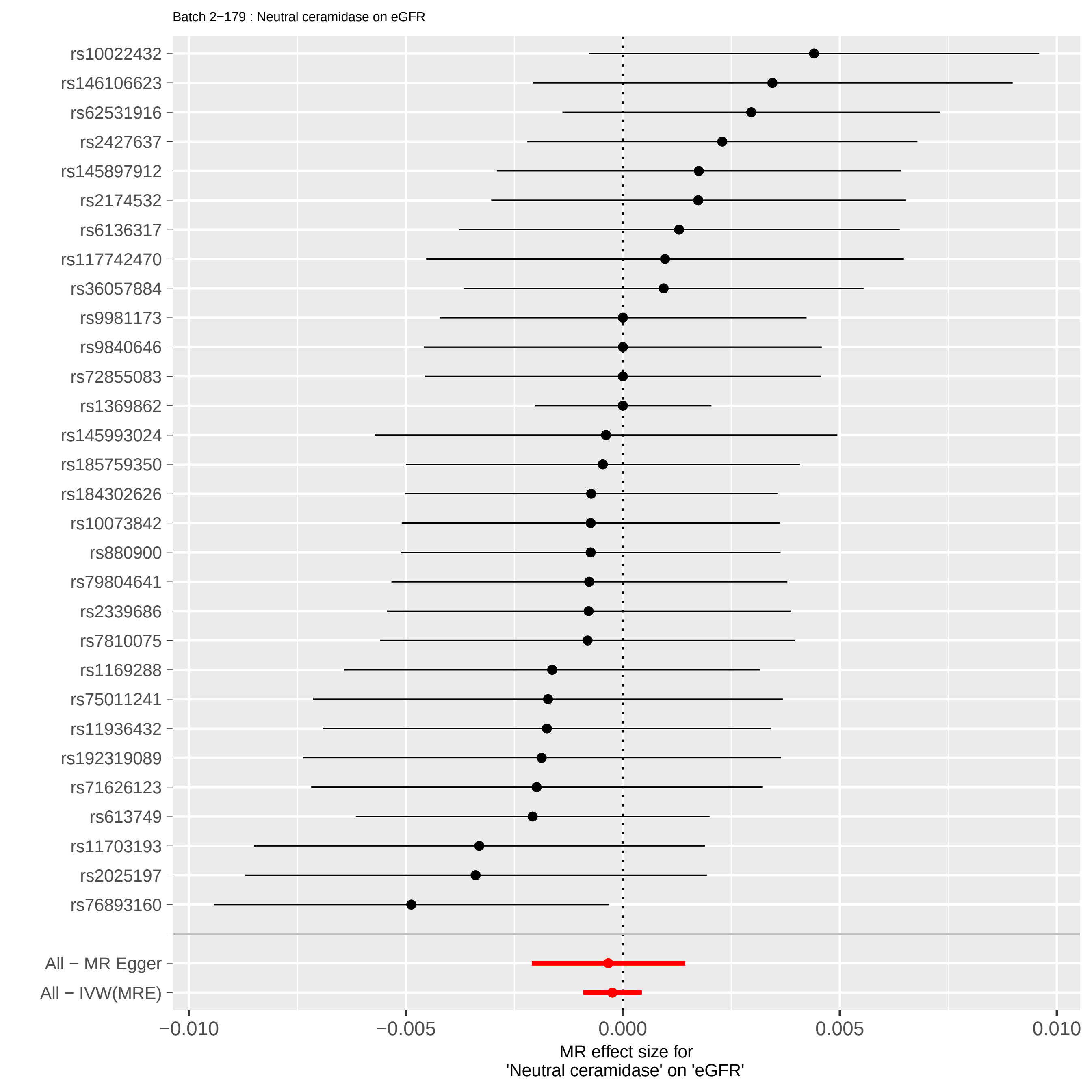

Batch 2−179 : Neutral ceramidase on eGFR
rs10022432
rs146106623
rs62531916
rs2427637
rs145897912
rs2174532
rs6136317
rs117742470
rs36057884
rs9981173
rs9840646
rs72855083
rs1369862
rs145993024
rs185759350
rs184302626
rs10073842
rs880900
rs79804641
rs2339686
rs7810075
rs1169288
rs75011241
rs11936432
rs192319089
rs71626123
rs613749
rs11703193
rs2025197
rs76893160
All − MR Egger
All − IVW(MRE)
−0.010
−0.005
0.000
0.005
0.010
MR effect size for
'Neutral ceramidase' on 'eGFR'

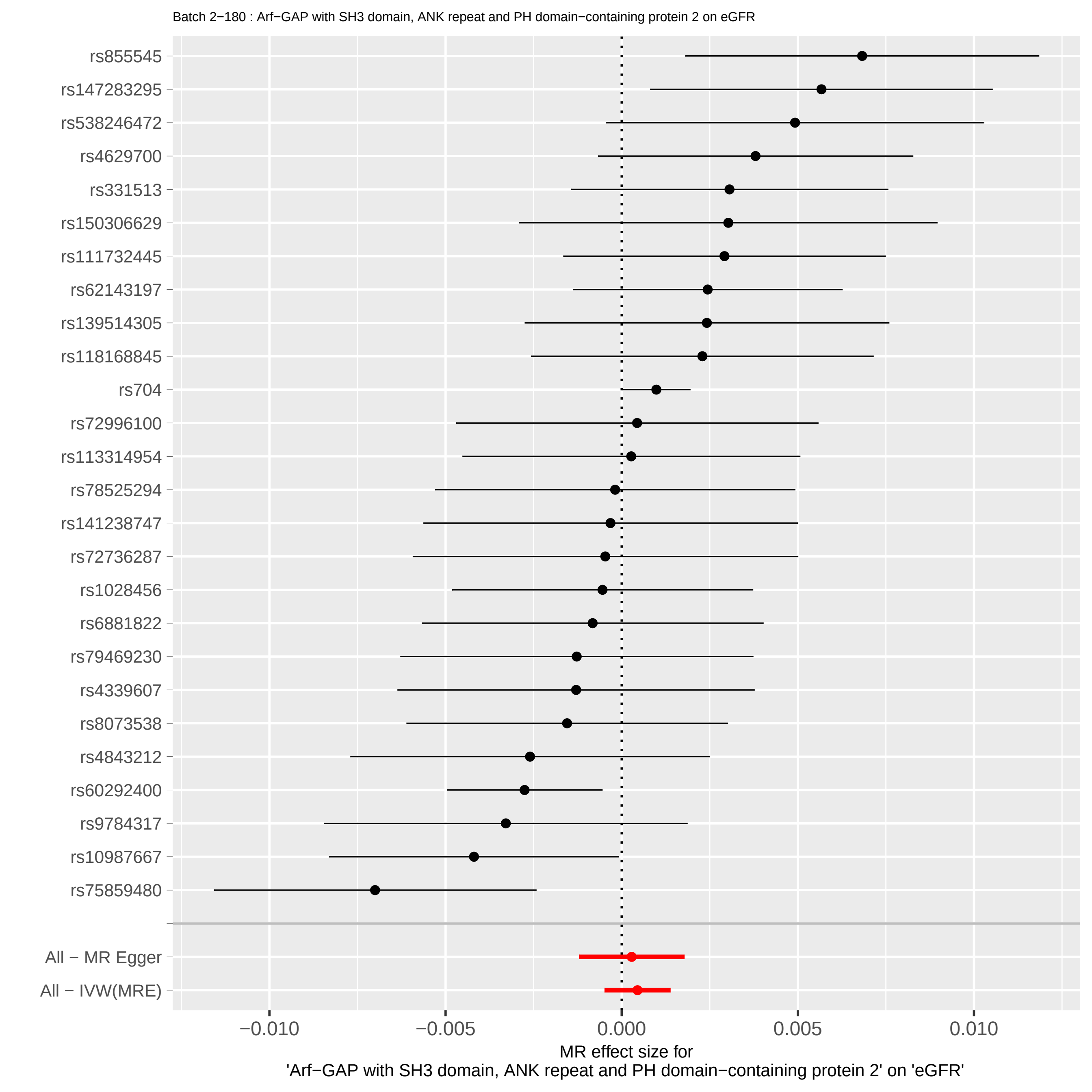

Batch 2−180 : Arf−GAP with SH3 domain, ANK repeat and PH domain−containing protein 2 on eGFR
rs855545
rs147283295
rs538246472
rs4629700
rs331513
rs150306629
rs111732445
rs62143197
rs139514305
rs118168845
rs704
rs72996100
rs113314954
rs78525294
rs141238747
rs72736287
rs1028456
rs6881822
rs79469230
rs4339607
rs8073538
rs4843212
rs60292400
rs9784317
rs10987667
rs75859480
All − MR Egger
All − IVW(MRE)
−0.010
−0.005
0.000
0.005
0.010
MR effect size for
'Arf−GAP with SH3 domain, ANK repeat and PH domain−containing protein 2' on 'eGFR'

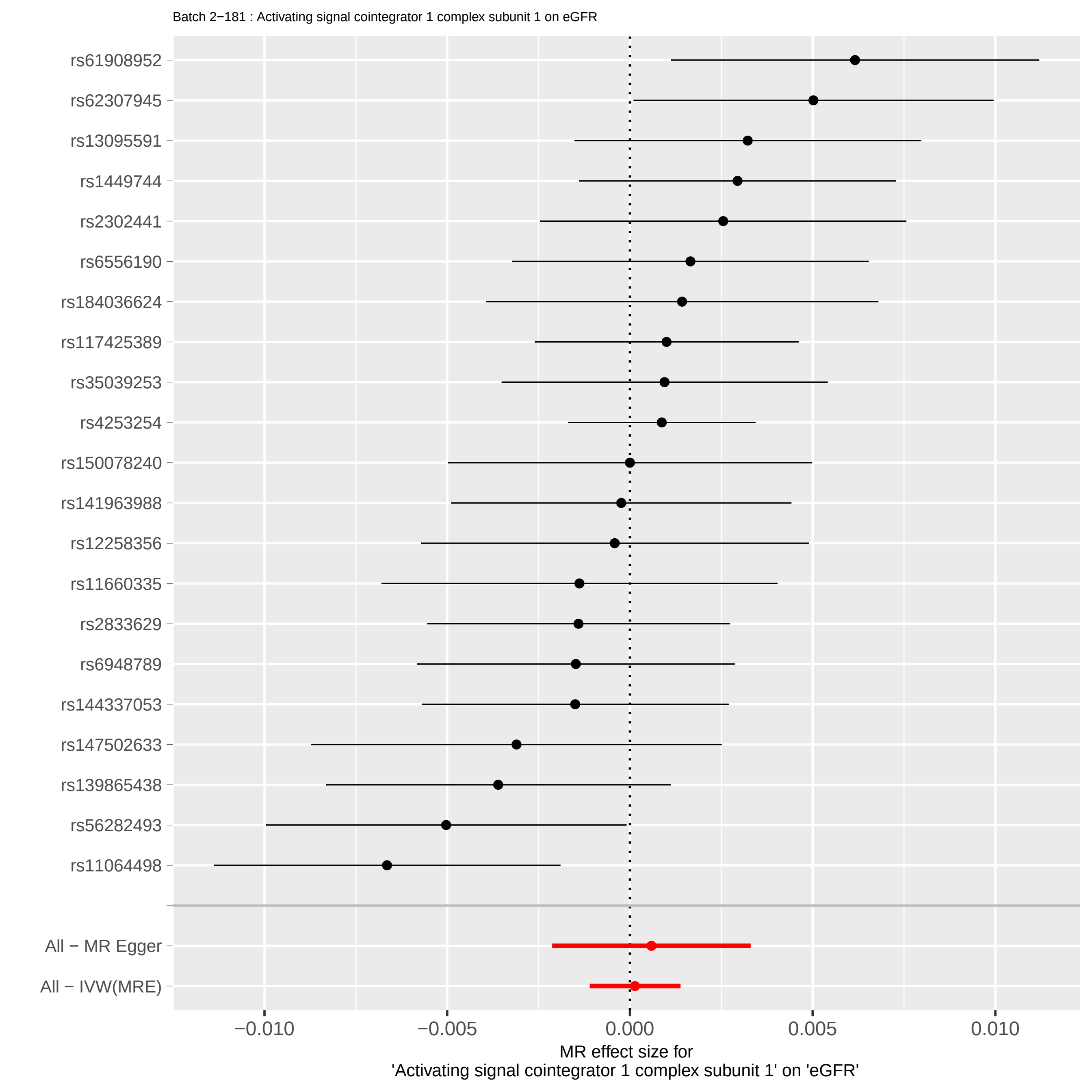

Batch 2−181 : Activating signal cointegrator 1 complex subunit 1 on eGFR
rs61908952
rs62307945
rs13095591
rs1449744
rs2302441
rs6556190
rs184036624
rs117425389
rs35039253
rs4253254
rs150078240
rs141963988
rs12258356
rs11660335
rs2833629
rs6948789
rs144337053
rs147502633
rs139865438
rs56282493
rs11064498
All − MR Egger
All − IVW(MRE)
−0.010
−0.005
0.000
0.005
0.010
MR effect size for
'Activating signal cointegrator 1 complex subunit 1' on 'eGFR'

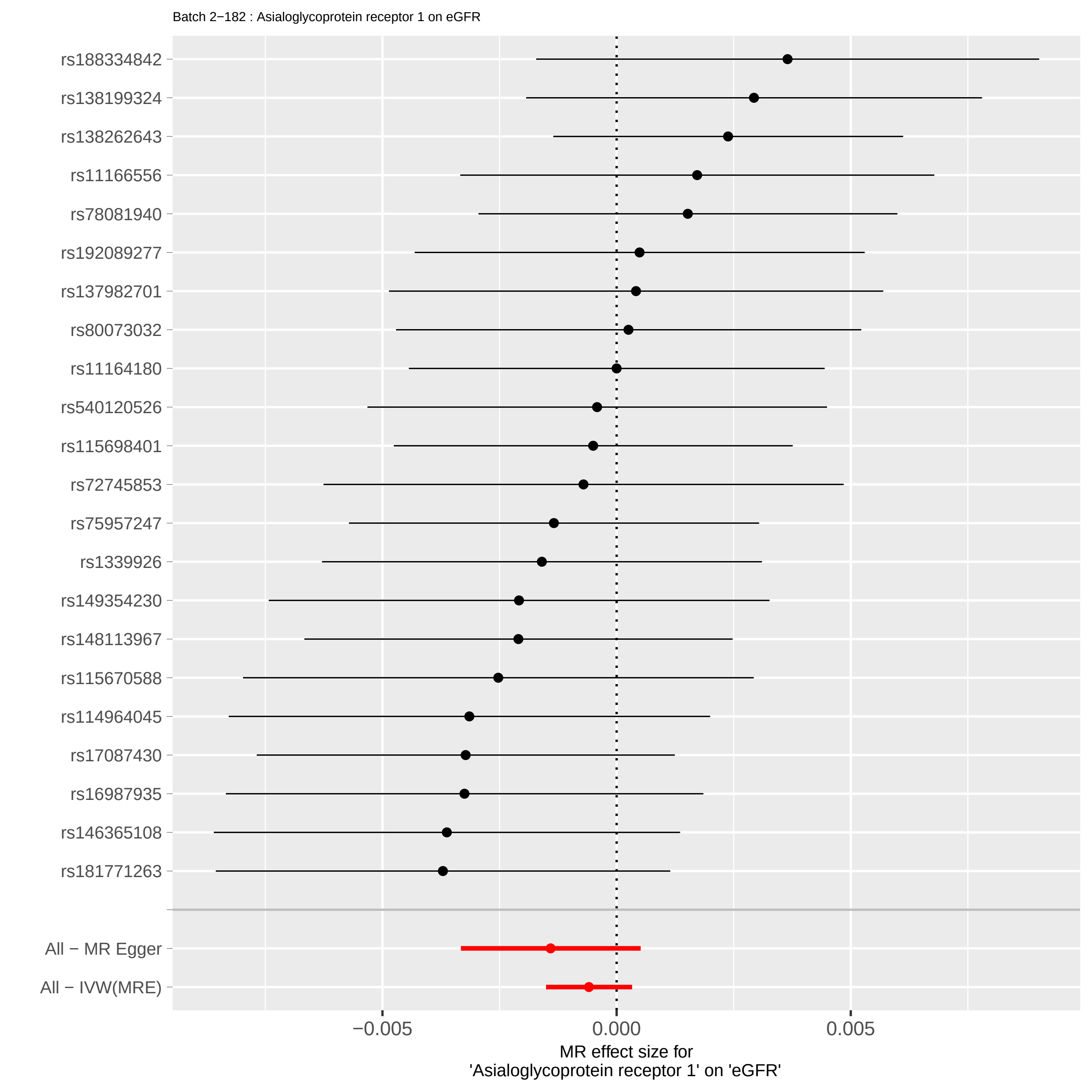

Batch 2−182 : Asialoglycoprotein receptor 1 on eGFR
rs188334842
rs138199324
rs138262643
rs11166556
rs78081940
rs192089277
rs137982701
rs80073032
rs11164180
rs540120526
rs115698401
rs72745853
rs75957247
rs1339926
rs149354230
rs148113967
rs115670588
rs114964045
rs17087430
rs16987935
rs146365108
rs181771263
All − MR Egger
All − IVW(MRE)
−0.005
0.000
0.005
MR effect size for
'Asialoglycoprotein receptor 1' on 'eGFR'

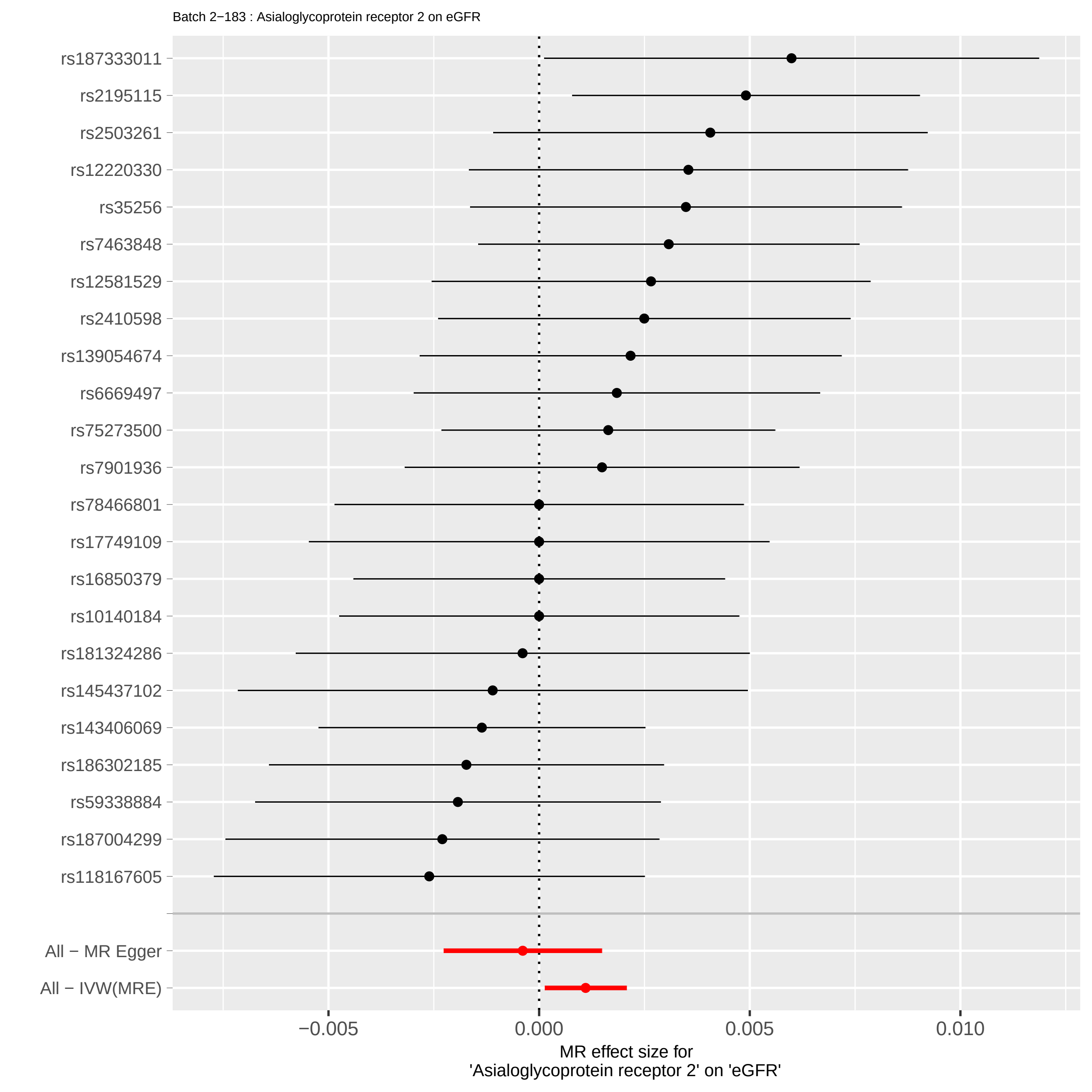

Batch 2−183 : Asialoglycoprotein receptor 2 on eGFR
rs187333011
rs2195115
rs2503261
rs12220330
rs35256
rs7463848
rs12581529
rs2410598
rs139054674
rs6669497
rs75273500
rs7901936
rs78466801
rs17749109
rs16850379
rs10140184
rs181324286
rs145437102
rs143406069
rs186302185
rs59338884
rs187004299
rs118167605
All − MR Egger
All − IVW(MRE)
−0.005
0.000
0.005
0.010
MR effect size for
'Asialoglycoprotein receptor 2' on 'eGFR'

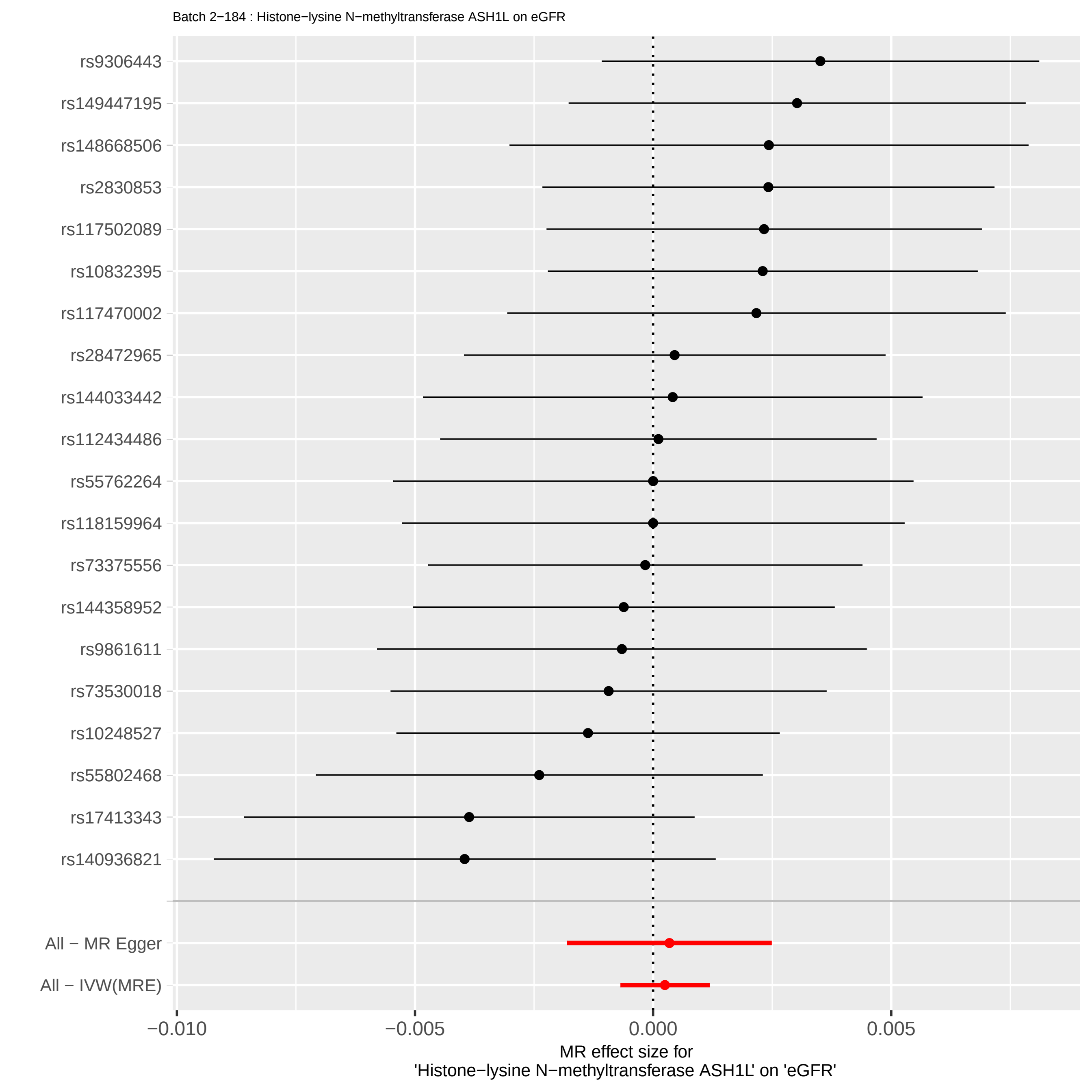

Batch 2−184 : Histone−lysine N−methyltransferase ASH1L on eGFR
rs9306443
rs149447195
rs148668506
rs2830853
rs117502089
rs10832395
rs117470002
rs28472965
rs144033442
rs112434486
rs55762264
rs118159964
rs73375556
rs144358952
rs9861611
rs73530018
rs10248527
rs55802468
rs17413343
rs140936821
All − MR Egger
All − IVW(MRE)
−0.010
−0.005
0.000
0.005
MR effect size for
'Histone−lysine N−methyltransferase ASH1L' on 'eGFR'

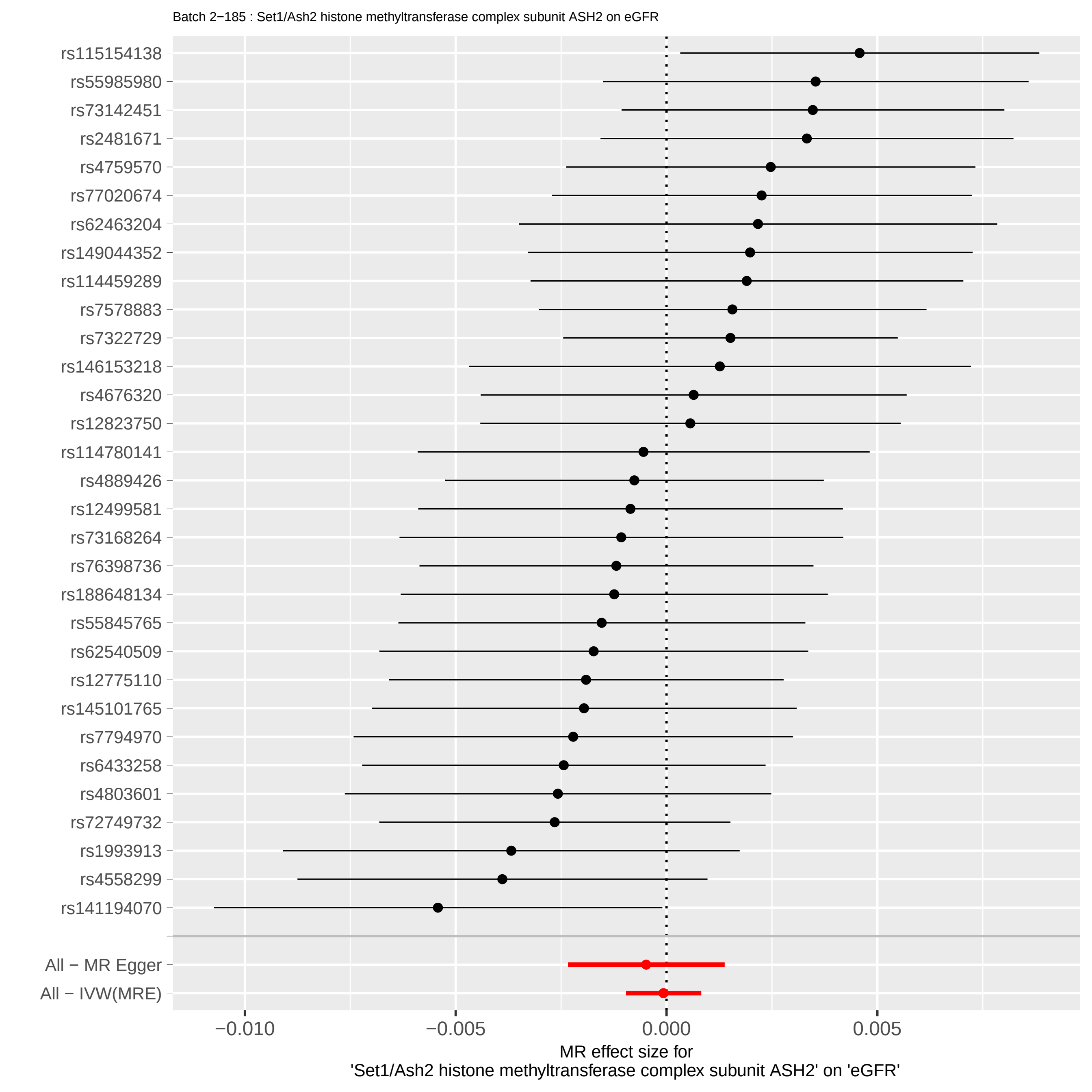
Batch 2−185 : Set1/Ash2 histone methyltransferase complex subunit ASH2 on eGFR
rs115154138
rs55985980
rs73142451
rs2481671
rs4759570
rs77020674
rs62463204
rs149044352
rs114459289
rs7578883
rs7322729
rs146153218
rs4676320
rs12823750
rs114780141
rs4889426
rs12499581
rs73168264
rs76398736
rs188648134
rs55845765
rs62540509
rs12775110
rs145101765
rs7794970
rs6433258
rs4803601
rs72749732
rs1993913
rs4558299
rs141194070
All − MR Egger
All − IVW(MRE)
−0.010
−0.005
0.000
0.005
MR effect size for
'Set1/Ash2 histone methyltransferase complex subunit ASH2' on 'eGFR'

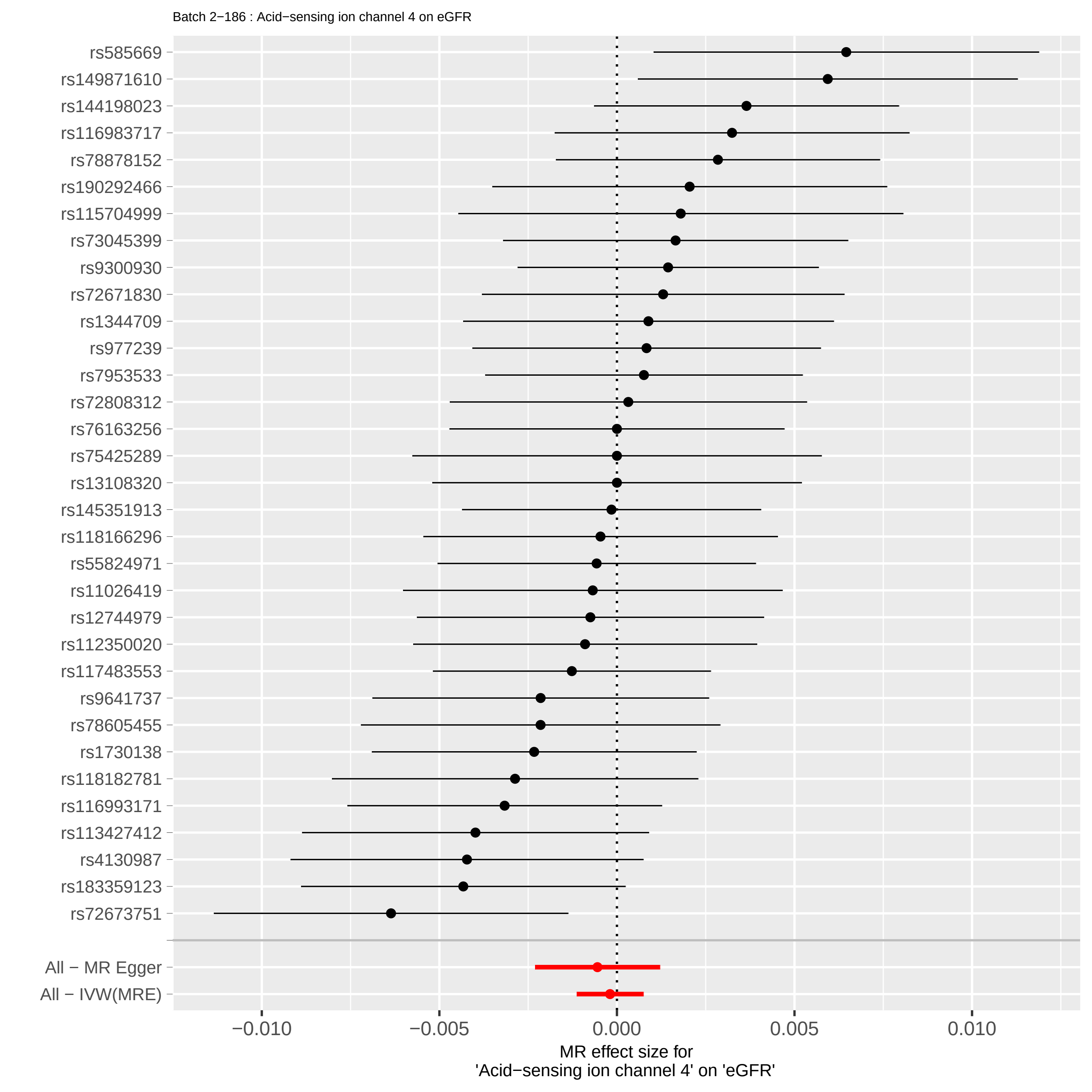

Batch 2−186 : Acid−sensing ion channel 4 on eGFR
rs585669
rs149871610
rs144198023
rs116983717
rs78878152
rs190292466
rs115704999
rs73045399
rs9300930
rs72671830
rs1344709
rs977239
rs7953533
rs72808312
rs76163256
rs75425289
rs13108320
rs145351913
rs118166296
rs55824971
rs11026419
rs12744979
rs112350020
rs117483553
rs9641737
rs78605455
rs1730138
rs118182781
rs116993171
rs113427412
rs4130987
rs183359123
rs72673751
All − MR Egger
All − IVW(MRE)
−0.010
−0.005
0.000
0.005
0.010
MR effect size for
'Acid−sensing ion channel 4' on 'eGFR'

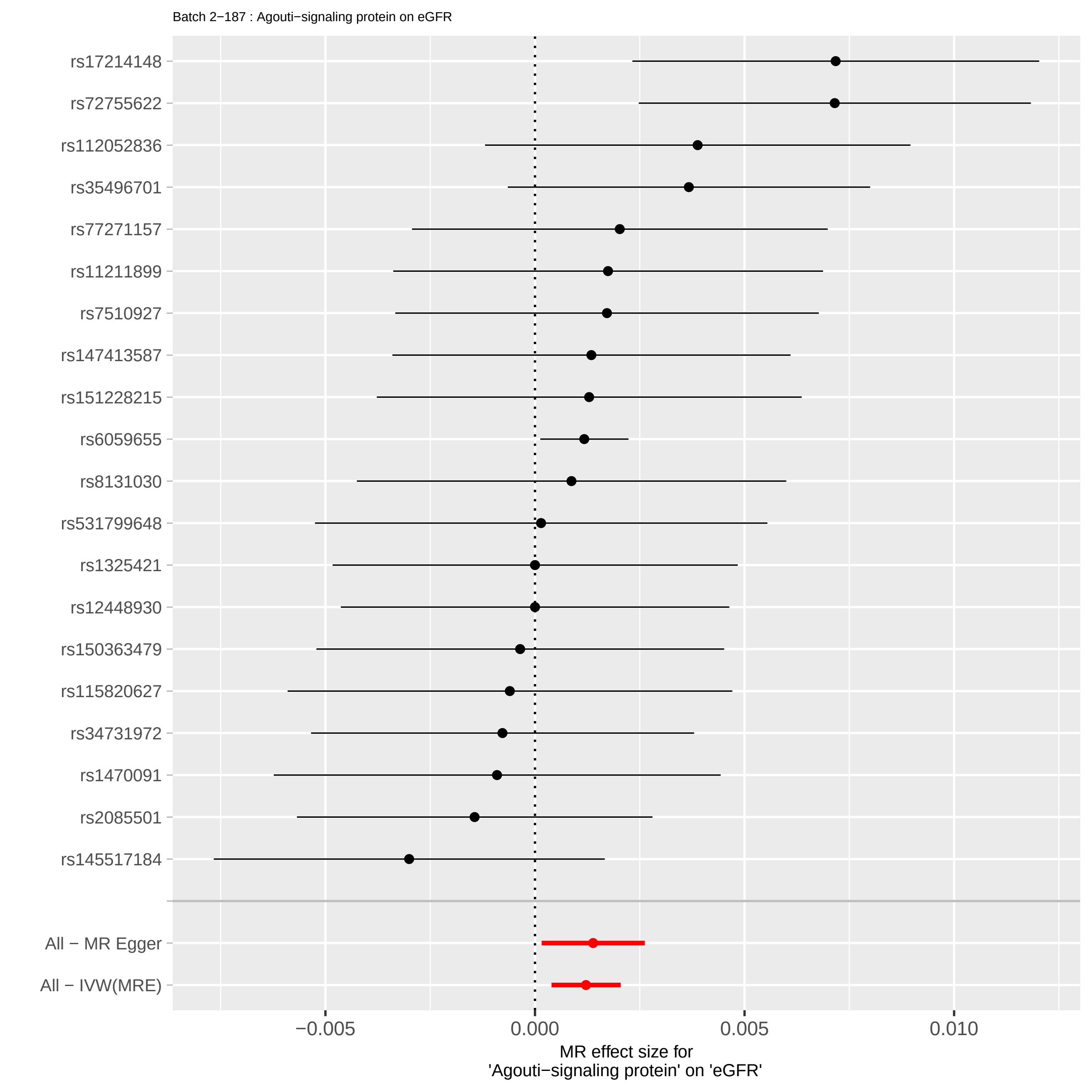
Batch 2−187 : Agouti−signaling protein on eGFR
rs17214148
rs72755622
rs112052836
rs35496701
rs77271157
rs11211899
rs7510927
rs147413587
rs151228215
rs6059655
rs8131030
rs531799648
rs1325421
rs12448930
rs150363479
rs115820627
rs34731972
rs1470091
rs2085501
rs145517184
All − MR Egger
All − IVW(MRE)
−0.005
0.000
0.005
0.010
MR effect size for
'Agouti−signaling protein' on 'eGFR'

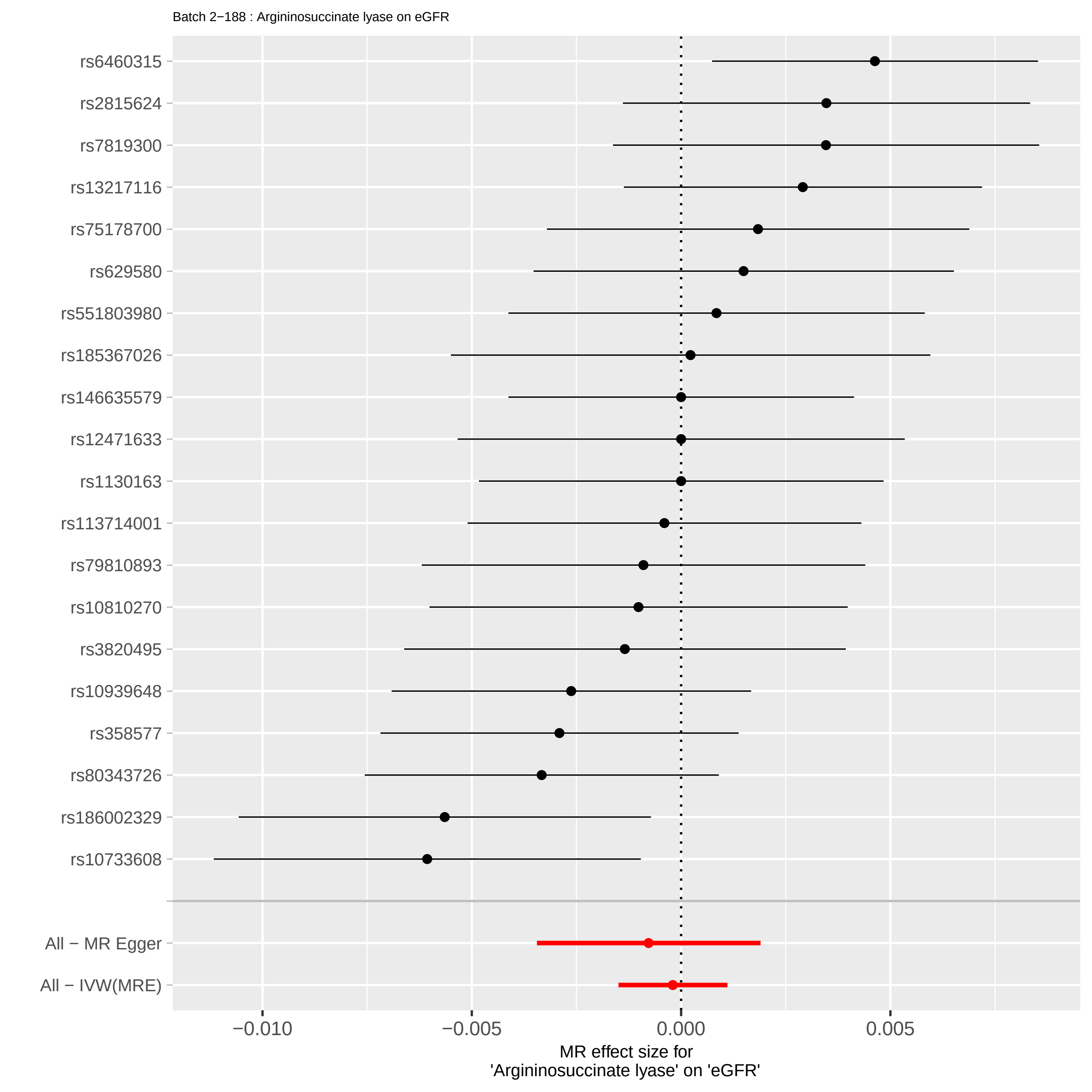
Batch 2−188 : Argininosuccinate lyase on eGFR
rs6460315
rs2815624
rs7819300
rs13217116
rs75178700
rs629580
rs551803980
rs185367026
rs146635579
rs12471633
rs1130163
rs113714001
rs79810893
rs10810270
rs3820495
rs10939648
rs358577
rs80343726
rs186002329
rs10733608
All − MR Egger
All − IVW(MRE)
−0.010
−0.005
0.000
0.005
MR effect size for
'Argininosuccinate lyase' on 'eGFR'

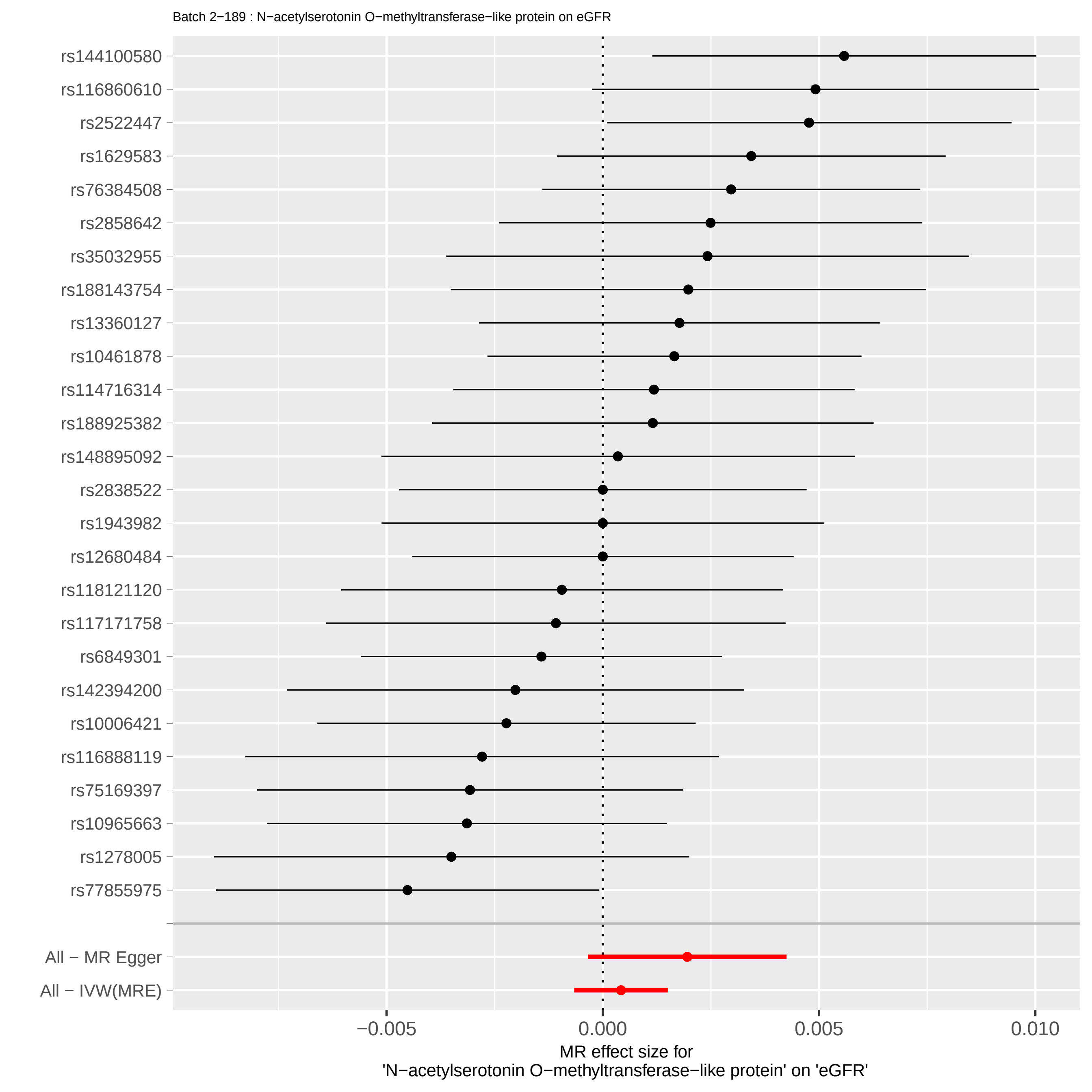
Batch 2−189 : N−acetylserotonin O−methyltransferase−like protein on eGFR
rs144100580
rs116860610
rs2522447
rs1629583
rs76384508
rs2858642
rs35032955
rs188143754
rs13360127
rs10461878
rs114716314
rs188925382
rs148895092
rs2838522
rs1943982
rs12680484
rs118121120
rs117171758
rs6849301
rs142394200
rs10006421
rs116888119
rs75169397
rs10965663
rs1278005
rs77855975
All − MR Egger
All − IVW(MRE)
−0.005
0.000
0.005
0.010
MR effect size for
'N−acetylserotonin O−methyltransferase−like protein' on 'eGFR'

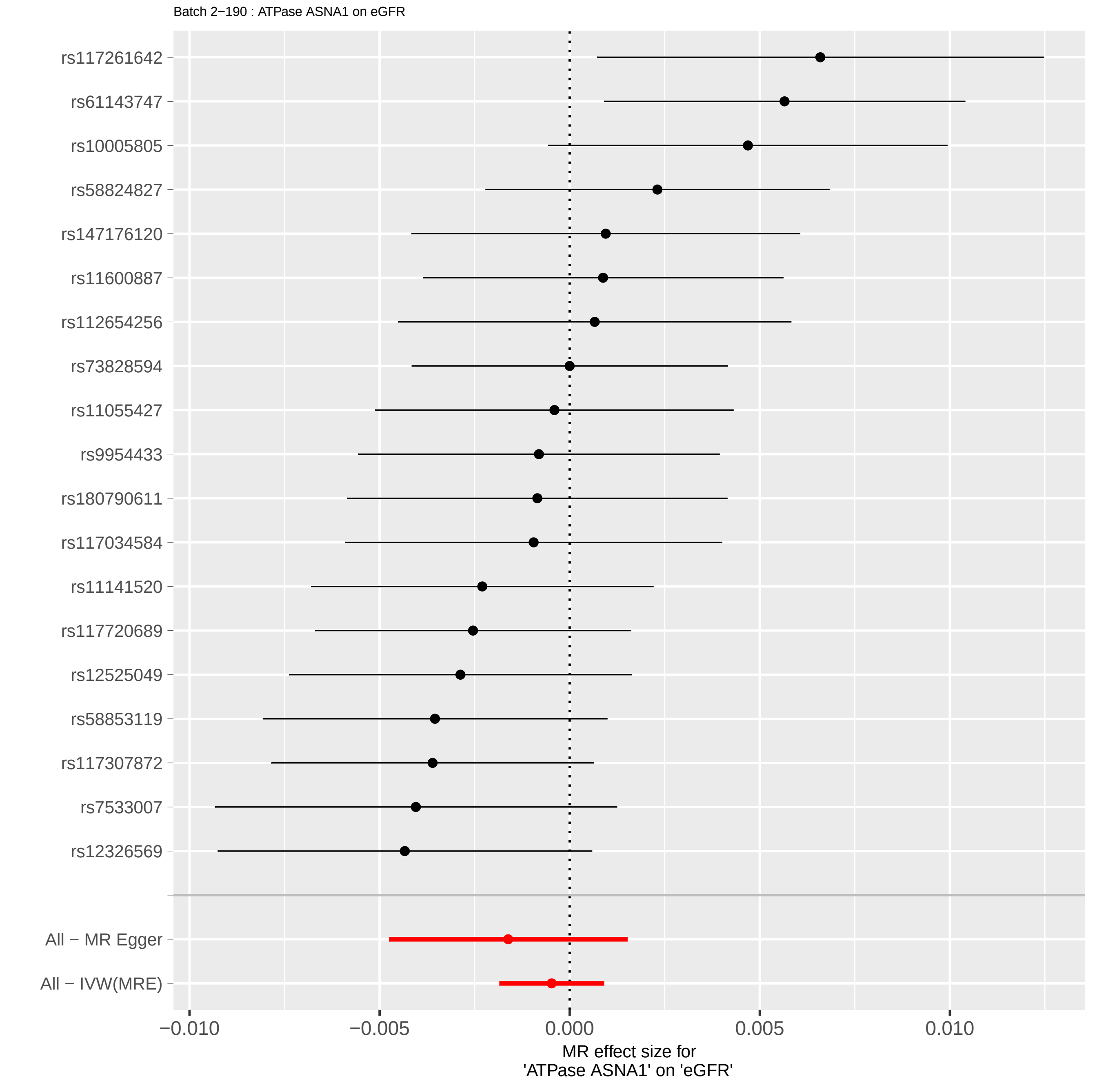

Batch 2−190 : ATPase ASNA1 on eGFR
rs117261642
rs61143747
rs10005805
rs58824827
rs147176120
rs11600887
rs112654256
rs73828594
rs11055427
rs9954433
rs180790611
rs117034584
rs11141520
rs117720689
rs12525049
rs58853119
rs117307872
rs7533007
rs12326569
All − MR Egger
All − IVW(MRE)
−0.010
−0.005
0.000
0.005
0.010
MR effect size for
'ATPase ASNA1' on 'eGFR'

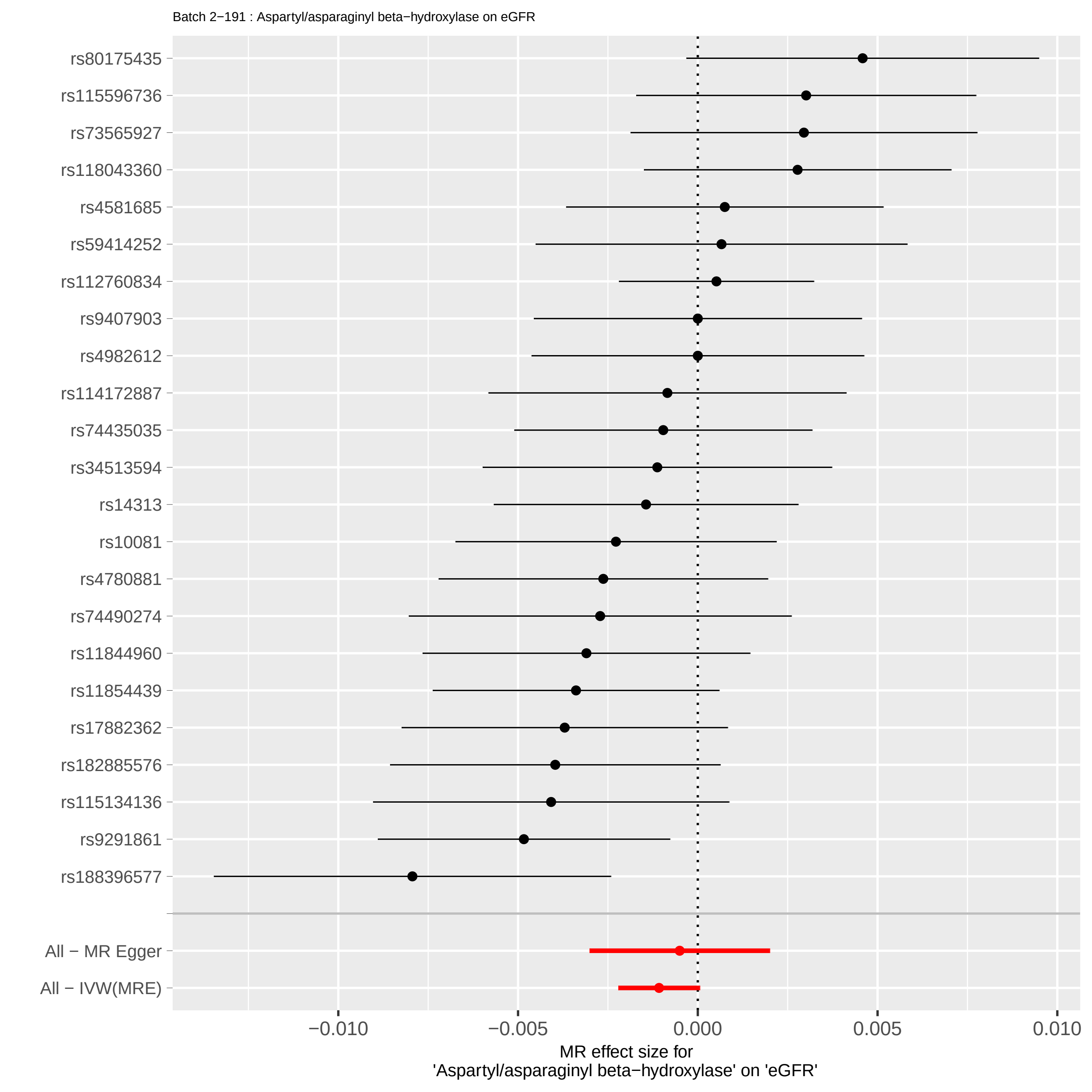
Batch 2−191 : Aspartyl/asparaginyl beta−hydroxylase on eGFR
rs80175435
rs115596736
rs73565927
rs118043360
rs4581685
rs59414252
rs112760834
rs9407903
rs4982612
rs114172887
rs74435035
rs34513594
rs14313
rs10081
rs4780881
rs74490274
rs11844960
rs11854439
rs17882362
rs182885576
rs115134136
rs9291861
rs188396577
All − MR Egger
All − IVW(MRE)
−0.010
−0.005
0.000
0.005
0.010
MR effect size for
'Aspartyl/asparaginyl beta−hydroxylase' on 'eGFR'

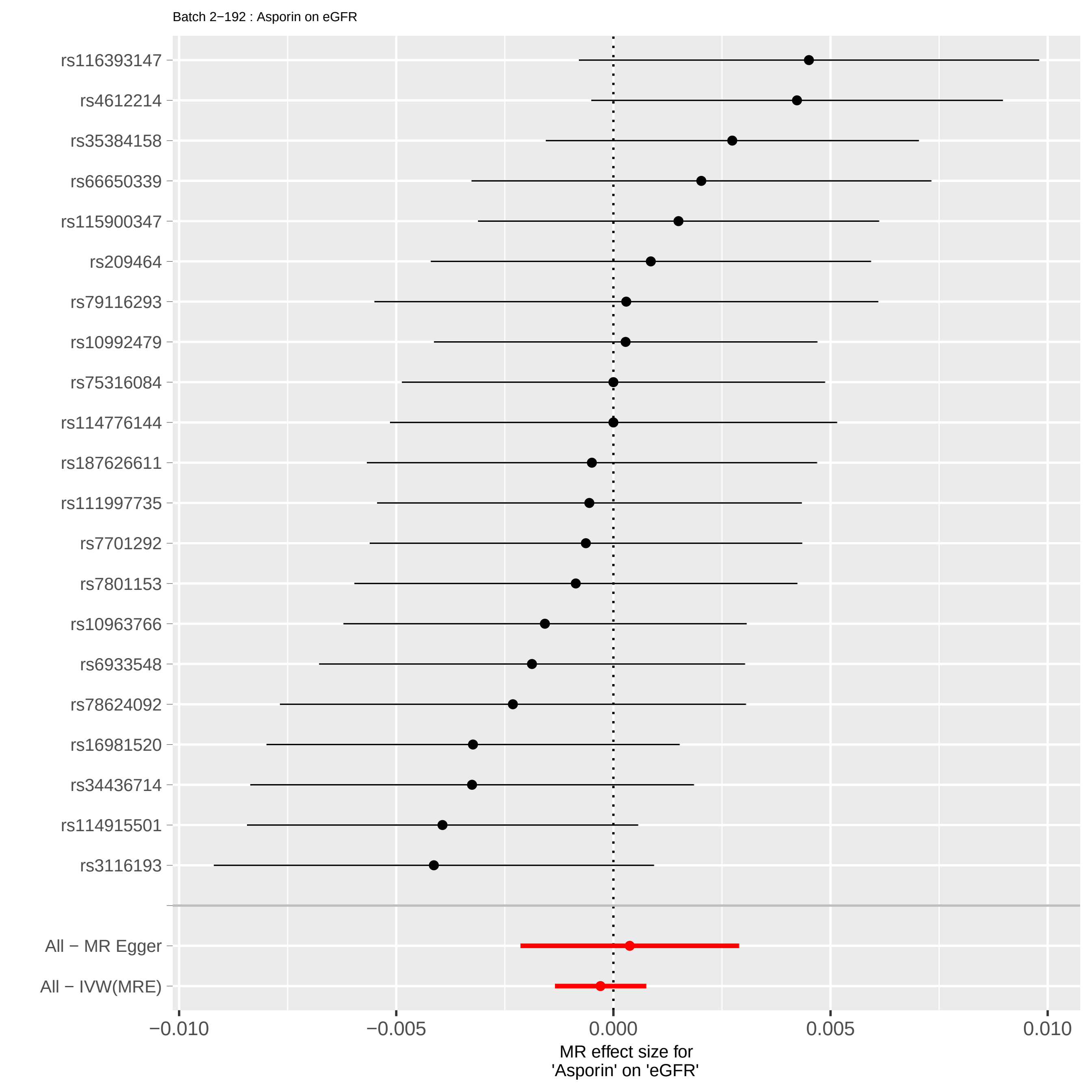

Batch 2−192 : Asporin on eGFR
rs116393147
rs4612214
rs35384158
rs66650339
rs115900347
rs209464
rs79116293
rs10992479
rs75316084
rs114776144
rs187626611
rs111997735
rs7701292
rs7801153
rs10963766
rs6933548
rs78624092
rs16981520
rs34436714
rs114915501
rs3116193
All − MR Egger
All − IVW(MRE)
−0.010
−0.005
0.000
0.005
0.010
MR effect size for
'Asporin' on 'eGFR'

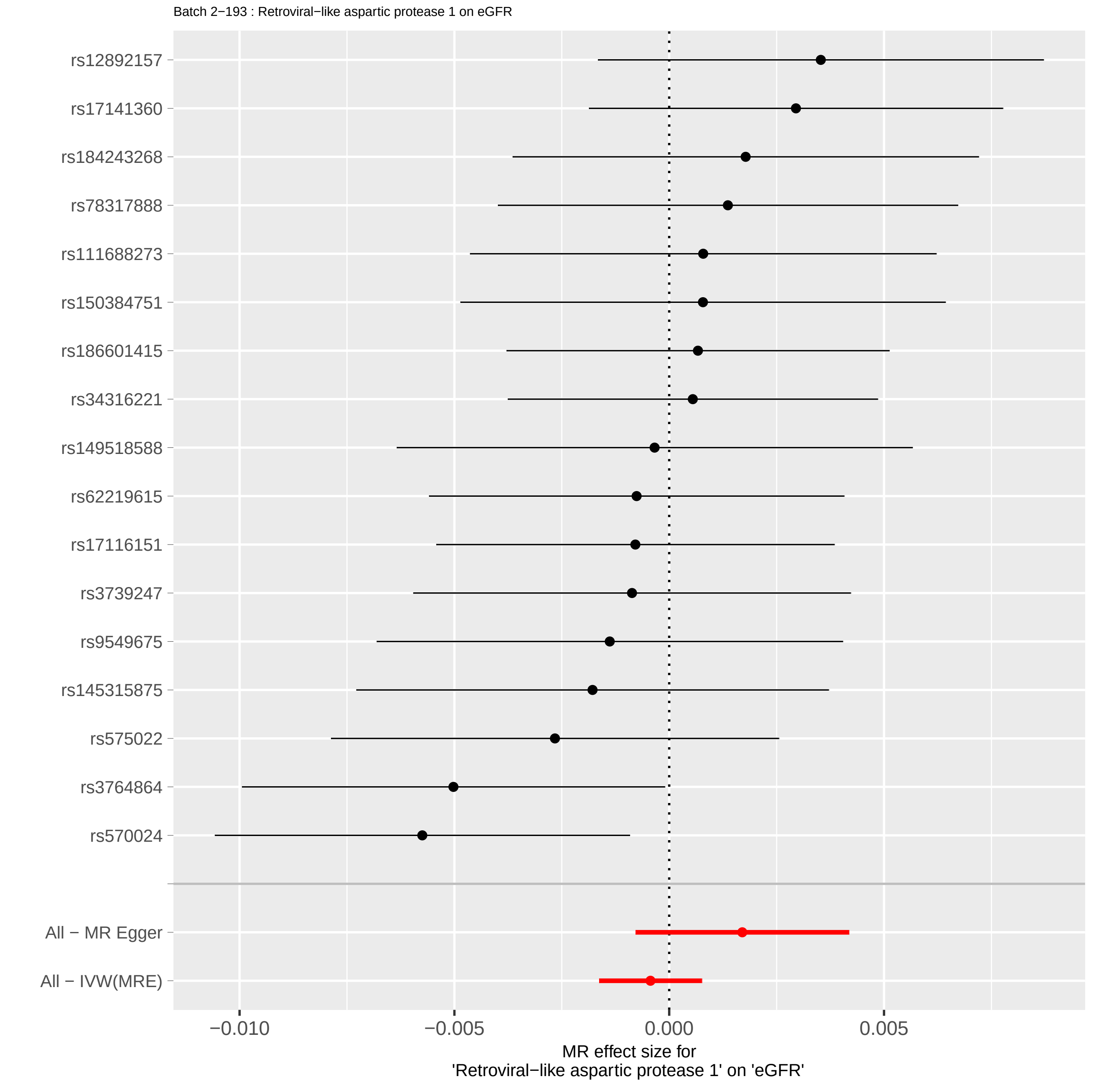

Batch 2−193 : Retroviral−like aspartic protease 1 on eGFR
rs12892157
rs17141360
rs184243268
rs78317888
rs111688273
rs150384751
rs186601415
rs34316221
rs149518588
rs62219615
rs17116151
rs3739247
rs9549675
rs145315875
rs575022
rs3764864
rs570024
All − MR Egger
All − IVW(MRE)
−0.010
−0.005
0.000
0.005
MR effect size for
'Retroviral−like aspartic protease 1' on 'eGFR'

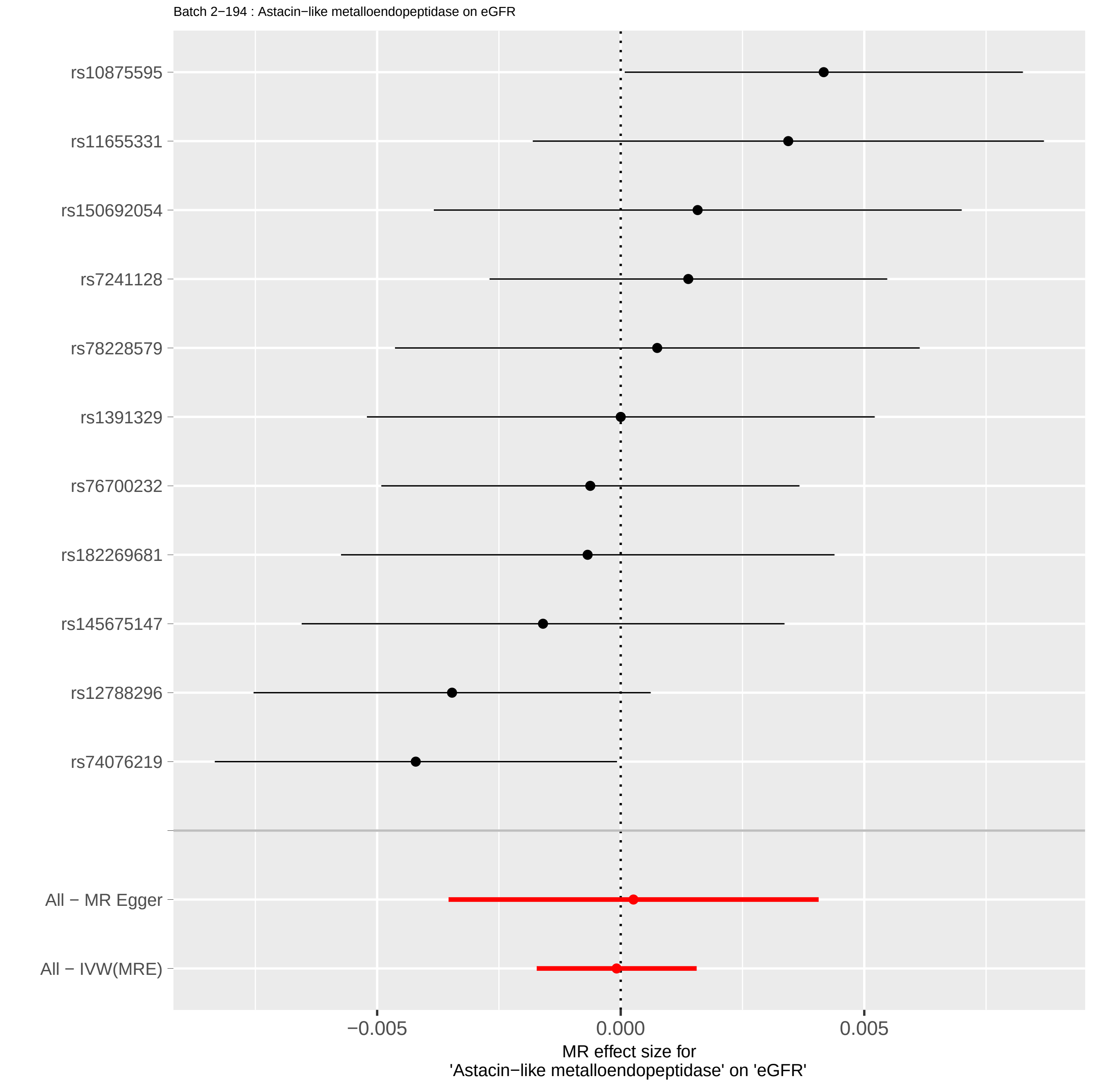

Batch 2−194 : Astacin−like metalloendopeptidase on eGFR
rs10875595
rs11655331
rs150692054
rs7241128
rs78228579
rs1391329
rs76700232
rs182269681
rs145675147
rs12788296
rs74076219
All − MR Egger
All − IVW(MRE)
−0.005
0.000
0.005
MR effect size for
'Astacin−like metalloendopeptidase' on 'eGFR'

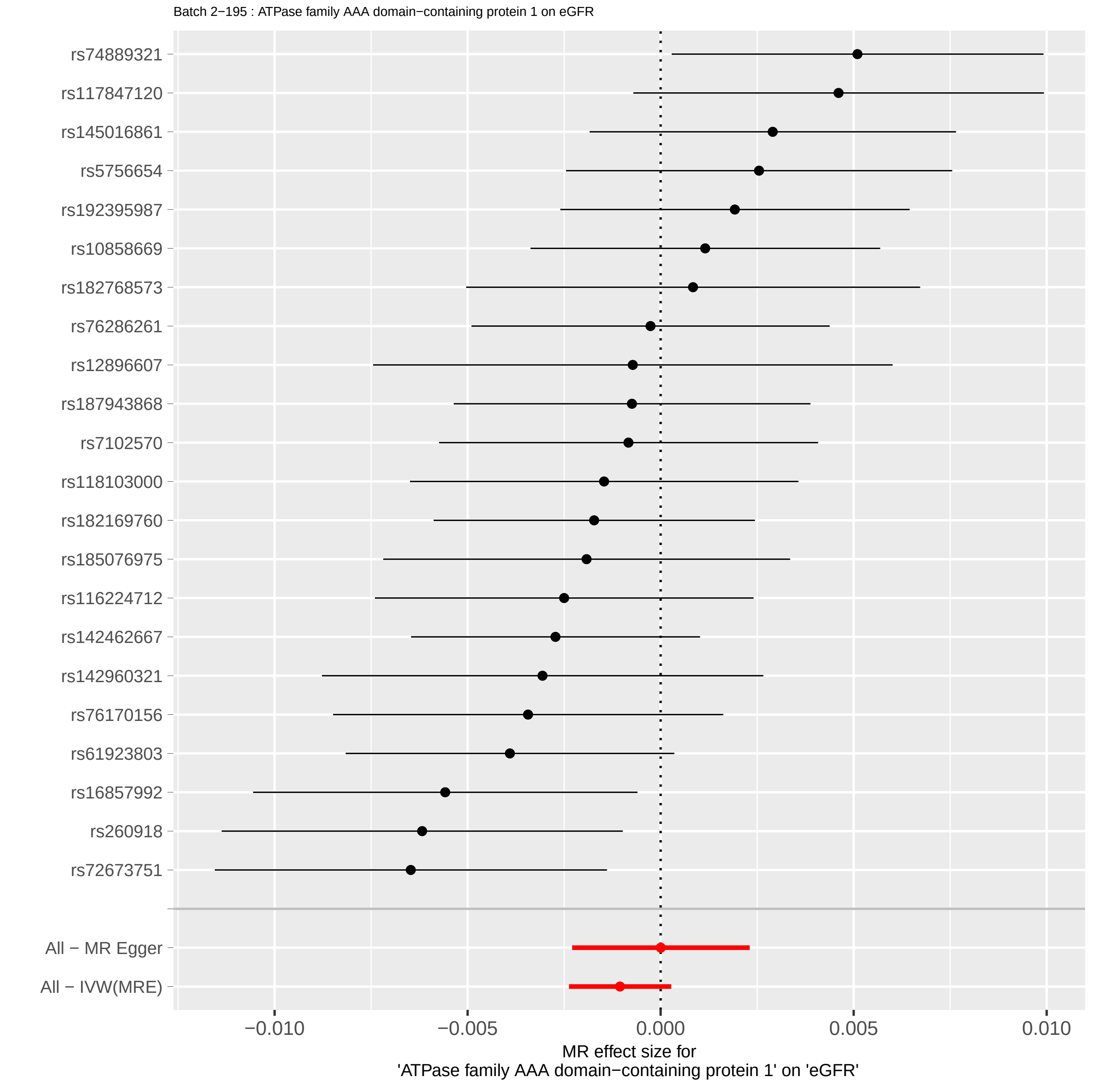

Batch 2−195 : ATPase family AAA domain−containing protein 1 on eGFR
rs74889321
rs117847120
rs145016861
rs5756654
rs192395987
rs10858669
rs182768573
rs76286261
rs12896607
rs187943868
rs7102570
rs118103000
rs182169760
rs185076975
rs116224712
rs142462667
rs142960321
rs76170156
rs61923803
rs16857992
rs260918
rs72673751
All − MR Egger
All − IVW(MRE)
−0.010
−0.005
0.000
0.005
0.010
MR effect size for
'ATPase family AAA domain−containing protein 1' on 'eGFR'

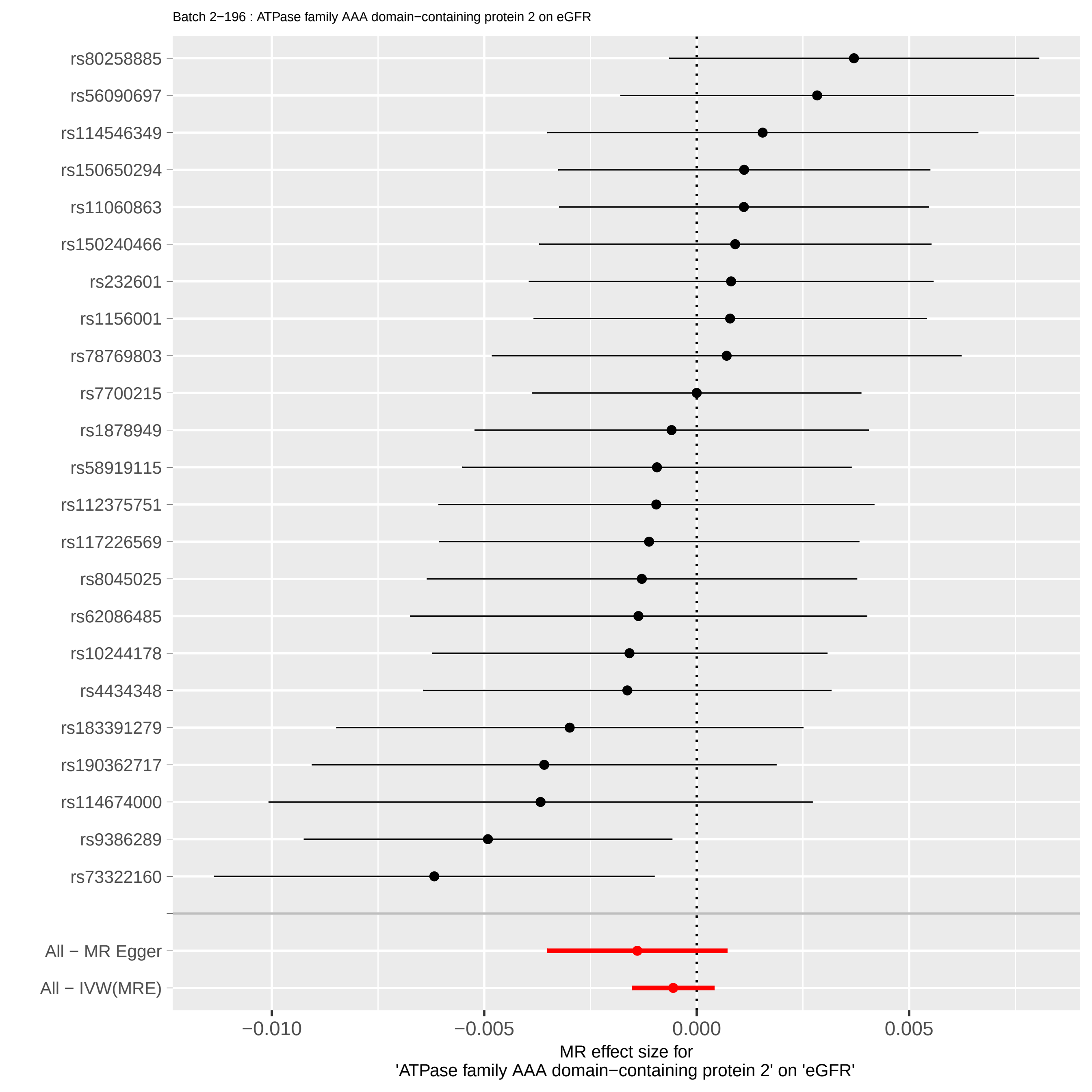

Batch 2−196 : ATPase family AAA domain−containing protein 2 on eGFR
rs80258885
rs56090697
rs114546349
rs150650294
rs11060863
rs150240466
rs232601
rs1156001
rs78769803
rs7700215
rs1878949
rs58919115
rs112375751
rs117226569
rs8045025
rs62086485
rs10244178
rs4434348
rs183391279
rs190362717
rs114674000
rs9386289
rs73322160
All − MR Egger
All − IVW(MRE)
−0.010
−0.005
0.000
0.005
MR effect size for
'ATPase family AAA domain−containing protein 2' on 'eGFR'

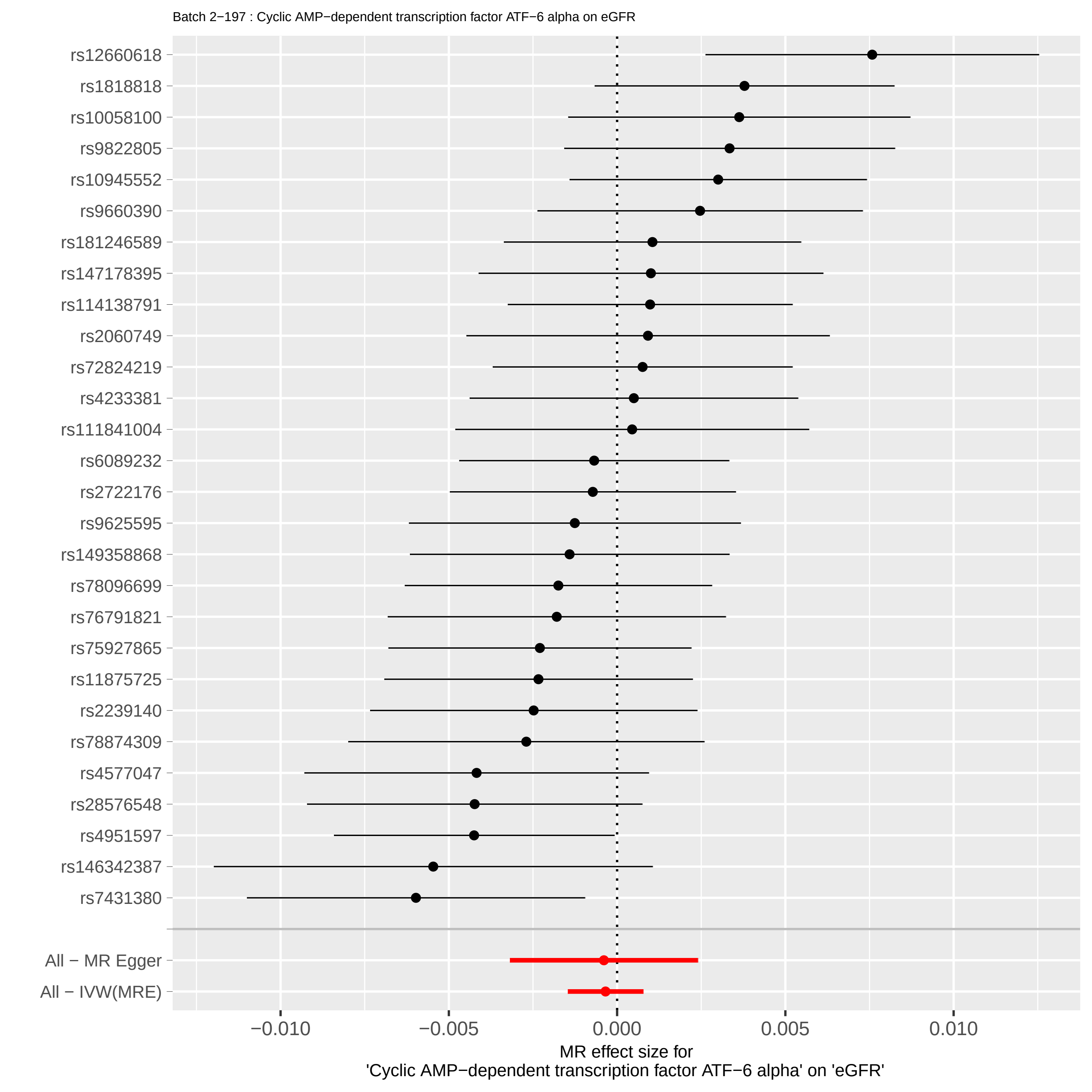

Batch 2−197 : Cyclic AMP−dependent transcription factor ATF−6 alpha on eGFR
rs12660618
rs1818818
rs10058100
rs9822805
rs10945552
rs9660390
rs181246589
rs147178395
rs114138791
rs2060749
rs72824219
rs4233381
rs111841004
rs6089232
rs2722176
rs9625595
rs149358868
rs78096699
rs76791821
rs75927865
rs11875725
rs2239140
rs78874309
rs4577047
rs28576548
rs4951597
rs146342387
rs7431380
All − MR Egger
All − IVW(MRE)
−0.010
−0.005
0.000
0.005
0.010
MR effect size for
'Cyclic AMP−dependent transcription factor ATF−6 alpha' on 'eGFR'

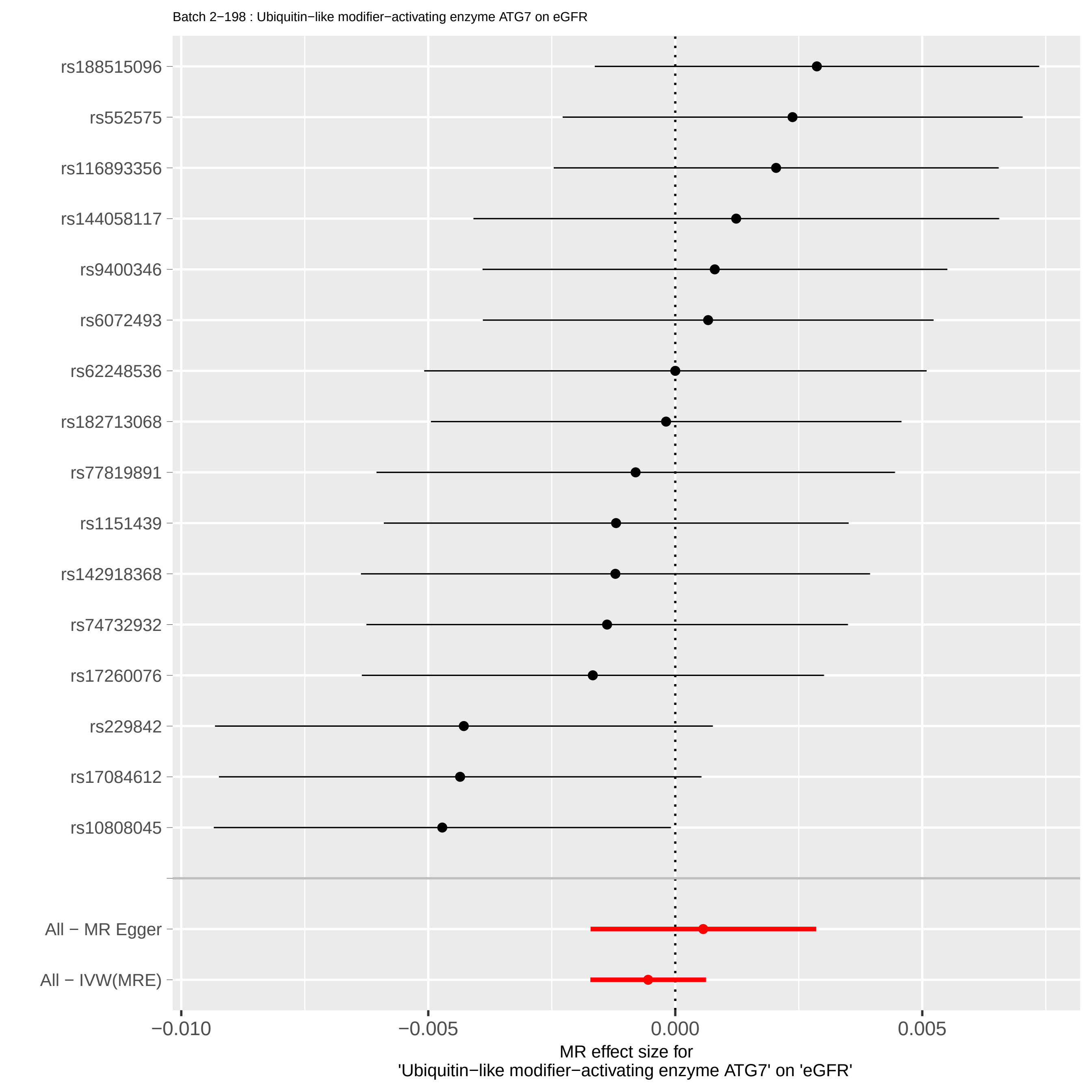

Batch 2−198 : Ubiquitin−like modifier−activating enzyme ATG7 on eGFR
rs188515096
rs552575
rs116893356
rs144058117
rs9400346
rs6072493
rs62248536
rs182713068
rs77819891
rs1151439
rs142918368
rs74732932
rs17260076
rs229842
rs17084612
rs10808045
All − MR Egger
All − IVW(MRE)
−0.010
−0.005
0.000
0.005
MR effect size for
'Ubiquitin−like modifier−activating enzyme ATG7' on 'eGFR'

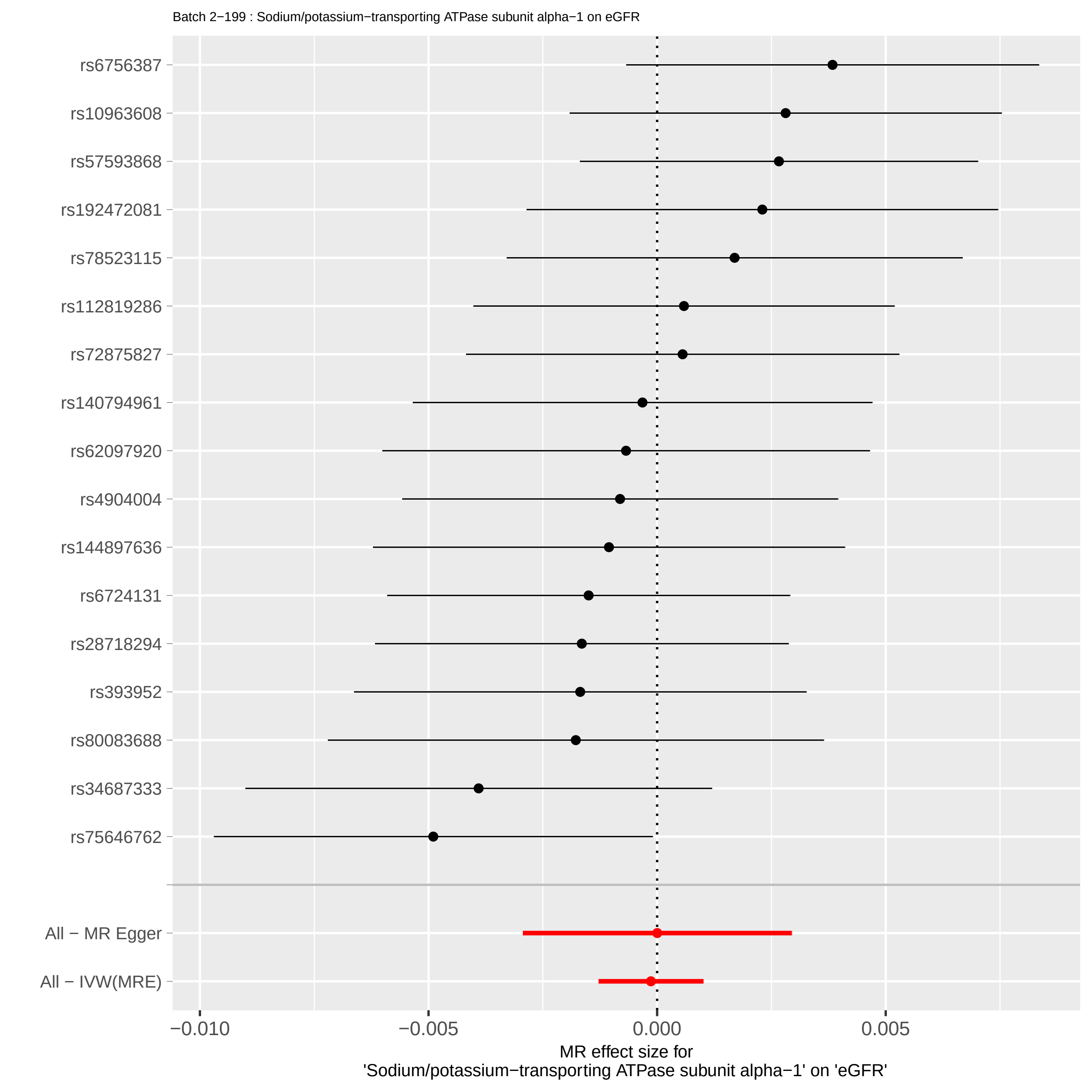

Batch 2−199 : Sodium/potassium−transporting ATPase subunit alpha−1 on eGFR
rs6756387
rs10963608
rs57593868
rs192472081
rs78523115
rs112819286
rs72875827
rs140794961
rs62097920
rs4904004
rs144897636
rs6724131
rs28718294
rs393952
rs80083688
rs34687333
rs75646762
All − MR Egger
All − IVW(MRE)
−0.010
−0.005
0.000
0.005
MR effect size for
'Sodium/potassium−transporting ATPase subunit alpha−1' on 'eGFR'

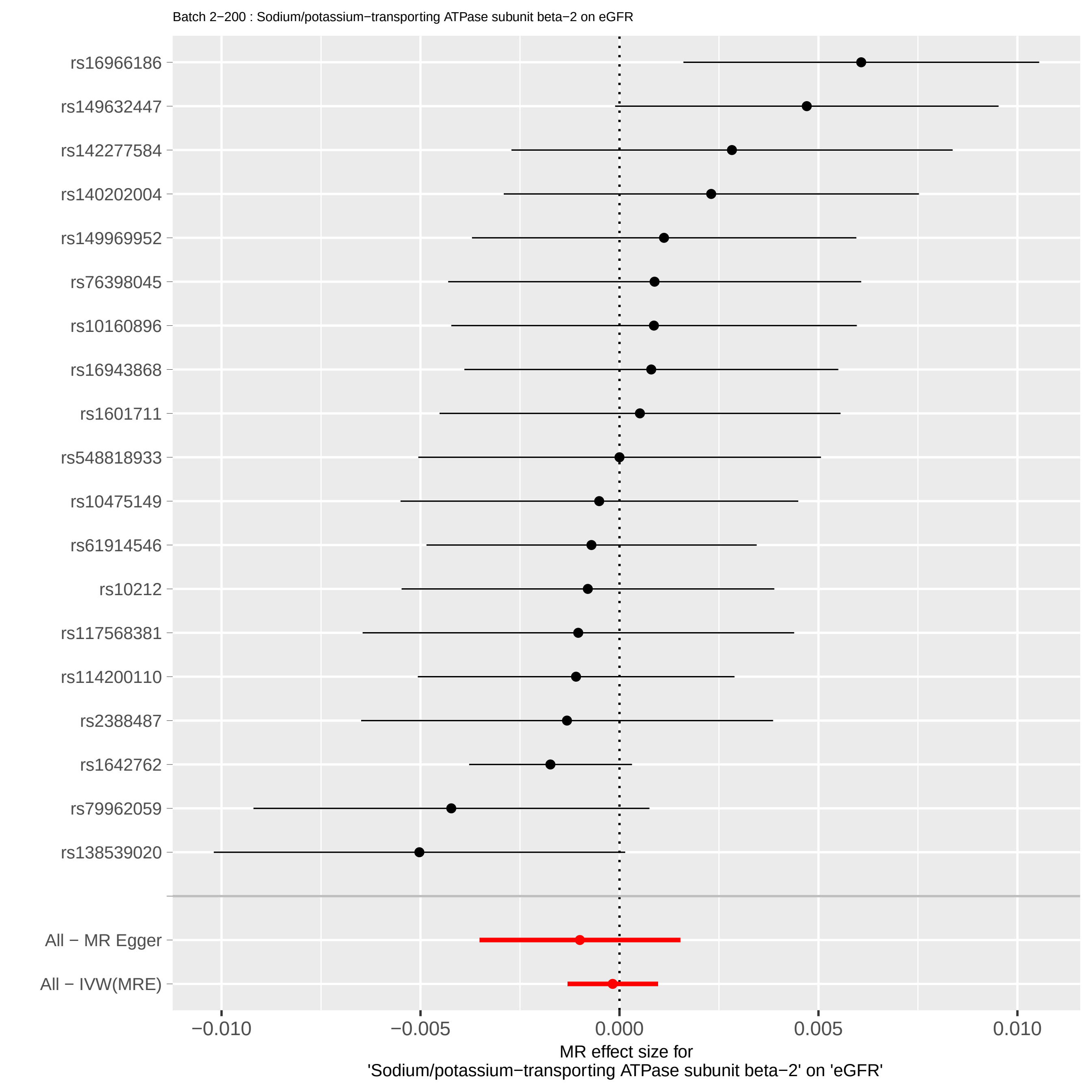
Batch 2−200 : Sodium/potassium−transporting ATPase subunit beta−2 on eGFR
rs16966186
rs149632447
rs142277584
rs140202004
rs149969952
rs76398045
rs10160896
rs16943868
rs1601711
rs548818933
rs10475149
rs61914546
rs10212
rs117568381
rs114200110
rs2388487
rs1642762
rs79962059
rs138539020
All − MR Egger
All − IVW(MRE)
−0.010
−0.005
0.000
0.005
0.010
MR effect size for
'Sodium/potassium−transporting ATPase subunit beta−2' on 'eGFR'

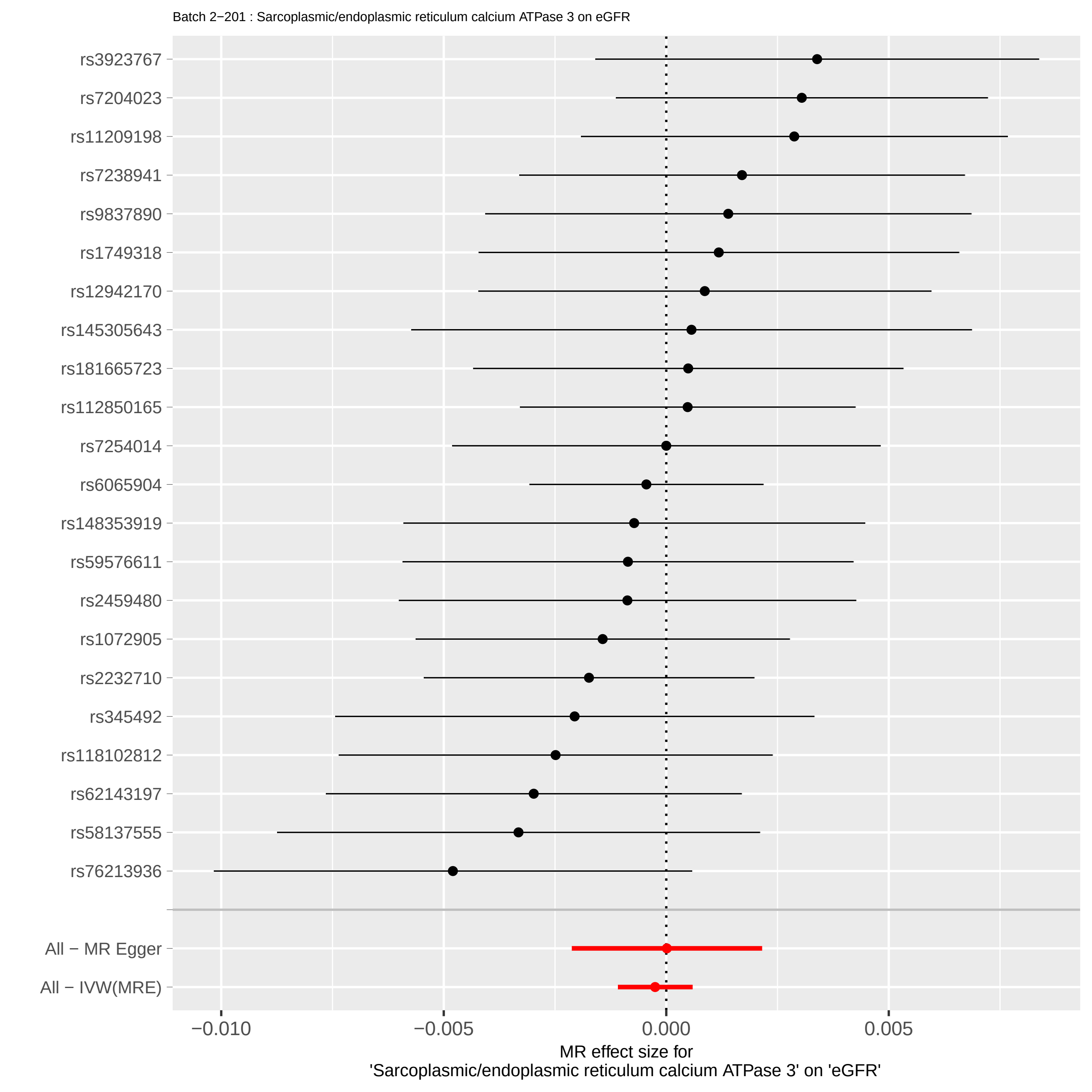
Batch 2−201 : Sarcoplasmic/endoplasmic reticulum calcium ATPase 3 on eGFR
rs3923767
rs7204023
rs11209198
rs7238941
rs9837890
rs1749318
rs12942170
rs145305643
rs181665723
rs112850165
rs7254014
rs6065904
rs148353919
rs59576611
rs2459480
rs1072905
rs2232710
rs345492
rs118102812
rs62143197
rs58137555
rs76213936
All − MR Egger
All − IVW(MRE)
−0.010
−0.005
0.000
0.005
MR effect size for
'Sarcoplasmic/endoplasmic reticulum calcium ATPase 3' on 'eGFR'

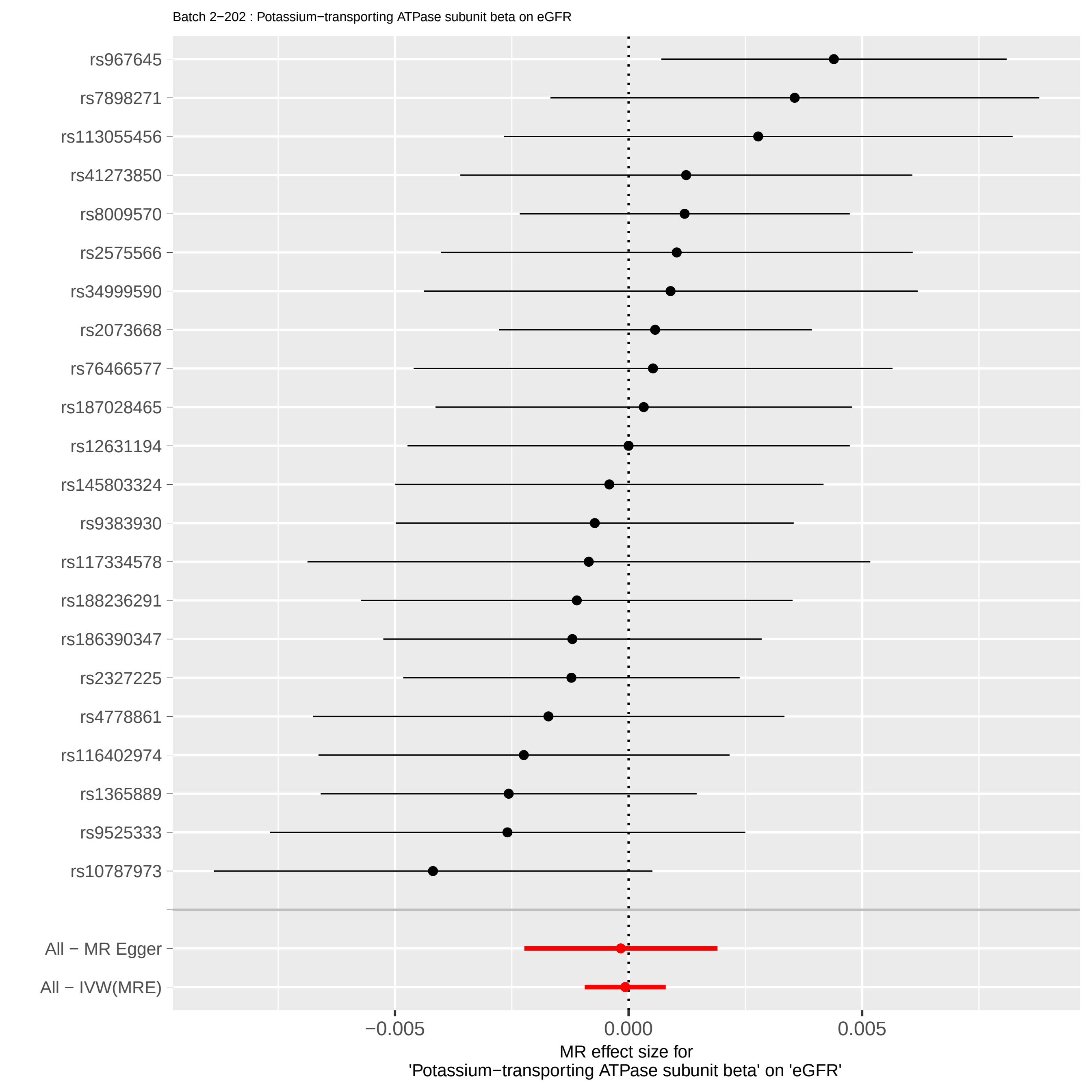

Batch 2−202 : Potassium−transporting ATPase subunit beta on eGFR
rs967645
rs7898271
rs113055456
rs41273850
rs8009570
rs2575566
rs34999590
rs2073668
rs76466577
rs187028465
rs12631194
rs145803324
rs9383930
rs117334578
rs188236291
rs186390347
rs2327225
rs4778861
rs116402974
rs1365889
rs9525333
rs10787973
All − MR Egger
All − IVW(MRE)
−0.005
0.000
0.005
MR effect size for
'Potassium−transporting ATPase subunit beta' on 'eGFR'

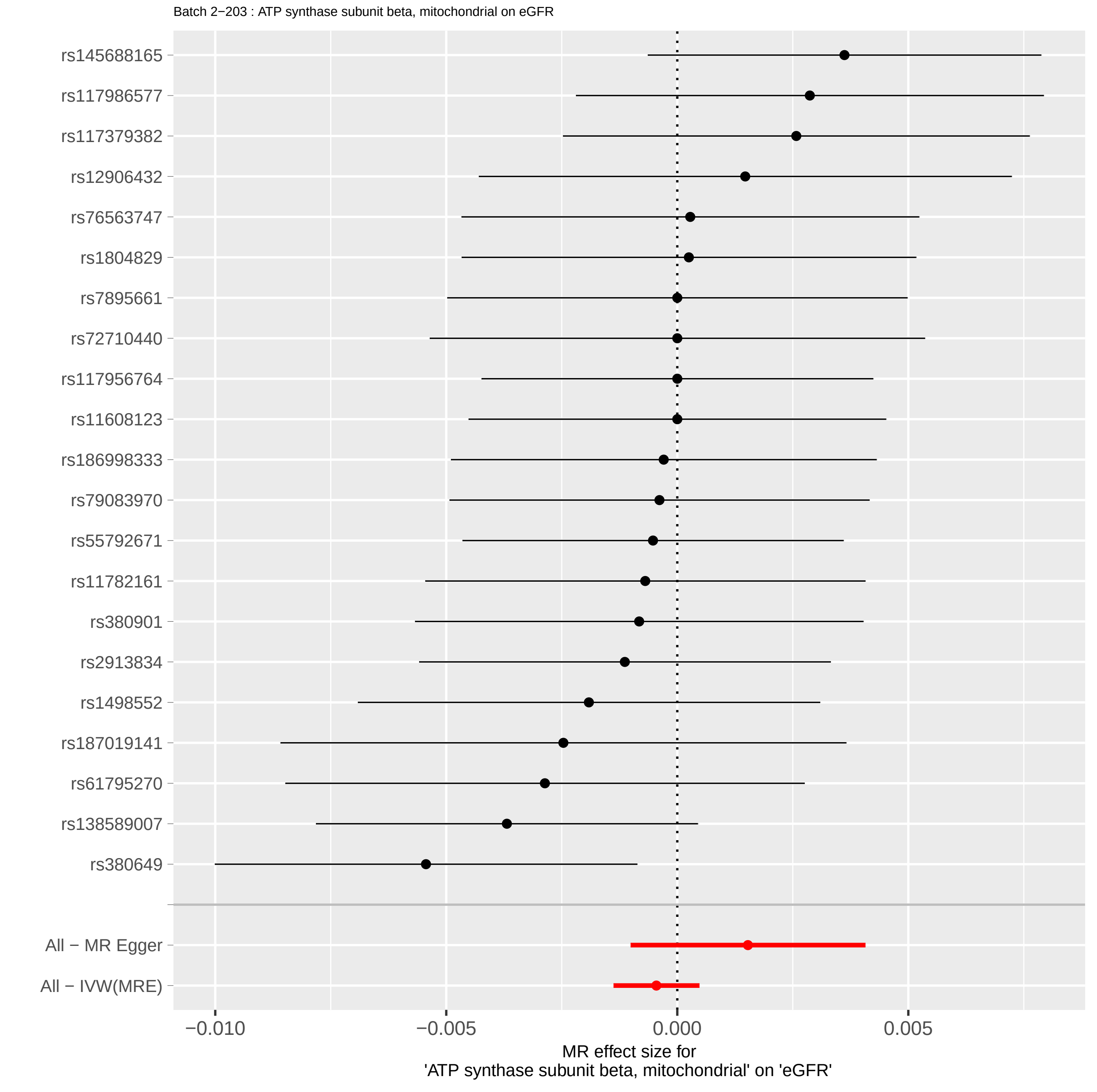

Batch 2−203 : ATP synthase subunit beta, mitochondrial on eGFR
rs145688165
rs117986577
rs117379382
rs12906432
rs76563747
rs1804829
rs7895661
rs72710440
rs117956764
rs11608123
rs186998333
rs79083970
rs55792671
rs11782161
rs380901
rs2913834
rs1498552
rs187019141
rs61795270
rs138589007
rs380649
All − MR Egger
All − IVW(MRE)
−0.010
−0.005
0.000
0.005
MR effect size for
'ATP synthase subunit beta, mitochondrial' on 'eGFR'

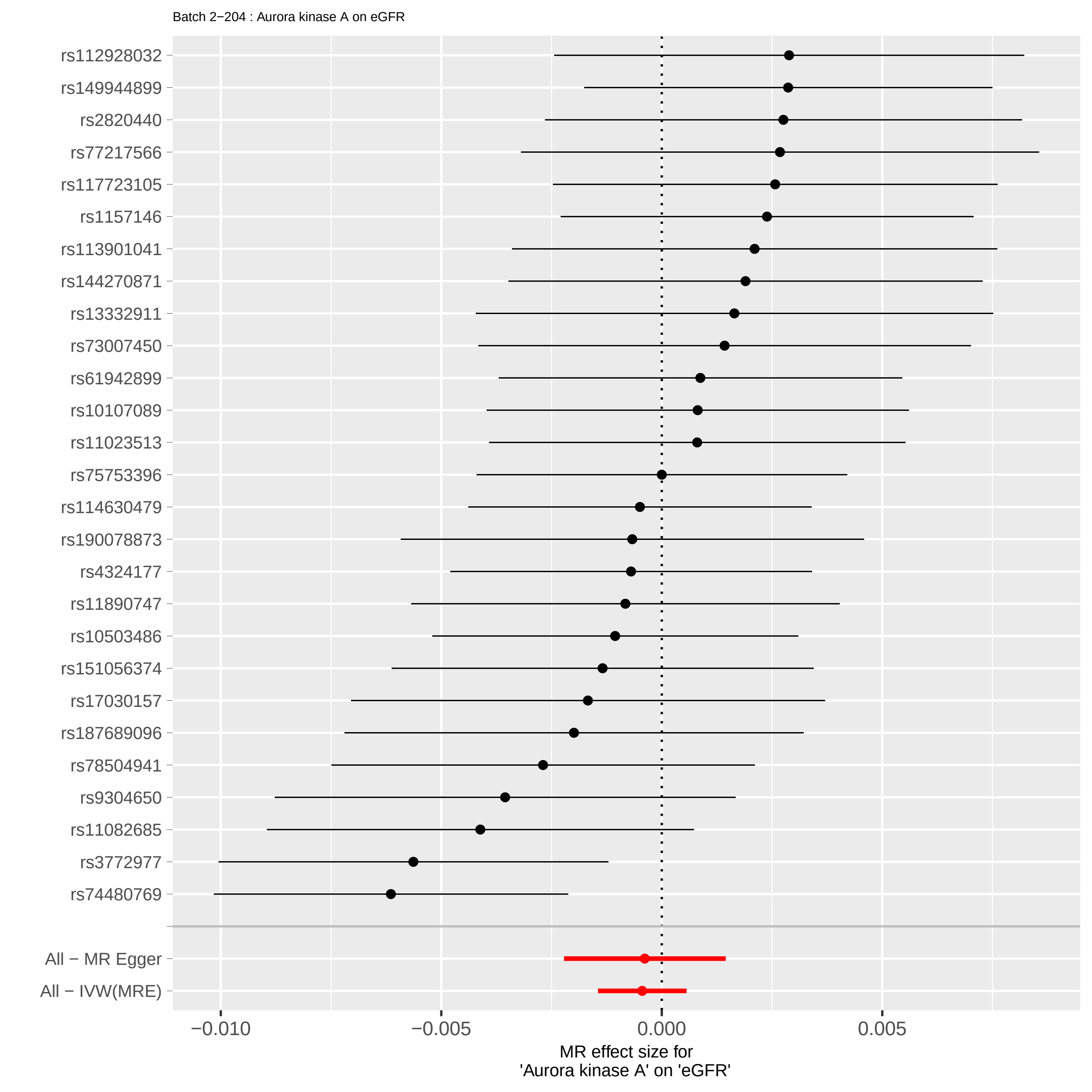

Batch 2−204 : Aurora kinase A on eGFR
rs112928032
rs149944899
rs2820440
rs77217566
rs117723105
rs1157146
rs113901041
rs144270871
rs13332911
rs73007450
rs61942899
rs10107089
rs11023513
rs75753396
rs114630479
rs190078873
rs4324177
rs11890747
rs10503486
rs151056374
rs17030157
rs187689096
rs78504941
rs9304650
rs11082685
rs3772977
rs74480769
All − MR Egger
All − IVW(MRE)
−0.010
−0.005
0.000
0.005
MR effect size for
'Aurora kinase A' on 'eGFR'

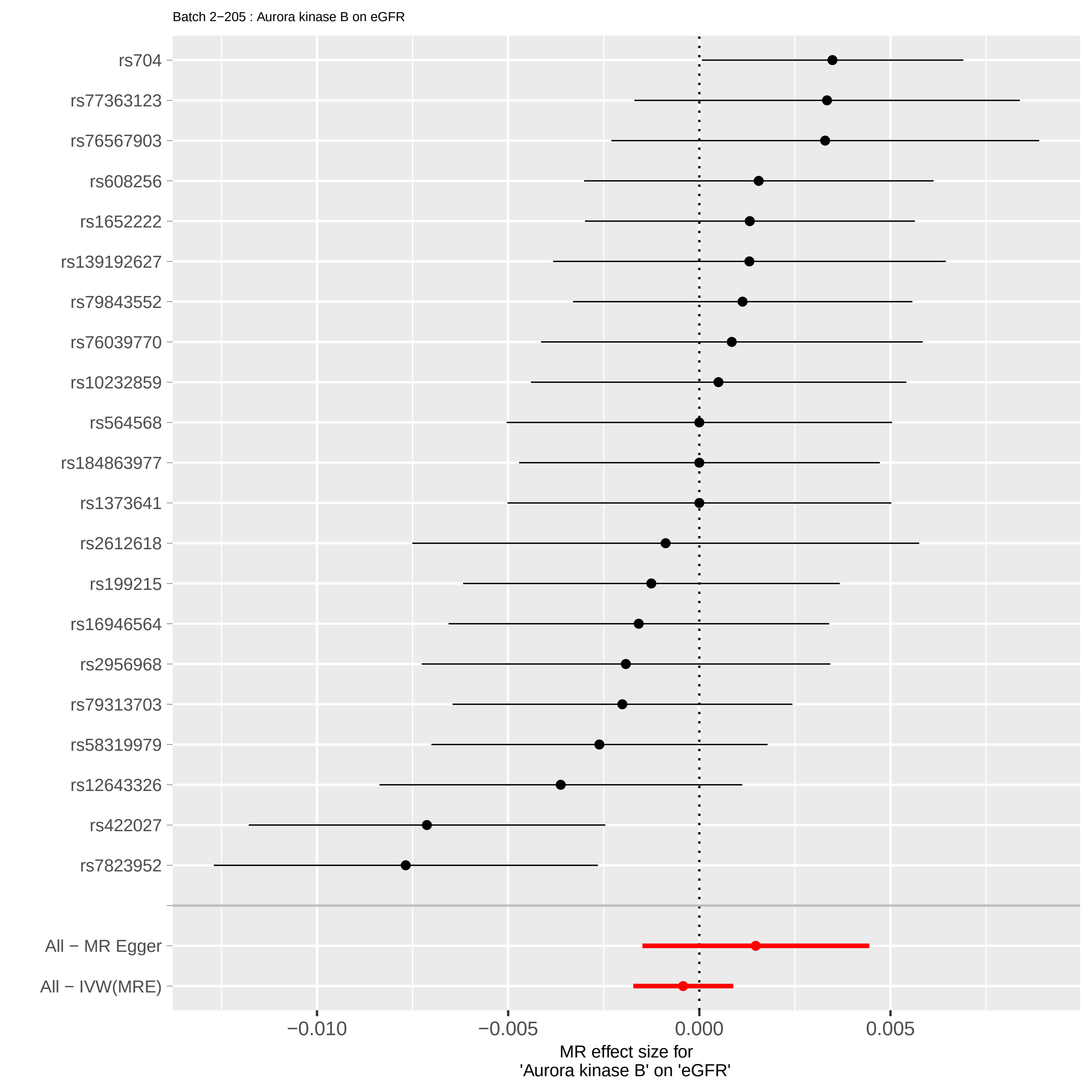
Batch 2−205 : Aurora kinase B on eGFR
rs704
rs77363123
rs76567903
rs608256
rs1652222
rs139192627
rs79843552
rs76039770
rs10232859
rs564568
rs184863977
rs1373641
rs2612618
rs199215
rs16946564
rs2956968
rs79313703
rs58319979
rs12643326
rs422027
rs7823952
All − MR Egger
All − IVW(MRE)
−0.010
−0.005
0.000
0.005
MR effect size for
'Aurora kinase B' on 'eGFR'

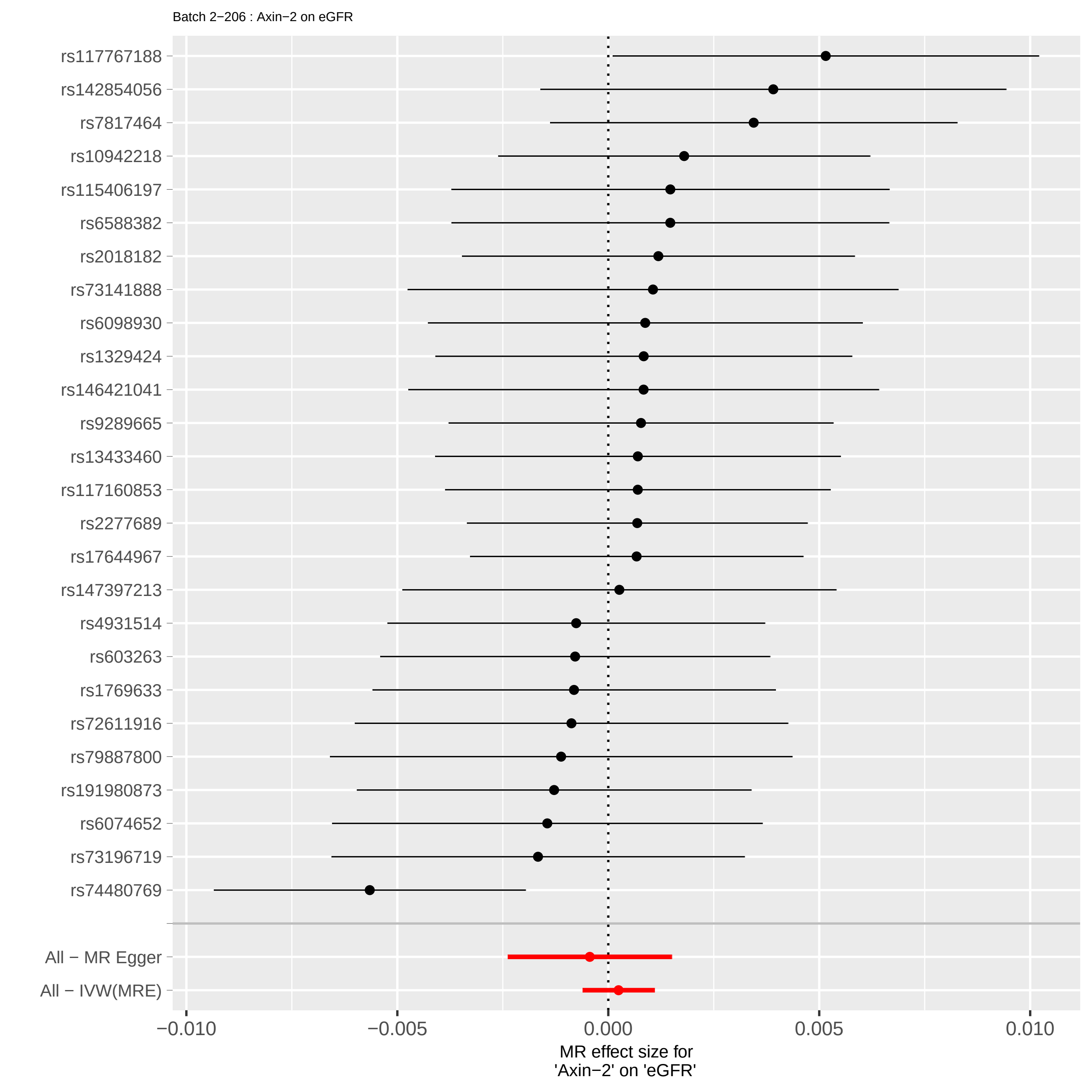

Batch 2−206 : Axin−2 on eGFR
rs117767188
rs142854056
rs7817464
rs10942218
rs115406197
rs6588382
rs2018182
rs73141888
rs6098930
rs1329424
rs146421041
rs9289665
rs13433460
rs117160853
rs2277689
rs17644967
rs147397213
rs4931514
rs603263
rs1769633
rs72611916
rs79887800
rs191980873
rs6074652
rs73196719
rs74480769
All − MR Egger
All − IVW(MRE)
−0.010
−0.005
0.000
0.005
0.010
MR effect size for
'Axin−2' on 'eGFR'

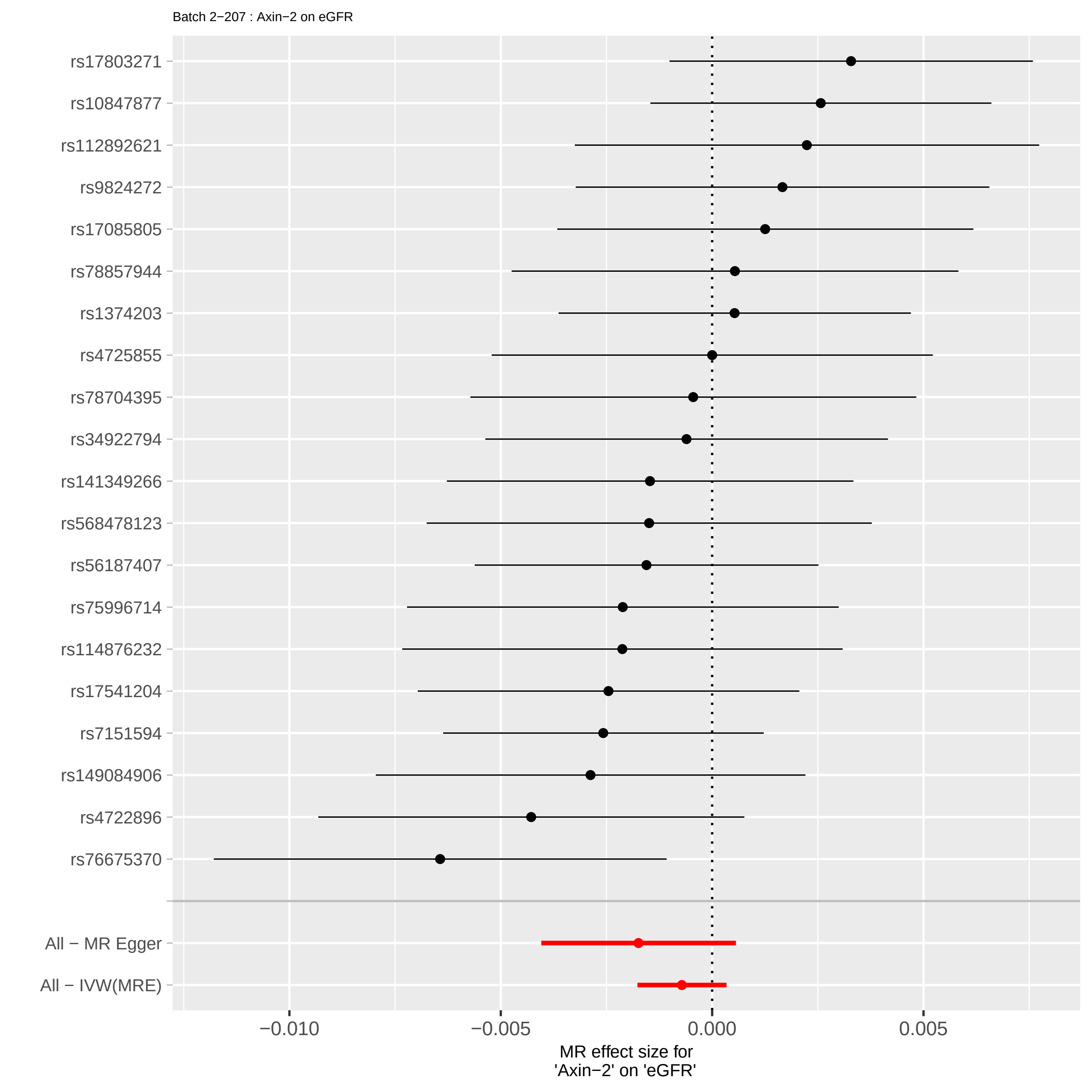
Batch 2−207 : Axin−2 on eGFR
rs17803271
rs10847877
rs112892621
rs9824272
rs17085805
rs78857944
rs1374203
rs4725855
rs78704395
rs34922794
rs141349266
rs568478123
rs56187407
rs75996714
rs114876232
rs17541204
rs7151594
rs149084906
rs4722896
rs76675370
All − MR Egger
All − IVW(MRE)
−0.010
−0.005
0.000
0.005
MR effect size for
'Axin−2' on 'eGFR'

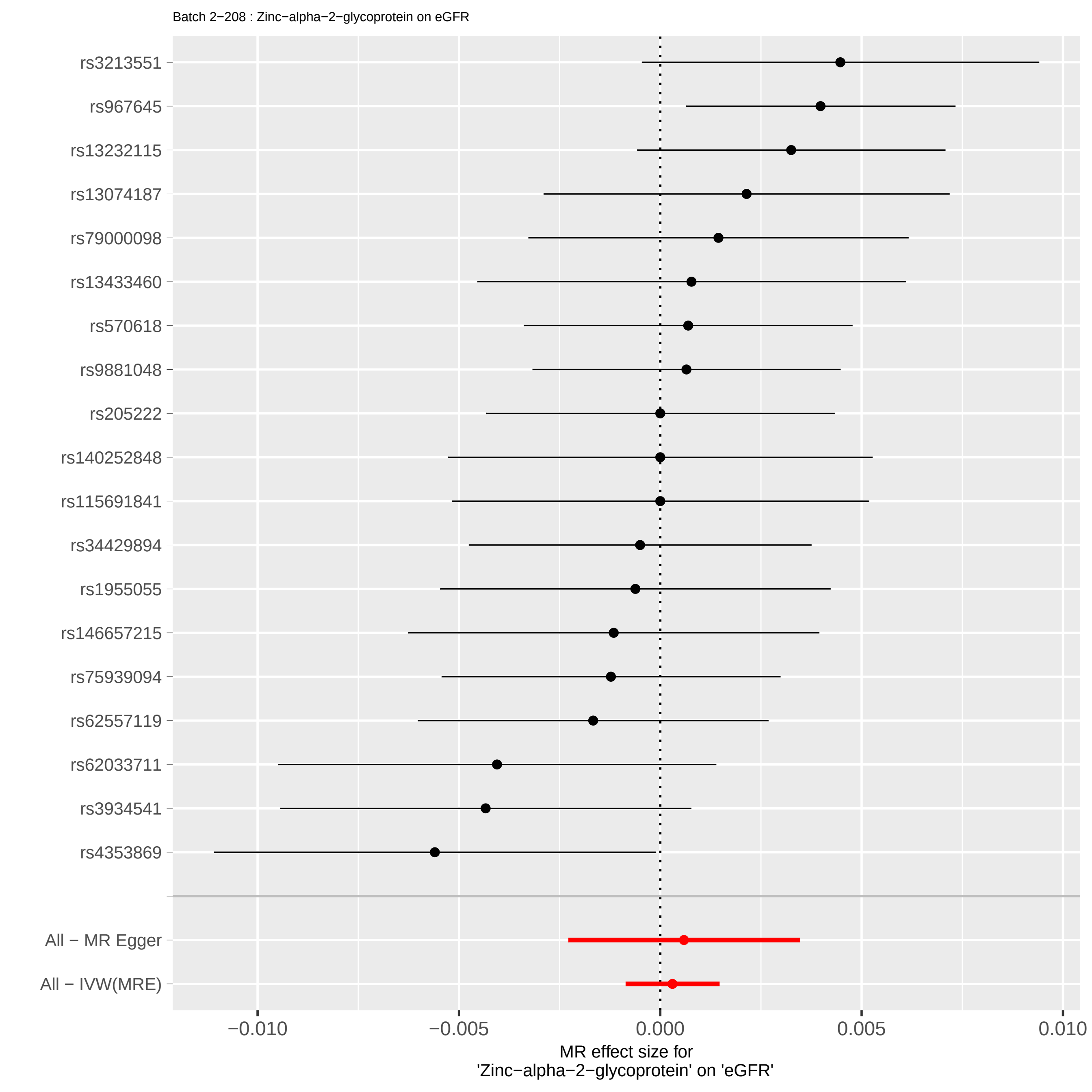
Batch 2−208 : Zinc−alpha−2−glycoprotein on eGFR
rs3213551
rs967645
rs13232115
rs13074187
rs79000098
rs13433460
rs570618
rs9881048
rs205222
rs140252848
rs115691841
rs34429894
rs1955055
rs146657215
rs75939094
rs62557119
rs62033711
rs3934541
rs4353869
All − MR Egger
All − IVW(MRE)
−0.010
−0.005
0.000
0.005
0.010
MR effect size for
'Zinc−alpha−2−glycoprotein' on 'eGFR'

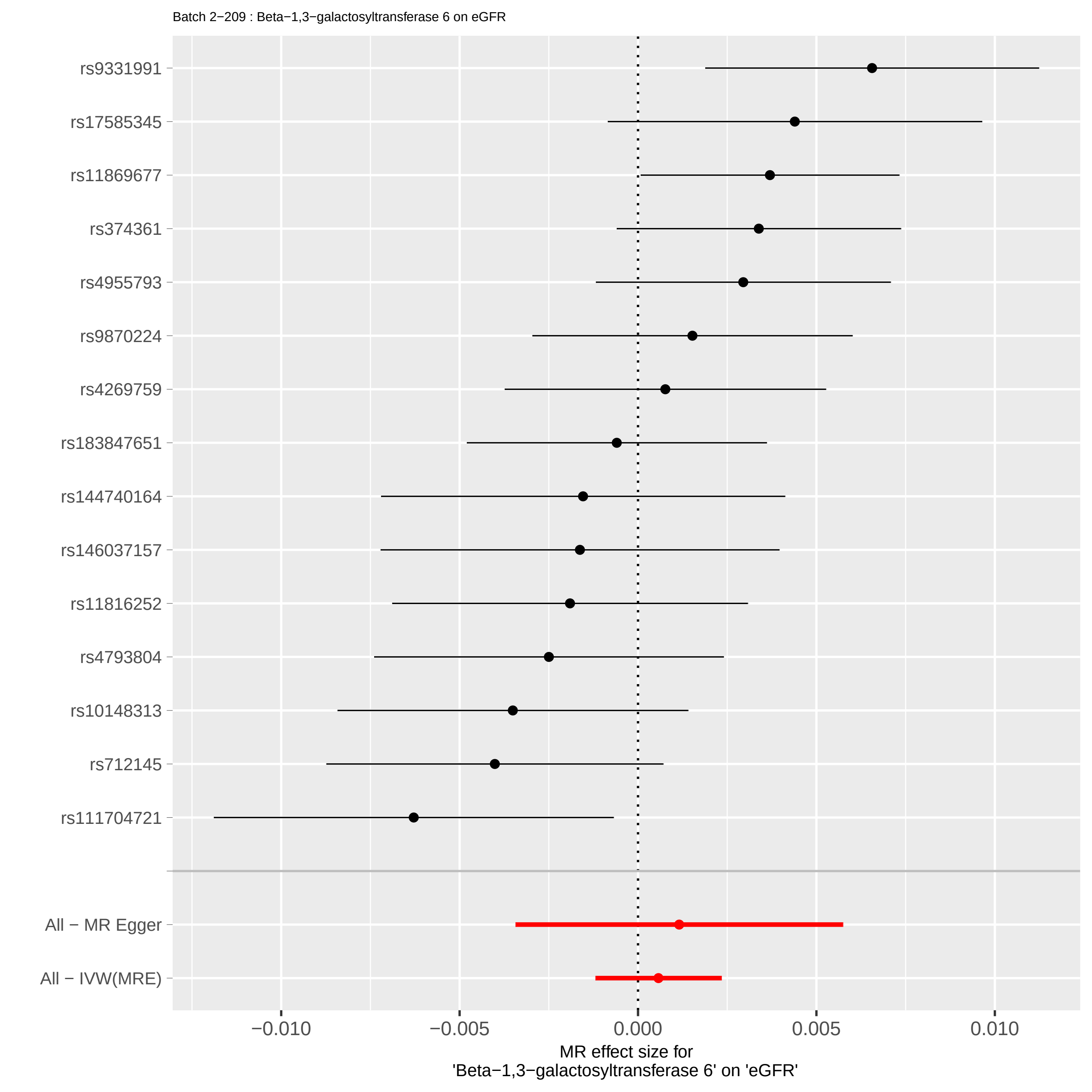
Batch 2−209 : Beta−1,3−galactosyltransferase 6 on eGFR
rs9331991
rs17585345
rs11869677
rs374361
rs4955793
rs9870224
rs4269759
rs183847651
rs144740164
rs146037157
rs11816252
rs4793804
rs10148313
rs712145
rs111704721
All − MR Egger
All − IVW(MRE)
−0.010
−0.005
0.000
0.005
0.010
MR effect size for
'Beta−1,3−galactosyltransferase 6' on 'eGFR'

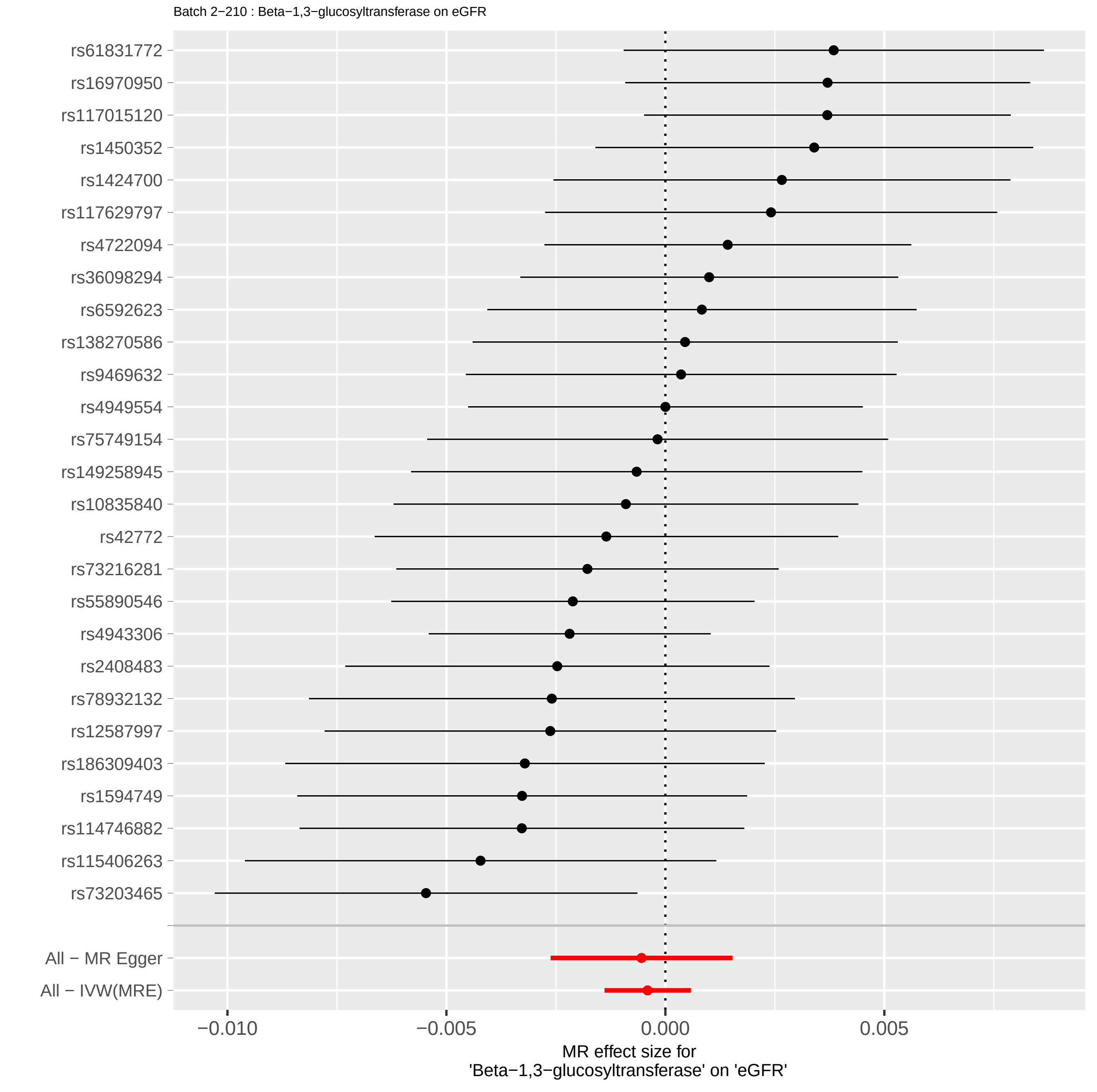

Batch 2−210 : Beta−1,3−glucosyltransferase on eGFR
rs61831772
rs16970950
rs117015120
rs1450352
rs1424700
rs117629797
rs4722094
rs36098294
rs6592623
rs138270586
rs9469632
rs4949554
rs75749154
rs149258945
rs10835840
rs42772
rs73216281
rs55890546
rs4943306
rs2408483
rs78932132
rs12587997
rs186309403
rs1594749
rs114746882
rs115406263
rs73203465
All − MR Egger
All − IVW(MRE)
−0.010
−0.005
0.000
0.005
MR effect size for
'Beta−1,3−glucosyltransferase' on 'eGFR'

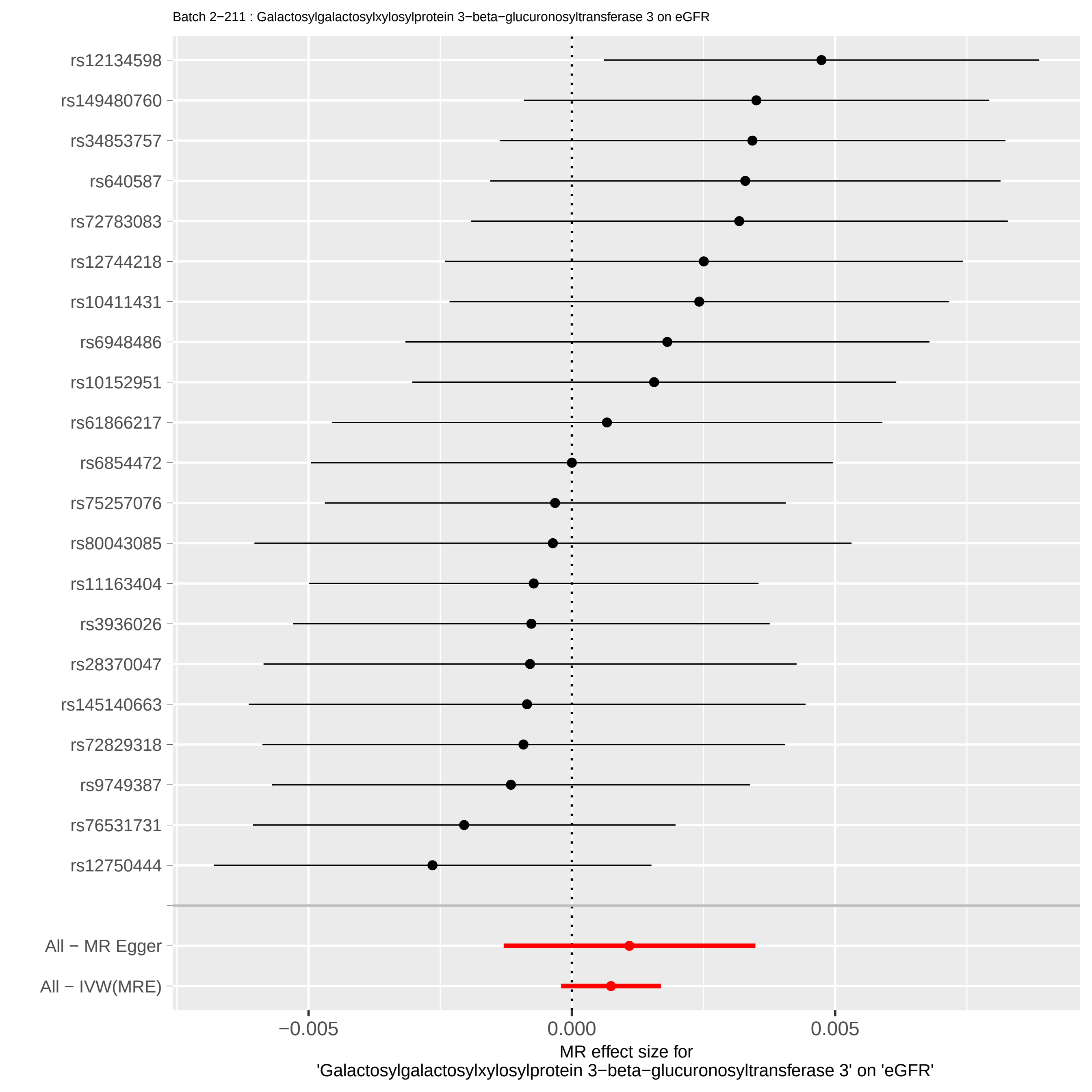

Batch 2−211 : Galactosylgalactosylxylosylprotein 3−beta−glucuronosyltransferase 3 on eGFR
rs12134598
rs149480760
rs34853757
rs640587
rs72783083
rs12744218
rs10411431
rs6948486
rs10152951
rs61866217
rs6854472
rs75257076
rs80043085
rs11163404
rs3936026
rs28370047
rs145140663
rs72829318
rs9749387
rs76531731
rs12750444
All − MR Egger
All − IVW(MRE)
−0.005
0.000
0.005
MR effect size for
'Galactosylgalactosylxylosylprotein 3−beta−glucuronosyltransferase 3' on 'eGFR'

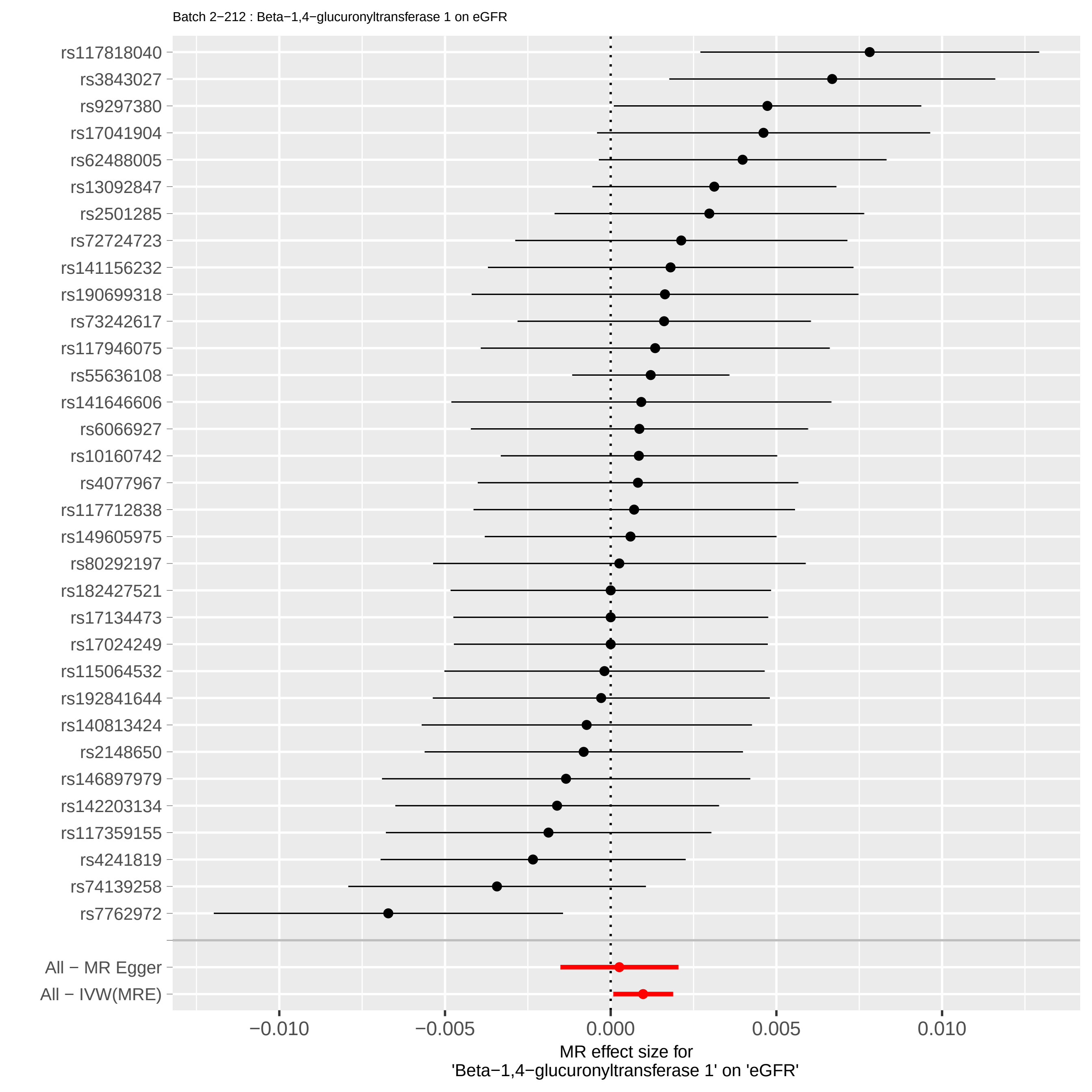
Batch 2−212 : Beta−1,4−glucuronyltransferase 1 on eGFR
rs117818040
rs3843027
rs9297380
rs17041904
rs62488005
rs13092847
rs2501285
rs72724723
rs141156232
rs190699318
rs73242617
rs117946075
rs55636108
rs141646606
rs6066927
rs10160742
rs4077967
rs117712838
rs149605975
rs80292197
rs182427521
rs17134473
rs17024249
rs115064532
rs192841644
rs140813424
rs2148650
rs146897979
rs142203134
rs117359155
rs4241819
rs74139258
rs7762972
All − MR Egger
All − IVW(MRE)
−0.010
−0.005
0.000
0.005
0.010
MR effect size for
'Beta−1,4−glucuronyltransferase 1' on 'eGFR'

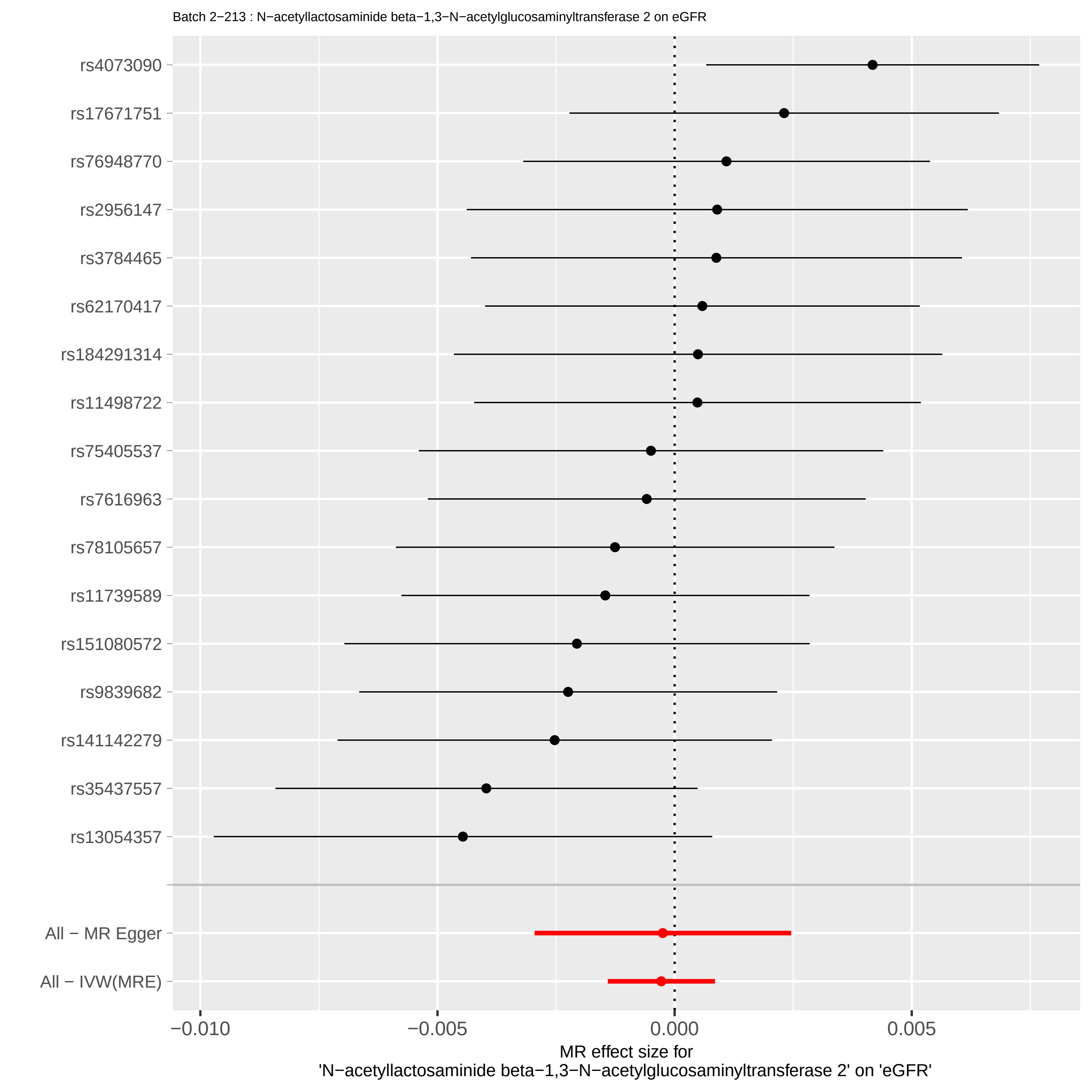

Batch 2−213 : N−acetyllactosaminide beta−1,3−N−acetylglucosaminyltransferase 2 on eGFR
rs4073090
rs17671751
rs76948770
rs2956147
rs3784465
rs62170417
rs184291314
rs11498722
rs75405537
rs7616963
rs78105657
rs11739589
rs151080572
rs9839682
rs141142279
rs35437557
rs13054357
All − MR Egger
All − IVW(MRE)
−0.010
−0.005
0.000
0.005
MR effect size for
'N−acetyllactosaminide beta−1,3−N−acetylglucosaminyltransferase 2' on 'eGFR'

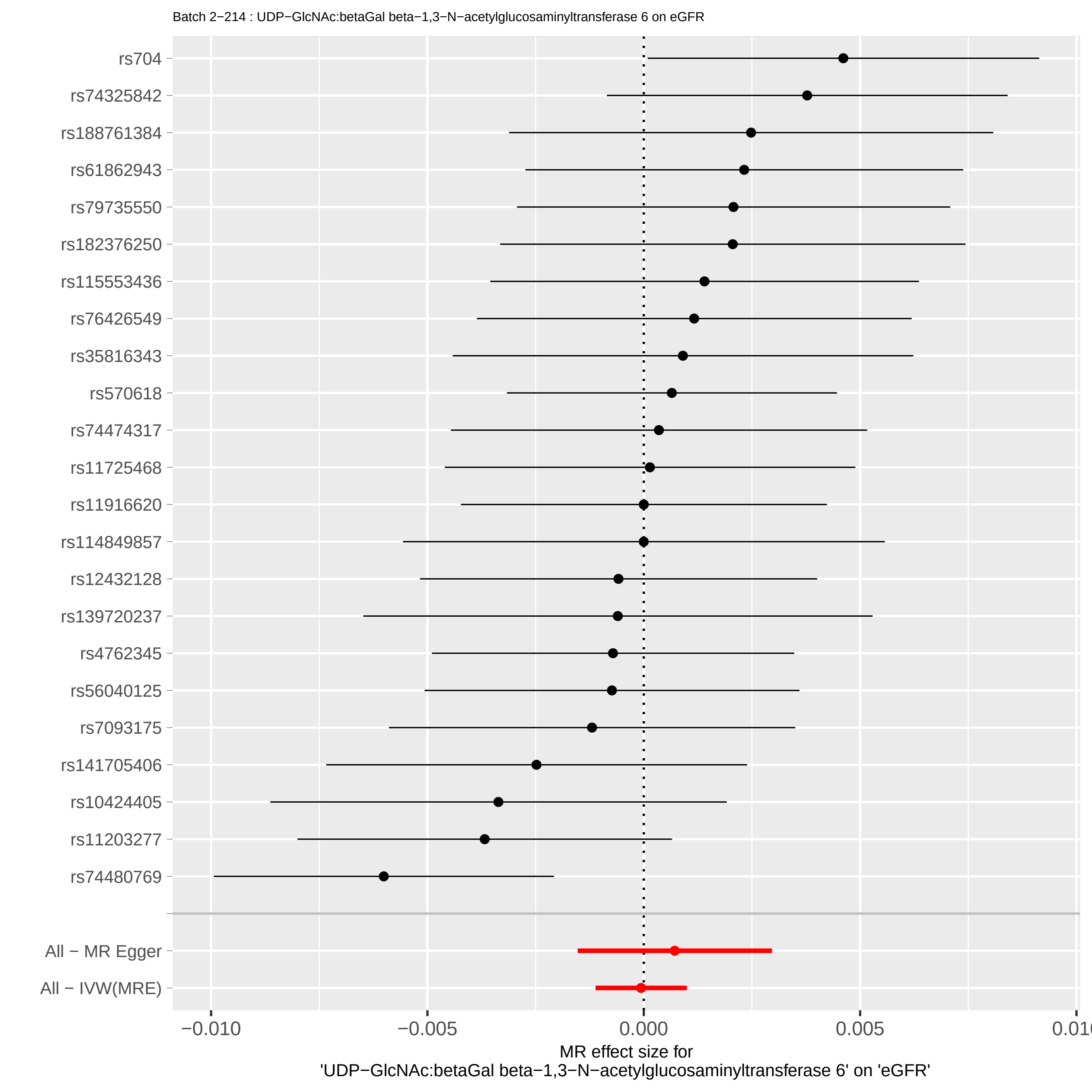

Batch 2−214 : UDP−GlcNAc:betaGal beta−1,3−N−acetylglucosaminyltransferase 6 on eGFR
rs704
rs74325842
rs188761384
rs61862943
rs79735550
rs182376250
rs115553436
rs76426549
rs35816343
rs570618
rs74474317
rs11725468
rs11916620
rs114849857
rs12432128
rs139720237
rs4762345
rs56040125
rs7093175
rs141705406
rs10424405
rs11203277
rs74480769
All − MR Egger
All − IVW(MRE)
−0.010
−0.005
0.000
0.005
MR effect size for
'UDP−GlcNAc:betaGal beta−1,3−N−acetylglucosaminyltransferase 6' on 'eGFR'

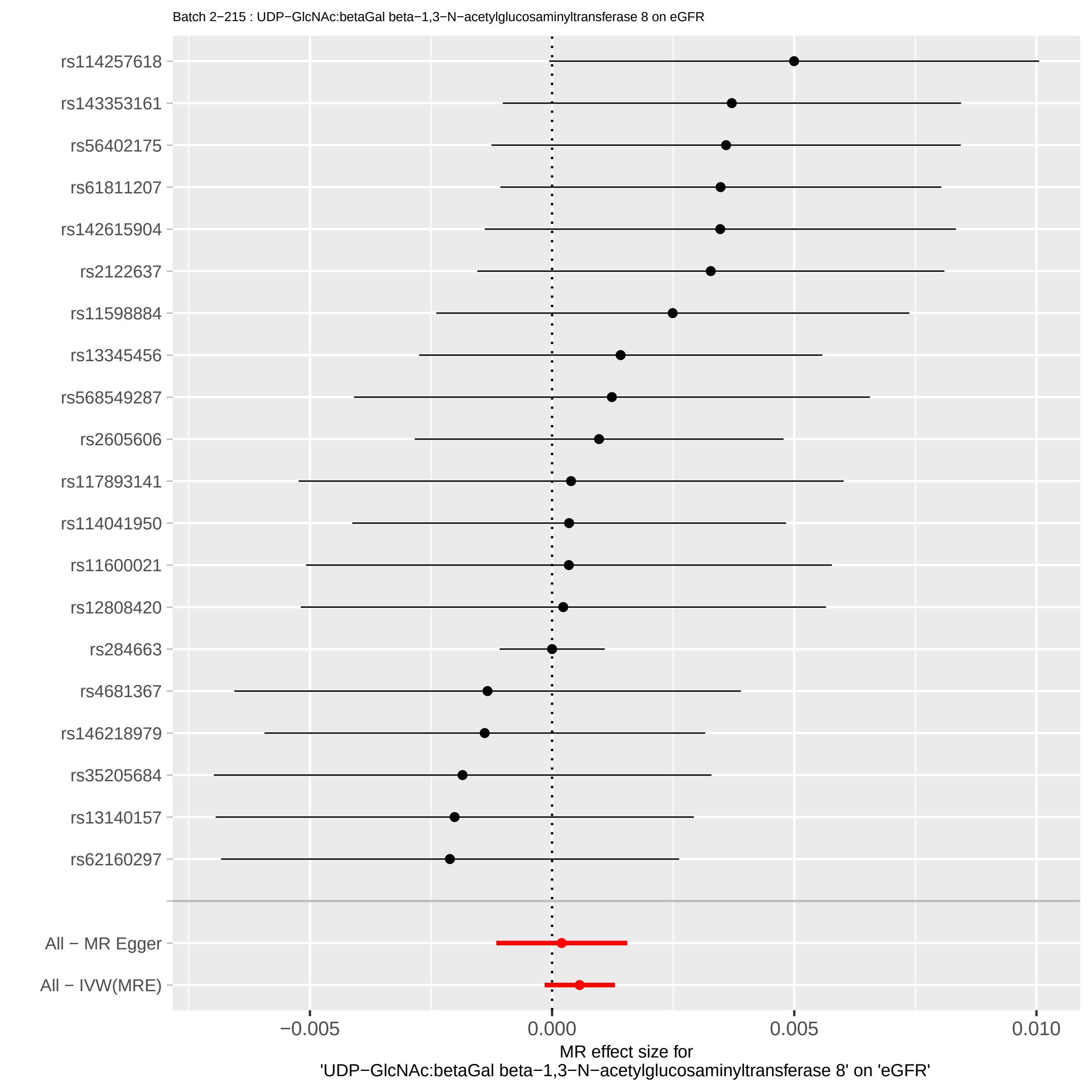

Batch 2−215 : UDP−GlcNAc:betaGal beta−1,3−N−acetylglucosaminyltransferase 8 on eGFR
rs114257618
rs143353161
rs56402175
rs61811207
rs142615904
rs2122637
rs11598884
rs13345456
rs568549287
rs2605606
rs117893141
rs114041950
rs11600021
rs12808420
rs284663
rs4681367
rs146218979
rs35205684
rs13140157
rs62160297
All − MR Egger
All − IVW(MRE)
−0.005
0.000
0.005
0.010
MR effect size for
'UDP−GlcNAc:betaGal beta−1,3−N−acetylglucosaminyltransferase 8' on 'eGFR'

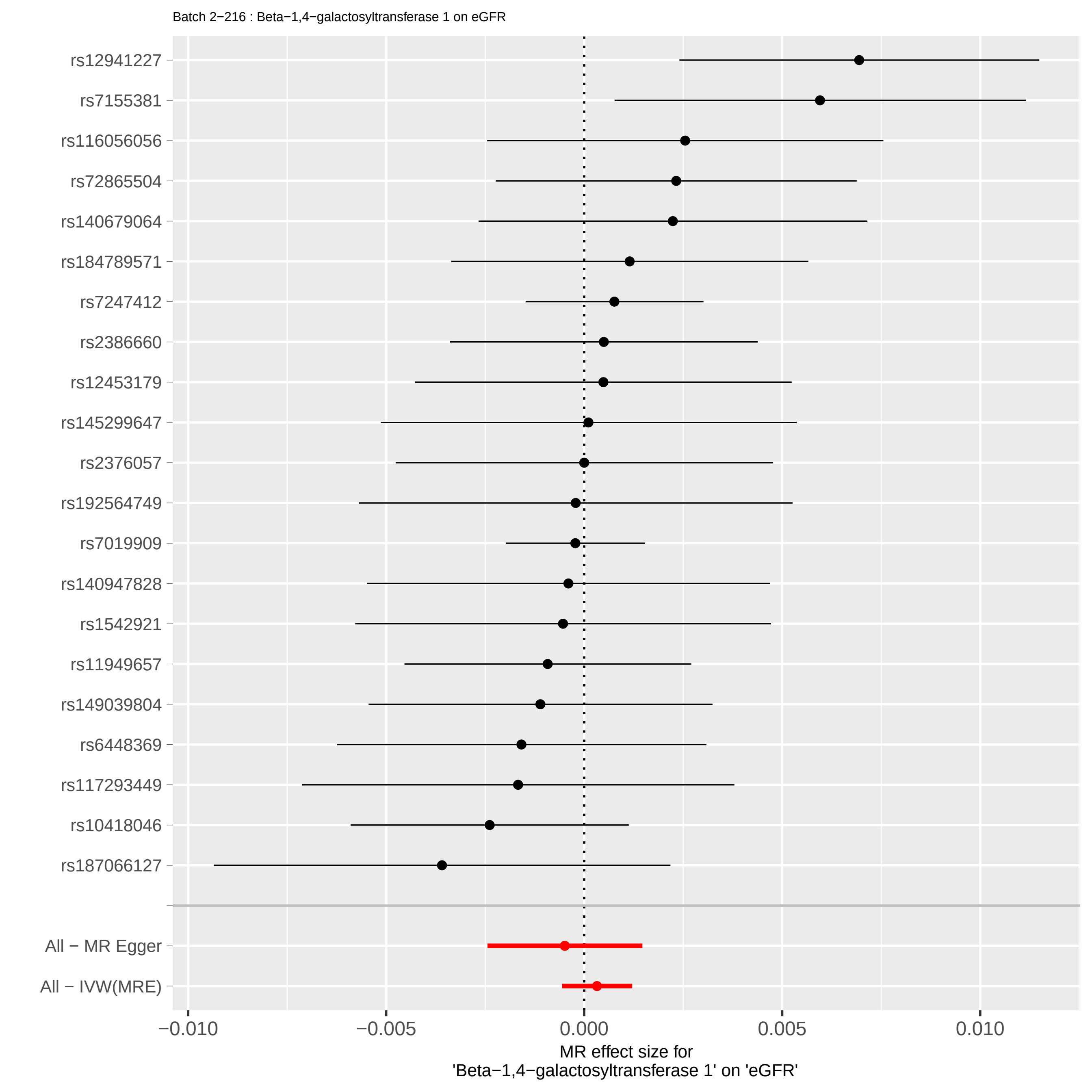

Batch 2−216 : Beta−1,4−galactosyltransferase 1 on eGFR
rs12941227
rs7155381
rs116056056
rs72865504
rs140679064
rs184789571
rs7247412
rs2386660
rs12453179
rs145299647
rs2376057
rs192564749
rs7019909
rs140947828
rs1542921
rs11949657
rs149039804
rs6448369
rs117293449
rs10418046
rs187066127
All − MR Egger
All − IVW(MRE)
−0.010
−0.005
0.000
0.005
0.010
MR effect size for
'Beta−1,4−galactosyltransferase 1' on 'eGFR'

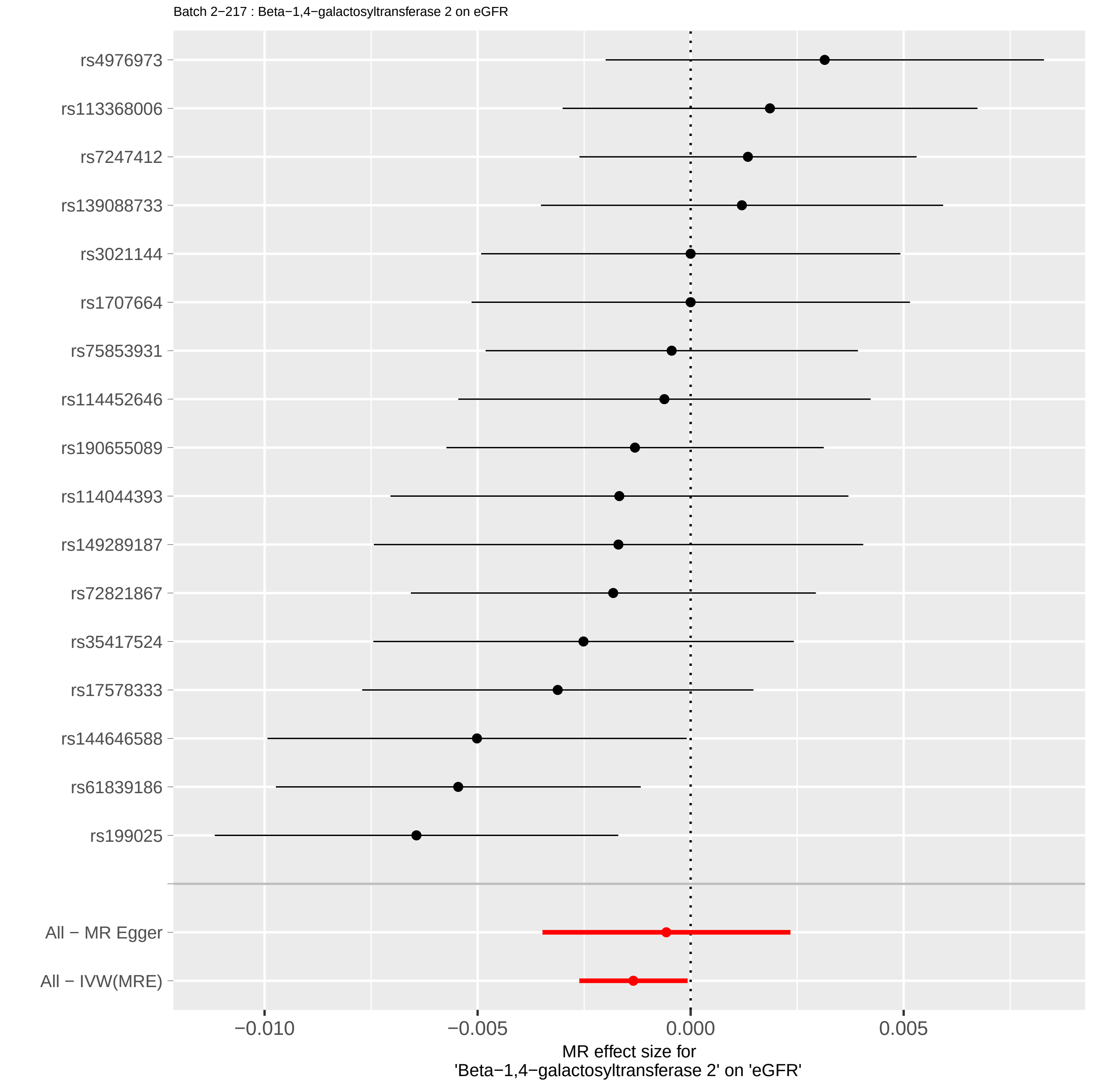
Batch 2−217 : Beta−1,4−galactosyltransferase 2 on eGFR
rs4976973
rs113368006
rs7247412
rs139088733
rs3021144
rs1707664
rs75853931
rs114452646
rs190655089
rs114044393
rs149289187
rs72821867
rs35417524
rs17578333
rs144646588
rs61839186
rs199025
All − MR Egger
All − IVW(MRE)
−0.010
−0.005
0.000
0.005
MR effect size for
'Beta−1,4−galactosyltransferase 2' on 'eGFR'

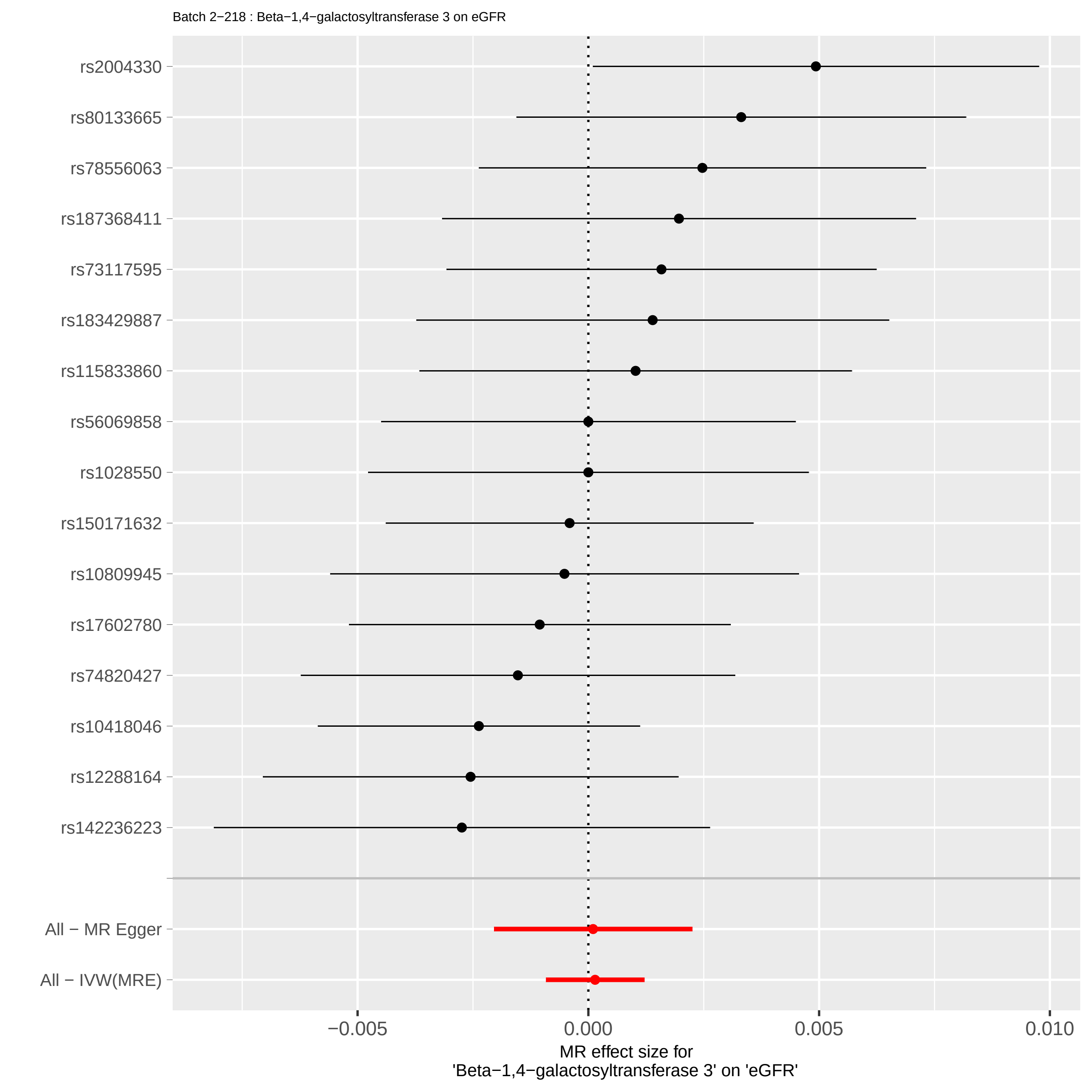

Batch 2−218 : Beta−1,4−galactosyltransferase 3 on eGFR
rs2004330
rs80133665
rs78556063
rs187368411
rs73117595
rs183429887
rs115833860
rs56069858
rs1028550
rs150171632
rs10809945
rs17602780
rs74820427
rs10418046
rs12288164
rs142236223
All − MR Egger
All − IVW(MRE)
−0.005
0.000
0.005
0.010
MR effect size for
'Beta−1,4−galactosyltransferase 3' on 'eGFR'

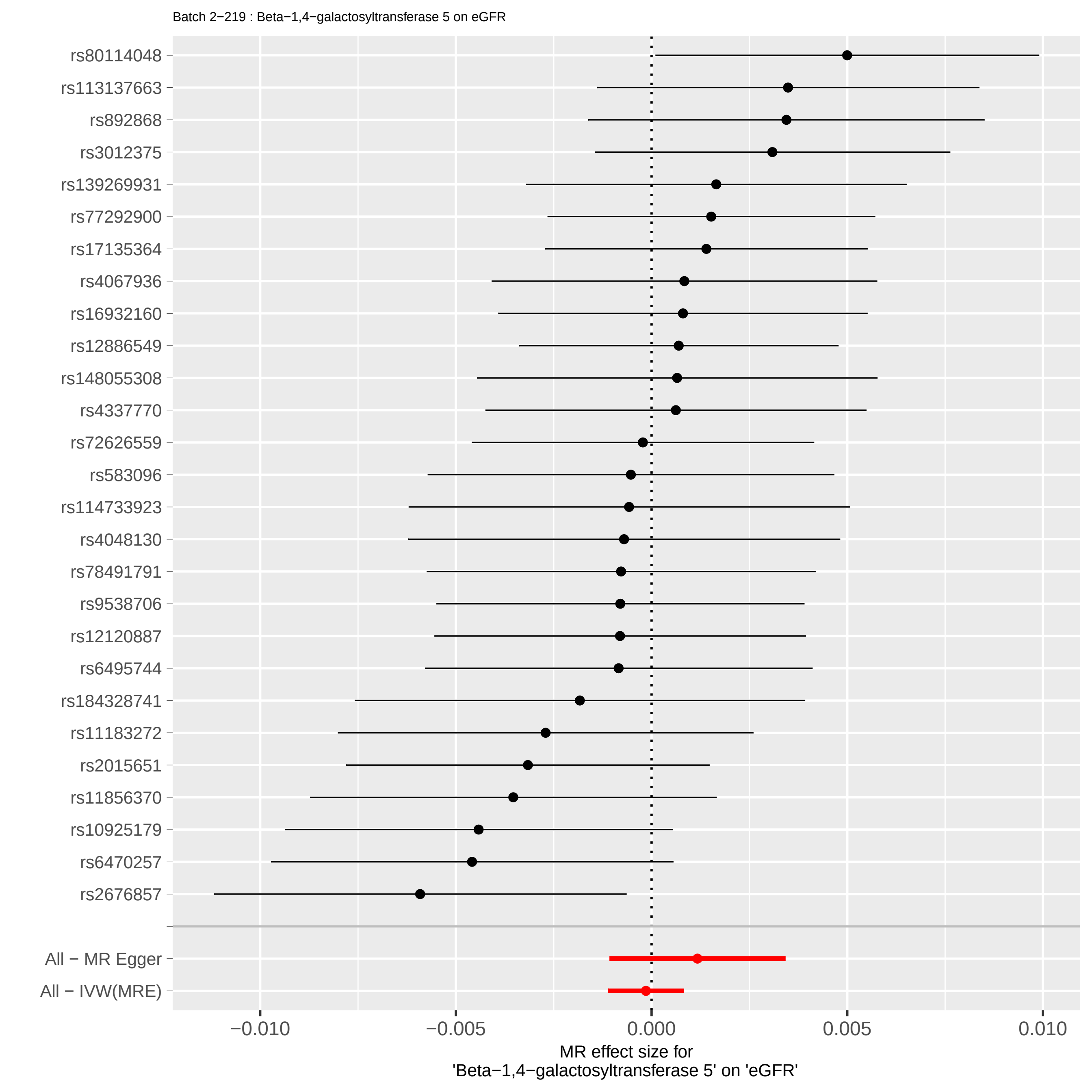

Batch 2−219 : Beta−1,4−galactosyltransferase 5 on eGFR
rs80114048
rs113137663
rs892868
rs3012375
rs139269931
rs77292900
rs17135364
rs4067936
rs16932160
rs12886549
rs148055308
rs4337770
rs72626559
rs583096
rs114733923
rs4048130
rs78491791
rs9538706
rs12120887
rs6495744
rs184328741
rs11183272
rs2015651
rs11856370
rs10925179
rs6470257
rs2676857
All − MR Egger
All − IVW(MRE)
−0.010
−0.005
0.000
0.005
0.010
MR effect size for
'Beta−1,4−galactosyltransferase 5' on 'eGFR'

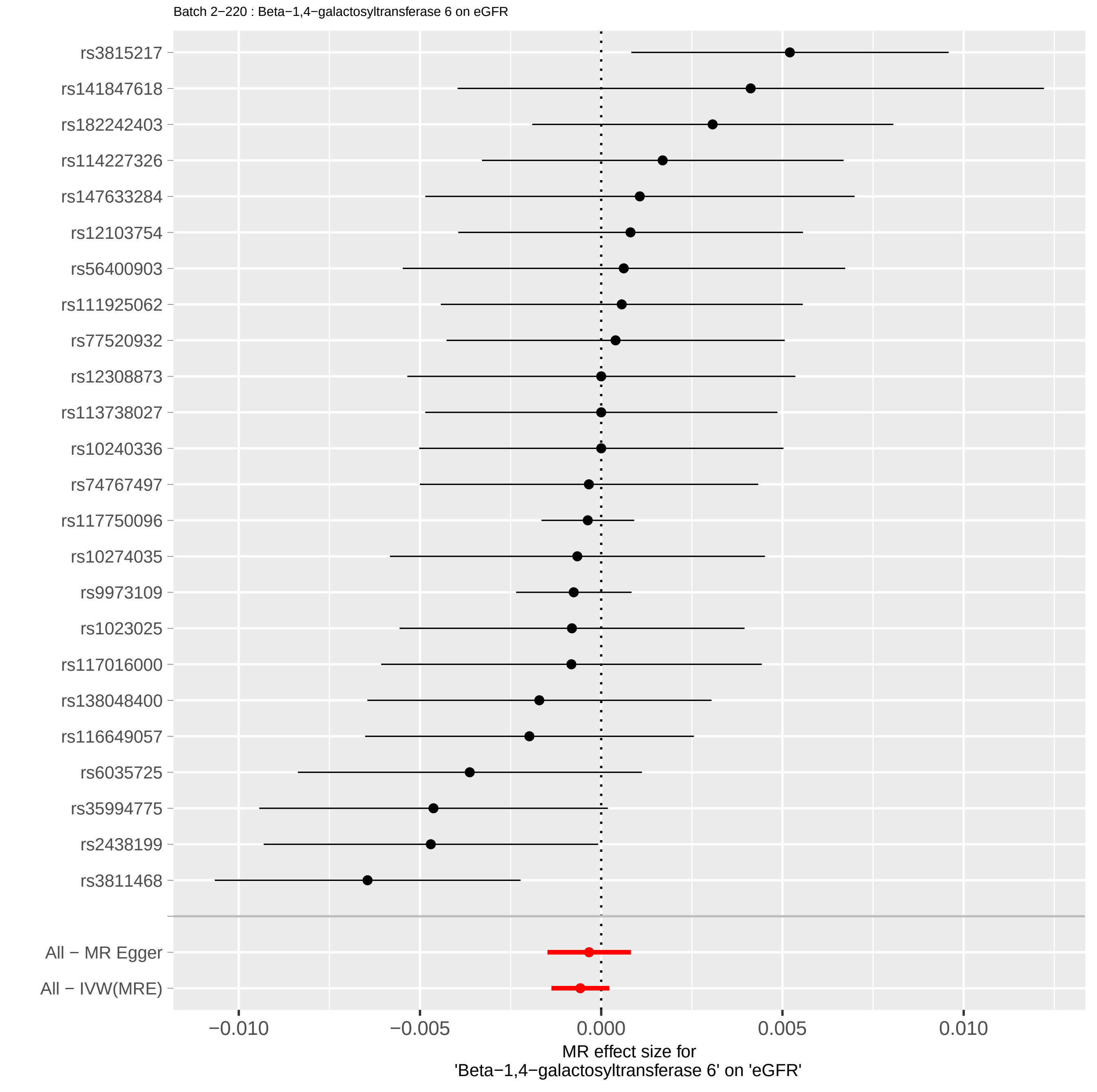

Batch 2−220 : Beta−1,4−galactosyltransferase 6 on eGFR
rs3815217
rs141847618
rs182242403
rs114227326
rs147633284
rs12103754
rs56400903
rs111925062
rs77520932
rs12308873
rs113738027
rs10240336
rs74767497
rs117750096
rs10274035
rs9973109
rs1023025
rs117016000
rs138048400
rs116649057
rs6035725
rs35994775
rs2438199
rs3811468
All − MR Egger
All − IVW(MRE)
−0.010
−0.005
0.000
0.005
0.010
MR effect size for
'Beta−1,4−galactosyltransferase 6' on 'eGFR'

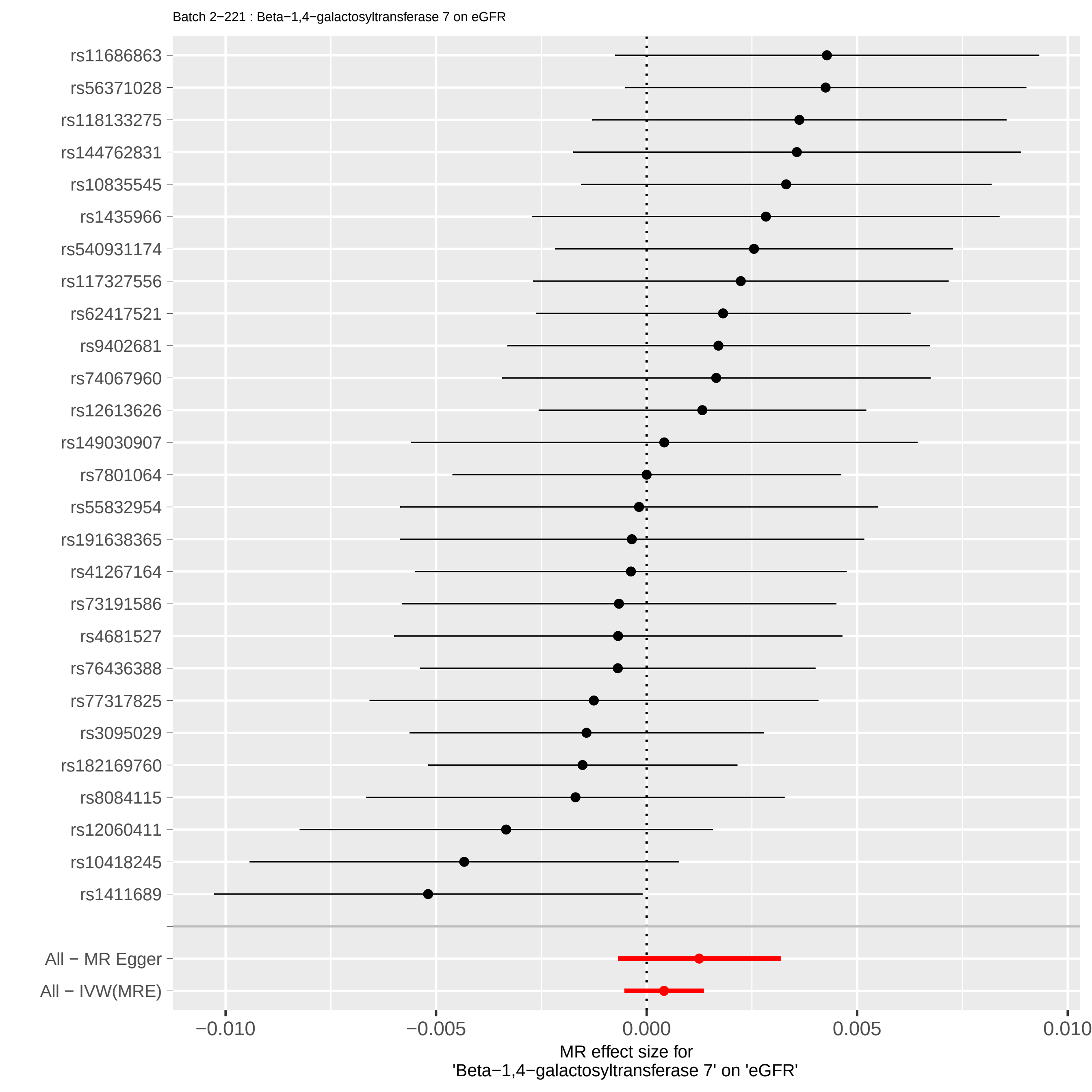
Batch 2−221 : Beta−1,4−galactosyltransferase 7 on eGFR
rs11686863
rs56371028
rs118133275
rs144762831
rs10835545
rs1435966
rs540931174
rs117327556
rs62417521
rs9402681
rs74067960
rs12613626
rs149030907
rs7801064
rs55832954
rs191638365
rs41267164
rs73191586
rs4681527
rs76436388
rs77317825
rs3095029
rs182169760
rs8084115
rs12060411
rs10418245
rs1411689
All − MR Egger
All − IVW(MRE)
−0.010
−0.005
0.000
0.005
0.010
MR effect size for
'Beta−1,4−galactosyltransferase 7' on 'eGFR'

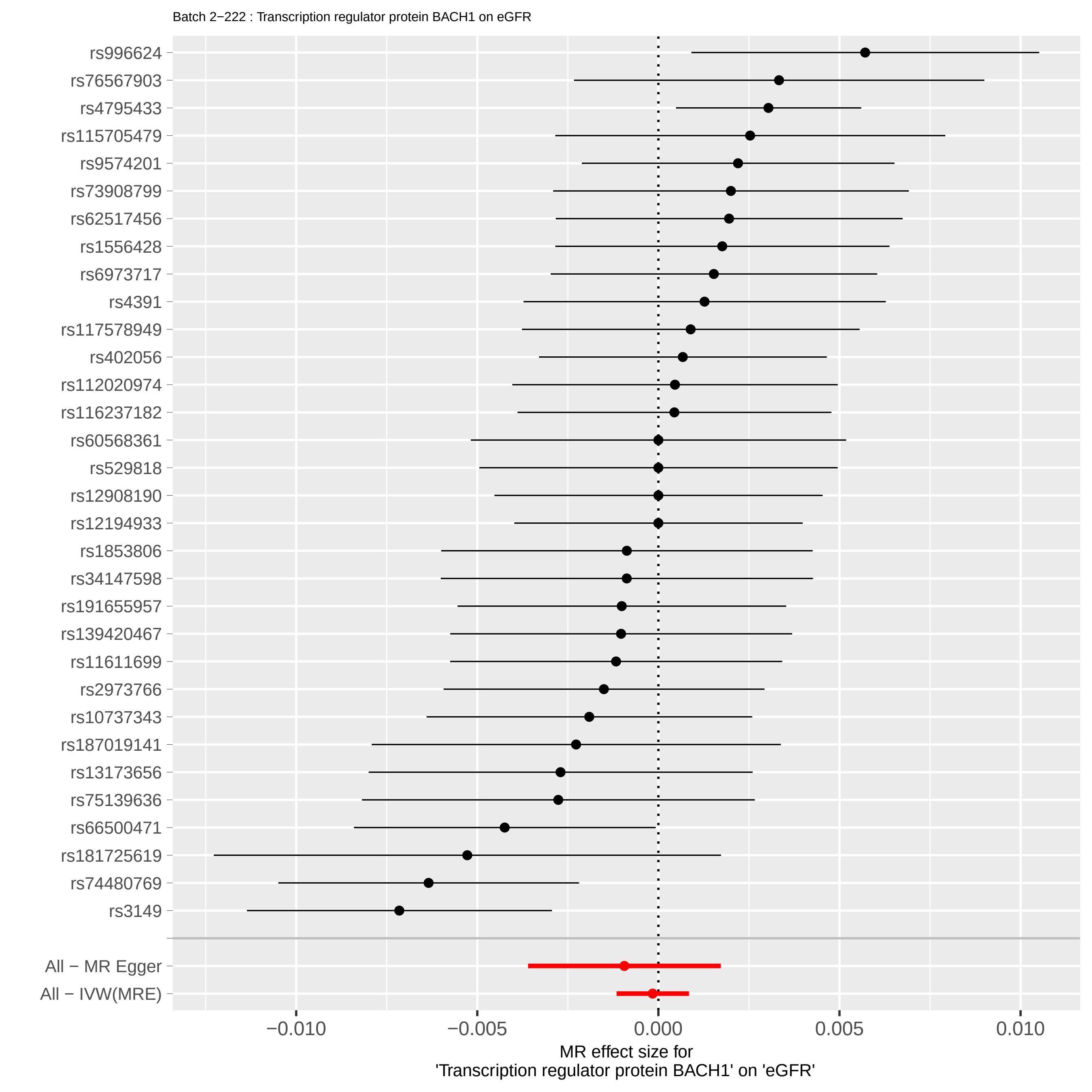

Batch 2−222 : Transcription regulator protein BACH1 on eGFR
rs996624
rs76567903
rs4795433
rs115705479
rs9574201
rs73908799
rs62517456
rs1556428
rs6973717
rs4391
rs117578949
rs402056
rs112020974
rs116237182
rs60568361
rs529818
rs12908190
rs12194933
rs1853806
rs34147598
rs191655957
rs139420467
rs11611699
rs2973766
rs10737343
rs187019141
rs13173656
rs75139636
rs66500471
rs181725619
rs74480769
rs3149
All − MR Egger
All − IVW(MRE)
−0.010
−0.005
0.000
0.005
0.010
MR effect size for
'Transcription regulator protein BACH1' on 'eGFR'

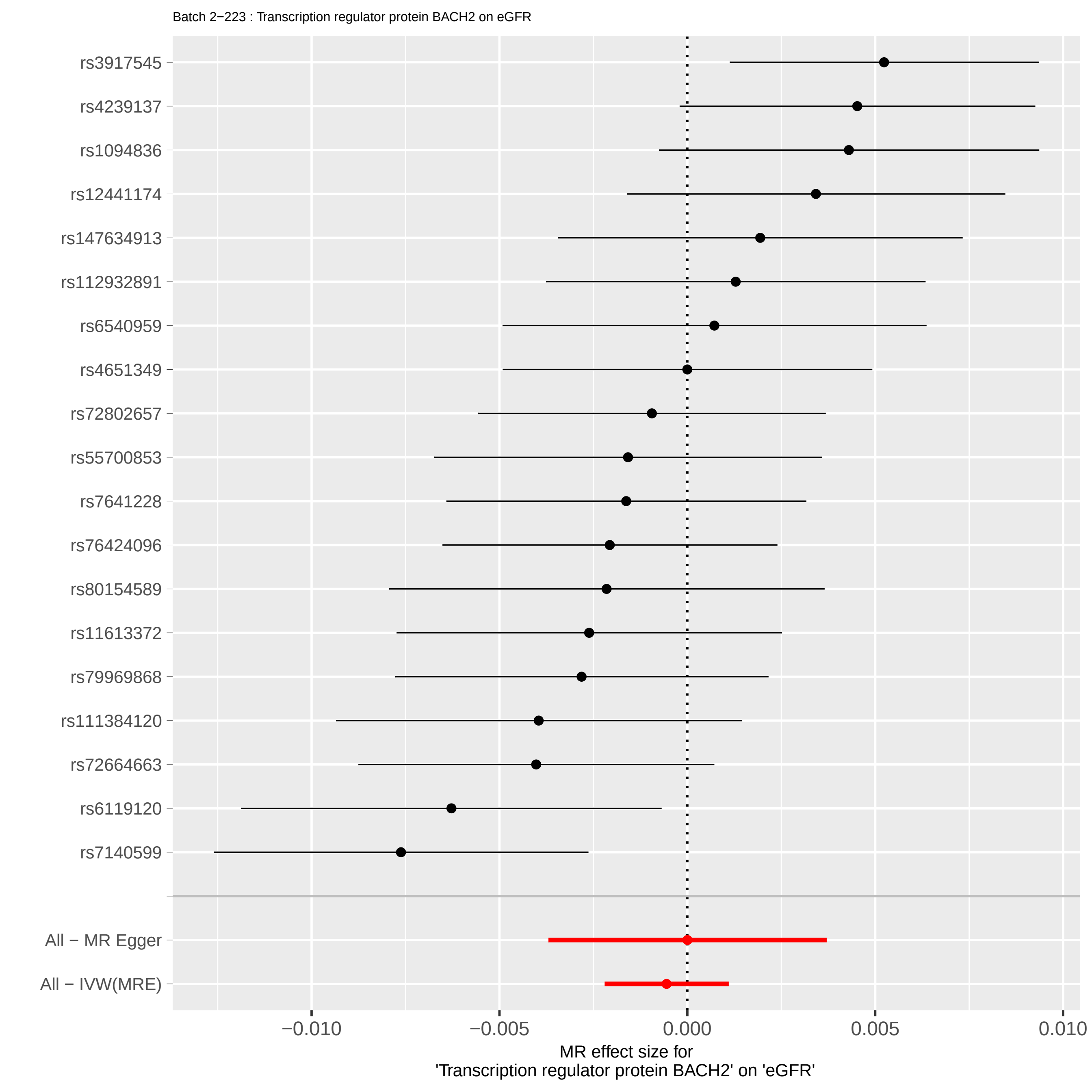
Batch 2−223 : Transcription regulator protein BACH2 on eGFR
rs3917545
rs4239137
rs1094836
rs12441174
rs147634913
rs112932891
rs6540959
rs4651349
rs72802657
rs55700853
rs7641228
rs76424096
rs80154589
rs11613372
rs79969868
rs111384120
rs72664663
rs6119120
rs7140599
All − MR Egger
All − IVW(MRE)
−0.010
−0.005
0.000
0.005
0.010
MR effect size for
'Transcription regulator protein BACH2' on 'eGFR'

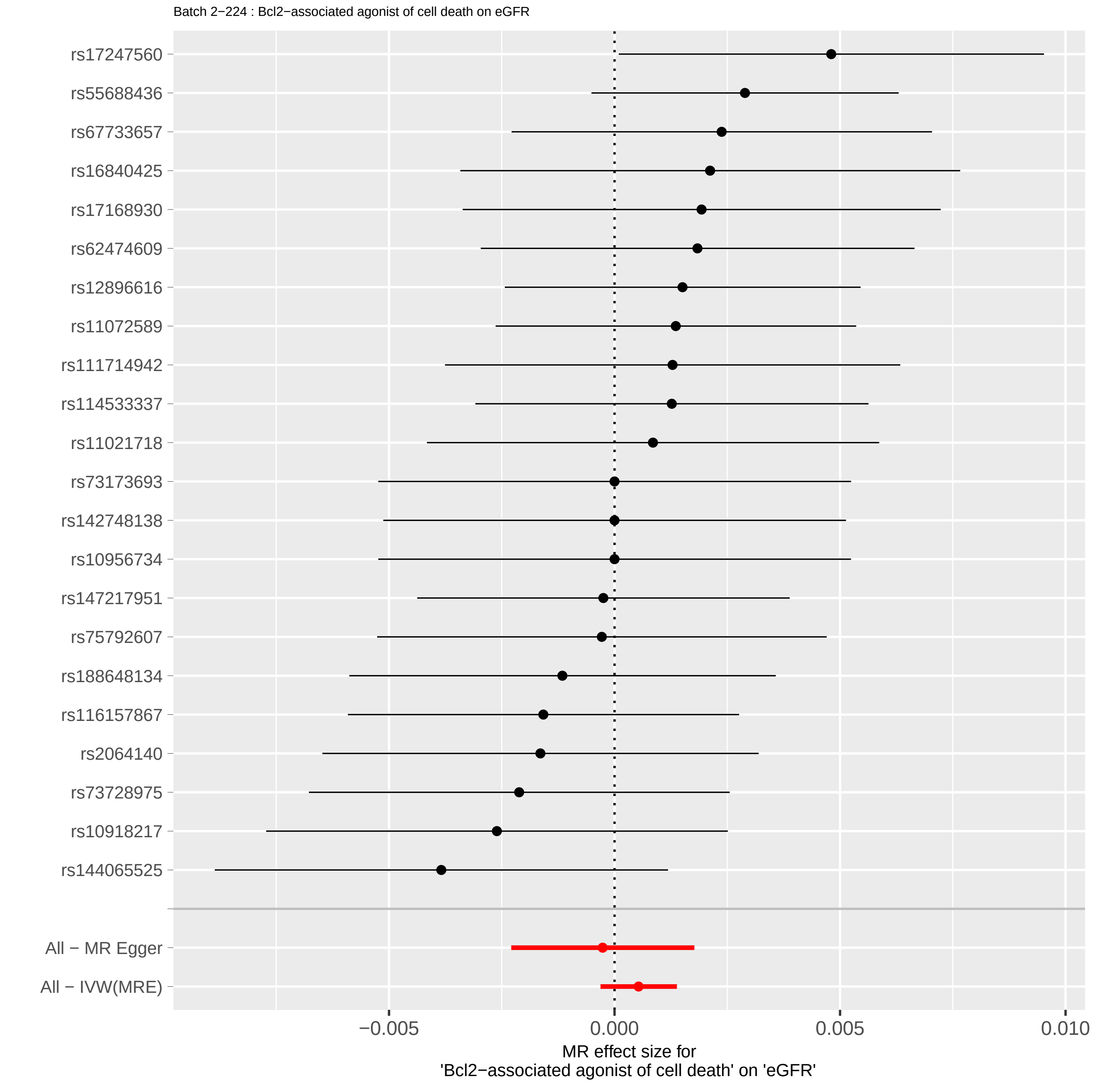

Batch 2−224 : Bcl2−associated agonist of cell death on eGFR
rs17247560
rs55688436
rs67733657
rs16840425
rs17168930
rs62474609
rs12896616
rs11072589
rs111714942
rs114533337
rs11021718
rs73173693
rs142748138
rs10956734
rs147217951
rs75792607
rs188648134
rs116157867
rs2064140
rs73728975
rs10918217
rs144065525
All − MR Egger
All − IVW(MRE)
−0.005
0.000
0.005
0.010
MR effect size for
'Bcl2−associated agonist of cell death' on 'eGFR'

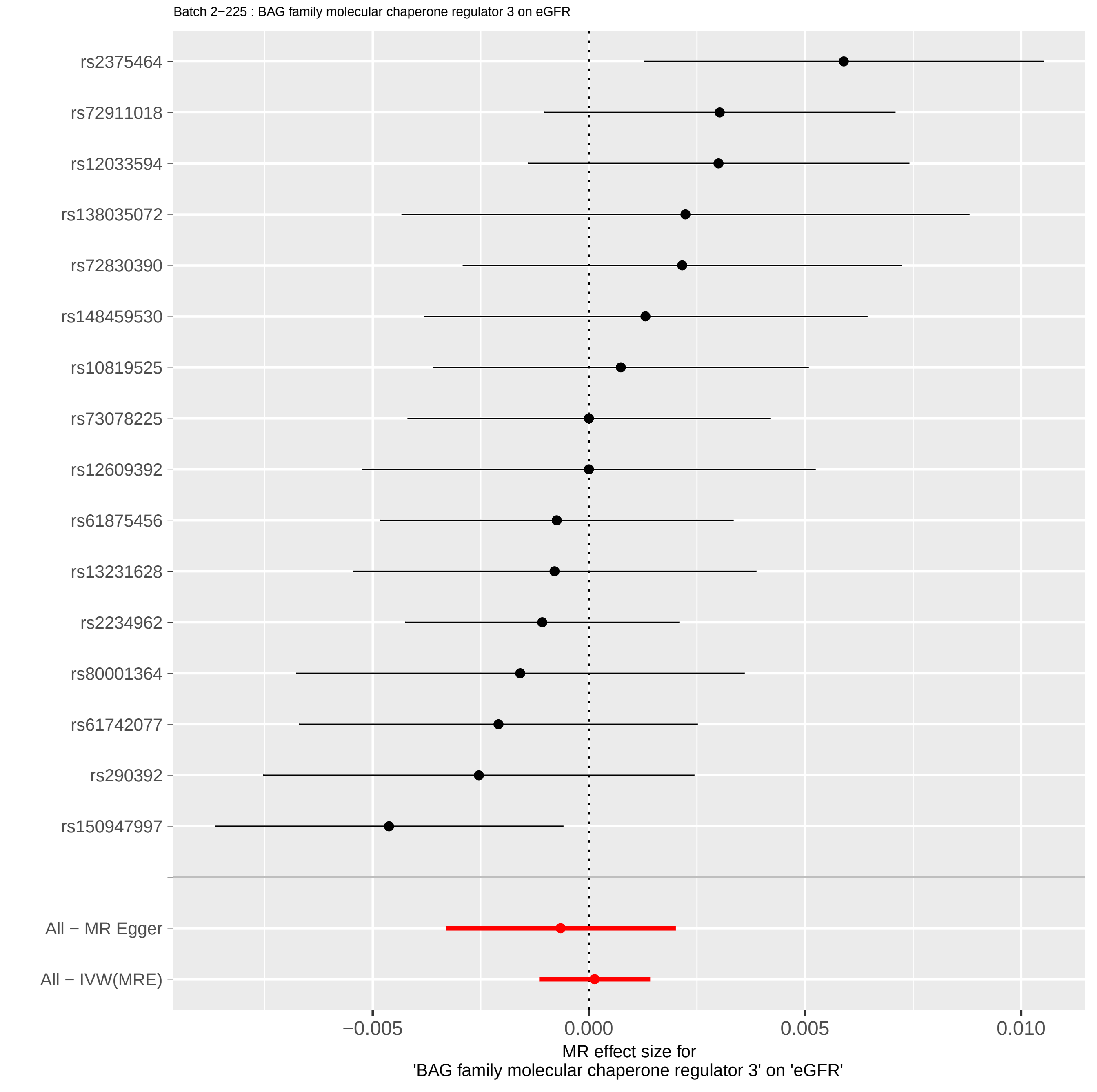
Batch 2−225 : BAG family molecular chaperone regulator 3 on eGFR
rs2375464
rs72911018
rs12033594
rs138035072
rs72830390
rs148459530
rs10819525
rs73078225
rs12609392
rs61875456
rs13231628
rs2234962
rs80001364
rs61742077
rs290392
rs150947997
All − MR Egger
All − IVW(MRE)
−0.005
0.000
0.005
0.010
MR effect size for
'BAG family molecular chaperone regulator 3' on 'eGFR'

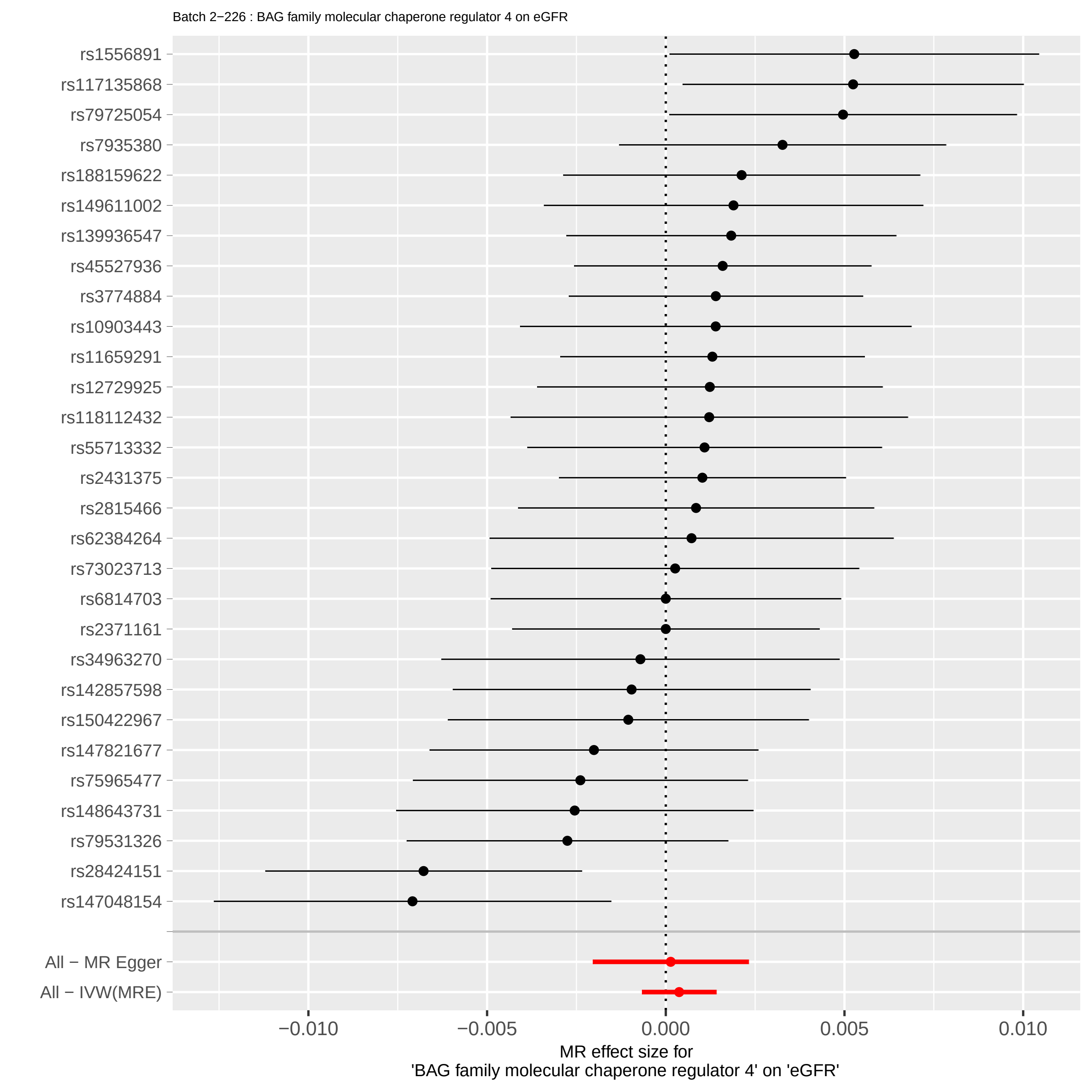
Batch 2−226 : BAG family molecular chaperone regulator 4 on eGFR
rs1556891
rs117135868
rs79725054
rs7935380
rs188159622
rs149611002
rs139936547
rs45527936
rs3774884
rs10903443
rs11659291
rs12729925
rs118112432
rs55713332
rs2431375
rs2815466
rs62384264
rs73023713
rs6814703
rs2371161
rs34963270
rs142857598
rs150422967
rs147821677
rs75965477
rs148643731
rs79531326
rs28424151
rs147048154
All − MR Egger
All − IVW(MRE)
−0.010
−0.005
0.000
0.005
0.010
MR effect size for
'BAG family molecular chaperone regulator 4' on 'eGFR'

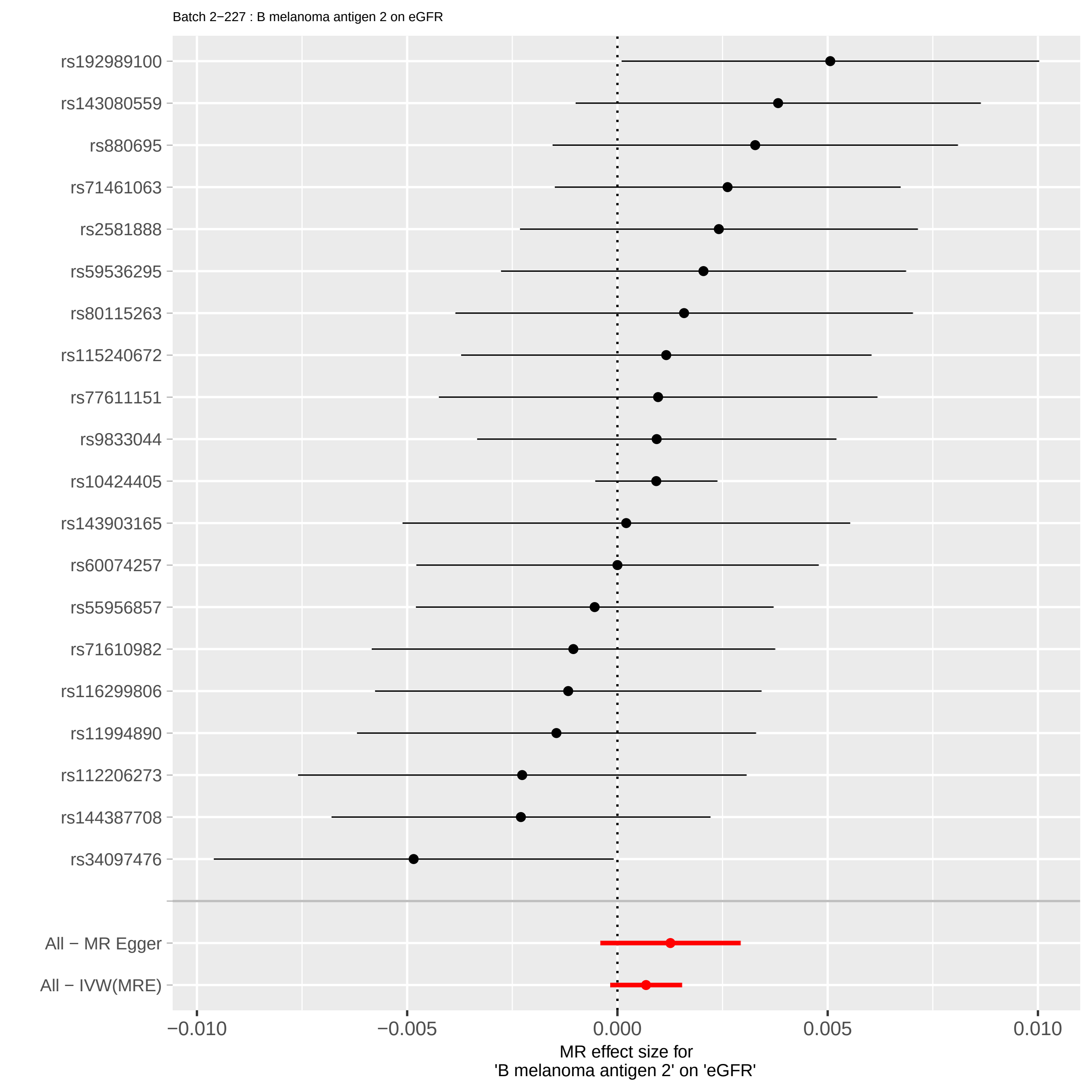

Batch 2−227 : B melanoma antigen 2 on eGFR
rs192989100
rs143080559
rs880695
rs71461063
rs2581888
rs59536295
rs80115263
rs115240672
rs77611151
rs9833044
rs10424405
rs143903165
rs60074257
rs55956857
rs71610982
rs116299806
rs11994890
rs112206273
rs144387708
rs34097476
All − MR Egger
All − IVW(MRE)
−0.010
−0.005
0.000
0.005
0.010
MR effect size for
'B melanoma antigen 2' on 'eGFR'

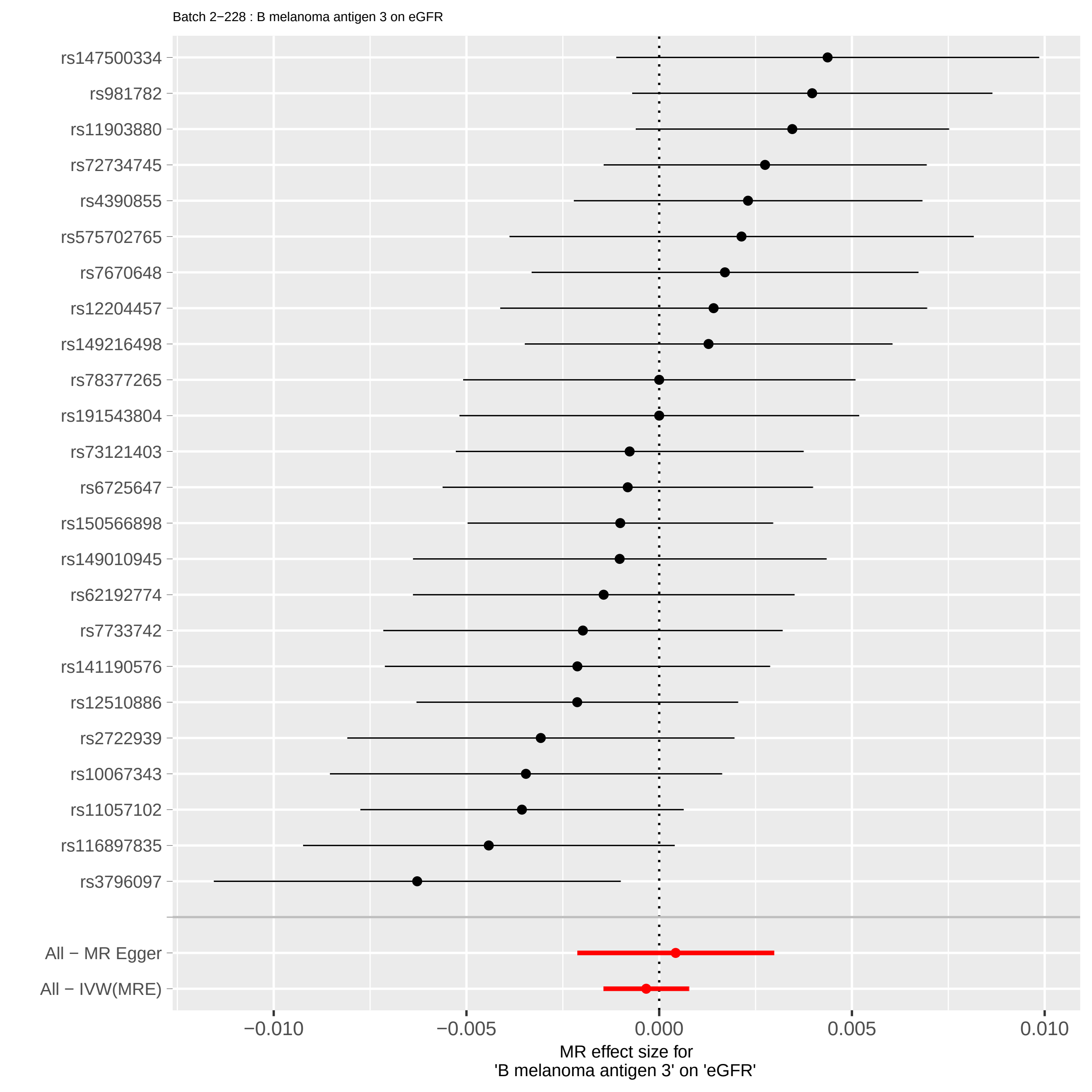

Batch 2−228 : B melanoma antigen 3 on eGFR
rs147500334
rs981782
rs11903880
rs72734745
rs4390855
rs575702765
rs7670648
rs12204457
rs149216498
rs78377265
rs191543804
rs73121403
rs6725647
rs150566898
rs149010945
rs62192774
rs7733742
rs141190576
rs12510886
rs2722939
rs10067343
rs11057102
rs116897835
rs3796097
All − MR Egger
All − IVW(MRE)
−0.010
−0.005
0.000
0.005
0.010
MR effect size for
'B melanoma antigen 3' on 'eGFR'

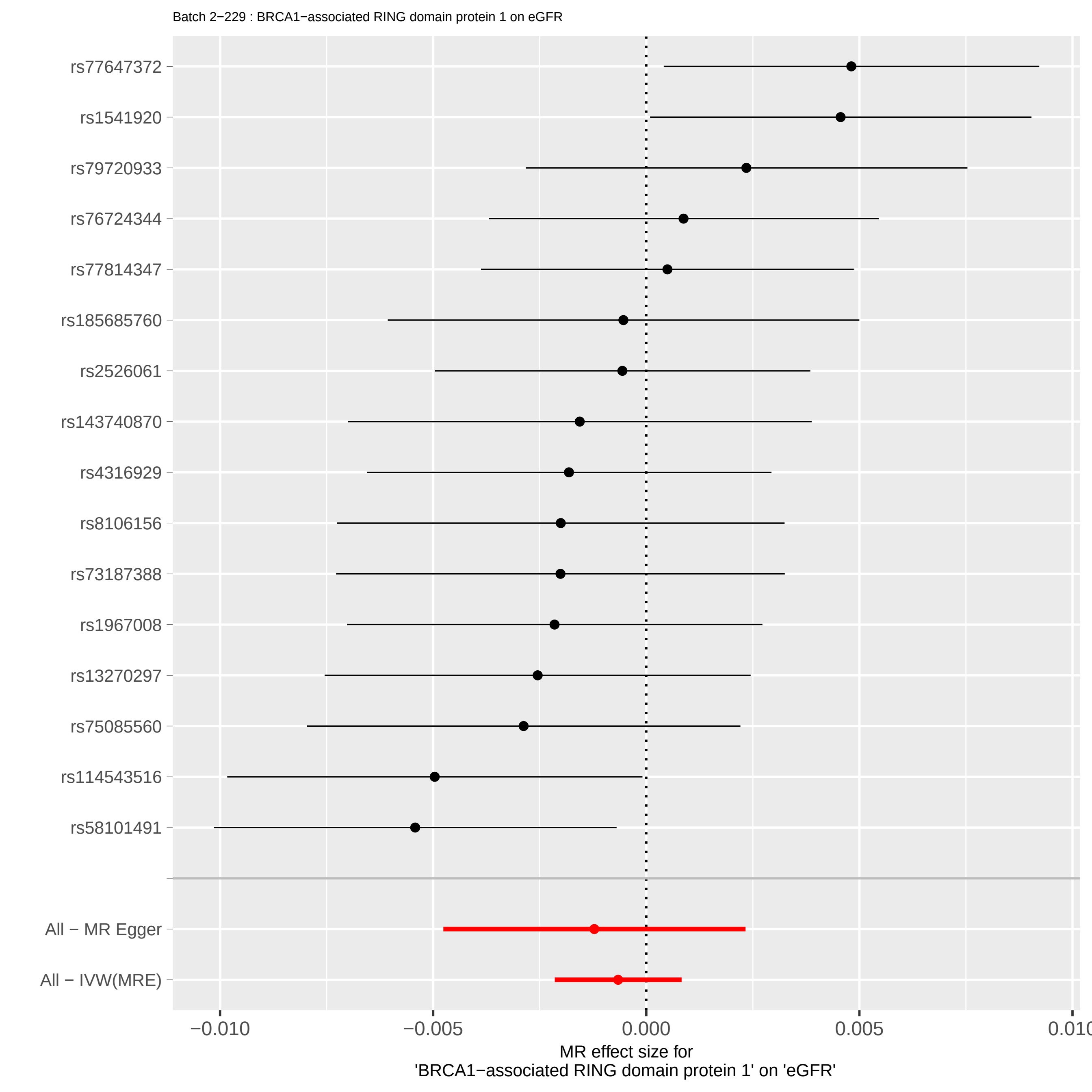

Batch 2−229 : BRCA1−associated RING domain protein 1 on eGFR
rs77647372
rs1541920
rs79720933
rs76724344
rs77814347
rs185685760
rs2526061
rs143740870
rs4316929
rs8106156
rs73187388
rs1967008
rs13270297
rs75085560
rs114543516
rs58101491
All − MR Egger
All − IVW(MRE)
−0.010
−0.005
0.000
0.005
0.010
MR effect size for
'BRCA1−associated RING domain protein 1' on 'eGFR'

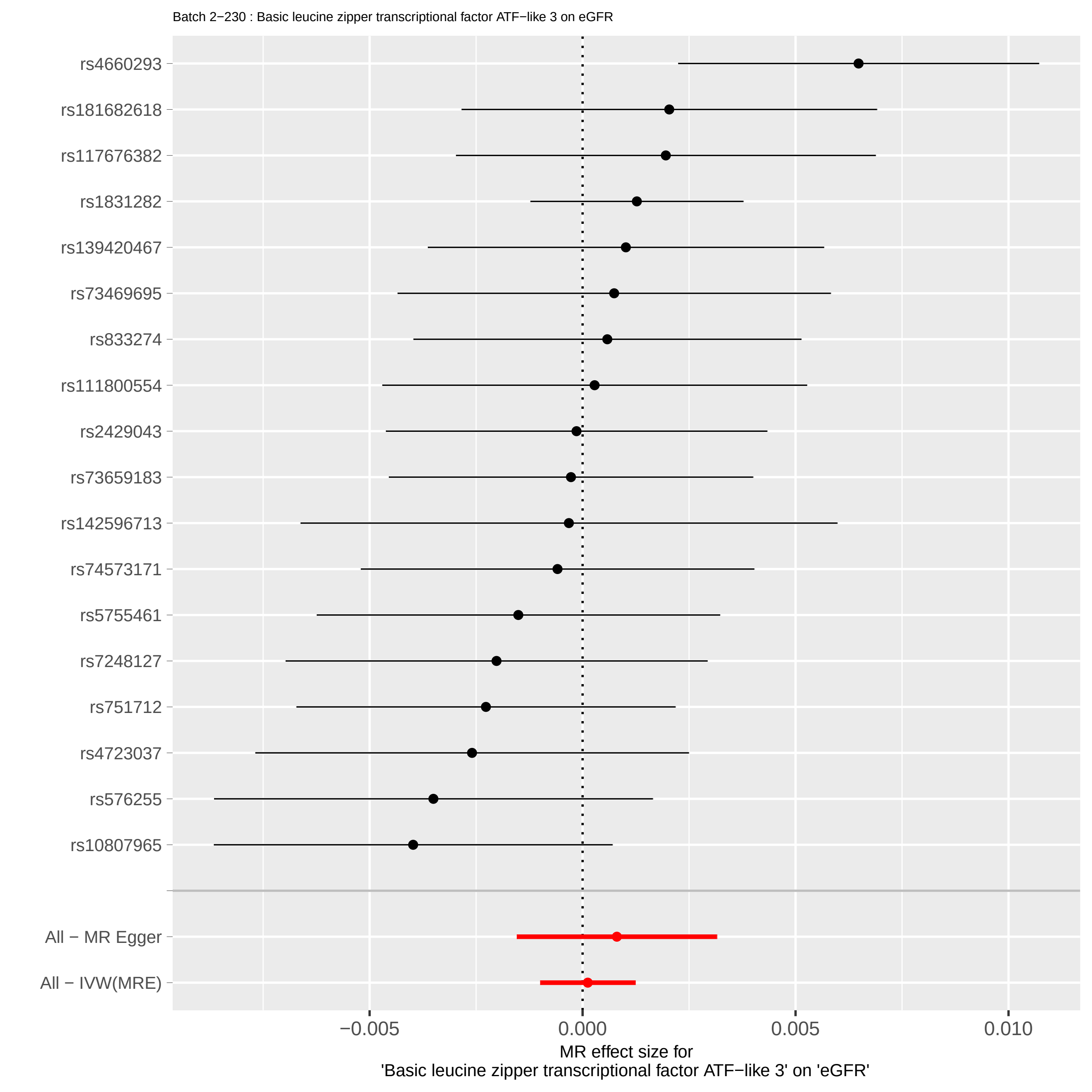
Batch 2−230 : Basic leucine zipper transcriptional factor ATF−like 3 on eGFR
rs4660293
rs181682618
rs117676382
rs1831282
rs139420467
rs73469695
rs833274
rs111800554
rs2429043
rs73659183
rs142596713
rs74573171
rs5755461
rs7248127
rs751712
rs4723037
rs576255
rs10807965
All − MR Egger
All − IVW(MRE)
−0.005
0.000
0.005
0.010
MR effect size for
'Basic leucine zipper transcriptional factor ATF−like 3' on 'eGFR'

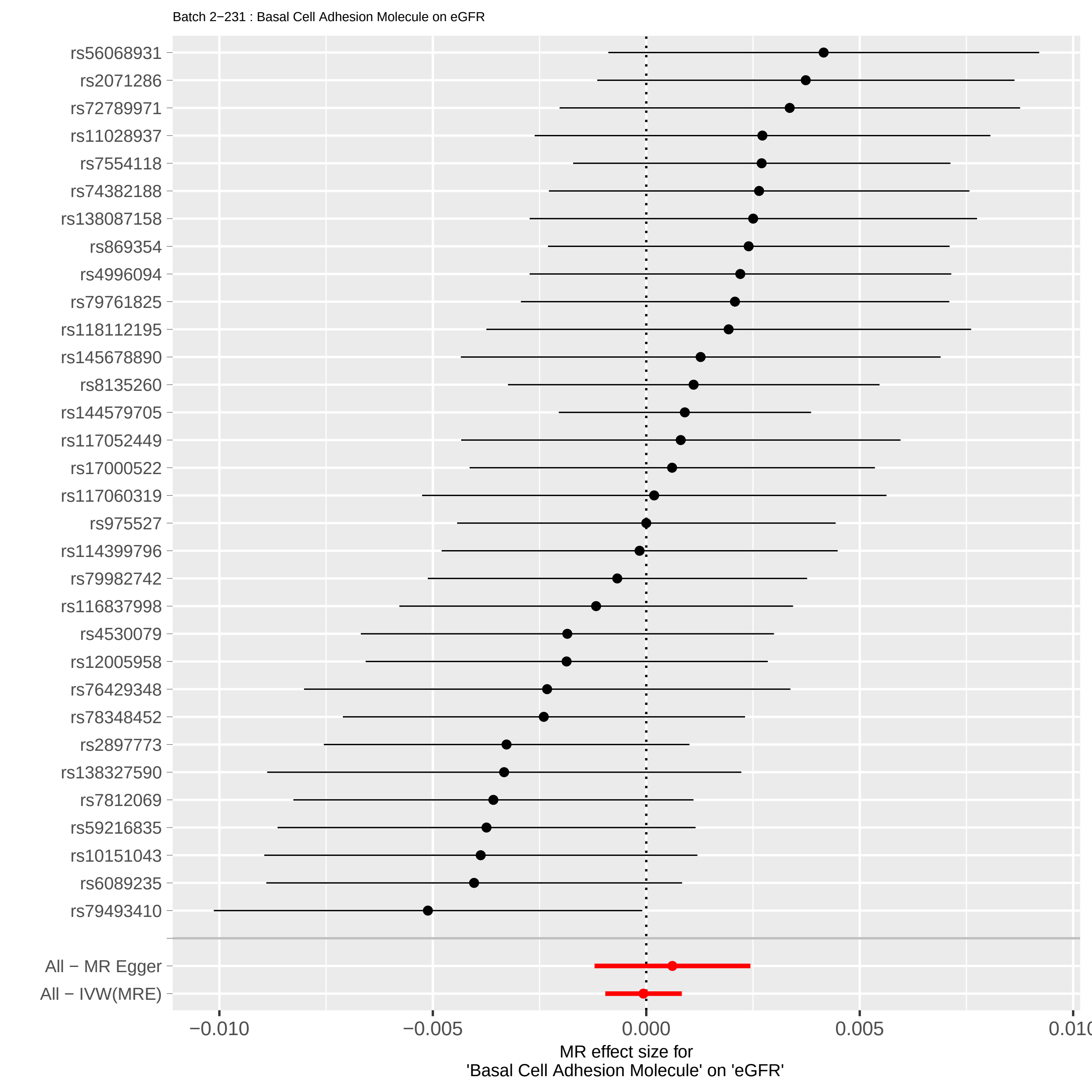

Batch 2−231 : Basal Cell Adhesion Molecule on eGFR
rs56068931
rs2071286
rs72789971
rs11028937
rs7554118
rs74382188
rs138087158
rs869354
rs4996094
rs79761825
rs118112195
rs145678890
rs8135260
rs144579705
rs117052449
rs17000522
rs117060319
rs975527
rs114399796
rs79982742
rs116837998
rs4530079
rs12005958
rs76429348
rs78348452
rs2897773
rs138327590
rs7812069
rs59216835
rs10151043
rs6089235
rs79493410
All − MR Egger
All − IVW(MRE)
−0.010
−0.005
0.000
0.005
0.010
MR effect size for
'Basal Cell Adhesion Molecule' on 'eGFR'

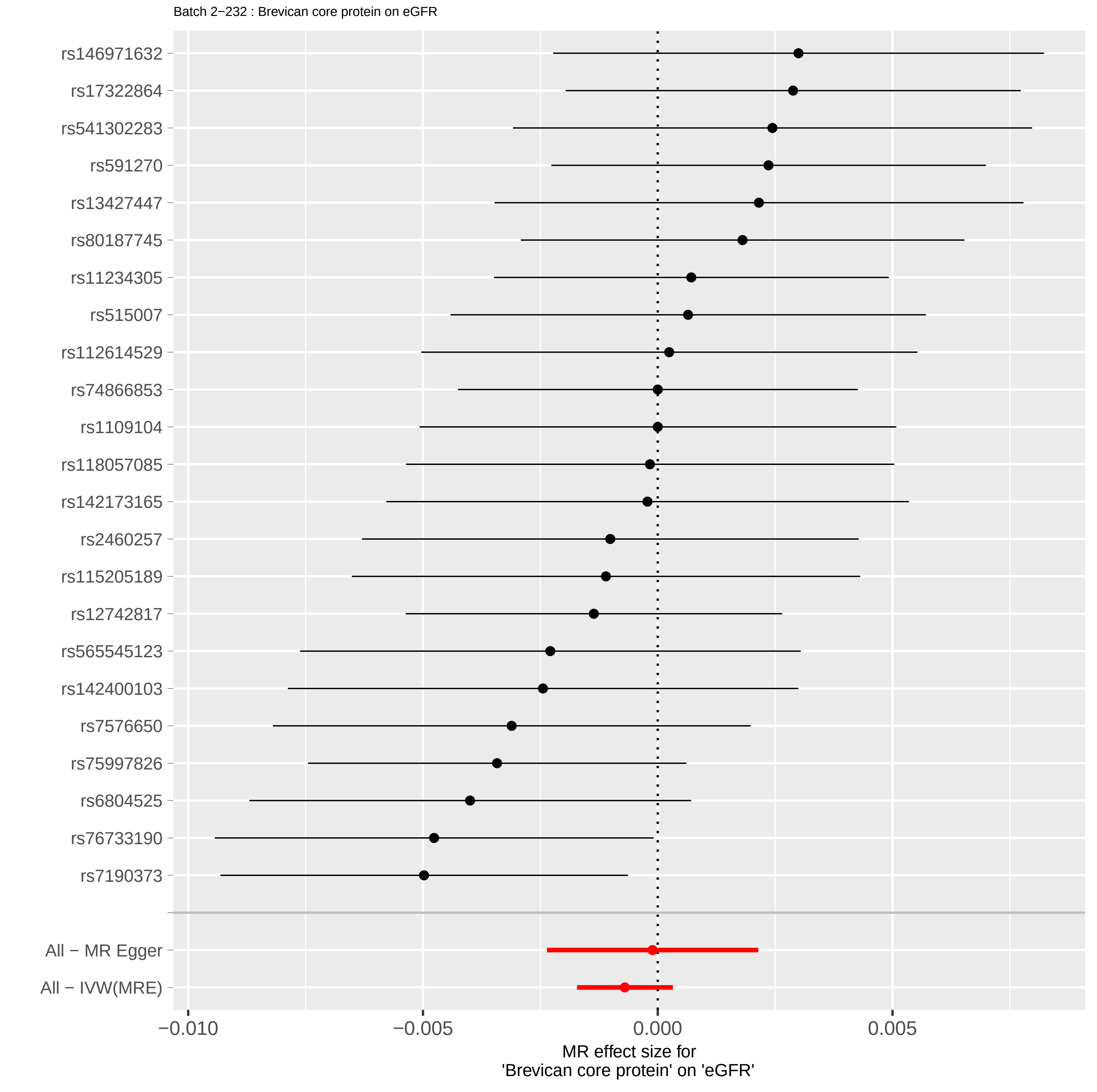

Batch 2−232 : Brevican core protein on eGFR
rs146971632
rs17322864
rs541302283
rs591270
rs13427447
rs80187745
rs11234305
rs515007
rs112614529
rs74866853
rs1109104
rs118057085
rs142173165
rs2460257
rs115205189
rs12742817
rs565545123
rs142400103
rs7576650
rs75997826
rs6804525
rs76733190
rs7190373
All − MR Egger
All − IVW(MRE)
−0.010
−0.005
0.000
0.005
MR effect size for
'Brevican core protein' on 'eGFR'

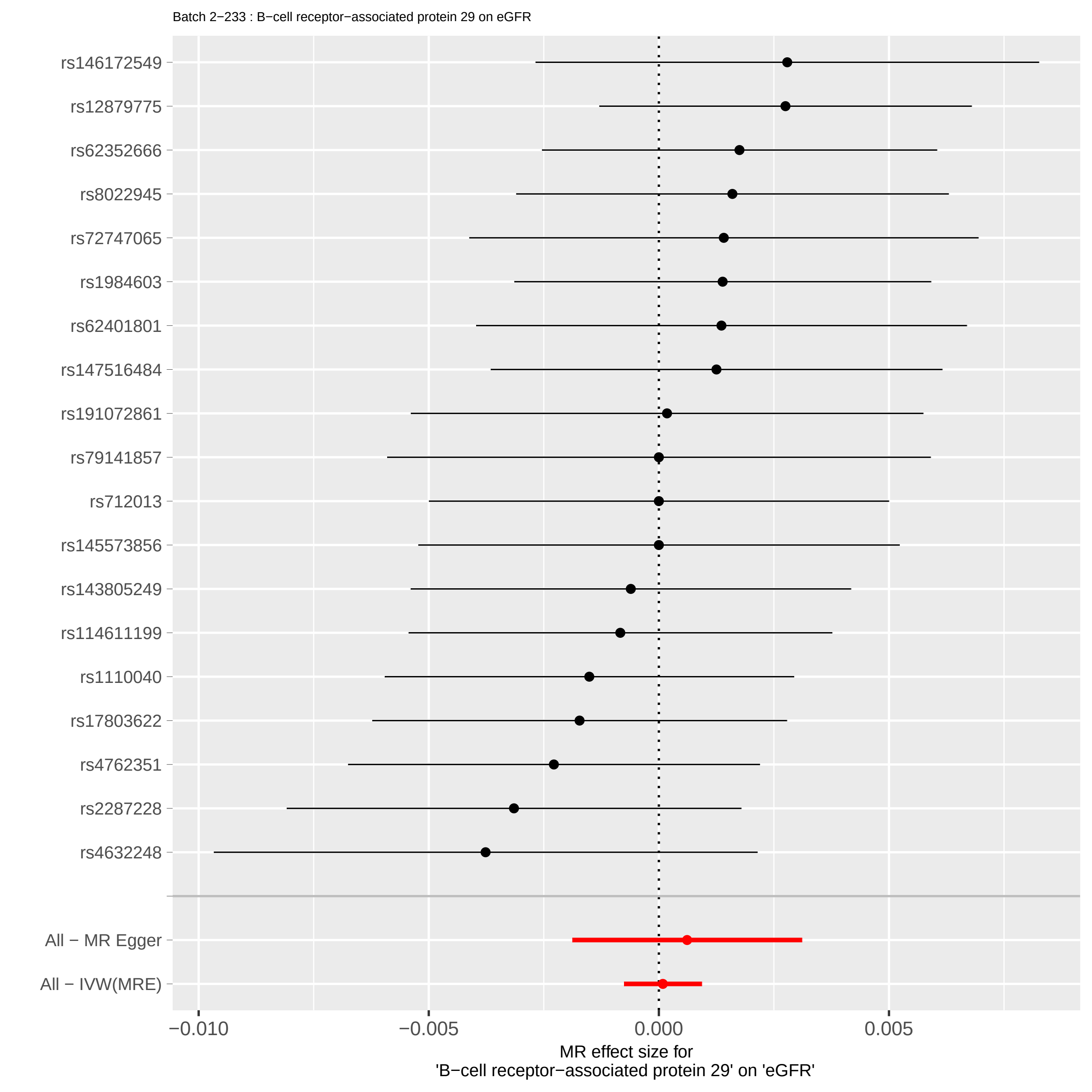
Batch 2−233 : B−cell receptor−associated protein 29 on eGFR
rs146172549
rs12879775
rs62352666
rs8022945
rs72747065
rs1984603
rs62401801
rs147516484
rs191072861
rs79141857
rs712013
rs145573856
rs143805249
rs114611199
rs1110040
rs17803622
rs4762351
rs2287228
rs4632248
All − MR Egger
All − IVW(MRE)
−0.010
−0.005
0.000
0.005
MR effect size for
'B−cell receptor−associated protein 29' on 'eGFR'

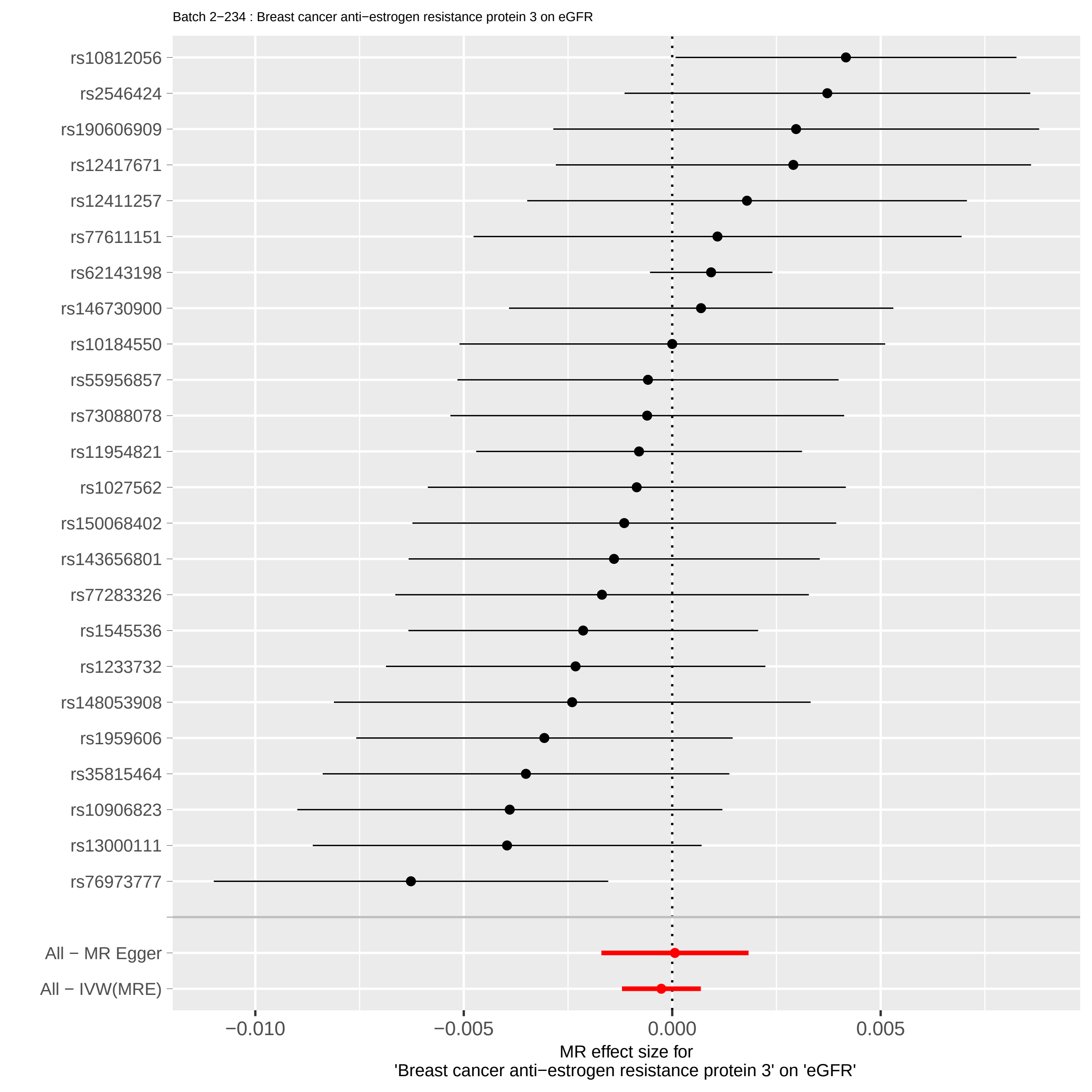

Batch 2−234 : Breast cancer anti−estrogen resistance protein 3 on eGFR
rs10812056
rs2546424
rs190606909
rs12417671
rs12411257
rs77611151
rs62143198
rs146730900
rs10184550
rs55956857
rs73088078
rs11954821
rs1027562
rs150068402
rs143656801
rs77283326
rs1545536
rs1233732
rs148053908
rs1959606
rs35815464
rs10906823
rs13000111
rs76973777
All − MR Egger
All − IVW(MRE)
−0.010
−0.005
0.000
0.005
MR effect size for
'Breast cancer anti−estrogen resistance protein 3' on 'eGFR'

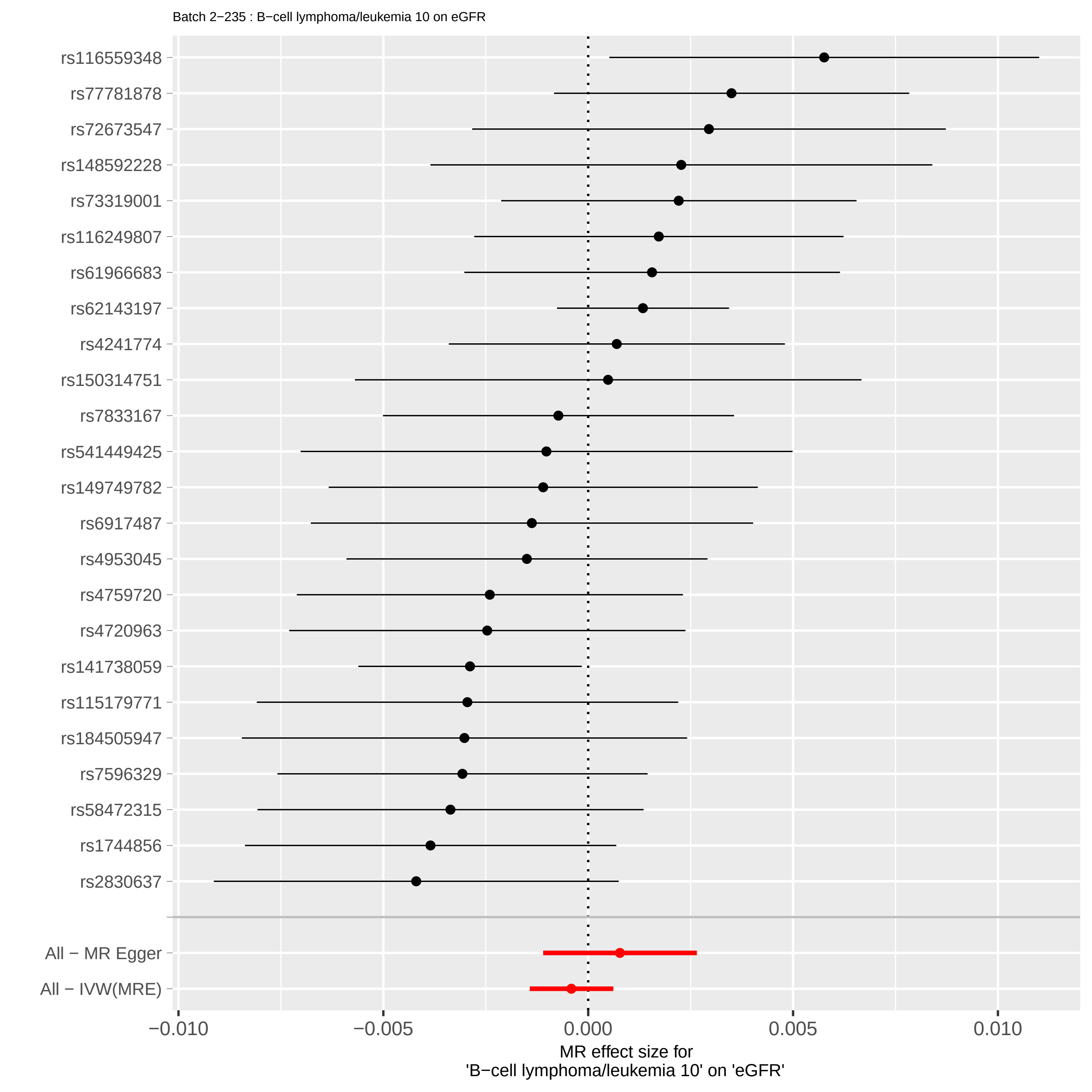
Batch 2−235 : B−cell lymphoma/leukemia 10 on eGFR
rs116559348
rs77781878
rs72673547
rs148592228
rs73319001
rs116249807
rs61966683
rs62143197
rs4241774
rs150314751
rs7833167
rs541449425
rs149749782
rs6917487
rs4953045
rs4759720
rs4720963
rs141738059
rs115179771
rs184505947
rs7596329
rs58472315
rs1744856
rs2830637
All − MR Egger
All − IVW(MRE)
−0.010
−0.005
0.000
0.005
0.010
MR effect size for
'B−cell lymphoma/leukemia 10' on 'eGFR'

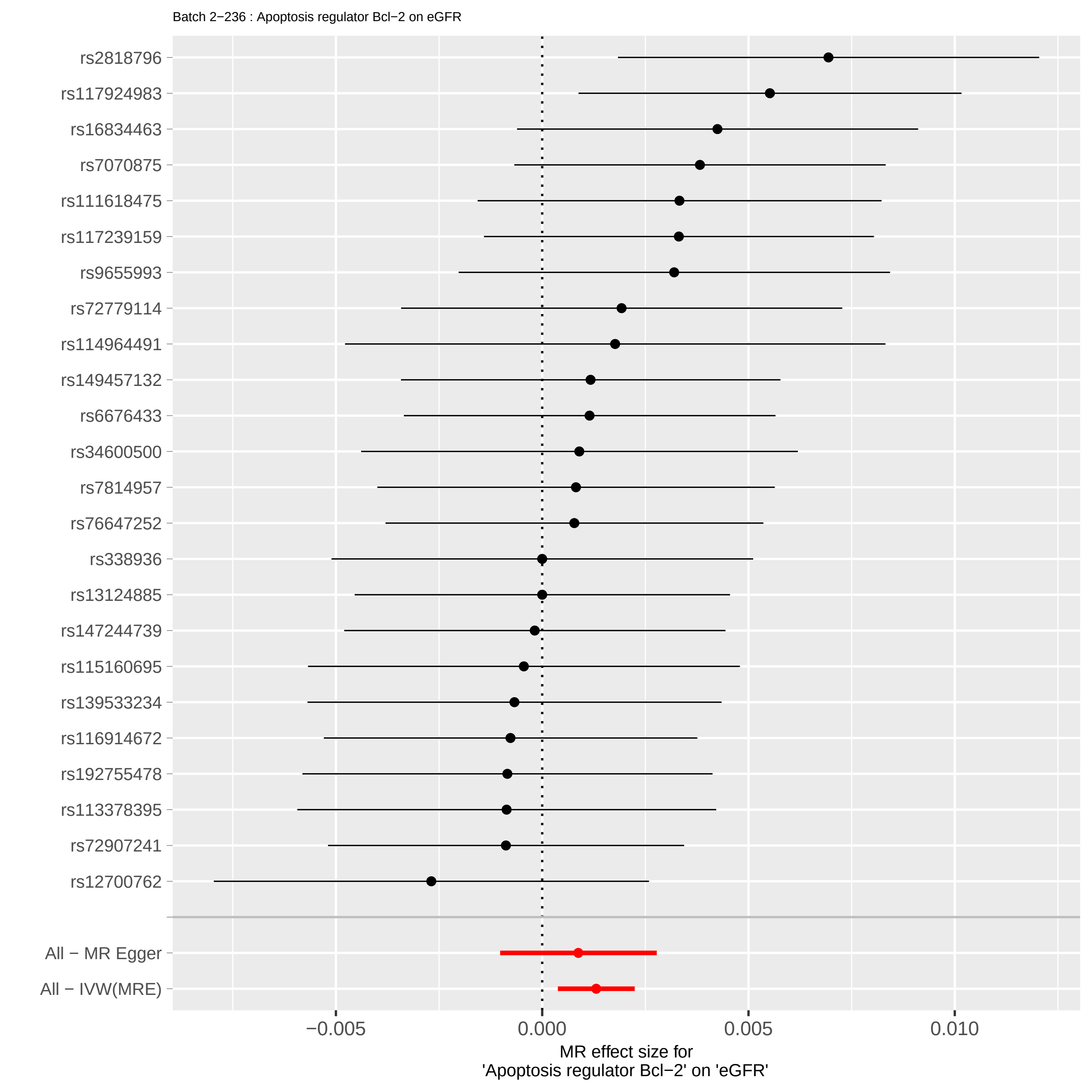

Batch 2−236 : Apoptosis regulator Bcl−2 on eGFR
rs2818796
rs117924983
rs16834463
rs7070875
rs111618475
rs117239159
rs9655993
rs72779114
rs114964491
rs149457132
rs6676433
rs34600500
rs7814957
rs76647252
rs338936
rs13124885
rs147244739
rs115160695
rs139533234
rs116914672
rs192755478
rs113378395
rs72907241
rs12700762
All − MR Egger
All − IVW(MRE)
−0.005
0.000
0.005
0.010
MR effect size for
'Apoptosis regulator Bcl−2' on 'eGFR'

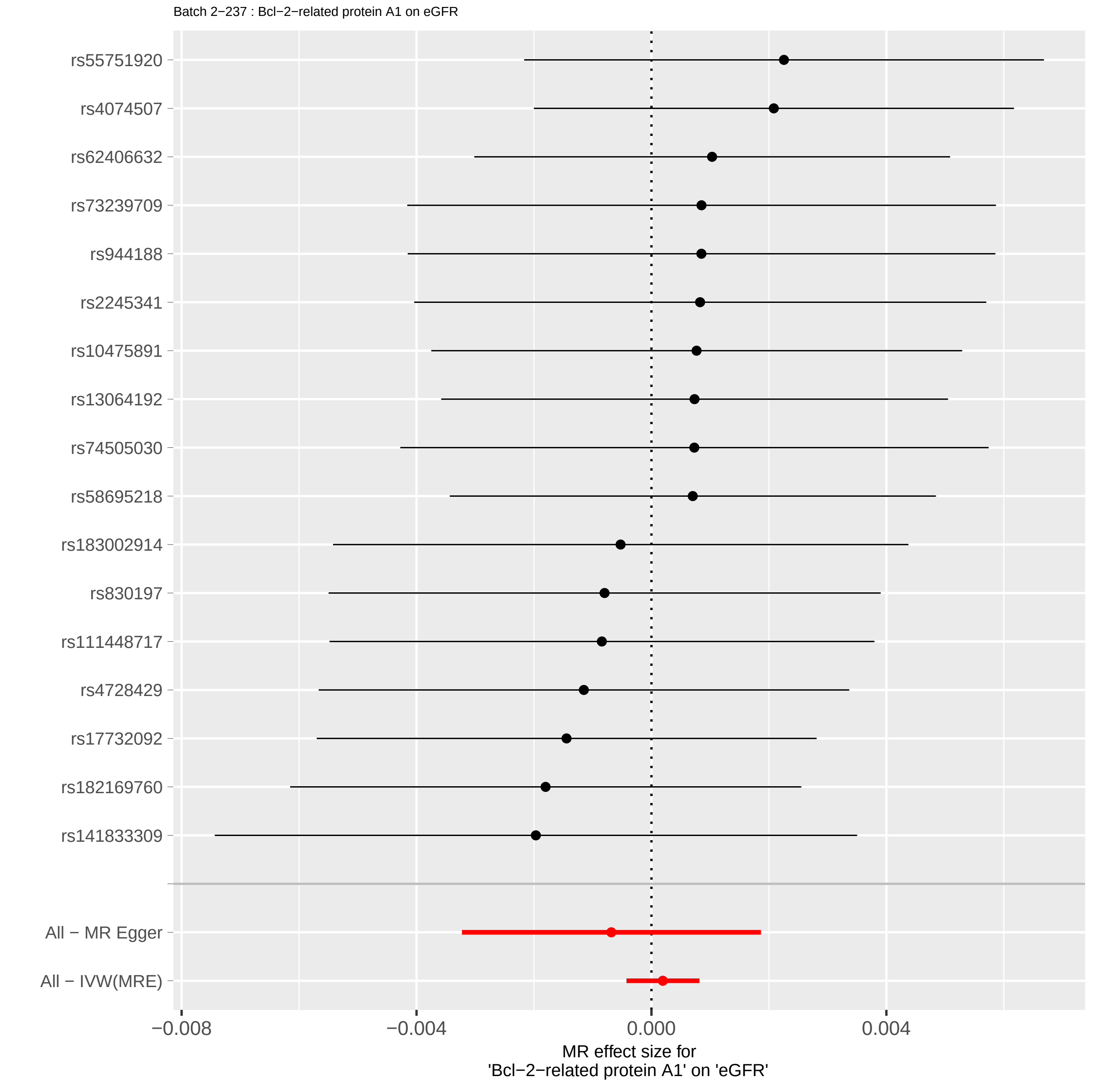

Batch 2−237 : Bcl−2−related protein A1 on eGFR
rs55751920
rs4074507
rs62406632
rs73239709
rs944188
rs2245341
rs10475891
rs13064192
rs74505030
rs58695218
rs183002914
rs830197
rs111448717
rs4728429
rs17732092
rs182169760
rs141833309
All − MR Egger
All − IVW(MRE)
−0.008
−0.004
0.000
0.004
MR effect size for
'Bcl−2−related protein A1' on 'eGFR'

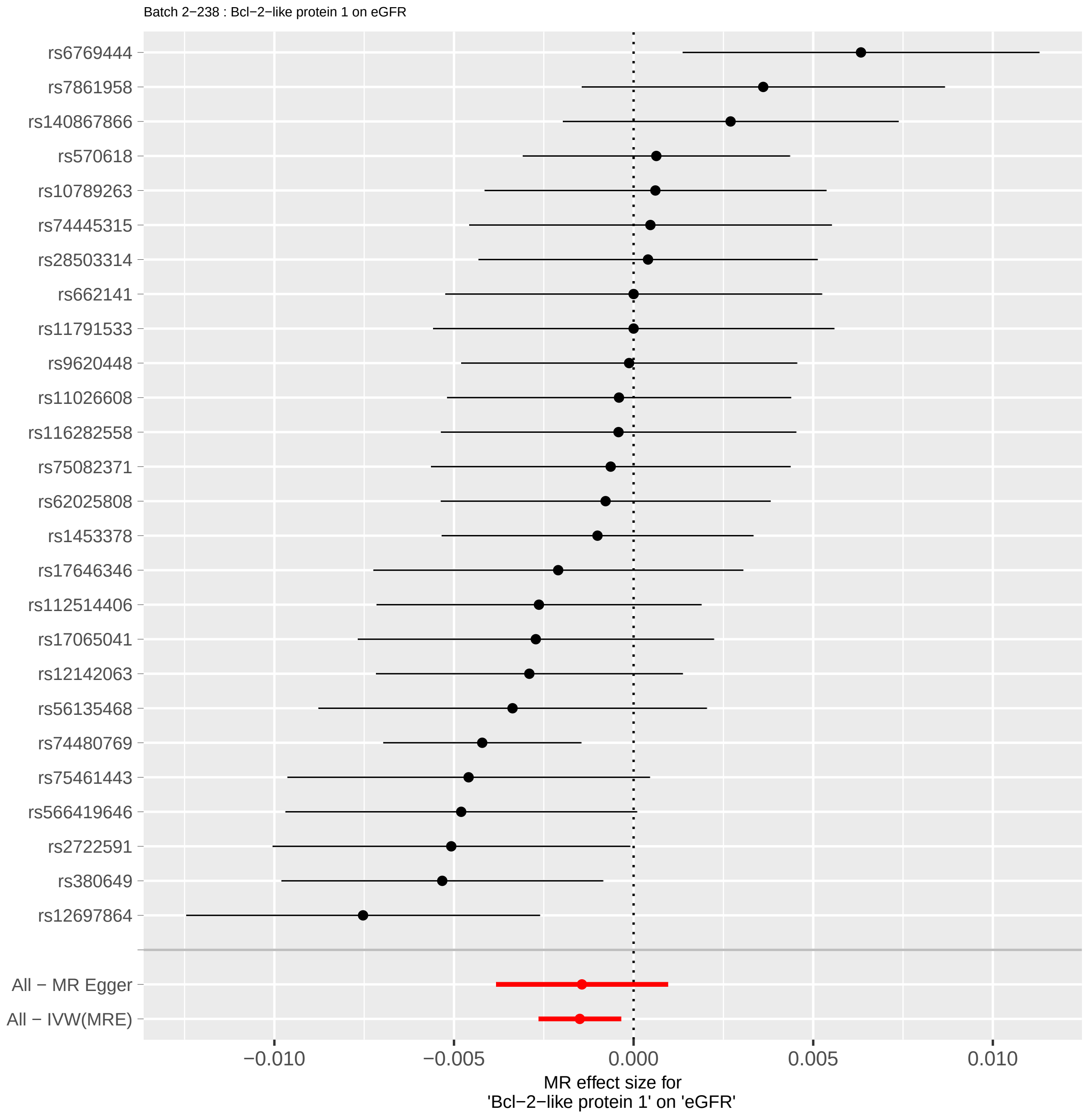

Batch 2−238 : Bcl−2−like protein 1 on eGFR
rs6769444
rs7861958
rs140867866
rs570618
rs10789263
rs74445315
rs28503314
rs662141
rs11791533
rs9620448
rs11026608
rs116282558
rs75082371
rs62025808
rs1453378
rs17646346
rs112514406
rs17065041
rs12142063
rs56135468
rs74480769
rs75461443
rs566419646
rs2722591
rs380649
rs12697864
All − MR Egger
All − IVW(MRE)
−0.010
−0.005
0.000
0.005
0.010
MR effect size for
'Bcl−2−like protein 1' on 'eGFR'

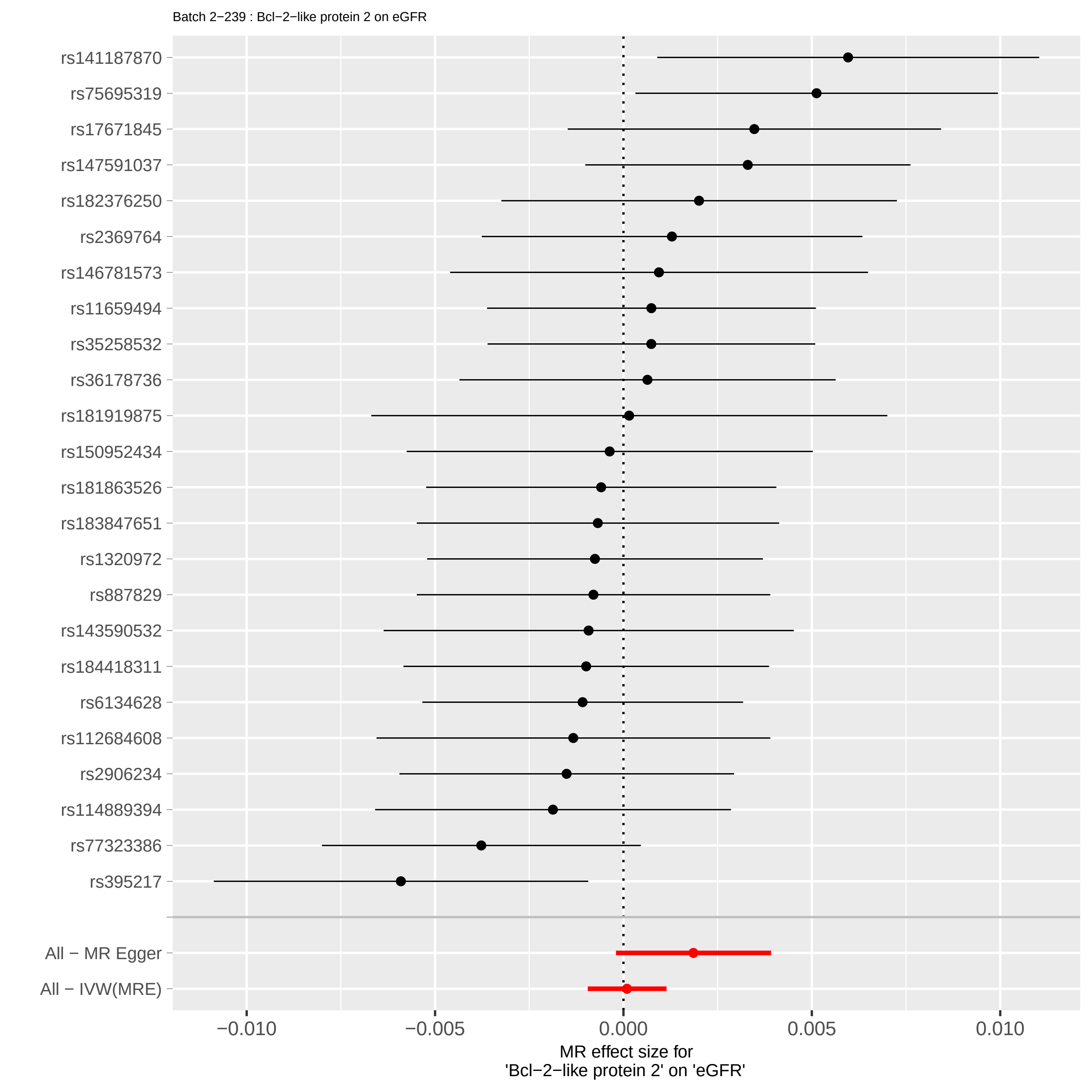

Batch 2−239 : Bcl−2−like protein 2 on eGFR
rs141187870
rs75695319
rs17671845
rs147591037
rs182376250
rs2369764
rs146781573
rs11659494
rs35258532
rs36178736
rs181919875
rs150952434
rs181863526
rs183847651
rs1320972
rs887829
rs143590532
rs184418311
rs6134628
rs112684608
rs2906234
rs114889394
rs77323386
rs395217
All − MR Egger
All − IVW(MRE)
−0.010
−0.005
0.000
0.005
0.010
MR effect size for
'Bcl−2−like protein 2' on 'eGFR'

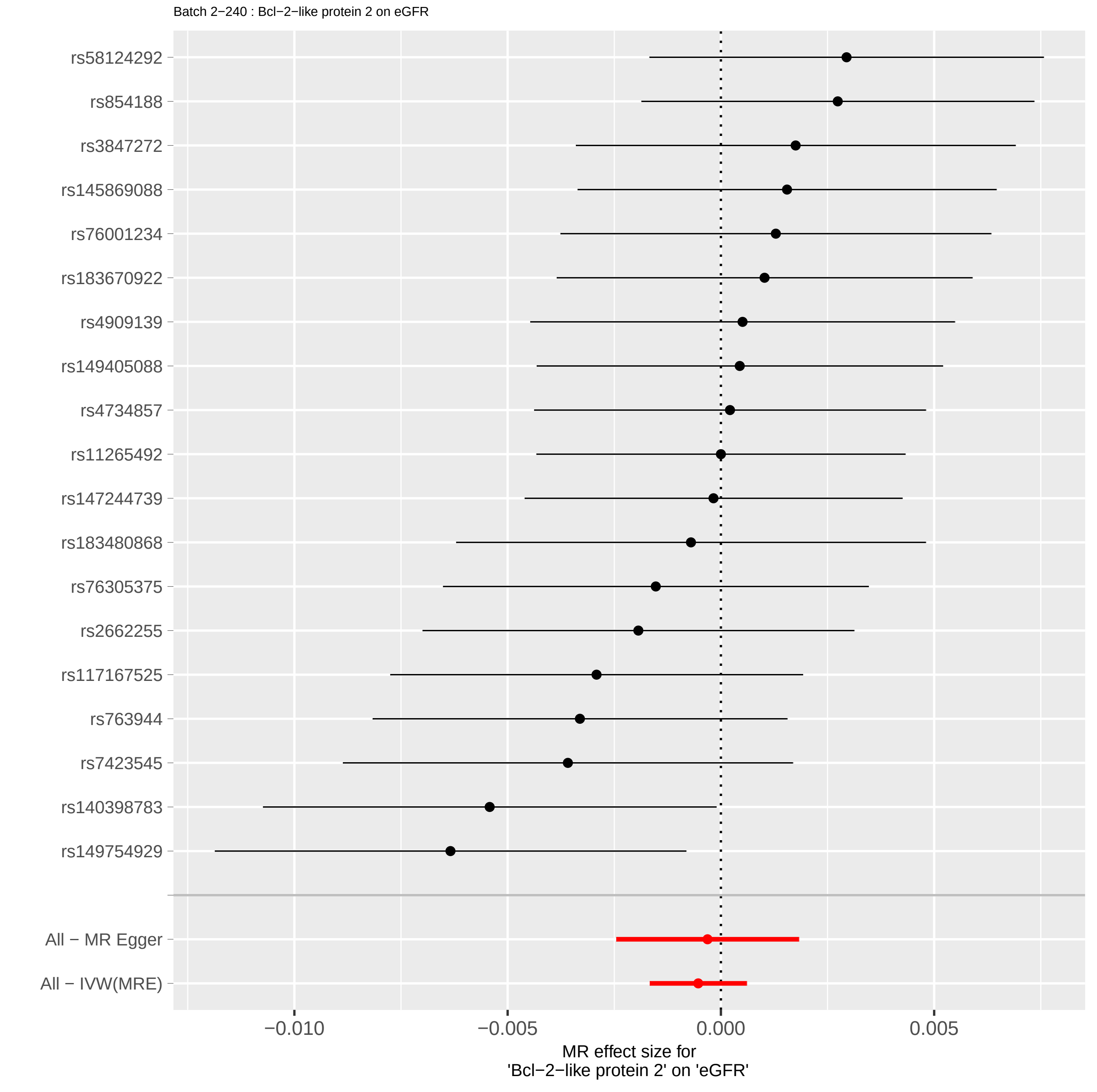

Batch 2−240 : Bcl−2−like protein 2 on eGFR
rs58124292
rs854188
rs3847272
rs145869088
rs76001234
rs183670922
rs4909139
rs149405088
rs4734857
rs11265492
rs147244739
rs183480868
rs76305375
rs2662255
rs117167525
rs763944
rs7423545
rs140398783
rs149754929
All − MR Egger
All − IVW(MRE)
−0.010
−0.005
0.000
0.005
MR effect size for
'Bcl−2−like protein 2' on 'eGFR'

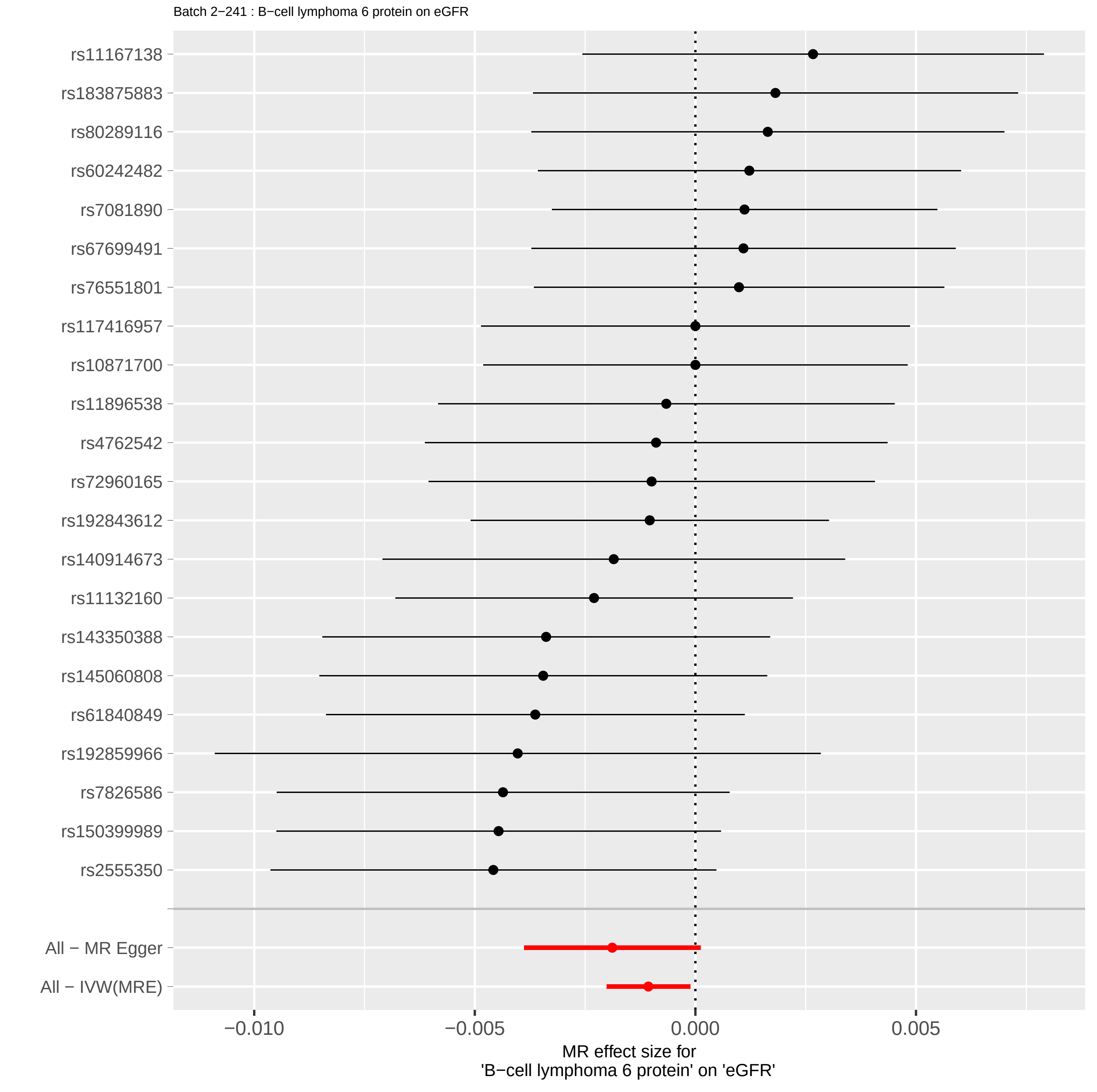

Batch 2−241 : B−cell lymphoma 6 protein on eGFR
rs11167138
rs183875883
rs80289116
rs60242482
rs7081890
rs67699491
rs76551801
rs117416957
rs10871700
rs11896538
rs4762542
rs72960165
rs192843612
rs140914673
rs11132160
rs143350388
rs145060808
rs61840849
rs192859966
rs7826586
rs150399989
rs2555350
All − MR Egger
All − IVW(MRE)
−0.010
−0.005
0.000
0.005
MR effect size for
'B−cell lymphoma 6 protein' on 'eGFR'

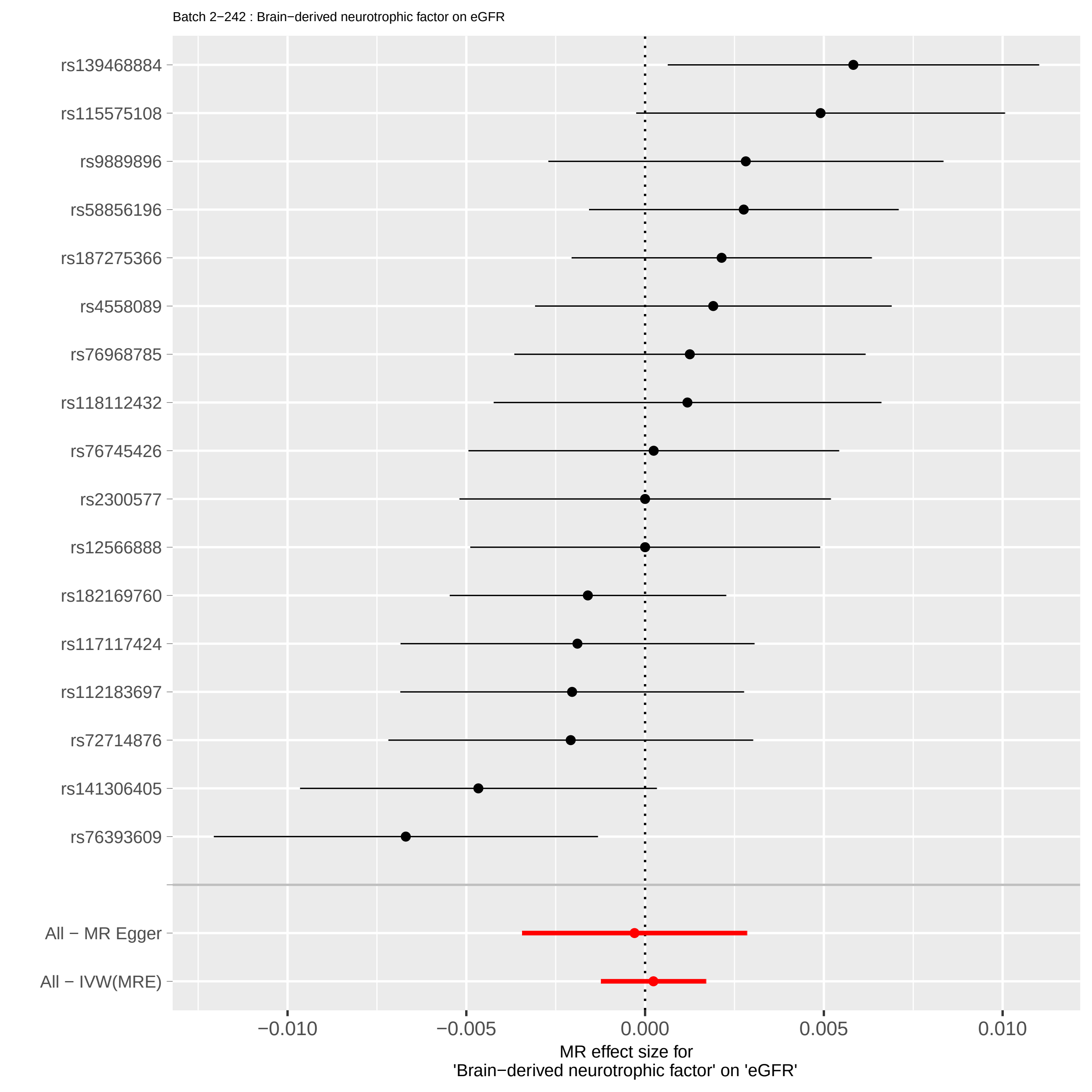
Batch 2−242 : Brain−derived neurotrophic factor on eGFR
rs139468884
rs115575108
rs9889896
rs58856196
rs187275366
rs4558089
rs76968785
rs118112432
rs76745426
rs2300577
rs12566888
rs182169760
rs117117424
rs112183697
rs72714876
rs141306405
rs76393609
All − MR Egger
All − IVW(MRE)
−0.010
−0.005
0.000
0.005
0.010
MR effect size for
'Brain−derived neurotrophic factor' on 'eGFR'

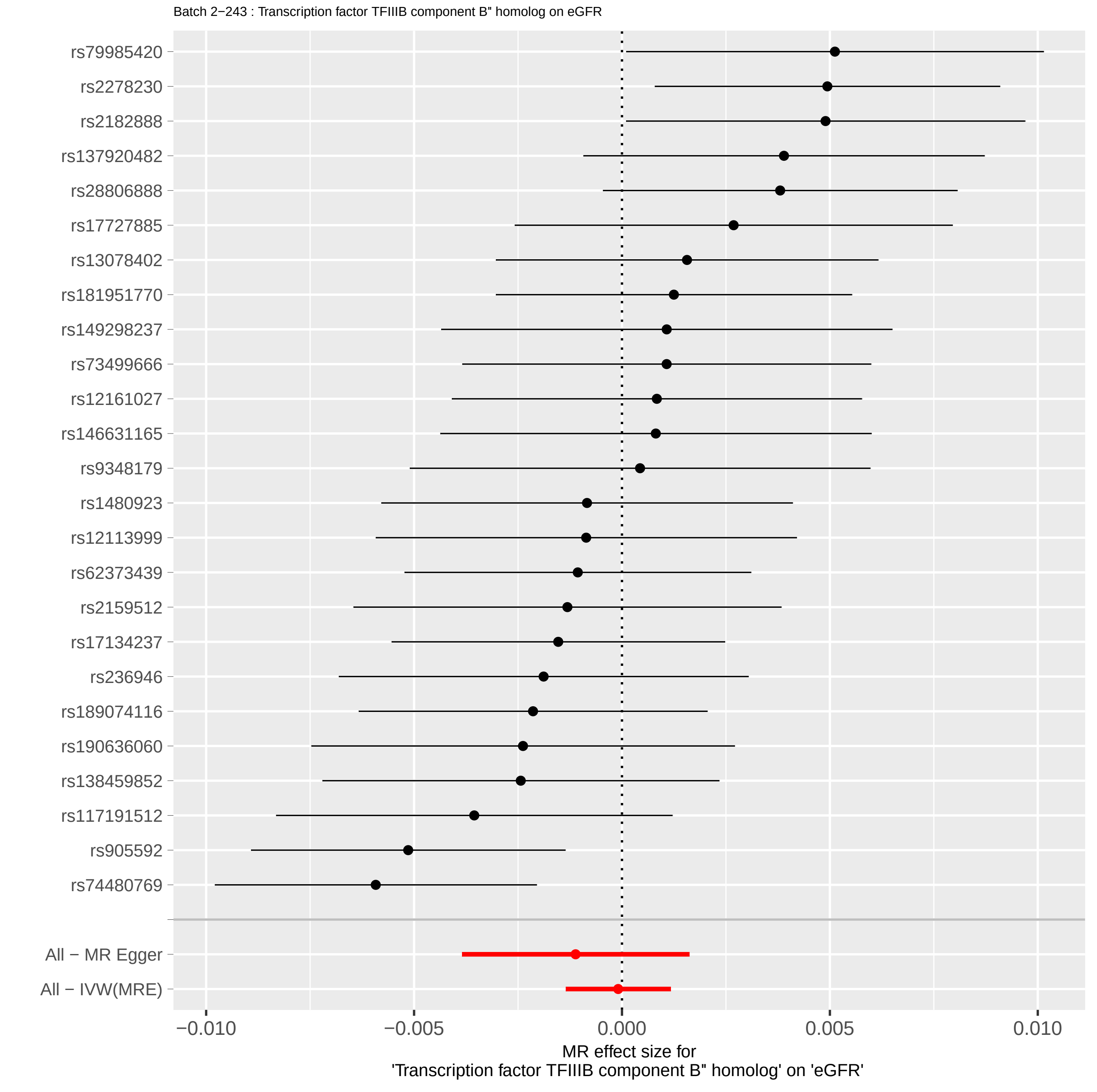

Batch 2−243 : Transcription factor TFIIIB component B" homolog on eGFR
rs79985420
rs2278230
rs2182888
rs137920482
rs28806888
rs17727885
rs13078402
rs181951770
rs149298237
rs73499666
rs12161027
rs146631165
rs9348179
rs1480923
rs12113999
rs62373439
rs2159512
rs17134237
rs236946
rs189074116
rs190636060
rs138459852
rs117191512
rs905592
rs74480769
All − MR Egger
All − IVW(MRE)
−0.010
−0.005
0.000
0.005
0.010
MR effect size for
'Transcription factor TFIIIB component B" homolog' on 'eGFR'

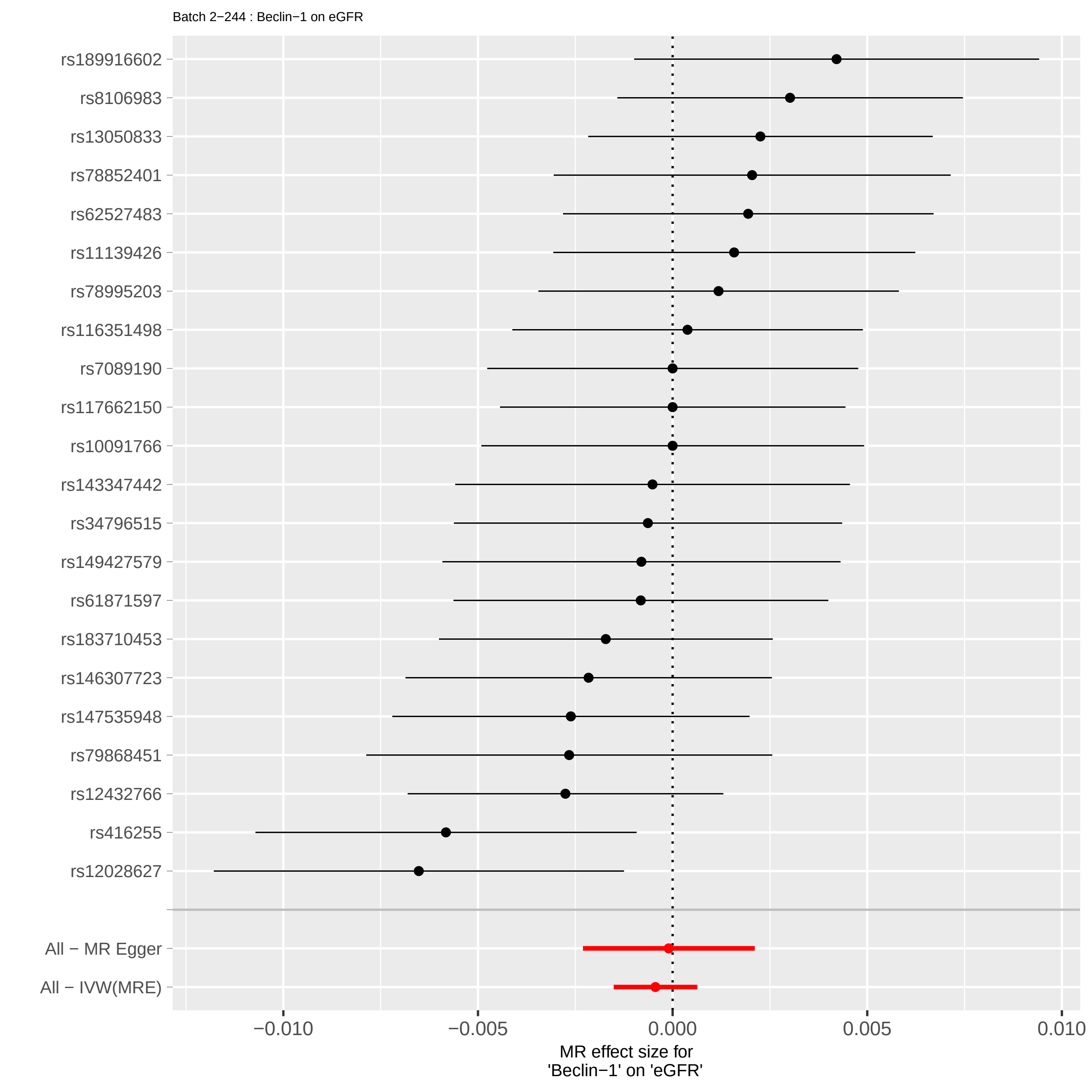

Batch 2−244 : Beclin−1 on eGFR
rs189916602
rs8106983
rs13050833
rs78852401
rs62527483
rs11139426
rs78995203
rs116351498
rs7089190
rs117662150
rs10091766
rs143347442
rs34796515
rs149427579
rs61871597
rs183710453
rs146307723
rs147535948
rs79868451
rs12432766
rs416255
rs12028627
All − MR Egger
All − IVW(MRE)
−0.010
−0.005
0.000
0.005
0.010
MR effect size for
'Beclin−1' on 'eGFR'

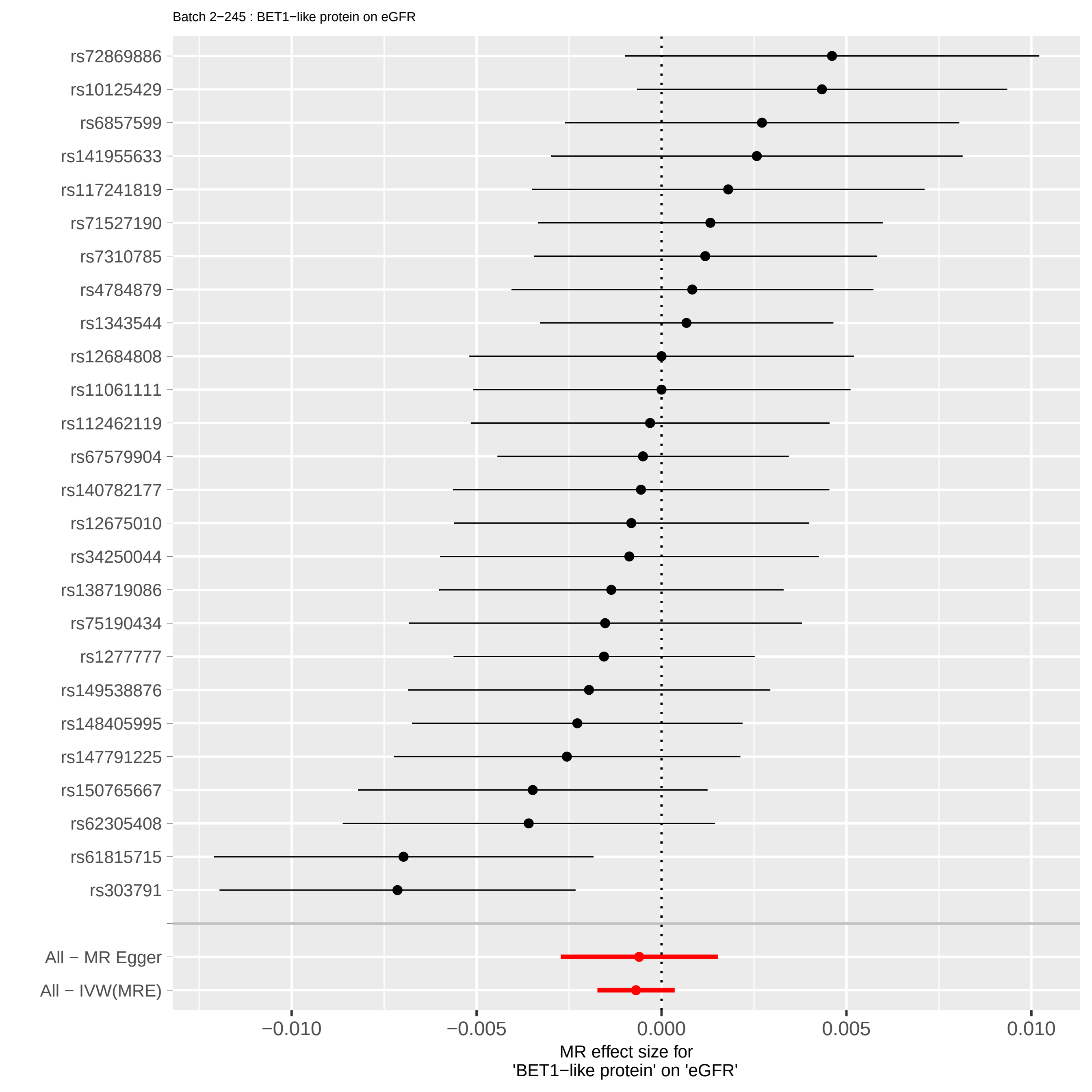

Batch 2−245 : BET1−like protein on eGFR
rs72869886
rs10125429
rs6857599
rs141955633
rs117241819
rs71527190
rs7310785
rs4784879
rs1343544
rs12684808
rs11061111
rs112462119
rs67579904
rs140782177
rs12675010
rs34250044
rs138719086
rs75190434
rs1277777
rs149538876
rs148405995
rs147791225
rs150765667
rs62305408
rs61815715
rs303791
All − MR Egger
All − IVW(MRE)
−0.010
−0.005
0.000
0.005
0.010
MR effect size for
'BET1−like protein' on 'eGFR'

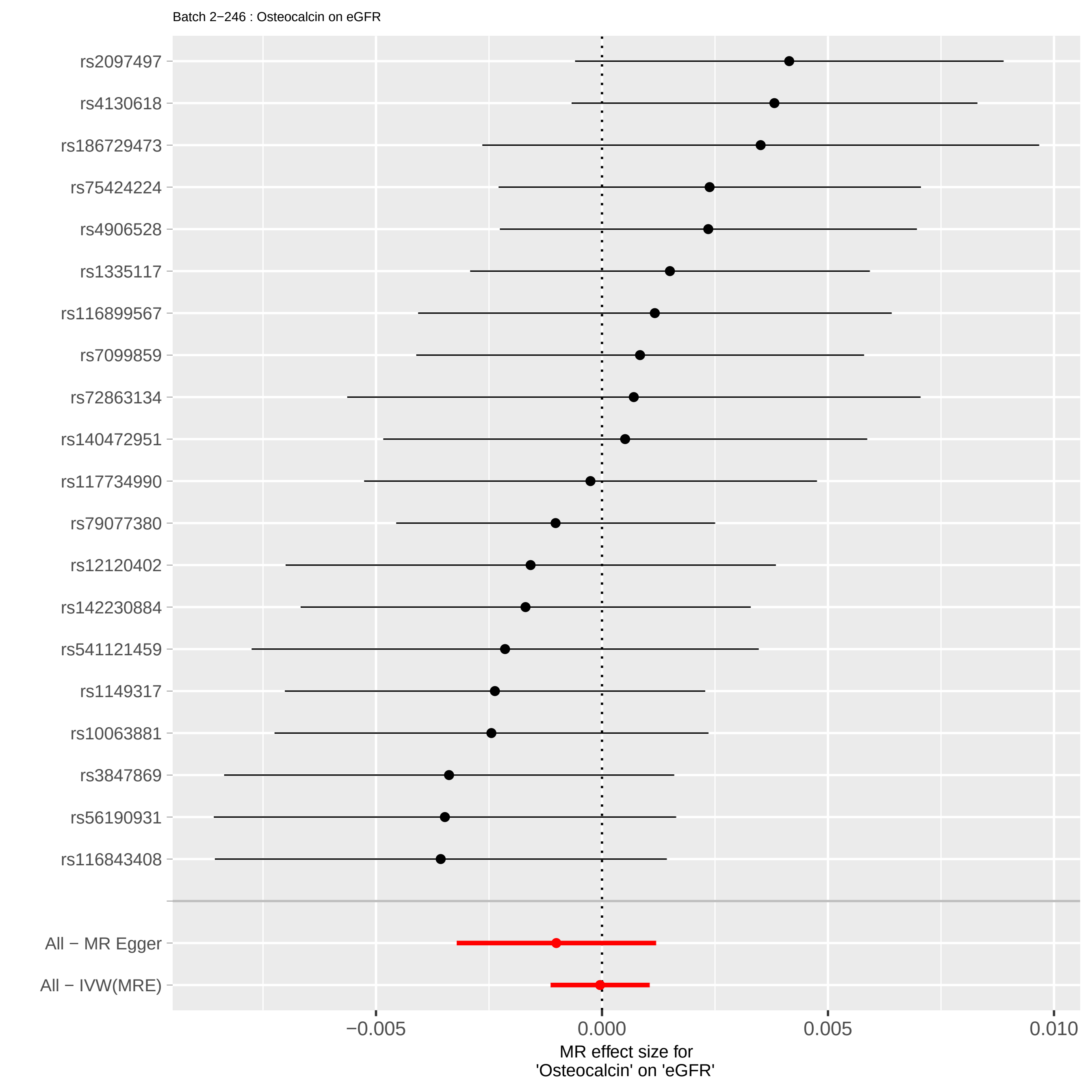

Batch 2−246 : Osteocalcin on eGFR
rs2097497
rs4130618
rs186729473
rs75424224
rs4906528
rs1335117
rs116899567
rs7099859
rs72863134
rs140472951
rs117734990
rs79077380
rs12120402
rs142230884
rs541121459
rs1149317
rs10063881
rs3847869
rs56190931
rs116843408
All − MR Egger
All − IVW(MRE)
−0.005
0.000
0.005
0.010
MR effect size for
'Osteocalcin' on 'eGFR'

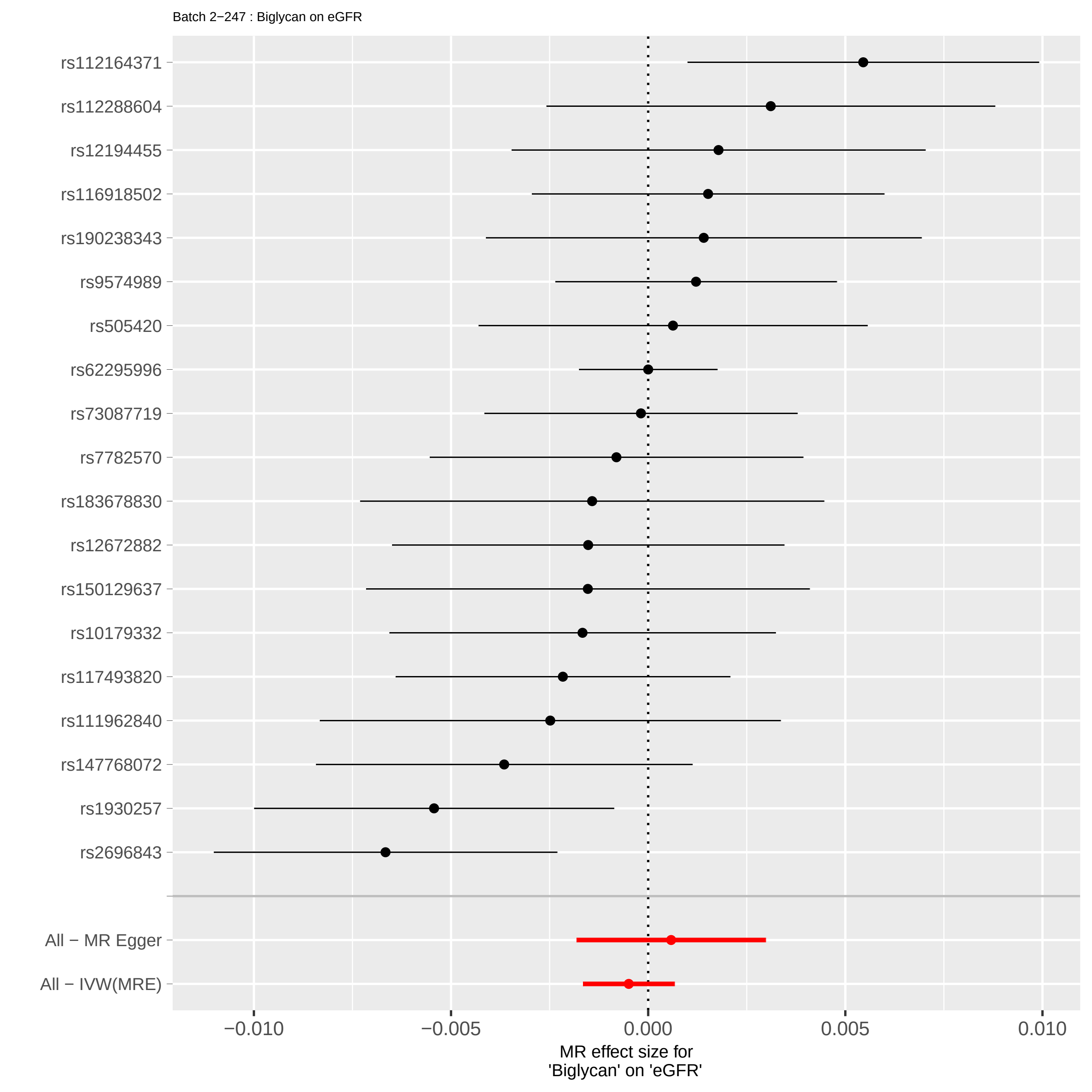

Batch 2−247 : Biglycan on eGFR
rs112164371
rs112288604
rs12194455
rs116918502
rs190238343
rs9574989
rs505420
rs62295996
rs73087719
rs7782570
rs183678830
rs12672882
rs150129637
rs10179332
rs117493820
rs111962840
rs147768072
rs1930257
rs2696843
All − MR Egger
All − IVW(MRE)
−0.010
−0.005
0.000
0.005
0.010
MR effect size for
'Biglycan' on 'eGFR'

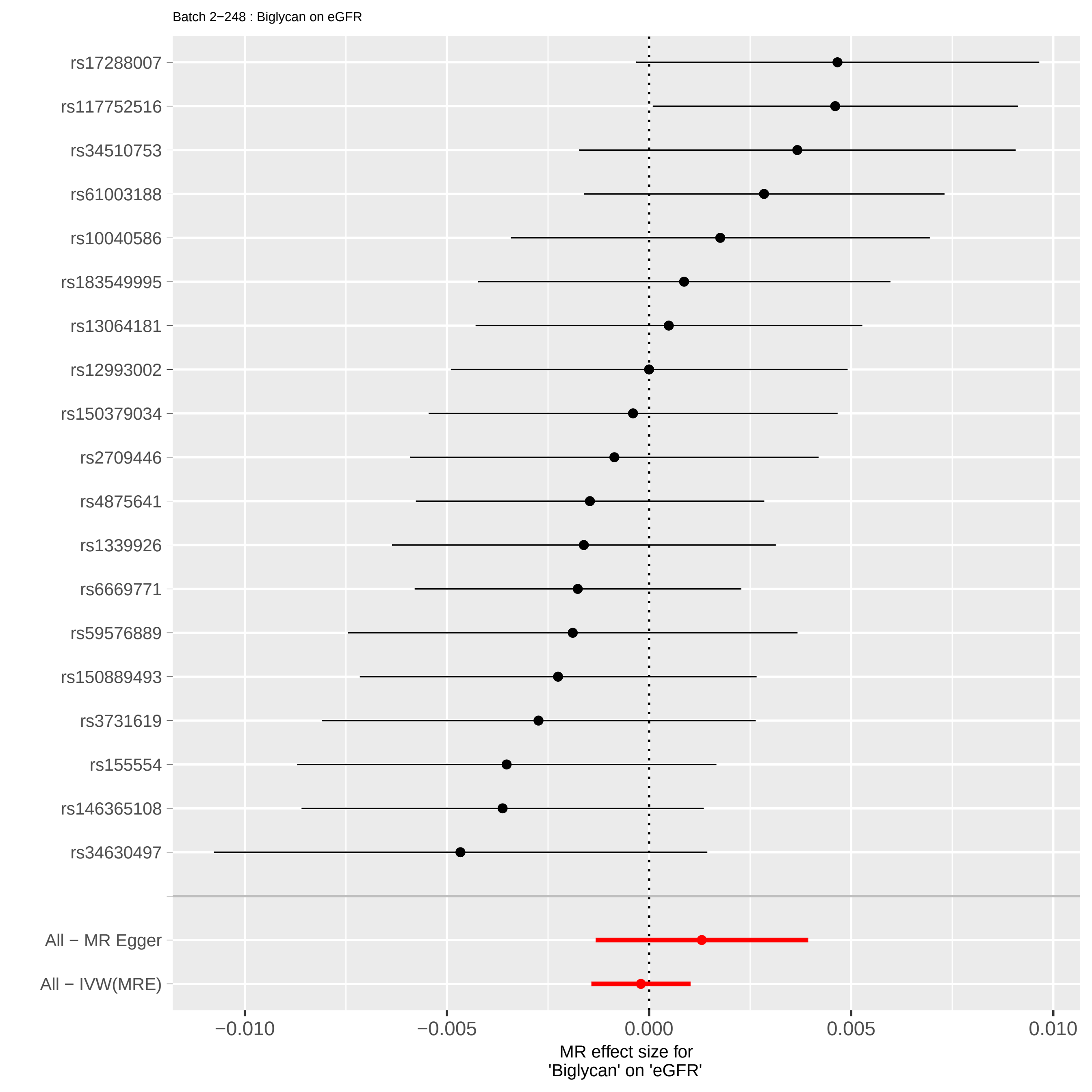

Batch 2−248 : Biglycan on eGFR
rs17288007
rs117752516
rs34510753
rs61003188
rs10040586
rs183549995
rs13064181
rs12993002
rs150379034
rs2709446
rs4875641
rs1339926
rs6669771
rs59576889
rs150889493
rs3731619
rs155554
rs146365108
rs34630497
All − MR Egger
All − IVW(MRE)
−0.010
−0.005
0.000
0.005
0.010
MR effect size for
'Biglycan' on 'eGFR'

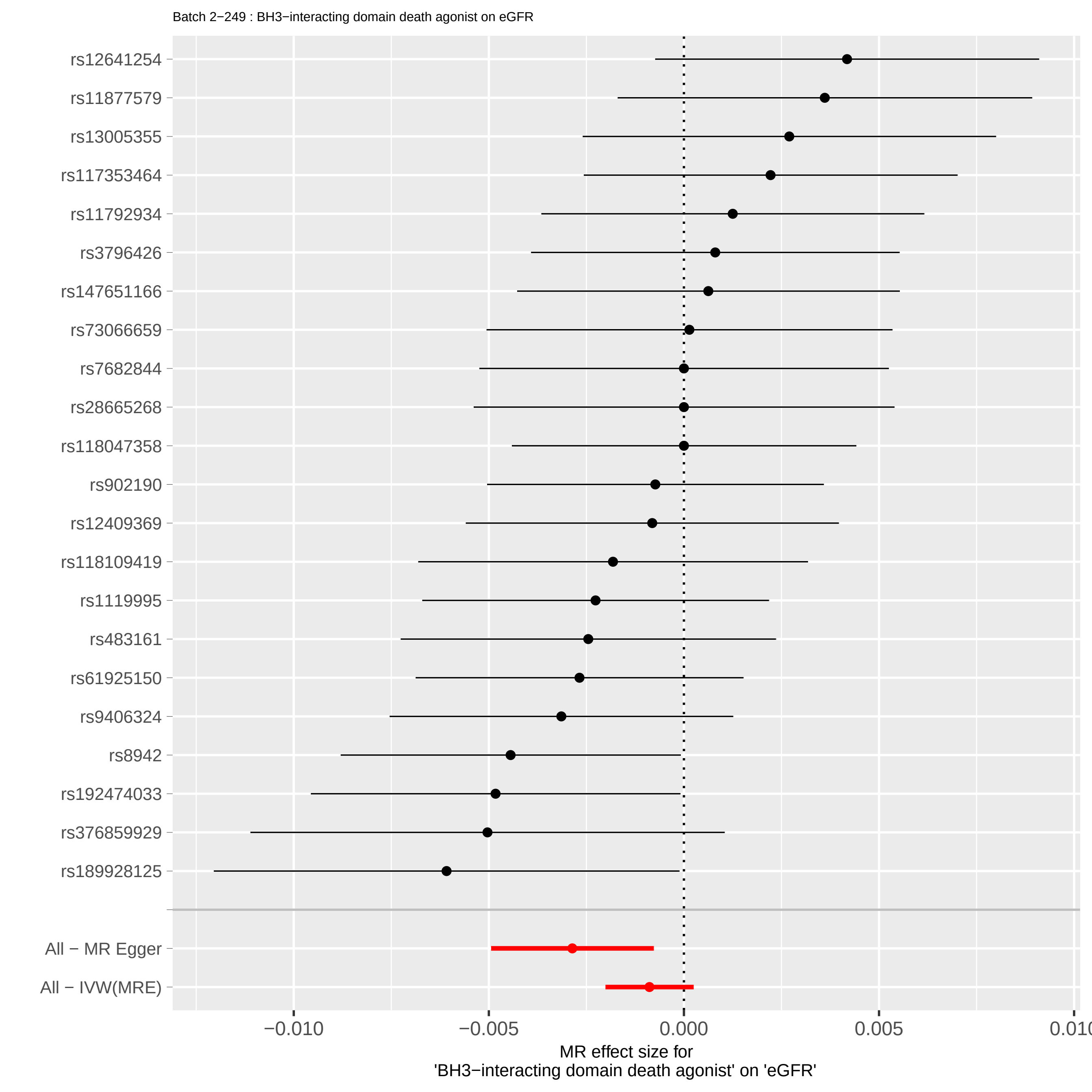

Batch 2−249 : BH3−interacting domain death agonist on eGFR
rs12641254
rs11877579
rs13005355
rs117353464
rs11792934
rs3796426
rs147651166
rs73066659
rs7682844
rs28665268
rs118047358
rs902190
rs12409369
rs118109419
rs1119995
rs483161
rs61925150
rs9406324
rs8942
rs192474033
rs376859929
rs189928125
All − MR Egger
All − IVW(MRE)
−0.010
−0.005
0.000
0.005
0.010
MR effect size for
'BH3−interacting domain death agonist' on 'eGFR'

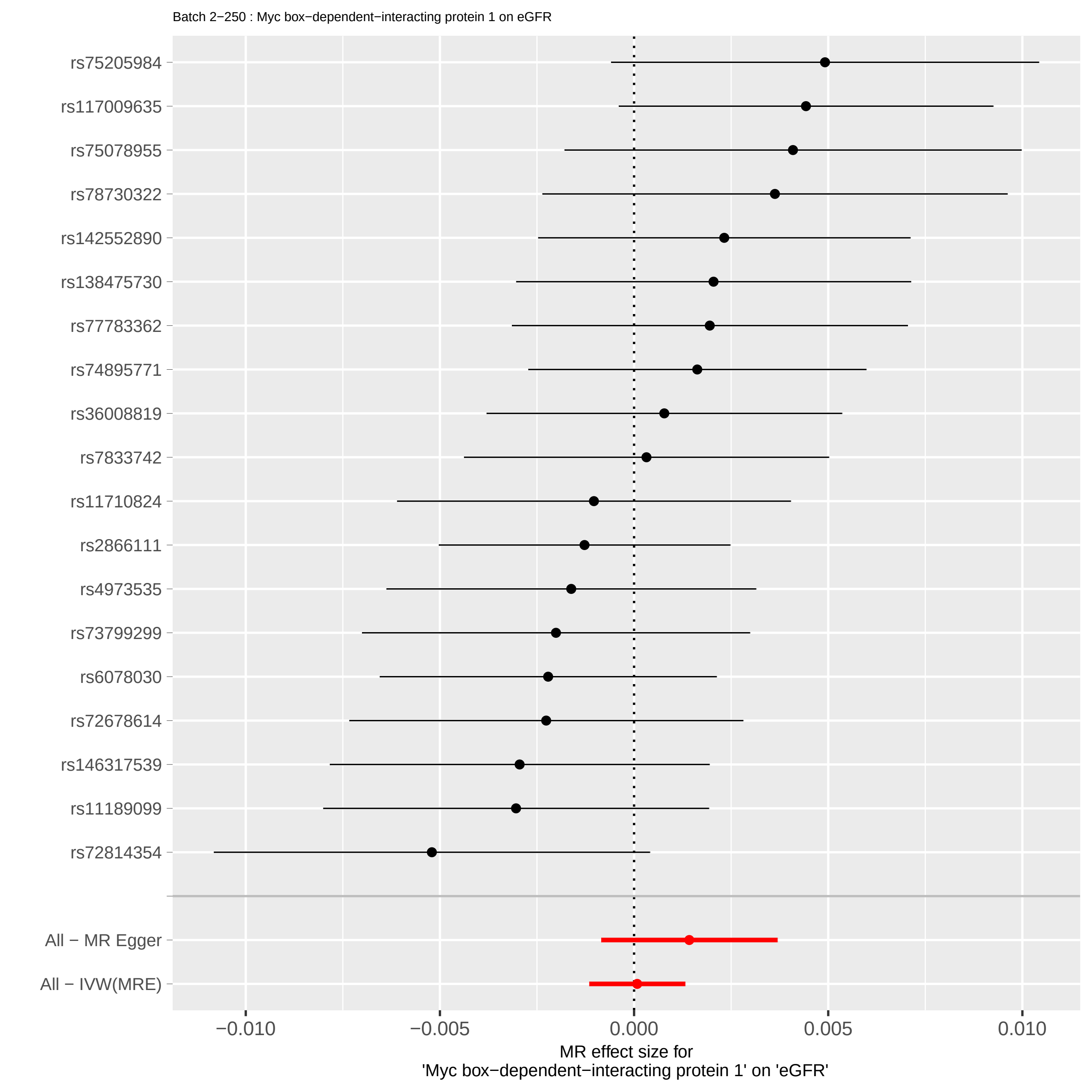
Batch 2−250 : Myc box−dependent−interacting protein 1 on eGFR
rs75205984
rs117009635
rs75078955
rs78730322
rs142552890
rs138475730
rs77783362
rs74895771
rs36008819
rs7833742
rs11710824
rs2866111
rs4973535
rs73799299
rs6078030
rs72678614
rs146317539
rs11189099
rs72814354
All − MR Egger
All − IVW(MRE)
−0.010
−0.005
0.000
0.005
0.010
MR effect size for
'Myc box−dependent−interacting protein 1' on 'eGFR'

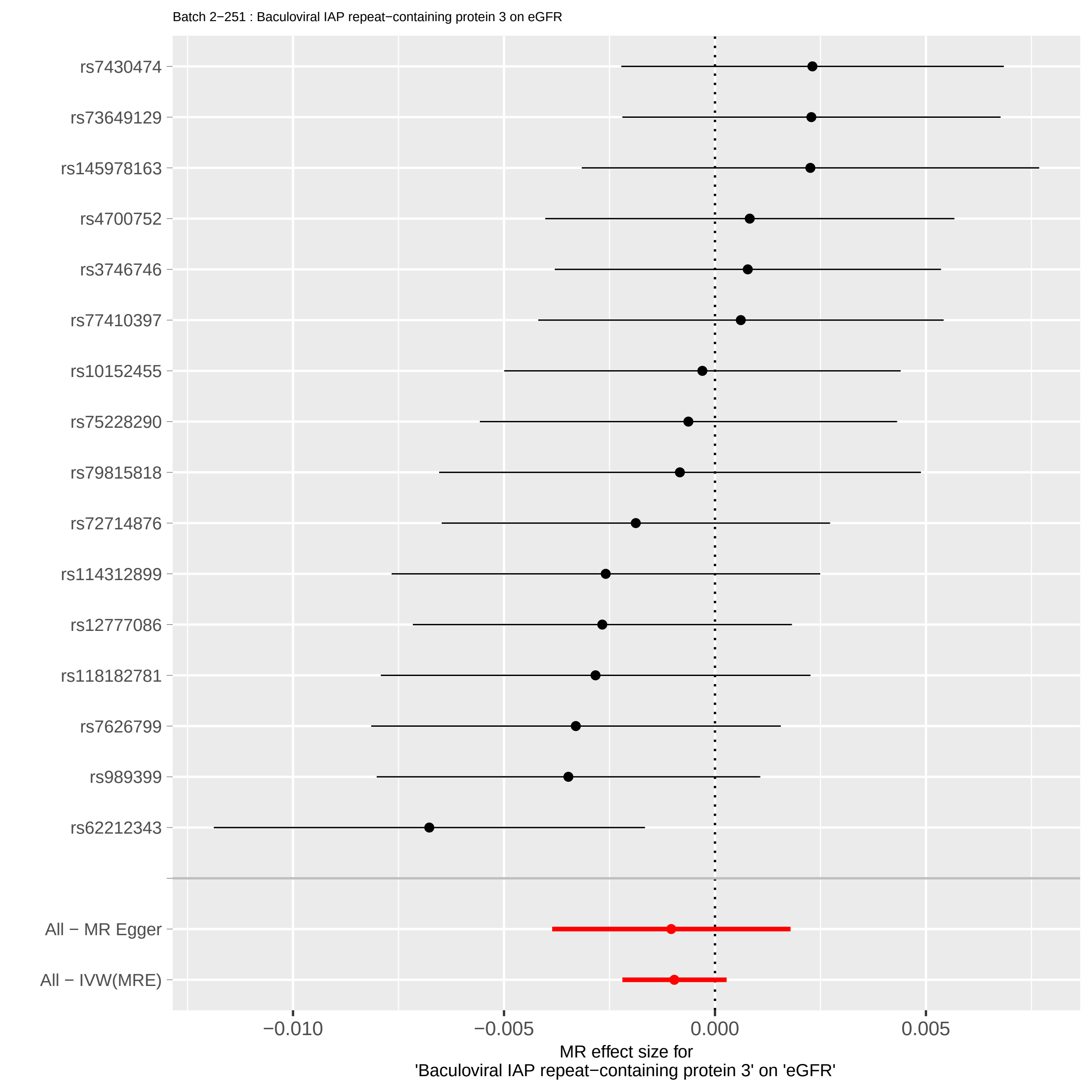

Batch 2−251 : Baculoviral IAP repeat−containing protein 3 on eGFR
rs7430474
rs73649129
rs145978163
rs4700752
rs3746746
rs77410397
rs10152455
rs75228290
rs79815818
rs72714876
rs114312899
rs12777086
rs118182781
rs7626799
rs989399
rs62212343
All − MR Egger
All − IVW(MRE)
−0.010
−0.005
0.000
0.005
MR effect size for
'Baculoviral IAP repeat−containing protein 3' on 'eGFR'

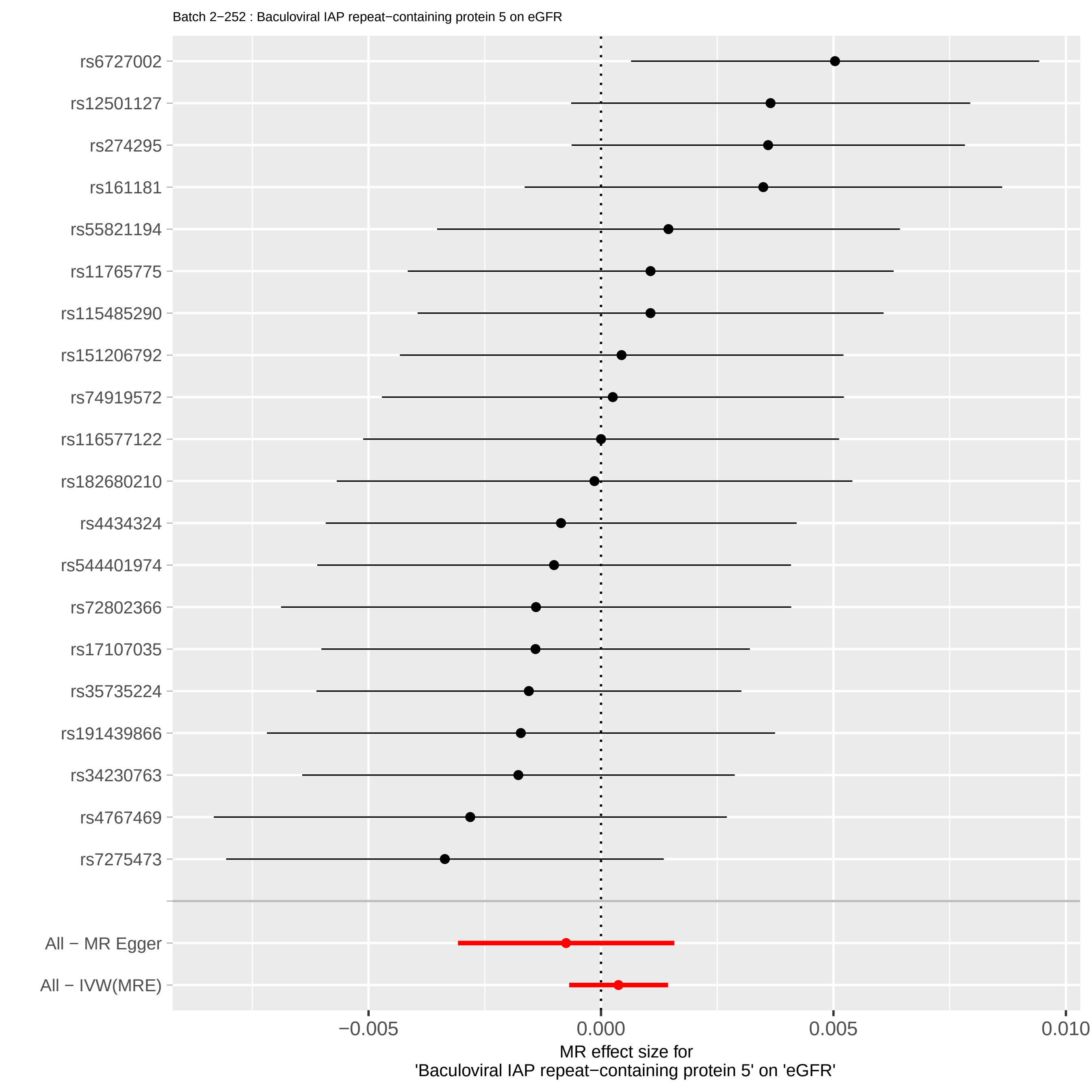

Batch 2−252 : Baculoviral IAP repeat−containing protein 5 on eGFR
rs6727002
rs12501127
rs274295
rs161181
rs55821194
rs11765775
rs115485290
rs151206792
rs74919572
rs116577122
rs182680210
rs4434324
rs544401974
rs72802366
rs17107035
rs35735224
rs191439866
rs34230763
rs4767469
rs7275473
All − MR Egger
All − IVW(MRE)
−0.005
0.000
0.005
0.010
MR effect size for
'Baculoviral IAP repeat−containing protein 5' on 'eGFR'

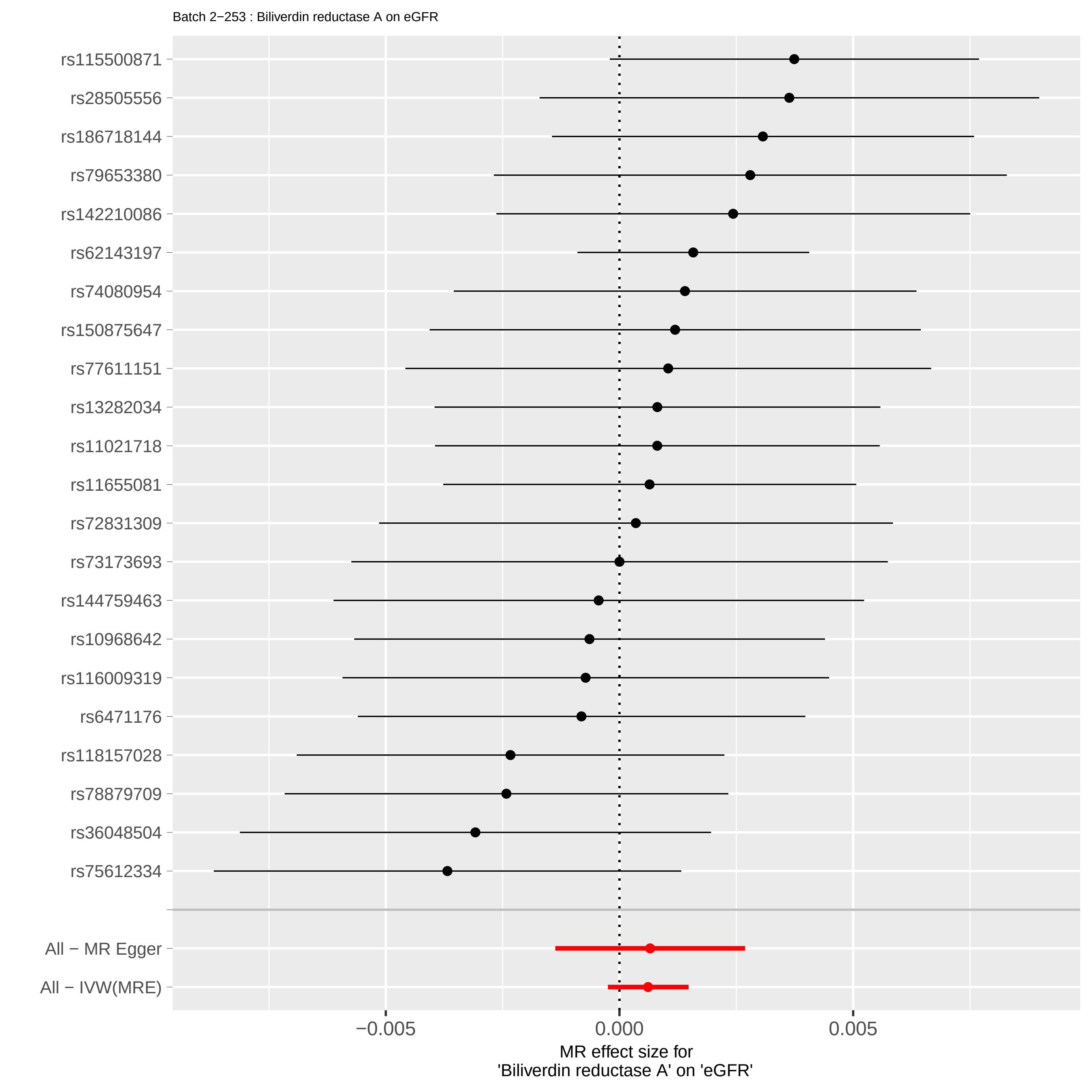
Batch 2−253 : Biliverdin reductase A on eGFR
rs115500871
rs28505556
rs186718144
rs79653380
rs142210086
rs62143197
rs74080954
rs150875647
rs77611151
rs13282034
rs11021718
rs11655081
rs72831309
rs73173693
rs144759463
rs10968642
rs116009319
rs6471176
rs118157028
rs78879709
rs36048504
rs75612334
All − MR Egger
All − IVW(MRE)
−0.005
0.000
0.005
MR effect size for
'Biliverdin reductase A' on 'eGFR'

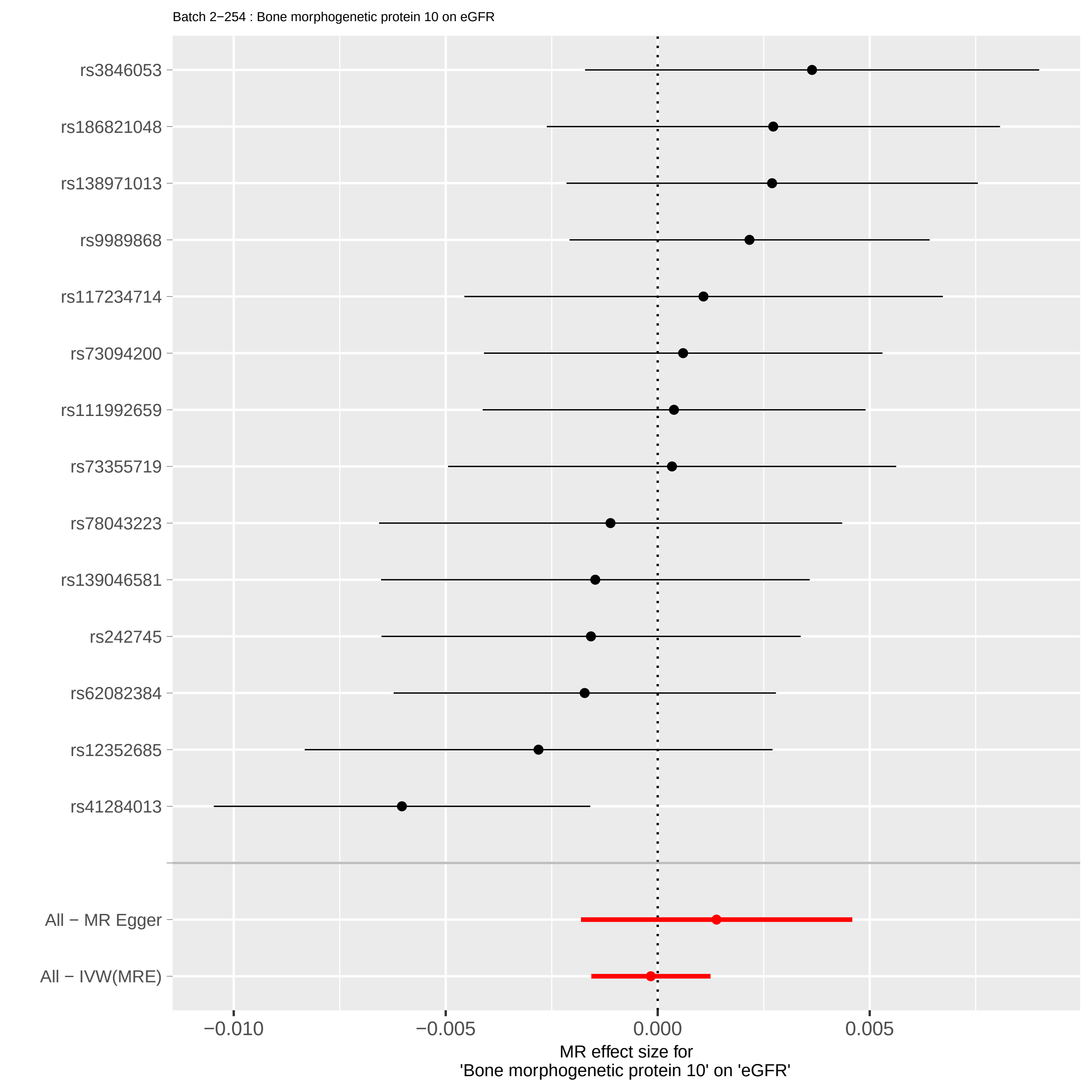

Batch 2−254 : Bone morphogenetic protein 10 on eGFR
rs3846053
rs186821048
rs138971013
rs9989868
rs117234714
rs73094200
rs111992659
rs73355719
rs78043223
rs139046581
rs242745
rs62082384
rs12352685
rs41284013
All − MR Egger
All − IVW(MRE)
−0.010
−0.005
0.000
0.005
MR effect size for
'Bone morphogenetic protein 10' on 'eGFR'

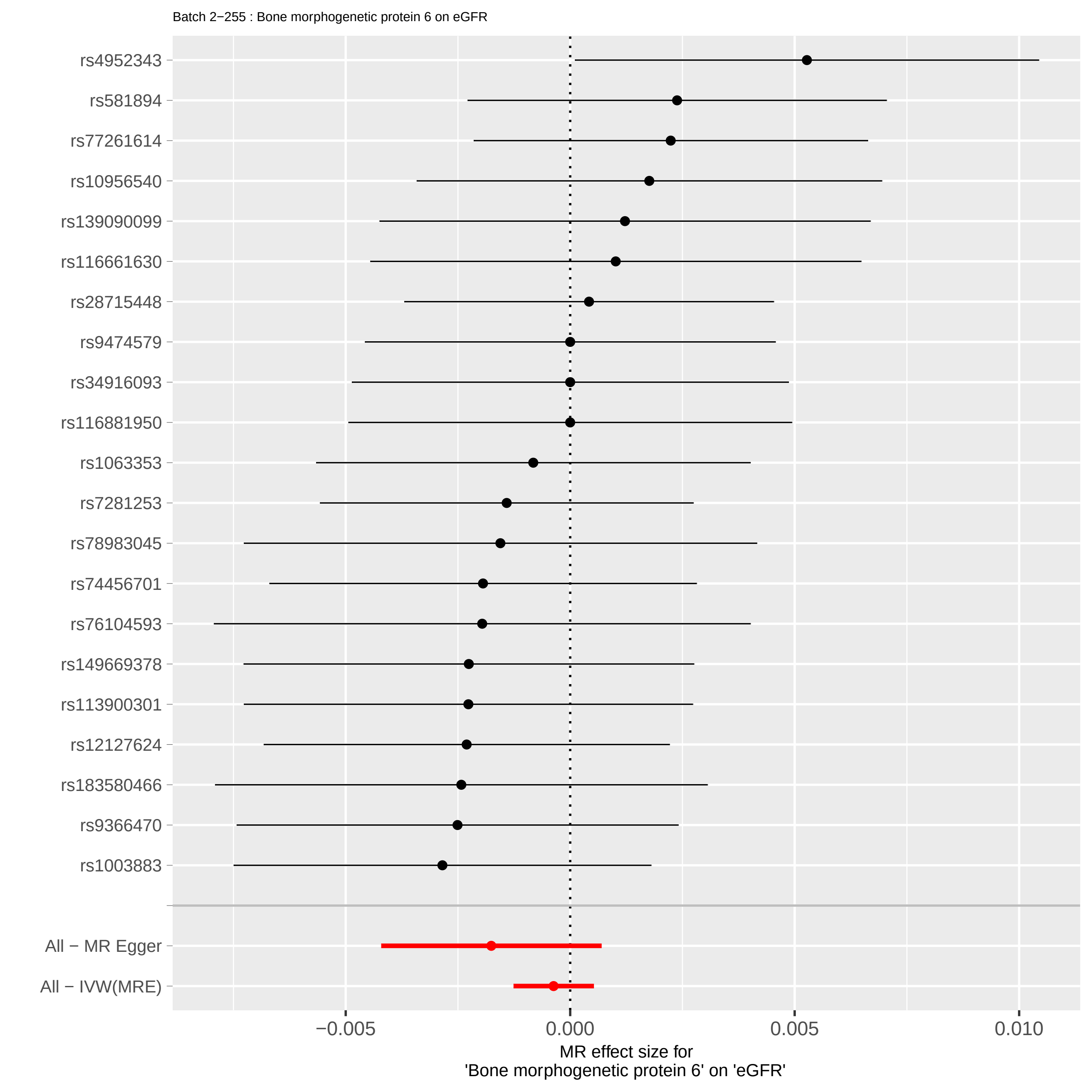

Batch 2−255 : Bone morphogenetic protein 6 on eGFR
rs4952343
rs581894
rs77261614
rs10956540
rs139090099
rs116661630
rs28715448
rs9474579
rs34916093
rs116881950
rs1063353
rs7281253
rs78983045
rs74456701
rs76104593
rs149669378
rs113900301
rs12127624
rs183580466
rs9366470
rs1003883
All − MR Egger
All − IVW(MRE)
−0.005
0.000
0.005
0.010
MR effect size for
'Bone morphogenetic protein 6' on 'eGFR'

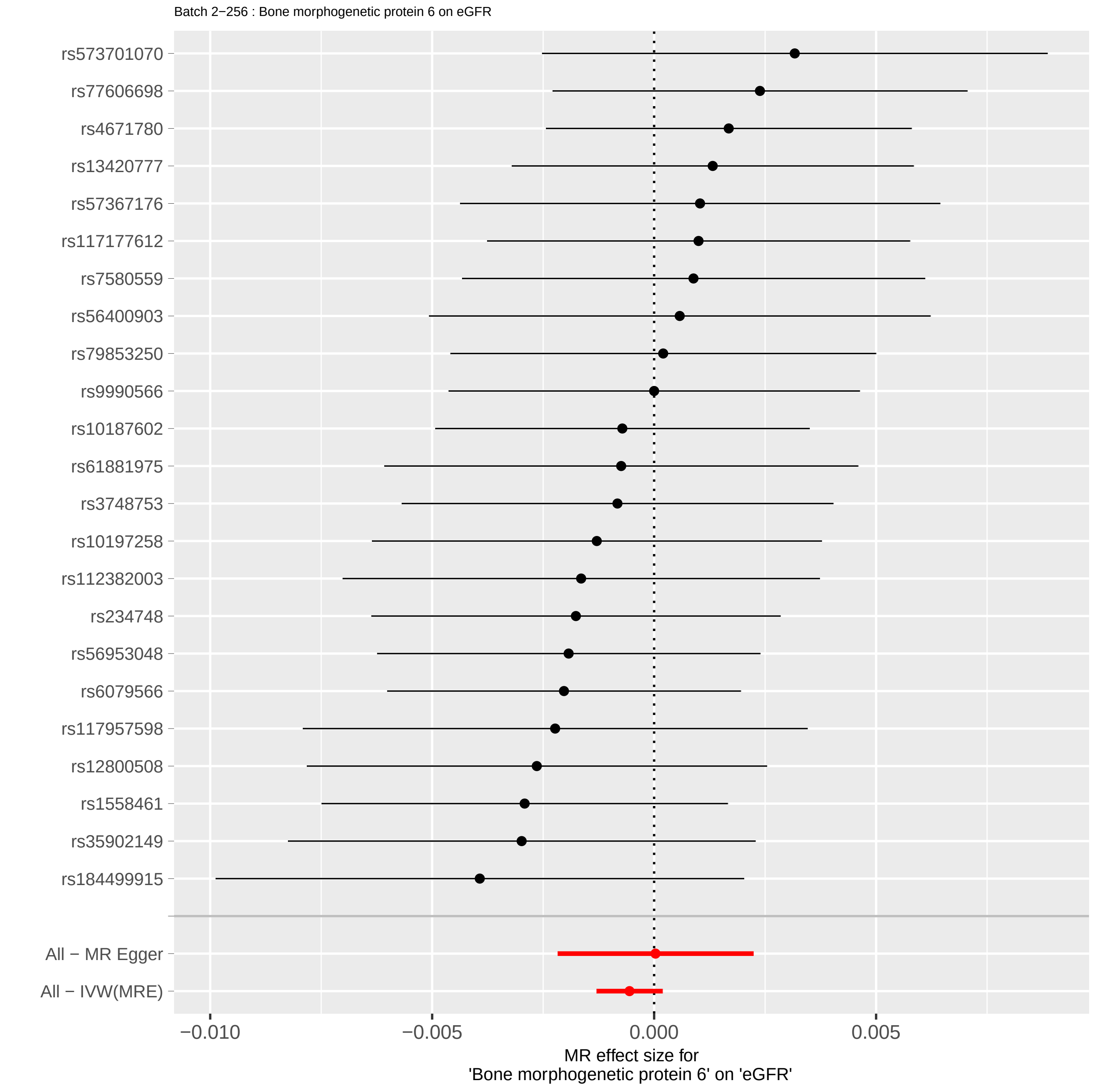

Batch 2−256 : Bone morphogenetic protein 6 on eGFR
rs573701070
rs77606698
rs4671780
rs13420777
rs57367176
rs117177612
rs7580559
rs56400903
rs79853250
rs9990566
rs10187602
rs61881975
rs3748753
rs10197258
rs112382003
rs234748
rs56953048
rs6079566
rs117957598
rs12800508
rs1558461
rs35902149
rs184499915
All − MR Egger
All − IVW(MRE)
−0.010
−0.005
0.000
0.005
MR effect size for
'Bone morphogenetic protein 6' on 'eGFR'

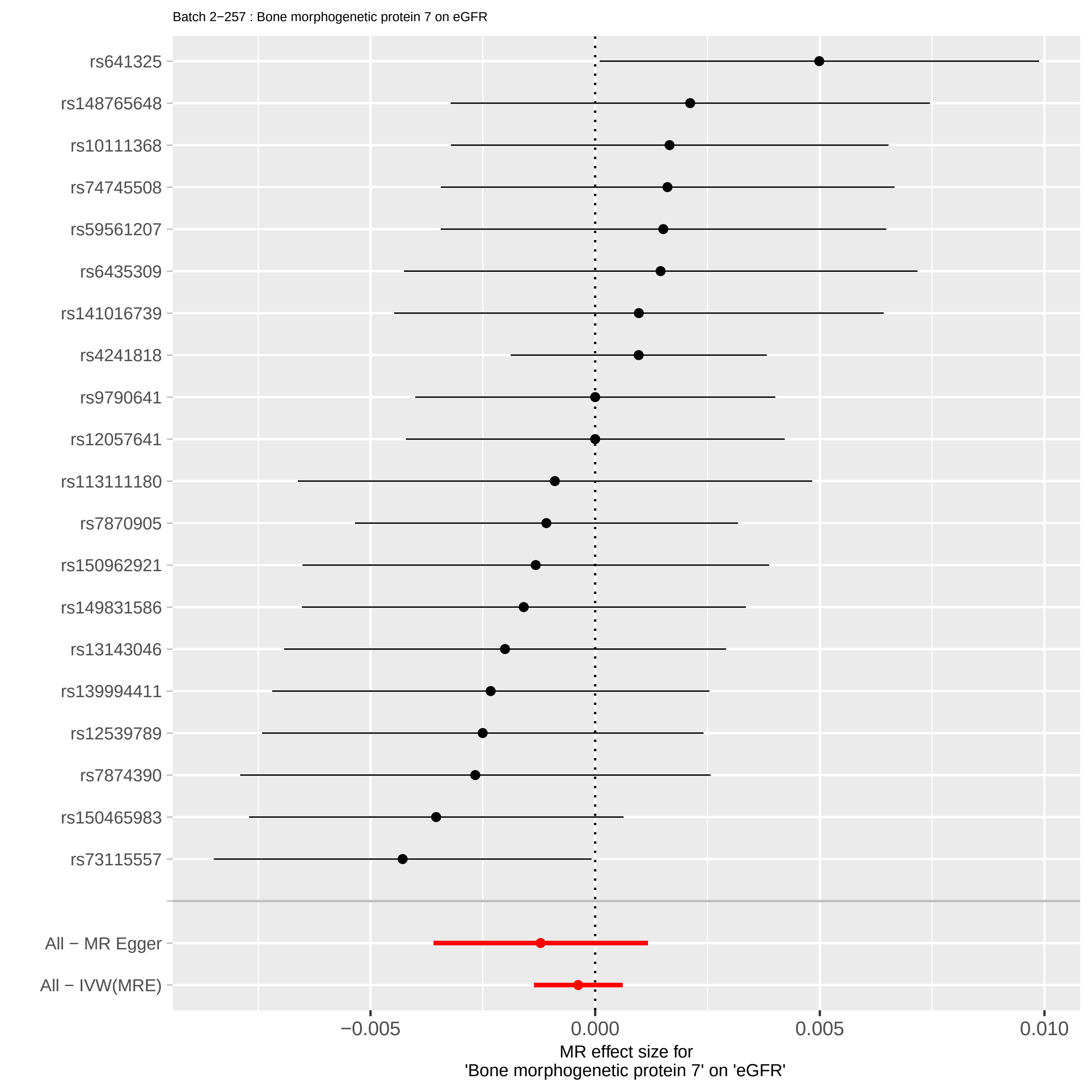

Batch 2−257 : Bone morphogenetic protein 7 on eGFR
rs641325
rs148765648
rs10111368
rs74745508
rs59561207
rs6435309
rs141016739
rs4241818
rs9790641
rs12057641
rs113111180
rs7870905
rs150962921
rs149831586
rs13143046
rs139994411
rs12539789
rs7874390
rs150465983
rs73115557
All − MR Egger
All − IVW(MRE)
−0.005
0.000
0.005
0.010
MR effect size for
'Bone morphogenetic protein 7' on 'eGFR'

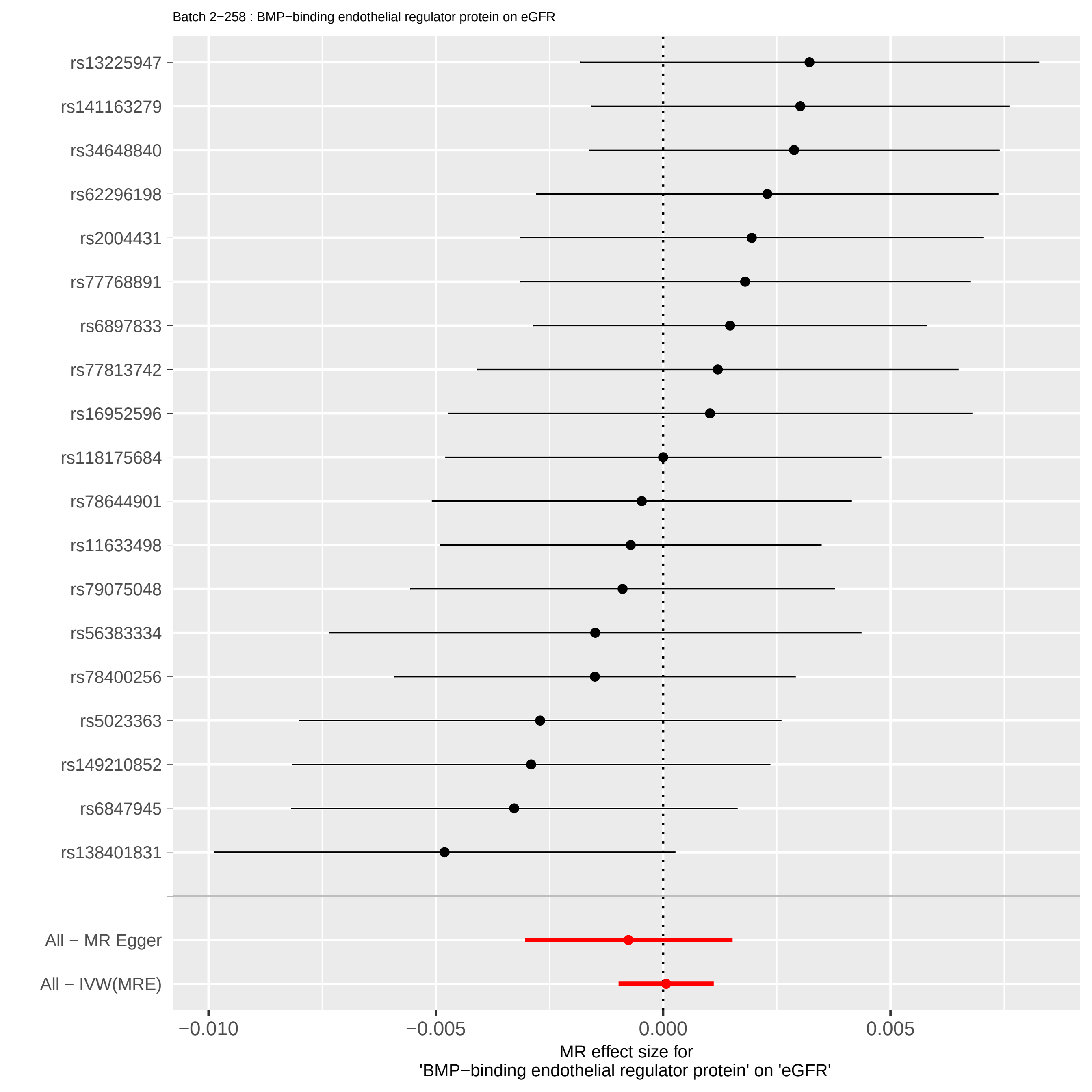
Batch 2−258 : BMP−binding endothelial regulator protein on eGFR
rs13225947
rs141163279
rs34648840
rs62296198
rs2004431
rs77768891
rs6897833
rs77813742
rs16952596
rs118175684
rs78644901
rs11633498
rs79075048
rs56383334
rs78400256
rs5023363
rs149210852
rs6847945
rs138401831
All − MR Egger
All − IVW(MRE)
−0.010
−0.005
0.000
0.005
MR effect size for
'BMP−binding endothelial regulator protein' on 'eGFR'

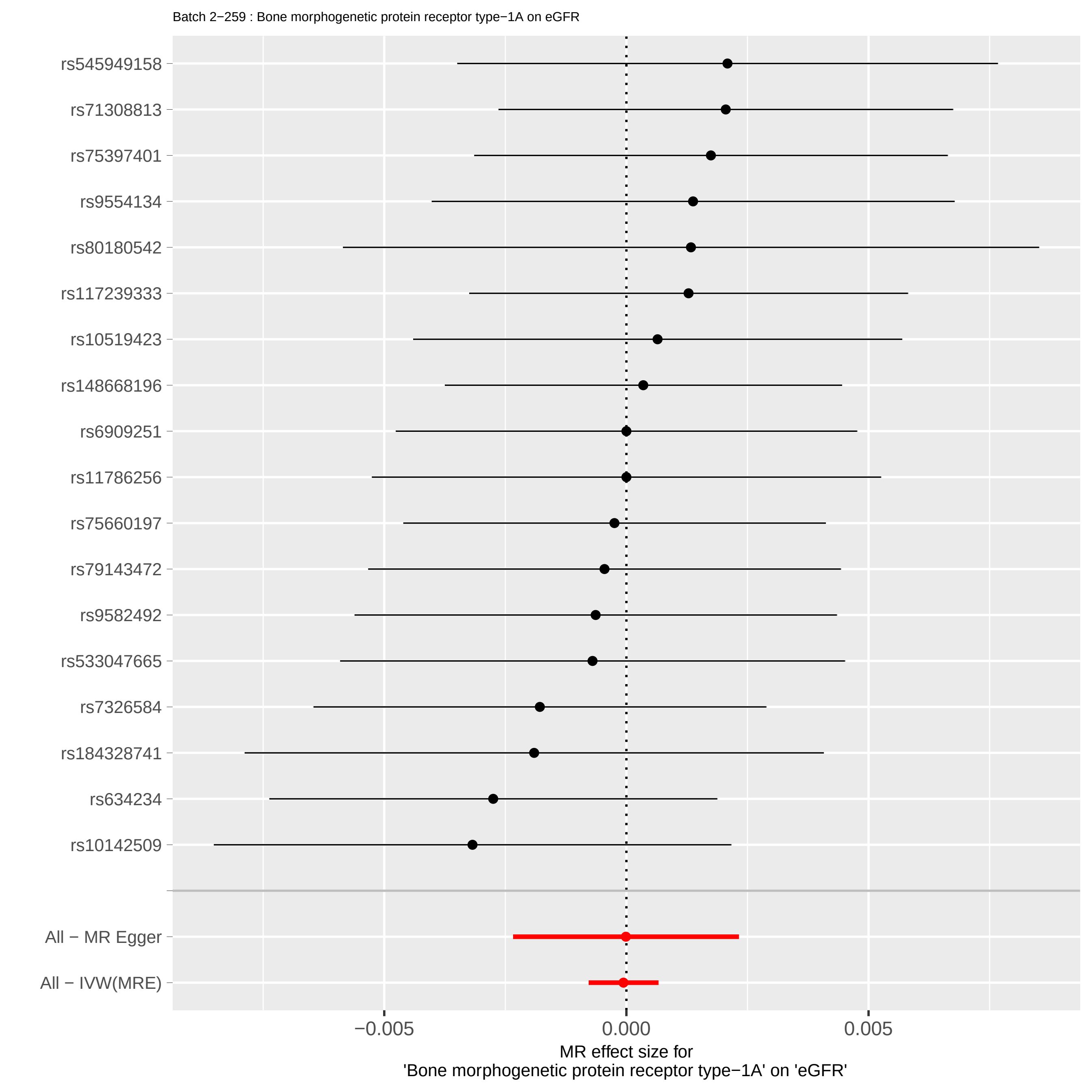

Batch 2−259 : Bone morphogenetic protein receptor type−1A on eGFR
rs545949158
rs71308813
rs75397401
rs9554134
rs80180542
rs117239333
rs10519423
rs148668196
rs6909251
rs11786256
rs75660197
rs79143472
rs9582492
rs533047665
rs7326584
rs184328741
rs634234
rs10142509
All − MR Egger
All − IVW(MRE)
−0.005
0.000
0.005
MR effect size for
'Bone morphogenetic protein receptor type−1A' on 'eGFR'

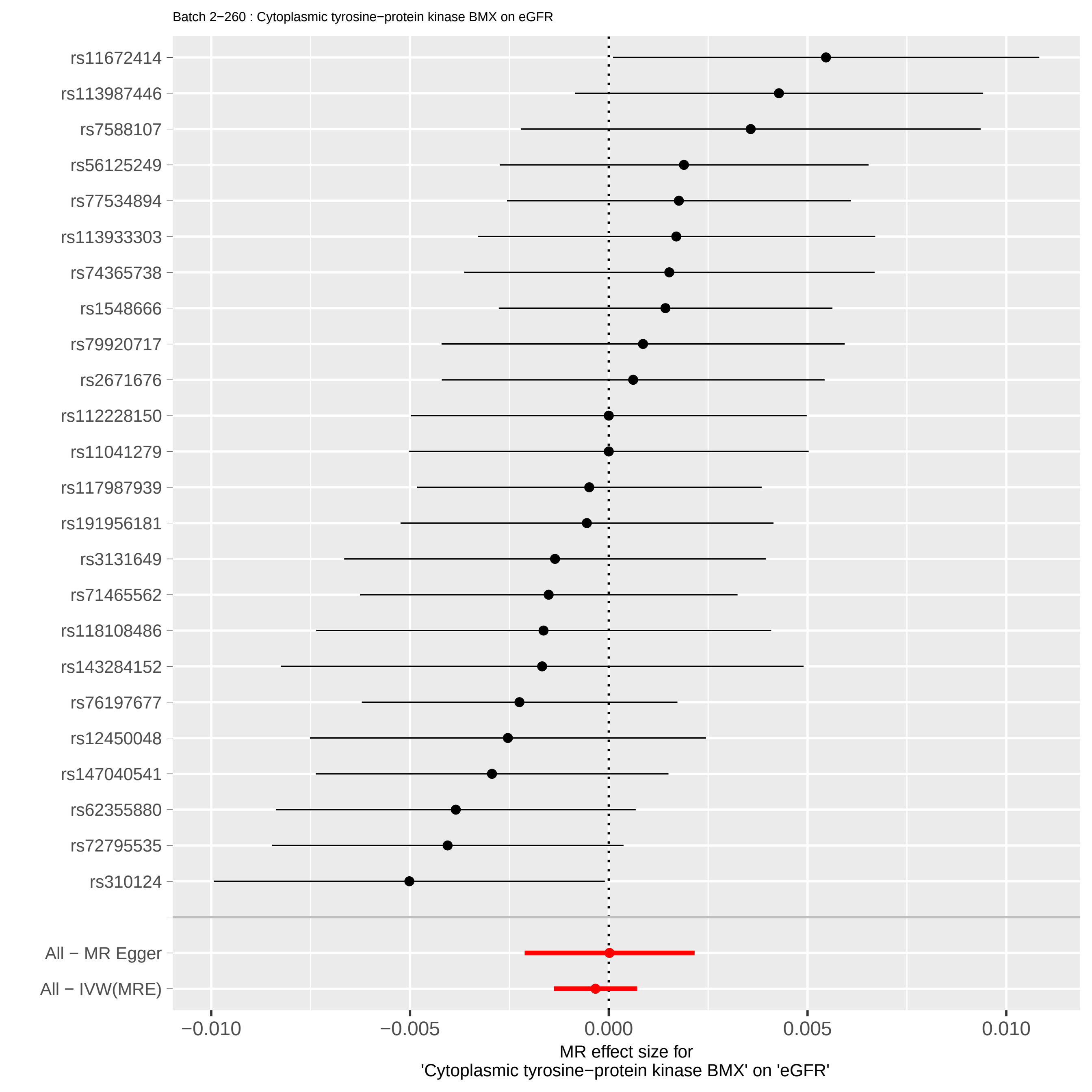
Batch 2−260 : Cytoplasmic tyrosine−protein kinase BMX on eGFR
rs11672414
rs113987446
rs7588107
rs56125249
rs77534894
rs113933303
rs74365738
rs1548666
rs79920717
rs2671676
rs112228150
rs11041279
rs117987939
rs191956181
rs3131649
rs71465562
rs118108486
rs143284152
rs76197677
rs12450048
rs147040541
rs62355880
rs72795535
rs310124
All − MR Egger
All − IVW(MRE)
−0.010
−0.005
0.000
0.005
0.010
MR effect size for
'Cytoplasmic tyrosine−protein kinase BMX' on 'eGFR'

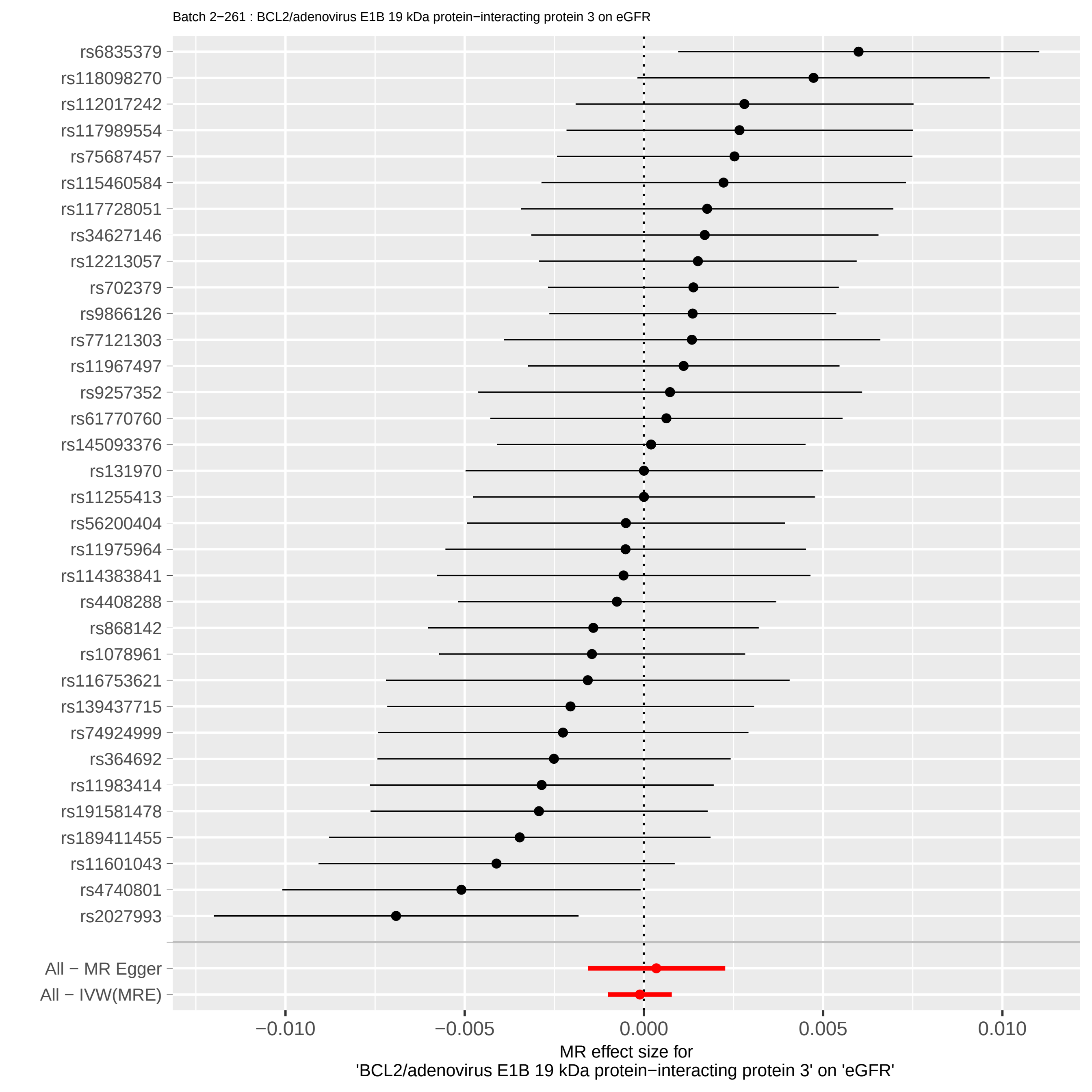

Batch 2−261 : BCL2/adenovirus E1B 19 kDa protein−interacting protein 3 on eGFR
rs6835379
rs118098270
rs112017242
rs117989554
rs75687457
rs115460584
rs117728051
rs34627146
rs12213057
rs702379
rs9866126
rs77121303
rs11967497
rs9257352
rs61770760
rs145093376
rs131970
rs11255413
rs56200404
rs11975964
rs114383841
rs4408288
rs868142
rs1078961
rs116753621
rs139437715
rs74924999
rs364692
rs11983414
rs191581478
rs189411455
rs11601043
rs4740801
rs2027993
All − MR Egger
All − IVW(MRE)
−0.010
−0.005
0.000
0.005
0.010
MR effect size for
'BCL2/adenovirus E1B 19 kDa protein−interacting protein 3' on 'eGFR'

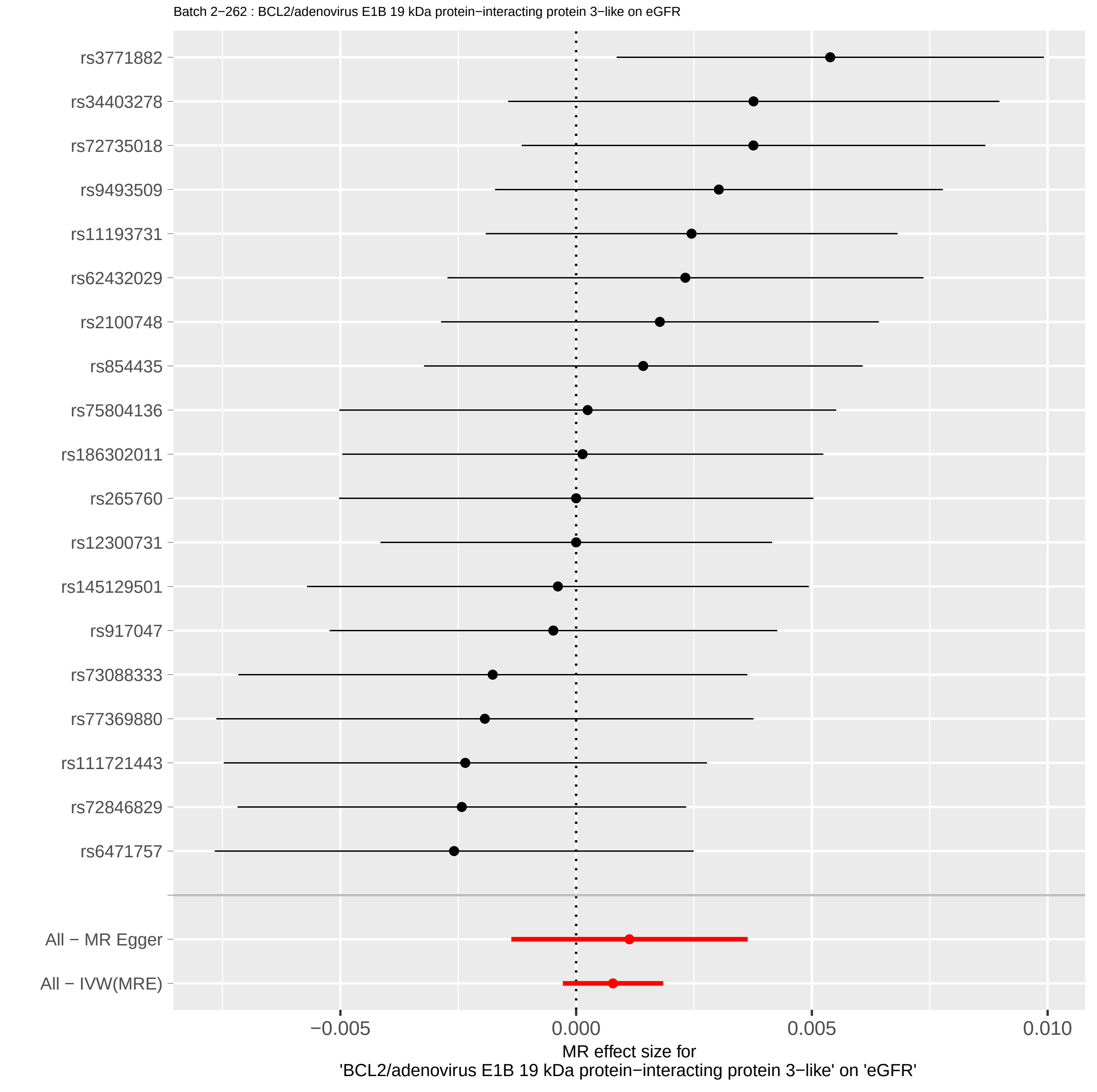

Batch 2−262 : BCL2/adenovirus E1B 19 kDa protein−interacting protein 3−like on eGFR
rs3771882
rs34403278
rs72735018
rs9493509
rs11193731
rs62432029
rs2100748
rs854435
rs75804136
rs186302011
rs265760
rs12300731
rs145129501
rs917047
rs73088333
rs77369880
rs111721443
rs72846829
rs6471757
All − MR Egger
All − IVW(MRE)
−0.005
0.000
0.005
0.010
MR effect size for
'BCL2/adenovirus E1B 19 kDa protein−interacting protein 3−like' on 'eGFR'

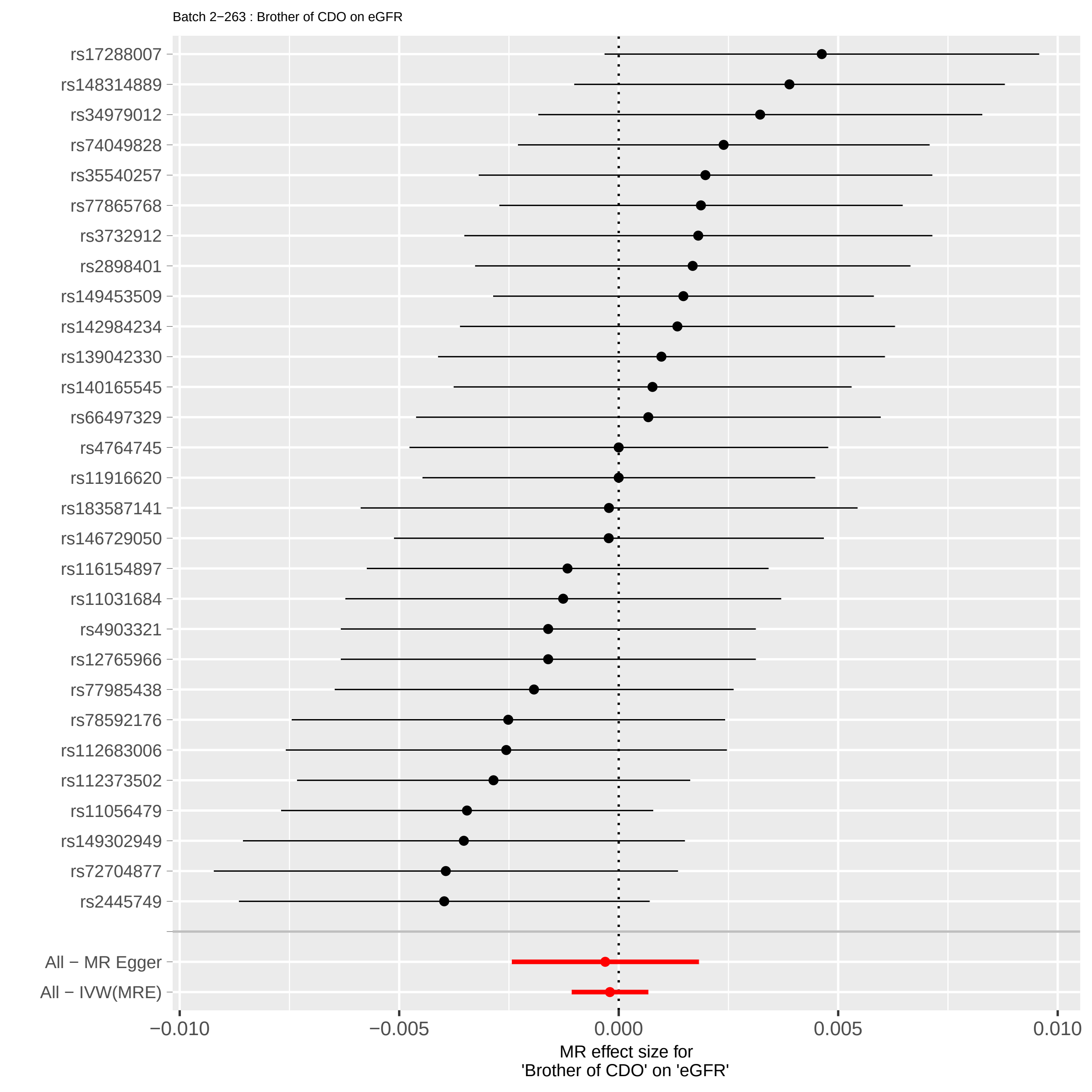

Batch 2−263 : Brother of CDO on eGFR
rs17288007
rs148314889
rs34979012
rs74049828
rs35540257
rs77865768
rs3732912
rs2898401
rs149453509
rs142984234
rs139042330
rs140165545
rs66497329
rs4764745
rs11916620
rs183587141
rs146729050
rs116154897
rs11031684
rs4903321
rs12765966
rs77985438
rs78592176
rs112683006
rs112373502
rs11056479
rs149302949
rs72704877
rs2445749
All − MR Egger
All − IVW(MRE)
−0.010
−0.005
0.000
0.005
0.010
MR effect size for
'Brother of CDO' on 'eGFR'

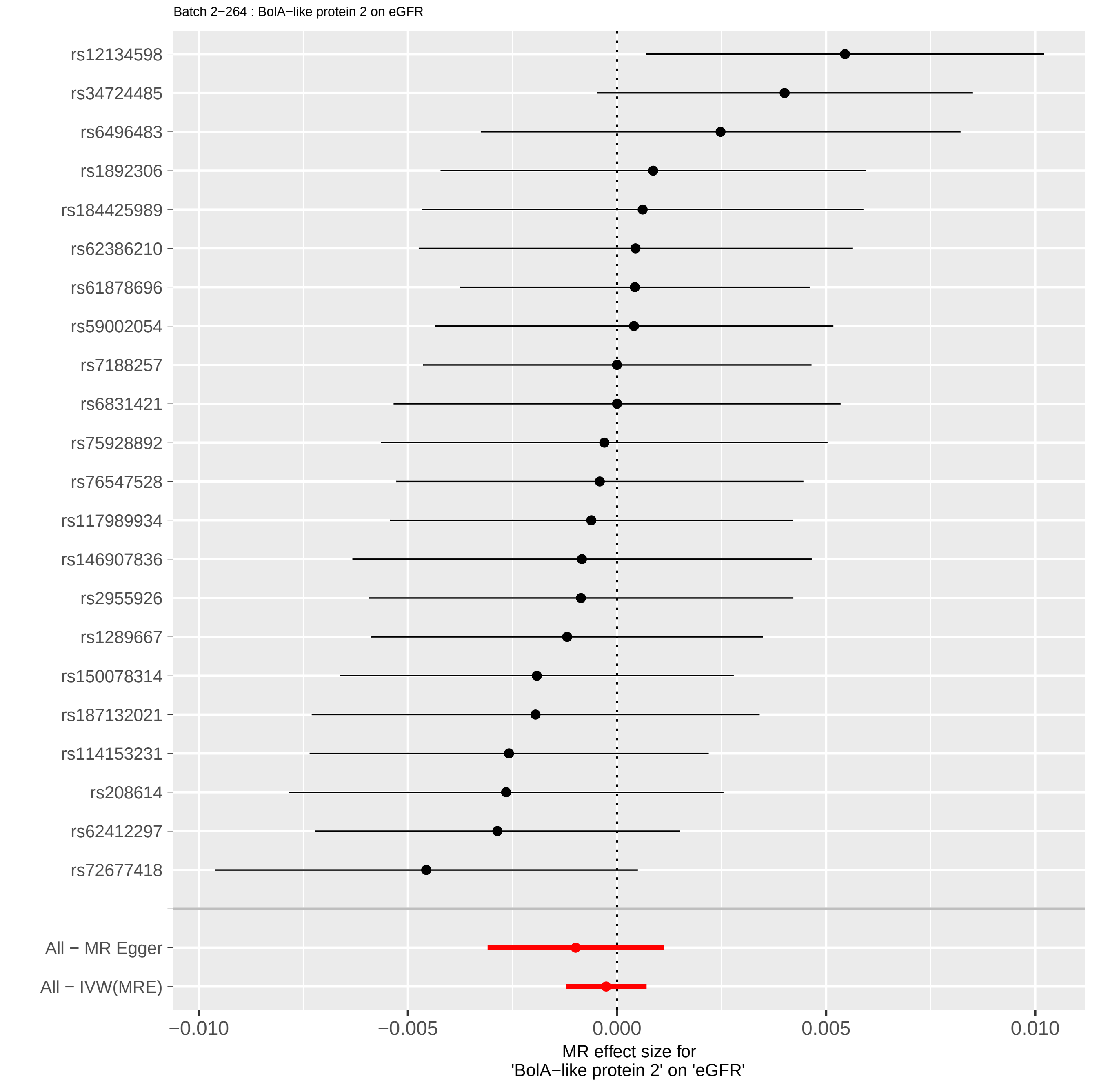

Batch 2−264 : BolA−like protein 2 on eGFR
rs12134598
rs34724485
rs6496483
rs1892306
rs184425989
rs62386210
rs61878696
rs59002054
rs7188257
rs6831421
rs75928892
rs76547528
rs117989934
rs146907836
rs2955926
rs1289667
rs150078314
rs187132021
rs114153231
rs208614
rs62412297
rs72677418
All − MR Egger
All − IVW(MRE)
−0.010
−0.005
0.000
0.005
0.010
MR effect size for
'BolA−like protein 2' on 'eGFR'

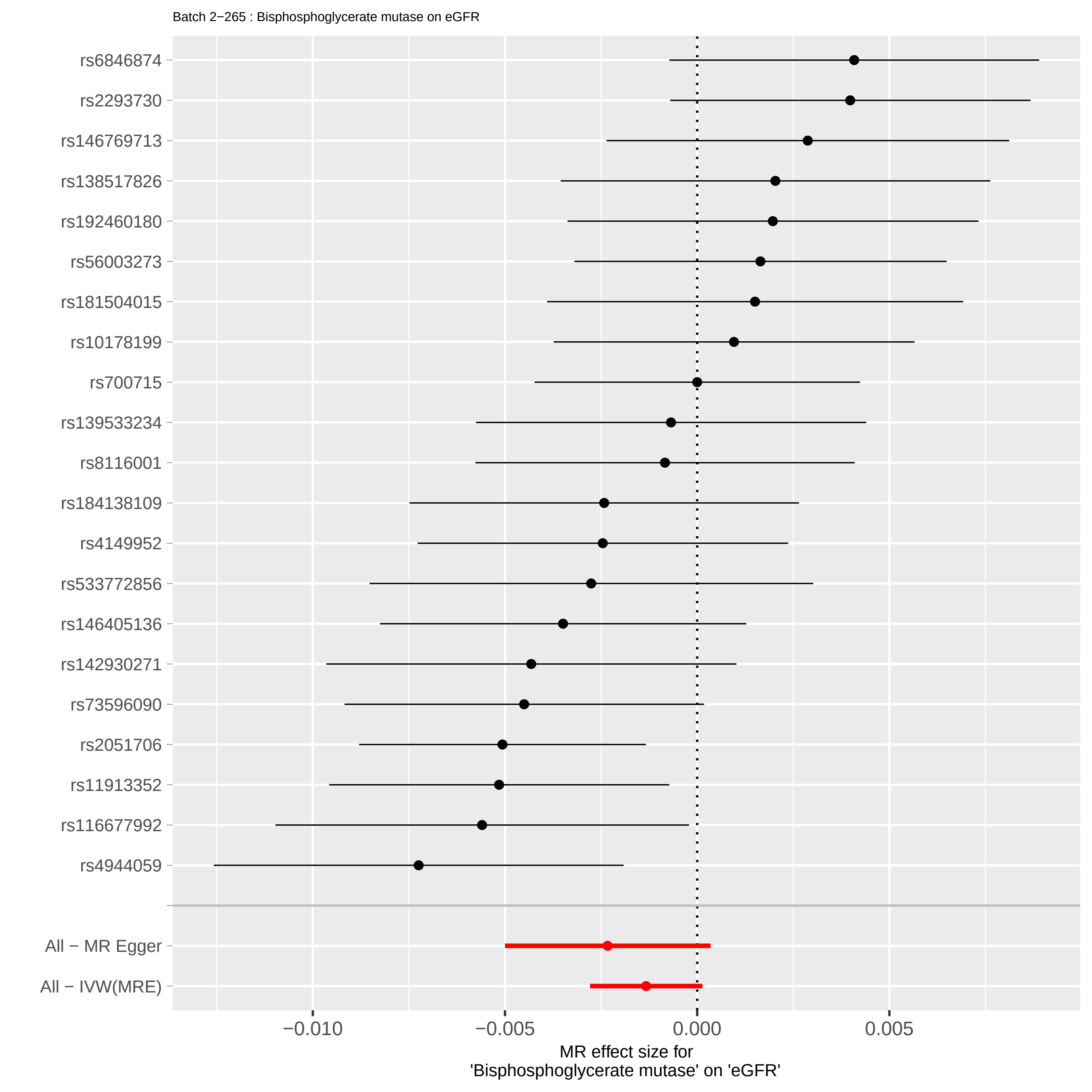

Batch 2−265 : Bisphosphoglycerate mutase on eGFR
rs6846874
rs2293730
rs146769713
rs138517826
rs192460180
rs56003273
rs181504015
rs10178199
rs700715
rs139533234
rs8116001
rs184138109
rs4149952
rs533772856
rs146405136
rs142930271
rs73596090
rs2051706
rs11913352
rs116677992
rs4944059
All − MR Egger
All − IVW(MRE)
−0.010
−0.005
0.000
0.005
MR effect size for
'Bisphosphoglycerate mutase' on 'eGFR'

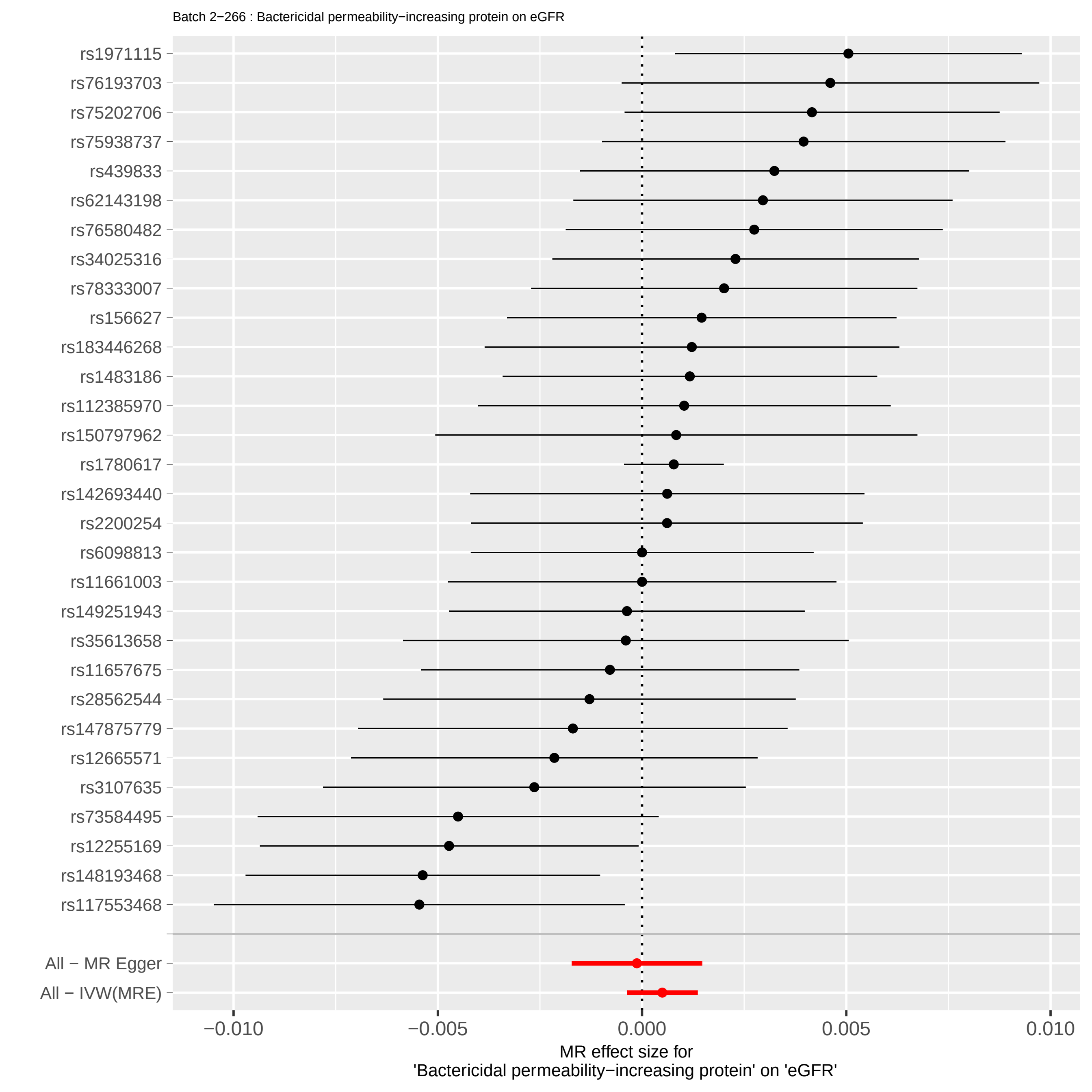
Batch 2−266 : Bactericidal permeability−increasing protein on eGFR
rs1971115
rs76193703
rs75202706
rs75938737
rs439833
rs62143198
rs76580482
rs34025316
rs78333007
rs156627
rs183446268
rs1483186
rs112385970
rs150797962
rs1780617
rs142693440
rs2200254
rs6098813
rs11661003
rs149251943
rs35613658
rs11657675
rs28562544
rs147875779
rs12665571
rs3107635
rs73584495
rs12255169
rs148193468
rs117553468
All − MR Egger
All − IVW(MRE)
−0.010
−0.005
0.000
0.005
0.010
MR effect size for
'Bactericidal permeability−increasing protein' on 'eGFR'

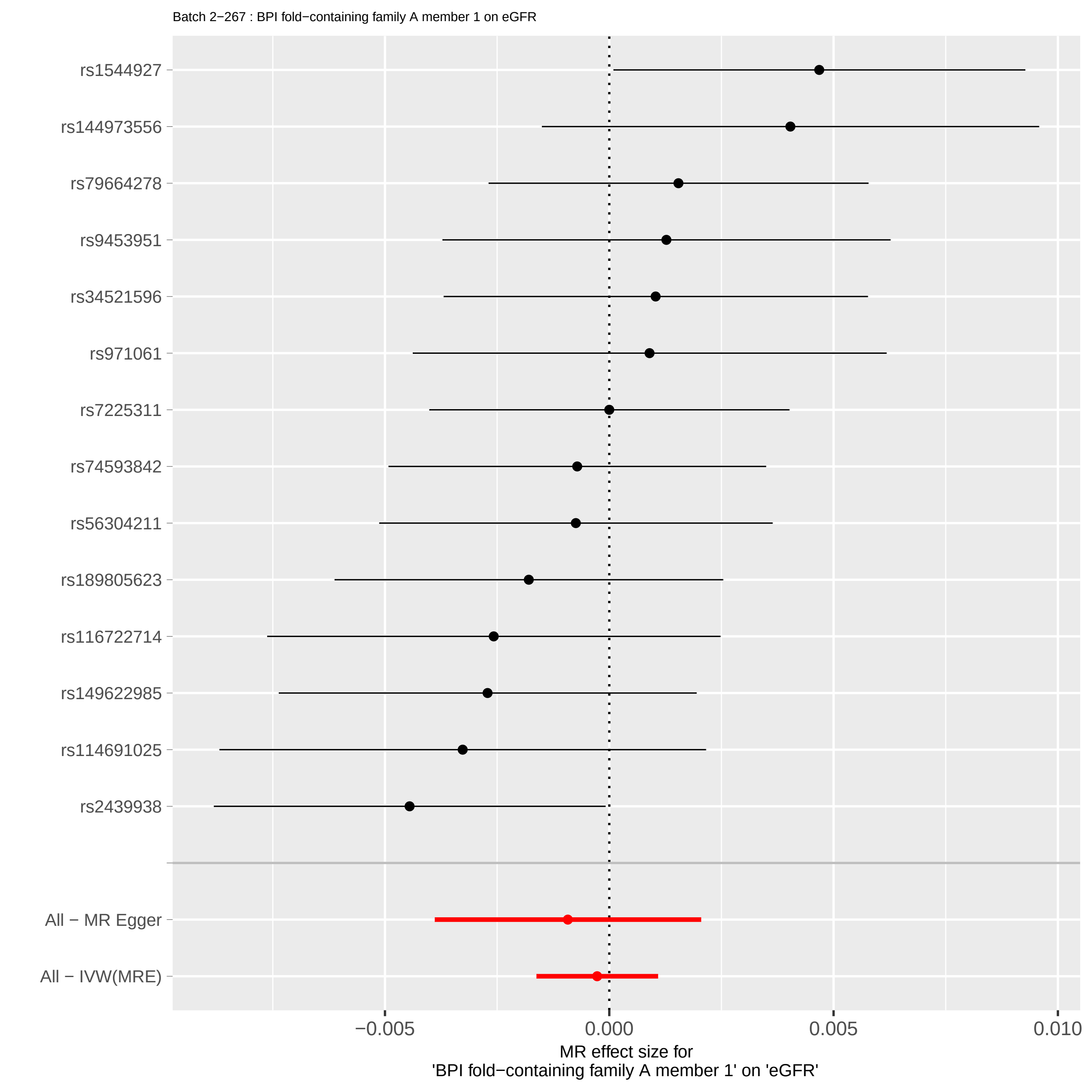

Batch 2−267 : BPI fold−containing family A member 1 on eGFR
rs1544927
rs144973556
rs79664278
rs9453951
rs34521596
rs971061
rs7225311
rs74593842
rs56304211
rs189805623
rs116722714
rs149622985
rs114691025
rs2439938
All − MR Egger
All − IVW(MRE)
−0.005
0.000
0.005
0.010
MR effect size for
'BPI fold−containing family A member 1' on 'eGFR'

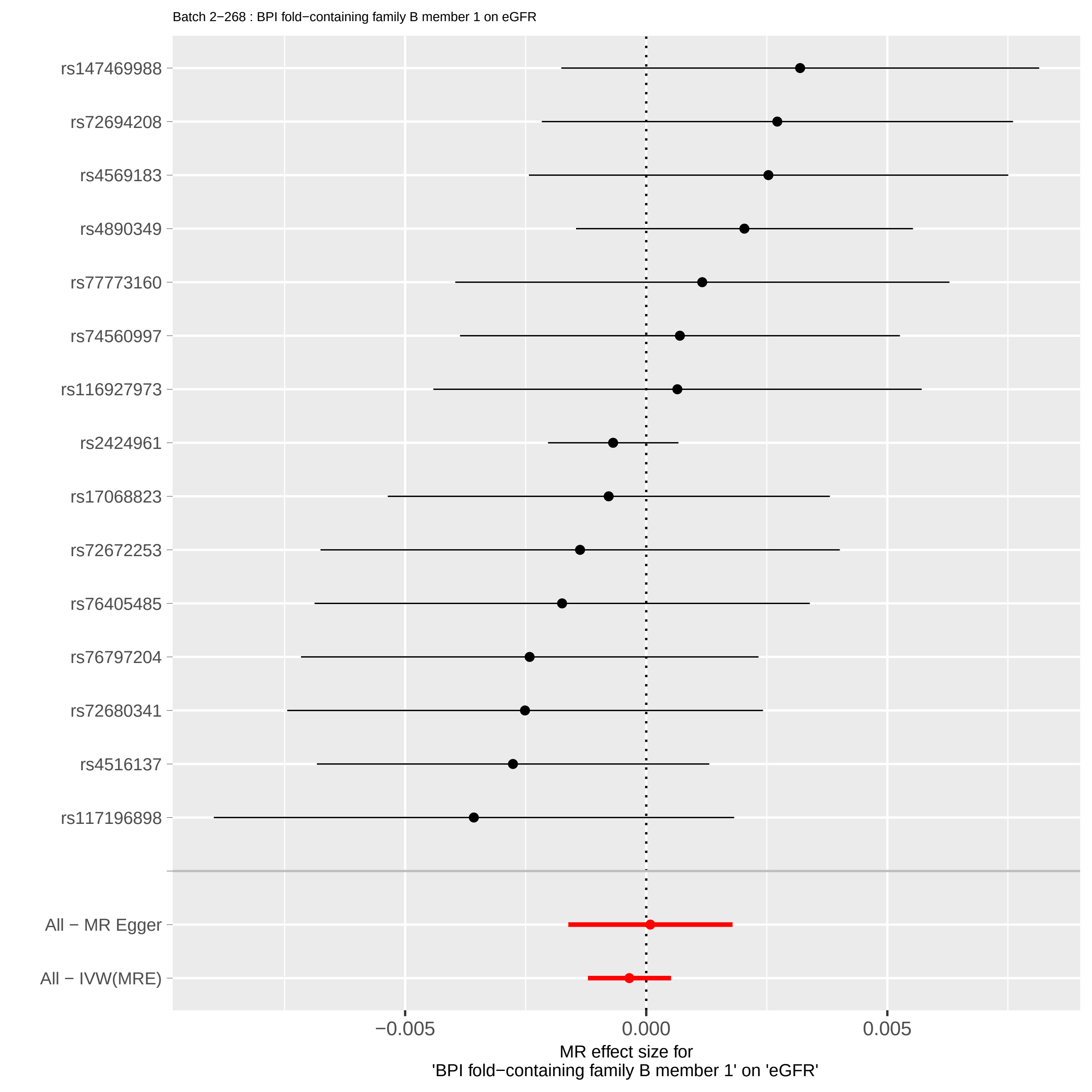

Batch 2−268 : BPI fold−containing family B member 1 on eGFR
rs147469988
rs72694208
rs4569183
rs4890349
rs77773160
rs74560997
rs116927973
rs2424961
rs17068823
rs72672253
rs76405485
rs76797204
rs72680341
rs4516137
rs117196898
All − MR Egger
All − IVW(MRE)
−0.005
0.000
0.005
MR effect size for
'BPI fold−containing family B member 1' on 'eGFR'

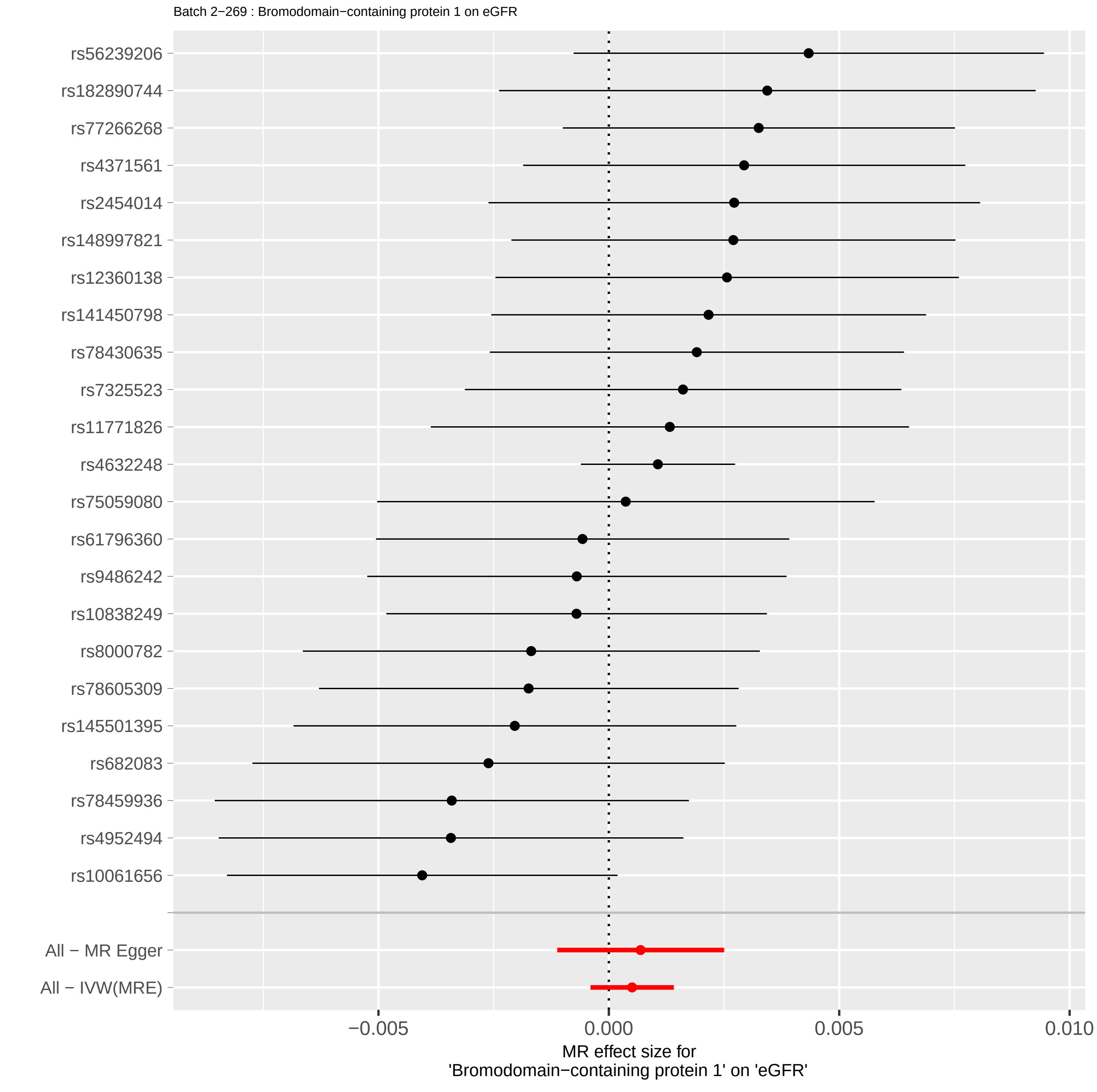

Batch 2−269 : Bromodomain−containing protein 1 on eGFR
rs56239206
rs182890744
rs77266268
rs4371561
rs2454014
rs148997821
rs12360138
rs141450798
rs78430635
rs7325523
rs11771826
rs4632248
rs75059080
rs61796360
rs9486242
rs10838249
rs8000782
rs78605309
rs145501395
rs682083
rs78459936
rs4952494
rs10061656
All − MR Egger
All − IVW(MRE)
−0.005
0.000
0.005
0.010
MR effect size for
'Bromodomain−containing protein 1' on 'eGFR'

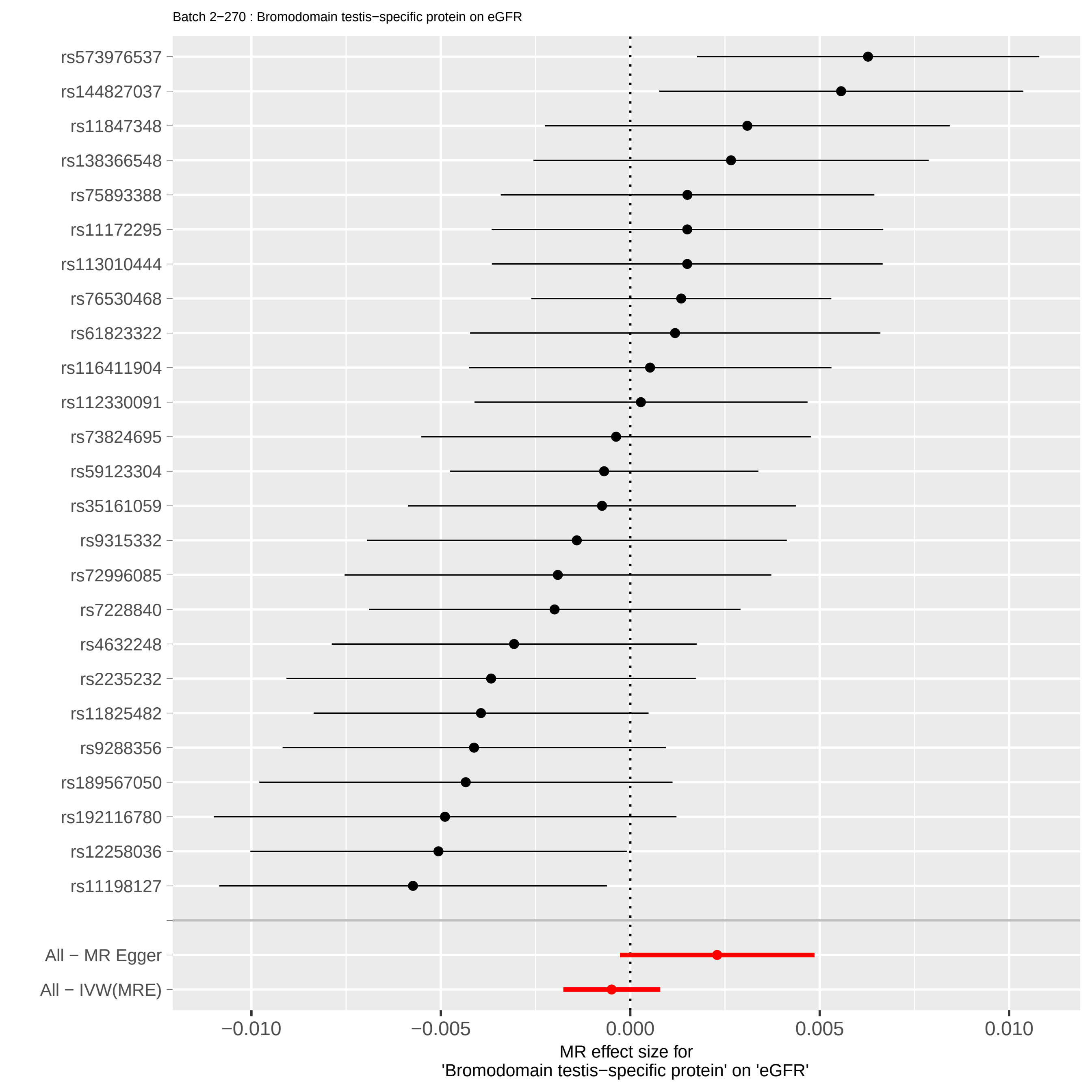

Batch 2−270 : Bromodomain testis−specific protein on eGFR
rs573976537
rs144827037
rs11847348
rs138366548
rs75893388
rs11172295
rs113010444
rs76530468
rs61823322
rs116411904
rs112330091
rs73824695
rs59123304
rs35161059
rs9315332
rs72996085
rs7228840
rs4632248
rs2235232
rs11825482
rs9288356
rs189567050
rs192116780
rs12258036
rs11198127
All − MR Egger
All − IVW(MRE)
−0.010
−0.005
0.000
0.005
0.010
MR effect size for
'Bromodomain testis−specific protein' on 'eGFR'

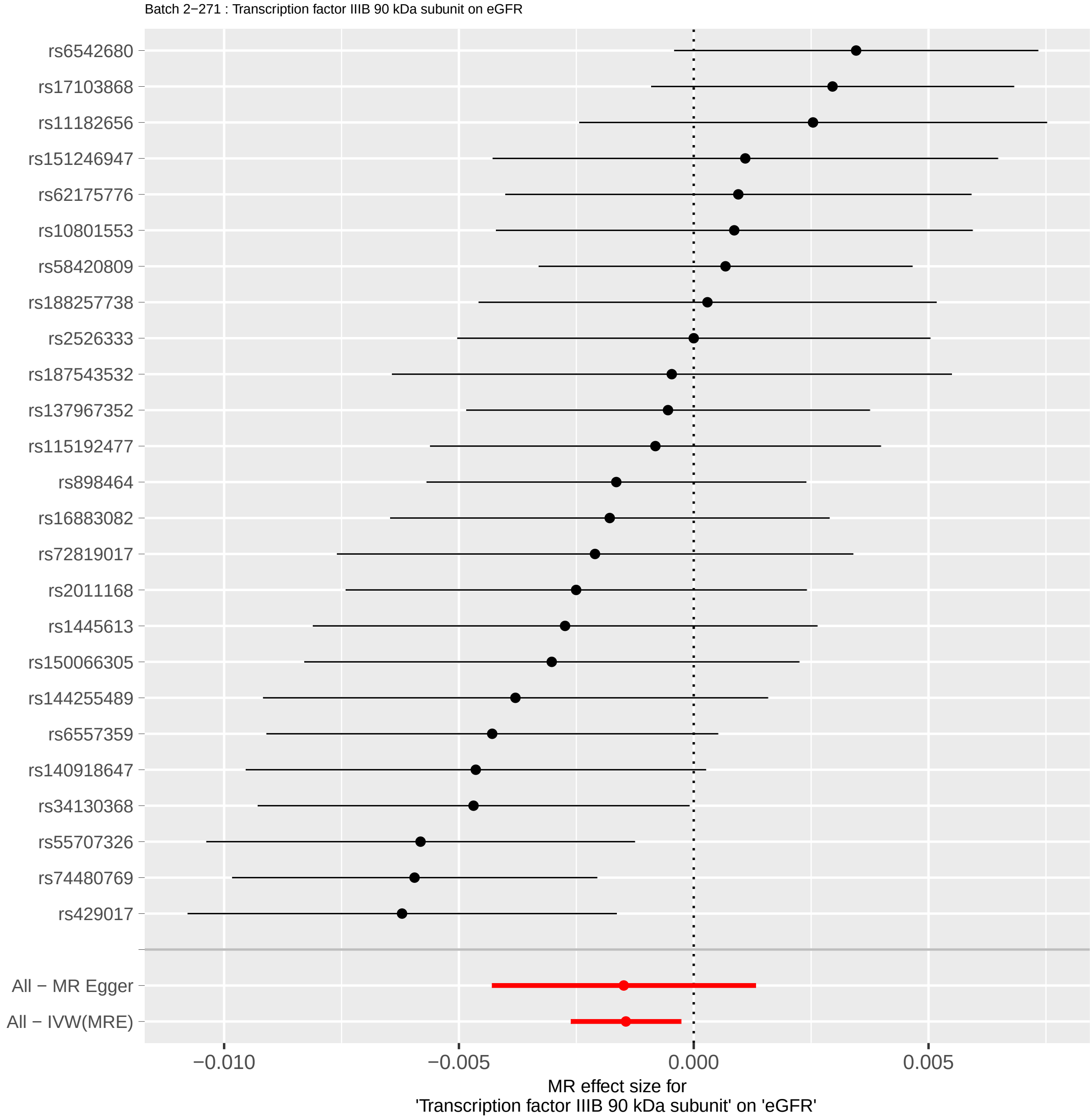

Batch 2−271 : Transcription factor IIIB 90 kDa subunit on eGFR
rs6542680
rs17103868
rs11182656
rs151246947
rs62175776
rs10801553
rs58420809
rs188257738
rs2526333
rs187543532
rs137967352
rs115192477
rs898464
rs16883082
rs72819017
rs2011168
rs1445613
rs150066305
rs144255489
rs6557359
rs140918647
rs34130368
rs55707326
rs74480769
rs429017
All − MR Egger
All − IVW(MRE)
−0.010
−0.005
0.000
0.005
MR effect size for
'Transcription factor IIIB 90 kDa subunit' on 'eGFR'

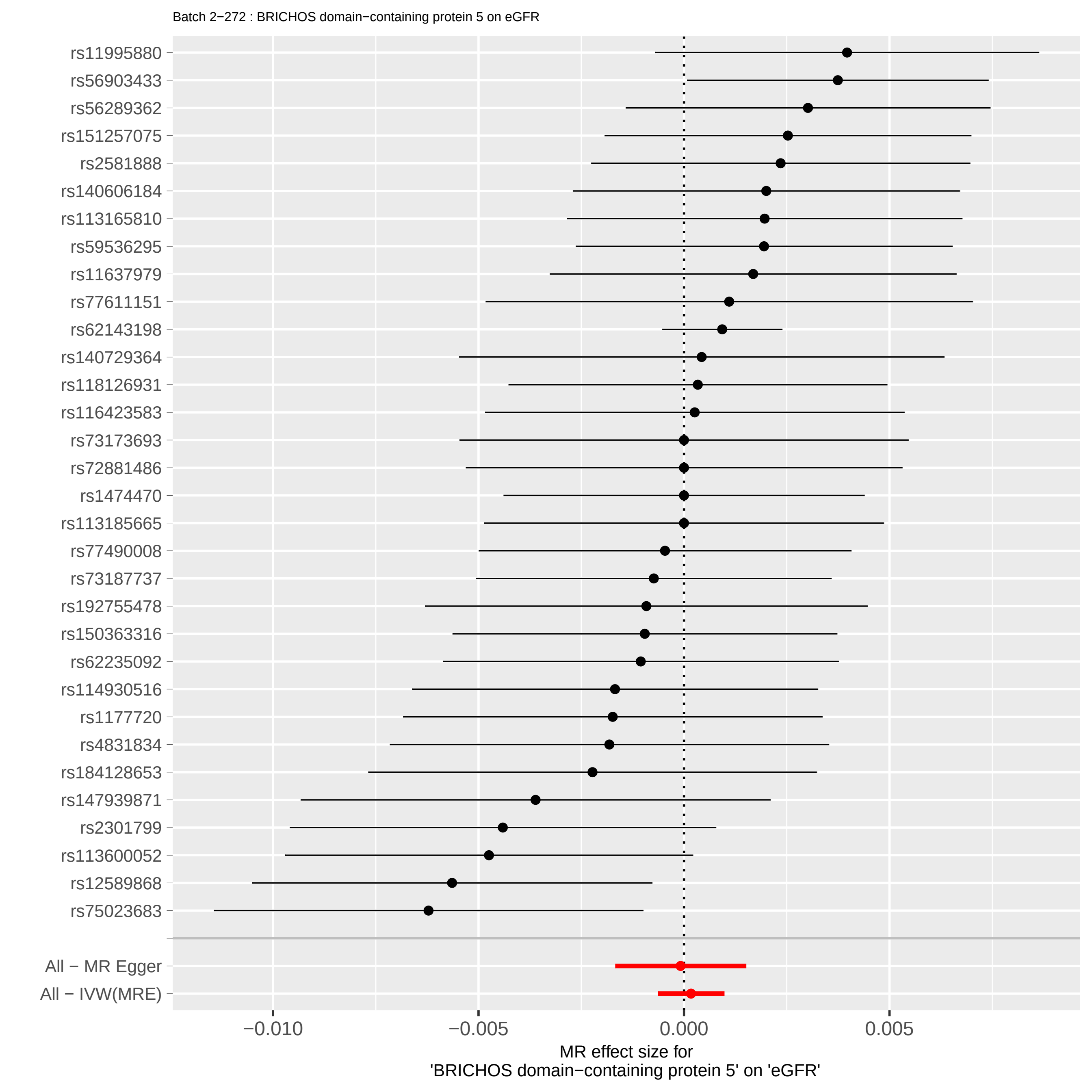

Batch 2−272 : BRICHOS domain−containing protein 5 on eGFR
rs11995880
rs56903433
rs56289362
rs151257075
rs2581888
rs140606184
rs113165810
rs59536295
rs11637979
rs77611151
rs62143198
rs140729364
rs118126931
rs116423583
rs73173693
rs72881486
rs1474470
rs113185665
rs77490008
rs73187737
rs192755478
rs150363316
rs62235092
rs114930516
rs1177720
rs4831834
rs184128653
rs147939871
rs2301799
rs113600052
rs12589868
rs75023683
All − MR Egger
All − IVW(MRE)
−0.010
−0.005
0.000
0.005
MR effect size for
'BRICHOS domain−containing protein 5' on 'eGFR'

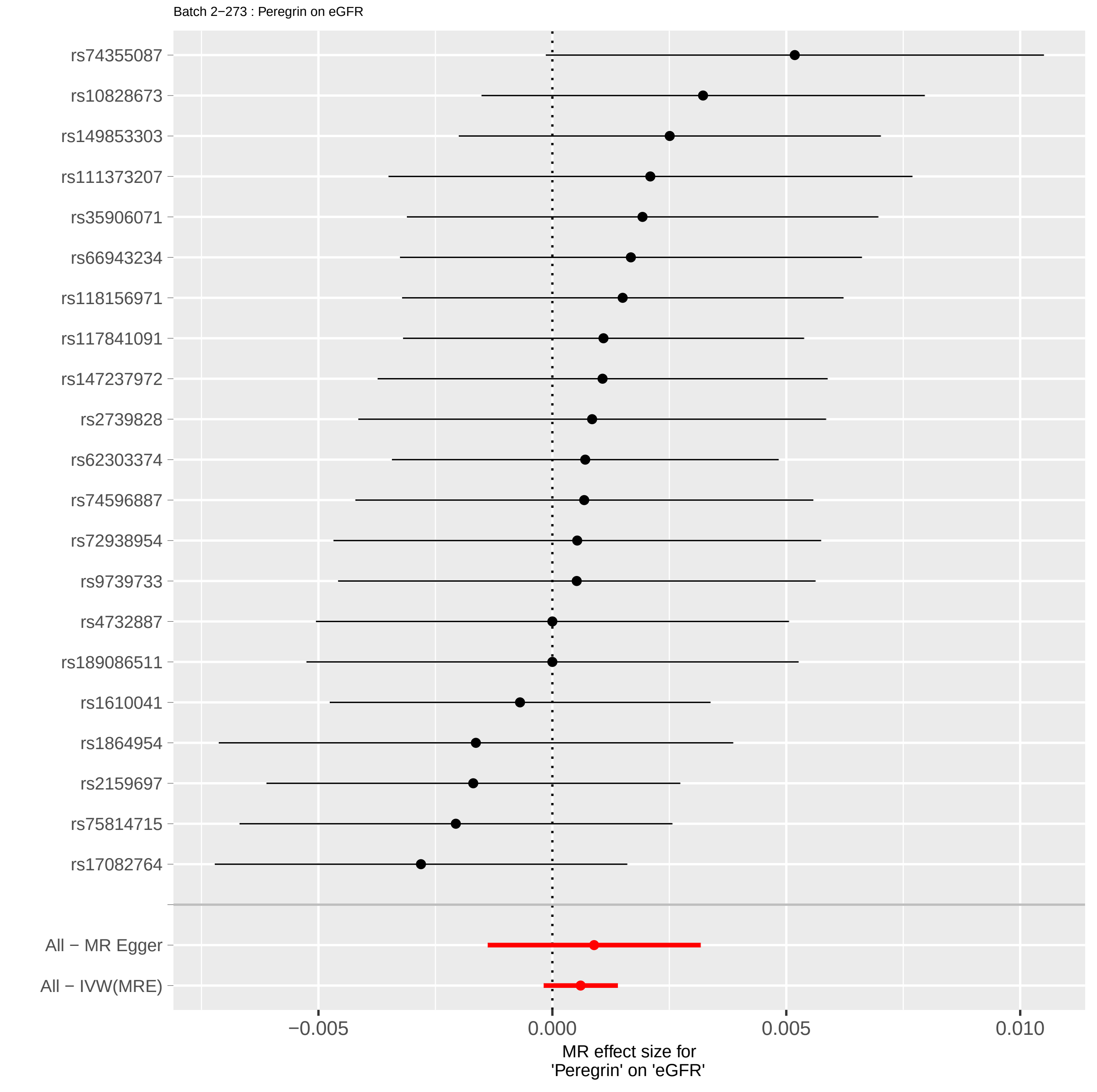

Batch 2−273 : Peregrin on eGFR
rs74355087
rs10828673
rs149853303
rs111373207
rs35906071
rs66943234
rs118156971
rs117841091
rs147237972
rs2739828
rs62303374
rs74596887
rs72938954
rs9739733
rs4732887
rs189086511
rs1610041
rs1864954
rs2159697
rs75814715
rs17082764
All − MR Egger
All − IVW(MRE)
−0.005
0.000
0.005
0.010
MR effect size for
'Peregrin' on 'eGFR'

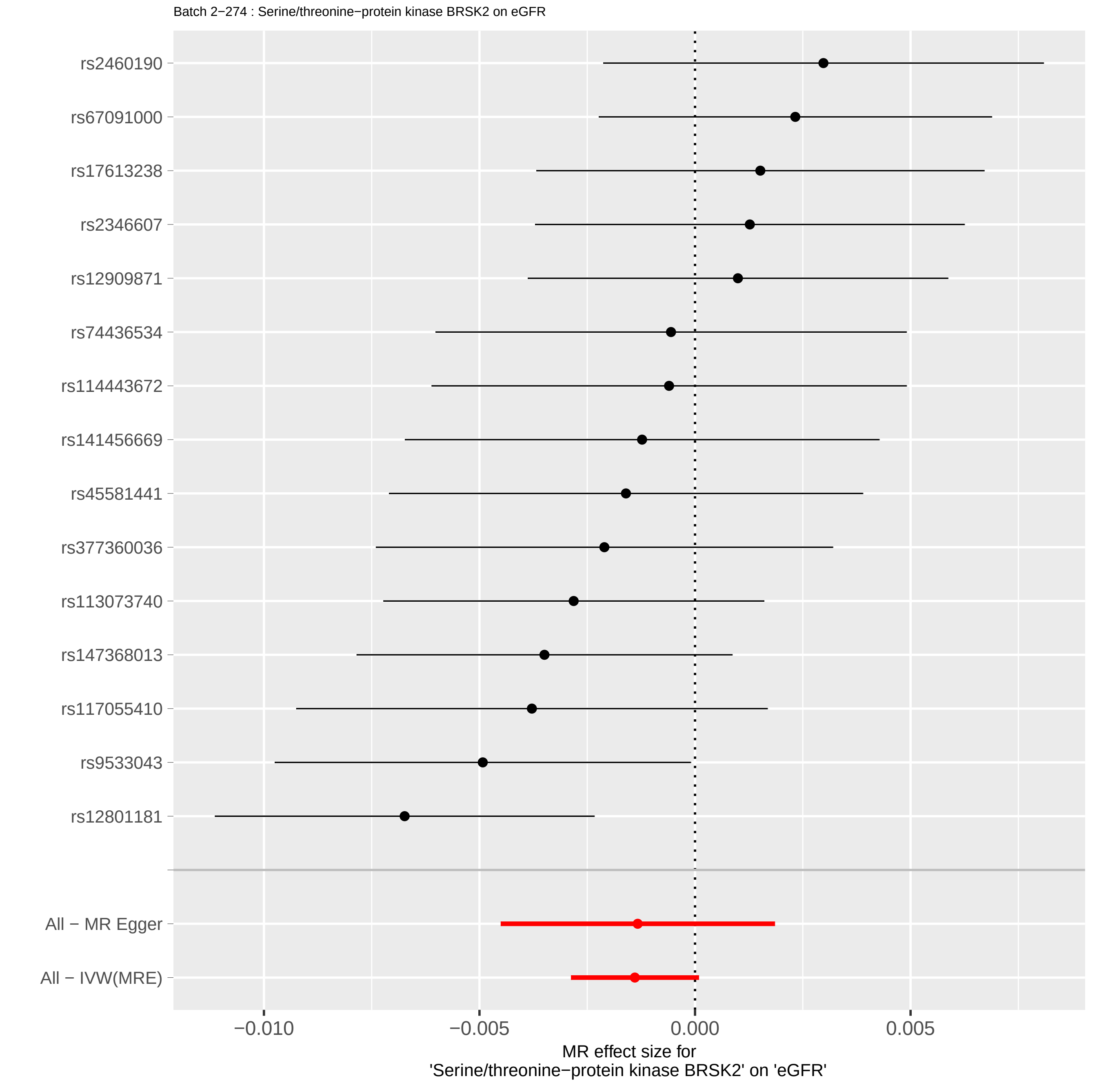

Batch 2−274 : Serine/threonine−protein kinase BRSK2 on eGFR
rs2460190
rs67091000
rs17613238
rs2346607
rs12909871
rs74436534
rs114443672
rs141456669
rs45581441
rs377360036
rs113073740
rs147368013
rs117055410
rs9533043
rs12801181
All − MR Egger
All − IVW(MRE)
−0.010
−0.005
0.000
0.005
MR effect size for
'Serine/threonine−protein kinase BRSK2' on 'eGFR'

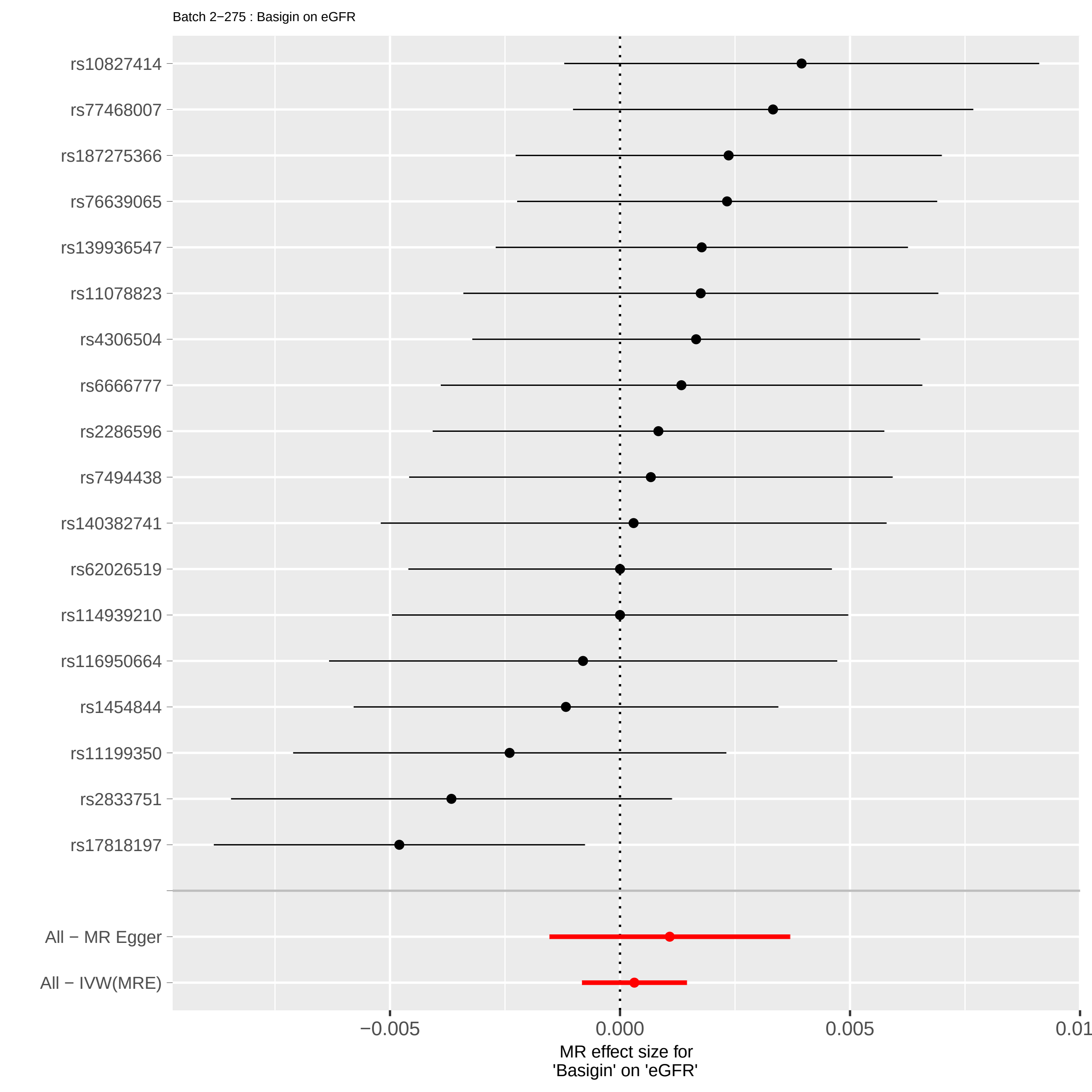

Batch 2−275 : Basigin on eGFR
rs10827414
rs77468007
rs187275366
rs76639065
rs139936547
rs11078823
rs4306504
rs6666777
rs2286596
rs7494438
rs140382741
rs62026519
rs114939210
rs116950664
rs1454844
rs11199350
rs2833751
rs17818197
All − MR Egger
All − IVW(MRE)
−0.005
0.000
0.005
0.01
MR effect size for
'Basigin' on 'eGFR'

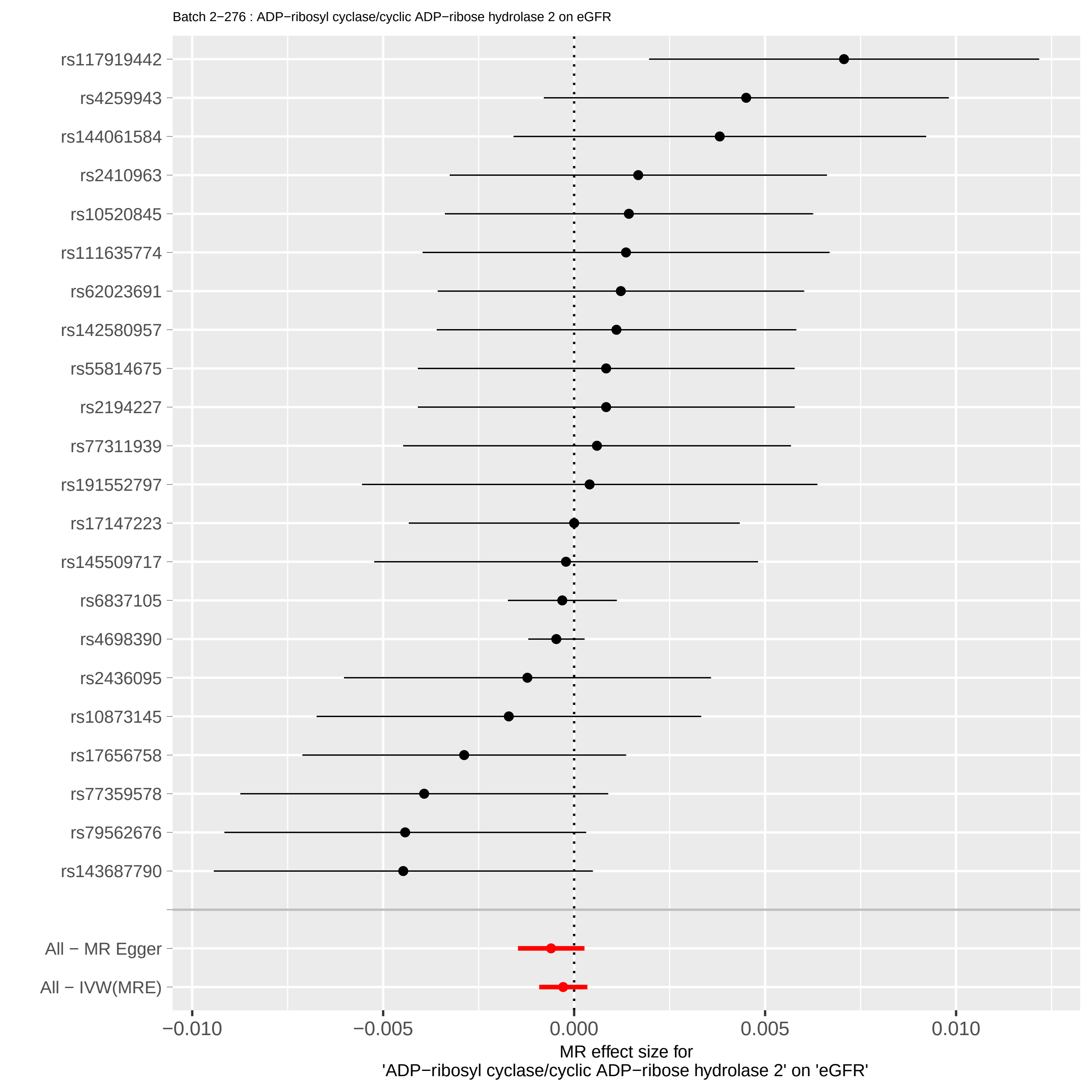
Batch 2−276 : ADP−ribosyl cyclase/cyclic ADP−ribose hydrolase 2 on eGFR
rs117919442
rs4259943
rs144061584
rs2410963
rs10520845
rs111635774
rs62023691
rs142580957
rs55814675
rs2194227
rs77311939
rs191552797
rs17147223
rs145509717
rs6837105
rs4698390
rs2436095
rs10873145
rs17656758
rs77359578
rs79562676
rs143687790
All − MR Egger
All − IVW(MRE)
−0.010
−0.005
0.000
0.005
0.010
MR effect size for
'ADP−ribosyl cyclase/cyclic ADP−ribose hydrolase 2' on 'eGFR'

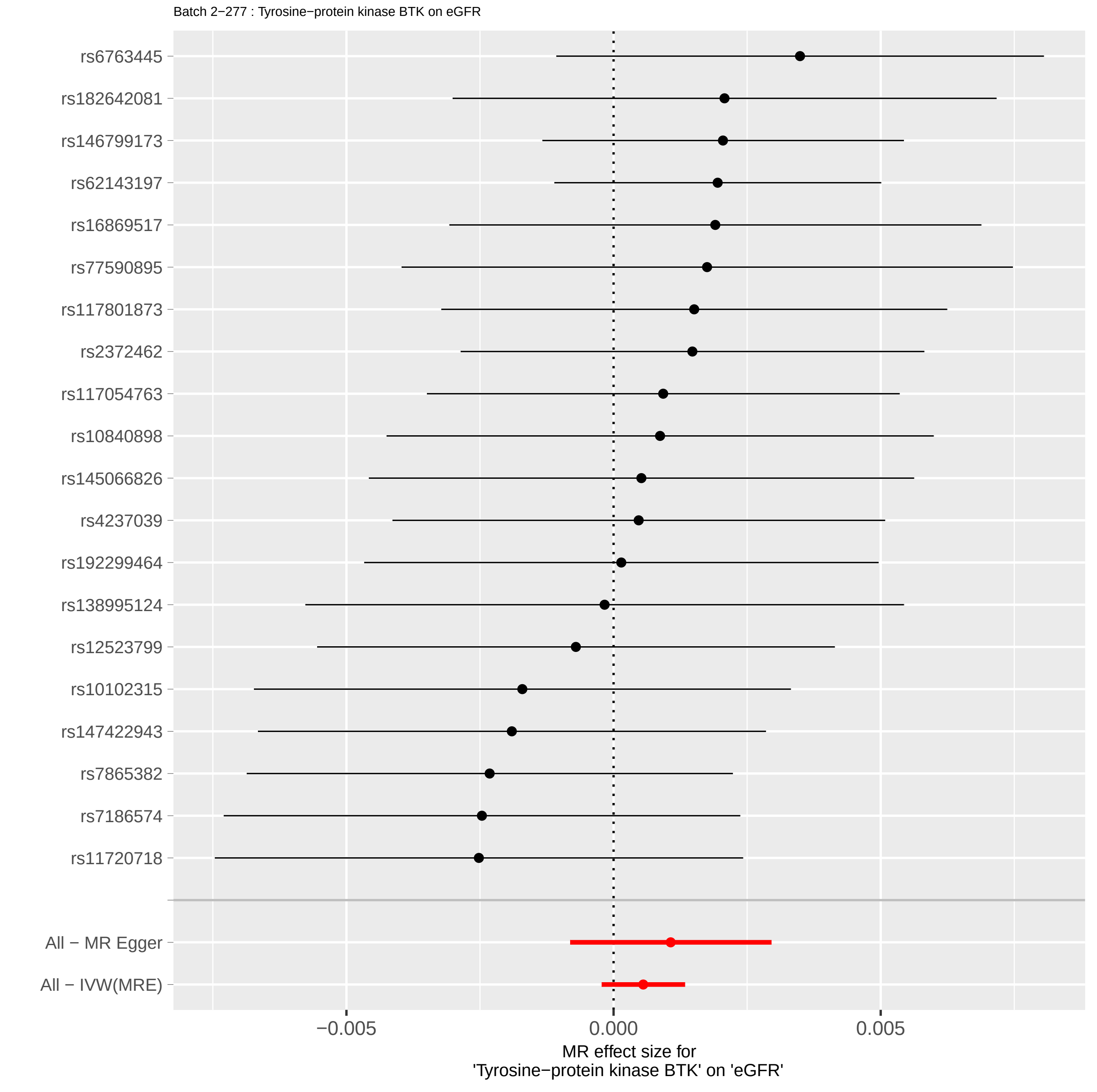

Batch 2−277 : Tyrosine−protein kinase BTK on eGFR
rs6763445
rs182642081
rs146799173
rs62143197
rs16869517
rs77590895
rs117801873
rs2372462
rs117054763
rs10840898
rs145066826
rs4237039
rs192299464
rs138995124
rs12523799
rs10102315
rs147422943
rs7865382
rs7186574
rs11720718
All − MR Egger
All − IVW(MRE)
−0.005
0.000
0.005
MR effect size for
'Tyrosine−protein kinase BTK' on 'eGFR'

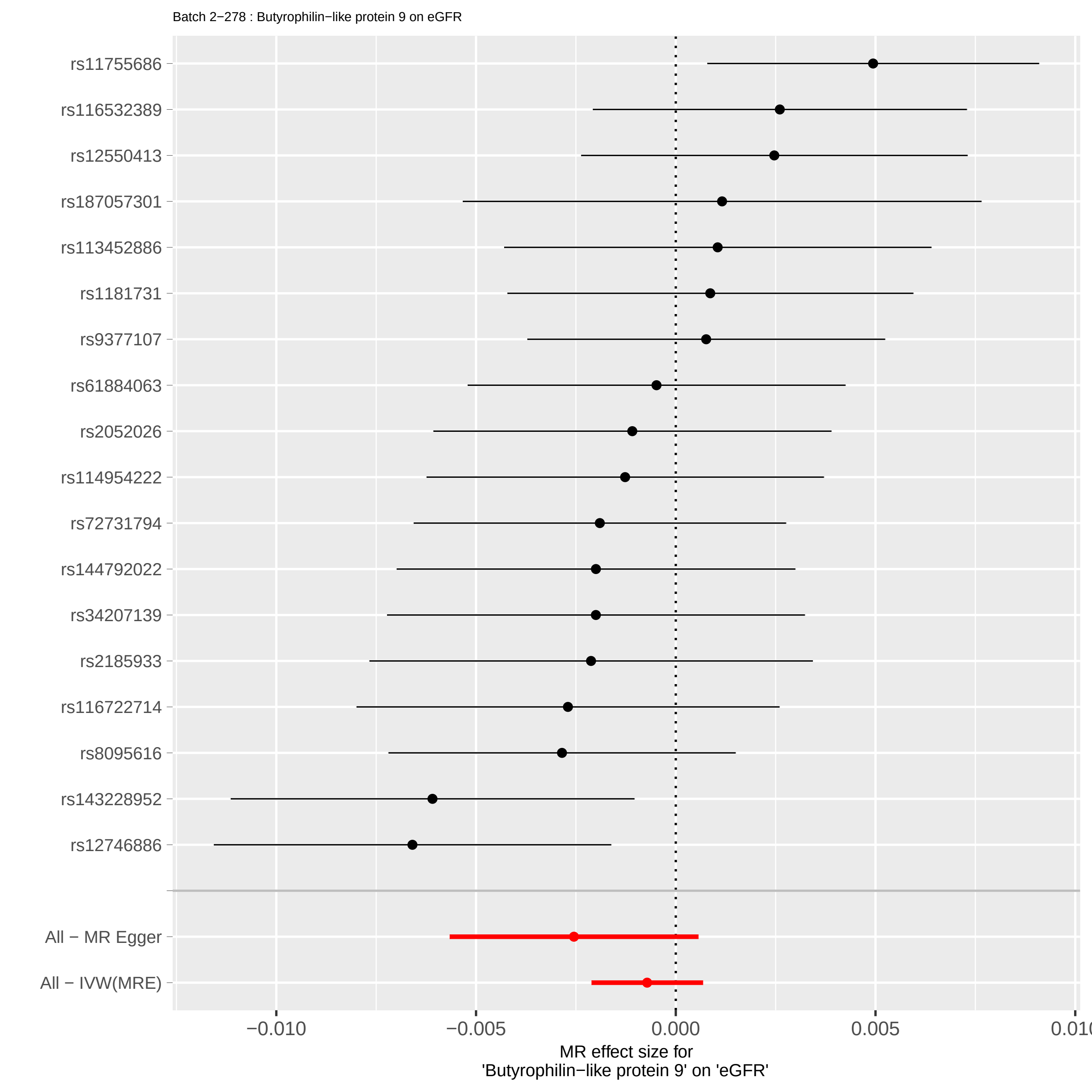

Batch 2−278 : Butyrophilin−like protein 9 on eGFR
rs11755686
rs116532389
rs12550413
rs187057301
rs113452886
rs1181731
rs9377107
rs61884063
rs2052026
rs114954222
rs72731794
rs144792022
rs34207139
rs2185933
rs116722714
rs8095616
rs143228952
rs12746886
All − MR Egger
All − IVW(MRE)
−0.010
−0.005
0.000
0.005
0.010
MR effect size for
'Butyrophilin−like protein 9' on 'eGFR'

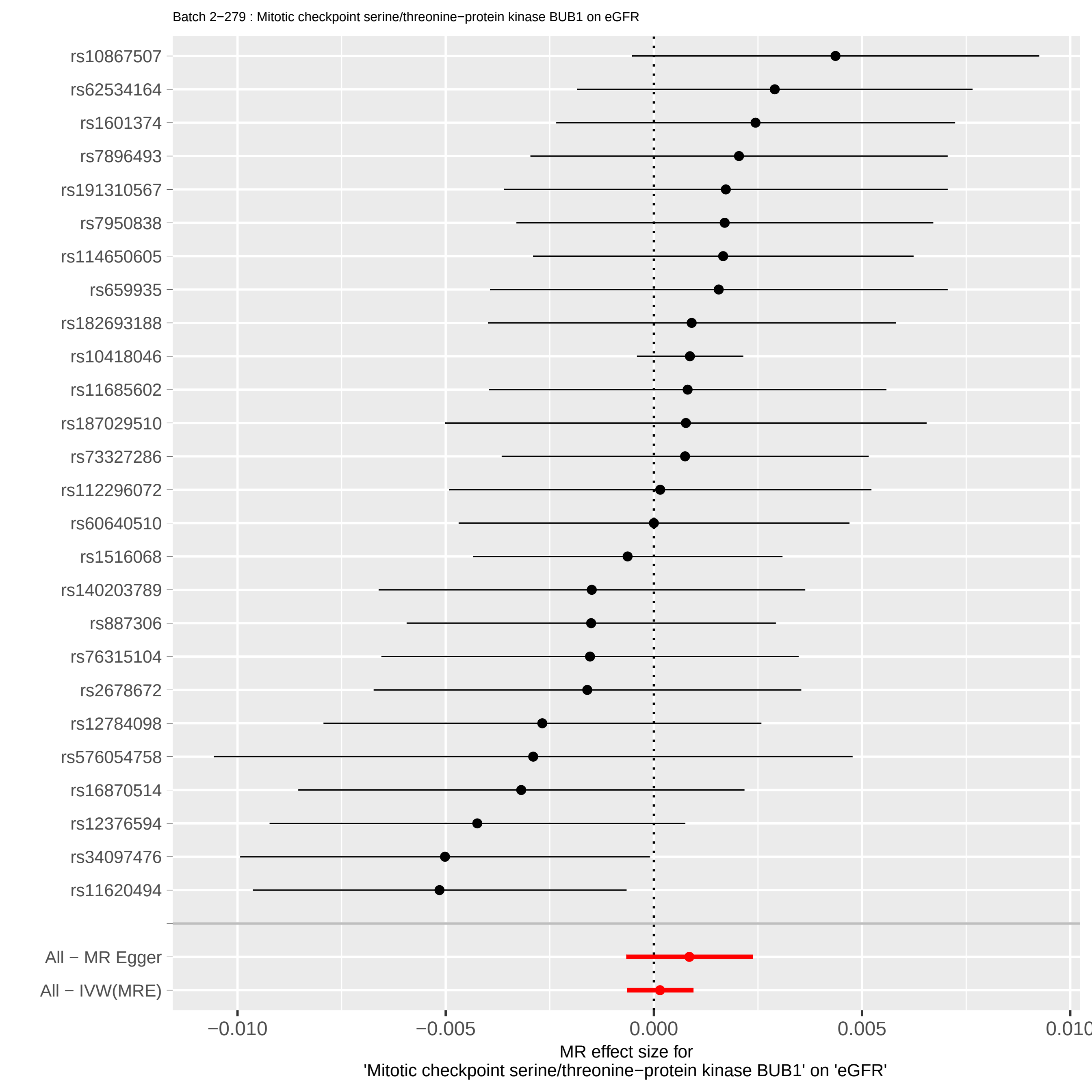

Batch 2−279 : Mitotic checkpoint serine/threonine−protein kinase BUB1 on eGFR
rs10867507
rs62534164
rs1601374
rs7896493
rs191310567
rs7950838
rs114650605
rs659935
rs182693188
rs10418046
rs11685602
rs187029510
rs73327286
rs112296072
rs60640510
rs1516068
rs140203789
rs887306
rs76315104
rs2678672
rs12784098
rs576054758
rs16870514
rs12376594
rs34097476
rs11620494
All − MR Egger
All − IVW(MRE)
−0.010
−0.005
0.000
0.005
0.010
MR effect size for
'Mitotic checkpoint serine/threonine−protein kinase BUB1' on 'eGFR'

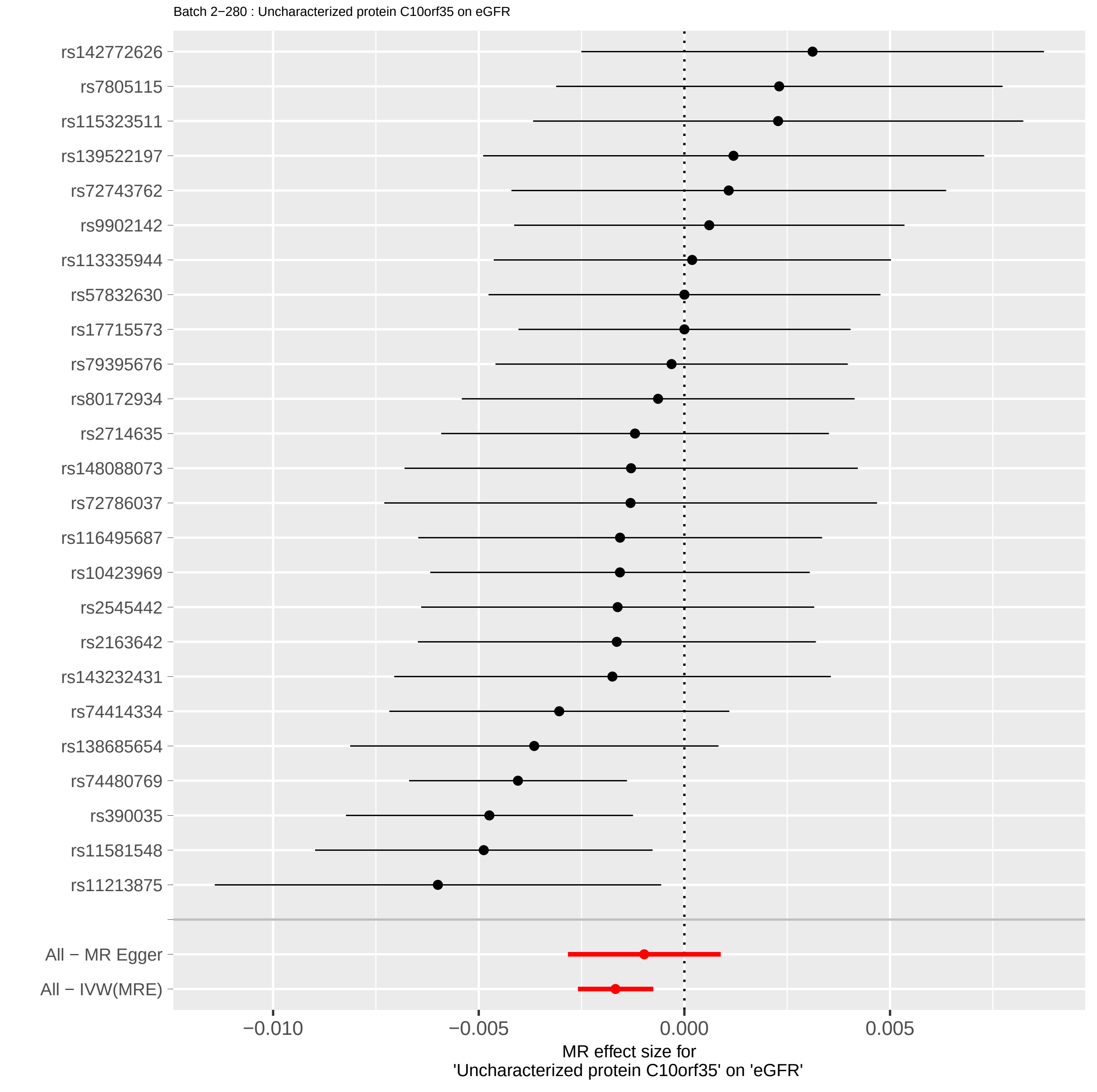

Batch 2−280 : Uncharacterized protein C10orf35 on eGFR
rs142772626
rs7805115
rs115323511
rs139522197
rs72743762
rs9902142
rs113335944
rs57832630
rs17715573
rs79395676
rs80172934
rs2714635
rs148088073
rs72786037
rs116495687
rs10423969
rs2545442
rs2163642
rs143232431
rs74414334
rs138685654
rs74480769
rs390035
rs11581548
rs11213875
All − MR Egger
All − IVW(MRE)
−0.010
−0.005
0.000
0.005
MR effect size for
'Uncharacterized protein C10orf35' on 'eGFR'

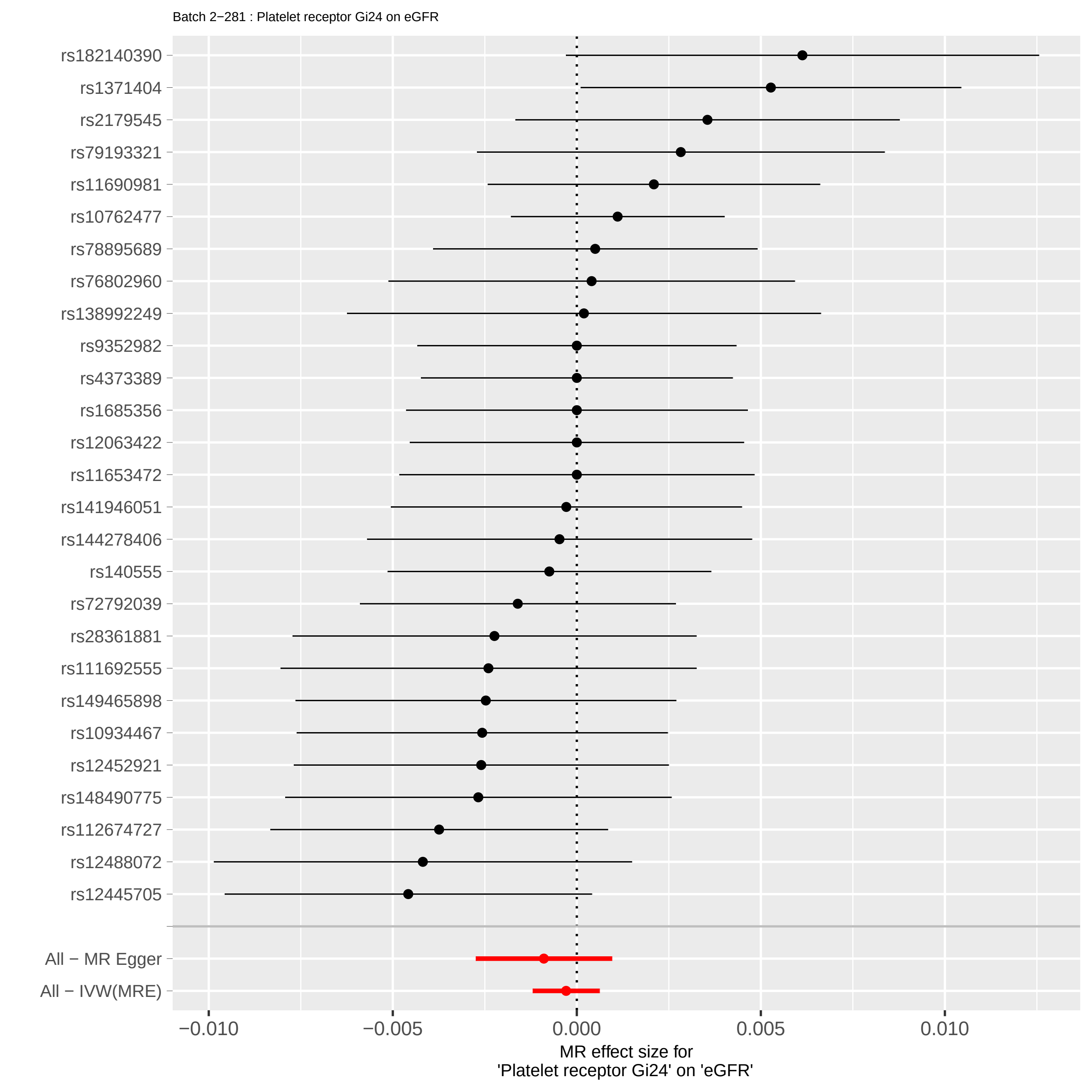
Batch 2−281 : Platelet receptor Gi24 on eGFR
rs182140390
rs1371404
rs2179545
rs79193321
rs11690981
rs10762477
rs78895689
rs76802960
rs138992249
rs9352982
rs4373389
rs1685356
rs12063422
rs11653472
rs141946051
rs144278406
rs140555
rs72792039
rs28361881
rs111692555
rs149465898
rs10934467
rs12452921
rs148490775
rs112674727
rs12488072
rs12445705
All − MR Egger
All − IVW(MRE)
−0.010
−0.005
0.000
0.005
0.010
MR effect size for
'Platelet receptor Gi24' on 'eGFR'

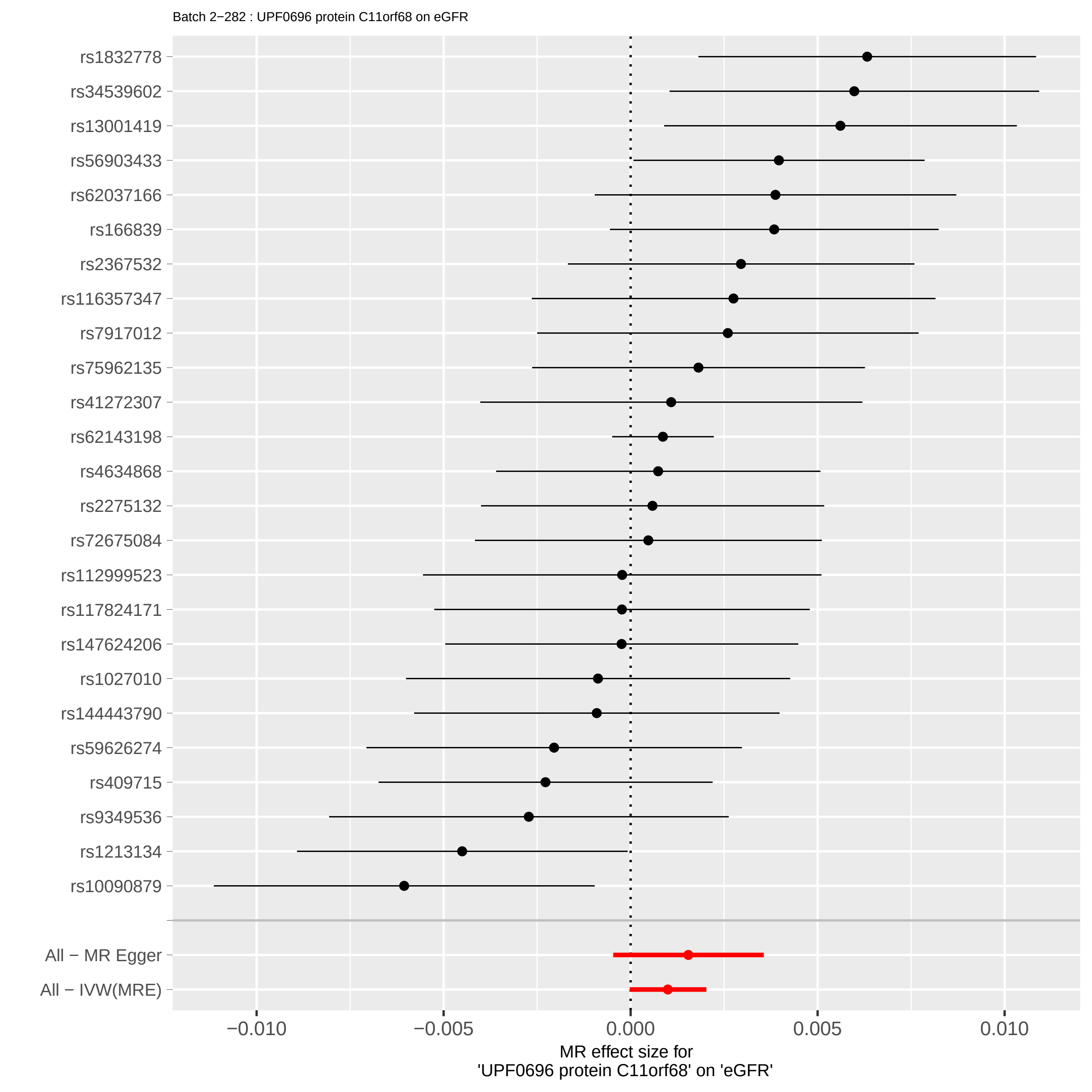
Batch 2−282 : UPF0696 protein C11orf68 on eGFR
rs1832778
rs34539602
rs13001419
rs56903433
rs62037166
rs166839
rs2367532
rs116357347
rs7917012
rs75962135
rs41272307
rs62143198
rs4634868
rs2275132
rs72675084
rs112999523
rs117824171
rs147624206
rs1027010
rs144443790
rs59626274
rs409715
rs9349536
rs1213134
rs10090879
All − MR Egger
All − IVW(MRE)
−0.010
−0.005
0.000
0.005
0.010
MR effect size for
'UPF0696 protein C11orf68' on 'eGFR'

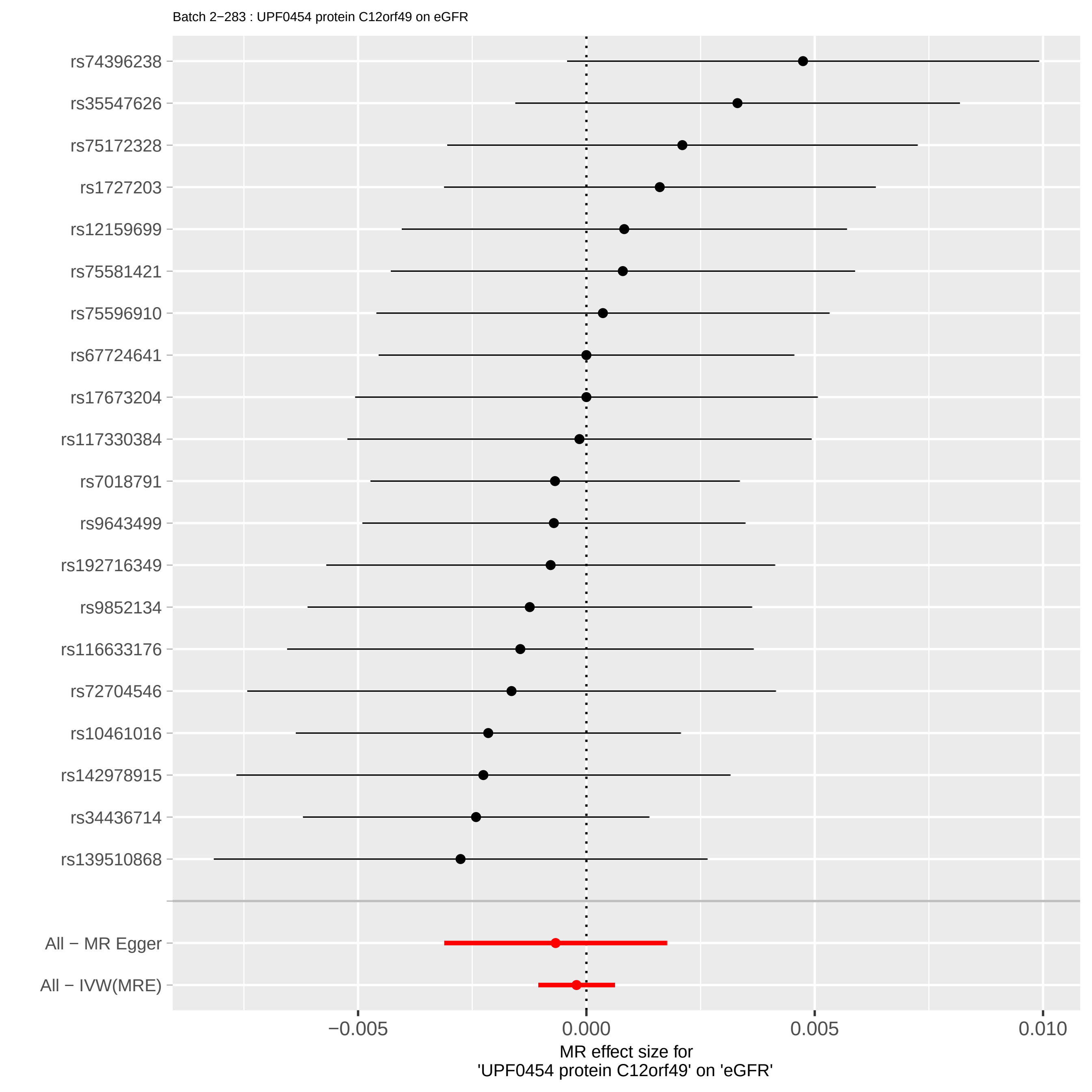

Batch 2−283 : UPF0454 protein C12orf49 on eGFR
rs74396238
rs35547626
rs75172328
rs1727203
rs12159699
rs75581421
rs75596910
rs67724641
rs17673204
rs117330384
rs7018791
rs9643499
rs192716349
rs9852134
rs116633176
rs72704546
rs10461016
rs142978915
rs34436714
rs139510868
All − MR Egger
All − IVW(MRE)
−0.005
0.000
0.005
0.010
MR effect size for
'UPF0454 protein C12orf49' on 'eGFR'

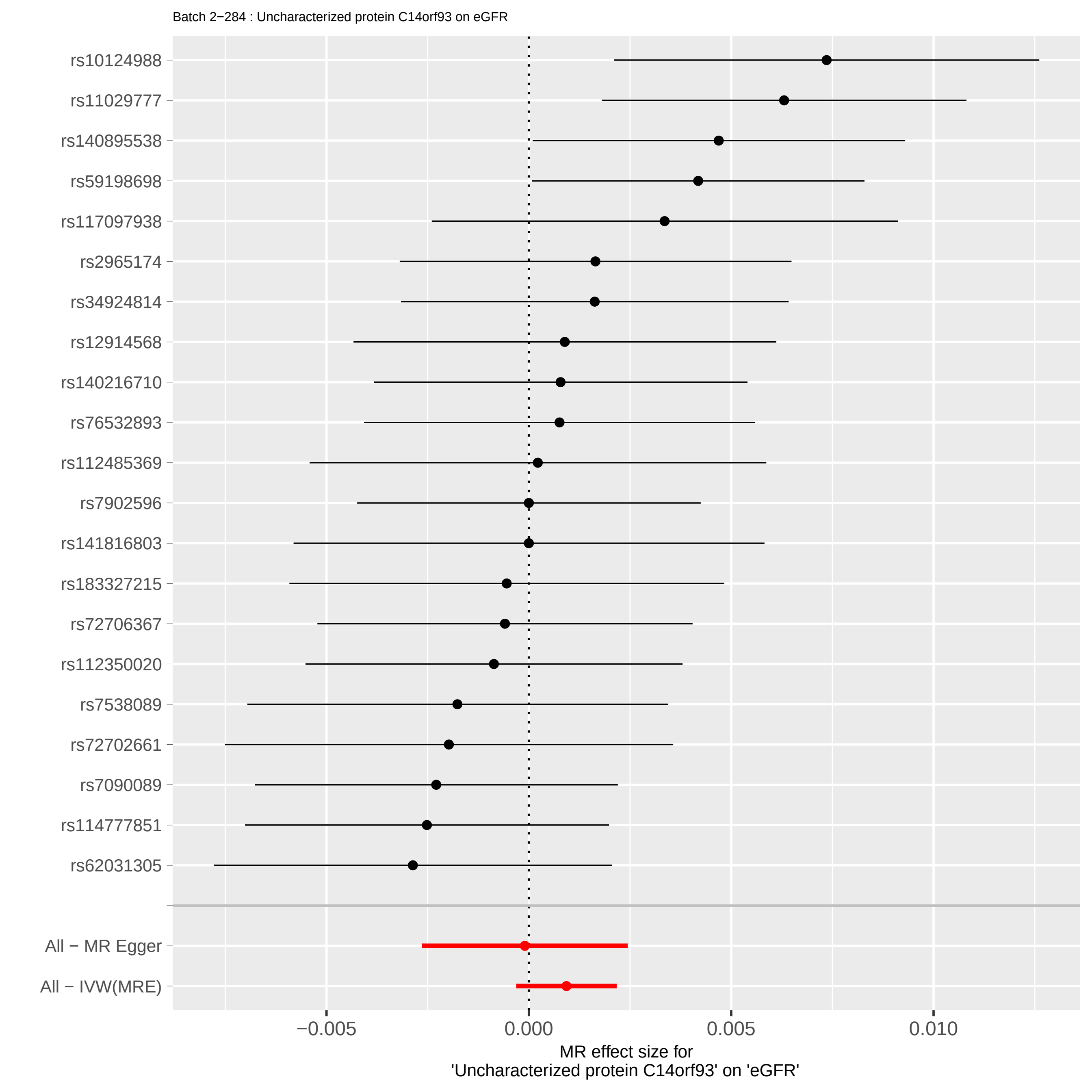

Batch 2−284 : Uncharacterized protein C14orf93 on eGFR
rs10124988
rs11029777
rs140895538
rs59198698
rs117097938
rs2965174
rs34924814
rs12914568
rs140216710
rs76532893
rs112485369
rs7902596
rs141816803
rs183327215
rs72706367
rs112350020
rs7538089
rs72702661
rs7090089
rs114777851
rs62031305
All − MR Egger
All − IVW(MRE)
−0.005
0.000
0.005
0.010
MR effect size for
'Uncharacterized protein C14orf93' on 'eGFR'

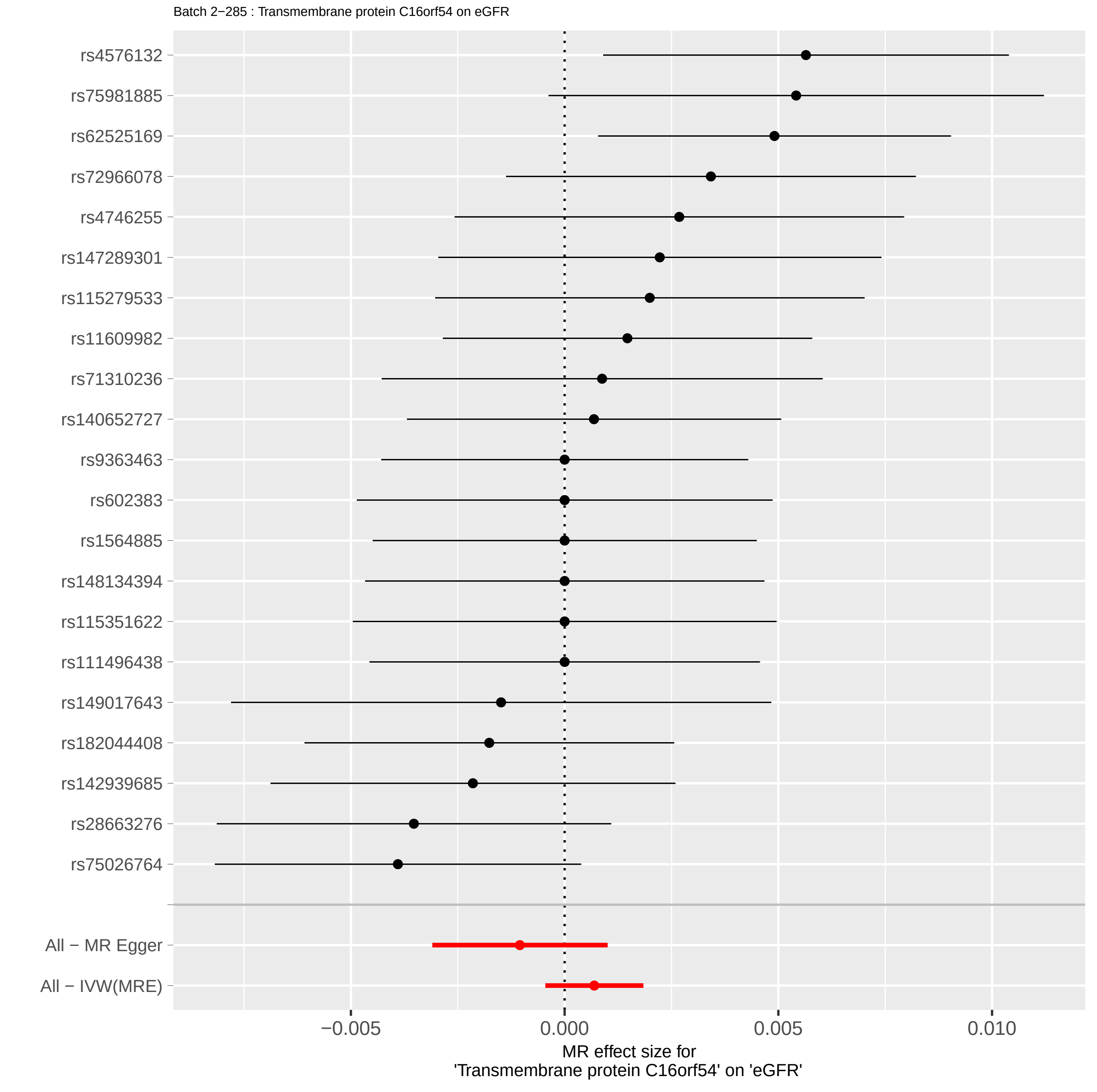

Batch 2−285 : Transmembrane protein C16orf54 on eGFR
rs4576132
rs75981885
rs62525169
rs72966078
rs4746255
rs147289301
rs115279533
rs11609982
rs71310236
rs140652727
rs9363463
rs602383
rs1564885
rs148134394
rs115351622
rs111496438
rs149017643
rs182044408
rs142939685
rs28663276
rs75026764
All − MR Egger
All − IVW(MRE)
−0.005
0.000
0.005
0.010
MR effect size for
'Transmembrane protein C16orf54' on 'eGFR'

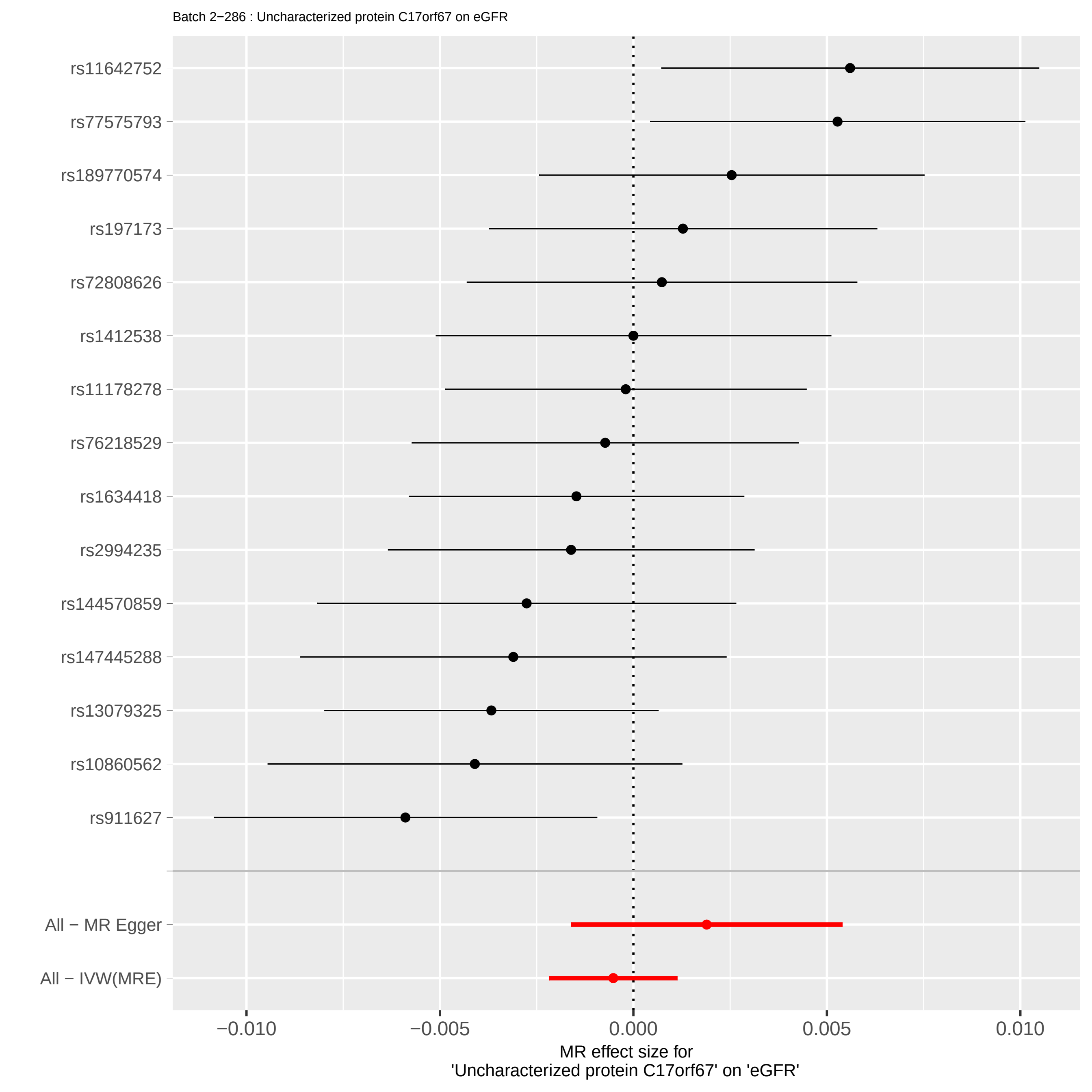
Batch 2−286 : Uncharacterized protein C17orf67 on eGFR
rs11642752
rs77575793
rs189770574
rs197173
rs72808626
rs1412538
rs11178278
rs76218529
rs1634418
rs2994235
rs144570859
rs147445288
rs13079325
rs10860562
rs911627
All − MR Egger
All − IVW(MRE)
−0.010
−0.005
0.000
0.005
0.010
MR effect size for
'Uncharacterized protein C17orf67' on 'eGFR'

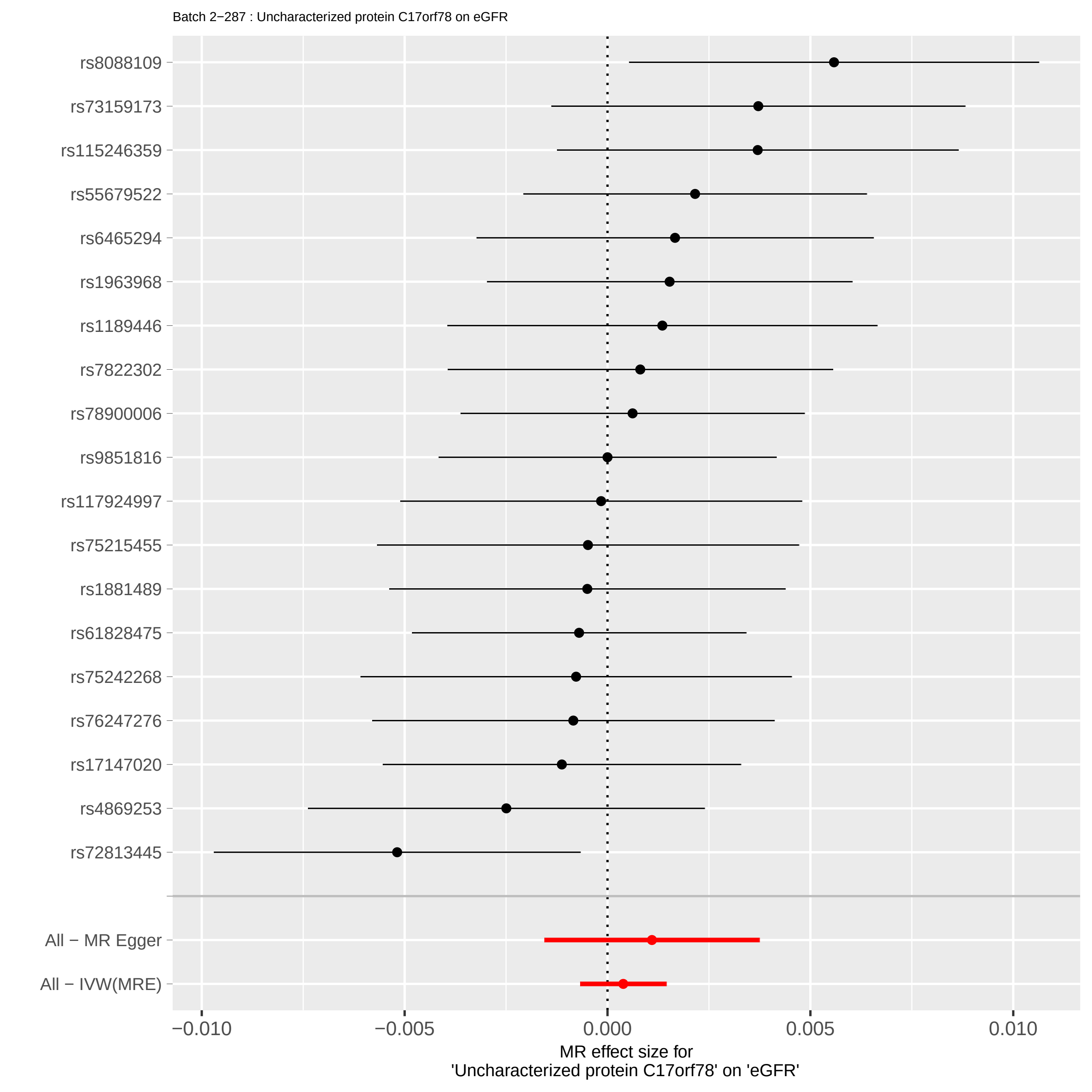
Batch 2−287 : Uncharacterized protein C17orf78 on eGFR
rs8088109
rs73159173
rs115246359
rs55679522
rs6465294
rs1963968
rs1189446
rs7822302
rs78900006
rs9851816
rs117924997
rs75215455
rs1881489
rs61828475
rs75242268
rs76247276
rs17147020
rs4869253
rs72813445
All − MR Egger
All − IVW(MRE)
−0.010
−0.005
0.000
0.005
0.010
MR effect size for
'Uncharacterized protein C17orf78' on 'eGFR'

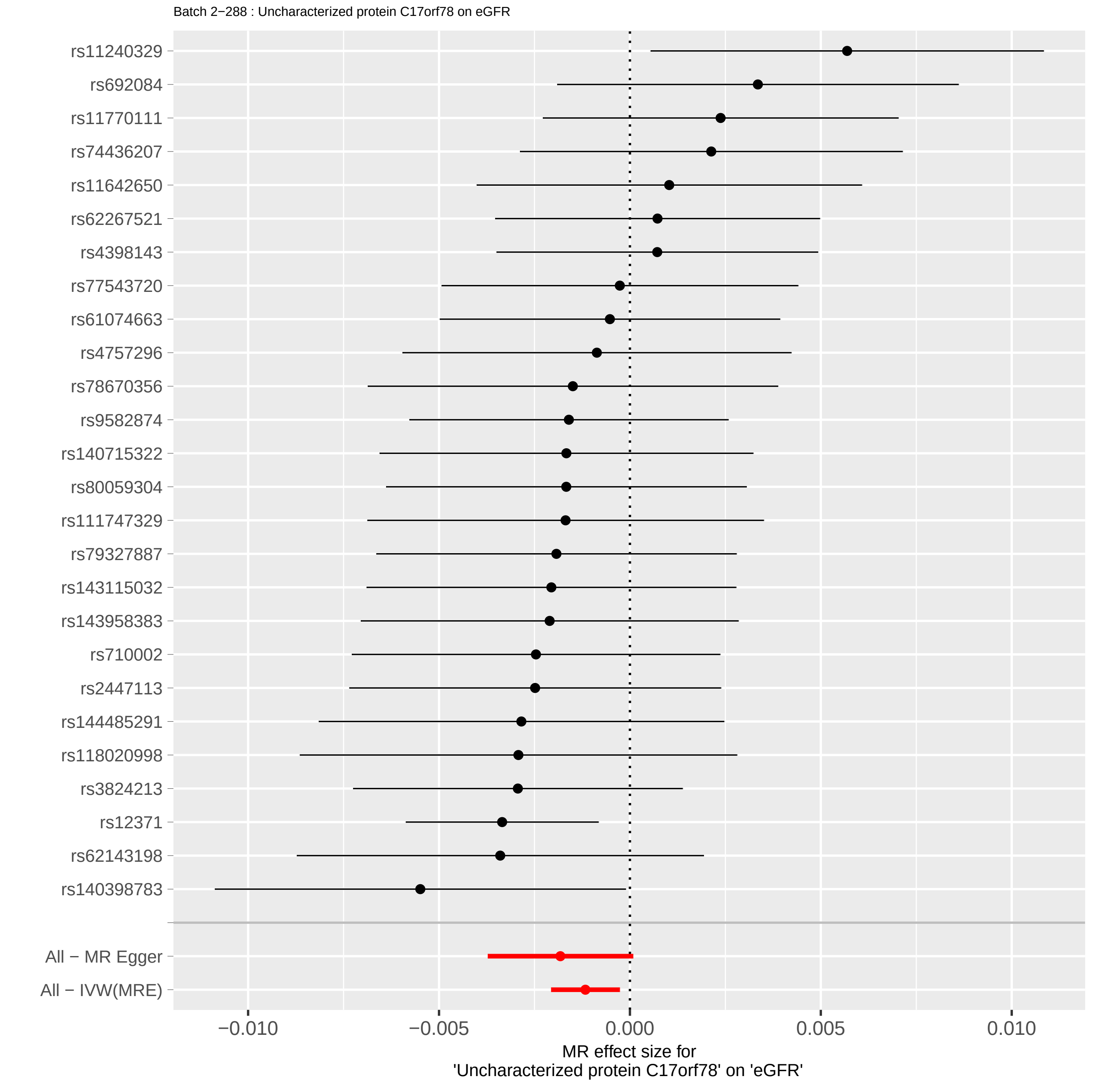
Batch 2−288 : Uncharacterized protein C17orf78 on eGFR
rs11240329
rs692084
rs11770111
rs74436207
rs11642650
rs62267521
rs4398143
rs77543720
rs61074663
rs4757296
rs78670356
rs9582874
rs140715322
rs80059304
rs111747329
rs79327887
rs143115032
rs143958383
rs710002
rs2447113
rs144485291
rs118020998
rs3824213
rs12371
rs62143198
rs140398783
All − MR Egger
All − IVW(MRE)
−0.010
−0.005
0.000
0.005
0.010
MR effect size for
'Uncharacterized protein C17orf78' on 'eGFR'

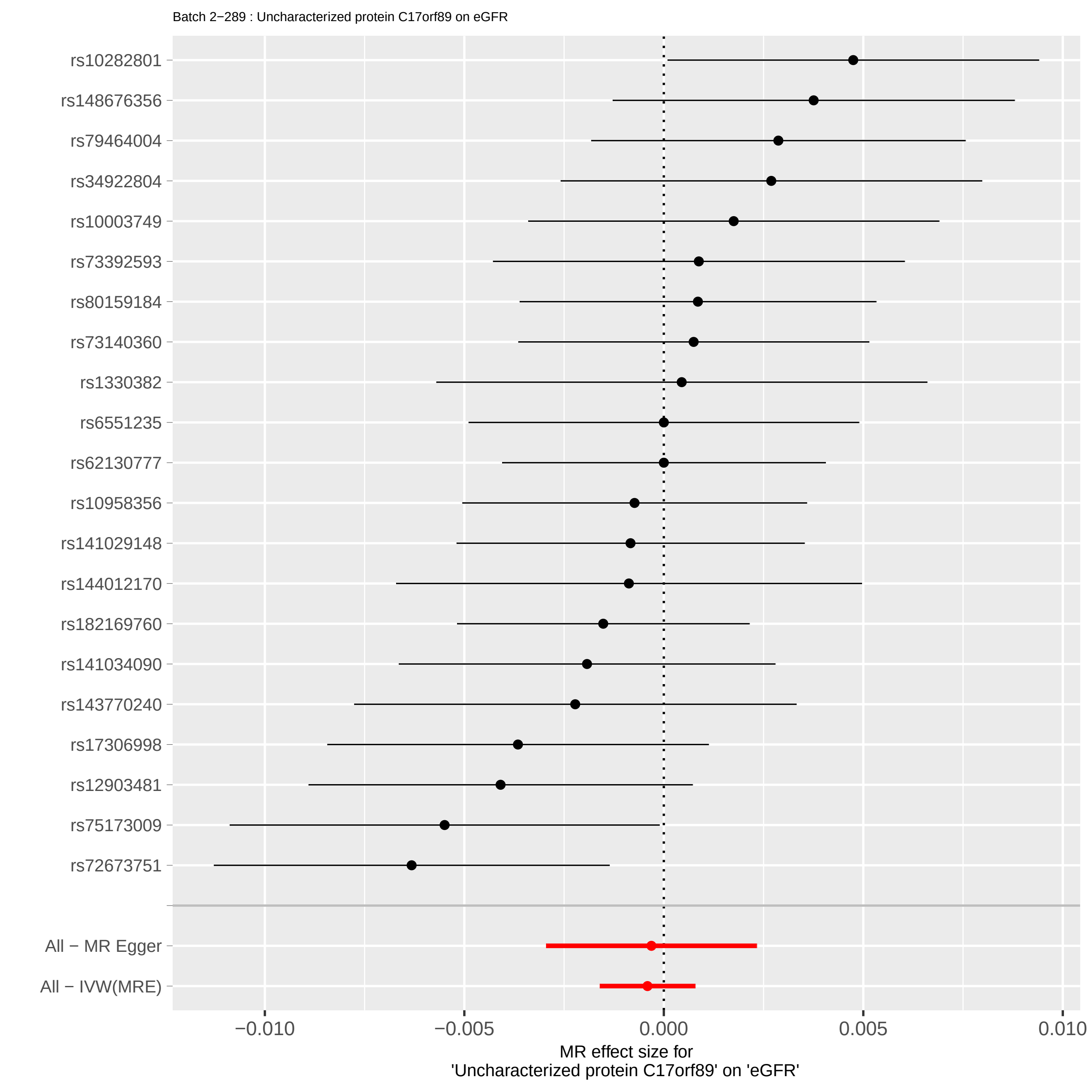

Batch 2−289 : Uncharacterized protein C17orf89 on eGFR
rs10282801
rs148676356
rs79464004
rs34922804
rs10003749
rs73392593
rs80159184
rs73140360
rs1330382
rs6551235
rs62130777
rs10958356
rs141029148
rs144012170
rs182169760
rs141034090
rs143770240
rs17306998
rs12903481
rs75173009
rs72673751
All − MR Egger
All − IVW(MRE)
−0.010
−0.005
0.000
0.005
0.010
MR effect size for
'Uncharacterized protein C17orf89' on 'eGFR'

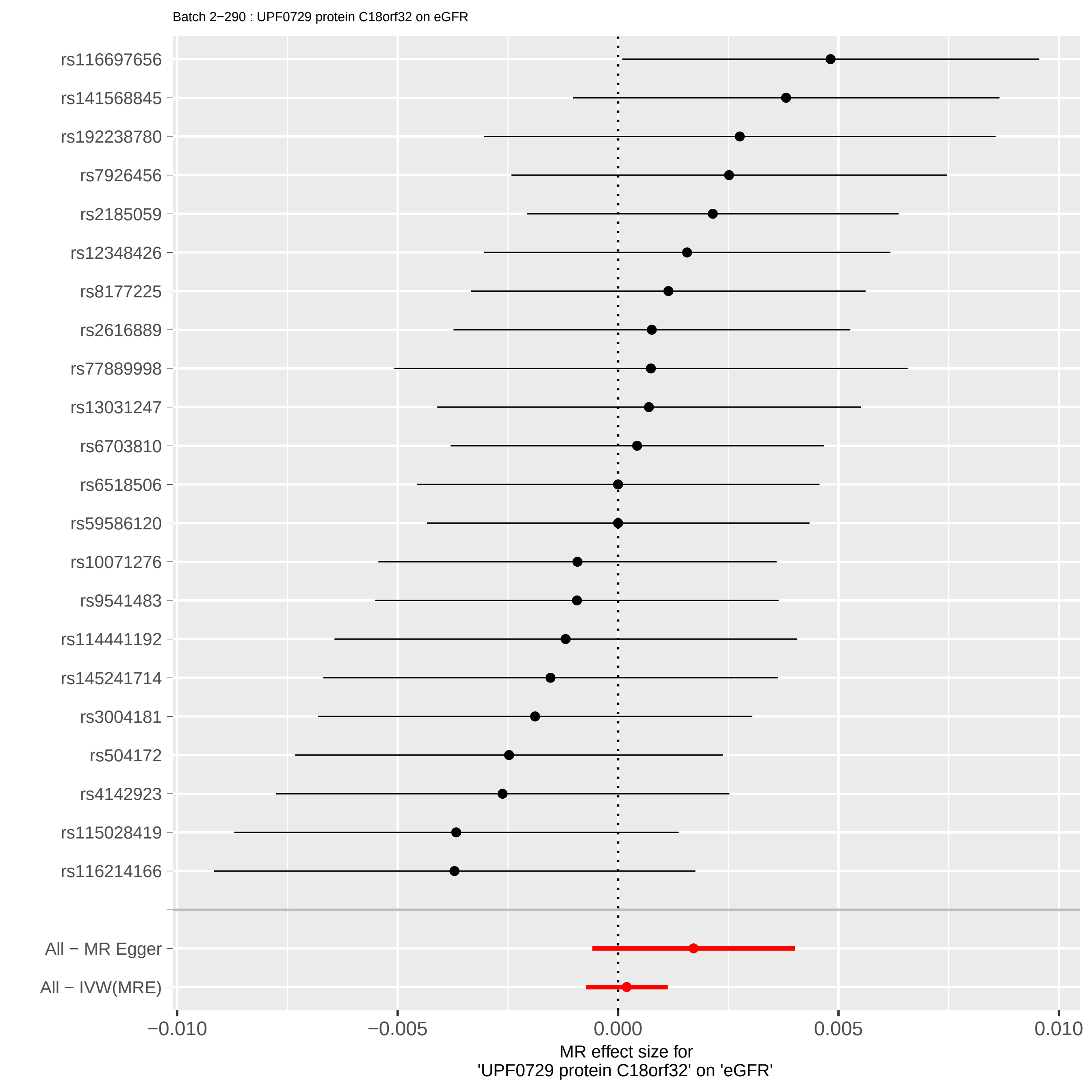
Batch 2−290 : UPF0729 protein C18orf32 on eGFR
rs116697656
rs141568845
rs192238780
rs7926456
rs2185059
rs12348426
rs8177225
rs2616889
rs77889998
rs13031247
rs6703810
rs6518506
rs59586120
rs10071276
rs9541483
rs114441192
rs145241714
rs3004181
rs504172
rs4142923
rs115028419
rs116214166
All − MR Egger
All − IVW(MRE)
−0.010
−0.005
0.000
0.005
0.010
MR effect size for
'UPF0729 protein C18orf32' on 'eGFR'

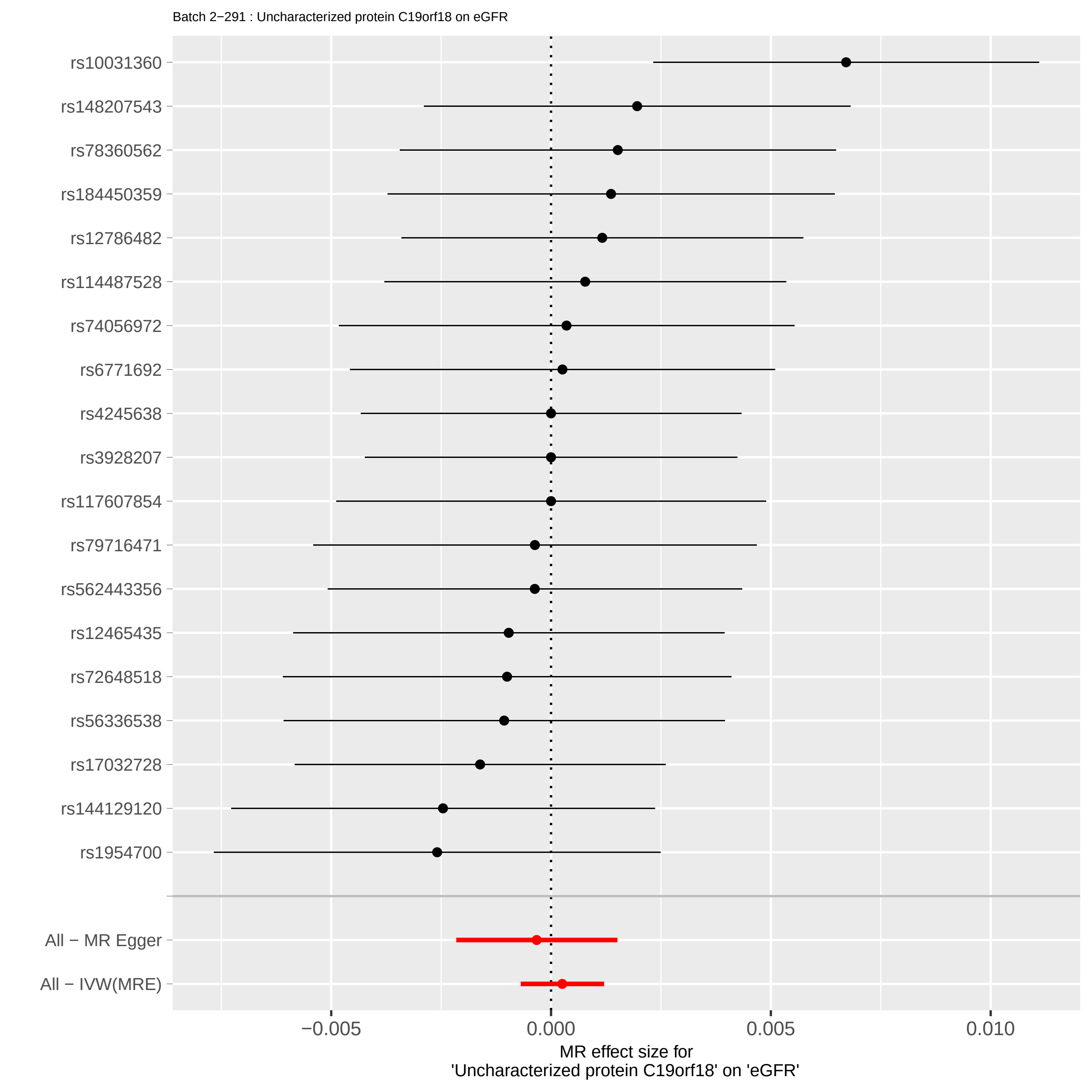

Batch 2−291 : Uncharacterized protein C19orf18 on eGFR
rs10031360
rs148207543
rs78360562
rs184450359
rs12786482
rs114487528
rs74056972
rs6771692
rs4245638
rs3928207
rs117607854
rs79716471
rs562443356
rs12465435
rs72648518
rs56336538
rs17032728
rs144129120
rs1954700
All − MR Egger
All − IVW(MRE)
−0.005
0.000
0.005
0.010
MR effect size for
'Uncharacterized protein C19orf18' on 'eGFR'

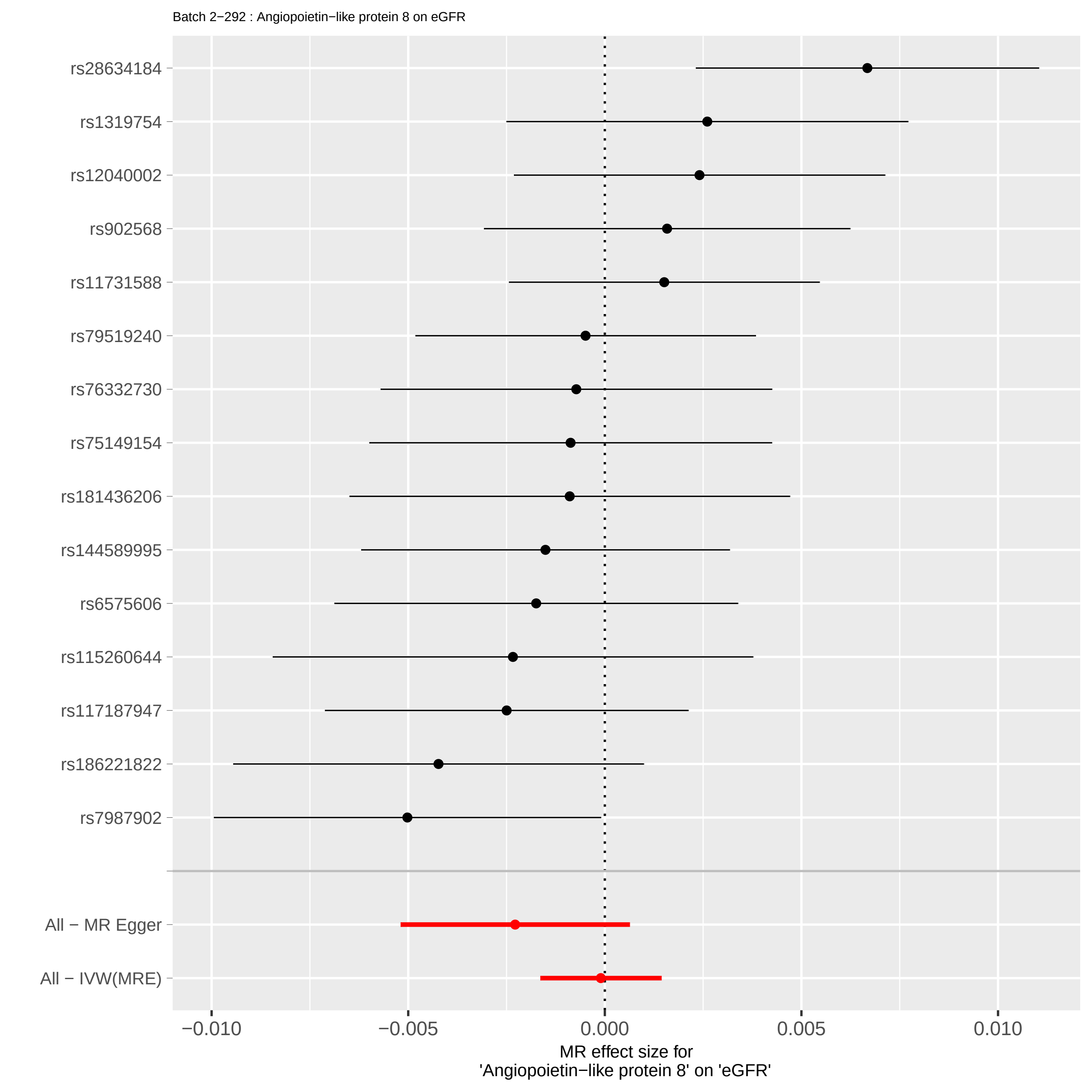
Batch 2−292 : Angiopoietin−like protein 8 on eGFR
rs28634184
rs1319754
rs12040002
rs902568
rs11731588
rs79519240
rs76332730
rs75149154
rs181436206
rs144589995
rs6575606
rs115260644
rs117187947
rs186221822
rs7987902
All − MR Egger
All − IVW(MRE)
−0.010
−0.005
0.000
0.005
0.010
MR effect size for
'Angiopoietin−like protein 8' on 'eGFR'

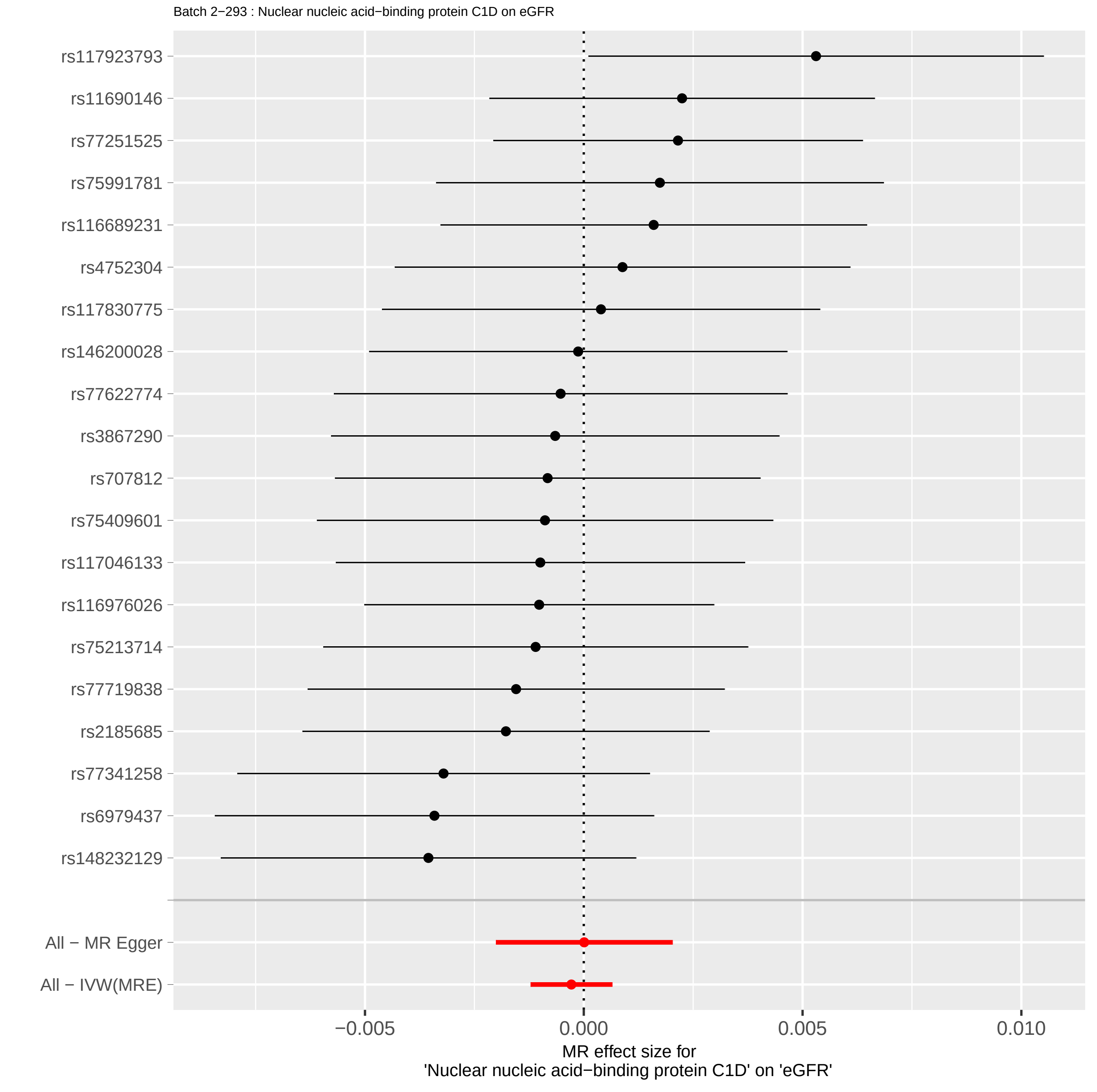

Batch 2−293 : Nuclear nucleic acid−binding protein C1D on eGFR
rs117923793
rs11690146
rs77251525
rs75991781
rs116689231
rs4752304
rs117830775
rs146200028
rs77622774
rs3867290
rs707812
rs75409601
rs117046133
rs116976026
rs75213714
rs77719838
rs2185685
rs77341258
rs6979437
rs148232129
All − MR Egger
All − IVW(MRE)
−0.005
0.000
0.005
0.010
MR effect size for
'Nuclear nucleic acid−binding protein C1D' on 'eGFR'

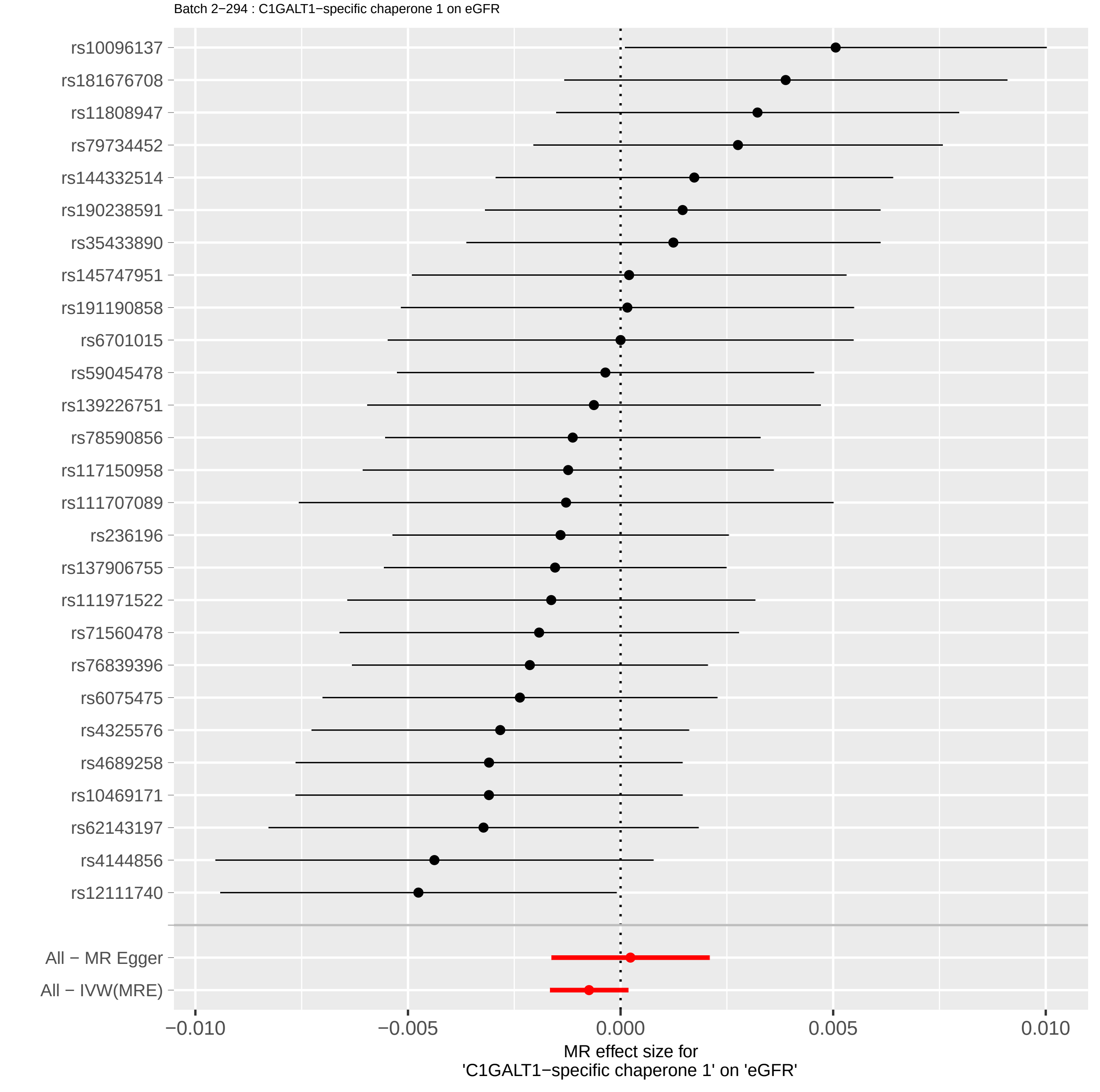
Batch 2−294 : C1GALT1−specific chaperone 1 on eGFR
rs10096137
rs181676708
rs11808947
rs79734452
rs144332514
rs190238591
rs35433890
rs145747951
rs191190858
rs6701015
rs59045478
rs139226751
rs78590856
rs117150958
rs111707089
rs236196
rs137906755
rs111971522
rs71560478
rs76839396
rs6075475
rs4325576
rs4689258
rs10469171
rs62143197
rs4144856
rs12111740
All − MR Egger
All − IVW(MRE)
−0.010
−0.005
0.000
0.005
0.010
MR effect size for
'C1GALT1−specific chaperone 1' on 'eGFR'

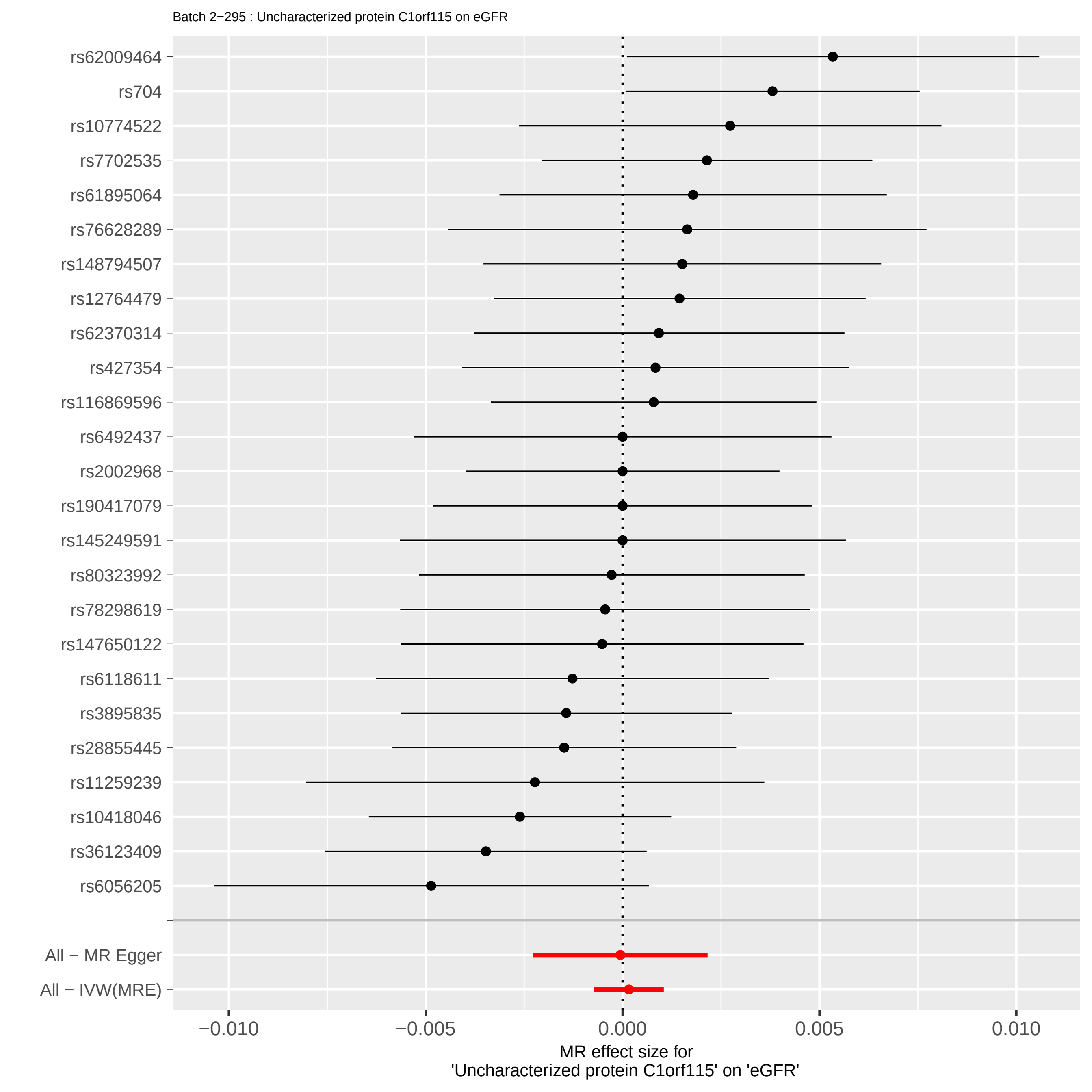

Batch 2−295 : Uncharacterized protein C1orf115 on eGFR
rs62009464
rs704
rs10774522
rs7702535
rs61895064
rs76628289
rs148794507
rs12764479
rs62370314
rs427354
rs116869596
rs6492437
rs2002968
rs190417079
rs145249591
rs80323992
rs78298619
rs147650122
rs6118611
rs3895835
rs28855445
rs11259239
rs10418046
rs36123409
rs6056205
All − MR Egger
All − IVW(MRE)
−0.010
−0.005
0.000
0.005
0.010
MR effect size for
'Uncharacterized protein C1orf115' on 'eGFR'

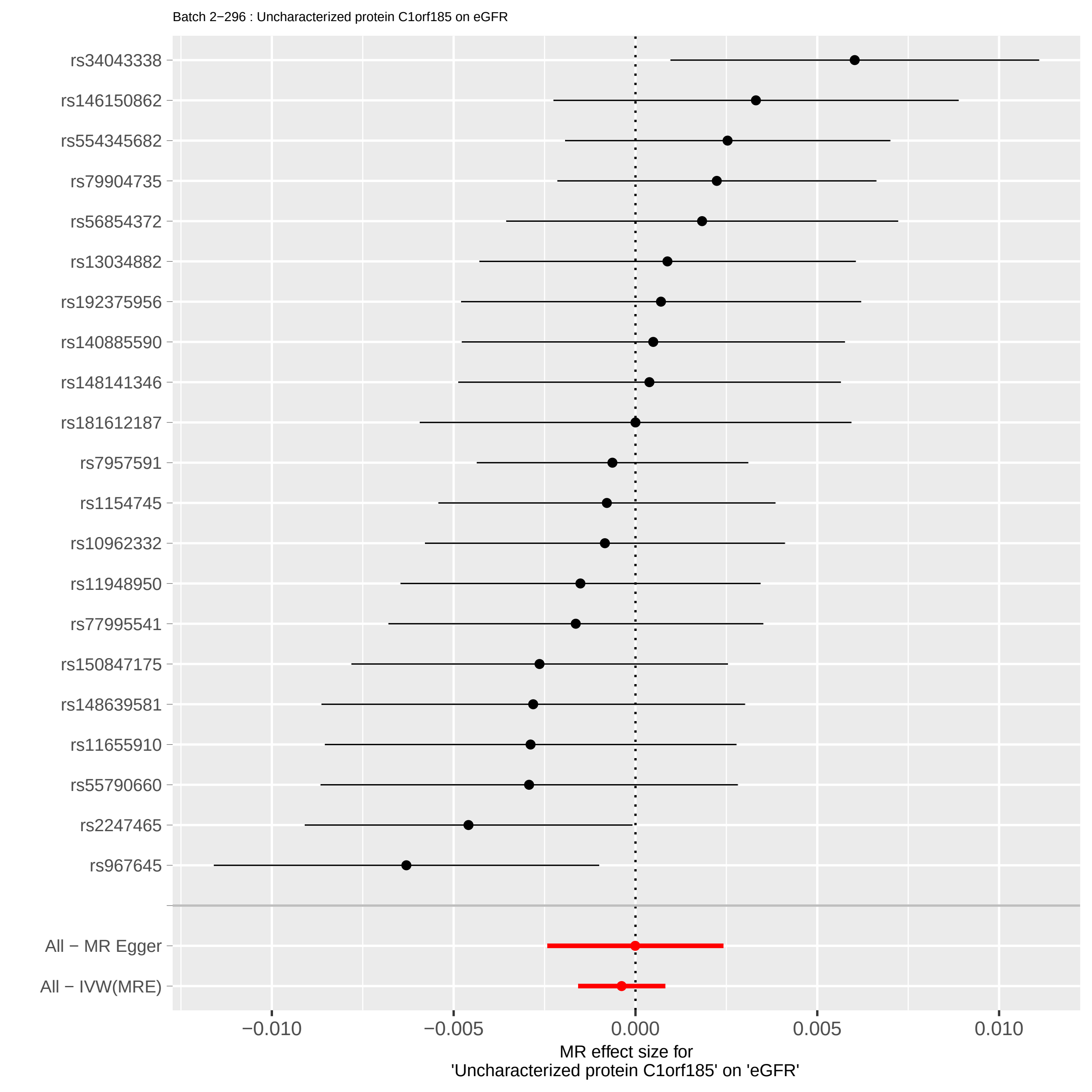

Batch 2−296 : Uncharacterized protein C1orf185 on eGFR
rs34043338
rs146150862
rs554345682
rs79904735
rs56854372
rs13034882
rs192375956
rs140885590
rs148141346
rs181612187
rs7957591
rs1154745
rs10962332
rs11948950
rs77995541
rs150847175
rs148639581
rs11655910
rs55790660
rs2247465
rs967645
All − MR Egger
All − IVW(MRE)
−0.010
−0.005
0.000
0.005
0.010
MR effect size for
'Uncharacterized protein C1orf185' on 'eGFR'

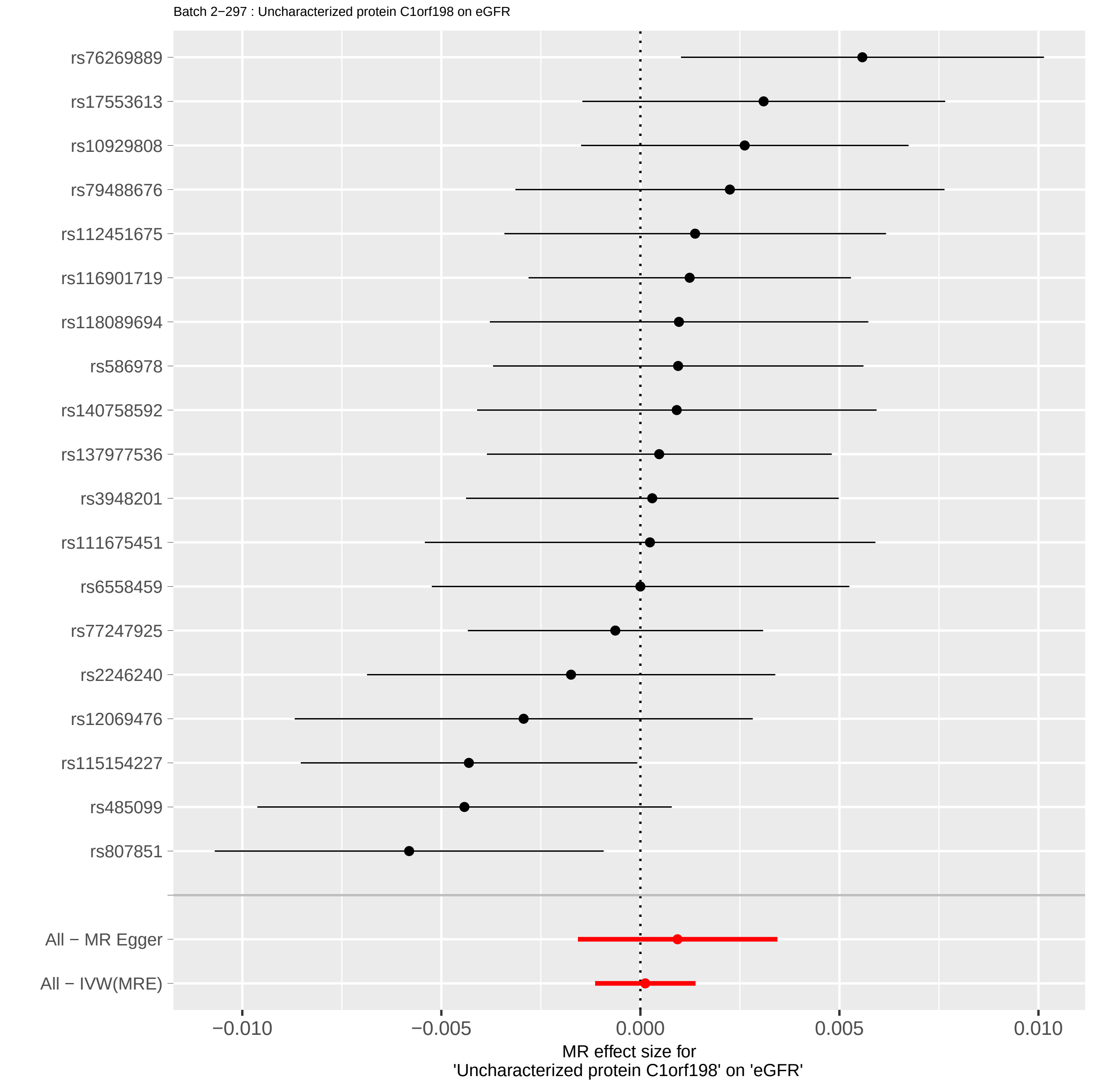

Batch 2−297 : Uncharacterized protein C1orf198 on eGFR
rs76269889
rs17553613
rs10929808
rs79488676
rs112451675
rs116901719
rs118089694
rs586978
rs140758592
rs137977536
rs3948201
rs111675451
rs6558459
rs77247925
rs2246240
rs12069476
rs115154227
rs485099
rs807851
All − MR Egger
All − IVW(MRE)
−0.010
−0.005
0.000
0.005
0.010
MR effect size for
'Uncharacterized protein C1orf198' on 'eGFR'

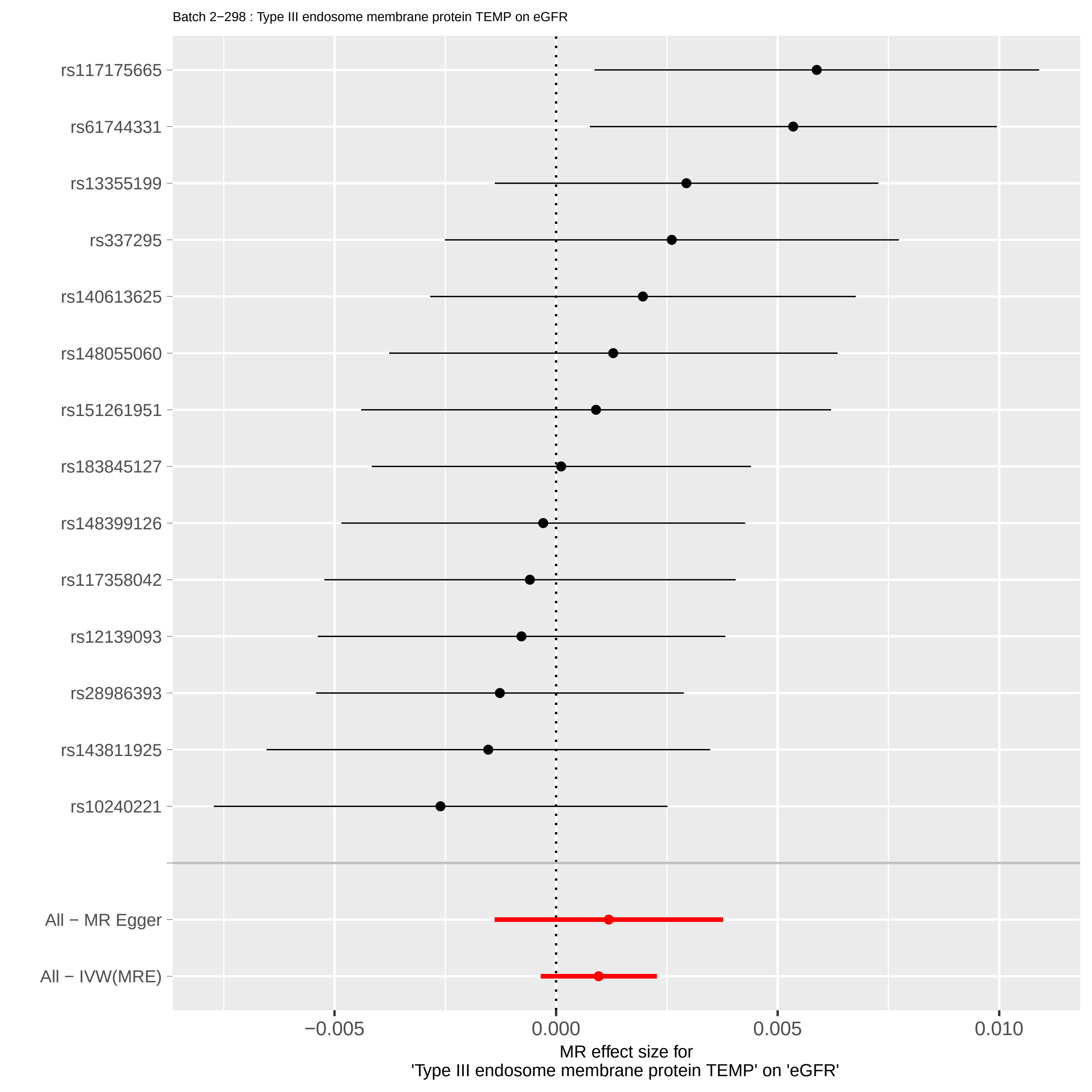

Batch 2−298 : Type III endosome membrane protein TEMP on eGFR
rs117175665
rs61744331
rs13355199
rs337295
rs140613625
rs148055060
rs151261951
rs183845127
rs148399126
rs117358042
rs12139093
rs28986393
rs143811925
rs10240221
All − MR Egger
All − IVW(MRE)
−0.005
0.000
0.005
0.010
MR effect size for
'Type III endosome membrane protein TEMP' on 'eGFR'

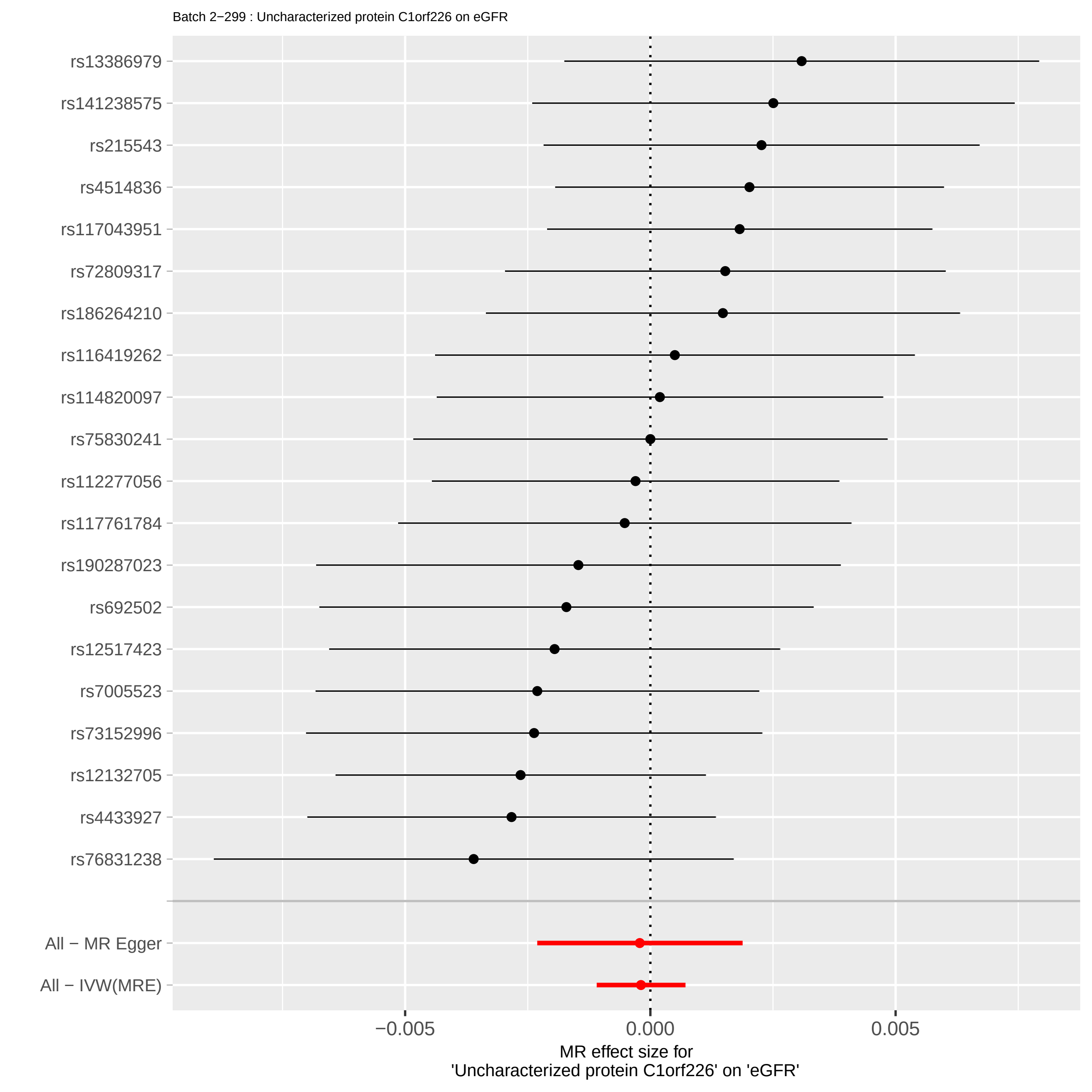
Batch 2−299 : Uncharacterized protein C1orf226 on eGFR
rs13386979
rs141238575
rs215543
rs4514836
rs117043951
rs72809317
rs186264210
rs116419262
rs114820097
rs75830241
rs112277056
rs117761784
rs190287023
rs692502
rs12517423
rs7005523
rs73152996
rs12132705
rs4433927
rs76831238
All − MR Egger
All − IVW(MRE)
−0.005
0.000
0.005
MR effect size for
'Uncharacterized protein C1orf226' on 'eGFR'

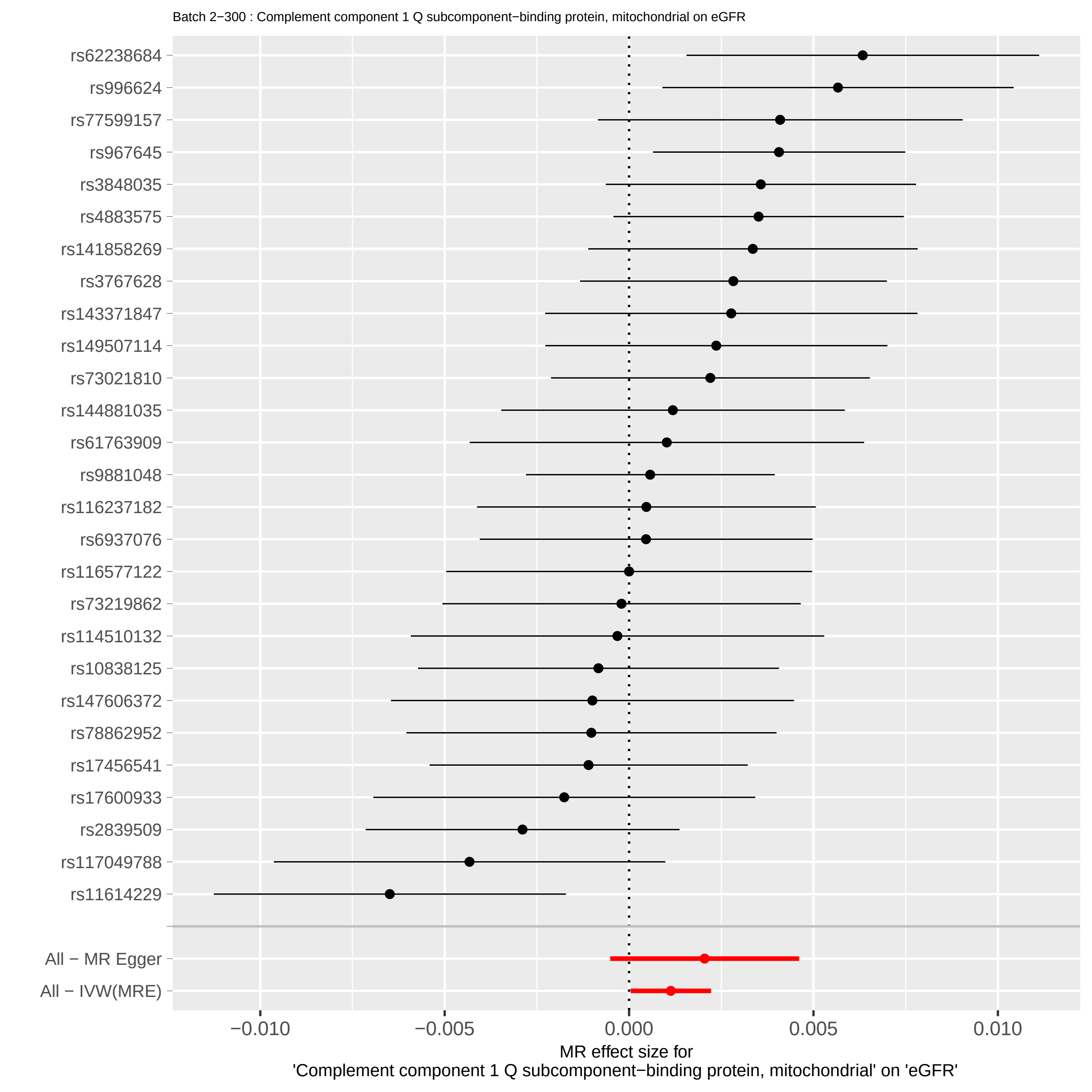

Batch 2−300 : Complement component 1 Q subcomponent−binding protein, mitochondrial on eGFR
rs62238684
rs996624
rs77599157
rs967645
rs3848035
rs4883575
rs141858269
rs3767628
rs143371847
rs149507114
rs73021810
rs144881035
rs61763909
rs9881048
rs116237182
rs6937076
rs116577122
rs73219862
rs114510132
rs10838125
rs147606372
rs78862952
rs17456541
rs17600933
rs2839509
rs117049788
rs11614229
All − MR Egger
All − IVW(MRE)
−0.010
−0.005
0.000
0.005
0.010
MR effect size for
'Complement component 1 Q subcomponent−binding protein, mitochondrial' on 'eGFR'

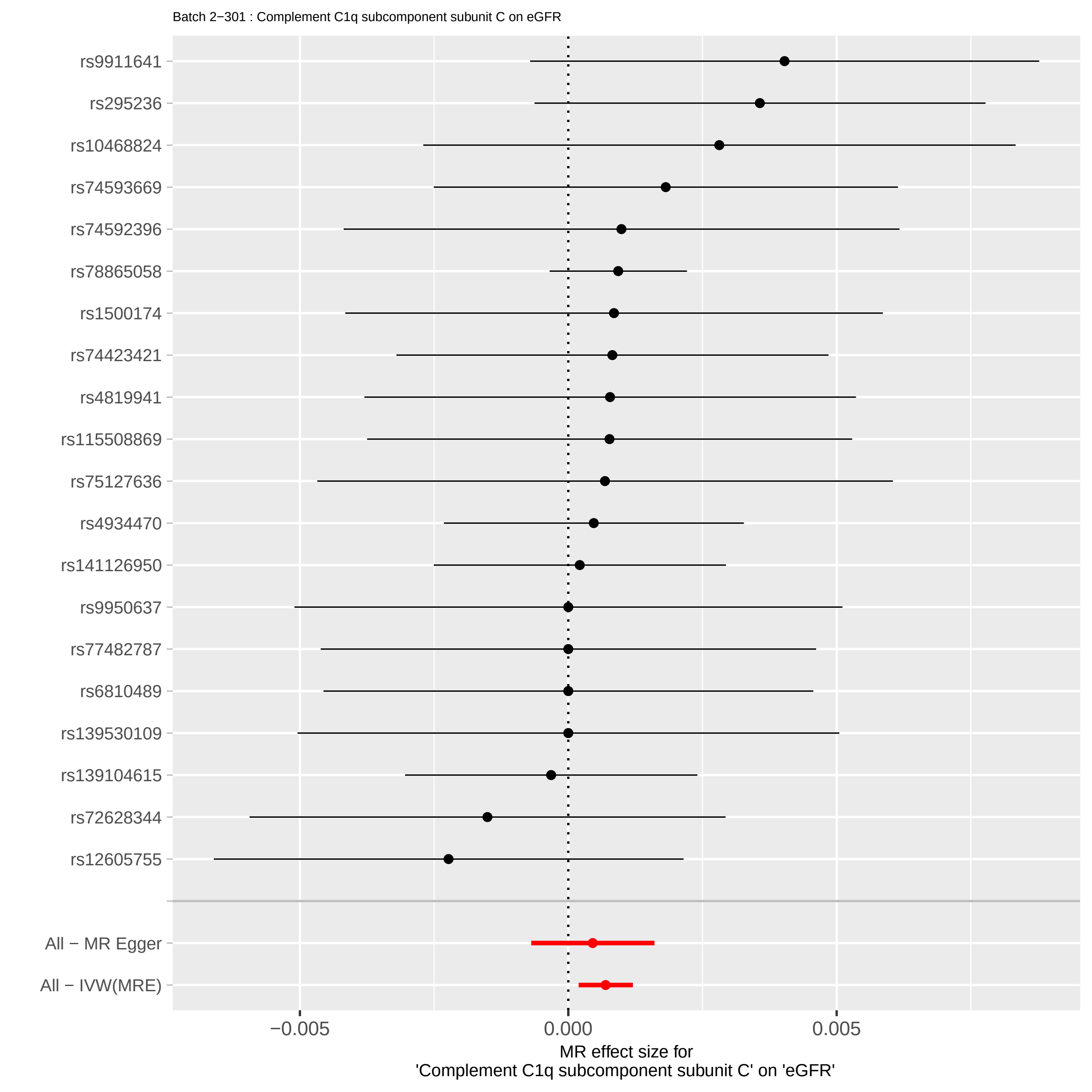

Batch 2−301 : Complement C1q subcomponent subunit C on eGFR
rs9911641
rs295236
rs10468824
rs74593669
rs74592396
rs78865058
rs1500174
rs74423421
rs4819941
rs115508869
rs75127636
rs4934470
rs141126950
rs9950637
rs77482787
rs6810489
rs139530109
rs139104615
rs72628344
rs12605755
All − MR Egger
All − IVW(MRE)
−0.005
0.000
0.005
MR effect size for
'Complement C1q subcomponent subunit C' on 'eGFR'

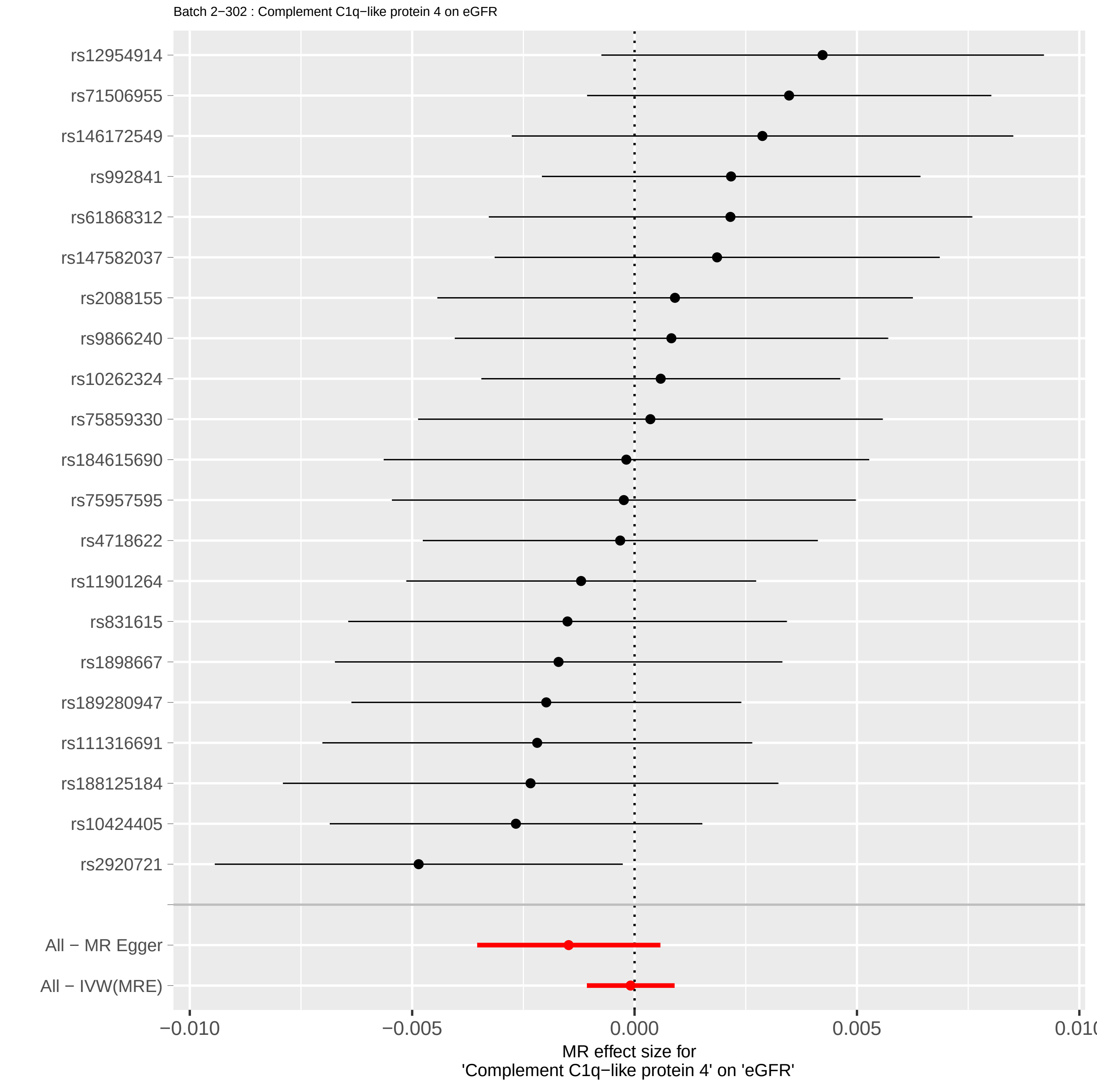

Batch 2−302 : Complement C1q−like protein 4 on eGFR
rs12954914
rs71506955
rs146172549
rs992841
rs61868312
rs147582037
rs2088155
rs9866240
rs10262324
rs75859330
rs184615690
rs75957595
rs4718622
rs11901264
rs831615
rs1898667
rs189280947
rs111316691
rs188125184
rs10424405
rs2920721
All − MR Egger
All − IVW(MRE)
−0.010
−0.005
0.000
0.005
MR effect size for
'Complement C1q−like protein 4' on 'eGFR'

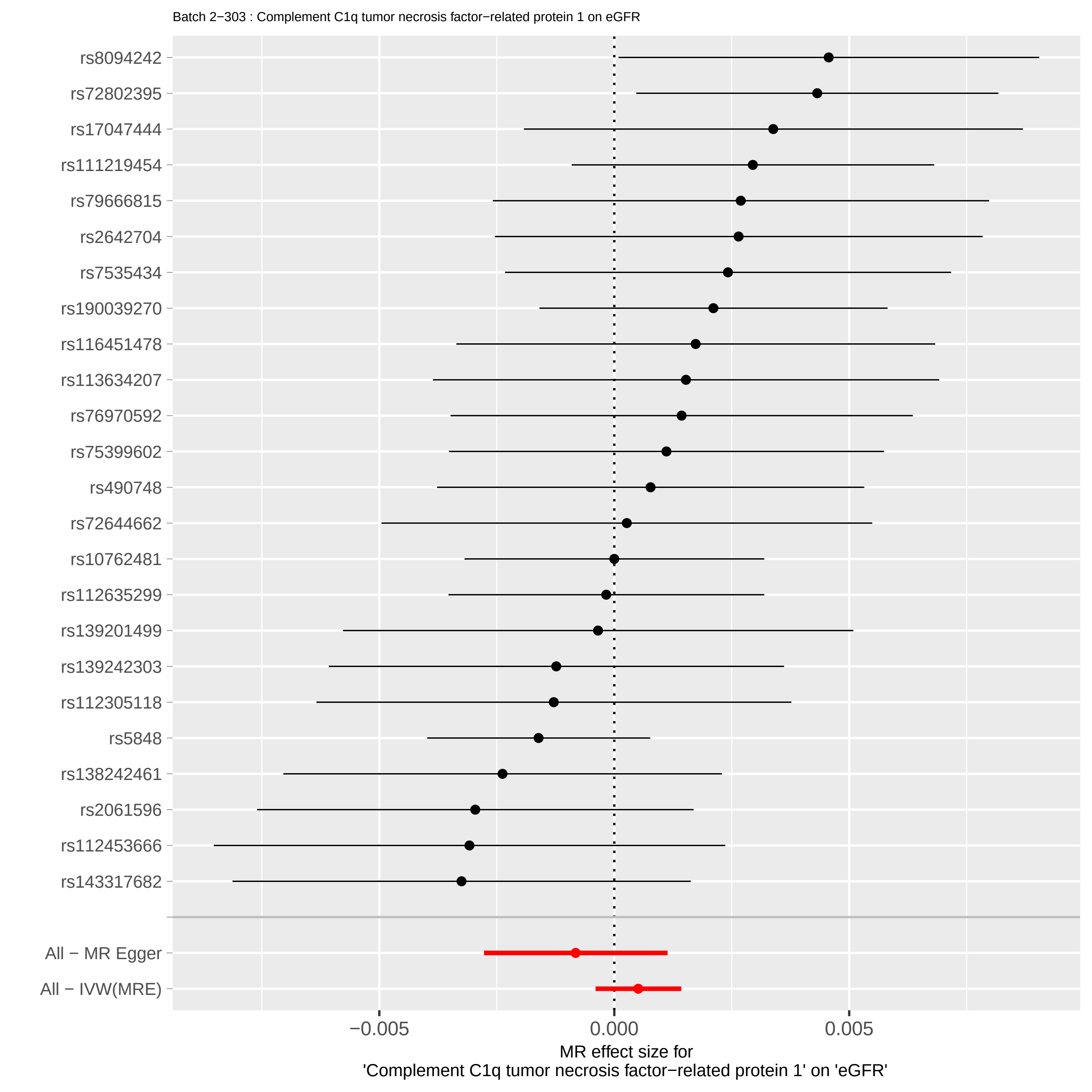
Batch 2−303 : Complement C1q tumor necrosis factor−related protein 1 on eGFR
rs8094242
rs72802395
rs17047444
rs111219454
rs79666815
rs2642704
rs7535434
rs190039270
rs116451478
rs113634207
rs76970592
rs75399602
rs490748
rs72644662
rs10762481
rs112635299
rs139201499
rs139242303
rs112305118
rs5848
rs138242461
rs2061596
rs112453666
rs143317682
All − MR Egger
All − IVW(MRE)
−0.005
0.000
0.005
MR effect size for
'Complement C1q tumor necrosis factor−related protein 1' on 'eGFR'

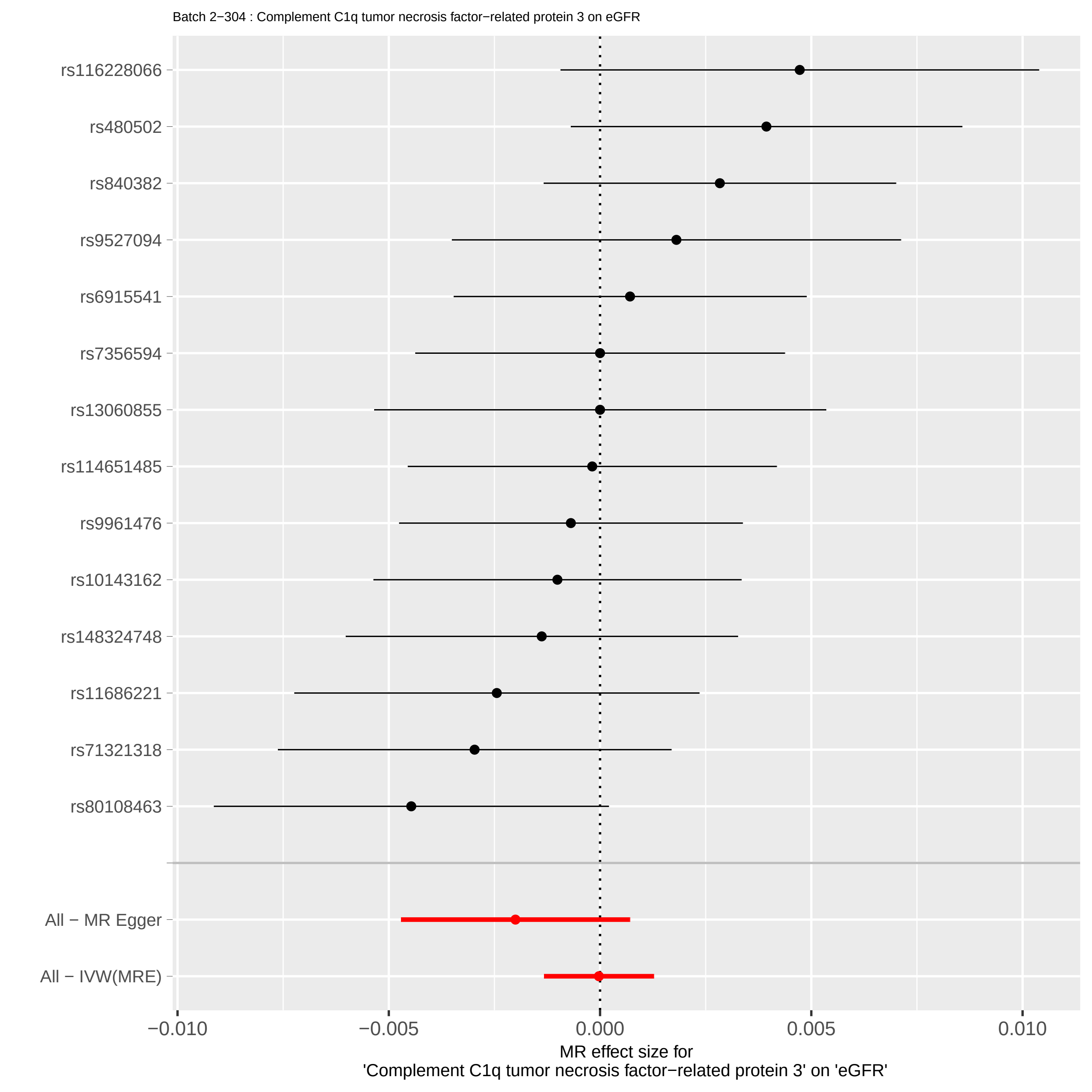
Batch 2−304 : Complement C1q tumor necrosis factor−related protein 3 on eGFR
rs116228066
rs480502
rs840382
rs9527094
rs6915541
rs7356594
rs13060855
rs114651485
rs9961476
rs10143162
rs148324748
rs11686221
rs71321318
rs80108463
All − MR Egger
All − IVW(MRE)
−0.010
−0.005
0.000
0.005
0.010
MR effect size for
'Complement C1q tumor necrosis factor−related protein 3' on 'eGFR'

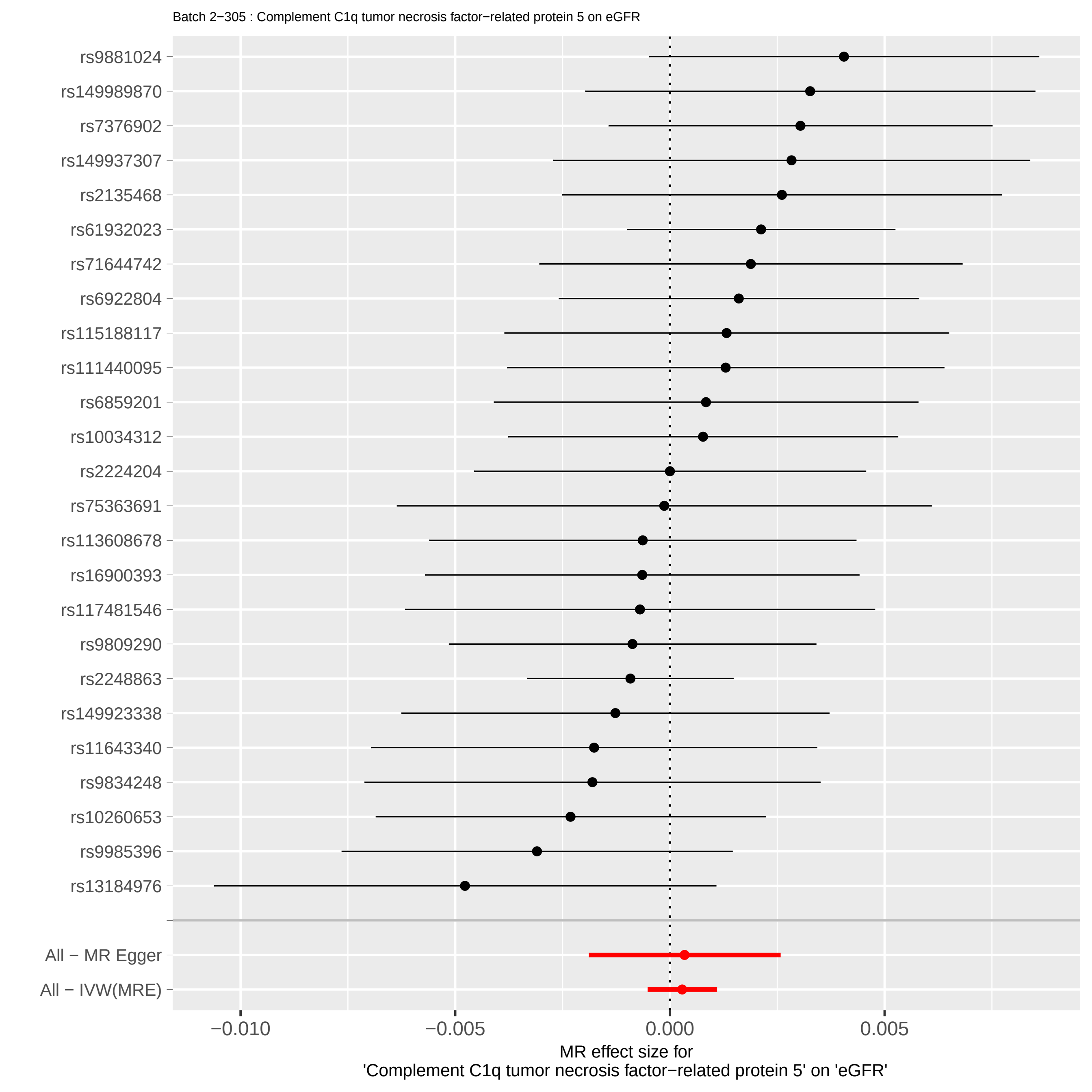

Batch 2−305 : Complement C1q tumor necrosis factor−related protein 5 on eGFR
rs9881024
rs149989870
rs7376902
rs149937307
rs2135468
rs61932023
rs71644742
rs6922804
rs115188117
rs111440095
rs6859201
rs10034312
rs2224204
rs75363691
rs113608678
rs16900393
rs117481546
rs9809290
rs2248863
rs149923338
rs11643340
rs9834248
rs10260653
rs9985396
rs13184976
All − MR Egger
All − IVW(MRE)
−0.010
−0.005
0.000
0.005
MR effect size for
'Complement C1q tumor necrosis factor−related protein 5' on 'eGFR'

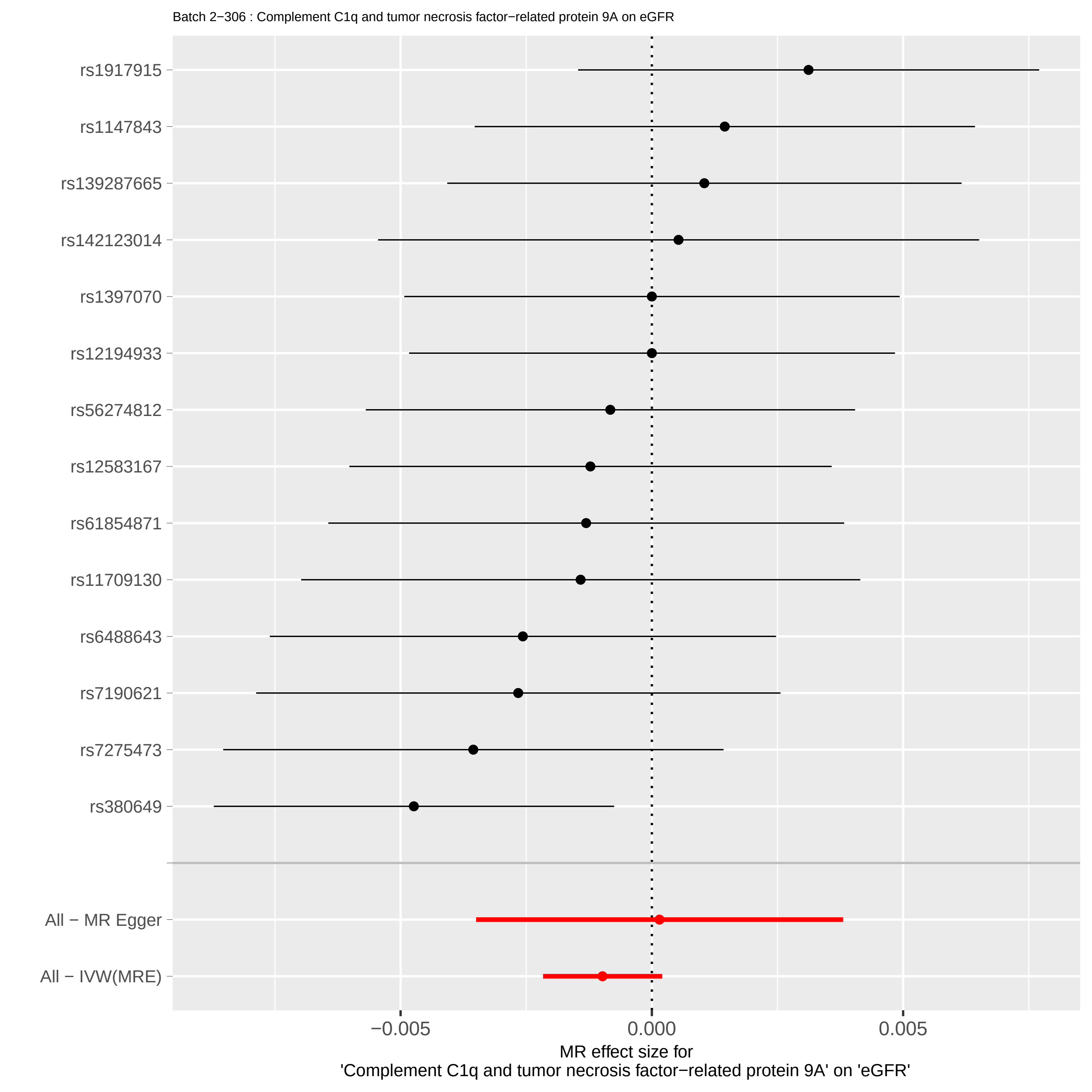
Batch 2−306 : Complement C1q and tumor necrosis factor−related protein 9A on eGFR
rs1917915
rs1147843
rs139287665
rs142123014
rs1397070
rs12194933
rs56274812
rs12583167
rs61854871
rs11709130
rs6488643
rs7190621
rs7275473
rs380649
All − MR Egger
All − IVW(MRE)
−0.005
0.000
0.005
MR effect size for
'Complement C1q and tumor necrosis factor−related protein 9A' on 'eGFR'

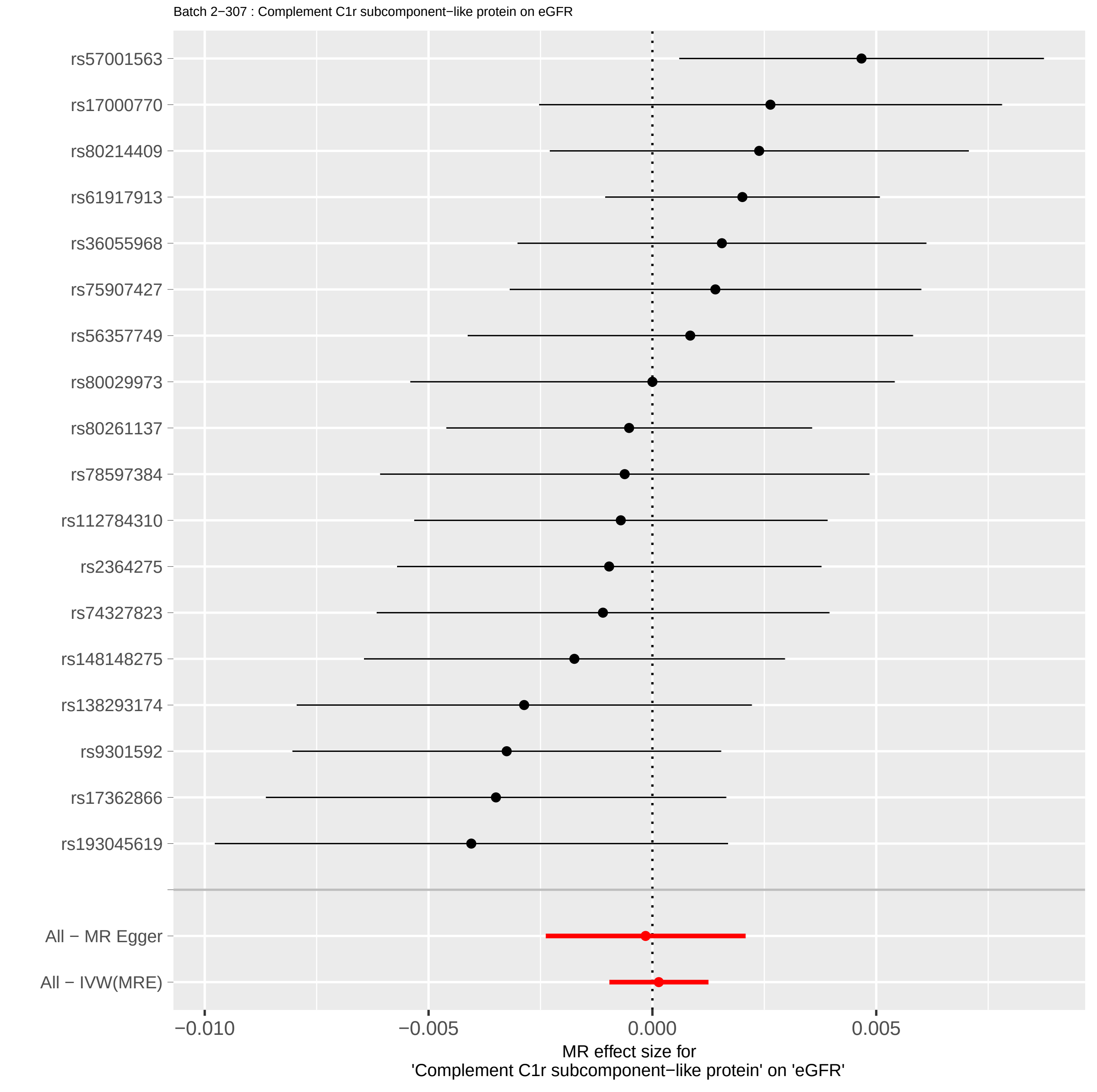

Batch 2−307 : Complement C1r subcomponent−like protein on eGFR
rs57001563
rs17000770
rs80214409
rs61917913
rs36055968
rs75907427
rs56357749
rs80029973
rs80261137
rs78597384
rs112784310
rs2364275
rs74327823
rs148148275
rs138293174
rs9301592
rs17362866
rs193045619
All − MR Egger
All − IVW(MRE)
−0.010
−0.005
0.000
0.005
MR effect size for
'Complement C1r subcomponent−like protein' on 'eGFR'

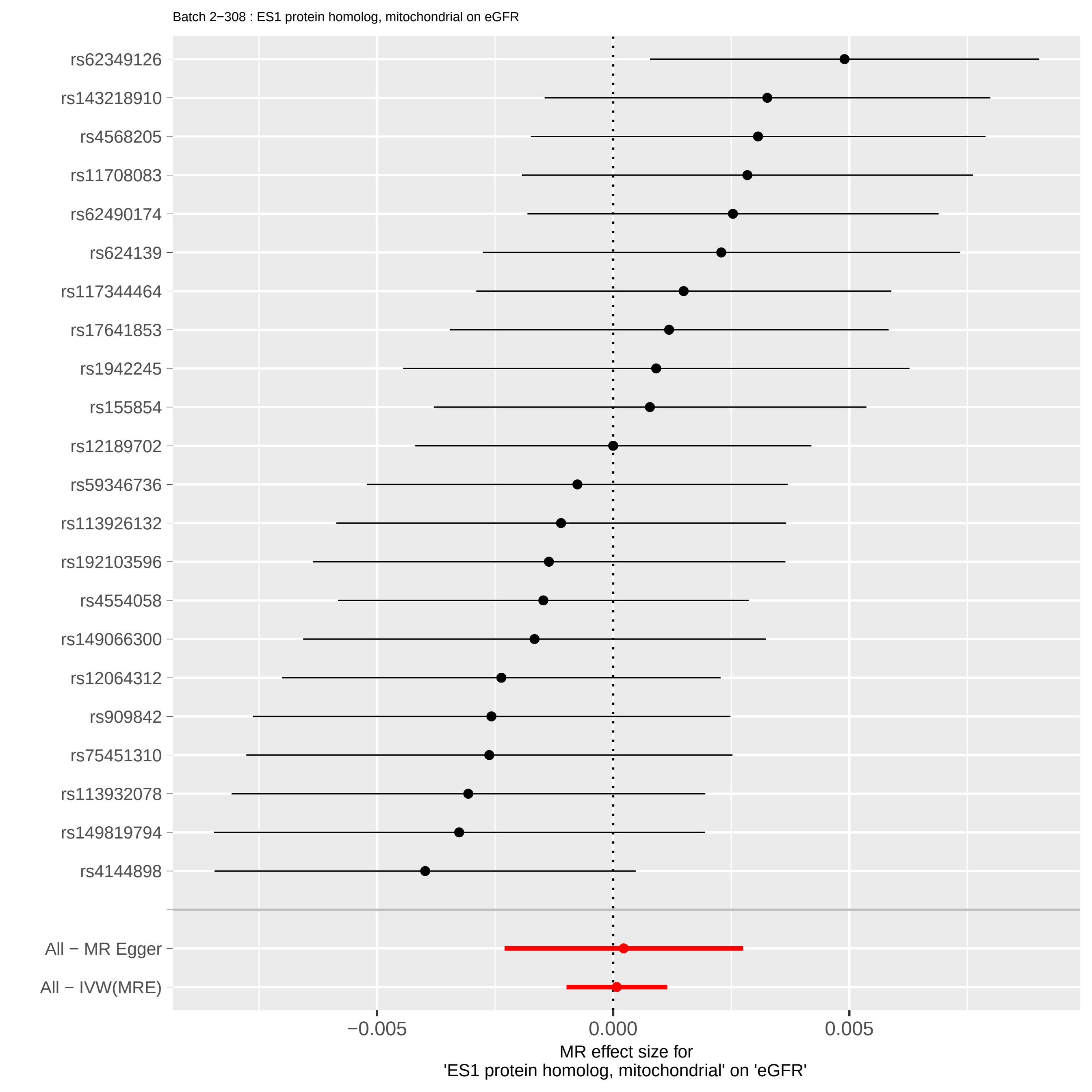
Batch 2−308 : ES1 protein homolog, mitochondrial on eGFR
rs62349126
rs143218910
rs4568205
rs11708083
rs62490174
rs624139
rs117344464
rs17641853
rs1942245
rs155854
rs12189702
rs59346736
rs113926132
rs192103596
rs4554058
rs149066300
rs12064312
rs909842
rs75451310
rs113932078
rs149819794
rs4144898
All − MR Egger
All − IVW(MRE)
−0.005
0.000
0.005
MR effect size for
'ES1 protein homolog, mitochondrial' on 'eGFR'

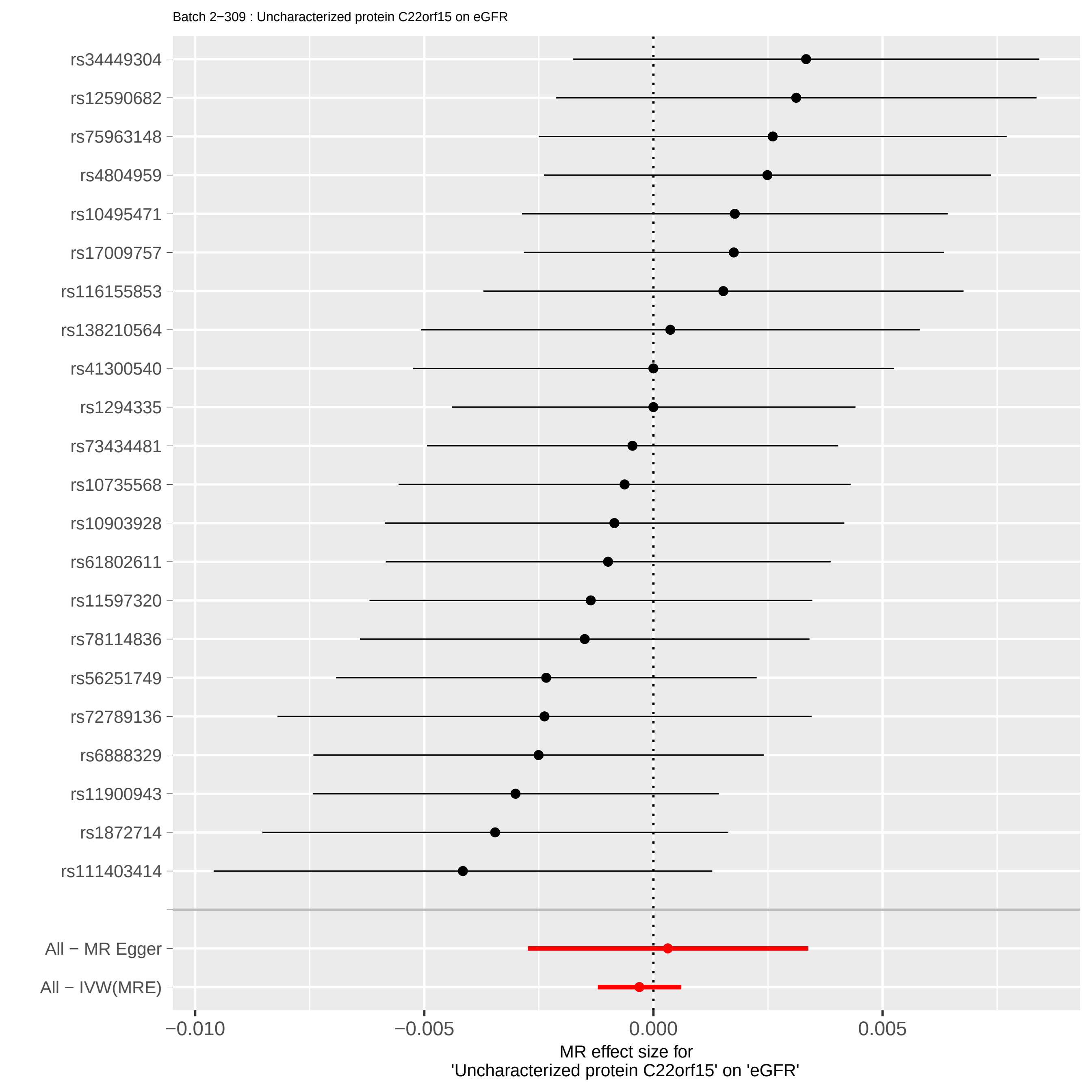
Batch 2−309 : Uncharacterized protein C22orf15 on eGFR
rs34449304
rs12590682
rs75963148
rs4804959
rs10495471
rs17009757
rs116155853
rs138210564
rs41300540
rs1294335
rs73434481
rs10735568
rs10903928
rs61802611
rs11597320
rs78114836
rs56251749
rs72789136
rs6888329
rs11900943
rs1872714
rs111403414
All − MR Egger
All − IVW(MRE)
−0.010
−0.005
0.000
0.005
MR effect size for
'Uncharacterized protein C22orf15' on 'eGFR'

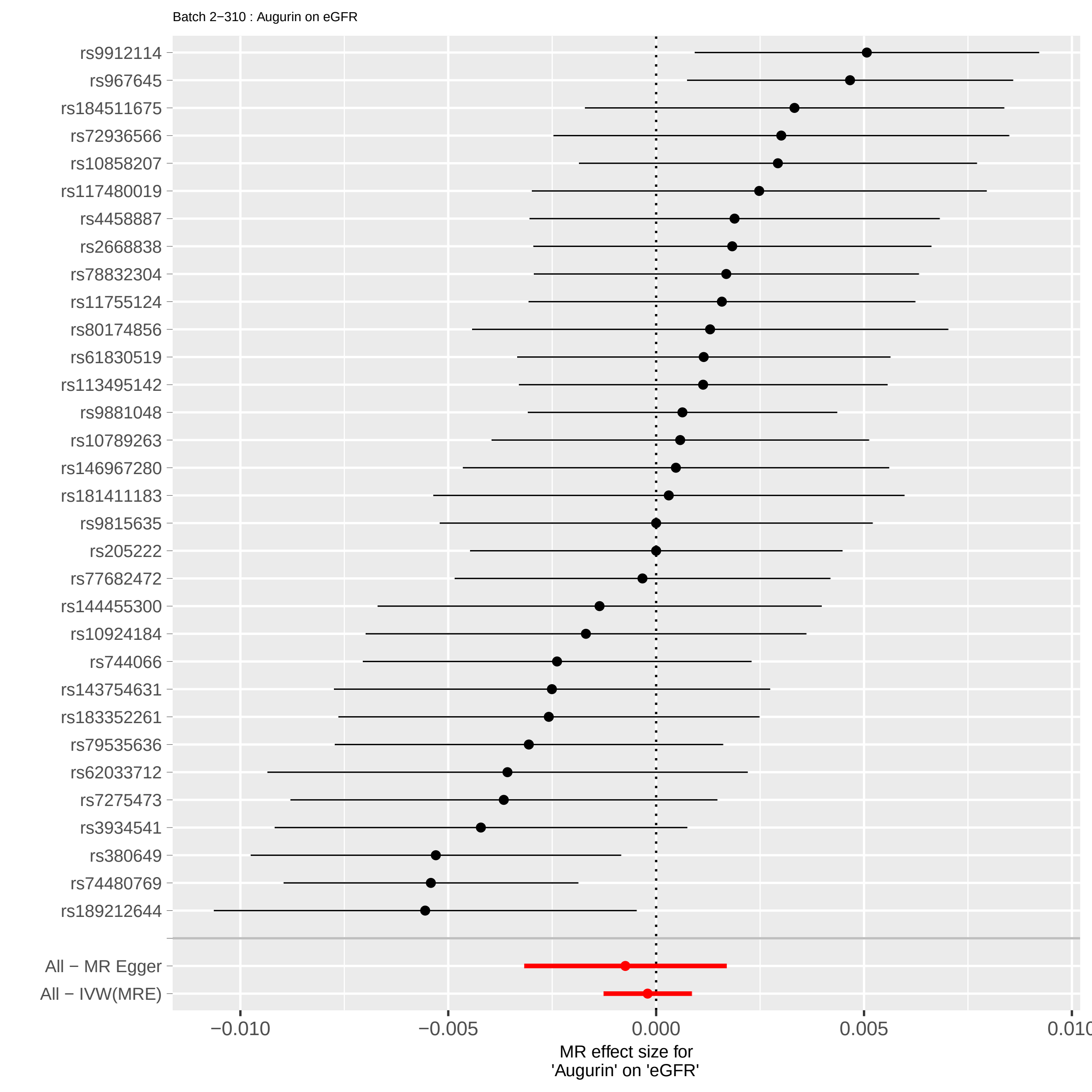

Batch 2−310 : Augurin on eGFR
rs9912114
rs967645
rs184511675
rs72936566
rs10858207
rs117480019
rs4458887
rs2668838
rs78832304
rs11755124
rs80174856
rs61830519
rs113495142
rs9881048
rs10789263
rs146967280
rs181411183
rs9815635
rs205222
rs77682472
rs144455300
rs10924184
rs744066
rs143754631
rs183352261
rs79535636
rs62033712
rs7275473
rs3934541
rs380649
rs74480769
rs189212644
All − MR Egger
All − IVW(MRE)
−0.010
−0.005
0.000
0.005
0.010
MR effect size for
'Augurin' on 'eGFR'

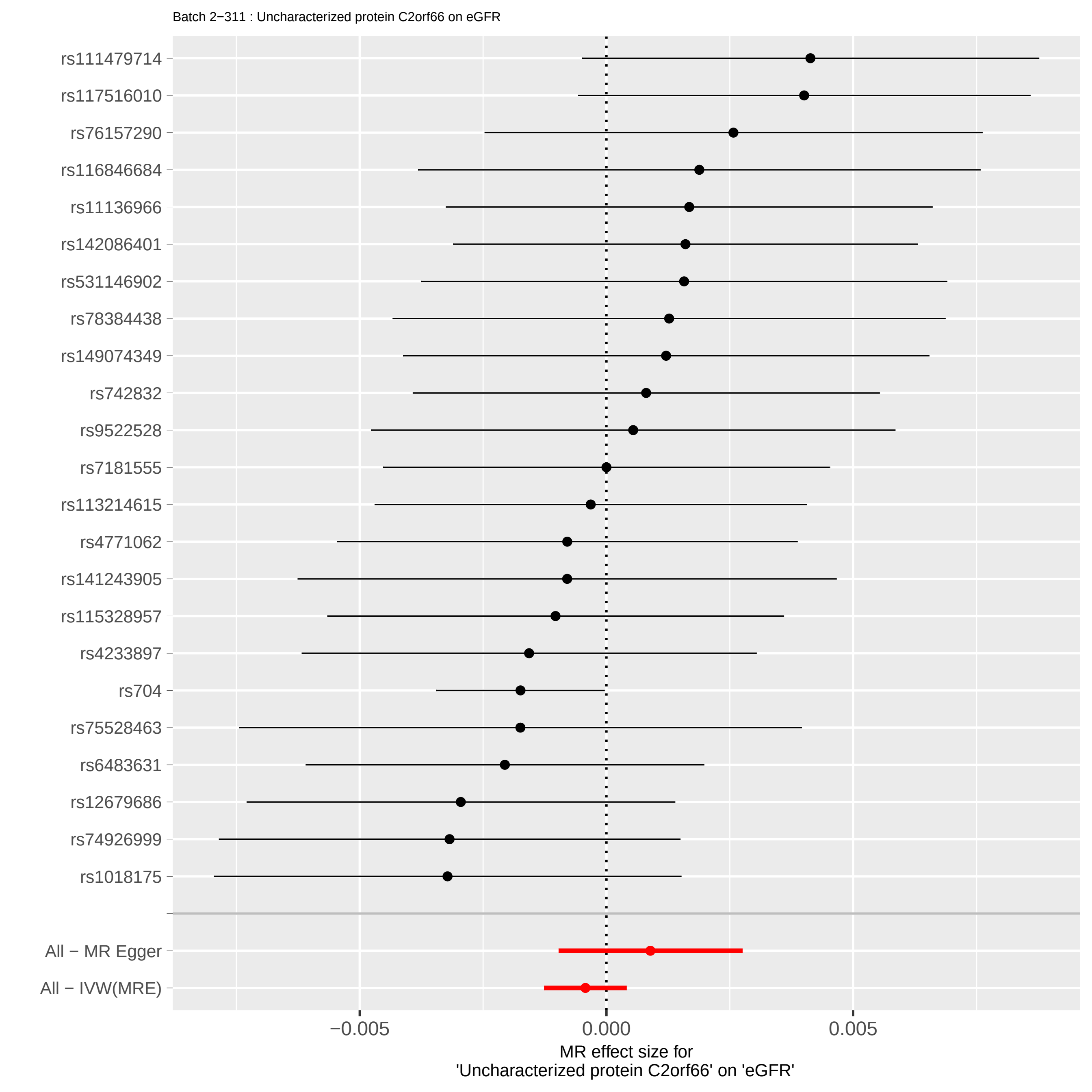
Batch 2−311 : Uncharacterized protein C2orf66 on eGFR
rs111479714
rs117516010
rs76157290
rs116846684
rs11136966
rs142086401
rs531146902
rs78384438
rs149074349
rs742832
rs9522528
rs7181555
rs113214615
rs4771062
rs141243905
rs115328957
rs4233897
rs704
rs75528463
rs6483631
rs12679686
rs74926999
rs1018175
All − MR Egger
All − IVW(MRE)
−0.005
0.000
0.005
MR effect size for
'Uncharacterized protein C2orf66' on 'eGFR'

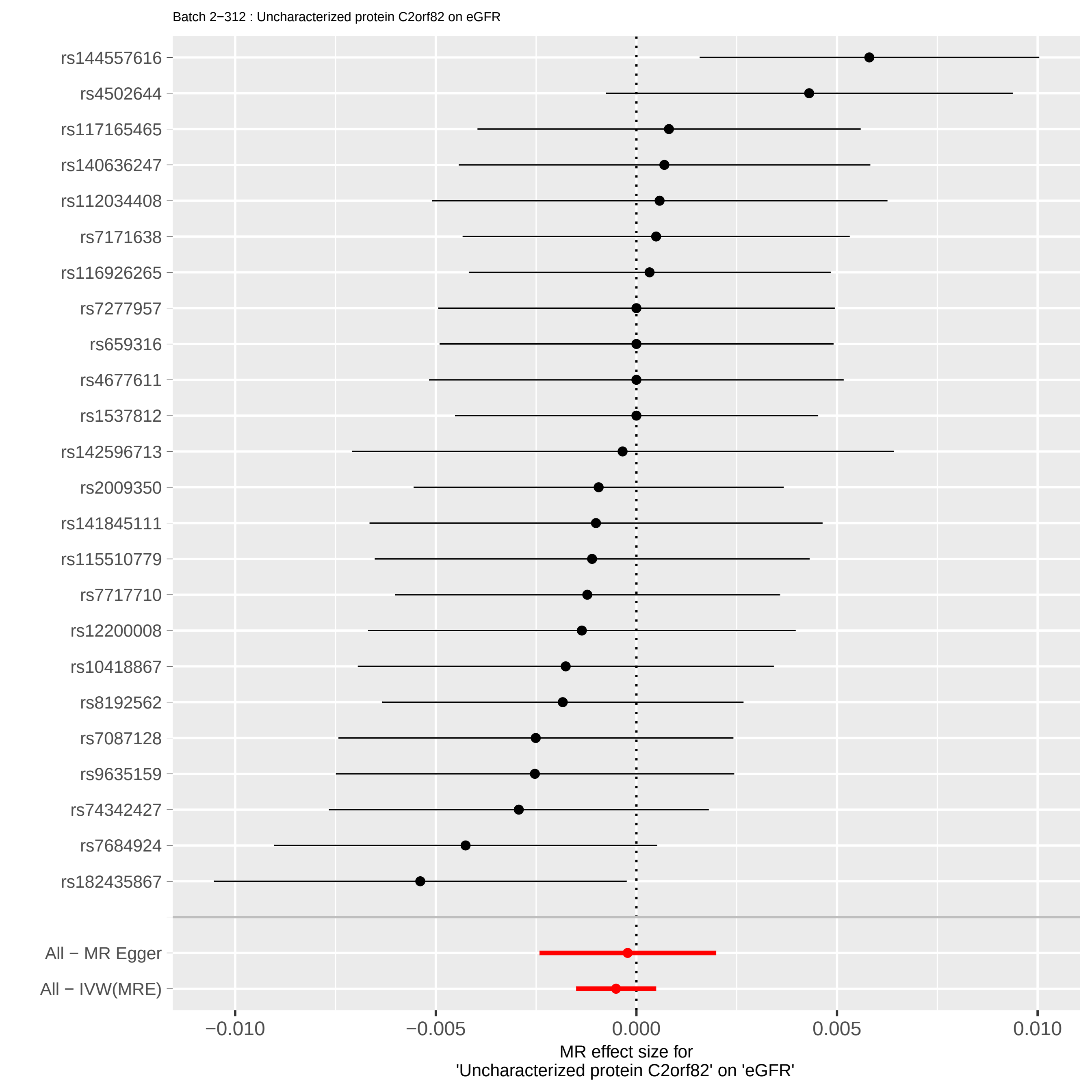

Batch 2−312 : Uncharacterized protein C2orf82 on eGFR
rs144557616
rs4502644
rs117165465
rs140636247
rs112034408
rs7171638
rs116926265
rs7277957
rs659316
rs4677611
rs1537812
rs142596713
rs2009350
rs141845111
rs115510779
rs7717710
rs12200008
rs10418867
rs8192562
rs7087128
rs9635159
rs74342427
rs7684924
rs182435867
All − MR Egger
All − IVW(MRE)
−0.010
−0.005
0.000
0.005
0.010
MR effect size for
'Uncharacterized protein C2orf82' on 'eGFR'

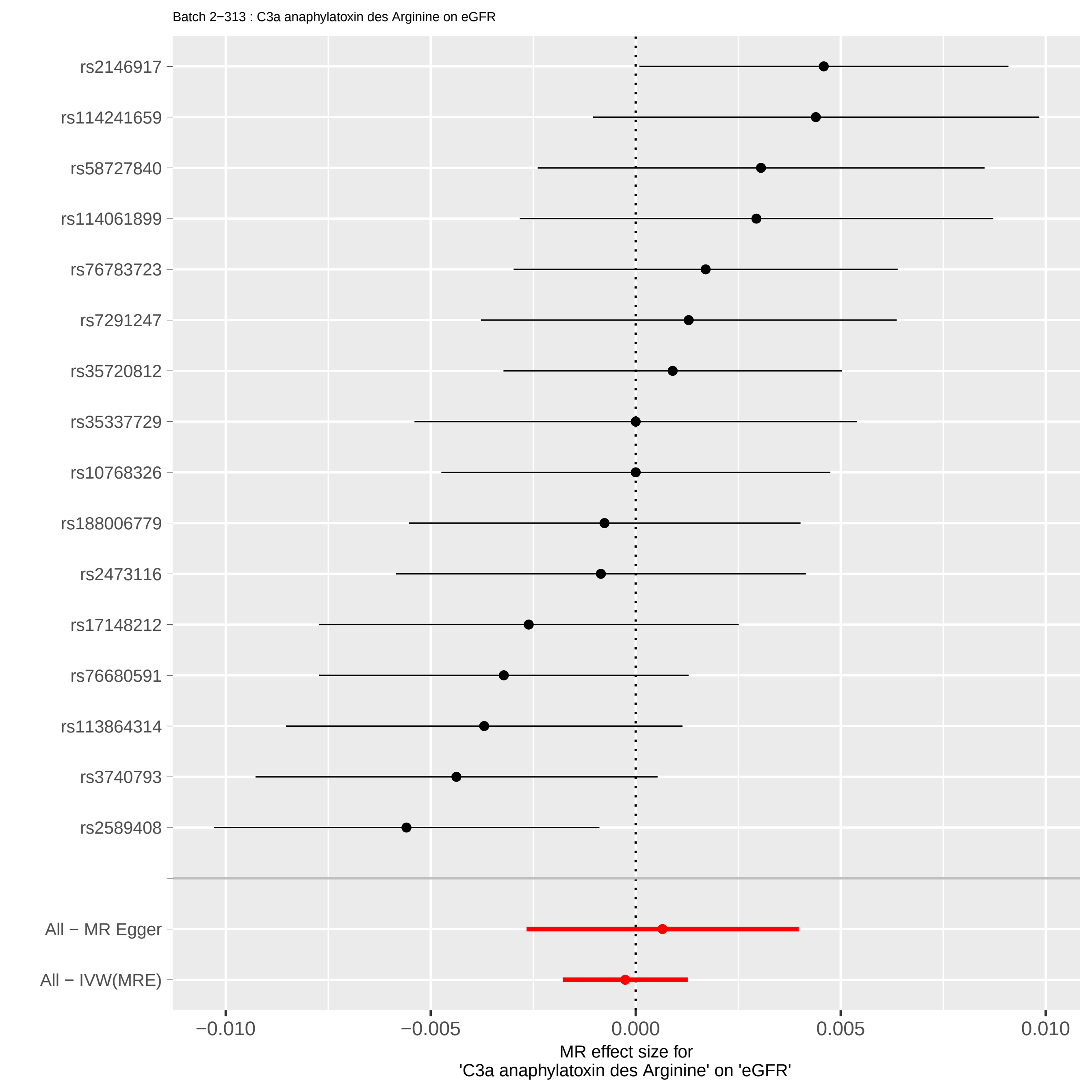

Batch 2−313 : C3a anaphylatoxin des Arginine on eGFR
rs2146917
rs114241659
rs58727840
rs114061899
rs76783723
rs7291247
rs35720812
rs35337729
rs10768326
rs188006779
rs2473116
rs17148212
rs76680591
rs113864314
rs3740793
rs2589408
All − MR Egger
All − IVW(MRE)
−0.010
−0.005
0.000
0.005
0.010
MR effect size for
'C3a anaphylatoxin des Arginine' on 'eGFR'

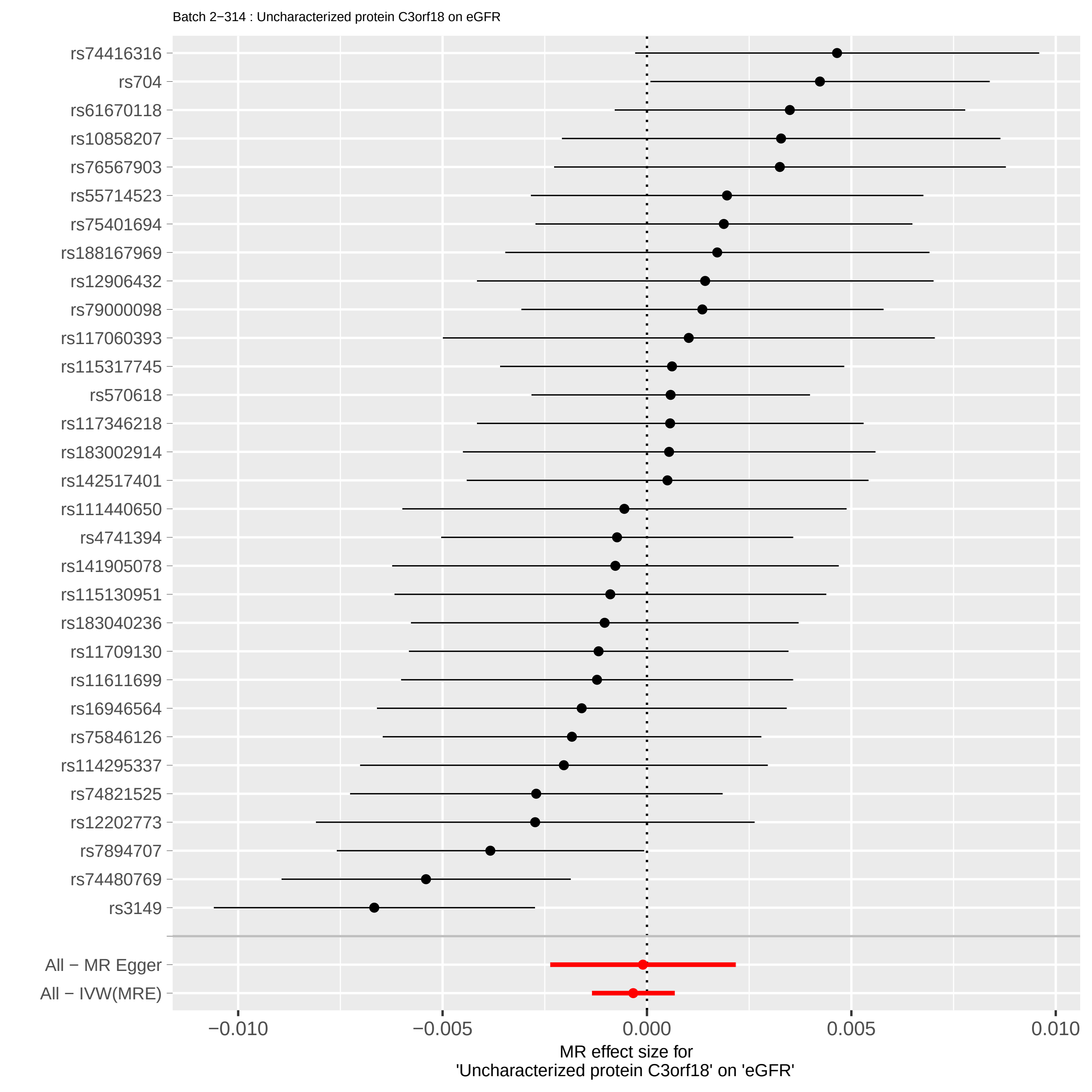

Batch 2−314 : Uncharacterized protein C3orf18 on eGFR
rs74416316
rs704
rs61670118
rs10858207
rs76567903
rs55714523
rs75401694
rs188167969
rs12906432
rs79000098
rs117060393
rs115317745
rs570618
rs117346218
rs183002914
rs142517401
rs111440650
rs4741394
rs141905078
rs115130951
rs183040236
rs11709130
rs11611699
rs16946564
rs75846126
rs114295337
rs74821525
rs12202773
rs7894707
rs74480769
rs3149
All − MR Egger
All − IVW(MRE)
−0.010
−0.005
0.000
0.005
0.010
MR effect size for
'Uncharacterized protein C3orf18' on 'eGFR'

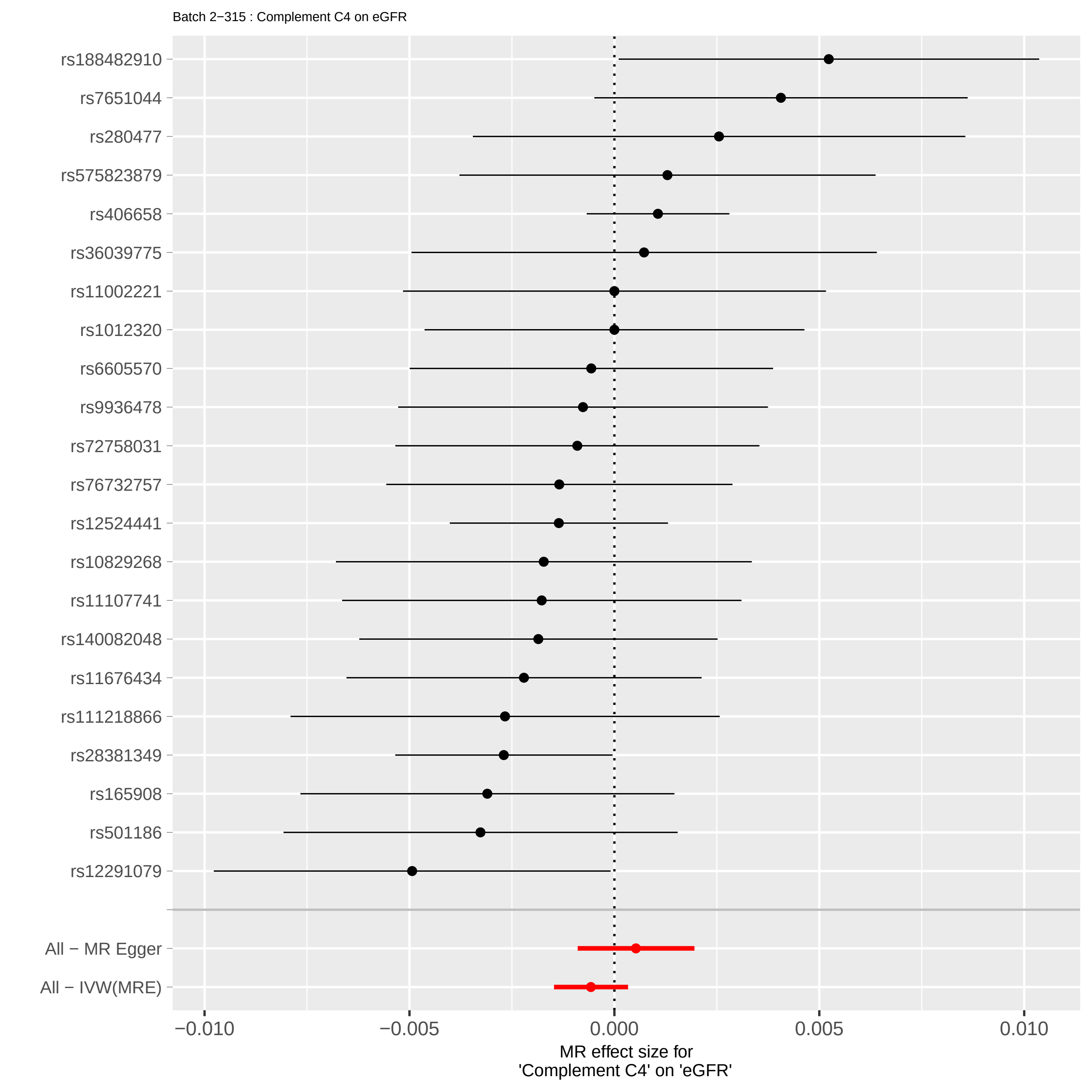

Batch 2−315 : Complement C4 on eGFR
rs188482910
rs7651044
rs280477
rs575823879
rs406658
rs36039775
rs11002221
rs1012320
rs6605570
rs9936478
rs72758031
rs76732757
rs12524441
rs10829268
rs11107741
rs140082048
rs11676434
rs111218866
rs28381349
rs165908
rs501186
rs12291079
All − MR Egger
All − IVW(MRE)
−0.010
−0.005
0.000
0.005
0.010
MR effect size for
'Complement C4' on 'eGFR'

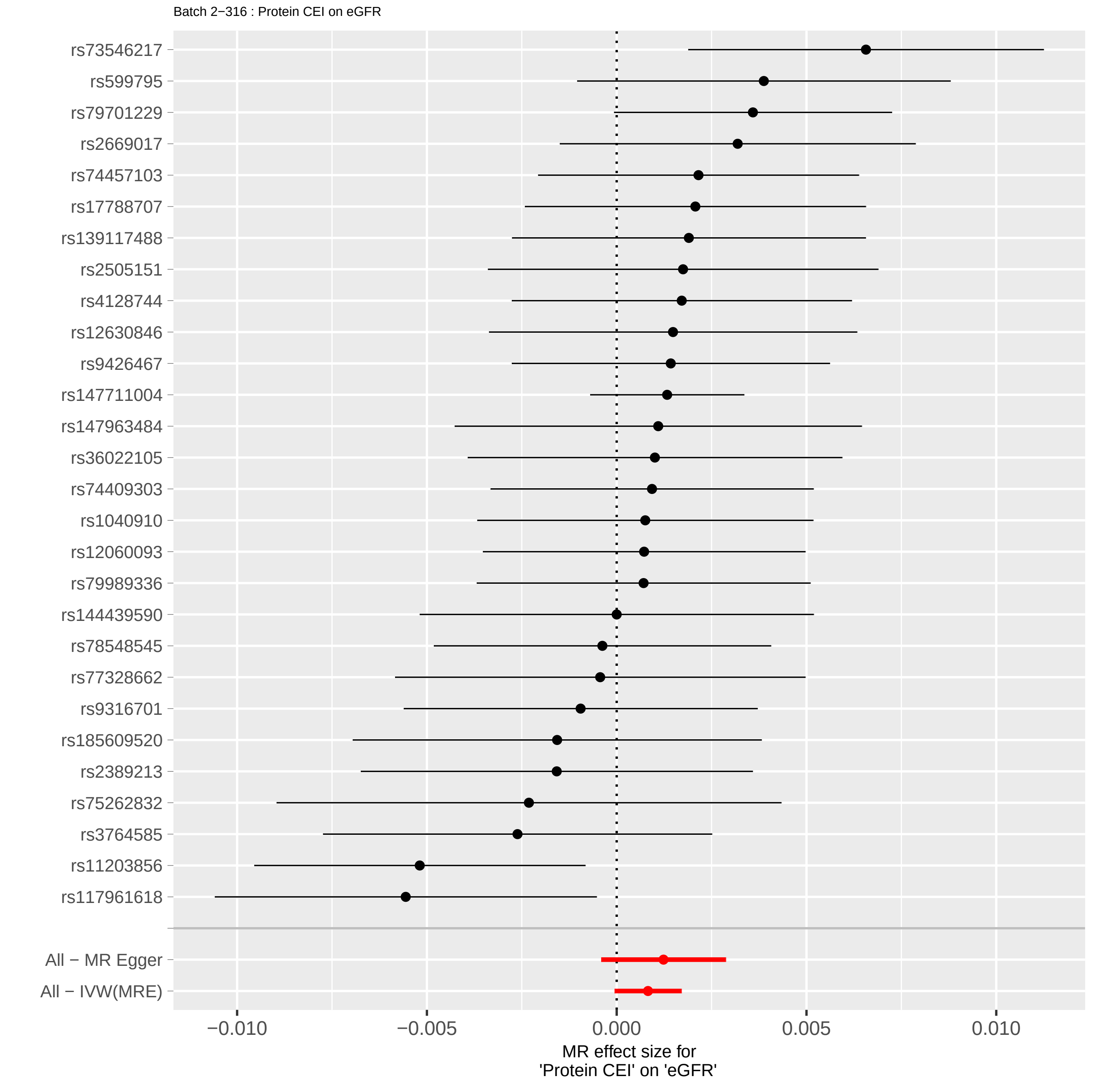

Batch 2−316 : Protein CEI on eGFR
rs73546217
rs599795
rs79701229
rs2669017
rs74457103
rs17788707
rs139117488
rs2505151
rs4128744
rs12630846
rs9426467
rs147711004
rs147963484
rs36022105
rs74409303
rs1040910
rs12060093
rs79989336
rs144439590
rs78548545
rs77328662
rs9316701
rs185609520
rs2389213
rs75262832
rs3764585
rs11203856
rs117961618
All − MR Egger
All − IVW(MRE)
−0.010
−0.005
0.000
0.005
0.010
MR effect size for
'Protein CEI' on 'eGFR'

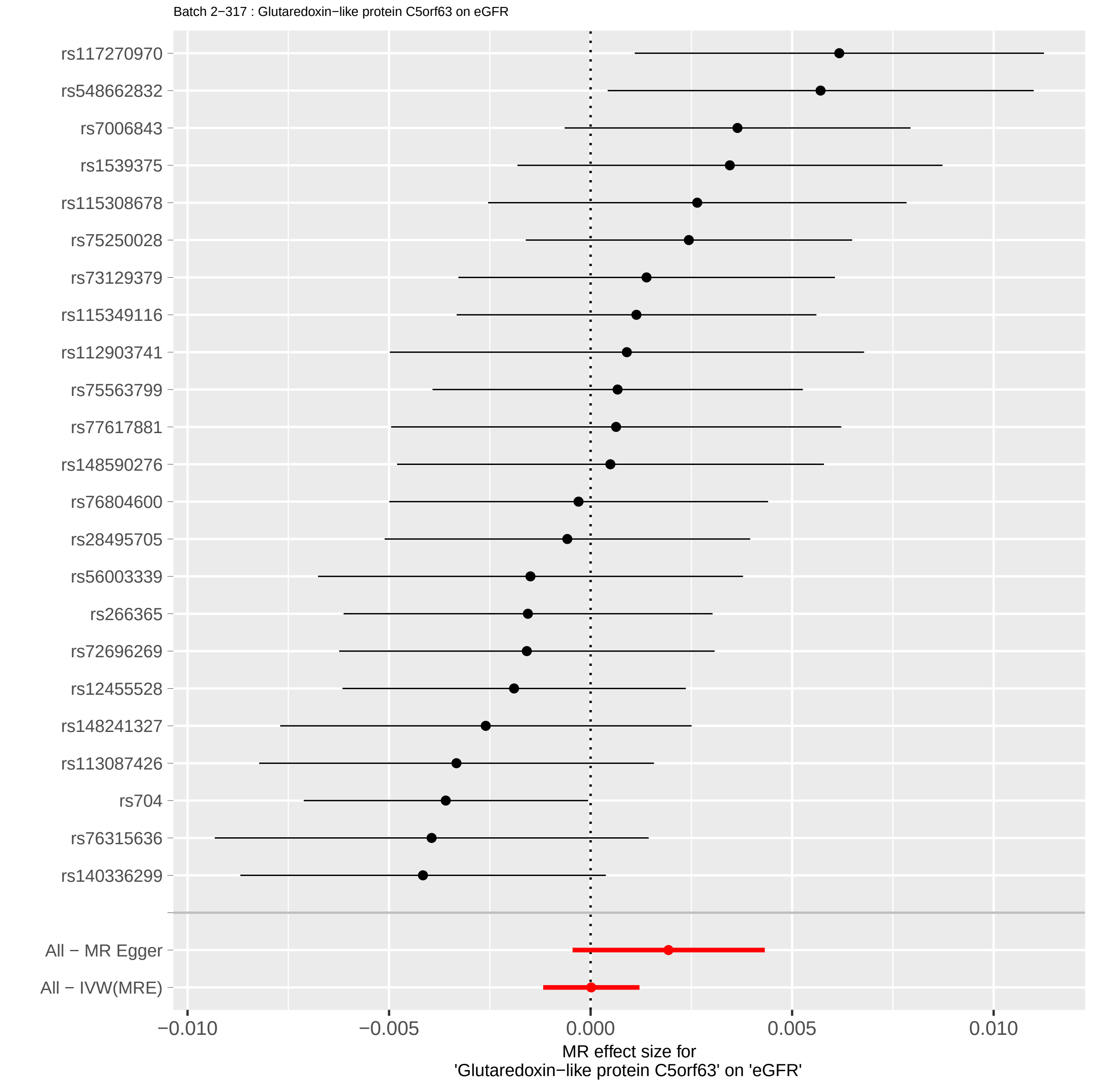

Batch 2−317 : Glutaredoxin−like protein C5orf63 on eGFR
rs117270970
rs548662832
rs7006843
rs1539375
rs115308678
rs75250028
rs73129379
rs115349116
rs112903741
rs75563799
rs77617881
rs148590276
rs76804600
rs28495705
rs56003339
rs266365
rs72696269
rs12455528
rs148241327
rs113087426
rs704
rs76315636
rs140336299
All − MR Egger
All − IVW(MRE)
−0.010
−0.005
0.000
0.005
0.010
MR effect size for
'Glutaredoxin−like protein C5orf63' on 'eGFR'

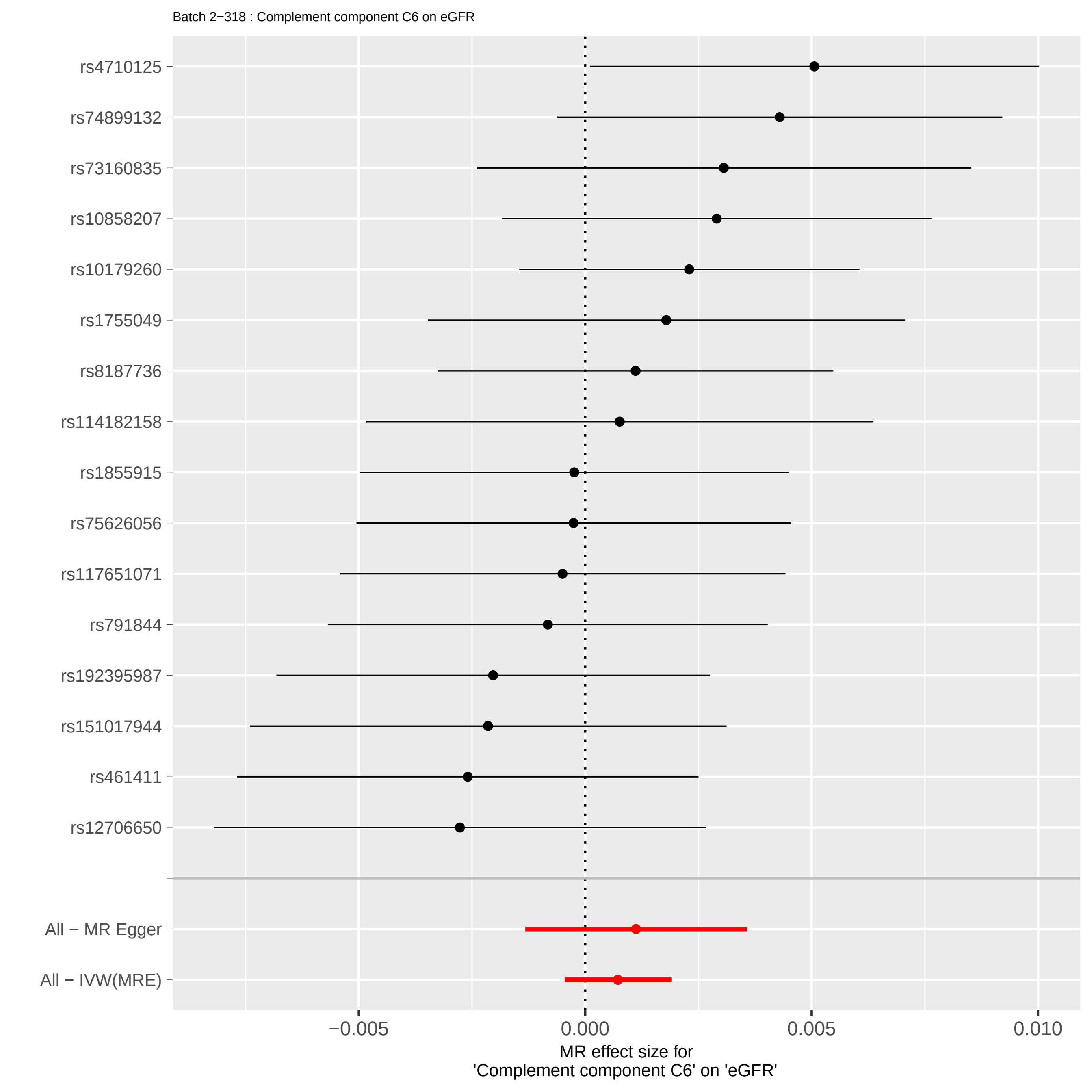
Batch 2−318 : Complement component C6 on eGFR
rs4710125
rs74899132
rs73160835
rs10858207
rs10179260
rs1755049
rs8187736
rs114182158
rs1855915
rs75626056
rs117651071
rs791844
rs192395987
rs151017944
rs461411
rs12706650
All − MR Egger
All − IVW(MRE)
−0.005
0.000
0.005
0.010
MR effect size for
'Complement component C6' on 'eGFR'

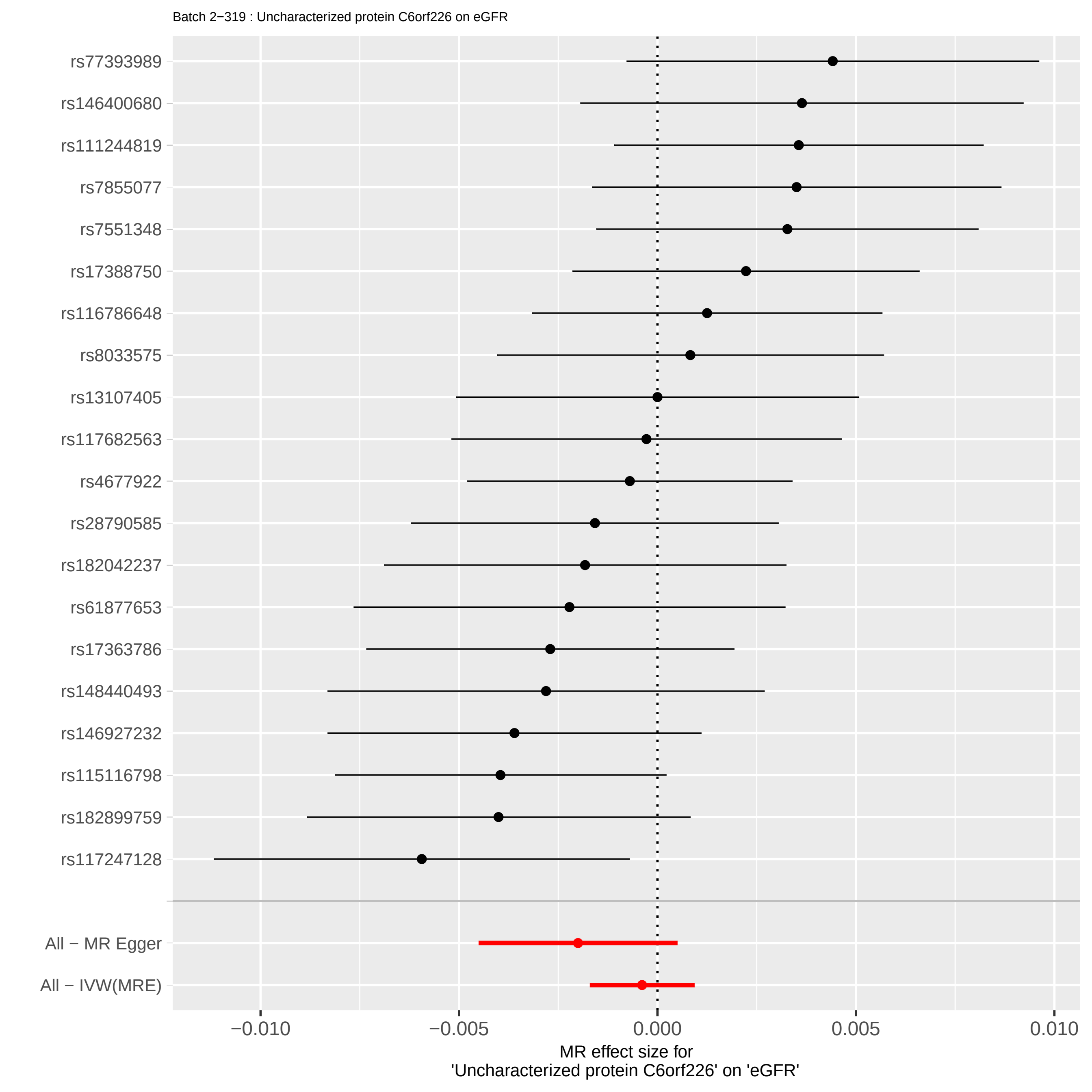

Batch 2−319 : Uncharacterized protein C6orf226 on eGFR
rs77393989
rs146400680
rs111244819
rs7855077
rs7551348
rs17388750
rs116786648
rs8033575
rs13107405
rs117682563
rs4677922
rs28790585
rs182042237
rs61877653
rs17363786
rs148440493
rs146927232
rs115116798
rs182899759
rs117247128
All − MR Egger
All − IVW(MRE)
−0.010
−0.005
0.000
0.005
0.010
MR effect size for
'Uncharacterized protein C6orf226' on 'eGFR'

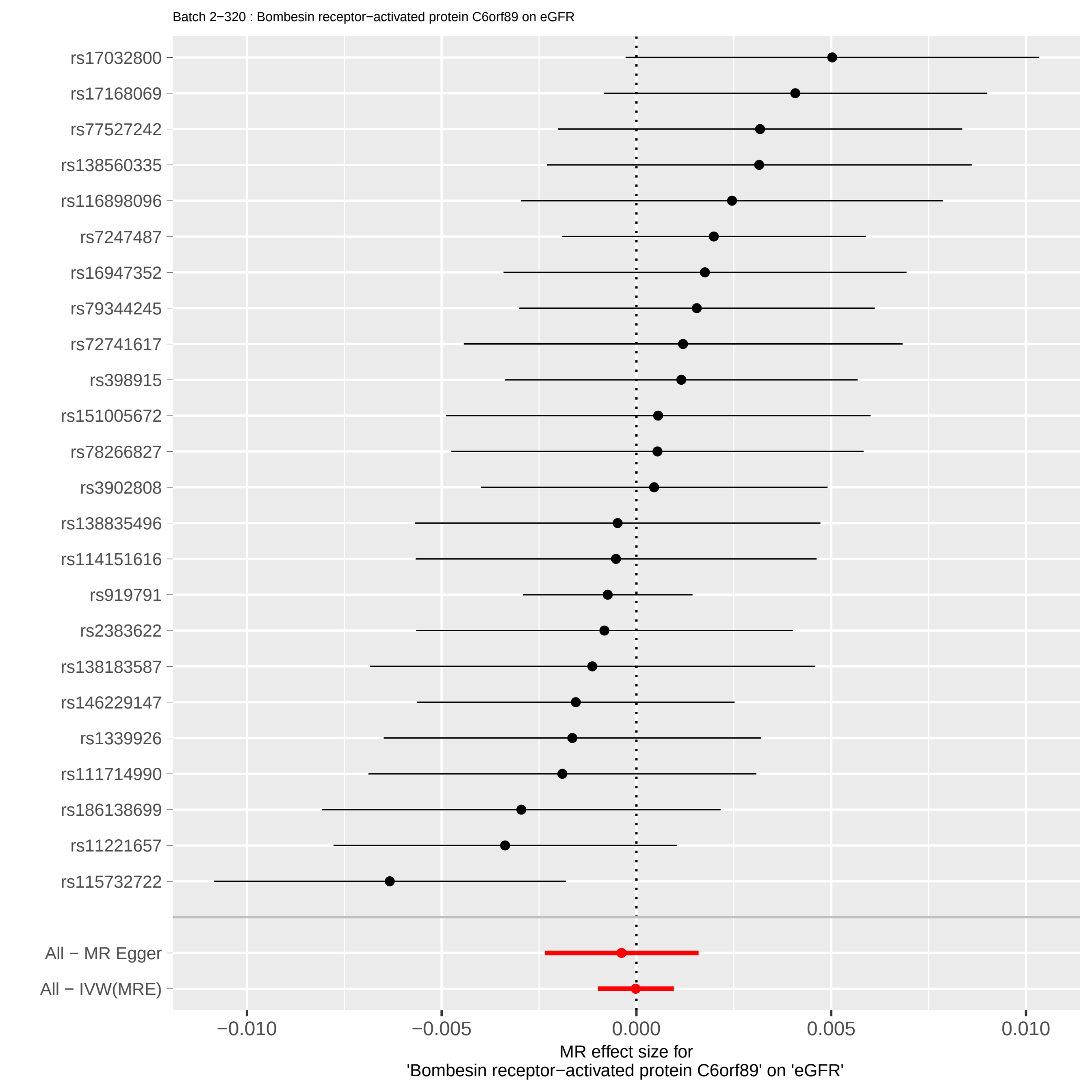
Batch 2−320 : Bombesin receptor−activated protein C6orf89 on eGFR
rs17032800
rs17168069
rs77527242
rs138560335
rs116898096
rs7247487
rs16947352
rs79344245
rs72741617
rs398915
rs151005672
rs78266827
rs3902808
rs138835496
rs114151616
rs919791
rs2383622
rs138183587
rs146229147
rs1339926
rs111714990
rs186138699
rs11221657
rs115732722
All − MR Egger
All − IVW(MRE)
−0.010
−0.005
0.000
0.005
0.010
MR effect size for
'Bombesin receptor−activated protein C6orf89' on 'eGFR'

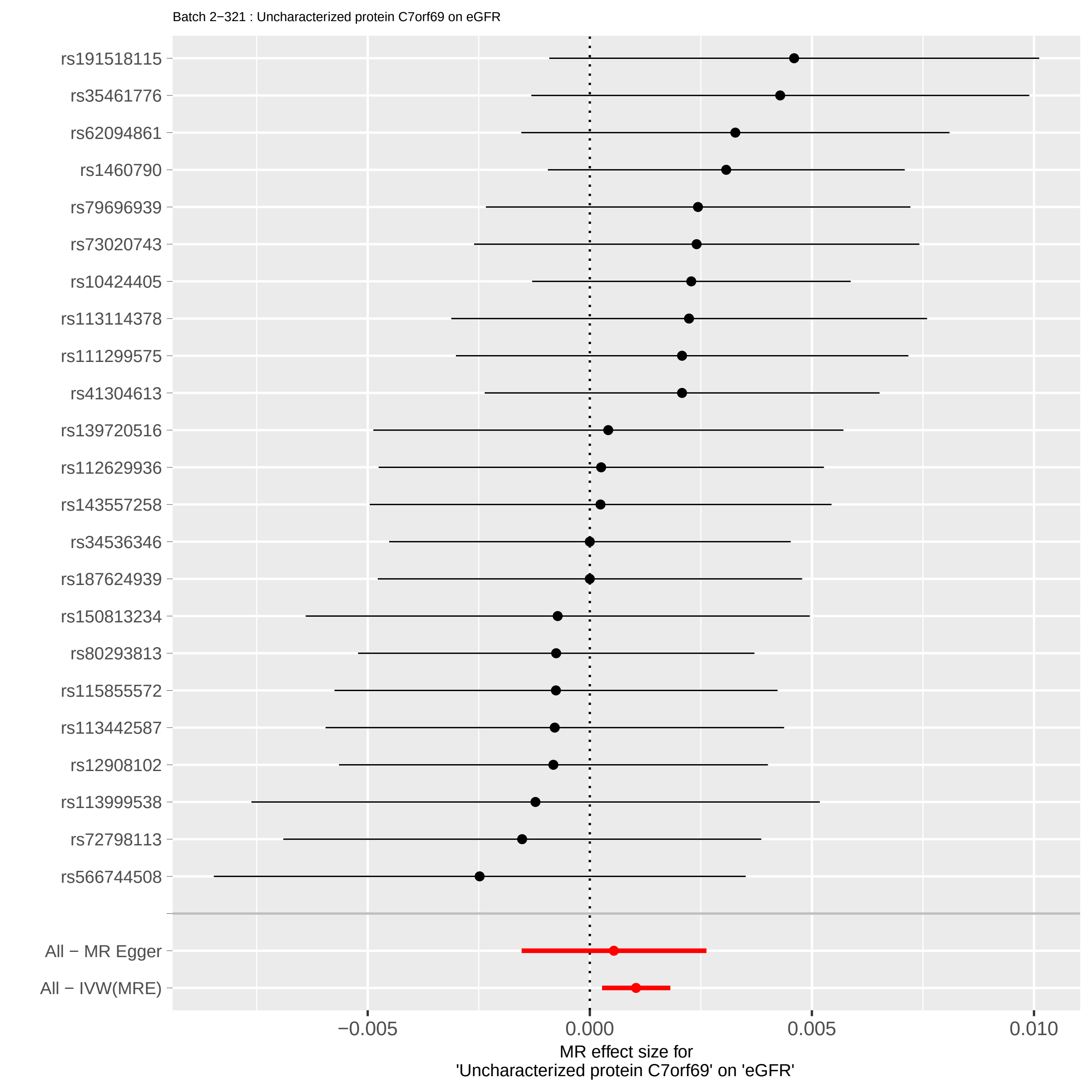

Batch 2−321 : Uncharacterized protein C7orf69 on eGFR
rs191518115
rs35461776
rs62094861
rs1460790
rs79696939
rs73020743
rs10424405
rs113114378
rs111299575
rs41304613
rs139720516
rs112629936
rs143557258
rs34536346
rs187624939
rs150813234
rs80293813
rs115855572
rs113442587
rs12908102
rs113999538
rs72798113
rs566744508
All − MR Egger
All − IVW(MRE)
−0.005
0.000
0.005
0.010
MR effect size for
'Uncharacterized protein C7orf69' on 'eGFR'

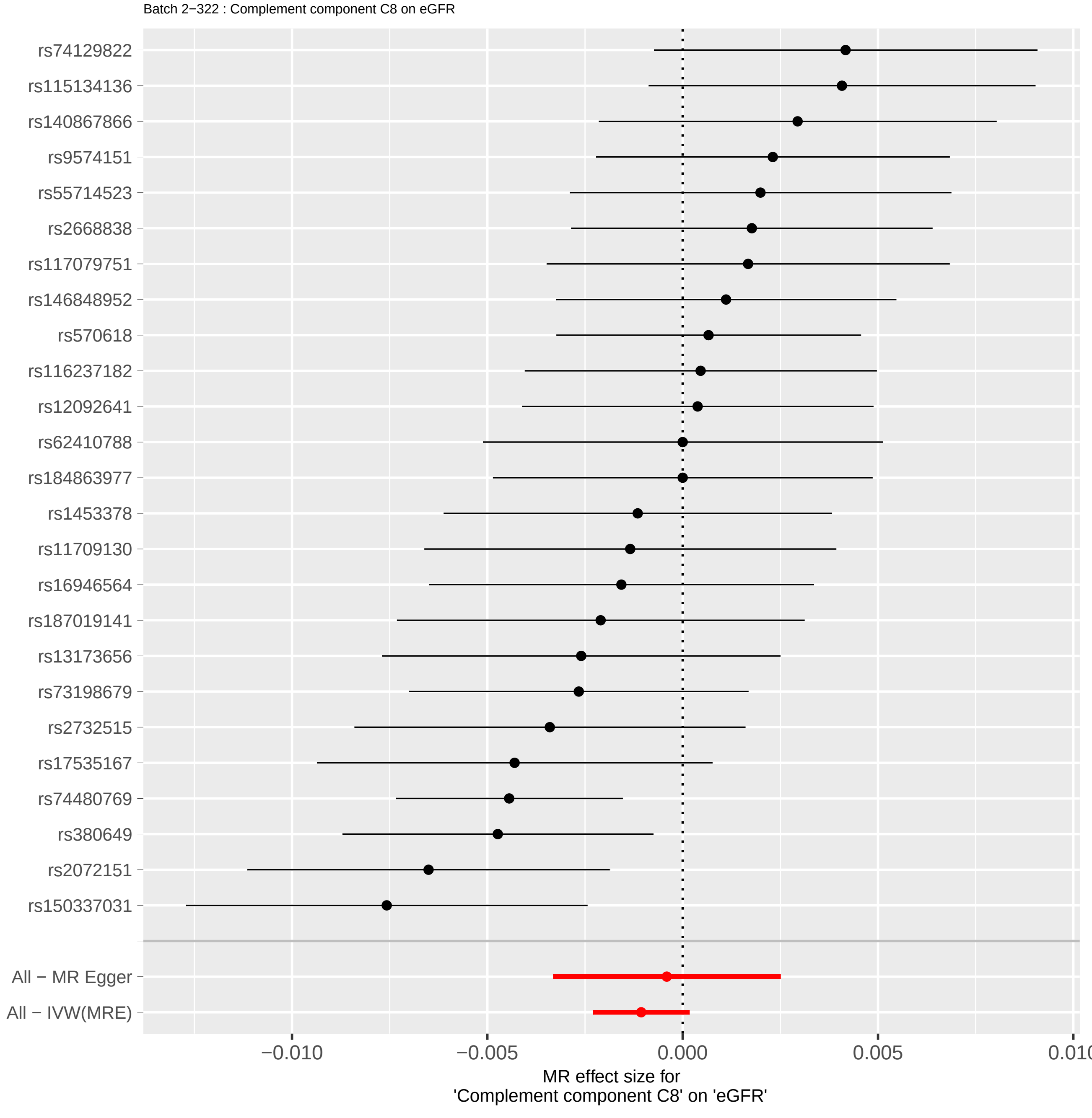
Batch 2−322 : Complement component C8 on eGFR
rs74129822
rs115134136
rs140867866
rs9574151
rs55714523
rs2668838
rs117079751
rs146848952
rs570618
rs116237182
rs12092641
rs62410788
rs184863977
rs1453378
rs11709130
rs16946564
rs187019141
rs13173656
rs73198679
rs2732515
rs17535167
rs74480769
rs380649
rs2072151
rs150337031
All − MR Egger
All − IVW(MRE)
−0.010
−0.005
0.000
0.005
0.010
MR effect size for
'Complement component C8' on 'eGFR'

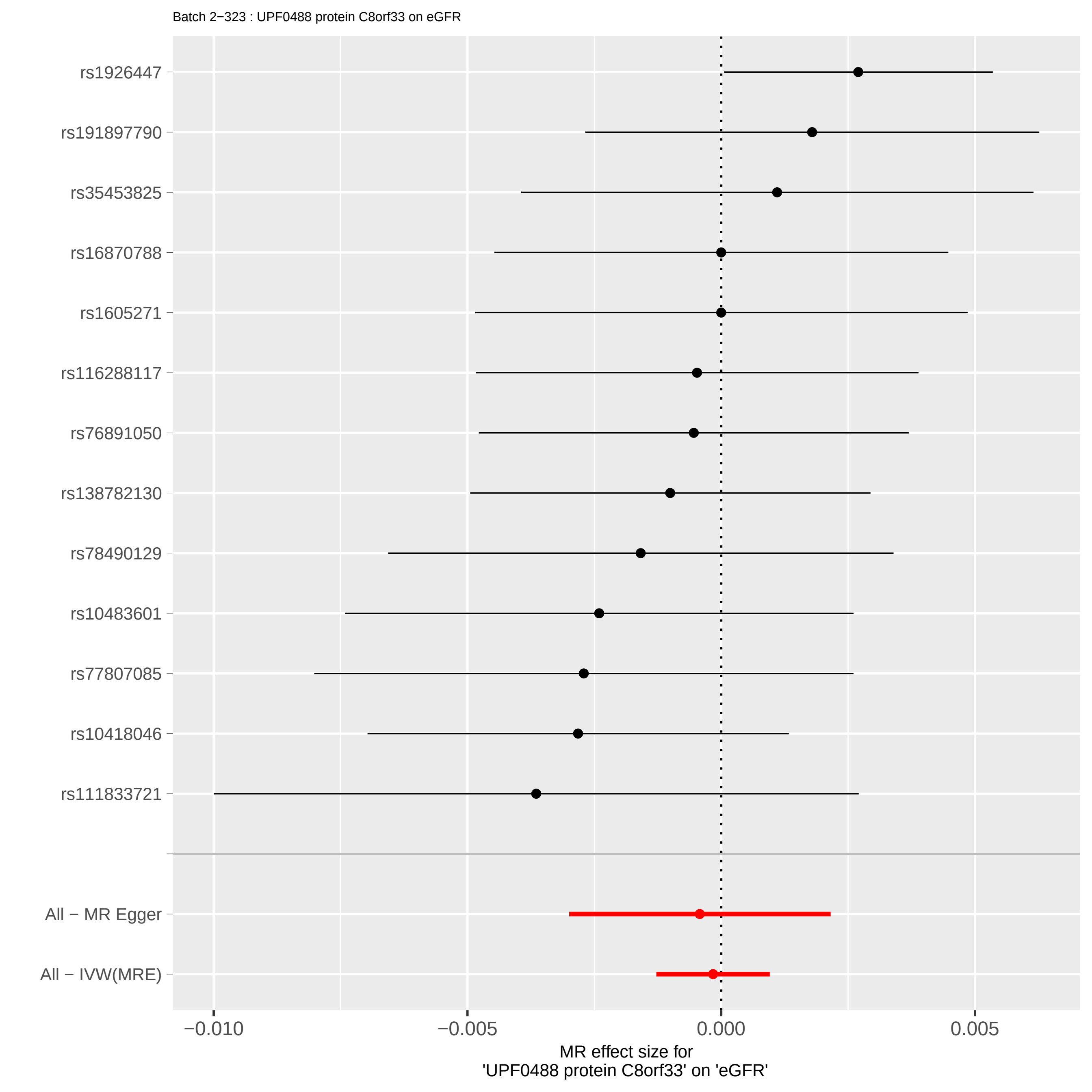

Batch 2−323 : UPF0488 protein C8orf33 on eGFR
rs1926447
rs191897790
rs35453825
rs16870788
rs1605271
rs116288117
rs76891050
rs138782130
rs78490129
rs10483601
rs77807085
rs10418046
rs111833721
All − MR Egger
All − IVW(MRE)
−0.010
−0.005
0.000
0.005
MR effect size for
'UPF0488 protein C8orf33' on 'eGFR'

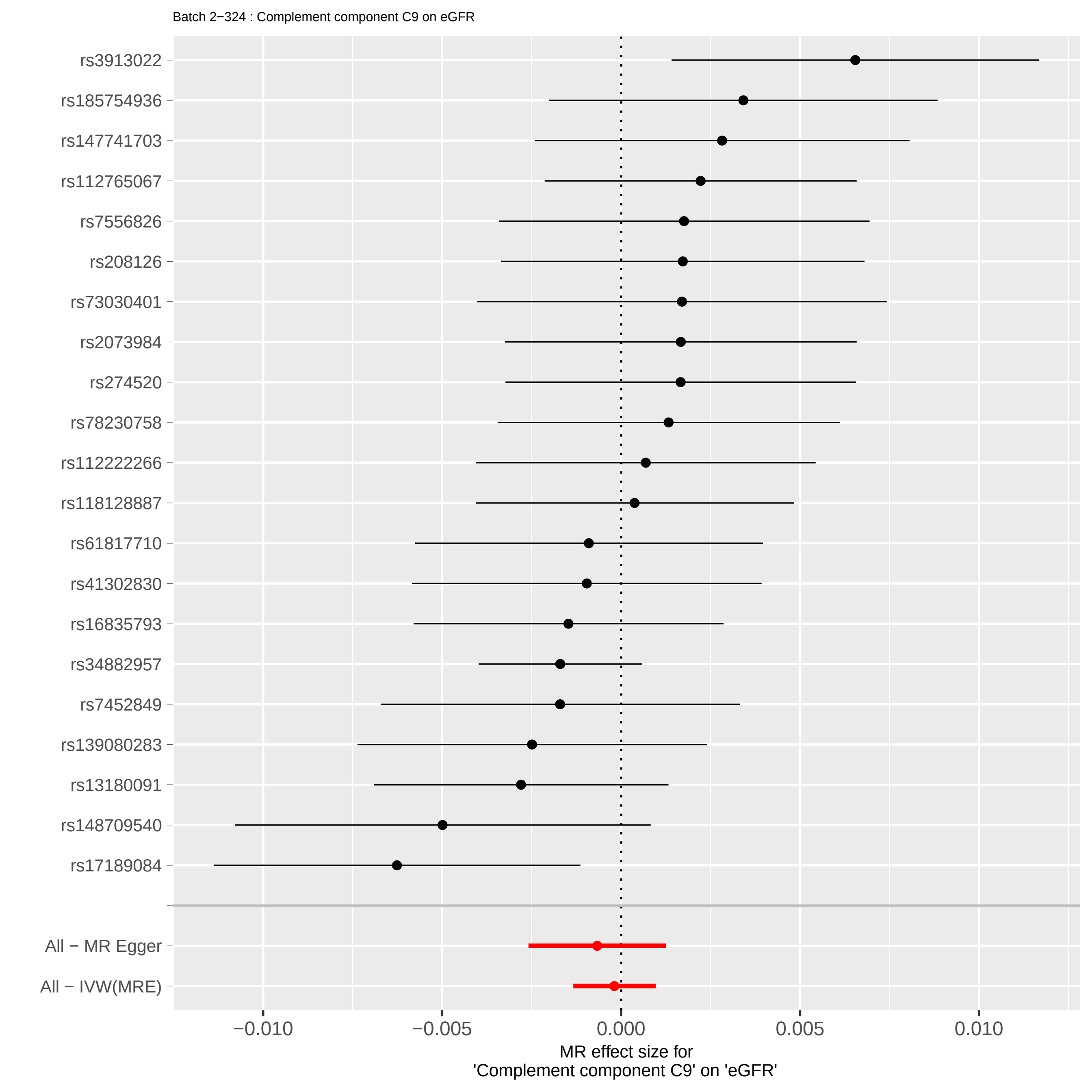

Batch 2−324 : Complement component C9 on eGFR
rs3913022
rs185754936
rs147741703
rs112765067
rs7556826
rs208126
rs73030401
rs2073984
rs274520
rs78230758
rs112222266
rs118128887
rs61817710
rs41302830
rs16835793
rs34882957
rs7452849
rs139080283
rs13180091
rs148709540
rs17189084
All − MR Egger
All − IVW(MRE)
−0.010
−0.005
0.000
0.005
0.010
MR effect size for
'Complement component C9' on 'eGFR'

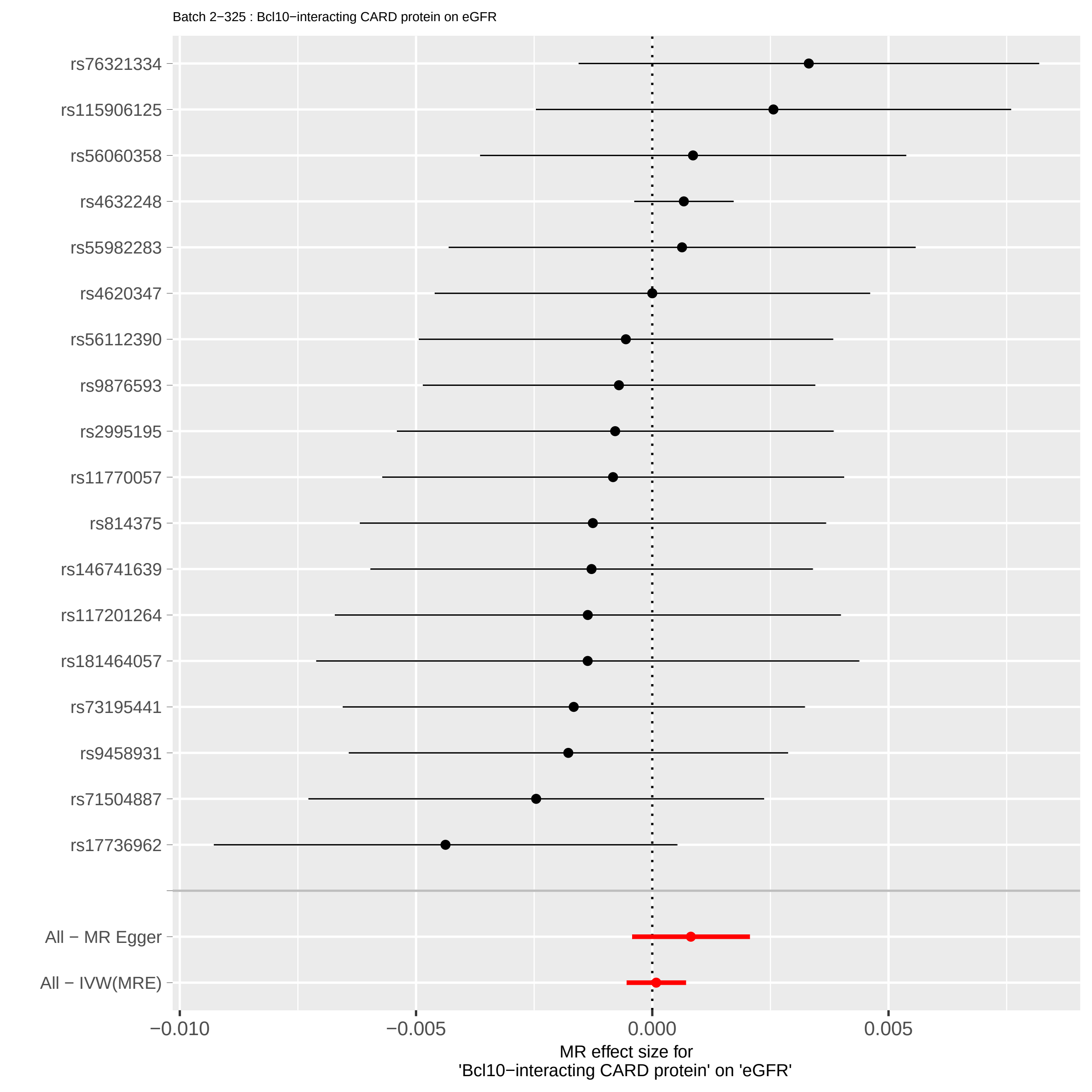
Batch 2−325 : Bcl10−interacting CARD protein on eGFR
rs76321334
rs115906125
rs56060358
rs4632248
rs55982283
rs4620347
rs56112390
rs9876593
rs2995195
rs11770057
rs814375
rs146741639
rs117201264
rs181464057
rs73195441
rs9458931
rs71504887
rs17736962
All − MR Egger
All − IVW(MRE)
−0.010
−0.005
0.000
0.005
MR effect size for
'Bcl10−interacting CARD protein' on 'eGFR'

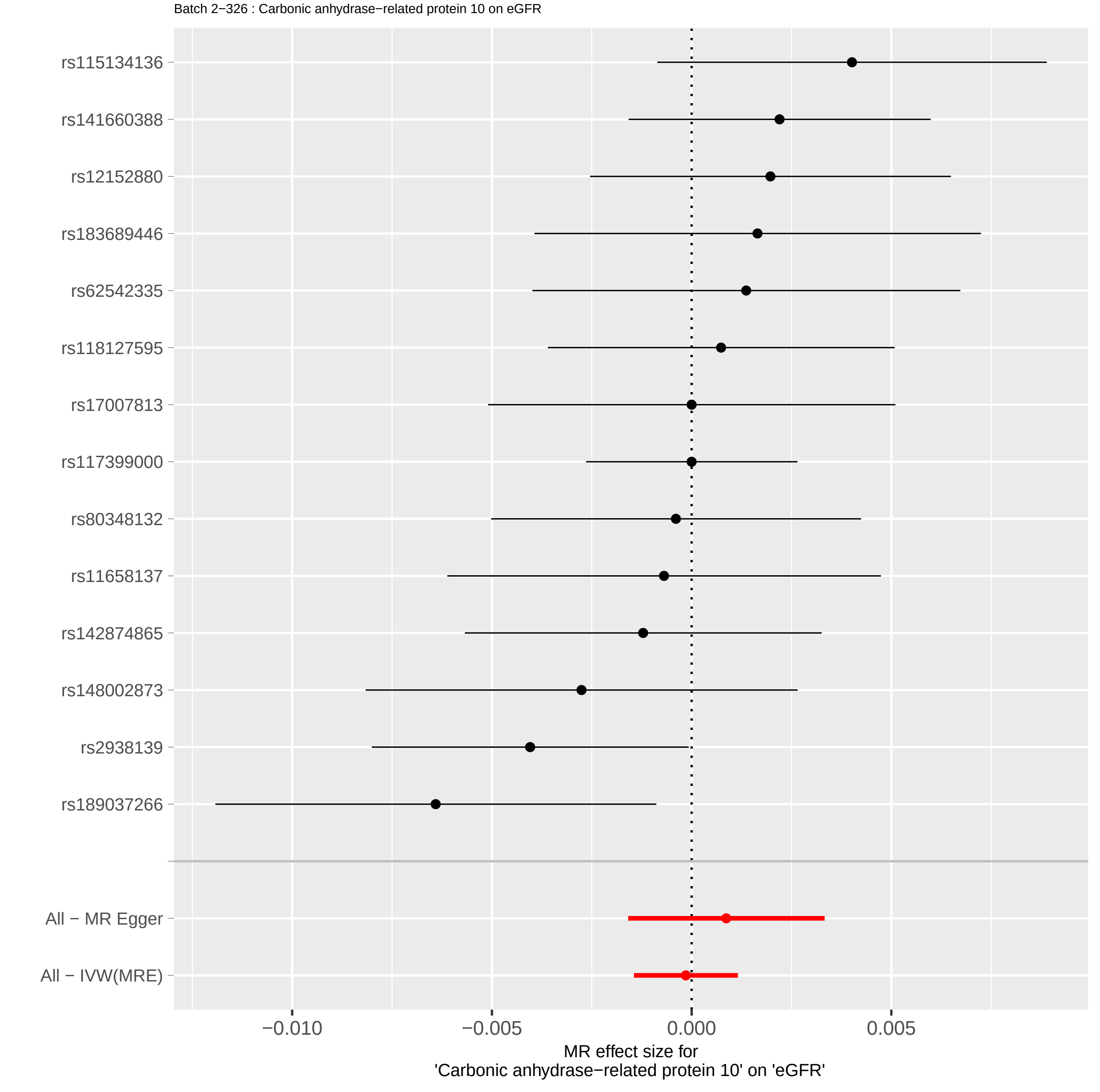

Batch 2−326 : Carbonic anhydrase−related protein 10 on eGFR
rs115134136
rs141660388
rs12152880
rs183689446
rs62542335
rs118127595
rs17007813
rs117399000
rs80348132
rs11658137
rs142874865
rs148002873
rs2938139
rs189037266
All − MR Egger
All − IVW(MRE)
−0.010
−0.005
0.000
0.005
MR effect size for
'Carbonic anhydrase−related protein 10' on 'eGFR'

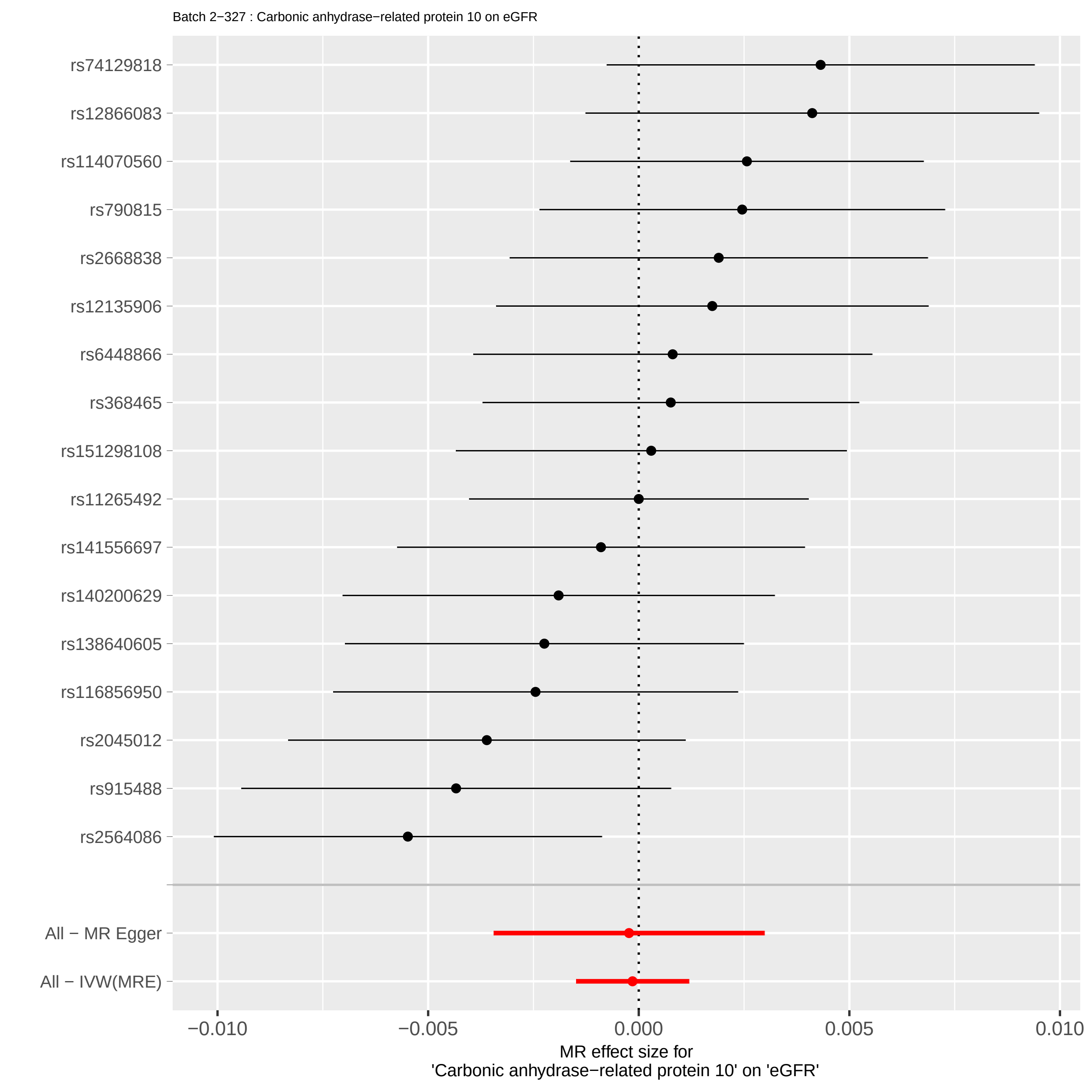

Batch 2−327 : Carbonic anhydrase−related protein 10 on eGFR
rs74129818
rs12866083
rs114070560
rs790815
rs2668838
rs12135906
rs6448866
rs368465
rs151298108
rs11265492
rs141556697
rs140200629
rs138640605
rs116856950
rs2045012
rs915488
rs2564086
All − MR Egger
All − IVW(MRE)
−0.010
−0.005
0.000
0.005
0.010
MR effect size for
'Carbonic anhydrase−related protein 10' on 'eGFR'

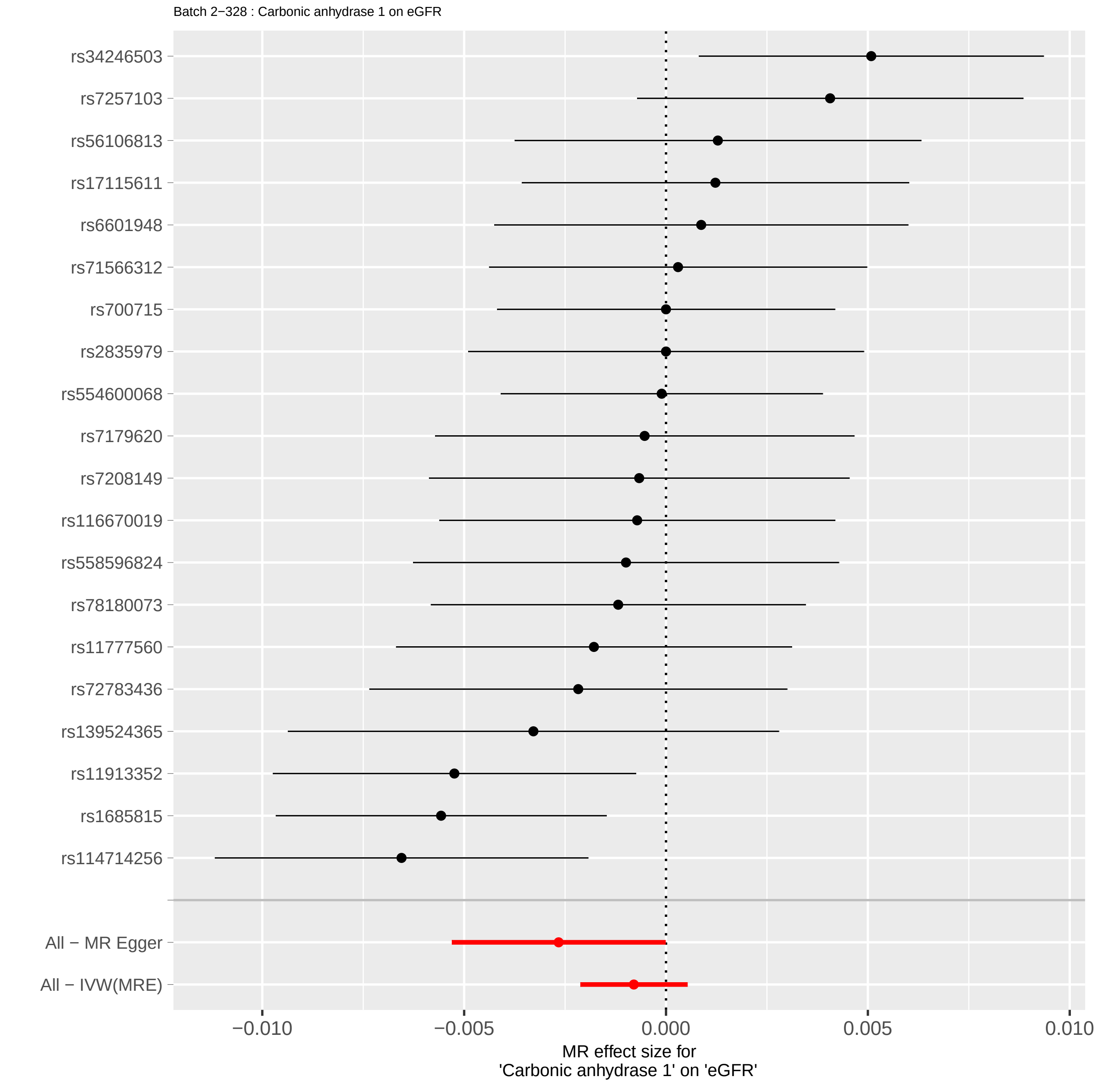
Batch 2−328 : Carbonic anhydrase 1 on eGFR
rs34246503
rs7257103
rs56106813
rs17115611
rs6601948
rs71566312
rs700715
rs2835979
rs554600068
rs7179620
rs7208149
rs116670019
rs558596824
rs78180073
rs11777560
rs72783436
rs139524365
rs11913352
rs1685815
rs114714256
All − MR Egger
All − IVW(MRE)
−0.010
−0.005
0.000
0.005
0.010
MR effect size for
'Carbonic anhydrase 1' on 'eGFR'

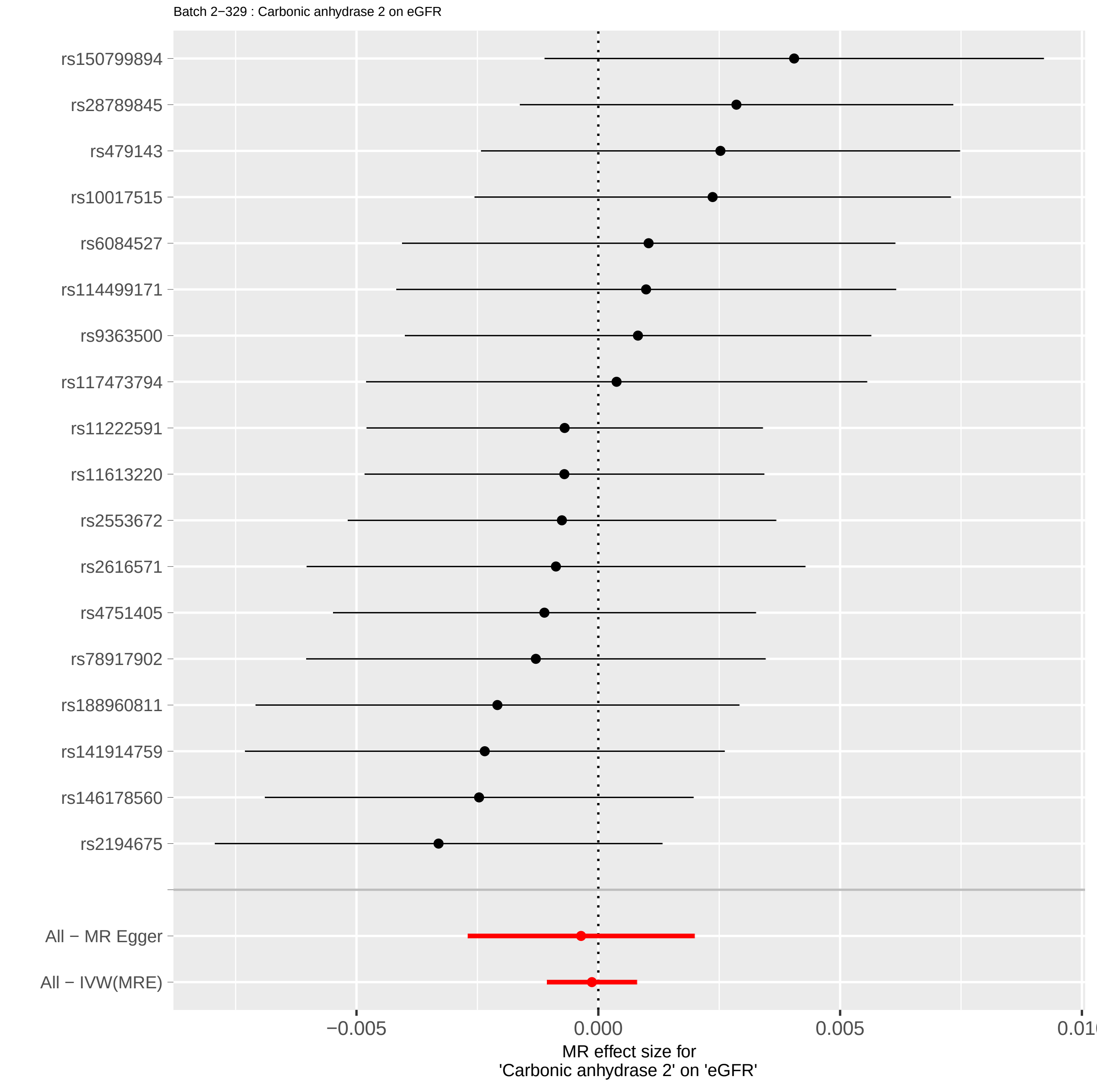
Batch 2−329 : Carbonic anhydrase 2 on eGFR
rs150799894
rs28789845
rs479143
rs10017515
rs6084527
rs114499171
rs9363500
rs117473794
rs11222591
rs11613220
rs2553672
rs2616571
rs4751405
rs78917902
rs188960811
rs141914759
rs146178560
rs2194675
All − MR Egger
All − IVW(MRE)
−0.005
0.000
0.005
MR effect size for
'Carbonic anhydrase 2' on 'eGFR'

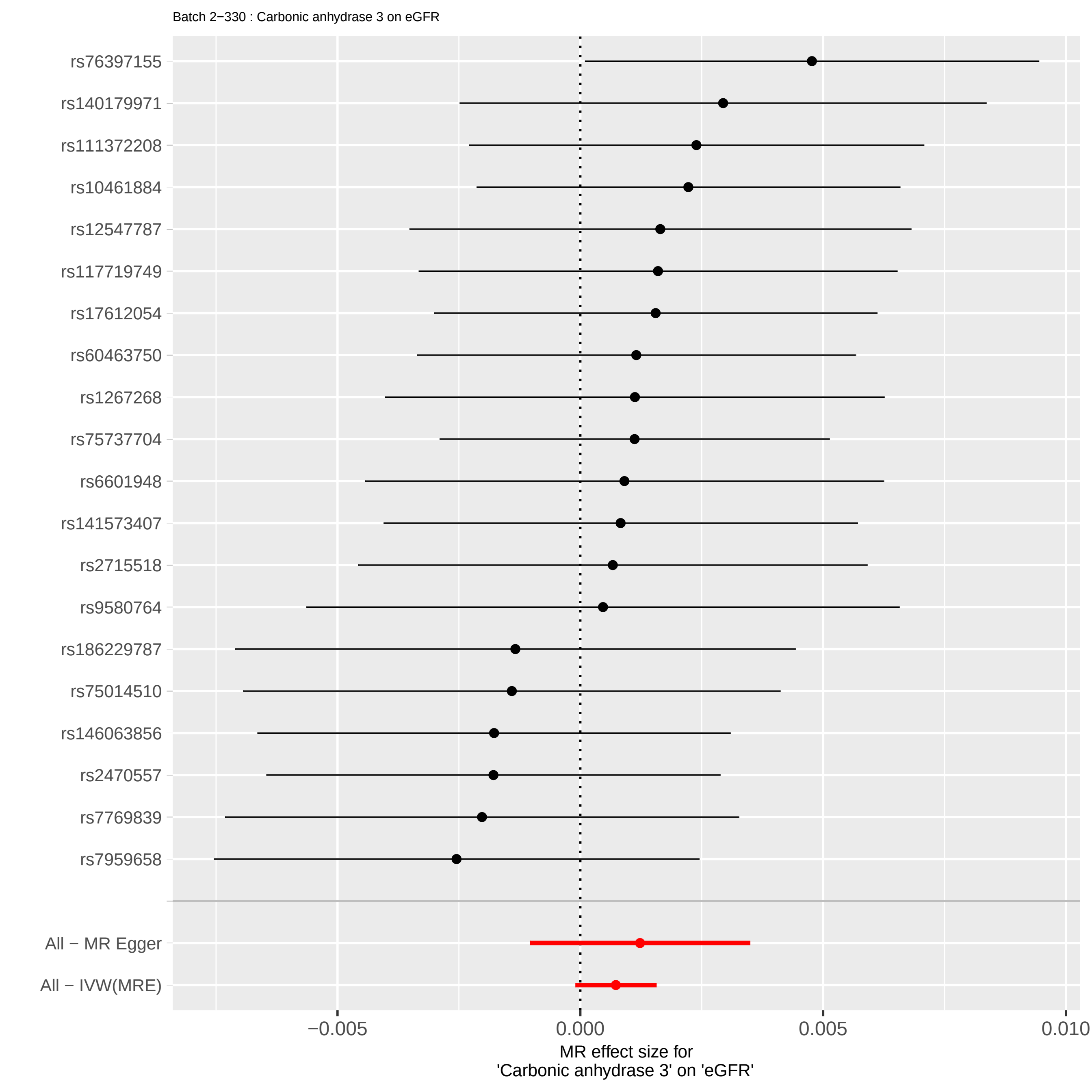
Batch 2−330 : Carbonic anhydrase 3 on eGFR
rs76397155
rs140179971
rs111372208
rs10461884
rs12547787
rs117719749
rs17612054
rs60463750
rs1267268
rs75737704
rs6601948
rs141573407
rs2715518
rs9580764
rs186229787
rs75014510
rs146063856
rs2470557
rs7769839
rs7959658
All − MR Egger
All − IVW(MRE)
−0.005
0.000
0.005
0.010
MR effect size for
'Carbonic anhydrase 3' on 'eGFR'

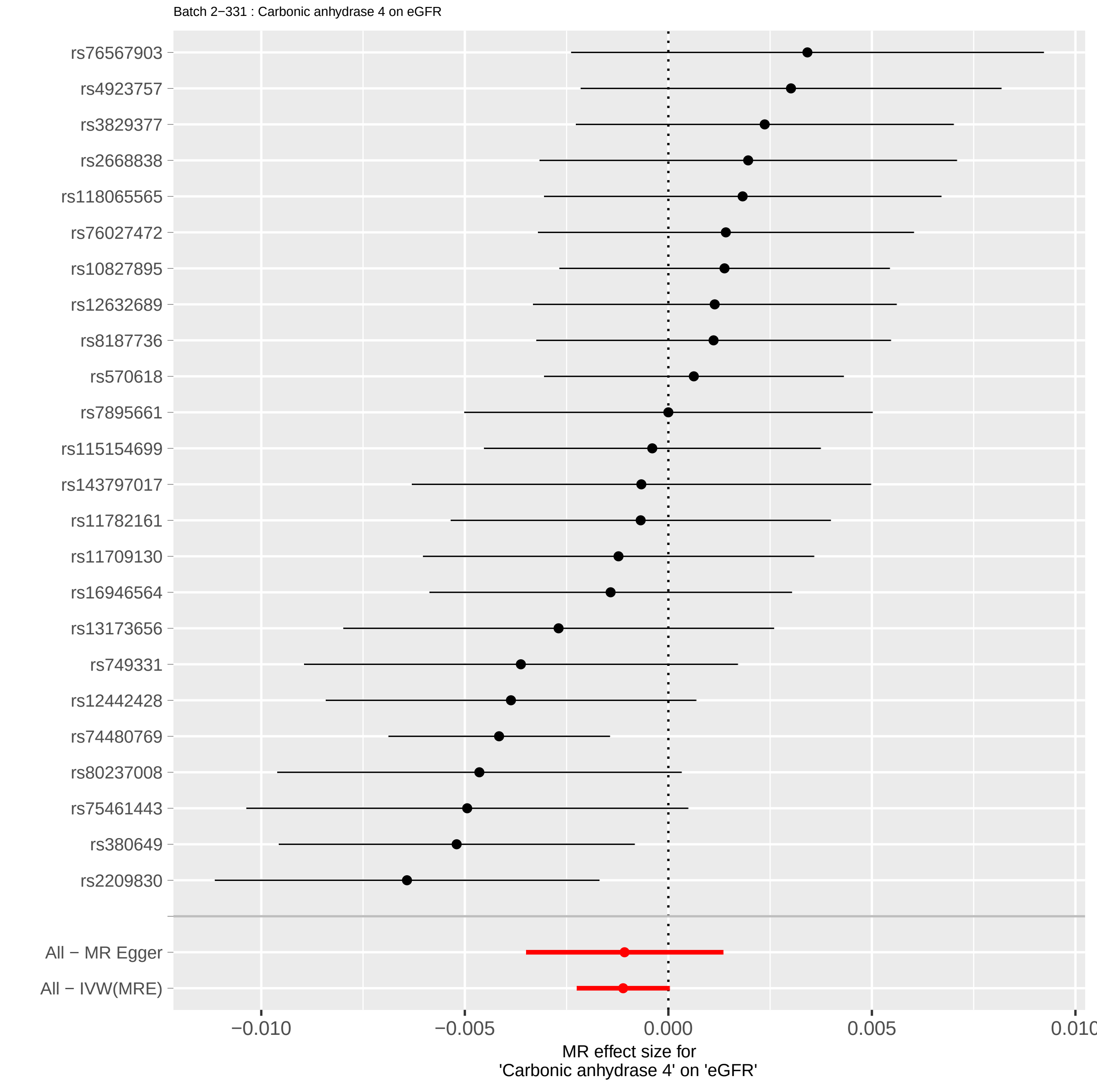

Batch 2−331 : Carbonic anhydrase 4 on eGFR
rs76567903
rs4923757
rs3829377
rs2668838
rs118065565
rs76027472
rs10827895
rs12632689
rs8187736
rs570618
rs7895661
rs115154699
rs143797017
rs11782161
rs11709130
rs16946564
rs13173656
rs749331
rs12442428
rs74480769
rs80237008
rs75461443
rs380649
rs2209830
All − MR Egger
All − IVW(MRE)
−0.010
−0.005
0.000
0.005
0.010
MR effect size for
'Carbonic anhydrase 4' on 'eGFR'

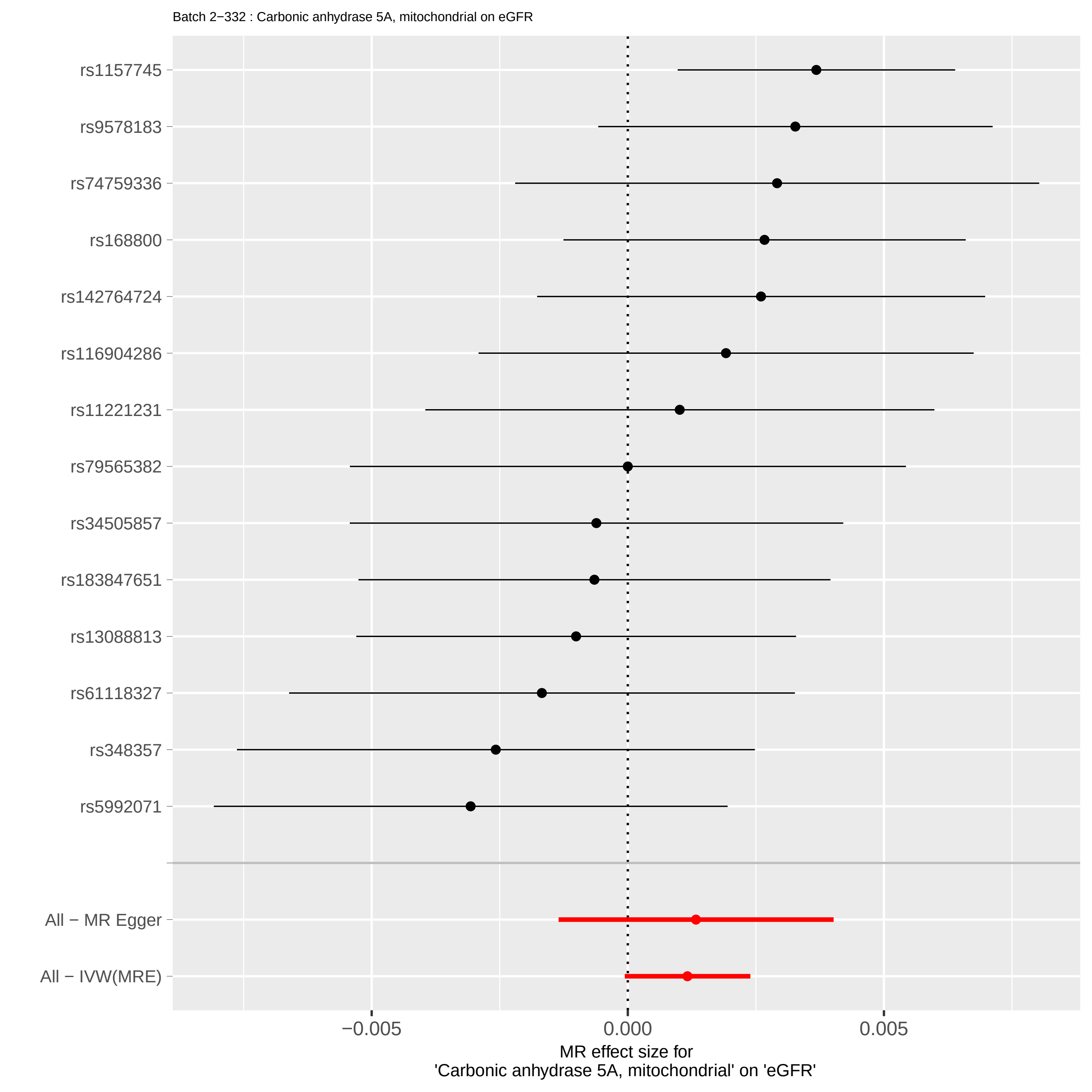

Batch 2−332 : Carbonic anhydrase 5A, mitochondrial on eGFR
rs1157745
rs9578183
rs74759336
rs168800
rs142764724
rs116904286
rs11221231
rs79565382
rs34505857
rs183847651
rs13088813
rs61118327
rs348357
rs5992071
All − MR Egger
All − IVW(MRE)
−0.005
0.000
0.005
MR effect size for
'Carbonic anhydrase 5A, mitochondrial' on 'eGFR'

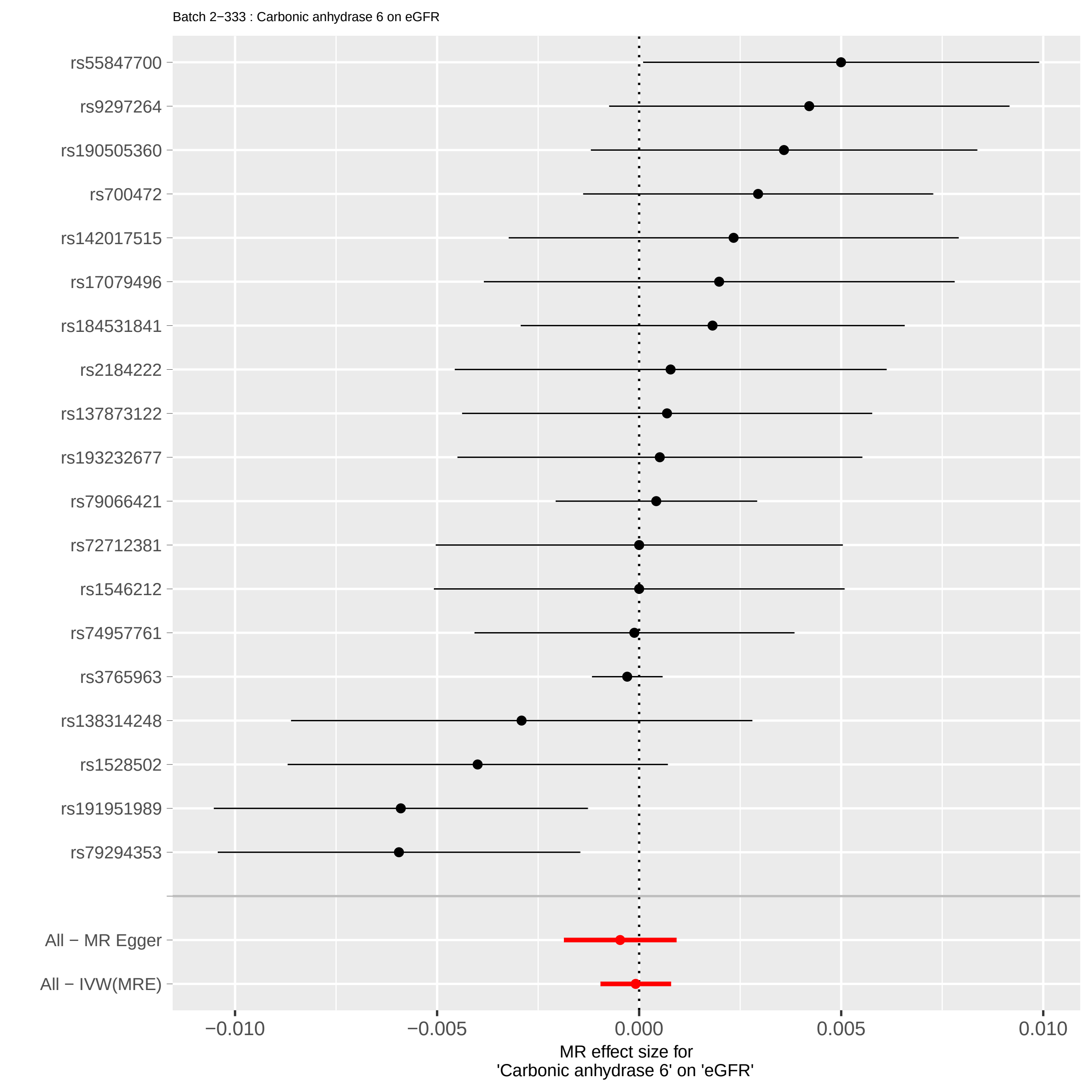

Batch 2−333 : Carbonic anhydrase 6 on eGFR
rs55847700
rs9297264
rs190505360
rs700472
rs142017515
rs17079496
rs184531841
rs2184222
rs137873122
rs193232677
rs79066421
rs72712381
rs1546212
rs74957761
rs3765963
rs138314248
rs1528502
rs191951989
rs79294353
All − MR Egger
All − IVW(MRE)
−0.010
−0.005
0.000
0.005
0.010
MR effect size for
'Carbonic anhydrase 6' on 'eGFR'

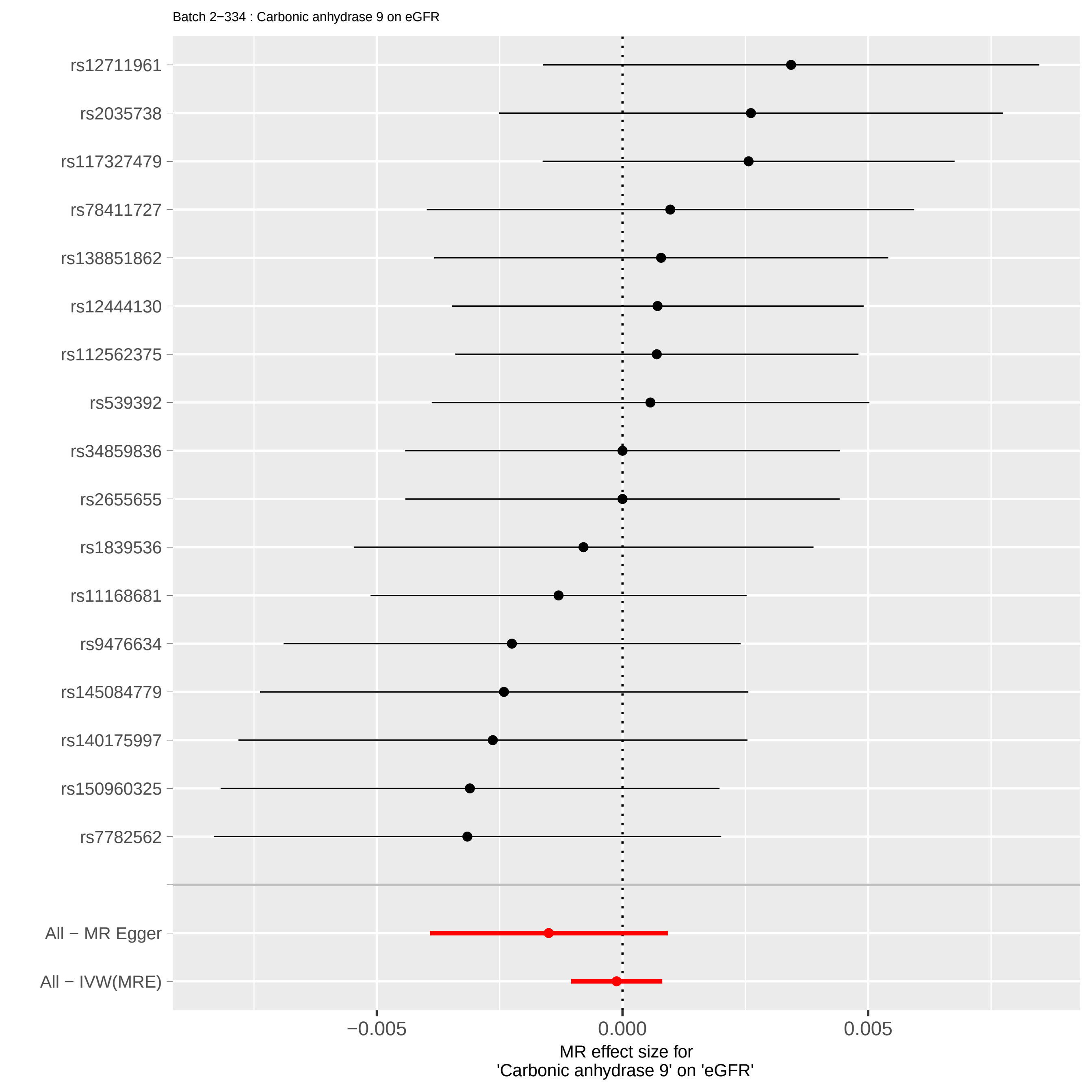
Batch 2−334 : Carbonic anhydrase 9 on eGFR
rs12711961
rs2035738
rs117327479
rs78411727
rs138851862
rs12444130
rs112562375
rs539392
rs34859836
rs2655655
rs1839536
rs11168681
rs9476634
rs145084779
rs140175997
rs150960325
rs7782562
All − MR Egger
All − IVW(MRE)
−0.005
0.000
0.005
MR effect size for
'Carbonic anhydrase 9' on 'eGFR'

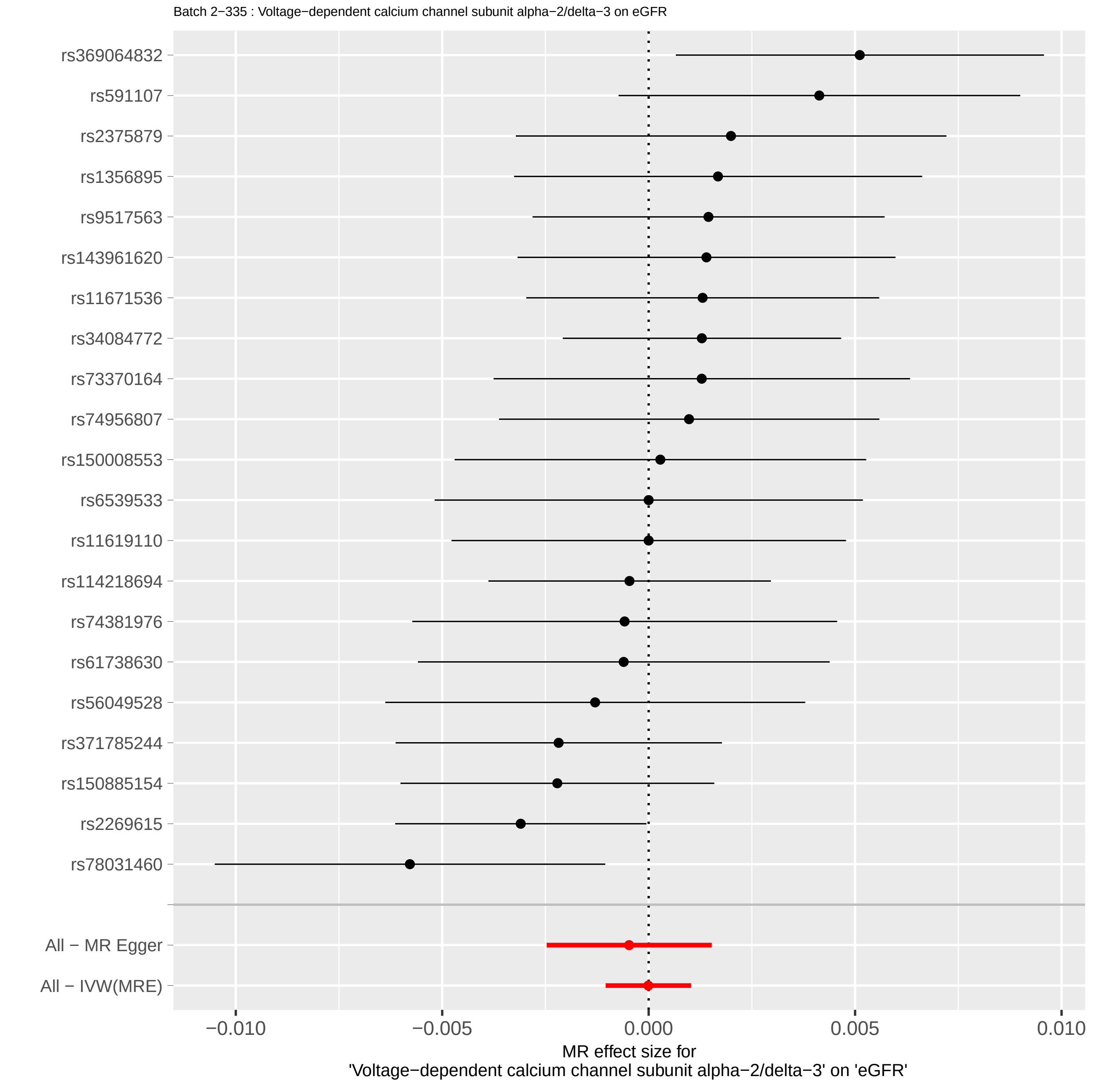

Batch 2−335 : Voltage−dependent calcium channel subunit alpha−2/delta−3 on eGFR
rs369064832
rs591107
rs2375879
rs1356895
rs9517563
rs143961620
rs11671536
rs34084772
rs73370164
rs74956807
rs150008553
rs6539533
rs11619110
rs114218694
rs74381976
rs61738630
rs56049528
rs371785244
rs150885154
rs2269615
rs78031460
All − MR Egger
All − IVW(MRE)
−0.010
−0.005
0.000
0.005
0.010
MR effect size for
'Voltage−dependent calcium channel subunit alpha−2/delta−3' on 'eGFR'

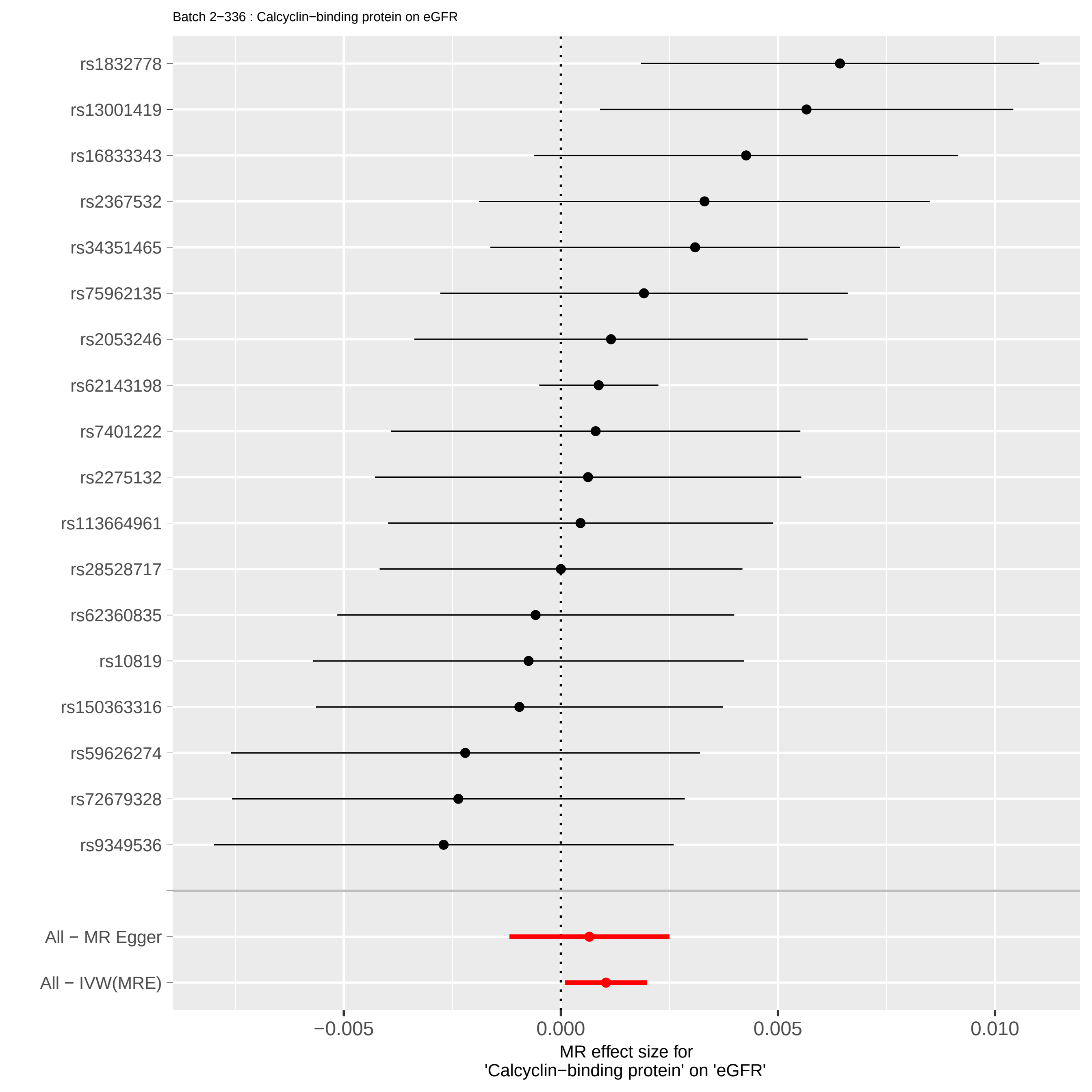

Batch 2−336 : Calcyclin−binding protein on eGFR
rs1832778
rs13001419
rs16833343
rs2367532
rs34351465
rs75962135
rs2053246
rs62143198
rs7401222
rs2275132
rs113664961
rs28528717
rs62360835
rs10819
rs150363316
rs59626274
rs72679328
rs9349536
All − MR Egger
All − IVW(MRE)
−0.005
0.000
0.005
0.010
MR effect size for
'Calcyclin−binding protein' on 'eGFR'

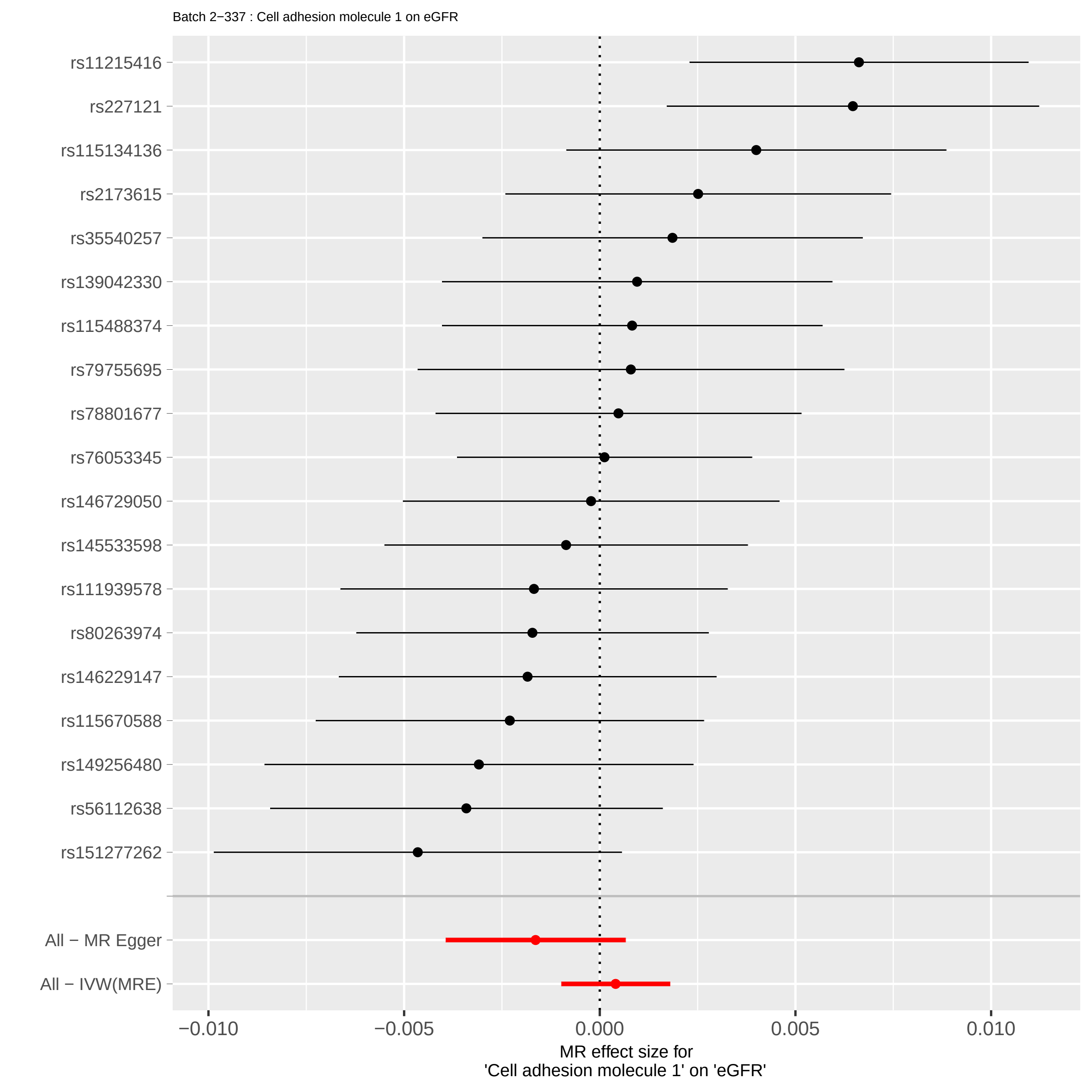
Batch 2−337 : Cell adhesion molecule 1 on eGFR
rs11215416
rs227121
rs115134136
rs2173615
rs35540257
rs139042330
rs115488374
rs79755695
rs78801677
rs76053345
rs146729050
rs145533598
rs111939578
rs80263974
rs146229147
rs115670588
rs149256480
rs56112638
rs151277262
All − MR Egger
All − IVW(MRE)
−0.010
−0.005
0.000
0.005
0.010
MR effect size for
'Cell adhesion molecule 1' on 'eGFR'

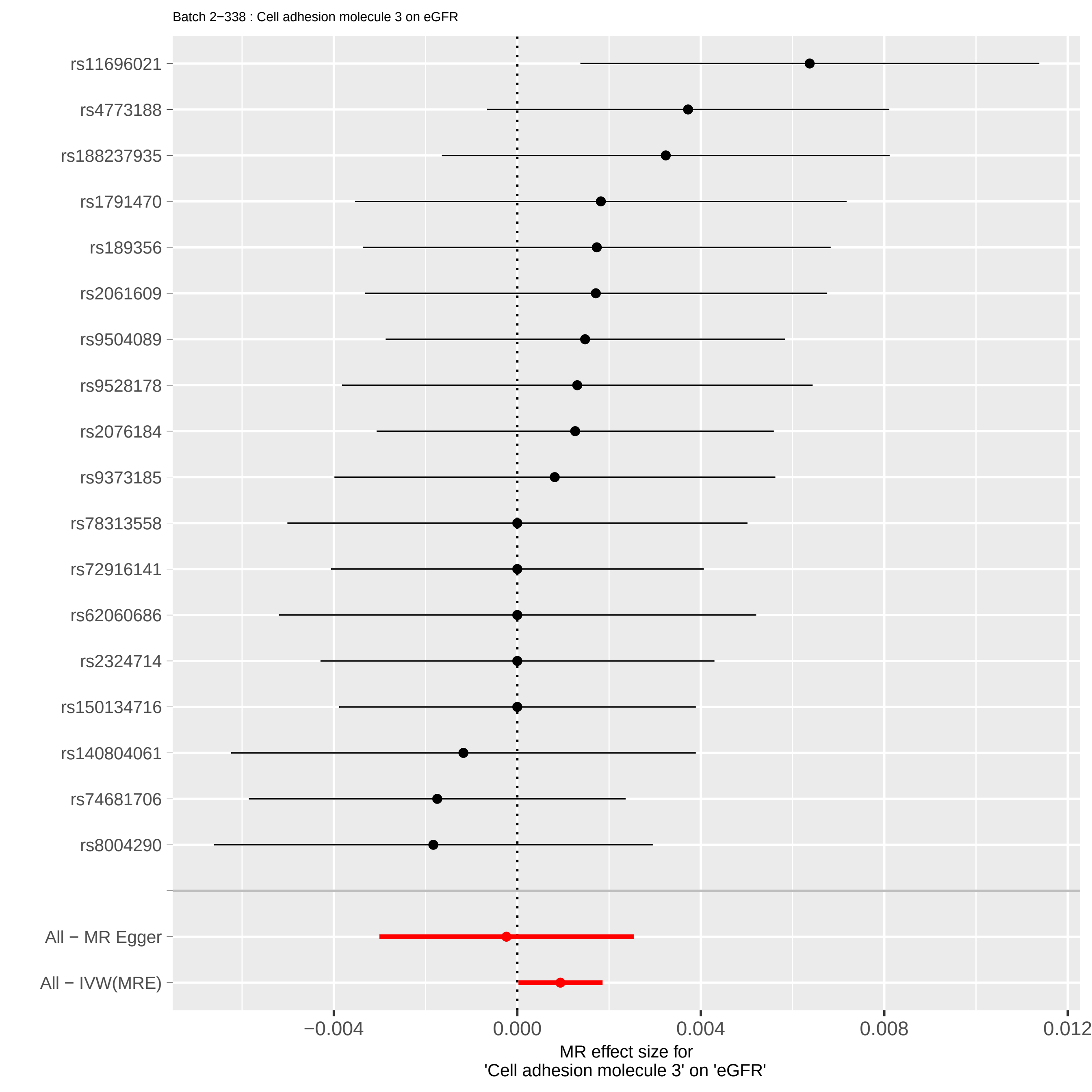
Batch 2−338 : Cell adhesion molecule 3 on eGFR
rs11696021
rs4773188
rs188237935
rs1791470
rs189356
rs2061609
rs9504089
rs9528178
rs2076184
rs9373185
rs78313558
rs72916141
rs62060686
rs2324714
rs150134716
rs140804061
rs74681706
rs8004290
All − MR Egger
All − IVW(MRE)
−0.004
0.000
0.004
0.008
0.012
MR effect size for
'Cell adhesion molecule 3' on 'eGFR'

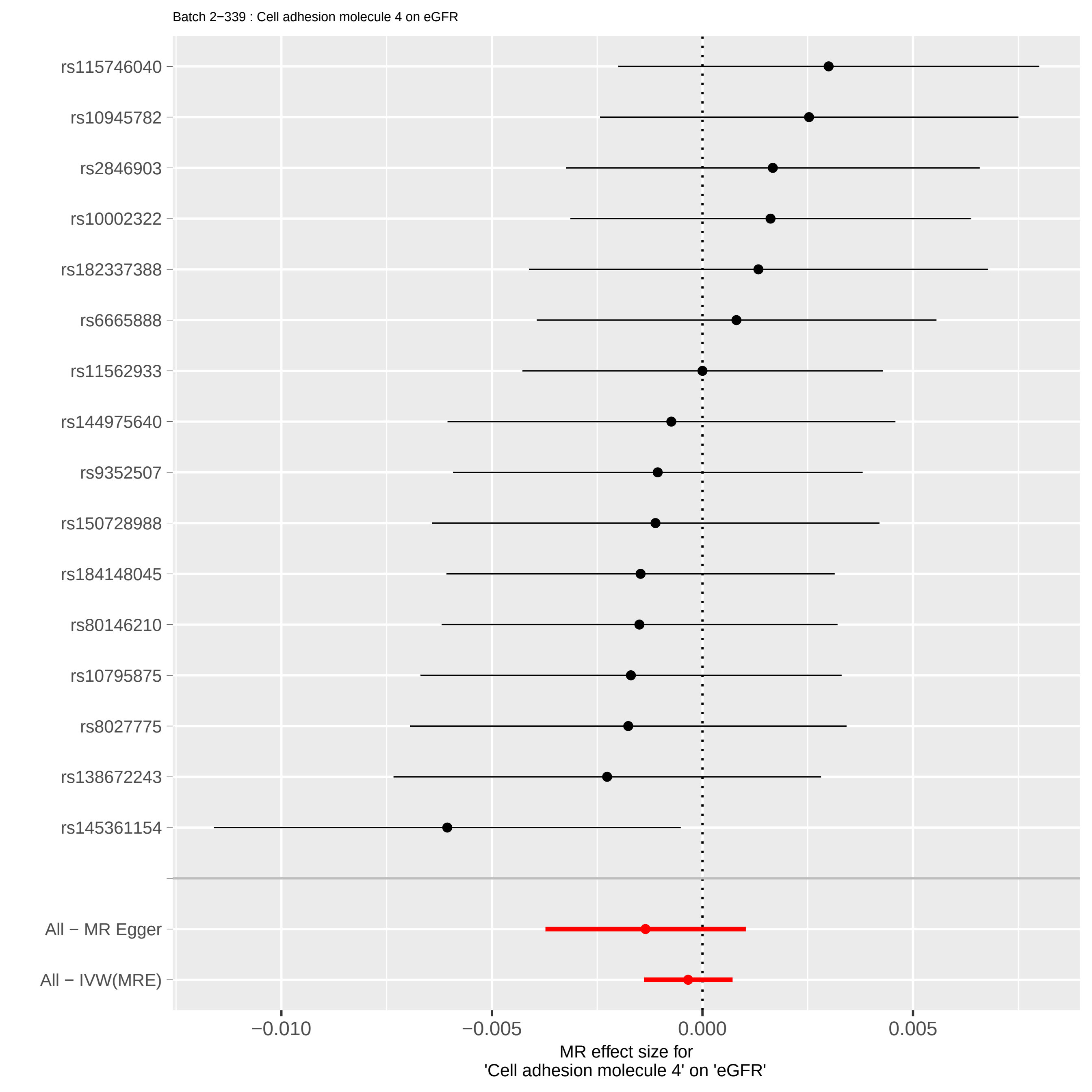
Batch 2−339 : Cell adhesion molecule 4 on eGFR
rs115746040
rs10945782
rs2846903
rs10002322
rs182337388
rs6665888
rs11562933
rs144975640
rs9352507
rs150728988
rs184148045
rs80146210
rs10795875
rs8027775
rs138672243
rs145361154
All − MR Egger
All − IVW(MRE)
−0.010
−0.005
0.000
0.005
MR effect size for
'Cell adhesion molecule 4' on 'eGFR'

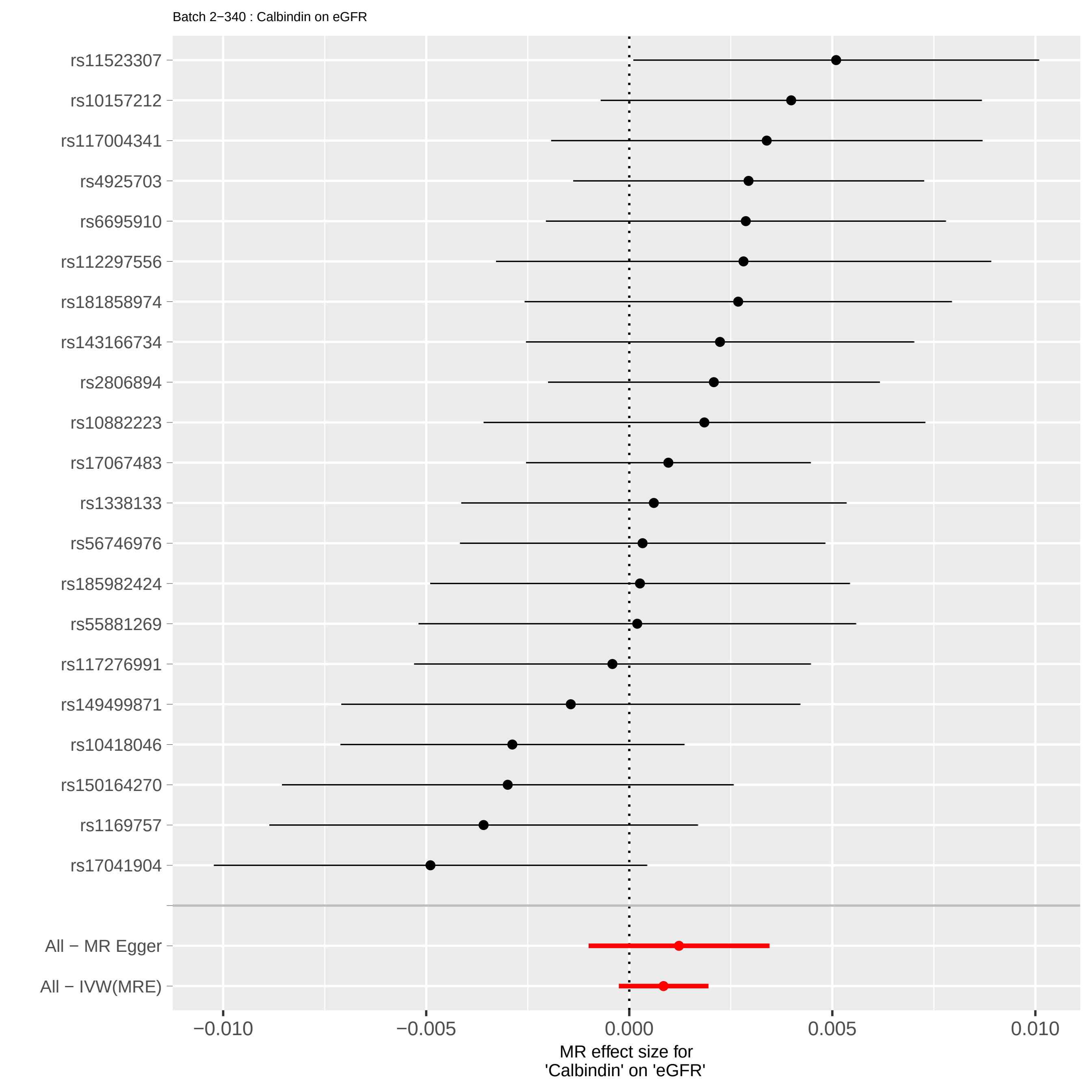
Batch 2−340 : Calbindin on eGFR
rs11523307
rs10157212
rs117004341
rs4925703
rs6695910
rs112297556
rs181858974
rs143166734
rs2806894
rs10882223
rs17067483
rs1338133
rs56746976
rs185982424
rs55881269
rs117276991
rs149499871
rs10418046
rs150164270
rs1169757
rs17041904
All − MR Egger
All − IVW(MRE)
−0.010
−0.005
0.000
0.005
0.010
MR effect size for
'Calbindin' on 'eGFR'

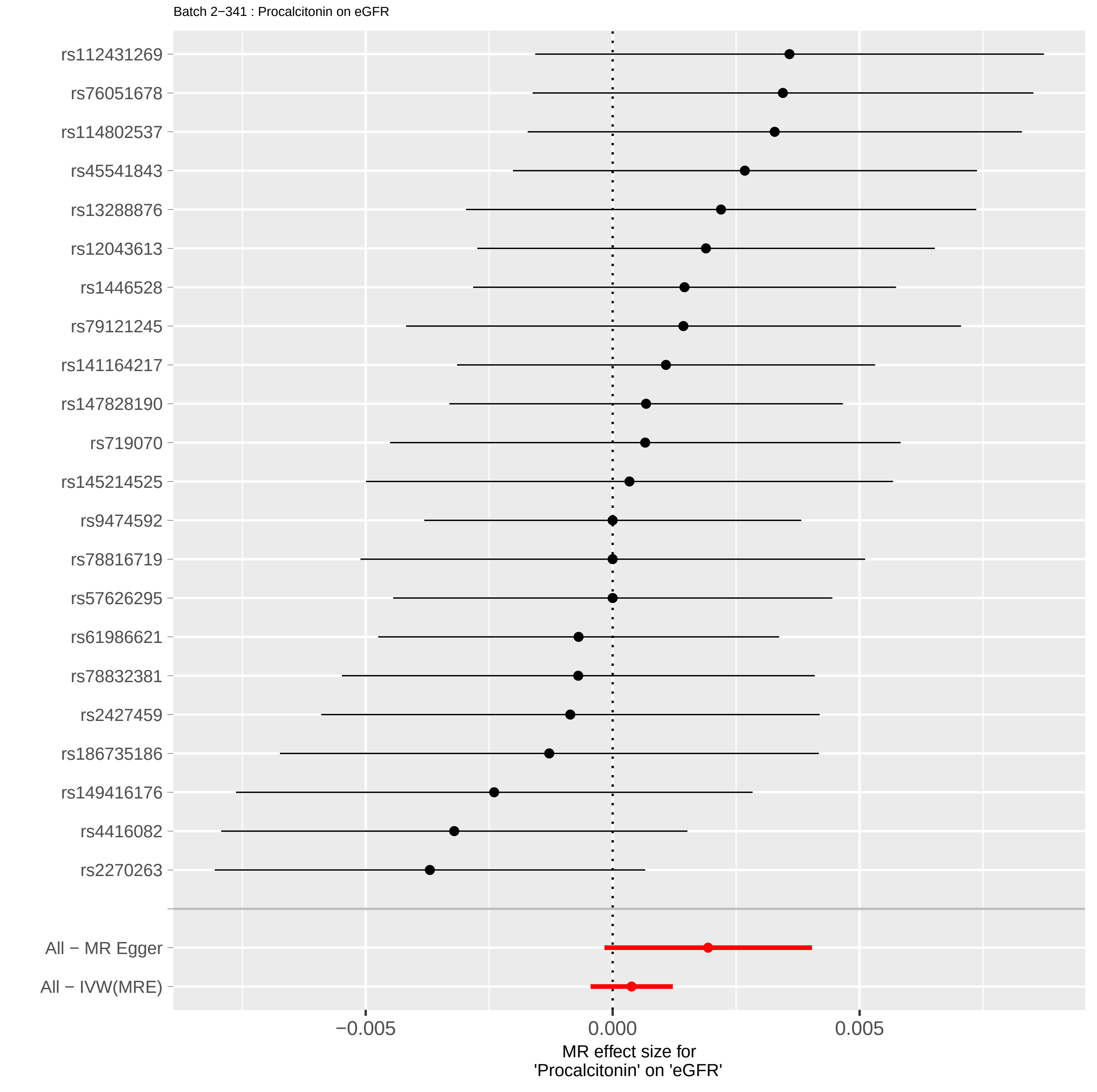

Batch 2−341 : Procalcitonin on eGFR
rs112431269
rs76051678
rs114802537
rs45541843
rs13288876
rs12043613
rs1446528
rs79121245
rs141164217
rs147828190
rs719070
rs145214525
rs9474592
rs78816719
rs57626295
rs61986621
rs78832381
rs2427459
rs186735186
rs149416176
rs4416082
rs2270263
All − MR Egger
All − IVW(MRE)
−0.005
0.000
0.005
MR effect size for
'Procalcitonin' on 'eGFR'

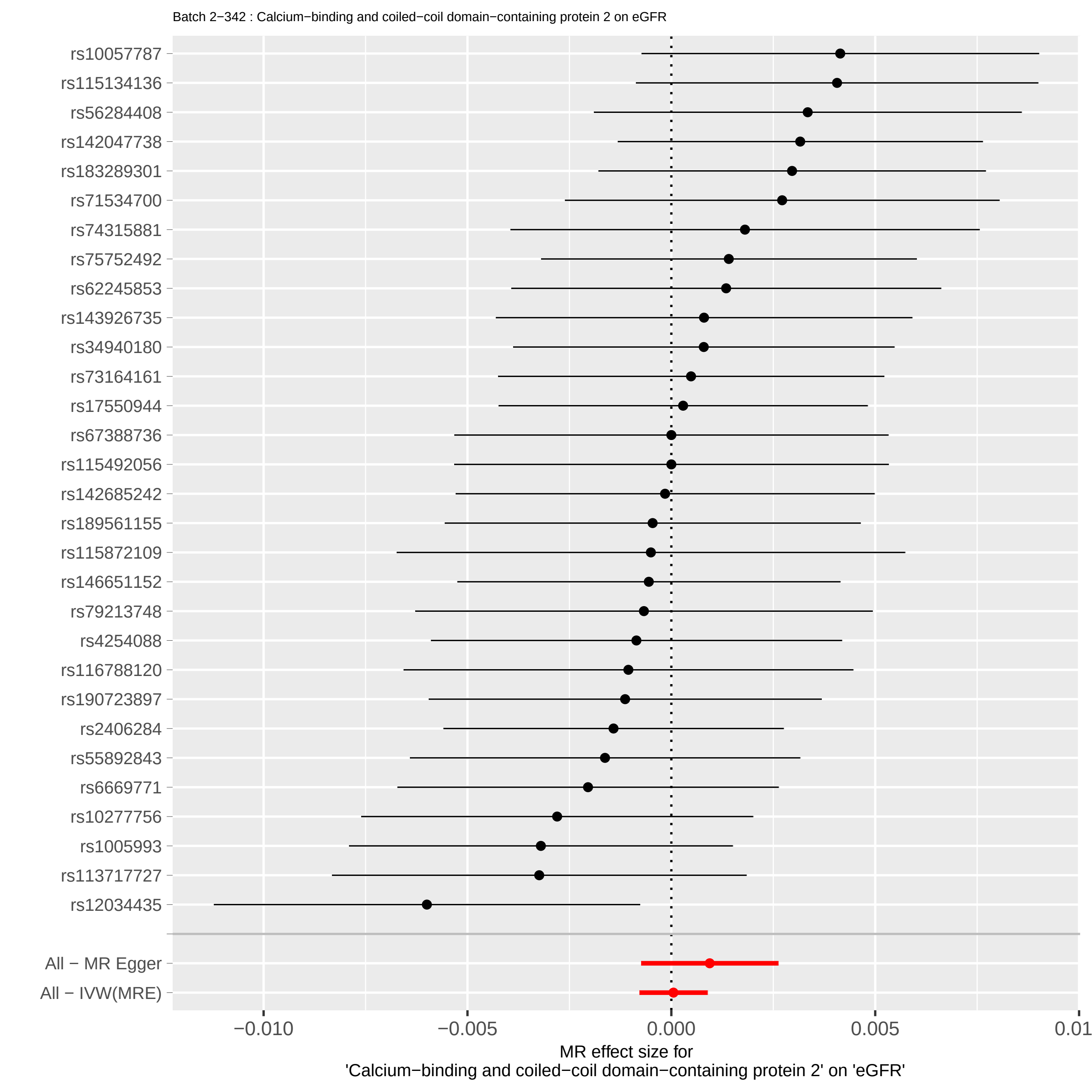

Batch 2−342 : Calcium−binding and coiled−coil domain−containing protein 2 on eGFR
rs10057787
rs115134136
rs56284408
rs142047738
rs183289301
rs71534700
rs74315881
rs75752492
rs62245853
rs143926735
rs34940180
rs73164161
rs17550944
rs67388736
rs115492056
rs142685242
rs189561155
rs115872109
rs146651152
rs79213748
rs4254088
rs116788120
rs190723897
rs2406284
rs55892843
rs6669771
rs10277756
rs1005993
rs113717727
rs12034435
All − MR Egger
All − IVW(MRE)
−0.010
−0.005
0.000
0.005
0.01
MR effect size for
'Calcium−binding and coiled−coil domain−containing protein 2' on 'eGFR'

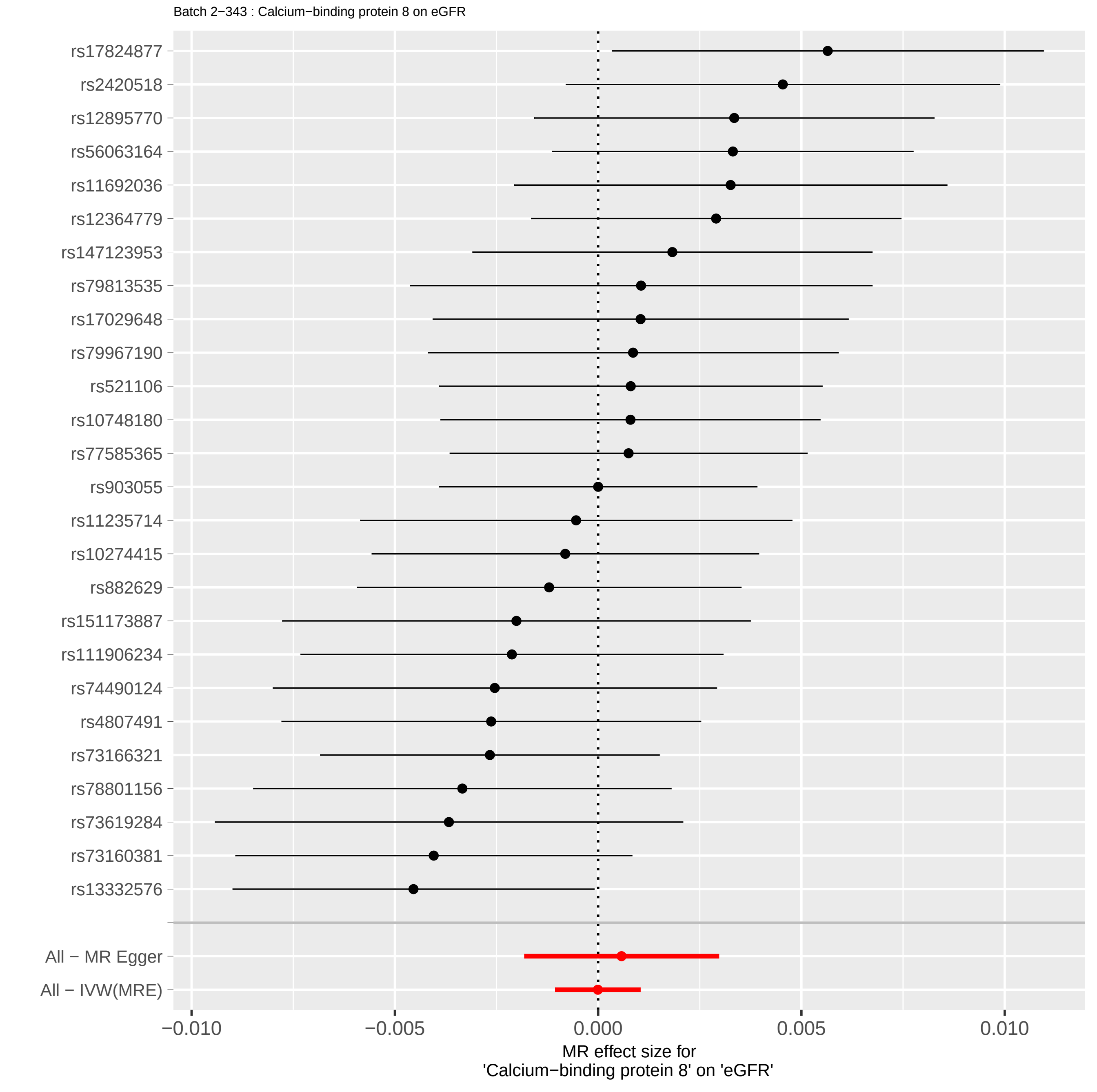

Batch 2−343 : Calcium−binding protein 8 on eGFR
rs17824877
rs2420518
rs12895770
rs56063164
rs11692036
rs12364779
rs147123953
rs79813535
rs17029648
rs79967190
rs521106
rs10748180
rs77585365
rs903055
rs11235714
rs10274415
rs882629
rs151173887
rs111906234
rs74490124
rs4807491
rs73166321
rs78801156
rs73619284
rs73160381
rs13332576
All − MR Egger
All − IVW(MRE)
−0.010
−0.005
0.000
0.005
0.010
MR effect size for
'Calcium−binding protein 8' on 'eGFR'

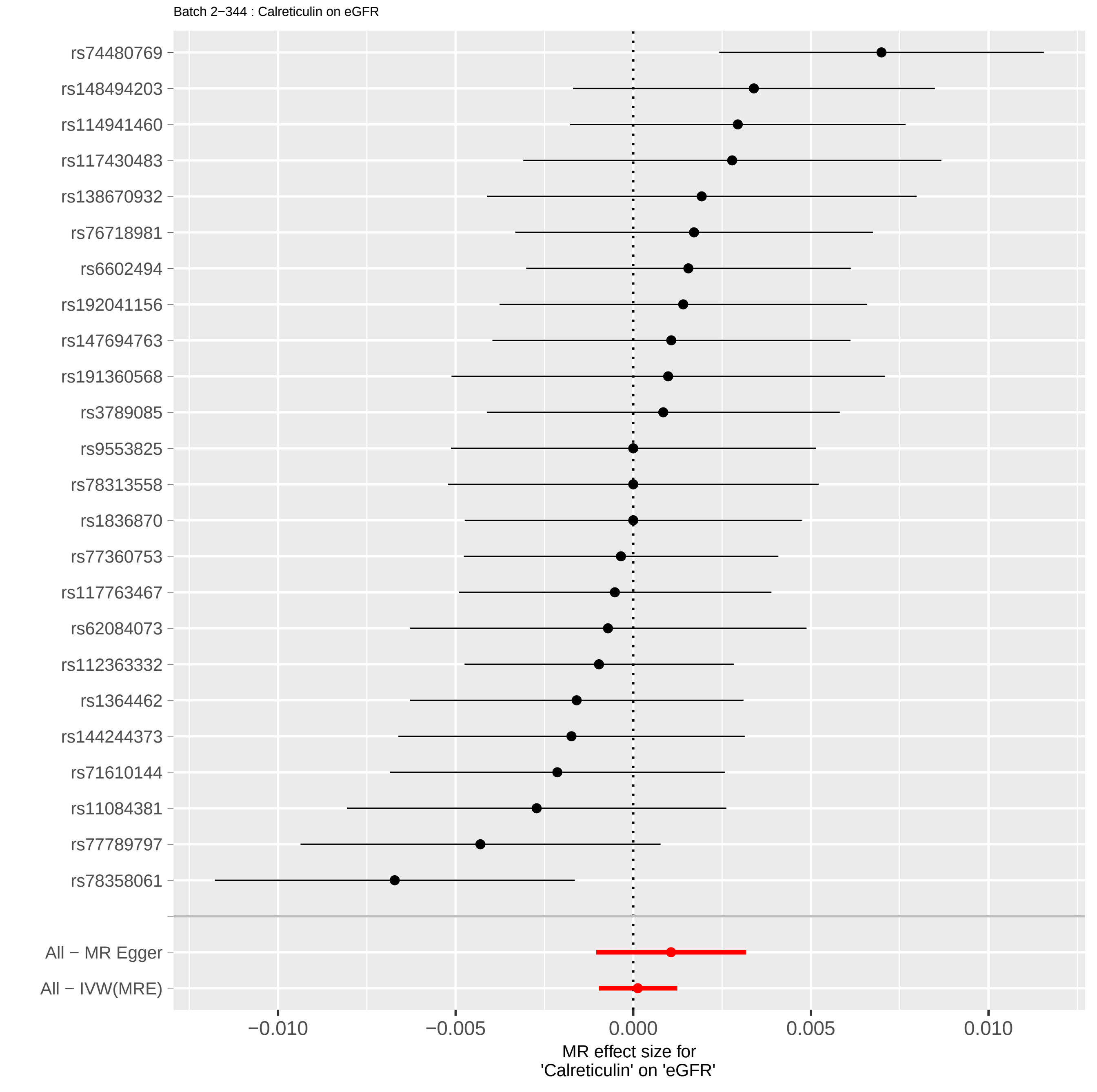

Batch 2−344 : Calreticulin on eGFR
rs74480769
rs148494203
rs114941460
rs117430483
rs138670932
rs76718981
rs6602494
rs192041156
rs147694763
rs191360568
rs3789085
rs9553825
rs78313558
rs1836870
rs77360753
rs117763467
rs62084073
rs112363332
rs1364462
rs144244373
rs71610144
rs11084381
rs77789797
rs78358061
All − MR Egger
All − IVW(MRE)
−0.010
−0.005
0.000
0.005
0.010
MR effect size for
'Calreticulin' on 'eGFR'

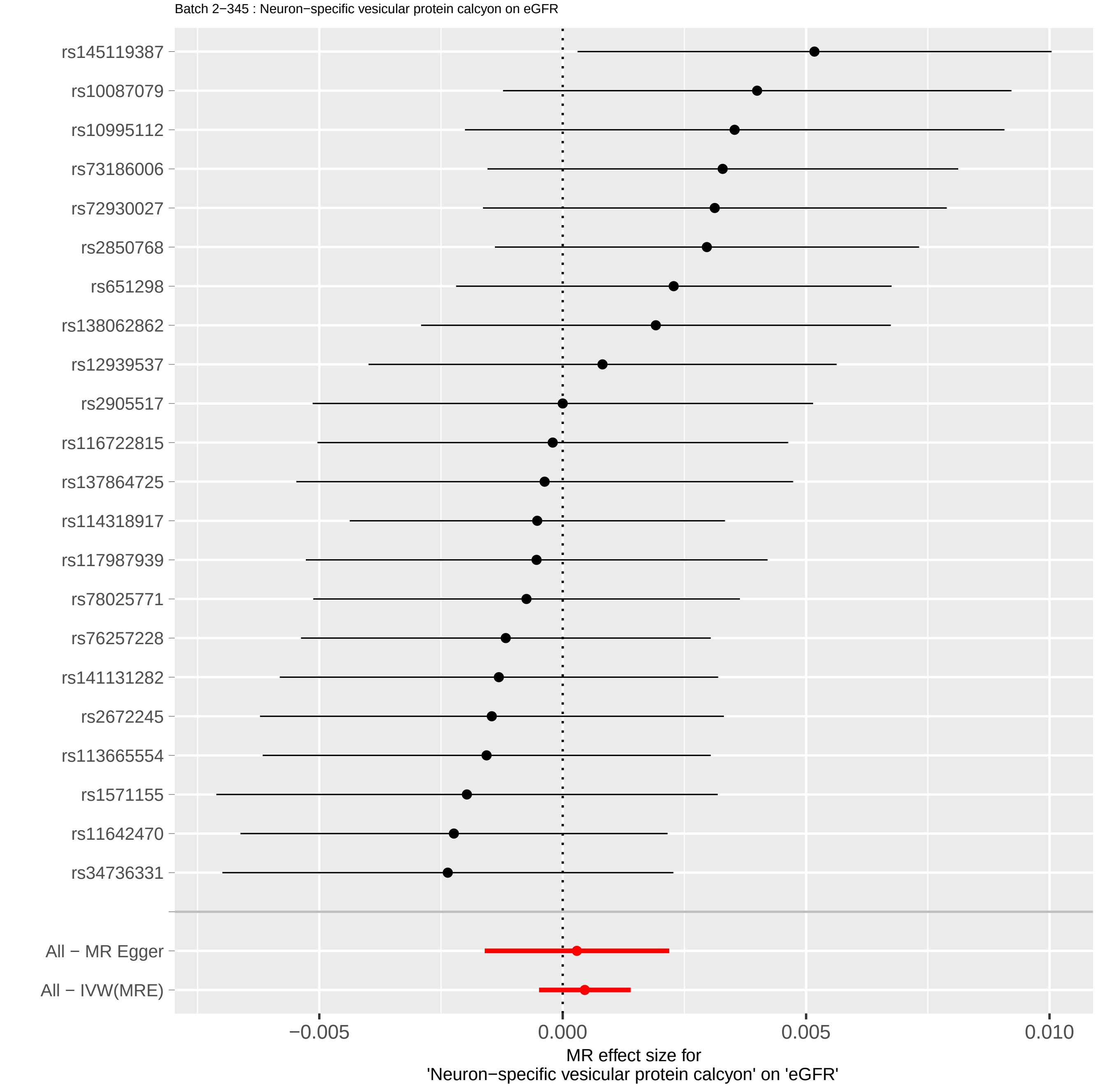

Batch 2−345 : Neuron−specific vesicular protein calcyon on eGFR
rs145119387
rs10087079
rs10995112
rs73186006
rs72930027
rs2850768
rs651298
rs138062862
rs12939537
rs2905517
rs116722815
rs137864725
rs114318917
rs117987939
rs78025771
rs76257228
rs141131282
rs2672245
rs113665554
rs1571155
rs11642470
rs34736331
All − MR Egger
All − IVW(MRE)
−0.005
0.000
0.005
0.010
MR effect size for
'Neuron−specific vesicular protein calcyon' on 'eGFR'

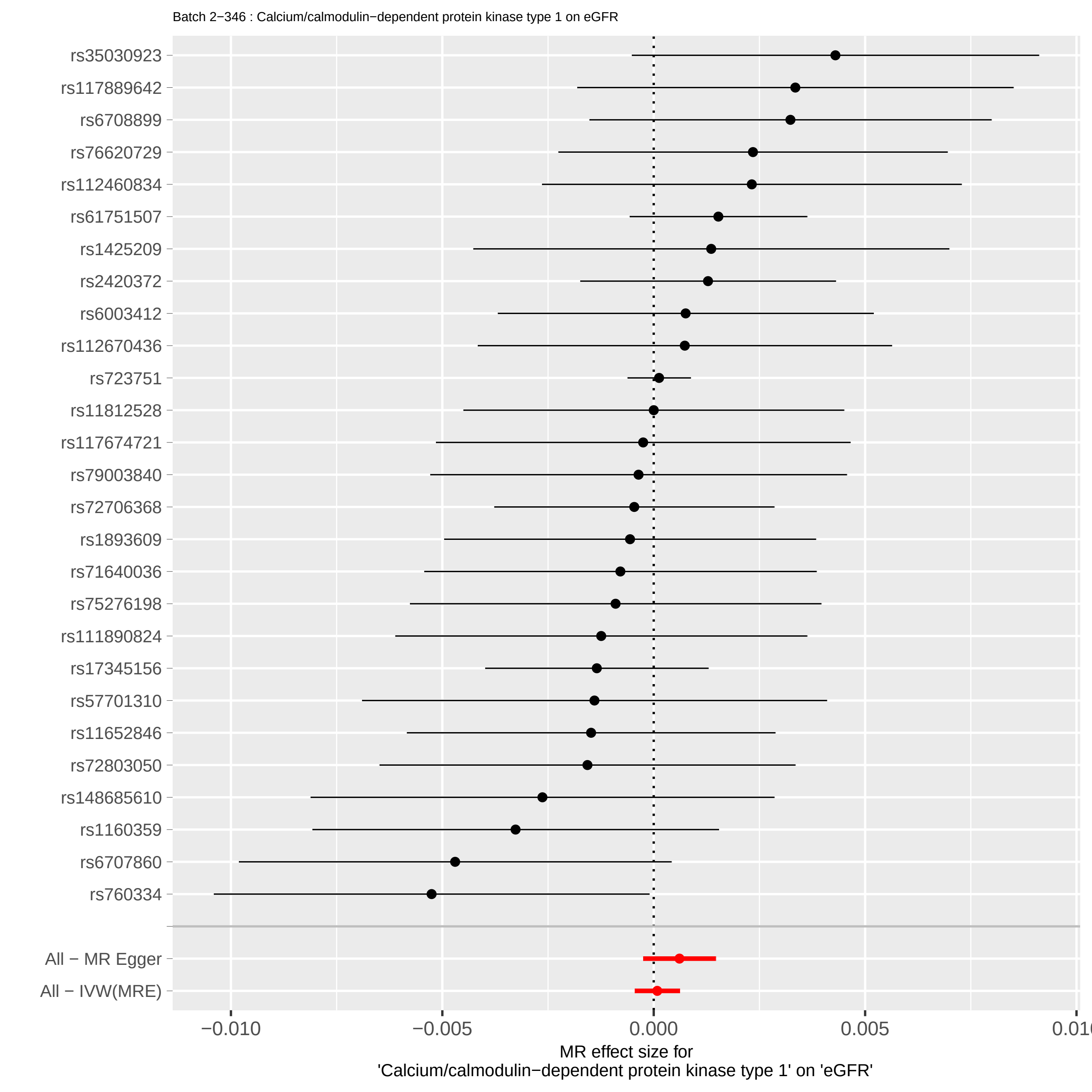

Batch 2−346 : Calcium/calmodulin−dependent protein kinase type 1 on eGFR
rs35030923
rs117889642
rs6708899
rs76620729
rs112460834
rs61751507
rs1425209
rs2420372
rs6003412
rs112670436
rs723751
rs11812528
rs117674721
rs79003840
rs72706368
rs1893609
rs71640036
rs75276198
rs111890824
rs17345156
rs57701310
rs11652846
rs72803050
rs148685610
rs1160359
rs6707860
rs760334
All − MR Egger
All − IVW(MRE)
−0.010
−0.005
0.000
0.005
0.010
MR effect size for
'Calcium/calmodulin−dependent protein kinase type 1' on 'eGFR'

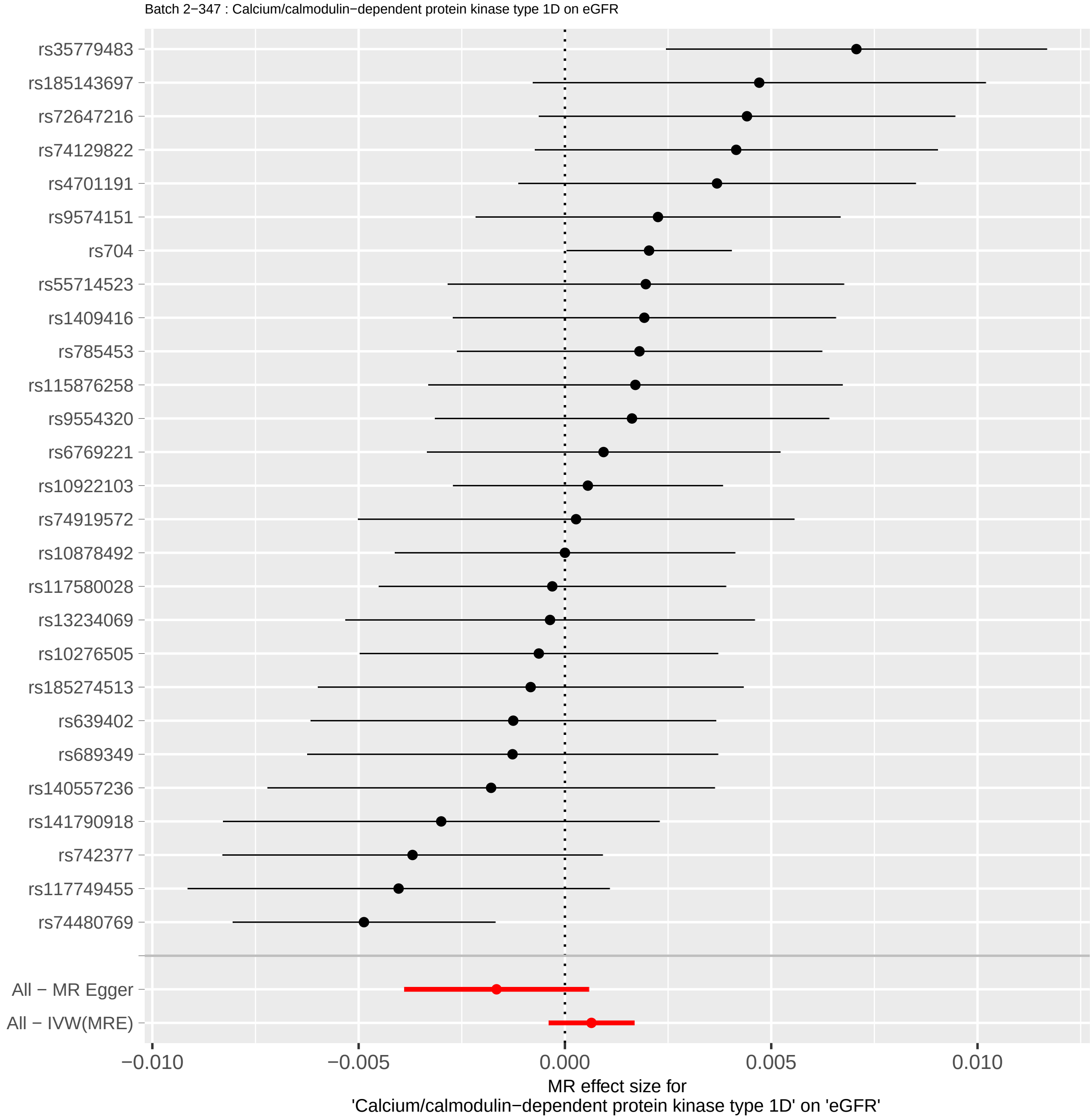

Batch 2−347 : Calcium/calmodulin−dependent protein kinase type 1D on eGFR
rs35779483
rs185143697
rs72647216
rs74129822
rs4701191
rs9574151
rs704
rs55714523
rs1409416
rs785453
rs115876258
rs9554320
rs6769221
rs10922103
rs74919572
rs10878492
rs117580028
rs13234069
rs10276505
rs185274513
rs639402
rs689349
rs140557236
rs141790918
rs742377
rs117749455
rs74480769
All − MR Egger
All − IVW(MRE)
−0.010
−0.005
0.000
0.005
0.010
MR effect size for
'Calcium/calmodulin−dependent protein kinase type 1D' on 'eGFR'

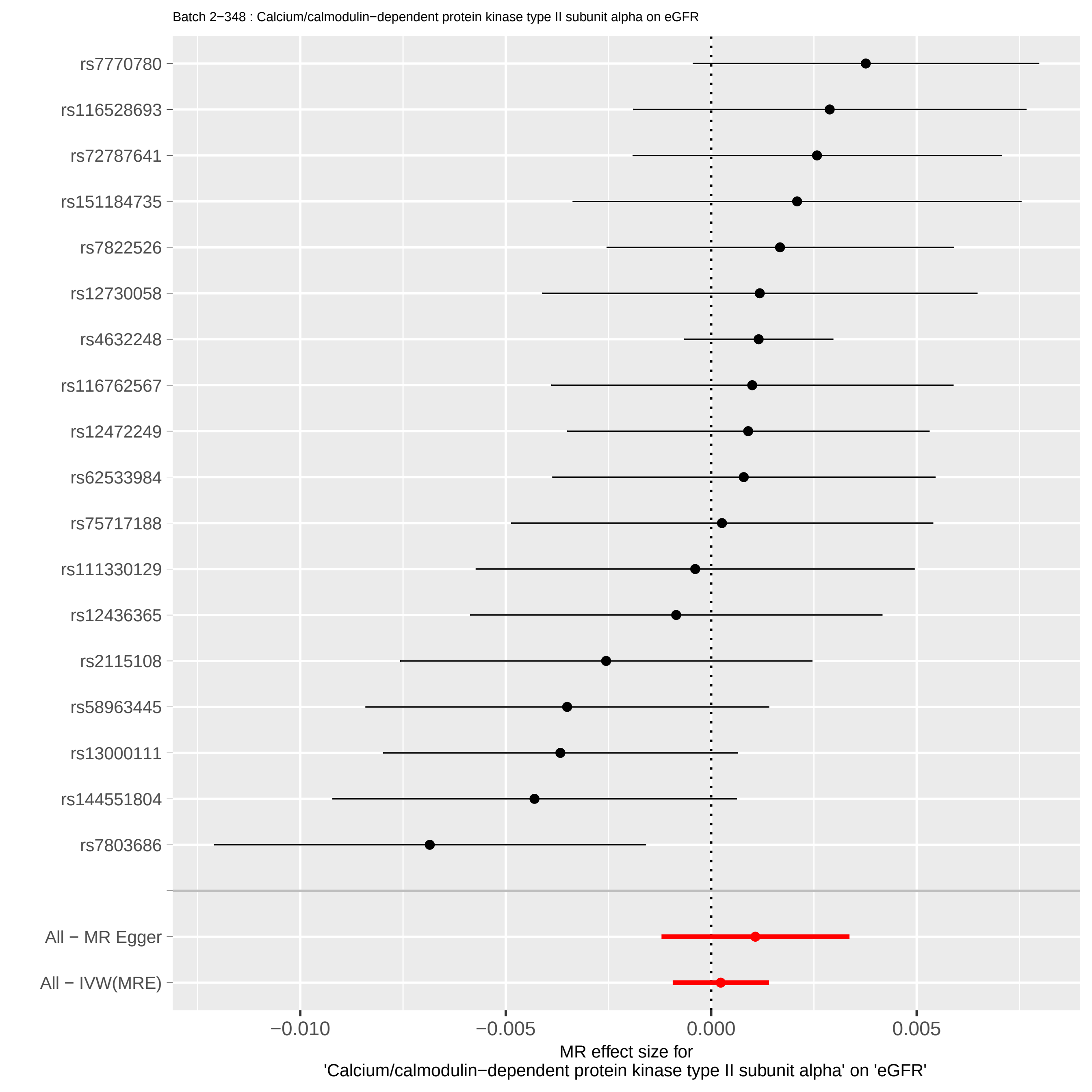

Batch 2−348 : Calcium/calmodulin−dependent protein kinase type II subunit alpha on eGFR
rs7770780
rs116528693
rs72787641
rs151184735
rs7822526
rs12730058
rs4632248
rs116762567
rs12472249
rs62533984
rs75717188
rs111330129
rs12436365
rs2115108
rs58963445
rs13000111
rs144551804
rs7803686
All − MR Egger
All − IVW(MRE)
−0.010
−0.005
0.000
0.005
MR effect size for
'Calcium/calmodulin−dependent protein kinase type II subunit alpha' on 'eGFR'

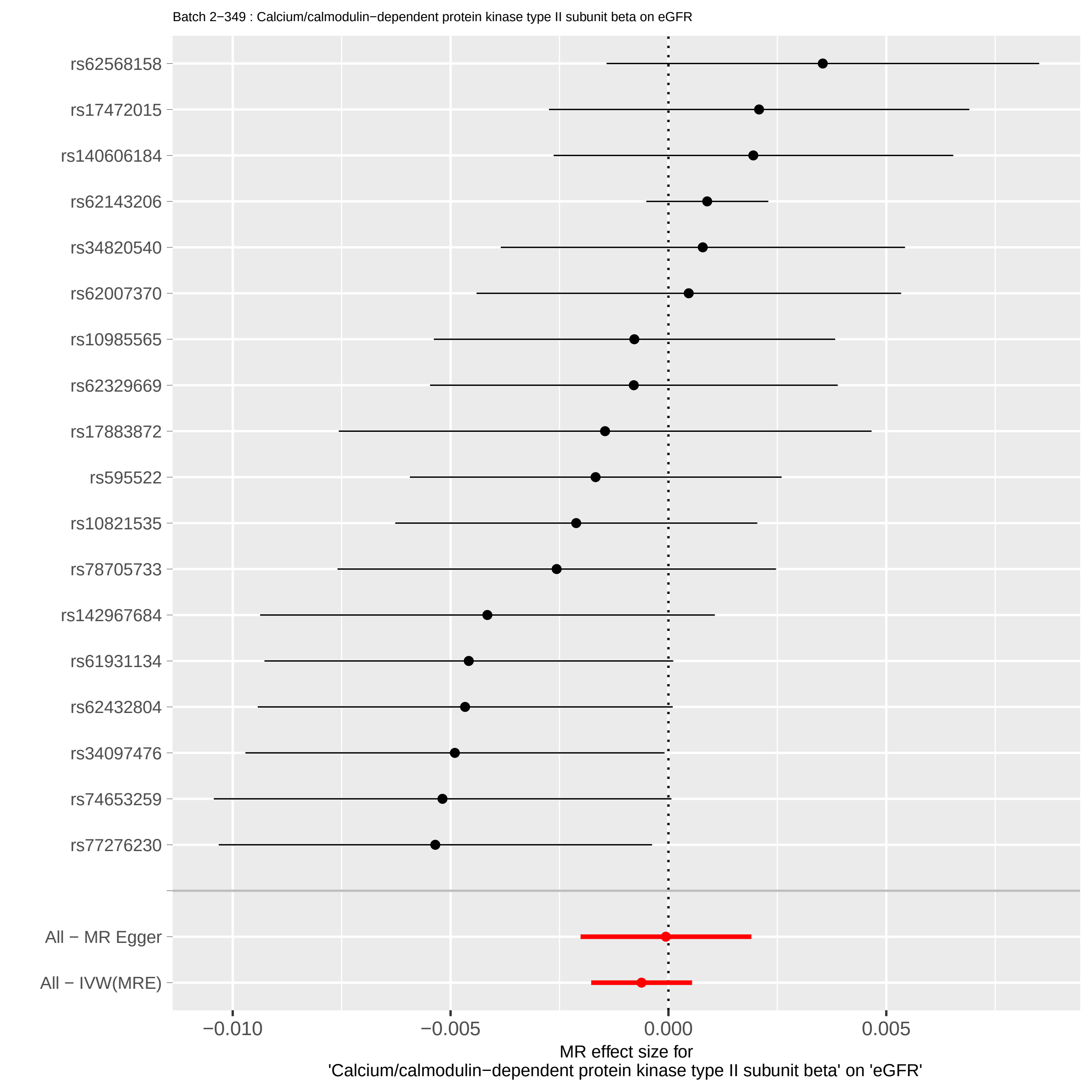
Batch 2−349 : Calcium/calmodulin−dependent protein kinase type II subunit beta on eGFR
rs62568158
rs17472015
rs140606184
rs62143206
rs34820540
rs62007370
rs10985565
rs62329669
rs17883872
rs595522
rs10821535
rs78705733
rs142967684
rs61931134
rs62432804
rs34097476
rs74653259
rs77276230
All − MR Egger
All − IVW(MRE)
−0.010
−0.005
0.000
0.005
MR effect size for
'Calcium/calmodulin−dependent protein kinase type II subunit beta' on 'eGFR'

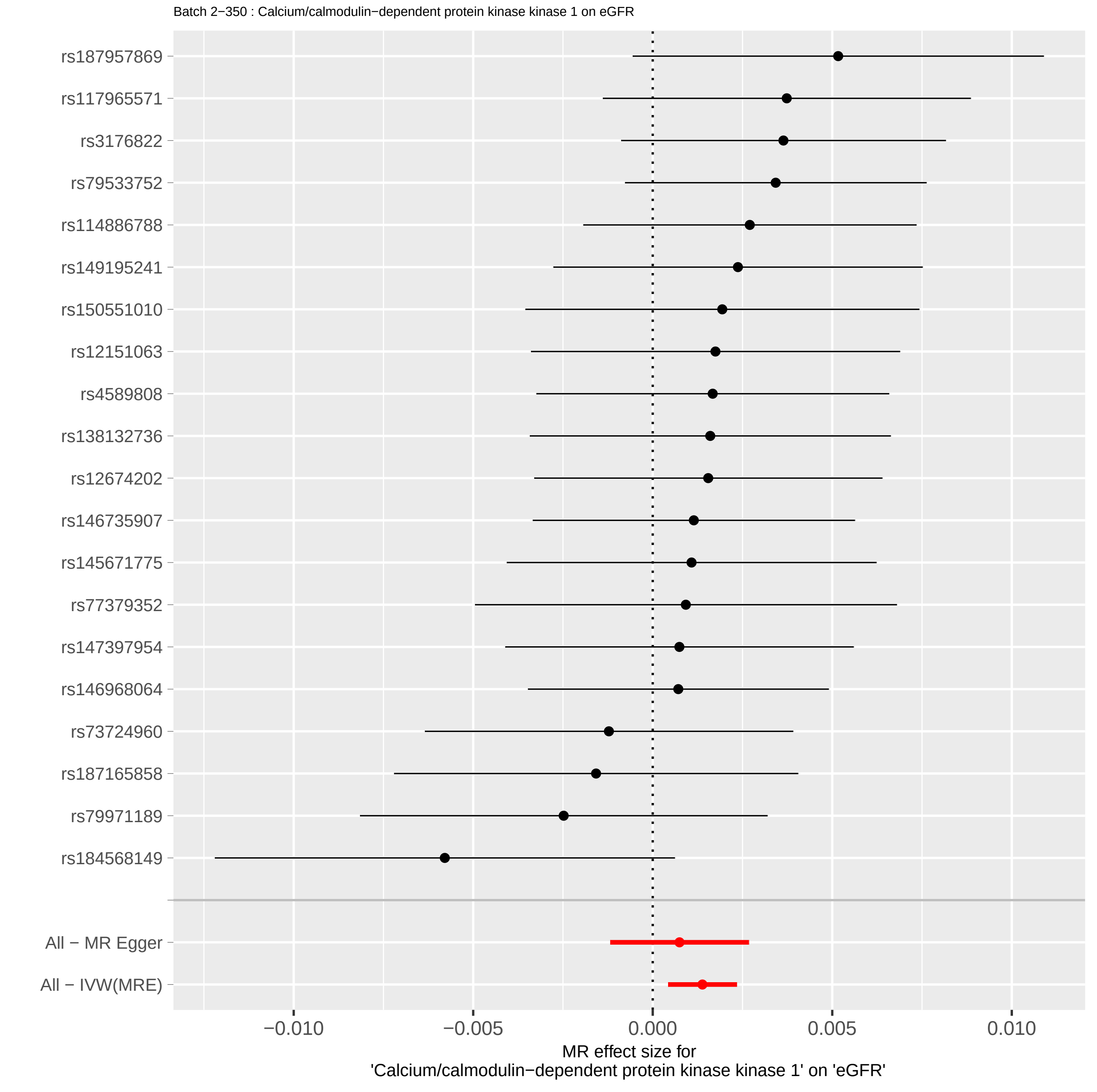

Batch 2−350 : Calcium/calmodulin−dependent protein kinase kinase 1 on eGFR
rs187957869
rs117965571
rs3176822
rs79533752
rs114886788
rs149195241
rs150551010
rs12151063
rs4589808
rs138132736
rs12674202
rs146735907
rs145671775
rs77379352
rs147397954
rs146968064
rs73724960
rs187165858
rs79971189
rs184568149
All − MR Egger
All − IVW(MRE)
−0.010
−0.005
0.000
0.005
0.010
MR effect size for
'Calcium/calmodulin−dependent protein kinase kinase 1' on 'eGFR'

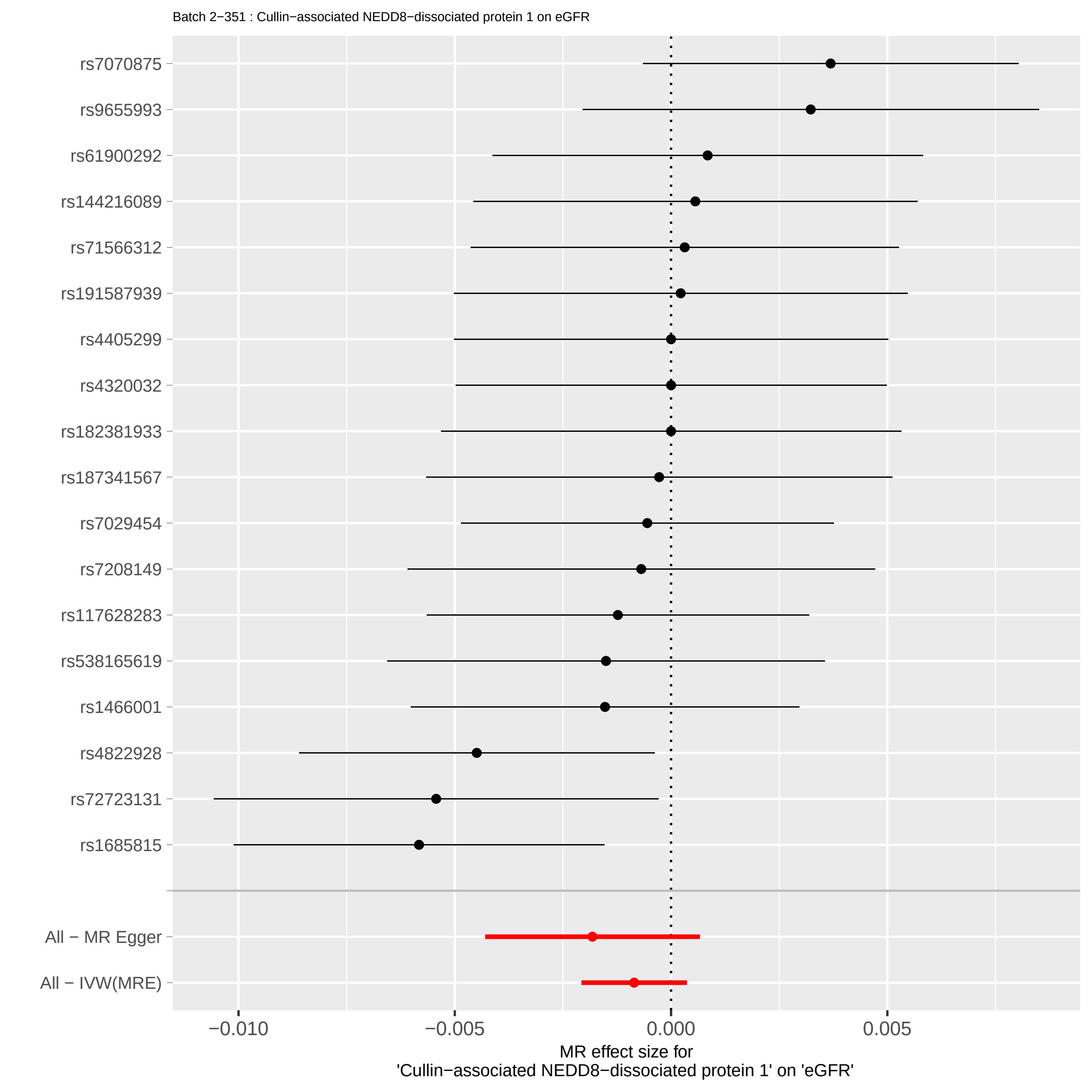
Batch 2−351 : Cullin−associated NEDD8−dissociated protein 1 on eGFR
rs7070875
rs9655993
rs61900292
rs144216089
rs71566312
rs191587939
rs4405299
rs4320032
rs182381933
rs187341567
rs7029454
rs7208149
rs117628283
rs538165619
rs1466001
rs4822928
rs72723131
rs1685815
All − MR Egger
All − IVW(MRE)
−0.010
−0.005
0.000
0.005
MR effect size for
'Cullin−associated NEDD8−dissociated protein 1' on 'eGFR'

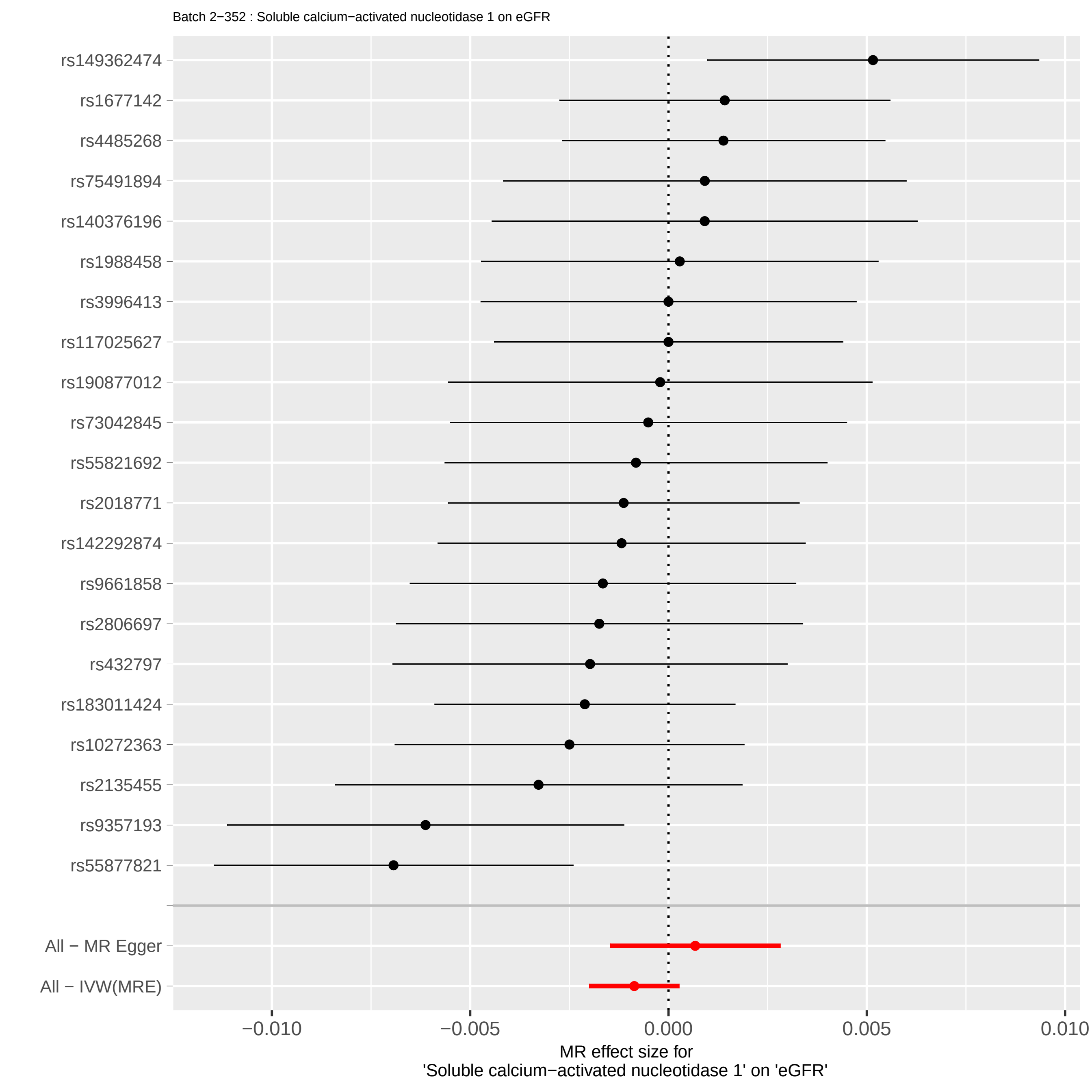

Batch 2−352 : Soluble calcium−activated nucleotidase 1 on eGFR
rs149362474
rs1677142
rs4485268
rs75491894
rs140376196
rs1988458
rs3996413
rs117025627
rs190877012
rs73042845
rs55821692
rs2018771
rs142292874
rs9661858
rs2806697
rs432797
rs183011424
rs10272363
rs2135455
rs9357193
rs55877821
All − MR Egger
All − IVW(MRE)
−0.010
−0.005
0.000
0.005
0.010
MR effect size for
'Soluble calcium−activated nucleotidase 1' on 'eGFR'

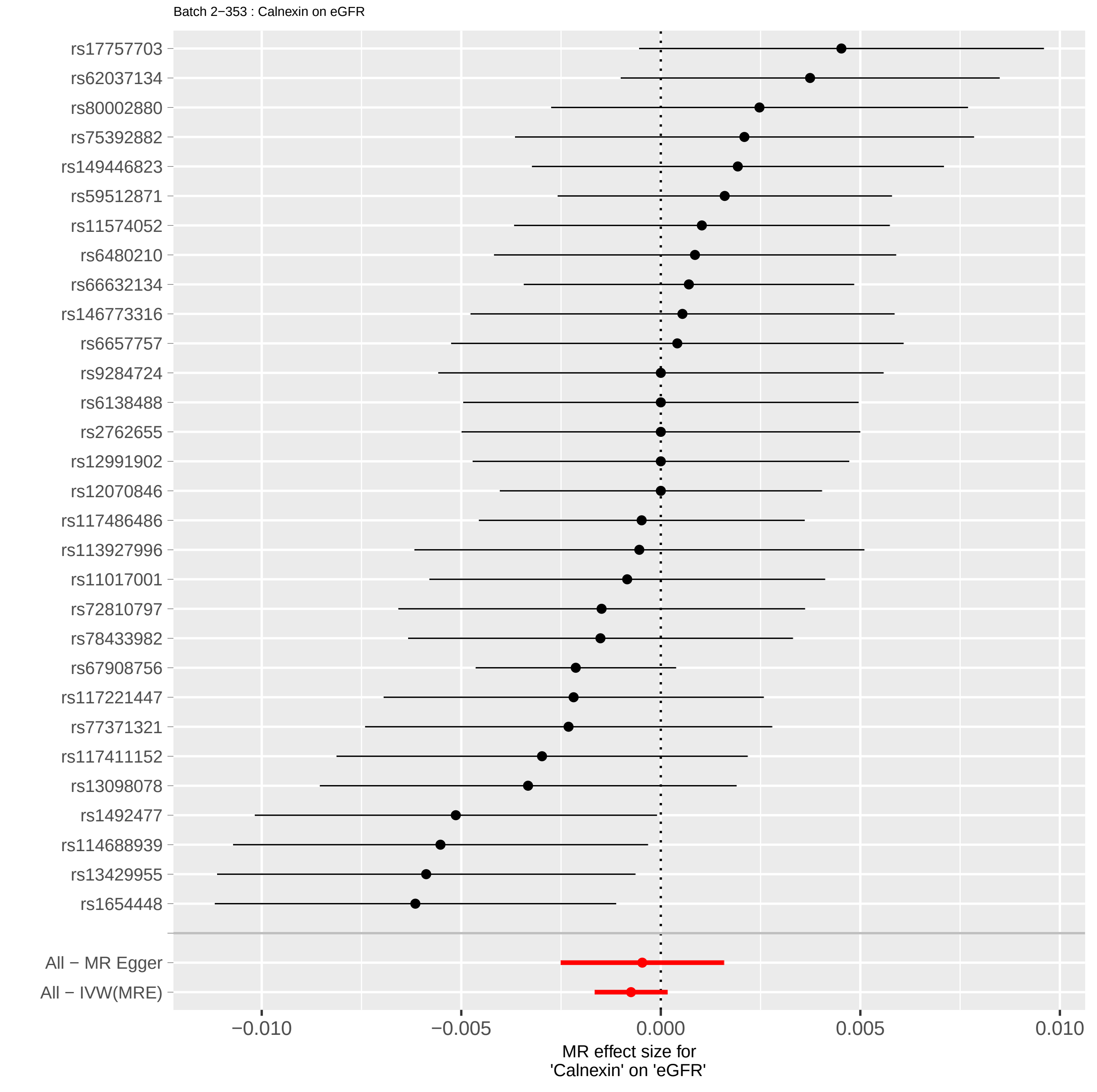

Batch 2−353 : Calnexin on eGFR
rs17757703
rs62037134
rs80002880
rs75392882
rs149446823
rs59512871
rs11574052
rs6480210
rs66632134
rs146773316
rs6657757
rs9284724
rs6138488
rs2762655
rs12991902
rs12070846
rs117486486
rs113927996
rs11017001
rs72810797
rs78433982
rs67908756
rs117221447
rs77371321
rs117411152
rs13098078
rs1492477
rs114688939
rs13429955
rs1654448
All − MR Egger
All − IVW(MRE)
−0.010
−0.005
0.000
0.005
0.010
MR effect size for
'Calnexin' on 'eGFR'

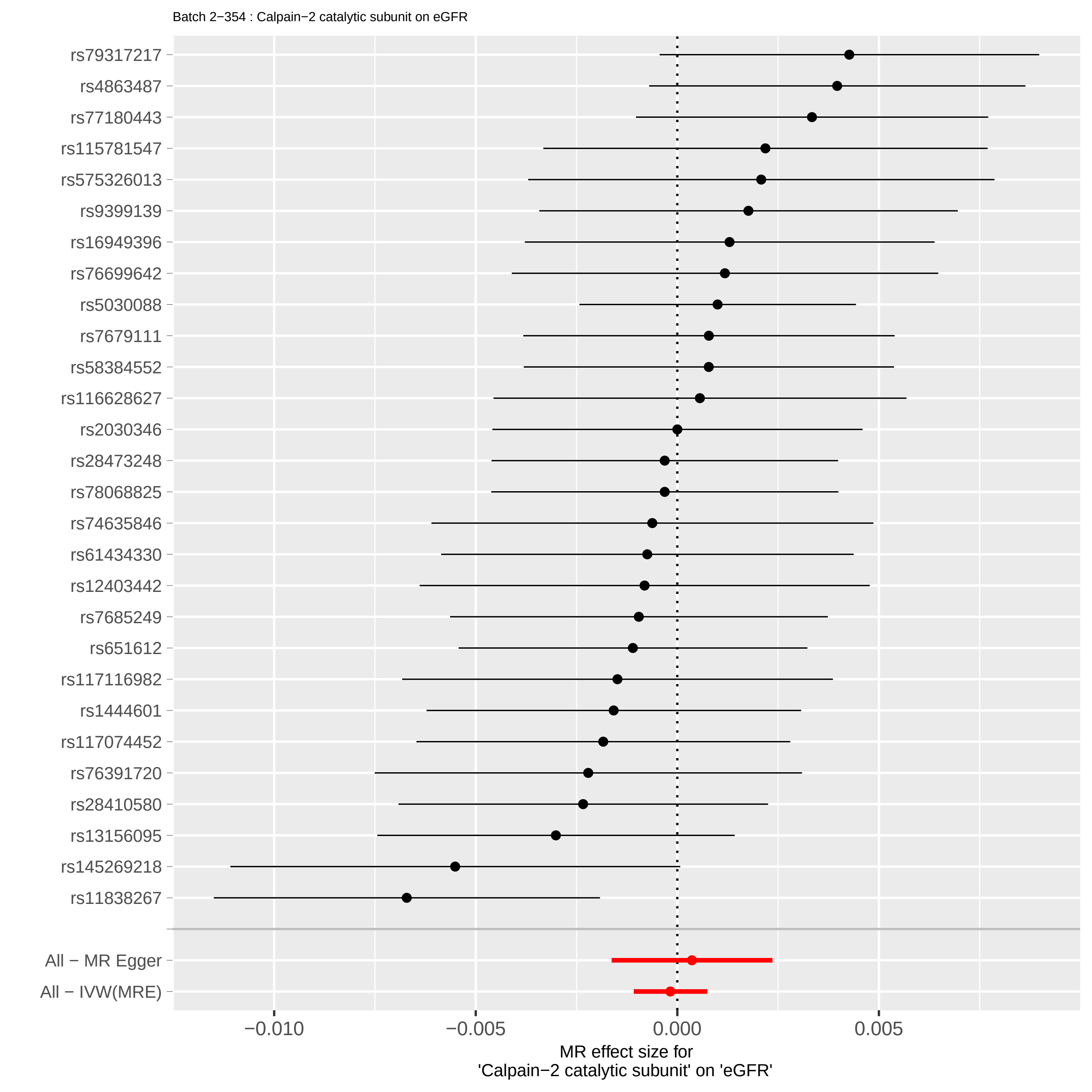
Batch 2−354 : Calpain−2 catalytic subunit on eGFR
rs79317217
rs4863487
rs77180443
rs115781547
rs575326013
rs9399139
rs16949396
rs76699642
rs5030088
rs7679111
rs58384552
rs116628627
rs2030346
rs28473248
rs78068825
rs74635846
rs61434330
rs12403442
rs7685249
rs651612
rs117116982
rs1444601
rs117074452
rs76391720
rs28410580
rs13156095
rs145269218
rs11838267
All − MR Egger
All − IVW(MRE)
−0.010
−0.005
0.000
0.005
MR effect size for
'Calpain−2 catalytic subunit' on 'eGFR'

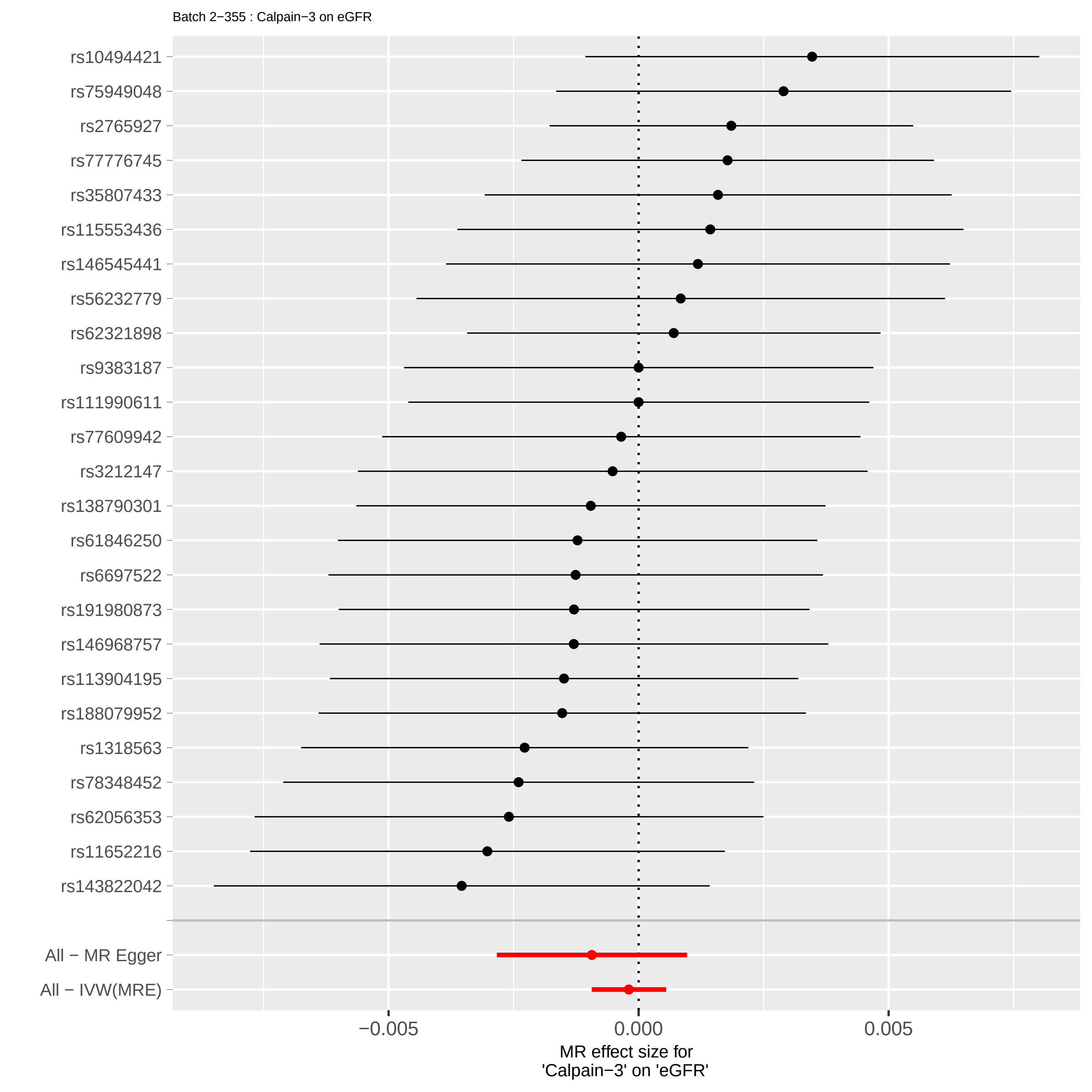

Batch 2−355 : Calpain−3 on eGFR
rs10494421
rs75949048
rs2765927
rs77776745
rs35807433
rs115553436
rs146545441
rs56232779
rs62321898
rs9383187
rs111990611
rs77609942
rs3212147
rs138790301
rs61846250
rs6697522
rs191980873
rs146968757
rs113904195
rs188079952
rs1318563
rs78348452
rs62056353
rs11652216
rs143822042
All − MR Egger
All − IVW(MRE)
−0.005
0.000
0.005
MR effect size for
'Calpain−3' on 'eGFR'

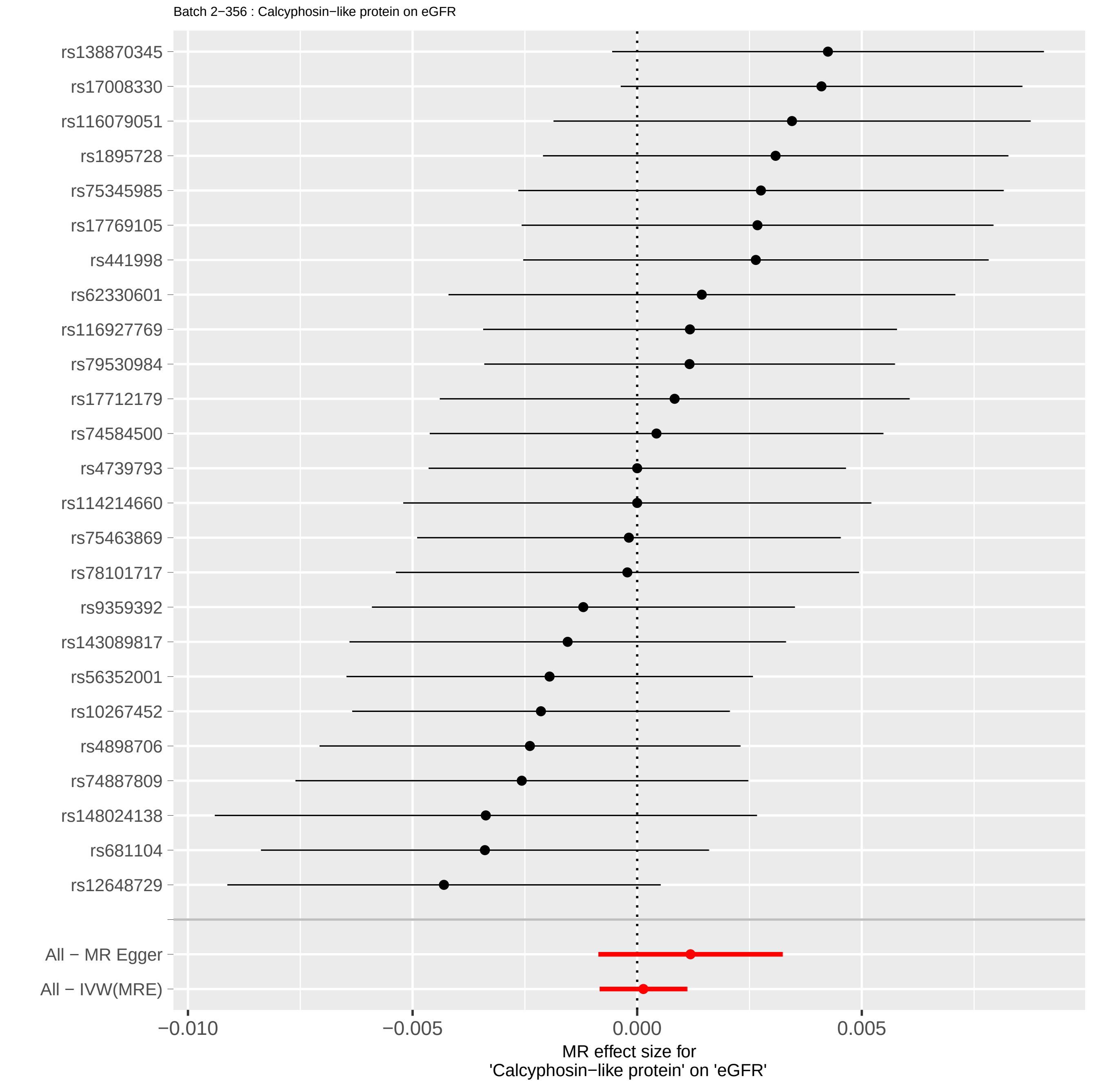

Batch 2−356 : Calcyphosin−like protein on eGFR
rs138870345
rs17008330
rs116079051
rs1895728
rs75345985
rs17769105
rs441998
rs62330601
rs116927769
rs79530984
rs17712179
rs74584500
rs4739793
rs114214660
rs75463869
rs78101717
rs9359392
rs143089817
rs56352001
rs10267452
rs4898706
rs74887809
rs148024138
rs681104
rs12648729
All − MR Egger
All − IVW(MRE)
−0.010
−0.005
0.000
0.005
MR effect size for
'Calcyphosin−like protein' on 'eGFR'

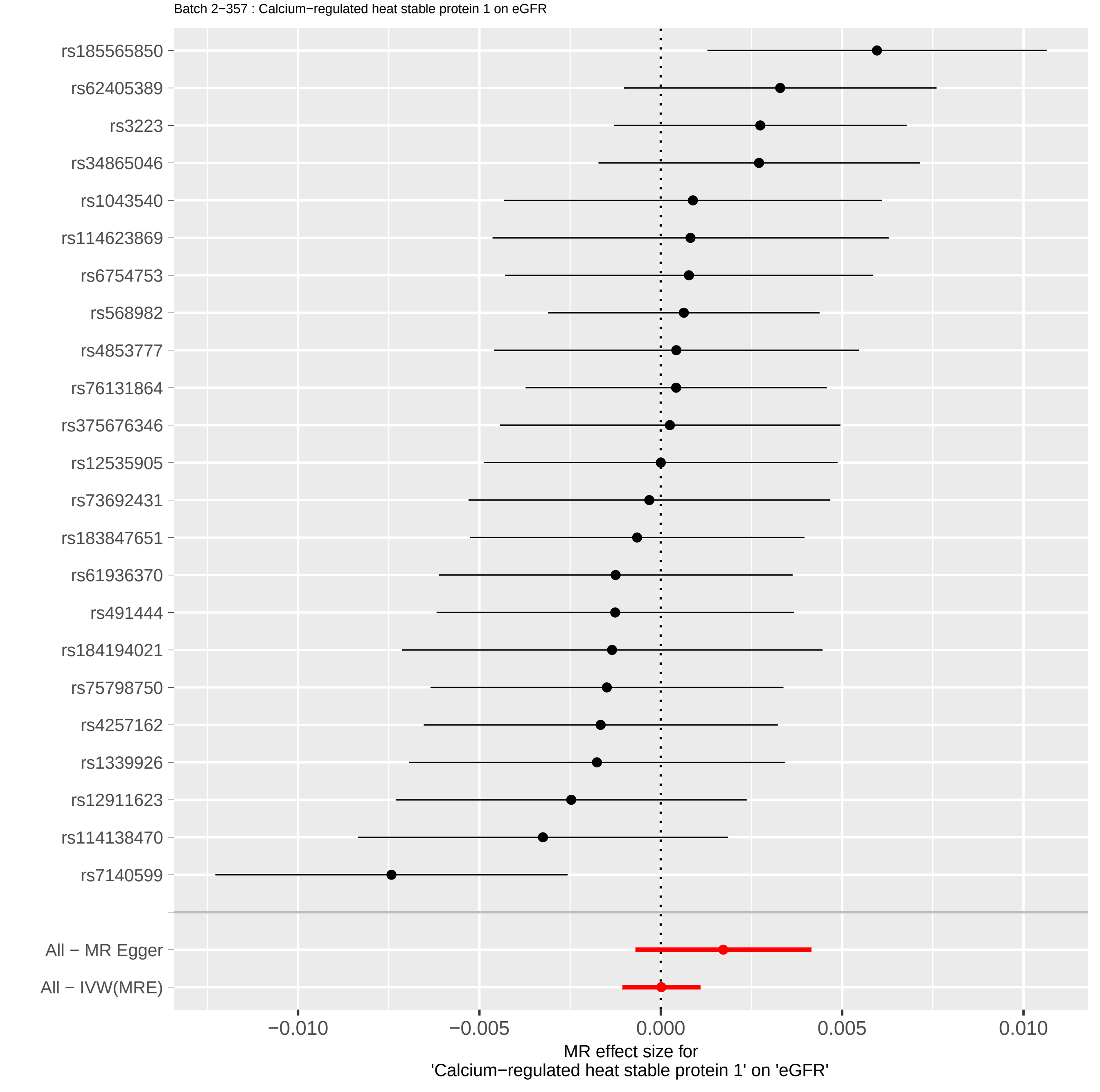
Batch 2−357 : Calcium−regulated heat stable protein 1 on eGFR
rs185565850
rs62405389
rs3223
rs34865046
rs1043540
rs114623869
rs6754753
rs568982
rs4853777
rs76131864
rs375676346
rs12535905
rs73692431
rs183847651
rs61936370
rs491444
rs184194021
rs75798750
rs4257162
rs1339926
rs12911623
rs114138470
rs7140599
All − MR Egger
All − IVW(MRE)
−0.010
−0.005
0.000
0.005
0.010
MR effect size for
'Calcium−regulated heat stable protein 1' on 'eGFR'

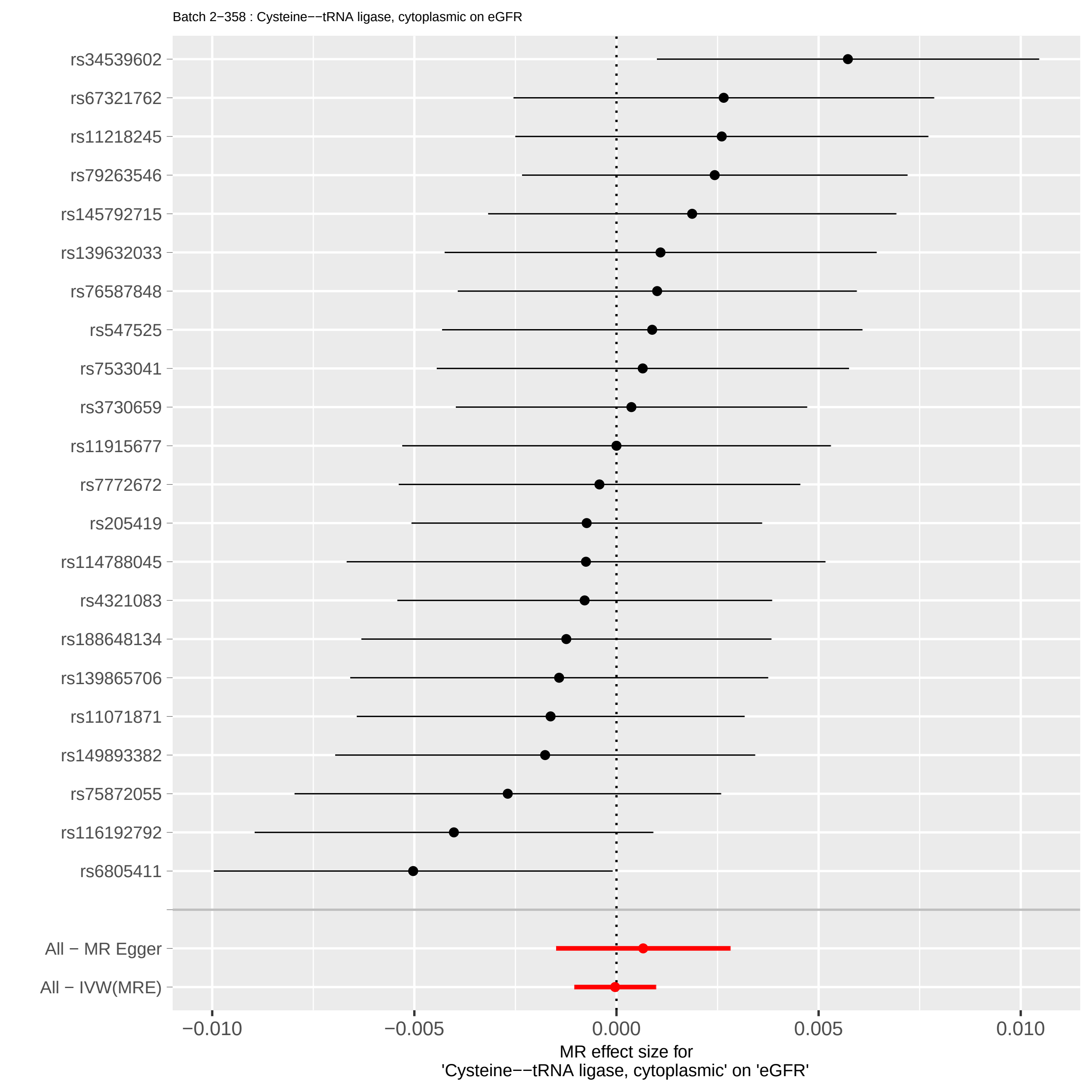
Batch 2−358 : Cysteine−−tRNA ligase, cytoplasmic on eGFR
rs34539602
rs67321762
rs11218245
rs79263546
rs145792715
rs139632033
rs76587848
rs547525
rs7533041
rs3730659
rs11915677
rs7772672
rs205419
rs114788045
rs4321083
rs188648134
rs139865706
rs11071871
rs149893382
rs75872055
rs116192792
rs6805411
All − MR Egger
All − IVW(MRE)
−0.010
−0.005
0.000
0.005
0.010
MR effect size for
'Cysteine−−tRNA ligase, cytoplasmic' on 'eGFR'

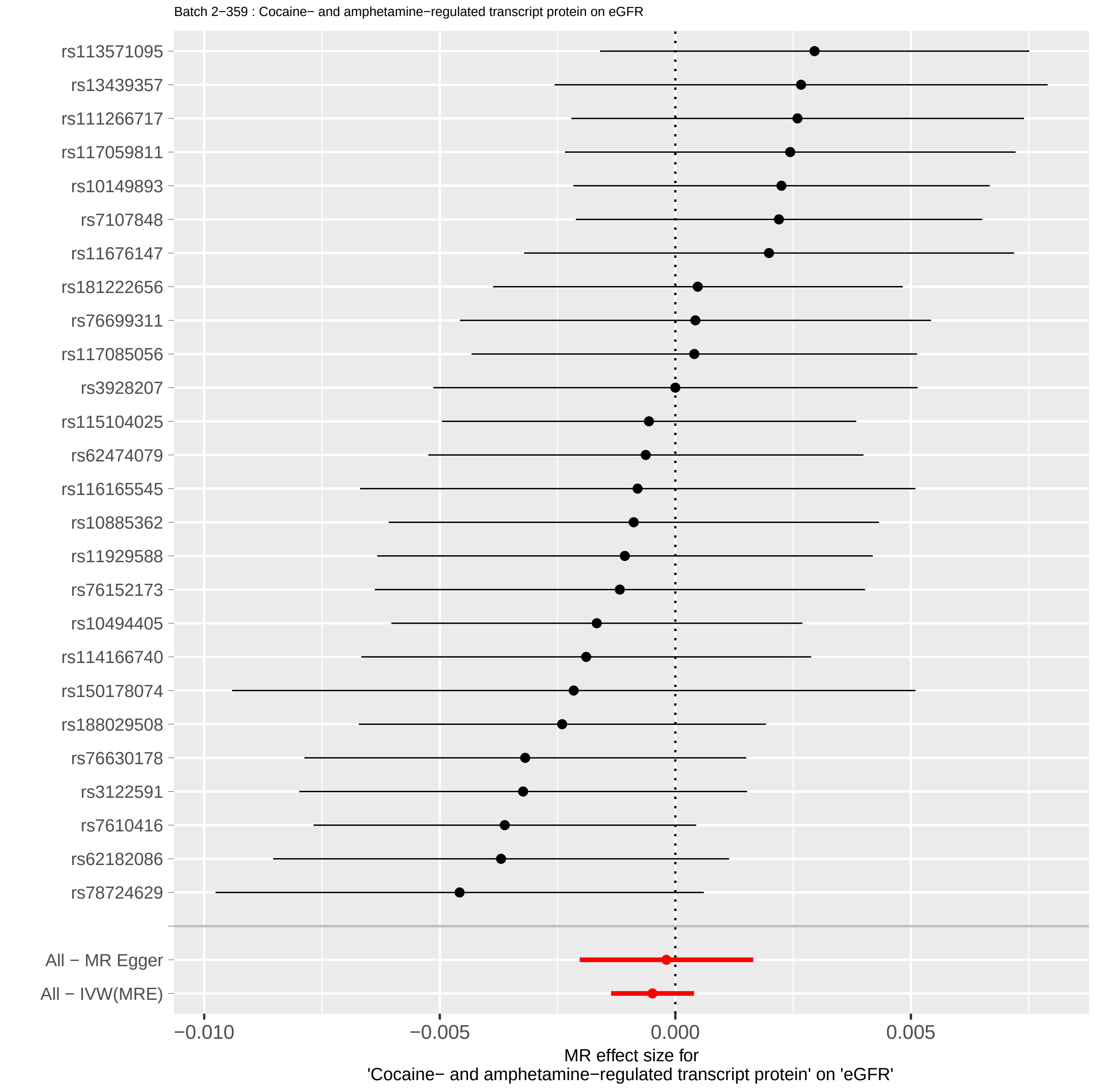
Batch 2−359 : Cocaine− and amphetamine−regulated transcript protein on eGFR
rs113571095
rs13439357
rs111266717
rs117059811
rs10149893
rs7107848
rs11676147
rs181222656
rs76699311
rs117085056
rs3928207
rs115104025
rs62474079
rs116165545
rs10885362
rs11929588
rs76152173
rs10494405
rs114166740
rs150178074
rs188029508
rs76630178
rs3122591
rs7610416
rs62182086
rs78724629
All − MR Egger
All − IVW(MRE)
−0.010
−0.005
0.000
0.005
MR effect size for
'Cocaine− and amphetamine−regulated transcript protein' on 'eGFR'

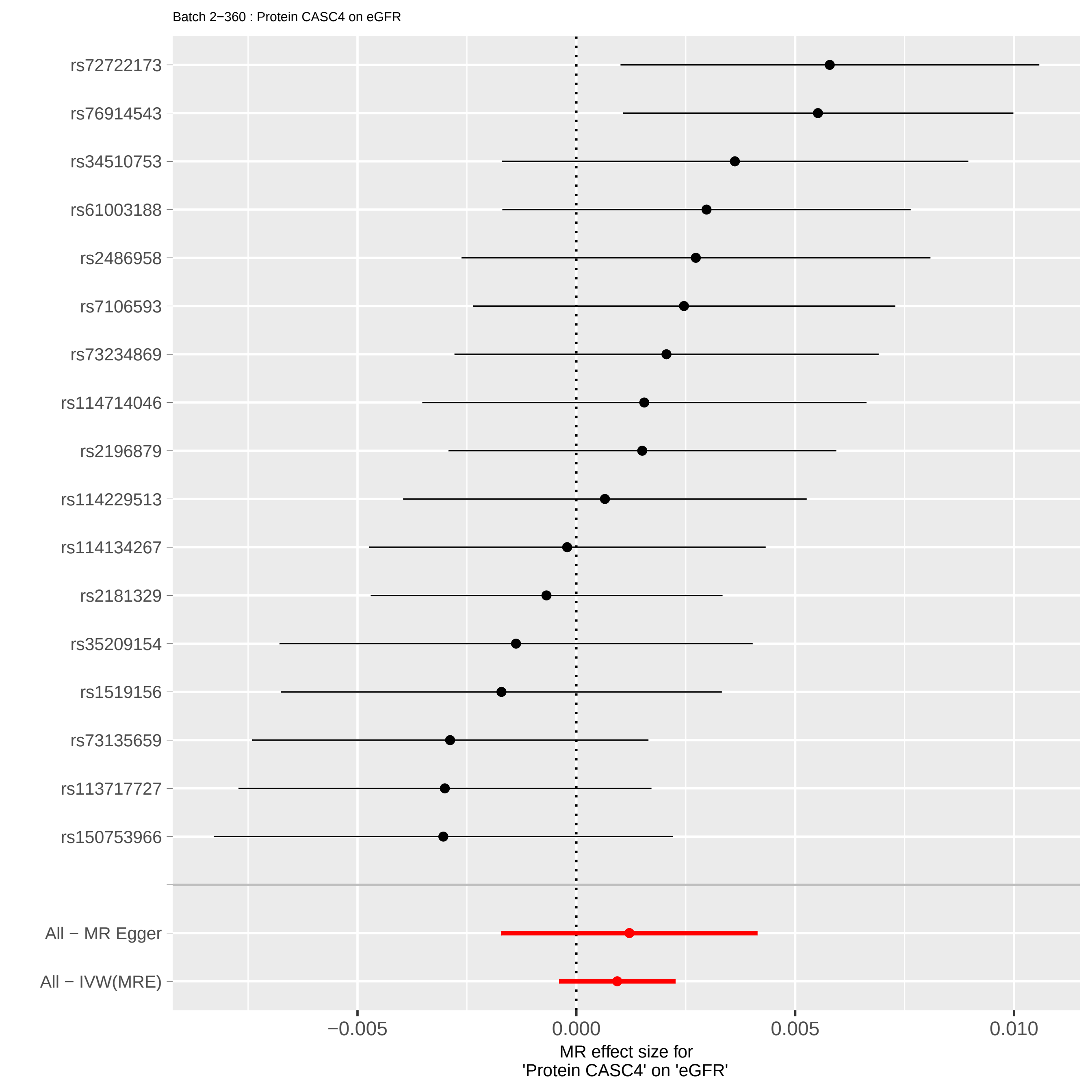
Batch 2−360 : Protein CASC4 on eGFR
rs72722173
rs76914543
rs34510753
rs61003188
rs2486958
rs7106593
rs73234869
rs114714046
rs2196879
rs114229513
rs114134267
rs2181329
rs35209154
rs1519156
rs73135659
rs113717727
rs150753966
All − MR Egger
All − IVW(MRE)
−0.005
0.000
0.005
0.010
MR effect size for
'Protein CASC4' on 'eGFR'

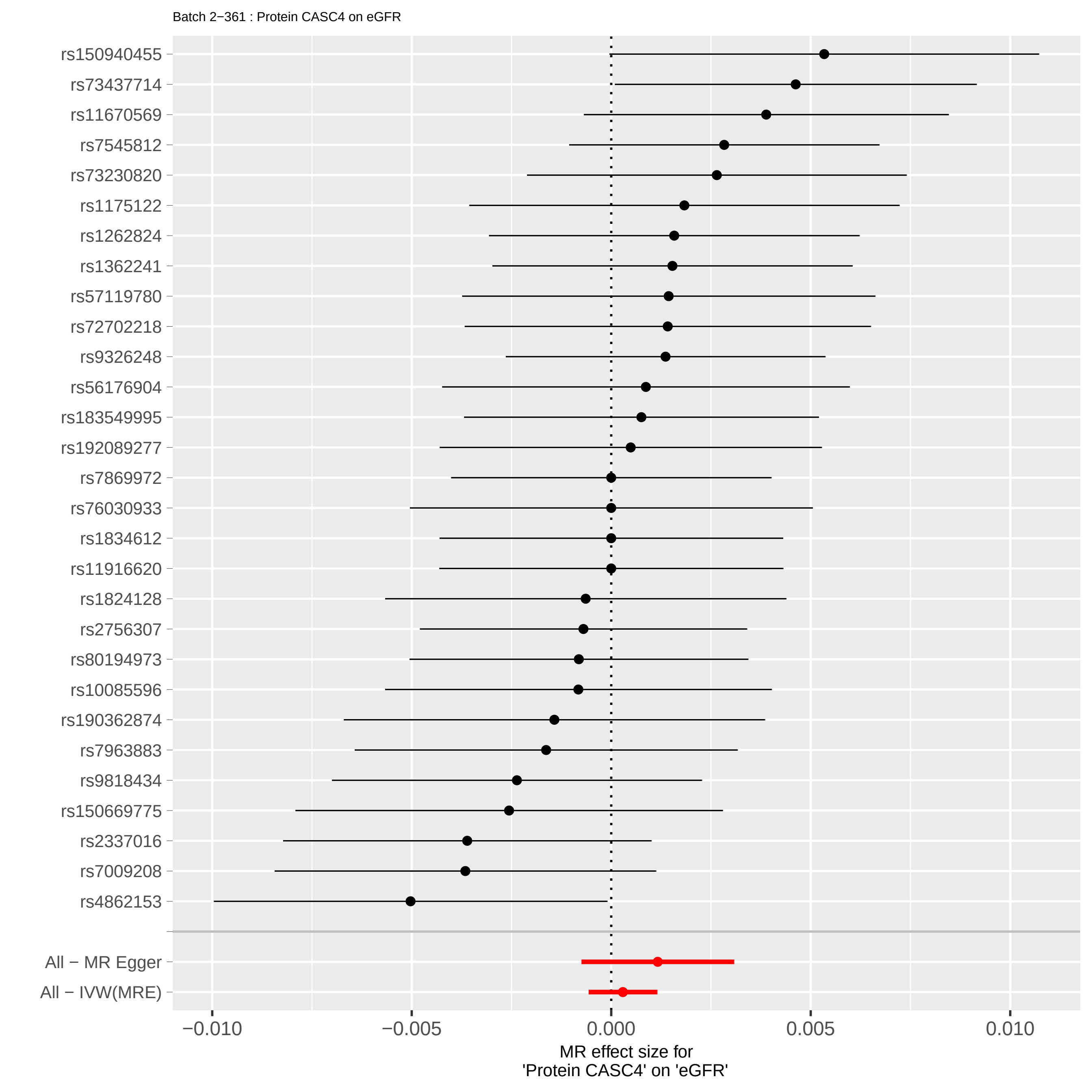

Batch 2−361 : Protein CASC4 on eGFR
rs150940455
rs73437714
rs11670569
rs7545812
rs73230820
rs1175122
rs1262824
rs1362241
rs57119780
rs72702218
rs9326248
rs56176904
rs183549995
rs192089277
rs7869972
rs76030933
rs1834612
rs11916620
rs1824128
rs2756307
rs80194973
rs10085596
rs190362874
rs7963883
rs9818434
rs150669775
rs2337016
rs7009208
rs4862153
All − MR Egger
All − IVW(MRE)
−0.010
−0.005
0.000
0.005
0.010
MR effect size for
'Protein CASC4' on 'eGFR'

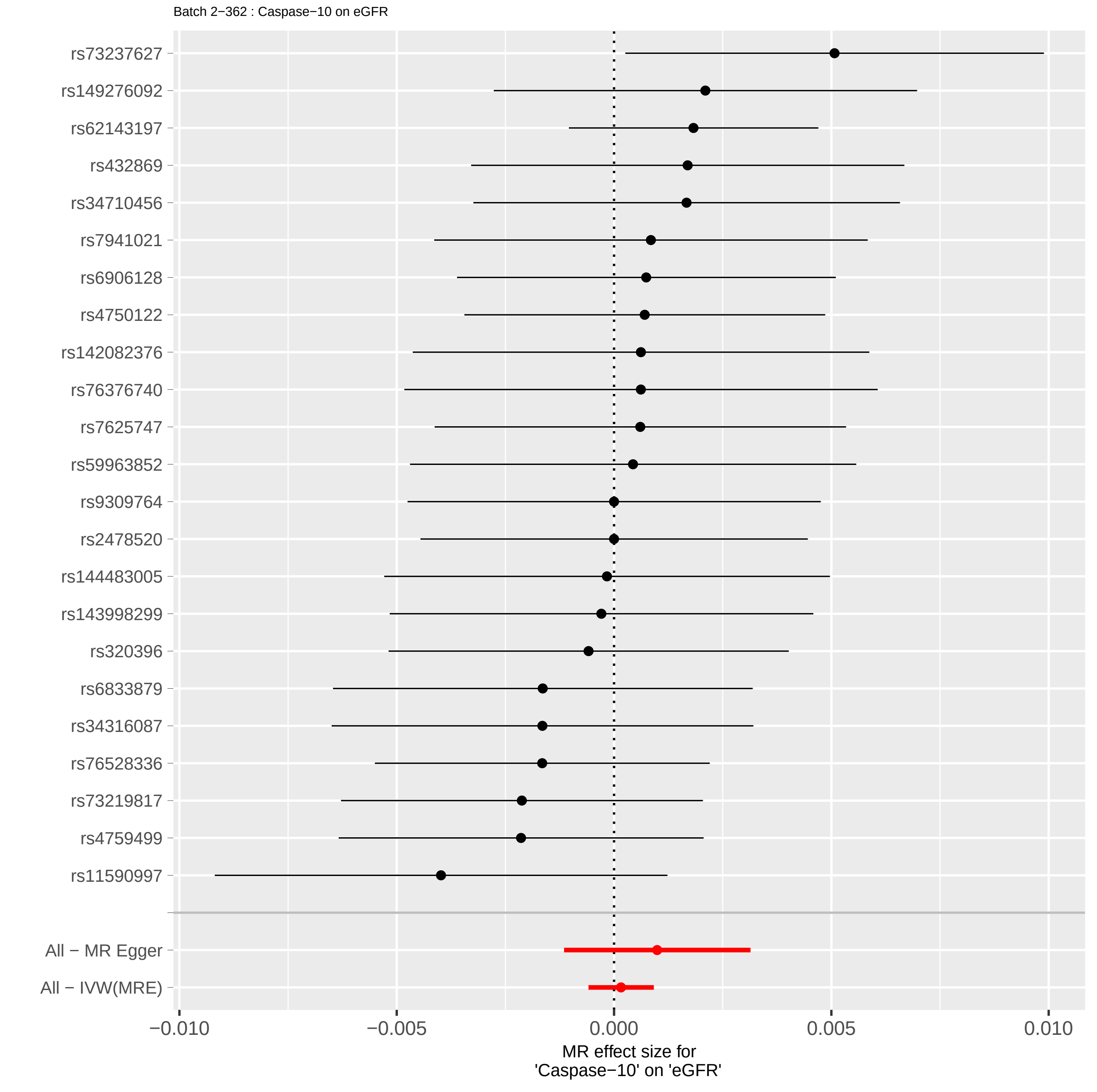

Batch 2−362 : Caspase−10 on eGFR
rs73237627
rs149276092
rs62143197
rs432869
rs34710456
rs7941021
rs6906128
rs4750122
rs142082376
rs76376740
rs7625747
rs59963852
rs9309764
rs2478520
rs144483005
rs143998299
rs320396
rs6833879
rs34316087
rs76528336
rs73219817
rs4759499
rs11590997
All − MR Egger
All − IVW(MRE)
−0.010
−0.005
0.000
0.005
0.010
MR effect size for
'Caspase−10' on 'eGFR'

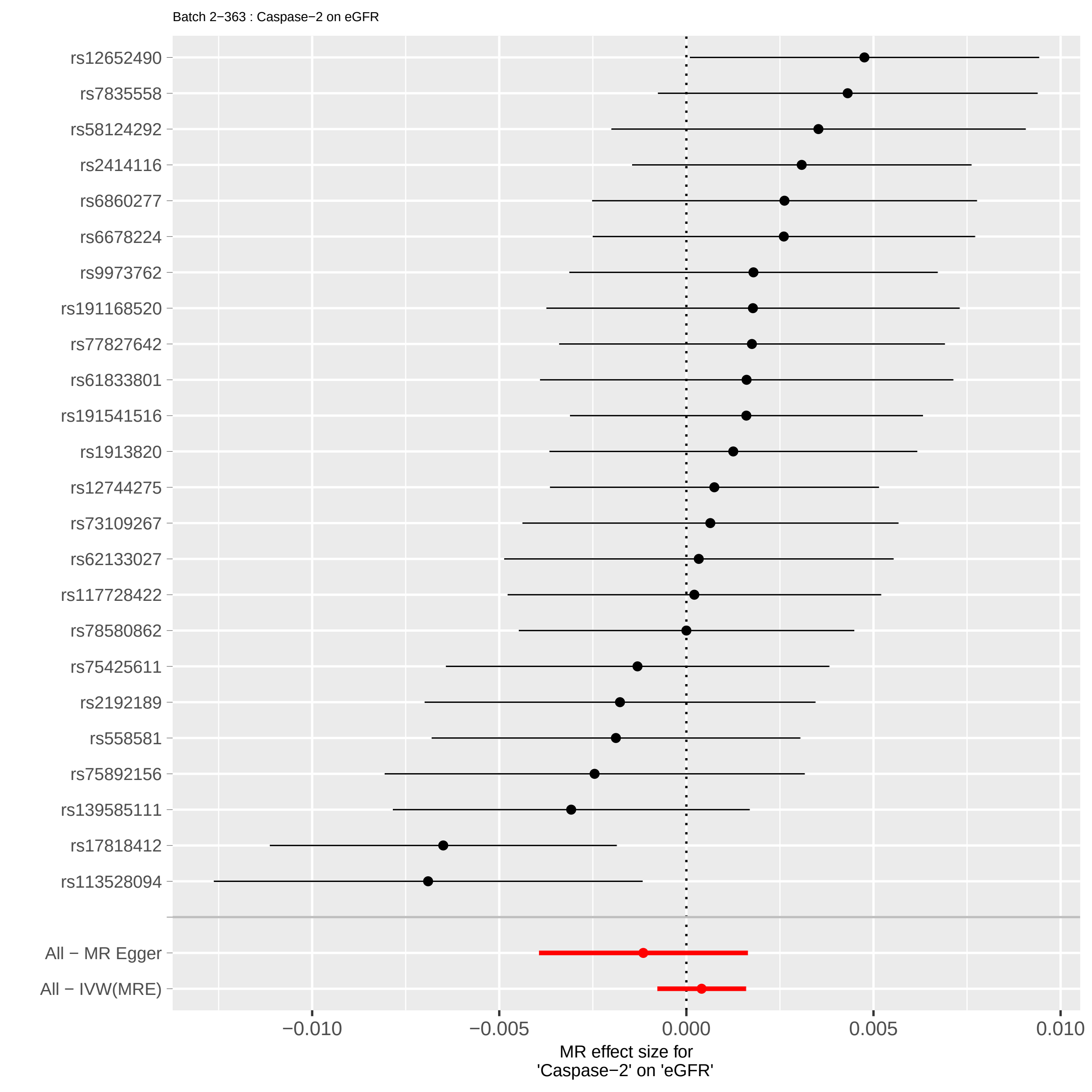
Batch 2−363 : Caspase−2 on eGFR
rs12652490
rs7835558
rs58124292
rs2414116
rs6860277
rs6678224
rs9973762
rs191168520
rs77827642
rs61833801
rs191541516
rs1913820
rs12744275
rs73109267
rs62133027
rs117728422
rs78580862
rs75425611
rs2192189
rs558581
rs75892156
rs139585111
rs17818412
rs113528094
All − MR Egger
All − IVW(MRE)
−0.010
−0.005
0.000
0.005
0.010
MR effect size for
'Caspase−2' on 'eGFR'

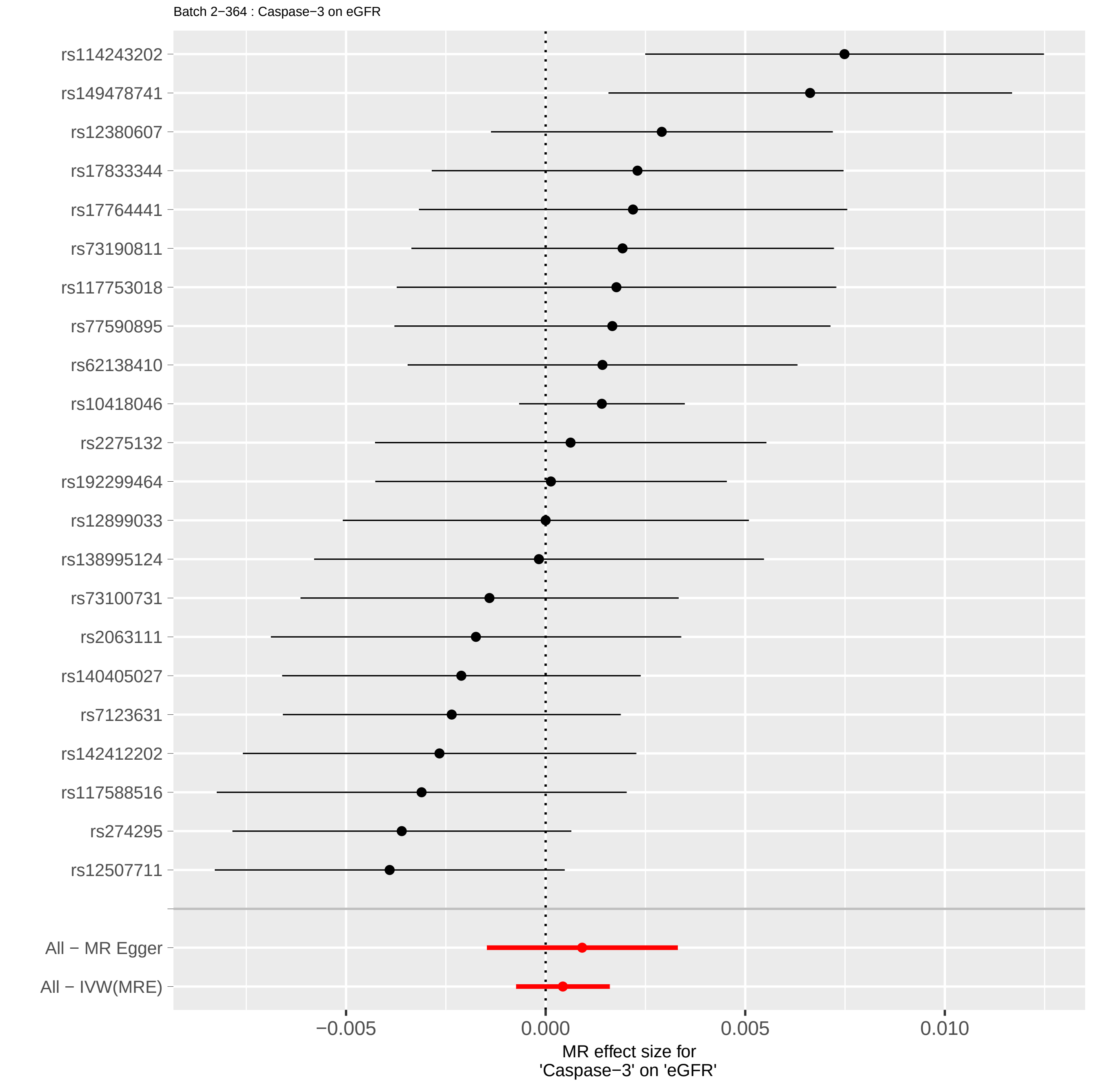

Batch 2−364 : Caspase−3 on eGFR
rs114243202
rs149478741
rs12380607
rs17833344
rs17764441
rs73190811
rs117753018
rs77590895
rs62138410
rs10418046
rs2275132
rs192299464
rs12899033
rs138995124
rs73100731
rs2063111
rs140405027
rs7123631
rs142412202
rs117588516
rs274295
rs12507711
All − MR Egger
All − IVW(MRE)
−0.005
0.000
0.005
0.010
MR effect size for
'Caspase−3' on 'eGFR'

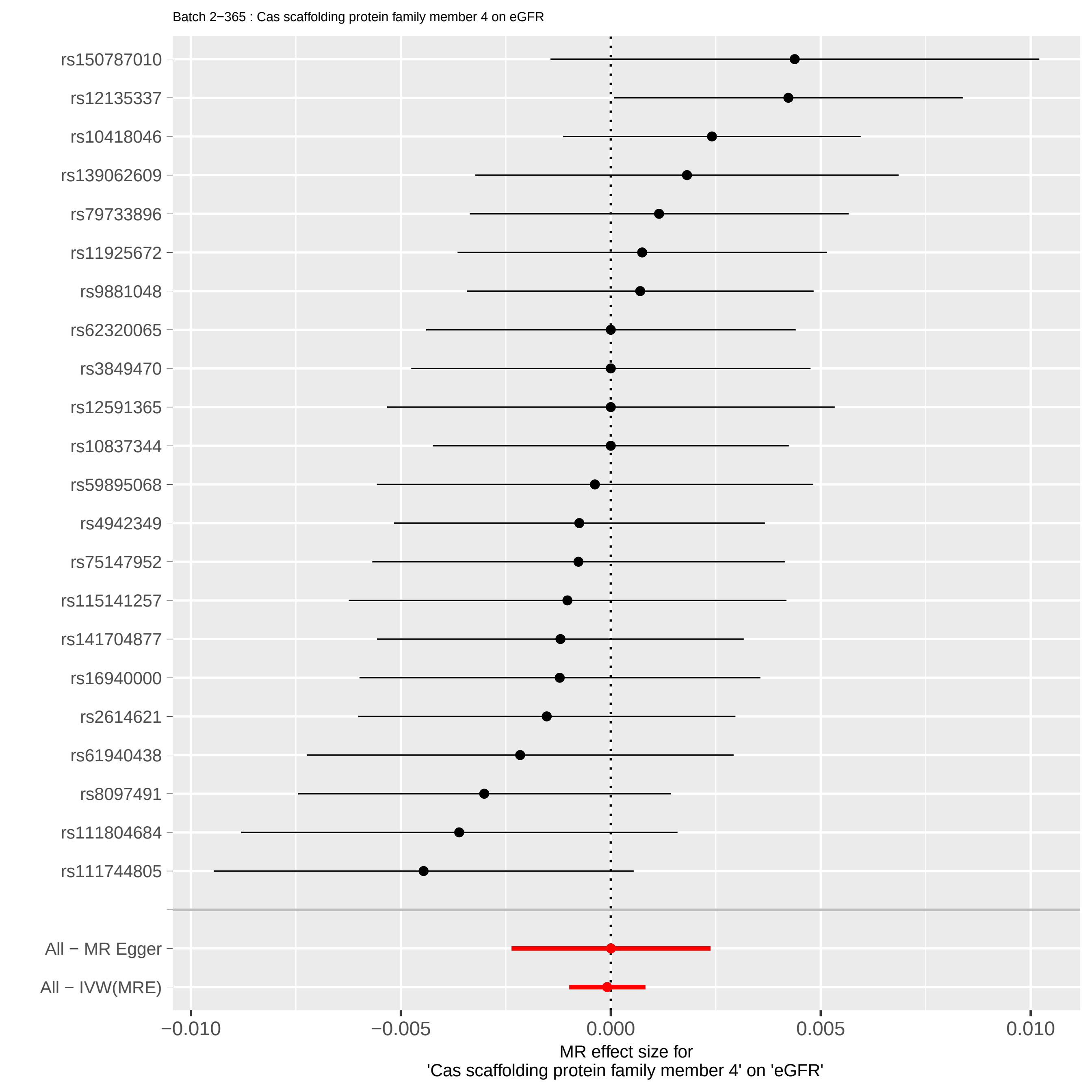

Batch 2−365 : Cas scaffolding protein family member 4 on eGFR
rs150787010
rs12135337
rs10418046
rs139062609
rs79733896
rs11925672
rs9881048
rs62320065
rs3849470
rs12591365
rs10837344
rs59895068
rs4942349
rs75147952
rs115141257
rs141704877
rs16940000
rs2614621
rs61940438
rs8097491
rs111804684
rs111744805
All − MR Egger
All − IVW(MRE)
−0.010
−0.005
0.000
0.005
0.010
MR effect size for
'Cas scaffolding protein family member 4' on 'eGFR'

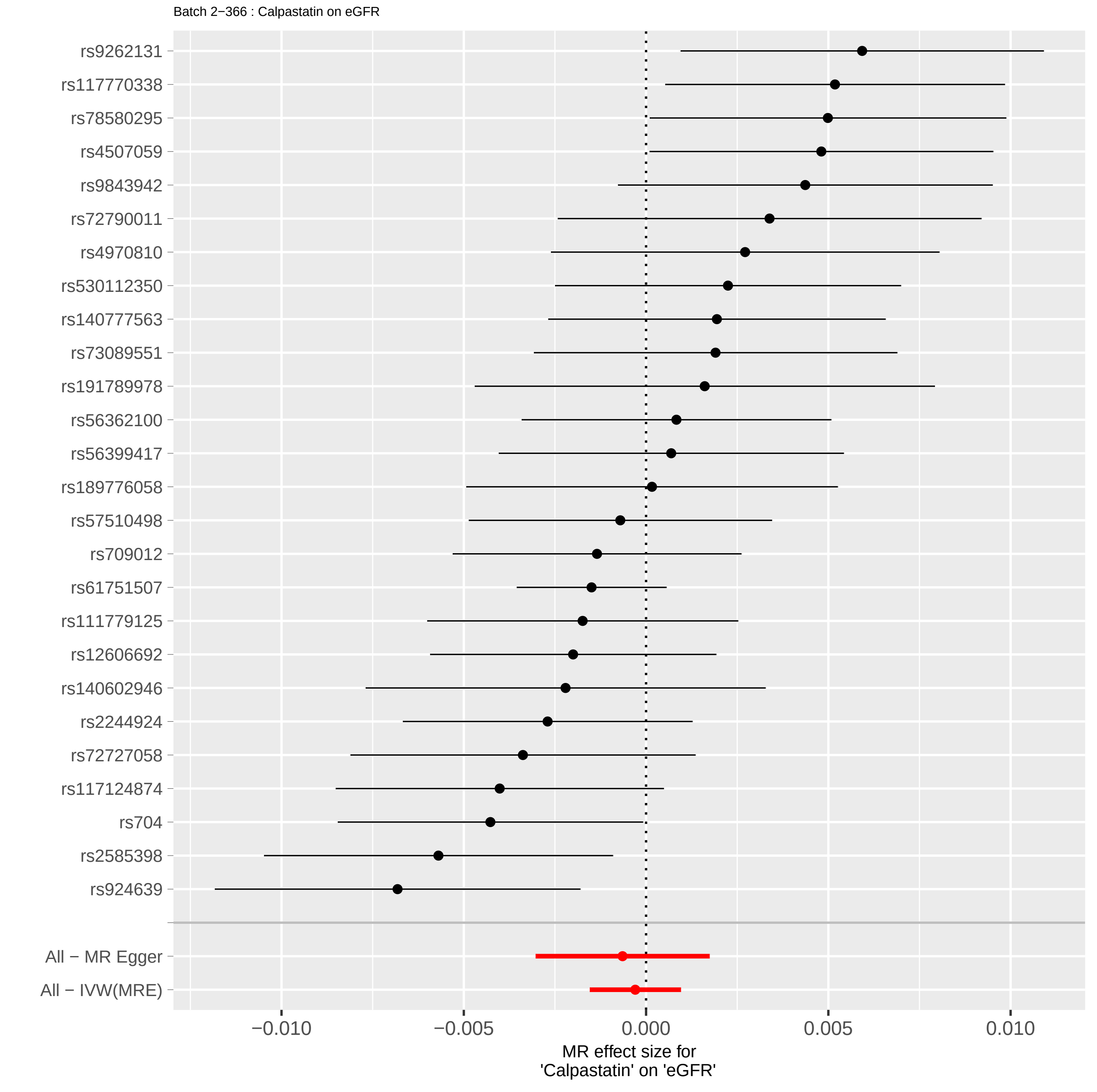

Batch 2−366 : Calpastatin on eGFR
rs9262131
rs117770338
rs78580295
rs4507059
rs9843942
rs72790011
rs4970810
rs530112350
rs140777563
rs73089551
rs191789978
rs56362100
rs56399417
rs189776058
rs57510498
rs709012
rs61751507
rs111779125
rs12606692
rs140602946
rs2244924
rs72727058
rs117124874
rs704
rs2585398
rs924639
All − MR Egger
All − IVW(MRE)
−0.010
−0.005
0.000
0.005
0.010
MR effect size for
'Calpastatin' on 'eGFR'

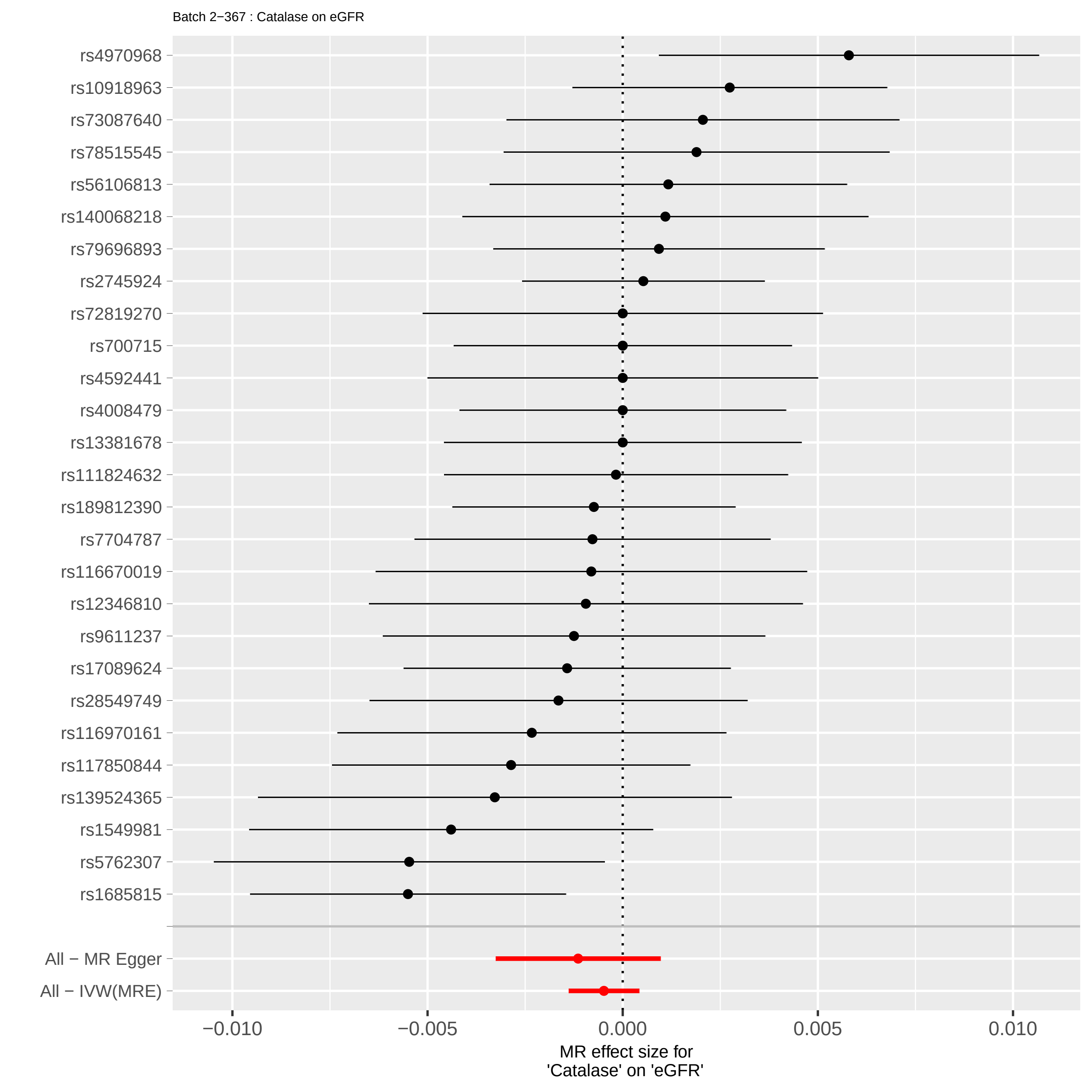

Batch 2−367 : Catalase on eGFR
rs4970968
rs10918963
rs73087640
rs78515545
rs56106813
rs140068218
rs79696893
rs2745924
rs72819270
rs700715
rs4592441
rs4008479
rs13381678
rs111824632
rs189812390
rs7704787
rs116670019
rs12346810
rs9611237
rs17089624
rs28549749
rs116970161
rs117850844
rs139524365
rs1549981
rs5762307
rs1685815
All − MR Egger
All − IVW(MRE)
−0.010
−0.005
0.000
0.005
0.010
MR effect size for
'Catalase' on 'eGFR'

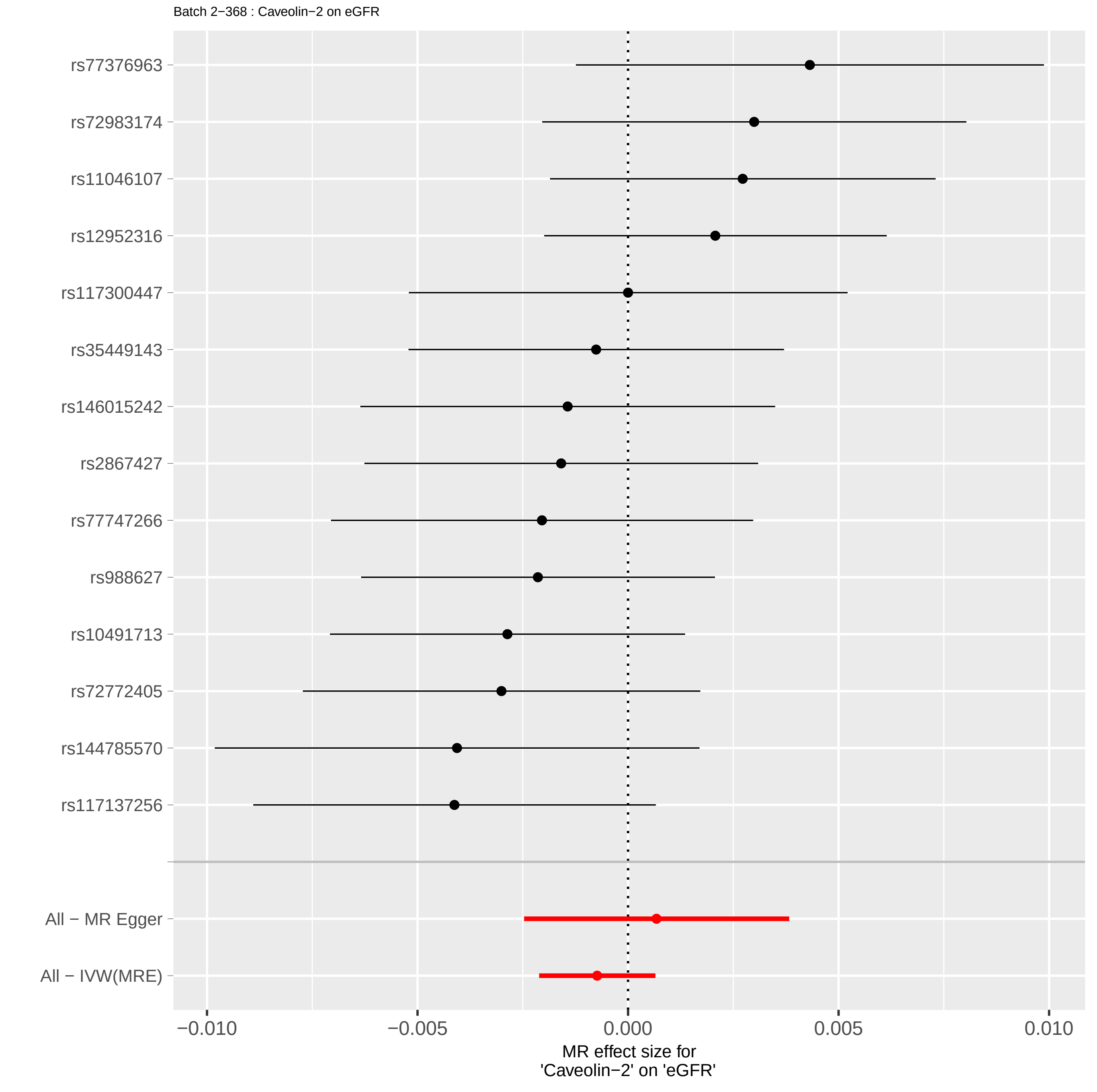

Batch 2−368 : Caveolin−2 on eGFR
rs77376963
rs72983174
rs11046107
rs12952316
rs117300447
rs35449143
rs146015242
rs2867427
rs77747266
rs988627
rs10491713
rs72772405
rs144785570
rs117137256
All − MR Egger
All − IVW(MRE)
−0.010
−0.005
0.000
0.005
0.010
MR effect size for
'Caveolin−2' on 'eGFR'

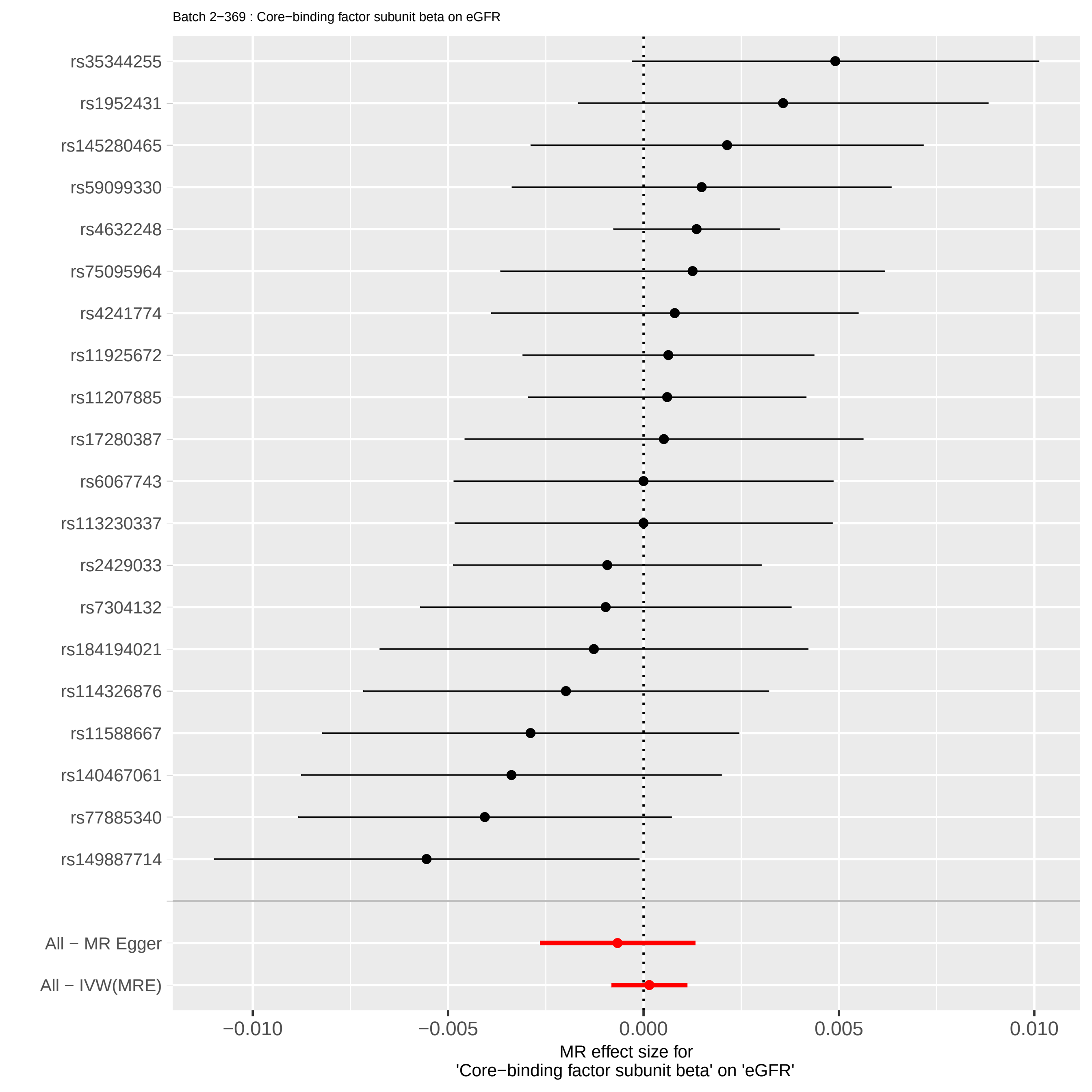

Batch 2−369 : Core−binding factor subunit beta on eGFR
rs35344255
rs1952431
rs145280465
rs59099330
rs4632248
rs75095964
rs4241774
rs11925672
rs11207885
rs17280387
rs6067743
rs113230337
rs2429033
rs7304132
rs184194021
rs114326876
rs11588667
rs140467061
rs77885340
rs149887714
All − MR Egger
All − IVW(MRE)
−0.010
−0.005
0.000
0.005
0.010
MR effect size for
'Core−binding factor subunit beta' on 'eGFR'

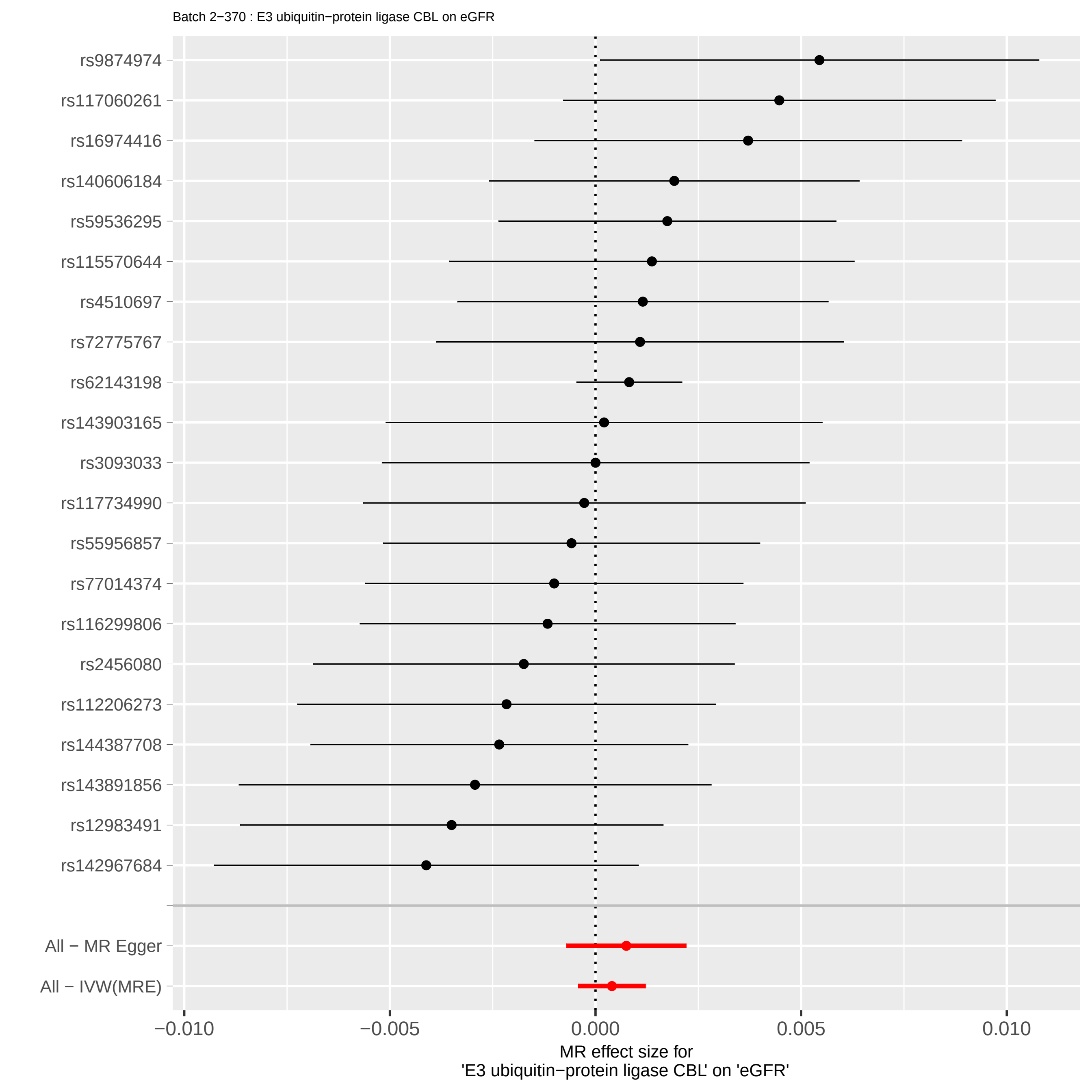
Batch 2−370 : E3 ubiquitin−protein ligase CBL on eGFR
rs9874974
rs117060261
rs16974416
rs140606184
rs59536295
rs115570644
rs4510697
rs72775767
rs62143198
rs143903165
rs3093033
rs117734990
rs55956857
rs77014374
rs116299806
rs2456080
rs112206273
rs144387708
rs143891856
rs12983491
rs142967684
All − MR Egger
All − IVW(MRE)
−0.010
−0.005
0.000
0.005
0.010
MR effect size for
'E3 ubiquitin−protein ligase CBL' on 'eGFR'

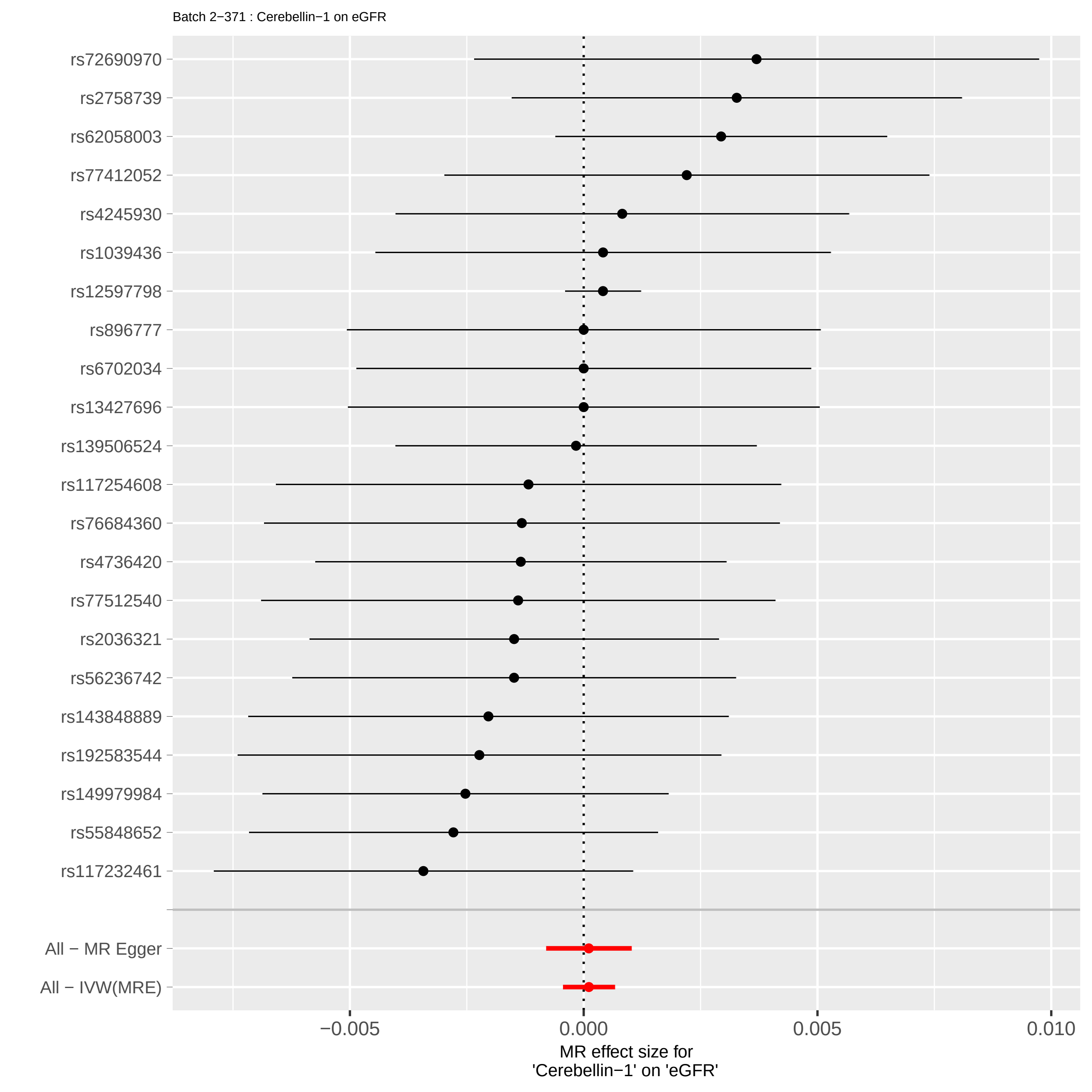
Batch 2−371 : Cerebellin−1 on eGFR
rs72690970
rs2758739
rs62058003
rs77412052
rs4245930
rs1039436
rs12597798
rs896777
rs6702034
rs13427696
rs139506524
rs117254608
rs76684360
rs4736420
rs77512540
rs2036321
rs56236742
rs143848889
rs192583544
rs149979984
rs55848652
rs117232461
All − MR Egger
All − IVW(MRE)
−0.005
0.000
0.005
0.010
MR effect size for
'Cerebellin−1' on 'eGFR'

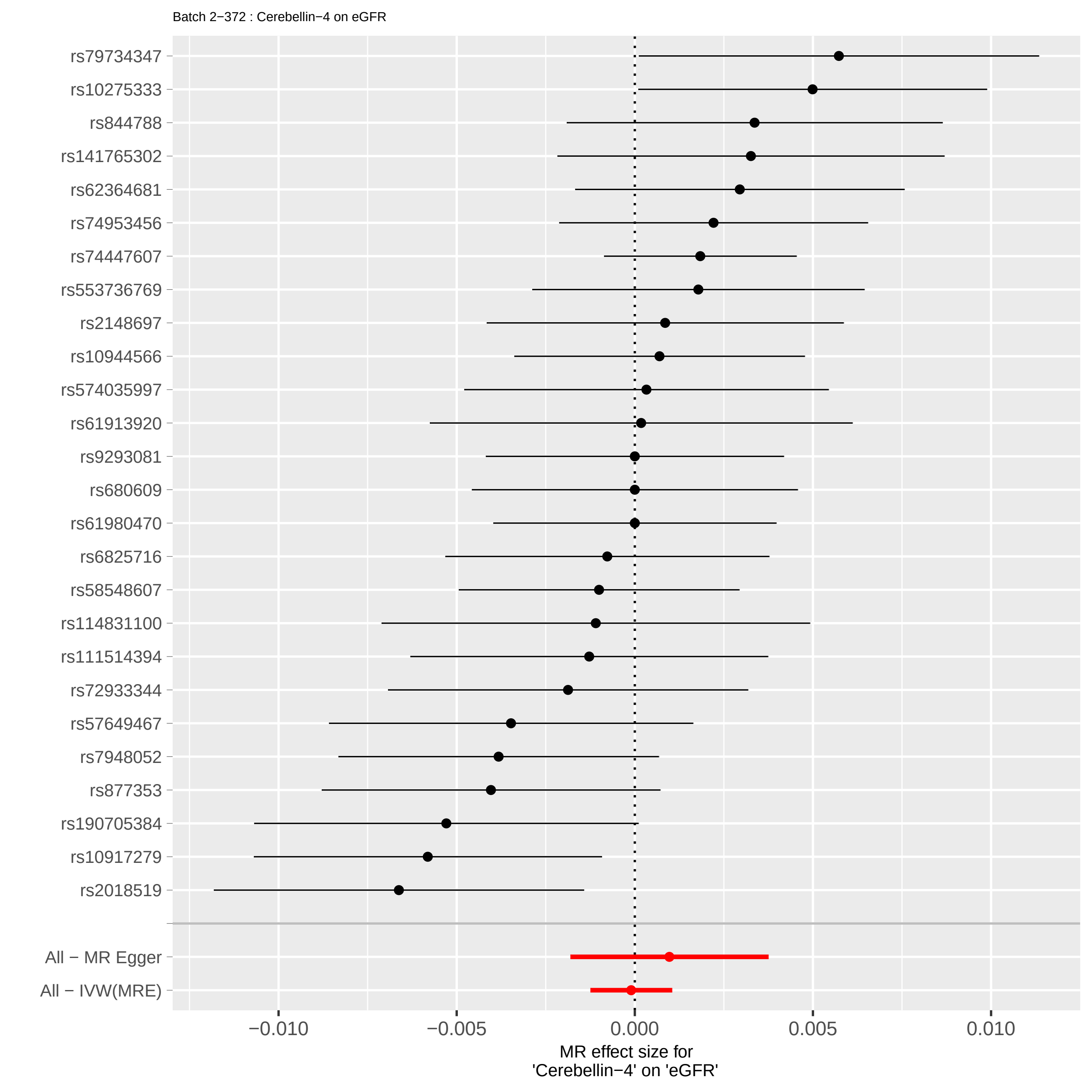

Batch 2−372 : Cerebellin−4 on eGFR
rs79734347
rs10275333
rs844788
rs141765302
rs62364681
rs74953456
rs74447607
rs553736769
rs2148697
rs10944566
rs574035997
rs61913920
rs9293081
rs680609
rs61980470
rs6825716
rs58548607
rs114831100
rs111514394
rs72933344
rs57649467
rs7948052
rs877353
rs190705384
rs10917279
rs2018519
All − MR Egger
All − IVW(MRE)
−0.010
−0.005
0.000
0.005
0.010
MR effect size for
'Cerebellin−4' on 'eGFR'

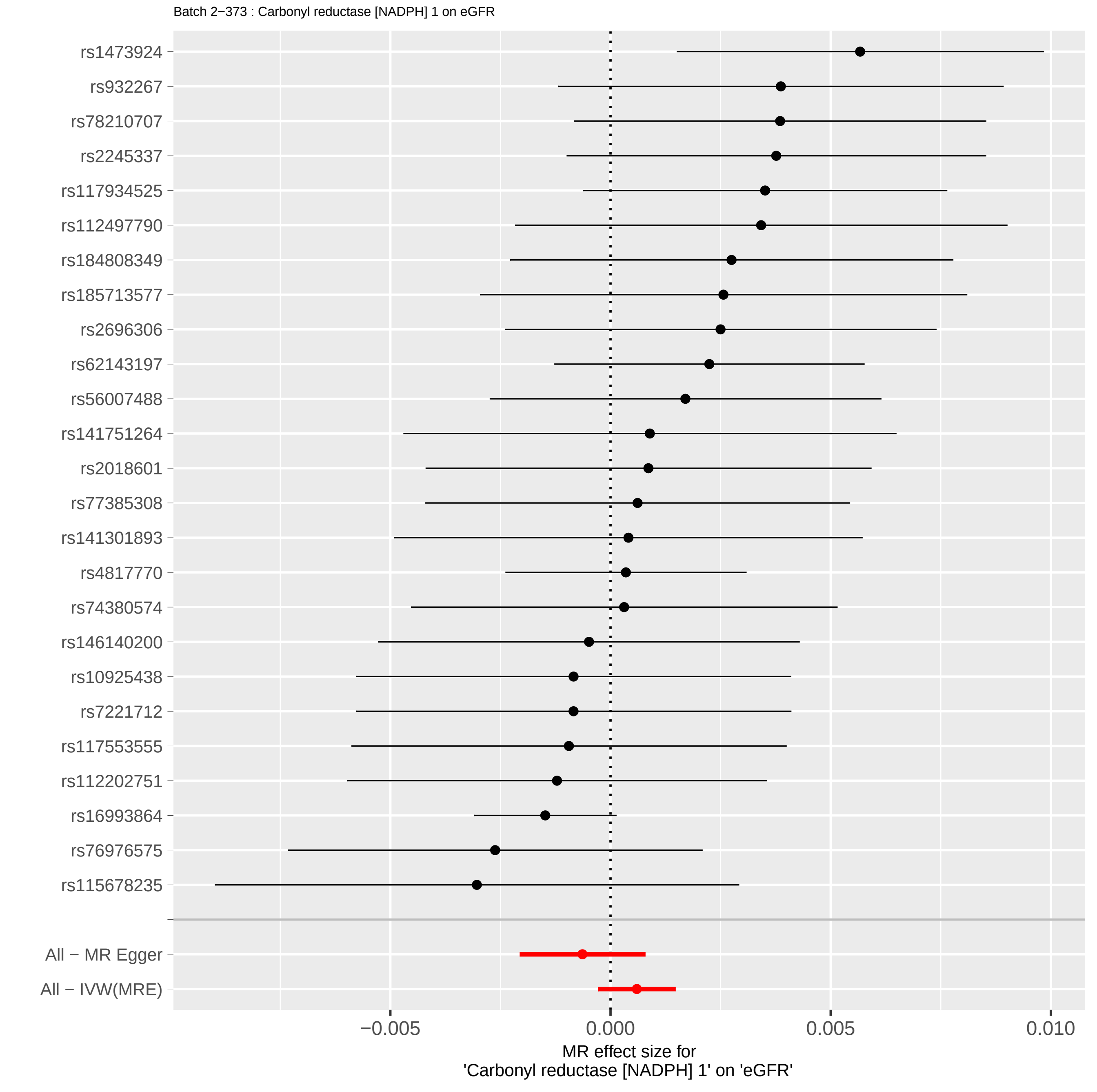

Batch 2−373 : Carbonyl reductase [NADPH] 1 on eGFR
rs1473924
rs932267
rs78210707
rs2245337
rs117934525
rs112497790
rs184808349
rs185713577
rs2696306
rs62143197
rs56007488
rs141751264
rs2018601
rs77385308
rs141301893
rs4817770
rs74380574
rs146140200
rs10925438
rs7221712
rs117553555
rs112202751
rs16993864
rs76976575
rs115678235
All − MR Egger
All − IVW(MRE)
−0.005
0.000
0.005
0.010
MR effect size for
'Carbonyl reductase [NADPH] 1' on 'eGFR'

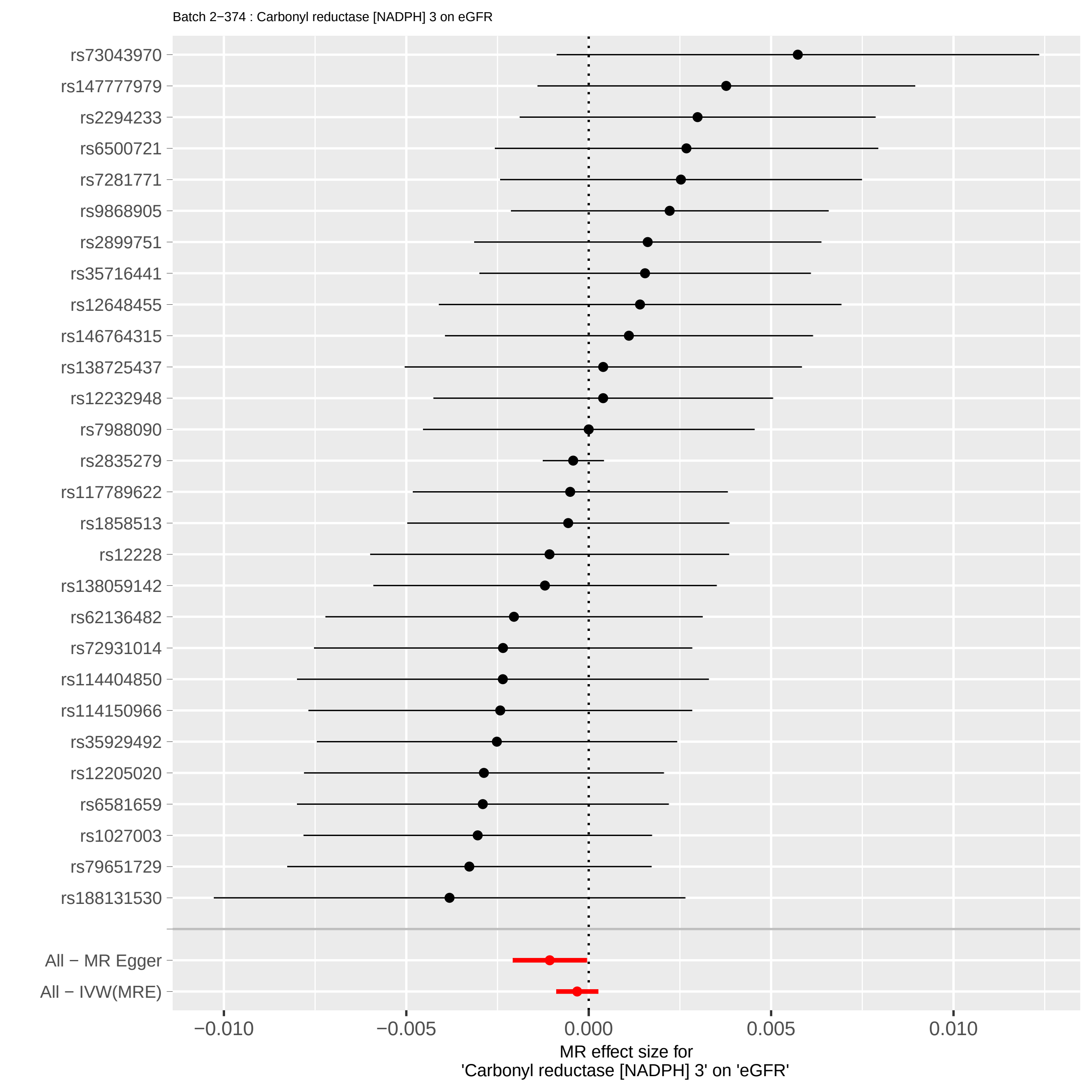

Batch 2−374 : Carbonyl reductase [NADPH] 3 on eGFR
rs73043970
rs147777979
rs2294233
rs6500721
rs7281771
rs9868905
rs2899751
rs35716441
rs12648455
rs146764315
rs138725437
rs12232948
rs7988090
rs2835279
rs117789622
rs1858513
rs12228
rs138059142
rs62136482
rs72931014
rs114404850
rs114150966
rs35929492
rs12205020
rs6581659
rs1027003
rs79651729
rs188131530
All − MR Egger
All − IVW(MRE)
−0.010
−0.005
0.000
0.005
0.010
MR effect size for
'Carbonyl reductase [NADPH] 3' on 'eGFR'

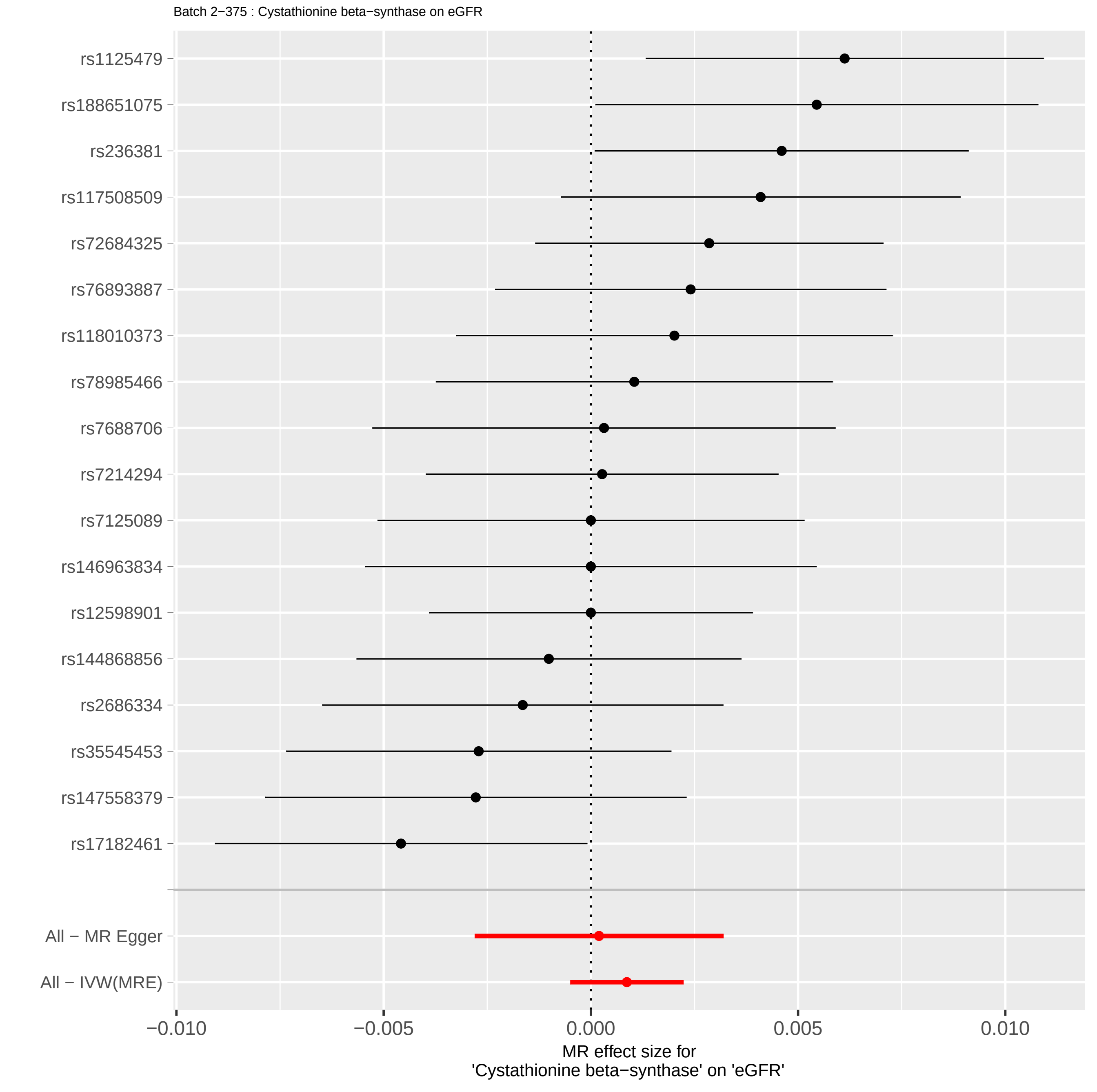
Batch 2−375 : Cystathionine beta−synthase on eGFR
rs1125479
rs188651075
rs236381
rs117508509
rs72684325
rs76893887
rs118010373
rs78985466
rs7688706
rs7214294
rs7125089
rs146963834
rs12598901
rs144868856
rs2686334
rs35545453
rs147558379
rs17182461
All − MR Egger
All − IVW(MRE)
−0.010
−0.005
0.000
0.005
0.010
MR effect size for
'Cystathionine beta−synthase' on 'eGFR'

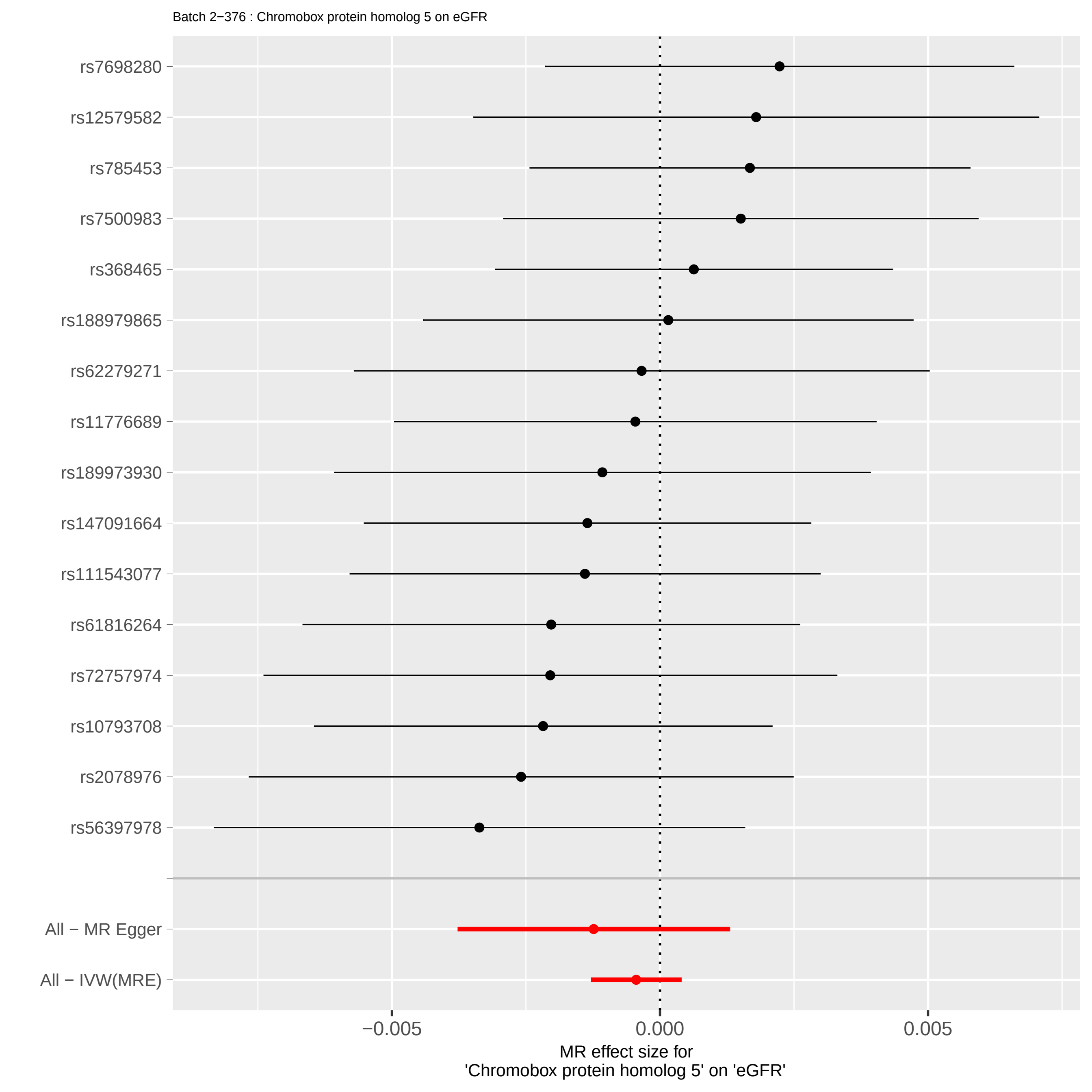
Batch 2−376 : Chromobox protein homolog 5 on eGFR
rs7698280
rs12579582
rs785453
rs7500983
rs368465
rs188979865
rs62279271
rs11776689
rs189973930
rs147091664
rs111543077
rs61816264
rs72757974
rs10793708
rs2078976
rs56397978
All − MR Egger
All − IVW(MRE)
−0.005
0.000
0.005
MR effect size for
'Chromobox protein homolog 5' on 'eGFR'

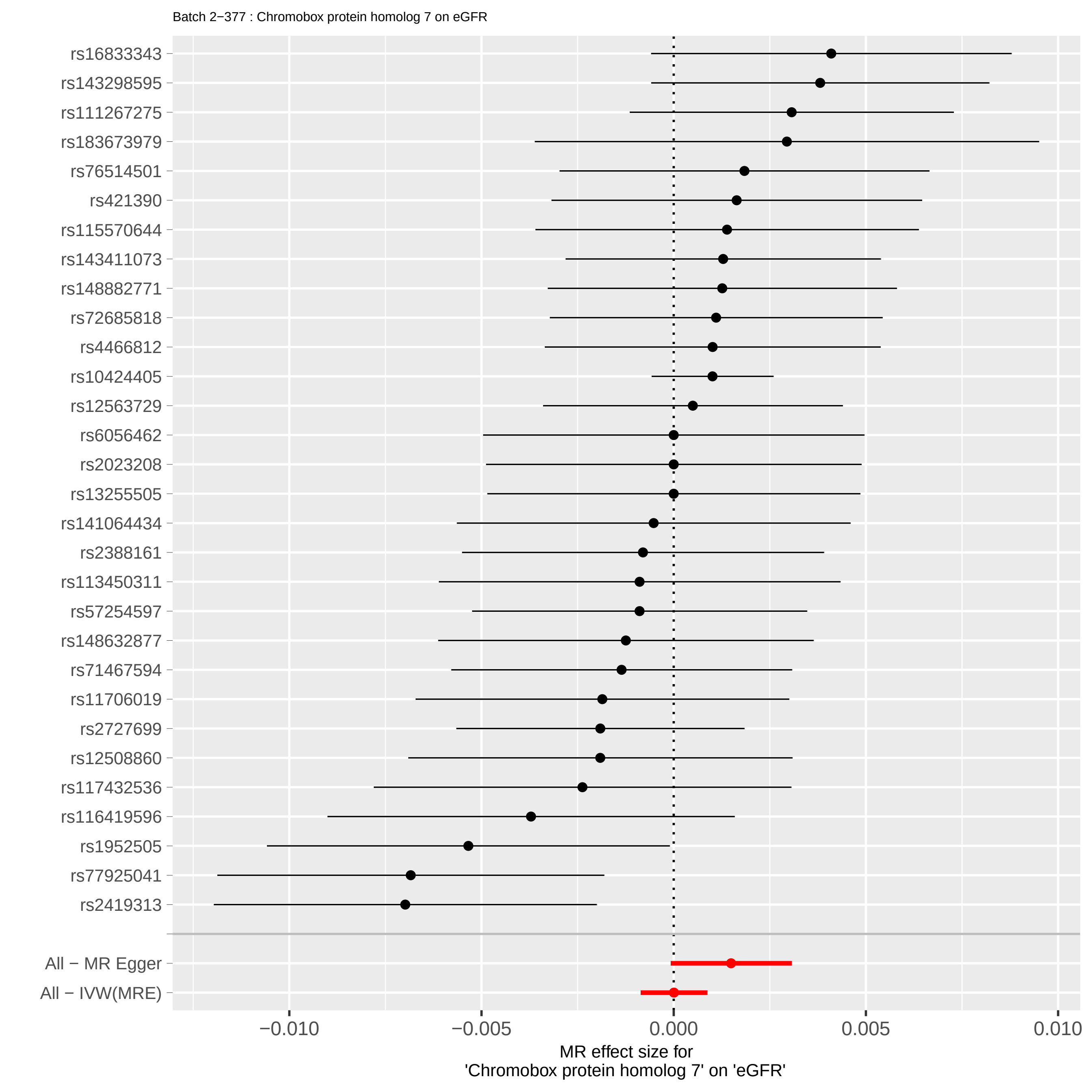

Batch 2−377 : Chromobox protein homolog 7 on eGFR
rs16833343
rs143298595
rs111267275
rs183673979
rs76514501
rs421390
rs115570644
rs143411073
rs148882771
rs72685818
rs4466812
rs10424405
rs12563729
rs6056462
rs2023208
rs13255505
rs141064434
rs2388161
rs113450311
rs57254597
rs148632877
rs71467594
rs11706019
rs2727699
rs12508860
rs117432536
rs116419596
rs1952505
rs77925041
rs2419313
All − MR Egger
All − IVW(MRE)
−0.010
−0.005
0.000
0.005
0.010
MR effect size for
'Chromobox protein homolog 7' on 'eGFR'

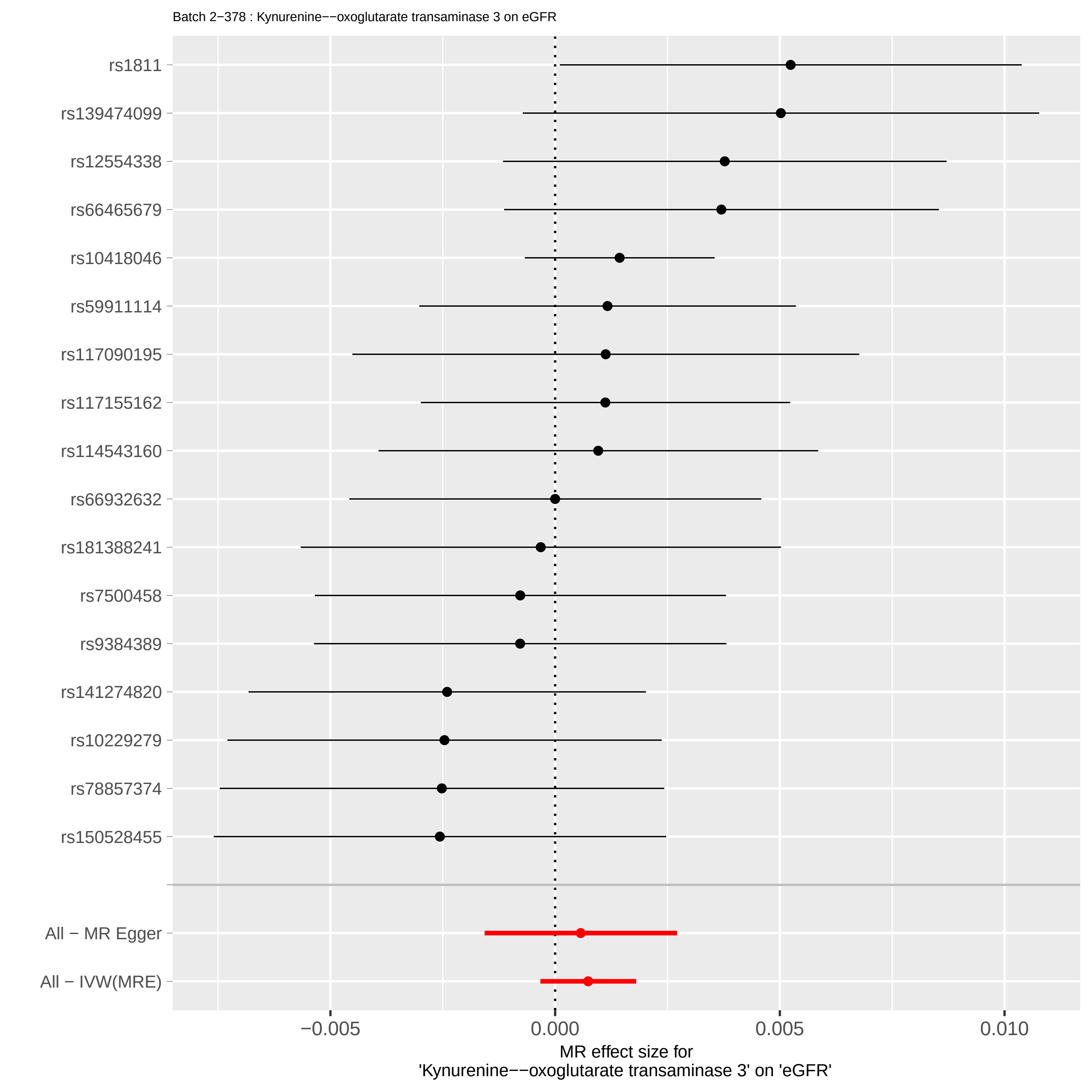

Batch 2−378 : Kynurenine−−oxoglutarate transaminase 3 on eGFR
rs1811
rs139474099
rs12554338
rs66465679
rs10418046
rs59911114
rs117090195
rs117155162
rs114543160
rs66932632
rs181388241
rs7500458
rs9384389
rs141274820
rs10229279
rs78857374
rs150528455
All − MR Egger
All − IVW(MRE)
−0.005
0.000
0.005
0.010
MR effect size for
'Kynurenine−−oxoglutarate transaminase 3' on 'eGFR'

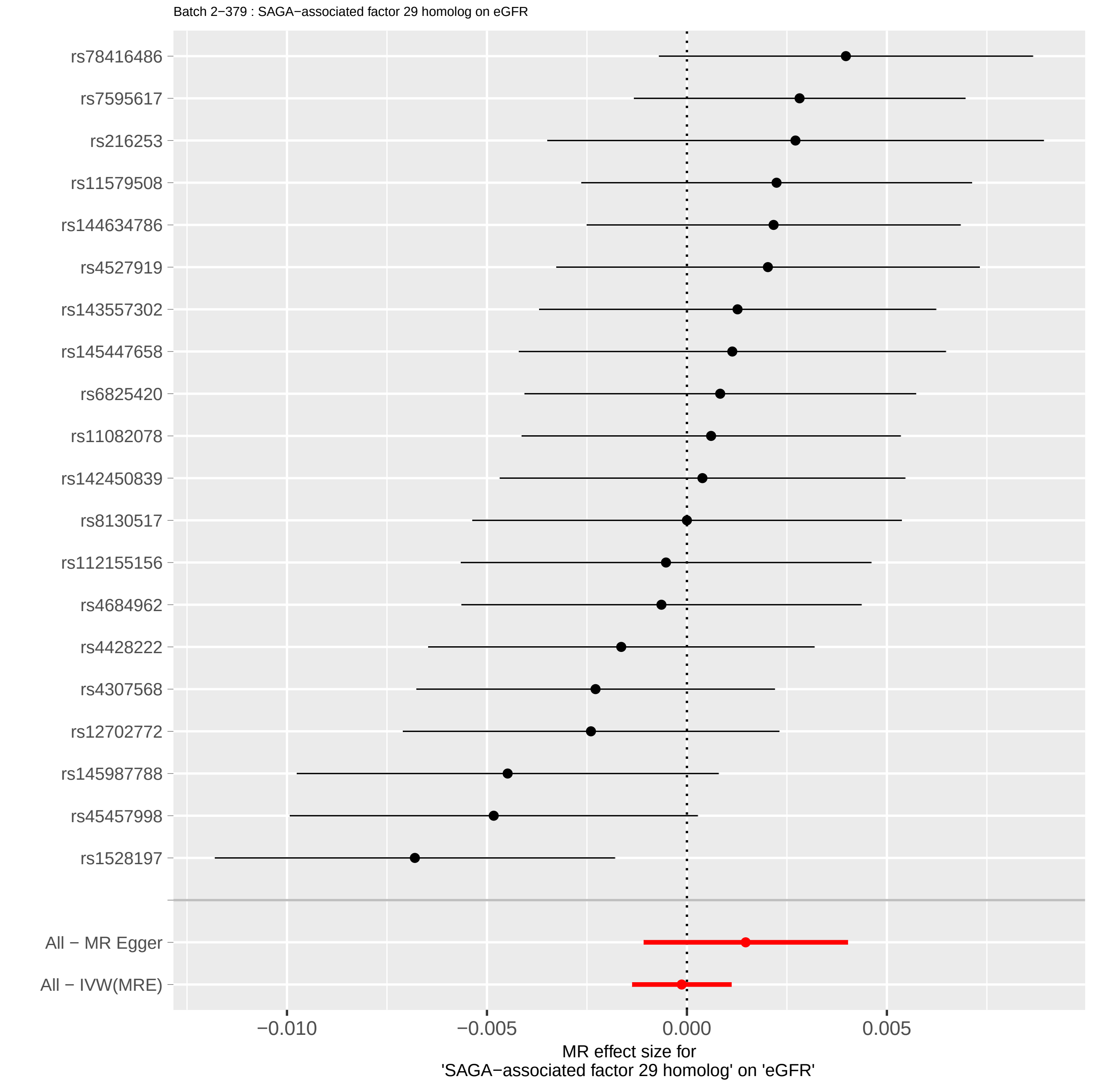

Batch 2−379 : SAGA−associated factor 29 homolog on eGFR
rs78416486
rs7595617
rs216253
rs11579508
rs144634786
rs4527919
rs143557302
rs145447658
rs6825420
rs11082078
rs142450839
rs8130517
rs112155156
rs4684962
rs4428222
rs4307568
rs12702772
rs145987788
rs45457998
rs1528197
All − MR Egger
All − IVW(MRE)
−0.010
−0.005
0.000
0.005
MR effect size for
'SAGA−associated factor 29 homolog' on 'eGFR'

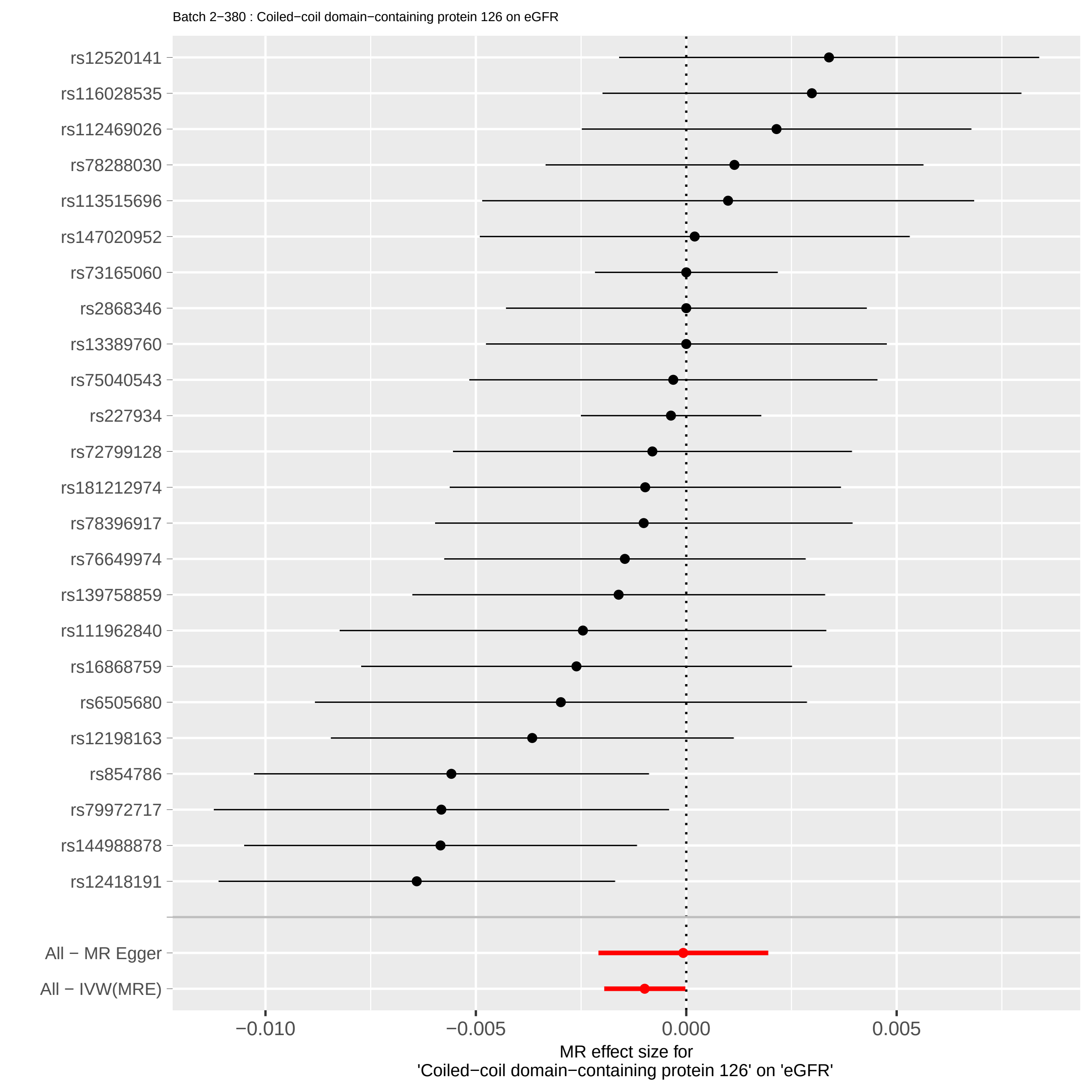
Batch 2−380 : Coiled−coil domain−containing protein 126 on eGFR
rs12520141
rs116028535
rs112469026
rs78288030
rs113515696
rs147020952
rs73165060
rs2868346
rs13389760
rs75040543
rs227934
rs72799128
rs181212974
rs78396917
rs76649974
rs139758859
rs111962840
rs16868759
rs6505680
rs12198163
rs854786
rs79972717
rs144988878
rs12418191
All − MR Egger
All − IVW(MRE)
−0.010
−0.005
0.000
0.005
MR effect size for
'Coiled−coil domain−containing protein 126' on 'eGFR'

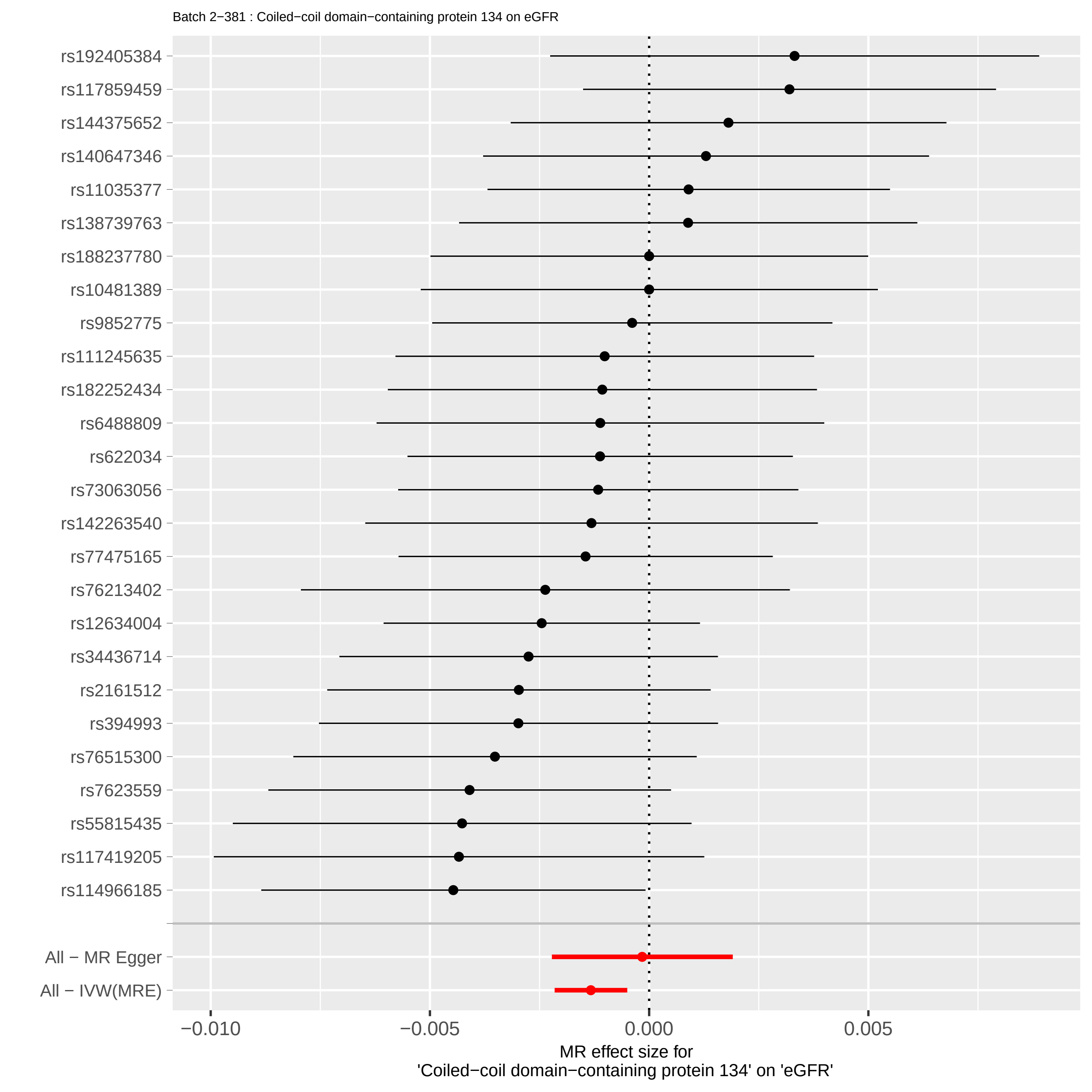

Batch 2−381 : Coiled−coil domain−containing protein 134 on eGFR
rs192405384
rs117859459
rs144375652
rs140647346
rs11035377
rs138739763
rs188237780
rs10481389
rs9852775
rs111245635
rs182252434
rs6488809
rs622034
rs73063056
rs142263540
rs77475165
rs76213402
rs12634004
rs34436714
rs2161512
rs394993
rs76515300
rs7623559
rs55815435
rs117419205
rs114966185
All − MR Egger
All − IVW(MRE)
−0.010
−0.005
0.000
0.005
MR effect size for
'Coiled−coil domain−containing protein 134' on 'eGFR'

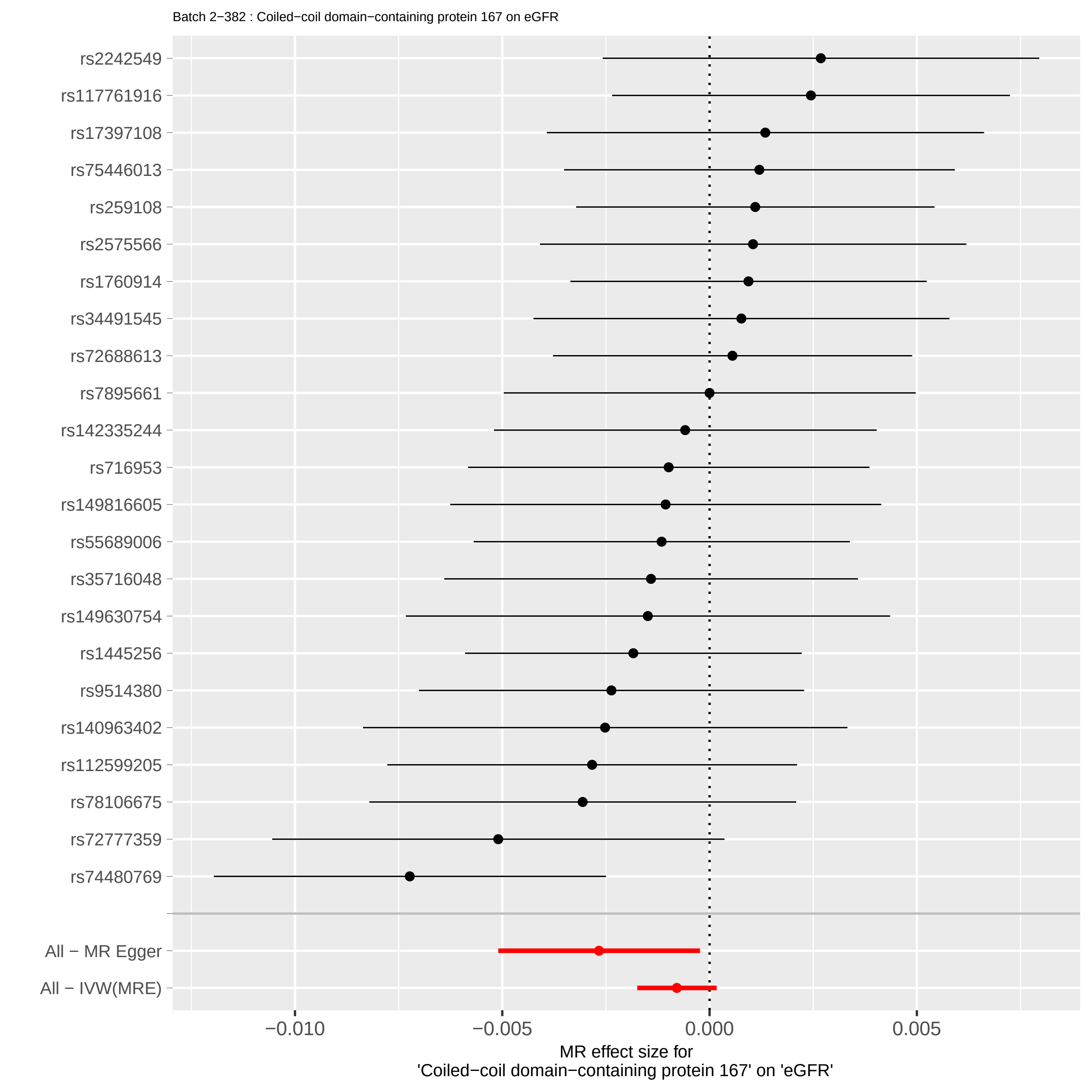
Batch 2−382 : Coiled−coil domain−containing protein 167 on eGFR
rs2242549
rs117761916
rs17397108
rs75446013
rs259108
rs2575566
rs1760914
rs34491545
rs72688613
rs7895661
rs142335244
rs716953
rs149816605
rs55689006
rs35716048
rs149630754
rs1445256
rs9514380
rs140963402
rs112599205
rs78106675
rs72777359
rs74480769
All − MR Egger
All − IVW(MRE)
−0.010
−0.005
0.000
0.005
MR effect size for
'Coiled−coil domain−containing protein 167' on 'eGFR'

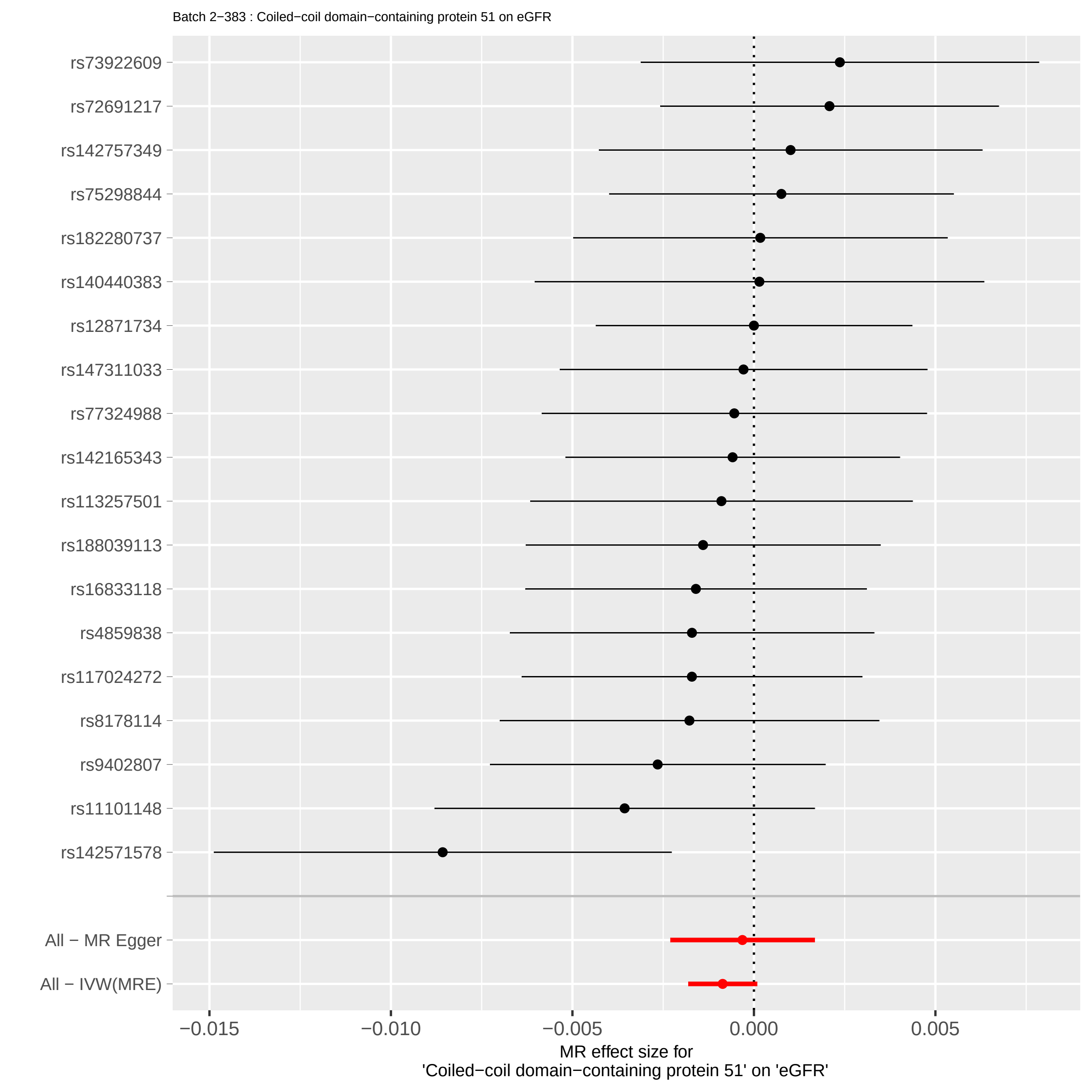

Batch 2−383 : Coiled−coil domain−containing protein 51 on eGFR
rs73922609
rs72691217
rs142757349
rs75298844
rs182280737
rs140440383
rs12871734
rs147311033
rs77324988
rs142165343
rs113257501
rs188039113
rs16833118
rs4859838
rs117024272
rs8178114
rs9402807
rs11101148
rs142571578
All − MR Egger
All − IVW(MRE)
−0.015
−0.010
−0.005
0.000
0.005
MR effect size for
'Coiled−coil domain−containing protein 51' on 'eGFR'

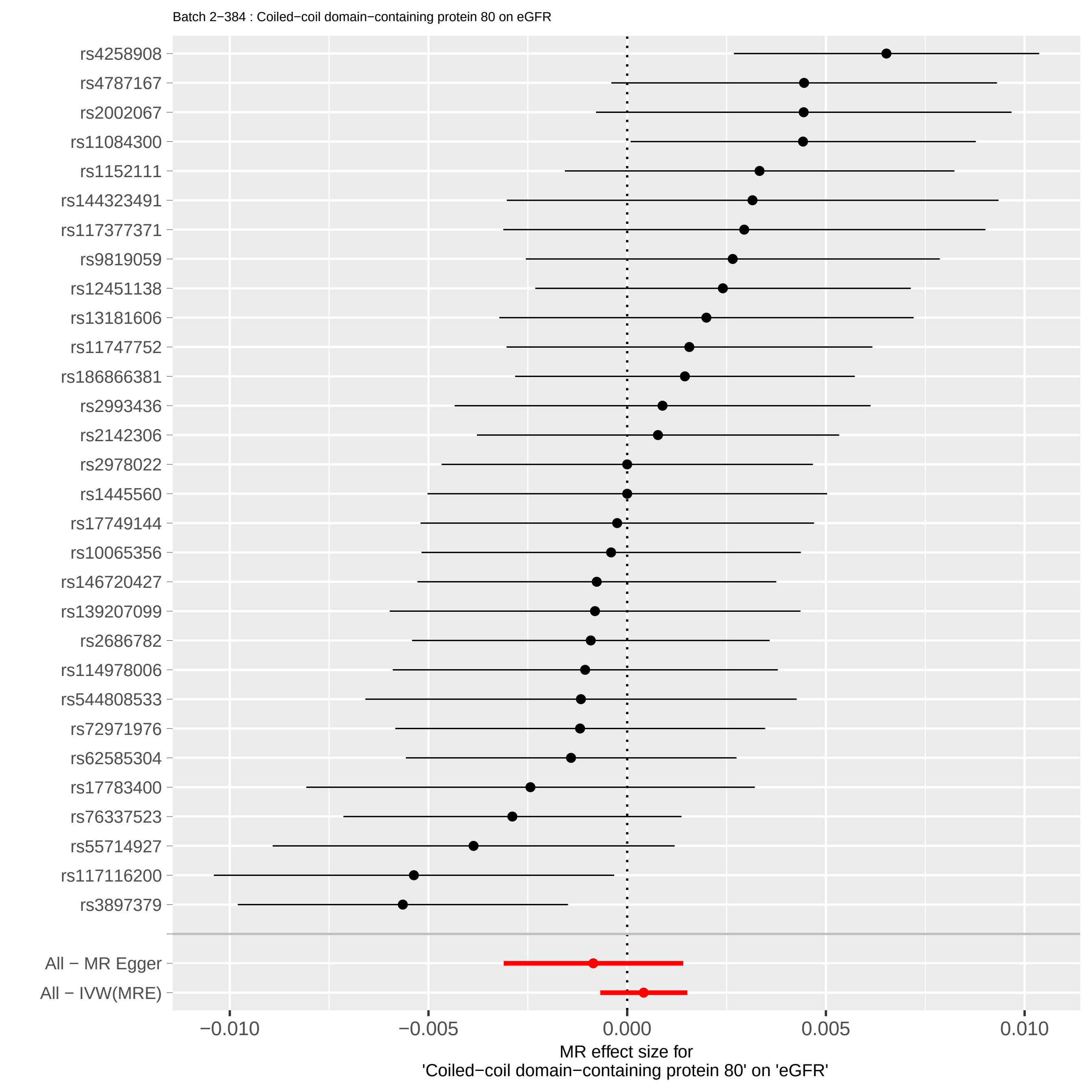
Batch 2−384 : Coiled−coil domain−containing protein 80 on eGFR
rs4258908
rs4787167
rs2002067
rs11084300
rs1152111
rs144323491
rs117377371
rs9819059
rs12451138
rs13181606
rs11747752
rs186866381
rs2993436
rs2142306
rs2978022
rs1445560
rs17749144
rs10065356
rs146720427
rs139207099
rs2686782
rs114978006
rs544808533
rs72971976
rs62585304
rs17783400
rs76337523
rs55714927
rs117116200
rs3897379
All − MR Egger
All − IVW(MRE)
−0.010
−0.005
0.000
0.005
0.010
MR effect size for
'Coiled−coil domain−containing protein 80' on 'eGFR'

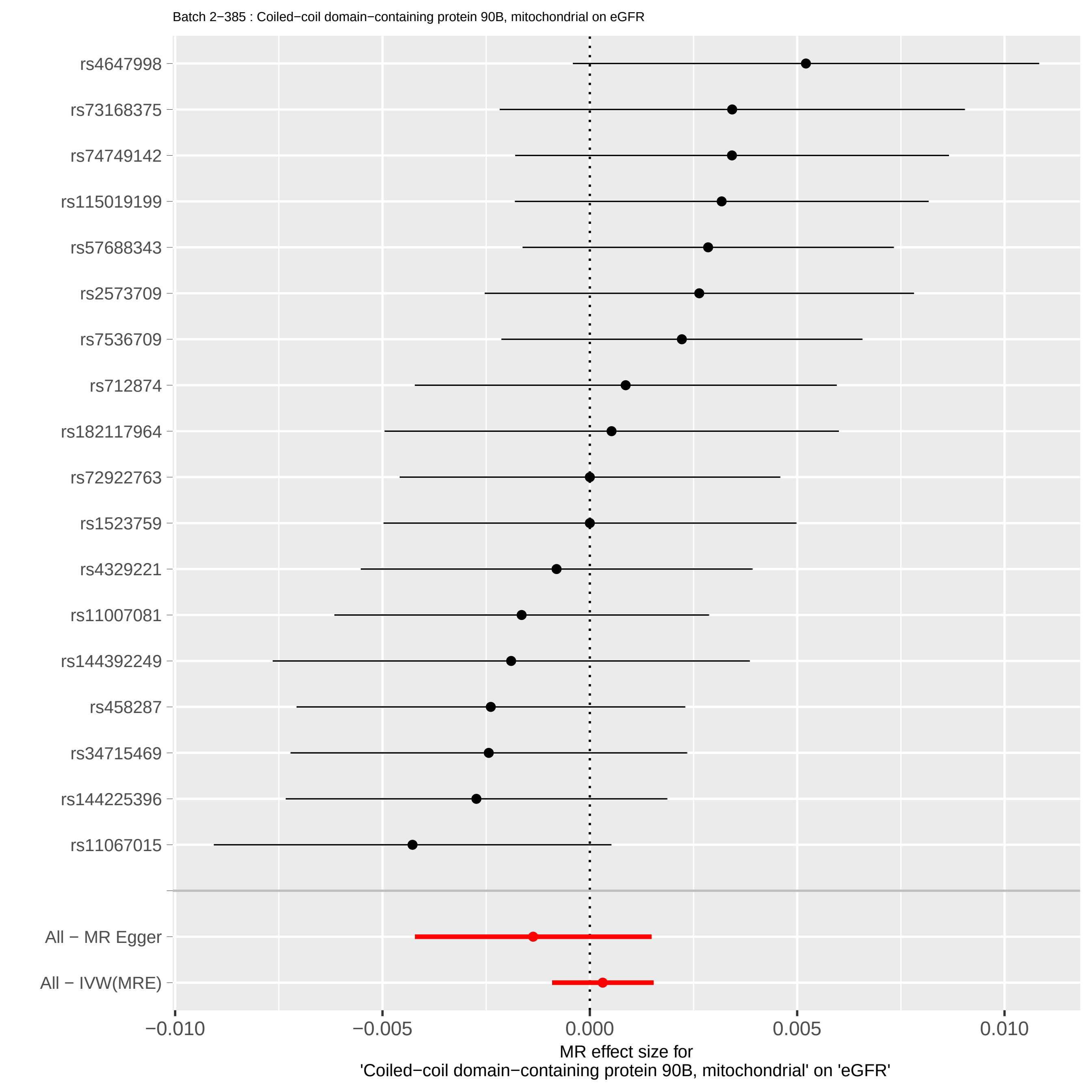

Batch 2−385 : Coiled−coil domain−containing protein 90B, mitochondrial on eGFR
rs4647998
rs73168375
rs74749142
rs115019199
rs57688343
rs2573709
rs7536709
rs712874
rs182117964
rs72922763
rs1523759
rs4329221
rs11007081
rs144392249
rs458287
rs34715469
rs144225396
rs11067015
All − MR Egger
All − IVW(MRE)
−0.010
−0.005
0.000
0.005
0.010
MR effect size for
'Coiled−coil domain−containing protein 90B, mitochondrial' on 'eGFR'

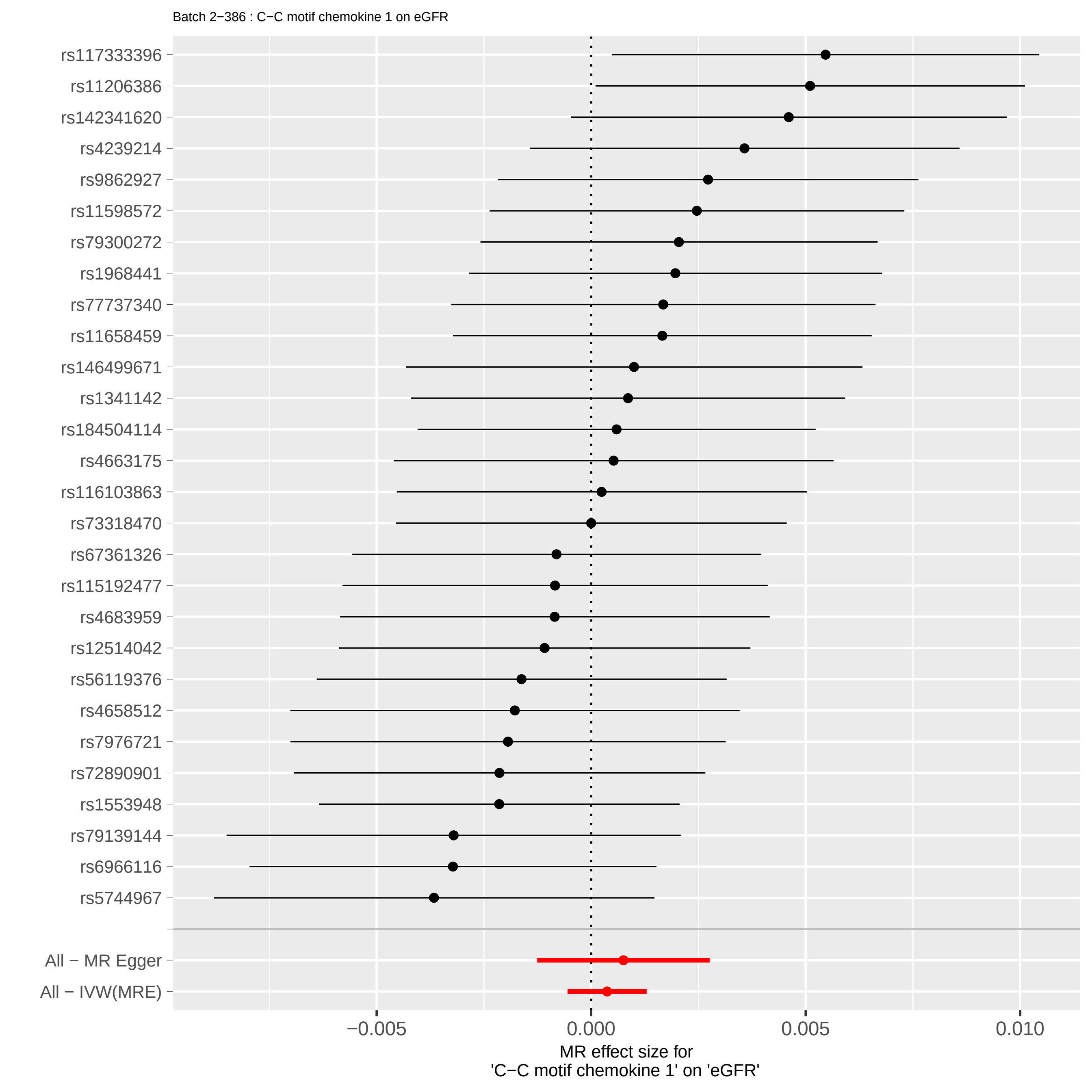

Batch 2−386 : C−C motif chemokine 1 on eGFR
rs117333396
rs11206386
rs142341620
rs4239214
rs9862927
rs11598572
rs79300272
rs1968441
rs77737340
rs11658459
rs146499671
rs1341142
rs184504114
rs4663175
rs116103863
rs73318470
rs67361326
rs115192477
rs4683959
rs12514042
rs56119376
rs4658512
rs7976721
rs72890901
rs1553948
rs79139144
rs6966116
rs5744967
All − MR Egger
All − IVW(MRE)
−0.005
0.000
0.005
0.010
MR effect size for
'C−C motif chemokine 1' on 'eGFR'

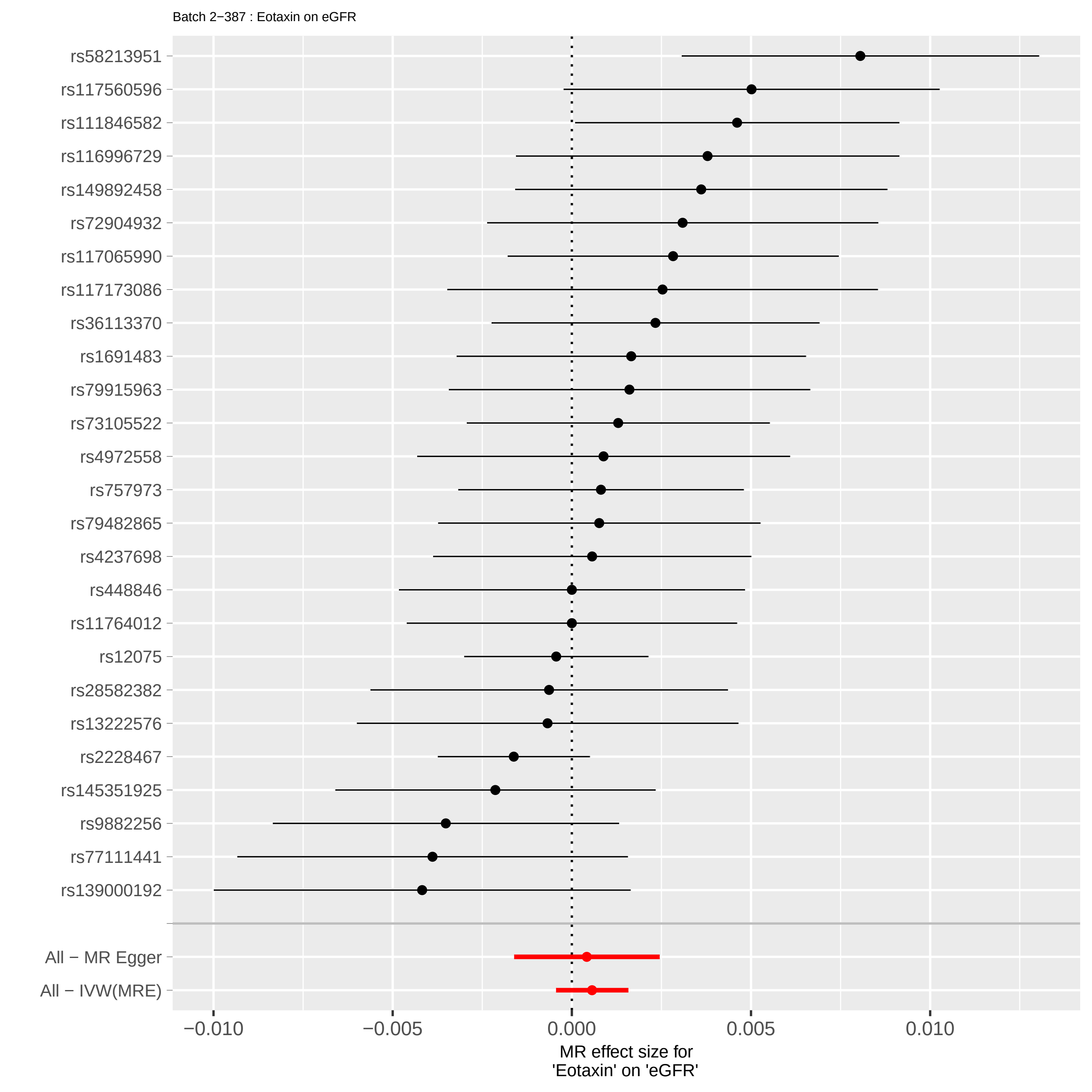

Batch 2−387 : Eotaxin on eGFR
rs58213951
rs117560596
rs111846582
rs116996729
rs149892458
rs72904932
rs117065990
rs117173086
rs36113370
rs1691483
rs79915963
rs73105522
rs4972558
rs757973
rs79482865
rs4237698
rs448846
rs11764012
rs12075
rs28582382
rs13222576
rs2228467
rs145351925
rs9882256
rs77111441
rs139000192
All − MR Egger
All − IVW(MRE)
−0.010
−0.005
0.000
0.005
0.010
MR effect size for
'Eotaxin' on 'eGFR'

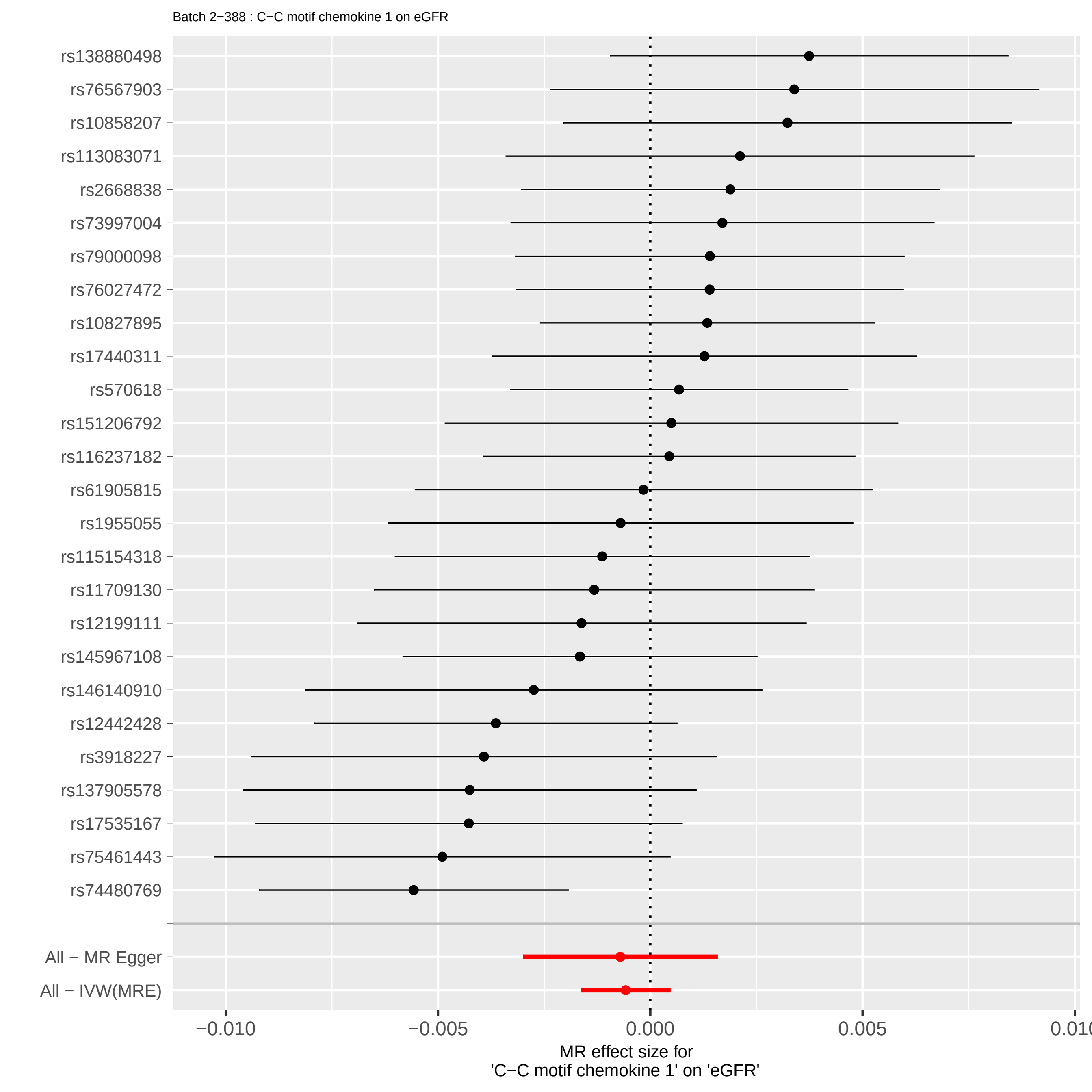

Batch 2−388 : C−C motif chemokine 1 on eGFR
rs138880498
rs76567903
rs10858207
rs113083071
rs2668838
rs73997004
rs79000098
rs76027472
rs10827895
rs17440311
rs570618
rs151206792
rs116237182
rs61905815
rs1955055
rs115154318
rs11709130
rs12199111
rs145967108
rs146140910
rs12442428
rs3918227
rs137905578
rs17535167
rs75461443
rs74480769
All − MR Egger
All − IVW(MRE)
−0.010
−0.005
0.000
0.005
0.010
MR effect size for
'C−C motif chemokine 1' on 'eGFR'

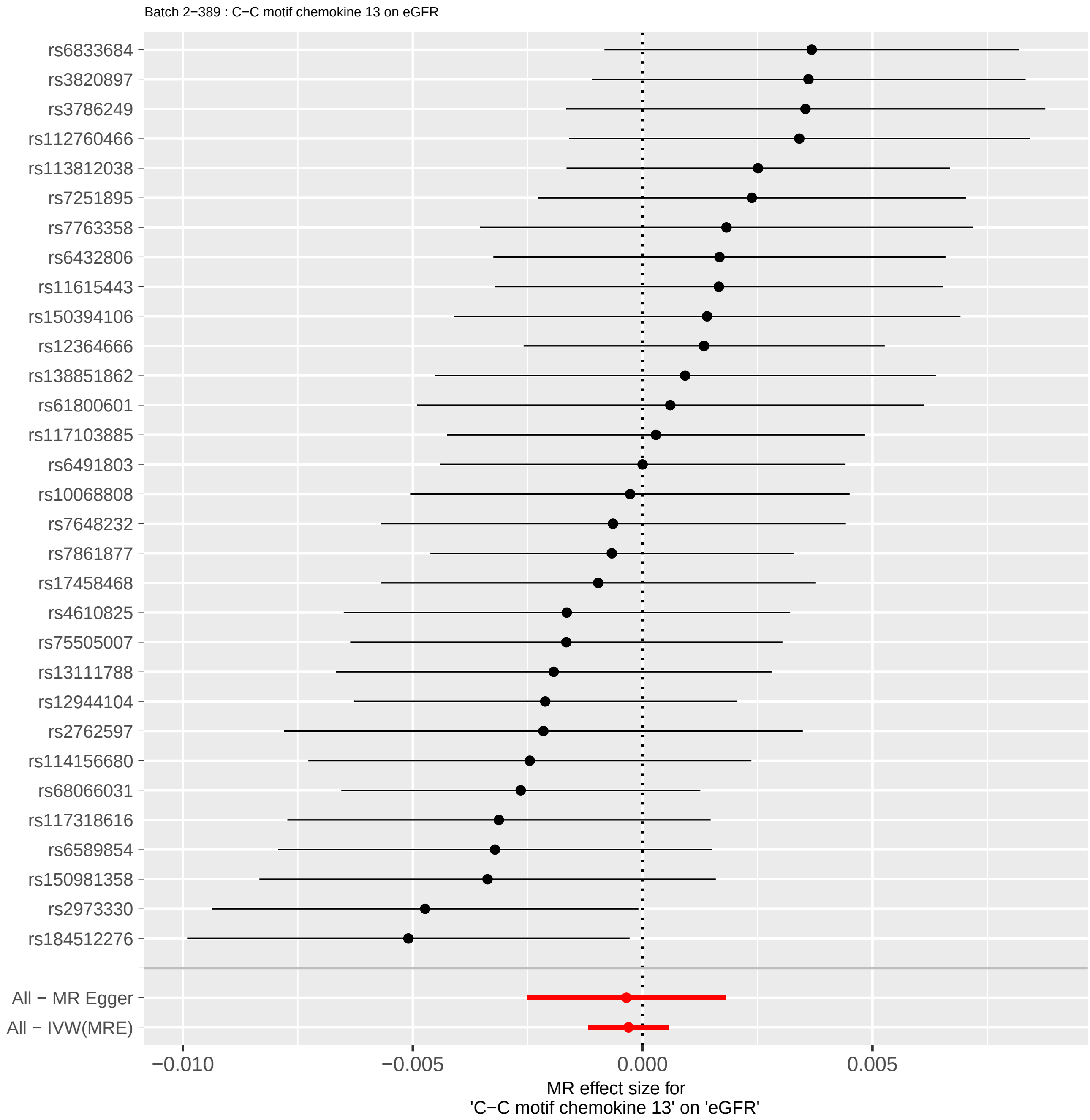

Batch 2−389 : C−C motif chemokine 13 on eGFR
rs6833684
rs3820897
rs3786249
rs112760466
rs113812038
rs7251895
rs7763358
rs6432806
rs11615443
rs150394106
rs12364666
rs138851862
rs61800601
rs117103885
rs6491803
rs10068808
rs7648232
rs7861877
rs17458468
rs4610825
rs75505007
rs13111788
rs12944104
rs2762597
rs114156680
rs68066031
rs117318616
rs6589854
rs150981358
rs2973330
rs184512276
All − MR Egger
All − IVW(MRE)
−0.010
−0.005
0.000
0.005
MR effect size for
'C−C motif chemokine 13' on 'eGFR'

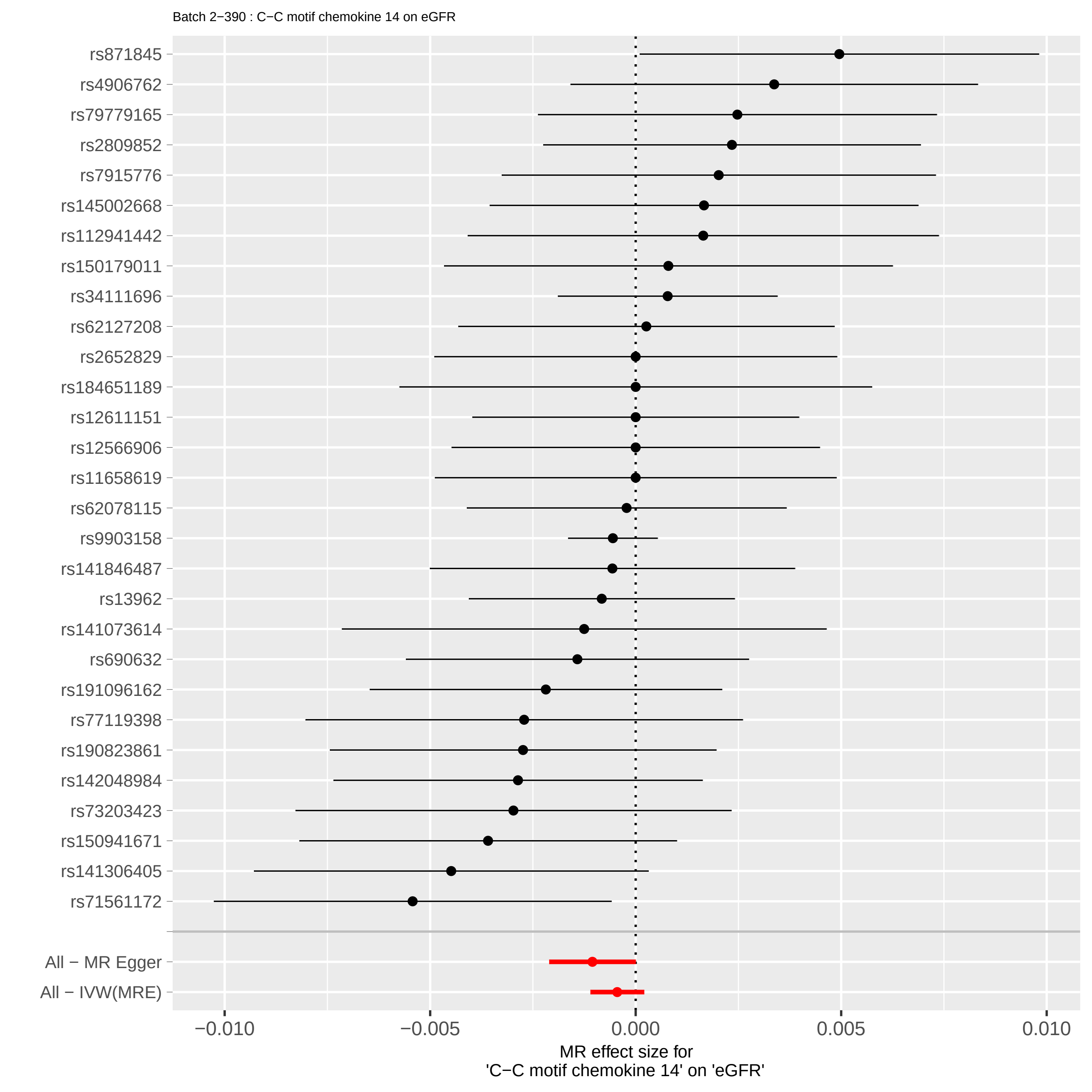
Batch 2−390 : C−C motif chemokine 14 on eGFR
rs871845
rs4906762
rs79779165
rs2809852
rs7915776
rs145002668
rs112941442
rs150179011
rs34111696
rs62127208
rs2652829
rs184651189
rs12611151
rs12566906
rs11658619
rs62078115
rs9903158
rs141846487
rs13962
rs141073614
rs690632
rs191096162
rs77119398
rs190823861
rs142048984
rs73203423
rs150941671
rs141306405
rs71561172
All − MR Egger
All − IVW(MRE)
−0.010
−0.005
0.000
0.005
0.010
MR effect size for
'C−C motif chemokine 14' on 'eGFR'

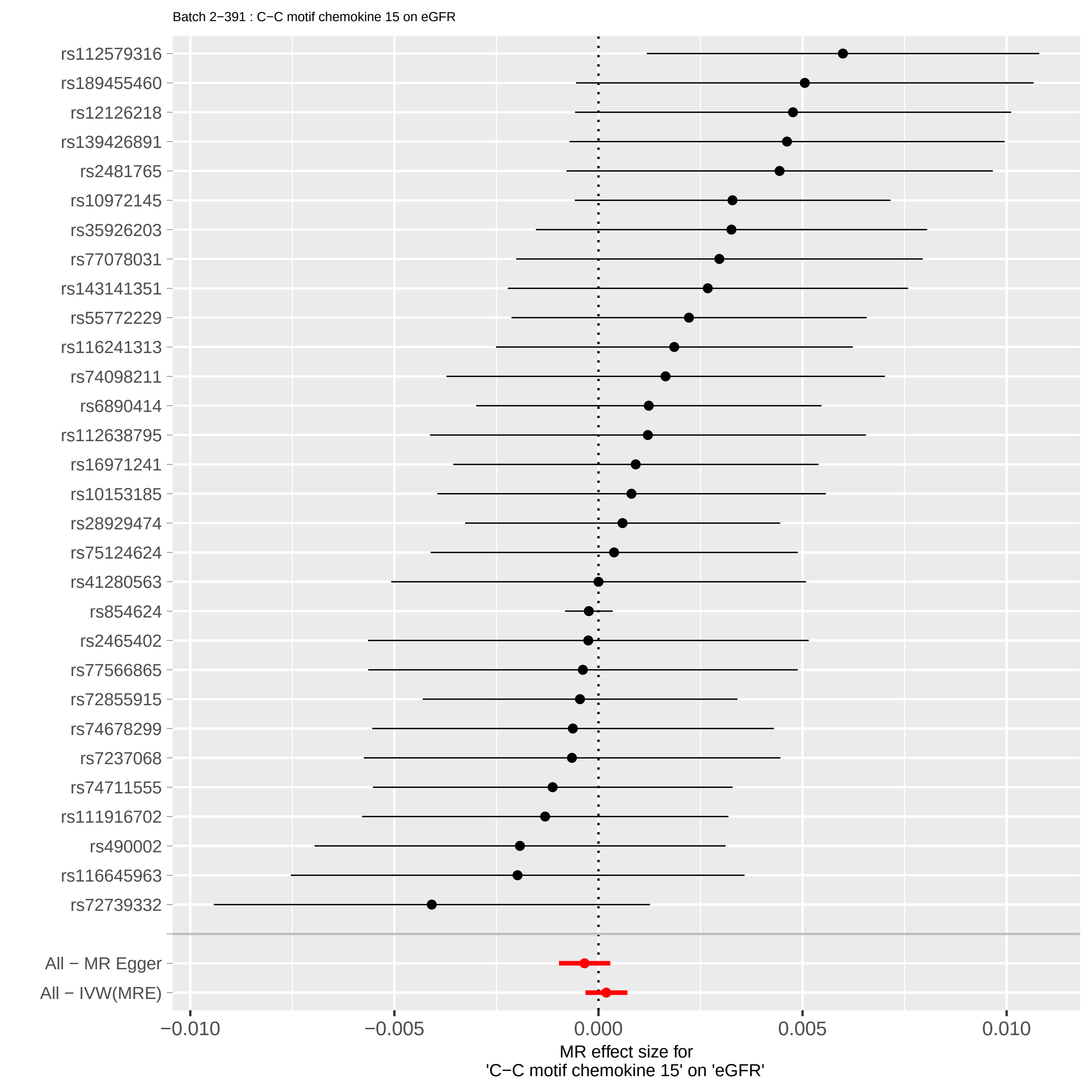

Batch 2−391 : C−C motif chemokine 15 on eGFR
rs112579316
rs189455460
rs12126218
rs139426891
rs2481765
rs10972145
rs35926203
rs77078031
rs143141351
rs55772229
rs116241313
rs74098211
rs6890414
rs112638795
rs16971241
rs10153185
rs28929474
rs75124624
rs41280563
rs854624
rs2465402
rs77566865
rs72855915
rs74678299
rs7237068
rs74711555
rs111916702
rs490002
rs116645963
rs72739332
All − MR Egger
All − IVW(MRE)
−0.010
−0.005
0.000
0.005
0.010
MR effect size for
'C−C motif chemokine 15' on 'eGFR'

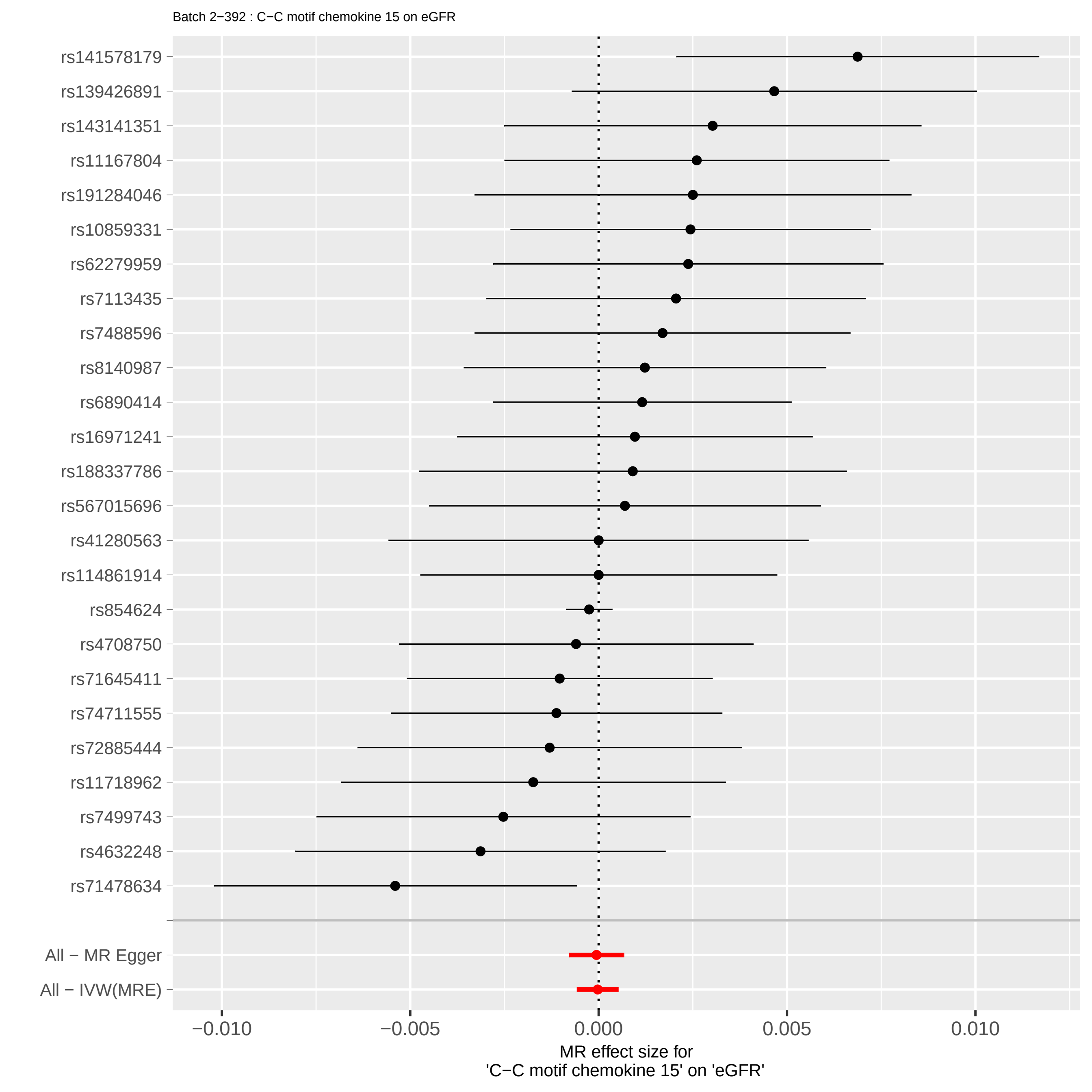

Batch 2−392 : C−C motif chemokine 15 on eGFR
rs141578179
rs139426891
rs143141351
rs11167804
rs191284046
rs10859331
rs62279959
rs7113435
rs7488596
rs8140987
rs6890414
rs16971241
rs188337786
rs567015696
rs41280563
rs114861914
rs854624
rs4708750
rs71645411
rs74711555
rs72885444
rs11718962
rs7499743
rs4632248
rs71478634
All − MR Egger
All − IVW(MRE)
−0.010
−0.005
0.000
0.005
0.010
MR effect size for
'C−C motif chemokine 15' on 'eGFR'

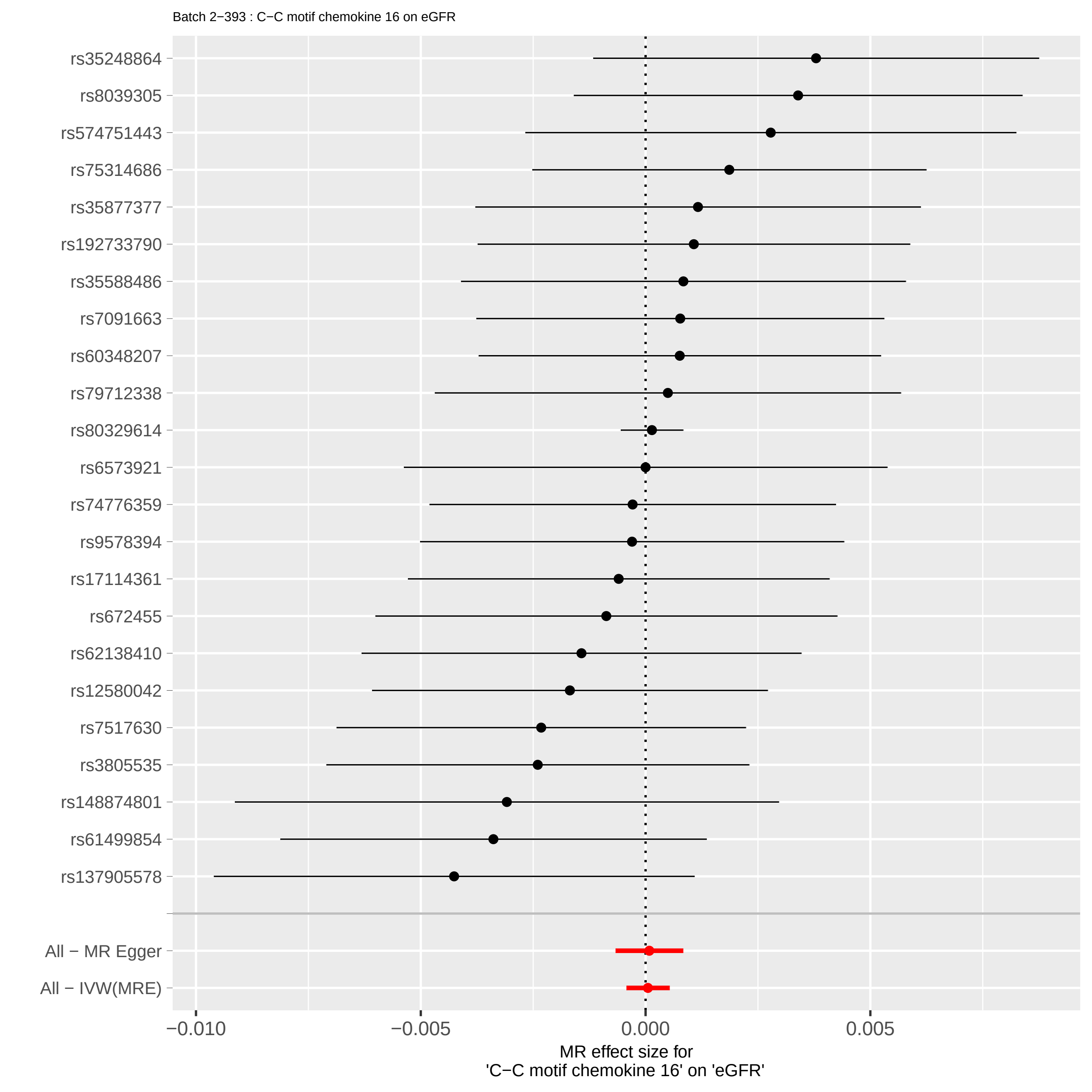
Batch 2−393 : C−C motif chemokine 16 on eGFR
rs35248864
rs8039305
rs574751443
rs75314686
rs35877377
rs192733790
rs35588486
rs7091663
rs60348207
rs79712338
rs80329614
rs6573921
rs74776359
rs9578394
rs17114361
rs672455
rs62138410
rs12580042
rs7517630
rs3805535
rs148874801
rs61499854
rs137905578
All − MR Egger
All − IVW(MRE)
−0.010
−0.005
0.000
0.005
MR effect size for
'C−C motif chemokine 16' on 'eGFR'

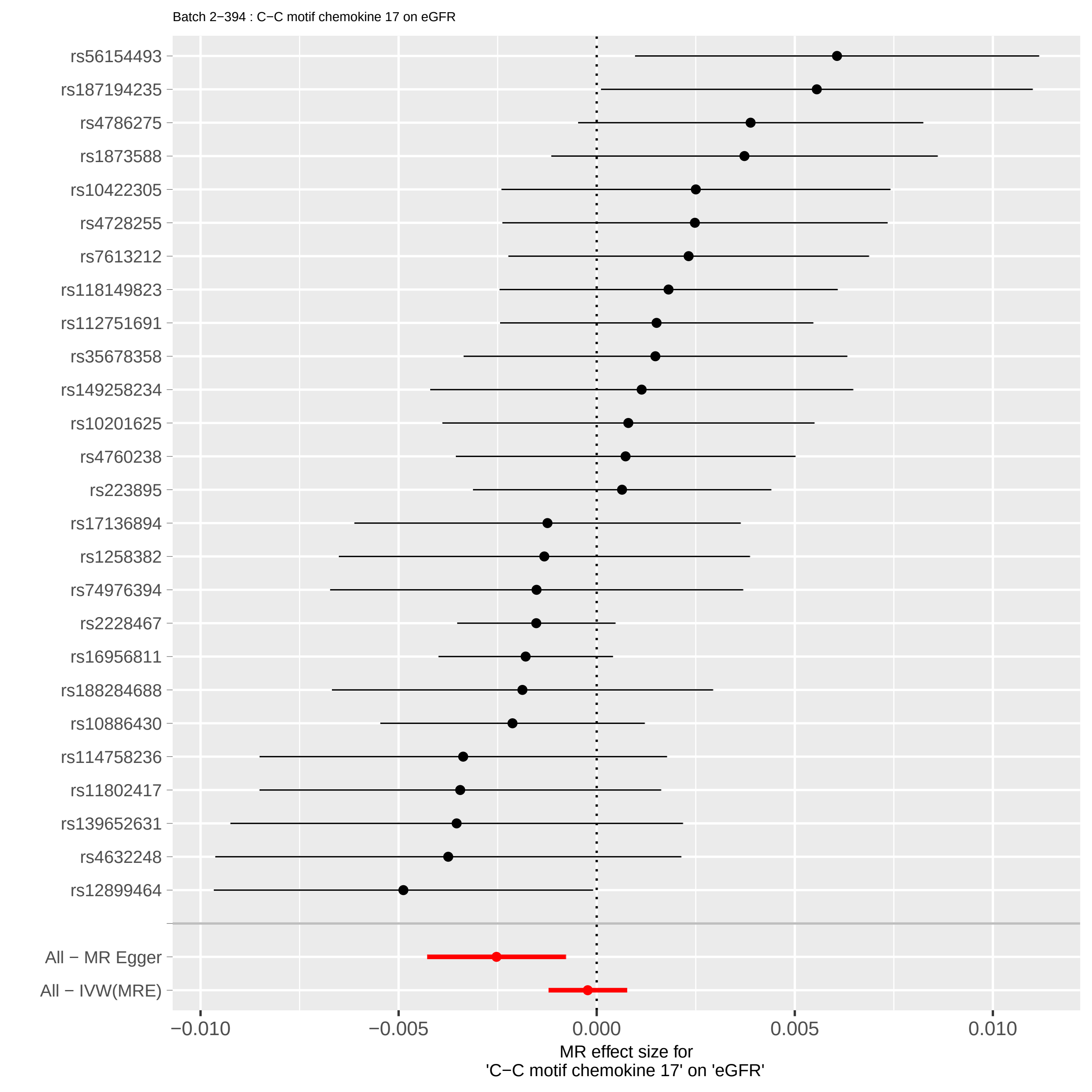

Batch 2−394 : C−C motif chemokine 17 on eGFR
rs56154493
rs187194235
rs4786275
rs1873588
rs10422305
rs4728255
rs7613212
rs118149823
rs112751691
rs35678358
rs149258234
rs10201625
rs4760238
rs223895
rs17136894
rs1258382
rs74976394
rs2228467
rs16956811
rs188284688
rs10886430
rs114758236
rs11802417
rs139652631
rs4632248
rs12899464
All − MR Egger
All − IVW(MRE)
−0.010
−0.005
0.000
0.005
0.010
MR effect size for
'C−C motif chemokine 17' on 'eGFR'

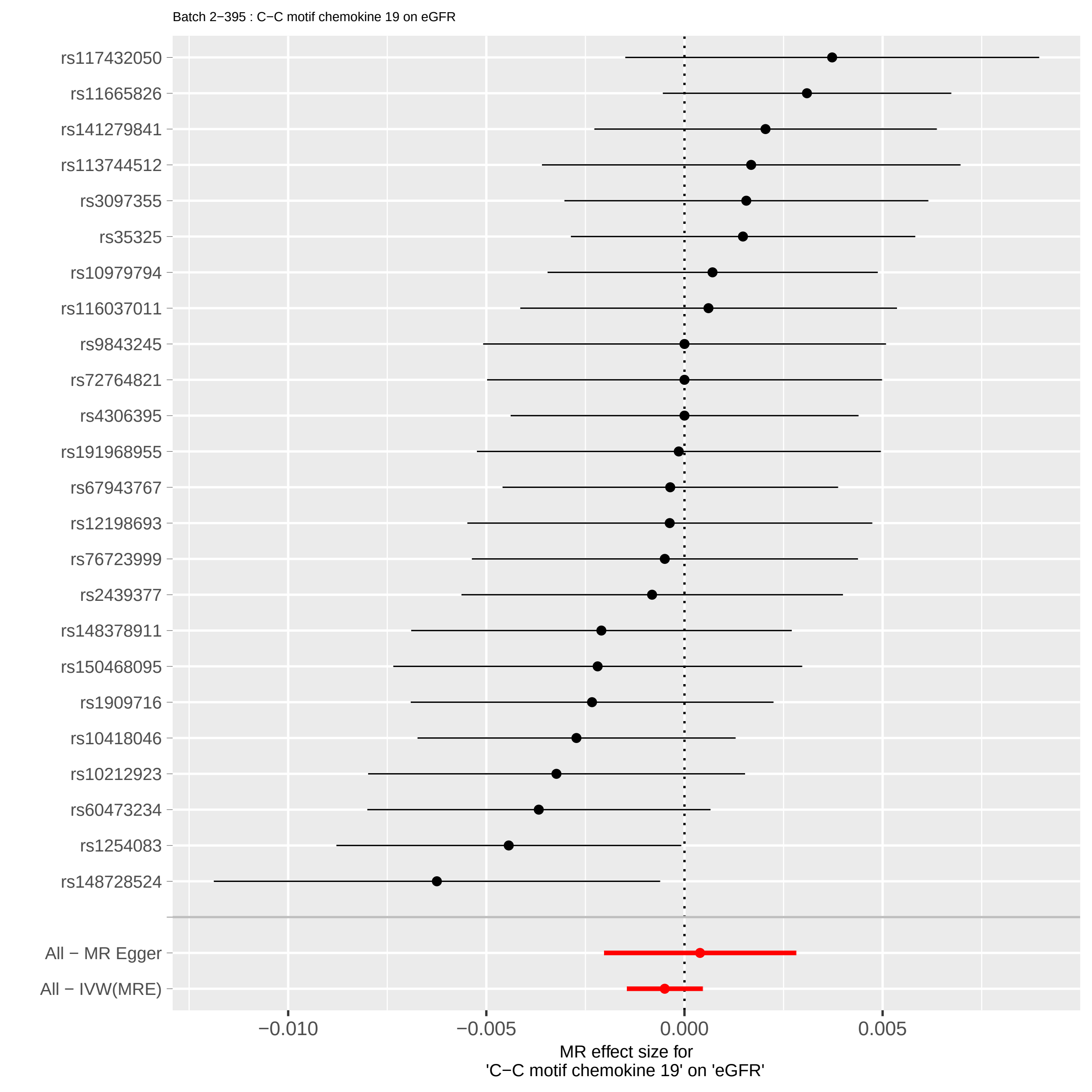

Batch 2−395 : C−C motif chemokine 19 on eGFR
rs117432050
rs11665826
rs141279841
rs113744512
rs3097355
rs35325
rs10979794
rs116037011
rs9843245
rs72764821
rs4306395
rs191968955
rs67943767
rs12198693
rs76723999
rs2439377
rs148378911
rs150468095
rs1909716
rs10418046
rs10212923
rs60473234
rs1254083
rs148728524
All − MR Egger
All − IVW(MRE)
−0.010
−0.005
0.000
0.005
MR effect size for
'C−C motif chemokine 19' on 'eGFR'

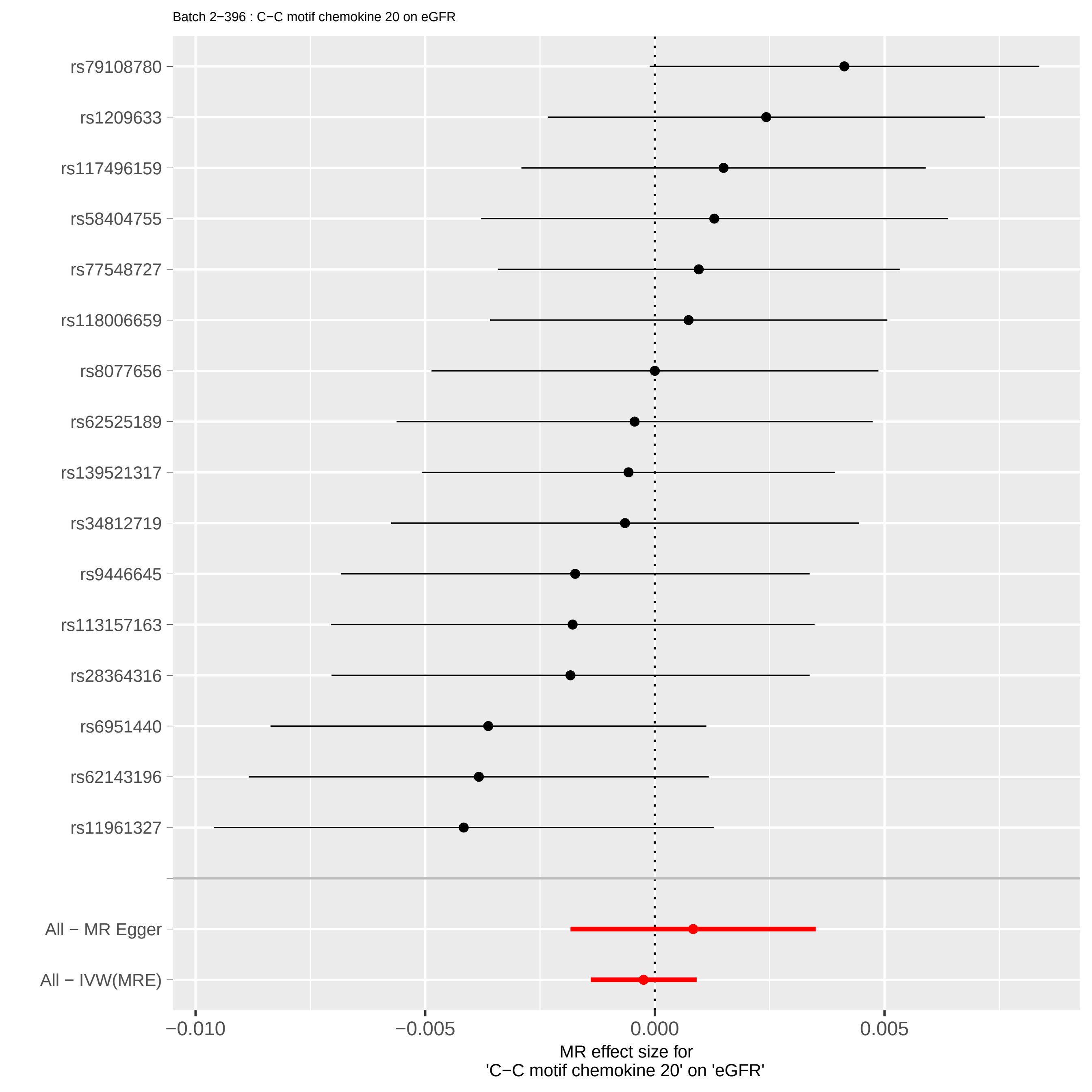
Batch 2−396 : C−C motif chemokine 20 on eGFR
rs79108780
rs1209633
rs117496159
rs58404755
rs77548727
rs118006659
rs8077656
rs62525189
rs139521317
rs34812719
rs9446645
rs113157163
rs28364316
rs6951440
rs62143196
rs11961327
All − MR Egger
All − IVW(MRE)
−0.010
−0.005
0.000
0.005
MR effect size for
'C−C motif chemokine 20' on 'eGFR'

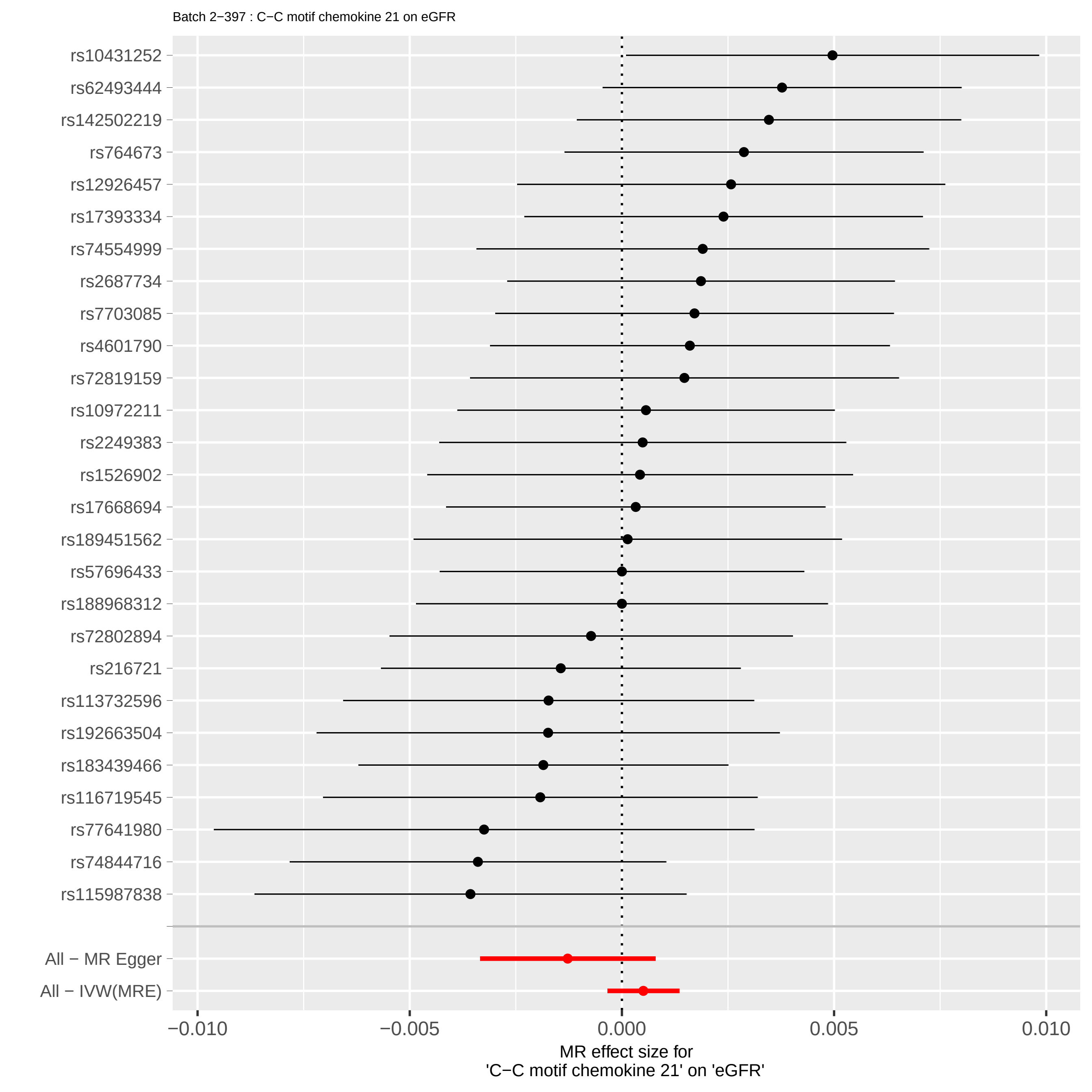

Batch 2−397 : C−C motif chemokine 21 on eGFR
rs10431252
rs62493444
rs142502219
rs764673
rs12926457
rs17393334
rs74554999
rs2687734
rs7703085
rs4601790
rs72819159
rs10972211
rs2249383
rs1526902
rs17668694
rs189451562
rs57696433
rs188968312
rs72802894
rs216721
rs113732596
rs192663504
rs183439466
rs116719545
rs77641980
rs74844716
rs115987838
All − MR Egger
All − IVW(MRE)
−0.010
−0.005
0.000
0.005
0.010
MR effect size for
'C−C motif chemokine 21' on 'eGFR'

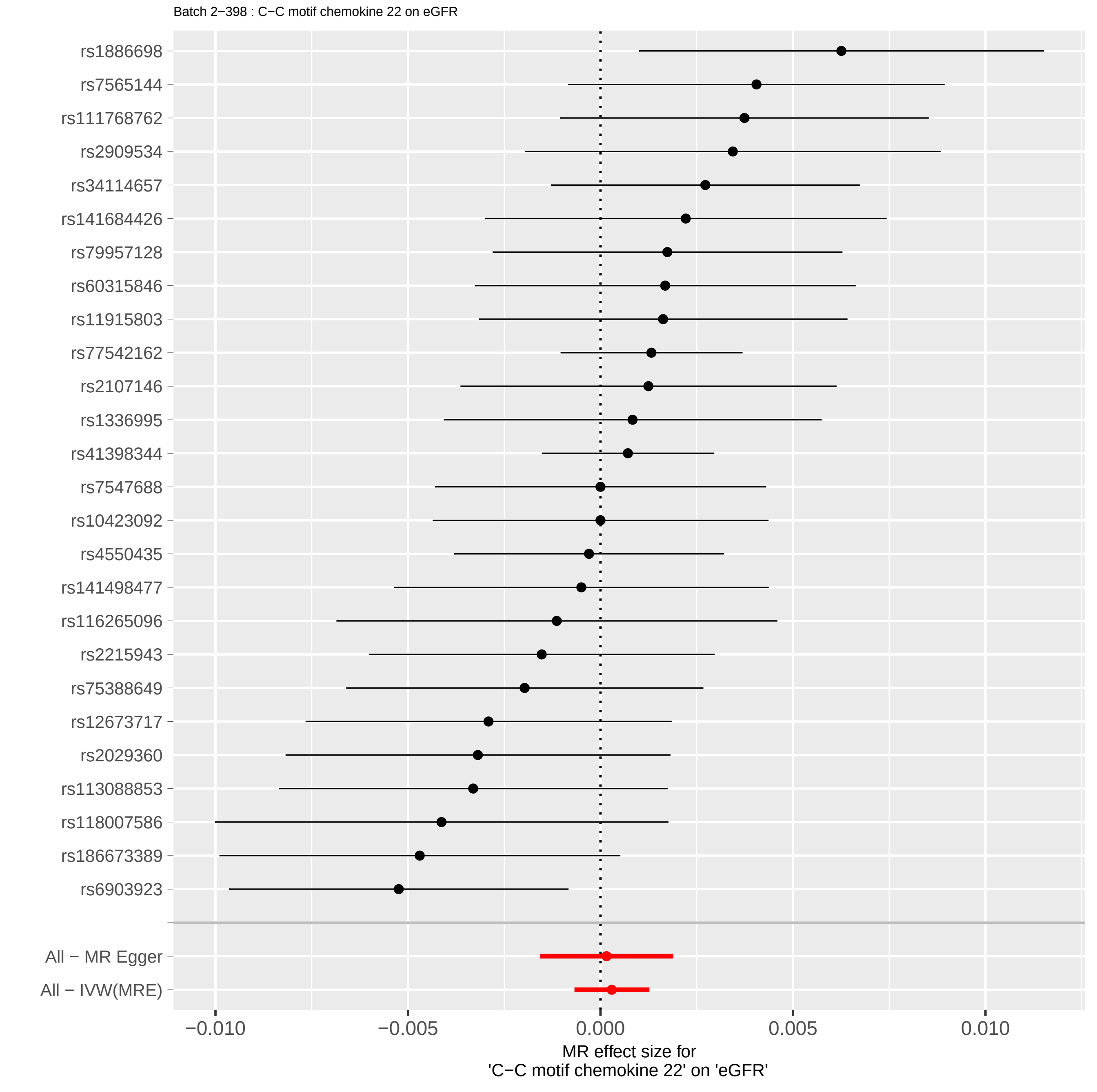

Batch 2−398 : C−C motif chemokine 22 on eGFR
rs1886698
rs7565144
rs111768762
rs2909534
rs34114657
rs141684426
rs79957128
rs60315846
rs11915803
rs77542162
rs2107146
rs1336995
rs41398344
rs7547688
rs10423092
rs4550435
rs141498477
rs116265096
rs2215943
rs75388649
rs12673717
rs2029360
rs113088853
rs118007586
rs186673389
rs6903923
All − MR Egger
All − IVW(MRE)
−0.010
−0.005
0.000
0.005
0.010
MR effect size for
'C−C motif chemokine 22' on 'eGFR'

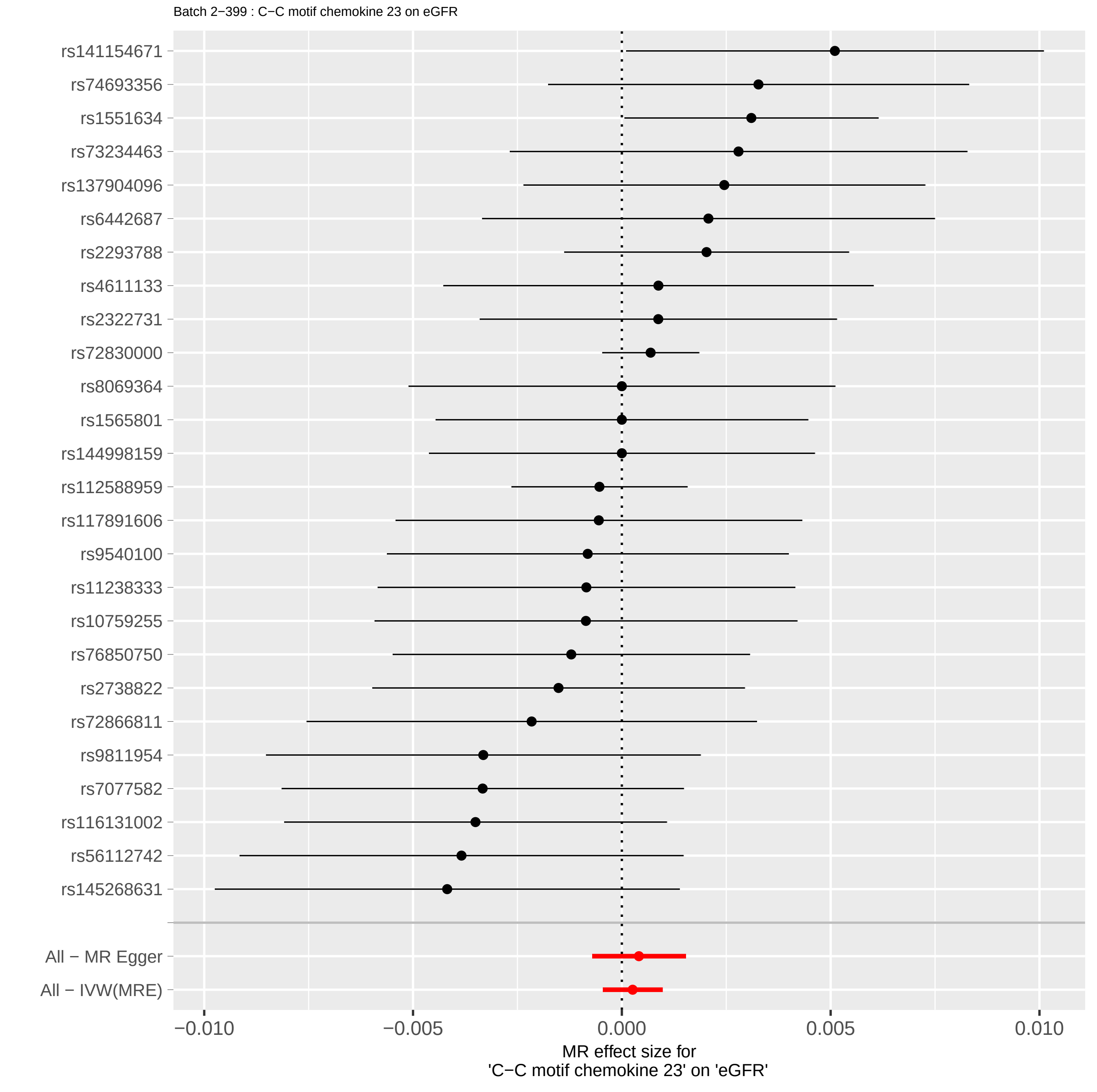

Batch 2−399 : C−C motif chemokine 23 on eGFR
rs141154671
rs74693356
rs1551634
rs73234463
rs137904096
rs6442687
rs2293788
rs4611133
rs2322731
rs72830000
rs8069364
rs1565801
rs144998159
rs112588959
rs117891606
rs9540100
rs11238333
rs10759255
rs76850750
rs2738822
rs72866811
rs9811954
rs7077582
rs116131002
rs56112742
rs145268631
All − MR Egger
All − IVW(MRE)
−0.010
−0.005
0.000
0.005
0.010
MR effect size for
'C−C motif chemokine 23' on 'eGFR'

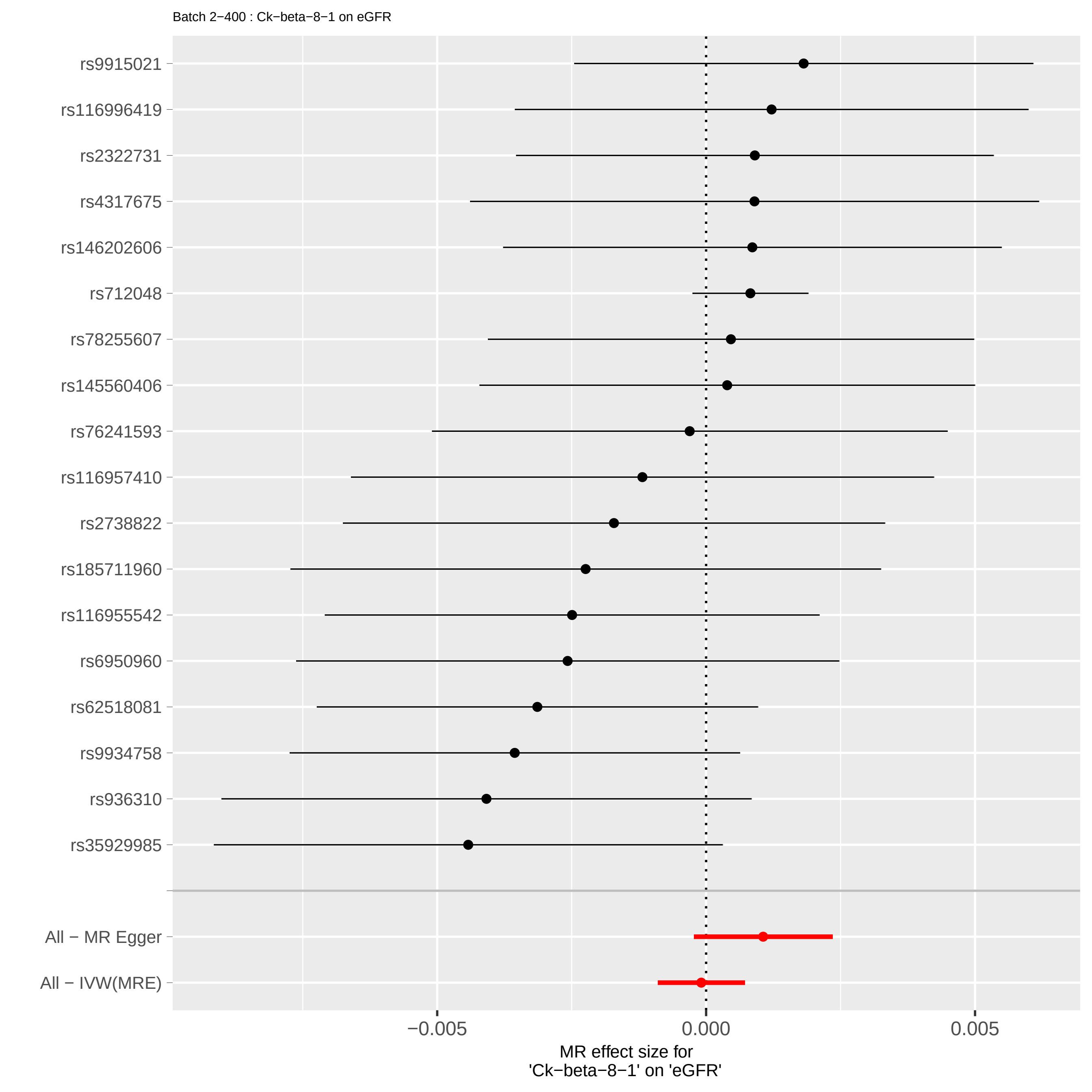

Batch 2−400 : Ck−beta−8−1 on eGFR
rs9915021
rs116996419
rs2322731
rs4317675
rs146202606
rs712048
rs78255607
rs145560406
rs76241593
rs116957410
rs2738822
rs185711960
rs116955542
rs6950960
rs62518081
rs9934758
rs936310
rs35929985
All − MR Egger
All − IVW(MRE)
−0.005
0.000
0.005
MR effect size for
'Ck−beta−8−1' on 'eGFR'

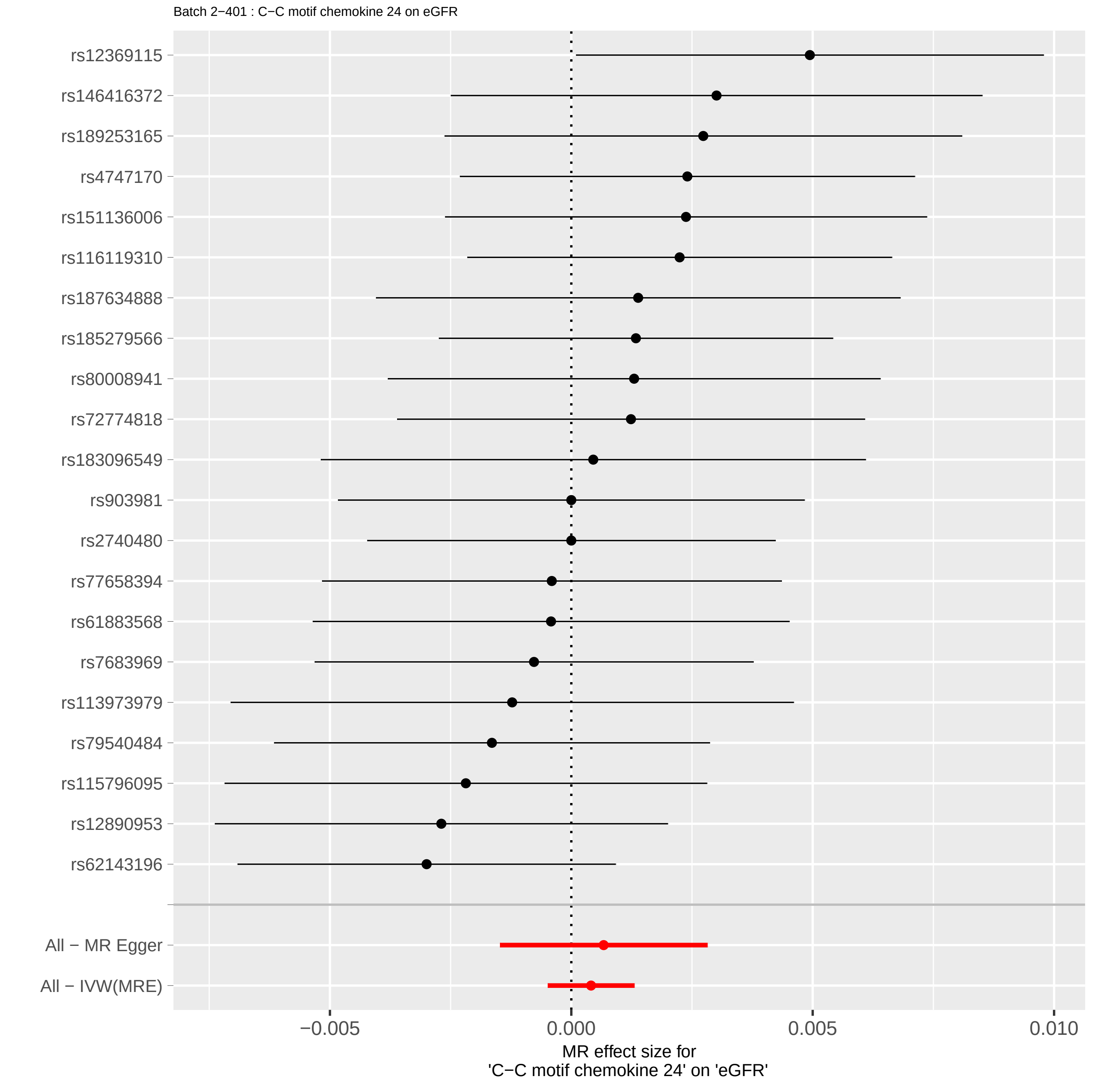
Batch 2−401 : C−C motif chemokine 24 on eGFR
rs12369115
rs146416372
rs189253165
rs4747170
rs151136006
rs116119310
rs187634888
rs185279566
rs80008941
rs72774818
rs183096549
rs903981
rs2740480
rs77658394
rs61883568
rs7683969
rs113973979
rs79540484
rs115796095
rs12890953
rs62143196
All − MR Egger
All − IVW(MRE)
−0.005
0.000
0.005
0.010
MR effect size for
'C−C motif chemokine 24' on 'eGFR'

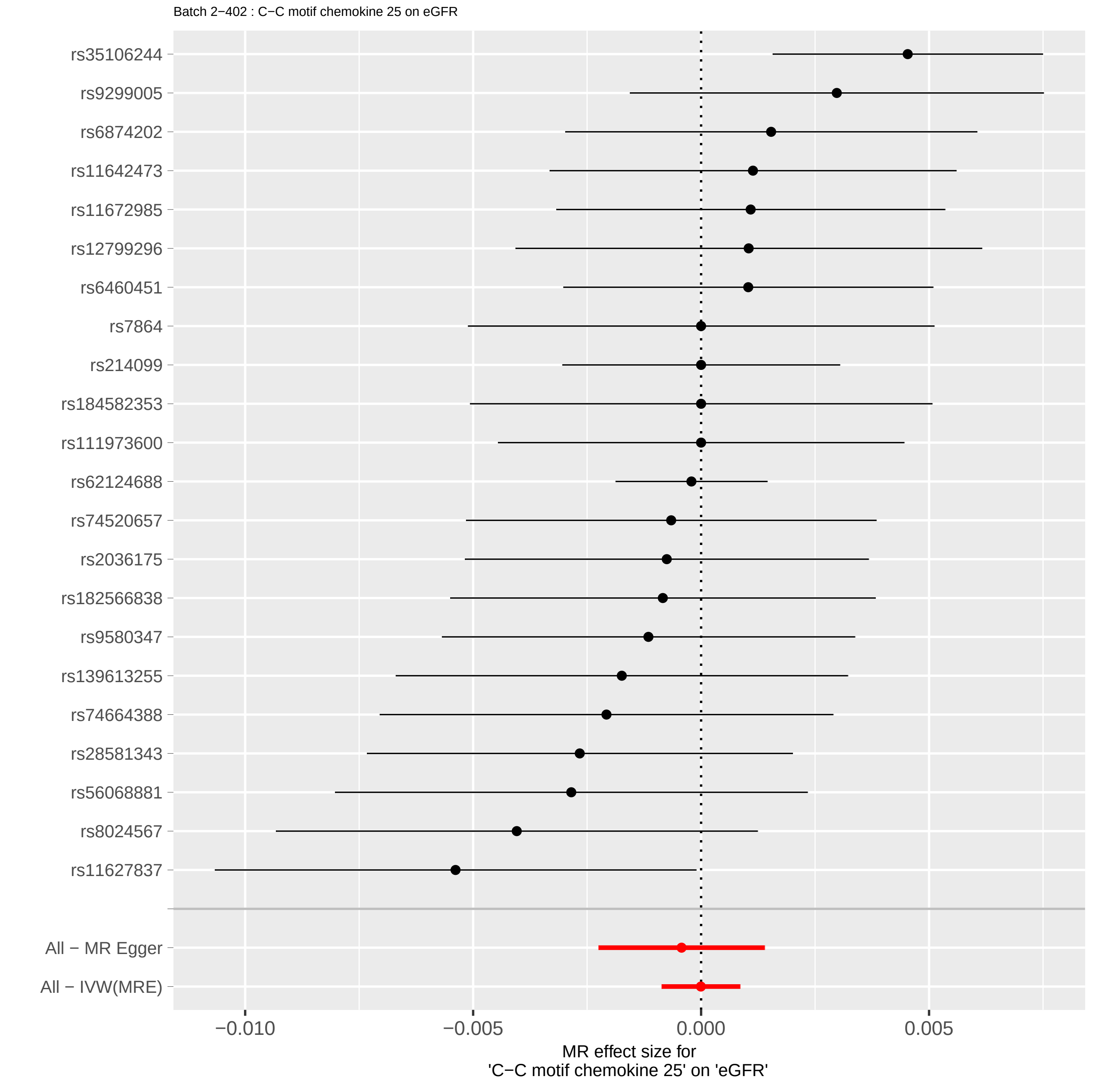

Batch 2−402 : C−C motif chemokine 25 on eGFR
rs35106244
rs9299005
rs6874202
rs11642473
rs11672985
rs12799296
rs6460451
rs7864
rs214099
rs184582353
rs111973600
rs62124688
rs74520657
rs2036175
rs182566838
rs9580347
rs139613255
rs74664388
rs28581343
rs56068881
rs8024567
rs11627837
All − MR Egger
All − IVW(MRE)
−0.010
−0.005
0.000
0.005
MR effect size for
'C−C motif chemokine 25' on 'eGFR'

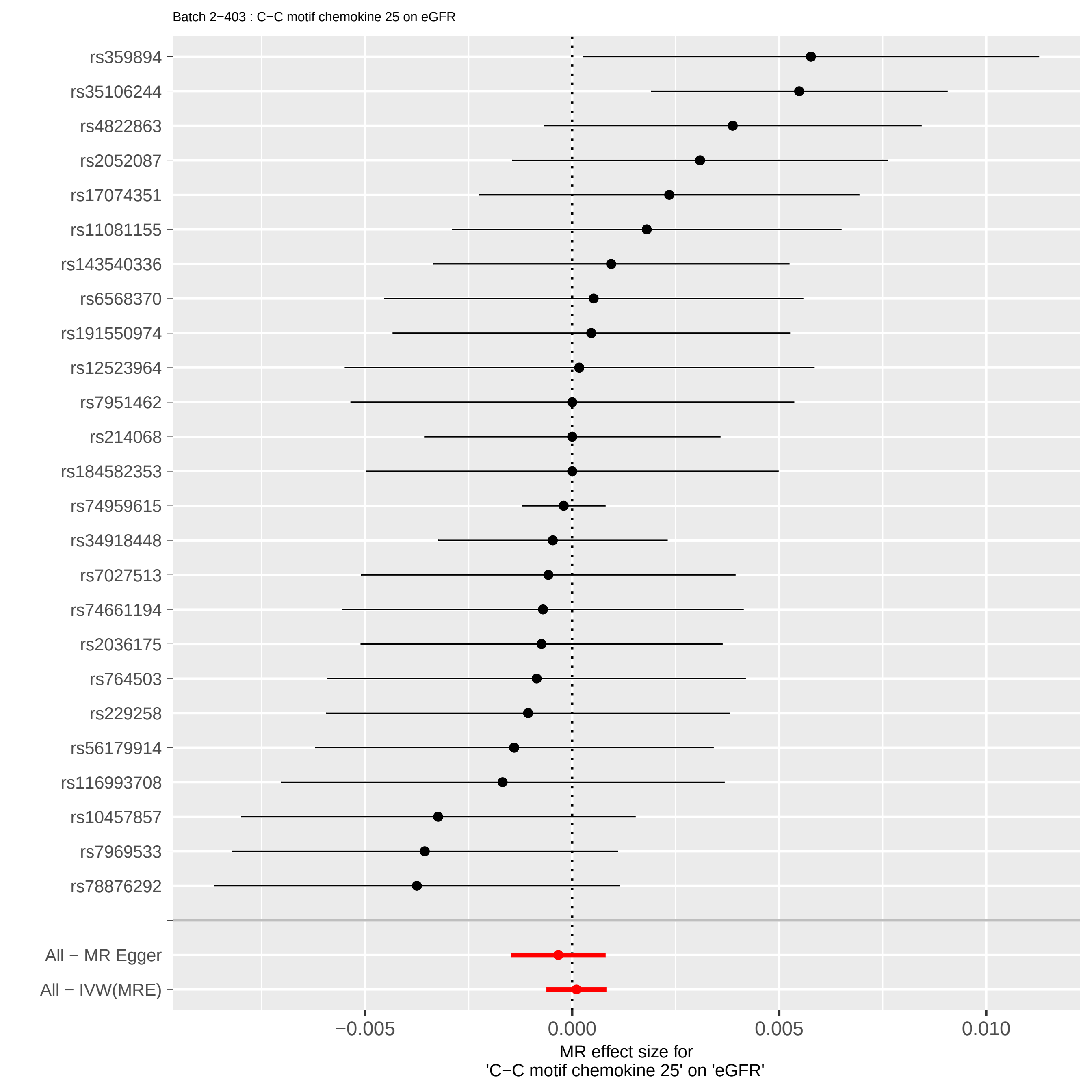

Batch 2−403 : C−C motif chemokine 25 on eGFR
rs359894
rs35106244
rs4822863
rs2052087
rs17074351
rs11081155
rs143540336
rs6568370
rs191550974
rs12523964
rs7951462
rs214068
rs184582353
rs74959615
rs34918448
rs7027513
rs74661194
rs2036175
rs764503
rs229258
rs56179914
rs116993708
rs10457857
rs7969533
rs78876292
All − MR Egger
All − IVW(MRE)
−0.005
0.000
0.005
0.010
MR effect size for
'C−C motif chemokine 25' on 'eGFR'

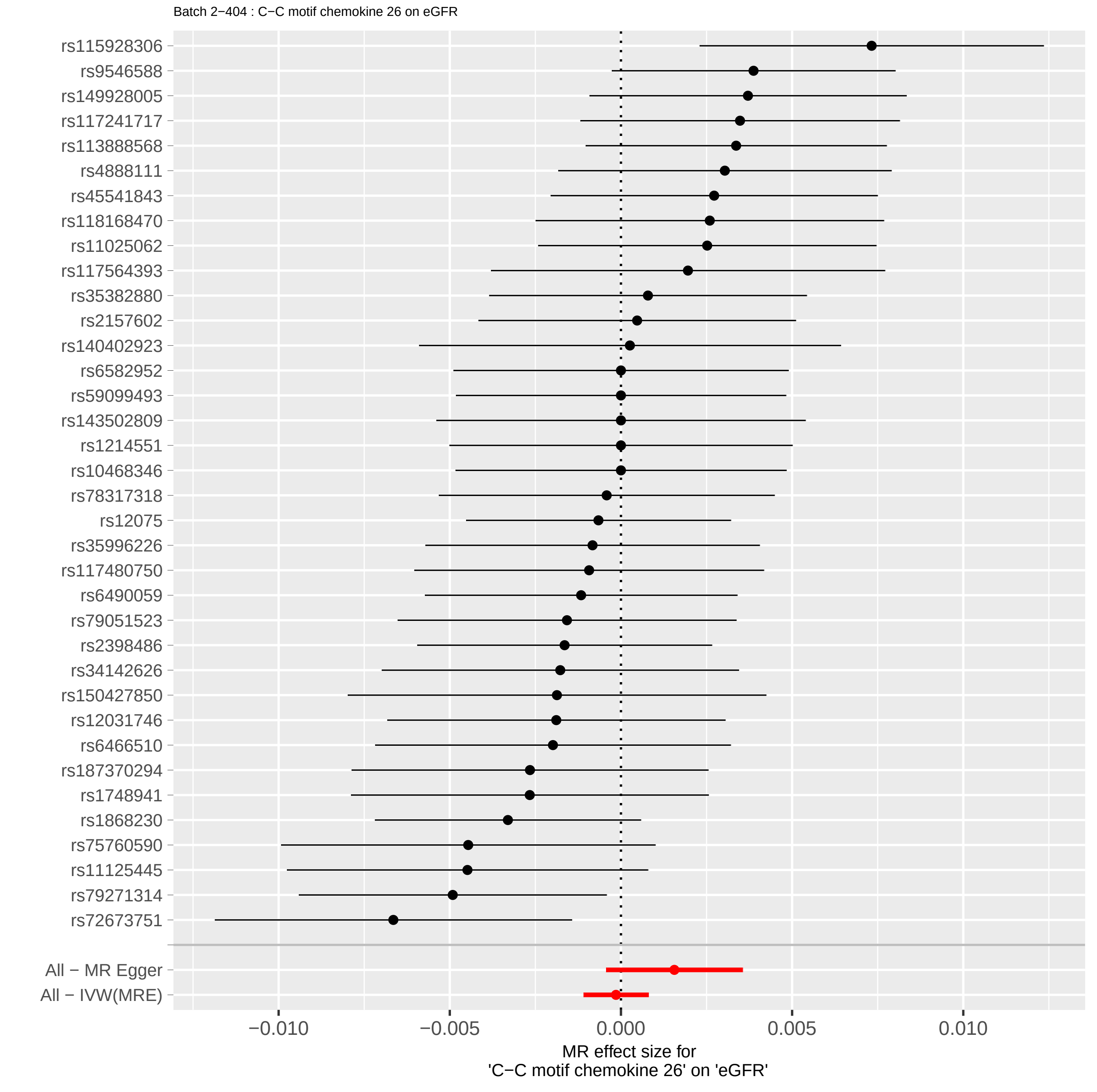

Batch 2−404 : C−C motif chemokine 26 on eGFR
rs115928306
rs9546588
rs149928005
rs117241717
rs113888568
rs4888111
rs45541843
rs118168470
rs11025062
rs117564393
rs35382880
rs2157602
rs140402923
rs6582952
rs59099493
rs143502809
rs1214551
rs10468346
rs78317318
rs12075
rs35996226
rs117480750
rs6490059
rs79051523
rs2398486
rs34142626
rs150427850
rs12031746
rs6466510
rs187370294
rs1748941
rs1868230
rs75760590
rs11125445
rs79271314
rs72673751
All − MR Egger
All − IVW(MRE)
−0.010
−0.005
0.000
0.005
0.010
MR effect size for
'C−C motif chemokine 26' on 'eGFR'

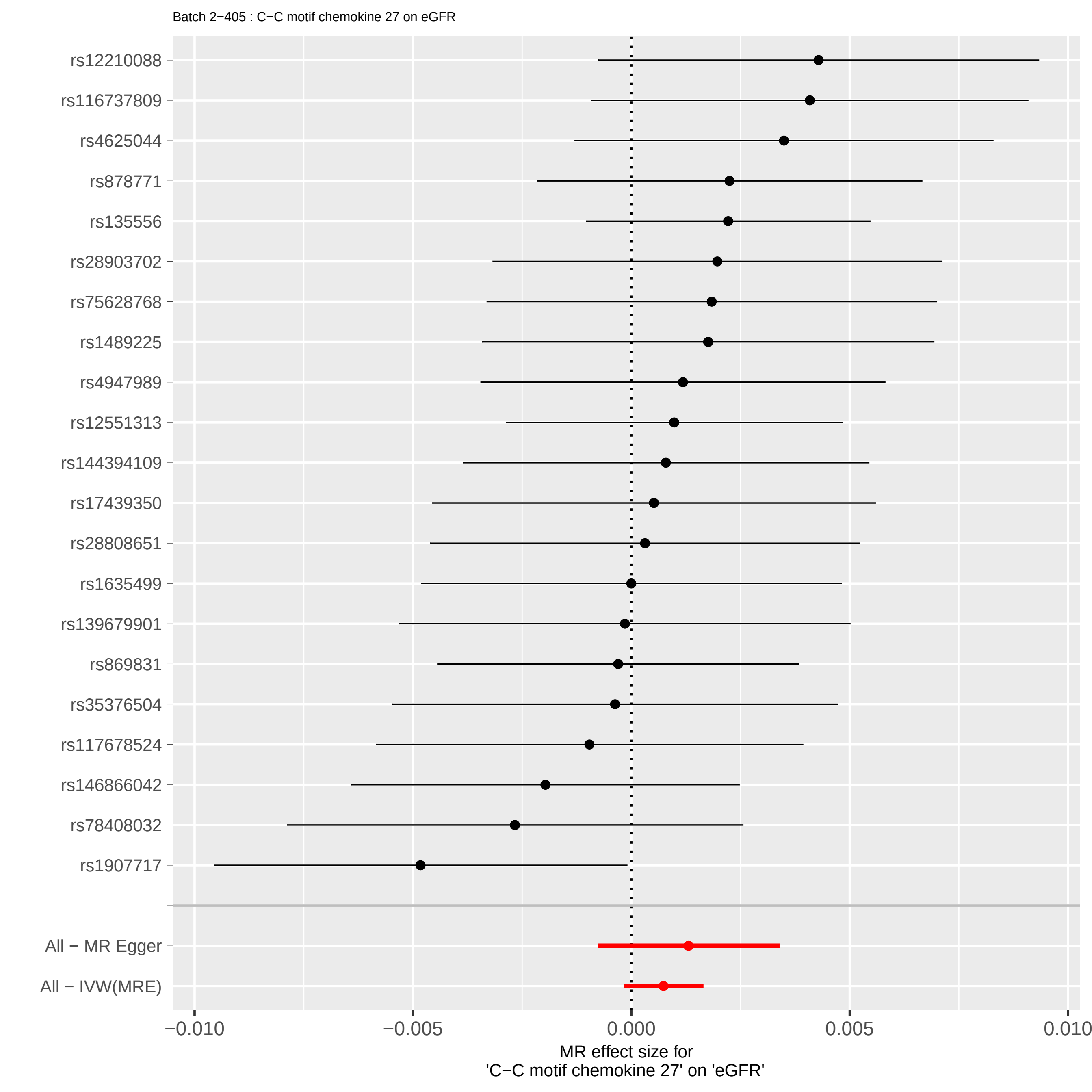
Batch 2−405 : C−C motif chemokine 27 on eGFR
rs12210088
rs116737809
rs4625044
rs878771
rs135556
rs28903702
rs75628768
rs1489225
rs4947989
rs12551313
rs144394109
rs17439350
rs28808651
rs1635499
rs139679901
rs869831
rs35376504
rs117678524
rs146866042
rs78408032
rs1907717
All − MR Egger
All − IVW(MRE)
−0.010
−0.005
0.000
0.005
0.010
MR effect size for
'C−C motif chemokine 27' on 'eGFR'

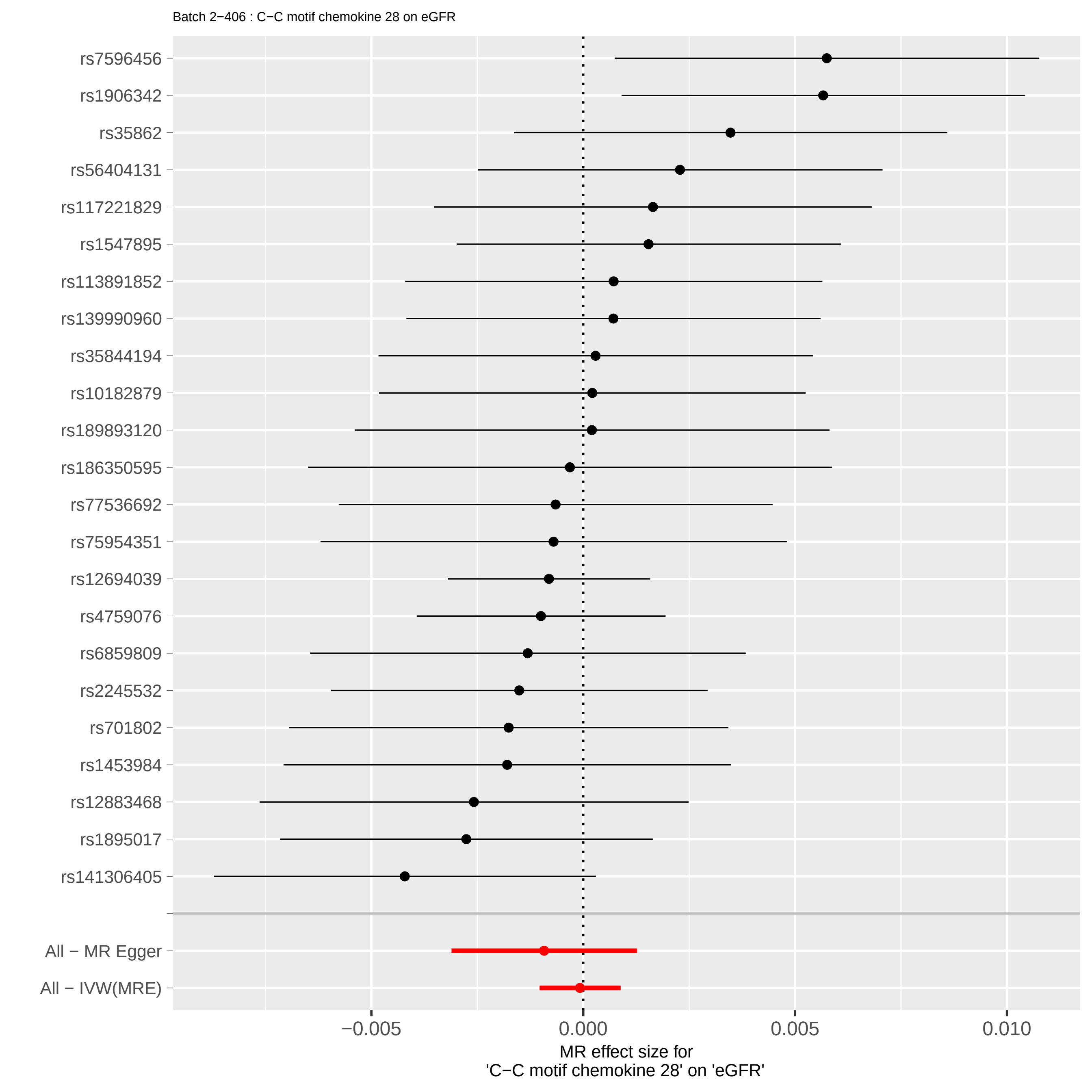

Batch 2−406 : C−C motif chemokine 28 on eGFR
rs7596456
rs1906342
rs35862
rs56404131
rs117221829
rs1547895
rs113891852
rs139990960
rs35844194
rs10182879
rs189893120
rs186350595
rs77536692
rs75954351
rs12694039
rs4759076
rs6859809
rs2245532
rs701802
rs1453984
rs12883468
rs1895017
rs141306405
All − MR Egger
All − IVW(MRE)
−0.005
0.000
0.005
0.010
MR effect size for
'C−C motif chemokine 28' on 'eGFR'

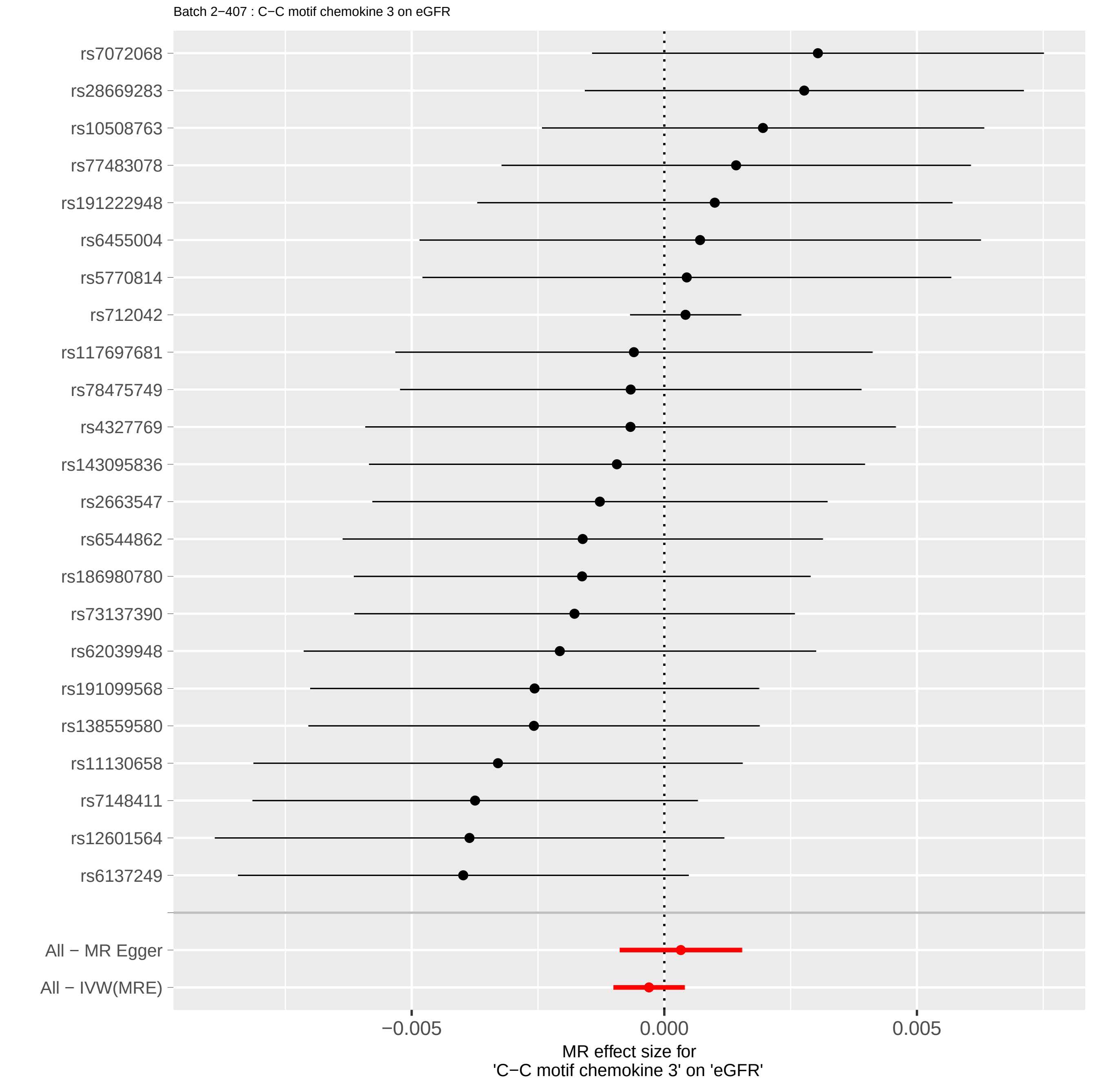
Batch 2−407 : C−C motif chemokine 3 on eGFR
rs7072068
rs28669283
rs10508763
rs77483078
rs191222948
rs6455004
rs5770814
rs712042
rs117697681
rs78475749
rs4327769
rs143095836
rs2663547
rs6544862
rs186980780
rs73137390
rs62039948
rs191099568
rs138559580
rs11130658
rs7148411
rs12601564
rs6137249
All − MR Egger
All − IVW(MRE)
−0.005
0.000
0.005
MR effect size for
'C−C motif chemokine 3' on 'eGFR'

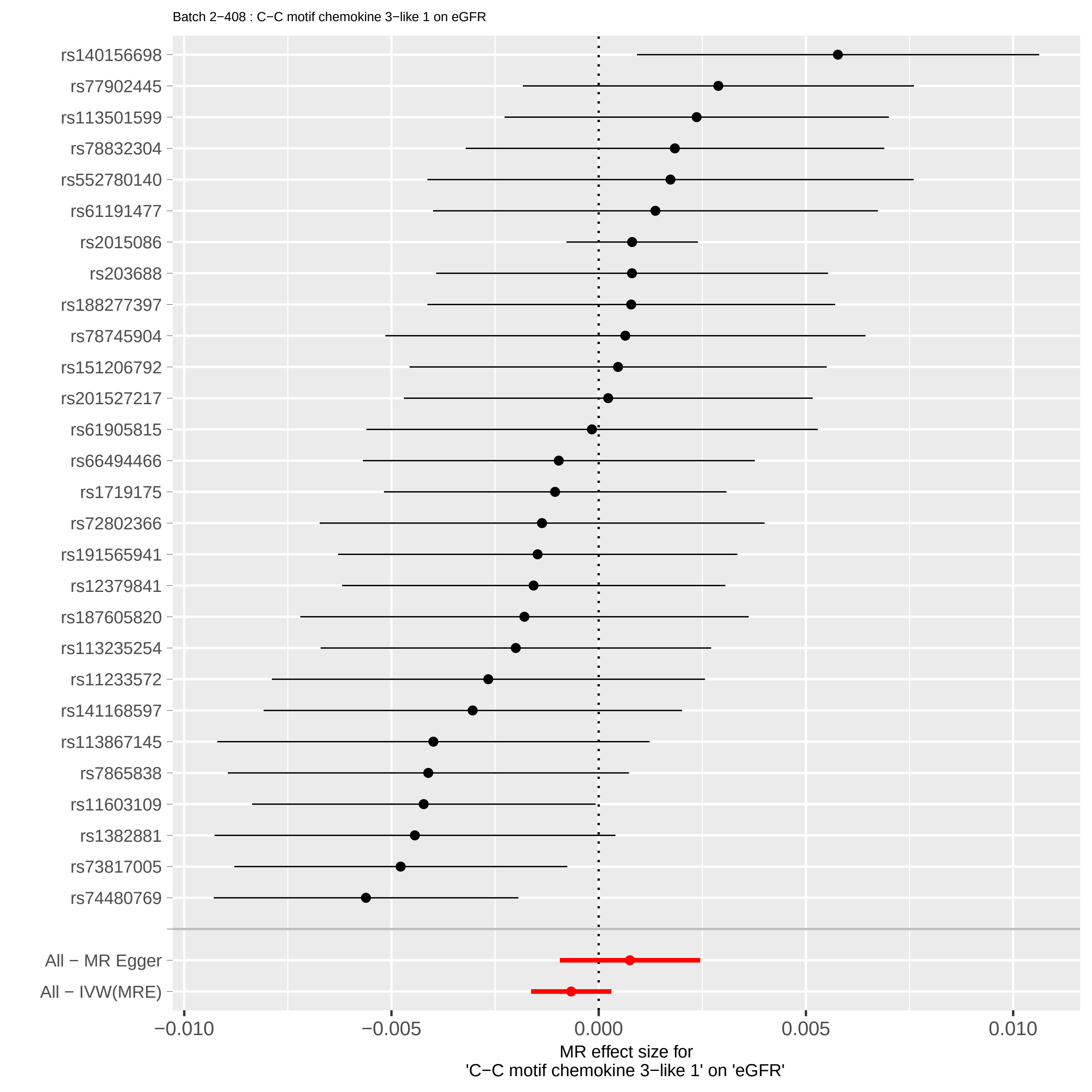
Batch 2−408 : C−C motif chemokine 3−like 1 on eGFR
rs140156698
rs77902445
rs113501599
rs78832304
rs552780140
rs61191477
rs2015086
rs203688
rs188277397
rs78745904
rs151206792
rs201527217
rs61905815
rs66494466
rs1719175
rs72802366
rs191565941
rs12379841
rs187605820
rs113235254
rs11233572
rs141168597
rs113867145
rs7865838
rs11603109
rs1382881
rs73817005
rs74480769
All − MR Egger
All − IVW(MRE)
−0.010
−0.005
0.000
0.005
0.010
MR effect size for
'C−C motif chemokine 3−like 1' on 'eGFR'

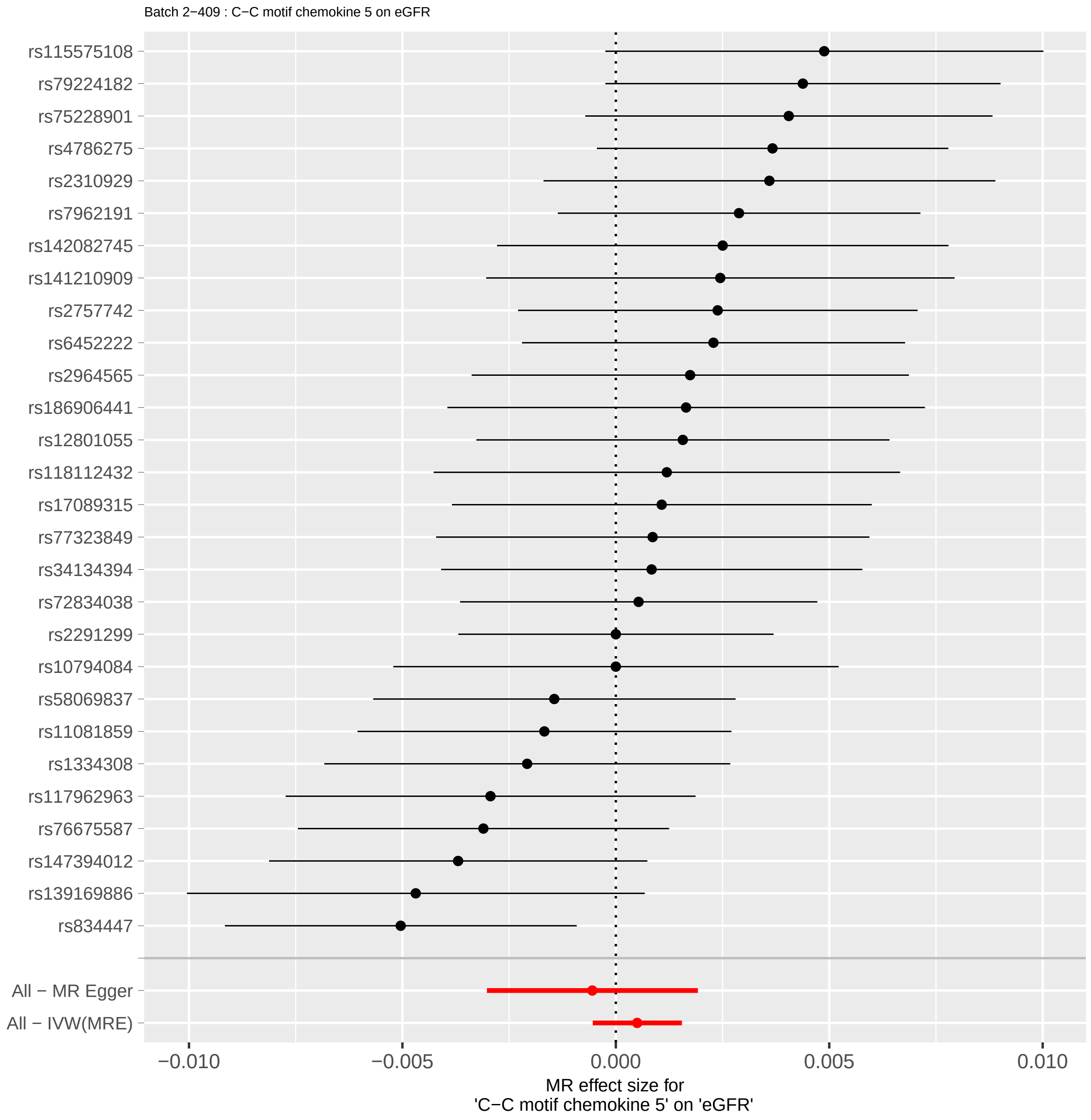

Batch 2−409 : C−C motif chemokine 5 on eGFR
rs115575108
rs79224182
rs75228901
rs4786275
rs2310929
rs7962191
rs142082745
rs141210909
rs2757742
rs6452222
rs2964565
rs186906441
rs12801055
rs118112432
rs17089315
rs77323849
rs34134394
rs72834038
rs2291299
rs10794084
rs58069837
rs11081859
rs1334308
rs117962963
rs76675587
rs147394012
rs139169886
rs834447
All − MR Egger
All − IVW(MRE)
−0.010
−0.005
0.000
0.005
0.010
MR effect size for
'C−C motif chemokine 5' on 'eGFR'

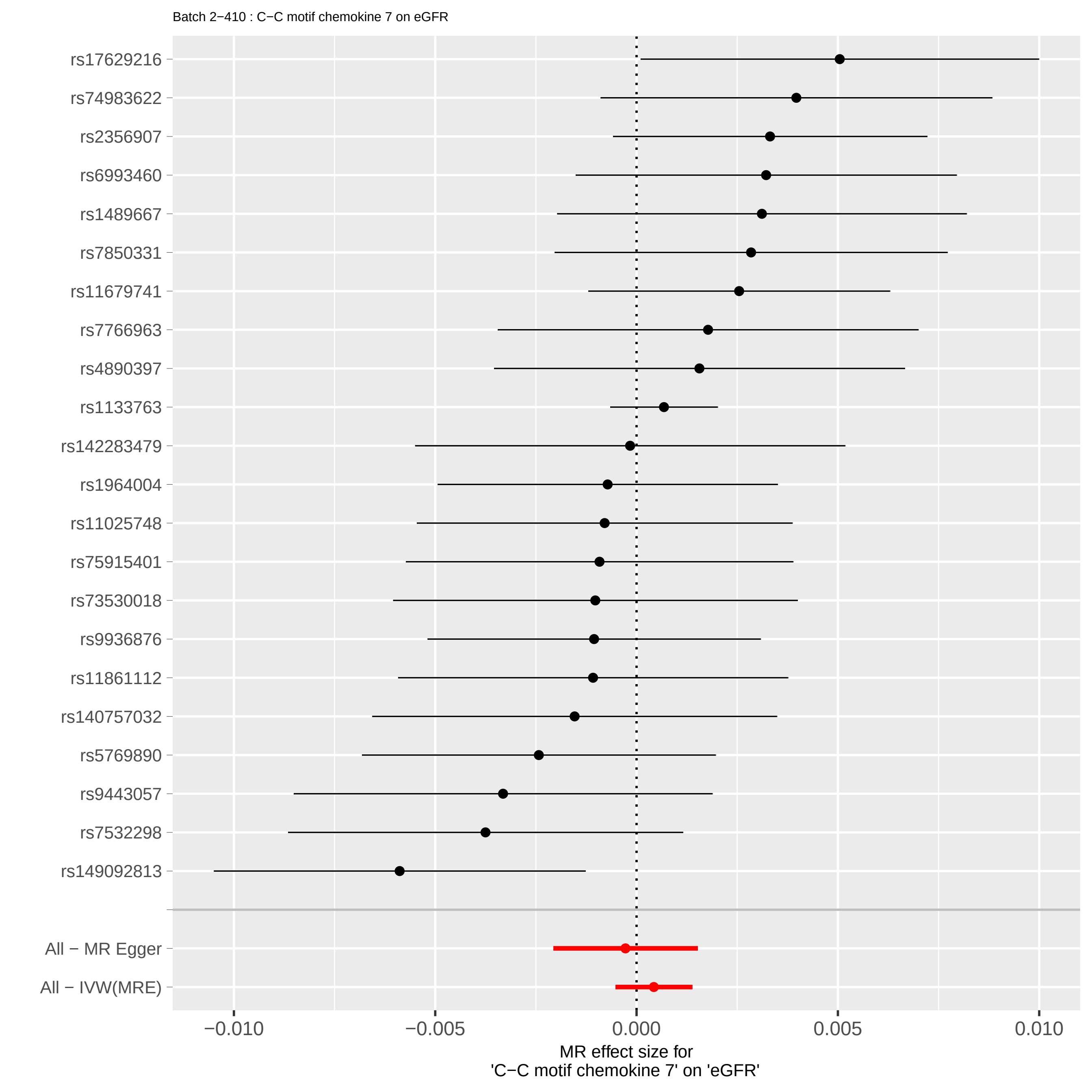

Batch 2−410 : C−C motif chemokine 7 on eGFR
rs17629216
rs74983622
rs2356907
rs6993460
rs1489667
rs7850331
rs11679741
rs7766963
rs4890397
rs1133763
rs142283479
rs1964004
rs11025748
rs75915401
rs73530018
rs9936876
rs11861112
rs140757032
rs5769890
rs9443057
rs7532298
rs149092813
All − MR Egger
All − IVW(MRE)
−0.010
−0.005
0.000
0.005
0.010
MR effect size for
'C−C motif chemokine 7' on 'eGFR'

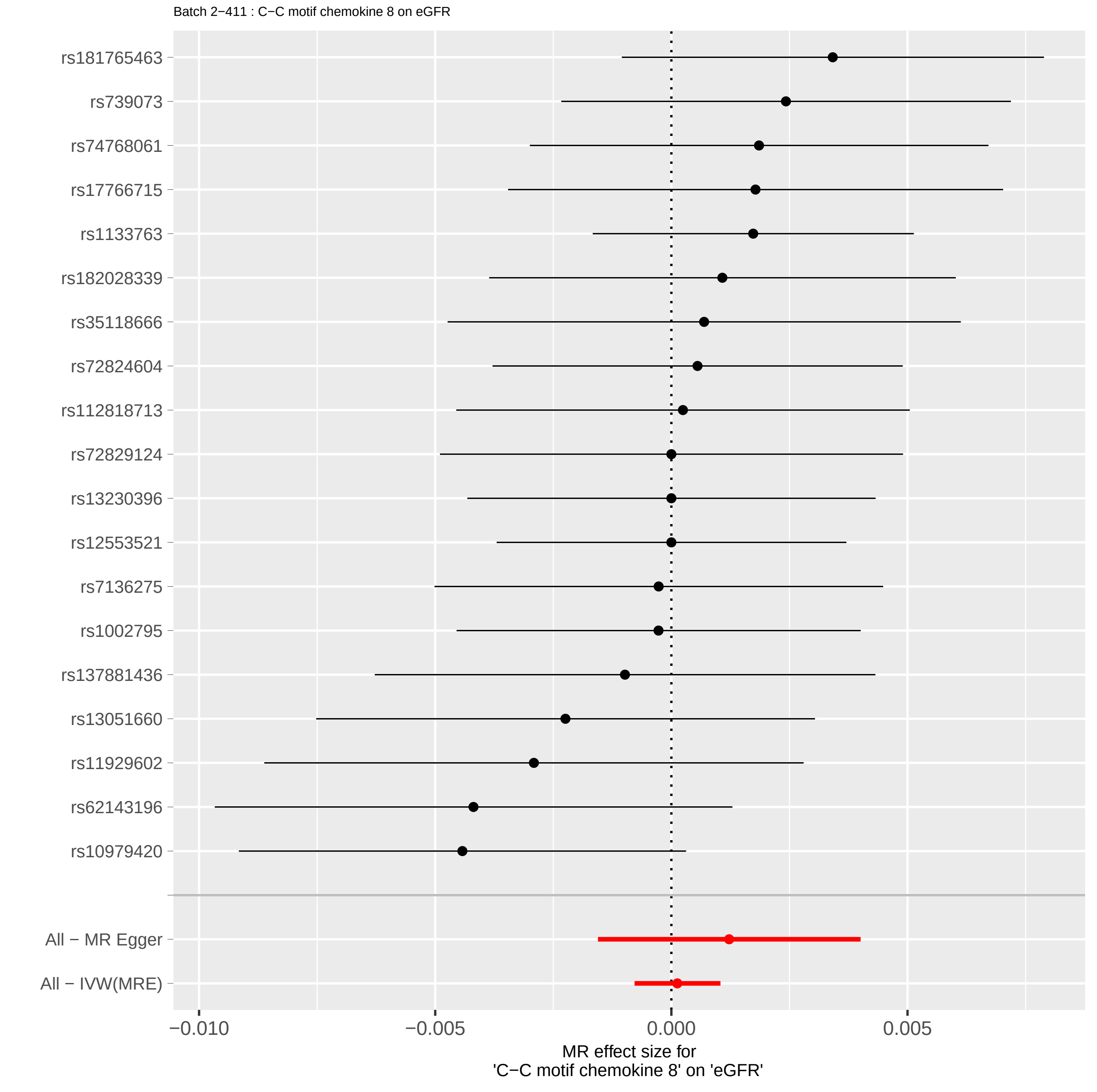

Batch 2−411 : C−C motif chemokine 8 on eGFR
rs181765463
rs739073
rs74768061
rs17766715
rs1133763
rs182028339
rs35118666
rs72824604
rs112818713
rs72829124
rs13230396
rs12553521
rs7136275
rs1002795
rs137881436
rs13051660
rs11929602
rs62143196
rs10979420
All − MR Egger
All − IVW(MRE)
−0.010
−0.005
0.000
0.005
MR effect size for
'C−C motif chemokine 8' on 'eGFR'

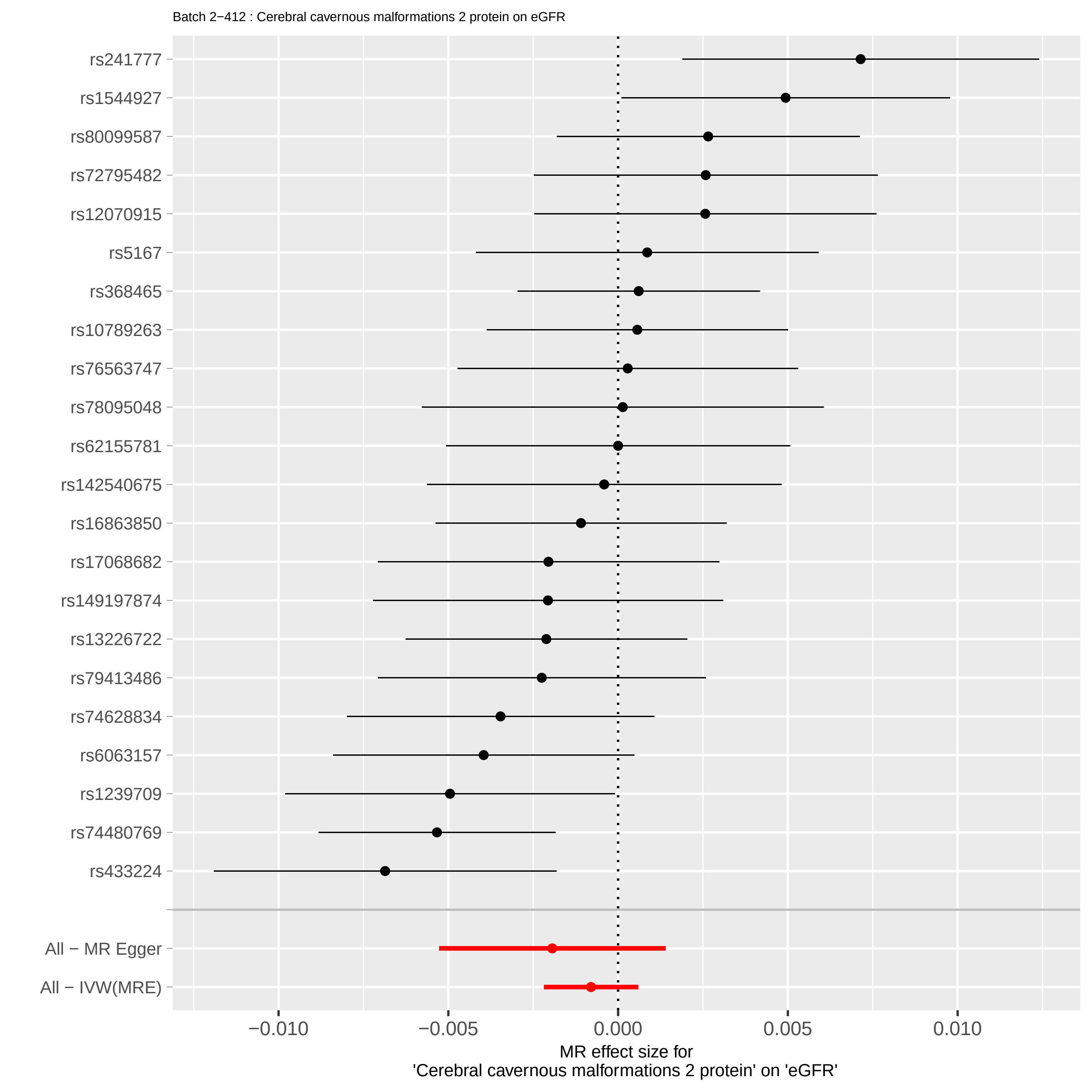

Batch 2−412 : Cerebral cavernous malformations 2 protein on eGFR
rs241777
rs1544927
rs80099587
rs72795482
rs12070915
rs5167
rs368465
rs10789263
rs76563747
rs78095048
rs62155781
rs142540675
rs16863850
rs17068682
rs149197874
rs13226722
rs79413486
rs74628834
rs6063157
rs1239709
rs74480769
rs433224
All − MR Egger
All − IVW(MRE)
−0.010
−0.005
0.000
0.005
0.010
MR effect size for
'Cerebral cavernous malformations 2 protein' on 'eGFR'

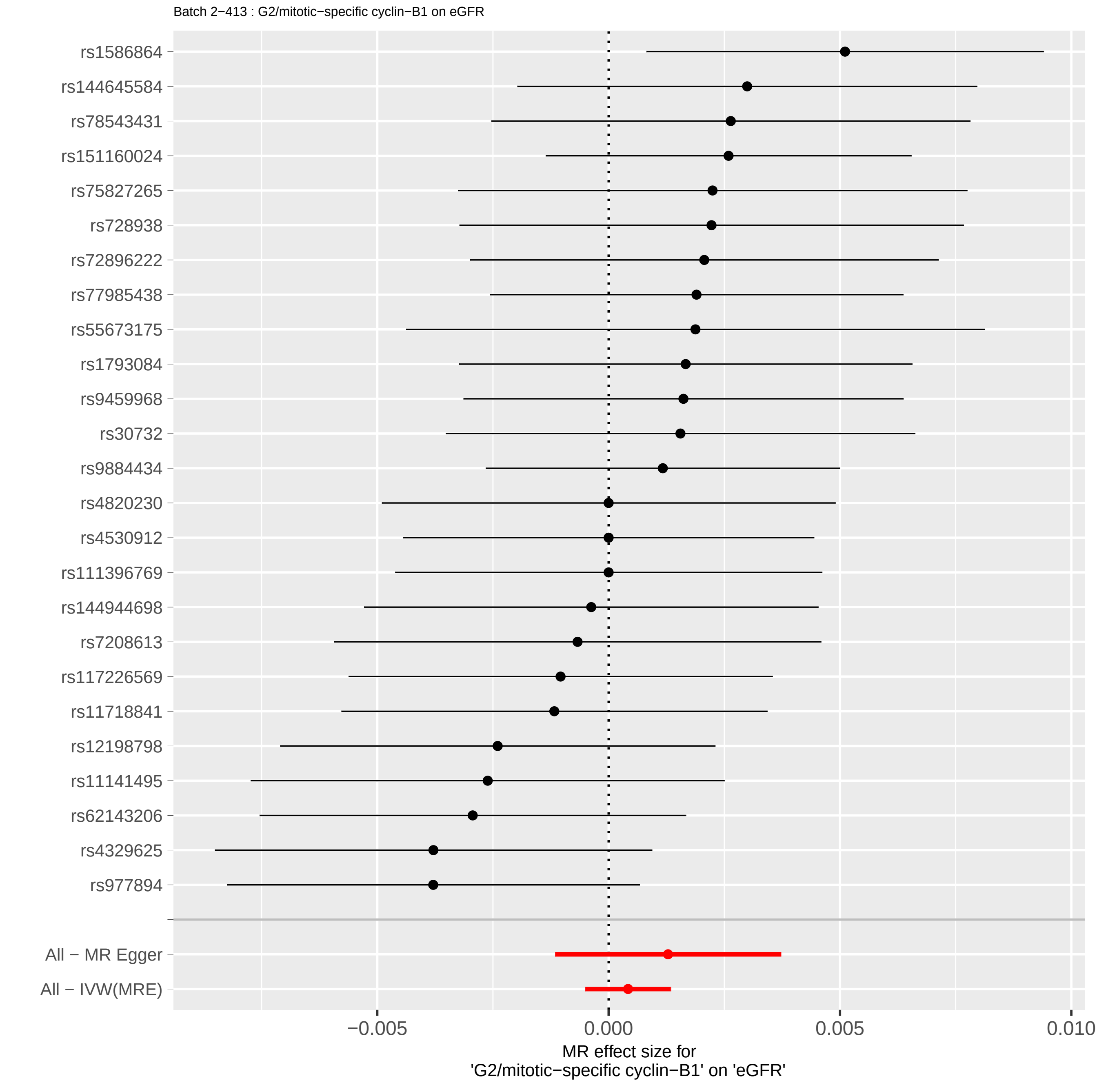
Batch 2−413 : G2/mitotic−specific cyclin−B1 on eGFR
rs1586864
rs144645584
rs78543431
rs151160024
rs75827265
rs728938
rs72896222
rs77985438
rs55673175
rs1793084
rs9459968
rs30732
rs9884434
rs4820230
rs4530912
rs111396769
rs144944698
rs7208613
rs117226569
rs11718841
rs12198798
rs11141495
rs62143206
rs4329625
rs977894
All − MR Egger
All − IVW(MRE)
−0.005
0.000
0.005
0.010
MR effect size for
'G2/mitotic−specific cyclin−B1' on 'eGFR'

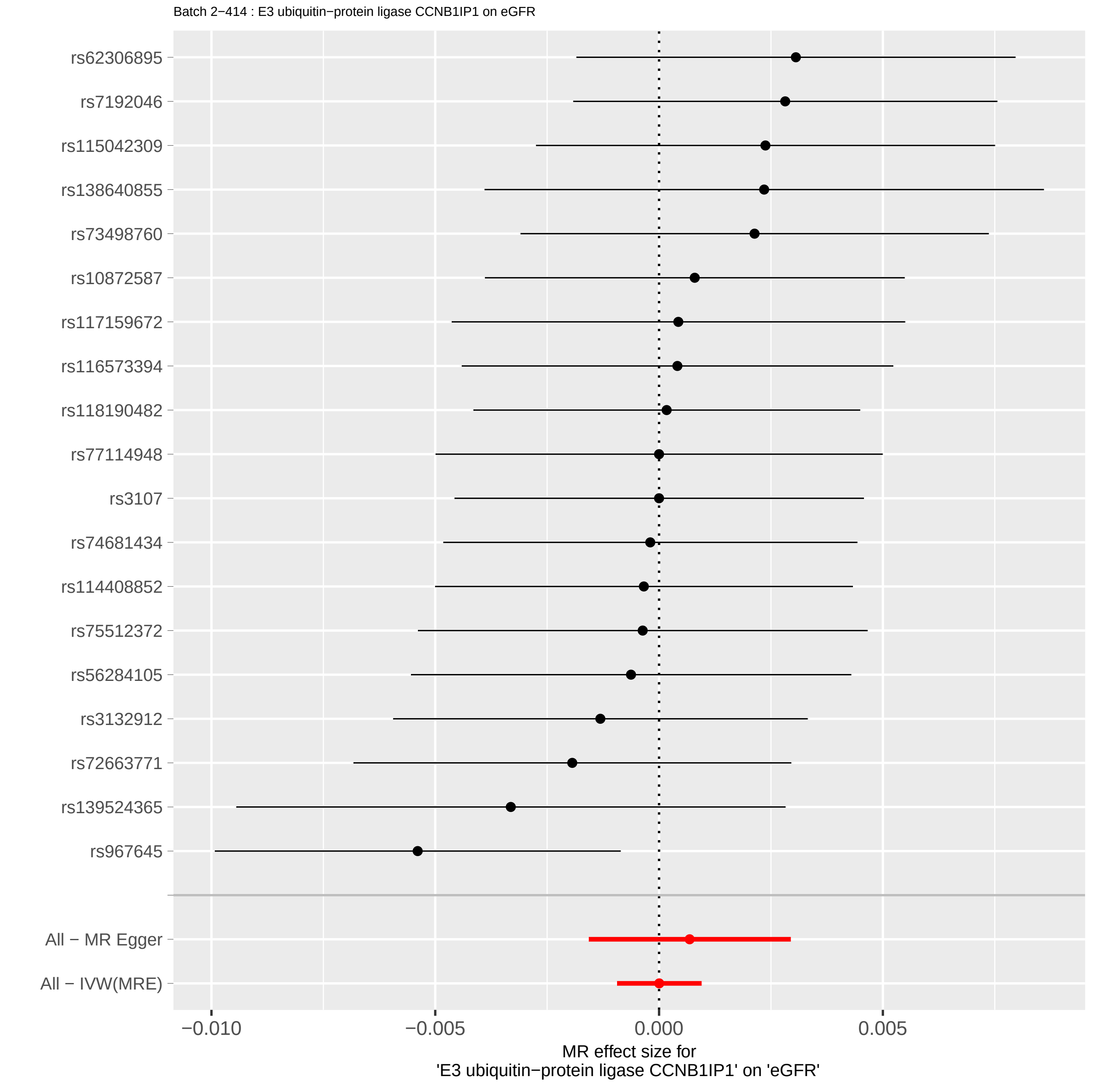

Batch 2−414 : E3 ubiquitin−protein ligase CCNB1IP1 on eGFR
rs62306895
rs7192046
rs115042309
rs138640855
rs73498760
rs10872587
rs117159672
rs116573394
rs118190482
rs77114948
rs3107
rs74681434
rs114408852
rs75512372
rs56284105
rs3132912
rs72663771
rs139524365
rs967645
All − MR Egger
All − IVW(MRE)
−0.010
−0.005
0.000
0.005
MR effect size for
'E3 ubiquitin−protein ligase CCNB1IP1' on 'eGFR'

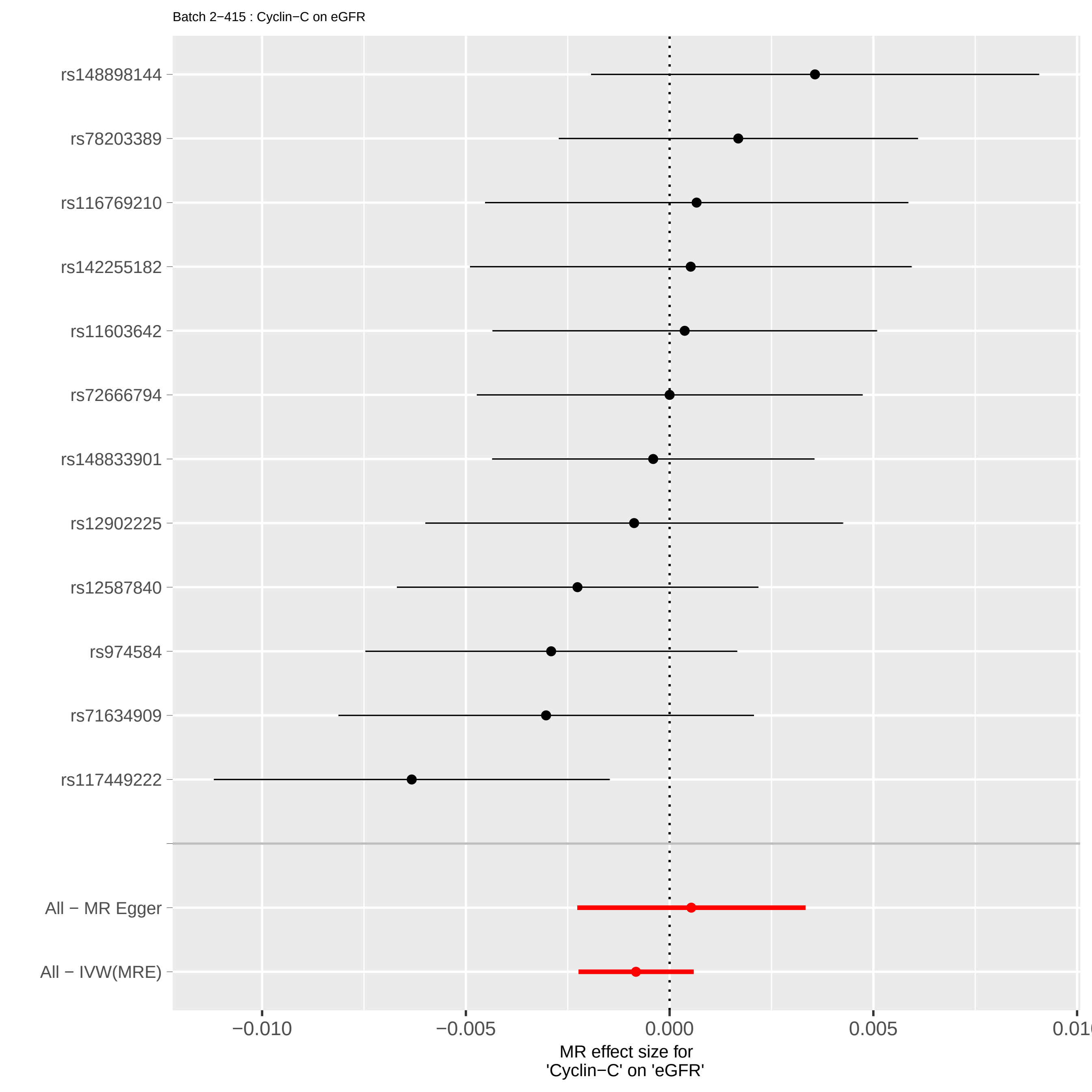

Batch 2−415 : Cyclin−C on eGFR
rs148898144
rs78203389
rs116769210
rs142255182
rs11603642
rs72666794
rs148833901
rs12902225
rs12587840
rs974584
rs71634909
rs117449222
All − MR Egger
All − IVW(MRE)
−0.010
−0.005
0.000
0.005
MR effect size for
'Cyclin−C' on 'eGFR'

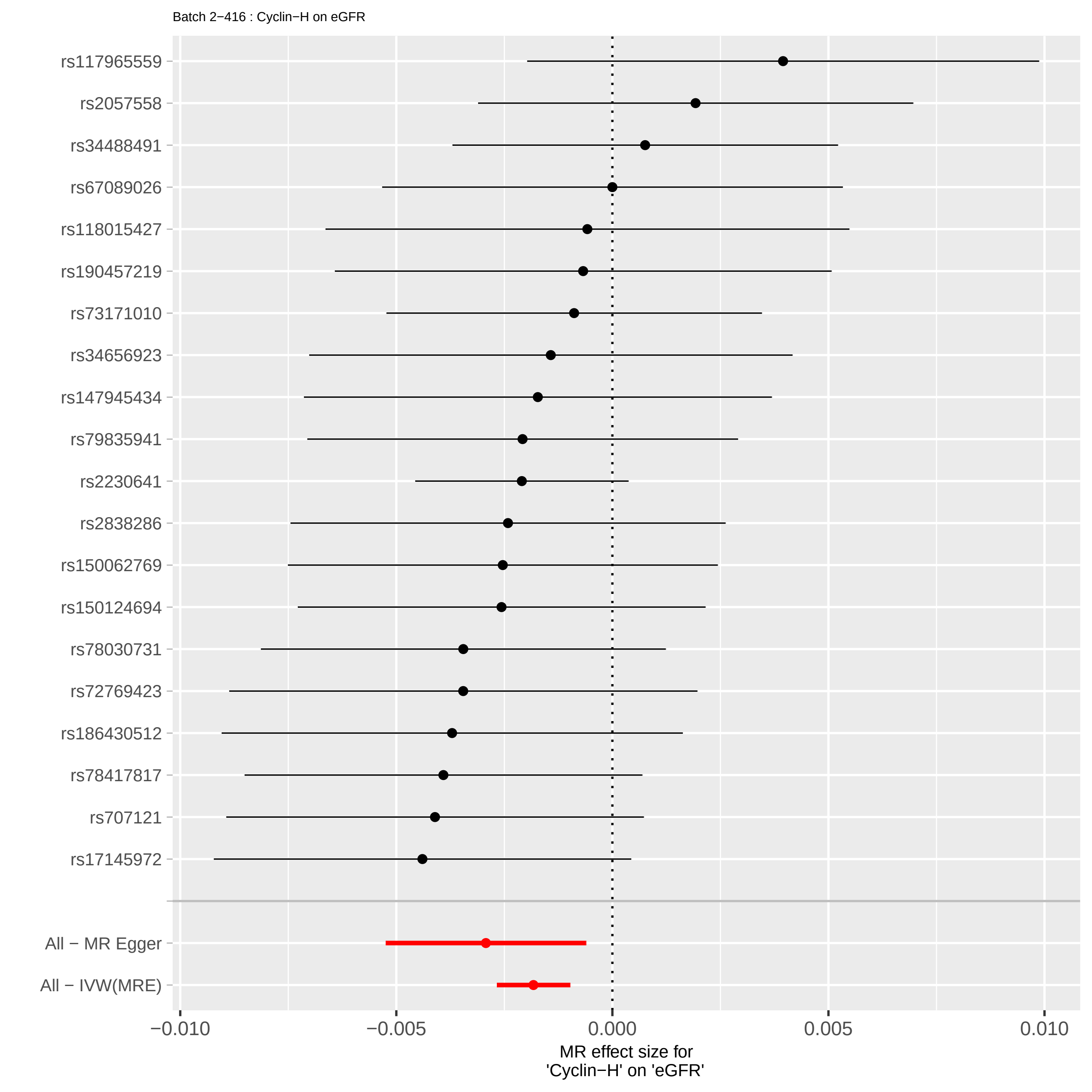

Batch 2−416 : Cyclin−H on eGFR
rs117965559
rs2057558
rs34488491
rs67089026
rs118015427
rs190457219
rs73171010
rs34656923
rs147945434
rs79835941
rs2230641
rs2838286
rs150062769
rs150124694
rs78030731
rs72769423
rs186430512
rs78417817
rs707121
rs17145972
All − MR Egger
All − IVW(MRE)
−0.010
−0.005
0.000
0.005
0.010
MR effect size for
'Cyclin−H' on 'eGFR'

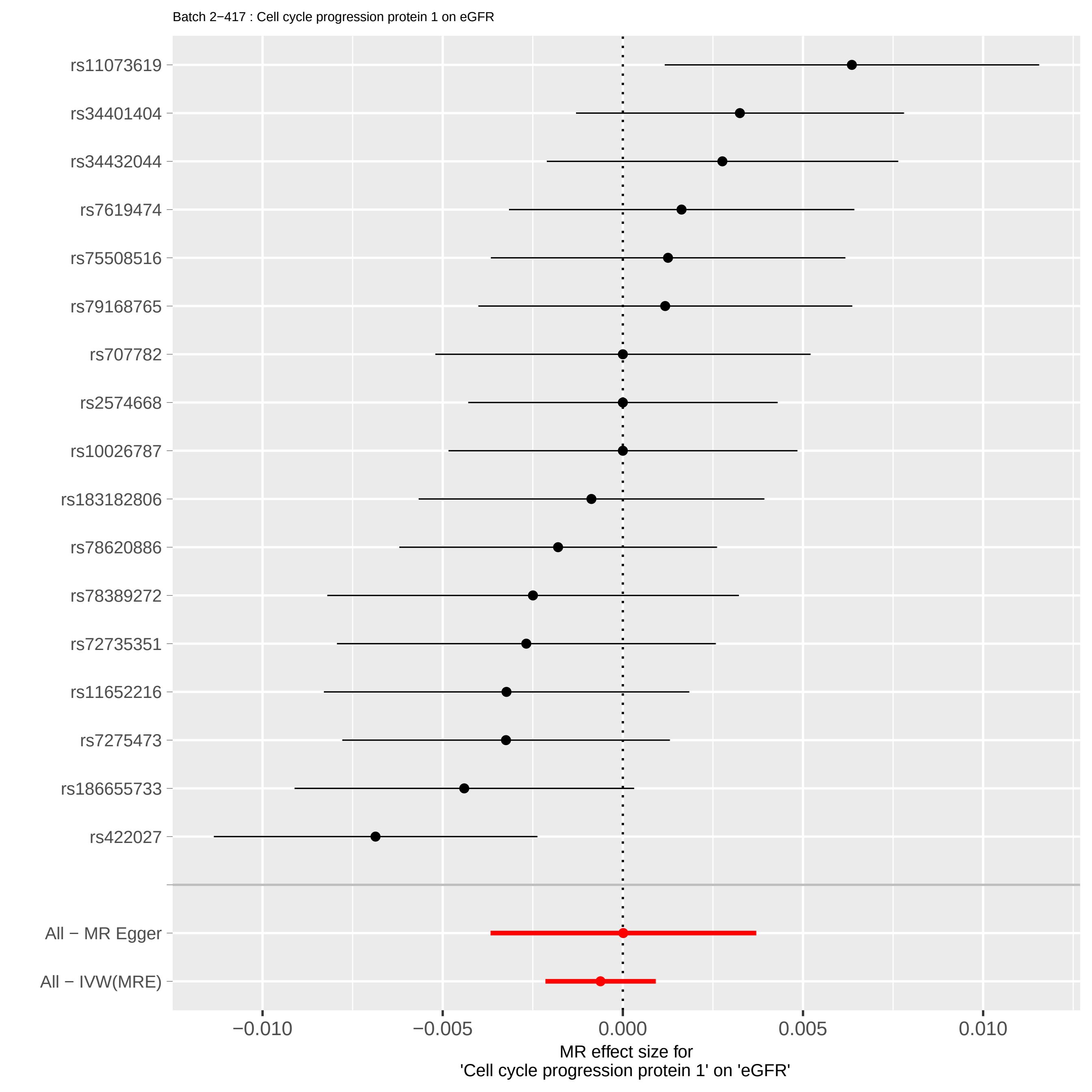

Batch 2−417 : Cell cycle progression protein 1 on eGFR
rs11073619
rs34401404
rs34432044
rs7619474
rs75508516
rs79168765
rs707782
rs2574668
rs10026787
rs183182806
rs78620886
rs78389272
rs72735351
rs11652216
rs7275473
rs186655733
rs422027
All − MR Egger
All − IVW(MRE)
−0.010
−0.005
0.000
0.005
0.010
MR effect size for
'Cell cycle progression protein 1' on 'eGFR'

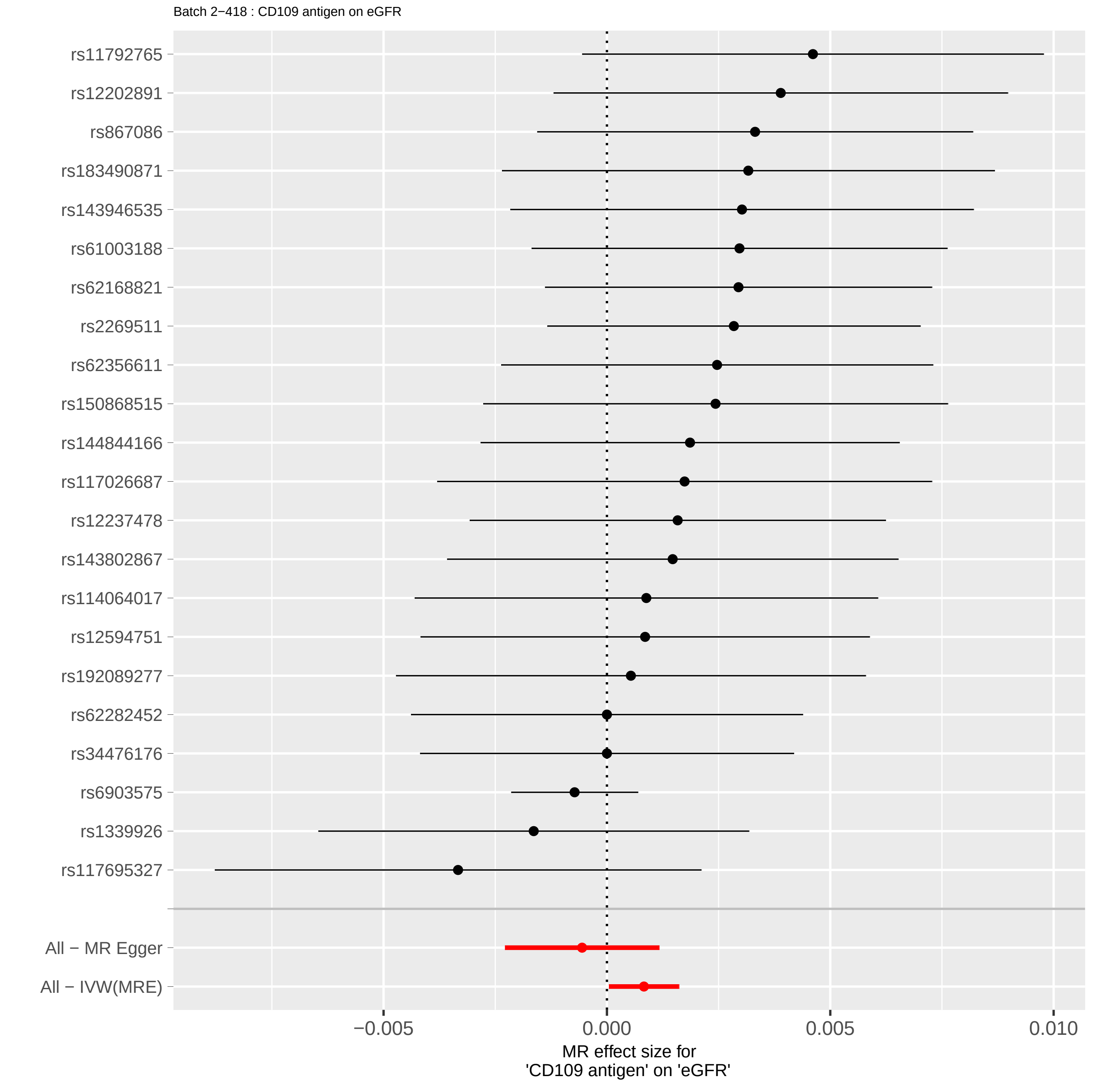

Batch 2−418 : CD109 antigen on eGFR
rs11792765
rs12202891
rs867086
rs183490871
rs143946535
rs61003188
rs62168821
rs2269511
rs62356611
rs150868515
rs144844166
rs117026687
rs12237478
rs143802867
rs114064017
rs12594751
rs192089277
rs62282452
rs34476176
rs6903575
rs1339926
rs117695327
All − MR Egger
All − IVW(MRE)
−0.005
0.000
0.005
0.010
MR effect size for
'CD109 antigen' on 'eGFR'

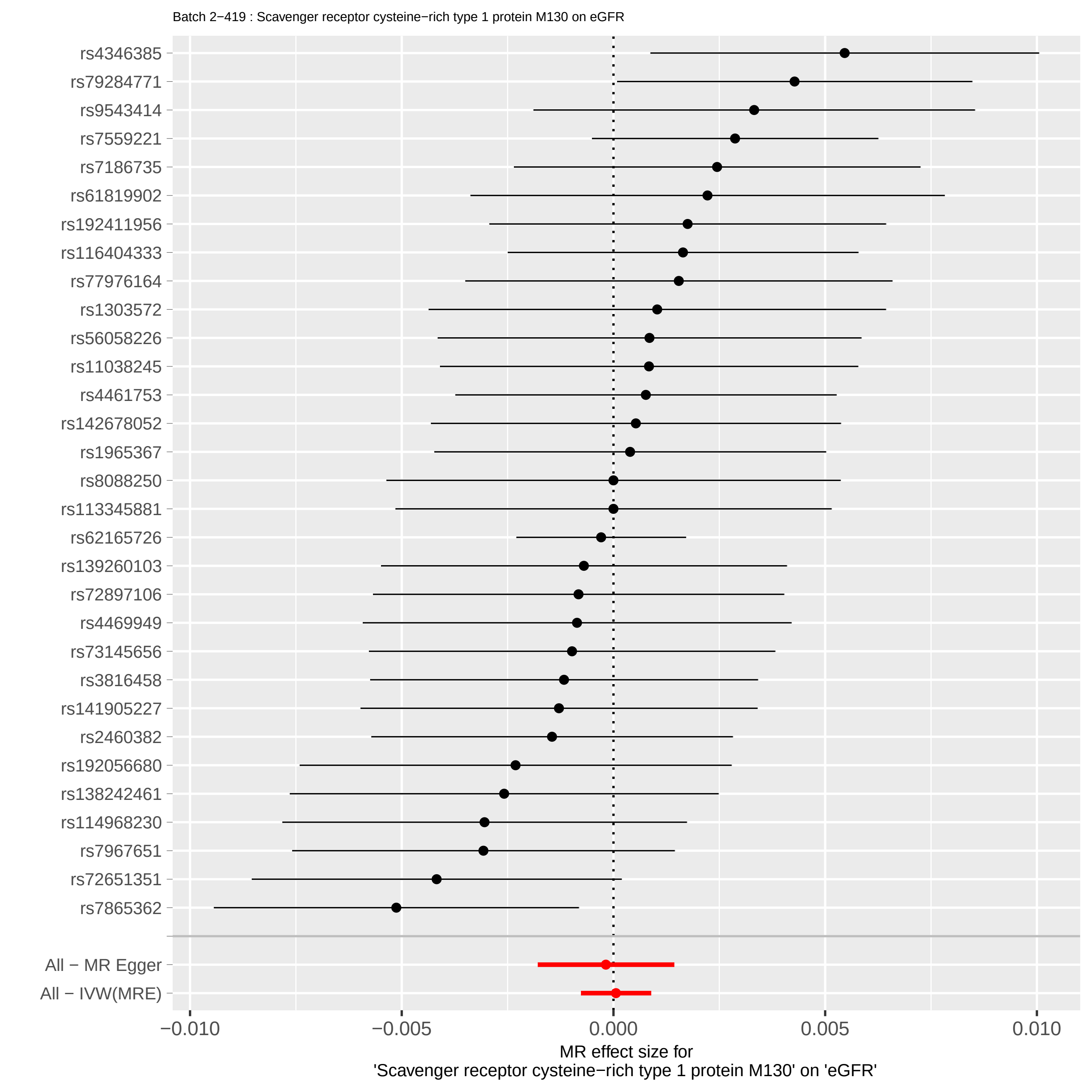
Batch 2−419 : Scavenger receptor cysteine−rich type 1 protein M130 on eGFR
rs4346385
rs79284771
rs9543414
rs7559221
rs7186735
rs61819902
rs192411956
rs116404333
rs77976164
rs1303572
rs56058226
rs11038245
rs4461753
rs142678052
rs1965367
rs8088250
rs113345881
rs62165726
rs139260103
rs72897106
rs4469949
rs73145656
rs3816458
rs141905227
rs2460382
rs192056680
rs138242461
rs114968230
rs7967651
rs72651351
rs7865362
All − MR Egger
All − IVW(MRE)
−0.010
−0.005
0.000
0.005
0.010
MR effect size for
'Scavenger receptor cysteine−rich type 1 protein M130' on 'eGFR'

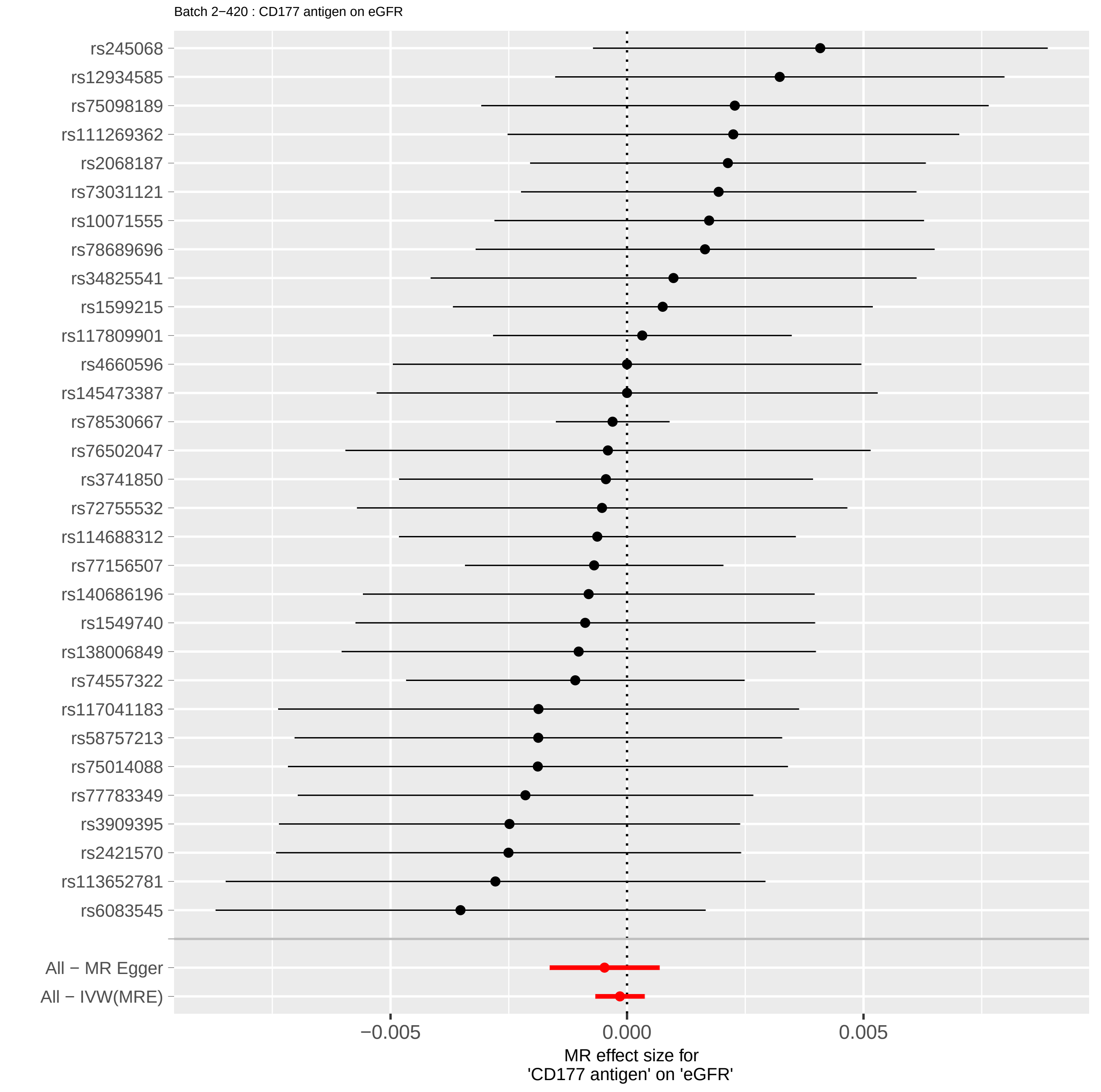

Batch 2−420 : CD177 antigen on eGFR
rs245068
rs12934585
rs75098189
rs111269362
rs2068187
rs73031121
rs10071555
rs78689696
rs34825541
rs1599215
rs117809901
rs4660596
rs145473387
rs78530667
rs76502047
rs3741850
rs72755532
rs114688312
rs77156507
rs140686196
rs1549740
rs138006849
rs74557322
rs117041183
rs58757213
rs75014088
rs77783349
rs3909395
rs2421570
rs113652781
rs6083545
All − MR Egger
All − IVW(MRE)
−0.005
0.000
0.005
MR effect size for
'CD177 antigen' on 'eGFR'

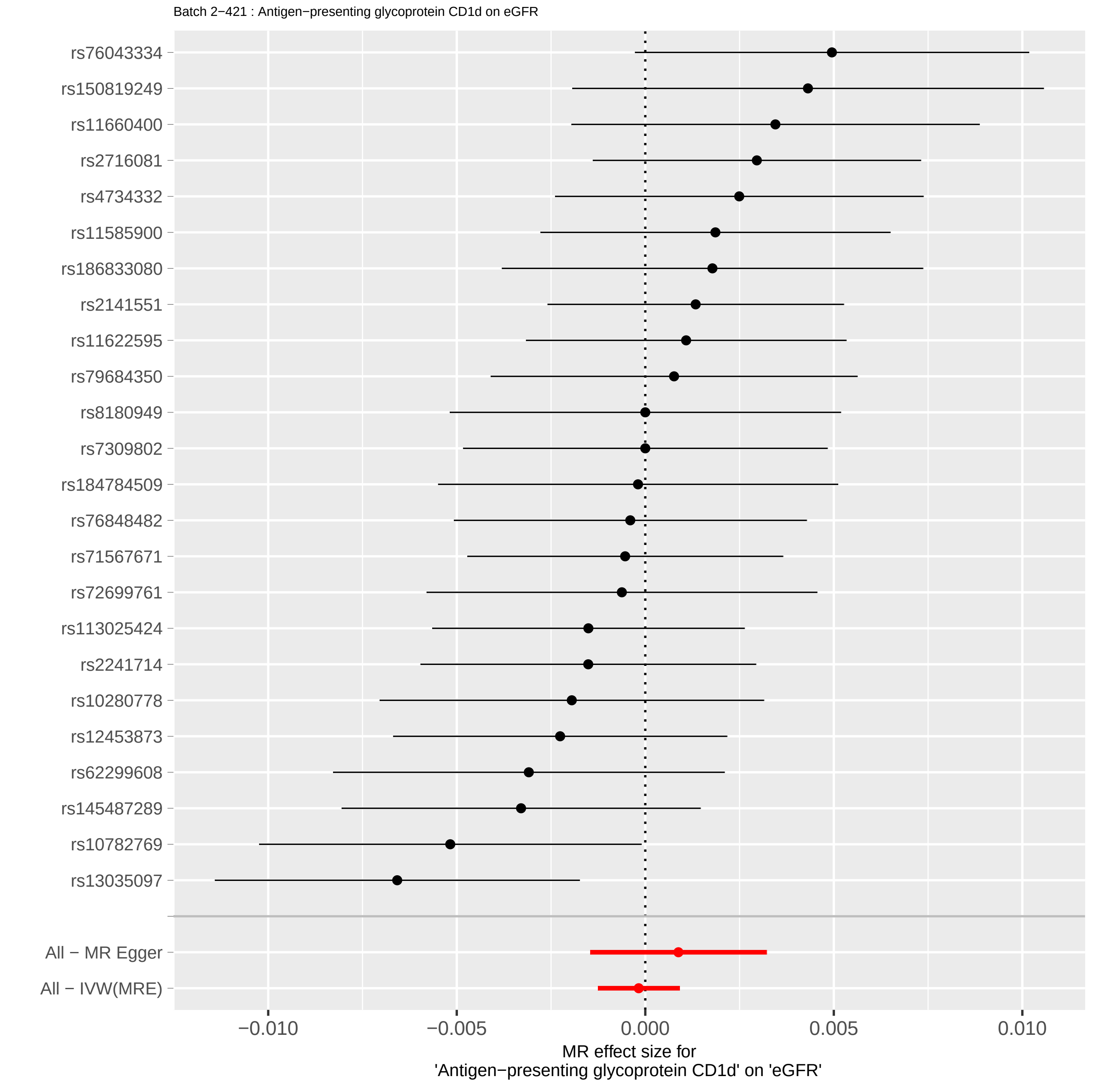
Batch 2−421 : Antigen−presenting glycoprotein CD1d on eGFR
rs76043334
rs150819249
rs11660400
rs2716081
rs4734332
rs11585900
rs186833080
rs2141551
rs11622595
rs79684350
rs8180949
rs7309802
rs184784509
rs76848482
rs71567671
rs72699761
rs113025424
rs2241714
rs10280778
rs12453873
rs62299608
rs145487289
rs10782769
rs13035097
All − MR Egger
All − IVW(MRE)
−0.010
−0.005
0.000
0.005
0.010
MR effect size for
'Antigen−presenting glycoprotein CD1d' on 'eGFR'

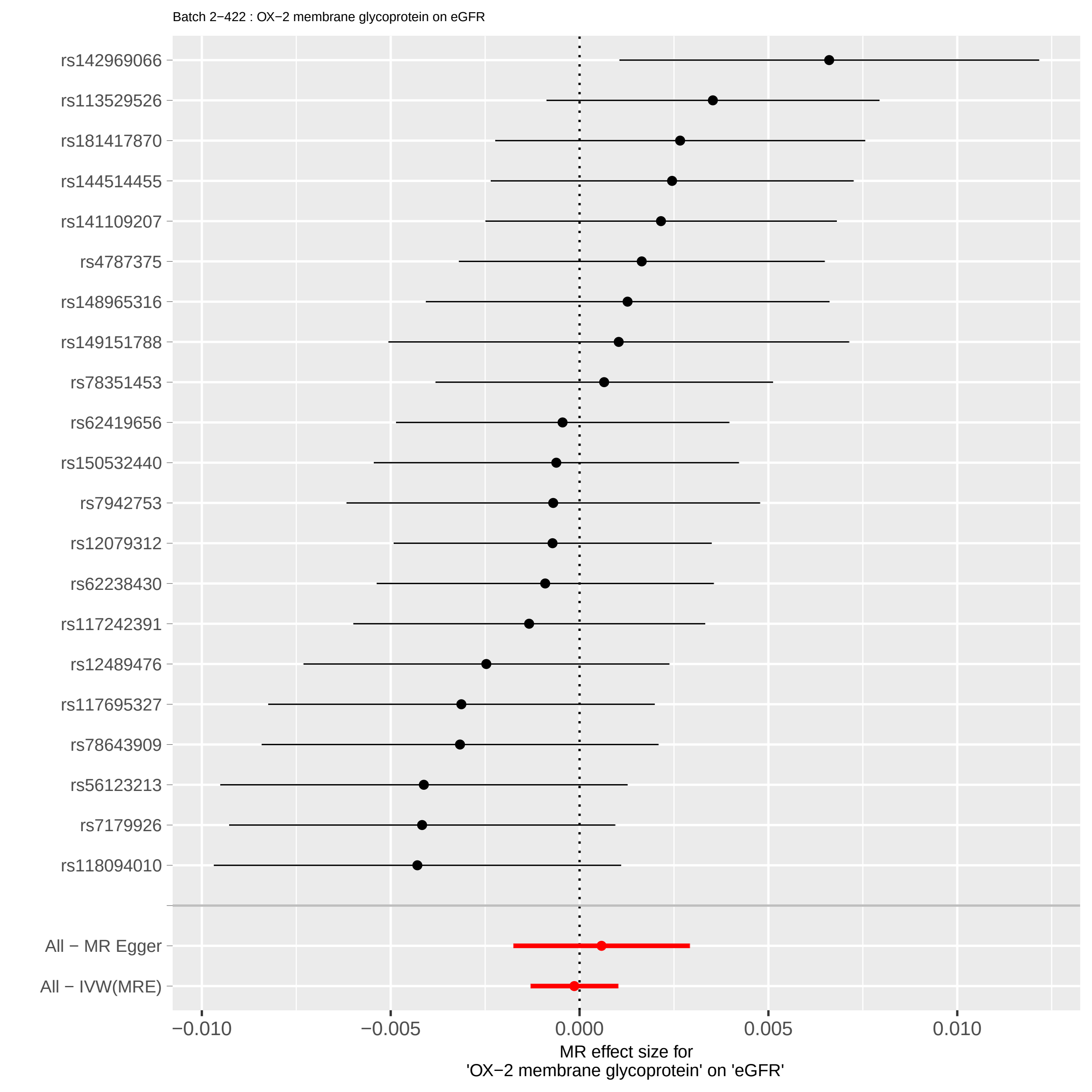
Batch 2−422 : OX−2 membrane glycoprotein on eGFR
rs142969066
rs113529526
rs181417870
rs144514455
rs141109207
rs4787375
rs148965316
rs149151788
rs78351453
rs62419656
rs150532440
rs7942753
rs12079312
rs62238430
rs117242391
rs12489476
rs117695327
rs78643909
rs56123213
rs7179926
rs118094010
All − MR Egger
All − IVW(MRE)
−0.010
−0.005
0.000
0.005
0.010
MR effect size for
'OX−2 membrane glycoprotein' on 'eGFR'

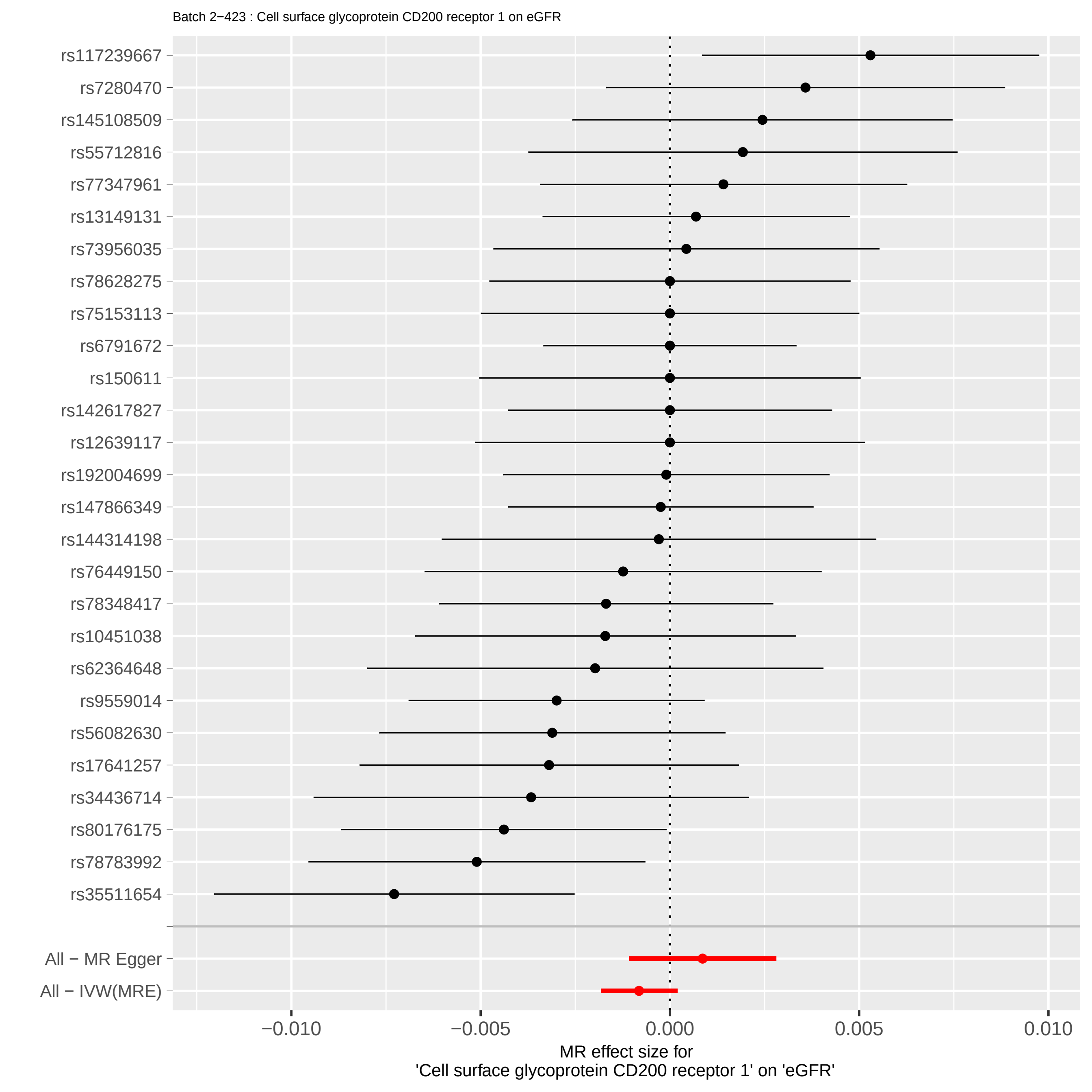

Batch 2−423 : Cell surface glycoprotein CD200 receptor 1 on eGFR
rs117239667
rs7280470
rs145108509
rs55712816
rs77347961
rs13149131
rs73956035
rs78628275
rs75153113
rs6791672
rs150611
rs142617827
rs12639117
rs192004699
rs147866349
rs144314198
rs76449150
rs78348417
rs10451038
rs62364648
rs9559014
rs56082630
rs17641257
rs34436714
rs80176175
rs78783992
rs35511654
All − MR Egger
All − IVW(MRE)
−0.010
−0.005
0.000
0.005
0.010
MR effect size for
'Cell surface glycoprotein CD200 receptor 1' on 'eGFR'

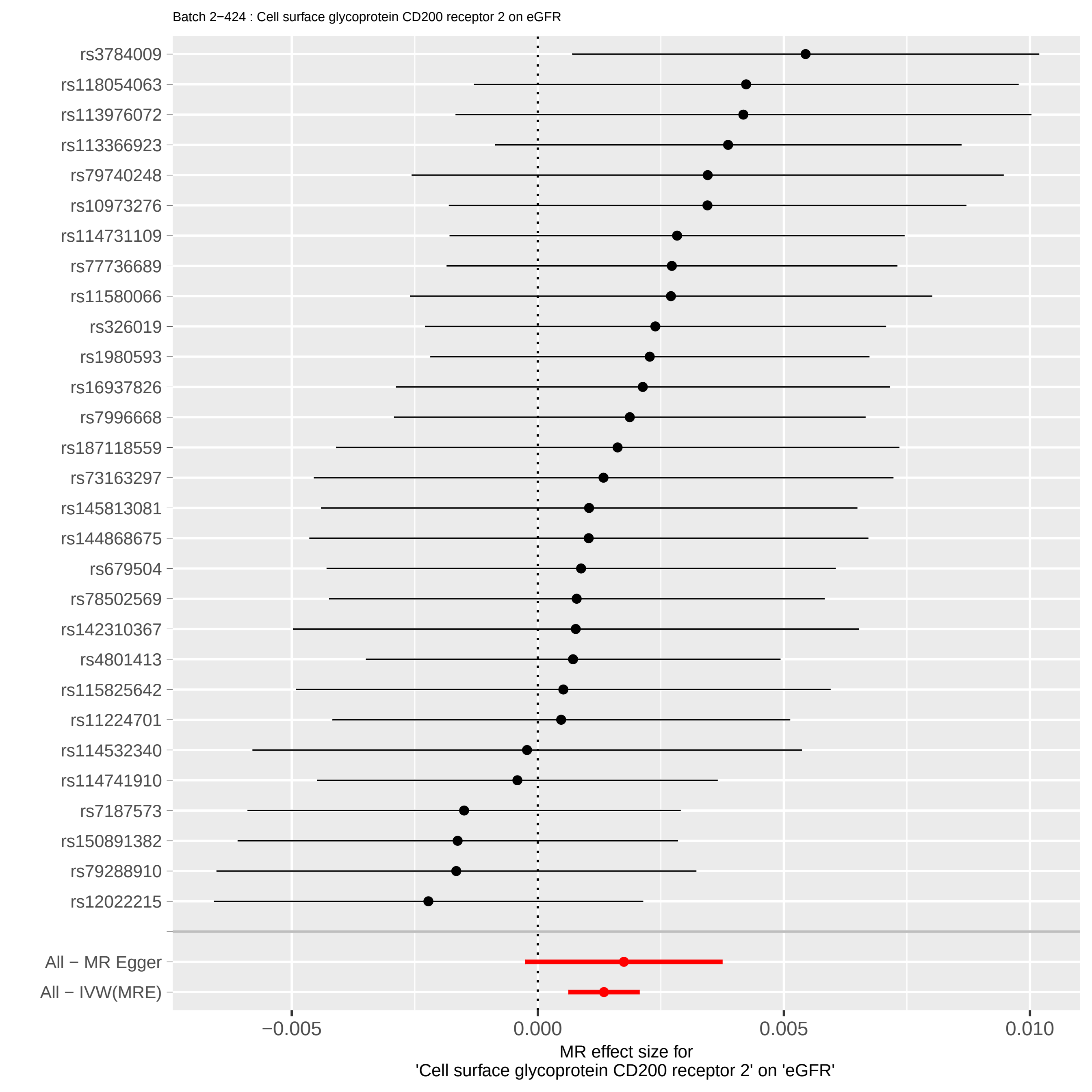

Batch 2−424 : Cell surface glycoprotein CD200 receptor 2 on eGFR
rs3784009
rs118054063
rs113976072
rs113366923
rs79740248
rs10973276
rs114731109
rs77736689
rs11580066
rs326019
rs1980593
rs16937826
rs7996668
rs187118559
rs73163297
rs145813081
rs144868675
rs679504
rs78502569
rs142310367
rs4801413
rs115825642
rs11224701
rs114532340
rs114741910
rs7187573
rs150891382
rs79288910
rs12022215
All − MR Egger
All − IVW(MRE)
−0.005
0.000
0.005
0.010
MR effect size for
'Cell surface glycoprotein CD200 receptor 2' on 'eGFR'

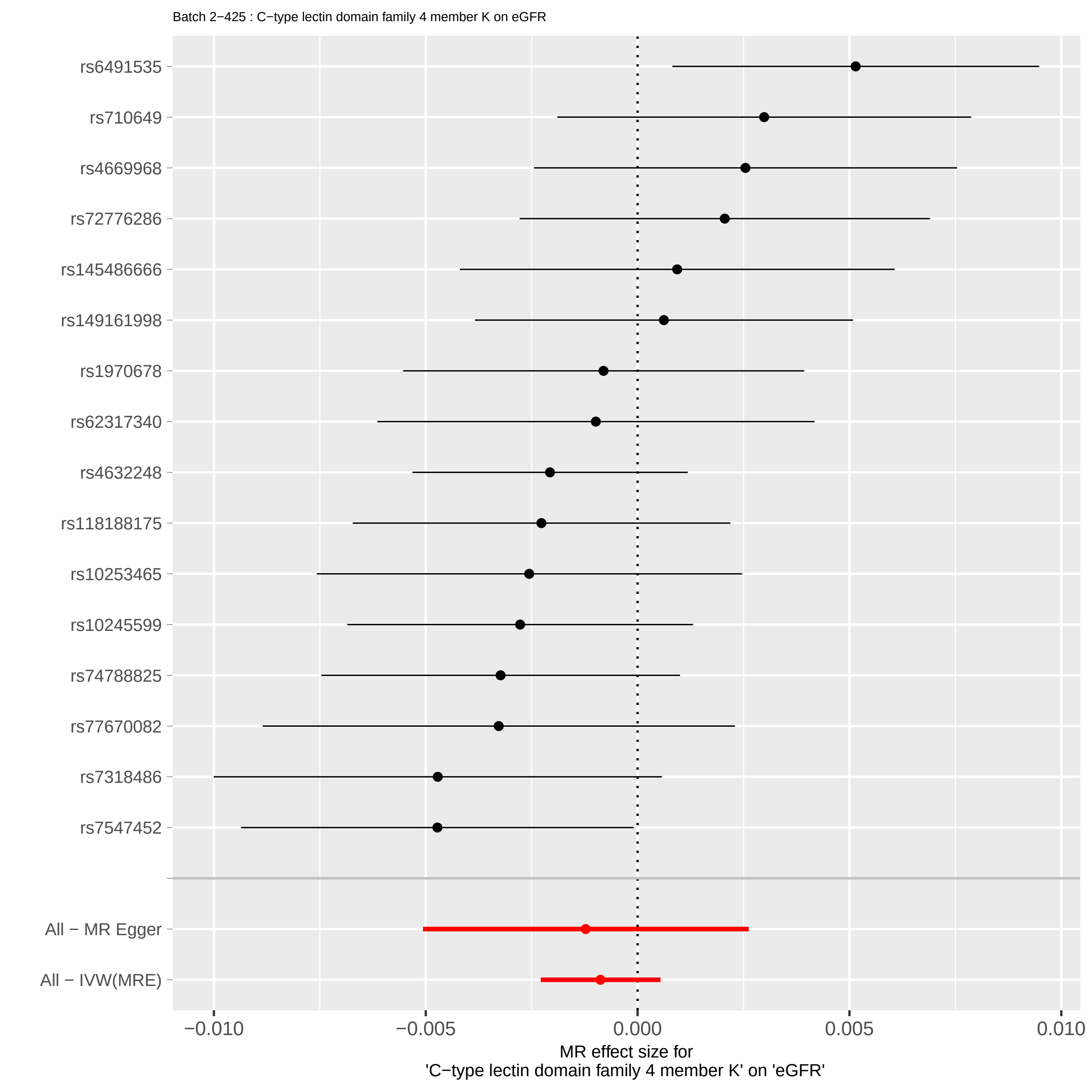

Batch 2−425 : C−type lectin domain family 4 member K on eGFR
rs6491535
rs710649
rs4669968
rs72776286
rs145486666
rs149161998
rs1970678
rs62317340
rs4632248
rs118188175
rs10253465
rs10245599
rs74788825
rs77670082
rs7318486
rs7547452
All − MR Egger
All − IVW(MRE)
−0.010
−0.005
0.000
0.005
0.010
MR effect size for
'C−type lectin domain family 4 member K' on 'eGFR'

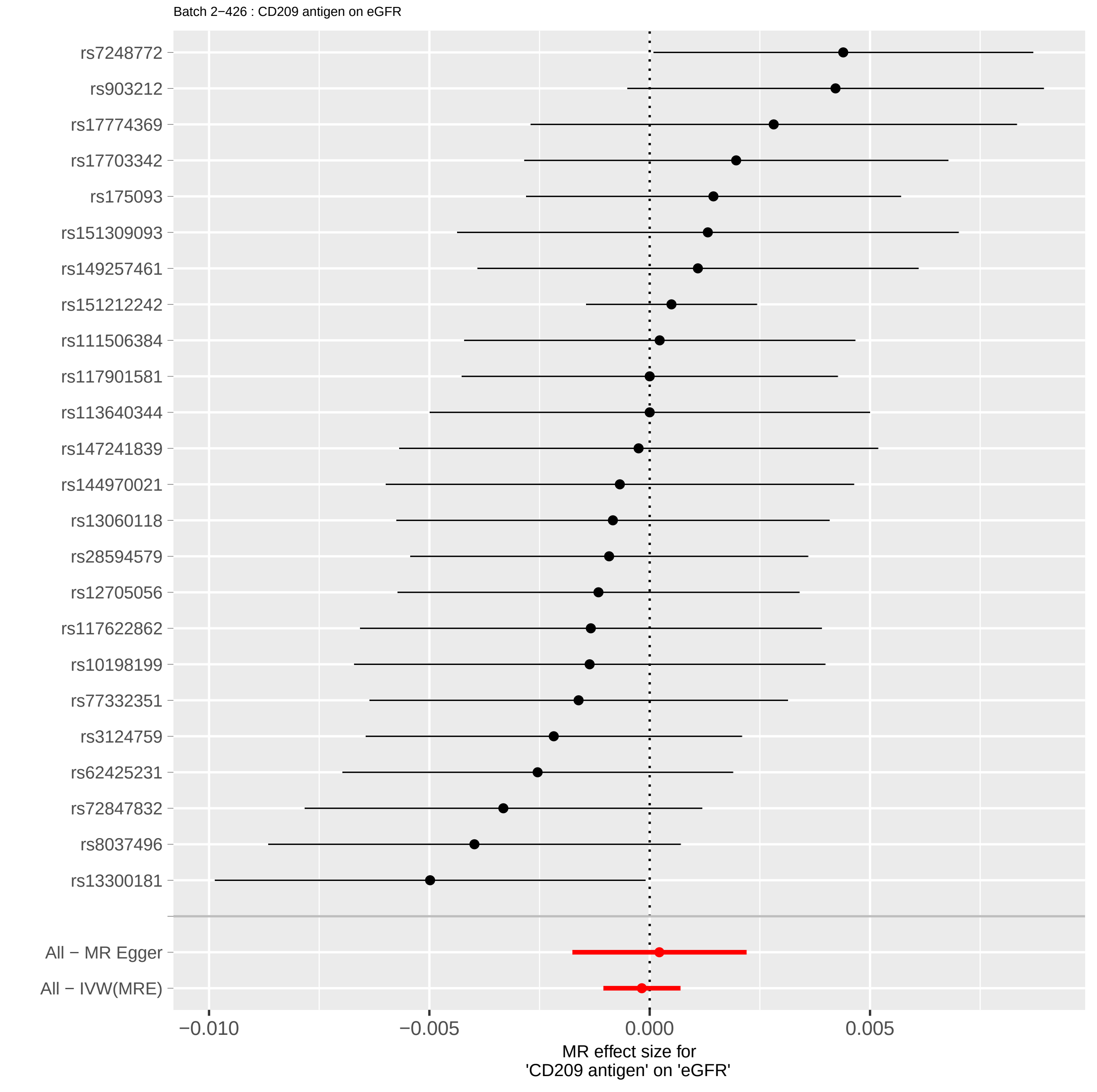
Batch 2−426 : CD209 antigen on eGFR
rs7248772
rs903212
rs17774369
rs17703342
rs175093
rs151309093
rs149257461
rs151212242
rs111506384
rs117901581
rs113640344
rs147241839
rs144970021
rs13060118
rs28594579
rs12705056
rs117622862
rs10198199
rs77332351
rs3124759
rs62425231
rs72847832
rs8037496
rs13300181
All − MR Egger
All − IVW(MRE)
−0.010
−0.005
0.000
0.005
MR effect size for
'CD209 antigen' on 'eGFR'

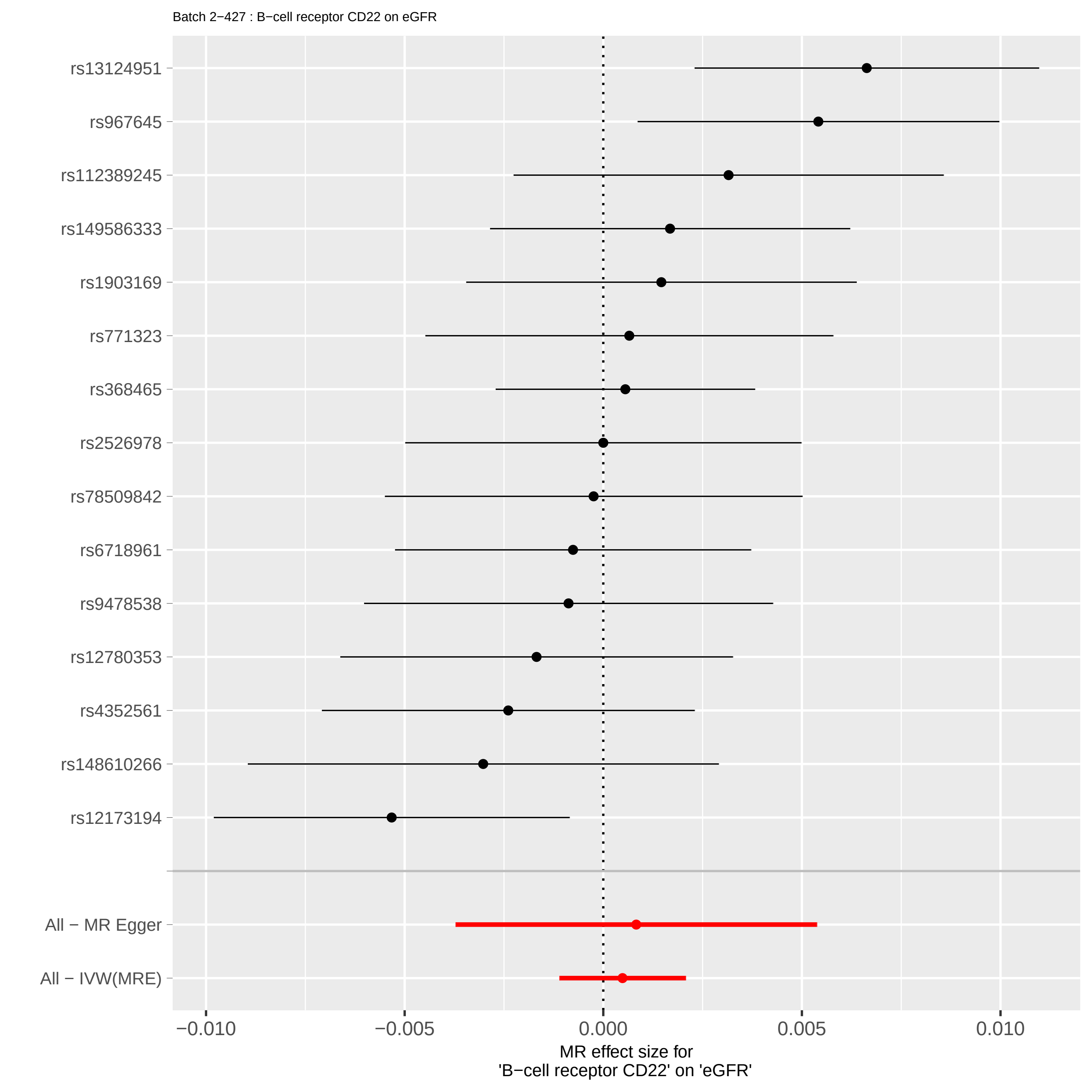

Batch 2−427 : B−cell receptor CD22 on eGFR
rs13124951
rs967645
rs112389245
rs149586333
rs1903169
rs771323
rs368465
rs2526978
rs78509842
rs6718961
rs9478538
rs12780353
rs4352561
rs148610266
rs12173194
All − MR Egger
All − IVW(MRE)
−0.010
−0.005
0.000
0.005
0.010
MR effect size for
'B−cell receptor CD22' on 'eGFR'

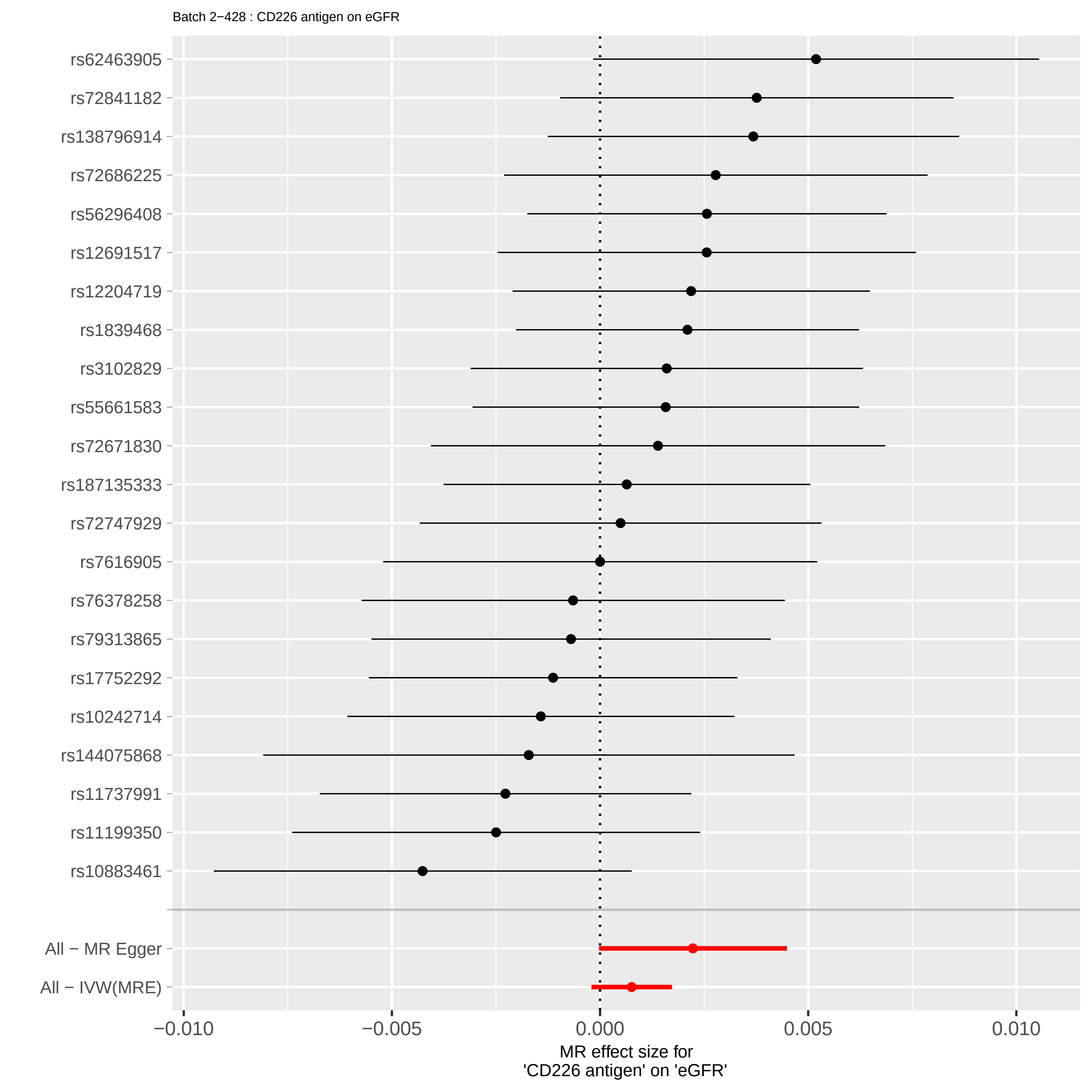
Batch 2−428 : CD226 antigen on eGFR
rs62463905
rs72841182
rs138796914
rs72686225
rs56296408
rs12691517
rs12204719
rs1839468
rs3102829
rs55661583
rs72671830
rs187135333
rs72747929
rs7616905
rs76378258
rs79313865
rs17752292
rs10242714
rs144075868
rs11737991
rs11199350
rs10883461
All − MR Egger
All − IVW(MRE)
−0.010
−0.005
0.000
0.005
0.010
MR effect size for
'CD226 antigen' on 'eGFR'

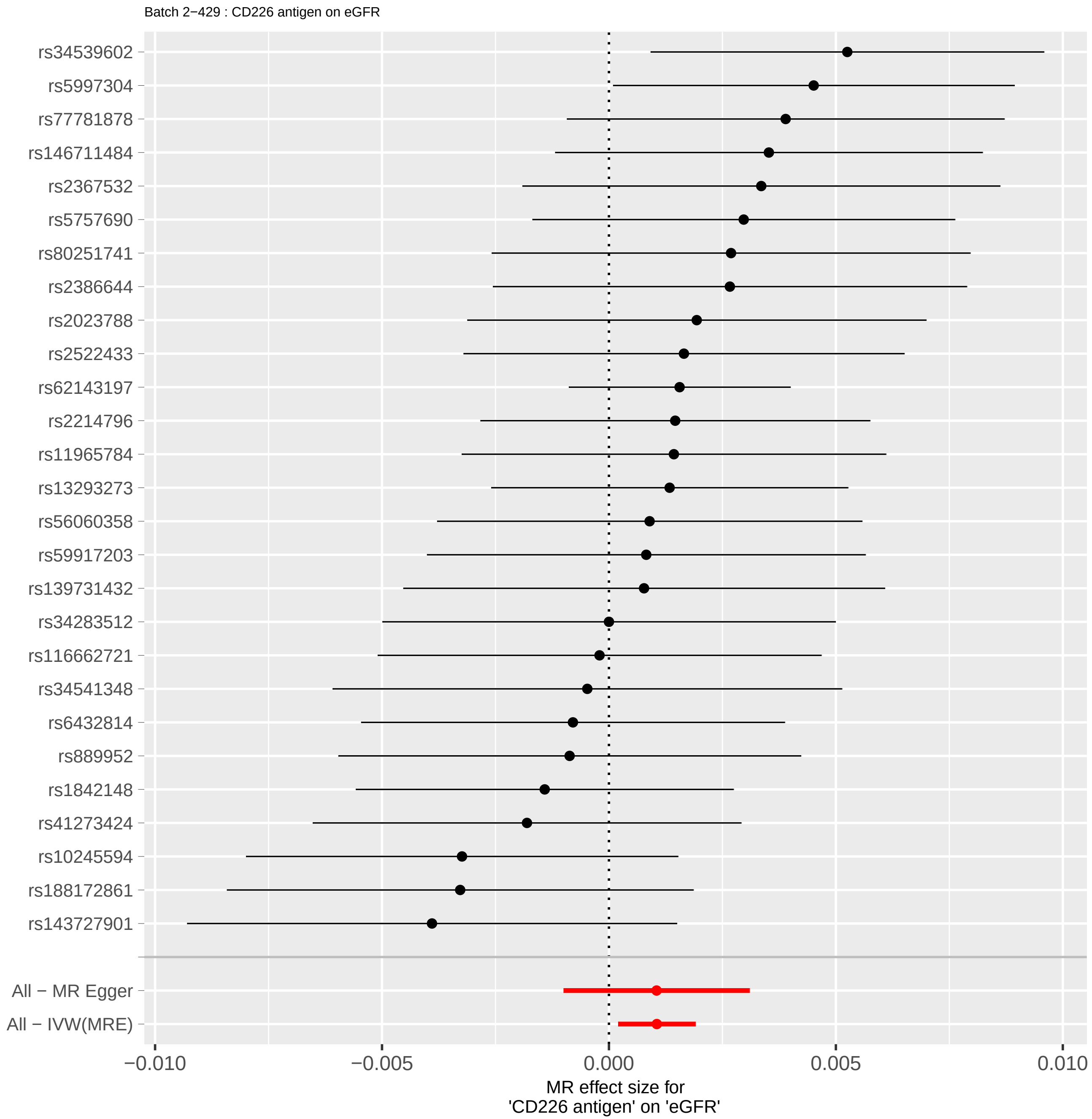
Batch 2−429 : CD226 antigen on eGFR
rs34539602
rs5997304
rs77781878
rs146711484
rs2367532
rs5757690
rs80251741
rs2386644
rs2023788
rs2522433
rs62143197
rs2214796
rs11965784
rs13293273
rs56060358
rs59917203
rs139731432
rs34283512
rs116662721
rs34541348
rs6432814
rs889952
rs1842148
rs41273424
rs10245594
rs188172861
rs143727901
All − MR Egger
All − IVW(MRE)
−0.010
−0.005
0.000
0.005
0.010
MR effect size for
'CD226 antigen' on 'eGFR'

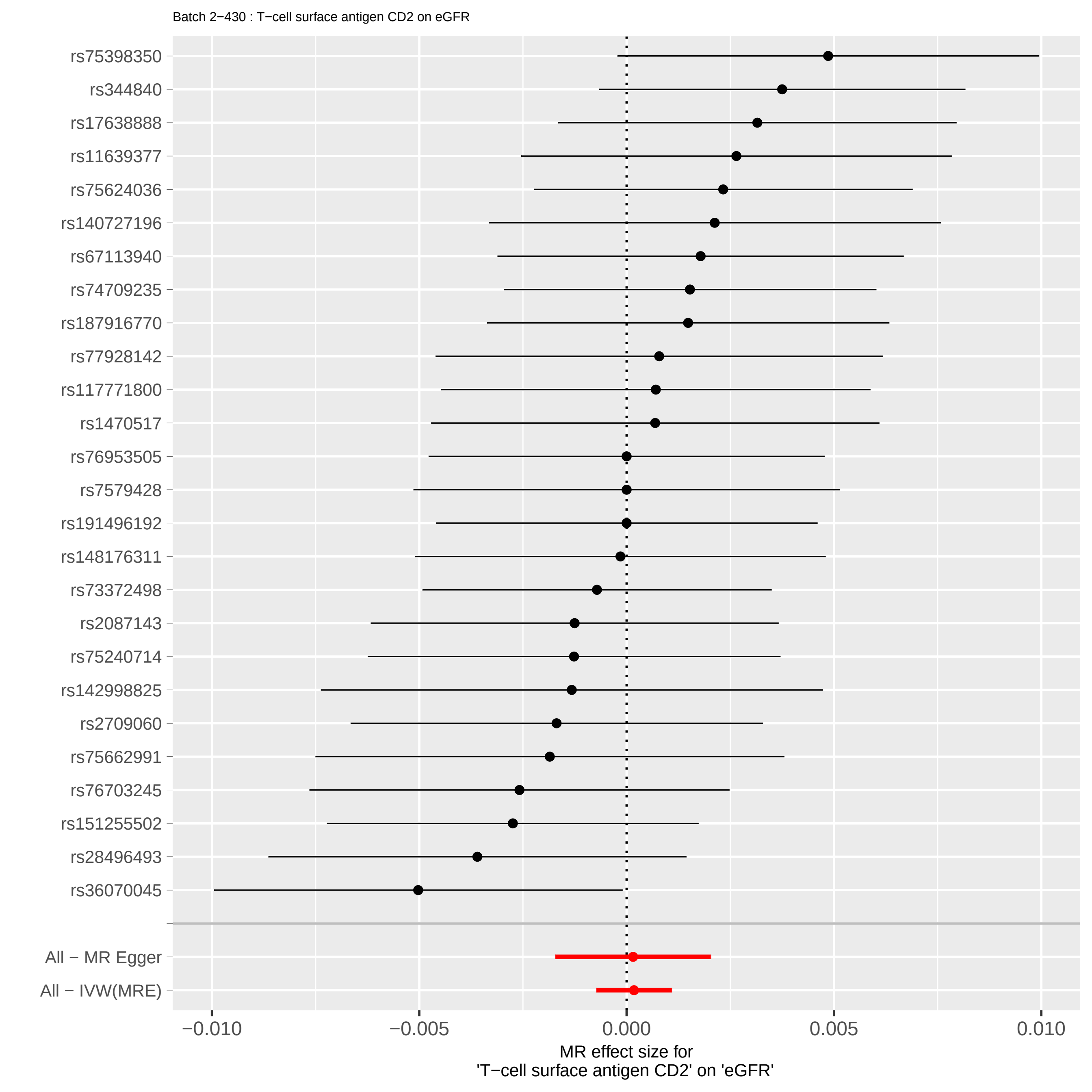

Batch 2−430 : T−cell surface antigen CD2 on eGFR
rs75398350
rs344840
rs17638888
rs11639377
rs75624036
rs140727196
rs67113940
rs74709235
rs187916770
rs77928142
rs117771800
rs1470517
rs76953505
rs7579428
rs191496192
rs148176311
rs73372498
rs2087143
rs75240714
rs142998825
rs2709060
rs75662991
rs76703245
rs151255502
rs28496493
rs36070045
All − MR Egger
All − IVW(MRE)
−0.010
−0.005
0.000
0.005
0.010
MR effect size for
'T−cell surface antigen CD2' on 'eGFR'

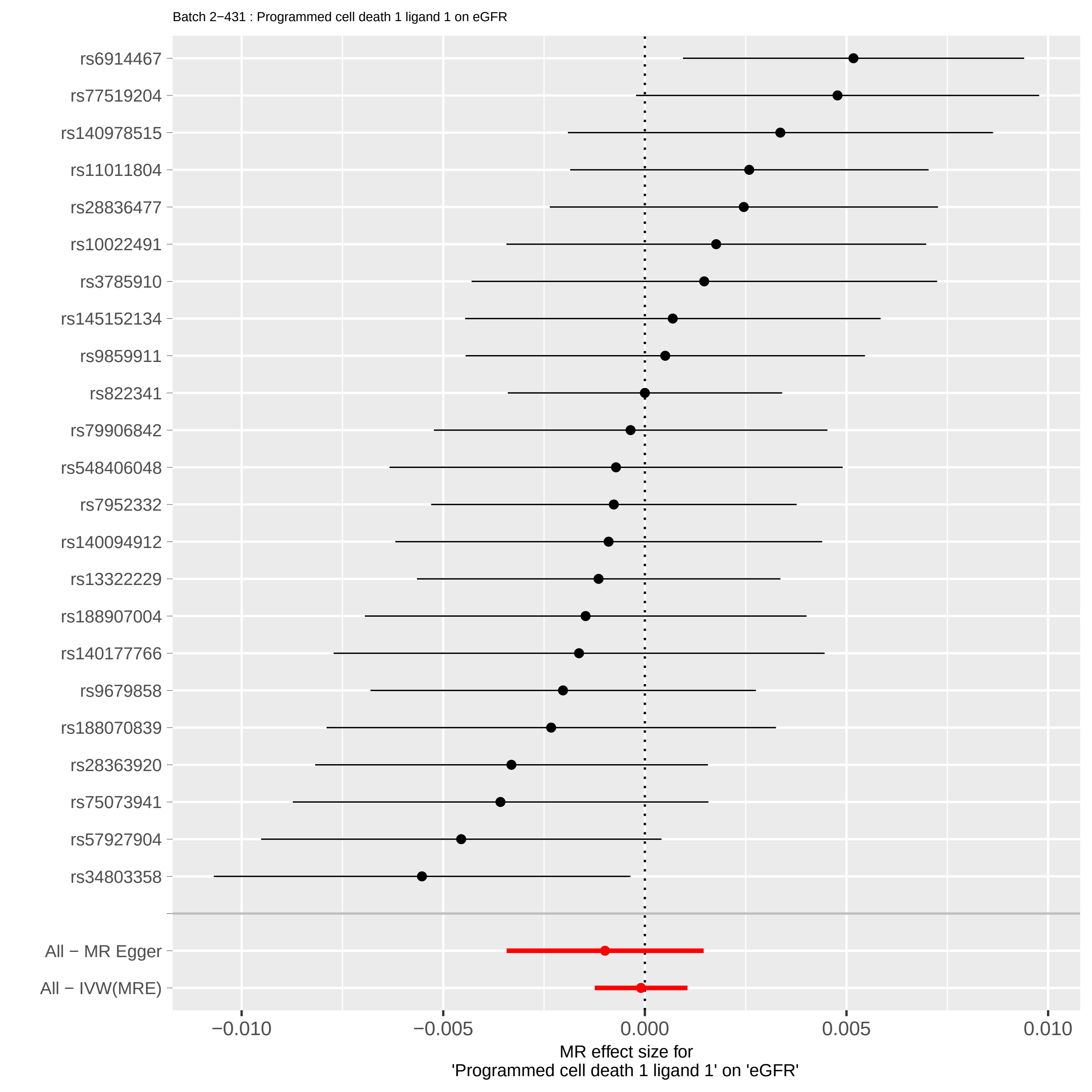

Batch 2−431 : Programmed cell death 1 ligand 1 on eGFR
rs6914467
rs77519204
rs140978515
rs11011804
rs28836477
rs10022491
rs3785910
rs145152134
rs9859911
rs822341
rs79906842
rs548406048
rs7952332
rs140094912
rs13322229
rs188907004
rs140177766
rs9679858
rs188070839
rs28363920
rs75073941
rs57927904
rs34803358
All − MR Egger
All − IVW(MRE)
−0.010
−0.005
0.000
0.005
0.010
MR effect size for
'Programmed cell death 1 ligand 1' on 'eGFR'

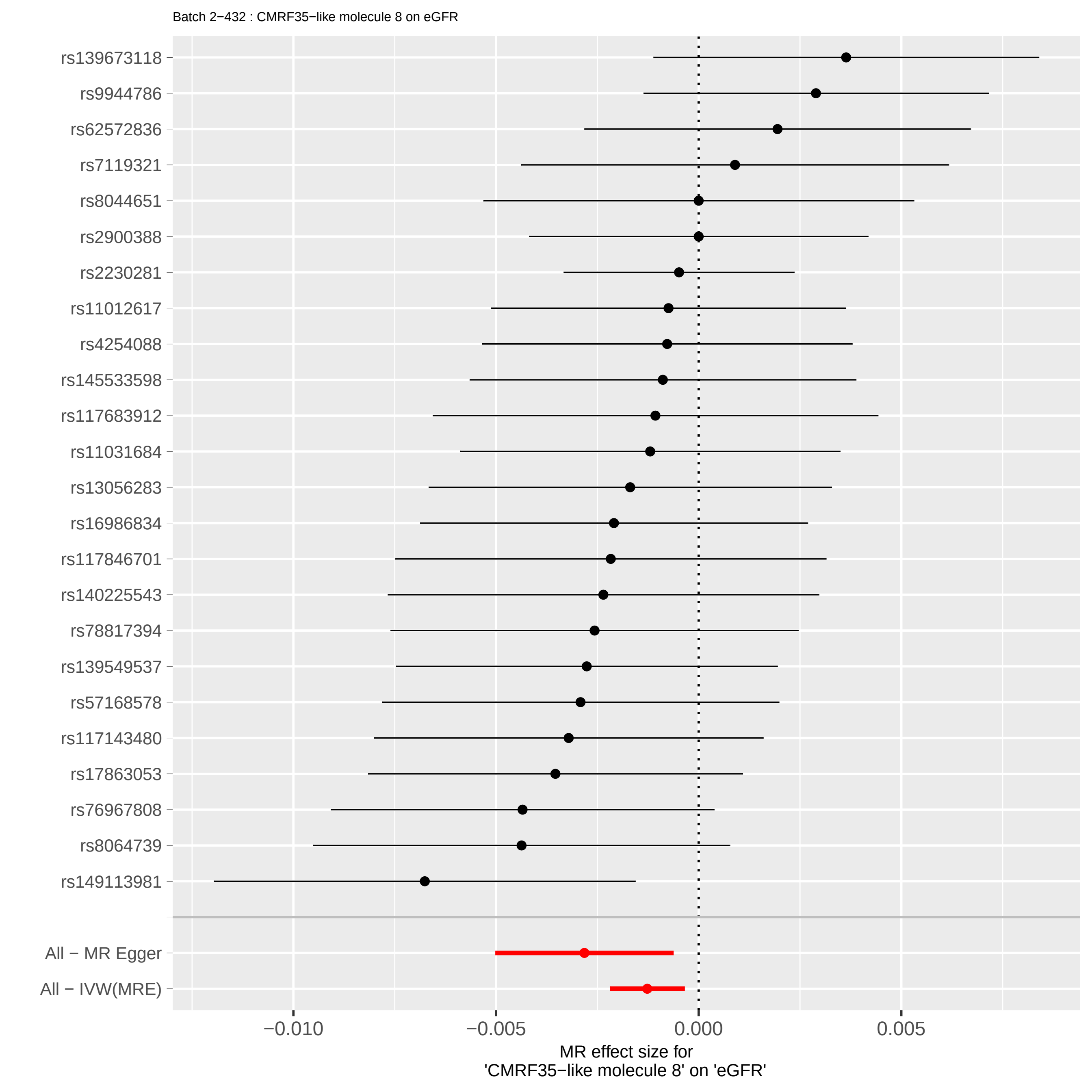
Batch 2−432 : CMRF35−like molecule 8 on eGFR
rs139673118
rs9944786
rs62572836
rs7119321
rs8044651
rs2900388
rs2230281
rs11012617
rs4254088
rs145533598
rs117683912
rs11031684
rs13056283
rs16986834
rs117846701
rs140225543
rs78817394
rs139549537
rs57168578
rs117143480
rs17863053
rs76967808
rs8064739
rs149113981
All − MR Egger
All − IVW(MRE)
−0.010
−0.005
0.000
0.005
MR effect size for
'CMRF35−like molecule 8' on 'eGFR'

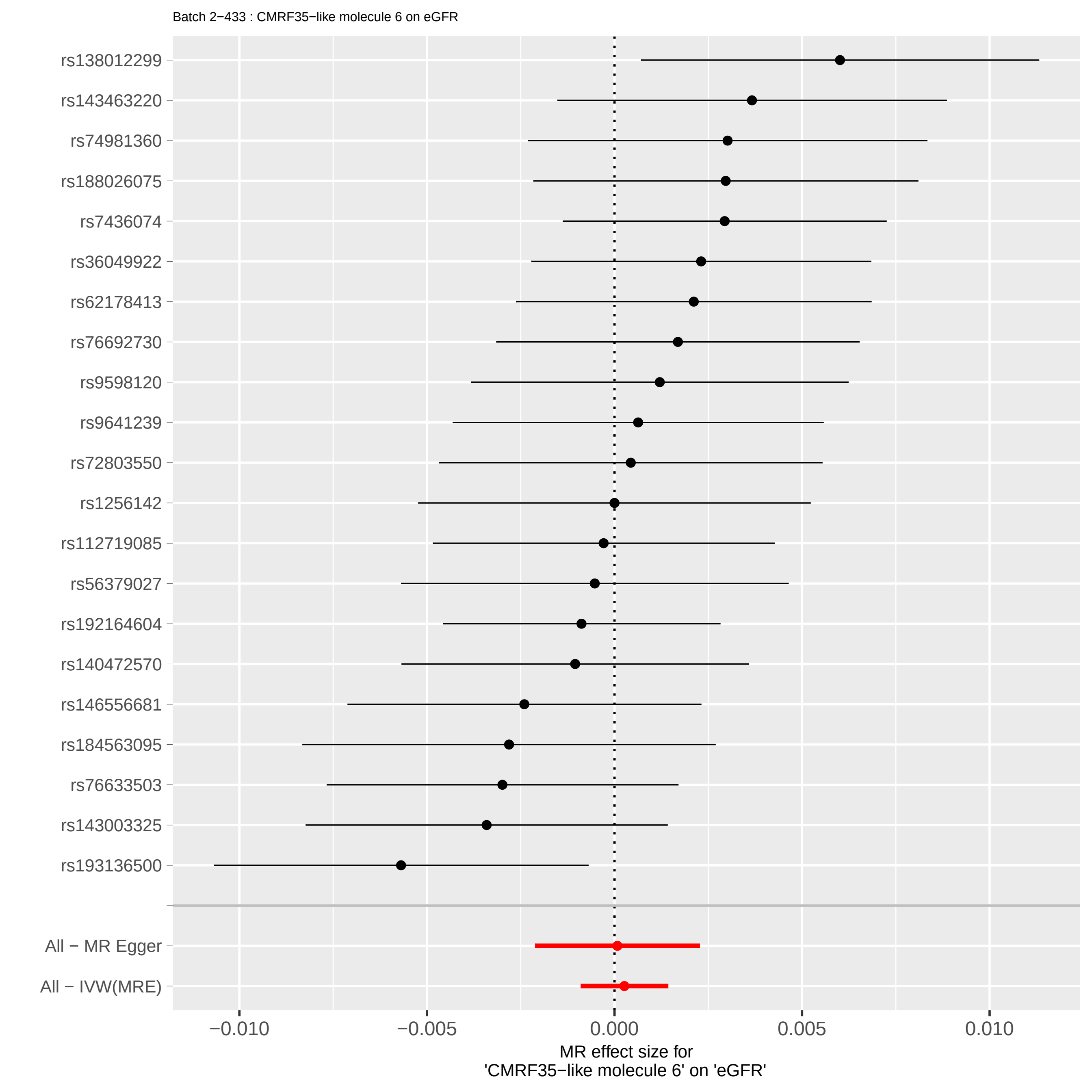

Batch 2−433 : CMRF35−like molecule 6 on eGFR
rs138012299
rs143463220
rs74981360
rs188026075
rs7436074
rs36049922
rs62178413
rs76692730
rs9598120
rs9641239
rs72803550
rs1256142
rs112719085
rs56379027
rs192164604
rs140472570
rs146556681
rs184563095
rs76633503
rs143003325
rs193136500
All − MR Egger
All − IVW(MRE)
−0.010
−0.005
0.000
0.005
0.010
MR effect size for
'CMRF35−like molecule 6' on 'eGFR'

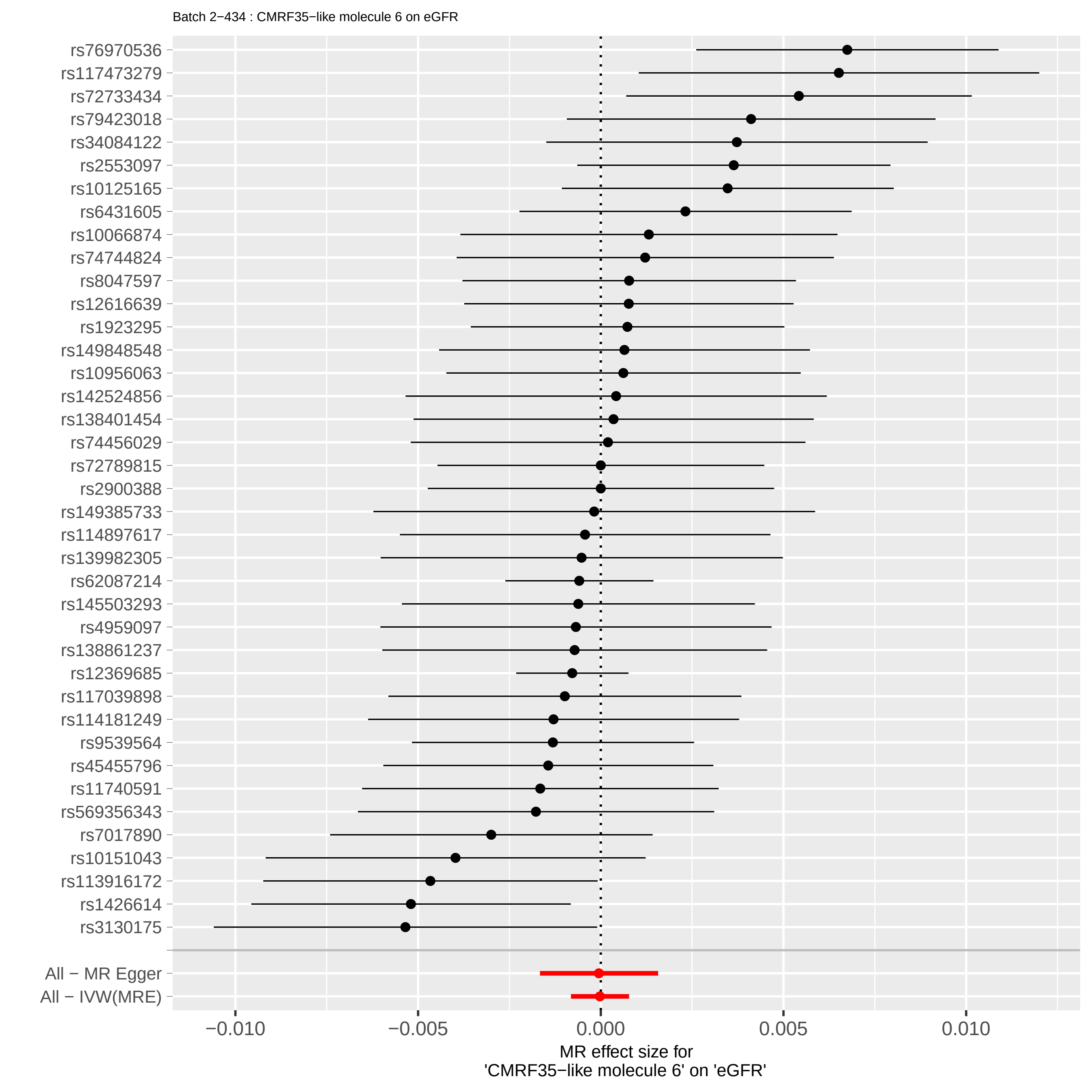

Batch 2−434 : CMRF35−like molecule 6 on eGFR
rs76970536
rs117473279
rs72733434
rs79423018
rs34084122
rs2553097
rs10125165
rs6431605
rs10066874
rs74744824
rs8047597
rs12616639
rs1923295
rs149848548
rs10956063
rs142524856
rs138401454
rs74456029
rs72789815
rs2900388
rs149385733
rs114897617
rs139982305
rs62087214
rs145503293
rs4959097
rs138861237
rs12369685
rs117039898
rs114181249
rs9539564
rs45455796
rs11740591
rs569356343
rs7017890
rs10151043
rs113916172
rs1426614
rs3130175
All − MR Egger
All − IVW(MRE)
−0.010
−0.005
0.000
0.005
0.010
MR effect size for
'CMRF35−like molecule 6' on 'eGFR'

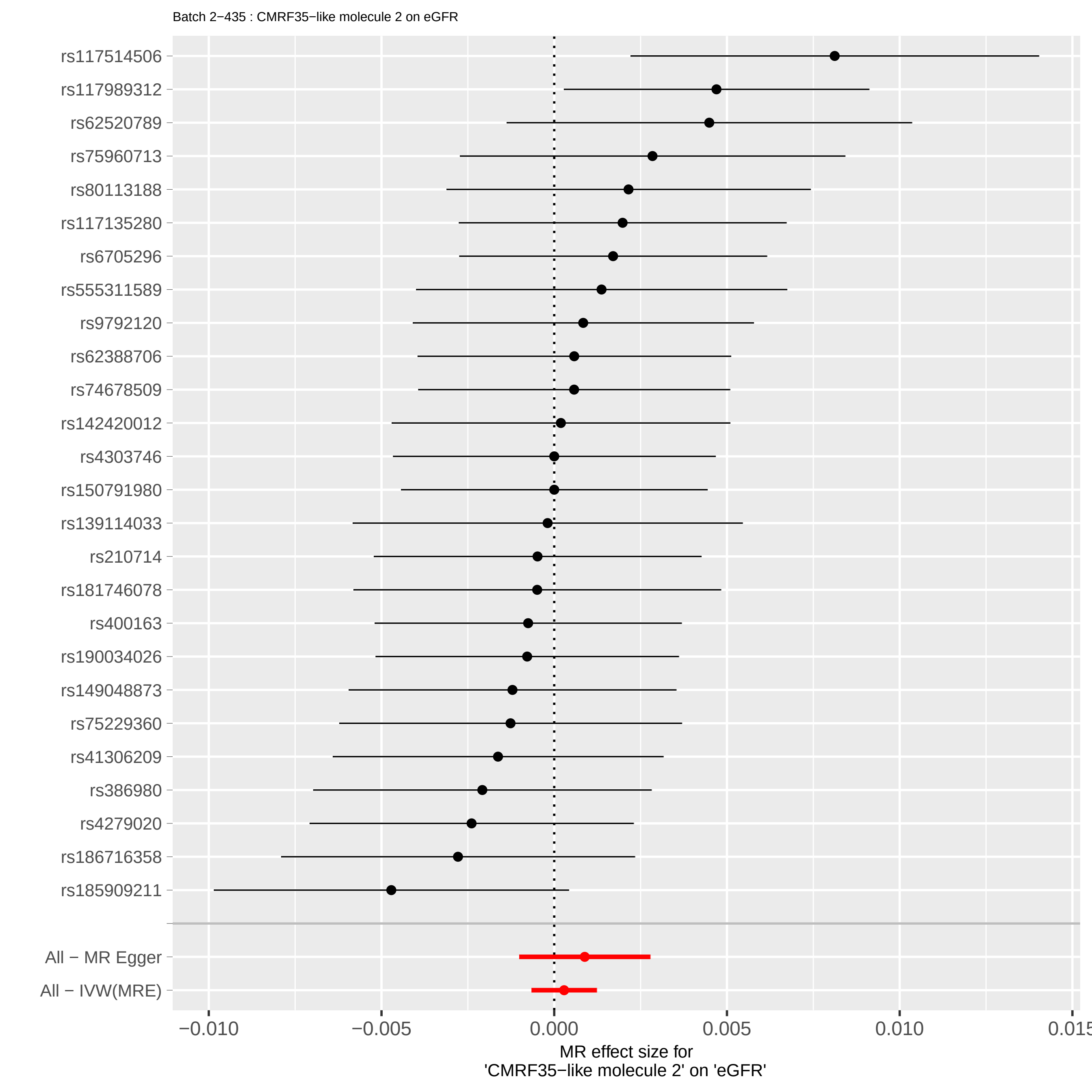
Batch 2−435 : CMRF35−like molecule 2 on eGFR
rs117514506
rs117989312
rs62520789
rs75960713
rs80113188
rs117135280
rs6705296
rs555311589
rs9792120
rs62388706
rs74678509
rs142420012
rs4303746
rs150791980
rs139114033
rs210714
rs181746078
rs400163
rs190034026
rs149048873
rs75229360
rs41306209
rs386980
rs4279020
rs186716358
rs185909211
All − MR Egger
All − IVW(MRE)
−0.010
−0.005
0.000
0.005
0.010
0.015
MR effect size for
'CMRF35−like molecule 2' on 'eGFR'

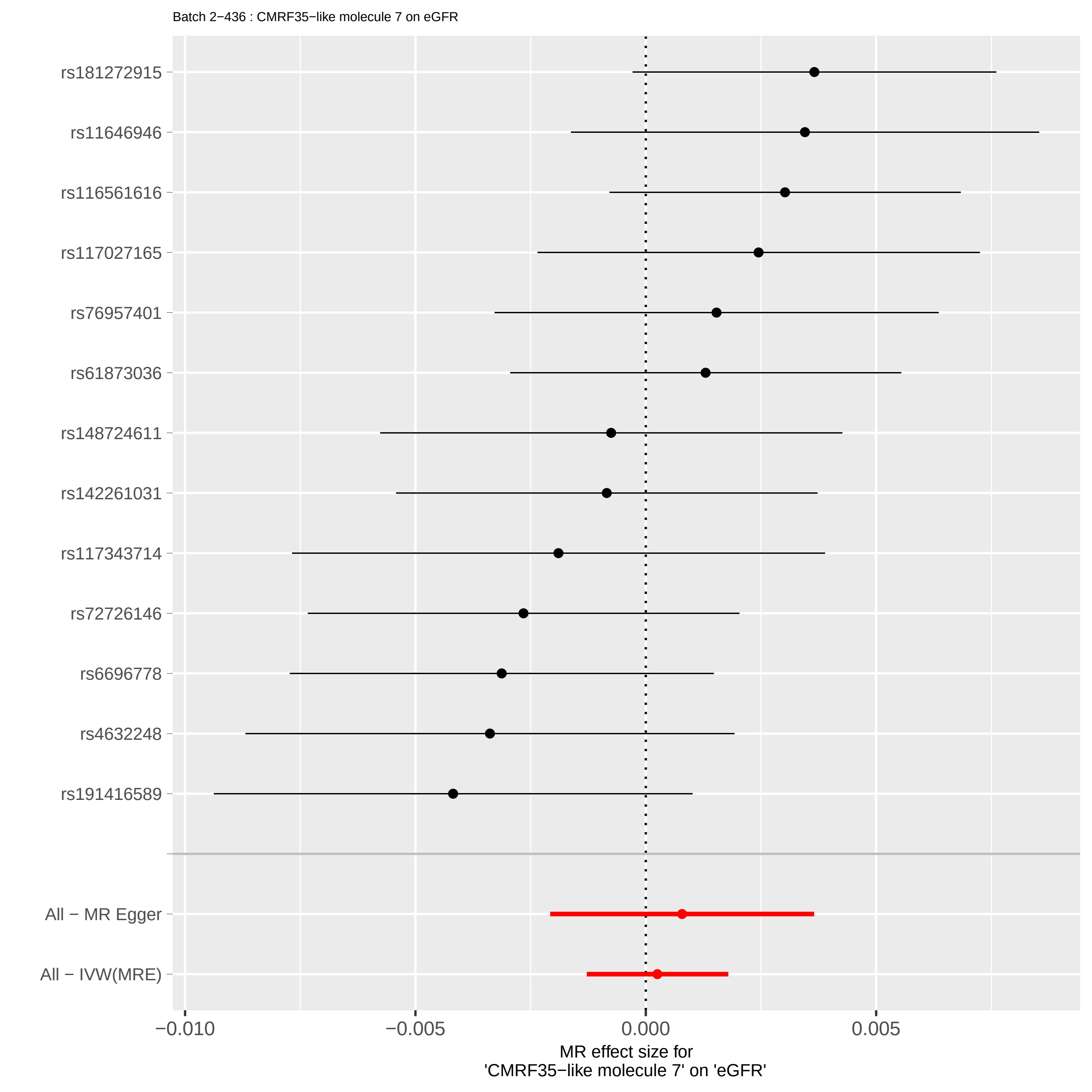

Batch 2−436 : CMRF35−like molecule 7 on eGFR
rs181272915
rs11646946
rs116561616
rs117027165
rs76957401
rs61873036
rs148724611
rs142261031
rs117343714
rs72726146
rs6696778
rs4632248
rs191416589
All − MR Egger
All − IVW(MRE)
−0.010
−0.005
0.000
0.005
MR effect size for
'CMRF35−like molecule 7' on 'eGFR'

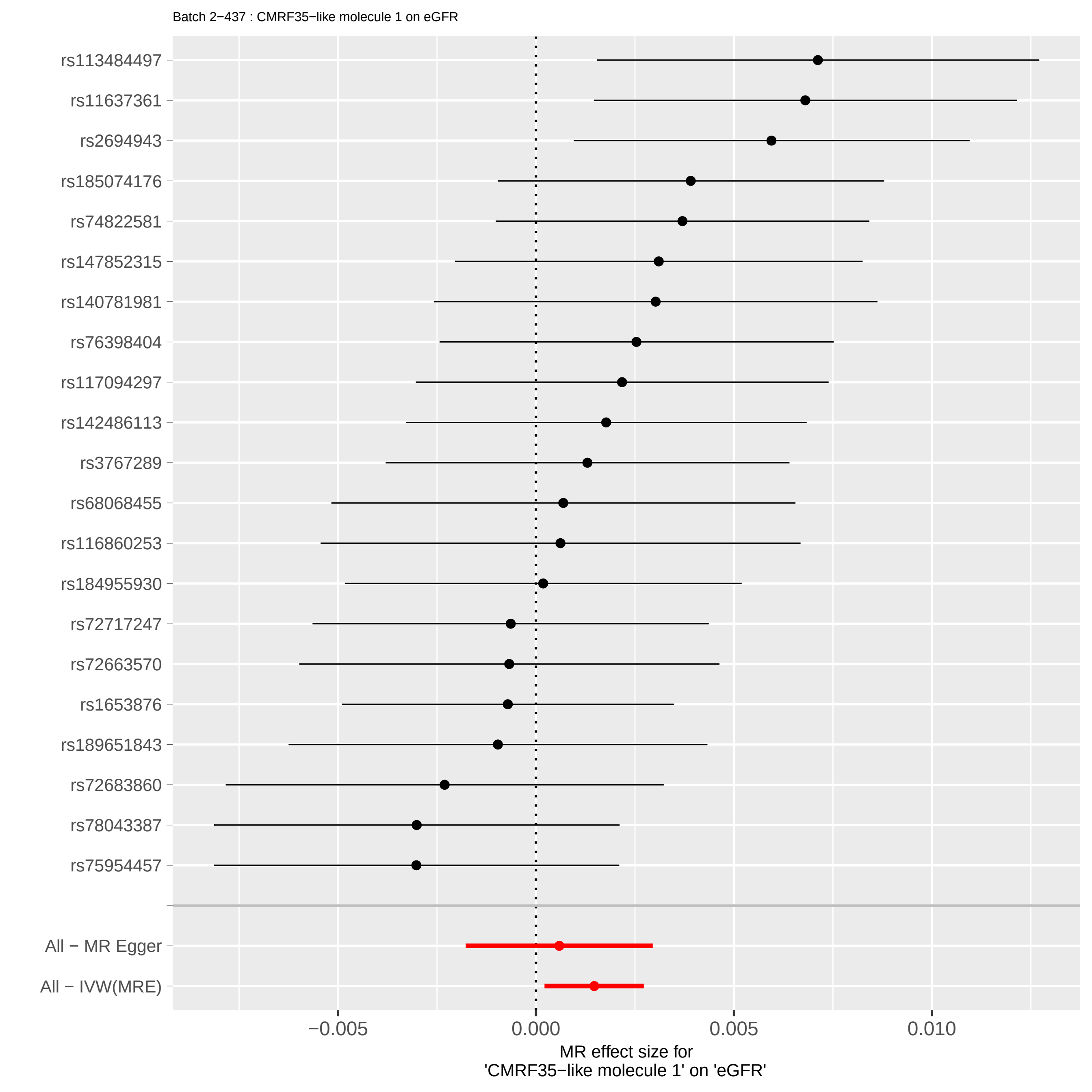

Batch 2−437 : CMRF35−like molecule 1 on eGFR
rs113484497
rs11637361
rs2694943
rs185074176
rs74822581
rs147852315
rs140781981
rs76398404
rs117094297
rs142486113
rs3767289
rs68068455
rs116860253
rs184955930
rs72717247
rs72663570
rs1653876
rs189651843
rs72683860
rs78043387
rs75954457
All − MR Egger
All − IVW(MRE)
−0.005
0.000
0.005
0.010
MR effect size for
'CMRF35−like molecule 1' on 'eGFR'

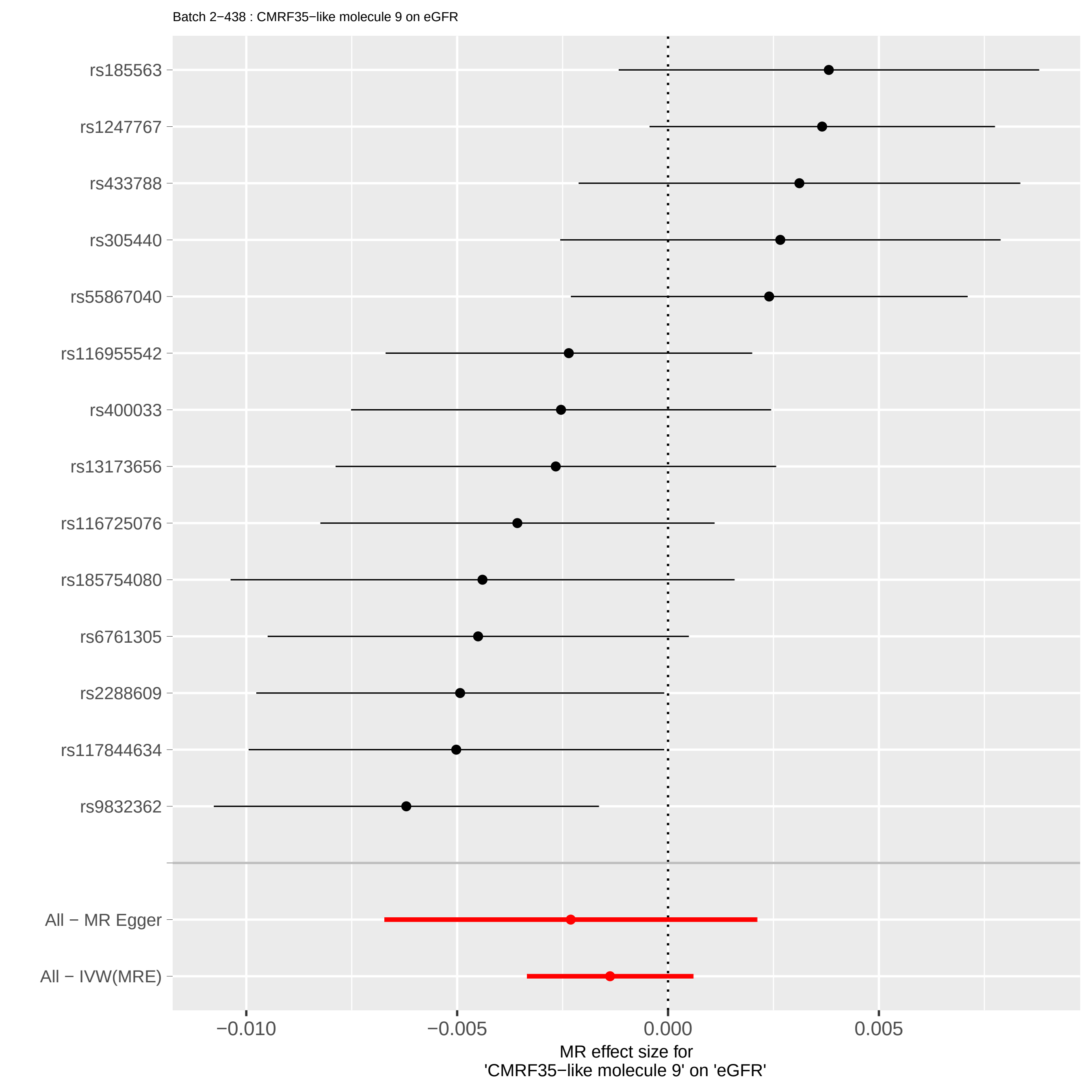

Batch 2−438 : CMRF35−like molecule 9 on eGFR
rs185563
rs1247767
rs433788
rs305440
rs55867040
rs116955542
rs400033
rs13173656
rs116725076
rs185754080
rs6761305
rs2288609
rs117844634
rs9832362
All − MR Egger
All − IVW(MRE)
−0.010
−0.005
0.000
0.005
MR effect size for
'CMRF35−like molecule 9' on 'eGFR'

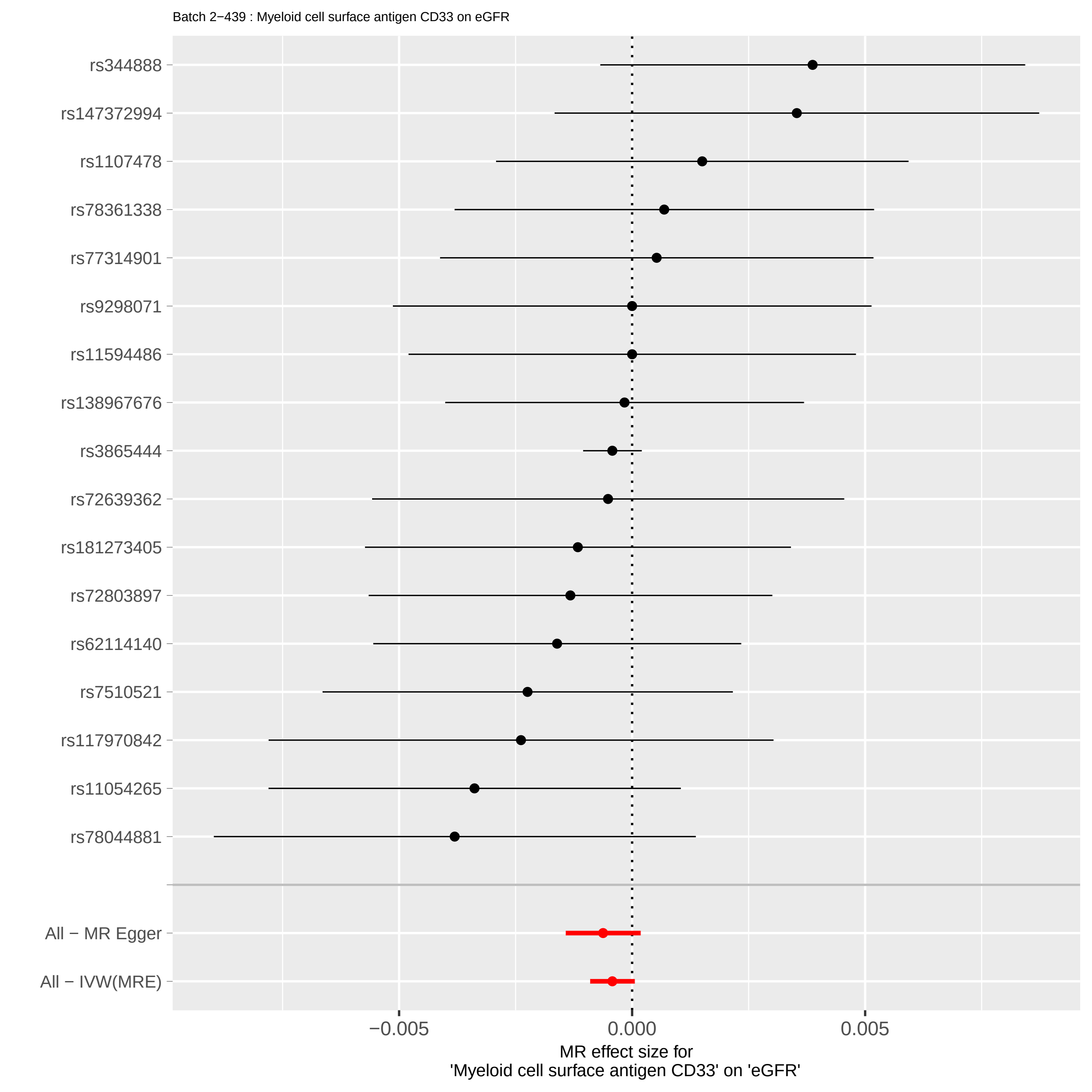

Batch 2−439 : Myeloid cell surface antigen CD33 on eGFR
rs344888
rs147372994
rs1107478
rs78361338
rs77314901
rs9298071
rs11594486
rs138967676
rs3865444
rs72639362
rs181273405
rs72803897
rs62114140
rs7510521
rs117970842
rs11054265
rs78044881
All − MR Egger
All − IVW(MRE)
−0.005
0.000
0.005
MR effect size for
'Myeloid cell surface antigen CD33' on 'eGFR'

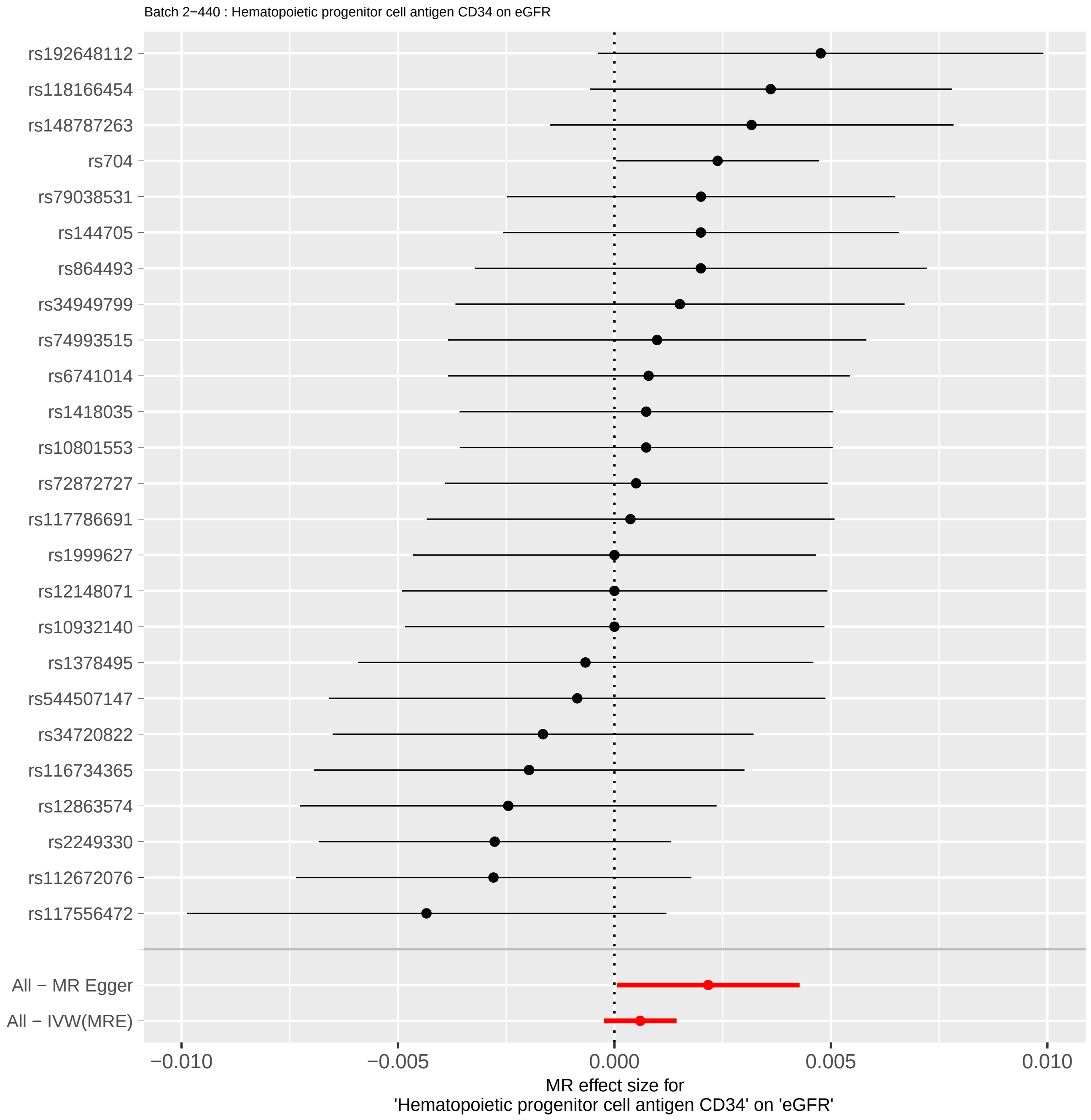

Batch 2−440 : Hematopoietic progenitor cell antigen CD34 on eGFR
rs192648112
rs118166454
rs148787263
rs704
rs79038531
rs144705
rs864493
rs34949799
rs74993515
rs6741014
rs1418035
rs10801553
rs72872727
rs117786691
rs1999627
rs12148071
rs10932140
rs1378495
rs544507147
rs34720822
rs116734365
rs12863574
rs2249330
rs112672076
rs117556472
All − MR Egger
All − IVW(MRE)
−0.010
−0.005
0.000
0.005
0.010
MR effect size for
'Hematopoietic progenitor cell antigen CD34' on 'eGFR'

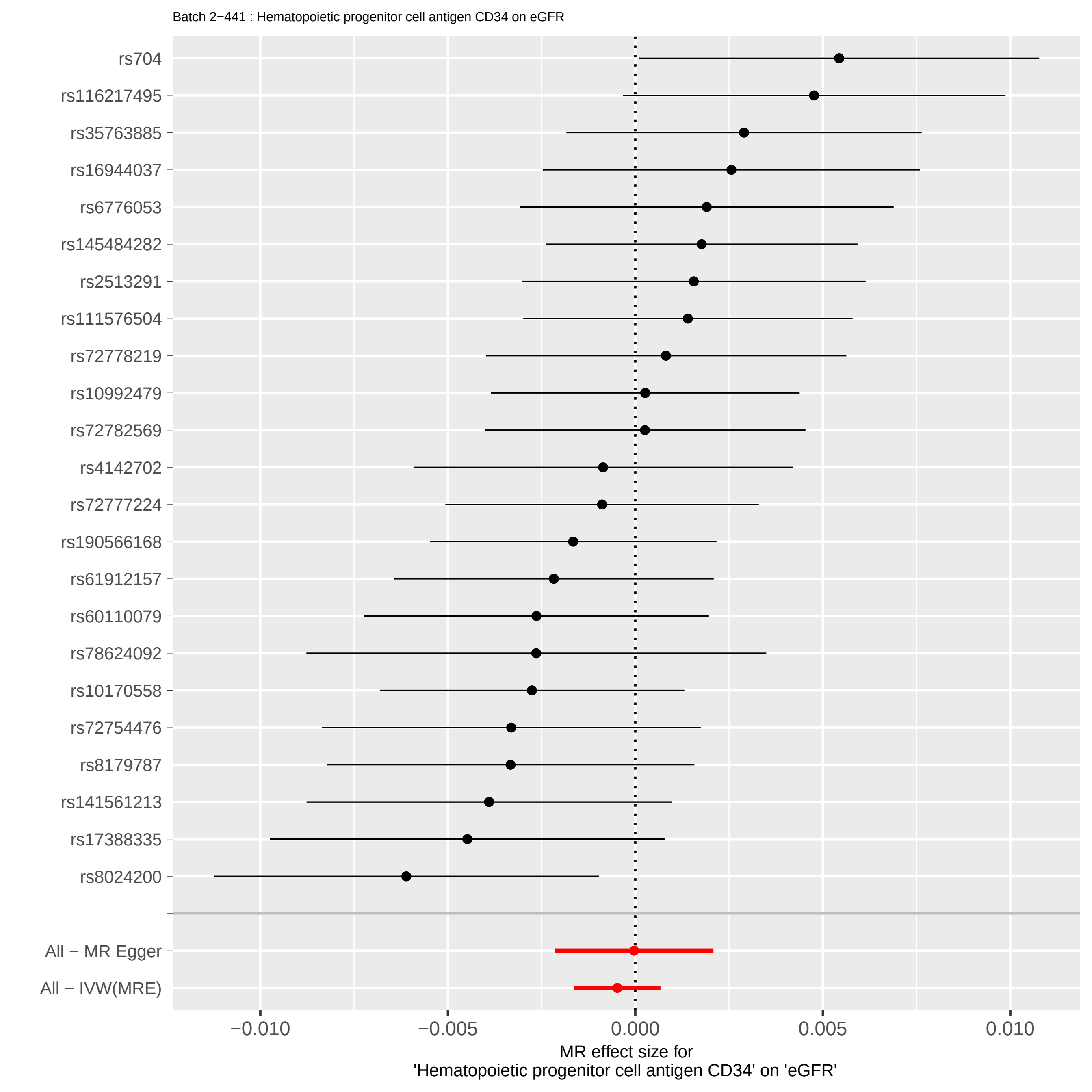
Batch 2−441 : Hematopoietic progenitor cell antigen CD34 on eGFR
rs704
rs116217495
rs35763885
rs16944037
rs6776053
rs145484282
rs2513291
rs111576504
rs72778219
rs10992479
rs72782569
rs4142702
rs72777224
rs190566168
rs61912157
rs60110079
rs78624092
rs10170558
rs72754476
rs8179787
rs141561213
rs17388335
rs8024200
All − MR Egger
All − IVW(MRE)
−0.010
−0.005
0.000
0.005
0.010
MR effect size for
'Hematopoietic progenitor cell antigen CD34' on 'eGFR'

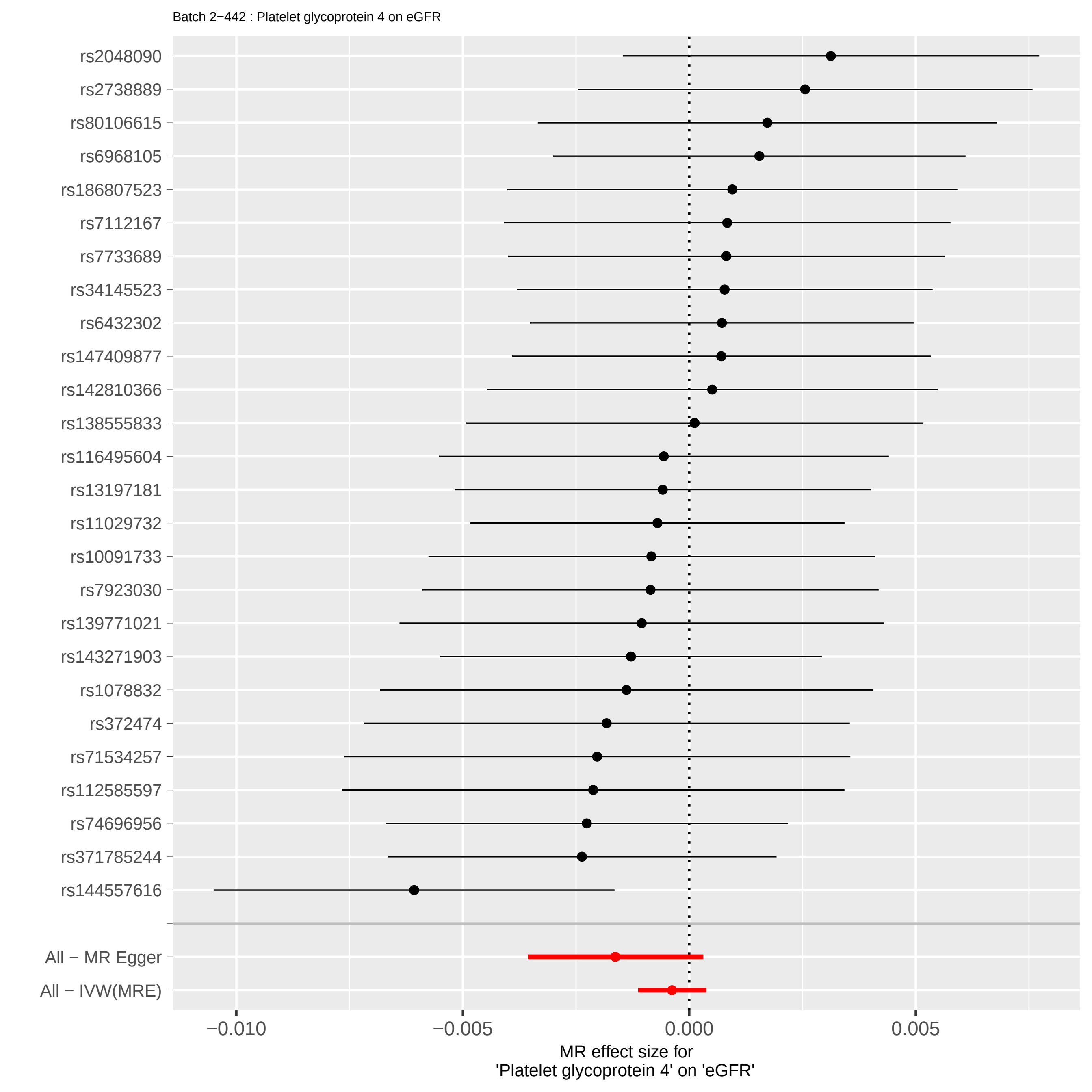

Batch 2−442 : Platelet glycoprotein 4 on eGFR
rs2048090
rs2738889
rs80106615
rs6968105
rs186807523
rs7112167
rs7733689
rs34145523
rs6432302
rs147409877
rs142810366
rs138555833
rs116495604
rs13197181
rs11029732
rs10091733
rs7923030
rs139771021
rs143271903
rs1078832
rs372474
rs71534257
rs112585597
rs74696956
rs371785244
rs144557616
All − MR Egger
All − IVW(MRE)
−0.010
−0.005
0.000
0.005
MR effect size for
'Platelet glycoprotein 4' on 'eGFR'

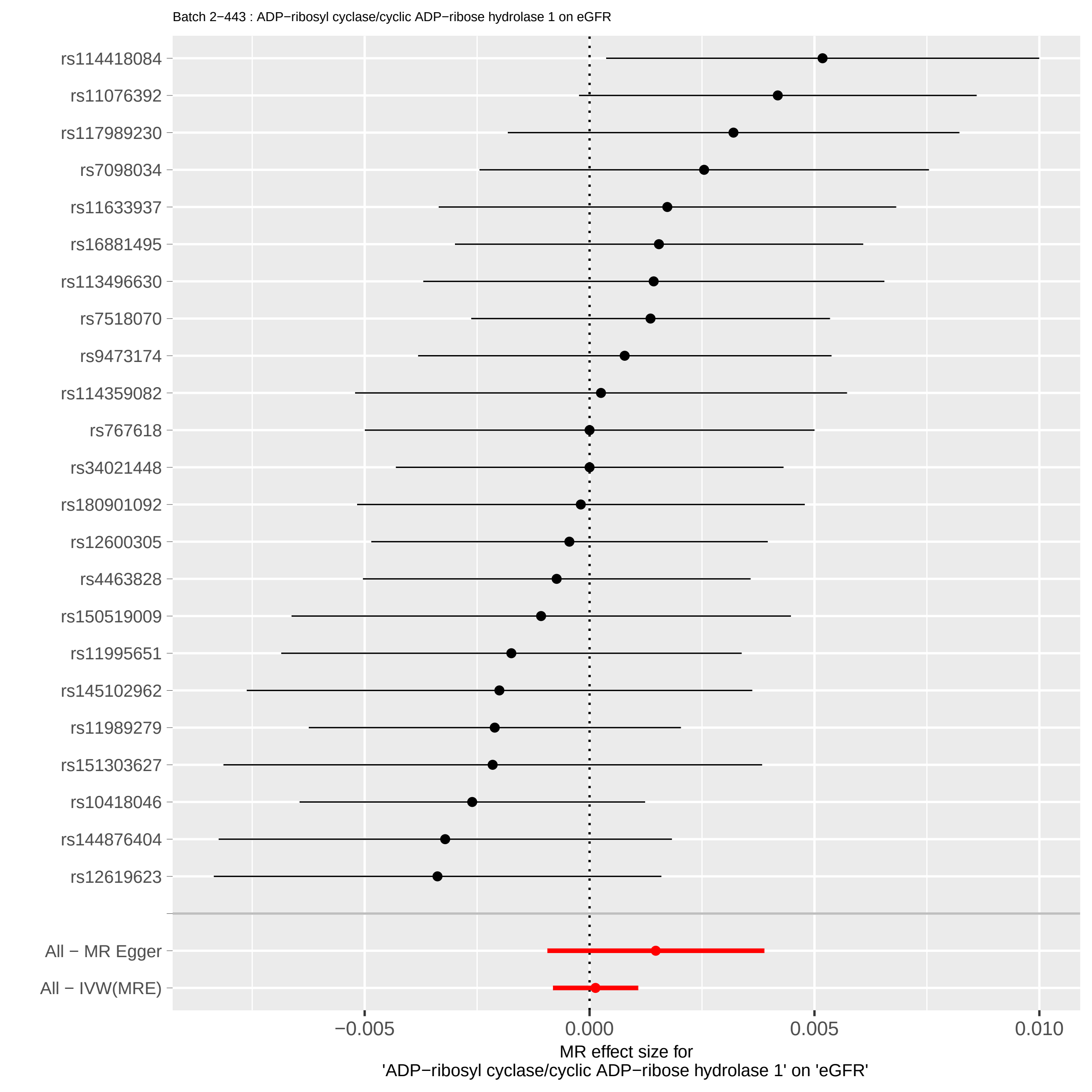

Batch 2−443 : ADP−ribosyl cyclase/cyclic ADP−ribose hydrolase 1 on eGFR
rs114418084
rs11076392
rs117989230
rs7098034
rs11633937
rs16881495
rs113496630
rs7518070
rs9473174
rs114359082
rs767618
rs34021448
rs180901092
rs12600305
rs4463828
rs150519009
rs11995651
rs145102962
rs11989279
rs151303627
rs10418046
rs144876404
rs12619623
All − MR Egger
All − IVW(MRE)
−0.005
0.000
0.005
0.010
MR effect size for
'ADP−ribosyl cyclase/cyclic ADP−ribose hydrolase 1' on 'eGFR'

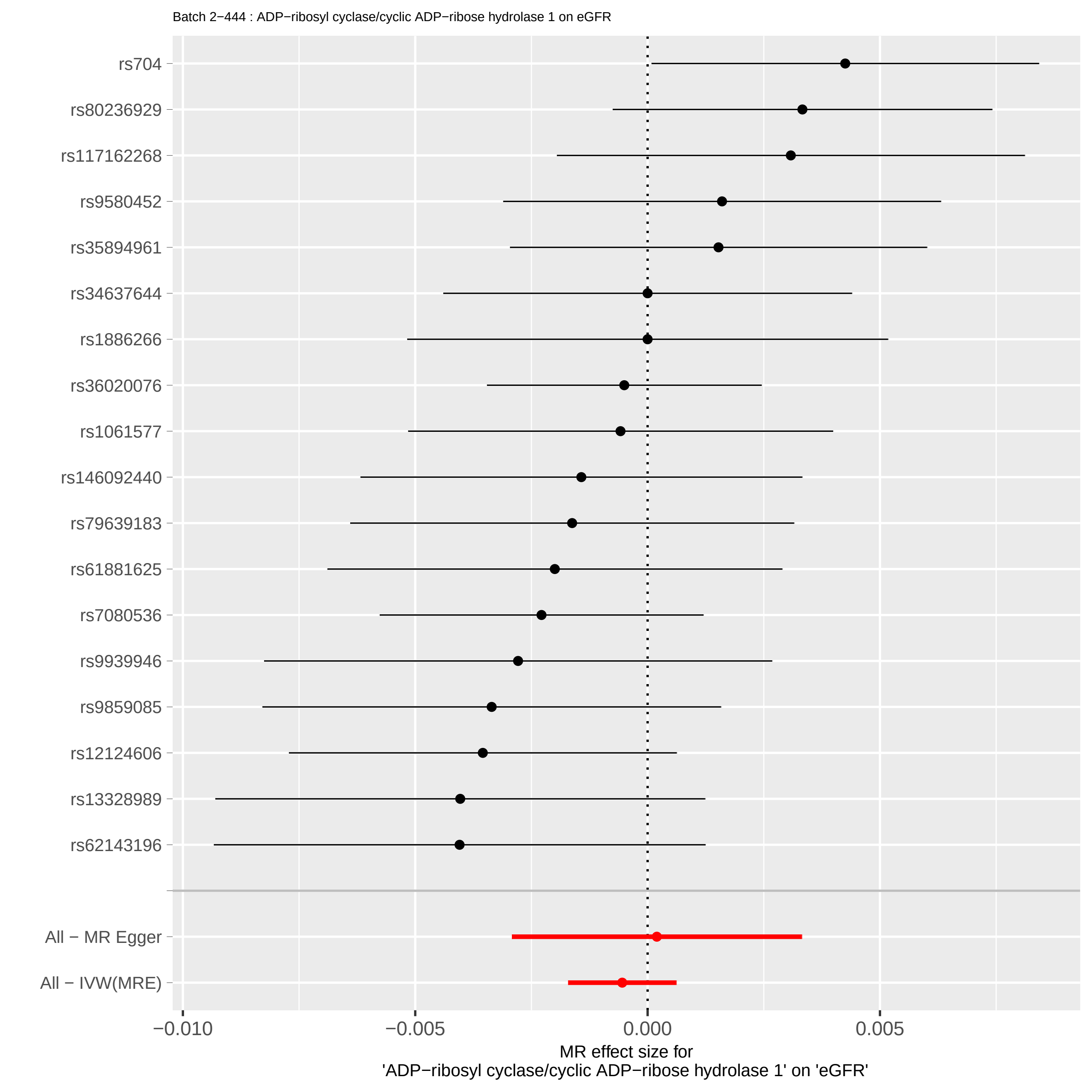
Batch 2−444 : ADP−ribosyl cyclase/cyclic ADP−ribose hydrolase 1 on eGFR
rs704
rs80236929
rs117162268
rs9580452
rs35894961
rs34637644
rs1886266
rs36020076
rs1061577
rs146092440
rs79639183
rs61881625
rs7080536
rs9939946
rs9859085
rs12124606
rs13328989
rs62143196
All − MR Egger
All − IVW(MRE)
−0.010
−0.005
0.000
0.005
MR effect size for
'ADP−ribosyl cyclase/cyclic ADP−ribose hydrolase 1' on 'eGFR'

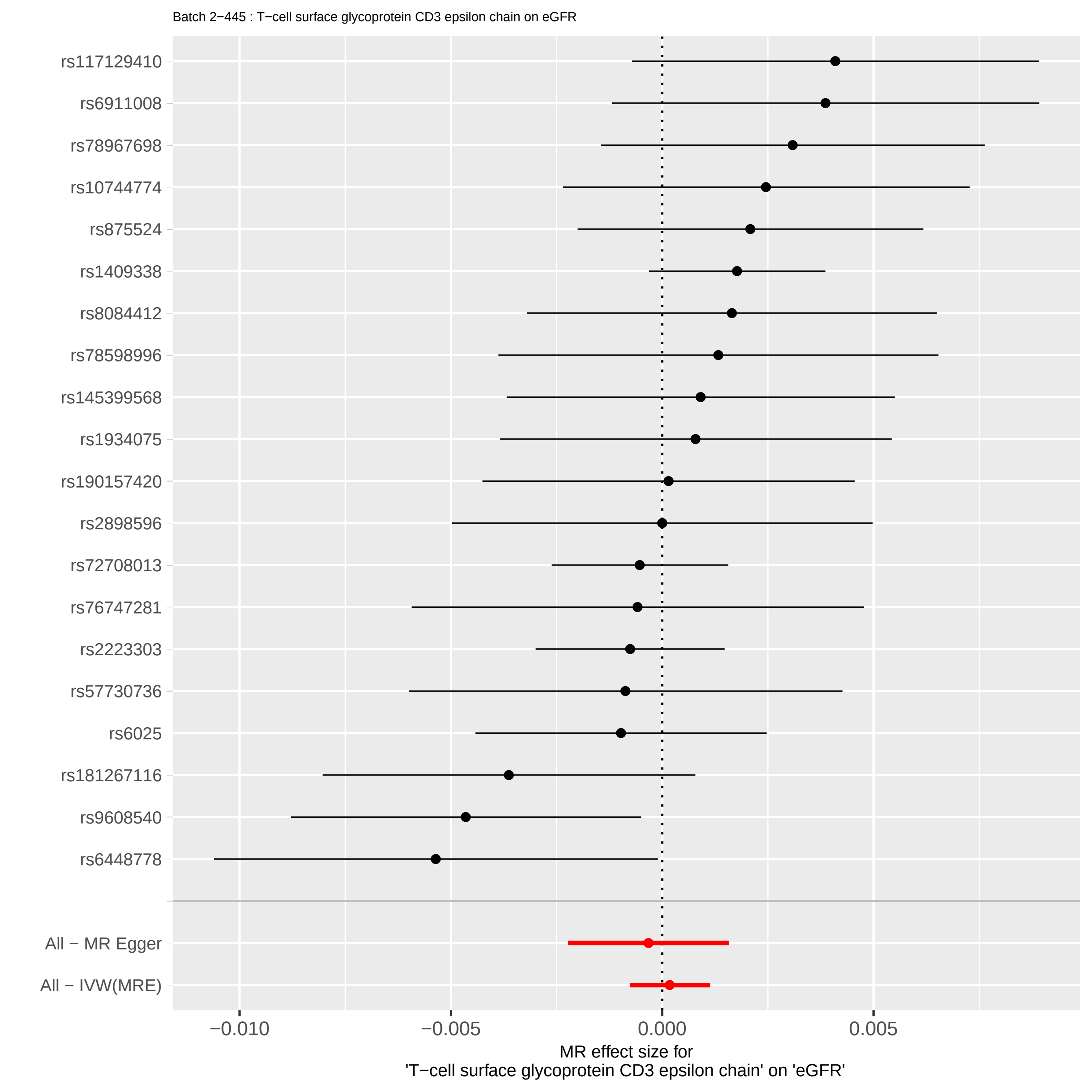

Batch 2−445 : T−cell surface glycoprotein CD3 epsilon chain on eGFR
rs117129410
rs6911008
rs78967698
rs10744774
rs875524
rs1409338
rs8084412
rs78598996
rs145399568
rs1934075
rs190157420
rs2898596
rs72708013
rs76747281
rs2223303
rs57730736
rs6025
rs181267116
rs9608540
rs6448778
All − MR Egger
All − IVW(MRE)
−0.010
−0.005
0.000
0.005
MR effect size for
'T−cell surface glycoprotein CD3 epsilon chain' on 'eGFR'

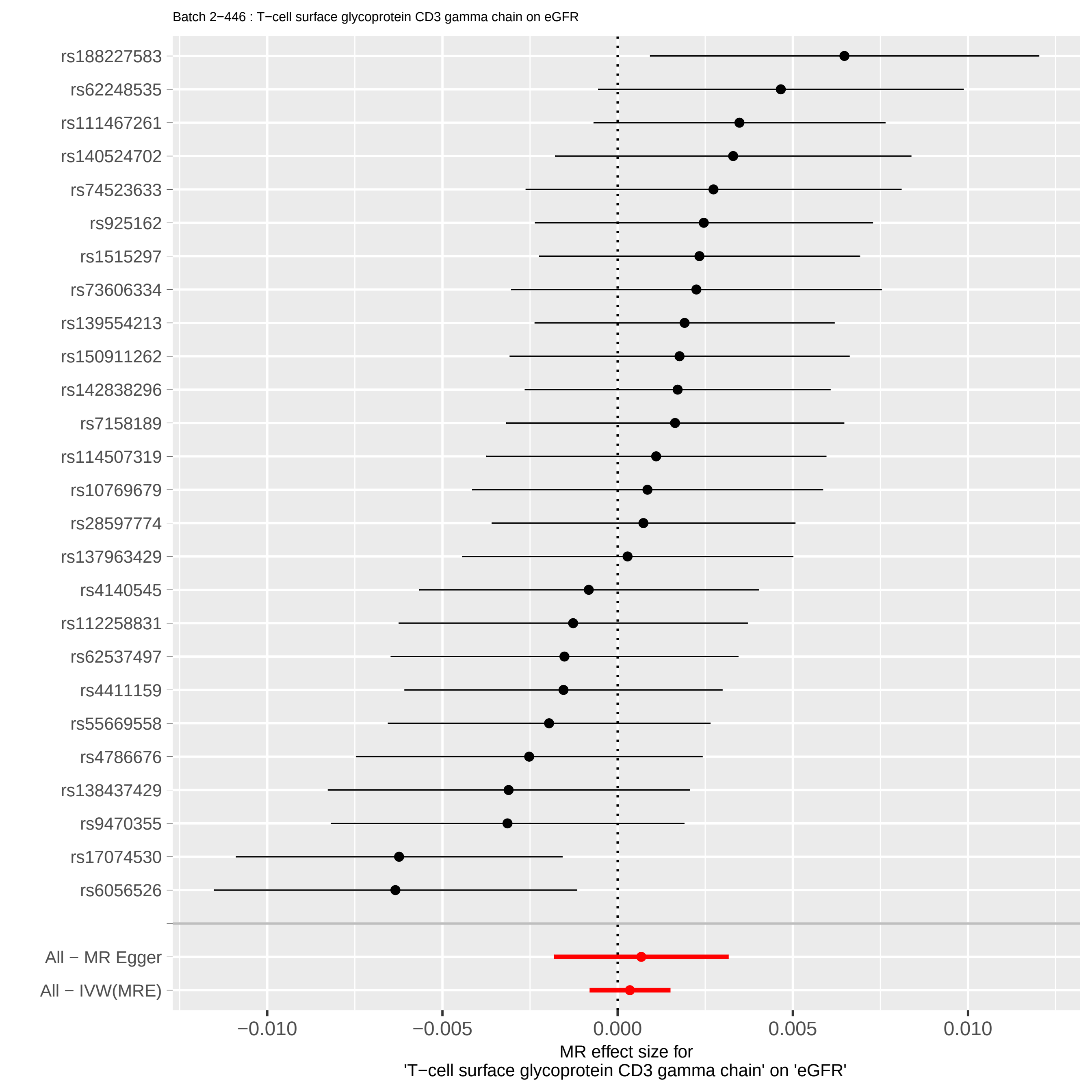

Batch 2−446 : T−cell surface glycoprotein CD3 gamma chain on eGFR
rs188227583
rs62248535
rs111467261
rs140524702
rs74523633
rs925162
rs1515297
rs73606334
rs139554213
rs150911262
rs142838296
rs7158189
rs114507319
rs10769679
rs28597774
rs137963429
rs4140545
rs112258831
rs62537497
rs4411159
rs55669558
rs4786676
rs138437429
rs9470355
rs17074530
rs6056526
All − MR Egger
All − IVW(MRE)
−0.010
−0.005
0.000
0.005
0.010
MR effect size for
'T−cell surface glycoprotein CD3 gamma chain' on 'eGFR'

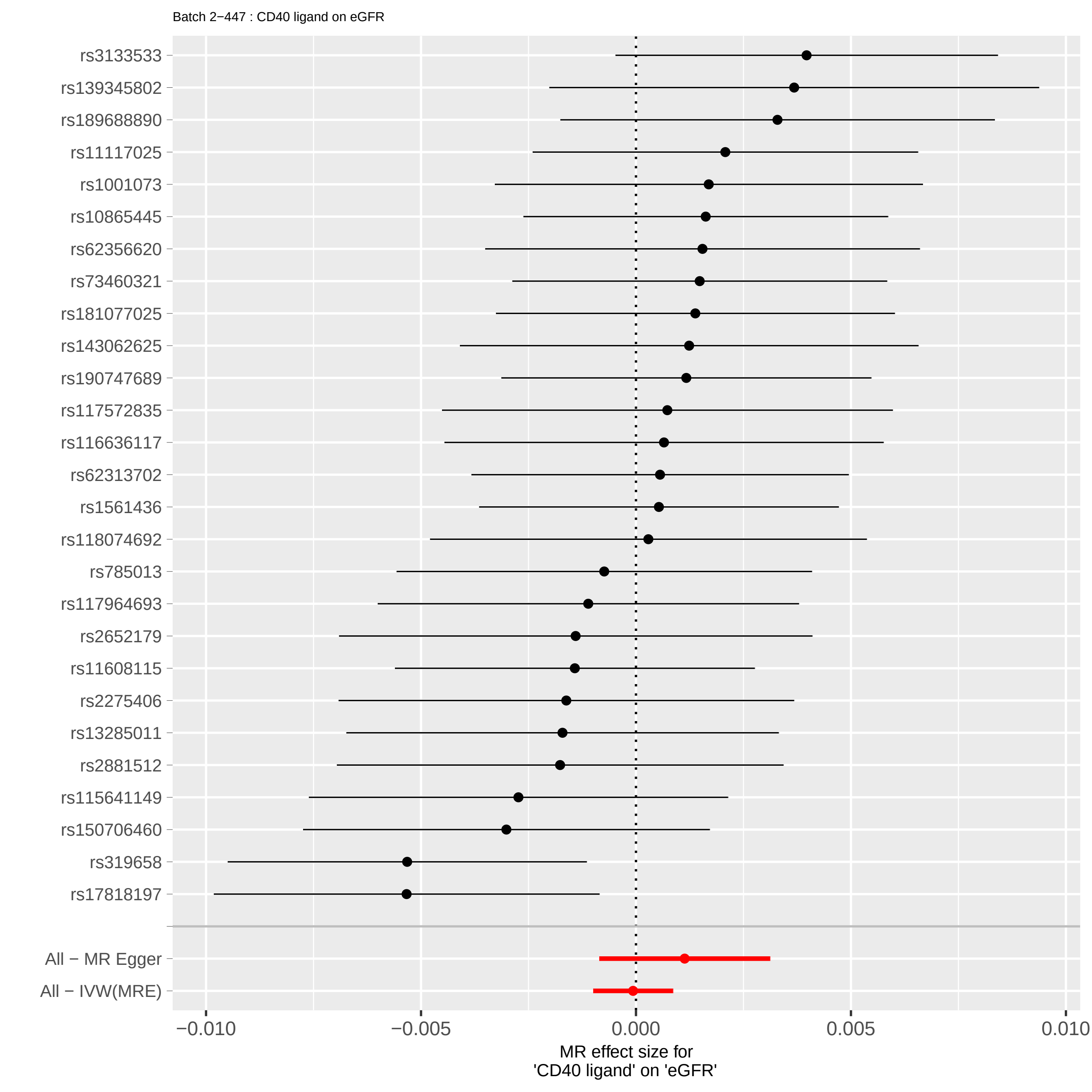

Batch 2−447 : CD40 ligand on eGFR
rs3133533
rs139345802
rs189688890
rs11117025
rs1001073
rs10865445
rs62356620
rs73460321
rs181077025
rs143062625
rs190747689
rs117572835
rs116636117
rs62313702
rs1561436
rs118074692
rs785013
rs117964693
rs2652179
rs11608115
rs2275406
rs13285011
rs2881512
rs115641149
rs150706460
rs319658
rs17818197
All − MR Egger
All − IVW(MRE)
−0.010
−0.005
0.000
0.005
0.010
MR effect size for
'CD40 ligand' on 'eGFR'

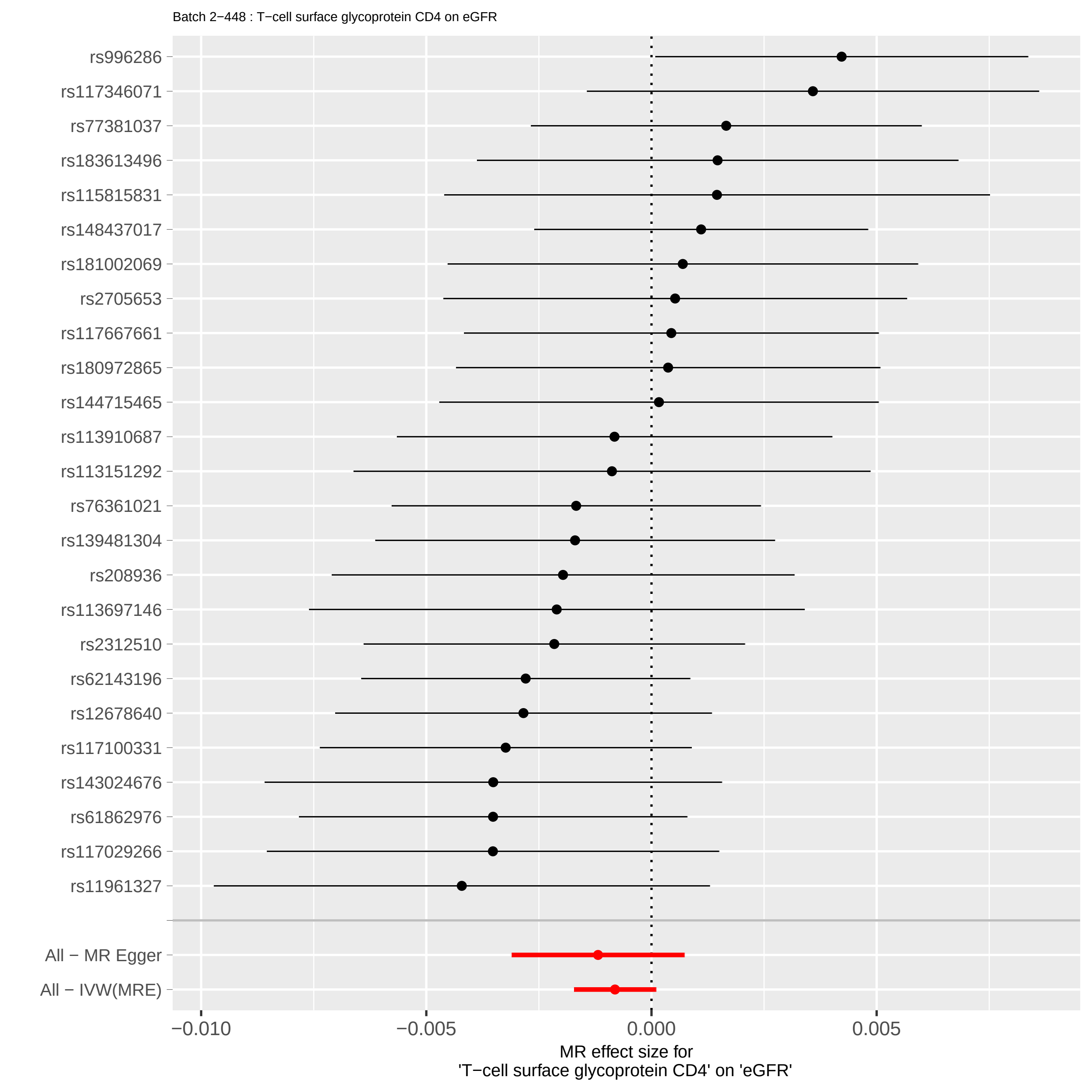

Batch 2−448 : T−cell surface glycoprotein CD4 on eGFR
rs996286
rs117346071
rs77381037
rs183613496
rs115815831
rs148437017
rs181002069
rs2705653
rs117667661
rs180972865
rs144715465
rs113910687
rs113151292
rs76361021
rs139481304
rs208936
rs113697146
rs2312510
rs62143196
rs12678640
rs117100331
rs143024676
rs61862976
rs117029266
rs11961327
All − MR Egger
All − IVW(MRE)
−0.010
−0.005
0.000
0.005
MR effect size for
'T−cell surface glycoprotein CD4' on 'eGFR'

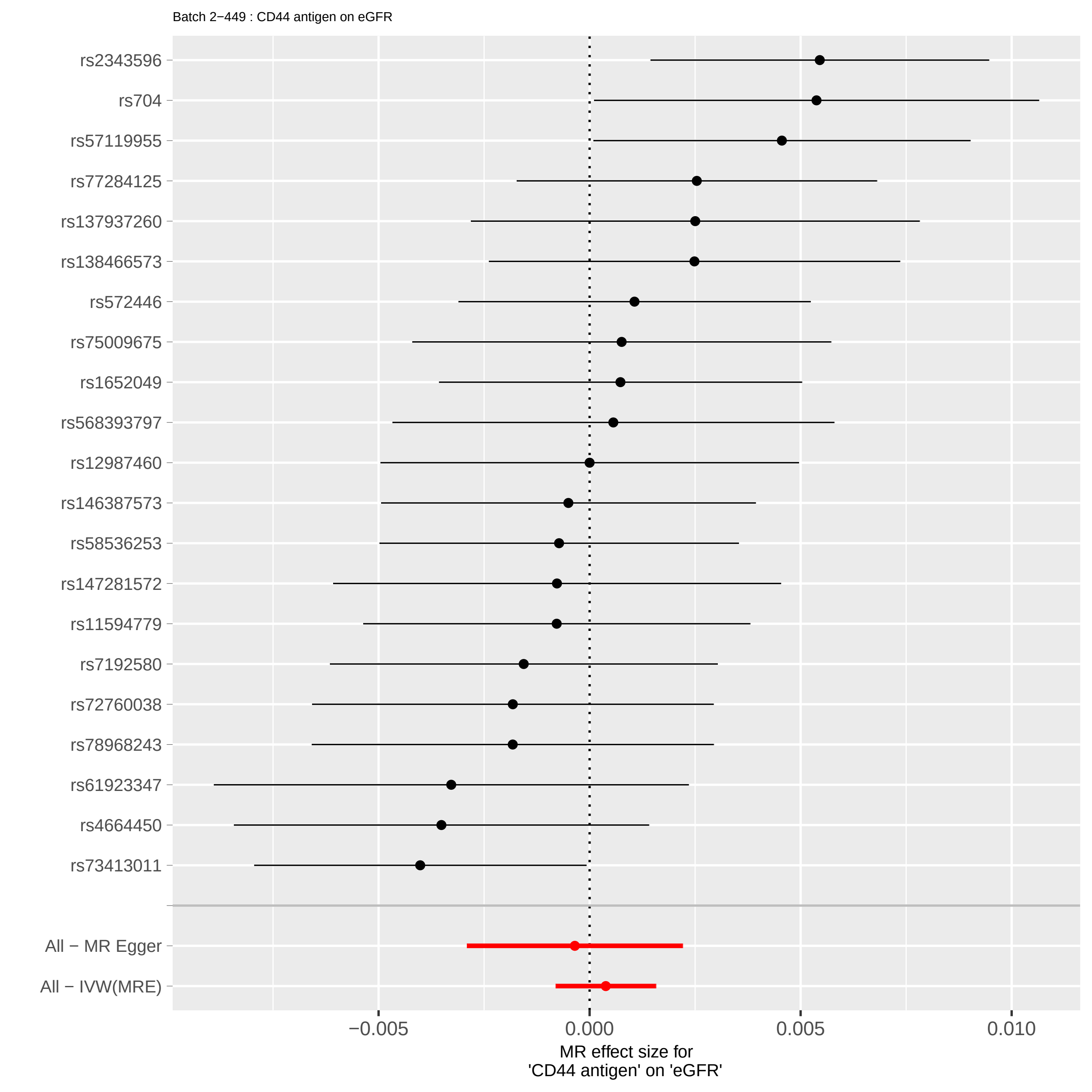

Batch 2−449 : CD44 antigen on eGFR
rs2343596
rs704
rs57119955
rs77284125
rs137937260
rs138466573
rs572446
rs75009675
rs1652049
rs568393797
rs12987460
rs146387573
rs58536253
rs147281572
rs11594779
rs7192580
rs72760038
rs78968243
rs61923347
rs4664450
rs73413011
All − MR Egger
All − IVW(MRE)
−0.005
0.000
0.005
0.010
MR effect size for
'CD44 antigen' on 'eGFR'

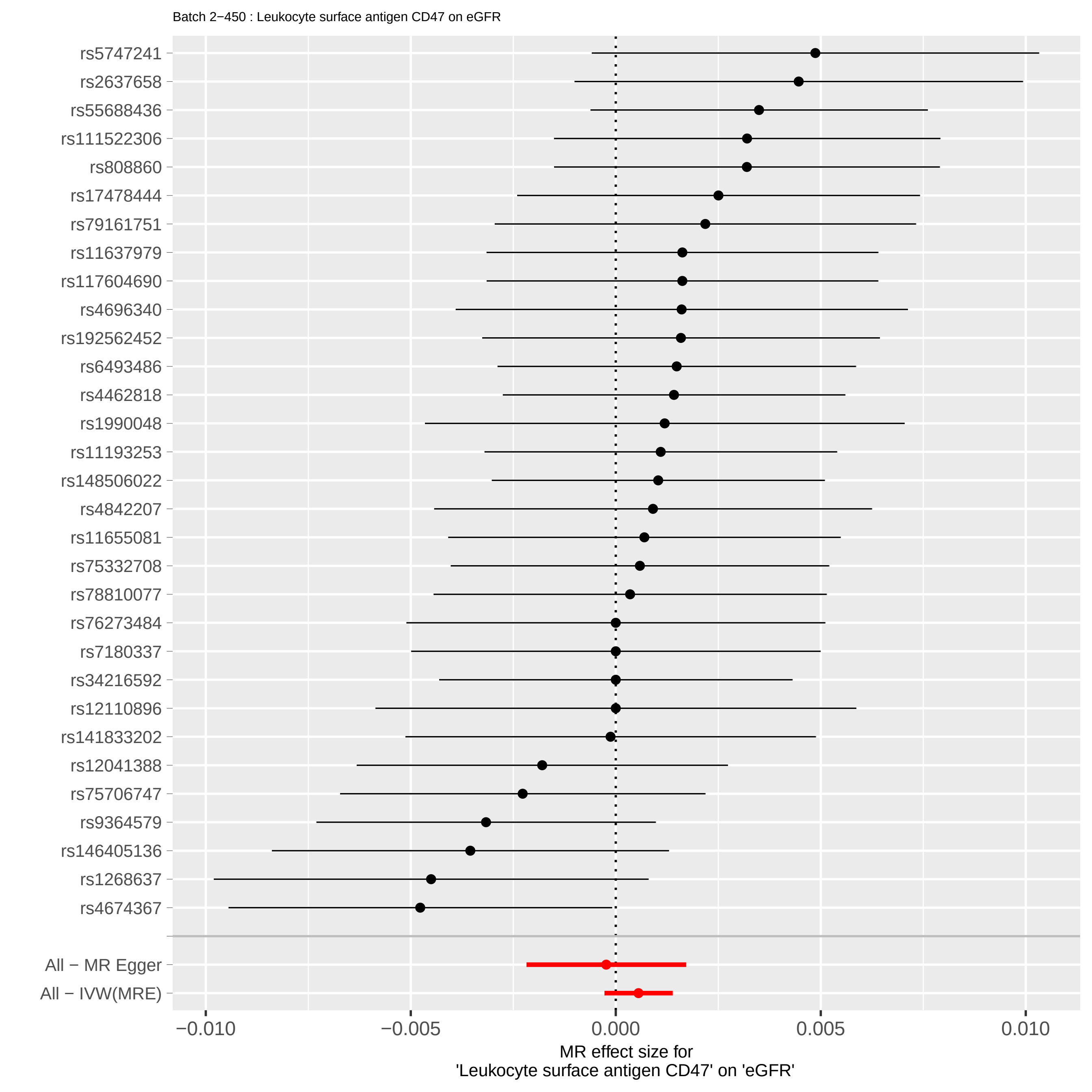

Batch 2−450 : Leukocyte surface antigen CD47 on eGFR
rs5747241
rs2637658
rs55688436
rs111522306
rs808860
rs17478444
rs79161751
rs11637979
rs117604690
rs4696340
rs192562452
rs6493486
rs4462818
rs1990048
rs11193253
rs148506022
rs4842207
rs11655081
rs75332708
rs78810077
rs76273484
rs7180337
rs34216592
rs12110896
rs141833202
rs12041388
rs75706747
rs9364579
rs146405136
rs1268637
rs4674367
All − MR Egger
All − IVW(MRE)
−0.010
−0.005
0.000
0.005
0.010
MR effect size for
'Leukocyte surface antigen CD47' on 'eGFR'

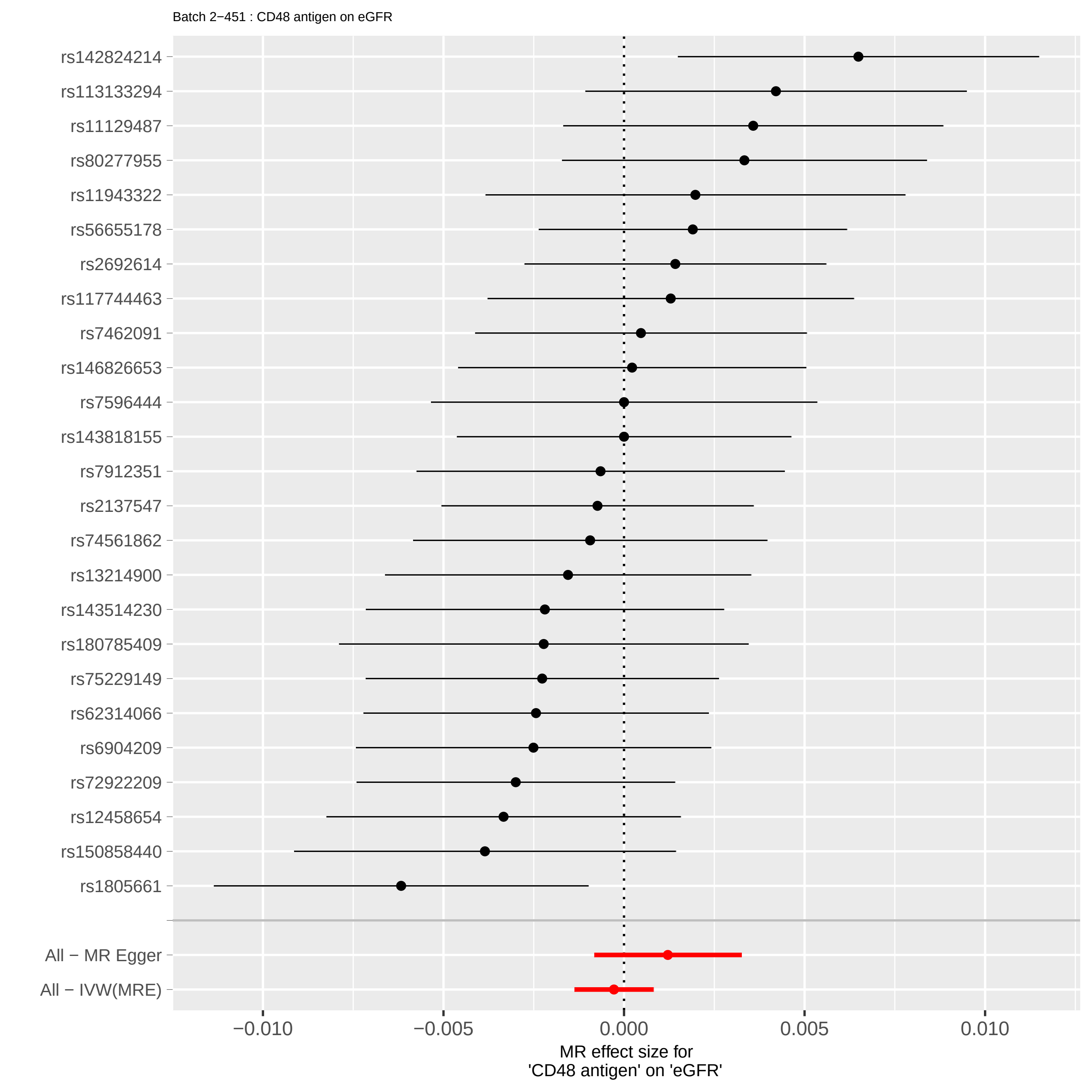

Batch 2−451 : CD48 antigen on eGFR
rs142824214
rs113133294
rs11129487
rs80277955
rs11943322
rs56655178
rs2692614
rs117744463
rs7462091
rs146826653
rs7596444
rs143818155
rs7912351
rs2137547
rs74561862
rs13214900
rs143514230
rs180785409
rs75229149
rs62314066
rs6904209
rs72922209
rs12458654
rs150858440
rs1805661
All − MR Egger
All − IVW(MRE)
−0.010
−0.005
0.000
0.005
0.010
MR effect size for
'CD48 antigen' on 'eGFR'

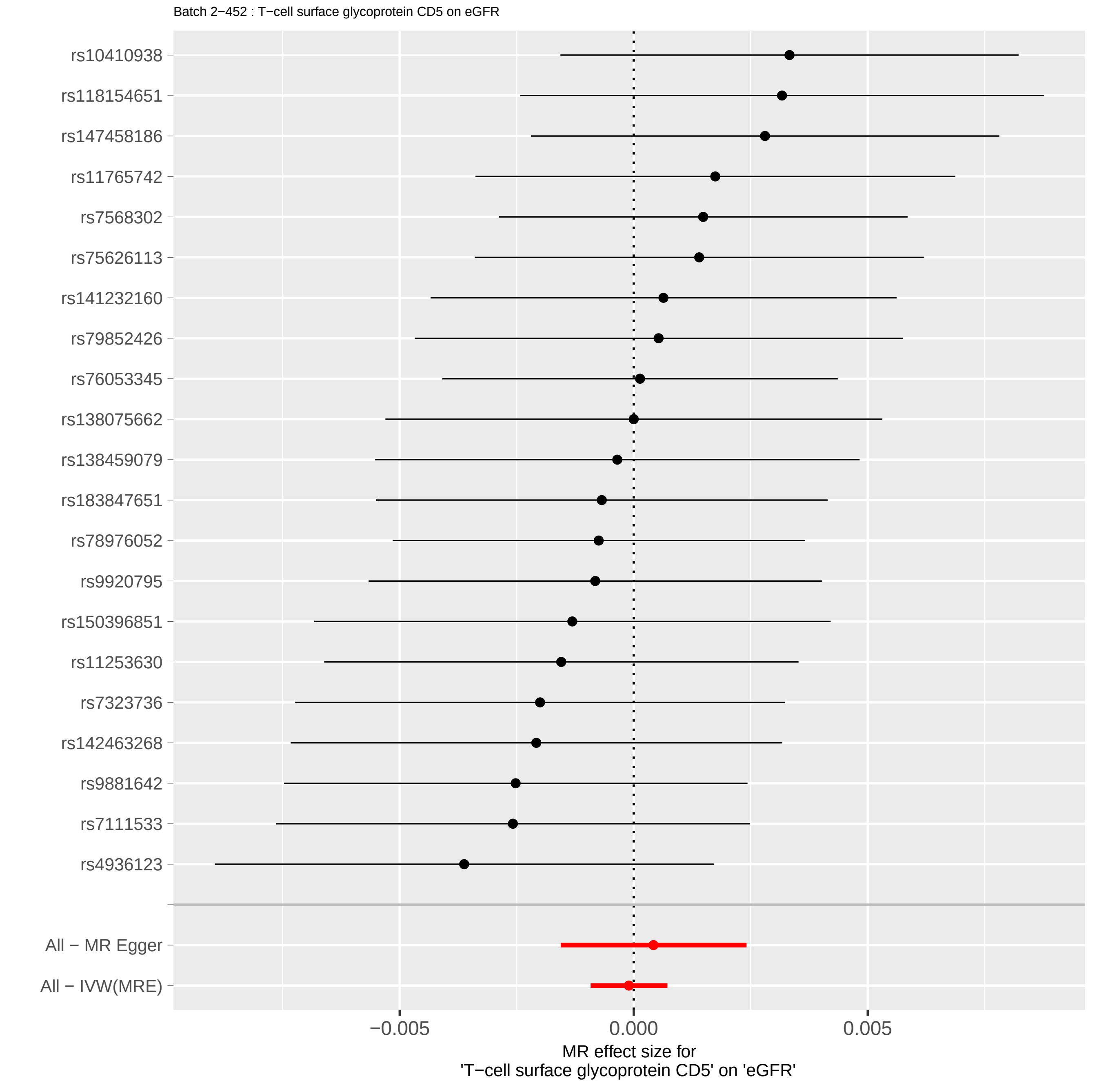

Batch 2−452 : T−cell surface glycoprotein CD5 on eGFR
rs10410938
rs118154651
rs147458186
rs11765742
rs7568302
rs75626113
rs141232160
rs79852426
rs76053345
rs138075662
rs138459079
rs183847651
rs78976052
rs9920795
rs150396851
rs11253630
rs7323736
rs142463268
rs9881642
rs7111533
rs4936123
All − MR Egger
All − IVW(MRE)
−0.005
0.000
0.005
MR effect size for
'T−cell surface glycoprotein CD5' on 'eGFR'

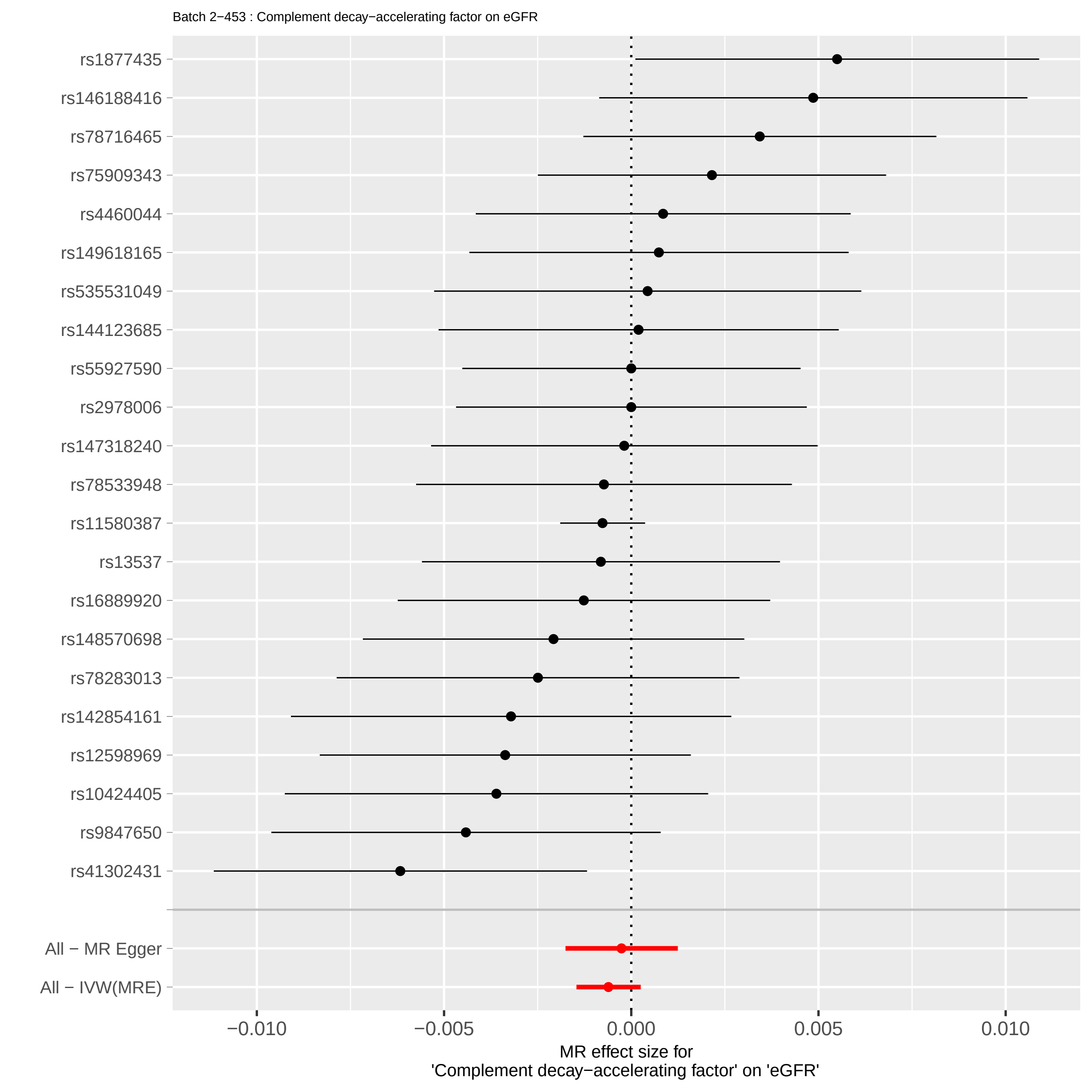

Batch 2−453 : Complement decay−accelerating factor on eGFR
rs1877435
rs146188416
rs78716465
rs75909343
rs4460044
rs149618165
rs535531049
rs144123685
rs55927590
rs2978006
rs147318240
rs78533948
rs11580387
rs13537
rs16889920
rs148570698
rs78283013
rs142854161
rs12598969
rs10424405
rs9847650
rs41302431
All − MR Egger
All − IVW(MRE)
−0.010
−0.005
0.000
0.005
0.010
MR effect size for
'Complement decay−accelerating factor' on 'eGFR'

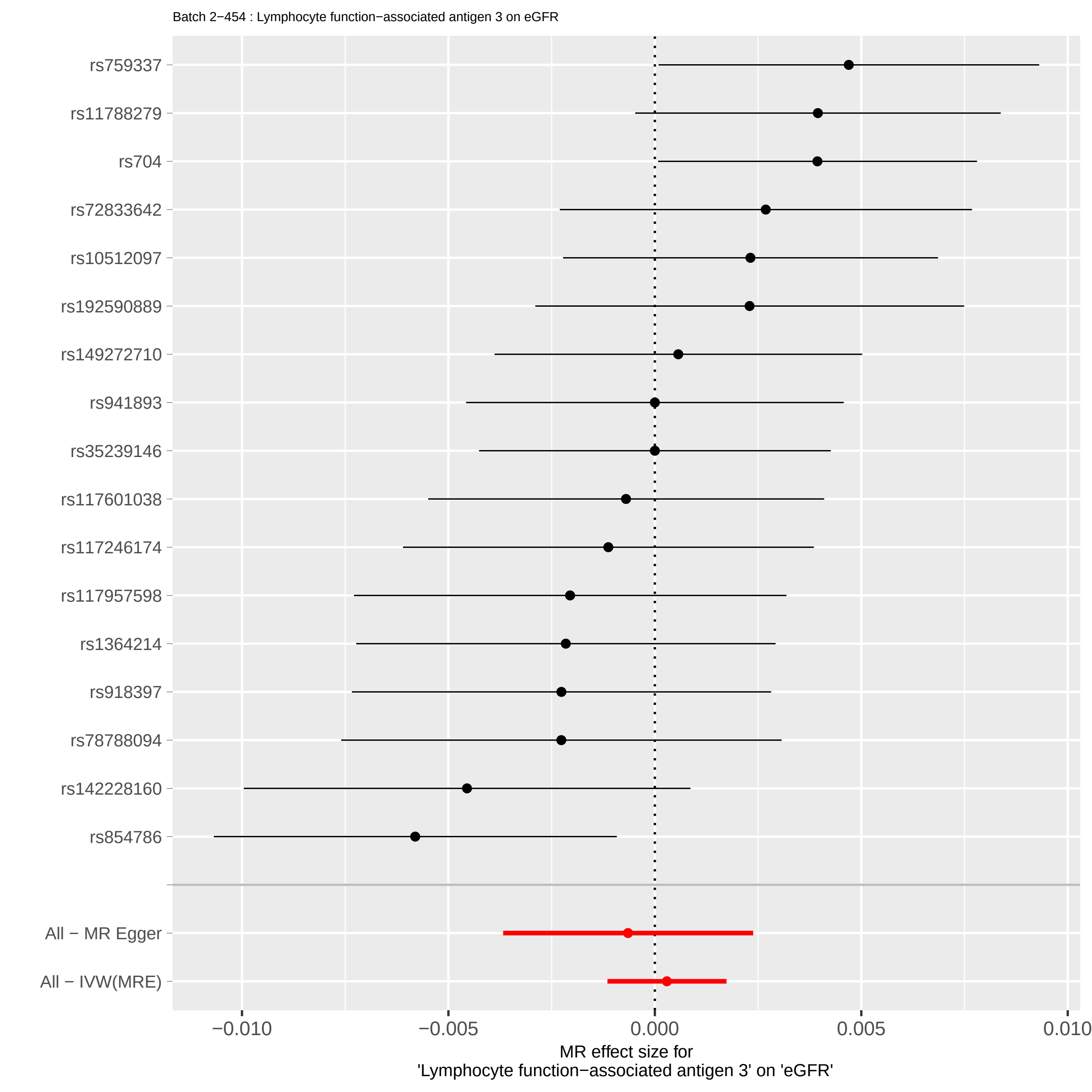

Batch 2−454 : Lymphocyte function−associated antigen 3 on eGFR
rs759337
rs11788279
rs704
rs72833642
rs10512097
rs192590889
rs149272710
rs941893
rs35239146
rs117601038
rs117246174
rs117957598
rs1364214
rs918397
rs78788094
rs142228160
rs854786
All − MR Egger
All − IVW(MRE)
−0.010
−0.005
0.000
0.005
0.010
MR effect size for
'Lymphocyte function−associated antigen 3' on 'eGFR'

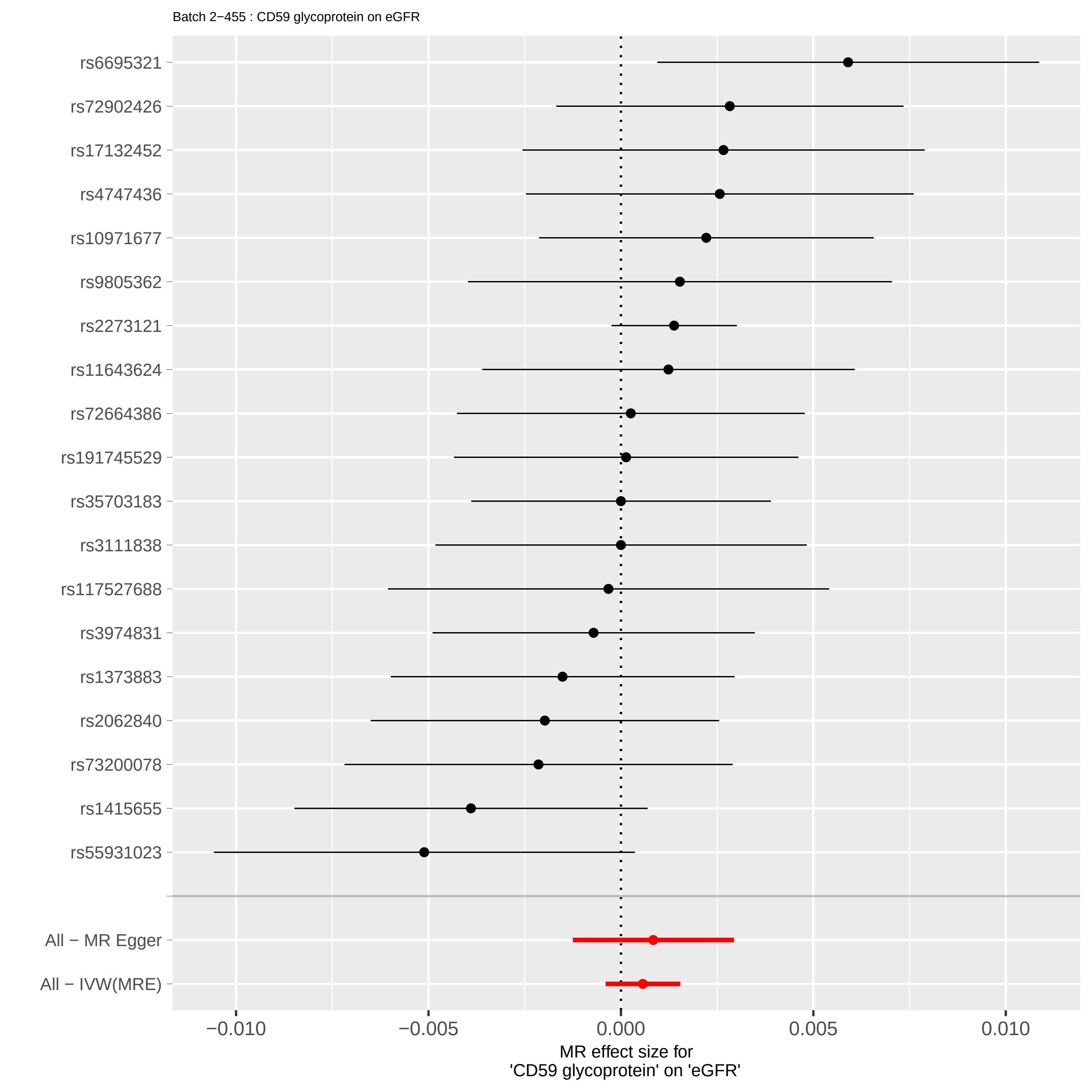

Batch 2−455 : CD59 glycoprotein on eGFR
rs6695321
rs72902426
rs17132452
rs4747436
rs10971677
rs9805362
rs2273121
rs11643624
rs72664386
rs191745529
rs35703183
rs3111838
rs117527688
rs3974831
rs1373883
rs2062840
rs73200078
rs1415655
rs55931023
All − MR Egger
All − IVW(MRE)
−0.010
−0.005
0.000
0.005
0.010
MR effect size for
'CD59 glycoprotein' on 'eGFR'

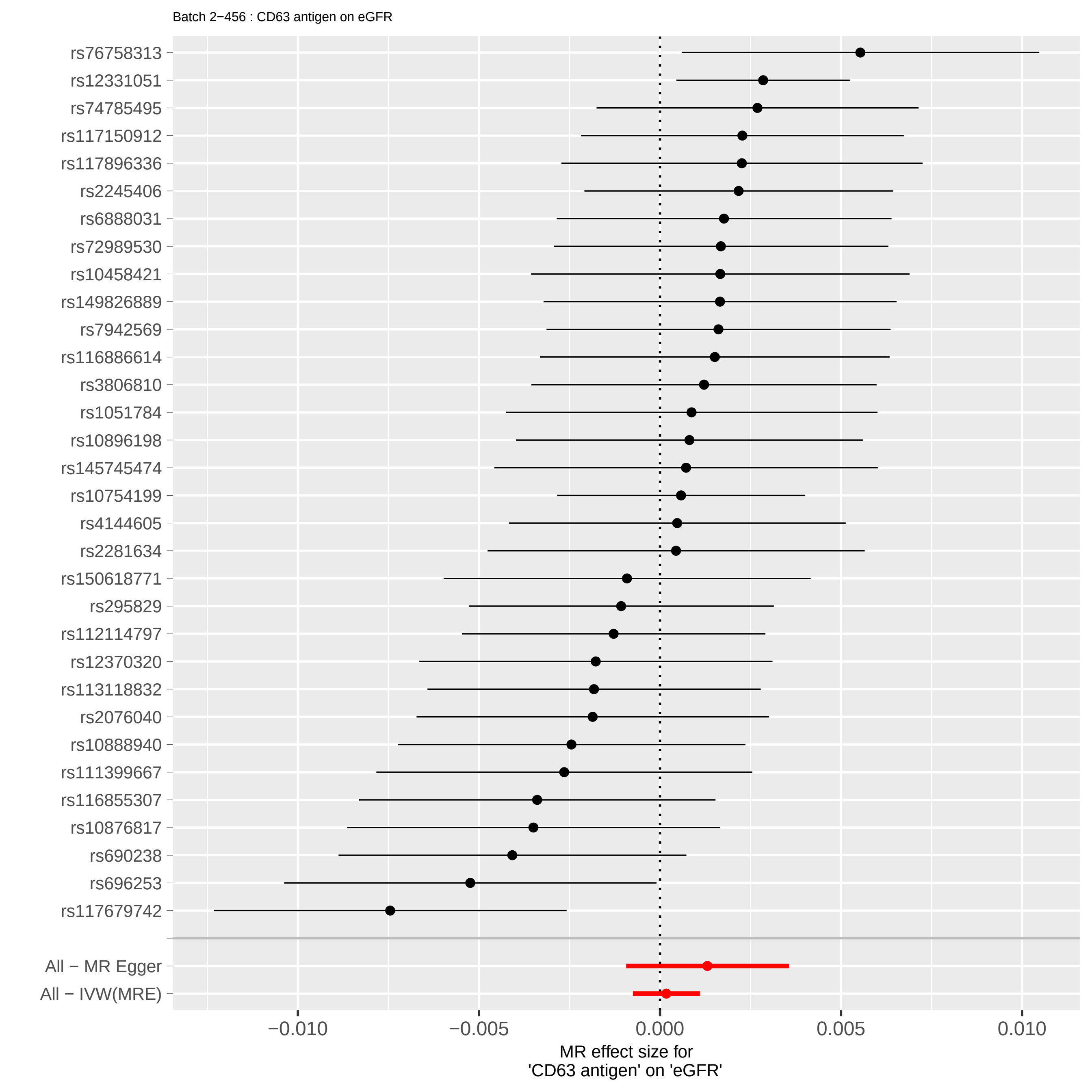
Batch 2−456 : CD63 antigen on eGFR
rs76758313
rs12331051
rs74785495
rs117150912
rs117896336
rs2245406
rs6888031
rs72989530
rs10458421
rs149826889
rs7942569
rs116886614
rs3806810
rs1051784
rs10896198
rs145745474
rs10754199
rs4144605
rs2281634
rs150618771
rs295829
rs112114797
rs12370320
rs113118832
rs2076040
rs10888940
rs111399667
rs116855307
rs10876817
rs690238
rs696253
rs117679742
All − MR Egger
All − IVW(MRE)
−0.010
−0.005
0.000
0.005
0.010
MR effect size for
'CD63 antigen' on 'eGFR'

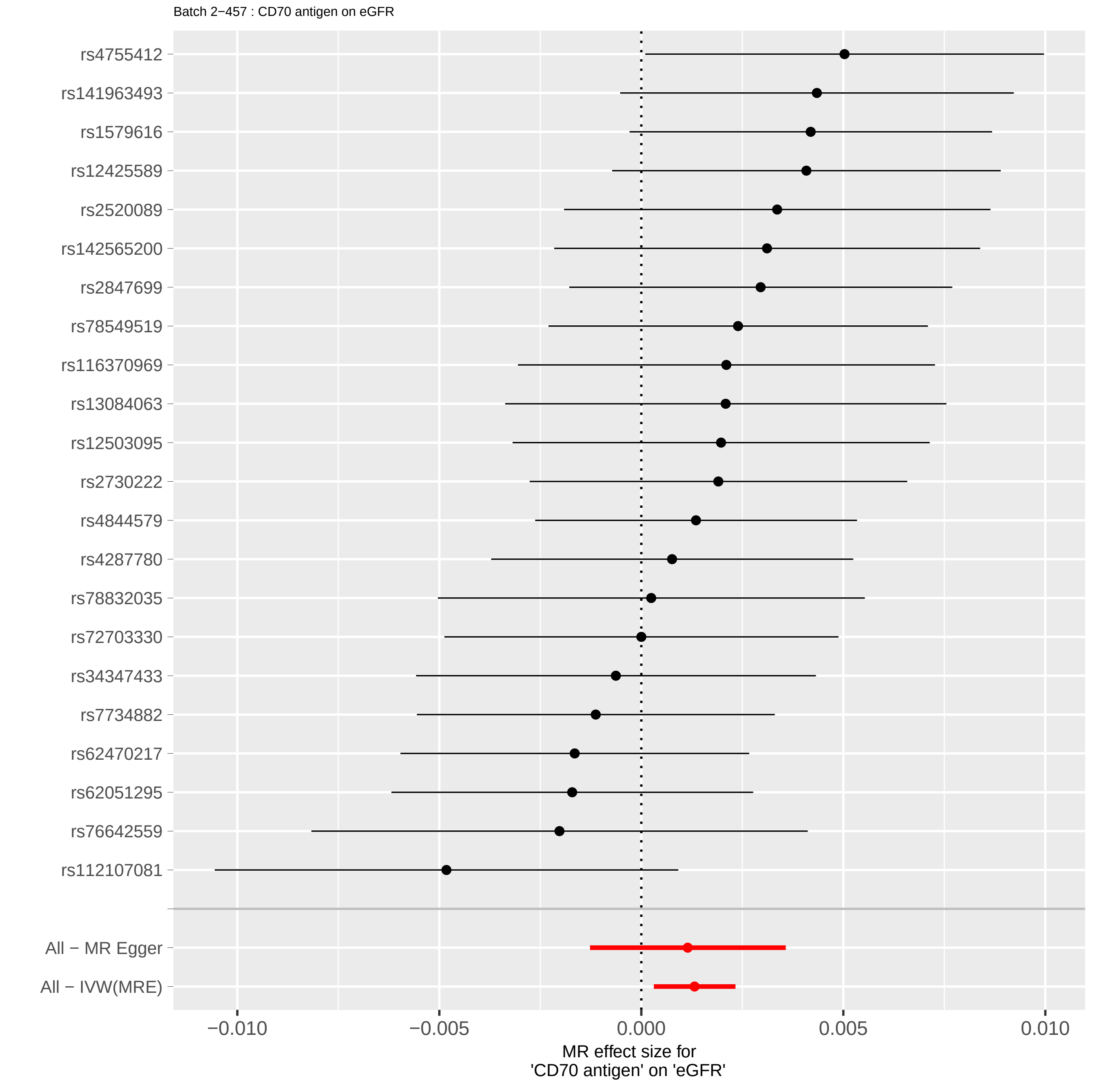
Batch 2−457 : CD70 antigen on eGFR
rs4755412
rs141963493
rs1579616
rs12425589
rs2520089
rs142565200
rs2847699
rs78549519
rs116370969
rs13084063
rs12503095
rs2730222
rs4844579
rs4287780
rs78832035
rs72703330
rs34347433
rs7734882
rs62470217
rs62051295
rs76642559
rs112107081
All − MR Egger
All − IVW(MRE)
−0.010
−0.005
0.000
0.005
0.010
MR effect size for
'CD70 antigen' on 'eGFR'

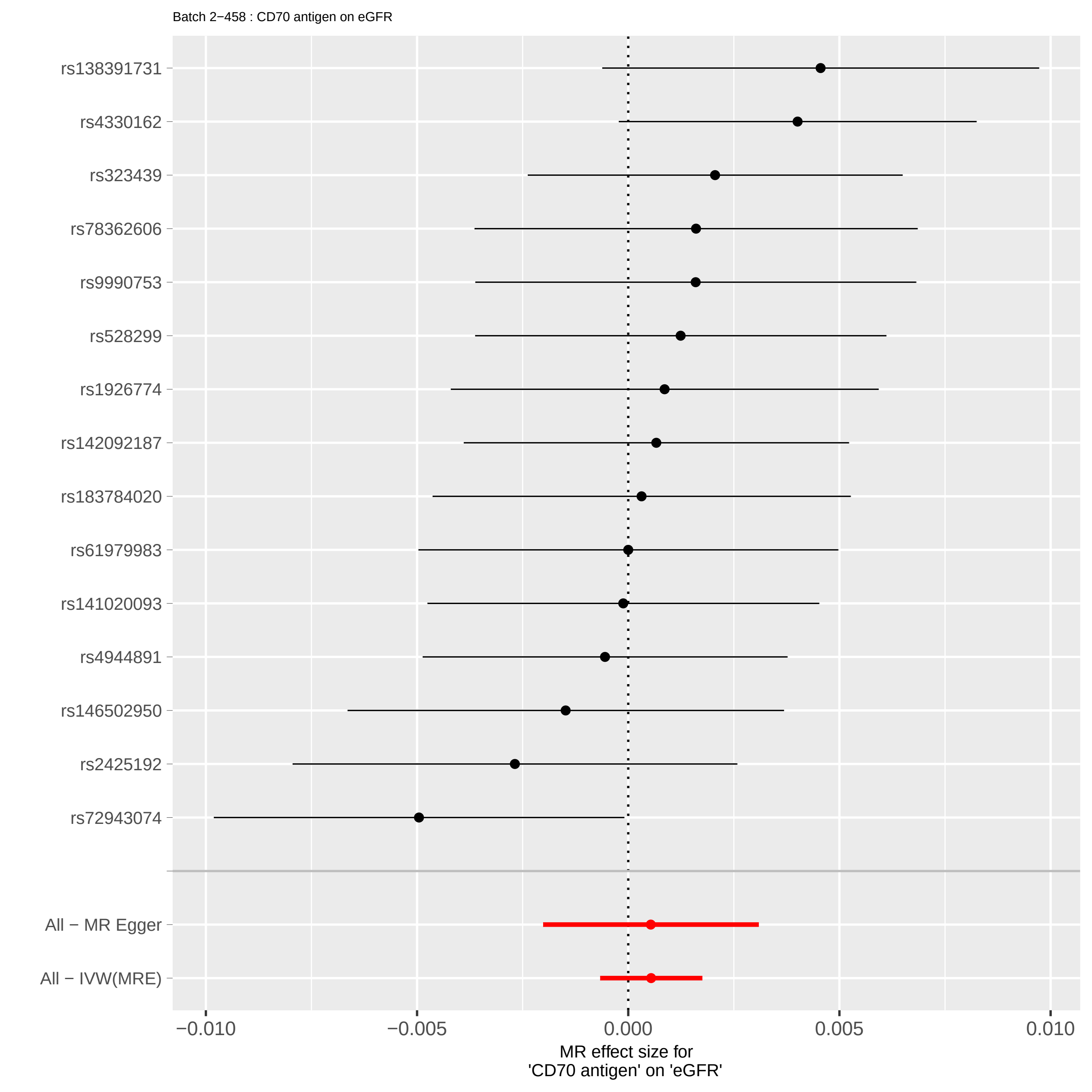

Batch 2−458 : CD70 antigen on eGFR
rs138391731
rs4330162
rs323439
rs78362606
rs9990753
rs528299
rs1926774
rs142092187
rs183784020
rs61979983
rs141020093
rs4944891
rs146502950
rs2425192
rs72943074
All − MR Egger
All − IVW(MRE)
−0.010
−0.005
0.000
0.005
0.010
MR effect size for
'CD70 antigen' on 'eGFR'

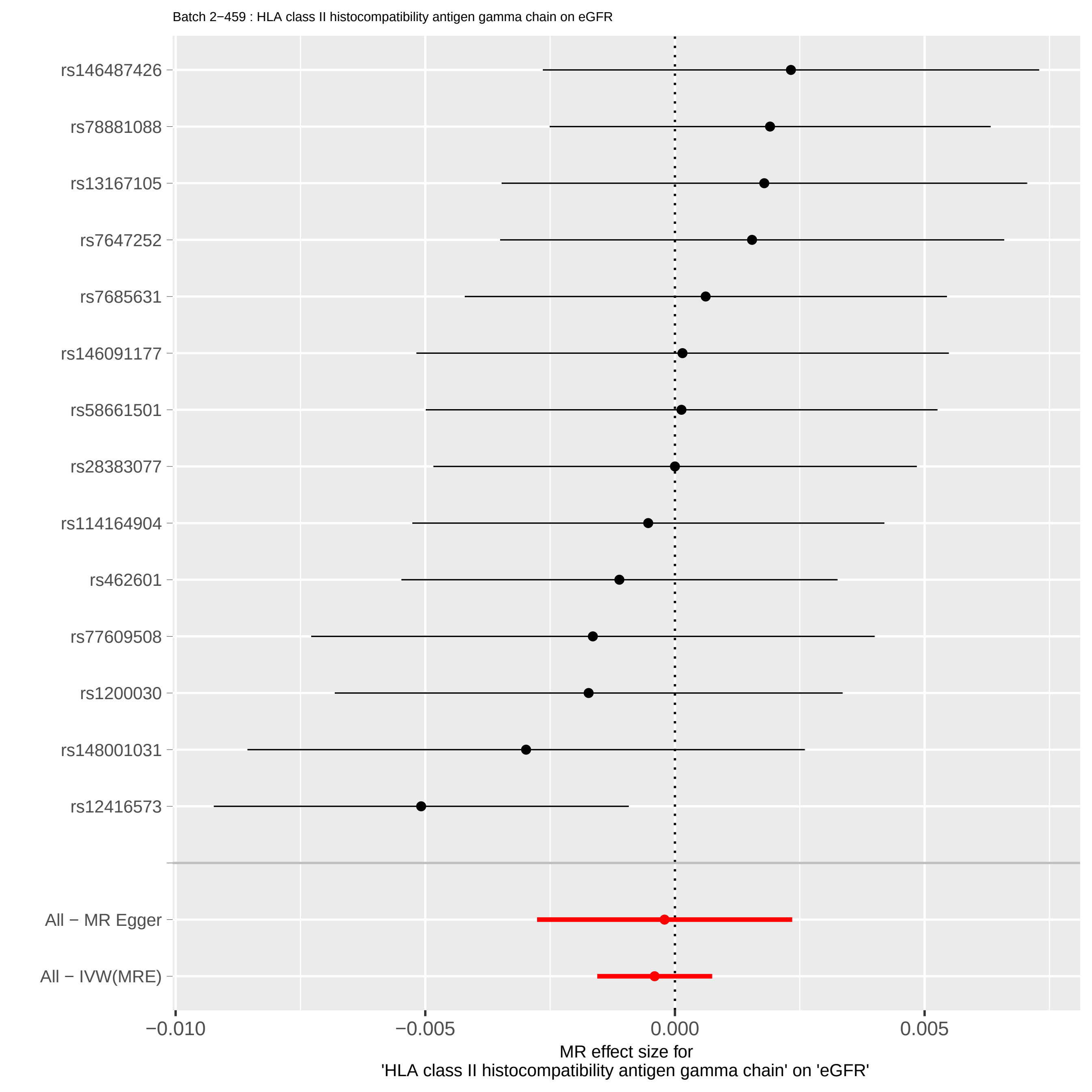

Batch 2−459 : HLA class II histocompatibility antigen gamma chain on eGFR
rs146487426
rs78881088
rs13167105
rs7647252
rs7685631
rs146091177
rs58661501
rs28383077
rs114164904
rs462601
rs77609508
rs1200030
rs148001031
rs12416573
All − MR Egger
All − IVW(MRE)
−0.010
−0.005
0.000
0.005
MR effect size for
'HLA class II histocompatibility antigen gamma chain' on 'eGFR'

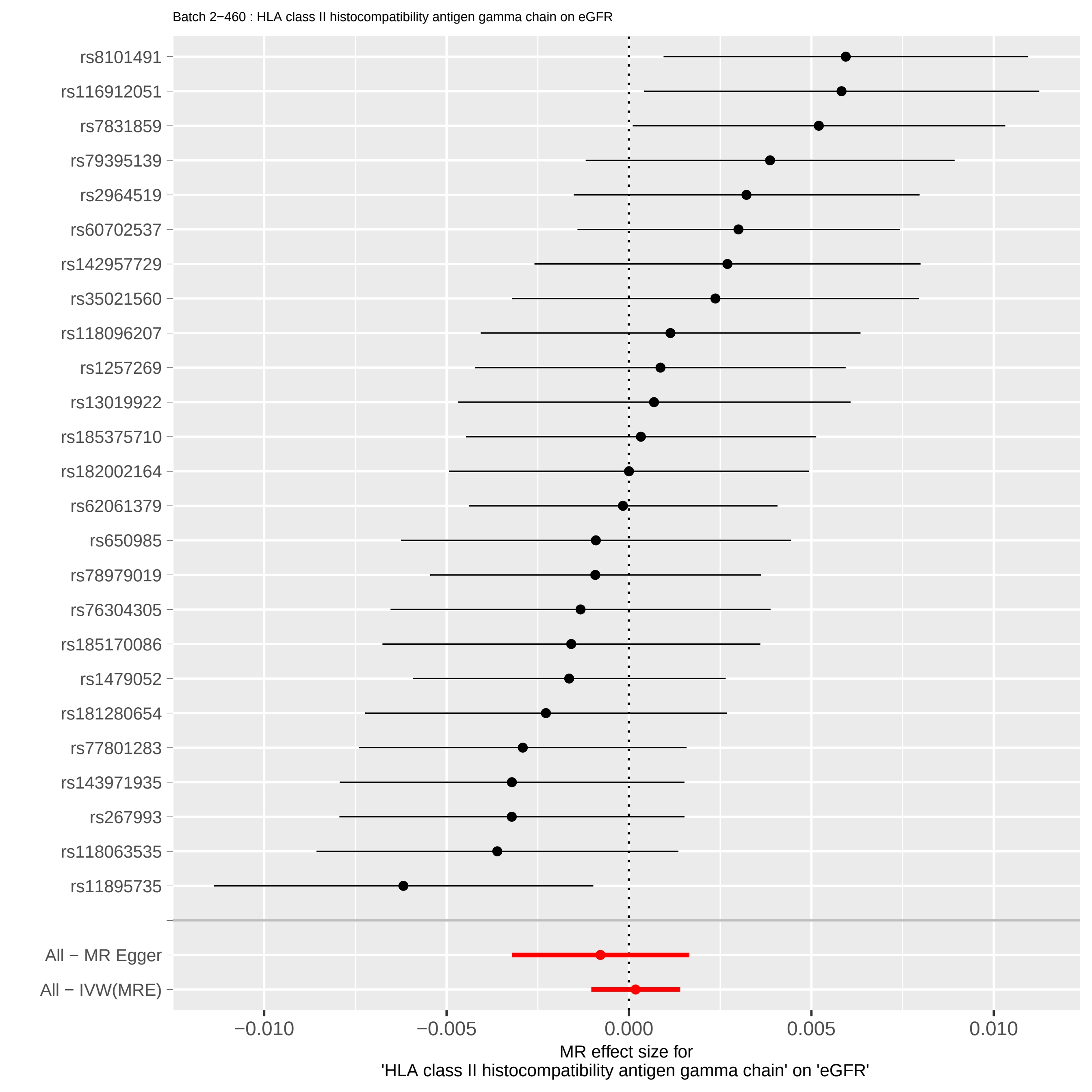

Batch 2−460 : HLA class II histocompatibility antigen gamma chain on eGFR
rs8101491
rs116912051
rs7831859
rs79395139
rs2964519
rs60702537
rs142957729
rs35021560
rs118096207
rs1257269
rs13019922
rs185375710
rs182002164
rs62061379
rs650985
rs78979019
rs76304305
rs185170086
rs1479052
rs181280654
rs77801283
rs143971935
rs267993
rs118063535
rs11895735
All − MR Egger
All − IVW(MRE)
−0.010
−0.005
0.000
0.005
0.010
MR effect size for
'HLA class II histocompatibility antigen gamma chain' on 'eGFR'

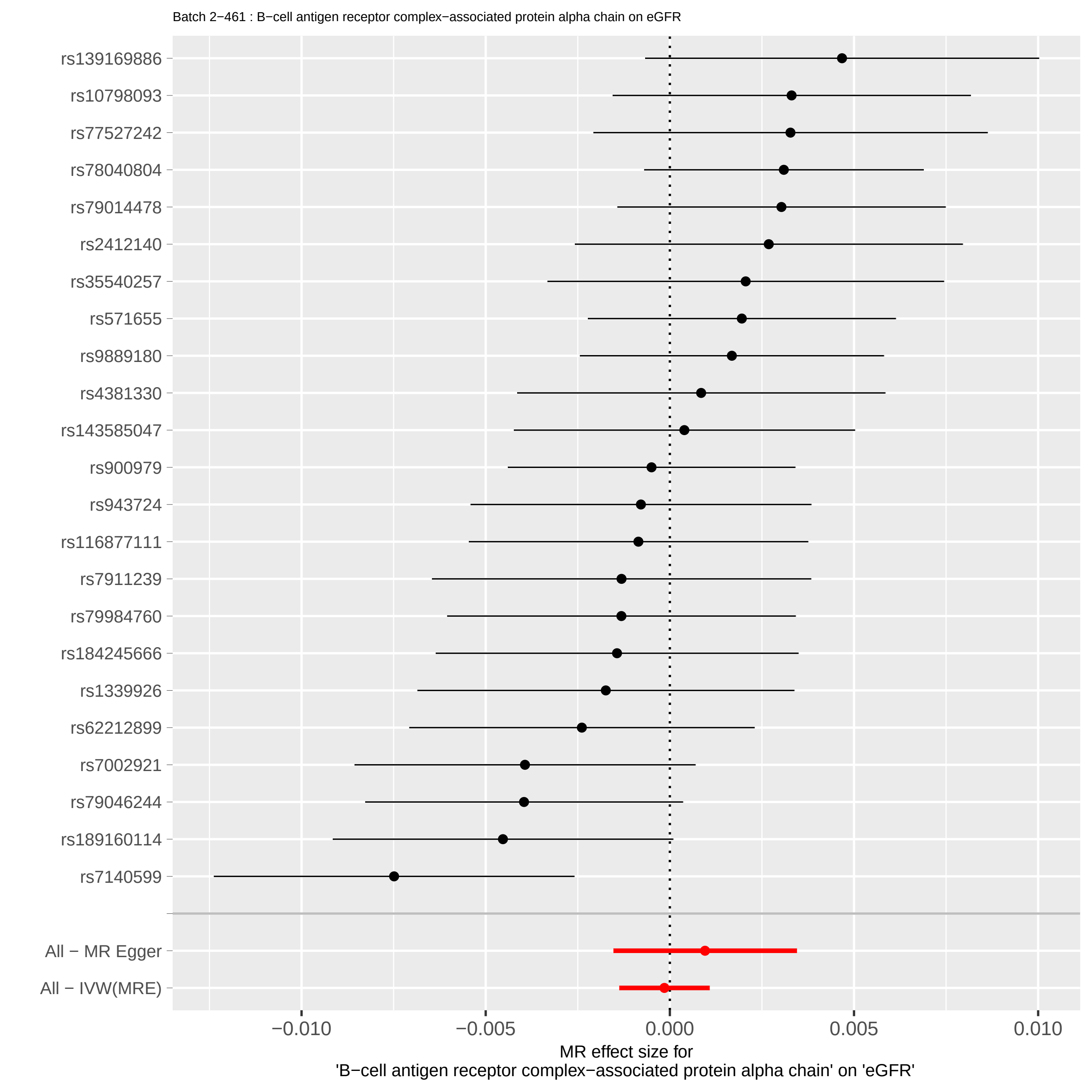

Batch 2−461 : B−cell antigen receptor complex−associated protein alpha chain on eGFR
rs139169886
rs10798093
rs77527242
rs78040804
rs79014478
rs2412140
rs35540257
rs571655
rs9889180
rs4381330
rs143585047
rs900979
rs943724
rs116877111
rs7911239
rs79984760
rs184245666
rs1339926
rs62212899
rs7002921
rs79046244
rs189160114
rs7140599
All − MR Egger
All − IVW(MRE)
−0.010
−0.005
0.000
0.005
0.010
MR effect size for
'B−cell antigen receptor complex−associated protein alpha chain' on 'eGFR'

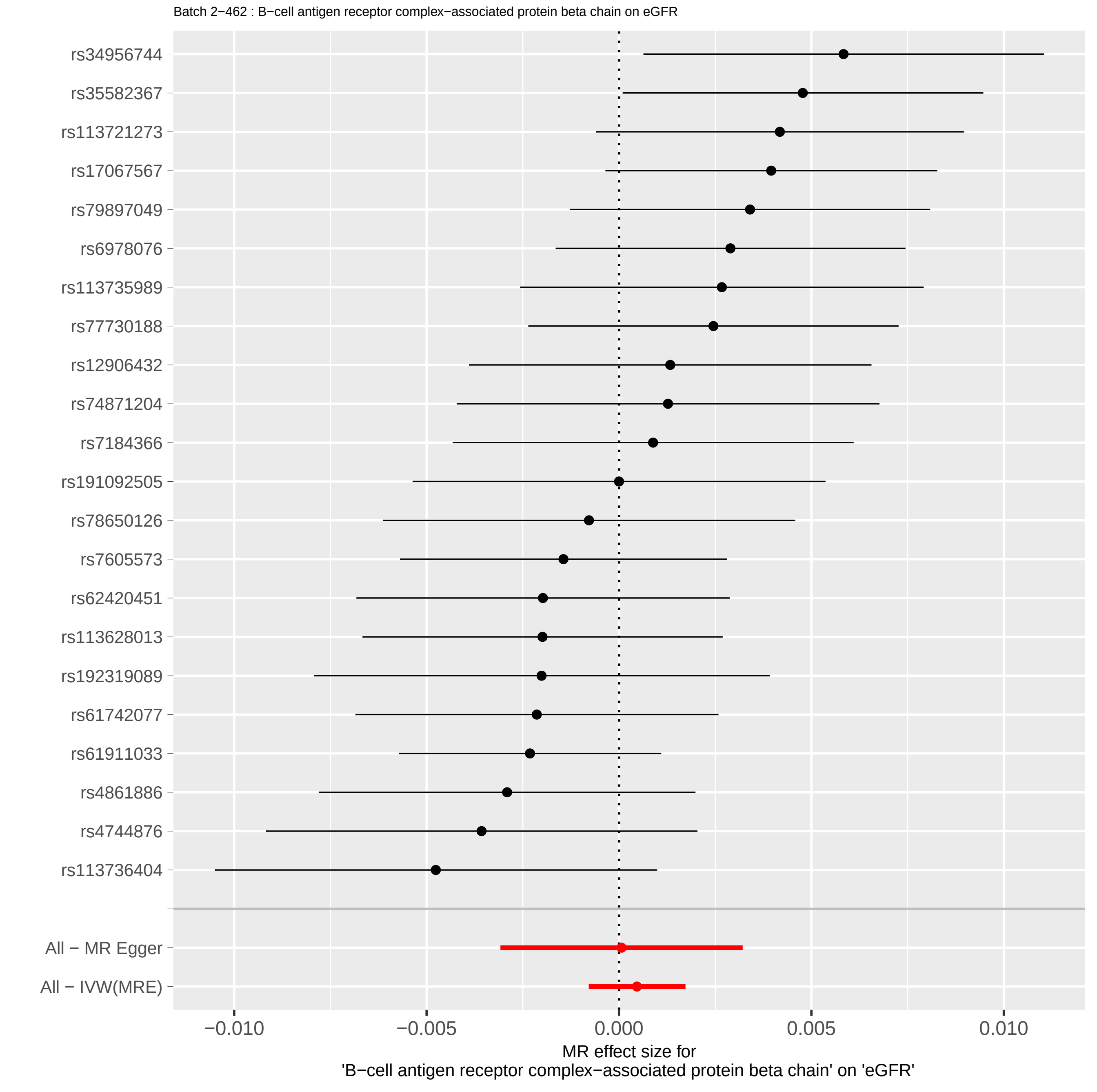

Batch 2−462 : B−cell antigen receptor complex−associated protein beta chain on eGFR
rs34956744
rs35582367
rs113721273
rs17067567
rs79897049
rs6978076
rs113735989
rs77730188
rs12906432
rs74871204
rs7184366
rs191092505
rs78650126
rs7605573
rs62420451
rs113628013
rs192319089
rs61742077
rs61911033
rs4861886
rs4744876
rs113736404
All − MR Egger
All − IVW(MRE)
−0.010
−0.005
0.000
0.005
0.010
MR effect size for
'B−cell antigen receptor complex−associated protein beta chain' on 'eGFR'

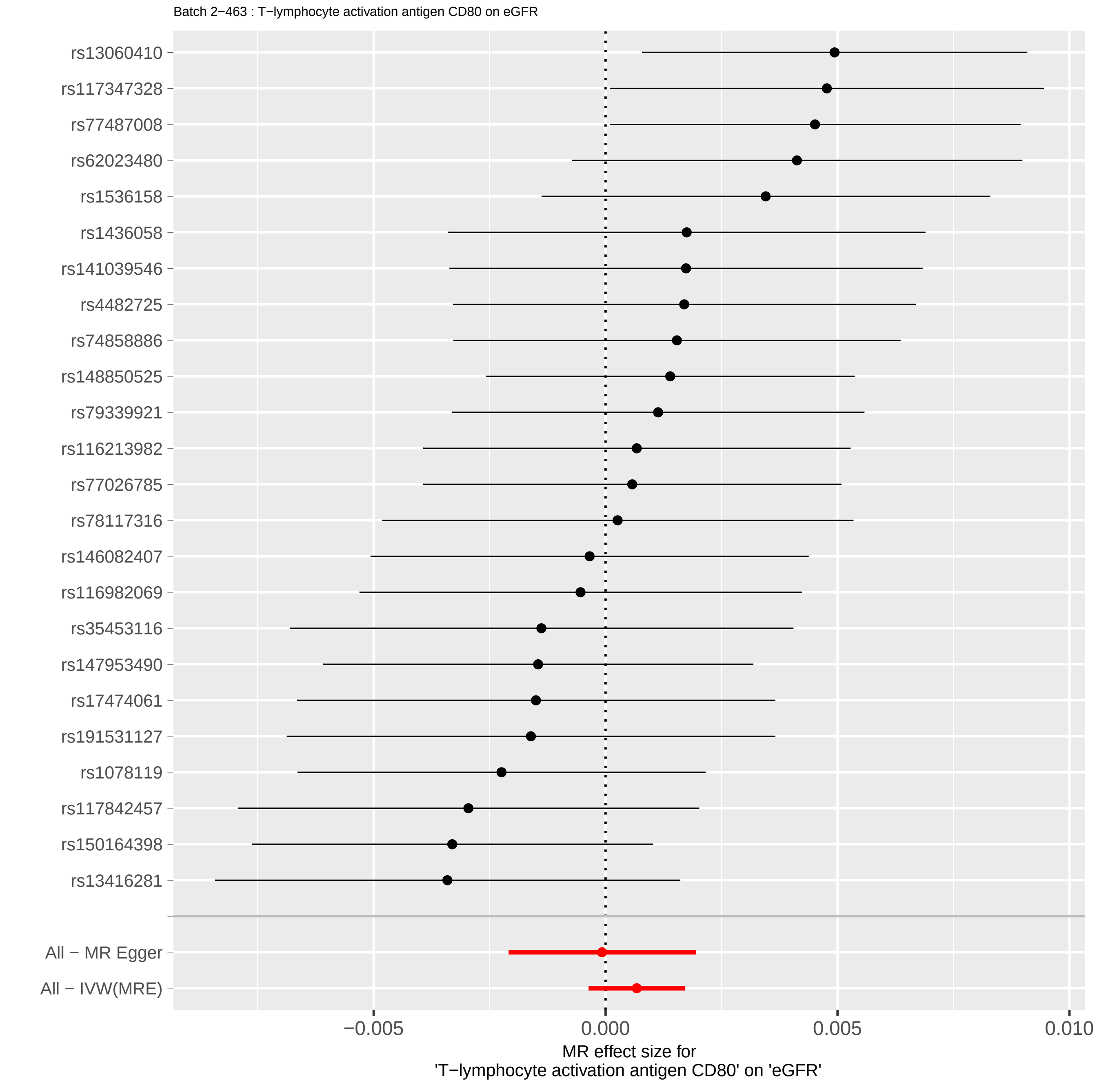

Batch 2−463 : T−lymphocyte activation antigen CD80 on eGFR
rs13060410
rs117347328
rs77487008
rs62023480
rs1536158
rs1436058
rs141039546
rs4482725
rs74858886
rs148850525
rs79339921
rs116213982
rs77026785
rs78117316
rs146082407
rs116982069
rs35453116
rs147953490
rs17474061
rs191531127
rs1078119
rs117842457
rs150164398
rs13416281
All − MR Egger
All − IVW(MRE)
−0.005
0.000
0.005
0.010
MR effect size for
'T−lymphocyte activation antigen CD80' on 'eGFR'

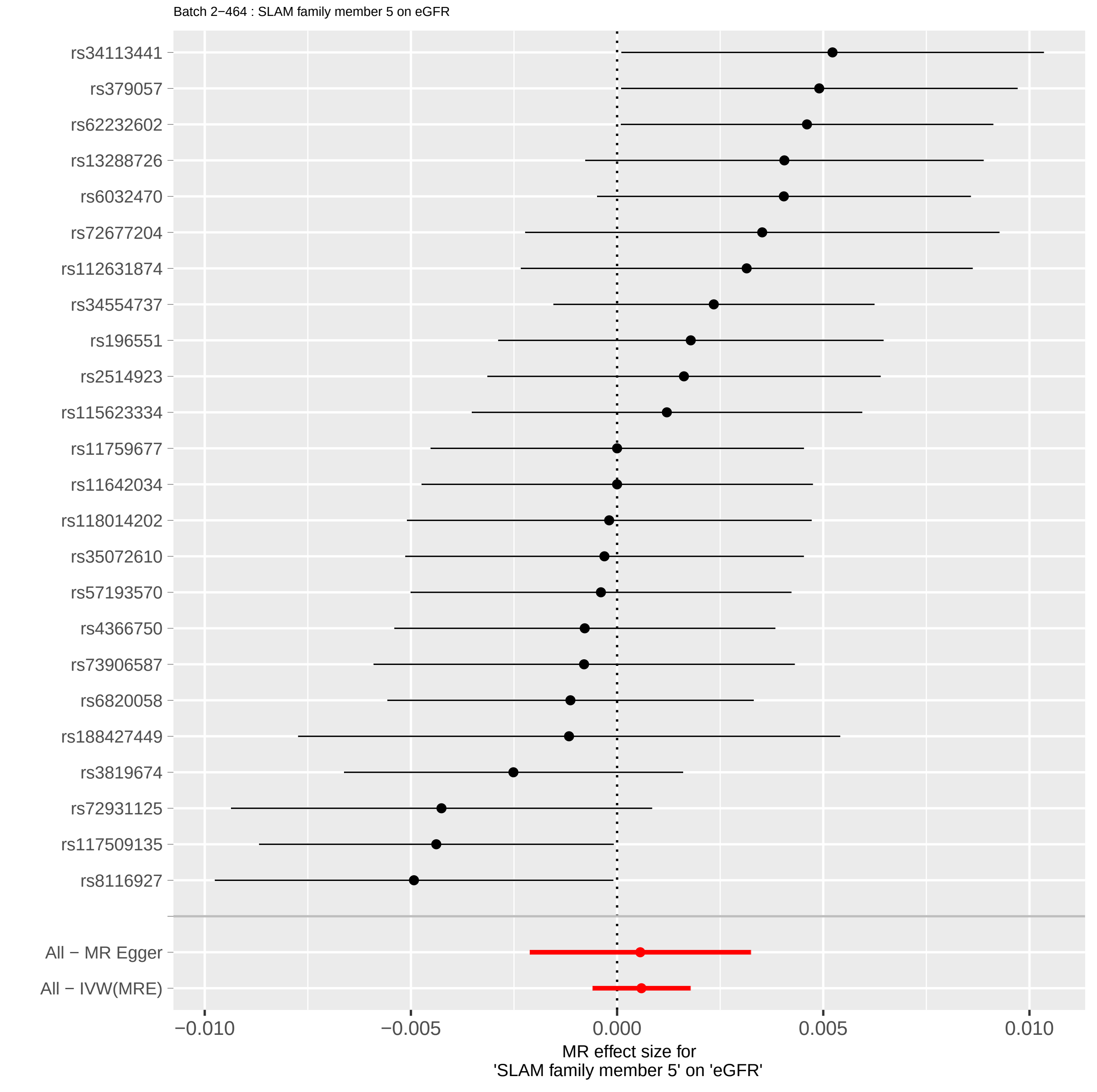

Batch 2−464 : SLAM family member 5 on eGFR
rs34113441
rs379057
rs62232602
rs13288726
rs6032470
rs72677204
rs112631874
rs34554737
rs196551
rs2514923
rs115623334
rs11759677
rs11642034
rs118014202
rs35072610
rs57193570
rs4366750
rs73906587
rs6820058
rs188427449
rs3819674
rs72931125
rs117509135
rs8116927
All − MR Egger
All − IVW(MRE)
−0.010
−0.005
0.000
0.005
0.010
MR effect size for
'SLAM family member 5' on 'eGFR'

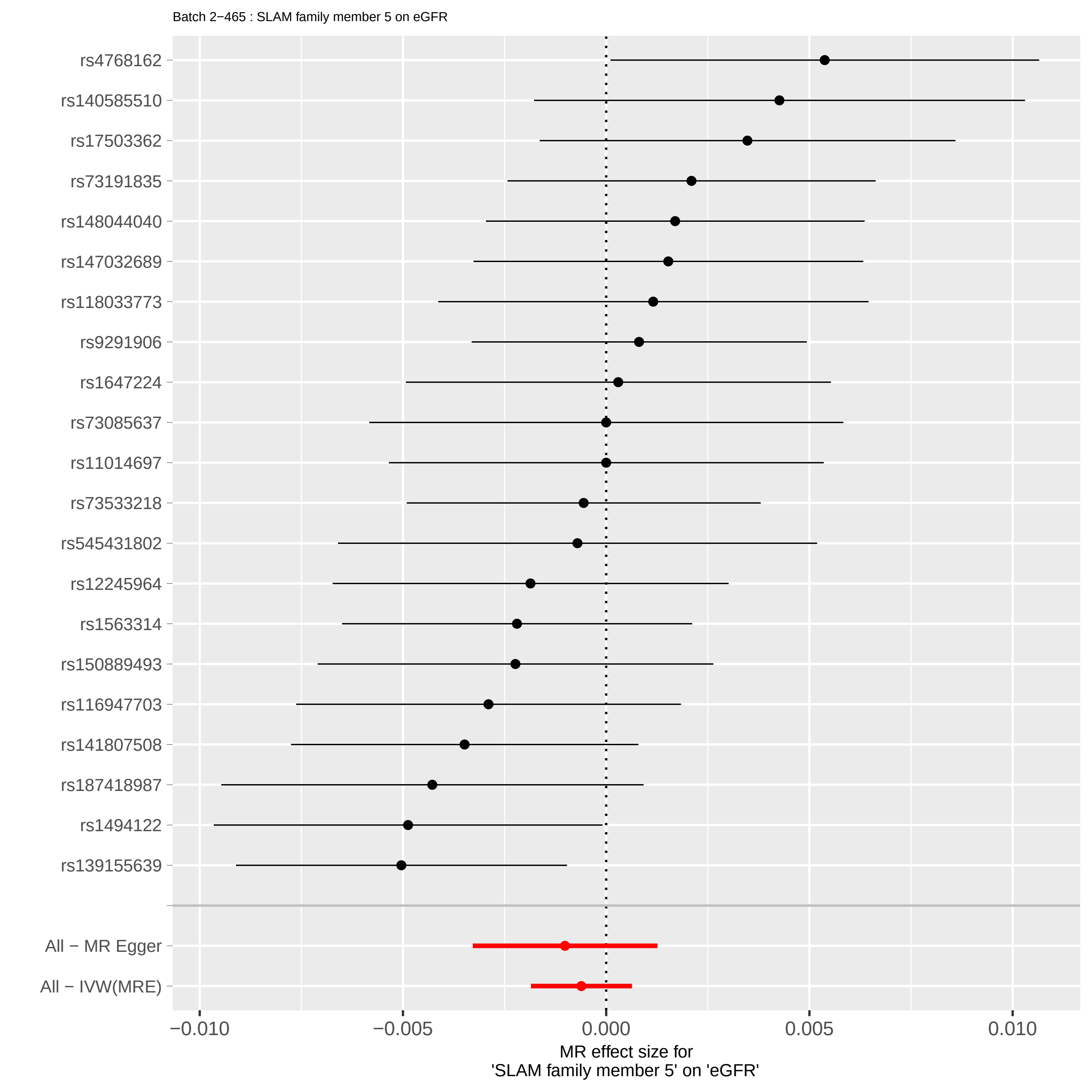
Batch 2−465 : SLAM family member 5 on eGFR
rs4768162
rs140585510
rs17503362
rs73191835
rs148044040
rs147032689
rs118033773
rs9291906
rs1647224
rs73085637
rs11014697
rs73533218
rs545431802
rs12245964
rs1563314
rs150889493
rs116947703
rs141807508
rs187418987
rs1494122
rs139155639
All − MR Egger
All − IVW(MRE)
−0.010
−0.005
0.000
0.005
0.010
MR effect size for
'SLAM family member 5' on 'eGFR'

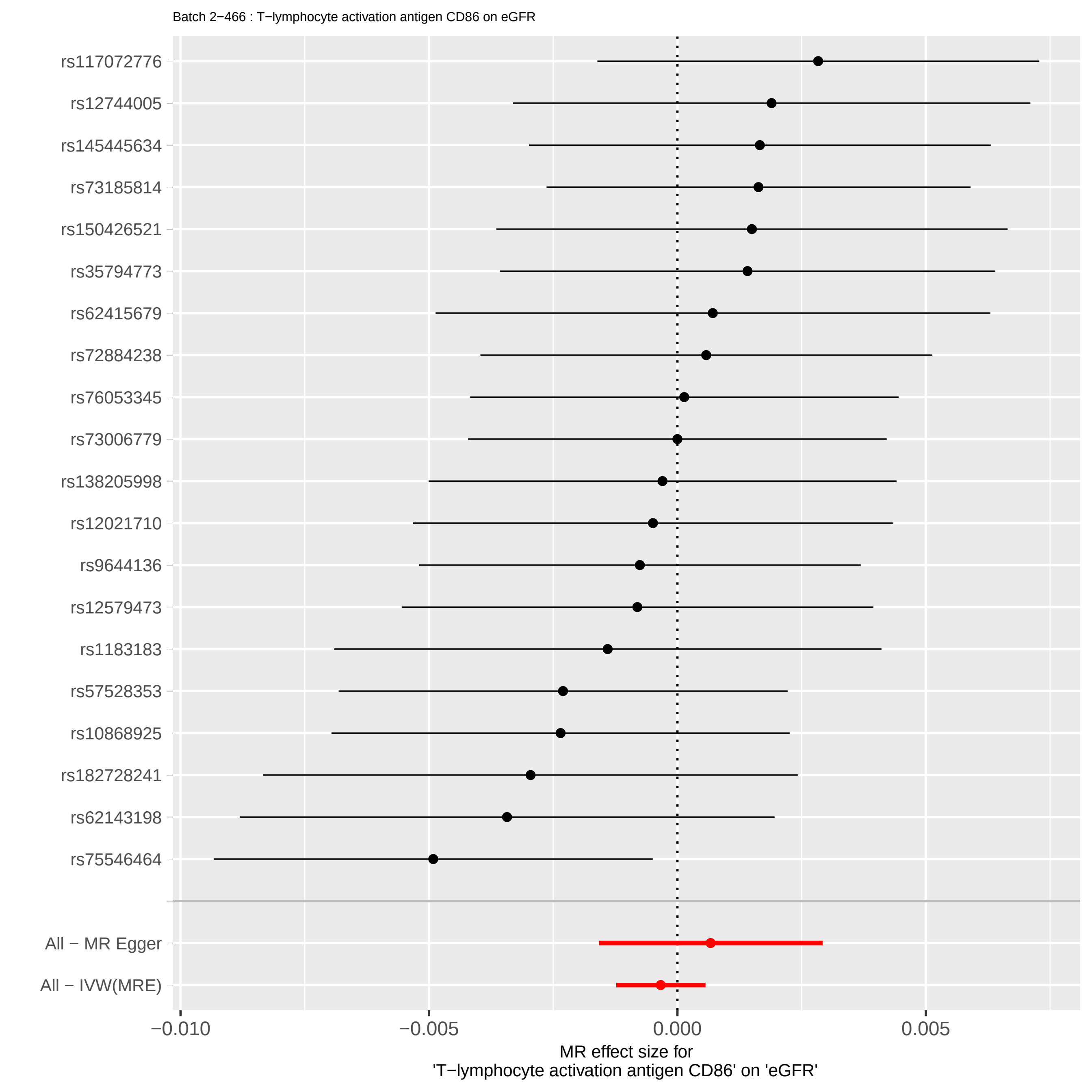

Batch 2−466 : T−lymphocyte activation antigen CD86 on eGFR
rs117072776
rs12744005
rs145445634
rs73185814
rs150426521
rs35794773
rs62415679
rs72884238
rs76053345
rs73006779
rs138205998
rs12021710
rs9644136
rs12579473
rs1183183
rs57528353
rs10868925
rs182728241
rs62143198
rs75546464
All − MR Egger
All − IVW(MRE)
−0.010
−0.005
0.000
0.005
MR effect size for
'T−lymphocyte activation antigen CD86' on 'eGFR'

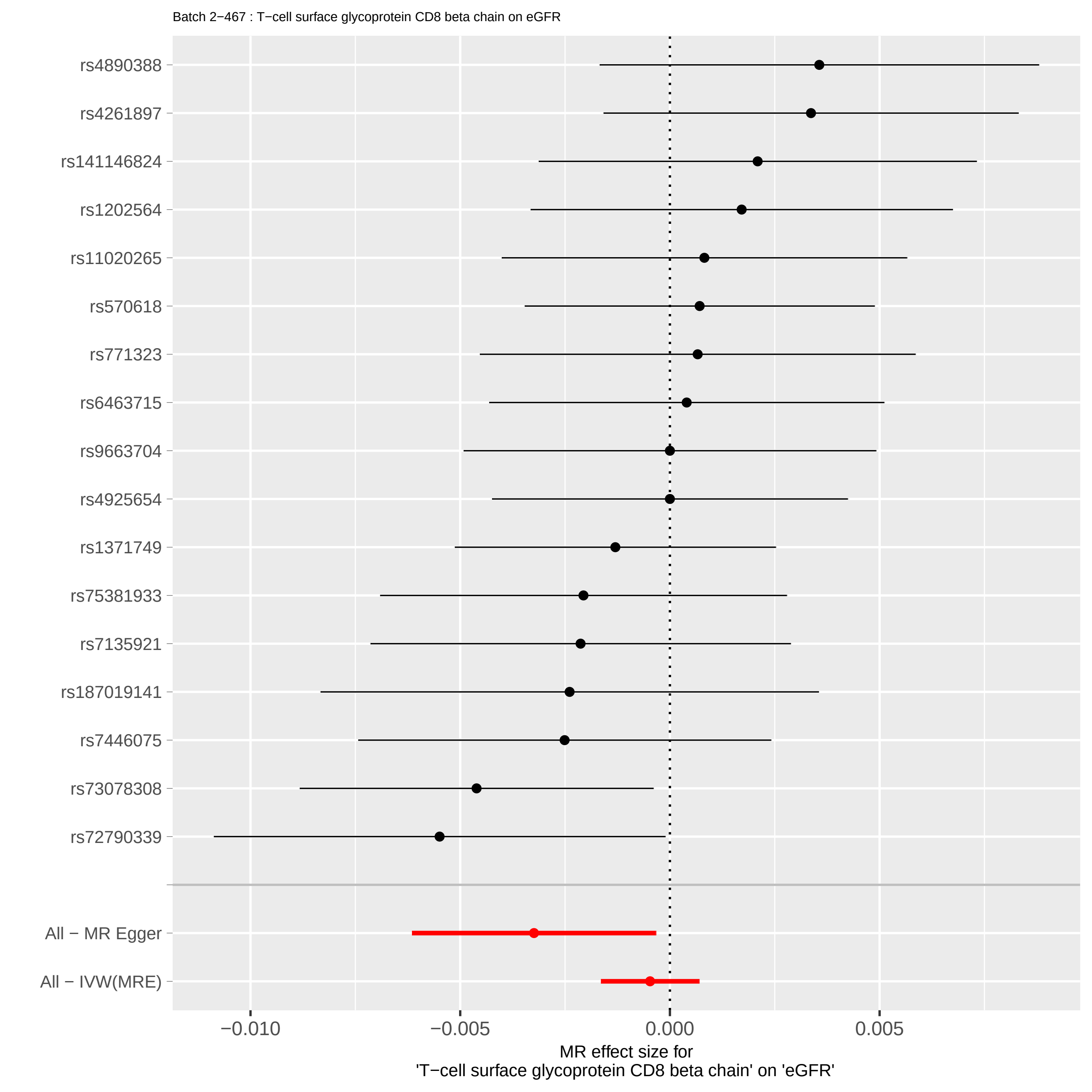

Batch 2−467 : T−cell surface glycoprotein CD8 beta chain on eGFR
rs4890388
rs4261897
rs141146824
rs1202564
rs11020265
rs570618
rs771323
rs6463715
rs9663704
rs4925654
rs1371749
rs75381933
rs7135921
rs187019141
rs7446075
rs73078308
rs72790339
All − MR Egger
All − IVW(MRE)
−0.010
−0.005
0.000
0.005
MR effect size for
'T−cell surface glycoprotein CD8 beta chain' on 'eGFR'

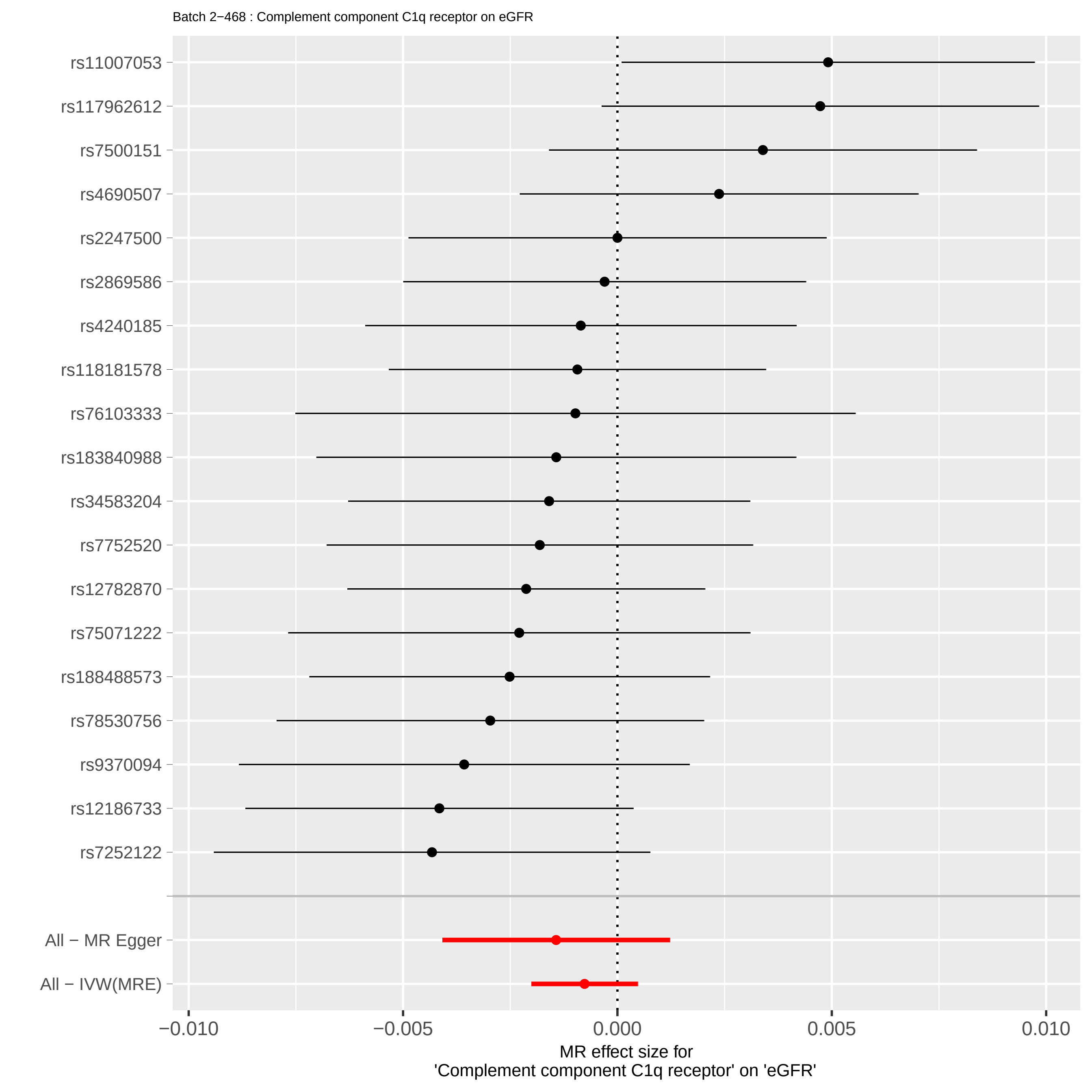
Batch 2−468 : Complement component C1q receptor on eGFR
rs11007053
rs117962612
rs7500151
rs4690507
rs2247500
rs2869586
rs4240185
rs118181578
rs76103333
rs183840988
rs34583204
rs7752520
rs12782870
rs75071222
rs188488573
rs78530756
rs9370094
rs12186733
rs7252122
All − MR Egger
All − IVW(MRE)
−0.010
−0.005
0.000
0.005
0.010
MR effect size for
'Complement component C1q receptor' on 'eGFR'

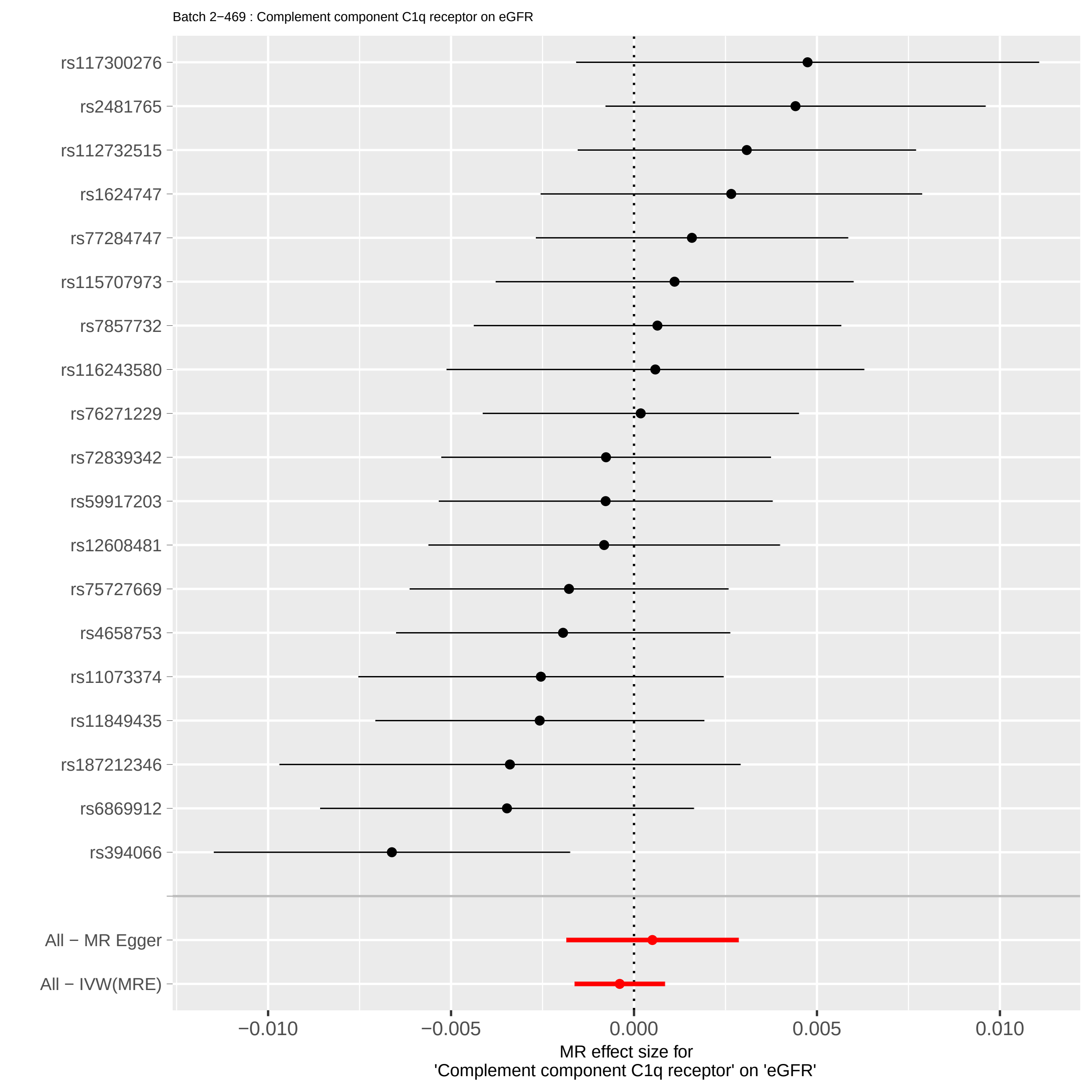

Batch 2−469 : Complement component C1q receptor on eGFR
rs117300276
rs2481765
rs112732515
rs1624747
rs77284747
rs115707973
rs7857732
rs116243580
rs76271229
rs72839342
rs59917203
rs12608481
rs75727669
rs4658753
rs11073374
rs11849435
rs187212346
rs6869912
rs394066
All − MR Egger
All − IVW(MRE)
−0.010
−0.005
0.000
0.005
0.010
MR effect size for
'Complement component C1q receptor' on 'eGFR'

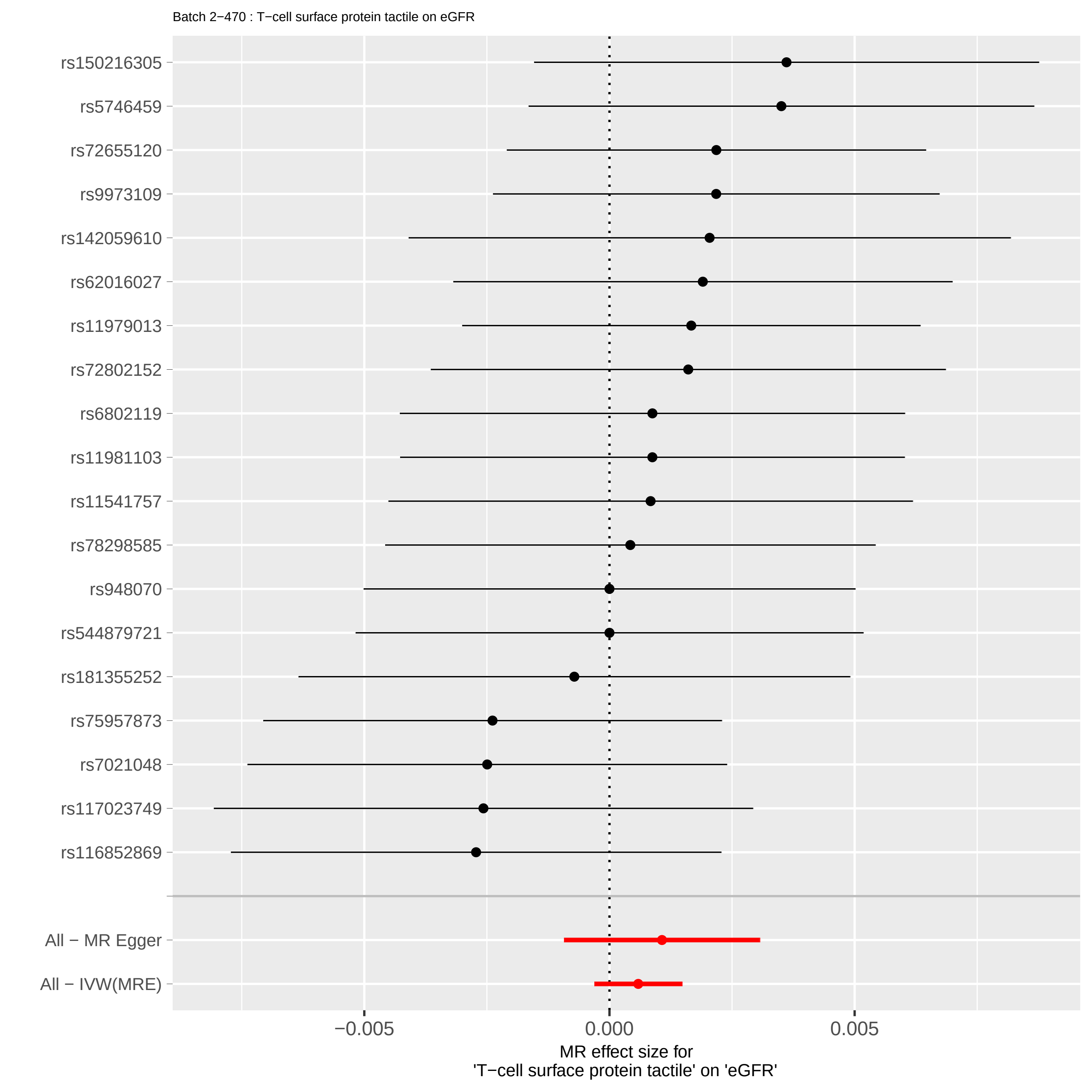
Batch 2−470 : T−cell surface protein tactile on eGFR
rs150216305
rs5746459
rs72655120
rs9973109
rs142059610
rs62016027
rs11979013
rs72802152
rs6802119
rs11981103
rs11541757
rs78298585
rs948070
rs544879721
rs181355252
rs75957873
rs7021048
rs117023749
rs116852869
All − MR Egger
All − IVW(MRE)
−0.005
0.000
0.005
MR effect size for
'T−cell surface protein tactile' on 'eGFR'

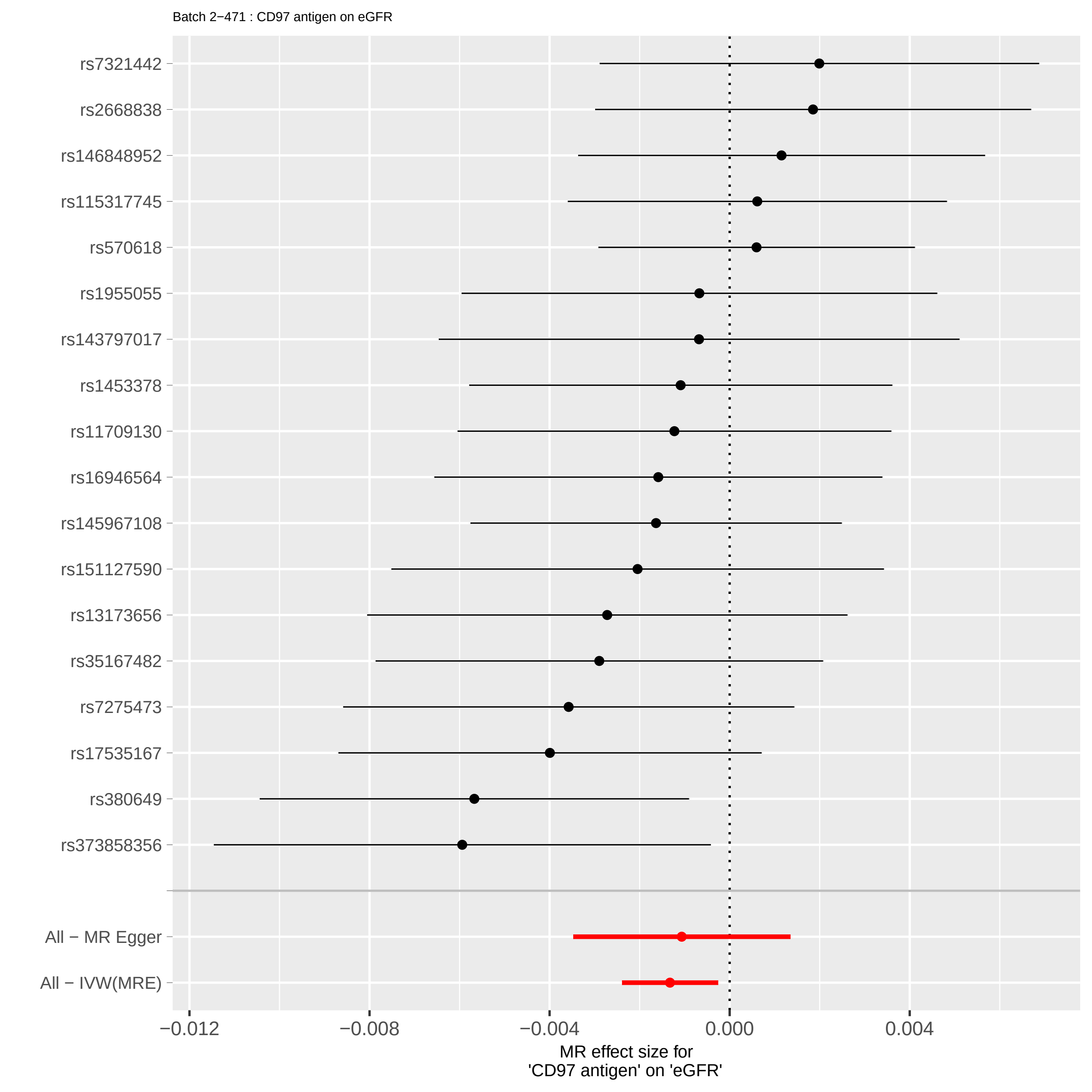

Batch 2−471 : CD97 antigen on eGFR
rs7321442
rs2668838
rs146848952
rs115317745
rs570618
rs1955055
rs143797017
rs1453378
rs11709130
rs16946564
rs145967108
rs151127590
rs13173656
rs35167482
rs7275473
rs17535167
rs380649
rs373858356
All − MR Egger
All − IVW(MRE)
−0.012
−0.008
−0.004
0.000
0.004
MR effect size for
'CD97 antigen' on 'eGFR'

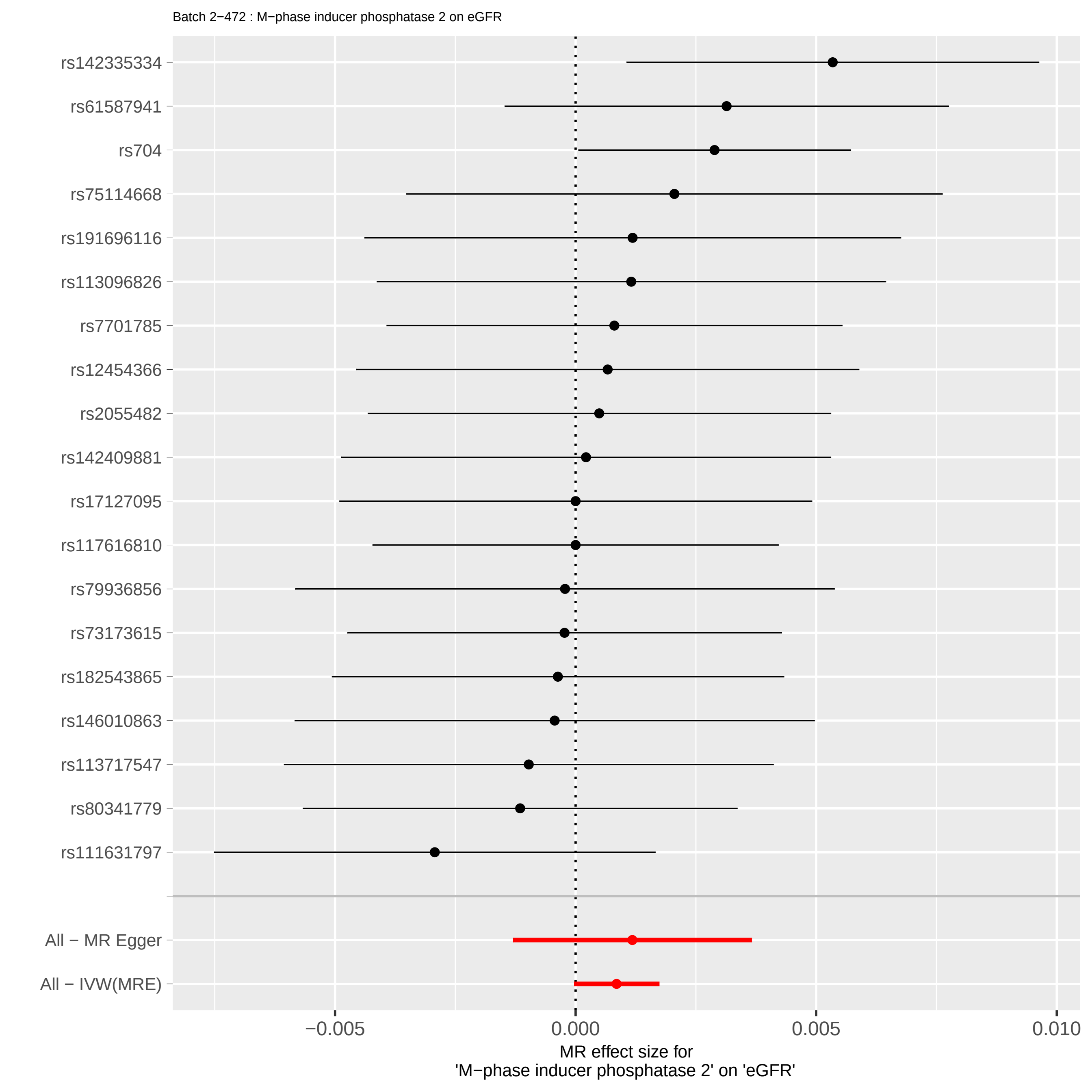

Batch 2−472 : M−phase inducer phosphatase 2 on eGFR
rs142335334
rs61587941
rs704
rs75114668
rs191696116
rs113096826
rs7701785
rs12454366
rs2055482
rs142409881
rs17127095
rs117616810
rs79936856
rs73173615
rs182543865
rs146010863
rs113717547
rs80341779
rs111631797
All − MR Egger
All − IVW(MRE)
−0.005
0.000
0.005
0.010
MR effect size for
'M−phase inducer phosphatase 2' on 'eGFR'

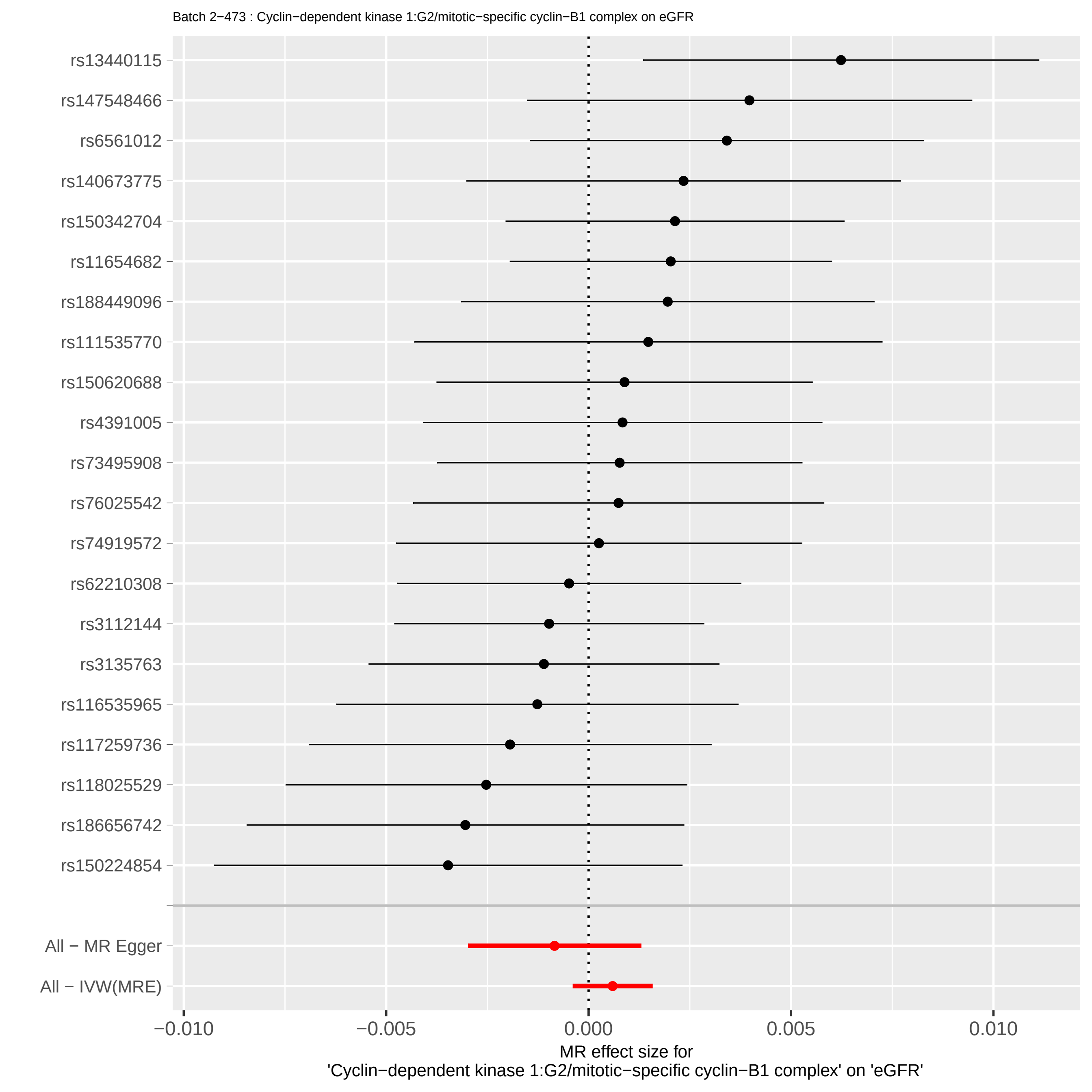

Batch 2−473 : Cyclin−dependent kinase 1:G2/mitotic−specific cyclin−B1 complex on eGFR
rs13440115
rs147548466
rs6561012
rs140673775
rs150342704
rs11654682
rs188449096
rs111535770
rs150620688
rs4391005
rs73495908
rs76025542
rs74919572
rs62210308
rs3112144
rs3135763
rs116535965
rs117259736
rs118025529
rs186656742
rs150224854
All − MR Egger
All − IVW(MRE)
−0.010
−0.005
0.000
0.005
0.010
MR effect size for
'Cyclin−dependent kinase 1:G2/mitotic−specific cyclin−B1 complex' on 'eGFR'

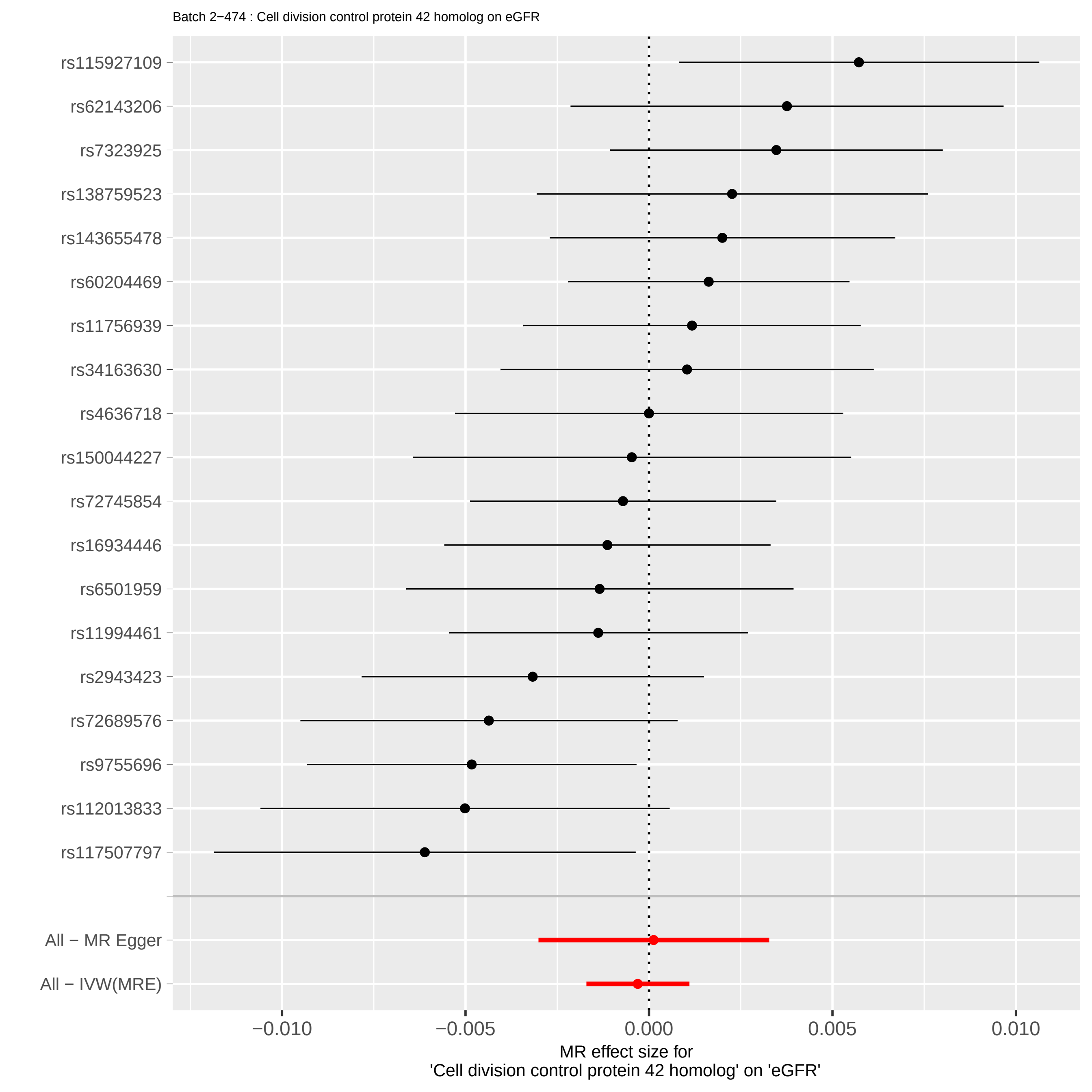
Batch 2−474 : Cell division control protein 42 homolog on eGFR
rs115927109
rs62143206
rs7323925
rs138759523
rs143655478
rs60204469
rs11756939
rs34163630
rs4636718
rs150044227
rs72745854
rs16934446
rs6501959
rs11994461
rs2943423
rs72689576
rs9755696
rs112013833
rs117507797
All − MR Egger
All − IVW(MRE)
−0.010
−0.005
0.000
0.005
0.010
MR effect size for
'Cell division control protein 42 homolog' on 'eGFR'

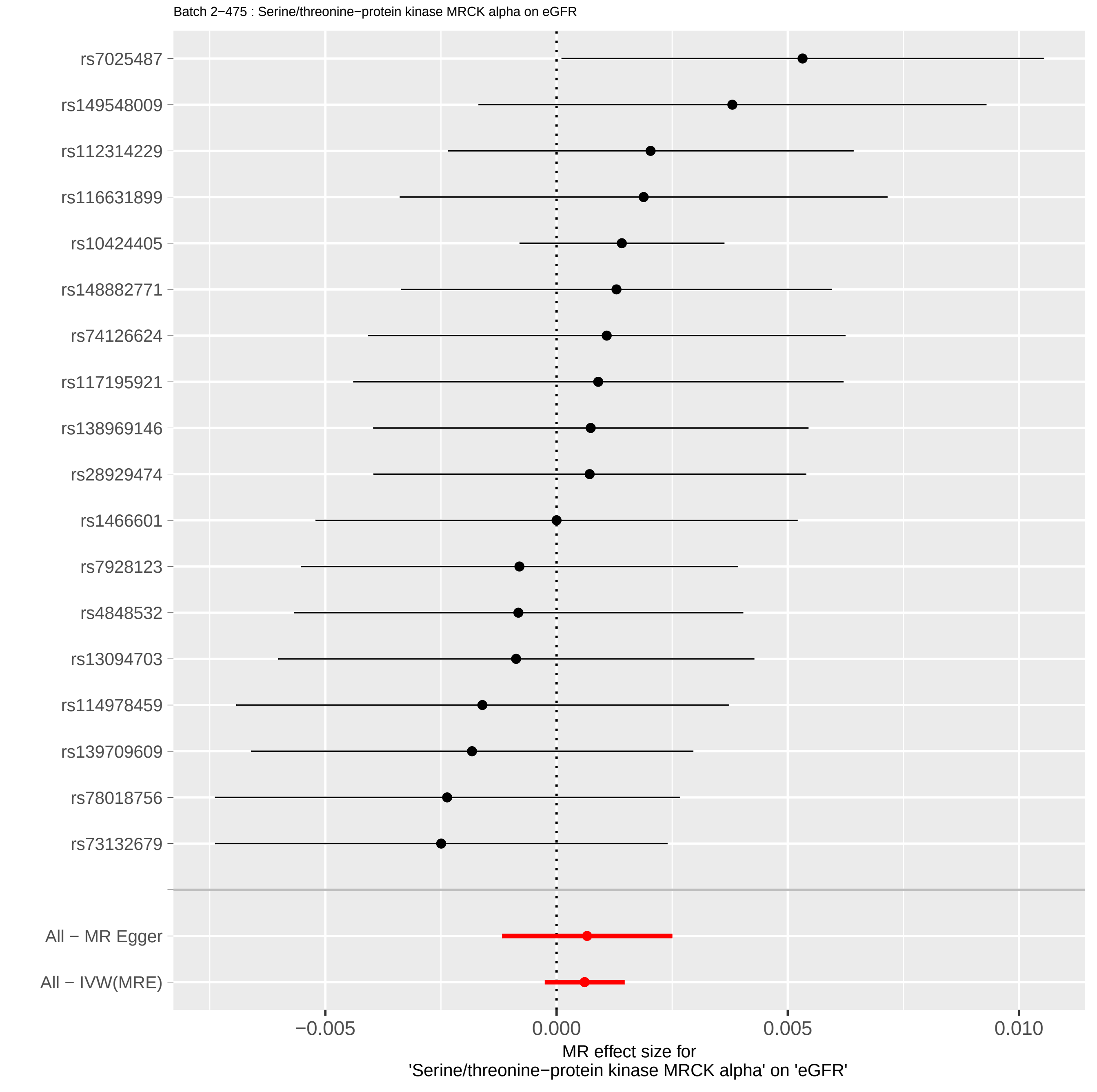

Batch 2−475 : Serine/threonine−protein kinase MRCK alpha on eGFR
rs7025487
rs149548009
rs112314229
rs116631899
rs10424405
rs148882771
rs74126624
rs117195921
rs138969146
rs28929474
rs1466601
rs7928123
rs4848532
rs13094703
rs114978459
rs139709609
rs78018756
rs73132679
All − MR Egger
All − IVW(MRE)
−0.005
0.000
0.005
0.010
MR effect size for
'Serine/threonine−protein kinase MRCK alpha' on 'eGFR'

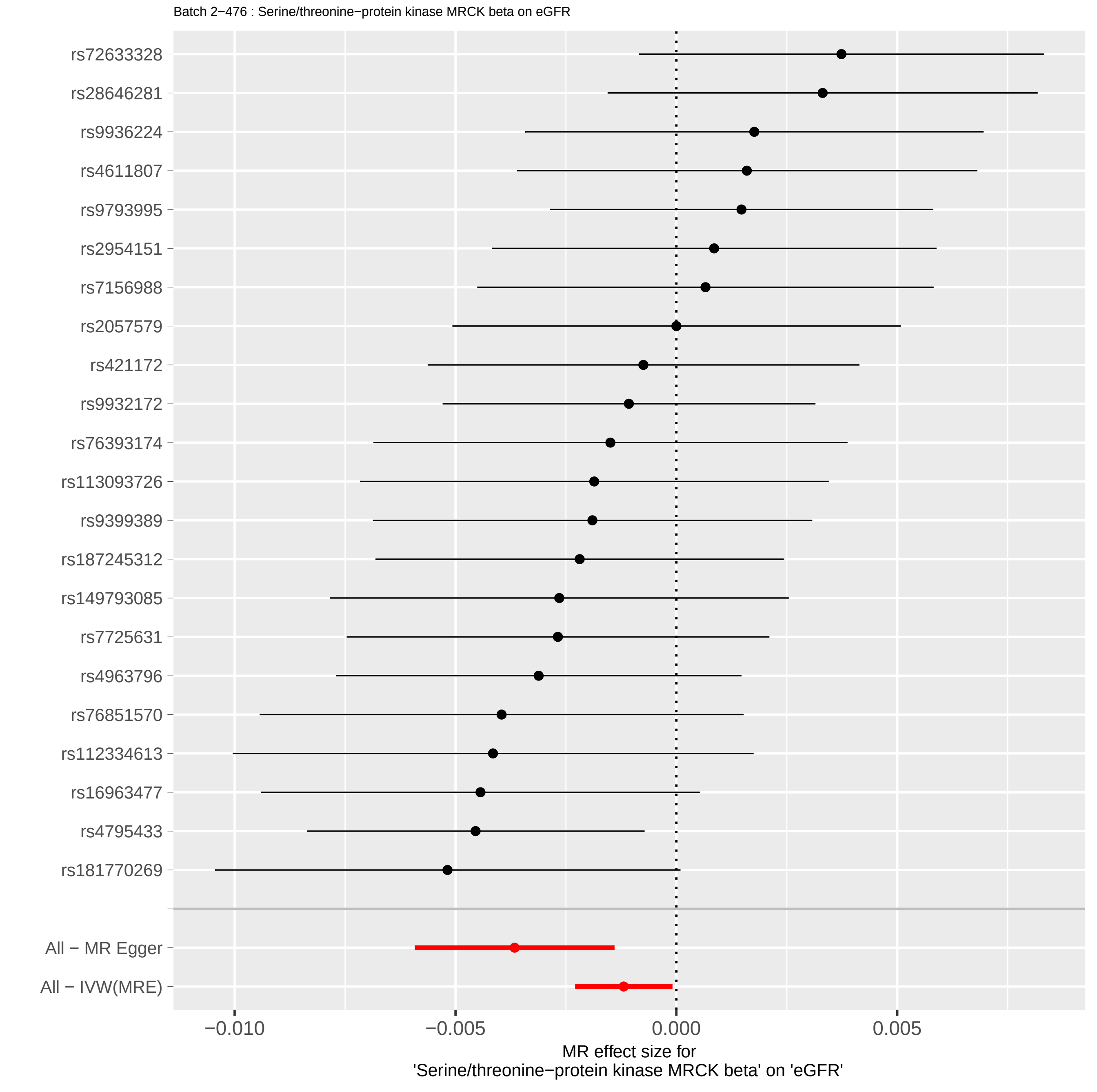
Batch 2−476 : Serine/threonine−protein kinase MRCK beta on eGFR
rs72633328
rs28646281
rs9936224
rs4611807
rs9793995
rs2954151
rs7156988
rs2057579
rs421172
rs9932172
rs76393174
rs113093726
rs9399389
rs187245312
rs149793085
rs7725631
rs4963796
rs76851570
rs112334613
rs16963477
rs4795433
rs181770269
All − MR Egger
All − IVW(MRE)
−0.010
−0.005
0.000
0.005
MR effect size for
'Serine/threonine−protein kinase MRCK beta' on 'eGFR'

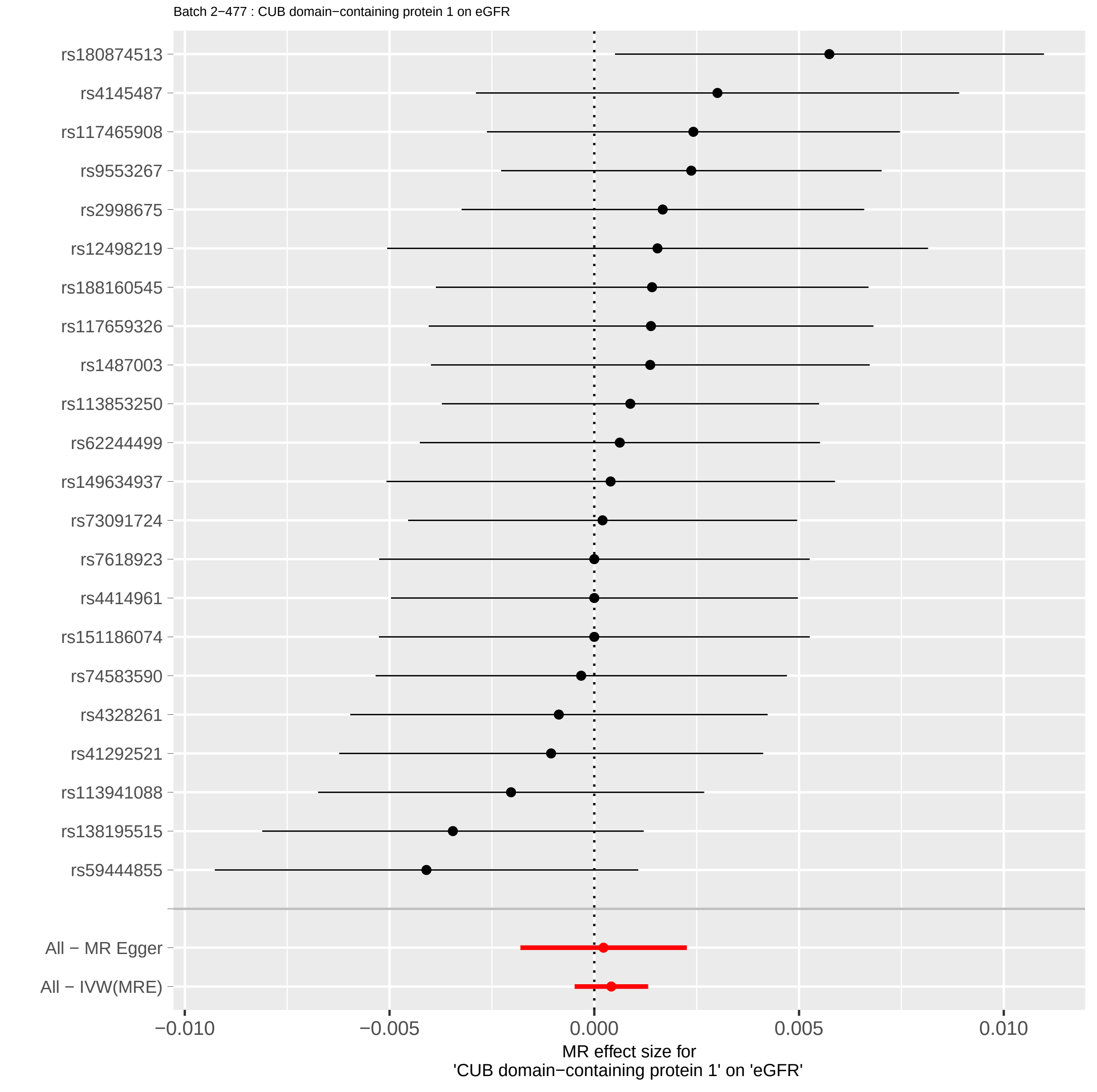
Batch 2−477 : CUB domain−containing protein 1 on eGFR
rs180874513
rs4145487
rs117465908
rs9553267
rs2998675
rs12498219
rs188160545
rs117659326
rs1487003
rs113853250
rs62244499
rs149634937
rs73091724
rs7618923
rs4414961
rs151186074
rs74583590
rs4328261
rs41292521
rs113941088
rs138195515
rs59444855
All − MR Egger
All − IVW(MRE)
−0.010
−0.005
0.000
0.005
0.010
MR effect size for
'CUB domain−containing protein 1' on 'eGFR'

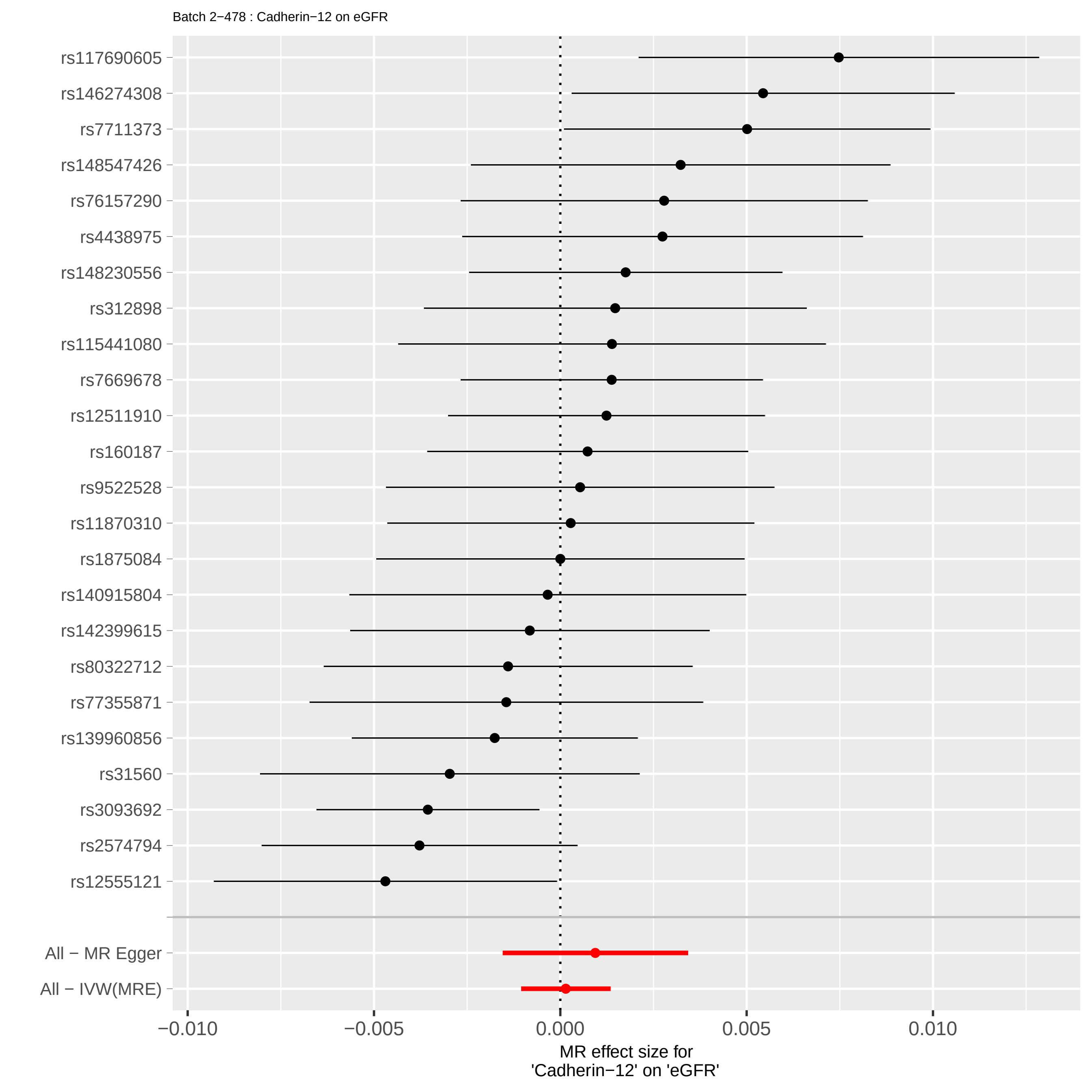
Batch 2−478 : Cadherin−12 on eGFR
rs117690605
rs146274308
rs7711373
rs148547426
rs76157290
rs4438975
rs148230556
rs312898
rs115441080
rs7669678
rs12511910
rs160187
rs9522528
rs11870310
rs1875084
rs140915804
rs142399615
rs80322712
rs77355871
rs139960856
rs31560
rs3093692
rs2574794
rs12555121
All − MR Egger
All − IVW(MRE)
−0.010
−0.005
0.000
0.005
0.010
MR effect size for
'Cadherin−12' on 'eGFR'

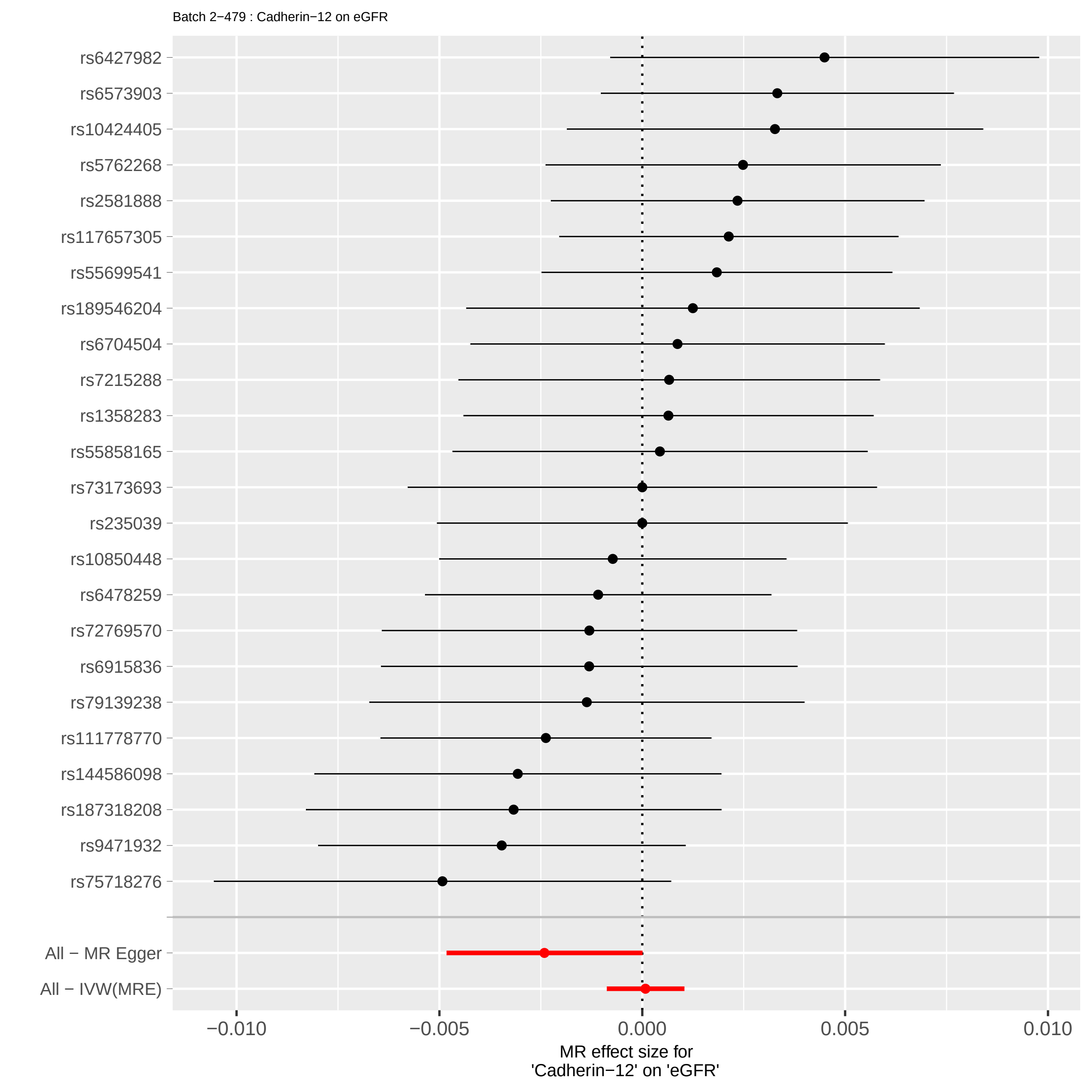

Batch 2−479 : Cadherin−12 on eGFR
rs6427982
rs6573903
rs10424405
rs5762268
rs2581888
rs117657305
rs55699541
rs189546204
rs6704504
rs7215288
rs1358283
rs55858165
rs73173693
rs235039
rs10850448
rs6478259
rs72769570
rs6915836
rs79139238
rs111778770
rs144586098
rs187318208
rs9471932
rs75718276
All − MR Egger
All − IVW(MRE)
−0.010
−0.005
0.000
0.005
0.010
MR effect size for
'Cadherin−12' on 'eGFR'

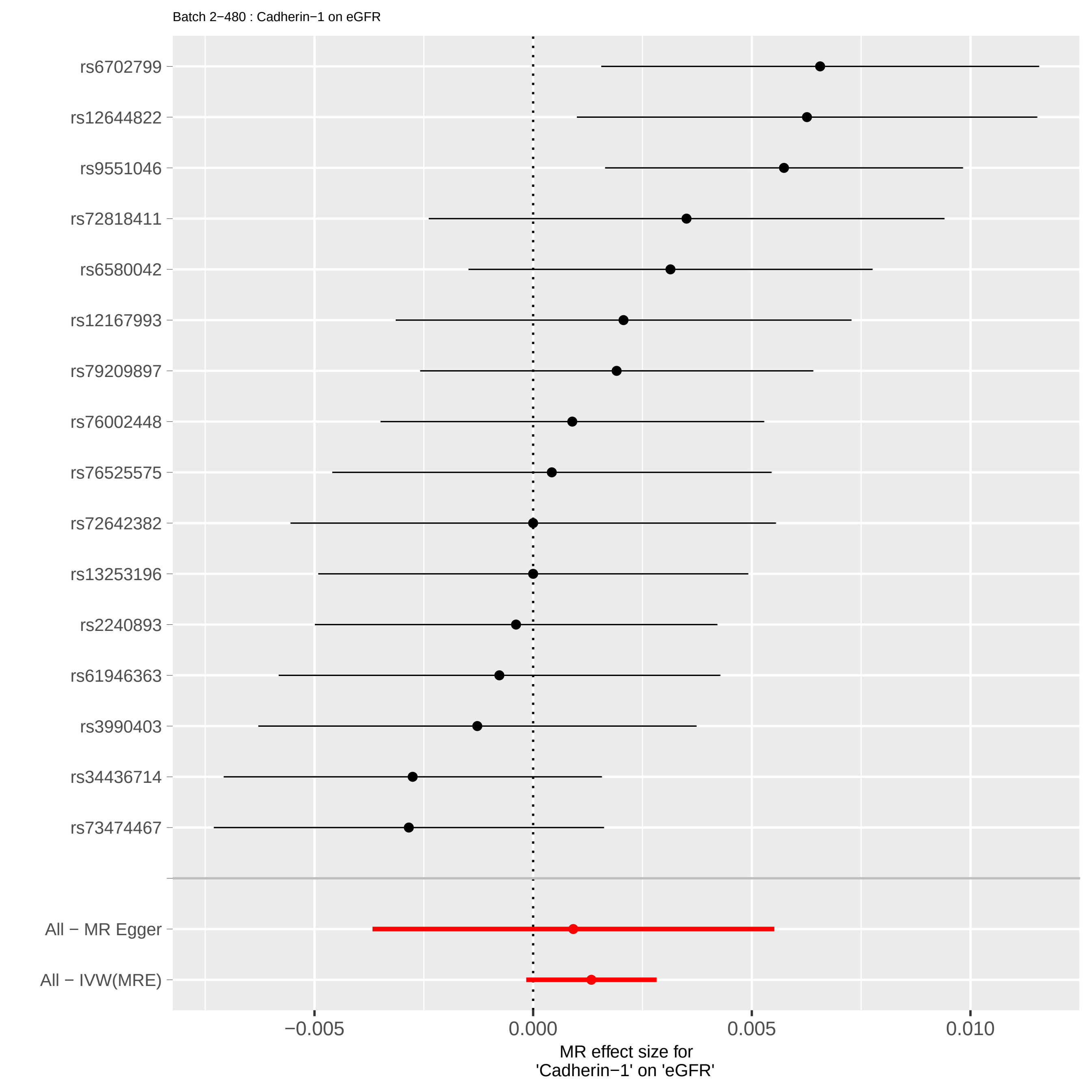

Batch 2−480 : Cadherin−1 on eGFR
rs6702799
rs12644822
rs9551046
rs72818411
rs6580042
rs12167993
rs79209897
rs76002448
rs76525575
rs72642382
rs13253196
rs2240893
rs61946363
rs3990403
rs34436714
rs73474467
All − MR Egger
All − IVW(MRE)
−0.005
0.000
0.005
0.010
MR effect size for
'Cadherin−1' on 'eGFR'

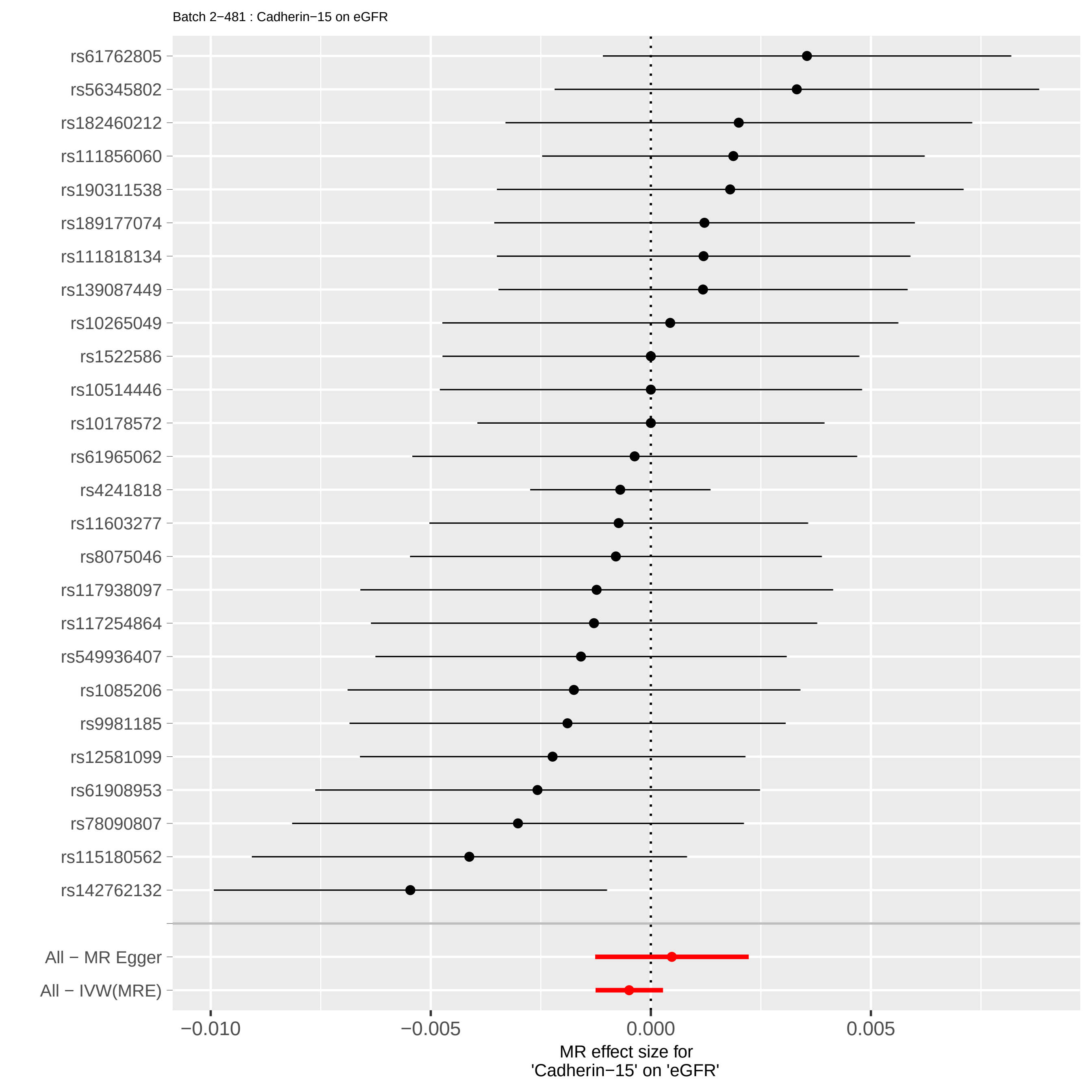
Batch 2−481 : Cadherin−15 on eGFR
rs61762805
rs56345802
rs182460212
rs111856060
rs190311538
rs189177074
rs111818134
rs139087449
rs10265049
rs1522586
rs10514446
rs10178572
rs61965062
rs4241818
rs11603277
rs8075046
rs117938097
rs117254864
rs549936407
rs1085206
rs9981185
rs12581099
rs61908953
rs78090807
rs115180562
rs142762132
All − MR Egger
All − IVW(MRE)
−0.010
−0.005
0.000
0.005
MR effect size for
'Cadherin−15' on 'eGFR'

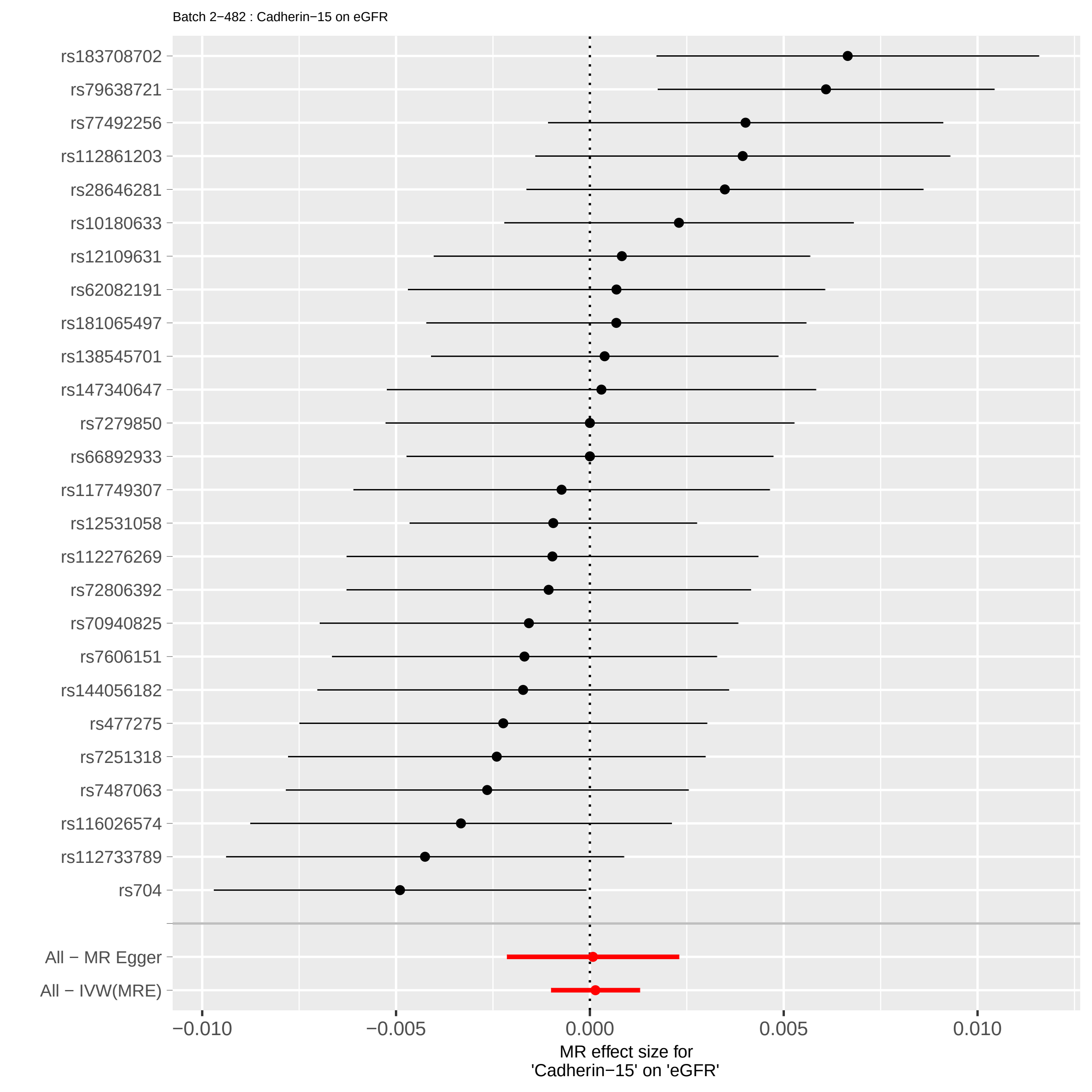

Batch 2−482 : Cadherin−15 on eGFR
rs183708702
rs79638721
rs77492256
rs112861203
rs28646281
rs10180633
rs12109631
rs62082191
rs181065497
rs138545701
rs147340647
rs7279850
rs66892933
rs117749307
rs12531058
rs112276269
rs72806392
rs70940825
rs7606151
rs144056182
rs477275
rs7251318
rs7487063
rs116026574
rs112733789
rs704
All − MR Egger
All − IVW(MRE)
−0.010
−0.005
0.000
0.005
0.010
MR effect size for
'Cadherin−15' on 'eGFR'

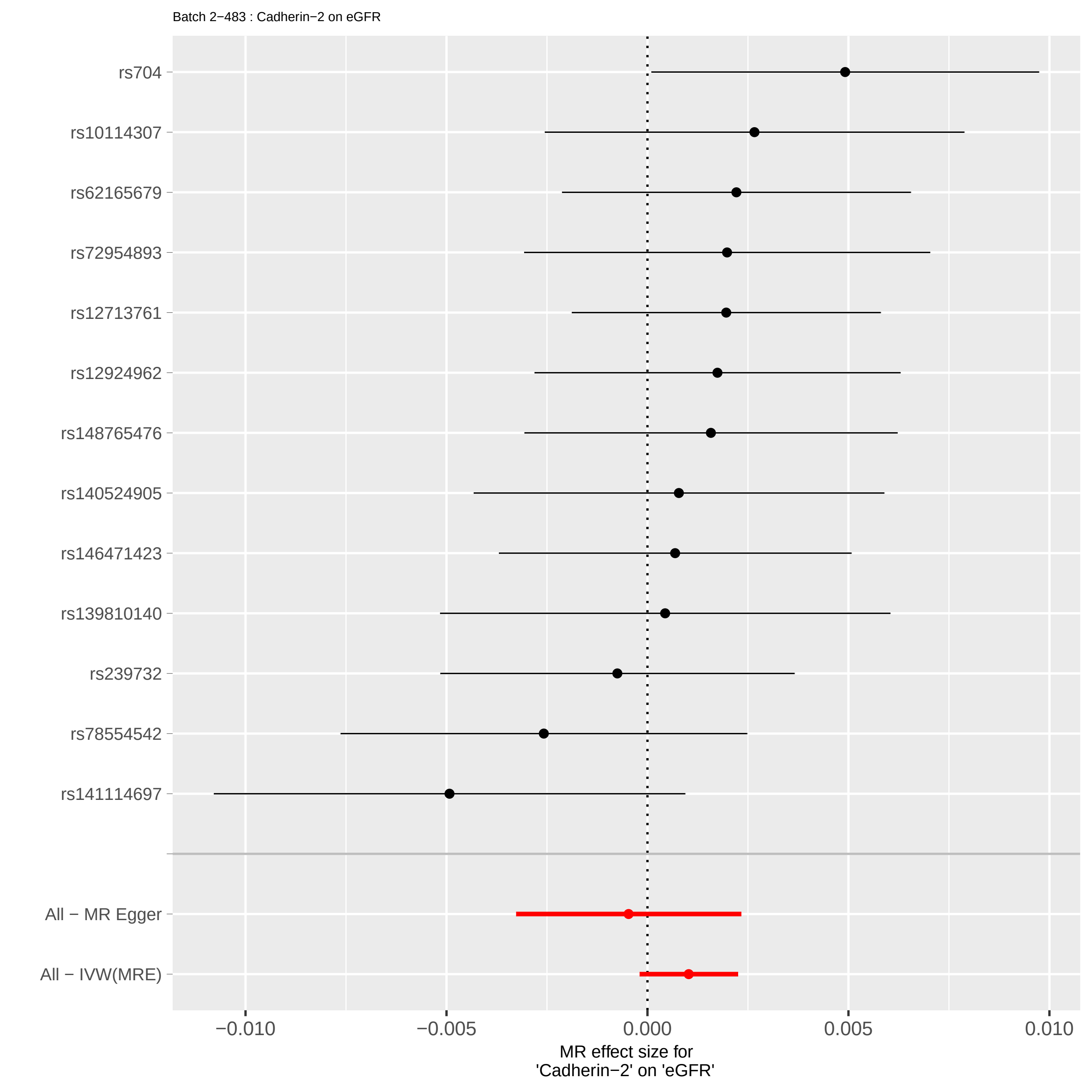
Batch 2−483 : Cadherin−2 on eGFR
rs704
rs10114307
rs62165679
rs72954893
rs12713761
rs12924962
rs148765476
rs140524905
rs146471423
rs139810140
rs239732
rs78554542
rs141114697
All − MR Egger
All − IVW(MRE)
−0.010
−0.005
0.000
0.005
0.010
MR effect size for
'Cadherin−2' on 'eGFR'

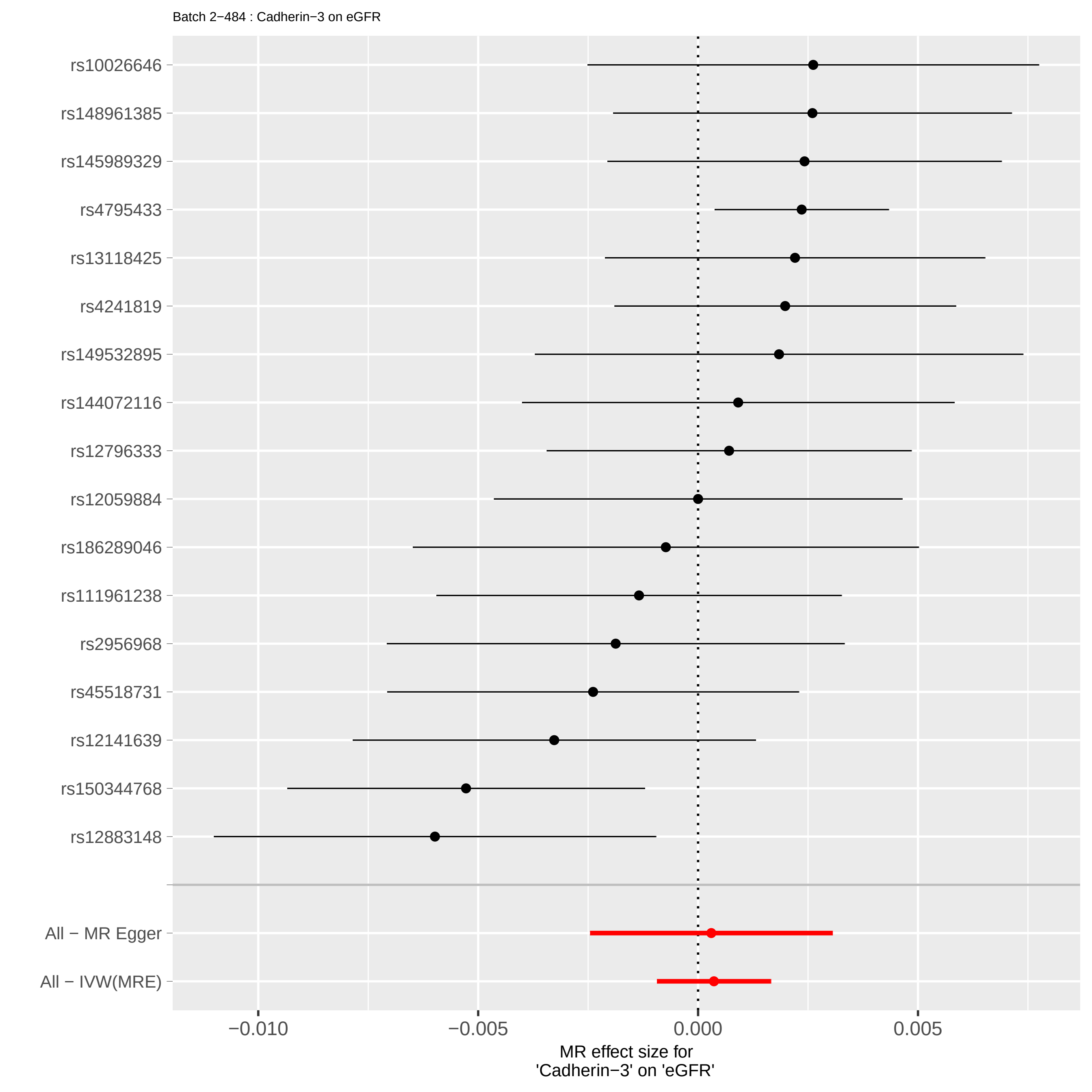

Batch 2−484 : Cadherin−3 on eGFR
rs10026646
rs148961385
rs145989329
rs4795433
rs13118425
rs4241819
rs149532895
rs144072116
rs12796333
rs12059884
rs186289046
rs111961238
rs2956968
rs45518731
rs12141639
rs150344768
rs12883148
All − MR Egger
All − IVW(MRE)
−0.010
−0.005
0.000
0.005
MR effect size for
'Cadherin−3' on 'eGFR'

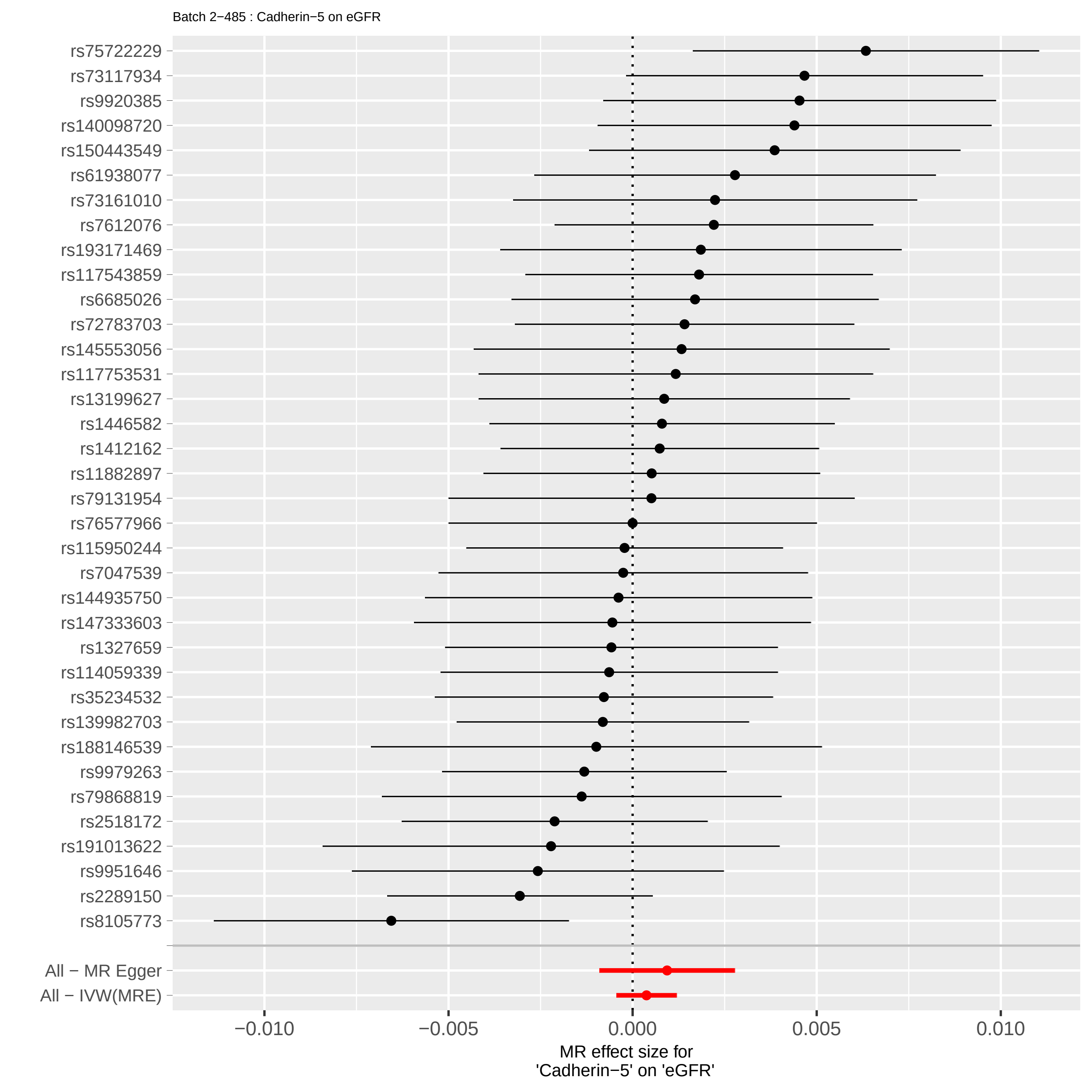
Batch 2−485 : Cadherin−5 on eGFR
rs75722229
rs73117934
rs9920385
rs140098720
rs150443549
rs61938077
rs73161010
rs7612076
rs193171469
rs117543859
rs6685026
rs72783703
rs145553056
rs117753531
rs13199627
rs1446582
rs1412162
rs11882897
rs79131954
rs76577966
rs115950244
rs7047539
rs144935750
rs147333603
rs1327659
rs114059339
rs35234532
rs139982703
rs188146539
rs9979263
rs79868819
rs2518172
rs191013622
rs9951646
rs2289150
rs8105773
All − MR Egger
All − IVW(MRE)
−0.010
−0.005
0.000
0.005
0.010
MR effect size for
'Cadherin−5' on 'eGFR'

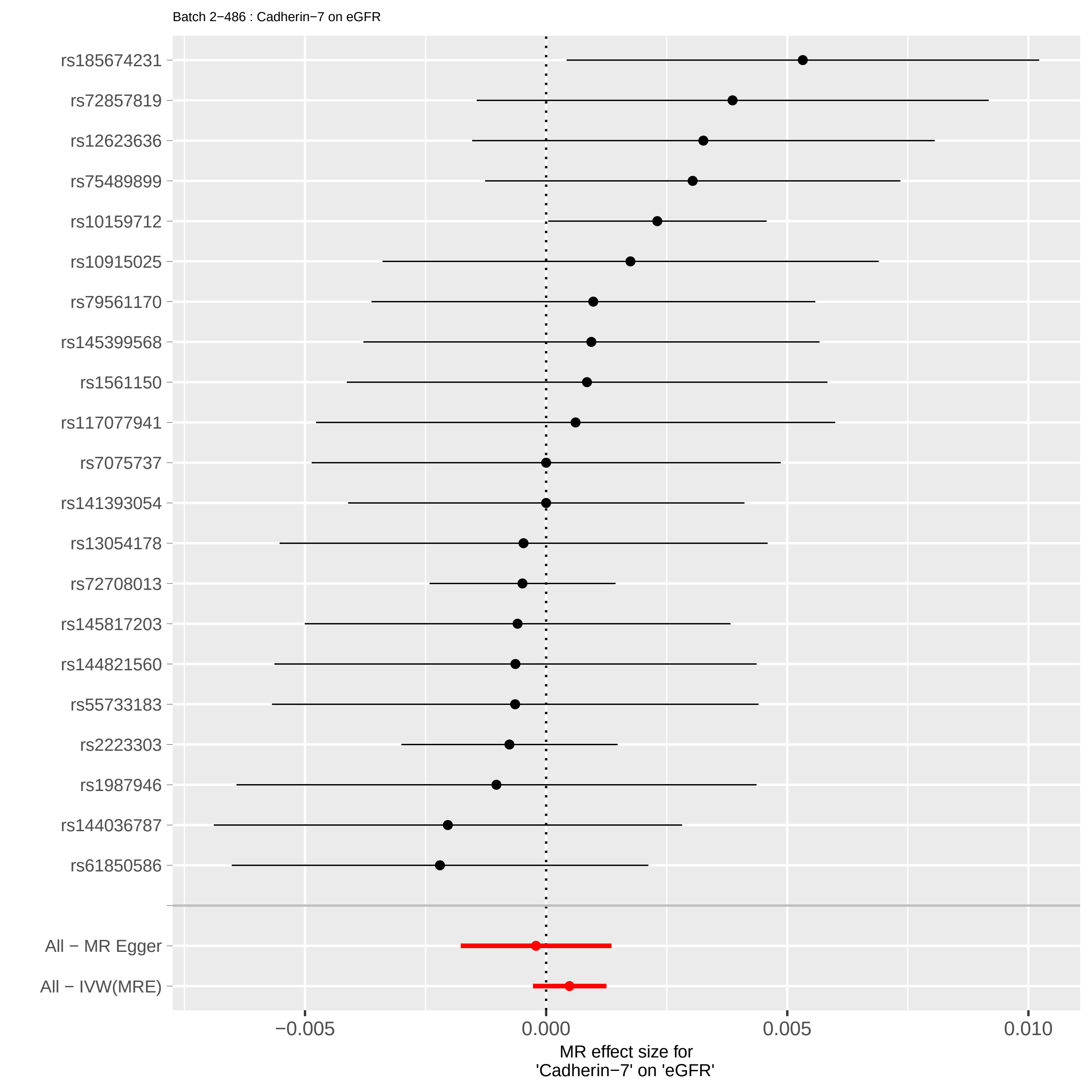
Batch 2−486 : Cadherin−7 on eGFR
rs185674231
rs72857819
rs12623636
rs75489899
rs10159712
rs10915025
rs79561170
rs145399568
rs1561150
rs117077941
rs7075737
rs141393054
rs13054178
rs72708013
rs145817203
rs144821560
rs55733183
rs2223303
rs1987946
rs144036787
rs61850586
All − MR Egger
All − IVW(MRE)
−0.005
0.000
0.005
0.010
MR effect size for
'Cadherin−7' on 'eGFR'

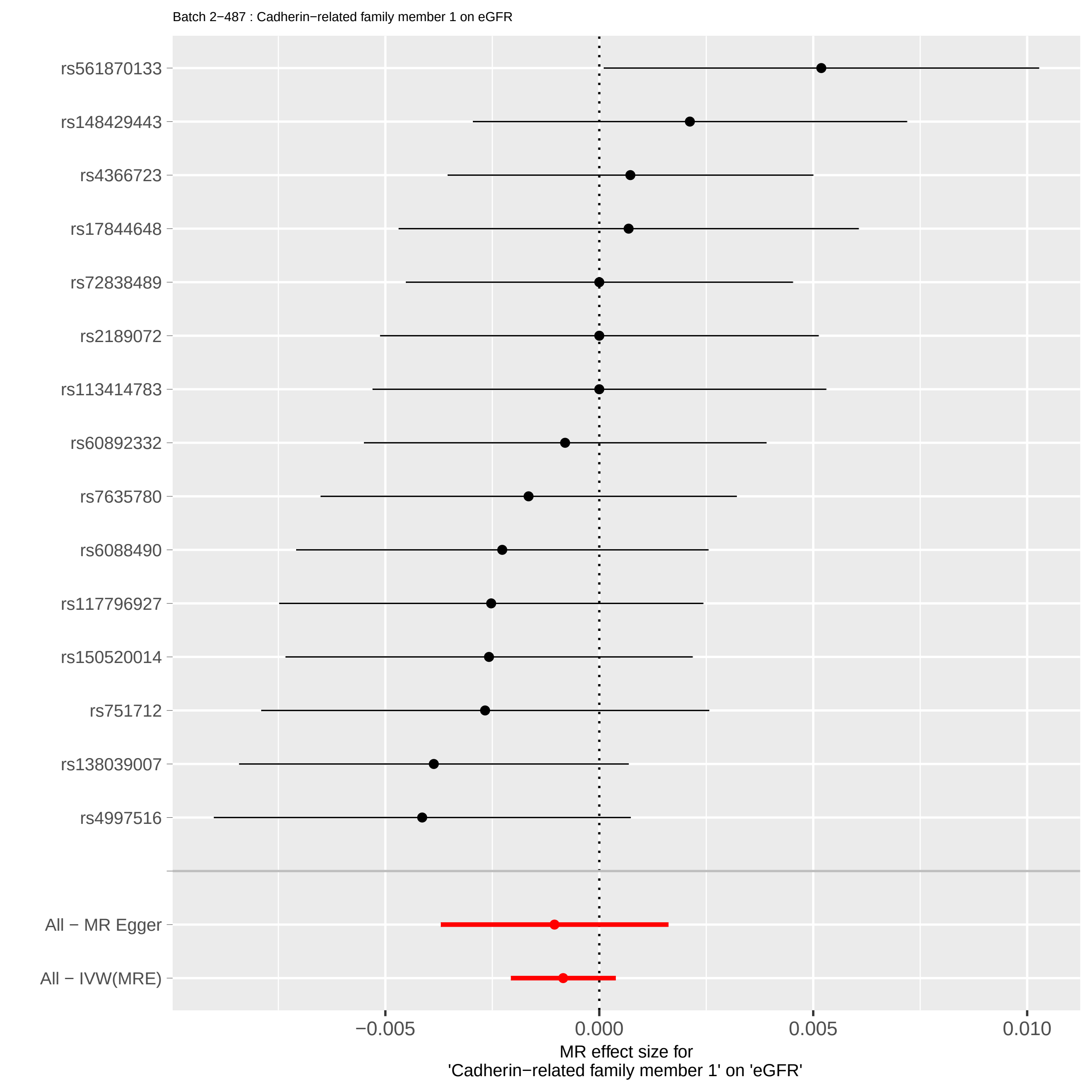

Batch 2−487 : Cadherin−related family member 1 on eGFR
rs561870133
rs148429443
rs4366723
rs17844648
rs72838489
rs2189072
rs113414783
rs60892332
rs7635780
rs6088490
rs117796927
rs150520014
rs751712
rs138039007
rs4997516
All − MR Egger
All − IVW(MRE)
−0.005
0.000
0.005
0.010
MR effect size for
'Cadherin−related family member 1' on 'eGFR'

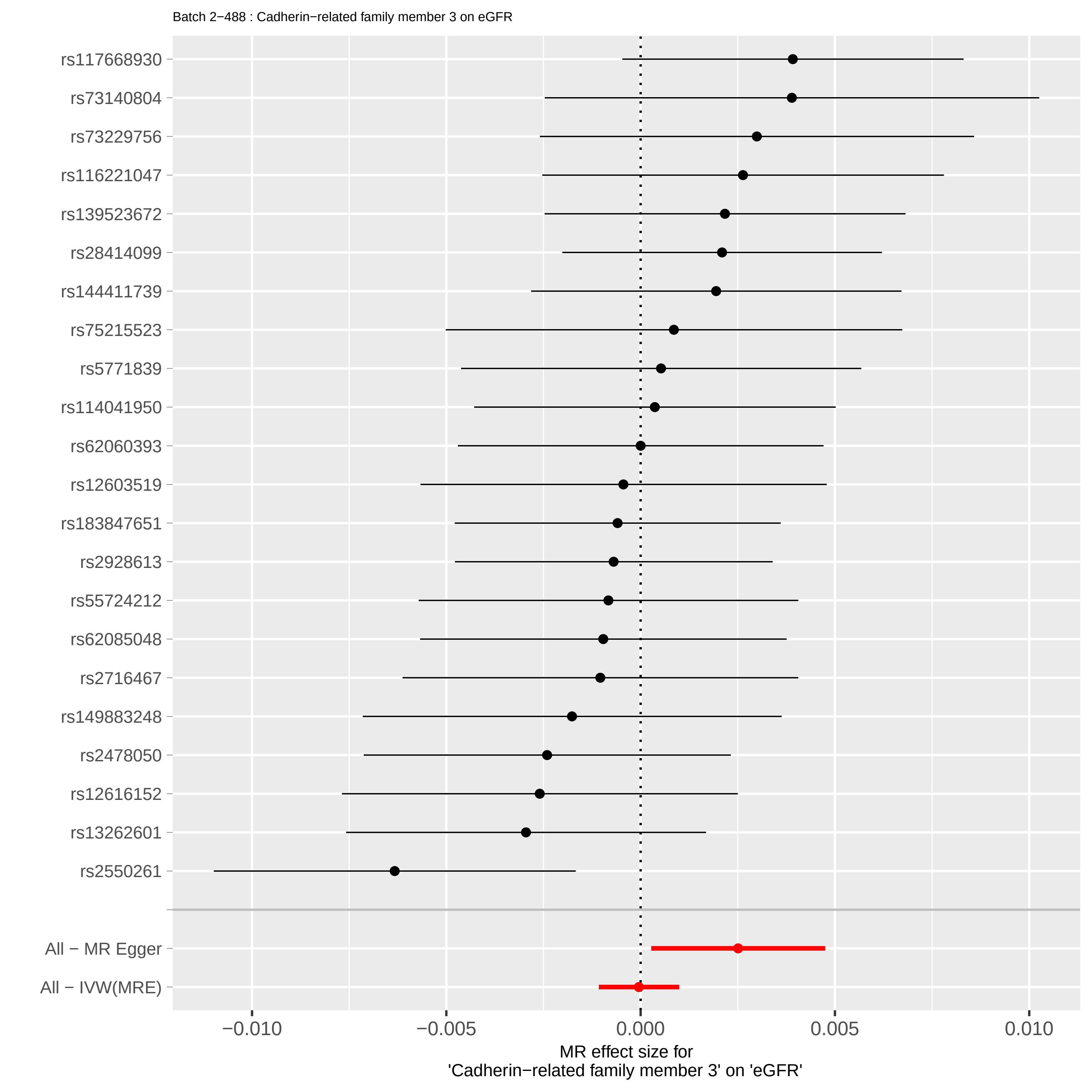
Batch 2−488 : Cadherin−related family member 3 on eGFR
rs117668930
rs73140804
rs73229756
rs116221047
rs139523672
rs28414099
rs144411739
rs75215523
rs5771839
rs114041950
rs62060393
rs12603519
rs183847651
rs2928613
rs55724212
rs62085048
rs2716467
rs149883248
rs2478050
rs12616152
rs13262601
rs2550261
All − MR Egger
All − IVW(MRE)
−0.010
−0.005
0.000
0.005
0.010
MR effect size for
'Cadherin−related family member 3' on 'eGFR'

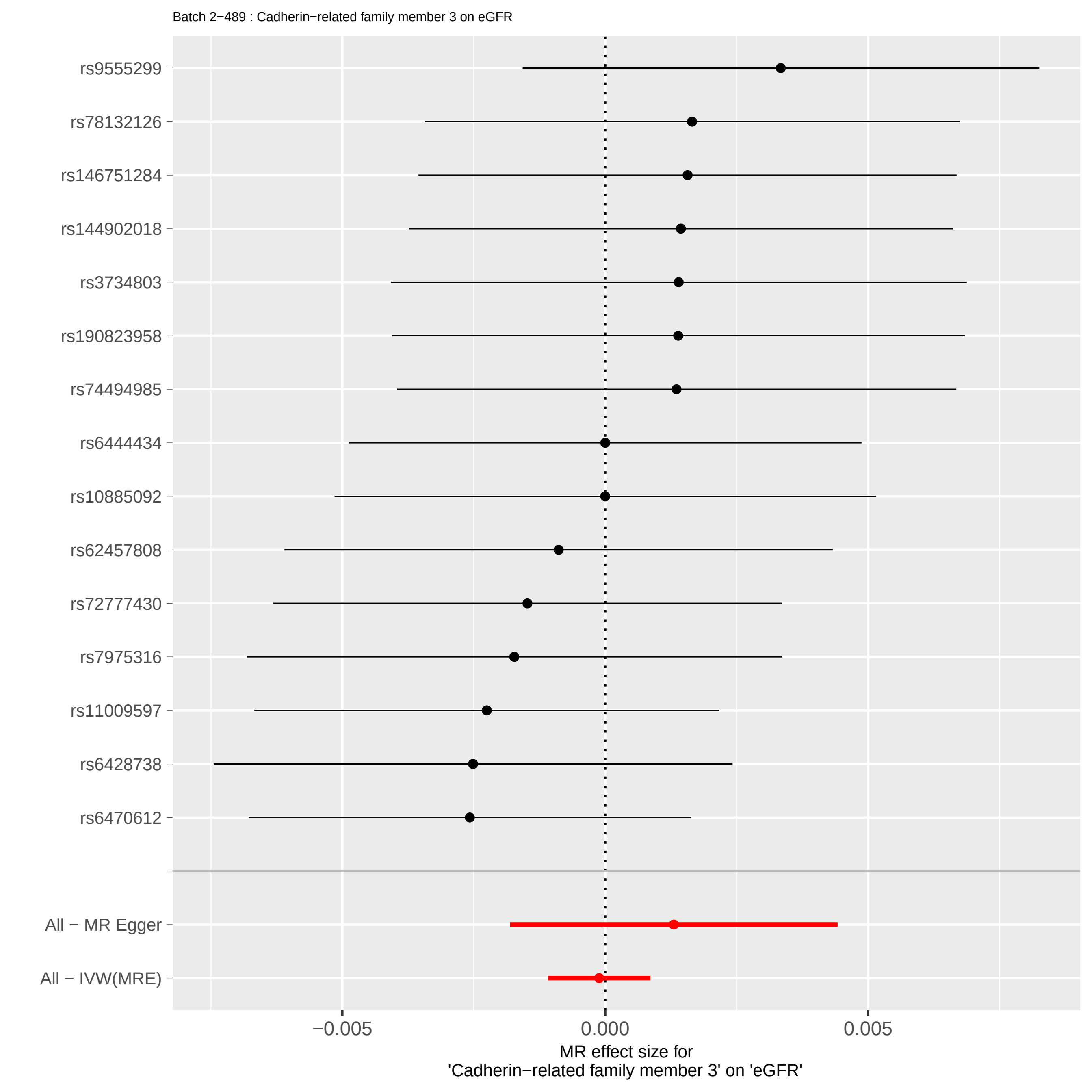
Batch 2−489 : Cadherin−related family member 3 on eGFR
rs9555299
rs78132126
rs146751284
rs144902018
rs3734803
rs190823958
rs74494985
rs6444434
rs10885092
rs62457808
rs72777430
rs7975316
rs11009597
rs6428738
rs6470612
All − MR Egger
All − IVW(MRE)
−0.005
0.000
0.005
MR effect size for
'Cadherin−related family member 3' on 'eGFR'

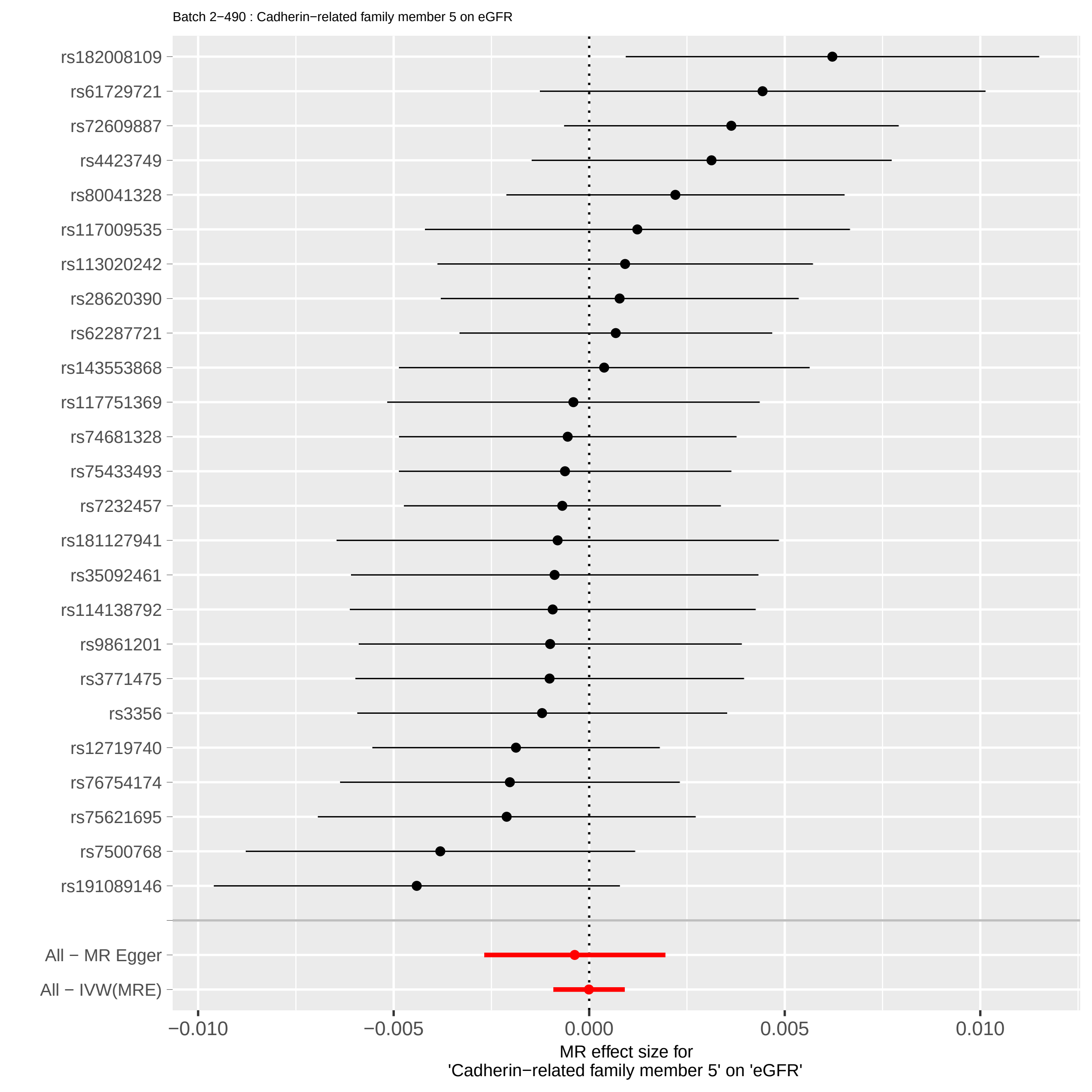
Batch 2−490 : Cadherin−related family member 5 on eGFR
rs182008109
rs61729721
rs72609887
rs4423749
rs80041328
rs117009535
rs113020242
rs28620390
rs62287721
rs143553868
rs117751369
rs74681328
rs75433493
rs7232457
rs181127941
rs35092461
rs114138792
rs9861201
rs3771475
rs3356
rs12719740
rs76754174
rs75621695
rs7500768
rs191089146
All − MR Egger
All − IVW(MRE)
−0.010
−0.005
0.000
0.005
0.010
MR effect size for
'Cadherin−related family member 5' on 'eGFR'

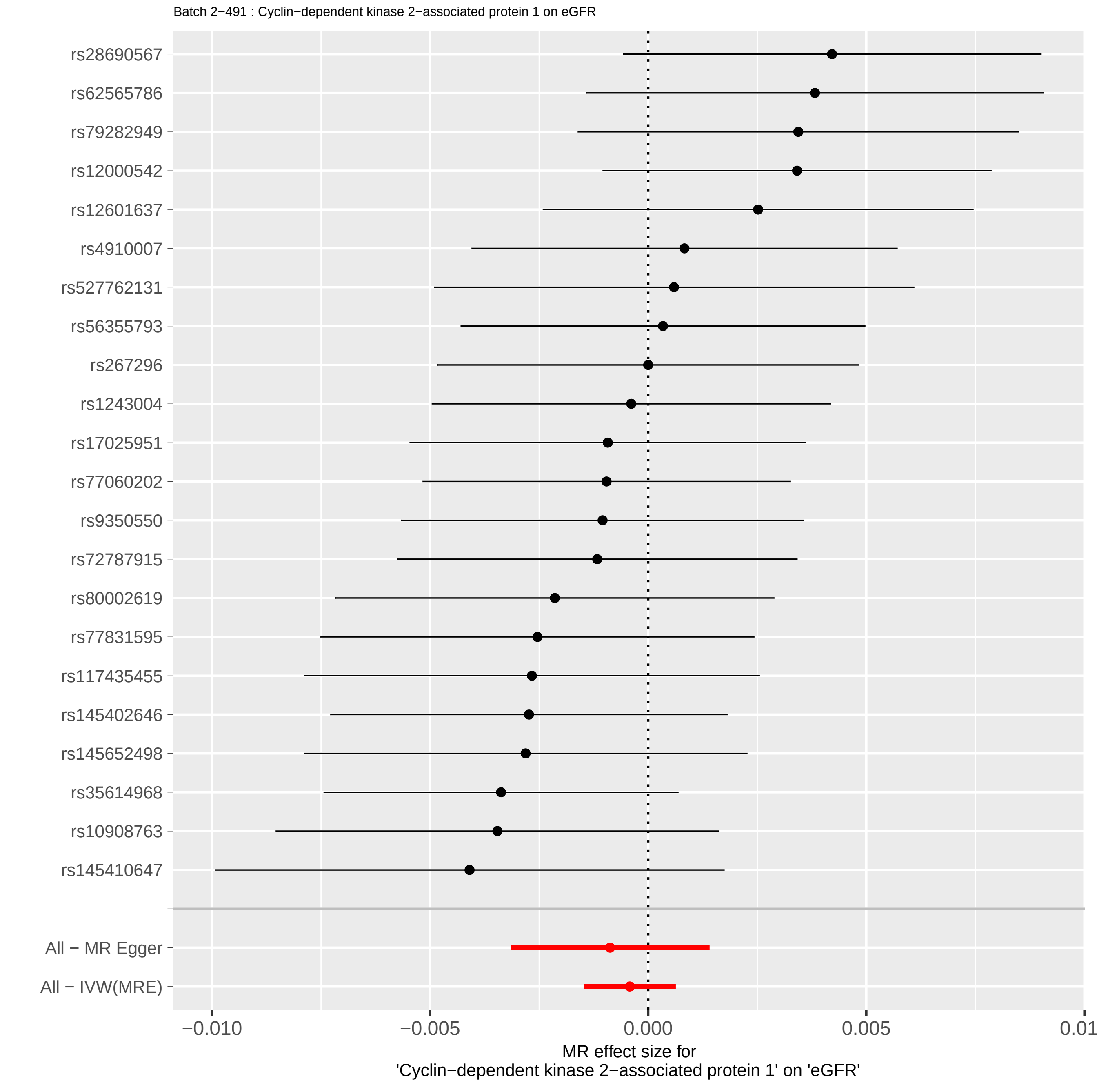

Batch 2−491 : Cyclin−dependent kinase 2−associated protein 1 on eGFR
rs28690567
rs62565786
rs79282949
rs12000542
rs12601637
rs4910007
rs527762131
rs56355793
rs267296
rs1243004
rs17025951
rs77060202
rs9350550
rs72787915
rs80002619
rs77831595
rs117435455
rs145402646
rs145652498
rs35614968
rs10908763
rs145410647
All − MR Egger
All − IVW(MRE)
−0.010
−0.005
0.000
0.005
0.01
MR effect size for
'Cyclin−dependent kinase 2−associated protein 1' on 'eGFR'

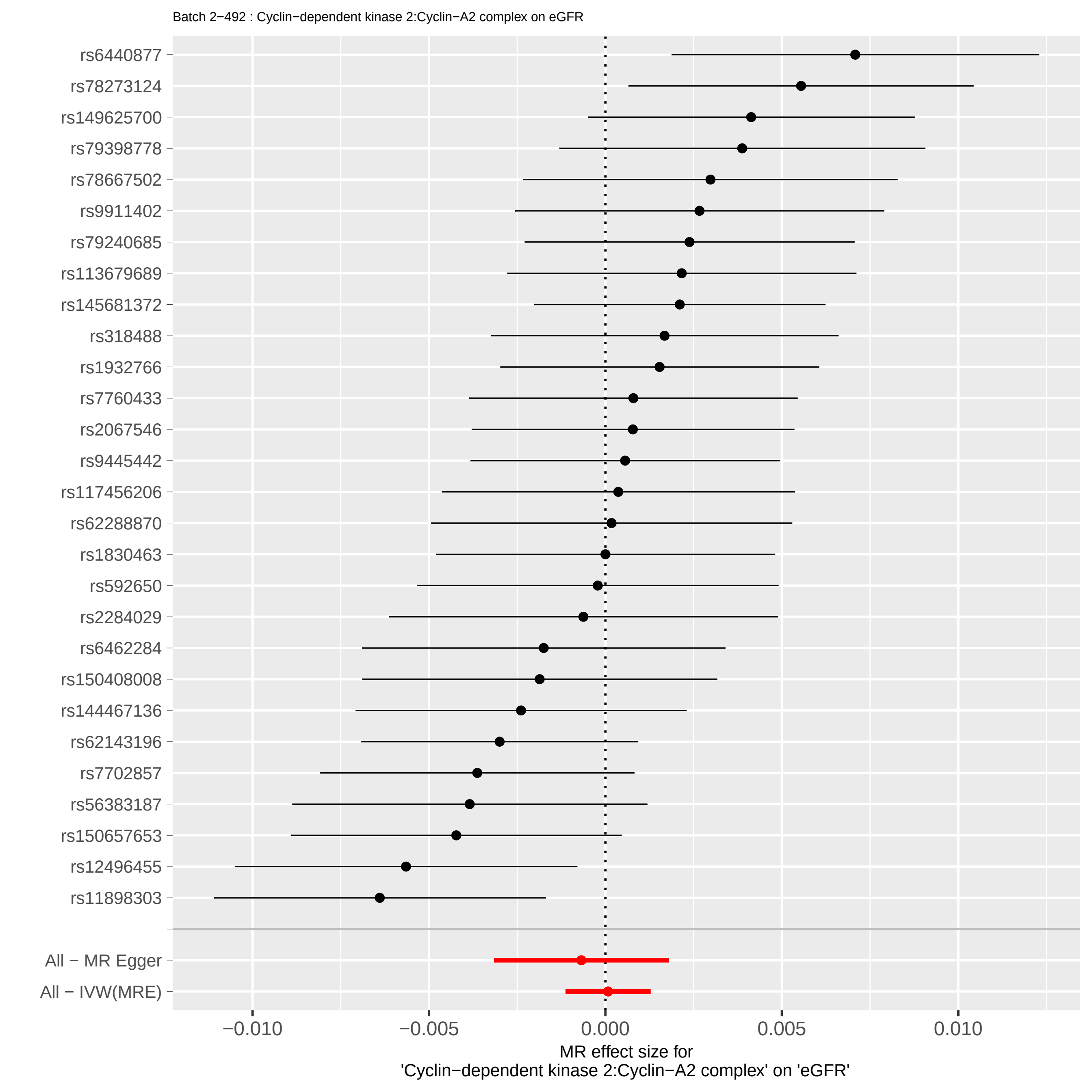
Batch 2−492 : Cyclin−dependent kinase 2:Cyclin−A2 complex on eGFR
rs6440877
rs78273124
rs149625700
rs79398778
rs78667502
rs9911402
rs79240685
rs113679689
rs145681372
rs318488
rs1932766
rs7760433
rs2067546
rs9445442
rs117456206
rs62288870
rs1830463
rs592650
rs2284029
rs6462284
rs150408008
rs144467136
rs62143196
rs7702857
rs56383187
rs150657653
rs12496455
rs11898303
All − MR Egger
All − IVW(MRE)
−0.010
−0.005
0.000
0.005
0.010
MR effect size for
'Cyclin−dependent kinase 2:Cyclin−A2 complex' on 'eGFR'

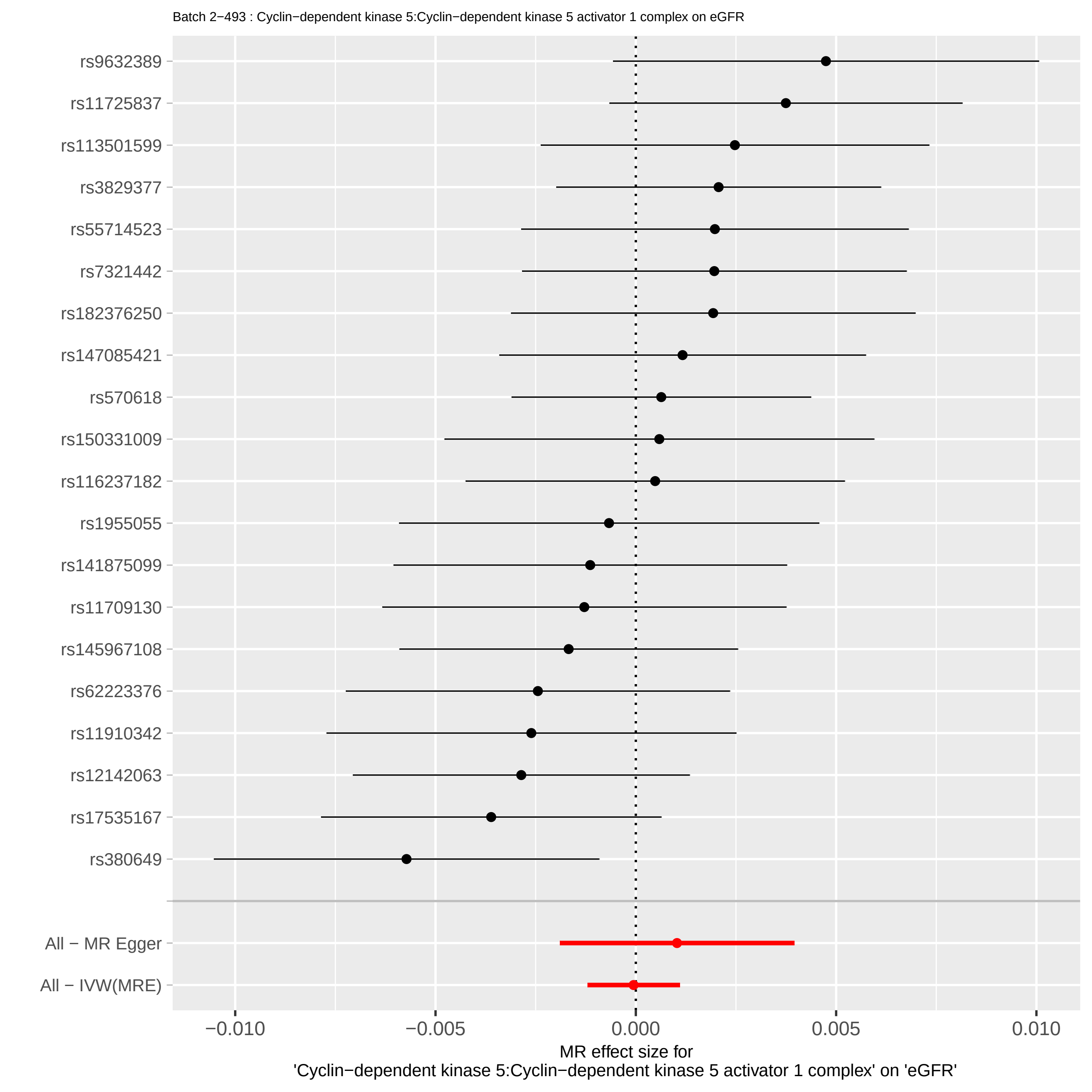
Batch 2−493 : Cyclin−dependent kinase 5:Cyclin−dependent kinase 5 activator 1 complex on eGFR
rs9632389
rs11725837
rs113501599
rs3829377
rs55714523
rs7321442
rs182376250
rs147085421
rs570618
rs150331009
rs116237182
rs1955055
rs141875099
rs11709130
rs145967108
rs62223376
rs11910342
rs12142063
rs17535167
rs380649
All − MR Egger
All − IVW(MRE)
−0.010
−0.005
0.000
0.005
0.010
MR effect size for
'Cyclin−dependent kinase 5:Cyclin−dependent kinase 5 activator 1 complex' on 'eGFR'

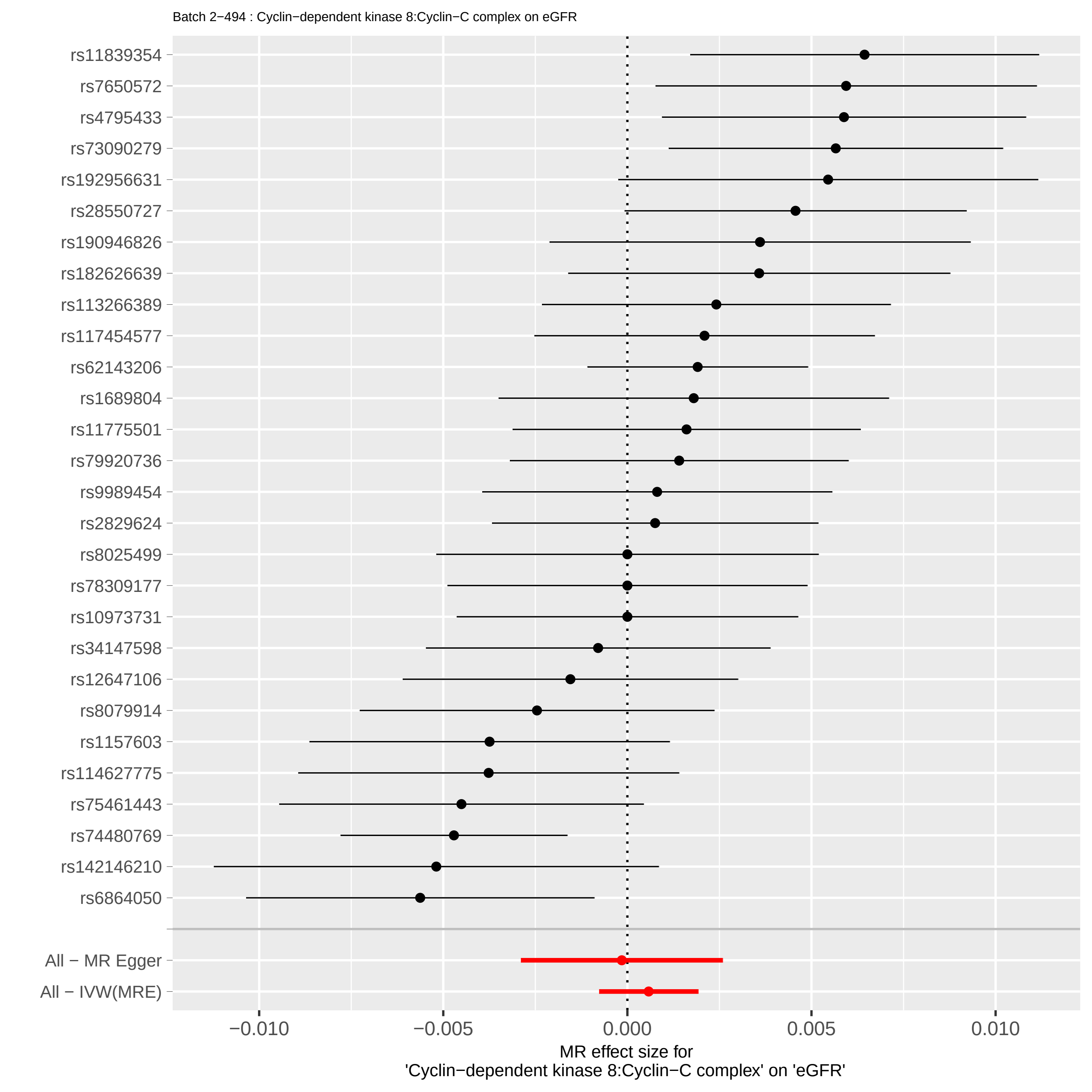
Batch 2−494 : Cyclin−dependent kinase 8:Cyclin−C complex on eGFR
rs11839354
rs7650572
rs4795433
rs73090279
rs192956631
rs28550727
rs190946826
rs182626639
rs113266389
rs117454577
rs62143206
rs1689804
rs11775501
rs79920736
rs9989454
rs2829624
rs8025499
rs78309177
rs10973731
rs34147598
rs12647106
rs8079914
rs1157603
rs114627775
rs75461443
rs74480769
rs142146210
rs6864050
All − MR Egger
All − IVW(MRE)
−0.010
−0.005
0.000
0.005
0.010
MR effect size for
'Cyclin−dependent kinase 8:Cyclin−C complex' on 'eGFR'

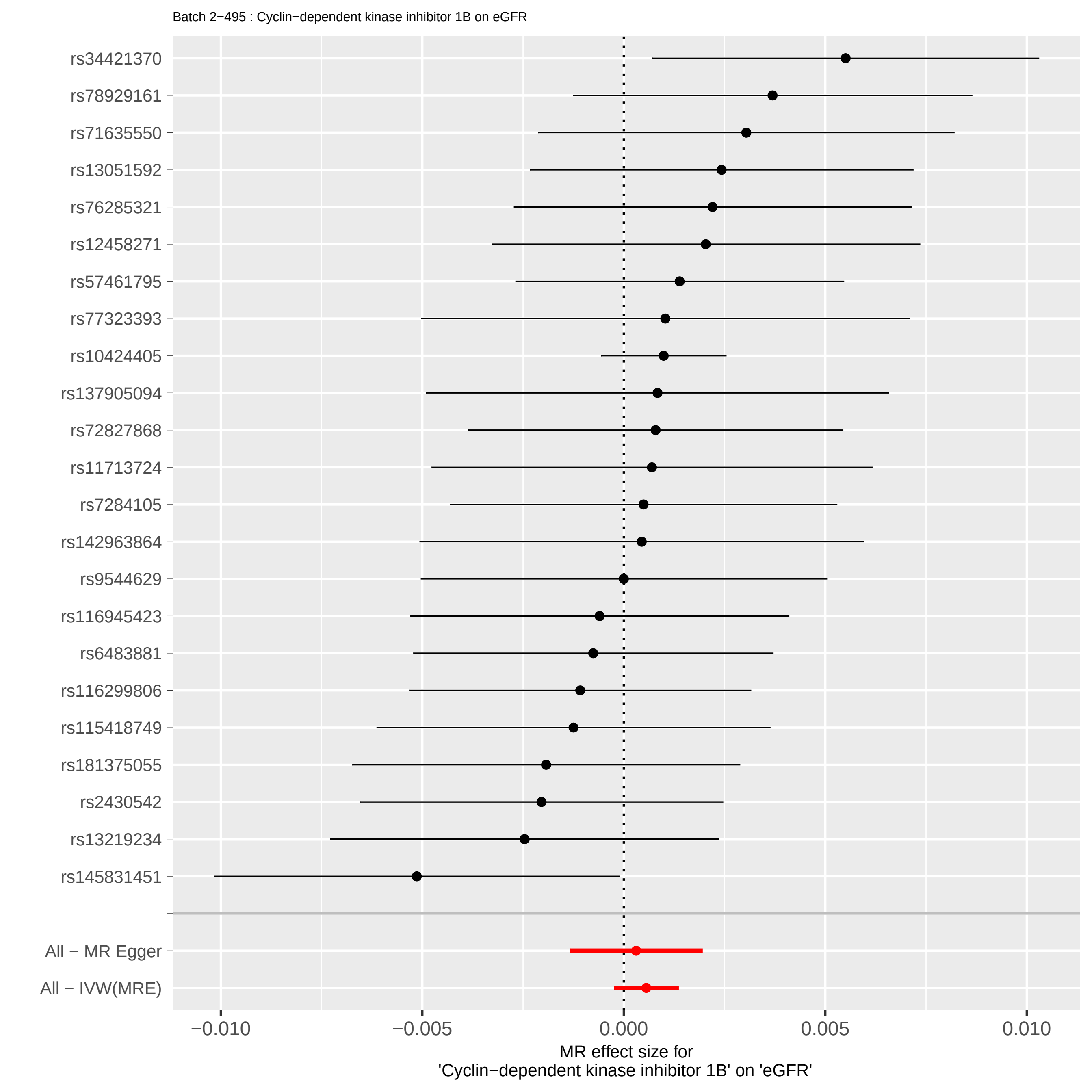

Batch 2−495 : Cyclin−dependent kinase inhibitor 1B on eGFR
rs34421370
rs78929161
rs71635550
rs13051592
rs76285321
rs12458271
rs57461795
rs77323393
rs10424405
rs137905094
rs72827868
rs11713724
rs7284105
rs142963864
rs9544629
rs116945423
rs6483881
rs116299806
rs115418749
rs181375055
rs2430542
rs13219234
rs145831451
All − MR Egger
All − IVW(MRE)
−0.010
−0.005
0.000
0.005
0.010
MR effect size for
'Cyclin−dependent kinase inhibitor 1B' on 'eGFR'

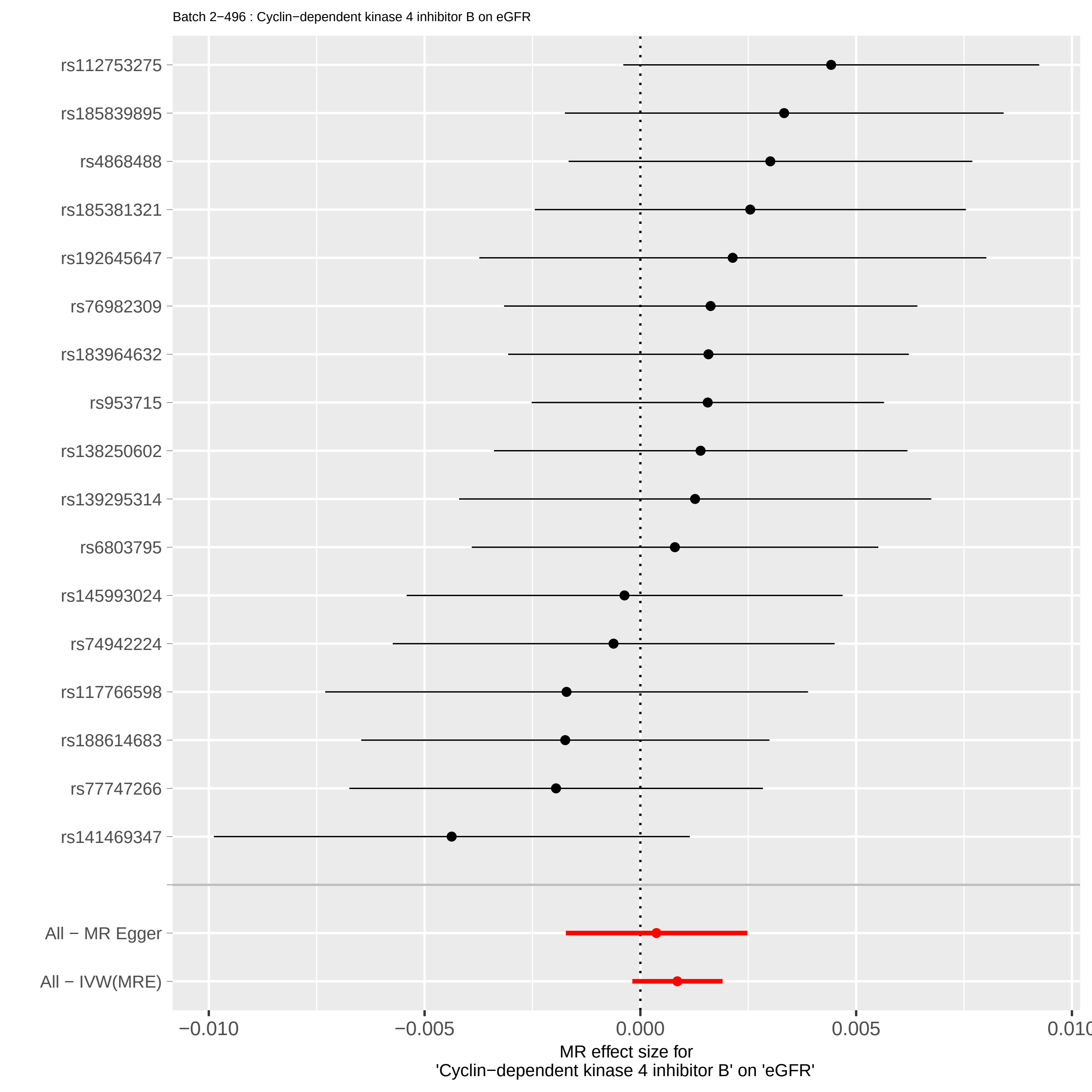
Batch 2−496 : Cyclin−dependent kinase 4 inhibitor B on eGFR
rs112753275
rs185839895
rs4868488
rs185381321
rs192645647
rs76982309
rs183964632
rs953715
rs138250602
rs139295314
rs6803795
rs145993024
rs74942224
rs117766598
rs188614683
rs77747266
rs141469347
All − MR Egger
All − IVW(MRE)
−0.010
−0.005
0.000
0.005
0.010
MR effect size for
'Cyclin−dependent kinase 4 inhibitor B' on 'eGFR'

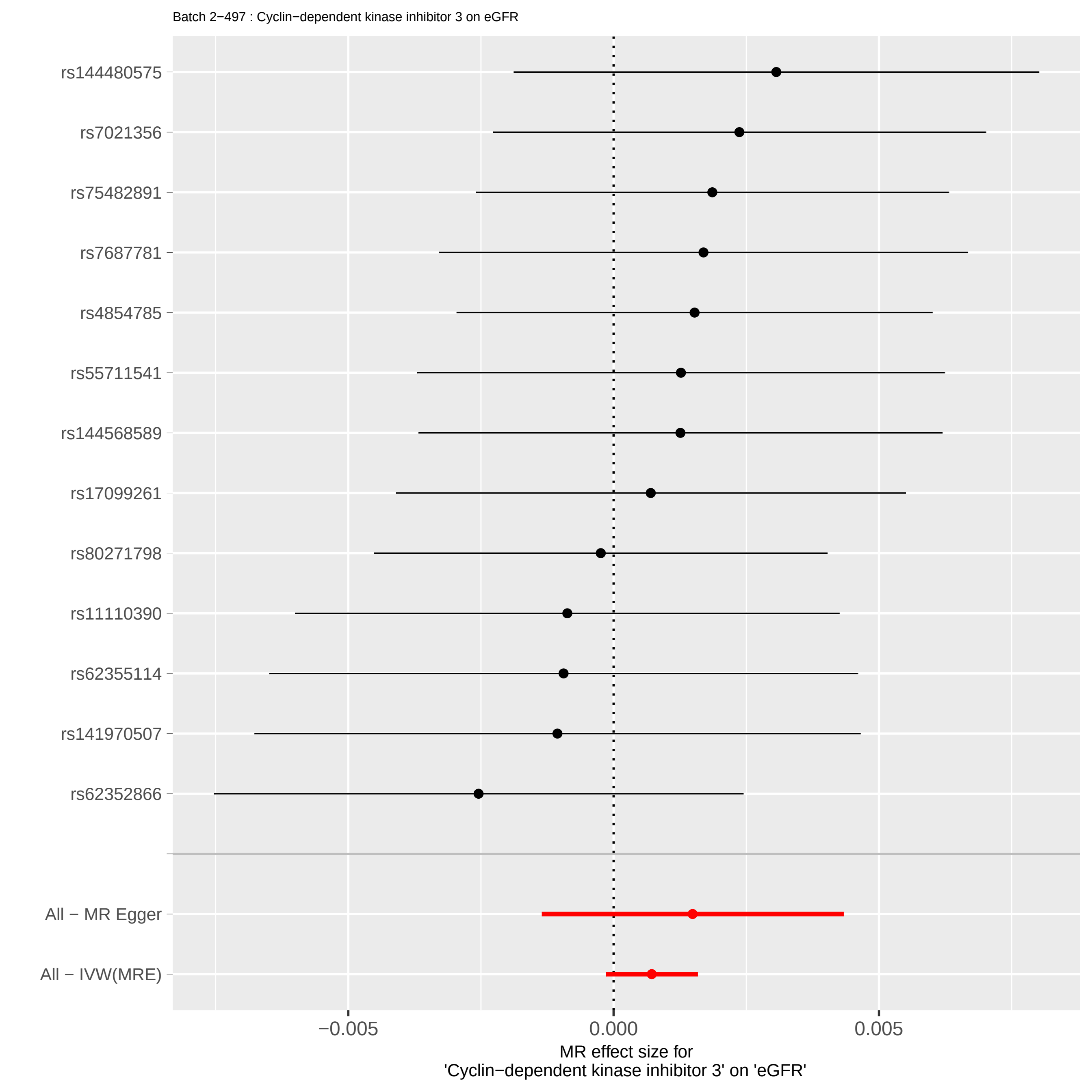
Batch 2−497 : Cyclin−dependent kinase inhibitor 3 on eGFR
rs144480575
rs7021356
rs75482891
rs7687781
rs4854785
rs55711541
rs144568589
rs17099261
rs80271798
rs11110390
rs62355114
rs141970507
rs62352866
All − MR Egger
All − IVW(MRE)
−0.005
0.000
0.005
MR effect size for
'Cyclin−dependent kinase inhibitor 3' on 'eGFR'

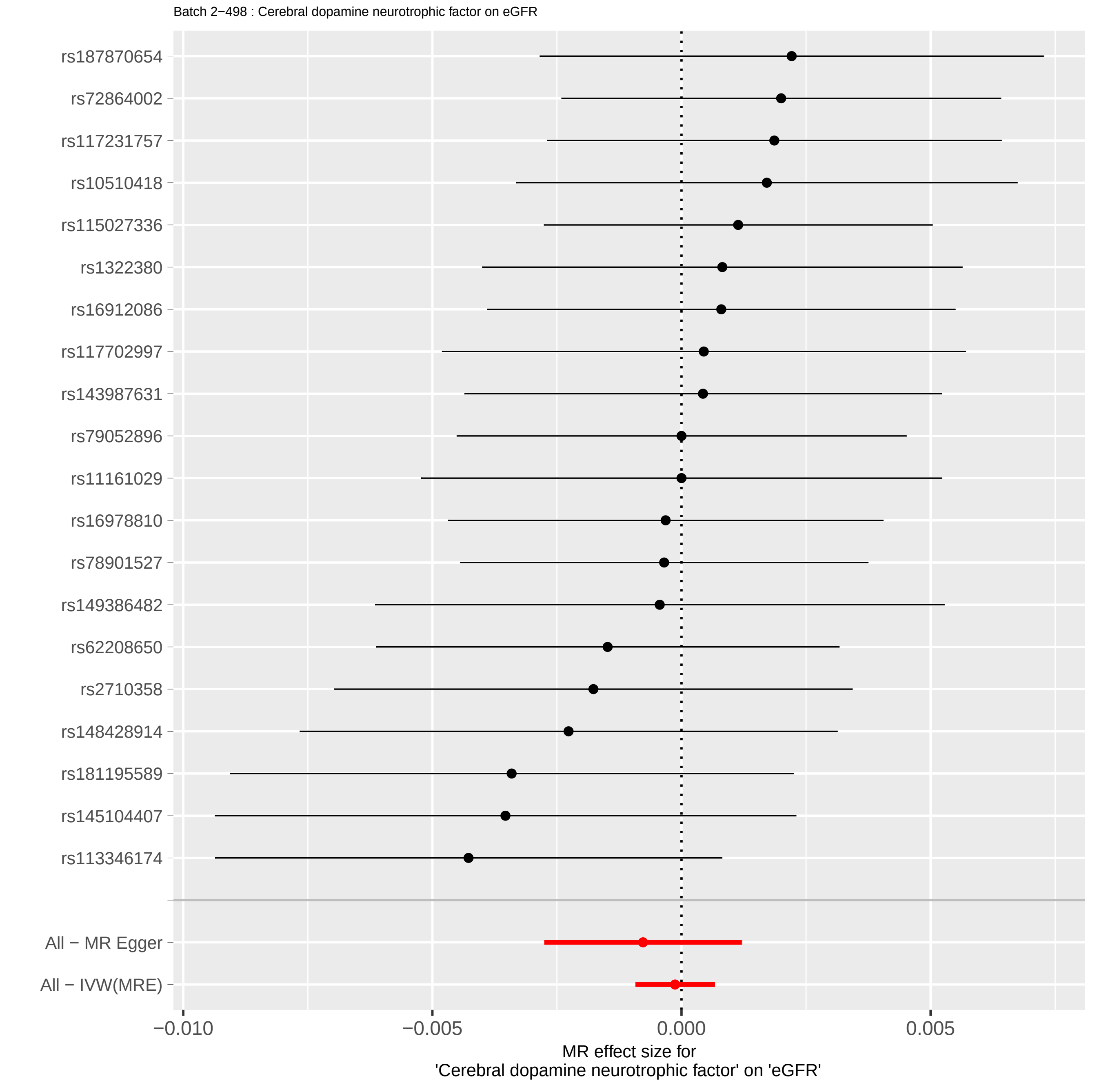
Batch 2−498 : Cerebral dopamine neurotrophic factor on eGFR
rs187870654
rs72864002
rs117231757
rs10510418
rs115027336
rs1322380
rs16912086
rs117702997
rs143987631
rs79052896
rs11161029
rs16978810
rs78901527
rs149386482
rs62208650
rs2710358
rs148428914
rs181195589
rs145104407
rs113346174
All − MR Egger
All − IVW(MRE)
−0.010
−0.005
0.000
0.005
MR effect size for
'Cerebral dopamine neurotrophic factor' on 'eGFR'

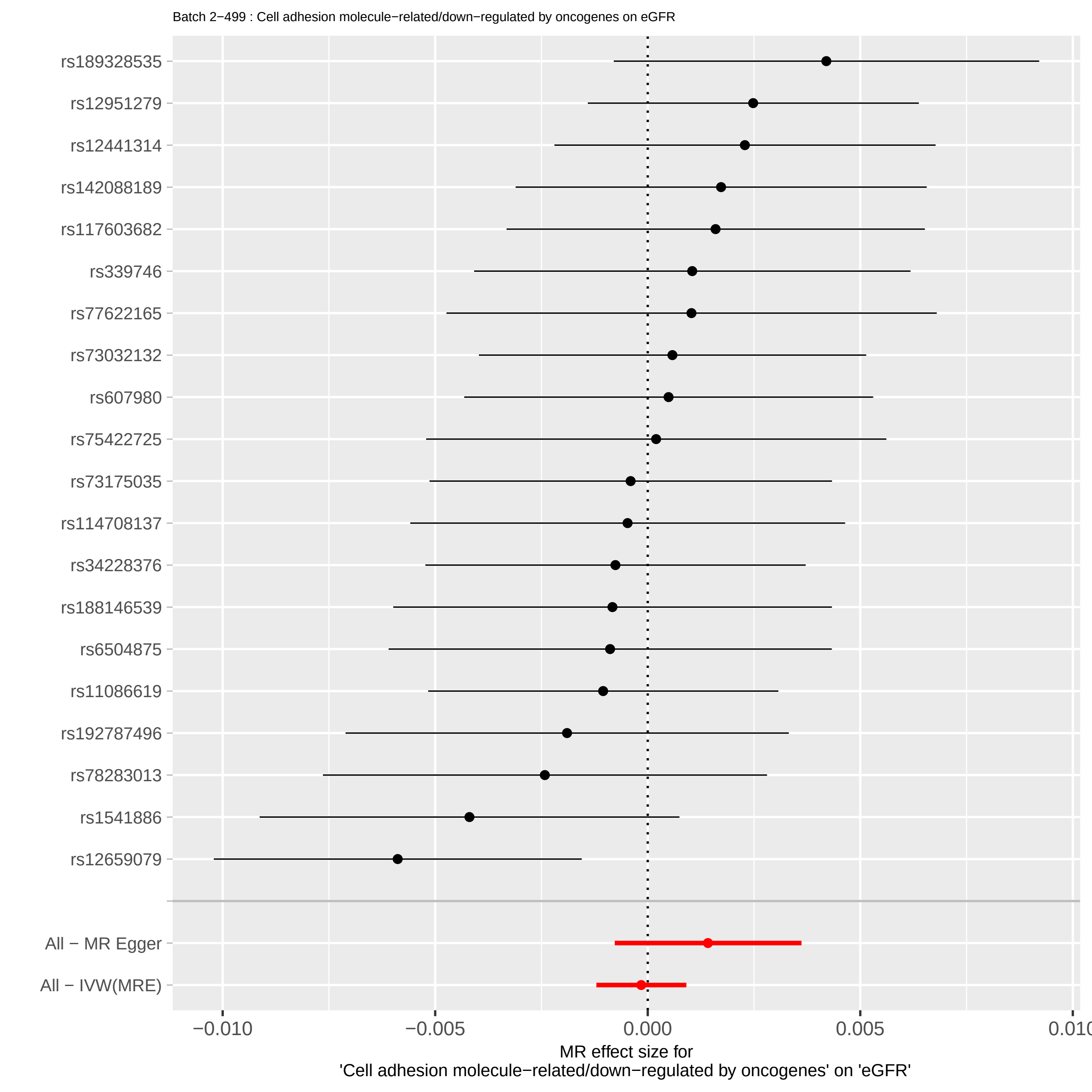

Batch 2−499 : Cell adhesion molecule−related/down−regulated by oncogenes on eGFR
rs189328535
rs12951279
rs12441314
rs142088189
rs117603682
rs339746
rs77622165
rs73032132
rs607980
rs75422725
rs73175035
rs114708137
rs34228376
rs188146539
rs6504875
rs11086619
rs192787496
rs78283013
rs1541886
rs12659079
All − MR Egger
All − IVW(MRE)
−0.010
−0.005
0.000
0.005
0.010
MR effect size for
'Cell adhesion molecule−related/down−regulated by oncogenes' on 'eGFR'

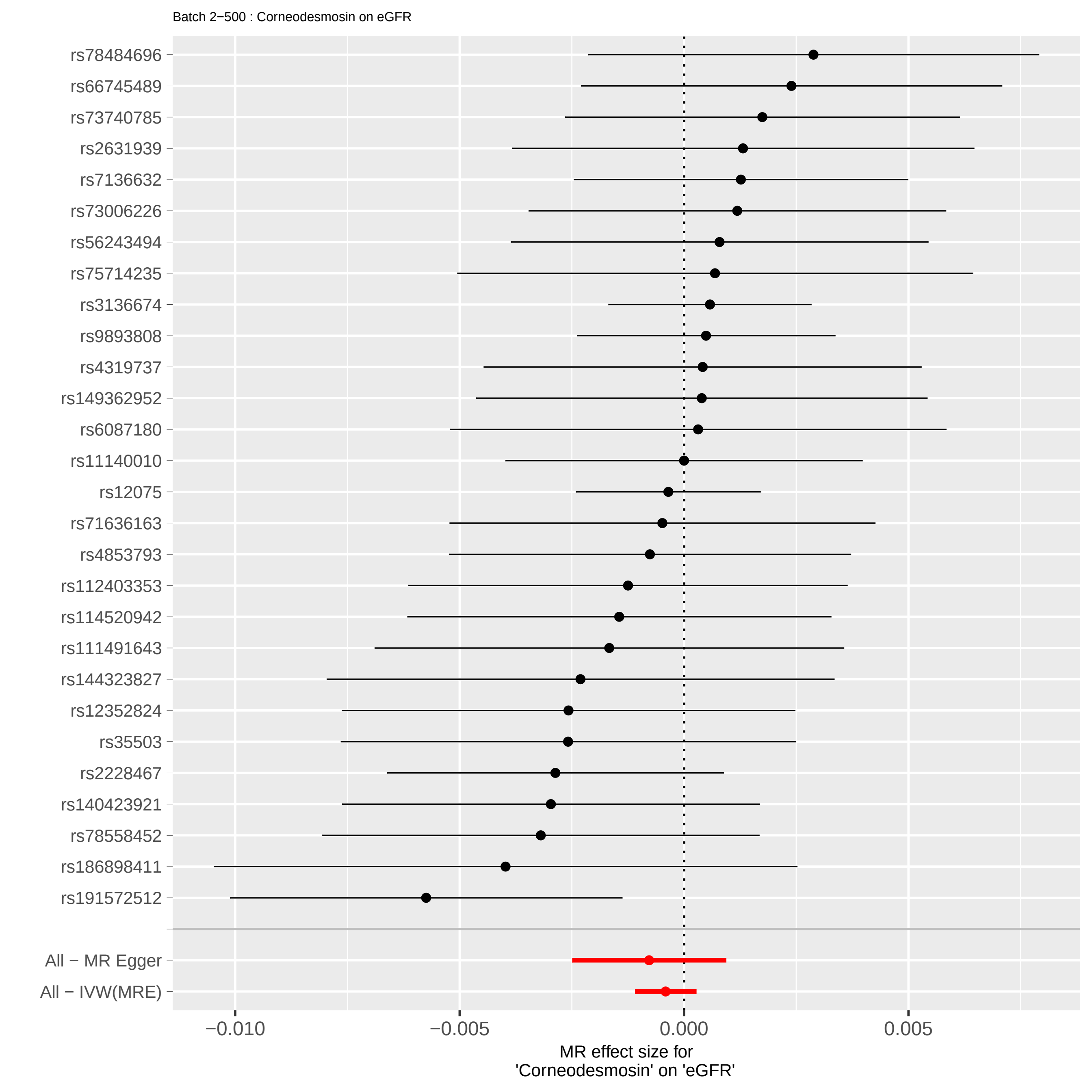

Batch 2−500 : Corneodesmosin on eGFR
rs78484696
rs66745489
rs73740785
rs2631939
rs7136632
rs73006226
rs56243494
rs75714235
rs3136674
rs9893808
rs4319737
rs149362952
rs6087180
rs11140010
rs12075
rs71636163
rs4853793
rs112403353
rs114520942
rs111491643
rs144323827
rs12352824
rs35503
rs2228467
rs140423921
rs78558452
rs186898411
rs191572512
All − MR Egger
All − IVW(MRE)
−0.010
−0.005
0.000
0.005
MR effect size for
'Corneodesmosin' on 'eGFR'

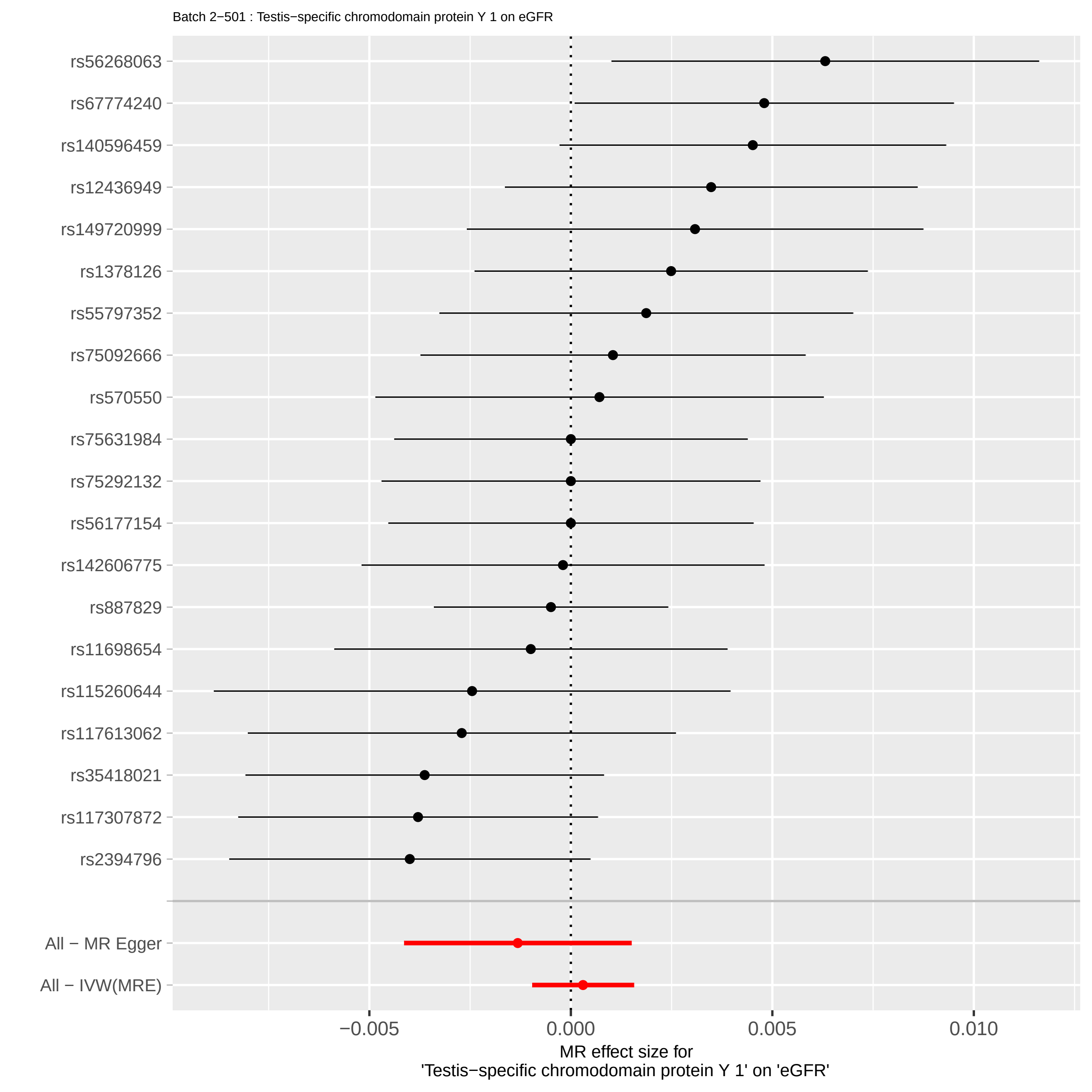
Batch 2−501 : Testis−specific chromodomain protein Y 1 on eGFR
rs56268063
rs67774240
rs140596459
rs12436949
rs149720999
rs1378126
rs55797352
rs75092666
rs570550
rs75631984
rs75292132
rs56177154
rs142606775
rs887829
rs11698654
rs115260644
rs117613062
rs35418021
rs117307872
rs2394796
All − MR Egger
All − IVW(MRE)
−0.005
0.000
0.005
0.010
MR effect size for
'Testis−specific chromodomain protein Y 1' on 'eGFR'

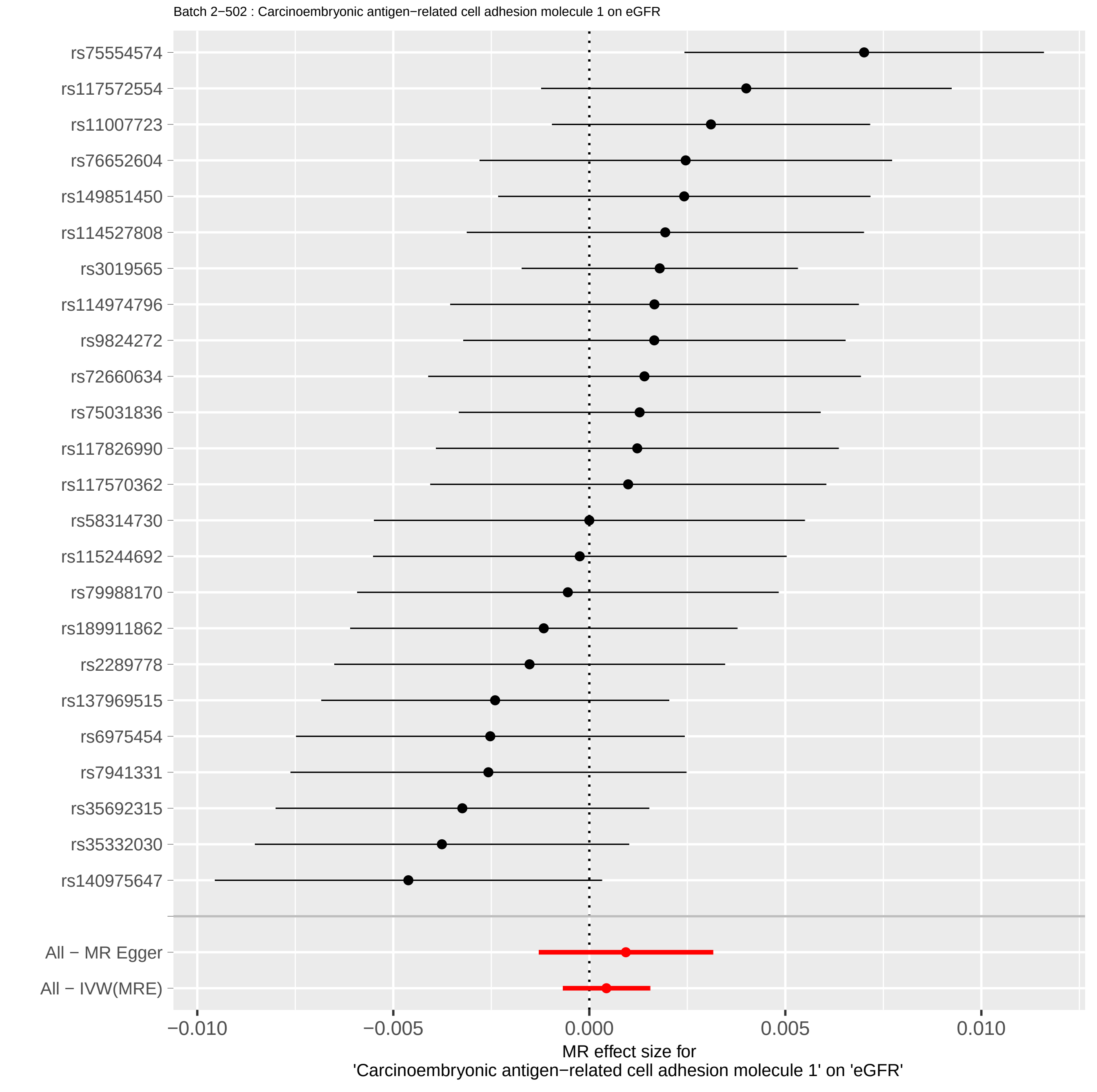

Batch 2−502 : Carcinoembryonic antigen−related cell adhesion molecule 1 on eGFR
rs75554574
rs117572554
rs11007723
rs76652604
rs149851450
rs114527808
rs3019565
rs114974796
rs9824272
rs72660634
rs75031836
rs117826990
rs117570362
rs58314730
rs115244692
rs79988170
rs189911862
rs2289778
rs137969515
rs6975454
rs7941331
rs35692315
rs35332030
rs140975647
All − MR Egger
All − IVW(MRE)
−0.010
−0.005
0.000
0.005
0.010
MR effect size for
'Carcinoembryonic antigen−related cell adhesion molecule 1' on 'eGFR'

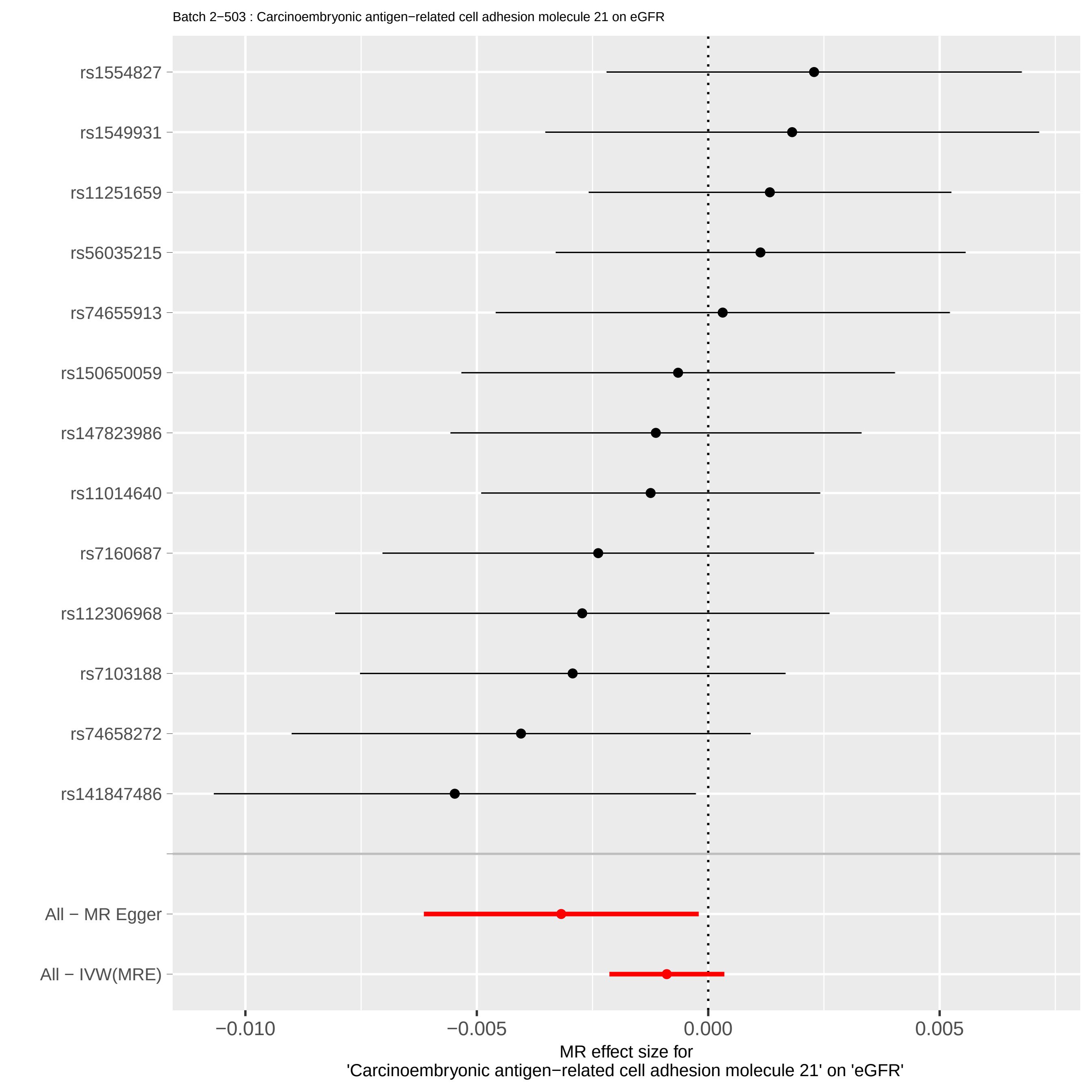
Batch 2−503 : Carcinoembryonic antigen−related cell adhesion molecule 21 on eGFR
rs1554827
rs1549931
rs11251659
rs56035215
rs74655913
rs150650059
rs147823986
rs11014640
rs7160687
rs112306968
rs7103188
rs74658272
rs141847486
All − MR Egger
All − IVW(MRE)
−0.010
−0.005
0.000
0.005
MR effect size for
'Carcinoembryonic antigen−related cell adhesion molecule 21' on 'eGFR'

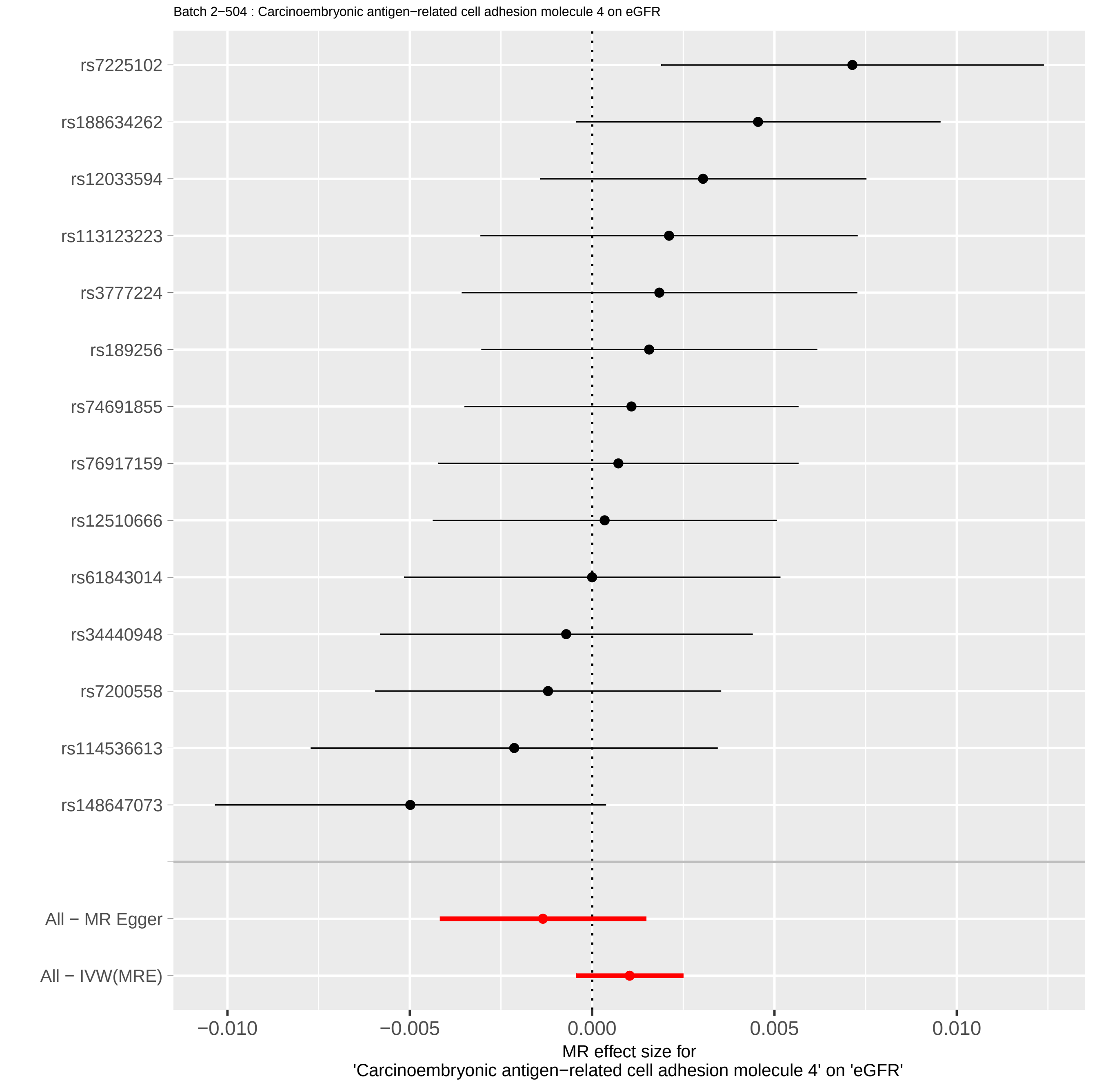

Batch 2−504 : Carcinoembryonic antigen−related cell adhesion molecule 4 on eGFR
rs7225102
rs188634262
rs12033594
rs113123223
rs3777224
rs189256
rs74691855
rs76917159
rs12510666
rs61843014
rs34440948
rs7200558
rs114536613
rs148647073
All − MR Egger
All − IVW(MRE)
−0.010
−0.005
0.000
0.005
0.010
MR effect size for
'Carcinoembryonic antigen−related cell adhesion molecule 4' on 'eGFR'

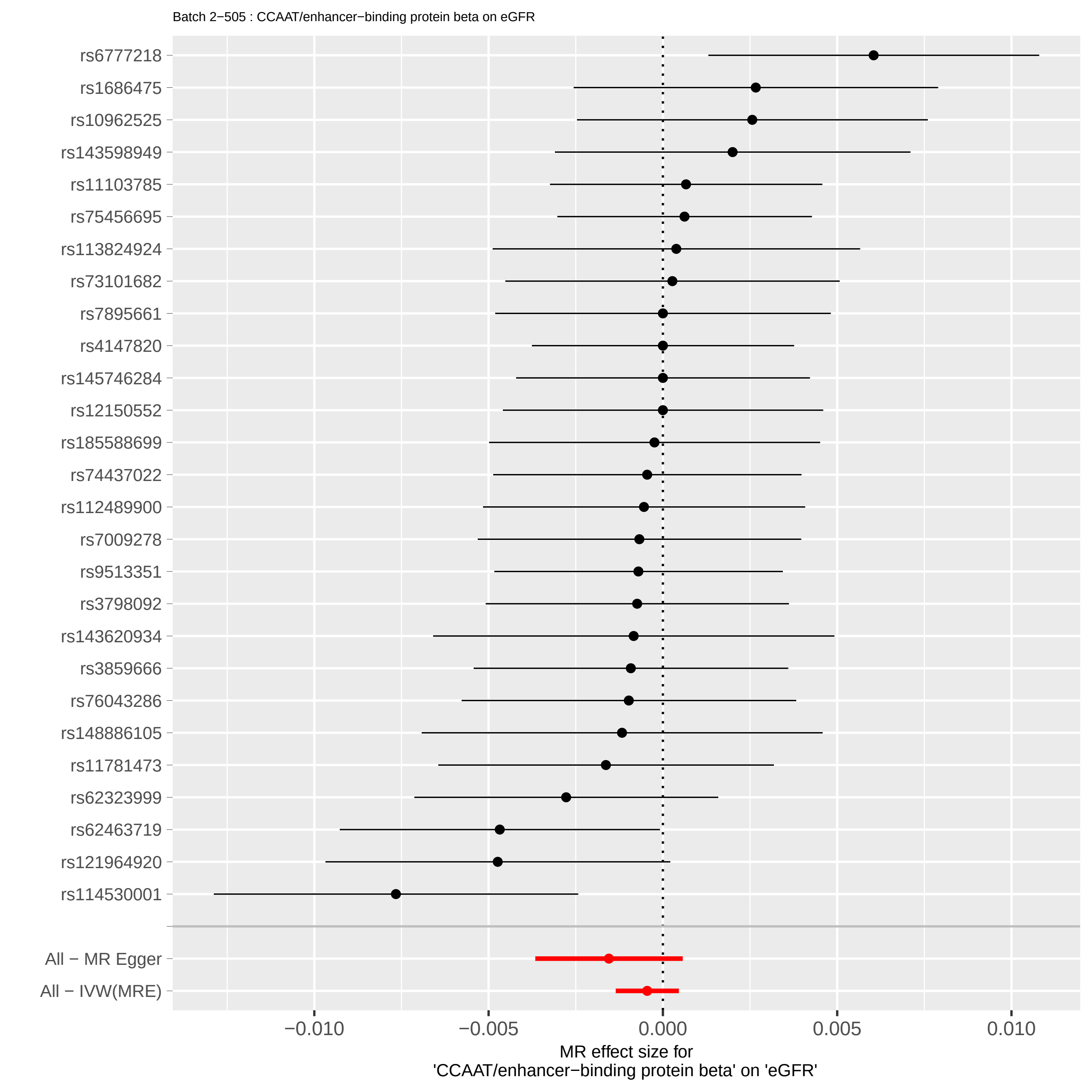

Batch 2−505 : CCAAT/enhancer−binding protein beta on eGFR
rs6777218
rs1686475
rs10962525
rs143598949
rs11103785
rs75456695
rs113824924
rs73101682
rs7895661
rs4147820
rs145746284
rs12150552
rs185588699
rs74437022
rs112489900
rs7009278
rs9513351
rs3798092
rs143620934
rs3859666
rs76043286
rs148886105
rs11781473
rs62323999
rs62463719
rs121964920
rs114530001
All − MR Egger
All − IVW(MRE)
−0.010
−0.005
0.000
0.005
0.010
MR effect size for
'CCAAT/enhancer−binding protein beta' on 'eGFR'

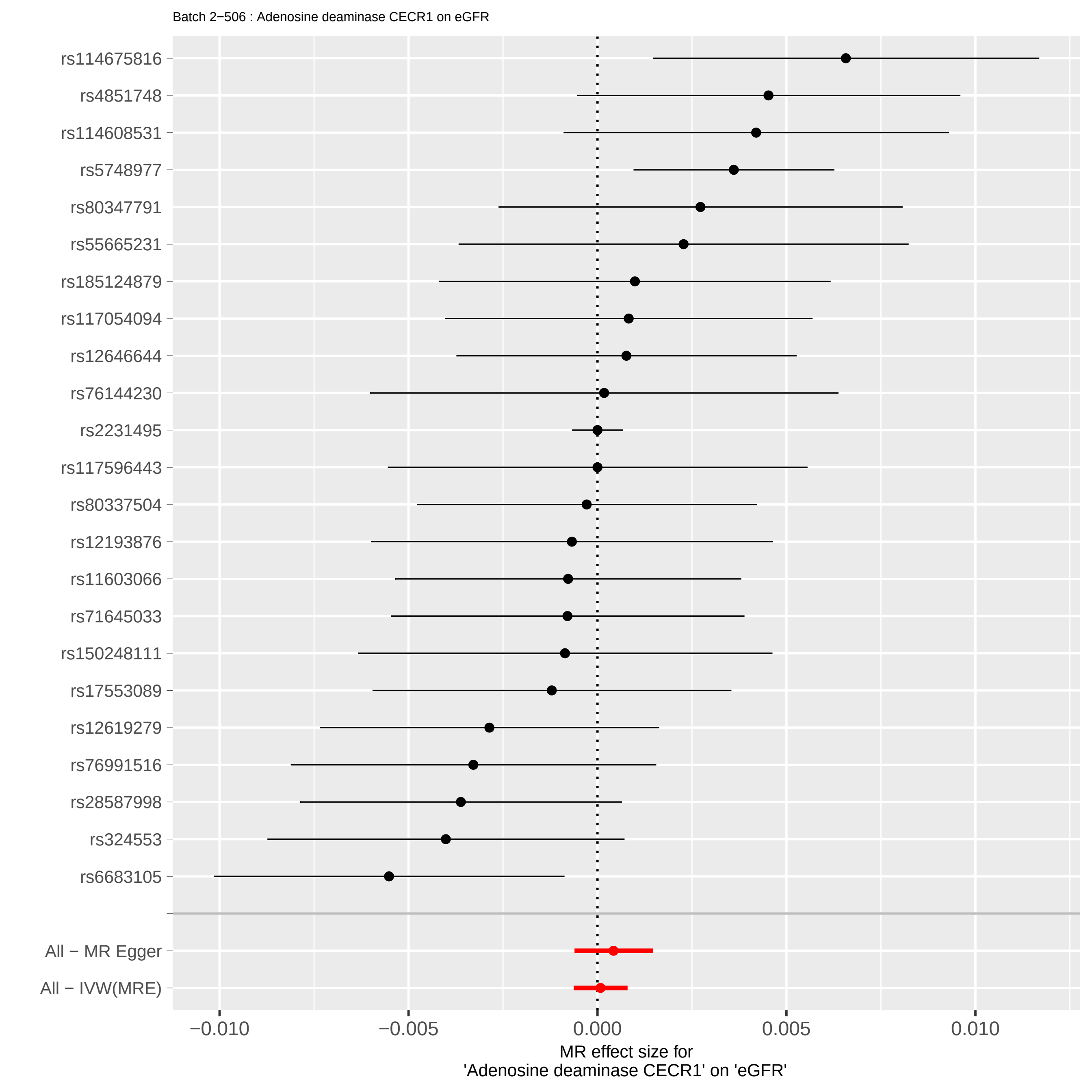

Batch 2−506 : Adenosine deaminase CECR1 on eGFR
rs114675816
rs4851748
rs114608531
rs5748977
rs80347791
rs55665231
rs185124879
rs117054094
rs12646644
rs76144230
rs2231495
rs117596443
rs80337504
rs12193876
rs11603066
rs71645033
rs150248111
rs17553089
rs12619279
rs76991516
rs28587998
rs324553
rs6683105
All − MR Egger
All − IVW(MRE)
−0.010
−0.005
0.000
0.005
0.010
MR effect size for
'Adenosine deaminase CECR1' on 'eGFR'

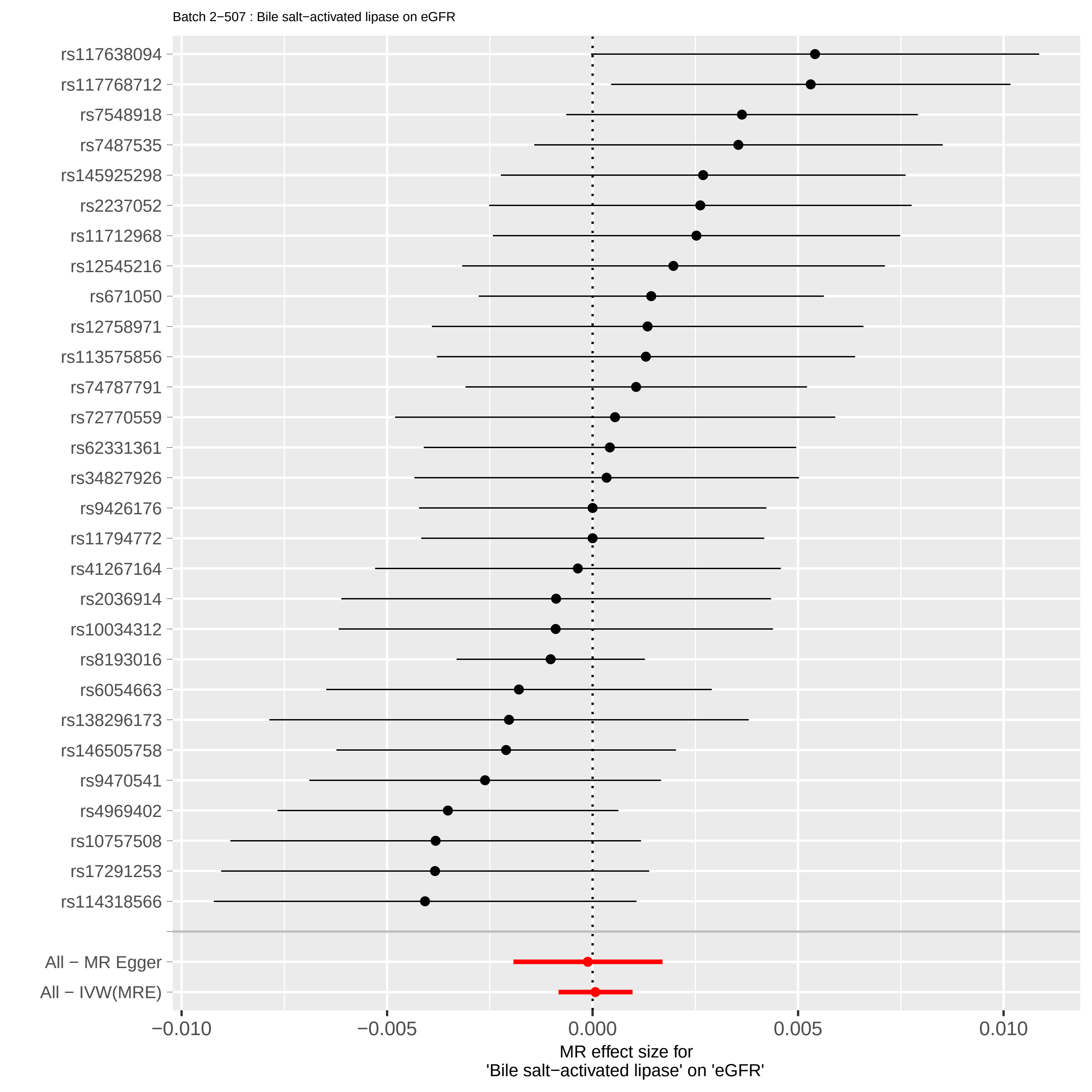

Batch 2−507 : Bile salt−activated lipase on eGFR
rs117638094
rs117768712
rs7548918
rs7487535
rs145925298
rs2237052
rs11712968
rs12545216
rs671050
rs12758971
rs113575856
rs74787791
rs72770559
rs62331361
rs34827926
rs9426176
rs11794772
rs41267164
rs2036914
rs10034312
rs8193016
rs6054663
rs138296173
rs146505758
rs9470541
rs4969402
rs10757508
rs17291253
rs114318566
All − MR Egger
All − IVW(MRE)
−0.010
−0.005
0.000
0.005
0.010
MR effect size for
'Bile salt−activated lipase' on 'eGFR'

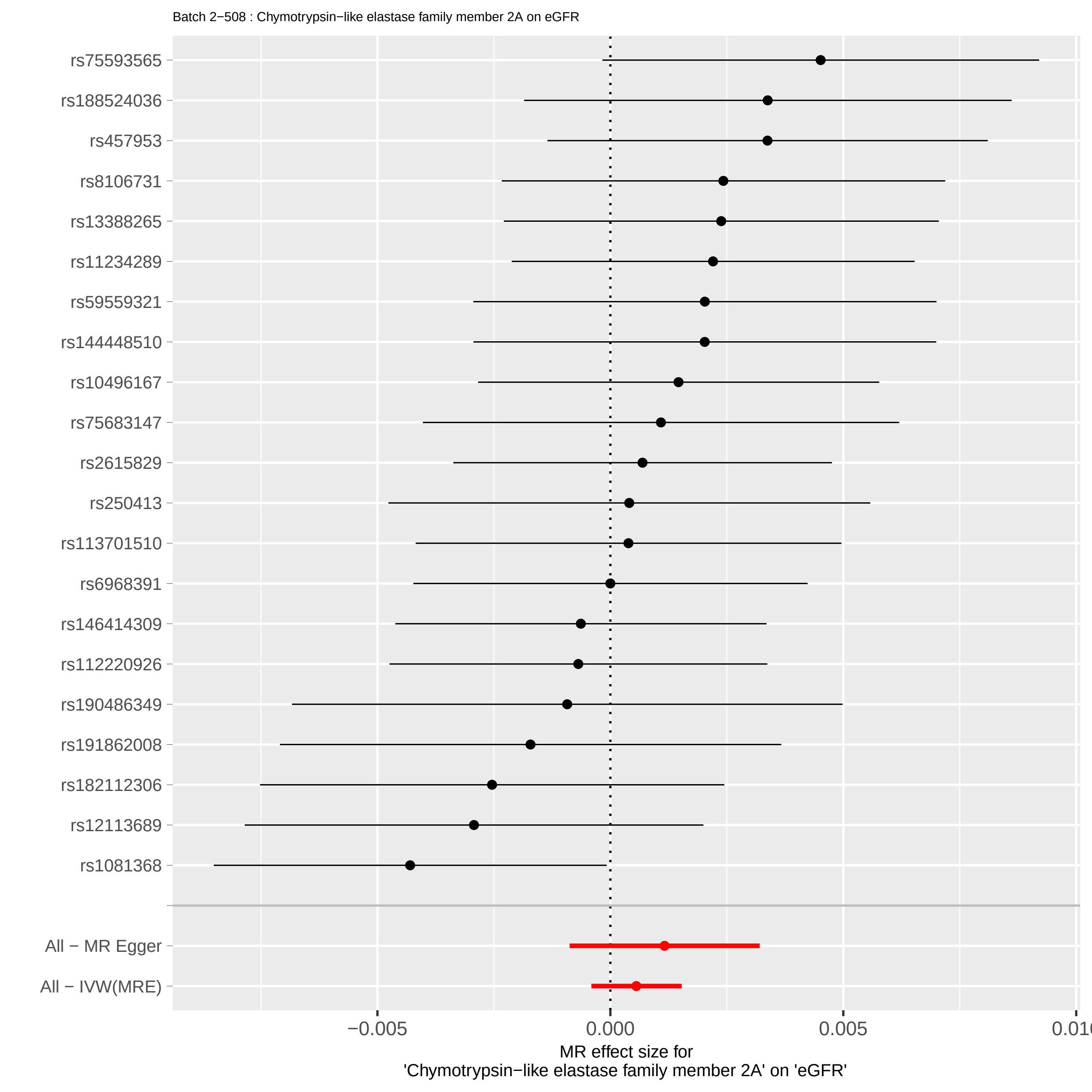

Batch 2−508 : Chymotrypsin−like elastase family member 2A on eGFR
rs75593565
rs188524036
rs457953
rs8106731
rs13388265
rs11234289
rs59559321
rs144448510
rs10496167
rs75683147
rs2615829
rs250413
rs113701510
rs6968391
rs146414309
rs112220926
rs190486349
rs191862008
rs182112306
rs12113689
rs1081368
All − MR Egger
All − IVW(MRE)
−0.005
0.000
0.005
MR effect size for
'Chymotrypsin−like elastase family member 2A' on 'eGFR'

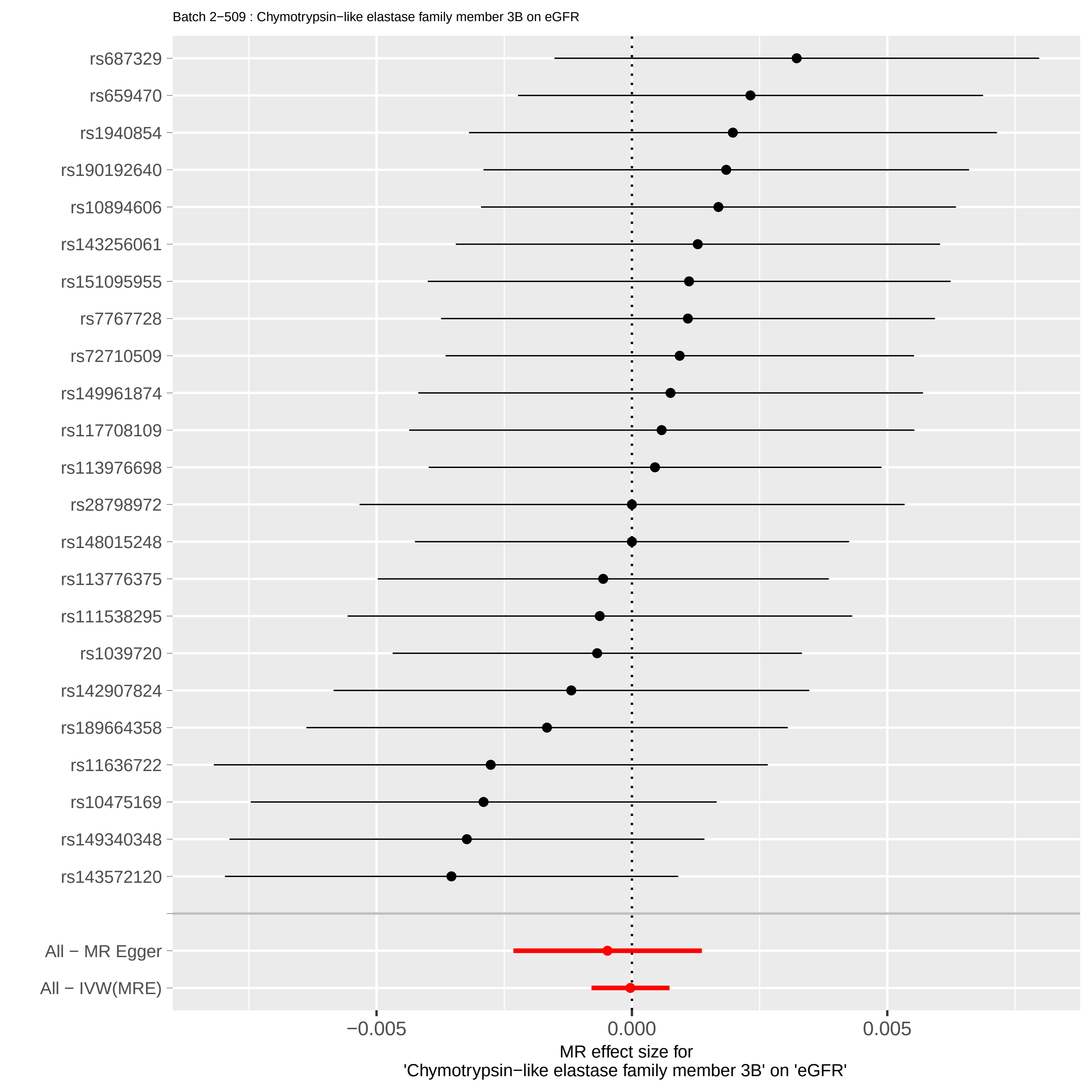

Batch 2−509 : Chymotrypsin−like elastase family member 3B on eGFR
rs687329
rs659470
rs1940854
rs190192640
rs10894606
rs143256061
rs151095955
rs7767728
rs72710509
rs149961874
rs117708109
rs113976698
rs28798972
rs148015248
rs113776375
rs111538295
rs1039720
rs142907824
rs189664358
rs11636722
rs10475169
rs149340348
rs143572120
All − MR Egger
All − IVW(MRE)
−0.005
0.000
0.005
MR effect size for
'Chymotrypsin−like elastase family member 3B' on 'eGFR'

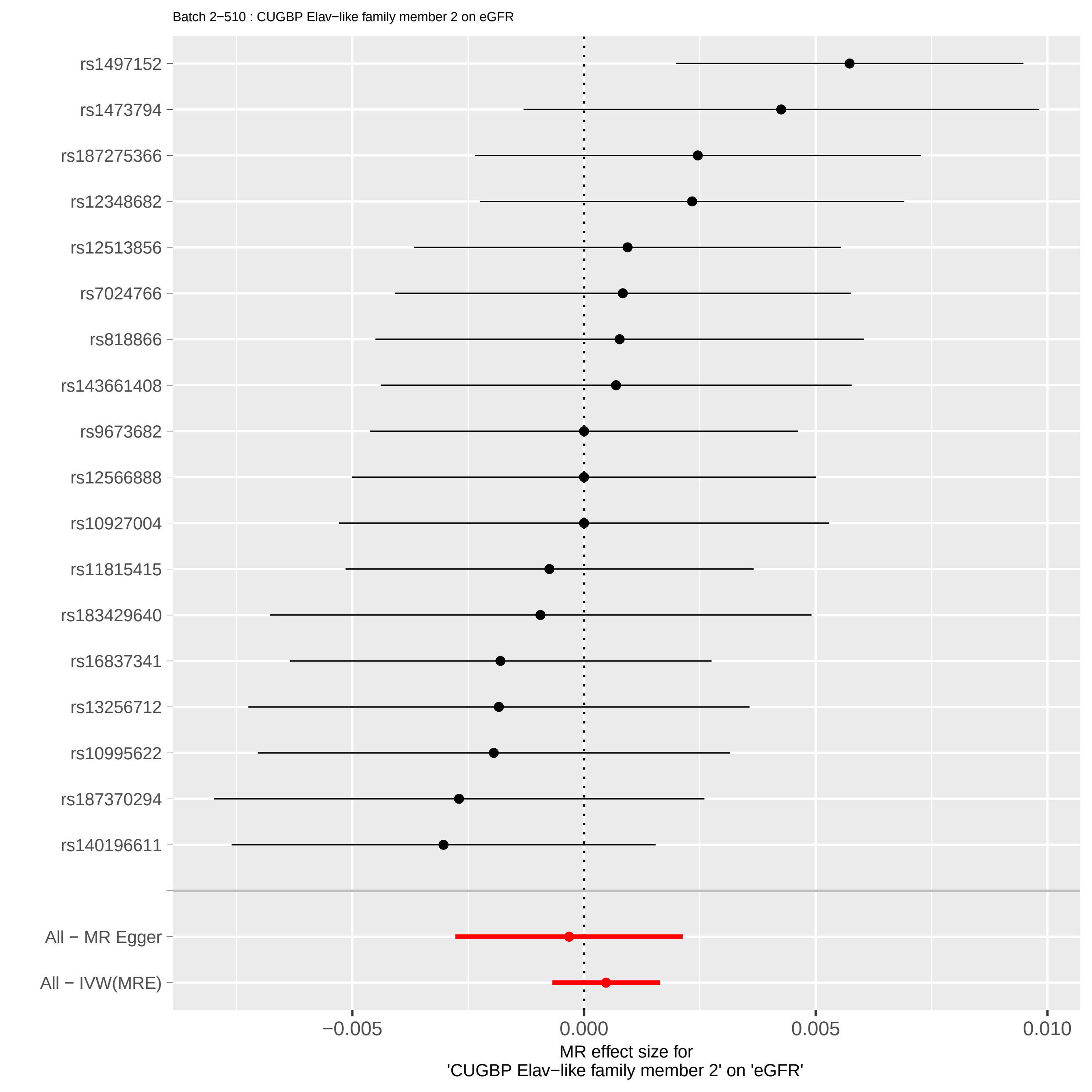
Batch 2−510 : CUGBP Elav−like family member 2 on eGFR
rs1497152
rs1473794
rs187275366
rs12348682
rs12513856
rs7024766
rs818866
rs143661408
rs9673682
rs12566888
rs10927004
rs11815415
rs183429640
rs16837341
rs13256712
rs10995622
rs187370294
rs140196611
All − MR Egger
All − IVW(MRE)
−0.005
0.000
0.005
0.010
MR effect size for
'CUGBP Elav−like family member 2' on 'eGFR'

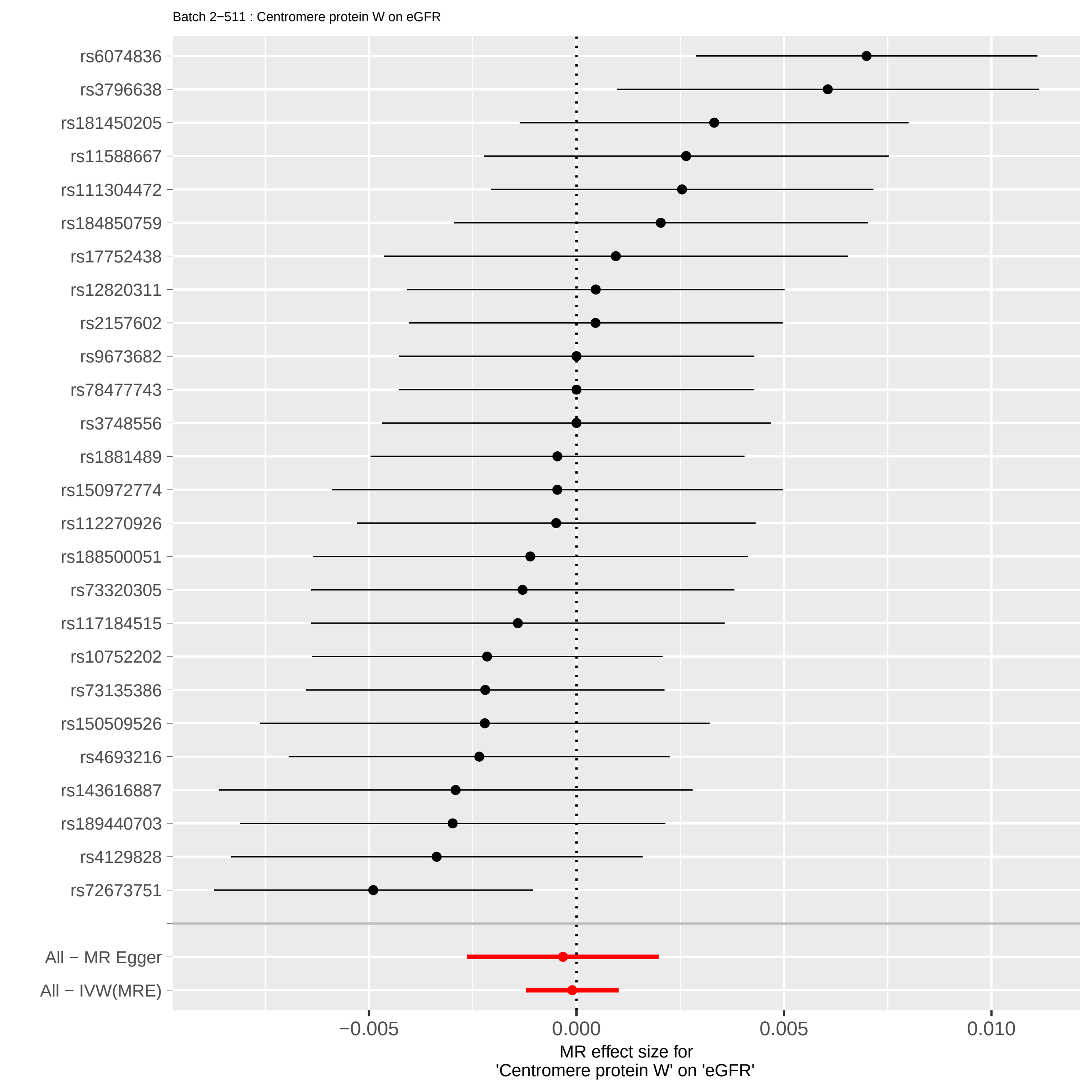
Batch 2−511 : Centromere protein W on eGFR
rs6074836
rs3796638
rs181450205
rs11588667
rs111304472
rs184850759
rs17752438
rs12820311
rs2157602
rs9673682
rs78477743
rs3748556
rs1881489
rs150972774
rs112270926
rs188500051
rs73320305
rs117184515
rs10752202
rs73135386
rs150509526
rs4693216
rs143616887
rs189440703
rs4129828
rs72673751
All − MR Egger
All − IVW(MRE)
−0.005
0.000
0.005
0.010
MR effect size for
'Centromere protein W' on 'eGFR'

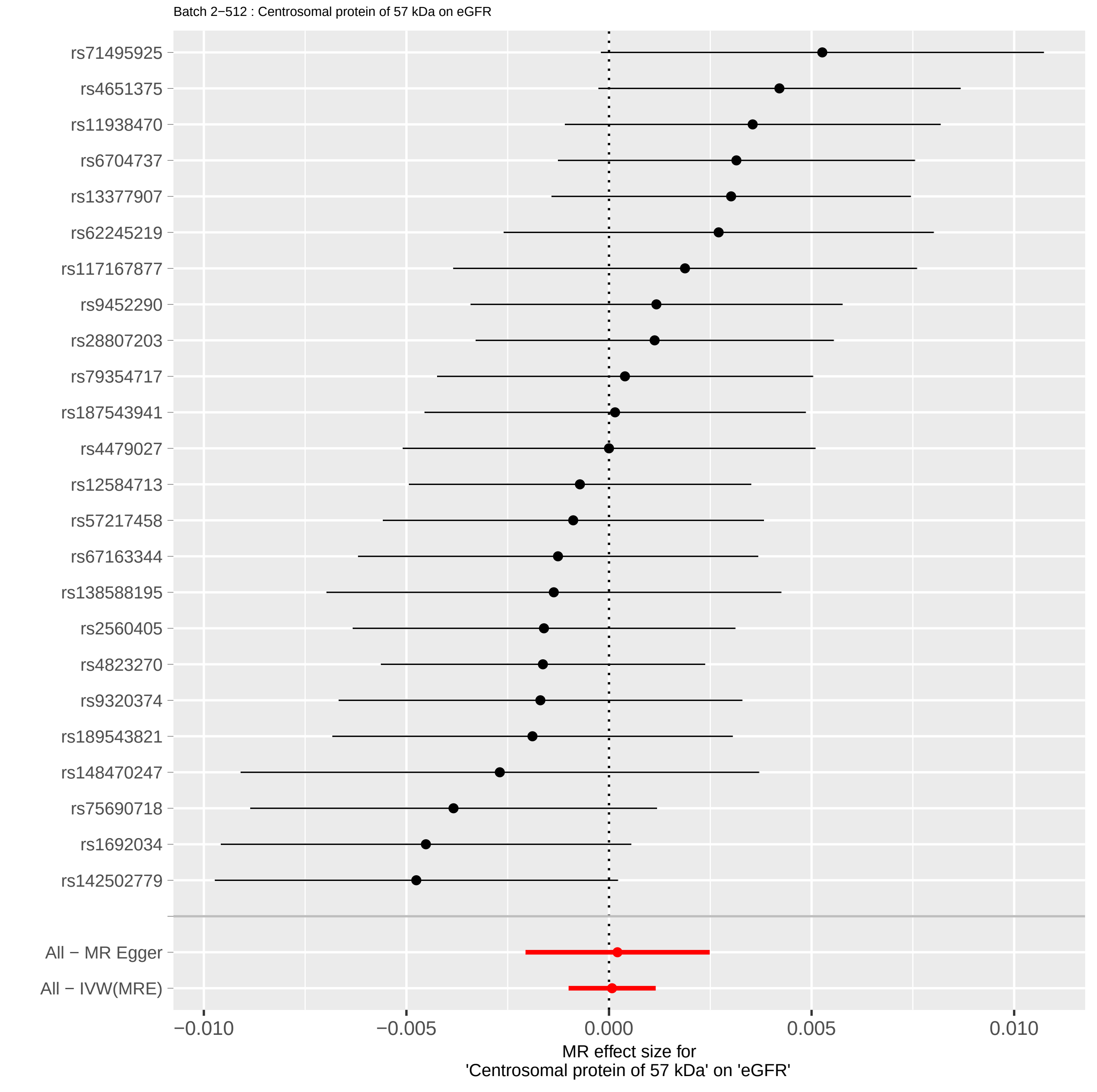

Batch 2−512 : Centrosomal protein of 57 kDa on eGFR
rs71495925
rs4651375
rs11938470
rs6704737
rs13377907
rs62245219
rs117167877
rs9452290
rs28807203
rs79354717
rs187543941
rs4479027
rs12584713
rs57217458
rs67163344
rs138588195
rs2560405
rs4823270
rs9320374
rs189543821
rs148470247
rs75690718
rs1692034
rs142502779
All − MR Egger
All − IVW(MRE)
−0.010
−0.005
0.000
0.005
0.010
MR effect size for
'Centrosomal protein of 57 kDa' on 'eGFR'

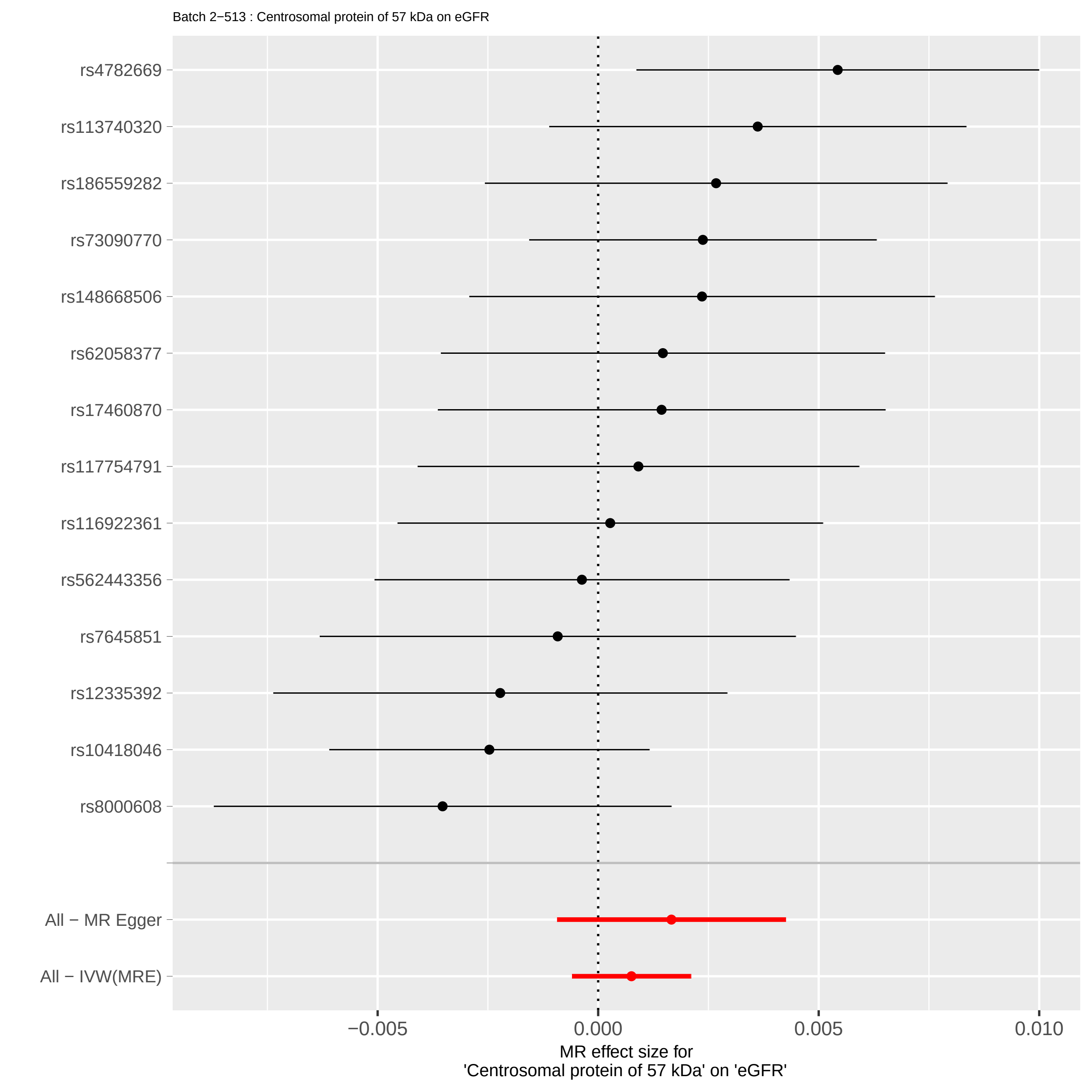

Batch 2−513 : Centrosomal protein of 57 kDa on eGFR
rs4782669
rs113740320
rs186559282
rs73090770
rs148668506
rs62058377
rs17460870
rs117754791
rs116922361
rs562443356
rs7645851
rs12335392
rs10418046
rs8000608
All − MR Egger
All − IVW(MRE)
−0.005
0.000
0.005
0.010
MR effect size for
'Centrosomal protein of 57 kDa' on 'eGFR'

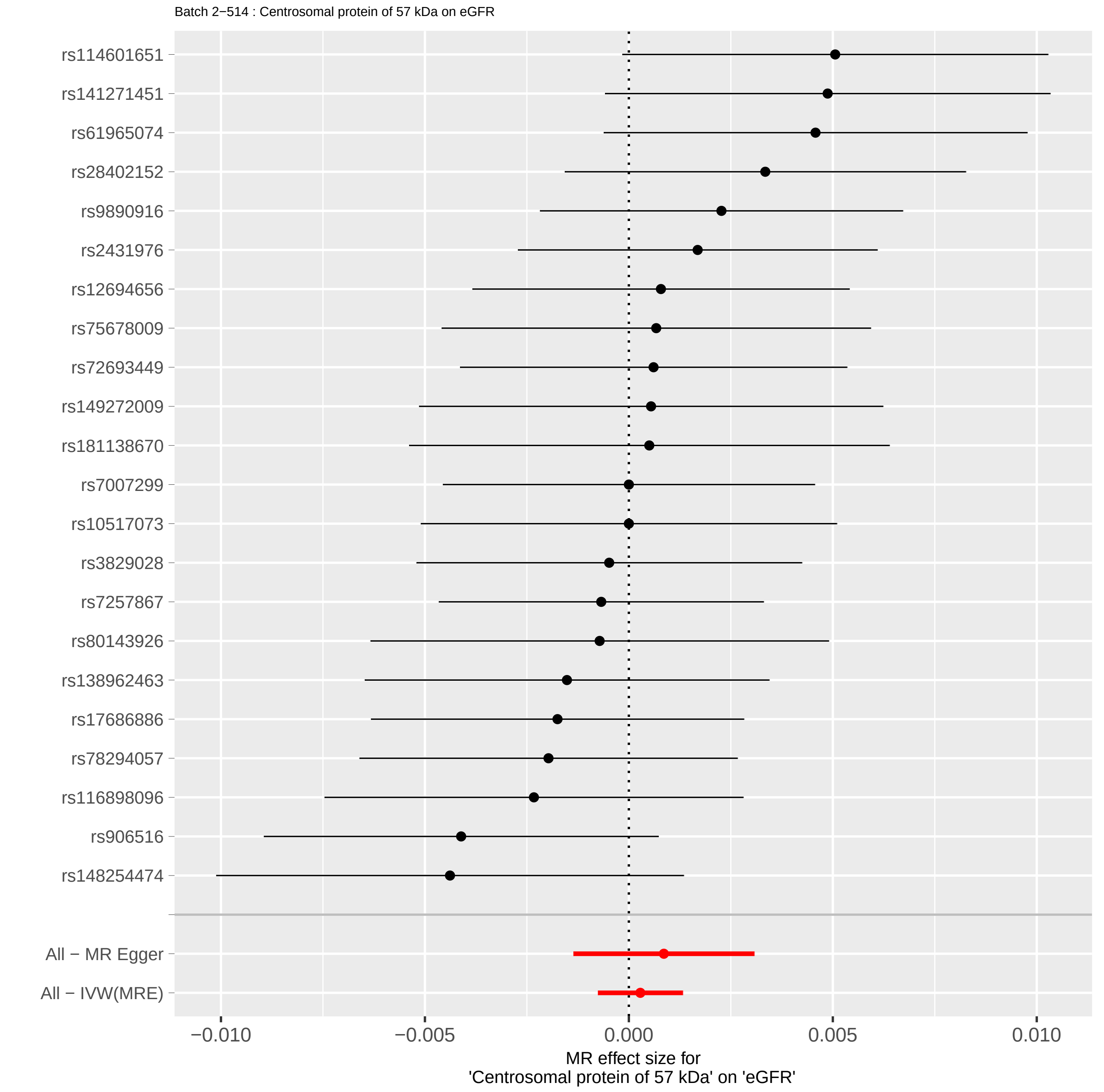

Batch 2−514 : Centrosomal protein of 57 kDa on eGFR
rs114601651
rs141271451
rs61965074
rs28402152
rs9890916
rs2431976
rs12694656
rs75678009
rs72693449
rs149272009
rs181138670
rs7007299
rs10517073
rs3829028
rs7257867
rs80143926
rs138962463
rs17686886
rs78294057
rs116898096
rs906516
rs148254474
All − MR Egger
All − IVW(MRE)
−0.010
−0.005
0.000
0.005
0.010
MR effect size for
'Centrosomal protein of 57 kDa' on 'eGFR'

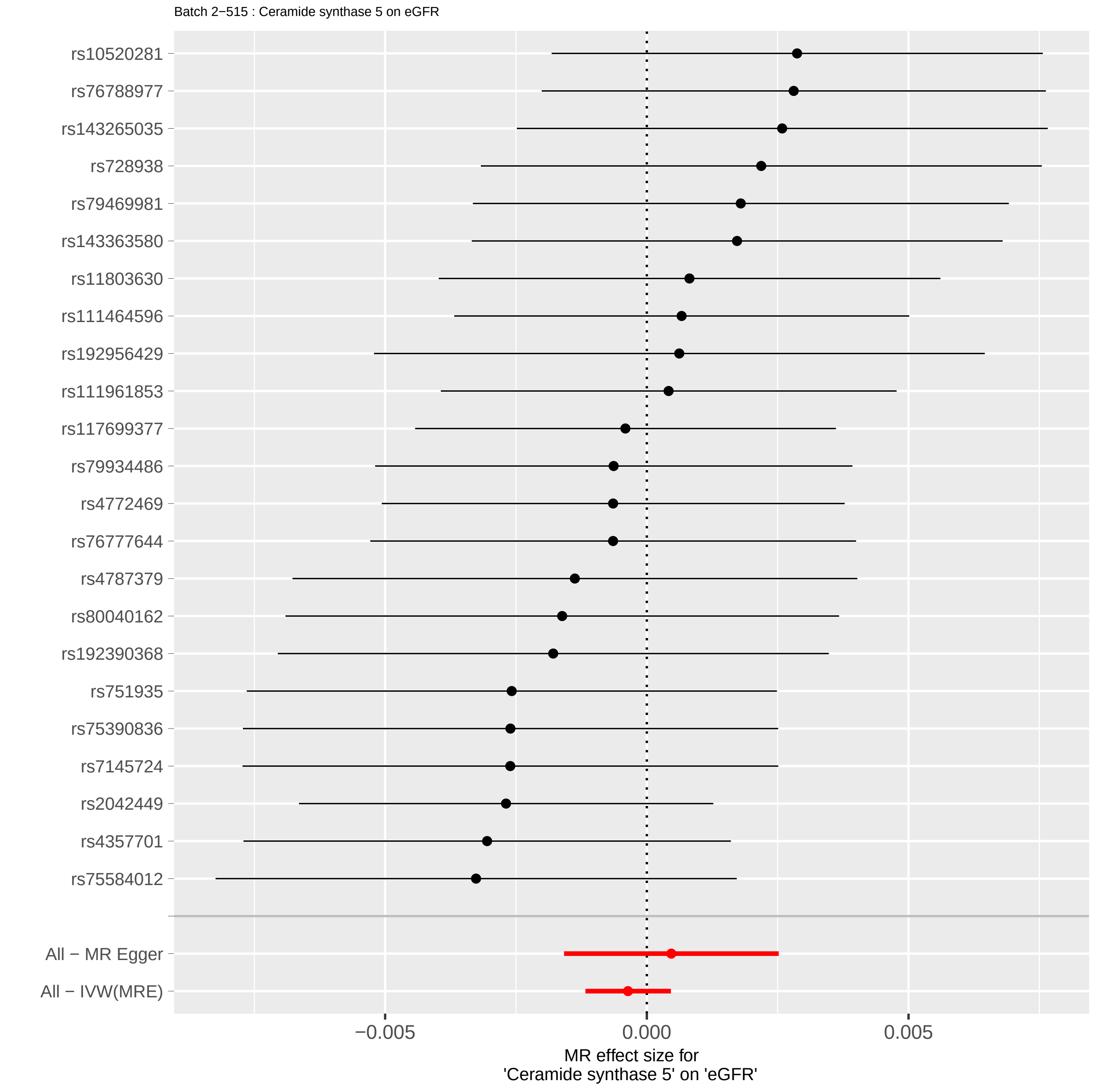

Batch 2−515 : Ceramide synthase 5 on eGFR
rs10520281
rs76788977
rs143265035
rs728938
rs79469981
rs143363580
rs11803630
rs111464596
rs192956429
rs111961853
rs117699377
rs79934486
rs4772469
rs76777644
rs4787379
rs80040162
rs192390368
rs751935
rs75390836
rs7145724
rs2042449
rs4357701
rs75584012
All − MR Egger
All − IVW(MRE)
−0.005
0.000
0.005
MR effect size for
'Ceramide synthase 5' on 'eGFR'

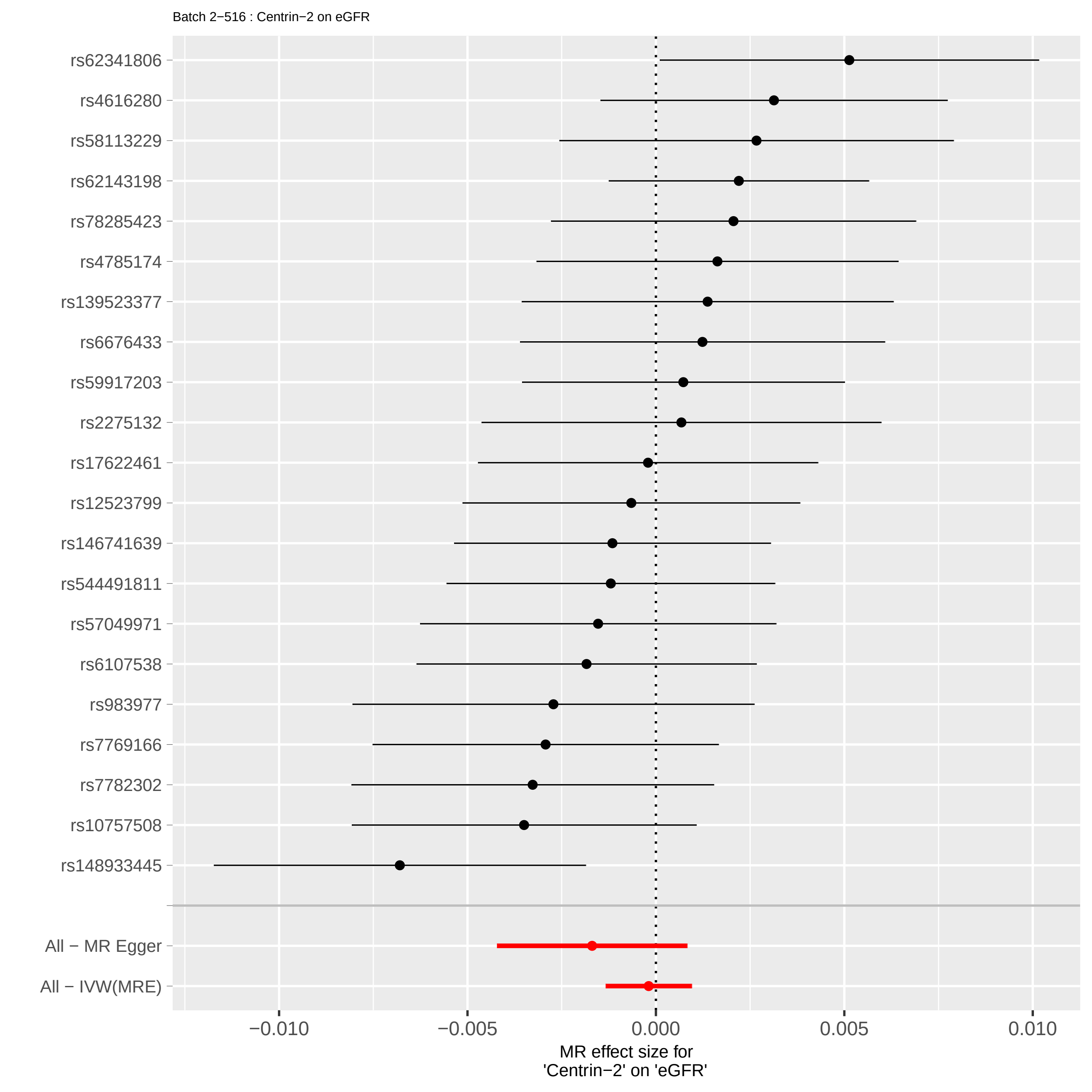
Batch 2−516 : Centrin−2 on eGFR
rs62341806
rs4616280
rs58113229
rs62143198
rs78285423
rs4785174
rs139523377
rs6676433
rs59917203
rs2275132
rs17622461
rs12523799
rs146741639
rs544491811
rs57049971
rs6107538
rs983977
rs7769166
rs7782302
rs10757508
rs148933445
All − MR Egger
All − IVW(MRE)
−0.010
−0.005
0.000
0.005
0.010
MR effect size for
'Centrin−2' on 'eGFR'

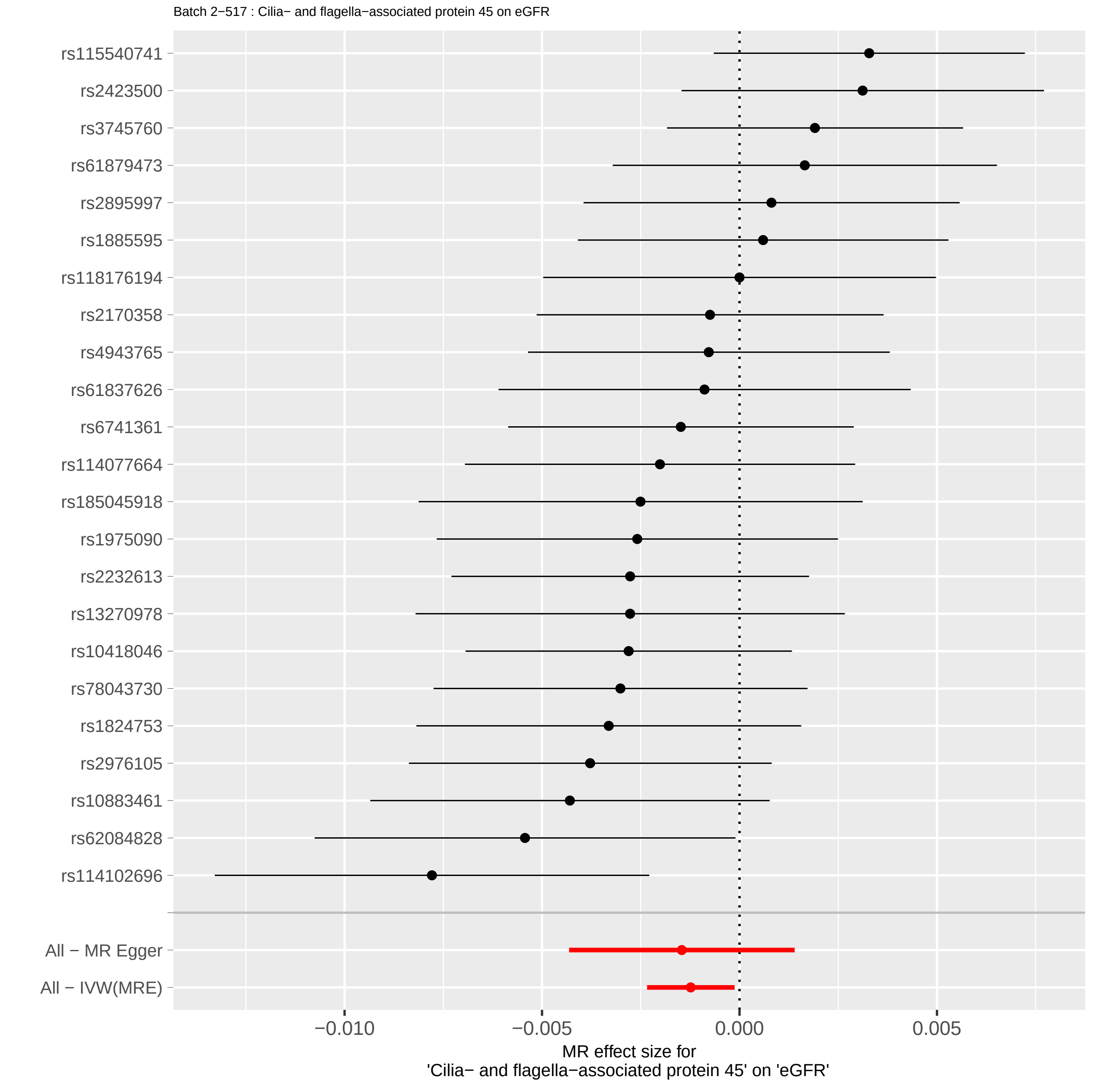
Batch 2−517 : Cilia− and flagella−associated protein 45 on eGFR
rs115540741
rs2423500
rs3745760
rs61879473
rs2895997
rs1885595
rs118176194
rs2170358
rs4943765
rs61837626
rs6741361
rs114077664
rs185045918
rs1975090
rs2232613
rs13270978
rs10418046
rs78043730
rs1824753
rs2976105
rs10883461
rs62084828
rs114102696
All − MR Egger
All − IVW(MRE)
−0.010
−0.005
0.000
0.005
MR effect size for
'Cilia− and flagella−associated protein 45' on 'eGFR'

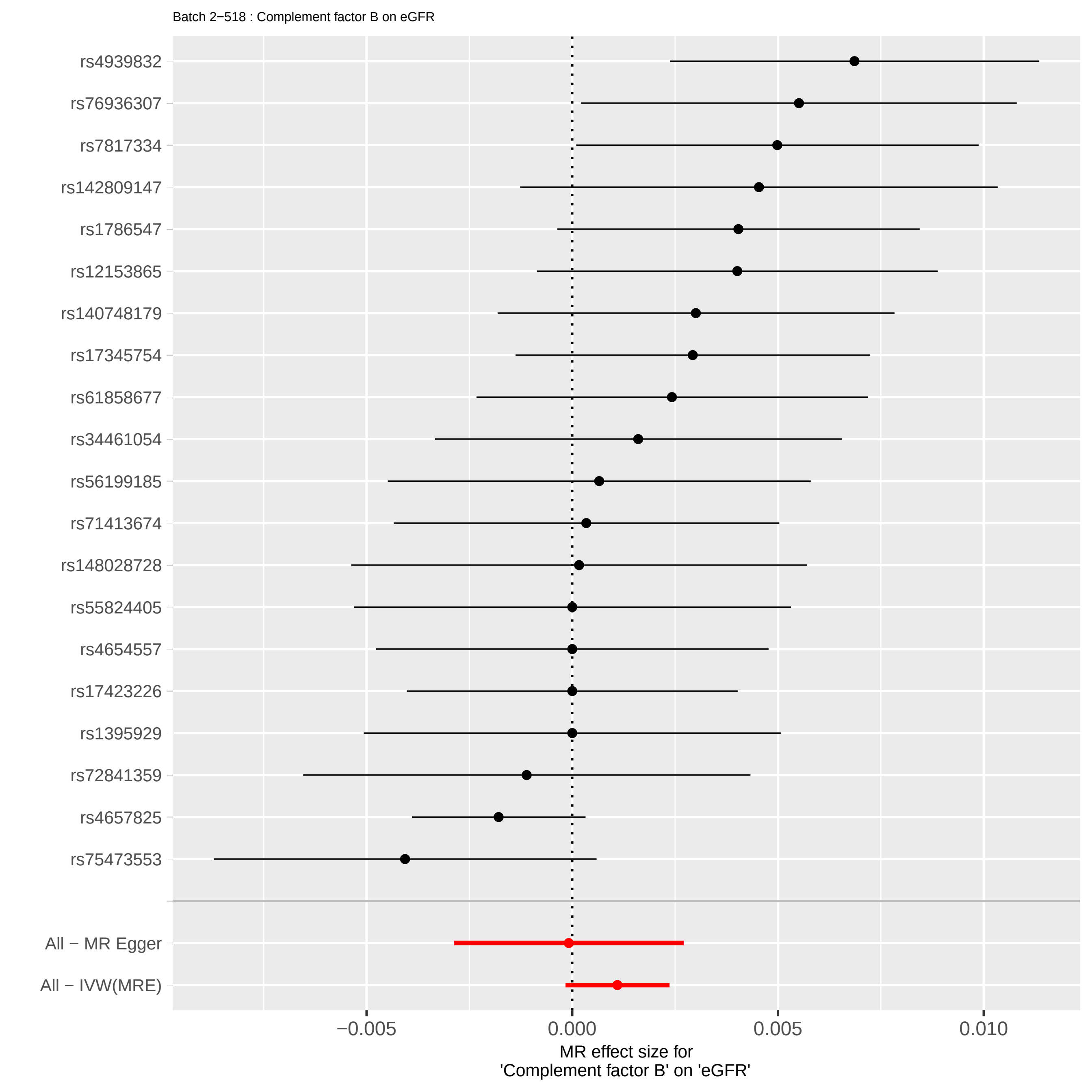

Batch 2−518 : Complement factor B on eGFR
rs4939832
rs76936307
rs7817334
rs142809147
rs1786547
rs12153865
rs140748179
rs17345754
rs61858677
rs34461054
rs56199185
rs71413674
rs148028728
rs55824405
rs4654557
rs17423226
rs1395929
rs72841359
rs4657825
rs75473553
All − MR Egger
All − IVW(MRE)
−0.005
0.000
0.005
0.010
MR effect size for
'Complement factor B' on 'eGFR'

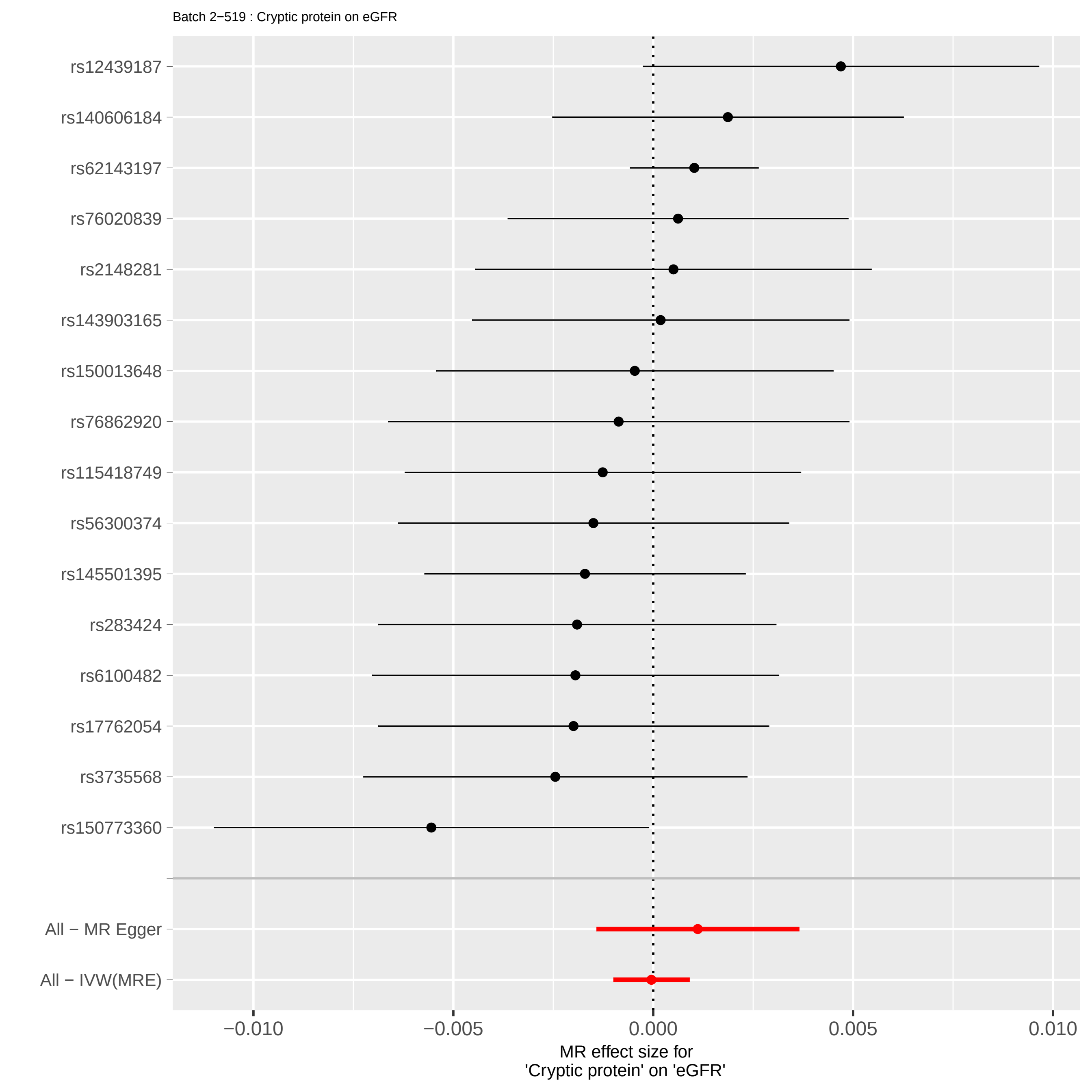
Batch 2−519 : Cryptic protein on eGFR
rs12439187
rs140606184
rs62143197
rs76020839
rs2148281
rs143903165
rs150013648
rs76862920
rs115418749
rs56300374
rs145501395
rs283424
rs6100482
rs17762054
rs3735568
rs150773360
All − MR Egger
All − IVW(MRE)
−0.010
−0.005
0.000
0.005
0.010
MR effect size for
'Cryptic protein' on 'eGFR'

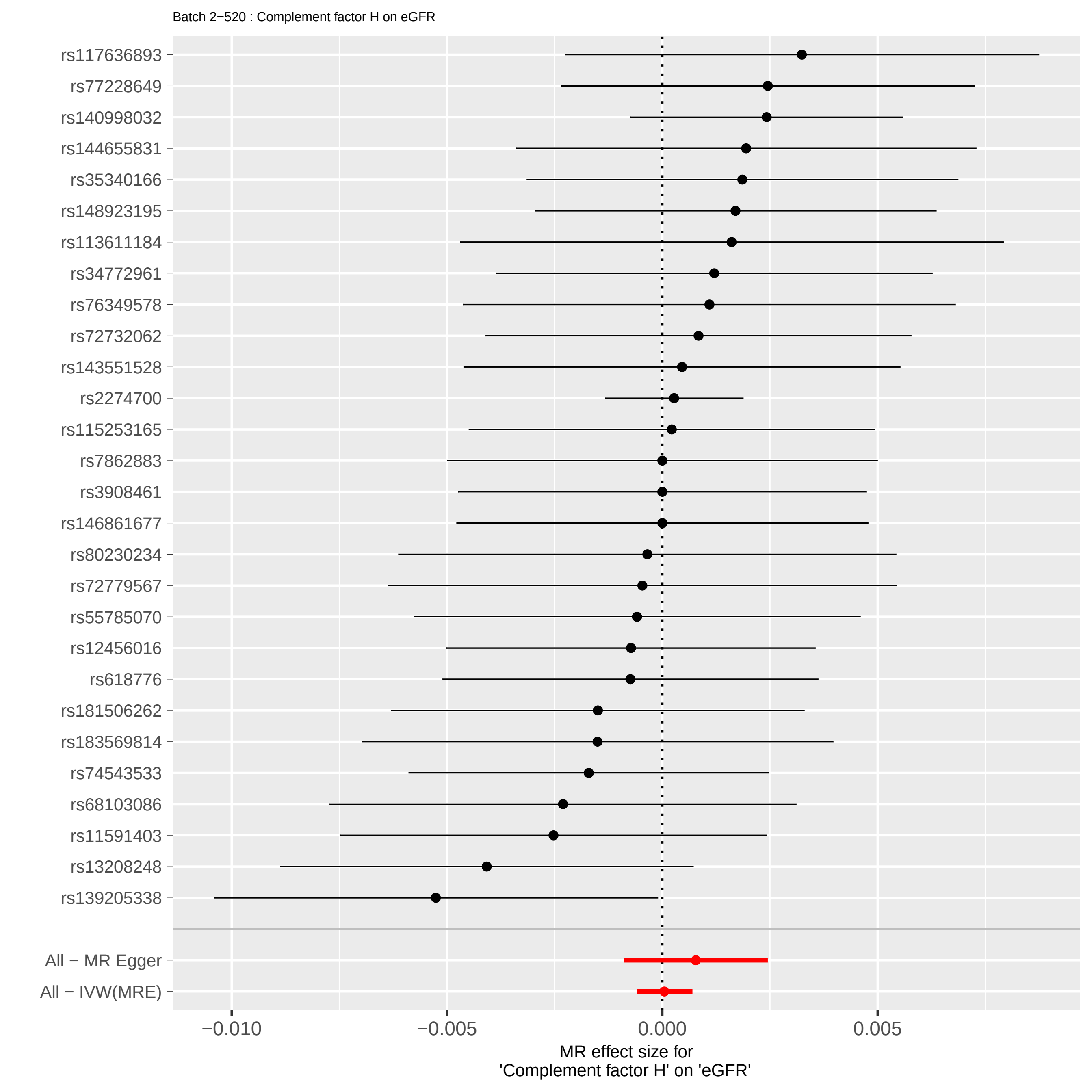

Batch 2−520 : Complement factor H on eGFR
rs117636893
rs77228649
rs140998032
rs144655831
rs35340166
rs148923195
rs113611184
rs34772961
rs76349578
rs72732062
rs143551528
rs2274700
rs115253165
rs7862883
rs3908461
rs146861677
rs80230234
rs72779567
rs55785070
rs12456016
rs618776
rs181506262
rs183569814
rs74543533
rs68103086
rs11591403
rs13208248
rs139205338
All − MR Egger
All − IVW(MRE)
−0.010
−0.005
0.000
0.005
MR effect size for
'Complement factor H' on 'eGFR'

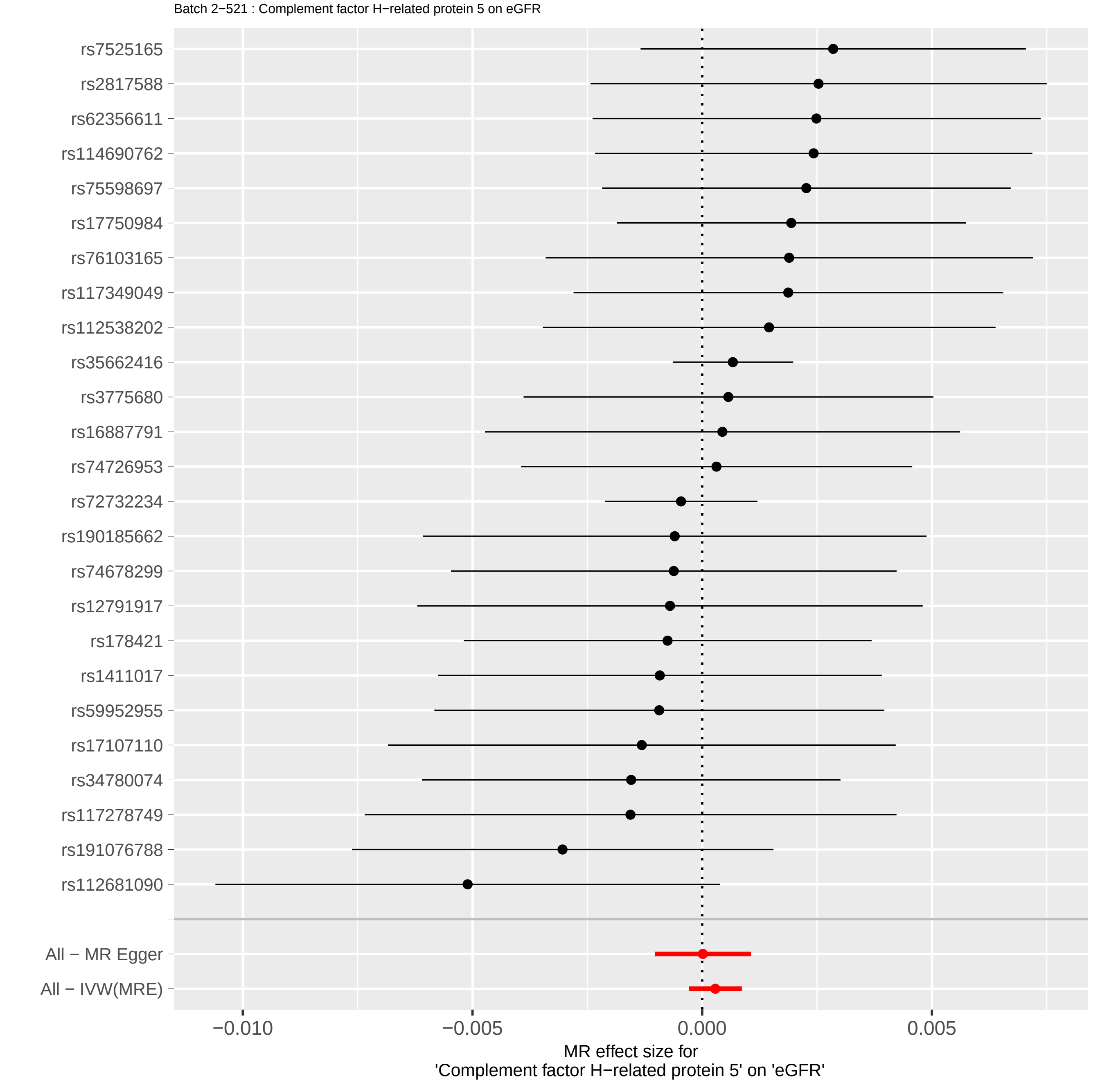

Batch 2−521 : Complement factor H−related protein 5 on eGFR
rs7525165
rs2817588
rs62356611
rs114690762
rs75598697
rs17750984
rs76103165
rs117349049
rs112538202
rs35662416
rs3775680
rs16887791
rs74726953
rs72732234
rs190185662
rs74678299
rs12791917
rs178421
rs1411017
rs59952955
rs17107110
rs34780074
rs117278749
rs191076788
rs112681090
All − MR Egger
All − IVW(MRE)
−0.010
−0.005
0.000
0.005
MR effect size for
'Complement factor H−related protein 5' on 'eGFR'

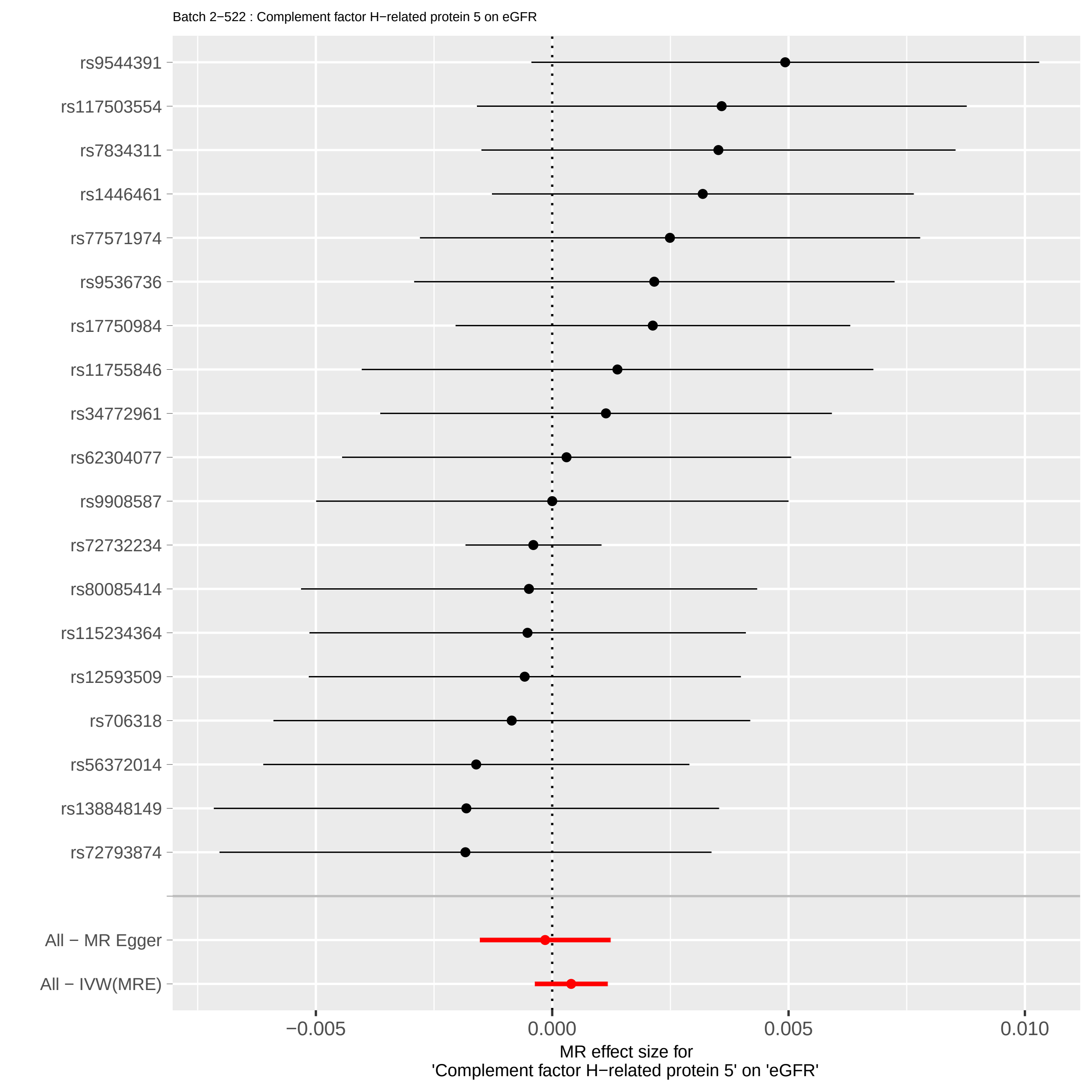

Batch 2−522 : Complement factor H−related protein 5 on eGFR
rs9544391
rs117503554
rs7834311
rs1446461
rs77571974
rs9536736
rs17750984
rs11755846
rs34772961
rs62304077
rs9908587
rs72732234
rs80085414
rs115234364
rs12593509
rs706318
rs56372014
rs138848149
rs72793874
All − MR Egger
All − IVW(MRE)
−0.005
0.000
0.005
0.010
MR effect size for
'Complement factor H−related protein 5' on 'eGFR'

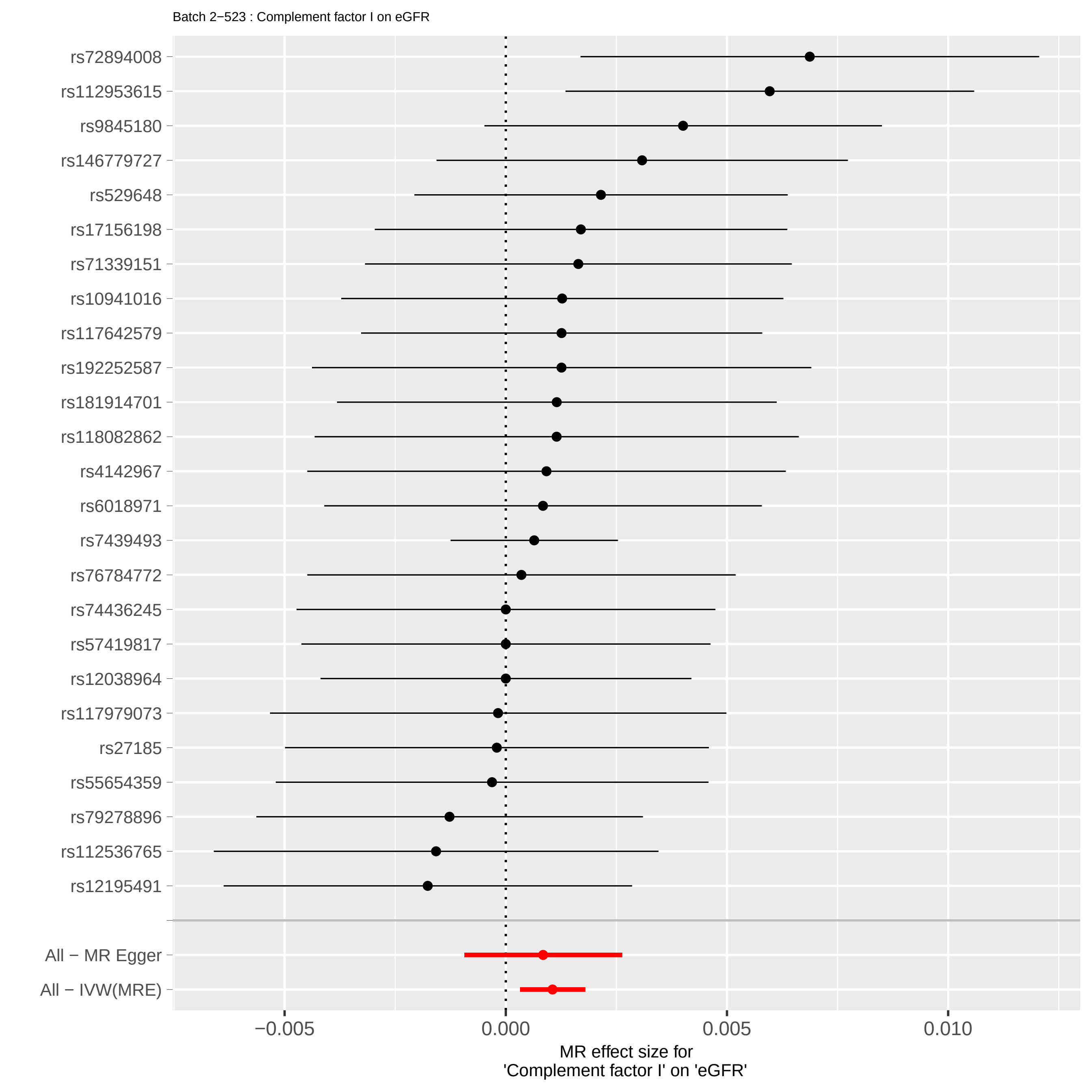

Batch 2−523 : Complement factor I on eGFR
rs72894008
rs112953615
rs9845180
rs146779727
rs529648
rs17156198
rs71339151
rs10941016
rs117642579
rs192252587
rs181914701
rs118082862
rs4142967
rs6018971
rs7439493
rs76784772
rs74436245
rs57419817
rs12038964
rs117979073
rs27185
rs55654359
rs79278896
rs112536765
rs12195491
All − MR Egger
All − IVW(MRE)
−0.005
0.000
0.005
0.010
MR effect size for
'Complement factor I' on 'eGFR'

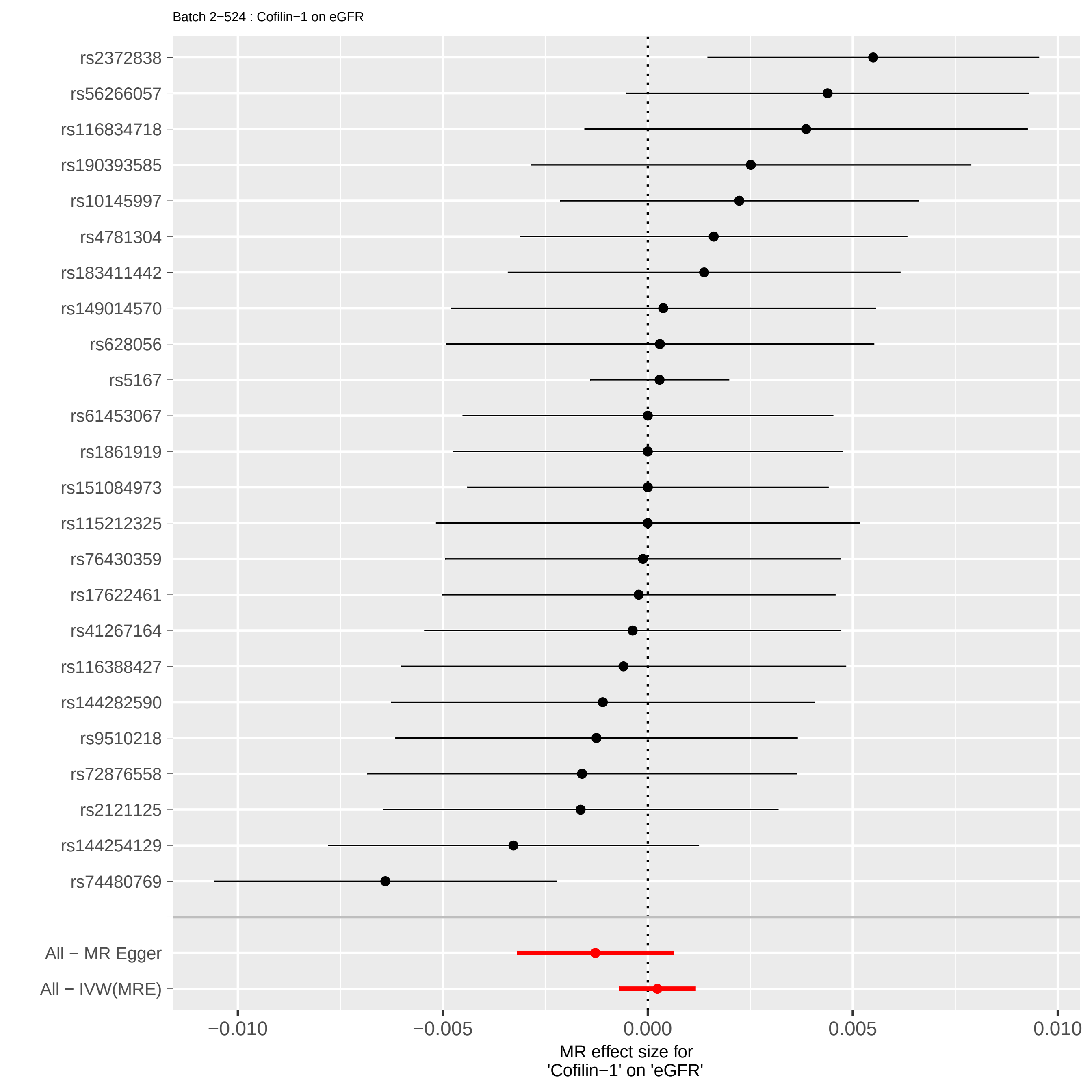

Batch 2−524 : Cofilin−1 on eGFR
rs2372838
rs56266057
rs116834718
rs190393585
rs10145997
rs4781304
rs183411442
rs149014570
rs628056
rs5167
rs61453067
rs1861919
rs151084973
rs115212325
rs76430359
rs17622461
rs41267164
rs116388427
rs144282590
rs9510218
rs72876558
rs2121125
rs144254129
rs74480769
All − MR Egger
All − IVW(MRE)
−0.010
−0.005
0.000
0.005
0.010
MR effect size for
'Cofilin−1' on 'eGFR'

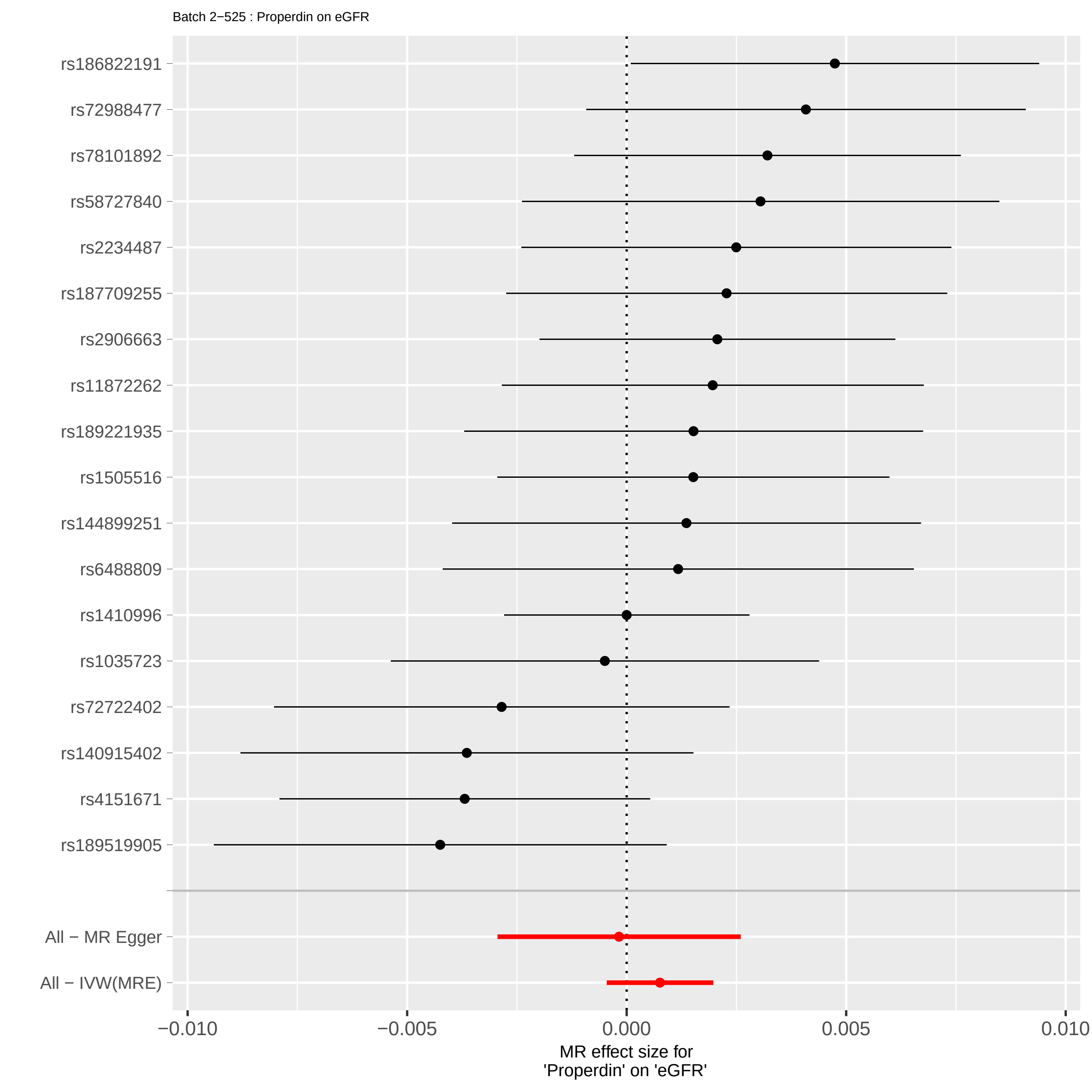
Batch 2−525 : Properdin on eGFR
rs186822191
rs72988477
rs78101892
rs58727840
rs2234487
rs187709255
rs2906663
rs11872262
rs189221935
rs1505516
rs144899251
rs6488809
rs1410996
rs1035723
rs72722402
rs140915402
rs4151671
rs189519905
All − MR Egger
All − IVW(MRE)
−0.010
−0.005
0.000
0.005
0.010
MR effect size for
'Properdin' on 'eGFR'

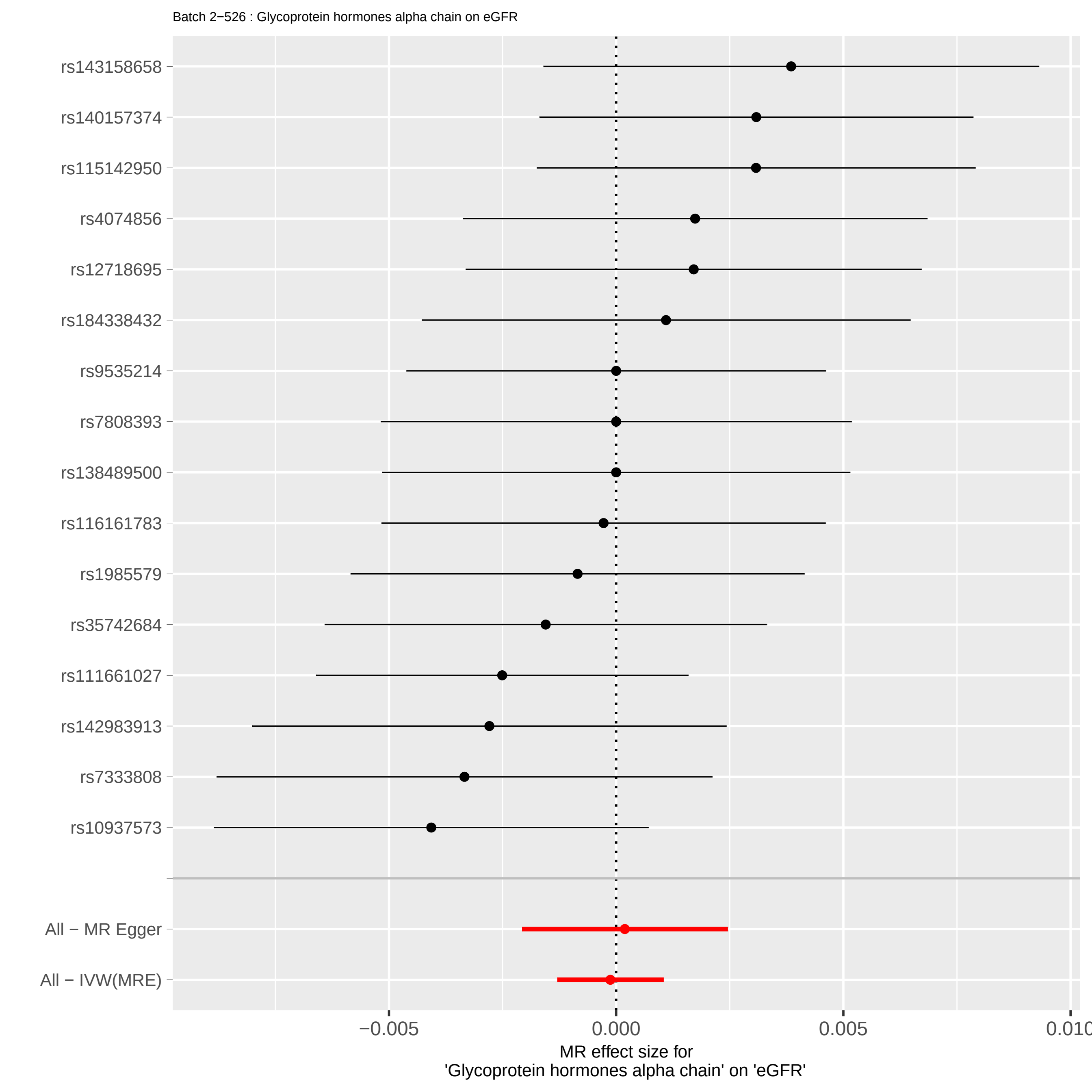

Batch 2−526 : Glycoprotein hormones alpha chain on eGFR
rs143158658
rs140157374
rs115142950
rs4074856
rs12718695
rs184338432
rs9535214
rs7808393
rs138489500
rs116161783
rs1985579
rs35742684
rs111661027
rs142983913
rs7333808
rs10937573
All − MR Egger
All − IVW(MRE)
−0.005
0.000
0.005
0.010
MR effect size for
'Glycoprotein hormones alpha chain' on 'eGFR'

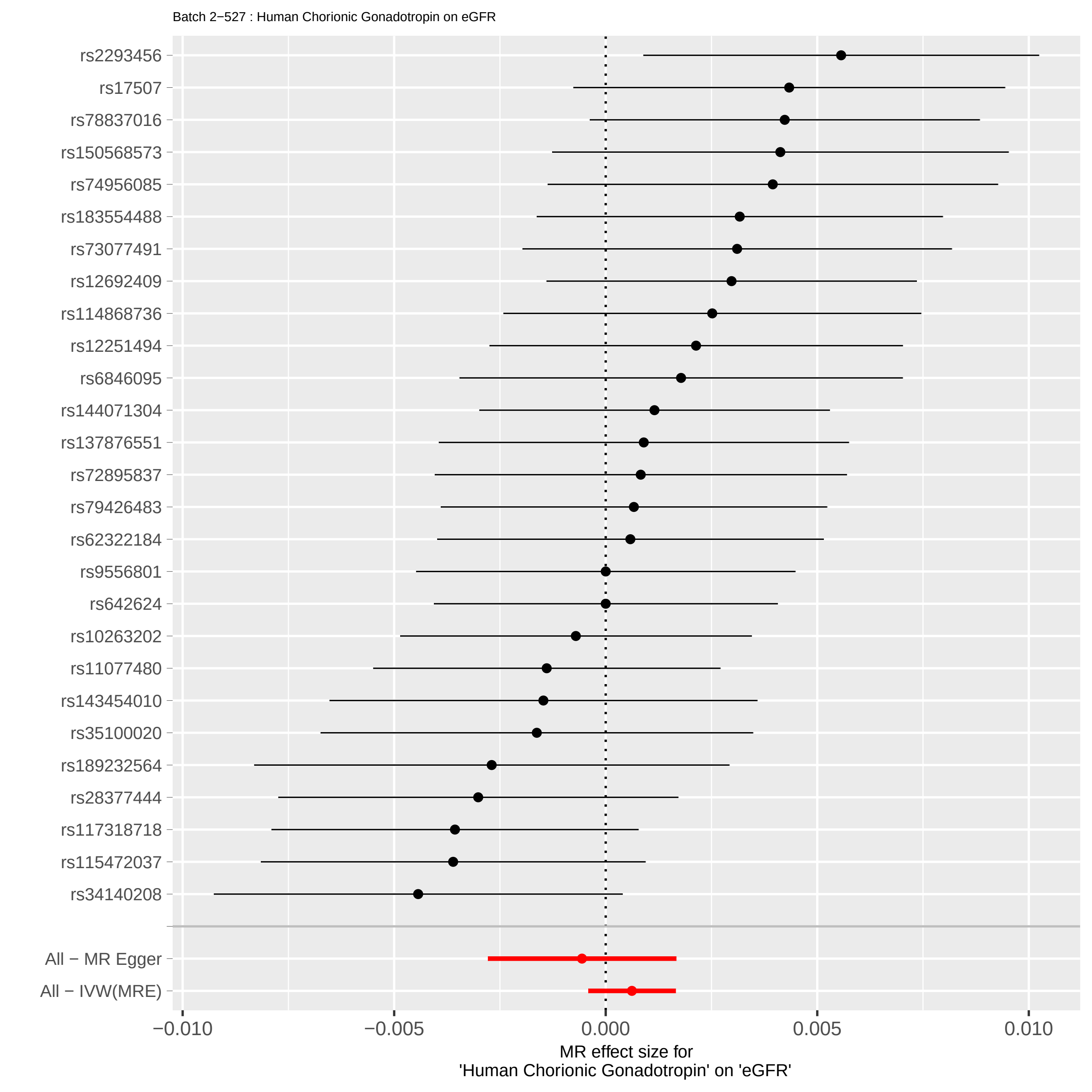
Batch 2−527 : Human Chorionic Gonadotropin on eGFR
rs2293456
rs17507
rs78837016
rs150568573
rs74956085
rs183554488
rs73077491
rs12692409
rs114868736
rs12251494
rs6846095
rs144071304
rs137876551
rs72895837
rs79426483
rs62322184
rs9556801
rs642624
rs10263202
rs11077480
rs143454010
rs35100020
rs189232564
rs28377444
rs117318718
rs115472037
rs34140208
All − MR Egger
All − IVW(MRE)
−0.010
−0.005
0.000
0.005
0.010
MR effect size for
'Human Chorionic Gonadotropin' on 'eGFR'

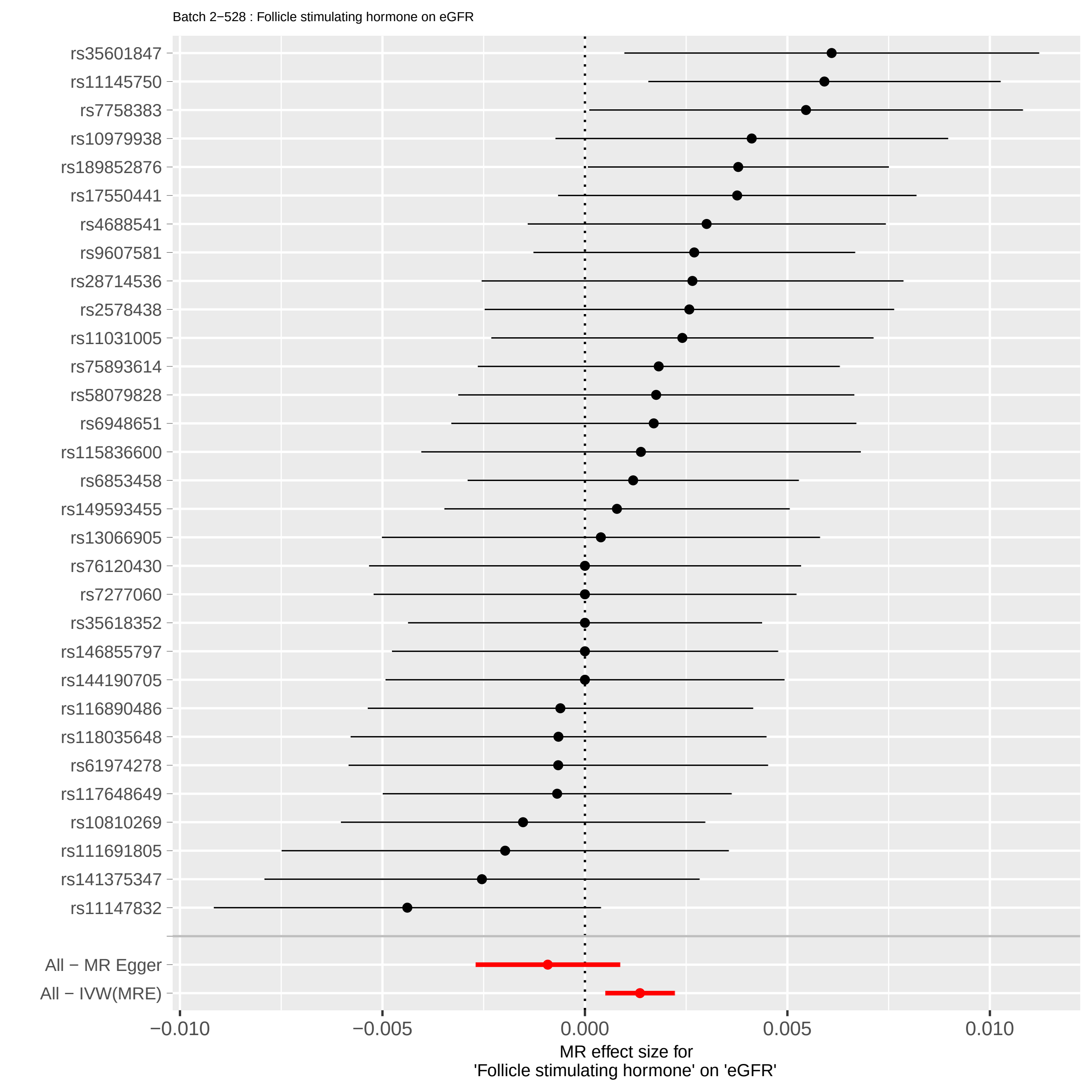

Batch 2−528 : Follicle stimulating hormone on eGFR
rs35601847
rs11145750
rs7758383
rs10979938
rs189852876
rs17550441
rs4688541
rs9607581
rs28714536
rs2578438
rs11031005
rs75893614
rs58079828
rs6948651
rs115836600
rs6853458
rs149593455
rs13066905
rs76120430
rs7277060
rs35618352
rs146855797
rs144190705
rs116890486
rs118035648
rs61974278
rs117648649
rs10810269
rs111691805
rs141375347
rs11147832
All − MR Egger
All − IVW(MRE)
−0.010
−0.005
0.000
0.005
0.010
MR effect size for
'Follicle stimulating hormone' on 'eGFR'

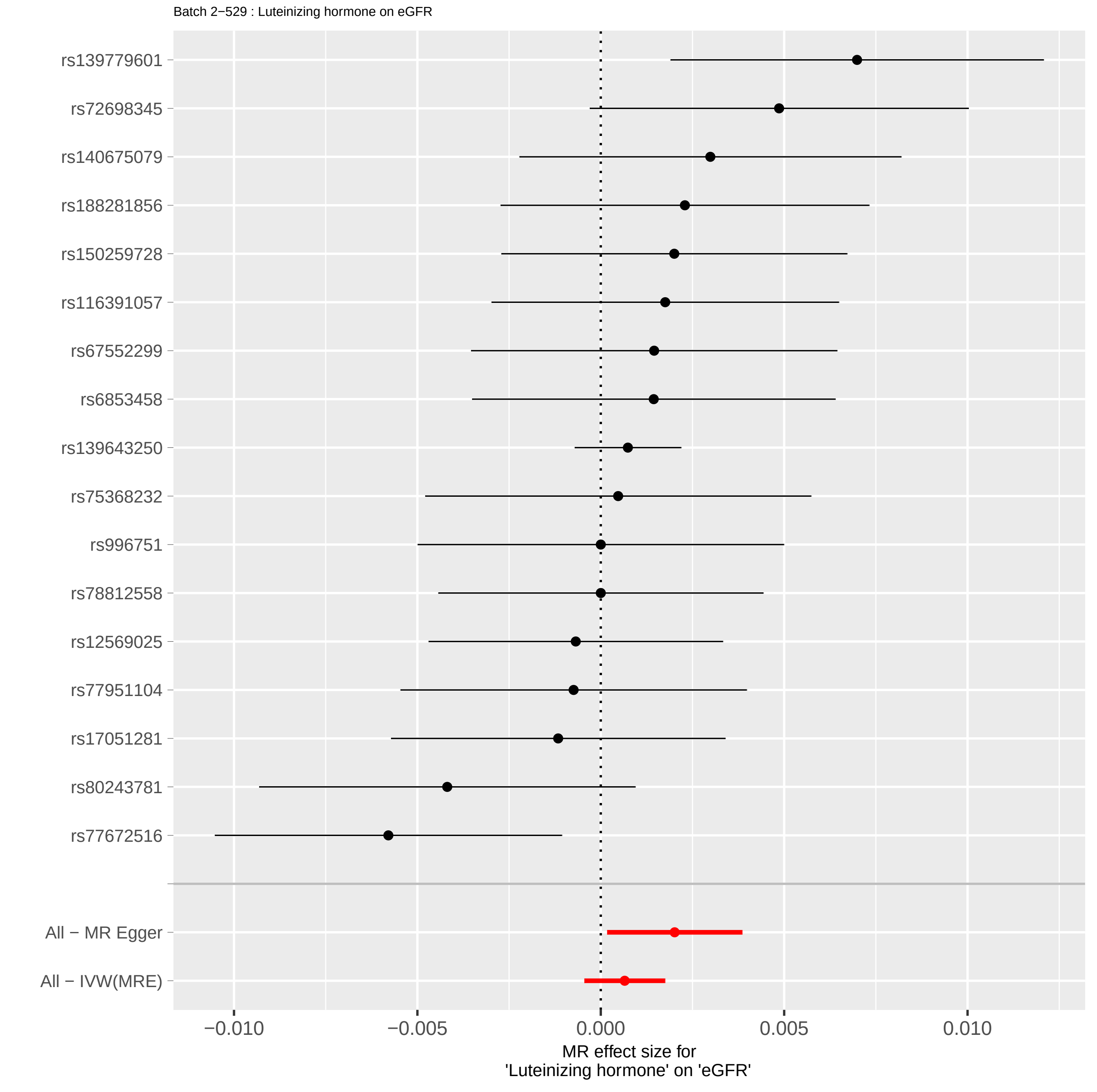
Batch 2−529 : Luteinizing hormone on eGFR
rs139779601
rs72698345
rs140675079
rs188281856
rs150259728
rs116391057
rs67552299
rs6853458
rs139643250
rs75368232
rs996751
rs78812558
rs12569025
rs77951104
rs17051281
rs80243781
rs77672516
All − MR Egger
All − IVW(MRE)
−0.010
−0.005
0.000
0.005
0.010
MR effect size for
'Luteinizing hormone' on 'eGFR'

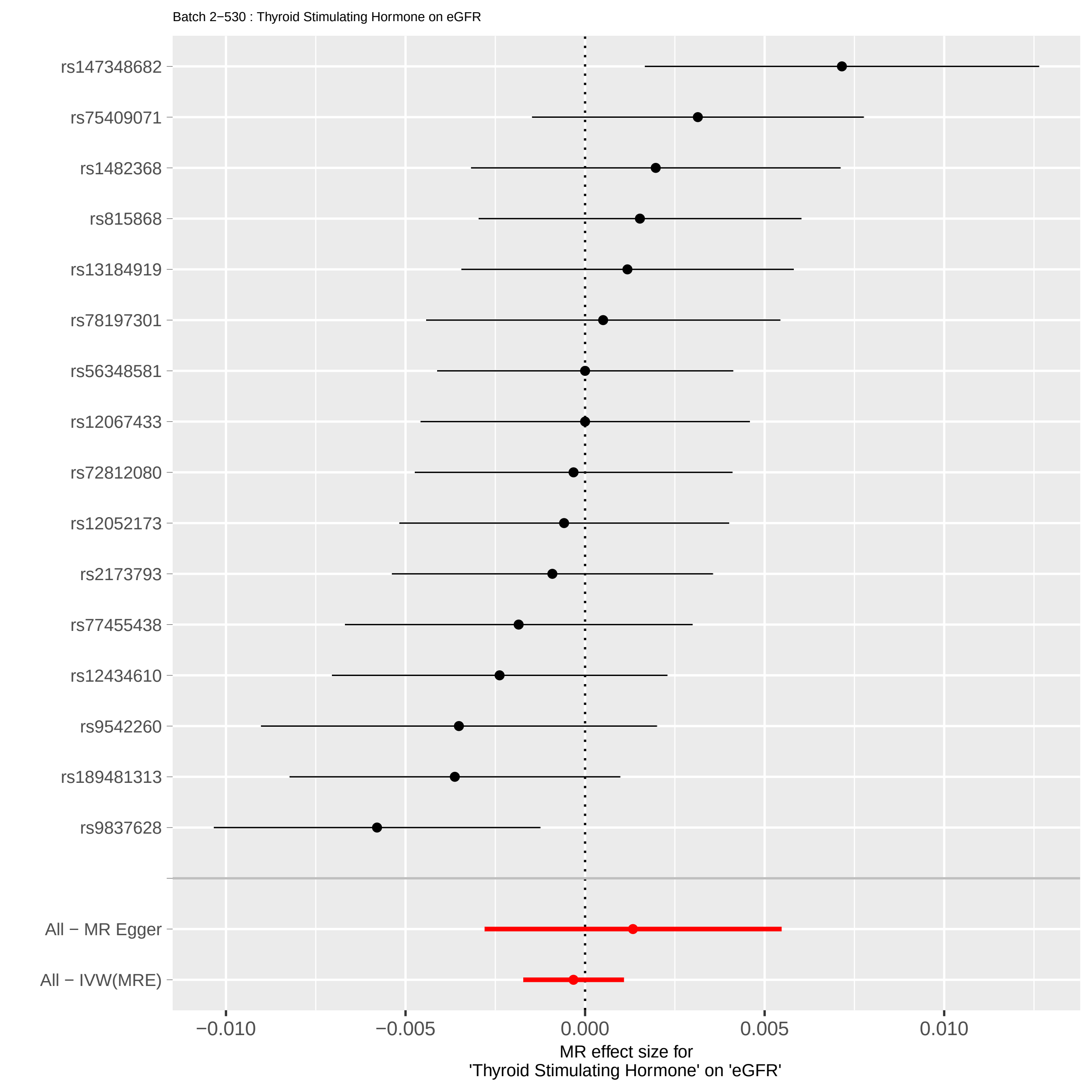

Batch 2−530 : Thyroid Stimulating Hormone on eGFR
rs147348682
rs75409071
rs1482368
rs815868
rs13184919
rs78197301
rs56348581
rs12067433
rs72812080
rs12052173
rs2173793
rs77455438
rs12434610
rs9542260
rs189481313
rs9837628
All − MR Egger
All − IVW(MRE)
−0.010
−0.005
0.000
0.005
0.010
MR effect size for
'Thyroid Stimulating Hormone' on 'eGFR'

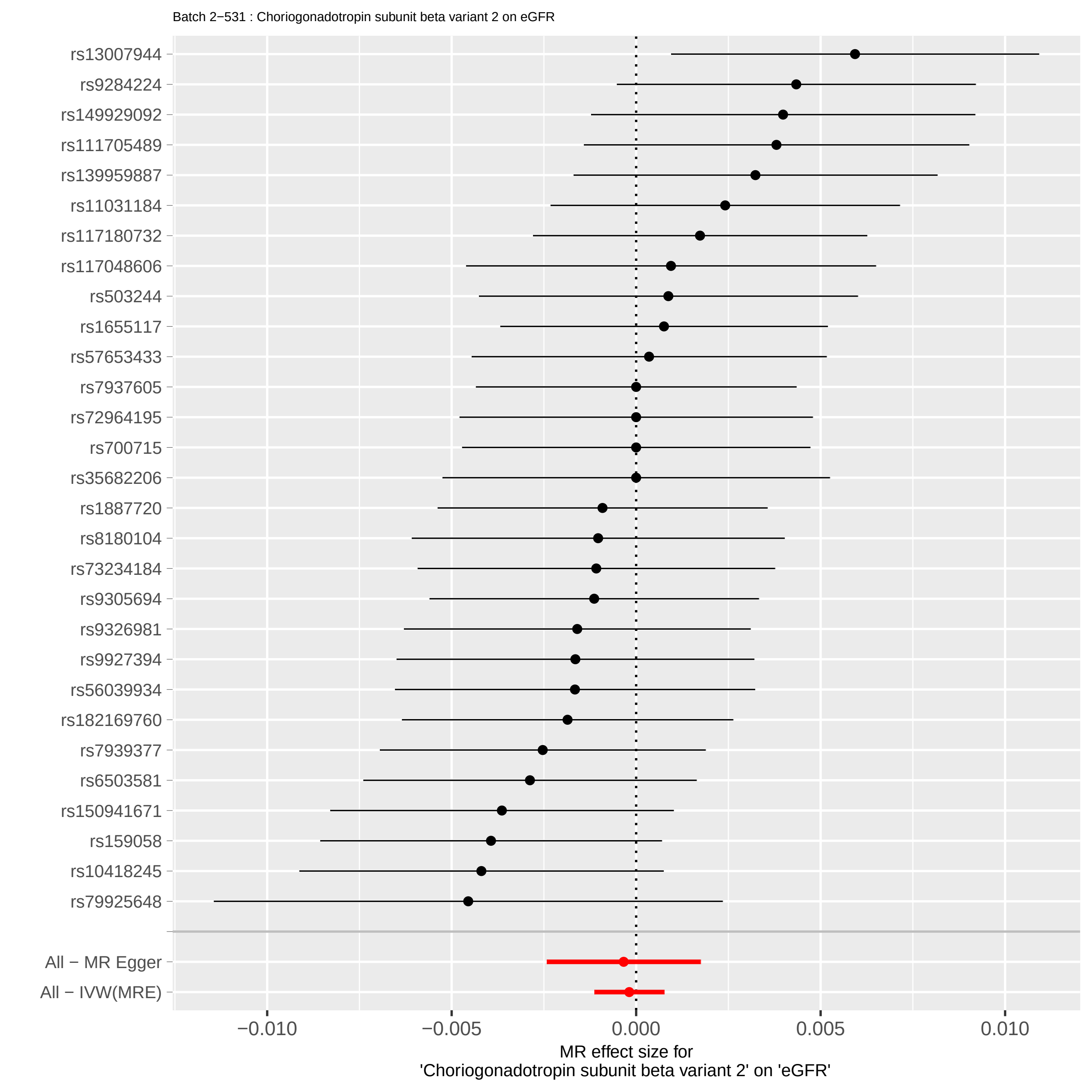

Batch 2−531 : Choriogonadotropin subunit beta variant 2 on eGFR
rs13007944
rs9284224
rs149929092
rs111705489
rs139959887
rs11031184
rs117180732
rs117048606
rs503244
rs1655117
rs57653433
rs7937605
rs72964195
rs700715
rs35682206
rs1887720
rs8180104
rs73234184
rs9305694
rs9326981
rs9927394
rs56039934
rs182169760
rs7939377
rs6503581
rs150941671
rs159058
rs10418245
rs79925648
All − MR Egger
All − IVW(MRE)
−0.010
−0.005
0.000
0.005
0.010
MR effect size for
'Choriogonadotropin subunit beta variant 2' on 'eGFR'

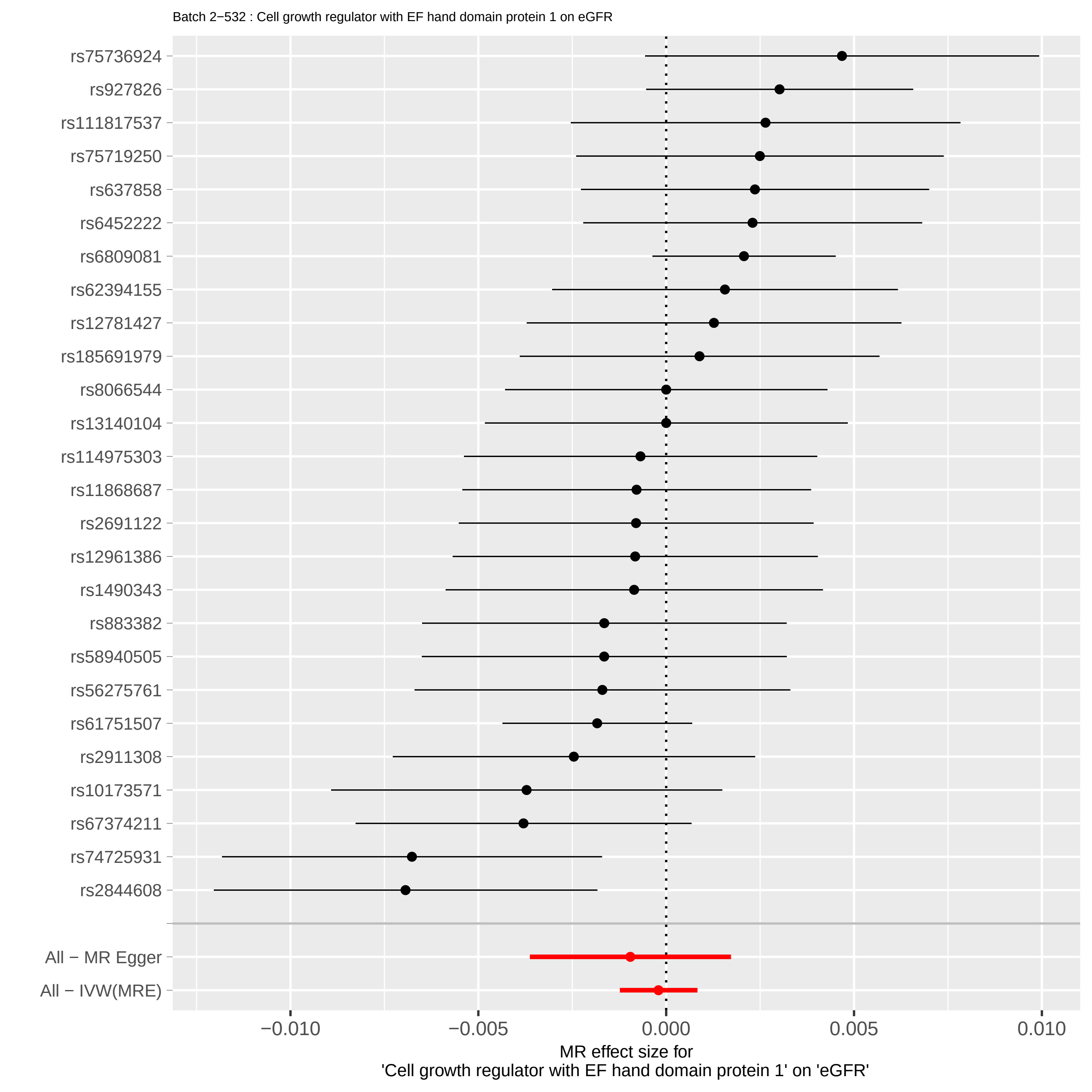

Batch 2−532 : Cell growth regulator with EF hand domain protein 1 on eGFR
rs75736924
rs927826
rs111817537
rs75719250
rs637858
rs6452222
rs6809081
rs62394155
rs12781427
rs185691979
rs8066544
rs13140104
rs114975303
rs11868687
rs2691122
rs12961386
rs1490343
rs883382
rs58940505
rs56275761
rs61751507
rs2911308
rs10173571
rs67374211
rs74725931
rs2844608
All − MR Egger
All − IVW(MRE)
−0.010
−0.005
0.000
0.005
0.010
MR effect size for
'Cell growth regulator with EF hand domain protein 1' on 'eGFR'

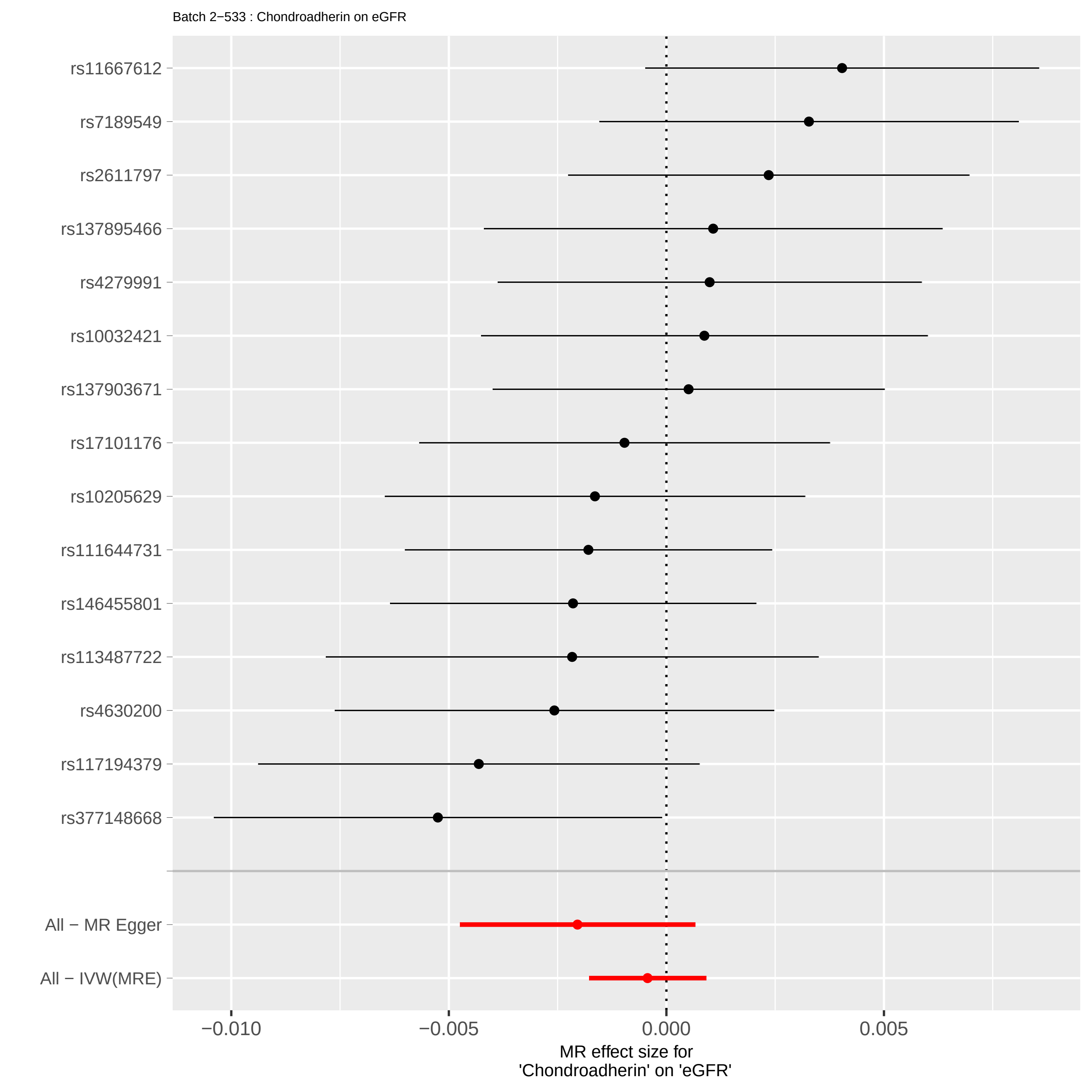
Batch 2−533 : Chondroadherin on eGFR
rs11667612
rs7189549
rs2611797
rs137895466
rs4279991
rs10032421
rs137903671
rs17101176
rs10205629
rs111644731
rs146455801
rs113487722
rs4630200
rs117194379
rs377148668
All − MR Egger
All − IVW(MRE)
−0.010
−0.005
0.000
0.005
MR effect size for
'Chondroadherin' on 'eGFR'

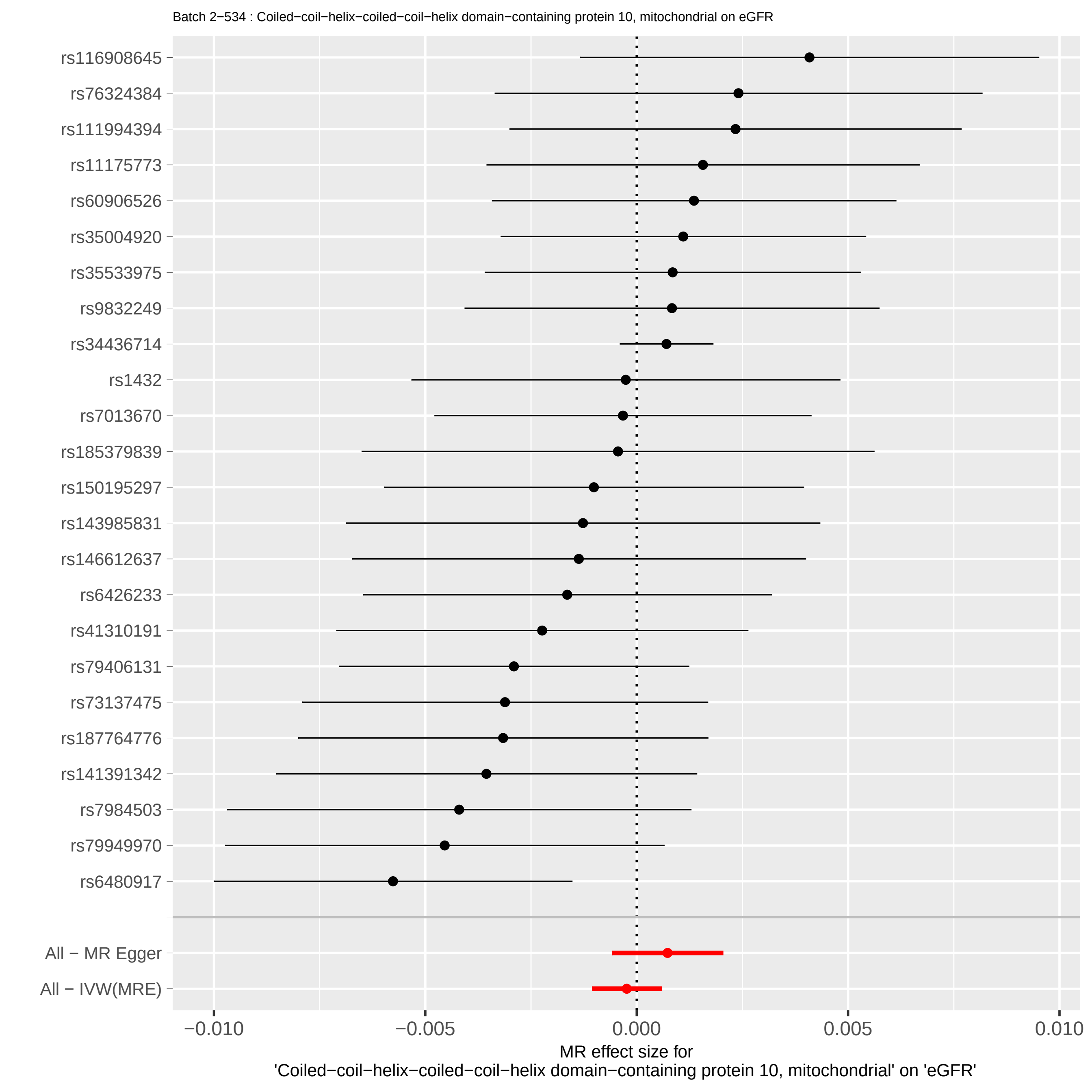
Batch 2−534 : Coiled−coil−helix−coiled−coil−helix domain−containing protein 10, mitochondrial on eGFR
rs116908645
rs76324384
rs111994394
rs11175773
rs60906526
rs35004920
rs35533975
rs9832249
rs34436714
rs1432
rs7013670
rs185379839
rs150195297
rs143985831
rs146612637
rs6426233
rs41310191
rs79406131
rs73137475
rs187764776
rs141391342
rs7984503
rs79949970
rs6480917
All − MR Egger
All − IVW(MRE)
−0.010
−0.005
0.000
0.005
0.010
MR effect size for
'Coiled−coil−helix−coiled−coil−helix domain−containing protein 10, mitochondrial' on 'eGFR'

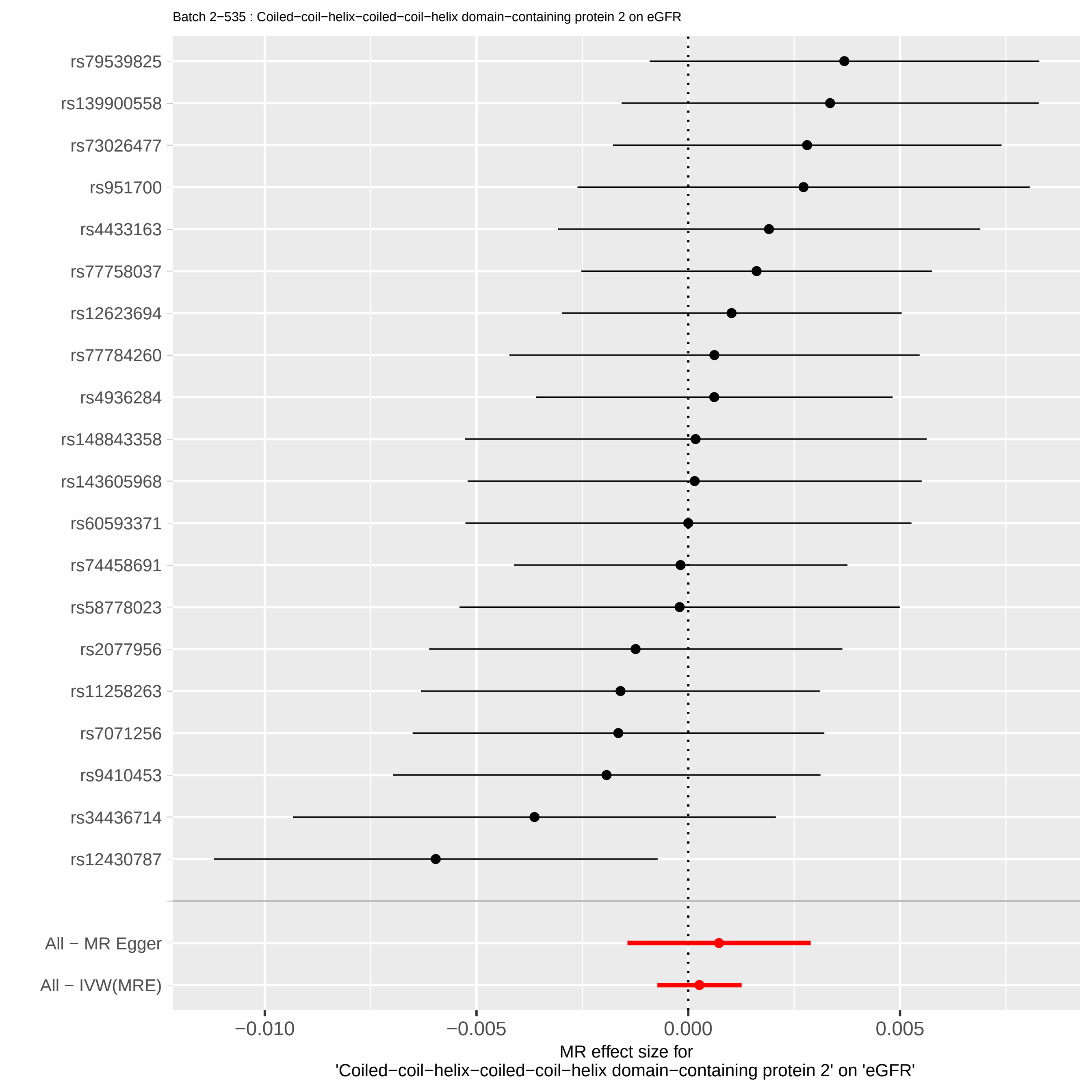

Batch 2−535 : Coiled−coil−helix−coiled−coil−helix domain−containing protein 2 on eGFR
rs79539825
rs139900558
rs73026477
rs951700
rs4433163
rs77758037
rs12623694
rs77784260
rs4936284
rs148843358
rs143605968
rs60593371
rs74458691
rs58778023
rs2077956
rs11258263
rs7071256
rs9410453
rs34436714
rs12430787
All − MR Egger
All − IVW(MRE)
−0.010
−0.005
0.000
0.005
MR effect size for
'Coiled−coil−helix−coiled−coil−helix domain−containing protein 2' on 'eGFR'

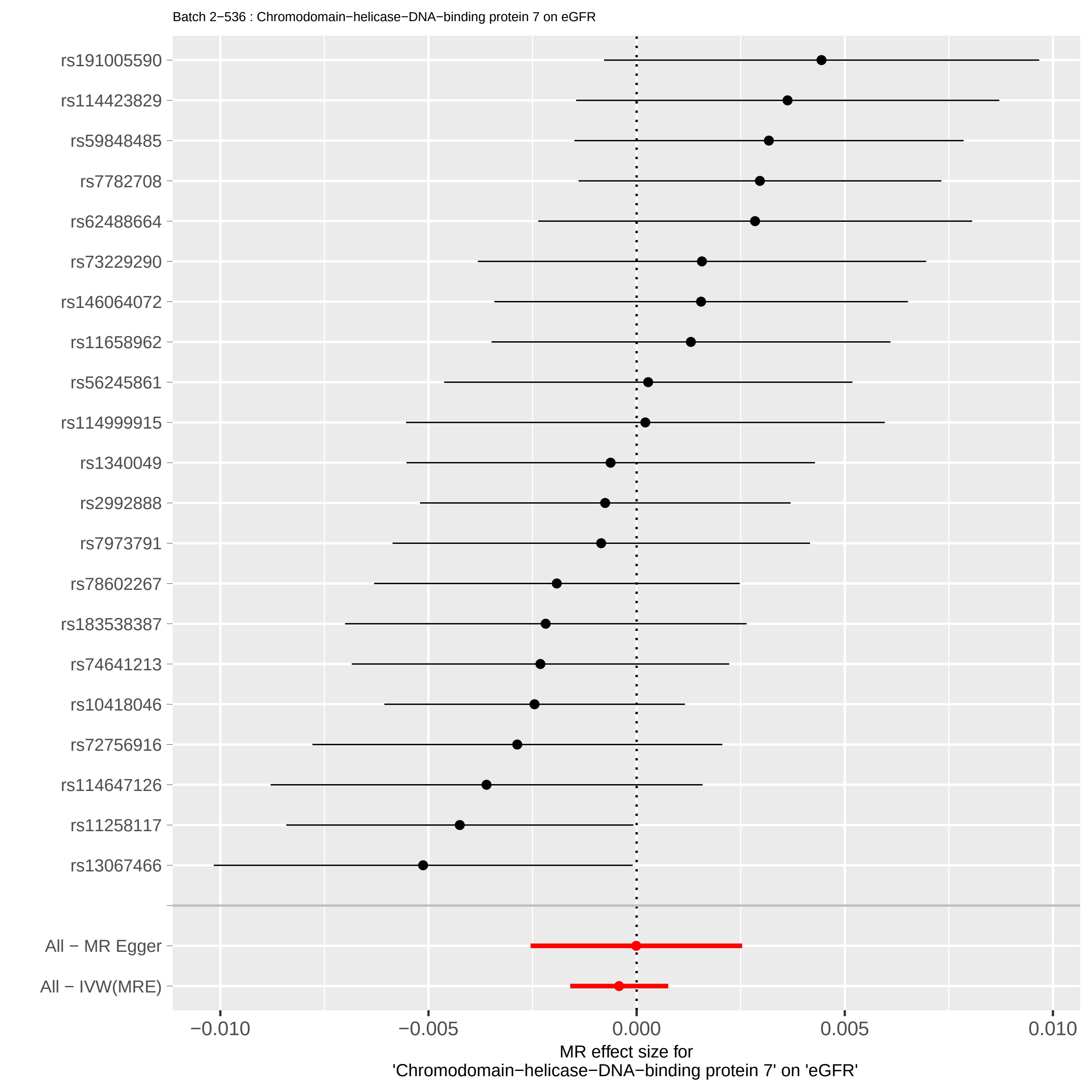
Batch 2−536 : Chromodomain−helicase−DNA−binding protein 7 on eGFR
rs191005590
rs114423829
rs59848485
rs7782708
rs62488664
rs73229290
rs146064072
rs11658962
rs56245861
rs114999915
rs1340049
rs2992888
rs7973791
rs78602267
rs183538387
rs74641213
rs10418046
rs72756916
rs114647126
rs11258117
rs13067466
All − MR Egger
All − IVW(MRE)
−0.010
−0.005
0.000
0.005
0.010
MR effect size for
'Chromodomain−helicase−DNA−binding protein 7' on 'eGFR'

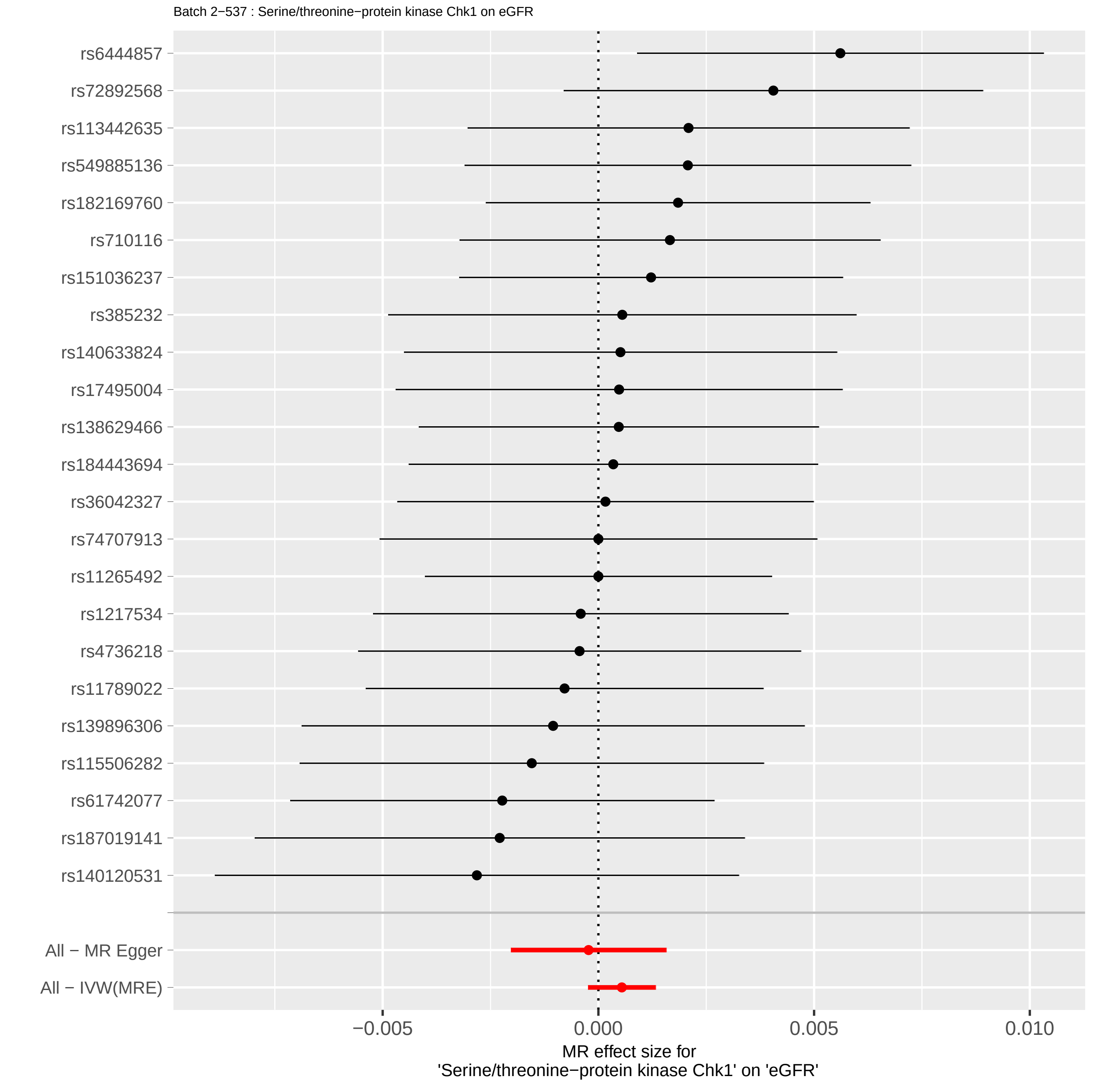

Batch 2−537 : Serine/threonine−protein kinase Chk1 on eGFR
rs6444857
rs72892568
rs113442635
rs549885136
rs182169760
rs710116
rs151036237
rs385232
rs140633824
rs17495004
rs138629466
rs184443694
rs36042327
rs74707913
rs11265492
rs1217534
rs4736218
rs11789022
rs139896306
rs115506282
rs61742077
rs187019141
rs140120531
All − MR Egger
All − IVW(MRE)
−0.005
0.000
0.005
0.010
MR effect size for
'Serine/threonine−protein kinase Chk1' on 'eGFR'

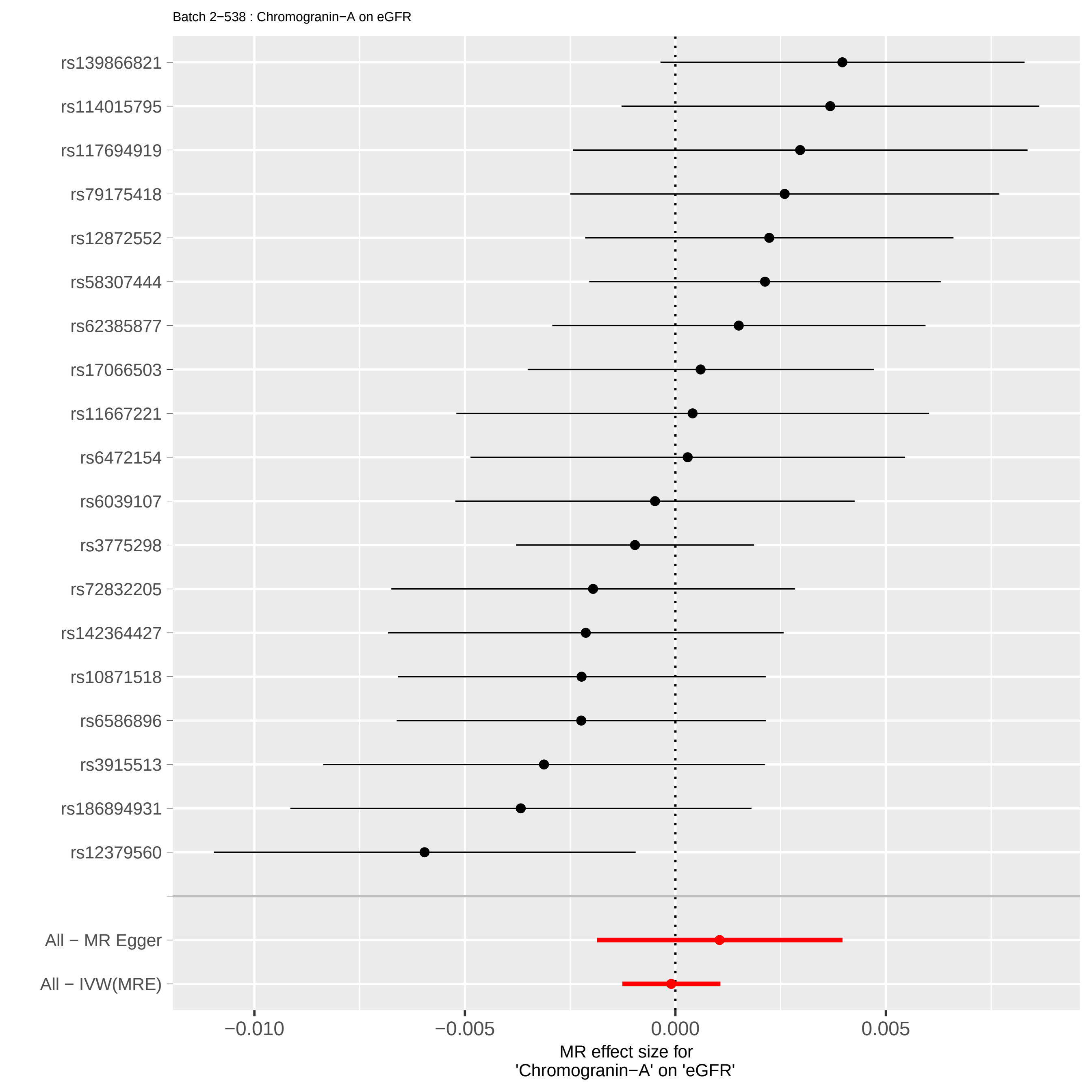
Batch 2−538 : Chromogranin−A on eGFR
rs139866821
rs114015795
rs117694919
rs79175418
rs12872552
rs58307444
rs62385877
rs17066503
rs11667221
rs6472154
rs6039107
rs3775298
rs72832205
rs142364427
rs10871518
rs6586896
rs3915513
rs186894931
rs12379560
All − MR Egger
All − IVW(MRE)
−0.010
−0.005
0.000
0.005
MR effect size for
'Chromogranin−A' on 'eGFR'

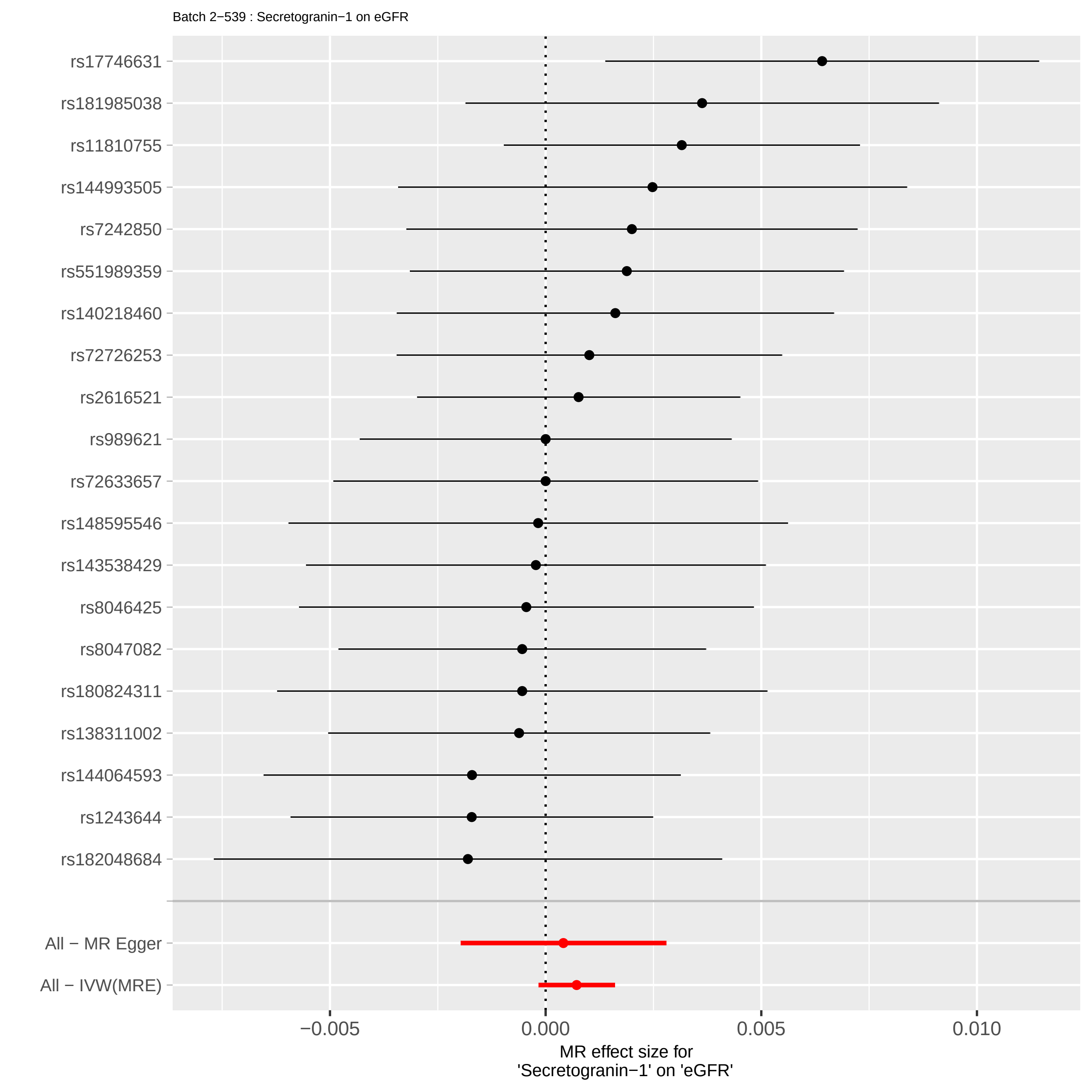
Batch 2−539 : Secretogranin−1 on eGFR
rs17746631
rs181985038
rs11810755
rs144993505
rs7242850
rs551989359
rs140218460
rs72726253
rs2616521
rs989621
rs72633657
rs148595546
rs143538429
rs8046425
rs8047082
rs180824311
rs138311002
rs144064593
rs1243644
rs182048684
All − MR Egger
All − IVW(MRE)
−0.005
0.000
0.005
0.010
MR effect size for
'Secretogranin−1' on 'eGFR'

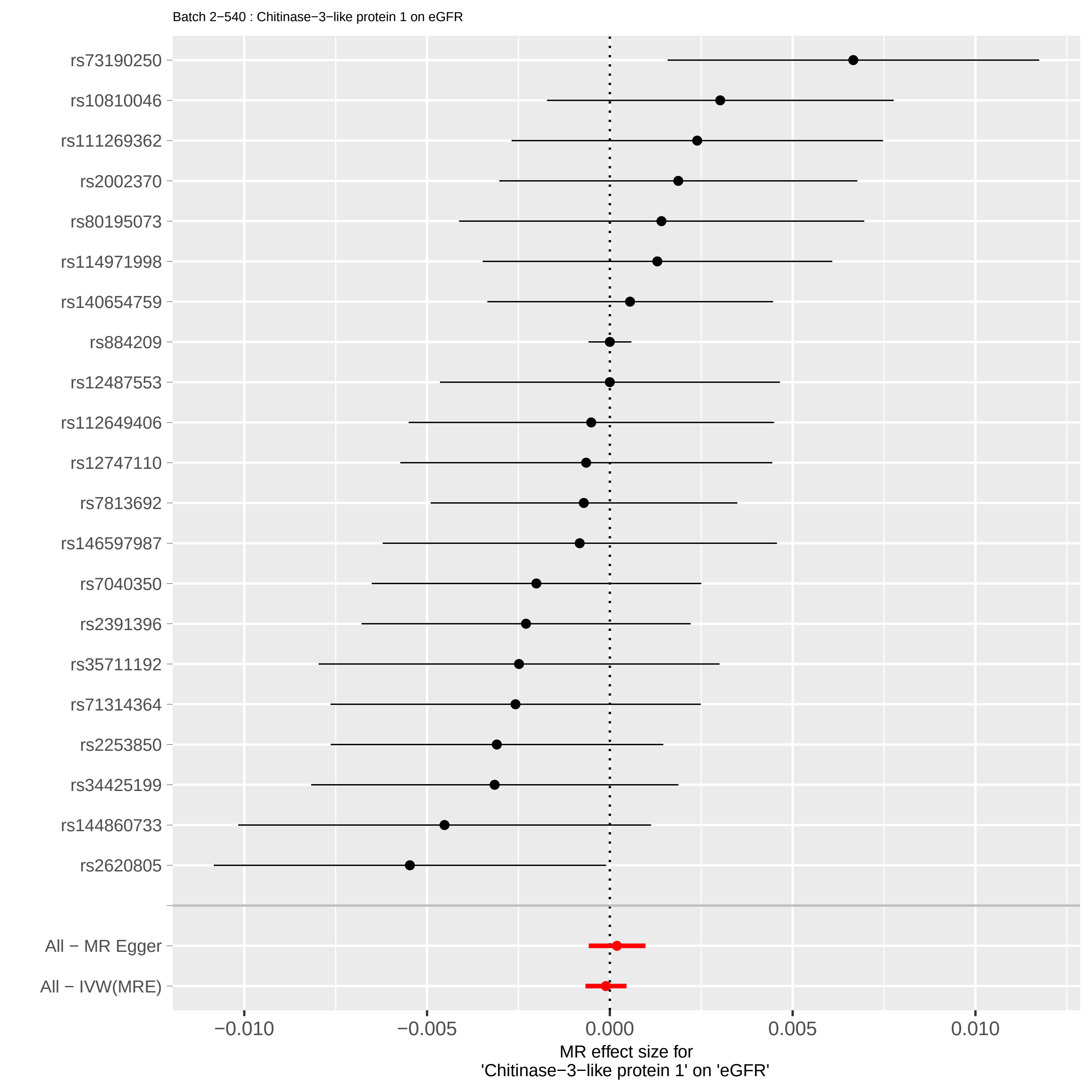

Batch 2−540 : Chitinase−3−like protein 1 on eGFR
rs73190250
rs10810046
rs111269362
rs2002370
rs80195073
rs114971998
rs140654759
rs884209
rs12487553
rs112649406
rs12747110
rs7813692
rs146597987
rs7040350
rs2391396
rs35711192
rs71314364
rs2253850
rs34425199
rs144860733
rs2620805
All − MR Egger
All − IVW(MRE)
−0.010
−0.005
0.000
0.005
0.010
MR effect size for
'Chitinase−3−like protein 1' on 'eGFR'

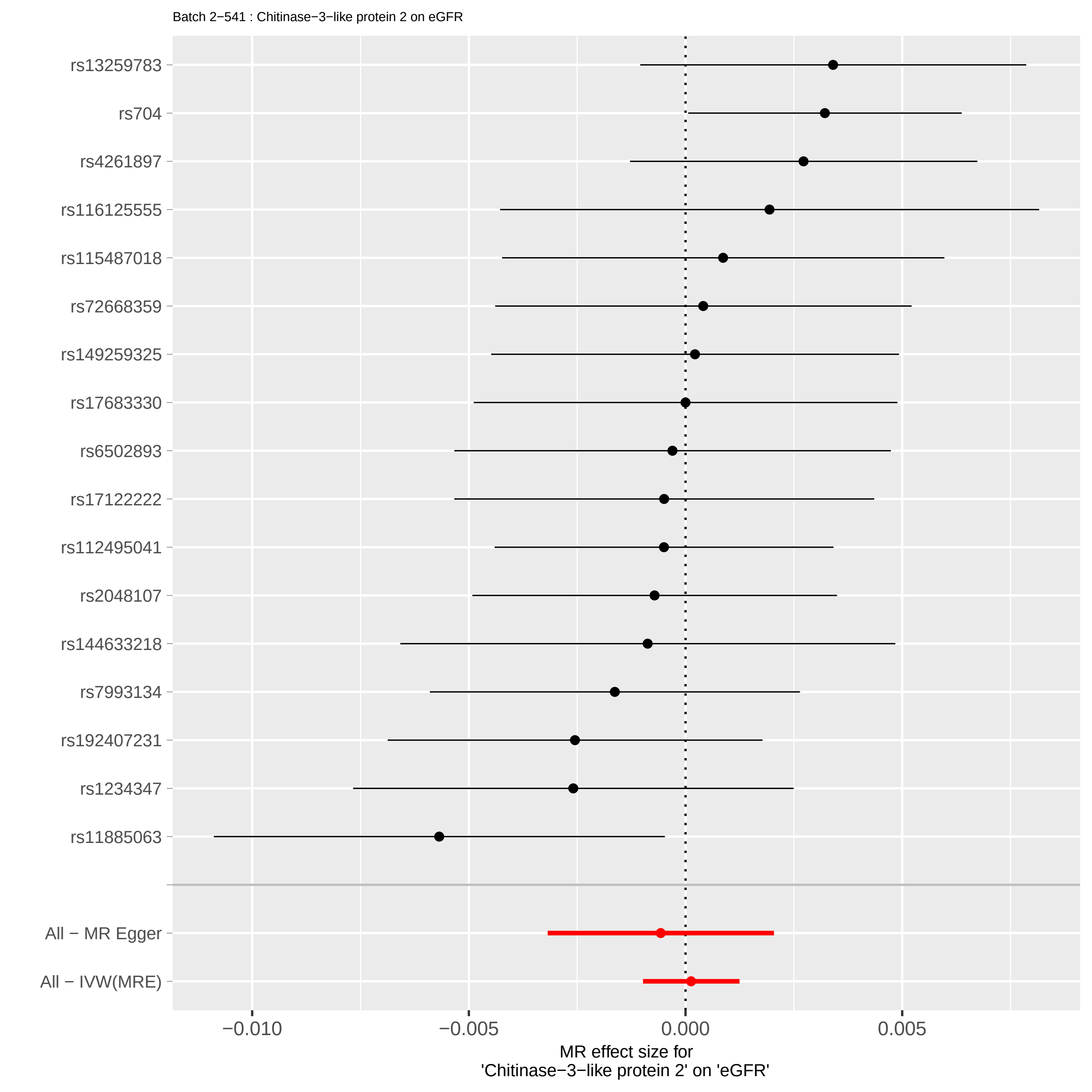

Batch 2−541 : Chitinase−3−like protein 2 on eGFR
rs13259783
rs704
rs4261897
rs116125555
rs115487018
rs72668359
rs149259325
rs17683330
rs6502893
rs17122222
rs112495041
rs2048107
rs144633218
rs7993134
rs192407231
rs1234347
rs11885063
All − MR Egger
All − IVW(MRE)
−0.010
−0.005
0.000
0.005
MR effect size for
'Chitinase−3−like protein 2' on 'eGFR'

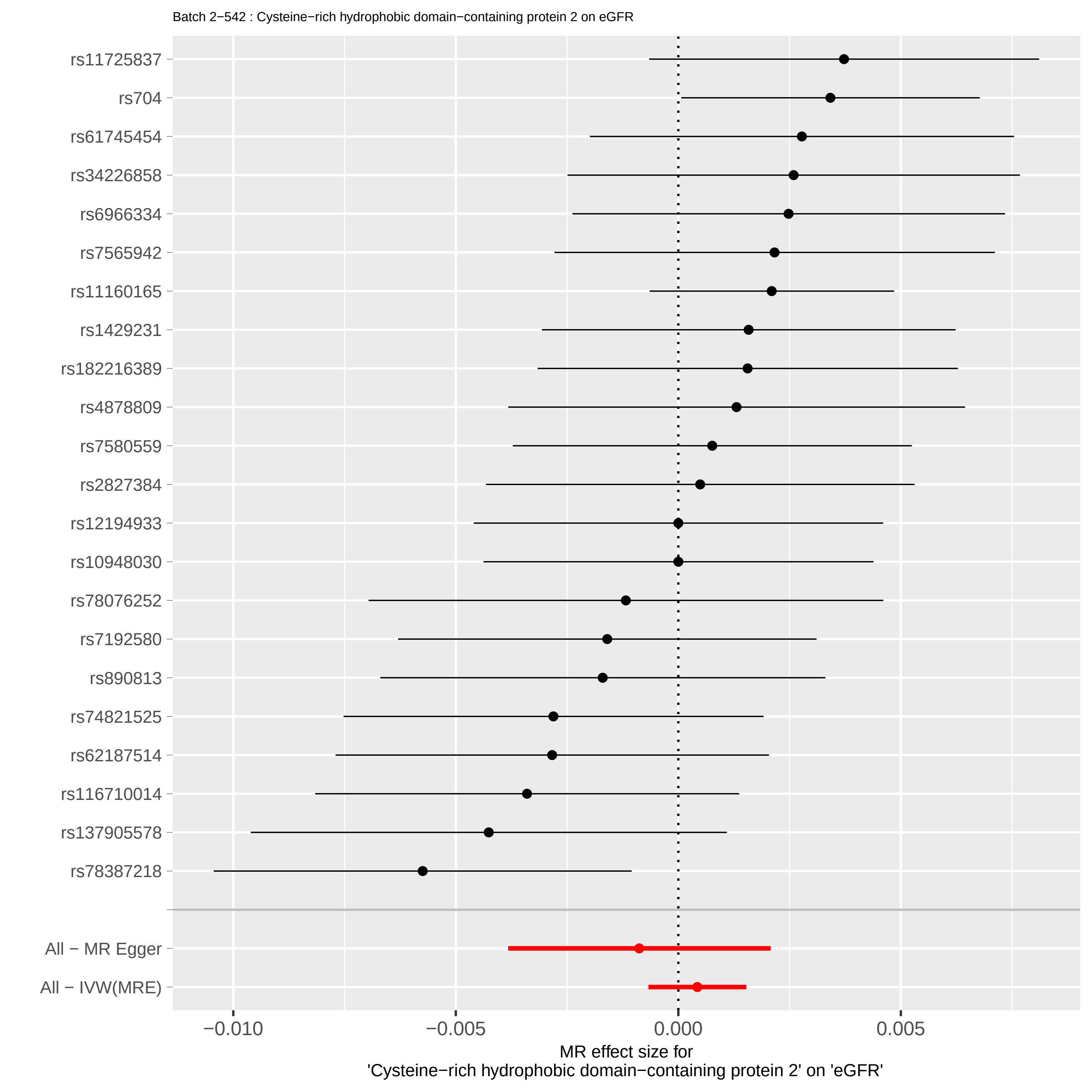

Batch 2−542 : Cysteine−rich hydrophobic domain−containing protein 2 on eGFR
rs11725837
rs704
rs61745454
rs34226858
rs6966334
rs7565942
rs11160165
rs1429231
rs182216389
rs4878809
rs7580559
rs2827384
rs12194933
rs10948030
rs78076252
rs7192580
rs890813
rs74821525
rs62187514
rs116710014
rs137905578
rs78387218
All − MR Egger
All − IVW(MRE)
−0.010
−0.005
0.000
0.005
MR effect size for
'Cysteine−rich hydrophobic domain−containing protein 2' on 'eGFR'

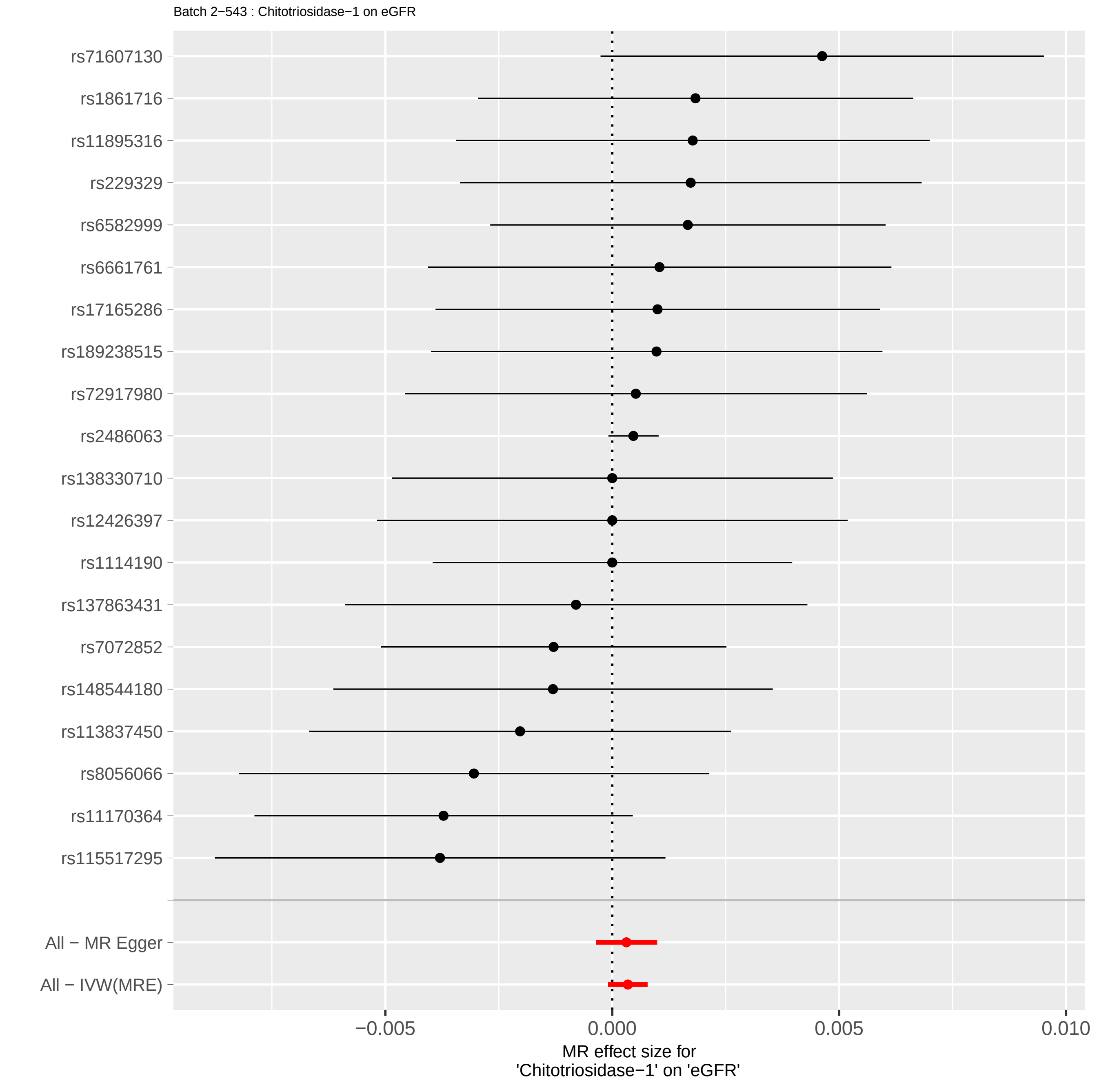

Batch 2−543 : Chitotriosidase−1 on eGFR
rs71607130
rs1861716
rs11895316
rs229329
rs6582999
rs6661761
rs17165286
rs189238515
rs72917980
rs2486063
rs138330710
rs12426397
rs1114190
rs137863431
rs7072852
rs148544180
rs113837450
rs8056066
rs11170364
rs115517295
All − MR Egger
All − IVW(MRE)
−0.005
0.000
0.005
0.010
MR effect size for
'Chitotriosidase−1' on 'eGFR'

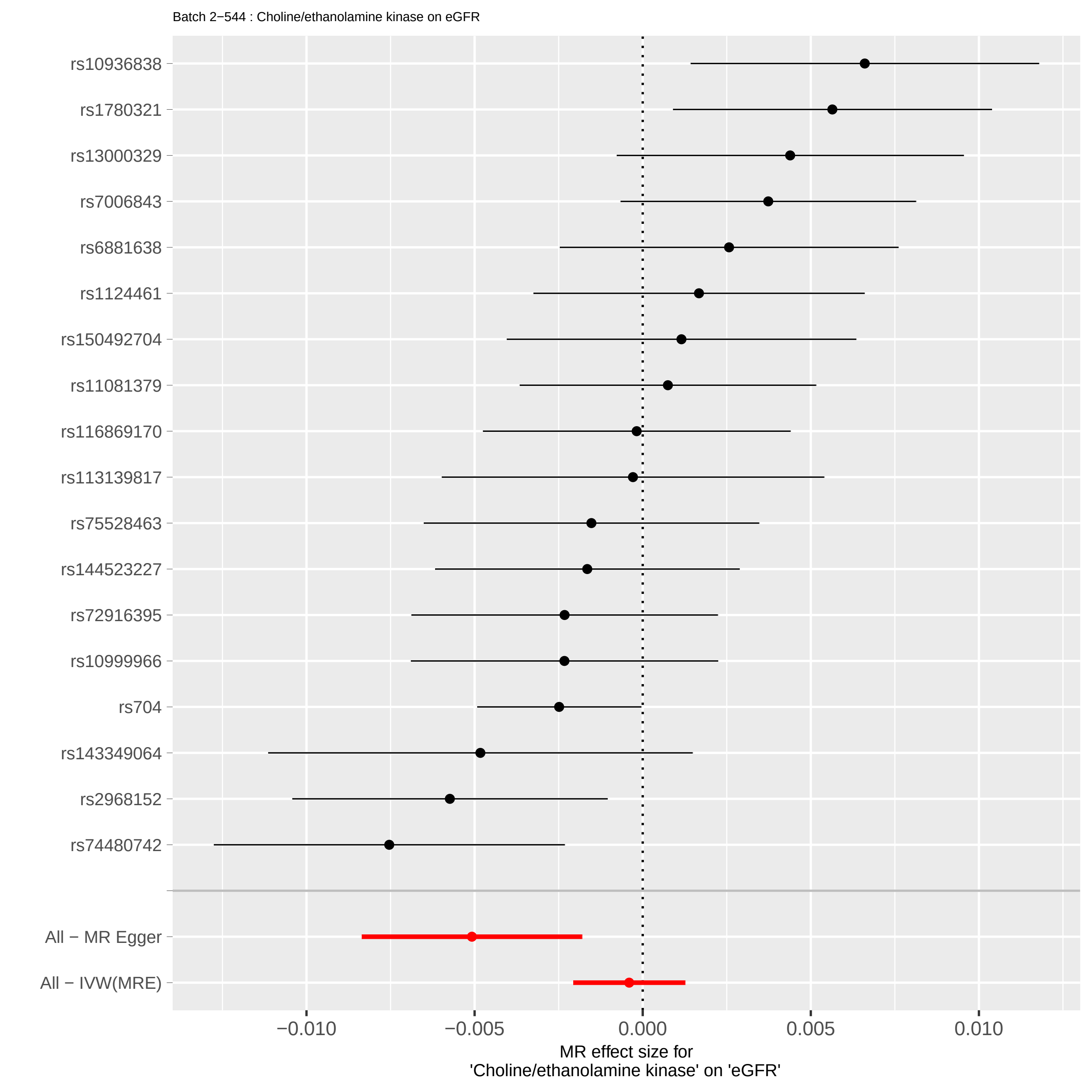

Batch 2−544 : Choline/ethanolamine kinase on eGFR
rs10936838
rs1780321
rs13000329
rs7006843
rs6881638
rs1124461
rs150492704
rs11081379
rs116869170
rs113139817
rs75528463
rs144523227
rs72916395
rs10999966
rs704
rs143349064
rs2968152
rs74480742
All − MR Egger
All − IVW(MRE)
−0.010
−0.005
0.000
0.005
0.010
MR effect size for
'Choline/ethanolamine kinase' on 'eGFR'

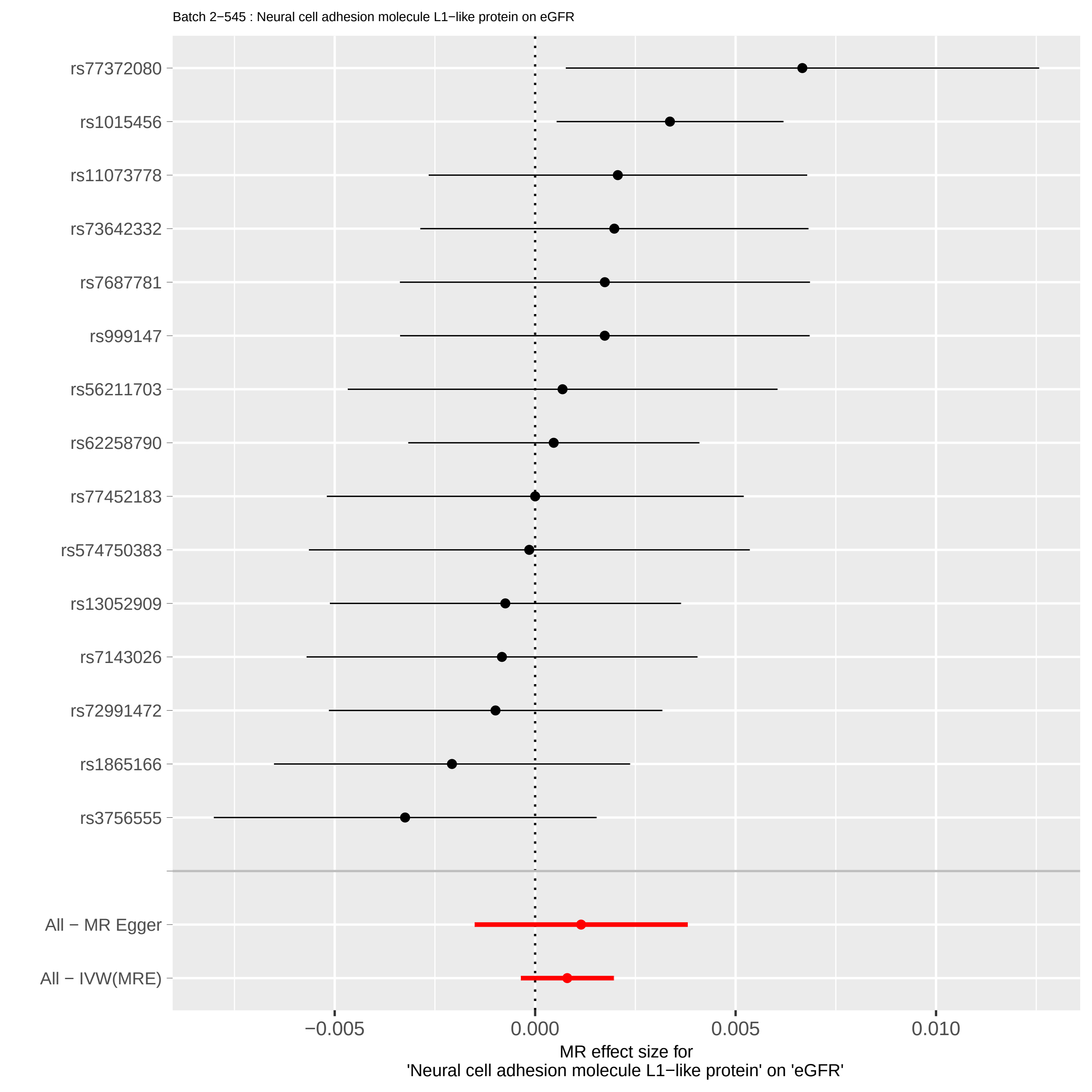

Batch 2−545 : Neural cell adhesion molecule L1−like protein on eGFR
rs77372080
rs1015456
rs11073778
rs73642332
rs7687781
rs999147
rs56211703
rs62258790
rs77452183
rs574750383
rs13052909
rs7143026
rs72991472
rs1865166
rs3756555
All − MR Egger
All − IVW(MRE)
−0.005
0.000
0.005
0.010
MR effect size for
'Neural cell adhesion molecule L1−like protein' on 'eGFR'

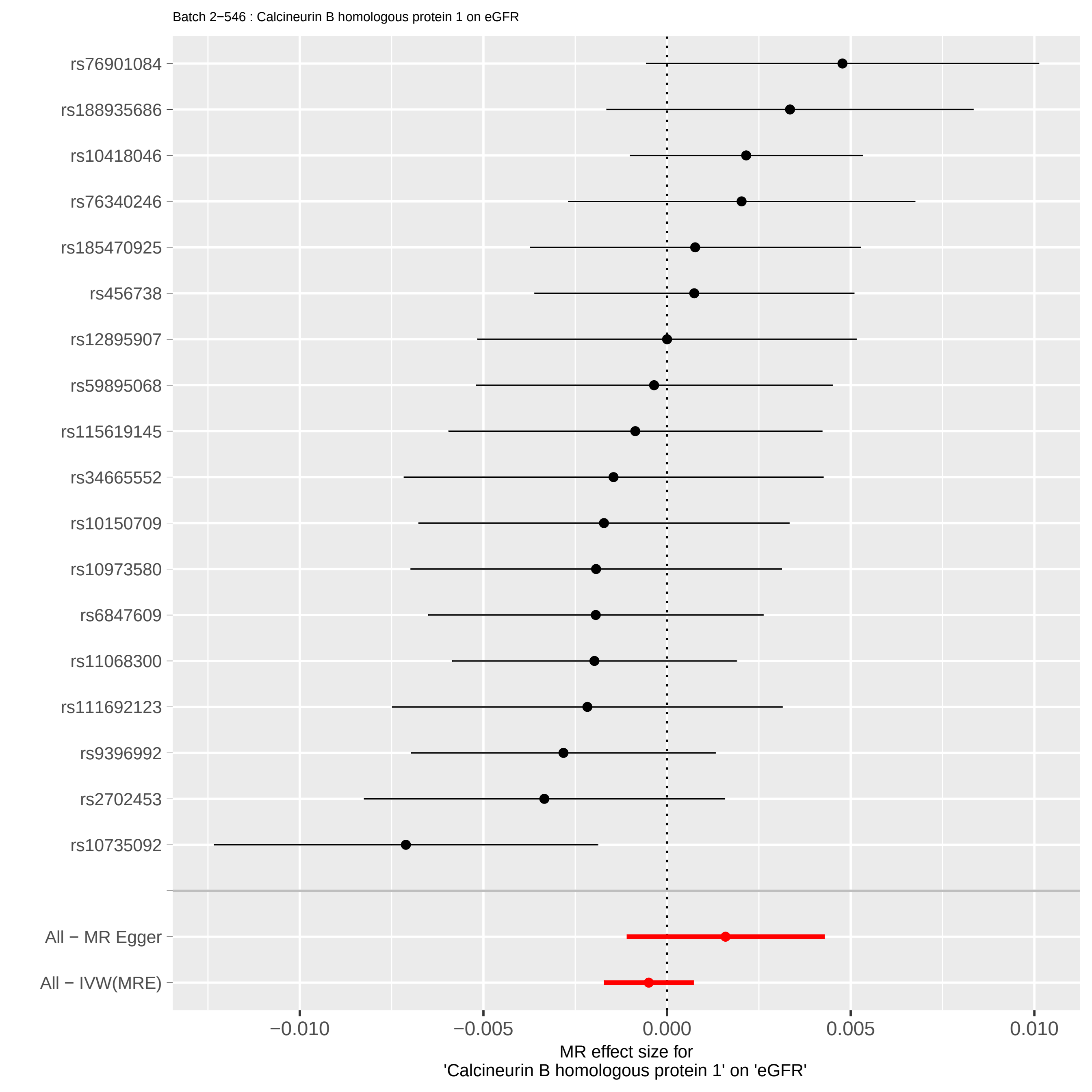
Batch 2−546 : Calcineurin B homologous protein 1 on eGFR
rs76901084
rs188935686
rs10418046
rs76340246
rs185470925
rs456738
rs12895907
rs59895068
rs115619145
rs34665552
rs10150709
rs10973580
rs6847609
rs11068300
rs111692123
rs9396992
rs2702453
rs10735092
All − MR Egger
All − IVW(MRE)
−0.010
−0.005
0.000
0.005
0.010
MR effect size for
'Calcineurin B homologous protein 1' on 'eGFR'

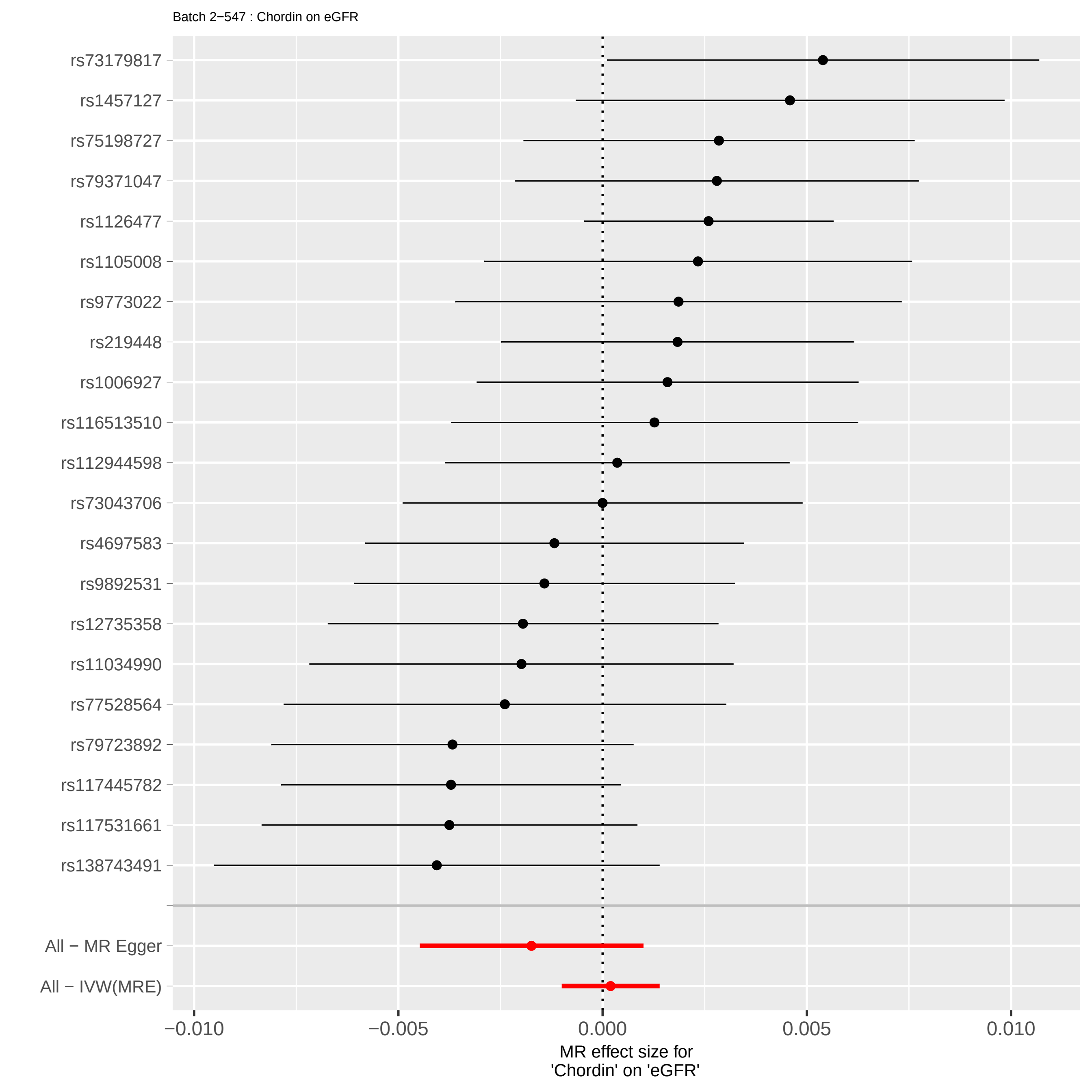

Batch 2−547 : Chordin on eGFR
rs73179817
rs1457127
rs75198727
rs79371047
rs1126477
rs1105008
rs9773022
rs219448
rs1006927
rs116513510
rs112944598
rs73043706
rs4697583
rs9892531
rs12735358
rs11034990
rs77528564
rs79723892
rs117445782
rs117531661
rs138743491
All − MR Egger
All − IVW(MRE)
−0.010
−0.005
0.000
0.005
0.010
MR effect size for
'Chordin' on 'eGFR'

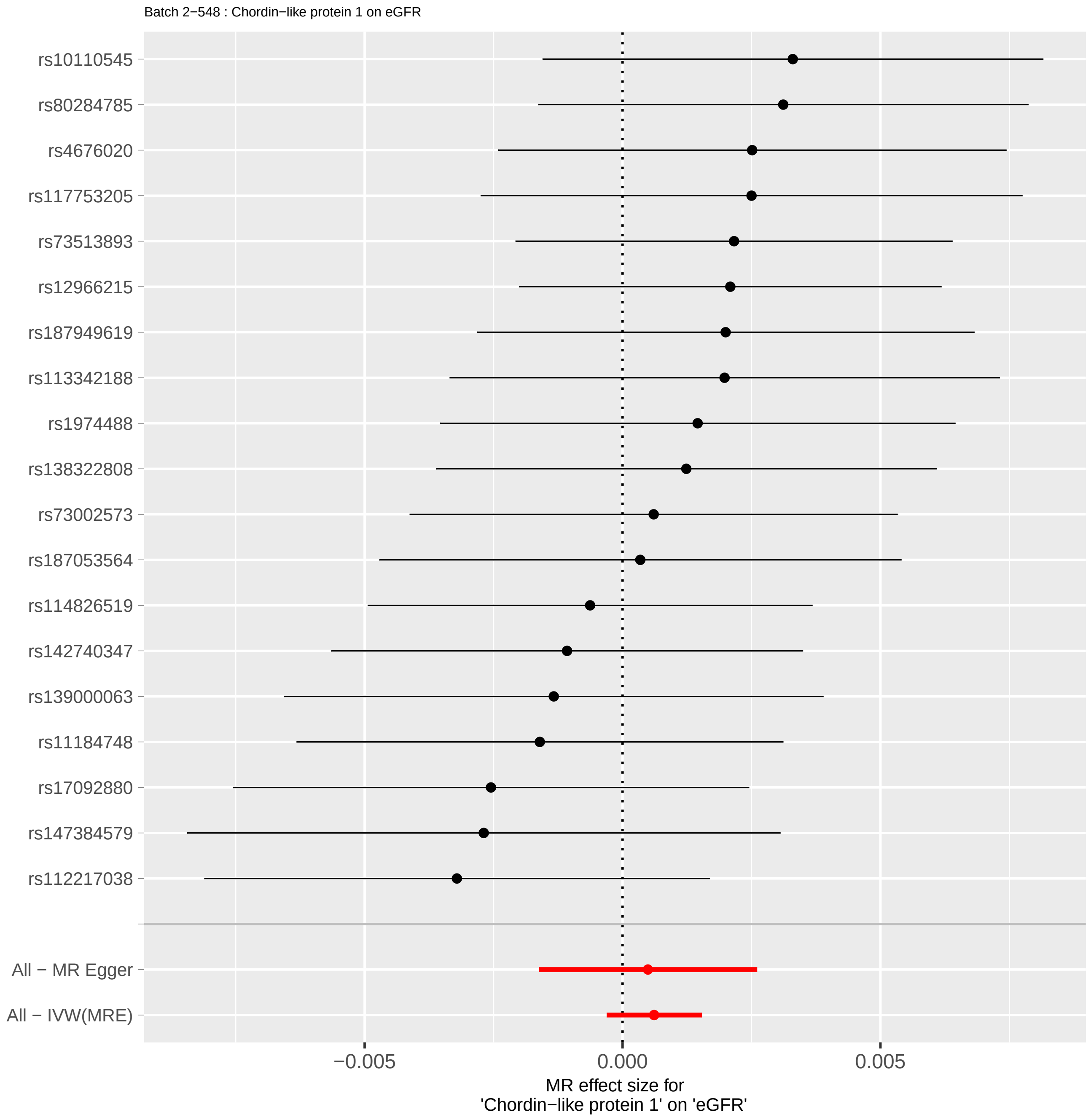

Batch 2−548 : Chordin−like protein 1 on eGFR
rs10110545
rs80284785
rs4676020
rs117753205
rs73513893
rs12966215
rs187949619
rs113342188
rs1974488
rs138322808
rs73002573
rs187053564
rs114826519
rs142740347
rs139000063
rs11184748
rs17092880
rs147384579
rs112217038
All − MR Egger
All − IVW(MRE)
−0.005
0.000
0.005
MR effect size for
'Chordin−like protein 1' on 'eGFR'

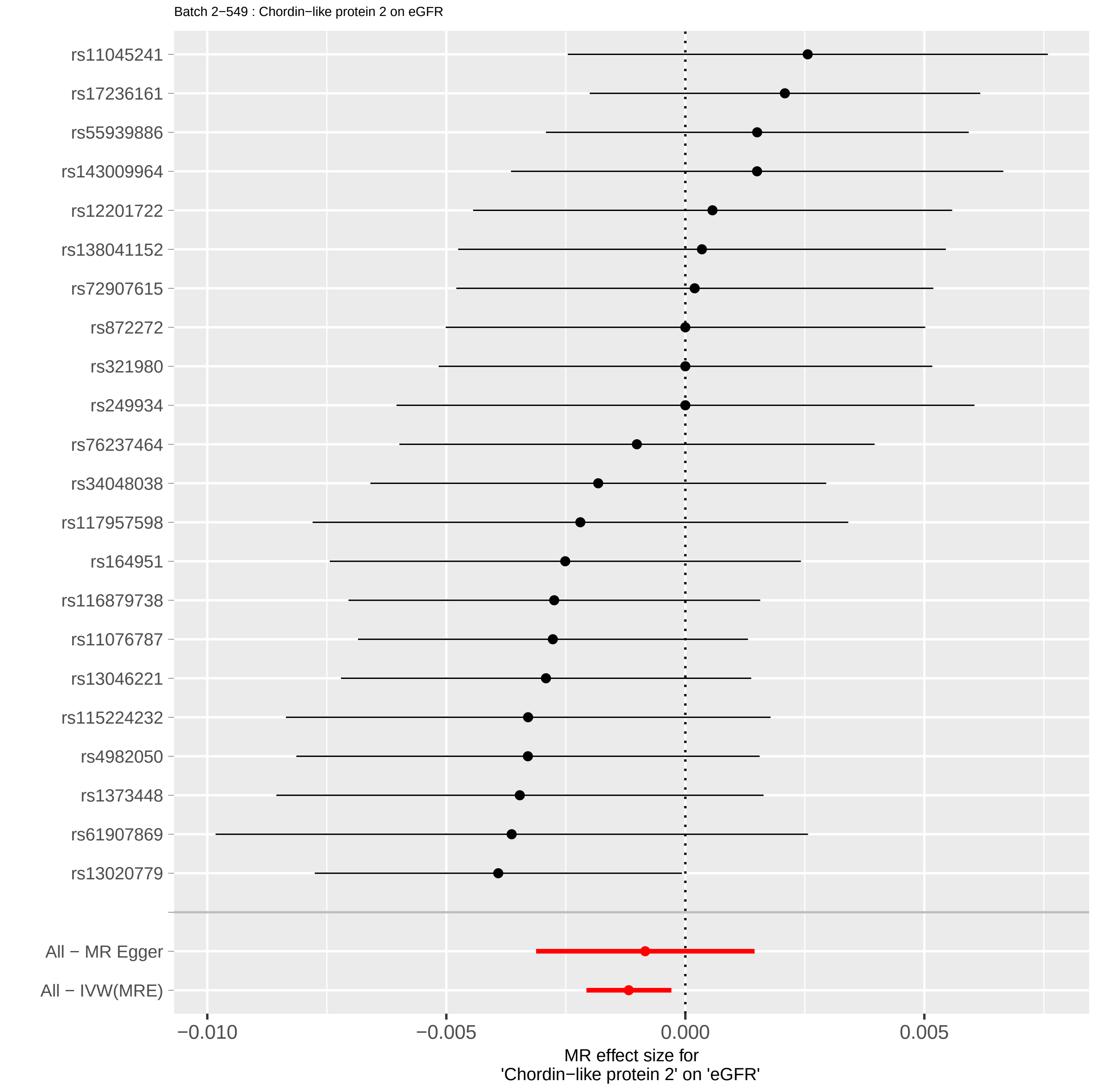

Batch 2−549 : Chordin−like protein 2 on eGFR
rs11045241
rs17236161
rs55939886
rs143009964
rs12201722
rs138041152
rs72907615
rs872272
rs321980
rs249934
rs76237464
rs34048038
rs117957598
rs164951
rs116879738
rs11076787
rs13046221
rs115224232
rs4982050
rs1373448
rs61907869
rs13020779
All − MR Egger
All − IVW(MRE)
−0.010
−0.005
0.000
0.005
MR effect size for
'Chordin−like protein 2' on 'eGFR'

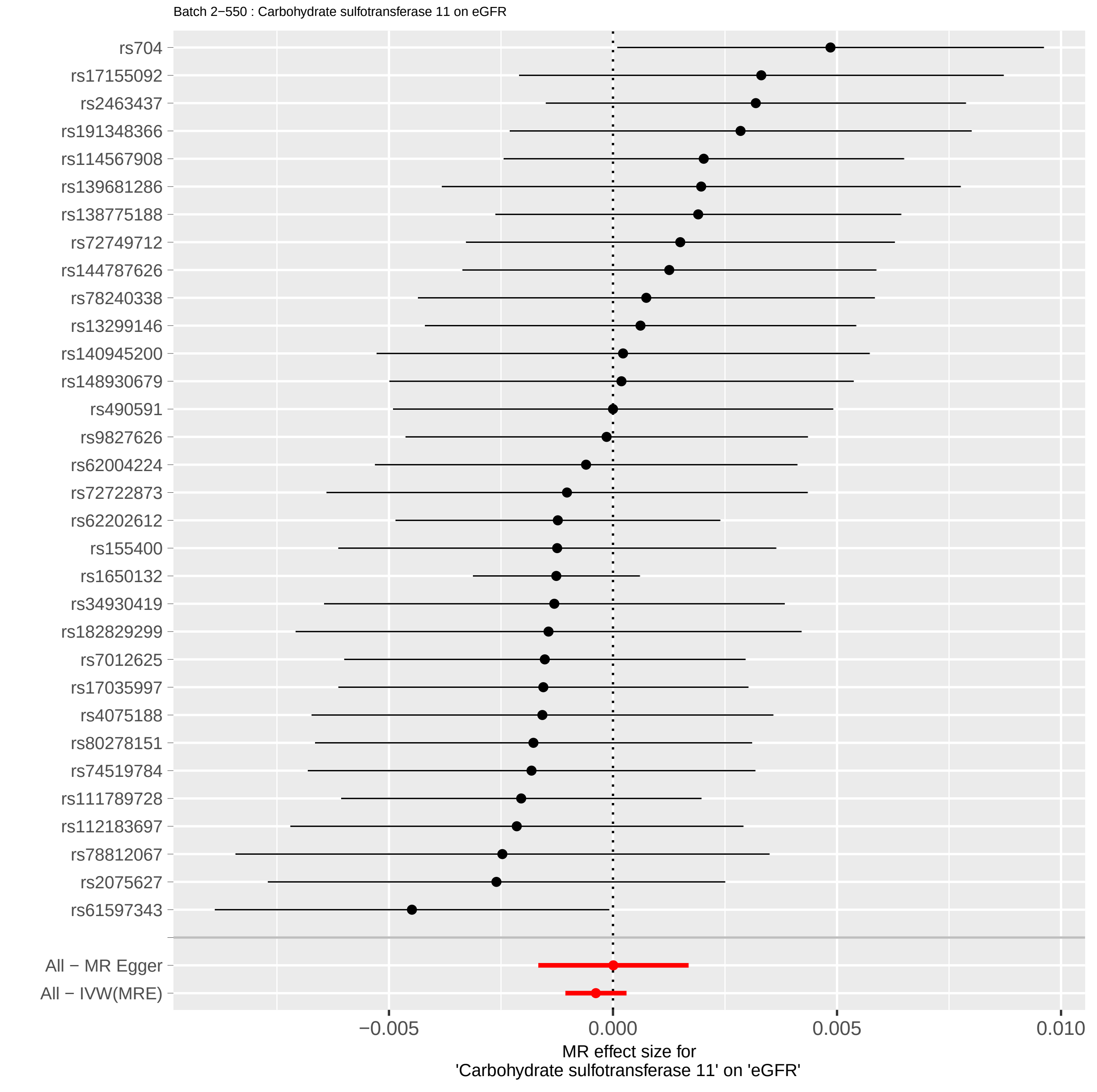

Batch 2−550 : Carbohydrate sulfotransferase 11 on eGFR
rs704
rs17155092
rs2463437
rs191348366
rs114567908
rs139681286
rs138775188
rs72749712
rs144787626
rs78240338
rs13299146
rs140945200
rs148930679
rs490591
rs9827626
rs62004224
rs72722873
rs62202612
rs155400
rs1650132
rs34930419
rs182829299
rs7012625
rs17035997
rs4075188
rs80278151
rs74519784
rs111789728
rs112183697
rs78812067
rs2075627
rs61597343
All − MR Egger
All − IVW(MRE)
−0.005
0.000
0.005
0.010
MR effect size for
'Carbohydrate sulfotransferase 11' on 'eGFR'

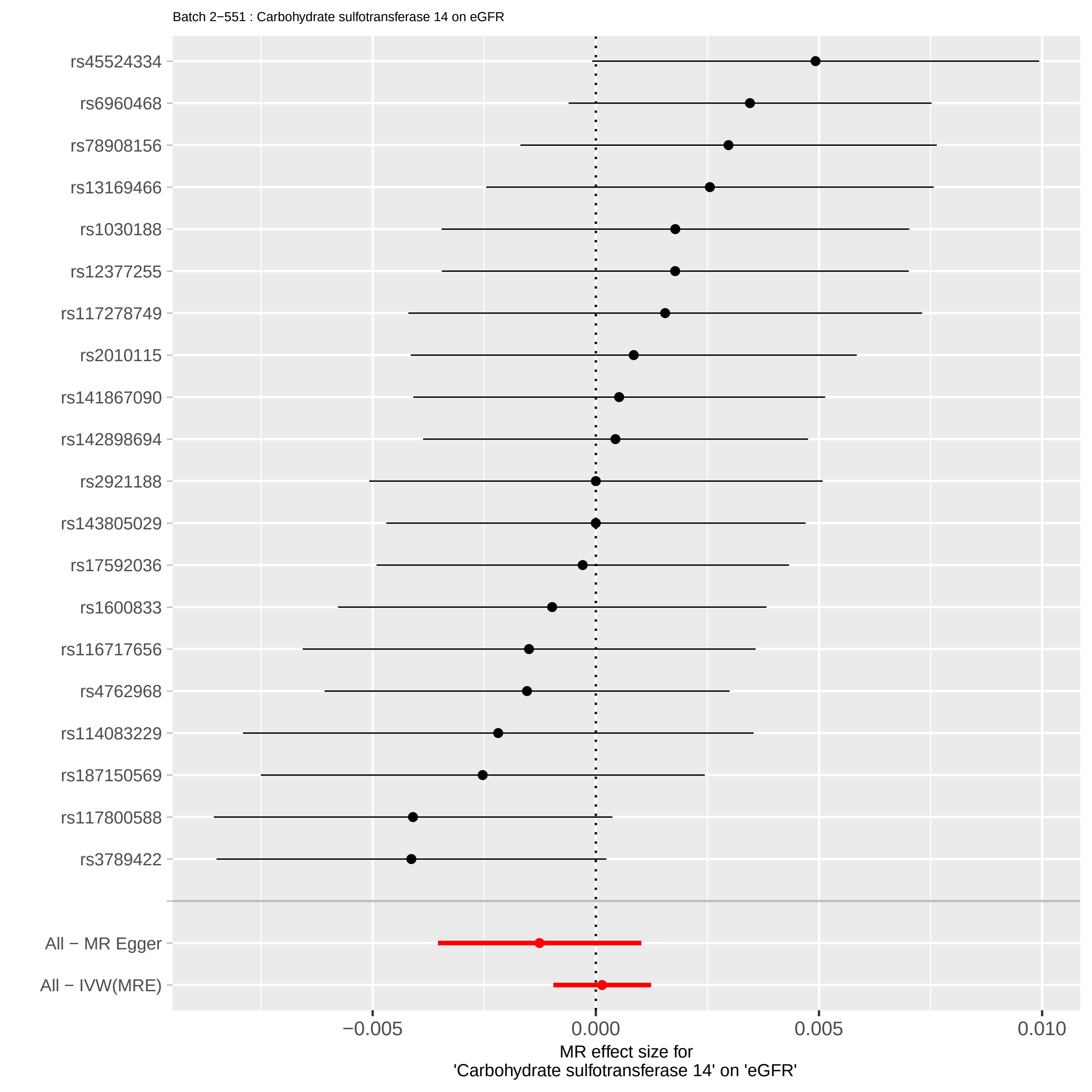

Batch 2−551 : Carbohydrate sulfotransferase 14 on eGFR
rs45524334
rs6960468
rs78908156
rs13169466
rs1030188
rs12377255
rs117278749
rs2010115
rs141867090
rs142898694
rs2921188
rs143805029
rs17592036
rs1600833
rs116717656
rs4762968
rs114083229
rs187150569
rs117800588
rs3789422
All − MR Egger
All − IVW(MRE)
−0.005
0.000
0.005
0.010
MR effect size for
'Carbohydrate sulfotransferase 14' on 'eGFR'

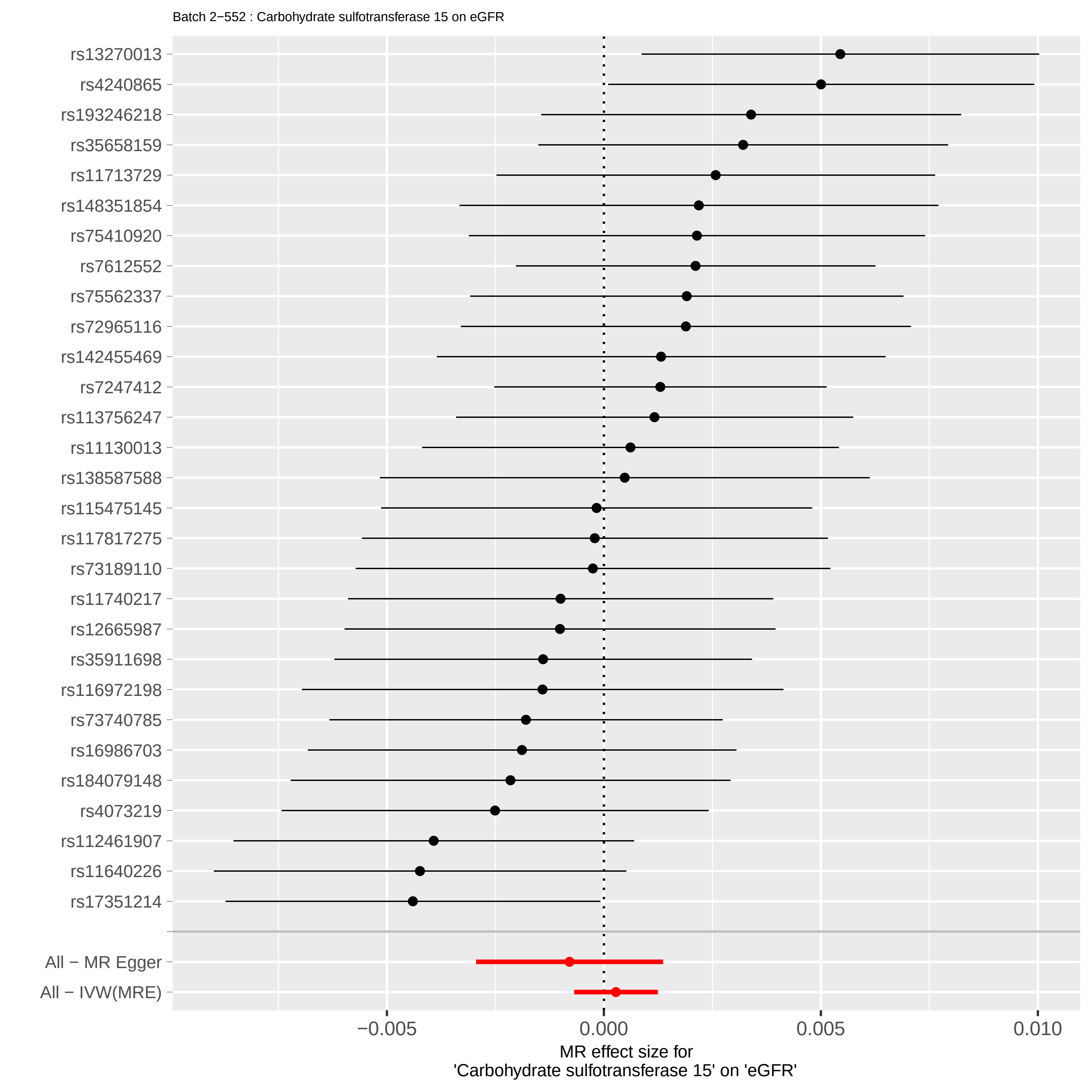
Batch 2−552 : Carbohydrate sulfotransferase 15 on eGFR
rs13270013
rs4240865
rs193246218
rs35658159
rs11713729
rs148351854
rs75410920
rs7612552
rs75562337
rs72965116
rs142455469
rs7247412
rs113756247
rs11130013
rs138587588
rs115475145
rs117817275
rs73189110
rs11740217
rs12665987
rs35911698
rs116972198
rs73740785
rs16986703
rs184079148
rs4073219
rs112461907
rs11640226
rs17351214
All − MR Egger
All − IVW(MRE)
−0.005
0.000
0.005
0.010
MR effect size for
'Carbohydrate sulfotransferase 15' on 'eGFR'

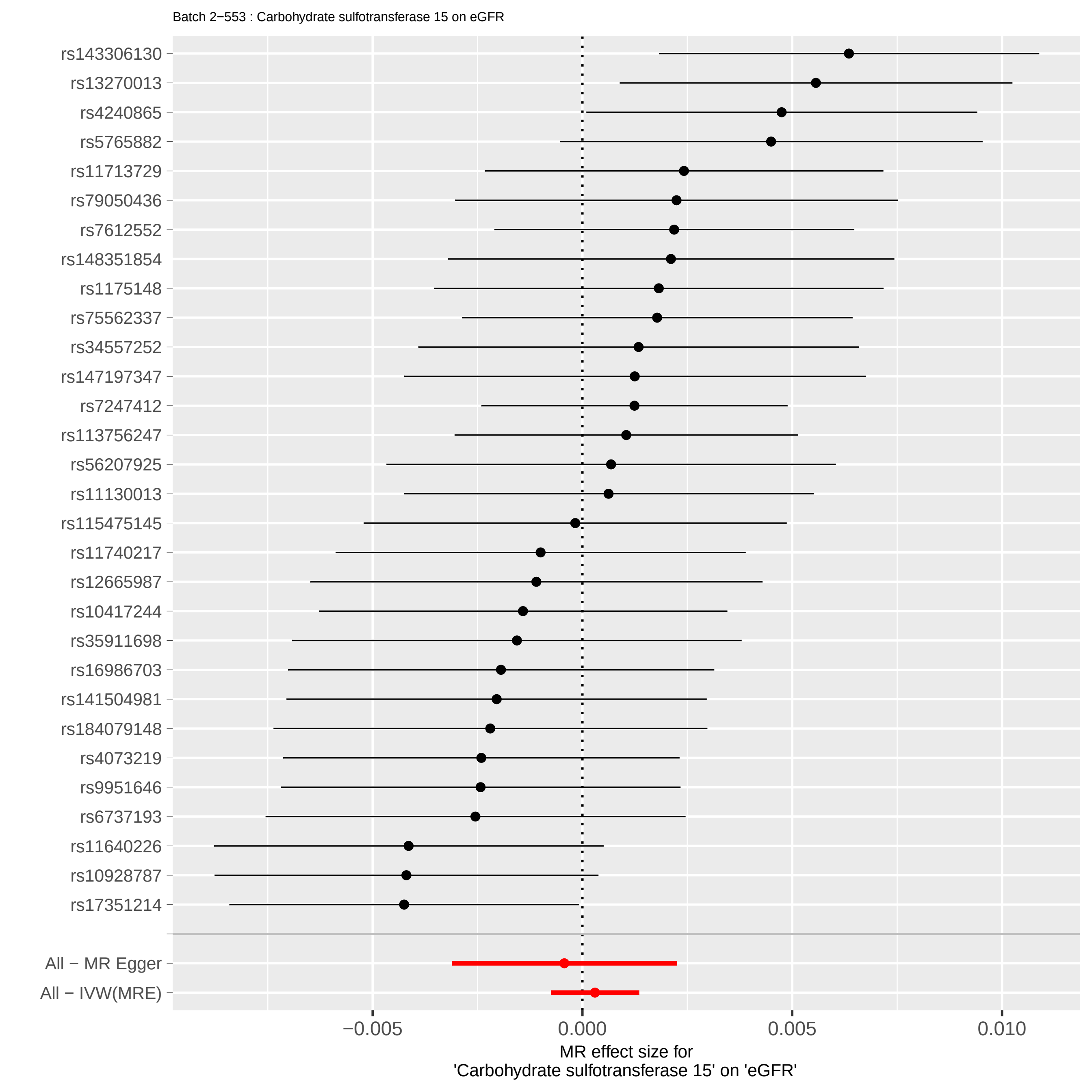

Batch 2−553 : Carbohydrate sulfotransferase 15 on eGFR
rs143306130
rs13270013
rs4240865
rs5765882
rs11713729
rs79050436
rs7612552
rs148351854
rs1175148
rs75562337
rs34557252
rs147197347
rs7247412
rs113756247
rs56207925
rs11130013
rs115475145
rs11740217
rs12665987
rs10417244
rs35911698
rs16986703
rs141504981
rs184079148
rs4073219
rs9951646
rs6737193
rs11640226
rs10928787
rs17351214
All − MR Egger
All − IVW(MRE)
−0.005
0.000
0.005
0.010
MR effect size for
'Carbohydrate sulfotransferase 15' on 'eGFR'

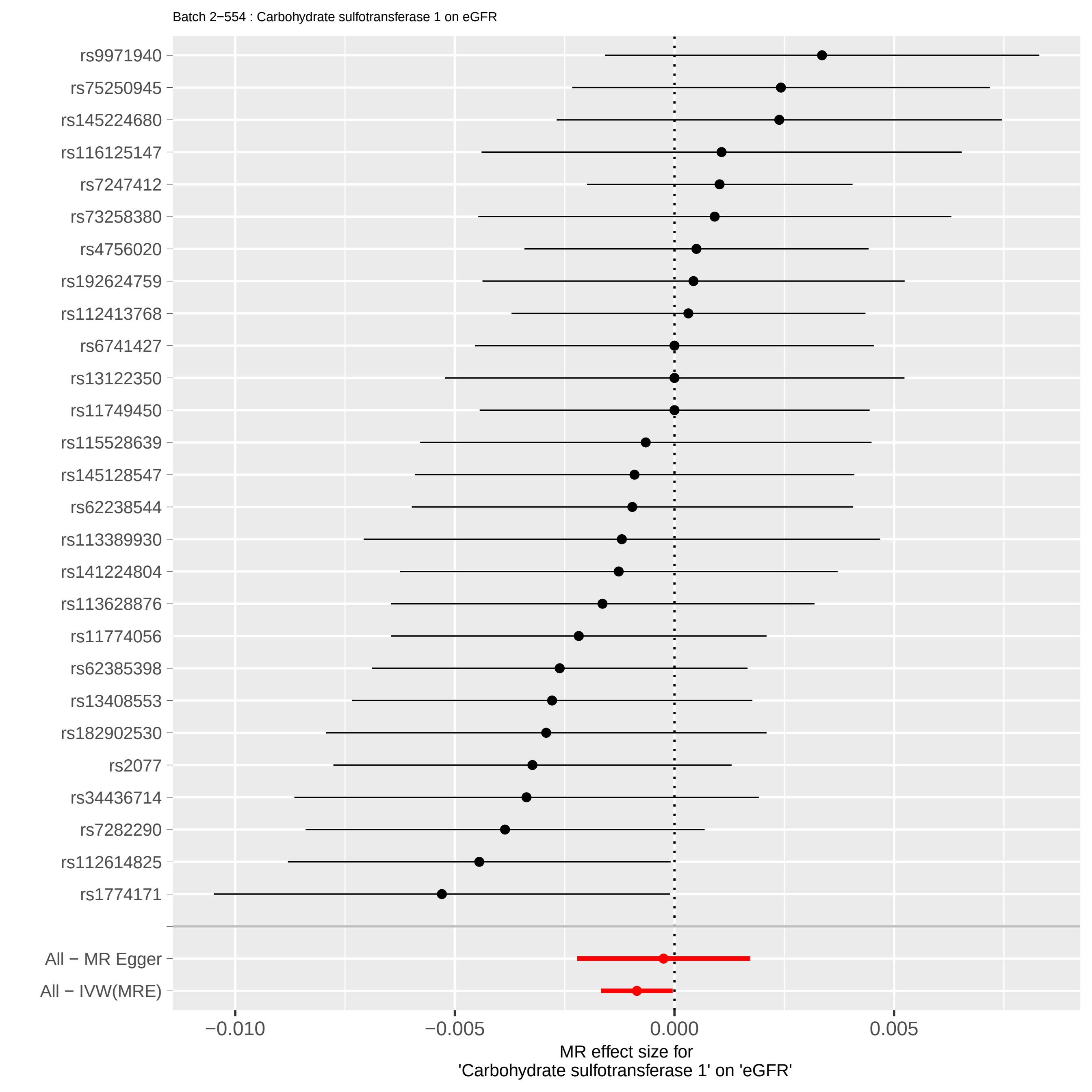

Batch 2−554 : Carbohydrate sulfotransferase 1 on eGFR
rs9971940
rs75250945
rs145224680
rs116125147
rs7247412
rs73258380
rs4756020
rs192624759
rs112413768
rs6741427
rs13122350
rs11749450
rs115528639
rs145128547
rs62238544
rs113389930
rs141224804
rs113628876
rs11774056
rs62385398
rs13408553
rs182902530
rs2077
rs34436714
rs7282290
rs112614825
rs1774171
All − MR Egger
All − IVW(MRE)
−0.010
−0.005
0.000
0.005
MR effect size for
'Carbohydrate sulfotransferase 1' on 'eGFR'

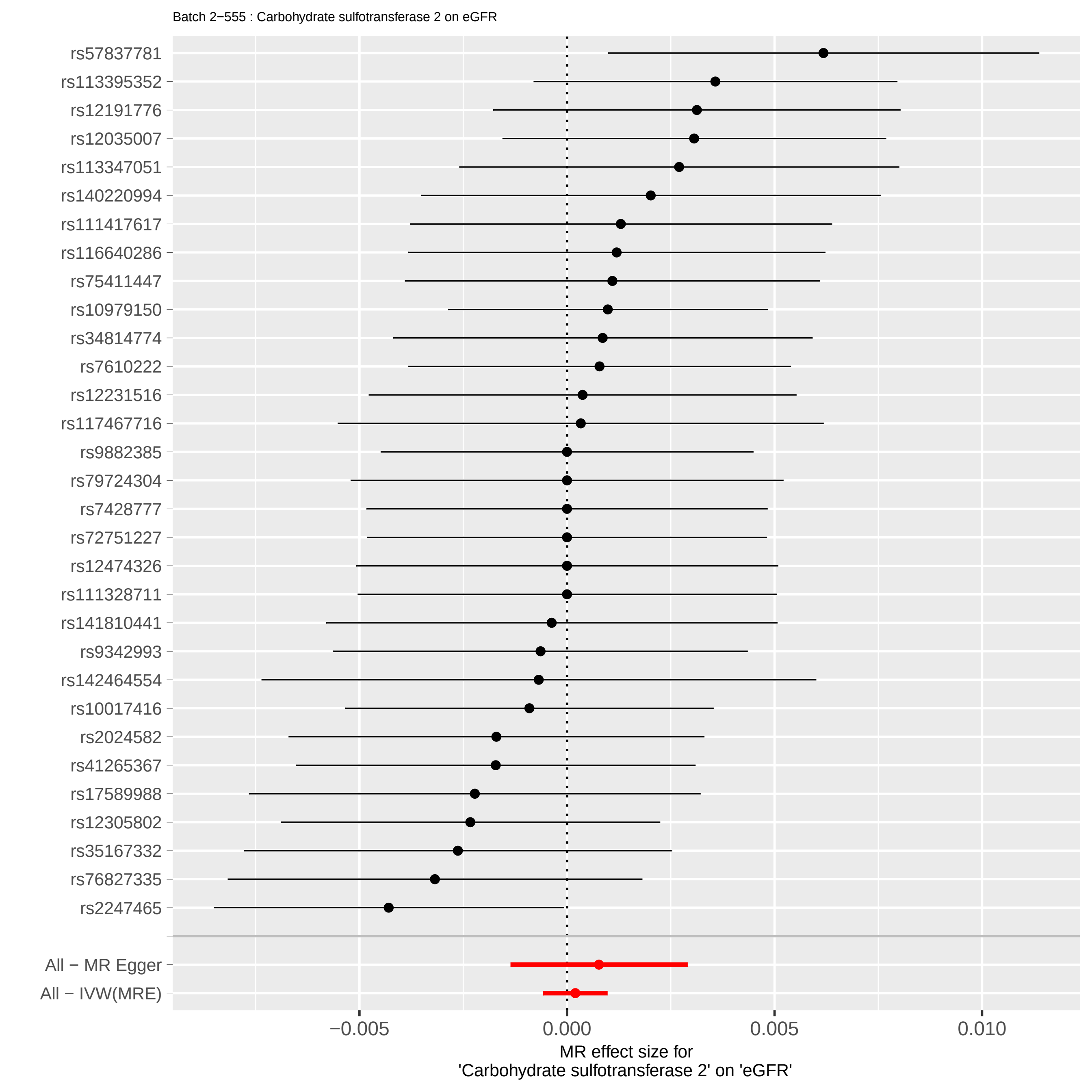
Batch 2−555 : Carbohydrate sulfotransferase 2 on eGFR
rs57837781
rs113395352
rs12191776
rs12035007
rs113347051
rs140220994
rs111417617
rs116640286
rs75411447
rs10979150
rs34814774
rs7610222
rs12231516
rs117467716
rs9882385
rs79724304
rs7428777
rs72751227
rs12474326
rs111328711
rs141810441
rs9342993
rs142464554
rs10017416
rs2024582
rs41265367
rs17589988
rs12305802
rs35167332
rs76827335
rs2247465
All − MR Egger
All − IVW(MRE)
−0.005
0.000
0.005
0.010
MR effect size for
'Carbohydrate sulfotransferase 2' on 'eGFR'

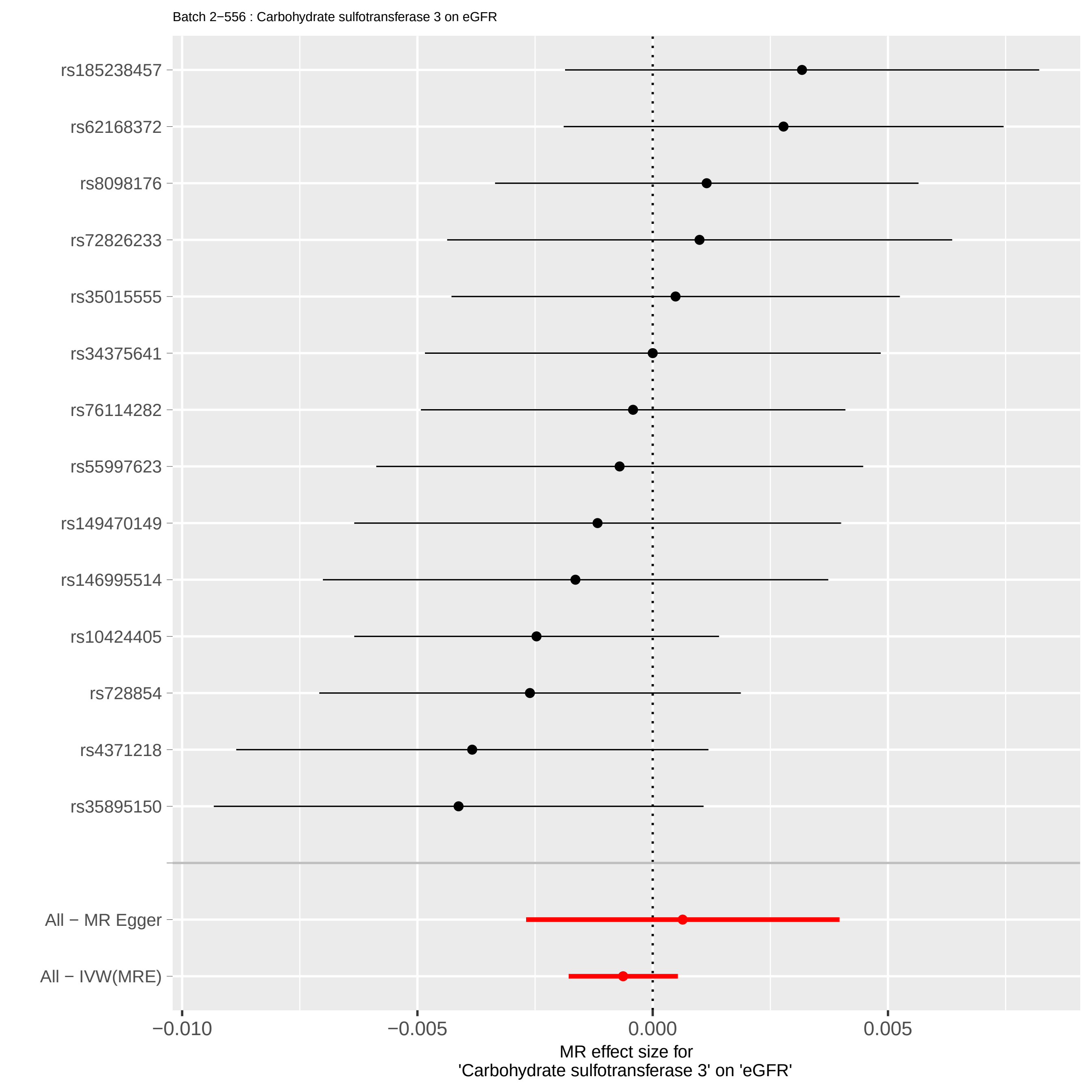

Batch 2−556 : Carbohydrate sulfotransferase 3 on eGFR
rs185238457
rs62168372
rs8098176
rs72826233
rs35015555
rs34375641
rs76114282
rs55997623
rs149470149
rs146995514
rs10424405
rs728854
rs4371218
rs35895150
All − MR Egger
All − IVW(MRE)
−0.010
−0.005
0.000
0.005
MR effect size for
'Carbohydrate sulfotransferase 3' on 'eGFR'

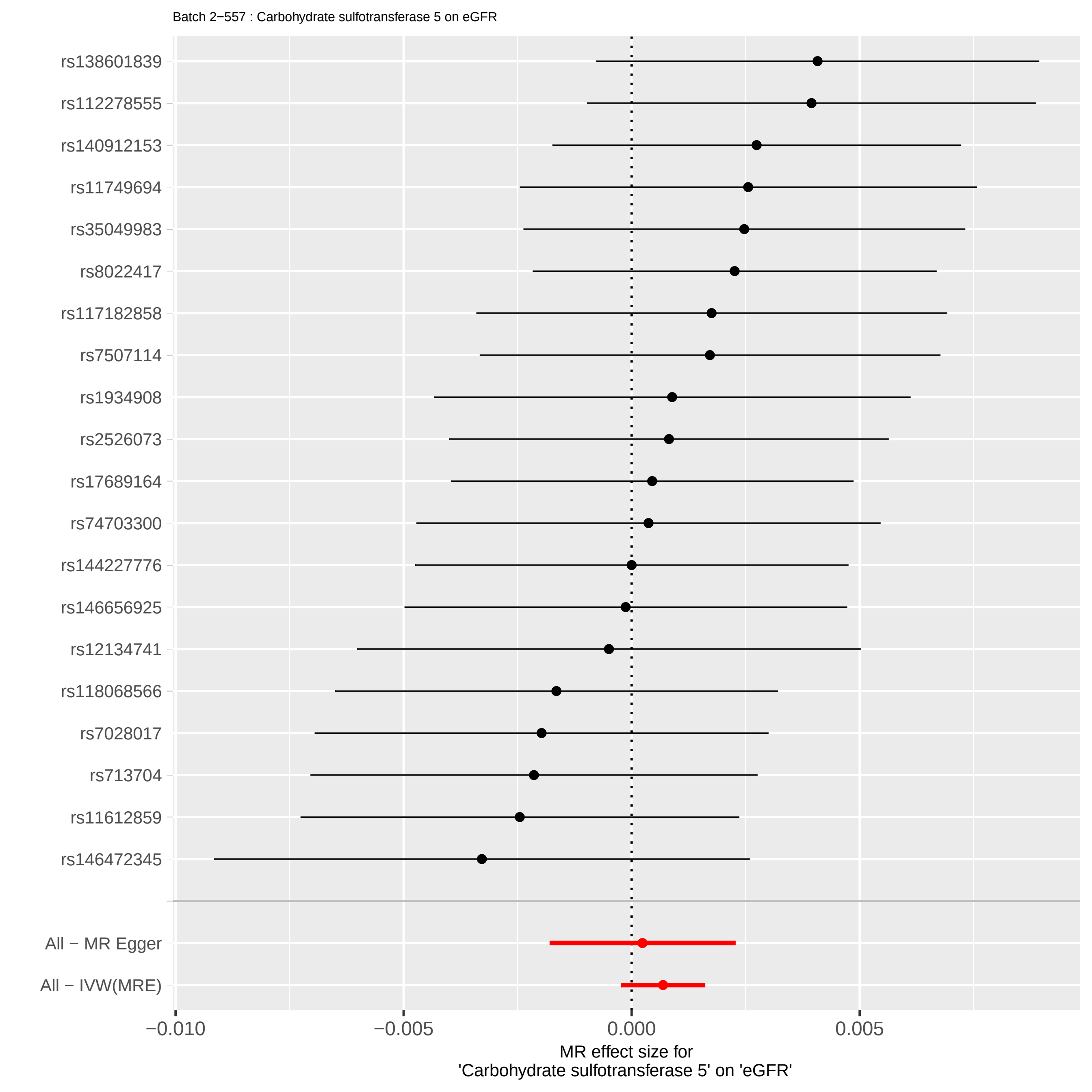

Batch 2−557 : Carbohydrate sulfotransferase 5 on eGFR
rs138601839
rs112278555
rs140912153
rs11749694
rs35049983
rs8022417
rs117182858
rs7507114
rs1934908
rs2526073
rs17689164
rs74703300
rs144227776
rs146656925
rs12134741
rs118068566
rs7028017
rs713704
rs11612859
rs146472345
All − MR Egger
All − IVW(MRE)
−0.010
−0.005
0.000
0.005
MR effect size for
'Carbohydrate sulfotransferase 5' on 'eGFR'

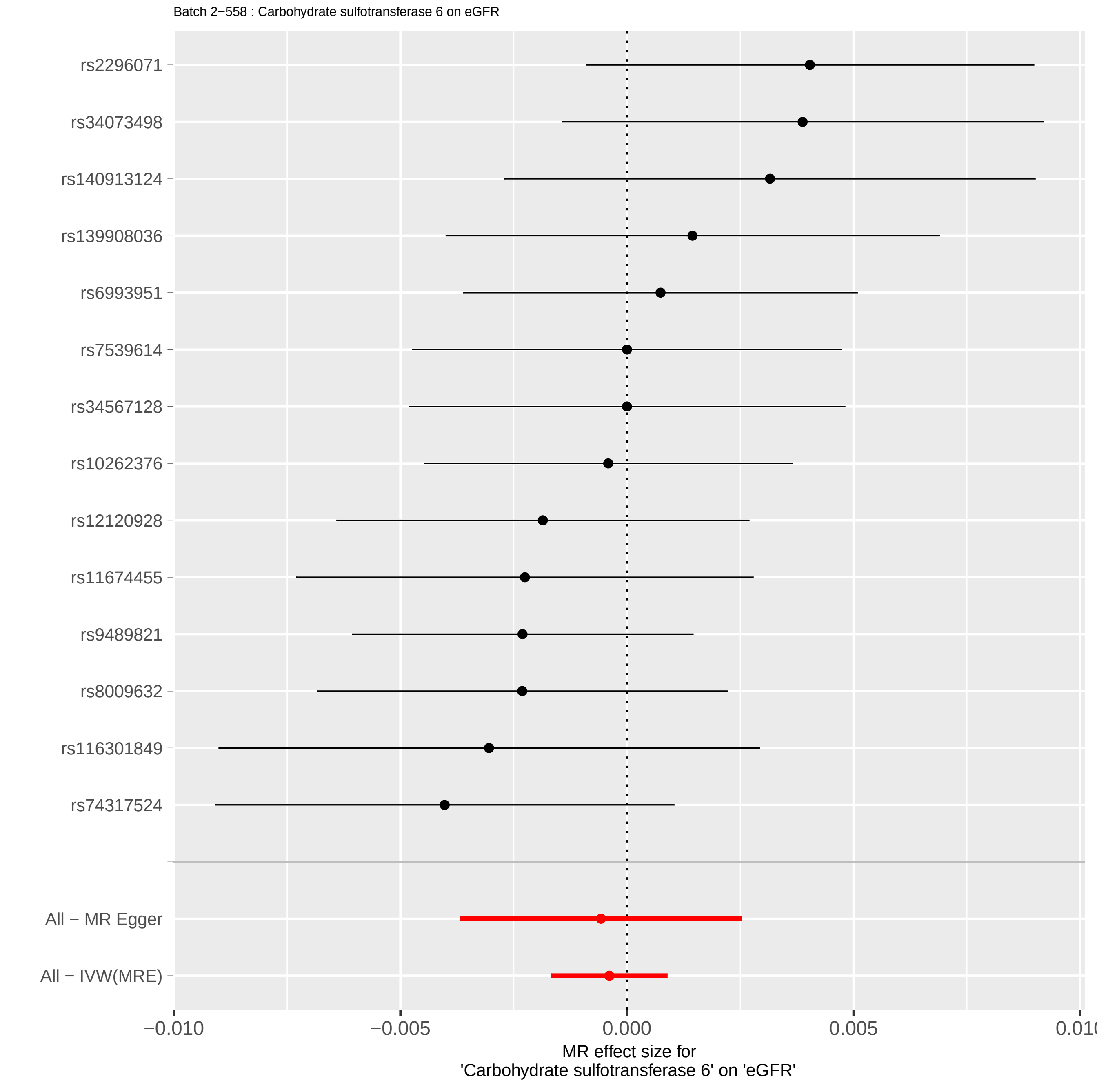
Batch 2−558 : Carbohydrate sulfotransferase 6 on eGFR
rs2296071
rs34073498
rs140913124
rs139908036
rs6993951
rs7539614
rs34567128
rs10262376
rs12120928
rs11674455
rs9489821
rs8009632
rs116301849
rs74317524
All − MR Egger
All − IVW(MRE)
−0.010
−0.005
0.000
0.005
0.010
MR effect size for
'Carbohydrate sulfotransferase 6' on 'eGFR'

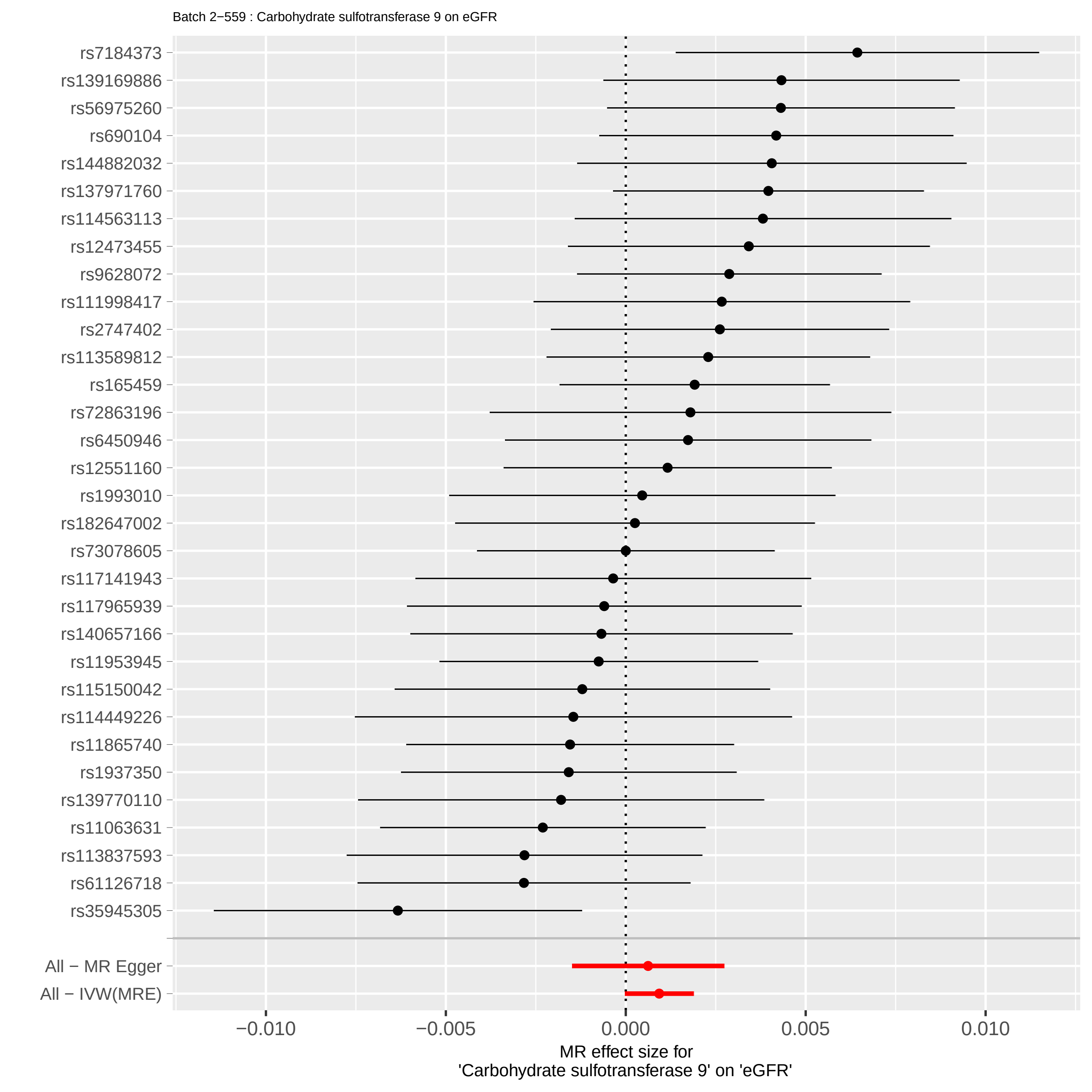

Batch 2−559 : Carbohydrate sulfotransferase 9 on eGFR
rs7184373
rs139169886
rs56975260
rs690104
rs144882032
rs137971760
rs114563113
rs12473455
rs9628072
rs111998417
rs2747402
rs113589812
rs165459
rs72863196
rs6450946
rs12551160
rs1993010
rs182647002
rs73078605
rs117141943
rs117965939
rs140657166
rs11953945
rs115150042
rs114449226
rs11865740
rs1937350
rs139770110
rs11063631
rs113837593
rs61126718
rs35945305
All − MR Egger
All − IVW(MRE)
−0.010
−0.005
0.000
0.005
0.010
MR effect size for
'Carbohydrate sulfotransferase 9' on 'eGFR'

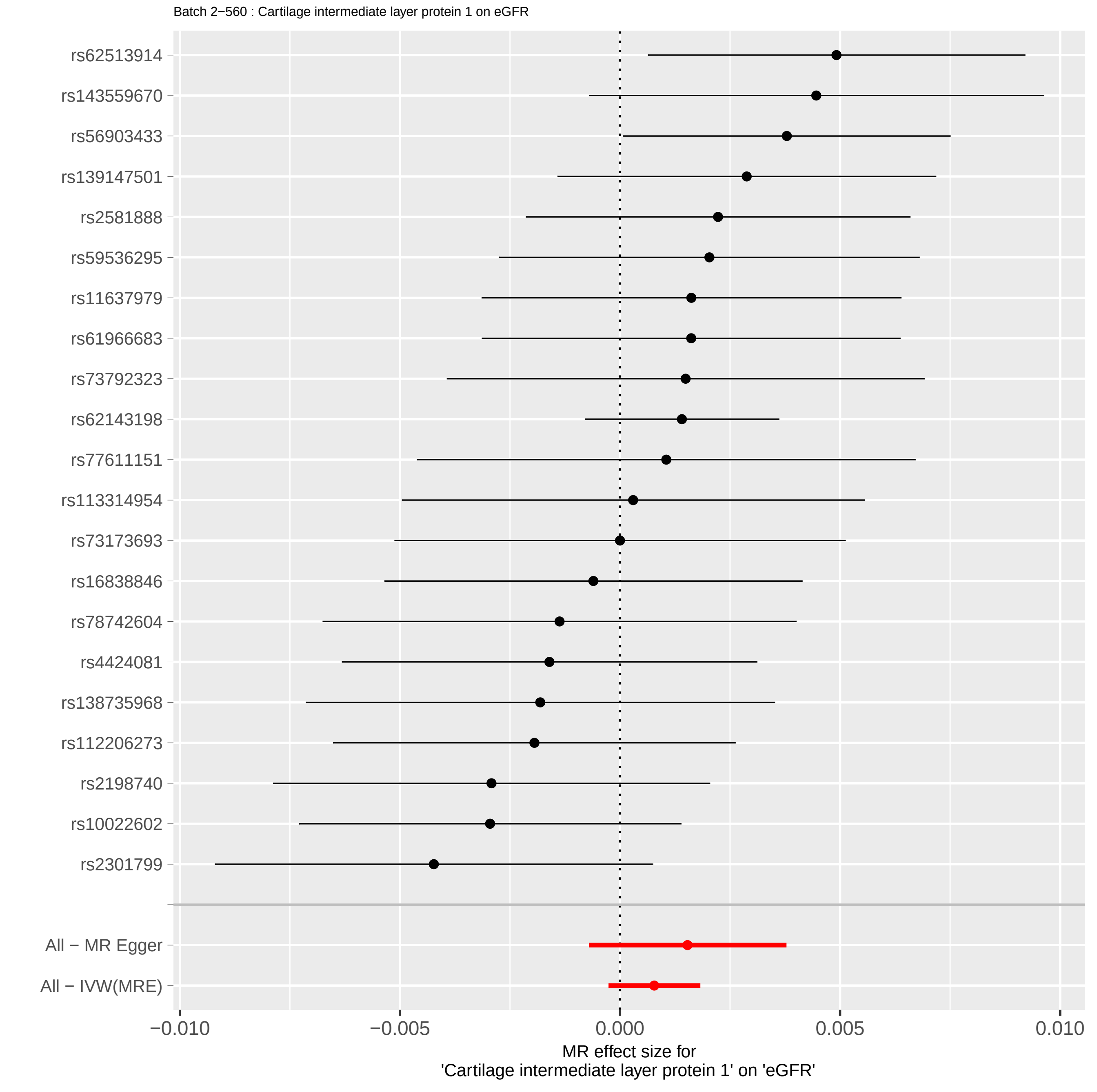

Batch 2−560 : Cartilage intermediate layer protein 1 on eGFR
rs62513914
rs143559670
rs56903433
rs139147501
rs2581888
rs59536295
rs11637979
rs61966683
rs73792323
rs62143198
rs77611151
rs113314954
rs73173693
rs16838846
rs78742604
rs4424081
rs138735968
rs112206273
rs2198740
rs10022602
rs2301799
All − MR Egger
All − IVW(MRE)
−0.010
−0.005
0.000
0.005
0.010
MR effect size for
'Cartilage intermediate layer protein 1' on 'eGFR'

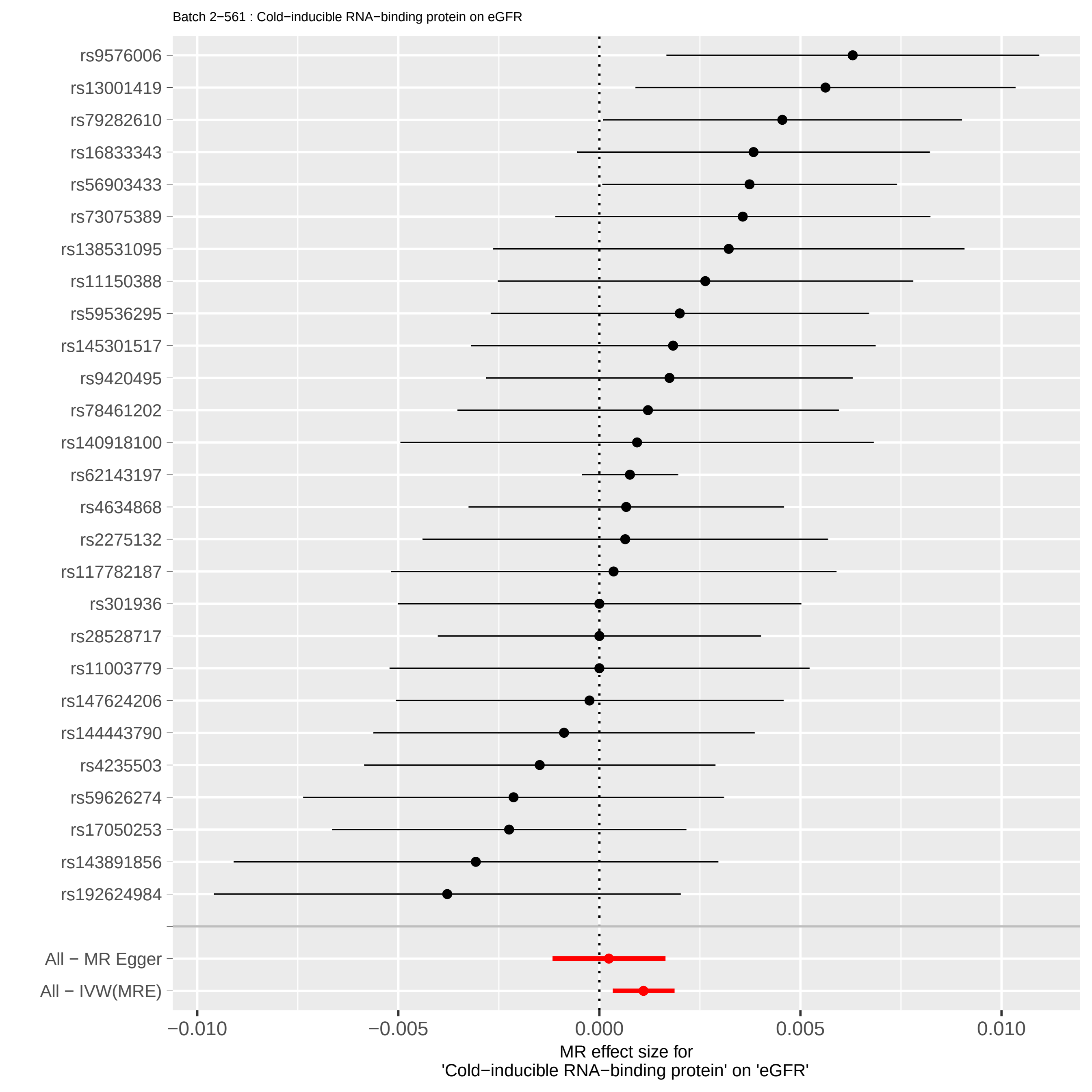

Batch 2−561 : Cold−inducible RNA−binding protein on eGFR
rs9576006
rs13001419
rs79282610
rs16833343
rs56903433
rs73075389
rs138531095
rs11150388
rs59536295
rs145301517
rs9420495
rs78461202
rs140918100
rs62143197
rs4634868
rs2275132
rs117782187
rs301936
rs28528717
rs11003779
rs147624206
rs144443790
rs4235503
rs59626274
rs17050253
rs143891856
rs192624984
All − MR Egger
All − IVW(MRE)
−0.010
−0.005
0.000
0.005
0.010
MR effect size for
'Cold−inducible RNA−binding protein' on 'eGFR'

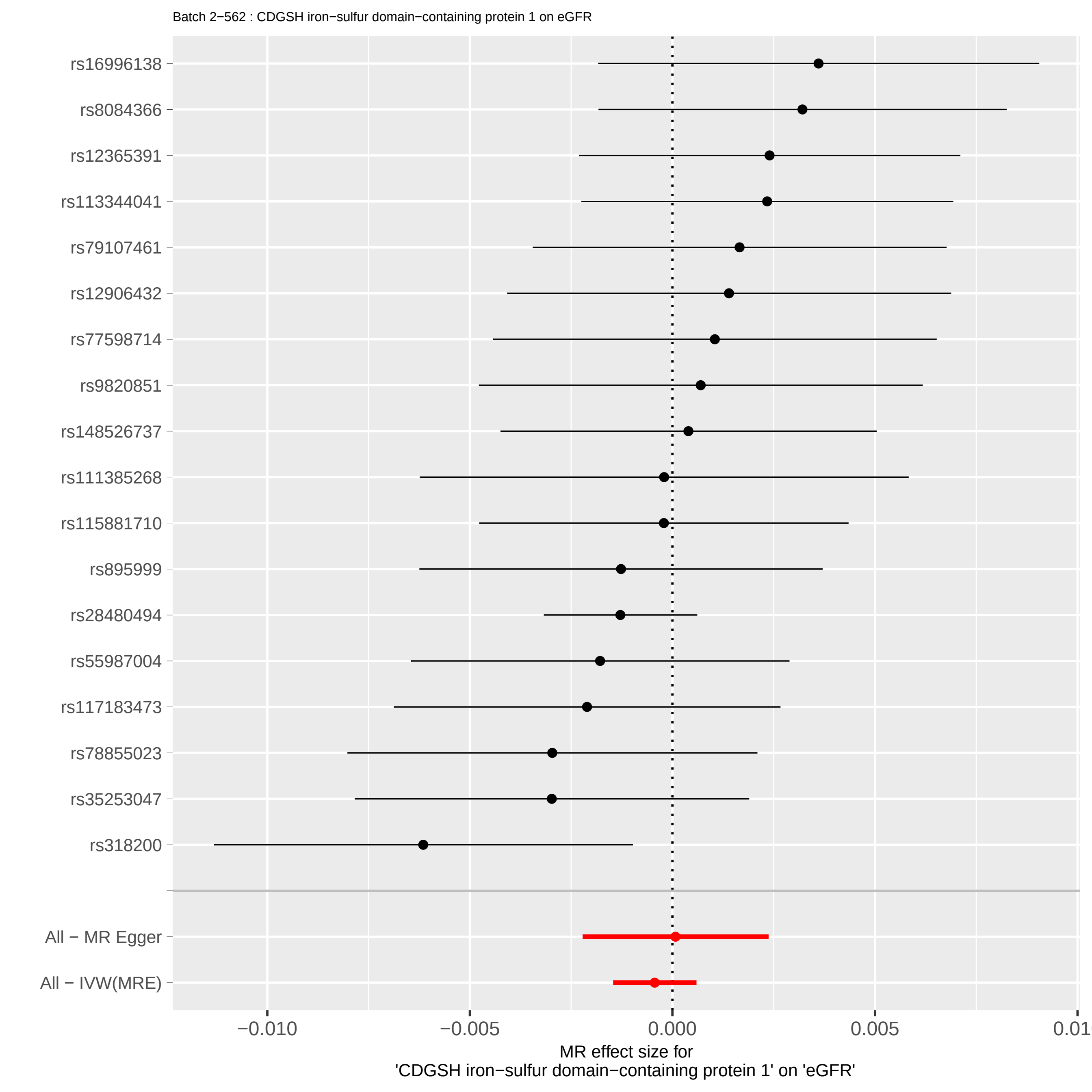
Batch 2−562 : CDGSH iron−sulfur domain−containing protein 1 on eGFR
rs16996138
rs8084366
rs12365391
rs113344041
rs79107461
rs12906432
rs77598714
rs9820851
rs148526737
rs111385268
rs115881710
rs895999
rs28480494
rs55987004
rs117183473
rs78855023
rs35253047
rs318200
All − MR Egger
All − IVW(MRE)
−0.010
−0.005
0.000
0.005
0.01
MR effect size for
'CDGSH iron−sulfur domain−containing protein 1' on 'eGFR'

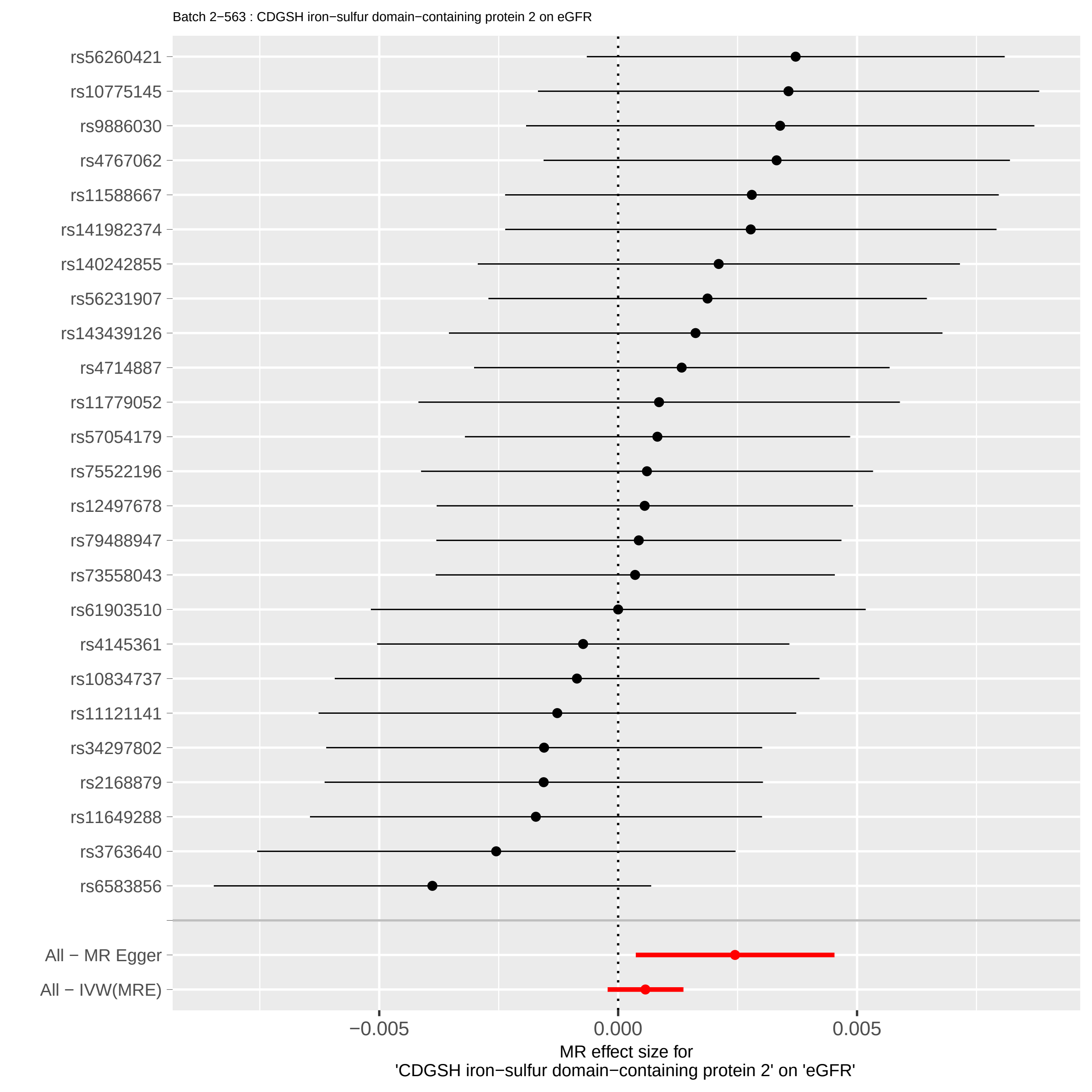

Batch 2−563 : CDGSH iron−sulfur domain−containing protein 2 on eGFR
rs56260421
rs10775145
rs9886030
rs4767062
rs11588667
rs141982374
rs140242855
rs56231907
rs143439126
rs4714887
rs11779052
rs57054179
rs75522196
rs12497678
rs79488947
rs73558043
rs61903510
rs4145361
rs10834737
rs11121141
rs34297802
rs2168879
rs11649288
rs3763640
rs6583856
All − MR Egger
All − IVW(MRE)
−0.005
0.000
0.005
MR effect size for
'CDGSH iron−sulfur domain−containing protein 2' on 'eGFR'

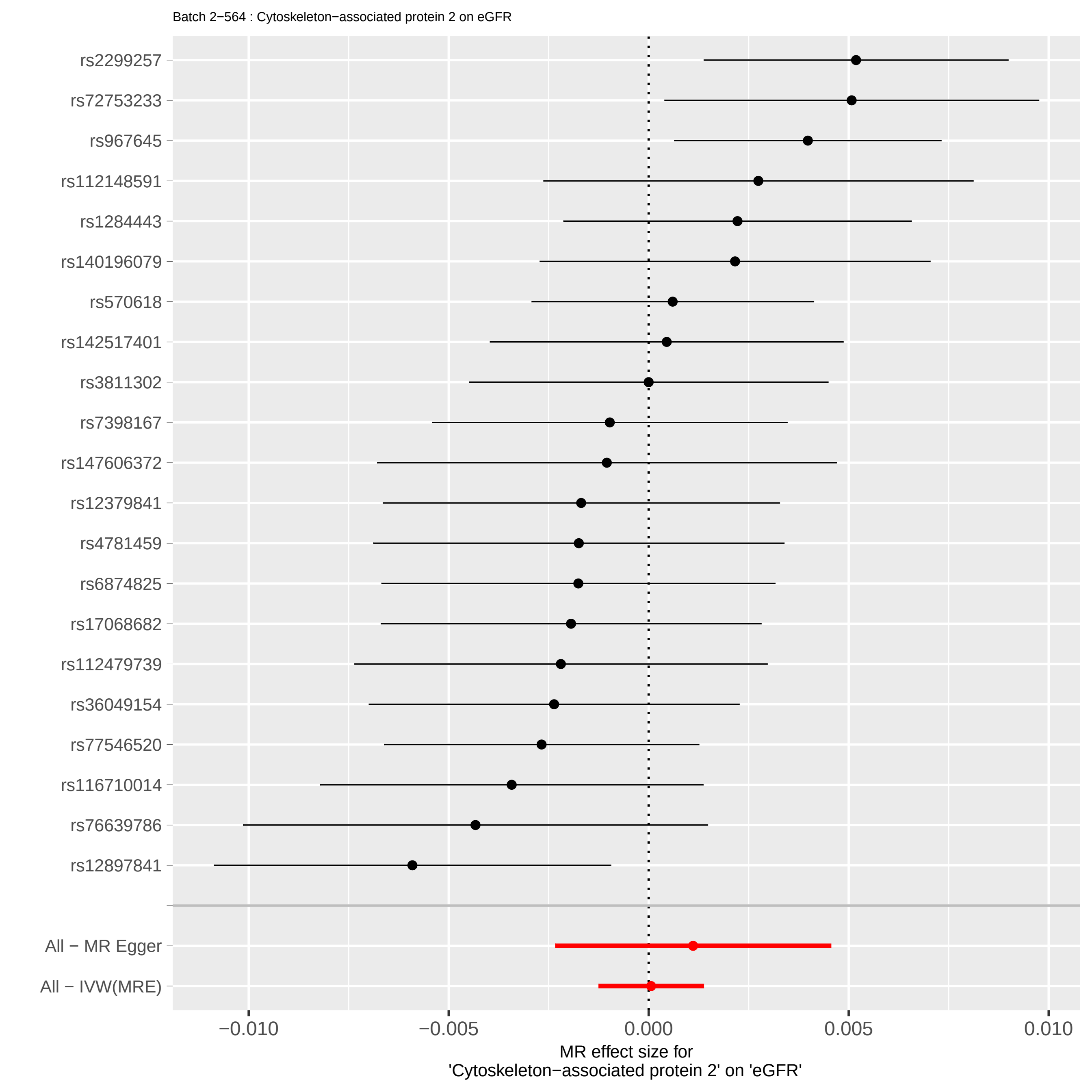
Batch 2−564 : Cytoskeleton−associated protein 2 on eGFR
rs2299257
rs72753233
rs967645
rs112148591
rs1284443
rs140196079
rs570618
rs142517401
rs3811302
rs7398167
rs147606372
rs12379841
rs4781459
rs6874825
rs17068682
rs112479739
rs36049154
rs77546520
rs116710014
rs76639786
rs12897841
All − MR Egger
All − IVW(MRE)
−0.010
−0.005
0.000
0.005
0.010
MR effect size for
'Cytoskeleton−associated protein 2' on 'eGFR'

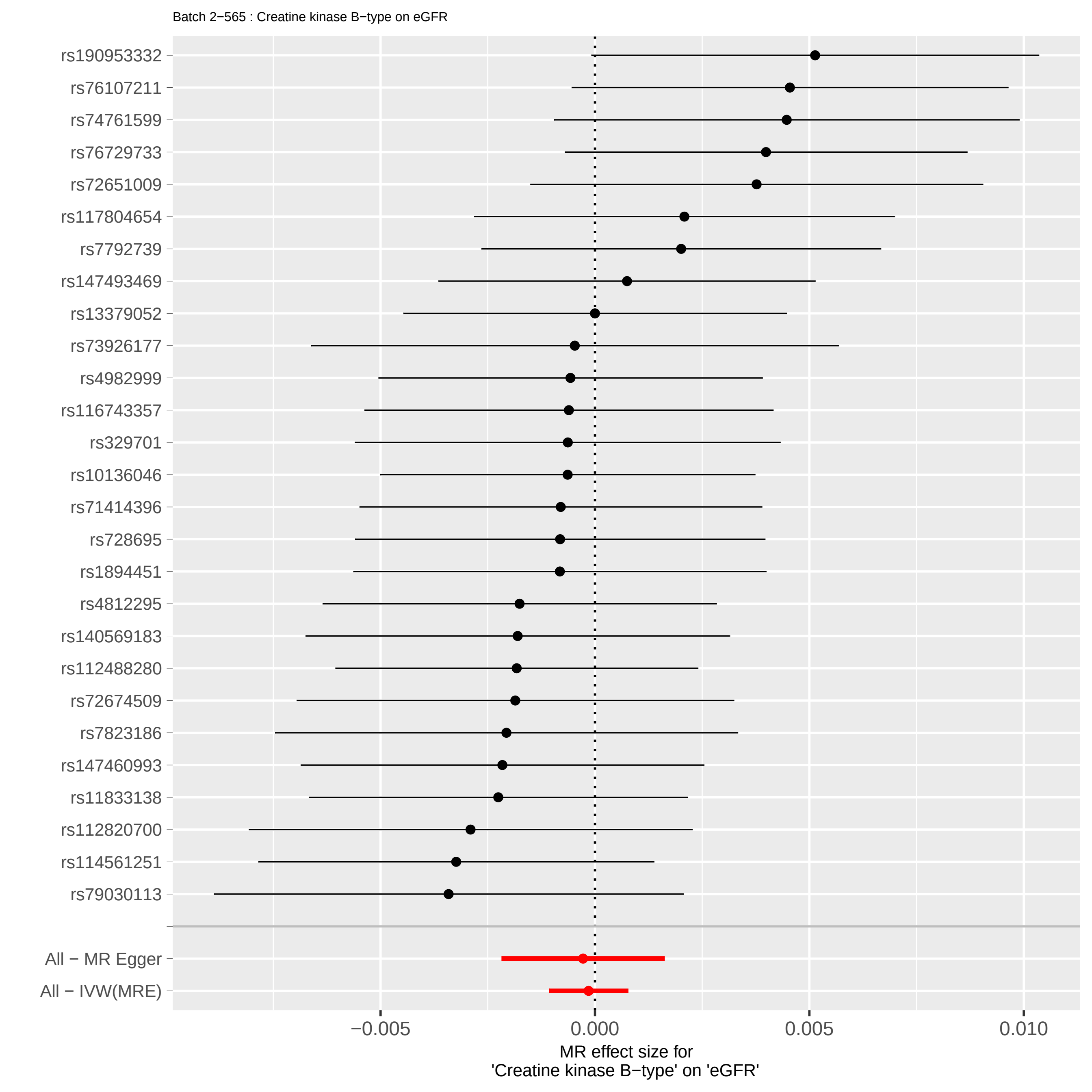

Batch 2−565 : Creatine kinase B−type on eGFR
rs190953332
rs76107211
rs74761599
rs76729733
rs72651009
rs117804654
rs7792739
rs147493469
rs13379052
rs73926177
rs4982999
rs116743357
rs329701
rs10136046
rs71414396
rs728695
rs1894451
rs4812295
rs140569183
rs112488280
rs72674509
rs7823186
rs147460993
rs11833138
rs112820700
rs114561251
rs79030113
All − MR Egger
All − IVW(MRE)
−0.005
0.000
0.005
0.010
MR effect size for
'Creatine kinase B−type' on 'eGFR'

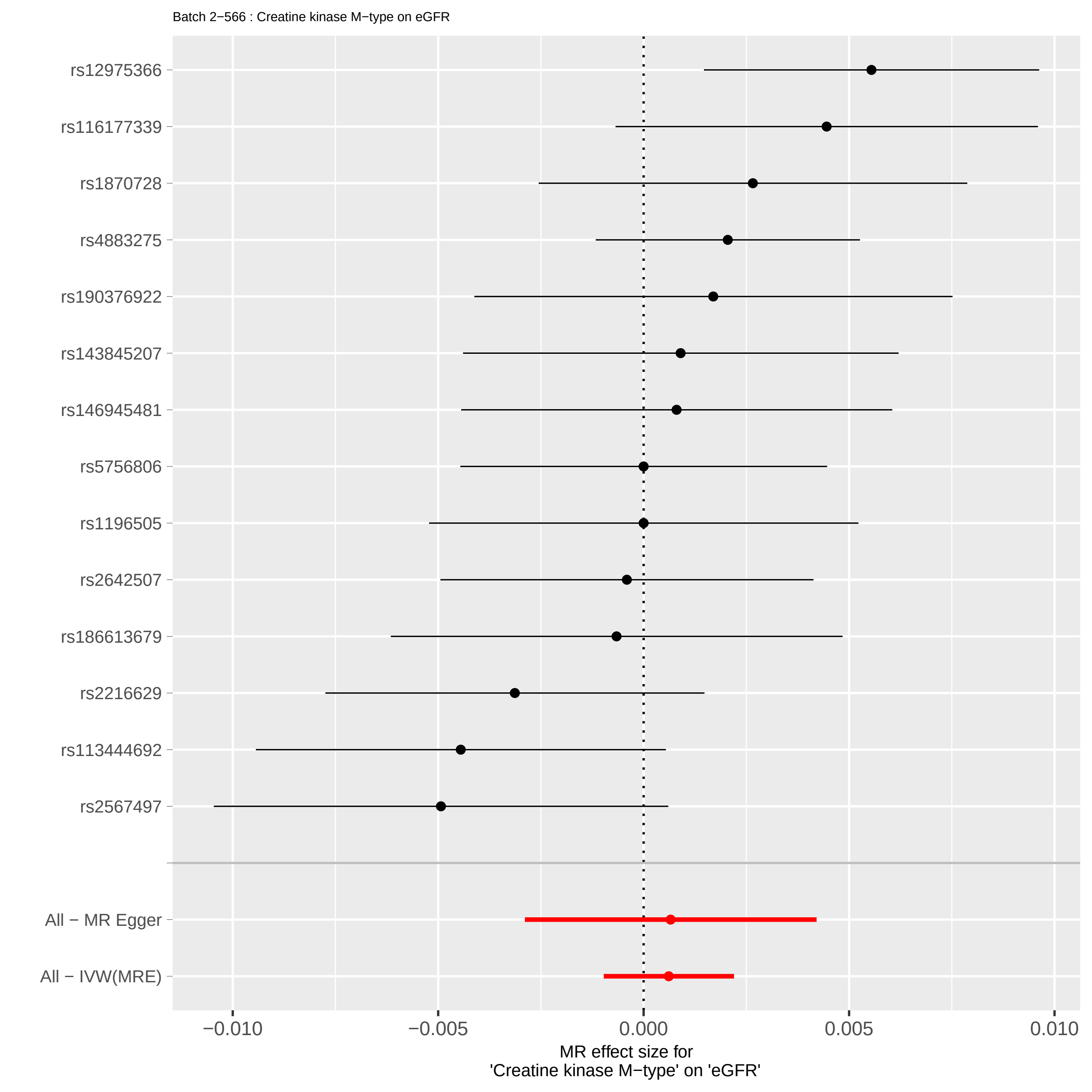

Batch 2−566 : Creatine kinase M−type on eGFR
rs12975366
rs116177339
rs1870728
rs4883275
rs190376922
rs143845207
rs146945481
rs5756806
rs1196505
rs2642507
rs186613679
rs2216629
rs113444692
rs2567497
All − MR Egger
All − IVW(MRE)
−0.010
−0.005
0.000
0.005
0.010
MR effect size for
'Creatine kinase M−type' on 'eGFR'

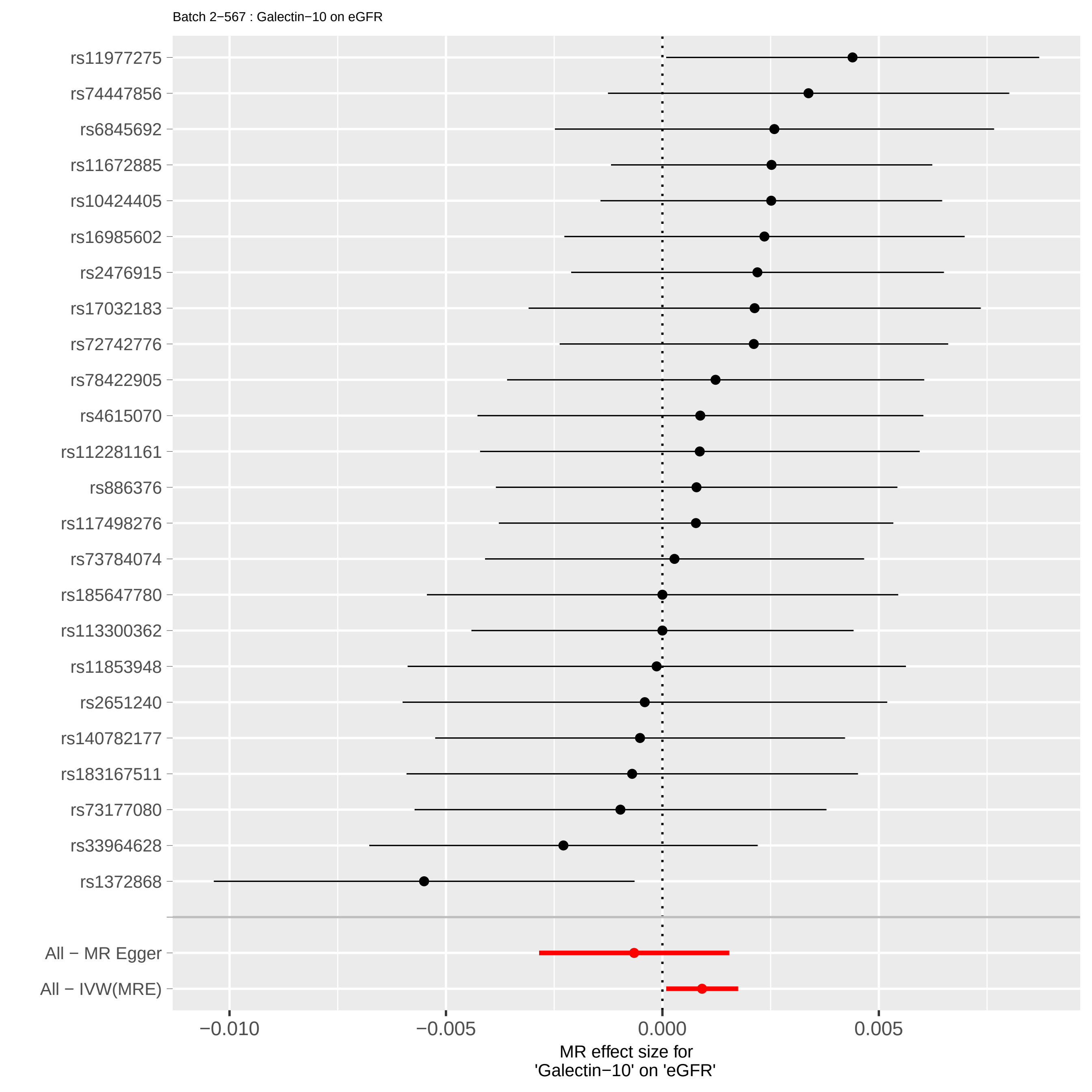

Batch 2−567 : Galectin−10 on eGFR
rs11977275
rs74447856
rs6845692
rs11672885
rs10424405
rs16985602
rs2476915
rs17032183
rs72742776
rs78422905
rs4615070
rs112281161
rs886376
rs117498276
rs73784074
rs185647780
rs113300362
rs11853948
rs2651240
rs140782177
rs183167511
rs73177080
rs33964628
rs1372868
All − MR Egger
All − IVW(MRE)
−0.010
−0.005
0.000
0.005
MR effect size for
'Galectin−10' on 'eGFR'

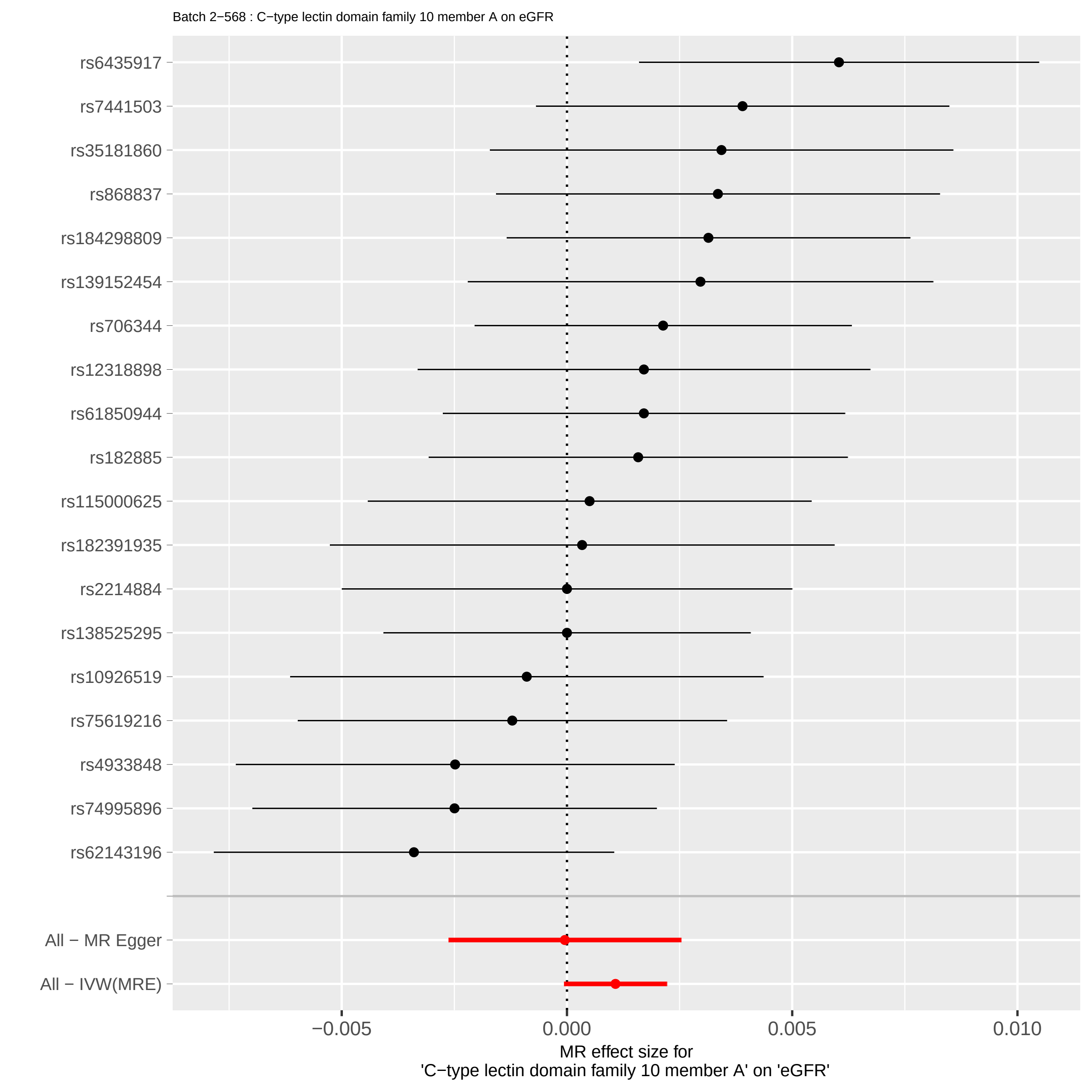

Batch 2−568 : C−type lectin domain family 10 member A on eGFR
rs6435917
rs7441503
rs35181860
rs868837
rs184298809
rs139152454
rs706344
rs12318898
rs61850944
rs182885
rs115000625
rs182391935
rs2214884
rs138525295
rs10926519
rs75619216
rs4933848
rs74995896
rs62143196
All − MR Egger
All − IVW(MRE)
−0.005
0.000
0.005
0.010
MR effect size for
'C−type lectin domain family 10 member A' on 'eGFR'

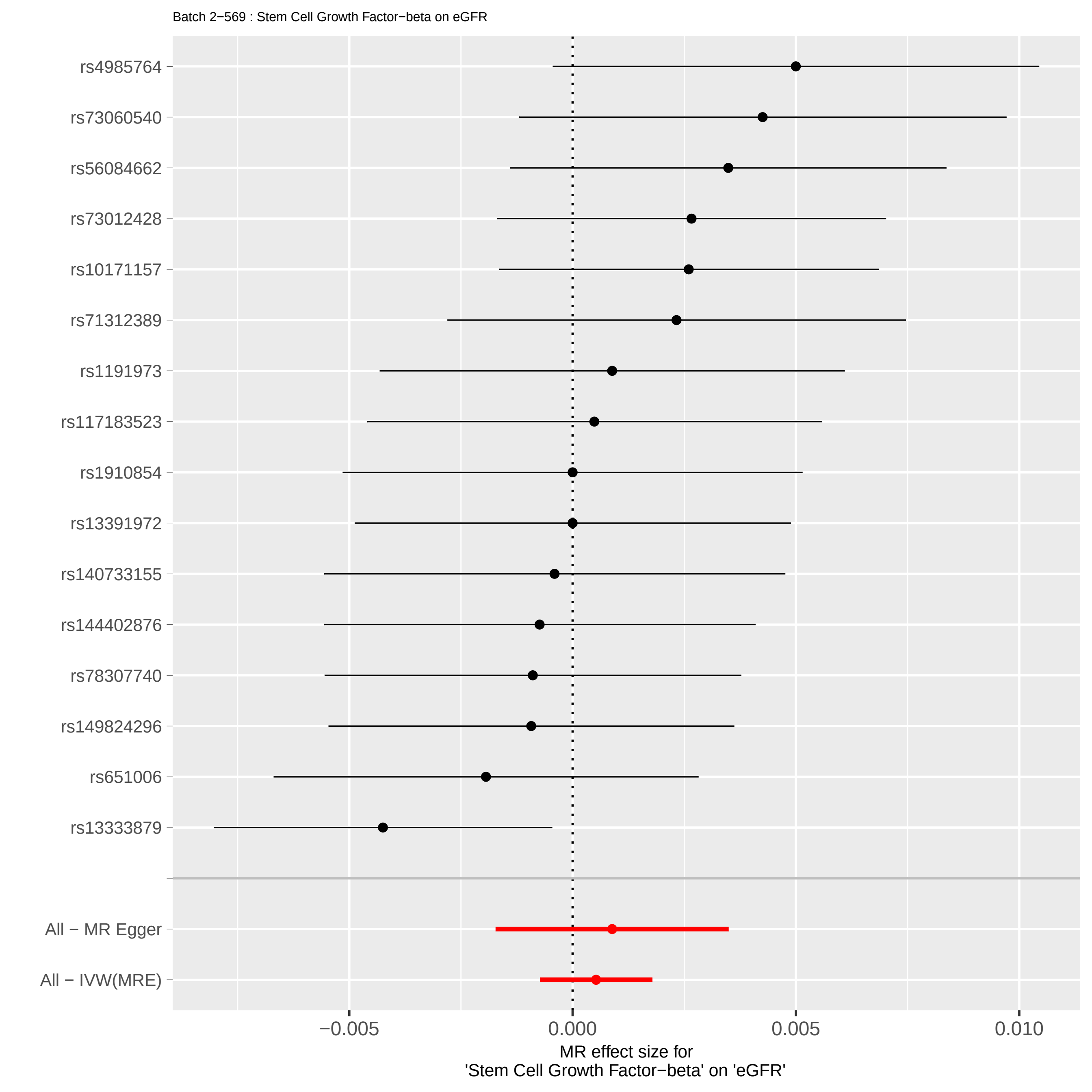

Batch 2−569 : Stem Cell Growth Factor−beta on eGFR
rs4985764
rs73060540
rs56084662
rs73012428
rs10171157
rs71312389
rs1191973
rs117183523
rs1910854
rs13391972
rs140733155
rs144402876
rs78307740
rs149824296
rs651006
rs13333879
All − MR Egger
All − IVW(MRE)
−0.005
0.000
0.005
0.010
MR effect size for
'Stem Cell Growth Factor−beta' on 'eGFR'

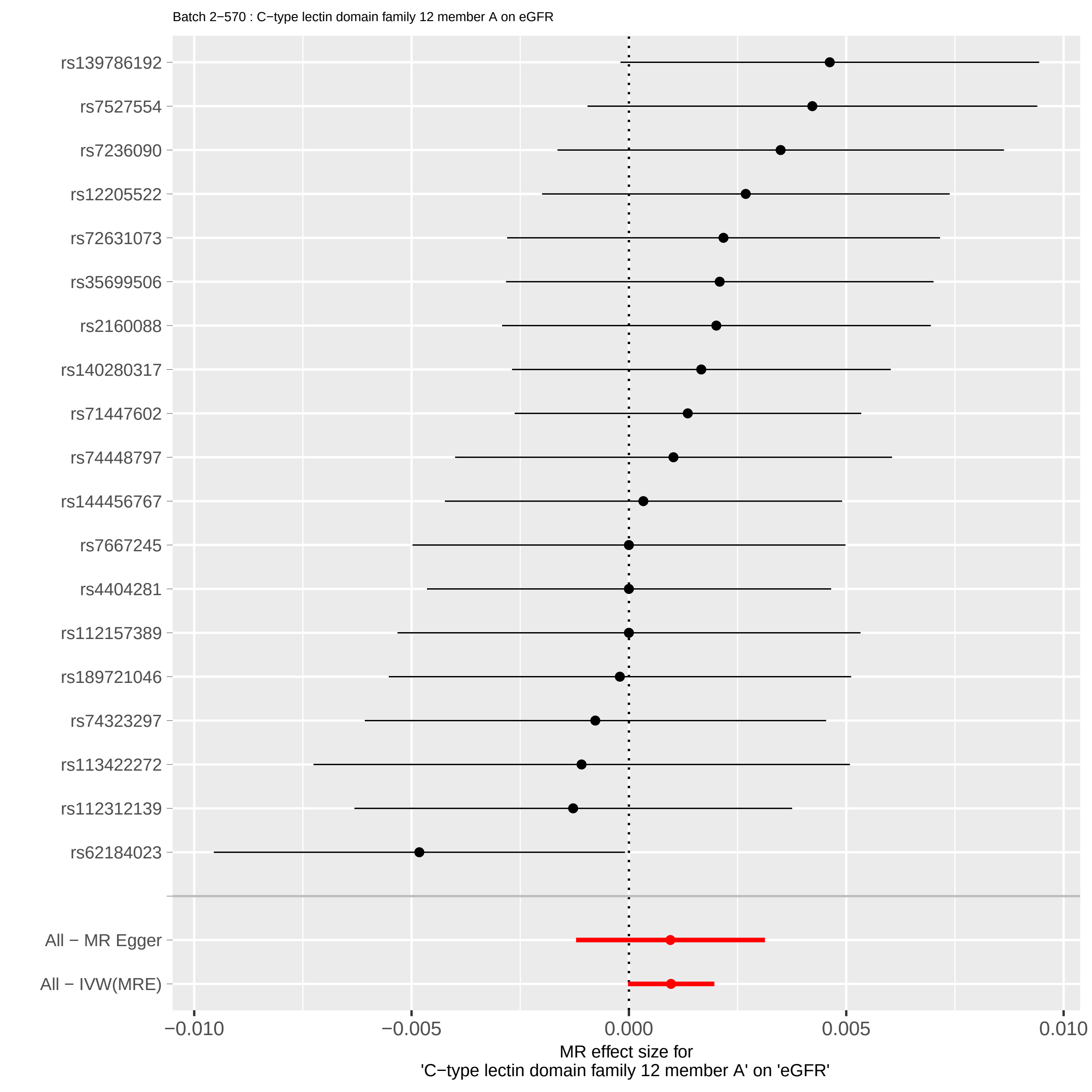
Batch 2−570 : C−type lectin domain family 12 member A on eGFR
rs139786192
rs7527554
rs7236090
rs12205522
rs72631073
rs35699506
rs2160088
rs140280317
rs71447602
rs74448797
rs144456767
rs7667245
rs4404281
rs112157389
rs189721046
rs74323297
rs113422272
rs112312139
rs62184023
All − MR Egger
All − IVW(MRE)
−0.010
−0.005
0.000
0.005
0.010
MR effect size for
'C−type lectin domain family 12 member A' on 'eGFR'

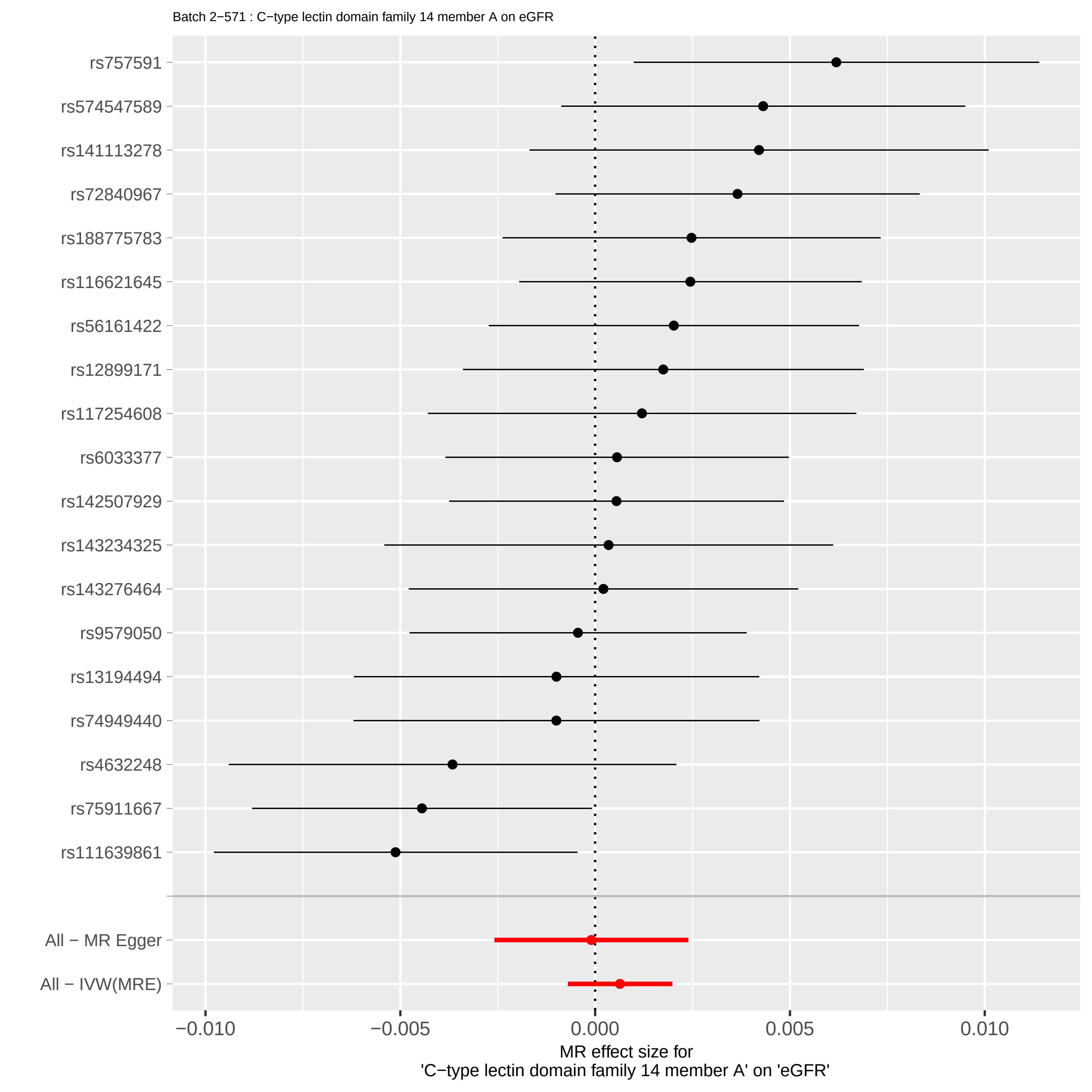

Batch 2−571 : C−type lectin domain family 14 member A on eGFR
rs757591
rs574547589
rs141113278
rs72840967
rs188775783
rs116621645
rs56161422
rs12899171
rs117254608
rs6033377
rs142507929
rs143234325
rs143276464
rs9579050
rs13194494
rs74949440
rs4632248
rs75911667
rs111639861
All − MR Egger
All − IVW(MRE)
−0.010
−0.005
0.000
0.005
0.010
MR effect size for
'C−type lectin domain family 14 member A' on 'eGFR'

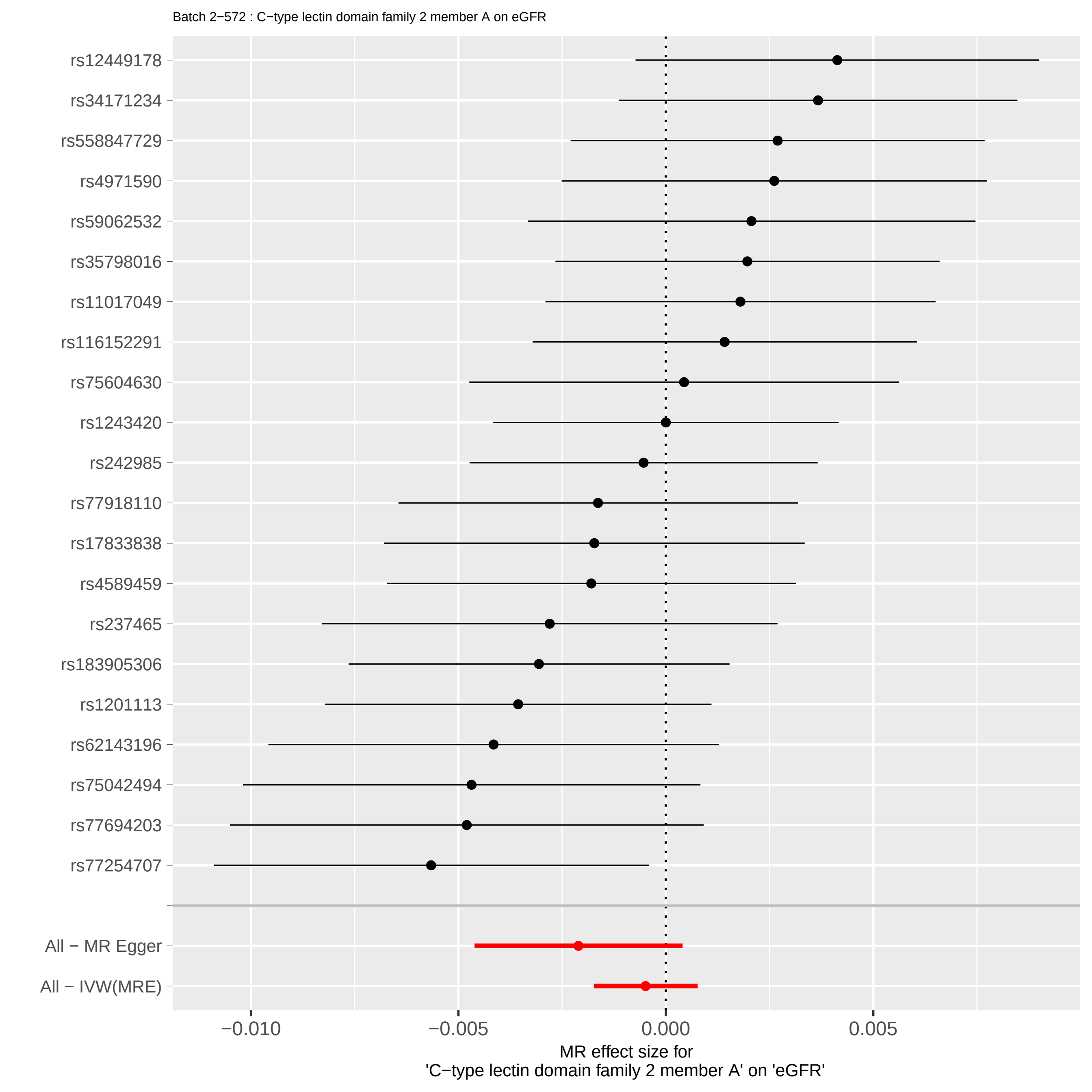
Batch 2−572 : C−type lectin domain family 2 member A on eGFR
rs12449178
rs34171234
rs558847729
rs4971590
rs59062532
rs35798016
rs11017049
rs116152291
rs75604630
rs1243420
rs242985
rs77918110
rs17833838
rs4589459
rs237465
rs183905306
rs1201113
rs62143196
rs75042494
rs77694203
rs77254707
All − MR Egger
All − IVW(MRE)
−0.010
−0.005
0.000
0.005
MR effect size for
'C−type lectin domain family 2 member A' on 'eGFR'

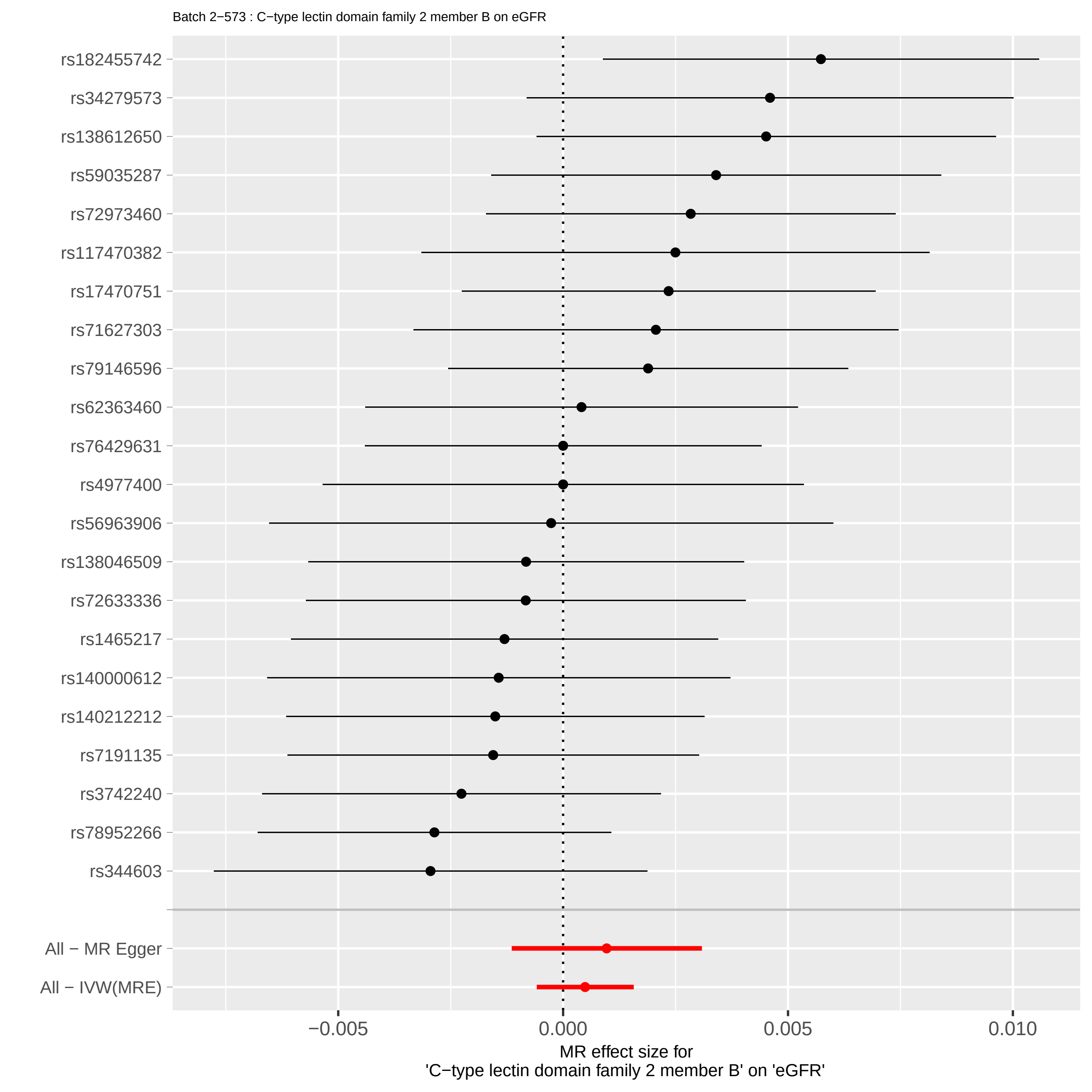

Batch 2−573 : C−type lectin domain family 2 member B on eGFR
rs182455742
rs34279573
rs138612650
rs59035287
rs72973460
rs117470382
rs17470751
rs71627303
rs79146596
rs62363460
rs76429631
rs4977400
rs56963906
rs138046509
rs72633336
rs1465217
rs140000612
rs140212212
rs7191135
rs3742240
rs78952266
rs344603
All − MR Egger
All − IVW(MRE)
−0.005
0.000
0.005
0.010
MR effect size for
'C−type lectin domain family 2 member B' on 'eGFR'

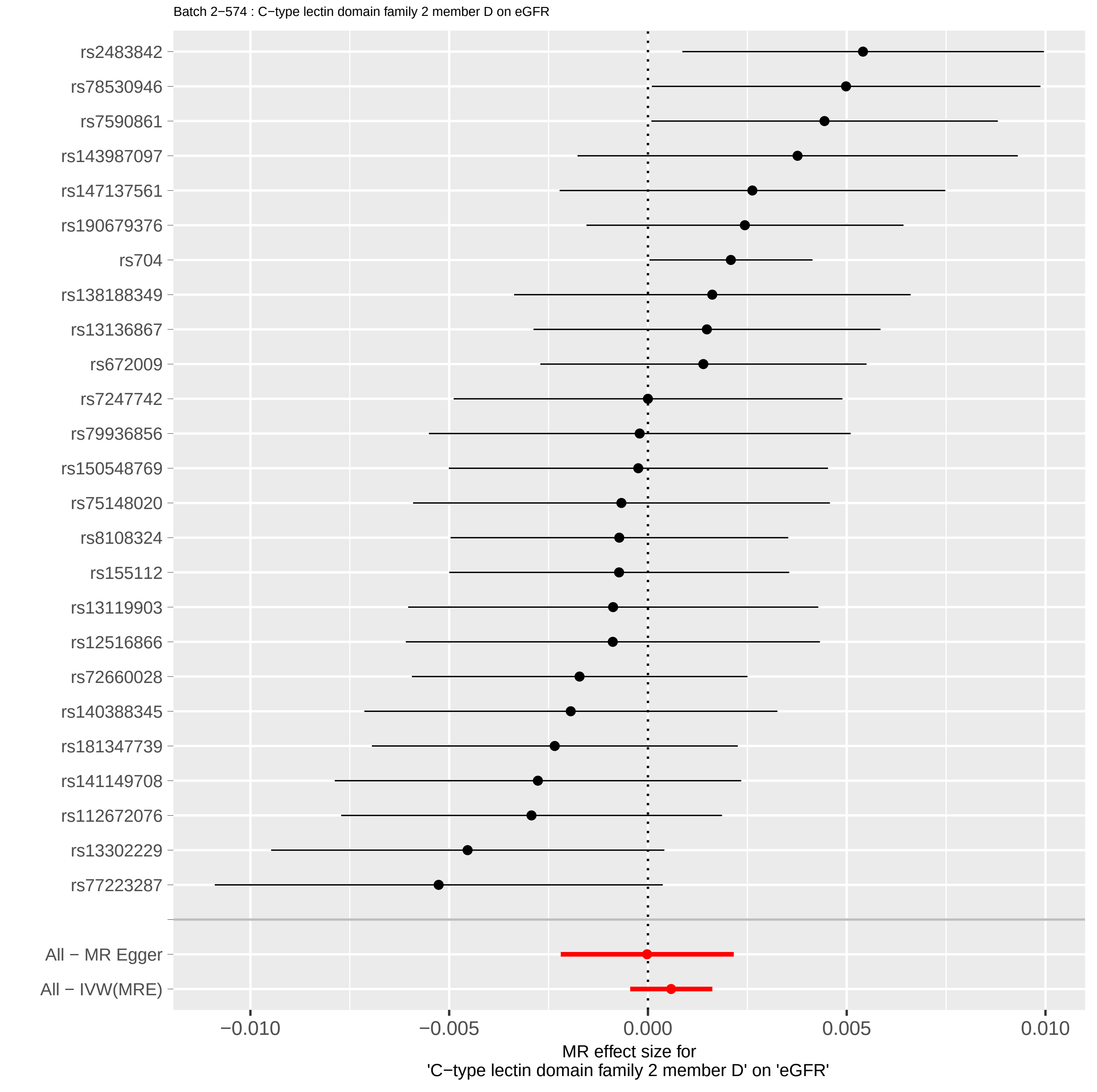

Batch 2−574 : C−type lectin domain family 2 member D on eGFR
rs2483842
rs78530946
rs7590861
rs143987097
rs147137561
rs190679376
rs704
rs138188349
rs13136867
rs672009
rs7247742
rs79936856
rs150548769
rs75148020
rs8108324
rs155112
rs13119903
rs12516866
rs72660028
rs140388345
rs181347739
rs141149708
rs112672076
rs13302229
rs77223287
All − MR Egger
All − IVW(MRE)
−0.010
−0.005
0.000
0.005
0.010
MR effect size for
'C−type lectin domain family 2 member D' on 'eGFR'

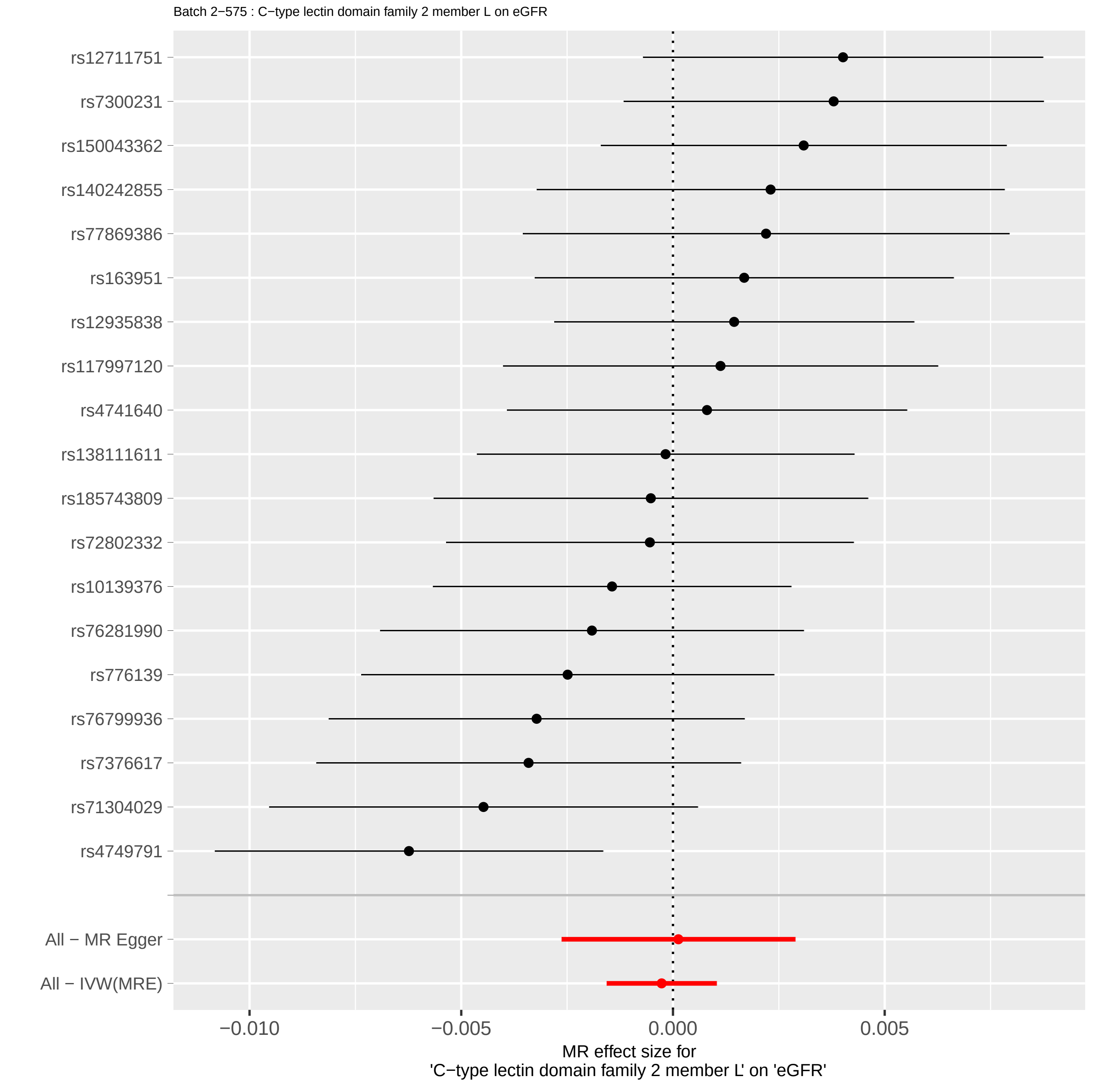

Batch 2−575 : C−type lectin domain family 2 member L on eGFR
rs12711751
rs7300231
rs150043362
rs140242855
rs77869386
rs163951
rs12935838
rs117997120
rs4741640
rs138111611
rs185743809
rs72802332
rs10139376
rs76281990
rs776139
rs76799936
rs7376617
rs71304029
rs4749791
All − MR Egger
All − IVW(MRE)
−0.010
−0.005
0.000
0.005
MR effect size for
'C−type lectin domain family 2 member L' on 'eGFR'

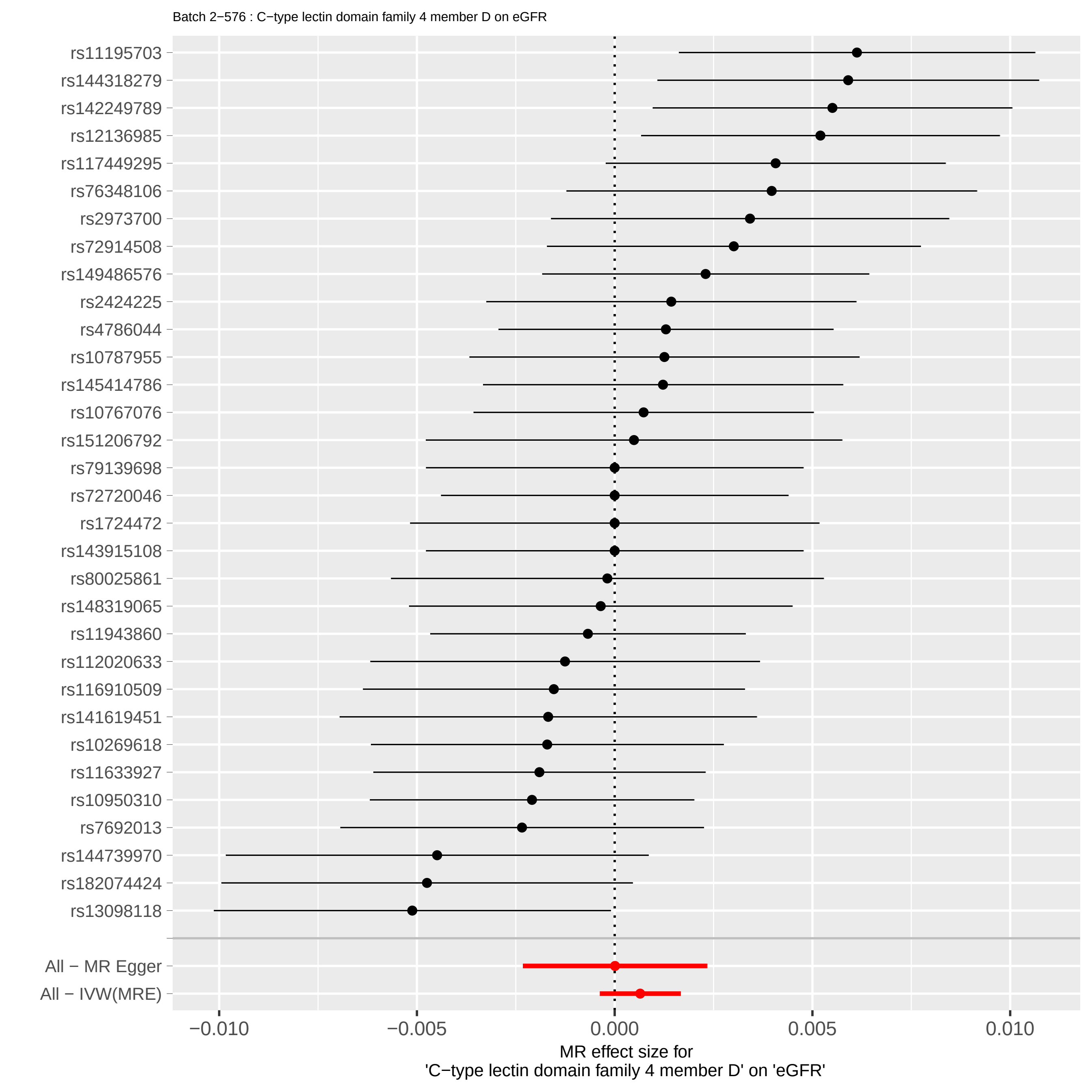

Batch 2−576 : C−type lectin domain family 4 member D on eGFR
rs11195703
rs144318279
rs142249789
rs12136985
rs117449295
rs76348106
rs2973700
rs72914508
rs149486576
rs2424225
rs4786044
rs10787955
rs145414786
rs10767076
rs151206792
rs79139698
rs72720046
rs1724472
rs143915108
rs80025861
rs148319065
rs11943860
rs112020633
rs116910509
rs141619451
rs10269618
rs11633927
rs10950310
rs7692013
rs144739970
rs182074424
rs13098118
All − MR Egger
All − IVW(MRE)
−0.010
−0.005
0.000
0.005
0.010
MR effect size for
'C−type lectin domain family 4 member D' on 'eGFR'

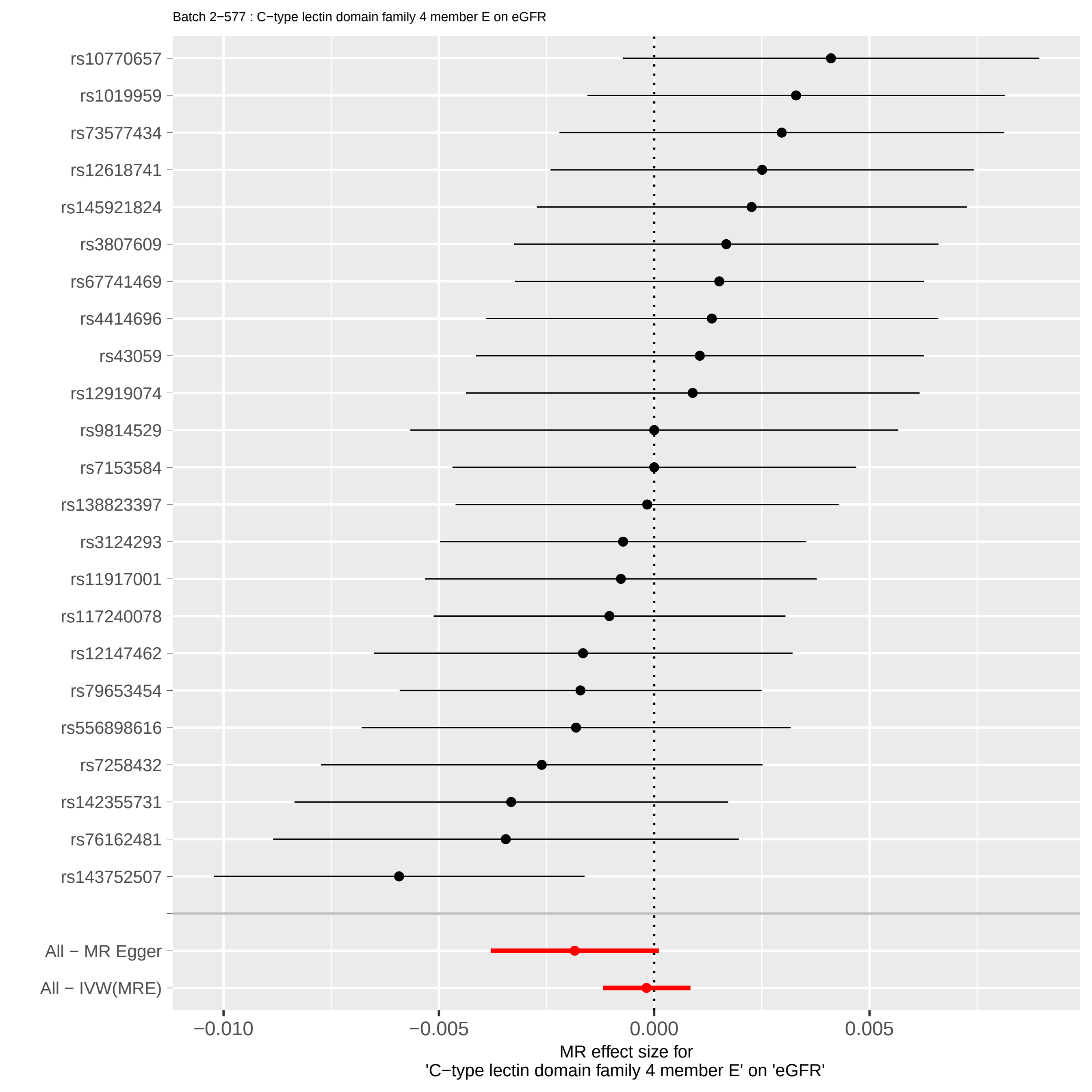

Batch 2−577 : C−type lectin domain family 4 member E on eGFR
rs10770657
rs1019959
rs73577434
rs12618741
rs145921824
rs3807609
rs67741469
rs4414696
rs43059
rs12919074
rs9814529
rs7153584
rs138823397
rs3124293
rs11917001
rs117240078
rs12147462
rs79653454
rs556898616
rs7258432
rs142355731
rs76162481
rs143752507
All − MR Egger
All − IVW(MRE)
−0.010
−0.005
0.000
0.005
MR effect size for
'C−type lectin domain family 4 member E' on 'eGFR'

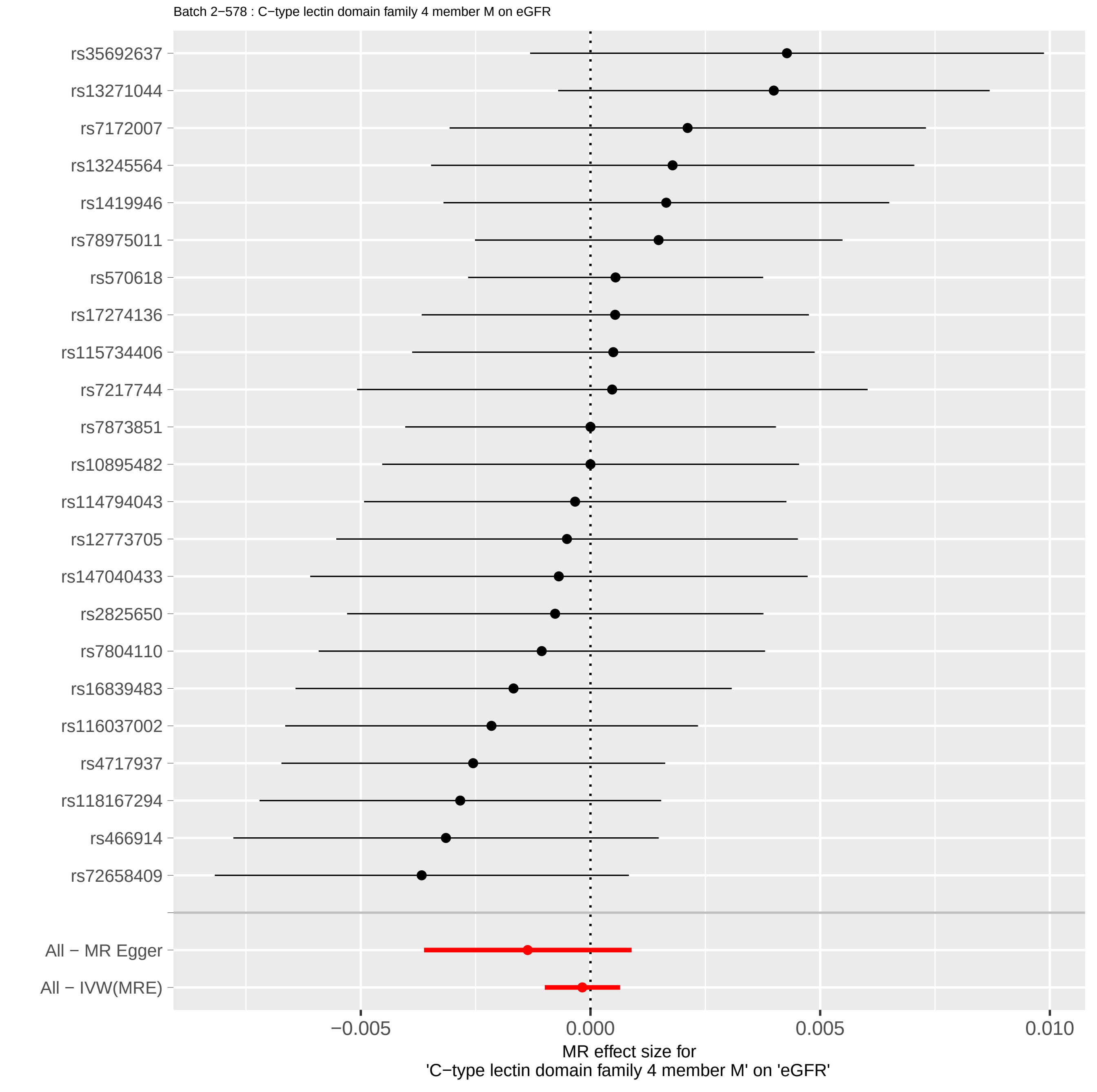
Batch 2−578 : C−type lectin domain family 4 member M on eGFR
rs35692637
rs13271044
rs7172007
rs13245564
rs1419946
rs78975011
rs570618
rs17274136
rs115734406
rs7217744
rs7873851
rs10895482
rs114794043
rs12773705
rs147040433
rs2825650
rs7804110
rs16839483
rs116037002
rs4717937
rs118167294
rs466914
rs72658409
All − MR Egger
All − IVW(MRE)
−0.005
0.000
0.005
0.010
MR effect size for
'C−type lectin domain family 4 member M' on 'eGFR'

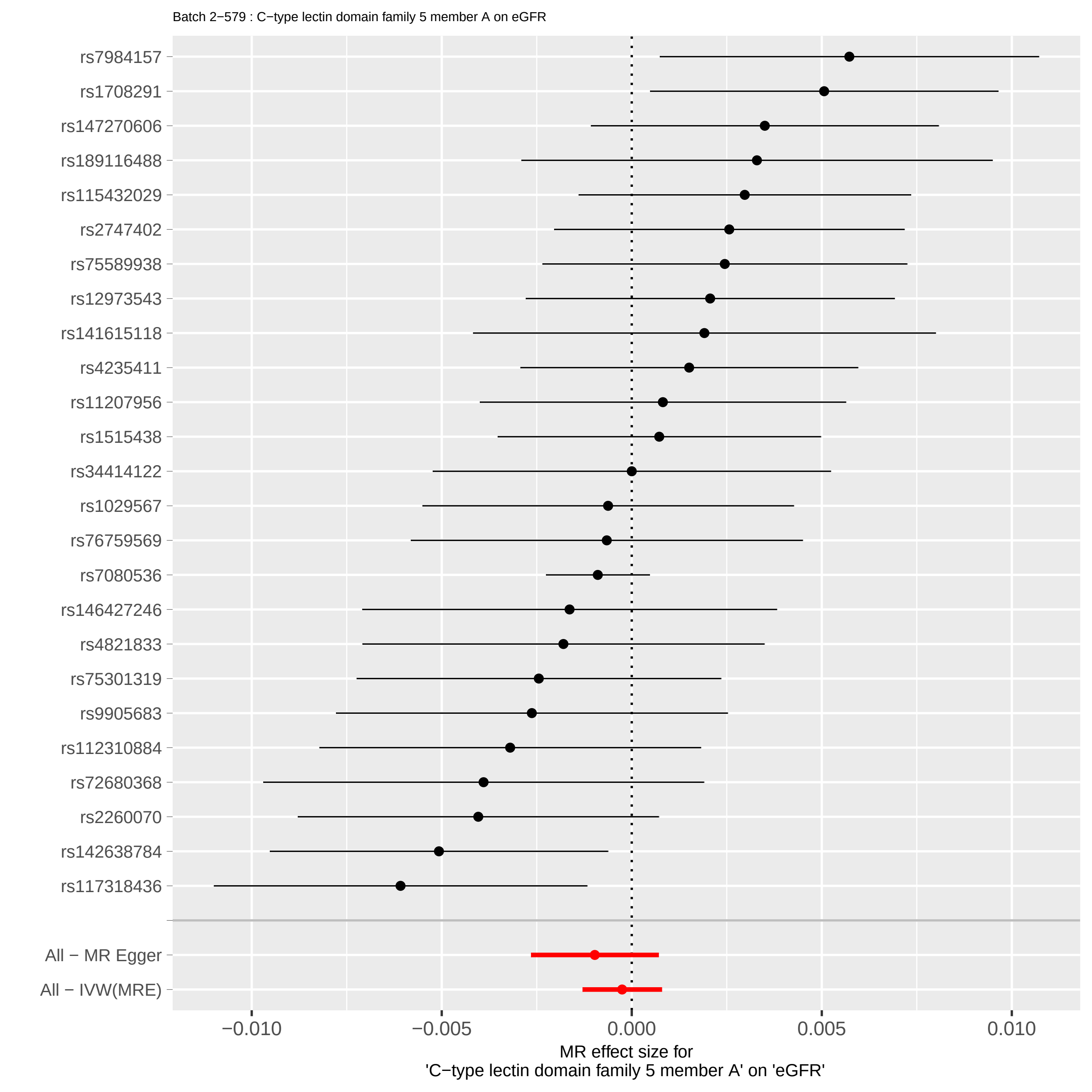
Batch 2−579 : C−type lectin domain family 5 member A on eGFR
rs7984157
rs1708291
rs147270606
rs189116488
rs115432029
rs2747402
rs75589938
rs12973543
rs141615118
rs4235411
rs11207956
rs1515438
rs34414122
rs1029567
rs76759569
rs7080536
rs146427246
rs4821833
rs75301319
rs9905683
rs112310884
rs72680368
rs2260070
rs142638784
rs117318436
All − MR Egger
All − IVW(MRE)
−0.010
−0.005
0.000
0.005
0.010
MR effect size for
'C−type lectin domain family 5 member A' on 'eGFR'

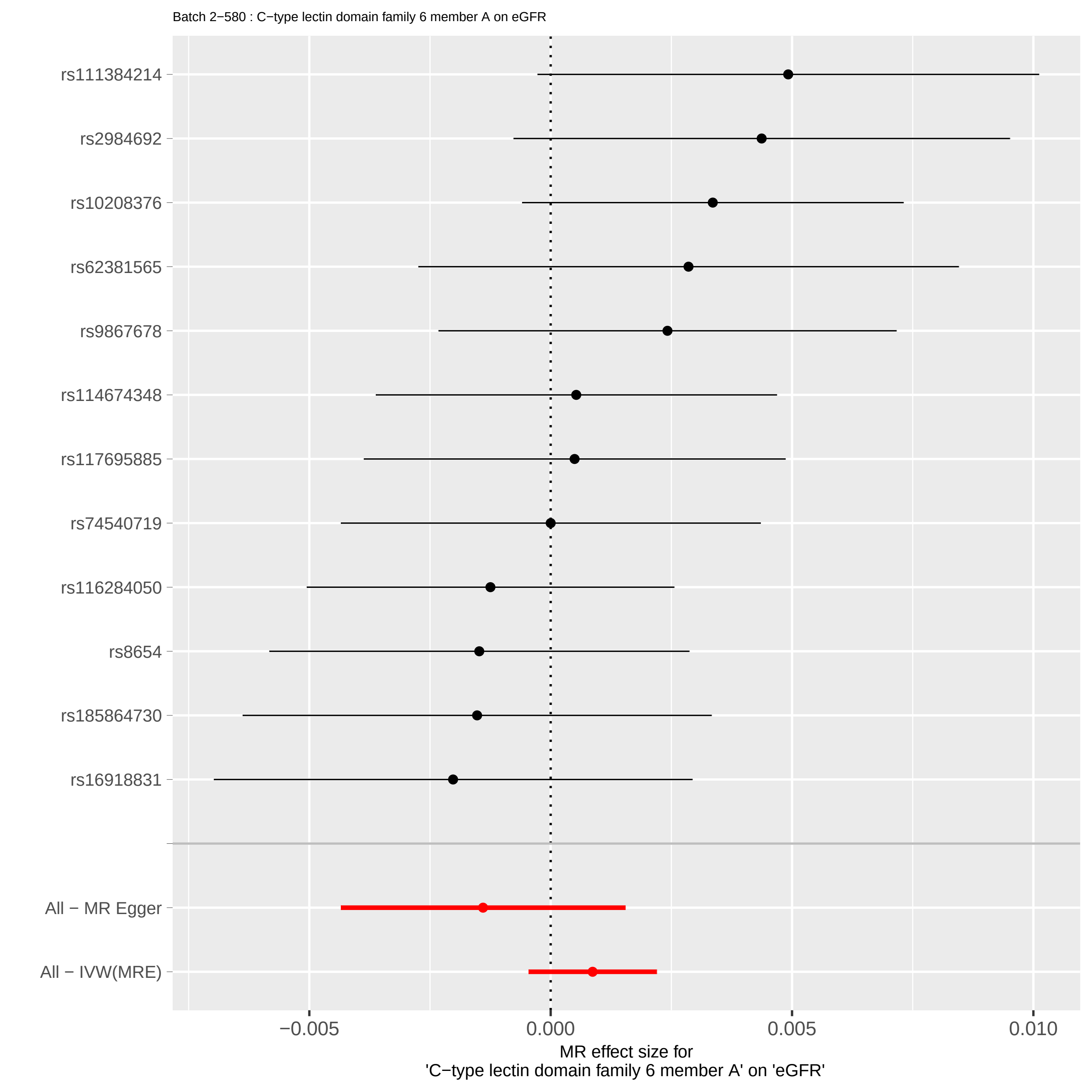
Batch 2−580 : C−type lectin domain family 6 member A on eGFR
rs111384214
rs2984692
rs10208376
rs62381565
rs9867678
rs114674348
rs117695885
rs74540719
rs116284050
rs8654
rs185864730
rs16918831
All − MR Egger
All − IVW(MRE)
−0.005
0.000
0.005
0.010
MR effect size for
'C−type lectin domain family 6 member A' on 'eGFR'

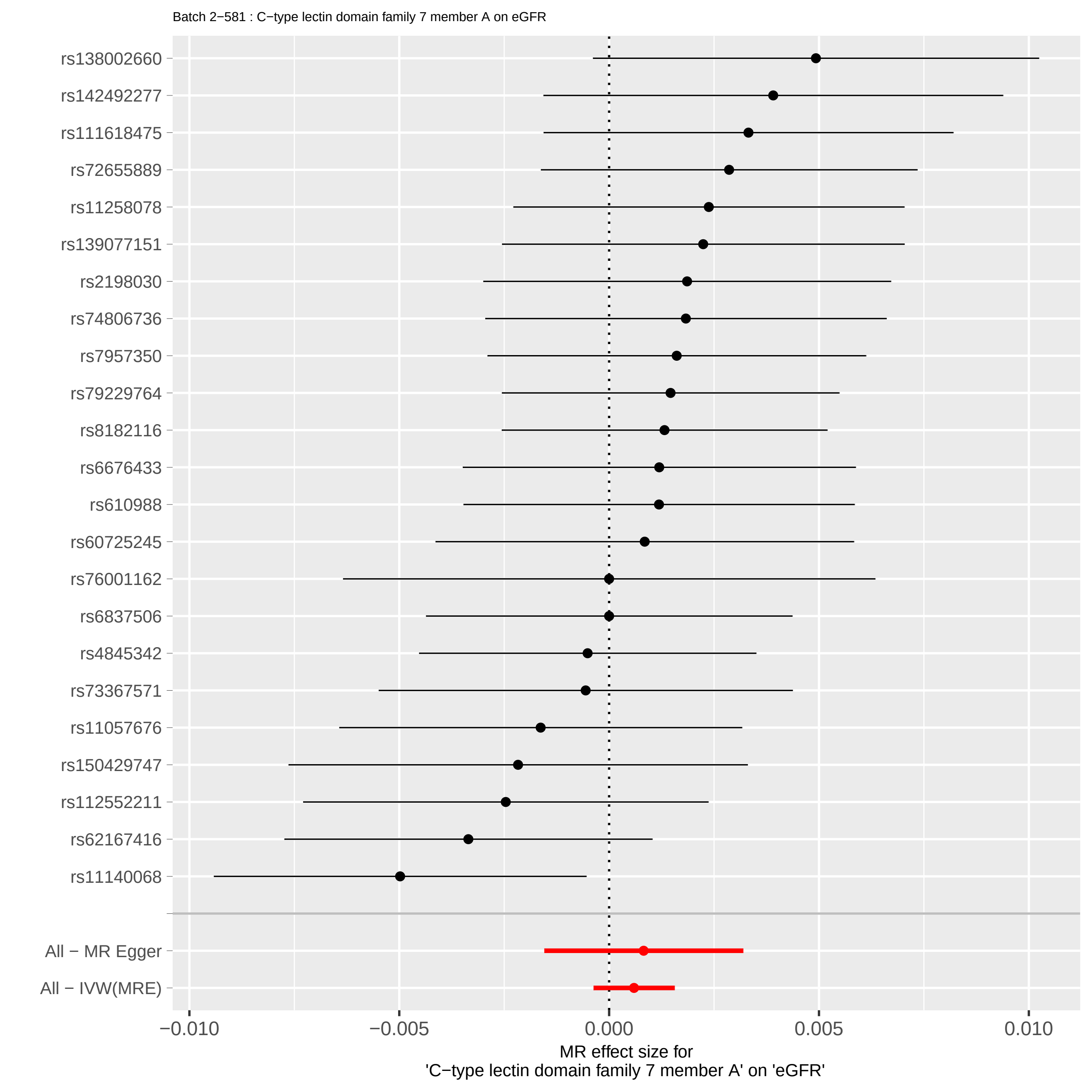

Batch 2−581 : C−type lectin domain family 7 member A on eGFR
rs138002660
rs142492277
rs111618475
rs72655889
rs11258078
rs139077151
rs2198030
rs74806736
rs7957350
rs79229764
rs8182116
rs6676433
rs610988
rs60725245
rs76001162
rs6837506
rs4845342
rs73367571
rs11057676
rs150429747
rs112552211
rs62167416
rs11140068
All − MR Egger
All − IVW(MRE)
−0.010
−0.005
0.000
0.005
0.010
MR effect size for
'C−type lectin domain family 7 member A' on 'eGFR'

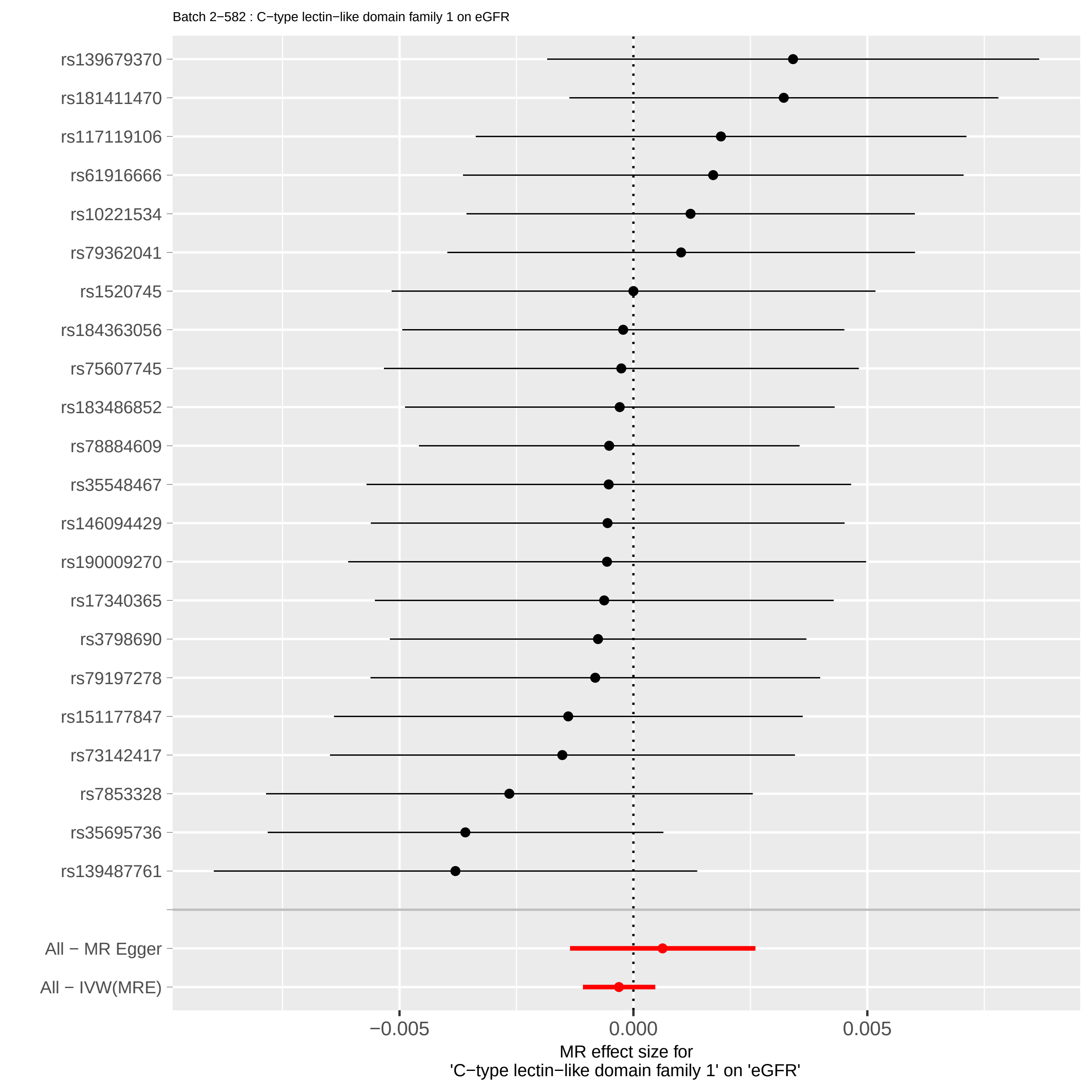

Batch 2−582 : C−type lectin−like domain family 1 on eGFR
rs139679370
rs181411470
rs117119106
rs61916666
rs10221534
rs79362041
rs1520745
rs184363056
rs75607745
rs183486852
rs78884609
rs35548467
rs146094429
rs190009270
rs17340365
rs3798690
rs79197278
rs151177847
rs73142417
rs7853328
rs35695736
rs139487761
All − MR Egger
All − IVW(MRE)
−0.005
0.000
0.005
MR effect size for
'C−type lectin−like domain family 1' on 'eGFR'

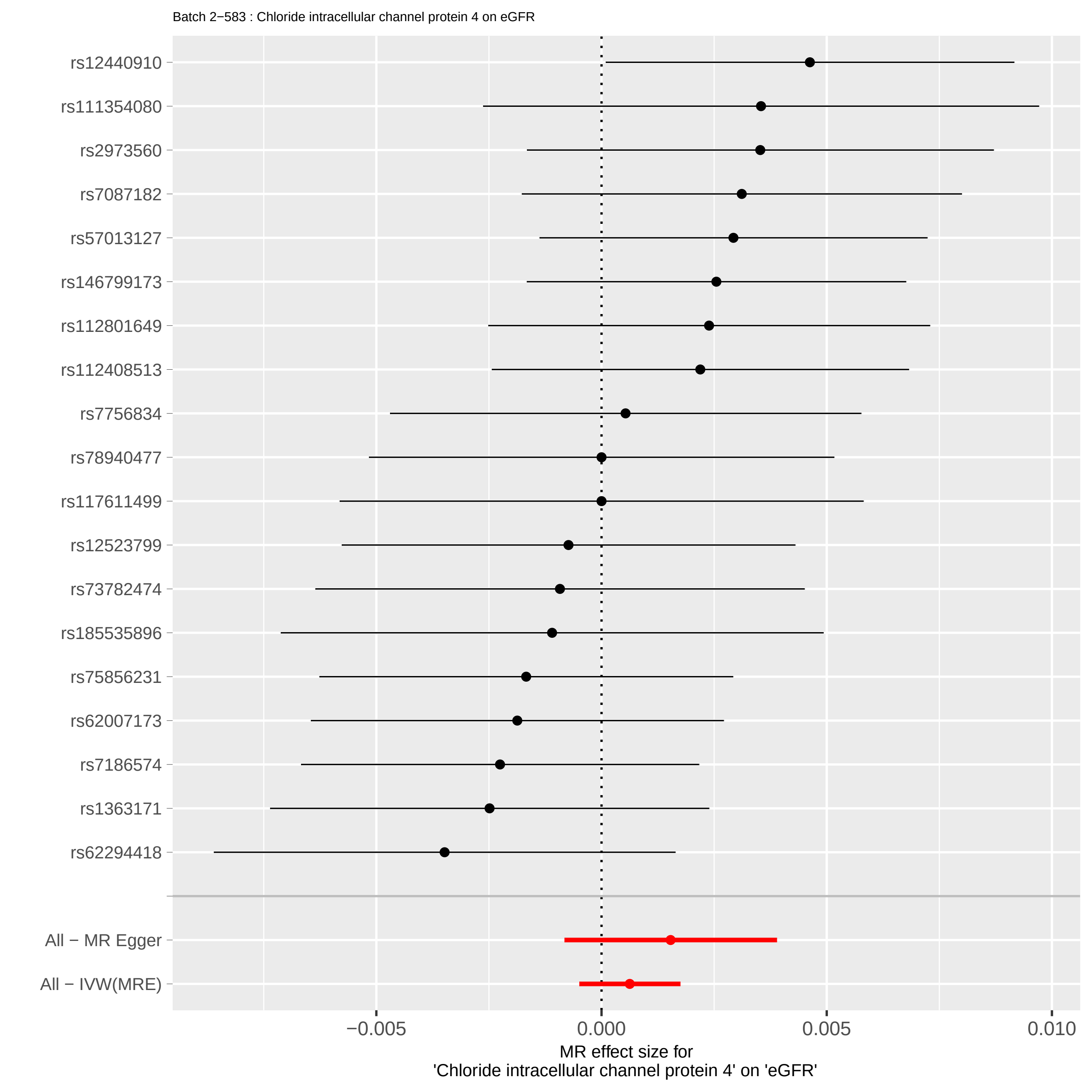
Batch 2−583 : Chloride intracellular channel protein 4 on eGFR
rs12440910
rs111354080
rs2973560
rs7087182
rs57013127
rs146799173
rs112801649
rs112408513
rs7756834
rs78940477
rs117611499
rs12523799
rs73782474
rs185535896
rs75856231
rs62007173
rs7186574
rs1363171
rs62294418
All − MR Egger
All − IVW(MRE)
−0.005
0.000
0.005
0.010
MR effect size for
'Chloride intracellular channel protein 4' on 'eGFR'

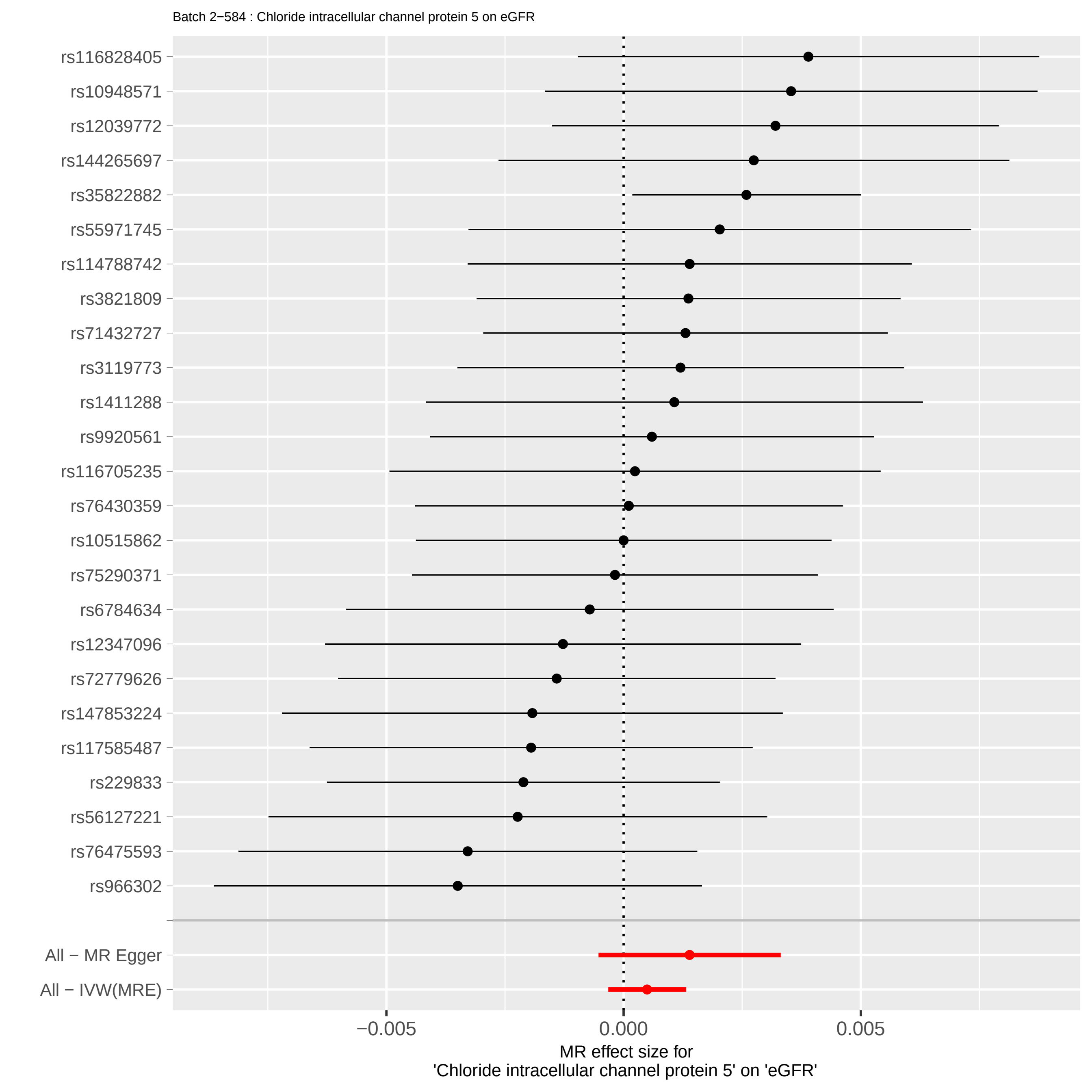

Batch 2−584 : Chloride intracellular channel protein 5 on eGFR
rs116828405
rs10948571
rs12039772
rs144265697
rs35822882
rs55971745
rs114788742
rs3821809
rs71432727
rs3119773
rs1411288
rs9920561
rs116705235
rs76430359
rs10515862
rs75290371
rs6784634
rs12347096
rs72779626
rs147853224
rs117585487
rs229833
rs56127221
rs76475593
rs966302
All − MR Egger
All − IVW(MRE)
−0.005
0.000
0.005
MR effect size for
'Chloride intracellular channel protein 5' on 'eGFR'

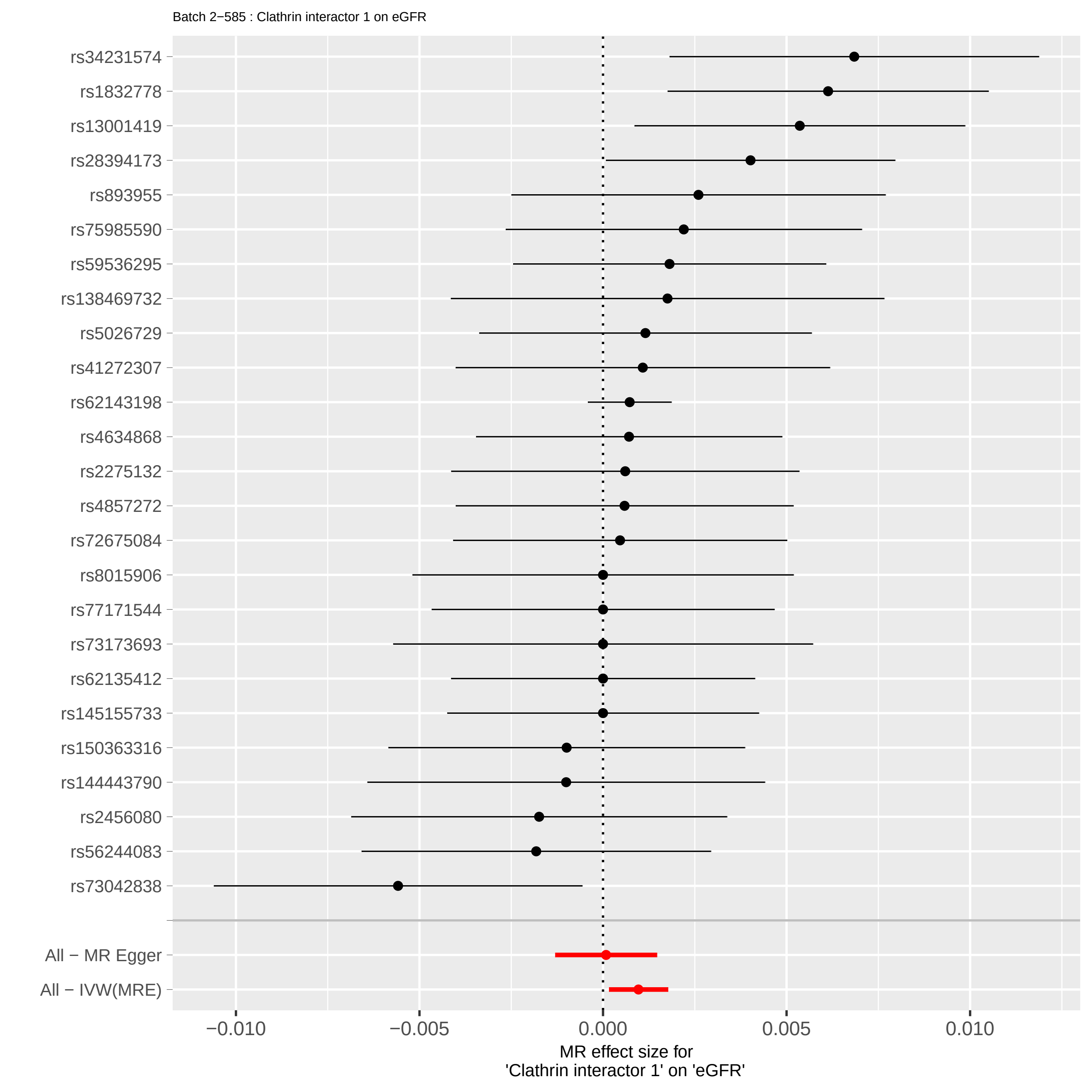
Batch 2−585 : Clathrin interactor 1 on eGFR
rs34231574
rs1832778
rs13001419
rs28394173
rs893955
rs75985590
rs59536295
rs138469732
rs5026729
rs41272307
rs62143198
rs4634868
rs2275132
rs4857272
rs72675084
rs8015906
rs77171544
rs73173693
rs62135412
rs145155733
rs150363316
rs144443790
rs2456080
rs56244083
rs73042838
All − MR Egger
All − IVW(MRE)
−0.010
−0.005
0.000
0.005
0.010
MR effect size for
'Clathrin interactor 1' on 'eGFR'

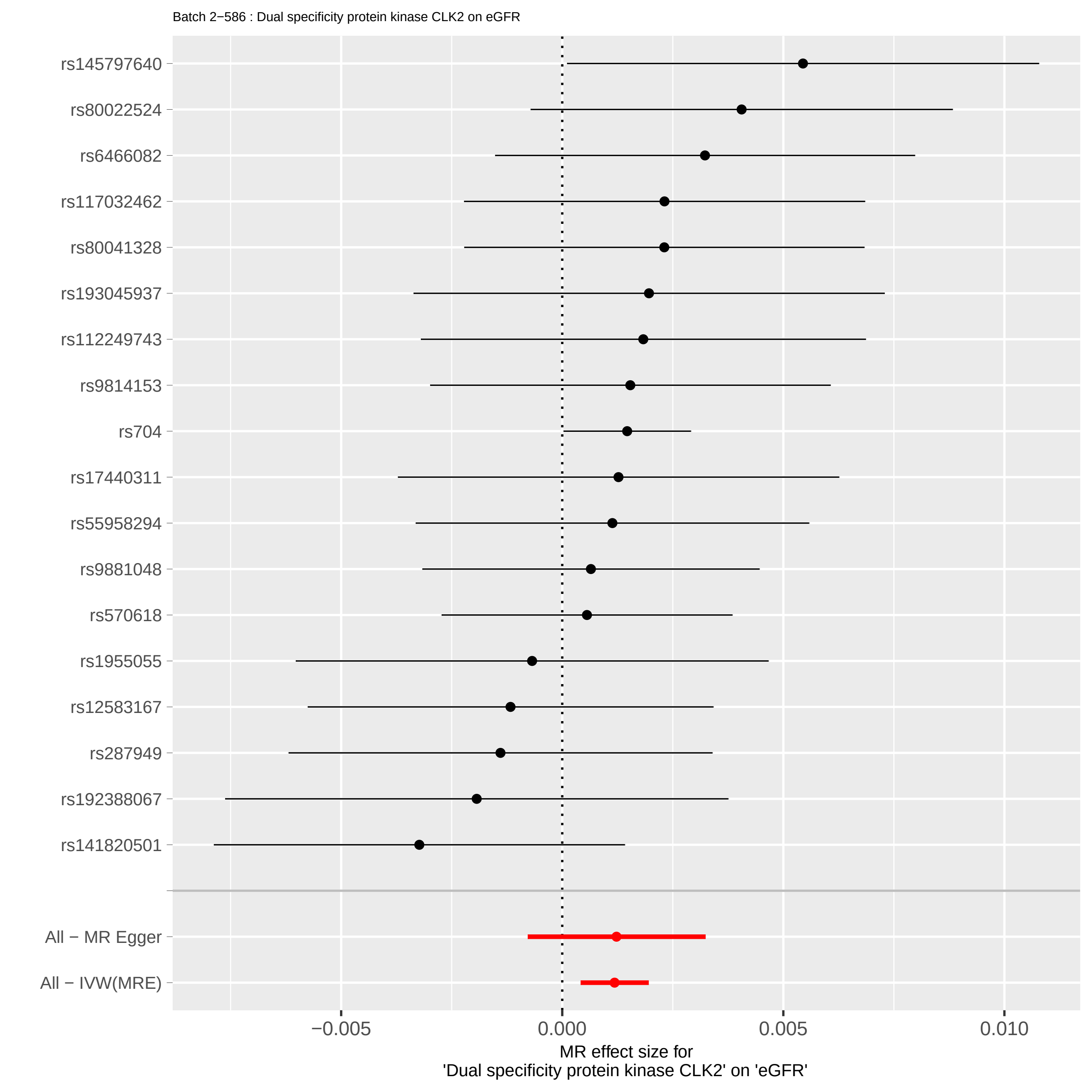

Batch 2−586 : Dual specificity protein kinase CLK2 on eGFR
rs145797640
rs80022524
rs6466082
rs117032462
rs80041328
rs193045937
rs112249743
rs9814153
rs704
rs17440311
rs55958294
rs9881048
rs570618
rs1955055
rs12583167
rs287949
rs192388067
rs141820501
All − MR Egger
All − IVW(MRE)
−0.005
0.000
0.005
0.010
MR effect size for
'Dual specificity protein kinase CLK2' on 'eGFR'

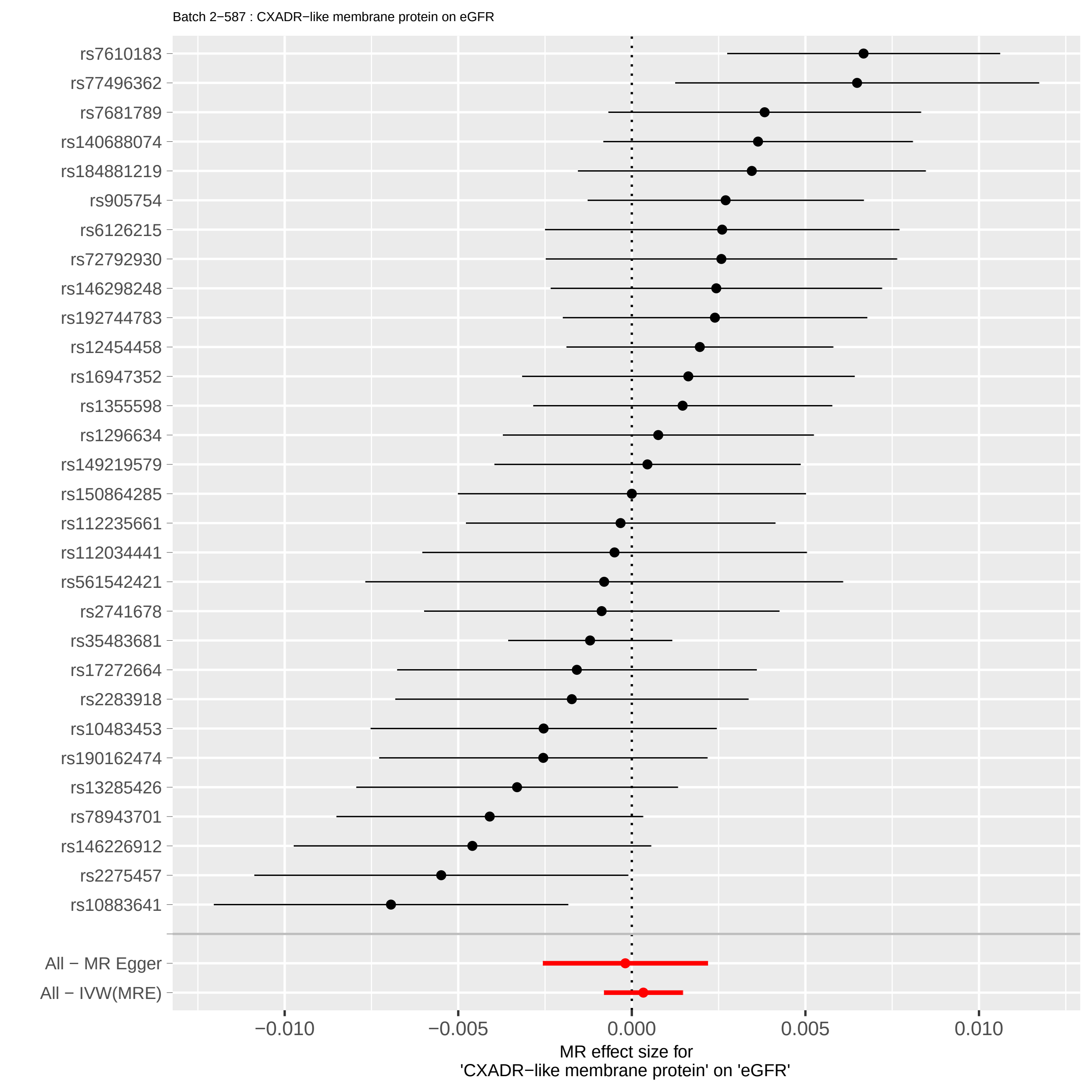
Batch 2−587 : CXADR−like membrane protein on eGFR
rs7610183
rs77496362
rs7681789
rs140688074
rs184881219
rs905754
rs6126215
rs72792930
rs146298248
rs192744783
rs12454458
rs16947352
rs1355598
rs1296634
rs149219579
rs150864285
rs112235661
rs112034441
rs561542421
rs2741678
rs35483681
rs17272664
rs2283918
rs10483453
rs190162474
rs13285426
rs78943701
rs146226912
rs2275457
rs10883641
All − MR Egger
All − IVW(MRE)
−0.010
−0.005
0.000
0.005
0.010
MR effect size for
'CXADR−like membrane protein' on 'eGFR'

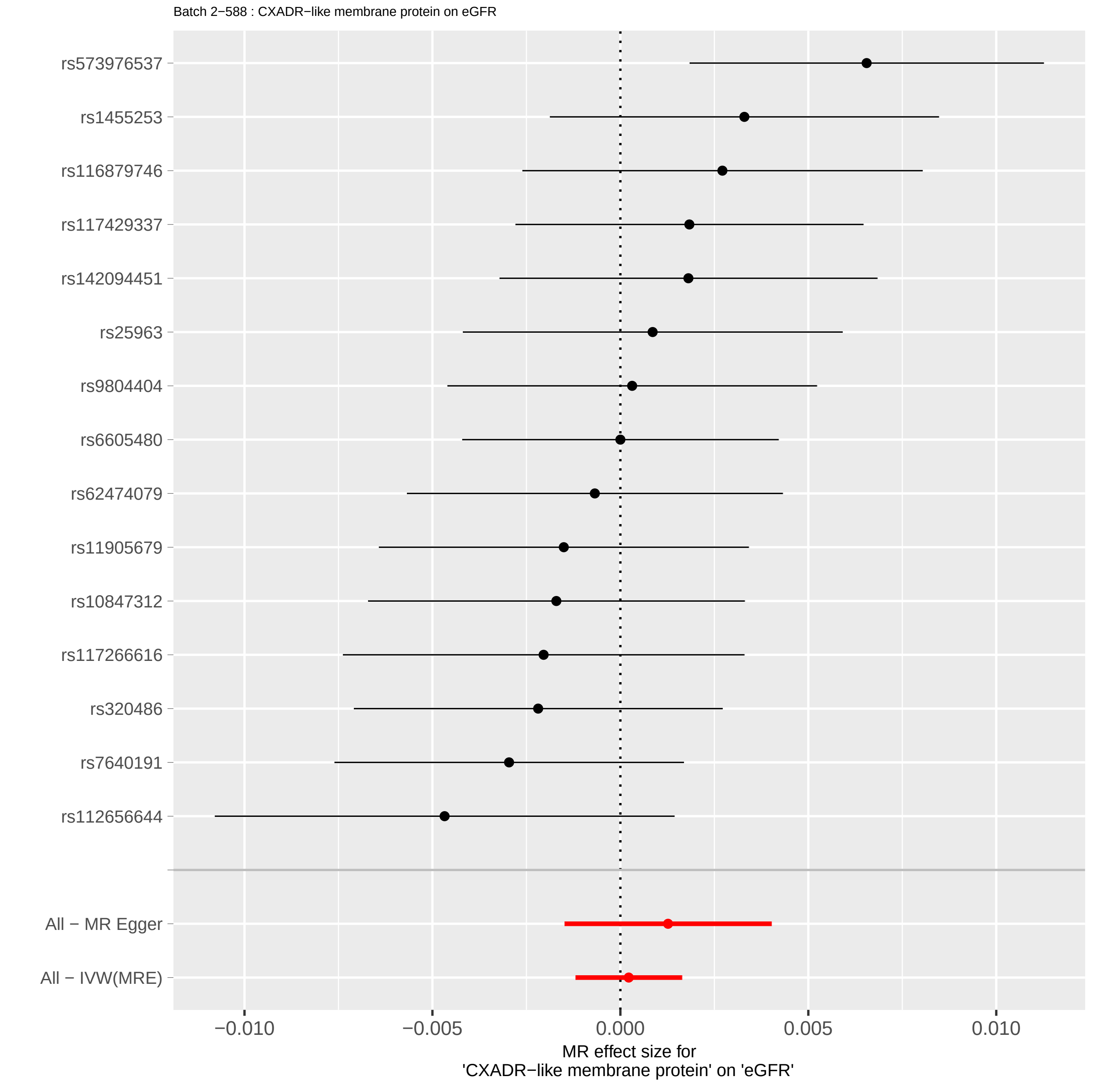
Batch 2−588 : CXADR−like membrane protein on eGFR
rs573976537
rs1455253
rs116879746
rs117429337
rs142094451
rs25963
rs9804404
rs6605480
rs62474079
rs11905679
rs10847312
rs117266616
rs320486
rs7640191
rs112656644
All − MR Egger
All − IVW(MRE)
−0.010
−0.005
0.000
0.005
0.010
MR effect size for
'CXADR−like membrane protein' on 'eGFR'

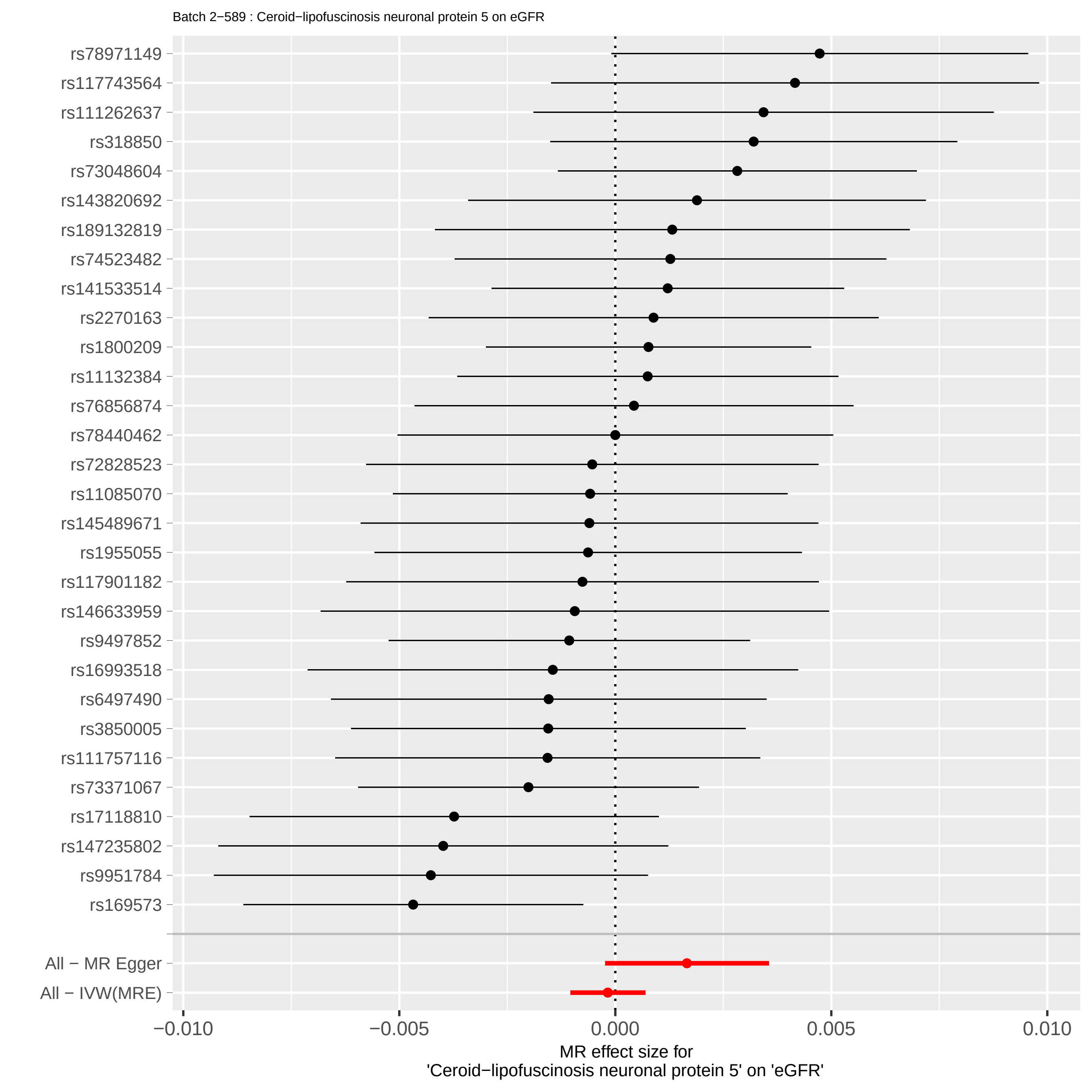

Batch 2−589 : Ceroid−lipofuscinosis neuronal protein 5 on eGFR
rs78971149
rs117743564
rs111262637
rs318850
rs73048604
rs143820692
rs189132819
rs74523482
rs141533514
rs2270163
rs1800209
rs11132384
rs76856874
rs78440462
rs72828523
rs11085070
rs145489671
rs1955055
rs117901182
rs146633959
rs9497852
rs16993518
rs6497490
rs3850005
rs111757116
rs73371067
rs17118810
rs147235802
rs9951784
rs169573
All − MR Egger
All − IVW(MRE)
−0.010
−0.005
0.000
0.005
0.010
MR effect size for
'Ceroid−lipofuscinosis neuronal protein 5' on 'eGFR'

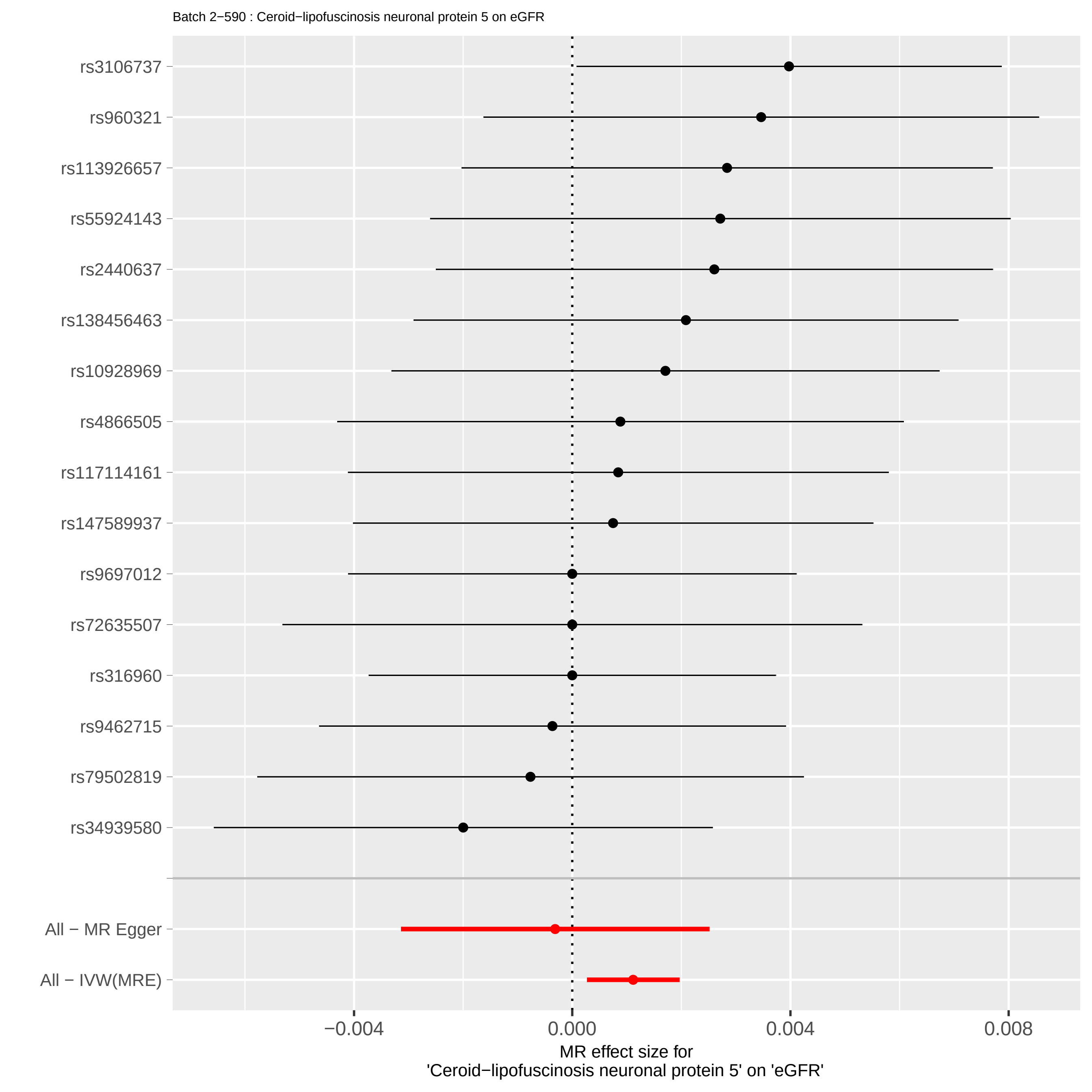

Batch 2−590 : Ceroid−lipofuscinosis neuronal protein 5 on eGFR
rs3106737
rs960321
rs113926657
rs55924143
rs2440637
rs138456463
rs10928969
rs4866505
rs117114161
rs147589937
rs9697012
rs72635507
rs316960
rs9462715
rs79502819
rs34939580
All − MR Egger
All − IVW(MRE)
−0.004
0.000
0.004
0.008
MR effect size for
'Ceroid−lipofuscinosis neuronal protein 5' on 'eGFR'

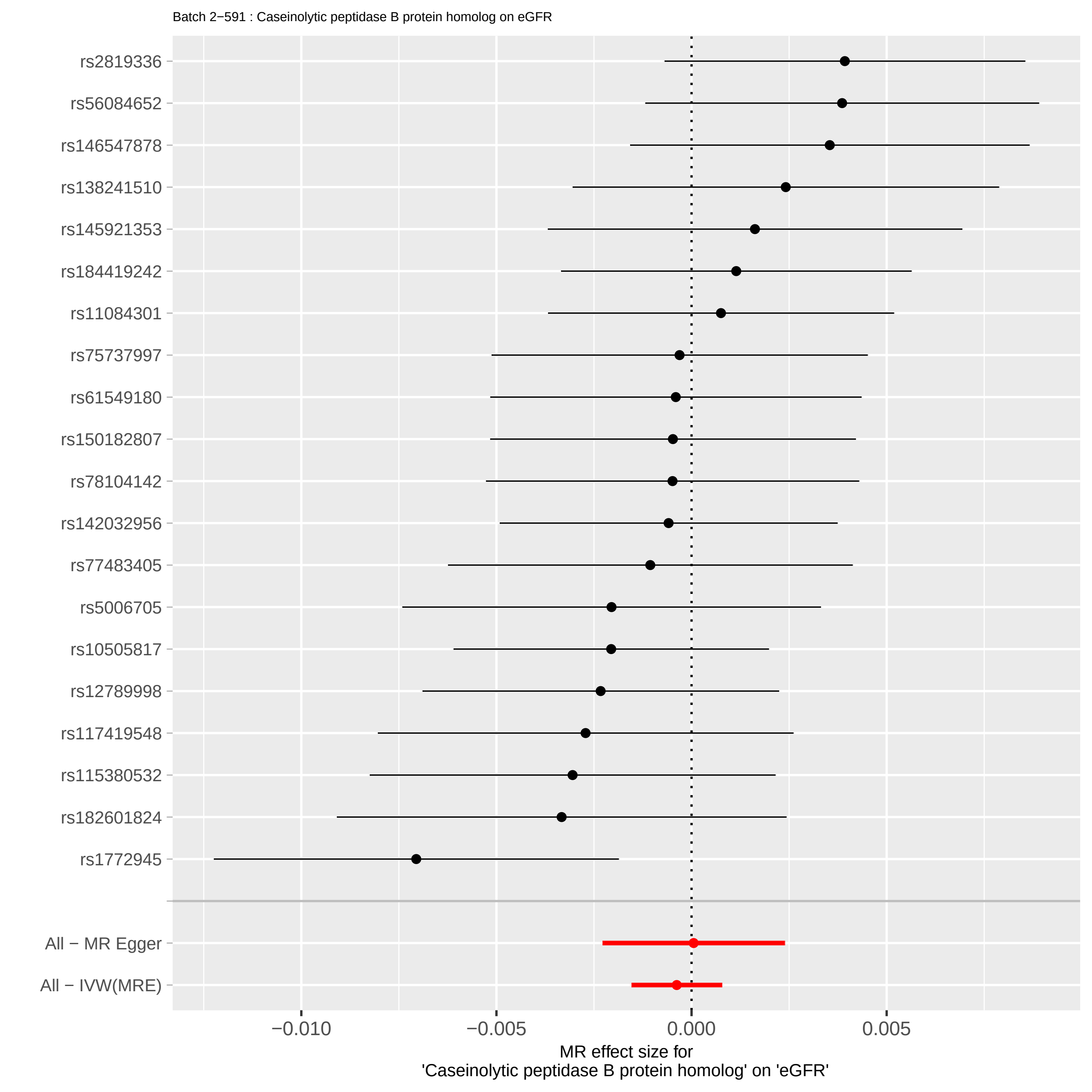
Batch 2−591 : Caseinolytic peptidase B protein homolog on eGFR
rs2819336
rs56084652
rs146547878
rs138241510
rs145921353
rs184419242
rs11084301
rs75737997
rs61549180
rs150182807
rs78104142
rs142032956
rs77483405
rs5006705
rs10505817
rs12789998
rs117419548
rs115380532
rs182601824
rs1772945
All − MR Egger
All − IVW(MRE)
−0.010
−0.005
0.000
0.005
MR effect size for
'Caseinolytic peptidase B protein homolog' on 'eGFR'

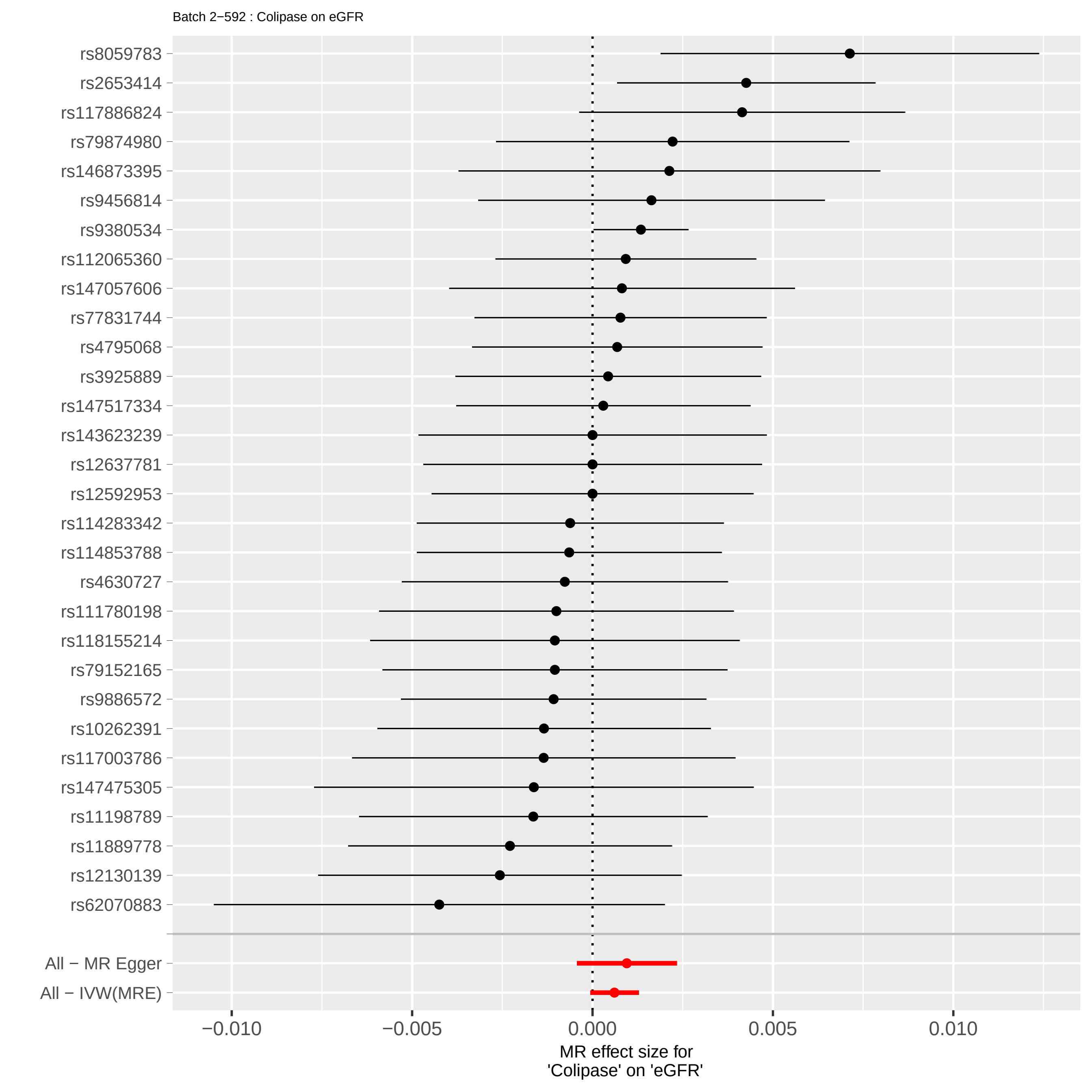

Batch 2−592 : Colipase on eGFR
rs8059783
rs2653414
rs117886824
rs79874980
rs146873395
rs9456814
rs9380534
rs112065360
rs147057606
rs77831744
rs4795068
rs3925889
rs147517334
rs143623239
rs12637781
rs12592953
rs114283342
rs114853788
rs4630727
rs111780198
rs118155214
rs79152165
rs9886572
rs10262391
rs117003786
rs147475305
rs11198789
rs11889778
rs12130139
rs62070883
All − MR Egger
All − IVW(MRE)
−0.010
−0.005
0.000
0.005
0.010
MR effect size for
'Colipase' on 'eGFR'

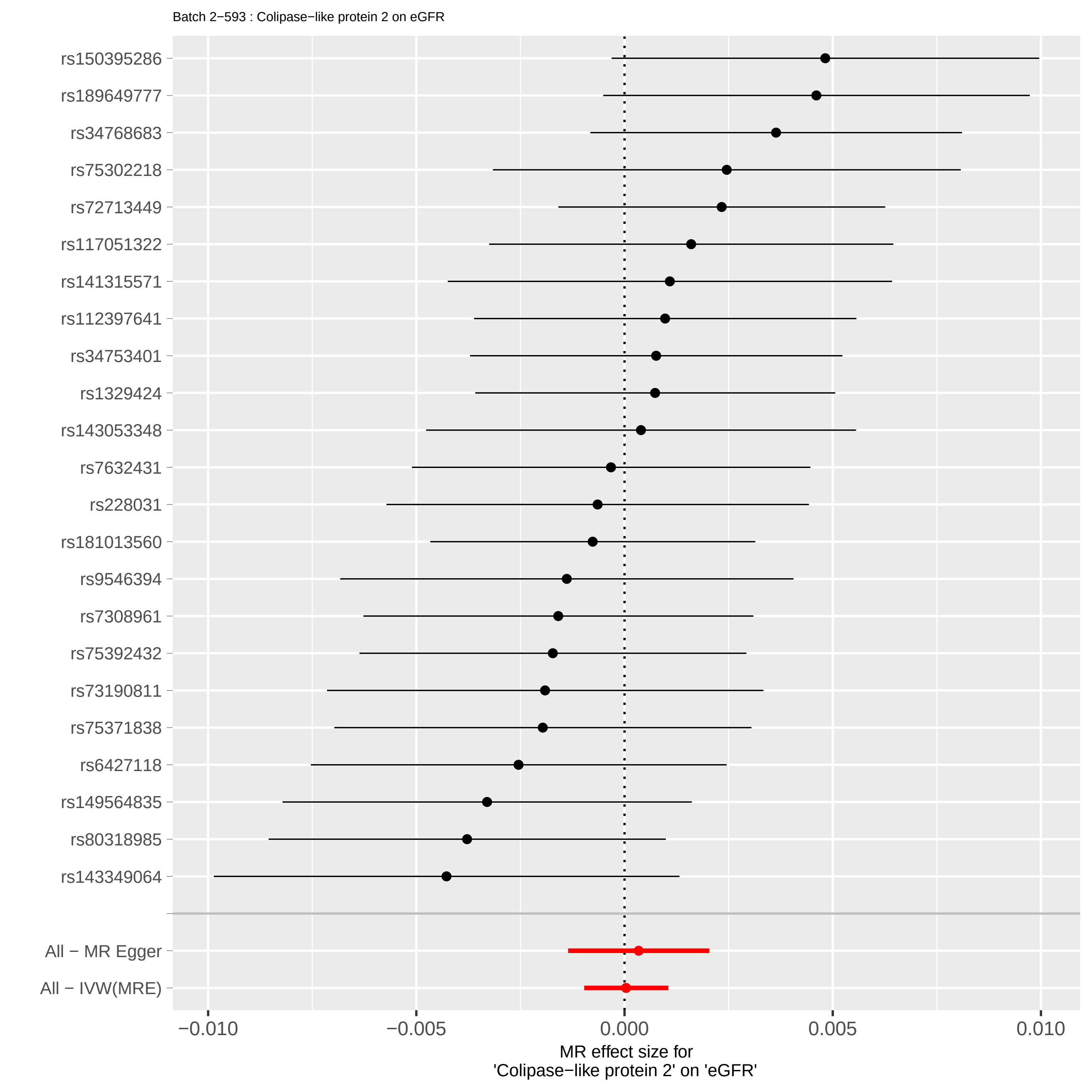
Batch 2−593 : Colipase−like protein 2 on eGFR
rs150395286
rs189649777
rs34768683
rs75302218
rs72713449
rs117051322
rs141315571
rs112397641
rs34753401
rs1329424
rs143053348
rs7632431
rs228031
rs181013560
rs9546394
rs7308961
rs75392432
rs73190811
rs75371838
rs6427118
rs149564835
rs80318985
rs143349064
All − MR Egger
All − IVW(MRE)
−0.010
−0.005
0.000
0.005
0.010
MR effect size for
'Colipase−like protein 2' on 'eGFR'

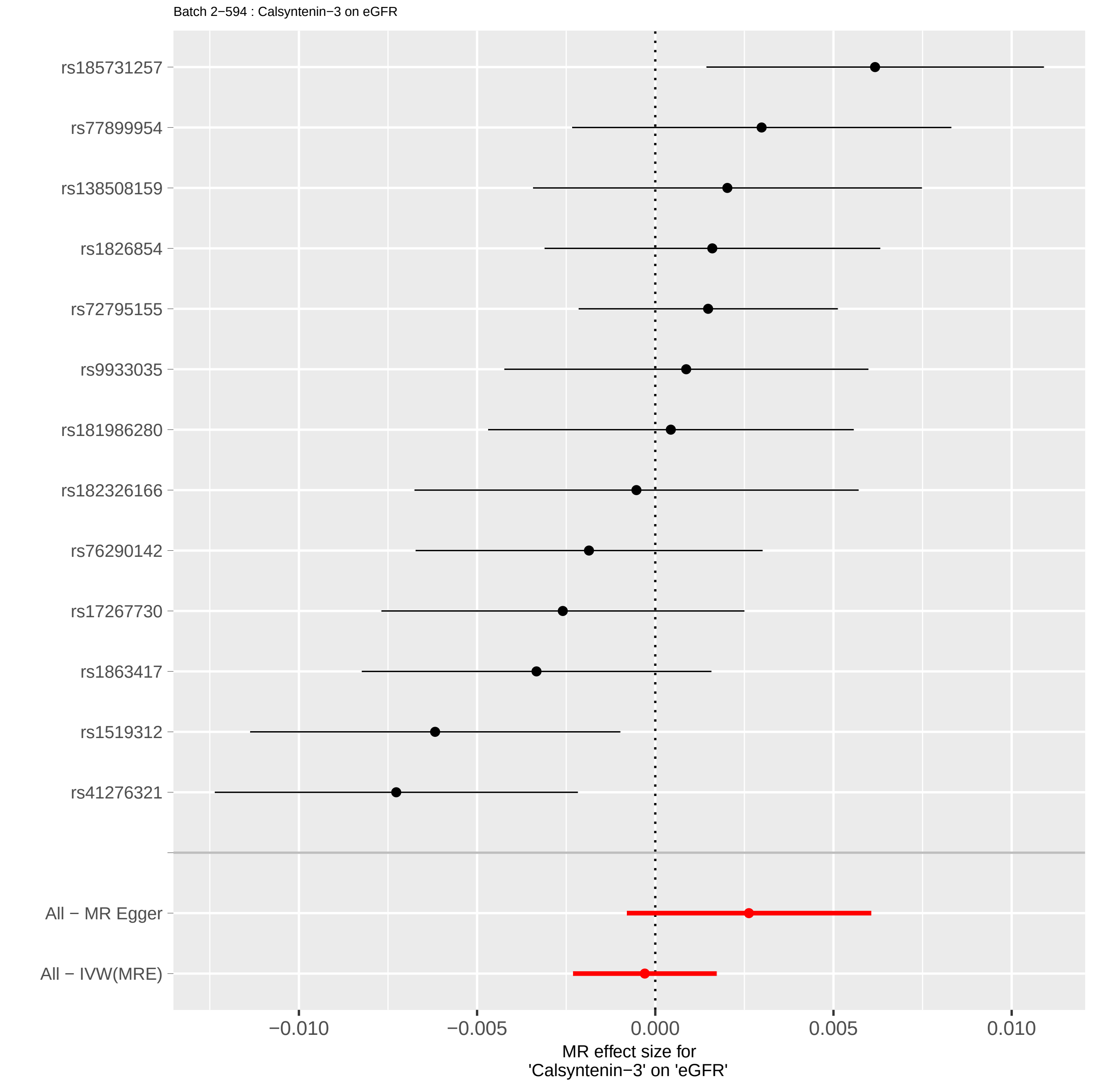

Batch 2−594 : Calsyntenin−3 on eGFR
rs185731257
rs77899954
rs138508159
rs1826854
rs72795155
rs9933035
rs181986280
rs182326166
rs76290142
rs17267730
rs1863417
rs1519312
rs41276321
All − MR Egger
All − IVW(MRE)
−0.010
−0.005
0.000
0.005
0.010
MR effect size for
'Calsyntenin−3' on 'eGFR'

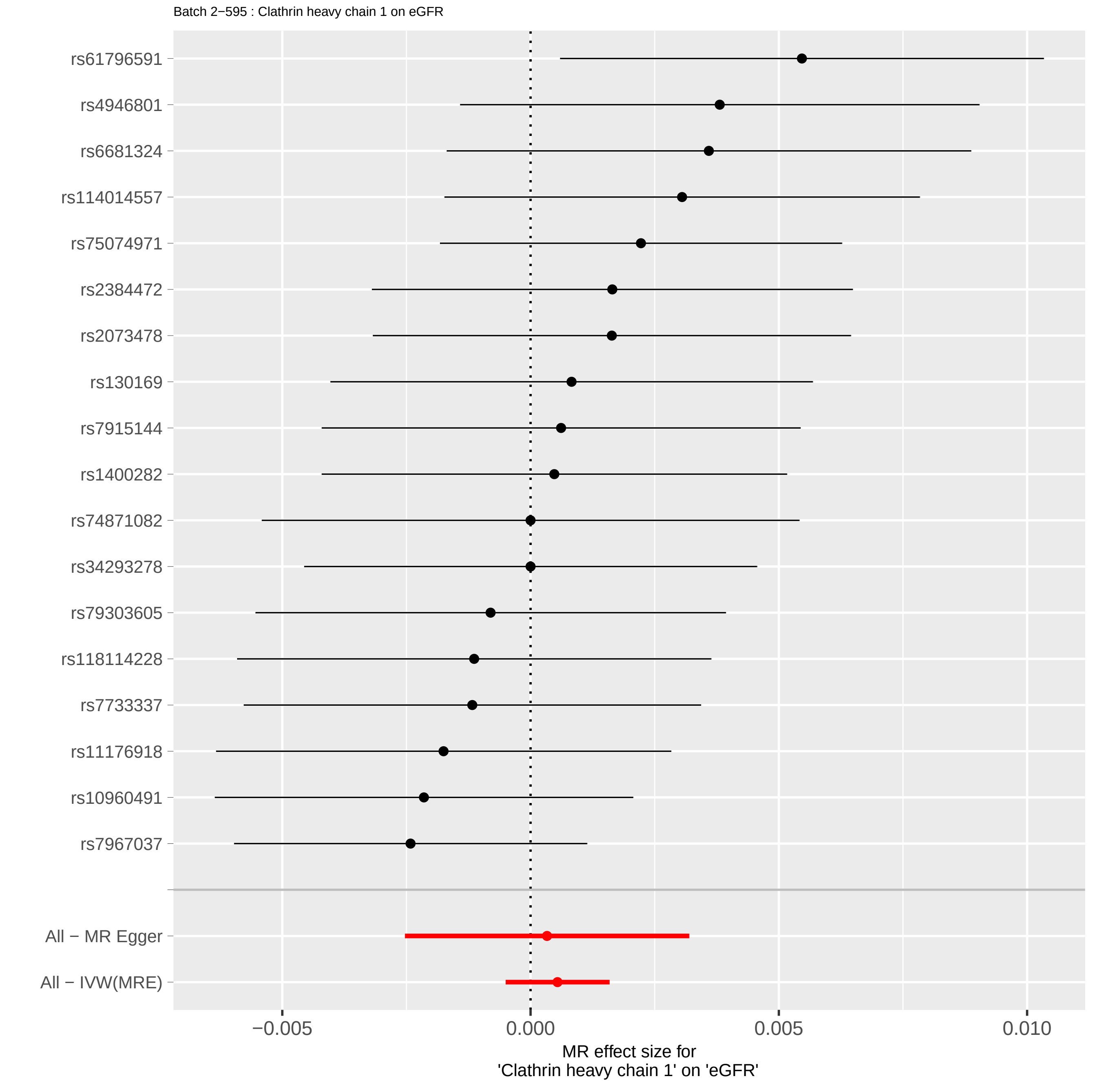
Batch 2−595 : Clathrin heavy chain 1 on eGFR
rs61796591
rs4946801
rs6681324
rs114014557
rs75074971
rs2384472
rs2073478
rs130169
rs7915144
rs1400282
rs74871082
rs34293278
rs79303605
rs118114228
rs7733337
rs11176918
rs10960491
rs7967037
All − MR Egger
All − IVW(MRE)
−0.005
0.000
0.005
0.010
MR effect size for
'Clathrin heavy chain 1' on 'eGFR'

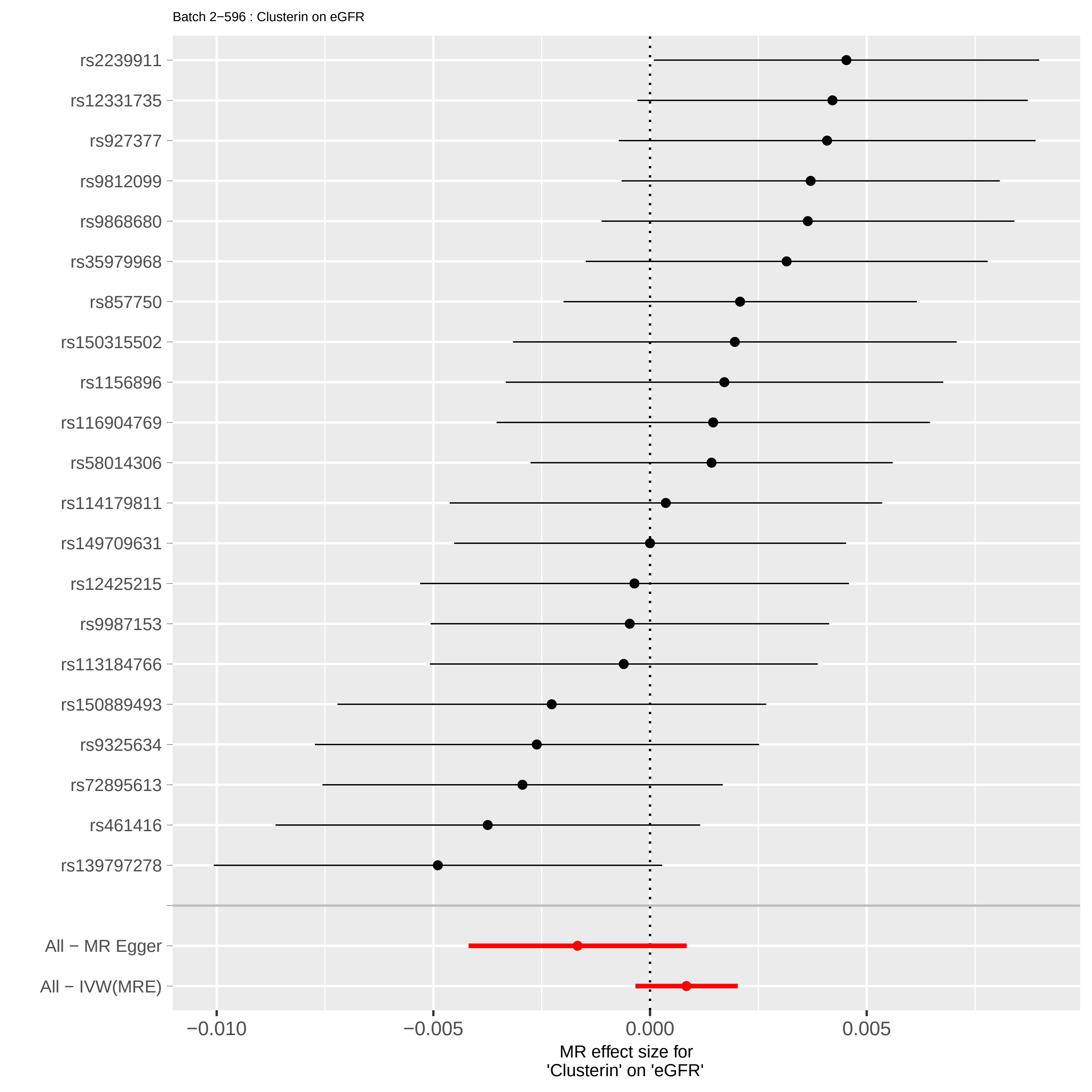

Batch 2−596 : Clusterin on eGFR
rs2239911
rs12331735
rs927377
rs9812099
rs9868680
rs35979968
rs857750
rs150315502
rs1156896
rs116904769
rs58014306
rs114179811
rs149709631
rs12425215
rs9987153
rs113184766
rs150889493
rs9325634
rs72895613
rs461416
rs139797278
All − MR Egger
All − IVW(MRE)
−0.010
−0.005
0.000
0.005
MR effect size for
'Clusterin' on 'eGFR'

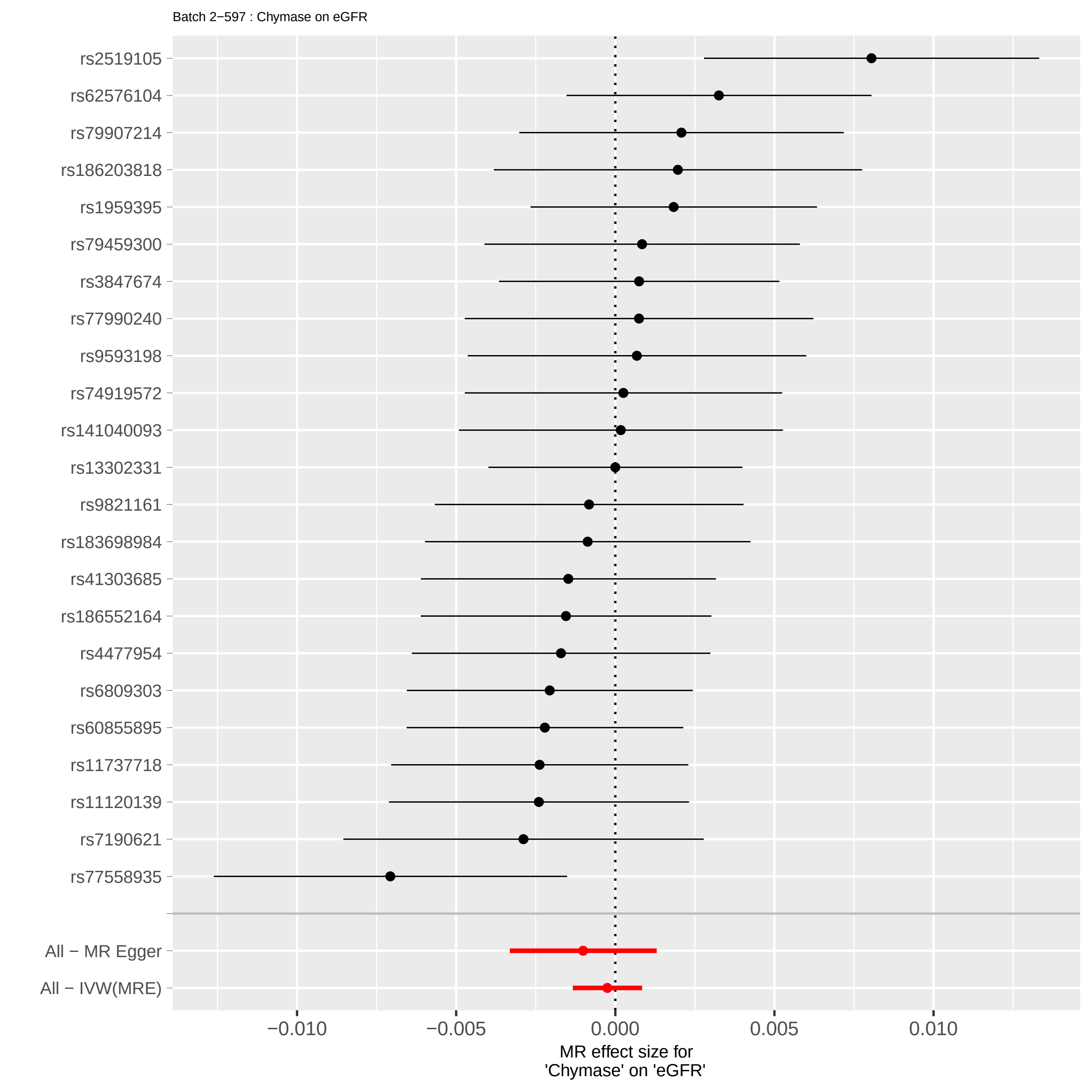

Batch 2−597 : Chymase on eGFR
rs2519105
rs62576104
rs79907214
rs186203818
rs1959395
rs79459300
rs3847674
rs77990240
rs9593198
rs74919572
rs141040093
rs13302331
rs9821161
rs183698984
rs41303685
rs186552164
rs4477954
rs6809303
rs60855895
rs11737718
rs11120139
rs7190621
rs77558935
All − MR Egger
All − IVW(MRE)
−0.010
−0.005
0.000
0.005
0.010
MR effect size for
'Chymase' on 'eGFR'

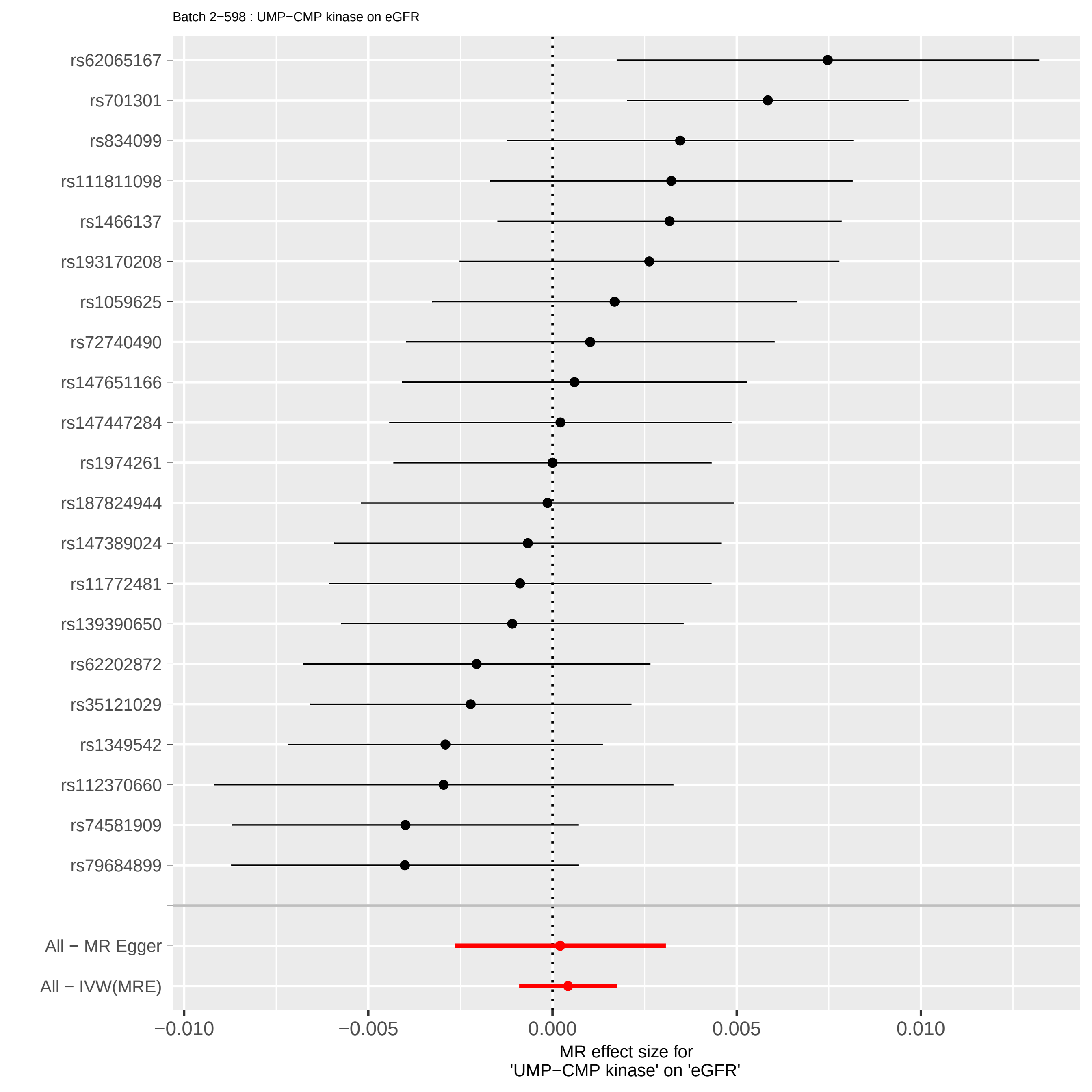

Batch 2−598 : UMP−CMP kinase on eGFR
rs62065167
rs701301
rs834099
rs111811098
rs1466137
rs193170208
rs1059625
rs72740490
rs147651166
rs147447284
rs1974261
rs187824944
rs147389024
rs11772481
rs139390650
rs62202872
rs35121029
rs1349542
rs112370660
rs74581909
rs79684899
All − MR Egger
All − IVW(MRE)
−0.010
−0.005
0.000
0.005
0.010
MR effect size for
'UMP−CMP kinase' on 'eGFR'

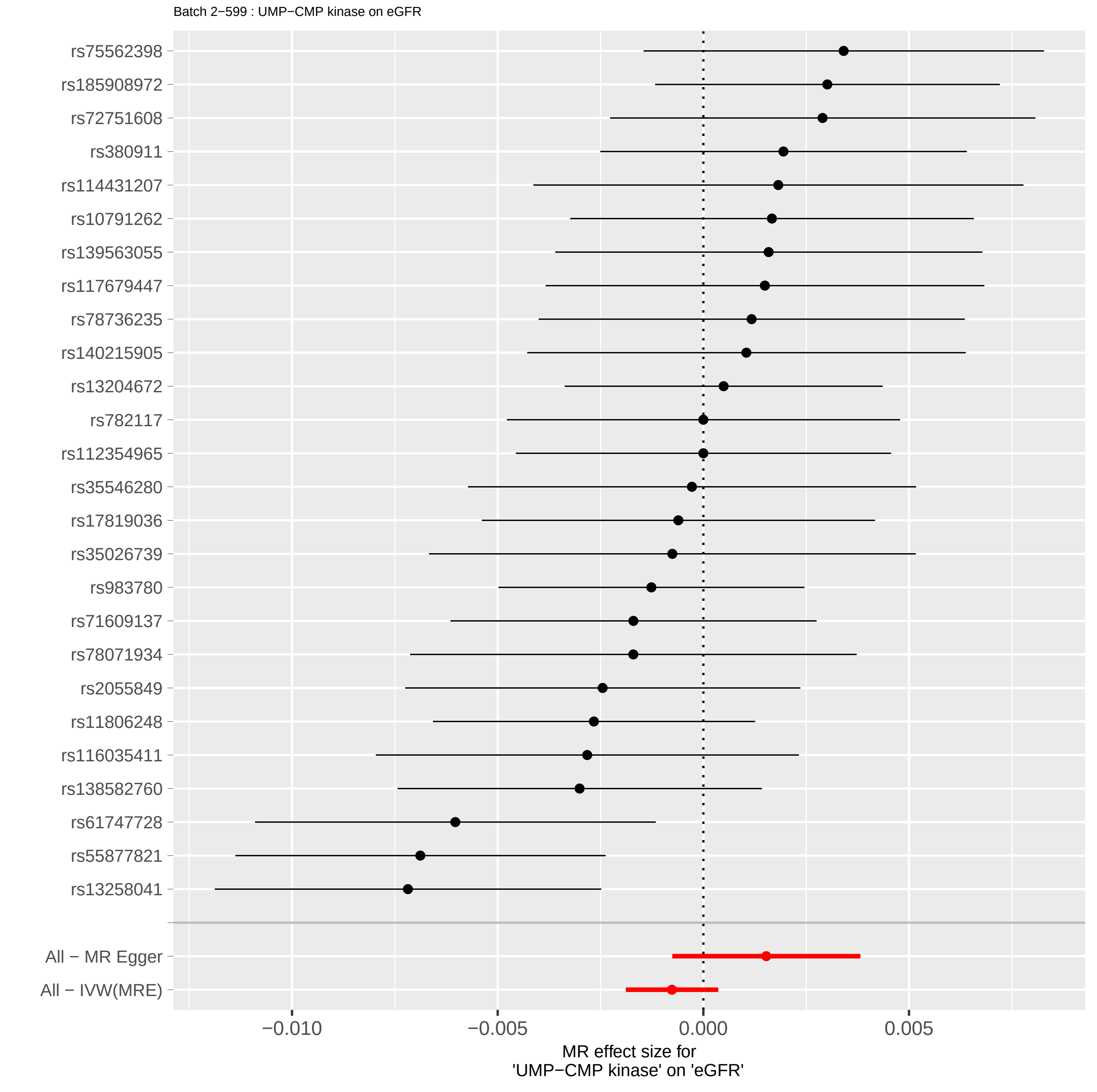

Batch 2−599 : UMP−CMP kinase on eGFR
rs75562398
rs185908972
rs72751608
rs380911
rs114431207
rs10791262
rs139563055
rs117679447
rs78736235
rs140215905
rs13204672
rs782117
rs112354965
rs35546280
rs17819036
rs35026739
rs983780
rs71609137
rs78071934
rs2055849
rs11806248
rs116035411
rs138582760
rs61747728
rs55877821
rs13258041
All − MR Egger
All − IVW(MRE)
−0.010
−0.005
0.000
0.005
MR effect size for
'UMP−CMP kinase' on 'eGFR'

Batch 2−600 : Cytosolic non−specific dipeptidase on eGFR

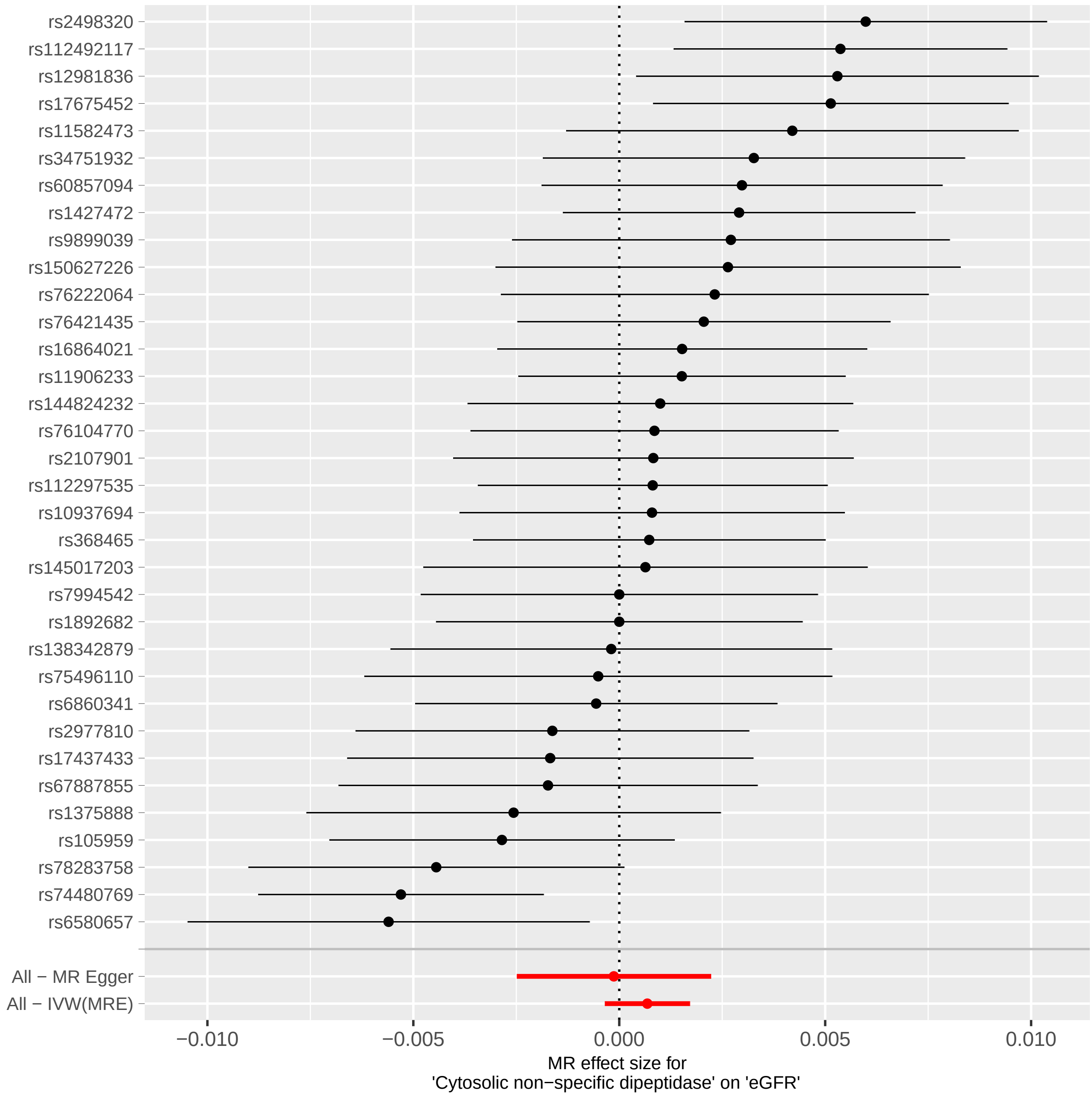

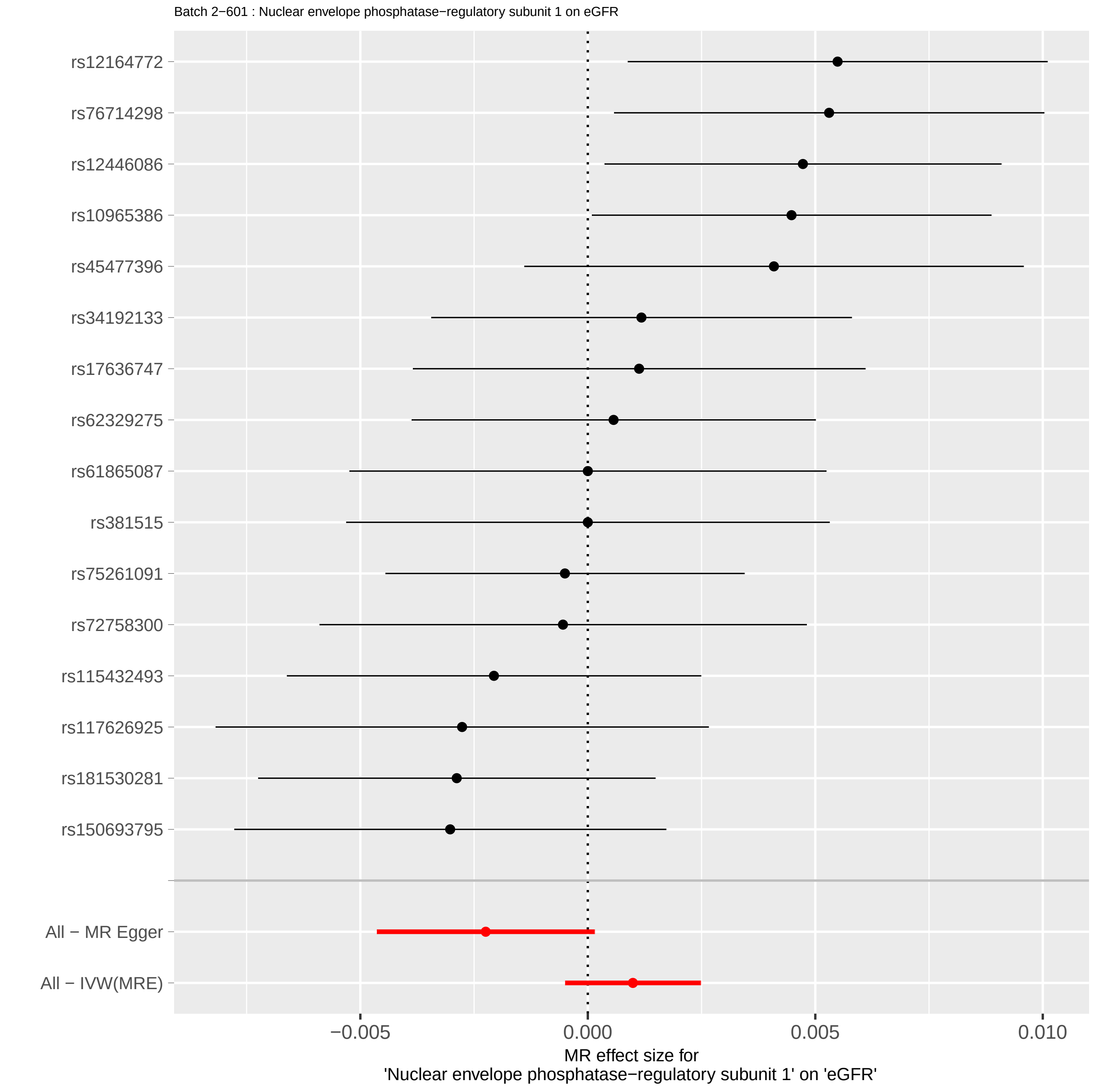
Batch 2−601 : Nuclear envelope phosphatase−regulatory subunit 1 on eGFR
rs12164772
rs76714298
rs12446086
rs10965386
rs45477396
rs34192133
rs17636747
rs62329275
rs61865087
rs381515
rs75261091
rs72758300
rs115432493
rs117626925
rs181530281
rs150693795
All − MR Egger
All − IVW(MRE)
−0.005
0.000
0.005
0.010
MR effect size for
'Nuclear envelope phosphatase−regulatory subunit 1' on 'eGFR'

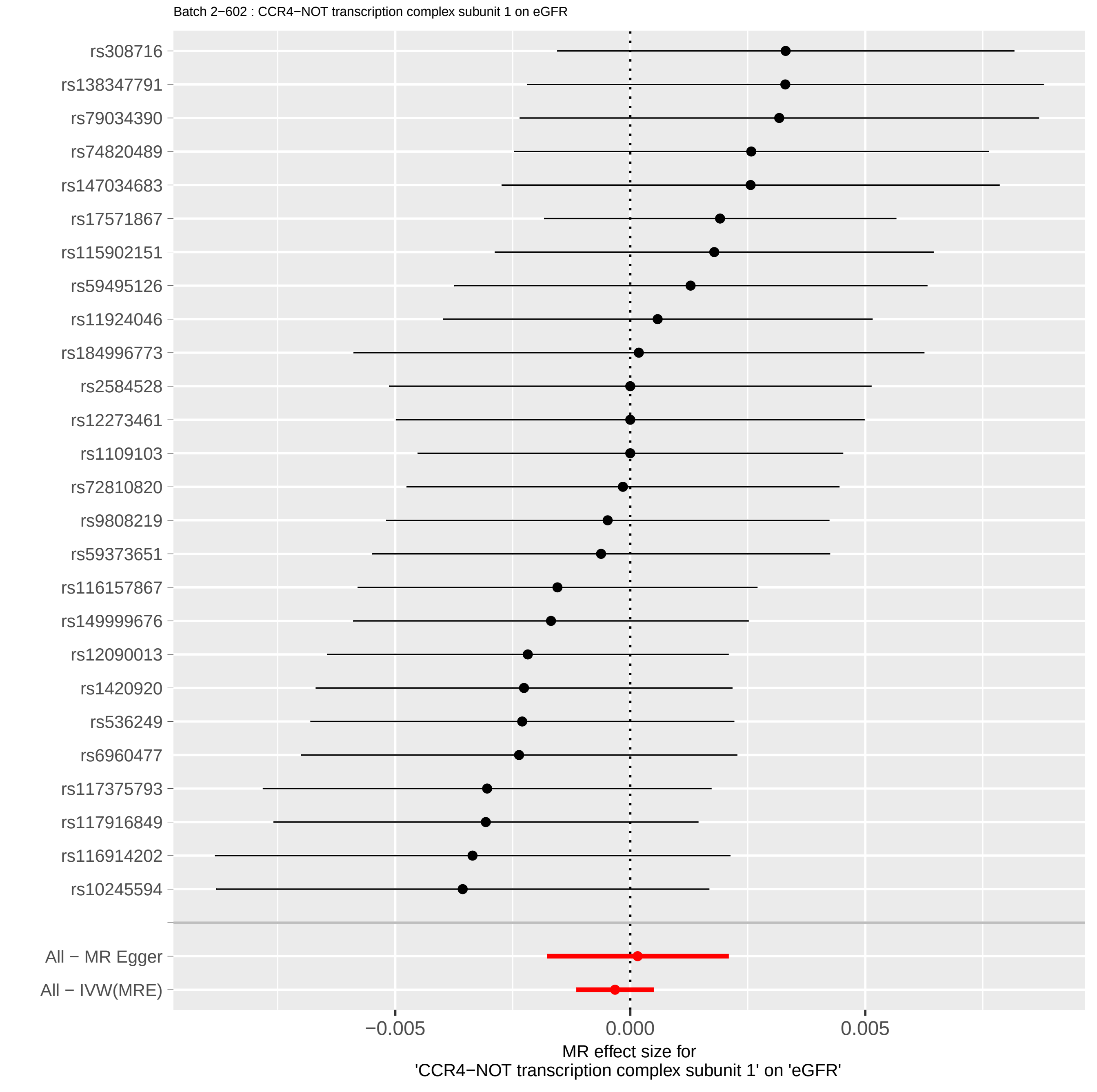

Batch 2−602 : CCR4−NOT transcription complex subunit 1 on eGFR
rs308716
rs138347791
rs79034390
rs74820489
rs147034683
rs17571867
rs115902151
rs59495126
rs11924046
rs184996773
rs2584528
rs12273461
rs1109103
rs72810820
rs9808219
rs59373651
rs116157867
rs149999676
rs12090013
rs1420920
rs536249
rs6960477
rs117375793
rs117916849
rs116914202
rs10245594
All − MR Egger
All − IVW(MRE)
−0.005
0.000
0.005
MR effect size for
'CCR4−NOT transcription complex subunit 1' on 'eGFR'

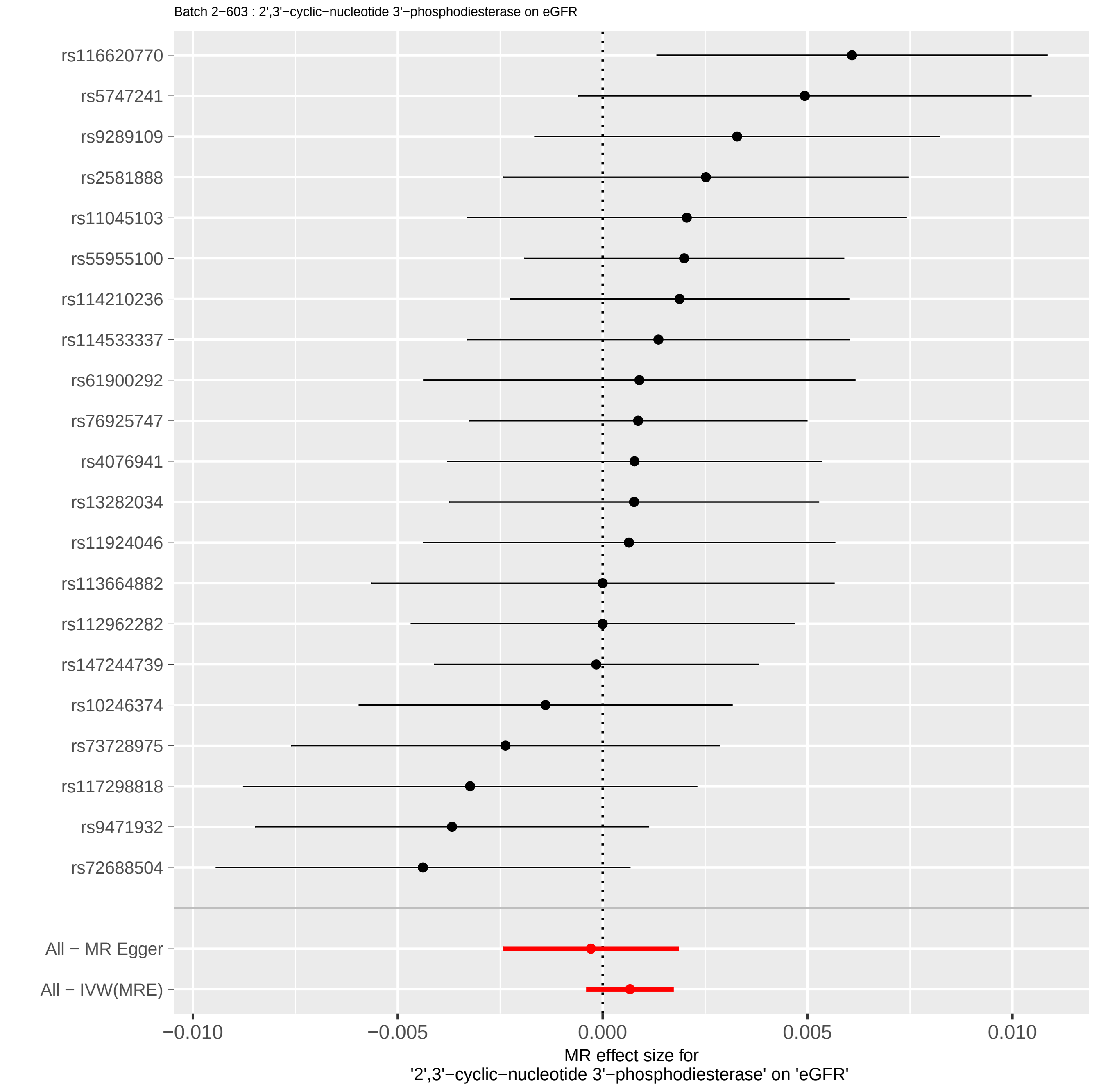

Batch 2−603 : 2',3'−cyclic−nucleotide 3'−phosphodiesterase on eGFR
rs116620770
rs5747241
rs9289109
rs2581888
rs11045103
rs55955100
rs114210236
rs114533337
rs61900292
rs76925747
rs4076941
rs13282034
rs11924046
rs113664882
rs112962282
rs147244739
rs10246374
rs73728975
rs117298818
rs9471932
rs72688504
All − MR Egger
All − IVW(MRE)
−0.010
−0.005
0.000
0.005
0.010
MR effect size for
'2',3'−cyclic−nucleotide 3'−phosphodiesterase' on 'eGFR'

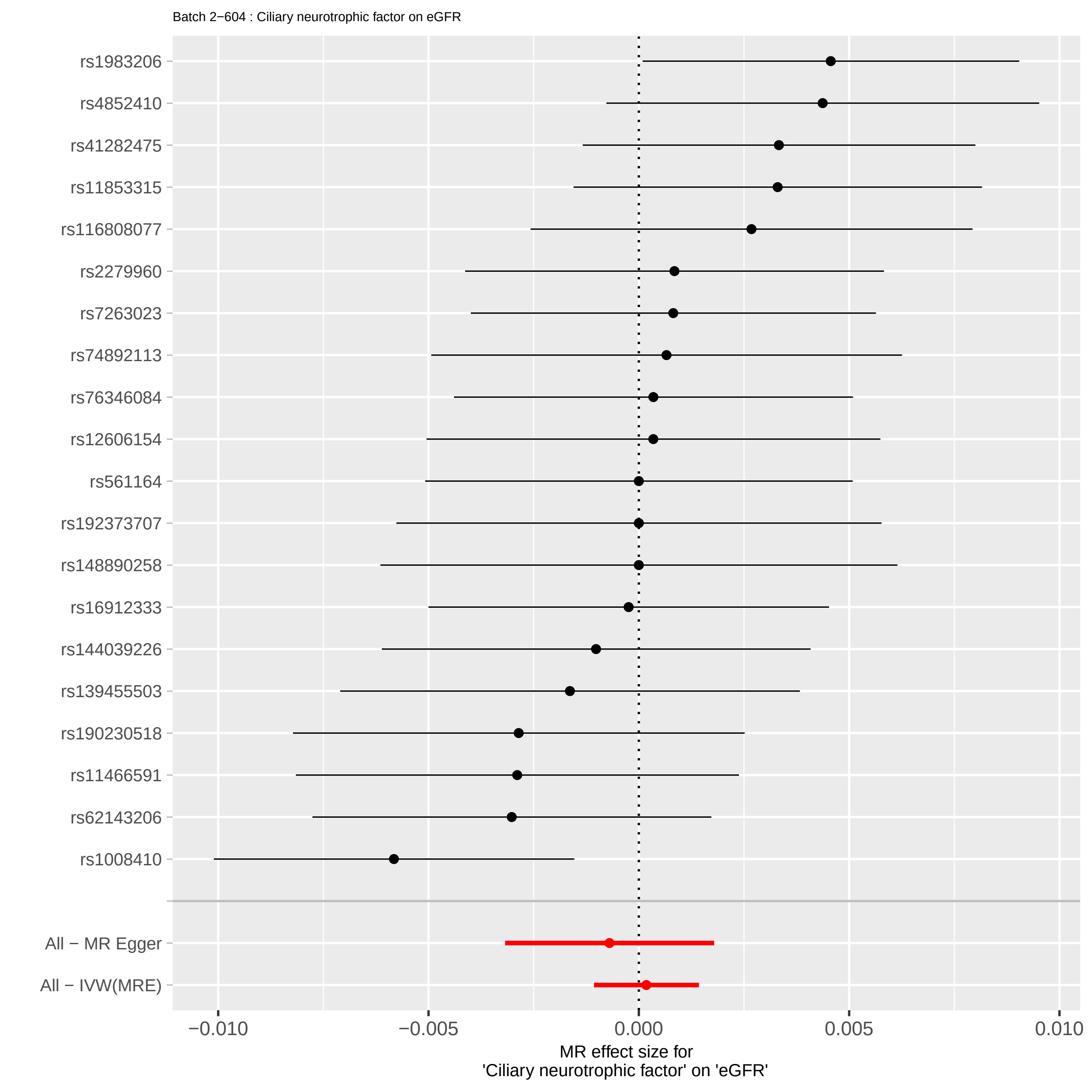

Batch 2−604 : Ciliary neurotrophic factor on eGFR
rs1983206
rs4852410
rs41282475
rs11853315
rs116808077
rs2279960
rs7263023
rs74892113
rs76346084
rs12606154
rs561164
rs192373707
rs148890258
rs16912333
rs144039226
rs139455503
rs190230518
rs11466591
rs62143206
rs1008410
All − MR Egger
All − IVW(MRE)
−0.010
−0.005
0.000
0.005
0.010
MR effect size for
'Ciliary neurotrophic factor' on 'eGFR'

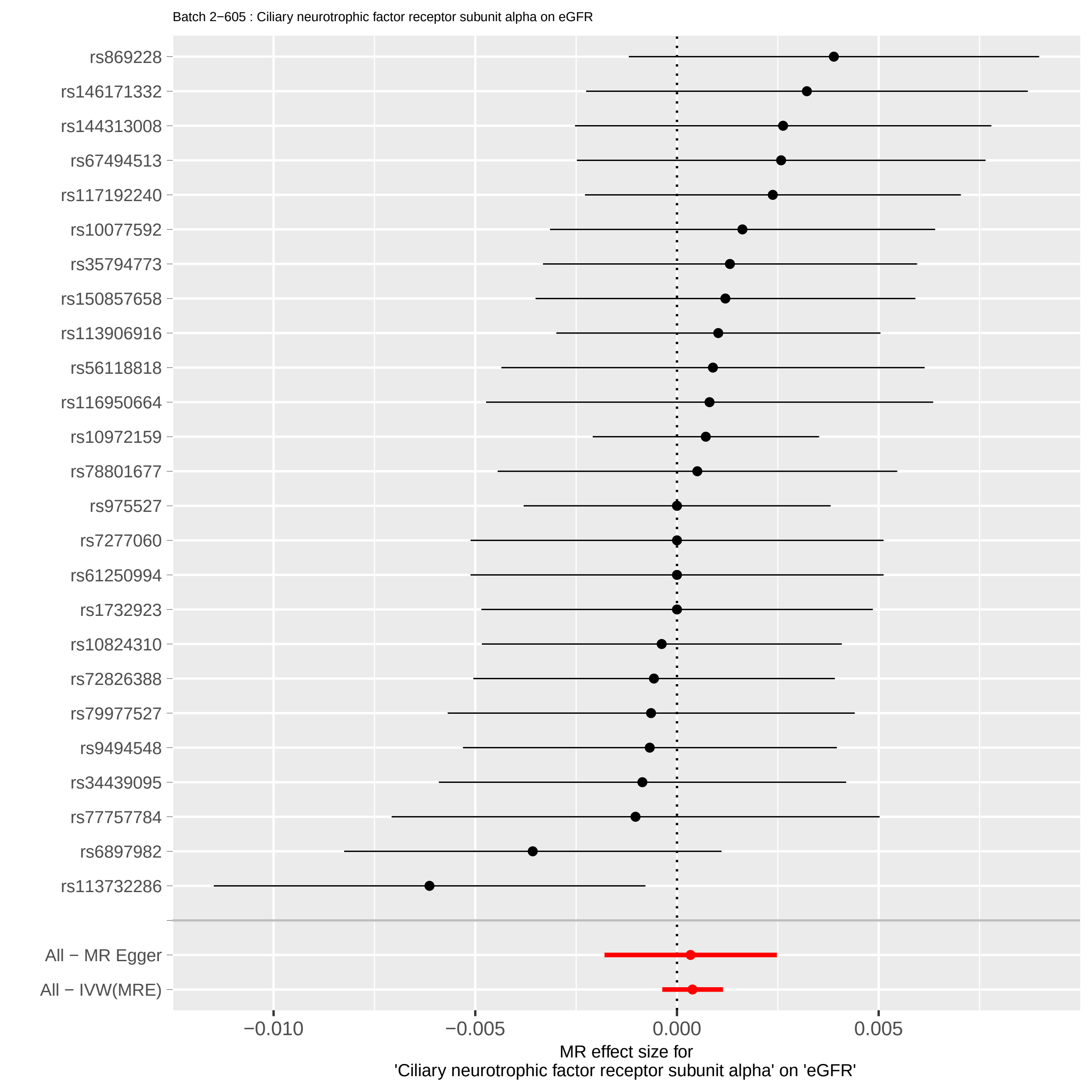
Batch 2−605 : Ciliary neurotrophic factor receptor subunit alpha on eGFR
rs869228
rs146171332
rs144313008
rs67494513
rs117192240
rs10077592
rs35794773
rs150857658
rs113906916
rs56118818
rs116950664
rs10972159
rs78801677
rs975527
rs7277060
rs61250994
rs1732923
rs10824310
rs72826388
rs79977527
rs9494548
rs34439095
rs77757784
rs6897982
rs113732286
All − MR Egger
All − IVW(MRE)
−0.010
−0.005
0.000
0.005
MR effect size for
'Ciliary neurotrophic factor receptor subunit alpha' on 'eGFR'

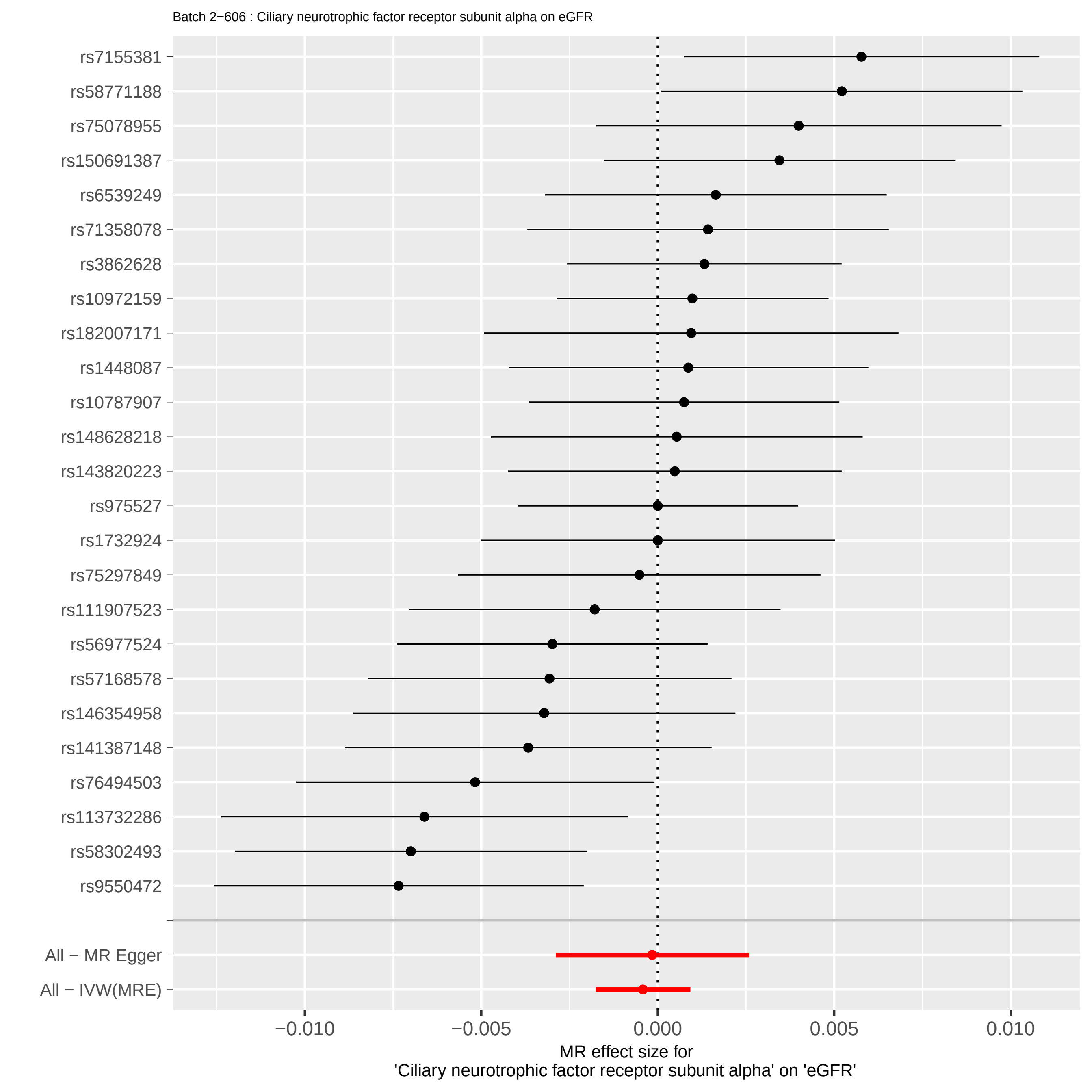

Batch 2−606 : Ciliary neurotrophic factor receptor subunit alpha on eGFR
rs7155381
rs58771188
rs75078955
rs150691387
rs6539249
rs71358078
rs3862628
rs10972159
rs182007171
rs1448087
rs10787907
rs148628218
rs143820223
rs975527
rs1732924
rs75297849
rs111907523
rs56977524
rs57168578
rs146354958
rs141387148
rs76494503
rs113732286
rs58302493
rs9550472
All − MR Egger
All − IVW(MRE)
−0.010
−0.005
0.000
0.005
0.010
MR effect size for
'Ciliary neurotrophic factor receptor subunit alpha' on 'eGFR'

Batch 2−607 : Contactin−2 on eGFR

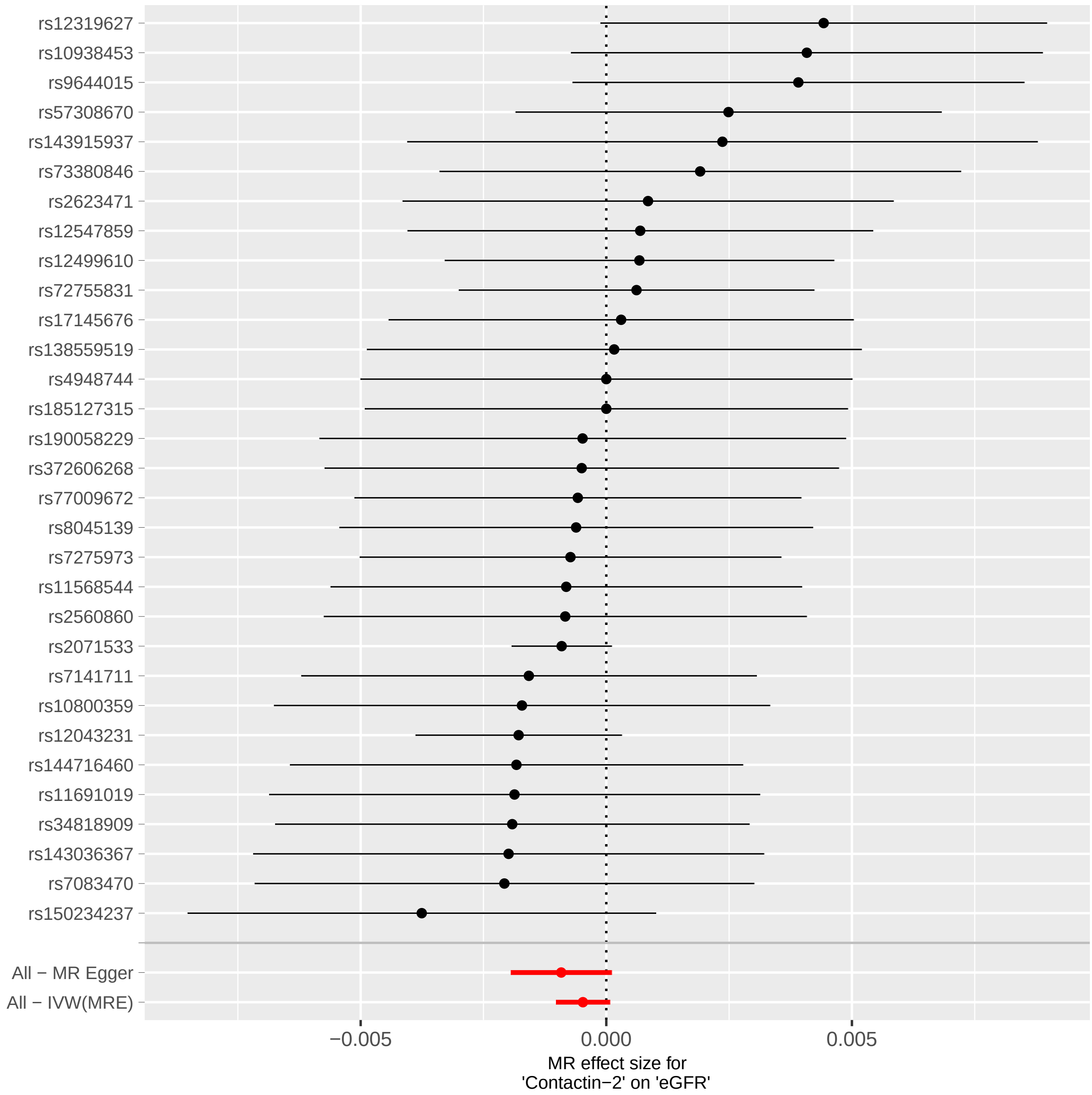

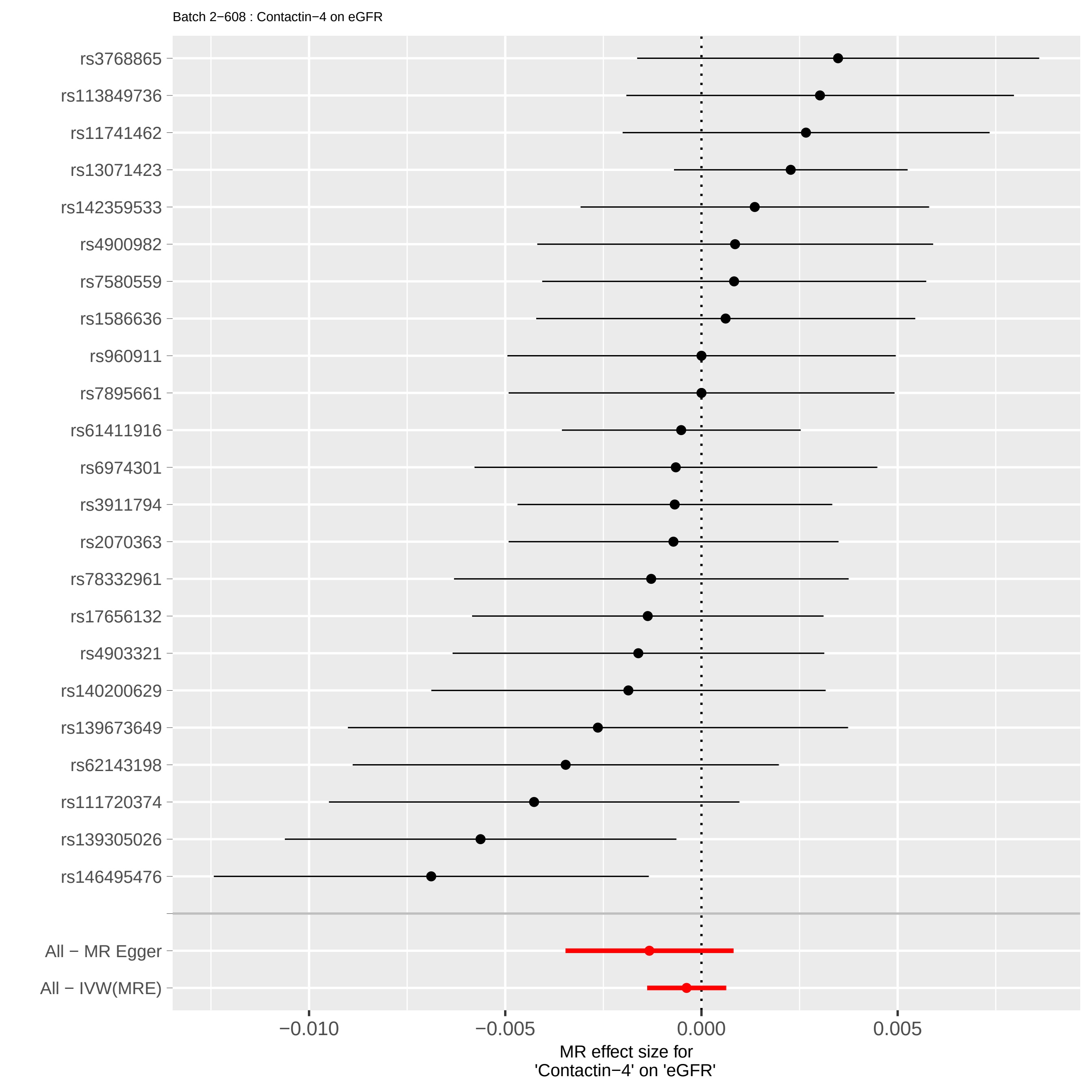

Batch 2−608 : Contactin−4 on eGFR
rs3768865
rs113849736
rs11741462
rs13071423
rs142359533
rs4900982
rs7580559
rs1586636
rs960911
rs7895661
rs61411916
rs6974301
rs3911794
rs2070363
rs78332961
rs17656132
rs4903321
rs140200629
rs139673649
rs62143198
rs111720374
rs139305026
rs146495476
All − MR Egger
All − IVW(MRE)
−0.010
−0.005
0.000
0.005
MR effect size for
'Contactin−4' on 'eGFR'

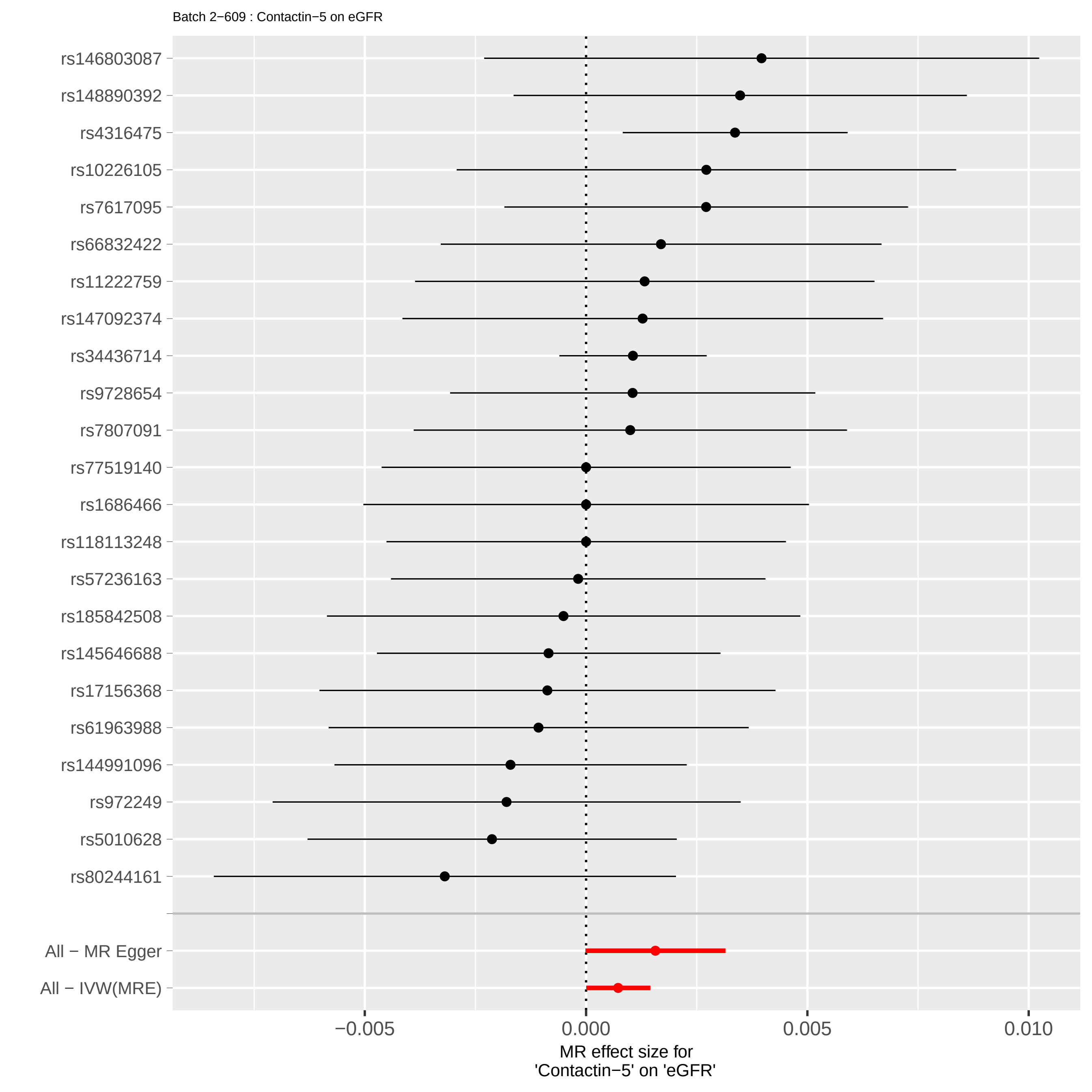

Batch 2−609 : Contactin−5 on eGFR
rs146803087
rs148890392
rs4316475
rs10226105
rs7617095
rs66832422
rs11222759
rs147092374
rs34436714
rs9728654
rs7807091
rs77519140
rs1686466
rs118113248
rs57236163
rs185842508
rs145646688
rs17156368
rs61963988
rs144991096
rs972249
rs5010628
rs80244161
All − MR Egger
All − IVW(MRE)
−0.005
0.000
0.005
0.010
MR effect size for
'Contactin−5' on 'eGFR'

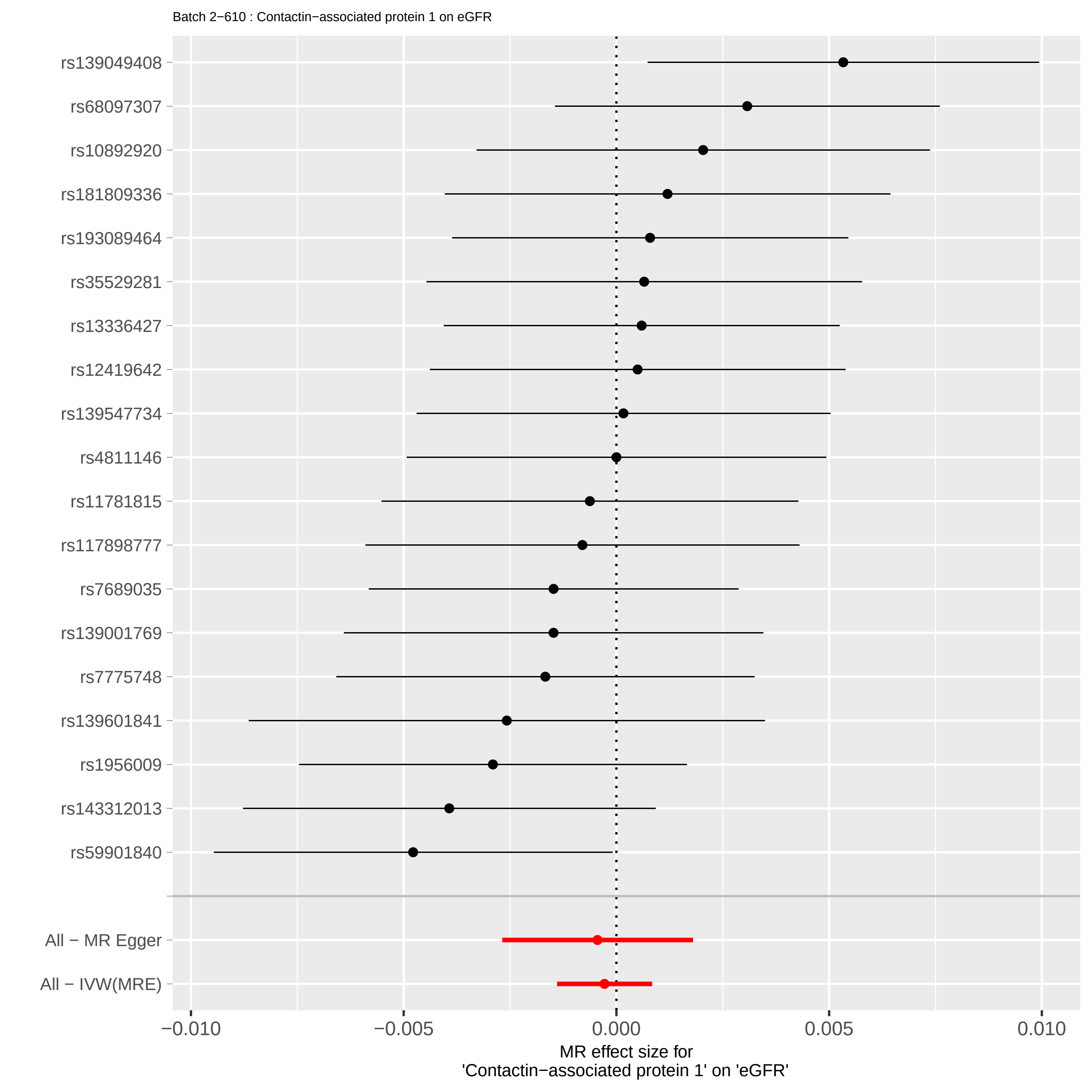

Batch 2−610 : Contactin−associated protein 1 on eGFR
rs139049408
rs68097307
rs10892920
rs181809336
rs193089464
rs35529281
rs13336427
rs12419642
rs139547734
rs4811146
rs11781815
rs117898777
rs7689035
rs139001769
rs7775748
rs139601841
rs1956009
rs143312013
rs59901840
All − MR Egger
All − IVW(MRE)
−0.010
−0.005
0.000
0.005
0.010
MR effect size for
'Contactin−associated protein 1' on 'eGFR'

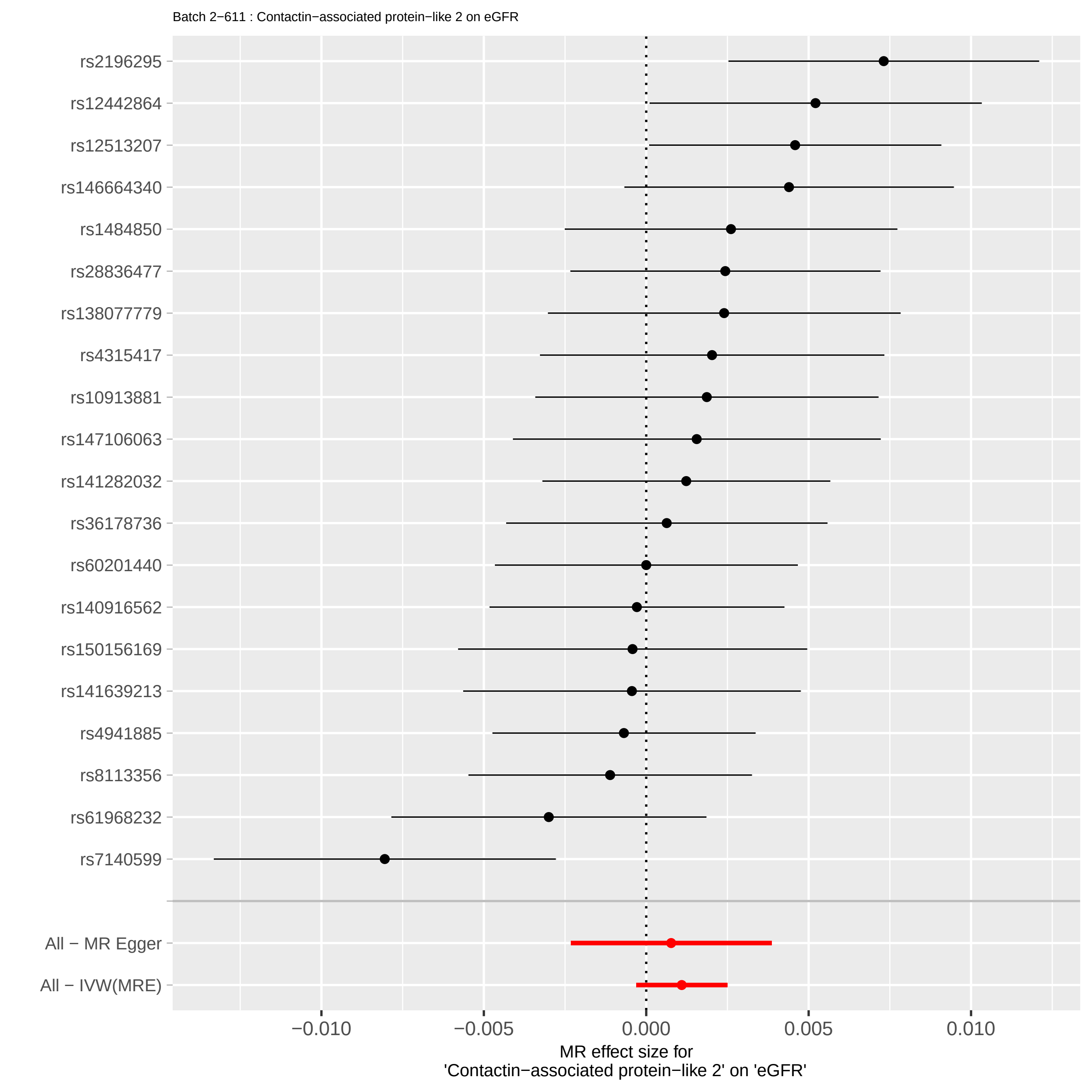

Batch 2−611 : Contactin−associated protein−like 2 on eGFR
rs2196295
rs12442864
rs12513207
rs146664340
rs1484850
rs28836477
rs138077779
rs4315417
rs10913881
rs147106063
rs141282032
rs36178736
rs60201440
rs140916562
rs150156169
rs141639213
rs4941885
rs8113356
rs61968232
rs7140599
All − MR Egger
All − IVW(MRE)
−0.010
−0.005
0.000
0.005
0.010
MR effect size for
'Contactin−associated protein−like 2' on 'eGFR'

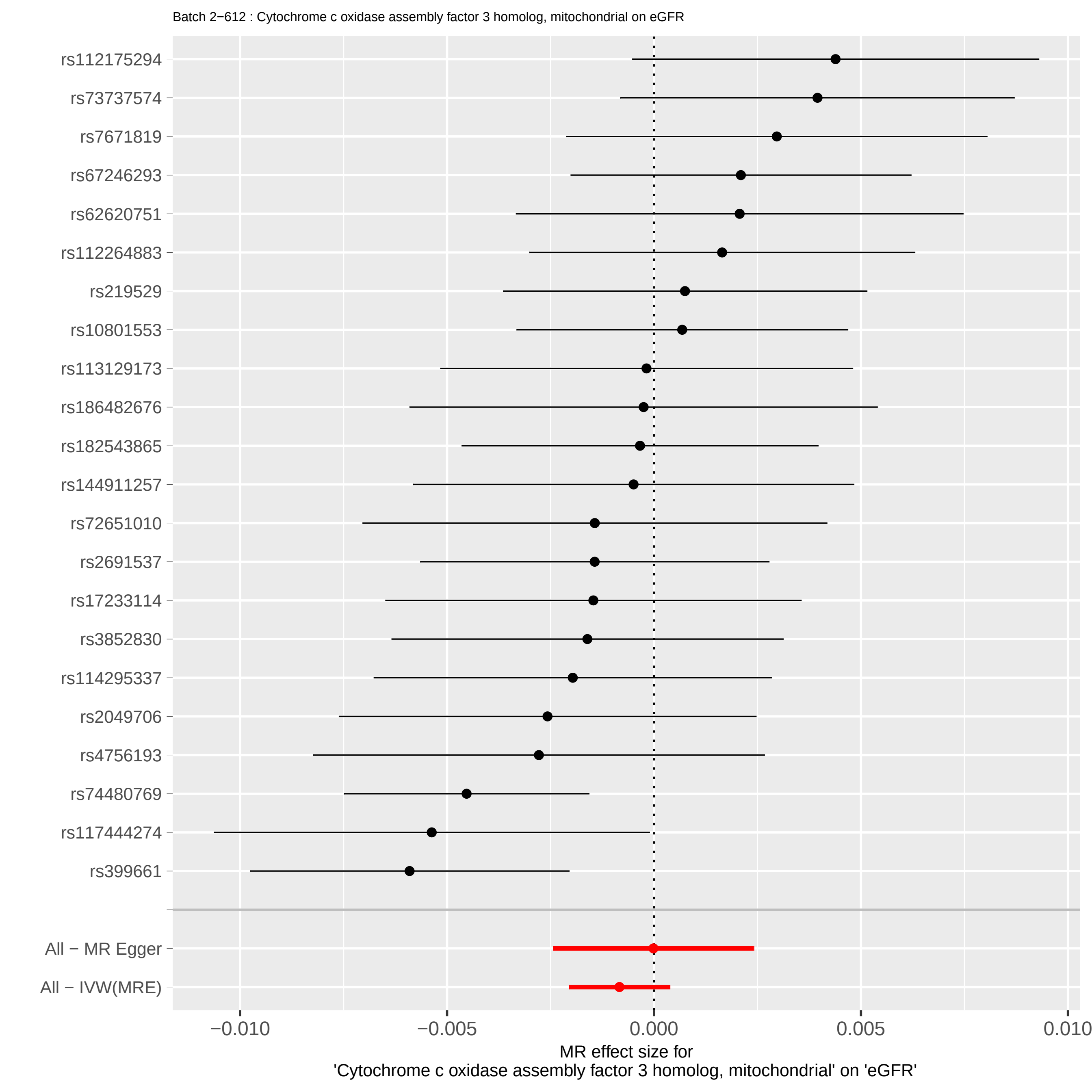

Batch 2−612 : Cytochrome c oxidase assembly factor 3 homolog, mitochondrial on eGFR
rs112175294
rs73737574
rs7671819
rs67246293
rs62620751
rs112264883
rs219529
rs10801553
rs113129173
rs186482676
rs182543865
rs144911257
rs72651010
rs2691537
rs17233114
rs3852830
rs114295337
rs2049706
rs4756193
rs74480769
rs117444274
rs399661
All − MR Egger
All − IVW(MRE)
−0.010
−0.005
0.000
0.005
0.010
MR effect size for
'Cytochrome c oxidase assembly factor 3 homolog, mitochondrial' on 'eGFR'

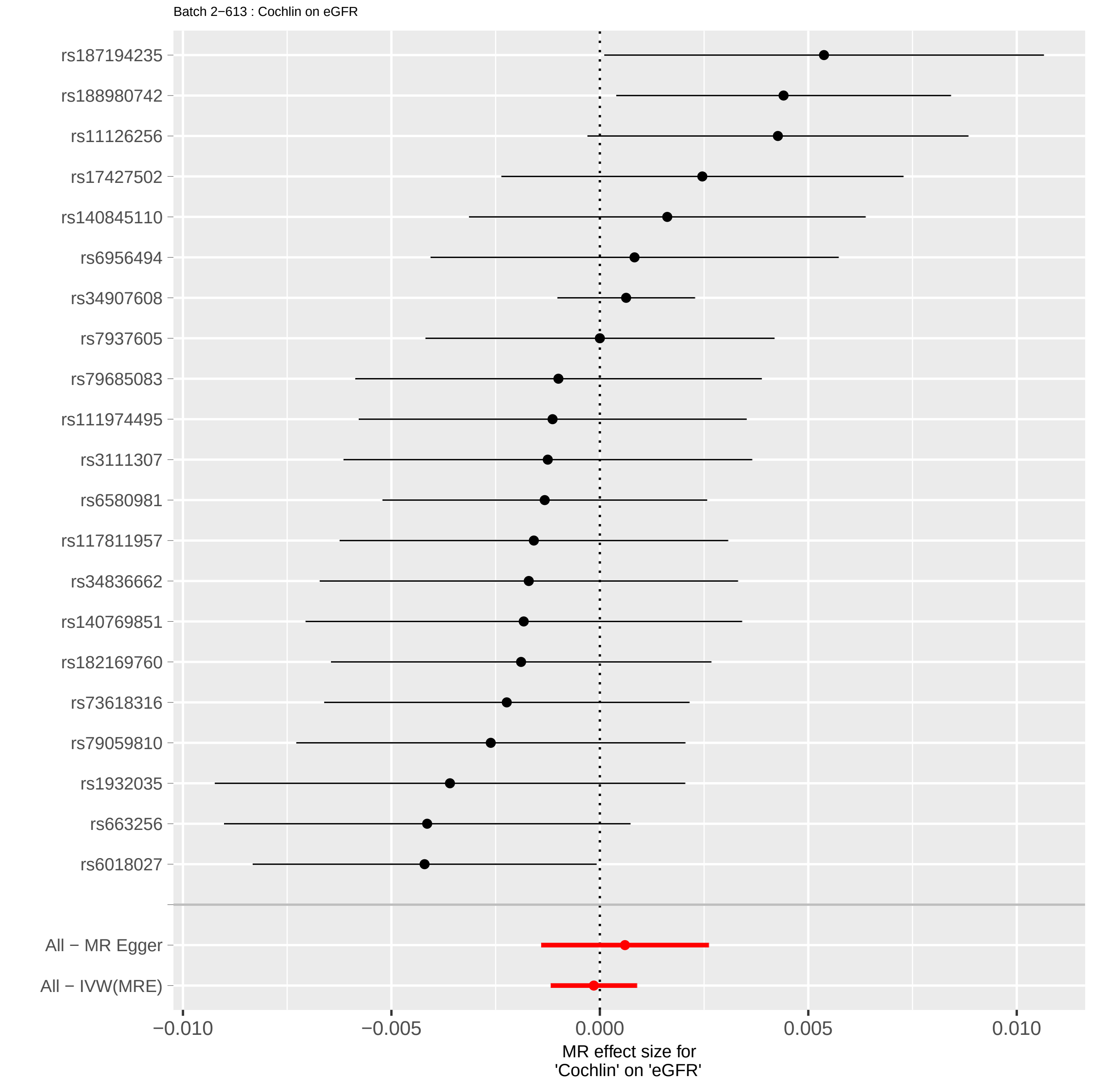
Batch 2−613 : Cochlin on eGFR
rs187194235
rs188980742
rs11126256
rs17427502
rs140845110
rs6956494
rs34907608
rs7937605
rs79685083
rs111974495
rs3111307
rs6580981
rs117811957
rs34836662
rs140769851
rs182169760
rs73618316
rs79059810
rs1932035
rs663256
rs6018027
All − MR Egger
All − IVW(MRE)
−0.010
−0.005
0.000
0.005
0.010
MR effect size for
'Cochlin' on 'eGFR'

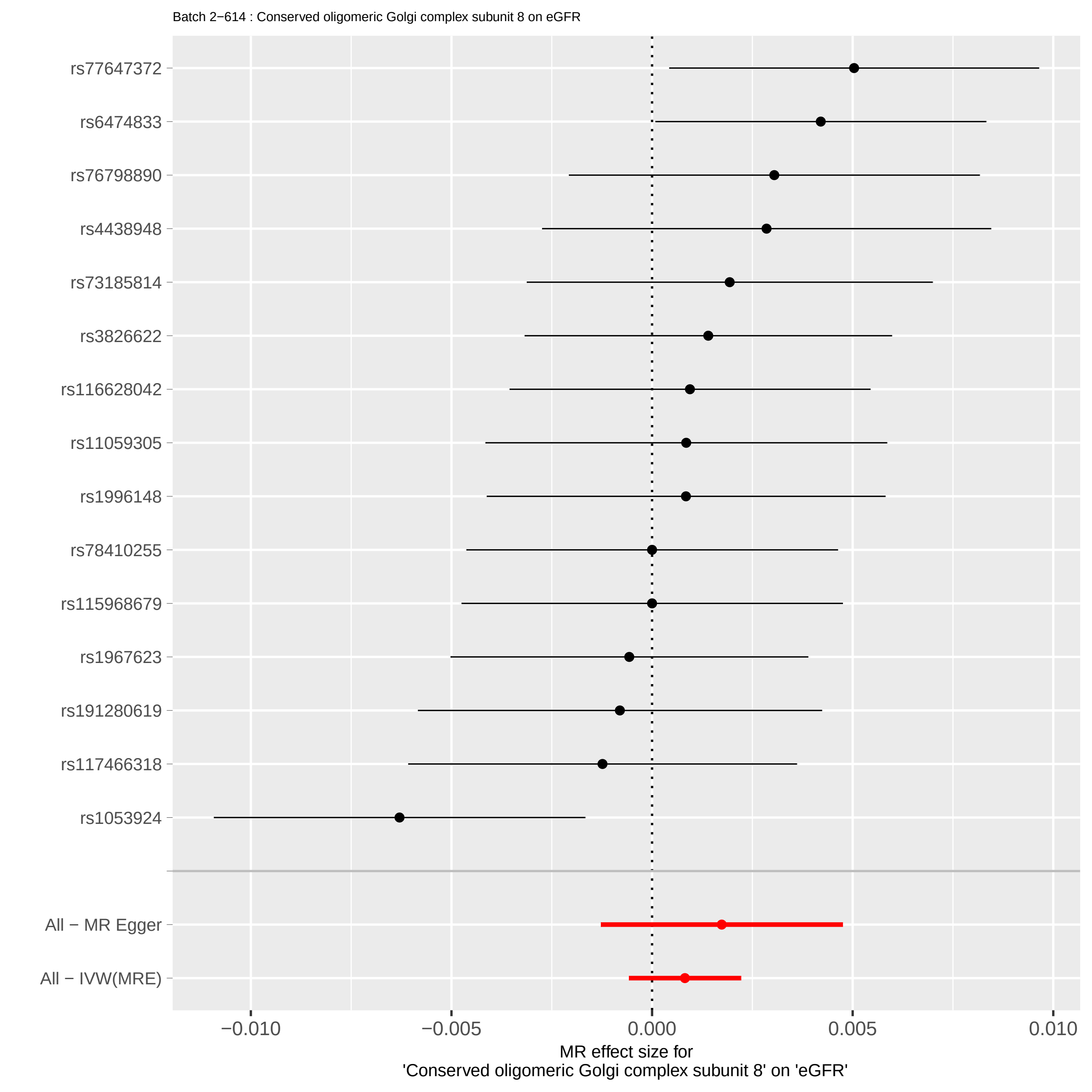
Batch 2−614 : Conserved oligomeric Golgi complex subunit 8 on eGFR
rs77647372
rs6474833
rs76798890
rs4438948
rs73185814
rs3826622
rs116628042
rs11059305
rs1996148
rs78410255
rs115968679
rs1967623
rs191280619
rs117466318
rs1053924
All − MR Egger
All − IVW(MRE)
−0.010
−0.005
0.000
0.005
0.010
MR effect size for
'Conserved oligomeric Golgi complex subunit 8' on 'eGFR'

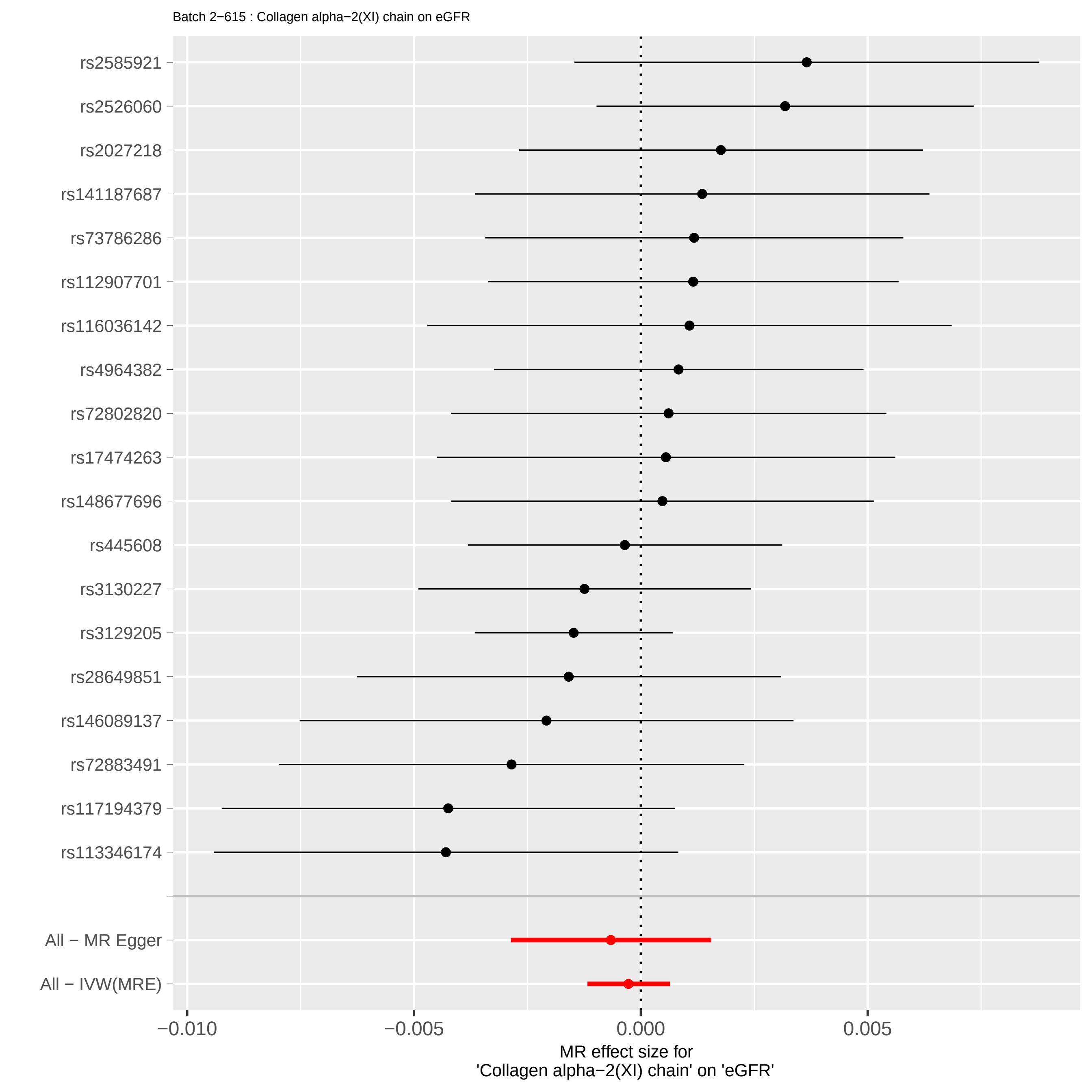
Batch 2−615 : Collagen alpha−2(XI) chain on eGFR
rs2585921
rs2526060
rs2027218
rs141187687
rs73786286
rs112907701
rs116036142
rs4964382
rs72802820
rs17474263
rs148677696
rs445608
rs3130227
rs3129205
rs28649851
rs146089137
rs72883491
rs117194379
rs113346174
All − MR Egger
All − IVW(MRE)
−0.010
−0.005
0.000
0.005
MR effect size for
'Collagen alpha−2(XI) chain' on 'eGFR'

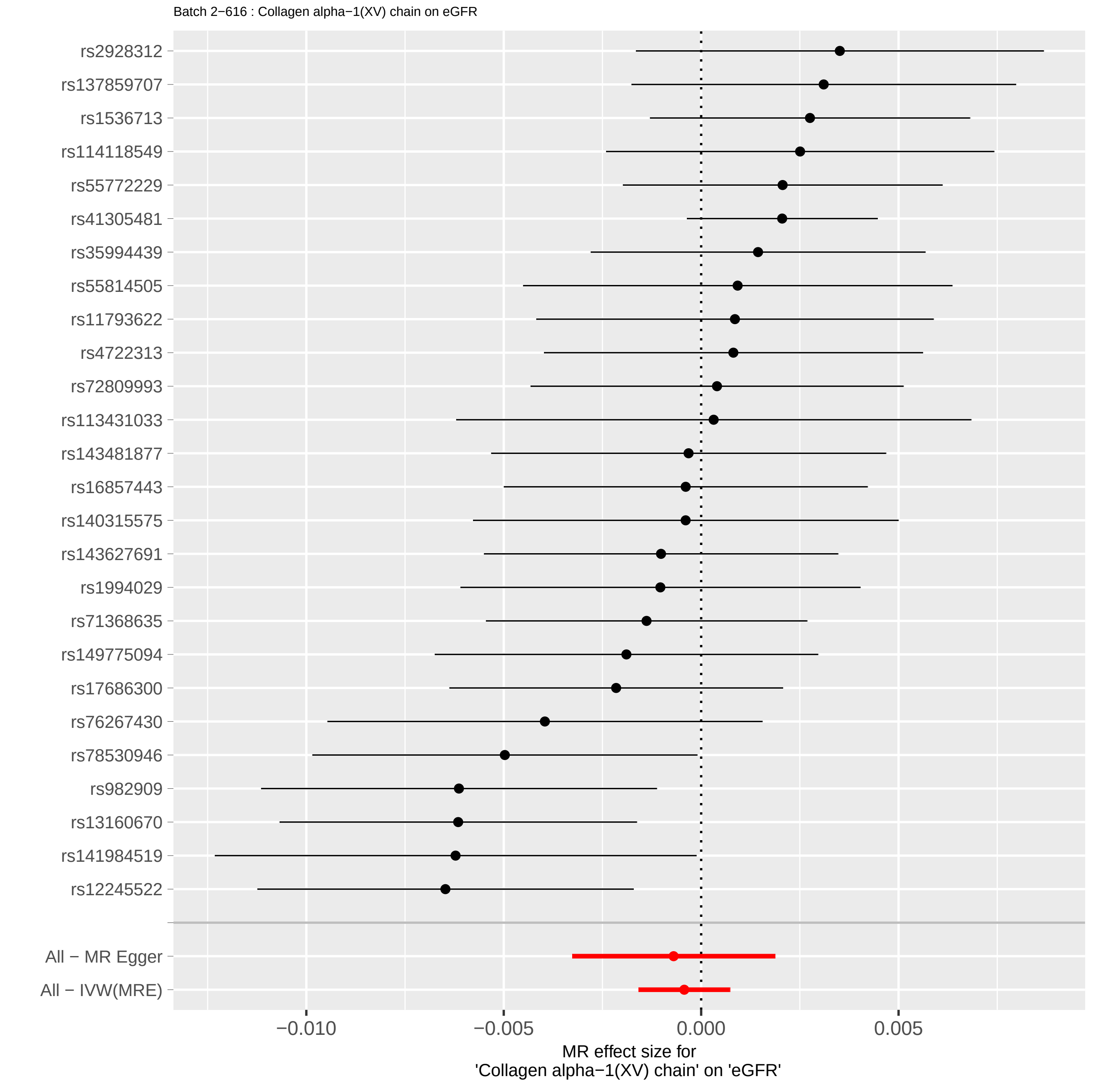

Batch 2−616 : Collagen alpha−1(XV) chain on eGFR
rs2928312
rs137859707
rs1536713
rs114118549
rs55772229
rs41305481
rs35994439
rs55814505
rs11793622
rs4722313
rs72809993
rs113431033
rs143481877
rs16857443
rs140315575
rs143627691
rs1994029
rs71368635
rs149775094
rs17686300
rs76267430
rs78530946
rs982909
rs13160670
rs141984519
rs12245522
All − MR Egger
All − IVW(MRE)
−0.010
−0.005
0.000
0.005
MR effect size for
'Collagen alpha−1(XV) chain' on 'eGFR'

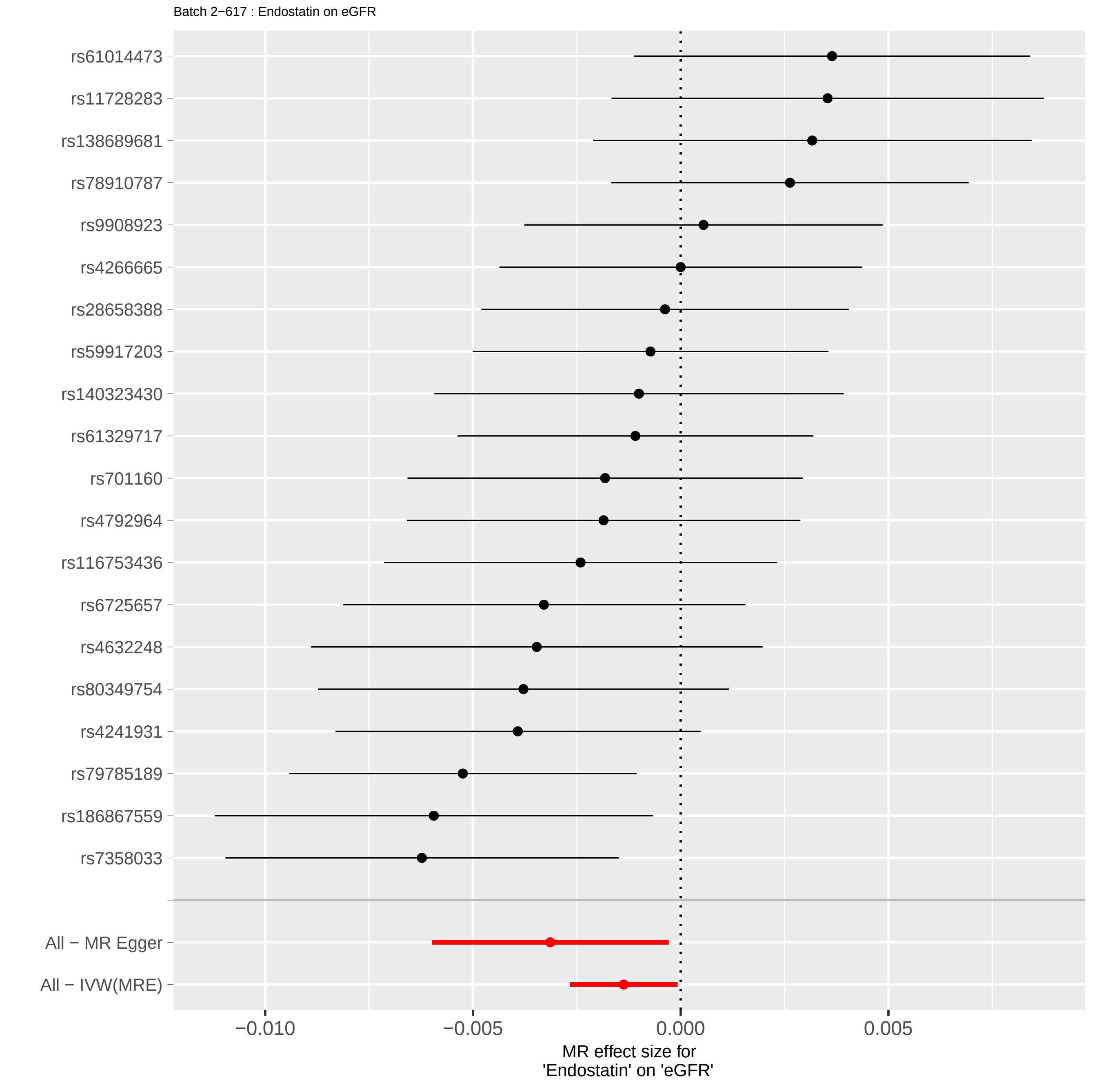
Batch 2−617 : Endostatin on eGFR
rs61014473
rs11728283
rs138689681
rs78910787
rs9908923
rs4266665
rs28658388
rs59917203
rs140323430
rs61329717
rs701160
rs4792964
rs116753436
rs6725657
rs4632248
rs80349754
rs4241931
rs79785189
rs186867559
rs7358033
All − MR Egger
All − IVW(MRE)
−0.010
−0.005
0.000
0.005
MR effect size for
'Endostatin' on 'eGFR'

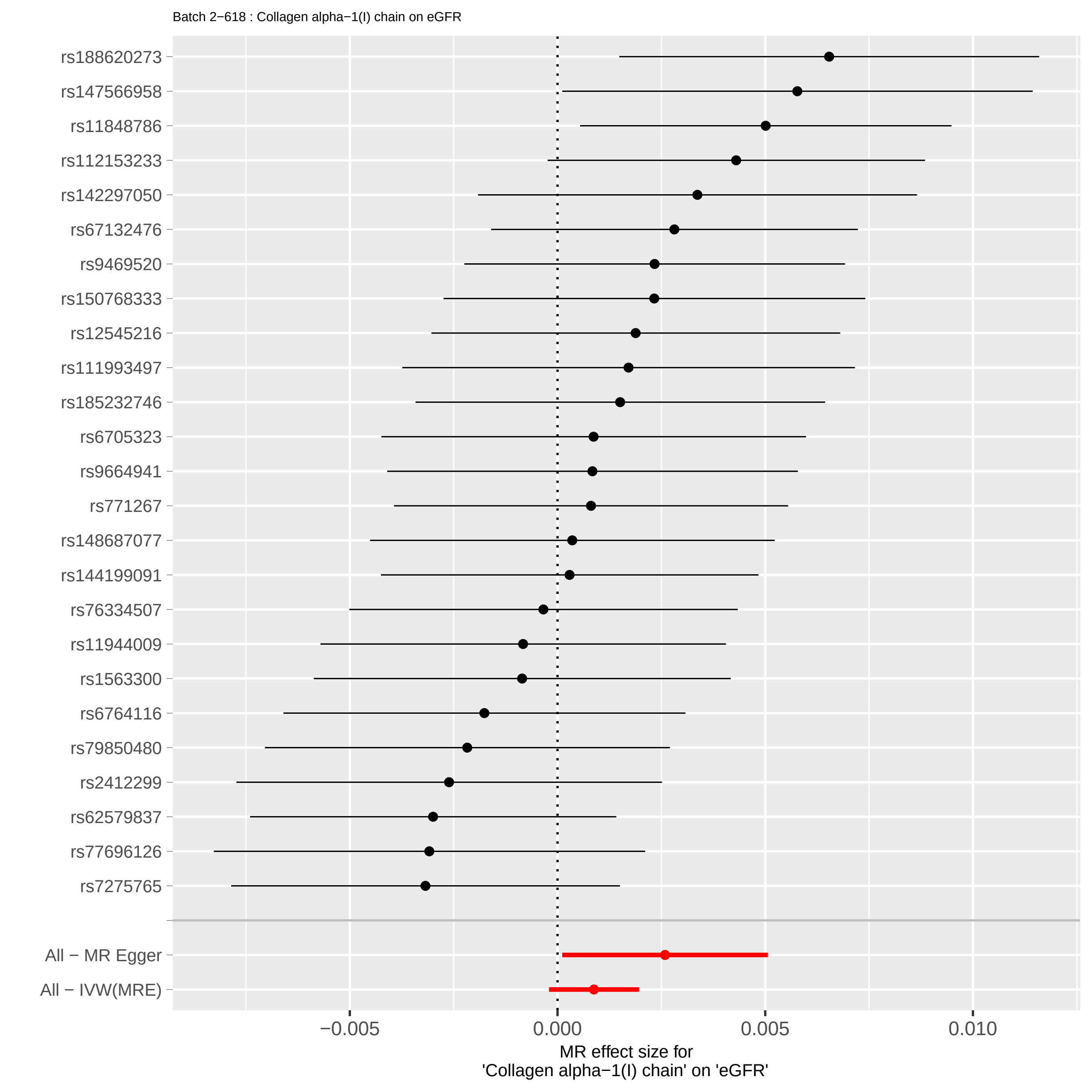

Batch 2−618 : Collagen alpha−1(I) chain on eGFR
rs188620273
rs147566958
rs11848786
rs112153233
rs142297050
rs67132476
rs9469520
rs150768333
rs12545216
rs111993497
rs185232746
rs6705323
rs9664941
rs771267
rs148687077
rs144199091
rs76334507
rs11944009
rs1563300
rs6764116
rs79850480
rs2412299
rs62579837
rs77696126
rs7275765
All − MR Egger
All − IVW(MRE)
−0.005
0.000
0.005
0.010
MR effect size for
'Collagen alpha−1(I) chain' on 'eGFR'

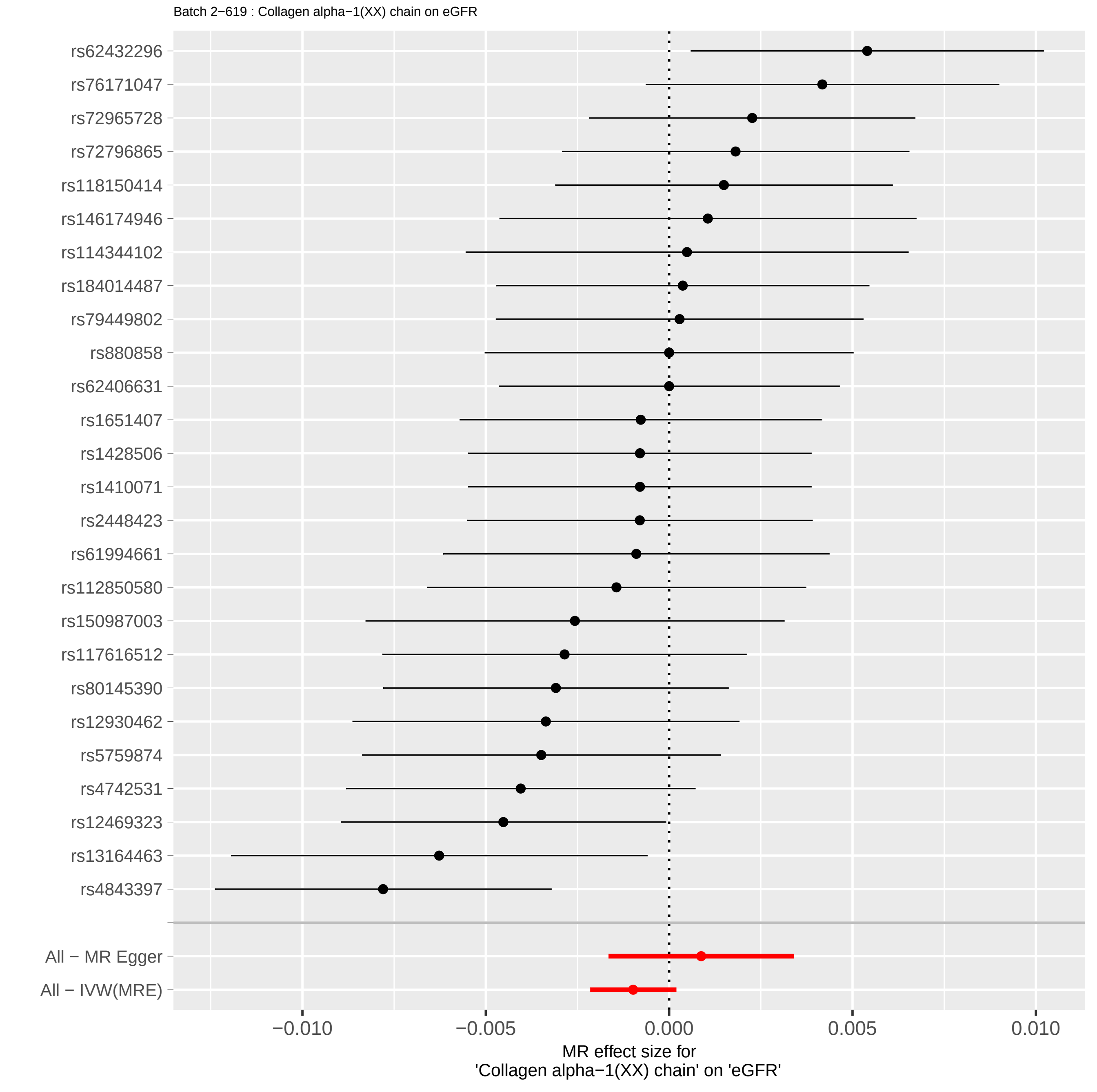

Batch 2−619 : Collagen alpha−1(XX) chain on eGFR
rs62432296
rs76171047
rs72965728
rs72796865
rs118150414
rs146174946
rs114344102
rs184014487
rs79449802
rs880858
rs62406631
rs1651407
rs1428506
rs1410071
rs2448423
rs61994661
rs112850580
rs150987003
rs117616512
rs80145390
rs12930462
rs5759874
rs4742531
rs12469323
rs13164463
rs4843397
All − MR Egger
All − IVW(MRE)
−0.010
−0.005
0.000
0.005
0.010
MR effect size for
'Collagen alpha−1(XX) chain' on 'eGFR'

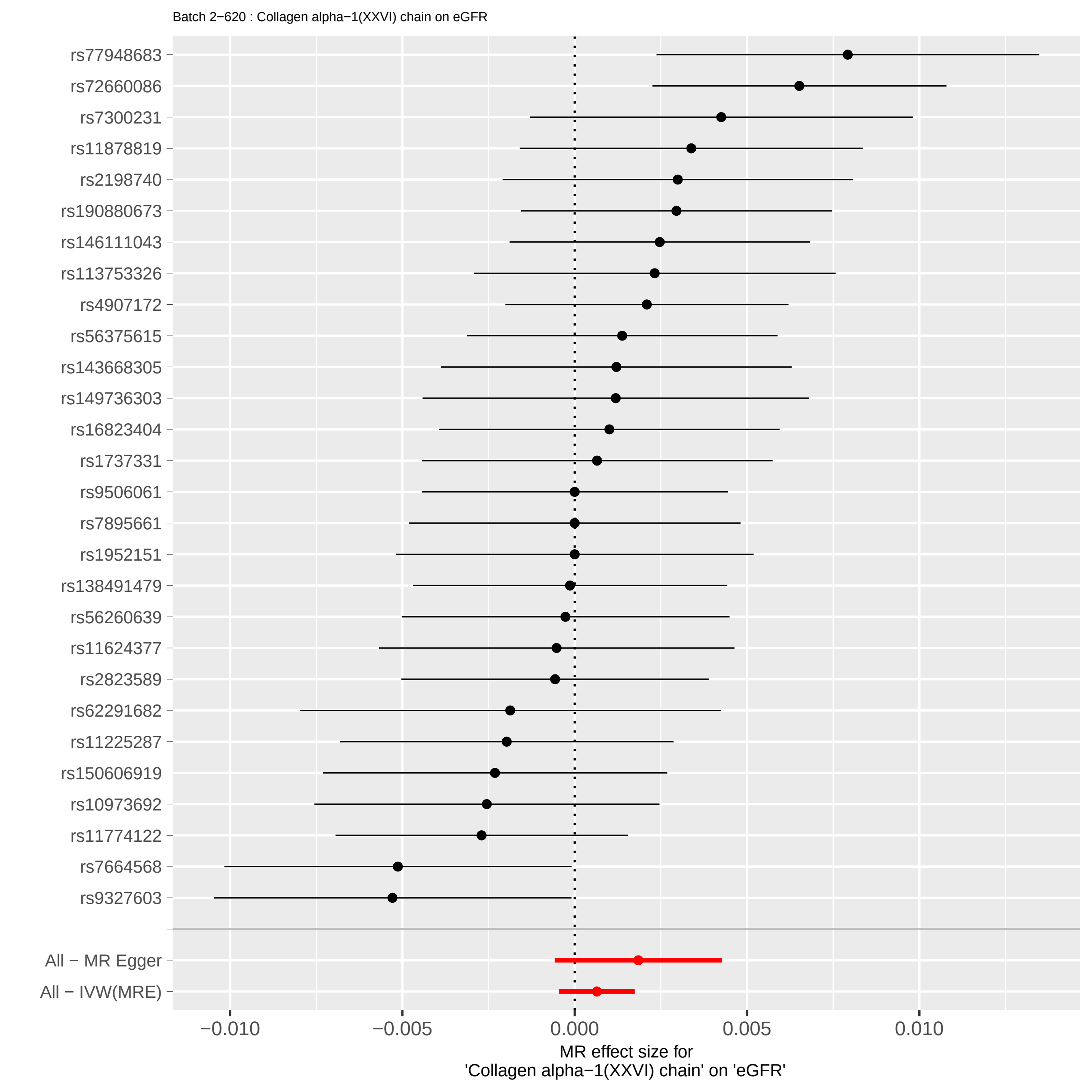

Batch 2−620 : Collagen alpha−1(XXVI) chain on eGFR
rs77948683
rs72660086
rs7300231
rs11878819
rs2198740
rs190880673
rs146111043
rs113753326
rs4907172
rs56375615
rs143668305
rs149736303
rs16823404
rs1737331
rs9506061
rs7895661
rs1952151
rs138491479
rs56260639
rs11624377
rs2823589
rs62291682
rs11225287
rs150606919
rs10973692
rs11774122
rs7664568
rs9327603
All − MR Egger
All − IVW(MRE)
−0.010
−0.005
0.000
0.005
0.010
MR effect size for
'Collagen alpha−1(XXVI) chain' on 'eGFR'

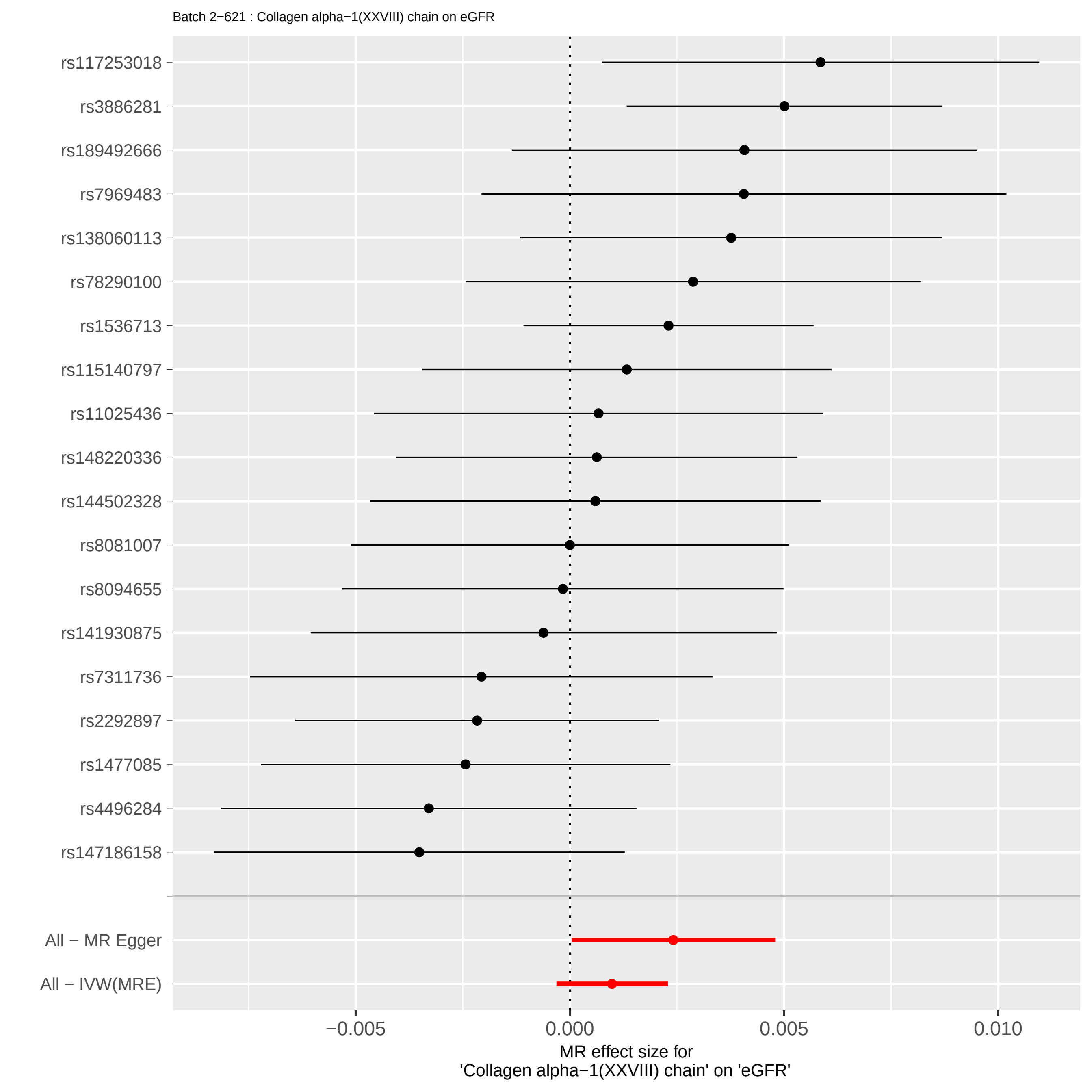

Batch 2−621 : Collagen alpha−1(XXVIII) chain on eGFR
rs117253018
rs3886281
rs189492666
rs7969483
rs138060113
rs78290100
rs1536713
rs115140797
rs11025436
rs148220336
rs144502328
rs8081007
rs8094655
rs141930875
rs7311736
rs2292897
rs1477085
rs4496284
rs147186158
All − MR Egger
All − IVW(MRE)
−0.005
0.000
0.005
0.010
MR effect size for
'Collagen alpha−1(XXVIII) chain' on 'eGFR'

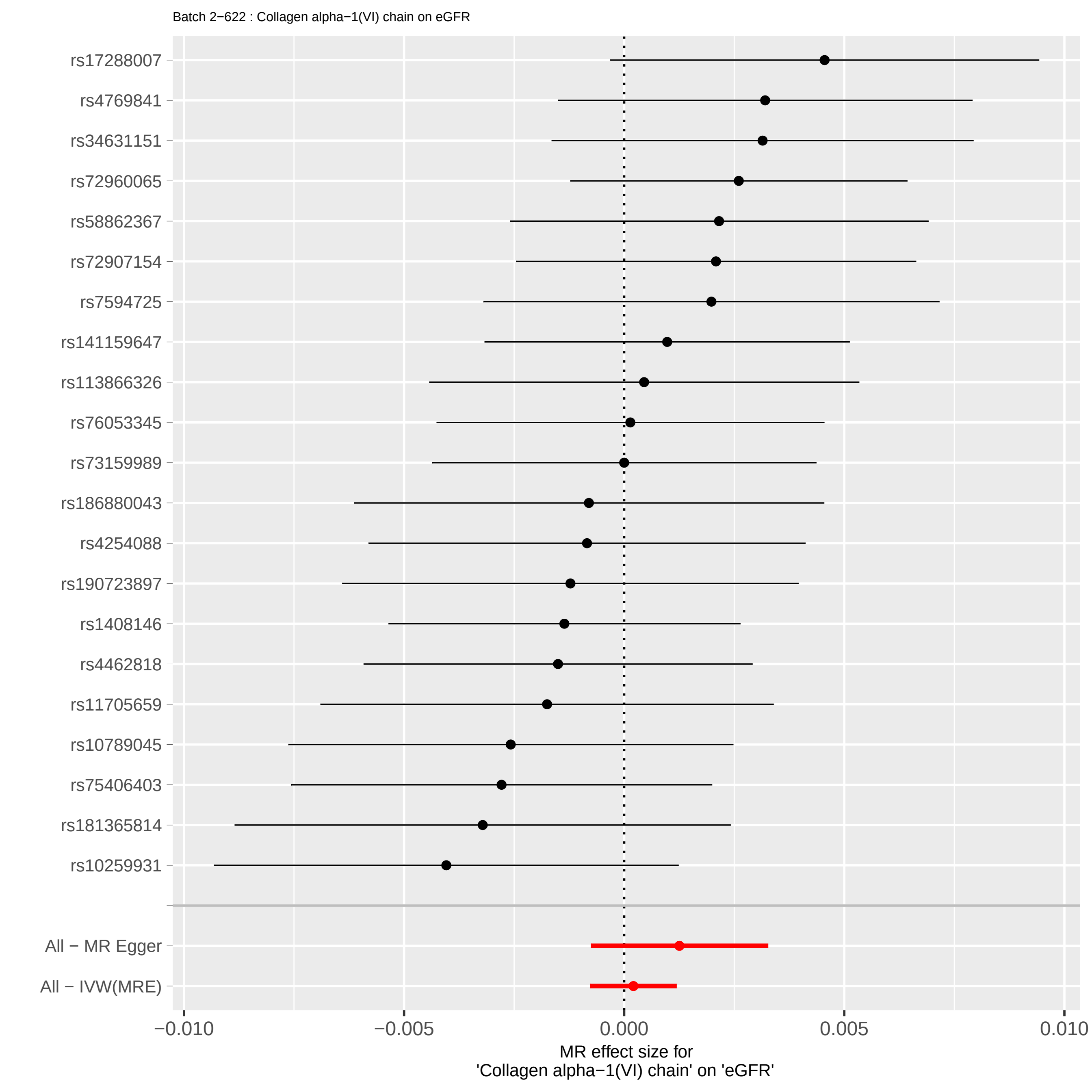

Batch 2−622 : Collagen alpha−1(VI) chain on eGFR
rs17288007
rs4769841
rs34631151
rs72960065
rs58862367
rs72907154
rs7594725
rs141159647
rs113866326
rs76053345
rs73159989
rs186880043
rs4254088
rs190723897
rs1408146
rs4462818
rs11705659
rs10789045
rs75406403
rs181365814
rs10259931
All − MR Egger
All − IVW(MRE)
−0.010
−0.005
0.000
0.005
0.010
MR effect size for
'Collagen alpha−1(VI) chain' on 'eGFR'

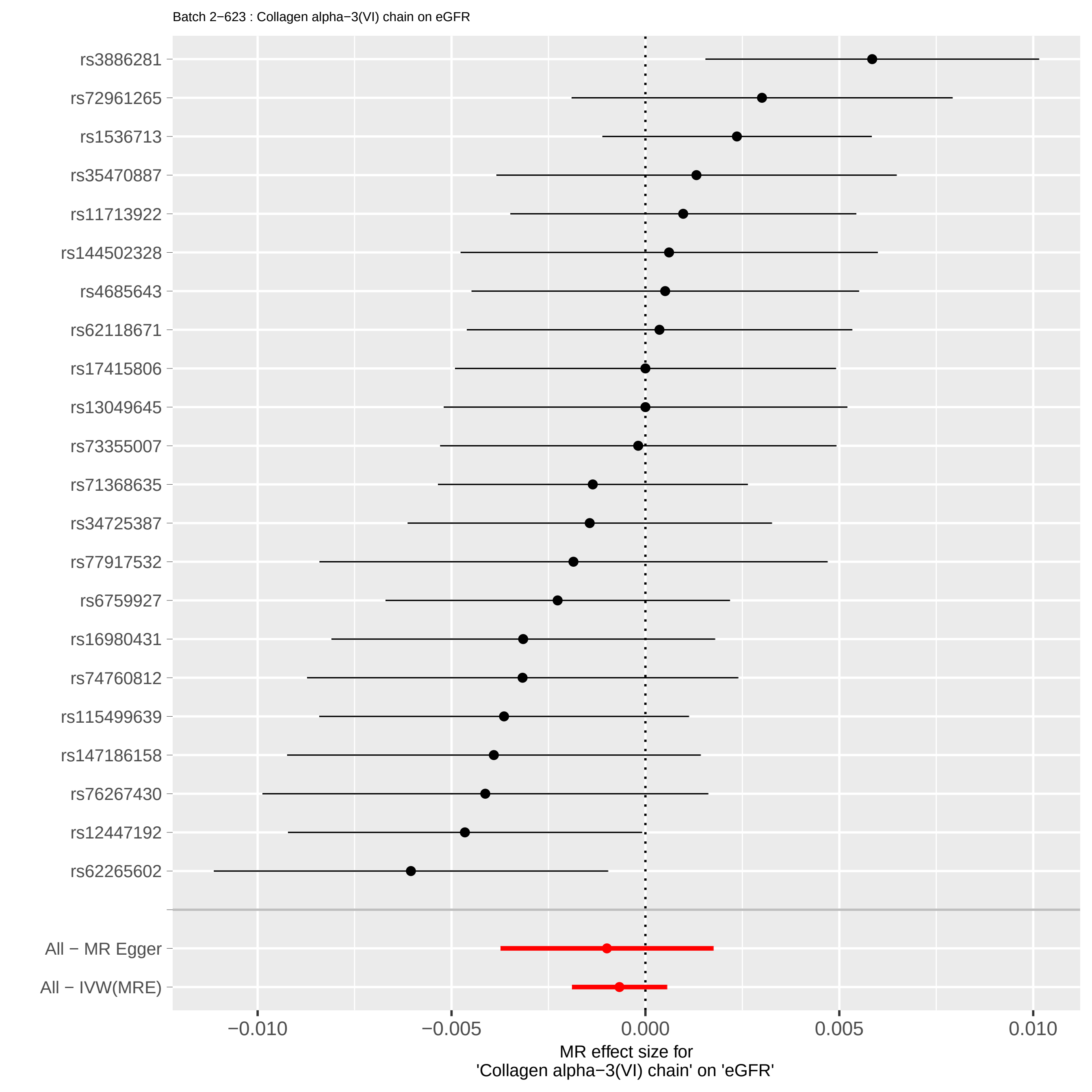
Batch 2−623 : Collagen alpha−3(VI) chain on eGFR
rs3886281
rs72961265
rs1536713
rs35470887
rs11713922
rs144502328
rs4685643
rs62118671
rs17415806
rs13049645
rs73355007
rs71368635
rs34725387
rs77917532
rs6759927
rs16980431
rs74760812
rs115499639
rs147186158
rs76267430
rs12447192
rs62265602
All − MR Egger
All − IVW(MRE)
−0.010
−0.005
0.000
0.005
0.010
MR effect size for
'Collagen alpha−3(VI) chain' on 'eGFR'

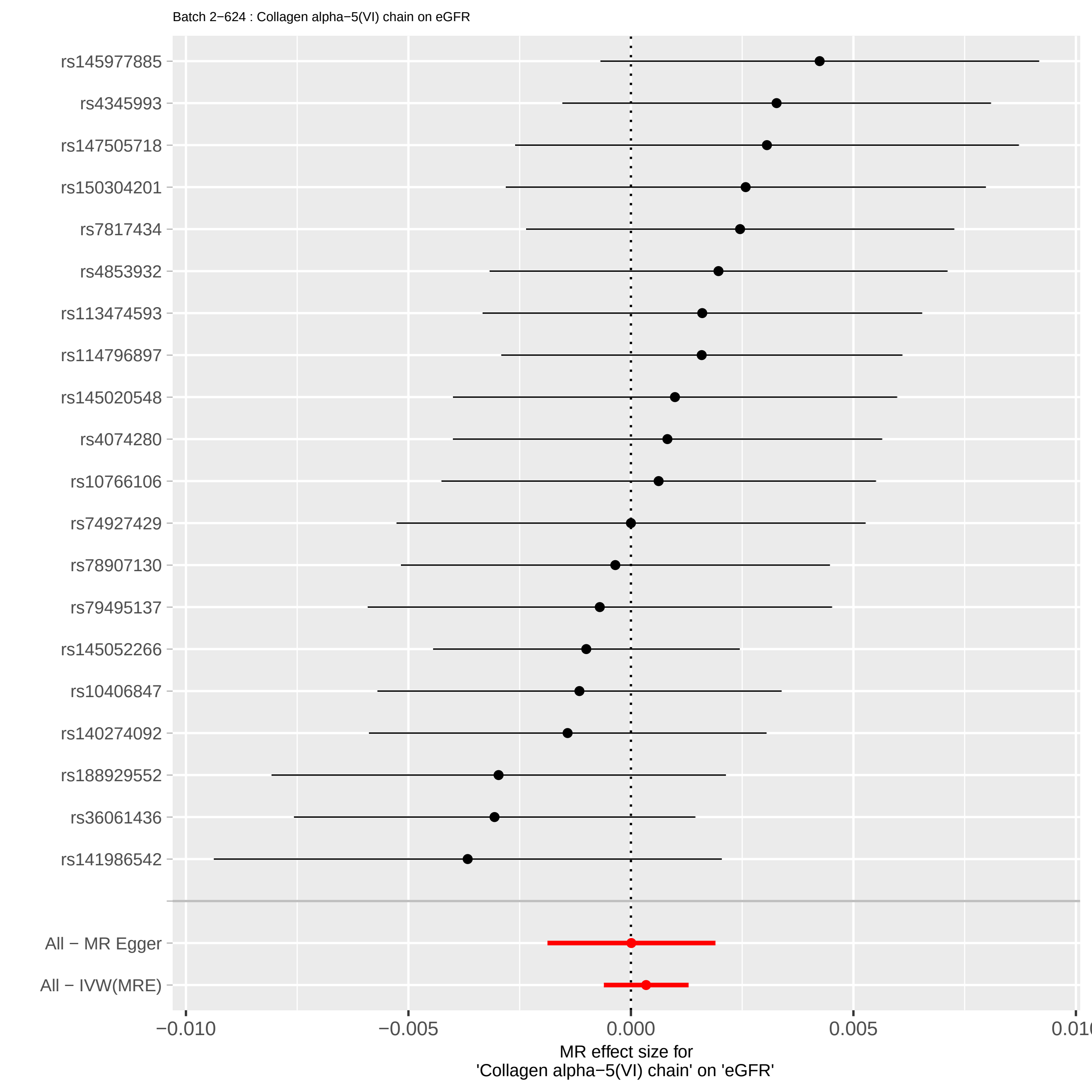

Batch 2−624 : Collagen alpha−5(VI) chain on eGFR
rs145977885
rs4345993
rs147505718
rs150304201
rs7817434
rs4853932
rs113474593
rs114796897
rs145020548
rs4074280
rs10766106
rs74927429
rs78907130
rs79495137
rs145052266
rs10406847
rs140274092
rs188929552
rs36061436
rs141986542
All − MR Egger
All − IVW(MRE)
−0.010
−0.005
0.000
0.005
MR effect size for
'Collagen alpha−5(VI) chain' on 'eGFR'

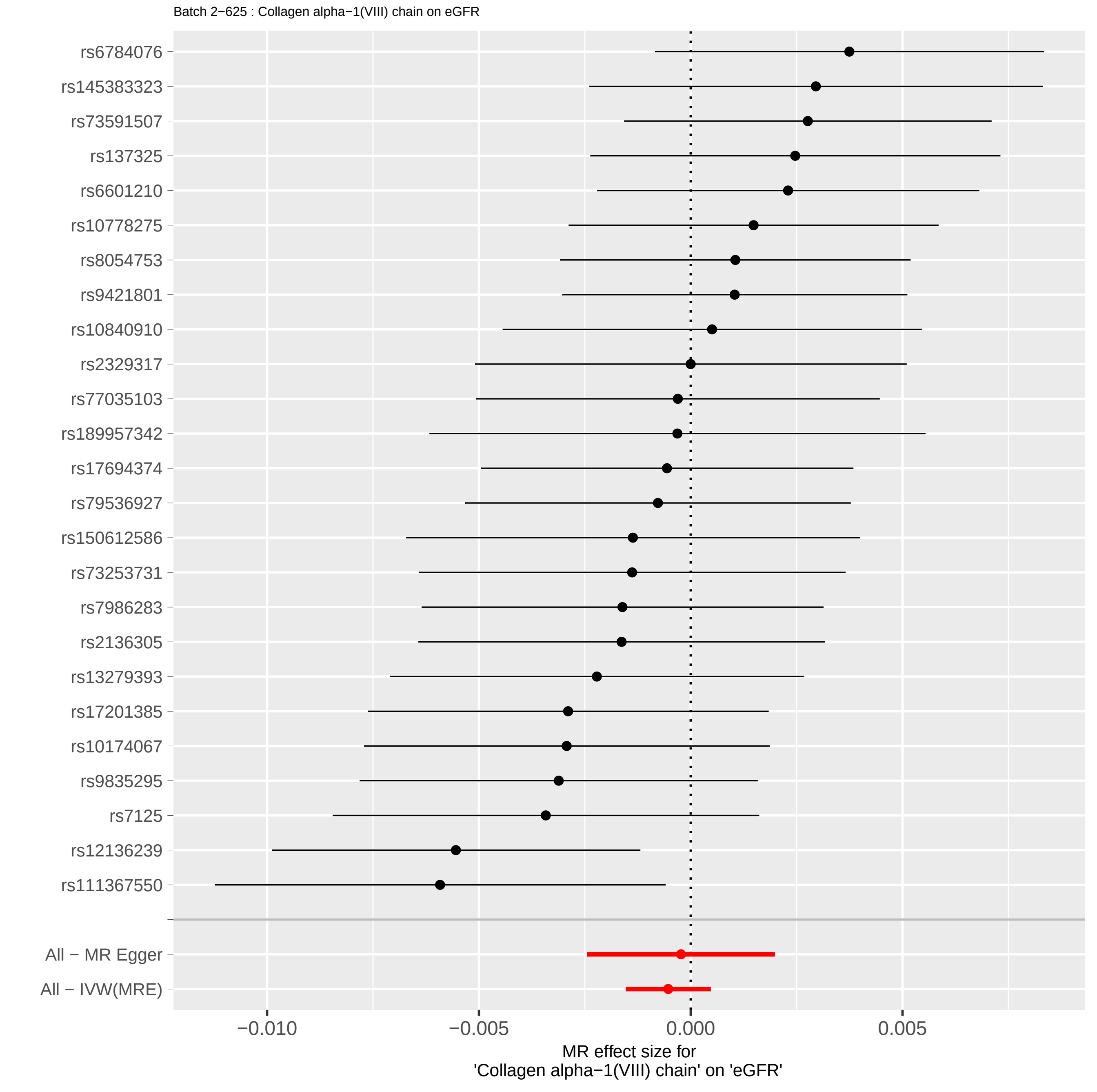

Batch 2−625 : Collagen alpha−1(VIII) chain on eGFR
rs6784076
rs145383323
rs73591507
rs137325
rs6601210
rs10778275
rs8054753
rs9421801
rs10840910
rs2329317
rs77035103
rs189957342
rs17694374
rs79536927
rs150612586
rs73253731
rs7986283
rs2136305
rs13279393
rs17201385
rs10174067
rs9835295
rs7125
rs12136239
rs111367550
All − MR Egger
All − IVW(MRE)
−0.010
−0.005
0.000
0.005
MR effect size for
'Collagen alpha−1(VIII) chain' on 'eGFR'

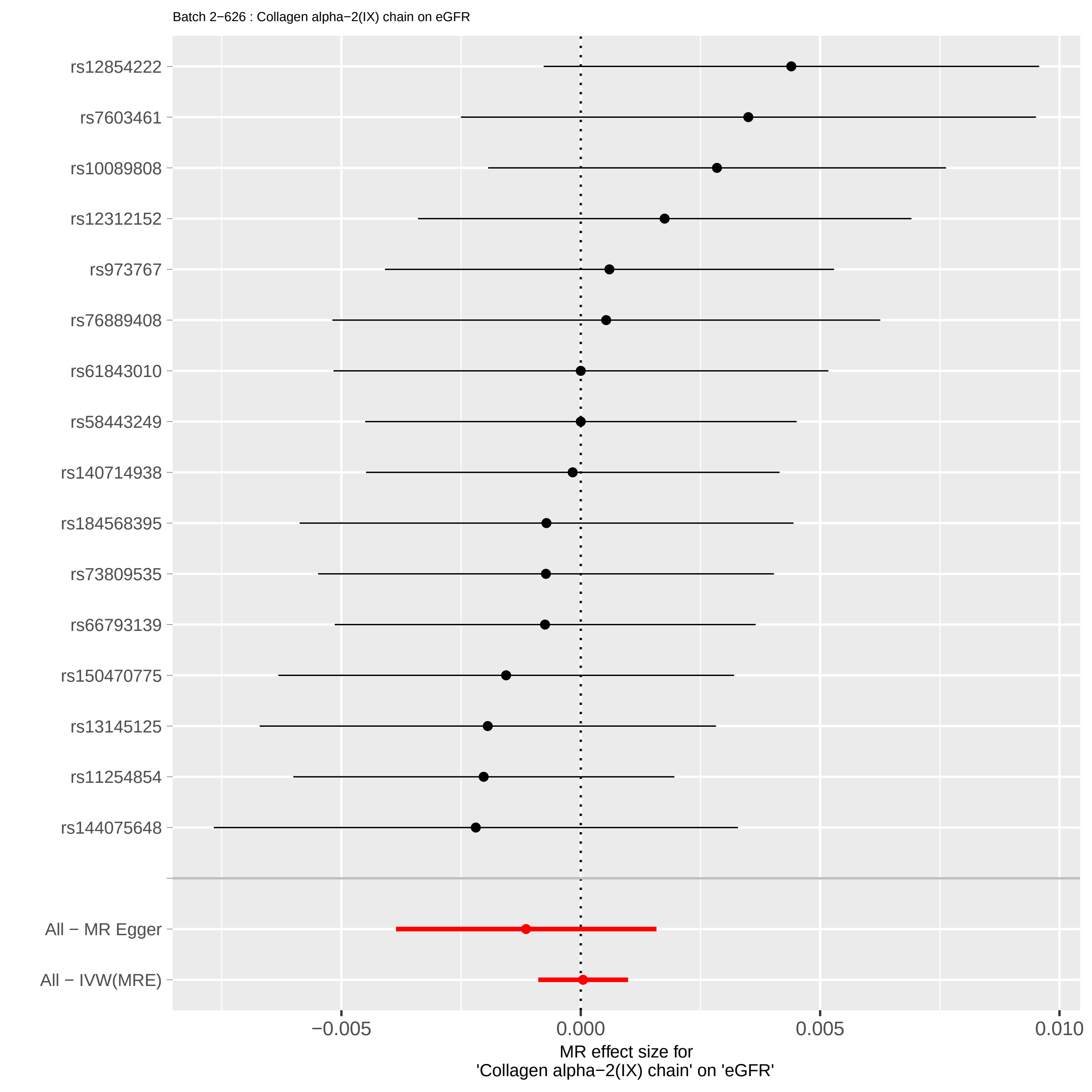
Batch 2−626 : Collagen alpha−2(IX) chain on eGFR
rs12854222
rs7603461
rs10089808
rs12312152
rs973767
rs76889408
rs61843010
rs58443249
rs140714938
rs184568395
rs73809535
rs66793139
rs150470775
rs13145125
rs11254854
rs144075648
All − MR Egger
All − IVW(MRE)
−0.005
0.000
0.005
0.010
MR effect size for
'Collagen alpha−2(IX) chain' on 'eGFR'

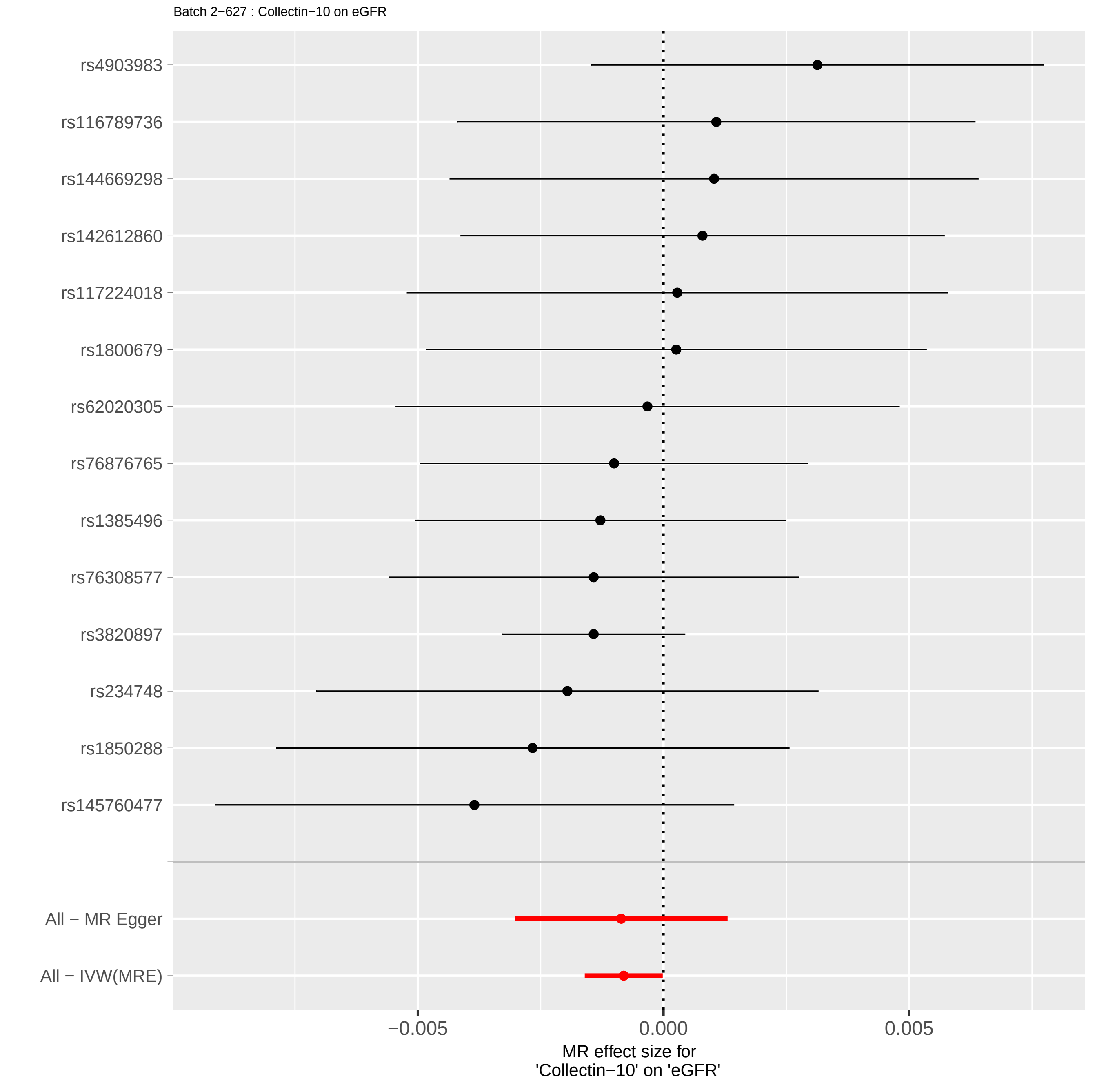

Batch 2−627 : Collectin−10 on eGFR
rs4903983
rs116789736
rs144669298
rs142612860
rs117224018
rs1800679
rs62020305
rs76876765
rs1385496
rs76308577
rs3820897
rs234748
rs1850288
rs145760477
All − MR Egger
All − IVW(MRE)
−0.005
0.000
0.005
MR effect size for
'Collectin−10' on 'eGFR'

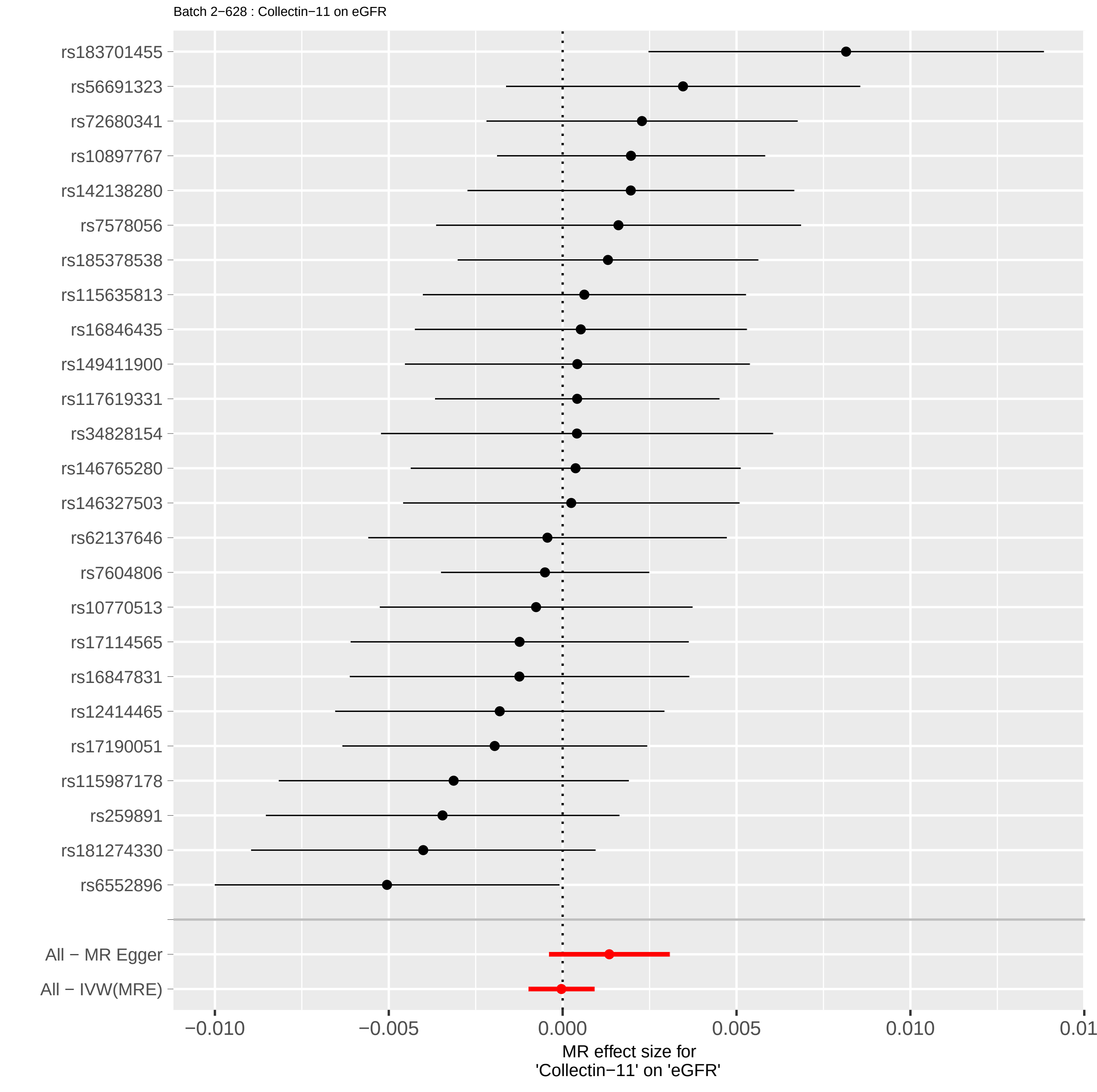

Batch 2−628 : Collectin−11 on eGFR
rs183701455
rs56691323
rs72680341
rs10897767
rs142138280
rs7578056
rs185378538
rs115635813
rs16846435
rs149411900
rs117619331
rs34828154
rs146765280
rs146327503
rs62137646
rs7604806
rs10770513
rs17114565
rs16847831
rs12414465
rs17190051
rs115987178
rs259891
rs181274330
rs6552896
All − MR Egger
All − IVW(MRE)
−0.010
−0.005
0.000
0.005
0.010
MR effect size for
'Collectin−11' on 'eGFR'

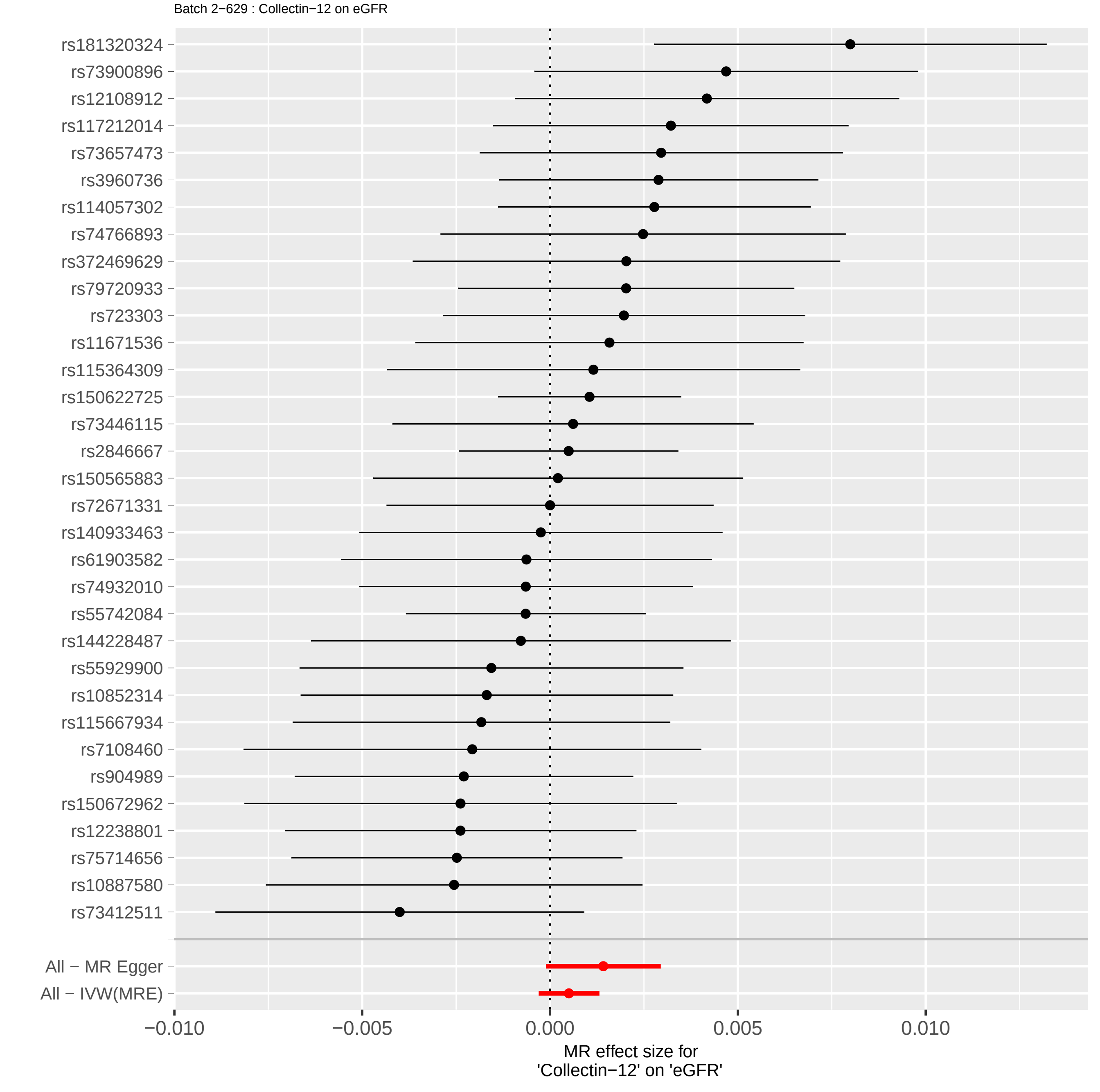

Batch 2−629 : Collectin−12 on eGFR
rs181320324
rs73900896
rs12108912
rs117212014
rs73657473
rs3960736
rs114057302
rs74766893
rs372469629
rs79720933
rs723303
rs11671536
rs115364309
rs150622725
rs73446115
rs2846667
rs150565883
rs72671331
rs140933463
rs61903582
rs74932010
rs55742084
rs144228487
rs55929900
rs10852314
rs115667934
rs7108460
rs904989
rs150672962
rs12238801
rs75714656
rs10887580
rs73412511
All − MR Egger
All − IVW(MRE)
−0.010
−0.005
0.000
0.005
0.010
MR effect size for
'Collectin−12' on 'eGFR'

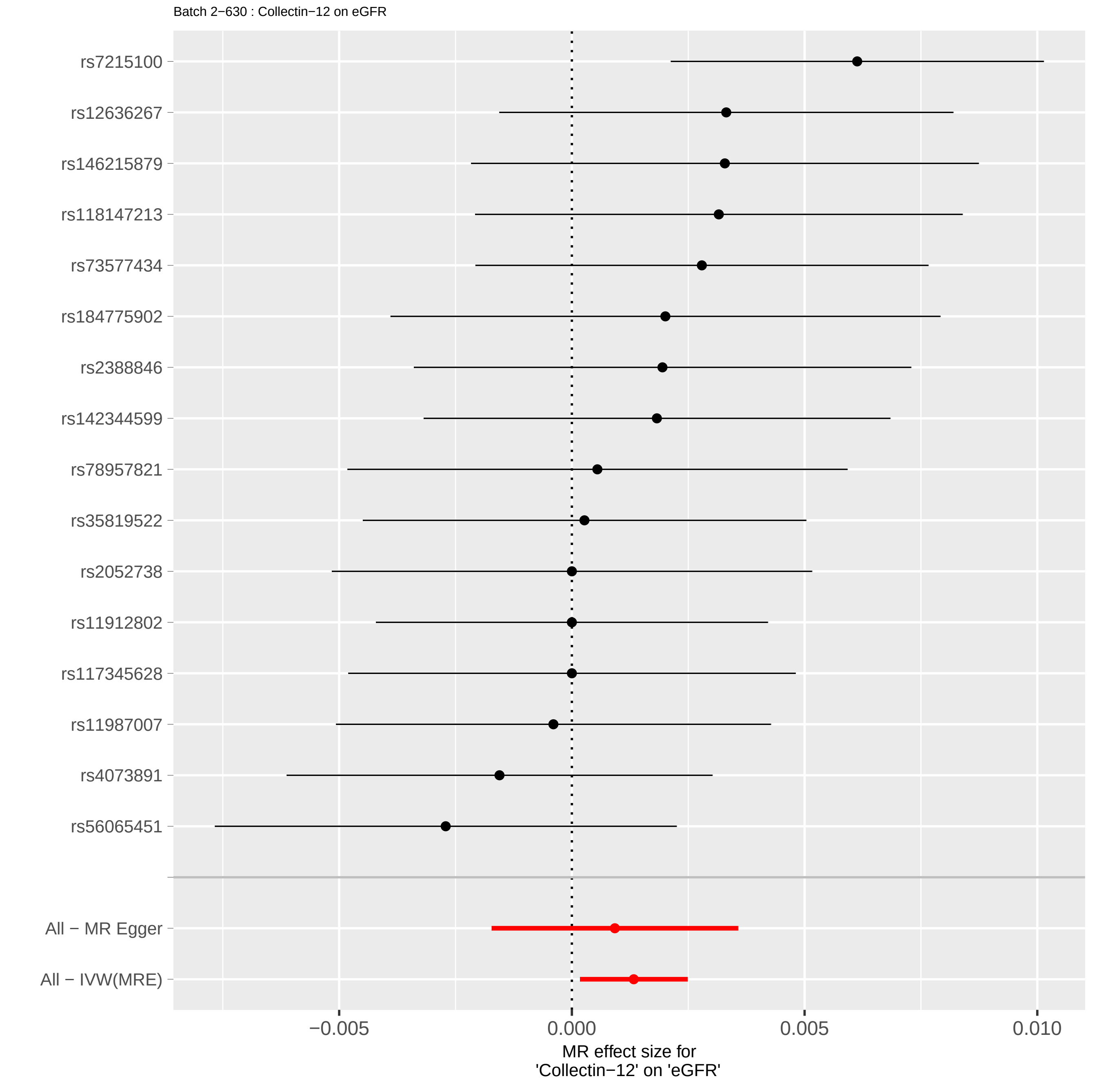

Batch 2−630 : Collectin−12 on eGFR
rs7215100
rs12636267
rs146215879
rs118147213
rs73577434
rs184775902
rs2388846
rs142344599
rs78957821
rs35819522
rs2052738
rs11912802
rs117345628
rs11987007
rs4073891
rs56065451
All − MR Egger
All − IVW(MRE)
−0.005
0.000
0.005
0.010
MR effect size for
'Collectin−12' on 'eGFR'

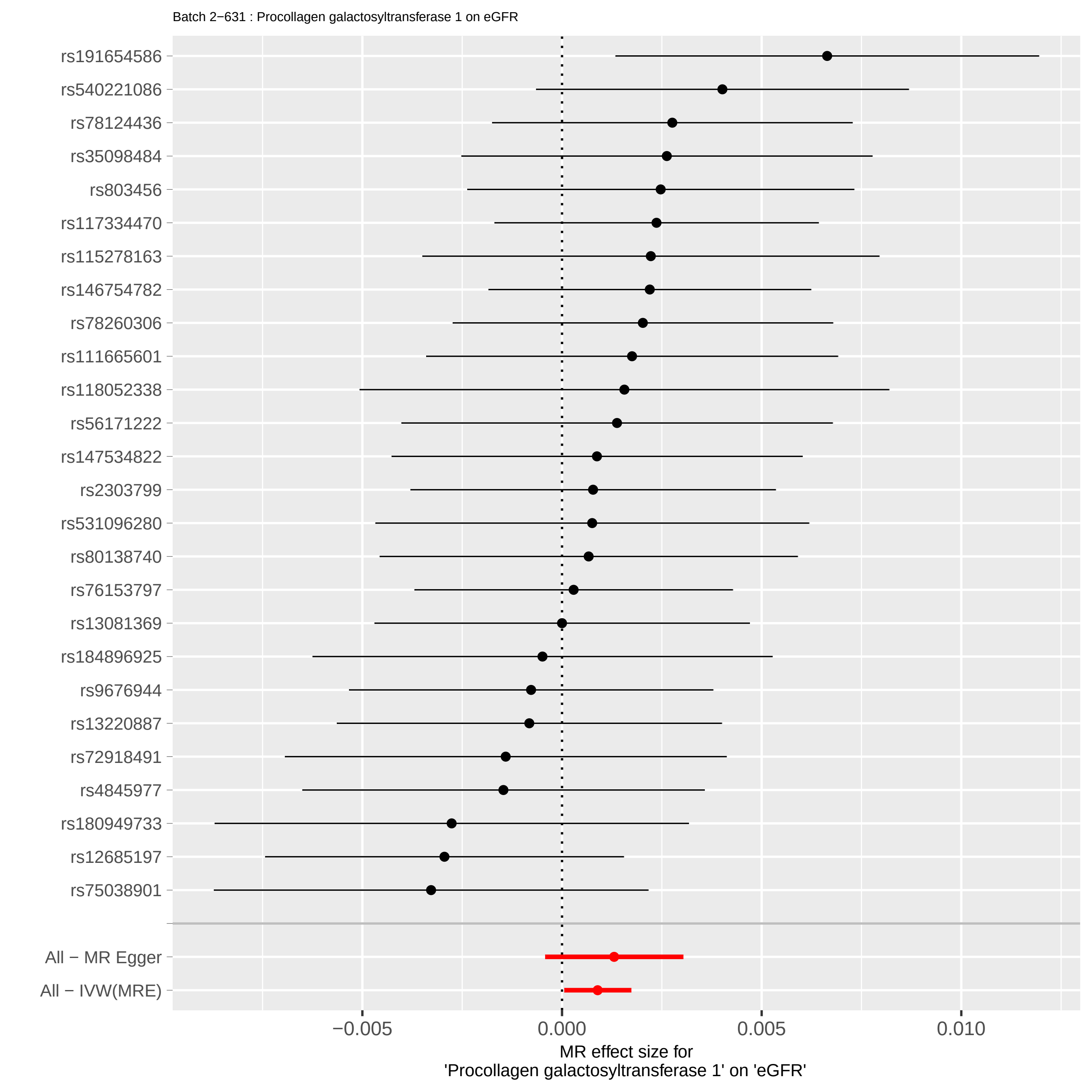

Batch 2−631 : Procollagen galactosyltransferase 1 on eGFR
rs191654586
rs540221086
rs78124436
rs35098484
rs803456
rs117334470
rs115278163
rs146754782
rs78260306
rs111665601
rs118052338
rs56171222
rs147534822
rs2303799
rs531096280
rs80138740
rs76153797
rs13081369
rs184896925
rs9676944
rs13220887
rs72918491
rs4845977
rs180949733
rs12685197
rs75038901
All − MR Egger
All − IVW(MRE)
−0.005
0.000
0.005
0.010
MR effect size for
'Procollagen galactosyltransferase 1' on 'eGFR'

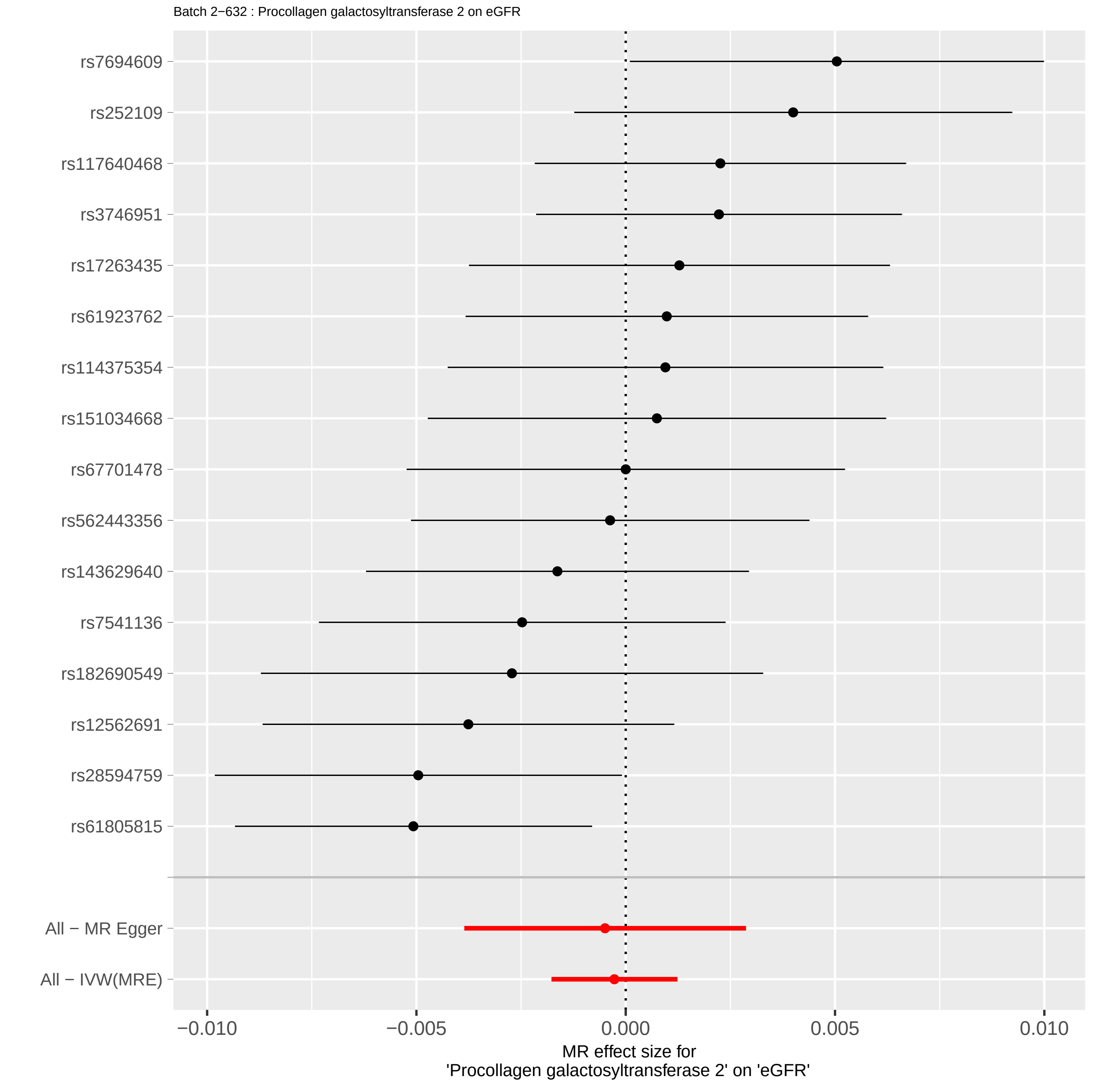

Batch 2−632 : Procollagen galactosyltransferase 2 on eGFR
rs7694609
rs252109
rs117640468
rs3746951
rs17263435
rs61923762
rs114375354
rs151034668
rs67701478
rs562443356
rs143629640
rs7541136
rs182690549
rs12562691
rs28594759
rs61805815
All − MR Egger
All − IVW(MRE)
−0.010
−0.005
0.000
0.005
0.010
MR effect size for
'Procollagen galactosyltransferase 2' on 'eGFR'

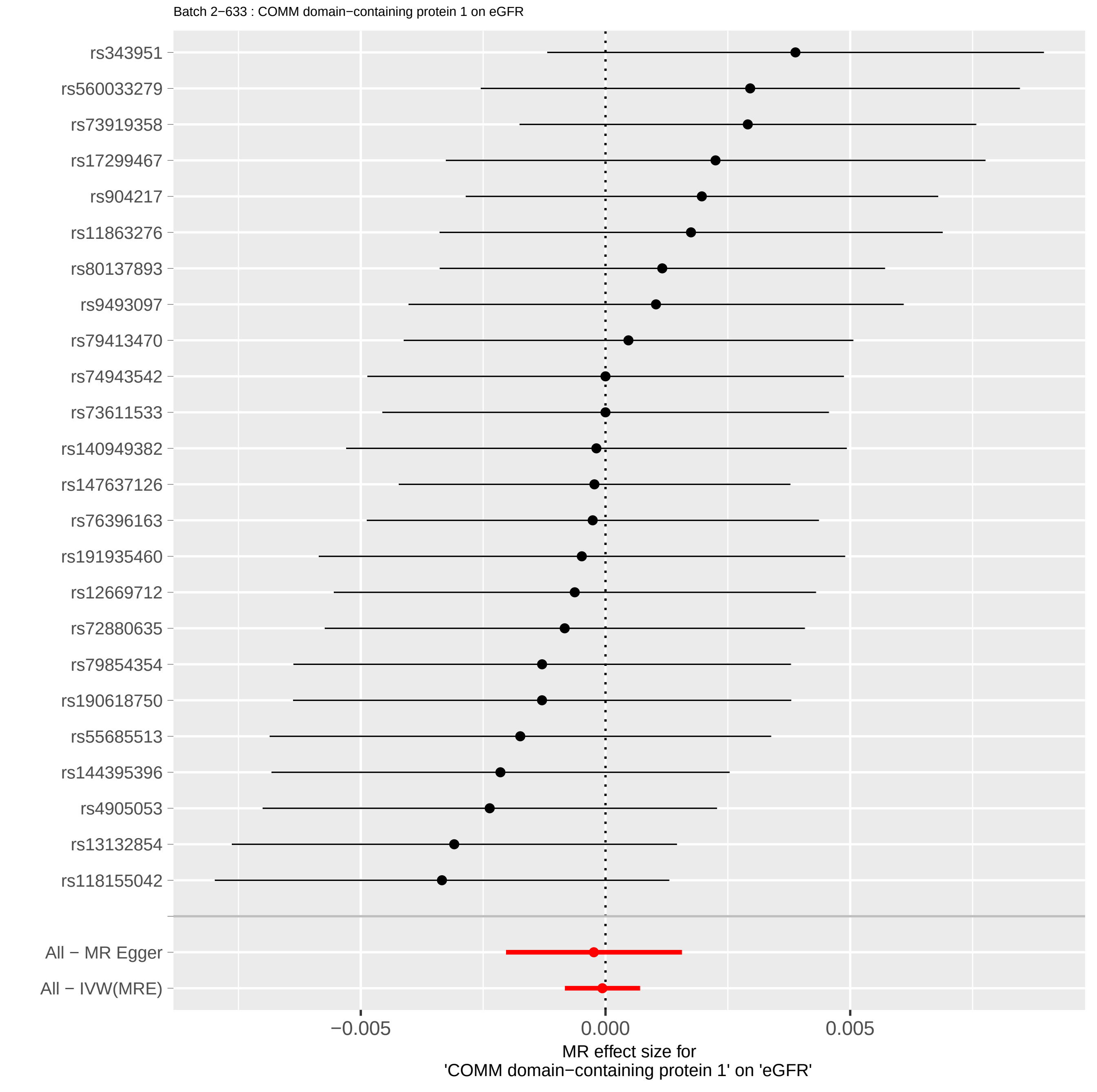

Batch 2−633 : COMM domain−containing protein 1 on eGFR
rs343951
rs560033279
rs73919358
rs17299467
rs904217
rs11863276
rs80137893
rs9493097
rs79413470
rs74943542
rs73611533
rs140949382
rs147637126
rs76396163
rs191935460
rs12669712
rs72880635
rs79854354
rs190618750
rs55685513
rs144395396
rs4905053
rs13132854
rs118155042
All − MR Egger
All − IVW(MRE)
−0.005
0.000
0.005
MR effect size for
'COMM domain−containing protein 1' on 'eGFR'

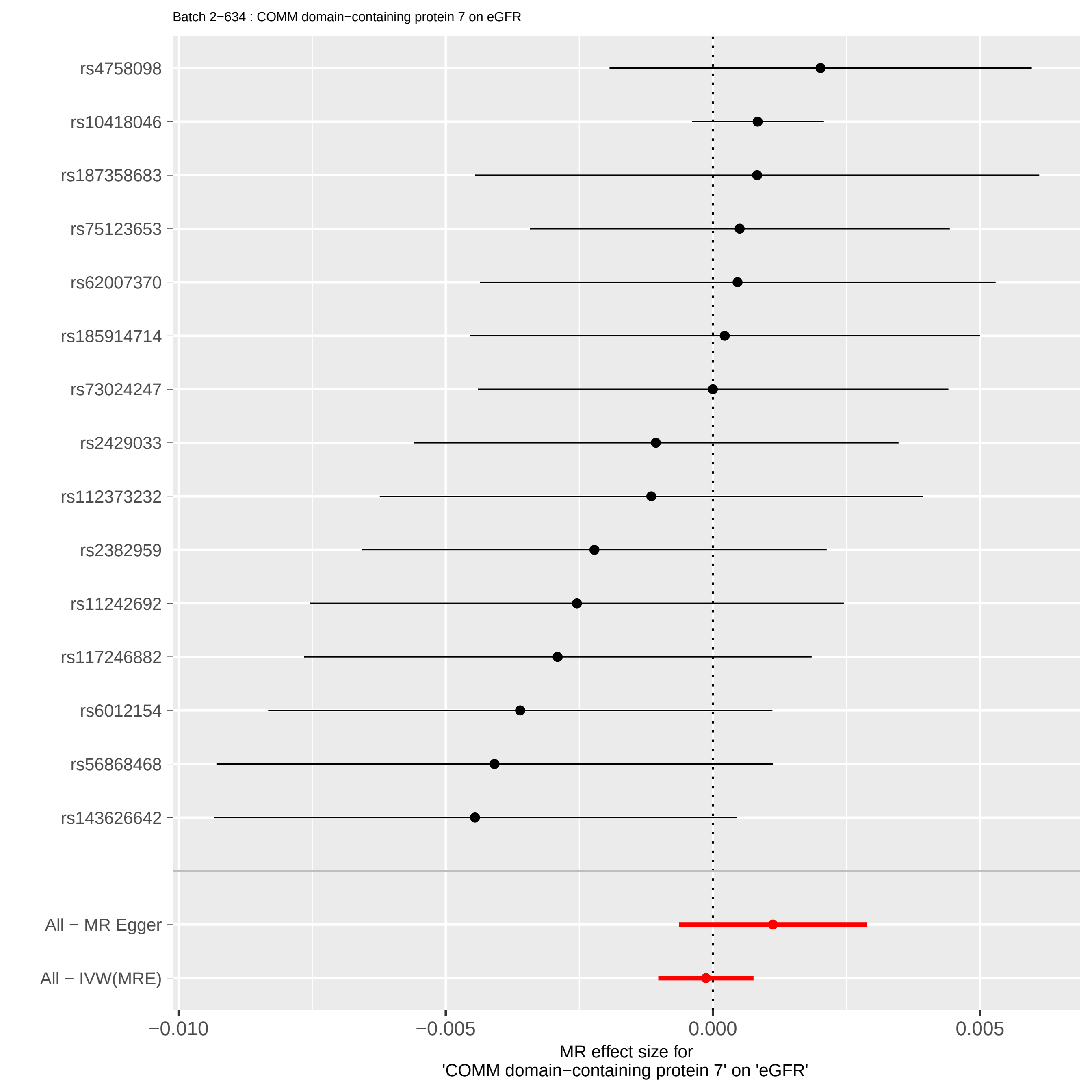

Batch 2−634 : COMM domain−containing protein 7 on eGFR
rs4758098
rs10418046
rs187358683
rs75123653
rs62007370
rs185914714
rs73024247
rs2429033
rs112373232
rs2382959
rs11242692
rs117246882
rs6012154
rs56868468
rs143626642
All − MR Egger
All − IVW(MRE)
−0.010
−0.005
0.000
0.005
MR effect size for
'COMM domain−containing protein 7' on 'eGFR'

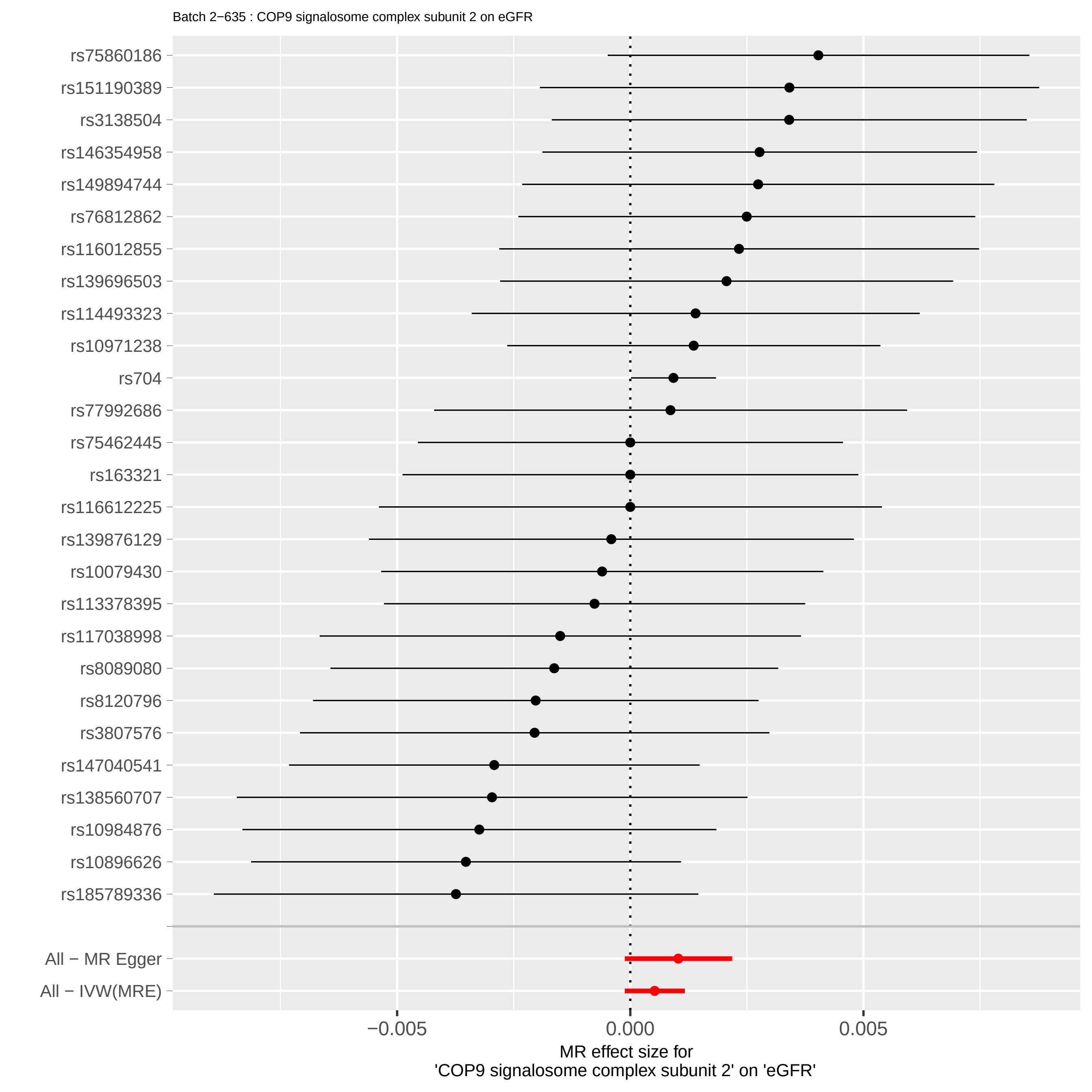

Batch 2−635 : COP9 signalosome complex subunit 2 on eGFR
rs75860186
rs151190389
rs3138504
rs146354958
rs149894744
rs76812862
rs116012855
rs139696503
rs114493323
rs10971238
rs704
rs77992686
rs75462445
rs163321
rs116612225
rs139876129
rs10079430
rs113378395
rs117038998
rs8089080
rs8120796
rs3807576
rs147040541
rs138560707
rs10984876
rs10896626
rs185789336
All − MR Egger
All − IVW(MRE)
−0.005
0.000
0.005
MR effect size for
'COP9 signalosome complex subunit 2' on 'eGFR'

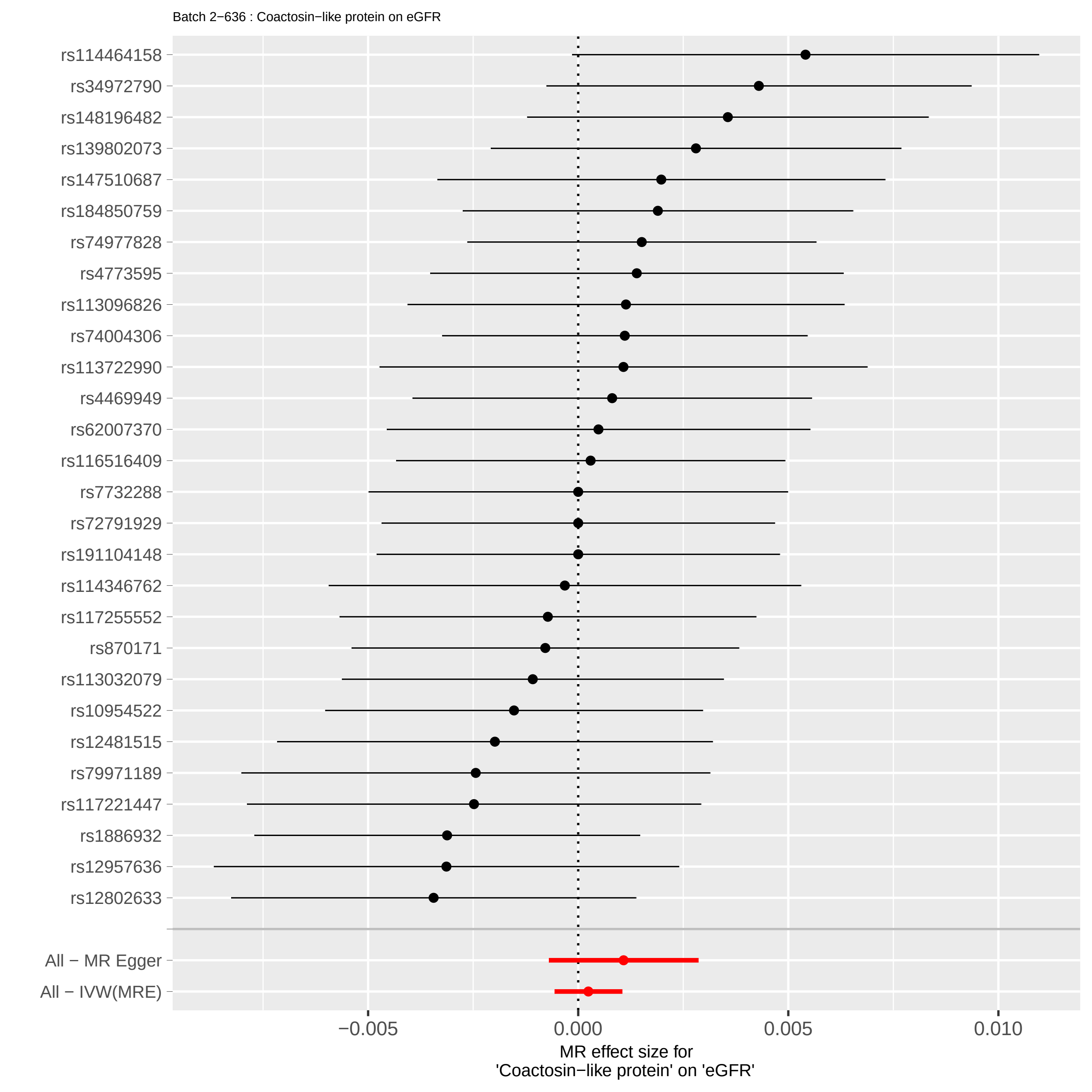

Batch 2−636 : Coactosin−like protein on eGFR
rs114464158
rs34972790
rs148196482
rs139802073
rs147510687
rs184850759
rs74977828
rs4773595
rs113096826
rs74004306
rs113722990
rs4469949
rs62007370
rs116516409
rs7732288
rs72791929
rs191104148
rs114346762
rs117255552
rs870171
rs113032079
rs10954522
rs12481515
rs79971189
rs117221447
rs1886932
rs12957636
rs12802633
All − MR Egger
All − IVW(MRE)
−0.005
0.000
0.005
0.010
MR effect size for
'Coactosin−like protein' on 'eGFR'

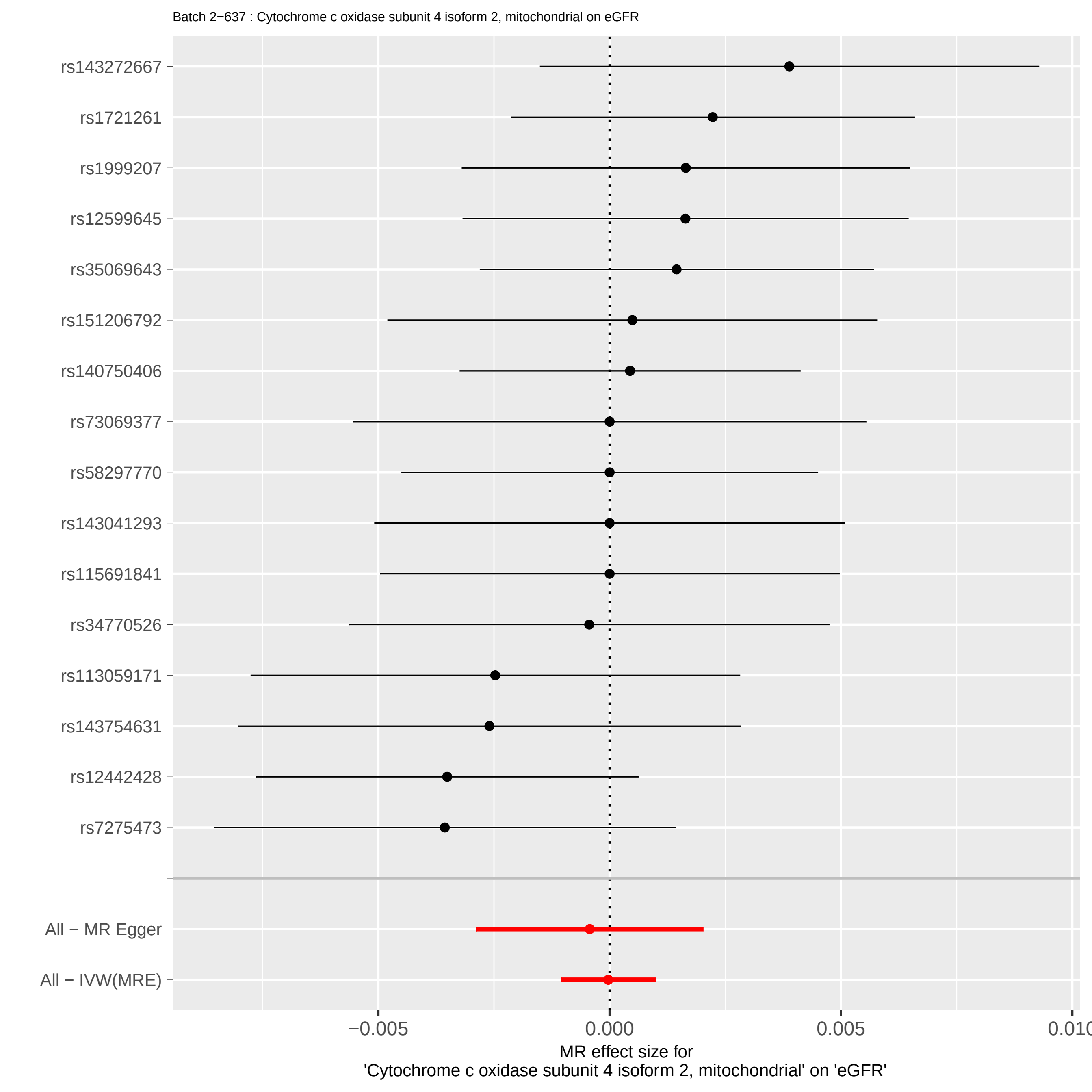
Batch 2−637 : Cytochrome c oxidase subunit 4 isoform 2, mitochondrial on eGFR
rs143272667
rs1721261
rs1999207
rs12599645
rs35069643
rs151206792
rs140750406
rs73069377
rs58297770
rs143041293
rs115691841
rs34770526
rs113059171
rs143754631
rs12442428
rs7275473
All − MR Egger
All − IVW(MRE)
−0.005
0.000
0.005
0.010
MR effect size for
'Cytochrome c oxidase subunit 4 isoform 2, mitochondrial' on 'eGFR'

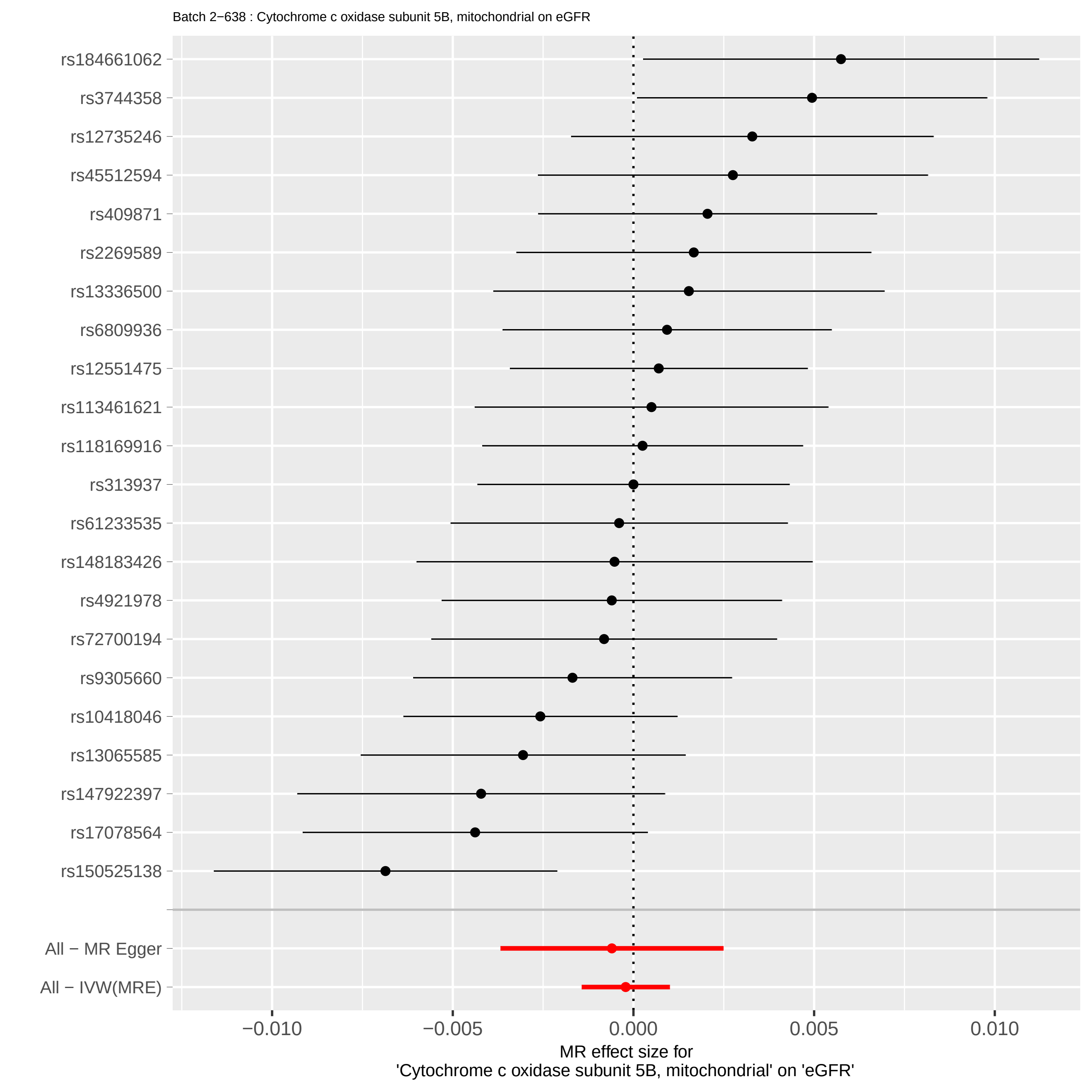

Batch 2−638 : Cytochrome c oxidase subunit 5B, mitochondrial on eGFR
rs184661062
rs3744358
rs12735246
rs45512594
rs409871
rs2269589
rs13336500
rs6809936
rs12551475
rs113461621
rs118169916
rs313937
rs61233535
rs148183426
rs4921978
rs72700194
rs9305660
rs10418046
rs13065585
rs147922397
rs17078564
rs150525138
All − MR Egger
All − IVW(MRE)
−0.010
−0.005
0.000
0.005
0.010
MR effect size for
'Cytochrome c oxidase subunit 5B, mitochondrial' on 'eGFR'

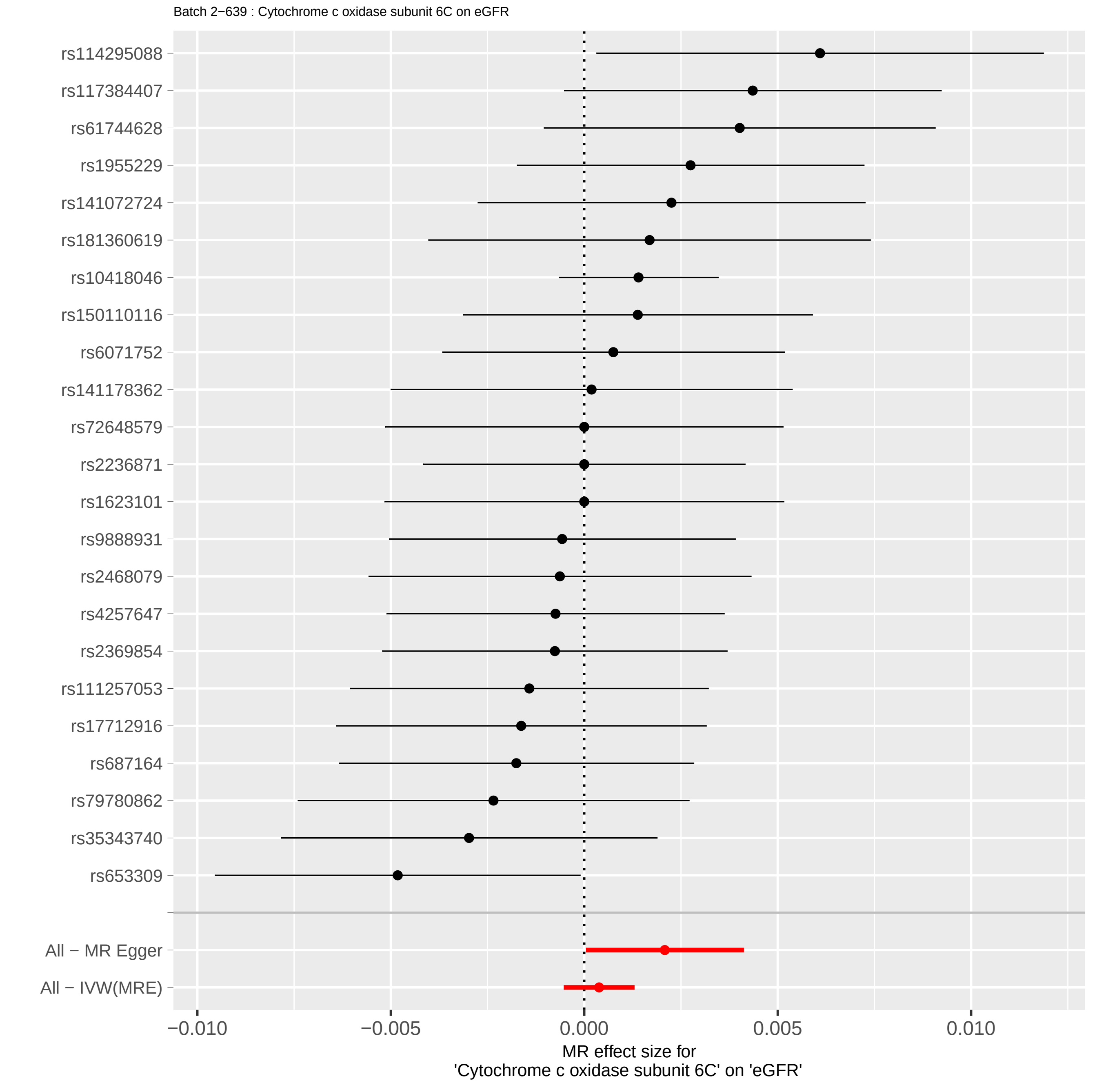

Batch 2−639 : Cytochrome c oxidase subunit 6C on eGFR
rs114295088
rs117384407
rs61744628
rs1955229
rs141072724
rs181360619
rs10418046
rs150110116
rs6071752
rs141178362
rs72648579
rs2236871
rs1623101
rs9888931
rs2468079
rs4257647
rs2369854
rs111257053
rs17712916
rs687164
rs79780862
rs35343740
rs653309
All − MR Egger
All − IVW(MRE)
−0.010
−0.005
0.000
0.005
0.010
MR effect size for
'Cytochrome c oxidase subunit 6C' on 'eGFR'

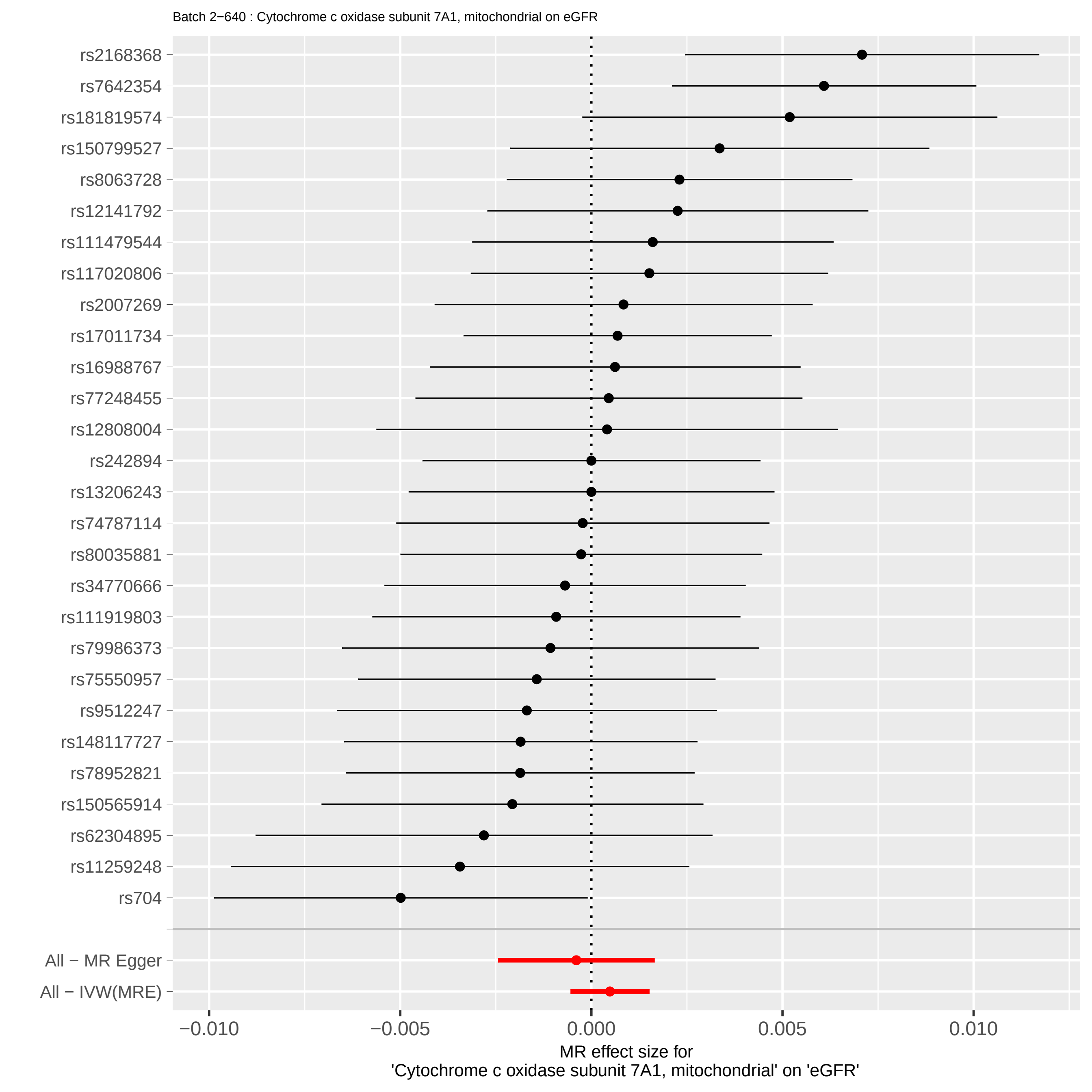

Batch 2−640 : Cytochrome c oxidase subunit 7A1, mitochondrial on eGFR
rs2168368
rs7642354
rs181819574
rs150799527
rs8063728
rs12141792
rs111479544
rs117020806
rs2007269
rs17011734
rs16988767
rs77248455
rs12808004
rs242894
rs13206243
rs74787114
rs80035881
rs34770666
rs111919803
rs79986373
rs75550957
rs9512247
rs148117727
rs78952821
rs150565914
rs62304895
rs11259248
rs704
All − MR Egger
All − IVW(MRE)
−0.010
−0.005
0.000
0.005
0.010
MR effect size for
'Cytochrome c oxidase subunit 7A1, mitochondrial' on 'eGFR'

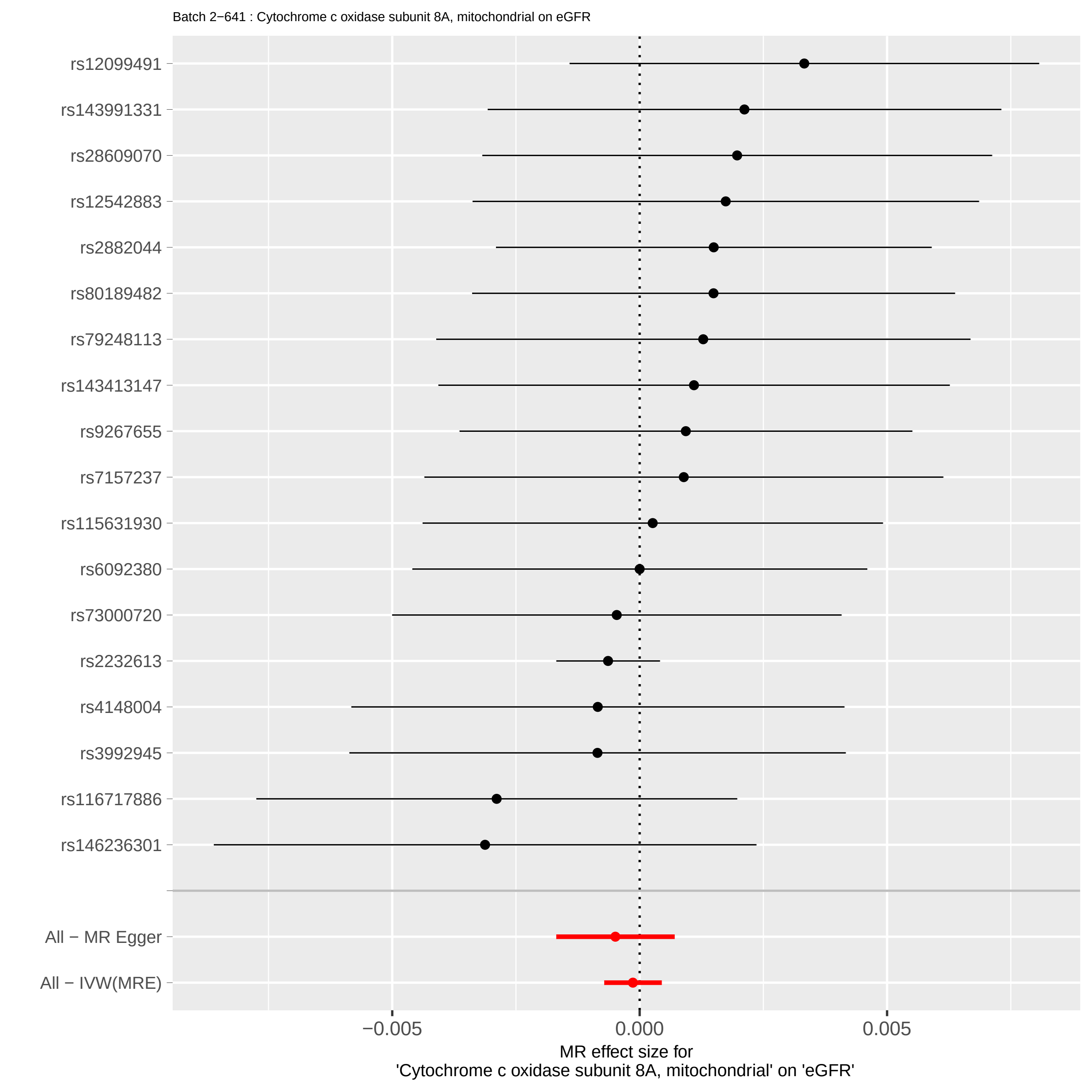

Batch 2−641 : Cytochrome c oxidase subunit 8A, mitochondrial on eGFR
rs12099491
rs143991331
rs28609070
rs12542883
rs2882044
rs80189482
rs79248113
rs143413147
rs9267655
rs7157237
rs115631930
rs6092380
rs73000720
rs2232613
rs4148004
rs3992945
rs116717886
rs146236301
All − MR Egger
All − IVW(MRE)
−0.005
0.000
0.005
MR effect size for
'Cytochrome c oxidase subunit 8A, mitochondrial' on 'eGFR'

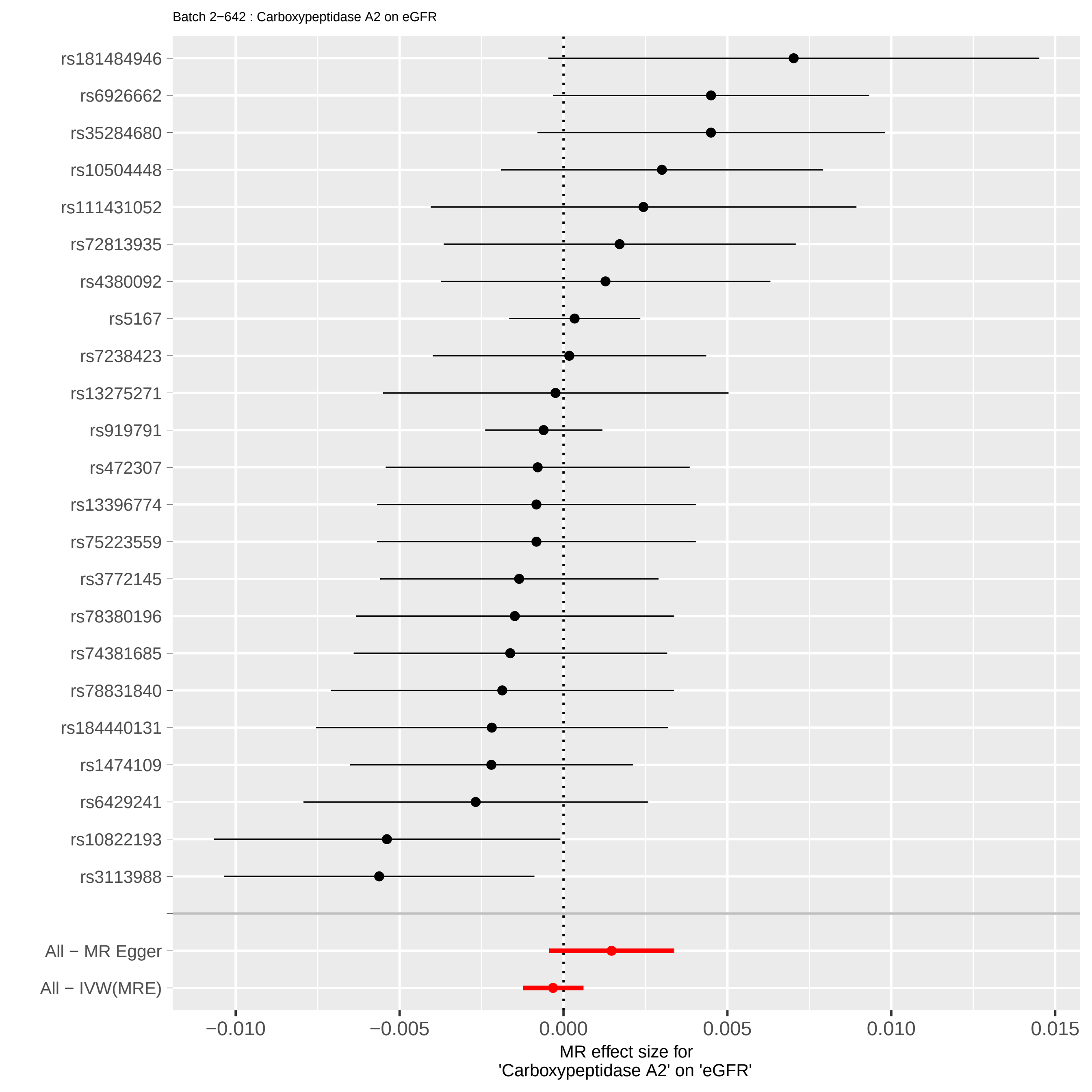

Batch 2−642 : Carboxypeptidase A2 on eGFR
rs181484946
rs6926662
rs35284680
rs10504448
rs111431052
rs72813935
rs4380092
rs5167
rs7238423
rs13275271
rs919791
rs472307
rs13396774
rs75223559
rs3772145
rs78380196
rs74381685
rs78831840
rs184440131
rs1474109
rs6429241
rs10822193
rs3113988
All − MR Egger
All − IVW(MRE)
−0.010
−0.005
0.000
0.005
0.010
0.015
MR effect size for
'Carboxypeptidase A2' on 'eGFR'

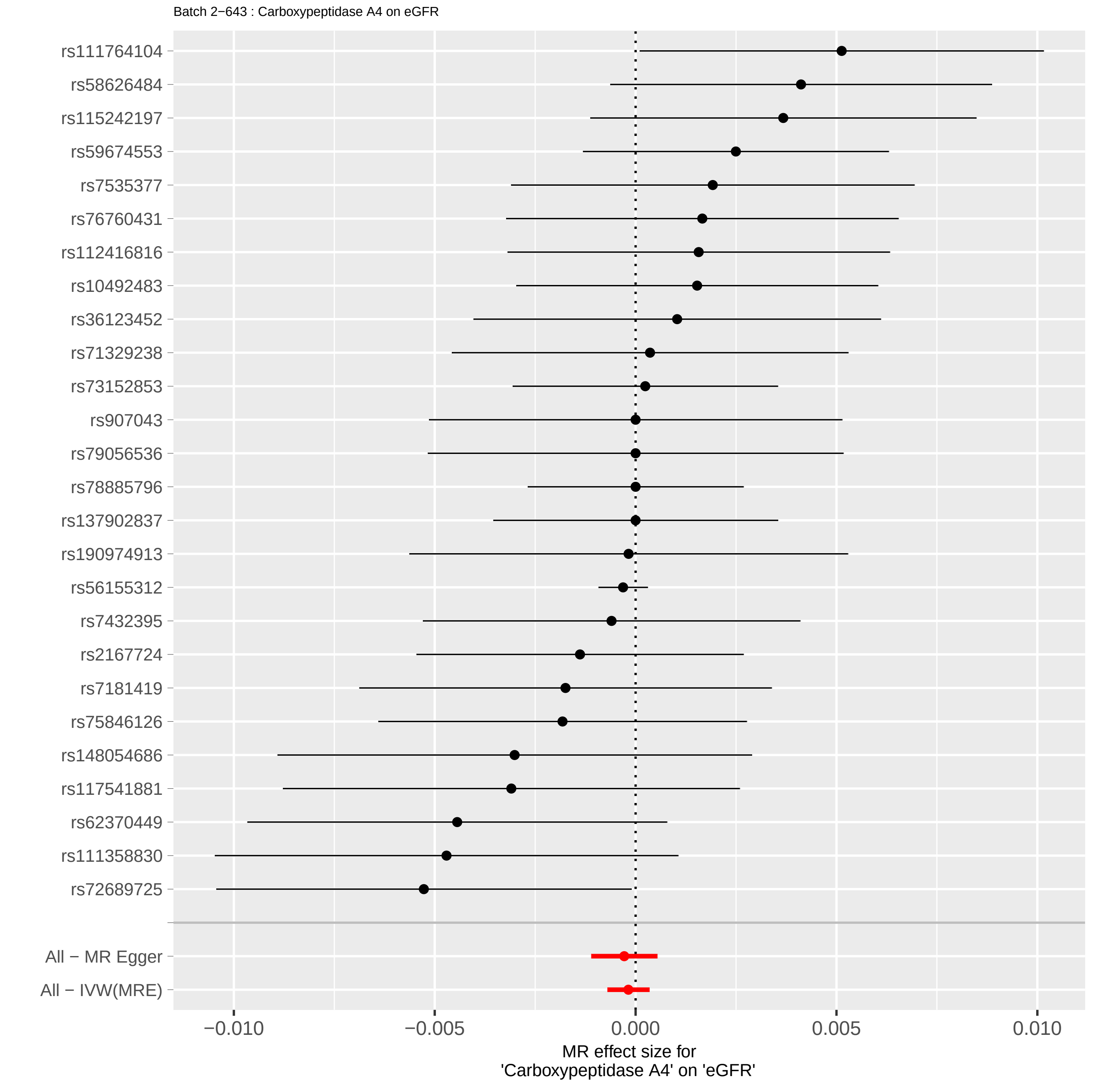

Batch 2−643 : Carboxypeptidase A4 on eGFR
rs111764104
rs58626484
rs115242197
rs59674553
rs7535377
rs76760431
rs112416816
rs10492483
rs36123452
rs71329238
rs73152853
rs907043
rs79056536
rs78885796
rs137902837
rs190974913
rs56155312
rs7432395
rs2167724
rs7181419
rs75846126
rs148054686
rs117541881
rs62370449
rs111358830
rs72689725
All − MR Egger
All − IVW(MRE)
−0.010
−0.005
0.000
0.005
0.010
MR effect size for
'Carboxypeptidase A4' on 'eGFR'

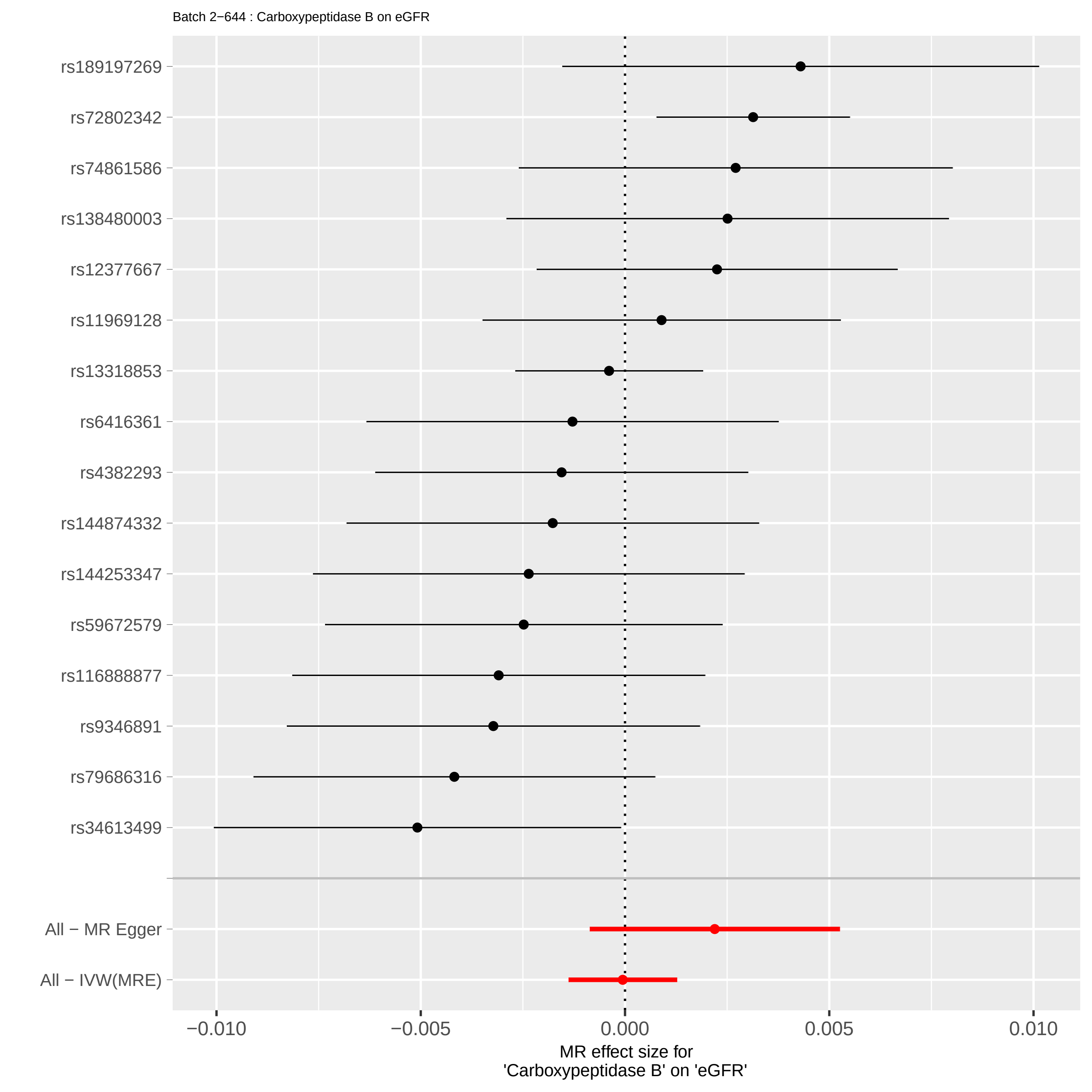
Batch 2−644 : Carboxypeptidase B on eGFR
rs189197269
rs72802342
rs74861586
rs138480003
rs12377667
rs11969128
rs13318853
rs6416361
rs4382293
rs144874332
rs144253347
rs59672579
rs116888877
rs9346891
rs79686316
rs34613499
All − MR Egger
All − IVW(MRE)
−0.010
−0.005
0.000
0.005
0.010
MR effect size for
'Carboxypeptidase B' on 'eGFR'

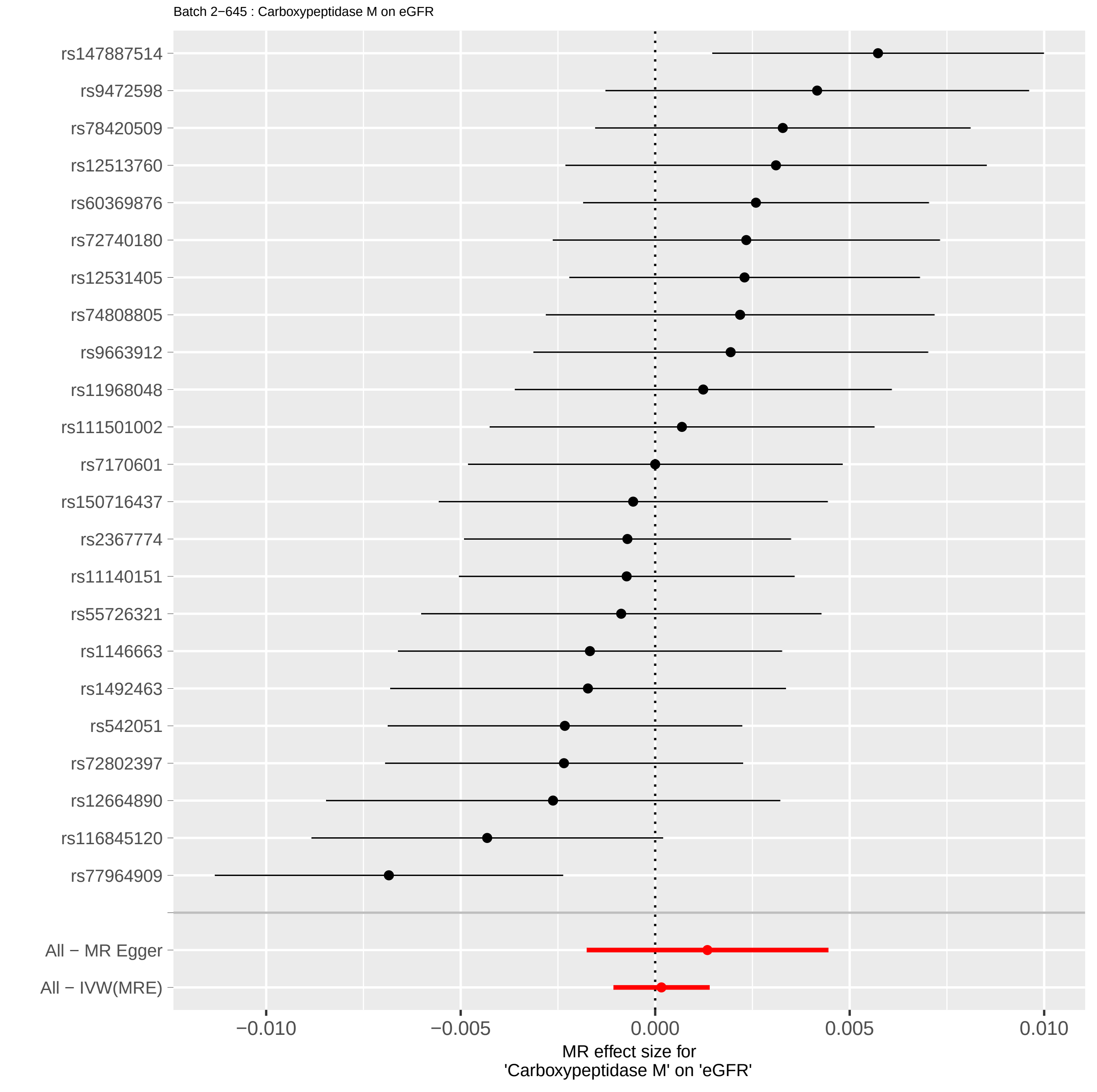
Batch 2−645 : Carboxypeptidase M on eGFR
rs147887514
rs9472598
rs78420509
rs12513760
rs60369876
rs72740180
rs12531405
rs74808805
rs9663912
rs11968048
rs111501002
rs7170601
rs150716437
rs2367774
rs11140151
rs55726321
rs1146663
rs1492463
rs542051
rs72802397
rs12664890
rs116845120
rs77964909
All − MR Egger
All − IVW(MRE)
−0.010
−0.005
0.000
0.005
0.010
MR effect size for
'Carboxypeptidase M' on 'eGFR'

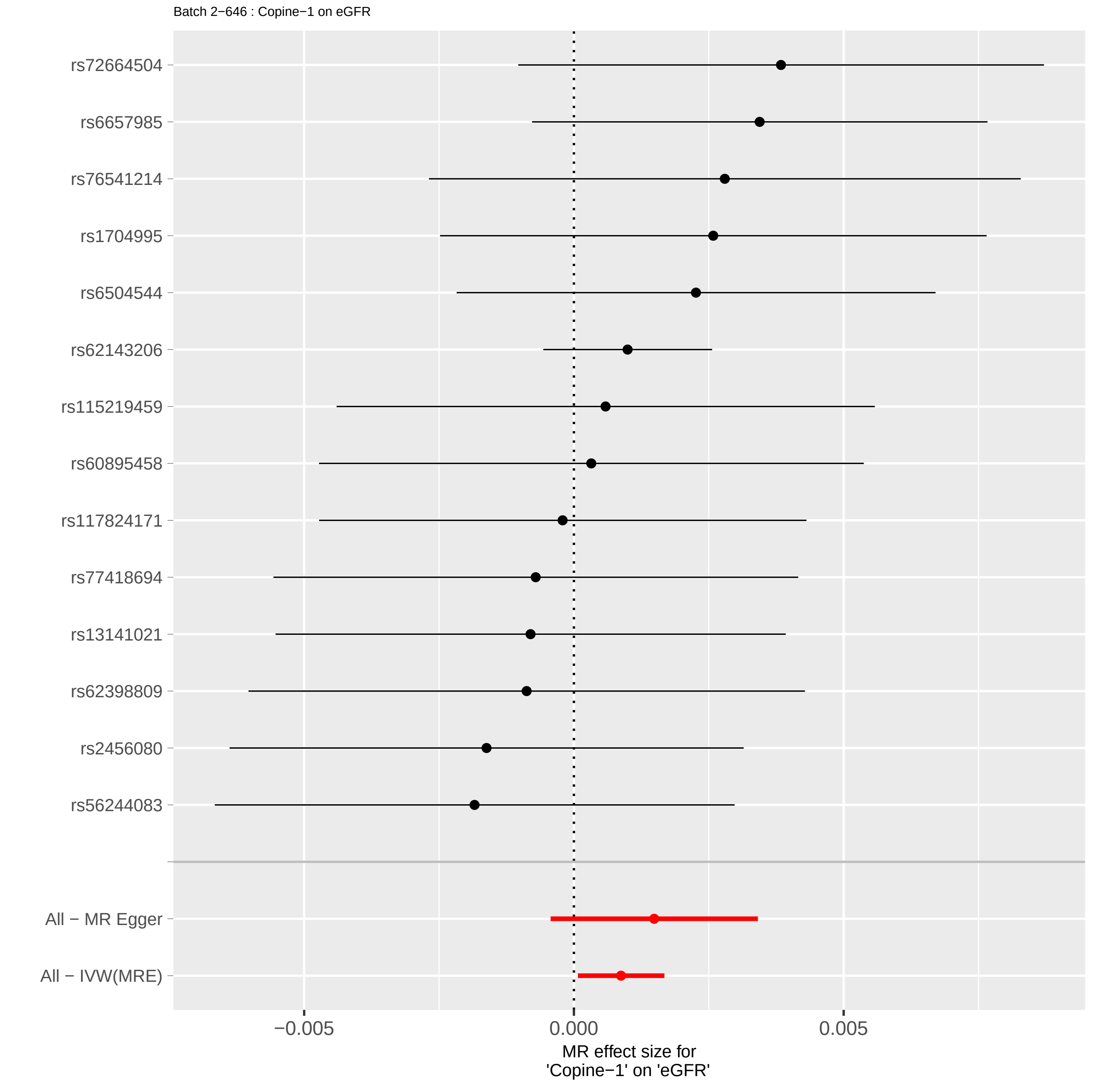

Batch 2−646 : Copine−1 on eGFR
rs72664504
rs6657985
rs76541214
rs1704995
rs6504544
rs62143206
rs115219459
rs60895458
rs117824171
rs77418694
rs13141021
rs62398809
rs2456080
rs56244083
All − MR Egger
All − IVW(MRE)
−0.005
0.000
0.005
MR effect size for
'Copine−1' on 'eGFR'

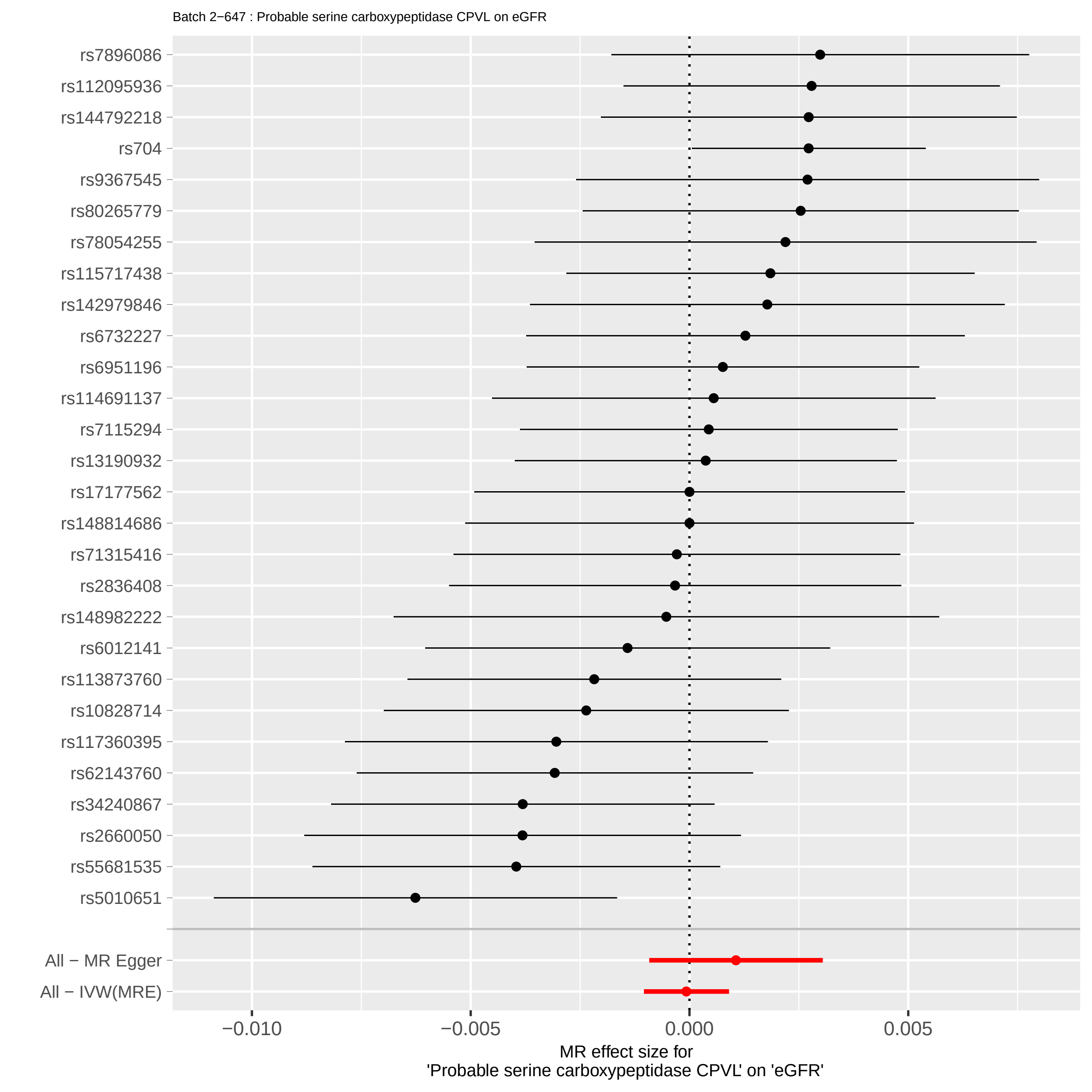

Batch 2−647 : Probable serine carboxypeptidase CPVL on eGFR
rs7896086
rs112095936
rs144792218
rs704
rs9367545
rs80265779
rs78054255
rs115717438
rs142979846
rs6732227
rs6951196
rs114691137
rs7115294
rs13190932
rs17177562
rs148814686
rs71315416
rs2836408
rs148982222
rs6012141
rs113873760
rs10828714
rs117360395
rs62143760
rs34240867
rs2660050
rs55681535
rs5010651
All − MR Egger
All − IVW(MRE)
−0.010
−0.005
0.000
0.005
MR effect size for
'Probable serine carboxypeptidase CPVL' on 'eGFR'

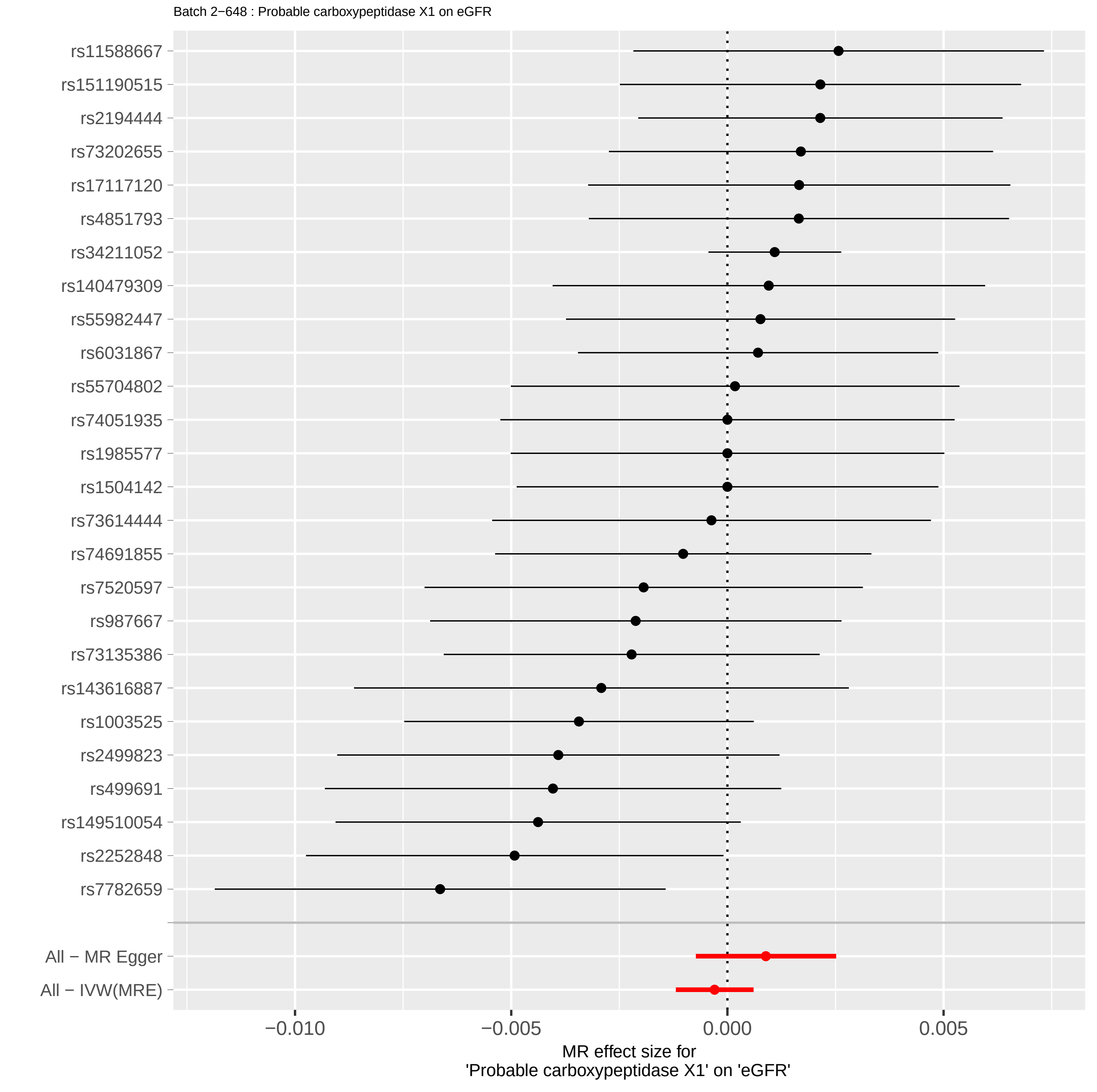

Batch 2−648 : Probable carboxypeptidase X1 on eGFR
rs11588667
rs151190515
rs2194444
rs73202655
rs17117120
rs4851793
rs34211052
rs140479309
rs55982447
rs6031867
rs55704802
rs74051935
rs1985577
rs1504142
rs73614444
rs74691855
rs7520597
rs987667
rs73135386
rs143616887
rs1003525
rs2499823
rs499691
rs149510054
rs2252848
rs7782659
All − MR Egger
All − IVW(MRE)
−0.010
−0.005
0.000
0.005
MR effect size for
'Probable carboxypeptidase X1' on 'eGFR'

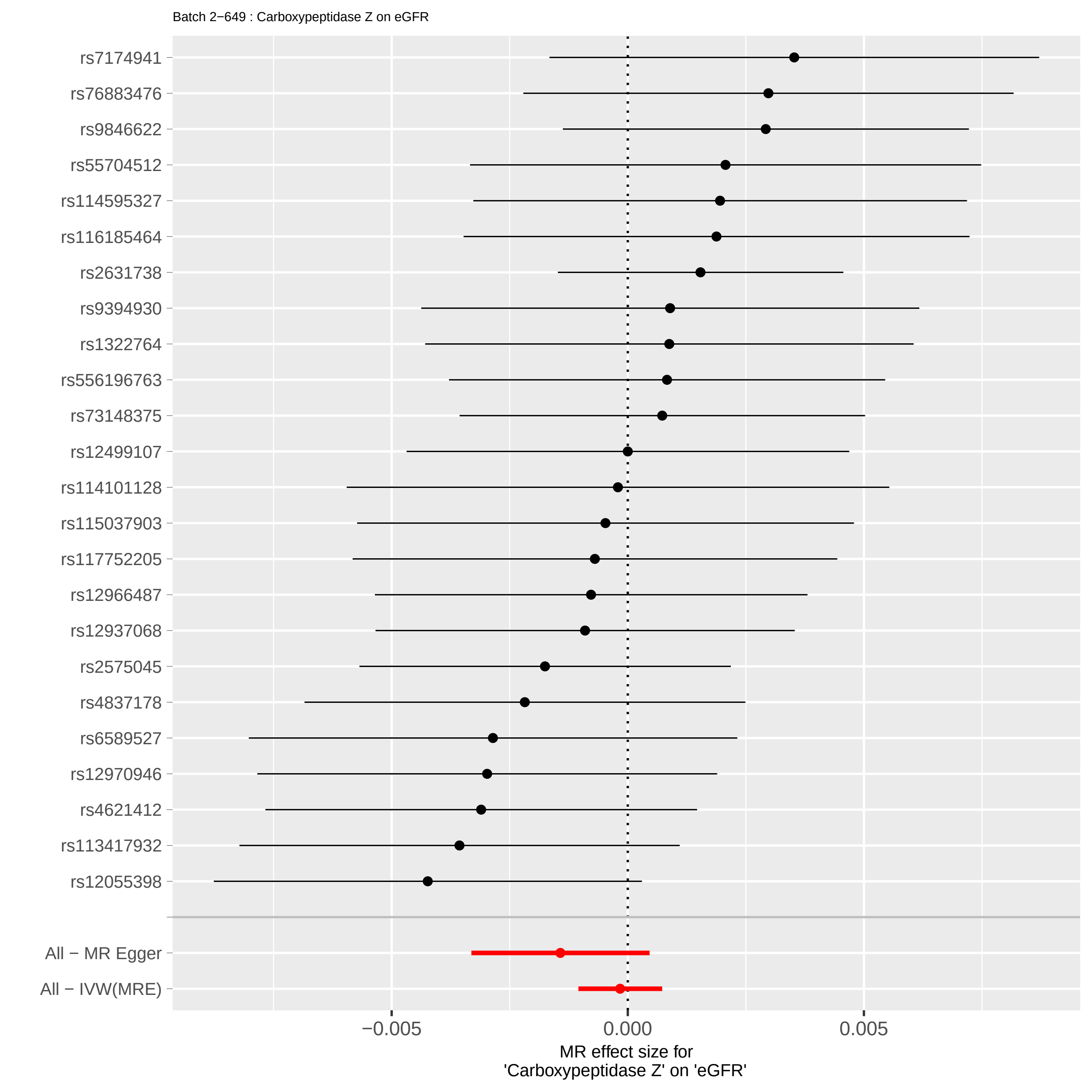

Batch 2−649 : Carboxypeptidase Z on eGFR
rs7174941
rs76883476
rs9846622
rs55704512
rs114595327
rs116185464
rs2631738
rs9394930
rs1322764
rs556196763
rs73148375
rs12499107
rs114101128
rs115037903
rs117752205
rs12966487
rs12937068
rs2575045
rs4837178
rs6589527
rs12970946
rs4621412
rs113417932
rs12055398
All − MR Egger
All − IVW(MRE)
−0.005
0.000
0.005
MR effect size for
'Carboxypeptidase Z' on 'eGFR'

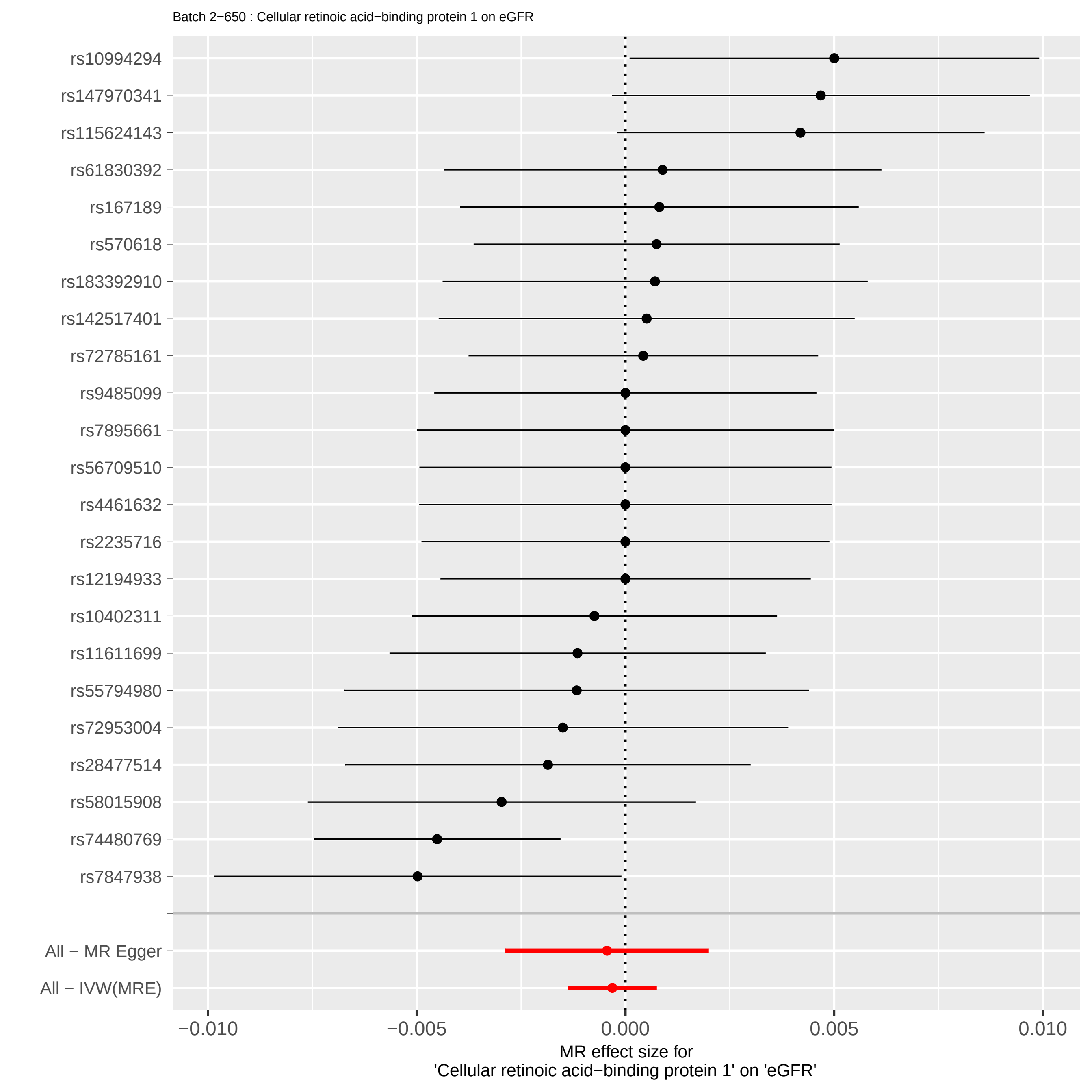

Batch 2−650 : Cellular retinoic acid−binding protein 1 on eGFR
rs10994294
rs147970341
rs115624143
rs61830392
rs167189
rs570618
rs183392910
rs142517401
rs72785161
rs9485099
rs7895661
rs56709510
rs4461632
rs2235716
rs12194933
rs10402311
rs11611699
rs55794980
rs72953004
rs28477514
rs58015908
rs74480769
rs7847938
All − MR Egger
All − IVW(MRE)
−0.010
−0.005
0.000
0.005
0.010
MR effect size for
'Cellular retinoic acid−binding protein 1' on 'eGFR'

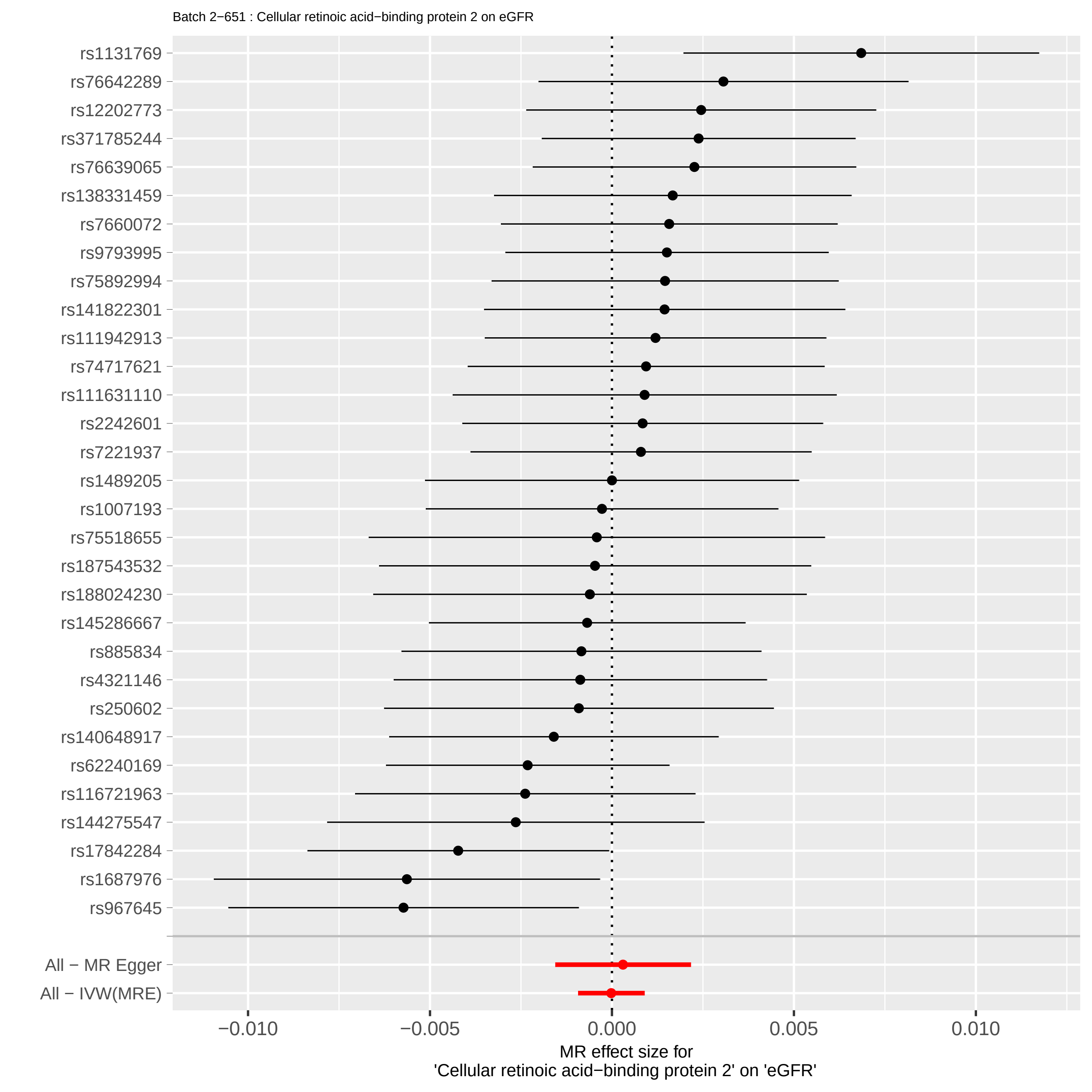

Batch 2−651 : Cellular retinoic acid−binding protein 2 on eGFR
rs1131769
rs76642289
rs12202773
rs371785244
rs76639065
rs138331459
rs7660072
rs9793995
rs75892994
rs141822301
rs111942913
rs74717621
rs111631110
rs2242601
rs7221937
rs1489205
rs1007193
rs75518655
rs187543532
rs188024230
rs145286667
rs885834
rs4321146
rs250602
rs140648917
rs62240169
rs116721963
rs144275547
rs17842284
rs1687976
rs967645
All − MR Egger
All − IVW(MRE)
−0.010
−0.005
0.000
0.005
0.010
MR effect size for
'Cellular retinoic acid−binding protein 2' on 'eGFR'

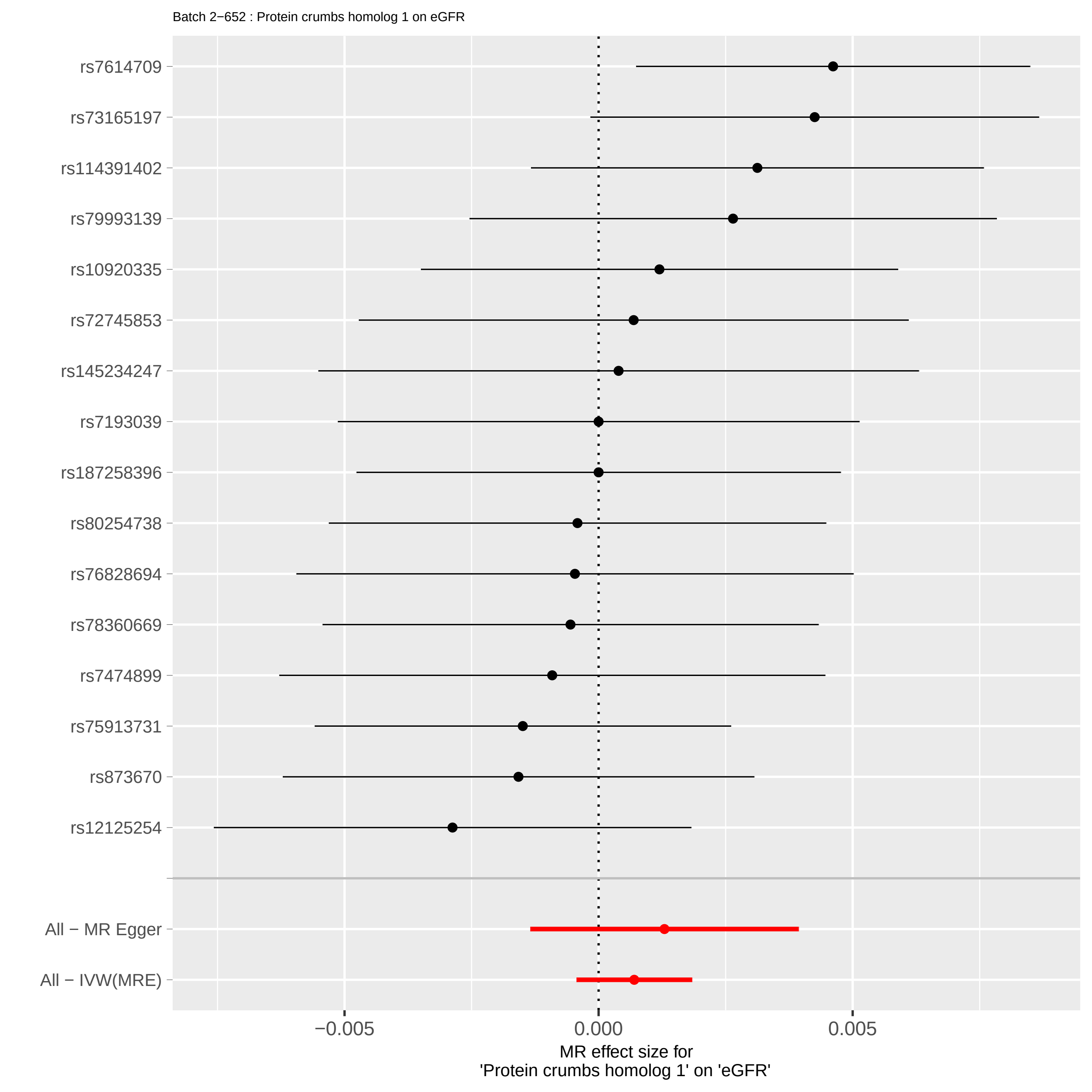

Batch 2−652 : Protein crumbs homolog 1 on eGFR
rs7614709
rs73165197
rs114391402
rs79993139
rs10920335
rs72745853
rs145234247
rs7193039
rs187258396
rs80254738
rs76828694
rs78360669
rs7474899
rs75913731
rs873670
rs12125254
All − MR Egger
All − IVW(MRE)
−0.005
0.000
0.005
MR effect size for
'Protein crumbs homolog 1' on 'eGFR'

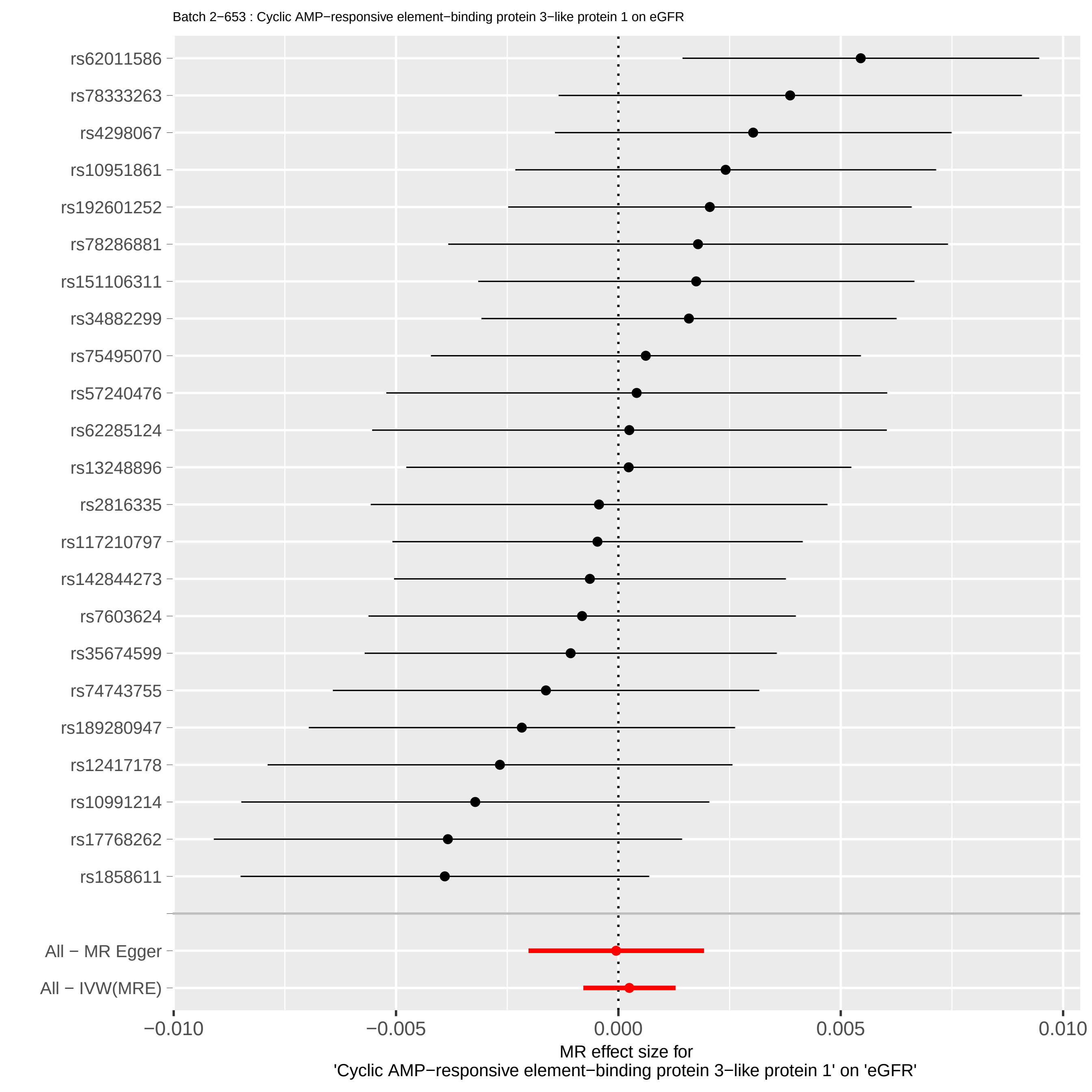
Batch 2−653 : Cyclic AMP−responsive element−binding protein 3−like protein 1 on eGFR
rs62011586
rs78333263
rs4298067
rs10951861
rs192601252
rs78286881
rs151106311
rs34882299
rs75495070
rs57240476
rs62285124
rs13248896
rs2816335
rs117210797
rs142844273
rs7603624
rs35674599
rs74743755
rs189280947
rs12417178
rs10991214
rs17768262
rs1858611
All − MR Egger
All − IVW(MRE)
−0.010
−0.005
0.000
0.005
0.010
MR effect size for
'Cyclic AMP−responsive element−binding protein 3−like protein 1' on 'eGFR'

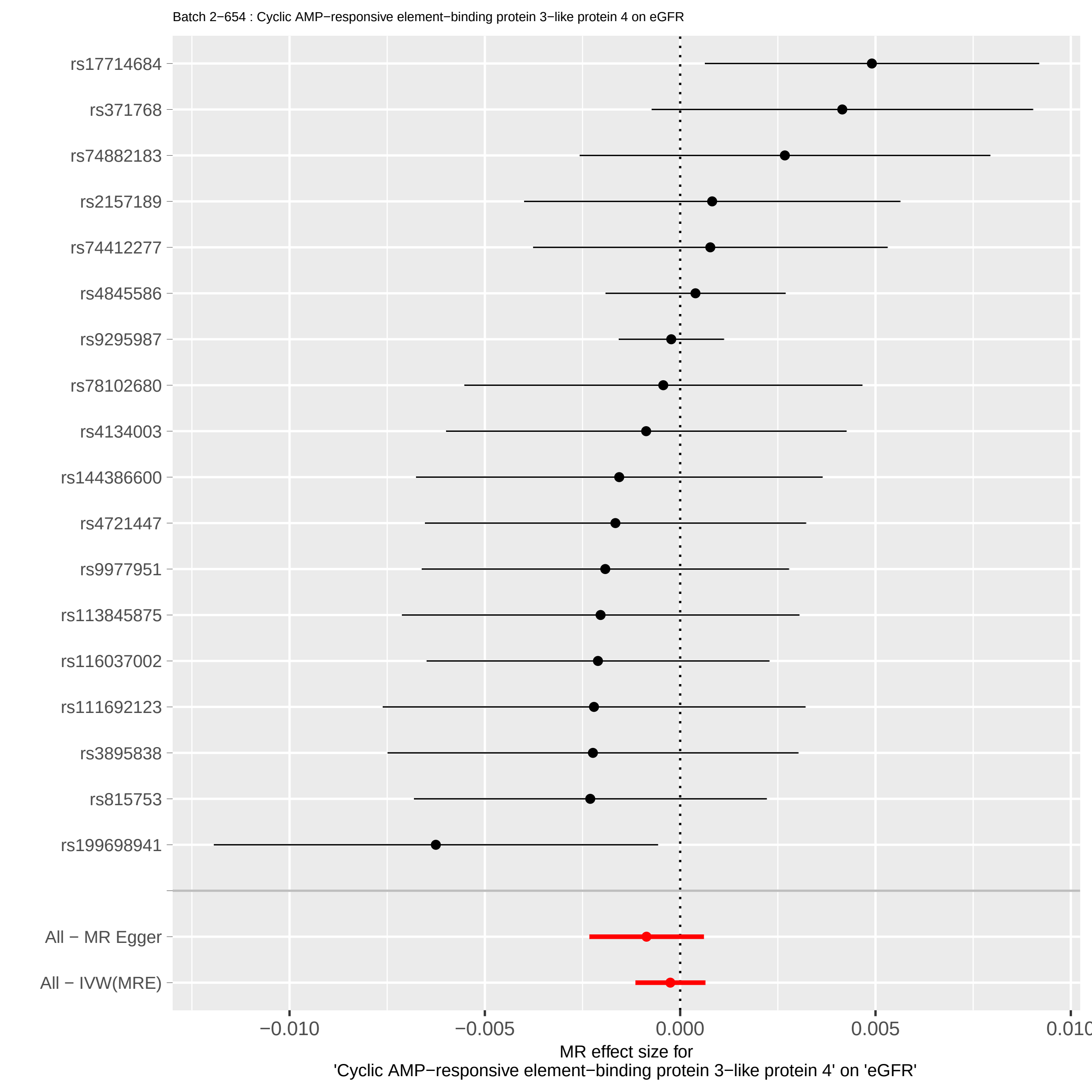
Batch 2−654 : Cyclic AMP−responsive element−binding protein 3−like protein 4 on eGFR
rs17714684
rs371768
rs74882183
rs2157189
rs74412277
rs4845586
rs9295987
rs78102680
rs4134003
rs144386600
rs4721447
rs9977951
rs113845875
rs116037002
rs111692123
rs3895838
rs815753
rs199698941
All − MR Egger
All − IVW(MRE)
−0.010
−0.005
0.000
0.005
0.010
MR effect size for
'Cyclic AMP−responsive element−binding protein 3−like protein 4' on 'eGFR'

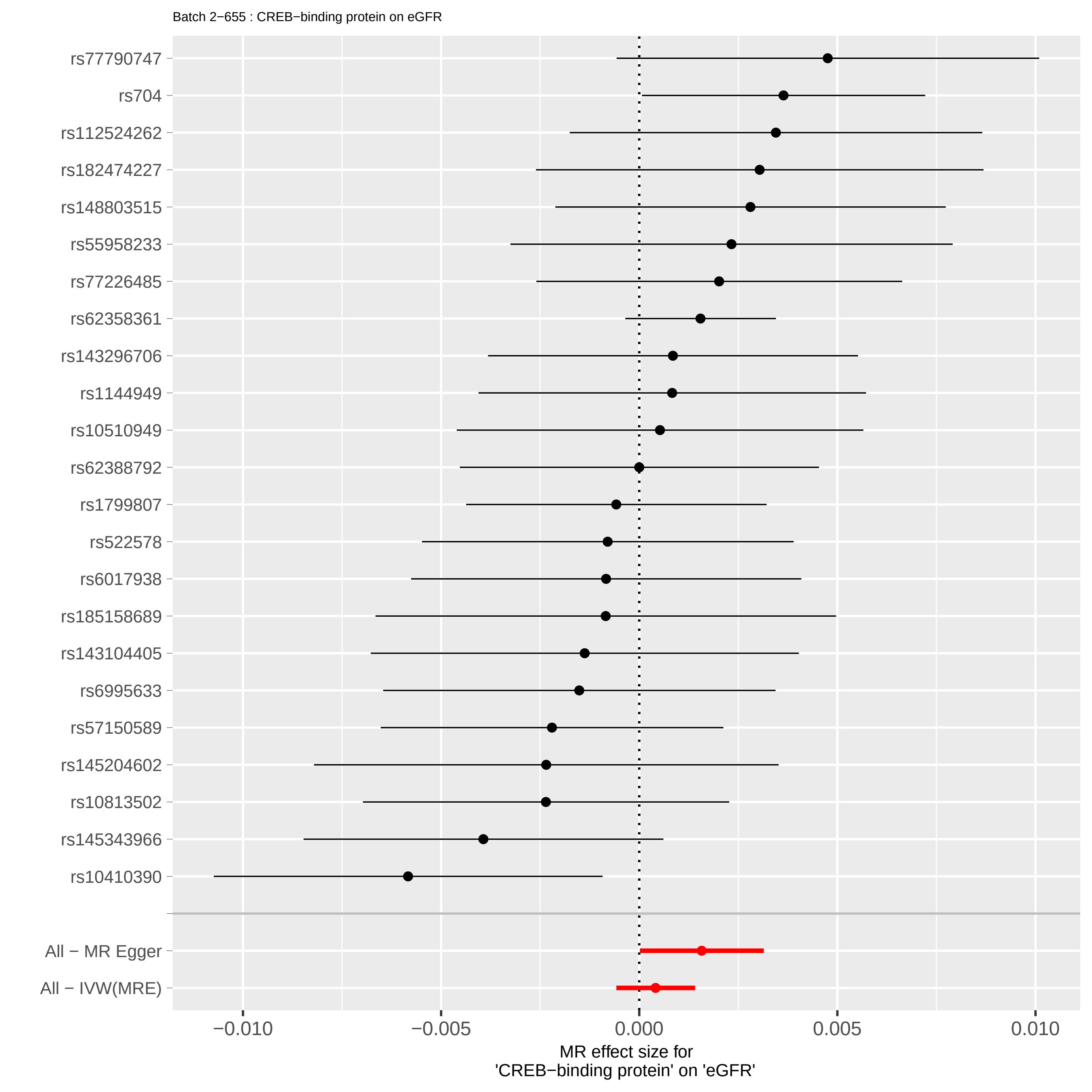

Batch 2−655 : CREB−binding protein on eGFR
rs77790747
rs704
rs112524262
rs182474227
rs148803515
rs55958233
rs77226485
rs62358361
rs143296706
rs1144949
rs10510949
rs62388792
rs1799807
rs522578
rs6017938
rs185158689
rs143104405
rs6995633
rs57150589
rs145204602
rs10813502
rs145343966
rs10410390
All − MR Egger
All − IVW(MRE)
−0.010
−0.005
0.000
0.005
0.010
MR effect size for
'CREB−binding protein' on 'eGFR'

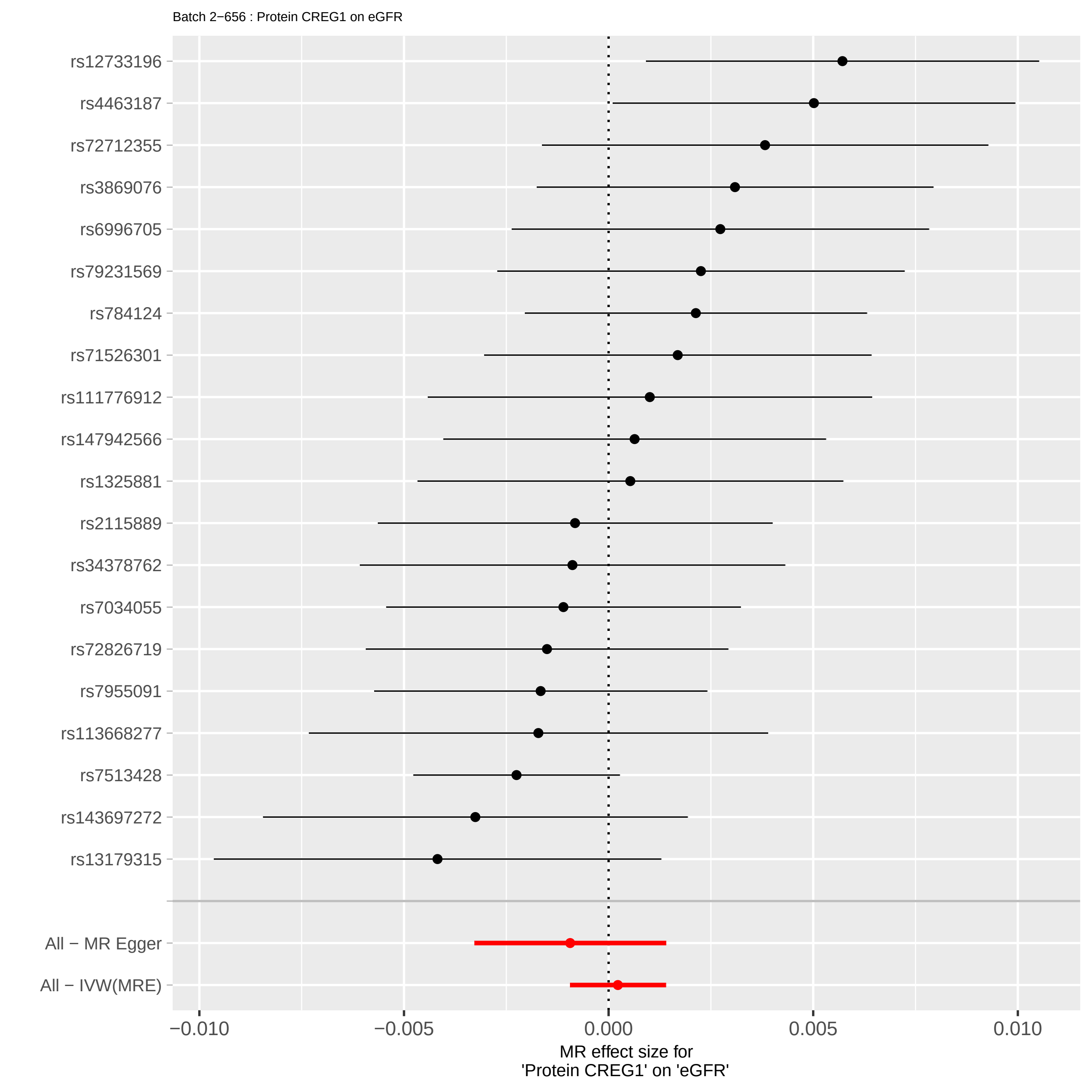

Batch 2−656 : Protein CREG1 on eGFR
rs12733196
rs4463187
rs72712355
rs3869076
rs6996705
rs79231569
rs784124
rs71526301
rs111776912
rs147942566
rs1325881
rs2115889
rs34378762
rs7034055
rs72826719
rs7955091
rs113668277
rs7513428
rs143697272
rs13179315
All − MR Egger
All − IVW(MRE)
−0.010
−0.005
0.000
0.005
0.010
MR effect size for
'Protein CREG1' on 'eGFR'

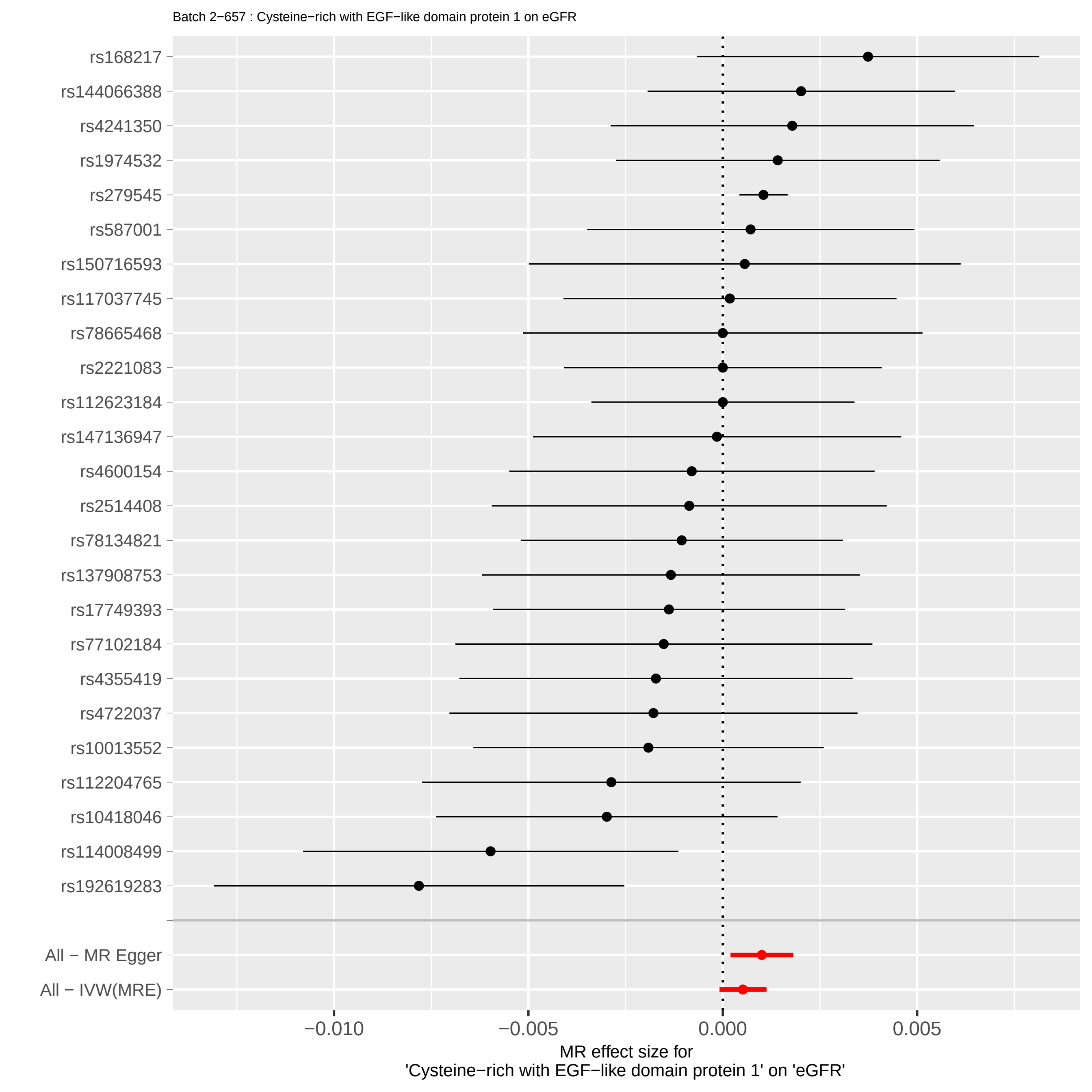

Batch 2−657 : Cysteine−rich with EGF−like domain protein 1 on eGFR
rs168217
rs144066388
rs4241350
rs1974532
rs279545
rs587001
rs150716593
rs117037745
rs78665468
rs2221083
rs112623184
rs147136947
rs4600154
rs2514408
rs78134821
rs137908753
rs17749393
rs77102184
rs4355419
rs4722037
rs10013552
rs112204765
rs10418046
rs114008499
rs192619283
All − MR Egger
All − IVW(MRE)
−0.010
−0.005
0.000
0.005
MR effect size for
'Cysteine−rich with EGF−like domain protein 1' on 'eGFR'

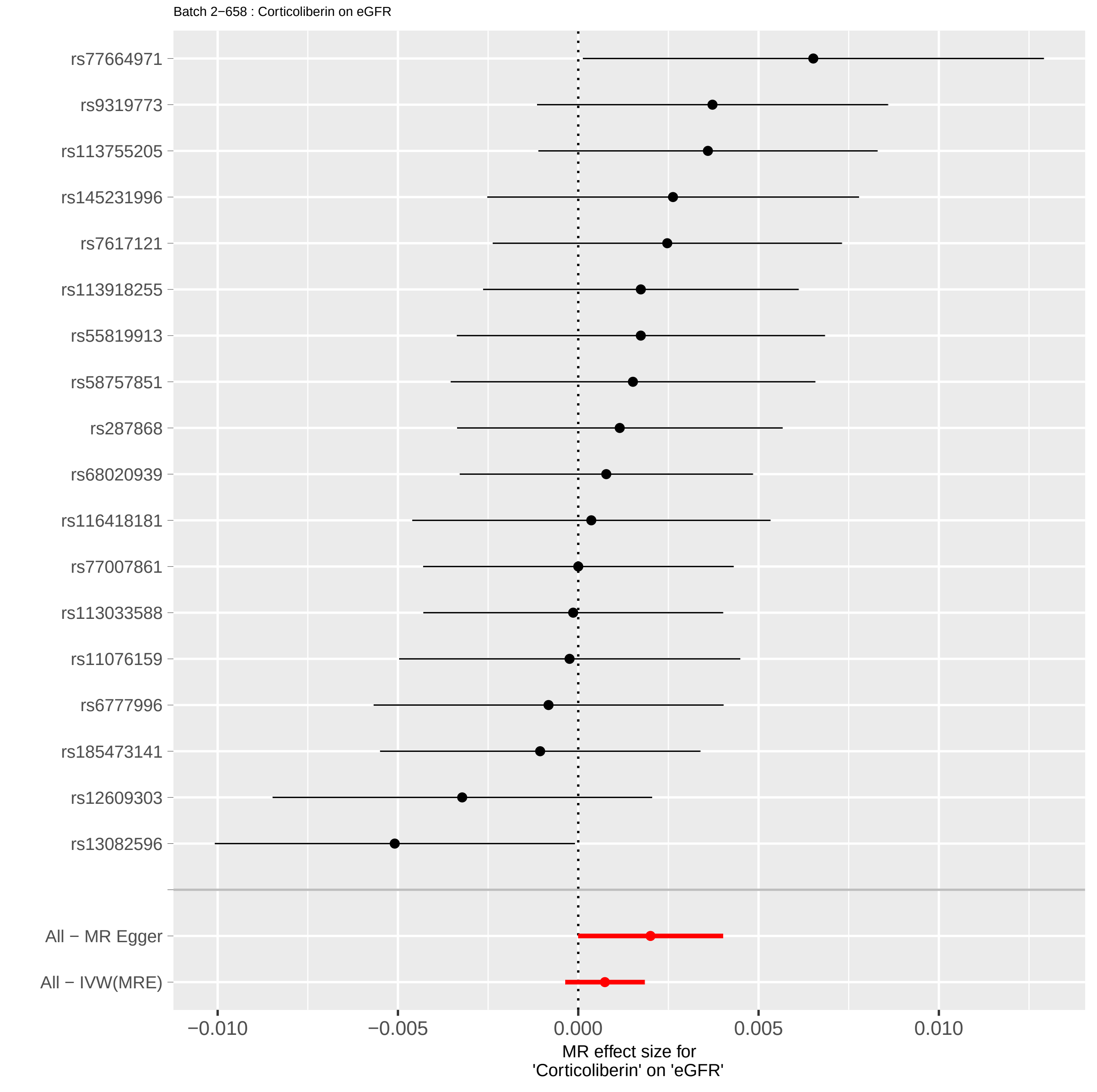

Batch 2−658 : Corticoliberin on eGFR
rs77664971
rs9319773
rs113755205
rs145231996
rs7617121
rs113918255
rs55819913
rs58757851
rs287868
rs68020939
rs116418181
rs77007861
rs113033588
rs11076159
rs6777996
rs185473141
rs12609303
rs13082596
All − MR Egger
All − IVW(MRE)
−0.010
−0.005
0.000
0.005
0.010
MR effect size for
'Corticoliberin' on 'eGFR'

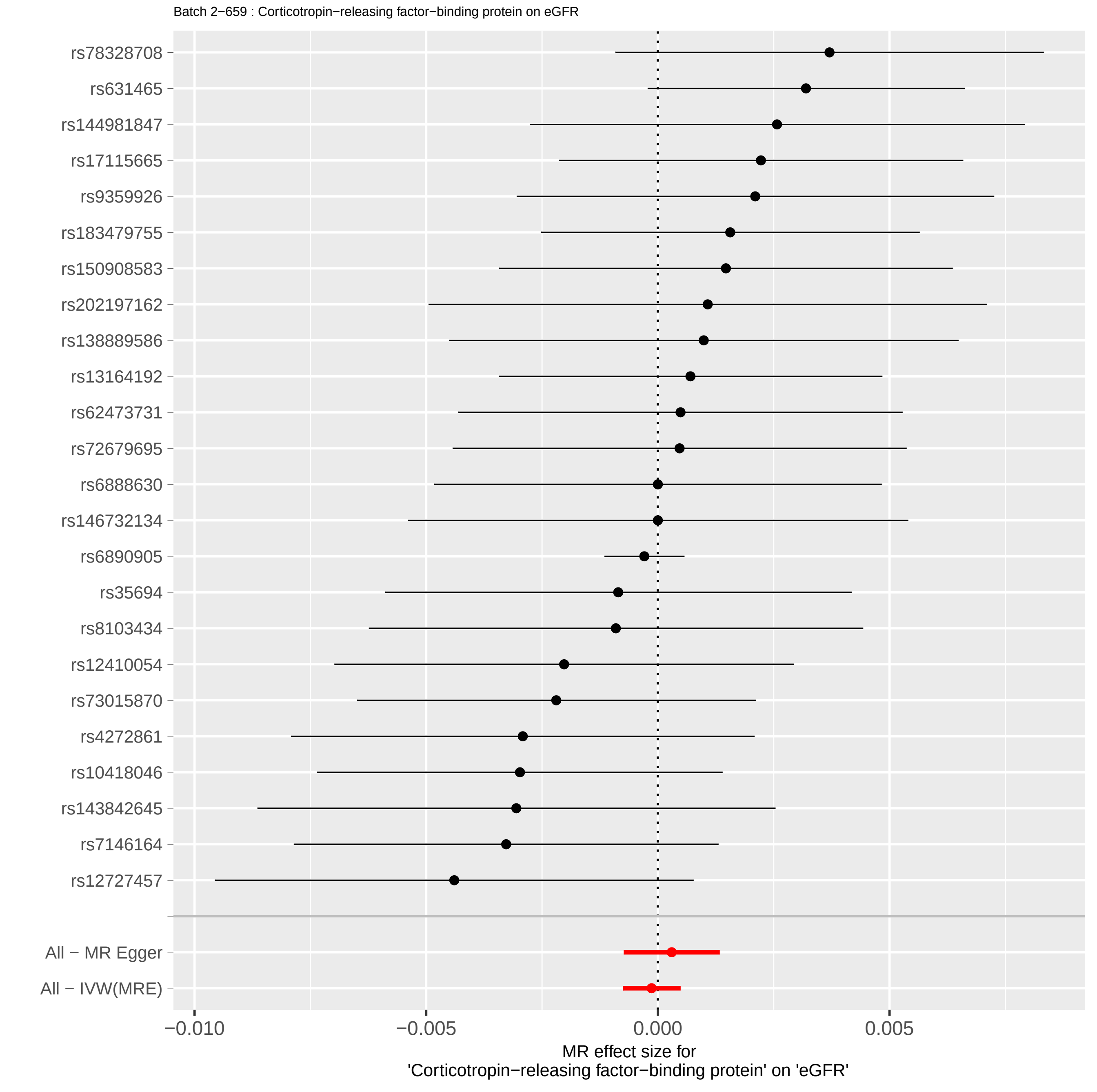

Batch 2−659 : Corticotropin−releasing factor−binding protein on eGFR
rs78328708
rs631465
rs144981847
rs17115665
rs9359926
rs183479755
rs150908583
rs202197162
rs138889586
rs13164192
rs62473731
rs72679695
rs6888630
rs146732134
rs6890905
rs35694
rs8103434
rs12410054
rs73015870
rs4272861
rs10418046
rs143842645
rs7146164
rs12727457
All − MR Egger
All − IVW(MRE)
−0.010
−0.005
0.000
0.005
MR effect size for
'Corticotropin−releasing factor−binding protein' on 'eGFR'

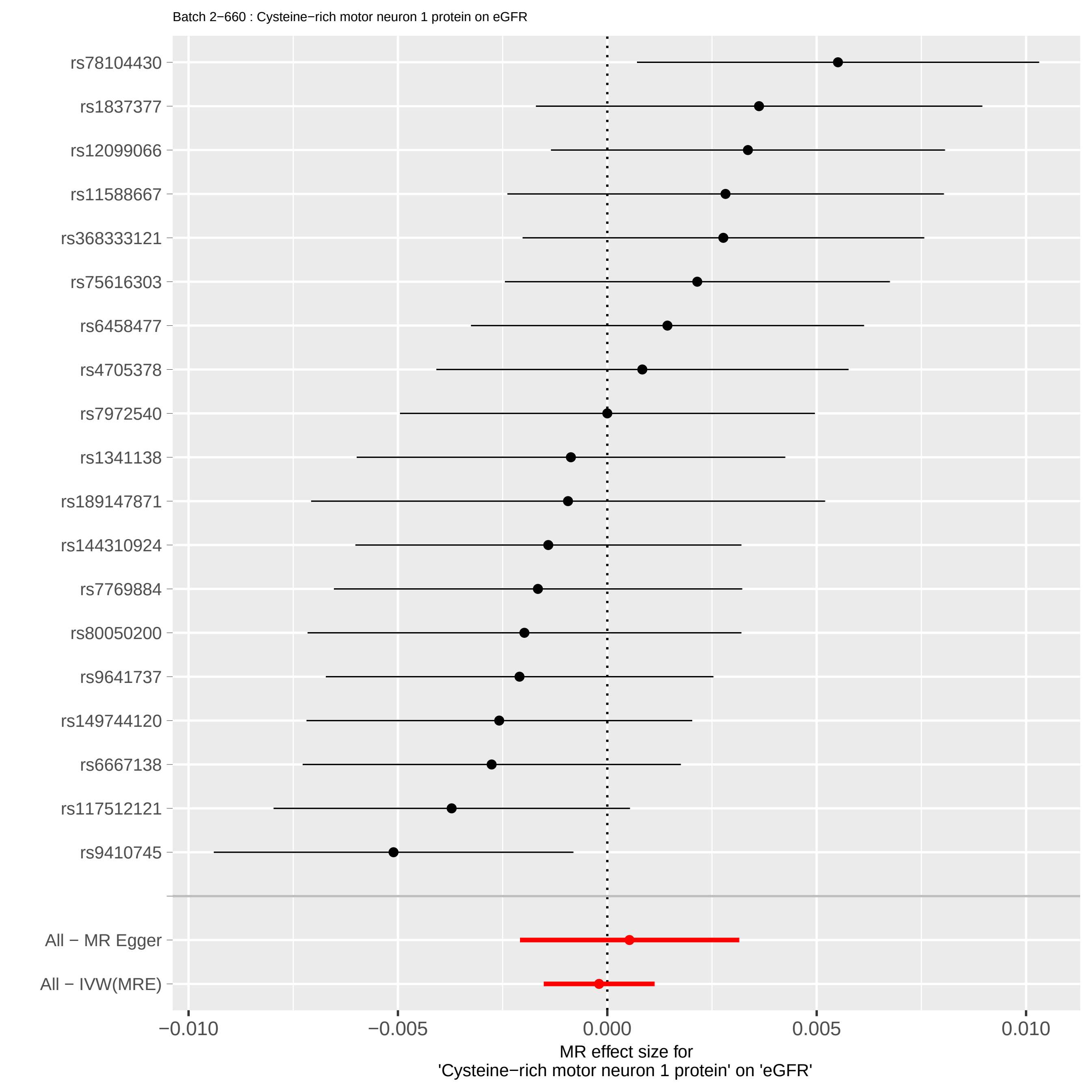

Batch 2−660 : Cysteine−rich motor neuron 1 protein on eGFR
rs78104430
rs1837377
rs12099066
rs11588667
rs368333121
rs75616303
rs6458477
rs4705378
rs7972540
rs1341138
rs189147871
rs144310924
rs7769884
rs80050200
rs9641737
rs149744120
rs6667138
rs117512121
rs9410745
All − MR Egger
All − IVW(MRE)
−0.010
−0.005
0.000
0.005
0.010
MR effect size for
'Cysteine−rich motor neuron 1 protein' on 'eGFR'

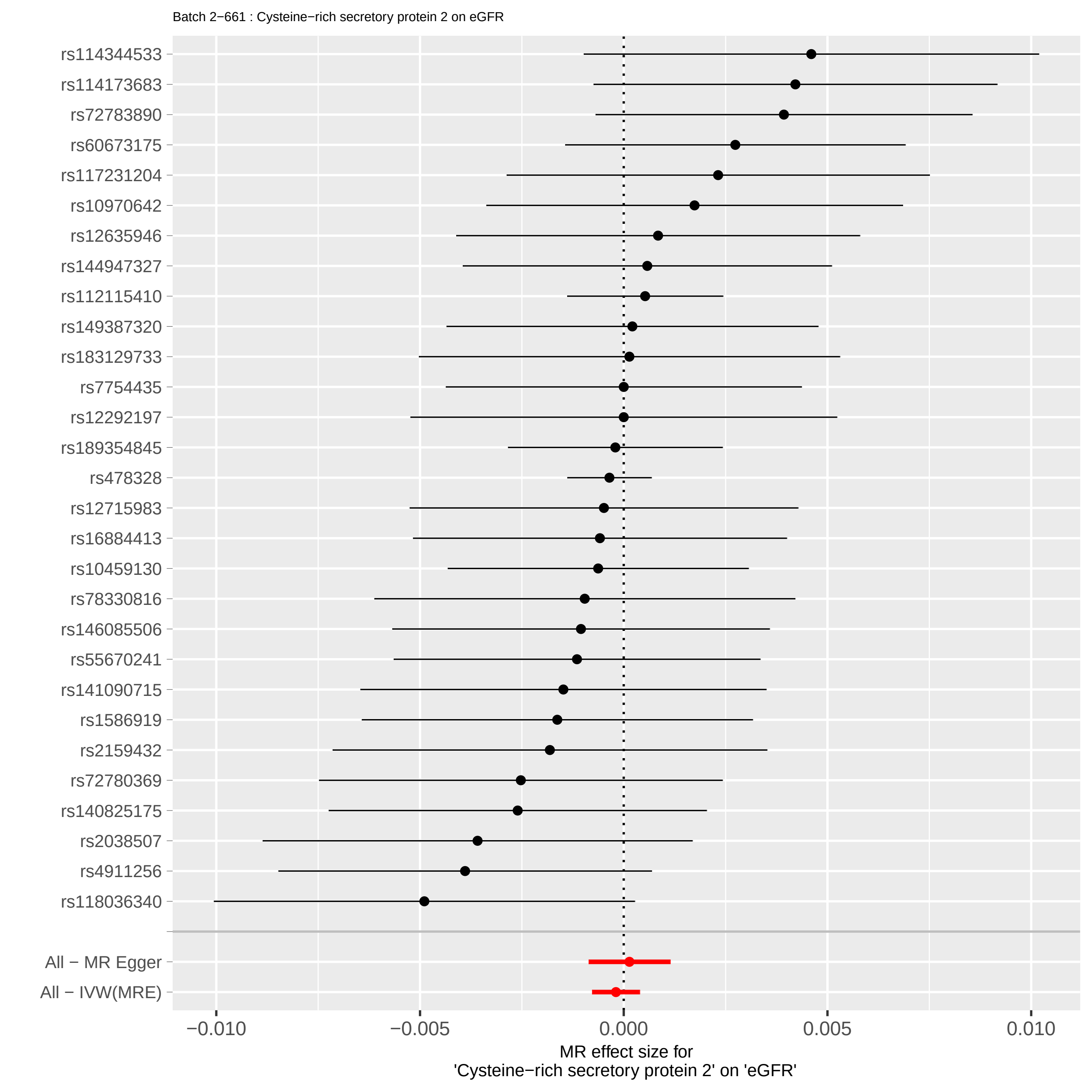

Batch 2−661 : Cysteine−rich secretory protein 2 on eGFR
rs114344533
rs114173683
rs72783890
rs60673175
rs117231204
rs10970642
rs12635946
rs144947327
rs112115410
rs149387320
rs183129733
rs7754435
rs12292197
rs189354845
rs478328
rs12715983
rs16884413
rs10459130
rs78330816
rs146085506
rs55670241
rs141090715
rs1586919
rs2159432
rs72780369
rs140825175
rs2038507
rs4911256
rs118036340
All − MR Egger
All − IVW(MRE)
−0.010
−0.005
0.000
0.005
0.010
MR effect size for
'Cysteine−rich secretory protein 2' on 'eGFR'

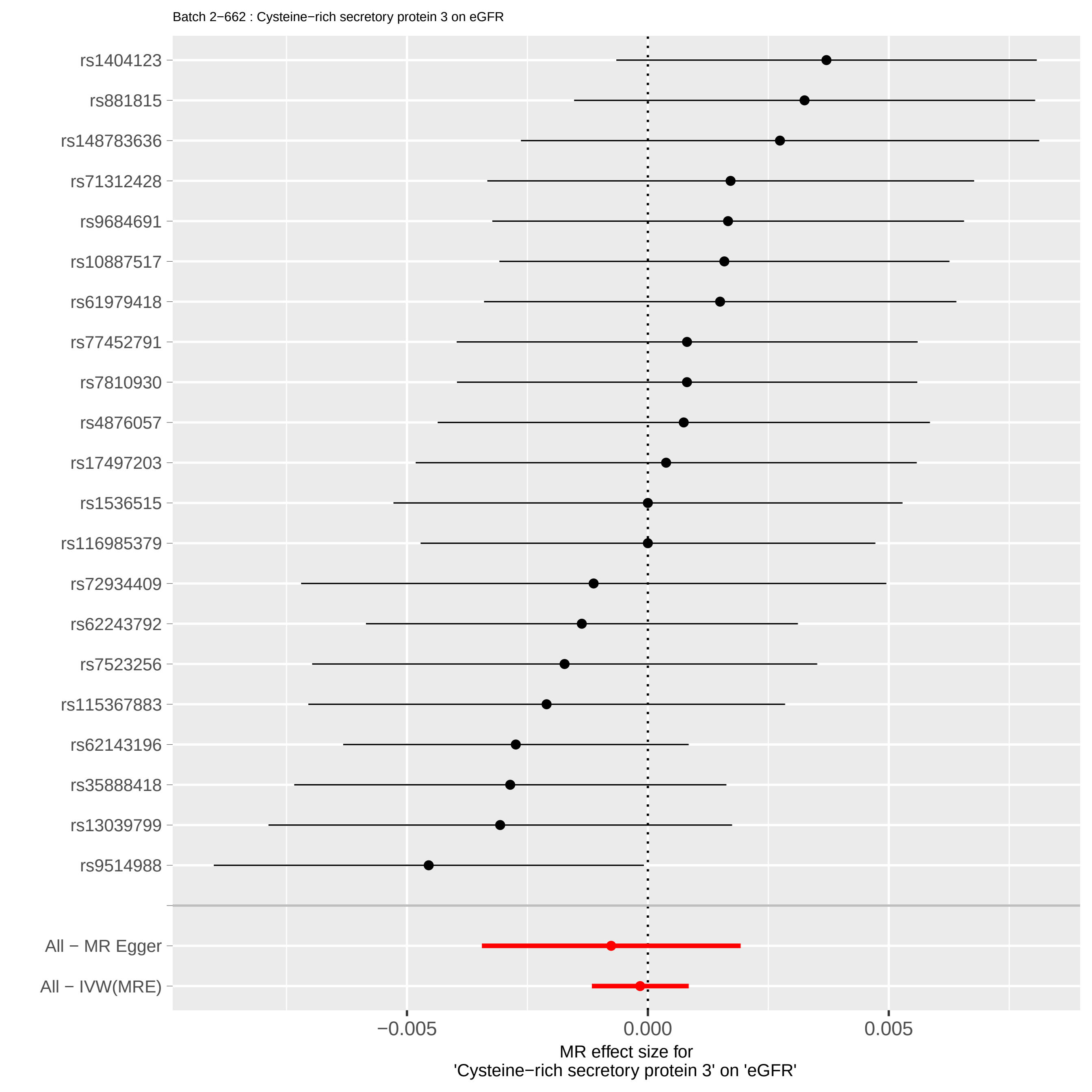

Batch 2−662 : Cysteine−rich secretory protein 3 on eGFR
rs1404123
rs881815
rs148783636
rs71312428
rs9684691
rs10887517
rs61979418
rs77452791
rs7810930
rs4876057
rs17497203
rs1536515
rs116985379
rs72934409
rs62243792
rs7523256
rs115367883
rs62143196
rs35888418
rs13039799
rs9514988
All − MR Egger
All − IVW(MRE)
−0.005
0.000
0.005
MR effect size for
'Cysteine−rich secretory protein 3' on 'eGFR'

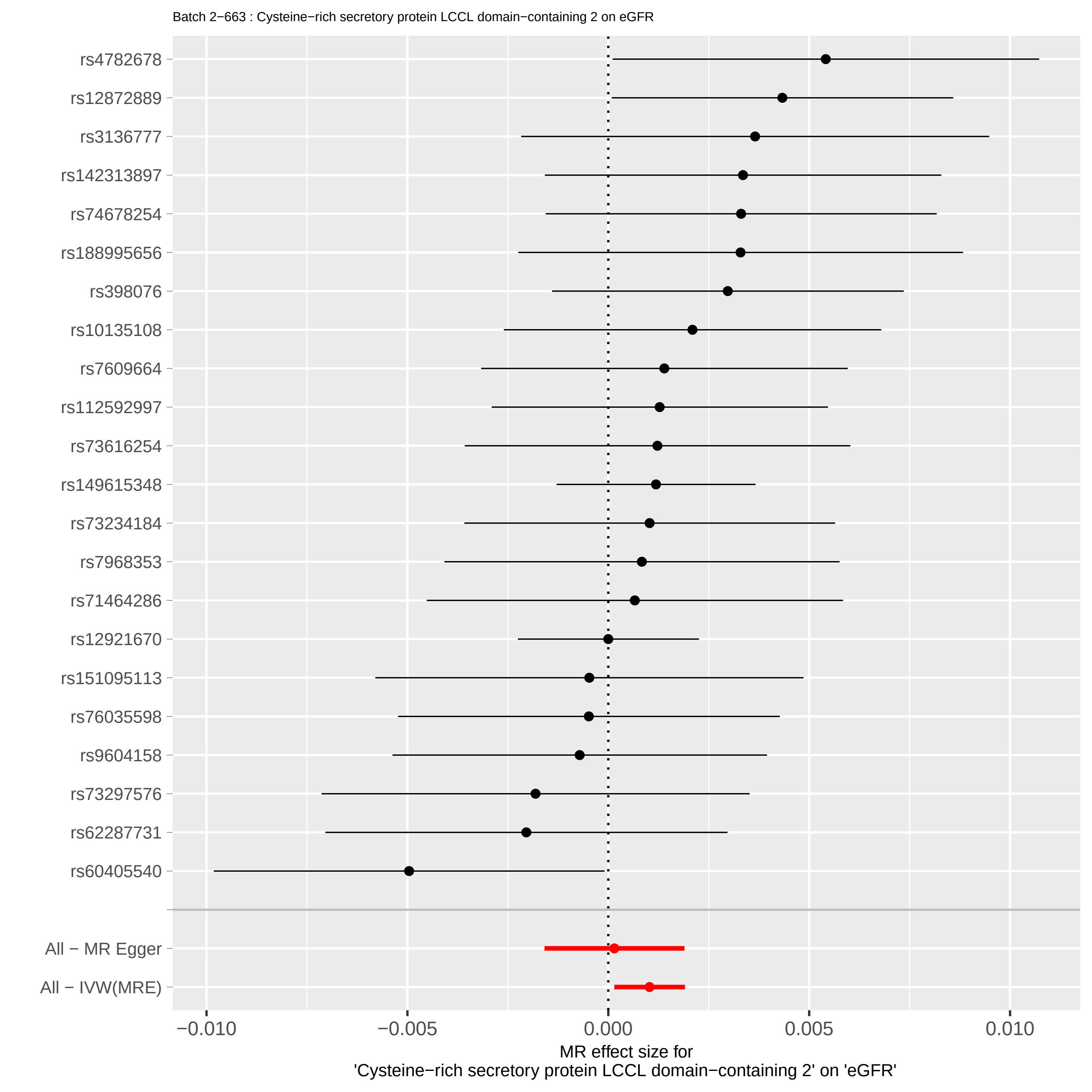

Batch 2−663 : Cysteine−rich secretory protein LCCL domain−containing 2 on eGFR
rs4782678
rs12872889
rs3136777
rs142313897
rs74678254
rs188995656
rs398076
rs10135108
rs7609664
rs112592997
rs73616254
rs149615348
rs73234184
rs7968353
rs71464286
rs12921670
rs151095113
rs76035598
rs9604158
rs73297576
rs62287731
rs60405540
All − MR Egger
All − IVW(MRE)
−0.010
−0.005
0.000
0.005
0.010
MR effect size for
'Cysteine−rich secretory protein LCCL domain−containing 2' on 'eGFR'

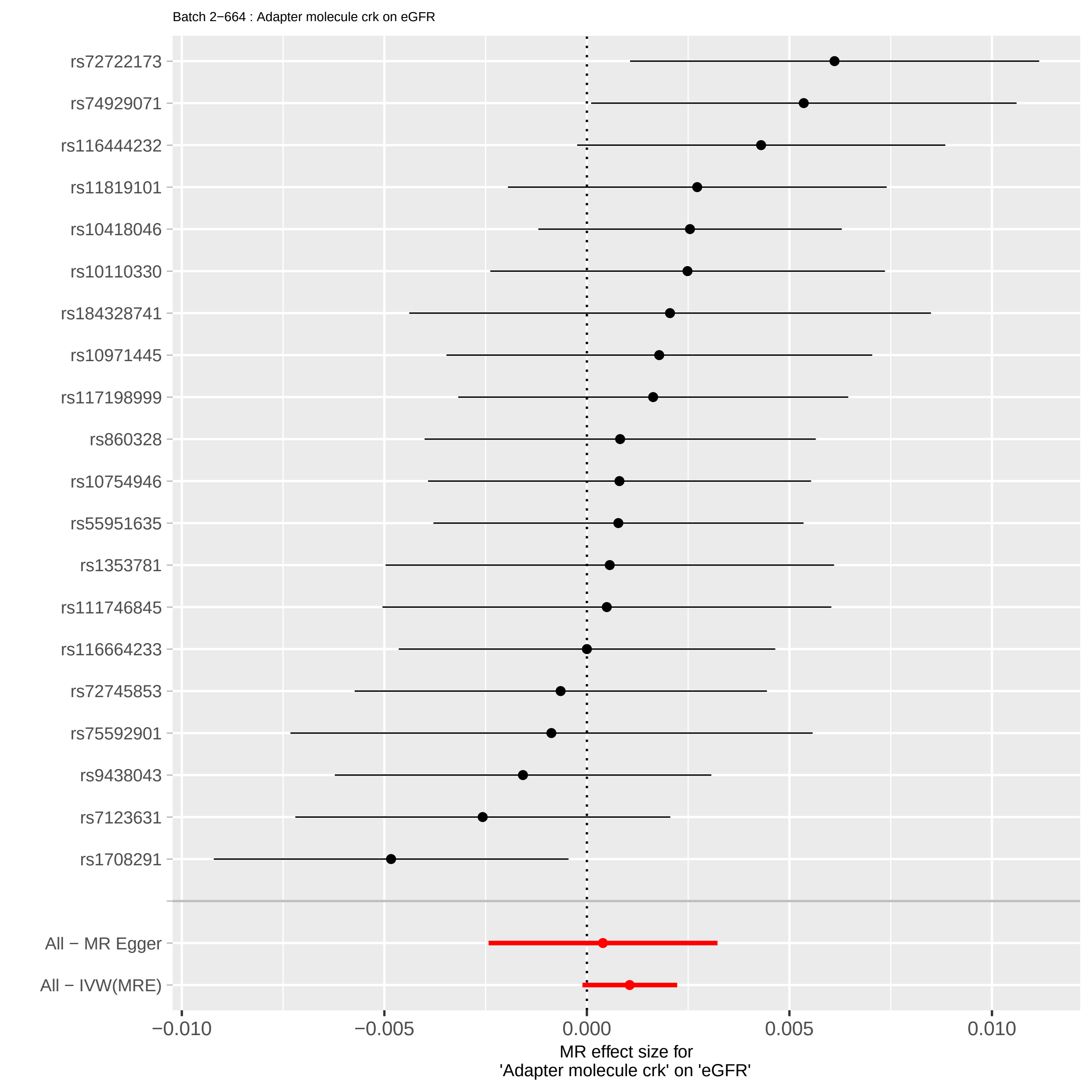

Batch 2−664 : Adapter molecule crk on eGFR
rs72722173
rs74929071
rs116444232
rs11819101
rs10418046
rs10110330
rs184328741
rs10971445
rs117198999
rs860328
rs10754946
rs55951635
rs1353781
rs111746845
rs116664233
rs72745853
rs75592901
rs9438043
rs7123631
rs1708291
All − MR Egger
All − IVW(MRE)
−0.010
−0.005
0.000
0.005
0.010
MR effect size for
'Adapter molecule crk' on 'eGFR'

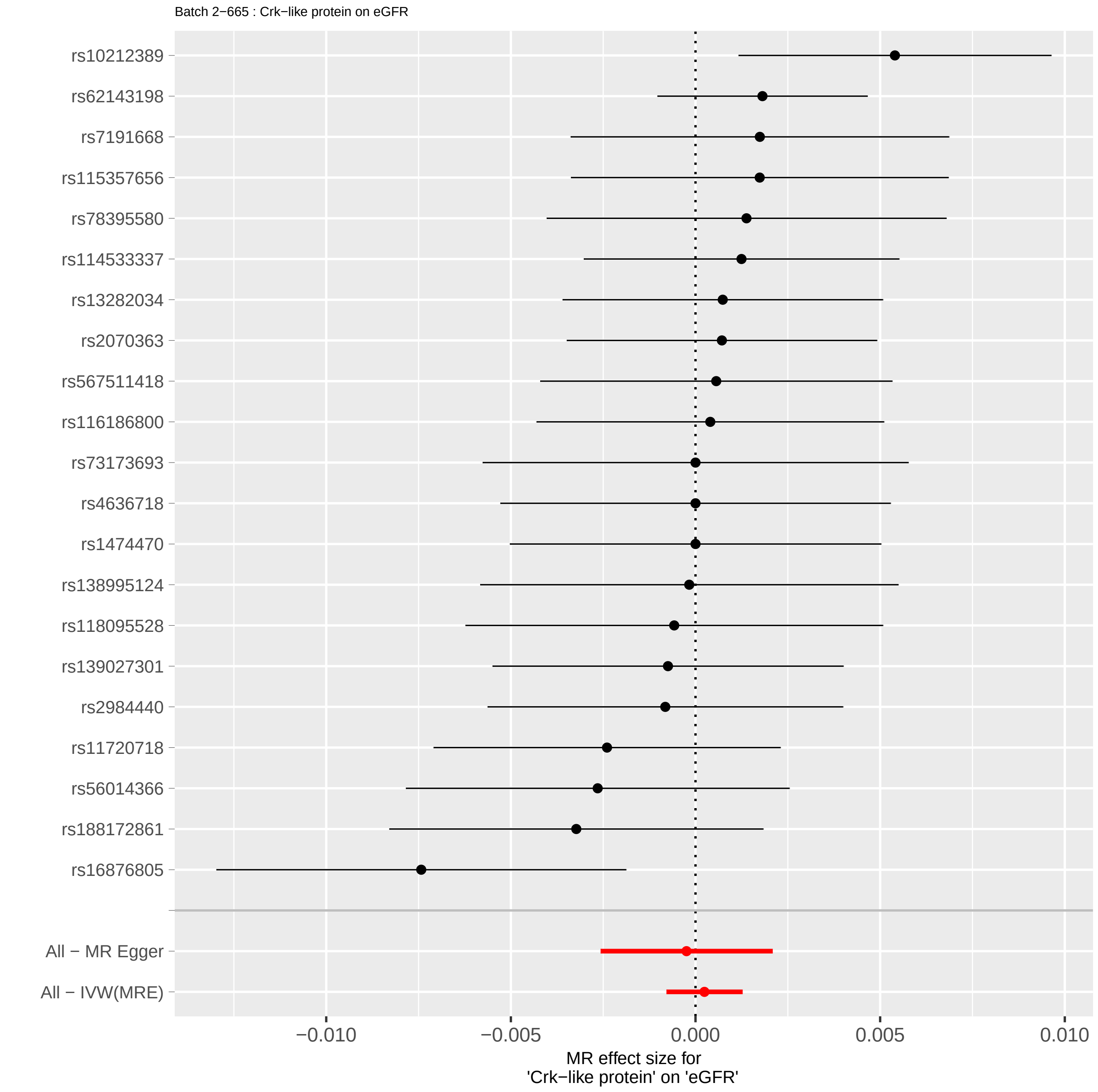
Batch 2−665 : Crk−like protein on eGFR
rs10212389
rs62143198
rs7191668
rs115357656
rs78395580
rs114533337
rs13282034
rs2070363
rs567511418
rs116186800
rs73173693
rs4636718
rs1474470
rs138995124
rs118095528
rs139027301
rs2984440
rs11720718
rs56014366
rs188172861
rs16876805
All − MR Egger
All − IVW(MRE)
−0.010
−0.005
0.000
0.005
0.010
MR effect size for
'Crk−like protein' on 'eGFR'

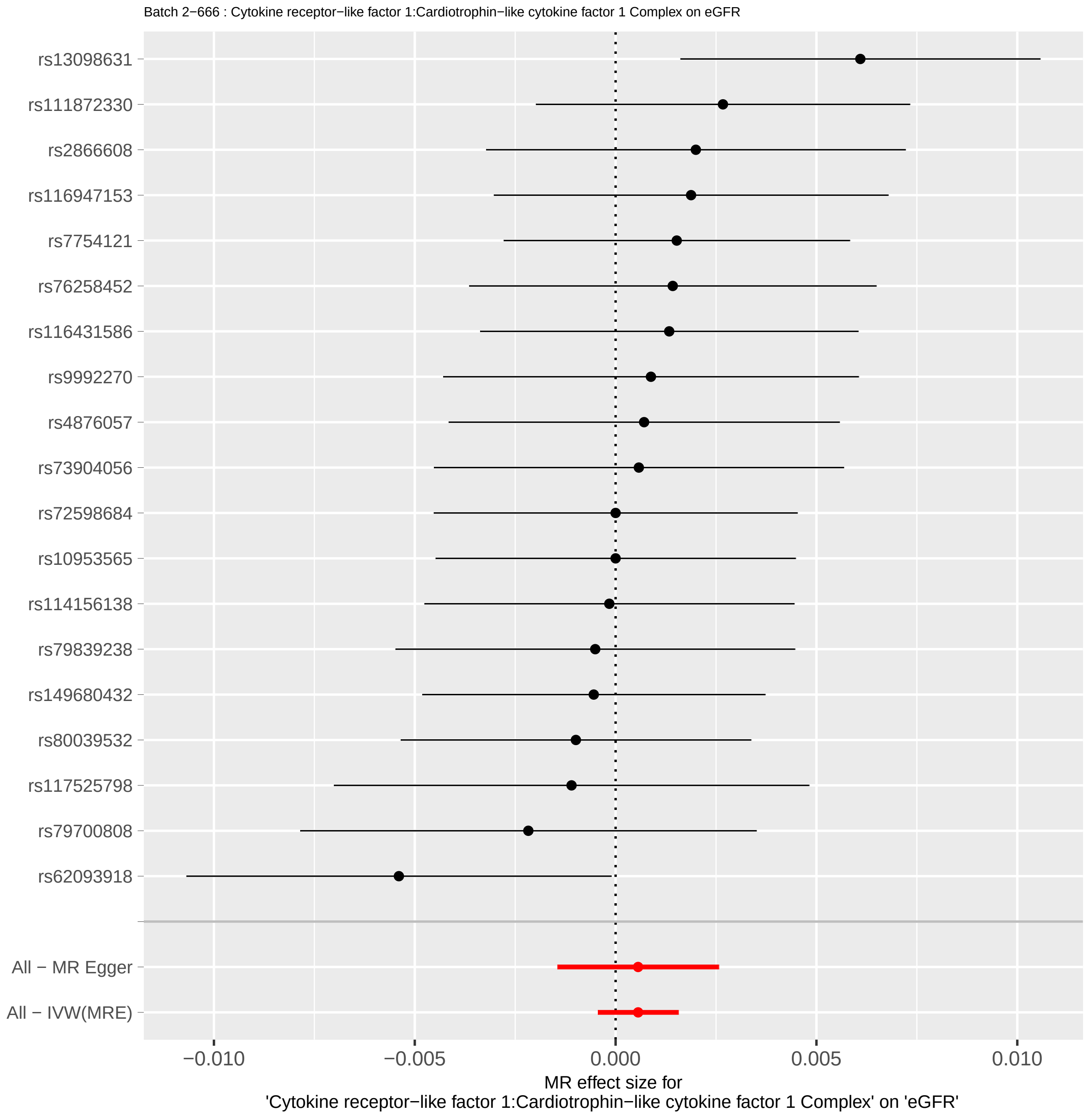
Batch 2−666 : Cytokine receptor−like factor 1:Cardiotrophin−like cytokine factor 1 Complex on eGFR
rs13098631
rs111872330
rs2866608
rs116947153
rs7754121
rs76258452
rs116431586
rs9992270
rs4876057
rs73904056
rs72598684
rs10953565
rs114156138
rs79839238
rs149680432
rs80039532
rs117525798
rs79700808
rs62093918
All − MR Egger
All − IVW(MRE)
−0.010
−0.005
0.000
0.005
0.010
MR effect size for
'Cytokine receptor−like factor 1:Cardiotrophin−like cytokine factor 1 Complex' on 'eGFR'

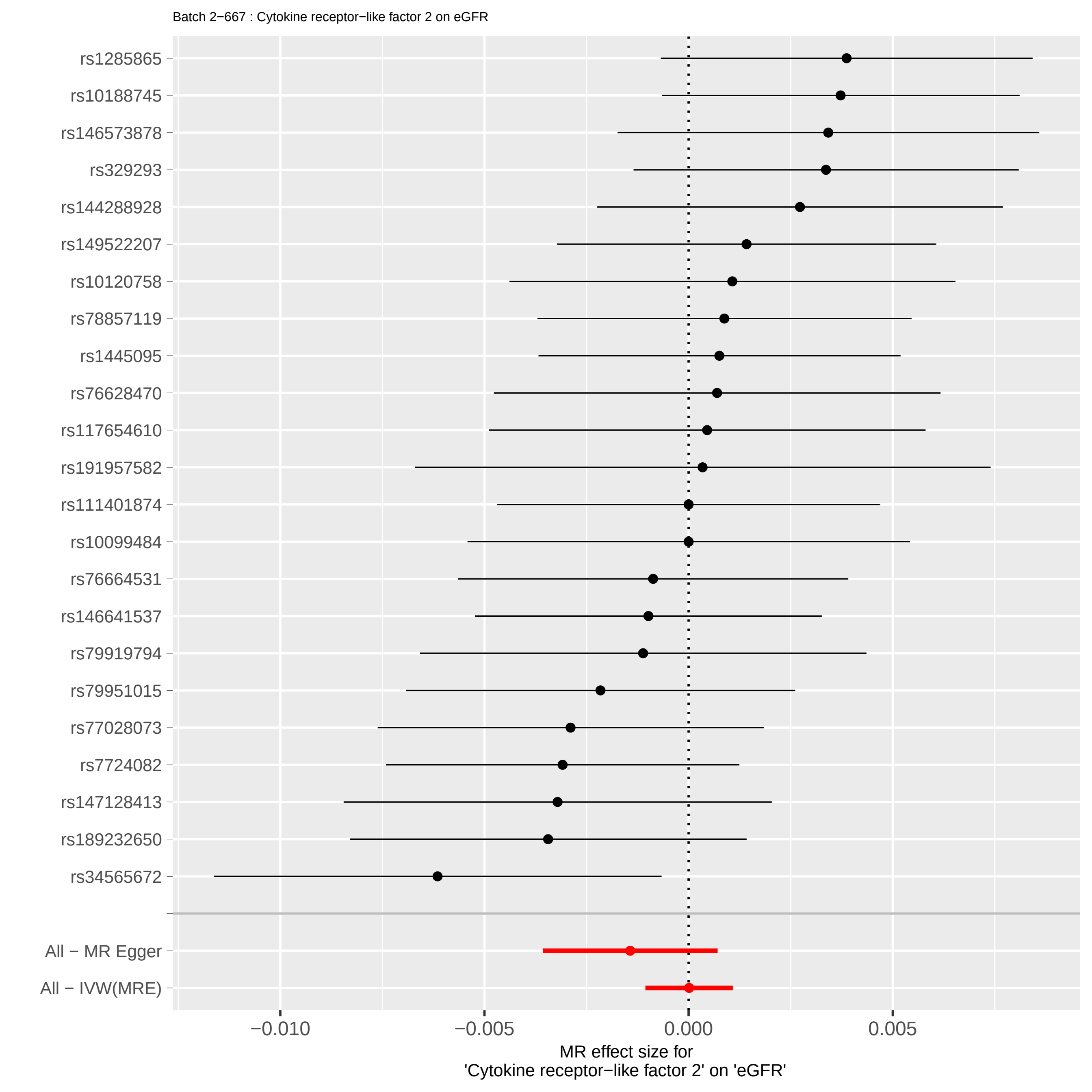

Batch 2−667 : Cytokine receptor−like factor 2 on eGFR
rs1285865
rs10188745
rs146573878
rs329293
rs144288928
rs149522207
rs10120758
rs78857119
rs1445095
rs76628470
rs117654610
rs191957582
rs111401874
rs10099484
rs76664531
rs146641537
rs79919794
rs79951015
rs77028073
rs7724082
rs147128413
rs189232650
rs34565672
All − MR Egger
All − IVW(MRE)
−0.010
−0.005
0.000
0.005
MR effect size for
'Cytokine receptor−like factor 2' on 'eGFR'

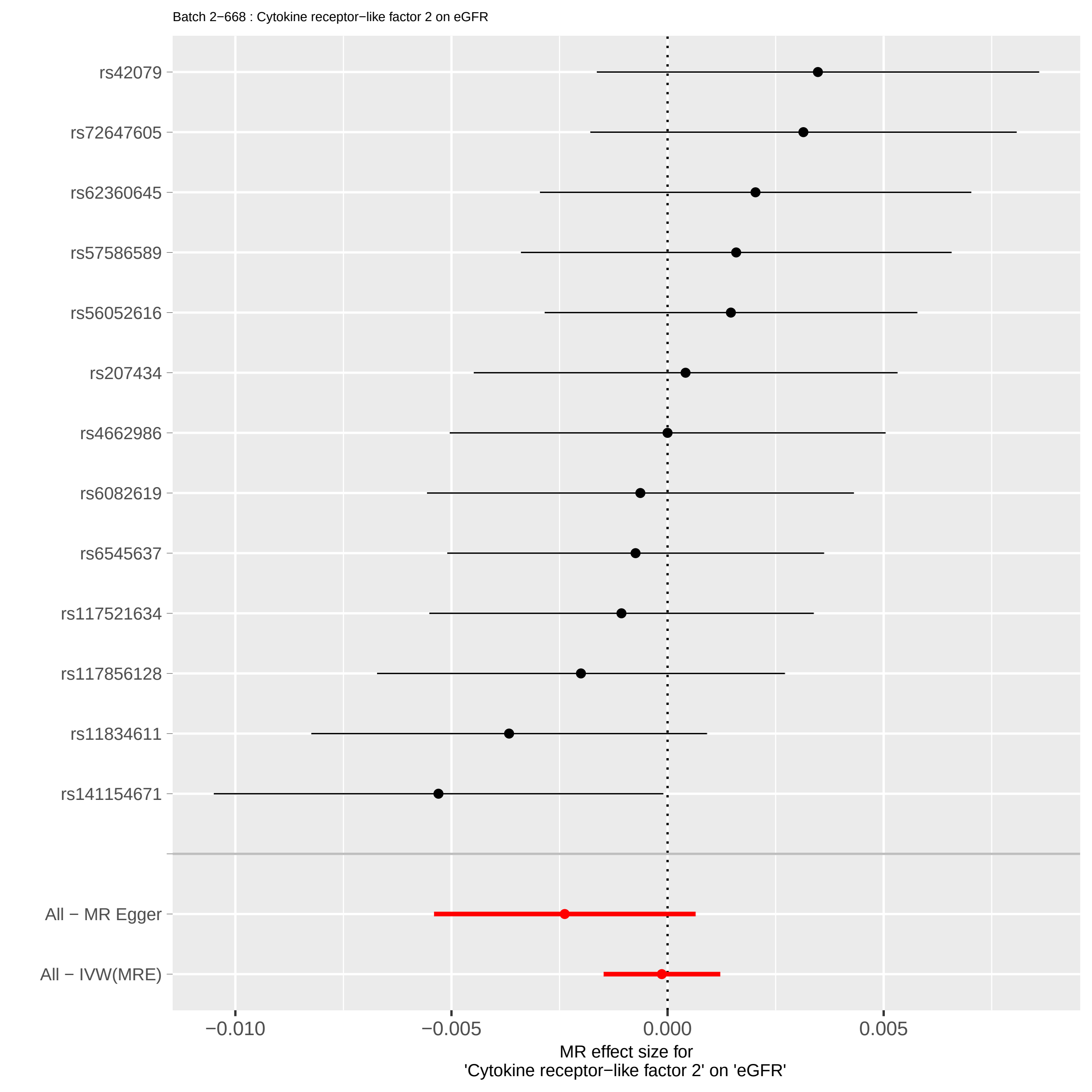
Batch 2−668 : Cytokine receptor−like factor 2 on eGFR
rs42079
rs72647605
rs62360645
rs57586589
rs56052616
rs207434
rs4662986
rs6082619
rs6545637
rs117521634
rs117856128
rs11834611
rs141154671
All − MR Egger
All − IVW(MRE)
−0.010
−0.005
0.000
0.005
MR effect size for
'Cytokine receptor−like factor 2' on 'eGFR'

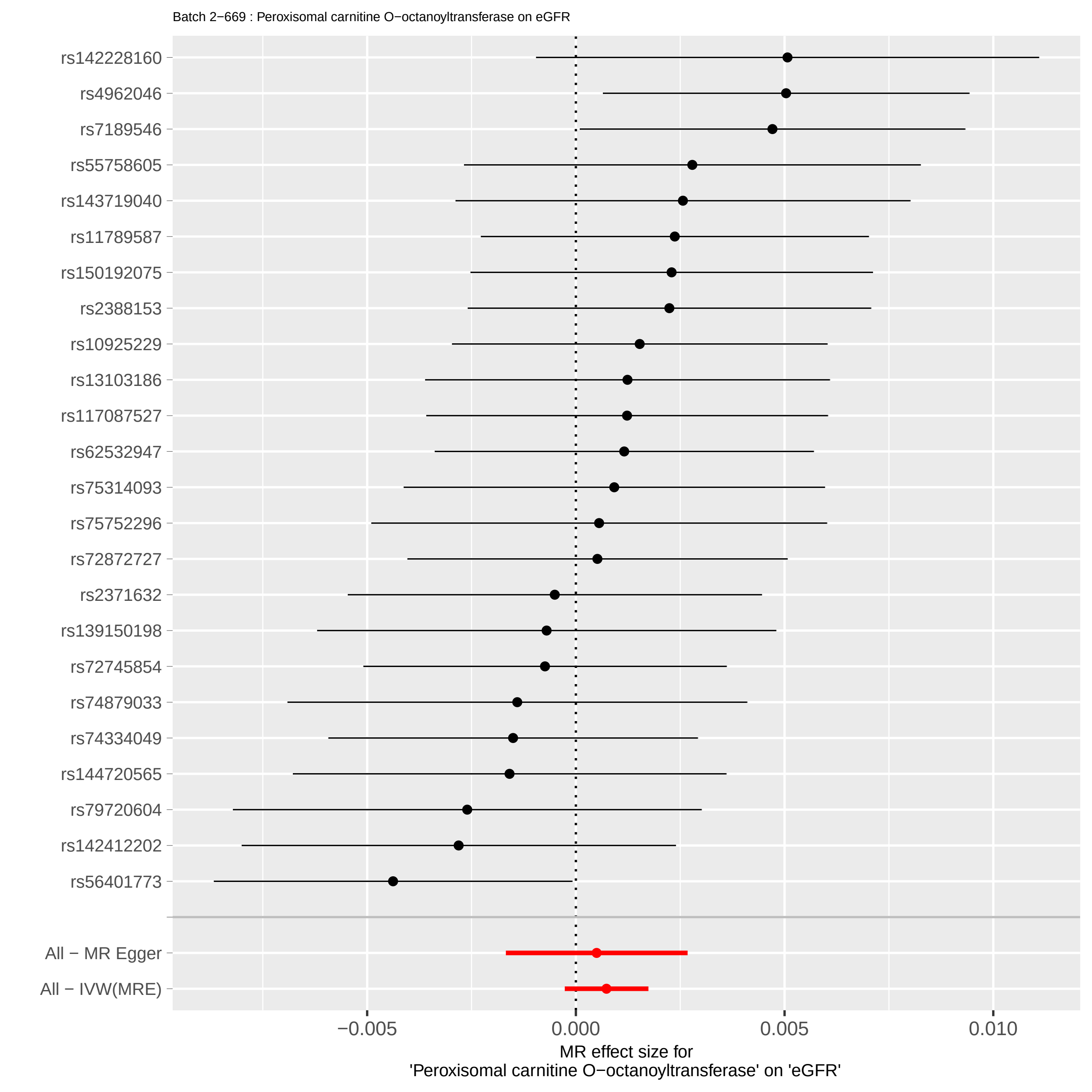

Batch 2−669 : Peroxisomal carnitine O−octanoyltransferase on eGFR
rs142228160
rs4962046
rs7189546
rs55758605
rs143719040
rs11789587
rs150192075
rs2388153
rs10925229
rs13103186
rs117087527
rs62532947
rs75314093
rs75752296
rs72872727
rs2371632
rs139150198
rs72745854
rs74879033
rs74334049
rs144720565
rs79720604
rs142412202
rs56401773
All − MR Egger
All − IVW(MRE)
−0.005
0.000
0.005
0.010
MR effect size for
'Peroxisomal carnitine O−octanoyltransferase' on 'eGFR'

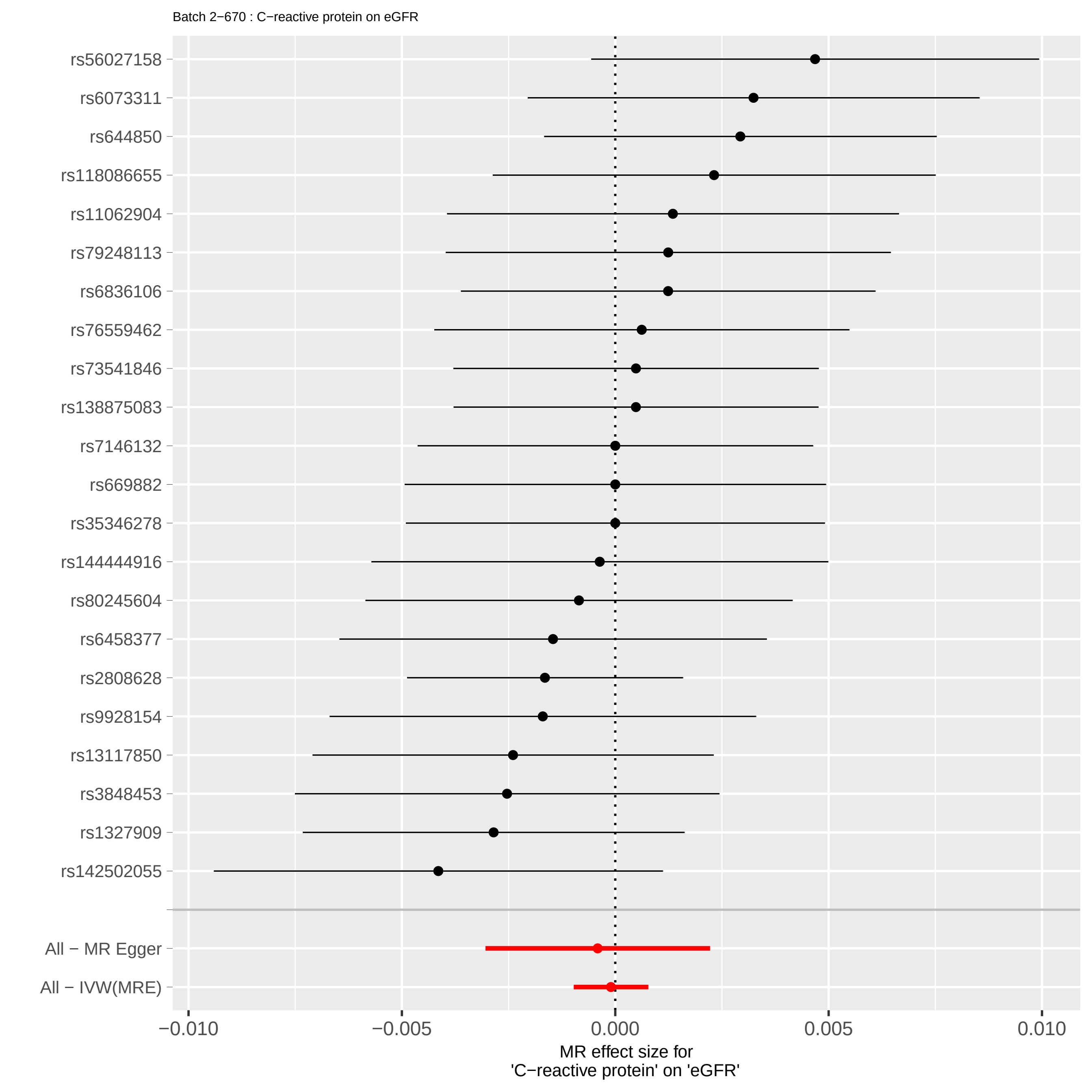
Batch 2−670 : C−reactive protein on eGFR
rs56027158
rs6073311
rs644850
rs118086655
rs11062904
rs79248113
rs6836106
rs76559462
rs73541846
rs138875083
rs7146132
rs669882
rs35346278
rs144444916
rs80245604
rs6458377
rs2808628
rs9928154
rs13117850
rs3848453
rs1327909
rs142502055
All − MR Egger
All − IVW(MRE)
−0.010
−0.005
0.000
0.005
0.010
MR effect size for
'C−reactive protein' on 'eGFR'

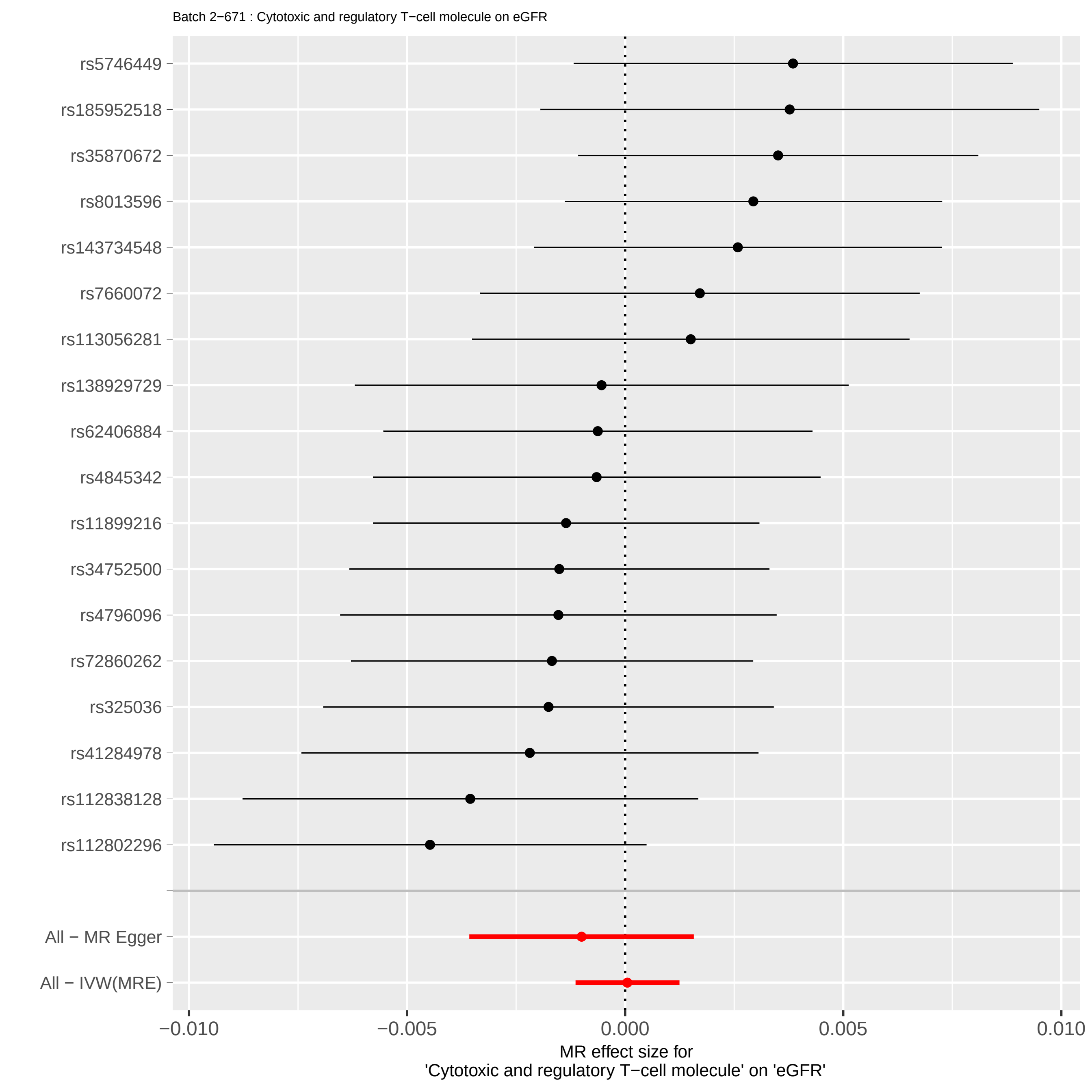

Batch 2−671 : Cytotoxic and regulatory T−cell molecule on eGFR
rs5746449
rs185952518
rs35870672
rs8013596
rs143734548
rs7660072
rs113056281
rs138929729
rs62406884
rs4845342
rs11899216
rs34752500
rs4796096
rs72860262
rs325036
rs41284978
rs112838128
rs112802296
All − MR Egger
All − IVW(MRE)
−0.010
−0.005
0.000
0.005
0.010
MR effect size for
'Cytotoxic and regulatory T−cell molecule' on 'eGFR'

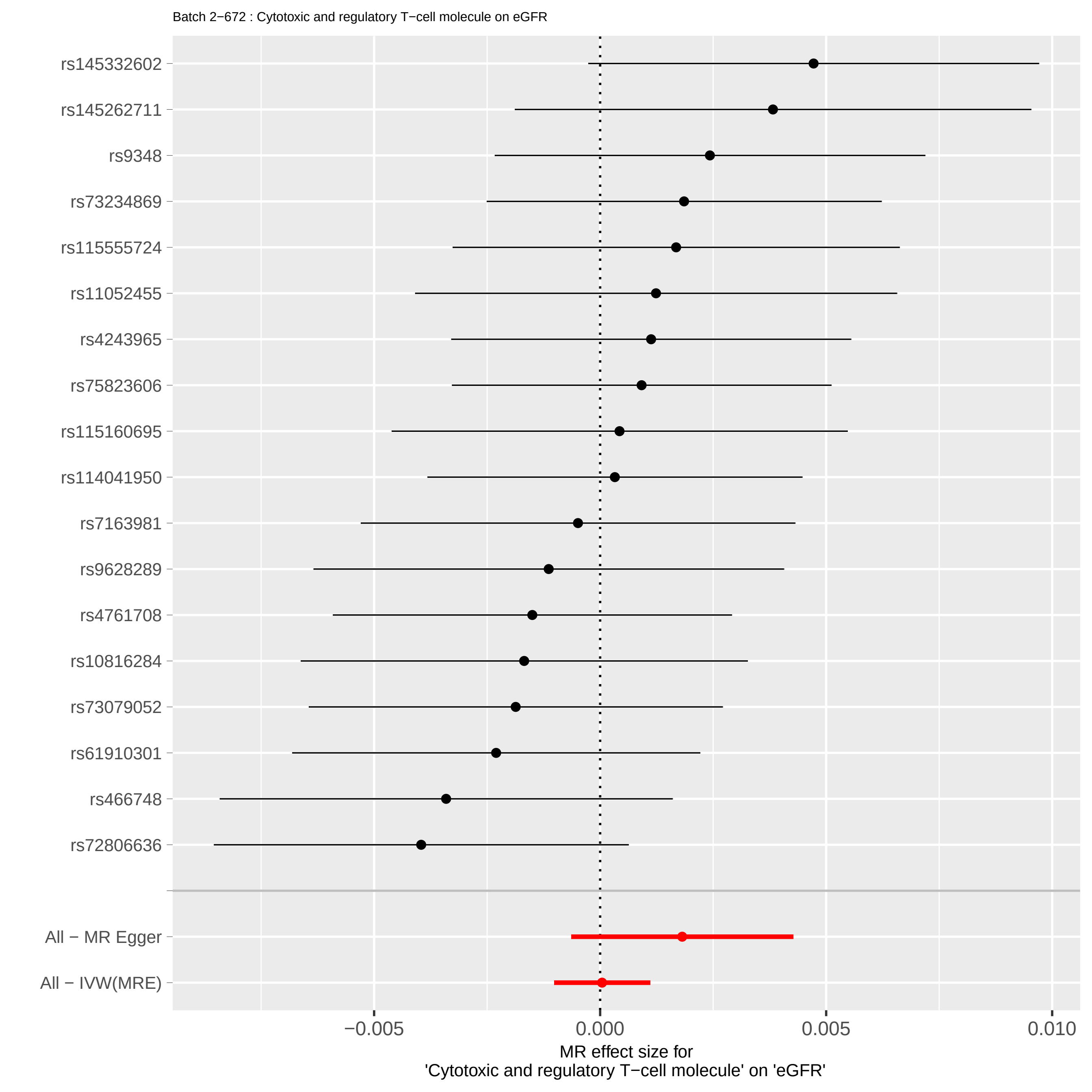

Batch 2−672 : Cytotoxic and regulatory T−cell molecule on eGFR
rs145332602
rs145262711
rs9348
rs73234869
rs115555724
rs11052455
rs4243965
rs75823606
rs115160695
rs114041950
rs7163981
rs9628289
rs4761708
rs10816284
rs73079052
rs61910301
rs466748
rs72806636
All − MR Egger
All − IVW(MRE)
−0.005
0.000
0.005
0.010
MR effect size for
'Cytotoxic and regulatory T−cell molecule' on 'eGFR'

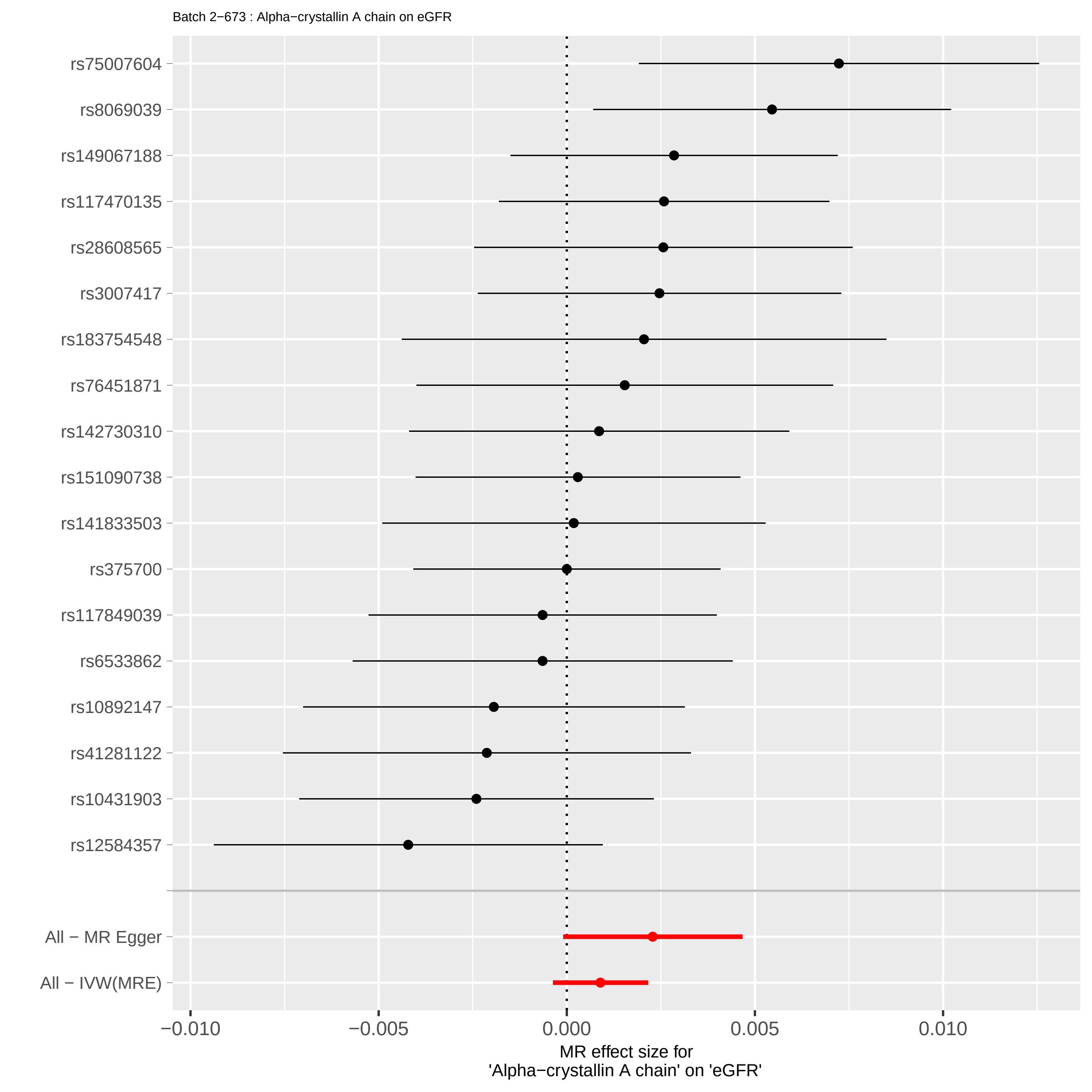

Batch 2−673 : Alpha−crystallin A chain on eGFR
rs75007604
rs8069039
rs149067188
rs117470135
rs28608565
rs3007417
rs183754548
rs76451871
rs142730310
rs151090738
rs141833503
rs375700
rs117849039
rs6533862
rs10892147
rs41281122
rs10431903
rs12584357
All − MR Egger
All − IVW(MRE)
−0.010
−0.005
0.000
0.005
0.010
MR effect size for
'Alpha−crystallin A chain' on 'eGFR'

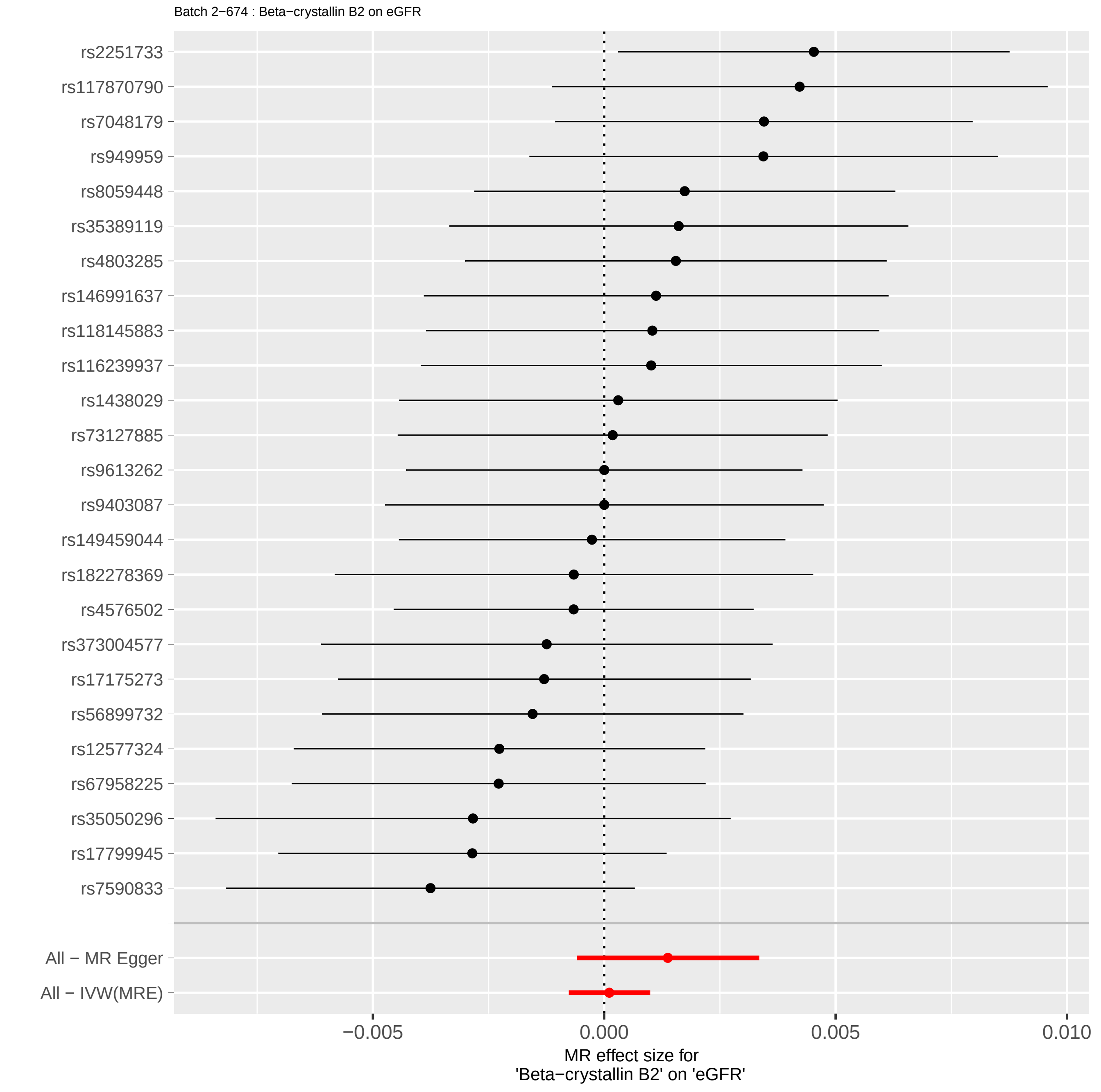

Batch 2−674 : Beta−crystallin B2 on eGFR
rs2251733
rs117870790
rs7048179
rs949959
rs8059448
rs35389119
rs4803285
rs146991637
rs118145883
rs116239937
rs1438029
rs73127885
rs9613262
rs9403087
rs149459044
rs182278369
rs4576502
rs373004577
rs17175273
rs56899732
rs12577324
rs67958225
rs35050296
rs17799945
rs7590833
All − MR Egger
All − IVW(MRE)
−0.005
0.000
0.005
0.010
MR effect size for
'Beta−crystallin B2' on 'eGFR'

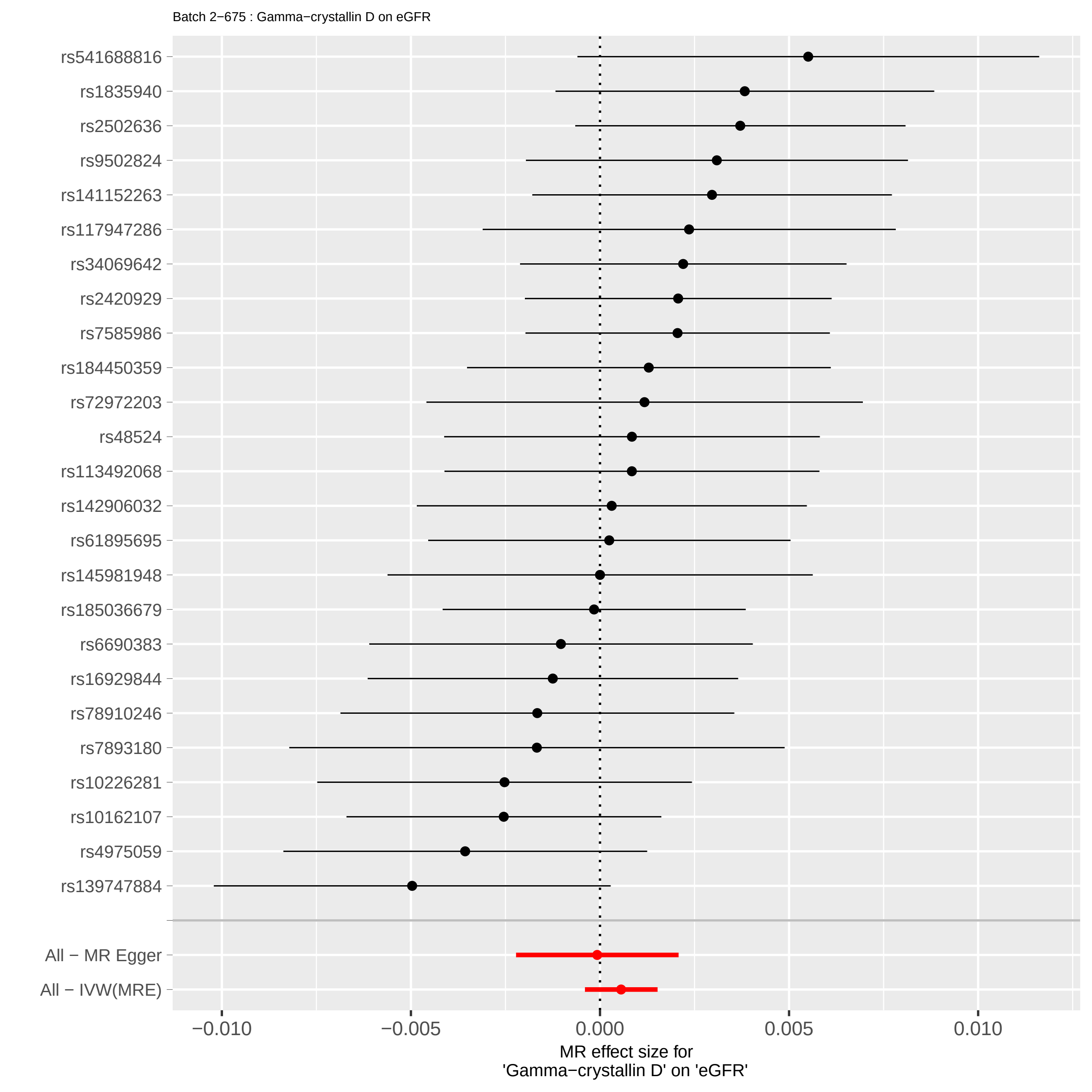

Batch 2−675 : Gamma−crystallin D on eGFR
rs541688816
rs1835940
rs2502636
rs9502824
rs141152263
rs117947286
rs34069642
rs2420929
rs7585986
rs184450359
rs72972203
rs48524
rs113492068
rs142906032
rs61895695
rs145981948
rs185036679
rs6690383
rs16929844
rs78910246
rs7893180
rs10226281
rs10162107
rs4975059
rs139747884
All − MR Egger
All − IVW(MRE)
−0.010
−0.005
0.000
0.005
0.010
MR effect size for
'Gamma−crystallin D' on 'eGFR'

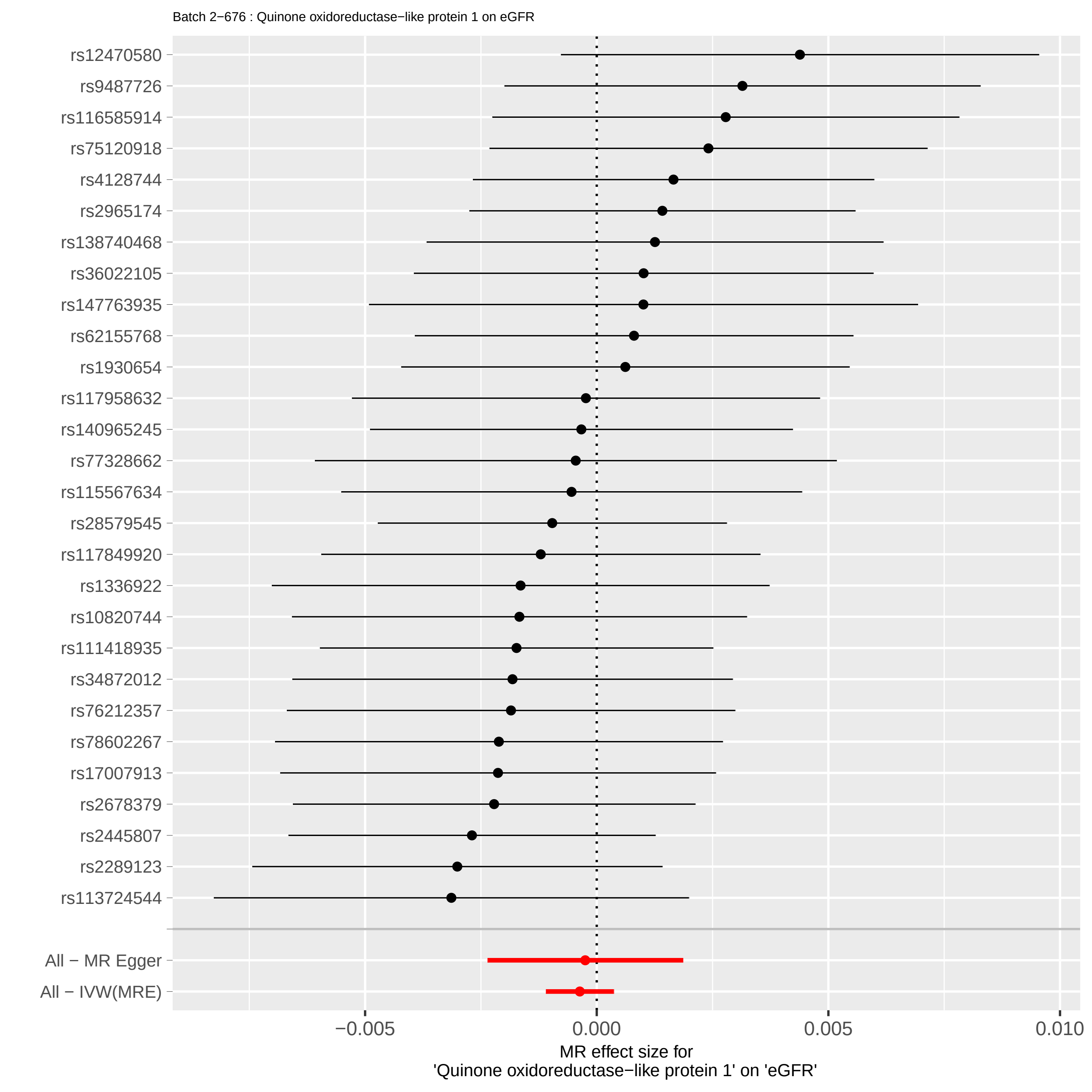

Batch 2−676 : Quinone oxidoreductase−like protein 1 on eGFR
rs12470580
rs9487726
rs116585914
rs75120918
rs4128744
rs2965174
rs138740468
rs36022105
rs147763935
rs62155768
rs1930654
rs117958632
rs140965245
rs77328662
rs115567634
rs28579545
rs117849920
rs1336922
rs10820744
rs111418935
rs34872012
rs76212357
rs78602267
rs17007913
rs2678379
rs2445807
rs2289123
rs113724544
All − MR Egger
All − IVW(MRE)
−0.005
0.000
0.005
0.010
MR effect size for
'Quinone oxidoreductase−like protein 1' on 'eGFR'

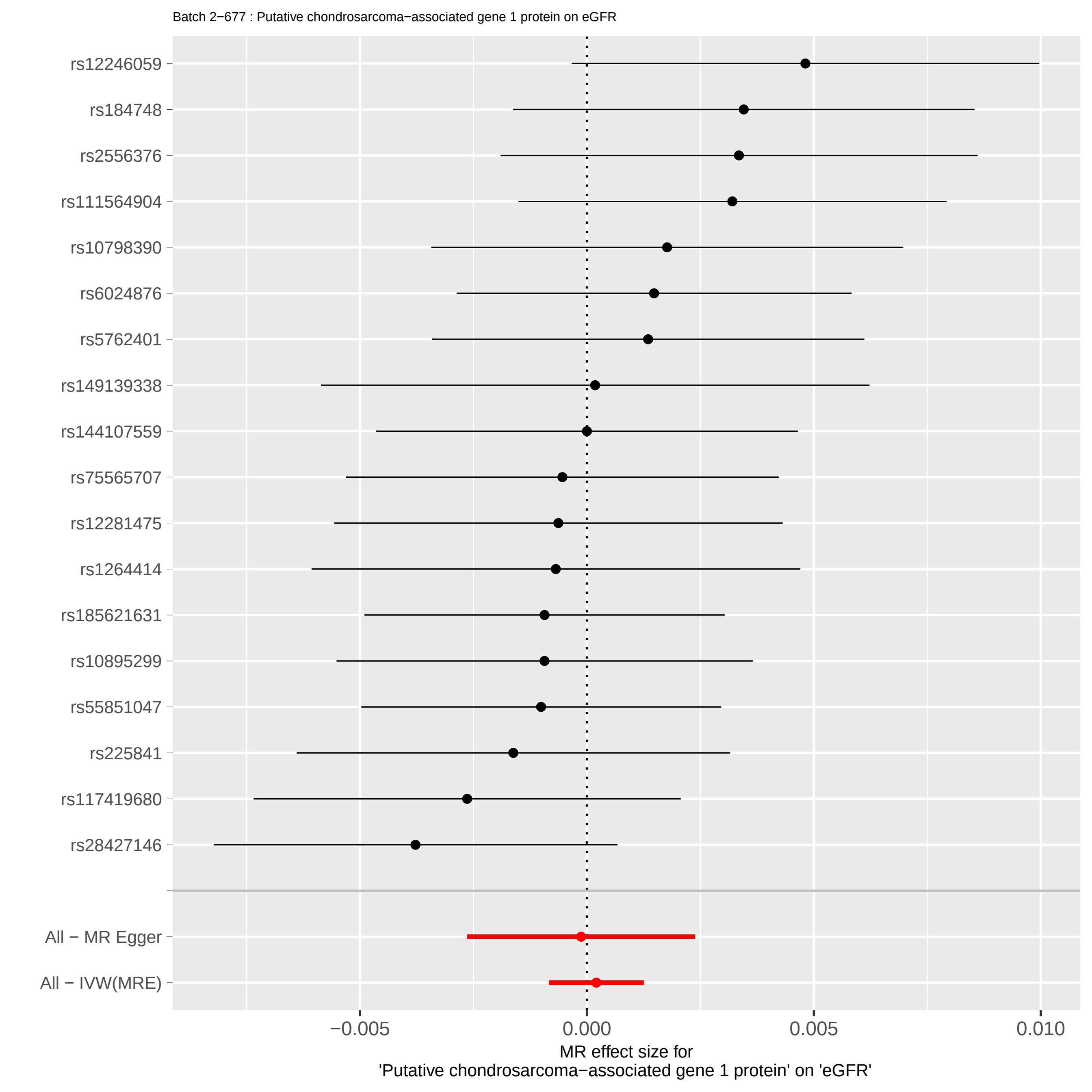

Batch 2−677 : Putative chondrosarcoma−associated gene 1 protein on eGFR
rs12246059
rs184748
rs2556376
rs111564904
rs10798390
rs6024876
rs5762401
rs149139338
rs144107559
rs75565707
rs12281475
rs1264414
rs185621631
rs10895299
rs55851047
rs225841
rs117419680
rs28427146
All − MR Egger
All − IVW(MRE)
−0.005
0.000
0.005
0.010
MR effect size for
'Putative chondrosarcoma−associated gene 1 protein' on 'eGFR'

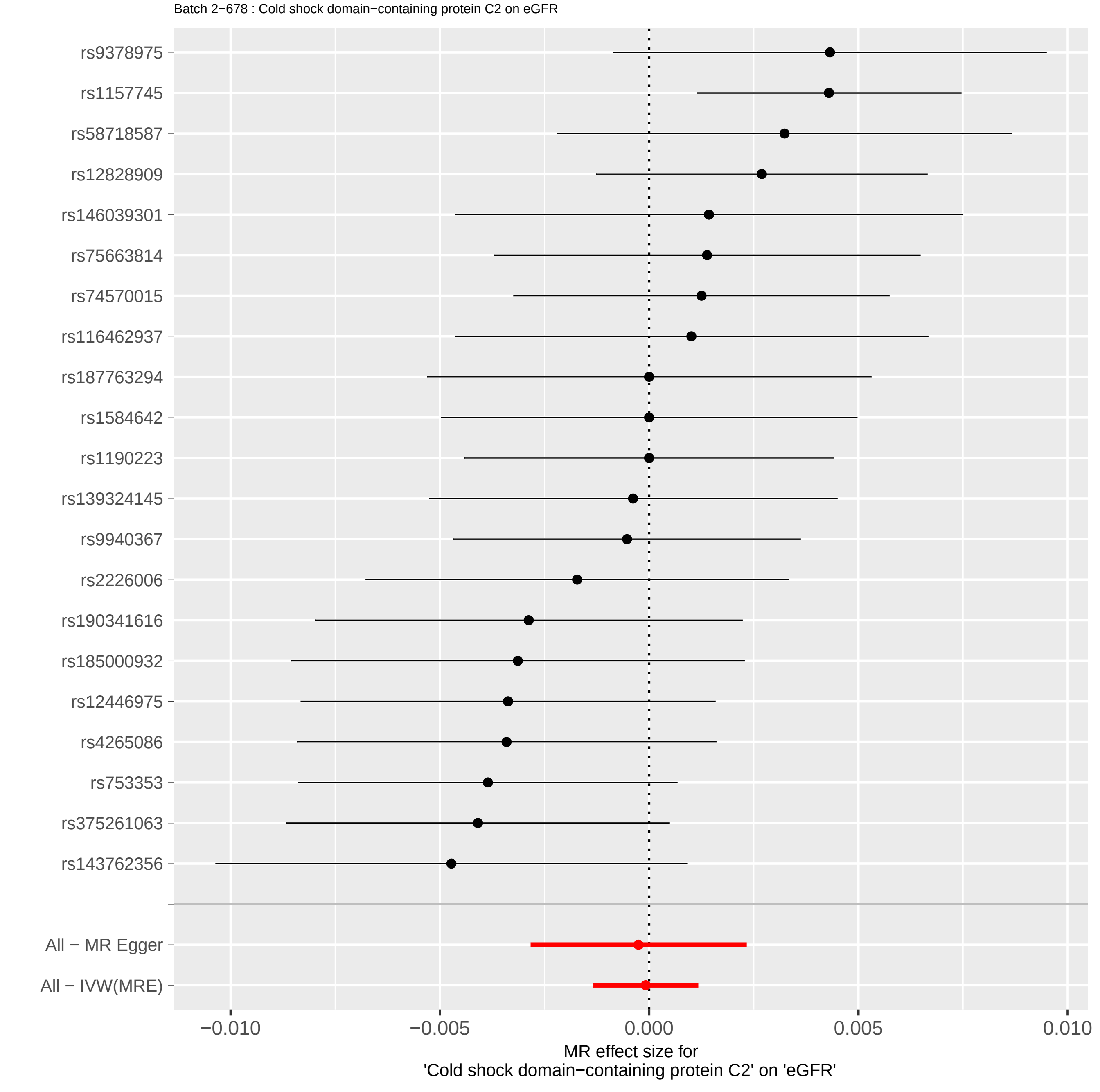

Batch 2−678 : Cold shock domain−containing protein C2 on eGFR
rs9378975
rs1157745
rs58718587
rs12828909
rs146039301
rs75663814
rs74570015
rs116462937
rs187763294
rs1584642
rs1190223
rs139324145
rs9940367
rs2226006
rs190341616
rs185000932
rs12446975
rs4265086
rs753353
rs375261063
rs143762356
All − MR Egger
All − IVW(MRE)
−0.010
−0.005
0.000
0.005
0.010
MR effect size for
'Cold shock domain−containing protein C2' on 'eGFR'

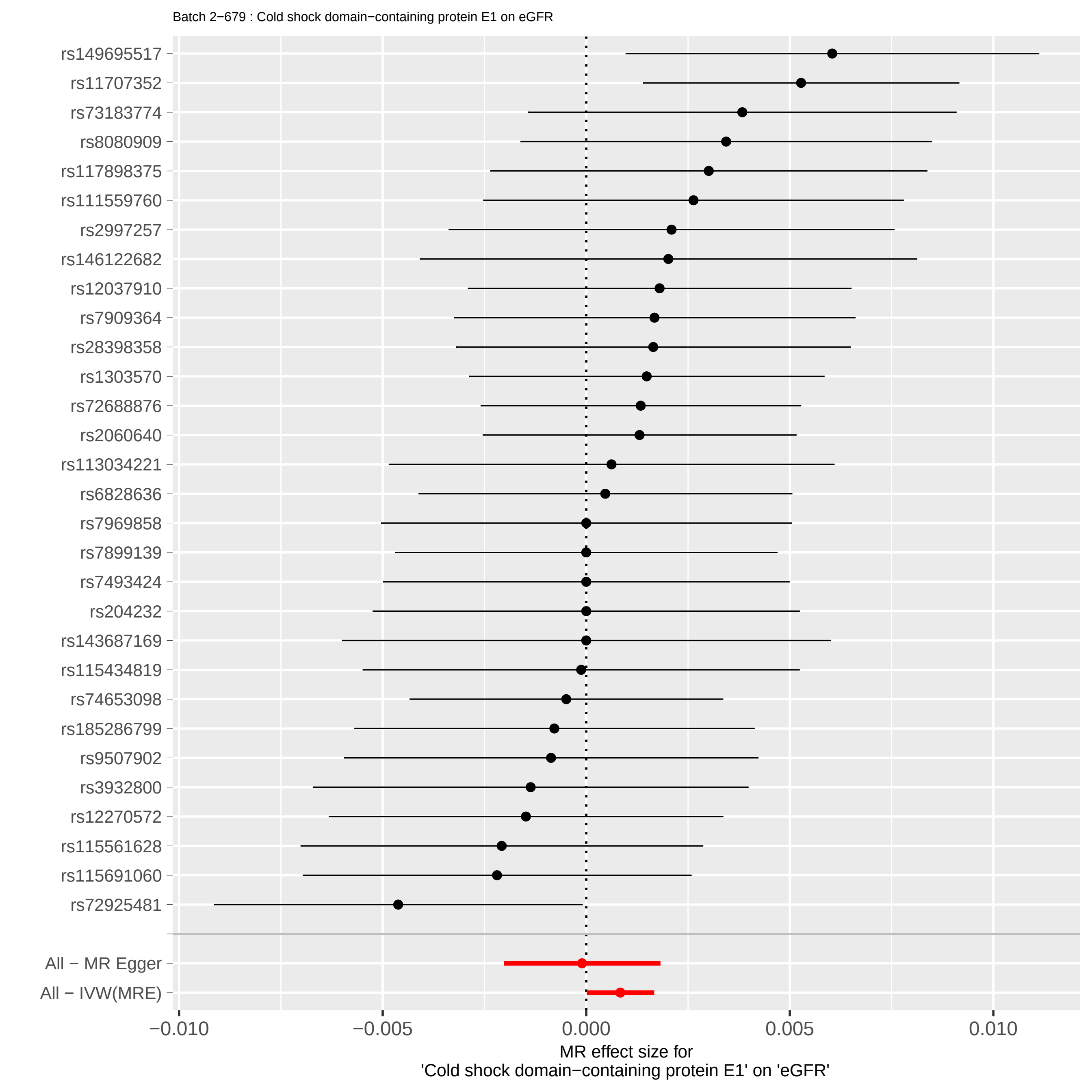
Batch 2−679 : Cold shock domain−containing protein E1 on eGFR
rs149695517
rs11707352
rs73183774
rs8080909
rs117898375
rs111559760
rs2997257
rs146122682
rs12037910
rs7909364
rs28398358
rs1303570
rs72688876
rs2060640
rs113034221
rs6828636
rs7969858
rs7899139
rs7493424
rs204232
rs143687169
rs115434819
rs74653098
rs185286799
rs9507902
rs3932800
rs12270572
rs115561628
rs115691060
rs72925481
All − MR Egger
All − IVW(MRE)
−0.010
−0.005
0.000
0.005
0.010
MR effect size for
'Cold shock domain−containing protein E1' on 'eGFR'

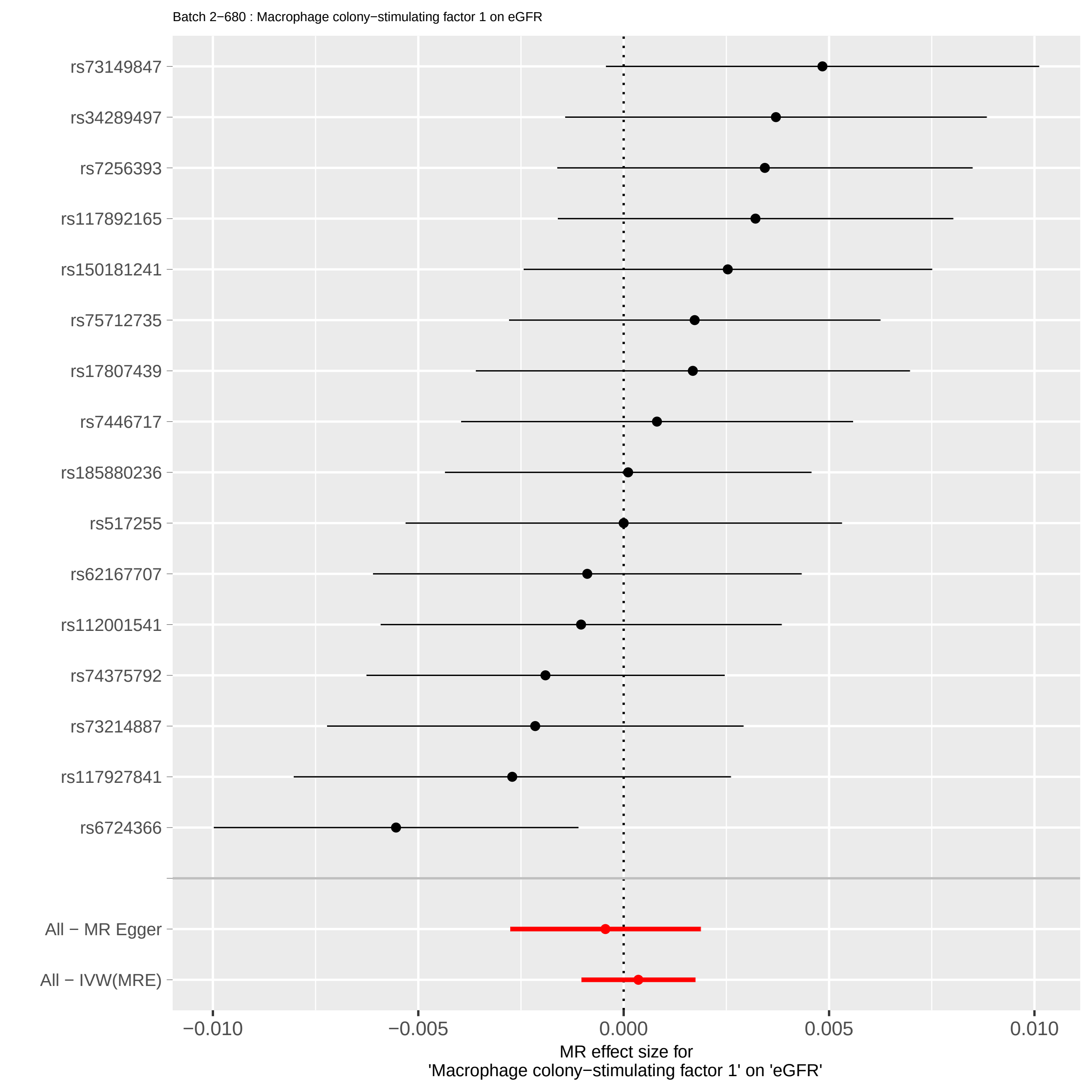

Batch 2−680 : Macrophage colony−stimulating factor 1 on eGFR
rs73149847
rs34289497
rs7256393
rs117892165
rs150181241
rs75712735
rs17807439
rs7446717
rs185880236
rs517255
rs62167707
rs112001541
rs74375792
rs73214887
rs117927841
rs6724366
All − MR Egger
All − IVW(MRE)
−0.010
−0.005
0.000
0.005
0.010
MR effect size for
'Macrophage colony−stimulating factor 1' on 'eGFR'

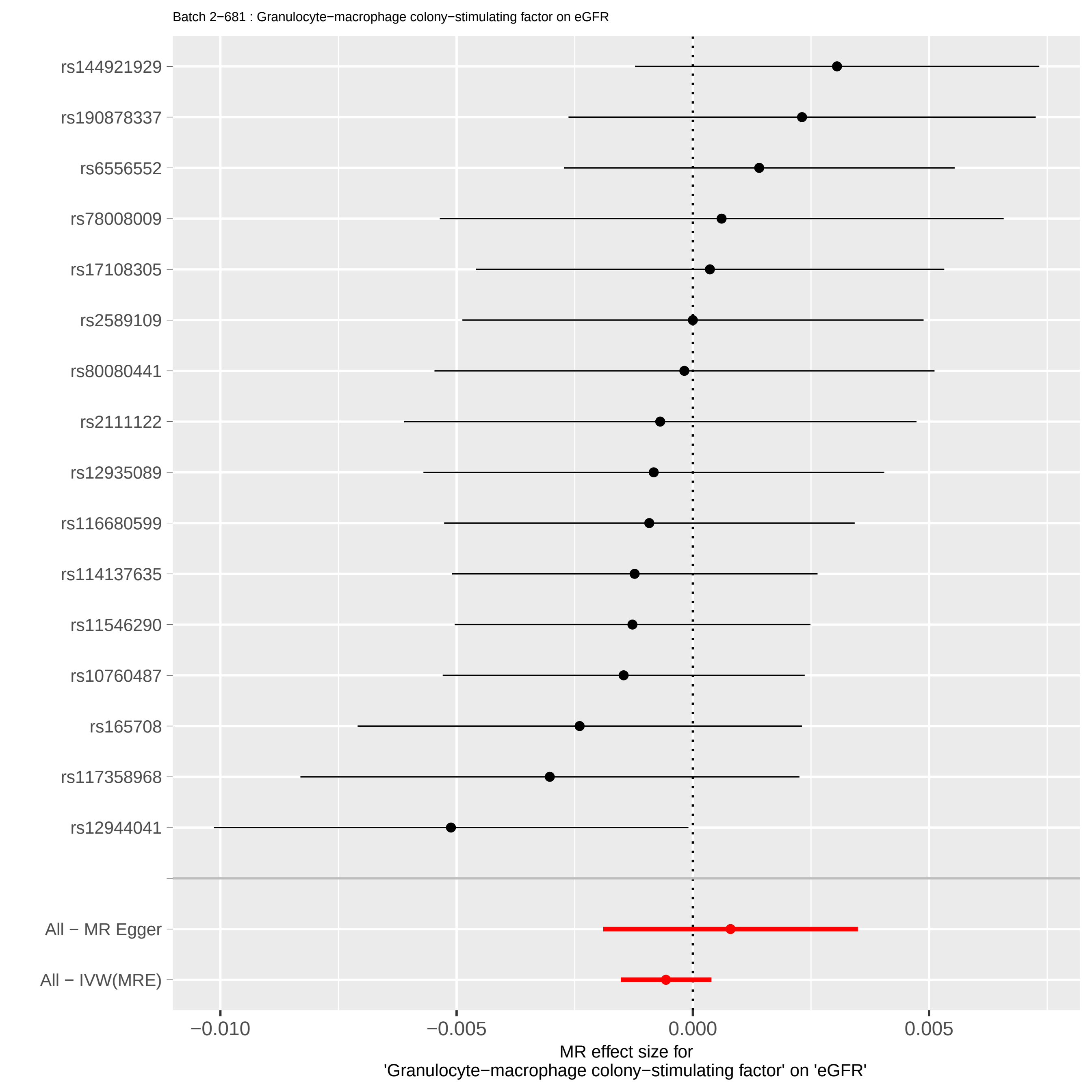

Batch 2−681 : Granulocyte−macrophage colony−stimulating factor on eGFR
rs144921929
rs190878337
rs6556552
rs78008009
rs17108305
rs2589109
rs80080441
rs2111122
rs12935089
rs116680599
rs114137635
rs11546290
rs10760487
rs165708
rs117358968
rs12944041
All − MR Egger
All − IVW(MRE)
−0.010
−0.005
0.000
0.005
MR effect size for
'Granulocyte−macrophage colony−stimulating factor' on 'eGFR'

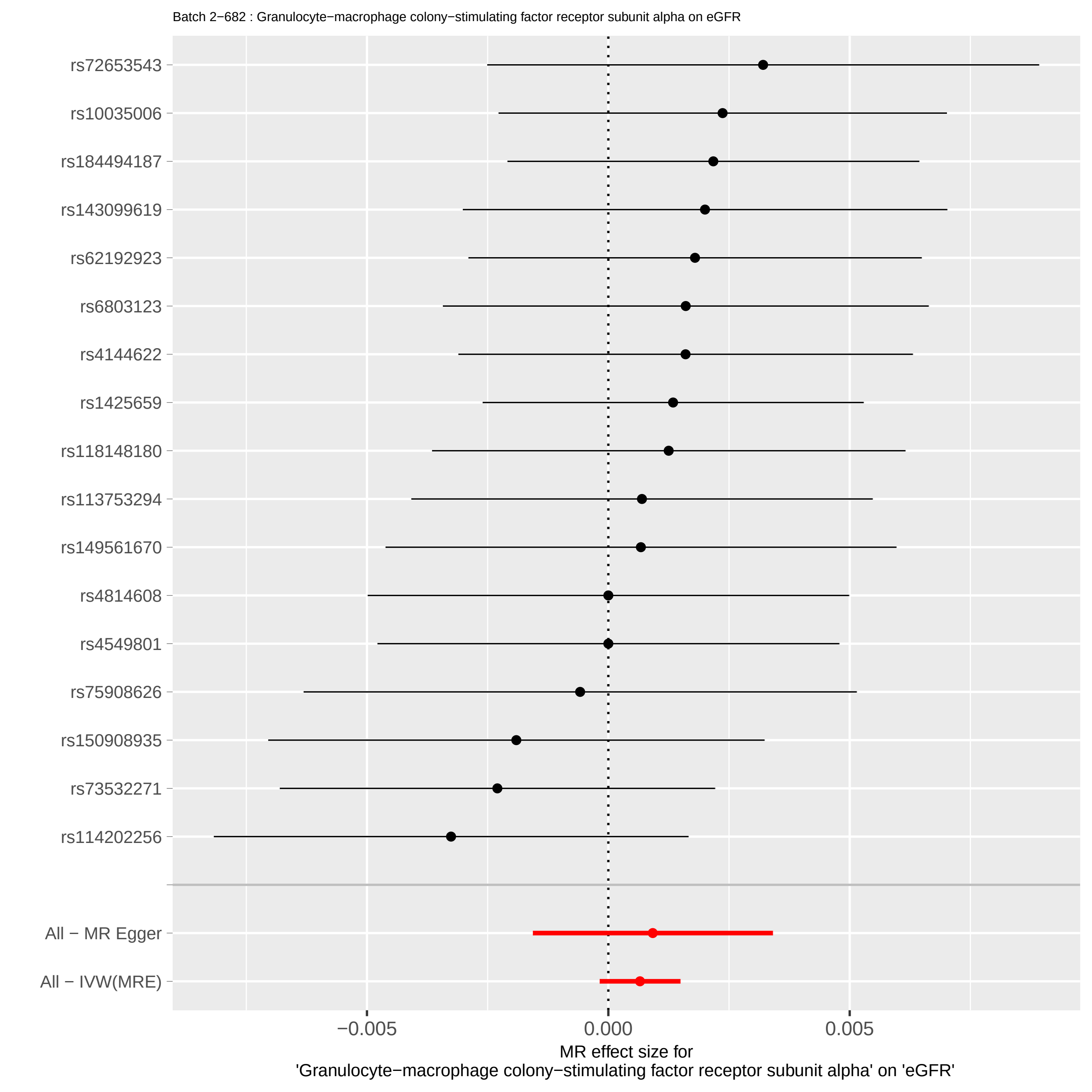

Batch 2−682 : Granulocyte−macrophage colony−stimulating factor receptor subunit alpha on eGFR
rs72653543
rs10035006
rs184494187
rs143099619
rs62192923
rs6803123
rs4144622
rs1425659
rs118148180
rs113753294
rs149561670
rs4814608
rs4549801
rs75908626
rs150908935
rs73532271
rs114202256
All − MR Egger
All − IVW(MRE)
−0.005
0.000
0.005
MR effect size for
'Granulocyte−macrophage colony−stimulating factor receptor subunit alpha' on 'eGFR'

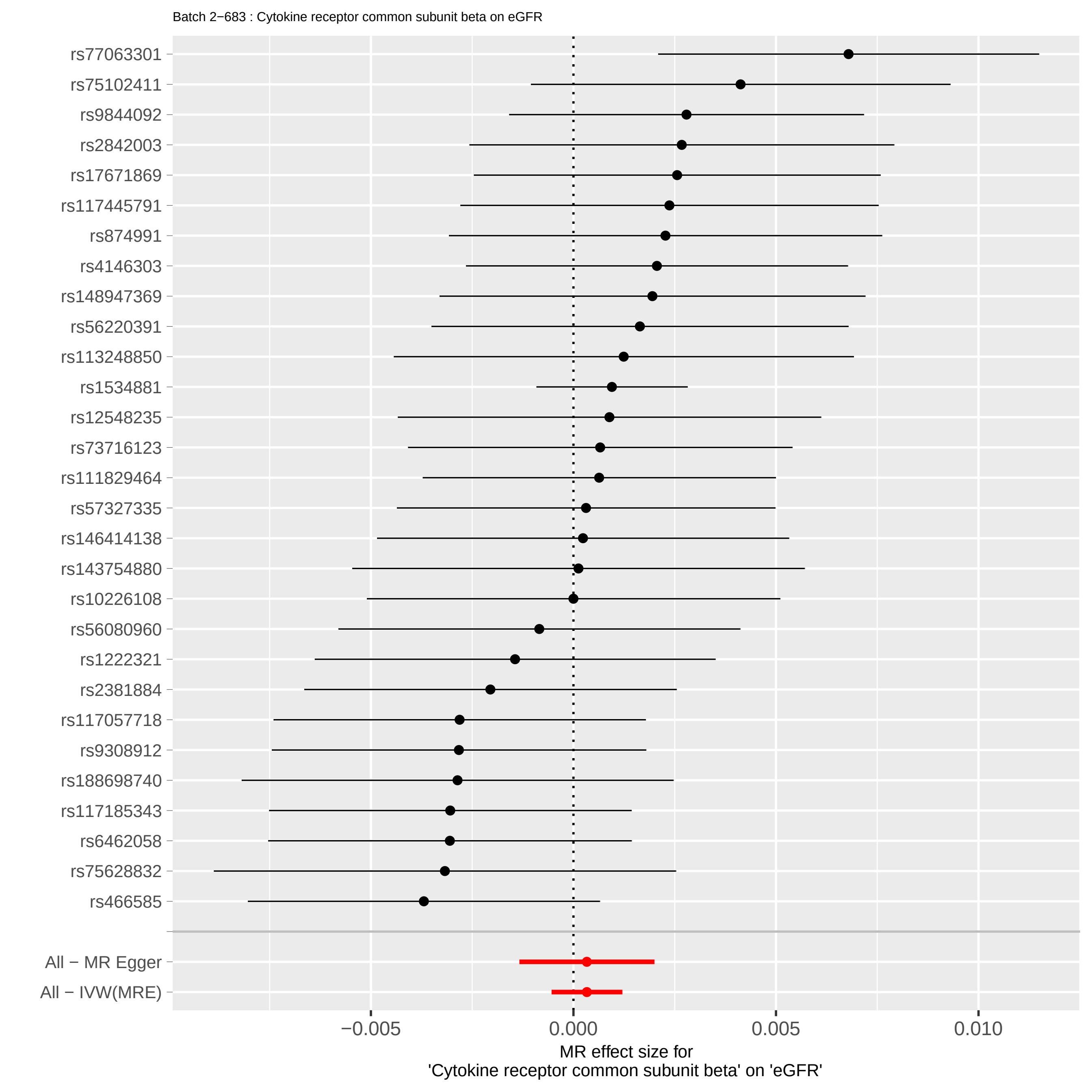
Batch 2−683 : Cytokine receptor common subunit beta on eGFR
rs77063301
rs75102411
rs9844092
rs2842003
rs17671869
rs117445791
rs874991
rs4146303
rs148947369
rs56220391
rs113248850
rs1534881
rs12548235
rs73716123
rs111829464
rs57327335
rs146414138
rs143754880
rs10226108
rs56080960
rs1222321
rs2381884
rs117057718
rs9308912
rs188698740
rs117185343
rs6462058
rs75628832
rs466585
All − MR Egger
All − IVW(MRE)
−0.005
0.000
0.005
0.010
MR effect size for
'Cytokine receptor common subunit beta' on 'eGFR'

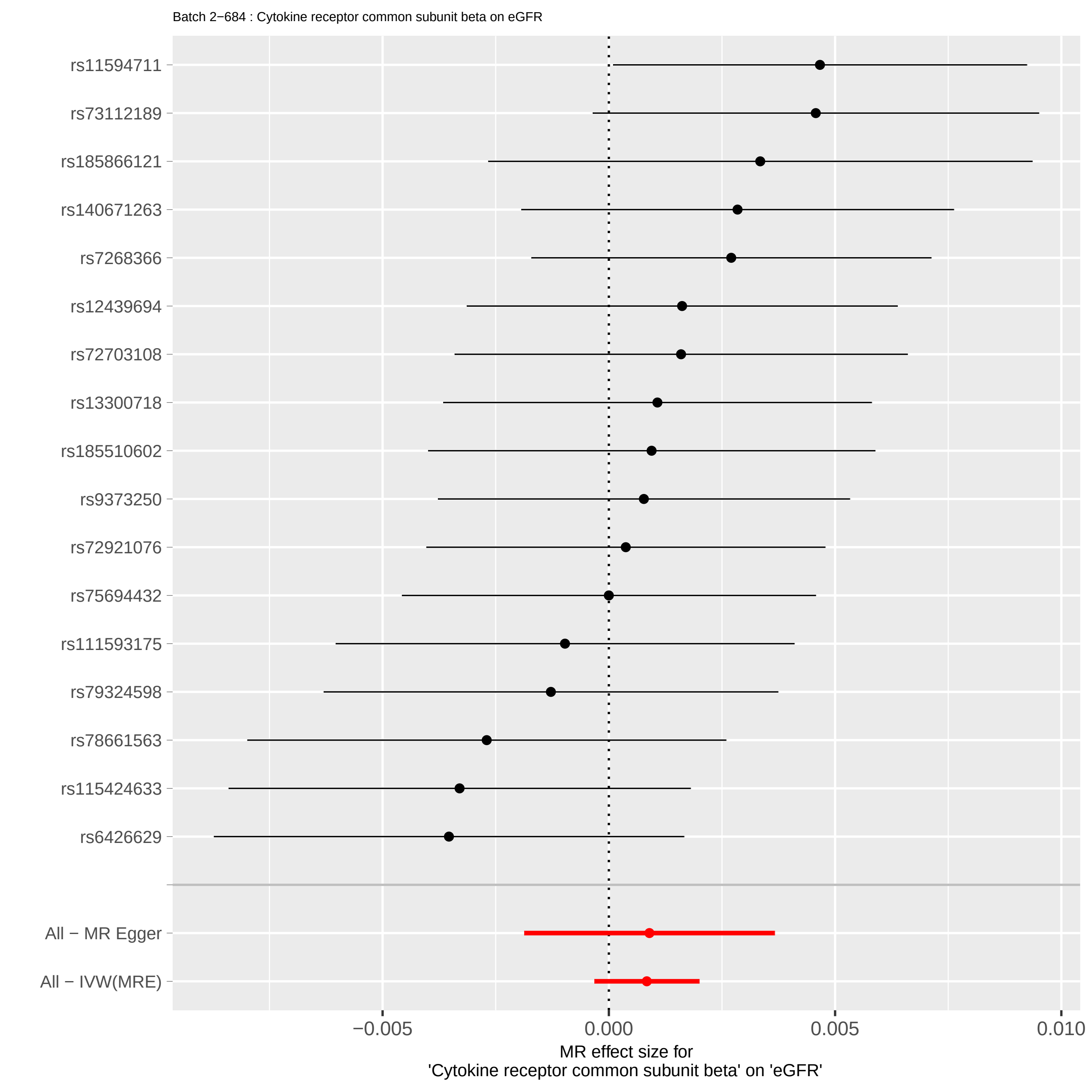
Batch 2−684 : Cytokine receptor common subunit beta on eGFR
rs11594711
rs73112189
rs185866121
rs140671263
rs7268366
rs12439694
rs72703108
rs13300718
rs185510602
rs9373250
rs72921076
rs75694432
rs111593175
rs79324598
rs78661563
rs115424633
rs6426629
All − MR Egger
All − IVW(MRE)
−0.005
0.000
0.005
0.010
MR effect size for
'Cytokine receptor common subunit beta' on 'eGFR'

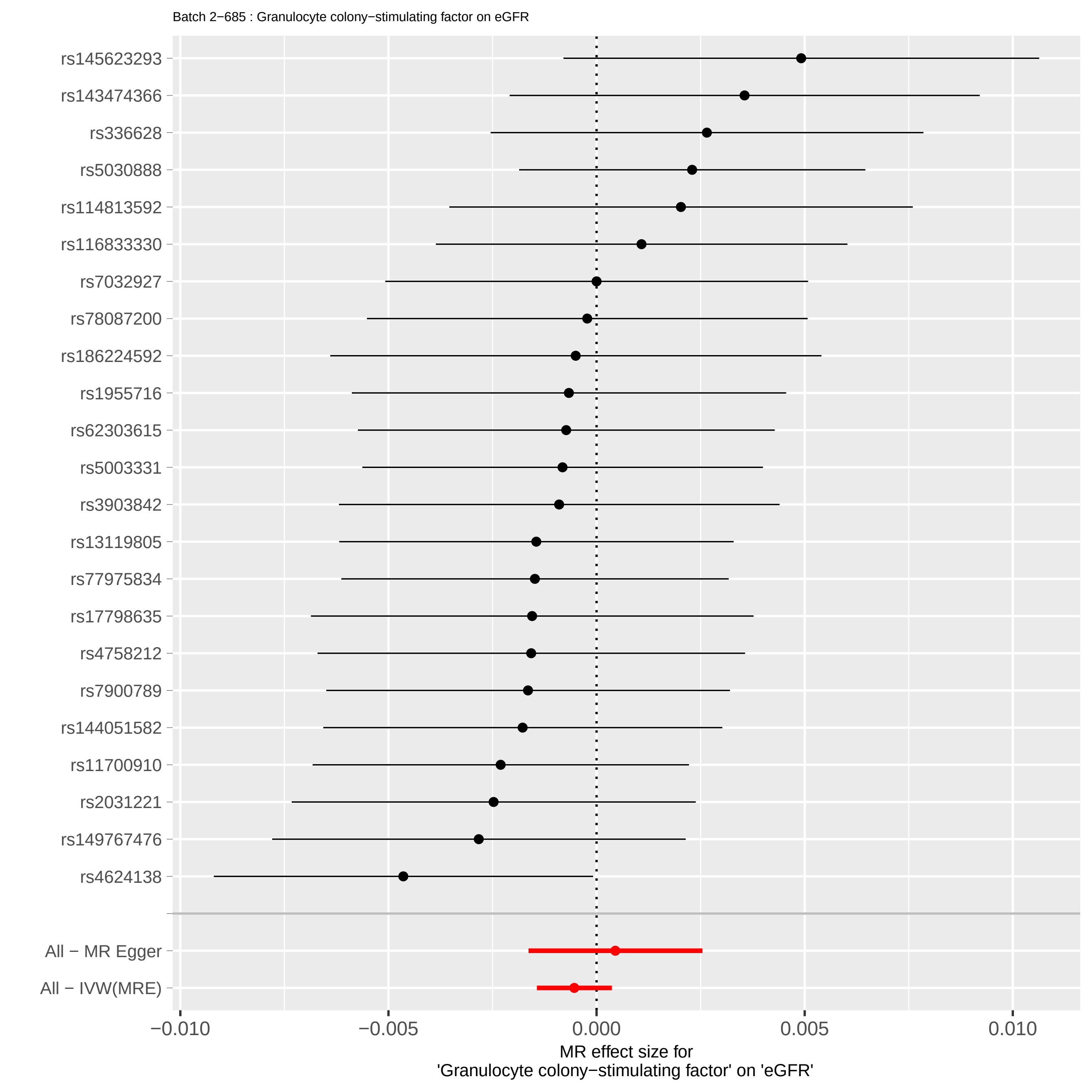

Batch 2−685 : Granulocyte colony−stimulating factor on eGFR
rs145623293
rs143474366
rs336628
rs5030888
rs114813592
rs116833330
rs7032927
rs78087200
rs186224592
rs1955716
rs62303615
rs5003331
rs3903842
rs13119805
rs77975834
rs17798635
rs4758212
rs7900789
rs144051582
rs11700910
rs2031221
rs149767476
rs4624138
All − MR Egger
All − IVW(MRE)
−0.010
−0.005
0.000
0.005
0.010
MR effect size for
'Granulocyte colony−stimulating factor' on 'eGFR'

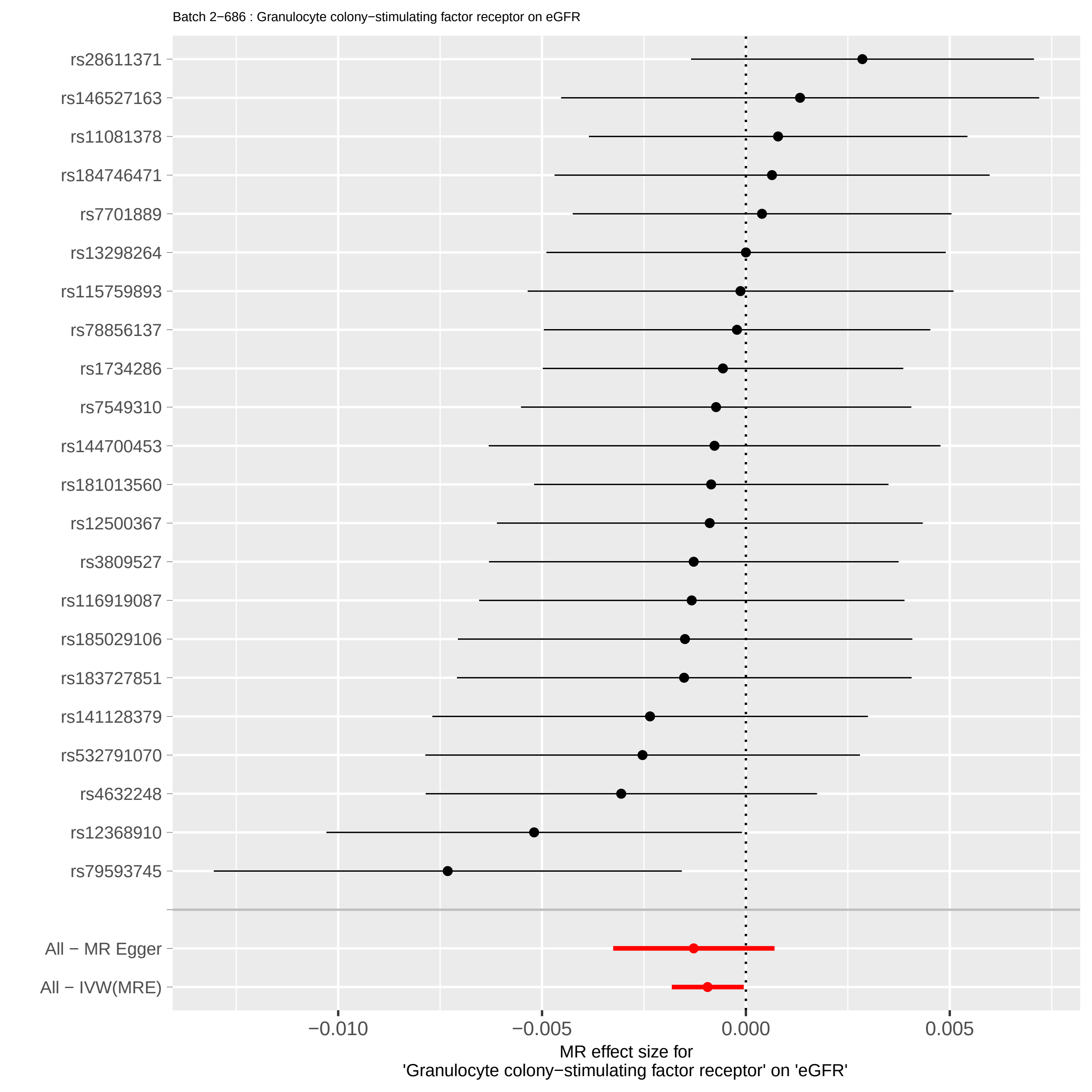

Batch 2−686 : Granulocyte colony−stimulating factor receptor on eGFR
rs28611371
rs146527163
rs11081378
rs184746471
rs7701889
rs13298264
rs115759893
rs78856137
rs1734286
rs7549310
rs144700453
rs181013560
rs12500367
rs3809527
rs116919087
rs185029106
rs183727851
rs141128379
rs532791070
rs4632248
rs12368910
rs79593745
All − MR Egger
All − IVW(MRE)
−0.010
−0.005
0.000
0.005
MR effect size for
'Granulocyte colony−stimulating factor receptor' on 'eGFR'

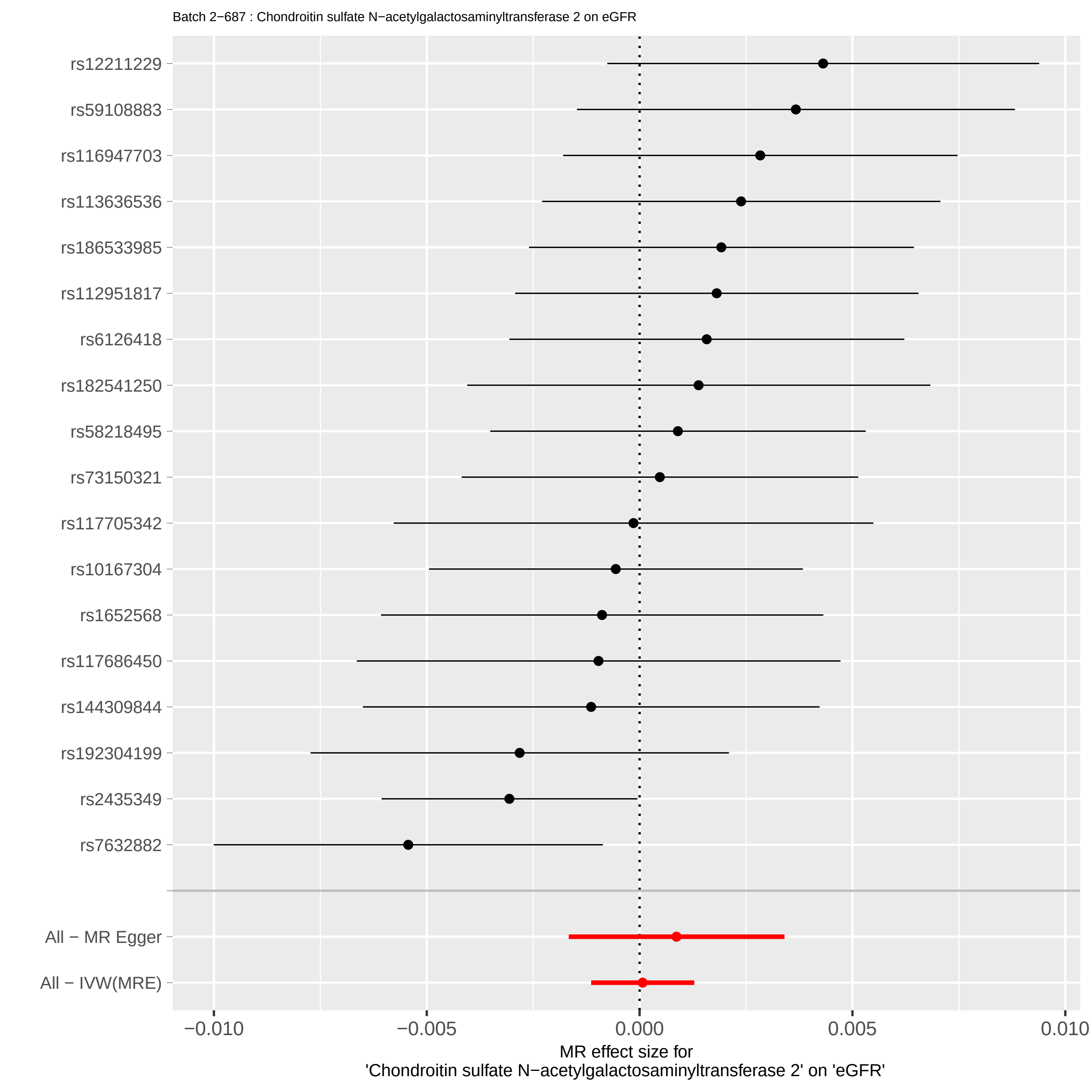

Batch 2−687 : Chondroitin sulfate N−acetylgalactosaminyltransferase 2 on eGFR
rs12211229
rs59108883
rs116947703
rs113636536
rs186533985
rs112951817
rs6126418
rs182541250
rs58218495
rs73150321
rs117705342
rs10167304
rs1652568
rs117686450
rs144309844
rs192304199
rs2435349
rs7632882
All − MR Egger
All − IVW(MRE)
−0.010
−0.005
0.000
0.005
0.010
MR effect size for
'Chondroitin sulfate N−acetylgalactosaminyltransferase 2' on 'eGFR'

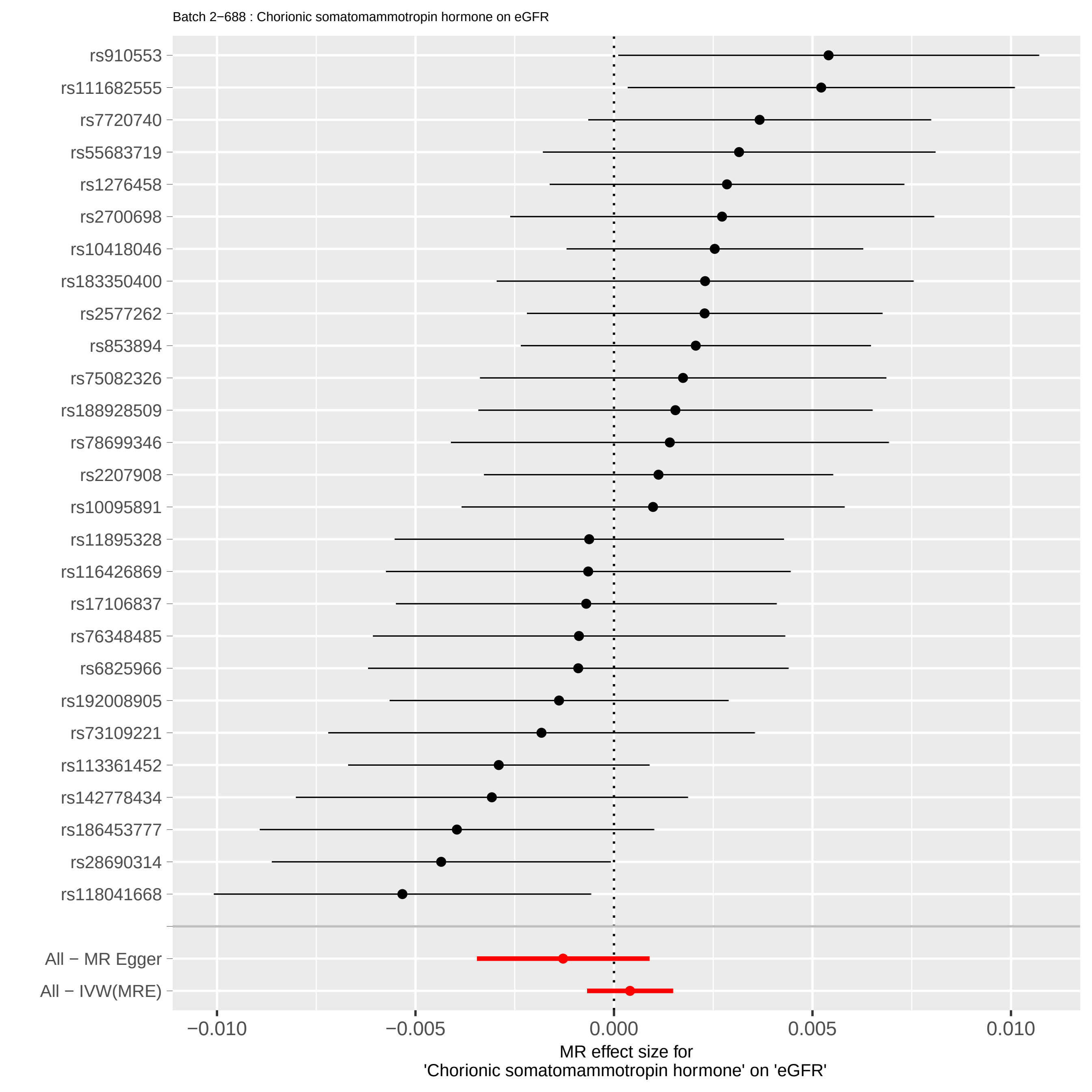
Batch 2−688 : Chorionic somatomammotropin hormone on eGFR
rs910553
rs111682555
rs7720740
rs55683719
rs1276458
rs2700698
rs10418046
rs183350400
rs2577262
rs853894
rs75082326
rs188928509
rs78699346
rs2207908
rs10095891
rs11895328
rs116426869
rs17106837
rs76348485
rs6825966
rs192008905
rs73109221
rs113361452
rs142778434
rs186453777
rs28690314
rs118041668
All − MR Egger
All − IVW(MRE)
−0.010
−0.005
0.000
0.005
0.010
MR effect size for
'Chorionic somatomammotropin hormone' on 'eGFR'

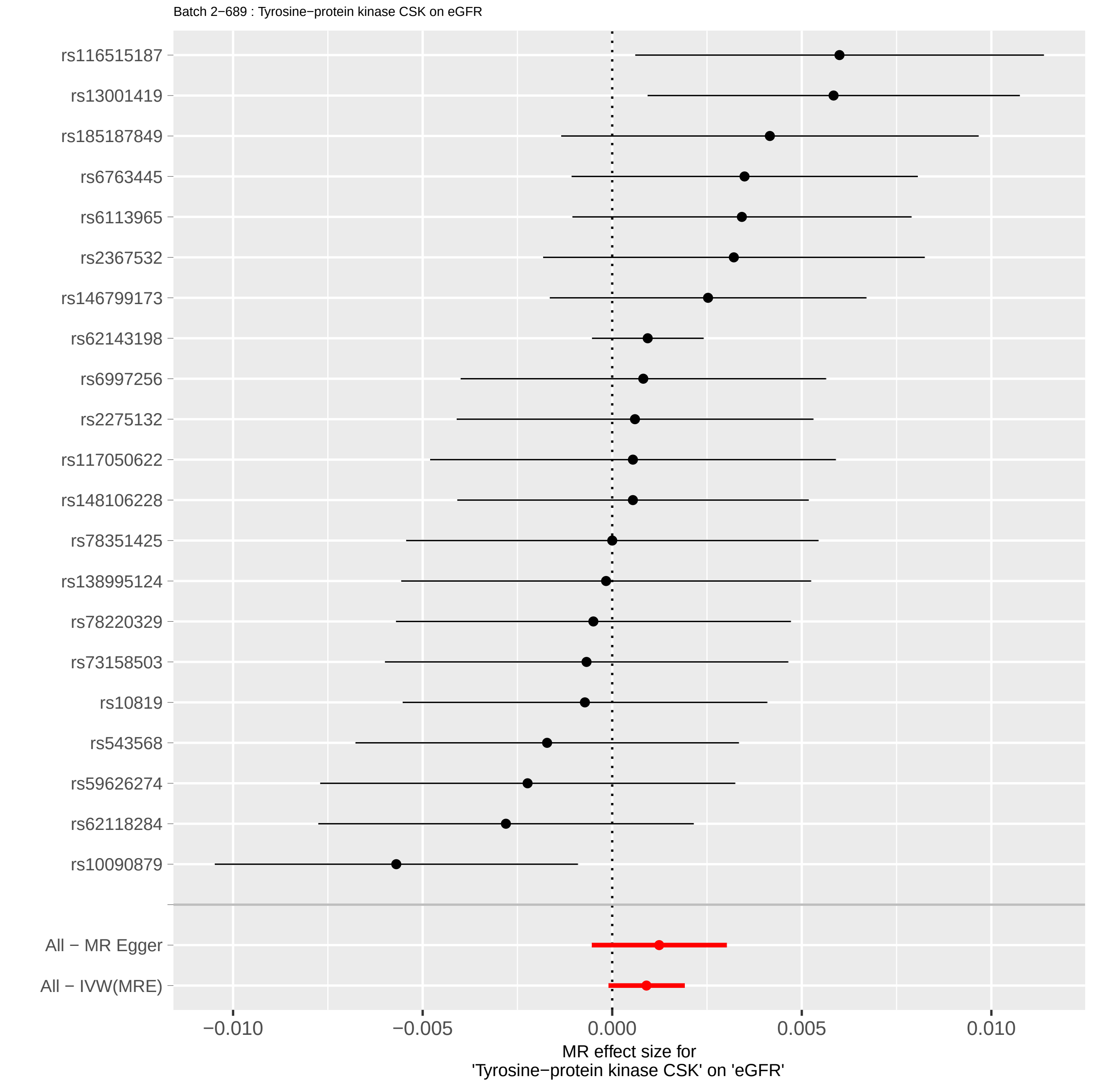

Batch 2−689 : Tyrosine−protein kinase CSK on eGFR
rs116515187
rs13001419
rs185187849
rs6763445
rs6113965
rs2367532
rs146799173
rs62143198
rs6997256
rs2275132
rs117050622
rs148106228
rs78351425
rs138995124
rs78220329
rs73158503
rs10819
rs543568
rs59626274
rs62118284
rs10090879
All − MR Egger
All − IVW(MRE)
−0.010
−0.005
0.000
0.005
0.010
MR effect size for
'Tyrosine−protein kinase CSK' on 'eGFR'

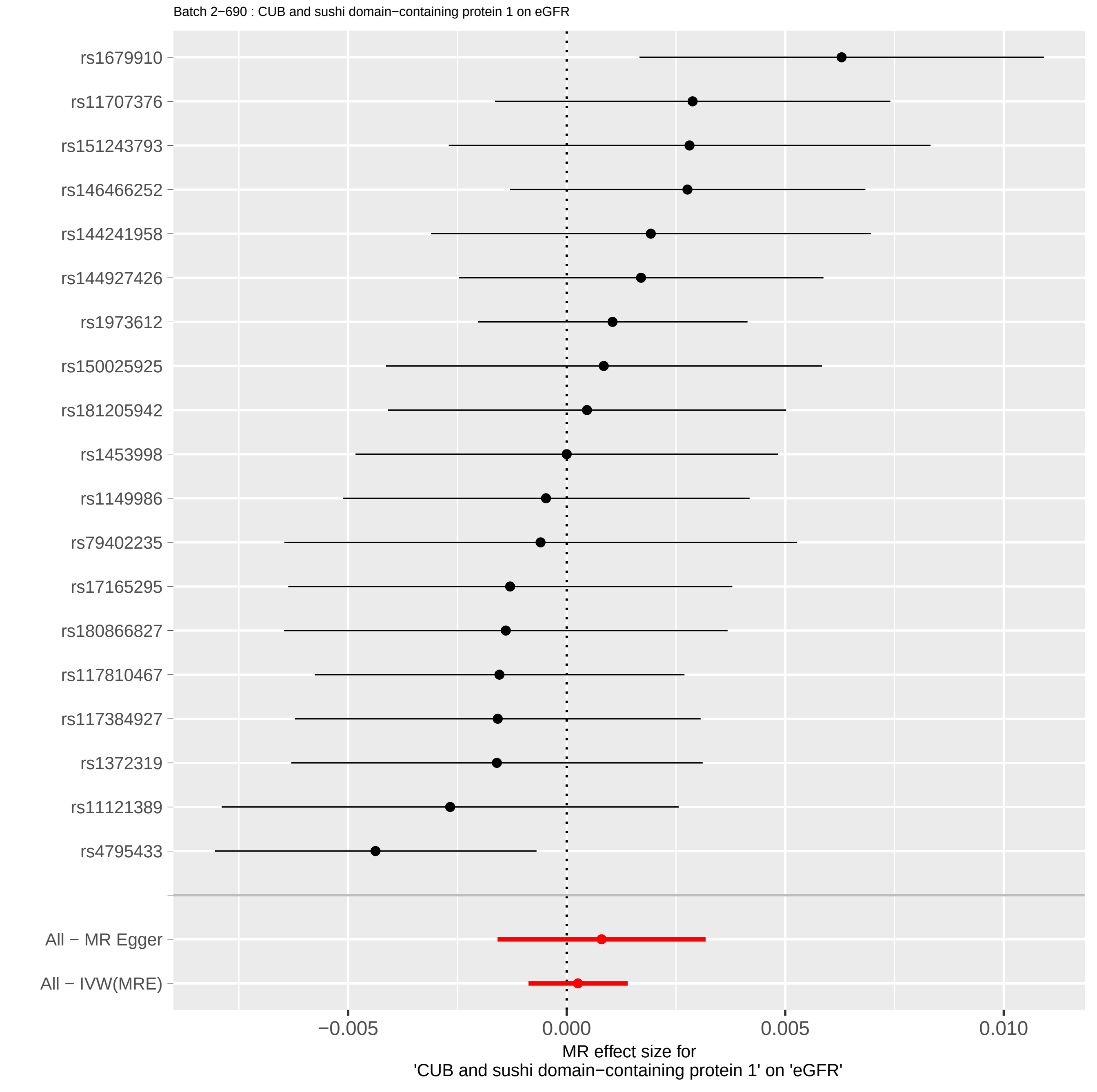

Batch 2−690 : CUB and sushi domain−containing protein 1 on eGFR
rs1679910
rs11707376
rs151243793
rs146466252
rs144241958
rs144927426
rs1973612
rs150025925
rs181205942
rs1453998
rs1149986
rs79402235
rs17165295
rs180866827
rs117810467
rs117384927
rs1372319
rs11121389
rs4795433
All − MR Egger
All − IVW(MRE)
−0.005
0.000
0.005
0.010
MR effect size for
'CUB and sushi domain−containing protein 1' on 'eGFR'

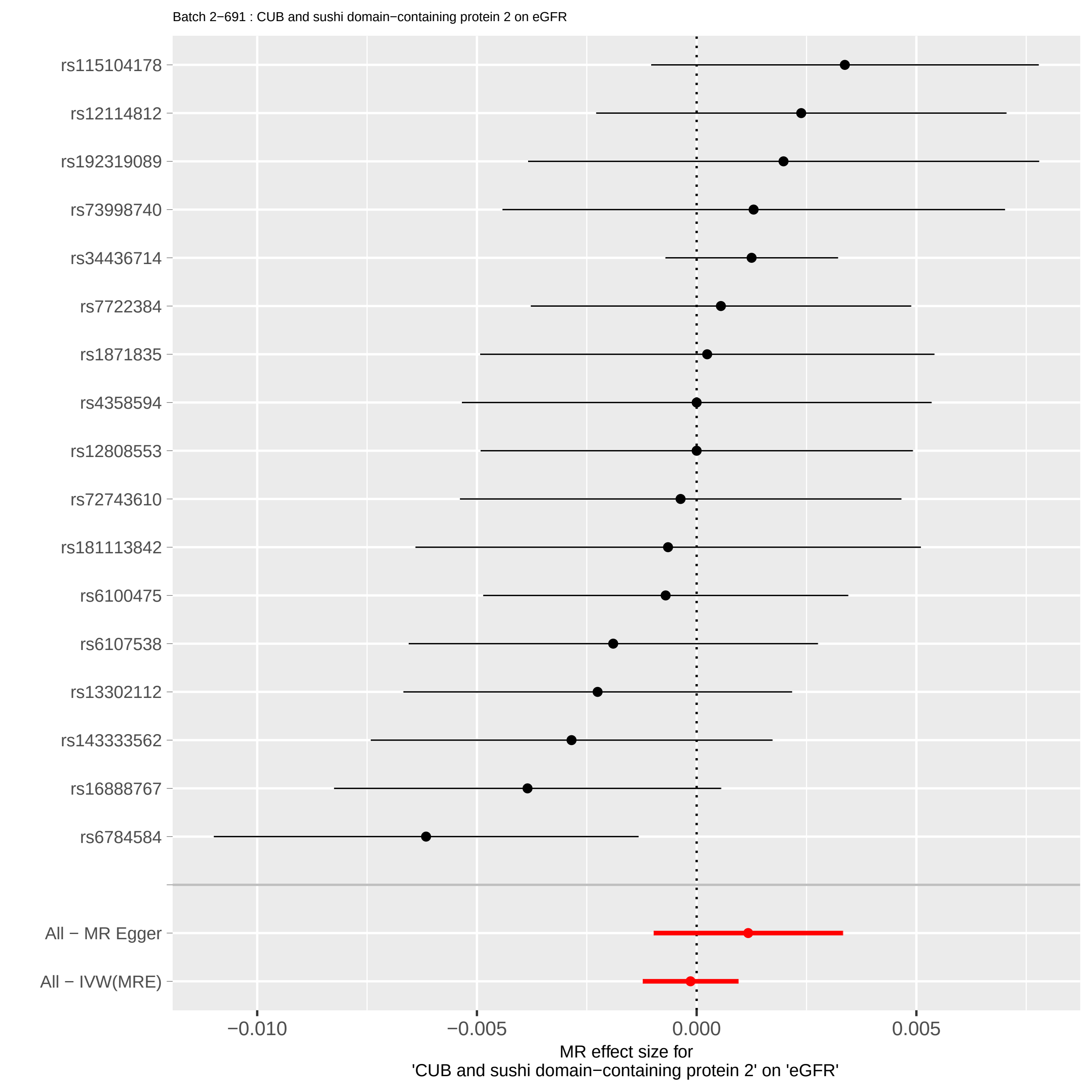

Batch 2−691 : CUB and sushi domain−containing protein 2 on eGFR
rs115104178
rs12114812
rs192319089
rs73998740
rs34436714
rs7722384
rs1871835
rs4358594
rs12808553
rs72743610
rs181113842
rs6100475
rs6107538
rs13302112
rs143333562
rs16888767
rs6784584
All − MR Egger
All − IVW(MRE)
−0.010
−0.005
0.000
0.005
MR effect size for
'CUB and sushi domain−containing protein 2' on 'eGFR'

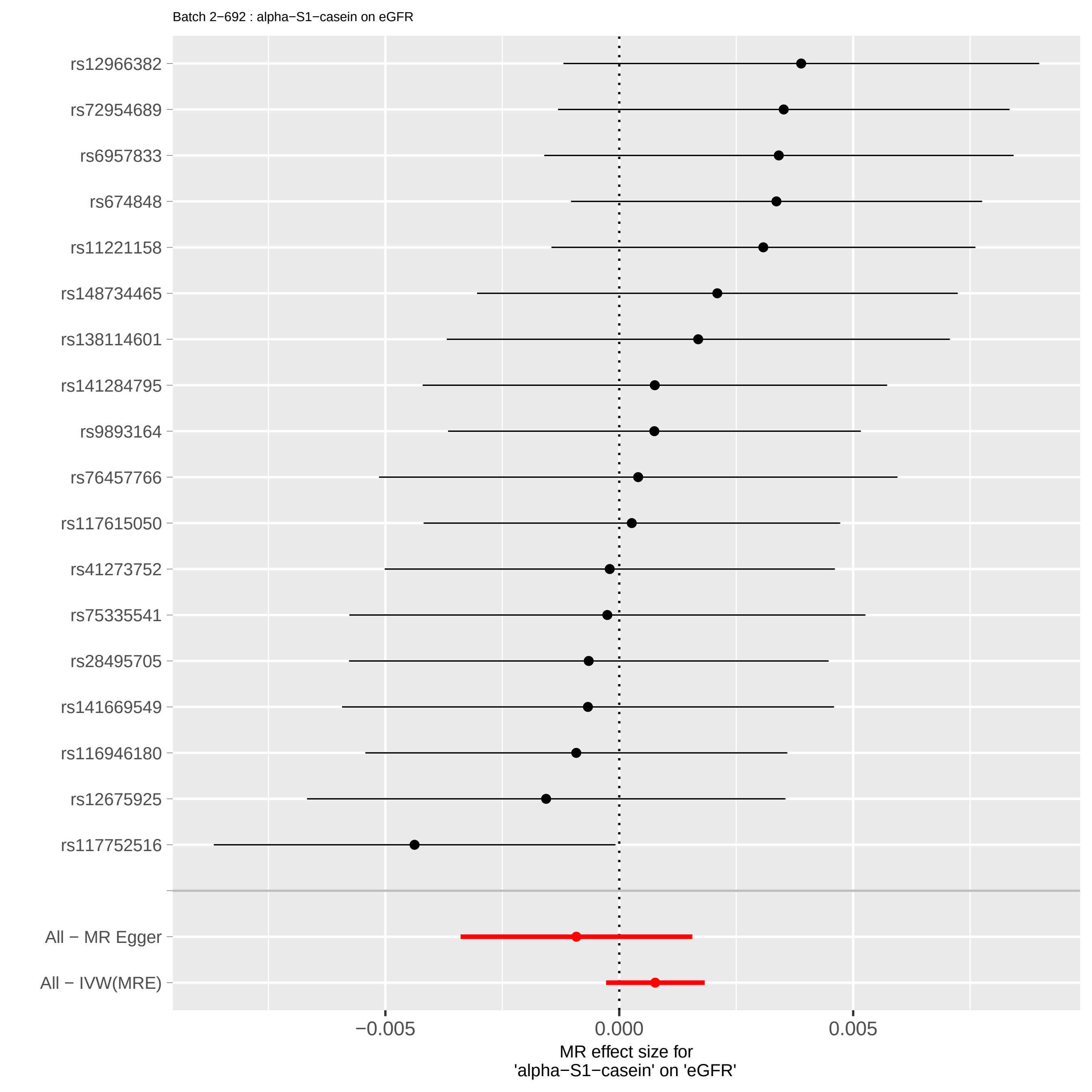

Batch 2−692 : alpha−S1−casein on eGFR
rs12966382
rs72954689
rs6957833
rs674848
rs11221158
rs148734465
rs138114601
rs141284795
rs9893164
rs76457766
rs117615050
rs41273752
rs75335541
rs28495705
rs141669549
rs116946180
rs12675925
rs117752516
All − MR Egger
All − IVW(MRE)
−0.005
0.000
0.005
MR effect size for
'alpha−S1−casein' on 'eGFR'

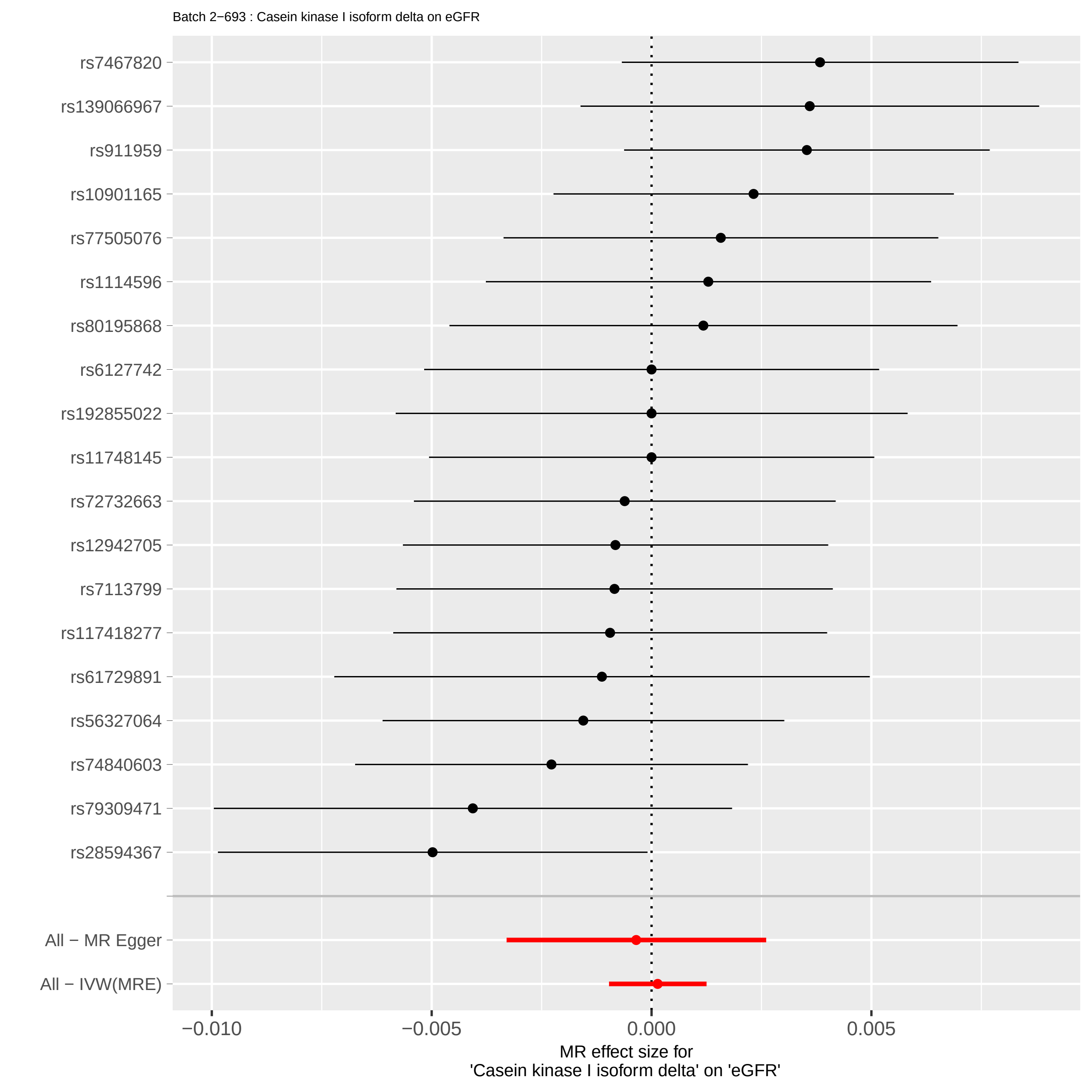

Batch 2−693 : Casein kinase I isoform delta on eGFR
rs7467820
rs139066967
rs911959
rs10901165
rs77505076
rs1114596
rs80195868
rs6127742
rs192855022
rs11748145
rs72732663
rs12942705
rs7113799
rs117418277
rs61729891
rs56327064
rs74840603
rs79309471
rs28594367
All − MR Egger
All − IVW(MRE)
−0.010
−0.005
0.000
0.005
MR effect size for
'Casein kinase I isoform delta' on 'eGFR'

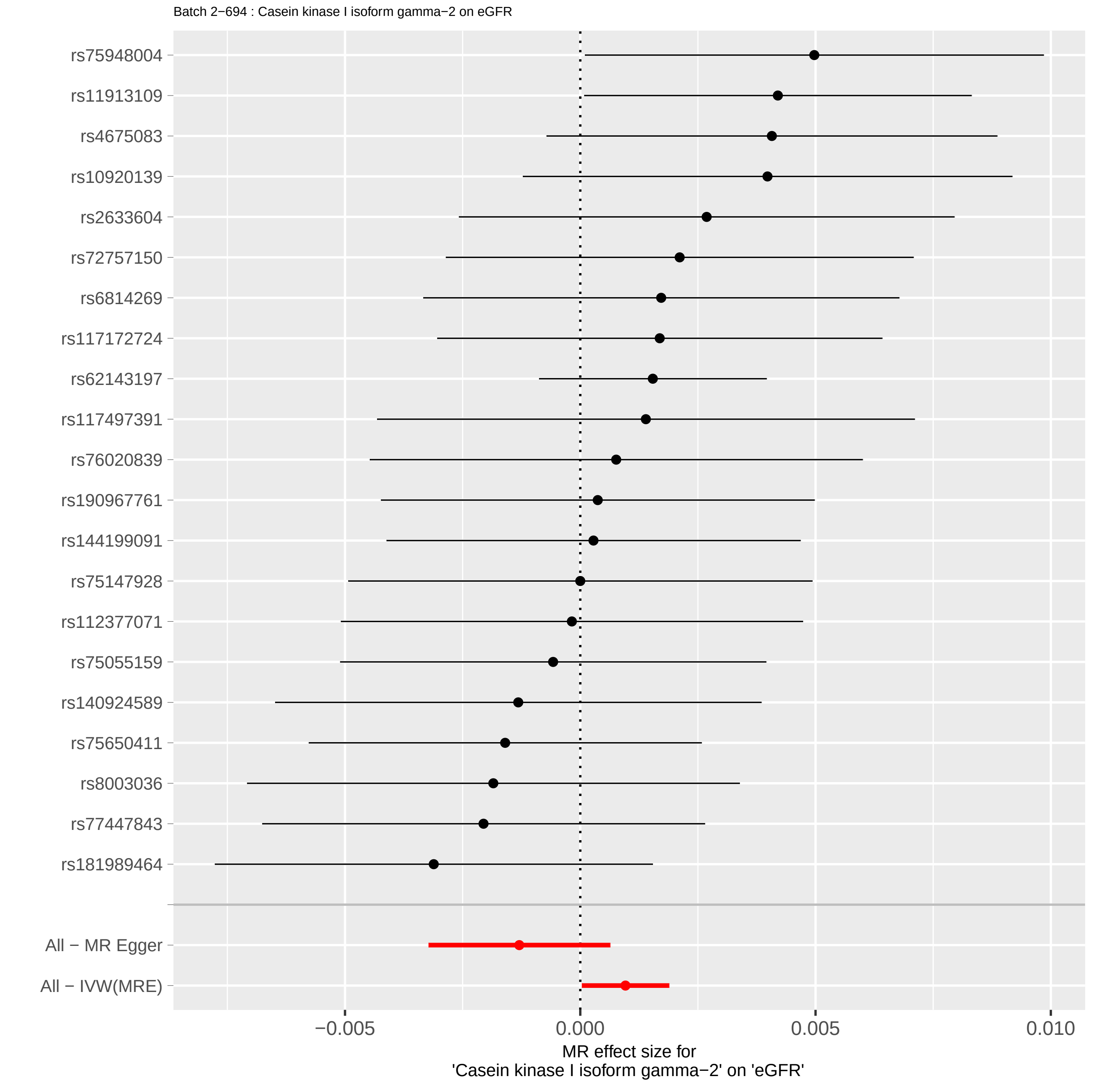

Batch 2−694 : Casein kinase I isoform gamma−2 on eGFR
rs75948004
rs11913109
rs4675083
rs10920139
rs2633604
rs72757150
rs6814269
rs117172724
rs62143197
rs117497391
rs76020839
rs190967761
rs144199091
rs75147928
rs112377071
rs75055159
rs140924589
rs75650411
rs8003036
rs77447843
rs181989464
All − MR Egger
All − IVW(MRE)
−0.005
0.000
0.005
0.010
MR effect size for
'Casein kinase I isoform gamma−2' on 'eGFR'

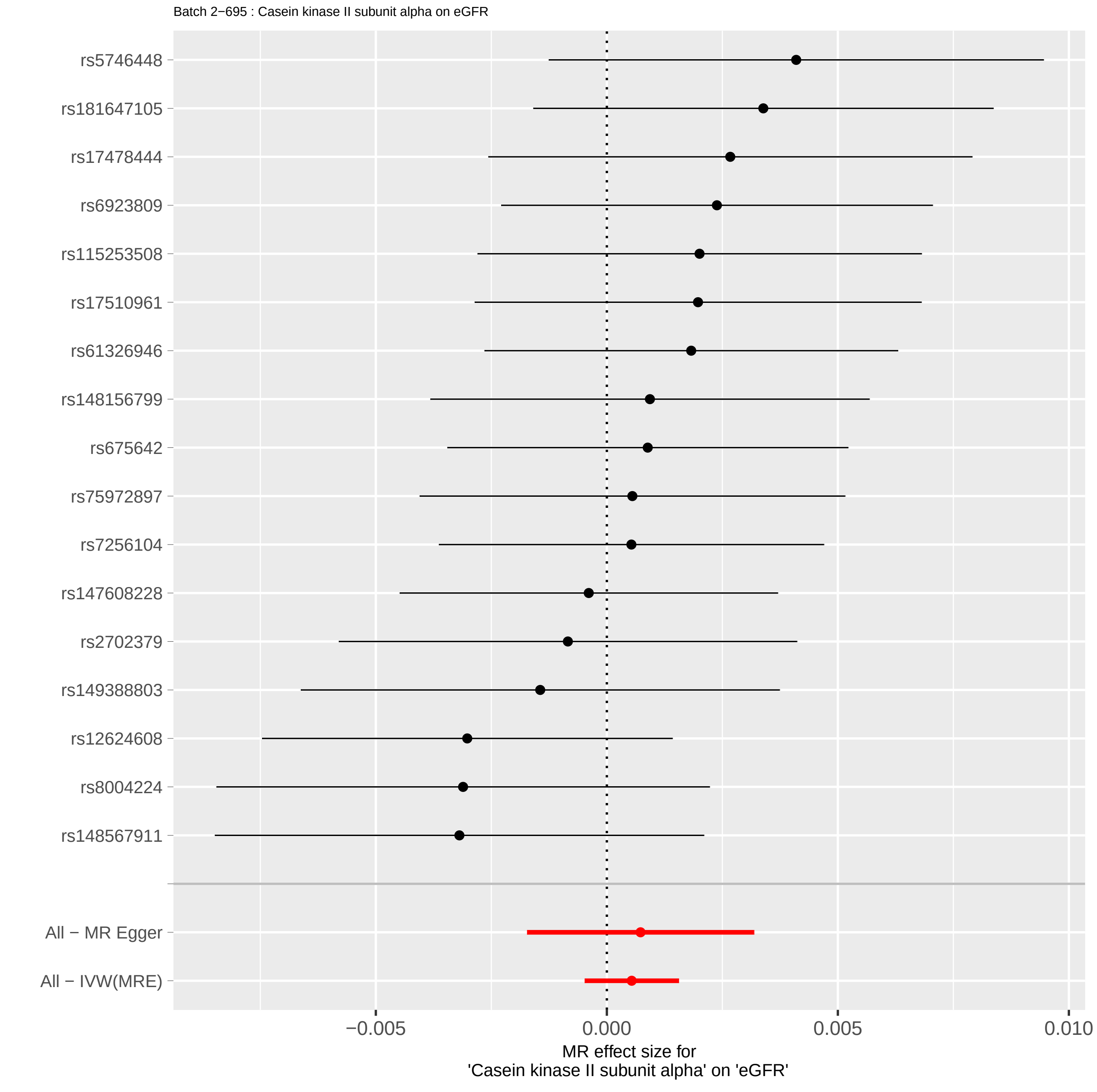

Batch 2−695 : Casein kinase II subunit alpha on eGFR
rs5746448
rs181647105
rs17478444
rs6923809
rs115253508
rs17510961
rs61326946
rs148156799
rs675642
rs75972897
rs7256104
rs147608228
rs2702379
rs149388803
rs12624608
rs8004224
rs148567911
All − MR Egger
All − IVW(MRE)
−0.005
0.000
0.005
0.010
MR effect size for
'Casein kinase II subunit alpha' on 'eGFR'

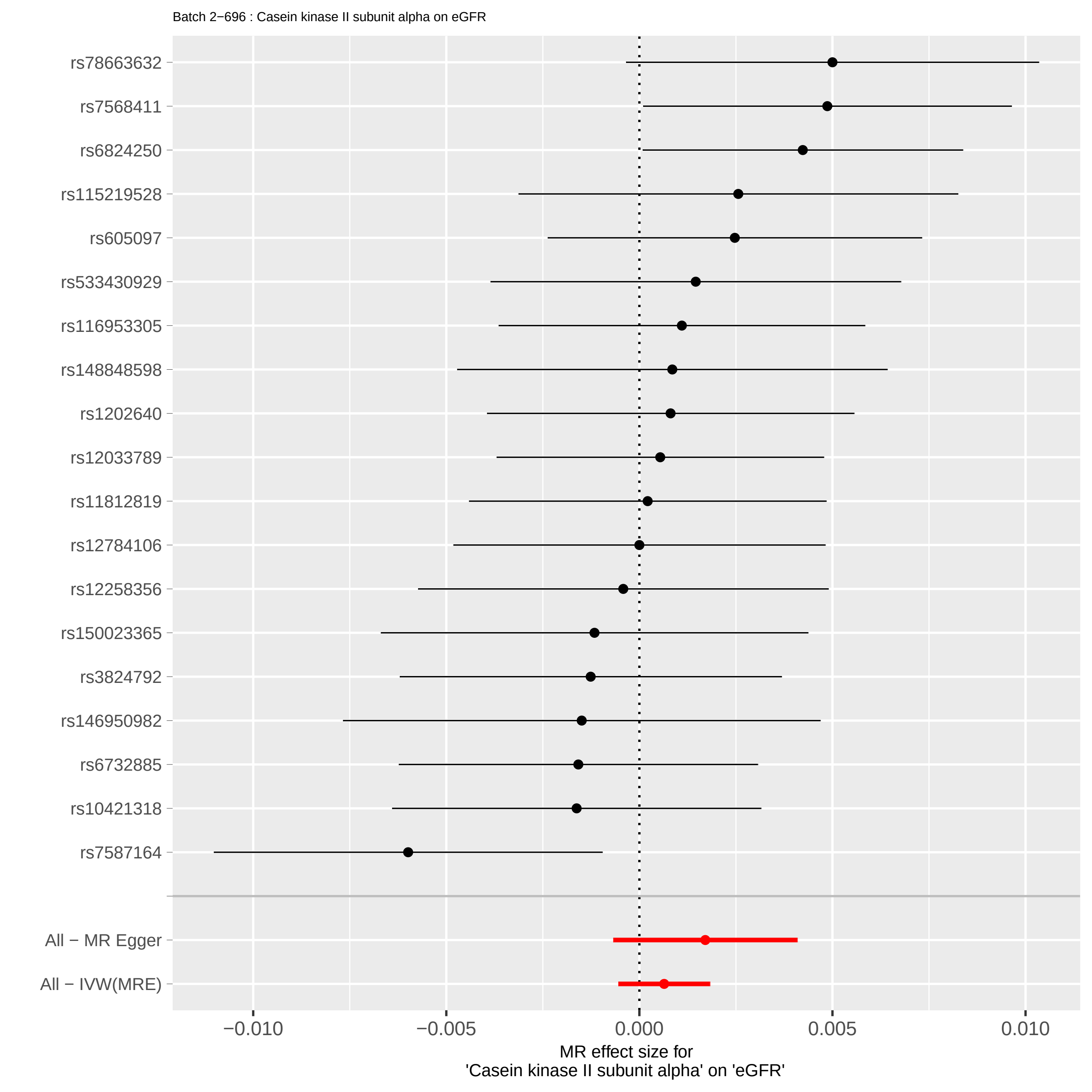

Batch 2−696 : Casein kinase II subunit alpha on eGFR
rs78663632
rs7568411
rs6824250
rs115219528
rs605097
rs533430929
rs116953305
rs148848598
rs1202640
rs12033789
rs11812819
rs12784106
rs12258356
rs150023365
rs3824792
rs146950982
rs6732885
rs10421318
rs7587164
All − MR Egger
All − IVW(MRE)
−0.010
−0.005
0.000
0.005
0.010
MR effect size for
'Casein kinase II subunit alpha' on 'eGFR'

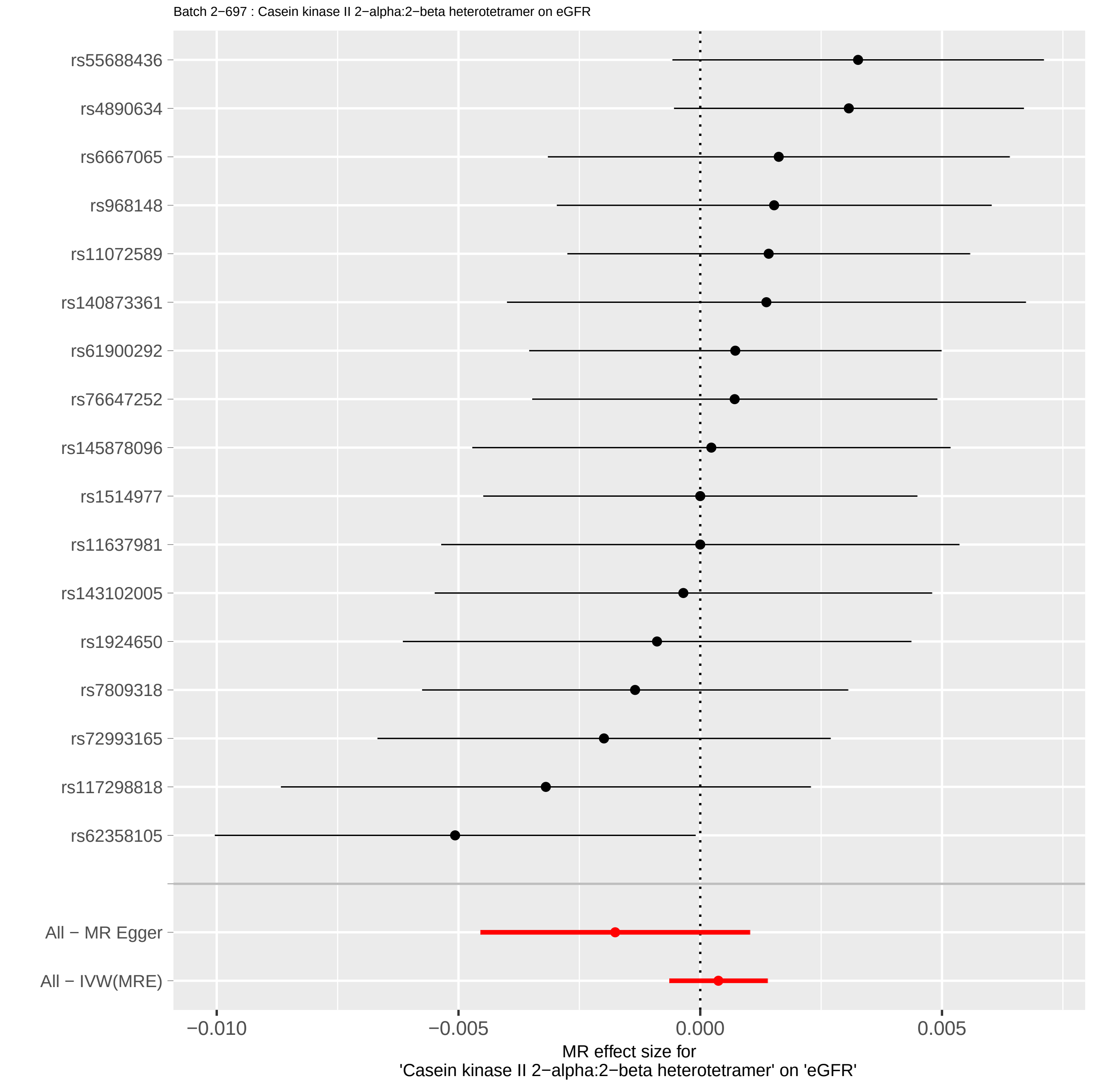

Batch 2−697 : Casein kinase II 2−alpha:2−beta heterotetramer on eGFR
rs55688436
rs4890634
rs6667065
rs968148
rs11072589
rs140873361
rs61900292
rs76647252
rs145878096
rs1514977
rs11637981
rs143102005
rs1924650
rs7809318
rs72993165
rs117298818
rs62358105
All − MR Egger
All − IVW(MRE)
−0.010
−0.005
0.000
0.005
MR effect size for
'Casein kinase II 2−alpha:2−beta heterotetramer' on 'eGFR'

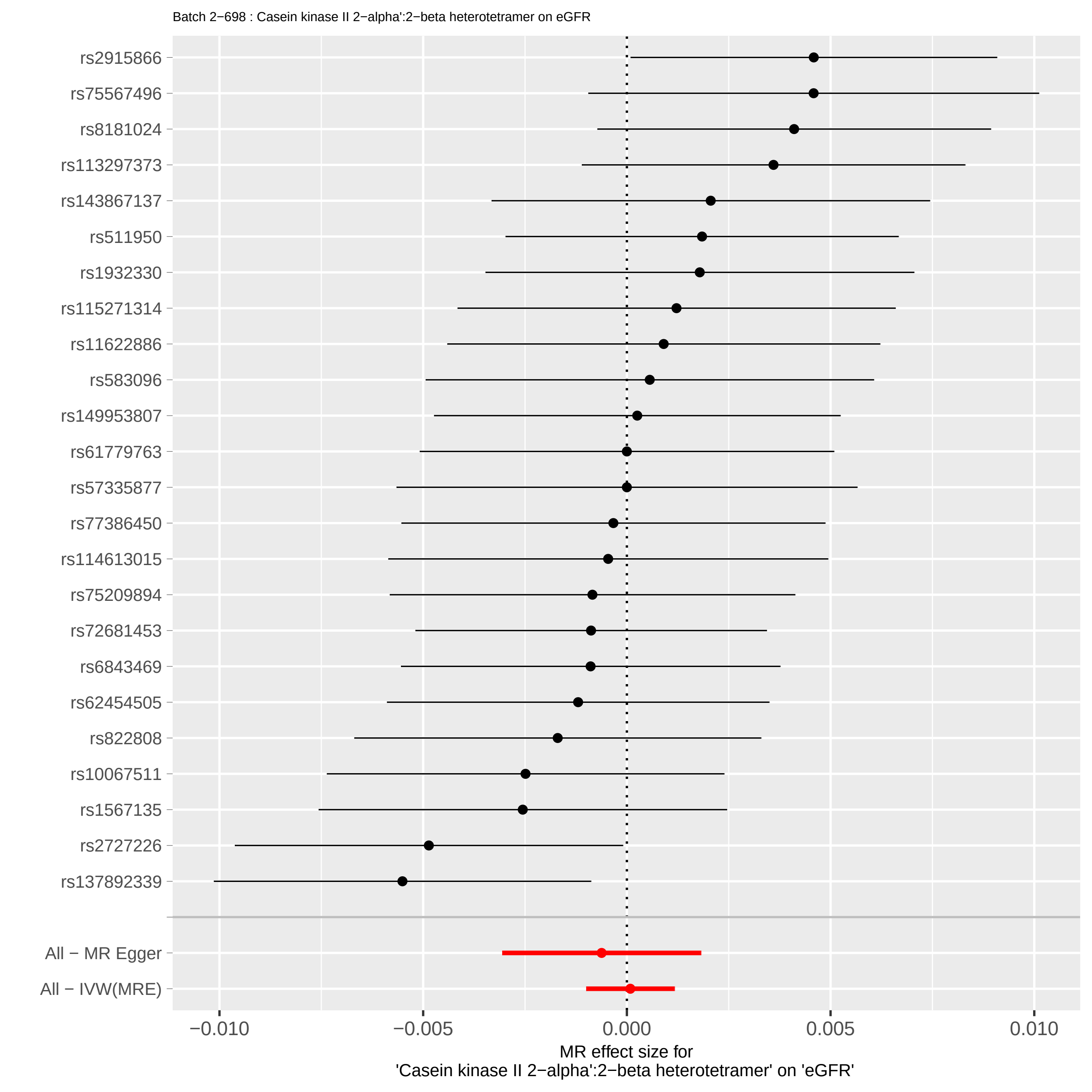

Batch 2−698 : Casein kinase II 2−alpha':2−beta heterotetramer on eGFR
rs2915866
rs75567496
rs8181024
rs113297373
rs143867137
rs511950
rs1932330
rs115271314
rs11622886
rs583096
rs149953807
rs61779763
rs57335877
rs77386450
rs114613015
rs75209894
rs72681453
rs6843469
rs62454505
rs822808
rs10067511
rs1567135
rs2727226
rs137892339
All − MR Egger
All − IVW(MRE)
−0.010
−0.005
0.000
0.005
0.010
MR effect size for
'Casein kinase II 2−alpha':2−beta heterotetramer' on 'eGFR'

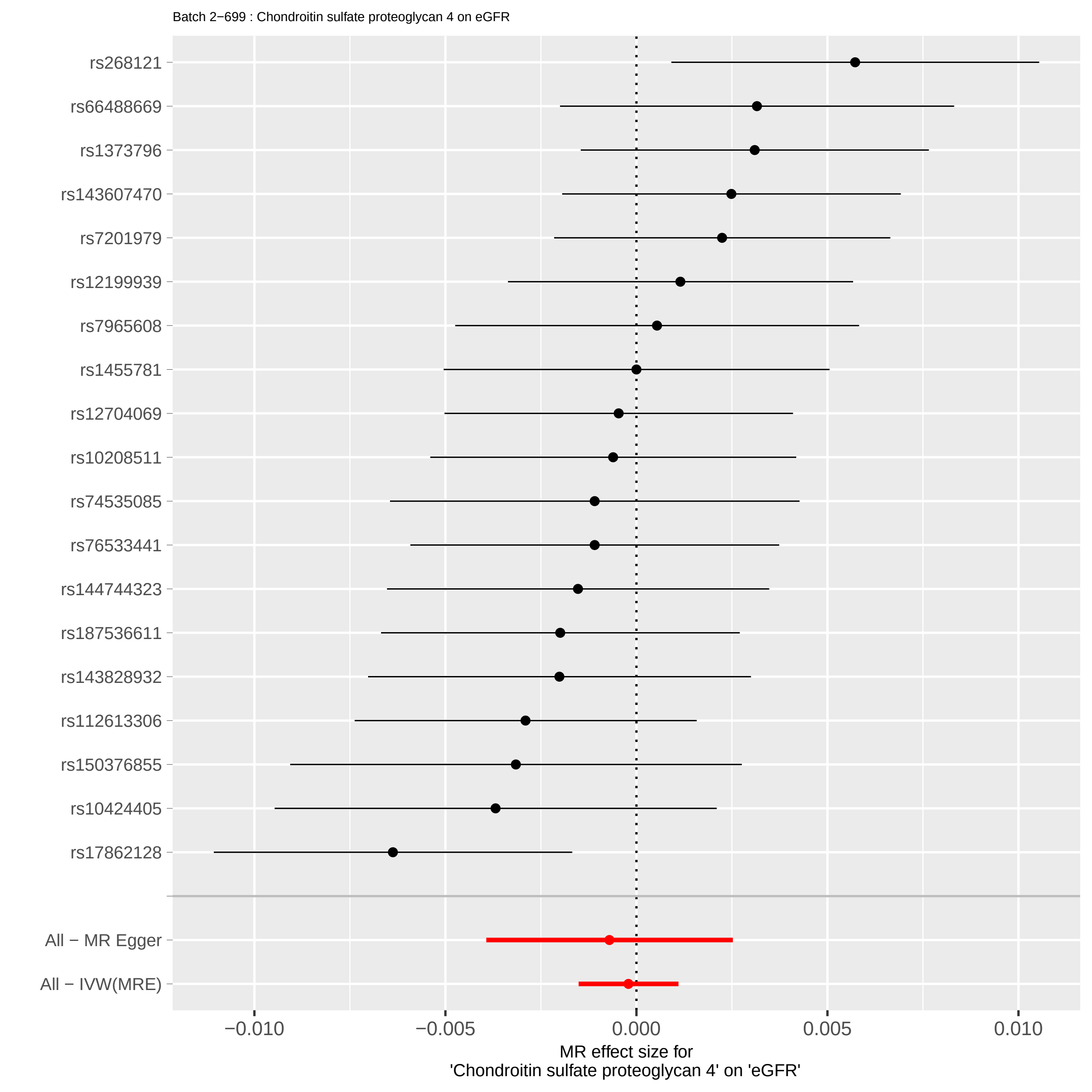

Batch 2−699 : Chondroitin sulfate proteoglycan 4 on eGFR
rs268121
rs66488669
rs1373796
rs143607470
rs7201979
rs12199939
rs7965608
rs1455781
rs12704069
rs10208511
rs74535085
rs76533441
rs144744323
rs187536611
rs143828932
rs112613306
rs150376855
rs10424405
rs17862128
All − MR Egger
All − IVW(MRE)
−0.010
−0.005
0.000
0.005
0.010
MR effect size for
'Chondroitin sulfate proteoglycan 4' on 'eGFR'

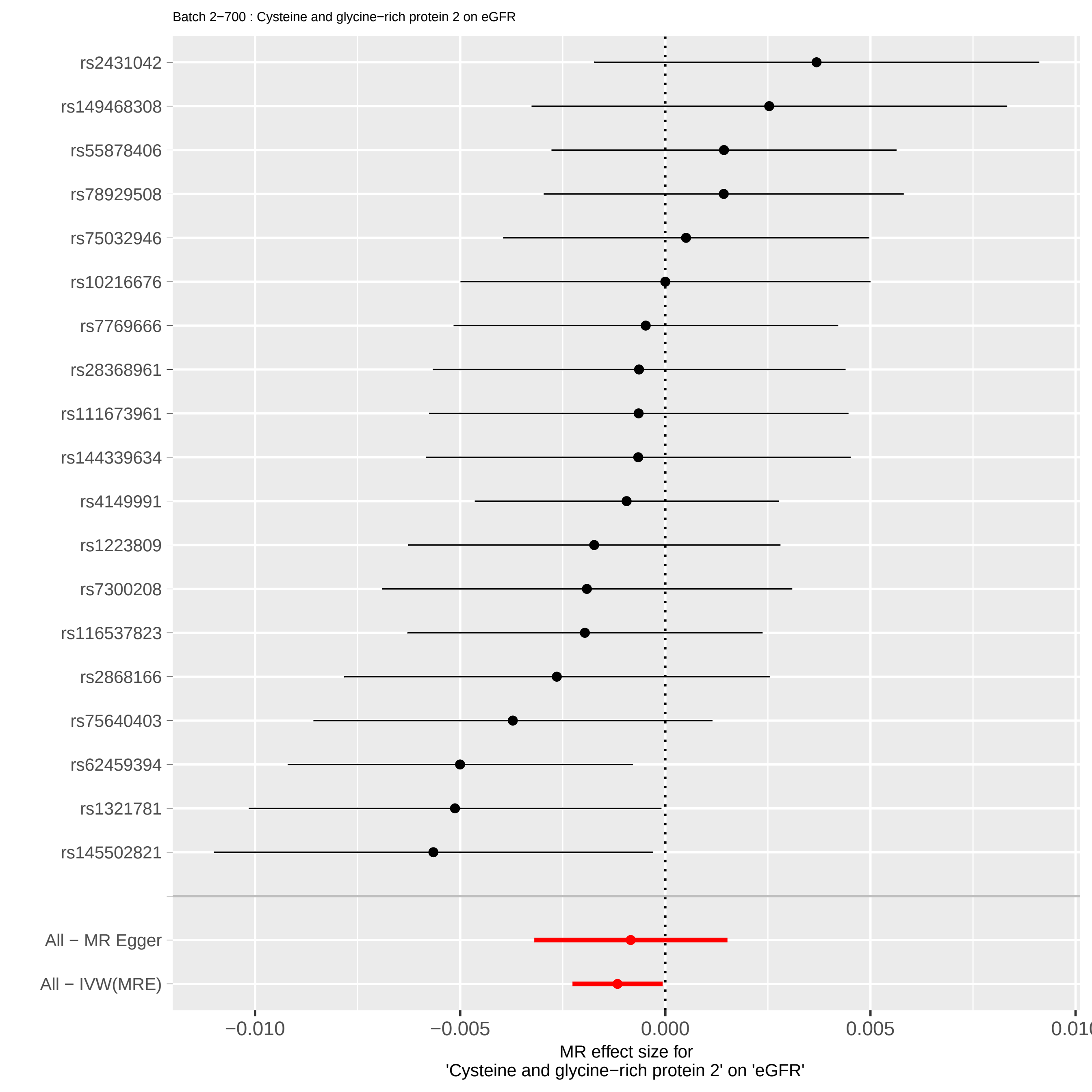

Batch 2−700 : Cysteine and glycine−rich protein 2 on eGFR
rs2431042
rs149468308
rs55878406
rs78929508
rs75032946
rs10216676
rs7769666
rs28368961
rs111673961
rs144339634
rs4149991
rs1223809
rs7300208
rs116537823
rs2868166
rs75640403
rs62459394
rs1321781
rs145502821
All − MR Egger
All − IVW(MRE)
−0.010
−0.005
0.000
0.005
0.010
MR effect size for
'Cysteine and glycine−rich protein 2' on 'eGFR'

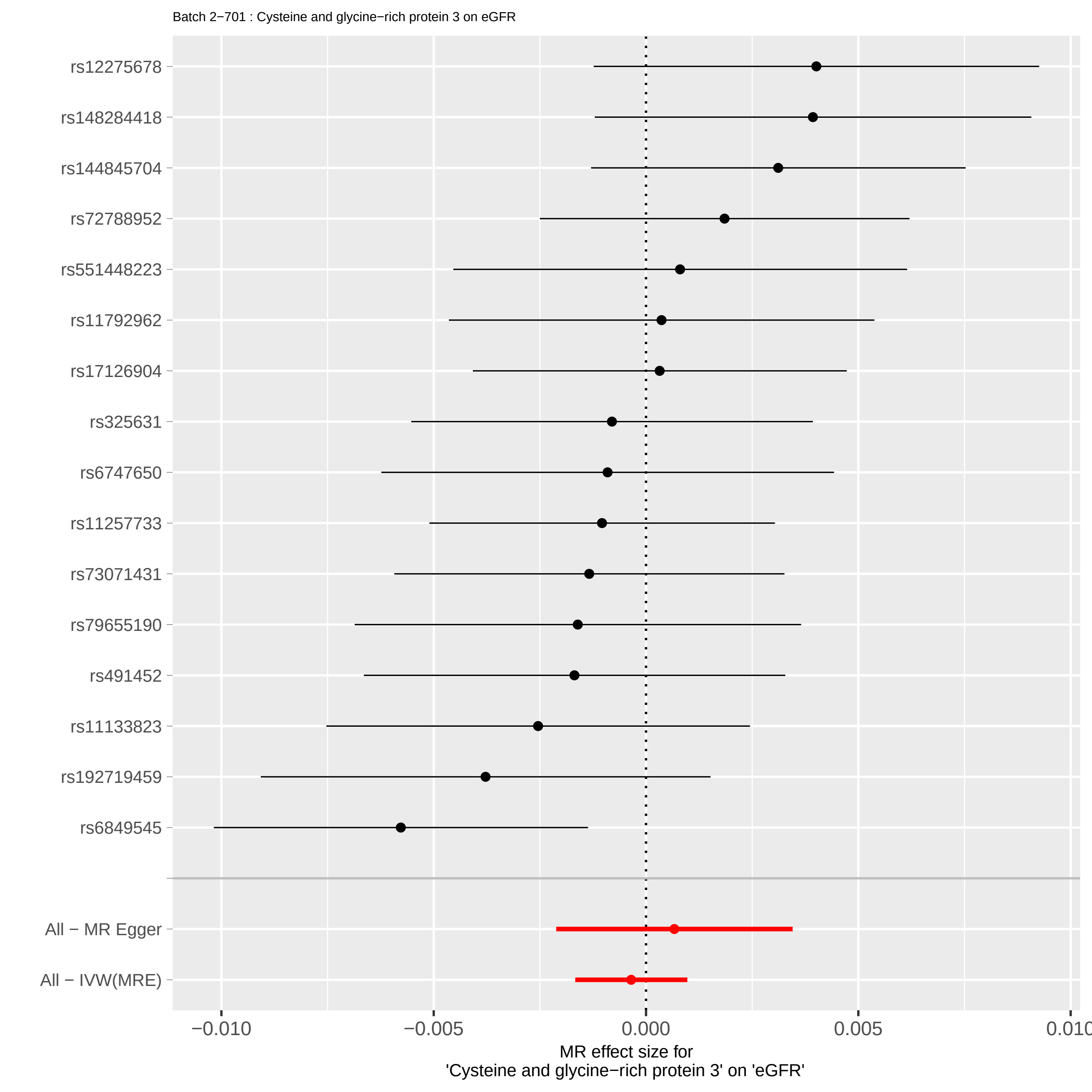

Batch 2−701 : Cysteine and glycine−rich protein 3 on eGFR
rs12275678
rs148284418
rs144845704
rs72788952
rs551448223
rs11792962
rs17126904
rs325631
rs6747650
rs11257733
rs73071431
rs79655190
rs491452
rs11133823
rs192719459
rs6849545
All − MR Egger
All − IVW(MRE)
−0.010
−0.005
0.000
0.005
0.010
MR effect size for
'Cysteine and glycine−rich protein 3' on 'eGFR'

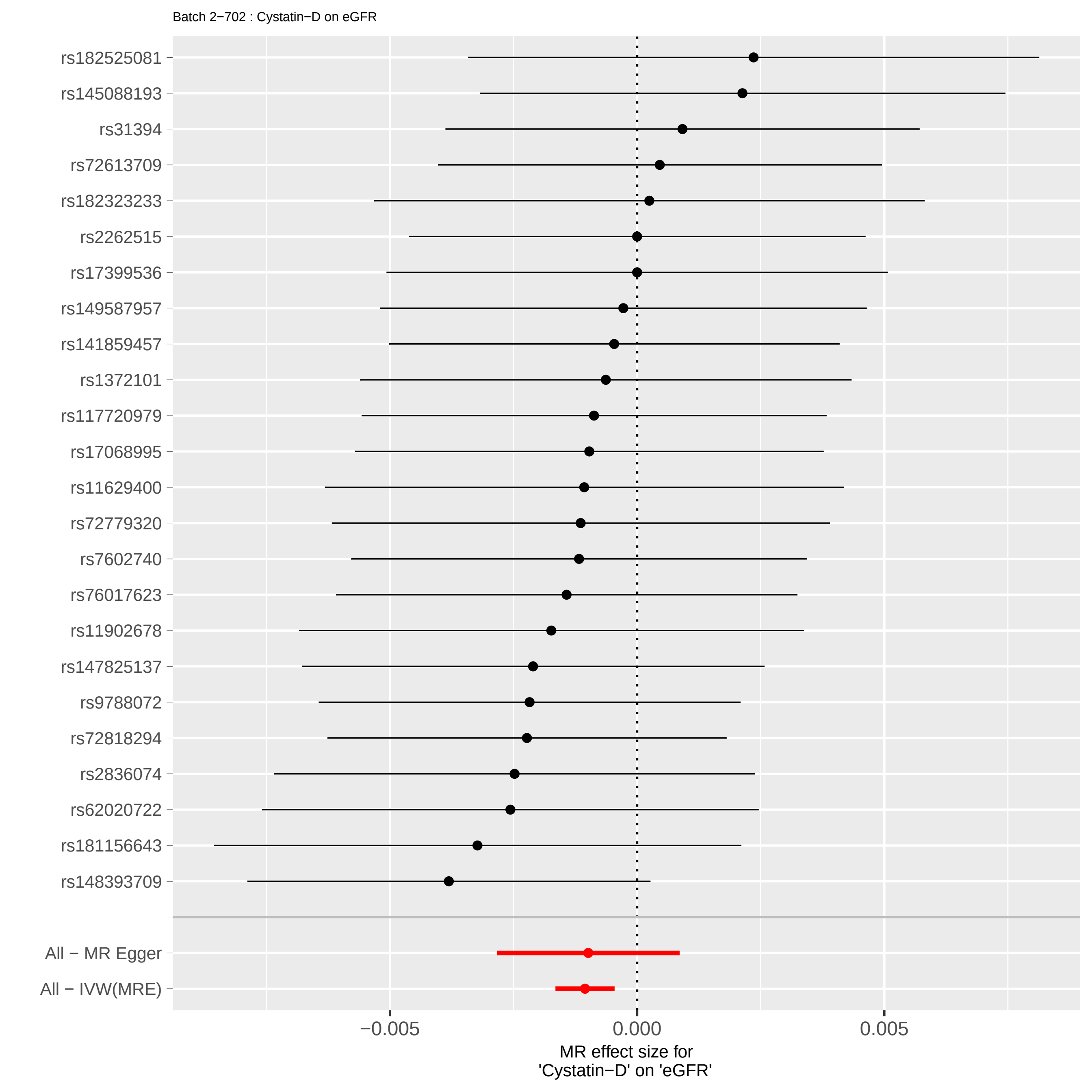
Batch 2−702 : Cystatin−D on eGFR
rs182525081
rs145088193
rs31394
rs72613709
rs182323233
rs2262515
rs17399536
rs149587957
rs141859457
rs1372101
rs117720979
rs17068995
rs11629400
rs72779320
rs7602740
rs76017623
rs11902678
rs147825137
rs9788072
rs72818294
rs2836074
rs62020722
rs181156643
rs148393709
All − MR Egger
All − IVW(MRE)
−0.005
0.000
0.005
MR effect size for
'Cystatin−D' on 'eGFR'

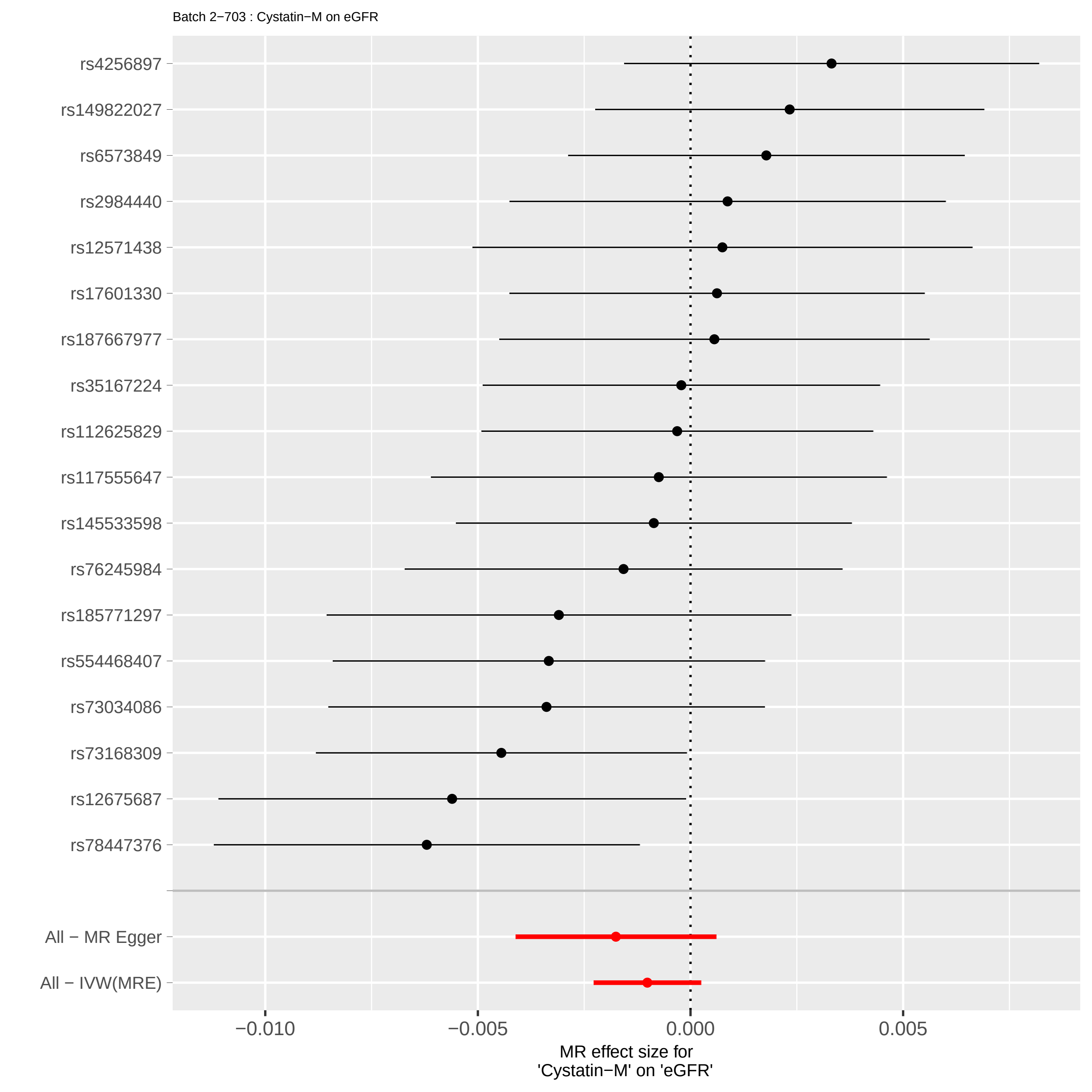

Batch 2−703 : Cystatin−M on eGFR
rs4256897
rs149822027
rs6573849
rs2984440
rs12571438
rs17601330
rs187667977
rs35167224
rs112625829
rs117555647
rs145533598
rs76245984
rs185771297
rs554468407
rs73034086
rs73168309
rs12675687
rs78447376
All − MR Egger
All − IVW(MRE)
−0.010
−0.005
0.000
0.005
MR effect size for
'Cystatin−M' on 'eGFR'

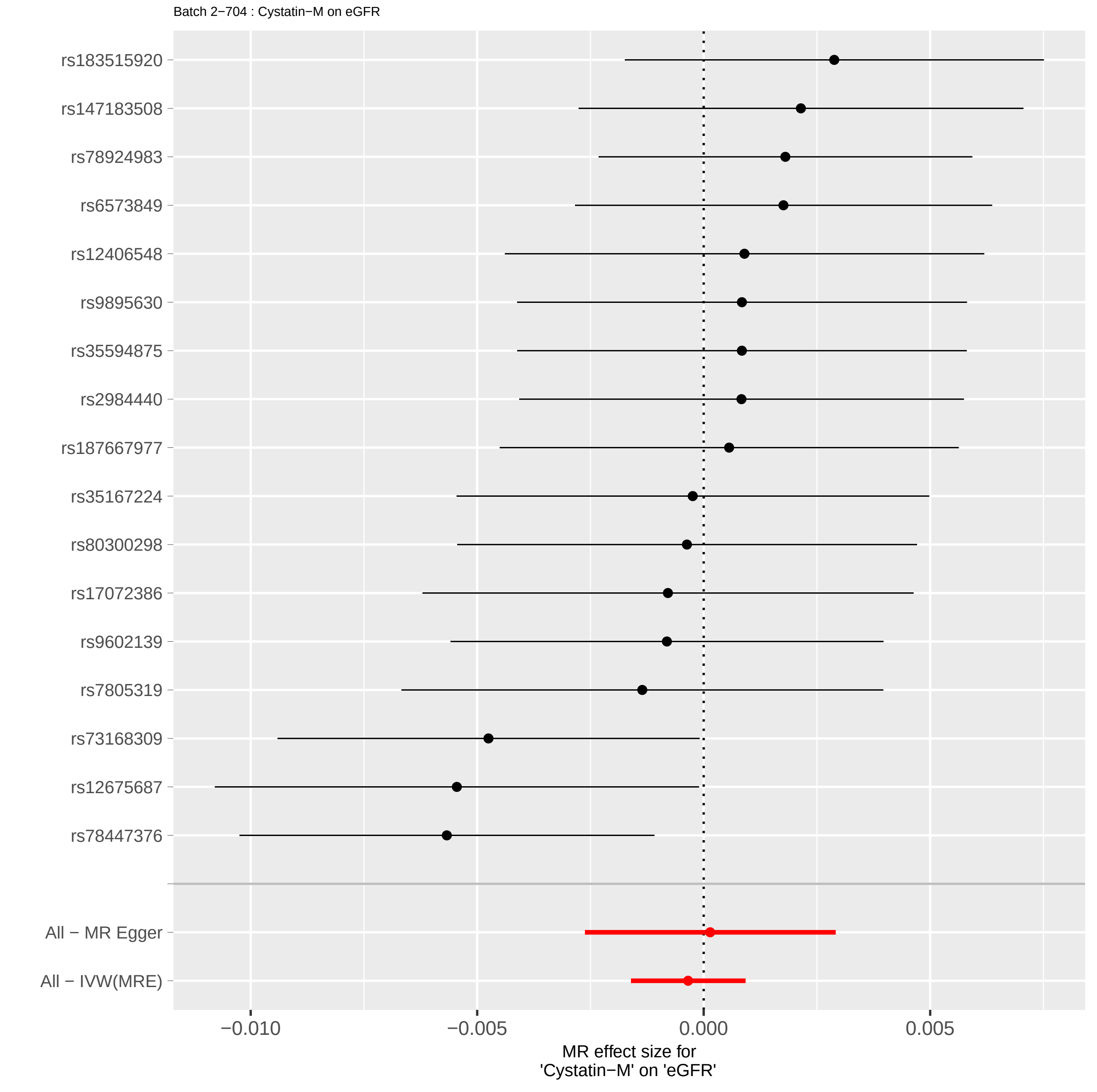

Batch 2−704 : Cystatin−M on eGFR
rs183515920
rs147183508
rs78924983
rs6573849
rs12406548
rs9895630
rs35594875
rs2984440
rs187667977
rs35167224
rs80300298
rs17072386
rs9602139
rs7805319
rs73168309
rs12675687
rs78447376
All − MR Egger
All − IVW(MRE)
−0.010
−0.005
0.000
0.005
MR effect size for
'Cystatin−M' on 'eGFR'

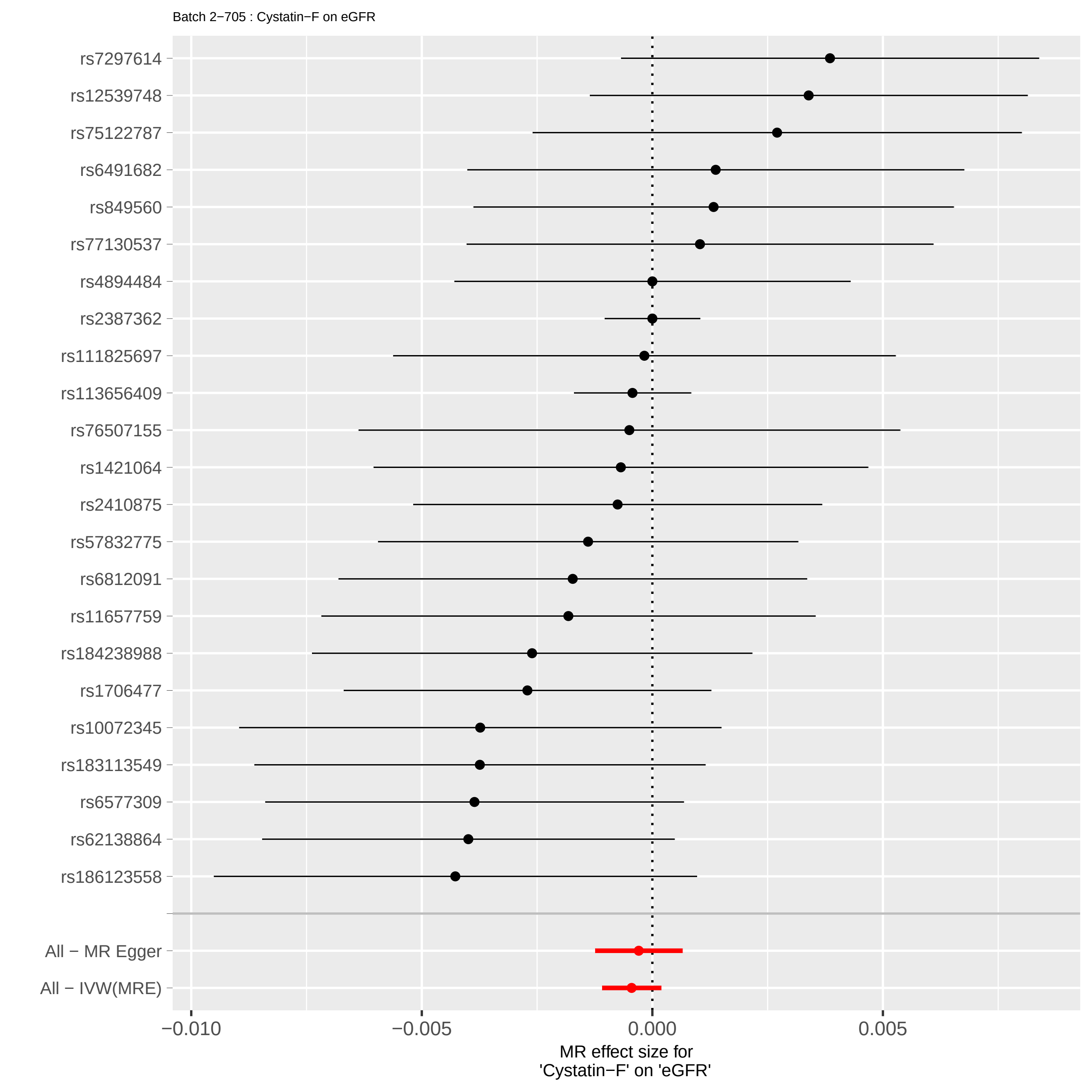

Batch 2−705 : Cystatin−F on eGFR
rs7297614
rs12539748
rs75122787
rs6491682
rs849560
rs77130537
rs4894484
rs2387362
rs111825697
rs113656409
rs76507155
rs1421064
rs2410875
rs57832775
rs6812091
rs11657759
rs184238988
rs1706477
rs10072345
rs183113549
rs6577309
rs62138864
rs186123558
All − MR Egger
All − IVW(MRE)
−0.010
−0.005
0.000
0.005
MR effect size for
'Cystatin−F' on 'eGFR'

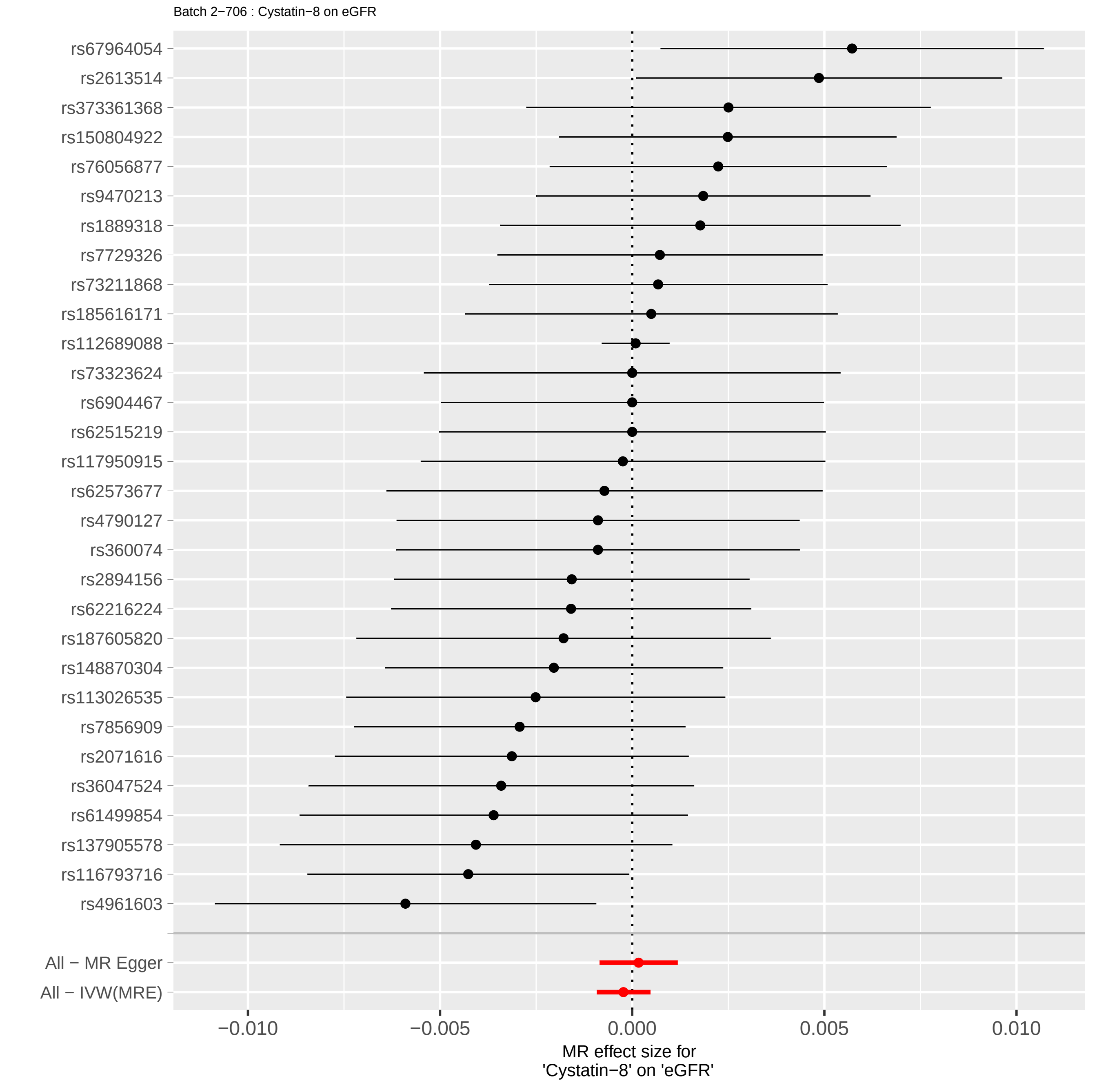

Batch 2−706 : Cystatin−8 on eGFR
rs67964054
rs2613514
rs373361368
rs150804922
rs76056877
rs9470213
rs1889318
rs7729326
rs73211868
rs185616171
rs112689088
rs73323624
rs6904467
rs62515219
rs117950915
rs62573677
rs4790127
rs360074
rs2894156
rs62216224
rs187605820
rs148870304
rs113026535
rs7856909
rs2071616
rs36047524
rs61499854
rs137905578
rs116793716
rs4961603
All − MR Egger
All − IVW(MRE)
−0.010
−0.005
0.000
0.005
0.010
MR effect size for
'Cystatin−8' on 'eGFR'

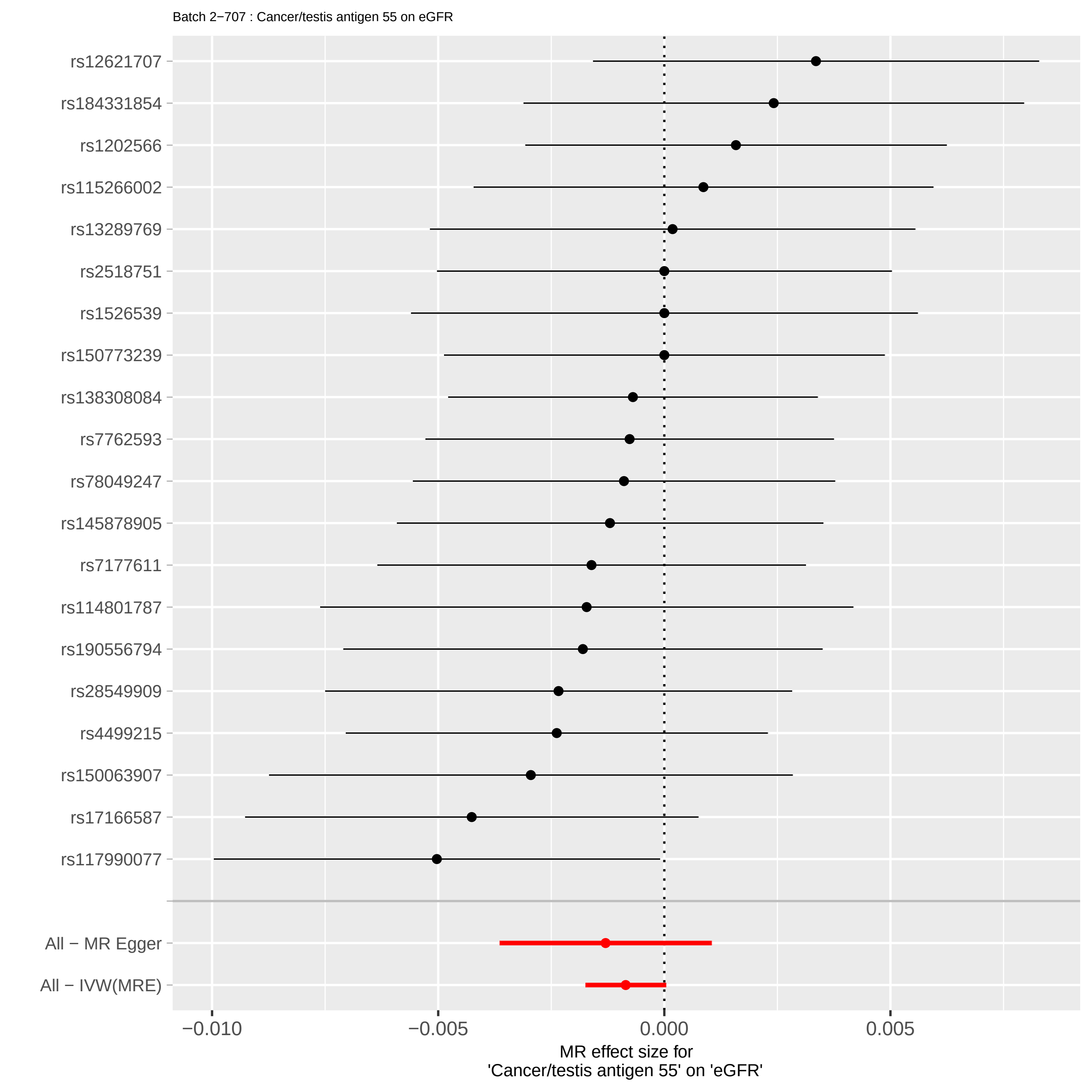

Batch 2−707 : Cancer/testis antigen 55 on eGFR
rs12621707
rs184331854
rs1202566
rs115266002
rs13289769
rs2518751
rs1526539
rs150773239
rs138308084
rs7762593
rs78049247
rs145878905
rs7177611
rs114801787
rs190556794
rs28549909
rs4499215
rs150063907
rs17166587
rs117990077
All − MR Egger
All − IVW(MRE)
−0.010
−0.005
0.000
0.005
MR effect size for
'Cancer/testis antigen 55' on 'eGFR'

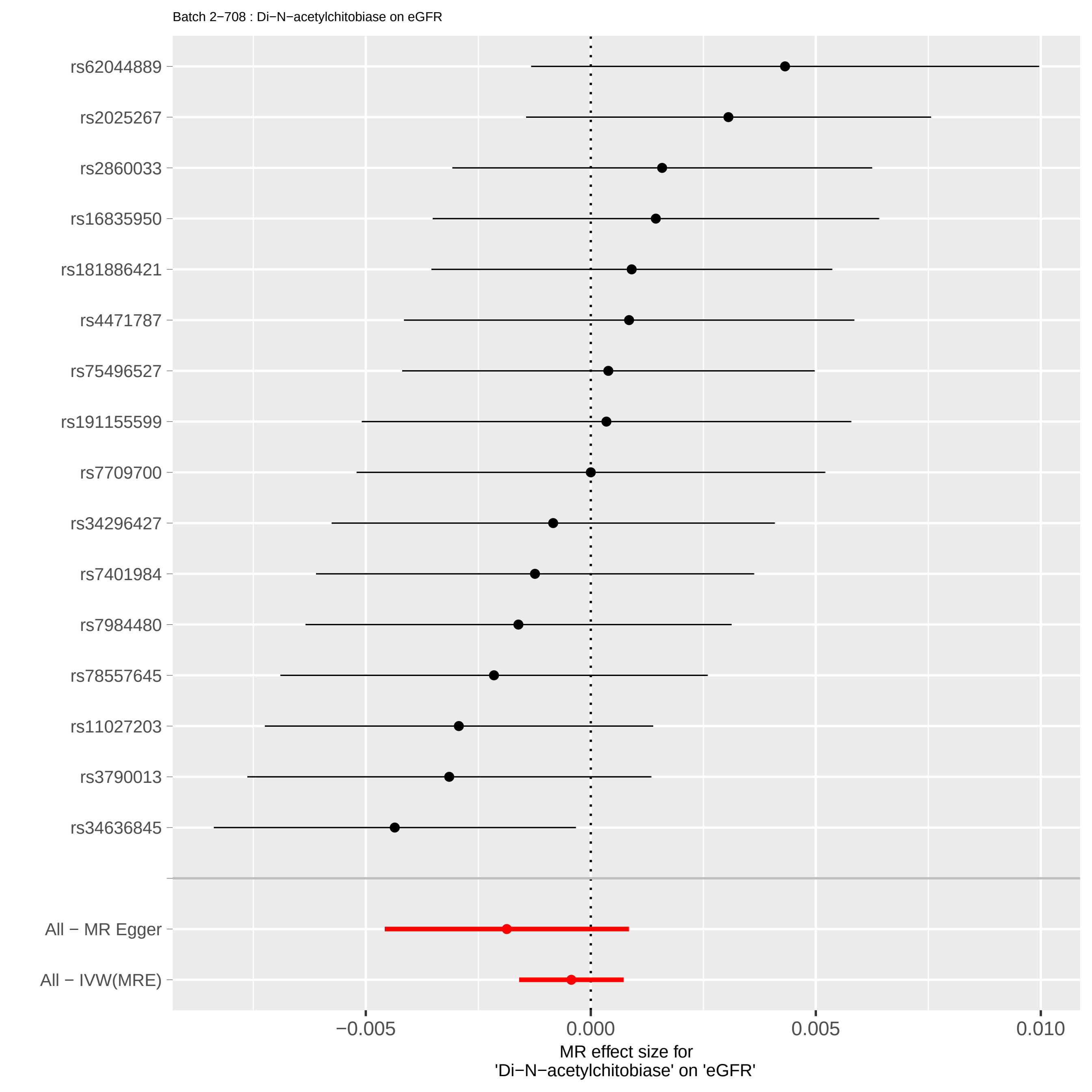
Batch 2−708 : Di−N−acetylchitobiase on eGFR
rs62044889
rs2025267
rs2860033
rs16835950
rs181886421
rs4471787
rs75496527
rs191155599
rs7709700
rs34296427
rs7401984
rs7984480
rs78557645
rs11027203
rs3790013
rs34636845
All − MR Egger
All − IVW(MRE)
−0.005
0.000
0.005
0.010
MR effect size for
'Di−N−acetylchitobiase' on 'eGFR'

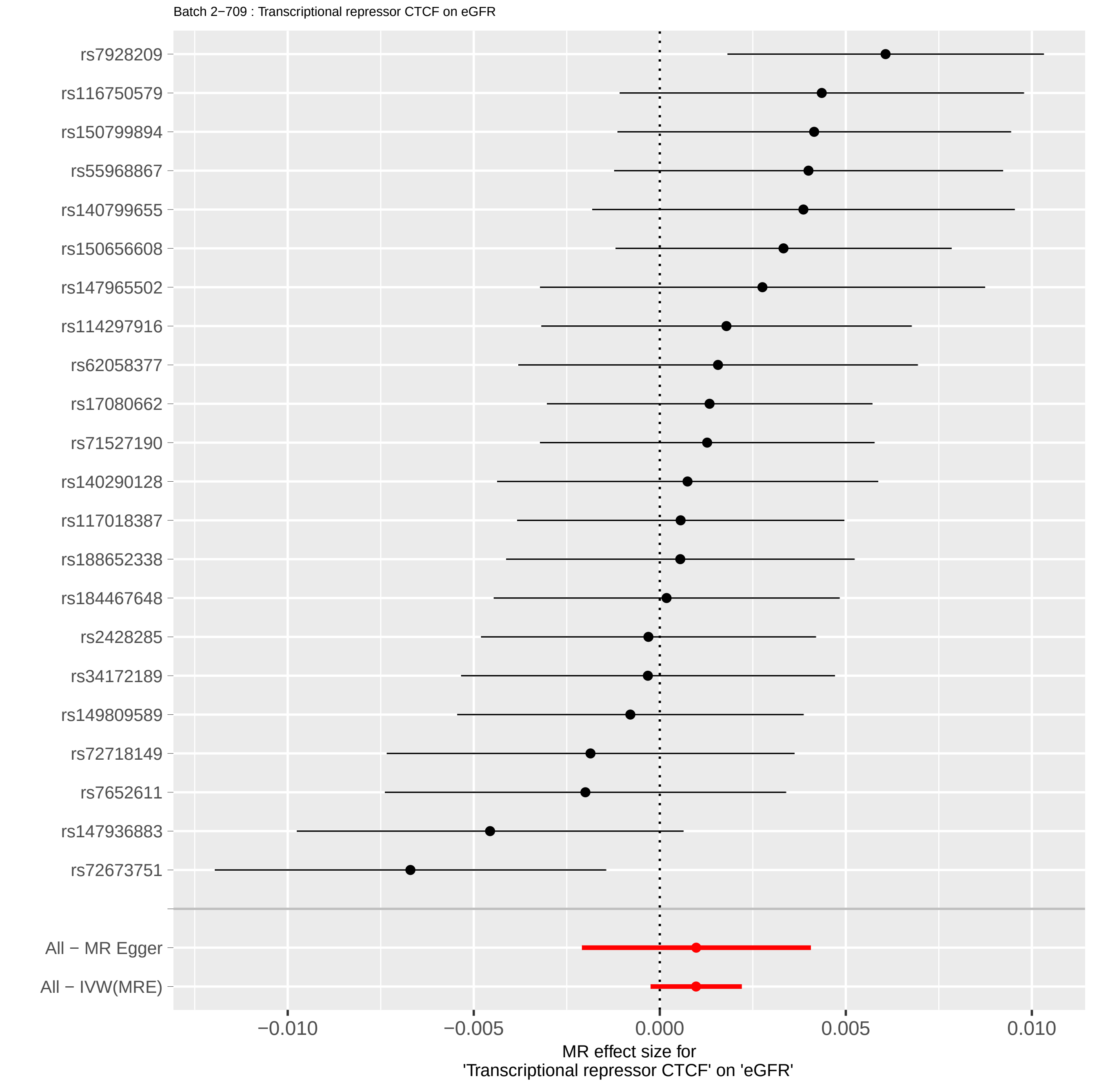
Batch 2−709 : Transcriptional repressor CTCF on eGFR
rs7928209
rs116750579
rs150799894
rs55968867
rs140799655
rs150656608
rs147965502
rs114297916
rs62058377
rs17080662
rs71527190
rs140290128
rs117018387
rs188652338
rs184467648
rs2428285
rs34172189
rs149809589
rs72718149
rs7652611
rs147936883
rs72673751
All − MR Egger
All − IVW(MRE)
−0.010
−0.005
0.000
0.005
0.010
MR effect size for
'Transcriptional repressor CTCF' on 'eGFR'

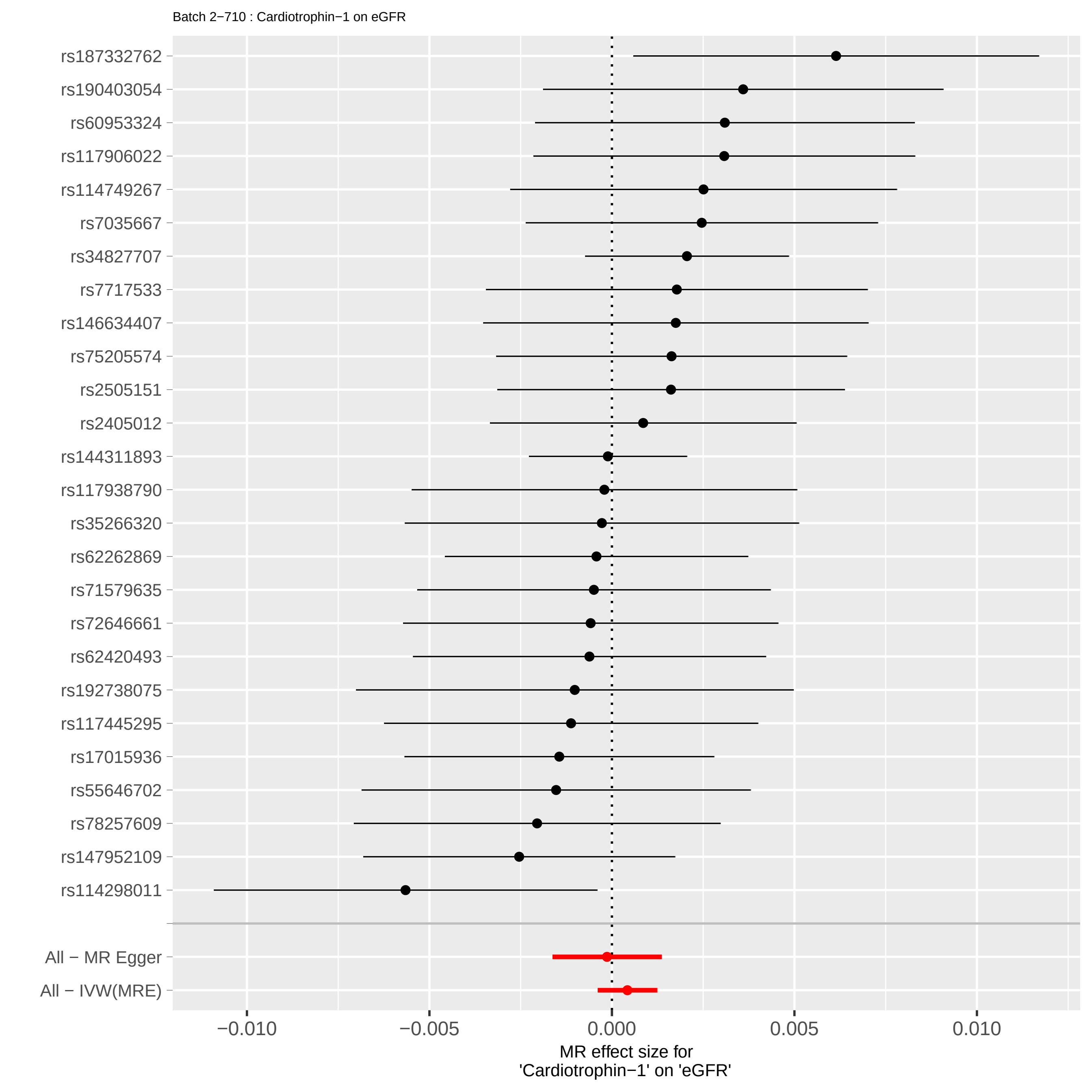

Batch 2−710 : Cardiotrophin−1 on eGFR
rs187332762
rs190403054
rs60953324
rs117906022
rs114749267
rs7035667
rs34827707
rs7717533
rs146634407
rs75205574
rs2505151
rs2405012
rs144311893
rs117938790
rs35266320
rs62262869
rs71579635
rs72646661
rs62420493
rs192738075
rs117445295
rs17015936
rs55646702
rs78257609
rs147952109
rs114298011
All − MR Egger
All − IVW(MRE)
−0.010
−0.005
0.000
0.005
0.010
MR effect size for
'Cardiotrophin−1' on 'eGFR'

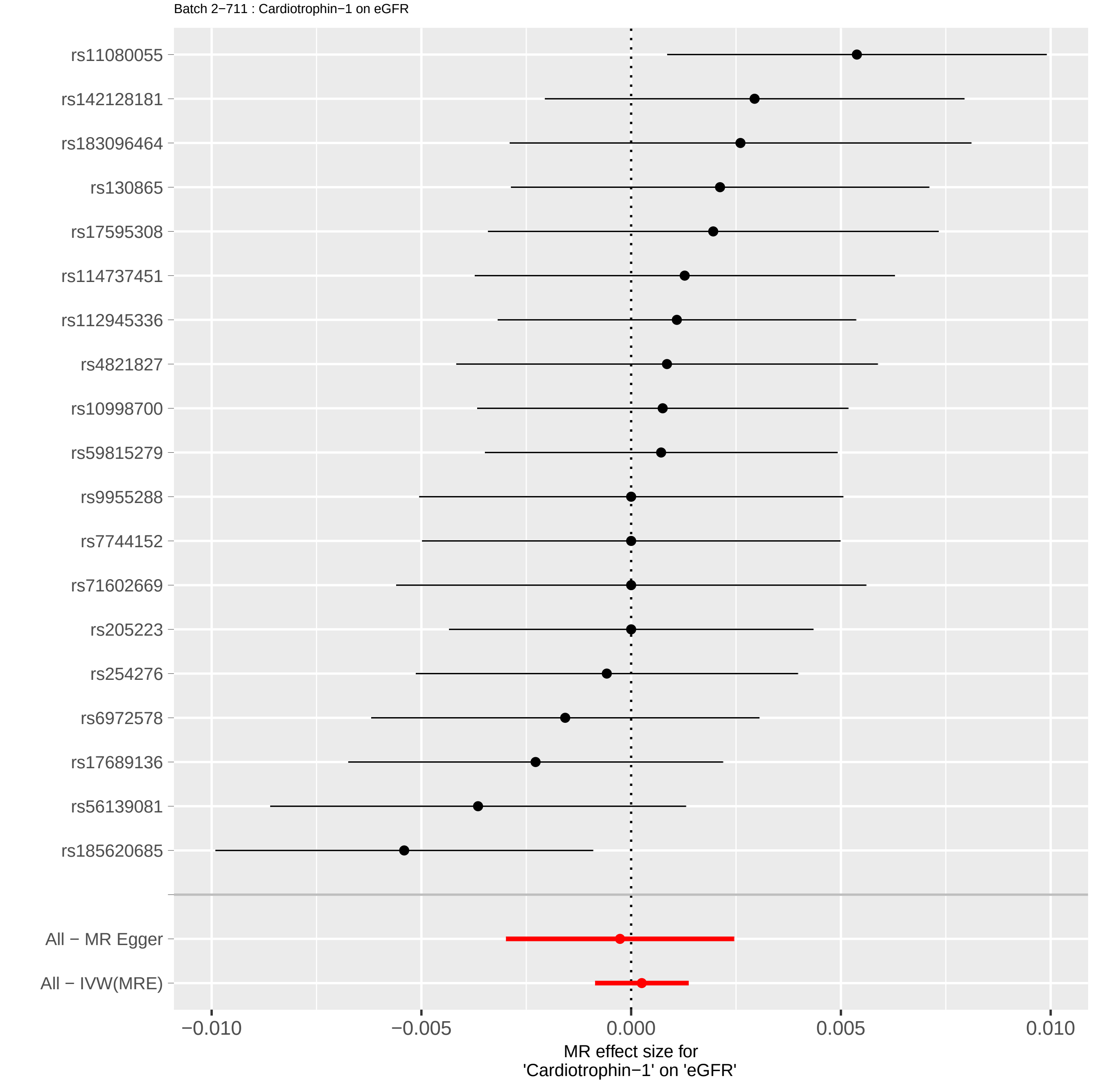
Batch 2−711 : Cardiotrophin−1 on eGFR
rs11080055
rs142128181
rs183096464
rs130865
rs17595308
rs114737451
rs112945336
rs4821827
rs10998700
rs59815279
rs9955288
rs7744152
rs71602669
rs205223
rs254276
rs6972578
rs17689136
rs56139081
rs185620685
All − MR Egger
All − IVW(MRE)
−0.010
−0.005
0.000
0.005
0.010
MR effect size for
'Cardiotrophin−1' on 'eGFR'

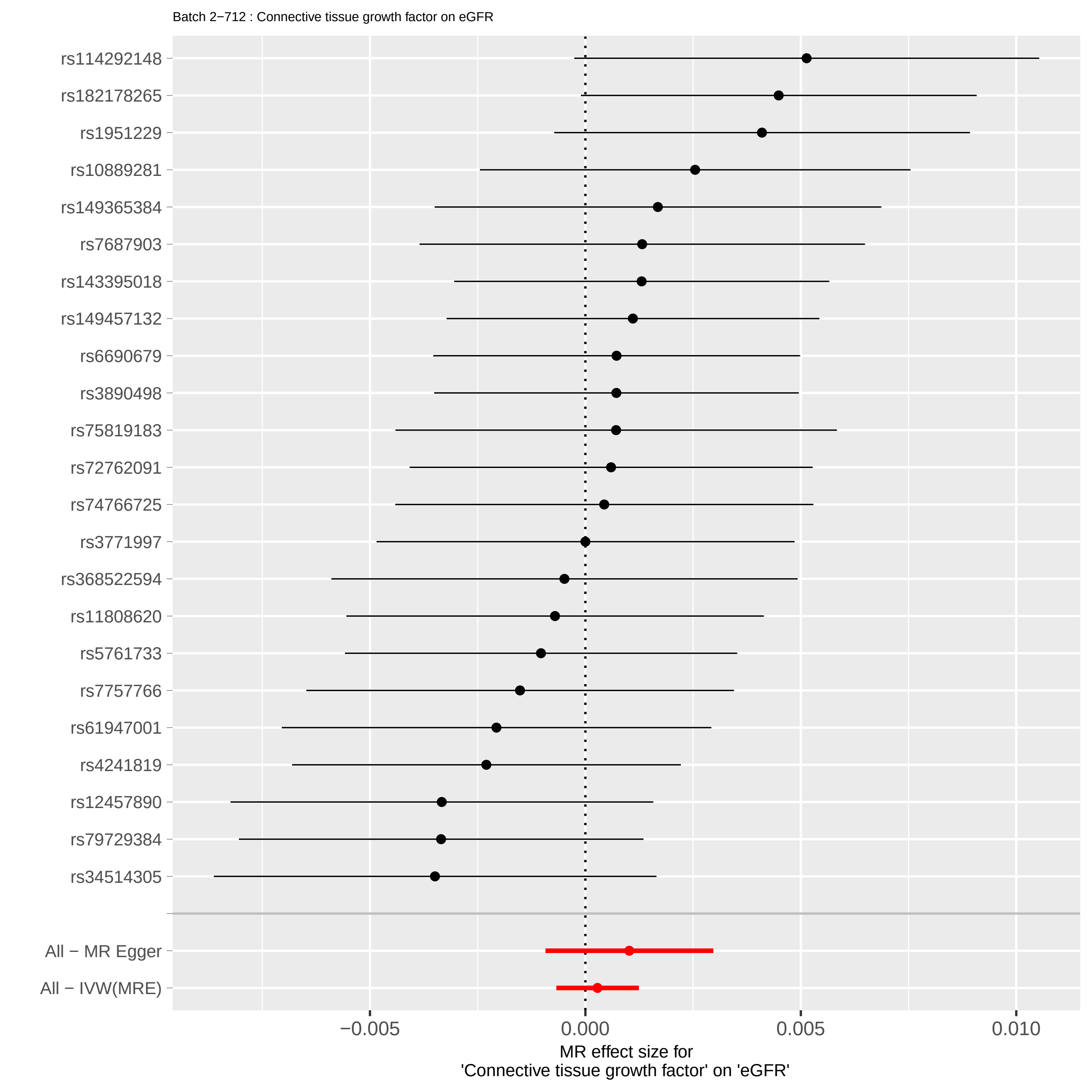

Batch 2−712 : Connective tissue growth factor on eGFR
rs114292148
rs182178265
rs1951229
rs10889281
rs149365384
rs7687903
rs143395018
rs149457132
rs6690679
rs3890498
rs75819183
rs72762091
rs74766725
rs3771997
rs368522594
rs11808620
rs5761733
rs7757766
rs61947001
rs4241819
rs12457890
rs79729384
rs34514305
All − MR Egger
All − IVW(MRE)
−0.005
0.000
0.005
0.010
MR effect size for
'Connective tissue growth factor' on 'eGFR'

Batch 2−713 : Catenin alpha−2 on eGFR

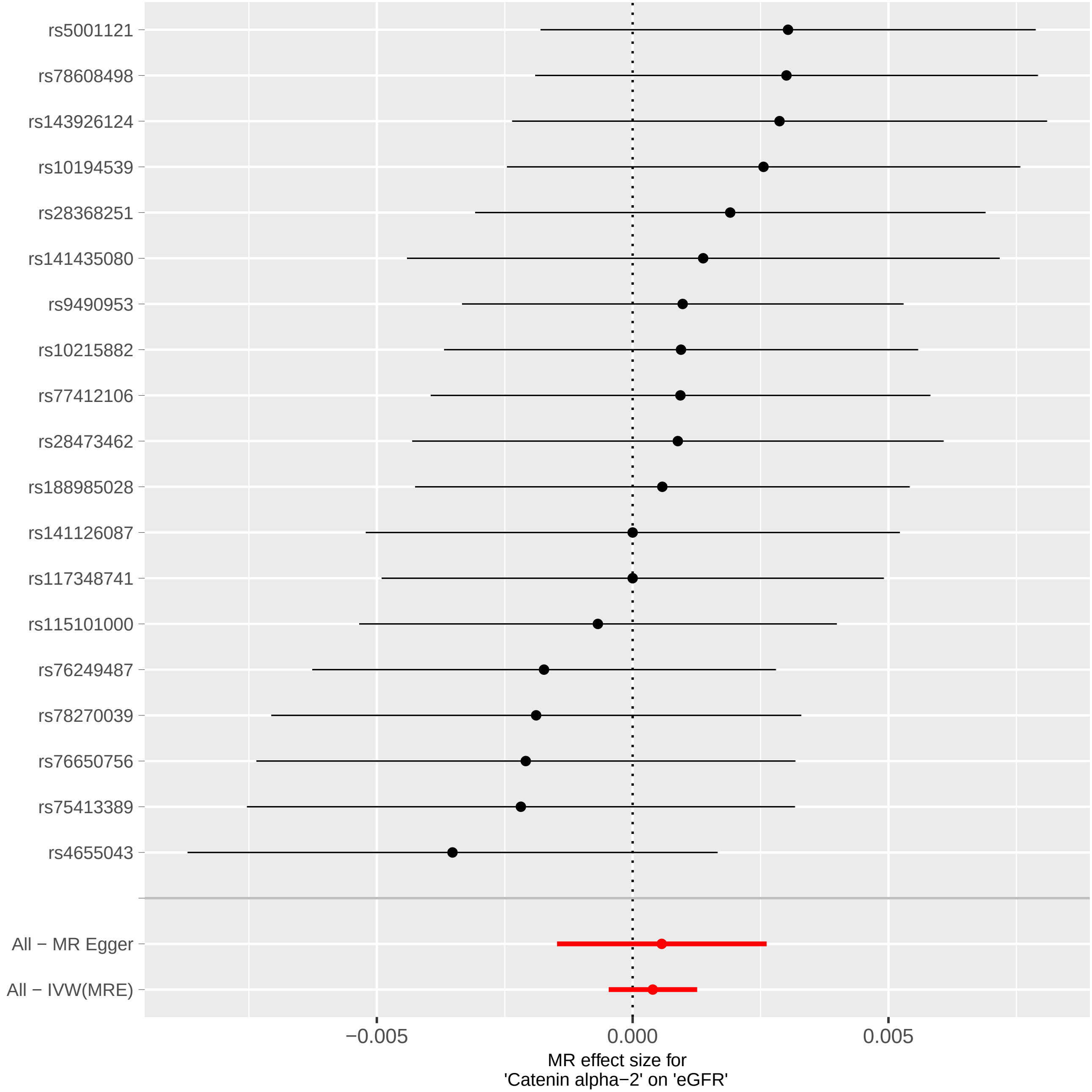

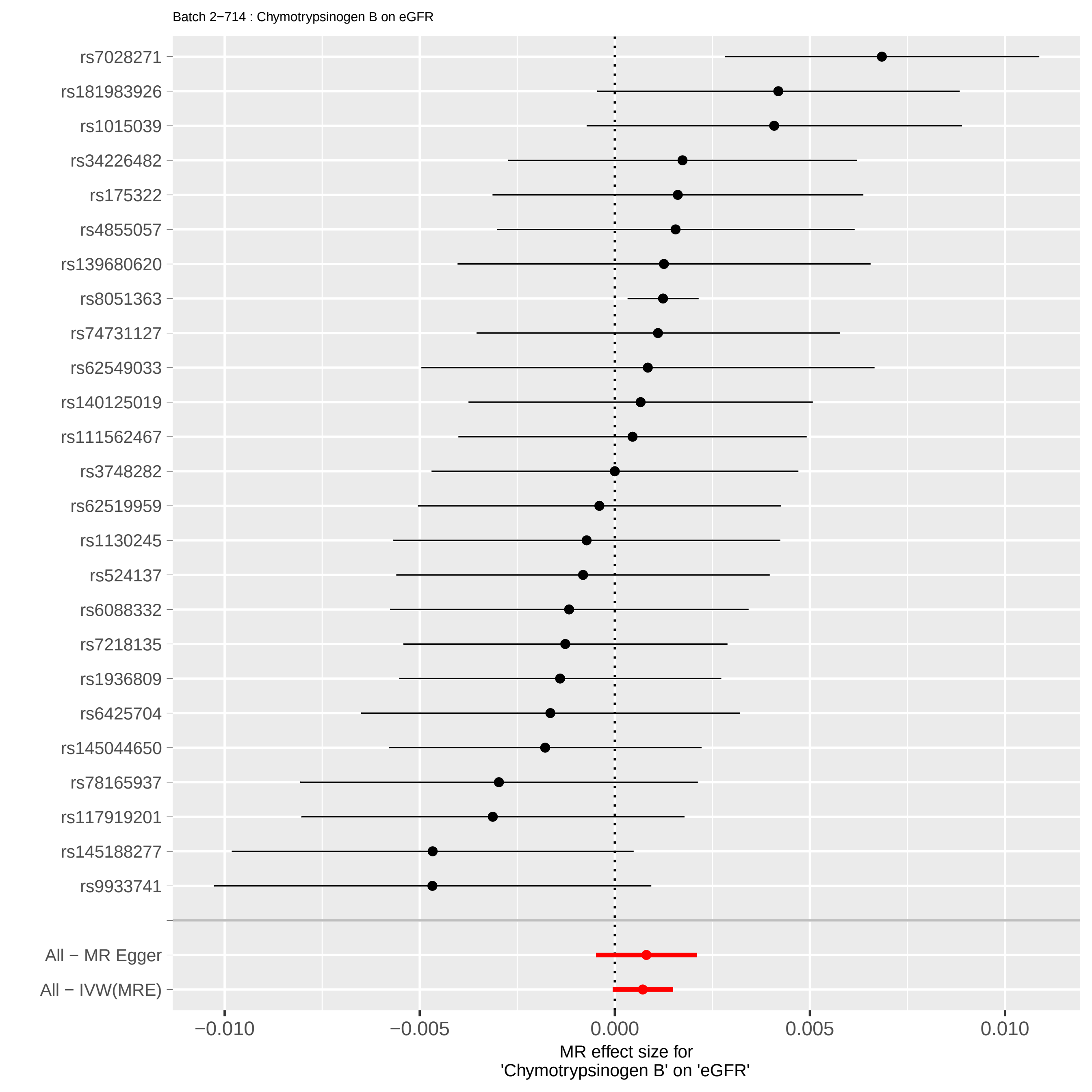

Batch 2−714 : Chymotrypsinogen B on eGFR
rs7028271
rs181983926
rs1015039
rs34226482
rs175322
rs4855057
rs139680620
rs8051363
rs74731127
rs62549033
rs140125019
rs111562467
rs3748282
rs62519959
rs1130245
rs524137
rs6088332
rs7218135
rs1936809
rs6425704
rs145044650
rs78165937
rs117919201
rs145188277
rs9933741
All − MR Egger
All − IVW(MRE)
−0.010
−0.005
0.000
0.005
0.010
MR effect size for
'Chymotrypsinogen B' on 'eGFR'

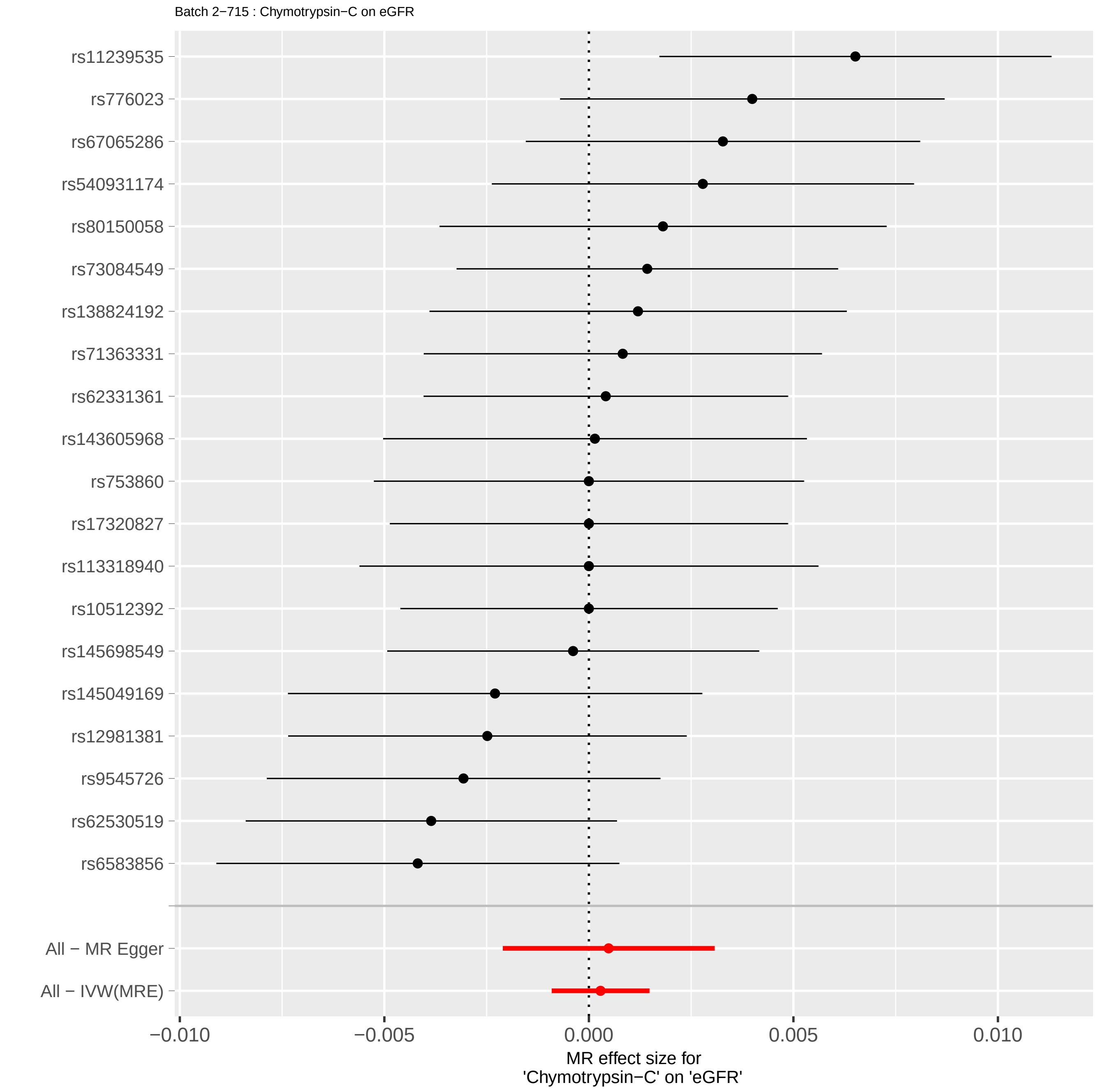
Batch 2−715 : Chymotrypsin−C on eGFR
rs11239535
rs776023
rs67065286
rs540931174
rs80150058
rs73084549
rs138824192
rs71363331
rs62331361
rs143605968
rs753860
rs17320827
rs113318940
rs10512392
rs145698549
rs145049169
rs12981381
rs9545726
rs62530519
rs6583856
All − MR Egger
All − IVW(MRE)
−0.010
−0.005
0.000
0.005
0.010
MR effect size for
'Chymotrypsin−C' on 'eGFR'

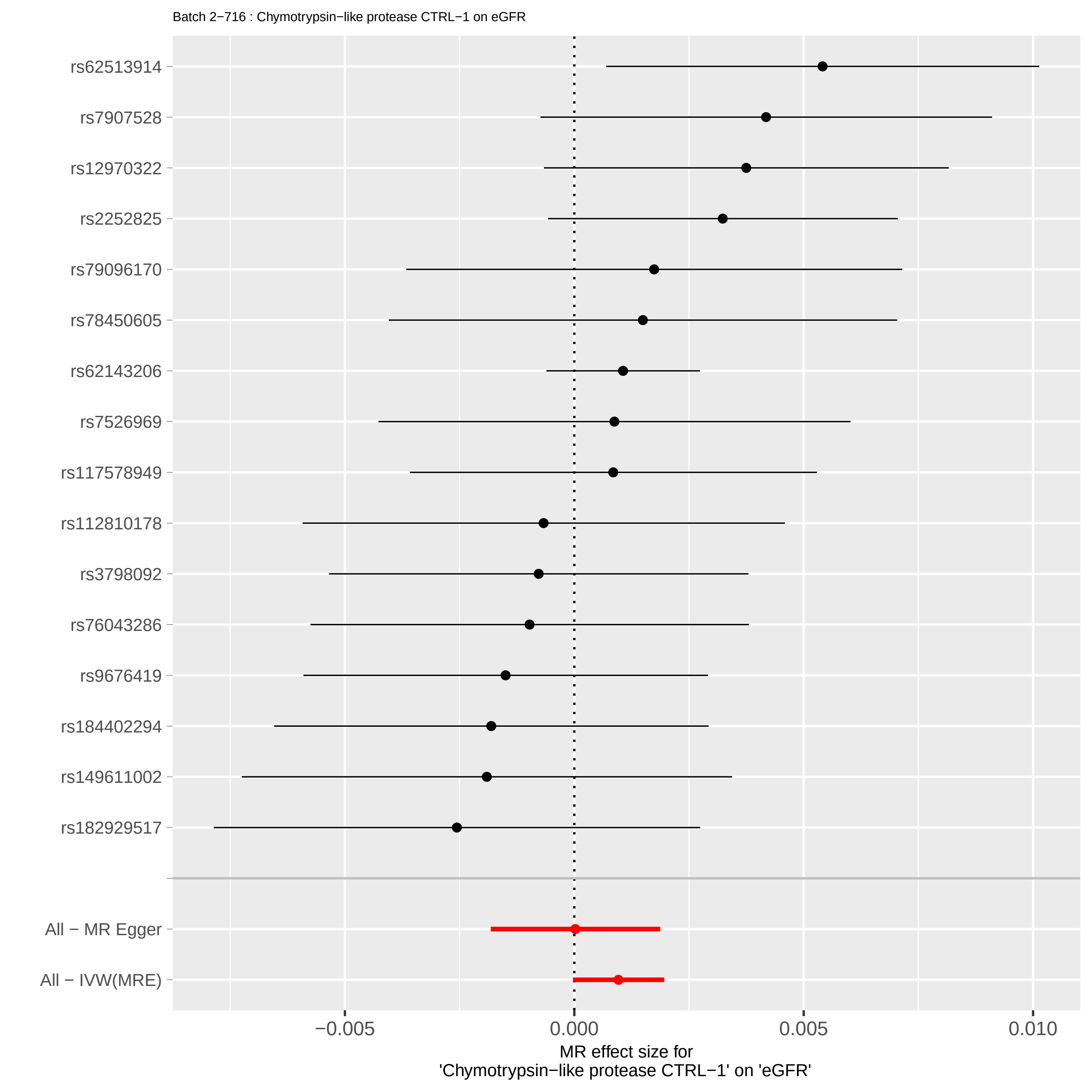

Batch 2−716 : Chymotrypsin−like protease CTRL−1 on eGFR
rs62513914
rs7907528
rs12970322
rs2252825
rs79096170
rs78450605
rs62143206
rs7526969
rs117578949
rs112810178
rs3798092
rs76043286
rs9676419
rs184402294
rs149611002
rs182929517
All − MR Egger
All − IVW(MRE)
−0.005
0.000
0.005
0.010
MR effect size for
'Chymotrypsin−like protease CTRL−1' on 'eGFR'

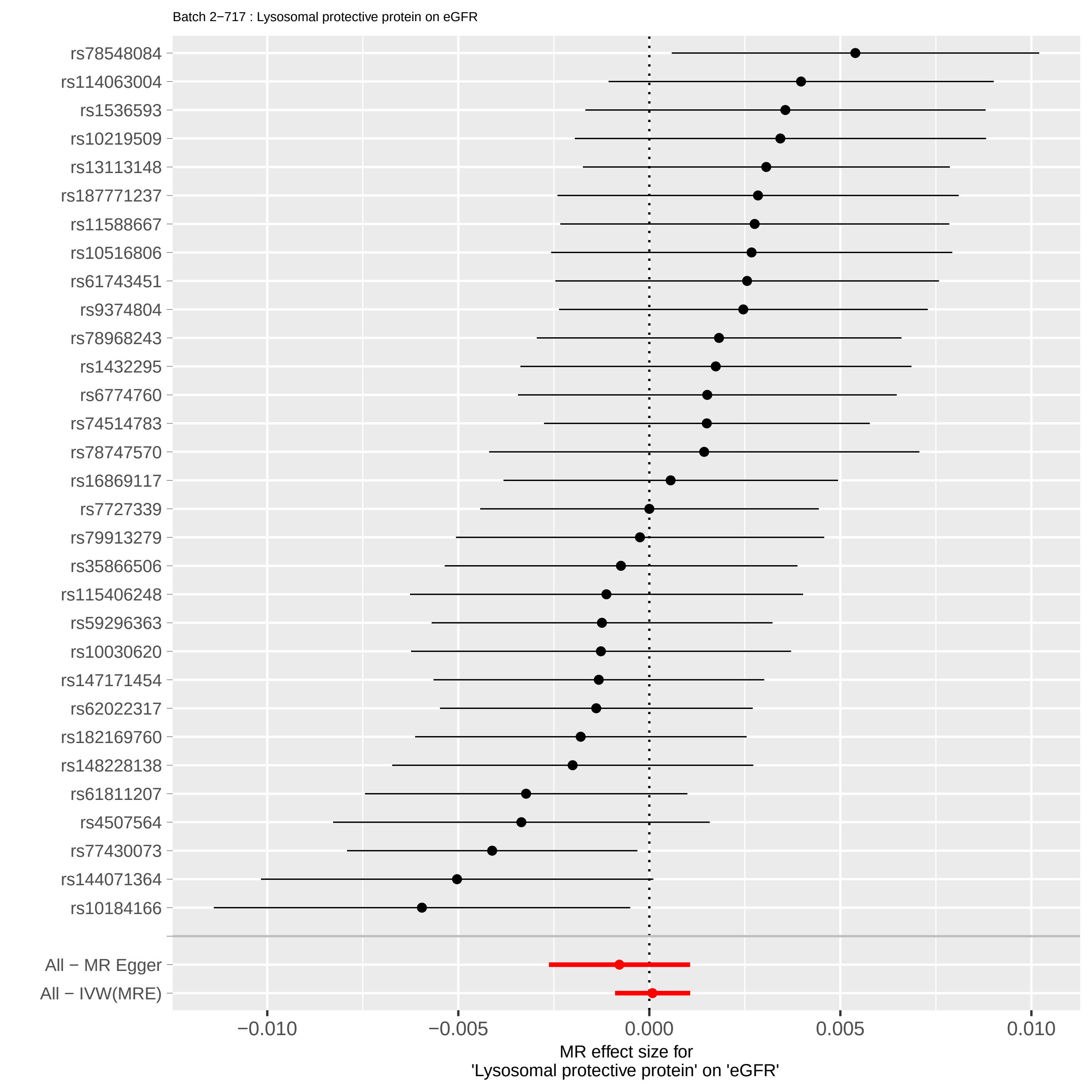
Batch 2−717 : Lysosomal protective protein on eGFR
rs78548084
rs114063004
rs1536593
rs10219509
rs13113148
rs187771237
rs11588667
rs10516806
rs61743451
rs9374804
rs78968243
rs1432295
rs6774760
rs74514783
rs78747570
rs16869117
rs7727339
rs79913279
rs35866506
rs115406248
rs59296363
rs10030620
rs147171454
rs62022317
rs182169760
rs148228138
rs61811207
rs4507564
rs77430073
rs144071364
rs10184166
All − MR Egger
All − IVW(MRE)
−0.010
−0.005
0.000
0.005
0.010
MR effect size for
'Lysosomal protective protein' on 'eGFR'

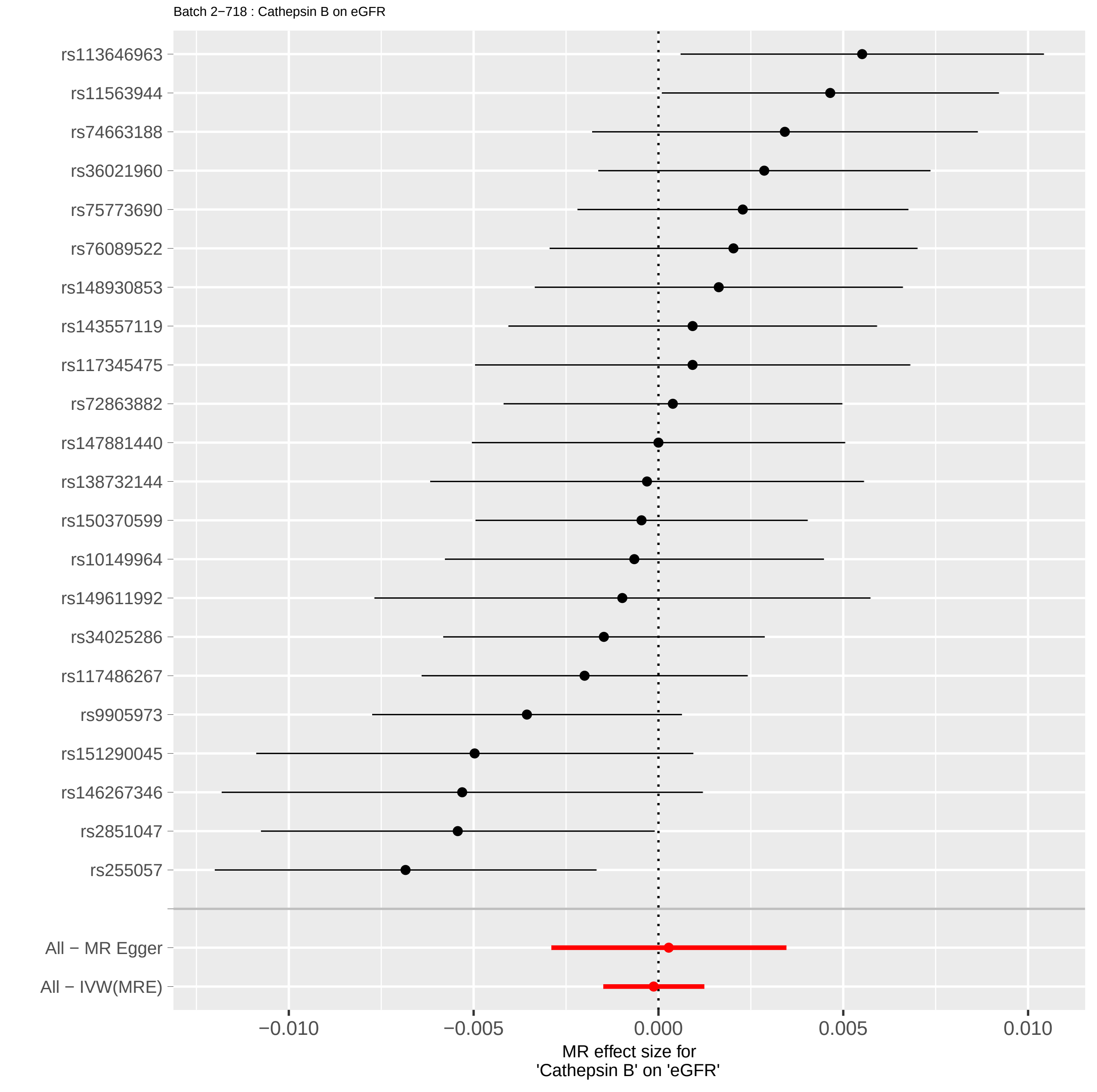
Batch 2−718 : Cathepsin B on eGFR
rs113646963
rs11563944
rs74663188
rs36021960
rs75773690
rs76089522
rs148930853
rs143557119
rs117345475
rs72863882
rs147881440
rs138732144
rs150370599
rs10149964
rs149611992
rs34025286
rs117486267
rs9905973
rs151290045
rs146267346
rs2851047
rs255057
All − MR Egger
All − IVW(MRE)
−0.010
−0.005
0.000
0.005
0.010
MR effect size for
'Cathepsin B' on 'eGFR'

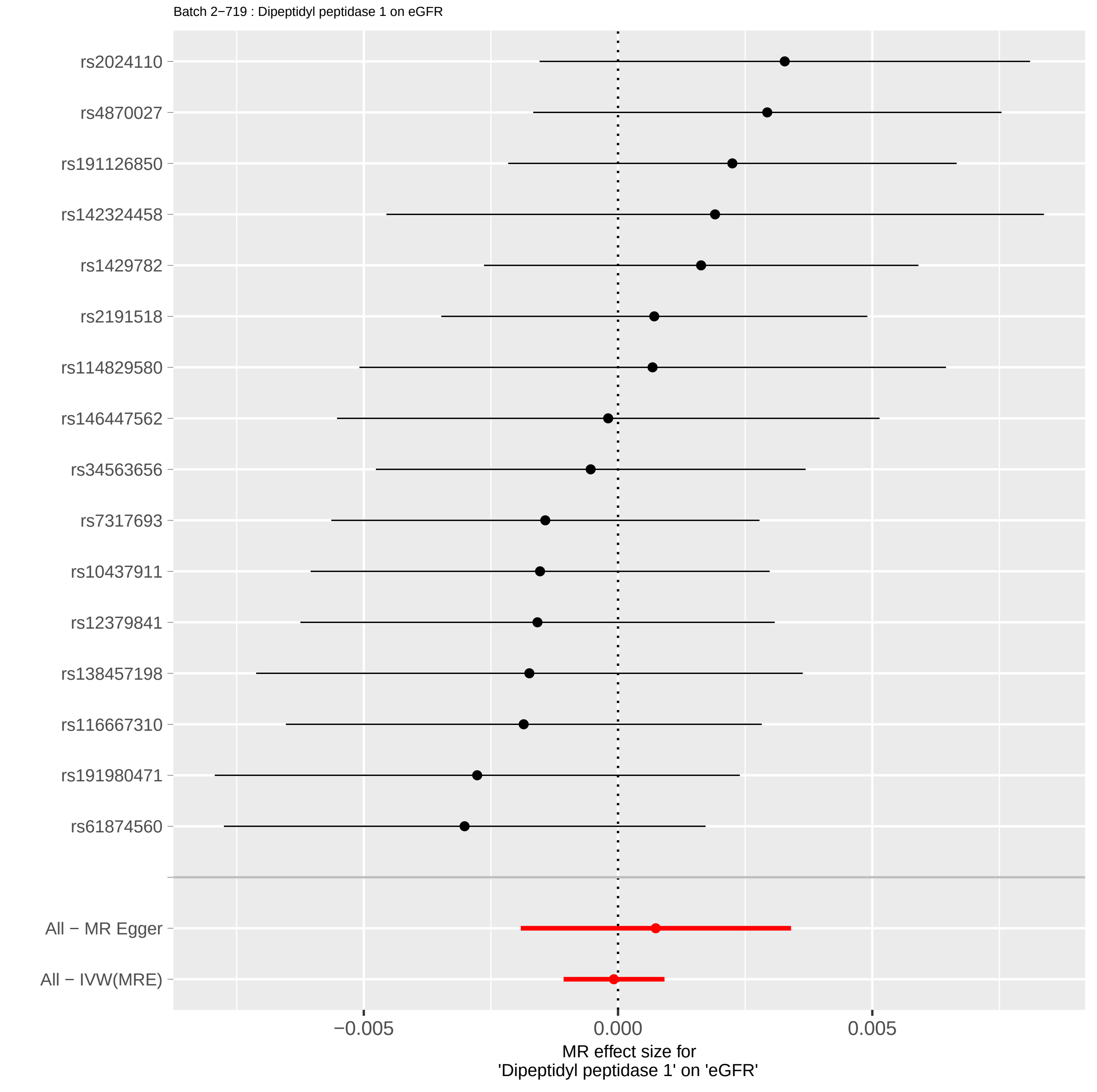

Batch 2−719 : Dipeptidyl peptidase 1 on eGFR
rs2024110
rs4870027
rs191126850
rs142324458
rs1429782
rs2191518
rs114829580
rs146447562
rs34563656
rs7317693
rs10437911
rs12379841
rs138457198
rs116667310
rs191980471
rs61874560
All − MR Egger
All − IVW(MRE)
−0.005
0.000
0.005
MR effect size for
'Dipeptidyl peptidase 1' on 'eGFR'

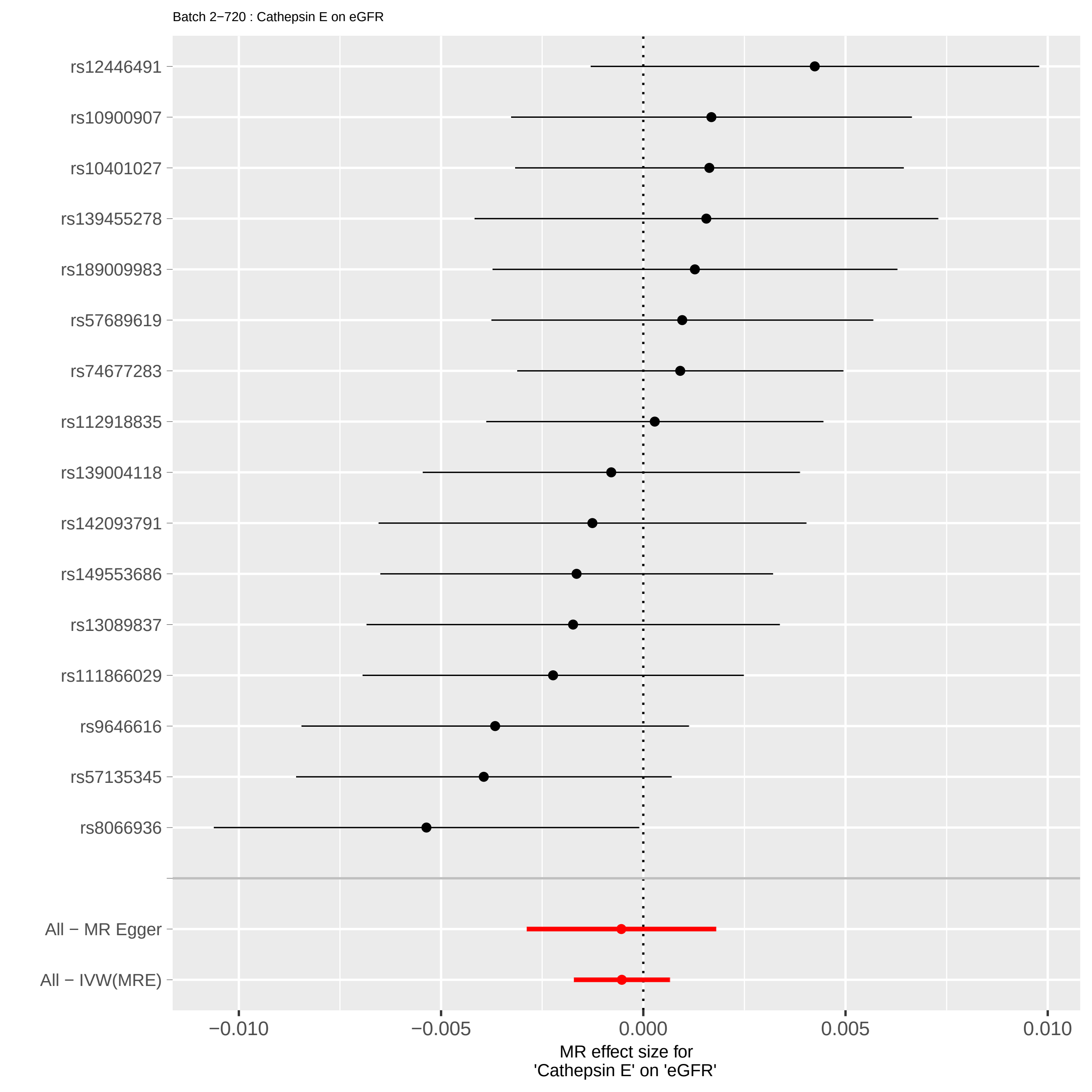

Batch 2−720 : Cathepsin E on eGFR
rs12446491
rs10900907
rs10401027
rs139455278
rs189009983
rs57689619
rs74677283
rs112918835
rs139004118
rs142093791
rs149553686
rs13089837
rs111866029
rs9646616
rs57135345
rs8066936
All − MR Egger
All − IVW(MRE)
−0.010
−0.005
0.000
0.005
0.010
MR effect size for
'Cathepsin E' on 'eGFR'

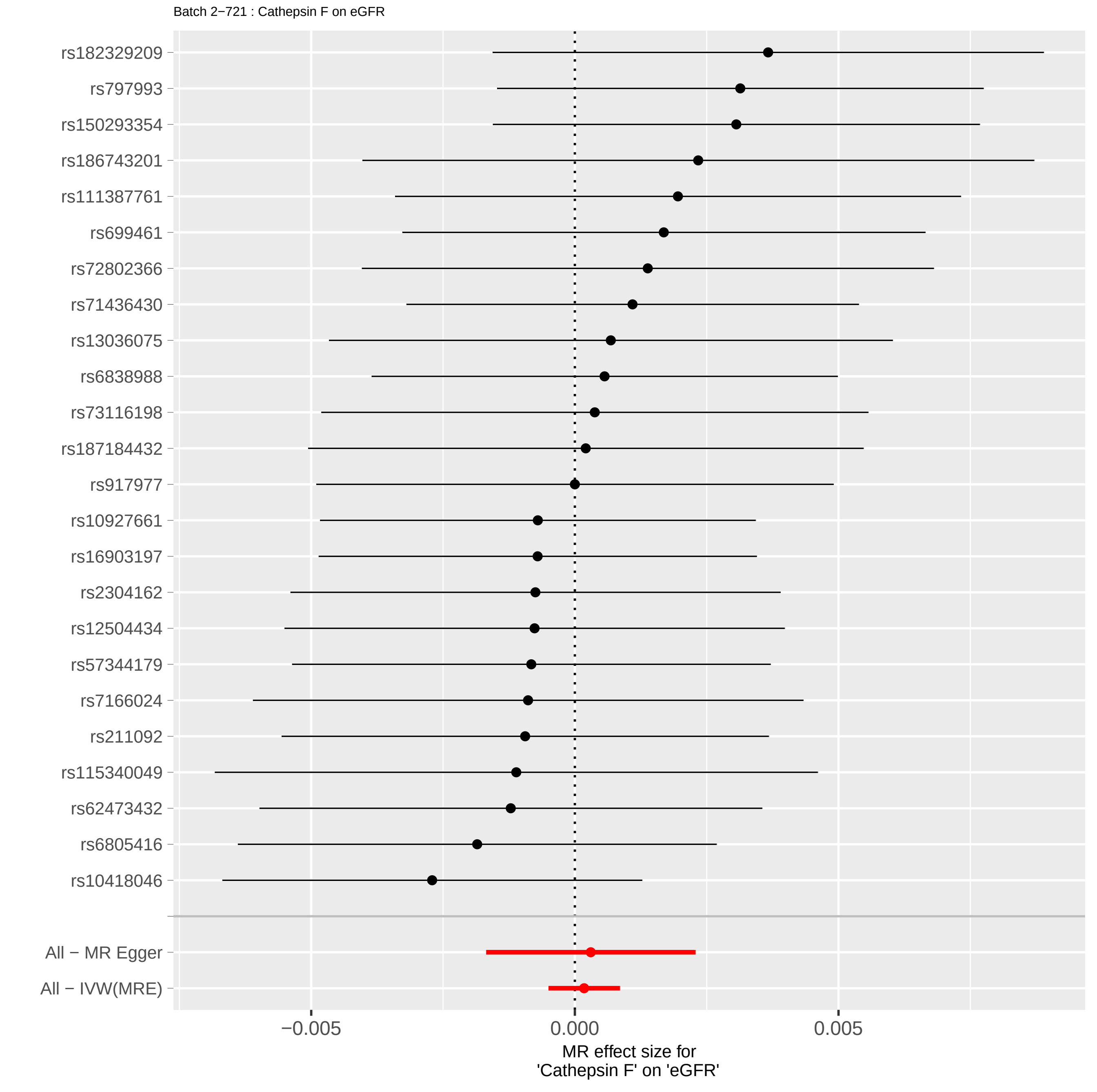

Batch 2−721 : Cathepsin F on eGFR
rs182329209
rs797993
rs150293354
rs186743201
rs111387761
rs699461
rs72802366
rs71436430
rs13036075
rs6838988
rs73116198
rs187184432
rs917977
rs10927661
rs16903197
rs2304162
rs12504434
rs57344179
rs7166024
rs211092
rs115340049
rs62473432
rs6805416
rs10418046
All − MR Egger
All − IVW(MRE)
−0.005
0.000
0.005
MR effect size for
'Cathepsin F' on 'eGFR'

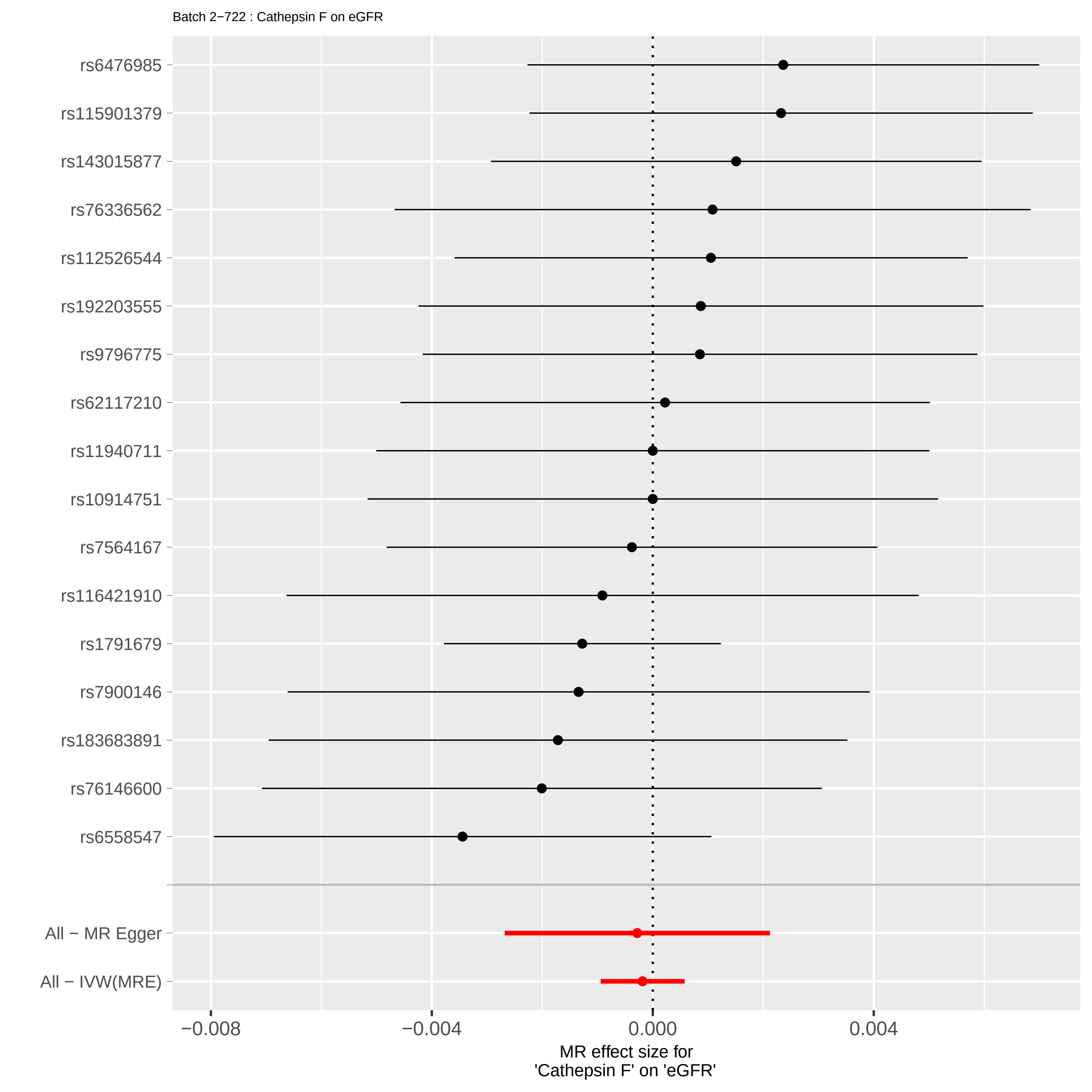

Batch 2−722 : Cathepsin F on eGFR
rs6476985
rs115901379
rs143015877
rs76336562
rs112526544
rs192203555
rs9796775
rs62117210
rs11940711
rs10914751
rs7564167
rs116421910
rs1791679
rs7900146
rs183683891
rs76146600
rs6558547
All − MR Egger
All − IVW(MRE)
−0.008
−0.004
0.000
0.004
MR effect size for
'Cathepsin F' on 'eGFR'

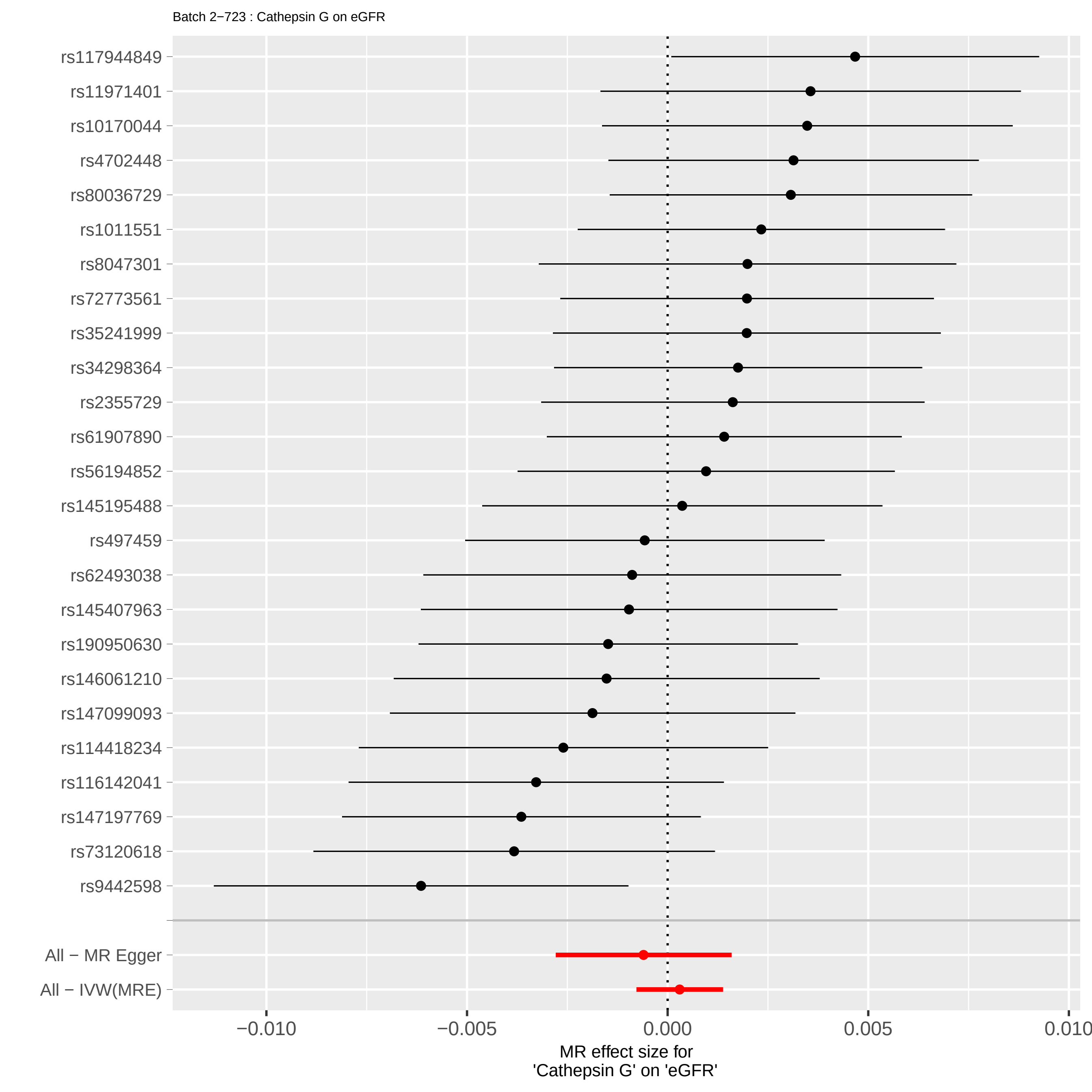

Batch 2−723 : Cathepsin G on eGFR
rs117944849
rs11971401
rs10170044
rs4702448
rs80036729
rs1011551
rs8047301
rs72773561
rs35241999
rs34298364
rs2355729
rs61907890
rs56194852
rs145195488
rs497459
rs62493038
rs145407963
rs190950630
rs146061210
rs147099093
rs114418234
rs116142041
rs147197769
rs73120618
rs9442598
All − MR Egger
All − IVW(MRE)
−0.010
−0.005
0.000
0.005
0.010
MR effect size for
'Cathepsin G' on 'eGFR'

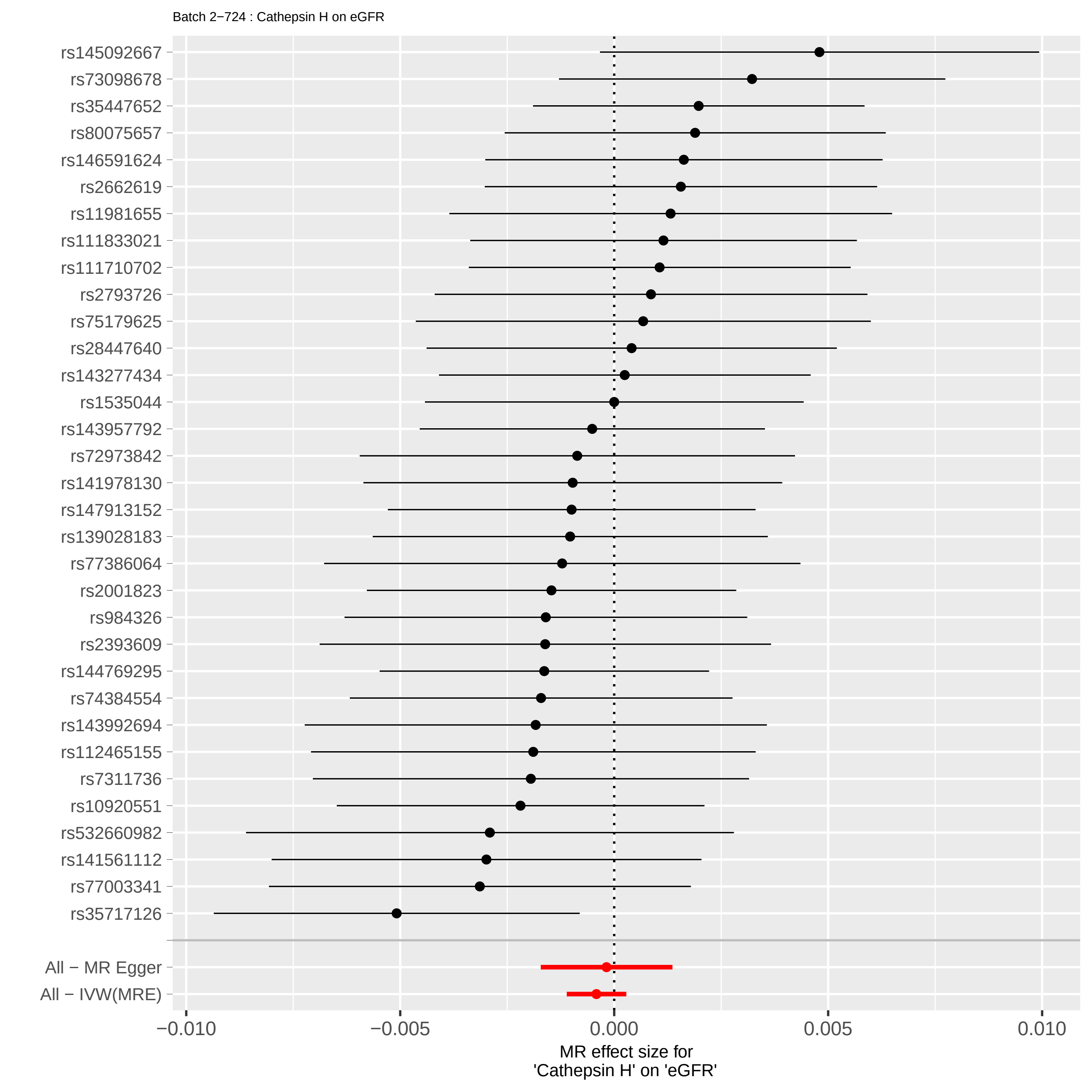

Batch 2−724 : Cathepsin H on eGFR
rs145092667
rs73098678
rs35447652
rs80075657
rs146591624
rs2662619
rs11981655
rs111833021
rs111710702
rs2793726
rs75179625
rs28447640
rs143277434
rs1535044
rs143957792
rs72973842
rs141978130
rs147913152
rs139028183
rs77386064
rs2001823
rs984326
rs2393609
rs144769295
rs74384554
rs143992694
rs112465155
rs7311736
rs10920551
rs532660982
rs141561112
rs77003341
rs35717126
All − MR Egger
All − IVW(MRE)
−0.010
−0.005
0.000
0.005
0.010
MR effect size for
'Cathepsin H' on 'eGFR'

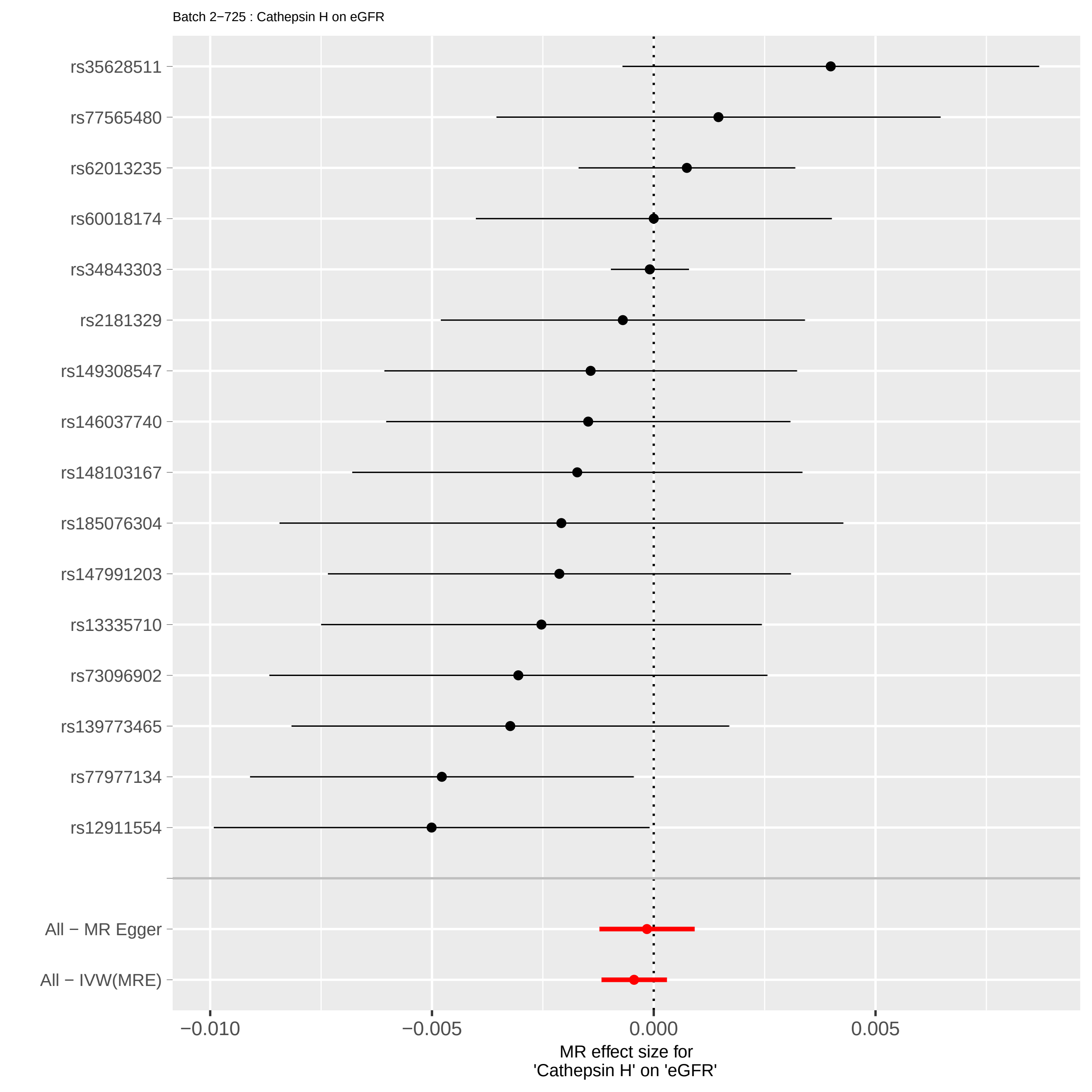

Batch 2−725 : Cathepsin H on eGFR
rs35628511
rs77565480
rs62013235
rs60018174
rs34843303
rs2181329
rs149308547
rs146037740
rs148103167
rs185076304
rs147991203
rs13335710
rs73096902
rs139773465
rs77977134
rs12911554
All − MR Egger
All − IVW(MRE)
−0.010
−0.005
0.000
0.005
MR effect size for
'Cathepsin H' on 'eGFR'

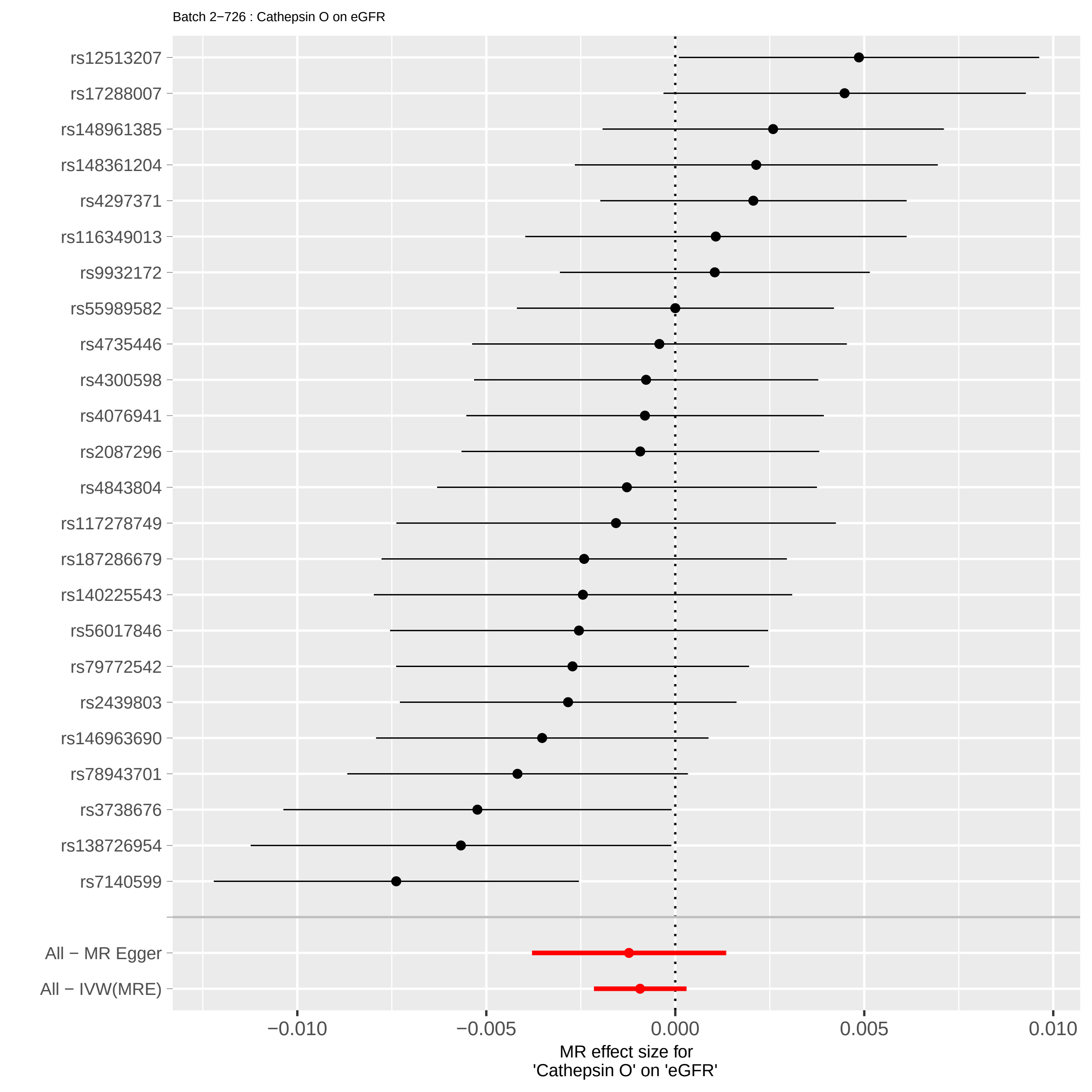

Batch 2−726 : Cathepsin O on eGFR
rs12513207
rs17288007
rs148961385
rs148361204
rs4297371
rs116349013
rs9932172
rs55989582
rs4735446
rs4300598
rs4076941
rs2087296
rs4843804
rs117278749
rs187286679
rs140225543
rs56017846
rs79772542
rs2439803
rs146963690
rs78943701
rs3738676
rs138726954
rs7140599
All − MR Egger
All − IVW(MRE)
−0.010
−0.005
0.000
0.005
0.010
MR effect size for
'Cathepsin O' on 'eGFR'

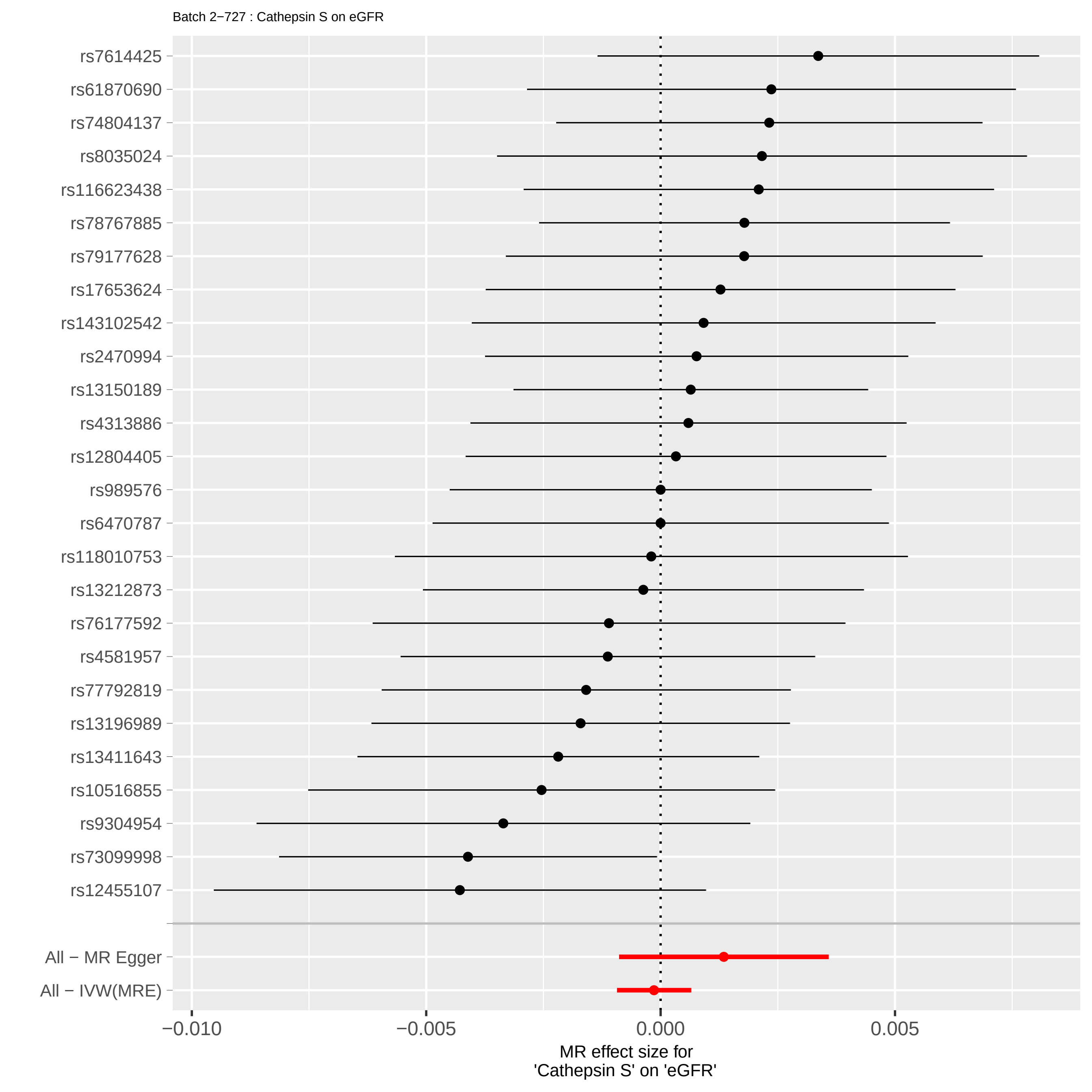

Batch 2−727 : Cathepsin S on eGFR
rs7614425
rs61870690
rs74804137
rs8035024
rs116623438
rs78767885
rs79177628
rs17653624
rs143102542
rs2470994
rs13150189
rs4313886
rs12804405
rs989576
rs6470787
rs118010753
rs13212873
rs76177592
rs4581957
rs77792819
rs13196989
rs13411643
rs10516855
rs9304954
rs73099998
rs12455107
All − MR Egger
All − IVW(MRE)
−0.010
−0.005
0.000
0.005
MR effect size for
'Cathepsin S' on 'eGFR'

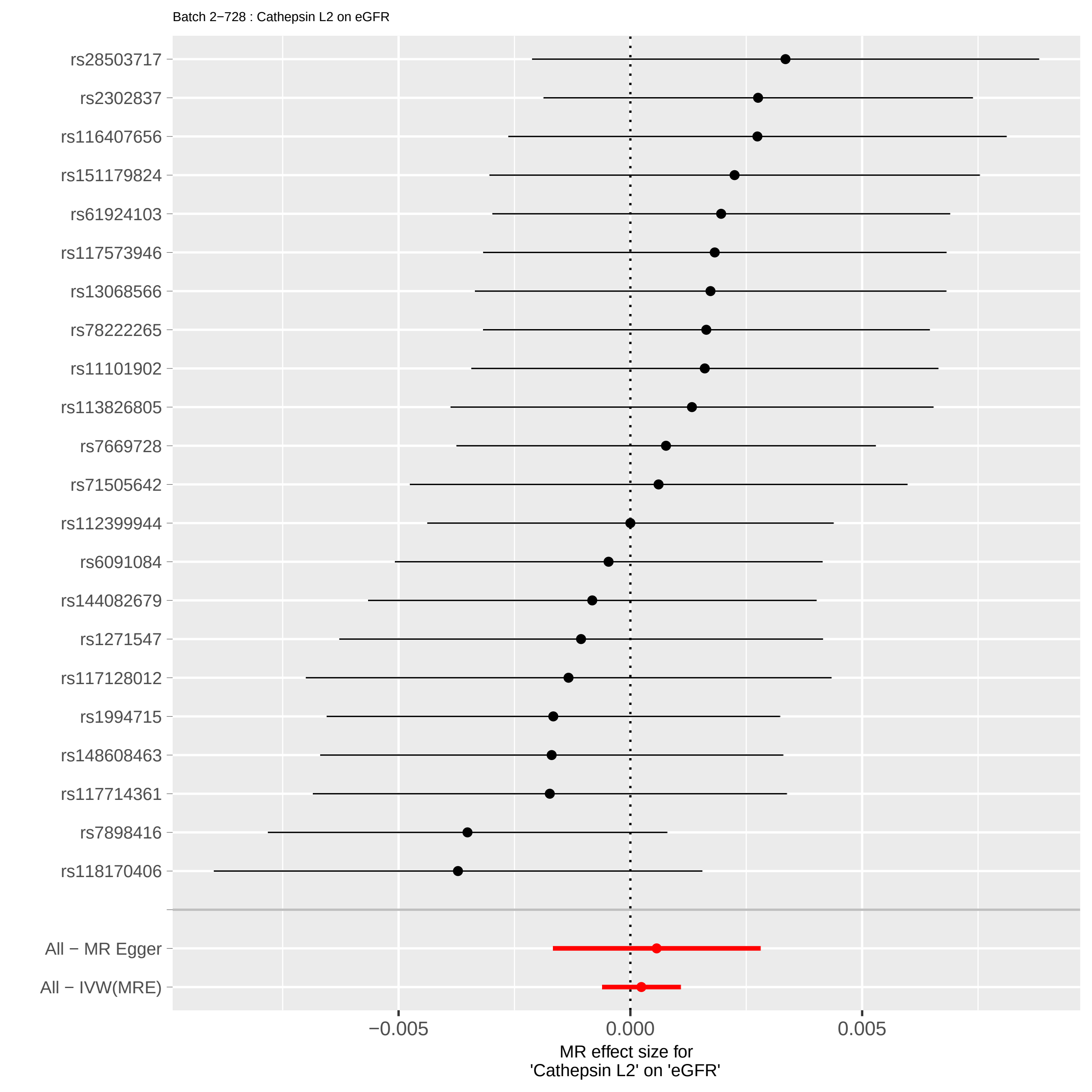

Batch 2−728 : Cathepsin L2 on eGFR
rs28503717
rs2302837
rs116407656
rs151179824
rs61924103
rs117573946
rs13068566
rs78222265
rs11101902
rs113826805
rs7669728
rs71505642
rs112399944
rs6091084
rs144082679
rs1271547
rs117128012
rs1994715
rs148608463
rs117714361
rs7898416
rs118170406
All − MR Egger
All − IVW(MRE)
−0.005
0.000
0.005
MR effect size for
'Cathepsin L2' on 'eGFR'

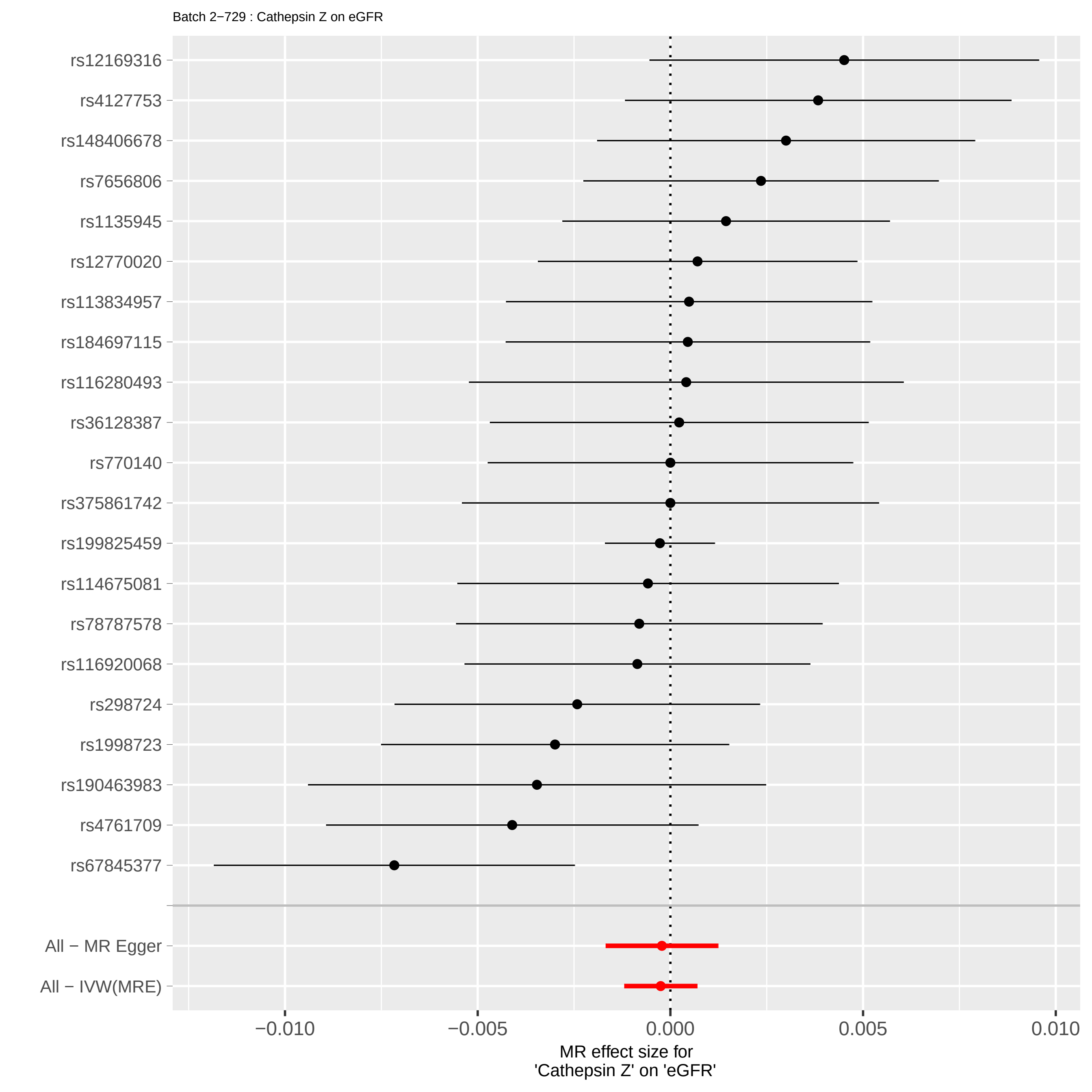

Batch 2−729 : Cathepsin Z on eGFR
rs12169316
rs4127753
rs148406678
rs7656806
rs1135945
rs12770020
rs113834957
rs184697115
rs116280493
rs36128387
rs770140
rs375861742
rs199825459
rs114675081
rs78787578
rs116920068
rs298724
rs1998723
rs190463983
rs4761709
rs67845377
All − MR Egger
All − IVW(MRE)
−0.010
−0.005
0.000
0.005
0.010
MR effect size for
'Cathepsin Z' on 'eGFR'

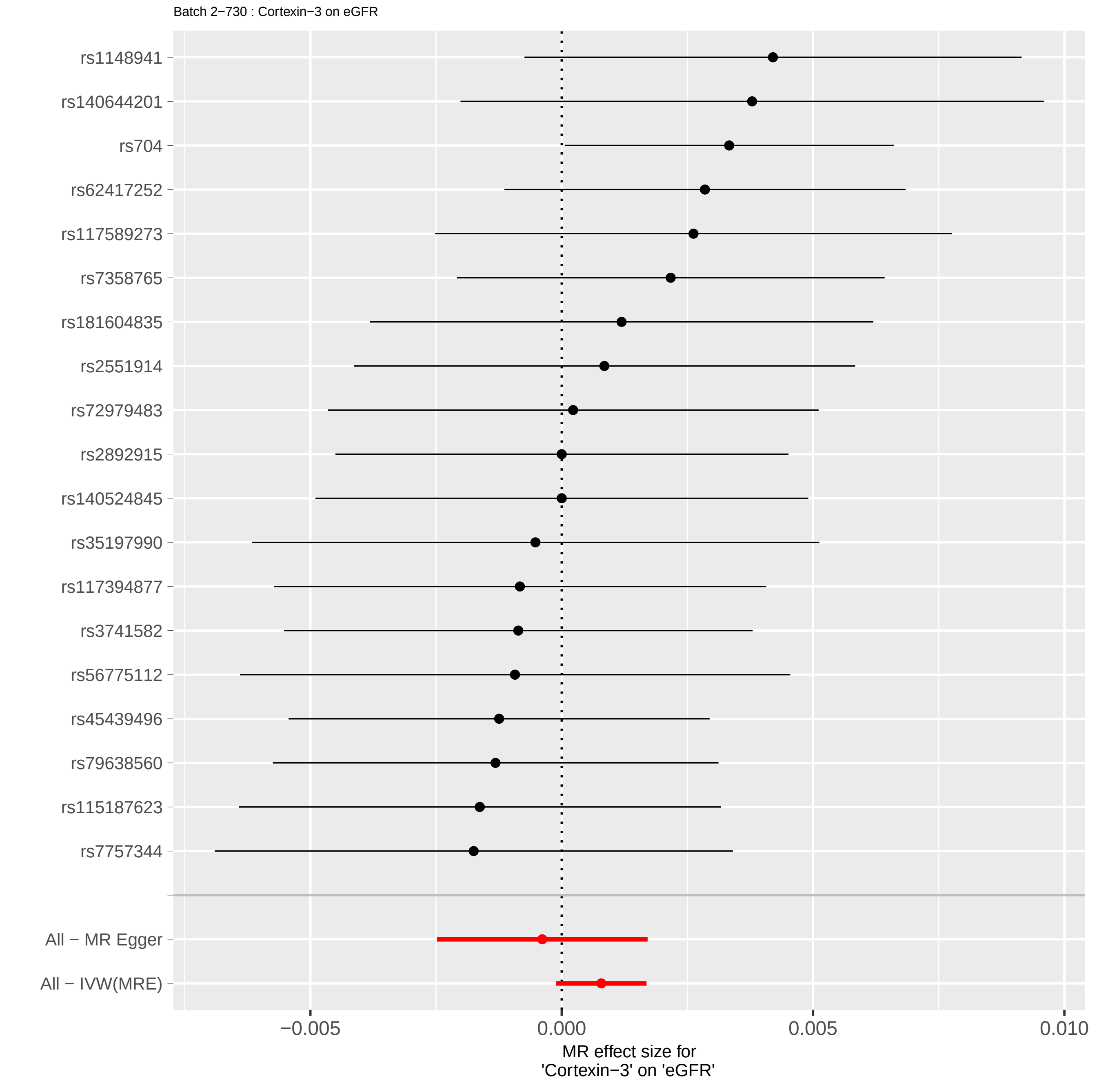

Batch 2−730 : Cortexin−3 on eGFR
rs1148941
rs140644201
rs704
rs62417252
rs117589273
rs7358765
rs181604835
rs2551914
rs72979483
rs2892915
rs140524845
rs35197990
rs117394877
rs3741582
rs56775112
rs45439496
rs79638560
rs115187623
rs7757344
All − MR Egger
All − IVW(MRE)
−0.005
0.000
0.005
0.010
MR effect size for
'Cortexin−3' on 'eGFR'

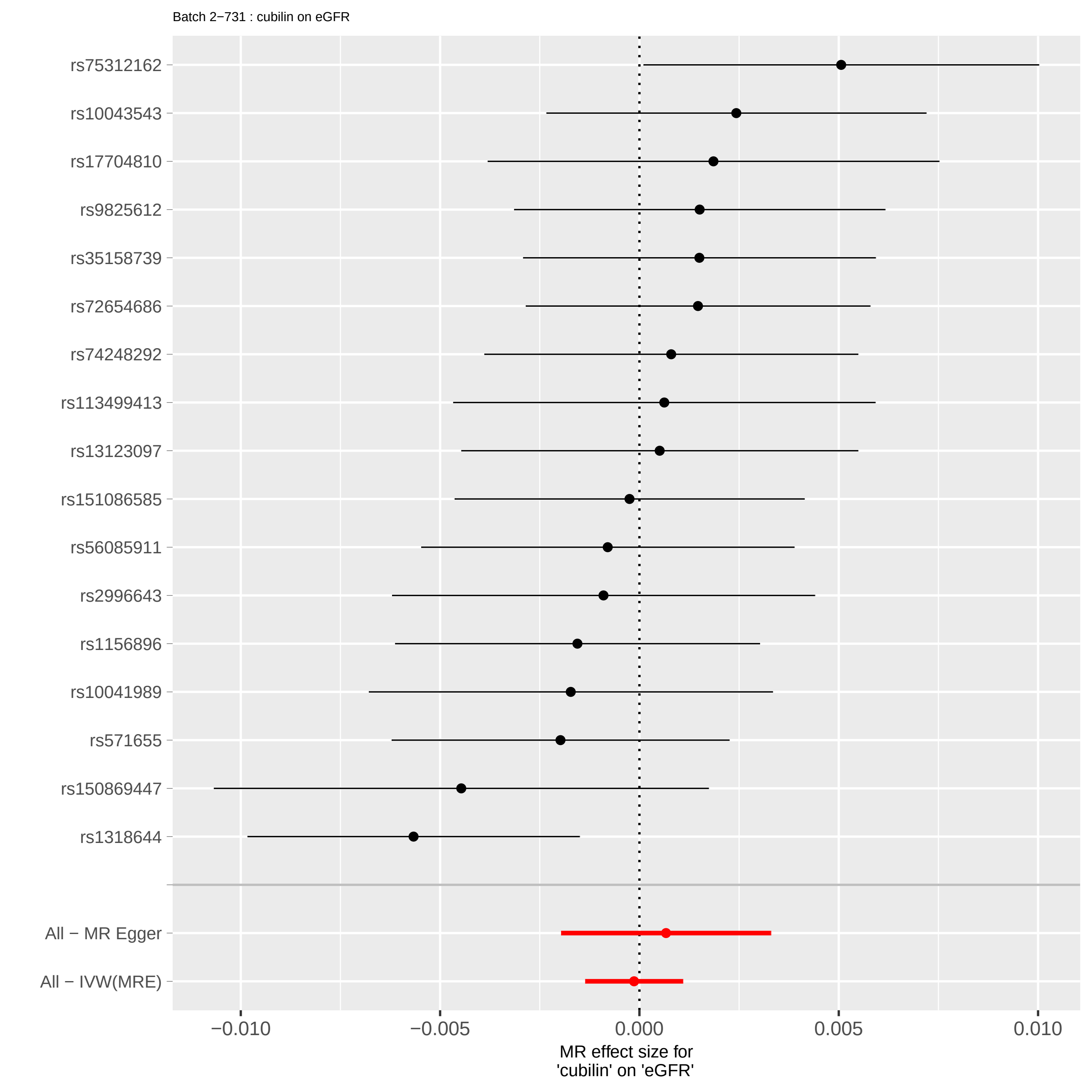

Batch 2−731 : cubilin on eGFR
rs75312162
rs10043543
rs17704810
rs9825612
rs35158739
rs72654686
rs74248292
rs113499413
rs13123097
rs151086585
rs56085911
rs2996643
rs1156896
rs10041989
rs571655
rs150869447
rs1318644
All − MR Egger
All − IVW(MRE)
−0.010
−0.005
0.000
0.005
0.010
MR effect size for
'cubilin' on 'eGFR'

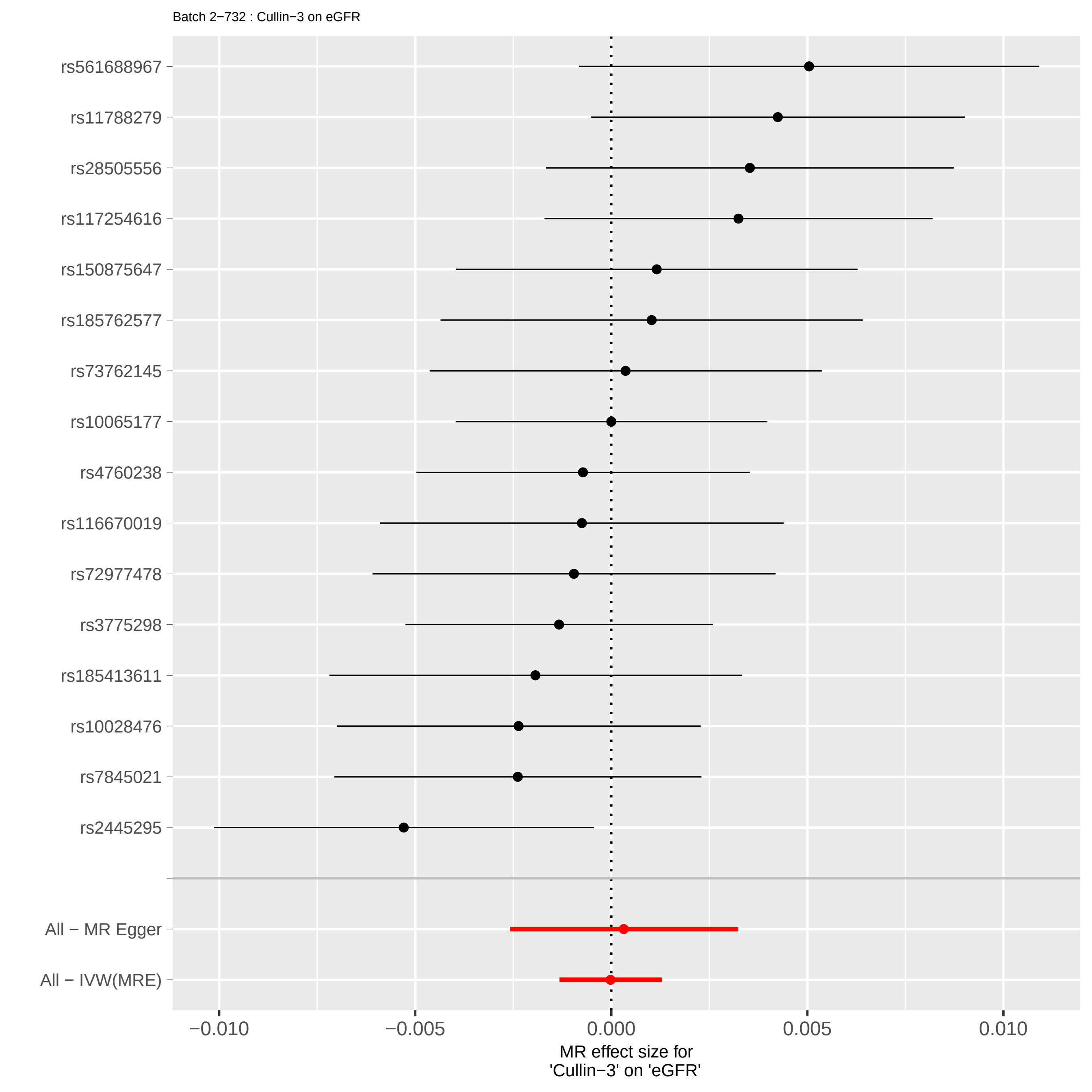

Batch 2−732 : Cullin−3 on eGFR
rs561688967
rs11788279
rs28505556
rs117254616
rs150875647
rs185762577
rs73762145
rs10065177
rs4760238
rs116670019
rs72977478
rs3775298
rs185413611
rs10028476
rs7845021
rs2445295
All − MR Egger
All − IVW(MRE)
−0.010
−0.005
0.000
0.005
0.010
MR effect size for
'Cullin−3' on 'eGFR'

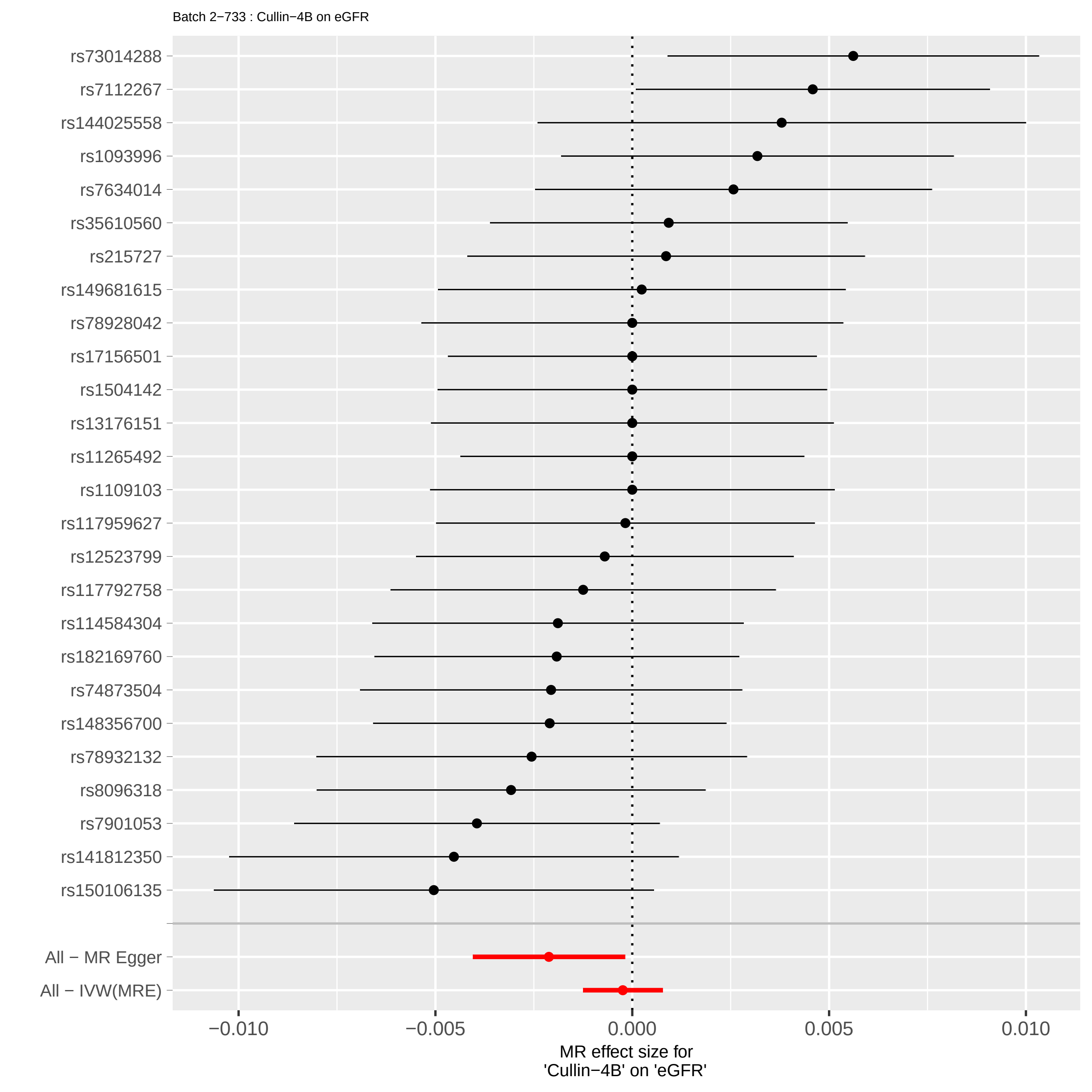
Batch 2−733 : Cullin−4B on eGFR
rs73014288
rs7112267
rs144025558
rs1093996
rs7634014
rs35610560
rs215727
rs149681615
rs78928042
rs17156501
rs1504142
rs13176151
rs11265492
rs1109103
rs117959627
rs12523799
rs117792758
rs114584304
rs182169760
rs74873504
rs148356700
rs78932132
rs8096318
rs7901053
rs141812350
rs150106135
All − MR Egger
All − IVW(MRE)
−0.010
−0.005
0.000
0.005
0.010
MR effect size for
'Cullin−4B' on 'eGFR'

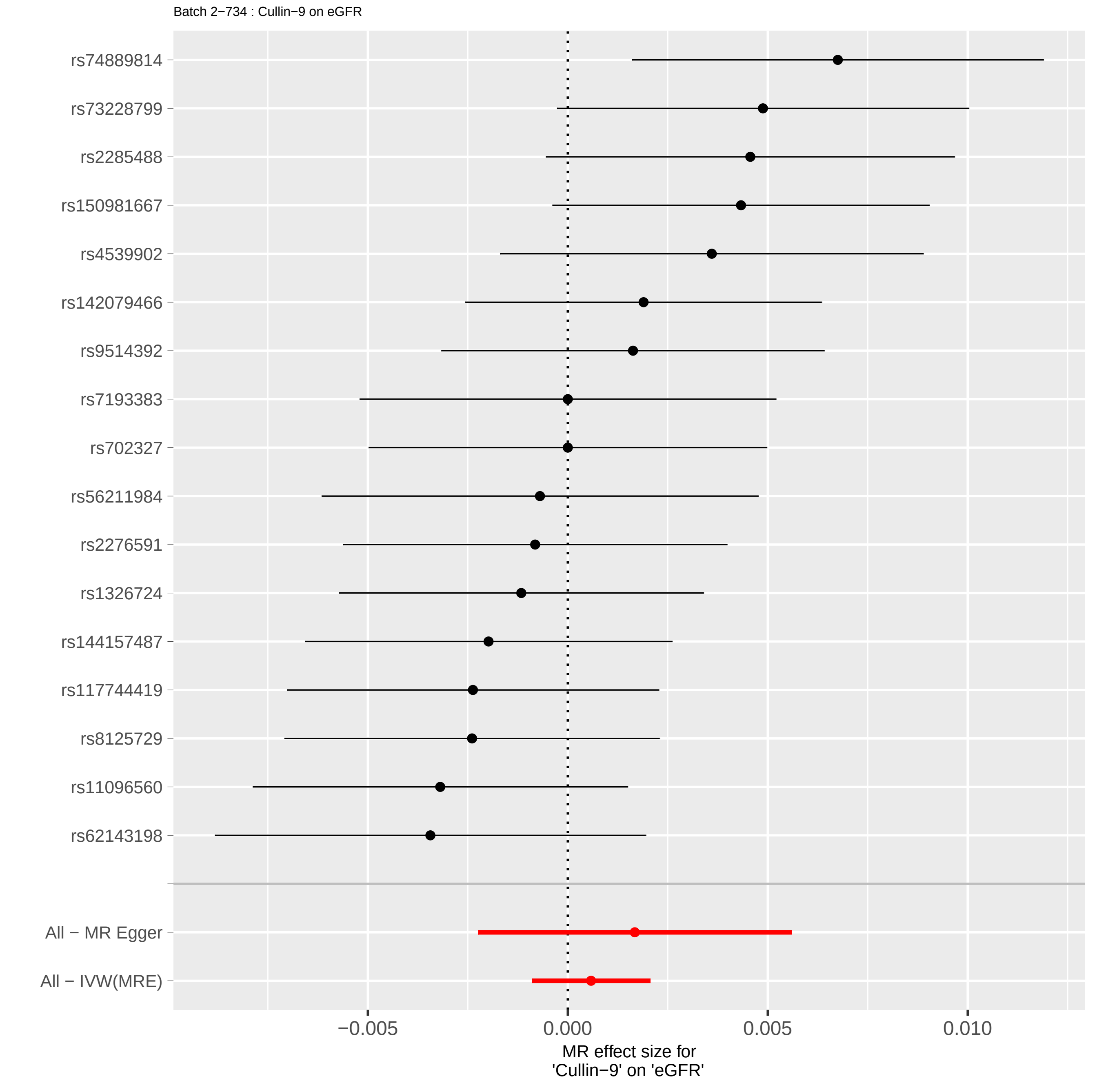

Batch 2−734 : Cullin−9 on eGFR
rs74889814
rs73228799
rs2285488
rs150981667
rs4539902
rs142079466
rs9514392
rs7193383
rs702327
rs56211984
rs2276591
rs1326724
rs144157487
rs117744419
rs8125729
rs11096560
rs62143198
All − MR Egger
All − IVW(MRE)
−0.005
0.000
0.005
0.010
MR effect size for
'Cullin−9' on 'eGFR'

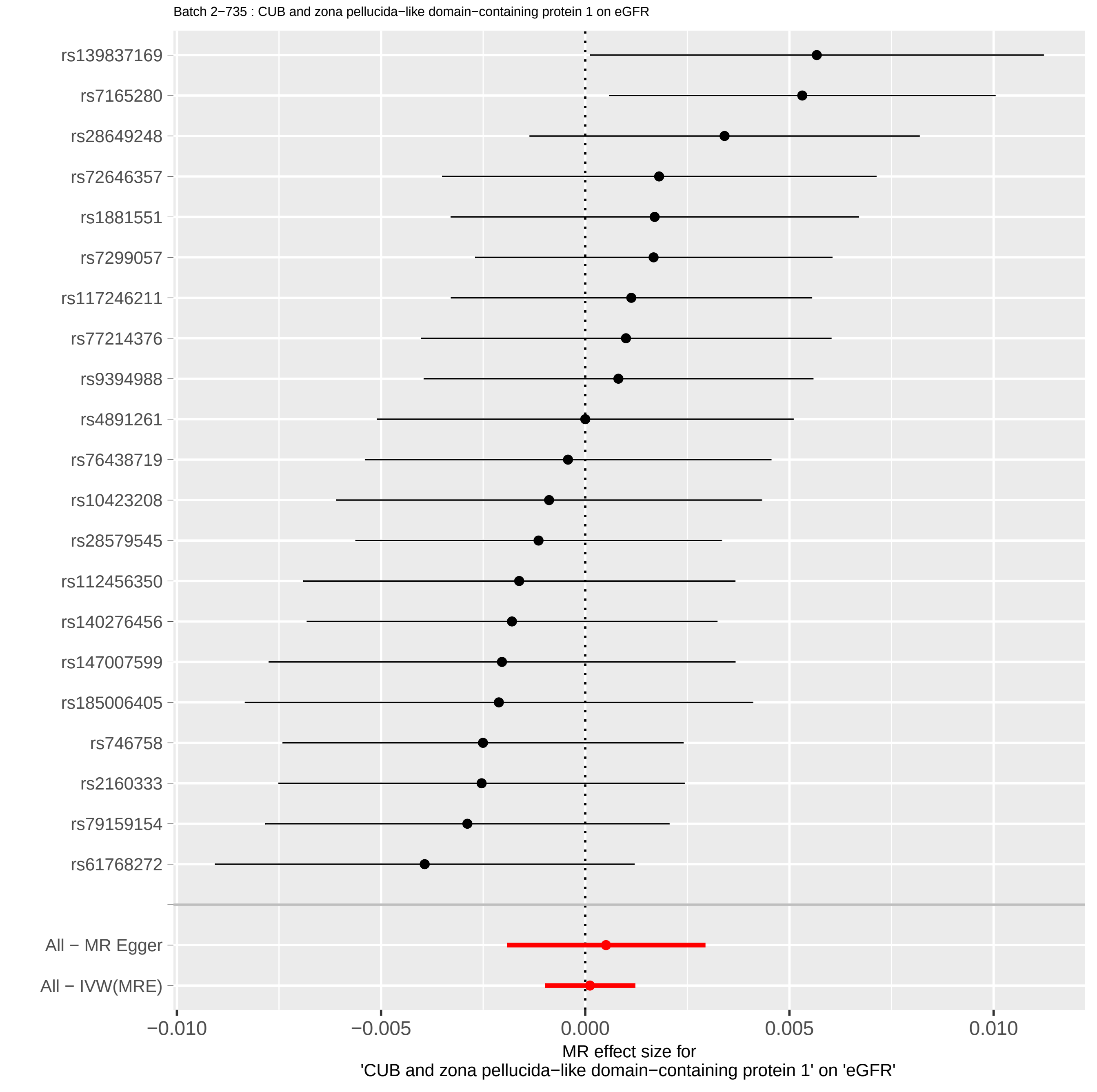

Batch 2−735 : CUB and zona pellucida−like domain−containing protein 1 on eGFR
rs139837169
rs7165280
rs28649248
rs72646357
rs1881551
rs7299057
rs117246211
rs77214376
rs9394988
rs4891261
rs76438719
rs10423208
rs28579545
rs112456350
rs140276456
rs147007599
rs185006405
rs746758
rs2160333
rs79159154
rs61768272
All − MR Egger
All − IVW(MRE)
−0.010
−0.005
0.000
0.005
0.010
MR effect size for
'CUB and zona pellucida−like domain−containing protein 1' on 'eGFR'

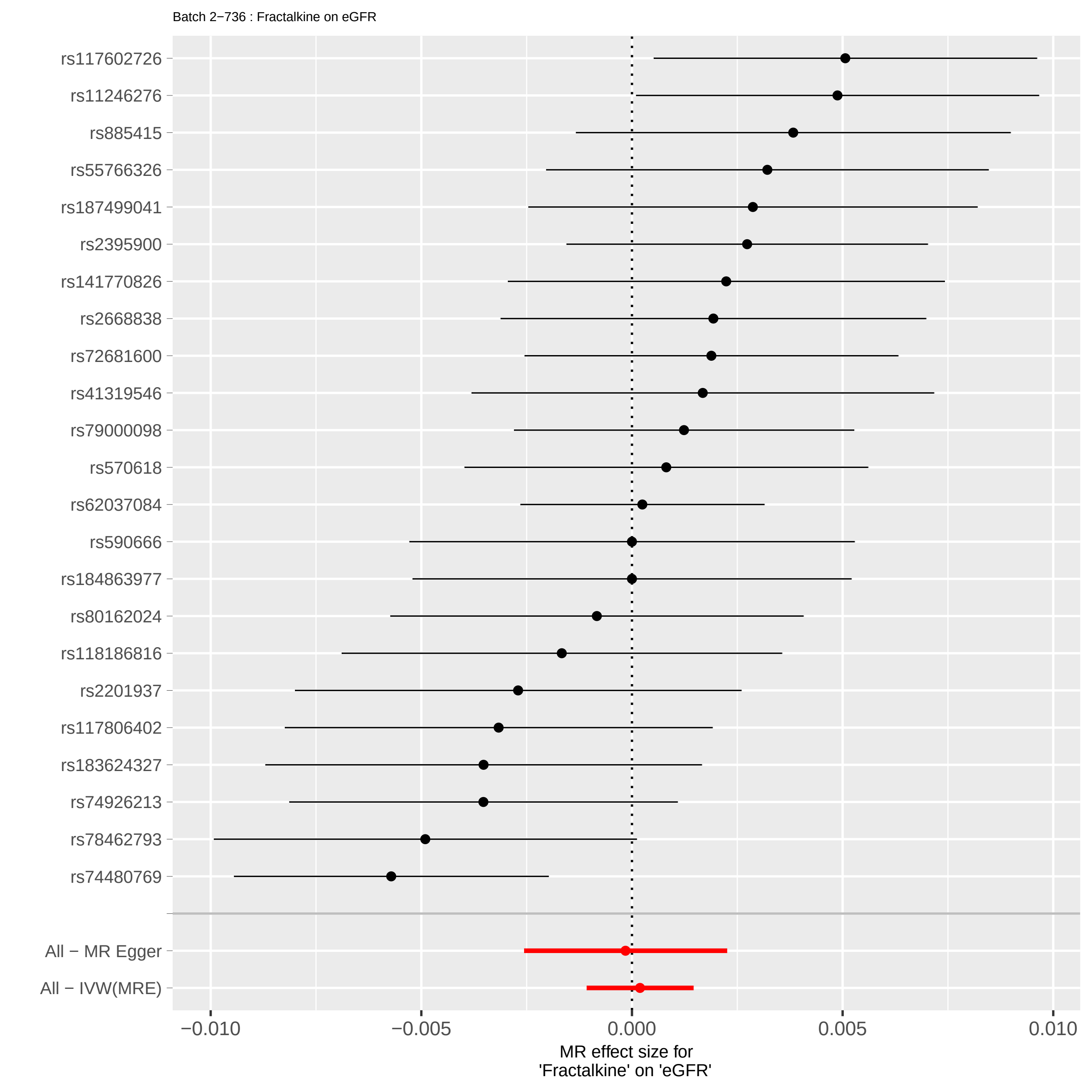

Batch 2−736 : Fractalkine on eGFR
rs117602726
rs11246276
rs885415
rs55766326
rs187499041
rs2395900
rs141770826
rs2668838
rs72681600
rs41319546
rs79000098
rs570618
rs62037084
rs590666
rs184863977
rs80162024
rs118186816
rs2201937
rs117806402
rs183624327
rs74926213
rs78462793
rs74480769
All − MR Egger
All − IVW(MRE)
−0.010
−0.005
0.000
0.005
0.010
MR effect size for
'Fractalkine' on 'eGFR'

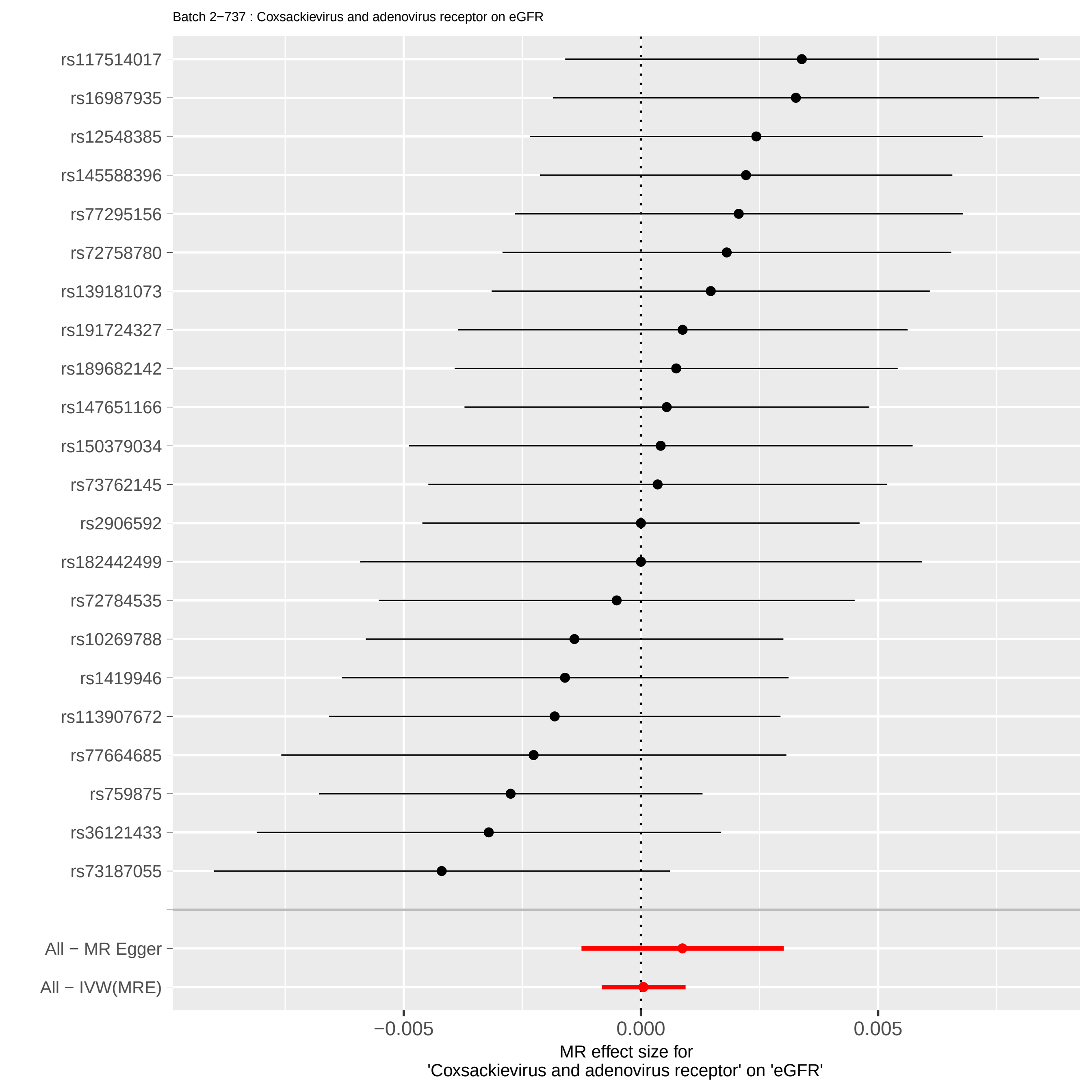
Batch 2−737 : Coxsackievirus and adenovirus receptor on eGFR
rs117514017
rs16987935
rs12548385
rs145588396
rs77295156
rs72758780
rs139181073
rs191724327
rs189682142
rs147651166
rs150379034
rs73762145
rs2906592
rs182442499
rs72784535
rs10269788
rs1419946
rs113907672
rs77664685
rs759875
rs36121433
rs73187055
All − MR Egger
All − IVW(MRE)
−0.005
0.000
0.005
MR effect size for
'Coxsackievirus and adenovirus receptor' on 'eGFR'

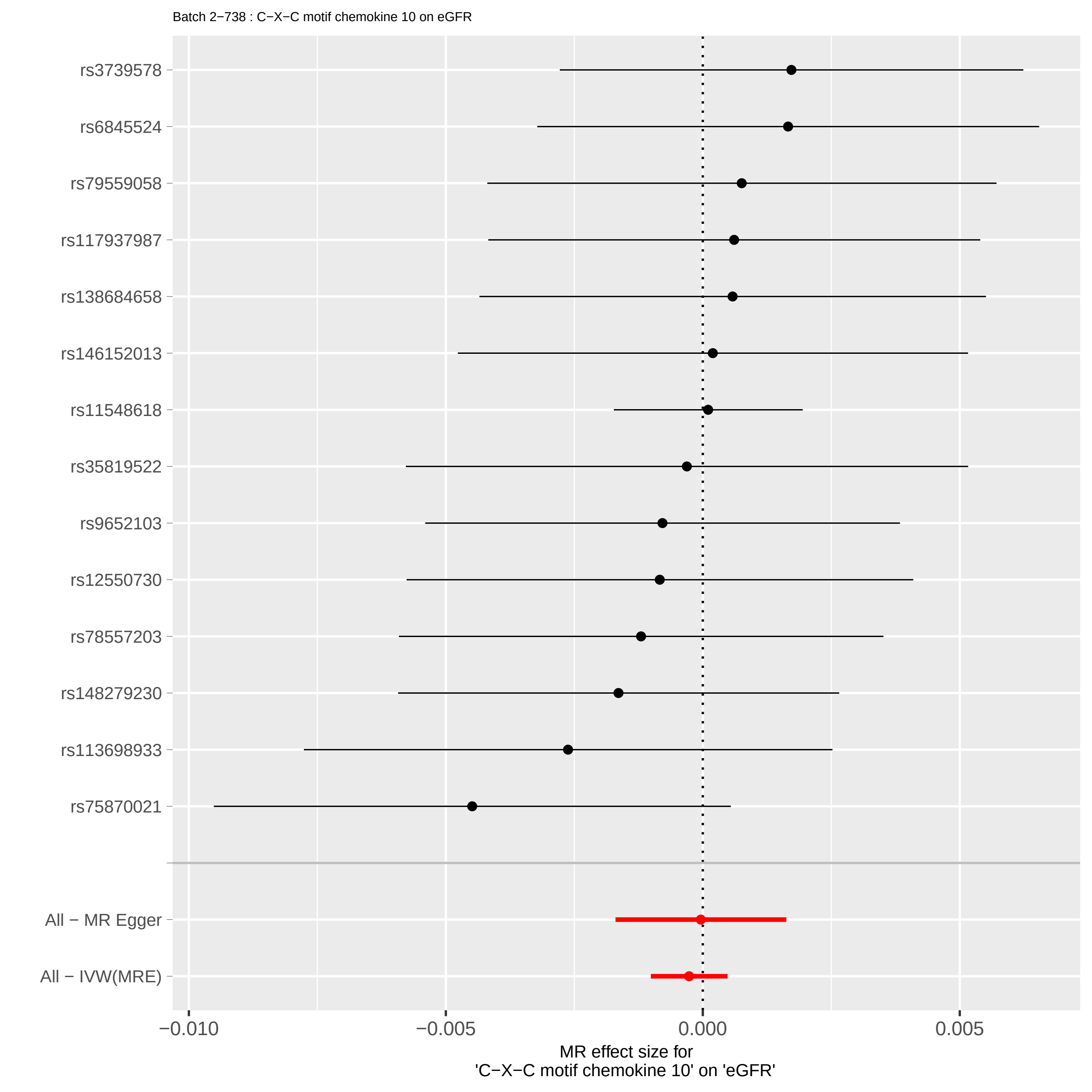
Batch 2−738 : C−X−C motif chemokine 10 on eGFR
rs3739578
rs6845524
rs79559058
rs117937987
rs138684658
rs146152013
rs11548618
rs35819522
rs9652103
rs12550730
rs78557203
rs148279230
rs113698933
rs75870021
All − MR Egger
All − IVW(MRE)
−0.010
−0.005
0.000
0.005
MR effect size for
'C−X−C motif chemokine 10' on 'eGFR'

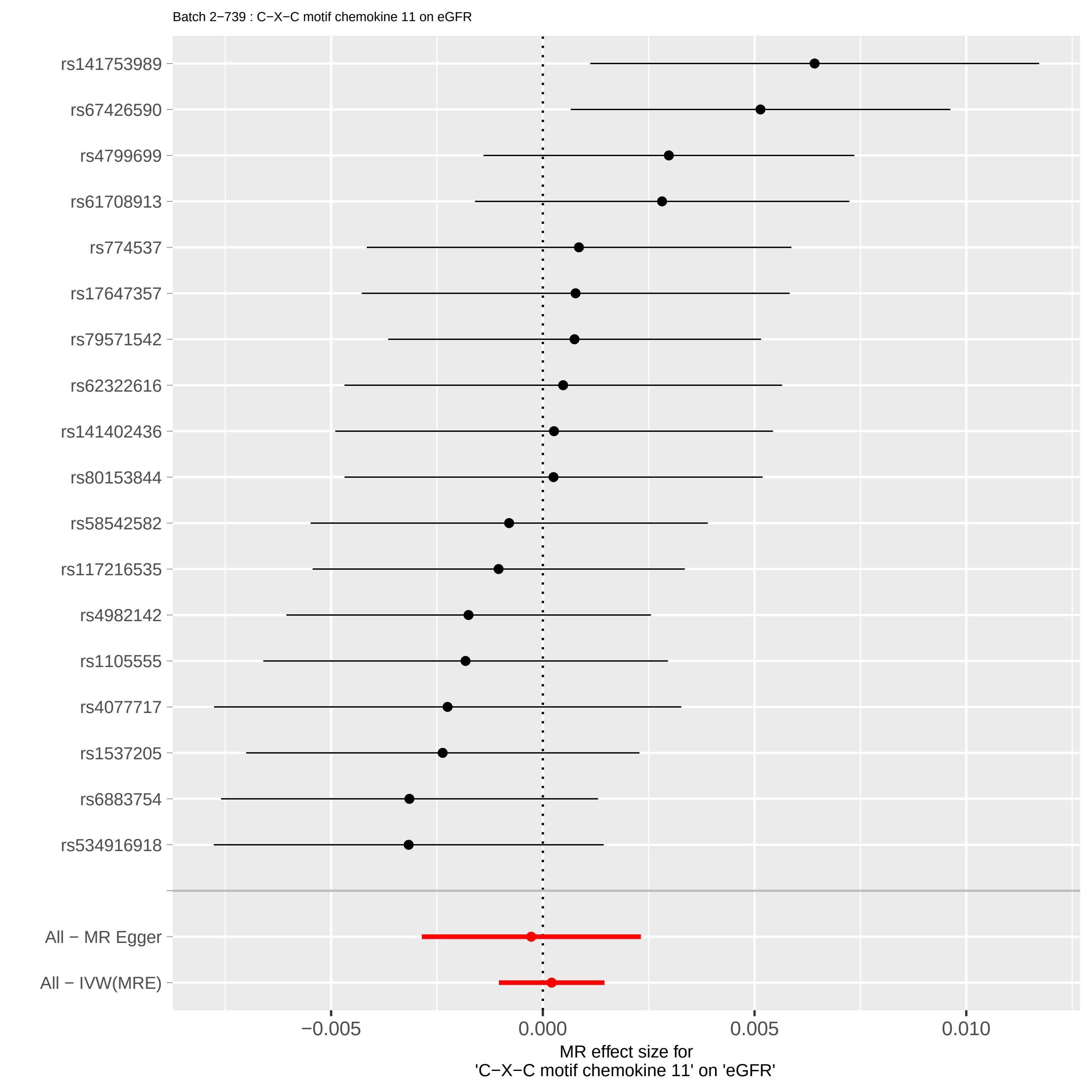

Batch 2−739 : C−X−C motif chemokine 11 on eGFR
rs141753989
rs67426590
rs4799699
rs61708913
rs774537
rs17647357
rs79571542
rs62322616
rs141402436
rs80153844
rs58542582
rs117216535
rs4982142
rs1105555
rs4077717
rs1537205
rs6883754
rs534916918
All − MR Egger
All − IVW(MRE)
−0.005
0.000
0.005
0.010
MR effect size for
'C−X−C motif chemokine 11' on 'eGFR'

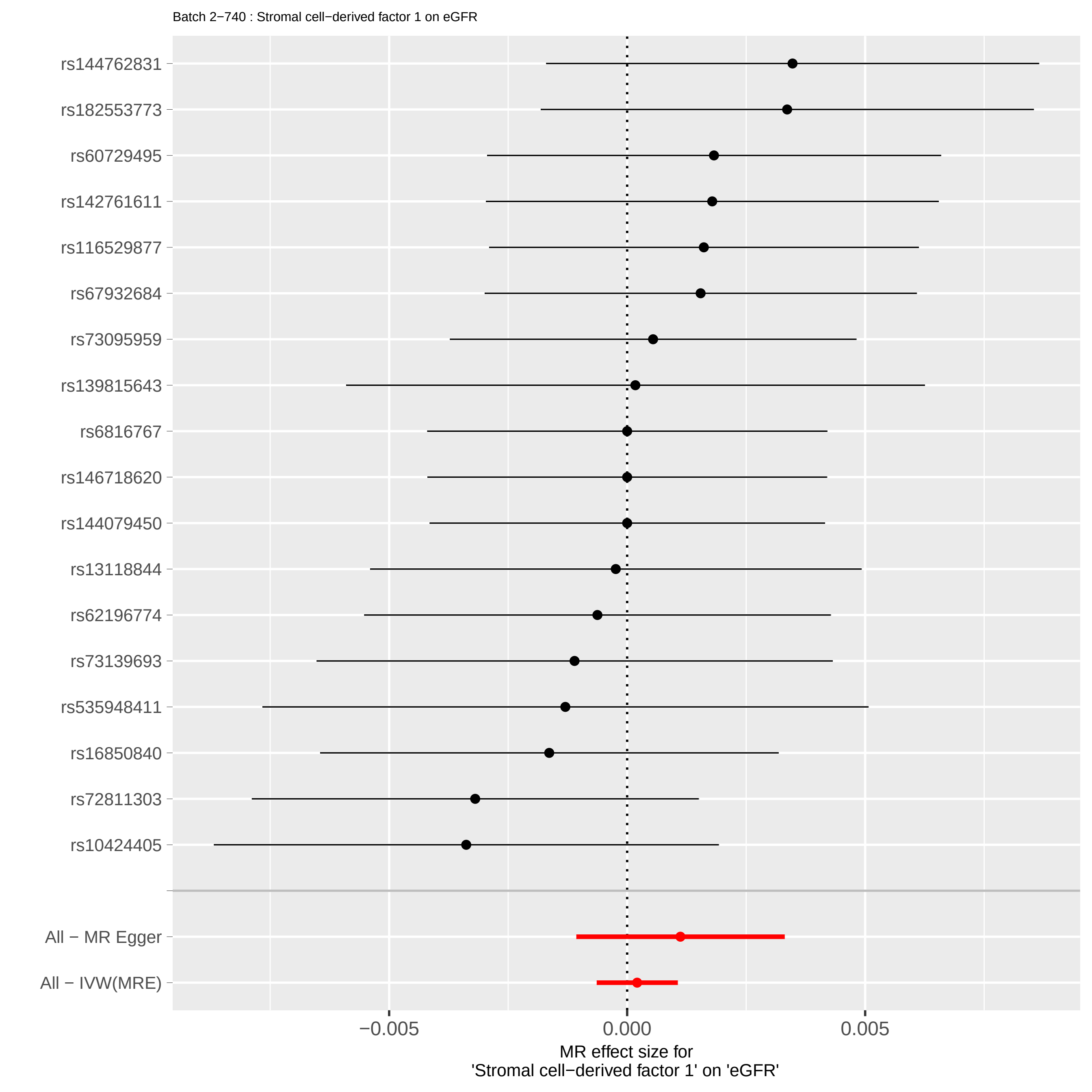

Batch 2−740 : Stromal cell−derived factor 1 on eGFR
rs144762831
rs182553773
rs60729495
rs142761611
rs116529877
rs67932684
rs73095959
rs139815643
rs6816767
rs146718620
rs144079450
rs13118844
rs62196774
rs73139693
rs535948411
rs16850840
rs72811303
rs10424405
All − MR Egger
All − IVW(MRE)
−0.005
0.000
0.005
MR effect size for
'Stromal cell−derived factor 1' on 'eGFR'

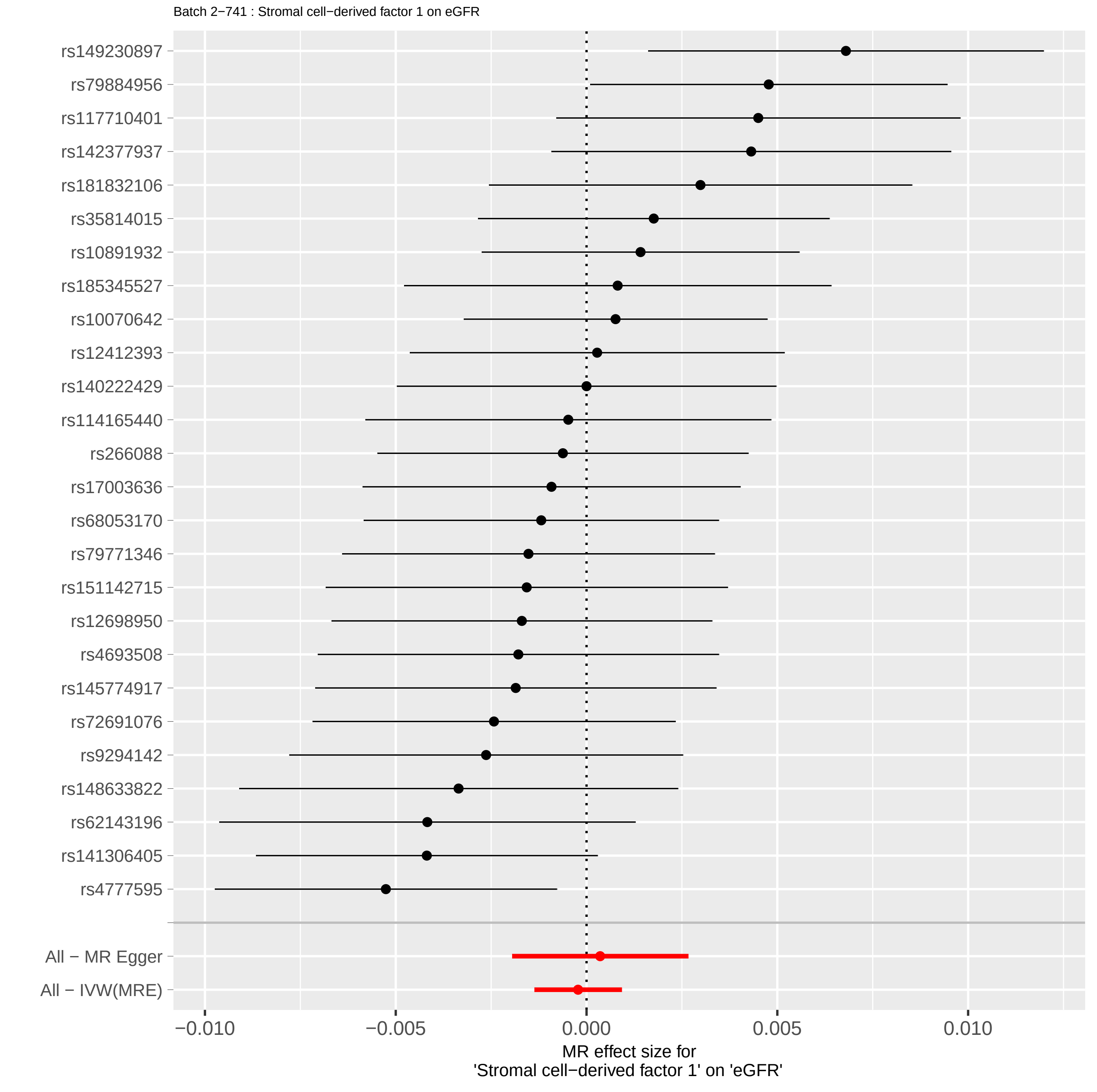
Batch 2−741 : Stromal cell−derived factor 1 on eGFR
rs149230897
rs79884956
rs117710401
rs142377937
rs181832106
rs35814015
rs10891932
rs185345527
rs10070642
rs12412393
rs140222429
rs114165440
rs266088
rs17003636
rs68053170
rs79771346
rs151142715
rs12698950
rs4693508
rs145774917
rs72691076
rs9294142
rs148633822
rs62143196
rs141306405
rs4777595
All − MR Egger
All − IVW(MRE)
−0.010
−0.005
0.000
0.005
0.010
MR effect size for
'Stromal cell−derived factor 1' on 'eGFR'

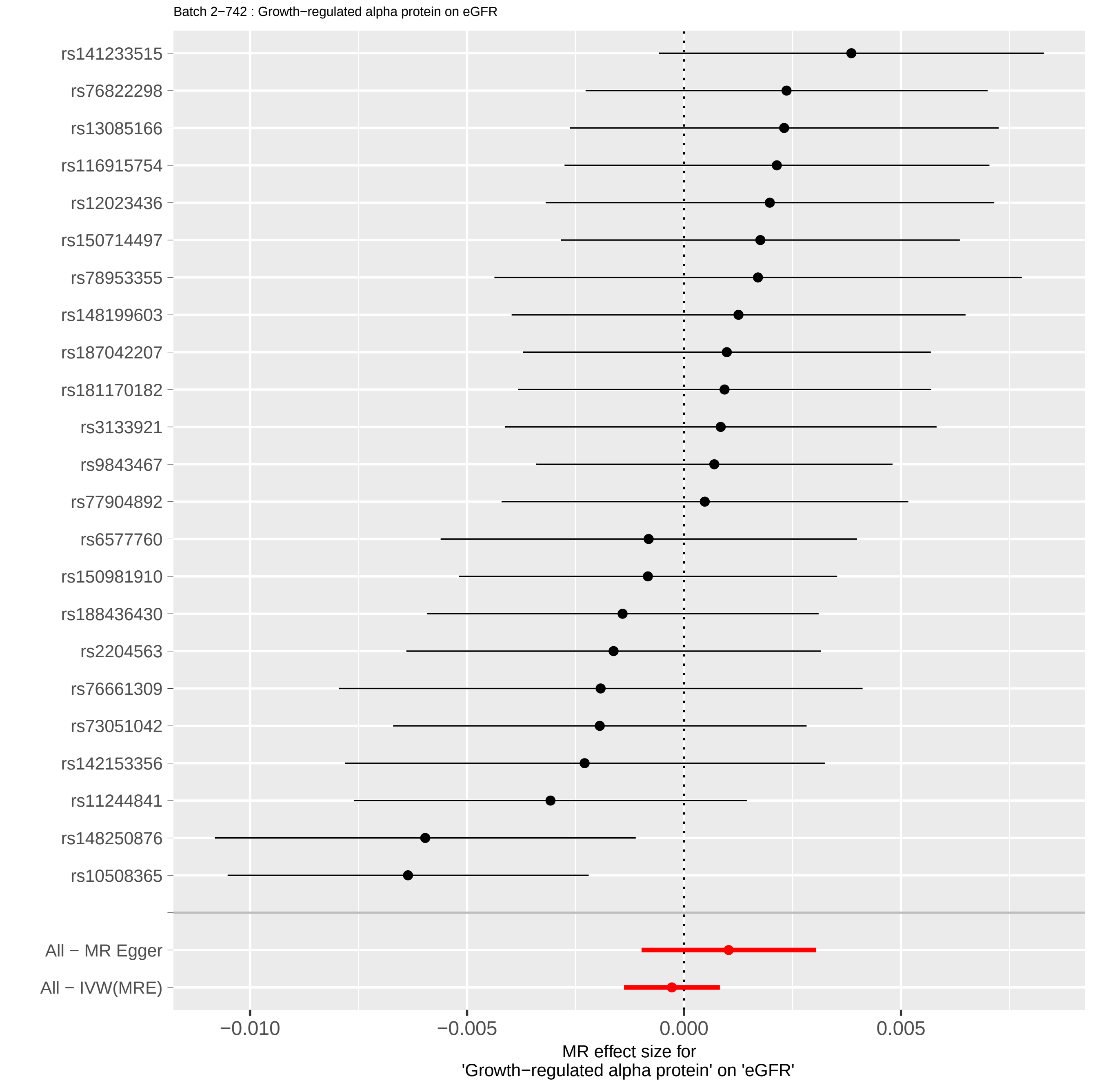

Batch 2−742 : Growth−regulated alpha protein on eGFR
rs141233515
rs76822298
rs13085166
rs116915754
rs12023436
rs150714497
rs78953355
rs148199603
rs187042207
rs181170182
rs3133921
rs9843467
rs77904892
rs6577760
rs150981910
rs188436430
rs2204563
rs76661309
rs73051042
rs142153356
rs11244841
rs148250876
rs10508365
All − MR Egger
All − IVW(MRE)
−0.010
−0.005
0.000
0.005
MR effect size for
'Growth−regulated alpha protein' on 'eGFR'

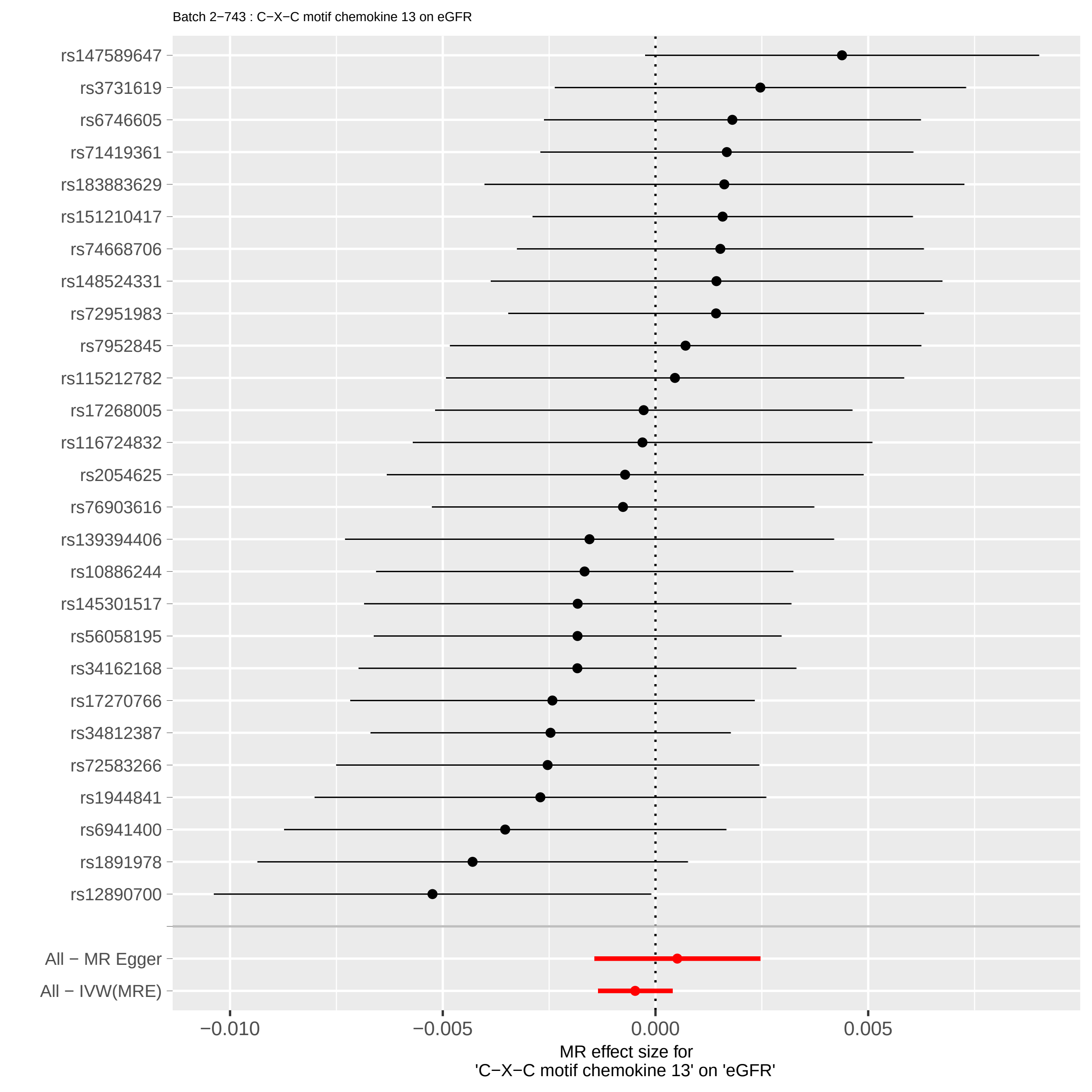

Batch 2−743 : C−X−C motif chemokine 13 on eGFR
rs147589647
rs3731619
rs6746605
rs71419361
rs183883629
rs151210417
rs74668706
rs148524331
rs72951983
rs7952845
rs115212782
rs17268005
rs116724832
rs2054625
rs76903616
rs139394406
rs10886244
rs145301517
rs56058195
rs34162168
rs17270766
rs34812387
rs72583266
rs1944841
rs6941400
rs1891978
rs12890700
All − MR Egger
All − IVW(MRE)
−0.010
−0.005
0.000
0.005
MR effect size for
'C−X−C motif chemokine 13' on 'eGFR'

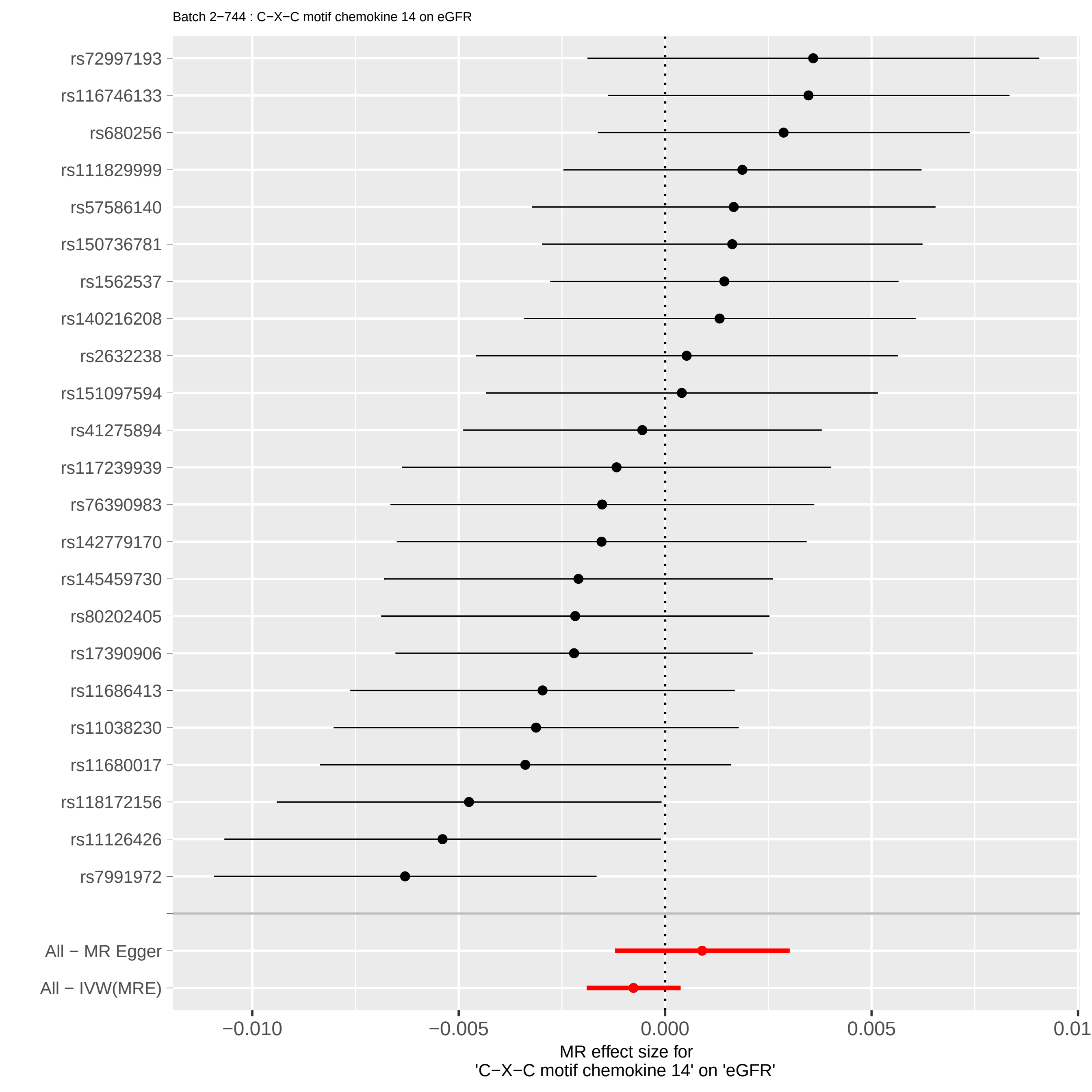

Batch 2−744 : C−X−C motif chemokine 14 on eGFR
rs72997193
rs116746133
rs680256
rs111829999
rs57586140
rs150736781
rs1562537
rs140216208
rs2632238
rs151097594
rs41275894
rs117239939
rs76390983
rs142779170
rs145459730
rs80202405
rs17390906
rs11686413
rs11038230
rs11680017
rs118172156
rs11126426
rs7991972
All − MR Egger
All − IVW(MRE)
−0.010
−0.005
0.000
0.005
0.01
MR effect size for
'C−X−C motif chemokine 14' on 'eGFR'

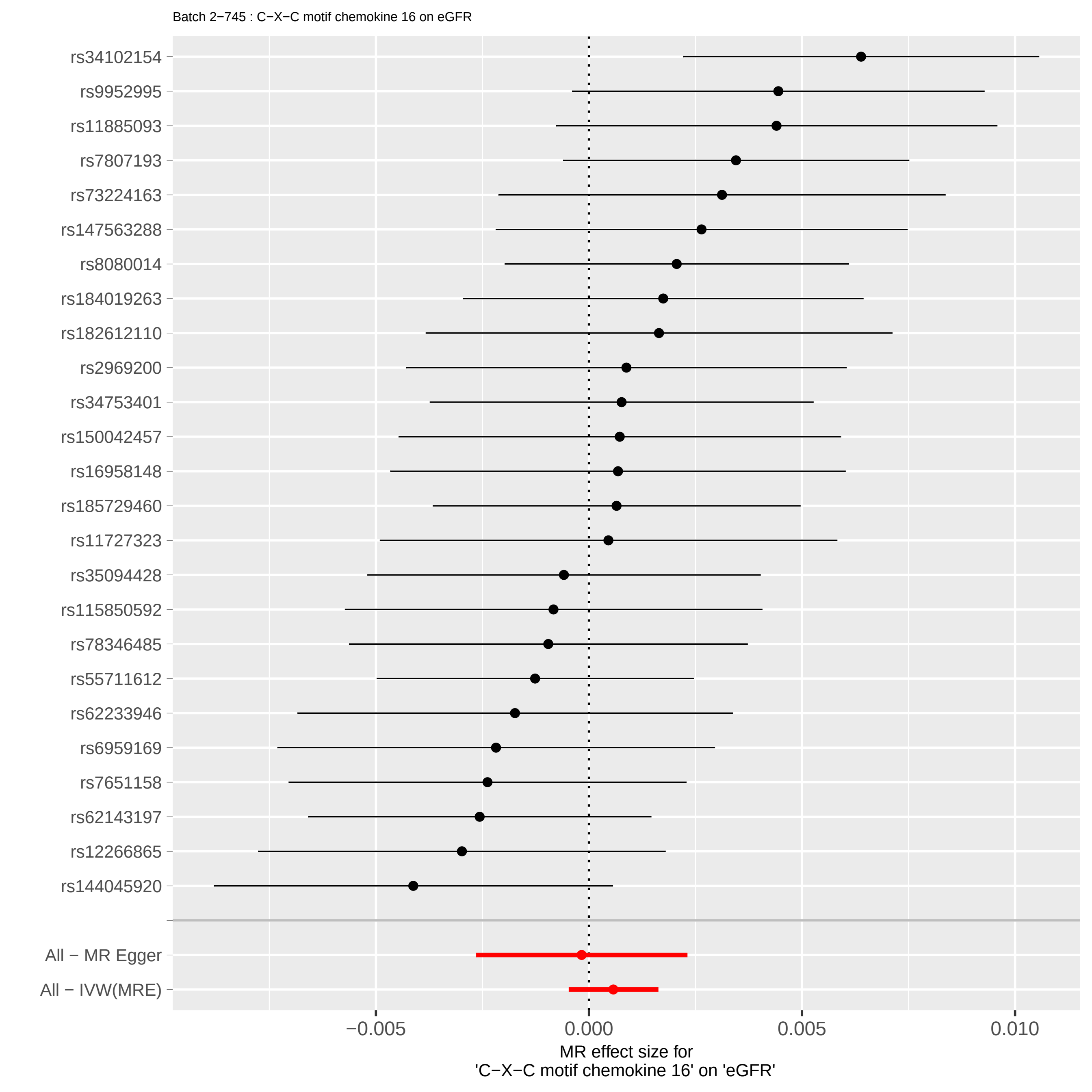
Batch 2−745 : C−X−C motif chemokine 16 on eGFR
rs34102154
rs9952995
rs11885093
rs7807193
rs73224163
rs147563288
rs8080014
rs184019263
rs182612110
rs2969200
rs34753401
rs150042457
rs16958148
rs185729460
rs11727323
rs35094428
rs115850592
rs78346485
rs55711612
rs62233946
rs6959169
rs7651158
rs62143197
rs12266865
rs144045920
All − MR Egger
All − IVW(MRE)
−0.005
0.000
0.005
0.010
MR effect size for
'C−X−C motif chemokine 16' on 'eGFR'

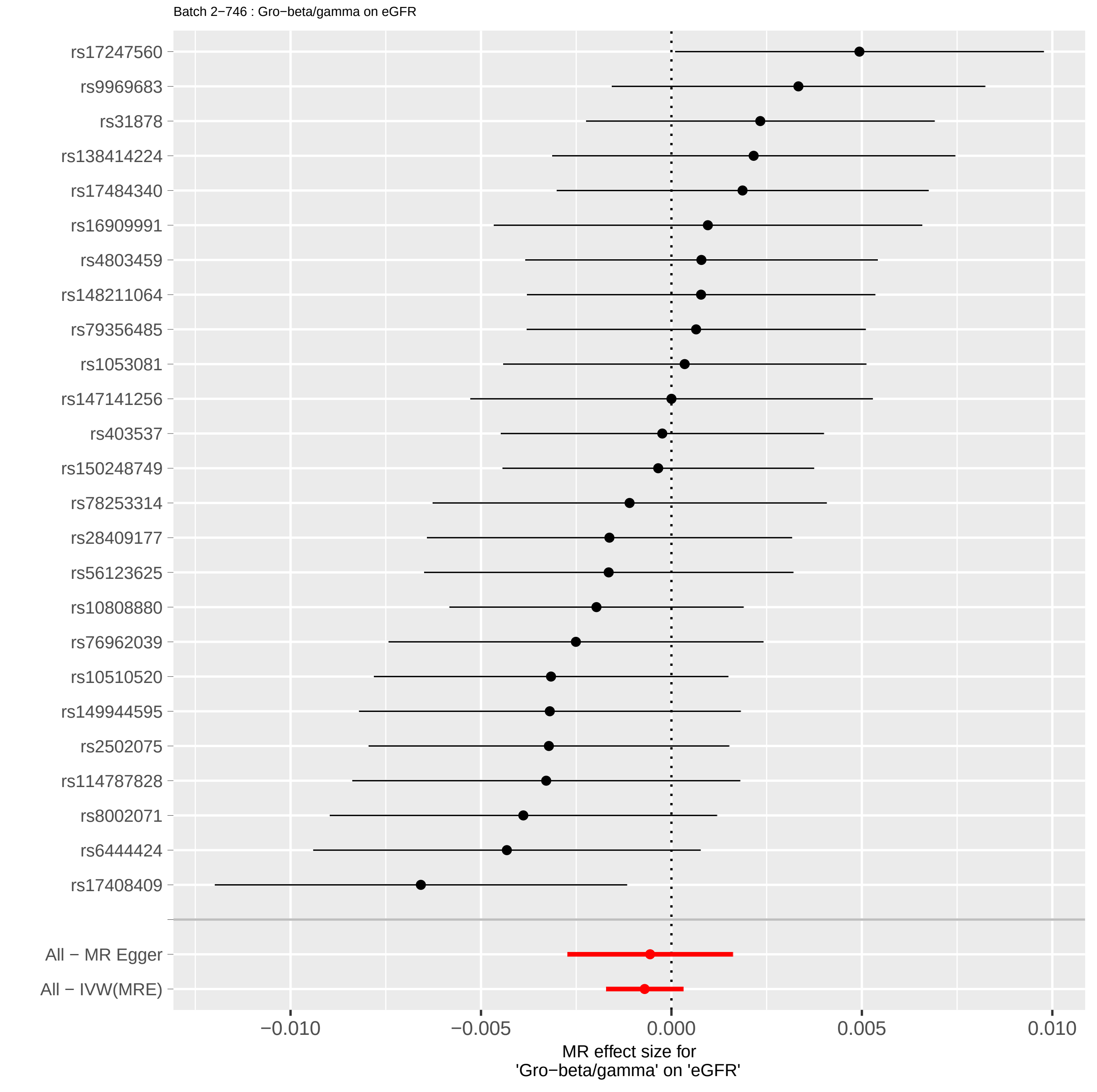

Batch 2−746 : Gro−beta/gamma on eGFR
rs17247560
rs9969683
rs31878
rs138414224
rs17484340
rs16909991
rs4803459
rs148211064
rs79356485
rs1053081
rs147141256
rs403537
rs150248749
rs78253314
rs28409177
rs56123625
rs10808880
rs76962039
rs10510520
rs149944595
rs2502075
rs114787828
rs8002071
rs6444424
rs17408409
All − MR Egger
All − IVW(MRE)
−0.010
−0.005
0.000
0.005
0.010
MR effect size for
'Gro−beta/gamma' on 'eGFR'

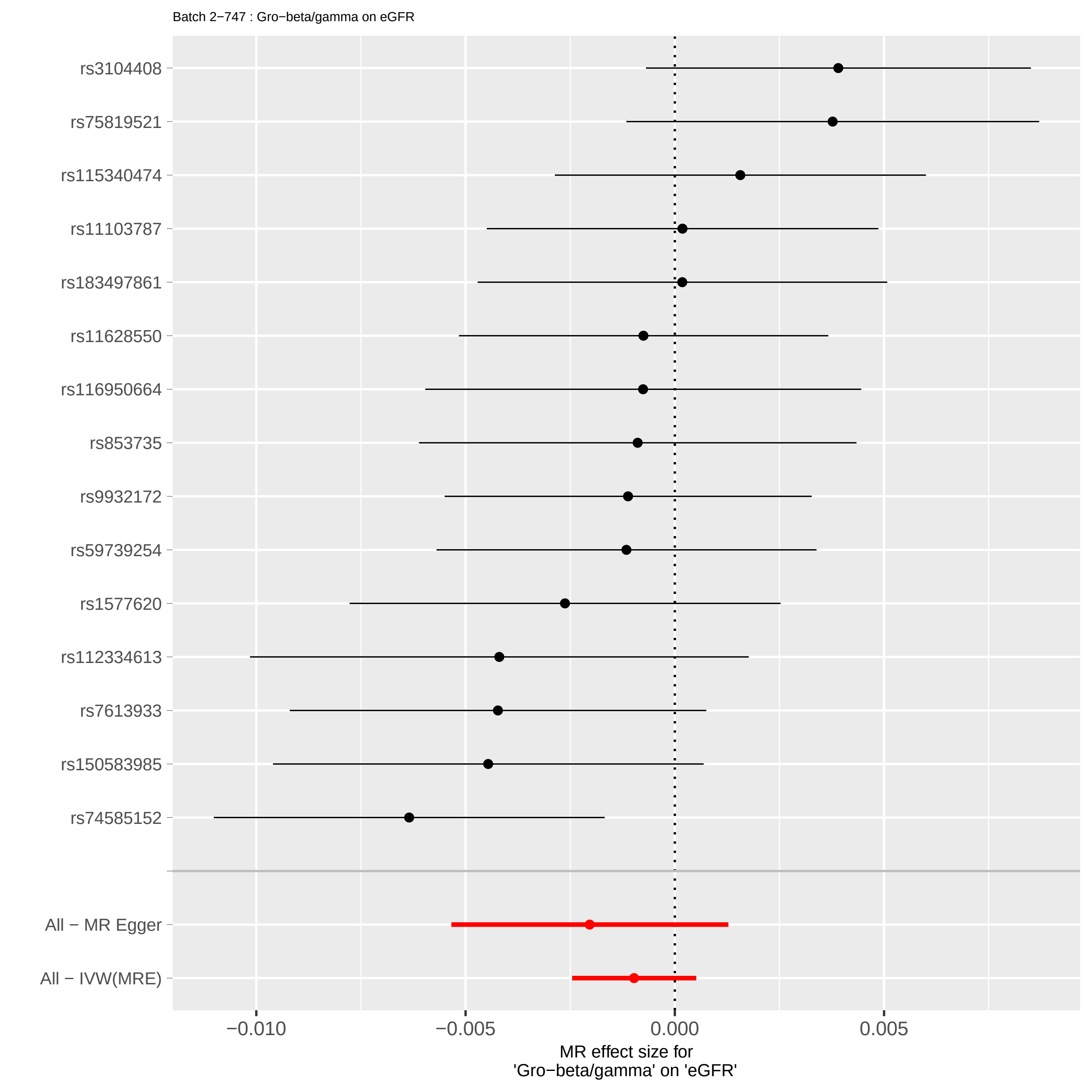
Batch 2−747 : Gro−beta/gamma on eGFR
rs3104408
rs75819521
rs115340474
rs11103787
rs183497861
rs11628550
rs116950664
rs853735
rs9932172
rs59739254
rs1577620
rs112334613
rs7613933
rs150583985
rs74585152
All − MR Egger
All − IVW(MRE)
−0.010
−0.005
0.000
0.005
MR effect size for
'Gro−beta/gamma' on 'eGFR'

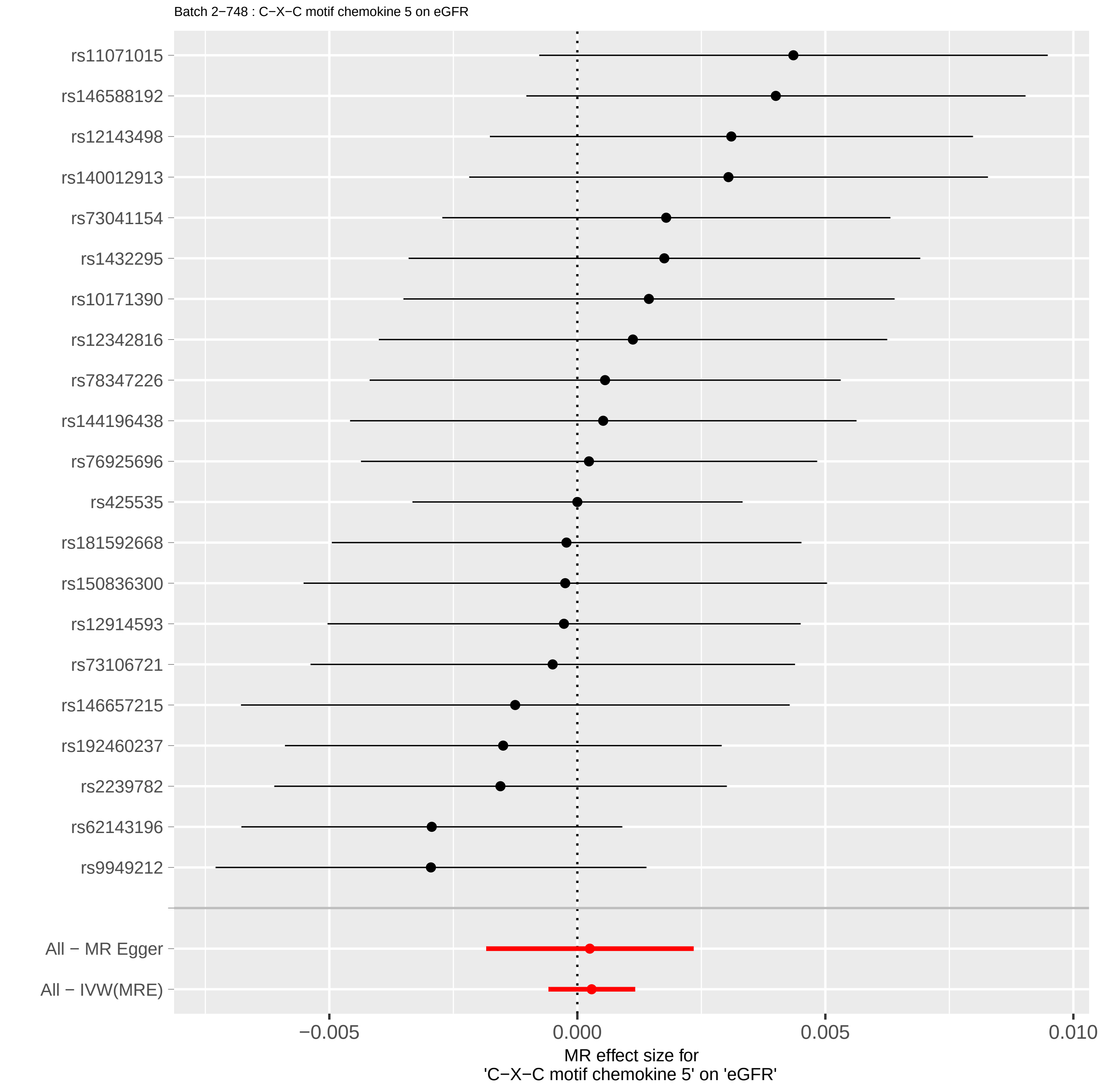
Batch 2−748 : C−X−C motif chemokine 5 on eGFR
rs11071015
rs146588192
rs12143498
rs140012913
rs73041154
rs1432295
rs10171390
rs12342816
rs78347226
rs144196438
rs76925696
rs425535
rs181592668
rs150836300
rs12914593
rs73106721
rs146657215
rs192460237
rs2239782
rs62143196
rs9949212
All − MR Egger
All − IVW(MRE)
−0.005
0.000
0.005
0.010
MR effect size for
'C−X−C motif chemokine 5' on 'eGFR'

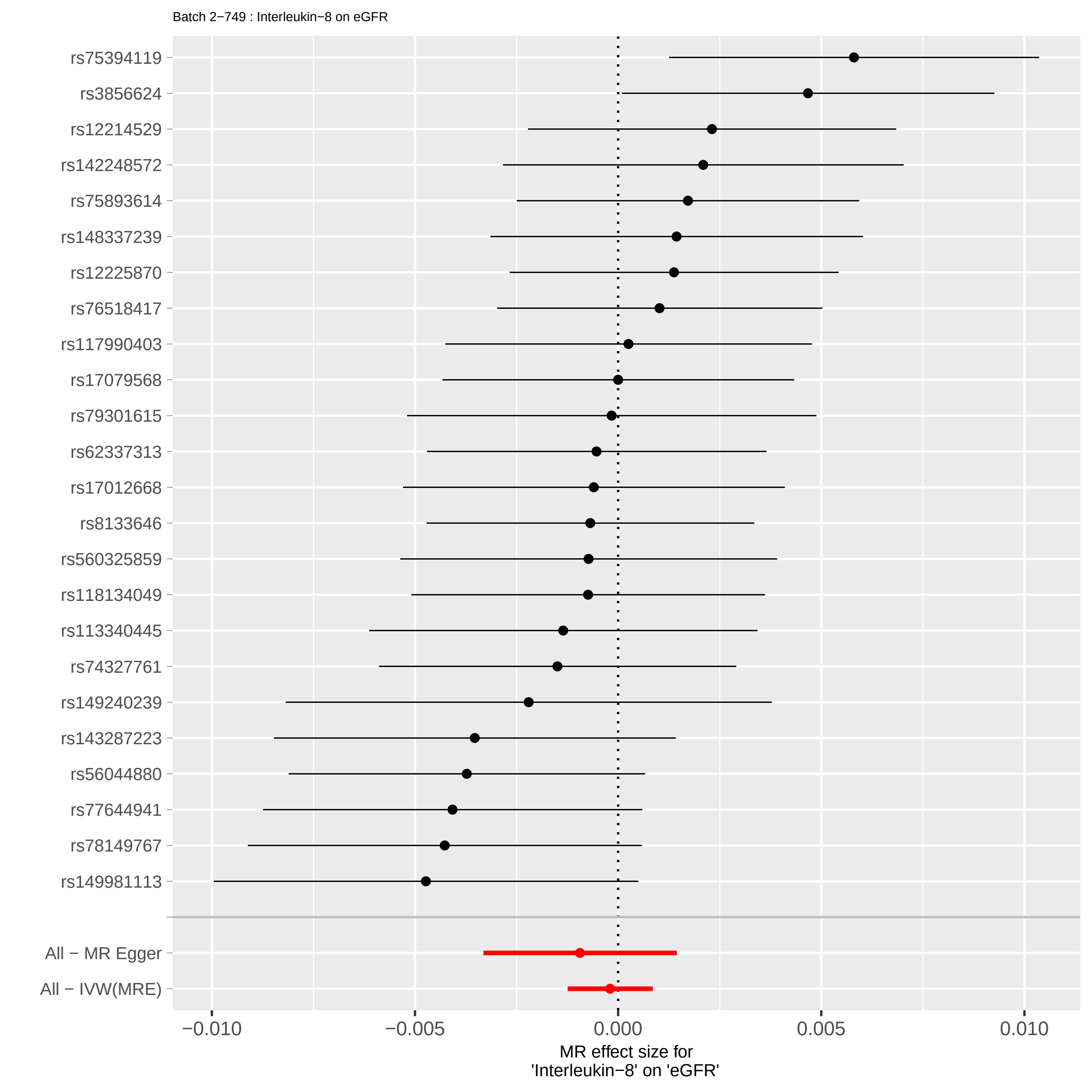
Batch 2−749 : Interleukin−8 on eGFR
rs75394119
rs3856624
rs12214529
rs142248572
rs75893614
rs148337239
rs12225870
rs76518417
rs117990403
rs17079568
rs79301615
rs62337313
rs17012668
rs8133646
rs560325859
rs118134049
rs113340445
rs74327761
rs149240239
rs143287223
rs56044880
rs77644941
rs78149767
rs149981113
All − MR Egger
All − IVW(MRE)
−0.010
−0.005
0.000
0.005
0.010
MR effect size for
'Interleukin−8' on 'eGFR'

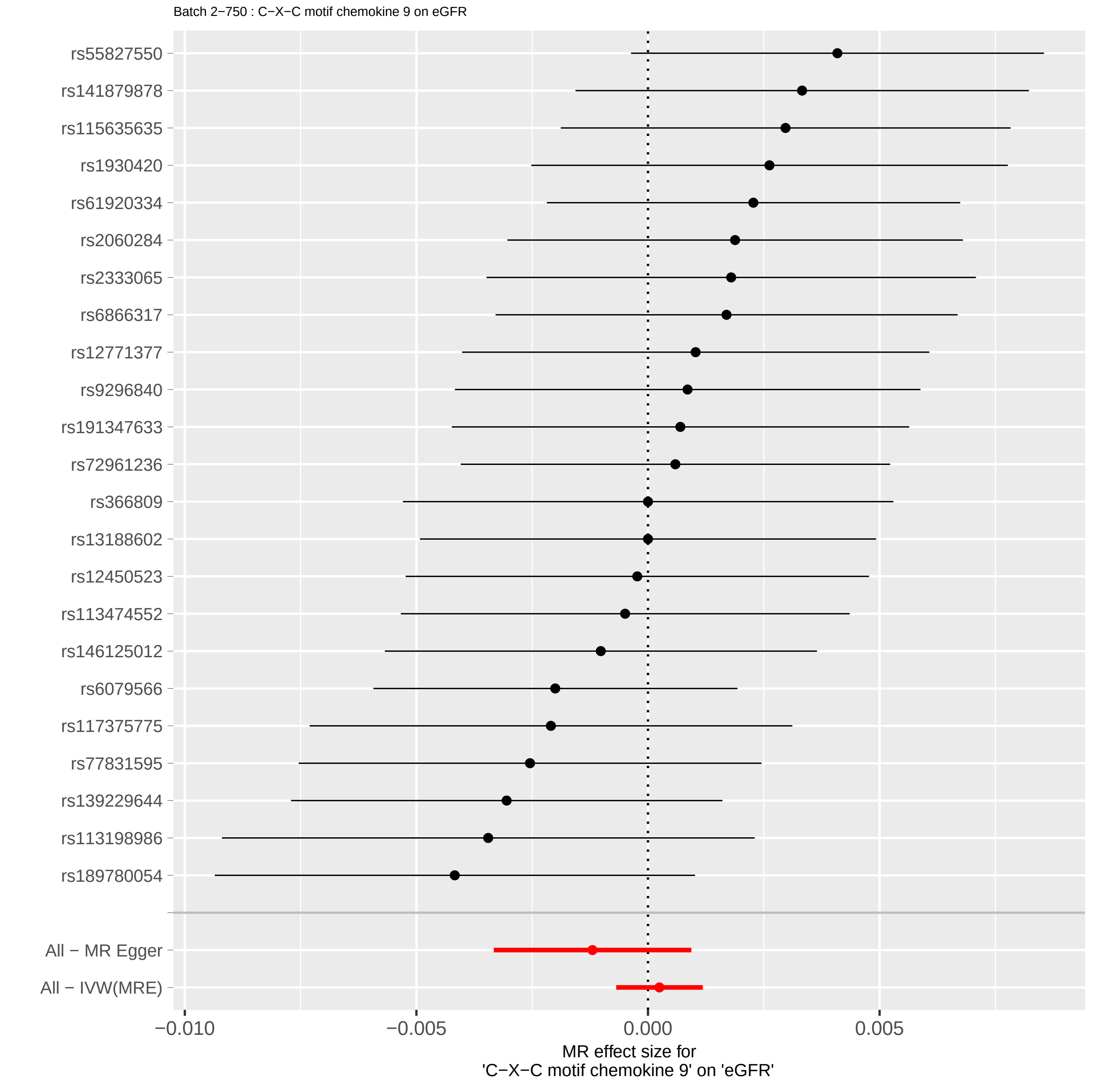

Batch 2−750 : C−X−C motif chemokine 9 on eGFR
rs55827550
rs141879878
rs115635635
rs1930420
rs61920334
rs2060284
rs2333065
rs6866317
rs12771377
rs9296840
rs191347633
rs72961236
rs366809
rs13188602
rs12450523
rs113474552
rs146125012
rs6079566
rs117375775
rs77831595
rs139229644
rs113198986
rs189780054
All − MR Egger
All − IVW(MRE)
−0.010
−0.005
0.000
0.005
MR effect size for
'C−X−C motif chemokine 9' on 'eGFR'

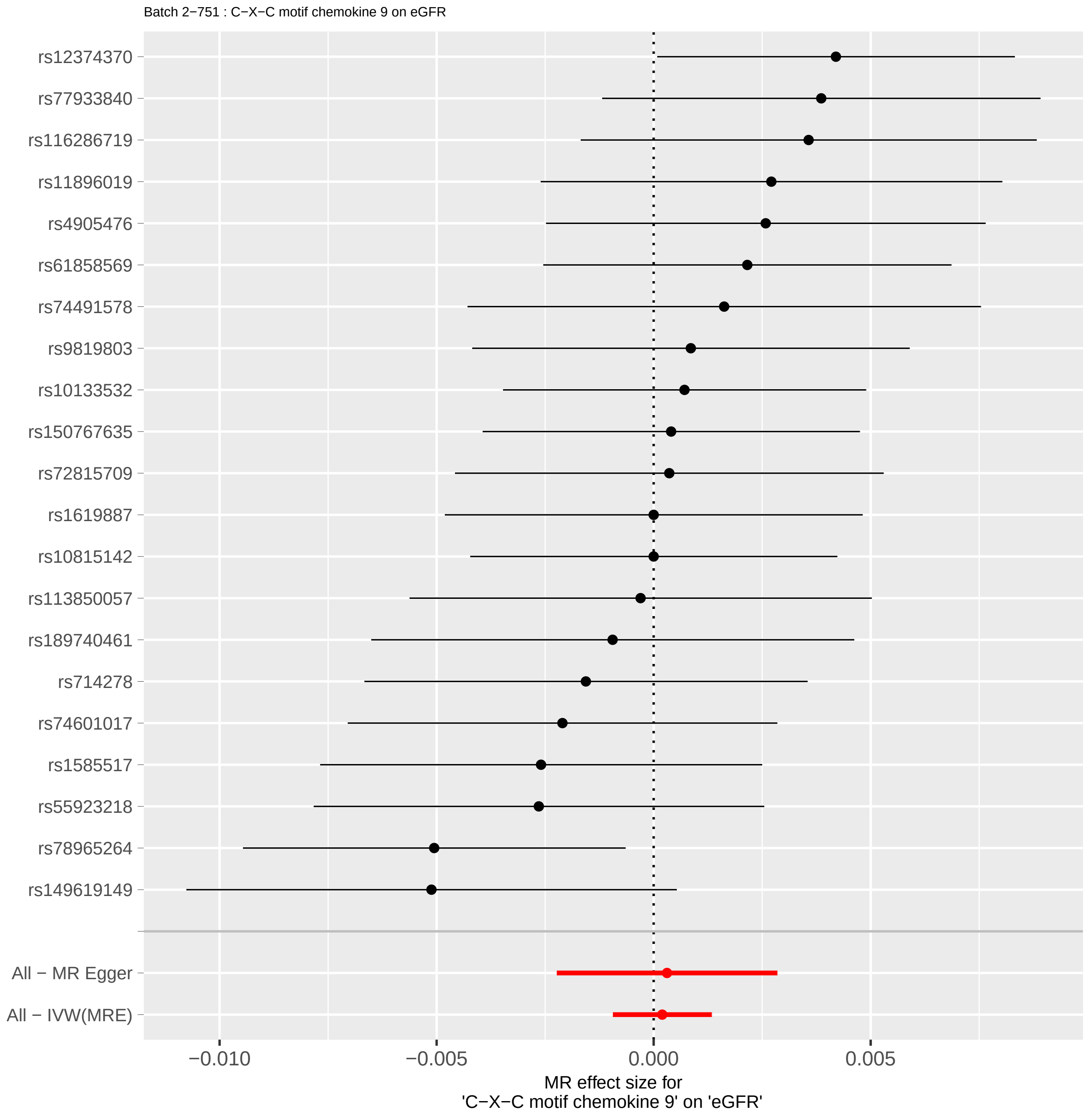

Batch 2−751 : C−X−C motif chemokine 9 on eGFR
rs12374370
rs77933840
rs116286719
rs11896019
rs4905476
rs61858569
rs74491578
rs9819803
rs10133532
rs150767635
rs72815709
rs1619887
rs10815142
rs113850057
rs189740461
rs714278
rs74601017
rs1585517
rs55923218
rs78965264
rs149619149
All − MR Egger
All − IVW(MRE)
−0.010
−0.005
0.000
0.005
MR effect size for
'C−X−C motif chemokine 9' on 'eGFR'

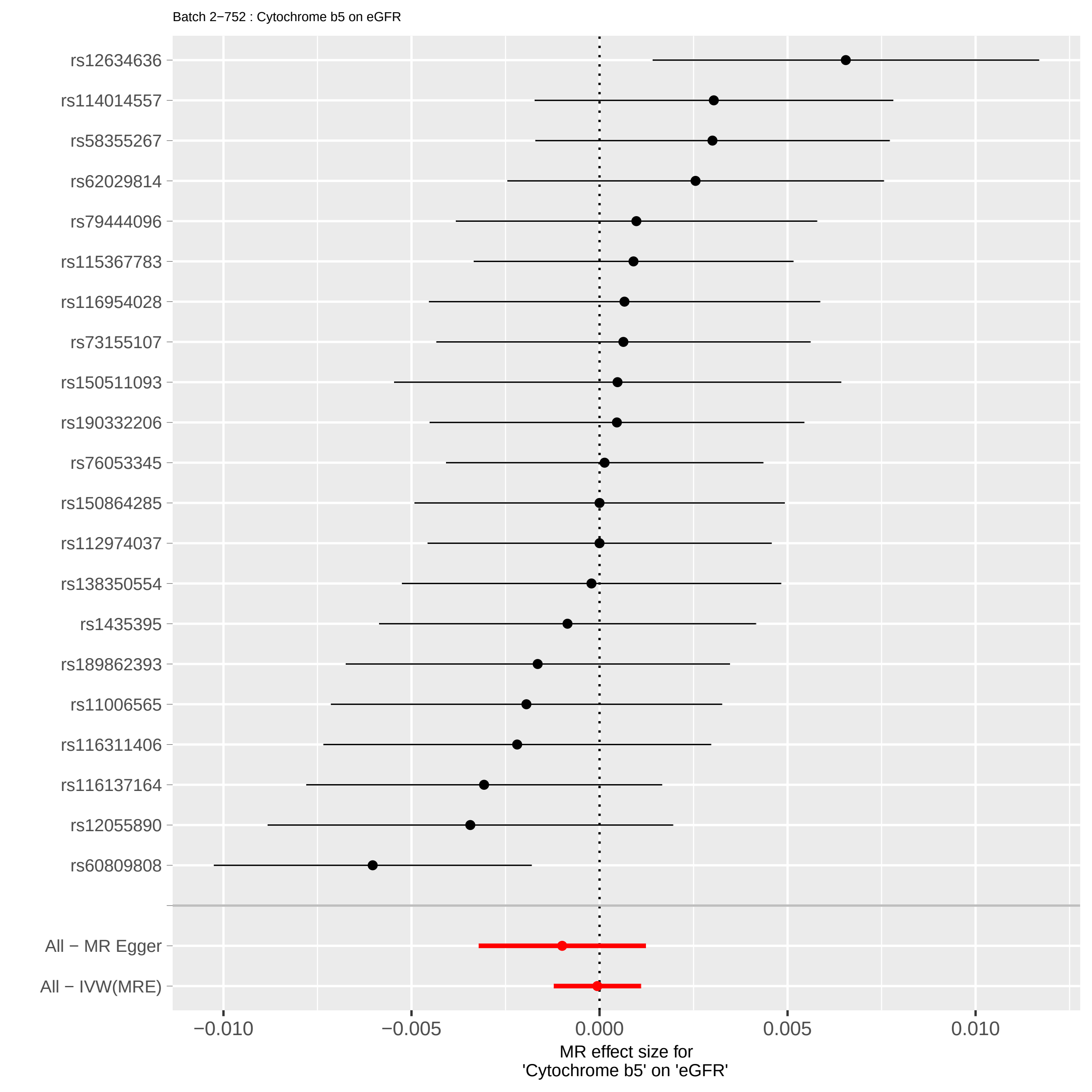

Batch 2−752 : Cytochrome b5 on eGFR
rs12634636
rs114014557
rs58355267
rs62029814
rs79444096
rs115367783
rs116954028
rs73155107
rs150511093
rs190332206
rs76053345
rs150864285
rs112974037
rs138350554
rs1435395
rs189862393
rs11006565
rs116311406
rs116137164
rs12055890
rs60809808
All − MR Egger
All − IVW(MRE)
−0.010
−0.005
0.000
0.005
0.010
MR effect size for
'Cytochrome b5' on 'eGFR'

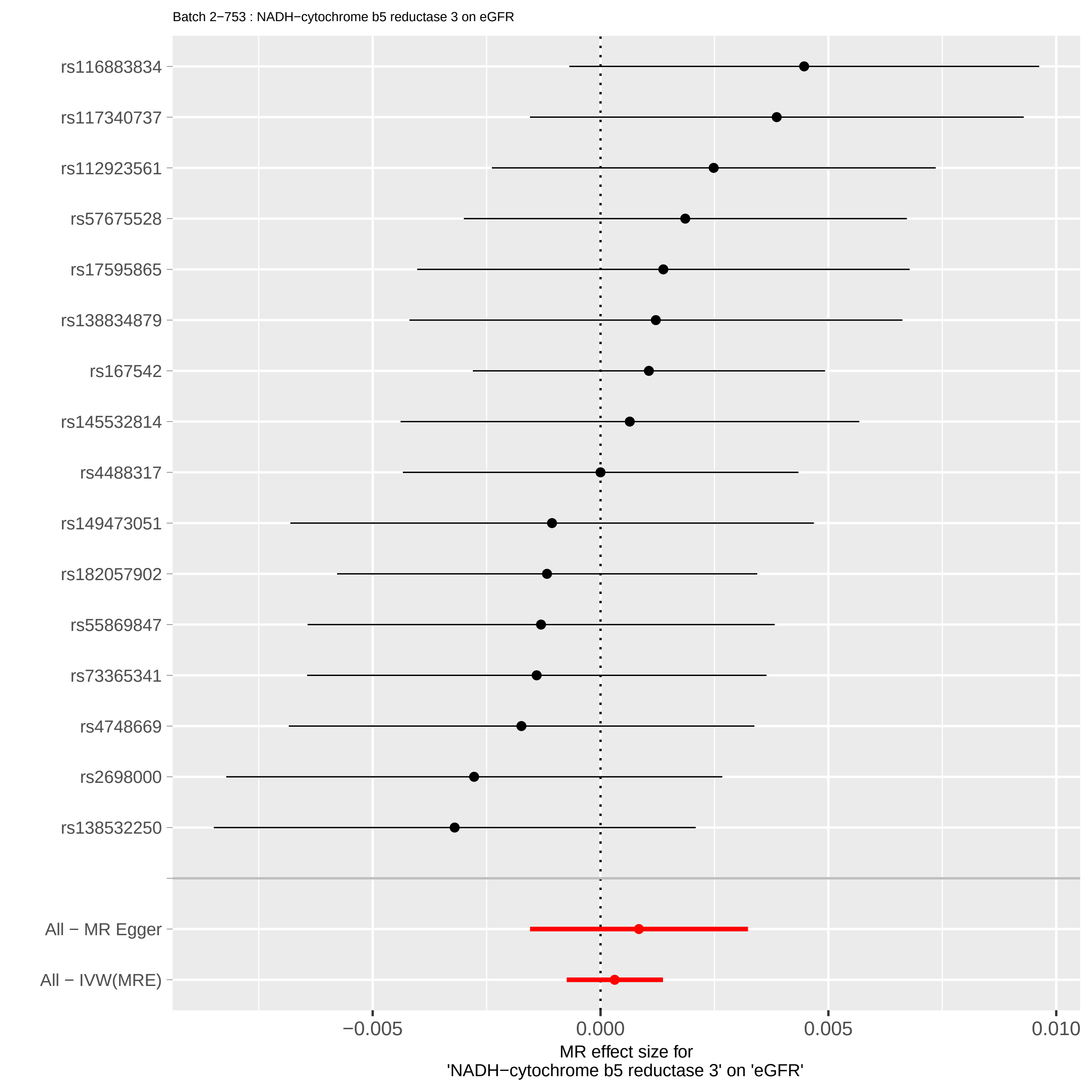

Batch 2−753 : NADH−cytochrome b5 reductase 3 on eGFR
rs116883834
rs117340737
rs112923561
rs57675528
rs17595865
rs138834879
rs167542
rs145532814
rs4488317
rs149473051
rs182057902
rs55869847
rs73365341
rs4748669
rs2698000
rs138532250
All − MR Egger
All − IVW(MRE)
−0.005
0.000
0.005
0.010
MR effect size for
'NADH−cytochrome b5 reductase 3' on 'eGFR'

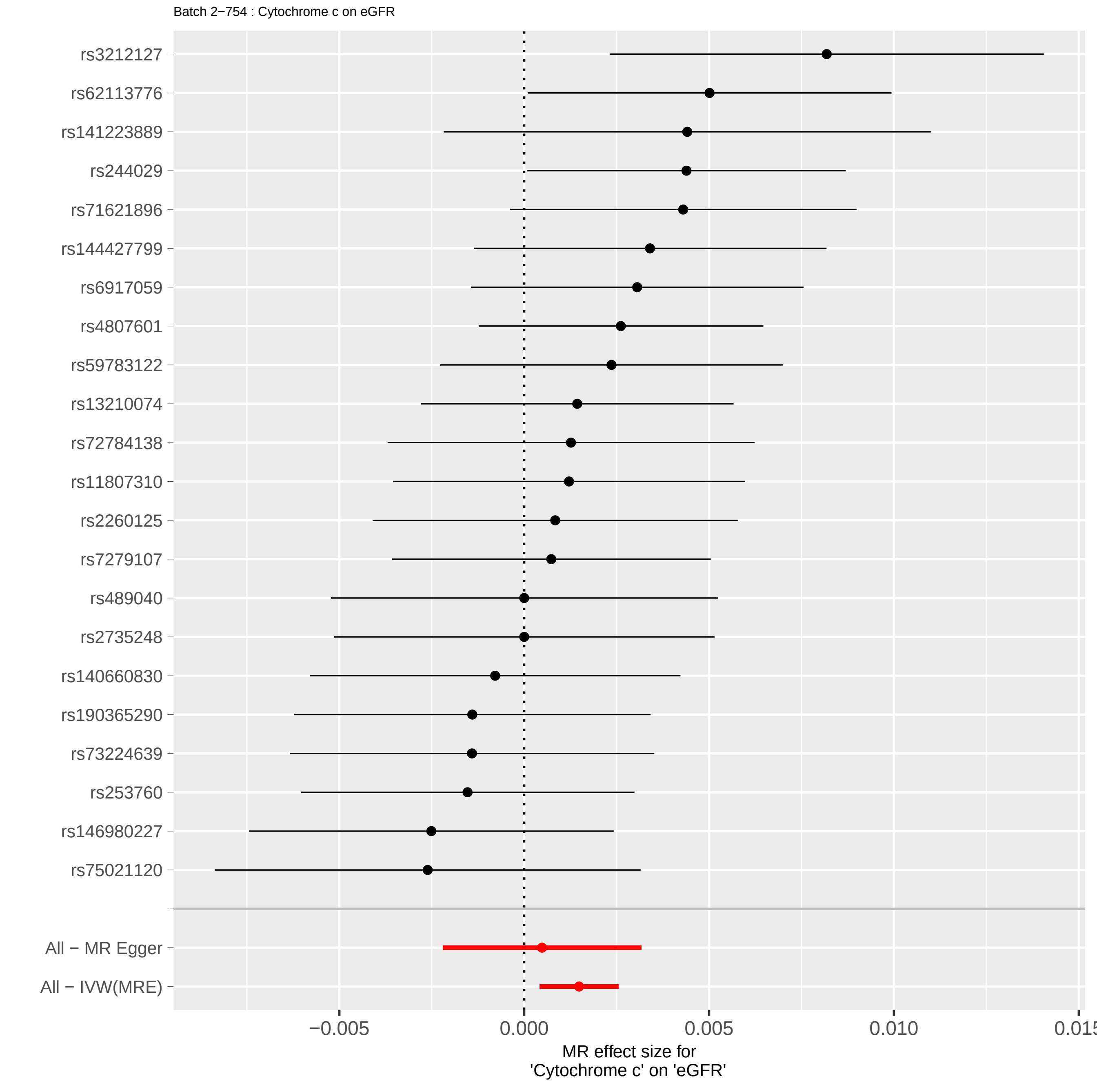

Batch 2−754 : Cytochrome c on eGFR
rs3212127
rs62113776
rs141223889
rs244029
rs71621896
rs144427799
rs6917059
rs4807601
rs59783122
rs13210074
rs72784138
rs11807310
rs2260125
rs7279107
rs489040
rs2735248
rs140660830
rs190365290
rs73224639
rs253760
rs146980227
rs75021120
All − MR Egger
All − IVW(MRE)
−0.005
0.000
0.005
0.010
0.015
MR effect size for
'Cytochrome c' on 'eGFR'

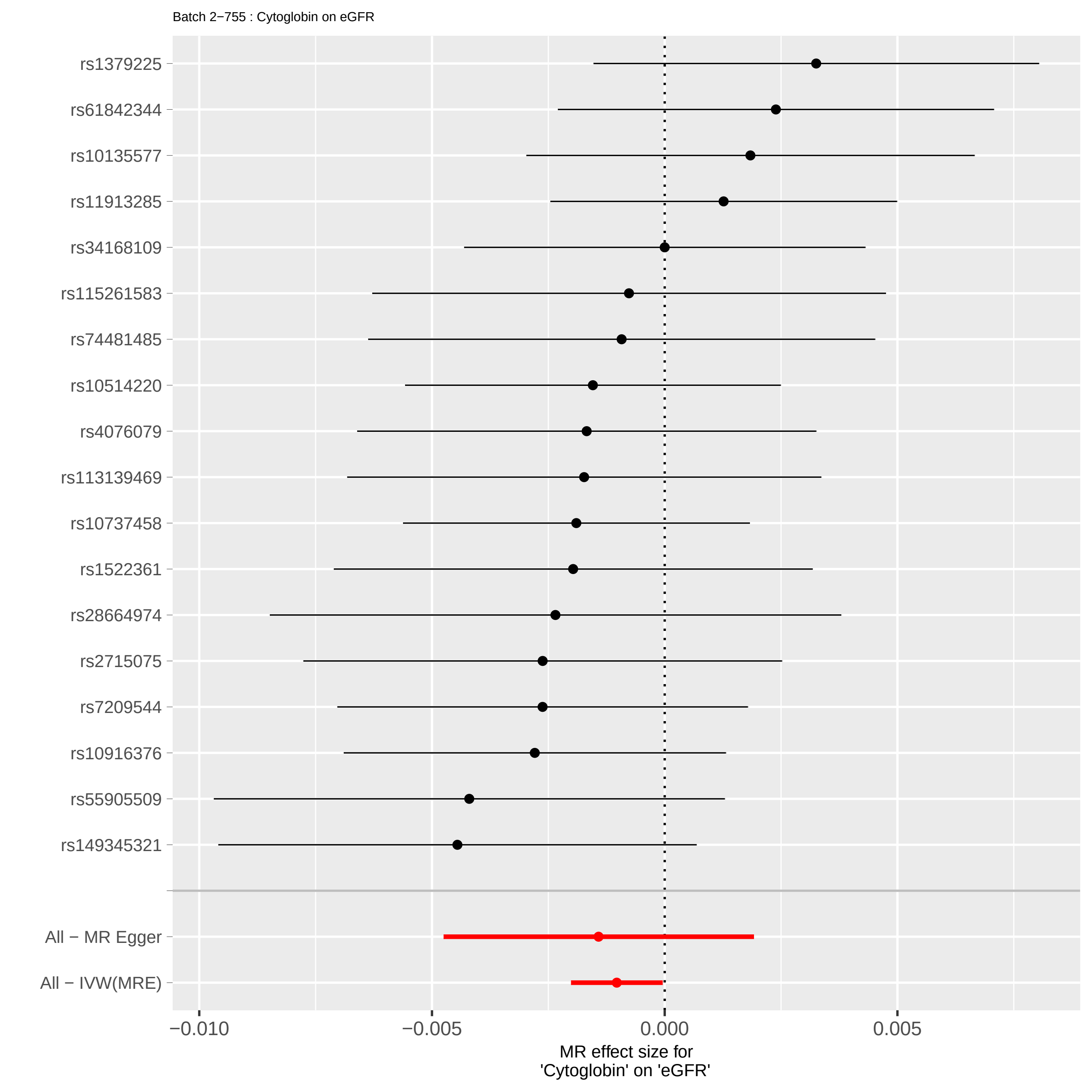

Batch 2−755 : Cytoglobin on eGFR
rs1379225
rs61842344
rs10135577
rs11913285
rs34168109
rs115261583
rs74481485
rs10514220
rs4076079
rs113139469
rs10737458
rs1522361
rs28664974
rs2715075
rs7209544
rs10916376
rs55905509
rs149345321
All − MR Egger
All − IVW(MRE)
−0.010
−0.005
0.000
0.005
MR effect size for
'Cytoglobin' on 'eGFR'

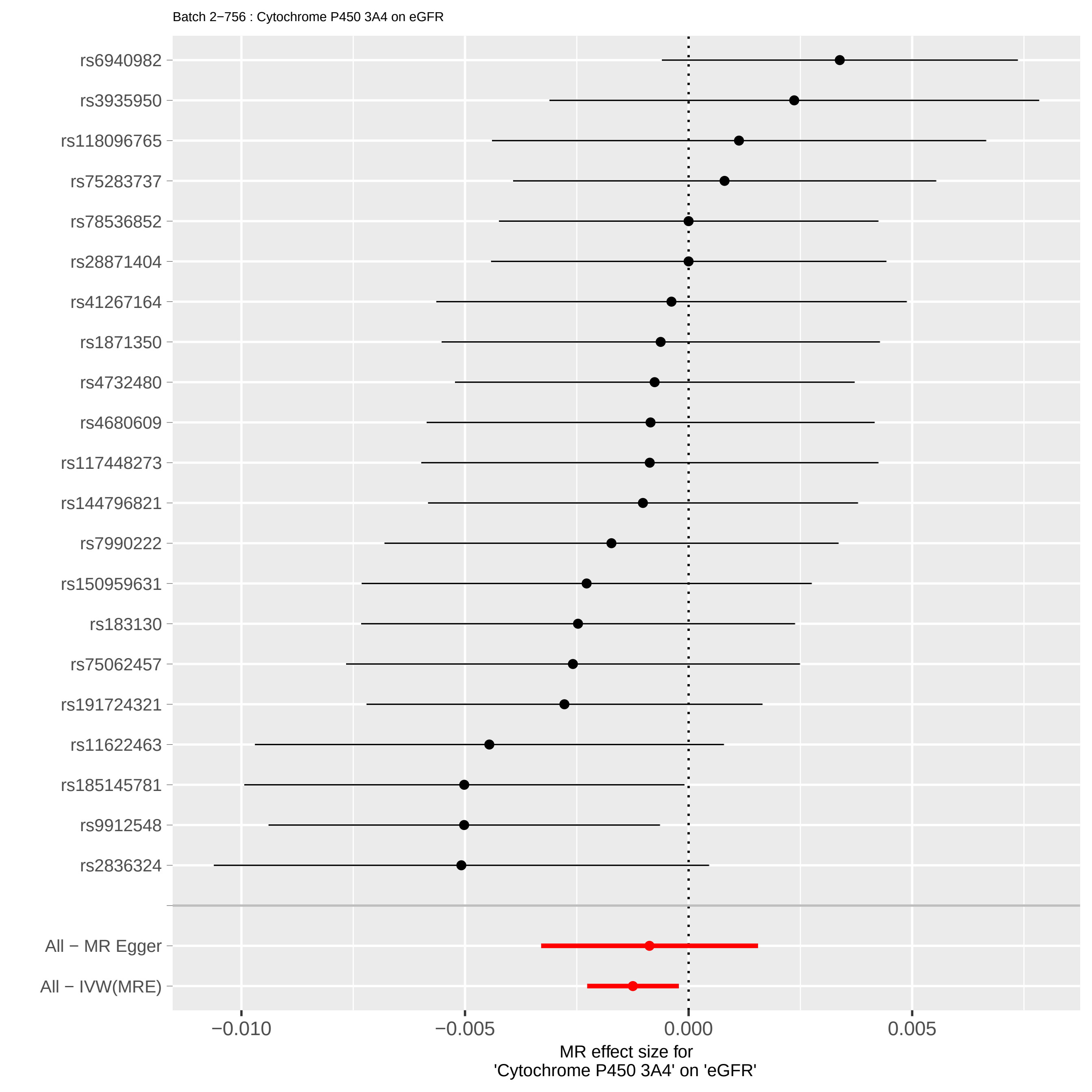

Batch 2−756 : Cytochrome P450 3A4 on eGFR
rs6940982
rs3935950
rs118096765
rs75283737
rs78536852
rs28871404
rs41267164
rs1871350
rs4732480
rs4680609
rs117448273
rs144796821
rs7990222
rs150959631
rs183130
rs75062457
rs191724321
rs11622463
rs185145781
rs9912548
rs2836324
All − MR Egger
All − IVW(MRE)
−0.010
−0.005
0.000
0.005
MR effect size for
'Cytochrome P450 3A4' on 'eGFR'

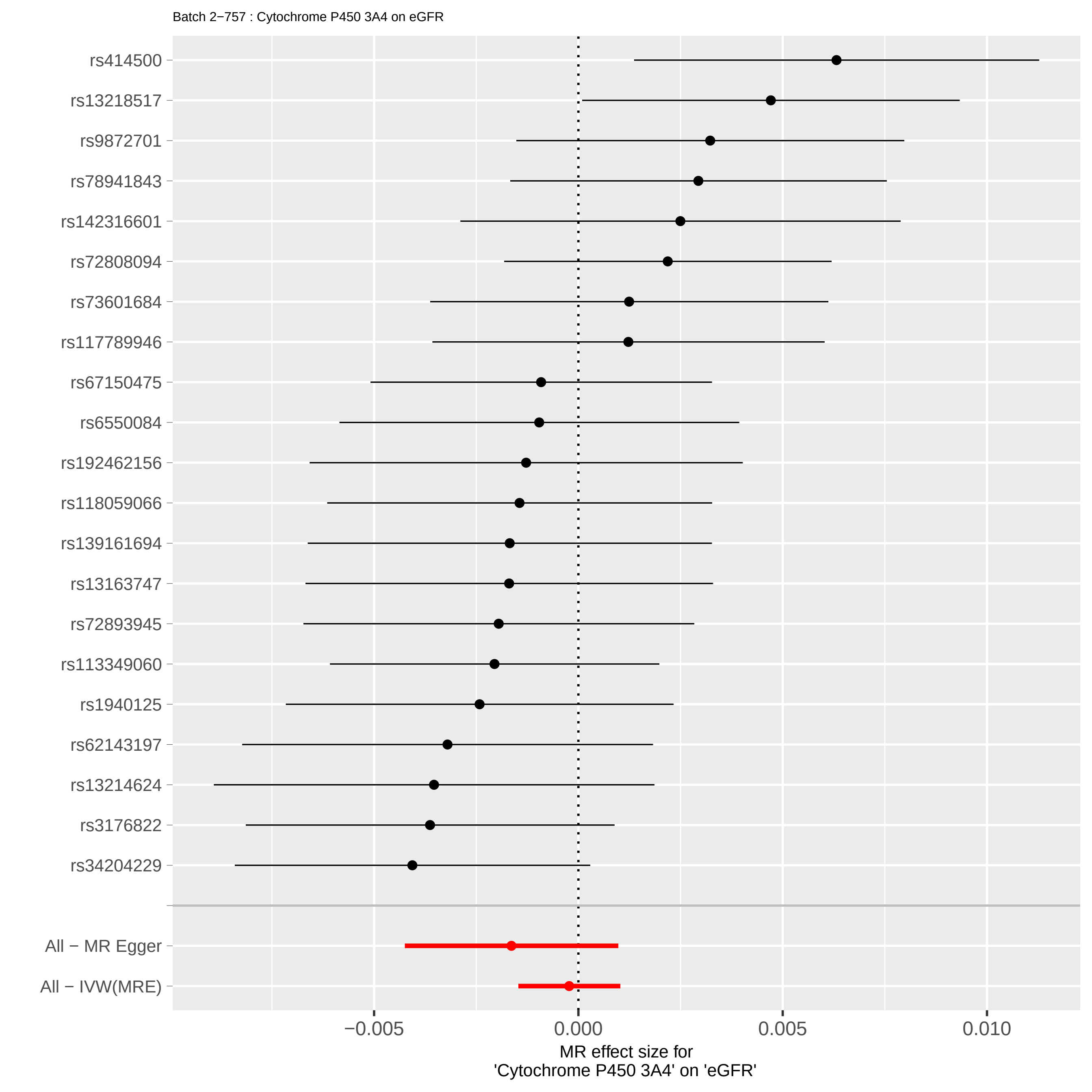
Batch 2−757 : Cytochrome P450 3A4 on eGFR
rs414500
rs13218517
rs9872701
rs78941843
rs142316601
rs72808094
rs73601684
rs117789946
rs67150475
rs6550084
rs192462156
rs118059066
rs139161694
rs13163747
rs72893945
rs113349060
rs1940125
rs62143197
rs13214624
rs3176822
rs34204229
All − MR Egger
All − IVW(MRE)
−0.005
0.000
0.005
0.010
MR effect size for
'Cytochrome P450 3A4' on 'eGFR'

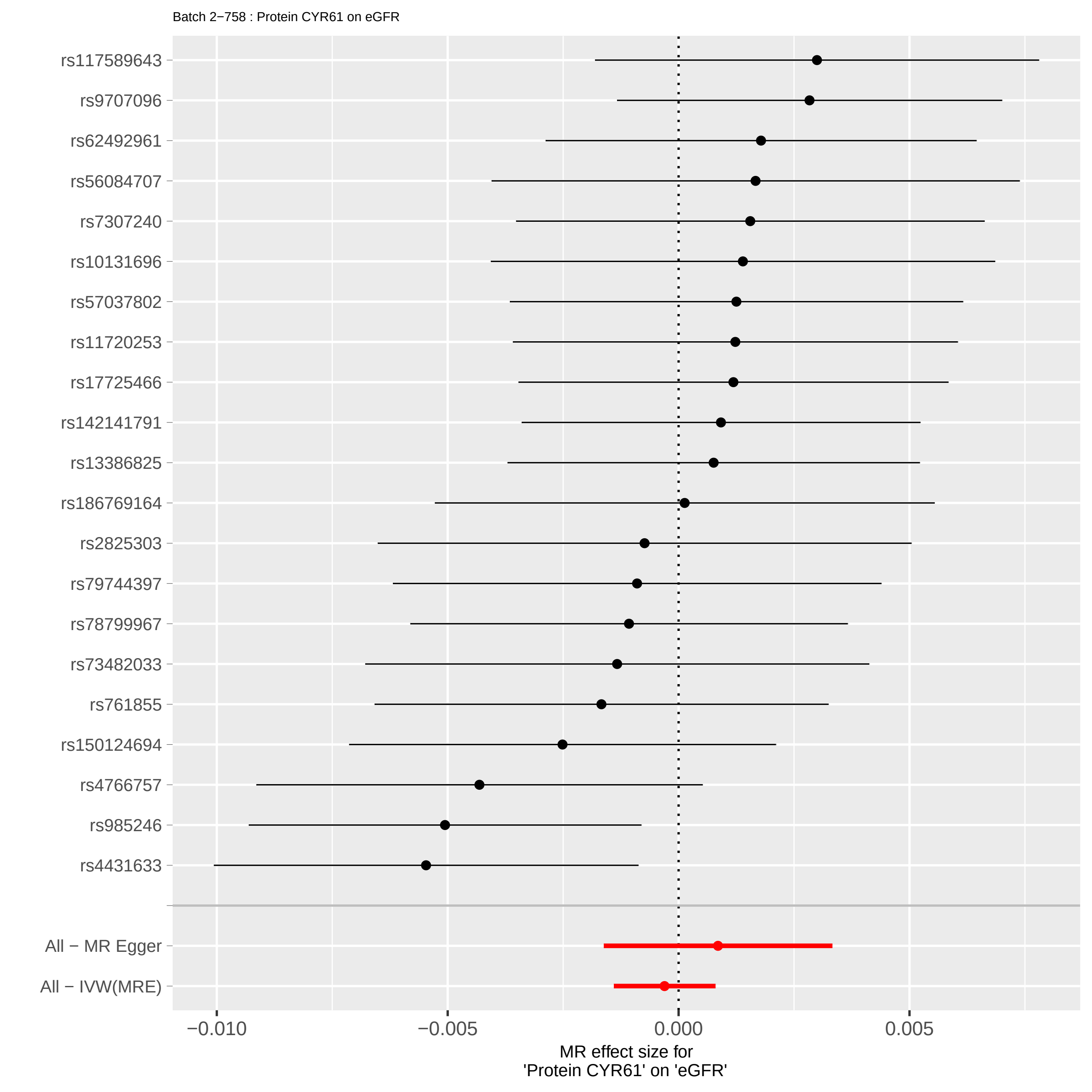

Batch 2−758 : Protein CYR61 on eGFR
rs117589643
rs9707096
rs62492961
rs56084707
rs7307240
rs10131696
rs57037802
rs11720253
rs17725466
rs142141791
rs13386825
rs186769164
rs2825303
rs79744397
rs78799967
rs73482033
rs761855
rs150124694
rs4766757
rs985246
rs4431633
All − MR Egger
All − IVW(MRE)
−0.010
−0.005
0.000
0.005
MR effect size for
'Protein CYR61' on 'eGFR'

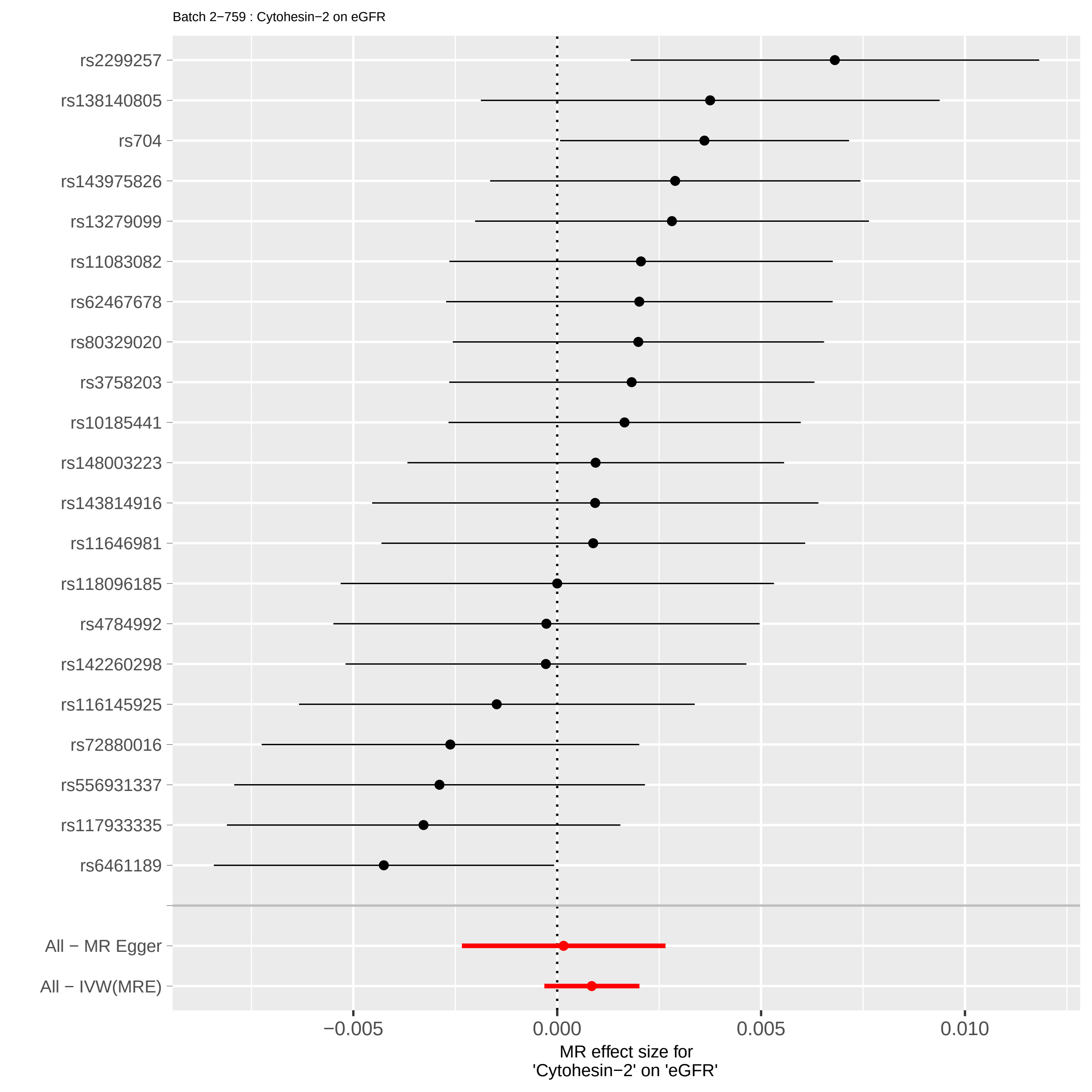
Batch 2−759 : Cytohesin−2 on eGFR
rs2299257
rs138140805
rs704
rs143975826
rs13279099
rs11083082
rs62467678
rs80329020
rs3758203
rs10185441
rs148003223
rs143814916
rs11646981
rs118096185
rs4784992
rs142260298
rs116145925
rs72880016
rs556931337
rs117933335
rs6461189
All − MR Egger
All − IVW(MRE)
−0.005
0.000
0.005
0.010
MR effect size for
'Cytohesin−2' on 'eGFR'

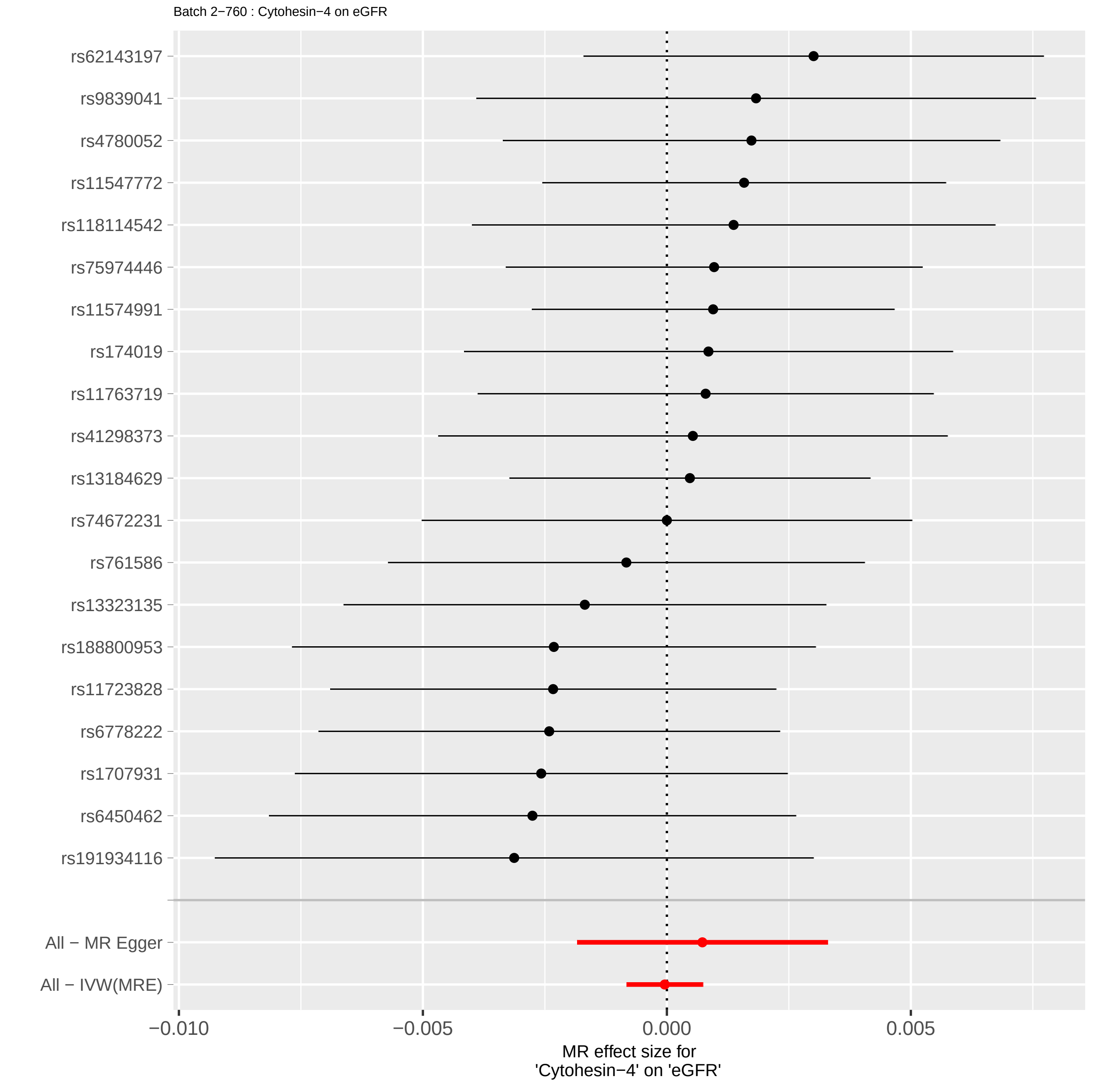

Batch 2−760 : Cytohesin−4 on eGFR
rs62143197
rs9839041
rs4780052
rs11547772
rs118114542
rs75974446
rs11574991
rs174019
rs11763719
rs41298373
rs13184629
rs74672231
rs761586
rs13323135
rs188800953
rs11723828
rs6778222
rs1707931
rs6450462
rs191934116
All − MR Egger
All − IVW(MRE)
−0.010
−0.005
0.000
0.005
MR effect size for
'Cytohesin−4' on 'eGFR'

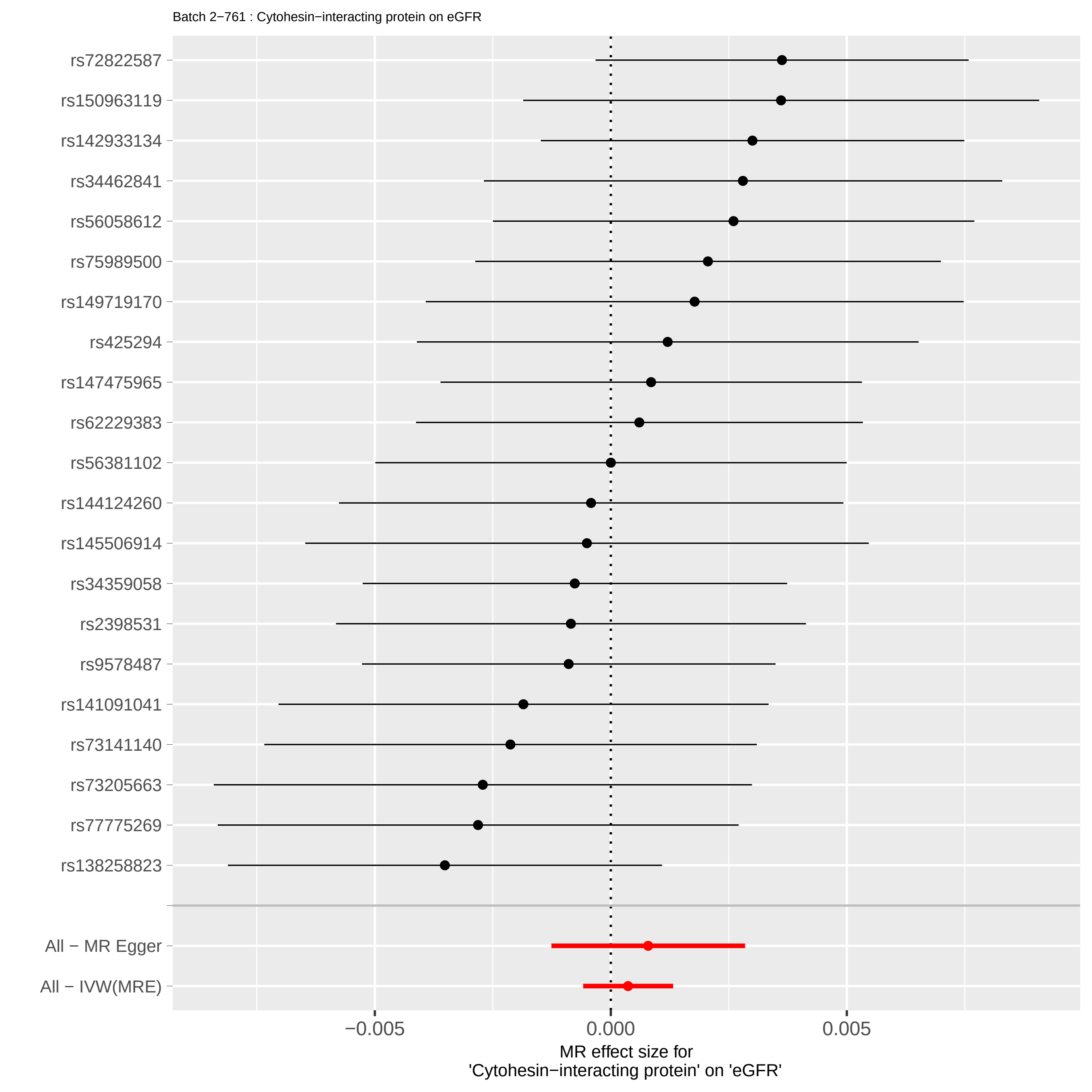

Batch 2−761 : Cytohesin−interacting protein on eGFR
rs72822587
rs150963119
rs142933134
rs34462841
rs56058612
rs75989500
rs149719170
rs425294
rs147475965
rs62229383
rs56381102
rs144124260
rs145506914
rs34359058
rs2398531
rs9578487
rs141091041
rs73141140
rs73205663
rs77775269
rs138258823
All − MR Egger
All − IVW(MRE)
−0.005
0.000
0.005
MR effect size for
'Cytohesin−interacting protein' on 'eGFR'

Batch 2−762 : Dystroglycan on eGFR

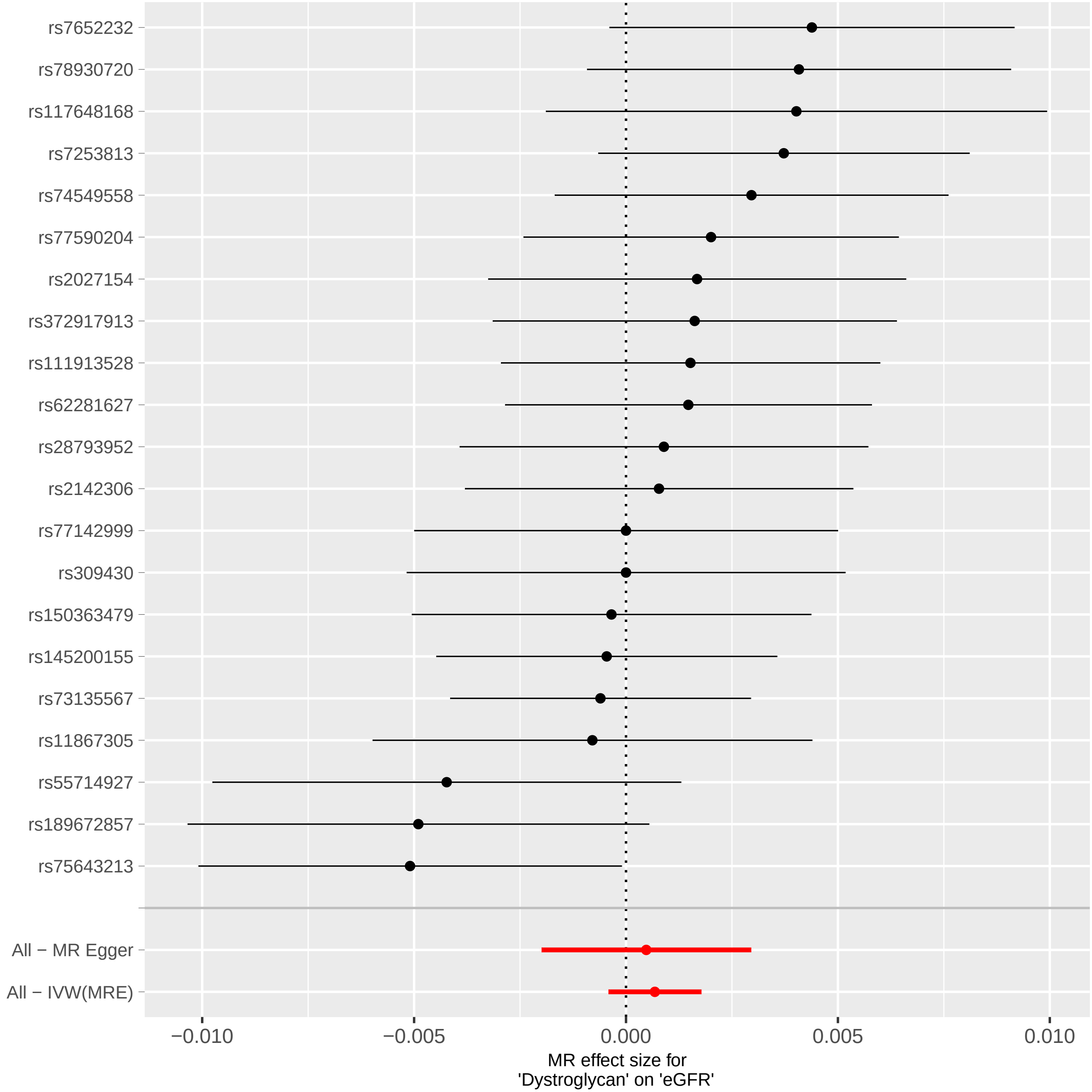

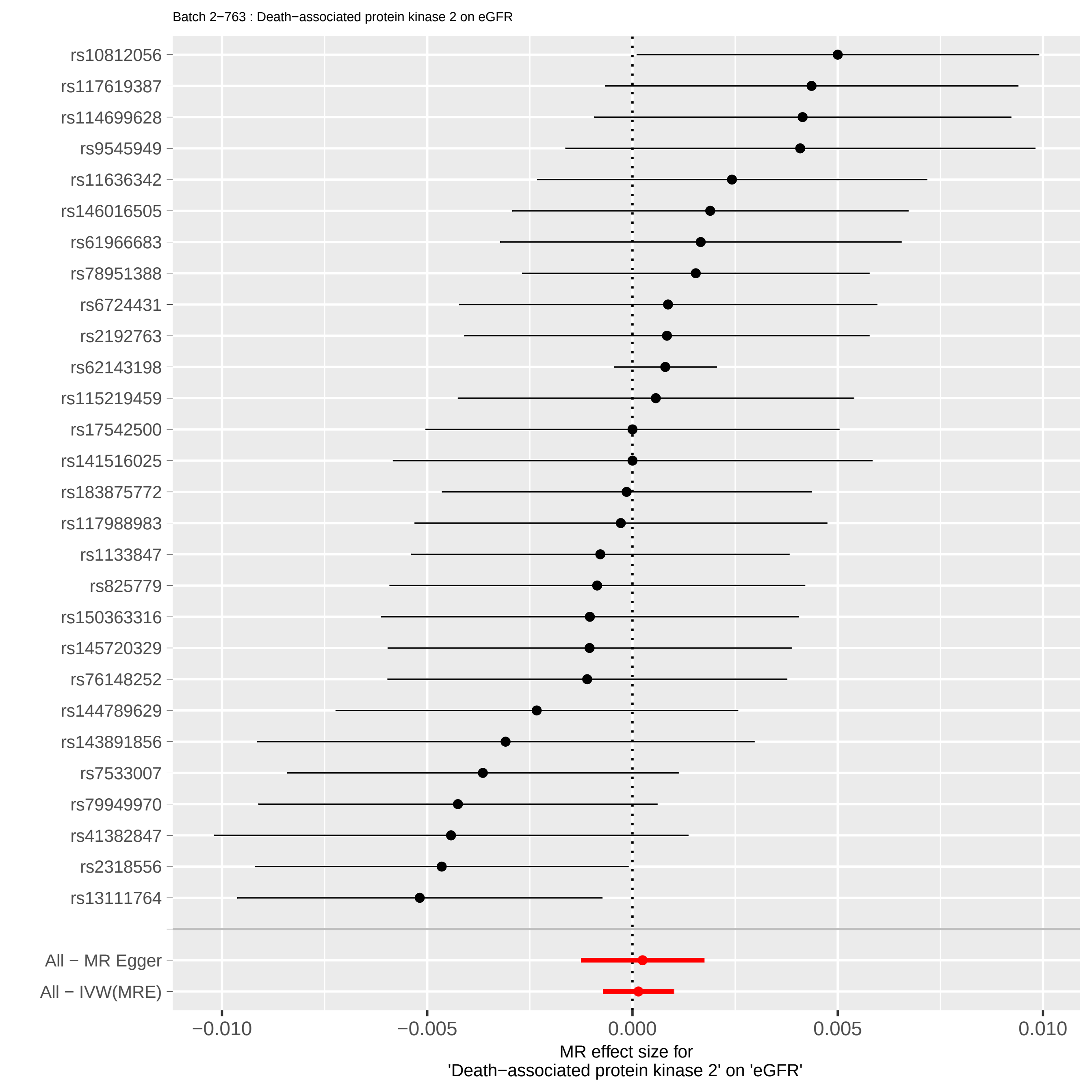

Batch 2−763 : Death−associated protein kinase 2 on eGFR
rs10812056
rs117619387
rs114699628
rs9545949
rs11636342
rs146016505
rs61966683
rs78951388
rs6724431
rs2192763
rs62143198
rs115219459
rs17542500
rs141516025
rs183875772
rs117988983
rs1133847
rs825779
rs150363316
rs145720329
rs76148252
rs144789629
rs143891856
rs7533007
rs79949970
rs41382847
rs2318556
rs13111764
All − MR Egger
All − IVW(MRE)
−0.010
−0.005
0.000
0.005
0.010
MR effect size for
'Death−associated protein kinase 2' on 'eGFR'

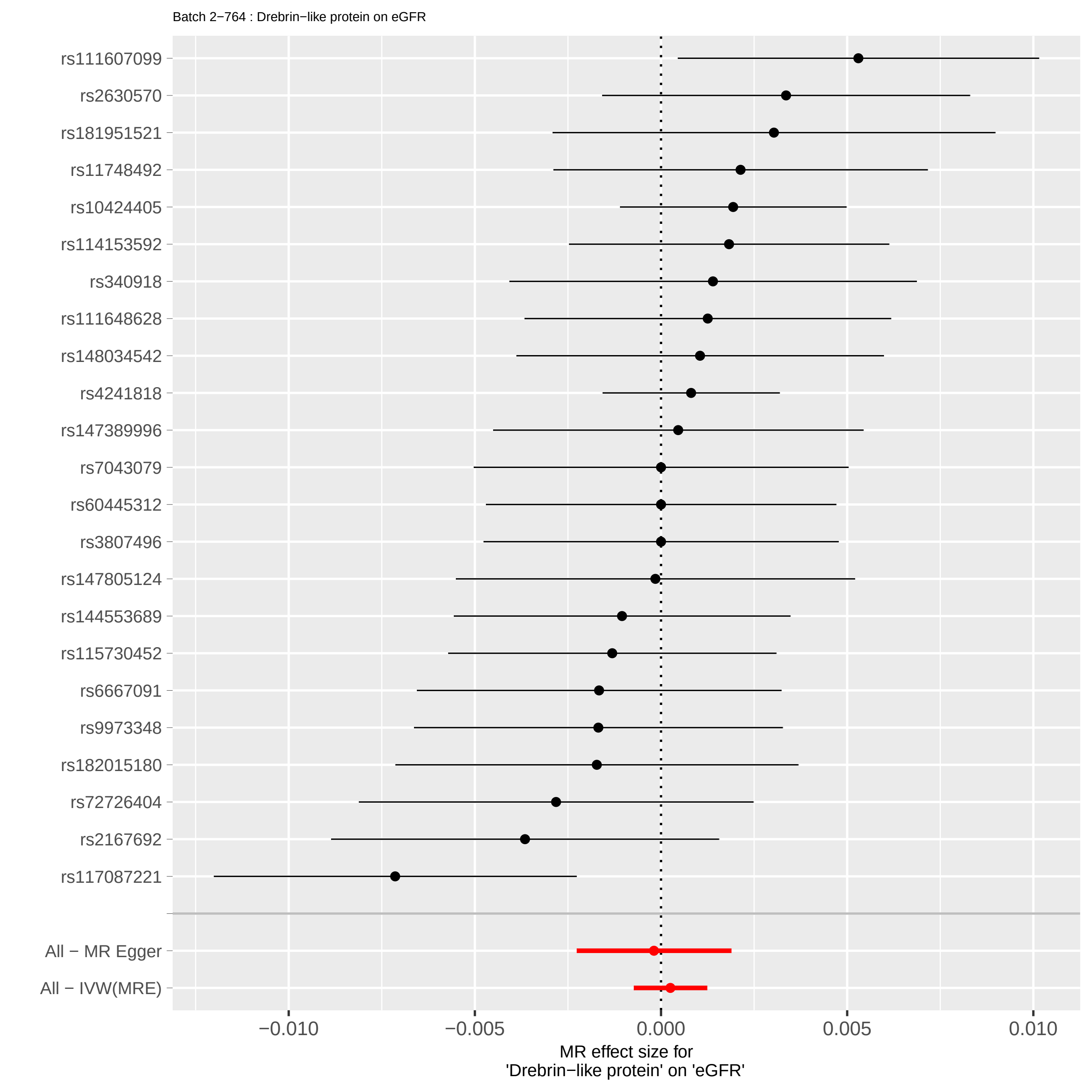

Batch 2−764 : Drebrin−like protein on eGFR
rs111607099
rs2630570
rs181951521
rs11748492
rs10424405
rs114153592
rs340918
rs111648628
rs148034542
rs4241818
rs147389996
rs7043079
rs60445312
rs3807496
rs147805124
rs144553689
rs115730452
rs6667091
rs9973348
rs182015180
rs72726404
rs2167692
rs117087221
All − MR Egger
All − IVW(MRE)
−0.010
−0.005
0.000
0.005
0.010
MR effect size for
'Drebrin−like protein' on 'eGFR'

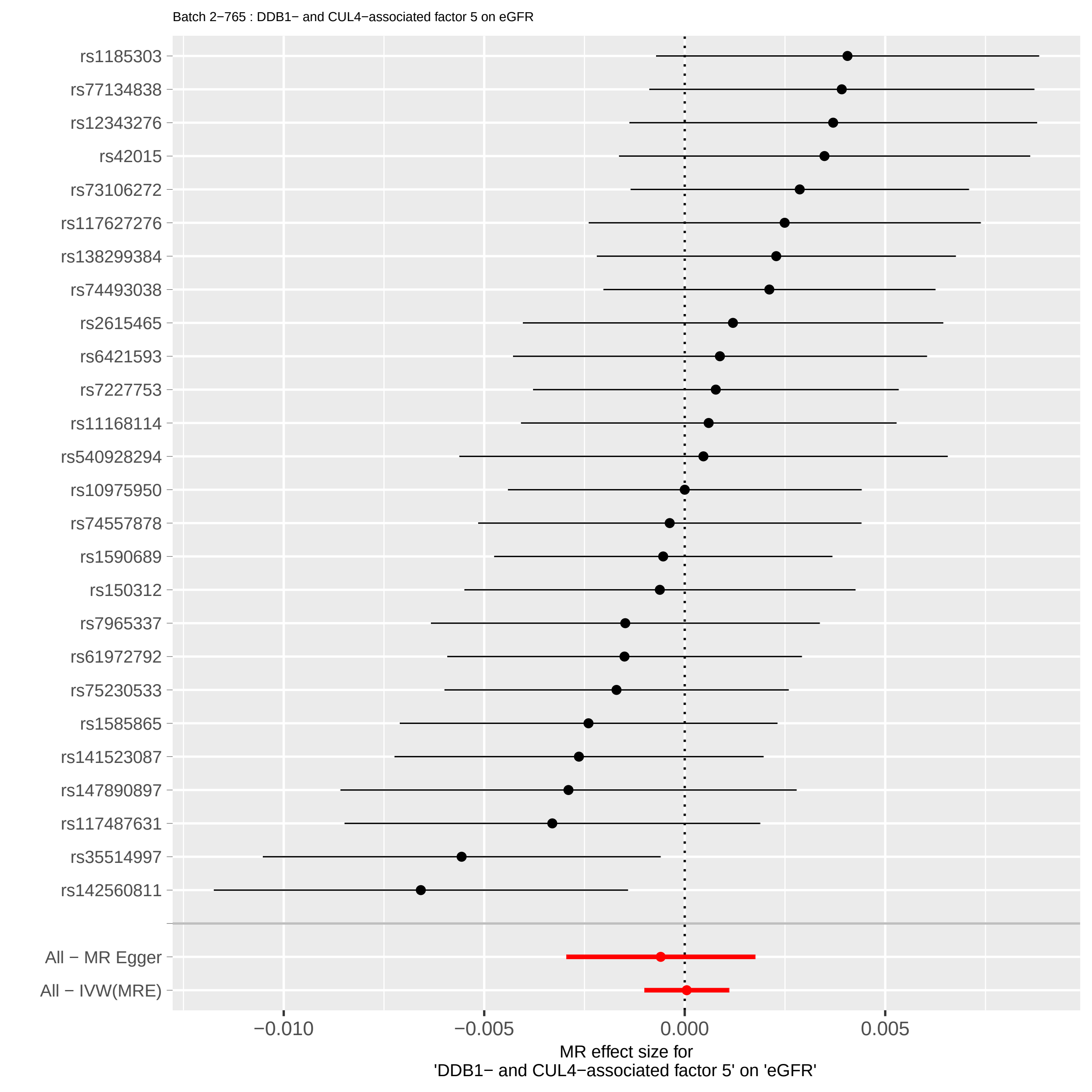

Batch 2−765 : DDB1− and CUL4−associated factor 5 on eGFR
rs1185303
rs77134838
rs12343276
rs42015
rs73106272
rs117627276
rs138299384
rs74493038
rs2615465
rs6421593
rs7227753
rs11168114
rs540928294
rs10975950
rs74557878
rs1590689
rs150312
rs7965337
rs61972792
rs75230533
rs1585865
rs141523087
rs147890897
rs117487631
rs35514997
rs142560811
All − MR Egger
All − IVW(MRE)
−0.010
−0.005
0.000
0.005
MR effect size for
'DDB1− and CUL4−associated factor 5' on 'eGFR'

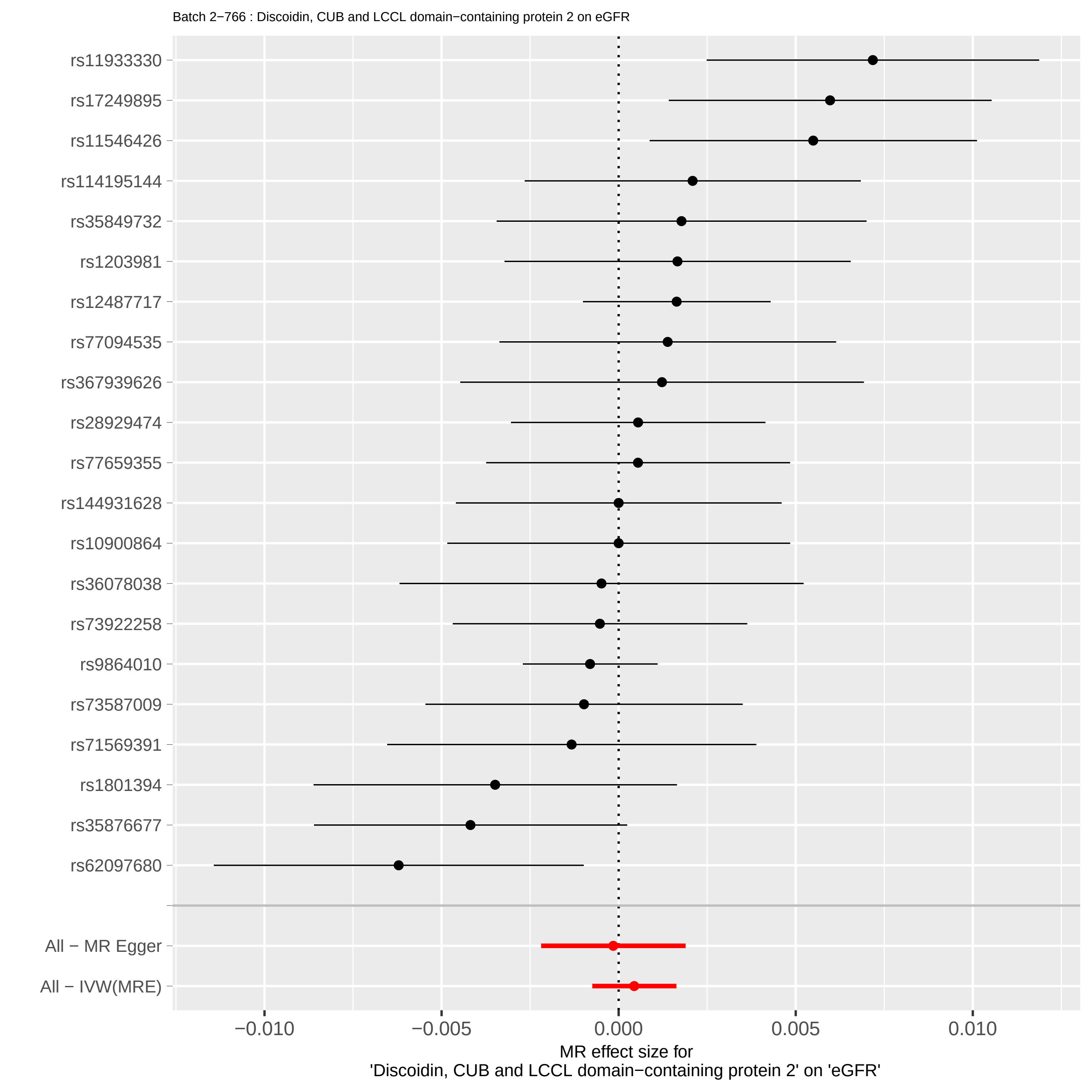

Batch 2−766 : Discoidin, CUB and LCCL domain−containing protein 2 on eGFR
rs11933330
rs17249895
rs11546426
rs114195144
rs35849732
rs1203981
rs12487717
rs77094535
rs367939626
rs28929474
rs77659355
rs144931628
rs10900864
rs36078038
rs73922258
rs9864010
rs73587009
rs71569391
rs1801394
rs35876677
rs62097680
All − MR Egger
All − IVW(MRE)
−0.010
−0.005
0.000
0.005
0.010
MR effect size for
'Discoidin, CUB and LCCL domain−containing protein 2' on 'eGFR'

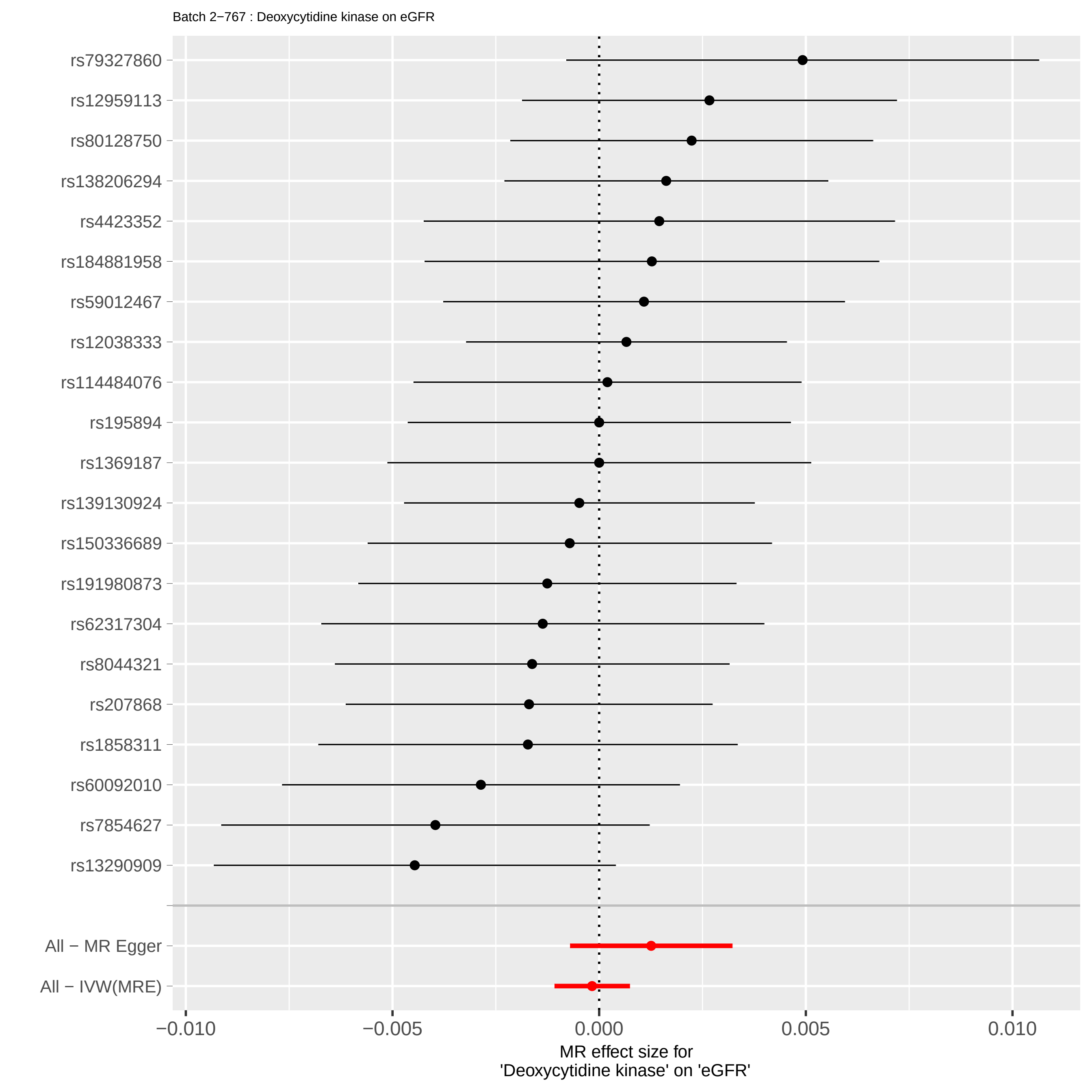

Batch 2−767 : Deoxycytidine kinase on eGFR
rs79327860
rs12959113
rs80128750
rs138206294
rs4423352
rs184881958
rs59012467
rs12038333
rs114484076
rs195894
rs1369187
rs139130924
rs150336689
rs191980873
rs62317304
rs8044321
rs207868
rs1858311
rs60092010
rs7854627
rs13290909
All − MR Egger
All − IVW(MRE)
−0.010
−0.005
0.000
0.005
0.010
MR effect size for
'Deoxycytidine kinase' on 'eGFR'

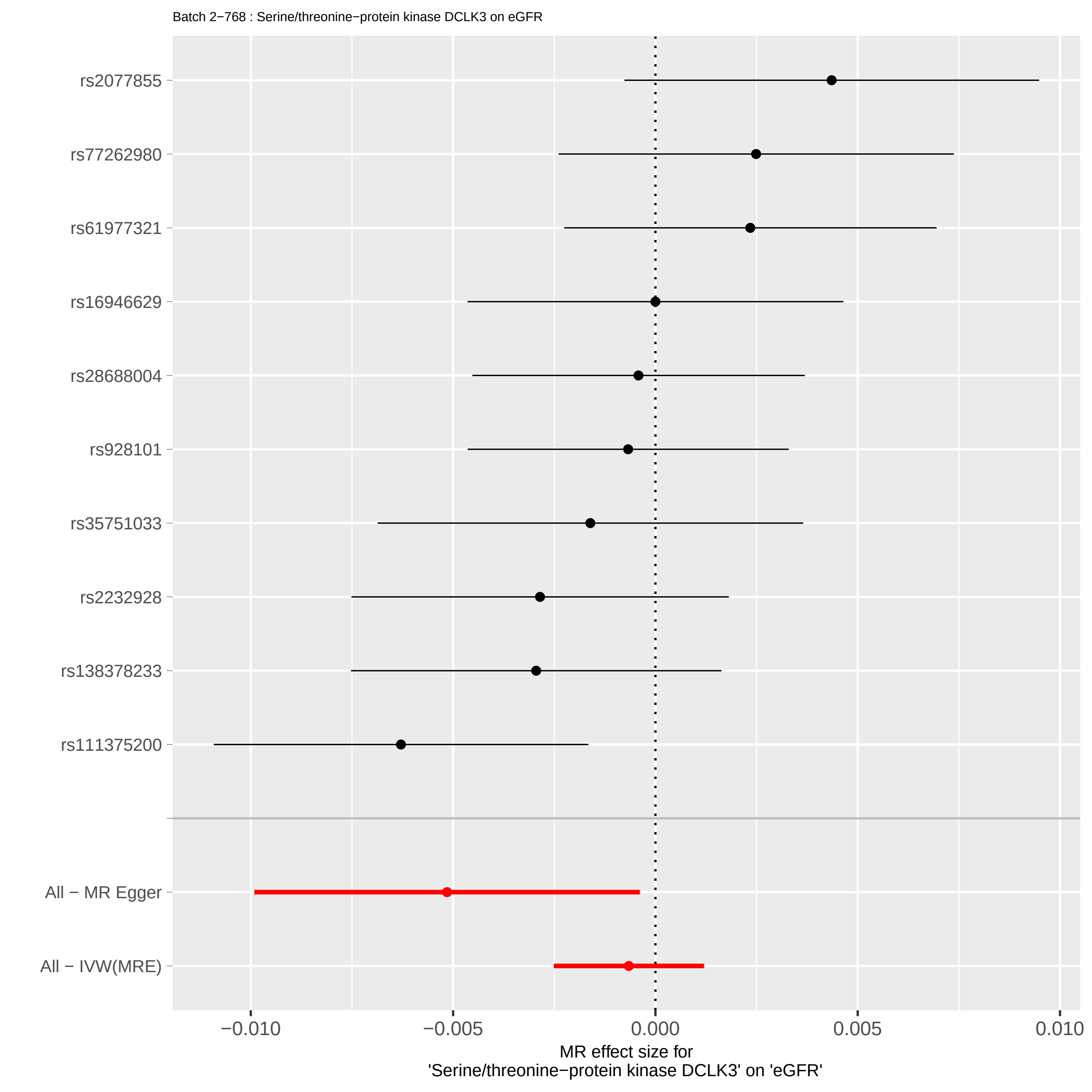
Batch 2−768 : Serine/threonine−protein kinase DCLK3 on eGFR
rs2077855
rs77262980
rs61977321
rs16946629
rs28688004
rs928101
rs35751033
rs2232928
rs138378233
rs111375200
All − MR Egger
All − IVW(MRE)
−0.010
−0.005
0.000
0.005
0.010
MR effect size for
'Serine/threonine−protein kinase DCLK3' on 'eGFR'

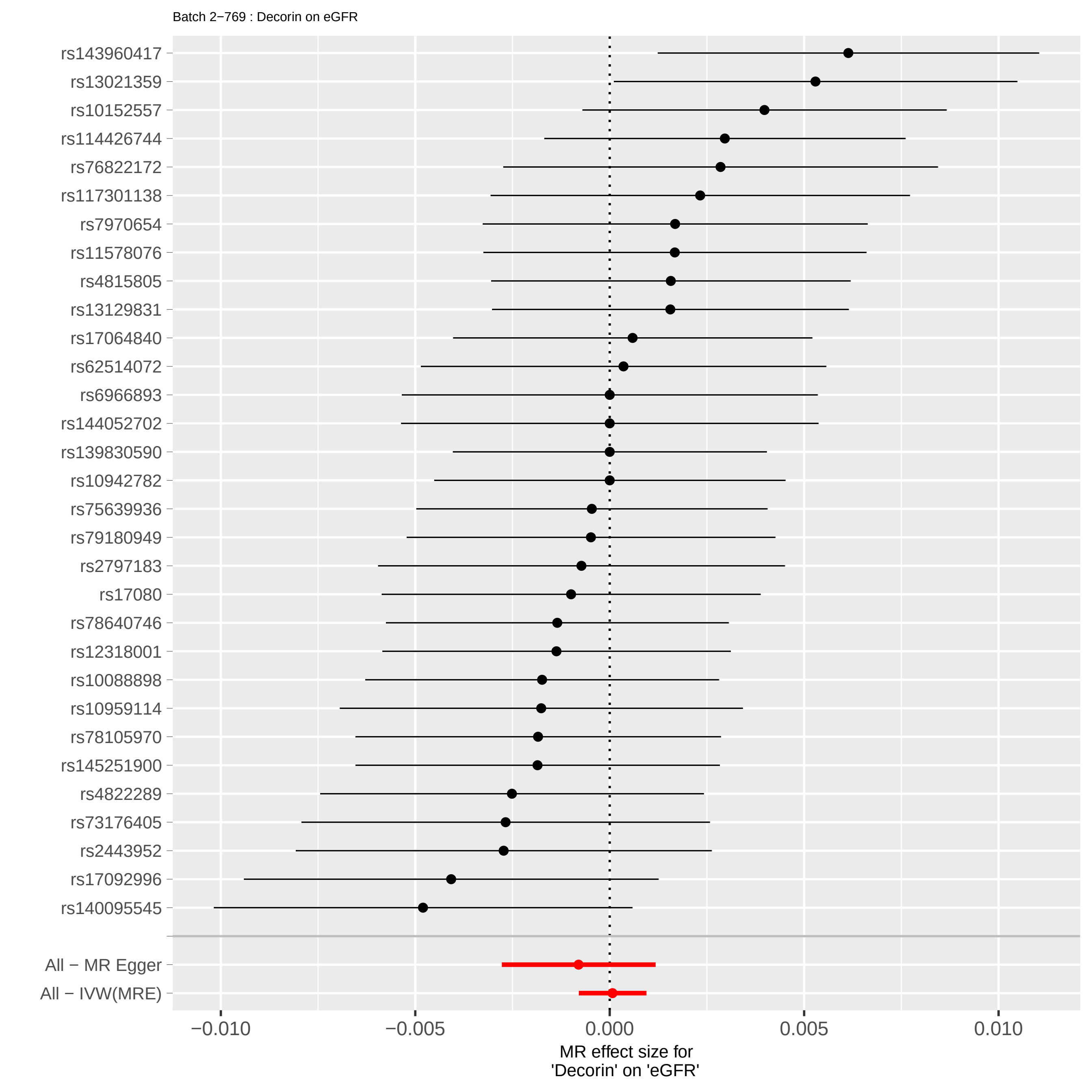

Batch 2−769 : Decorin on eGFR
rs143960417
rs13021359
rs10152557
rs114426744
rs76822172
rs117301138
rs7970654
rs11578076
rs4815805
rs13129831
rs17064840
rs62514072
rs6966893
rs144052702
rs139830590
rs10942782
rs75639936
rs79180949
rs2797183
rs17080
rs78640746
rs12318001
rs10088898
rs10959114
rs78105970
rs145251900
rs4822289
rs73176405
rs2443952
rs17092996
rs140095545
All − MR Egger
All − IVW(MRE)
−0.010
−0.005
0.000
0.005
0.010
MR effect size for
'Decorin' on 'eGFR'

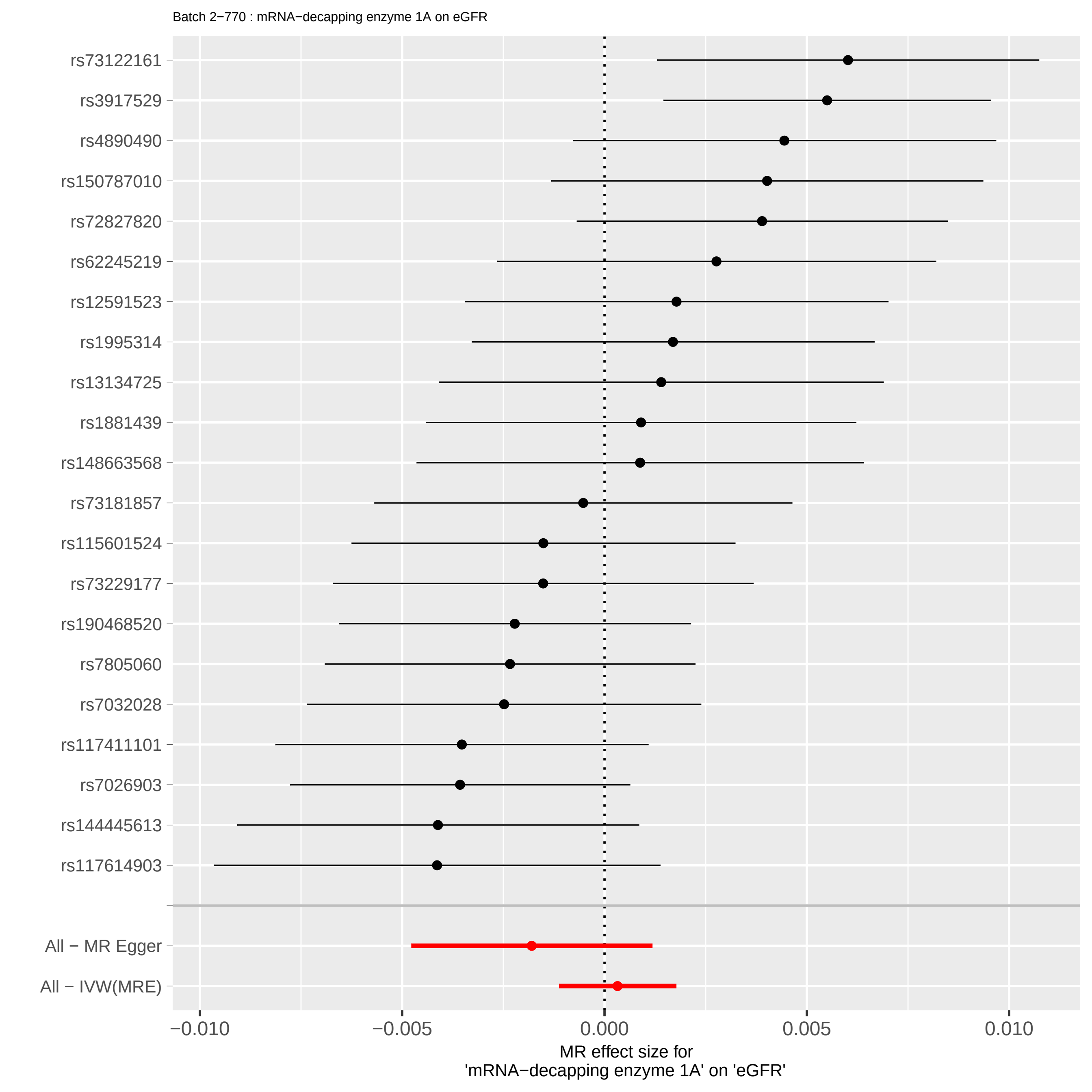

Batch 2−770 : mRNA−decapping enzyme 1A on eGFR
rs73122161
rs3917529
rs4890490
rs150787010
rs72827820
rs62245219
rs12591523
rs1995314
rs13134725
rs1881439
rs148663568
rs73181857
rs115601524
rs73229177
rs190468520
rs7805060
rs7032028
rs117411101
rs7026903
rs144445613
rs117614903
All − MR Egger
All − IVW(MRE)
−0.010
−0.005
0.000
0.005
0.010
MR effect size for
'mRNA−decapping enzyme 1A' on 'eGFR'

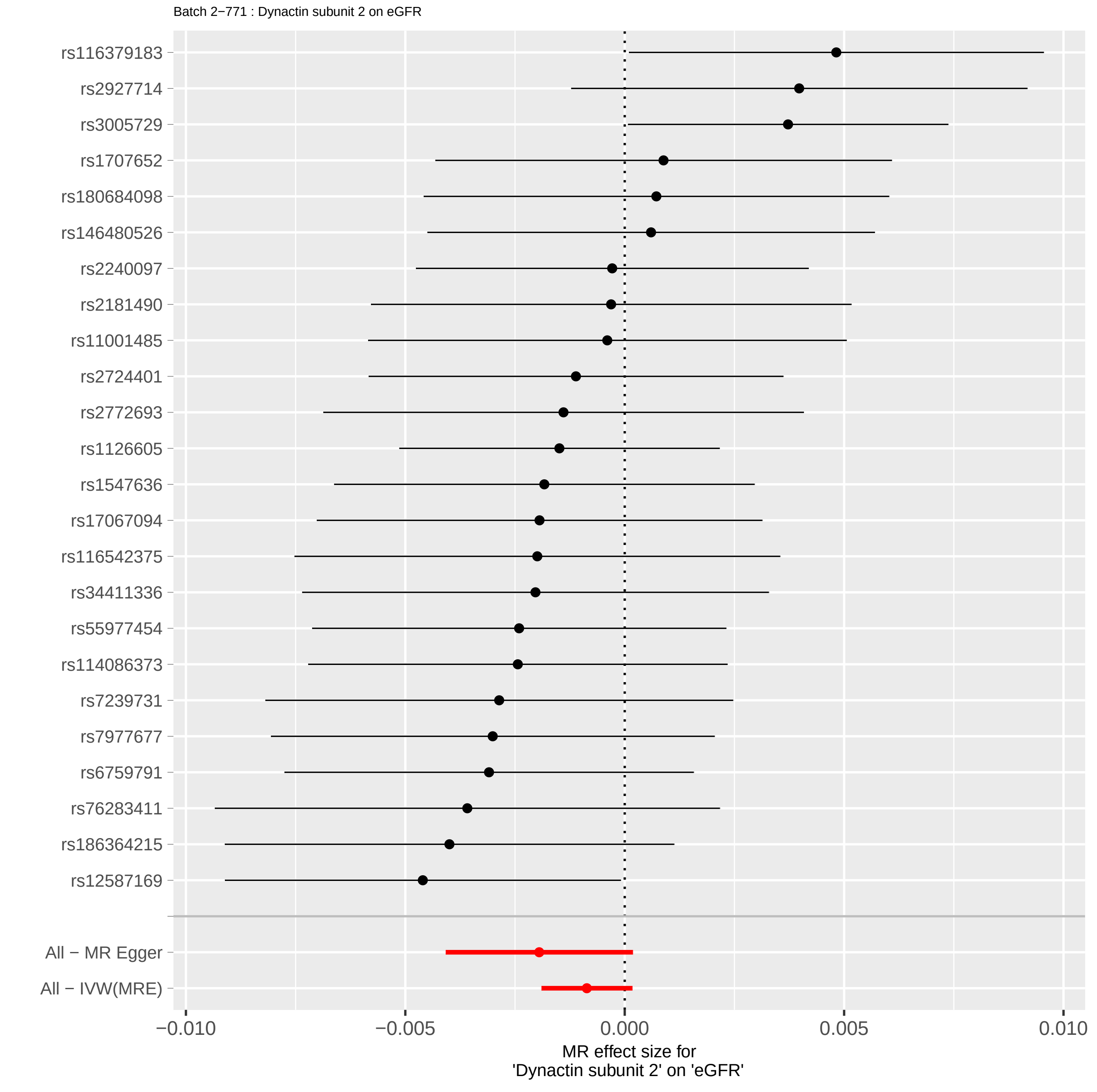

Batch 2−771 : Dynactin subunit 2 on eGFR
rs116379183
rs2927714
rs3005729
rs1707652
rs180684098
rs146480526
rs2240097
rs2181490
rs11001485
rs2724401
rs2772693
rs1126605
rs1547636
rs17067094
rs116542375
rs34411336
rs55977454
rs114086373
rs7239731
rs7977677
rs6759791
rs76283411
rs186364215
rs12587169
All − MR Egger
All − IVW(MRE)
−0.010
−0.005
0.000
0.005
0.010
MR effect size for
'Dynactin subunit 2' on 'eGFR'

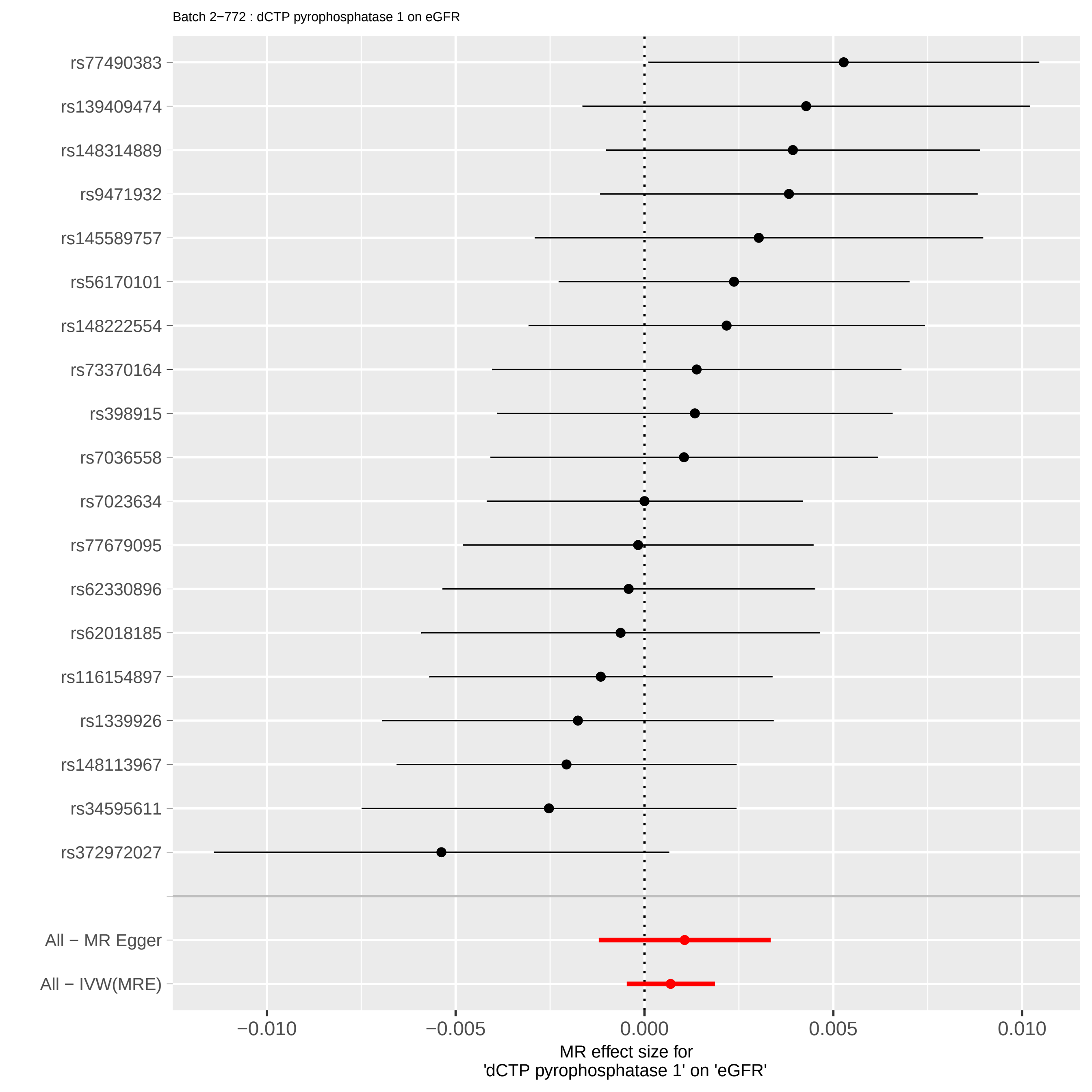

Batch 2−772 : dCTP pyrophosphatase 1 on eGFR
rs77490383
rs139409474
rs148314889
rs9471932
rs145589757
rs56170101
rs148222554
rs73370164
rs398915
rs7036558
rs7023634
rs77679095
rs62330896
rs62018185
rs116154897
rs1339926
rs148113967
rs34595611
rs372972027
All − MR Egger
All − IVW(MRE)
−0.010
−0.005
0.000
0.005
0.010
MR effect size for
'dCTP pyrophosphatase 1' on 'eGFR'

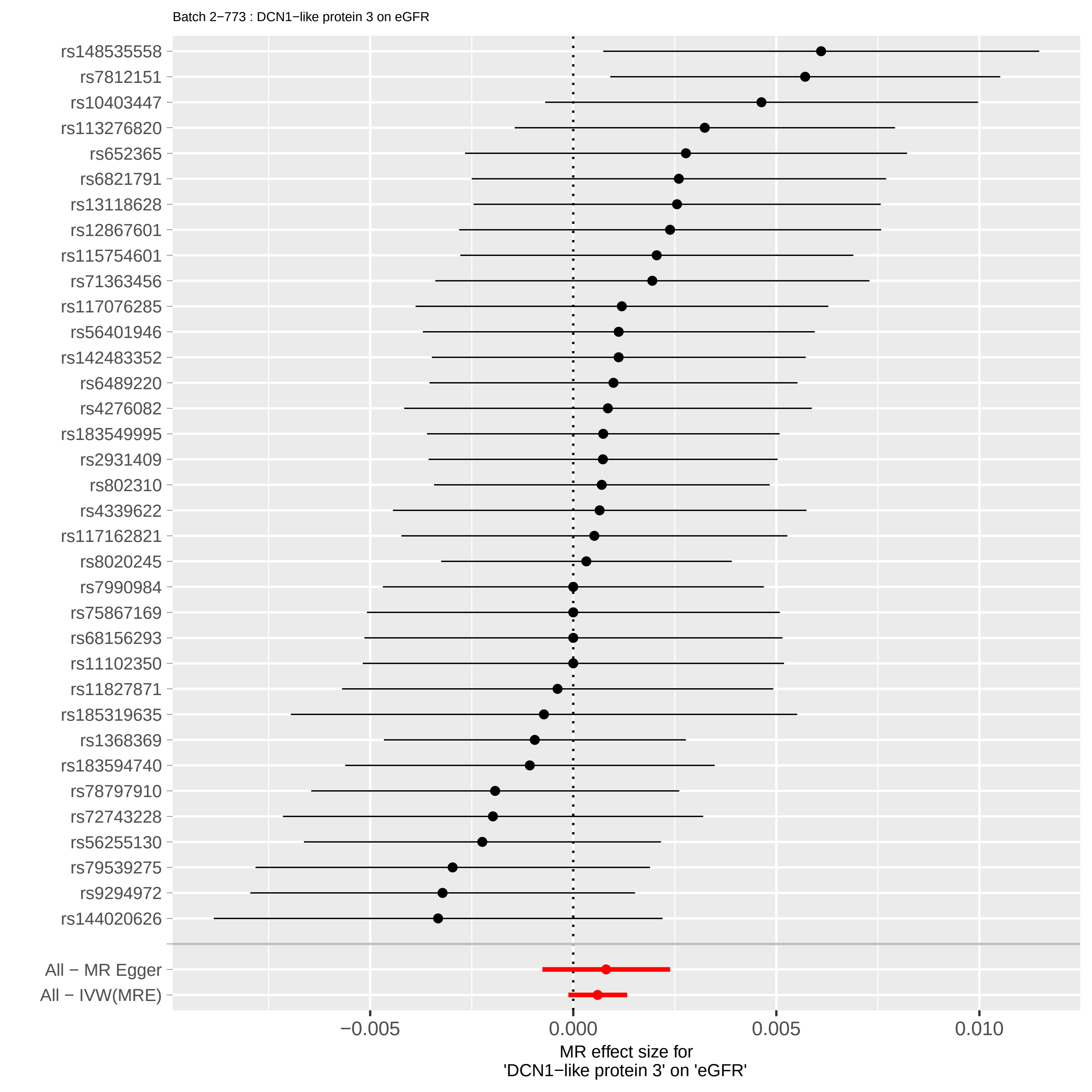

Batch 2−773 : DCN1−like protein 3 on eGFR
rs148535558
rs7812151
rs10403447
rs113276820
rs652365
rs6821791
rs13118628
rs12867601
rs115754601
rs71363456
rs117076285
rs56401946
rs142483352
rs6489220
rs4276082
rs183549995
rs2931409
rs802310
rs4339622
rs117162821
rs8020245
rs7990984
rs75867169
rs68156293
rs11102350
rs11827871
rs185319635
rs1368369
rs183594740
rs78797910
rs72743228
rs56255130
rs79539275
rs9294972
rs144020626
All − MR Egger
All − IVW(MRE)
−0.005
0.000
0.005
0.010
MR effect size for
'DCN1−like protein 3' on 'eGFR'

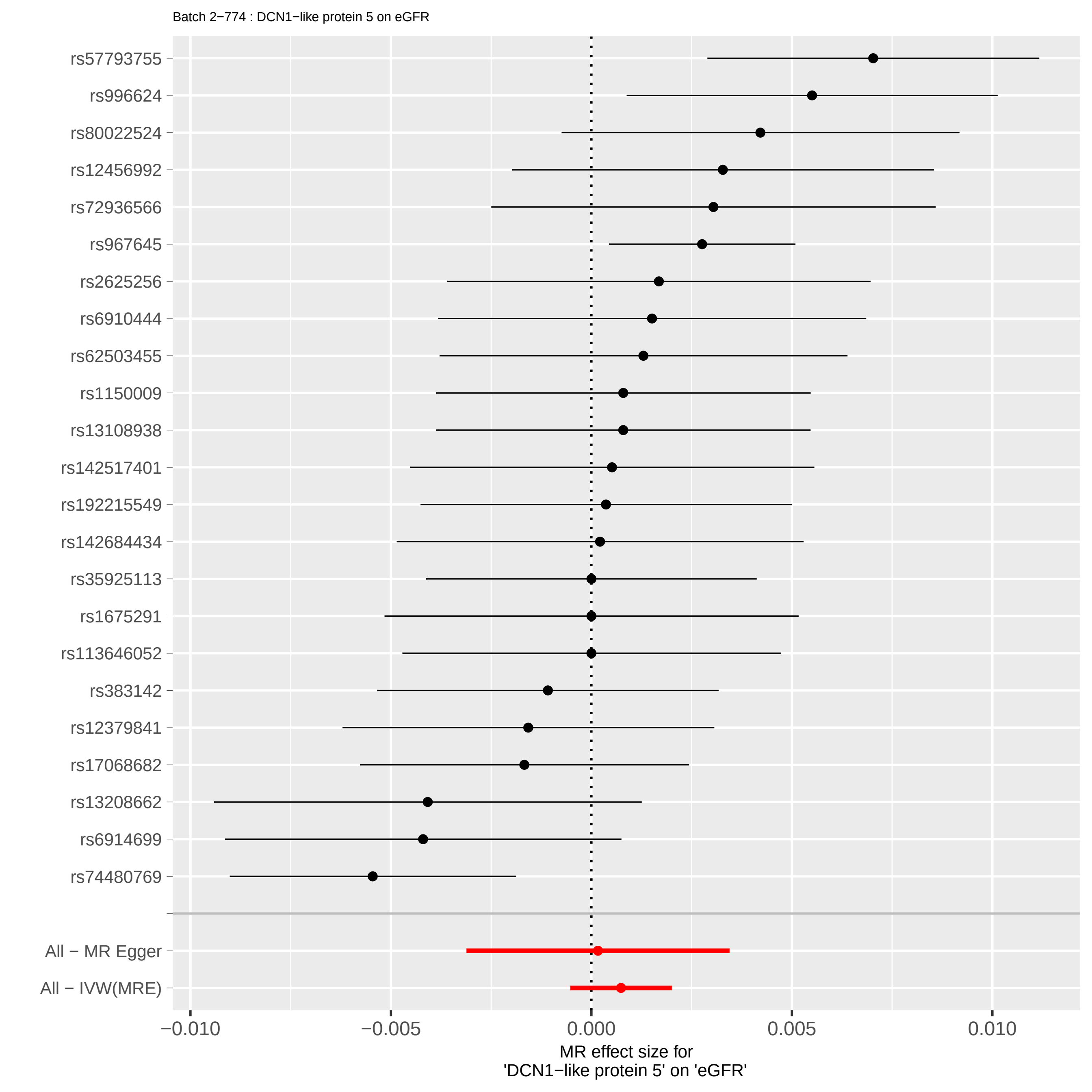
Batch 2−774 : DCN1−like protein 5 on eGFR
rs57793755
rs996624
rs80022524
rs12456992
rs72936566
rs967645
rs2625256
rs6910444
rs62503455
rs1150009
rs13108938
rs142517401
rs192215549
rs142684434
rs35925113
rs1675291
rs113646052
rs383142
rs12379841
rs17068682
rs13208662
rs6914699
rs74480769
All − MR Egger
All − IVW(MRE)
−0.010
−0.005
0.000
0.005
0.010
MR effect size for
'DCN1−like protein 5' on 'eGFR'

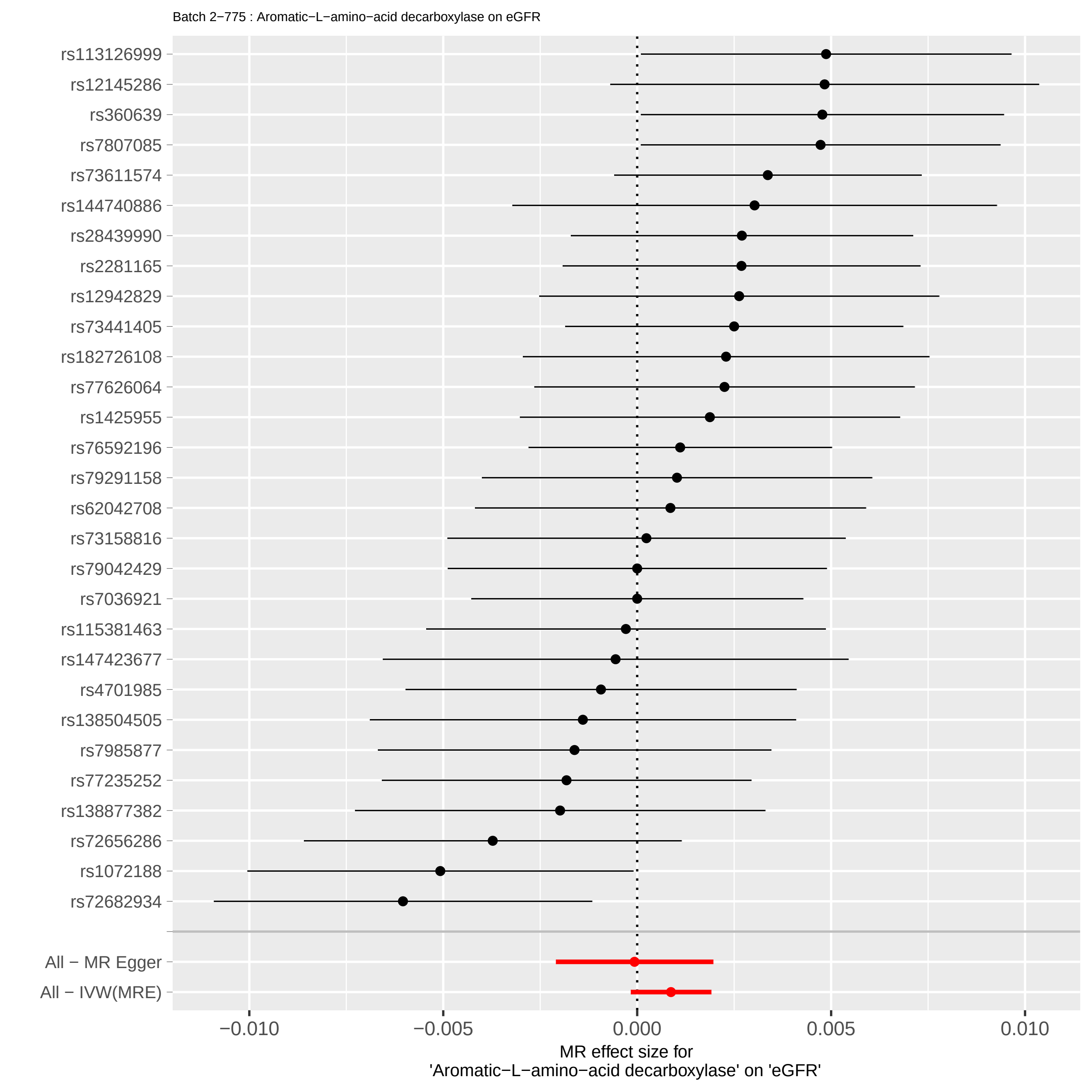

Batch 2−775 : Aromatic−L−amino−acid decarboxylase on eGFR
rs113126999
rs12145286
rs360639
rs7807085
rs73611574
rs144740886
rs28439990
rs2281165
rs12942829
rs73441405
rs182726108
rs77626064
rs1425955
rs76592196
rs79291158
rs62042708
rs73158816
rs79042429
rs7036921
rs115381463
rs147423677
rs4701985
rs138504505
rs7985877
rs77235252
rs138877382
rs72656286
rs1072188
rs72682934
All − MR Egger
All − IVW(MRE)
−0.010
−0.005
0.000
0.005
0.010
MR effect size for
'Aromatic−L−amino−acid decarboxylase' on 'eGFR'

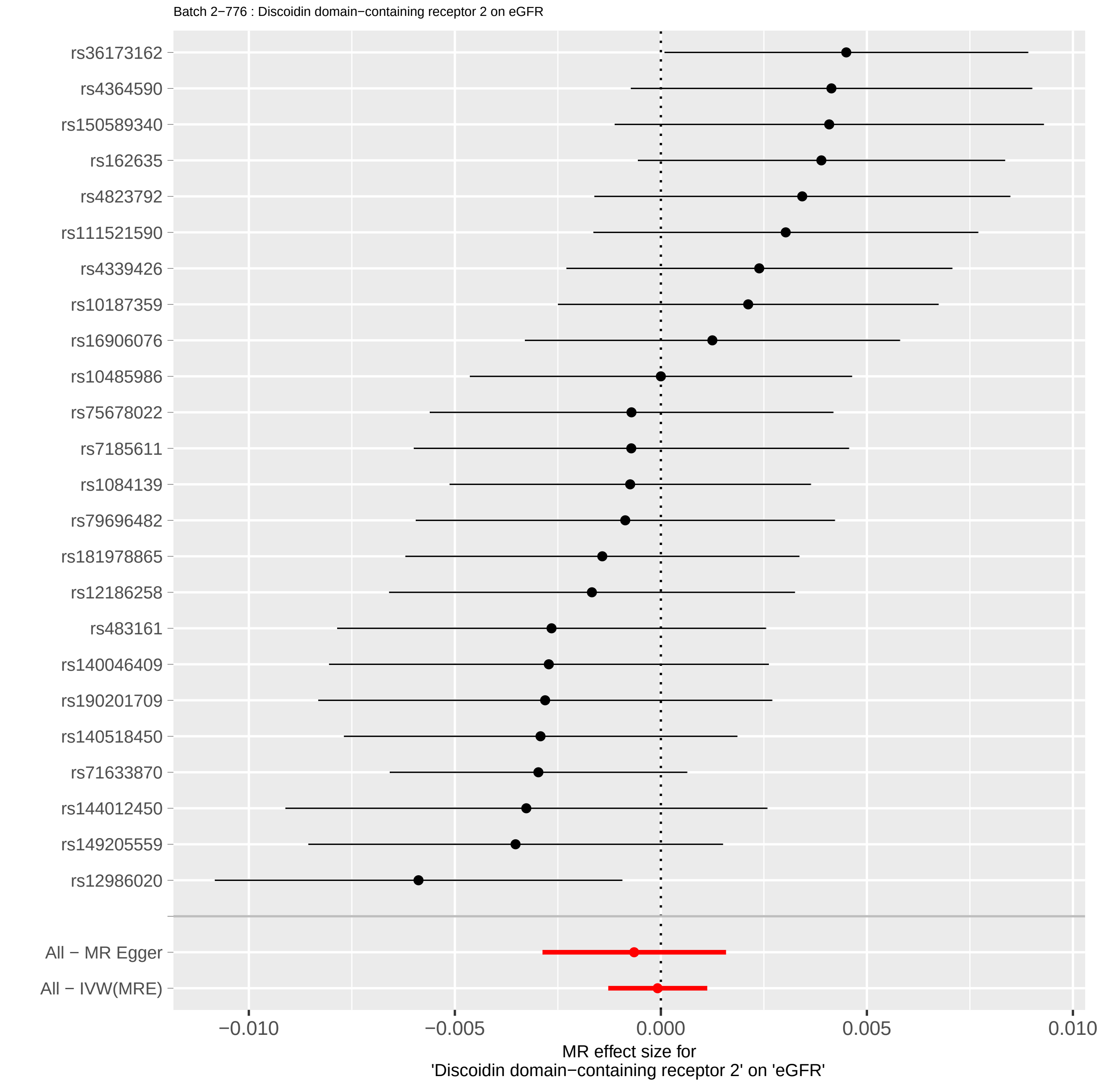

Batch 2−776 : Discoidin domain−containing receptor 2 on eGFR
rs36173162
rs4364590
rs150589340
rs162635
rs4823792
rs111521590
rs4339426
rs10187359
rs16906076
rs10485986
rs75678022
rs7185611
rs1084139
rs79696482
rs181978865
rs12186258
rs483161
rs140046409
rs190201709
rs140518450
rs71633870
rs144012450
rs149205559
rs12986020
All − MR Egger
All − IVW(MRE)
−0.010
−0.005
0.000
0.005
0.010
MR effect size for
'Discoidin domain−containing receptor 2' on 'eGFR'

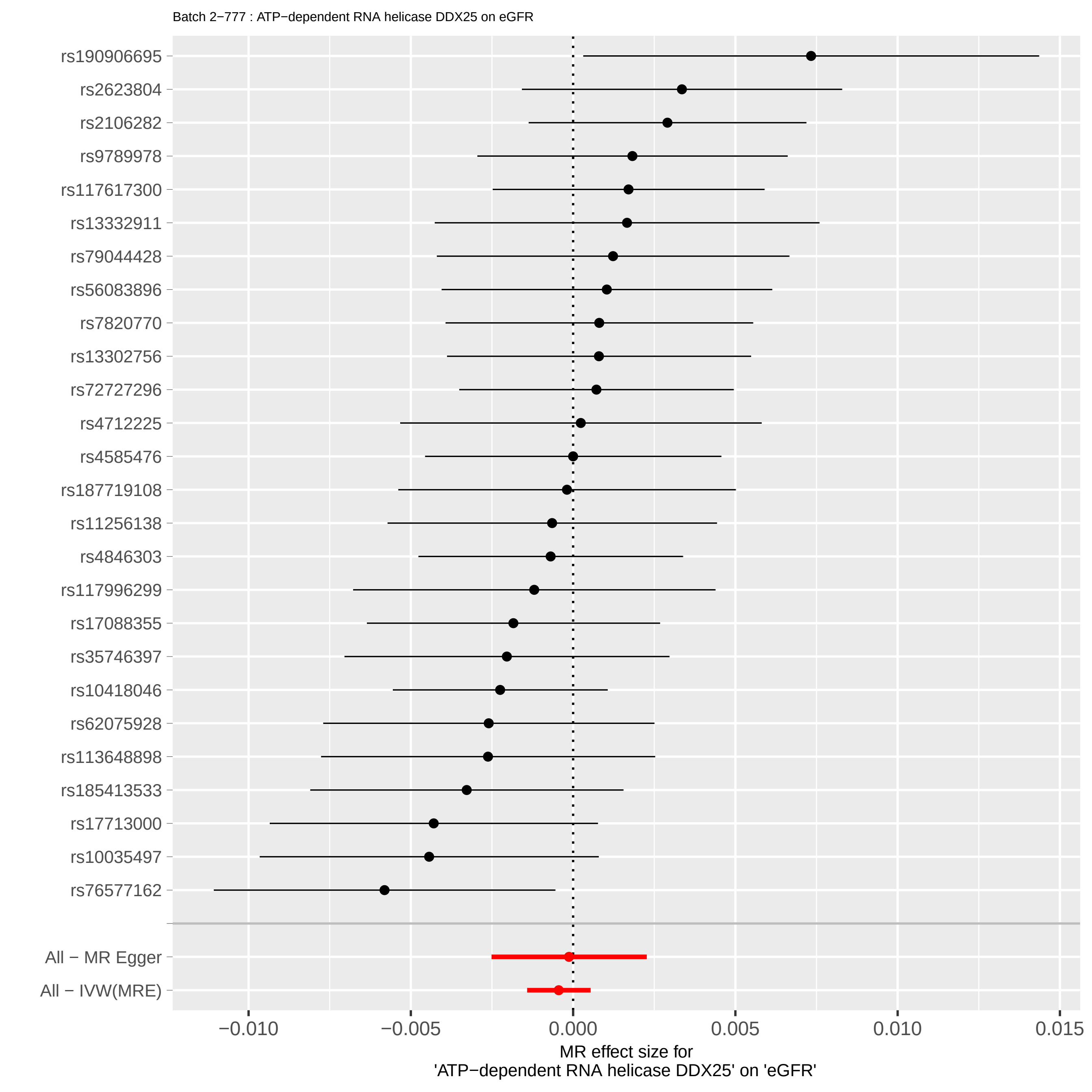
Batch 2−777 : ATP−dependent RNA helicase DDX25 on eGFR
rs190906695
rs2623804
rs2106282
rs9789978
rs117617300
rs13332911
rs79044428
rs56083896
rs7820770
rs13302756
rs72727296
rs4712225
rs4585476
rs187719108
rs11256138
rs4846303
rs117996299
rs17088355
rs35746397
rs10418046
rs62075928
rs113648898
rs185413533
rs17713000
rs10035497
rs76577162
All − MR Egger
All − IVW(MRE)
−0.010
−0.005
0.000
0.005
0.010
0.015
MR effect size for
'ATP−dependent RNA helicase DDX25' on 'eGFR'

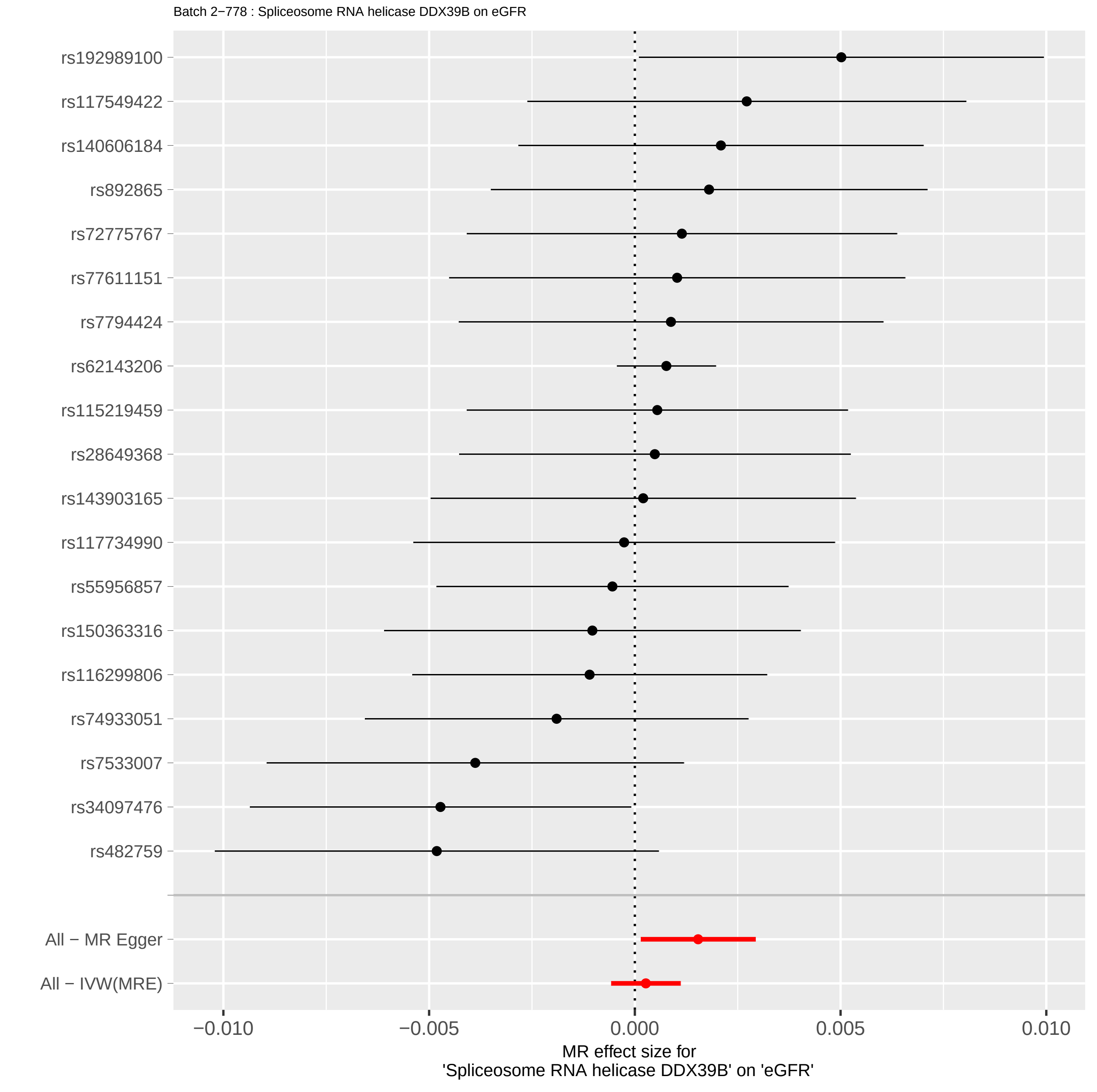
Batch 2−778 : Spliceosome RNA helicase DDX39B on eGFR
rs192989100
rs117549422
rs140606184
rs892865
rs72775767
rs77611151
rs7794424
rs62143206
rs115219459
rs28649368
rs143903165
rs117734990
rs55956857
rs150363316
rs116299806
rs74933051
rs7533007
rs34097476
rs482759
All − MR Egger
All − IVW(MRE)
−0.010
−0.005
0.000
0.005
0.010
MR effect size for
'Spliceosome RNA helicase DDX39B' on 'eGFR'

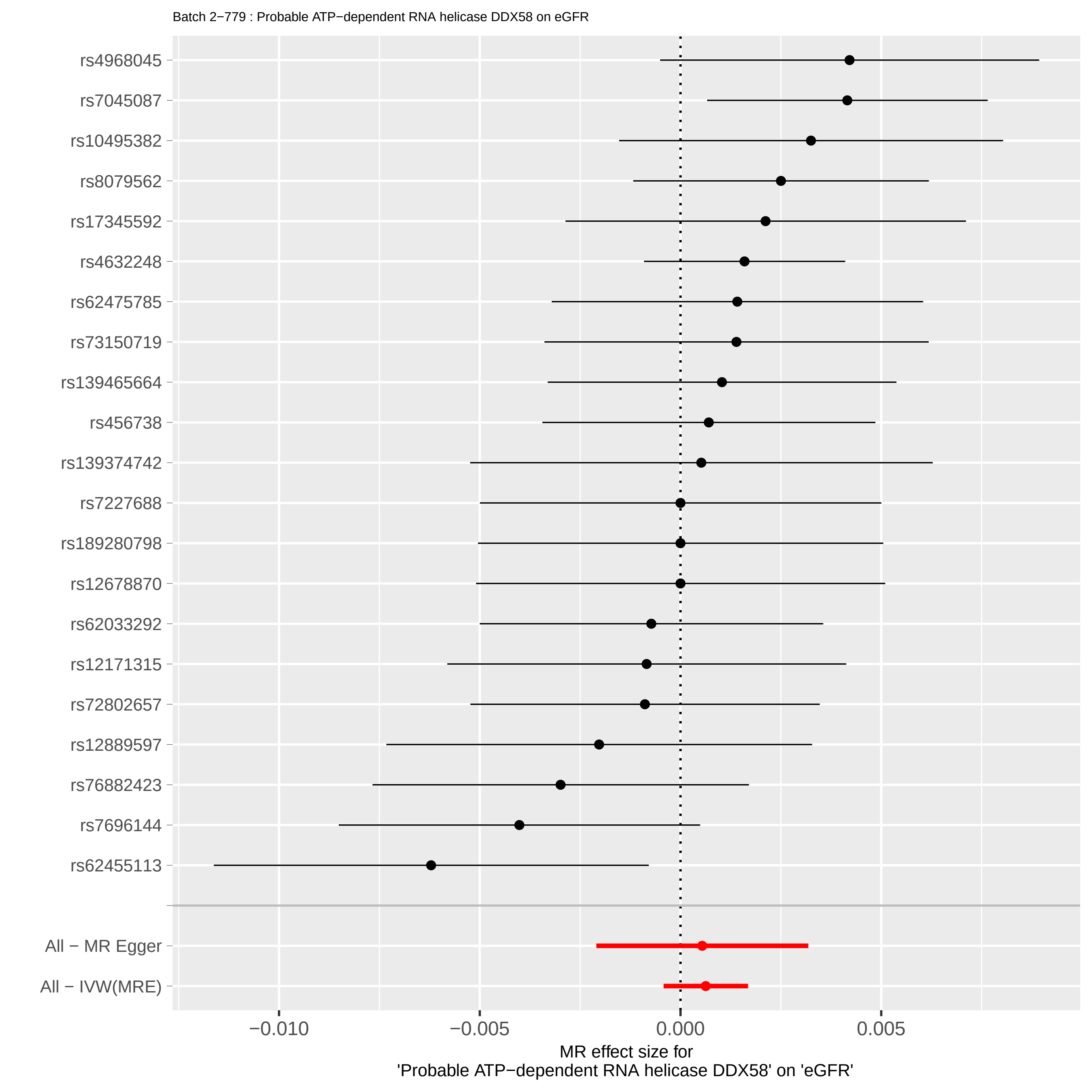
Batch 2−779 : Probable ATP−dependent RNA helicase DDX58 on eGFR
rs4968045
rs7045087
rs10495382
rs8079562
rs17345592
rs4632248
rs62475785
rs73150719
rs139465664
rs456738
rs139374742
rs7227688
rs189280798
rs12678870
rs62033292
rs12171315
rs72802657
rs12889597
rs76882423
rs7696144
rs62455113
All − MR Egger
All − IVW(MRE)
−0.010
−0.005
0.000
0.005
MR effect size for
'Probable ATP−dependent RNA helicase DDX58' on 'eGFR'

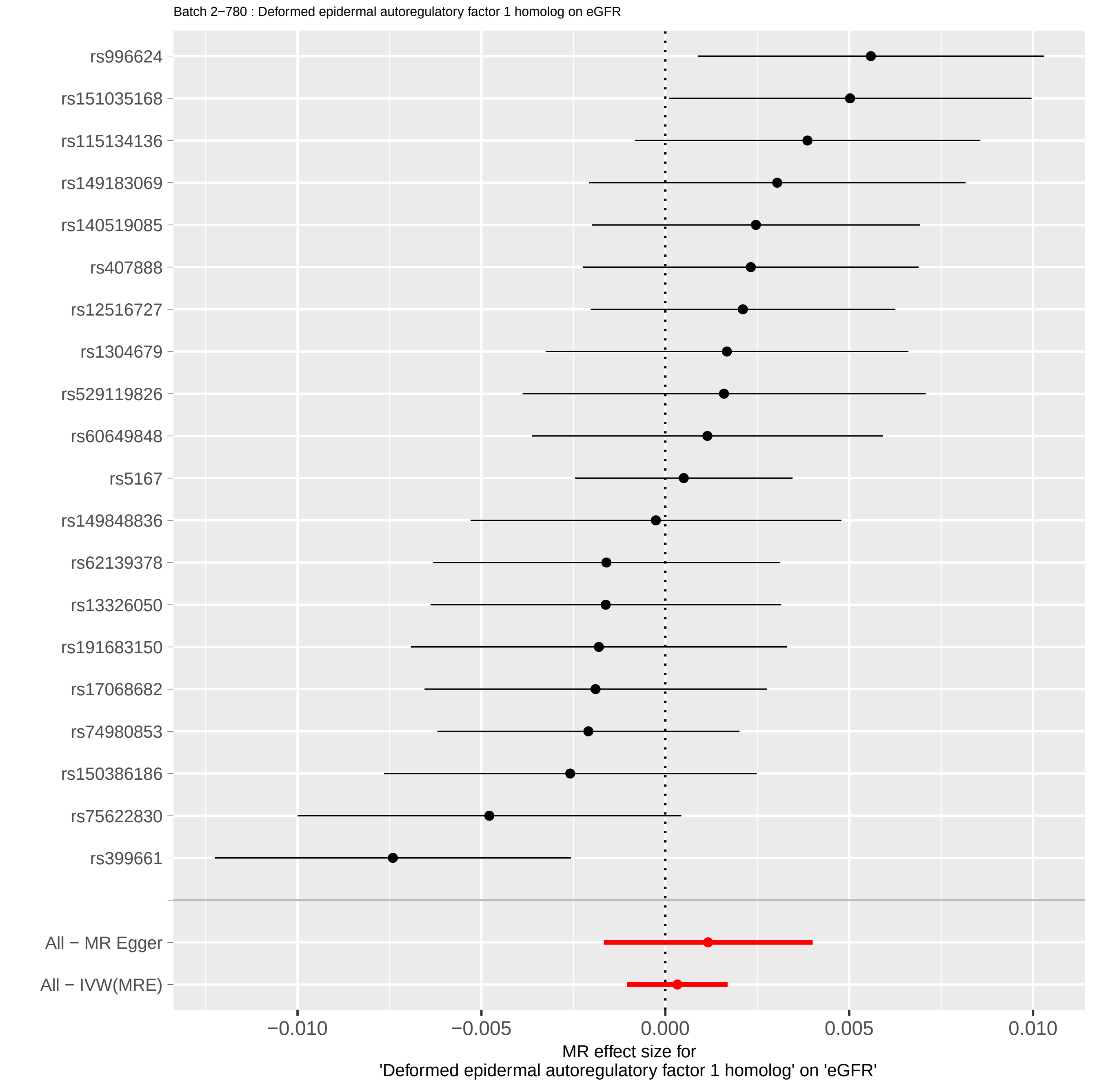

Batch 2−780 : Deformed epidermal autoregulatory factor 1 homolog on eGFR
rs996624
rs151035168
rs115134136
rs149183069
rs140519085
rs407888
rs12516727
rs1304679
rs529119826
rs60649848
rs5167
rs149848836
rs62139378
rs13326050
rs191683150
rs17068682
rs74980853
rs150386186
rs75622830
rs399661
All − MR Egger
All − IVW(MRE)
−0.010
−0.005
0.000
0.005
0.010
MR effect size for
'Deformed epidermal autoregulatory factor 1 homolog' on 'eGFR'

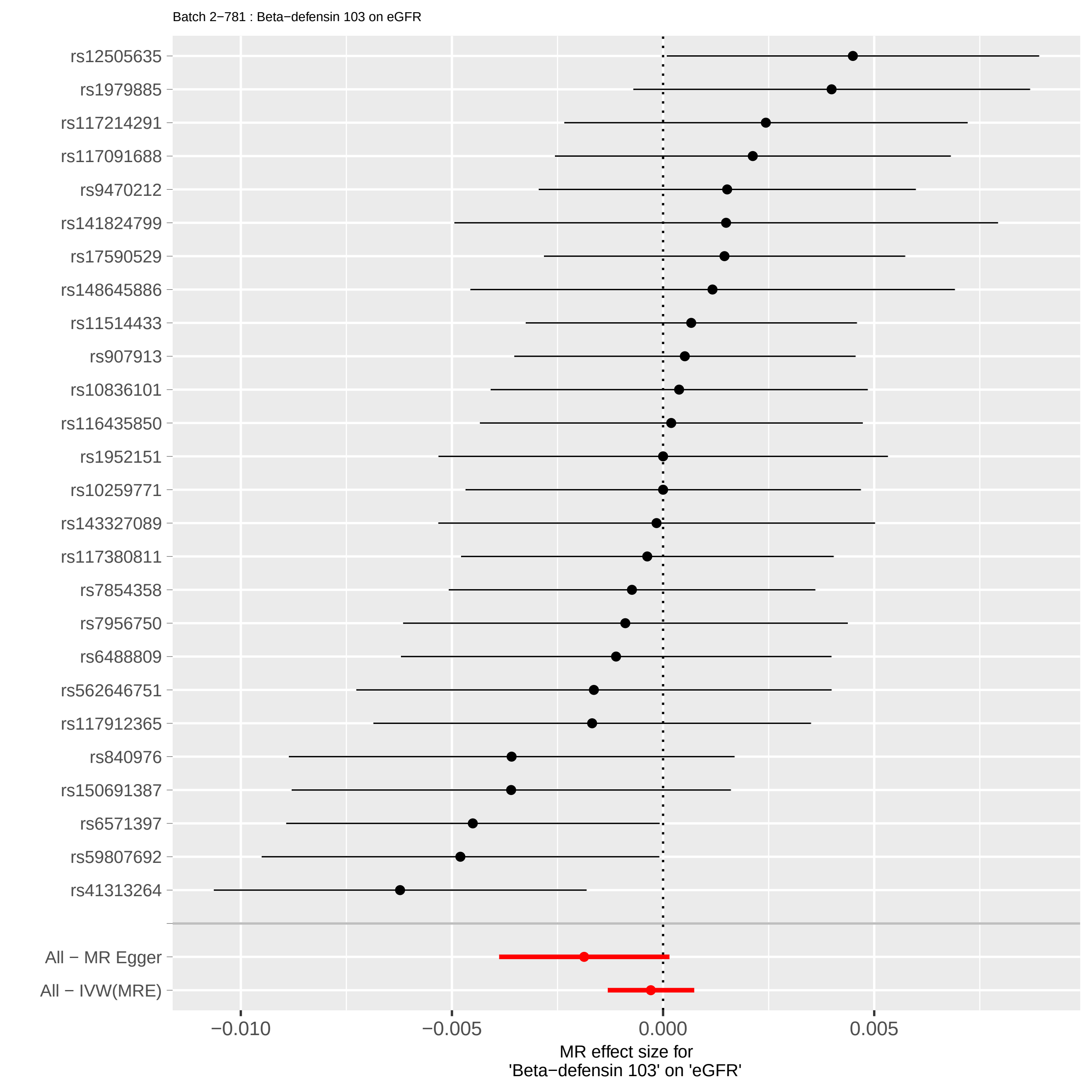

Batch 2−781 : Beta−defensin 103 on eGFR
rs12505635
rs1979885
rs117214291
rs117091688
rs9470212
rs141824799
rs17590529
rs148645886
rs11514433
rs907913
rs10836101
rs116435850
rs1952151
rs10259771
rs143327089
rs117380811
rs7854358
rs7956750
rs6488809
rs562646751
rs117912365
rs840976
rs150691387
rs6571397
rs59807692
rs41313264
All − MR Egger
All − IVW(MRE)
−0.010
−0.005
0.000
0.005
MR effect size for
'Beta−defensin 103' on 'eGFR'

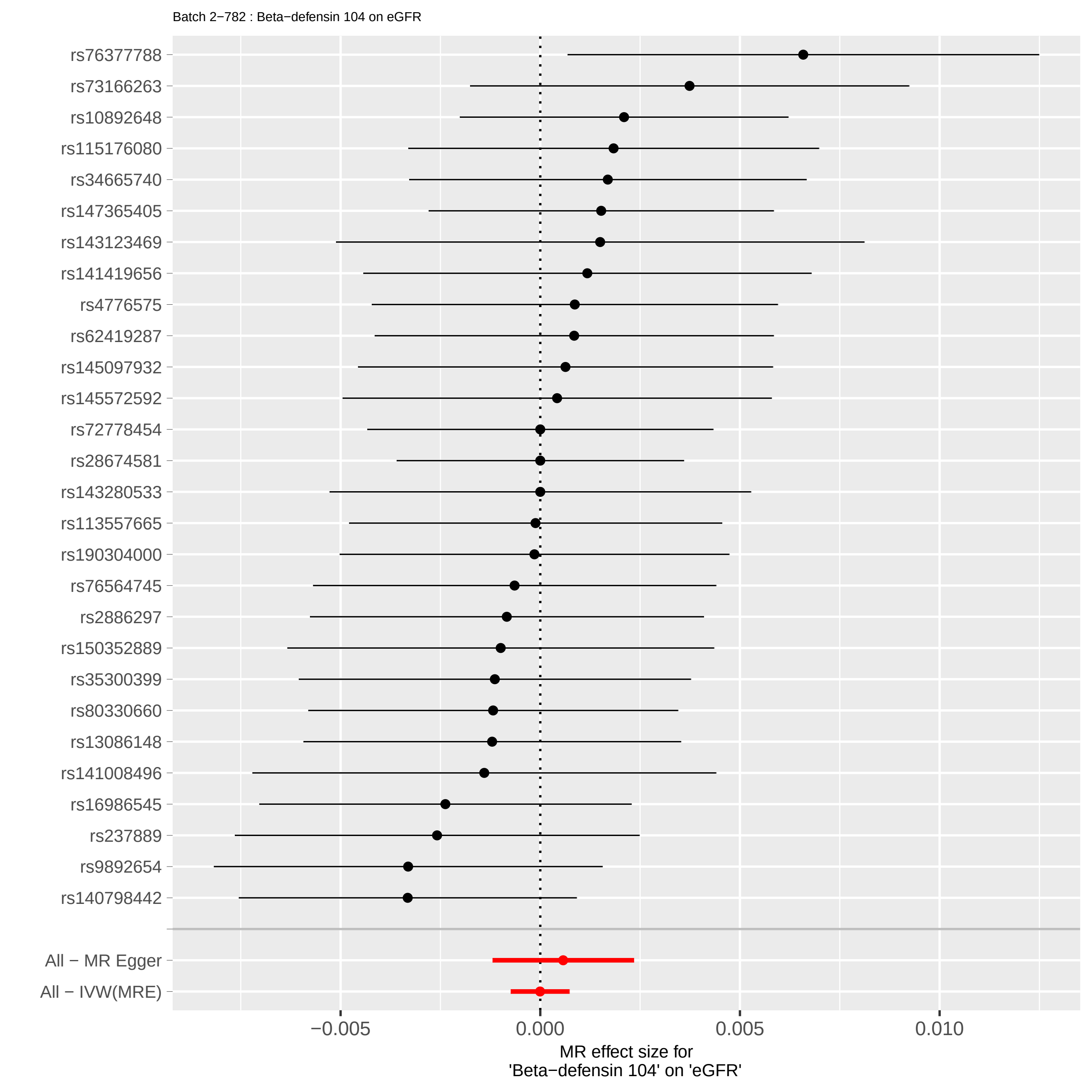
Batch 2−782 : Beta−defensin 104 on eGFR
rs76377788
rs73166263
rs10892648
rs115176080
rs34665740
rs147365405
rs143123469
rs141419656
rs4776575
rs62419287
rs145097932
rs145572592
rs72778454
rs28674581
rs143280533
rs113557665
rs190304000
rs76564745
rs2886297
rs150352889
rs35300399
rs80330660
rs13086148
rs141008496
rs16986545
rs237889
rs9892654
rs140798442
All − MR Egger
All − IVW(MRE)
−0.005
0.000
0.005
0.010
MR effect size for
'Beta−defensin 104' on 'eGFR'

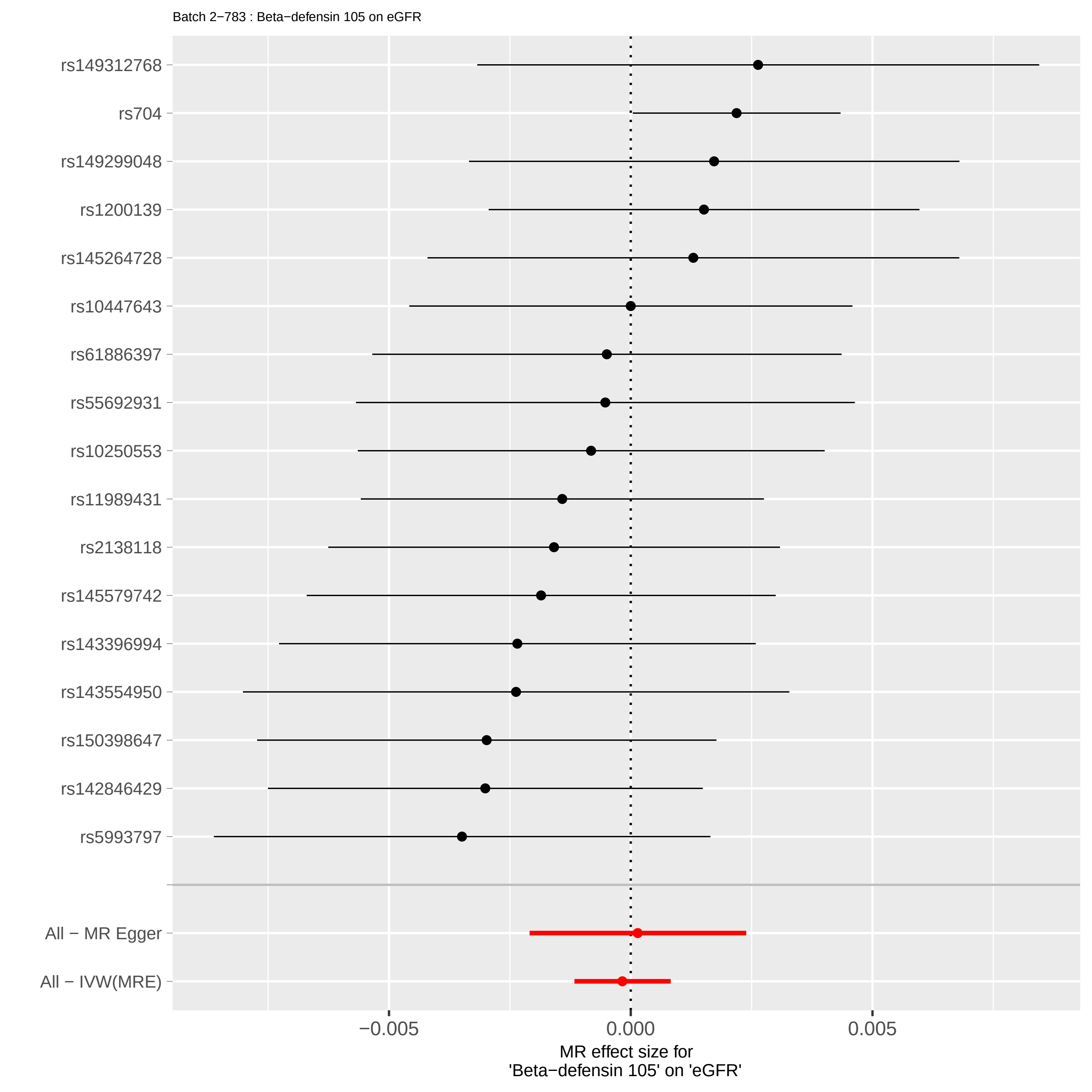

Batch 2−783 : Beta−defensin 105 on eGFR
rs149312768
rs704
rs149299048
rs1200139
rs145264728
rs10447643
rs61886397
rs55692931
rs10250553
rs11989431
rs2138118
rs145579742
rs143396994
rs143554950
rs150398647
rs142846429
rs5993797
All − MR Egger
All − IVW(MRE)
−0.005
0.000
0.005
MR effect size for
'Beta−defensin 105' on 'eGFR'

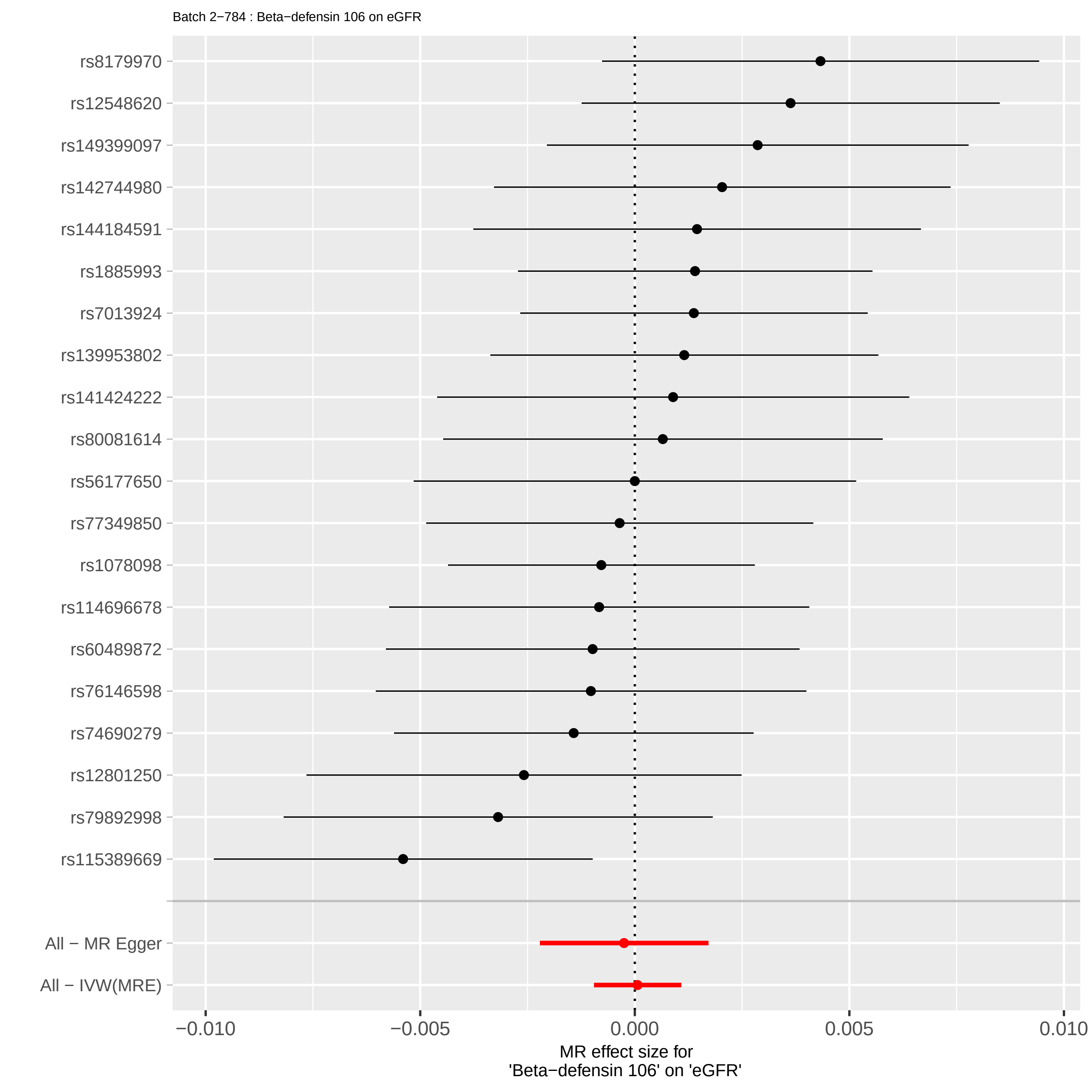

Batch 2−784 : Beta−defensin 106 on eGFR
rs8179970
rs12548620
rs149399097
rs142744980
rs144184591
rs1885993
rs7013924
rs139953802
rs141424222
rs80081614
rs56177650
rs77349850
rs1078098
rs114696678
rs60489872
rs76146598
rs74690279
rs12801250
rs79892998
rs115389669
All − MR Egger
All − IVW(MRE)
−0.010
−0.005
0.000
0.005
0.010
MR effect size for
'Beta−defensin 106' on 'eGFR'

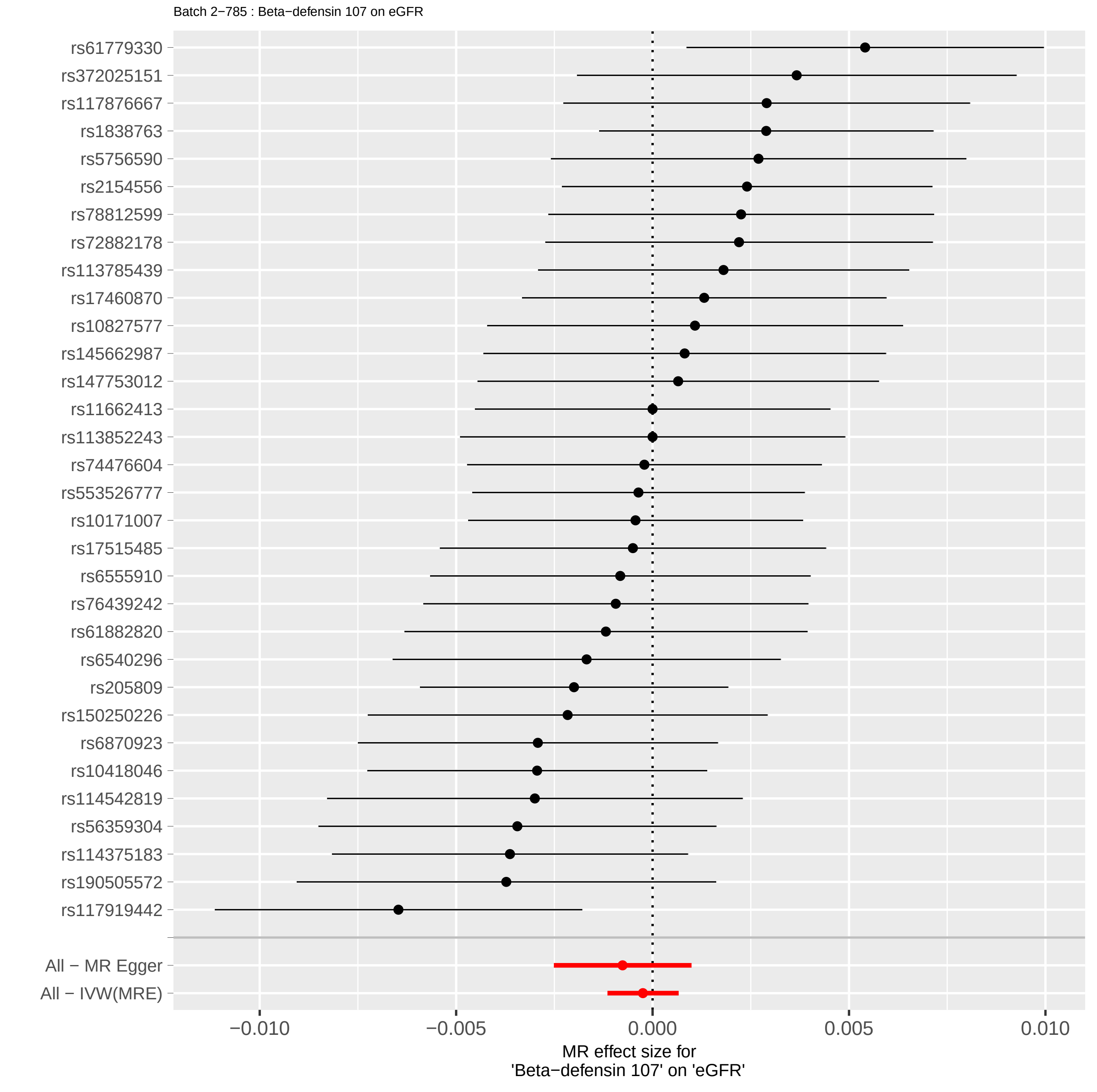

Batch 2−785 : Beta−defensin 107 on eGFR
rs61779330
rs372025151
rs117876667
rs1838763
rs5756590
rs2154556
rs78812599
rs72882178
rs113785439
rs17460870
rs10827577
rs145662987
rs147753012
rs11662413
rs113852243
rs74476604
rs553526777
rs10171007
rs17515485
rs6555910
rs76439242
rs61882820
rs6540296
rs205809
rs150250226
rs6870923
rs10418046
rs114542819
rs56359304
rs114375183
rs190505572
rs117919442
All − MR Egger
All − IVW(MRE)
−0.010
−0.005
0.000
0.005
0.010
MR effect size for
'Beta−defensin 107' on 'eGFR'

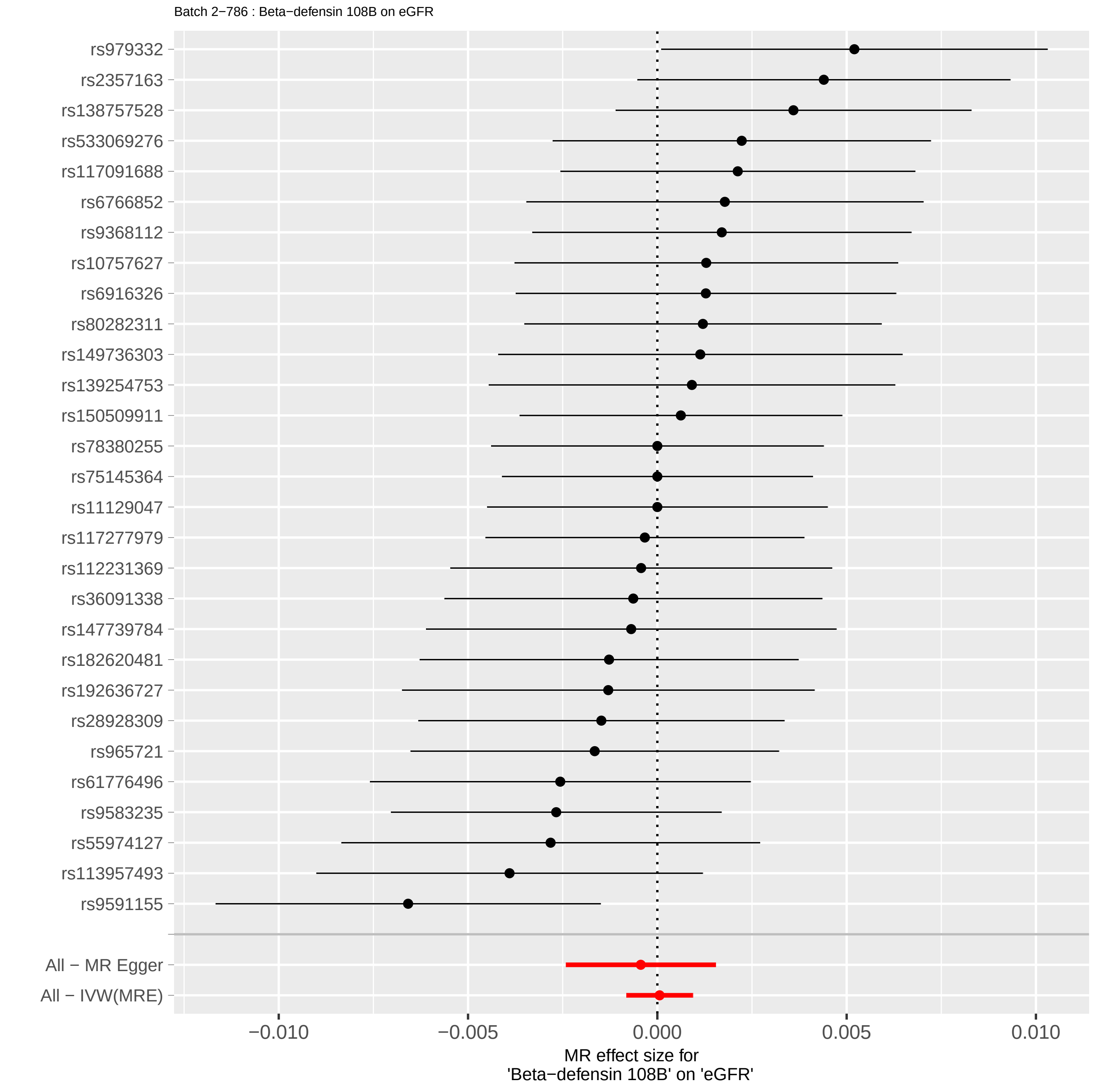

Batch 2−786 : Beta−defensin 108B on eGFR
rs979332
rs2357163
rs138757528
rs533069276
rs117091688
rs6766852
rs9368112
rs10757627
rs6916326
rs80282311
rs149736303
rs139254753
rs150509911
rs78380255
rs75145364
rs11129047
rs117277979
rs112231369
rs36091338
rs147739784
rs182620481
rs192636727
rs28928309
rs965721
rs61776496
rs9583235
rs55974127
rs113957493
rs9591155
All − MR Egger
All − IVW(MRE)
−0.010
−0.005
0.000
0.005
0.010
MR effect size for
'Beta−defensin 108B' on 'eGFR'

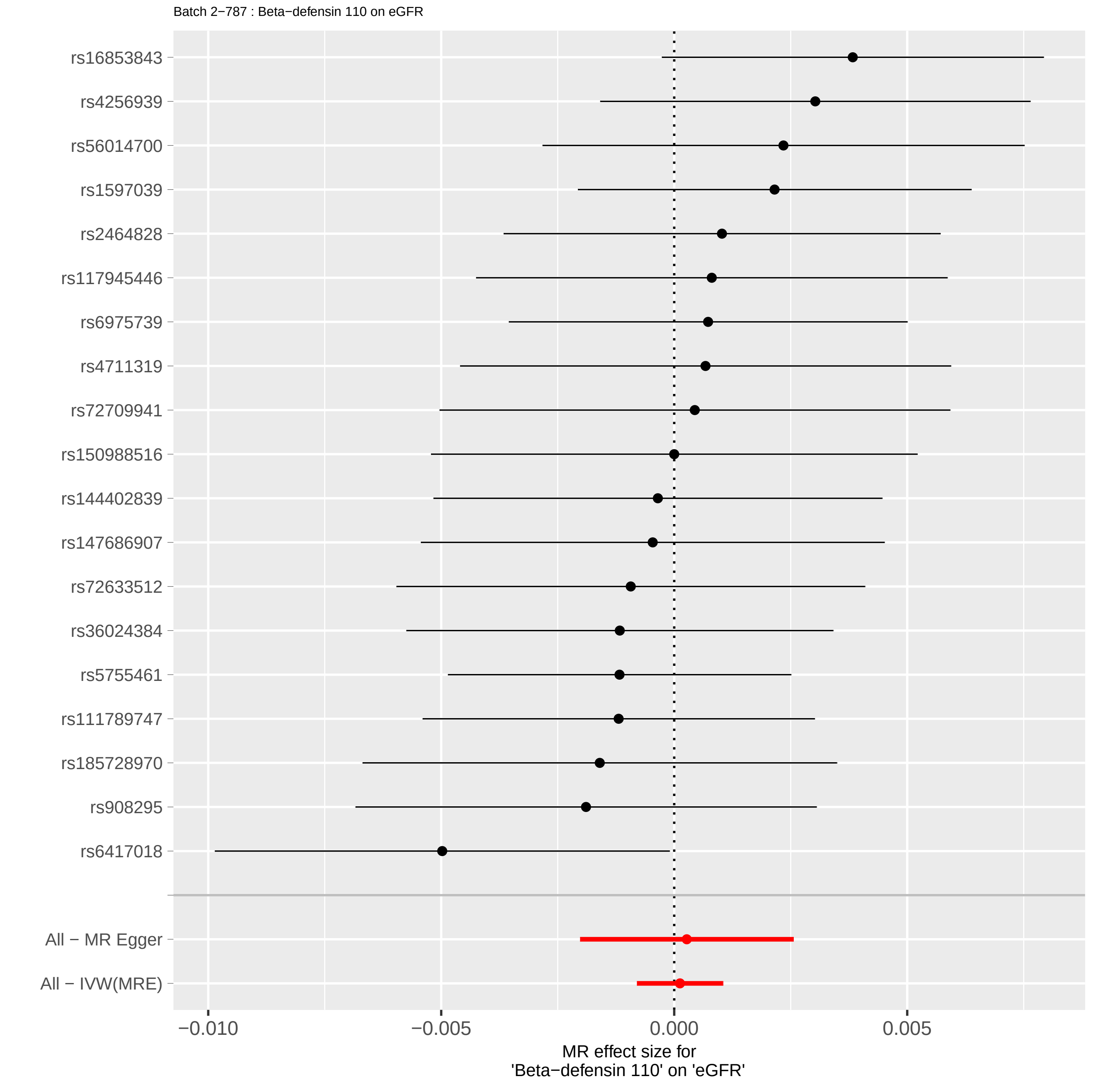

Batch 2−787 : Beta−defensin 110 on eGFR
rs16853843
rs4256939
rs56014700
rs1597039
rs2464828
rs117945446
rs6975739
rs4711319
rs72709941
rs150988516
rs144402839
rs147686907
rs72633512
rs36024384
rs5755461
rs111789747
rs185728970
rs908295
rs6417018
All − MR Egger
All − IVW(MRE)
−0.010
−0.005
0.000
0.005
MR effect size for
'Beta−defensin 110' on 'eGFR'

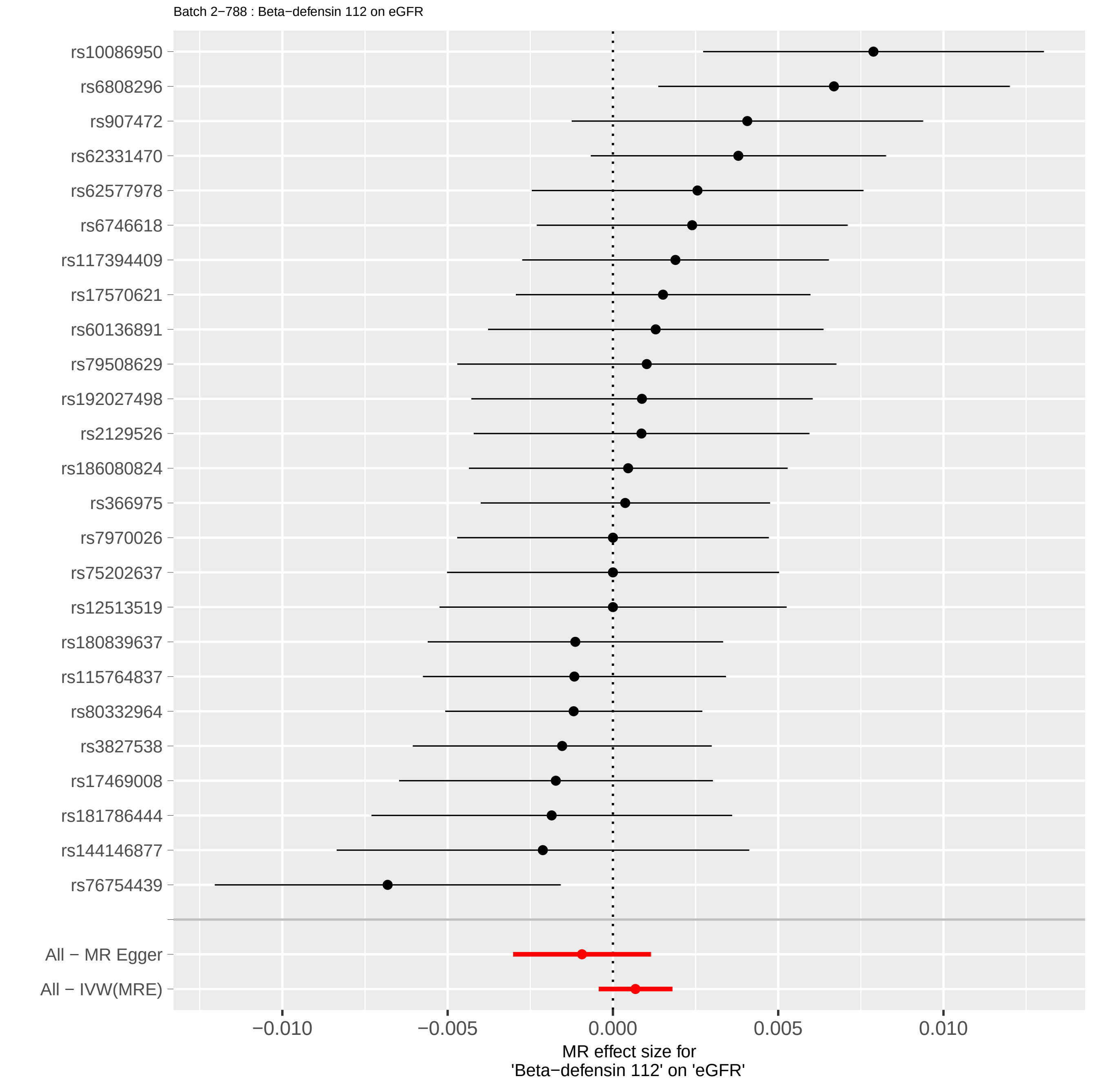

Batch 2−788 : Beta−defensin 112 on eGFR
rs10086950
rs6808296
rs907472
rs62331470
rs62577978
rs6746618
rs117394409
rs17570621
rs60136891
rs79508629
rs192027498
rs2129526
rs186080824
rs366975
rs7970026
rs75202637
rs12513519
rs180839637
rs115764837
rs80332964
rs3827538
rs17469008
rs181786444
rs144146877
rs76754439
All − MR Egger
All − IVW(MRE)
−0.010
−0.005
0.000
0.005
0.010
MR effect size for
'Beta−defensin 112' on 'eGFR'

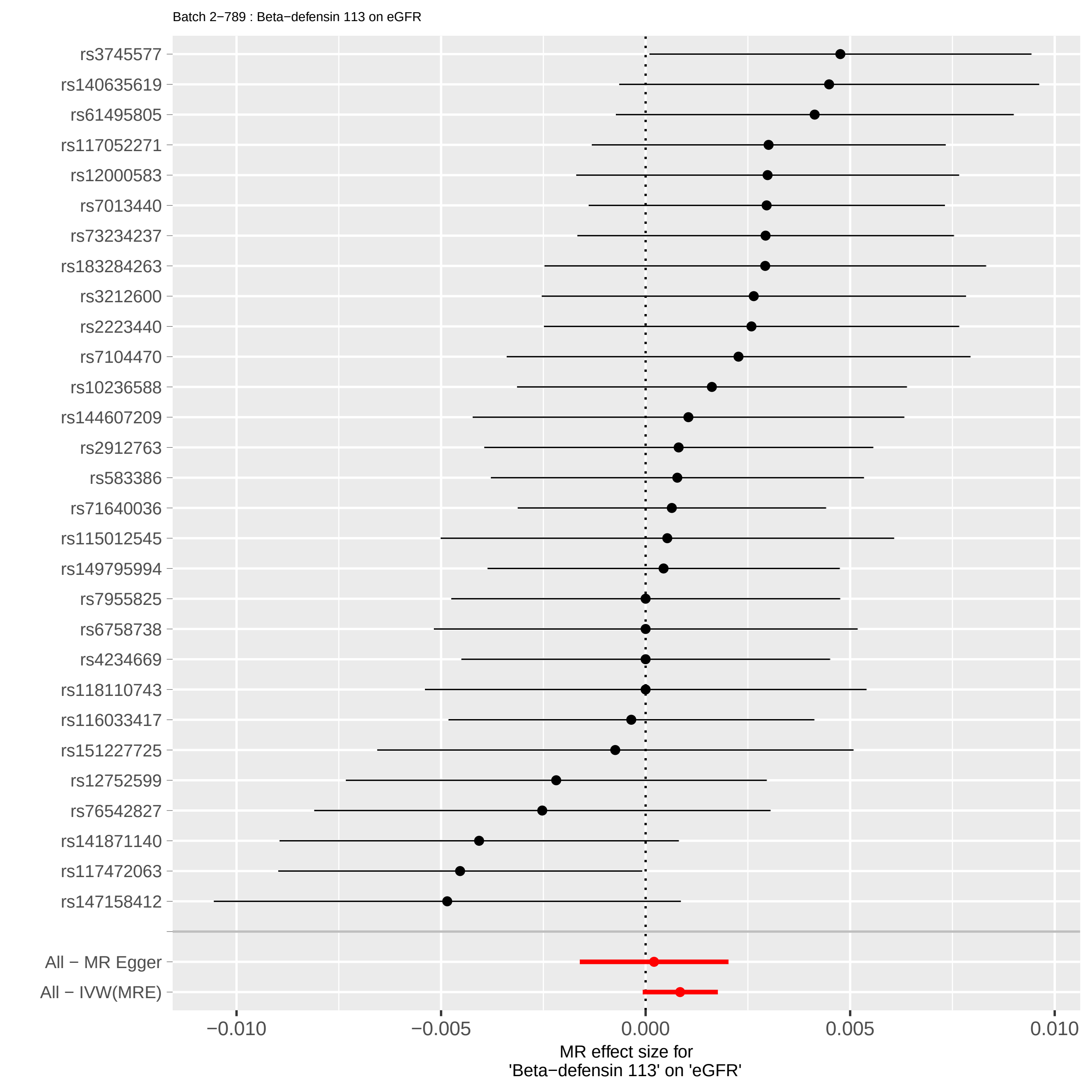

Batch 2−789 : Beta−defensin 113 on eGFR
rs3745577
rs140635619
rs61495805
rs117052271
rs12000583
rs7013440
rs73234237
rs183284263
rs3212600
rs2223440
rs7104470
rs10236588
rs144607209
rs2912763
rs583386
rs71640036
rs115012545
rs149795994
rs7955825
rs6758738
rs4234669
rs118110743
rs116033417
rs151227725
rs12752599
rs76542827
rs141871140
rs117472063
rs147158412
All − MR Egger
All − IVW(MRE)
−0.010
−0.005
0.000
0.005
0.010
MR effect size for
'Beta−defensin 113' on 'eGFR'

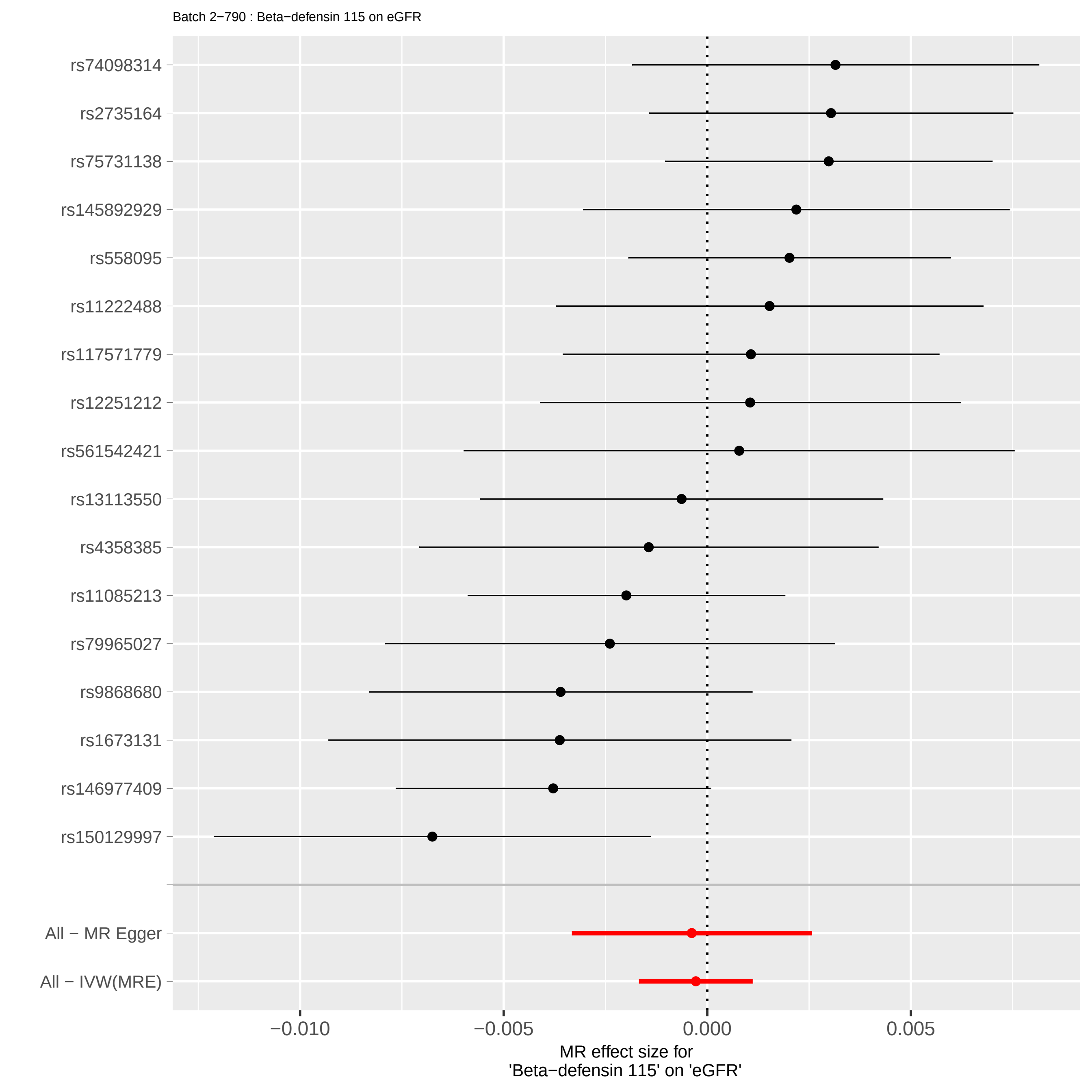

Batch 2−790 : Beta−defensin 115 on eGFR
rs74098314
rs2735164
rs75731138
rs145892929
rs558095
rs11222488
rs117571779
rs12251212
rs561542421
rs13113550
rs4358385
rs11085213
rs79965027
rs9868680
rs1673131
rs146977409
rs150129997
All − MR Egger
All − IVW(MRE)
−0.010
−0.005
0.000
0.005
MR effect size for
'Beta−defensin 115' on 'eGFR'

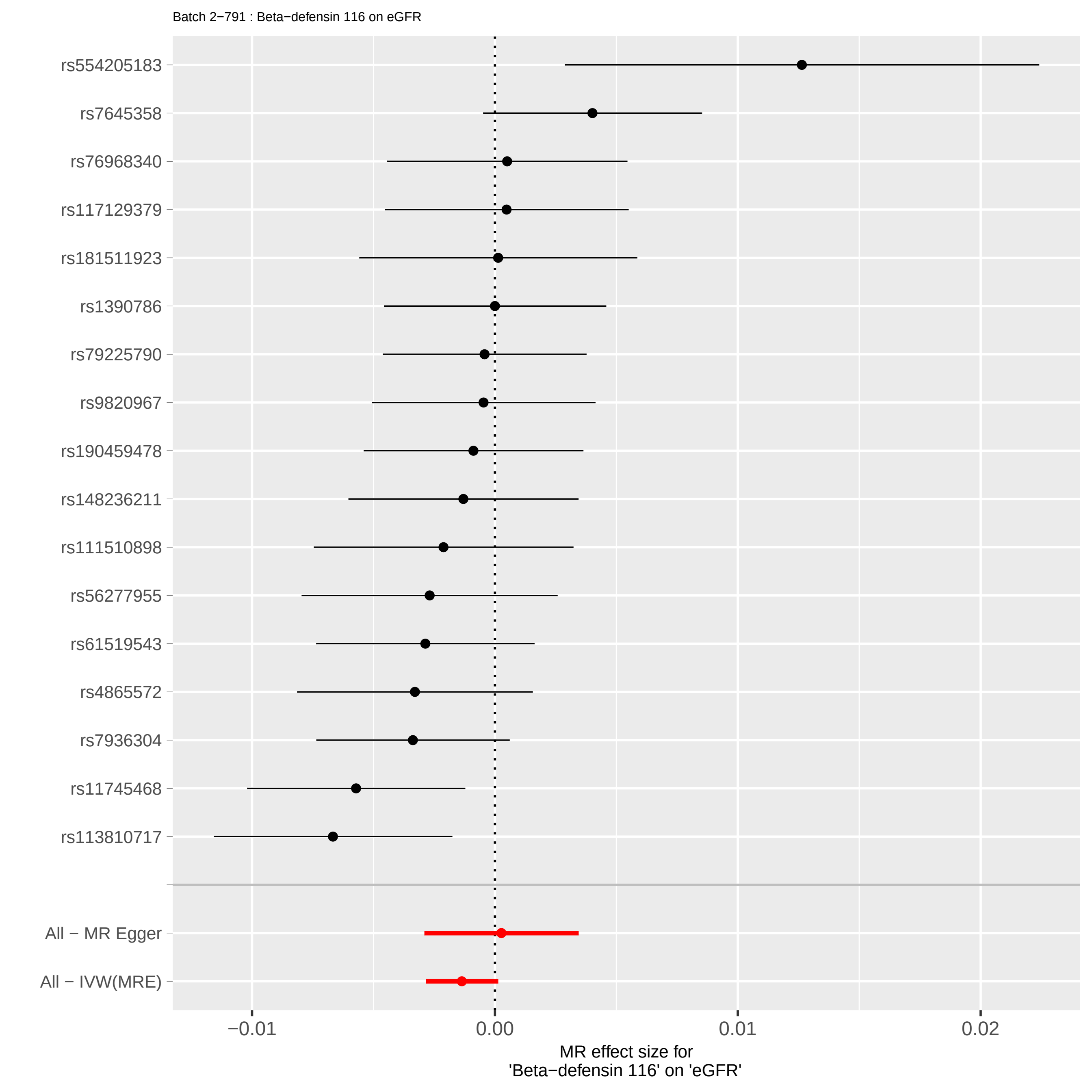

Batch 2−791 : Beta−defensin 116 on eGFR
rs554205183
rs7645358
rs76968340
rs117129379
rs181511923
rs1390786
rs79225790
rs9820967
rs190459478
rs148236211
rs111510898
rs56277955
rs61519543
rs4865572
rs7936304
rs11745468
rs113810717
All − MR Egger
All − IVW(MRE)
−0.01
0.00
0.01
0.02
MR effect size for
'Beta−defensin 116' on 'eGFR'

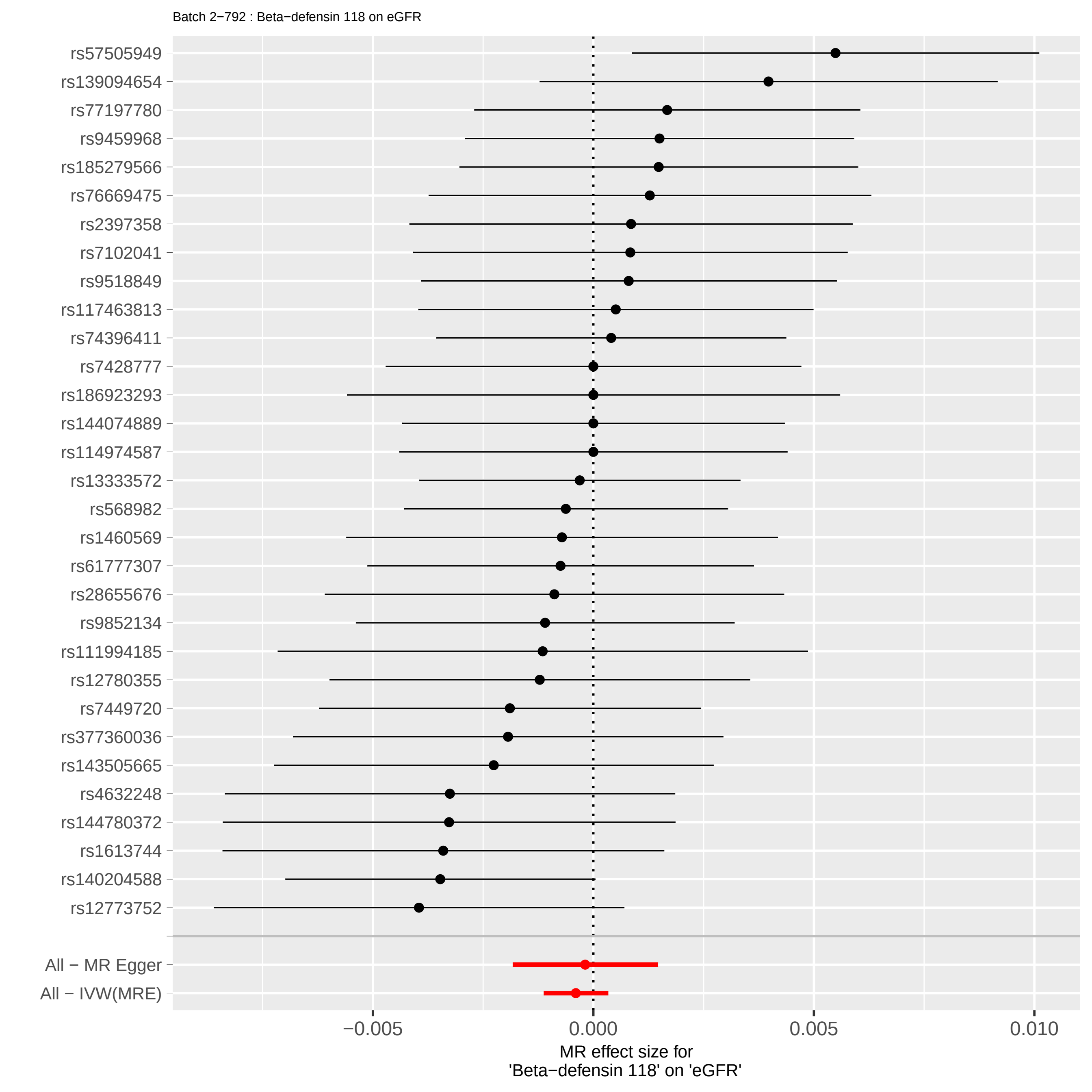

Batch 2−792 : Beta−defensin 118 on eGFR
rs57505949
rs139094654
rs77197780
rs9459968
rs185279566
rs76669475
rs2397358
rs7102041
rs9518849
rs117463813
rs74396411
rs7428777
rs186923293
rs144074889
rs114974587
rs13333572
rs568982
rs1460569
rs61777307
rs28655676
rs9852134
rs111994185
rs12780355
rs7449720
rs377360036
rs143505665
rs4632248
rs144780372
rs1613744
rs140204588
rs12773752
All − MR Egger
All − IVW(MRE)
−0.005
0.000
0.005
0.010
MR effect size for
'Beta−defensin 118' on 'eGFR'

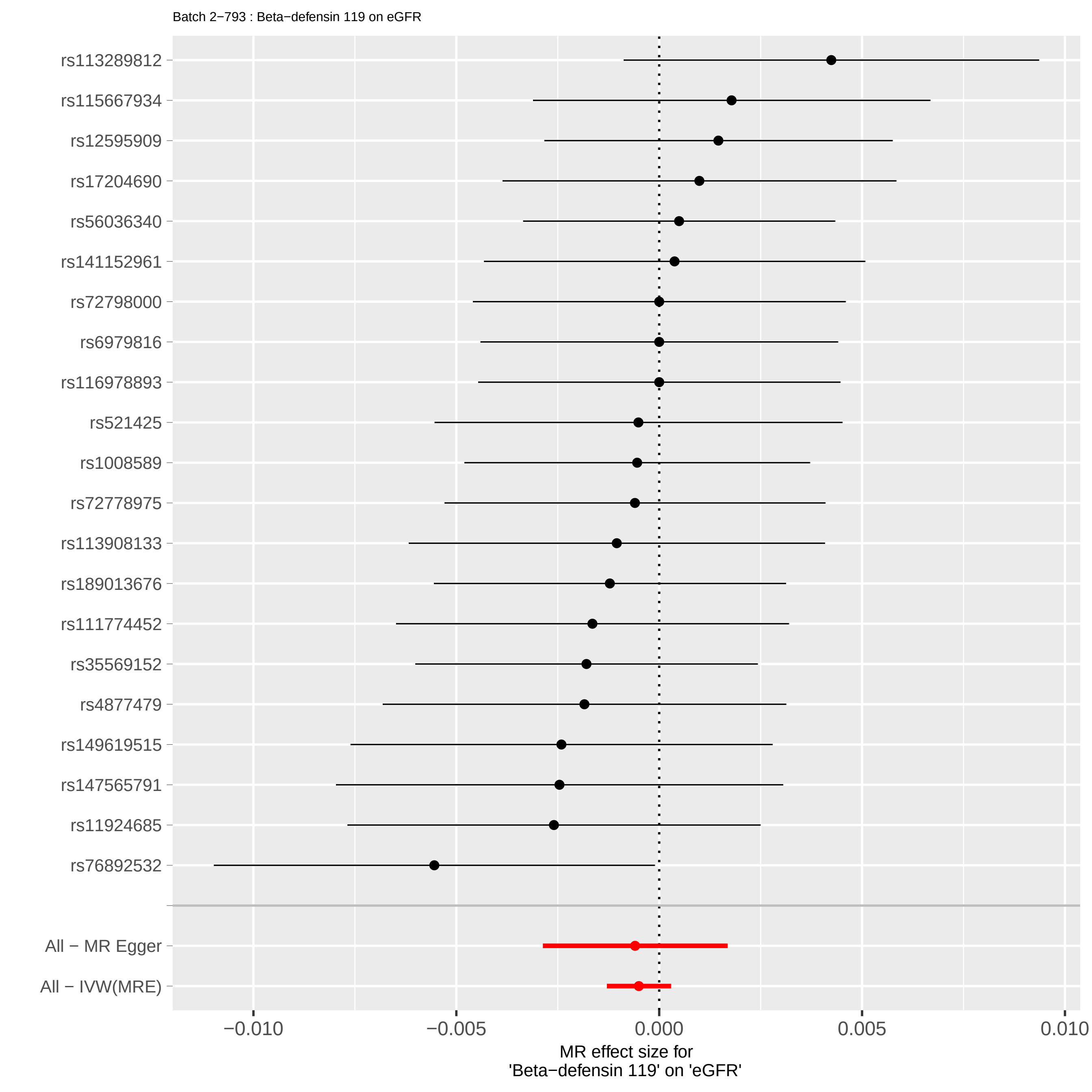

Batch 2−793 : Beta−defensin 119 on eGFR
rs113289812
rs115667934
rs12595909
rs17204690
rs56036340
rs141152961
rs72798000
rs6979816
rs116978893
rs521425
rs1008589
rs72778975
rs113908133
rs189013676
rs111774452
rs35569152
rs4877479
rs149619515
rs147565791
rs11924685
rs76892532
All − MR Egger
All − IVW(MRE)
−0.010
−0.005
0.000
0.005
0.010
MR effect size for
'Beta−defensin 119' on 'eGFR'

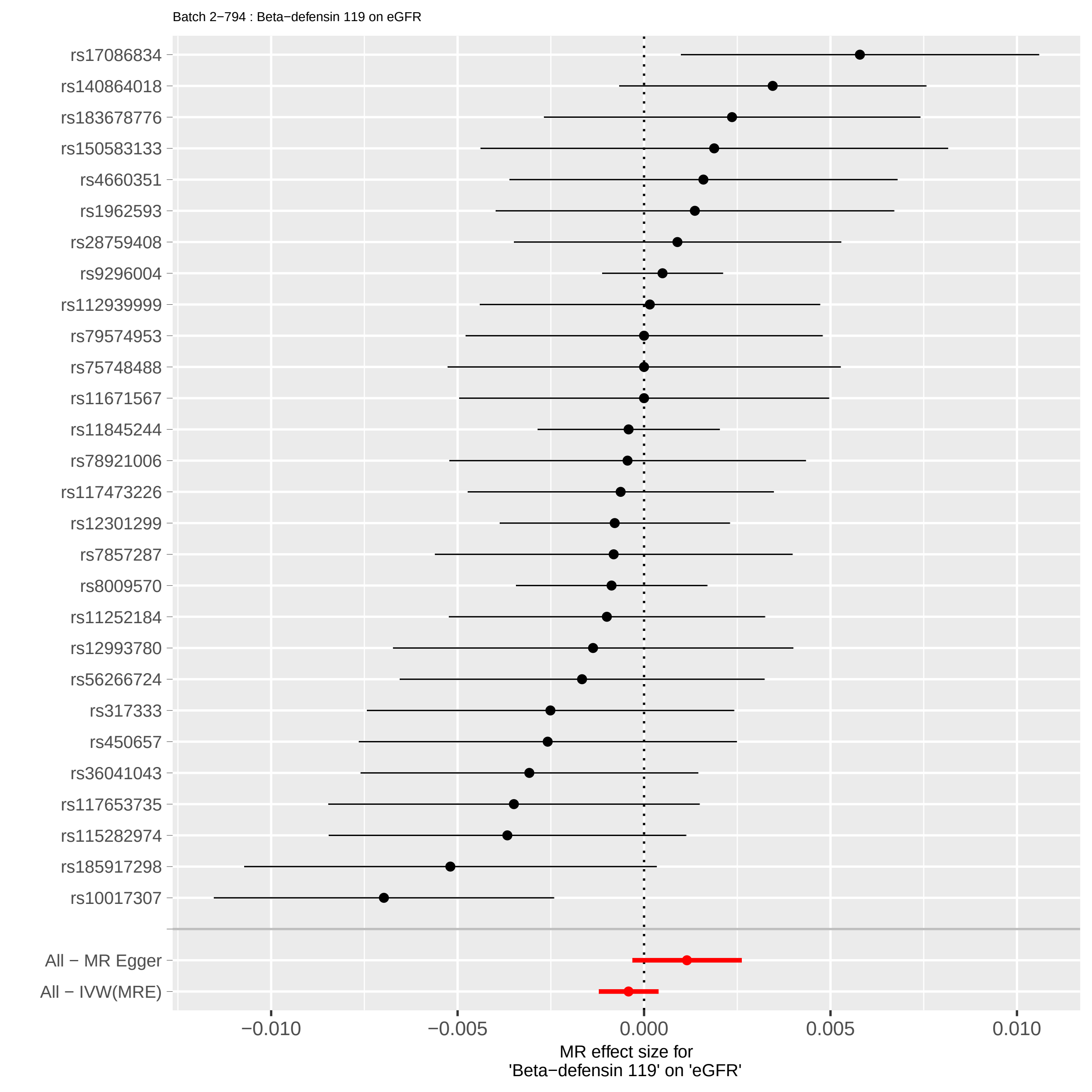

Batch 2−794 : Beta−defensin 119 on eGFR
rs17086834
rs140864018
rs183678776
rs150583133
rs4660351
rs1962593
rs28759408
rs9296004
rs112939999
rs79574953
rs75748488
rs11671567
rs11845244
rs78921006
rs117473226
rs12301299
rs7857287
rs8009570
rs11252184
rs12993780
rs56266724
rs317333
rs450657
rs36041043
rs117653735
rs115282974
rs185917298
rs10017307
All − MR Egger
All − IVW(MRE)
−0.010
−0.005
0.000
0.005
0.010
MR effect size for
'Beta−defensin 119' on 'eGFR'

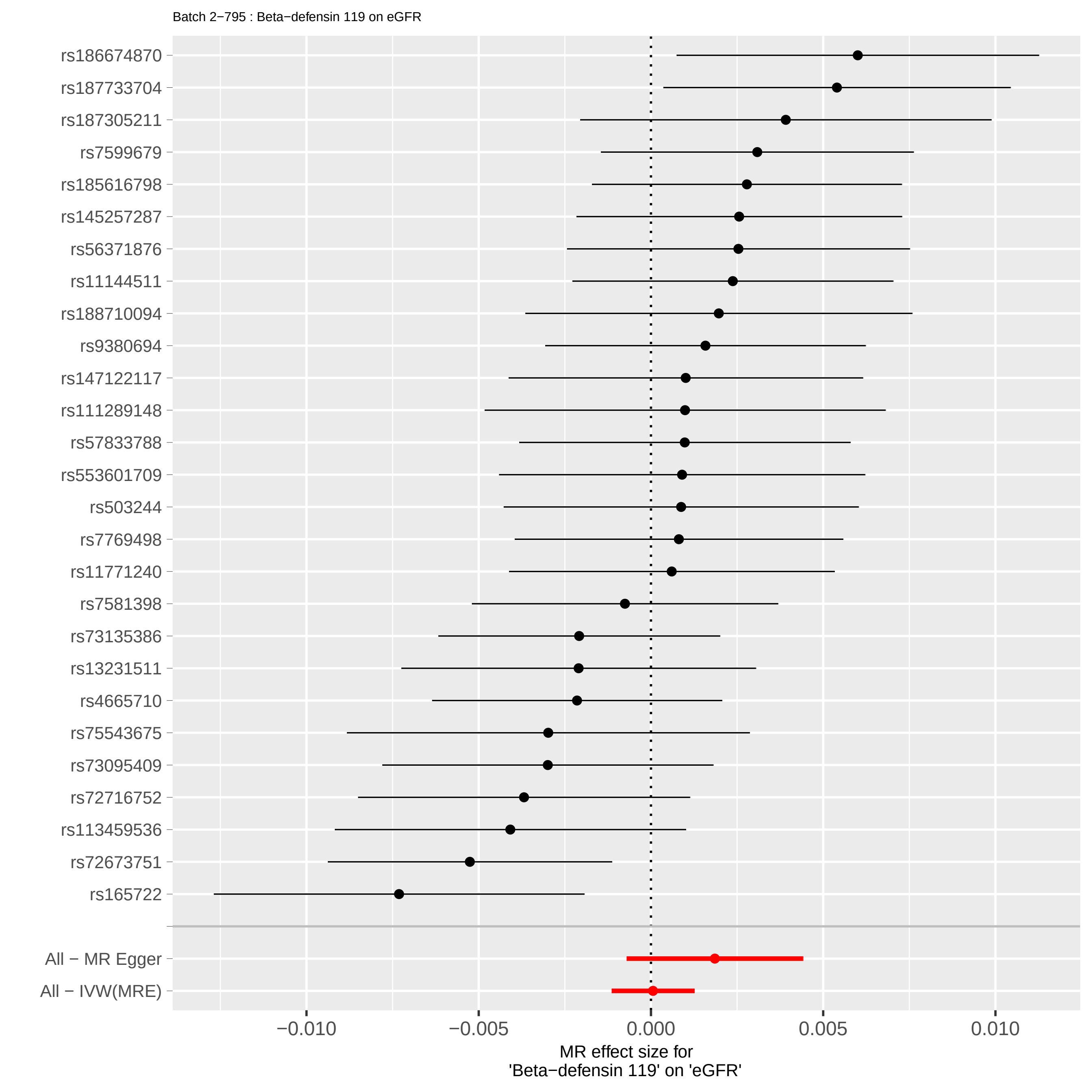

Batch 2−795 : Beta−defensin 119 on eGFR
rs186674870
rs187733704
rs187305211
rs7599679
rs185616798
rs145257287
rs56371876
rs11144511
rs188710094
rs9380694
rs147122117
rs111289148
rs57833788
rs553601709
rs503244
rs7769498
rs11771240
rs7581398
rs73135386
rs13231511
rs4665710
rs75543675
rs73095409
rs72716752
rs113459536
rs72673751
rs165722
All − MR Egger
All − IVW(MRE)
−0.010
−0.005
0.000
0.005
0.010
MR effect size for
'Beta−defensin 119' on 'eGFR'

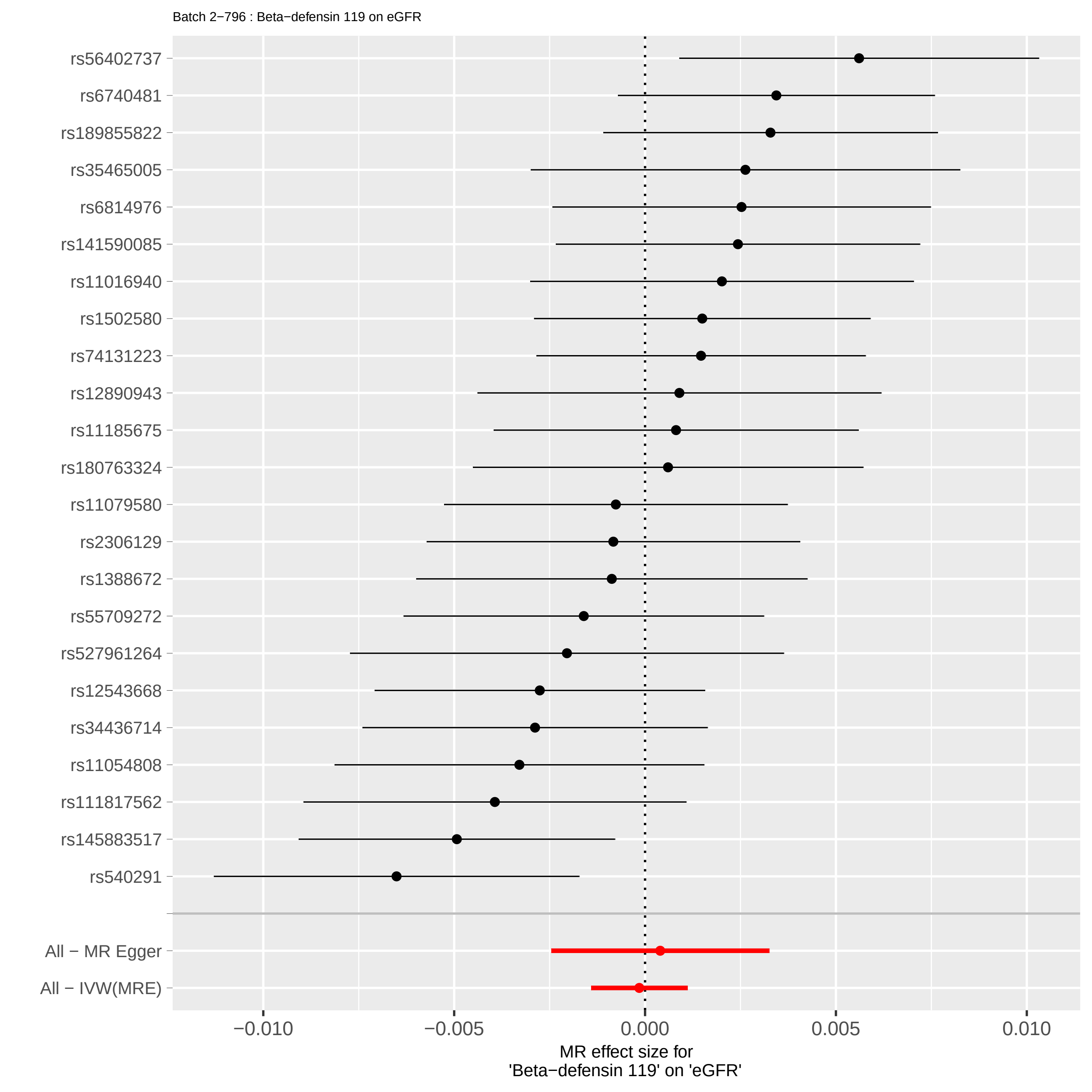

Batch 2−796 : Beta−defensin 119 on eGFR
rs56402737
rs6740481
rs189855822
rs35465005
rs6814976
rs141590085
rs11016940
rs1502580
rs74131223
rs12890943
rs11185675
rs180763324
rs11079580
rs2306129
rs1388672
rs55709272
rs527961264
rs12543668
rs34436714
rs11054808
rs111817562
rs145883517
rs540291
All − MR Egger
All − IVW(MRE)
−0.010
−0.005
0.000
0.005
0.010
MR effect size for
'Beta−defensin 119' on 'eGFR'

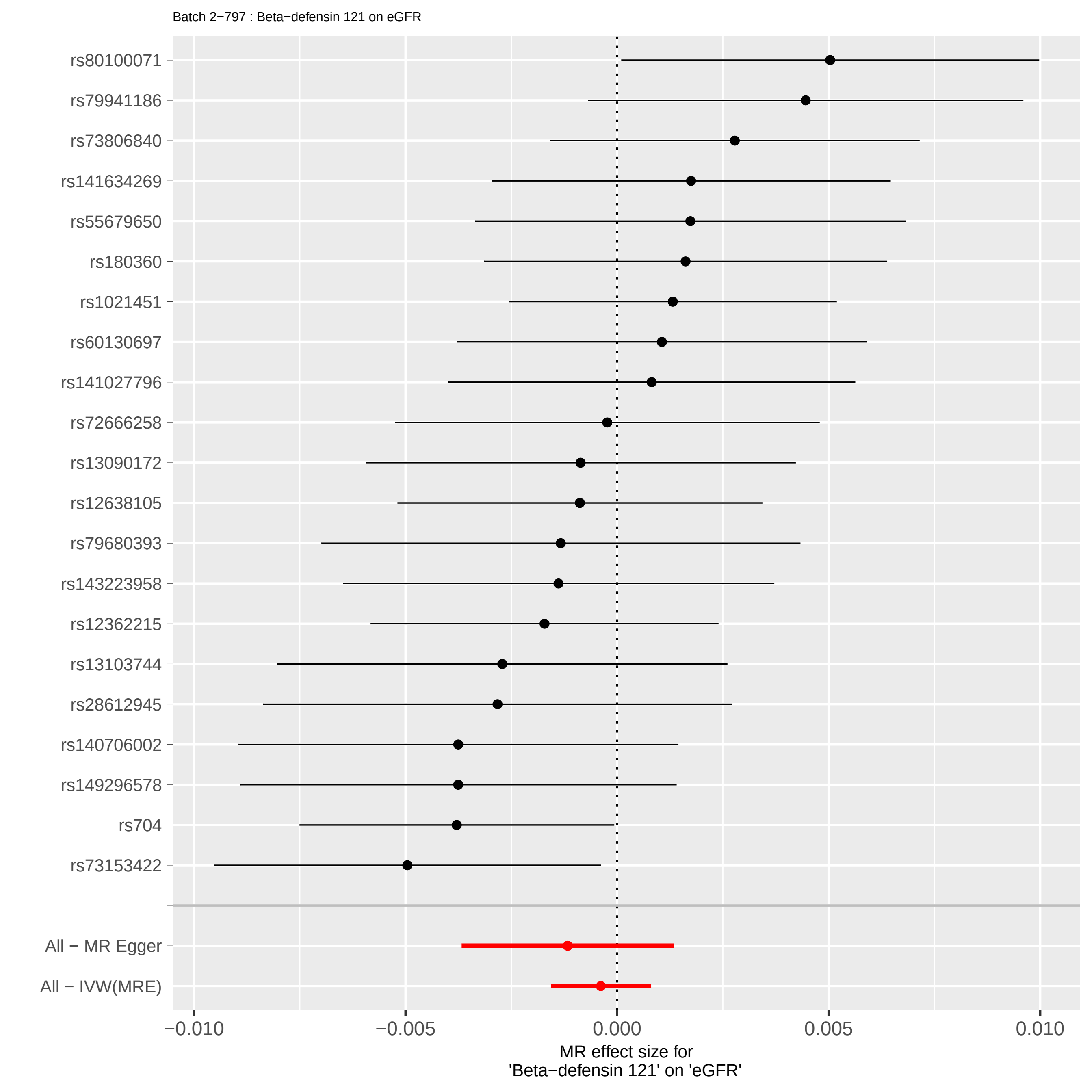

Batch 2−797 : Beta−defensin 121 on eGFR
rs80100071
rs79941186
rs73806840
rs141634269
rs55679650
rs180360
rs1021451
rs60130697
rs141027796
rs72666258
rs13090172
rs12638105
rs79680393
rs143223958
rs12362215
rs13103744
rs28612945
rs140706002
rs149296578
rs704
rs73153422
All − MR Egger
All − IVW(MRE)
−0.010
−0.005
0.000
0.005
0.010
MR effect size for
'Beta−defensin 121' on 'eGFR'

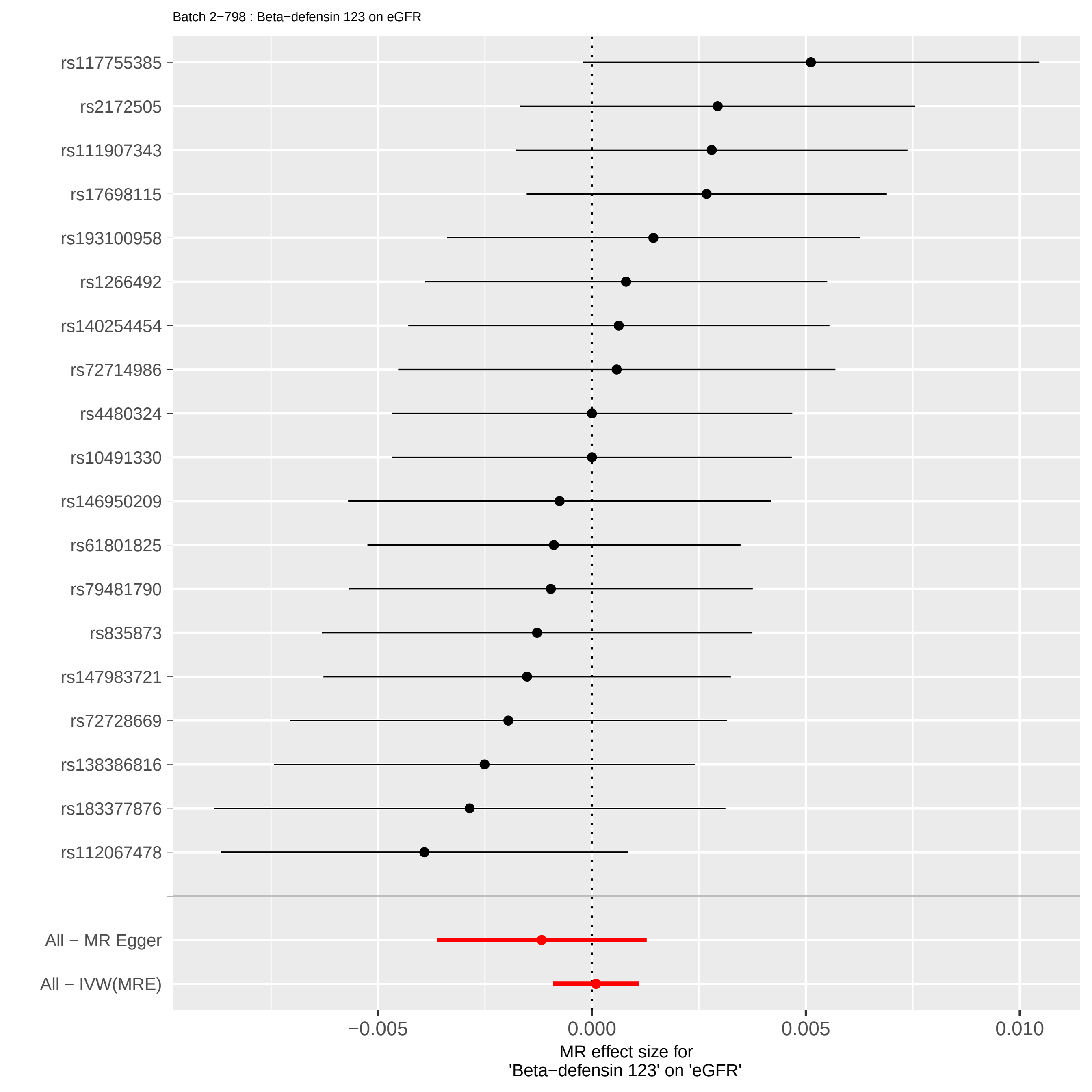

Batch 2−798 : Beta−defensin 123 on eGFR
rs117755385
rs2172505
rs111907343
rs17698115
rs193100958
rs1266492
rs140254454
rs72714986
rs4480324
rs10491330
rs146950209
rs61801825
rs79481790
rs835873
rs147983721
rs72728669
rs138386816
rs183377876
rs112067478
All − MR Egger
All − IVW(MRE)
−0.005
0.000
0.005
0.010
MR effect size for
'Beta−defensin 123' on 'eGFR'

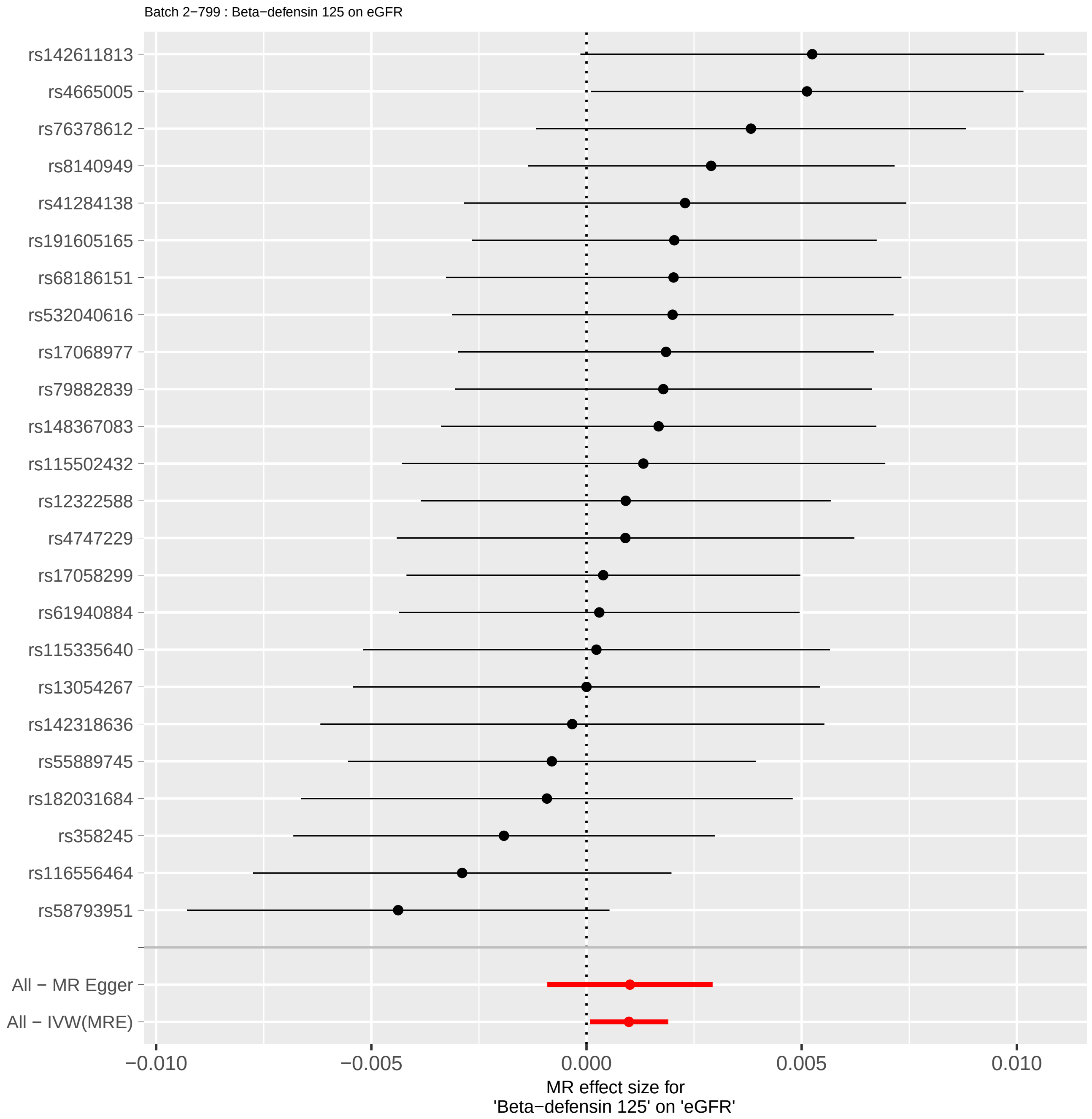

Batch 2−799 : Beta−defensin 125 on eGFR
rs142611813
rs4665005
rs76378612
rs8140949
rs41284138
rs191605165
rs68186151
rs532040616
rs17068977
rs79882839
rs148367083
rs115502432
rs12322588
rs4747229
rs17058299
rs61940884
rs115335640
rs13054267
rs142318636
rs55889745
rs182031684
rs358245
rs116556464
rs58793951
All − MR Egger
All − IVW(MRE)
−0.010
−0.005
0.000
0.005
0.010
MR effect size for
'Beta−defensin 125' on 'eGFR'

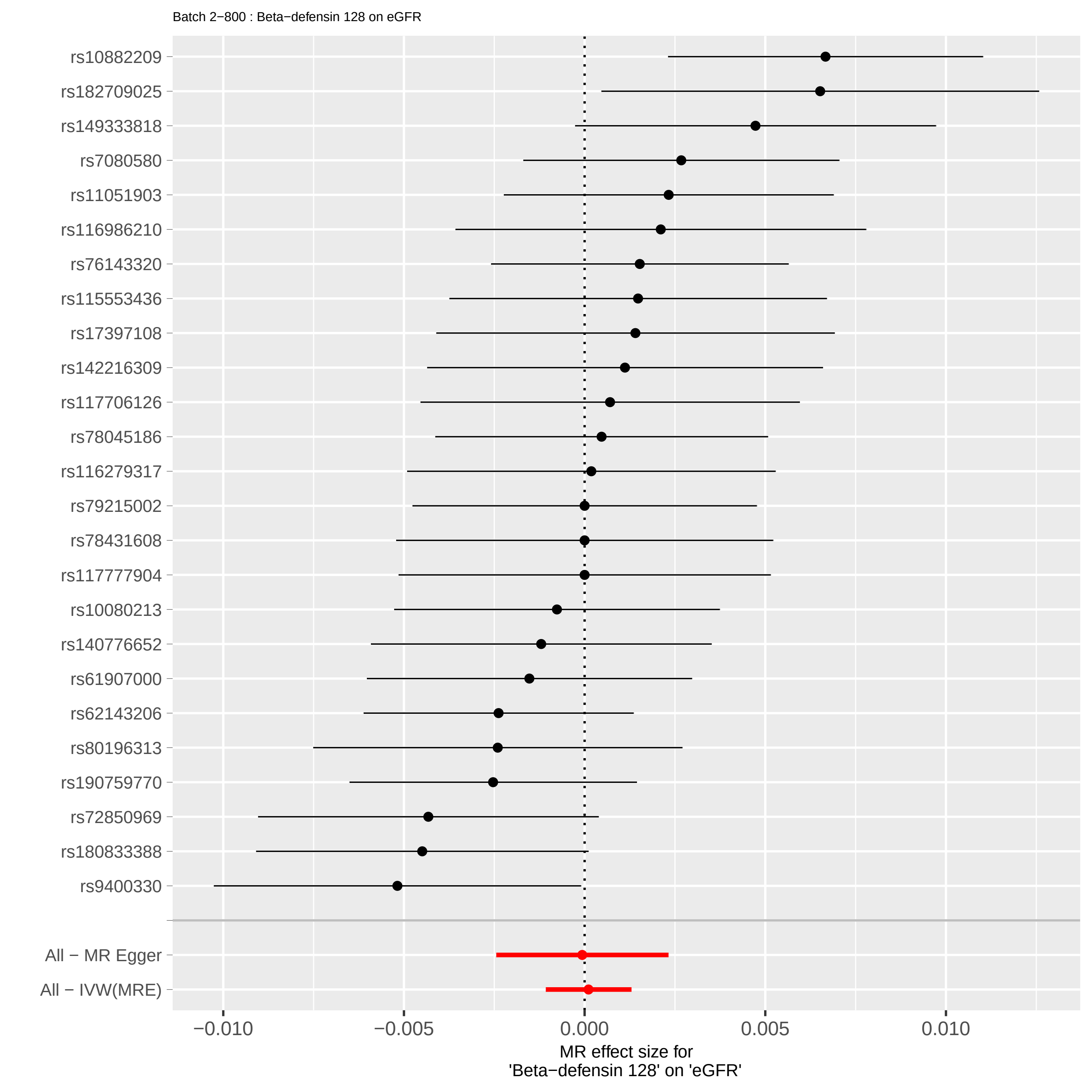

Batch 2−800 : Beta−defensin 128 on eGFR
rs10882209
rs182709025
rs149333818
rs7080580
rs11051903
rs116986210
rs76143320
rs115553436
rs17397108
rs142216309
rs117706126
rs78045186
rs116279317
rs79215002
rs78431608
rs117777904
rs10080213
rs140776652
rs61907000
rs62143206
rs80196313
rs190759770
rs72850969
rs180833388
rs9400330
All − MR Egger
All − IVW(MRE)
−0.010
−0.005
0.000
0.005
0.010
MR effect size for
'Beta−defensin 128' on 'eGFR'

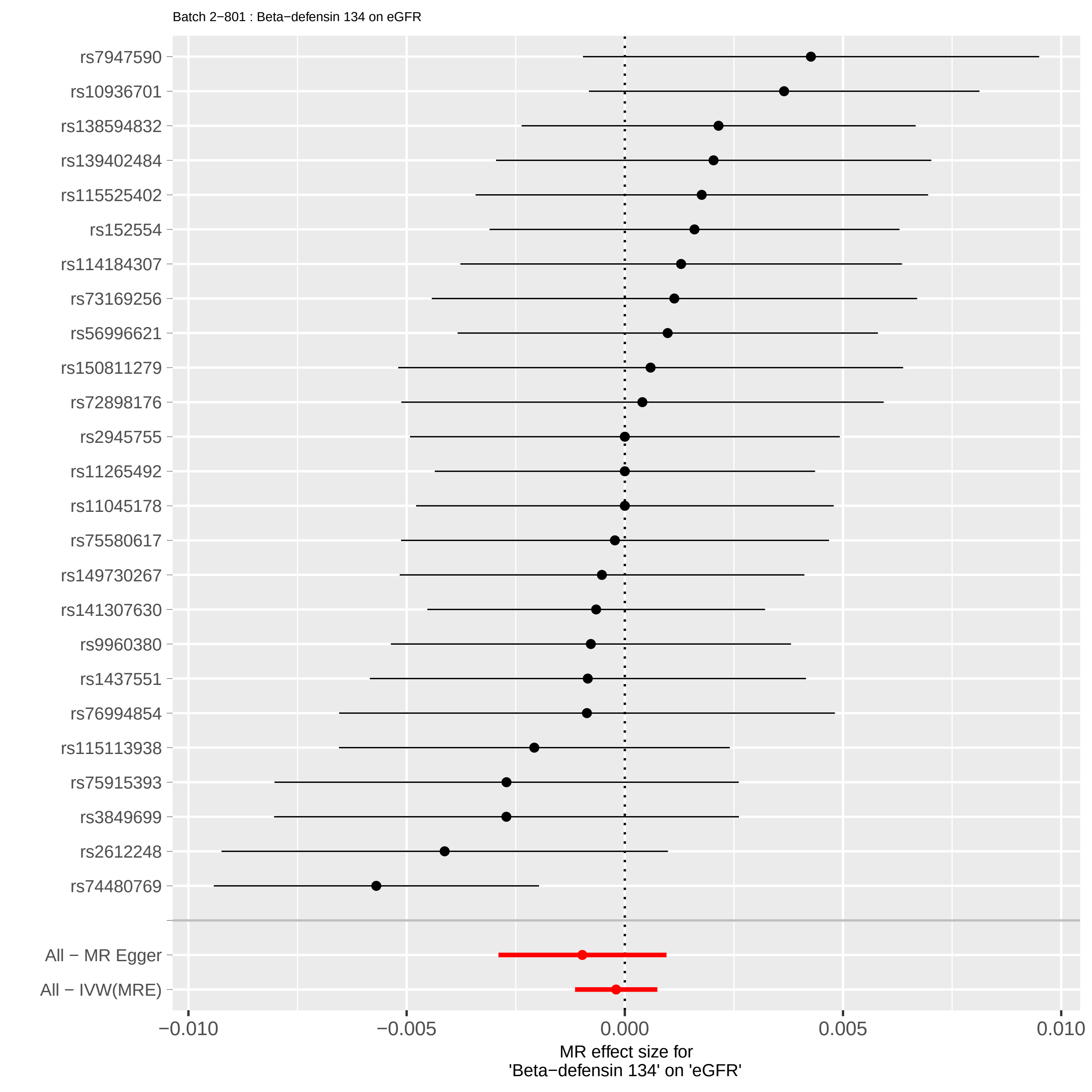

Batch 2−801 : Beta−defensin 134 on eGFR
rs7947590
rs10936701
rs138594832
rs139402484
rs115525402
rs152554
rs114184307
rs73169256
rs56996621
rs150811279
rs72898176
rs2945755
rs11265492
rs11045178
rs75580617
rs149730267
rs141307630
rs9960380
rs1437551
rs76994854
rs115113938
rs75915393
rs3849699
rs2612248
rs74480769
All − MR Egger
All − IVW(MRE)
−0.010
−0.005
0.000
0.005
0.010
MR effect size for
'Beta−defensin 134' on 'eGFR'

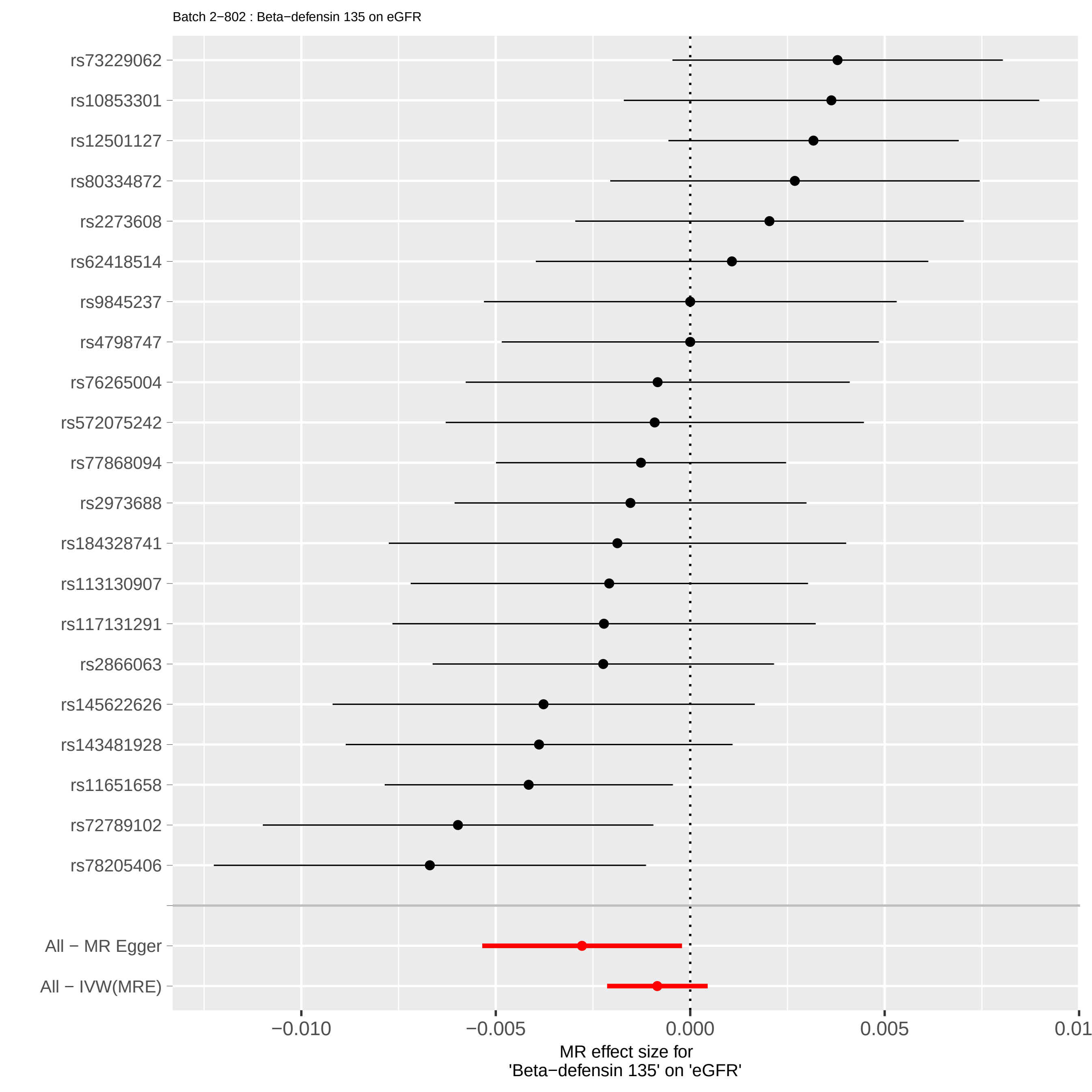

Batch 2−802 : Beta−defensin 135 on eGFR
rs73229062
rs10853301
rs12501127
rs80334872
rs2273608
rs62418514
rs9845237
rs4798747
rs76265004
rs572075242
rs77868094
rs2973688
rs184328741
rs113130907
rs117131291
rs2866063
rs145622626
rs143481928
rs11651658
rs72789102
rs78205406
All − MR Egger
All − IVW(MRE)
−0.010
−0.005
0.000
0.005
0.01
MR effect size for
'Beta−defensin 135' on 'eGFR'

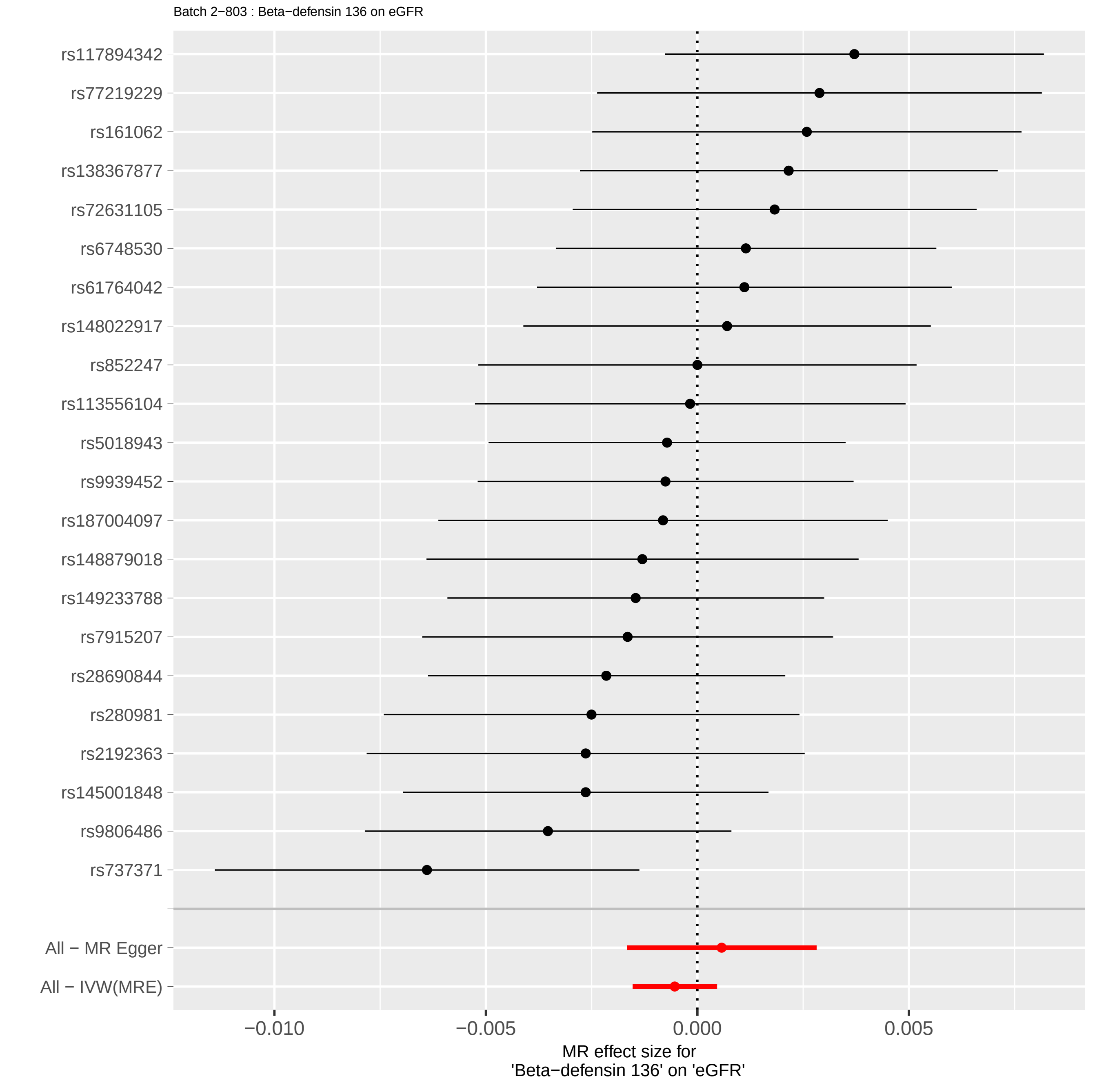

Batch 2−803 : Beta−defensin 136 on eGFR
rs117894342
rs77219229
rs161062
rs138367877
rs72631105
rs6748530
rs61764042
rs148022917
rs852247
rs113556104
rs5018943
rs9939452
rs187004097
rs148879018
rs149233788
rs7915207
rs28690844
rs280981
rs2192363
rs145001848
rs9806486
rs737371
All − MR Egger
All − IVW(MRE)
−0.010
−0.005
0.000
0.005
MR effect size for
'Beta−defensin 136' on 'eGFR'

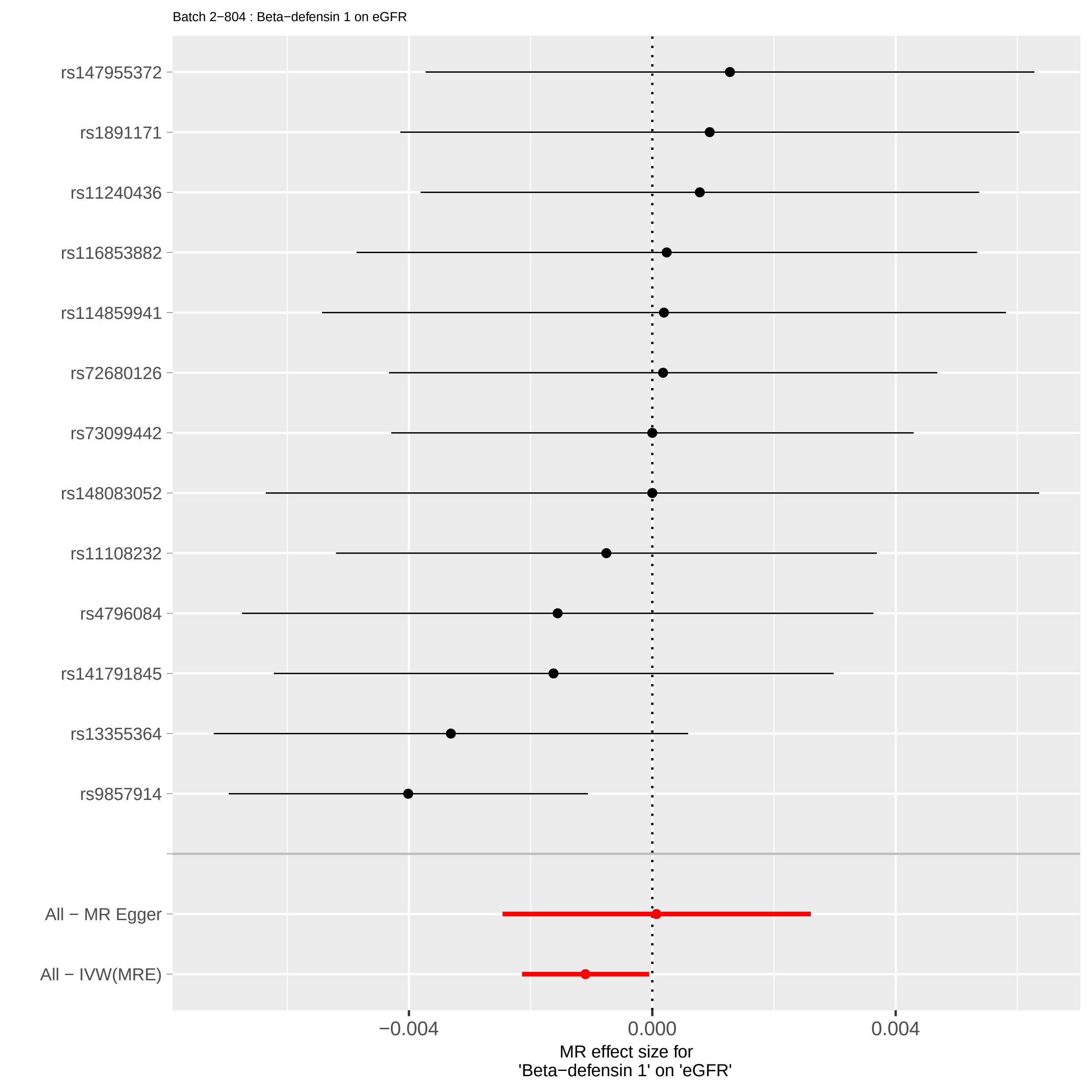

Batch 2−804 : Beta−defensin 1 on eGFR
rs147955372
rs1891171
rs11240436
rs116853882
rs114859941
rs72680126
rs73099442
rs148083052
rs11108232
rs4796084
rs141791845
rs13355364
rs9857914
All − MR Egger
All − IVW(MRE)
−0.004
0.000
0.004
MR effect size for
'Beta−defensin 1' on 'eGFR'

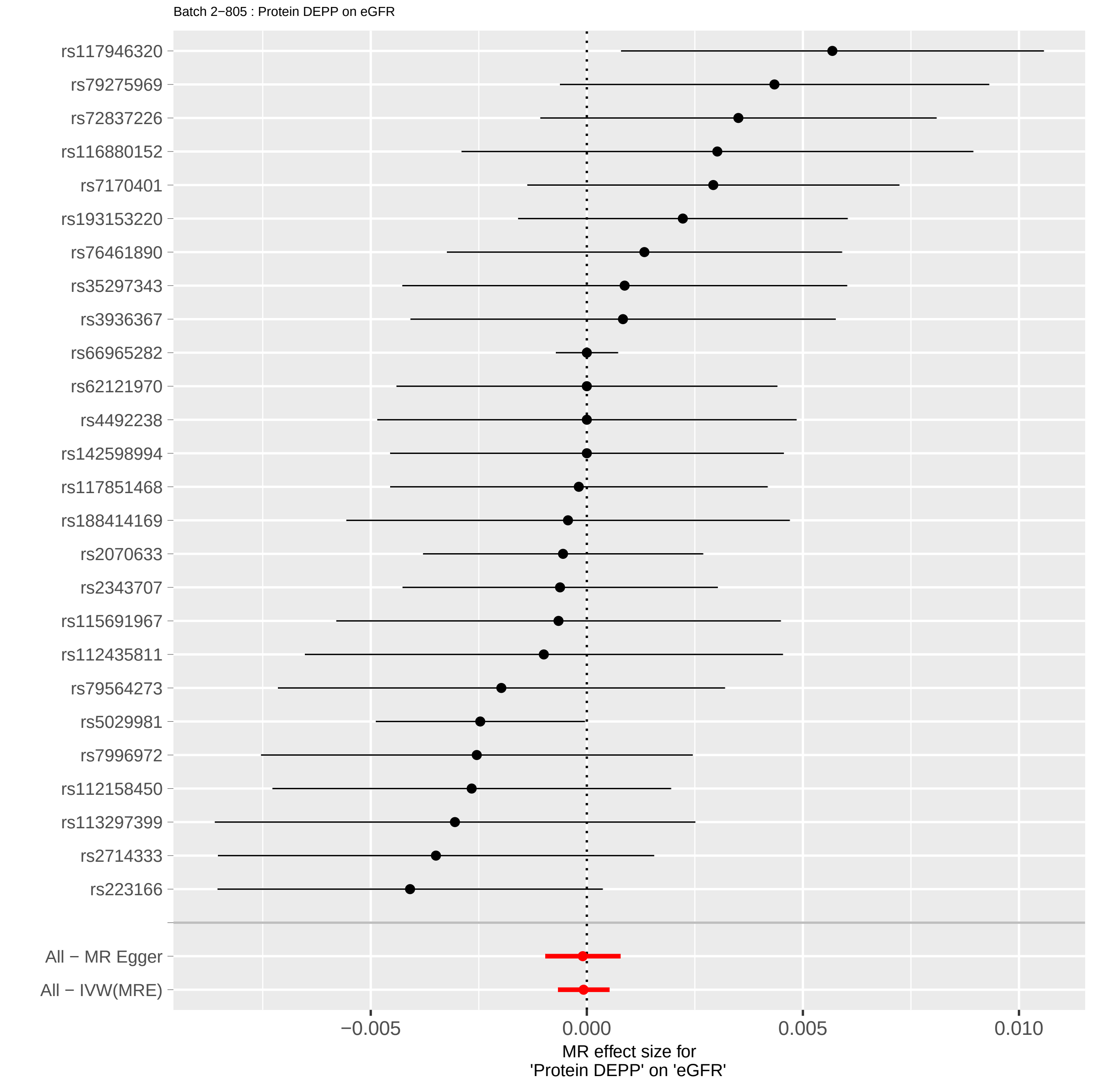
Batch 2−805 : Protein DEPP on eGFR
rs117946320
rs79275969
rs72837226
rs116880152
rs7170401
rs193153220
rs76461890
rs35297343
rs3936367
rs66965282
rs62121970
rs4492238
rs142598994
rs117851468
rs188414169
rs2070633
rs2343707
rs115691967
rs112435811
rs79564273
rs5029981
rs7996972
rs112158450
rs113297399
rs2714333
rs223166
All − MR Egger
All − IVW(MRE)
−0.005
0.000
0.005
0.010
MR effect size for
'Protein DEPP' on 'eGFR'

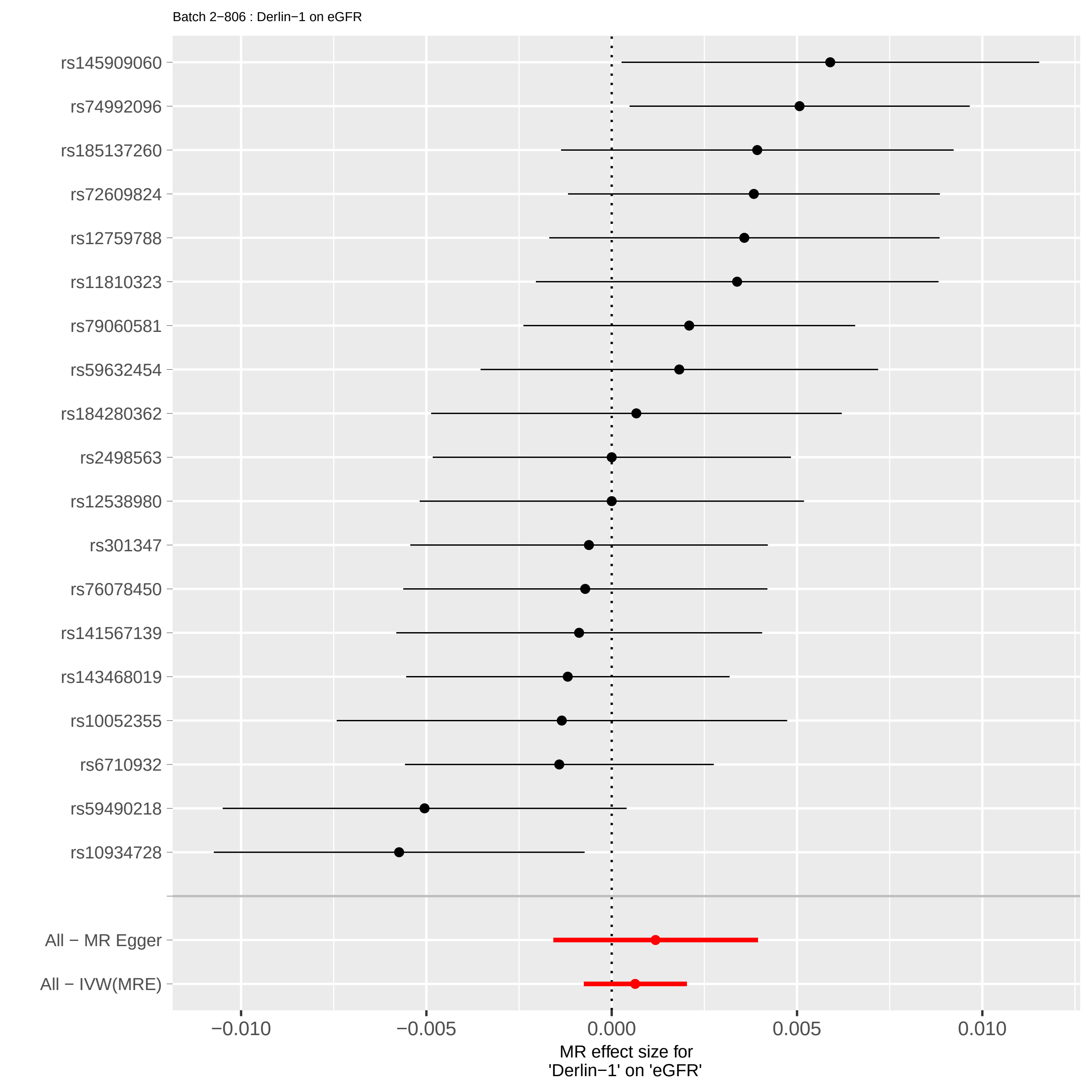

Batch 2−806 : Derlin−1 on eGFR
rs145909060
rs74992096
rs185137260
rs72609824
rs12759788
rs11810323
rs79060581
rs59632454
rs184280362
rs2498563
rs12538980
rs301347
rs76078450
rs141567139
rs143468019
rs10052355
rs6710932
rs59490218
rs10934728
All − MR Egger
All − IVW(MRE)
−0.010
−0.005
0.000
0.005
0.010
MR effect size for
'Derlin−1' on 'eGFR'

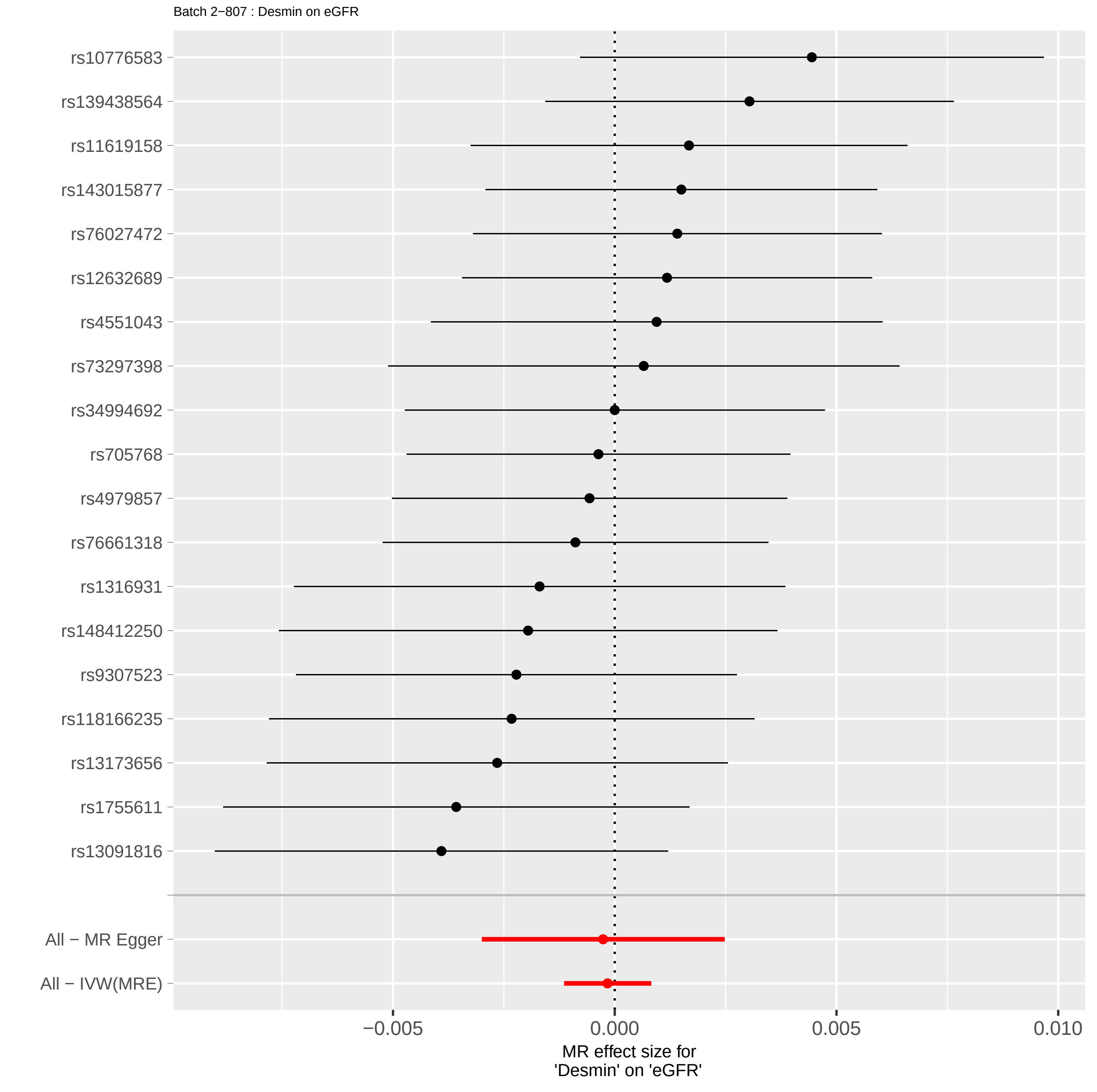

Batch 2−807 : Desmin on eGFR
rs10776583
rs139438564
rs11619158
rs143015877
rs76027472
rs12632689
rs4551043
rs73297398
rs34994692
rs705768
rs4979857
rs76661318
rs1316931
rs148412250
rs9307523
rs118166235
rs13173656
rs1755611
rs13091816
All − MR Egger
All − IVW(MRE)
−0.005
0.000
0.005
0.010
MR effect size for
'Desmin' on 'eGFR'

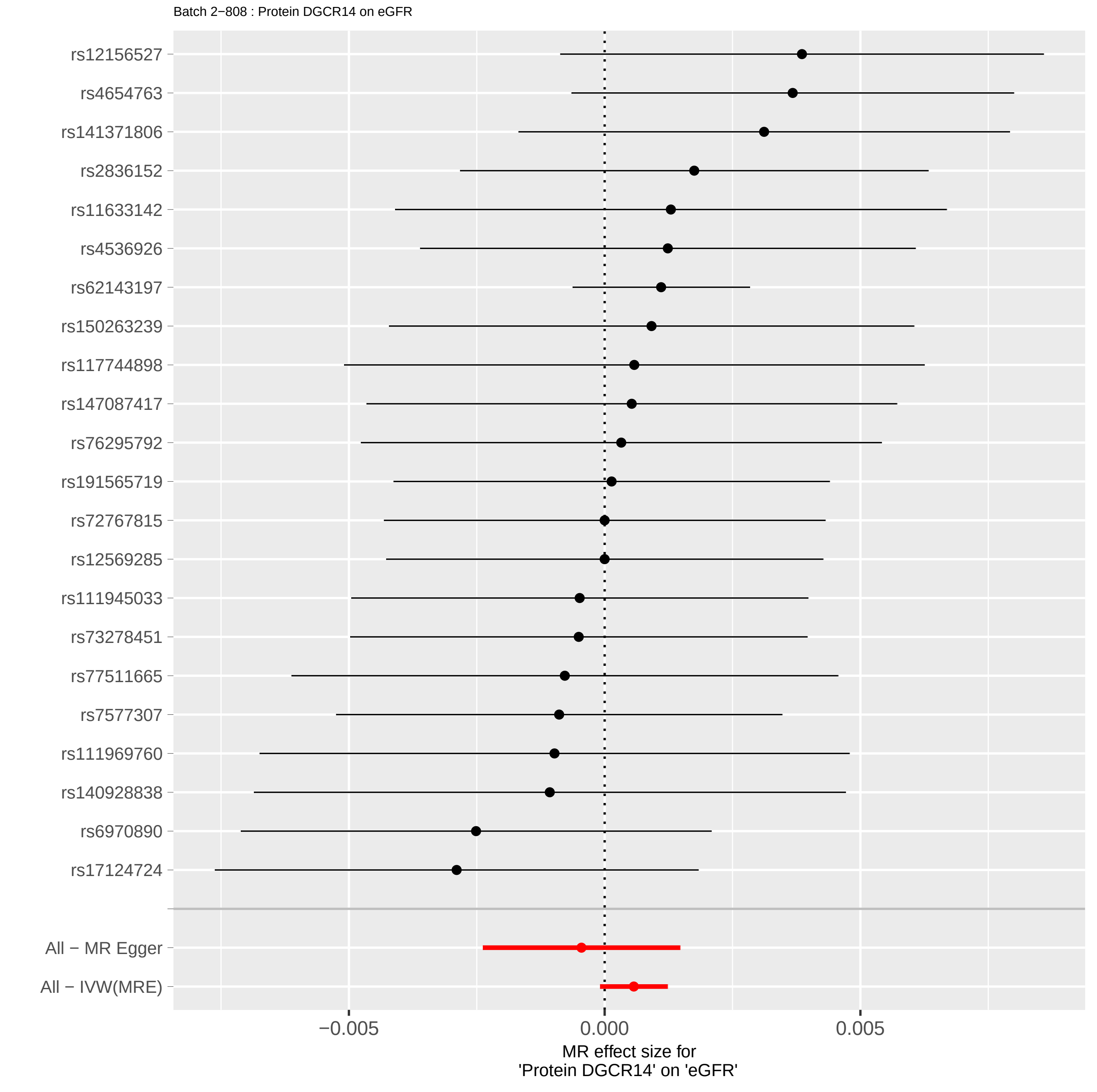

Batch 2−808 : Protein DGCR14 on eGFR
rs12156527
rs4654763
rs141371806
rs2836152
rs11633142
rs4536926
rs62143197
rs150263239
rs117744898
rs147087417
rs76295792
rs191565719
rs72767815
rs12569285
rs111945033
rs73278451
rs77511665
rs7577307
rs111969760
rs140928838
rs6970890
rs17124724
All − MR Egger
All − IVW(MRE)
−0.005
0.000
0.005
MR effect size for
'Protein DGCR14' on 'eGFR'

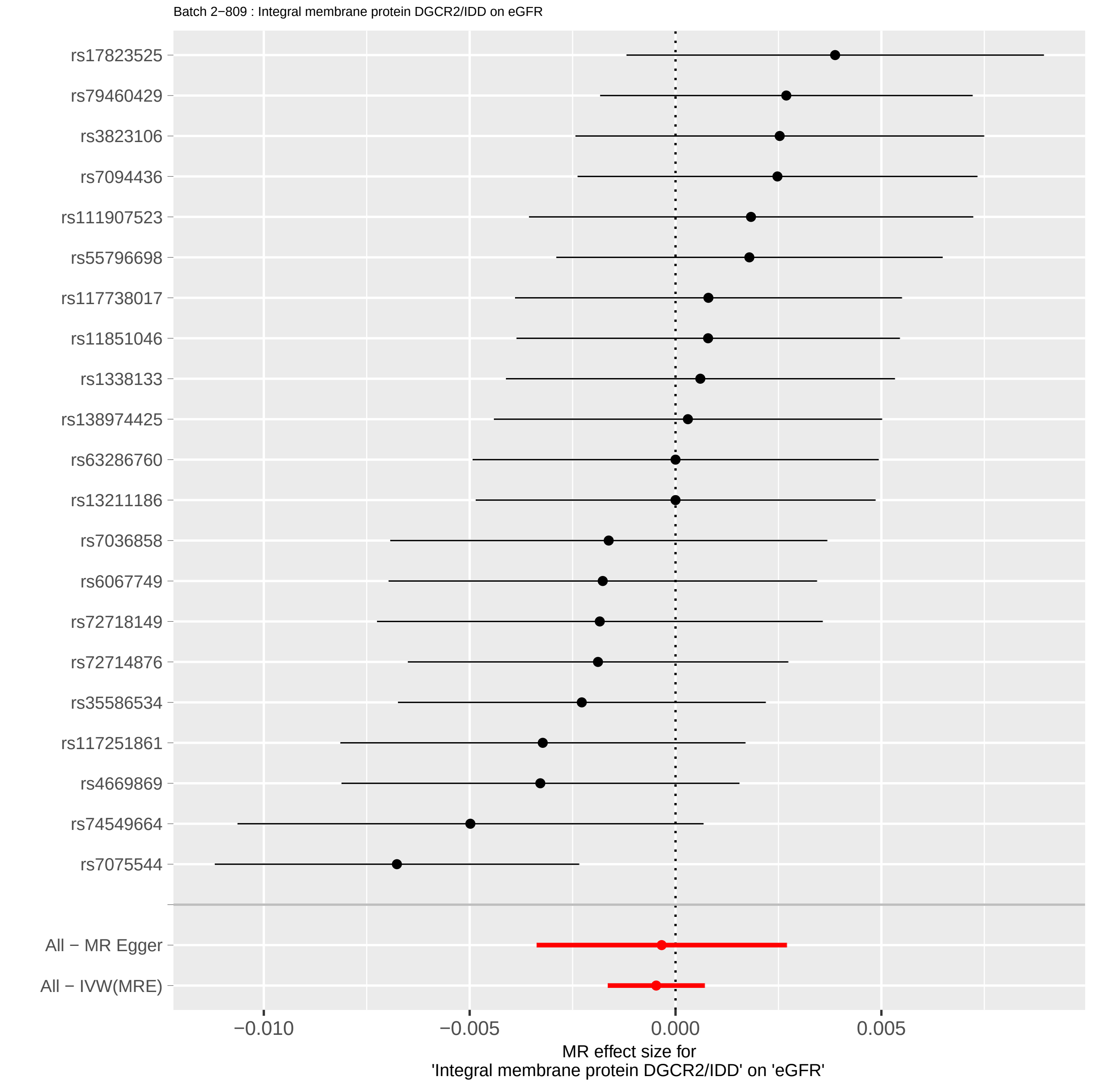

Batch 2−809 : Integral membrane protein DGCR2/IDD on eGFR
rs17823525
rs79460429
rs3823106
rs7094436
rs111907523
rs55796698
rs117738017
rs11851046
rs1338133
rs138974425
rs63286760
rs13211186
rs7036858
rs6067749
rs72718149
rs72714876
rs35586534
rs117251861
rs4669869
rs74549664
rs7075544
All − MR Egger
All − IVW(MRE)
−0.010
−0.005
0.000
0.005
MR effect size for
'Integral membrane protein DGCR2/IDD' on 'eGFR'

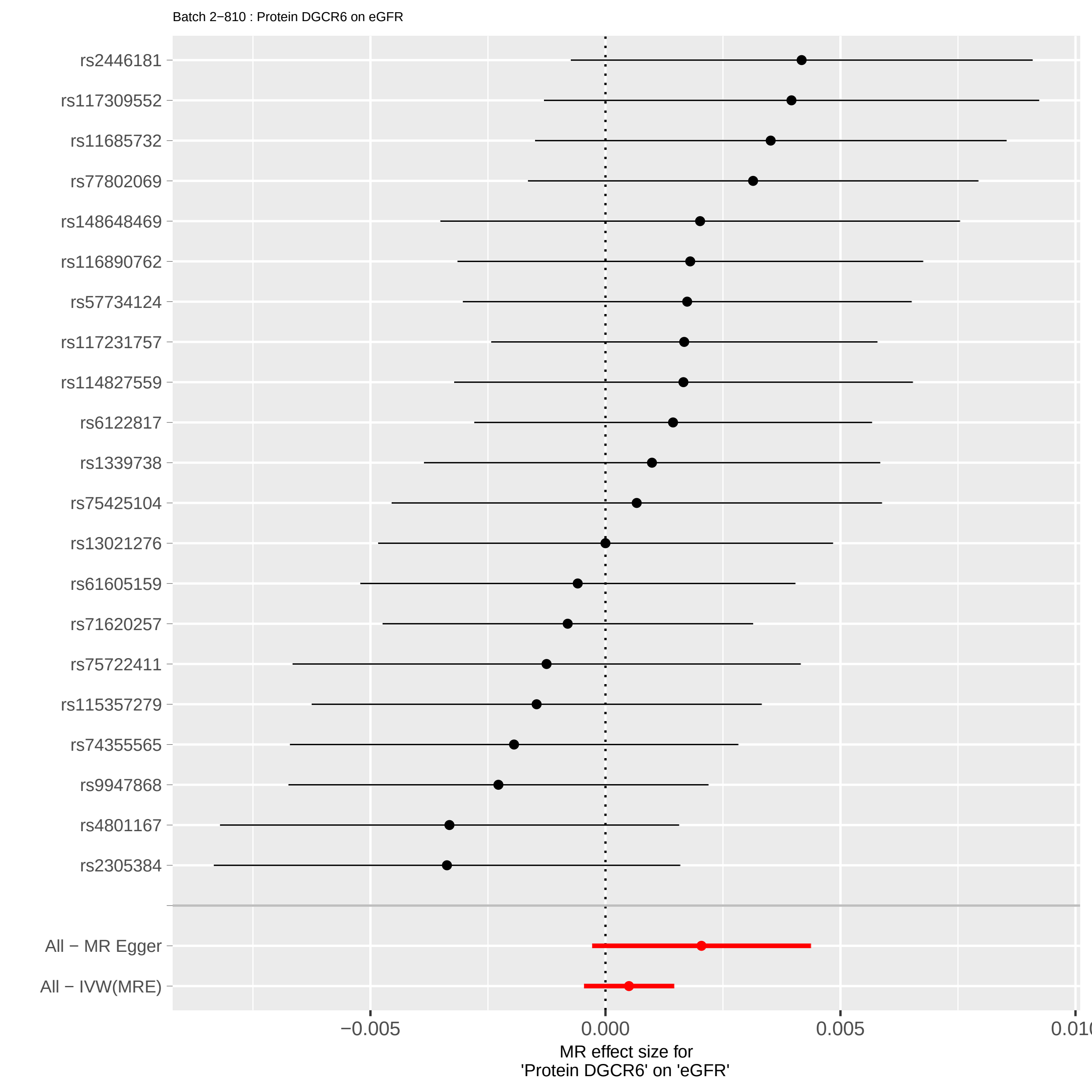

Batch 2−810 : Protein DGCR6 on eGFR
rs2446181
rs117309552
rs11685732
rs77802069
rs148648469
rs116890762
rs57734124
rs117231757
rs114827559
rs6122817
rs1339738
rs75425104
rs13021276
rs61605159
rs71620257
rs75722411
rs115357279
rs74355565
rs9947868
rs4801167
rs2305384
All − MR Egger
All − IVW(MRE)
−0.005
0.000
0.005
0.010
MR effect size for
'Protein DGCR6' on 'eGFR'

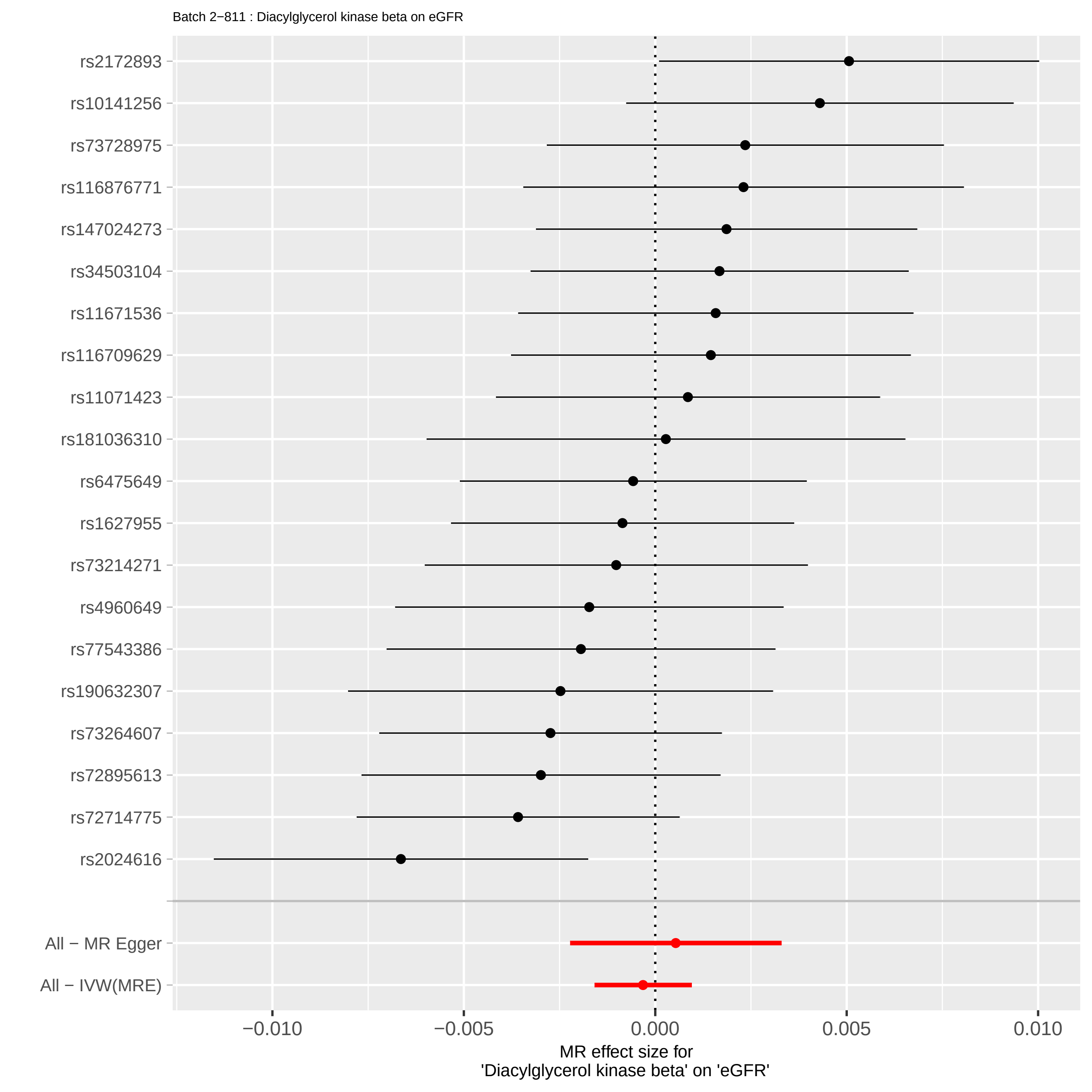

Batch 2−811 : Diacylglycerol kinase beta on eGFR
rs2172893
rs10141256
rs73728975
rs116876771
rs147024273
rs34503104
rs11671536
rs116709629
rs11071423
rs181036310
rs6475649
rs1627955
rs73214271
rs4960649
rs77543386
rs190632307
rs73264607
rs72895613
rs72714775
rs2024616
All − MR Egger
All − IVW(MRE)
−0.010
−0.005
0.000
0.005
0.010
MR effect size for
'Diacylglycerol kinase beta' on 'eGFR'

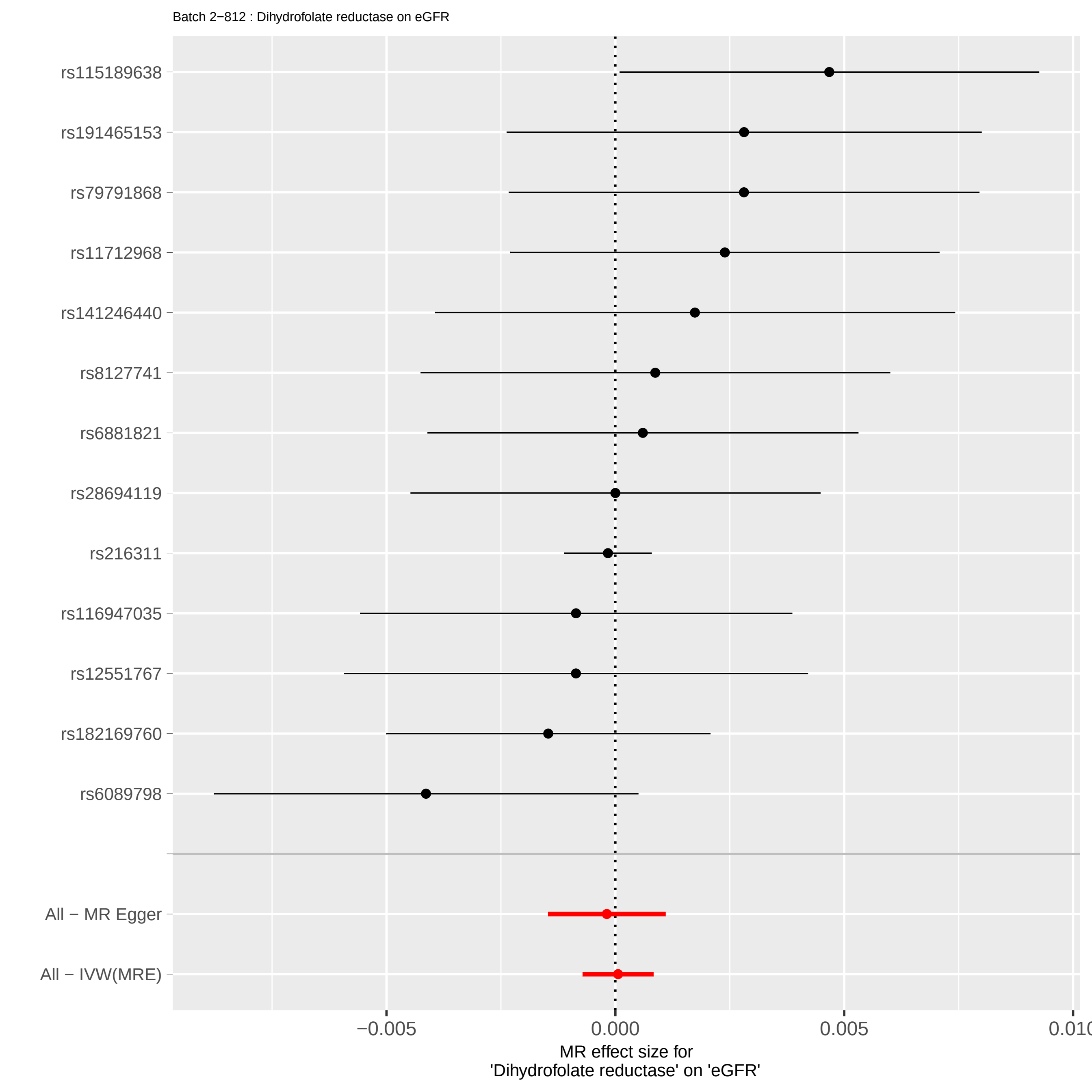

Batch 2−812 : Dihydrofolate reductase on eGFR
rs115189638
rs191465153
rs79791868
rs11712968
rs141246440
rs8127741
rs6881821
rs28694119
rs216311
rs116947035
rs12551767
rs182169760
rs6089798
All − MR Egger
All − IVW(MRE)
−0.005
0.000
0.005
0.010
MR effect size for
'Dihydrofolate reductase' on 'eGFR'

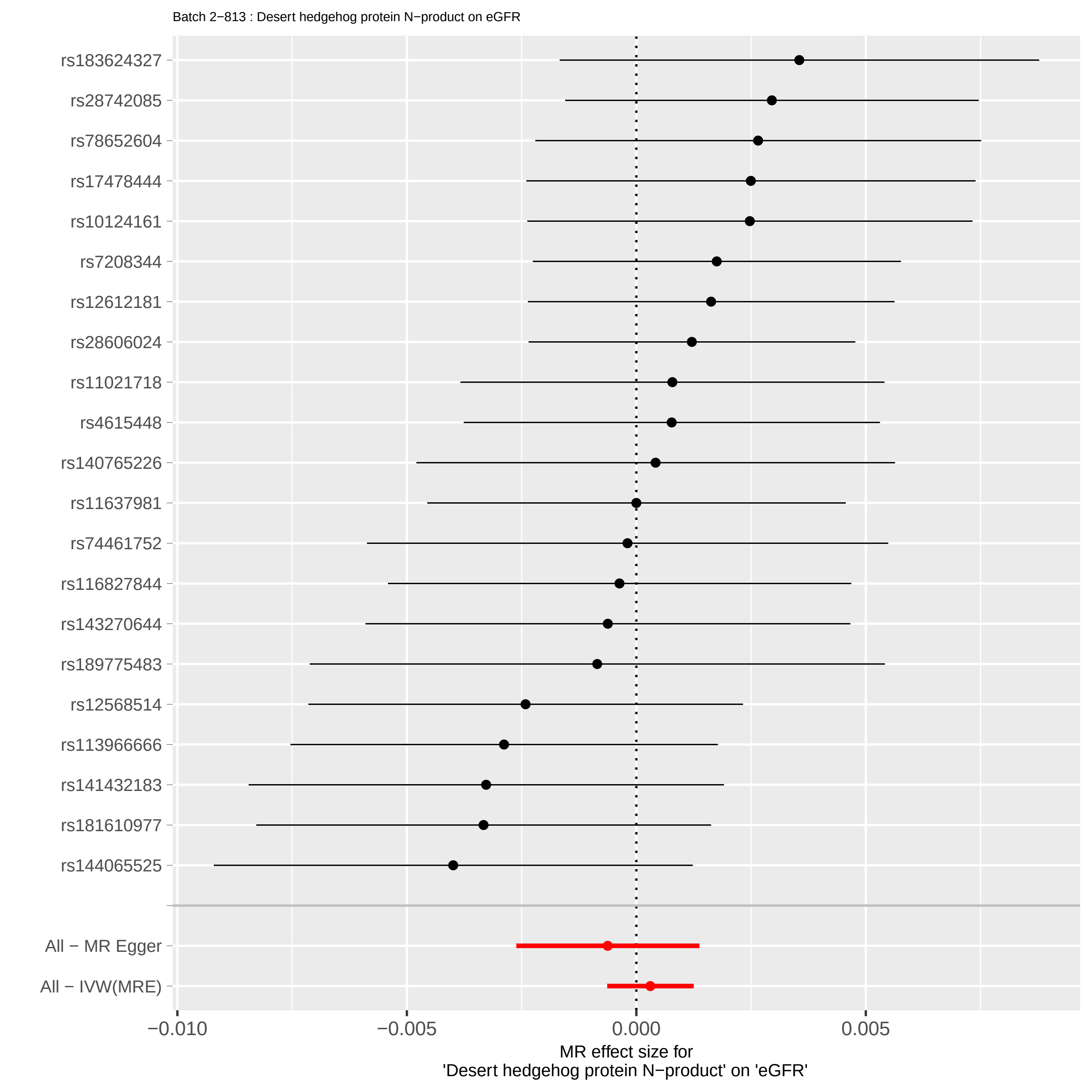

Batch 2−813 : Desert hedgehog protein N−product on eGFR
rs183624327
rs28742085
rs78652604
rs17478444
rs10124161
rs7208344
rs12612181
rs28606024
rs11021718
rs4615448
rs140765226
rs11637981
rs74461752
rs116827844
rs143270644
rs189775483
rs12568514
rs113966666
rs141432183
rs181610977
rs144065525
All − MR Egger
All − IVW(MRE)
−0.010
−0.005
0.000
0.005
MR effect size for
'Desert hedgehog protein N−product' on 'eGFR'

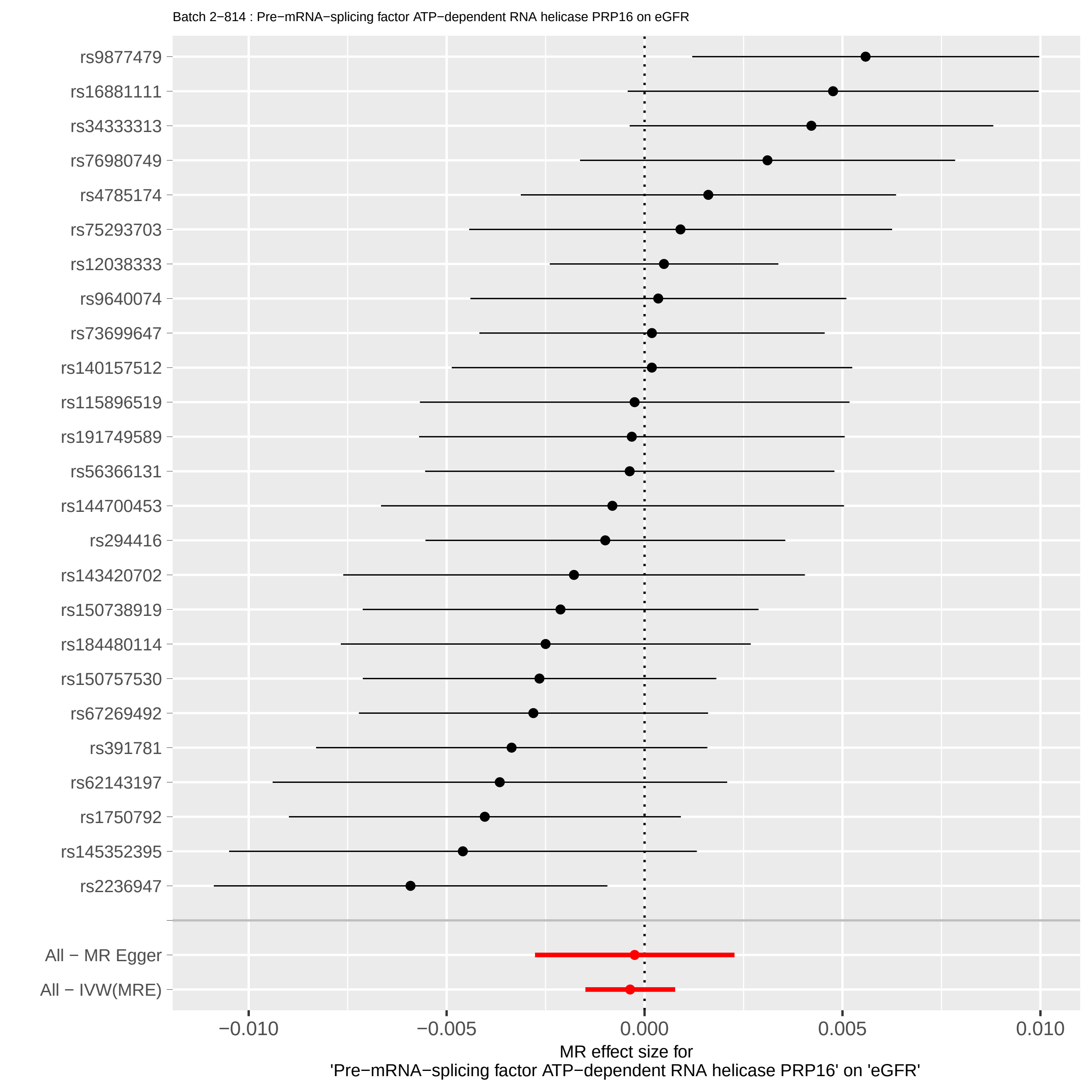
Batch 2−814 : Pre−mRNA−splicing factor ATP−dependent RNA helicase PRP16 on eGFR
rs9877479
rs16881111
rs34333313
rs76980749
rs4785174
rs75293703
rs12038333
rs9640074
rs73699647
rs140157512
rs115896519
rs191749589
rs56366131
rs144700453
rs294416
rs143420702
rs150738919
rs184480114
rs150757530
rs67269492
rs391781
rs62143197
rs1750792
rs145352395
rs2236947
All − MR Egger
All − IVW(MRE)
−0.010
−0.005
0.000
0.005
0.010
MR effect size for
'Pre−mRNA−splicing factor ATP−dependent RNA helicase PRP16' on 'eGFR'

Batch 2−815 : Probable ATP−dependent RNA helicase DHX58 on eGFR

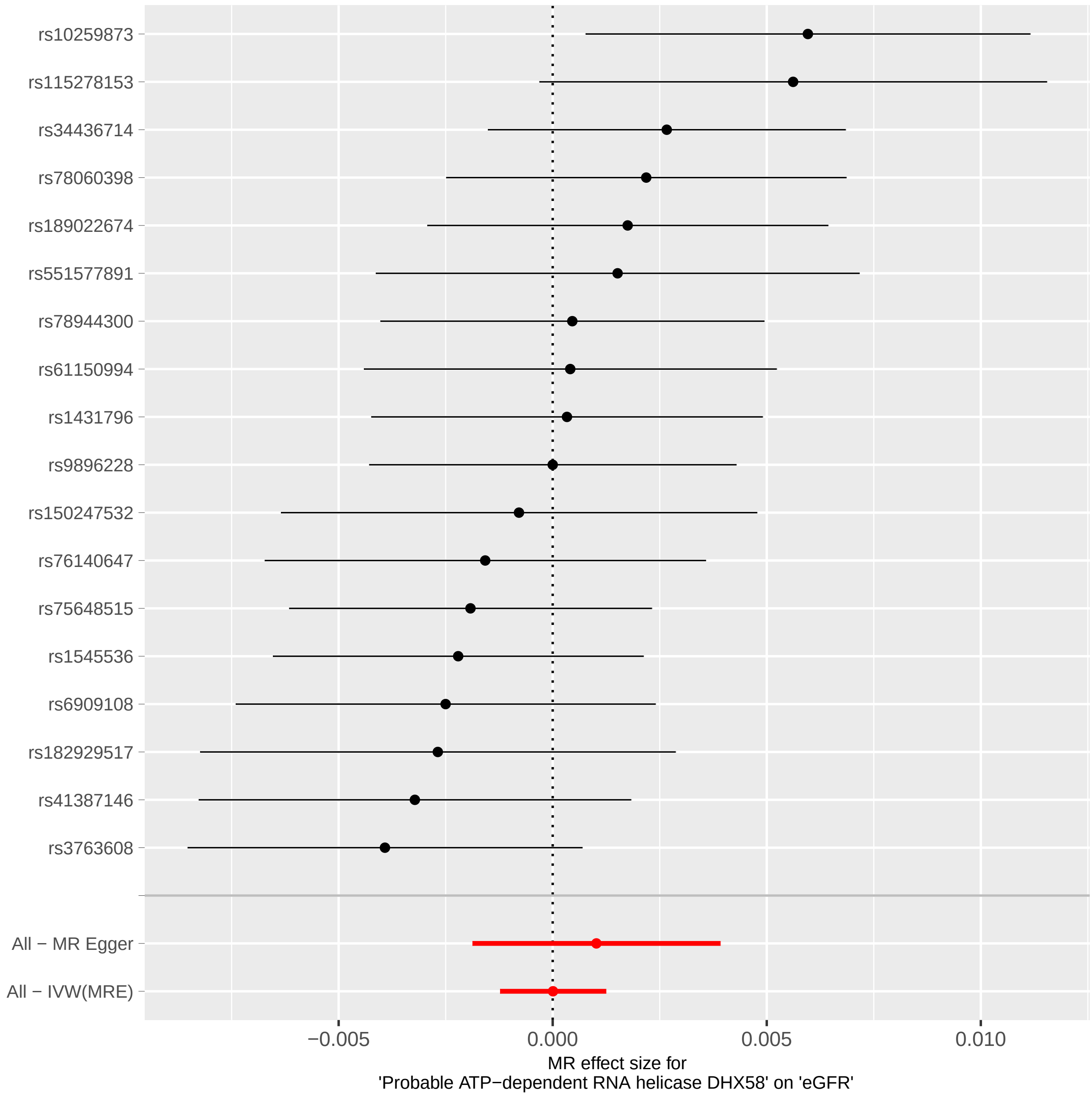

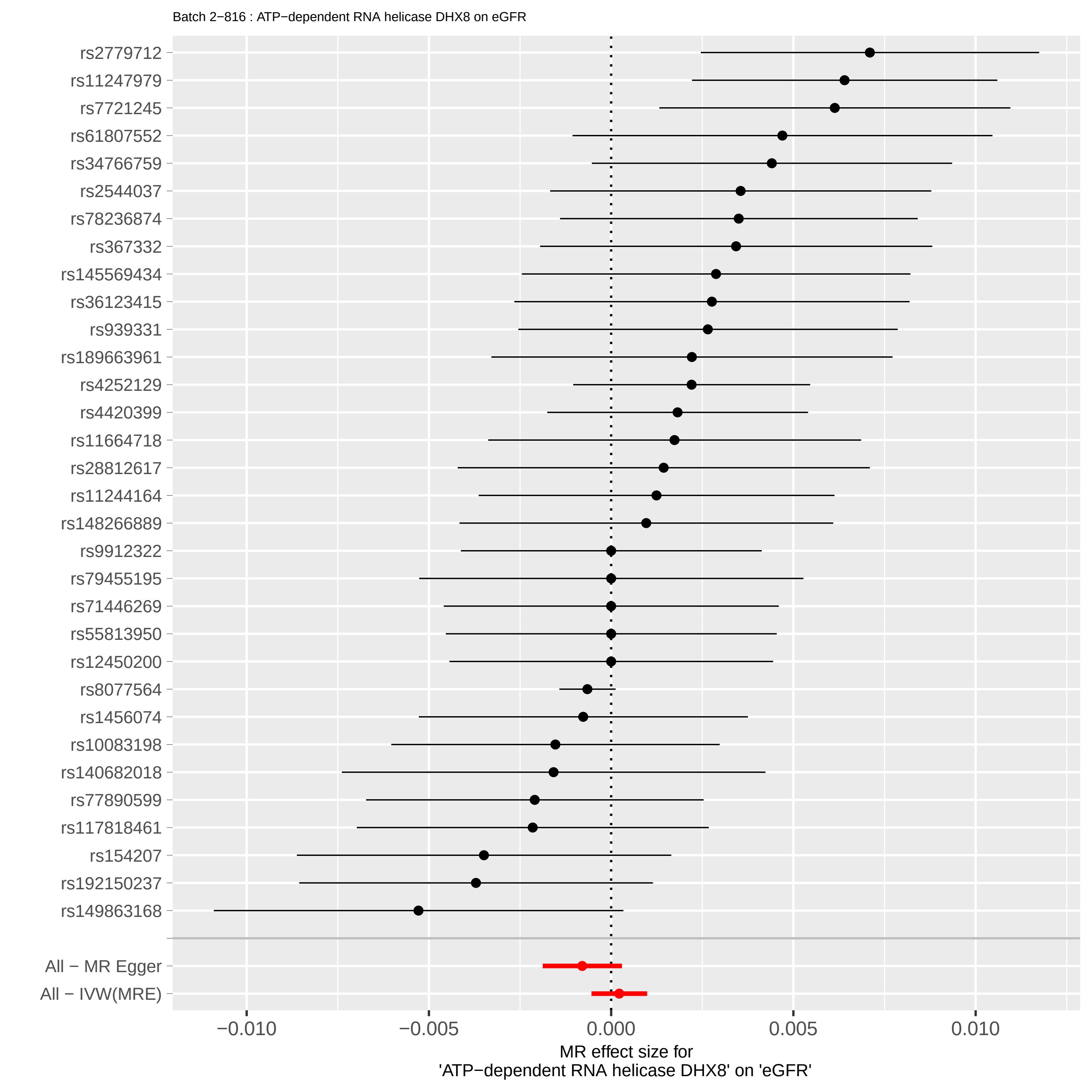

Batch 2−816 : ATP−dependent RNA helicase DHX8 on eGFR
rs2779712
rs11247979
rs7721245
rs61807552
rs34766759
rs2544037
rs78236874
rs367332
rs145569434
rs36123415
rs939331
rs189663961
rs4252129
rs4420399
rs11664718
rs28812617
rs11244164
rs148266889
rs9912322
rs79455195
rs71446269
rs55813950
rs12450200
rs8077564
rs1456074
rs10083198
rs140682018
rs77890599
rs117818461
rs154207
rs192150237
rs149863168
All − MR Egger
All − IVW(MRE)
−0.010
−0.005
0.000
0.005
0.010
MR effect size for
'ATP−dependent RNA helicase DHX8' on 'eGFR'

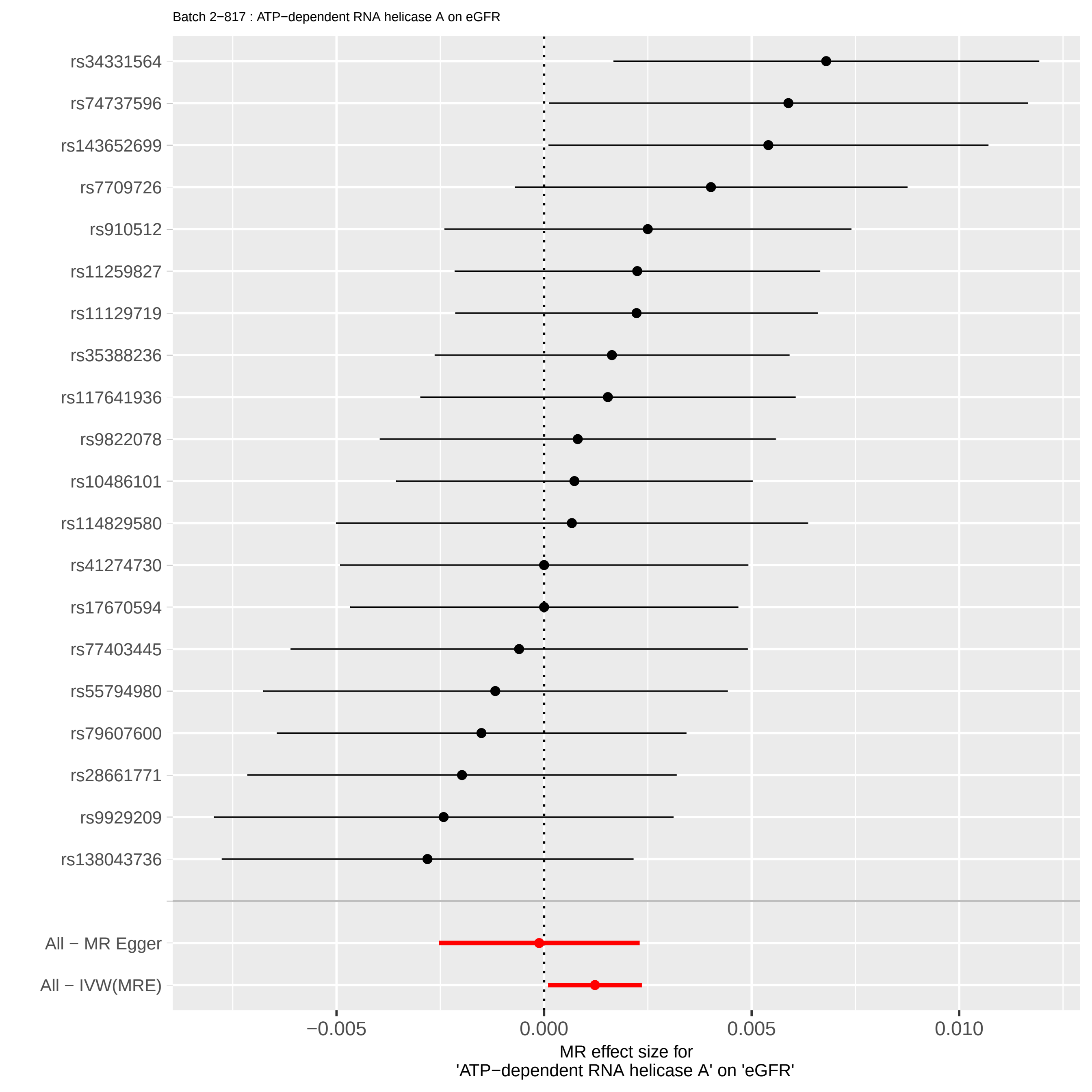

Batch 2−817 : ATP−dependent RNA helicase A on eGFR
rs34331564
rs74737596
rs143652699
rs7709726
rs910512
rs11259827
rs11129719
rs35388236
rs117641936
rs9822078
rs10486101
rs114829580
rs41274730
rs17670594
rs77403445
rs55794980
rs79607600
rs28661771
rs9929209
rs138043736
All − MR Egger
All − IVW(MRE)
−0.005
0.000
0.005
0.010
MR effect size for
'ATP−dependent RNA helicase A' on 'eGFR'

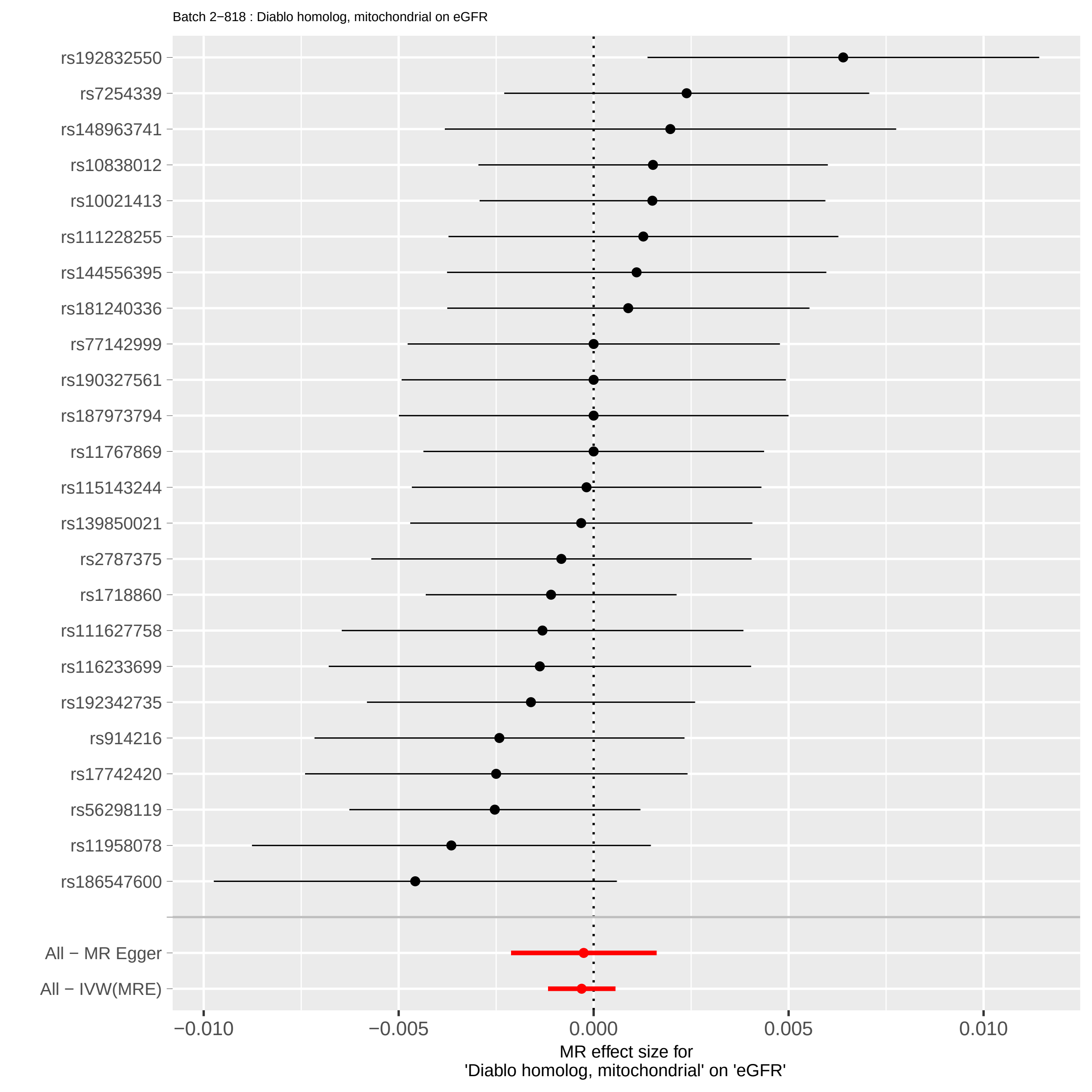

Batch 2−818 : Diablo homolog, mitochondrial on eGFR
rs192832550
rs7254339
rs148963741
rs10838012
rs10021413
rs111228255
rs144556395
rs181240336
rs77142999
rs190327561
rs187973794
rs11767869
rs115143244
rs139850021
rs2787375
rs1718860
rs111627758
rs116233699
rs192342735
rs914216
rs17742420
rs56298119
rs11958078
rs186547600
All − MR Egger
All − IVW(MRE)
−0.010
−0.005
0.000
0.005
0.010
MR effect size for
'Diablo homolog, mitochondrial' on 'eGFR'

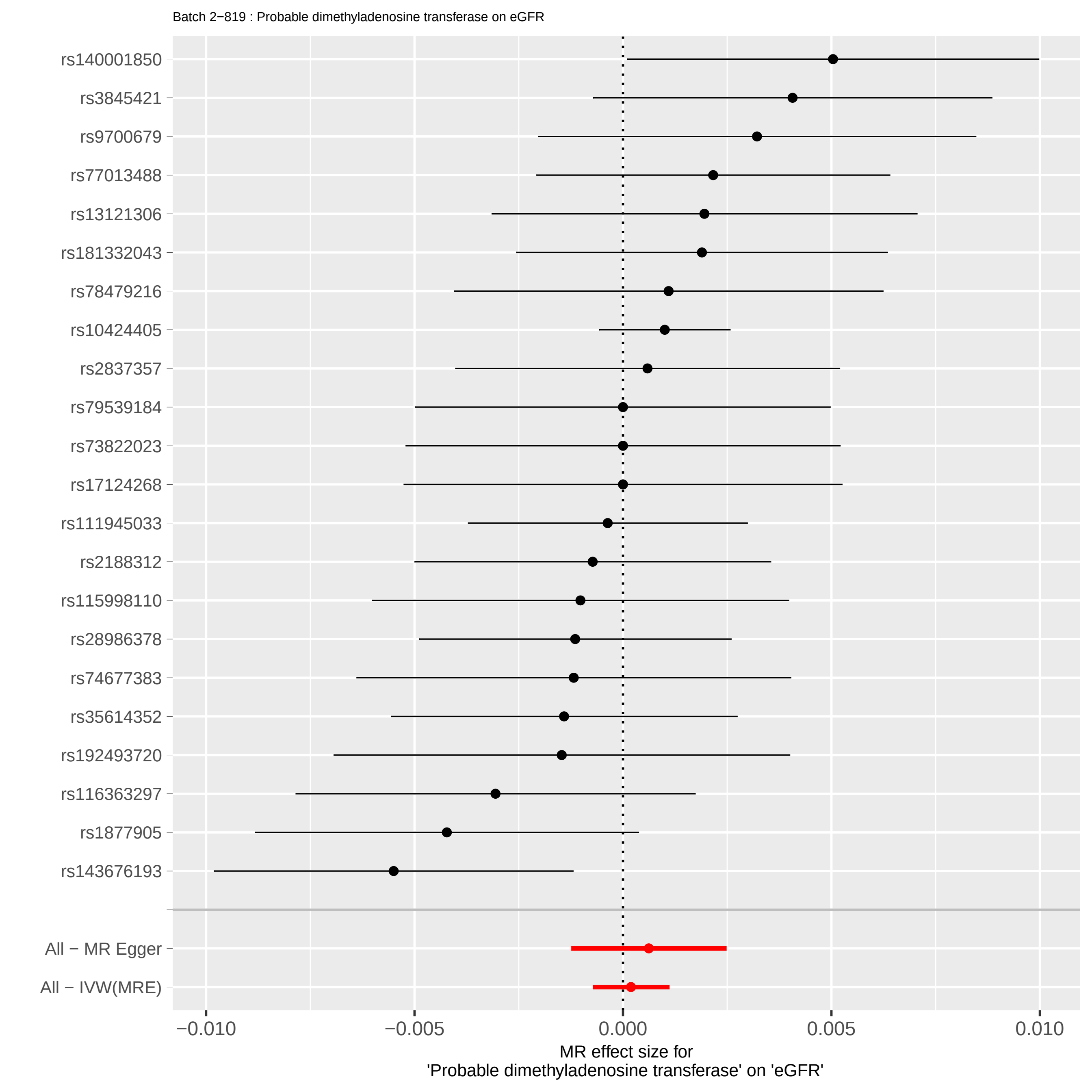
Batch 2−819 : Probable dimethyladenosine transferase on eGFR
rs140001850
rs3845421
rs9700679
rs77013488
rs13121306
rs181332043
rs78479216
rs10424405
rs2837357
rs79539184
rs73822023
rs17124268
rs111945033
rs2188312
rs115998110
rs28986378
rs74677383
rs35614352
rs192493720
rs116363297
rs1877905
rs143676193
All − MR Egger
All − IVW(MRE)
−0.010
−0.005
0.000
0.005
0.010
MR effect size for
'Probable dimethyladenosine transferase' on 'eGFR'

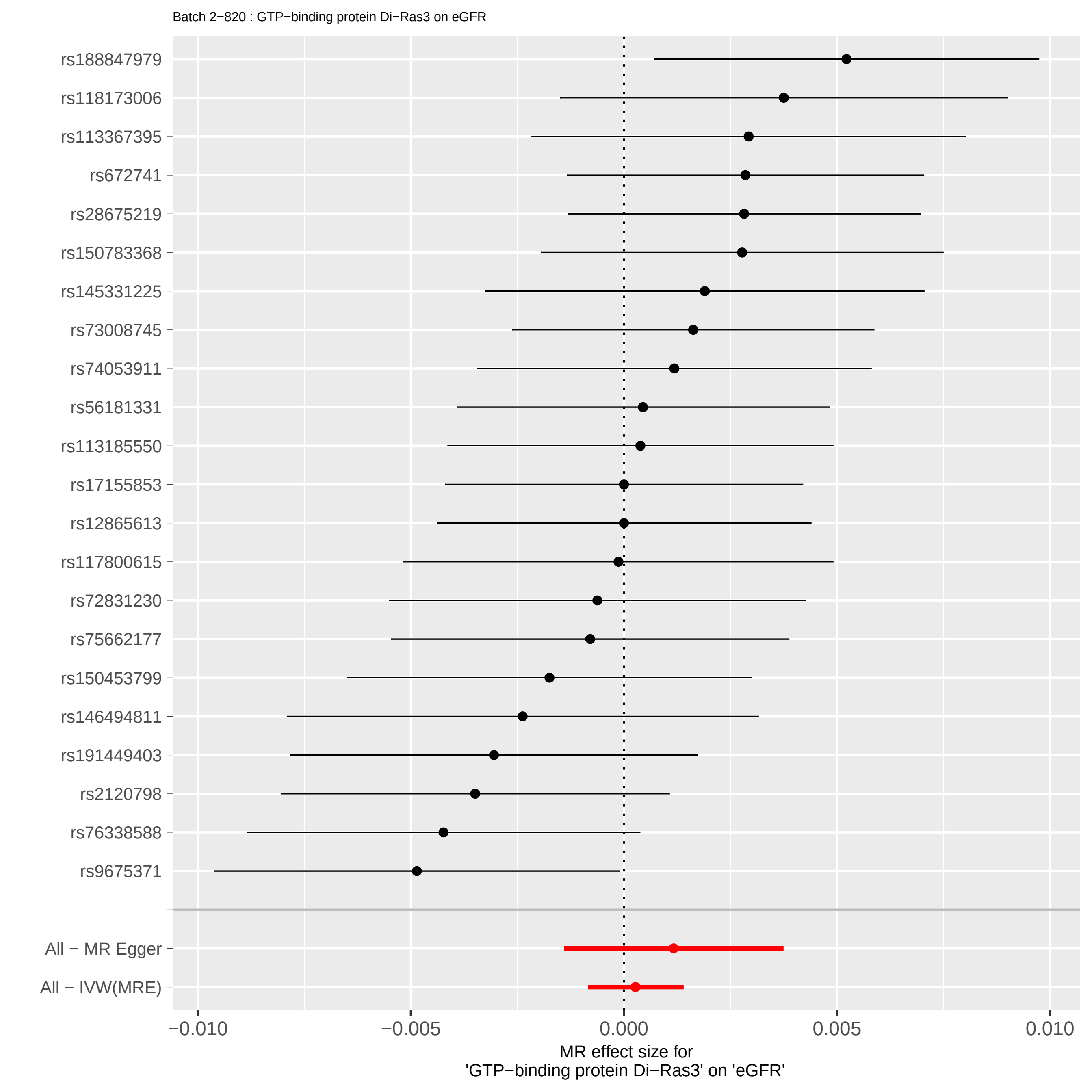

Batch 2−820 : GTP−binding protein Di−Ras3 on eGFR
rs188847979
rs118173006
rs113367395
rs672741
rs28675219
rs150783368
rs145331225
rs73008745
rs74053911
rs56181331
rs113185550
rs17155853
rs12865613
rs117800615
rs72831230
rs75662177
rs150453799
rs146494811
rs191449403
rs2120798
rs76338588
rs9675371
All − MR Egger
All − IVW(MRE)
−0.010
−0.005
0.000
0.005
0.010
MR effect size for
'GTP−binding protein Di−Ras3' on 'eGFR'

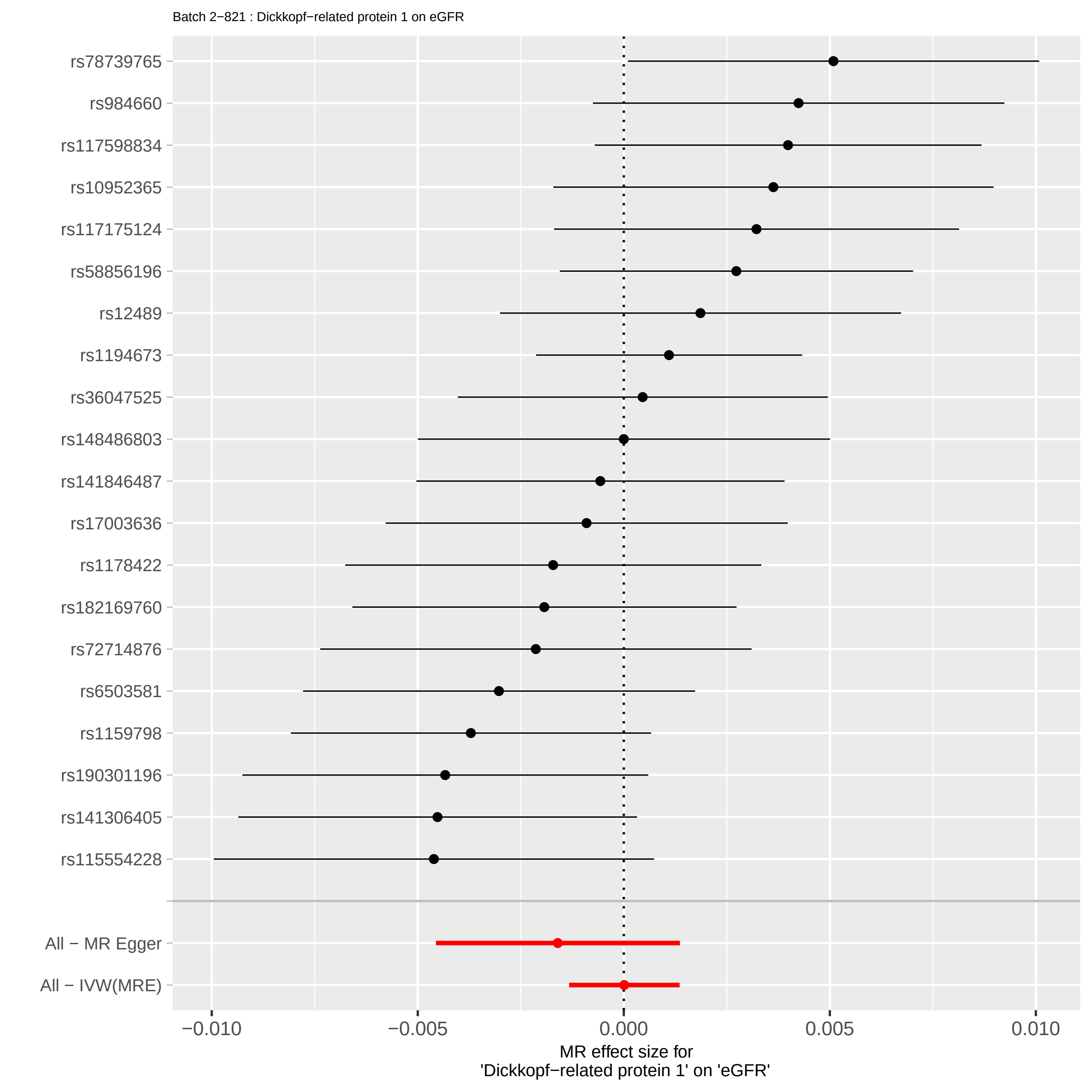

Batch 2−821 : Dickkopf−related protein 1 on eGFR
rs78739765
rs984660
rs117598834
rs10952365
rs117175124
rs58856196
rs12489
rs1194673
rs36047525
rs148486803
rs141846487
rs17003636
rs1178422
rs182169760
rs72714876
rs6503581
rs1159798
rs190301196
rs141306405
rs115554228
All − MR Egger
All − IVW(MRE)
−0.010
−0.005
0.000
0.005
0.010
MR effect size for
'Dickkopf−related protein 1' on 'eGFR'

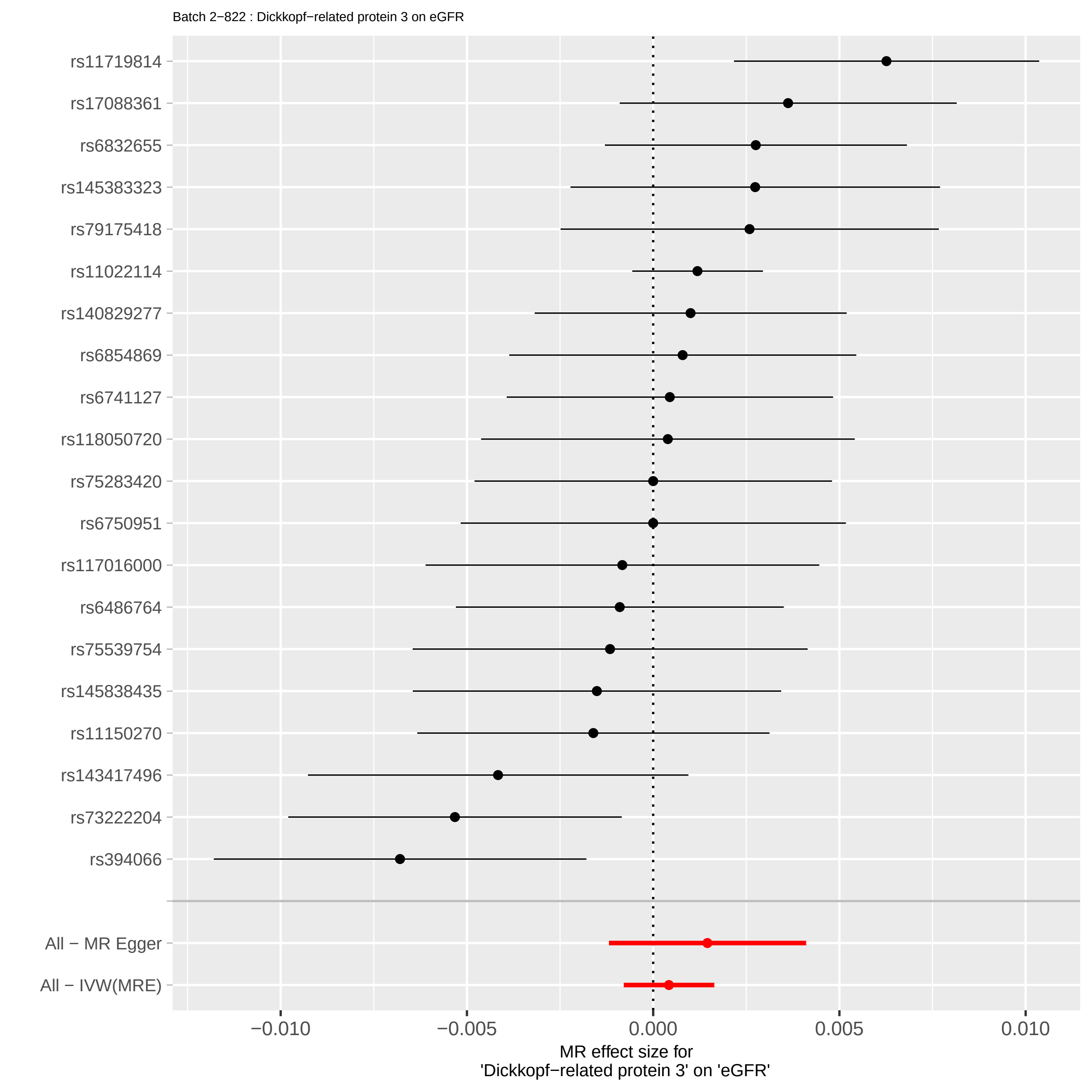

Batch 2−822 : Dickkopf−related protein 3 on eGFR
rs11719814
rs17088361
rs6832655
rs145383323
rs79175418
rs11022114
rs140829277
rs6854869
rs6741127
rs118050720
rs75283420
rs6750951
rs117016000
rs6486764
rs75539754
rs145838435
rs11150270
rs143417496
rs73222204
rs394066
All − MR Egger
All − IVW(MRE)
−0.010
−0.005
0.000
0.005
0.010
MR effect size for
'Dickkopf−related protein 3' on 'eGFR'

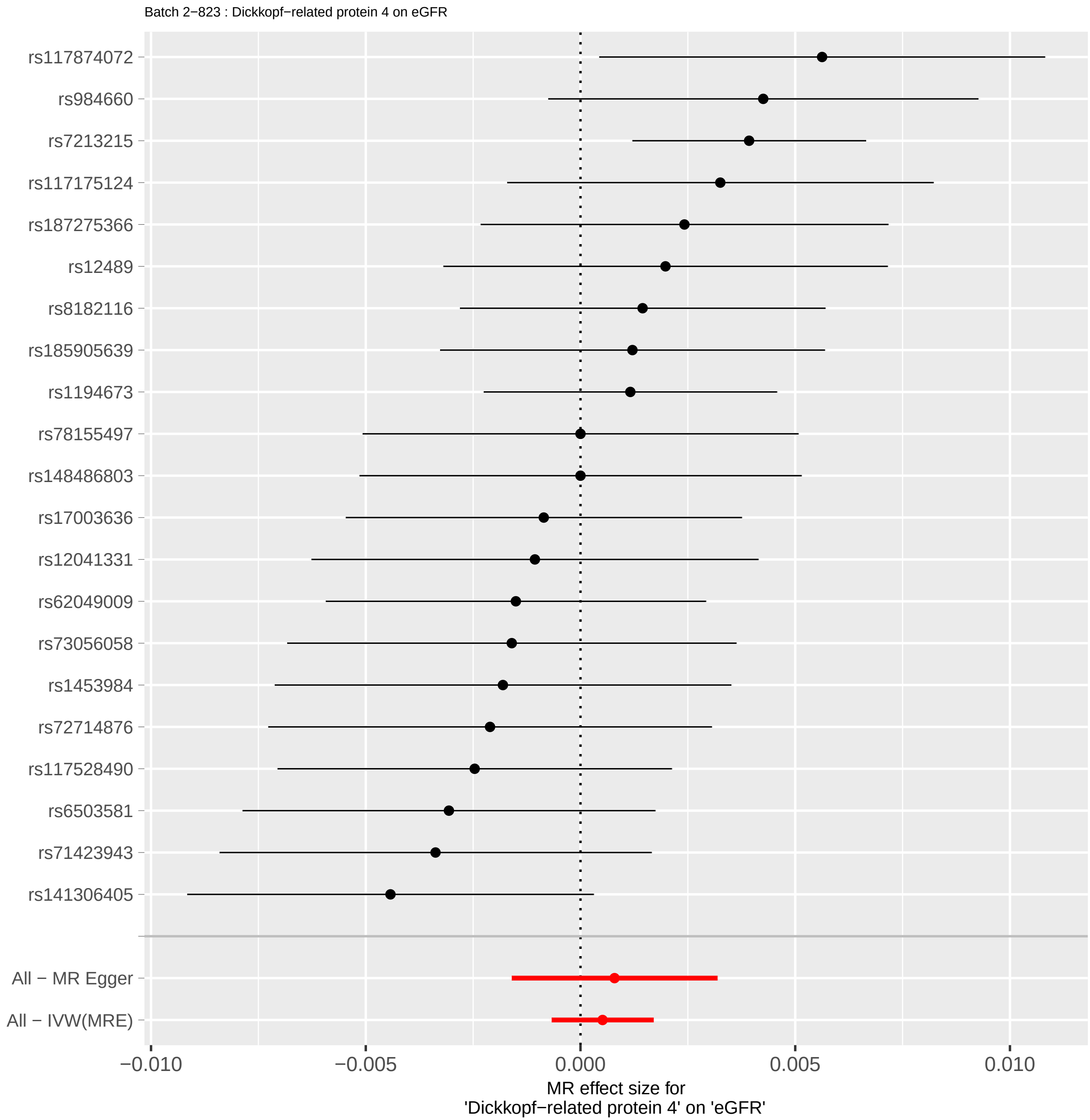

Batch 2−823 : Dickkopf−related protein 4 on eGFR
rs117874072
rs984660
rs7213215
rs117175124
rs187275366
rs12489
rs8182116
rs185905639
rs1194673
rs78155497
rs148486803
rs17003636
rs12041331
rs62049009
rs73056058
rs1453984
rs72714876
rs117528490
rs6503581
rs71423943
rs141306405
All − MR Egger
All − IVW(MRE)
−0.010
−0.005
0.000
0.005
0.010
MR effect size for
'Dickkopf−related protein 4' on 'eGFR'

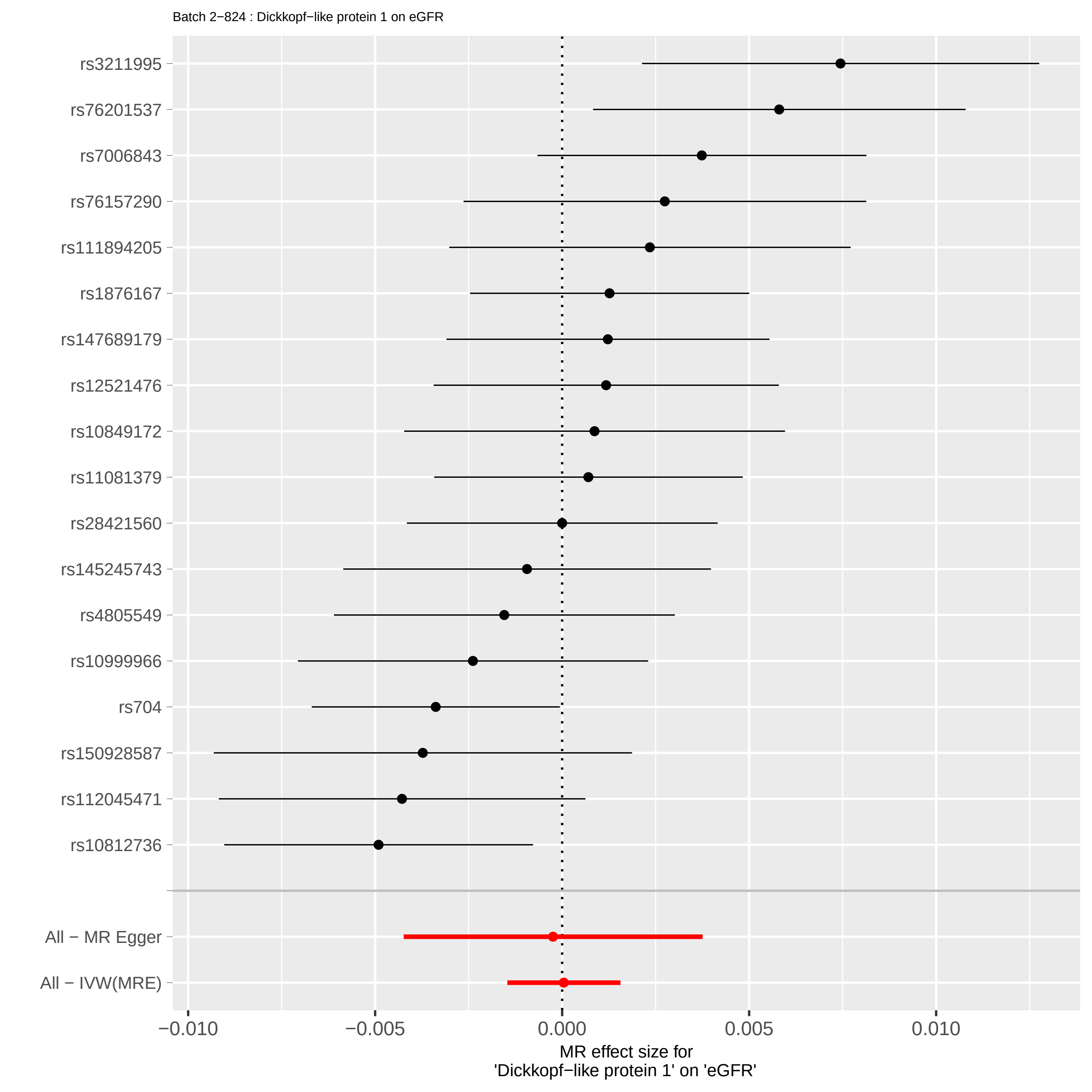

Batch 2−824 : Dickkopf−like protein 1 on eGFR
rs3211995
rs76201537
rs7006843
rs76157290
rs111894205
rs1876167
rs147689179
rs12521476
rs10849172
rs11081379
rs28421560
rs145245743
rs4805549
rs10999966
rs704
rs150928587
rs112045471
rs10812736
All − MR Egger
All − IVW(MRE)
−0.010
−0.005
0.000
0.005
0.010
MR effect size for
'Dickkopf−like protein 1' on 'eGFR'

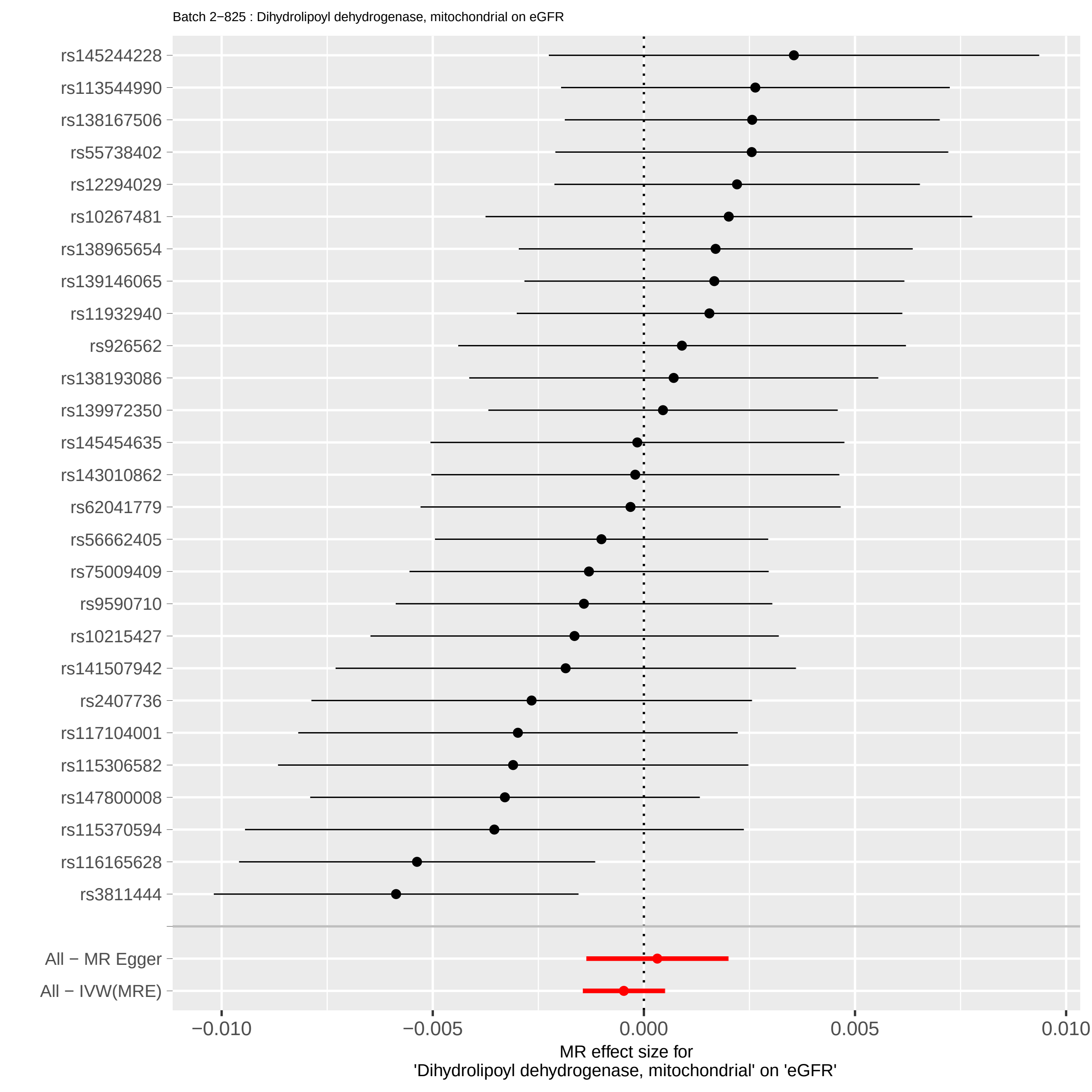

Batch 2−825 : Dihydrolipoyl dehydrogenase, mitochondrial on eGFR
rs145244228
rs113544990
rs138167506
rs55738402
rs12294029
rs10267481
rs138965654
rs139146065
rs11932940
rs926562
rs138193086
rs139972350
rs145454635
rs143010862
rs62041779
rs56662405
rs75009409
rs9590710
rs10215427
rs141507942
rs2407736
rs117104001
rs115306582
rs147800008
rs115370594
rs116165628
rs3811444
All − MR Egger
All − IVW(MRE)
−0.010
−0.005
0.000
0.005
0.010
MR effect size for
'Dihydrolipoyl dehydrogenase, mitochondrial' on 'eGFR'

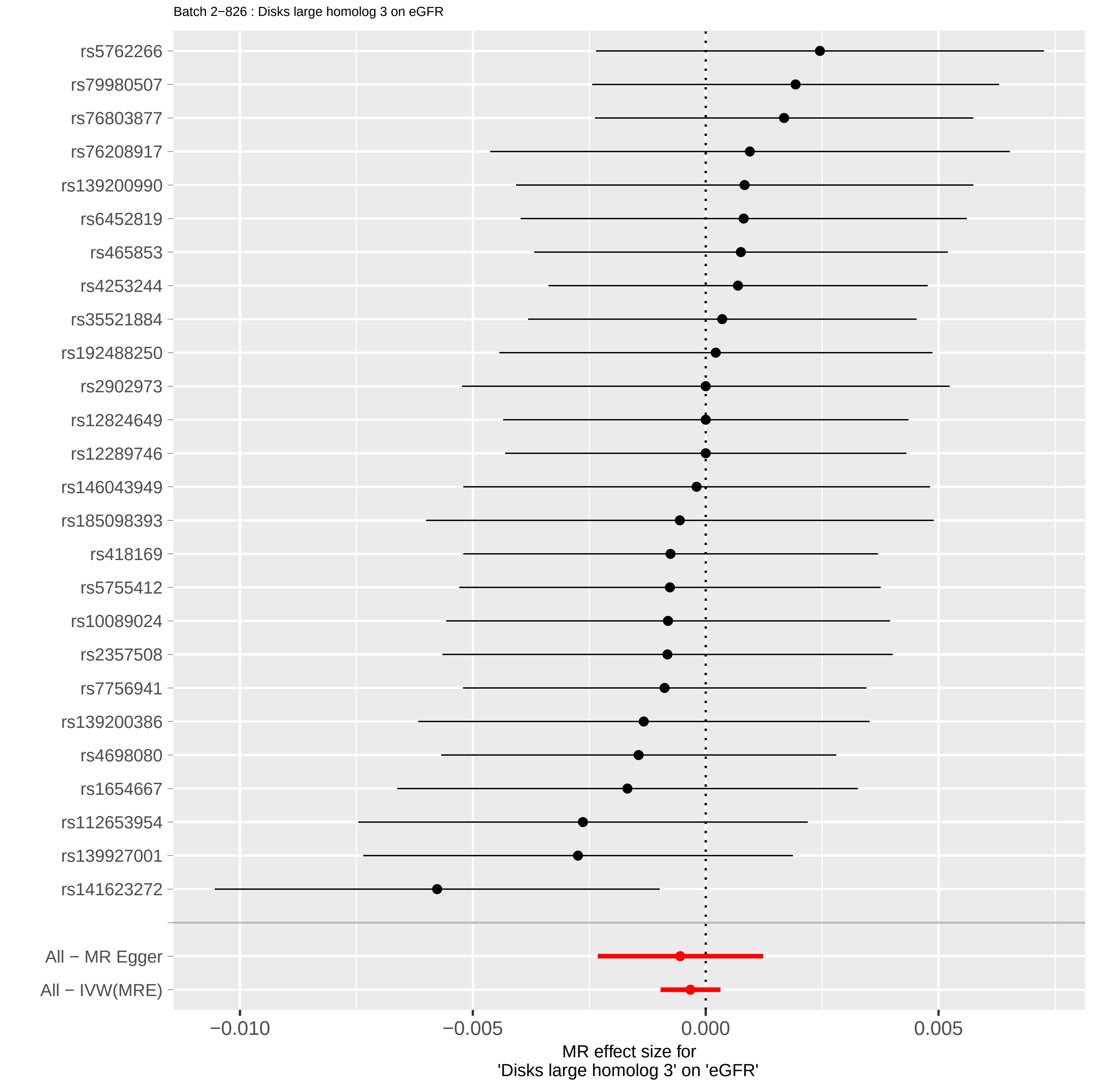

Batch 2−826 : Disks large homolog 3 on eGFR
rs5762266
rs79980507
rs76803877
rs76208917
rs139200990
rs6452819
rs465853
rs4253244
rs35521884
rs192488250
rs2902973
rs12824649
rs12289746
rs146043949
rs185098393
rs418169
rs5755412
rs10089024
rs2357508
rs7756941
rs139200386
rs4698080
rs1654667
rs112653954
rs139927001
rs141623272
All − MR Egger
All − IVW(MRE)
−0.010
−0.005
0.000
0.005
MR effect size for
'Disks large homolog 3' on 'eGFR'

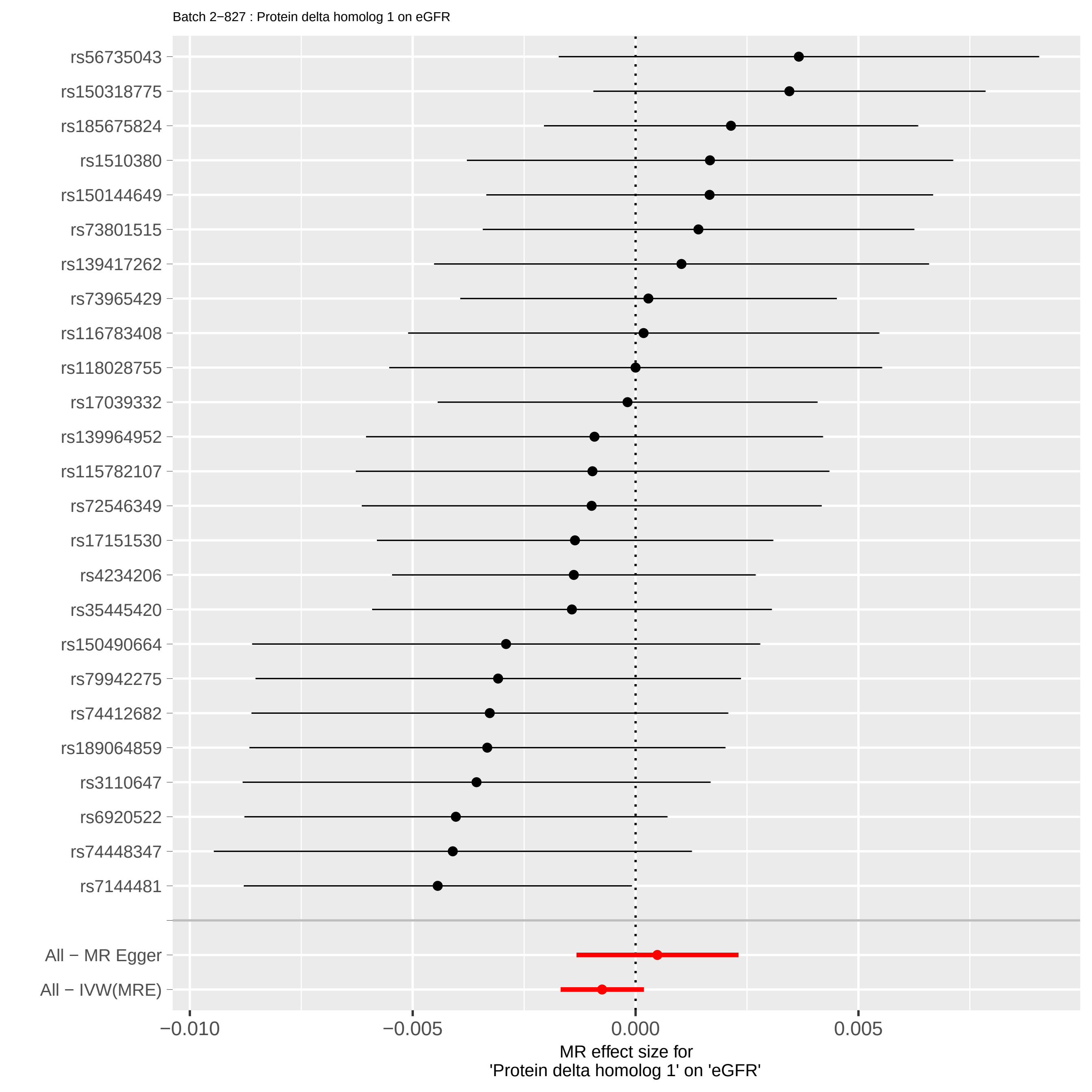
Batch 2−827 : Protein delta homolog 1 on eGFR
rs56735043
rs150318775
rs185675824
rs1510380
rs150144649
rs73801515
rs139417262
rs73965429
rs116783408
rs118028755
rs17039332
rs139964952
rs115782107
rs72546349
rs17151530
rs4234206
rs35445420
rs150490664
rs79942275
rs74412682
rs189064859
rs3110647
rs6920522
rs74448347
rs7144481
All − MR Egger
All − IVW(MRE)
−0.010
−0.005
0.000
0.005
MR effect size for
'Protein delta homolog 1' on 'eGFR'

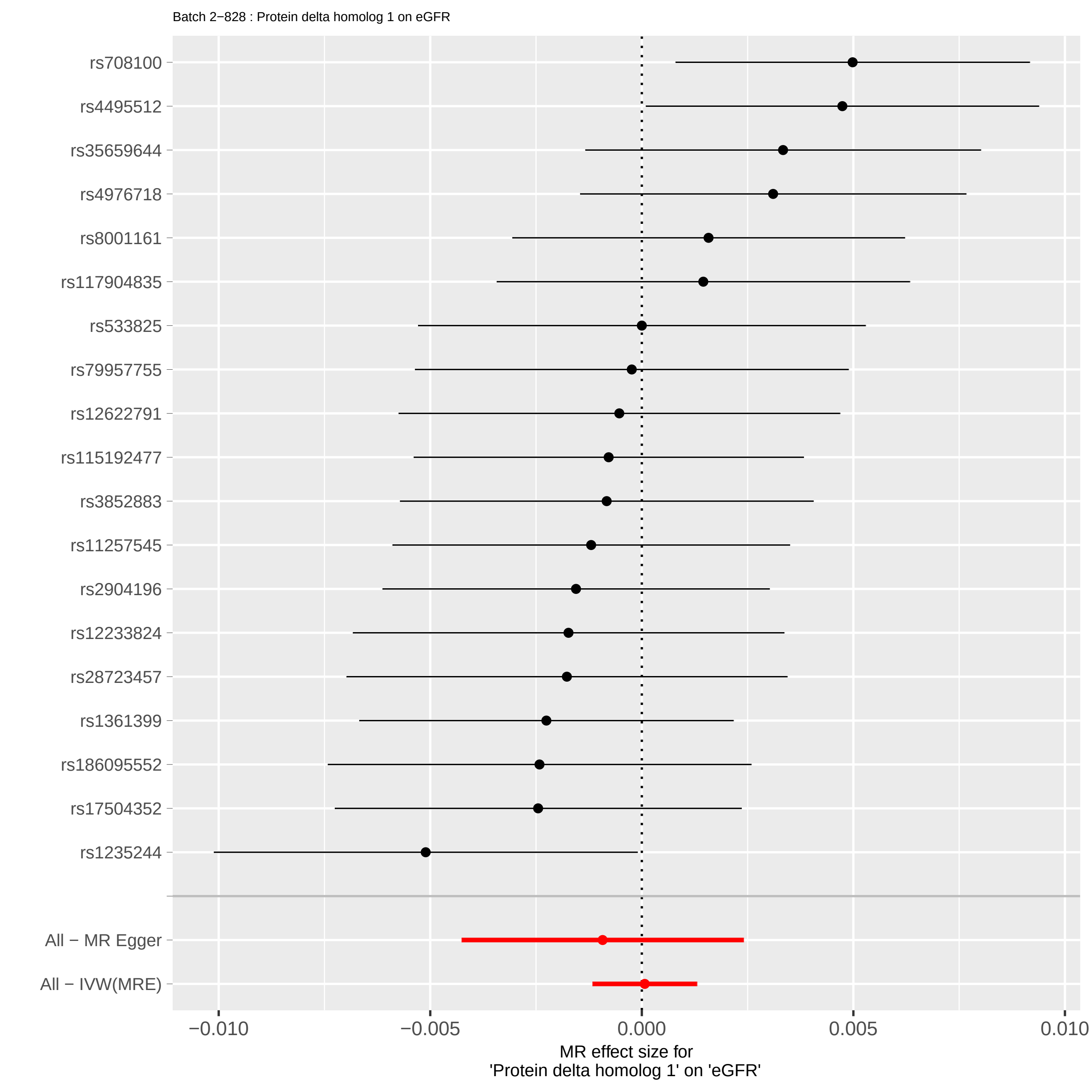
Batch 2−828 : Protein delta homolog 1 on eGFR
rs708100
rs4495512
rs35659644
rs4976718
rs8001161
rs117904835
rs533825
rs79957755
rs12622791
rs115192477
rs3852883
rs11257545
rs2904196
rs12233824
rs28723457
rs1361399
rs186095552
rs17504352
rs1235244
All − MR Egger
All − IVW(MRE)
−0.010
−0.005
0.000
0.005
0.010
MR effect size for
'Protein delta homolog 1' on 'eGFR'

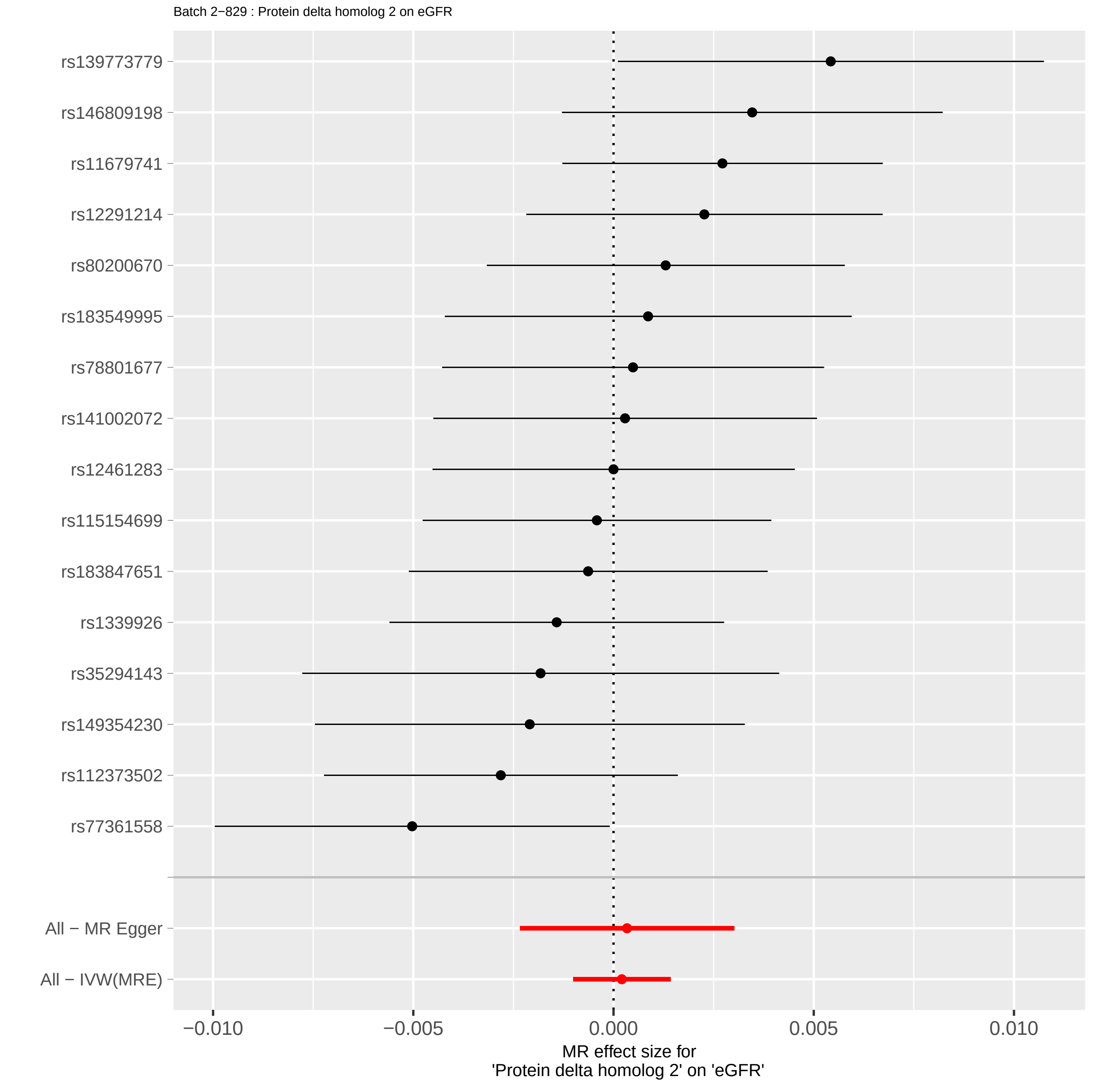

Batch 2−829 : Protein delta homolog 2 on eGFR
rs139773779
rs146809198
rs11679741
rs12291214
rs80200670
rs183549995
rs78801677
rs141002072
rs12461283
rs115154699
rs183847651
rs1339926
rs35294143
rs149354230
rs112373502
rs77361558
All − MR Egger
All − IVW(MRE)
−0.010
−0.005
0.000
0.005
0.010
MR effect size for
'Protein delta homolog 2' on 'eGFR'

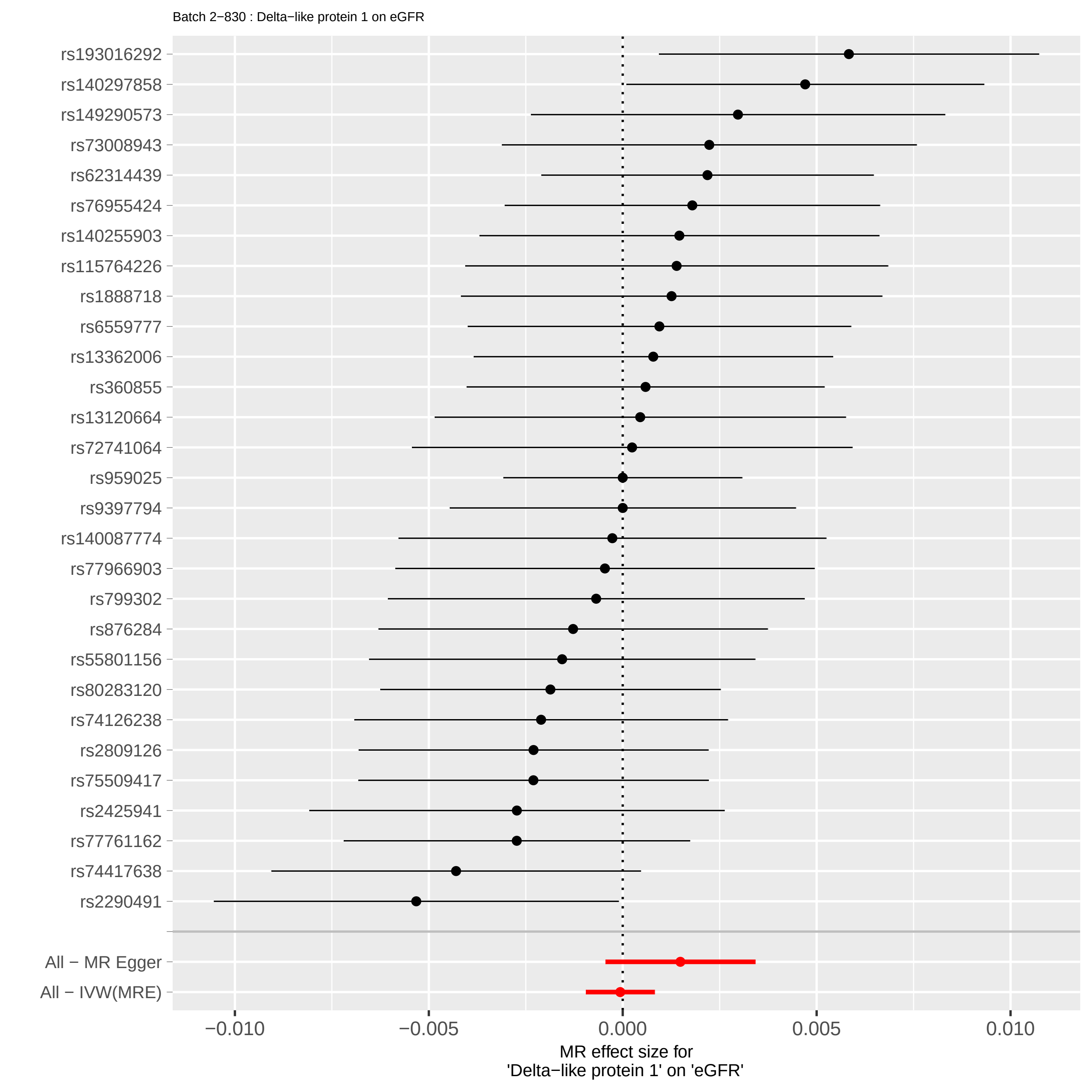

Batch 2−830 : Delta−like protein 1 on eGFR
rs193016292
rs140297858
rs149290573
rs73008943
rs62314439
rs76955424
rs140255903
rs115764226
rs1888718
rs6559777
rs13362006
rs360855
rs13120664
rs72741064
rs959025
rs9397794
rs140087774
rs77966903
rs799302
rs876284
rs55801156
rs80283120
rs74126238
rs2809126
rs75509417
rs2425941
rs77761162
rs74417638
rs2290491
All − MR Egger
All − IVW(MRE)
−0.010
−0.005
0.000
0.005
0.010
MR effect size for
'Delta−like protein 1' on 'eGFR'

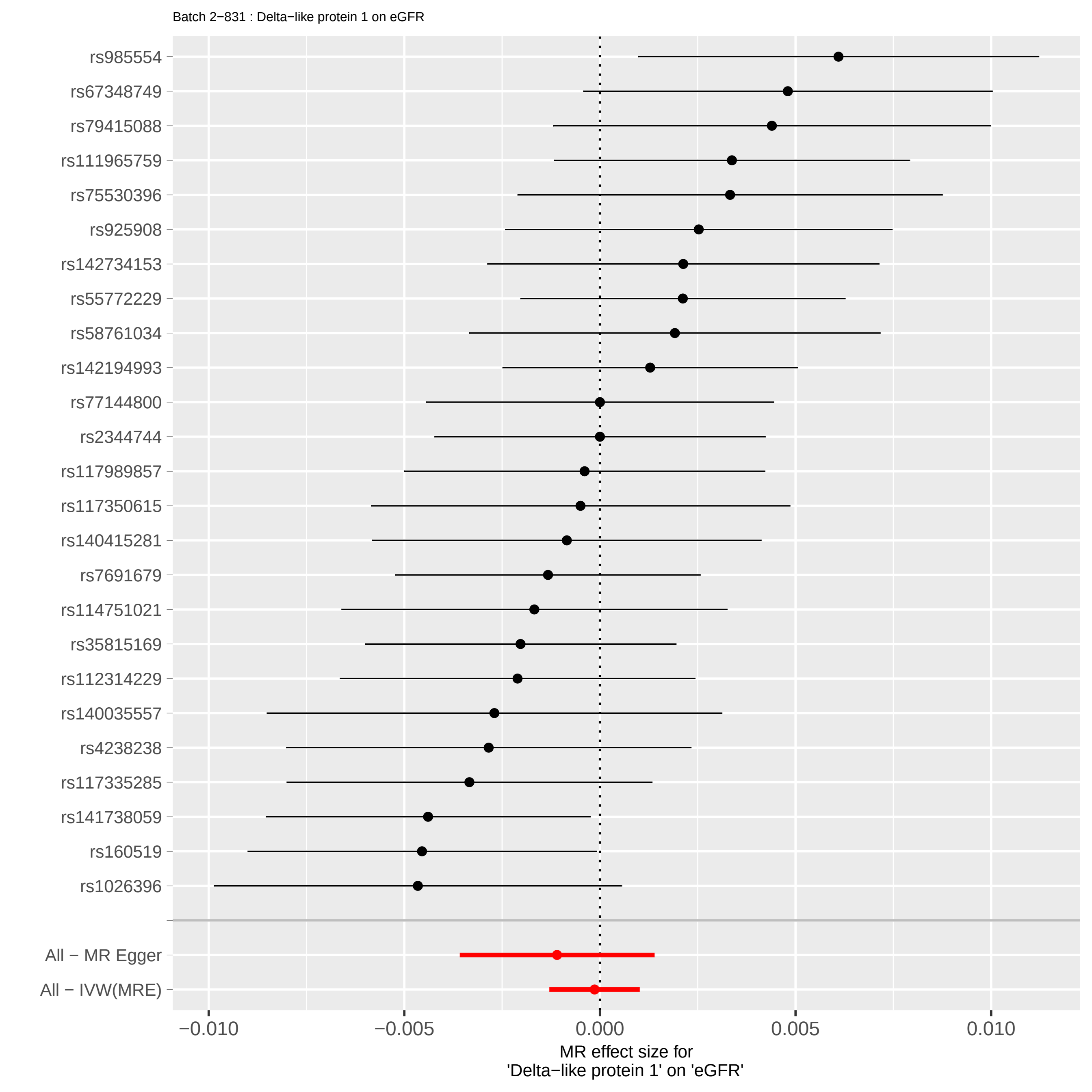

Batch 2−831 : Delta−like protein 1 on eGFR
rs985554
rs67348749
rs79415088
rs111965759
rs75530396
rs925908
rs142734153
rs55772229
rs58761034
rs142194993
rs77144800
rs2344744
rs117989857
rs117350615
rs140415281
rs7691679
rs114751021
rs35815169
rs112314229
rs140035557
rs4238238
rs117335285
rs141738059
rs160519
rs1026396
All − MR Egger
All − IVW(MRE)
−0.010
−0.005
0.000
0.005
0.010
MR effect size for
'Delta−like protein 1' on 'eGFR'

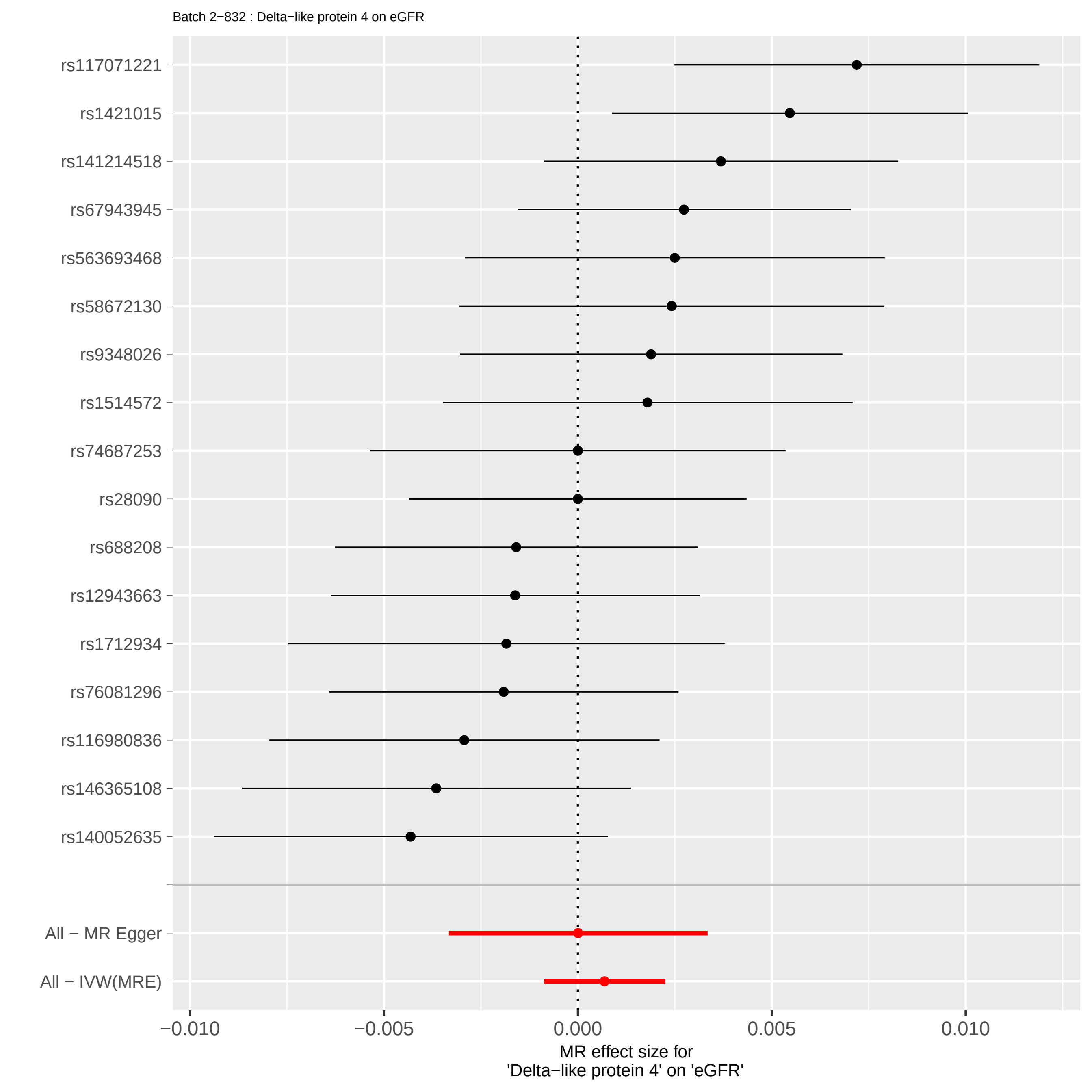
Batch 2−832 : Delta−like protein 4 on eGFR
rs117071221
rs1421015
rs141214518
rs67943945
rs563693468
rs58672130
rs9348026
rs1514572
rs74687253
rs28090
rs688208
rs12943663
rs1712934
rs76081296
rs116980836
rs146365108
rs140052635
All − MR Egger
All − IVW(MRE)
−0.010
−0.005
0.000
0.005
0.010
MR effect size for
'Delta−like protein 4' on 'eGFR'

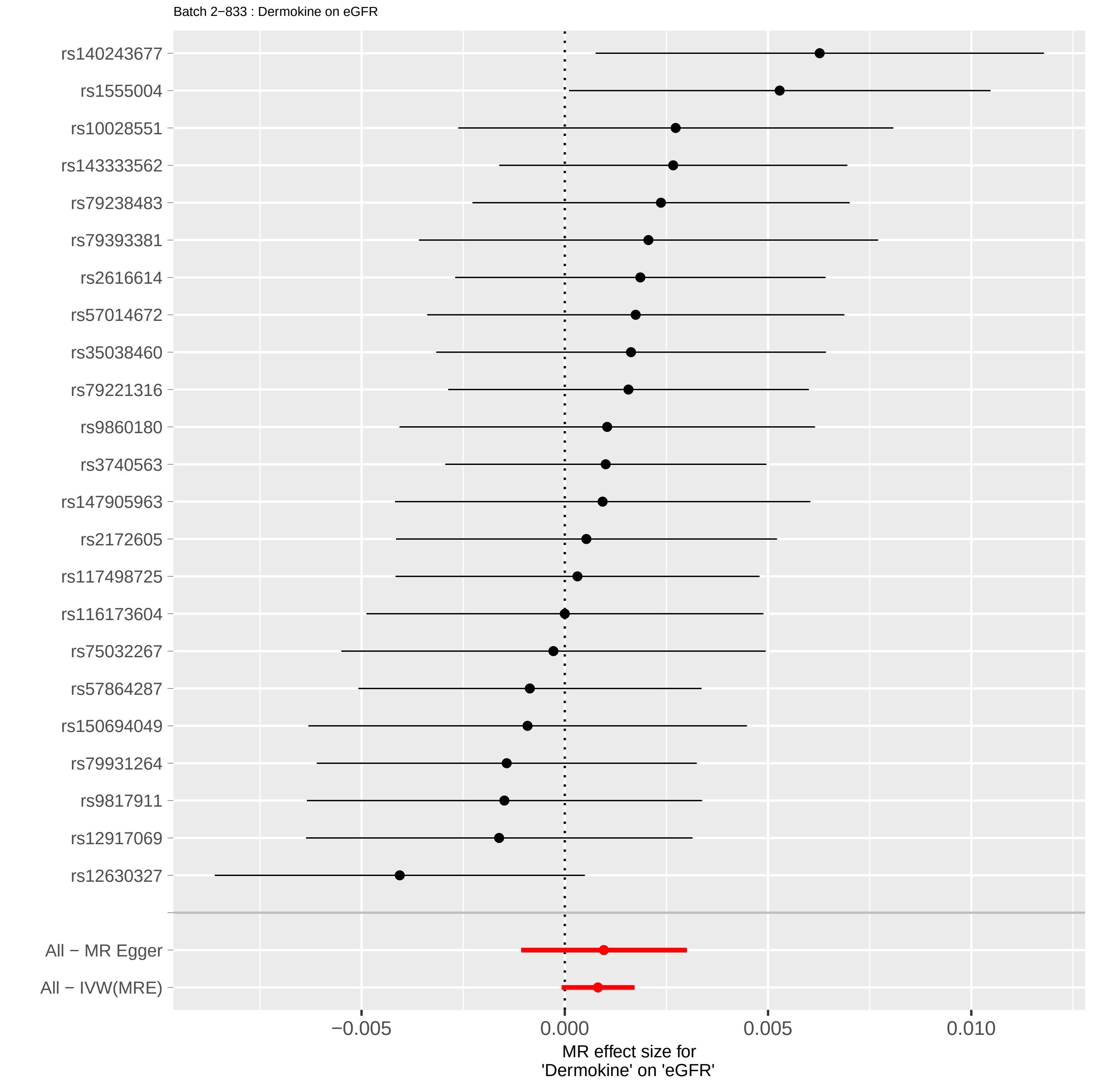

Batch 2−833 : Dermokine on eGFR
rs140243677
rs1555004
rs10028551
rs143333562
rs79238483
rs79393381
rs2616614
rs57014672
rs35038460
rs79221316
rs9860180
rs3740563
rs147905963
rs2172605
rs117498725
rs116173604
rs75032267
rs57864287
rs150694049
rs79931264
rs9817911
rs12917069
rs12630327
All − MR Egger
All − IVW(MRE)
−0.005
0.000
0.005
0.010
MR effect size for
'Dermokine' on 'eGFR'

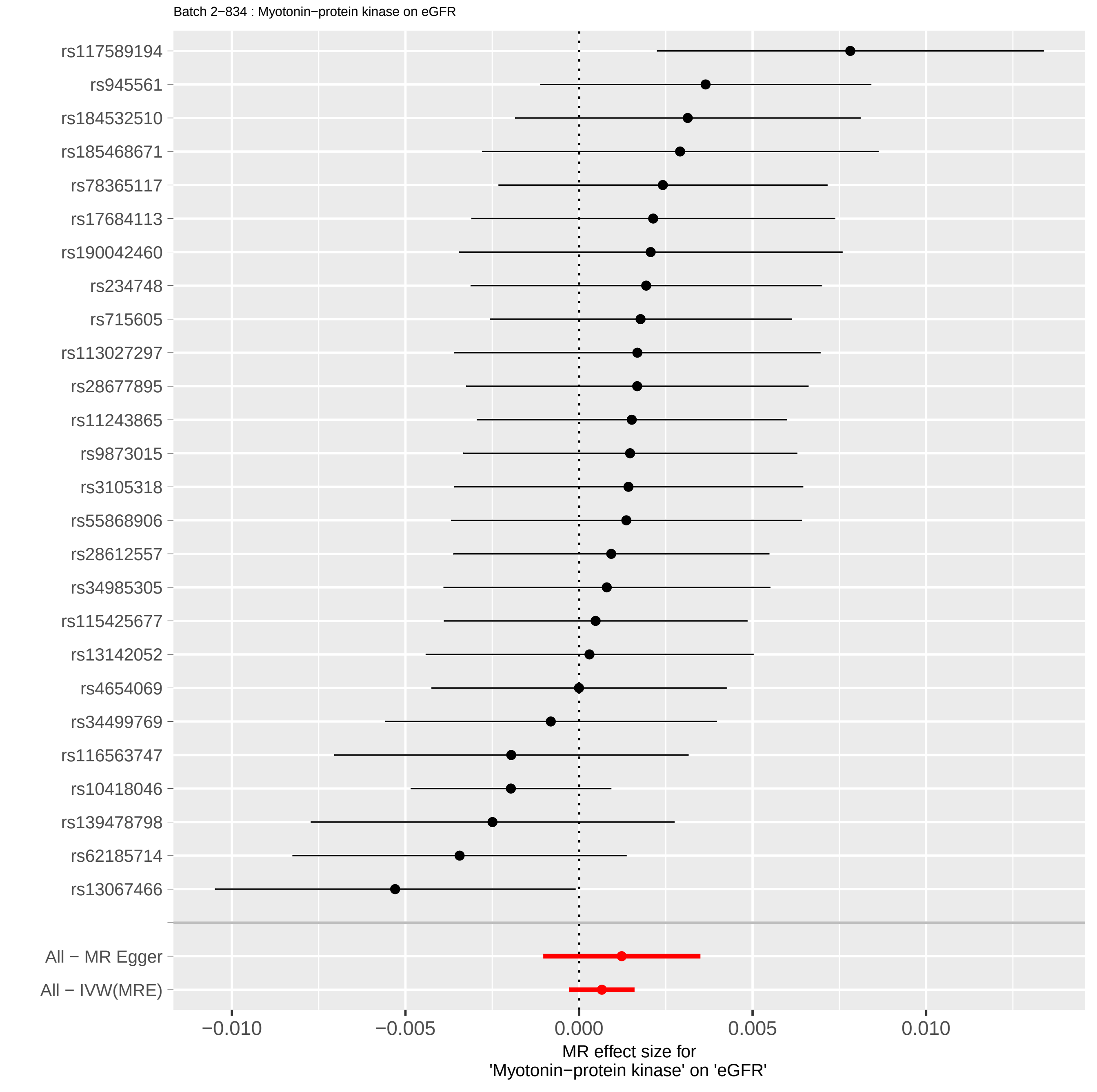
Batch 2−834 : Myotonin−protein kinase on eGFR
rs117589194
rs945561
rs184532510
rs185468671
rs78365117
rs17684113
rs190042460
rs234748
rs715605
rs113027297
rs28677895
rs11243865
rs9873015
rs3105318
rs55868906
rs28612557
rs34985305
rs115425677
rs13142052
rs4654069
rs34499769
rs116563747
rs10418046
rs139478798
rs62185714
rs13067466
All − MR Egger
All − IVW(MRE)
−0.010
−0.005
0.000
0.005
0.010
MR effect size for
'Myotonin−protein kinase' on 'eGFR'

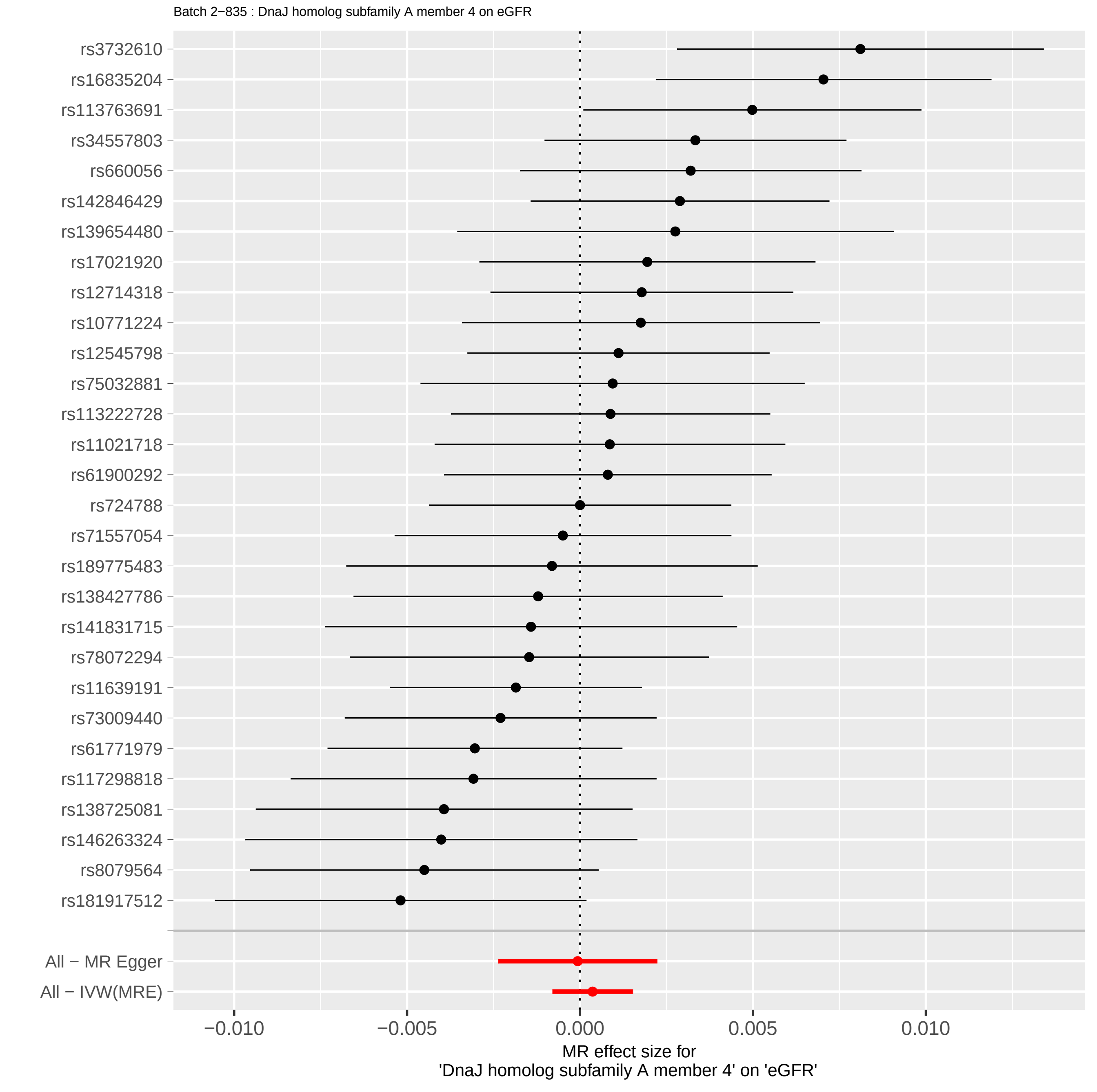
Batch 2−835 : DnaJ homolog subfamily A member 4 on eGFR
rs3732610
rs16835204
rs113763691
rs34557803
rs660056
rs142846429
rs139654480
rs17021920
rs12714318
rs10771224
rs12545798
rs75032881
rs113222728
rs11021718
rs61900292
rs724788
rs71557054
rs189775483
rs138427786
rs141831715
rs78072294
rs11639191
rs73009440
rs61771979
rs117298818
rs138725081
rs146263324
rs8079564
rs181917512
All − MR Egger
All − IVW(MRE)
−0.010
−0.005
0.000
0.005
0.010
MR effect size for
'DnaJ homolog subfamily A member 4' on 'eGFR'

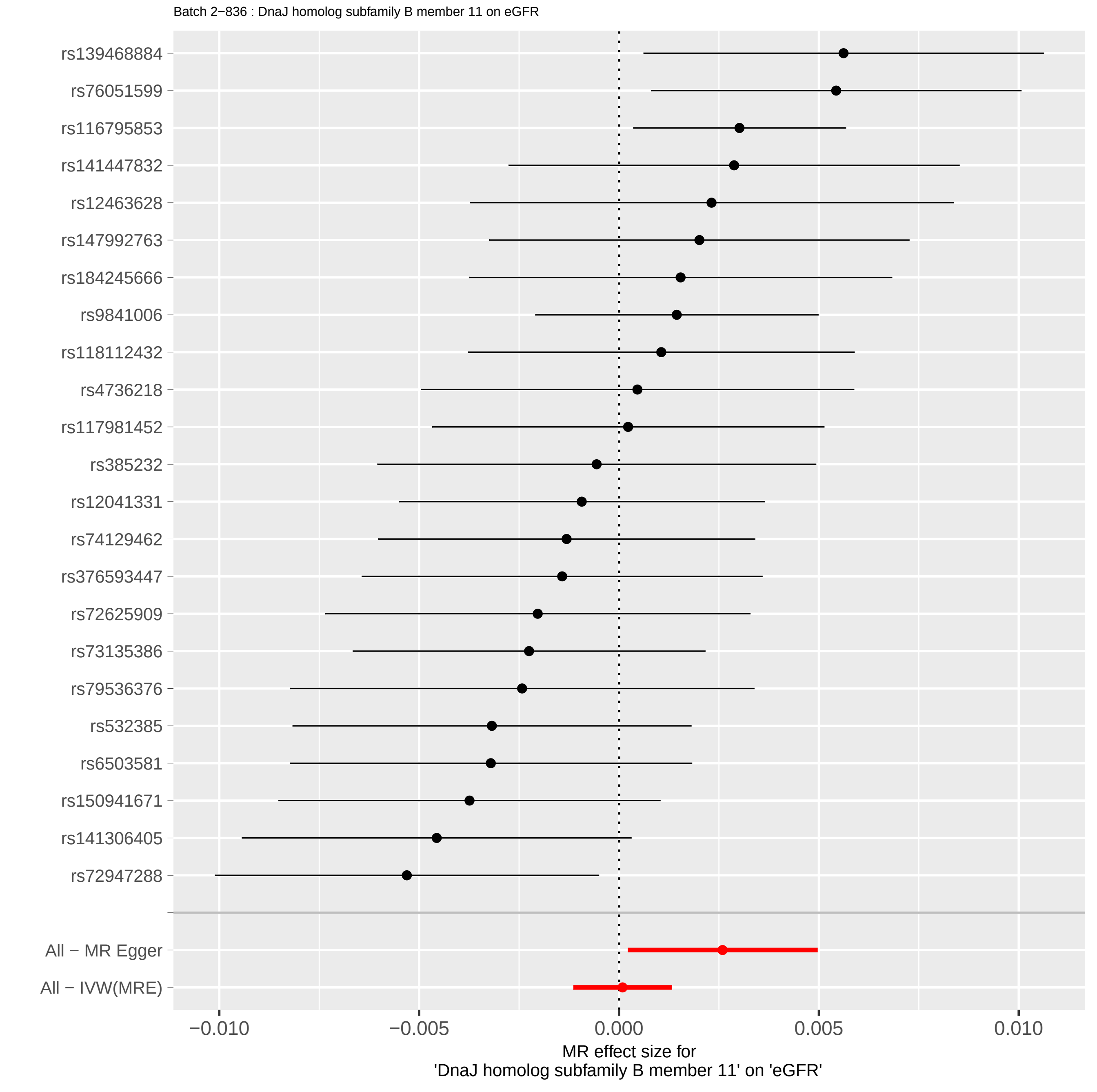

Batch 2−836 : DnaJ homolog subfamily B member 11 on eGFR
rs139468884
rs76051599
rs116795853
rs141447832
rs12463628
rs147992763
rs184245666
rs9841006
rs118112432
rs4736218
rs117981452
rs385232
rs12041331
rs74129462
rs376593447
rs72625909
rs73135386
rs79536376
rs532385
rs6503581
rs150941671
rs141306405
rs72947288
All − MR Egger
All − IVW(MRE)
−0.010
−0.005
0.000
0.005
0.010
MR effect size for
'DnaJ homolog subfamily B member 11' on 'eGFR'

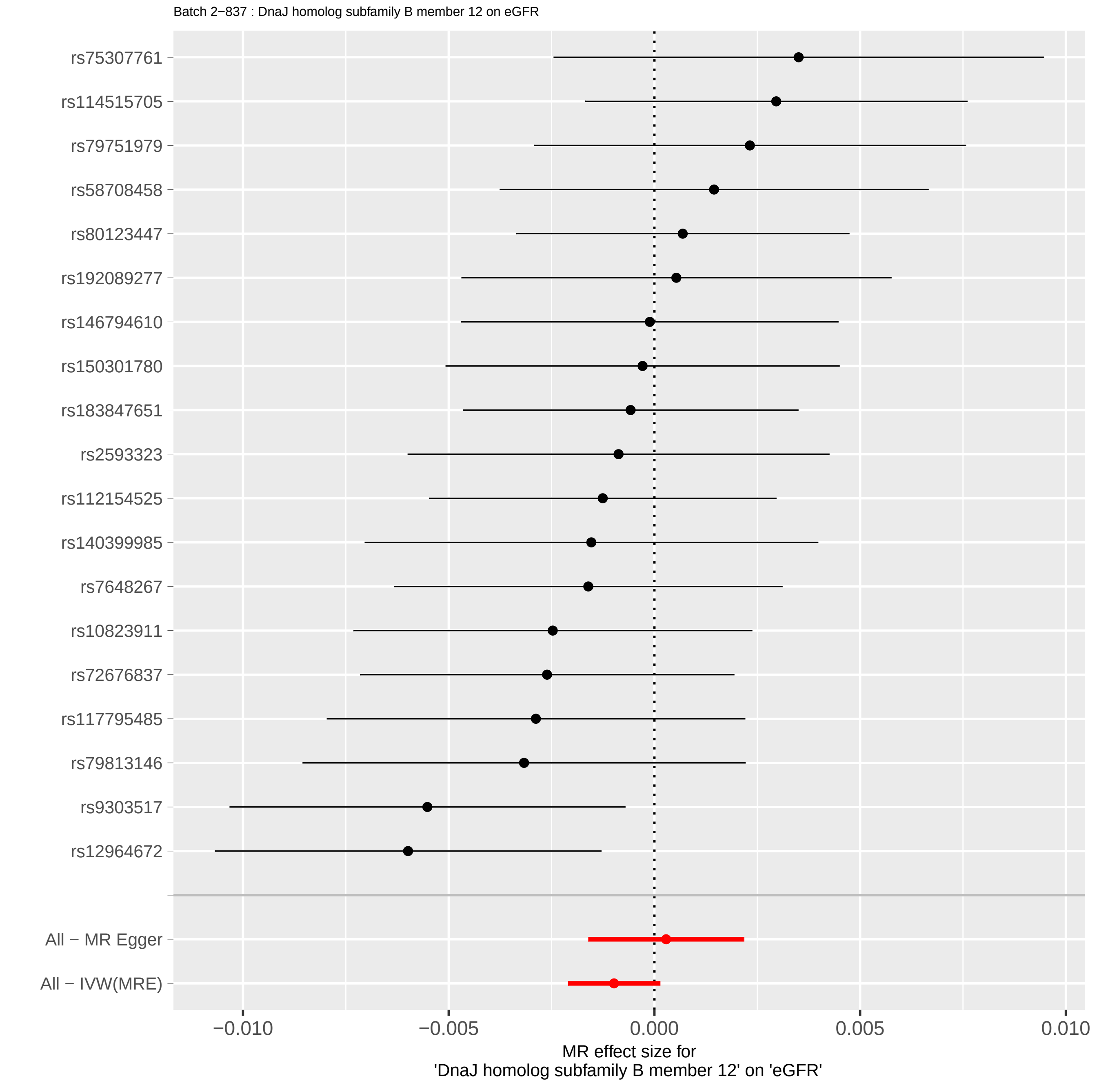
Batch 2−837 : DnaJ homolog subfamily B member 12 on eGFR
rs75307761
rs114515705
rs79751979
rs58708458
rs80123447
rs192089277
rs146794610
rs150301780
rs183847651
rs2593323
rs112154525
rs140399985
rs7648267
rs10823911
rs72676837
rs117795485
rs79813146
rs9303517
rs12964672
All − MR Egger
All − IVW(MRE)
−0.010
−0.005
0.000
0.005
0.010
MR effect size for
'DnaJ homolog subfamily B member 12' on 'eGFR'

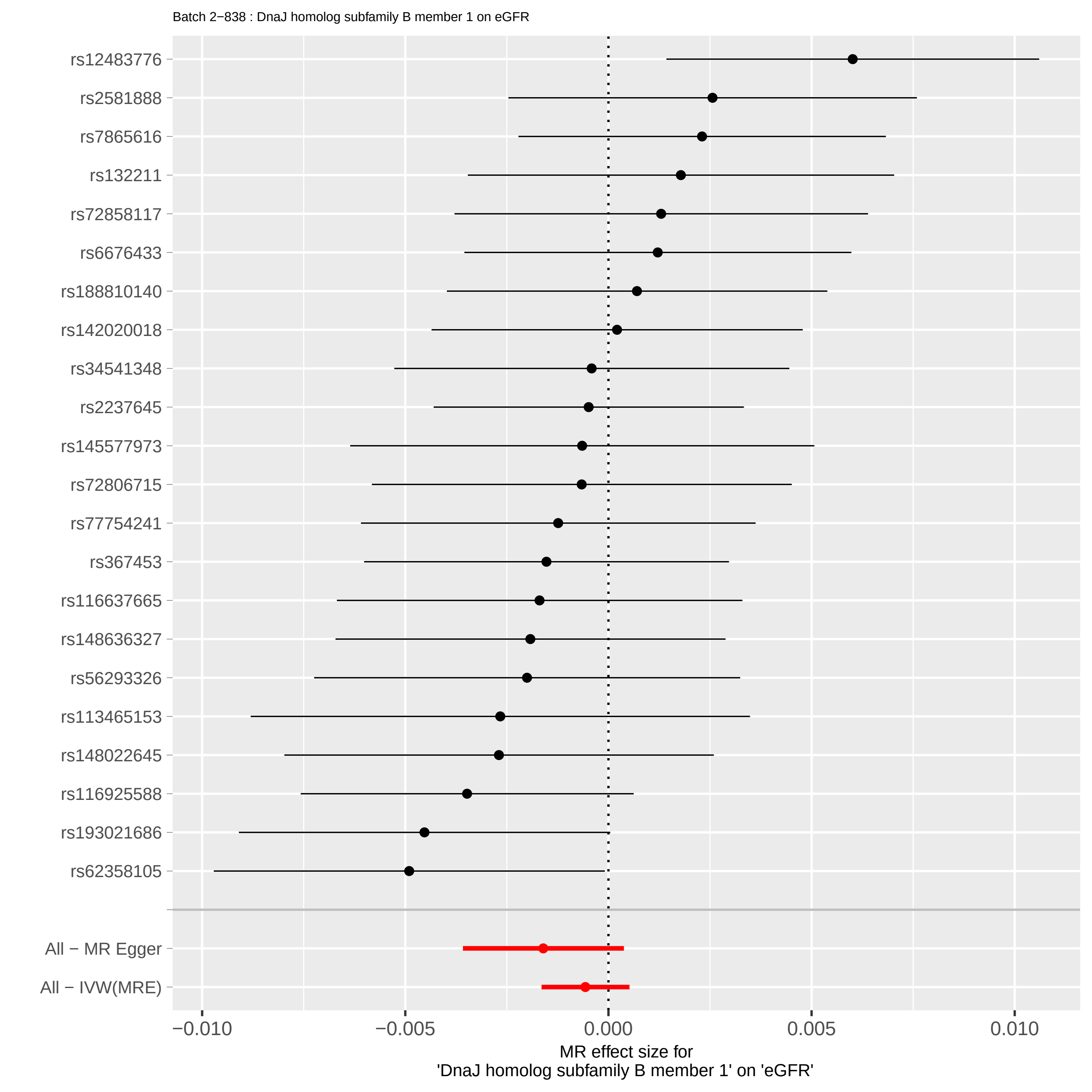

Batch 2−838 : DnaJ homolog subfamily B member 1 on eGFR
rs12483776
rs2581888
rs7865616
rs132211
rs72858117
rs6676433
rs188810140
rs142020018
rs34541348
rs2237645
rs145577973
rs72806715
rs77754241
rs367453
rs116637665
rs148636327
rs56293326
rs113465153
rs148022645
rs116925588
rs193021686
rs62358105
All − MR Egger
All − IVW(MRE)
−0.010
−0.005
0.000
0.005
0.010
MR effect size for
'DnaJ homolog subfamily B member 1' on 'eGFR'

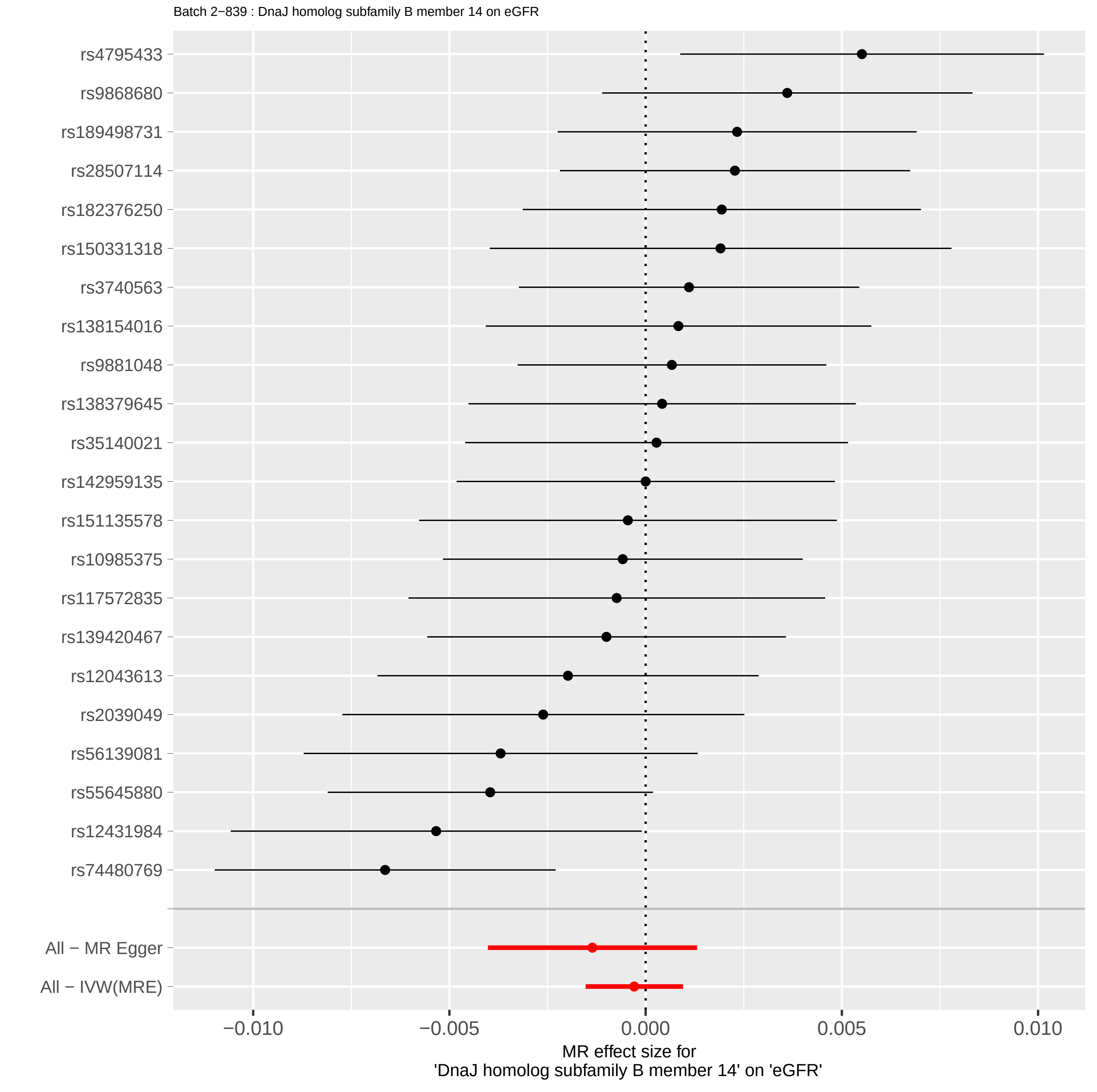

Batch 2−839 : DnaJ homolog subfamily B member 14 on eGFR
rs4795433
rs9868680
rs189498731
rs28507114
rs182376250
rs150331318
rs3740563
rs138154016
rs9881048
rs138379645
rs35140021
rs142959135
rs151135578
rs10985375
rs117572835
rs139420467
rs12043613
rs2039049
rs56139081
rs55645880
rs12431984
rs74480769
All − MR Egger
All − IVW(MRE)
−0.010
−0.005
0.000
0.005
0.010
MR effect size for
'DnaJ homolog subfamily B member 14' on 'eGFR'

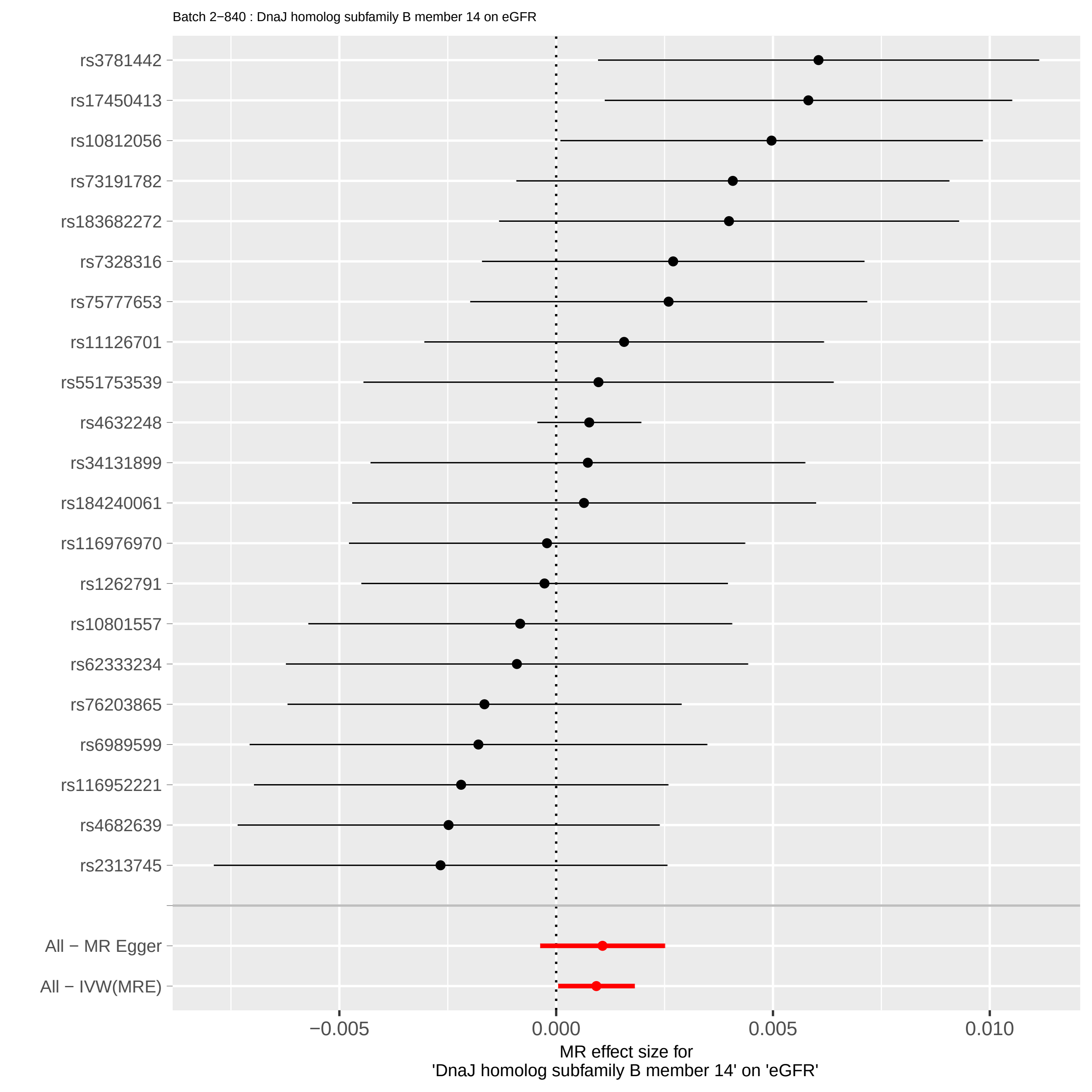

Batch 2−840 : DnaJ homolog subfamily B member 14 on eGFR
rs3781442
rs17450413
rs10812056
rs73191782
rs183682272
rs7328316
rs75777653
rs11126701
rs551753539
rs4632248
rs34131899
rs184240061
rs116976970
rs1262791
rs10801557
rs62333234
rs76203865
rs6989599
rs116952221
rs4682639
rs2313745
All − MR Egger
All − IVW(MRE)
−0.005
0.000
0.005
0.010
MR effect size for
'DnaJ homolog subfamily B member 14' on 'eGFR'

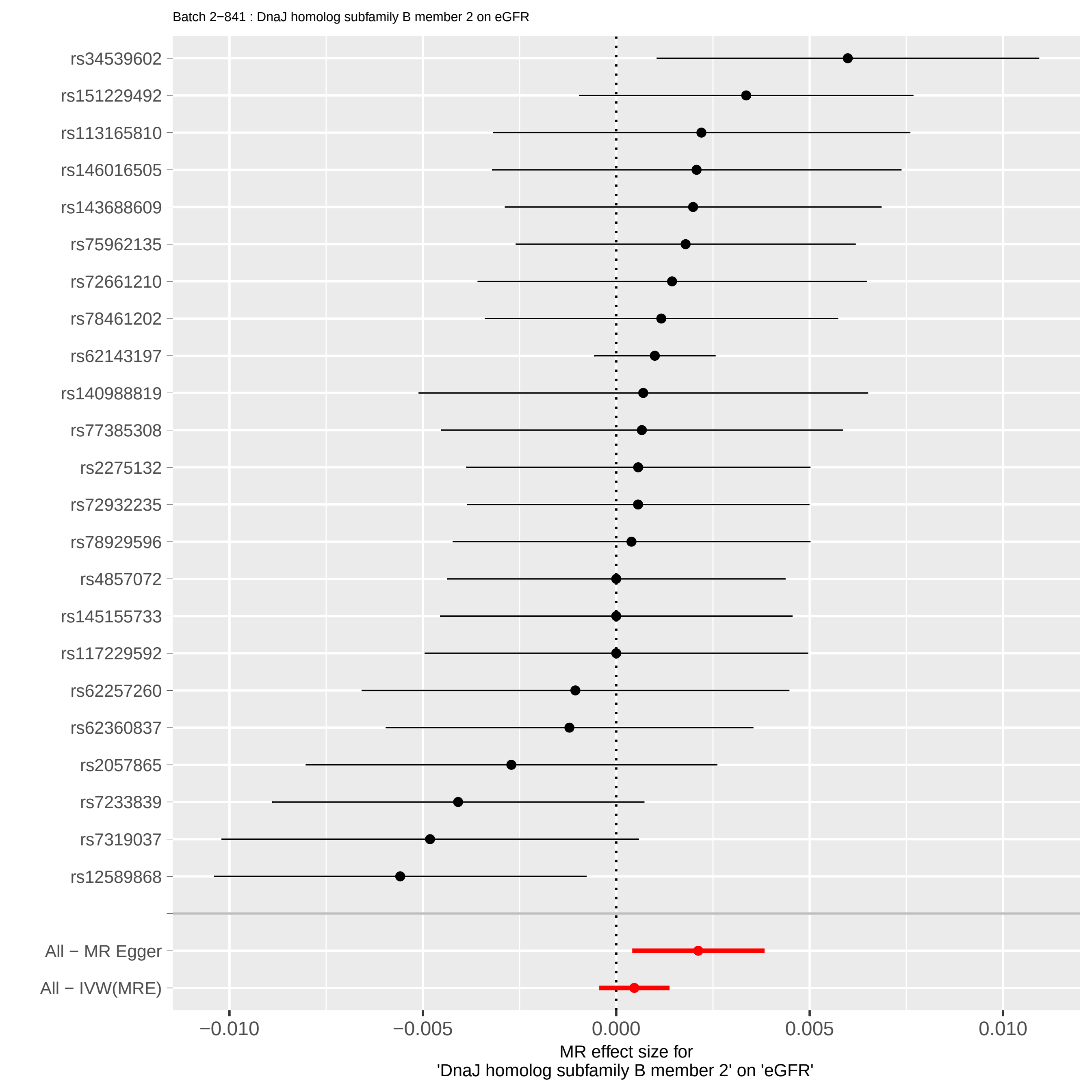

Batch 2−841 : DnaJ homolog subfamily B member 2 on eGFR
rs34539602
rs151229492
rs113165810
rs146016505
rs143688609
rs75962135
rs72661210
rs78461202
rs62143197
rs140988819
rs77385308
rs2275132
rs72932235
rs78929596
rs4857072
rs145155733
rs117229592
rs62257260
rs62360837
rs2057865
rs7233839
rs7319037
rs12589868
All − MR Egger
All − IVW(MRE)
−0.010
−0.005
0.000
0.005
0.010
MR effect size for
'DnaJ homolog subfamily B member 2' on 'eGFR'

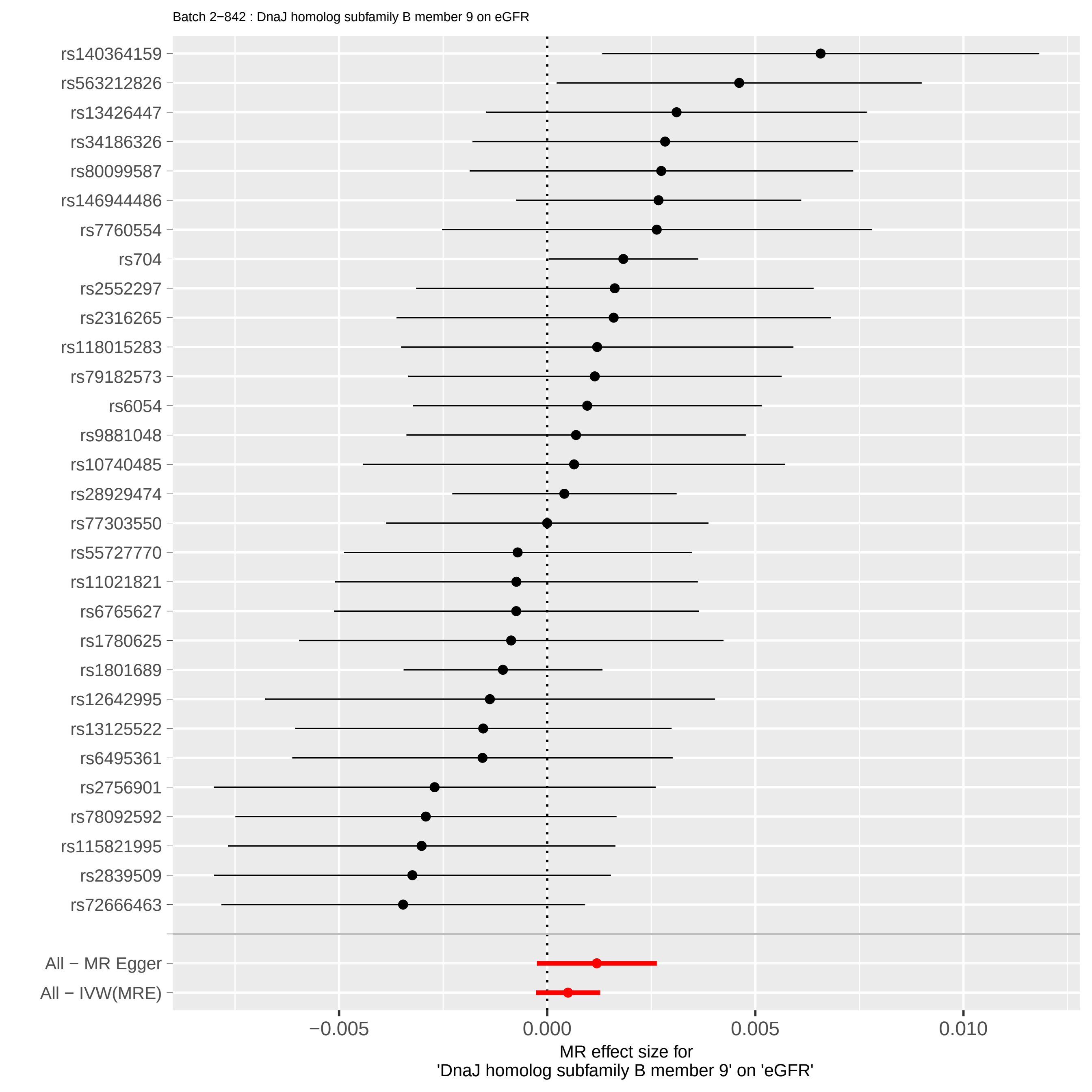

Batch 2−842 : DnaJ homolog subfamily B member 9 on eGFR
rs140364159
rs563212826
rs13426447
rs34186326
rs80099587
rs146944486
rs7760554
rs704
rs2552297
rs2316265
rs118015283
rs79182573
rs6054
rs9881048
rs10740485
rs28929474
rs77303550
rs55727770
rs11021821
rs6765627
rs1780625
rs1801689
rs12642995
rs13125522
rs6495361
rs2756901
rs78092592
rs115821995
rs2839509
rs72666463
All − MR Egger
All − IVW(MRE)
−0.005
0.000
0.005
0.010
MR effect size for
'DnaJ homolog subfamily B member 9' on 'eGFR'

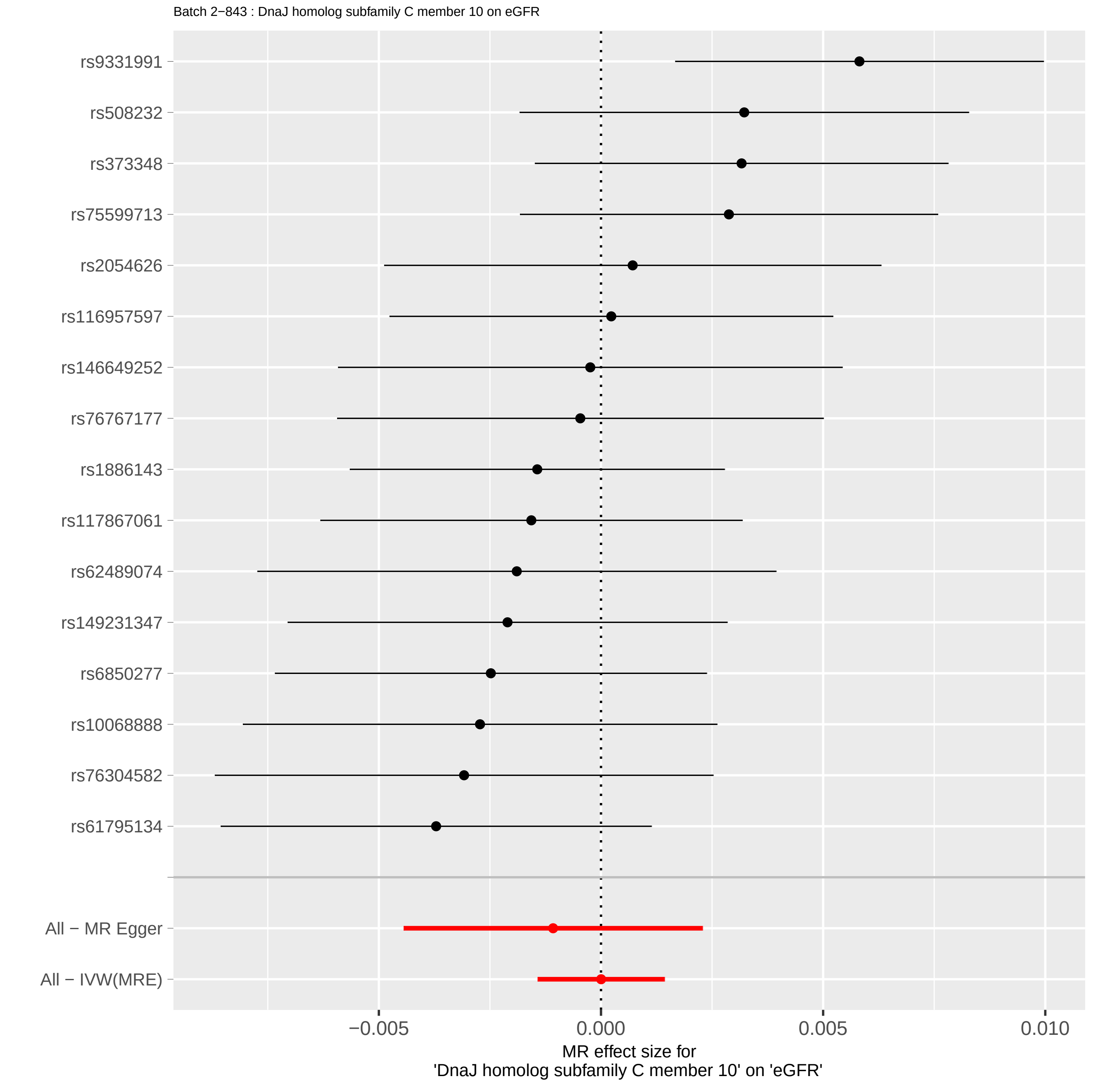

Batch 2−843 : DnaJ homolog subfamily C member 10 on eGFR
rs9331991
rs508232
rs373348
rs75599713
rs2054626
rs116957597
rs146649252
rs76767177
rs1886143
rs117867061
rs62489074
rs149231347
rs6850277
rs10068888
rs76304582
rs61795134
All − MR Egger
All − IVW(MRE)
−0.005
0.000
0.005
0.010
MR effect size for
'DnaJ homolog subfamily C member 10' on 'eGFR'

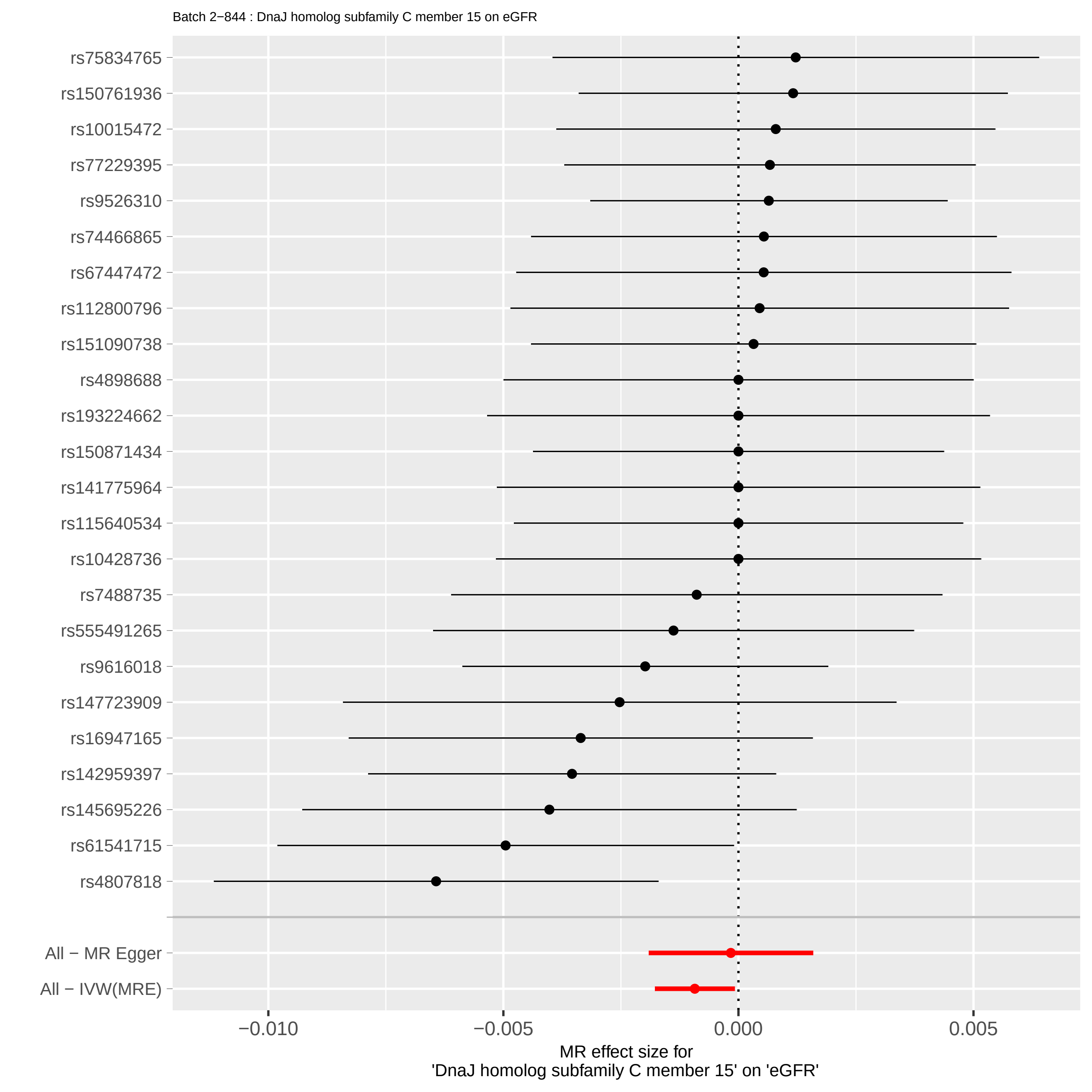

Batch 2−844 : DnaJ homolog subfamily C member 15 on eGFR
rs75834765
rs150761936
rs10015472
rs77229395
rs9526310
rs74466865
rs67447472
rs112800796
rs151090738
rs4898688
rs193224662
rs150871434
rs141775964
rs115640534
rs10428736
rs7488735
rs555491265
rs9616018
rs147723909
rs16947165
rs142959397
rs145695226
rs61541715
rs4807818
All − MR Egger
All − IVW(MRE)
−0.010
−0.005
0.000
0.005
MR effect size for
'DnaJ homolog subfamily C member 15' on 'eGFR'

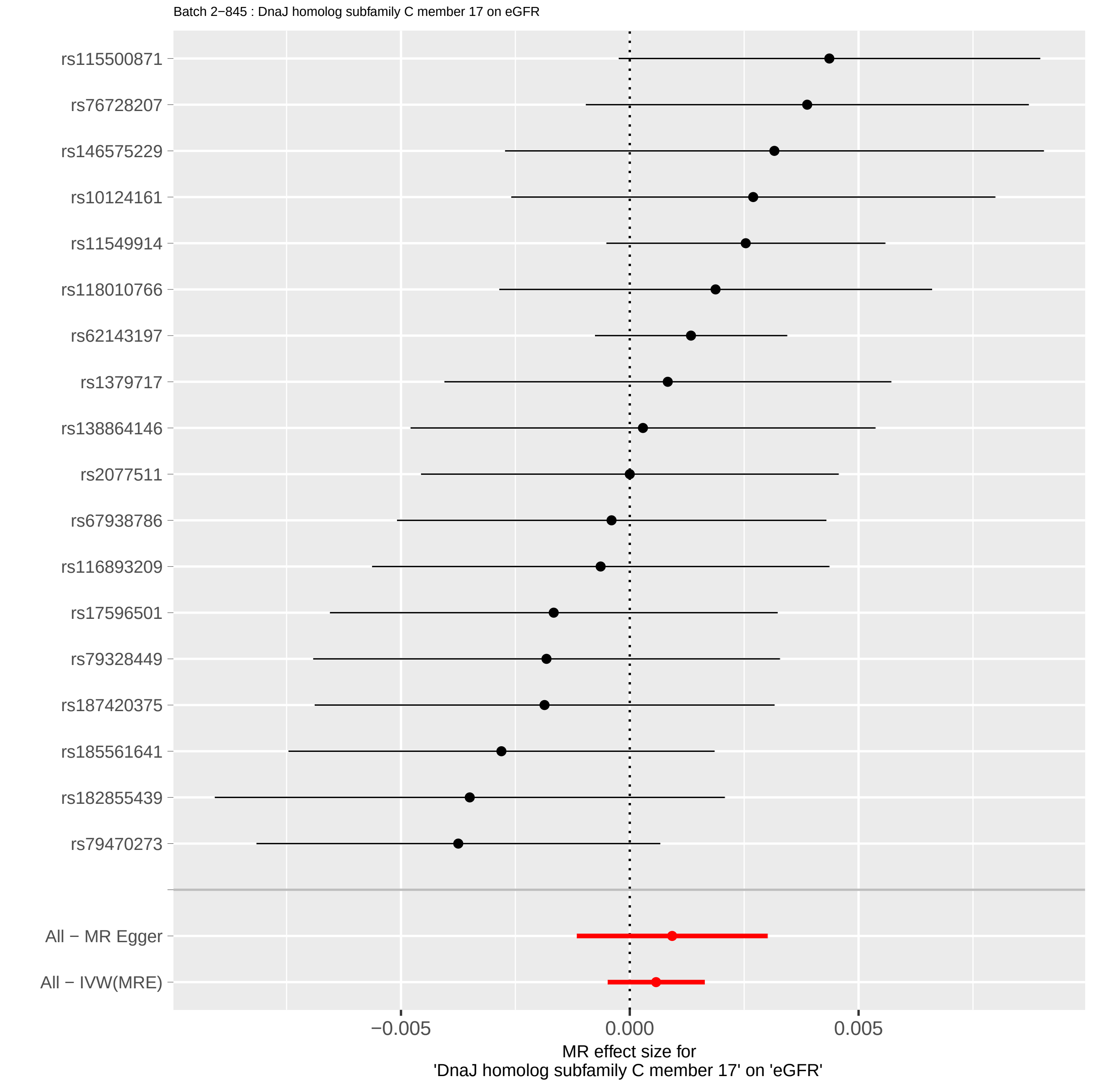
Batch 2−845 : DnaJ homolog subfamily C member 17 on eGFR
rs115500871
rs76728207
rs146575229
rs10124161
rs11549914
rs118010766
rs62143197
rs1379717
rs138864146
rs2077511
rs67938786
rs116893209
rs17596501
rs79328449
rs187420375
rs185561641
rs182855439
rs79470273
All − MR Egger
All − IVW(MRE)
−0.005
0.000
0.005
MR effect size for
'DnaJ homolog subfamily C member 17' on 'eGFR'

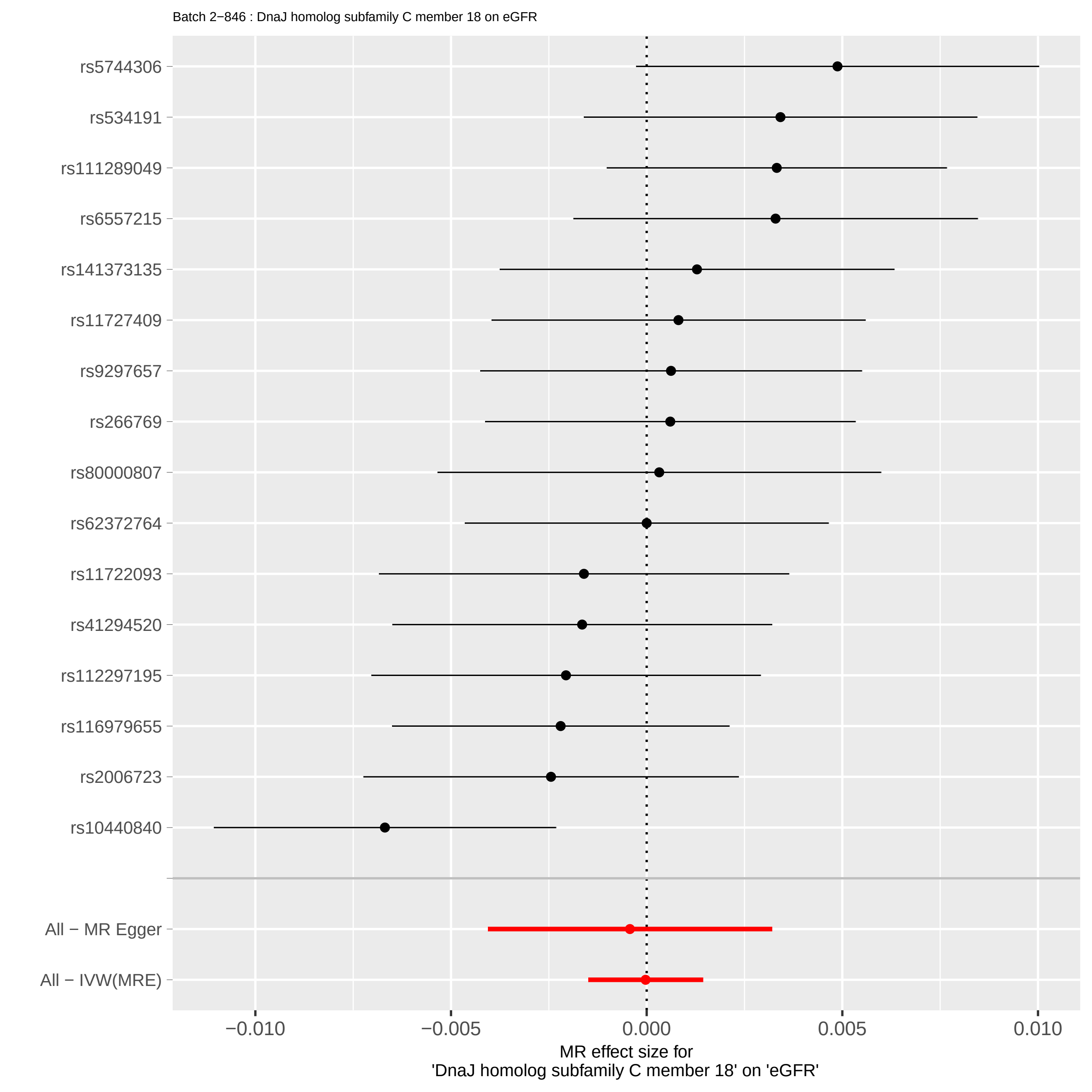

Batch 2−846 : DnaJ homolog subfamily C member 18 on eGFR
rs5744306
rs534191
rs111289049
rs6557215
rs141373135
rs11727409
rs9297657
rs266769
rs80000807
rs62372764
rs11722093
rs41294520
rs112297195
rs116979655
rs2006723
rs10440840
All − MR Egger
All − IVW(MRE)
−0.010
−0.005
0.000
0.005
0.010
MR effect size for
'DnaJ homolog subfamily C member 18' on 'eGFR'

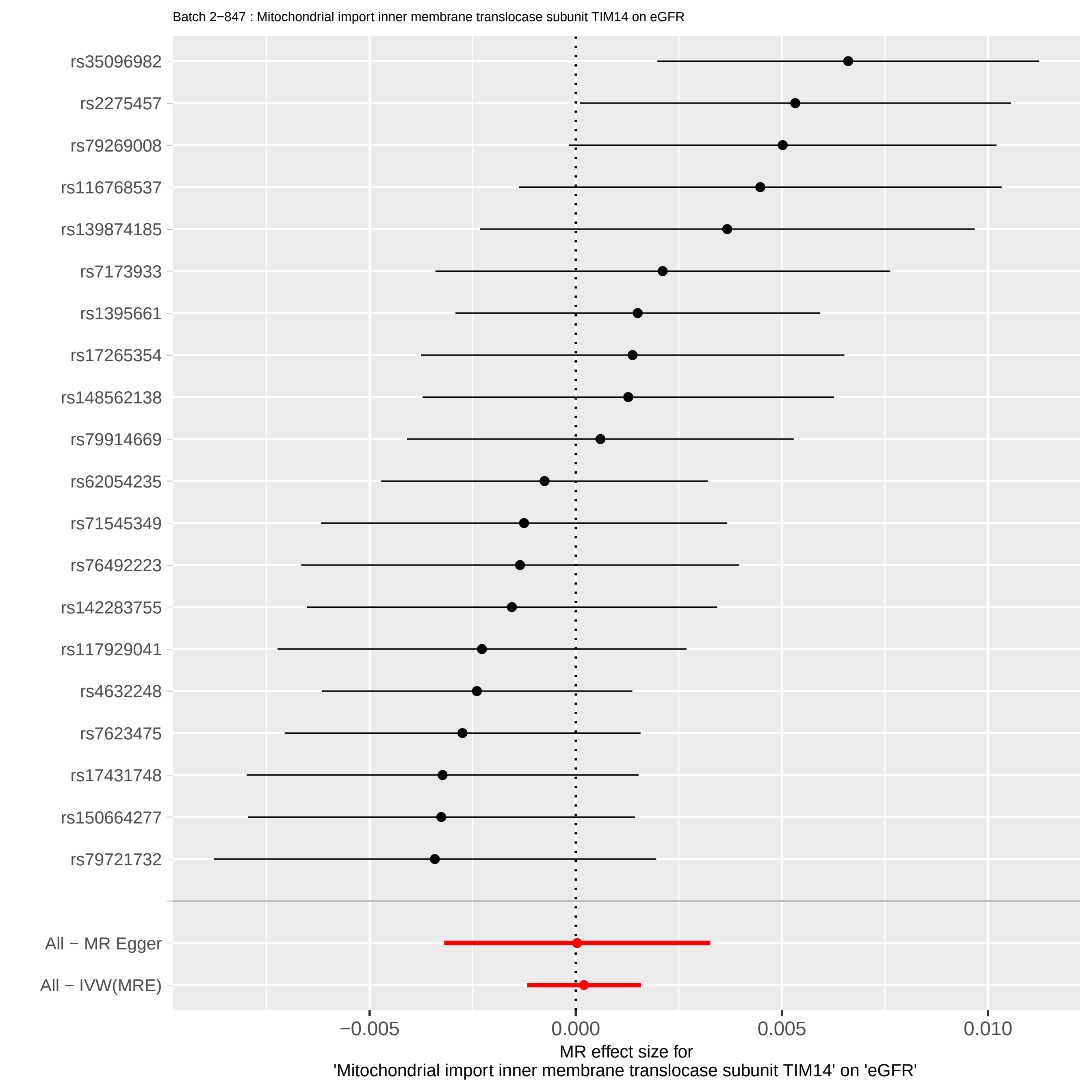
Batch 2−847 : Mitochondrial import inner membrane translocase subunit TIM14 on eGFR
rs35096982
rs2275457
rs79269008
rs116768537
rs139874185
rs7173933
rs1395661
rs17265354
rs148562138
rs79914669
rs62054235
rs71545349
rs76492223
rs142283755
rs117929041
rs4632248
rs7623475
rs17431748
rs150664277
rs79721732
All − MR Egger
All − IVW(MRE)
−0.005
0.000
0.005
0.010
MR effect size for
'Mitochondrial import inner membrane translocase subunit TIM14' on 'eGFR'

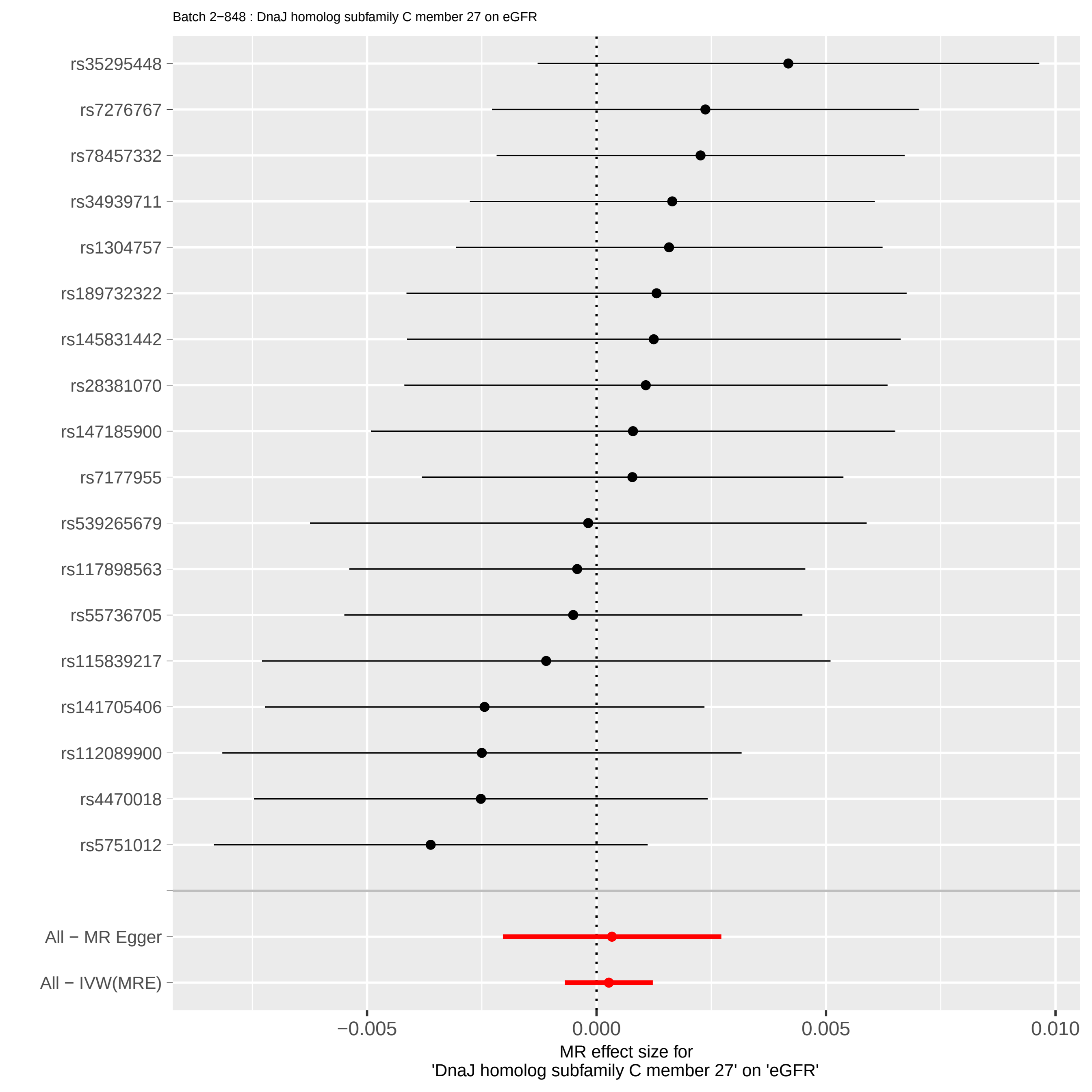

Batch 2−848 : DnaJ homolog subfamily C member 27 on eGFR
rs35295448
rs7276767
rs78457332
rs34939711
rs1304757
rs189732322
rs145831442
rs28381070
rs147185900
rs7177955
rs539265679
rs117898563
rs55736705
rs115839217
rs141705406
rs112089900
rs4470018
rs5751012
All − MR Egger
All − IVW(MRE)
−0.005
0.000
0.005
0.010
MR effect size for
'DnaJ homolog subfamily C member 27' on 'eGFR'

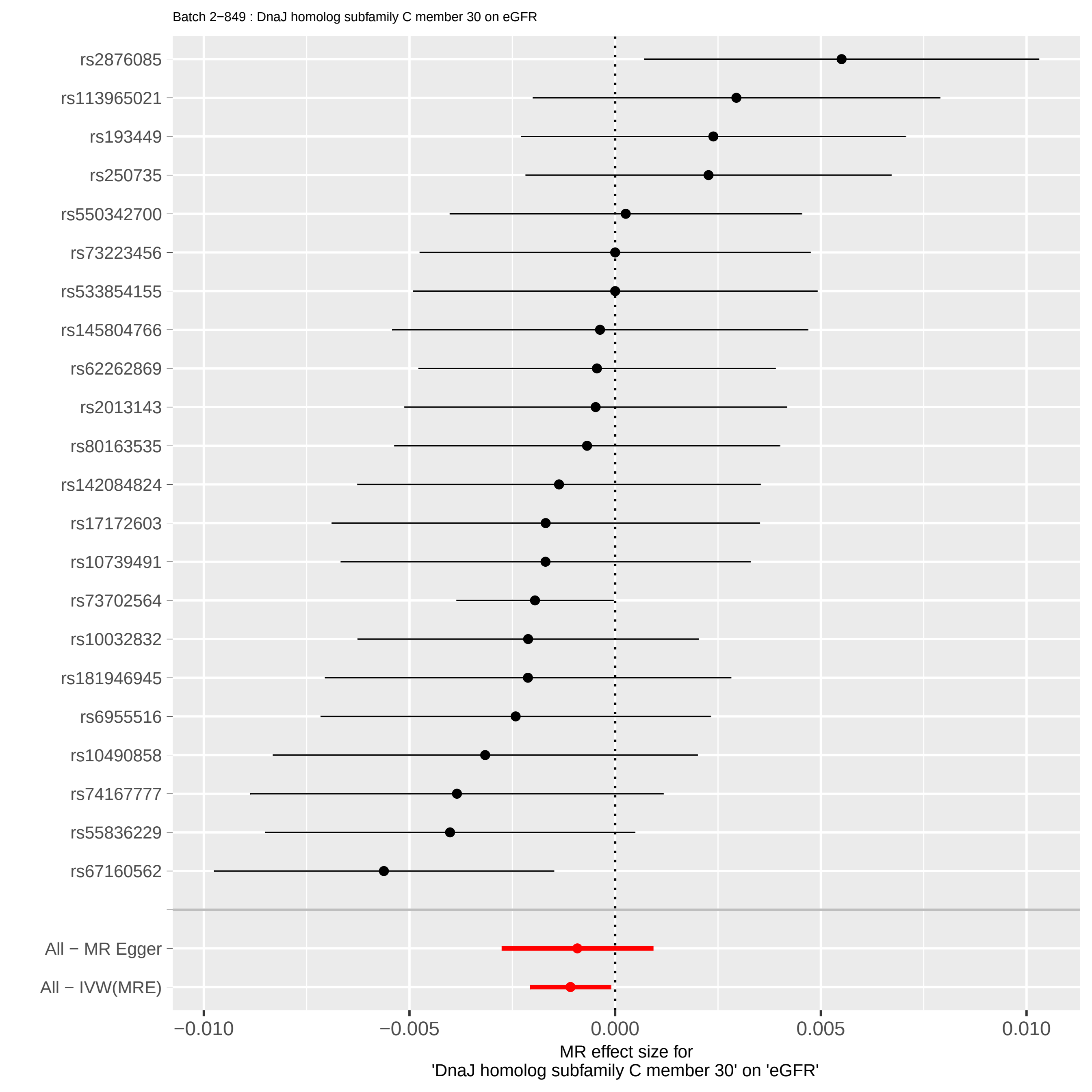

Batch 2−849 : DnaJ homolog subfamily C member 30 on eGFR
rs2876085
rs113965021
rs193449
rs250735
rs550342700
rs73223456
rs533854155
rs145804766
rs62262869
rs2013143
rs80163535
rs142084824
rs17172603
rs10739491
rs73702564
rs10032832
rs181946945
rs6955516
rs10490858
rs74167777
rs55836229
rs67160562
All − MR Egger
All − IVW(MRE)
−0.010
−0.005
0.000
0.005
0.010
MR effect size for
'DnaJ homolog subfamily C member 30' on 'eGFR'

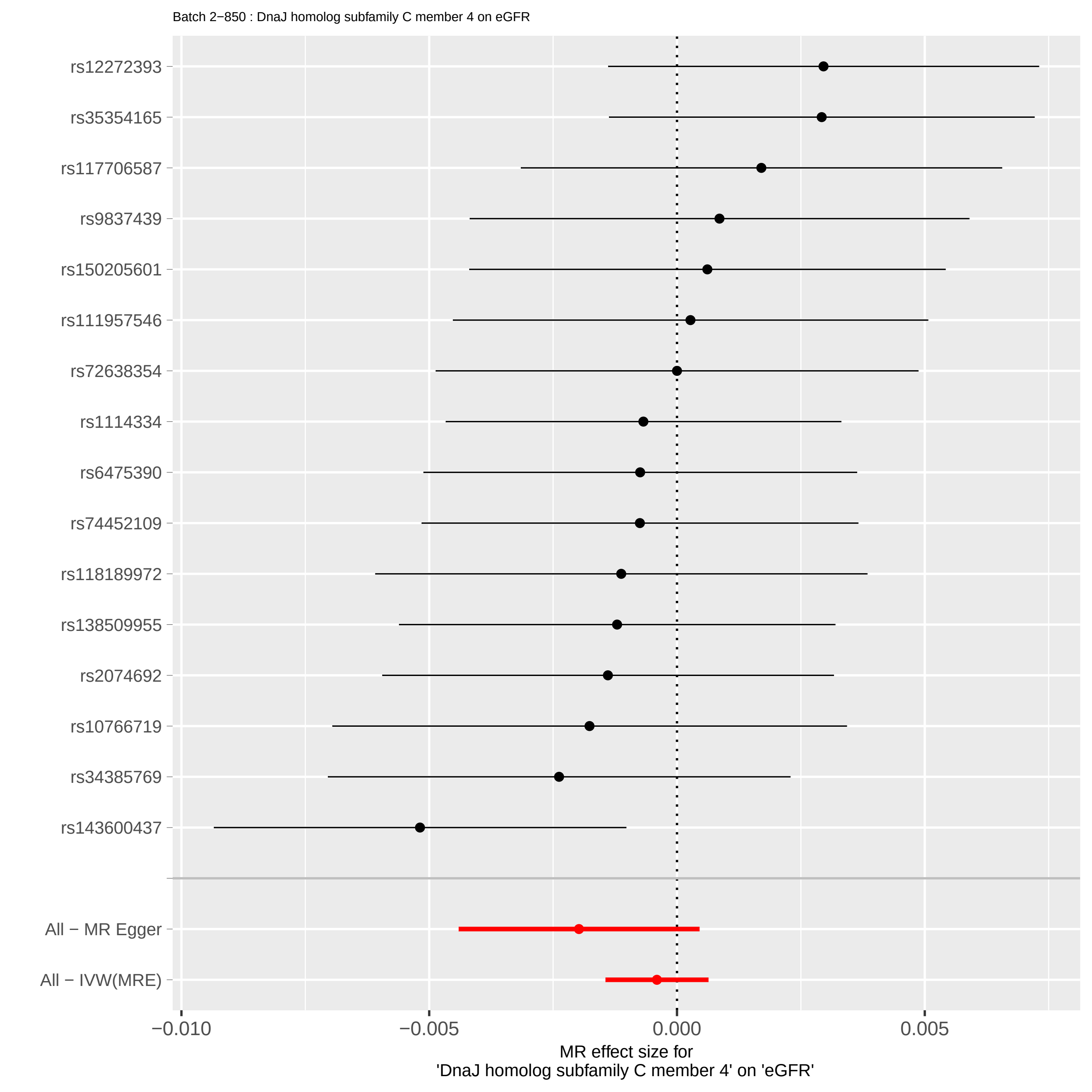

Batch 2−850 : DnaJ homolog subfamily C member 4 on eGFR
rs12272393
rs35354165
rs117706587
rs9837439
rs150205601
rs111957546
rs72638354
rs1114334
rs6475390
rs74452109
rs118189972
rs138509955
rs2074692
rs10766719
rs34385769
rs143600437
All − MR Egger
All − IVW(MRE)
−0.010
−0.005
0.000
0.005
MR effect size for
'DnaJ homolog subfamily C member 4' on 'eGFR'

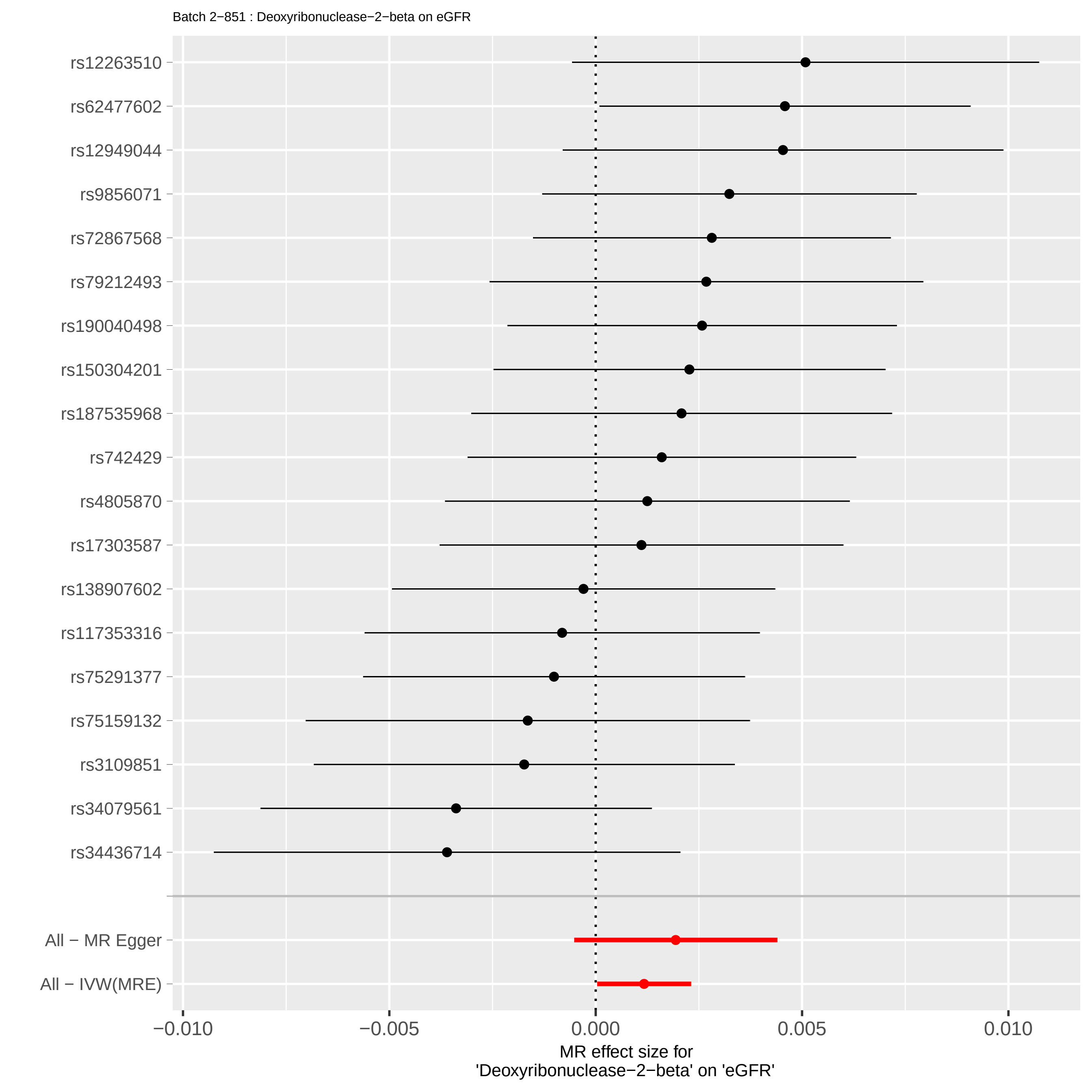

Batch 2−851 : Deoxyribonuclease−2−beta on eGFR
rs12263510
rs62477602
rs12949044
rs9856071
rs72867568
rs79212493
rs190040498
rs150304201
rs187535968
rs742429
rs4805870
rs17303587
rs138907602
rs117353316
rs75291377
rs75159132
rs3109851
rs34079561
rs34436714
All − MR Egger
All − IVW(MRE)
−0.010
−0.005
0.000
0.005
0.010
MR effect size for
'Deoxyribonuclease−2−beta' on 'eGFR'

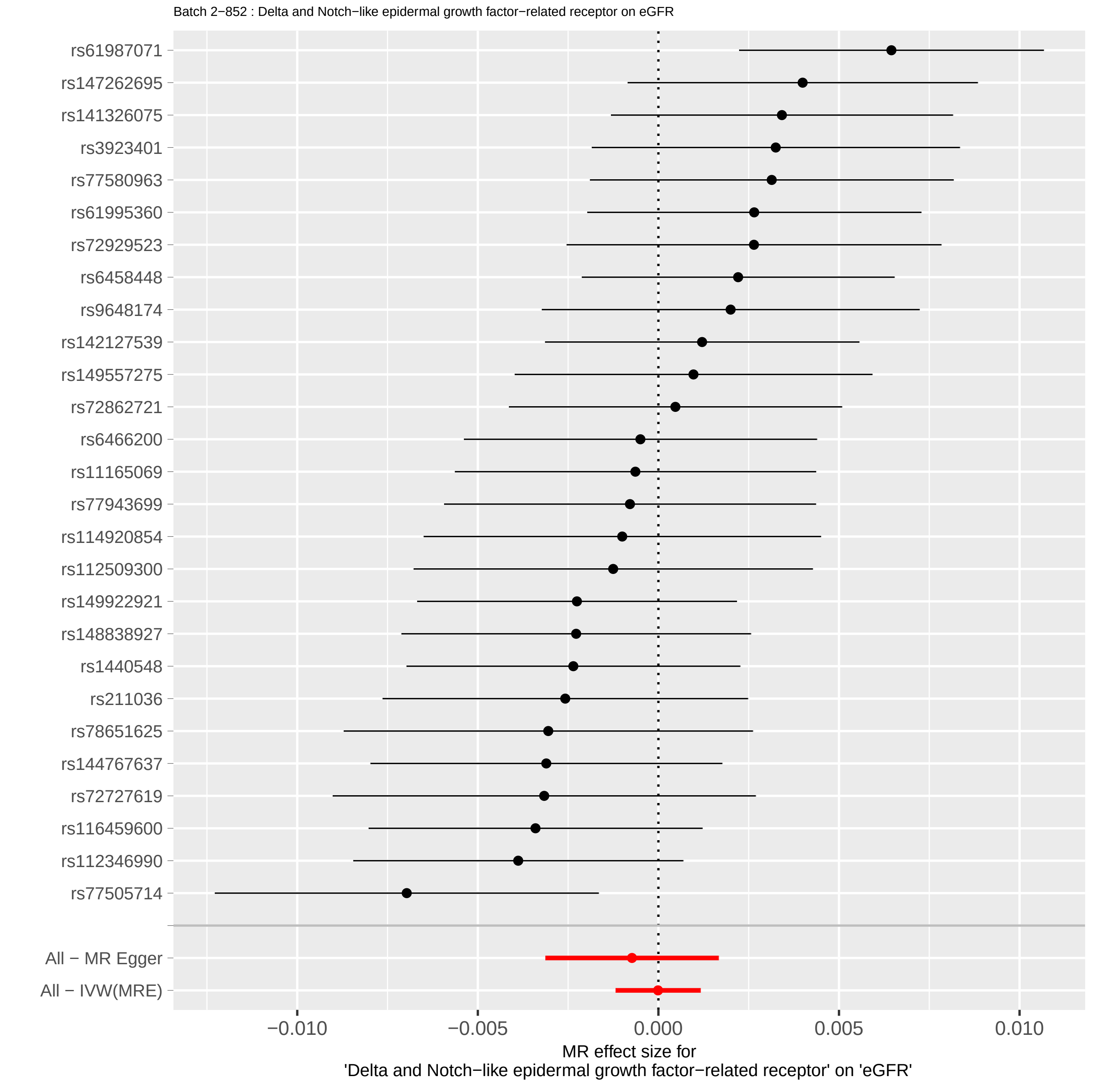

Batch 2−852 : Delta and Notch−like epidermal growth factor−related receptor on eGFR
rs61987071
rs147262695
rs141326075
rs3923401
rs77580963
rs61995360
rs72929523
rs6458448
rs9648174
rs142127539
rs149557275
rs72862721
rs6466200
rs11165069
rs77943699
rs114920854
rs112509300
rs149922921
rs148838927
rs1440548
rs211036
rs78651625
rs144767637
rs72727619
rs116459600
rs112346990
rs77505714
All − MR Egger
All − IVW(MRE)
−0.010
−0.005
0.000
0.005
0.010
MR effect size for
'Delta and Notch−like epidermal growth factor−related receptor' on 'eGFR'

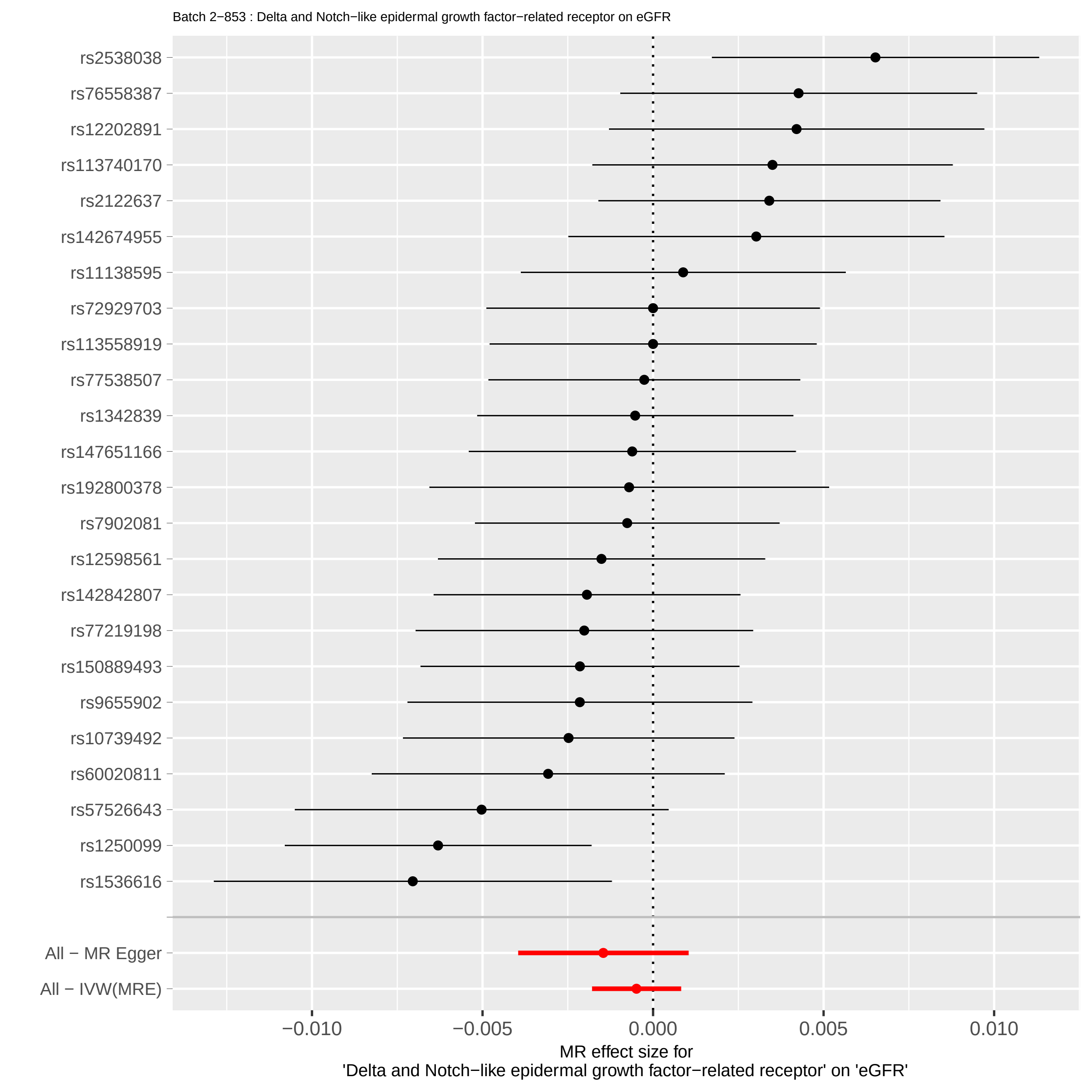

Batch 2−853 : Delta and Notch−like epidermal growth factor−related receptor on eGFR
rs2538038
rs76558387
rs12202891
rs113740170
rs2122637
rs142674955
rs11138595
rs72929703
rs113558919
rs77538507
rs1342839
rs147651166
rs192800378
rs7902081
rs12598561
rs142842807
rs77219198
rs150889493
rs9655902
rs10739492
rs60020811
rs57526643
rs1250099
rs1536616
All − MR Egger
All − IVW(MRE)
−0.010
−0.005
0.000
0.005
0.010
MR effect size for
'Delta and Notch−like epidermal growth factor−related receptor' on 'eGFR'

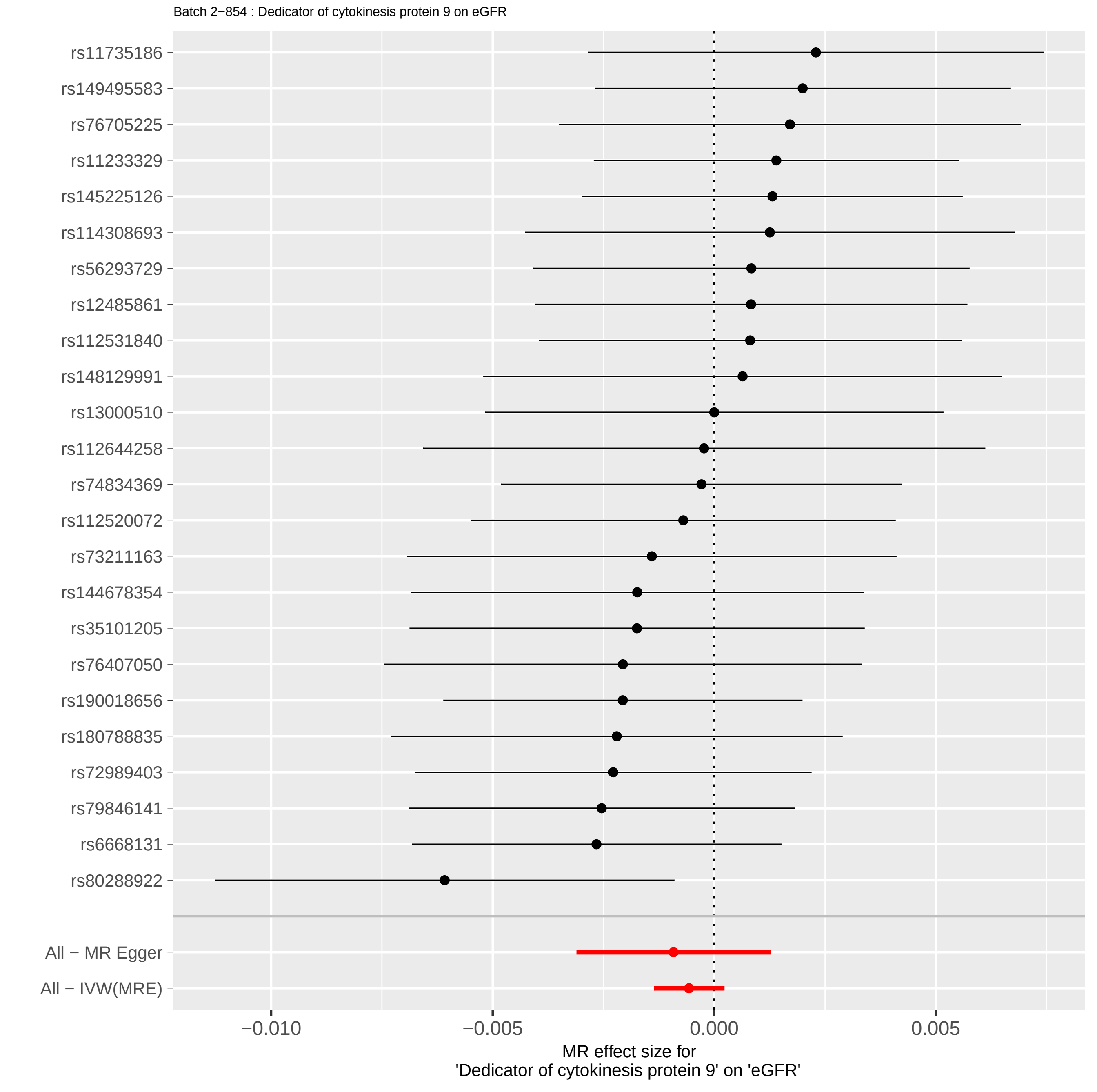

Batch 2−854 : Dedicator of cytokinesis protein 9 on eGFR
rs11735186
rs149495583
rs76705225
rs11233329
rs145225126
rs114308693
rs56293729
rs12485861
rs112531840
rs148129991
rs13000510
rs112644258
rs74834369
rs112520072
rs73211163
rs144678354
rs35101205
rs76407050
rs190018656
rs180788835
rs72989403
rs79846141
rs6668131
rs80288922
All − MR Egger
All − IVW(MRE)
−0.010
−0.005
0.000
0.005
MR effect size for
'Dedicator of cytokinesis protein 9' on 'eGFR'

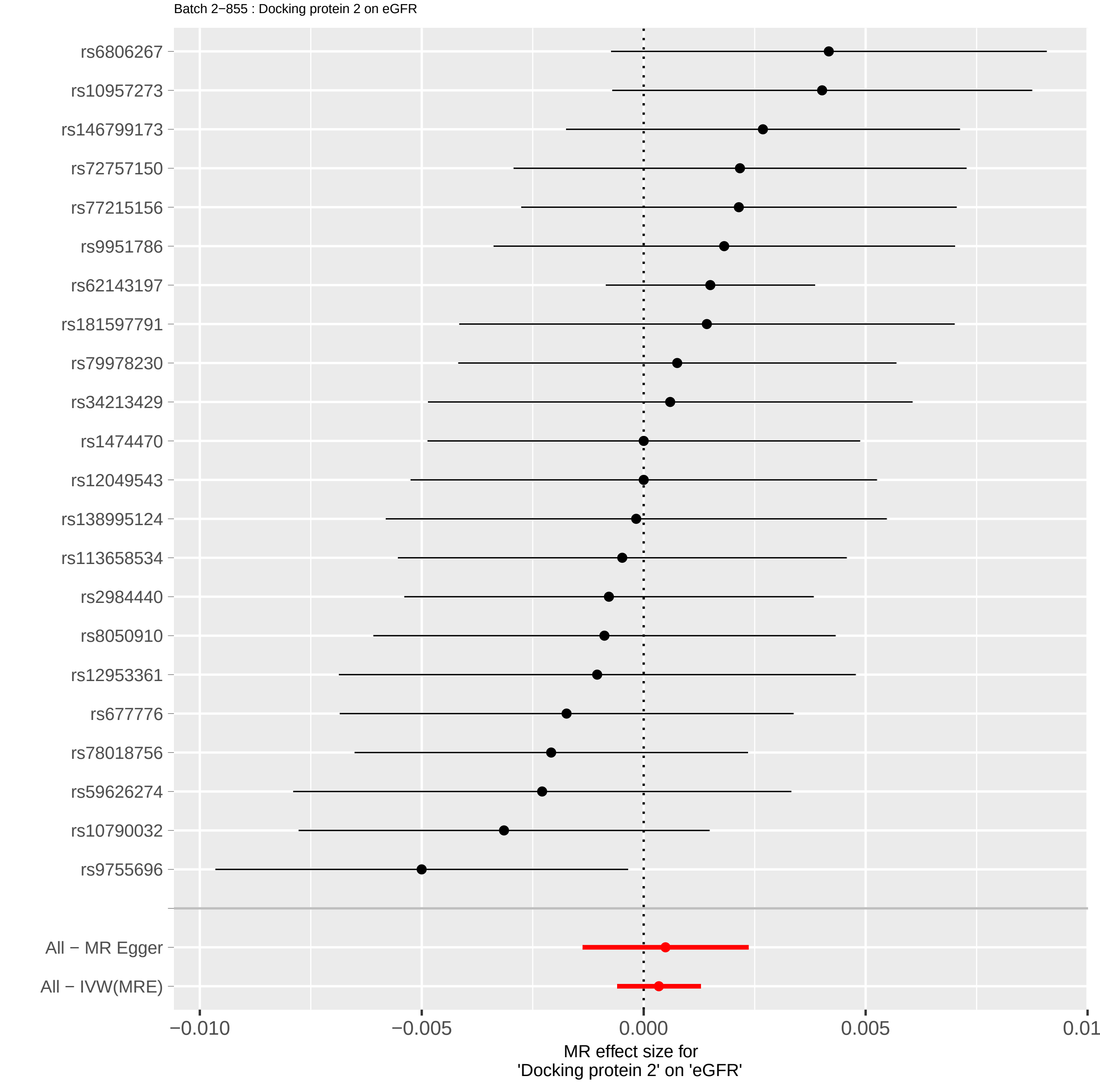

Batch 2−855 : Docking protein 2 on eGFR
rs6806267
rs10957273
rs146799173
rs72757150
rs77215156
rs9951786
rs62143197
rs181597791
rs79978230
rs34213429
rs1474470
rs12049543
rs138995124
rs113658534
rs2984440
rs8050910
rs12953361
rs677776
rs78018756
rs59626274
rs10790032
rs9755696
All − MR Egger
All − IVW(MRE)
−0.010
−0.005
0.000
0.005
0.01
MR effect size for
'Docking protein 2' on 'eGFR'

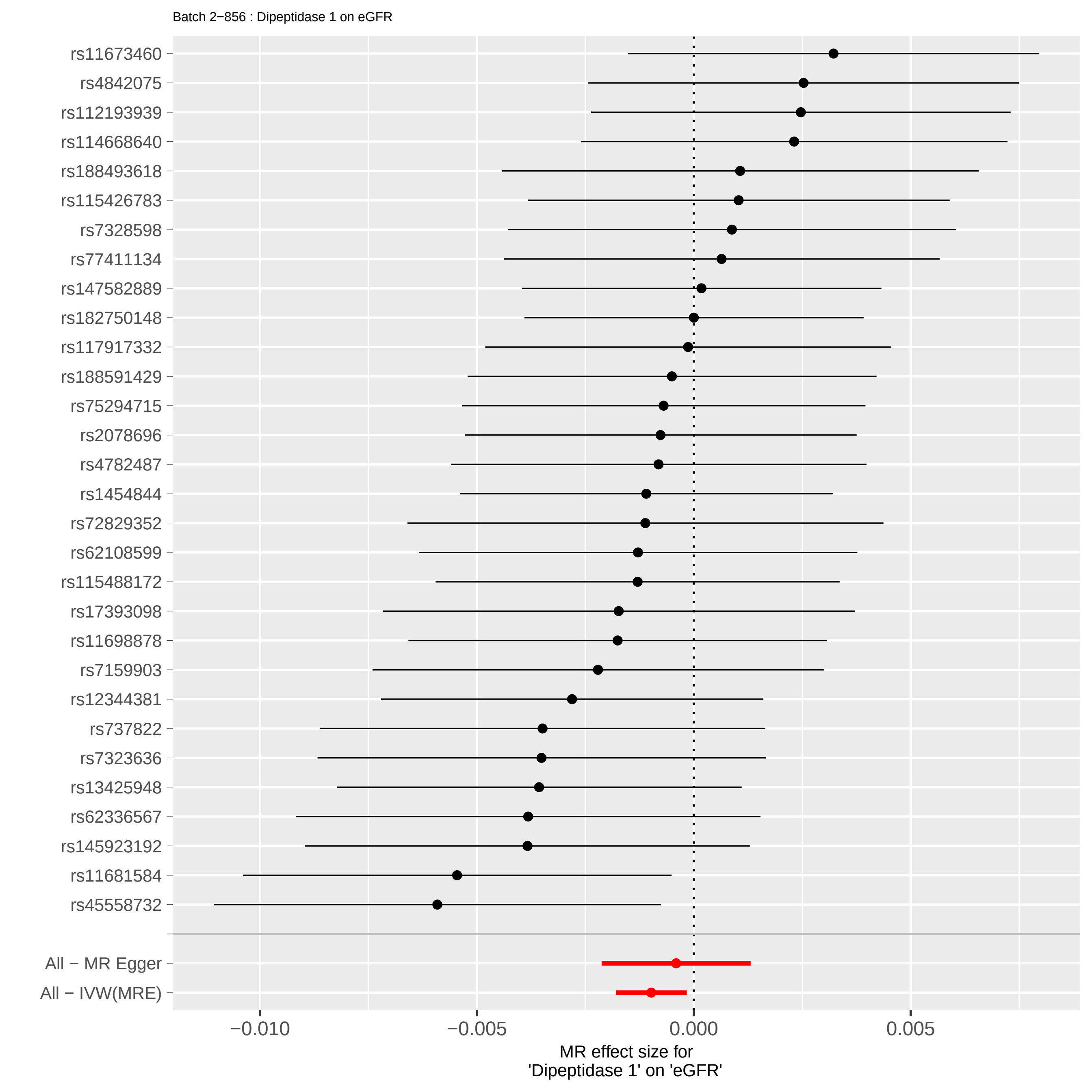

Batch 2−856 : Dipeptidase 1 on eGFR
rs11673460
rs4842075
rs112193939
rs114668640
rs188493618
rs115426783
rs7328598
rs77411134
rs147582889
rs182750148
rs117917332
rs188591429
rs75294715
rs2078696
rs4782487
rs1454844
rs72829352
rs62108599
rs115488172
rs17393098
rs11698878
rs7159903
rs12344381
rs737822
rs7323636
rs13425948
rs62336567
rs145923192
rs11681584
rs45558732
All − MR Egger
All − IVW(MRE)
−0.010
−0.005
0.000
0.005
MR effect size for
'Dipeptidase 1' on 'eGFR'

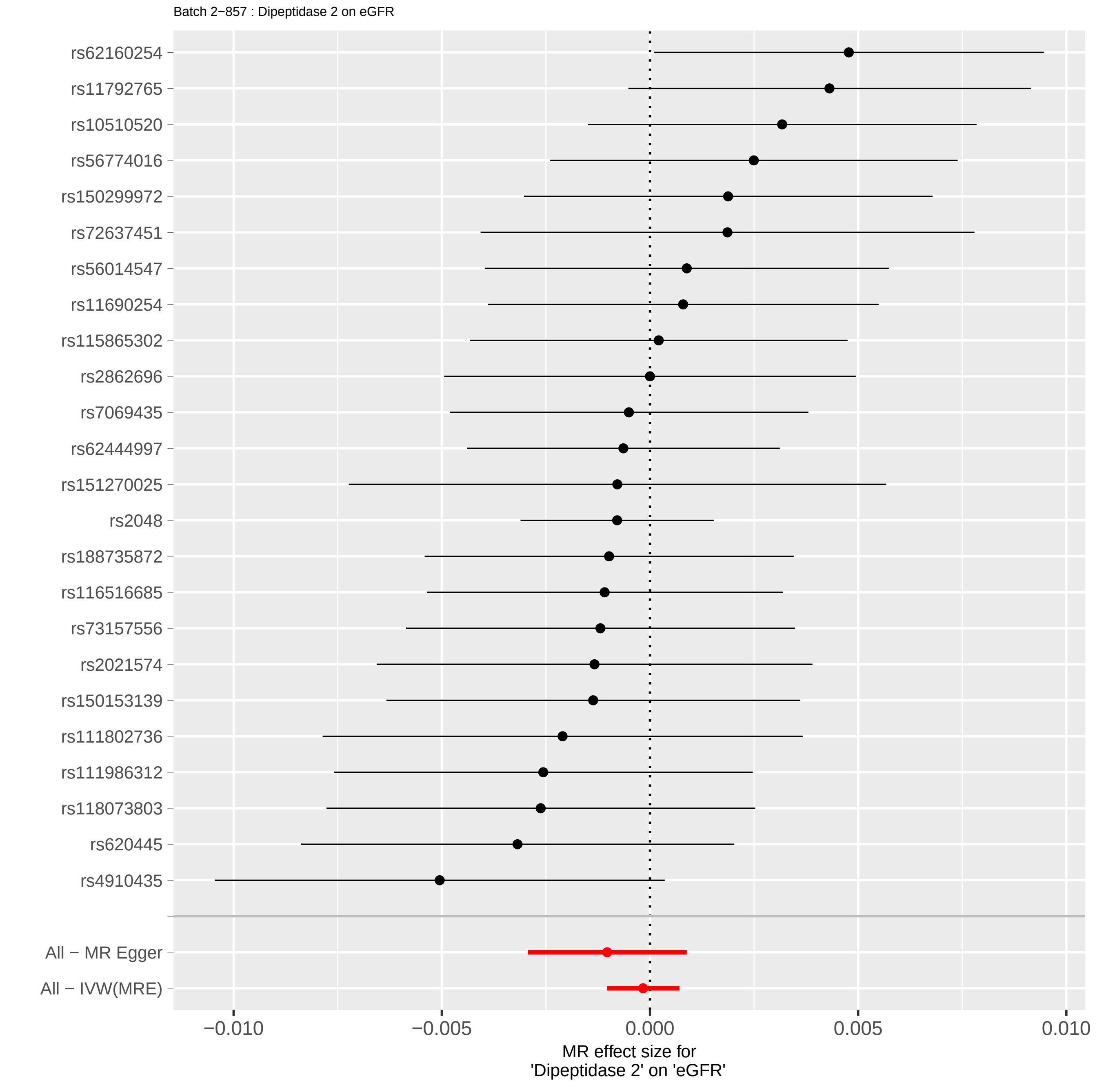

Batch 2−857 : Dipeptidase 2 on eGFR
rs62160254
rs11792765
rs10510520
rs56774016
rs150299972
rs72637451
rs56014547
rs11690254
rs115865302
rs2862696
rs7069435
rs62444997
rs151270025
rs2048
rs188735872
rs116516685
rs73157556
rs2021574
rs150153139
rs111802736
rs111986312
rs118073803
rs620445
rs4910435
All − MR Egger
All − IVW(MRE)
−0.010
−0.005
0.000
0.005
0.010
MR effect size for
'Dipeptidase 2' on 'eGFR'

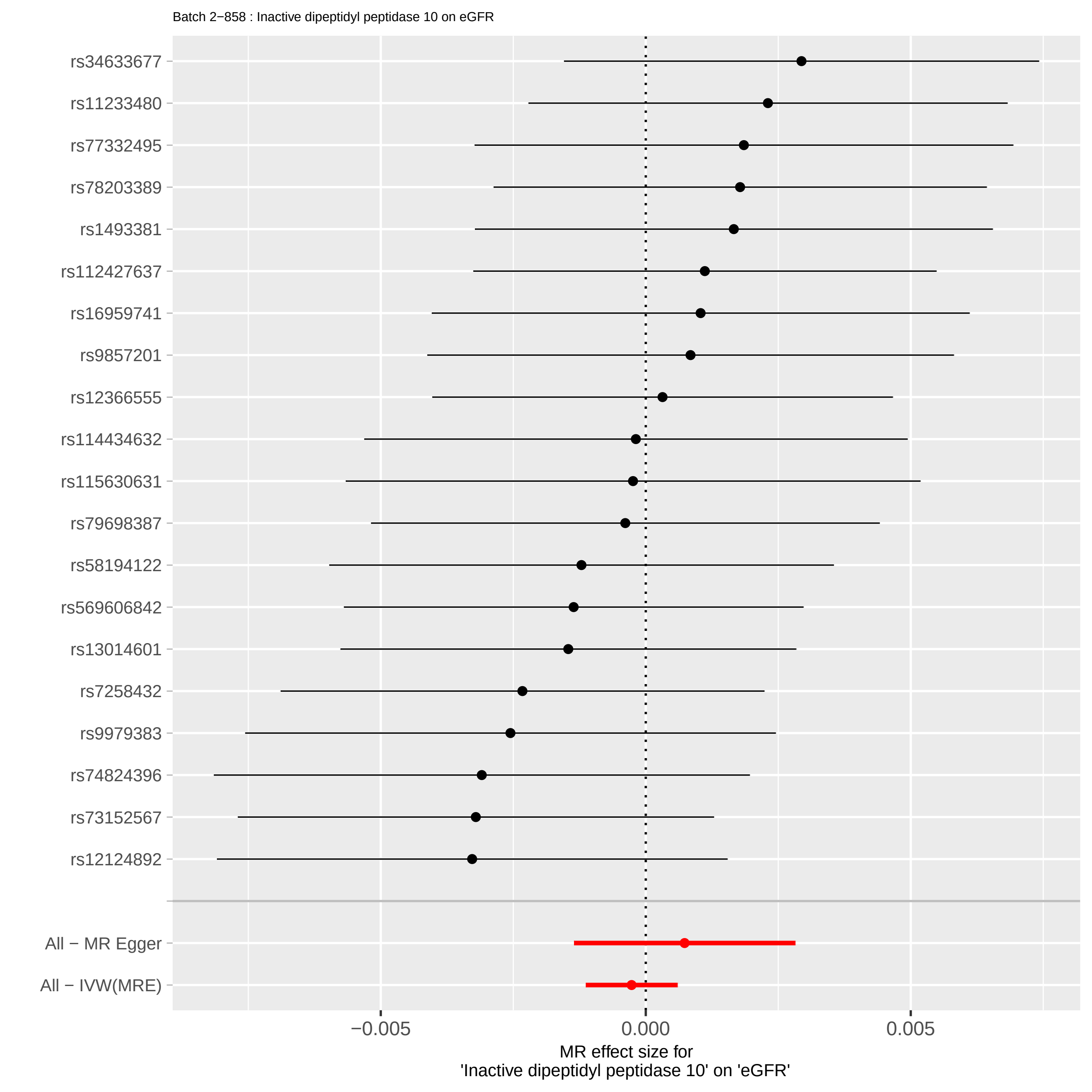

Batch 2−858 : Inactive dipeptidyl peptidase 10 on eGFR
rs34633677
rs11233480
rs77332495
rs78203389
rs1493381
rs112427637
rs16959741
rs9857201
rs12366555
rs114434632
rs115630631
rs79698387
rs58194122
rs569606842
rs13014601
rs7258432
rs9979383
rs74824396
rs73152567
rs12124892
All − MR Egger
All − IVW(MRE)
−0.005
0.000
0.005
MR effect size for
'Inactive dipeptidyl peptidase 10' on 'eGFR'

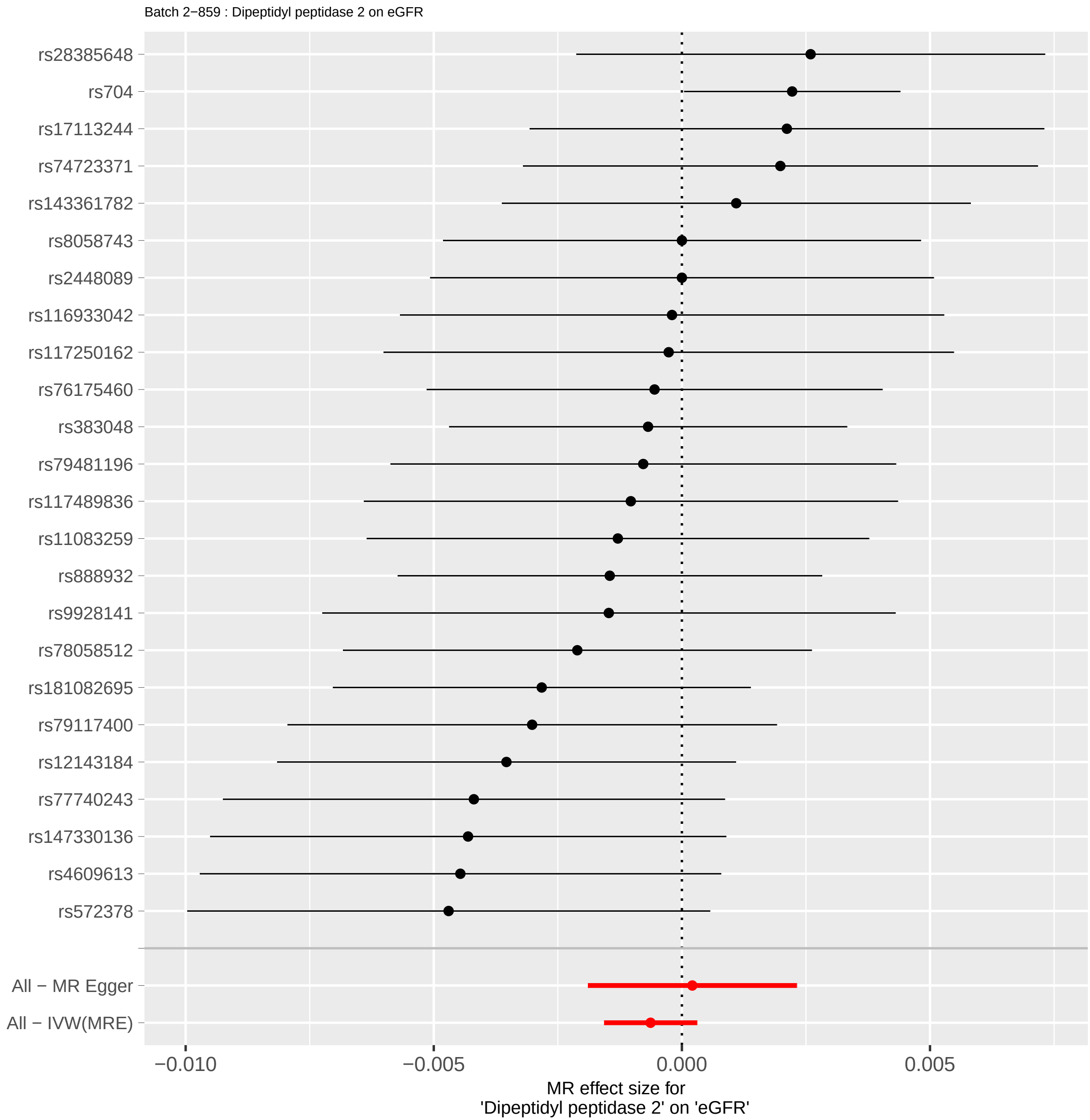

Batch 2−859 : Dipeptidyl peptidase 2 on eGFR
rs28385648
rs704
rs17113244
rs74723371
rs143361782
rs8058743
rs2448089
rs116933042
rs117250162
rs76175460
rs383048
rs79481196
rs117489836
rs11083259
rs888932
rs9928141
rs78058512
rs181082695
rs79117400
rs12143184
rs77740243
rs147330136
rs4609613
rs572378
All − MR Egger
All − IVW(MRE)
−0.010
−0.005
0.000
0.005
MR effect size for
'Dipeptidyl peptidase 2' on 'eGFR'

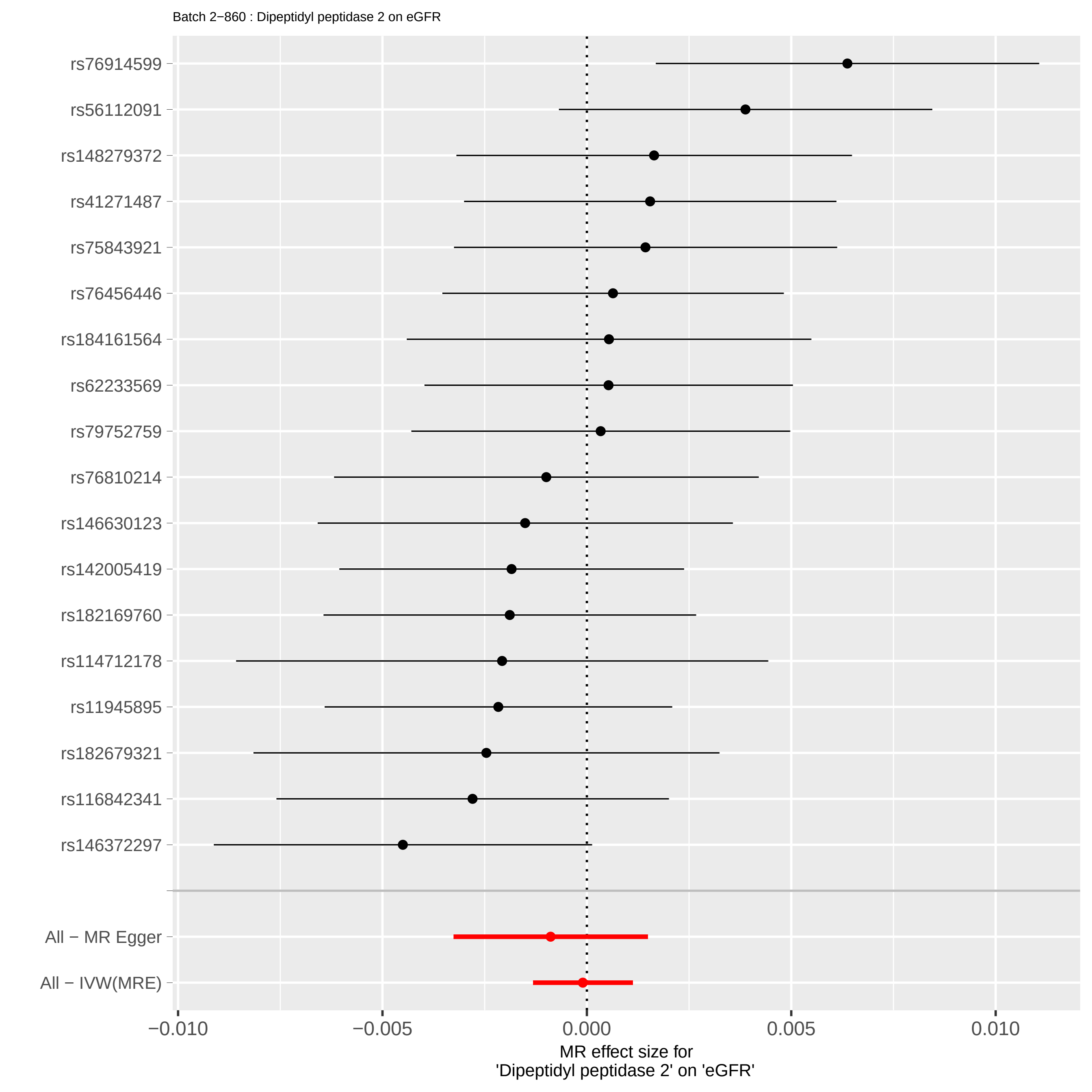

Batch 2−860 : Dipeptidyl peptidase 2 on eGFR
rs76914599
rs56112091
rs148279372
rs41271487
rs75843921
rs76456446
rs184161564
rs62233569
rs79752759
rs76810214
rs146630123
rs142005419
rs182169760
rs114712178
rs11945895
rs182679321
rs116842341
rs146372297
All − MR Egger
All − IVW(MRE)
−0.010
−0.005
0.000
0.005
0.010
MR effect size for
'Dipeptidyl peptidase 2' on 'eGFR'

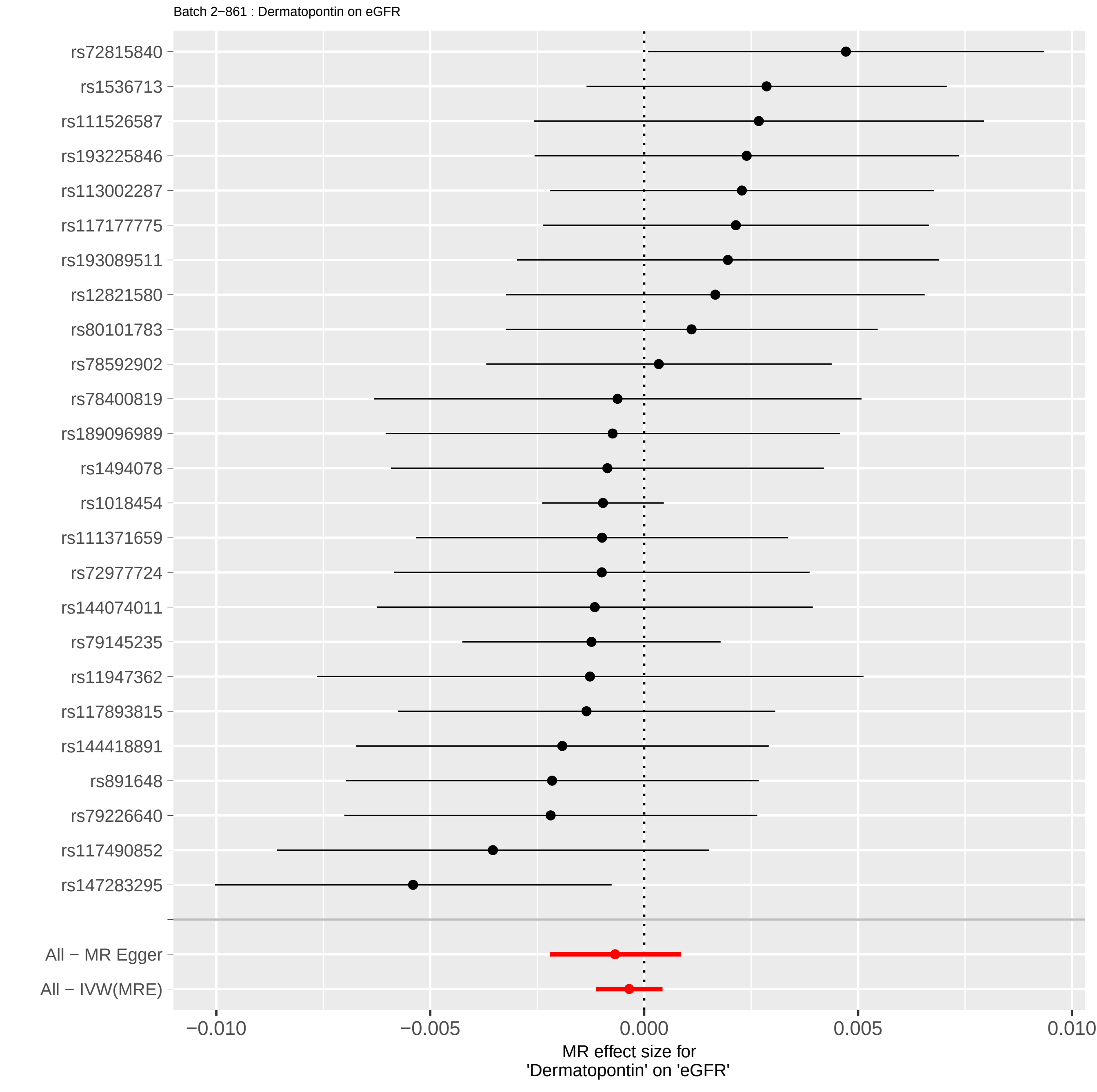
Batch 2−861 : Dermatopontin on eGFR
rs72815840
rs1536713
rs111526587
rs193225846
rs113002287
rs117177775
rs193089511
rs12821580
rs80101783
rs78592902
rs78400819
rs189096989
rs1494078
rs1018454
rs111371659
rs72977724
rs144074011
rs79145235
rs11947362
rs117893815
rs144418891
rs891648
rs79226640
rs117490852
rs147283295
All − MR Egger
All − IVW(MRE)
−0.010
−0.005
0.000
0.005
0.010
MR effect size for
'Dermatopontin' on 'eGFR'

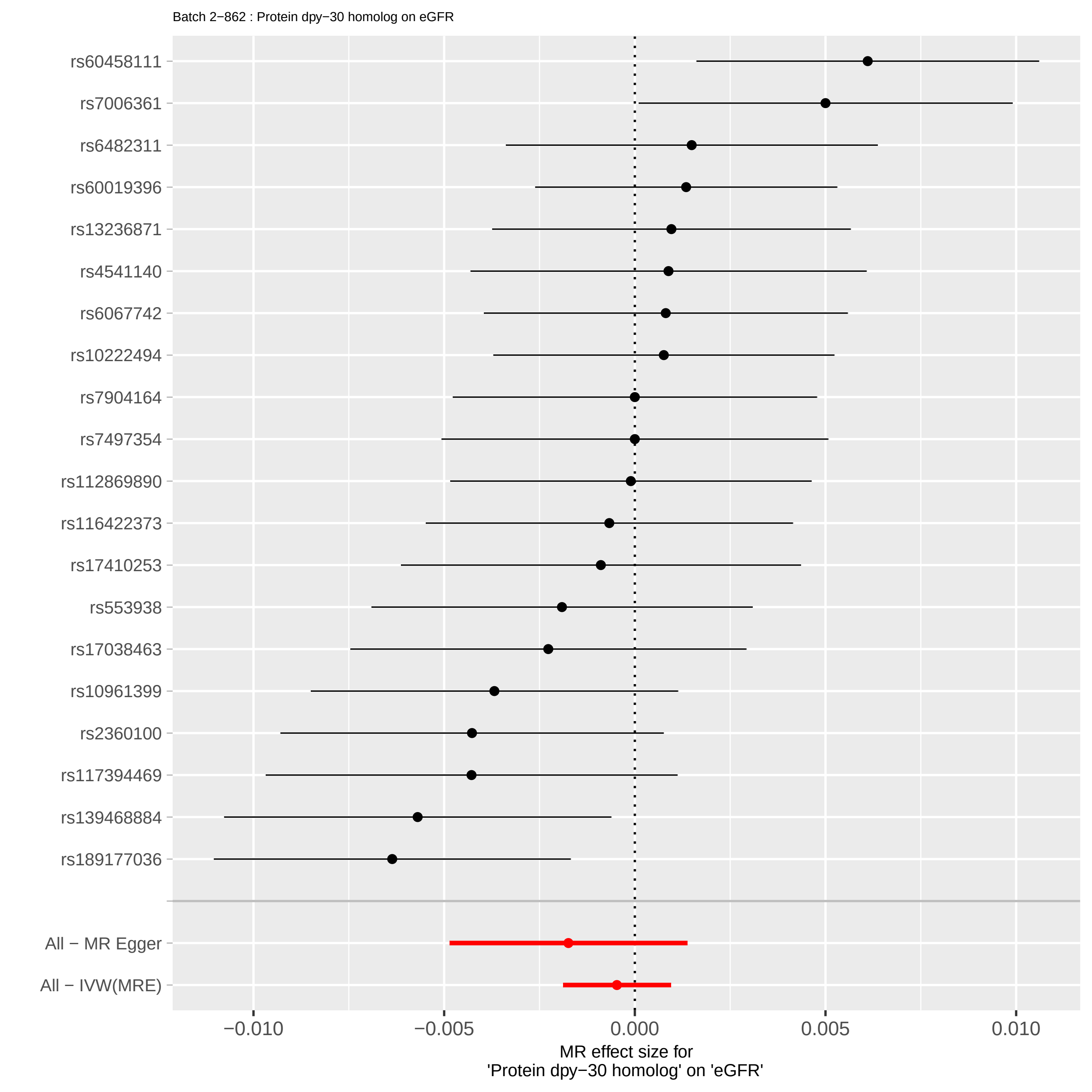

Batch 2−862 : Protein dpy−30 homolog on eGFR
rs60458111
rs7006361
rs6482311
rs60019396
rs13236871
rs4541140
rs6067742
rs10222494
rs7904164
rs7497354
rs112869890
rs116422373
rs17410253
rs553938
rs17038463
rs10961399
rs2360100
rs117394469
rs139468884
rs189177036
All − MR Egger
All − IVW(MRE)
−0.010
−0.005
0.000
0.005
0.010
MR effect size for
'Protein dpy−30 homolog' on 'eGFR'

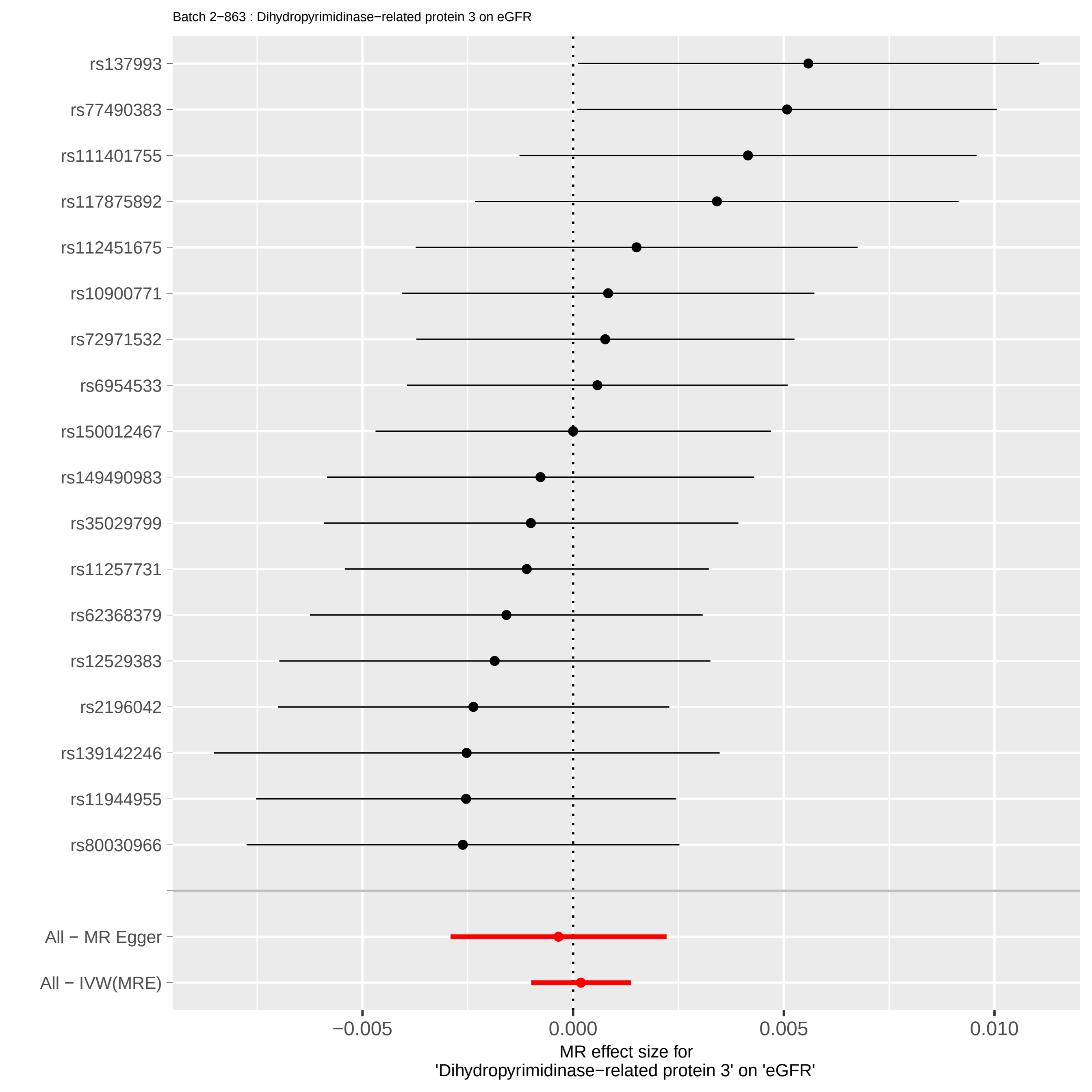

Batch 2−863 : Dihydropyrimidinase−related protein 3 on eGFR
rs137993
rs77490383
rs111401755
rs117875892
rs112451675
rs10900771
rs72971532
rs6954533
rs150012467
rs149490983
rs35029799
rs11257731
rs62368379
rs12529383
rs2196042
rs139142246
rs11944955
rs80030966
All − MR Egger
All − IVW(MRE)
−0.005
0.000
0.005
0.010
MR effect size for
'Dihydropyrimidinase−related protein 3' on 'eGFR'

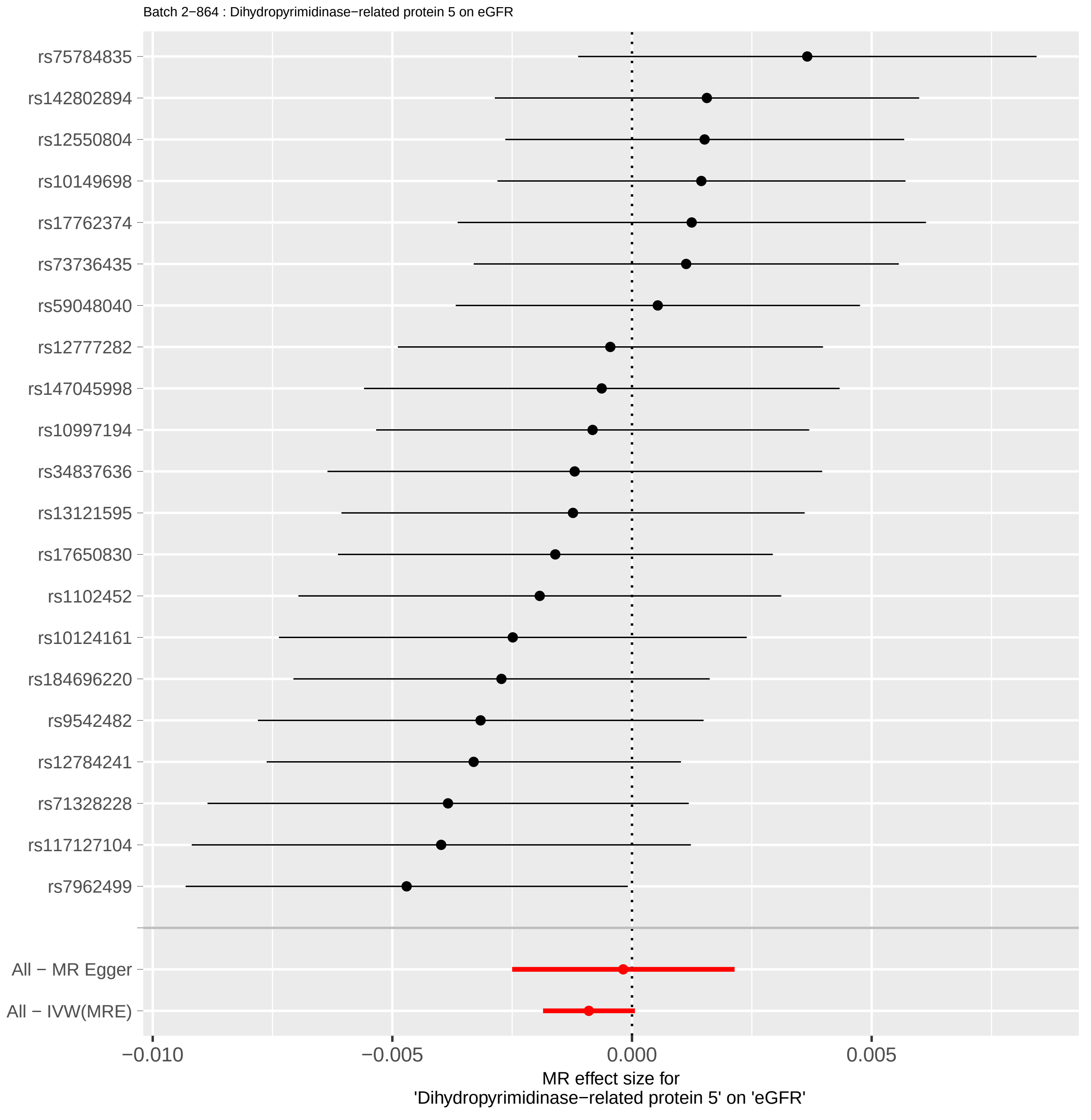
Batch 2−864 : Dihydropyrimidinase−related protein 5 on eGFR
rs75784835
rs142802894
rs12550804
rs10149698
rs17762374
rs73736435
rs59048040
rs12777282
rs147045998
rs10997194
rs34837636
rs13121595
rs17650830
rs1102452
rs10124161
rs184696220
rs9542482
rs12784241
rs71328228
rs117127104
rs7962499
All − MR Egger
All − IVW(MRE)
−0.010
−0.005
0.000
0.005
MR effect size for
'Dihydropyrimidinase−related protein 5' on 'eGFR'

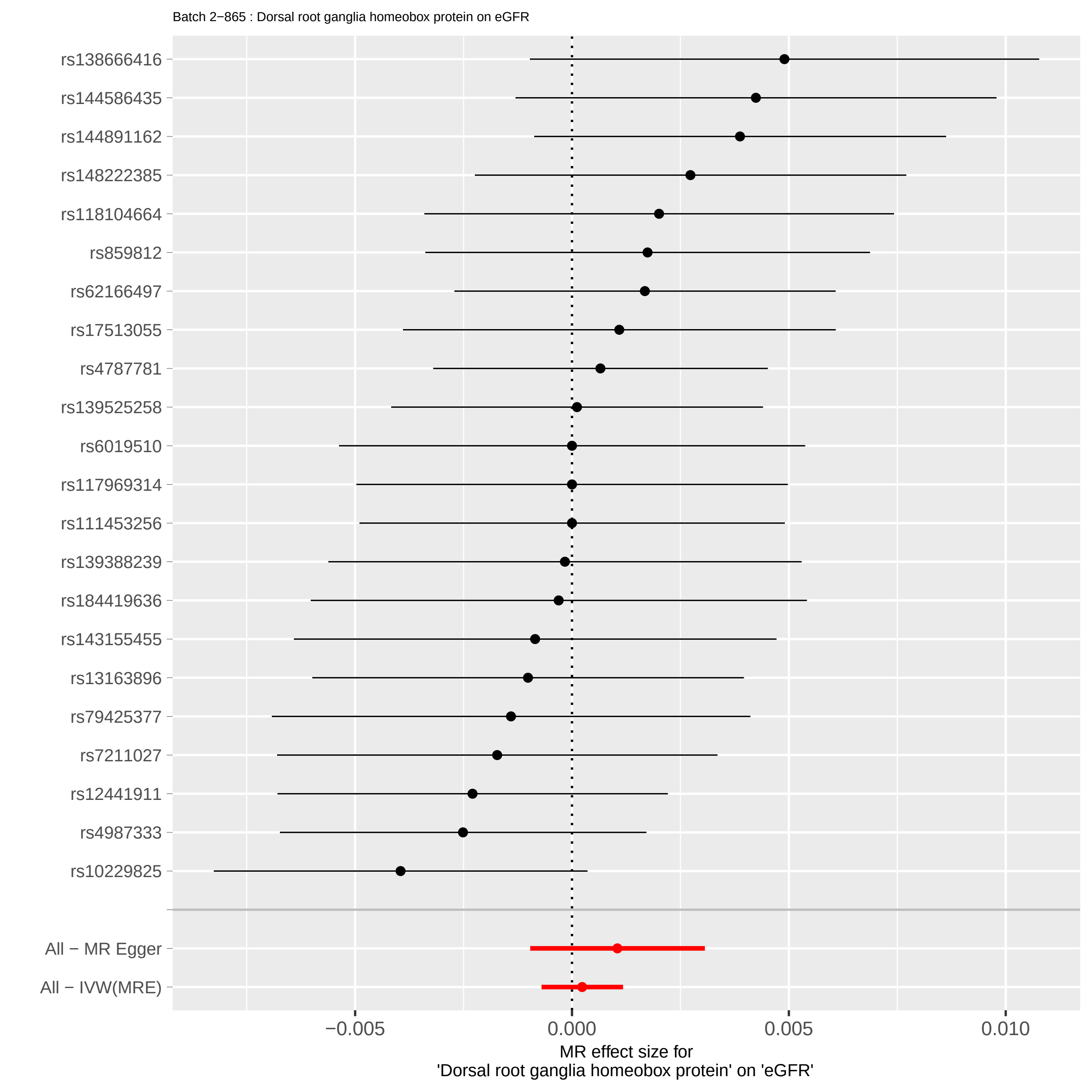
Batch 2−865 : Dorsal root ganglia homeobox protein on eGFR
rs138666416
rs144586435
rs144891162
rs148222385
rs118104664
rs859812
rs62166497
rs17513055
rs4787781
rs139525258
rs6019510
rs117969314
rs111453256
rs139388239
rs184419636
rs143155455
rs13163896
rs79425377
rs7211027
rs12441911
rs4987333
rs10229825
All − MR Egger
All − IVW(MRE)
−0.005
0.000
0.005
0.010
MR effect size for
'Dorsal root ganglia homeobox protein' on 'eGFR'

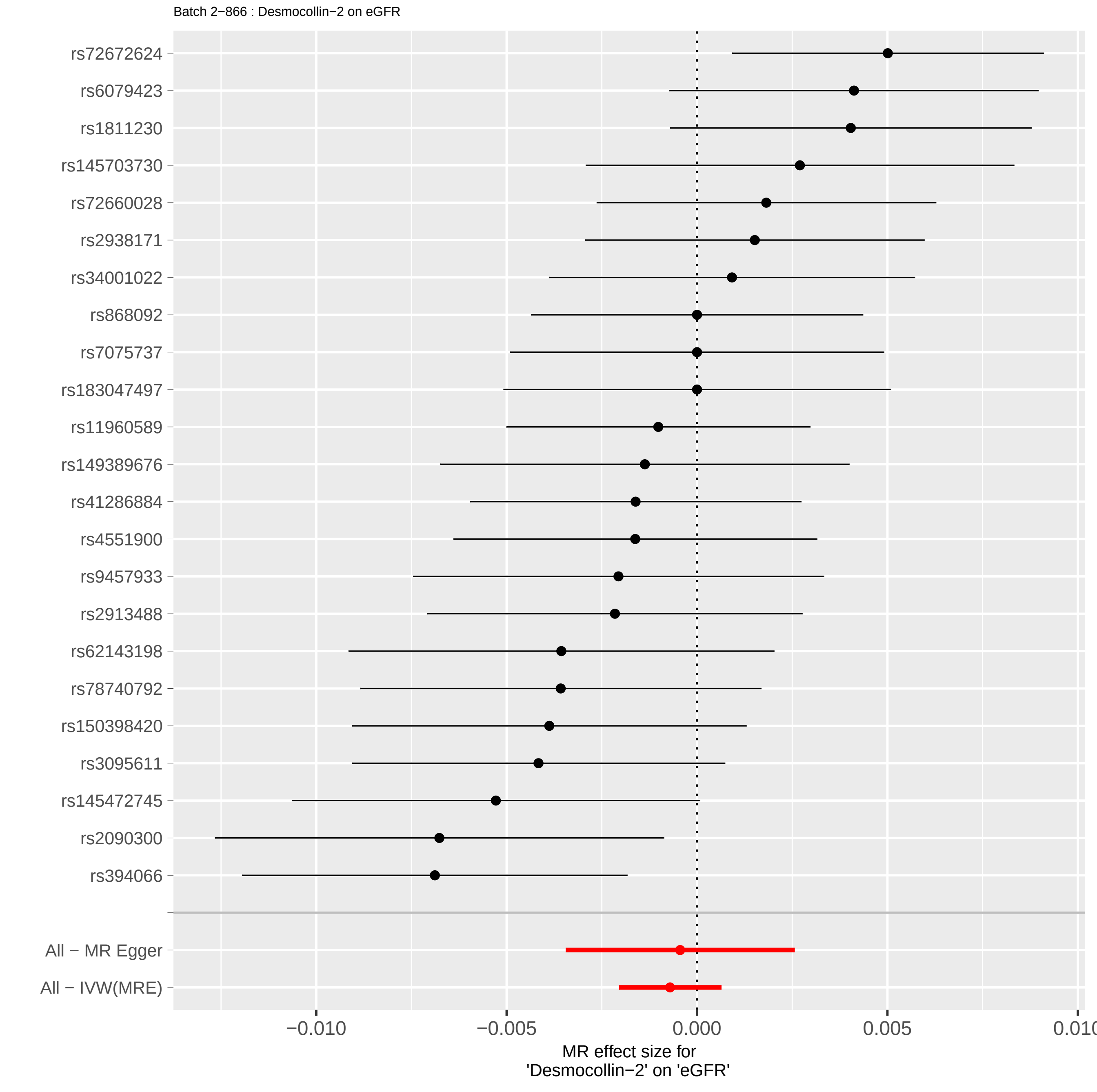
Batch 2−866 : Desmocollin−2 on eGFR
rs72672624
rs6079423
rs1811230
rs145703730
rs72660028
rs2938171
rs34001022
rs868092
rs7075737
rs183047497
rs11960589
rs149389676
rs41286884
rs4551900
rs9457933
rs2913488
rs62143198
rs78740792
rs150398420
rs3095611
rs145472745
rs2090300
rs394066
All − MR Egger
All − IVW(MRE)
−0.010
−0.005
0.000
0.005
0.010
MR effect size for
'Desmocollin−2' on 'eGFR'

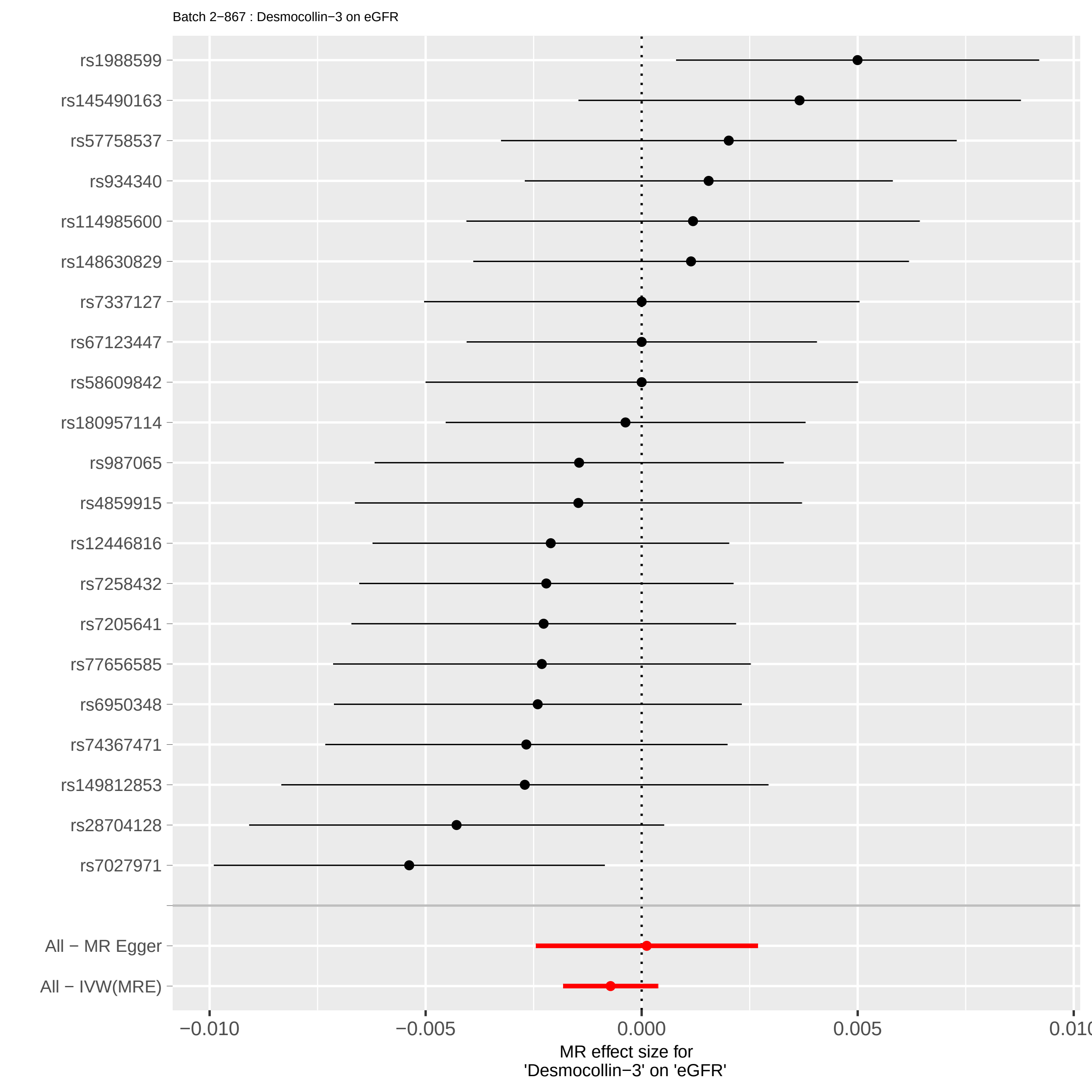

Batch 2−867 : Desmocollin−3 on eGFR
rs1988599
rs145490163
rs57758537
rs934340
rs114985600
rs148630829
rs7337127
rs67123447
rs58609842
rs180957114
rs987065
rs4859915
rs12446816
rs7258432
rs7205641
rs77656585
rs6950348
rs74367471
rs149812853
rs28704128
rs7027971
All − MR Egger
All − IVW(MRE)
−0.010
−0.005
0.000
0.005
0.010
MR effect size for
'Desmocollin−3' on 'eGFR'

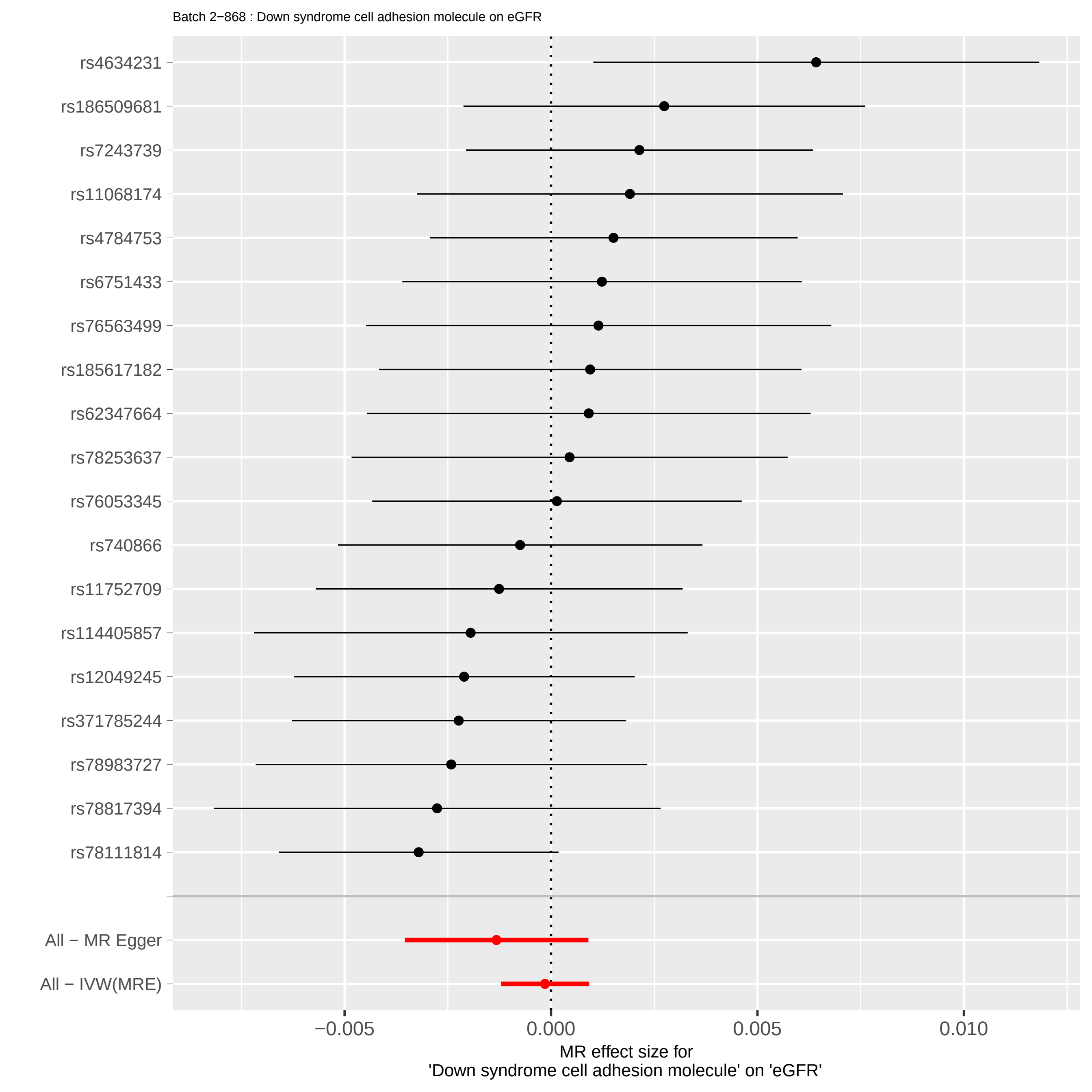

Batch 2−868 : Down syndrome cell adhesion molecule on eGFR
rs4634231
rs186509681
rs7243739
rs11068174
rs4784753
rs6751433
rs76563499
rs185617182
rs62347664
rs78253637
rs76053345
rs740866
rs11752709
rs114405857
rs12049245
rs371785244
rs78983727
rs78817394
rs78111814
All − MR Egger
All − IVW(MRE)
−0.005
0.000
0.005
0.010
MR effect size for
'Down syndrome cell adhesion molecule' on 'eGFR'

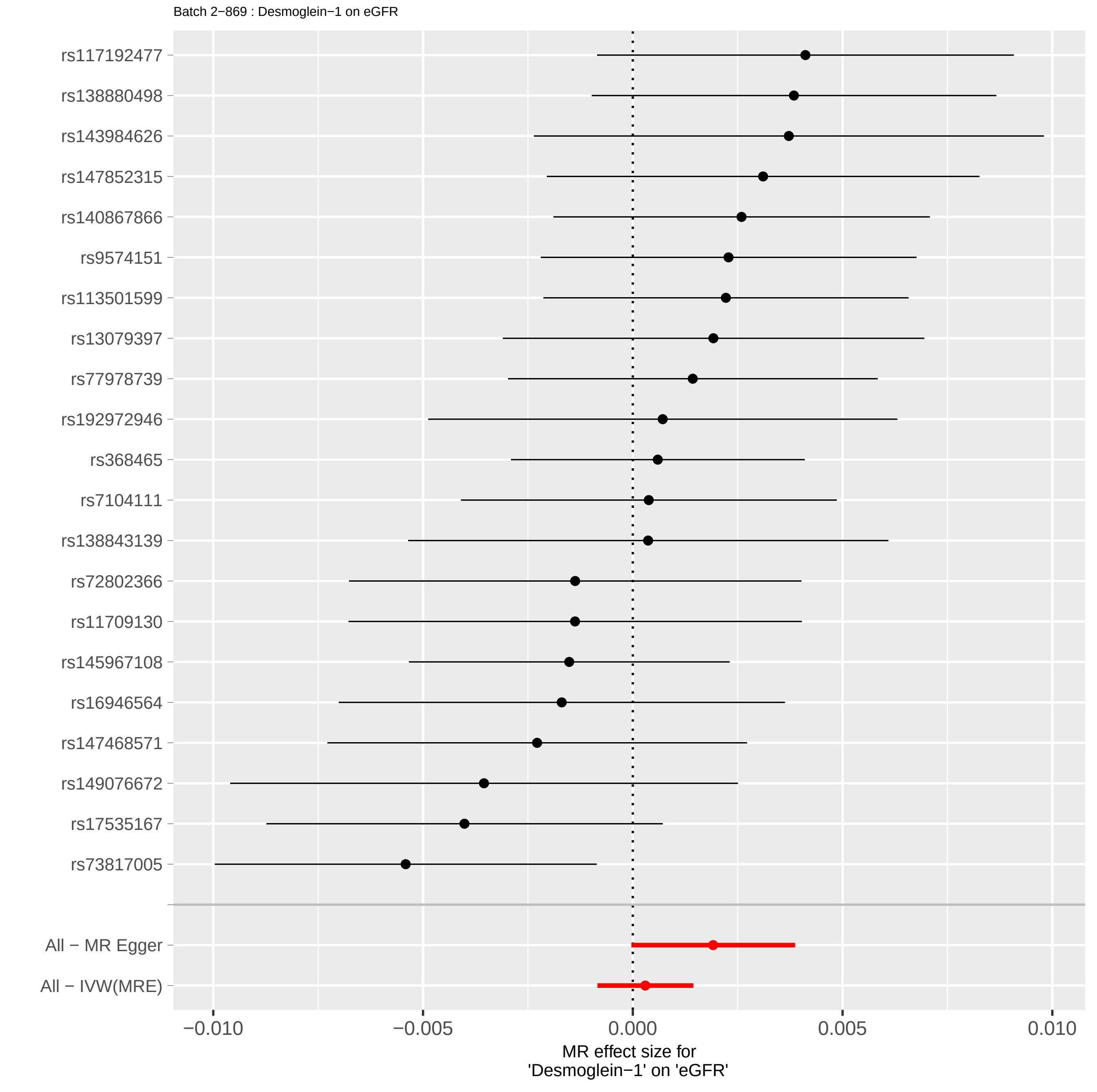
Batch 2−869 : Desmoglein−1 on eGFR
rs117192477
rs138880498
rs143984626
rs147852315
rs140867866
rs9574151
rs113501599
rs13079397
rs77978739
rs192972946
rs368465
rs7104111
rs138843139
rs72802366
rs11709130
rs145967108
rs16946564
rs147468571
rs149076672
rs17535167
rs73817005
All − MR Egger
All − IVW(MRE)
−0.010
−0.005
0.000
0.005
0.010
MR effect size for
'Desmoglein−1' on 'eGFR'

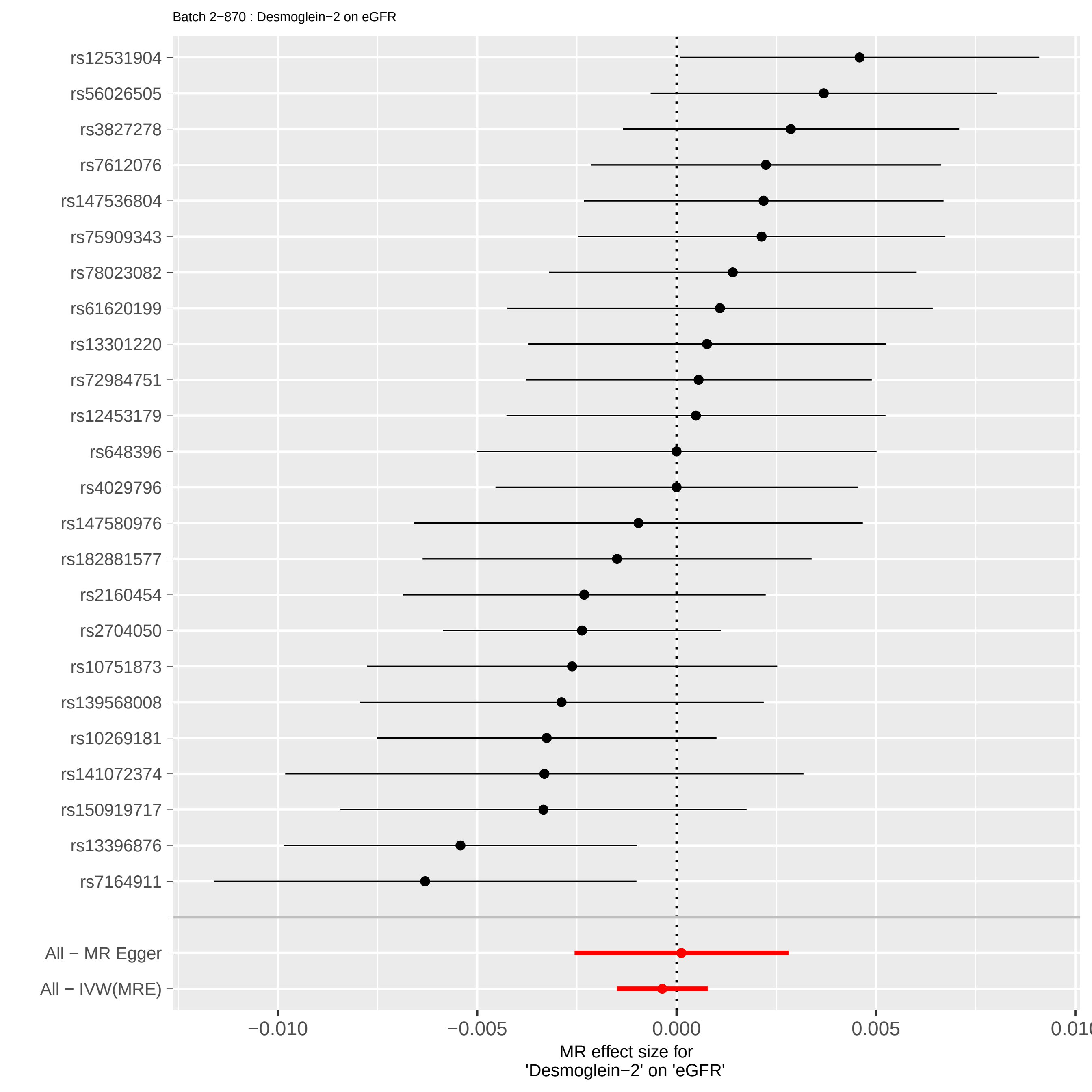

Batch 2−870 : Desmoglein−2 on eGFR
rs12531904
rs56026505
rs3827278
rs7612076
rs147536804
rs75909343
rs78023082
rs61620199
rs13301220
rs72984751
rs12453179
rs648396
rs4029796
rs147580976
rs182881577
rs2160454
rs2704050
rs10751873
rs139568008
rs10269181
rs141072374
rs150919717
rs13396876
rs7164911
All − MR Egger
All − IVW(MRE)
−0.010
−0.005
0.000
0.005
0.010
MR effect size for
'Desmoglein−2' on 'eGFR'

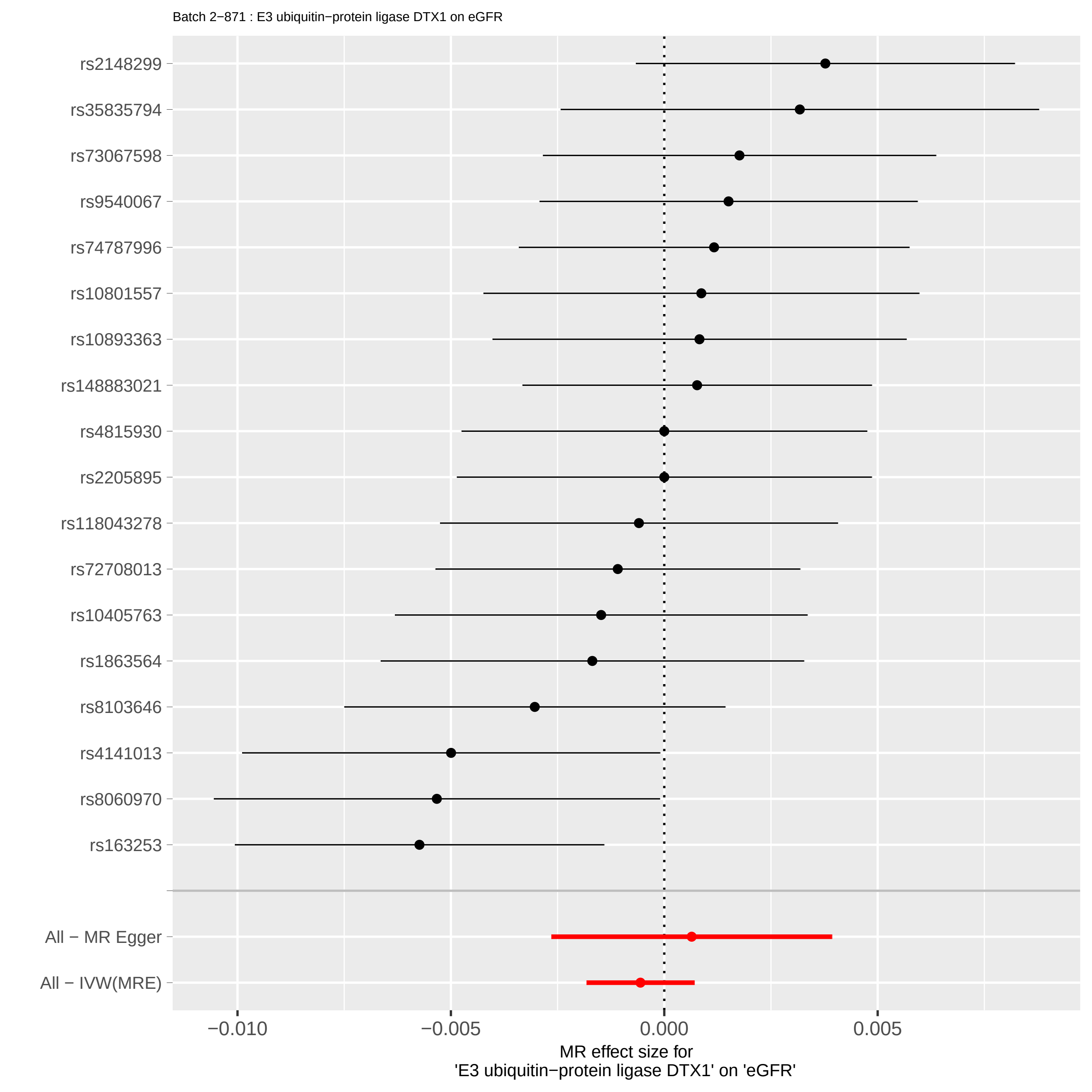

Batch 2−871 : E3 ubiquitin−protein ligase DTX1 on eGFR
rs2148299
rs35835794
rs73067598
rs9540067
rs74787996
rs10801557
rs10893363
rs148883021
rs4815930
rs2205895
rs118043278
rs72708013
rs10405763
rs1863564
rs8103646
rs4141013
rs8060970
rs163253
All − MR Egger
All − IVW(MRE)
−0.010
−0.005
0.000
0.005
MR effect size for
'E3 ubiquitin−protein ligase DTX1' on 'eGFR'

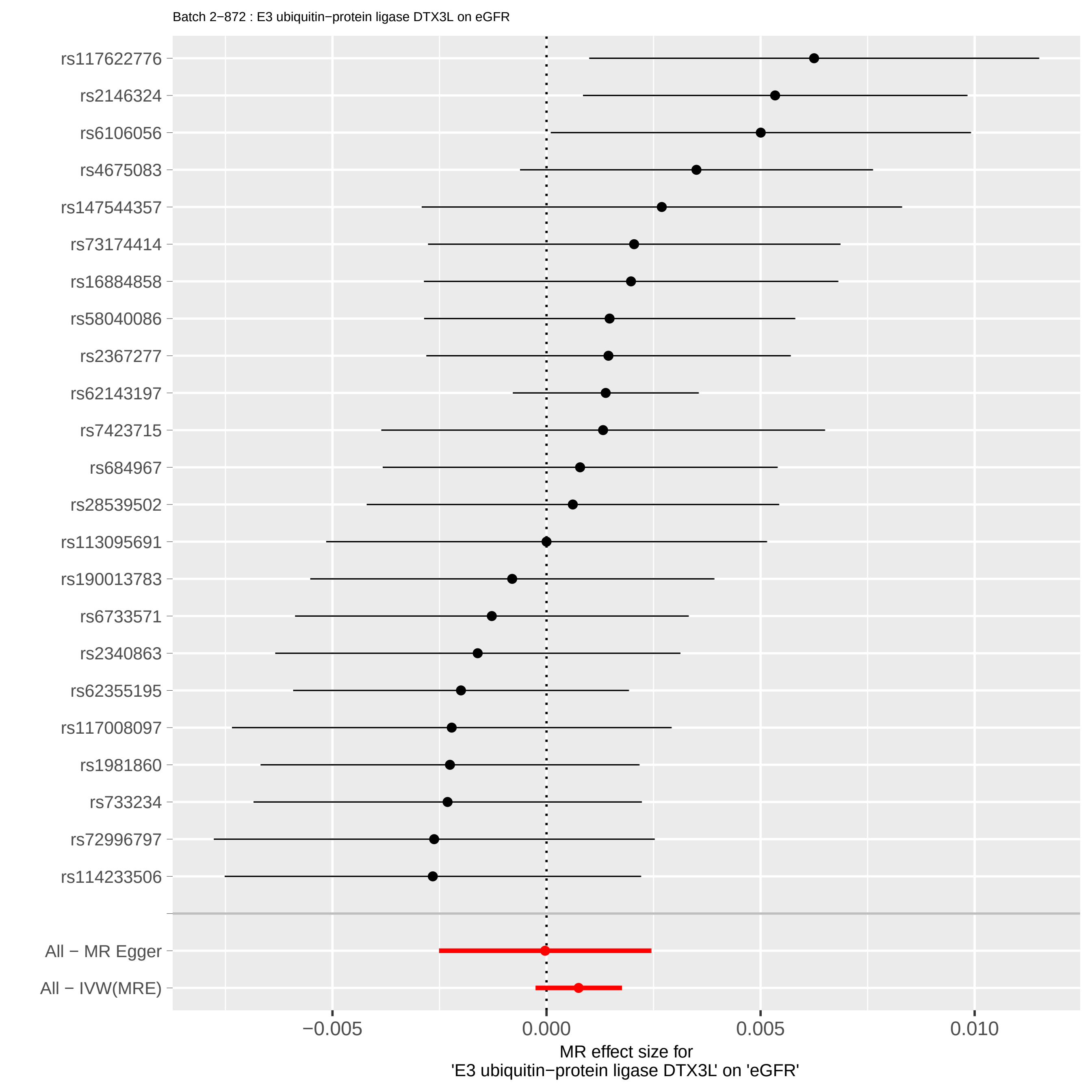

Batch 2−872 : E3 ubiquitin−protein ligase DTX3L on eGFR
rs117622776
rs2146324
rs6106056
rs4675083
rs147544357
rs73174414
rs16884858
rs58040086
rs2367277
rs62143197
rs7423715
rs684967
rs28539502
rs113095691
rs190013783
rs6733571
rs2340863
rs62355195
rs117008097
rs1981860
rs733234
rs72996797
rs114233506
All − MR Egger
All − IVW(MRE)
−0.005
0.000
0.005
0.010
MR effect size for
'E3 ubiquitin−protein ligase DTX3L' on 'eGFR'

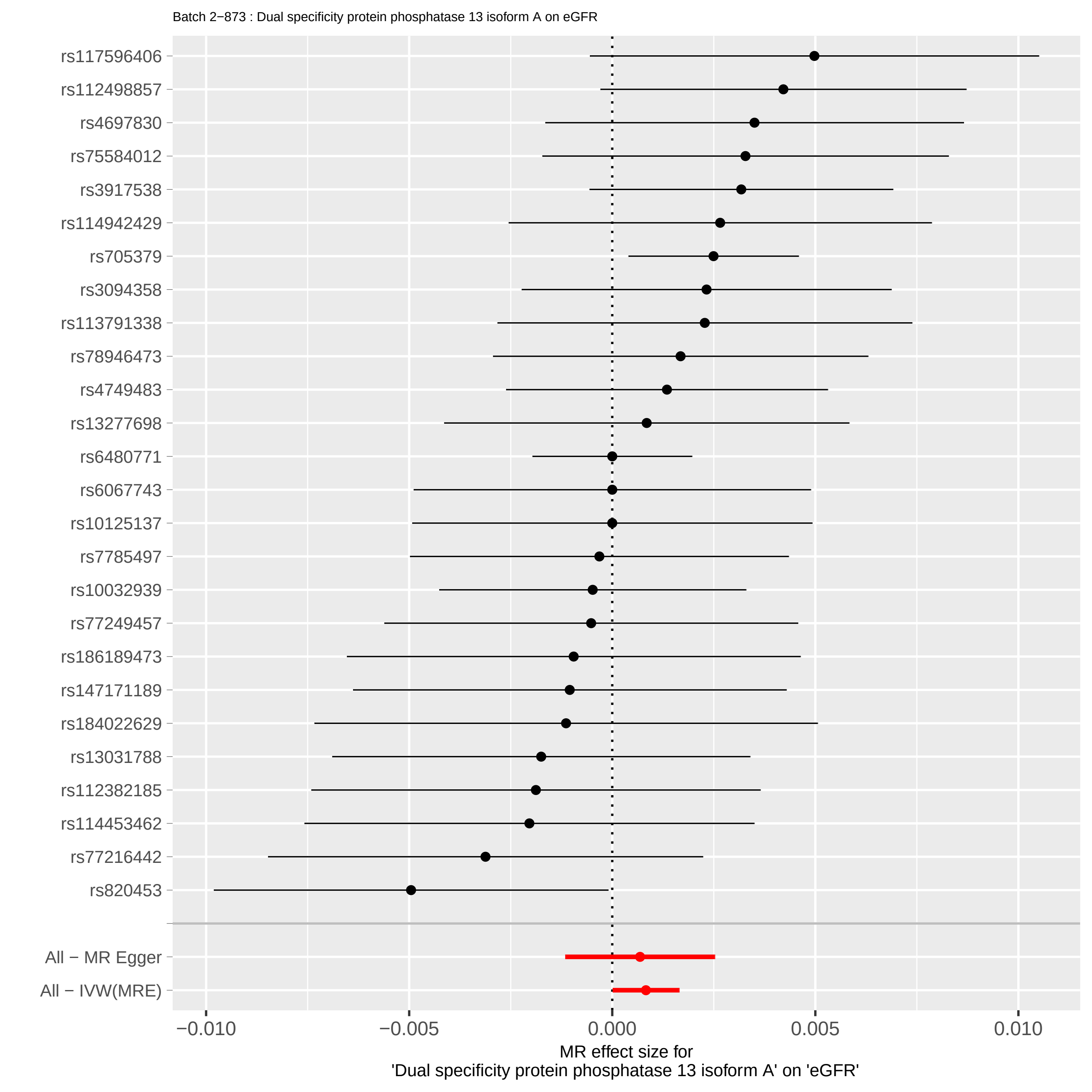
Batch 2−873 : Dual specificity protein phosphatase 13 isoform A on eGFR
rs117596406
rs112498857
rs4697830
rs75584012
rs3917538
rs114942429
rs705379
rs3094358
rs113791338
rs78946473
rs4749483
rs13277698
rs6480771
rs6067743
rs10125137
rs7785497
rs10032939
rs77249457
rs186189473
rs147171189
rs184022629
rs13031788
rs112382185
rs114453462
rs77216442
rs820453
All − MR Egger
All − IVW(MRE)
−0.010
−0.005
0.000
0.005
0.010
MR effect size for
'Dual specificity protein phosphatase 13 isoform A' on 'eGFR'

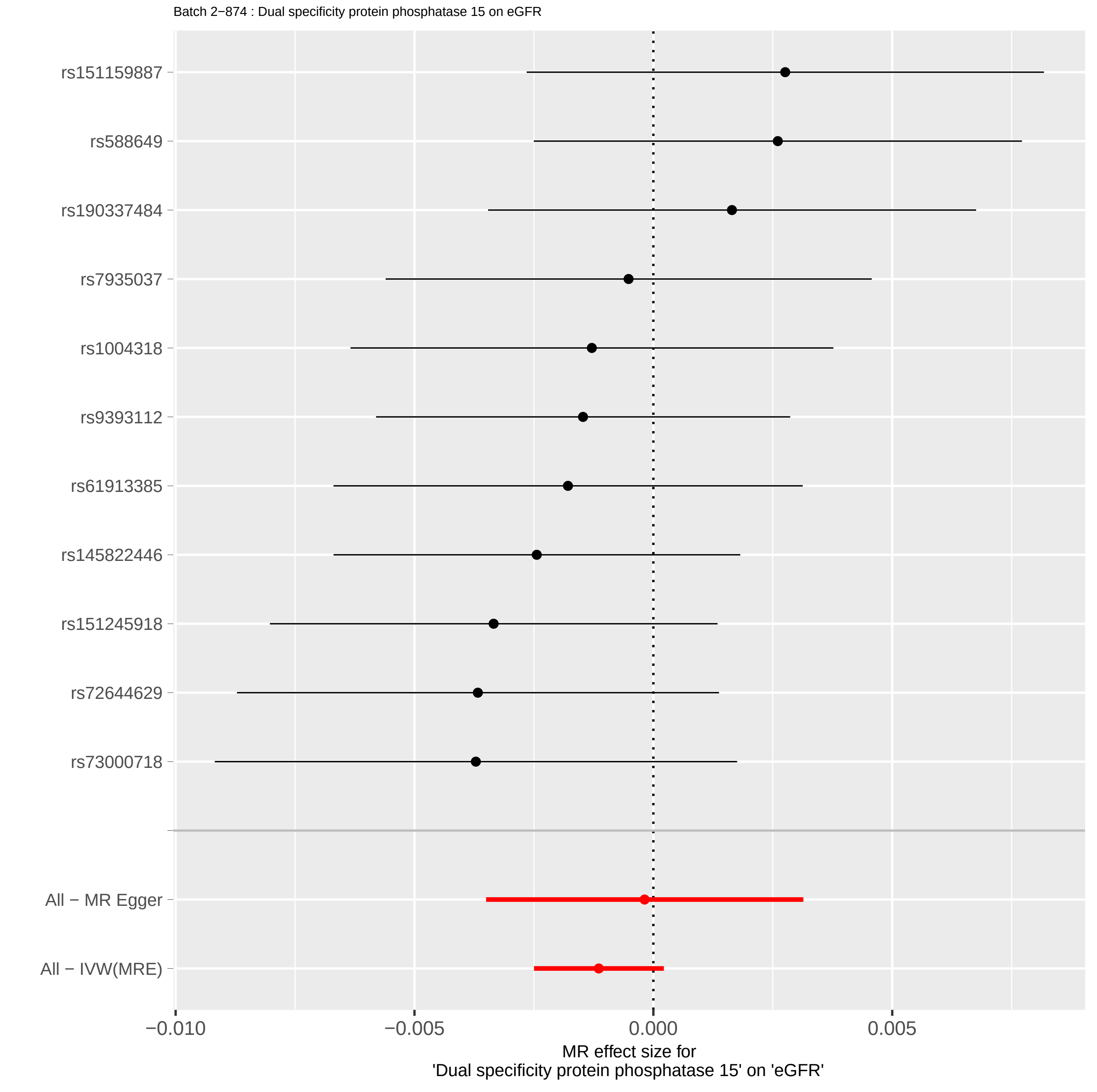

Batch 2−874 : Dual specificity protein phosphatase 15 on eGFR
rs151159887
rs588649
rs190337484
rs7935037
rs1004318
rs9393112
rs61913385
rs145822446
rs151245918
rs72644629
rs73000718
All − MR Egger
All − IVW(MRE)
−0.010
−0.005
0.000
0.005
MR effect size for
'Dual specificity protein phosphatase 15' on 'eGFR'

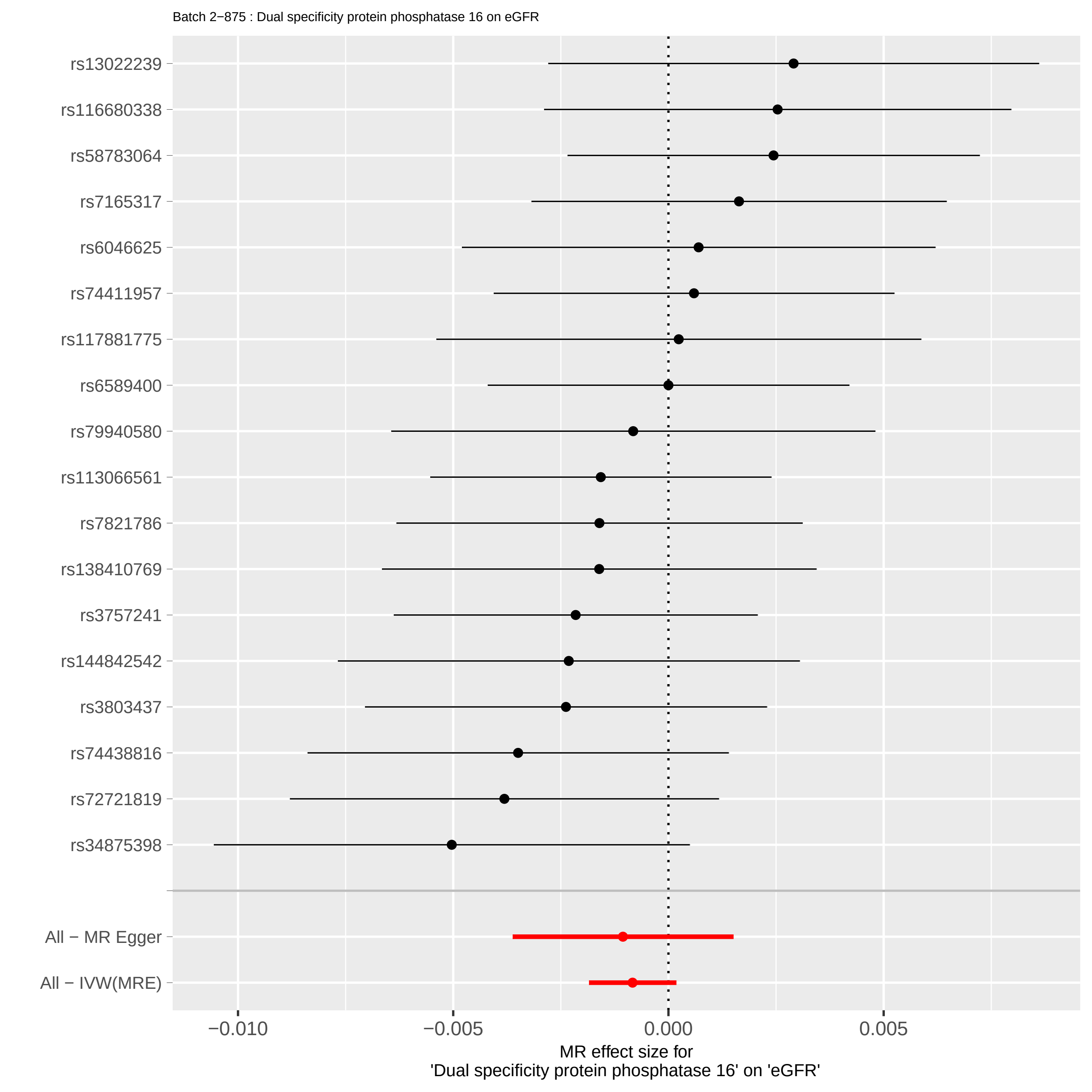

Batch 2−875 : Dual specificity protein phosphatase 16 on eGFR
rs13022239
rs116680338
rs58783064
rs7165317
rs6046625
rs74411957
rs117881775
rs6589400
rs79940580
rs113066561
rs7821786
rs138410769
rs3757241
rs144842542
rs3803437
rs74438816
rs72721819
rs34875398
All − MR Egger
All − IVW(MRE)
−0.010
−0.005
0.000
0.005
MR effect size for
'Dual specificity protein phosphatase 16' on 'eGFR'

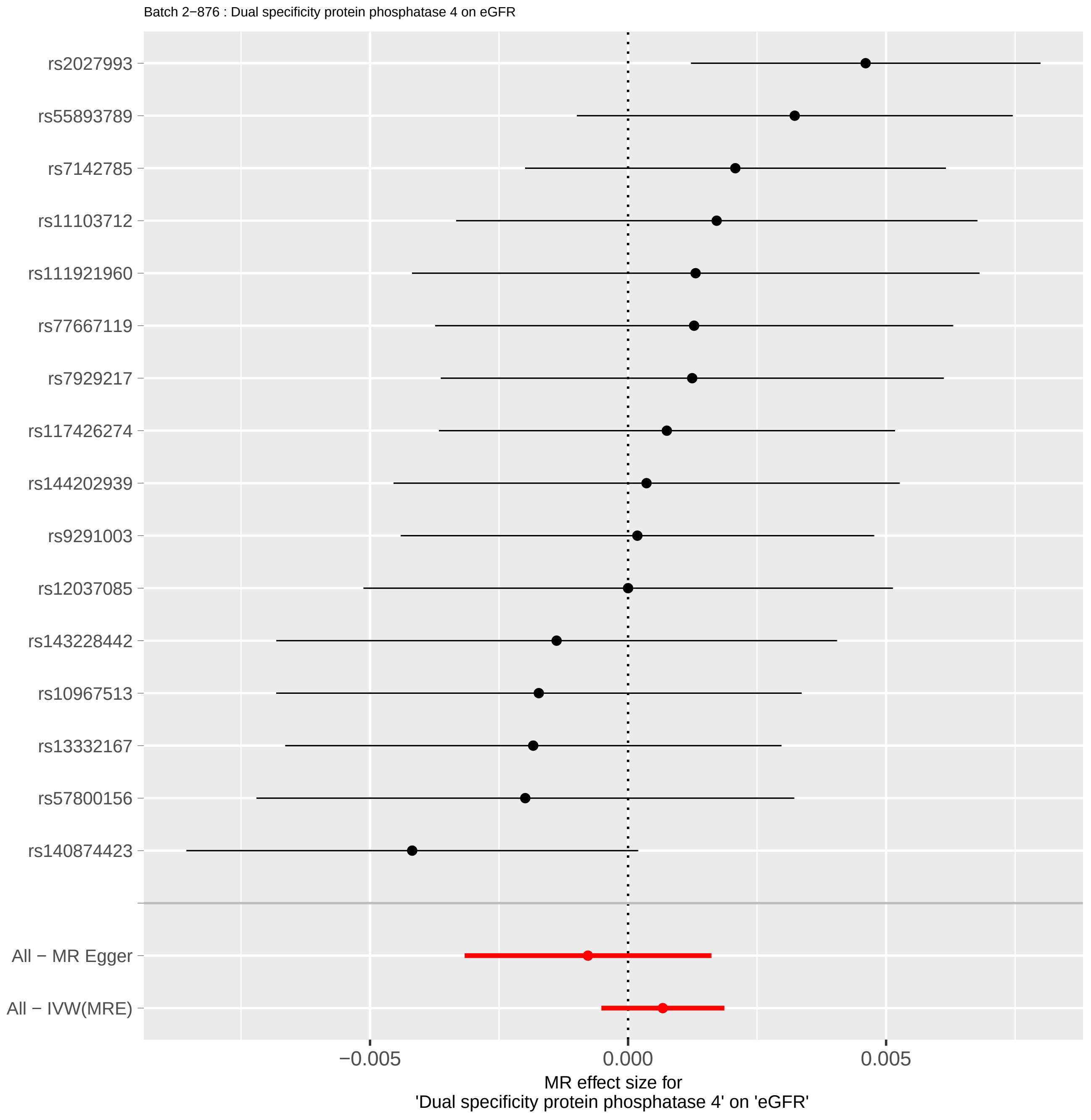

Batch 2−876 : Dual specificity protein phosphatase 4 on eGFR
rs2027993
rs55893789
rs7142785
rs11103712
rs111921960
rs77667119
rs7929217
rs117426274
rs144202939
rs9291003
rs12037085
rs143228442
rs10967513
rs13332167
rs57800156
rs140874423
All − MR Egger
All − IVW(MRE)
−0.005
0.000
0.005
MR effect size for
'Dual specificity protein phosphatase 4' on 'eGFR'

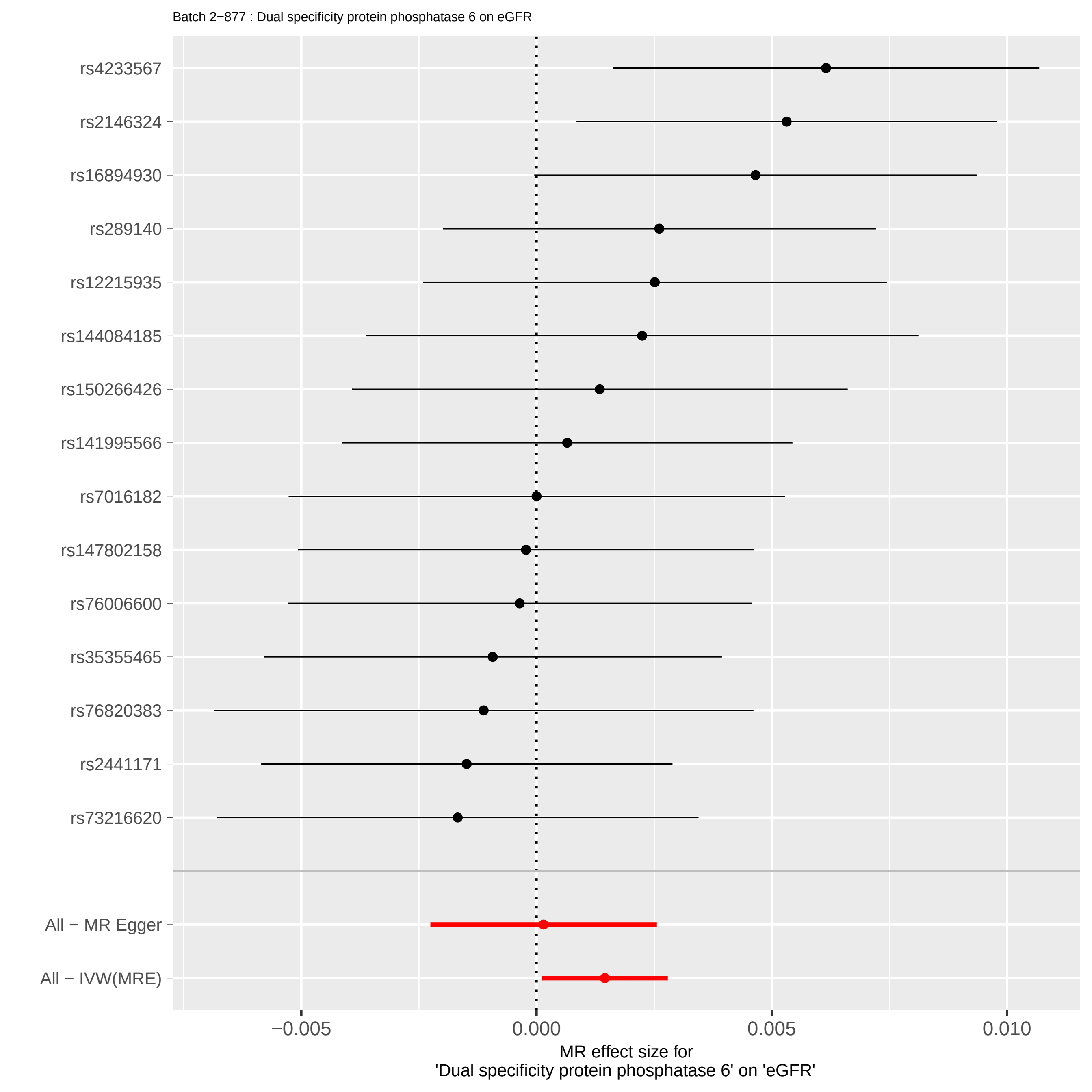

Batch 2−877 : Dual specificity protein phosphatase 6 on eGFR
rs4233567
rs2146324
rs16894930
rs289140
rs12215935
rs144084185
rs150266426
rs141995566
rs7016182
rs147802158
rs76006600
rs35355465
rs76820383
rs2441171
rs73216620
All − MR Egger
All − IVW(MRE)
−0.005
0.000
0.005
0.010
MR effect size for
'Dual specificity protein phosphatase 6' on 'eGFR'

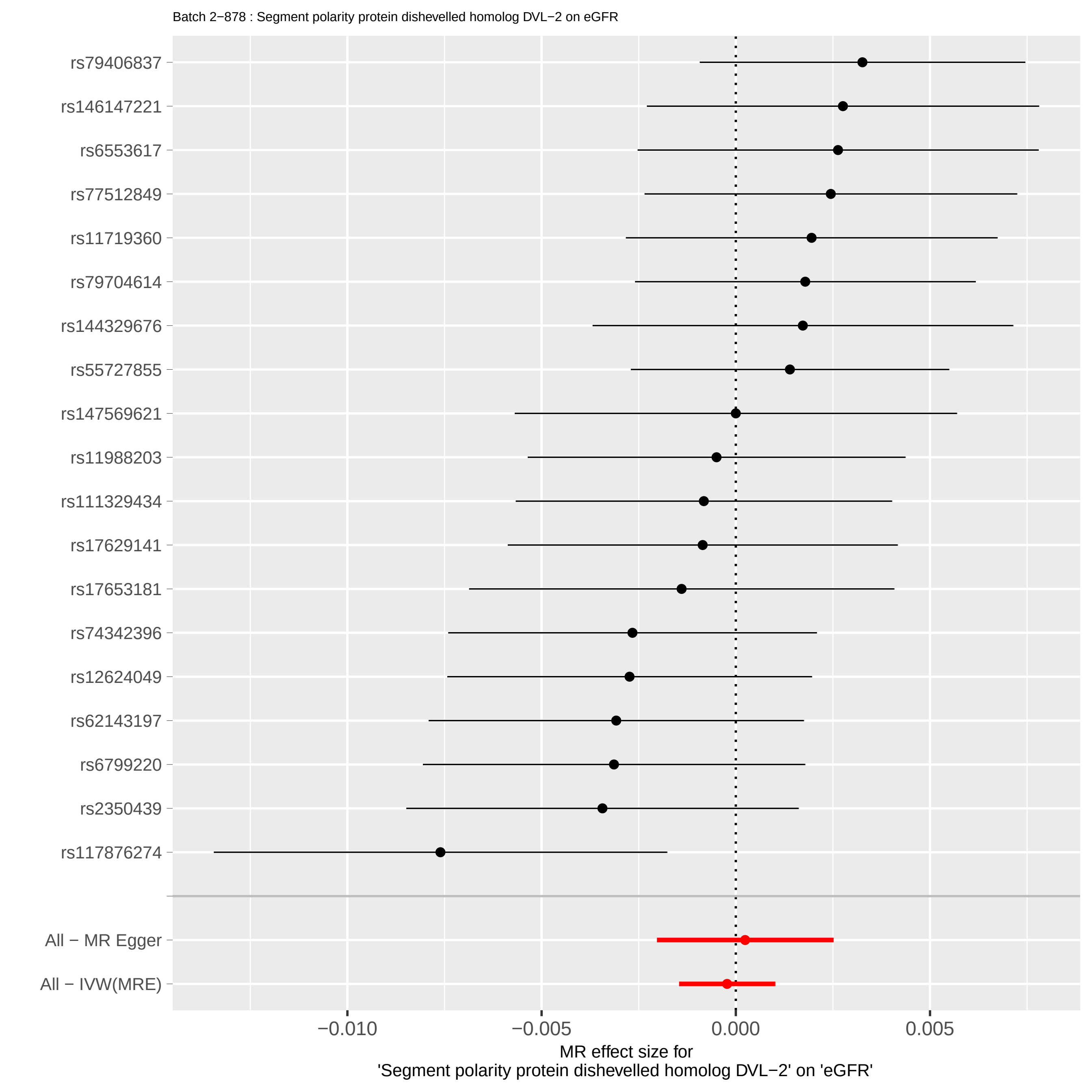
Batch 2−878 : Segment polarity protein dishevelled homolog DVL−2 on eGFR
rs79406837
rs146147221
rs6553617
rs77512849
rs11719360
rs79704614
rs144329676
rs55727855
rs147569621
rs11988203
rs111329434
rs17629141
rs17653181
rs74342396
rs12624049
rs62143197
rs6799220
rs2350439
rs117876274
All − MR Egger
All − IVW(MRE)
−0.010
−0.005
0.000
0.005
MR effect size for
'Segment polarity protein dishevelled homolog DVL−2' on 'eGFR'

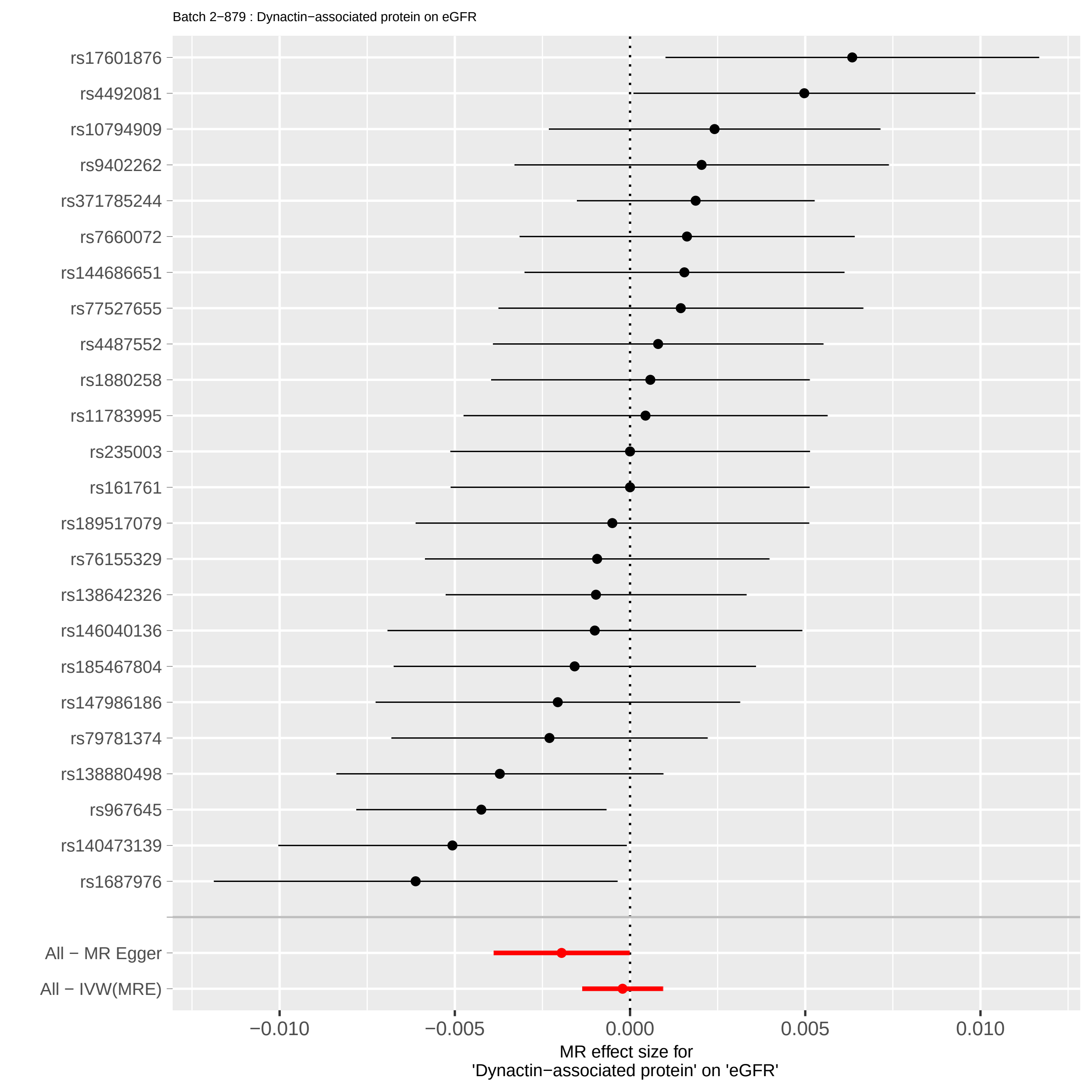

Batch 2−879 : Dynactin−associated protein on eGFR
rs17601876
rs4492081
rs10794909
rs9402262
rs371785244
rs7660072
rs144686651
rs77527655
rs4487552
rs1880258
rs11783995
rs235003
rs161761
rs189517079
rs76155329
rs138642326
rs146040136
rs185467804
rs147986186
rs79781374
rs138880498
rs967645
rs140473139
rs1687976
All − MR Egger
All − IVW(MRE)
−0.010
−0.005
0.000
0.005
0.010
MR effect size for
'Dynactin−associated protein' on 'eGFR'

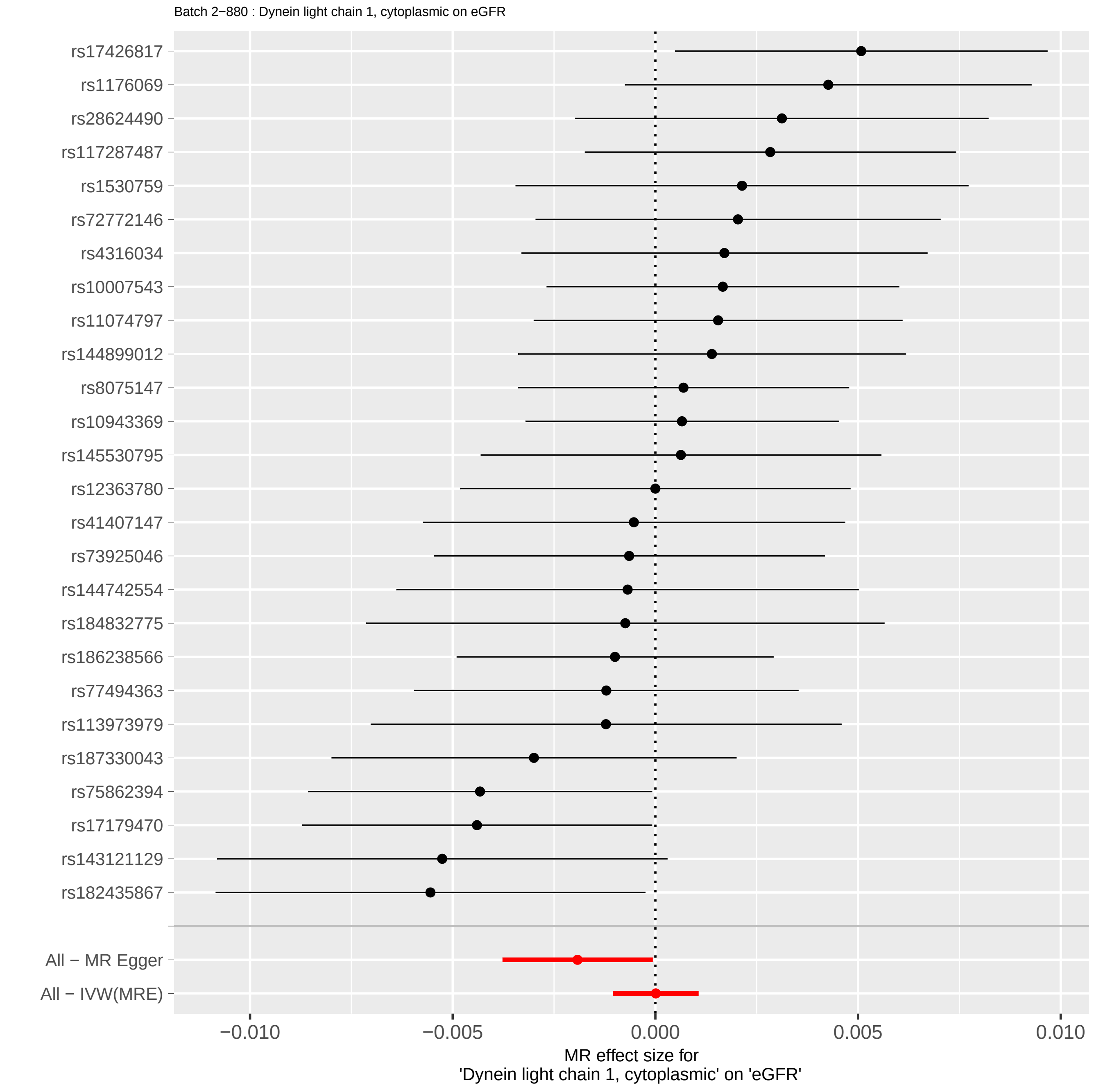

Batch 2−880 : Dynein light chain 1, cytoplasmic on eGFR
rs17426817
rs1176069
rs28624490
rs117287487
rs1530759
rs72772146
rs4316034
rs10007543
rs11074797
rs144899012
rs8075147
rs10943369
rs145530795
rs12363780
rs41407147
rs73925046
rs144742554
rs184832775
rs186238566
rs77494363
rs113973979
rs187330043
rs75862394
rs17179470
rs143121129
rs182435867
All − MR Egger
All − IVW(MRE)
−0.010
−0.005
0.000
0.005
0.010
MR effect size for
'Dynein light chain 1, cytoplasmic' on 'eGFR'

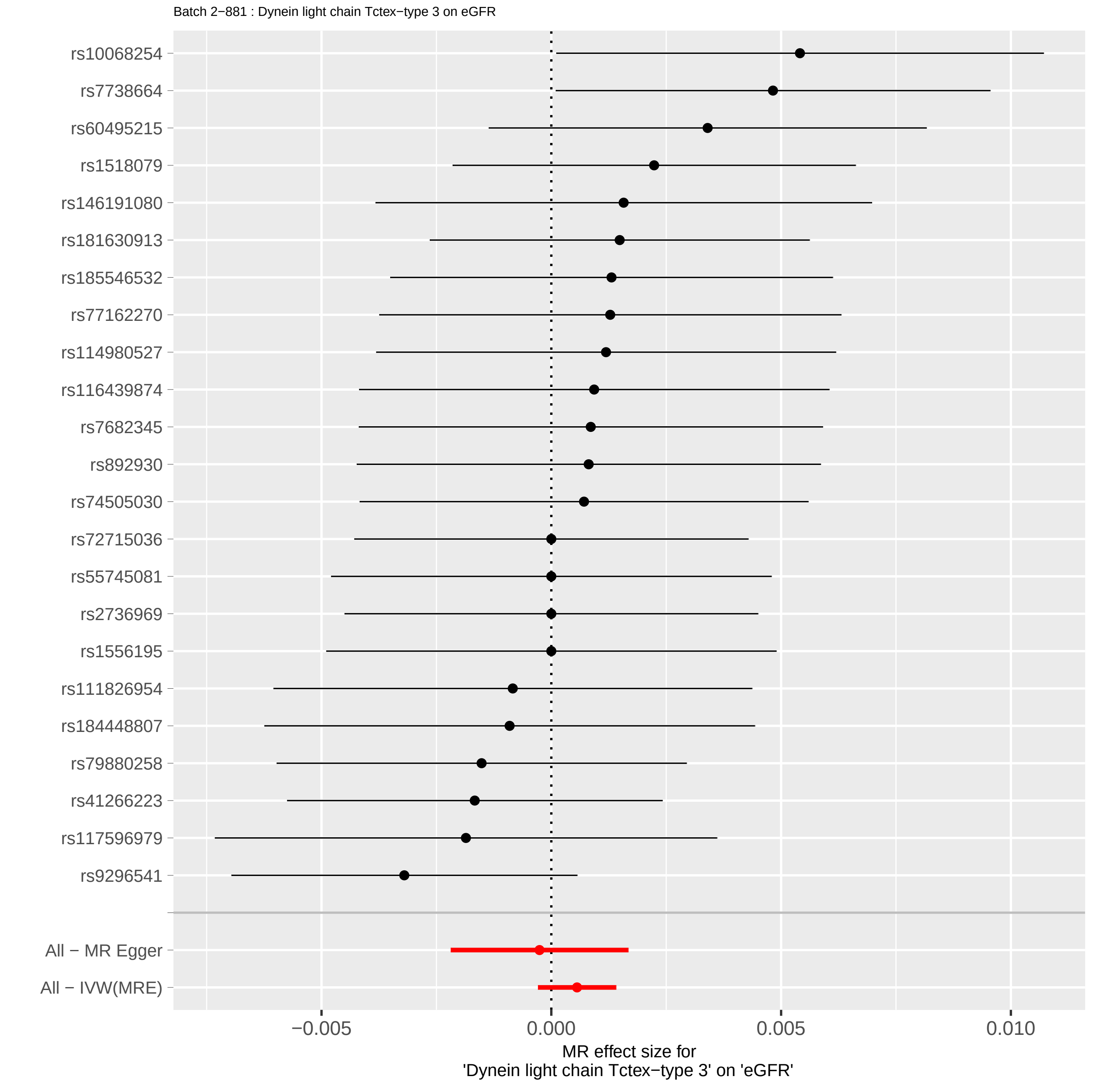

Batch 2−881 : Dynein light chain Tctex−type 3 on eGFR
rs10068254
rs7738664
rs60495215
rs1518079
rs146191080
rs181630913
rs185546532
rs77162270
rs114980527
rs116439874
rs7682345
rs892930
rs74505030
rs72715036
rs55745081
rs2736969
rs1556195
rs111826954
rs184448807
rs79880258
rs41266223
rs117596979
rs9296541
All − MR Egger
All − IVW(MRE)
−0.005
0.000
0.005
0.010
MR effect size for
'Dynein light chain Tctex−type 3' on 'eGFR'

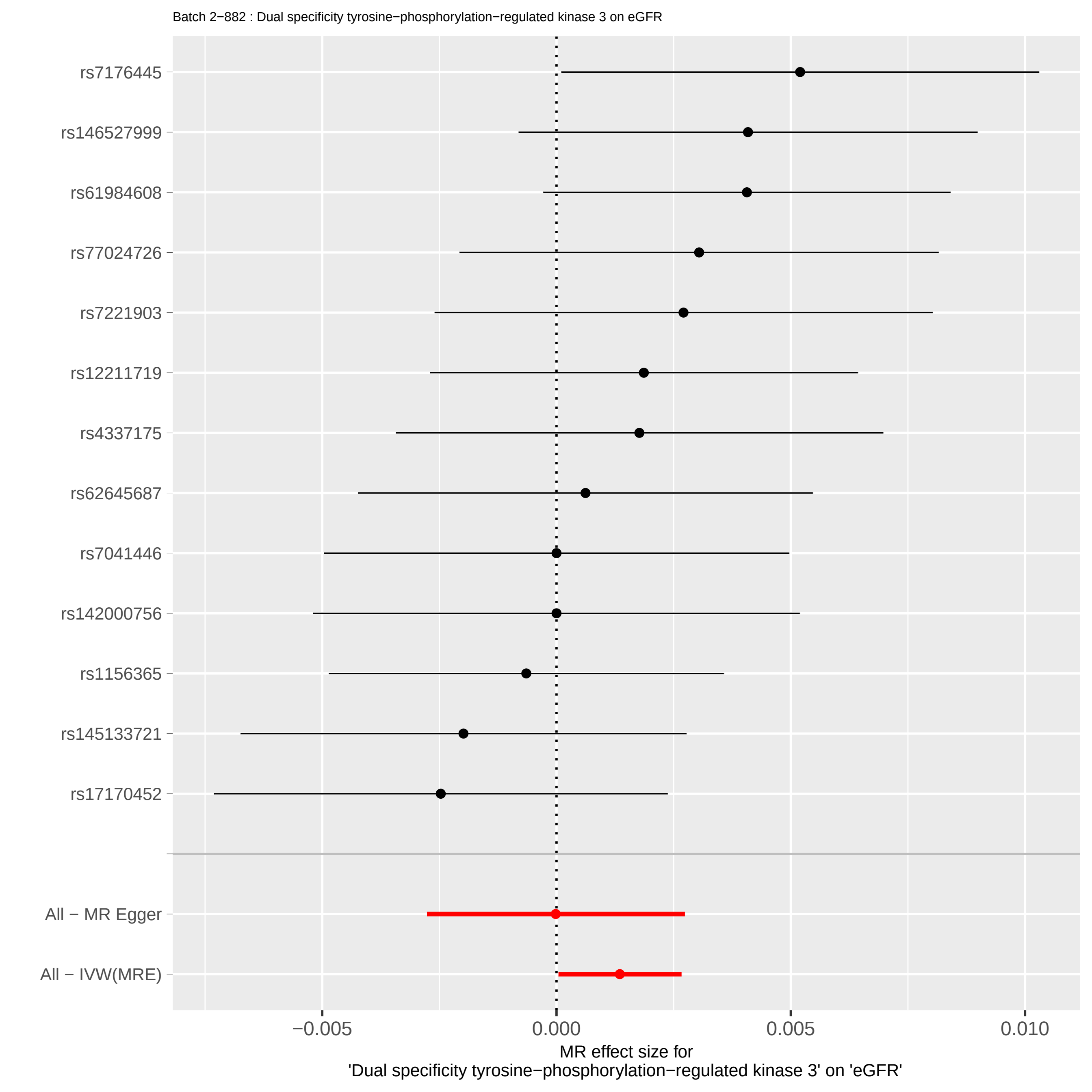

Batch 2−882 : Dual specificity tyrosine−phosphorylation−regulated kinase 3 on eGFR
rs7176445
rs146527999
rs61984608
rs77024726
rs7221903
rs12211719
rs4337175
rs62645687
rs7041446
rs142000756
rs1156365
rs145133721
rs17170452
All − MR Egger
All − IVW(MRE)
−0.005
0.000
0.005
0.010
MR effect size for
'Dual specificity tyrosine−phosphorylation−regulated kinase 3' on 'eGFR'

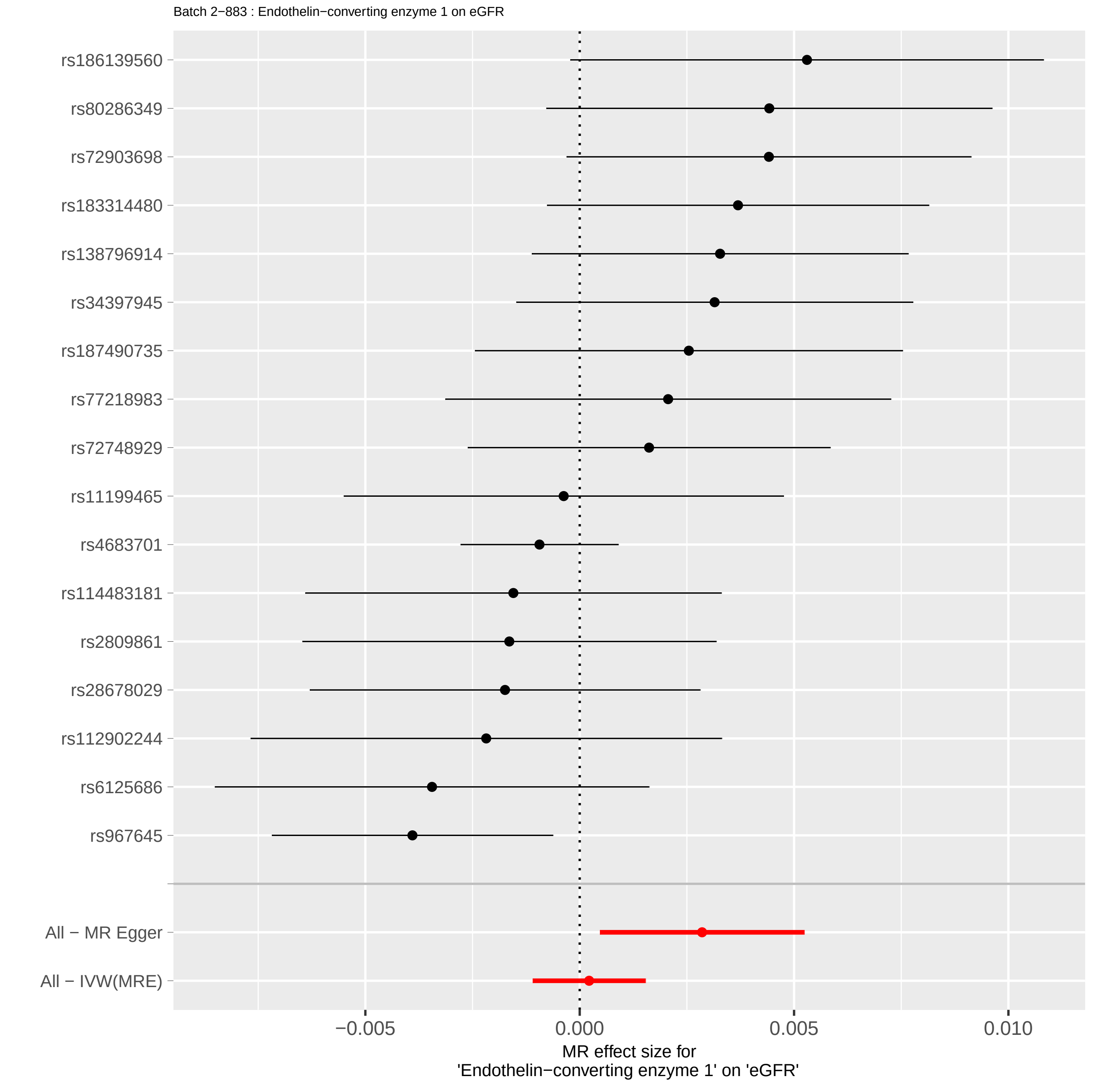
Batch 2−883 : Endothelin−converting enzyme 1 on eGFR
rs186139560
rs80286349
rs72903698
rs183314480
rs138796914
rs34397945
rs187490735
rs77218983
rs72748929
rs11199465
rs4683701
rs114483181
rs2809861
rs28678029
rs112902244
rs6125686
rs967645
All − MR Egger
All − IVW(MRE)
−0.005
0.000
0.005
0.010
MR effect size for
'Endothelin−converting enzyme 1' on 'eGFR'

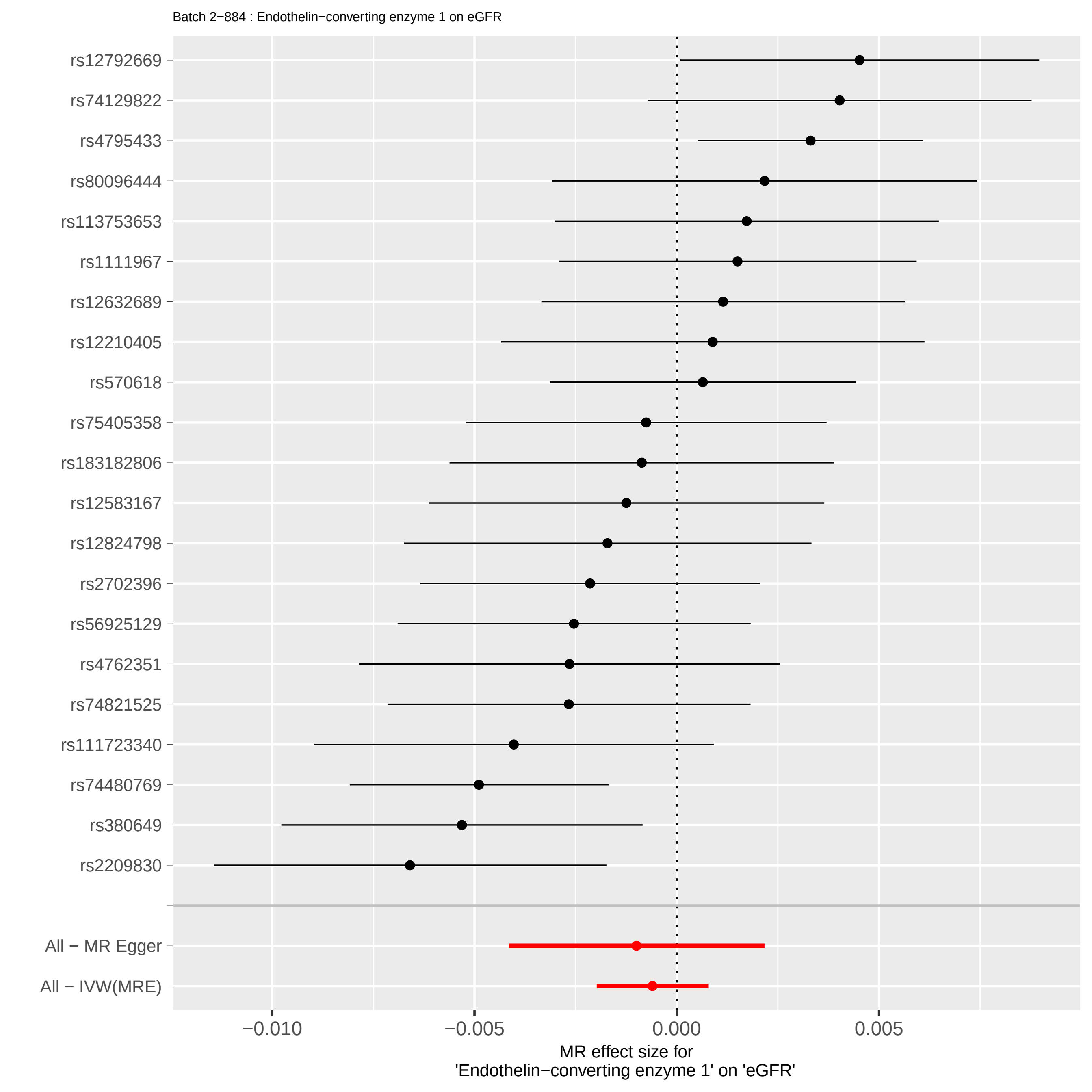
Batch 2−884 : Endothelin−converting enzyme 1 on eGFR
rs12792669
rs74129822
rs4795433
rs80096444
rs113753653
rs1111967
rs12632689
rs12210405
rs570618
rs75405358
rs183182806
rs12583167
rs12824798
rs2702396
rs56925129
rs4762351
rs74821525
rs111723340
rs74480769
rs380649
rs2209830
All − MR Egger
All − IVW(MRE)
−0.010
−0.005
0.000
0.005
MR effect size for
'Endothelin−converting enzyme 1' on 'eGFR'

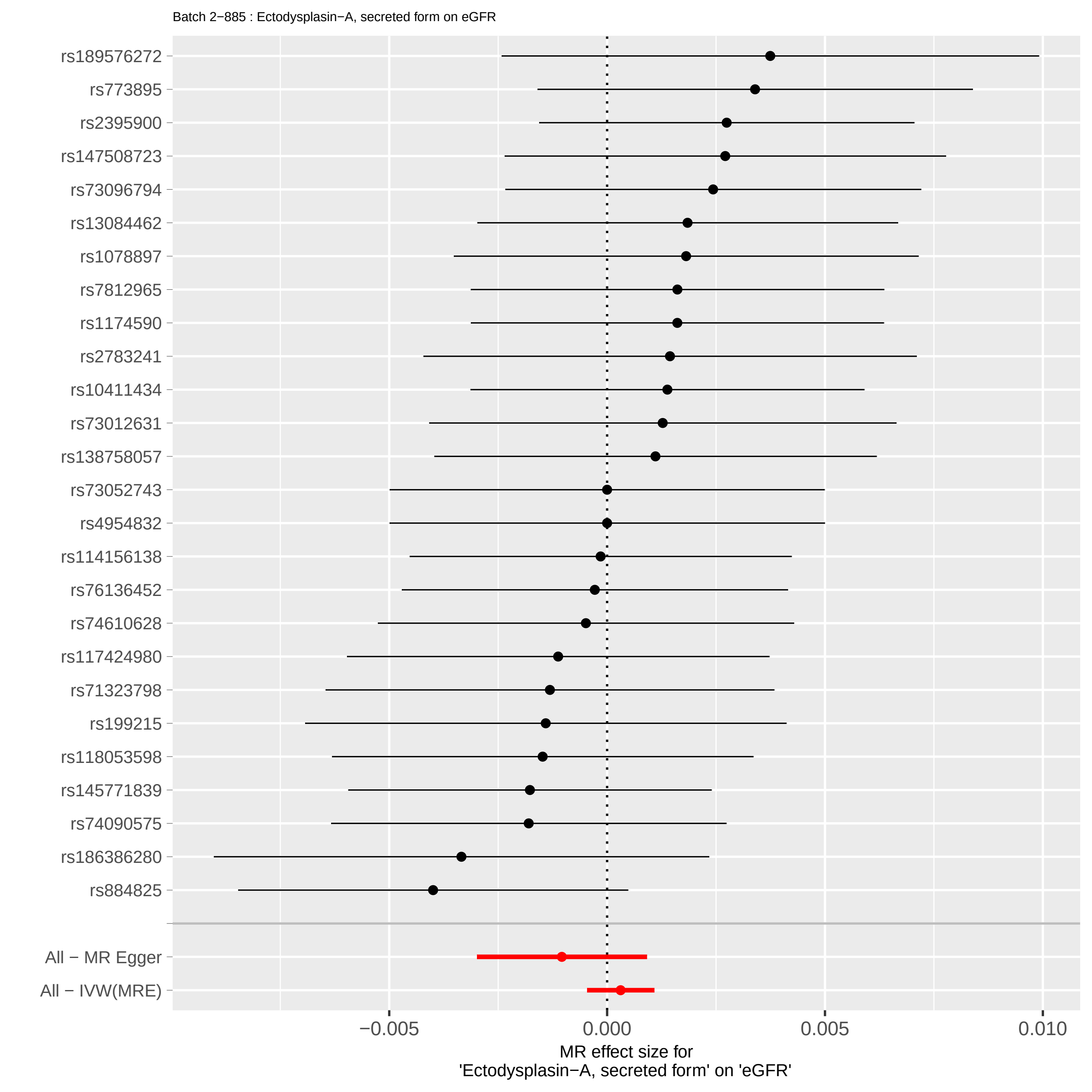

Batch 2−885 : Ectodysplasin−A, secreted form on eGFR
rs189576272
rs773895
rs2395900
rs147508723
rs73096794
rs13084462
rs1078897
rs7812965
rs1174590
rs2783241
rs10411434
rs73012631
rs138758057
rs73052743
rs4954832
rs114156138
rs76136452
rs74610628
rs117424980
rs71323798
rs199215
rs118053598
rs145771839
rs74090575
rs186386280
rs884825
All − MR Egger
All − IVW(MRE)
−0.005
0.000
0.005
0.010
MR effect size for
'Ectodysplasin−A, secreted form' on 'eGFR'

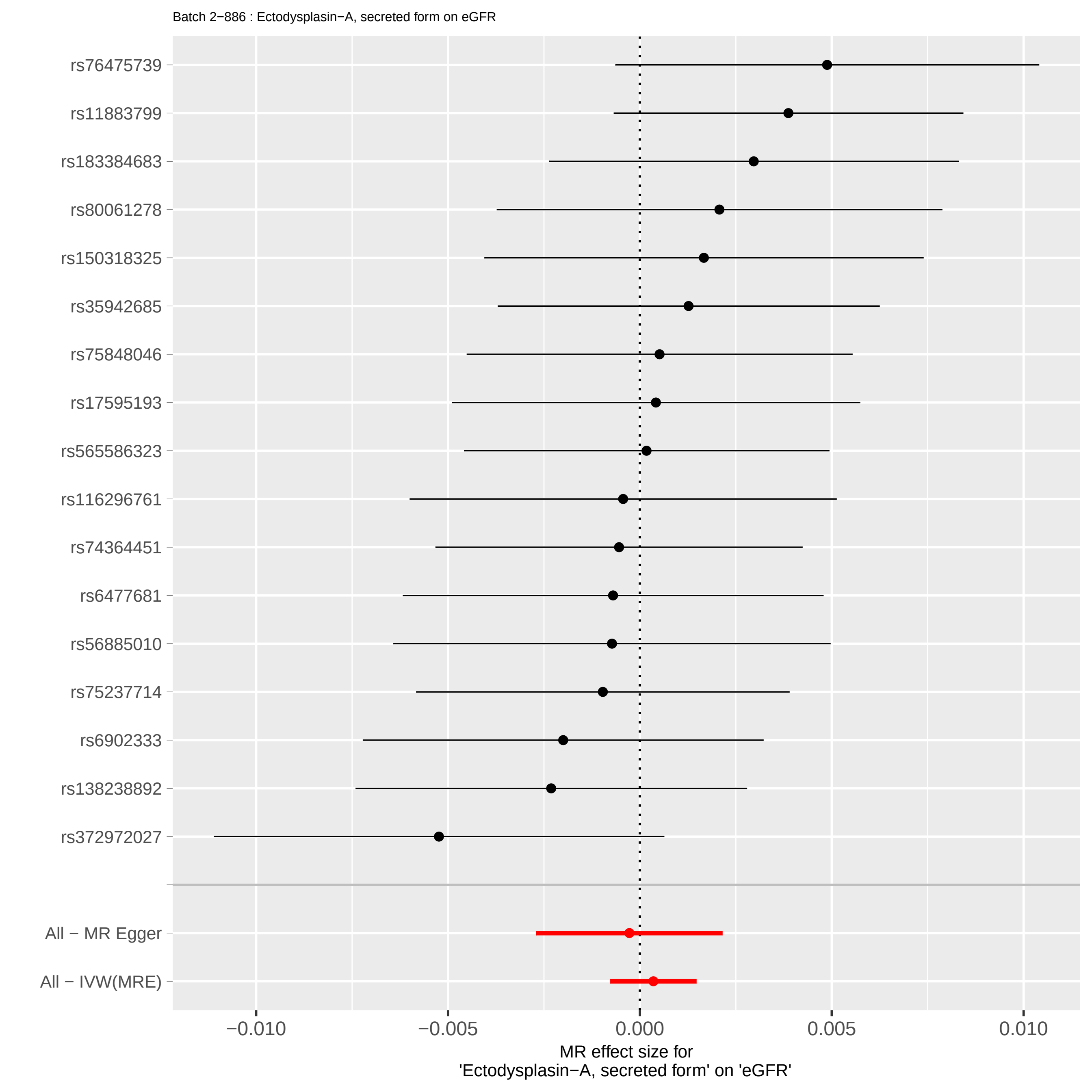
Batch 2−886 : Ectodysplasin−A, secreted form on eGFR
rs76475739
rs11883799
rs183384683
rs80061278
rs150318325
rs35942685
rs75848046
rs17595193
rs565586323
rs116296761
rs74364451
rs6477681
rs56885010
rs75237714
rs6902333
rs138238892
rs372972027
All − MR Egger
All − IVW(MRE)
−0.010
−0.005
0.000
0.005
0.010
MR effect size for
'Ectodysplasin−A, secreted form' on 'eGFR'

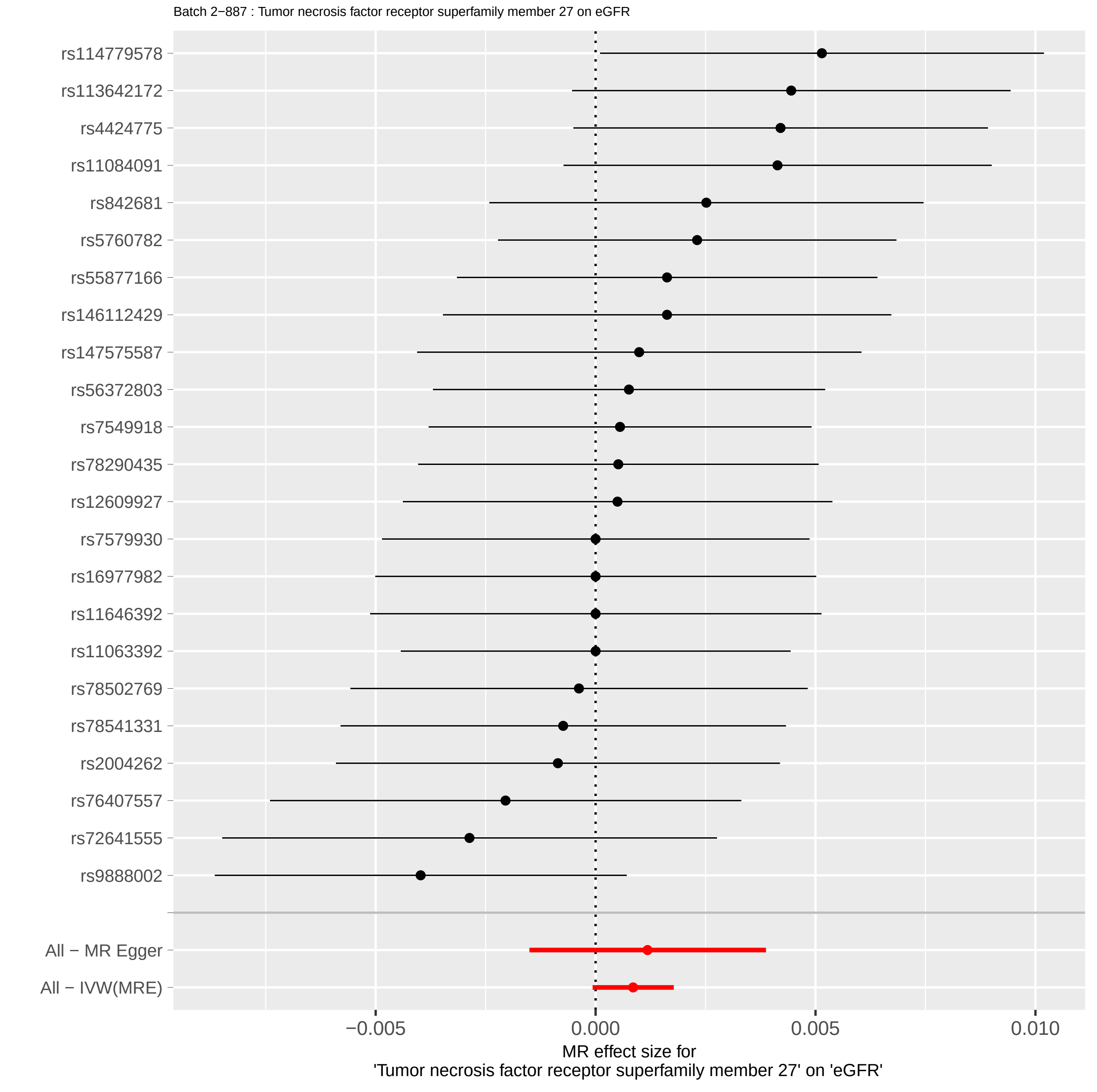
Batch 2−887 : Tumor necrosis factor receptor superfamily member 27 on eGFR
rs114779578
rs113642172
rs4424775
rs11084091
rs842681
rs5760782
rs55877166
rs146112429
rs147575587
rs56372803
rs7549918
rs78290435
rs12609927
rs7579930
rs16977982
rs11646392
rs11063392
rs78502769
rs78541331
rs2004262
rs76407557
rs72641555
rs9888002
All − MR Egger
All − IVW(MRE)
−0.005
0.000
0.005
0.010
MR effect size for
'Tumor necrosis factor receptor superfamily member 27' on 'eGFR'

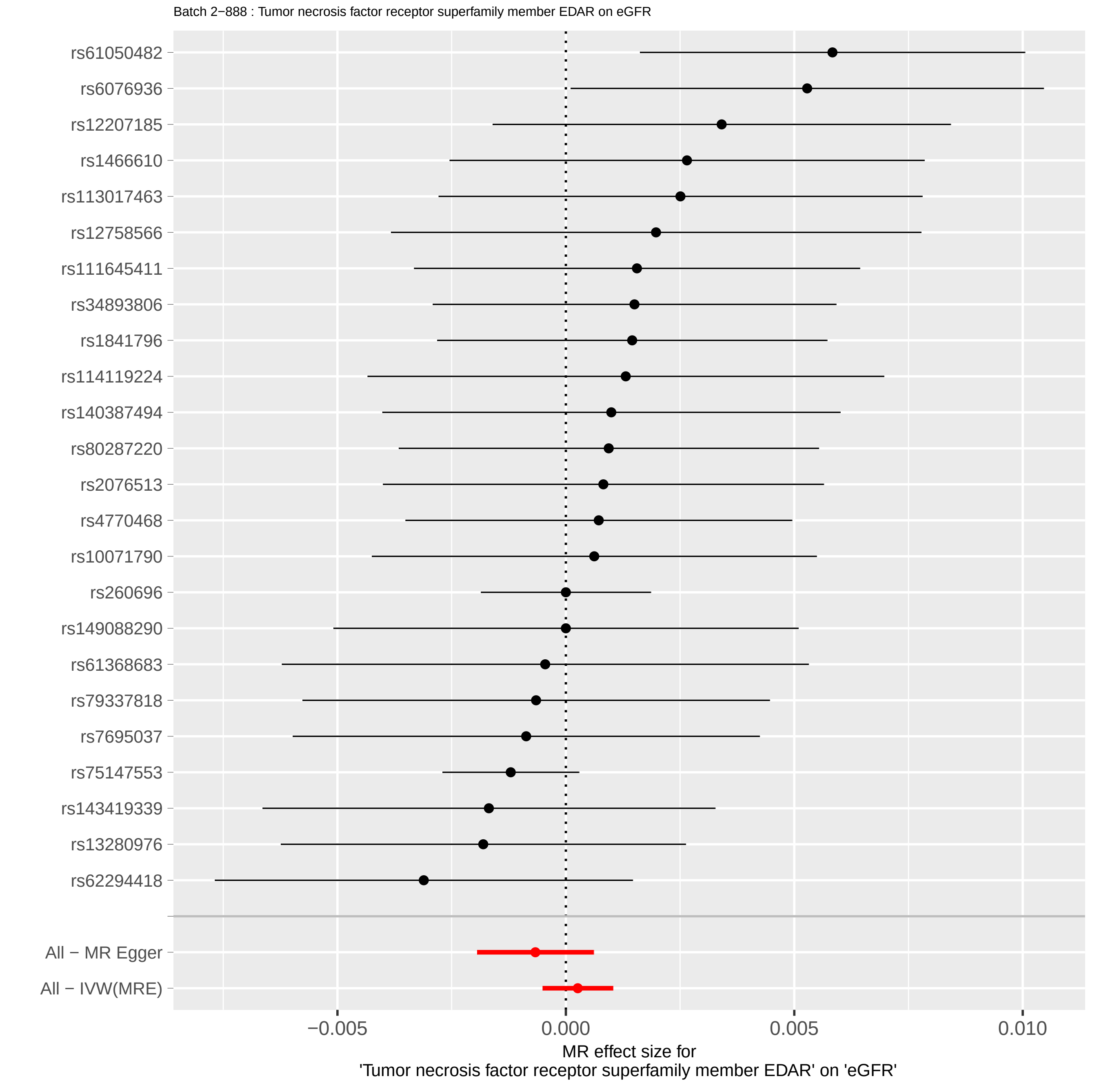
Batch 2−888 : Tumor necrosis factor receptor superfamily member EDAR on eGFR
rs61050482
rs6076936
rs12207185
rs1466610
rs113017463
rs12758566
rs111645411
rs34893806
rs1841796
rs114119224
rs140387494
rs80287220
rs2076513
rs4770468
rs10071790
rs260696
rs149088290
rs61368683
rs79337818
rs7695037
rs75147553
rs143419339
rs13280976
rs62294418
All − MR Egger
All − IVW(MRE)
−0.005
0.000
0.005
0.010
MR effect size for
'Tumor necrosis factor receptor superfamily member EDAR' on 'eGFR'

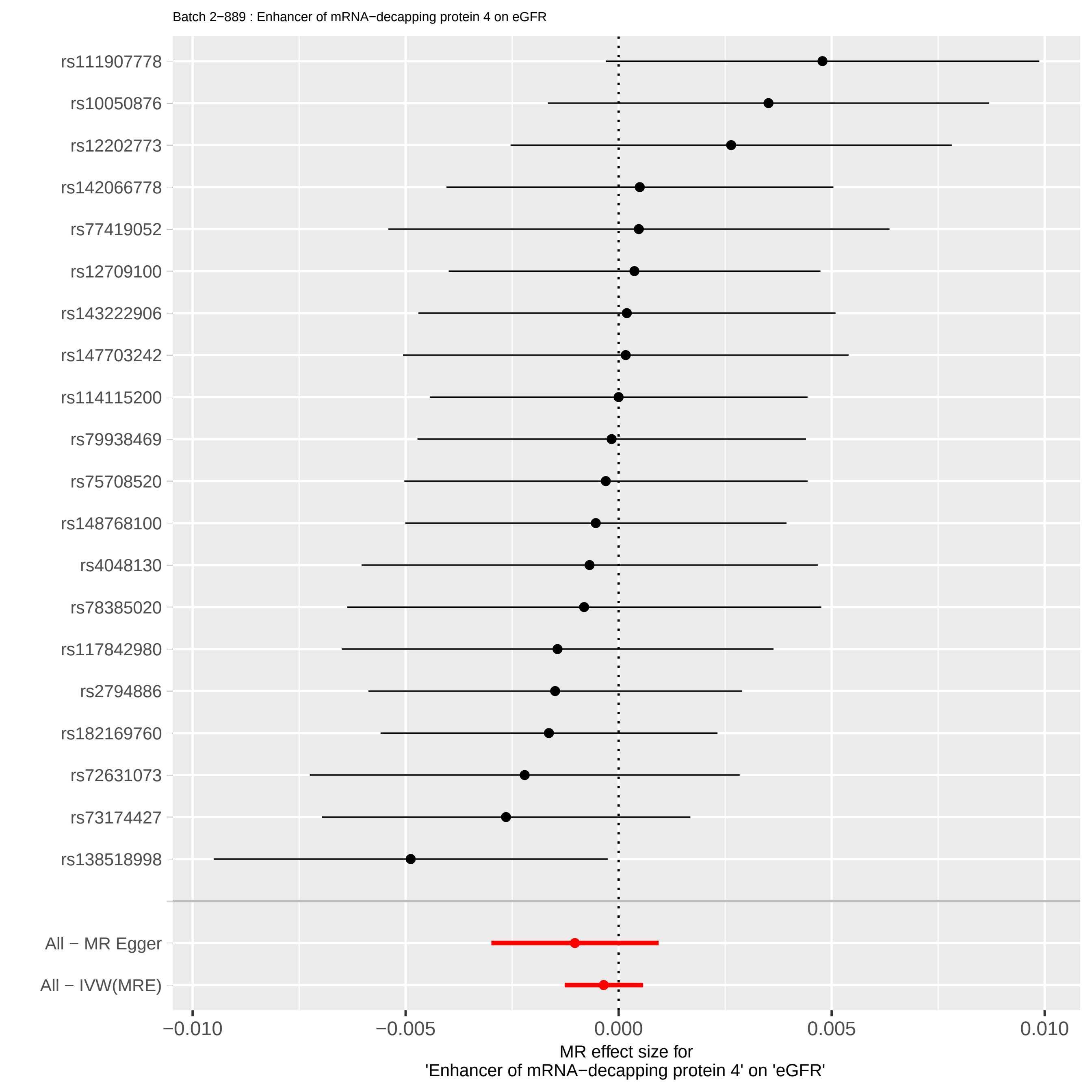

Batch 2−889 : Enhancer of mRNA−decapping protein 4 on eGFR
rs111907778
rs10050876
rs12202773
rs142066778
rs77419052
rs12709100
rs143222906
rs147703242
rs114115200
rs79938469
rs75708520
rs148768100
rs4048130
rs78385020
rs117842980
rs2794886
rs182169760
rs72631073
rs73174427
rs138518998
All − MR Egger
All − IVW(MRE)
−0.010
−0.005
0.000
0.005
0.010
MR effect size for
'Enhancer of mRNA−decapping protein 4' on 'eGFR'

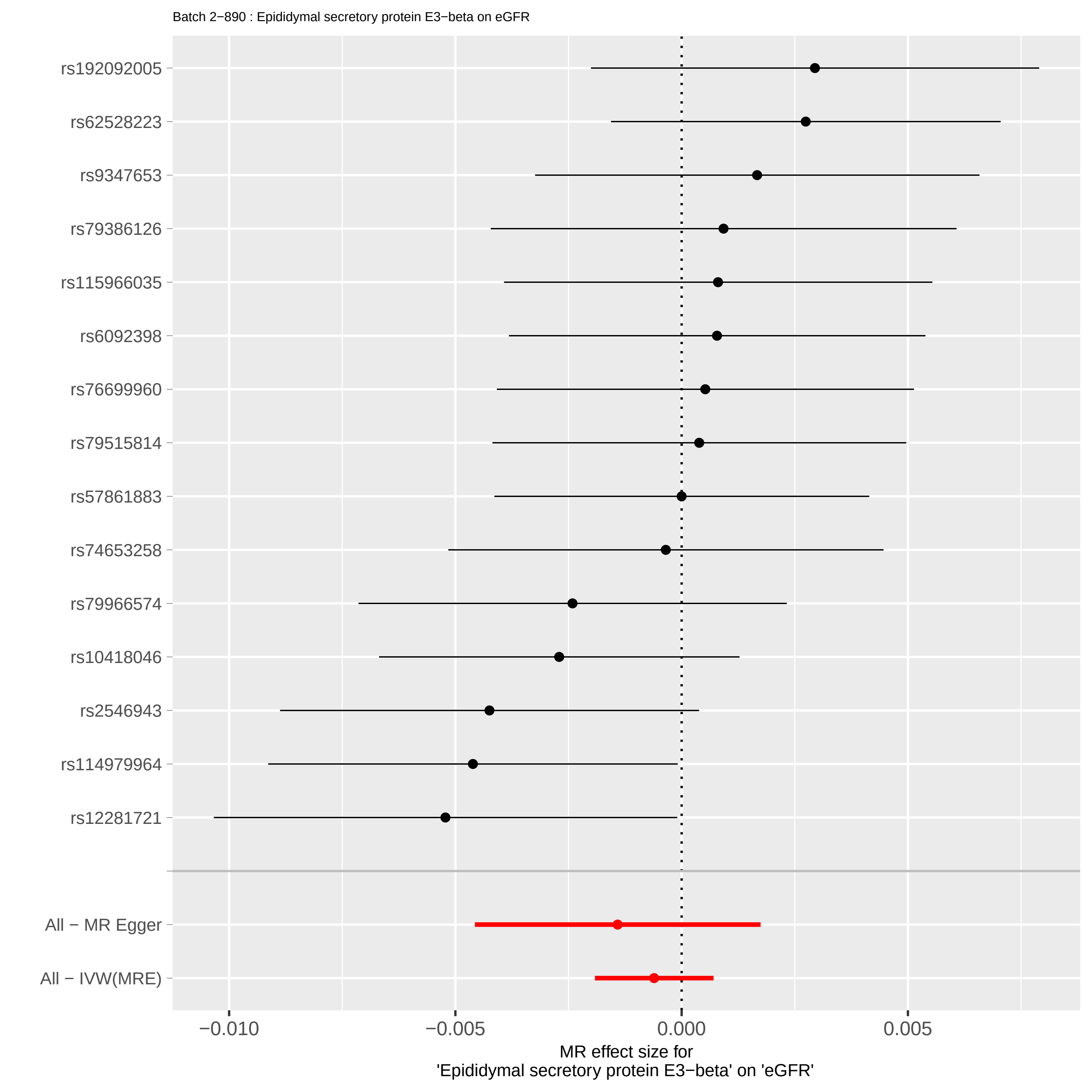

Batch 2−890 : Epididymal secretory protein E3−beta on eGFR
rs192092005
rs62528223
rs9347653
rs79386126
rs115966035
rs6092398
rs76699960
rs79515814
rs57861883
rs74653258
rs79966574
rs10418046
rs2546943
rs114979964
rs12281721
All − MR Egger
All − IVW(MRE)
−0.010
−0.005
0.000
0.005
MR effect size for
'Epididymal secretory protein E3−beta' on 'eGFR'

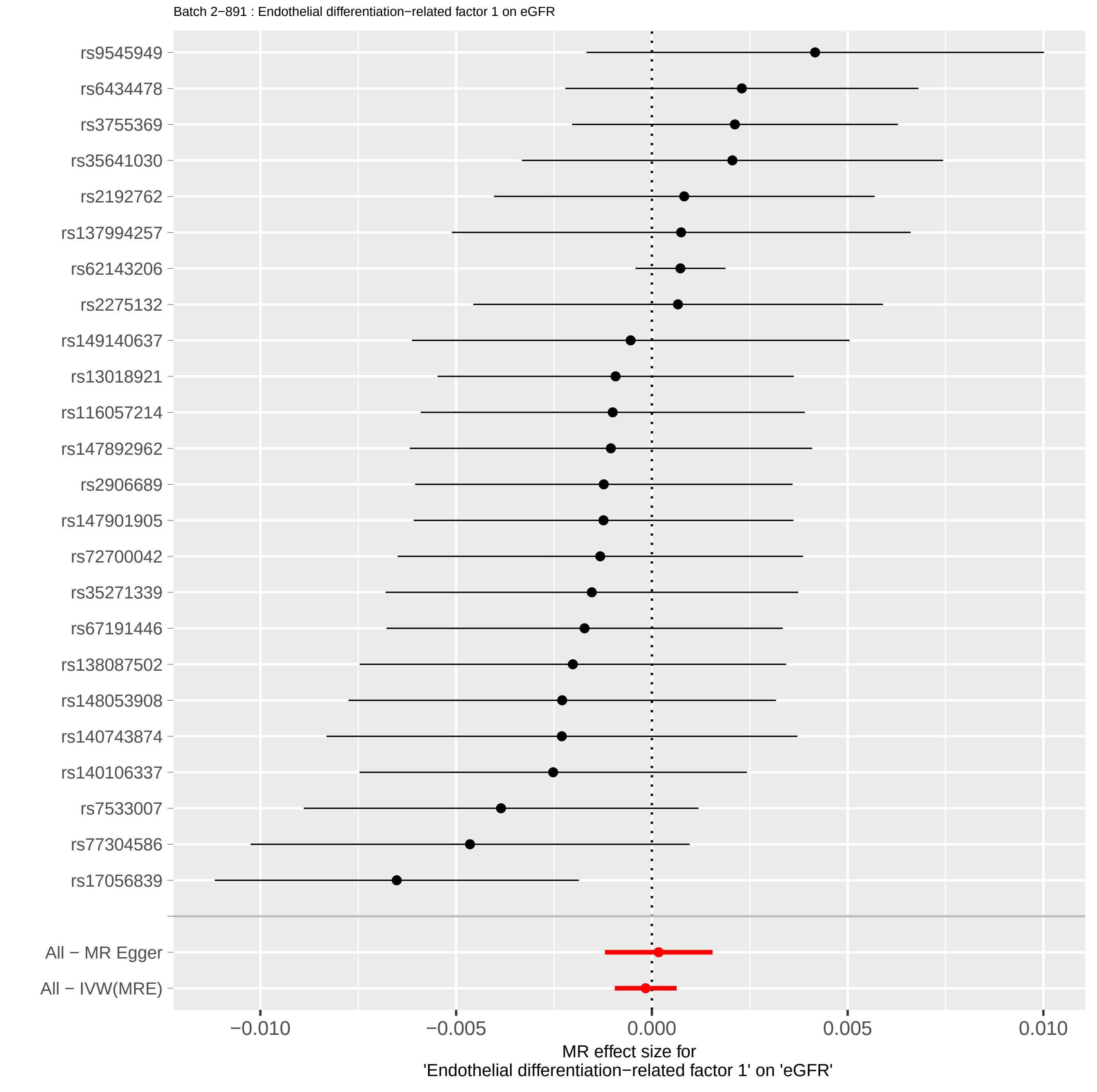
Batch 2−891 : Endothelial differentiation−related factor 1 on eGFR
rs9545949
rs6434478
rs3755369
rs35641030
rs2192762
rs137994257
rs62143206
rs2275132
rs149140637
rs13018921
rs116057214
rs147892962
rs2906689
rs147901905
rs72700042
rs35271339
rs67191446
rs138087502
rs148053908
rs140743874
rs140106337
rs7533007
rs77304586
rs17056839
All − MR Egger
All − IVW(MRE)
−0.010
−0.005
0.000
0.005
0.010
MR effect size for
'Endothelial differentiation−related factor 1' on 'eGFR'

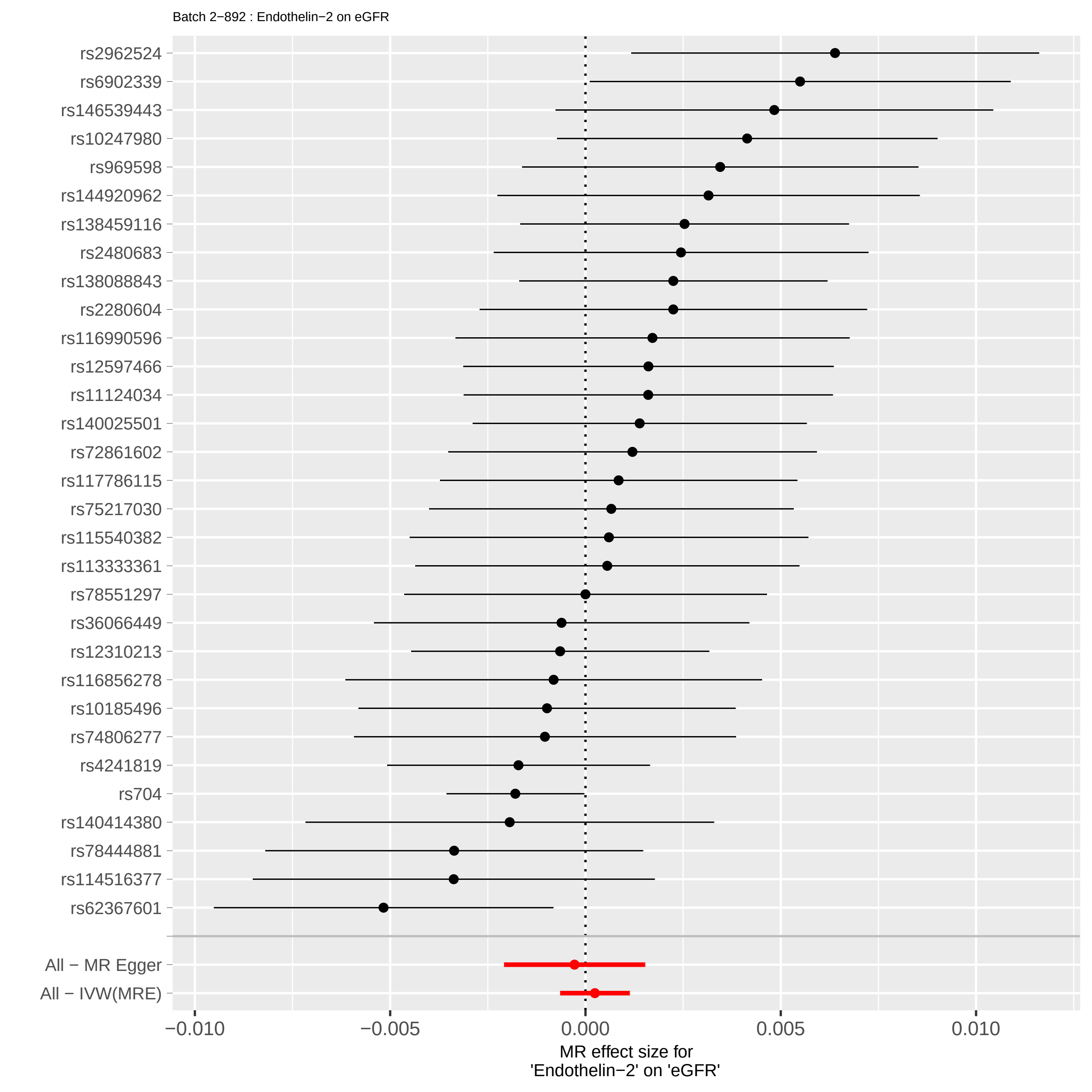

Batch 2−892 : Endothelin−2 on eGFR
rs2962524
rs6902339
rs146539443
rs10247980
rs969598
rs144920962
rs138459116
rs2480683
rs138088843
rs2280604
rs116990596
rs12597466
rs11124034
rs140025501
rs72861602
rs117786115
rs75217030
rs115540382
rs113333361
rs78551297
rs36066449
rs12310213
rs116856278
rs10185496
rs74806277
rs4241819
rs704
rs140414380
rs78444881
rs114516377
rs62367601
All − MR Egger
All − IVW(MRE)
−0.010
−0.005
0.000
0.005
0.010
MR effect size for
'Endothelin−2' on 'eGFR'

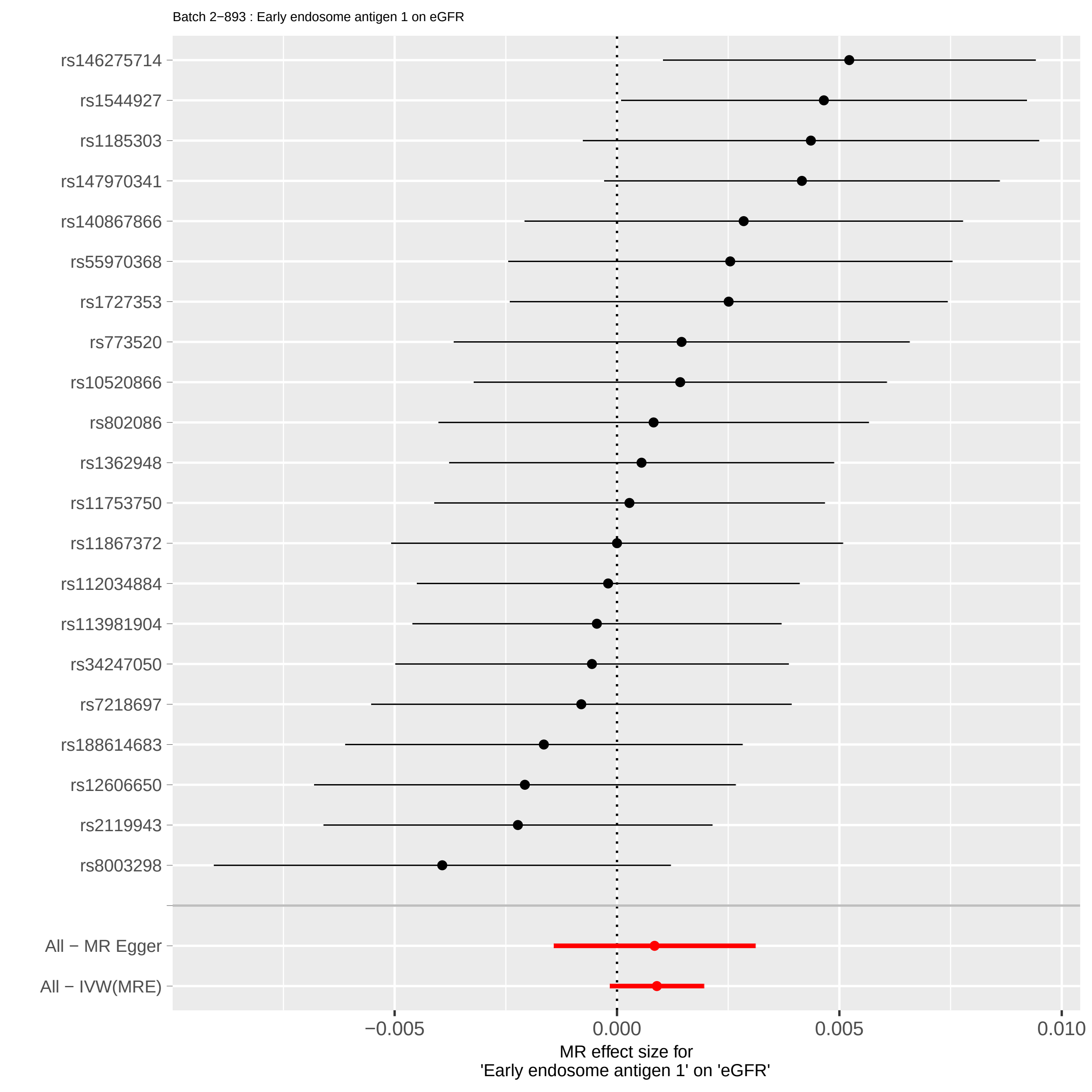
Batch 2−893 : Early endosome antigen 1 on eGFR
rs146275714
rs1544927
rs1185303
rs147970341
rs140867866
rs55970368
rs1727353
rs773520
rs10520866
rs802086
rs1362948
rs11753750
rs11867372
rs112034884
rs113981904
rs34247050
rs7218697
rs188614683
rs12606650
rs2119943
rs8003298
All − MR Egger
All − IVW(MRE)
−0.005
0.000
0.005
0.010
MR effect size for
'Early endosome antigen 1' on 'eGFR'

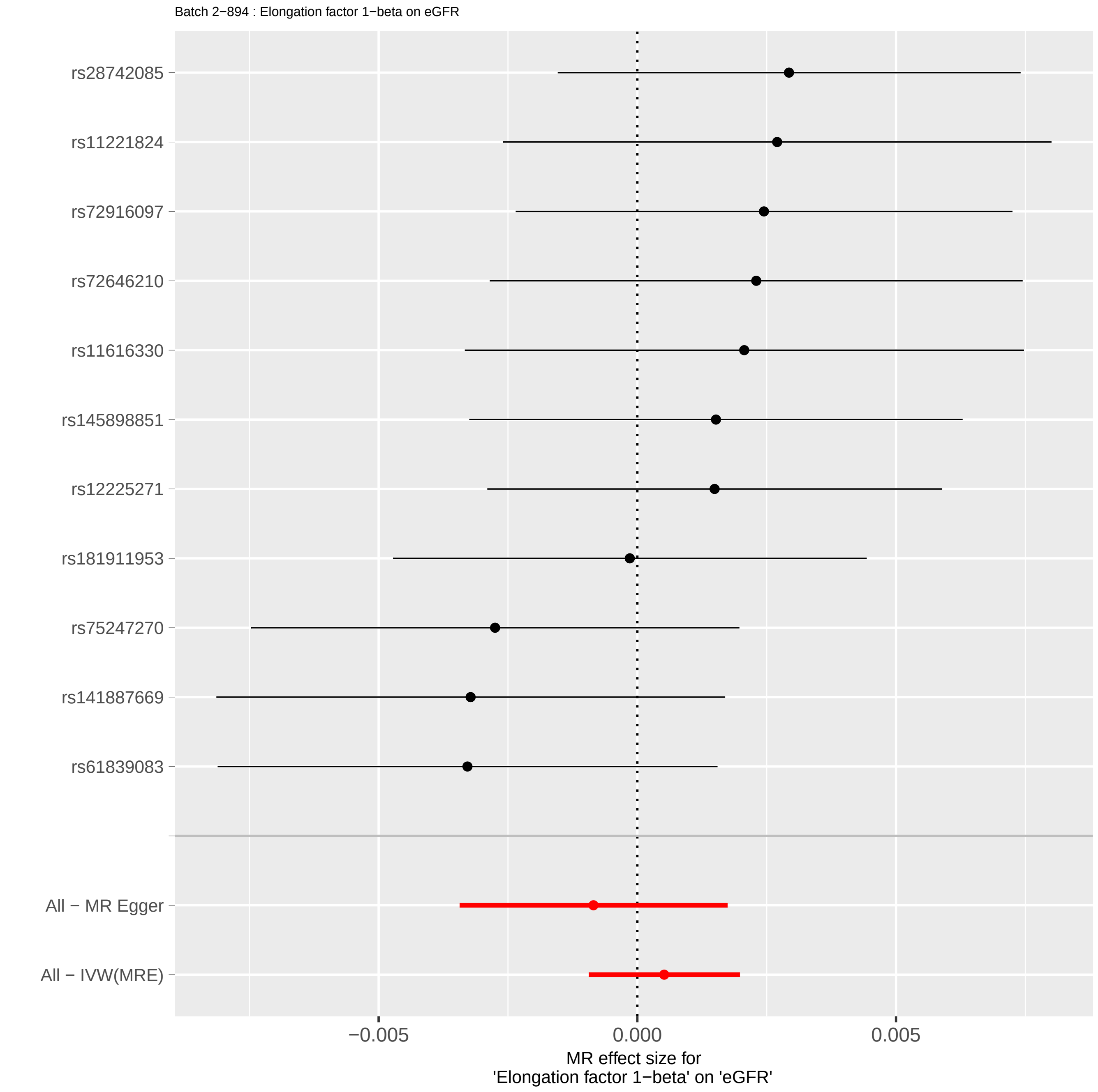

Batch 2−894 : Elongation factor 1−beta on eGFR
rs28742085
rs11221824
rs72916097
rs72646210
rs11616330
rs145898851
rs12225271
rs181911953
rs75247270
rs141887669
rs61839083
All − MR Egger
All − IVW(MRE)
−0.005
0.000
0.005
MR effect size for
'Elongation factor 1−beta' on 'eGFR'

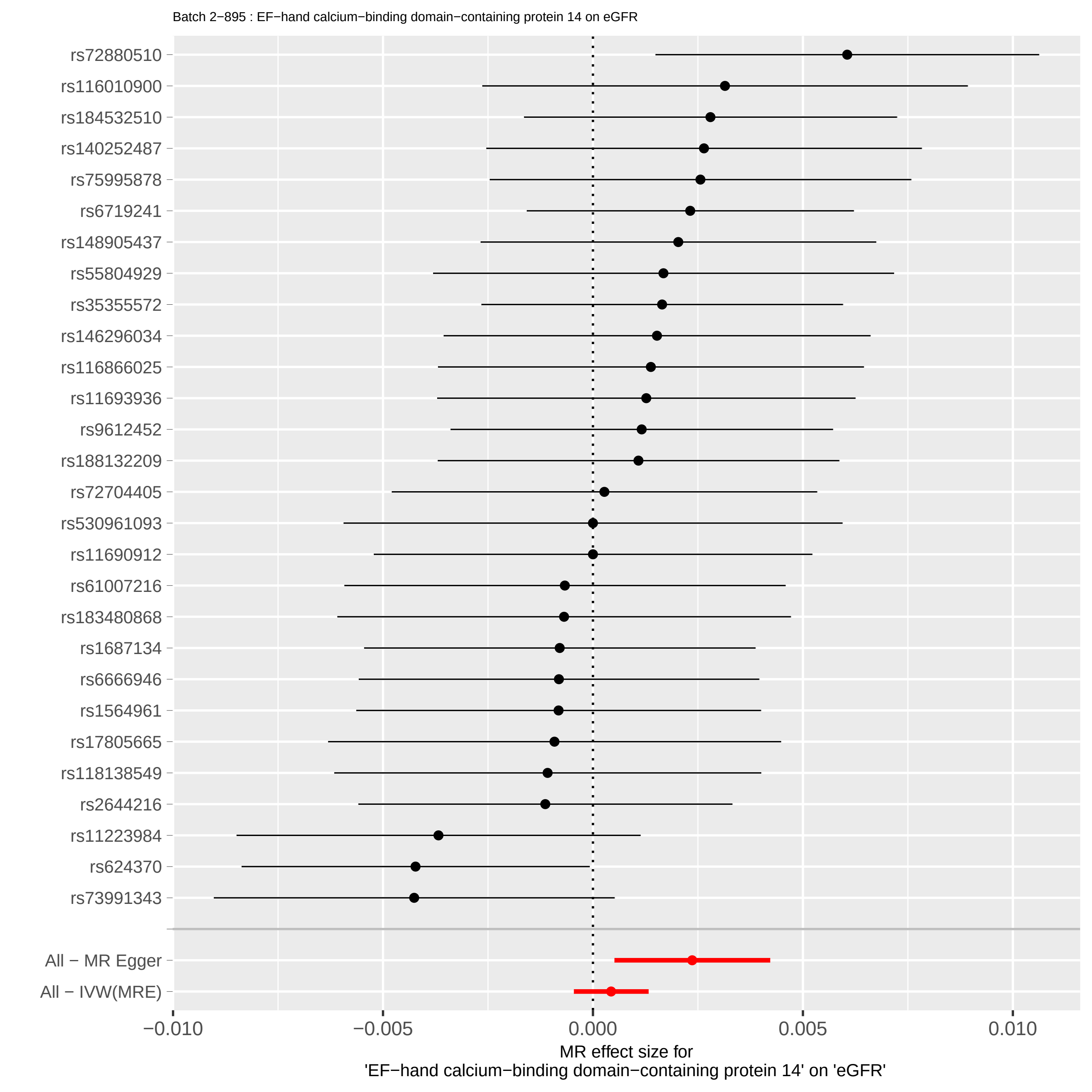

Batch 2−895 : EF−hand calcium−binding domain−containing protein 14 on eGFR
rs72880510
rs116010900
rs184532510
rs140252487
rs75995878
rs6719241
rs148905437
rs55804929
rs35355572
rs146296034
rs116866025
rs11693936
rs9612452
rs188132209
rs72704405
rs530961093
rs11690912
rs61007216
rs183480868
rs1687134
rs6666946
rs1564961
rs17805665
rs118138549
rs2644216
rs11223984
rs624370
rs73991343
All − MR Egger
All − IVW(MRE)
−0.010
−0.005
0.000
0.005
0.010
MR effect size for
'EF−hand calcium−binding domain−containing protein 14' on 'eGFR'

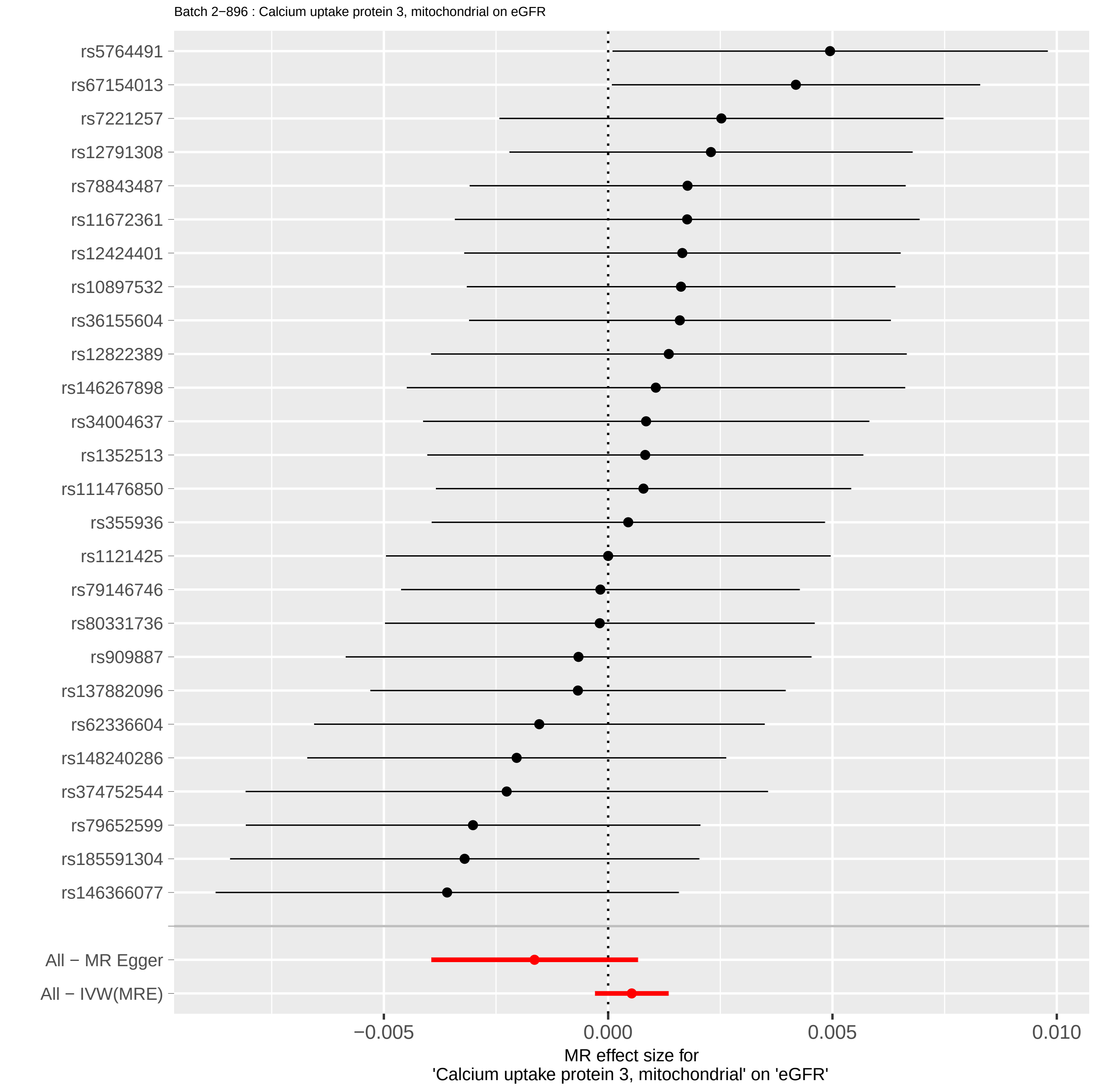

Batch 2−896 : Calcium uptake protein 3, mitochondrial on eGFR
rs5764491
rs67154013
rs7221257
rs12791308
rs78843487
rs11672361
rs12424401
rs10897532
rs36155604
rs12822389
rs146267898
rs34004637
rs1352513
rs111476850
rs355936
rs1121425
rs79146746
rs80331736
rs909887
rs137882096
rs62336604
rs148240286
rs374752544
rs79652599
rs185591304
rs146366077
All − MR Egger
All − IVW(MRE)
−0.005
0.000
0.005
0.010
MR effect size for
'Calcium uptake protein 3, mitochondrial' on 'eGFR'

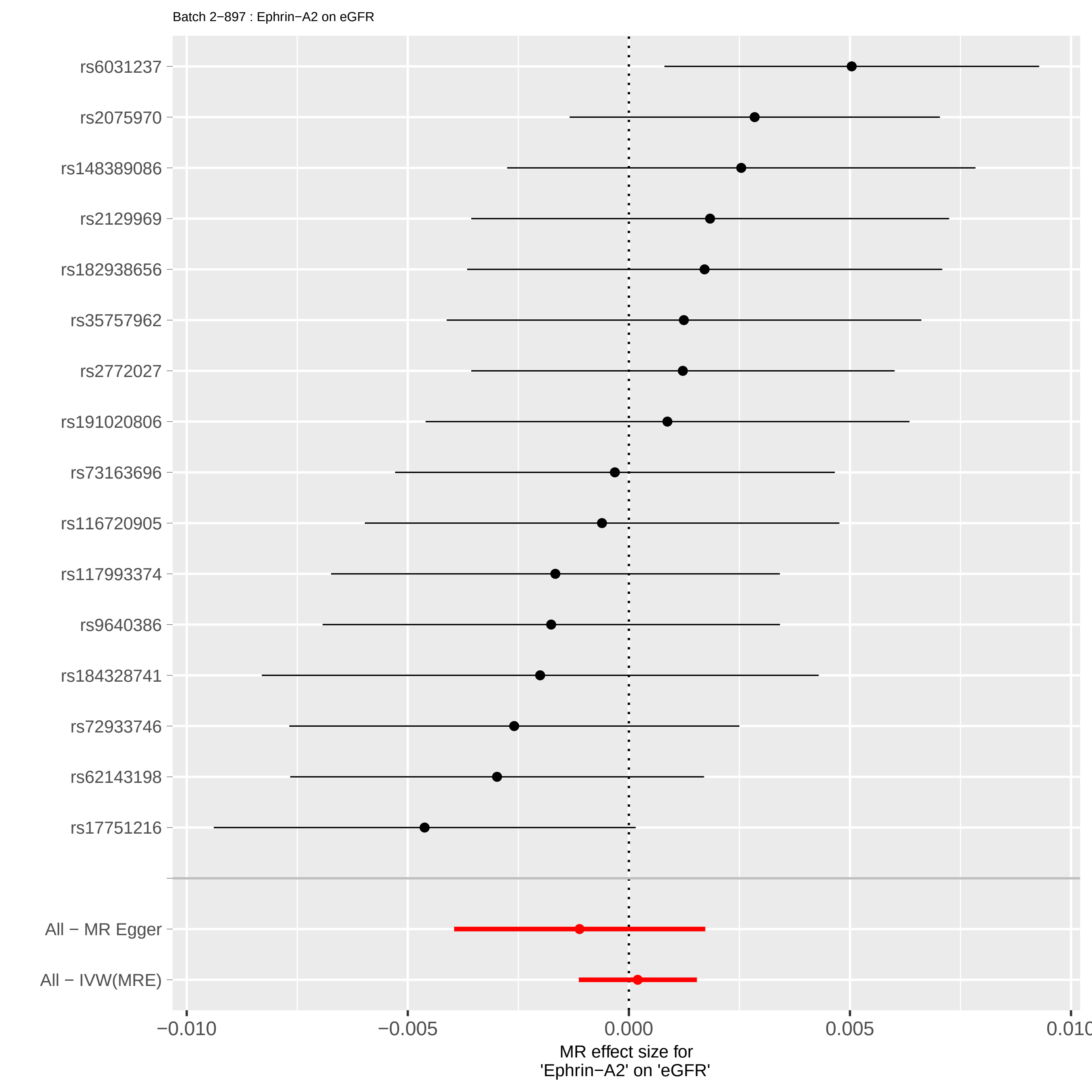

Batch 2−897 : Ephrin−A2 on eGFR
rs6031237
rs2075970
rs148389086
rs2129969
rs182938656
rs35757962
rs2772027
rs191020806
rs73163696
rs116720905
rs117993374
rs9640386
rs184328741
rs72933746
rs62143198
rs17751216
All − MR Egger
All − IVW(MRE)
−0.010
−0.005
0.000
0.005
0.010
MR effect size for
'Ephrin−A2' on 'eGFR'

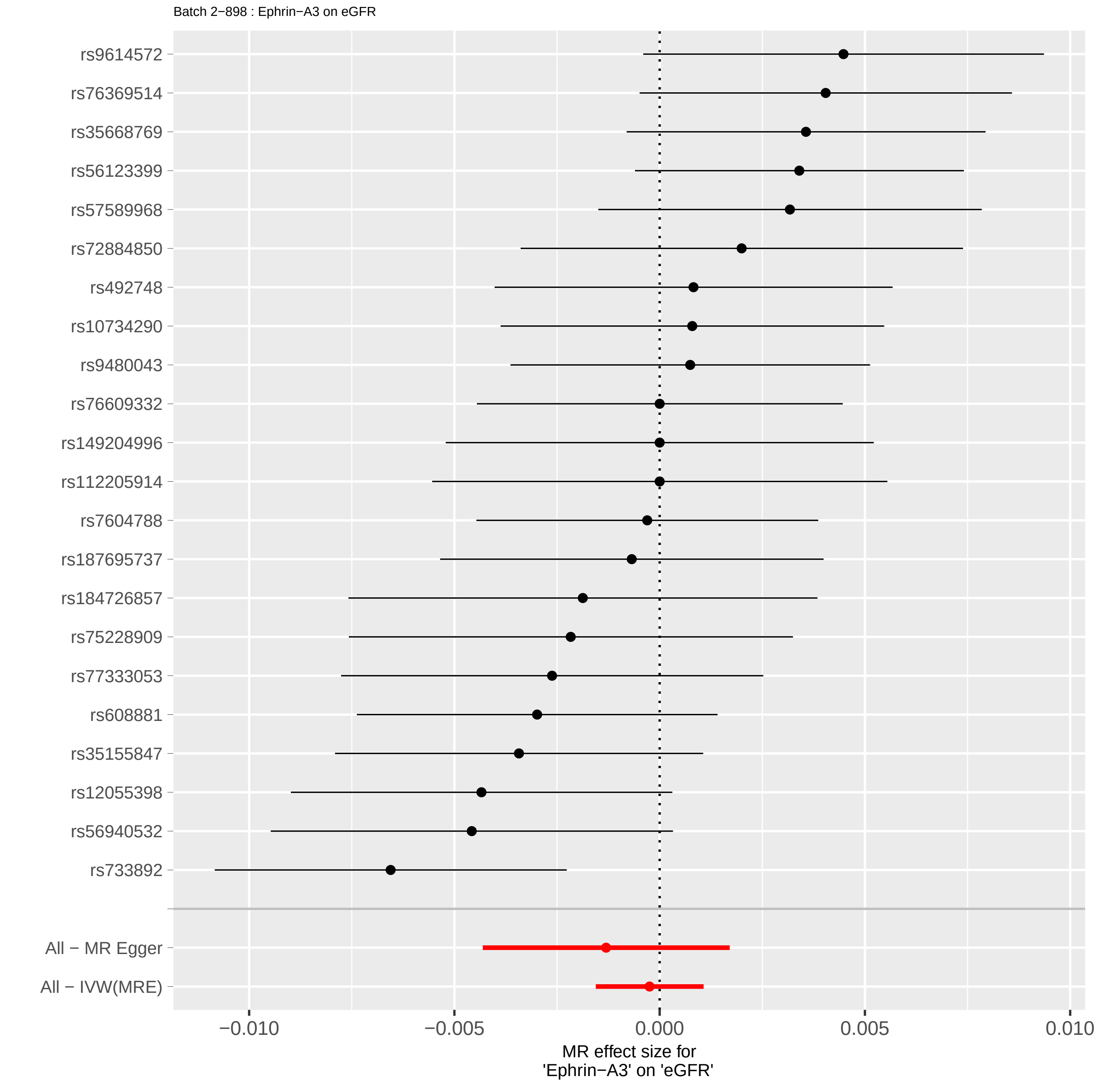

Batch 2−898 : Ephrin−A3 on eGFR
rs9614572
rs76369514
rs35668769
rs56123399
rs57589968
rs72884850
rs492748
rs10734290
rs9480043
rs76609332
rs149204996
rs112205914
rs7604788
rs187695737
rs184726857
rs75228909
rs77333053
rs608881
rs35155847
rs12055398
rs56940532
rs733892
All − MR Egger
All − IVW(MRE)
−0.010
−0.005
0.000
0.005
0.010
MR effect size for
'Ephrin−A3' on 'eGFR'

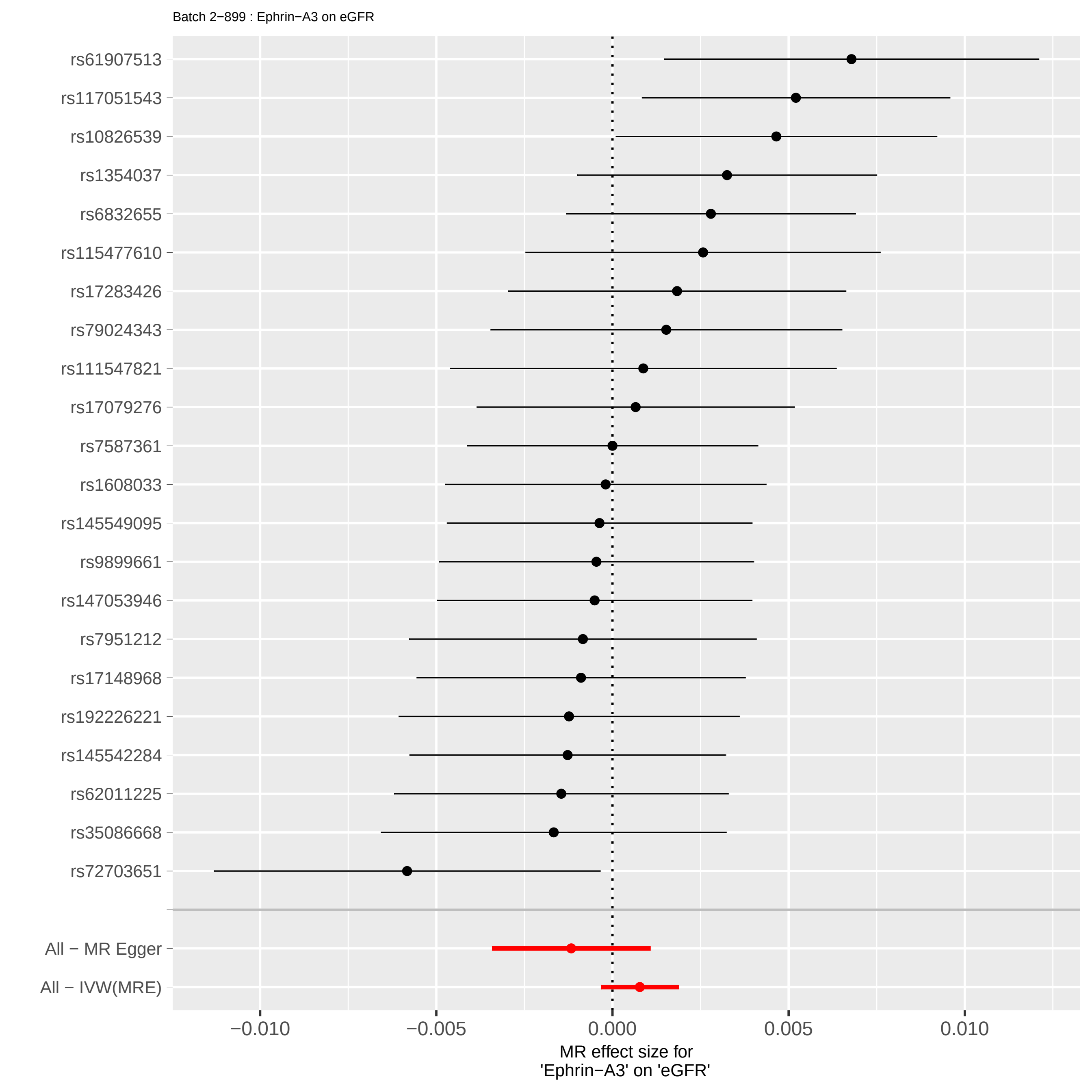

Batch 2−899 : Ephrin−A3 on eGFR
rs61907513
rs117051543
rs10826539
rs1354037
rs6832655
rs115477610
rs17283426
rs79024343
rs111547821
rs17079276
rs7587361
rs1608033
rs145549095
rs9899661
rs147053946
rs7951212
rs17148968
rs192226221
rs145542284
rs62011225
rs35086668
rs72703651
All − MR Egger
All − IVW(MRE)
−0.010
−0.005
0.000
0.005
0.010
MR effect size for
'Ephrin−A3' on 'eGFR'

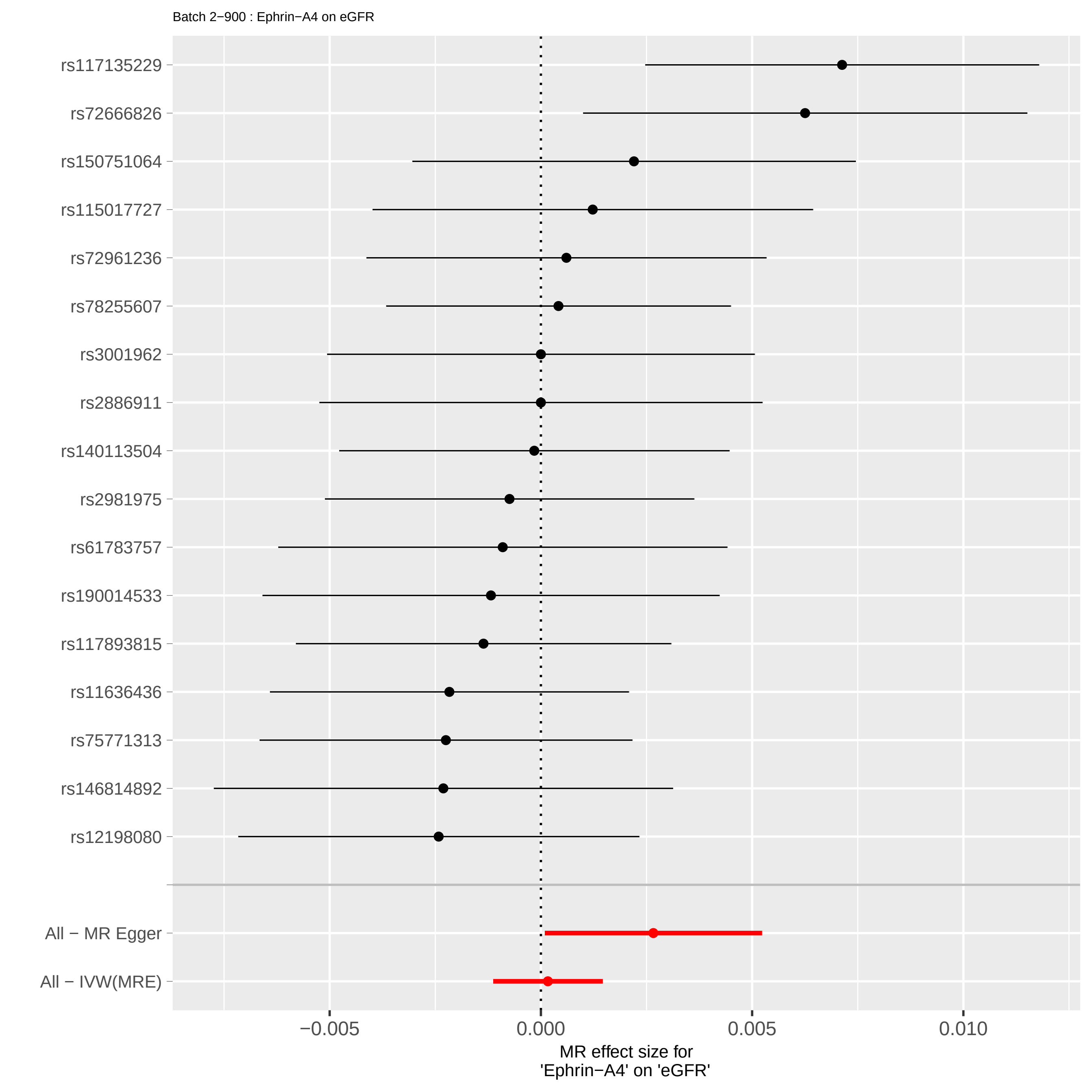

Batch 2−900 : Ephrin−A4 on eGFR
rs117135229
rs72666826
rs150751064
rs115017727
rs72961236
rs78255607
rs3001962
rs2886911
rs140113504
rs2981975
rs61783757
rs190014533
rs117893815
rs11636436
rs75771313
rs146814892
rs12198080
All − MR Egger
All − IVW(MRE)
−0.005
0.000
0.005
0.010
MR effect size for
'Ephrin−A4' on 'eGFR'

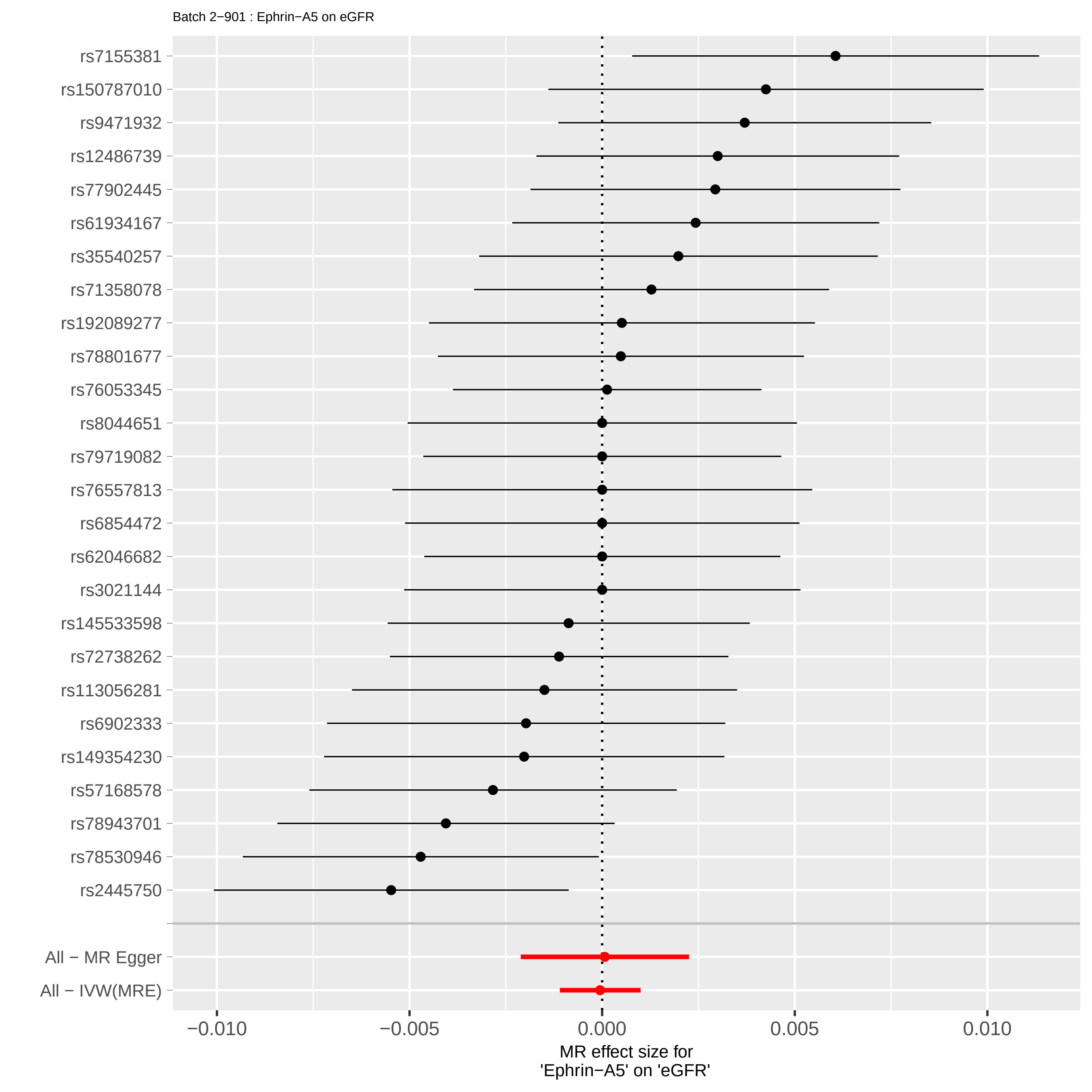

Batch 2−901 : Ephrin−A5 on eGFR
rs7155381
rs150787010
rs9471932
rs12486739
rs77902445
rs61934167
rs35540257
rs71358078
rs192089277
rs78801677
rs76053345
rs8044651
rs79719082
rs76557813
rs6854472
rs62046682
rs3021144
rs145533598
rs72738262
rs113056281
rs6902333
rs149354230
rs57168578
rs78943701
rs78530946
rs2445750
All − MR Egger
All − IVW(MRE)
−0.010
−0.005
0.000
0.005
0.010
MR effect size for
'Ephrin−A5' on 'eGFR'

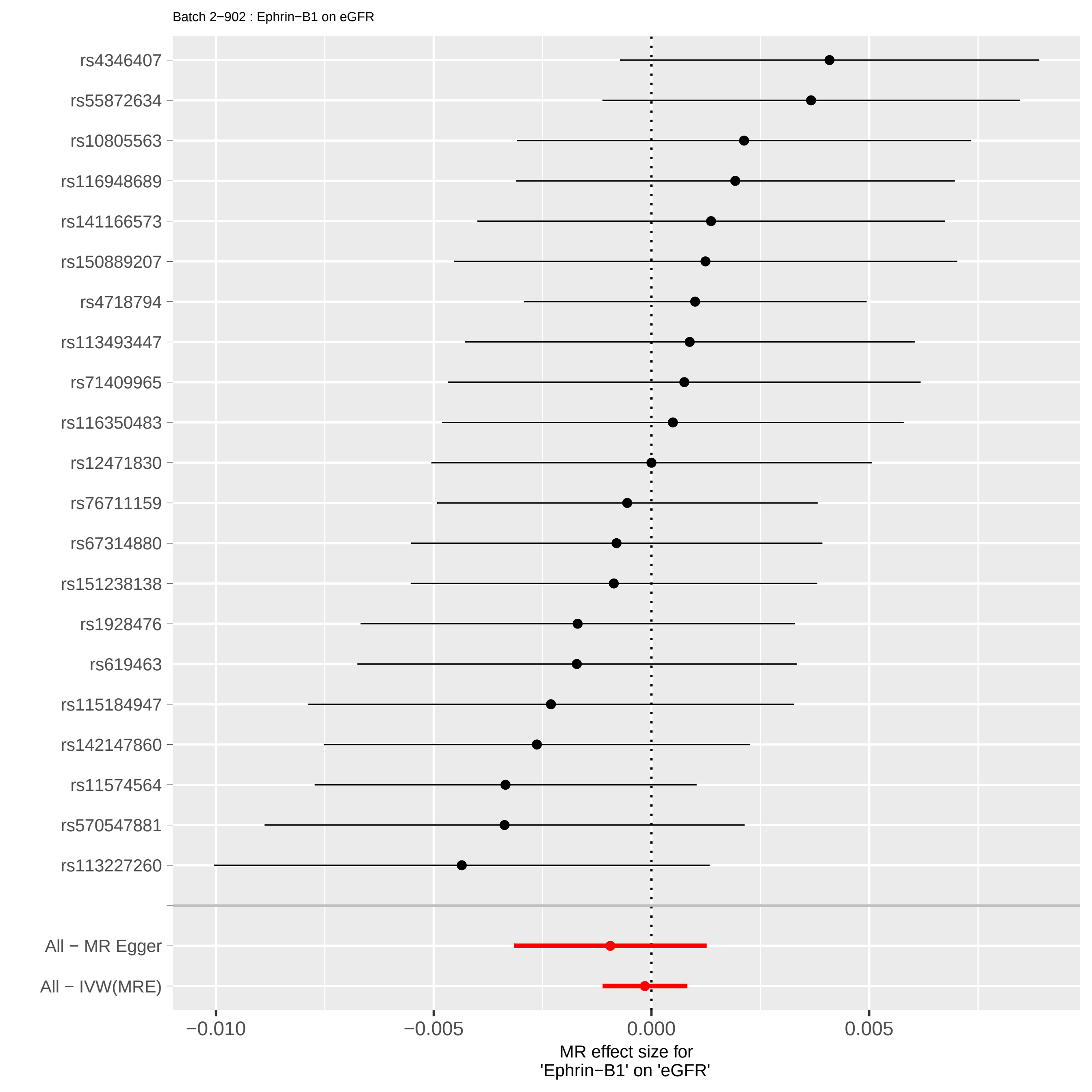

Batch 2−902 : Ephrin−B1 on eGFR
rs4346407
rs55872634
rs10805563
rs116948689
rs141166573
rs150889207
rs4718794
rs113493447
rs71409965
rs116350483
rs12471830
rs76711159
rs67314880
rs151238138
rs1928476
rs619463
rs115184947
rs142147860
rs11574564
rs570547881
rs113227260
All − MR Egger
All − IVW(MRE)
−0.010
−0.005
0.000
0.005
MR effect size for
'Ephrin−B1' on 'eGFR'

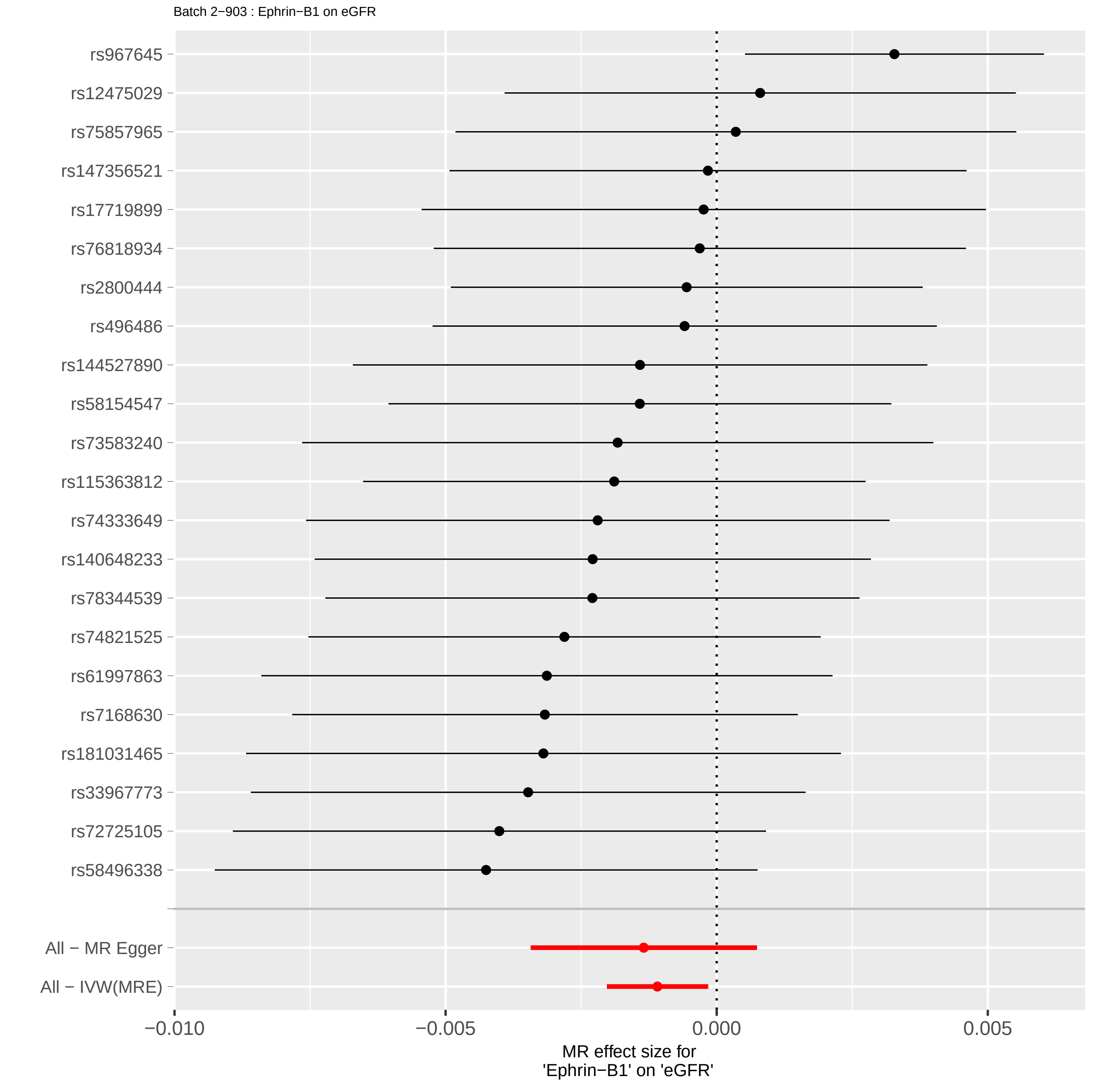

Batch 2−903 : Ephrin−B1 on eGFR
rs967645
rs12475029
rs75857965
rs147356521
rs17719899
rs76818934
rs2800444
rs496486
rs144527890
rs58154547
rs73583240
rs115363812
rs74333649
rs140648233
rs78344539
rs74821525
rs61997863
rs7168630
rs181031465
rs33967773
rs72725105
rs58496338
All − MR Egger
All − IVW(MRE)
−0.010
−0.005
0.000
0.005
MR effect size for
'Ephrin−B1' on 'eGFR'

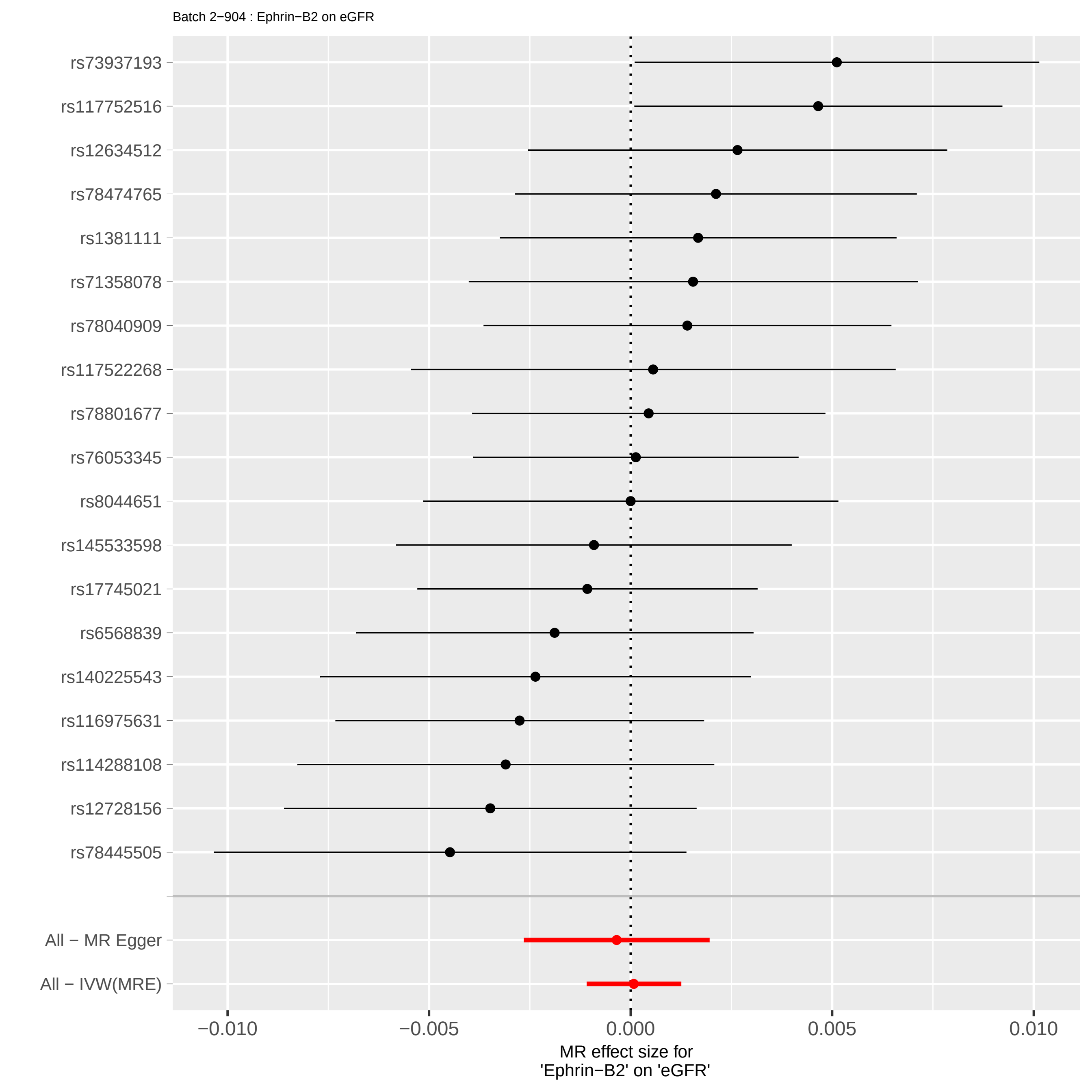

Batch 2−904 : Ephrin−B2 on eGFR
rs73937193
rs117752516
rs12634512
rs78474765
rs1381111
rs71358078
rs78040909
rs117522268
rs78801677
rs76053345
rs8044651
rs145533598
rs17745021
rs6568839
rs140225543
rs116975631
rs114288108
rs12728156
rs78445505
All − MR Egger
All − IVW(MRE)
−0.010
−0.005
0.000
0.005
0.010
MR effect size for
'Ephrin−B2' on 'eGFR'

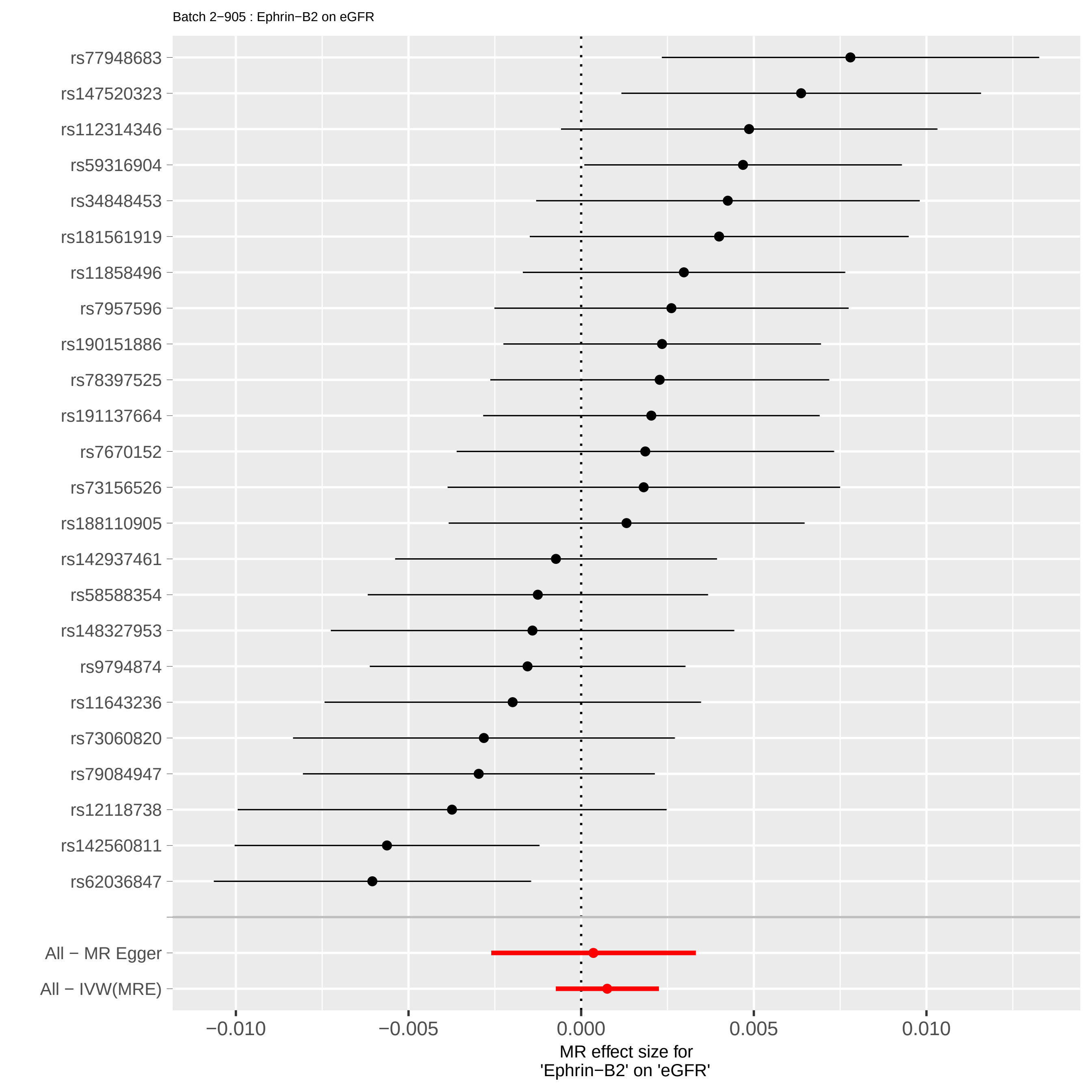

Batch 2−905 : Ephrin−B2 on eGFR
rs77948683
rs147520323
rs112314346
rs59316904
rs34848453
rs181561919
rs11858496
rs7957596
rs190151886
rs78397525
rs191137664
rs7670152
rs73156526
rs188110905
rs142937461
rs58588354
rs148327953
rs9794874
rs11643236
rs73060820
rs79084947
rs12118738
rs142560811
rs62036847
All − MR Egger
All − IVW(MRE)
−0.010
−0.005
0.000
0.005
0.010
MR effect size for
'Ephrin−B2' on 'eGFR'

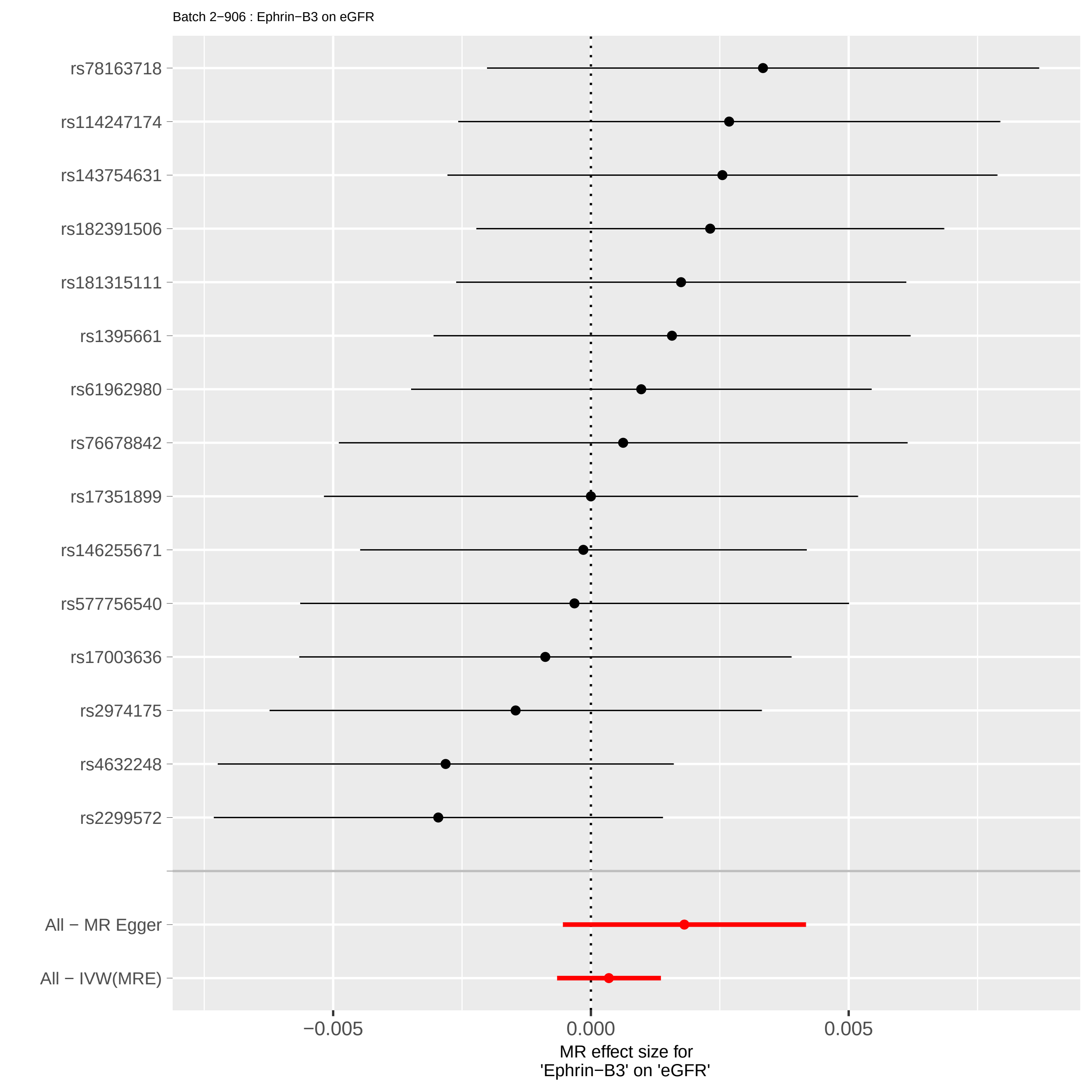

Batch 2−906 : Ephrin−B3 on eGFR
rs78163718
rs114247174
rs143754631
rs182391506
rs181315111
rs1395661
rs61962980
rs76678842
rs17351899
rs146255671
rs577756540
rs17003636
rs2974175
rs4632248
rs2299572
All − MR Egger
All − IVW(MRE)
−0.005
0.000
0.005
MR effect size for
'Ephrin−B3' on 'eGFR'

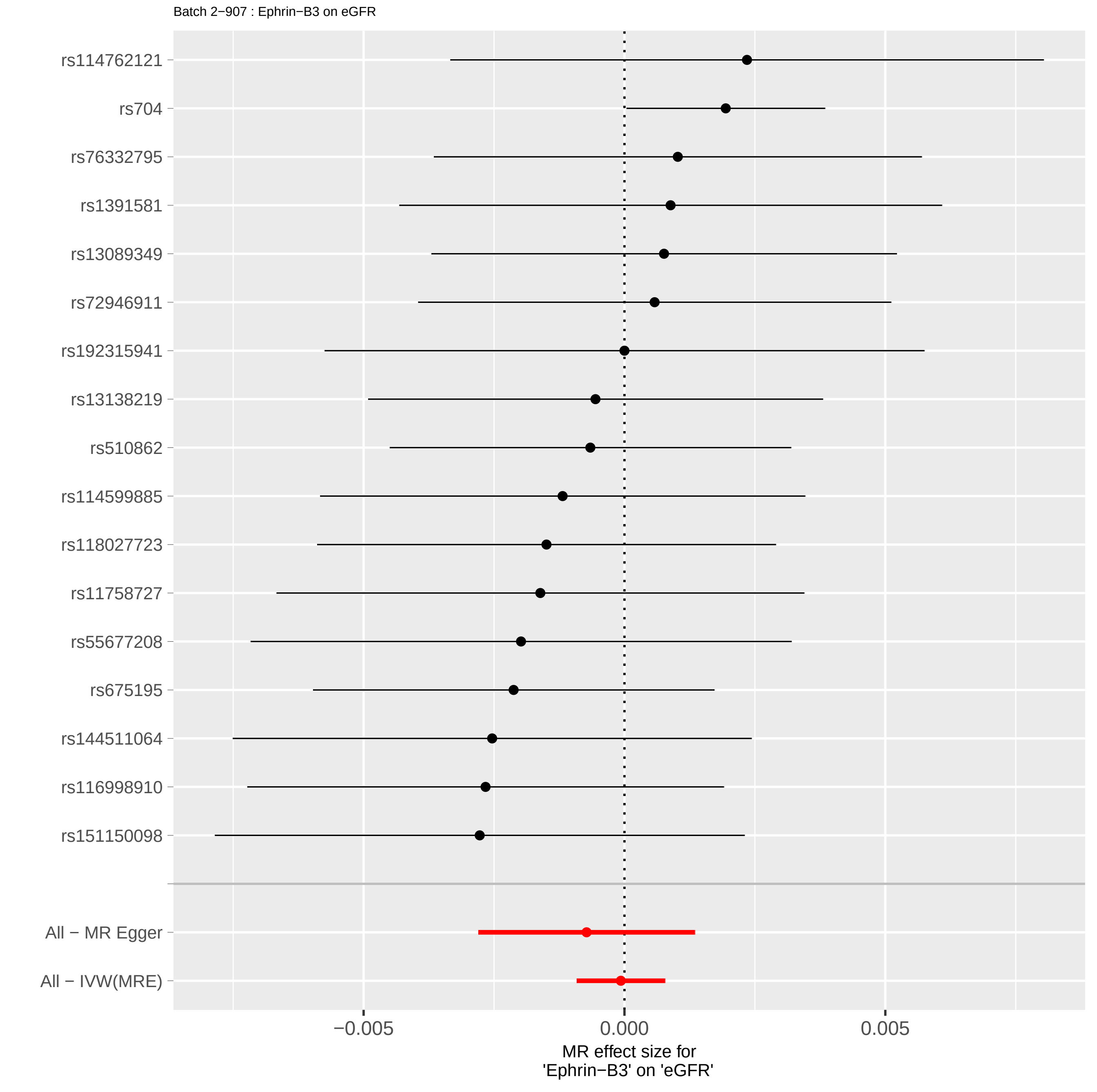

Batch 2−907 : Ephrin−B3 on eGFR
rs114762121
rs704
rs76332795
rs1391581
rs13089349
rs72946911
rs192315941
rs13138219
rs510862
rs114599885
rs118027723
rs11758727
rs55677208
rs675195
rs144511064
rs116998910
rs151150098
All − MR Egger
All − IVW(MRE)
−0.005
0.000
0.005
MR effect size for
'Ephrin−B3' on 'eGFR'

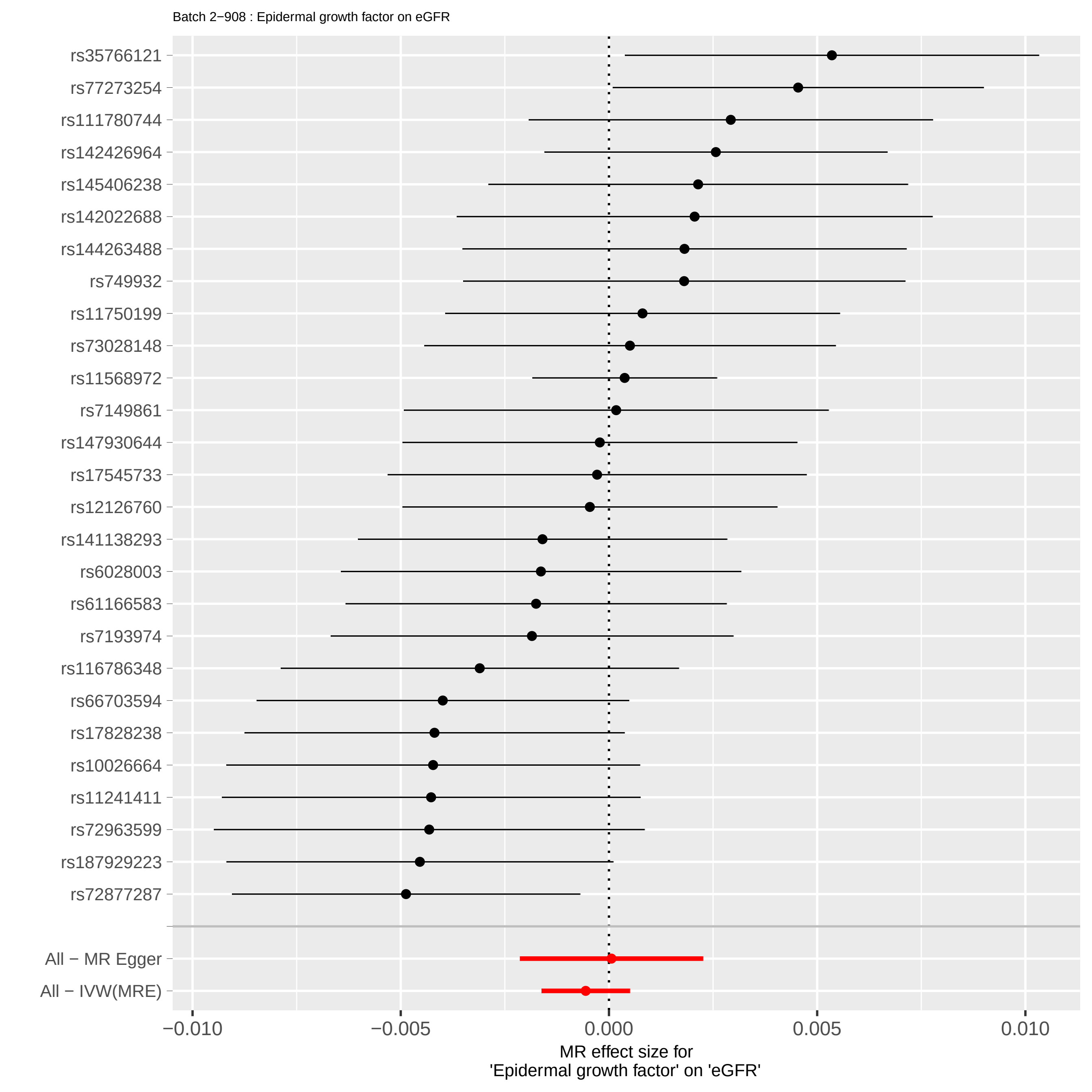

Batch 2−908 : Epidermal growth factor on eGFR
rs35766121
rs77273254
rs111780744
rs142426964
rs145406238
rs142022688
rs144263488
rs749932
rs11750199
rs73028148
rs11568972
rs7149861
rs147930644
rs17545733
rs12126760
rs141138293
rs6028003
rs61166583
rs7193974
rs116786348
rs66703594
rs17828238
rs10026664
rs11241411
rs72963599
rs187929223
rs72877287
All − MR Egger
All − IVW(MRE)
−0.010
−0.005
0.000
0.005
0.010
MR effect size for
'Epidermal growth factor' on 'eGFR'

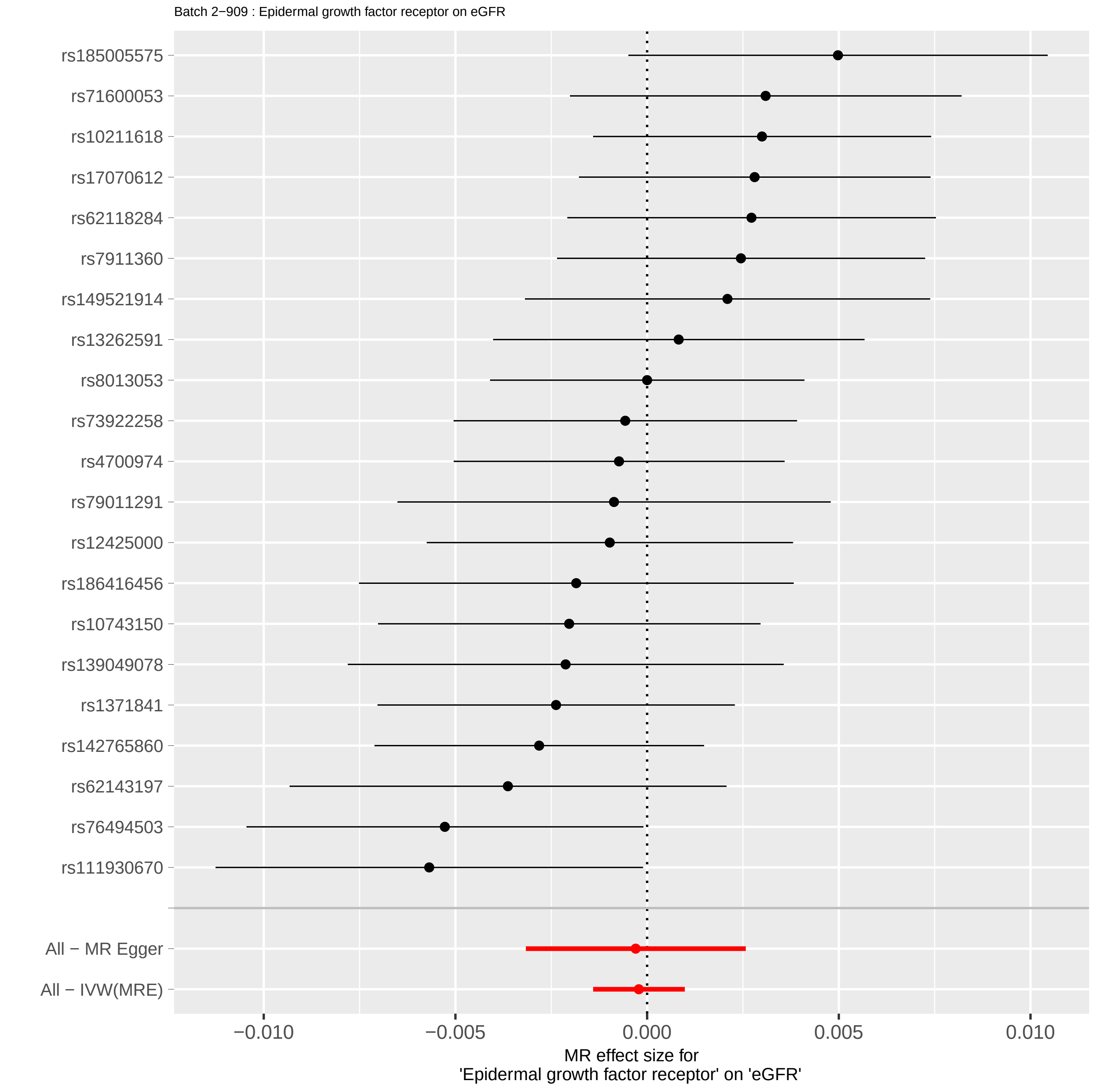

Batch 2−909 : Epidermal growth factor receptor on eGFR
rs185005575
rs71600053
rs10211618
rs17070612
rs62118284
rs7911360
rs149521914
rs13262591
rs8013053
rs73922258
rs4700974
rs79011291
rs12425000
rs186416456
rs10743150
rs139049078
rs1371841
rs142765860
rs62143197
rs76494503
rs111930670
All − MR Egger
All − IVW(MRE)
−0.010
−0.005
0.000
0.005
0.010
MR effect size for
'Epidermal growth factor receptor' on 'eGFR'

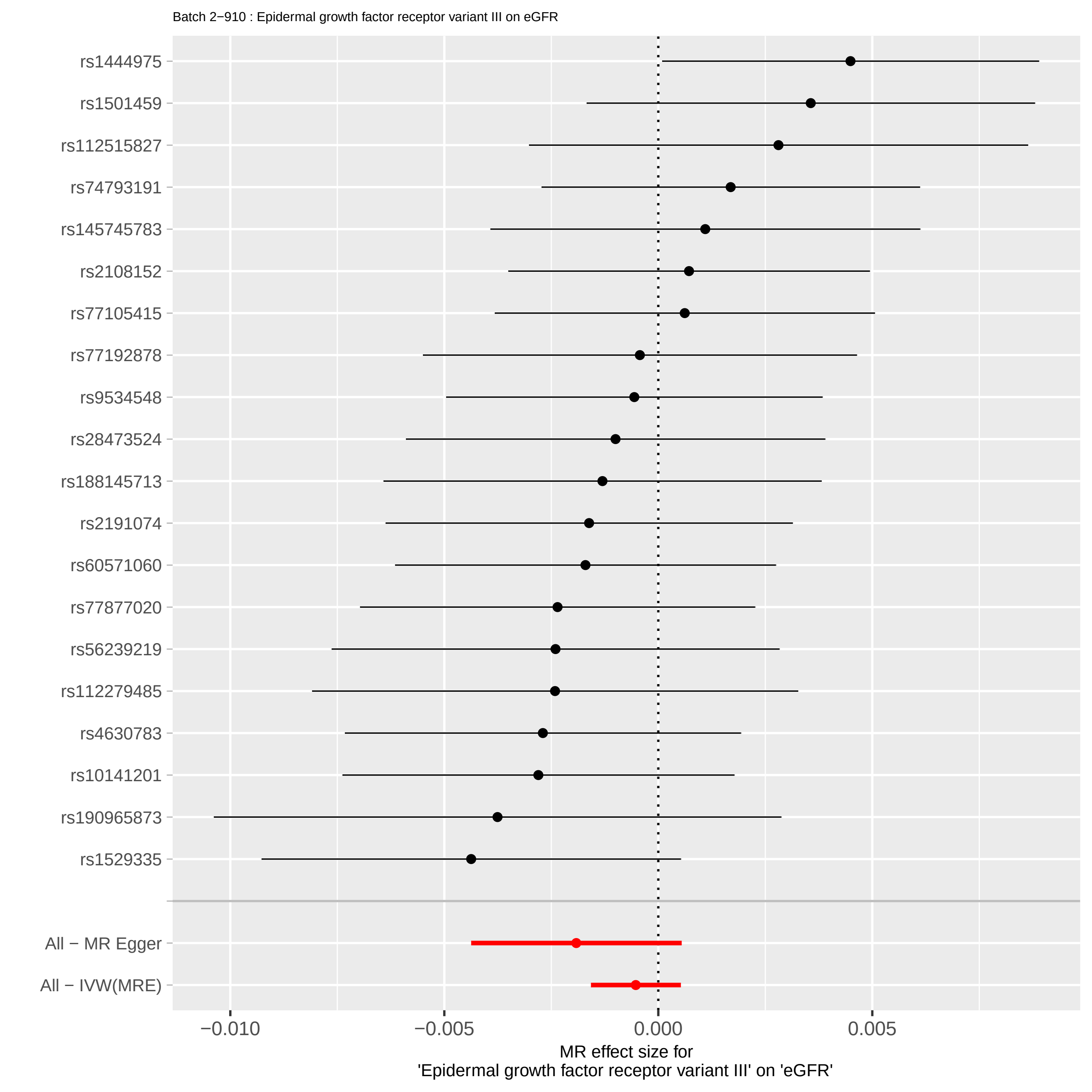

Batch 2−910 : Epidermal growth factor receptor variant III on eGFR
rs1444975
rs1501459
rs112515827
rs74793191
rs145745783
rs2108152
rs77105415
rs77192878
rs9534548
rs28473524
rs188145713
rs2191074
rs60571060
rs77877020
rs56239219
rs112279485
rs4630783
rs10141201
rs190965873
rs1529335
All − MR Egger
All − IVW(MRE)
−0.010
−0.005
0.000
0.005
MR effect size for
'Epidermal growth factor receptor variant III' on 'eGFR'

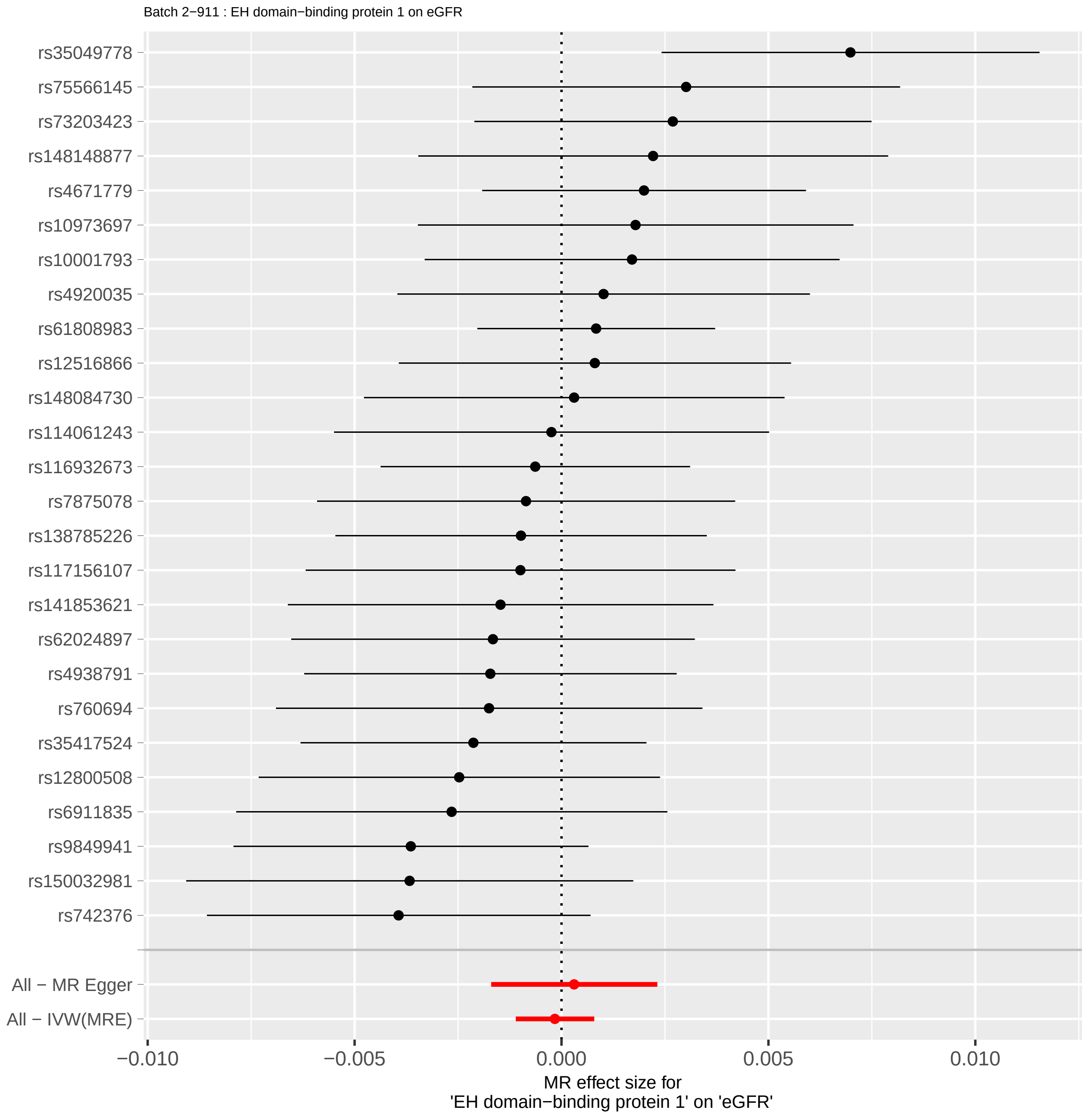

Batch 2−911 : EH domain−binding protein 1 on eGFR
rs35049778
rs75566145
rs73203423
rs148148877
rs4671779
rs10973697
rs10001793
rs4920035
rs61808983
rs12516866
rs148084730
rs114061243
rs116932673
rs7875078
rs138785226
rs117156107
rs141853621
rs62024897
rs4938791
rs760694
rs35417524
rs12800508
rs6911835
rs9849941
rs150032981
rs742376
All − MR Egger
All − IVW(MRE)
−0.010
−0.005
0.000
0.005
0.010
MR effect size for
'EH domain−binding protein 1' on 'eGFR'

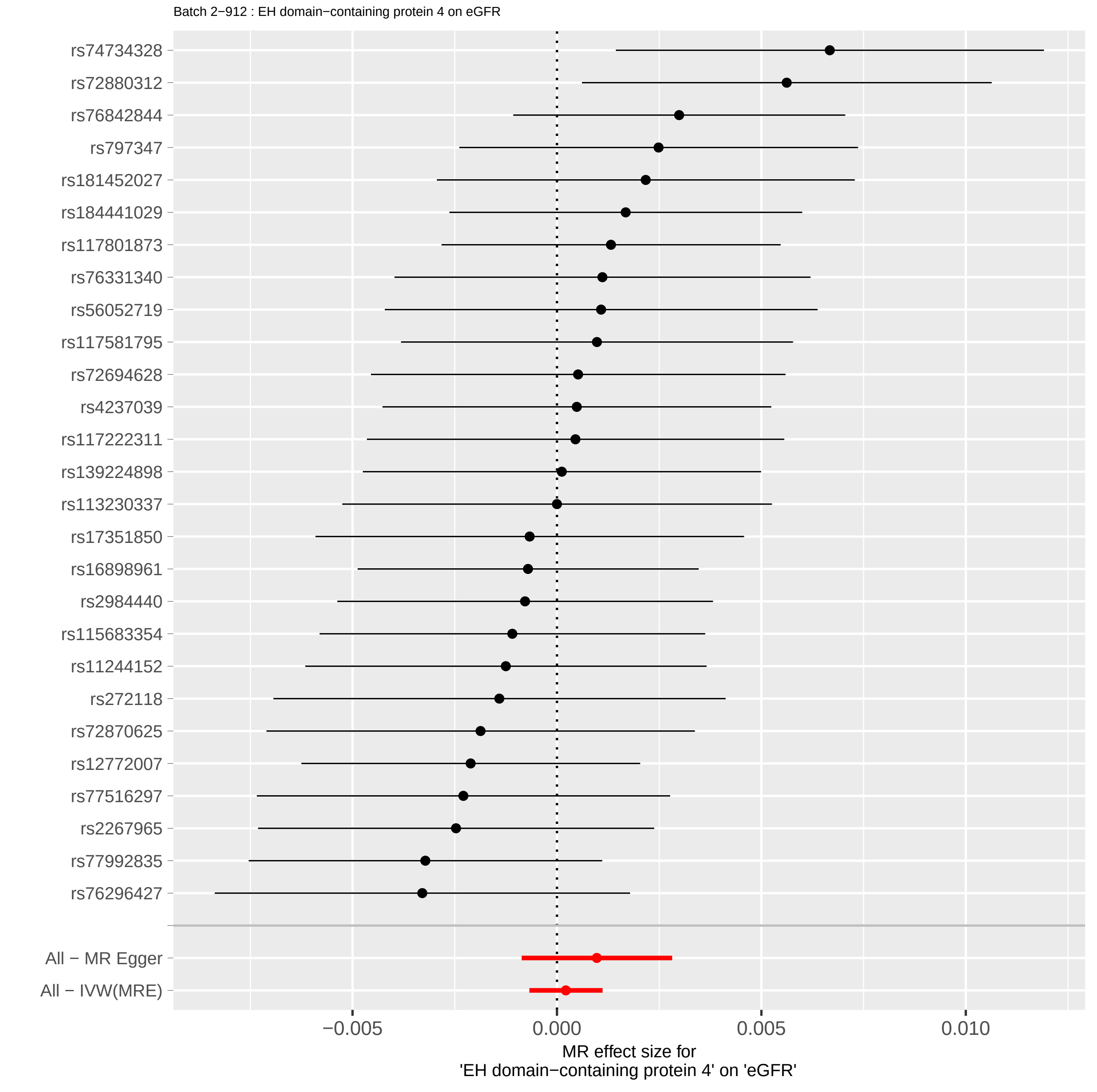

Batch 2−912 : EH domain−containing protein 4 on eGFR
rs74734328
rs72880312
rs76842844
rs797347
rs181452027
rs184441029
rs117801873
rs76331340
rs56052719
rs117581795
rs72694628
rs4237039
rs117222311
rs139224898
rs113230337
rs17351850
rs16898961
rs2984440
rs115683354
rs11244152
rs272118
rs72870625
rs12772007
rs77516297
rs2267965
rs77992835
rs76296427
All − MR Egger
All − IVW(MRE)
−0.005
0.000
0.005
0.010
MR effect size for
'EH domain−containing protein 4' on 'eGFR'

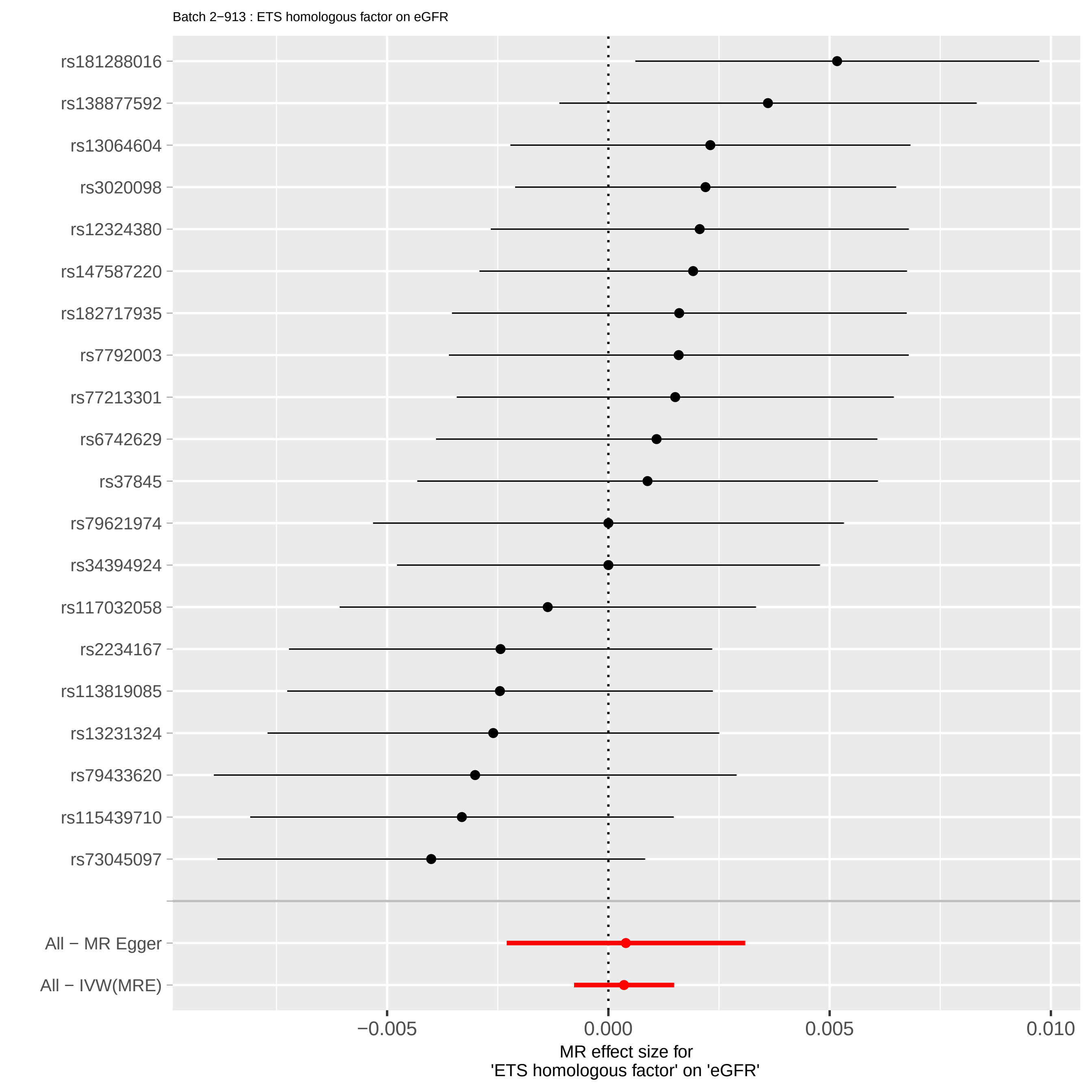
Batch 2−913 : ETS homologous factor on eGFR
rs181288016
rs138877592
rs13064604
rs3020098
rs12324380
rs147587220
rs182717935
rs7792003
rs77213301
rs6742629
rs37845
rs79621974
rs34394924
rs117032058
rs2234167
rs113819085
rs13231324
rs79433620
rs115439710
rs73045097
All − MR Egger
All − IVW(MRE)
−0.005
0.000
0.005
0.010
MR effect size for
'ETS homologous factor' on 'eGFR'

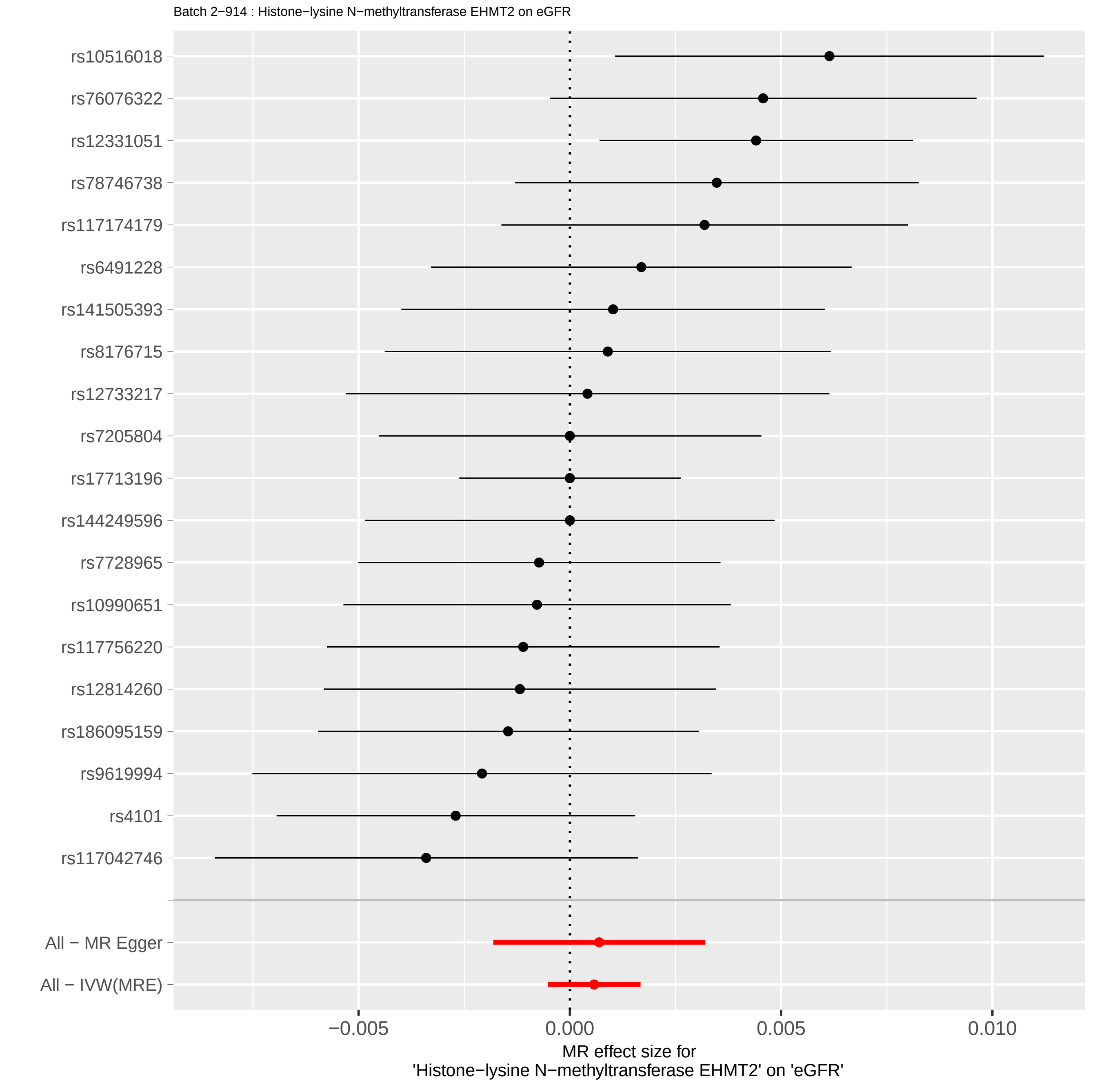

Batch 2−914 : Histone−lysine N−methyltransferase EHMT2 on eGFR
rs10516018
rs76076322
rs12331051
rs78746738
rs117174179
rs6491228
rs141505393
rs8176715
rs12733217
rs7205804
rs17713196
rs144249596
rs7728965
rs10990651
rs117756220
rs12814260
rs186095159
rs9619994
rs4101
rs117042746
All − MR Egger
All − IVW(MRE)
−0.005
0.000
0.005
0.010
MR effect size for
'Histone−lysine N−methyltransferase EHMT2' on 'eGFR'

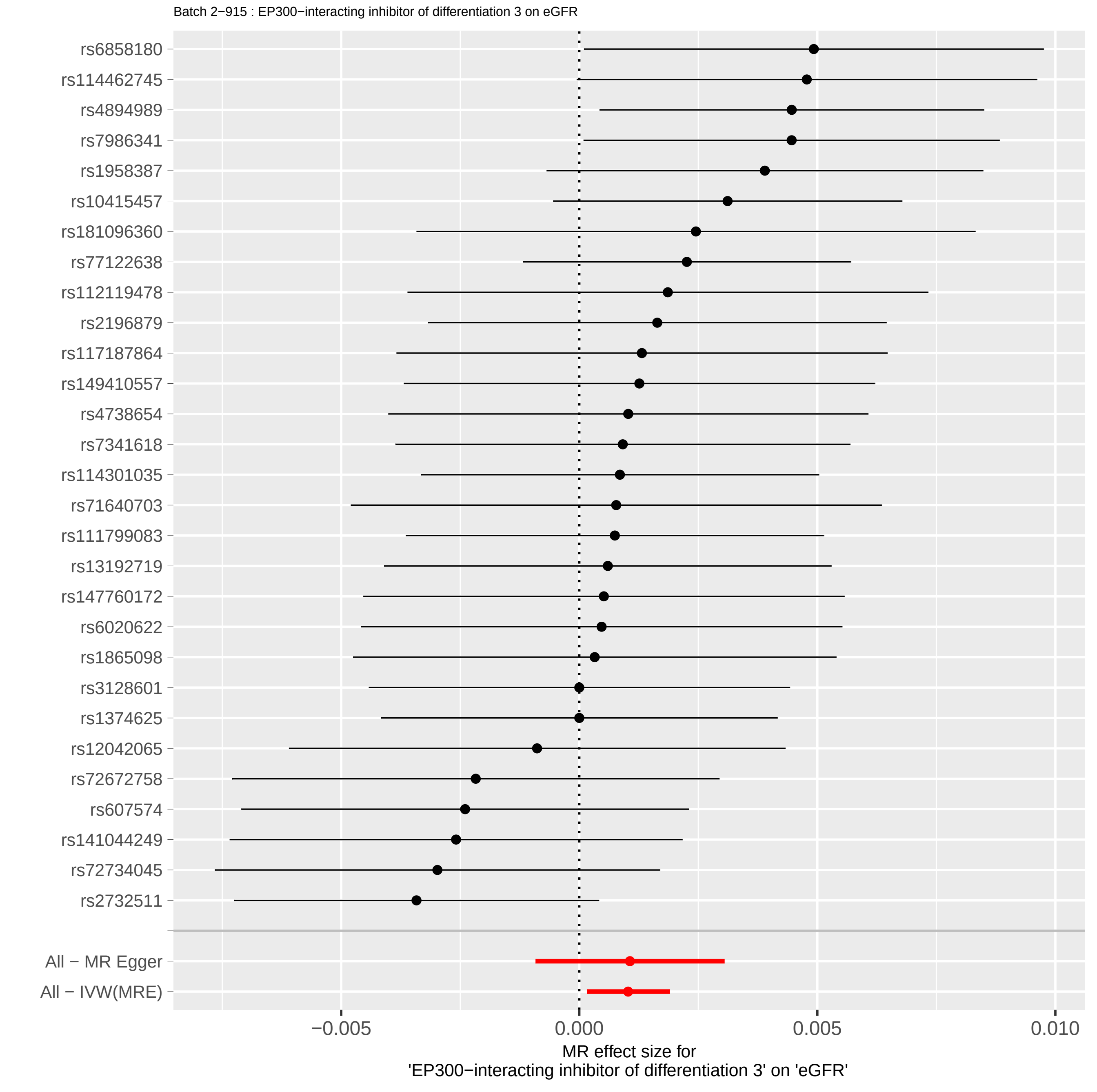

Batch 2−915 : EP300−interacting inhibitor of differentiation 3 on eGFR
rs6858180
rs114462745
rs4894989
rs7986341
rs1958387
rs10415457
rs181096360
rs77122638
rs112119478
rs2196879
rs117187864
rs149410557
rs4738654
rs7341618
rs114301035
rs71640703
rs111799083
rs13192719
rs147760172
rs6020622
rs1865098
rs3128601
rs1374625
rs12042065
rs72672758
rs607574
rs141044249
rs72734045
rs2732511
All − MR Egger
All − IVW(MRE)
−0.005
0.000
0.005
0.010
MR effect size for
'EP300−interacting inhibitor of differentiation 3' on 'eGFR'

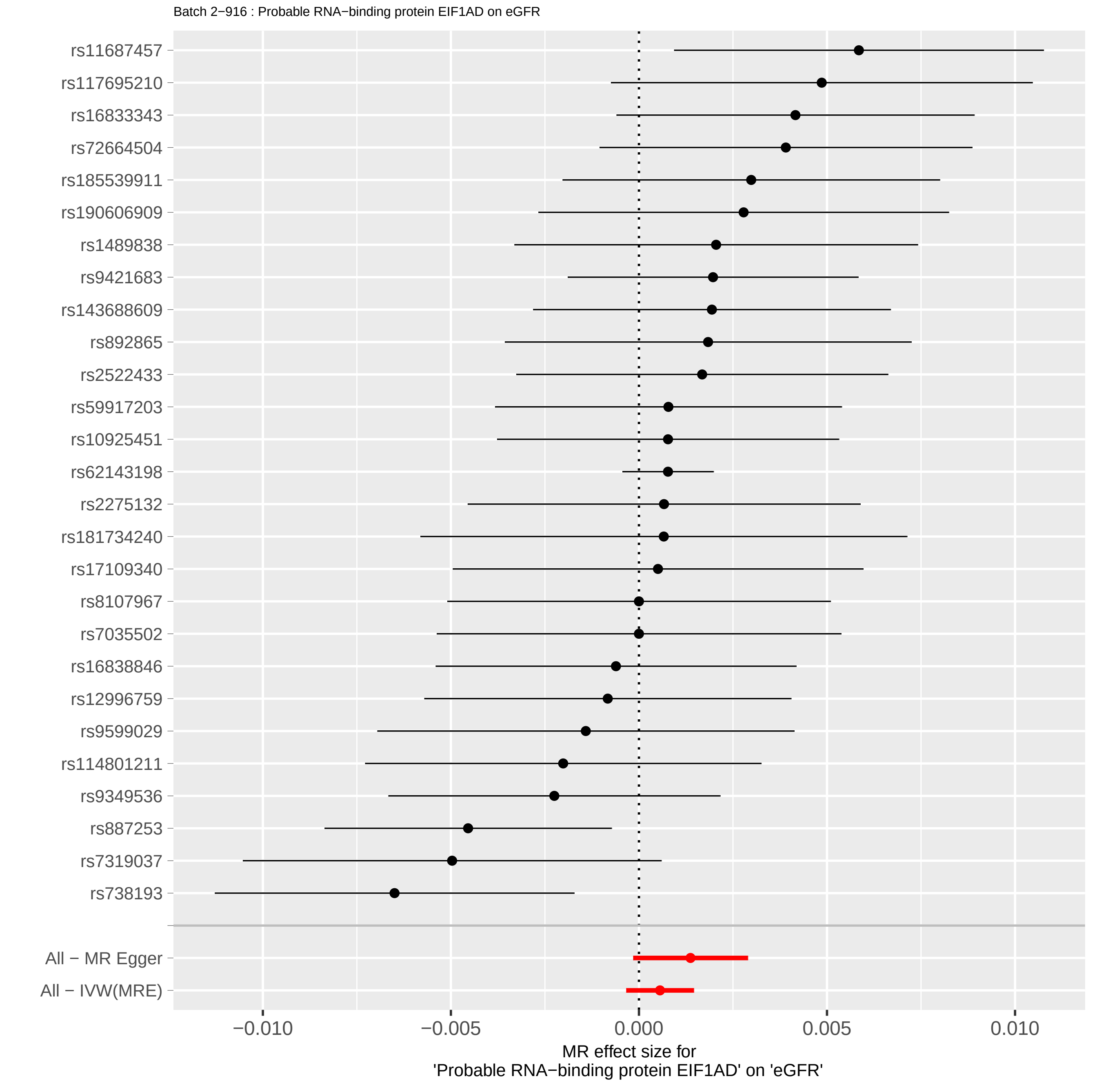

Batch 2−916 : Probable RNA−binding protein EIF1AD on eGFR
rs11687457
rs117695210
rs16833343
rs72664504
rs185539911
rs190606909
rs1489838
rs9421683
rs143688609
rs892865
rs2522433
rs59917203
rs10925451
rs62143198
rs2275132
rs181734240
rs17109340
rs8107967
rs7035502
rs16838846
rs12996759
rs9599029
rs114801211
rs9349536
rs887253
rs7319037
rs738193
All − MR Egger
All − IVW(MRE)
−0.010
−0.005
0.000
0.005
0.010
MR effect size for
'Probable RNA−binding protein EIF1AD' on 'eGFR'

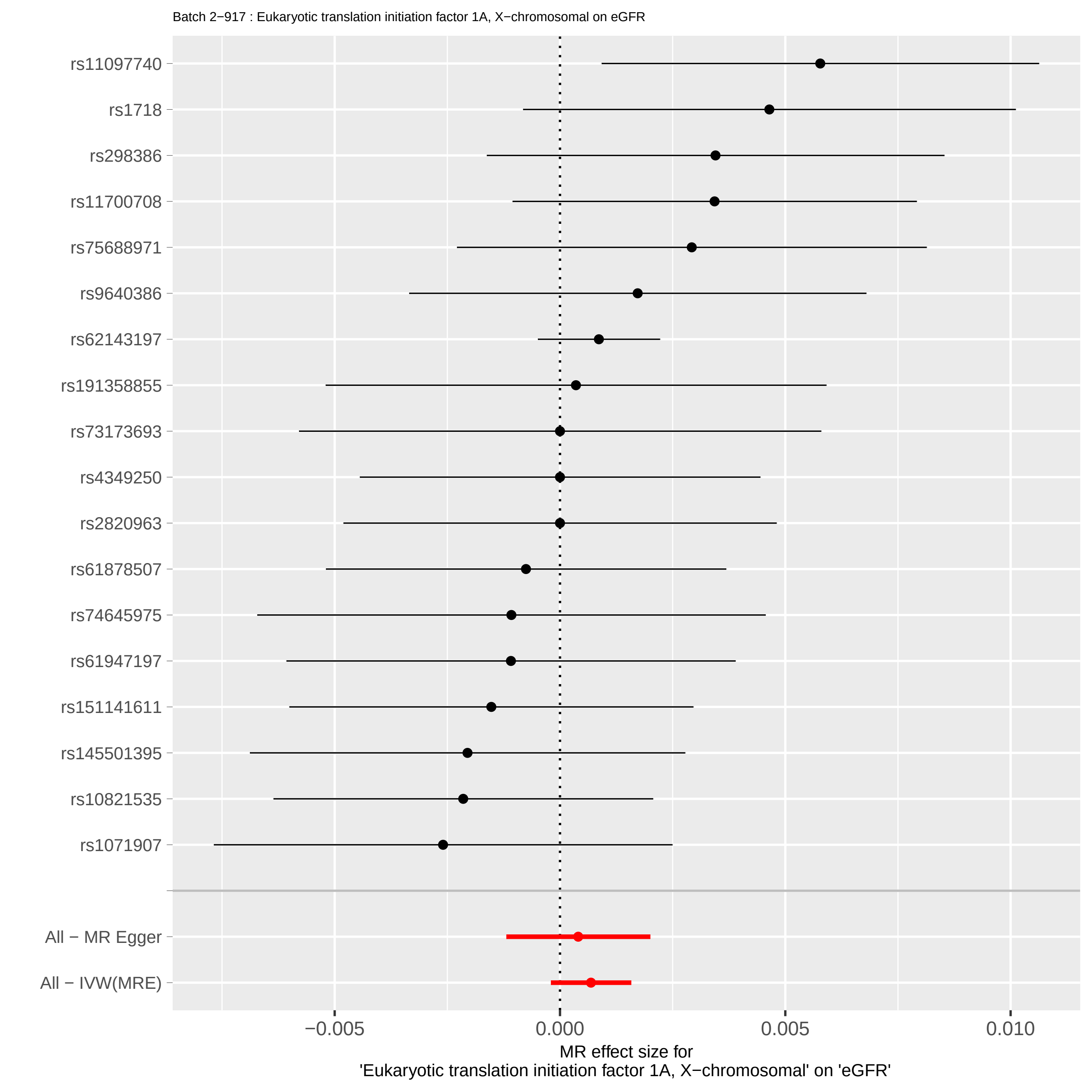
Batch 2−917 : Eukaryotic translation initiation factor 1A, X−chromosomal on eGFR
rs11097740
rs1718
rs298386
rs11700708
rs75688971
rs9640386
rs62143197
rs191358855
rs73173693
rs4349250
rs2820963
rs61878507
rs74645975
rs61947197
rs151141611
rs145501395
rs10821535
rs1071907
All − MR Egger
All − IVW(MRE)
−0.005
0.000
0.005
0.010
MR effect size for
'Eukaryotic translation initiation factor 1A, X−chromosomal' on 'eGFR'

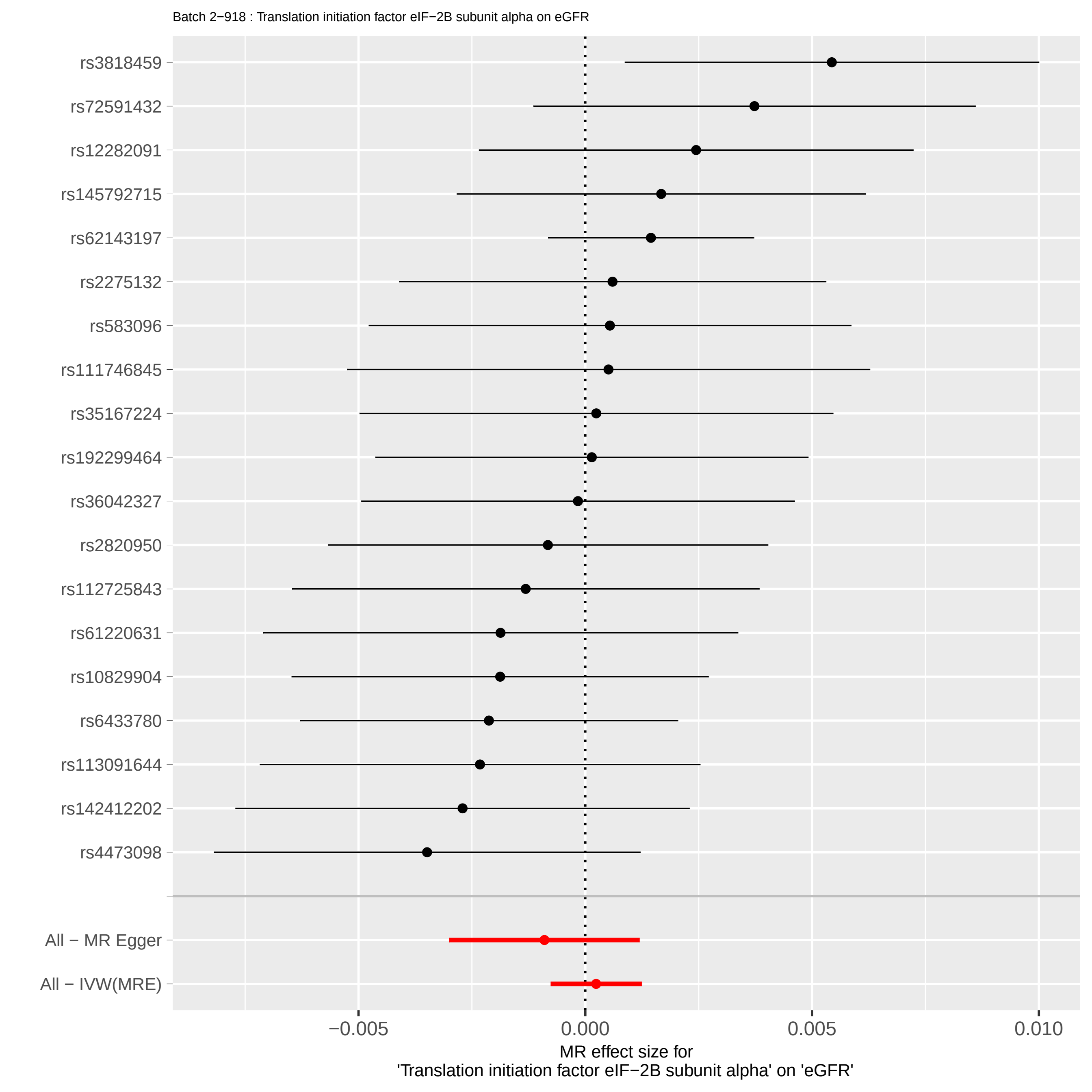
Batch 2−918 : Translation initiation factor eIF−2B subunit alpha on eGFR
rs3818459
rs72591432
rs12282091
rs145792715
rs62143197
rs2275132
rs583096
rs111746845
rs35167224
rs192299464
rs36042327
rs2820950
rs112725843
rs61220631
rs10829904
rs6433780
rs113091644
rs142412202
rs4473098
All − MR Egger
All − IVW(MRE)
−0.005
0.000
0.005
0.010
MR effect size for
'Translation initiation factor eIF−2B subunit alpha' on 'eGFR'

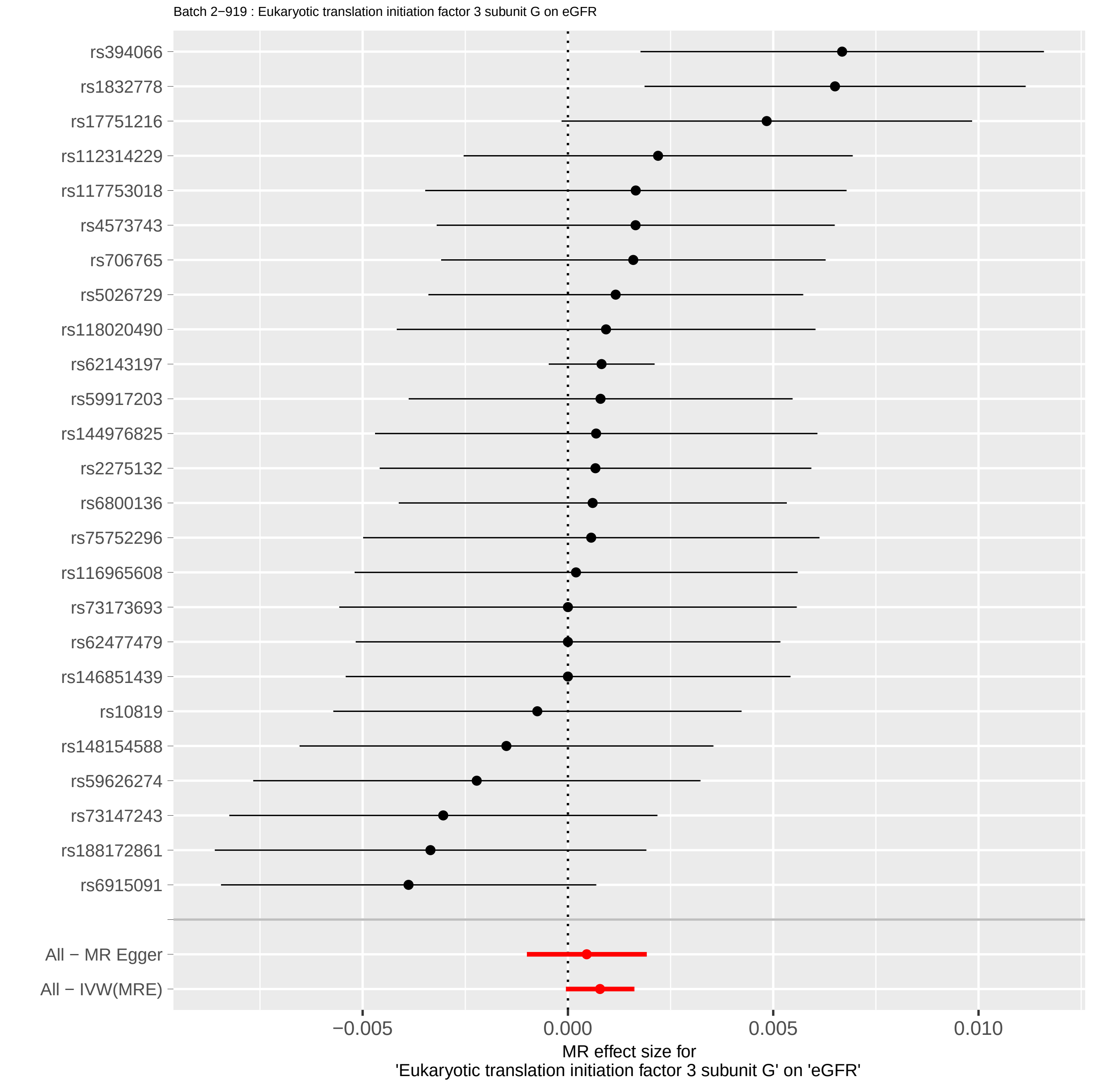
Batch 2−919 : Eukaryotic translation initiation factor 3 subunit G on eGFR
rs394066
rs1832778
rs17751216
rs112314229
rs117753018
rs4573743
rs706765
rs5026729
rs118020490
rs62143197
rs59917203
rs144976825
rs2275132
rs6800136
rs75752296
rs116965608
rs73173693
rs62477479
rs146851439
rs10819
rs148154588
rs59626274
rs73147243
rs188172861
rs6915091
All − MR Egger
All − IVW(MRE)
−0.005
0.000
0.005
0.010
MR effect size for
'Eukaryotic translation initiation factor 3 subunit G' on 'eGFR'

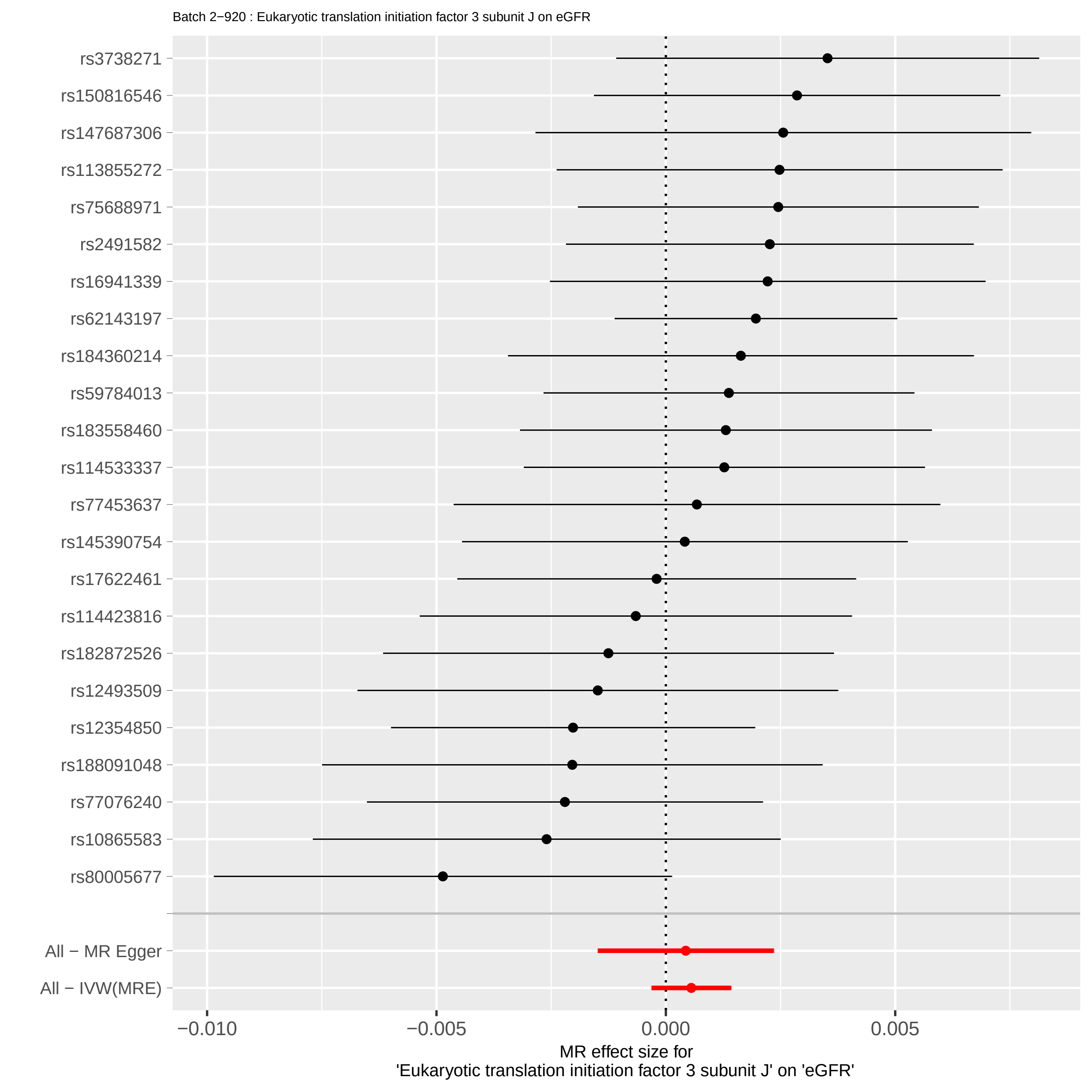

Batch 2−920 : Eukaryotic translation initiation factor 3 subunit J on eGFR
rs3738271
rs150816546
rs147687306
rs113855272
rs75688971
rs2491582
rs16941339
rs62143197
rs184360214
rs59784013
rs183558460
rs114533337
rs77453637
rs145390754
rs17622461
rs114423816
rs182872526
rs12493509
rs12354850
rs188091048
rs77076240
rs10865583
rs80005677
All − MR Egger
All − IVW(MRE)
−0.010
−0.005
0.000
0.005
MR effect size for
'Eukaryotic translation initiation factor 3 subunit J' on 'eGFR'

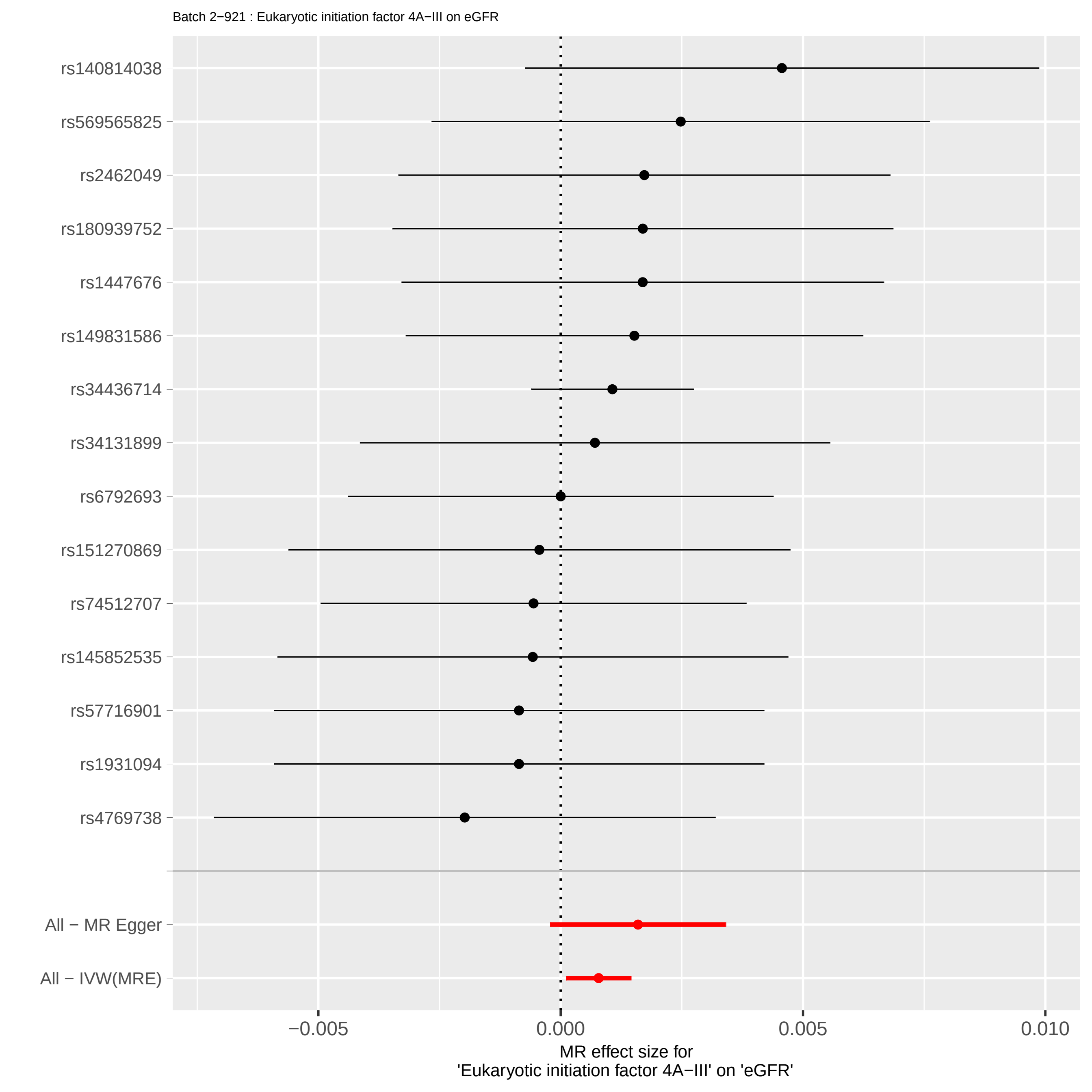

Batch 2−921 : Eukaryotic initiation factor 4A−III on eGFR
rs140814038
rs569565825
rs2462049
rs180939752
rs1447676
rs149831586
rs34436714
rs34131899
rs6792693
rs151270869
rs74512707
rs145852535
rs57716901
rs1931094
rs4769738
All − MR Egger
All − IVW(MRE)
−0.005
0.000
0.005
0.010
MR effect size for
'Eukaryotic initiation factor 4A−III' on 'eGFR'

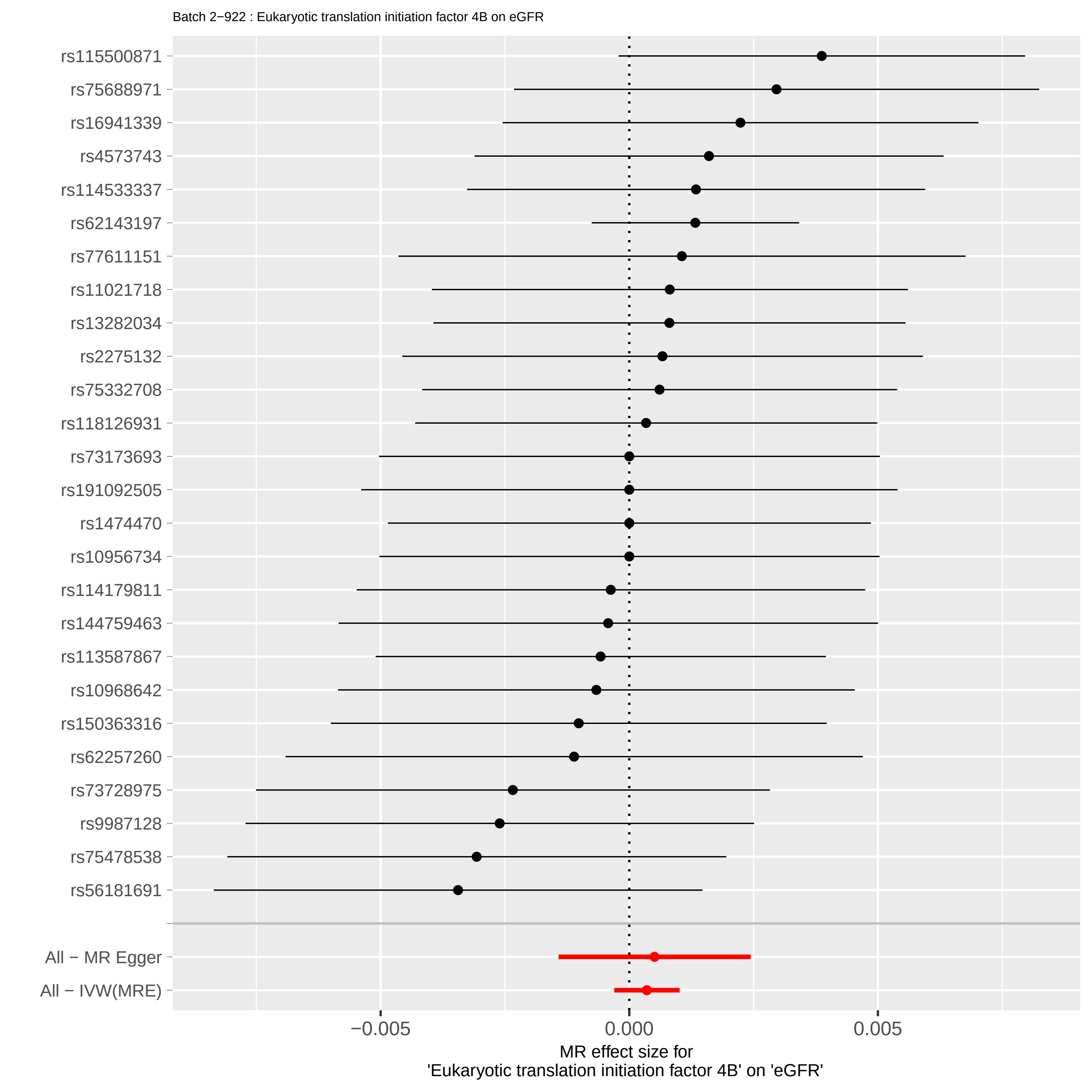

Batch 2−922 : Eukaryotic translation initiation factor 4B on eGFR
rs115500871
rs75688971
rs16941339
rs4573743
rs114533337
rs62143197
rs77611151
rs11021718
rs13282034
rs2275132
rs75332708
rs118126931
rs73173693
rs191092505
rs1474470
rs10956734
rs114179811
rs144759463
rs113587867
rs10968642
rs150363316
rs62257260
rs73728975
rs9987128
rs75478538
rs56181691
All − MR Egger
All − IVW(MRE)
−0.005
0.000
0.005
MR effect size for
'Eukaryotic translation initiation factor 4B' on 'eGFR'

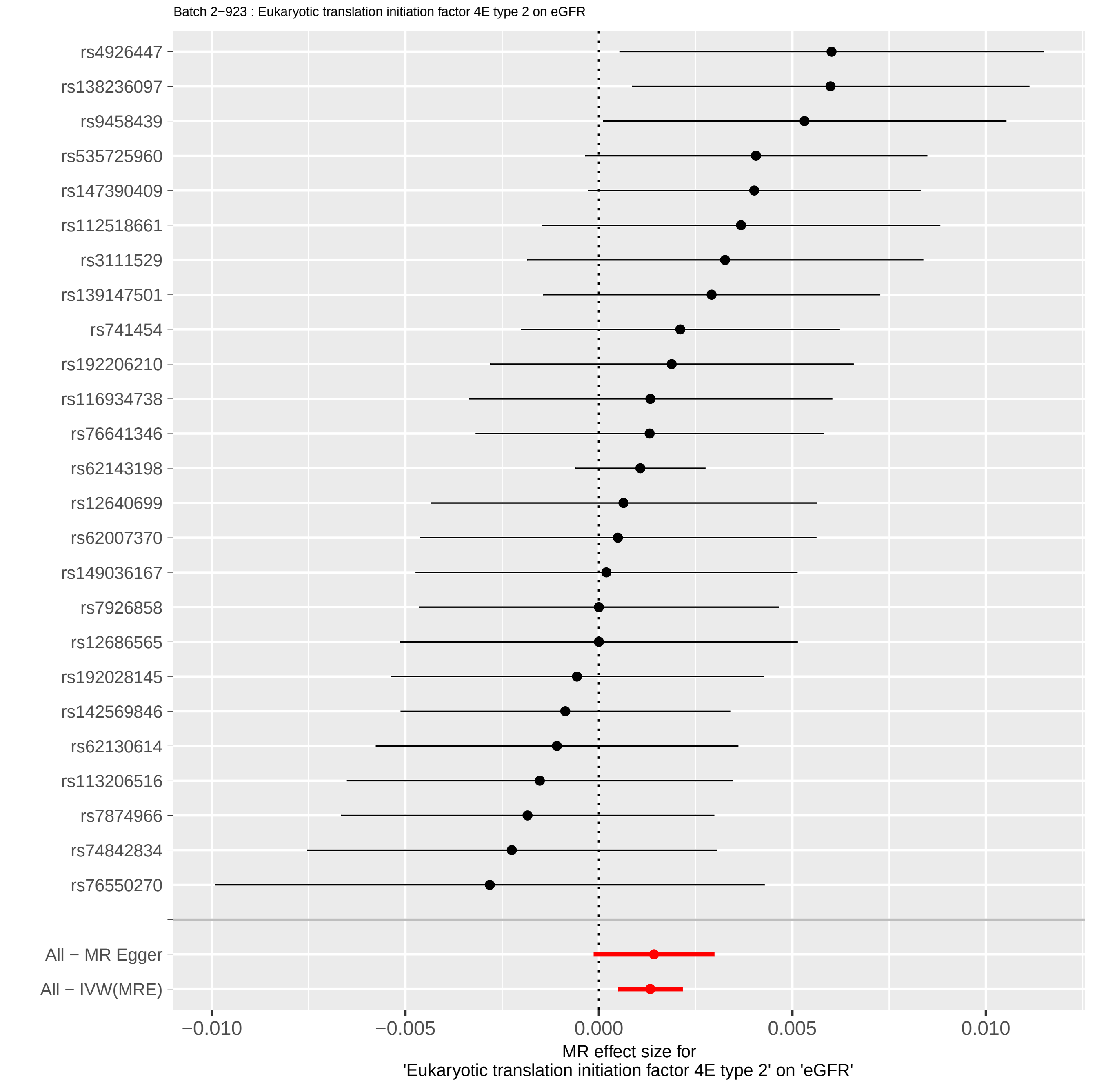

Batch 2−923 : Eukaryotic translation initiation factor 4E type 2 on eGFR
rs4926447
rs138236097
rs9458439
rs535725960
rs147390409
rs112518661
rs3111529
rs139147501
rs741454
rs192206210
rs116934738
rs76641346
rs62143198
rs12640699
rs62007370
rs149036167
rs7926858
rs12686565
rs192028145
rs142569846
rs62130614
rs113206516
rs7874966
rs74842834
rs76550270
All − MR Egger
All − IVW(MRE)
−0.010
−0.005
0.000
0.005
0.010
MR effect size for
'Eukaryotic translation initiation factor 4E type 2' on 'eGFR'

Batch 2−924 : Eukaryotic translation initiation factor 4E−binding protein 2 on eGFR

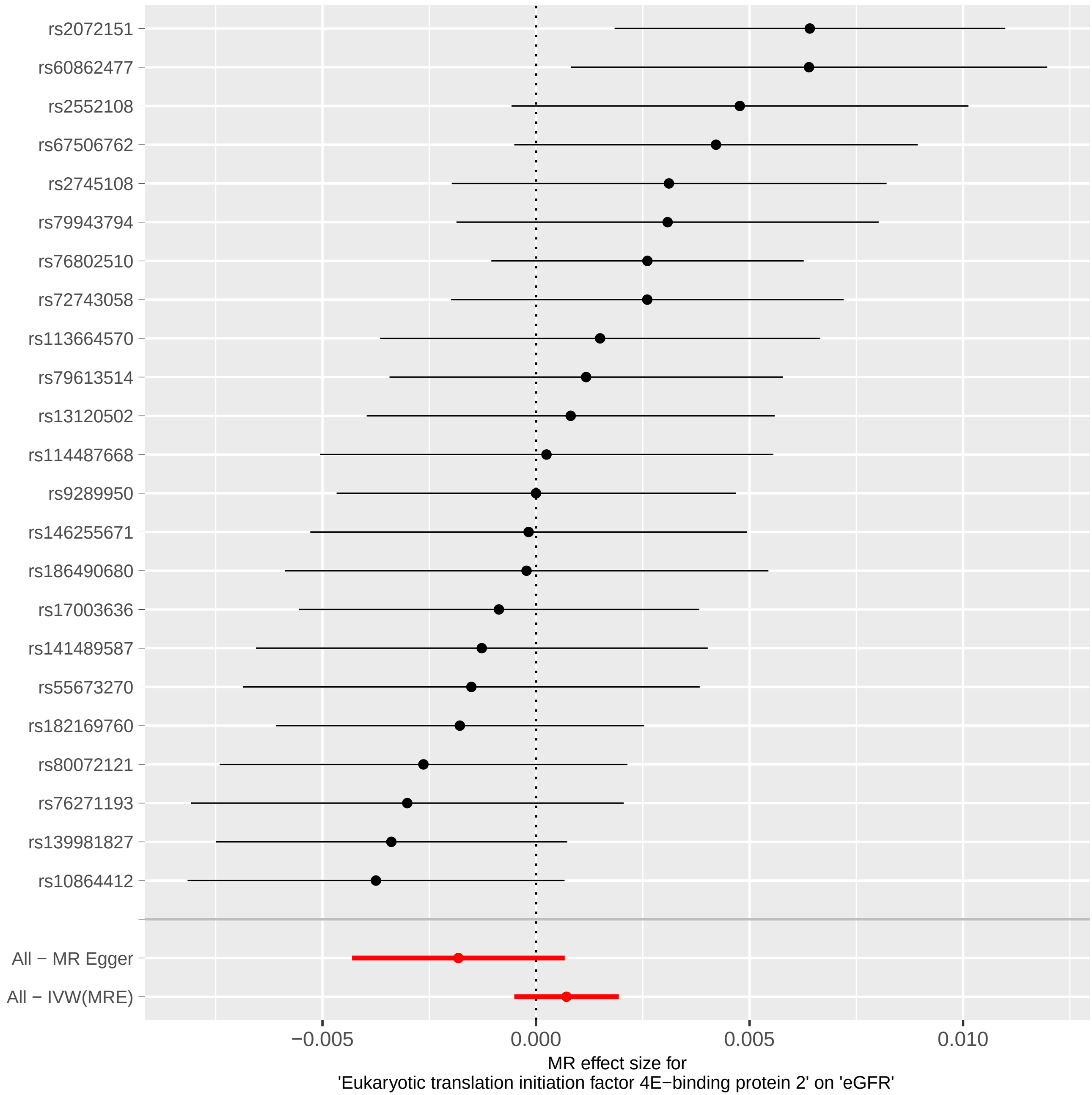

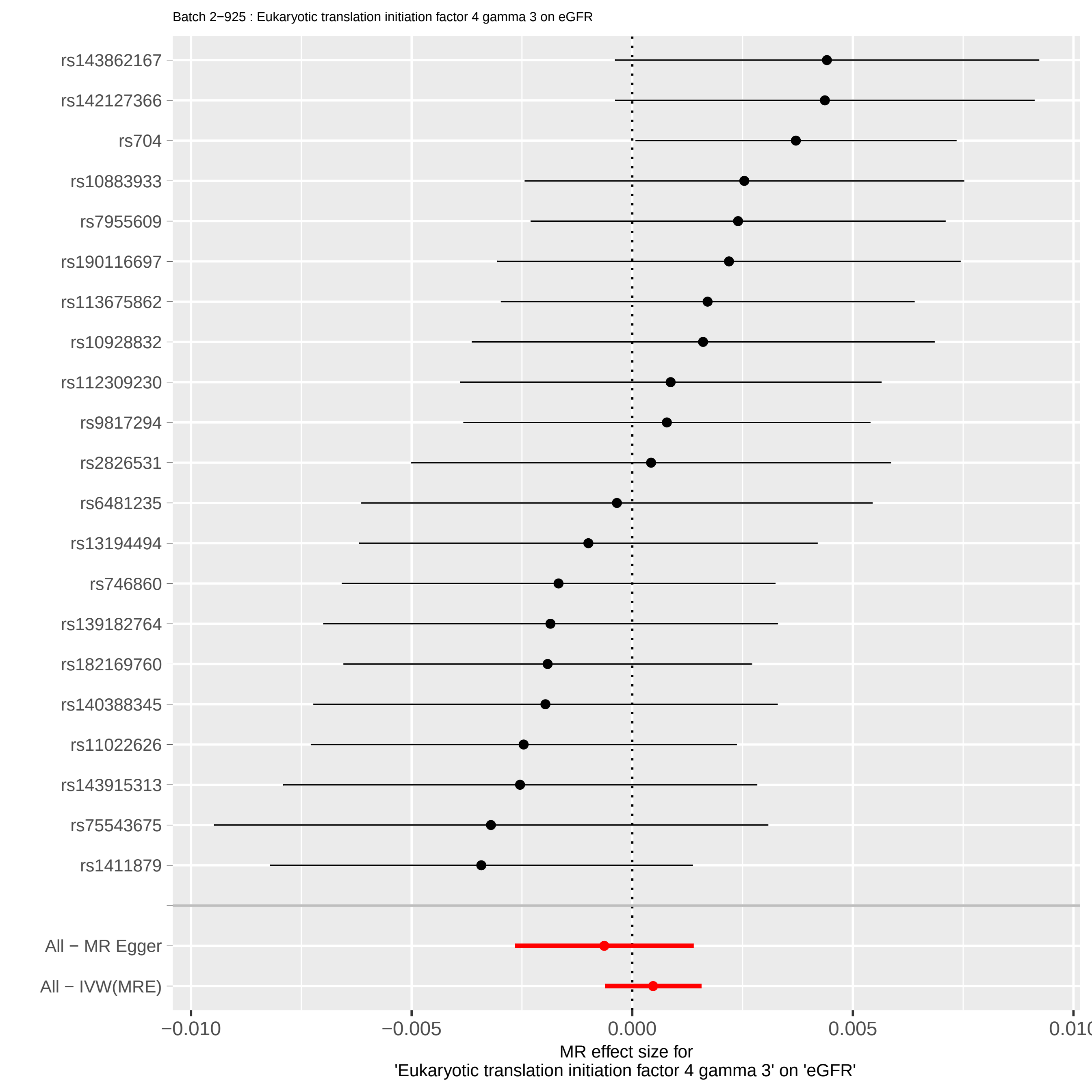

Batch 2−925 : Eukaryotic translation initiation factor 4 gamma 3 on eGFR
rs143862167
rs142127366
rs704
rs10883933
rs7955609
rs190116697
rs113675862
rs10928832
rs112309230
rs9817294
rs2826531
rs6481235
rs13194494
rs746860
rs139182764
rs182169760
rs140388345
rs11022626
rs143915313
rs75543675
rs1411879
All − MR Egger
All − IVW(MRE)
−0.010
−0.005
0.000
0.005
0.010
MR effect size for
'Eukaryotic translation initiation factor 4 gamma 3' on 'eGFR'

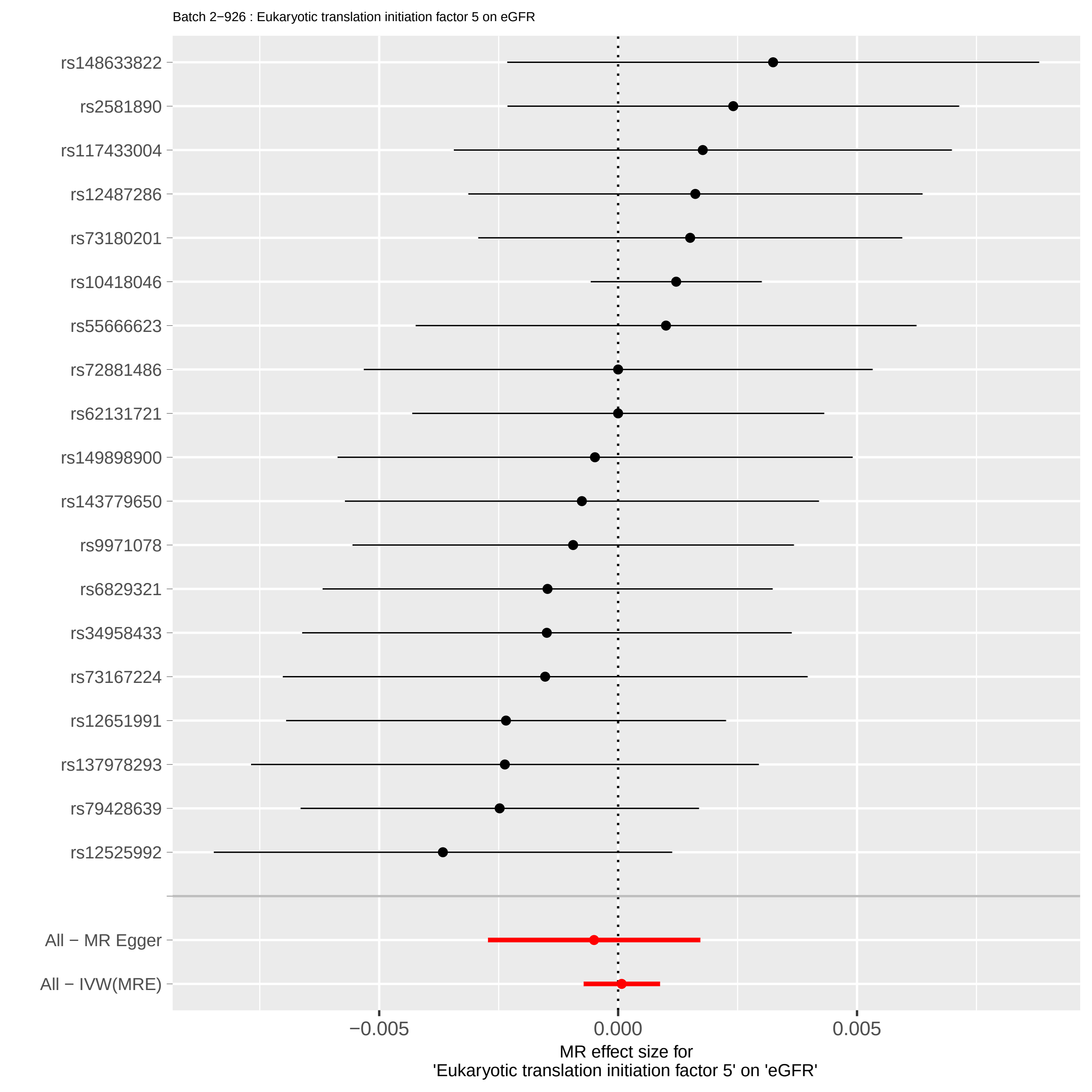
Batch 2−926 : Eukaryotic translation initiation factor 5 on eGFR
rs148633822
rs2581890
rs117433004
rs12487286
rs73180201
rs10418046
rs55666623
rs72881486
rs62131721
rs149898900
rs143779650
rs9971078
rs6829321
rs34958433
rs73167224
rs12651991
rs137978293
rs79428639
rs12525992
All − MR Egger
All − IVW(MRE)
−0.005
0.000
0.005
MR effect size for
'Eukaryotic translation initiation factor 5' on 'eGFR'

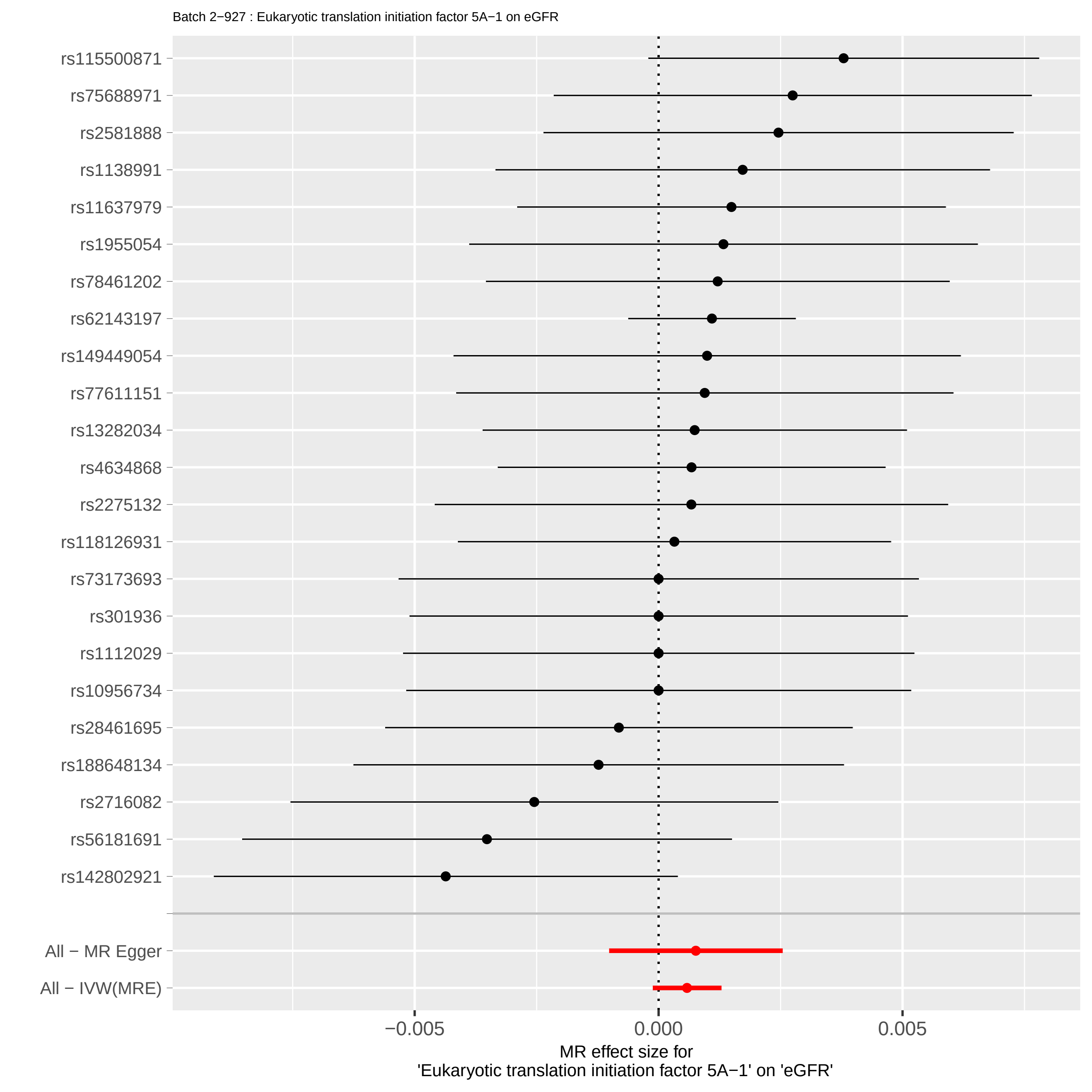

Batch 2−927 : Eukaryotic translation initiation factor 5A−1 on eGFR
rs115500871
rs75688971
rs2581888
rs1138991
rs11637979
rs1955054
rs78461202
rs62143197
rs149449054
rs77611151
rs13282034
rs4634868
rs2275132
rs118126931
rs73173693
rs301936
rs1112029
rs10956734
rs28461695
rs188648134
rs2716082
rs56181691
rs142802921
All − MR Egger
All − IVW(MRE)
−0.005
0.000
0.005
MR effect size for
'Eukaryotic translation initiation factor 5A−1' on 'eGFR'

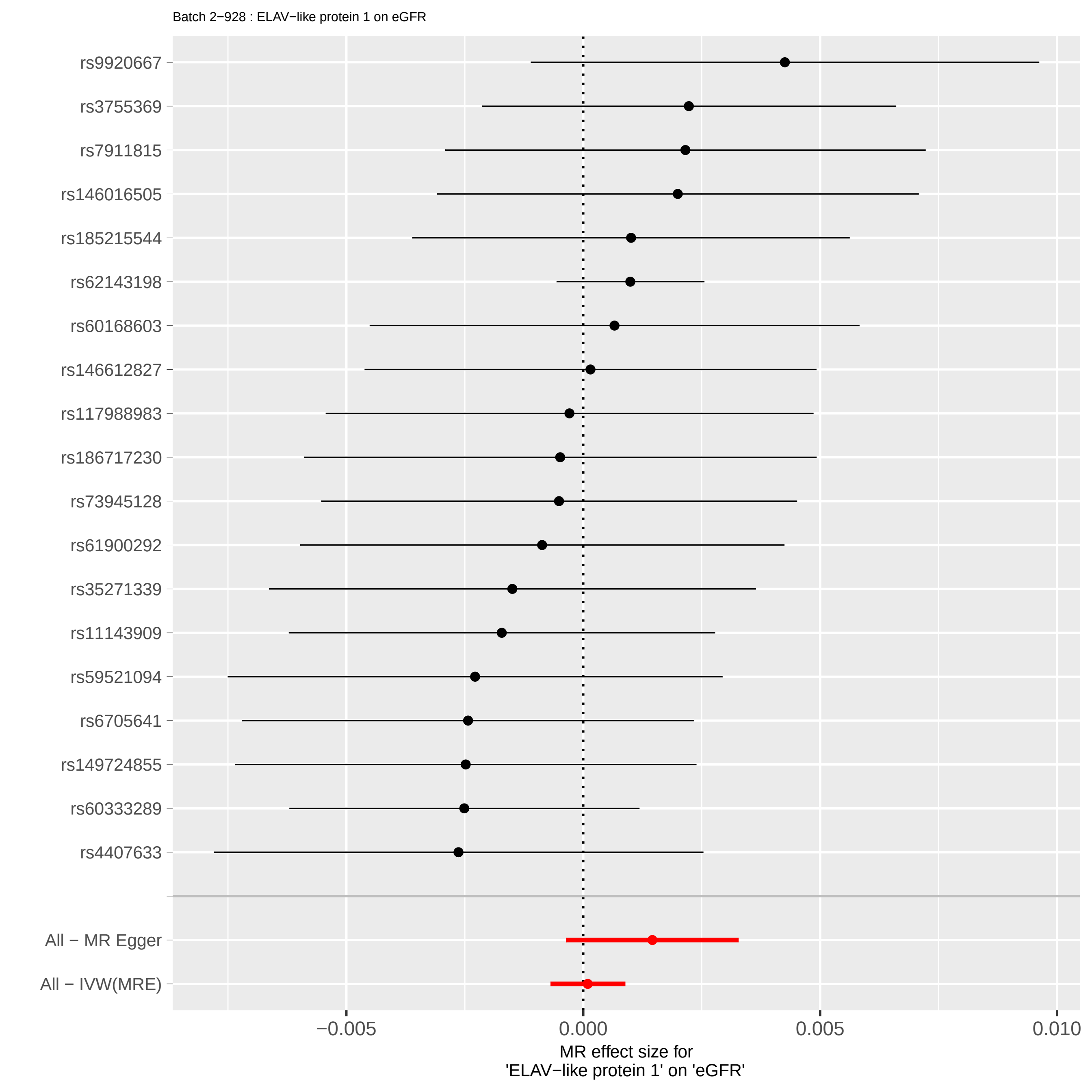
Batch 2−928 : ELAV−like protein 1 on eGFR
rs9920667
rs3755369
rs7911815
rs146016505
rs185215544
rs62143198
rs60168603
rs146612827
rs117988983
rs186717230
rs73945128
rs61900292
rs35271339
rs11143909
rs59521094
rs6705641
rs149724855
rs60333289
rs4407633
All − MR Egger
All − IVW(MRE)
−0.005
0.000
0.005
0.010
MR effect size for
'ELAV−like protein 1' on 'eGFR'

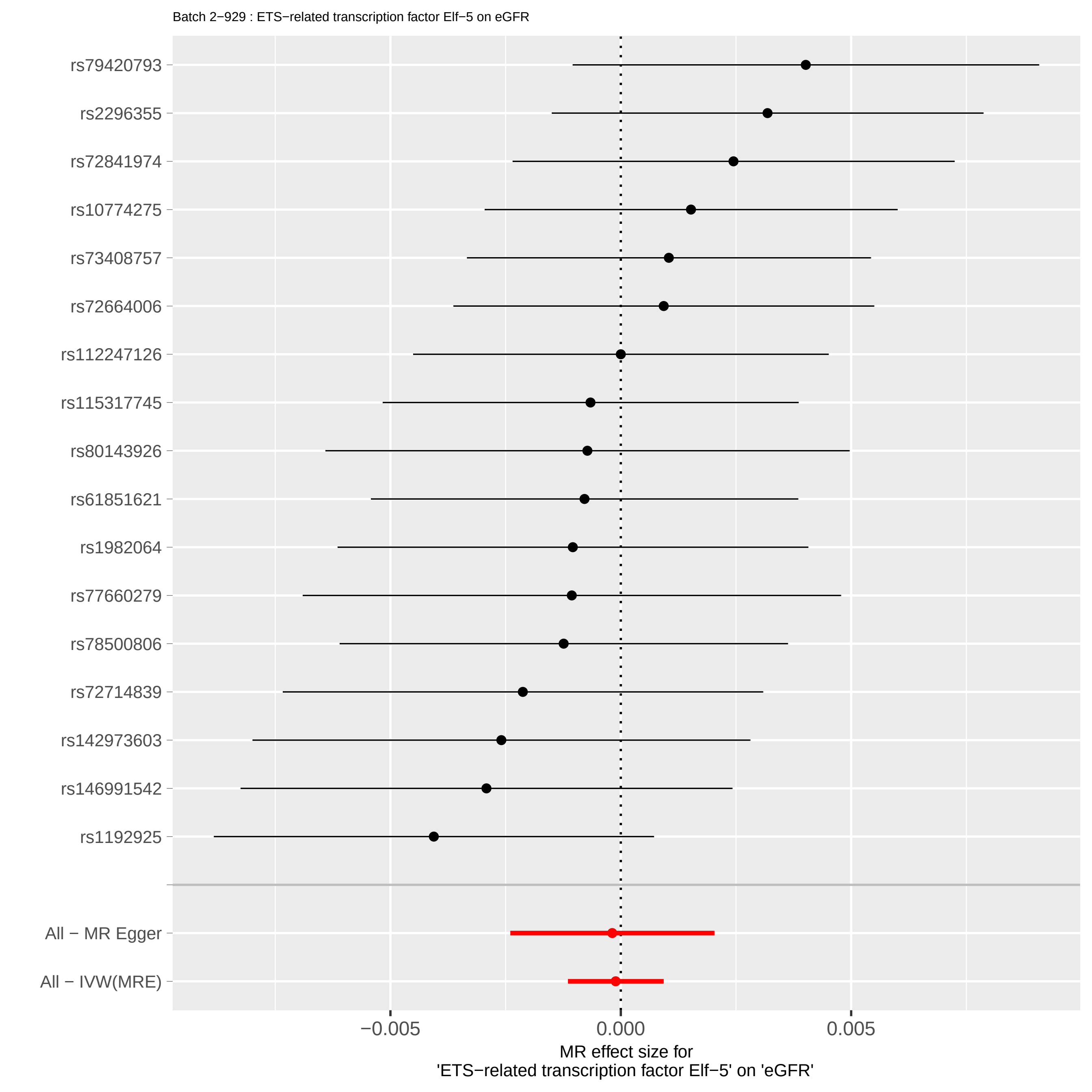

Batch 2−929 : ETS−related transcription factor Elf−5 on eGFR
rs79420793
rs2296355
rs72841974
rs10774275
rs73408757
rs72664006
rs112247126
rs115317745
rs80143926
rs61851621
rs1982064
rs77660279
rs78500806
rs72714839
rs142973603
rs146991542
rs1192925
All − MR Egger
All − IVW(MRE)
−0.005
0.000
0.005
MR effect size for
'ETS−related transcription factor Elf−5' on 'eGFR'

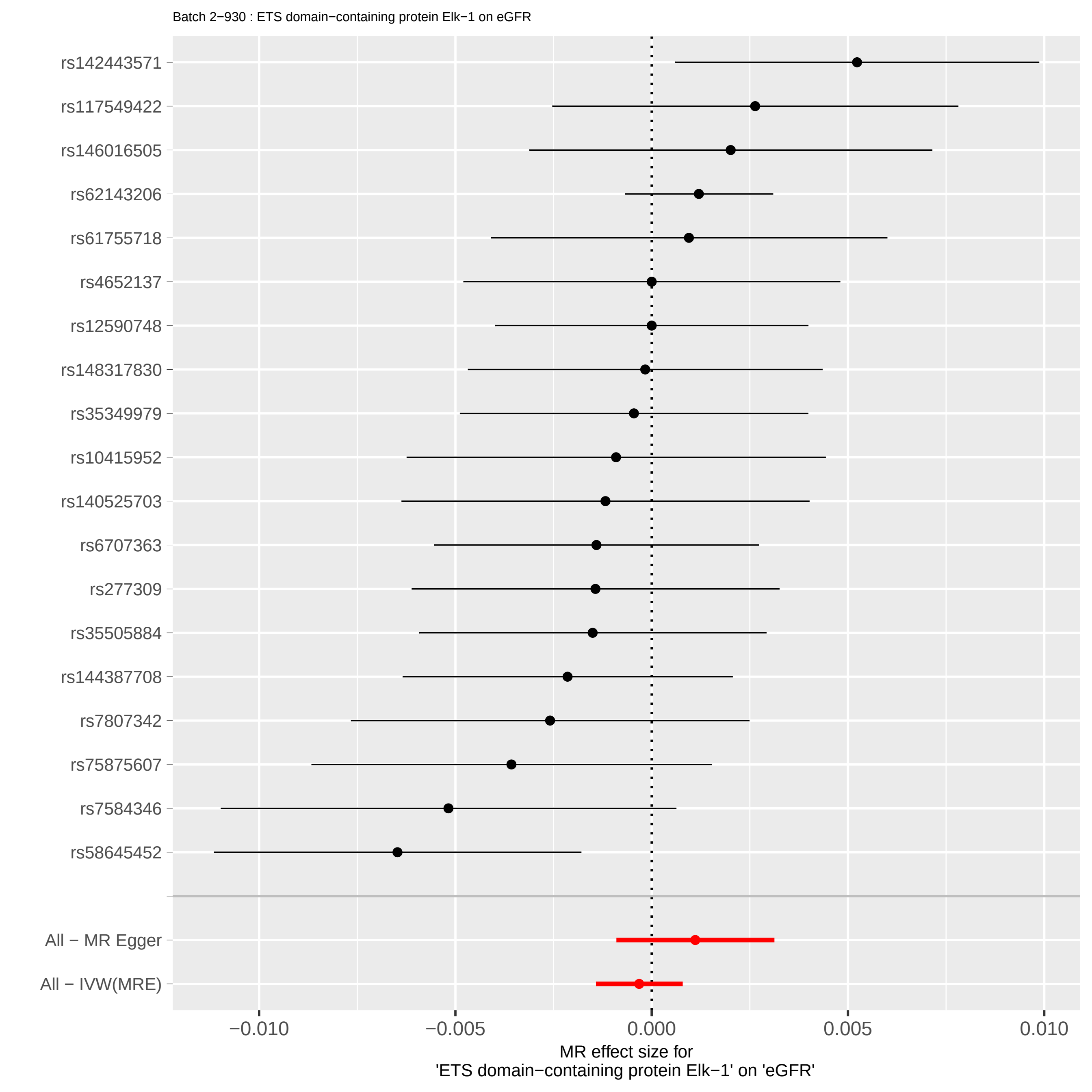

Batch 2−930 : ETS domain−containing protein Elk−1 on eGFR
rs142443571
rs117549422
rs146016505
rs62143206
rs61755718
rs4652137
rs12590748
rs148317830
rs35349979
rs10415952
rs140525703
rs6707363
rs277309
rs35505884
rs144387708
rs7807342
rs75875607
rs7584346
rs58645452
All − MR Egger
All − IVW(MRE)
−0.010
−0.005
0.000
0.005
0.010
MR effect size for
'ETS domain−containing protein Elk−1' on 'eGFR'

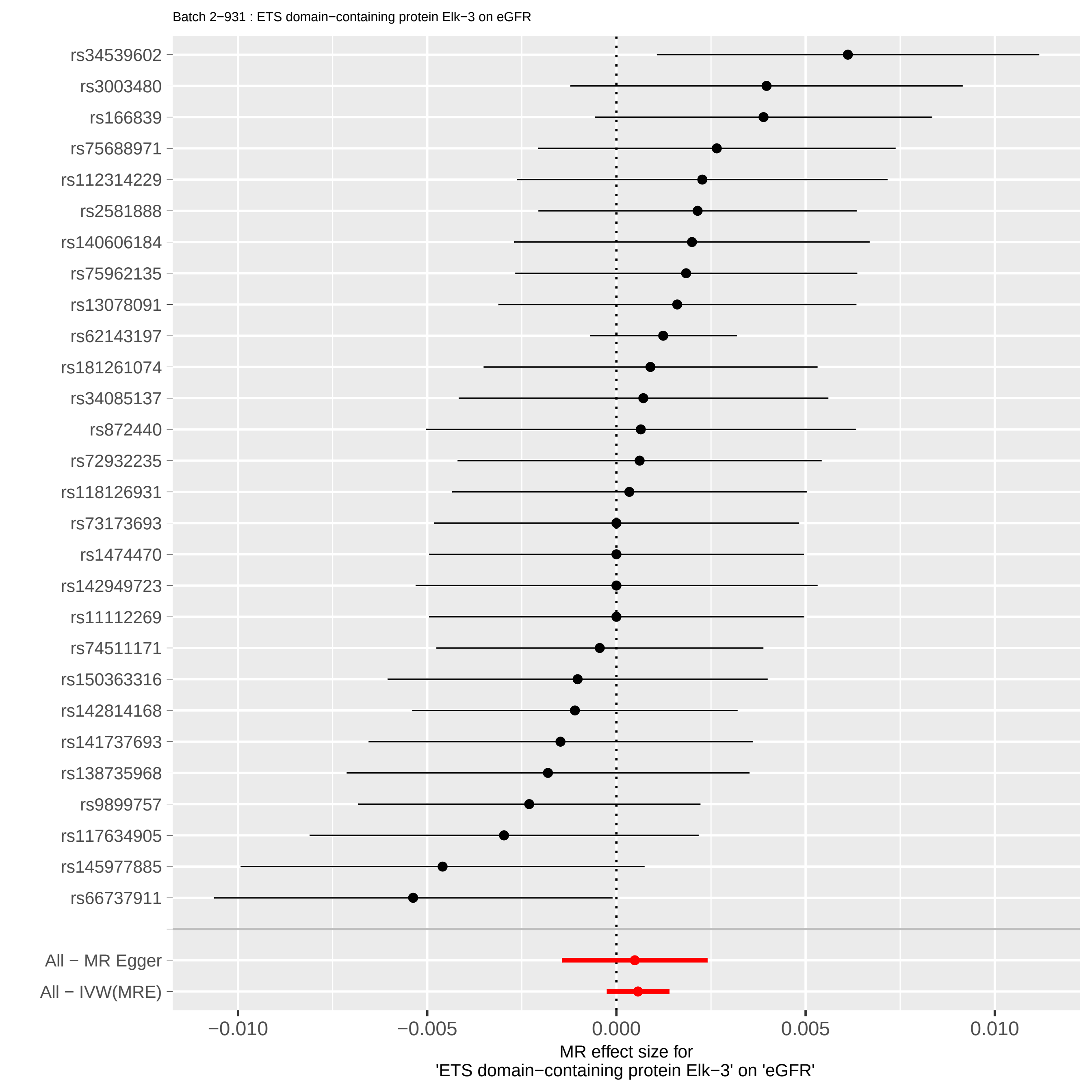

Batch 2−931 : ETS domain−containing protein Elk−3 on eGFR
rs34539602
rs3003480
rs166839
rs75688971
rs112314229
rs2581888
rs140606184
rs75962135
rs13078091
rs62143197
rs181261074
rs34085137
rs872440
rs72932235
rs118126931
rs73173693
rs1474470
rs142949723
rs11112269
rs74511171
rs150363316
rs142814168
rs141737693
rs138735968
rs9899757
rs117634905
rs145977885
rs66737911
All − MR Egger
All − IVW(MRE)
−0.010
−0.005
0.000
0.005
0.010
MR effect size for
'ETS domain−containing protein Elk−3' on 'eGFR'

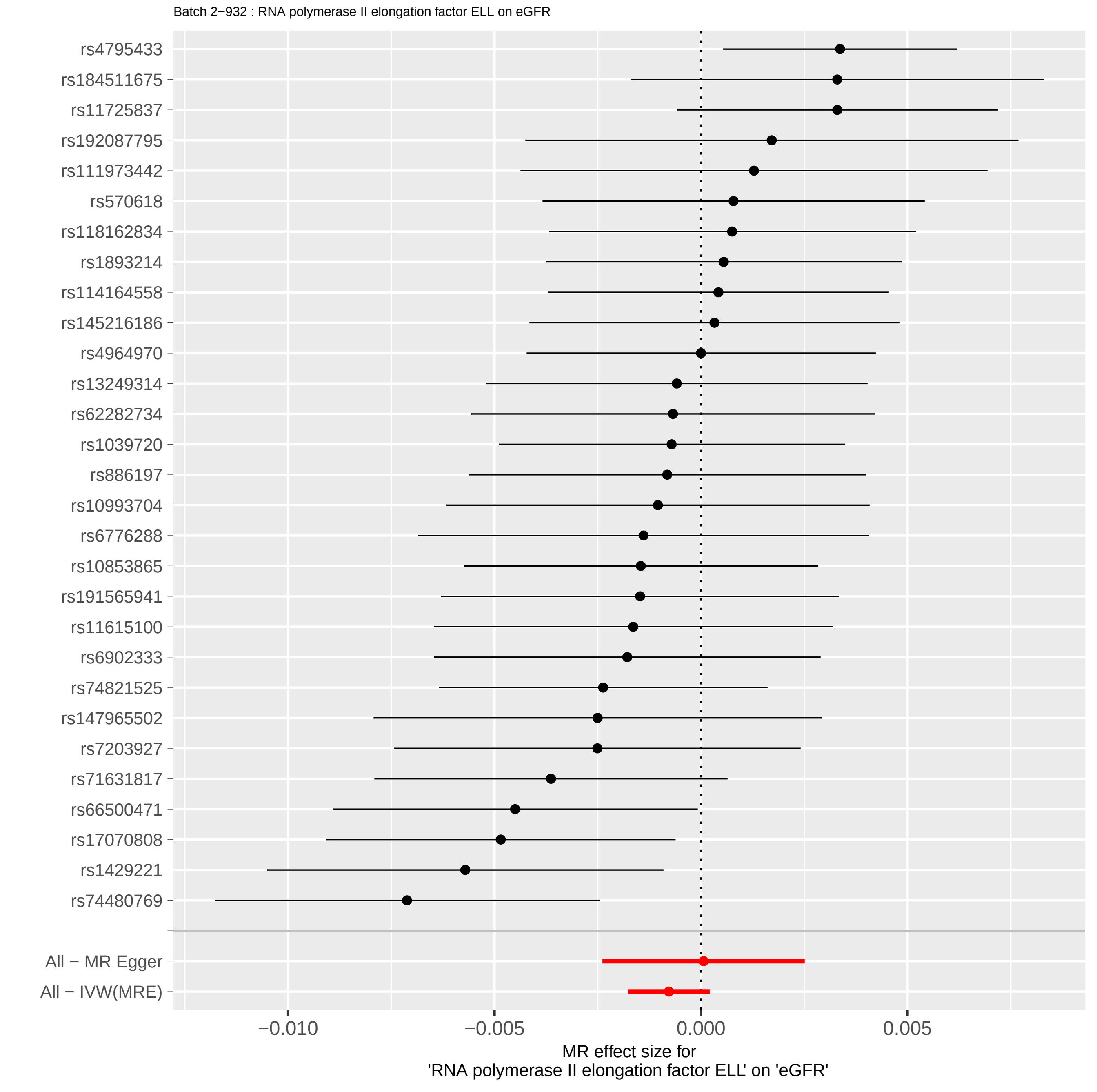

Batch 2−932 : RNA polymerase II elongation factor ELL on eGFR
rs4795433
rs184511675
rs11725837
rs192087795
rs111973442
rs570618
rs118162834
rs1893214
rs114164558
rs145216186
rs4964970
rs13249314
rs62282734
rs1039720
rs886197
rs10993704
rs6776288
rs10853865
rs191565941
rs11615100
rs6902333
rs74821525
rs147965502
rs7203927
rs71631817
rs66500471
rs17070808
rs1429221
rs74480769
All − MR Egger
All − IVW(MRE)
−0.010
−0.005
0.000
0.005
MR effect size for
'RNA polymerase II elongation factor ELL' on 'eGFR'

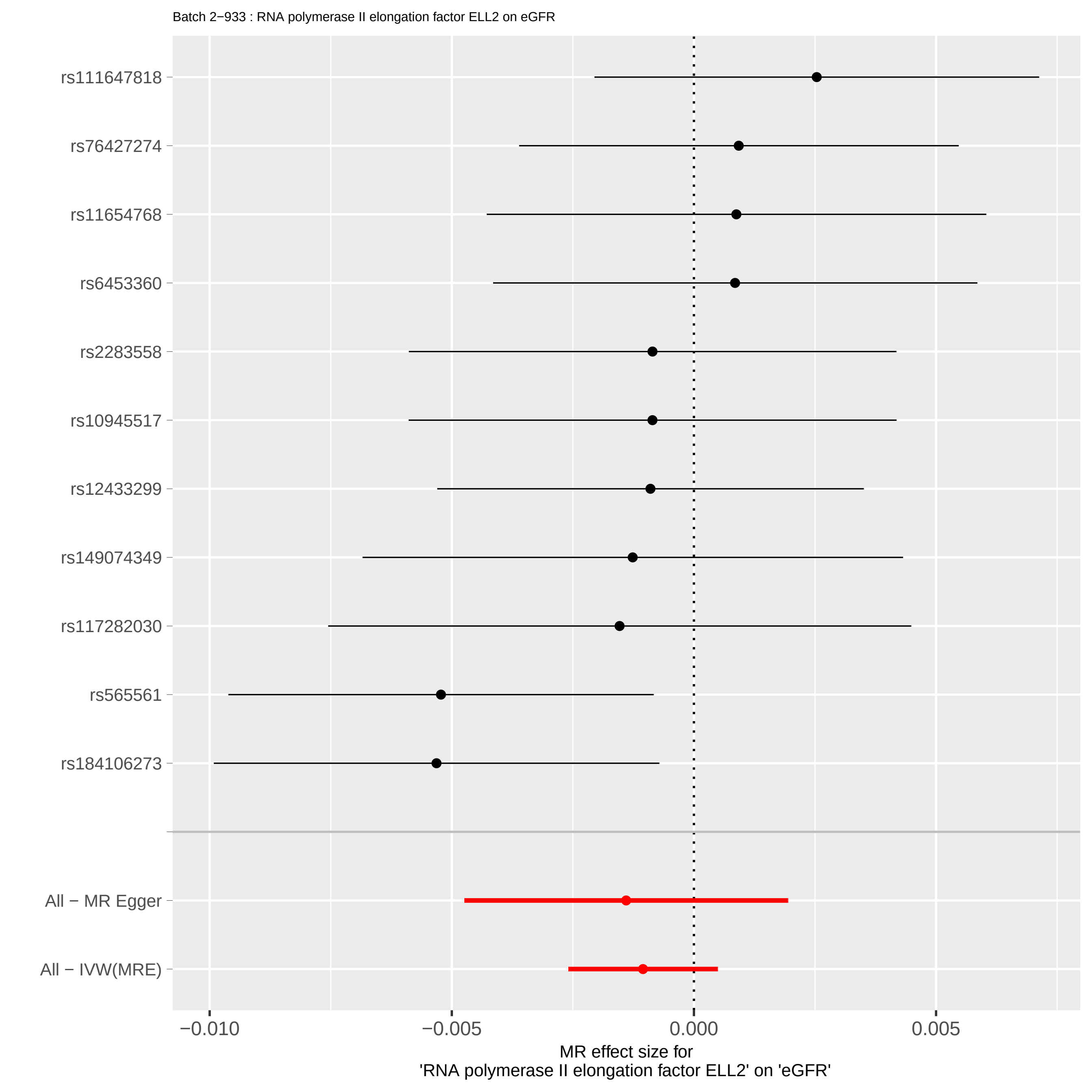

Batch 2−933 : RNA polymerase II elongation factor ELL2 on eGFR
rs111647818
rs76427274
rs11654768
rs6453360
rs2283558
rs10945517
rs12433299
rs149074349
rs117282030
rs565561
rs184106273
All − MR Egger
All − IVW(MRE)
−0.010
−0.005
0.000
0.005
MR effect size for
'RNA polymerase II elongation factor ELL2' on 'eGFR'

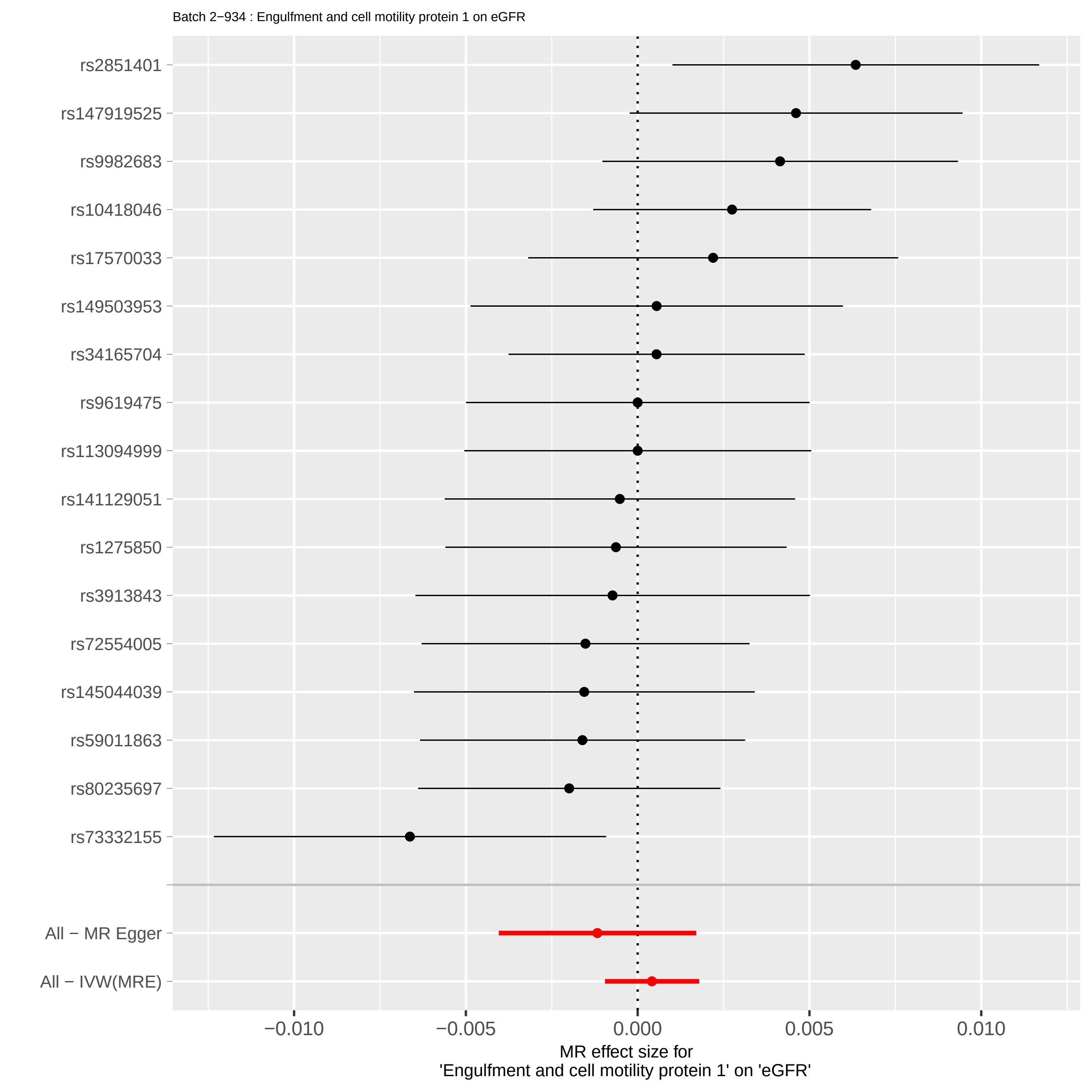

Batch 2−934 : Engulfment and cell motility protein 1 on eGFR
rs2851401
rs147919525
rs9982683
rs10418046
rs17570033
rs149503953
rs34165704
rs9619475
rs113094999
rs141129051
rs1275850
rs3913843
rs72554005
rs145044039
rs59011863
rs80235697
rs73332155
All − MR Egger
All − IVW(MRE)
−0.010
−0.005
0.000
0.005
0.010
MR effect size for
'Engulfment and cell motility protein 1' on 'eGFR'

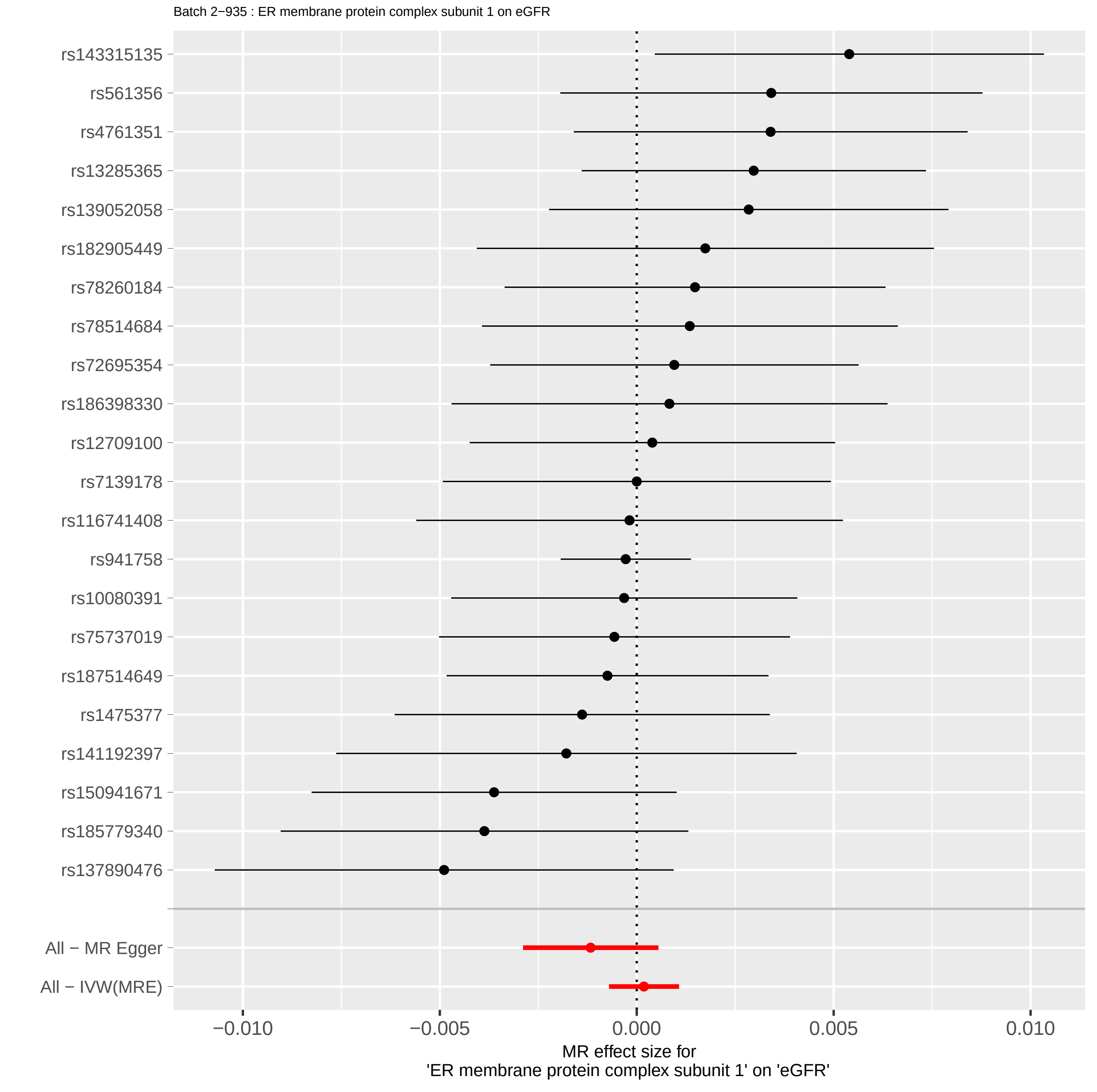
Batch 2−935 : ER membrane protein complex subunit 1 on eGFR
rs143315135
rs561356
rs4761351
rs13285365
rs139052058
rs182905449
rs78260184
rs78514684
rs72695354
rs186398330
rs12709100
rs7139178
rs116741408
rs941758
rs10080391
rs75737019
rs187514649
rs1475377
rs141192397
rs150941671
rs185779340
rs137890476
All − MR Egger
All − IVW(MRE)
−0.010
−0.005
0.000
0.005
0.010
MR effect size for
'ER membrane protein complex subunit 1' on 'eGFR'

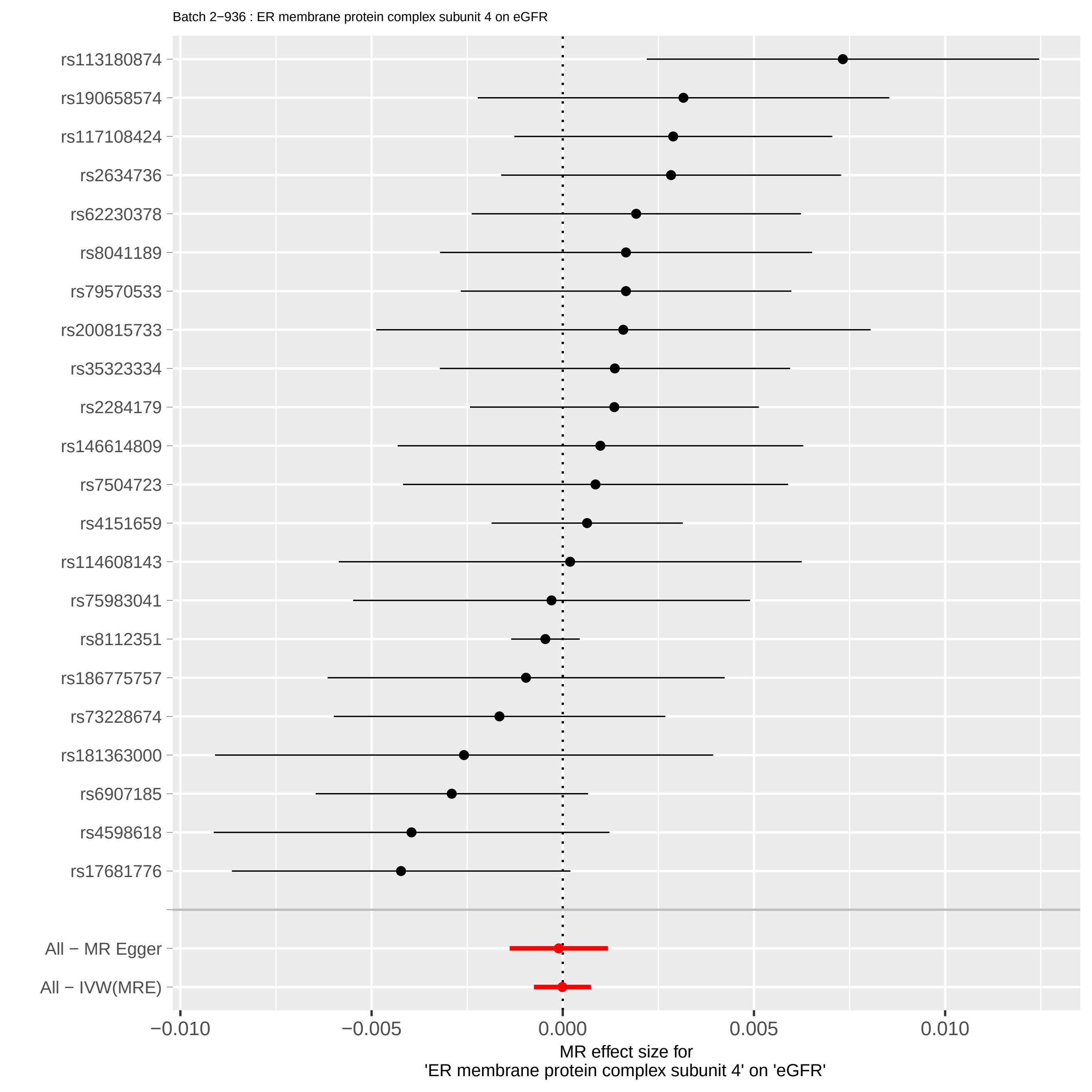

Batch 2−936 : ER membrane protein complex subunit 4 on eGFR
rs113180874
rs190658574
rs117108424
rs2634736
rs62230378
rs8041189
rs79570533
rs200815733
rs35323334
rs2284179
rs146614809
rs7504723
rs4151659
rs114608143
rs75983041
rs8112351
rs186775757
rs73228674
rs181363000
rs6907185
rs4598618
rs17681776
All − MR Egger
All − IVW(MRE)
−0.010
−0.005
0.000
0.005
0.010
MR effect size for
'ER membrane protein complex subunit 4' on 'eGFR'

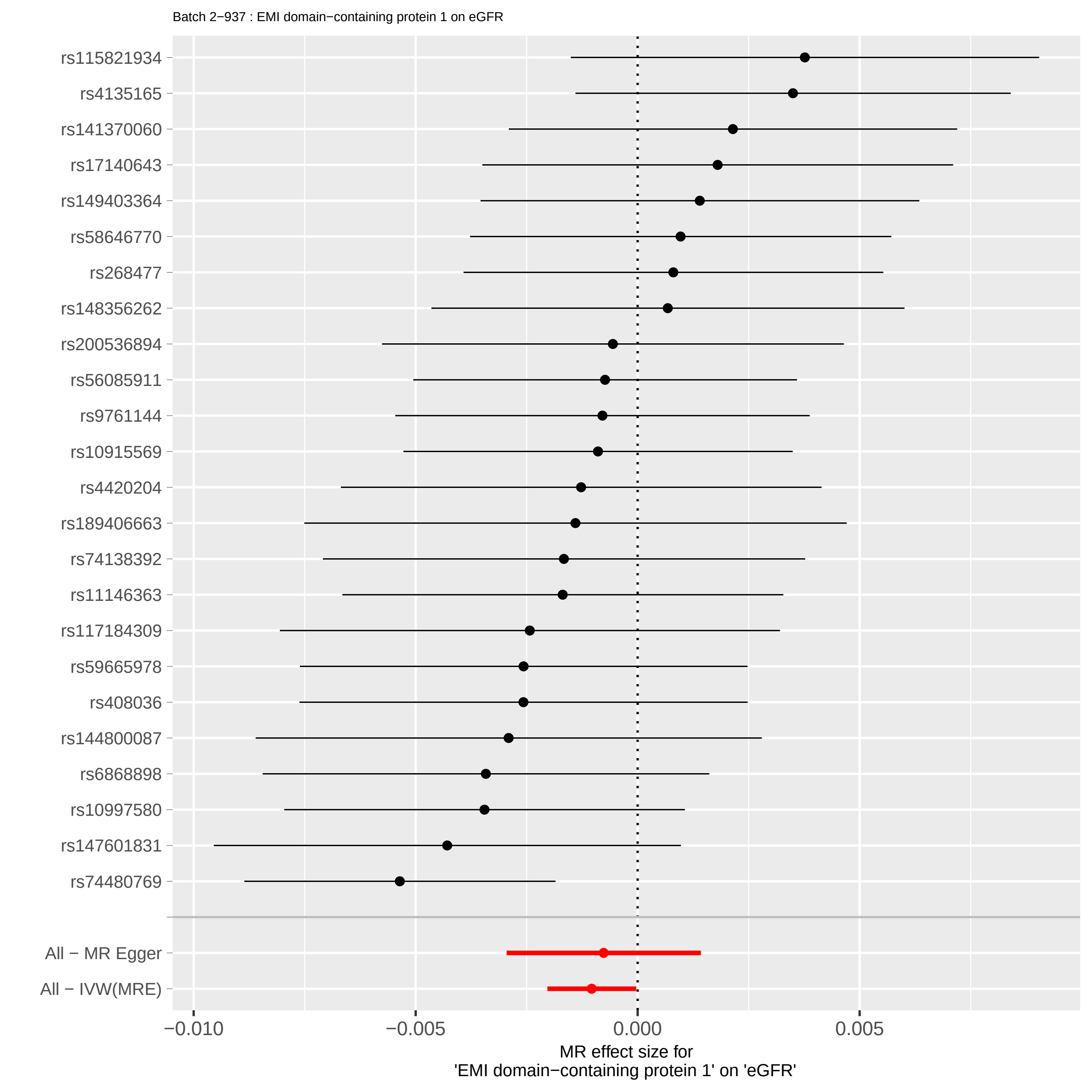

Batch 2−937 : EMI domain−containing protein 1 on eGFR
rs115821934
rs4135165
rs141370060
rs17140643
rs149403364
rs58646770
rs268477
rs148356262
rs200536894
rs56085911
rs9761144
rs10915569
rs4420204
rs189406663
rs74138392
rs11146363
rs117184309
rs59665978
rs408036
rs144800087
rs6868898
rs10997580
rs147601831
rs74480769
All − MR Egger
All − IVW(MRE)
−0.010
−0.005
0.000
0.005
MR effect size for
'EMI domain−containing protein 1' on 'eGFR'

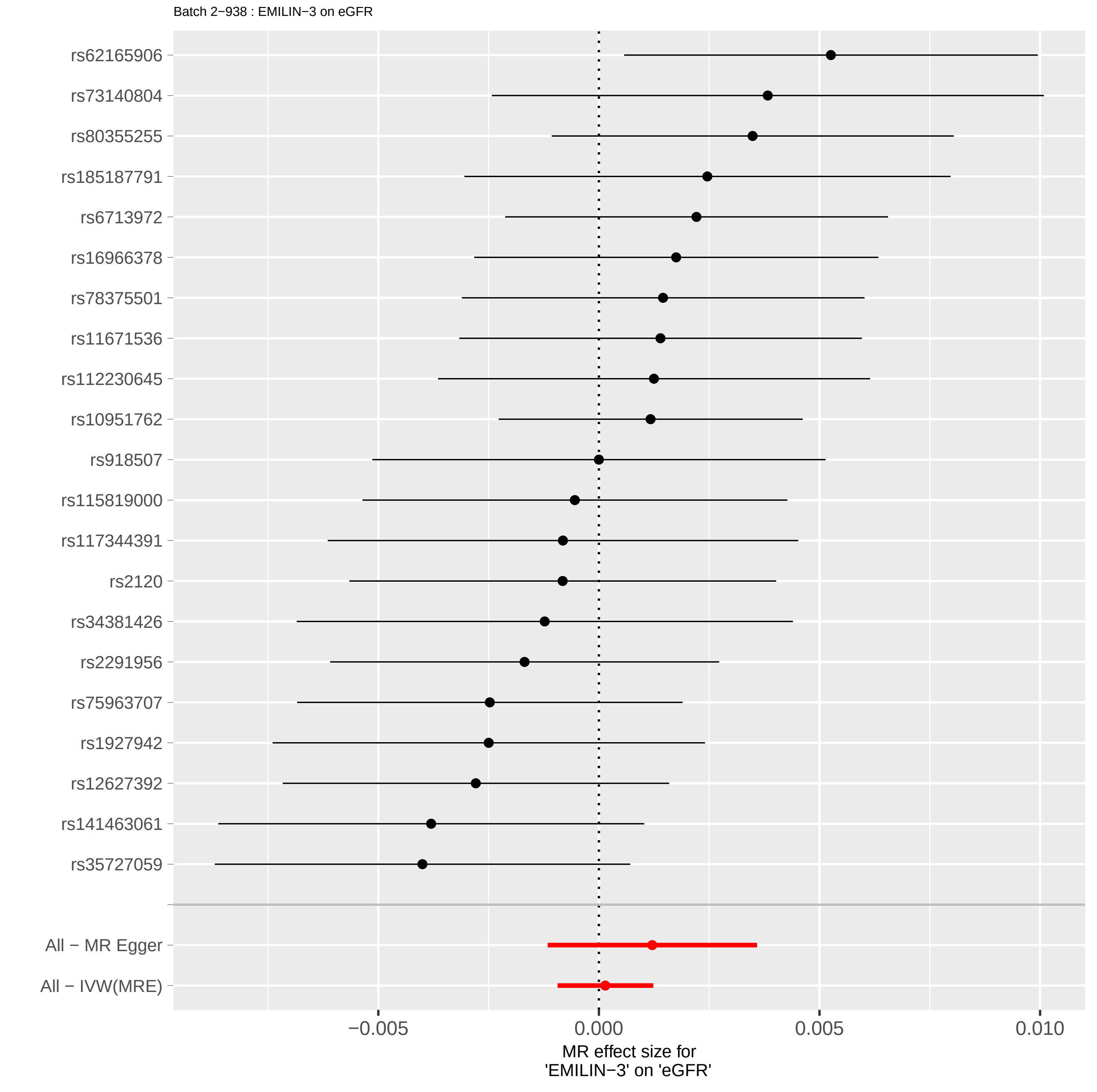
Batch 2−938 : EMILIN−3 on eGFR
rs62165906
rs73140804
rs80355255
rs185187791
rs6713972
rs16966378
rs78375501
rs11671536
rs112230645
rs10951762
rs918507
rs115819000
rs117344391
rs2120
rs34381426
rs2291956
rs75963707
rs1927942
rs12627392
rs141463061
rs35727059
All − MR Egger
All − IVW(MRE)
−0.005
0.000
0.005
0.010
MR effect size for
'EMILIN−3' on 'eGFR'

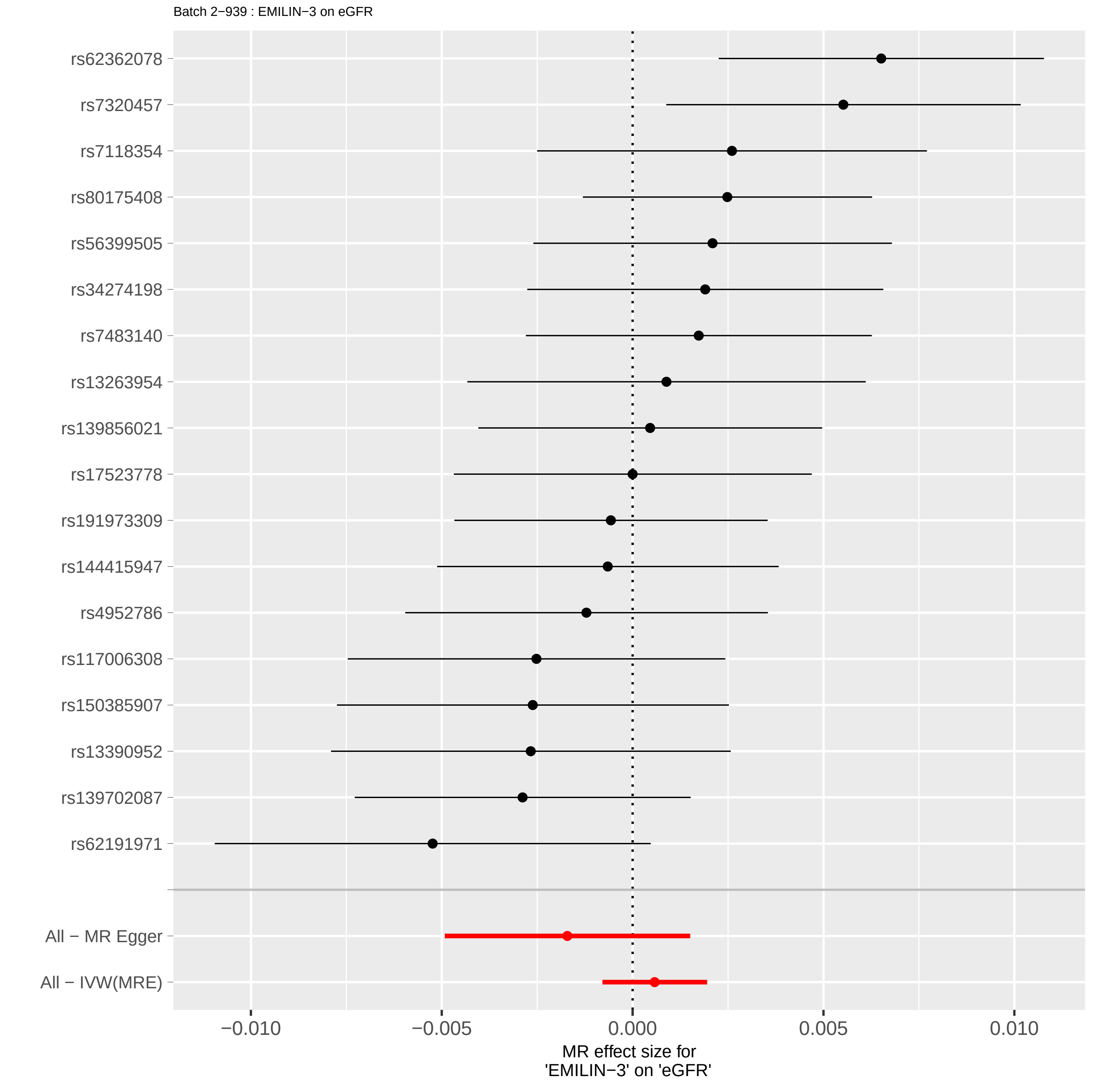

Batch 2−939 : EMILIN−3 on eGFR
rs62362078
rs7320457
rs7118354
rs80175408
rs56399505
rs34274198
rs7483140
rs13263954
rs139856021
rs17523778
rs191973309
rs144415947
rs4952786
rs117006308
rs150385907
rs13390952
rs139702087
rs62191971
All − MR Egger
All − IVW(MRE)
−0.010
−0.005
0.000
0.005
0.010
MR effect size for
'EMILIN−3' on 'eGFR'

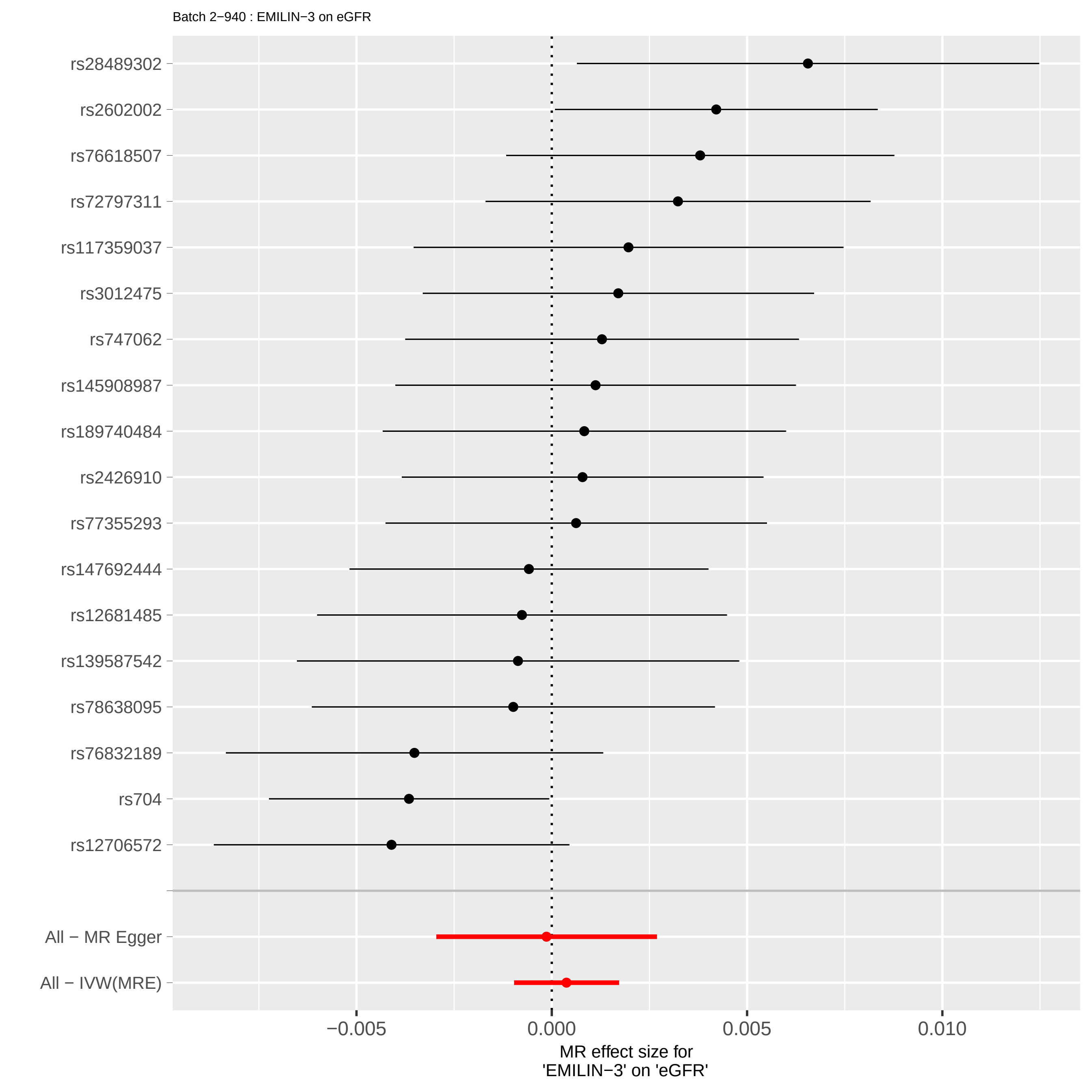

Batch 2−940 : EMILIN−3 on eGFR
rs28489302
rs2602002
rs76618507
rs72797311
rs117359037
rs3012475
rs747062
rs145908987
rs189740484
rs2426910
rs77355293
rs147692444
rs12681485
rs139587542
rs78638095
rs76832189
rs704
rs12706572
All − MR Egger
All − IVW(MRE)
−0.005
0.000
0.005
0.010
MR effect size for
'EMILIN−3' on 'eGFR'

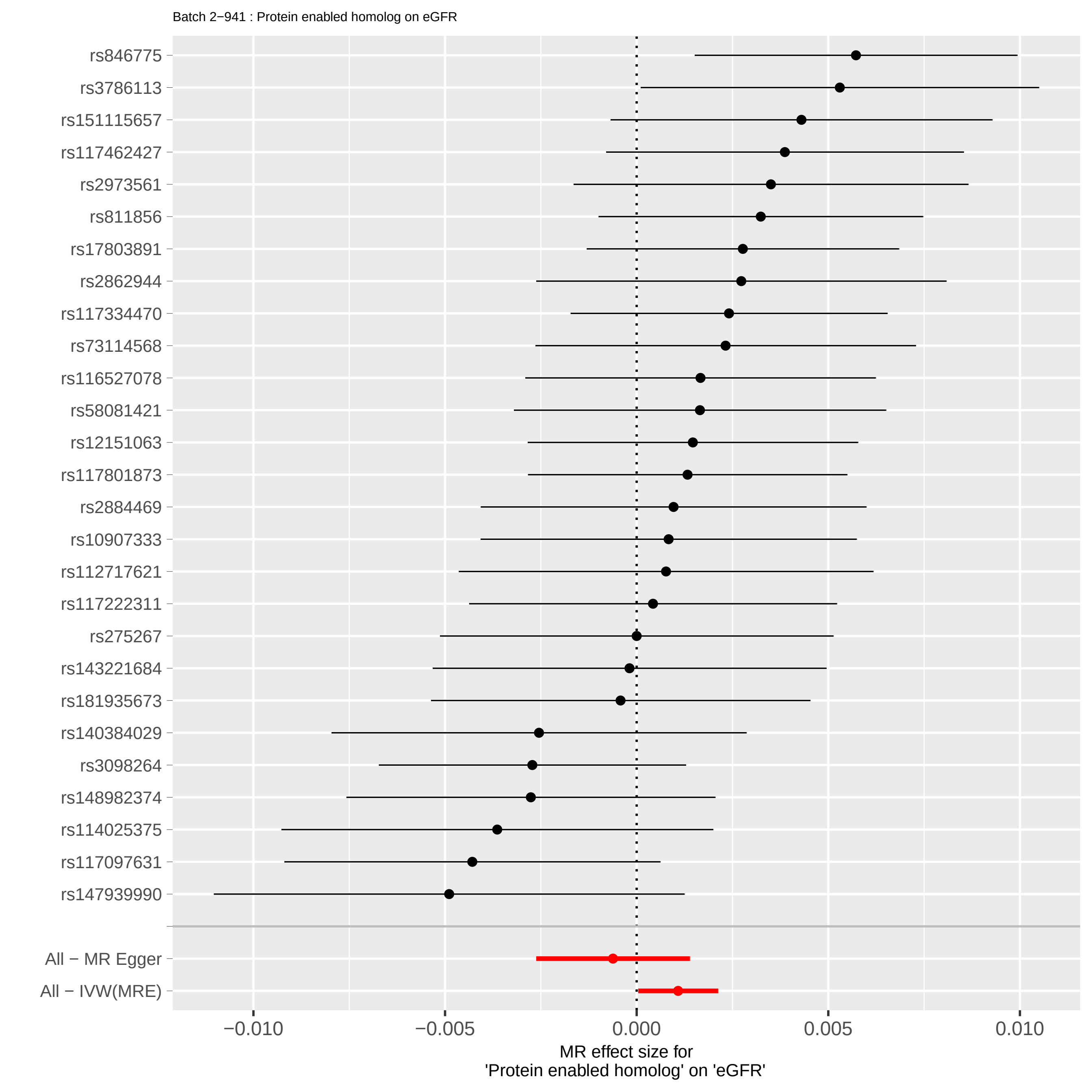

Batch 2−941 : Protein enabled homolog on eGFR
rs846775
rs3786113
rs151115657
rs117462427
rs2973561
rs811856
rs17803891
rs2862944
rs117334470
rs73114568
rs116527078
rs58081421
rs12151063
rs117801873
rs2884469
rs10907333
rs112717621
rs117222311
rs275267
rs143221684
rs181935673
rs140384029
rs3098264
rs148982374
rs114025375
rs117097631
rs147939990
All − MR Egger
All − IVW(MRE)
−0.010
−0.005
0.000
0.005
0.010
MR effect size for
'Protein enabled homolog' on 'eGFR'

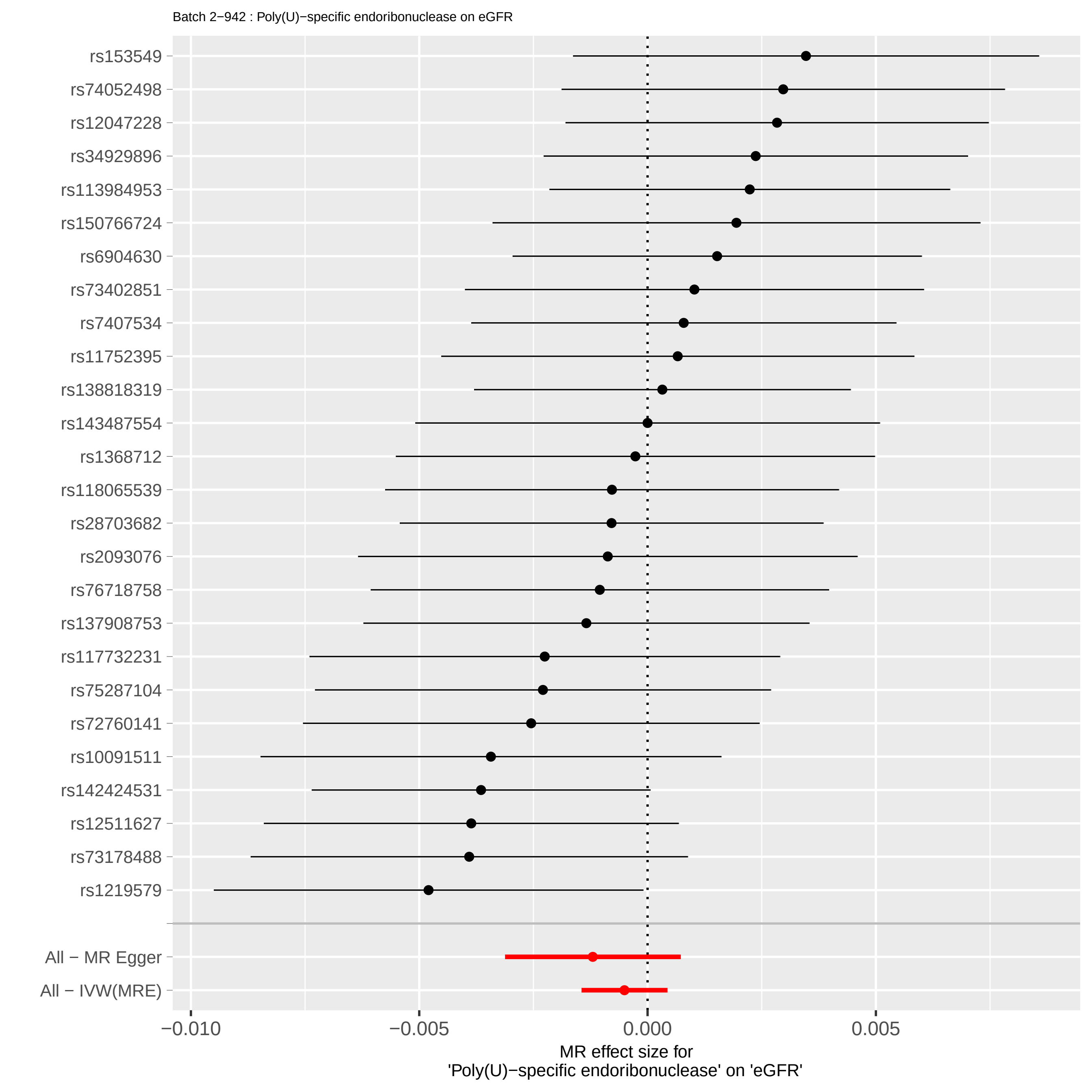
Batch 2−942 : Poly(U)−specific endoribonuclease on eGFR
rs153549
rs74052498
rs12047228
rs34929896
rs113984953
rs150766724
rs6904630
rs73402851
rs7407534
rs11752395
rs138818319
rs143487554
rs1368712
rs118065539
rs28703682
rs2093076
rs76718758
rs137908753
rs117732231
rs75287104
rs72760141
rs10091511
rs142424531
rs12511627
rs73178488
rs1219579
All − MR Egger
All − IVW(MRE)
−0.010
−0.005
0.000
0.005
MR effect size for
'Poly(U)−specific endoribonuclease' on 'eGFR'

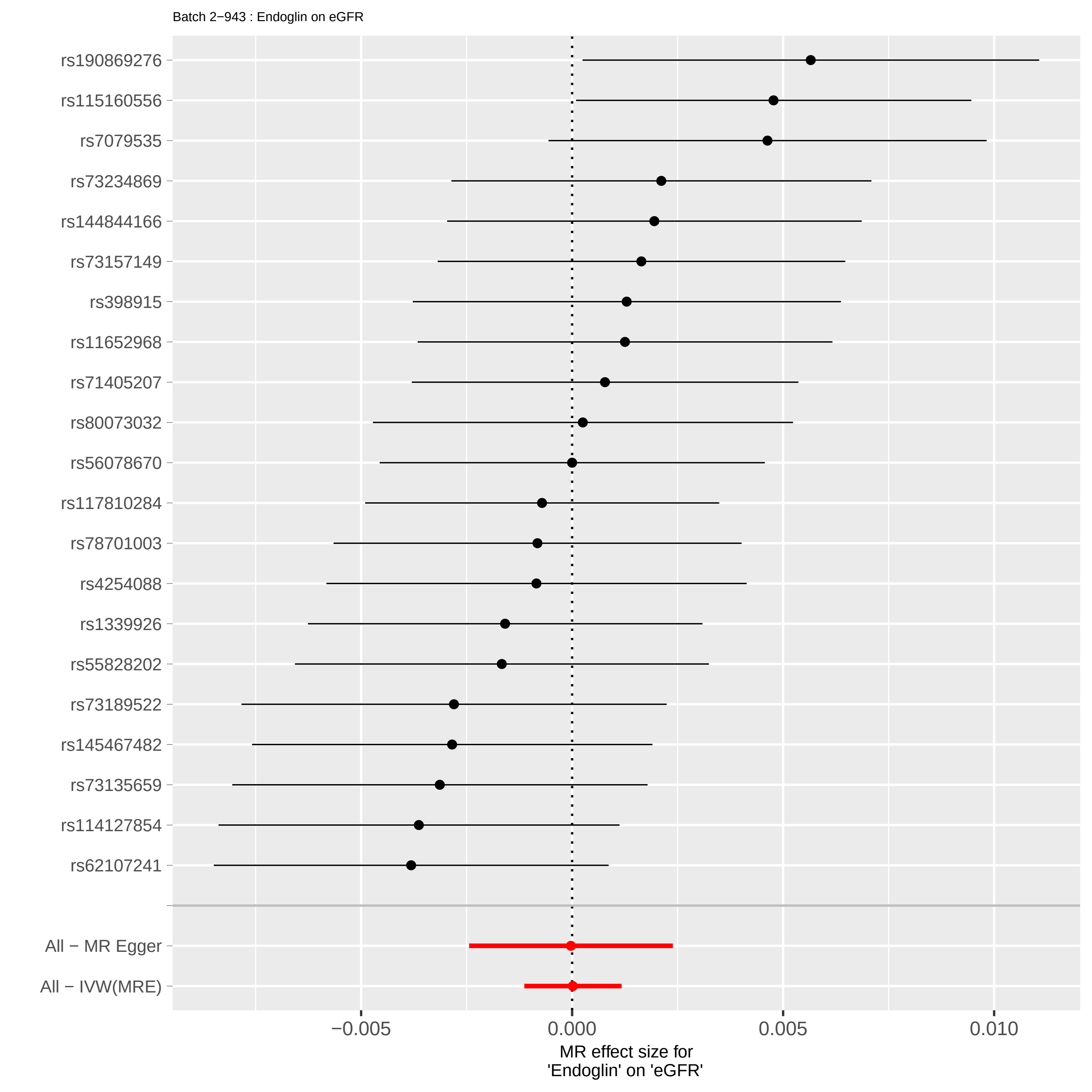

Batch 2−943 : Endoglin on eGFR
rs190869276
rs115160556
rs7079535
rs73234869
rs144844166
rs73157149
rs398915
rs11652968
rs71405207
rs80073032
rs56078670
rs117810284
rs78701003
rs4254088
rs1339926
rs55828202
rs73189522
rs145467482
rs73135659
rs114127854
rs62107241
All − MR Egger
All − IVW(MRE)
−0.005
0.000
0.005
0.010
MR effect size for
'Endoglin' on 'eGFR'

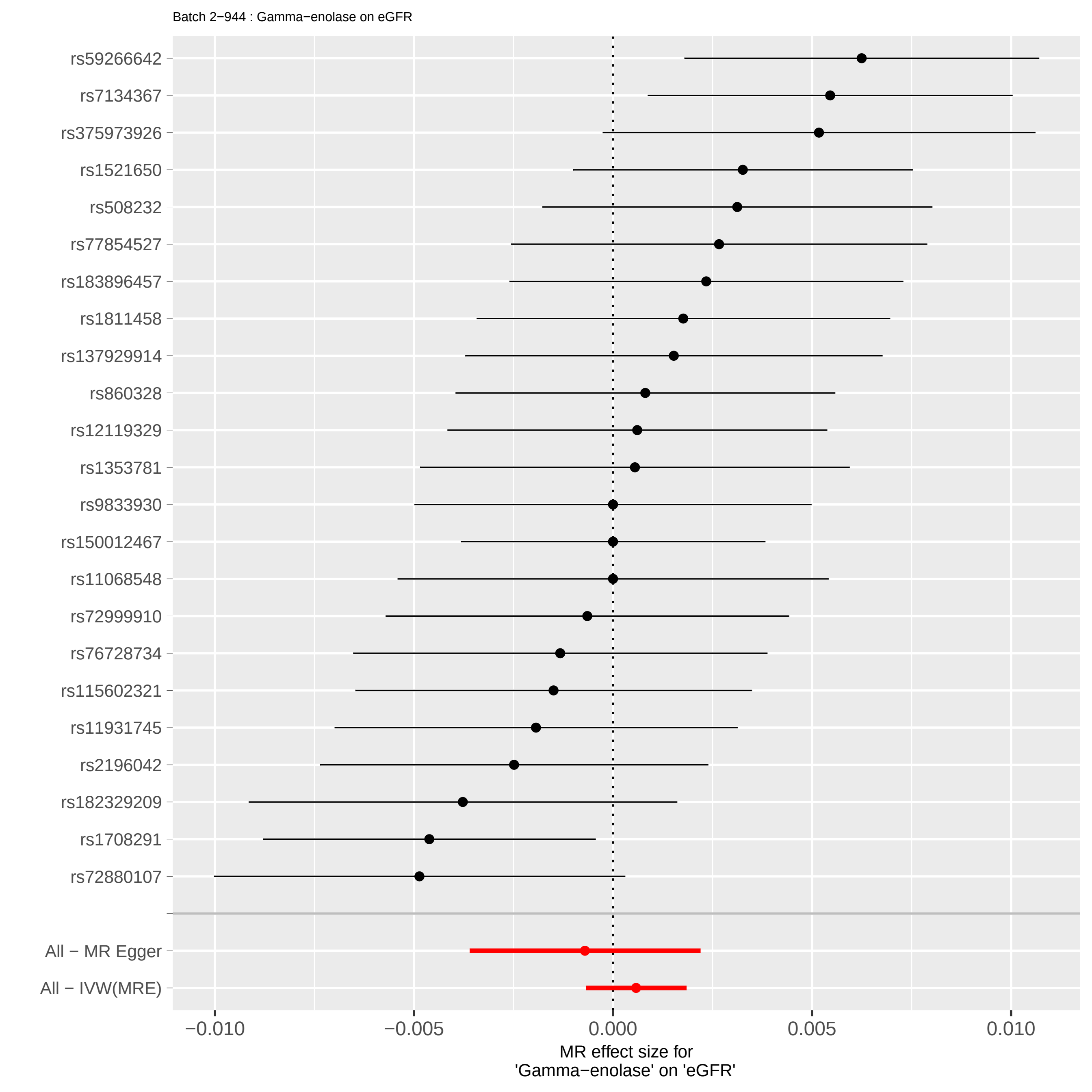

Batch 2−944 : Gamma−enolase on eGFR
rs59266642
rs7134367
rs375973926
rs1521650
rs508232
rs77854527
rs183896457
rs1811458
rs137929914
rs860328
rs12119329
rs1353781
rs9833930
rs150012467
rs11068548
rs72999910
rs76728734
rs115602321
rs11931745
rs2196042
rs182329209
rs1708291
rs72880107
All − MR Egger
All − IVW(MRE)
−0.010
−0.005
0.000
0.005
0.010
MR effect size for
'Gamma−enolase' on 'eGFR'

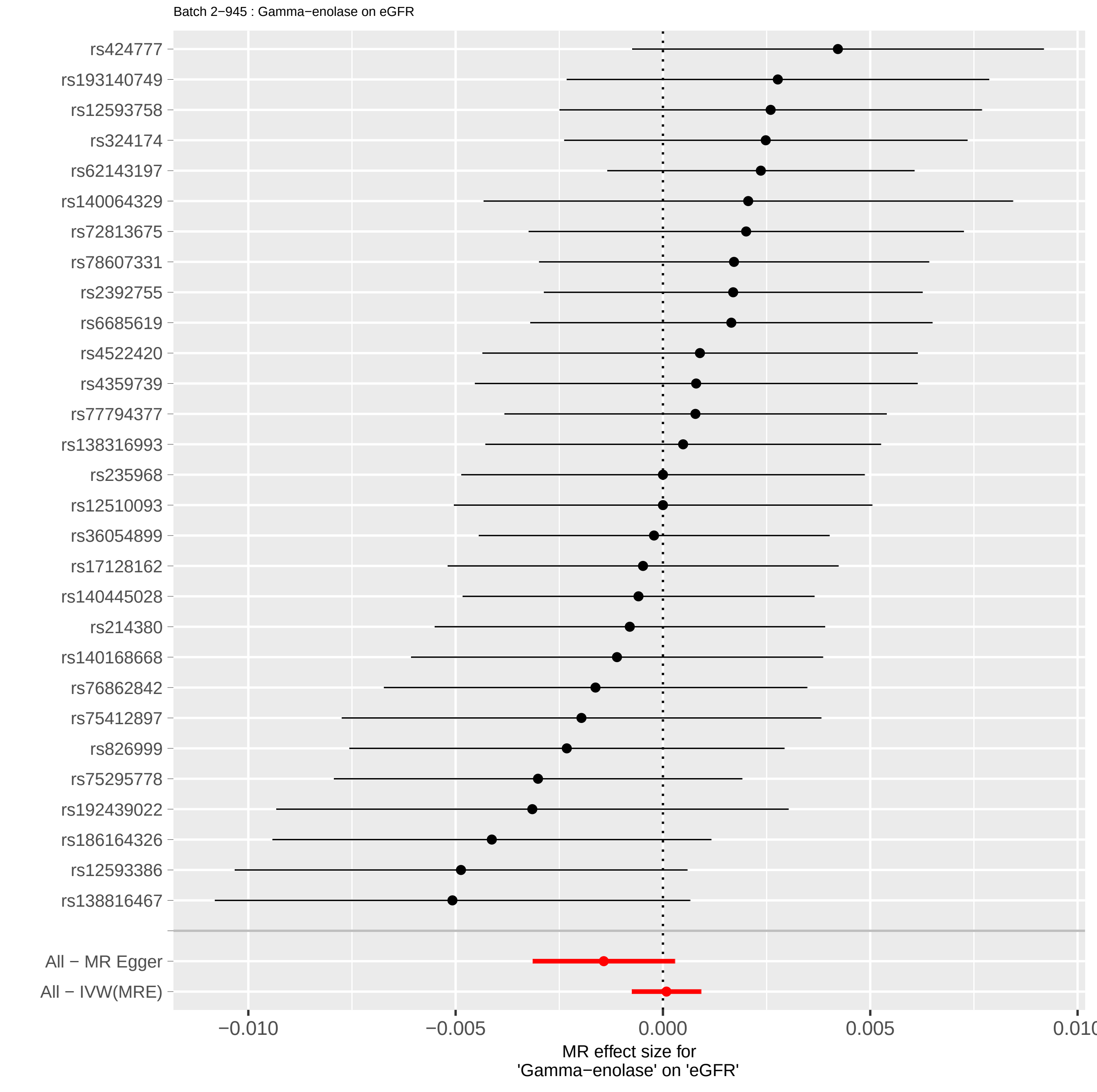

Batch 2−945 : Gamma−enolase on eGFR
rs424777
rs193140749
rs12593758
rs324174
rs62143197
rs140064329
rs72813675
rs78607331
rs2392755
rs6685619
rs4522420
rs4359739
rs77794377
rs138316993
rs235968
rs12510093
rs36054899
rs17128162
rs140445028
rs214380
rs140168668
rs76862842
rs75412897
rs826999
rs75295778
rs192439022
rs186164326
rs12593386
rs138816467
All − MR Egger
All − IVW(MRE)
−0.010
−0.005
0.000
0.005
0.010
MR effect size for
'Gamma−enolase' on 'eGFR'

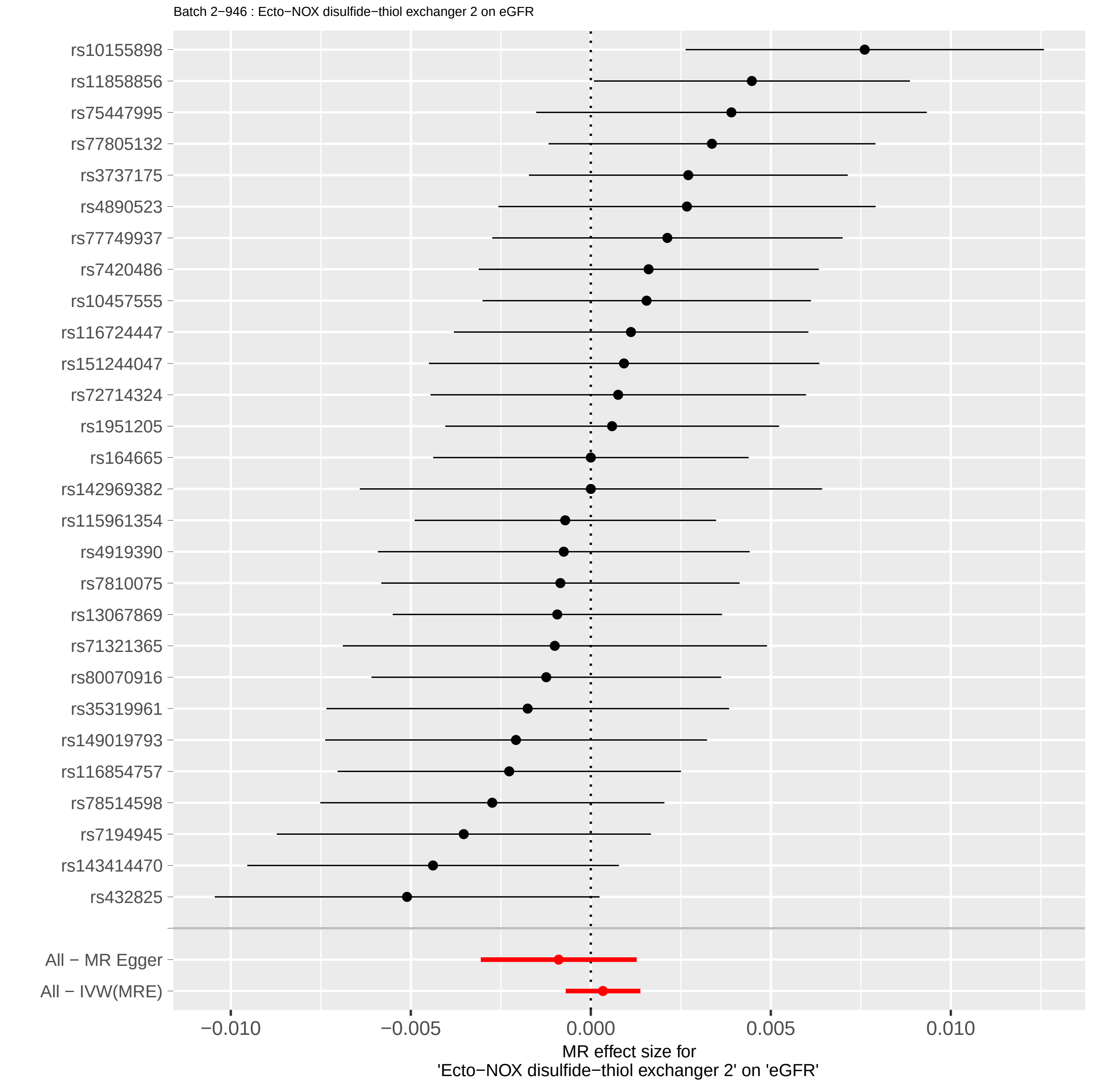
Batch 2−946 : Ecto−NOX disulfide−thiol exchanger 2 on eGFR
rs10155898
rs11858856
rs75447995
rs77805132
rs3737175
rs4890523
rs77749937
rs7420486
rs10457555
rs116724447
rs151244047
rs72714324
rs1951205
rs164665
rs142969382
rs115961354
rs4919390
rs7810075
rs13067869
rs71321365
rs80070916
rs35319961
rs149019793
rs116854757
rs78514598
rs7194945
rs143414470
rs432825
All − MR Egger
All − IVW(MRE)
−0.010
−0.005
0.000
0.005
0.010
MR effect size for
'Ecto−NOX disulfide−thiol exchanger 2' on 'eGFR'

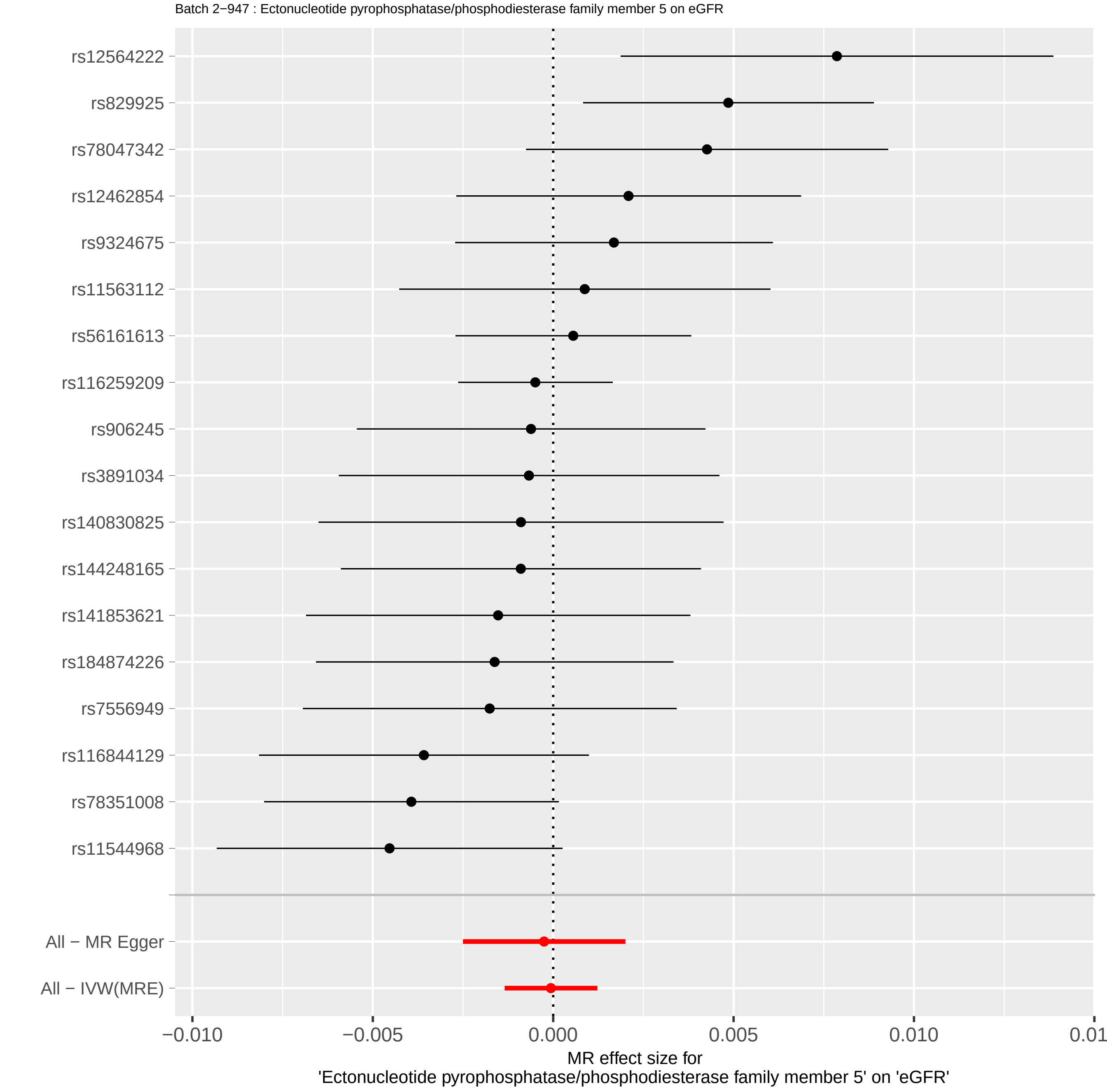

Batch 2−947 : Ectonucleotide pyrophosphatase/phosphodiesterase family member 5 on eGFR
rs12564222
rs829925
rs78047342
rs12462854
rs9324675
rs11563112
rs56161613
rs116259209
rs906245
rs3891034
rs140830825
rs144248165
rs141853621
rs184874226
rs7556949
rs116844129
rs78351008
rs11544968
All − MR Egger
All − IVW(MRE)
−0.010
−0.005
0.000
0.005
0.010
MR effect size for
'Ectonucleotide pyrophosphatase/phosphodiesterase family member 5' on 'eGFR'

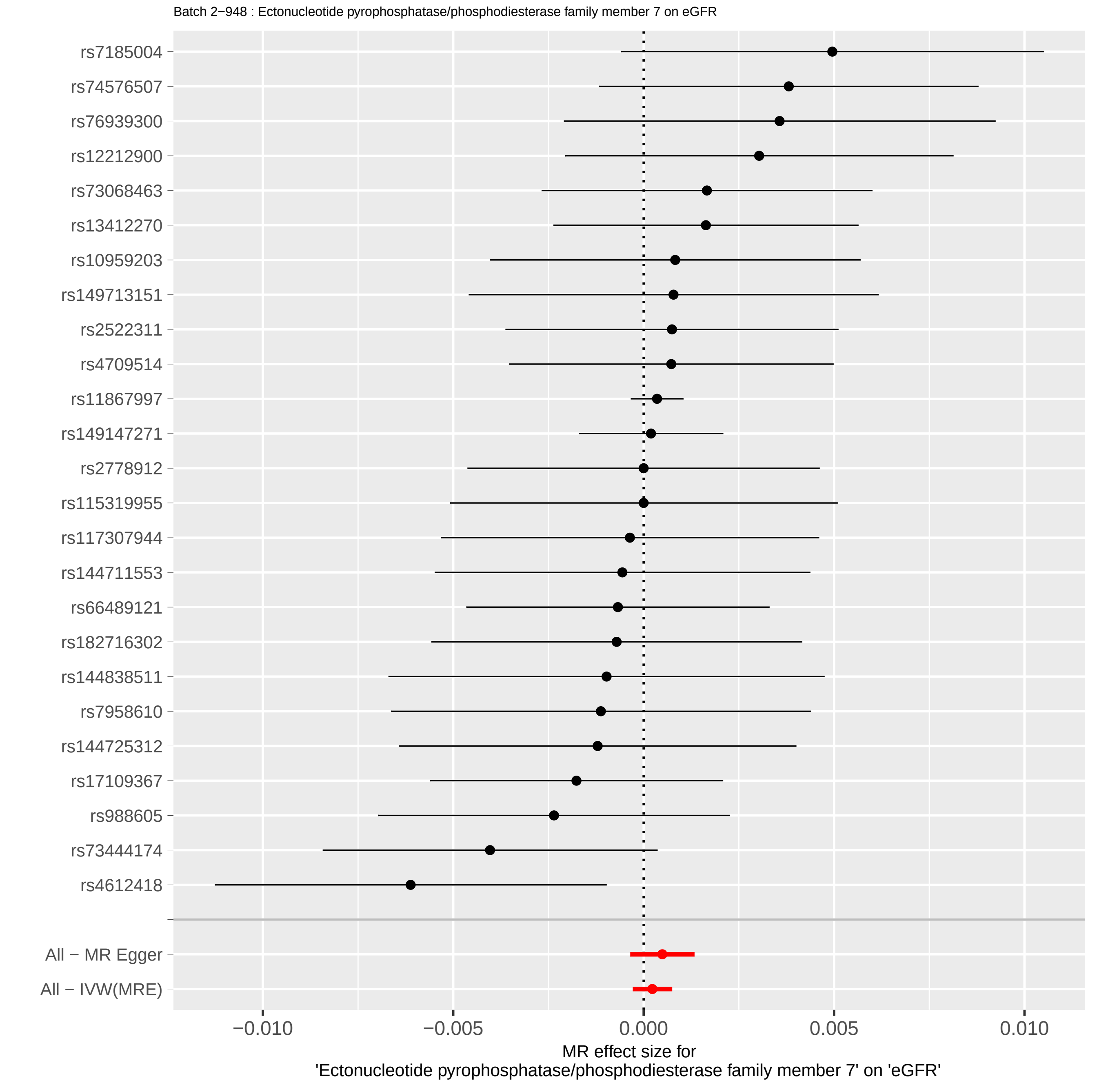

Batch 2−948 : Ectonucleotide pyrophosphatase/phosphodiesterase family member 7 on eGFR
rs7185004
rs74576507
rs76939300
rs12212900
rs73068463
rs13412270
rs10959203
rs149713151
rs2522311
rs4709514
rs11867997
rs149147271
rs2778912
rs115319955
rs117307944
rs144711553
rs66489121
rs182716302
rs144838511
rs7958610
rs144725312
rs17109367
rs988605
rs73444174
rs4612418
All − MR Egger
All − IVW(MRE)
−0.010
−0.005
0.000
0.005
0.010
MR effect size for
'Ectonucleotide pyrophosphatase/phosphodiesterase family member 7' on 'eGFR'

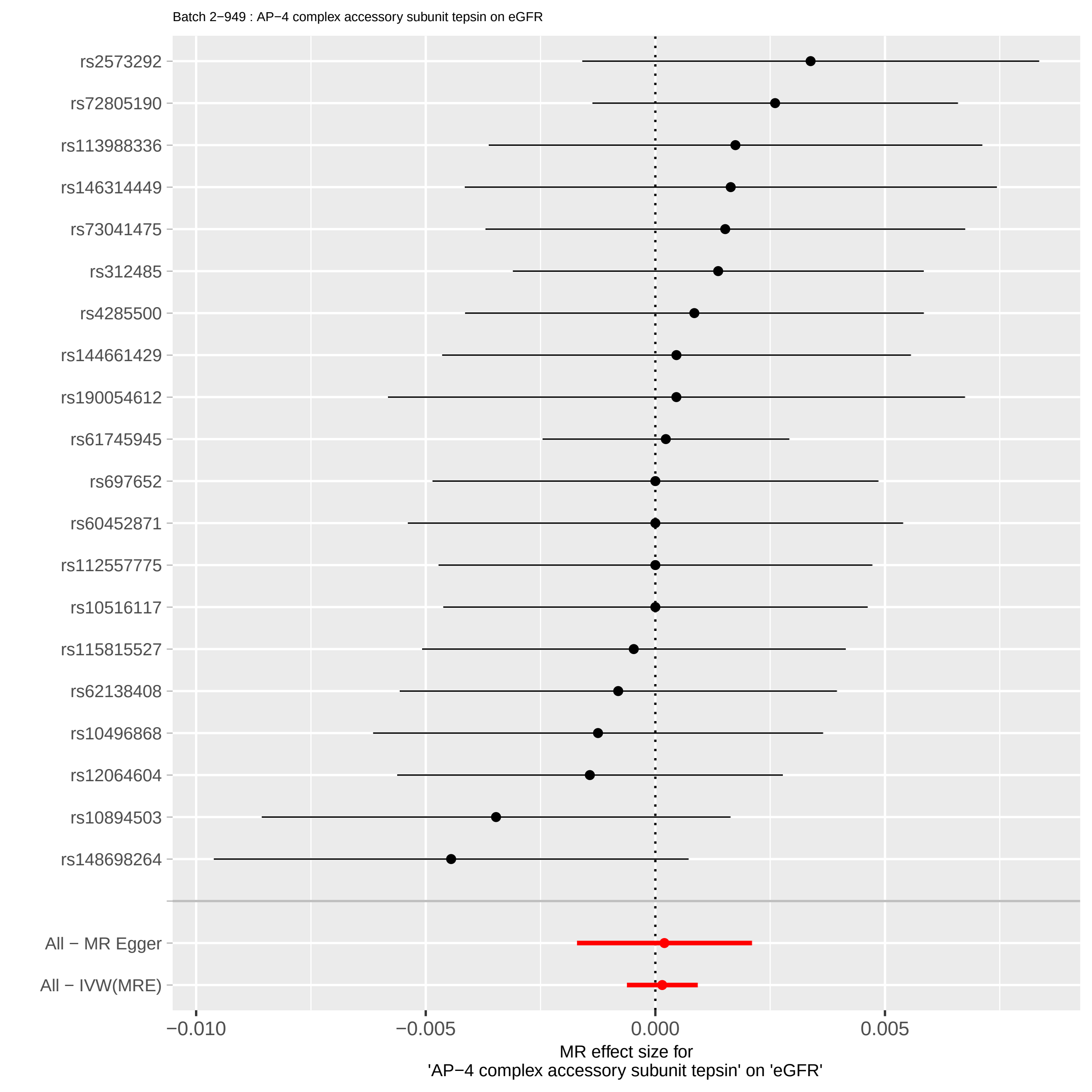

Batch 2−949 : AP−4 complex accessory subunit tepsin on eGFR
rs2573292
rs72805190
rs113988336
rs146314449
rs73041475
rs312485
rs4285500
rs144661429
rs190054612
rs61745945
rs697652
rs60452871
rs112557775
rs10516117
rs115815527
rs62138408
rs10496868
rs12064604
rs10894503
rs148698264
All − MR Egger
All − IVW(MRE)
−0.010
−0.005
0.000
0.005
MR effect size for
'AP−4 complex accessory subunit tepsin' on 'eGFR'

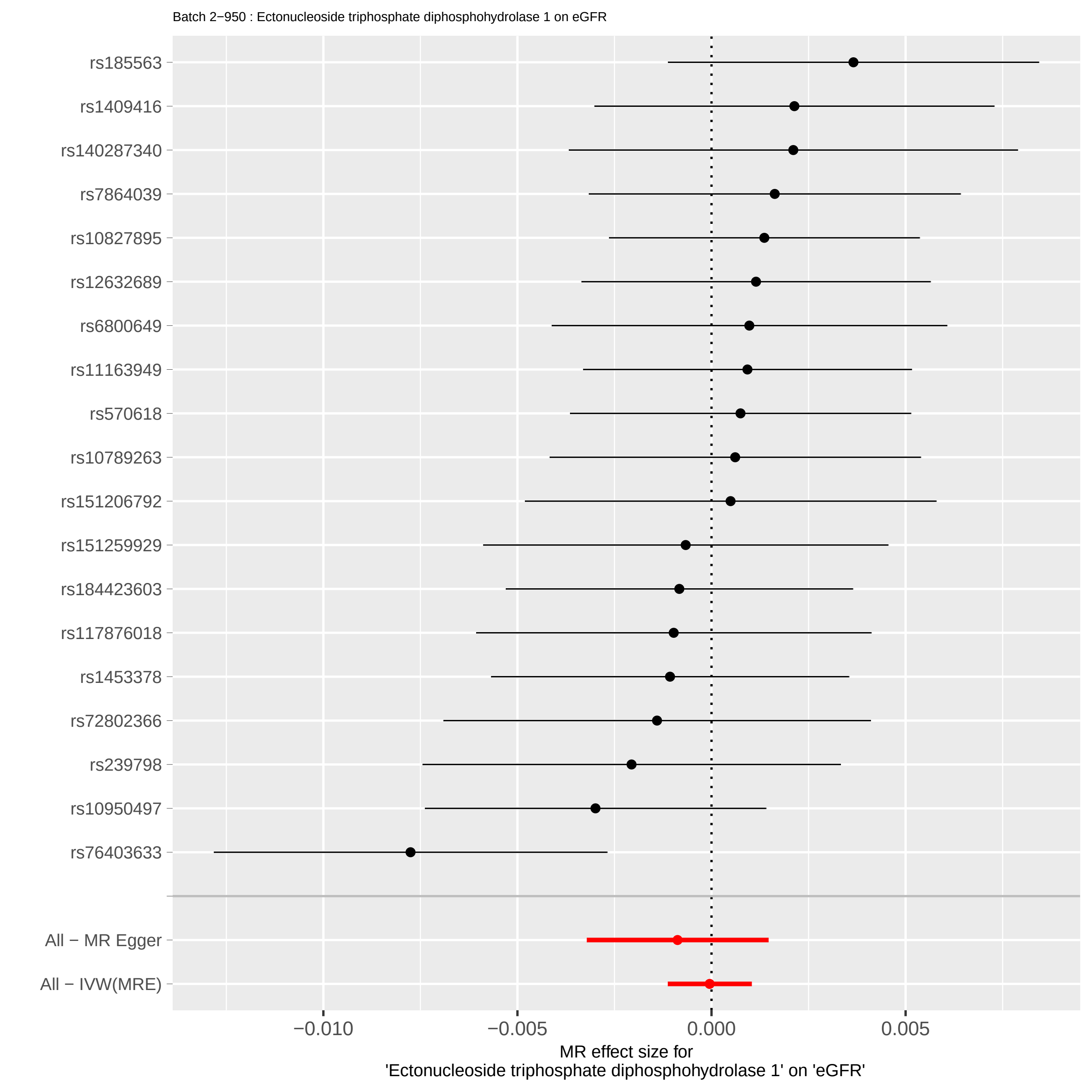
Batch 2−950 : Ectonucleoside triphosphate diphosphohydrolase 1 on eGFR
rs185563
rs1409416
rs140287340
rs7864039
rs10827895
rs12632689
rs6800649
rs11163949
rs570618
rs10789263
rs151206792
rs151259929
rs184423603
rs117876018
rs1453378
rs72802366
rs239798
rs10950497
rs76403633
All − MR Egger
All − IVW(MRE)
−0.010
−0.005
0.000
0.005
MR effect size for
'Ectonucleoside triphosphate diphosphohydrolase 1' on 'eGFR'

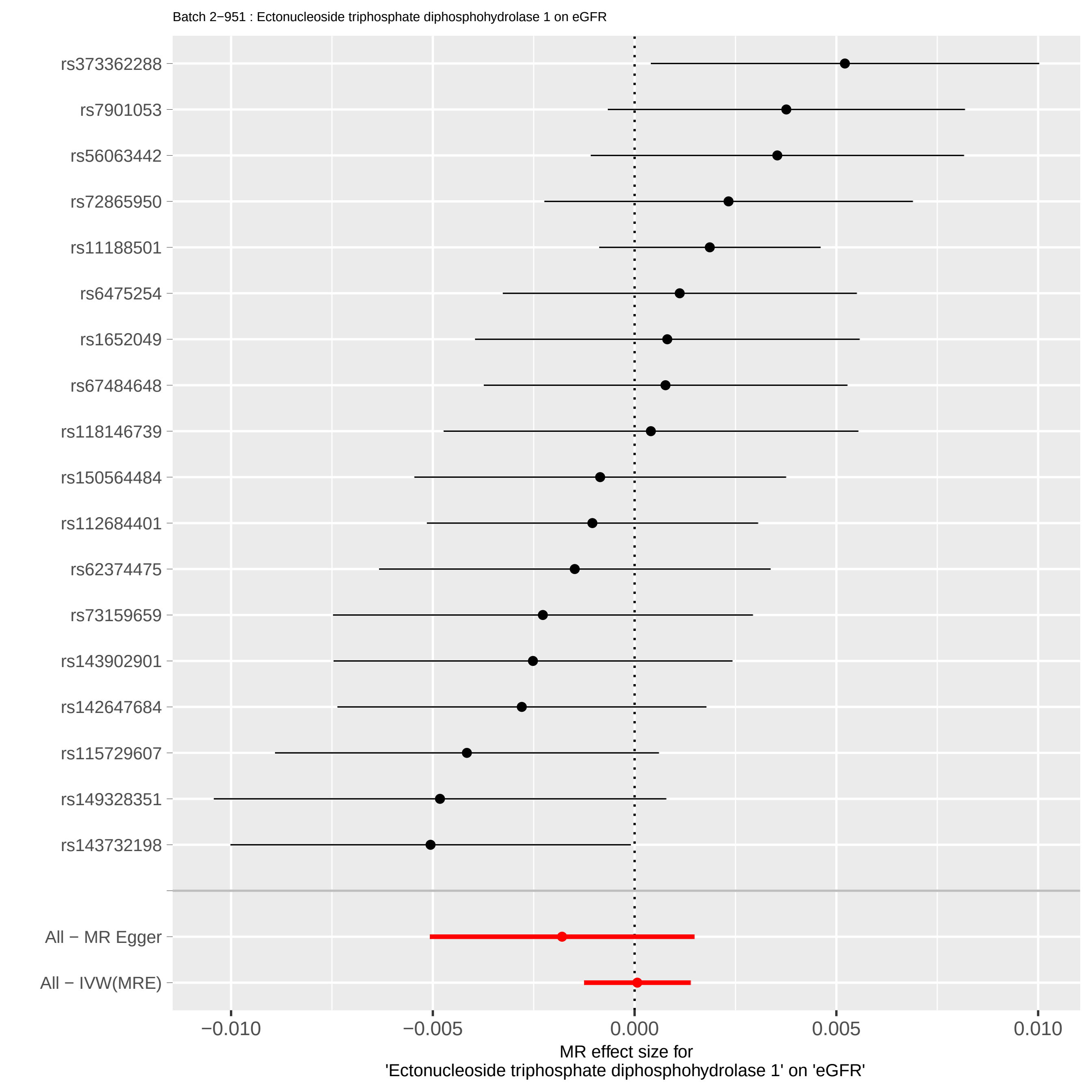

Batch 2−951 : Ectonucleoside triphosphate diphosphohydrolase 1 on eGFR
rs373362288
rs7901053
rs56063442
rs72865950
rs11188501
rs6475254
rs1652049
rs67484648
rs118146739
rs150564484
rs112684401
rs62374475
rs73159659
rs143902901
rs142647684
rs115729607
rs149328351
rs143732198
All − MR Egger
All − IVW(MRE)
−0.010
−0.005
0.000
0.005
0.010
MR effect size for
'Ectonucleoside triphosphate diphosphohydrolase 1' on 'eGFR'

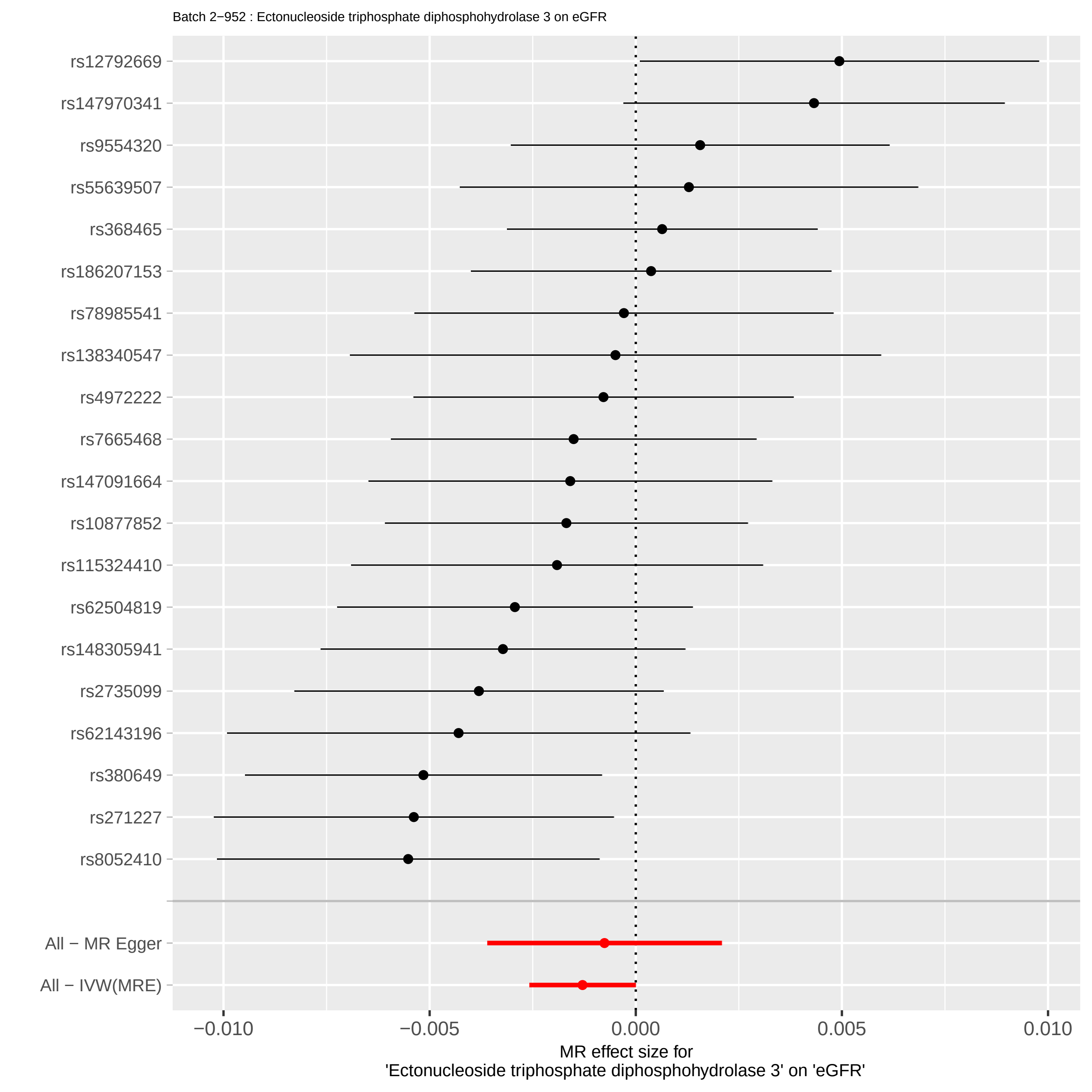

Batch 2−952 : Ectonucleoside triphosphate diphosphohydrolase 3 on eGFR
rs12792669
rs147970341
rs9554320
rs55639507
rs368465
rs186207153
rs78985541
rs138340547
rs4972222
rs7665468
rs147091664
rs10877852
rs115324410
rs62504819
rs148305941
rs2735099
rs62143196
rs380649
rs271227
rs8052410
All − MR Egger
All − IVW(MRE)
−0.010
−0.005
0.000
0.005
0.010
MR effect size for
'Ectonucleoside triphosphate diphosphohydrolase 3' on 'eGFR'

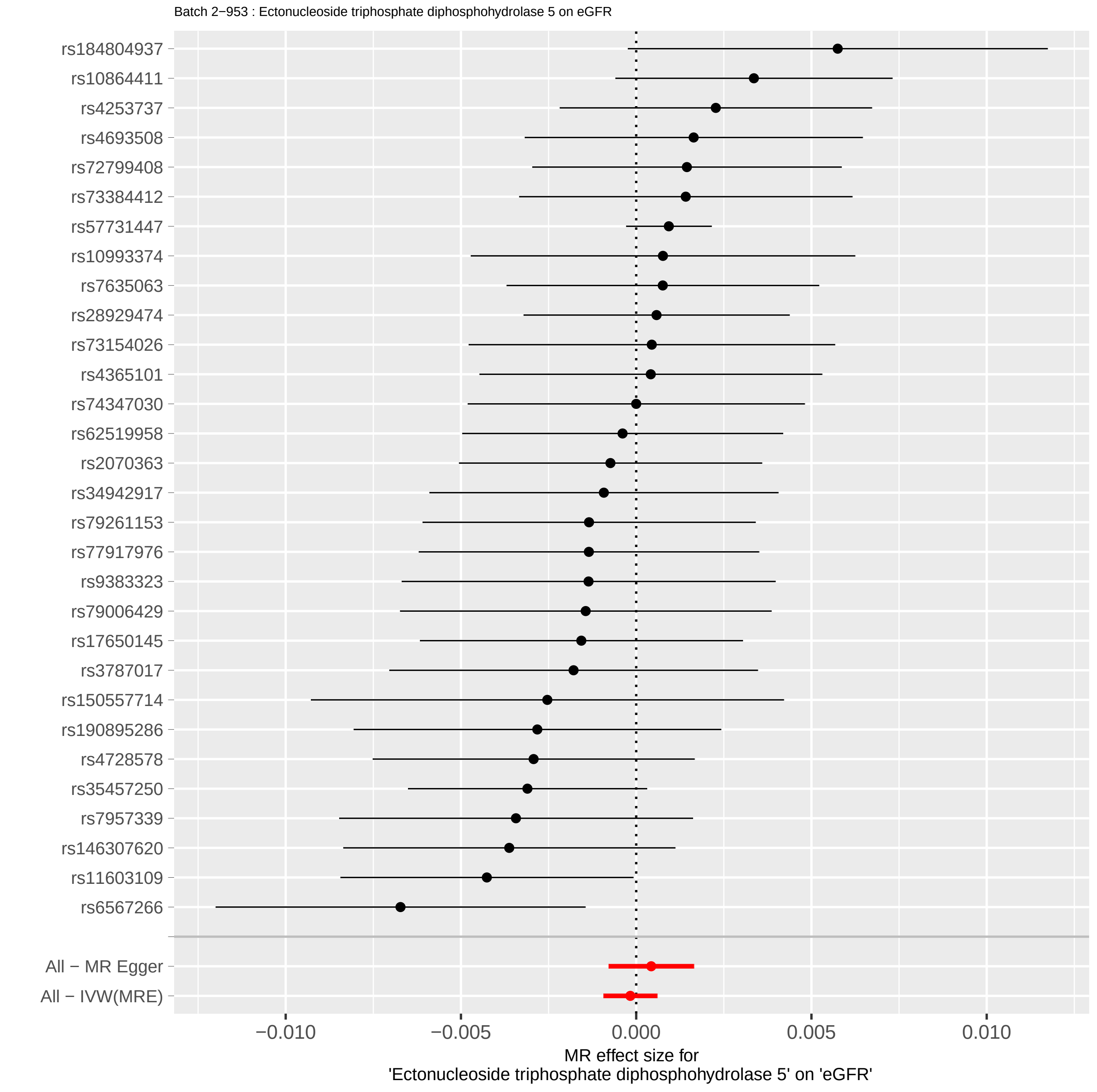

Batch 2−953 : Ectonucleoside triphosphate diphosphohydrolase 5 on eGFR
rs184804937
rs10864411
rs4253737
rs4693508
rs72799408
rs73384412
rs57731447
rs10993374
rs7635063
rs28929474
rs73154026
rs4365101
rs74347030
rs62519958
rs2070363
rs34942917
rs79261153
rs77917976
rs9383323
rs79006429
rs17650145
rs3787017
rs150557714
rs190895286
rs4728578
rs35457250
rs7957339
rs146307620
rs11603109
rs6567266
All − MR Egger
All − IVW(MRE)
−0.010
−0.005
0.000
0.005
0.010
MR effect size for
'Ectonucleoside triphosphate diphosphohydrolase 5' on 'eGFR'

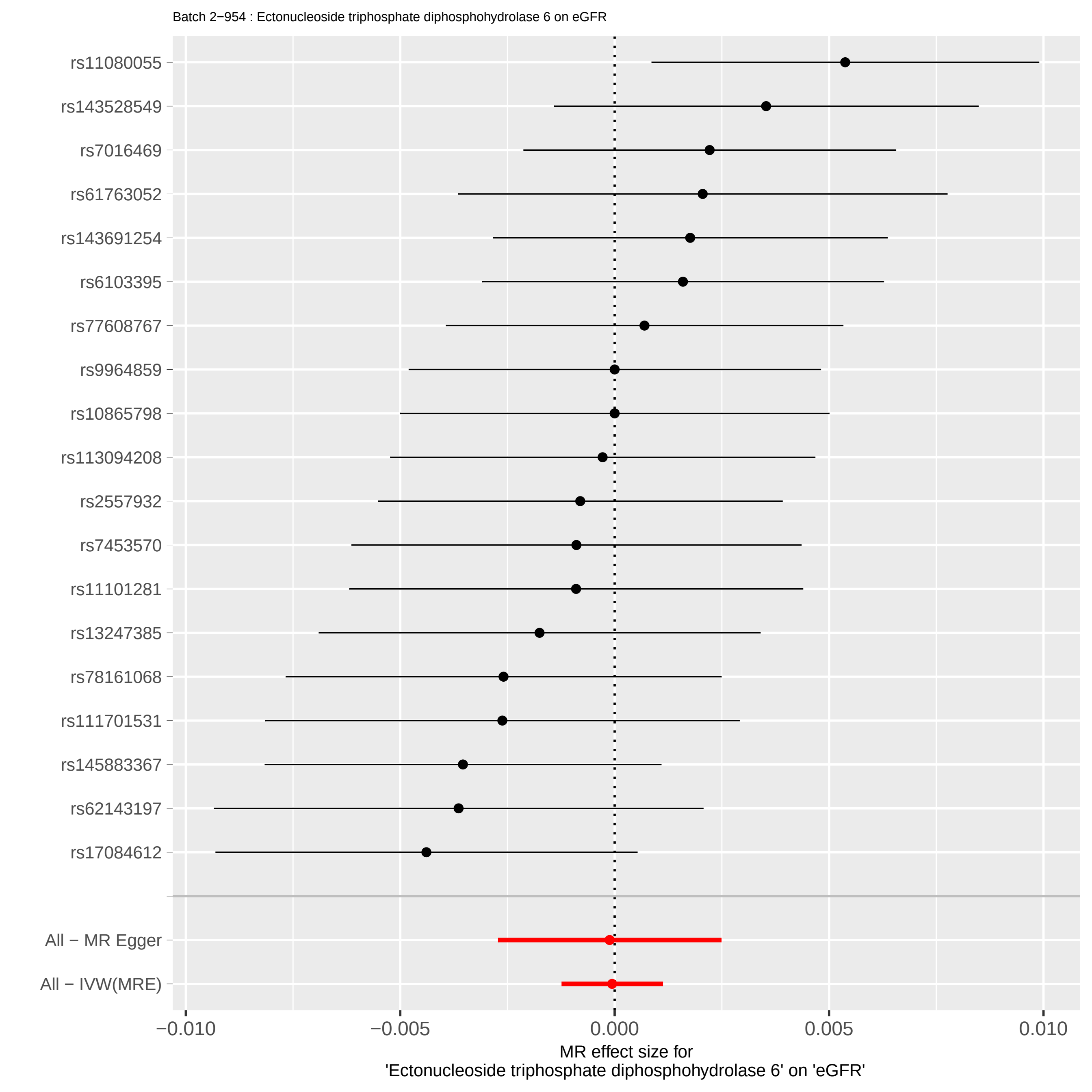
Batch 2−954 : Ectonucleoside triphosphate diphosphohydrolase 6 on eGFR
rs11080055
rs143528549
rs7016469
rs61763052
rs143691254
rs6103395
rs77608767
rs9964859
rs10865798
rs113094208
rs2557932
rs7453570
rs11101281
rs13247385
rs78161068
rs111701531
rs145883367
rs62143197
rs17084612
All − MR Egger
All − IVW(MRE)
−0.010
−0.005
0.000
0.005
0.010
MR effect size for
'Ectonucleoside triphosphate diphosphohydrolase 6' on 'eGFR'

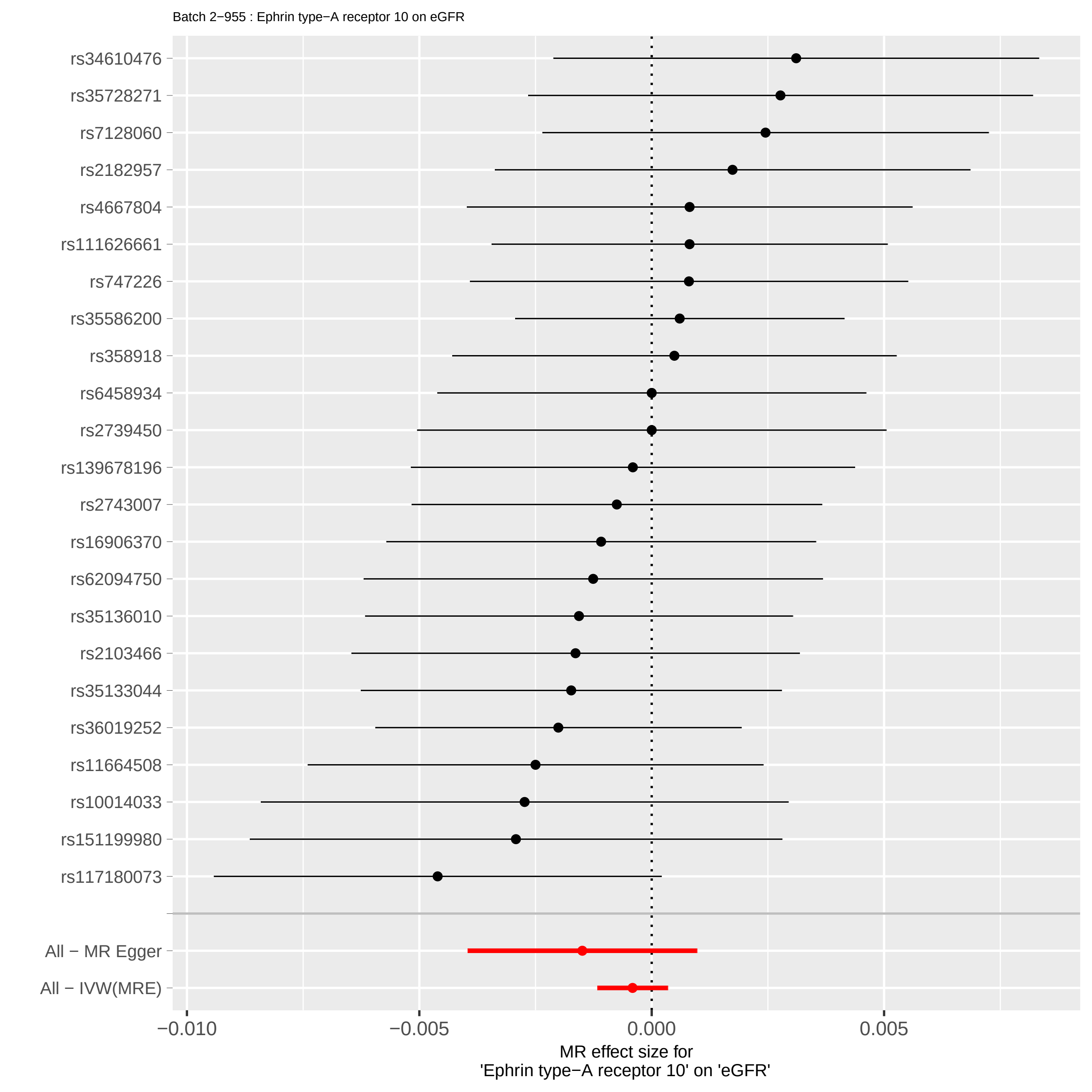

Batch 2−955 : Ephrin type−A receptor 10 on eGFR
rs34610476
rs35728271
rs7128060
rs2182957
rs4667804
rs111626661
rs747226
rs35586200
rs358918
rs6458934
rs2739450
rs139678196
rs2743007
rs16906370
rs62094750
rs35136010
rs2103466
rs35133044
rs36019252
rs11664508
rs10014033
rs151199980
rs117180073
All − MR Egger
All − IVW(MRE)
−0.010
−0.005
0.000
0.005
MR effect size for
'Ephrin type−A receptor 10' on 'eGFR'

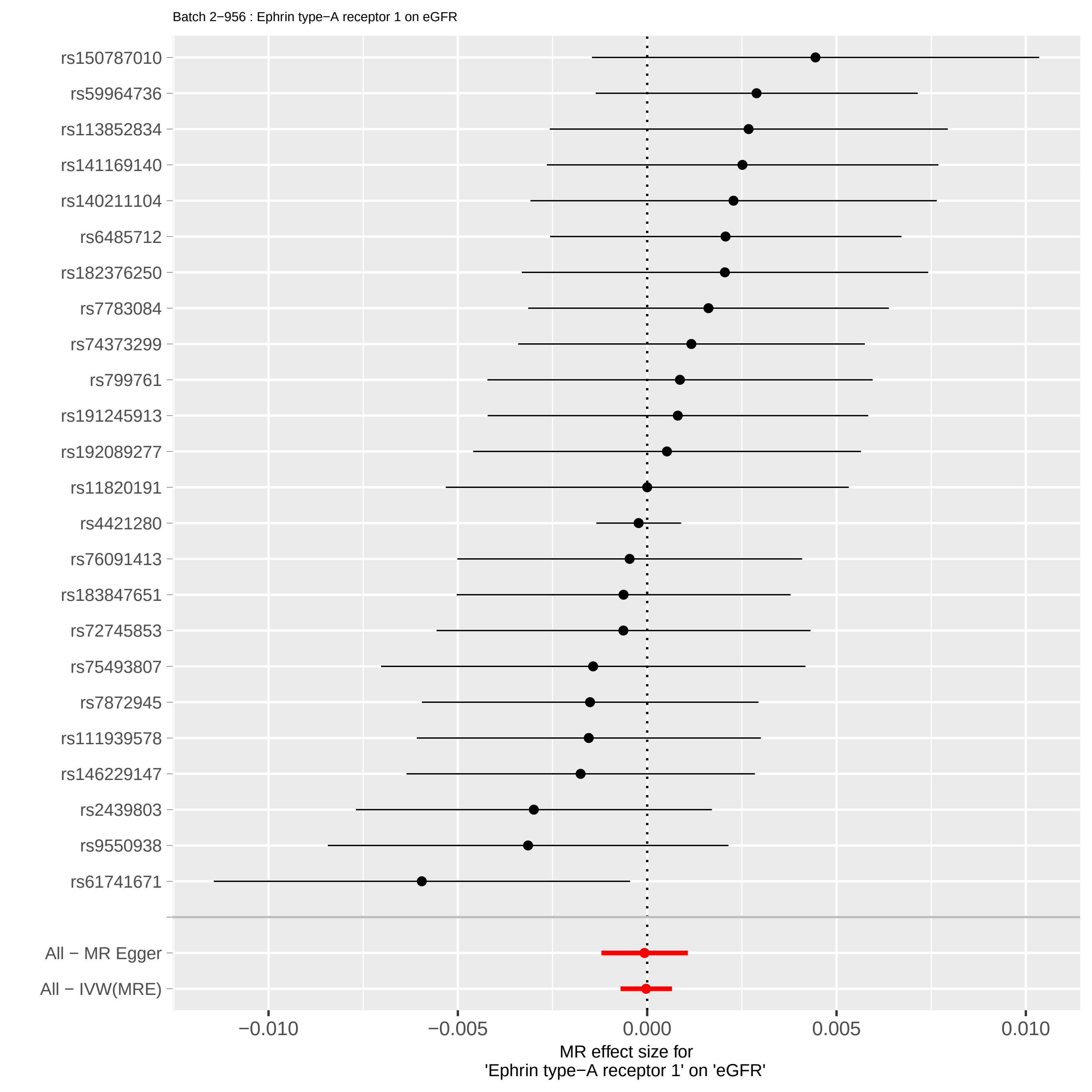
Batch 2−956 : Ephrin type−A receptor 1 on eGFR
rs150787010
rs59964736
rs113852834
rs141169140
rs140211104
rs6485712
rs182376250
rs7783084
rs74373299
rs799761
rs191245913
rs192089277
rs11820191
rs4421280
rs76091413
rs183847651
rs72745853
rs75493807
rs7872945
rs111939578
rs146229147
rs2439803
rs9550938
rs61741671
All − MR Egger
All − IVW(MRE)
−0.010
−0.005
0.000
0.005
0.010
MR effect size for
'Ephrin type−A receptor 1' on 'eGFR'

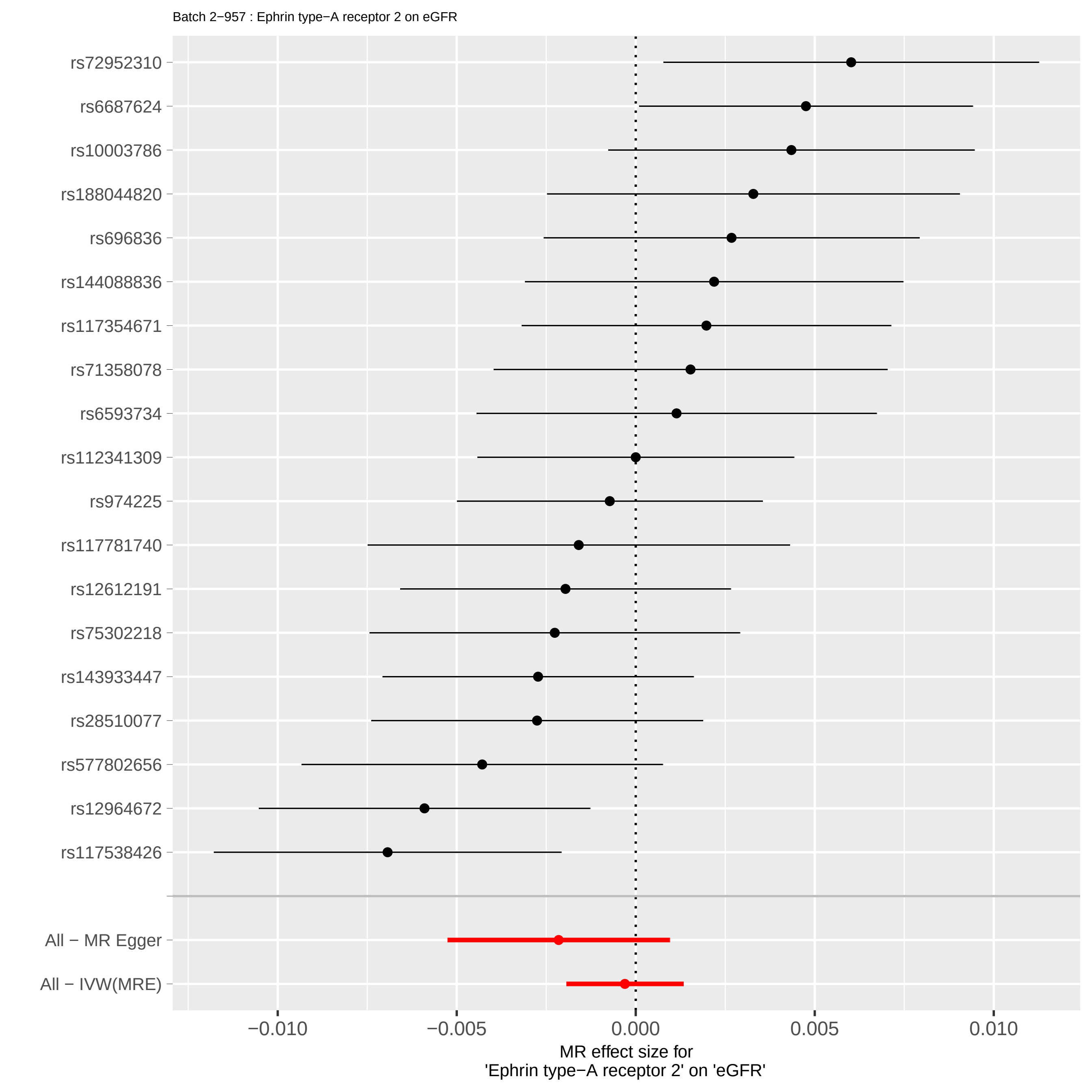

Batch 2−957 : Ephrin type−A receptor 2 on eGFR
rs72952310
rs6687624
rs10003786
rs188044820
rs696836
rs144088836
rs117354671
rs71358078
rs6593734
rs112341309
rs974225
rs117781740
rs12612191
rs75302218
rs143933447
rs28510077
rs577802656
rs12964672
rs117538426
All − MR Egger
All − IVW(MRE)
−0.010
−0.005
0.000
0.005
0.010
MR effect size for
'Ephrin type−A receptor 2' on 'eGFR'

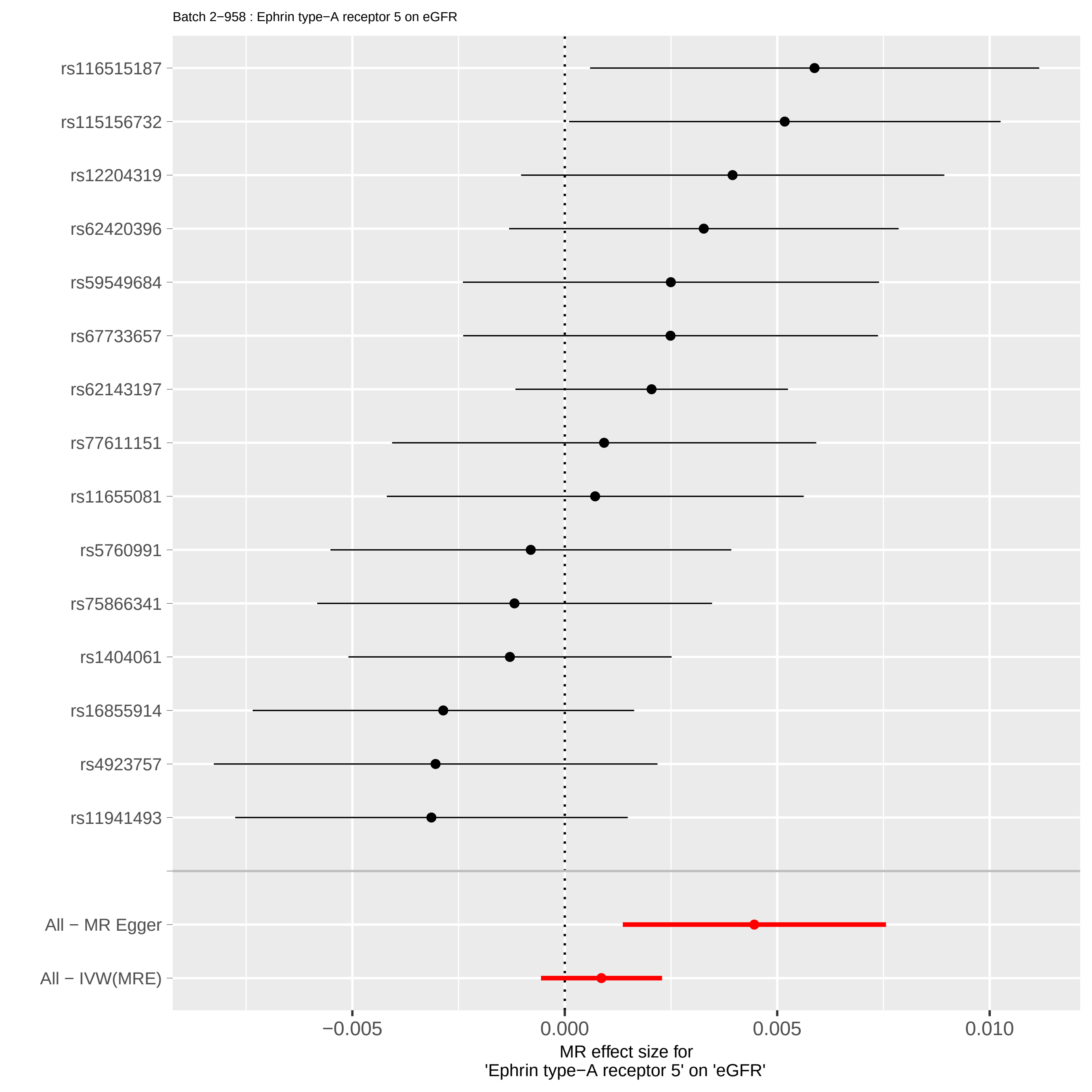
Batch 2−958 : Ephrin type−A receptor 5 on eGFR
rs116515187
rs115156732
rs12204319
rs62420396
rs59549684
rs67733657
rs62143197
rs77611151
rs11655081
rs5760991
rs75866341
rs1404061
rs16855914
rs4923757
rs11941493
All − MR Egger
All − IVW(MRE)
−0.005
0.000
0.005
0.010
MR effect size for
'Ephrin type−A receptor 5' on 'eGFR'

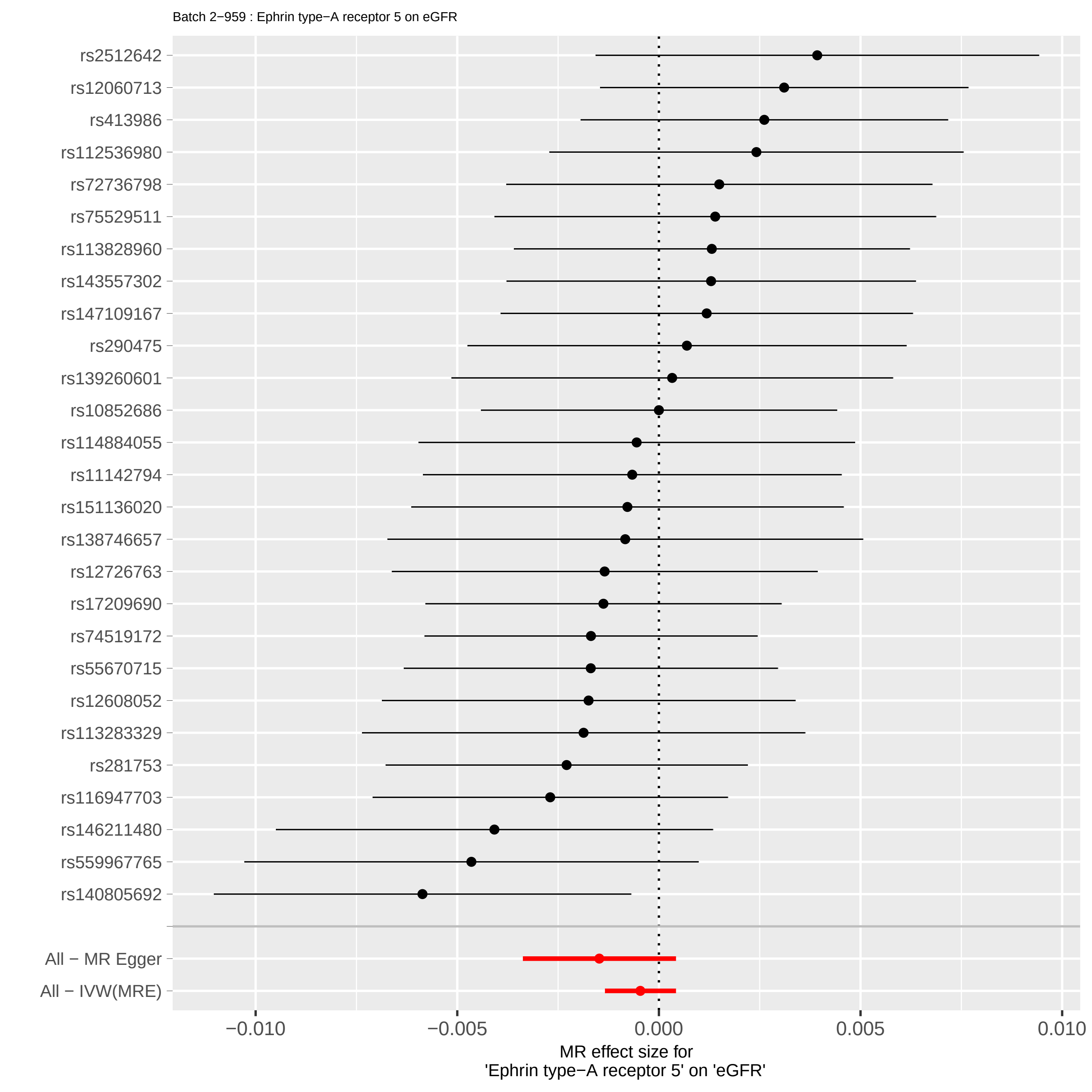

Batch 2−959 : Ephrin type−A receptor 5 on eGFR
rs2512642
rs12060713
rs413986
rs112536980
rs72736798
rs75529511
rs113828960
rs143557302
rs147109167
rs290475
rs139260601
rs10852686
rs114884055
rs11142794
rs151136020
rs138746657
rs12726763
rs17209690
rs74519172
rs55670715
rs12608052
rs113283329
rs281753
rs116947703
rs146211480
rs559967765
rs140805692
All − MR Egger
All − IVW(MRE)
−0.010
−0.005
0.000
0.005
0.010
MR effect size for
'Ephrin type−A receptor 5' on 'eGFR'

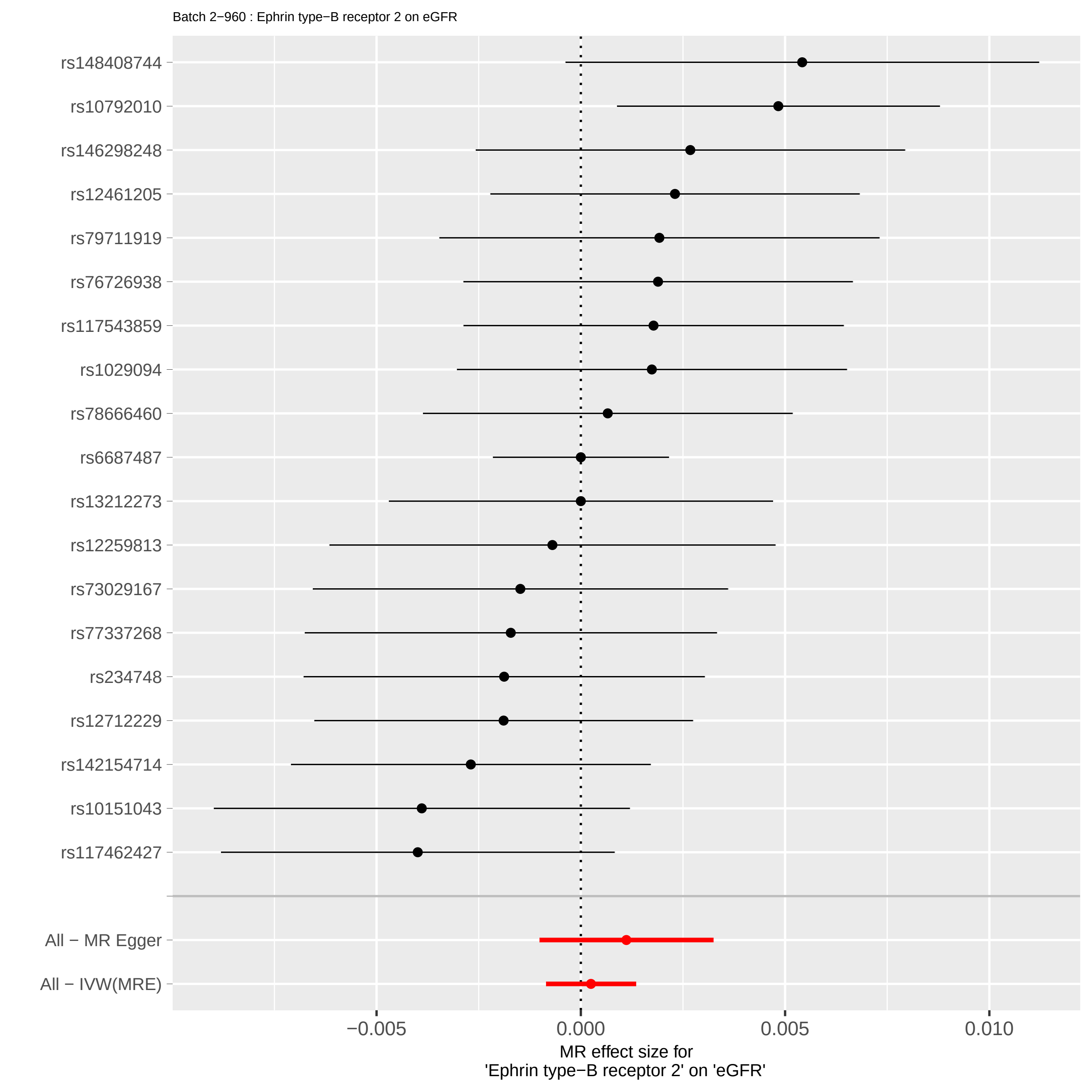
Batch 2−960 : Ephrin type−B receptor 2 on eGFR
rs148408744
rs10792010
rs146298248
rs12461205
rs79711919
rs76726938
rs117543859
rs1029094
rs78666460
rs6687487
rs13212273
rs12259813
rs73029167
rs77337268
rs234748
rs12712229
rs142154714
rs10151043
rs117462427
All − MR Egger
All − IVW(MRE)
−0.005
0.000
0.005
0.010
MR effect size for
'Ephrin type−B receptor 2' on 'eGFR'

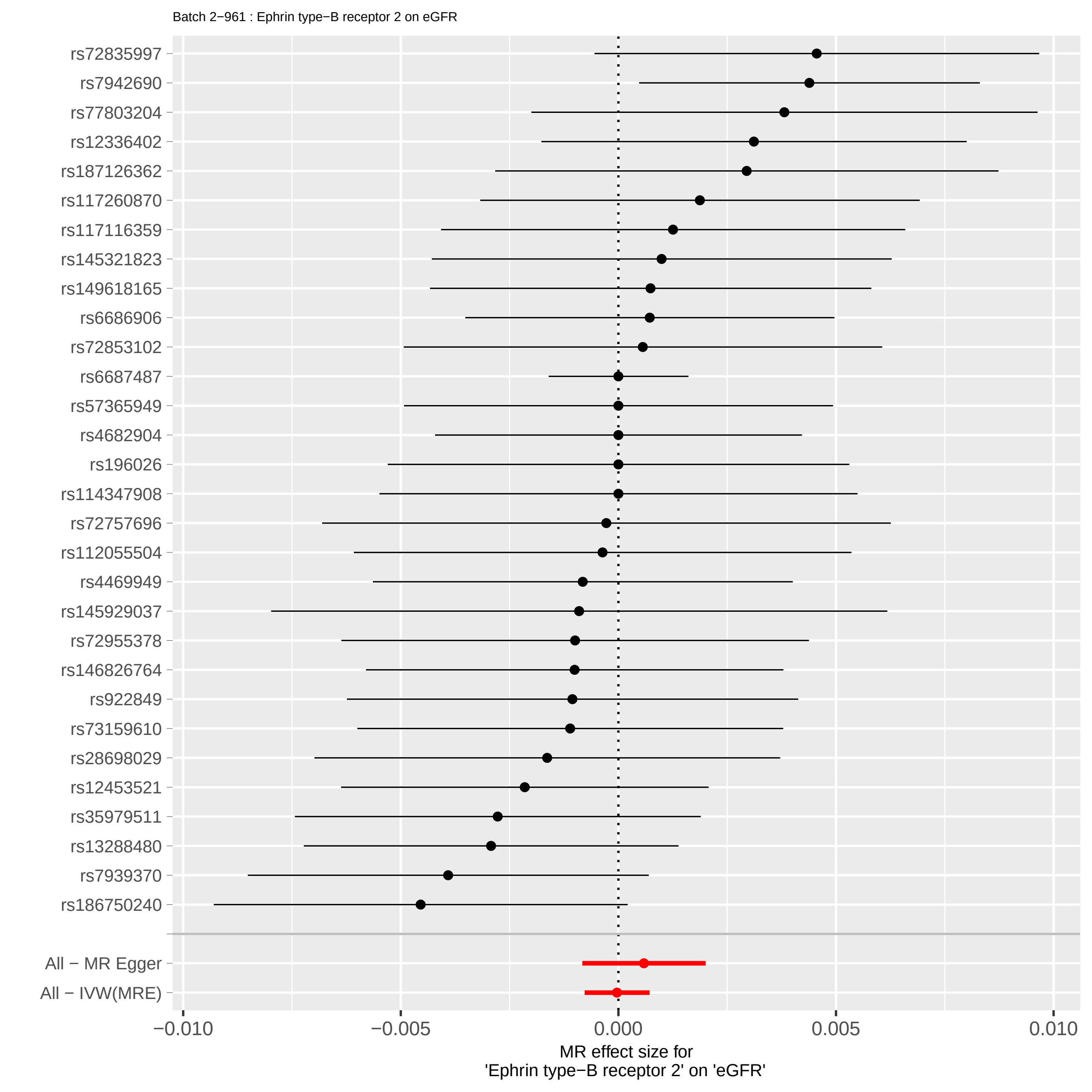
Batch 2−961 : Ephrin type−B receptor 2 on eGFR
rs72835997
rs7942690
rs77803204
rs12336402
rs187126362
rs117260870
rs117116359
rs145321823
rs149618165
rs6686906
rs72853102
rs6687487
rs57365949
rs4682904
rs196026
rs114347908
rs72757696
rs112055504
rs4469949
rs145929037
rs72955378
rs146826764
rs922849
rs73159610
rs28698029
rs12453521
rs35979511
rs13288480
rs7939370
rs186750240
All − MR Egger
All − IVW(MRE)
−0.010
−0.005
0.000
0.005
0.010
MR effect size for
'Ephrin type−B receptor 2' on 'eGFR'

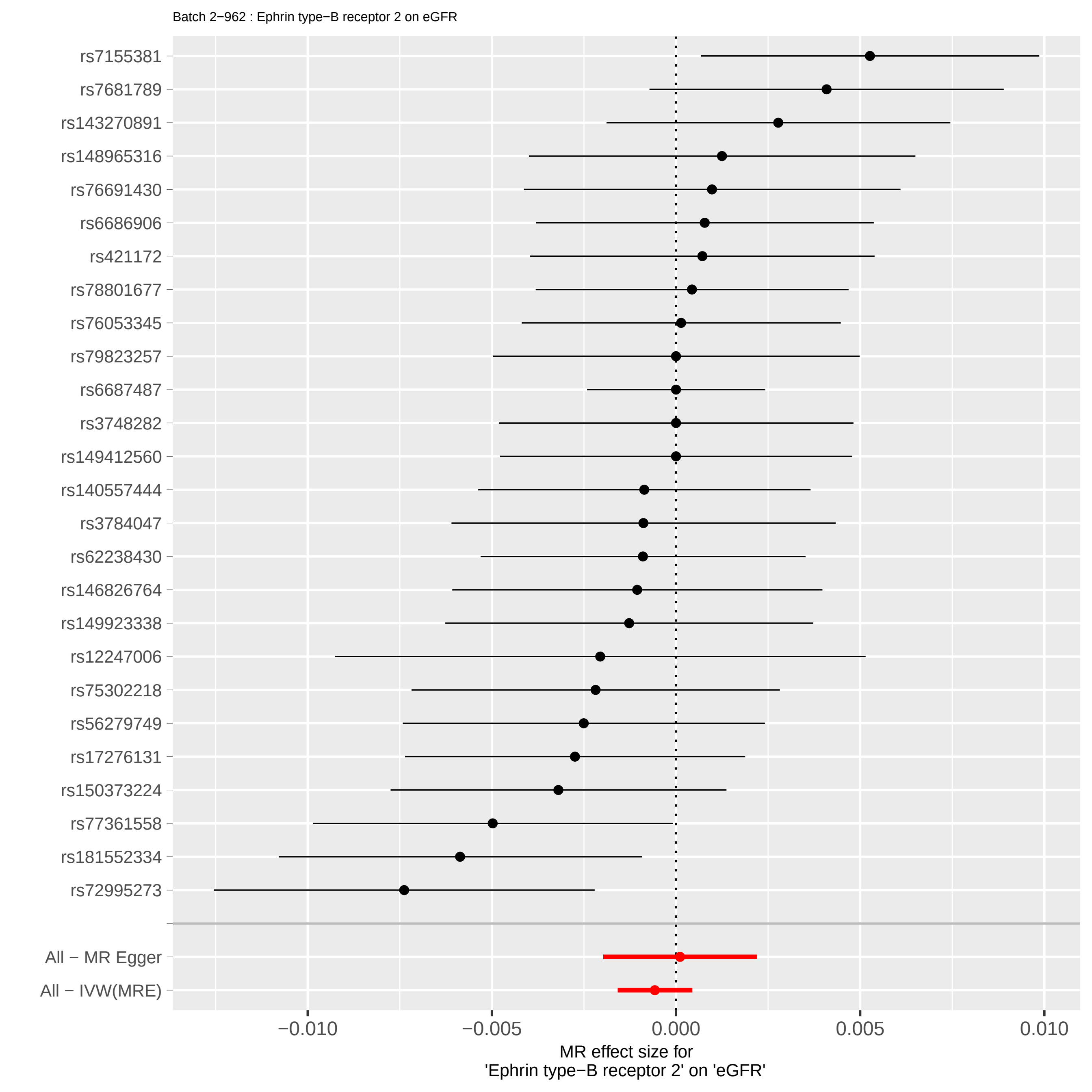

Batch 2−962 : Ephrin type−B receptor 2 on eGFR
rs7155381
rs7681789
rs143270891
rs148965316
rs76691430
rs6686906
rs421172
rs78801677
rs76053345
rs79823257
rs6687487
rs3748282
rs149412560
rs140557444
rs3784047
rs62238430
rs146826764
rs149923338
rs12247006
rs75302218
rs56279749
rs17276131
rs150373224
rs77361558
rs181552334
rs72995273
All − MR Egger
All − IVW(MRE)
−0.010
−0.005
0.000
0.005
0.010
MR effect size for
'Ephrin type−B receptor 2' on 'eGFR'

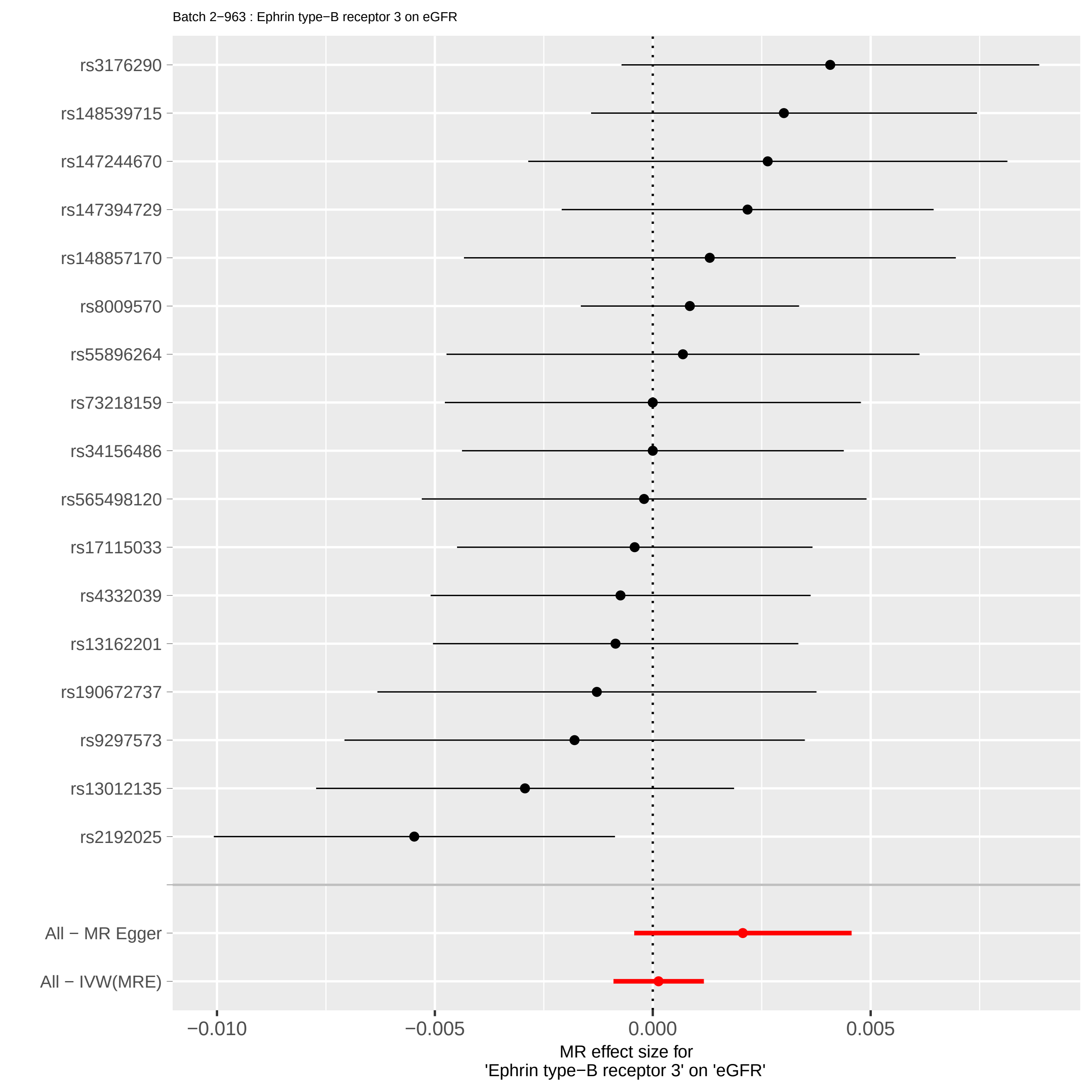
Batch 2−963 : Ephrin type−B receptor 3 on eGFR
rs3176290
rs148539715
rs147244670
rs147394729
rs148857170
rs8009570
rs55896264
rs73218159
rs34156486
rs565498120
rs17115033
rs4332039
rs13162201
rs190672737
rs9297573
rs13012135
rs2192025
All − MR Egger
All − IVW(MRE)
−0.010
−0.005
0.000
0.005
MR effect size for
'Ephrin type−B receptor 3' on 'eGFR'

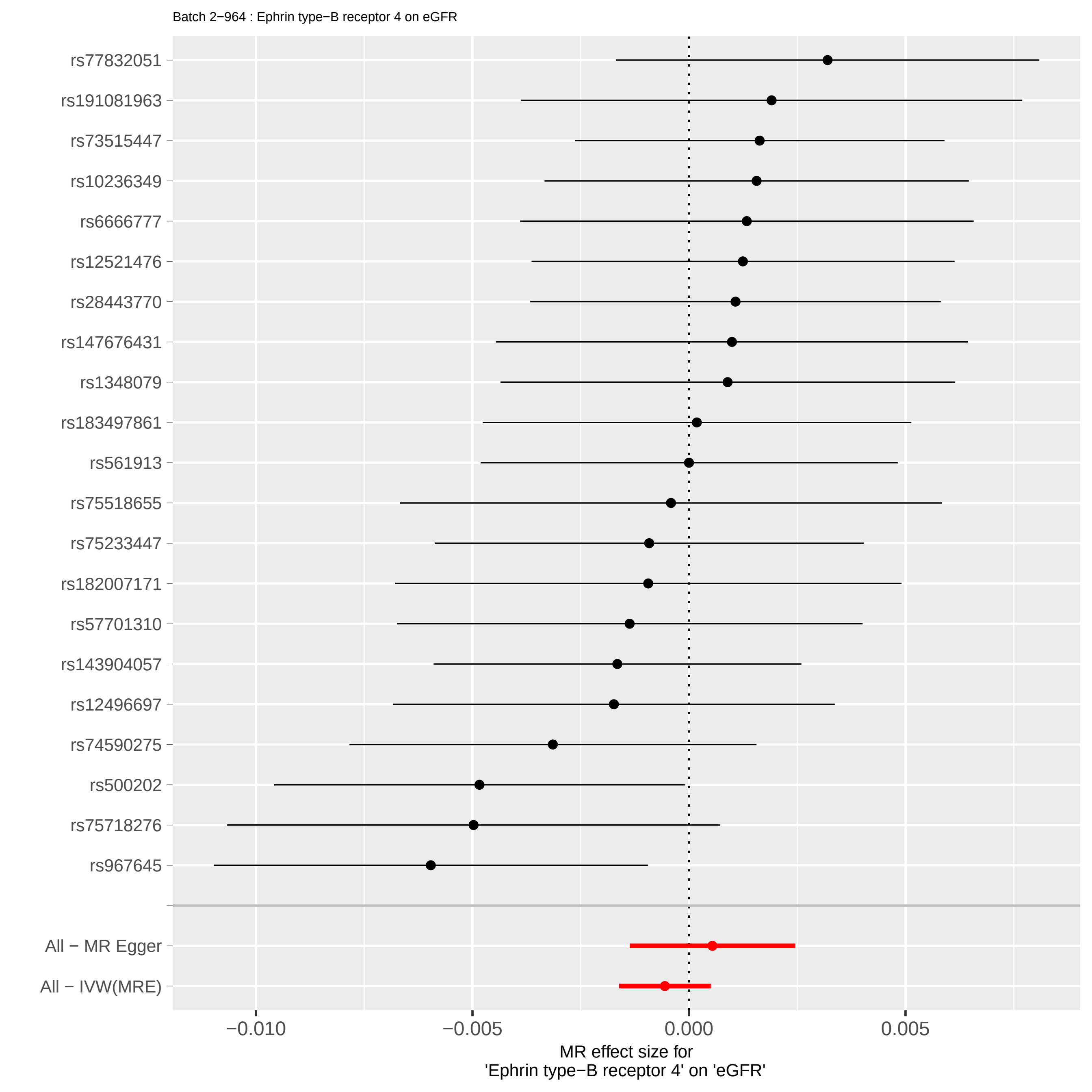
Batch 2−964 : Ephrin type−B receptor 4 on eGFR
rs77832051
rs191081963
rs73515447
rs10236349
rs6666777
rs12521476
rs28443770
rs147676431
rs1348079
rs183497861
rs561913
rs75518655
rs75233447
rs182007171
rs57701310
rs143904057
rs12496697
rs74590275
rs500202
rs75718276
rs967645
All − MR Egger
All − IVW(MRE)
−0.010
−0.005
0.000
0.005
MR effect size for
'Ephrin type−B receptor 4' on 'eGFR'

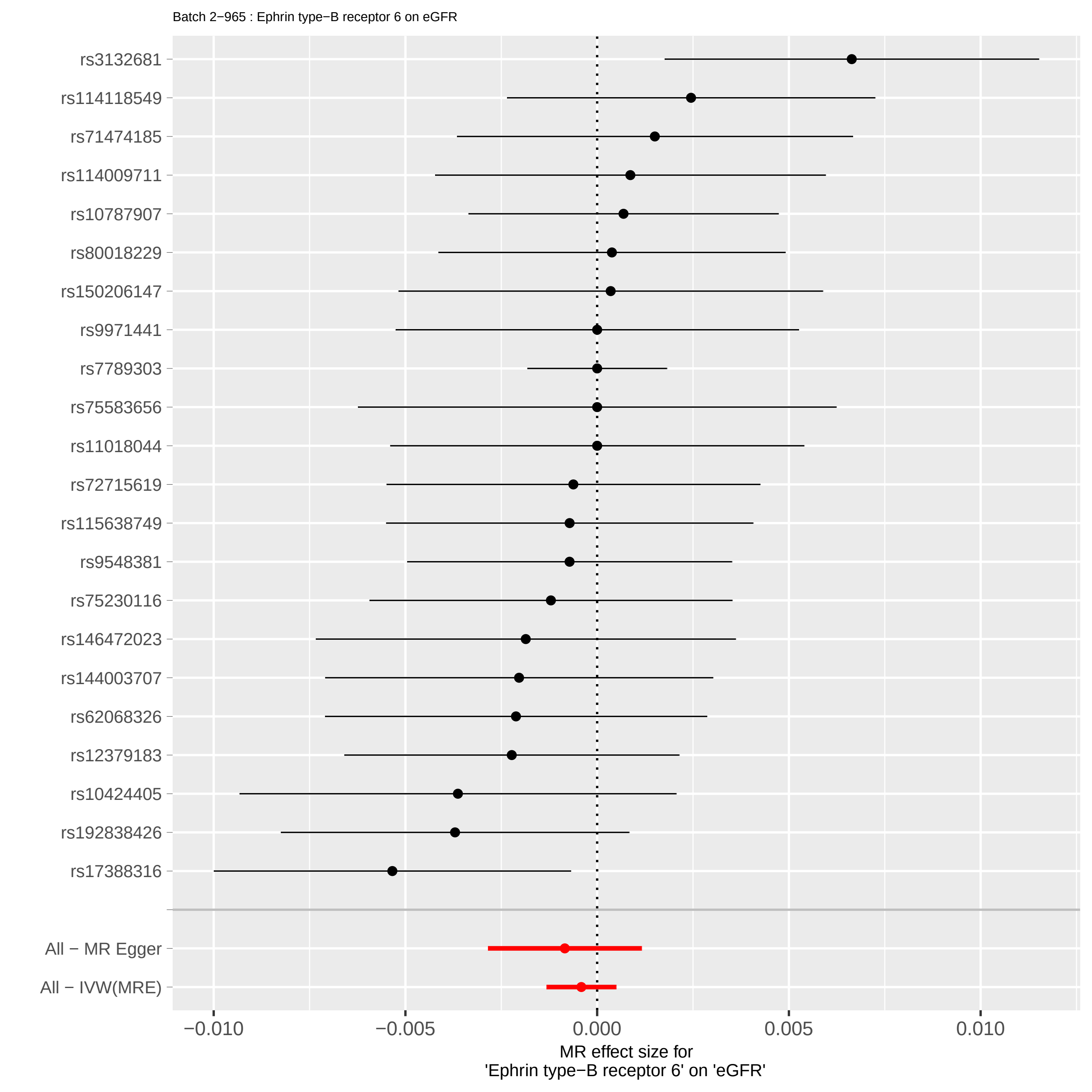

Batch 2−965 : Ephrin type−B receptor 6 on eGFR
rs3132681
rs114118549
rs71474185
rs114009711
rs10787907
rs80018229
rs150206147
rs9971441
rs7789303
rs75583656
rs11018044
rs72715619
rs115638749
rs9548381
rs75230116
rs146472023
rs144003707
rs62068326
rs12379183
rs10424405
rs192838426
rs17388316
All − MR Egger
All − IVW(MRE)
−0.010
−0.005
0.000
0.005
0.010
MR effect size for
'Ephrin type−B receptor 6' on 'eGFR'

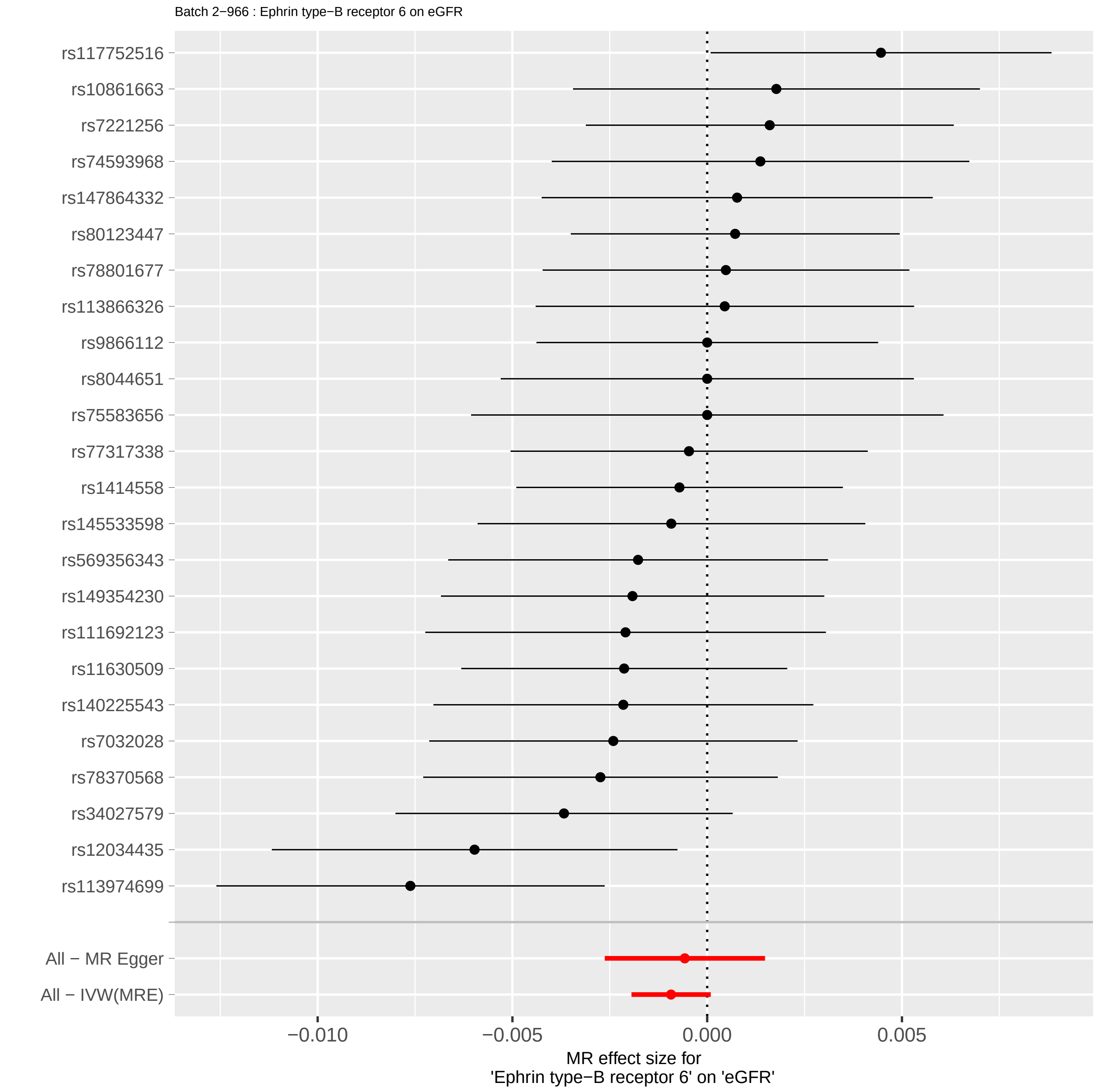

Batch 2−966 : Ephrin type−B receptor 6 on eGFR
rs117752516
rs10861663
rs7221256
rs74593968
rs147864332
rs80123447
rs78801677
rs113866326
rs9866112
rs8044651
rs75583656
rs77317338
rs1414558
rs145533598
rs569356343
rs149354230
rs111692123
rs11630509
rs140225543
rs7032028
rs78370568
rs34027579
rs12034435
rs113974699
All − MR Egger
All − IVW(MRE)
−0.010
−0.005
0.000
0.005
MR effect size for
'Ephrin type−B receptor 6' on 'eGFR'

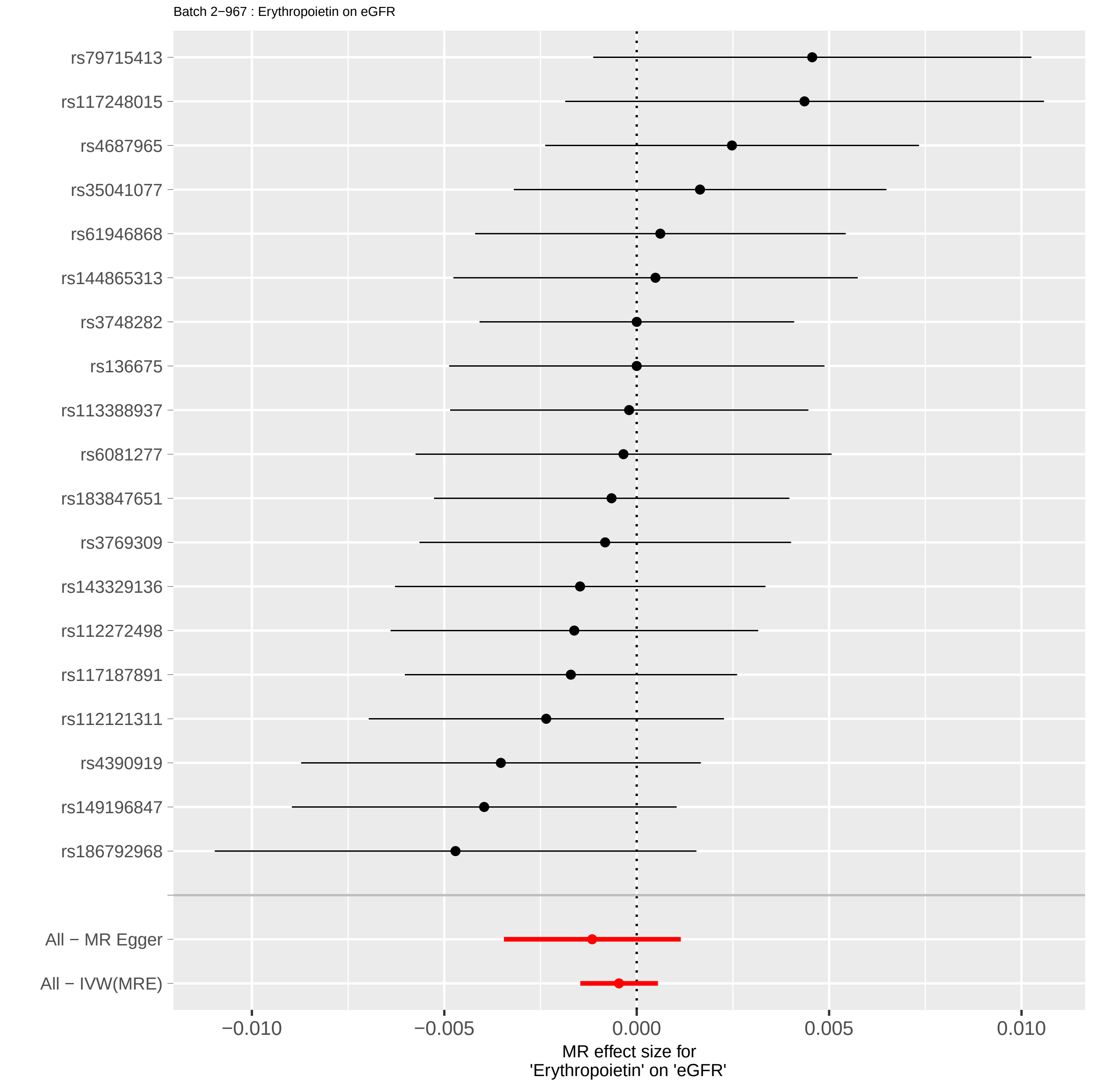

Batch 2−967 : Erythropoietin on eGFR
rs79715413
rs117248015
rs4687965
rs35041077
rs61946868
rs144865313
rs3748282
rs136675
rs113388937
rs6081277
rs183847651
rs3769309
rs143329136
rs112272498
rs117187891
rs112121311
rs4390919
rs149196847
rs186792968
All − MR Egger
All − IVW(MRE)
−0.010
−0.005
0.000
0.005
0.010
MR effect size for
'Erythropoietin' on 'eGFR'

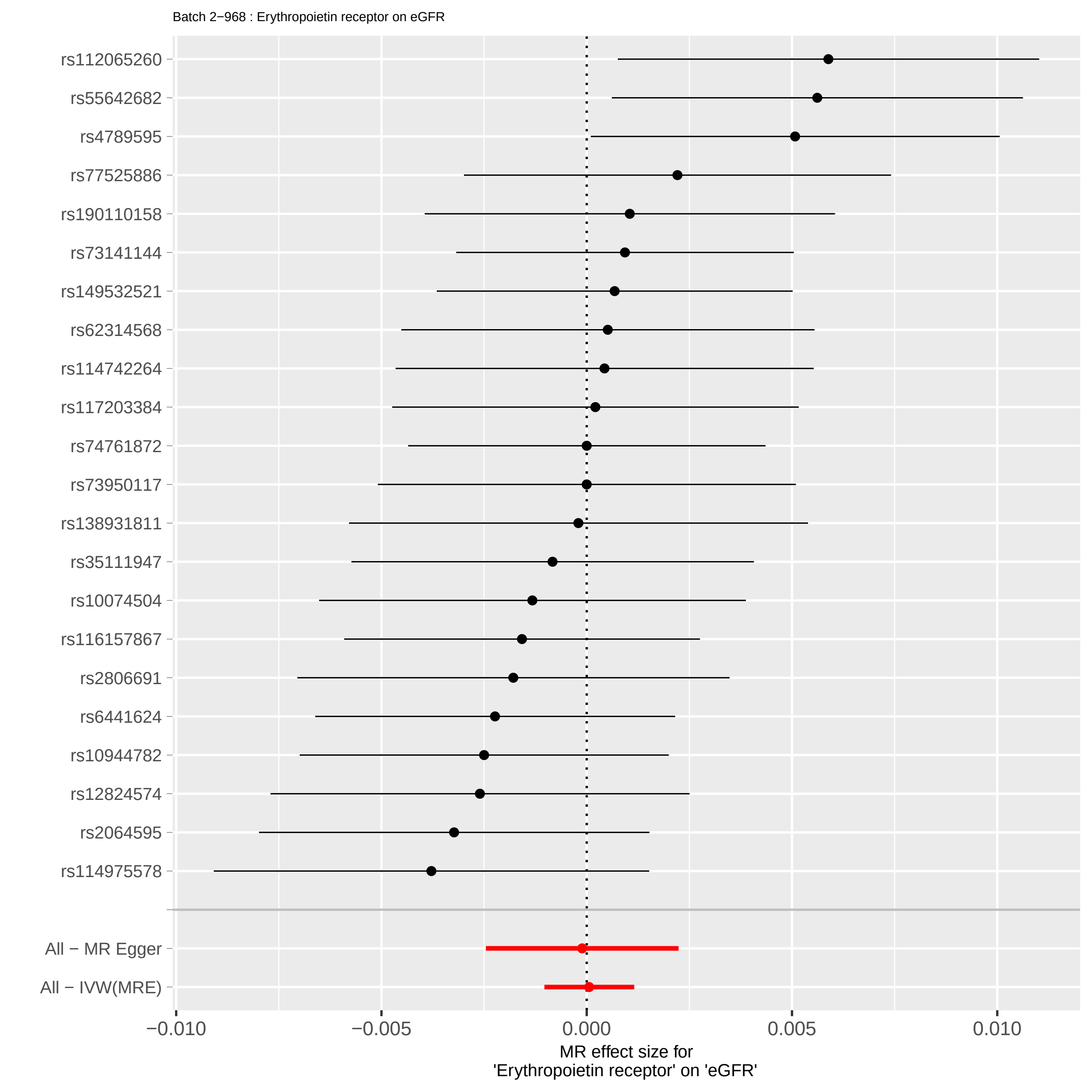
Batch 2−968 : Erythropoietin receptor on eGFR
rs112065260
rs55642682
rs4789595
rs77525886
rs190110158
rs73141144
rs149532521
rs62314568
rs114742264
rs117203384
rs74761872
rs73950117
rs138931811
rs35111947
rs10074504
rs116157867
rs2806691
rs6441624
rs10944782
rs12824574
rs2064595
rs114975578
All − MR Egger
All − IVW(MRE)
−0.010
−0.005
0.000
0.005
0.010
MR effect size for
'Erythropoietin receptor' on 'eGFR'

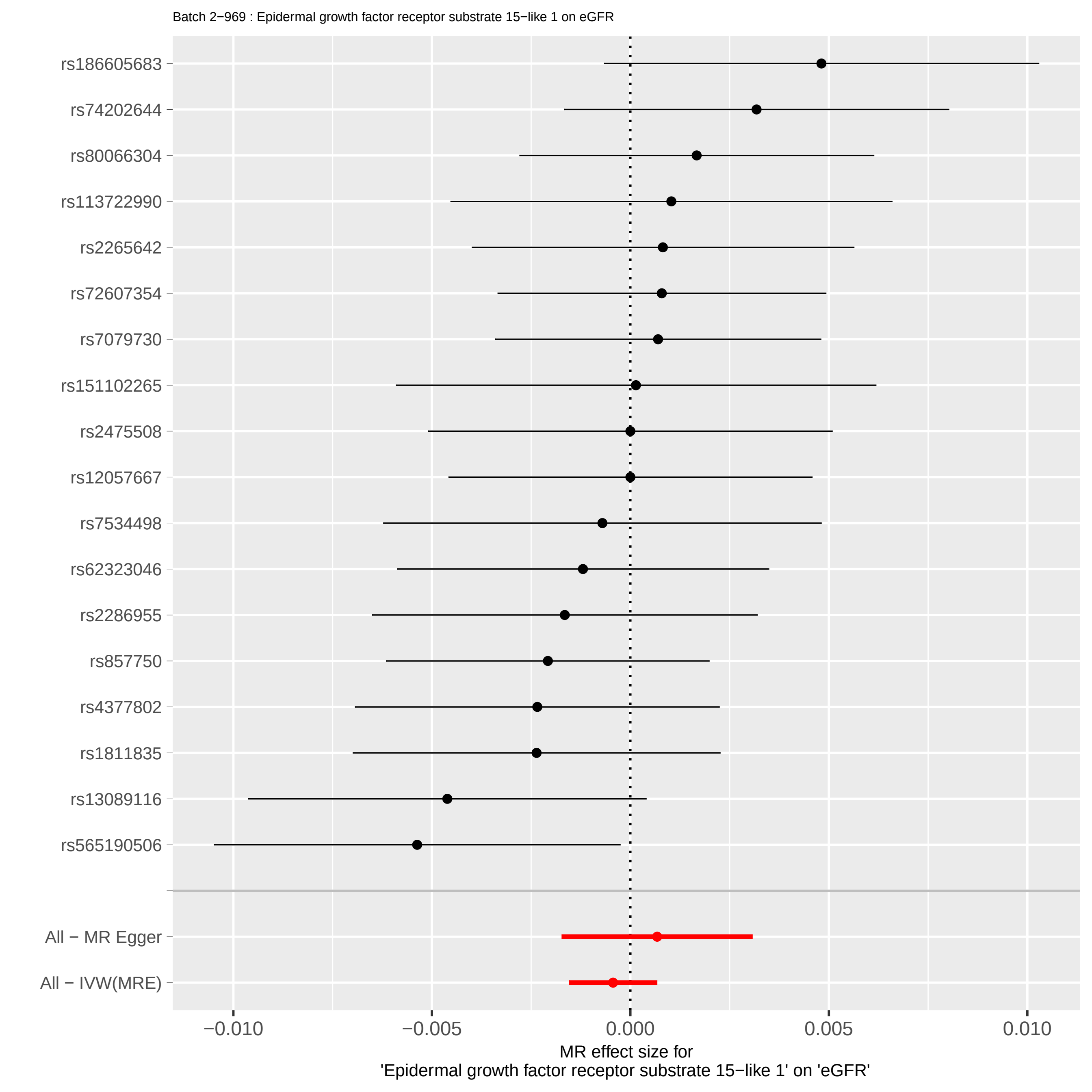

Batch 2−969 : Epidermal growth factor receptor substrate 15−like 1 on eGFR
rs186605683
rs74202644
rs80066304
rs113722990
rs2265642
rs72607354
rs7079730
rs151102265
rs2475508
rs12057667
rs7534498
rs62323046
rs2286955
rs857750
rs4377802
rs1811835
rs13089116
rs565190506
All − MR Egger
All − IVW(MRE)
−0.010
−0.005
0.000
0.005
0.010
MR effect size for
'Epidermal growth factor receptor substrate 15−like 1' on 'eGFR'

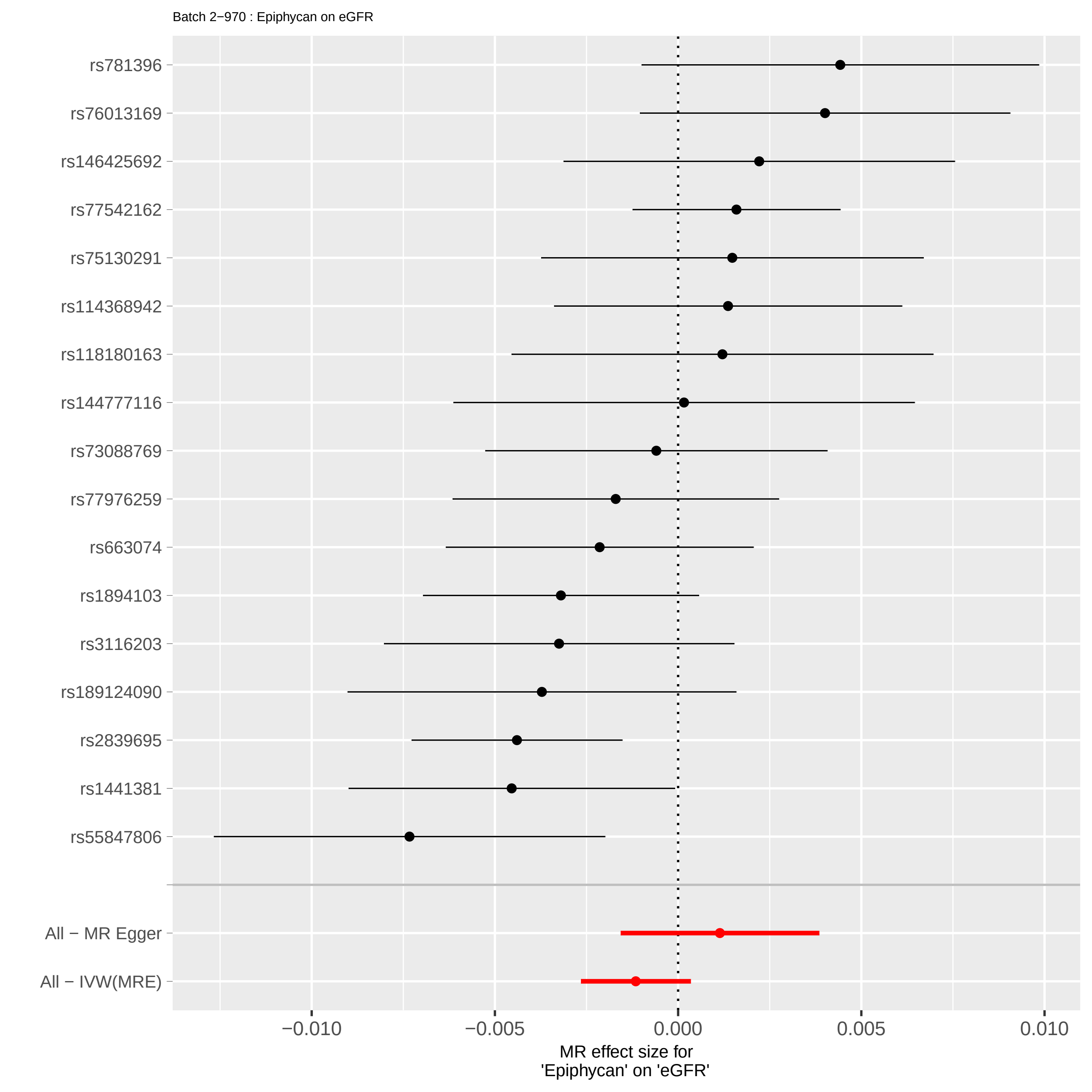

Batch 2−970 : Epiphycan on eGFR
rs781396
rs76013169
rs146425692
rs77542162
rs75130291
rs114368942
rs118180163
rs144777116
rs73088769
rs77976259
rs663074
rs1894103
rs3116203
rs189124090
rs2839695
rs1441381
rs55847806
All − MR Egger
All − IVW(MRE)
−0.010
−0.005
0.000
0.005
0.010
MR effect size for
'Epiphycan' on 'eGFR'

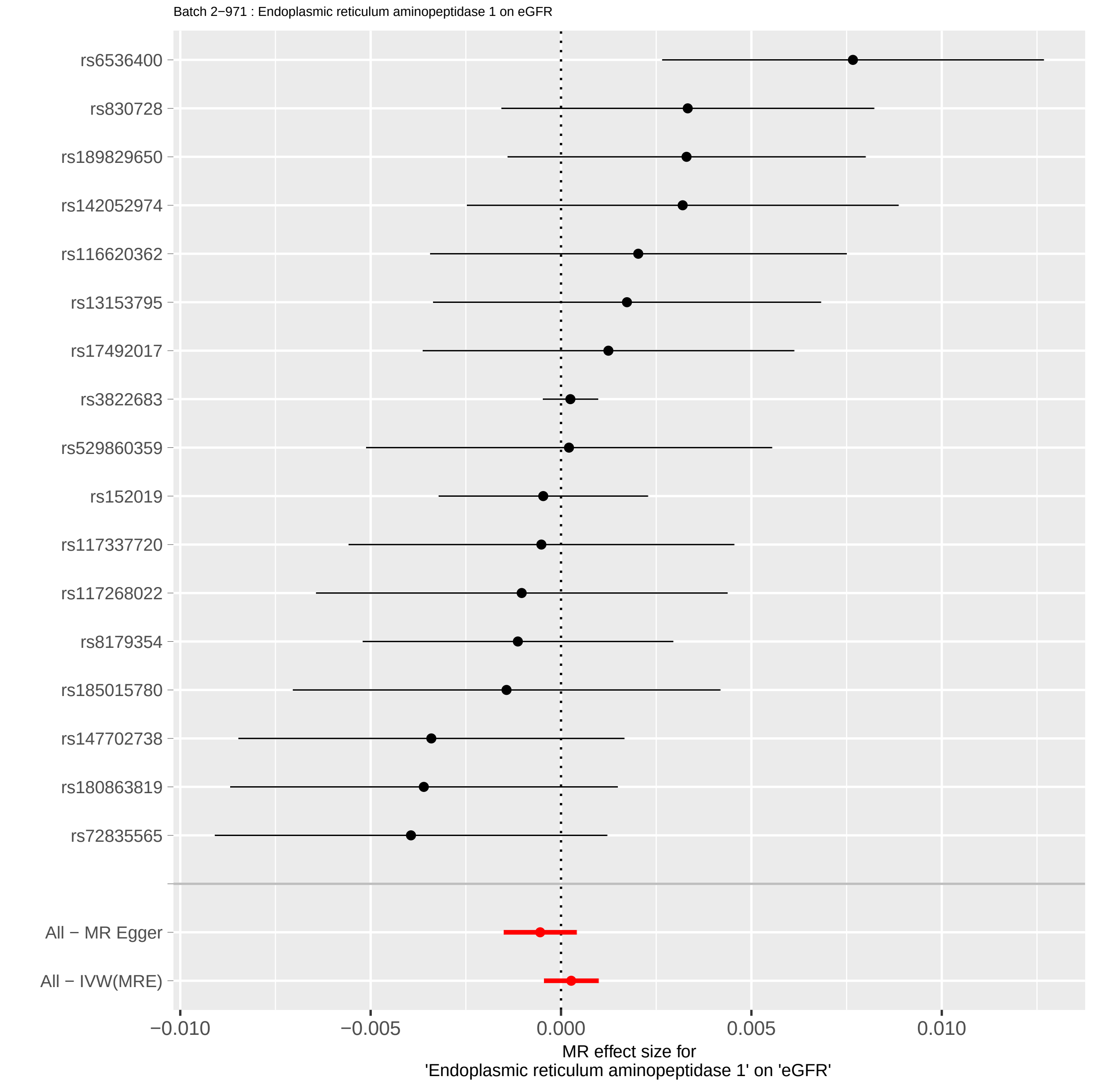
Batch 2−971 : Endoplasmic reticulum aminopeptidase 1 on eGFR
rs6536400
rs830728
rs189829650
rs142052974
rs116620362
rs13153795
rs17492017
rs3822683
rs529860359
rs152019
rs117337720
rs117268022
rs8179354
rs185015780
rs147702738
rs180863819
rs72835565
All − MR Egger
All − IVW(MRE)
−0.010
−0.005
0.000
0.005
0.010
MR effect size for
'Endoplasmic reticulum aminopeptidase 1' on 'eGFR'

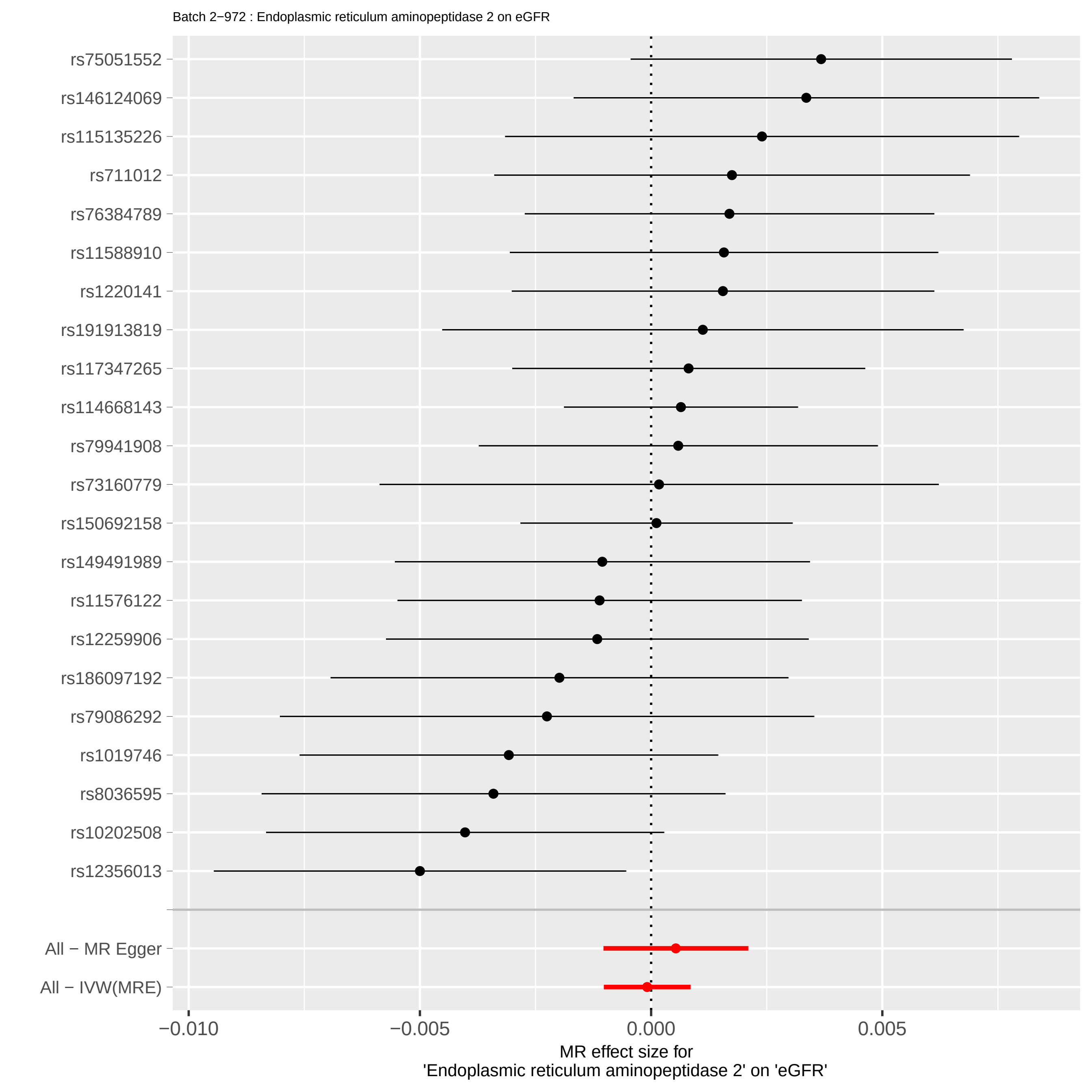

Batch 2−972 : Endoplasmic reticulum aminopeptidase 2 on eGFR
rs75051552
rs146124069
rs115135226
rs711012
rs76384789
rs11588910
rs1220141
rs191913819
rs117347265
rs114668143
rs79941908
rs73160779
rs150692158
rs149491989
rs11576122
rs12259906
rs186097192
rs79086292
rs1019746
rs8036595
rs10202508
rs12356013
All − MR Egger
All − IVW(MRE)
−0.010
−0.005
0.000
0.005
MR effect size for
'Endoplasmic reticulum aminopeptidase 2' on 'eGFR'

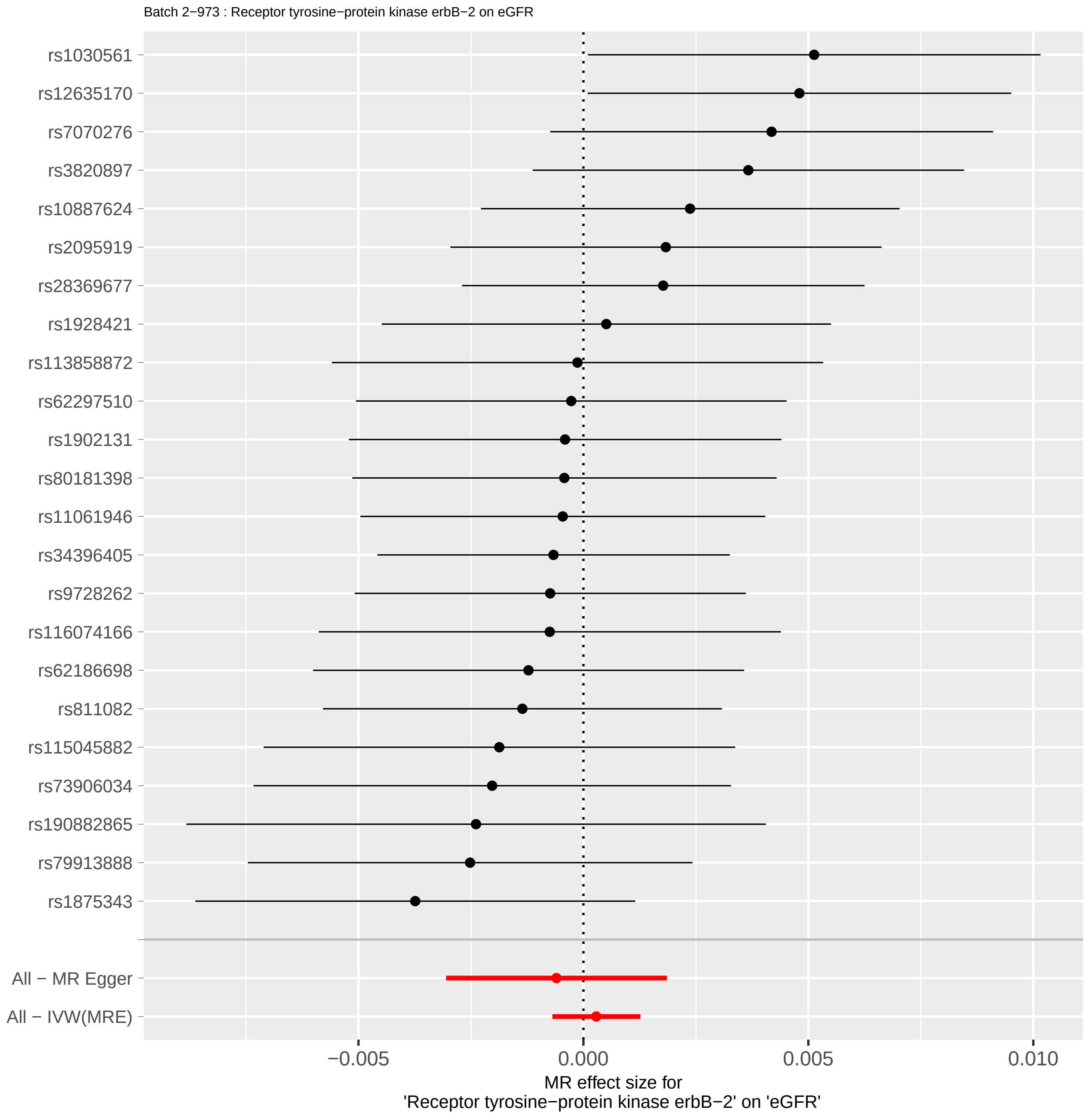

Batch 2−973 : Receptor tyrosine−protein kinase erbB−2 on eGFR
rs1030561
rs12635170
rs7070276
rs3820897
rs10887624
rs2095919
rs28369677
rs1928421
rs113858872
rs62297510
rs1902131
rs80181398
rs11061946
rs34396405
rs9728262
rs116074166
rs62186698
rs811082
rs115045882
rs73906034
rs190882865
rs79913888
rs1875343
All − MR Egger
All − IVW(MRE)
−0.005
0.000
0.005
0.010
MR effect size for
'Receptor tyrosine−protein kinase erbB−2' on 'eGFR'

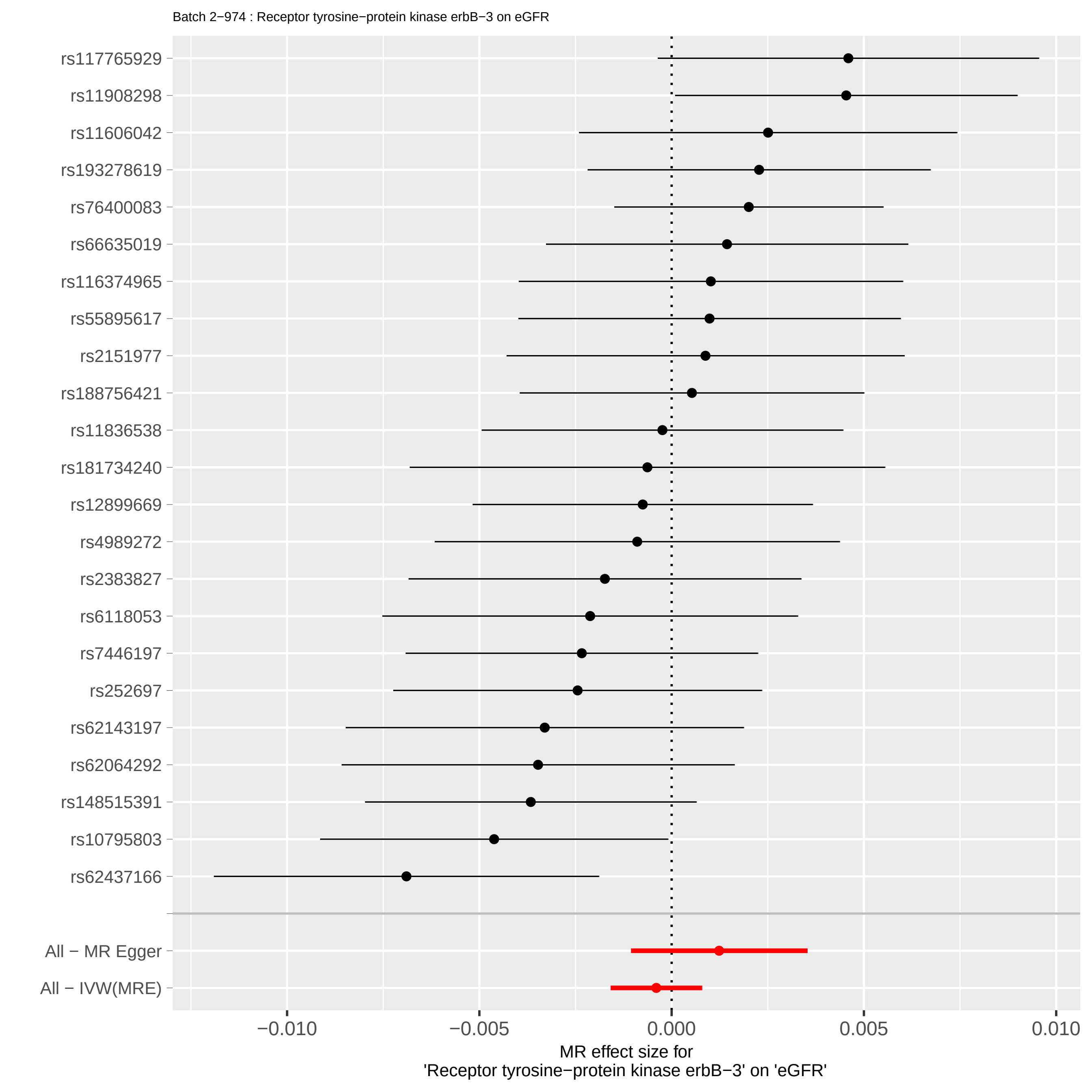

Batch 2−974 : Receptor tyrosine−protein kinase erbB−3 on eGFR
rs117765929
rs11908298
rs11606042
rs193278619
rs76400083
rs66635019
rs116374965
rs55895617
rs2151977
rs188756421
rs11836538
rs181734240
rs12899669
rs4989272
rs2383827
rs6118053
rs7446197
rs252697
rs62143197
rs62064292
rs148515391
rs10795803
rs62437166
All − MR Egger
All − IVW(MRE)
−0.010
−0.005
0.000
0.005
0.010
MR effect size for
'Receptor tyrosine−protein kinase erbB−3' on 'eGFR'

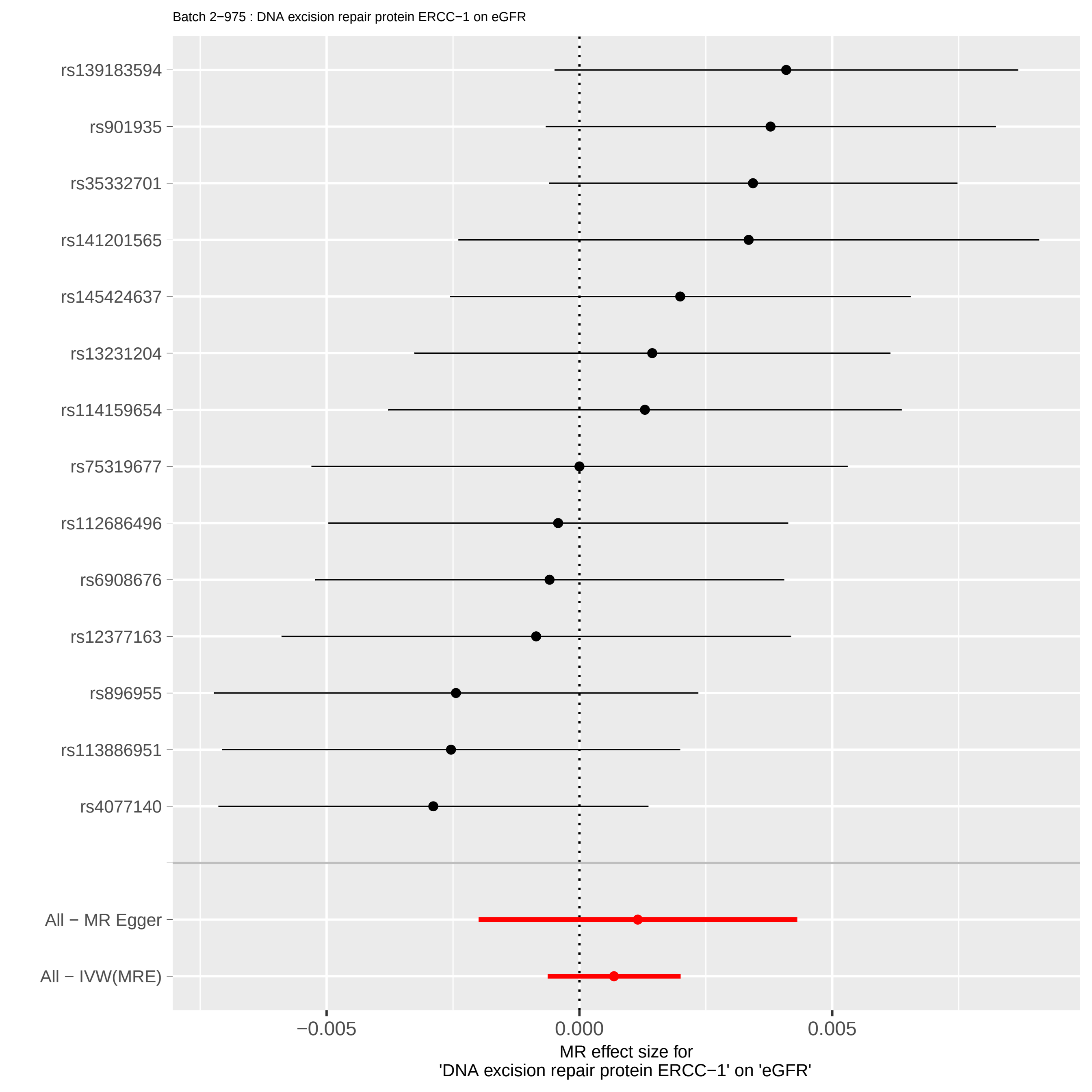

Batch 2−975 : DNA excision repair protein ERCC−1 on eGFR
rs139183594
rs901935
rs35332701
rs141201565
rs145424637
rs13231204
rs114159654
rs75319677
rs112686496
rs6908676
rs12377163
rs896955
rs113886951
rs4077140
All − MR Egger
All − IVW(MRE)
−0.005
0.000
0.005
MR effect size for
'DNA excision repair protein ERCC−1' on 'eGFR'

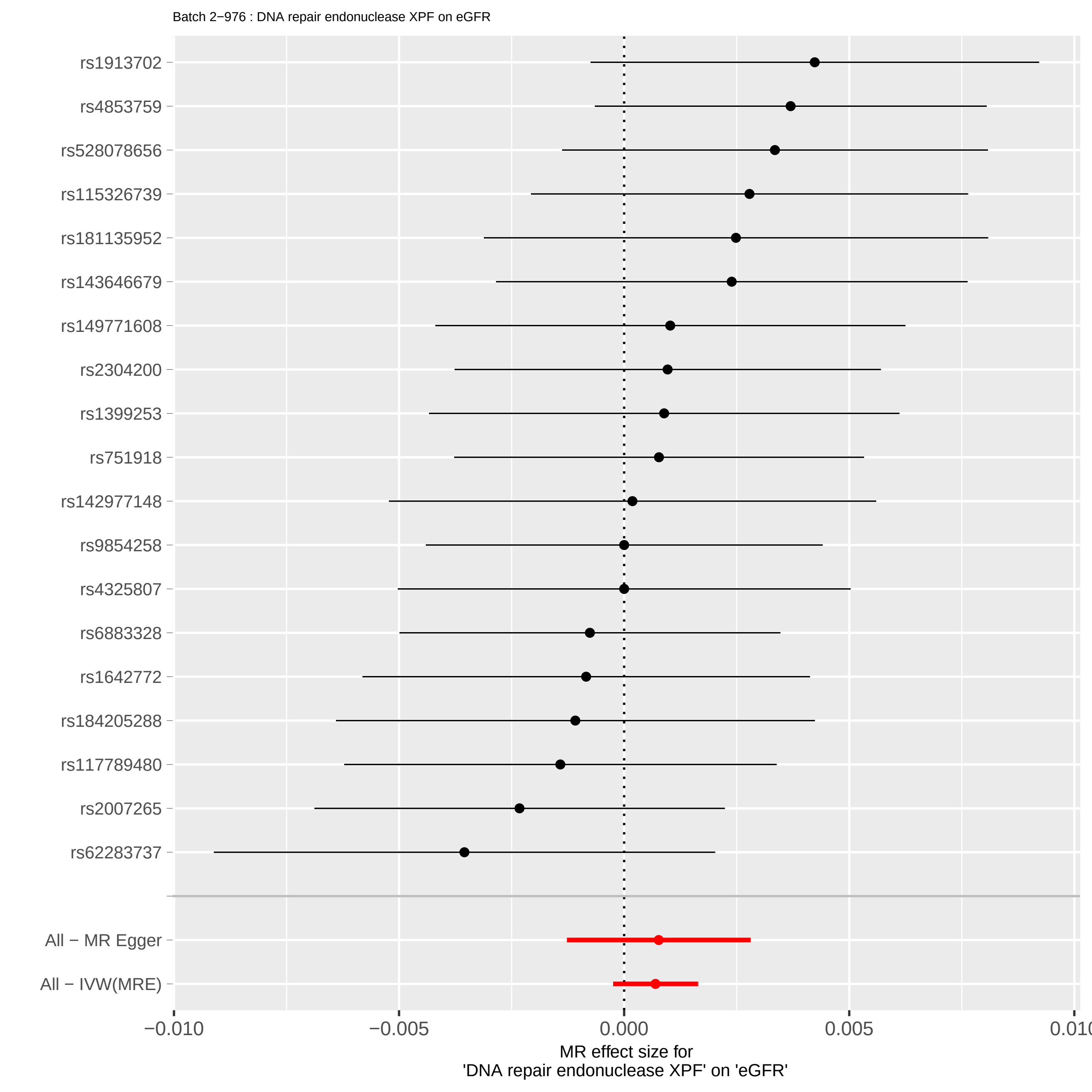

Batch 2−976 : DNA repair endonuclease XPF on eGFR
rs1913702
rs4853759
rs528078656
rs115326739
rs181135952
rs143646679
rs149771608
rs2304200
rs1399253
rs751918
rs142977148
rs9854258
rs4325807
rs6883328
rs1642772
rs184205288
rs117789480
rs2007265
rs62283737
All − MR Egger
All − IVW(MRE)
−0.010
−0.005
0.000
0.005
0.010
MR effect size for
'DNA repair endonuclease XPF' on 'eGFR'

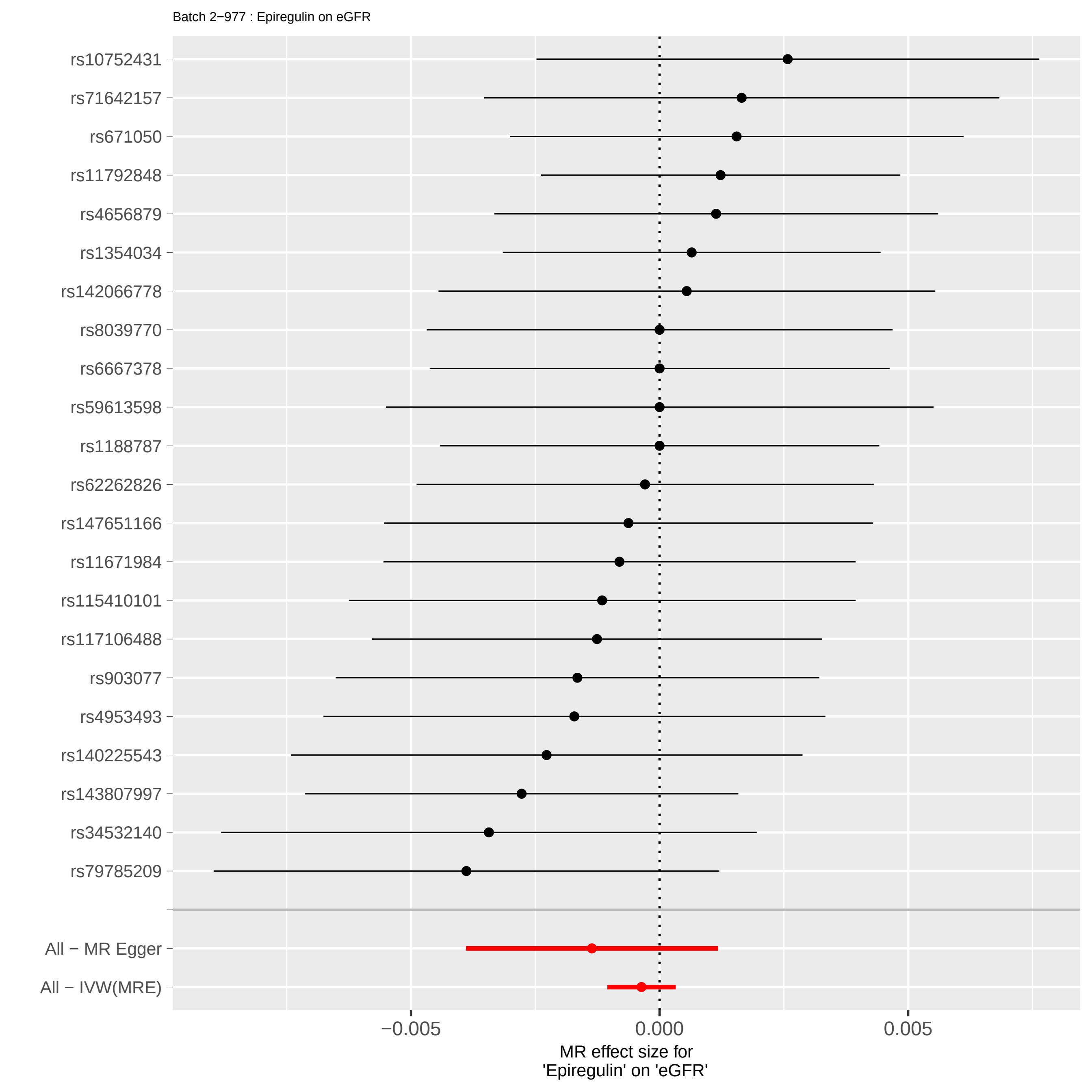

Batch 2−977 : Epiregulin on eGFR
rs10752431
rs71642157
rs671050
rs11792848
rs4656879
rs1354034
rs142066778
rs8039770
rs6667378
rs59613598
rs1188787
rs62262826
rs147651166
rs11671984
rs115410101
rs117106488
rs903077
rs4953493
rs140225543
rs143807997
rs34532140
rs79785209
All − MR Egger
All − IVW(MRE)
−0.005
0.000
0.005
MR effect size for
'Epiregulin' on 'eGFR'

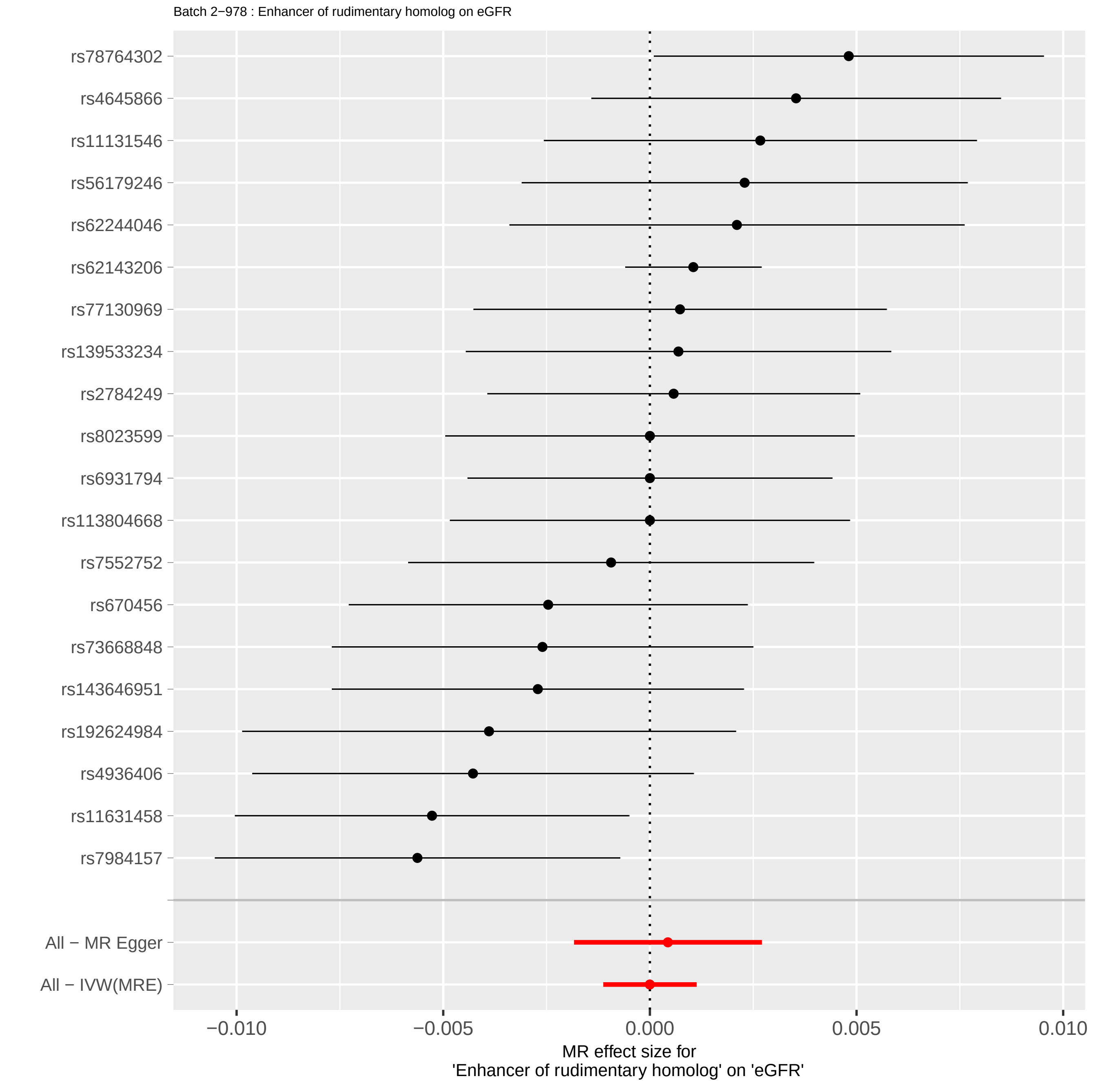

Batch 2−978 : Enhancer of rudimentary homolog on eGFR
rs78764302
rs4645866
rs11131546
rs56179246
rs62244046
rs62143206
rs77130969
rs139533234
rs2784249
rs8023599
rs6931794
rs113804668
rs7552752
rs670456
rs73668848
rs143646951
rs192624984
rs4936406
rs11631458
rs7984157
All − MR Egger
All − IVW(MRE)
−0.010
−0.005
0.000
0.005
0.010
MR effect size for
'Enhancer of rudimentary homolog' on 'eGFR'

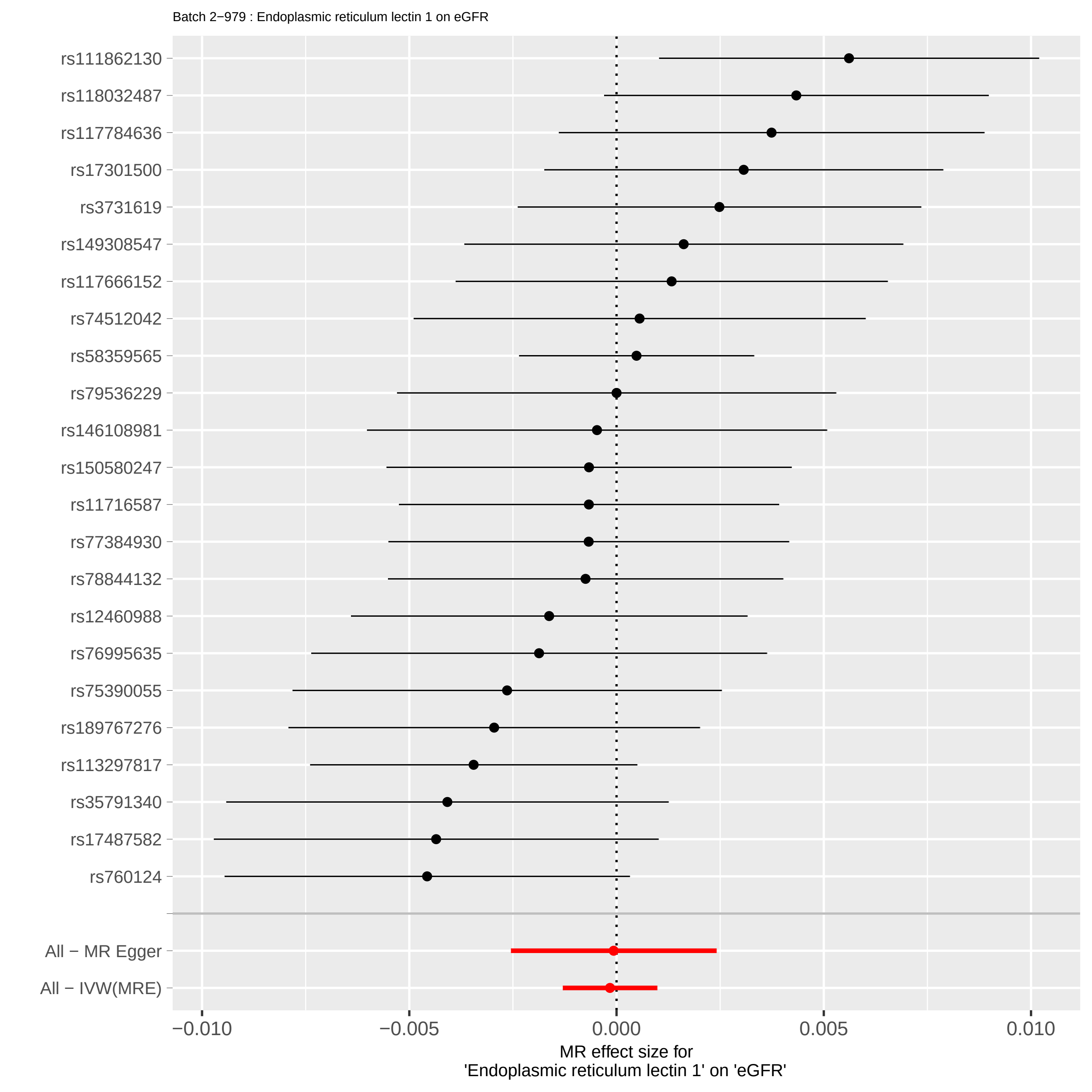

Batch 2−979 : Endoplasmic reticulum lectin 1 on eGFR
rs111862130
rs118032487
rs117784636
rs17301500
rs3731619
rs149308547
rs117666152
rs74512042
rs58359565
rs79536229
rs146108981
rs150580247
rs11716587
rs77384930
rs78844132
rs12460988
rs76995635
rs75390055
rs189767276
rs113297817
rs35791340
rs17487582
rs760124
All − MR Egger
All − IVW(MRE)
−0.010
−0.005
0.000
0.005
0.010
MR effect size for
'Endoplasmic reticulum lectin 1' on 'eGFR'

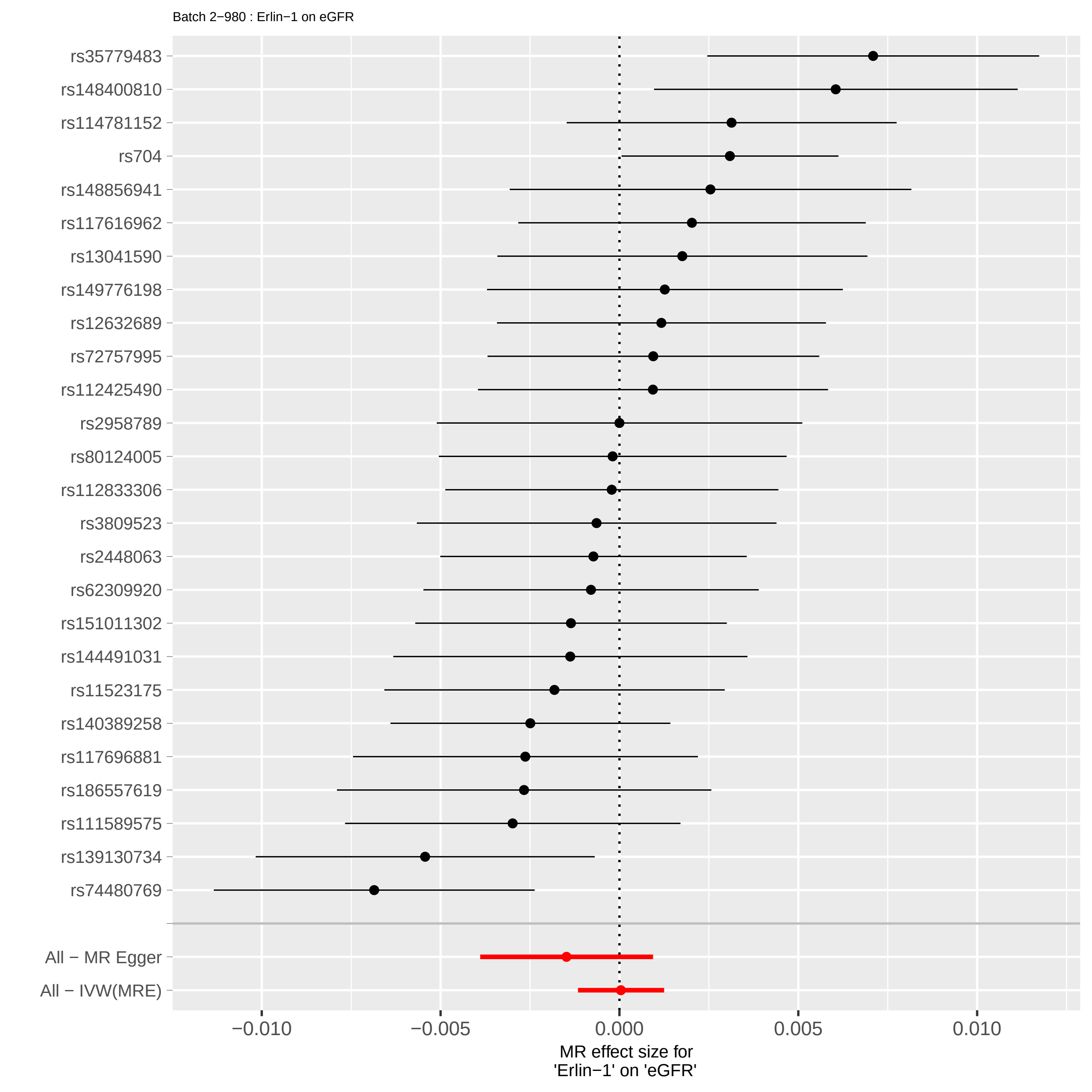

Batch 2−980 : Erlin−1 on eGFR
rs35779483
rs148400810
rs114781152
rs704
rs148856941
rs117616962
rs13041590
rs149776198
rs12632689
rs72757995
rs112425490
rs2958789
rs80124005
rs112833306
rs3809523
rs2448063
rs62309920
rs151011302
rs144491031
rs11523175
rs140389258
rs117696881
rs186557619
rs111589575
rs139130734
rs74480769
All − MR Egger
All − IVW(MRE)
−0.010
−0.005
0.000
0.005
0.010
MR effect size for
'Erlin−1' on 'eGFR'

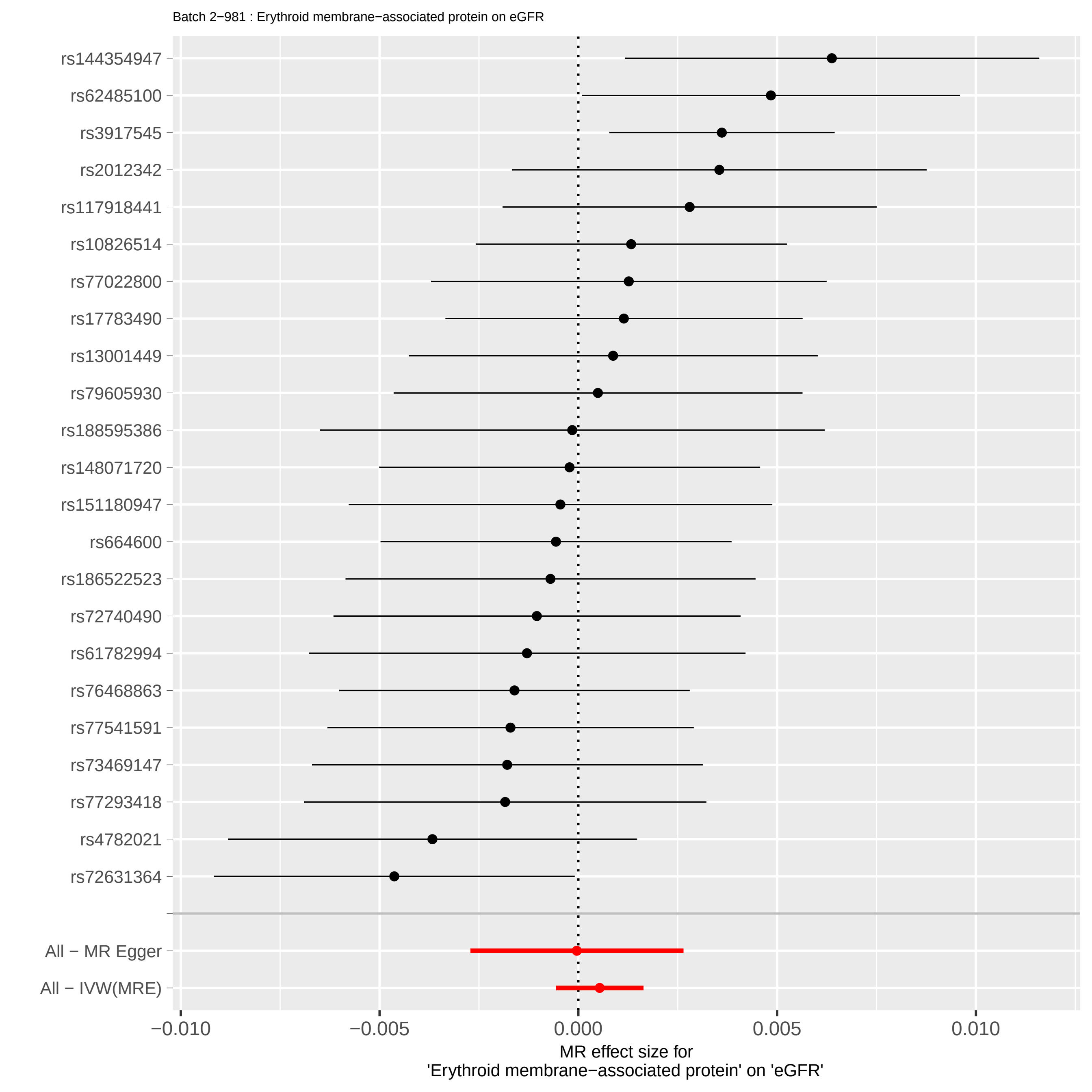

Batch 2−981 : Erythroid membrane−associated protein on eGFR
rs144354947
rs62485100
rs3917545
rs2012342
rs117918441
rs10826514
rs77022800
rs17783490
rs13001449
rs79605930
rs188595386
rs148071720
rs151180947
rs664600
rs186522523
rs72740490
rs61782994
rs76468863
rs77541591
rs73469147
rs77293418
rs4782021
rs72631364
All − MR Egger
All − IVW(MRE)
−0.010
−0.005
0.000
0.005
0.010
MR effect size for
'Erythroid membrane−associated protein' on 'eGFR'

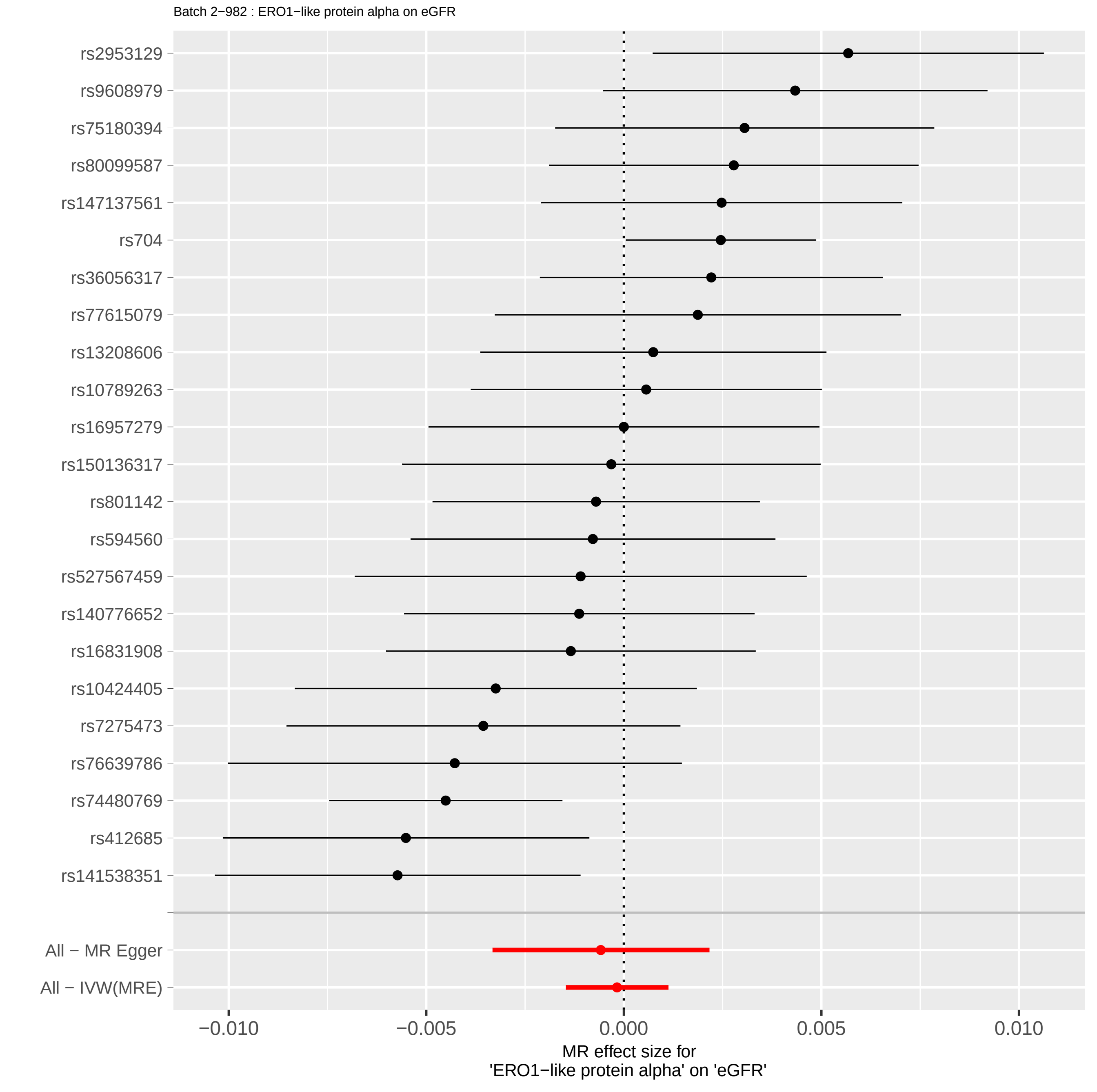

Batch 2−982 : ERO1−like protein alpha on eGFR
rs2953129
rs9608979
rs75180394
rs80099587
rs147137561
rs704
rs36056317
rs77615079
rs13208606
rs10789263
rs16957279
rs150136317
rs801142
rs594560
rs527567459
rs140776652
rs16831908
rs10424405
rs7275473
rs76639786
rs74480769
rs412685
rs141538351
All − MR Egger
All − IVW(MRE)
−0.010
−0.005
0.000
0.005
0.010
MR effect size for
'ERO1−like protein alpha' on 'eGFR'

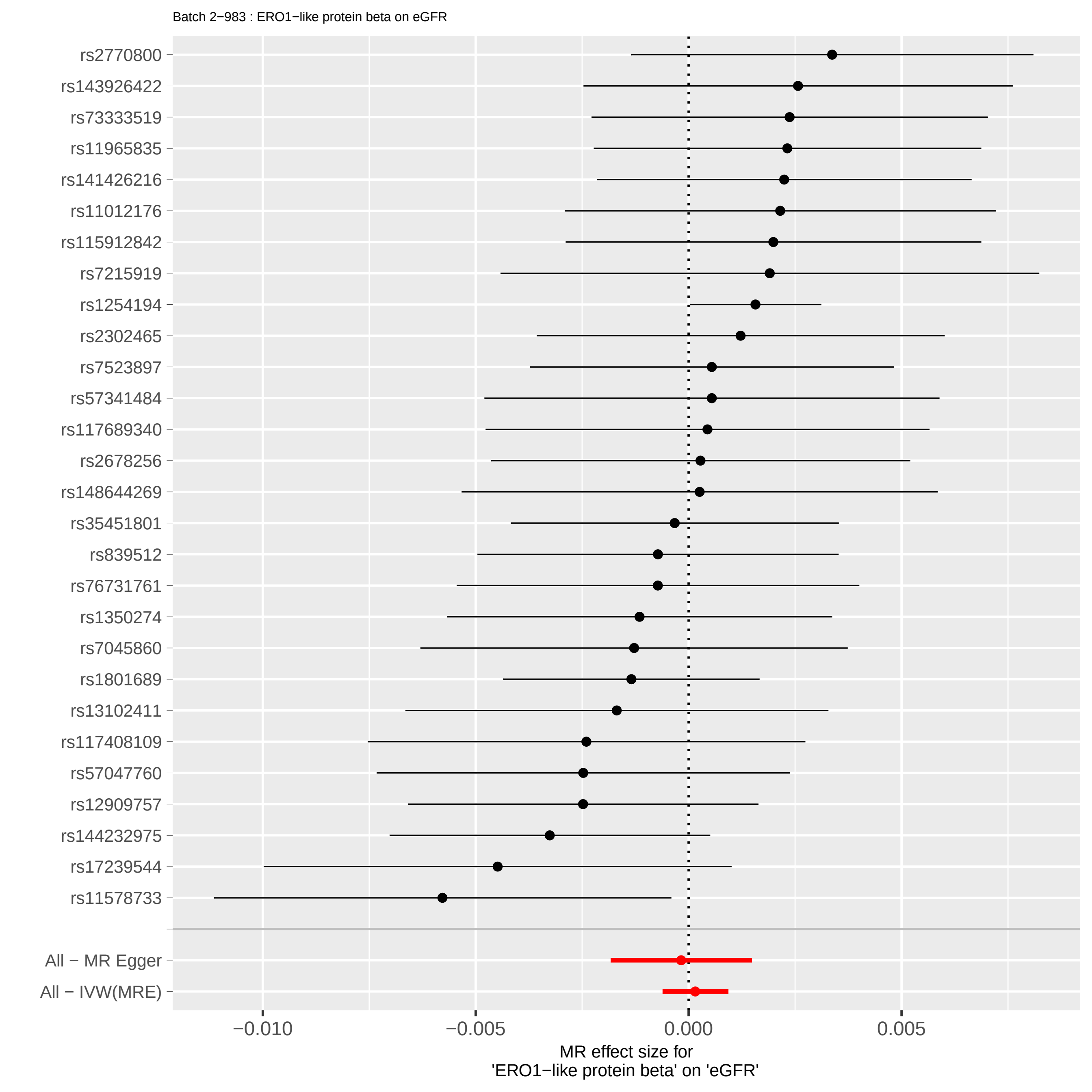

Batch 2−983 : ERO1−like protein beta on eGFR
rs2770800
rs143926422
rs73333519
rs11965835
rs141426216
rs11012176
rs115912842
rs7215919
rs1254194
rs2302465
rs7523897
rs57341484
rs117689340
rs2678256
rs148644269
rs35451801
rs839512
rs76731761
rs1350274
rs7045860
rs1801689
rs13102411
rs117408109
rs57047760
rs12909757
rs144232975
rs17239544
rs11578733
All − MR Egger
All − IVW(MRE)
−0.010
−0.005
0.000
0.005
MR effect size for
'ERO1−like protein beta' on 'eGFR'

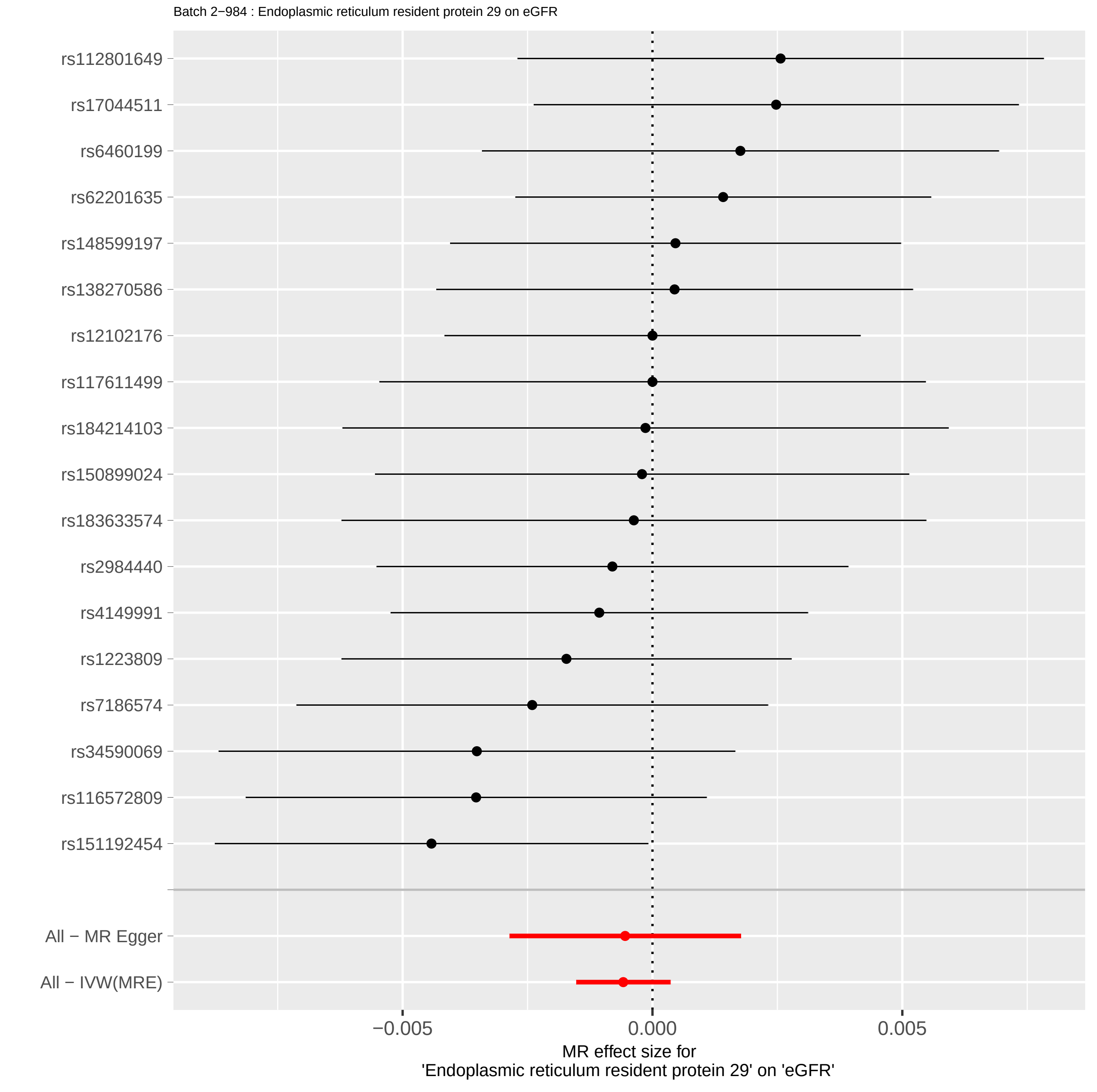

Batch 2−984 : Endoplasmic reticulum resident protein 29 on eGFR
rs112801649
rs17044511
rs6460199
rs62201635
rs148599197
rs138270586
rs12102176
rs117611499
rs184214103
rs150899024
rs183633574
rs2984440
rs4149991
rs1223809
rs7186574
rs34590069
rs116572809
rs151192454
All − MR Egger
All − IVW(MRE)
−0.005
0.000
0.005
MR effect size for
'Endoplasmic reticulum resident protein 29' on 'eGFR'

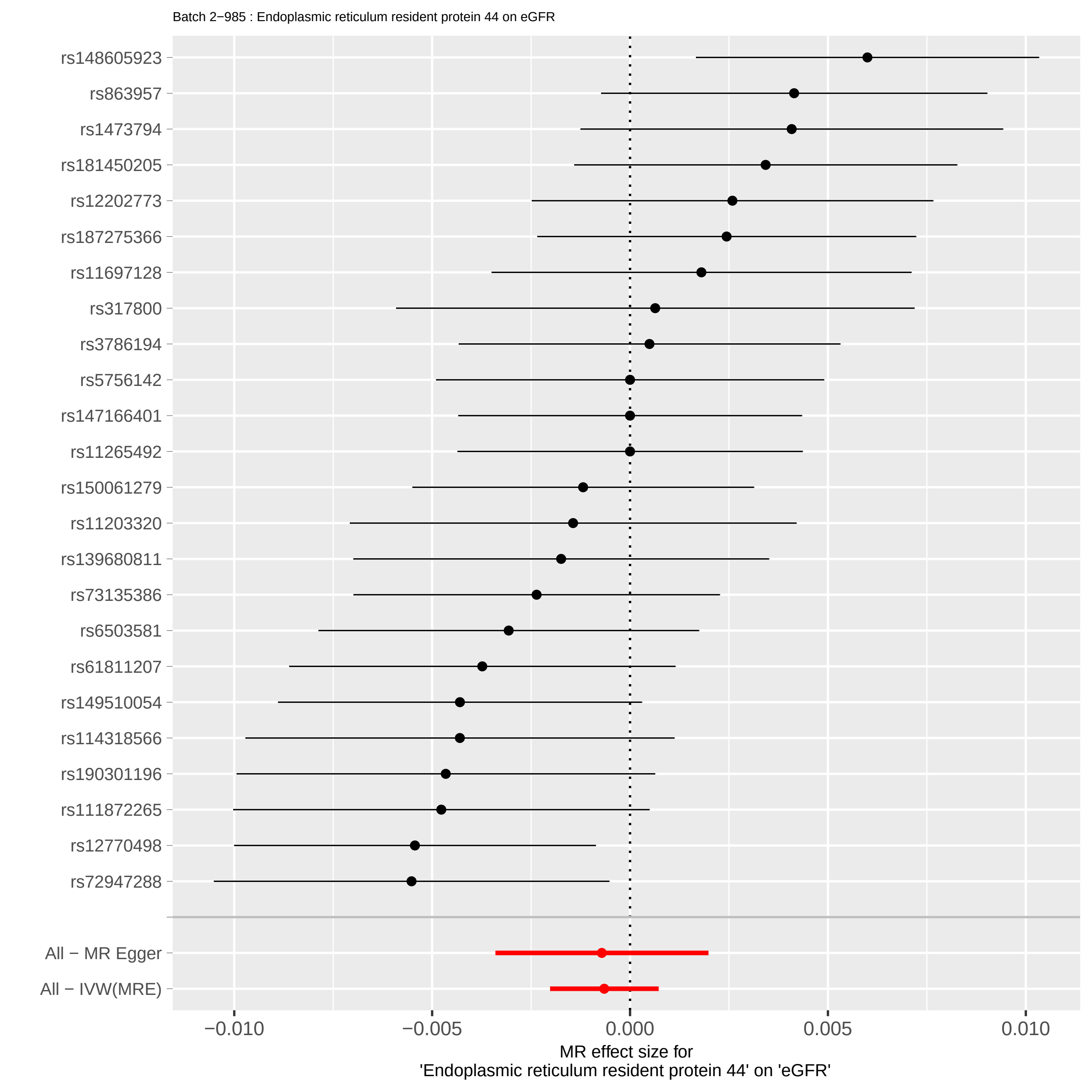

Batch 2−985 : Endoplasmic reticulum resident protein 44 on eGFR
rs148605923
rs863957
rs1473794
rs181450205
rs12202773
rs187275366
rs11697128
rs317800
rs3786194
rs5756142
rs147166401
rs11265492
rs150061279
rs11203320
rs139680811
rs73135386
rs6503581
rs61811207
rs149510054
rs114318566
rs190301196
rs111872265
rs12770498
rs72947288
All − MR Egger
All − IVW(MRE)
−0.010
−0.005
0.000
0.005
0.010
MR effect size for
'Endoplasmic reticulum resident protein 44' on 'eGFR'

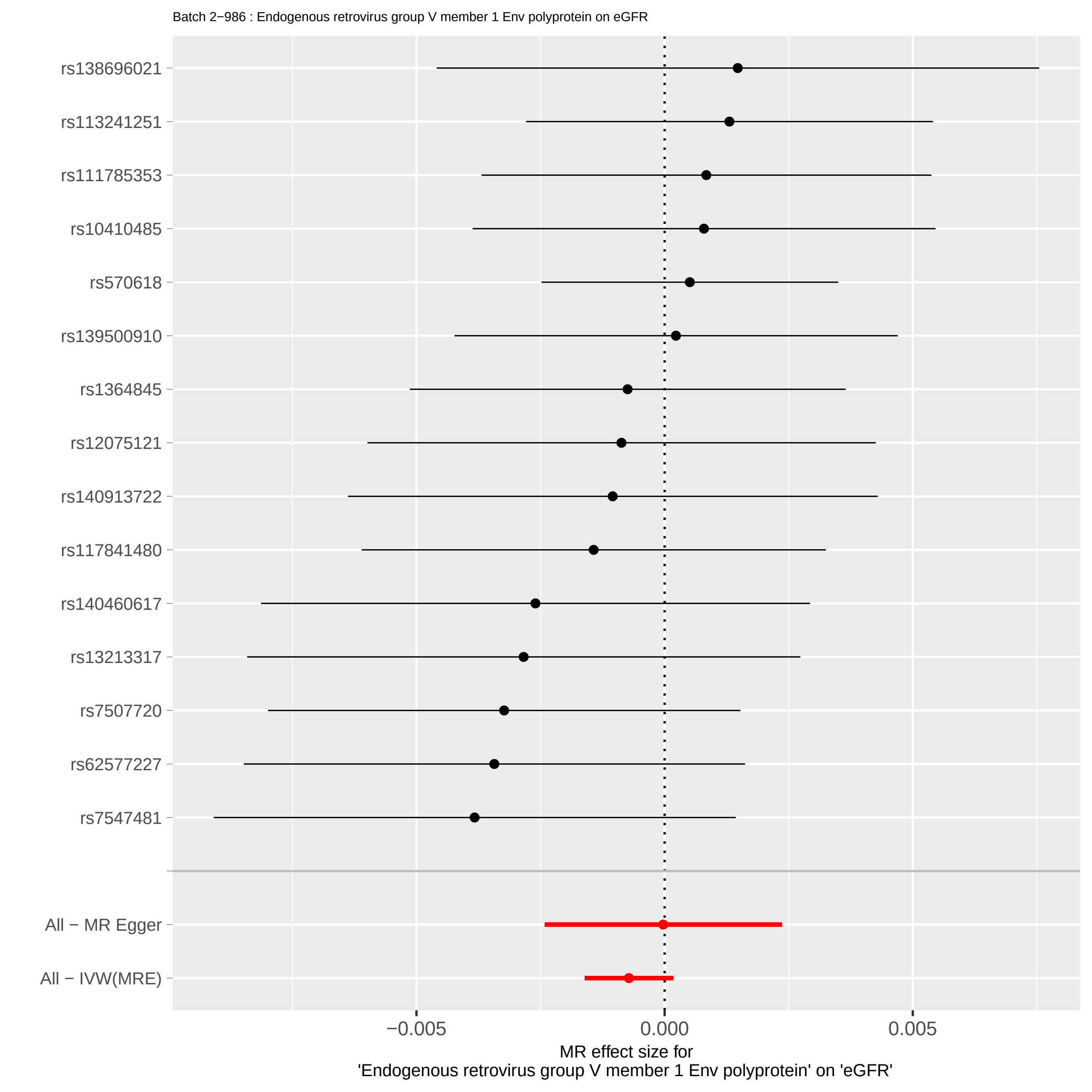
Batch 2−986 : Endogenous retrovirus group V member 1 Env polyprotein on eGFR
rs138696021
rs113241251
rs111785353
rs10410485
rs570618
rs139500910
rs1364845
rs12075121
rs140913722
rs117841480
rs140460617
rs13213317
rs7507720
rs62577227
rs7547481
All − MR Egger
All − IVW(MRE)
−0.005
0.000
0.005
MR effect size for
'Endogenous retrovirus group V member 1 Env polyprotein' on 'eGFR'

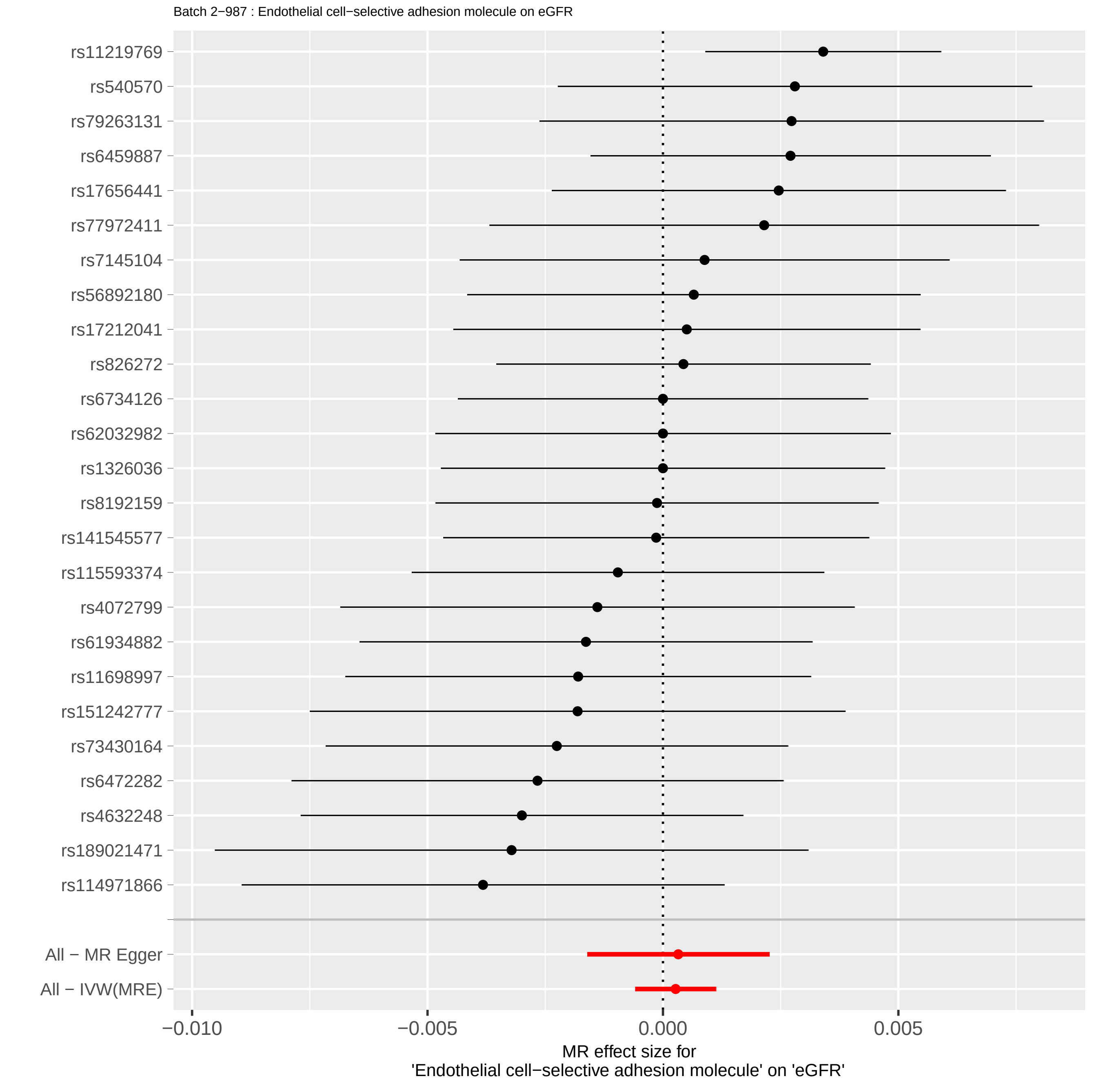

Batch 2−987 : Endothelial cell−selective adhesion molecule on eGFR
rs11219769
rs540570
rs79263131
rs6459887
rs17656441
rs77972411
rs7145104
rs56892180
rs17212041
rs826272
rs6734126
rs62032982
rs1326036
rs8192159
rs141545577
rs115593374
rs4072799
rs61934882
rs11698997
rs151242777
rs73430164
rs6472282
rs4632248
rs189021471
rs114971866
All − MR Egger
All − IVW(MRE)
−0.010
−0.005
0.000
0.005
MR effect size for
'Endothelial cell−selective adhesion molecule' on 'eGFR'

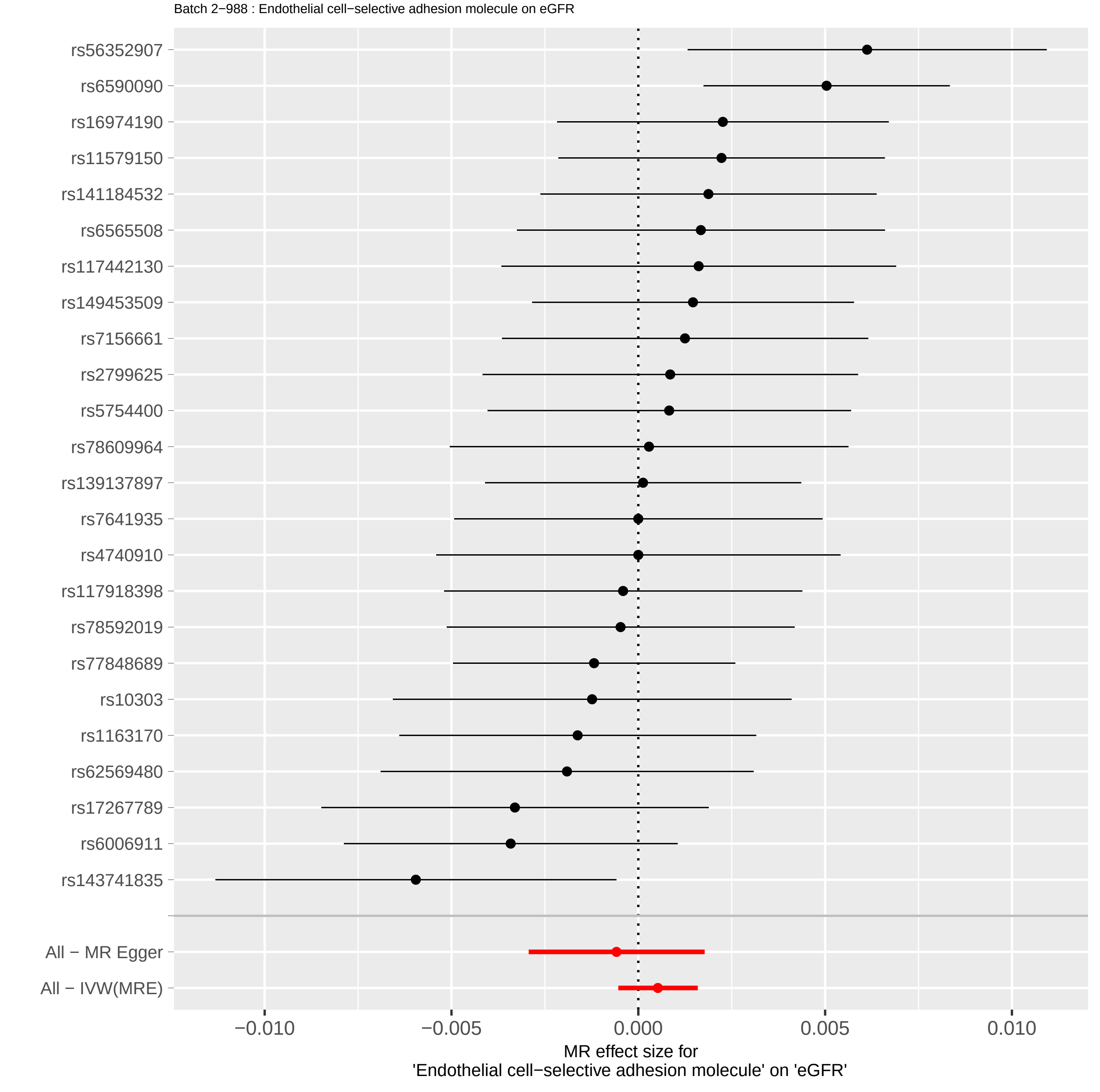

Batch 2−988 : Endothelial cell−selective adhesion molecule on eGFR
rs56352907
rs6590090
rs16974190
rs11579150
rs141184532
rs6565508
rs117442130
rs149453509
rs7156661
rs2799625
rs5754400
rs78609964
rs139137897
rs7641935
rs4740910
rs117918398
rs78592019
rs77848689
rs10303
rs1163170
rs62569480
rs17267789
rs6006911
rs143741835
All − MR Egger
All − IVW(MRE)
−0.010
−0.005
0.000
0.005
0.010
MR effect size for
'Endothelial cell−selective adhesion molecule' on 'eGFR'

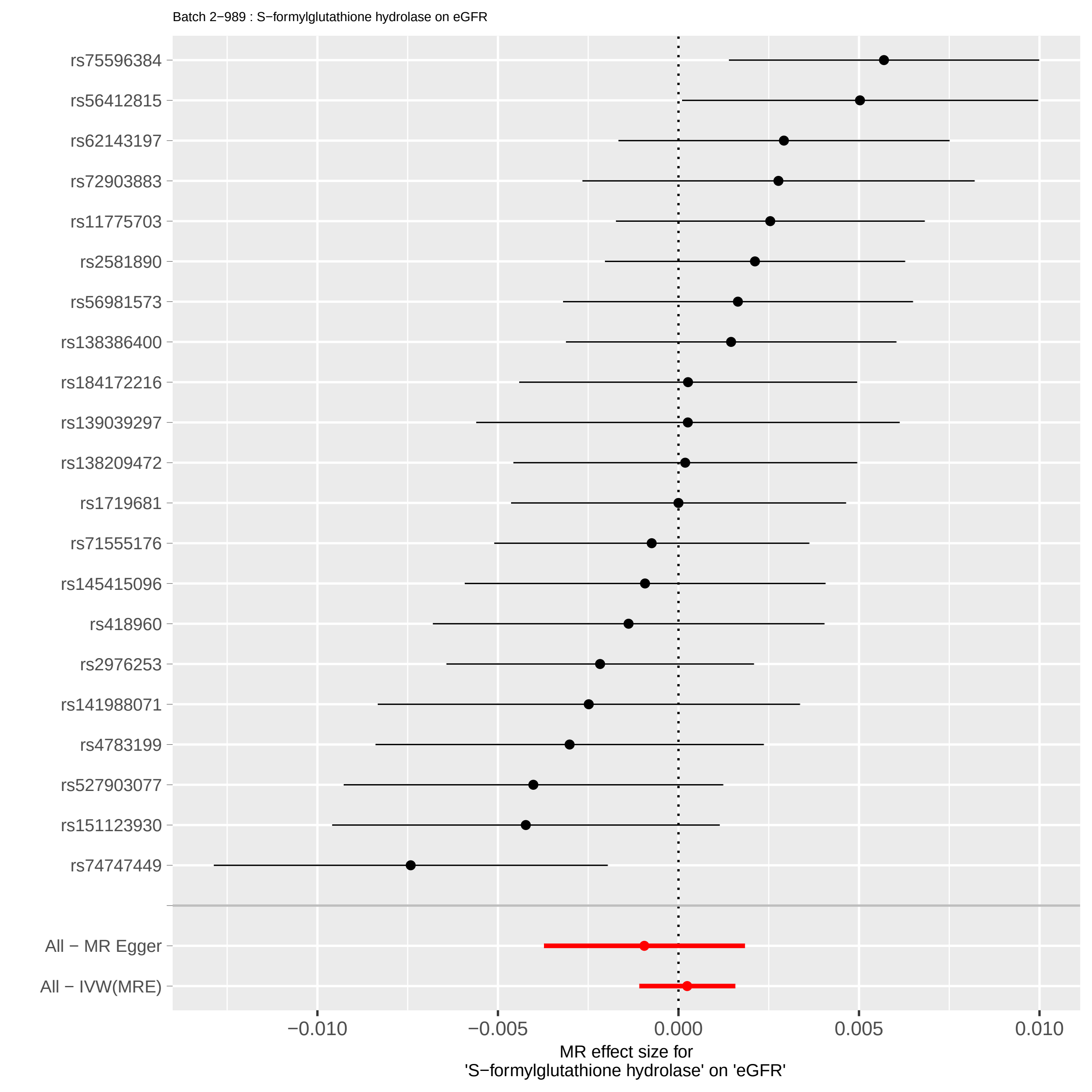
Batch 2−989 : S−formylglutathione hydrolase on eGFR
rs75596384
rs56412815
rs62143197
rs72903883
rs11775703
rs2581890
rs56981573
rs138386400
rs184172216
rs139039297
rs138209472
rs1719681
rs71555176
rs145415096
rs418960
rs2976253
rs141988071
rs4783199
rs527903077
rs151123930
rs74747449
All − MR Egger
All − IVW(MRE)
−0.010
−0.005
0.000
0.005
0.010
MR effect size for
'S−formylglutathione hydrolase' on 'eGFR'

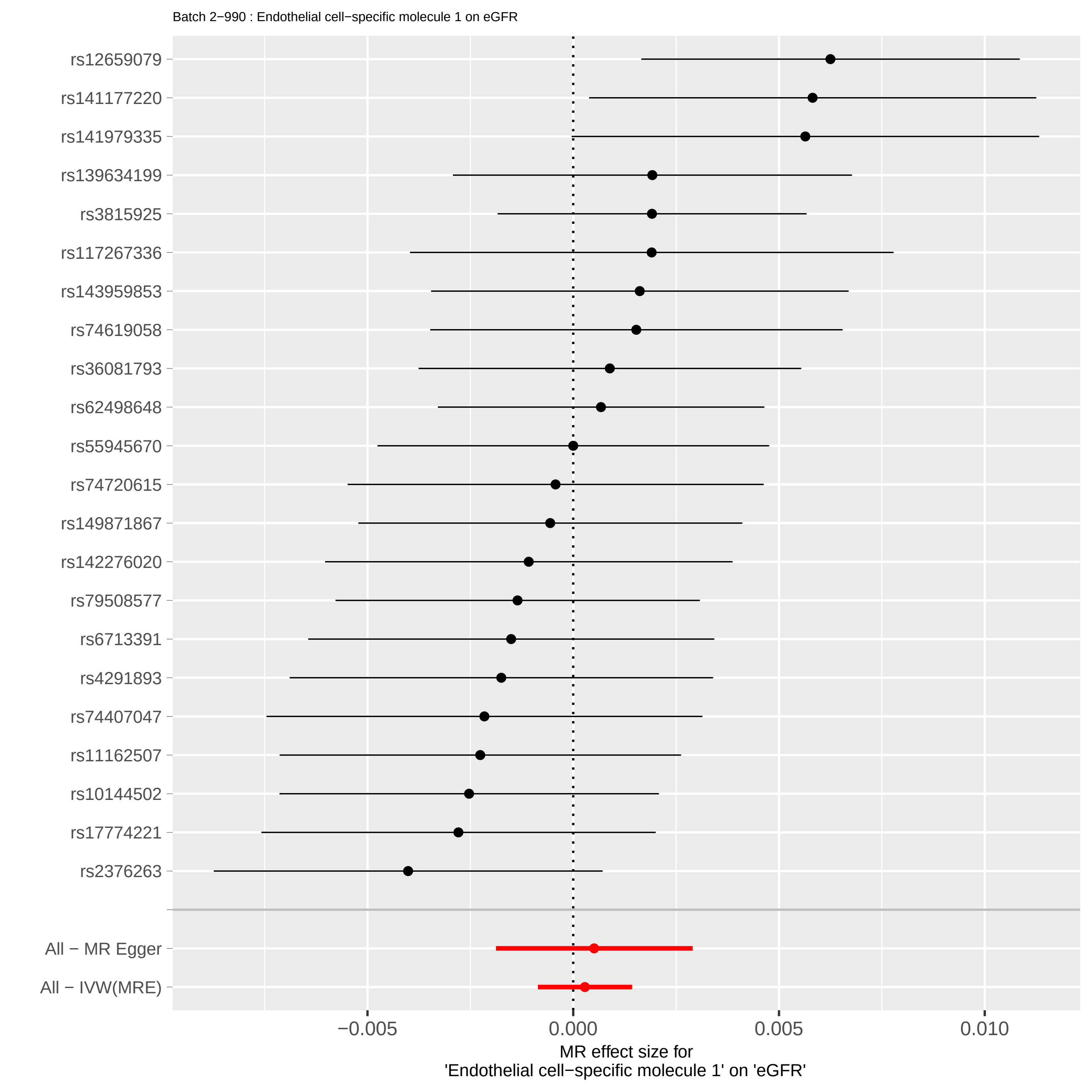

Batch 2−990 : Endothelial cell−specific molecule 1 on eGFR
rs12659079
rs141177220
rs141979335
rs139634199
rs3815925
rs117267336
rs143959853
rs74619058
rs36081793
rs62498648
rs55945670
rs74720615
rs149871867
rs142276020
rs79508577
rs6713391
rs4291893
rs74407047
rs11162507
rs10144502
rs17774221
rs2376263
All − MR Egger
All − IVW(MRE)
−0.005
0.000
0.005
0.010
MR effect size for
'Endothelial cell−specific molecule 1' on 'eGFR'

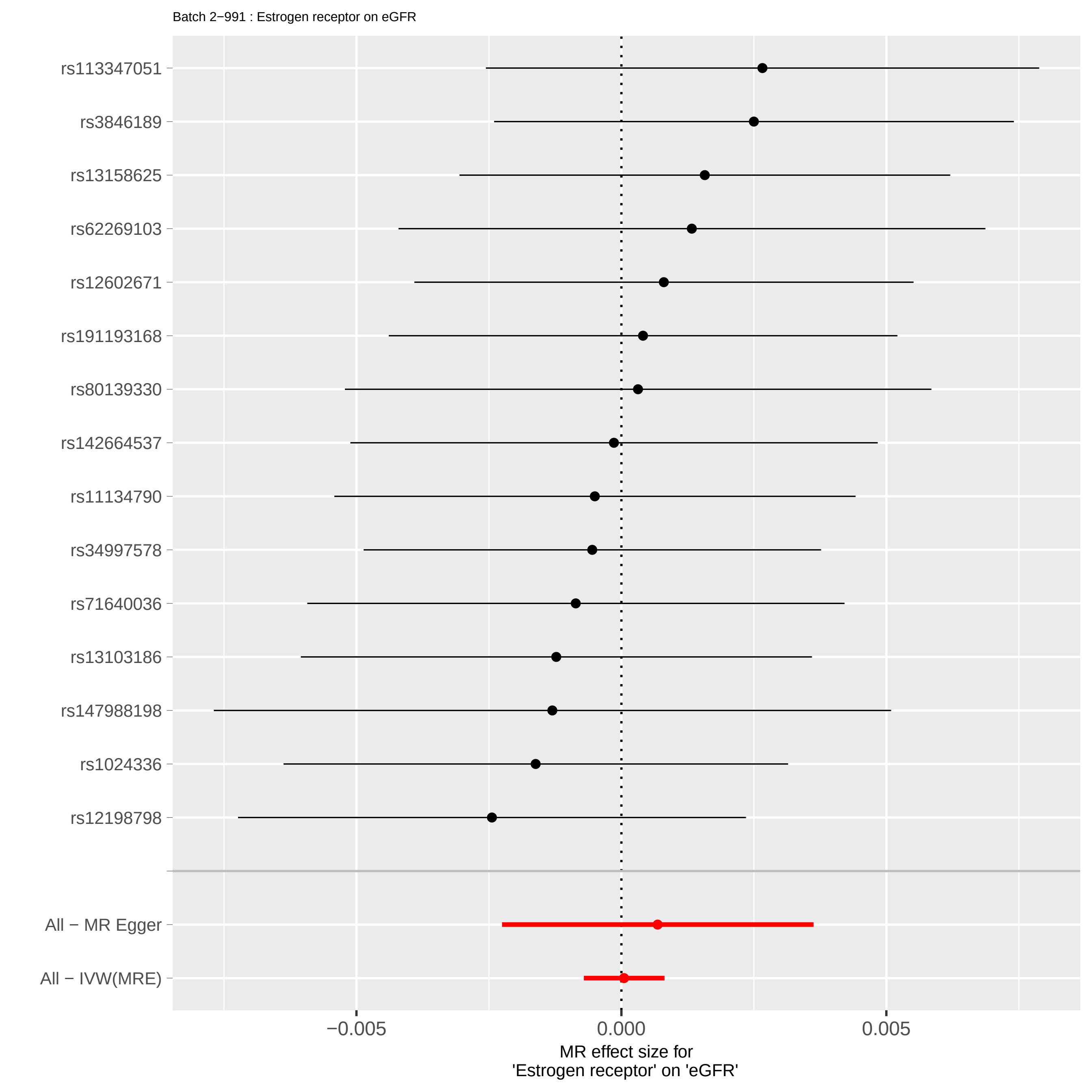
Batch 2−991 : Estrogen receptor on eGFR
rs113347051
rs3846189
rs13158625
rs62269103
rs12602671
rs191193168
rs80139330
rs142664537
rs11134790
rs34997578
rs71640036
rs13103186
rs147988198
rs1024336
rs12198798
All − MR Egger
All − IVW(MRE)
−0.005
0.000
0.005
MR effect size for
'Estrogen receptor' on 'eGFR'

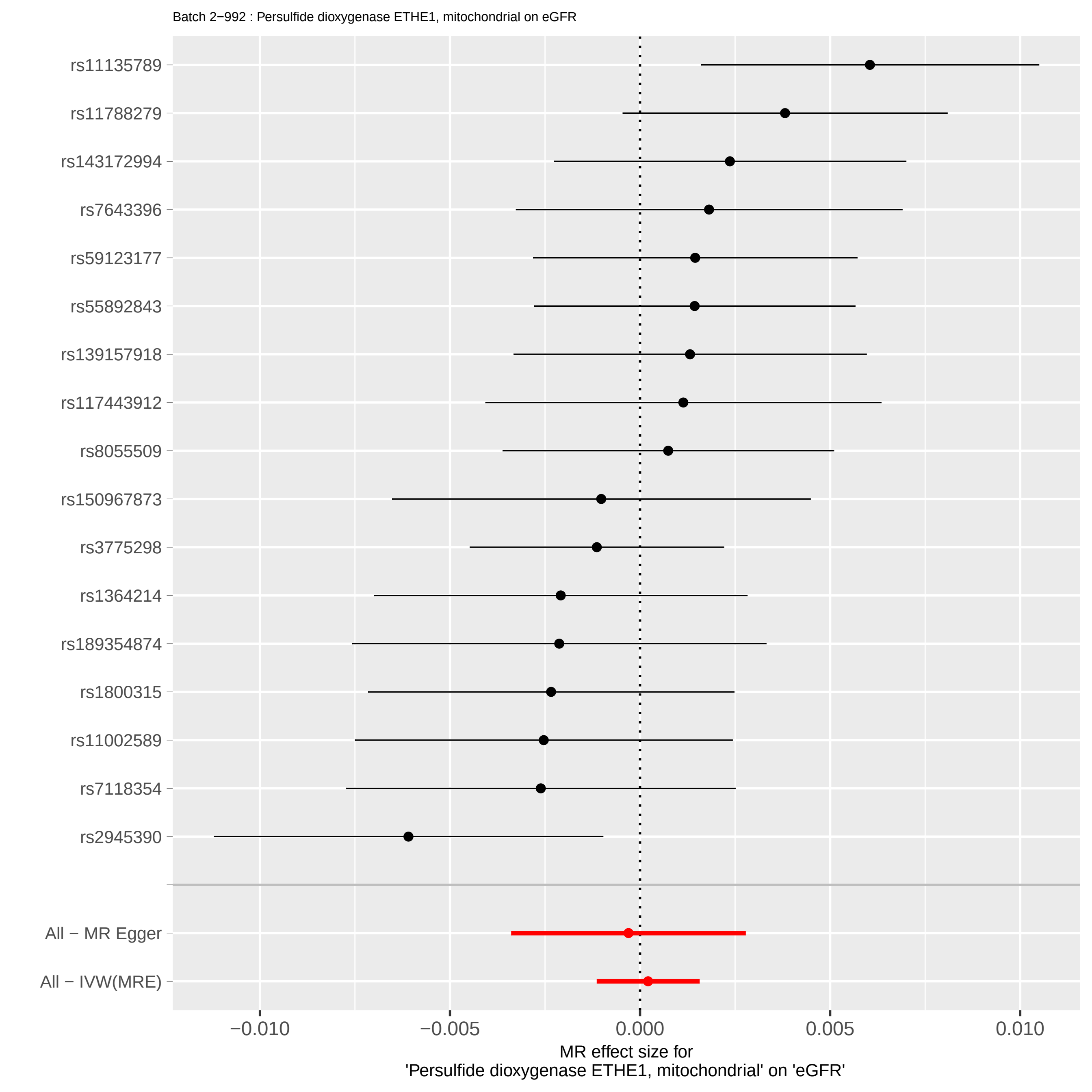

Batch 2−992 : Persulfide dioxygenase ETHE1, mitochondrial on eGFR
rs11135789
rs11788279
rs143172994
rs7643396
rs59123177
rs55892843
rs139157918
rs117443912
rs8055509
rs150967873
rs3775298
rs1364214
rs189354874
rs1800315
rs11002589
rs7118354
rs2945390
All − MR Egger
All − IVW(MRE)
−0.010
−0.005
0.000
0.005
0.010
MR effect size for
'Persulfide dioxygenase ETHE1, mitochondrial' on 'eGFR'

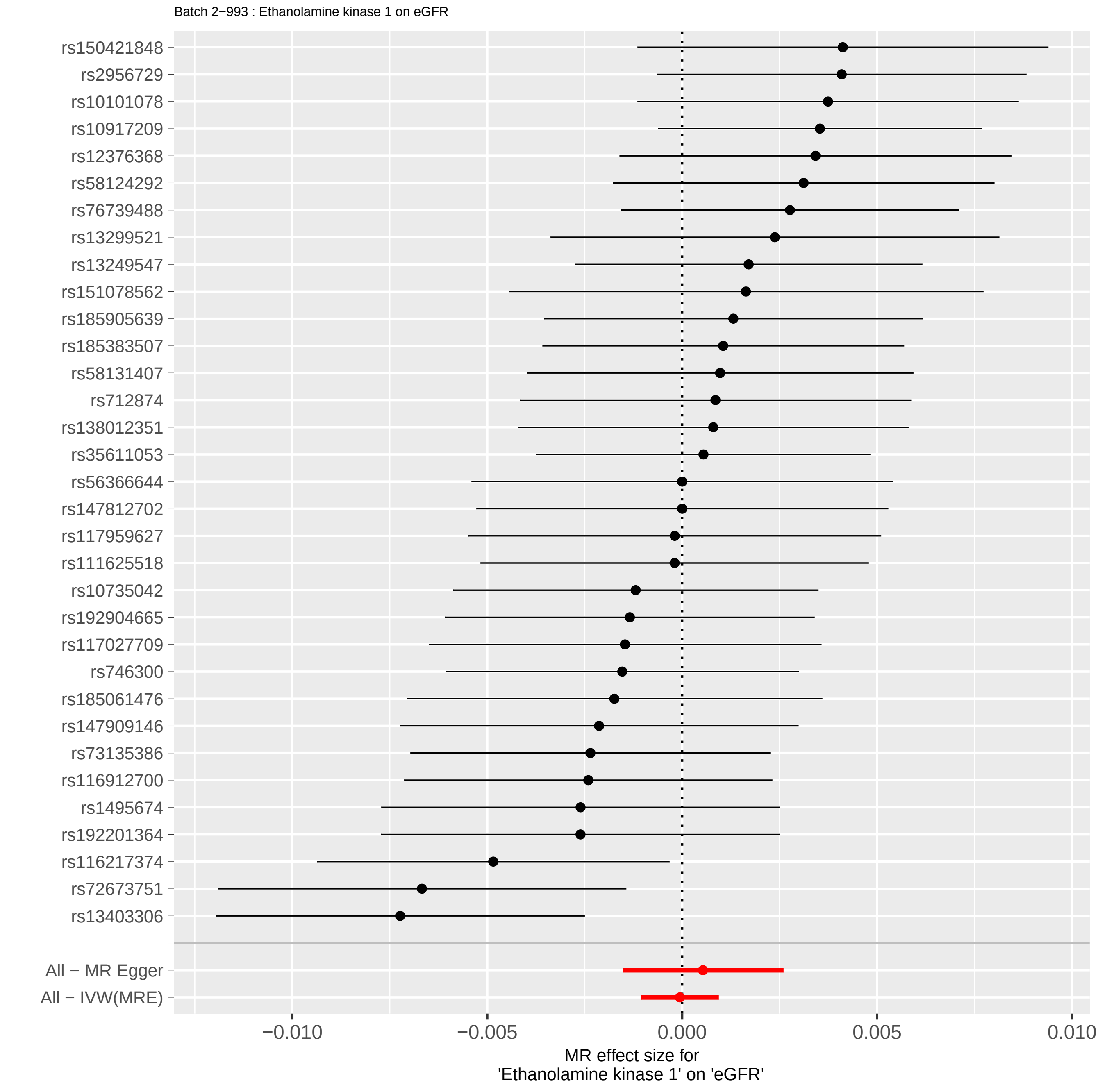

Batch 2−993 : Ethanolamine kinase 1 on eGFR
rs150421848
rs2956729
rs10101078
rs10917209
rs12376368
rs58124292
rs76739488
rs13299521
rs13249547
rs151078562
rs185905639
rs185383507
rs58131407
rs712874
rs138012351
rs35611053
rs56366644
rs147812702
rs117959627
rs111625518
rs10735042
rs192904665
rs117027709
rs746300
rs185061476
rs147909146
rs73135386
rs116912700
rs1495674
rs192201364
rs116217374
rs72673751
rs13403306
All − MR Egger
All − IVW(MRE)
−0.010
−0.005
0.000
0.005
0.010
MR effect size for
'Ethanolamine kinase 1' on 'eGFR'

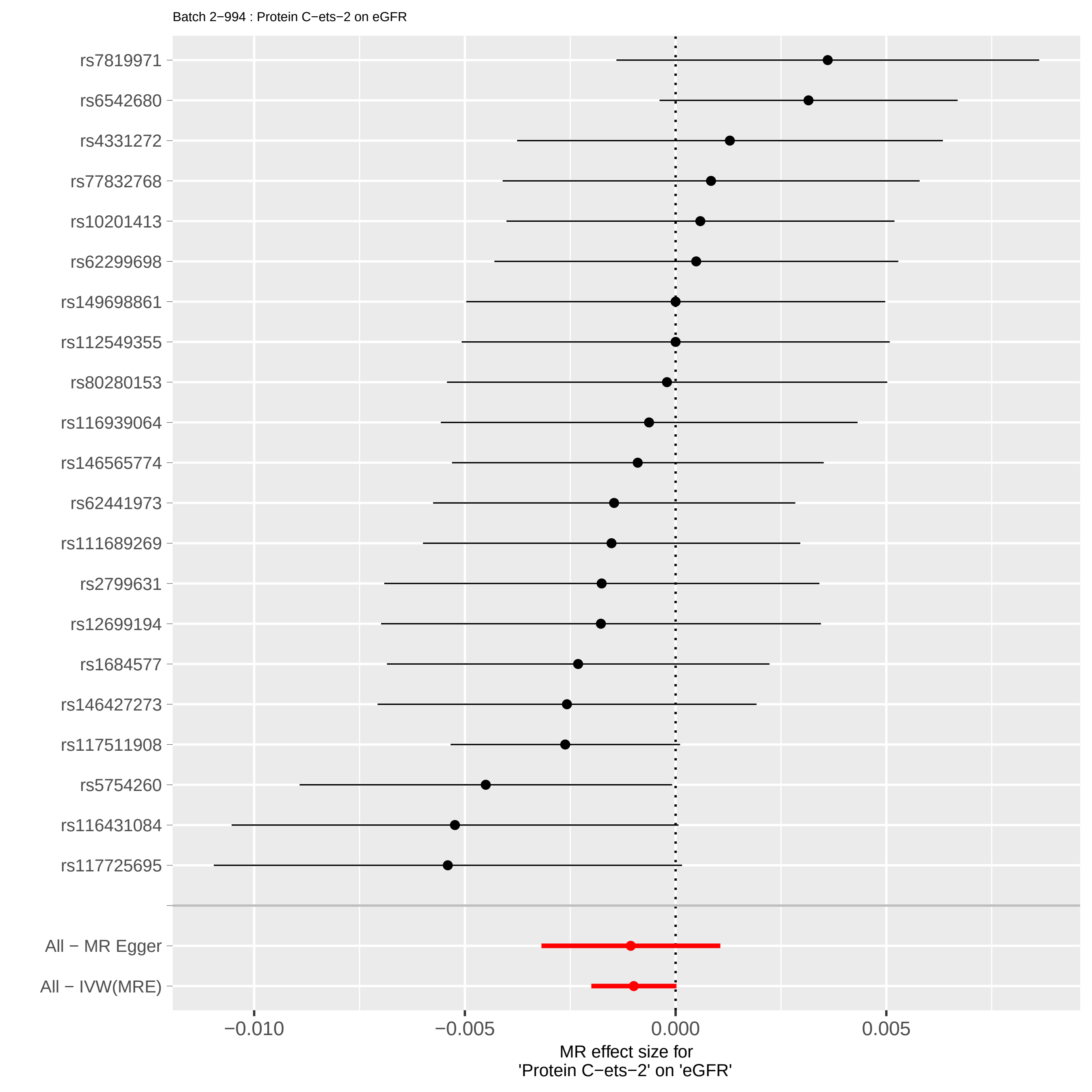
Batch 2−994 : Protein C−ets−2 on eGFR
rs7819971
rs6542680
rs4331272
rs77832768
rs10201413
rs62299698
rs149698861
rs112549355
rs80280153
rs116939064
rs146565774
rs62441973
rs111689269
rs2799631
rs12699194
rs1684577
rs146427273
rs117511908
rs5754260
rs116431084
rs117725695
All − MR Egger
All − IVW(MRE)
−0.010
−0.005
0.000
0.005
MR effect size for
'Protein C−ets−2' on 'eGFR'

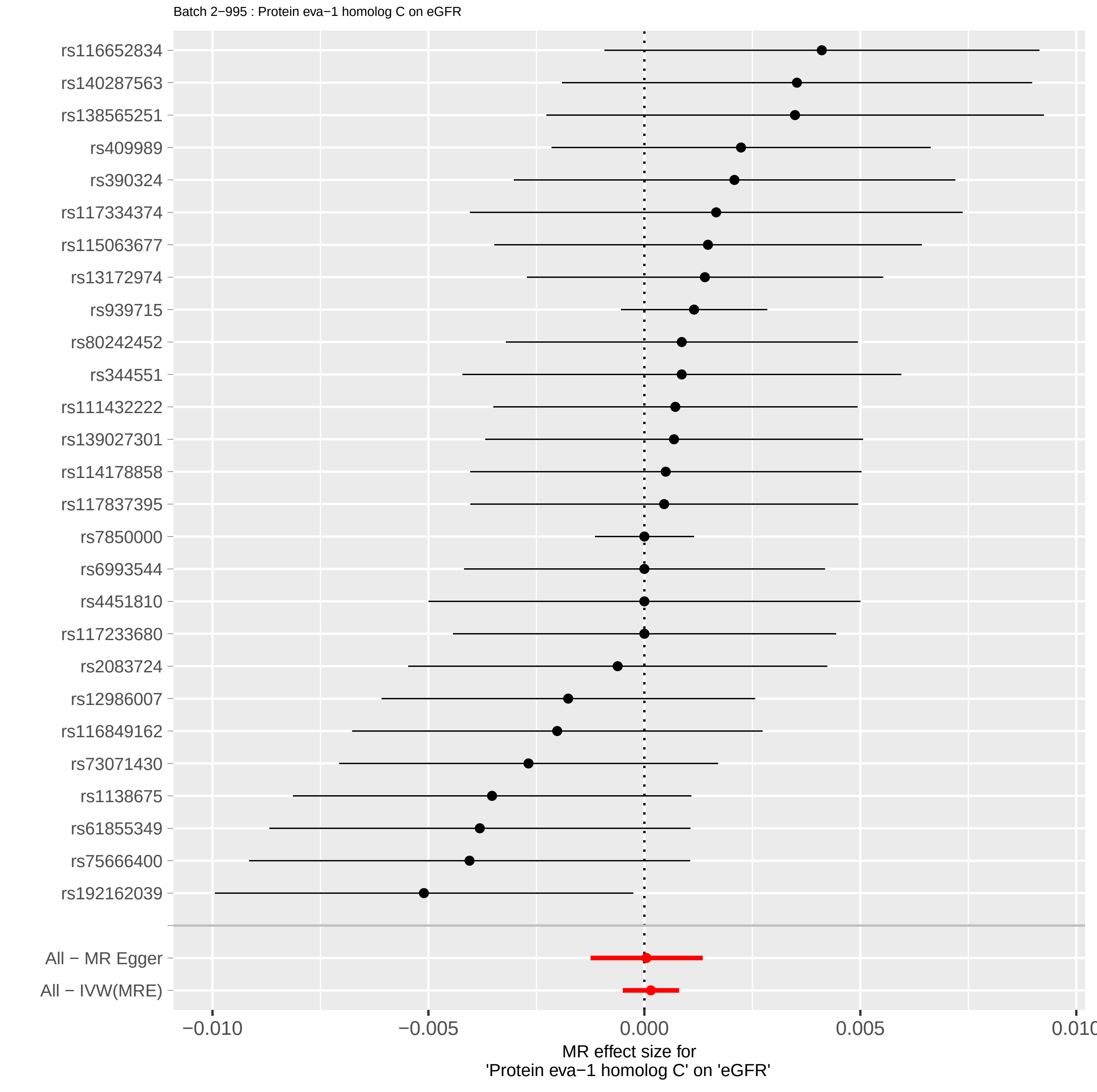
Batch 2−995 : Protein eva−1 homolog C on eGFR
rs116652834
rs140287563
rs138565251
rs409989
rs390324
rs117334374
rs115063677
rs13172974
rs939715
rs80242452
rs344551
rs111432222
rs139027301
rs114178858
rs117837395
rs7850000
rs6993544
rs4451810
rs117233680
rs2083724
rs12986007
rs116849162
rs73071430
rs1138675
rs61855349
rs75666400
rs192162039
All − MR Egger
All − IVW(MRE)
−0.010
−0.005
0.000
0.005
0.010
MR effect size for
'Protein eva−1 homolog C' on 'eGFR'

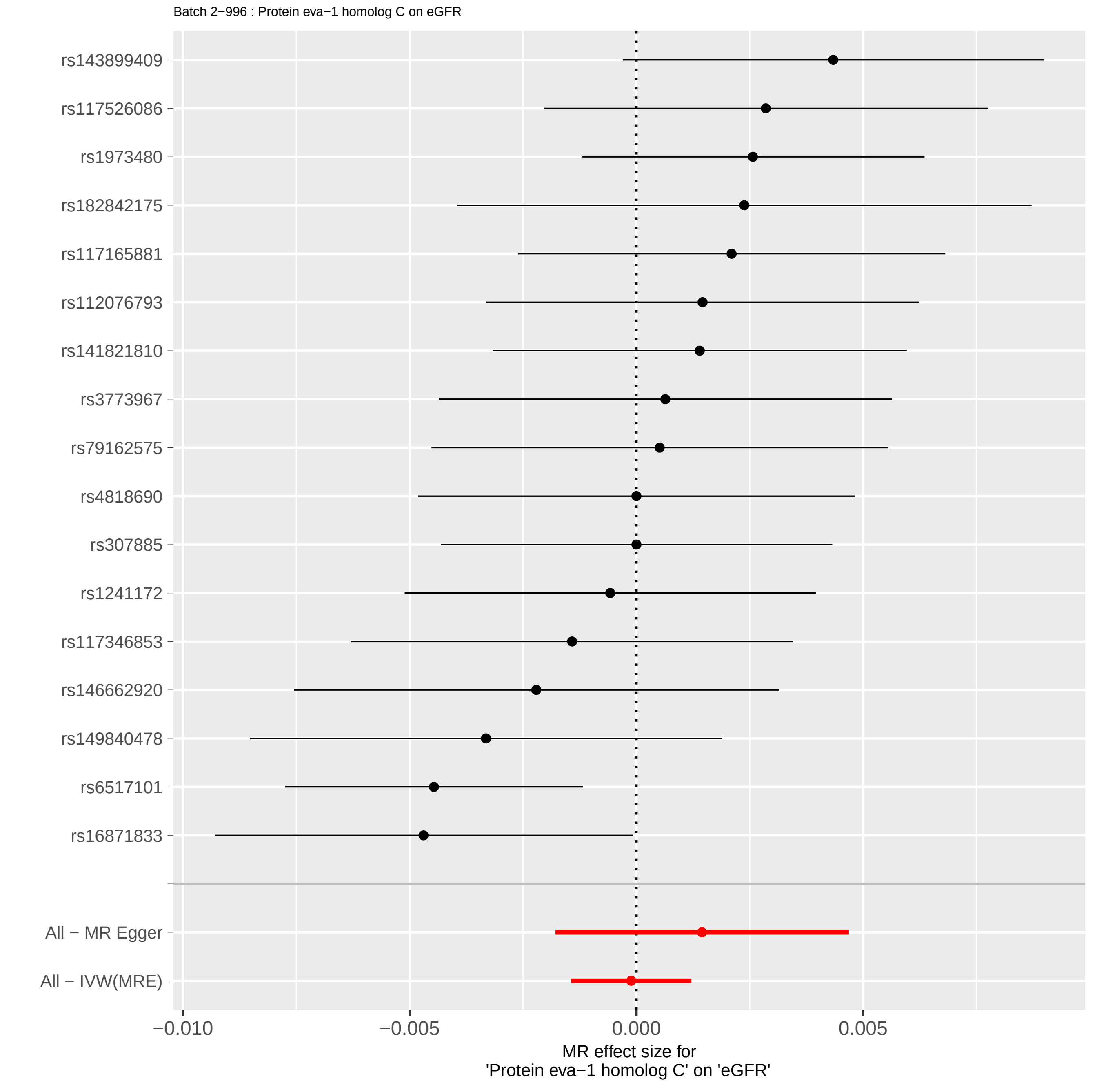

Batch 2−996 : Protein eva−1 homolog C on eGFR
rs143899409
rs117526086
rs1973480
rs182842175
rs117165881
rs112076793
rs141821810
rs3773967
rs79162575
rs4818690
rs307885
rs1241172
rs117346853
rs146662920
rs149840478
rs6517101
rs16871833
All − MR Egger
All − IVW(MRE)
−0.010
−0.005
0.000
0.005
MR effect size for
'Protein eva−1 homolog C' on 'eGFR'

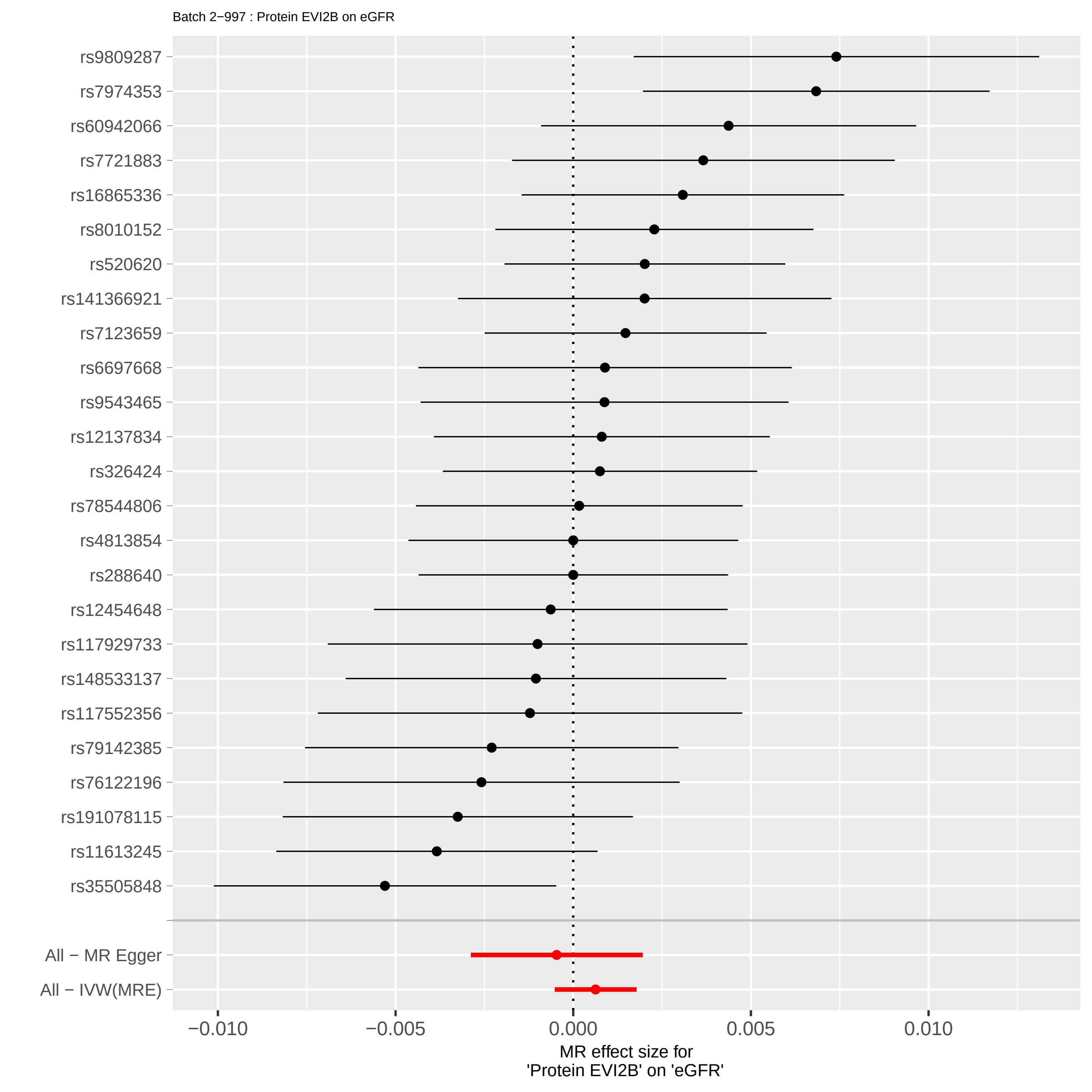
Batch 2−997 : Protein EVI2B on eGFR
rs9809287
rs7974353
rs60942066
rs7721883
rs16865336
rs8010152
rs520620
rs141366921
rs7123659
rs6697668
rs9543465
rs12137834
rs326424
rs78544806
rs4813854
rs288640
rs12454648
rs117929733
rs148533137
rs117552356
rs79142385
rs76122196
rs191078115
rs11613245
rs35505848
All − MR Egger
All − IVW(MRE)
−0.010
−0.005
0.000
0.005
0.010
MR effect size for
'Protein EVI2B' on 'eGFR'

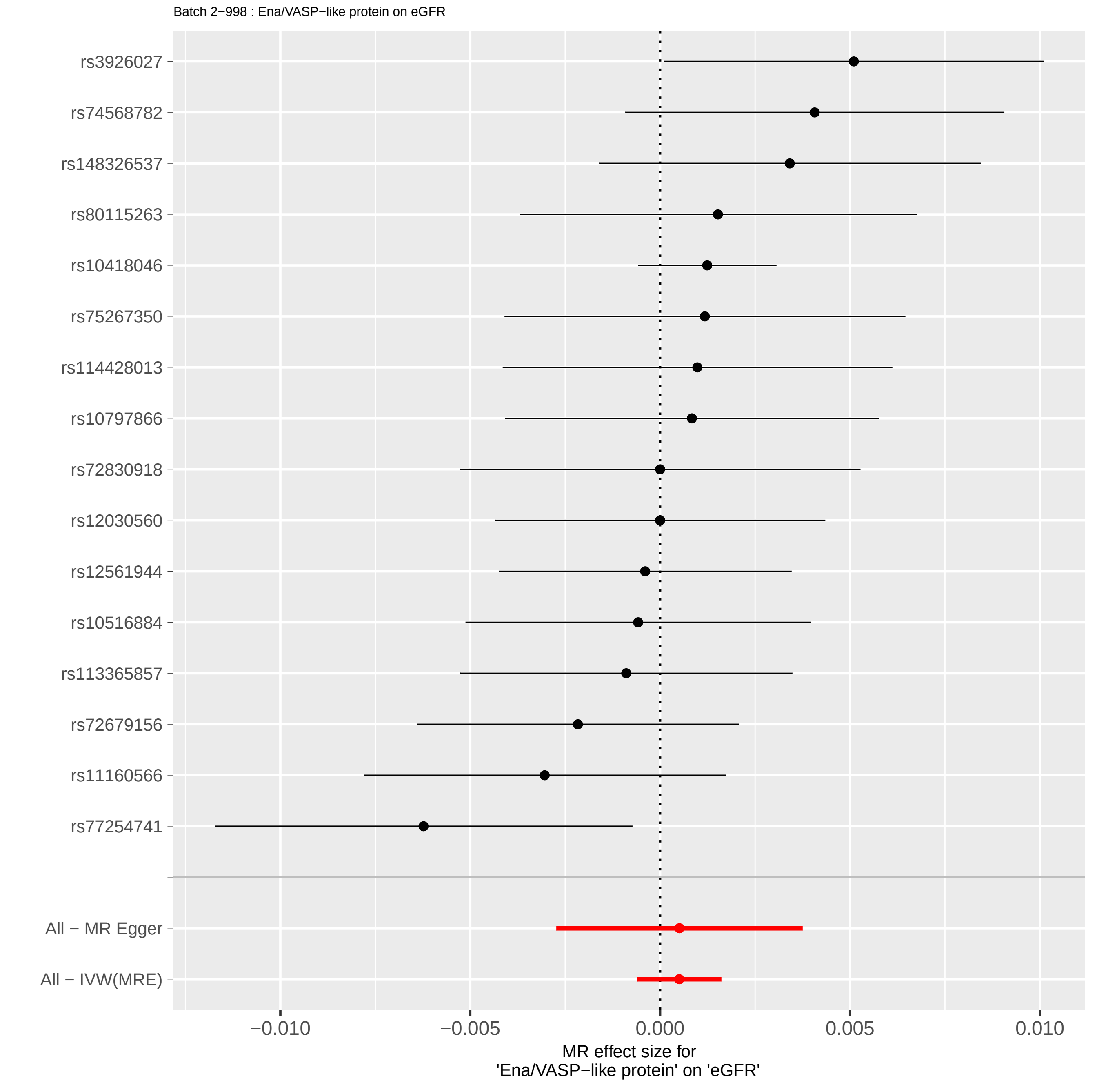

Batch 2−998 : Ena/VASP−like protein on eGFR
rs3926027
rs74568782
rs148326537
rs80115263
rs10418046
rs75267350
rs114428013
rs10797866
rs72830918
rs12030560
rs12561944
rs10516884
rs113365857
rs72679156
rs11160566
rs77254741
All − MR Egger
All − IVW(MRE)
−0.010
−0.005
0.000
0.005
0.010
MR effect size for
'Ena/VASP−like protein' on 'eGFR'

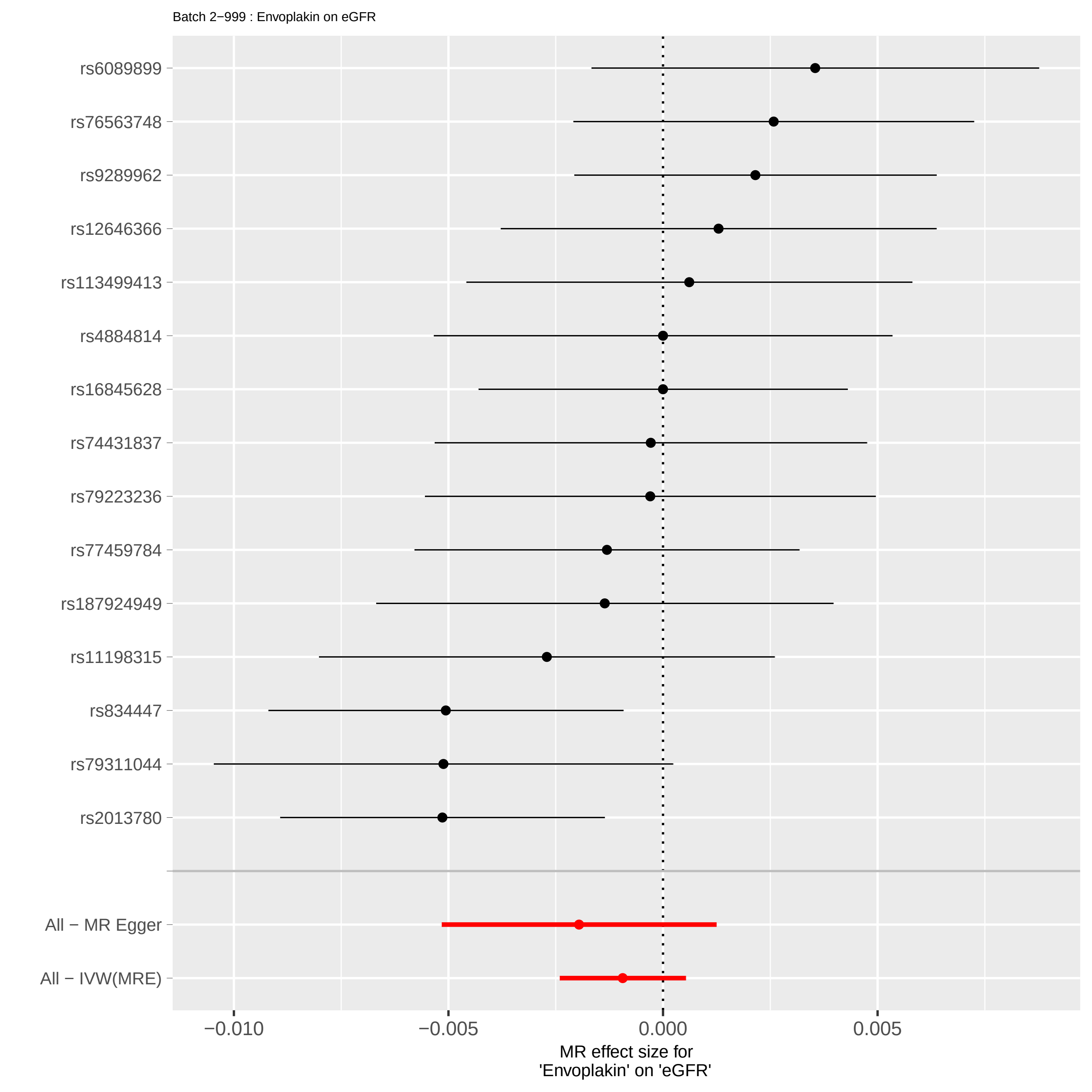
Batch 2−999 : Envoplakin on eGFR
rs6089899
rs76563748
rs9289962
rs12646366
rs113499413
rs4884814
rs16845628
rs74431837
rs79223236
rs77459784
rs187924949
rs11198315
rs834447
rs79311044
rs2013780
All − MR Egger
All − IVW(MRE)
−0.010
−0.005
0.000
0.005
MR effect size for
'Envoplakin' on 'eGFR'

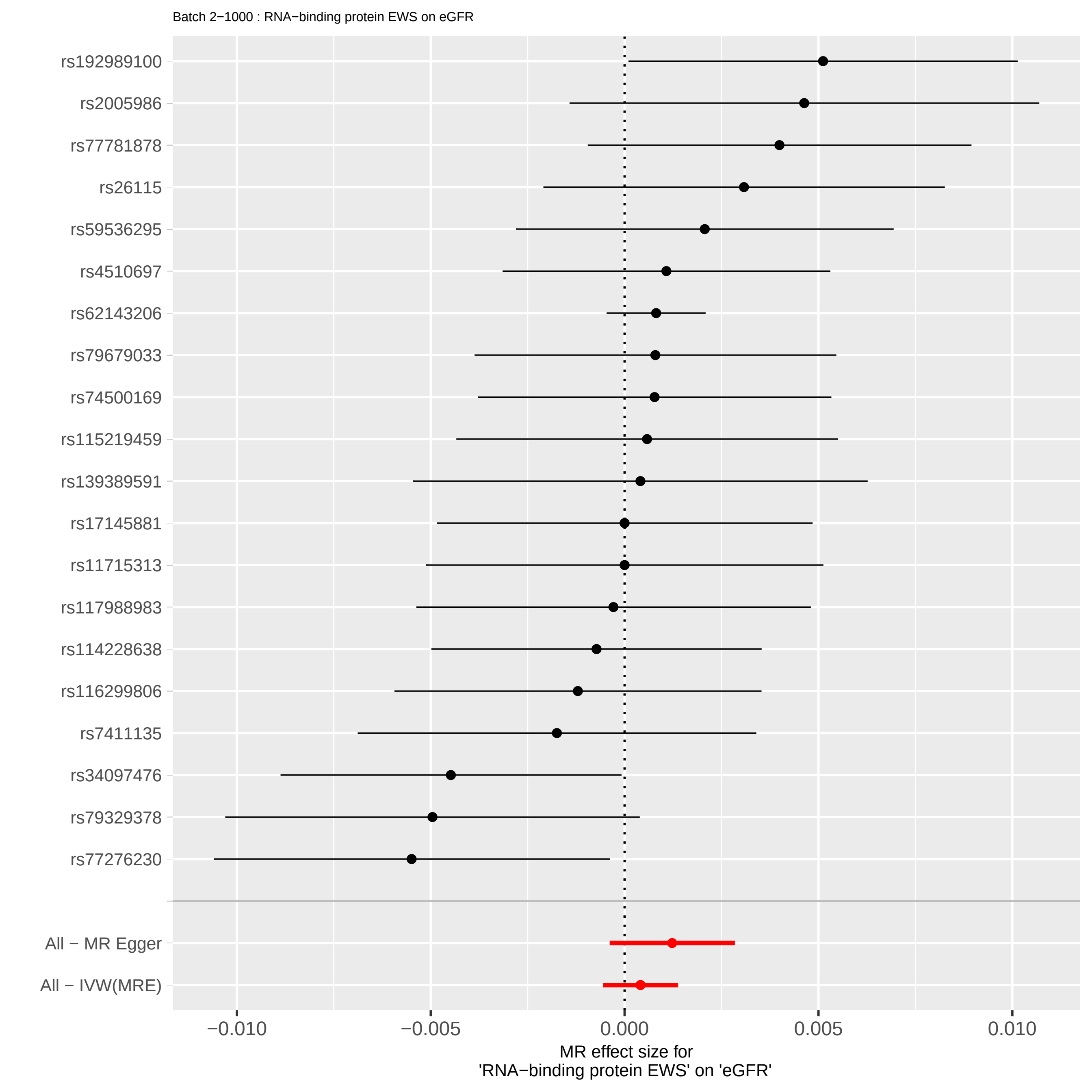

Batch 2−1000 : RNA−binding protein EWS on eGFR
rs192989100
rs2005986
rs77781878
rs26115
rs59536295
rs4510697
rs62143206
rs79679033
rs74500169
rs115219459
rs139389591
rs17145881
rs11715313
rs117988983
rs114228638
rs116299806
rs7411135
rs34097476
rs79329378
rs77276230
All − MR Egger
All − IVW(MRE)
−0.010
−0.005
0.000
0.005
0.010
MR effect size for
'RNA−binding protein EWS' on 'eGFR'

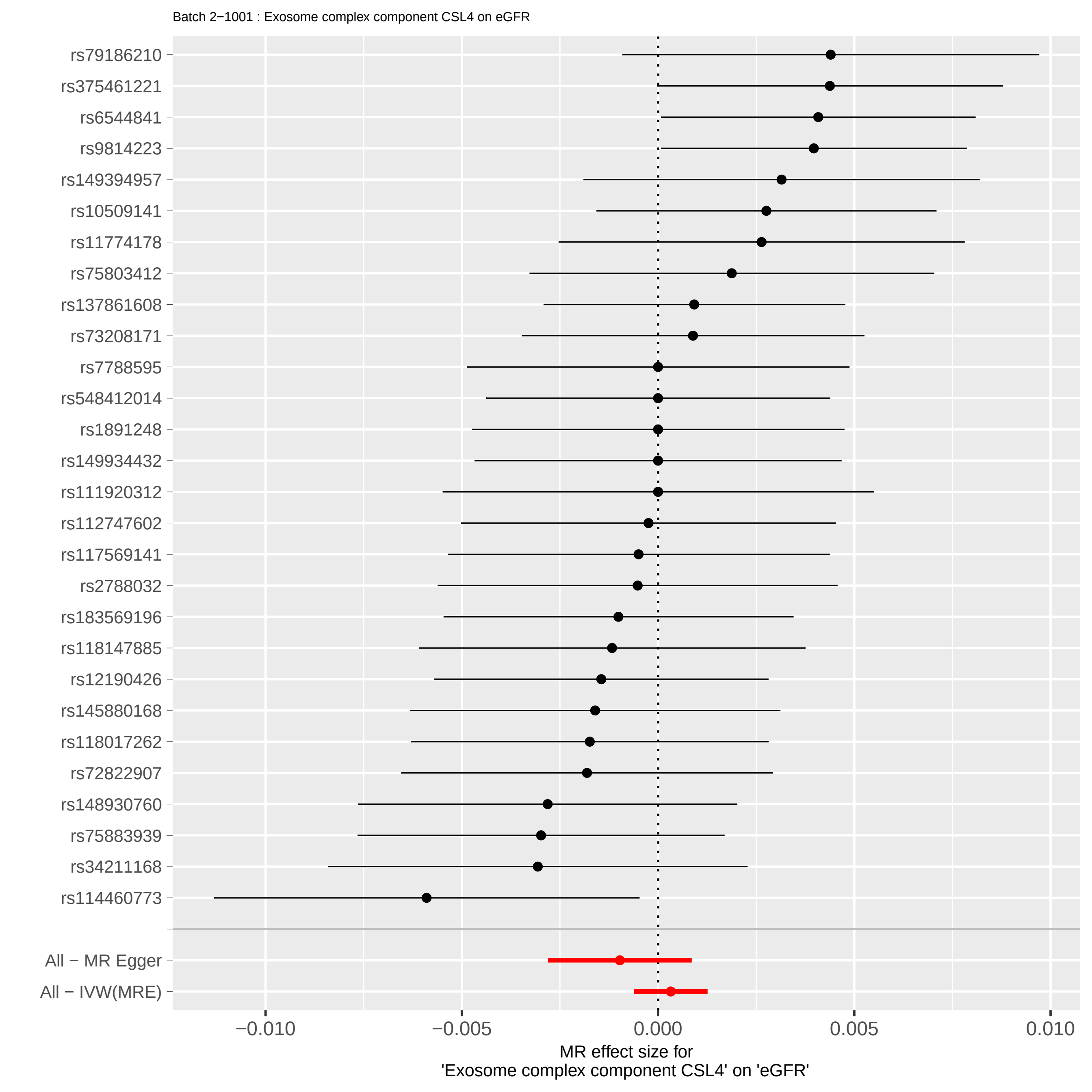

Batch 2−1001 : Exosome complex component CSL4 on eGFR
rs79186210
rs375461221
rs6544841
rs9814223
rs149394957
rs10509141
rs11774178
rs75803412
rs137861608
rs73208171
rs7788595
rs548412014
rs1891248
rs149934432
rs111920312
rs112747602
rs117569141
rs2788032
rs183569196
rs118147885
rs12190426
rs145880168
rs118017262
rs72822907
rs148930760
rs75883939
rs34211168
rs114460773
All − MR Egger
All − IVW(MRE)
−0.010
−0.005
0.000
0.005
0.010
MR effect size for
'Exosome complex component CSL4' on 'eGFR'

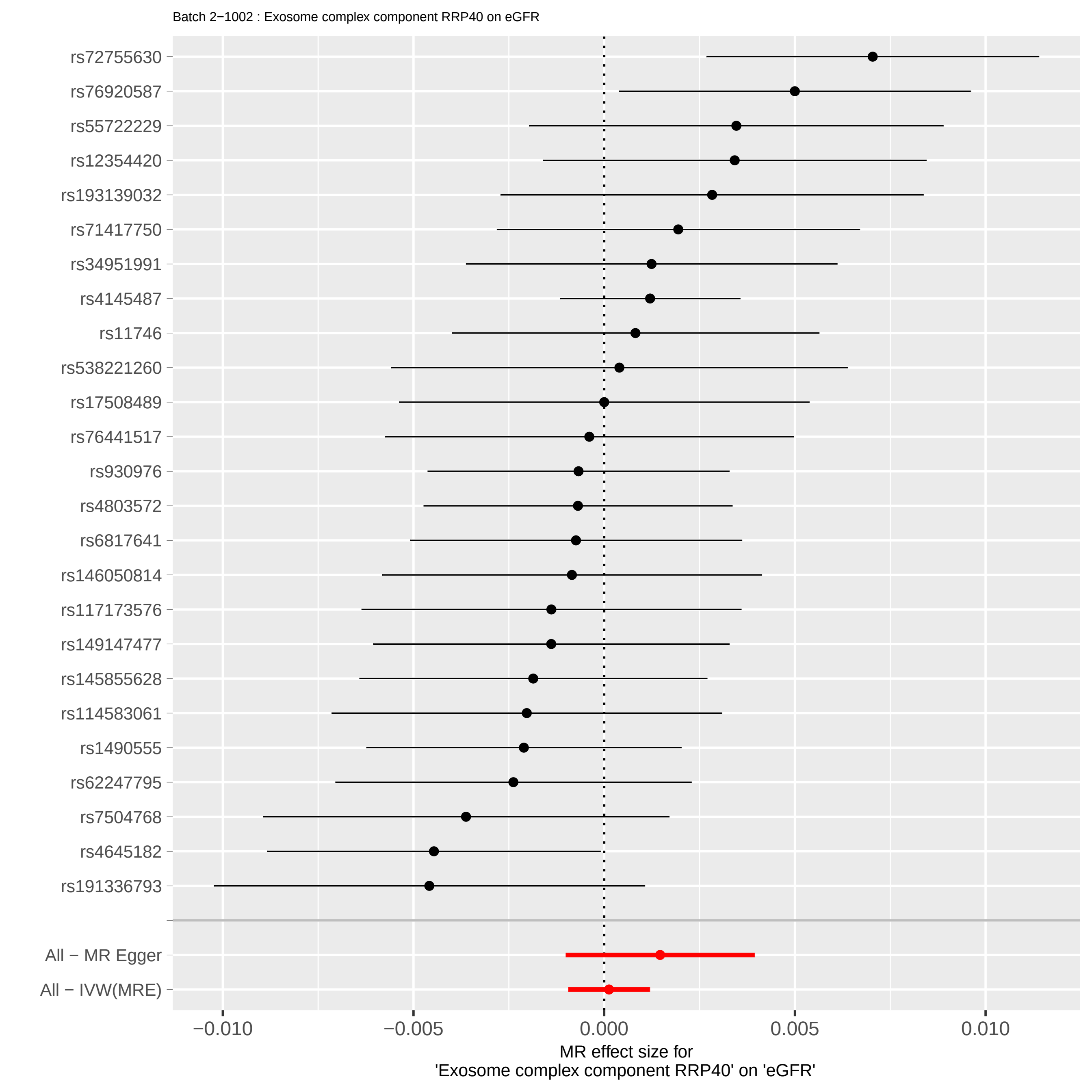
Batch 2−1002 : Exosome complex component RRP40 on eGFR
rs72755630
rs76920587
rs55722229
rs12354420
rs193139032
rs71417750
rs34951991
rs4145487
rs11746
rs538221260
rs17508489
rs76441517
rs930976
rs4803572
rs6817641
rs146050814
rs117173576
rs149147477
rs145855628
rs114583061
rs1490555
rs62247795
rs7504768
rs4645182
rs191336793
All − MR Egger
All − IVW(MRE)
−0.010
−0.005
0.000
0.005
0.010
MR effect size for
'Exosome complex component RRP40' on 'eGFR'

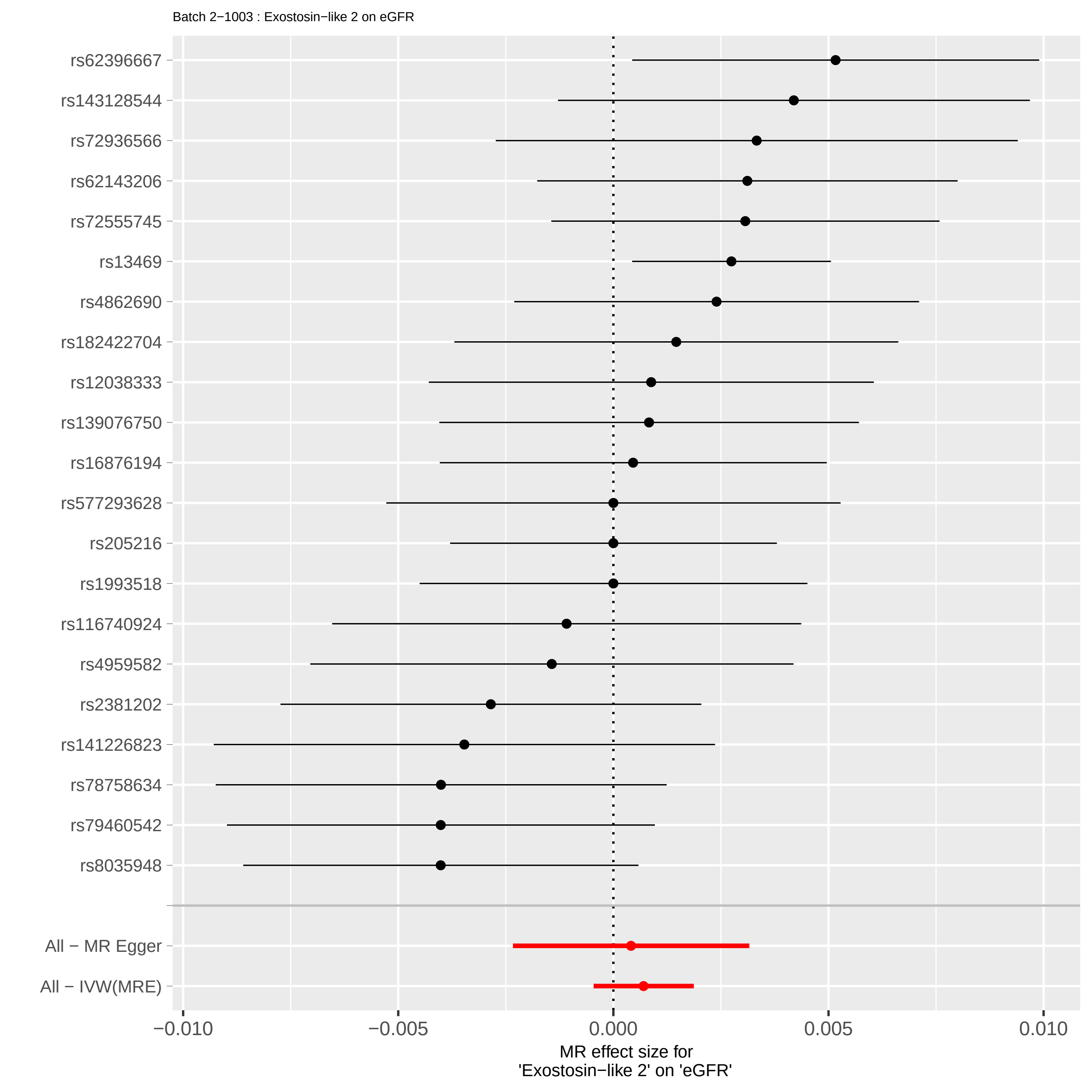

Batch 2−1003 : Exostosin−like 2 on eGFR
rs62396667
rs143128544
rs72936566
rs62143206
rs72555745
rs13469
rs4862690
rs182422704
rs12038333
rs139076750
rs16876194
rs577293628
rs205216
rs1993518
rs116740924
rs4959582
rs2381202
rs141226823
rs78758634
rs79460542
rs8035948
All − MR Egger
All − IVW(MRE)
−0.010
−0.005
0.000
0.005
0.010
MR effect size for
'Exostosin−like 2' on 'eGFR'

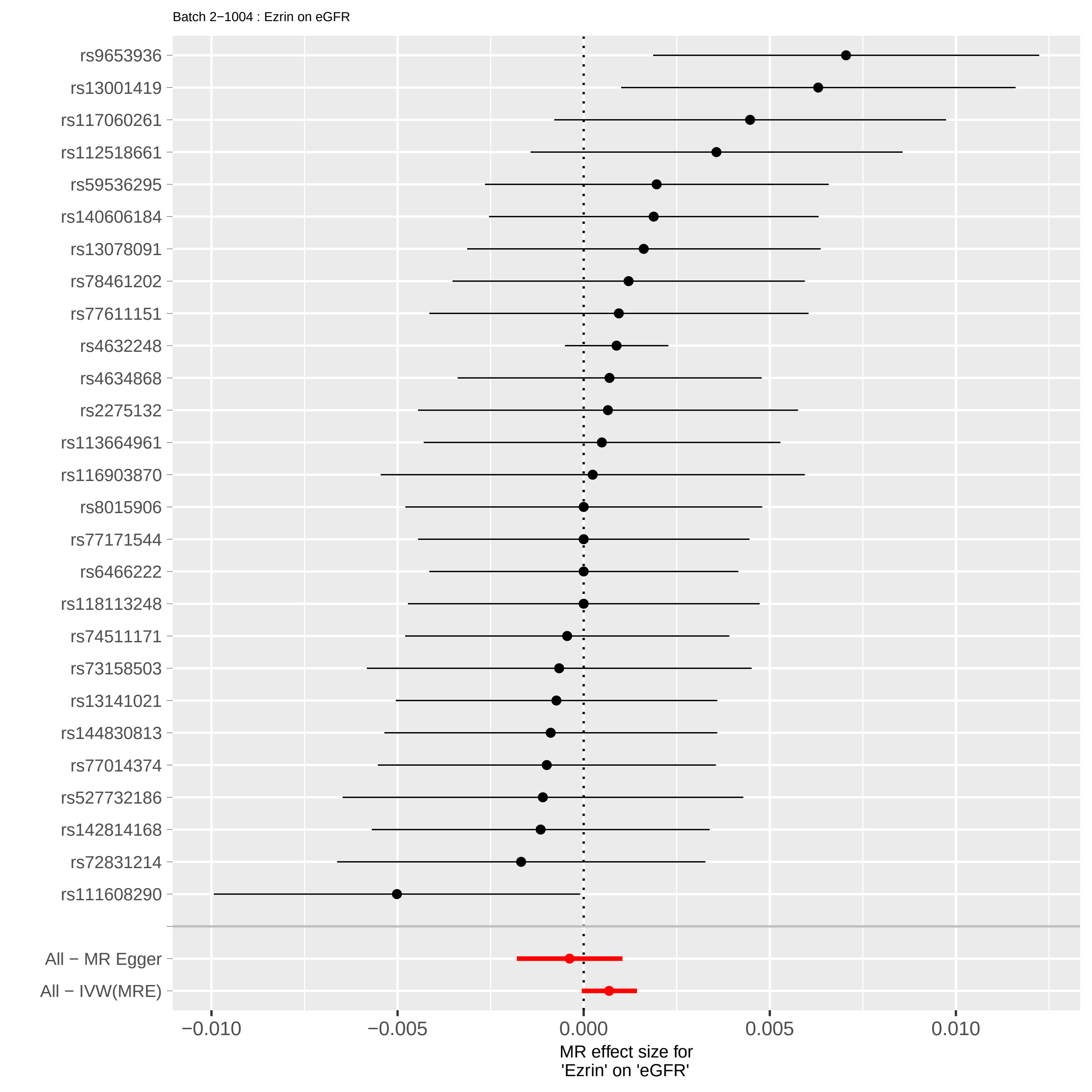

Batch 2−1004 : Ezrin on eGFR
rs9653936
rs13001419
rs117060261
rs112518661
rs59536295
rs140606184
rs13078091
rs78461202
rs77611151
rs4632248
rs4634868
rs2275132
rs113664961
rs116903870
rs8015906
rs77171544
rs6466222
rs118113248
rs74511171
rs73158503
rs13141021
rs144830813
rs77014374
rs527732186
rs142814168
rs72831214
rs111608290
All − MR Egger
All − IVW(MRE)
−0.010
−0.005
0.000
0.005
0.010
MR effect size for
'Ezrin' on 'eGFR'

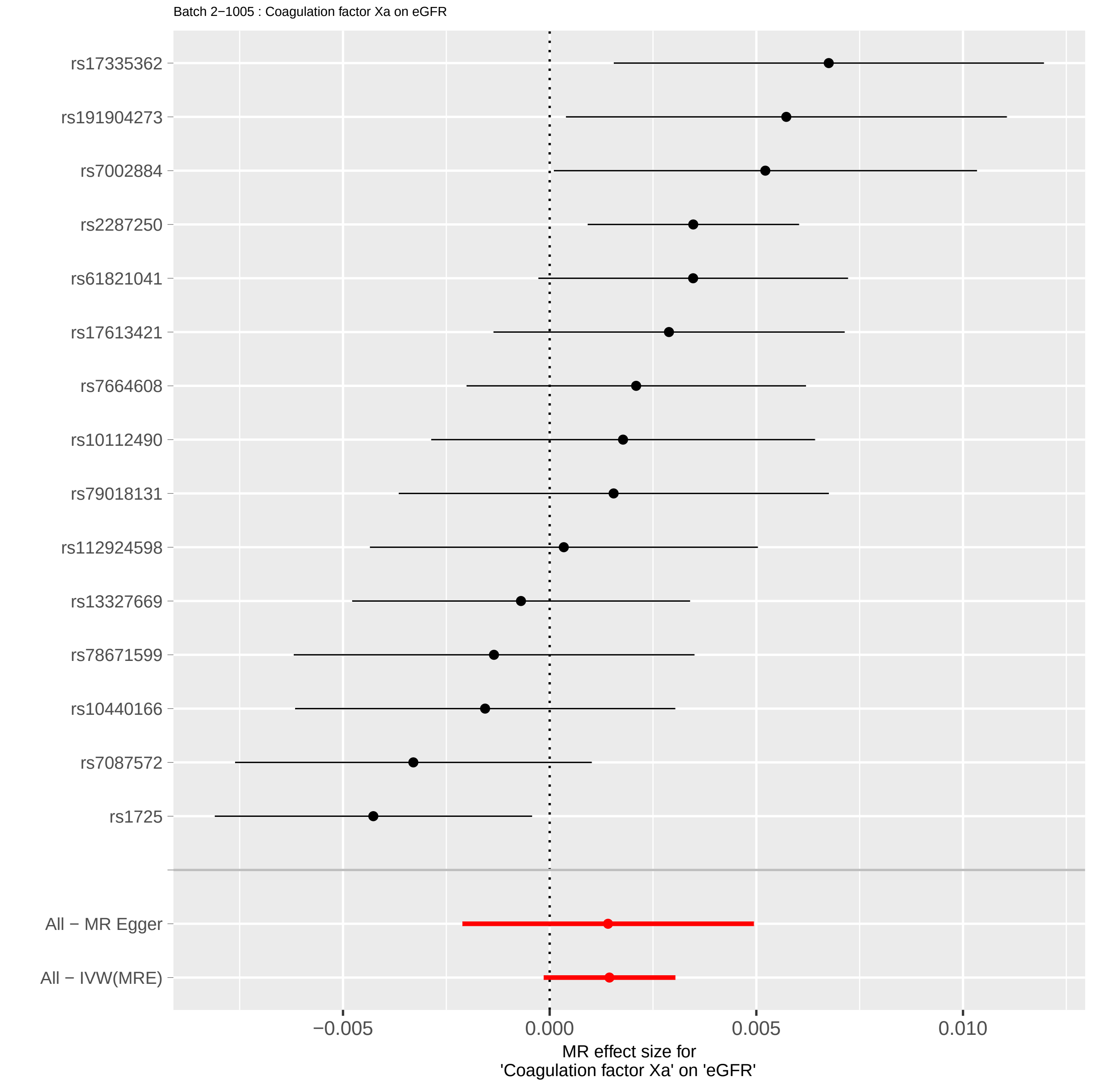

Batch 2−1005 : Coagulation factor Xa on eGFR
rs17335362
rs191904273
rs7002884
rs2287250
rs61821041
rs17613421
rs7664608
rs10112490
rs79018131
rs112924598
rs13327669
rs78671599
rs10440166
rs7087572
rs1725
All − MR Egger
All − IVW(MRE)
−0.005
0.000
0.005
0.010
MR effect size for
'Coagulation factor Xa' on 'eGFR'

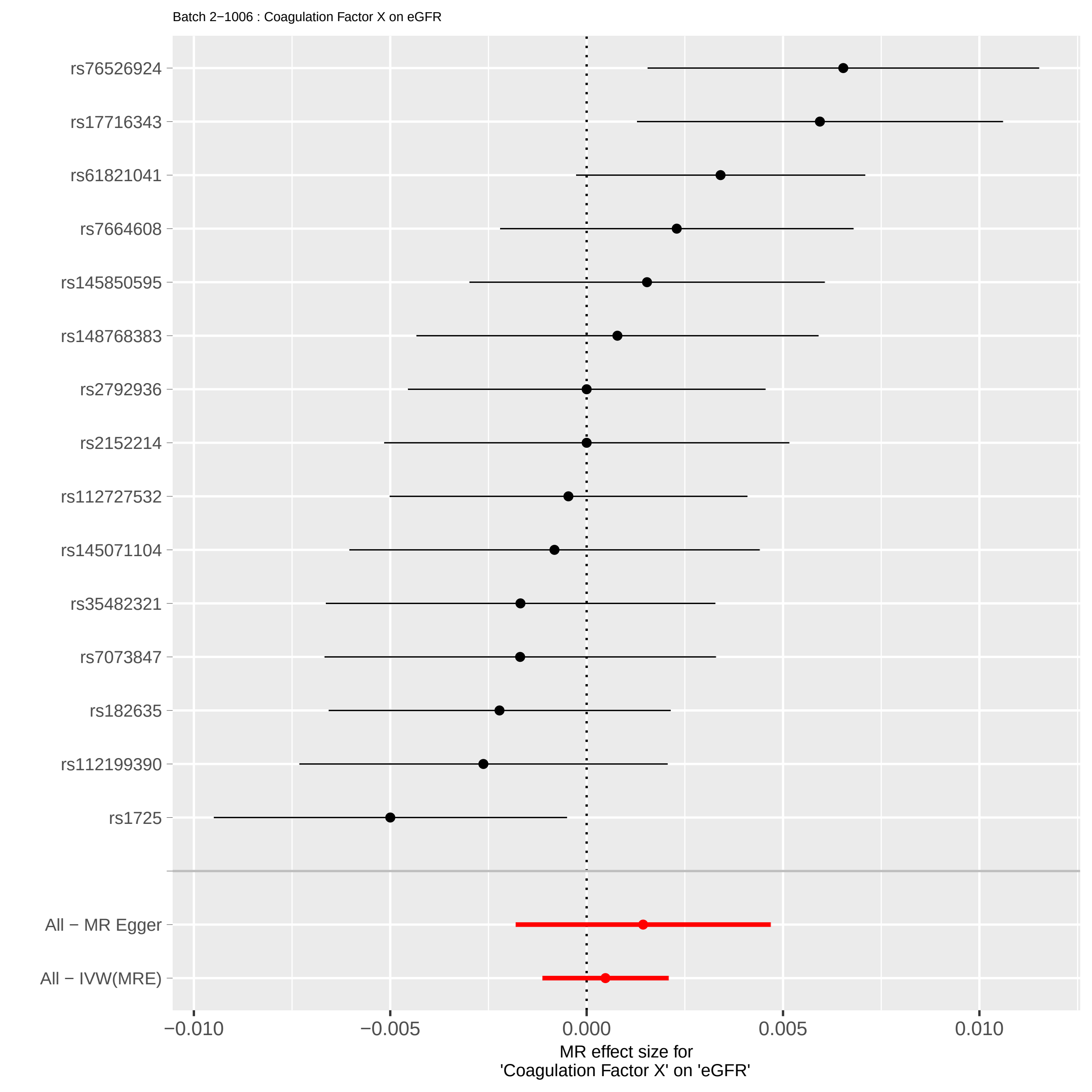

Batch 2−1006 : Coagulation Factor X on eGFR
rs76526924
rs17716343
rs61821041
rs7664608
rs145850595
rs148768383
rs2792936
rs2152214
rs112727532
rs145071104
rs35482321
rs7073847
rs182635
rs112199390
rs1725
All − MR Egger
All − IVW(MRE)
−0.010
−0.005
0.000
0.005
0.010
MR effect size for
'Coagulation Factor X' on 'eGFR'

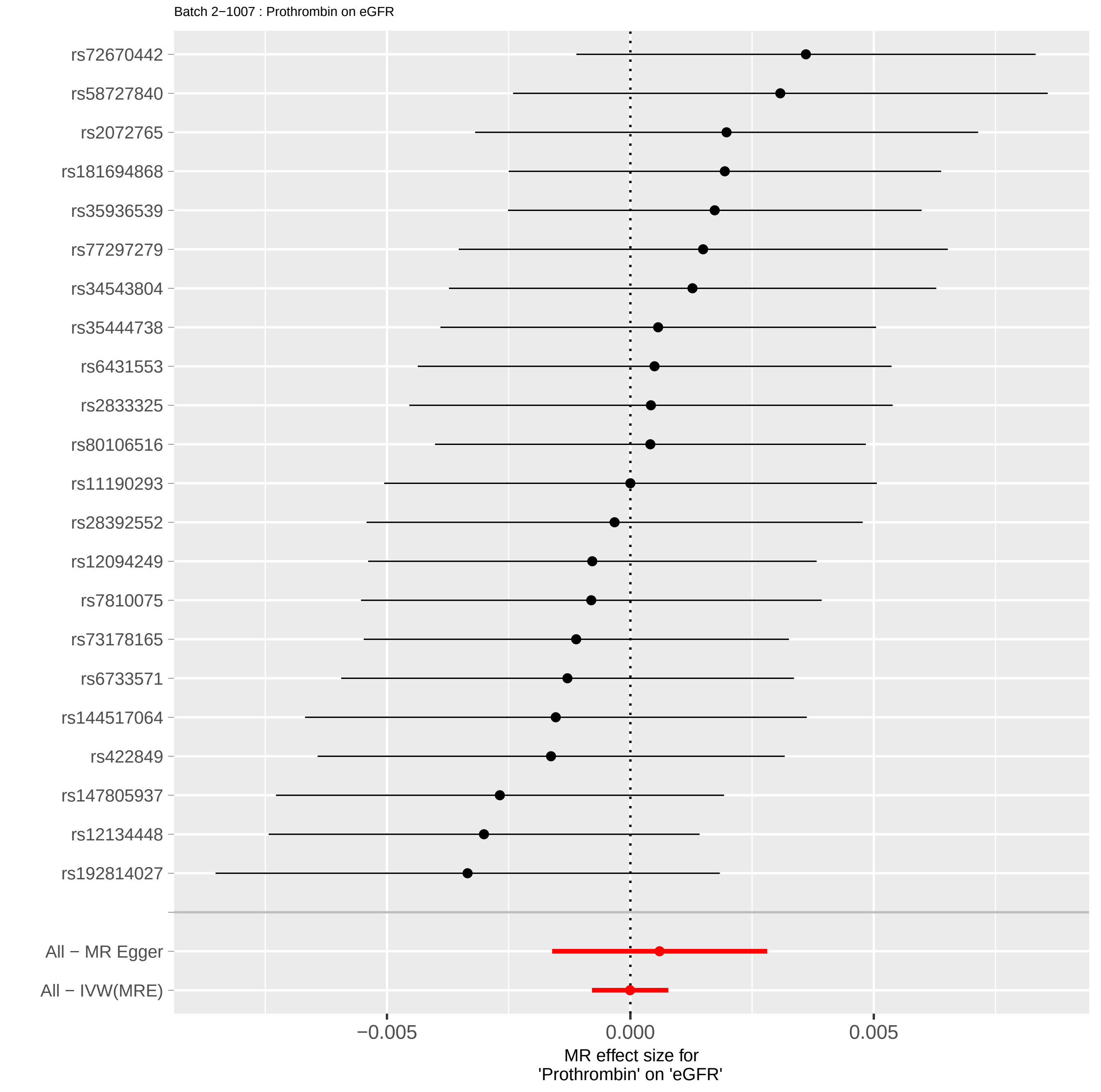

Batch 2−1007 : Prothrombin on eGFR
rs72670442
rs58727840
rs2072765
rs181694868
rs35936539
rs77297279
rs34543804
rs35444738
rs6431553
rs2833325
rs80106516
rs11190293
rs28392552
rs12094249
rs7810075
rs73178165
rs6733571
rs144517064
rs422849
rs147805937
rs12134448
rs192814027
All − MR Egger
All − IVW(MRE)
−0.005
0.000
0.005
MR effect size for
'Prothrombin' on 'eGFR'

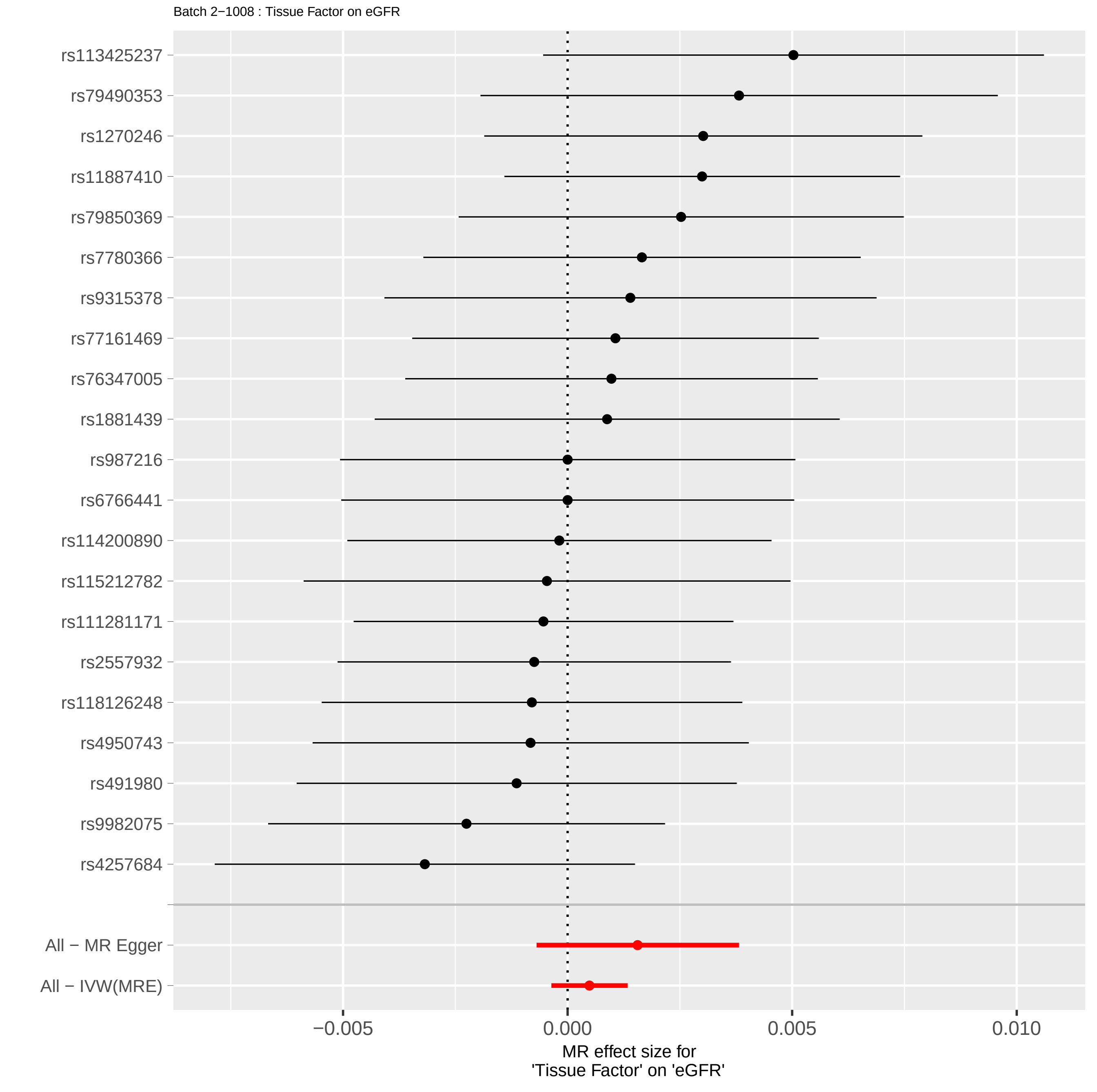

Batch 2−1008 : Tissue Factor on eGFR
rs113425237
rs79490353
rs1270246
rs11887410
rs79850369
rs7780366
rs9315378
rs77161469
rs76347005
rs1881439
rs987216
rs6766441
rs114200890
rs115212782
rs111281171
rs2557932
rs118126248
rs4950743
rs491980
rs9982075
rs4257684
All − MR Egger
All − IVW(MRE)
−0.005
0.000
0.005
0.010
MR effect size for
'Tissue Factor' on 'eGFR'

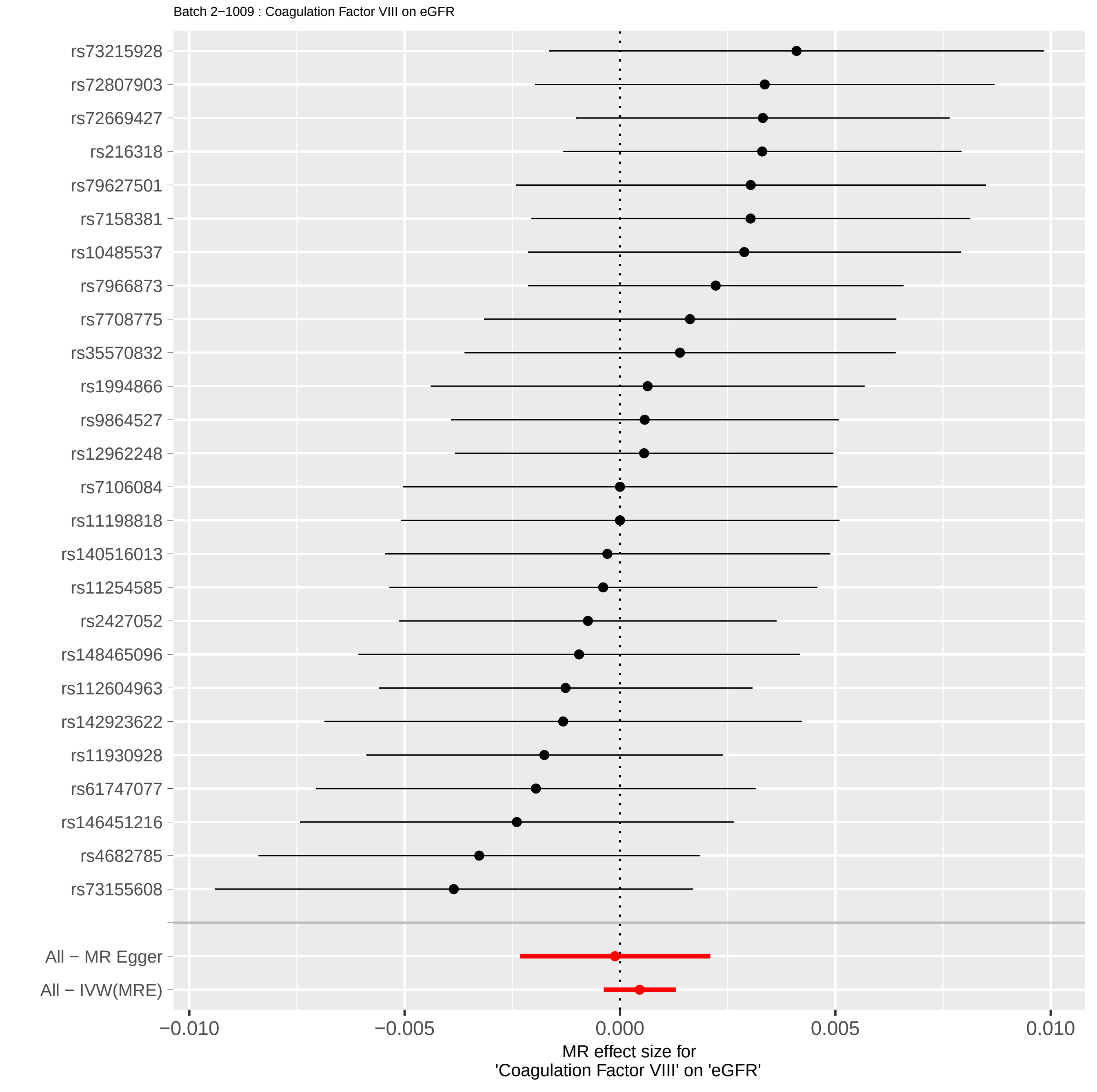
Batch 2−1009 : Coagulation Factor VIII on eGFR
rs73215928
rs72807903
rs72669427
rs216318
rs79627501
rs7158381
rs10485537
rs7966873
rs7708775
rs35570832
rs1994866
rs9864527
rs12962248
rs7106084
rs11198818
rs140516013
rs11254585
rs2427052
rs148465096
rs112604963
rs142923622
rs11930928
rs61747077
rs146451216
rs4682785
rs73155608
All − MR Egger
All − IVW(MRE)
−0.010
−0.005
0.000
0.005
0.010
MR effect size for
'Coagulation Factor VIII' on 'eGFR'

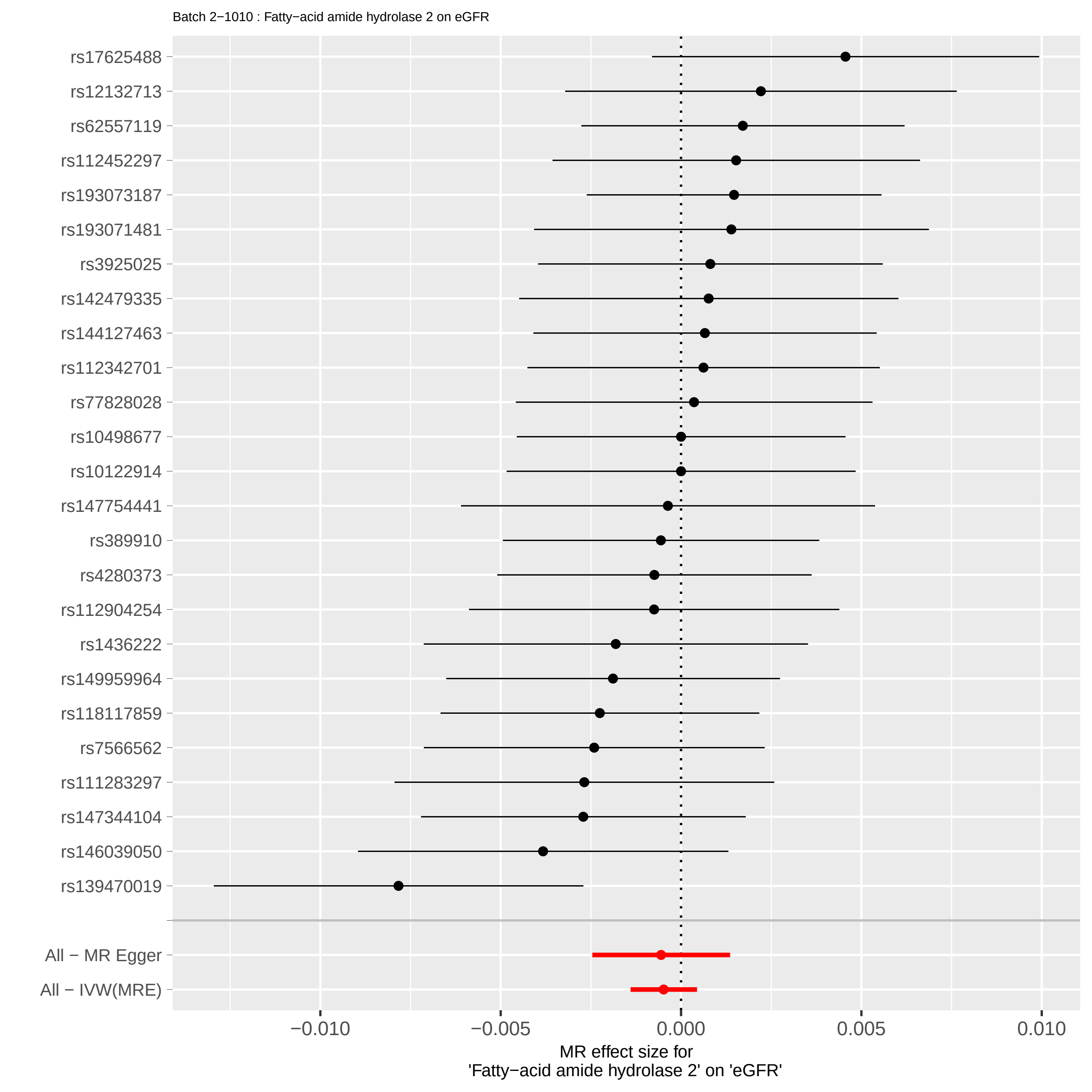

Batch 2−1010 : Fatty−acid amide hydrolase 2 on eGFR
rs17625488
rs12132713
rs62557119
rs112452297
rs193073187
rs193071481
rs3925025
rs142479335
rs144127463
rs112342701
rs77828028
rs10498677
rs10122914
rs147754441
rs389910
rs4280373
rs112904254
rs1436222
rs149959964
rs118117859
rs7566562
rs111283297
rs147344104
rs146039050
rs139470019
All − MR Egger
All − IVW(MRE)
−0.010
−0.005
0.000
0.005
0.010
MR effect size for
'Fatty−acid amide hydrolase 2' on 'eGFR'

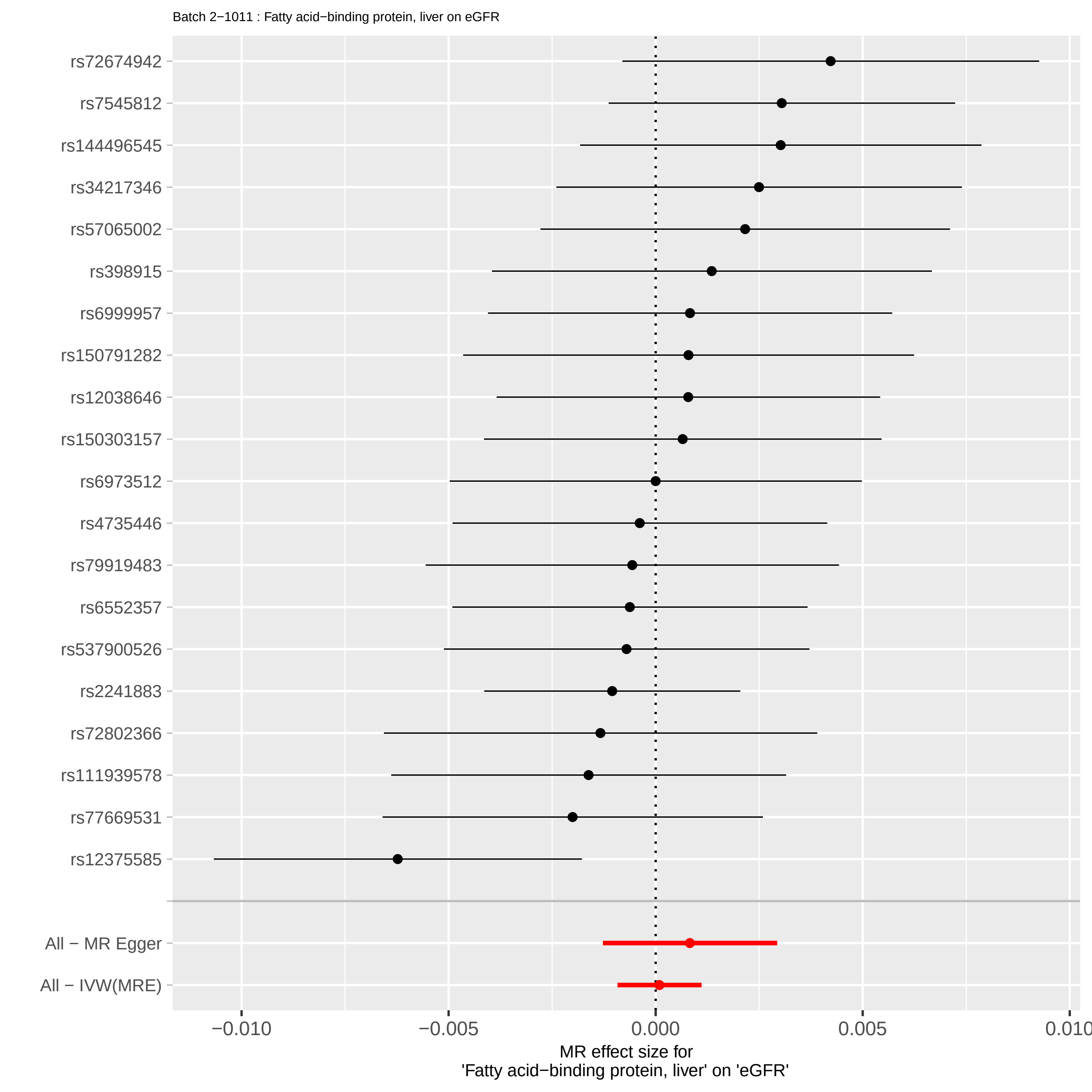

Batch 2−1011 : Fatty acid−binding protein, liver on eGFR
rs72674942
rs7545812
rs144496545
rs34217346
rs57065002
rs398915
rs6999957
rs150791282
rs12038646
rs150303157
rs6973512
rs4735446
rs79919483
rs6552357
rs537900526
rs2241883
rs72802366
rs111939578
rs77669531
rs12375585
All − MR Egger
All − IVW(MRE)
−0.010
−0.005
0.000
0.005
0.010
MR effect size for
'Fatty acid−binding protein, liver' on 'eGFR'

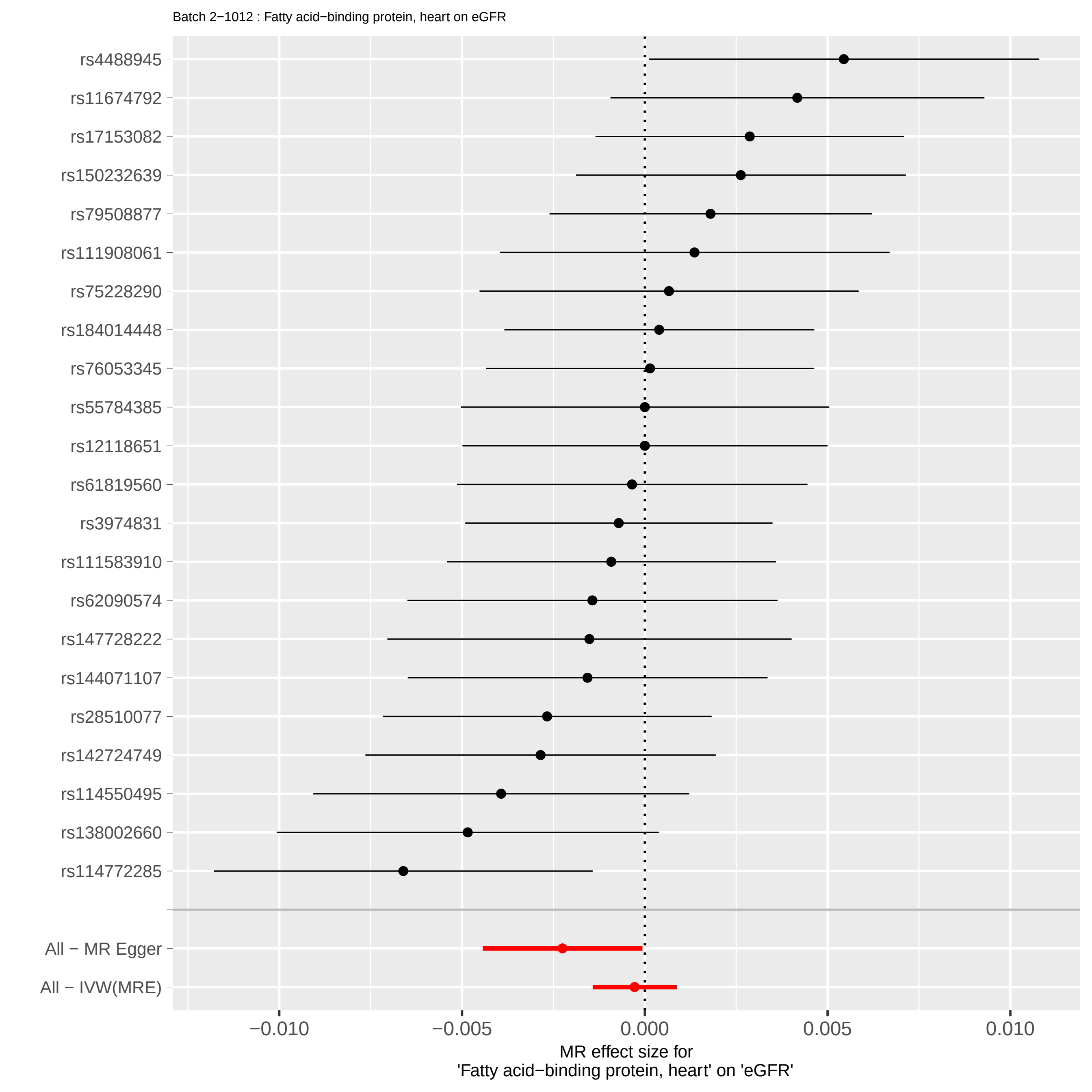

Batch 2−1012 : Fatty acid−binding protein, heart on eGFR
rs4488945
rs11674792
rs17153082
rs150232639
rs79508877
rs111908061
rs75228290
rs184014448
rs76053345
rs55784385
rs12118651
rs61819560
rs3974831
rs111583910
rs62090574
rs147728222
rs144071107
rs28510077
rs142724749
rs114550495
rs138002660
rs114772285
All − MR Egger
All − IVW(MRE)
−0.010
−0.005
0.000
0.005
0.010
MR effect size for
'Fatty acid−binding protein, heart' on 'eGFR'

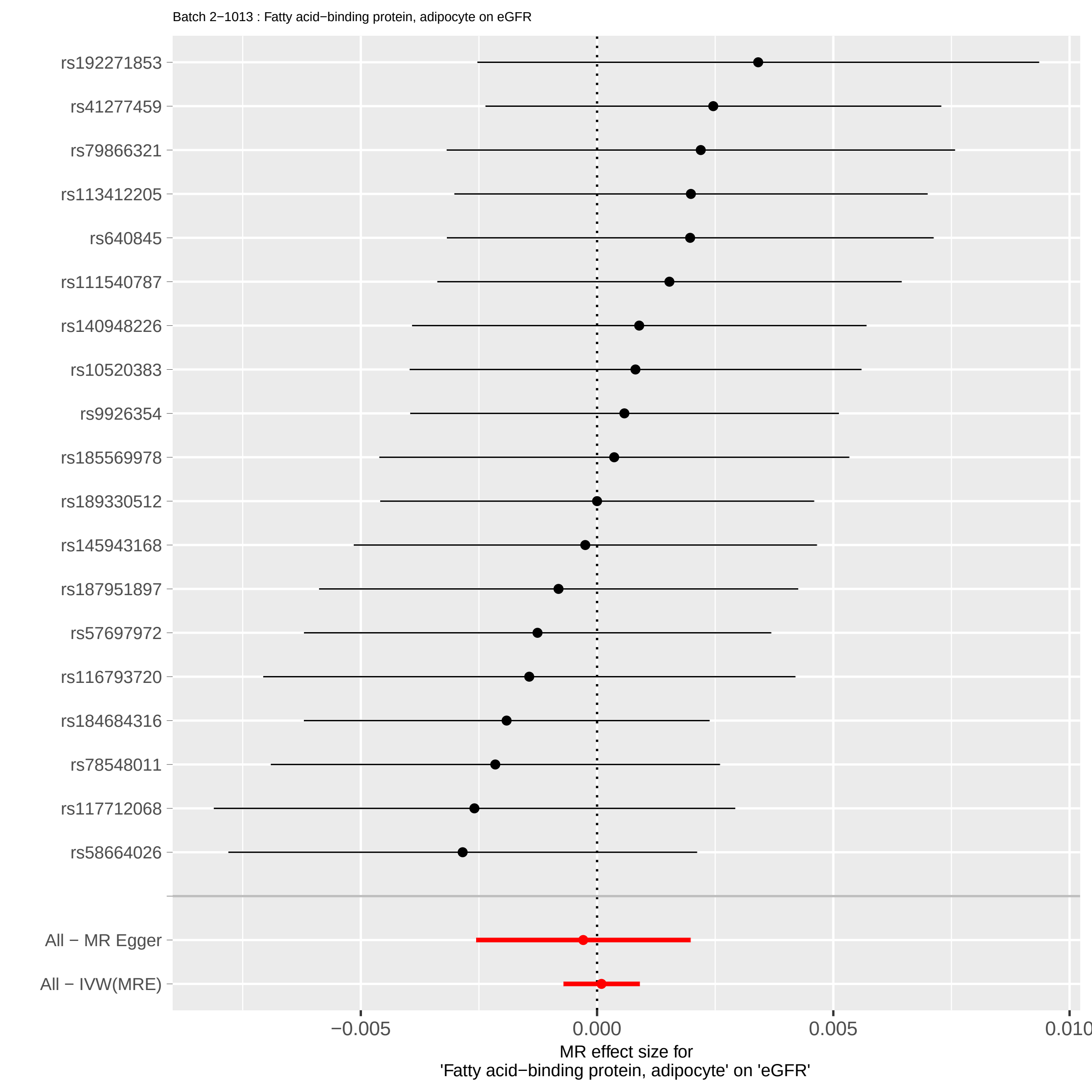

Batch 2−1013 : Fatty acid−binding protein, adipocyte on eGFR
rs192271853
rs41277459
rs79866321
rs113412205
rs640845
rs111540787
rs140948226
rs10520383
rs9926354
rs185569978
rs189330512
rs145943168
rs187951897
rs57697972
rs116793720
rs184684316
rs78548011
rs117712068
rs58664026
All − MR Egger
All − IVW(MRE)
−0.005
0.000
0.005
0.010
MR effect size for
'Fatty acid−binding protein, adipocyte' on 'eGFR'

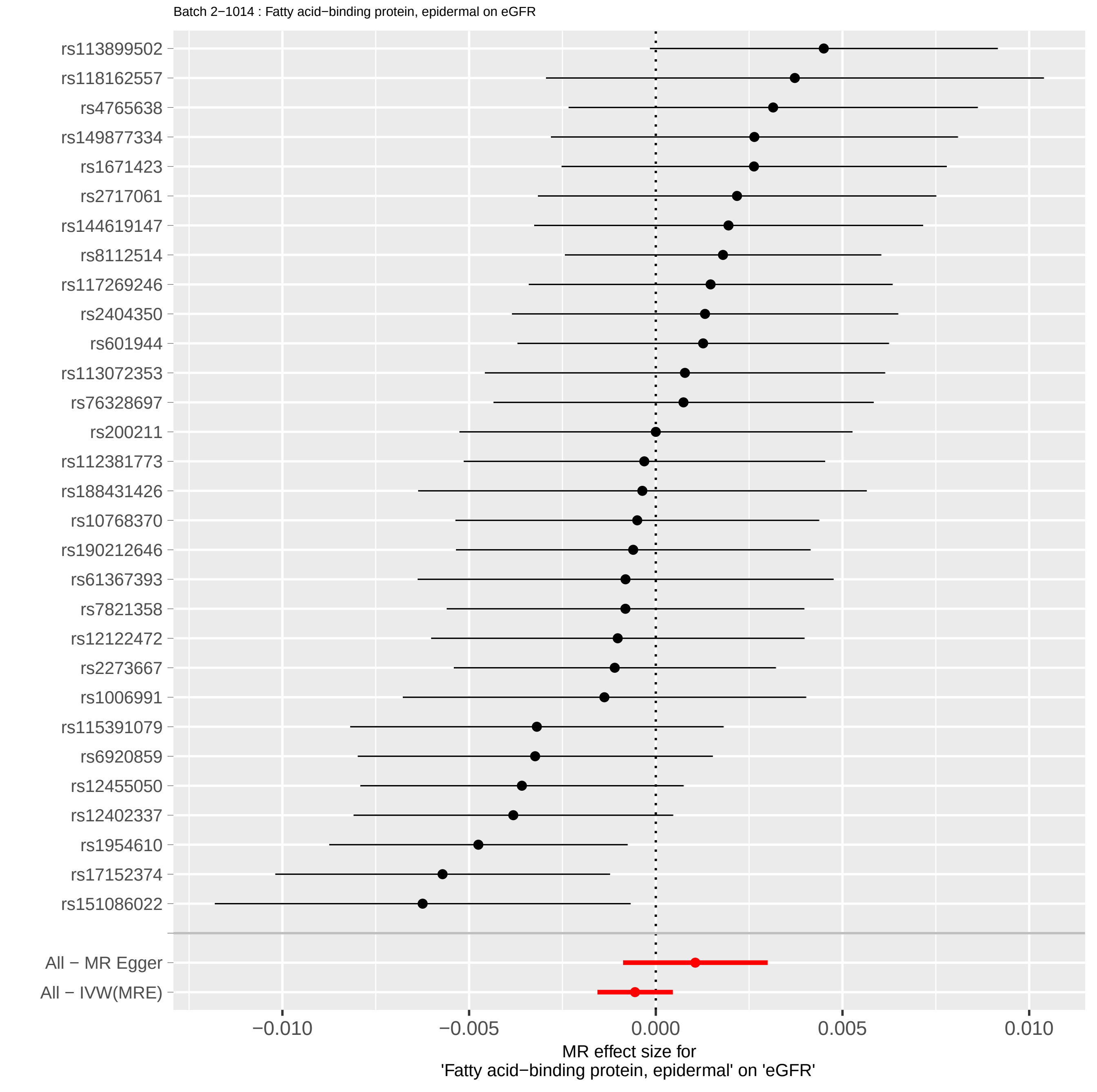

Batch 2−1014 : Fatty acid−binding protein, epidermal on eGFR
rs113899502
rs118162557
rs4765638
rs149877334
rs1671423
rs2717061
rs144619147
rs8112514
rs117269246
rs2404350
rs601944
rs113072353
rs76328697
rs200211
rs112381773
rs188431426
rs10768370
rs190212646
rs61367393
rs7821358
rs12122472
rs2273667
rs1006991
rs115391079
rs6920859
rs12455050
rs12402337
rs1954610
rs17152374
rs151086022
All − MR Egger
All − IVW(MRE)
−0.010
−0.005
0.000
0.005
0.010
MR effect size for
'Fatty acid−binding protein, epidermal' on 'eGFR'

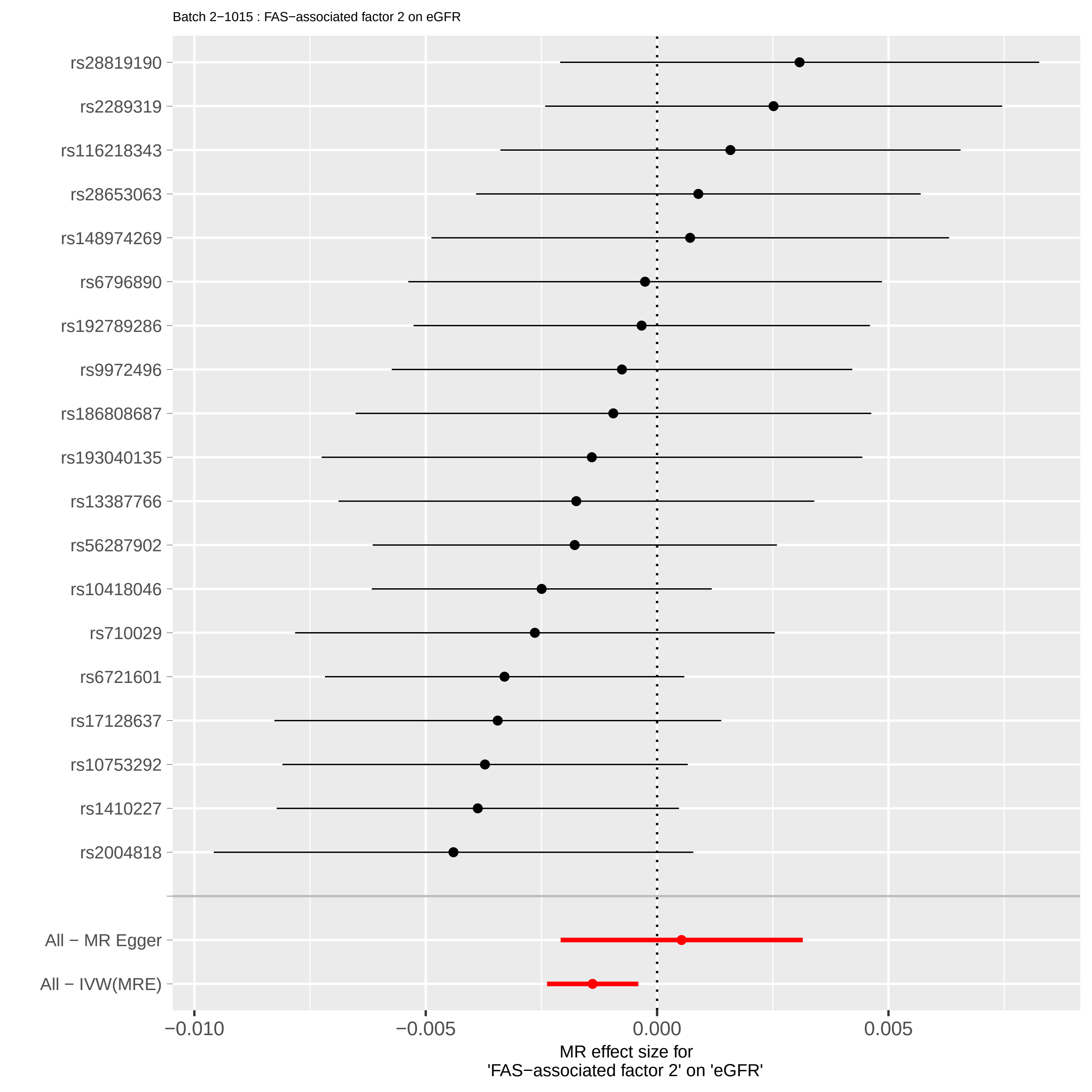

Batch 2−1015 : FAS−associated factor 2 on eGFR
rs28819190
rs2289319
rs116218343
rs28653063
rs148974269
rs6796890
rs192789286
rs9972496
rs186808687
rs193040135
rs13387766
rs56287902
rs10418046
rs710029
rs6721601
rs17128637
rs10753292
rs1410227
rs2004818
All − MR Egger
All − IVW(MRE)
−0.010
−0.005
0.000
0.005
MR effect size for
'FAS−associated factor 2' on 'eGFR'

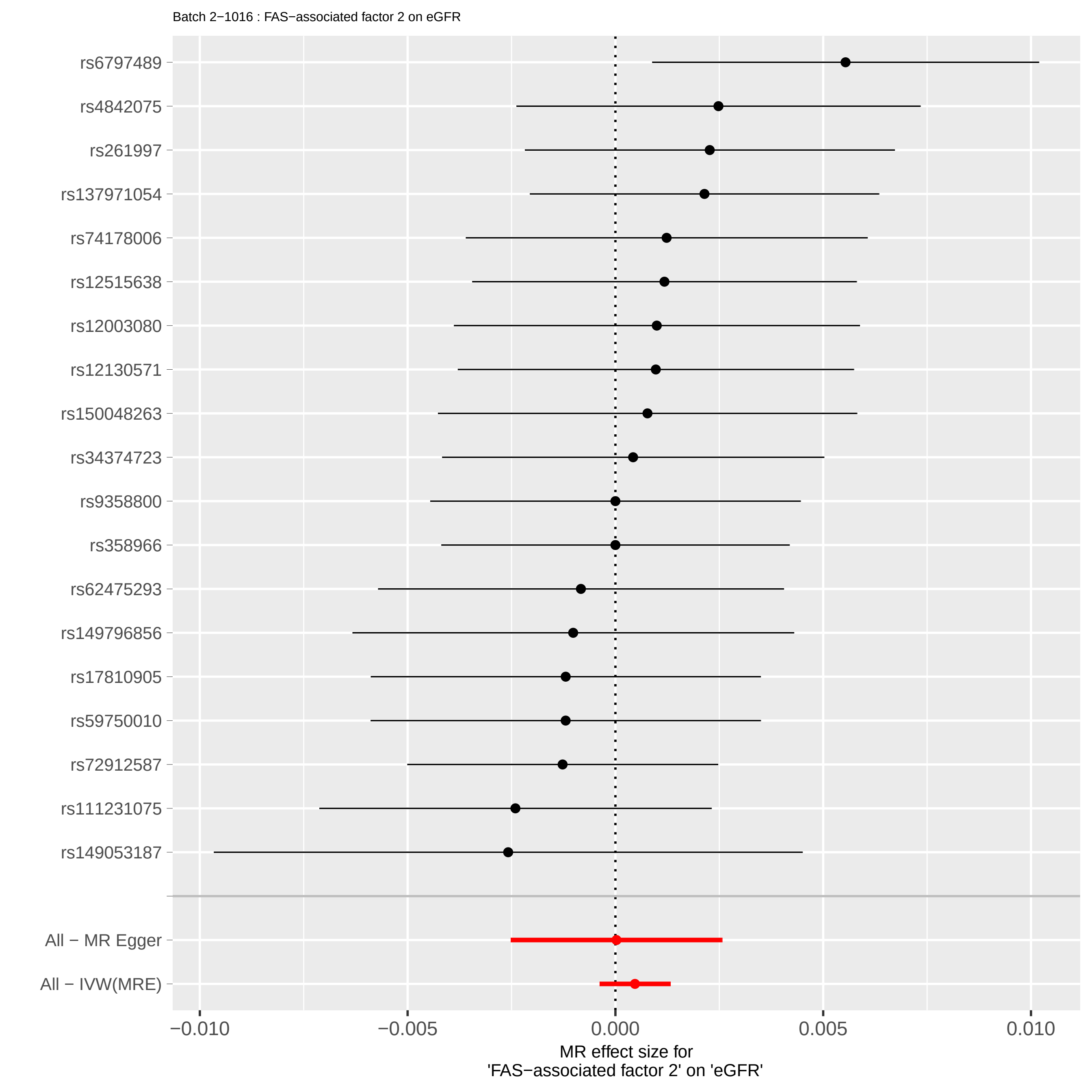
Batch 2−1016 : FAS−associated factor 2 on eGFR
rs6797489
rs4842075
rs261997
rs137971054
rs74178006
rs12515638
rs12003080
rs12130571
rs150048263
rs34374723
rs9358800
rs358966
rs62475293
rs149796856
rs17810905
rs59750010
rs72912587
rs111231075
rs149053187
All − MR Egger
All − IVW(MRE)
−0.010
−0.005
0.000
0.005
0.010
MR effect size for
'FAS−associated factor 2' on 'eGFR'

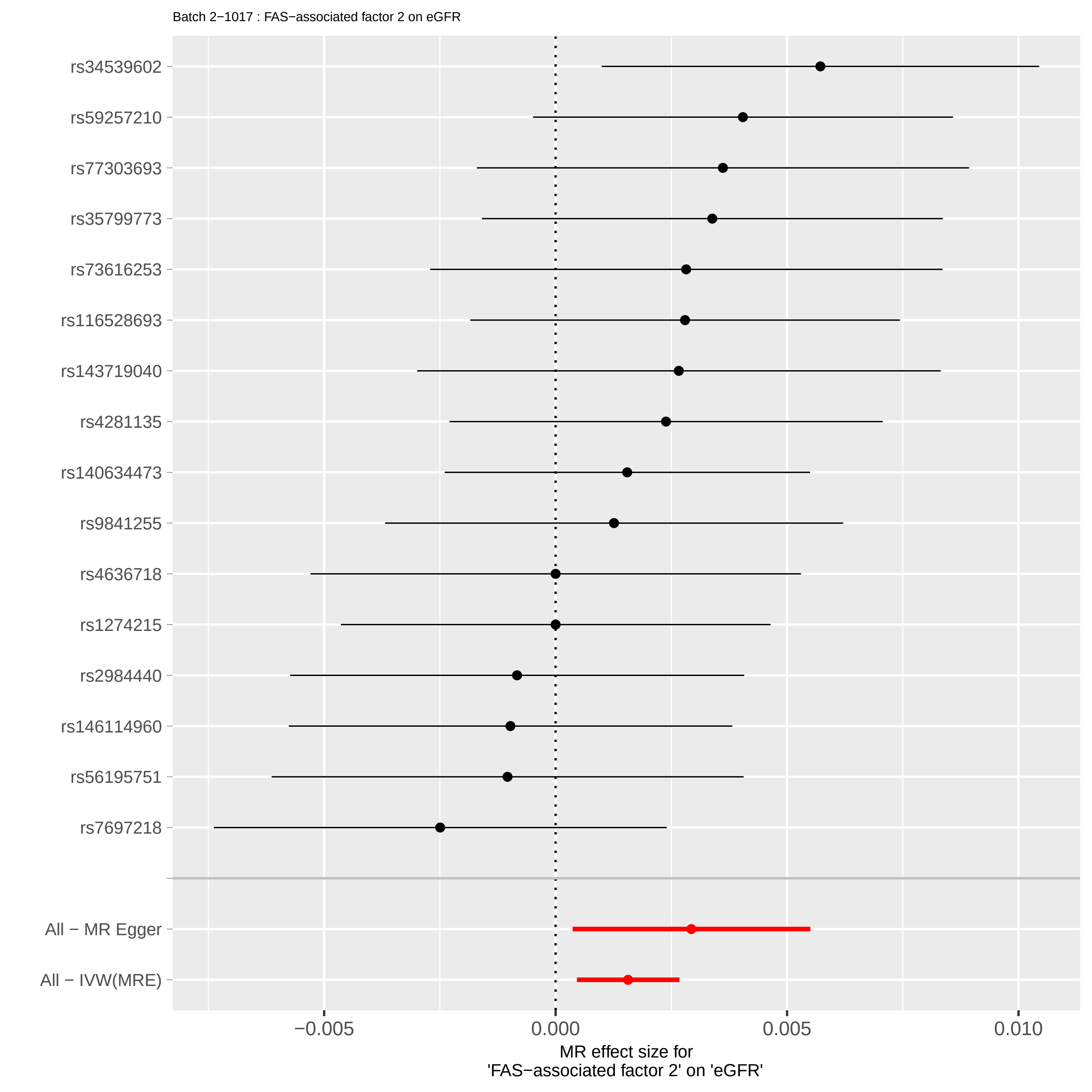
Batch 2−1017 : FAS−associated factor 2 on eGFR
rs34539602
rs59257210
rs77303693
rs35799773
rs73616253
rs116528693
rs143719040
rs4281135
rs140634473
rs9841255
rs4636718
rs1274215
rs2984440
rs146114960
rs56195751
rs7697218
All − MR Egger
All − IVW(MRE)
−0.005
0.000
0.005
0.010
MR effect size for
'FAS−associated factor 2' on 'eGFR'

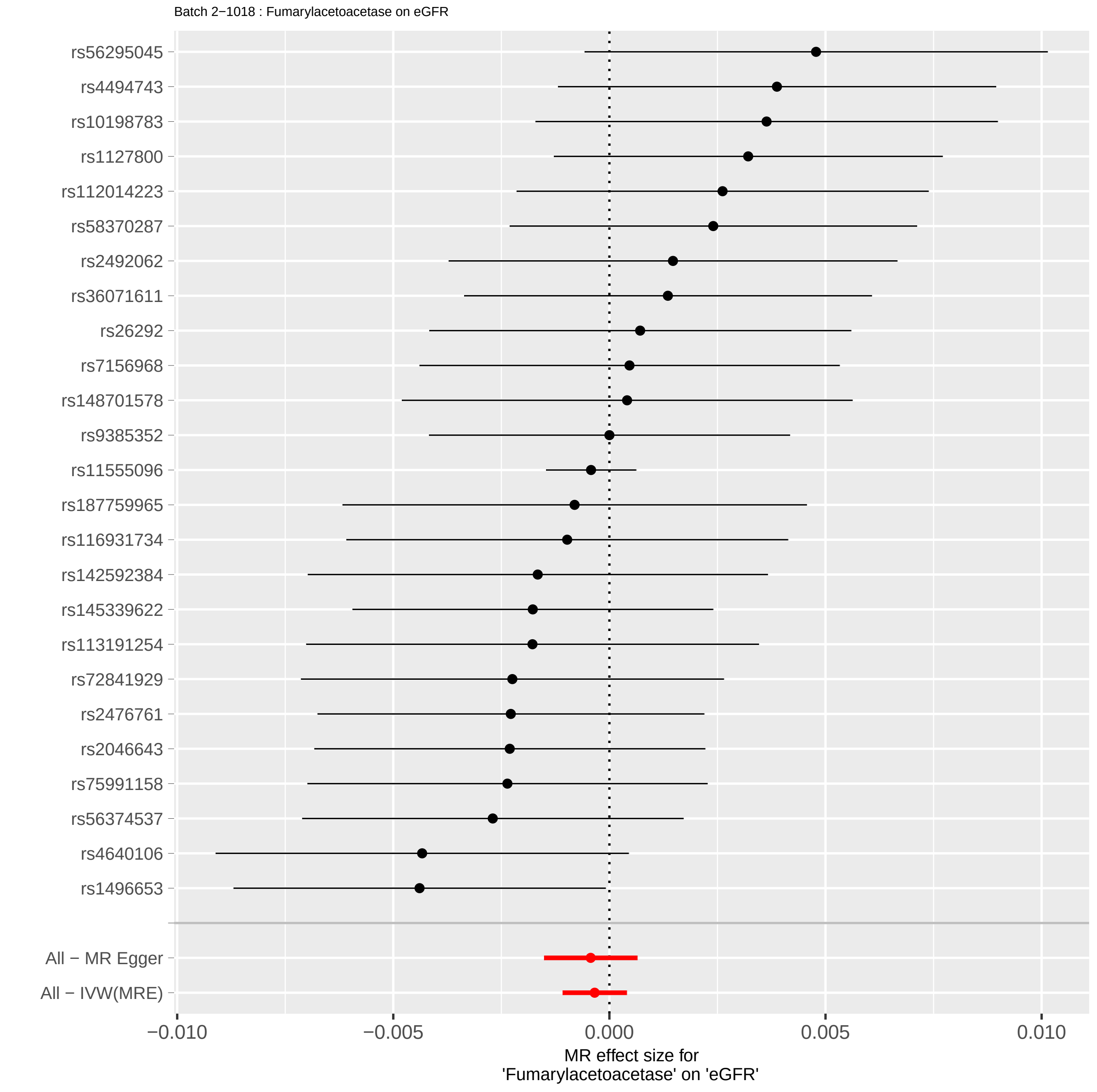

Batch 2−1018 : Fumarylacetoacetase on eGFR
rs56295045
rs4494743
rs10198783
rs1127800
rs112014223
rs58370287
rs2492062
rs36071611
rs26292
rs7156968
rs148701578
rs9385352
rs11555096
rs187759965
rs116931734
rs142592384
rs145339622
rs113191254
rs72841929
rs2476761
rs2046643
rs75991158
rs56374537
rs4640106
rs1496653
All − MR Egger
All − IVW(MRE)
−0.010
−0.005
0.000
0.005
0.010
MR effect size for
'Fumarylacetoacetase' on 'eGFR'

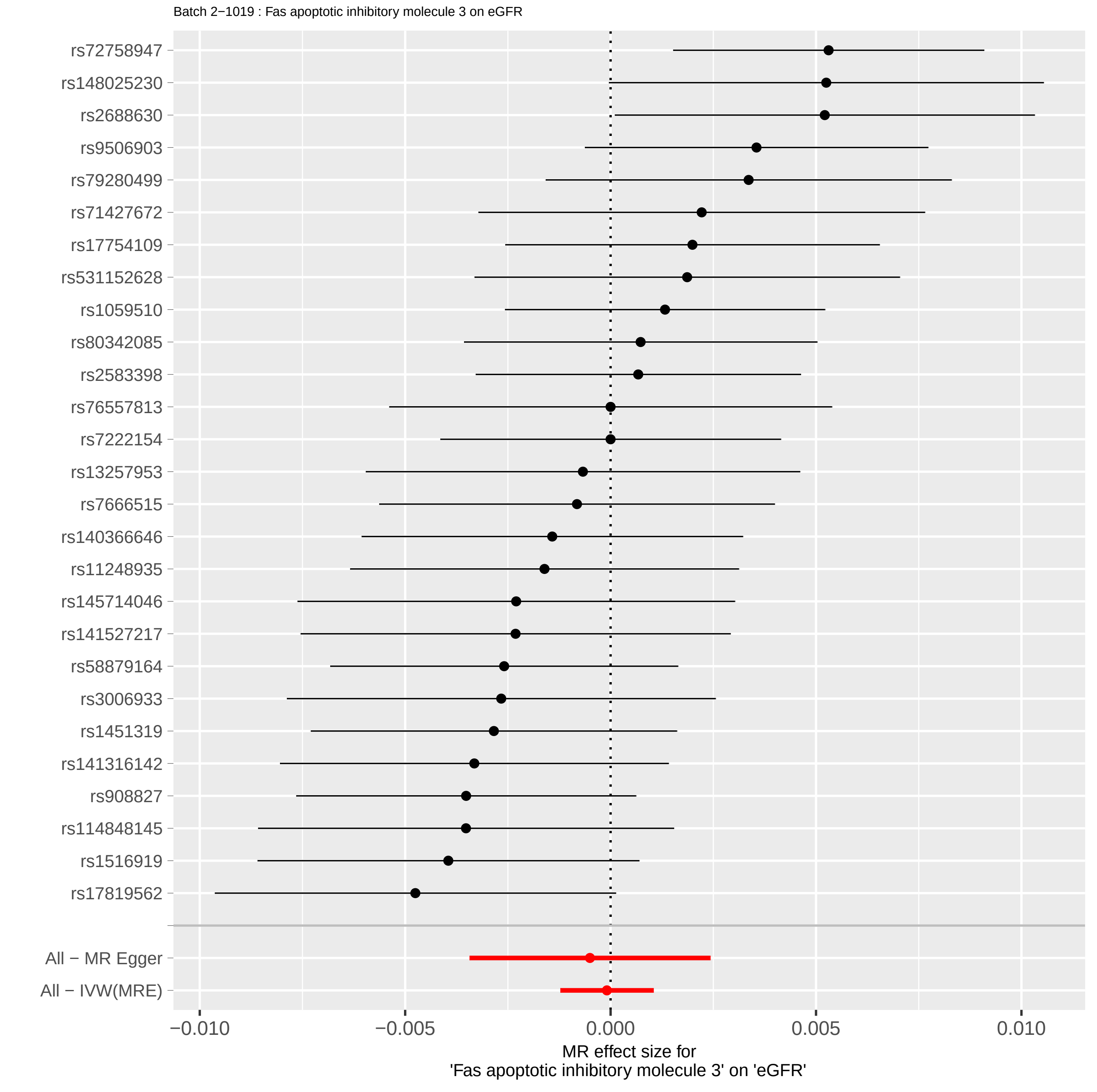

Batch 2−1019 : Fas apoptotic inhibitory molecule 3 on eGFR
rs72758947
rs148025230
rs2688630
rs9506903
rs79280499
rs71427672
rs17754109
rs531152628
rs1059510
rs80342085
rs2583398
rs76557813
rs7222154
rs13257953
rs7666515
rs140366646
rs11248935
rs145714046
rs141527217
rs58879164
rs3006933
rs1451319
rs141316142
rs908827
rs114848145
rs1516919
rs17819562
All − MR Egger
All − IVW(MRE)
−0.010
−0.005
0.000
0.005
0.010
MR effect size for
'Fas apoptotic inhibitory molecule 3' on 'eGFR'

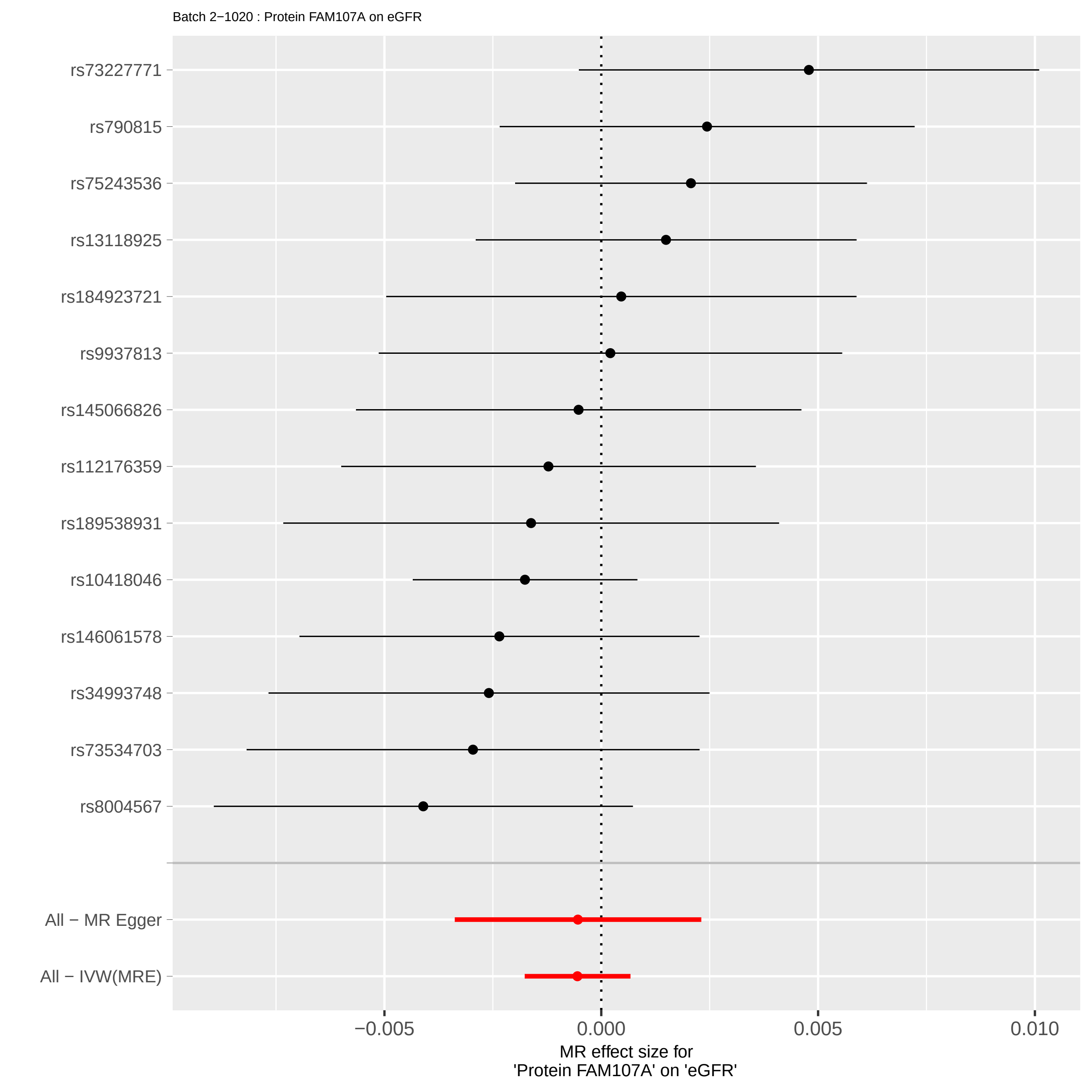

Batch 2−1020 : Protein FAM107A on eGFR
rs73227771
rs790815
rs75243536
rs13118925
rs184923721
rs9937813
rs145066826
rs112176359
rs189538931
rs10418046
rs146061578
rs34993748
rs73534703
rs8004567
All − MR Egger
All − IVW(MRE)
−0.005
0.000
0.005
0.010
MR effect size for
'Protein FAM107A' on 'eGFR'

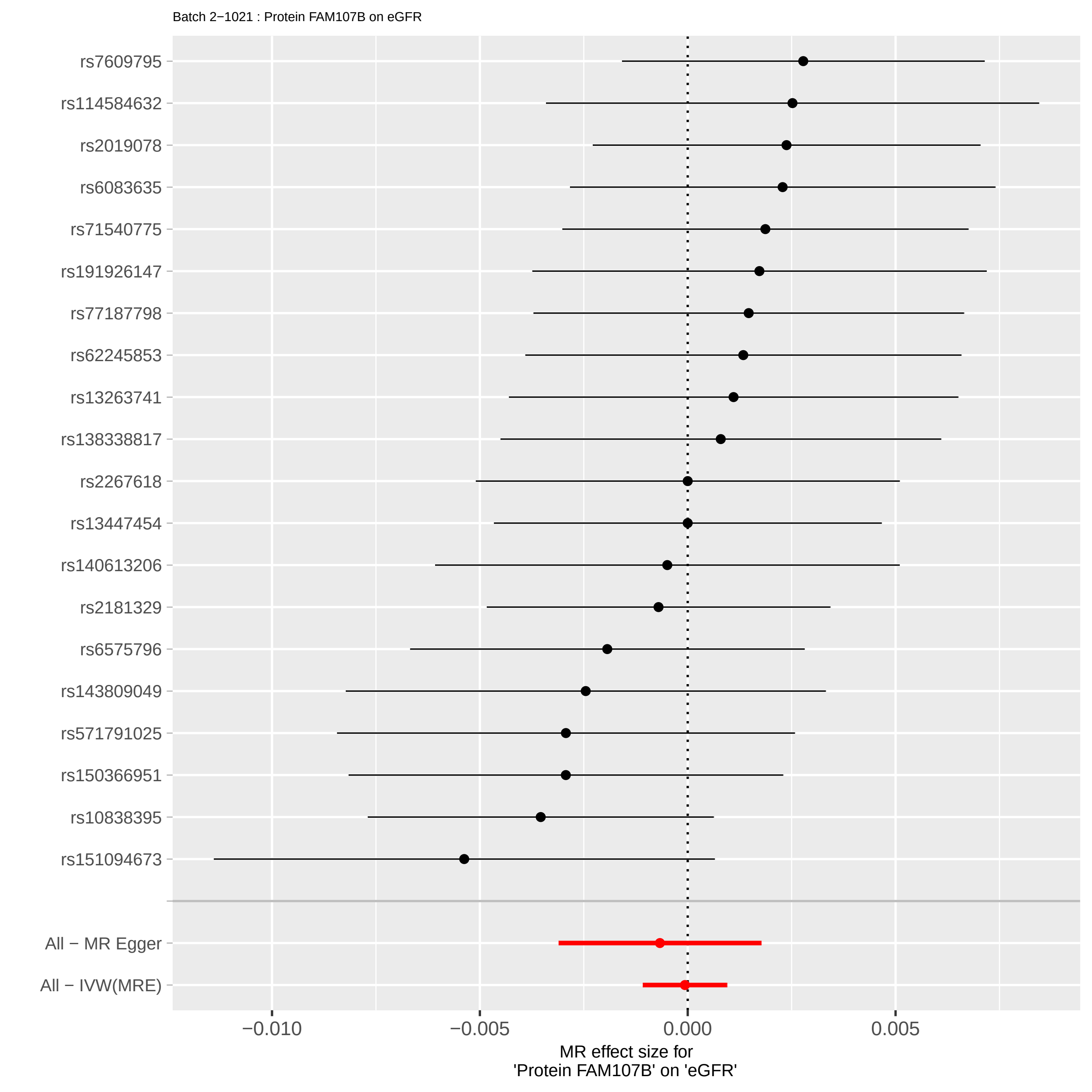

Batch 2−1021 : Protein FAM107B on eGFR
rs7609795
rs114584632
rs2019078
rs6083635
rs71540775
rs191926147
rs77187798
rs62245853
rs13263741
rs138338817
rs2267618
rs13447454
rs140613206
rs2181329
rs6575796
rs143809049
rs571791025
rs150366951
rs10838395
rs151094673
All − MR Egger
All − IVW(MRE)
−0.010
−0.005
0.000
0.005
MR effect size for
'Protein FAM107B' on 'eGFR'

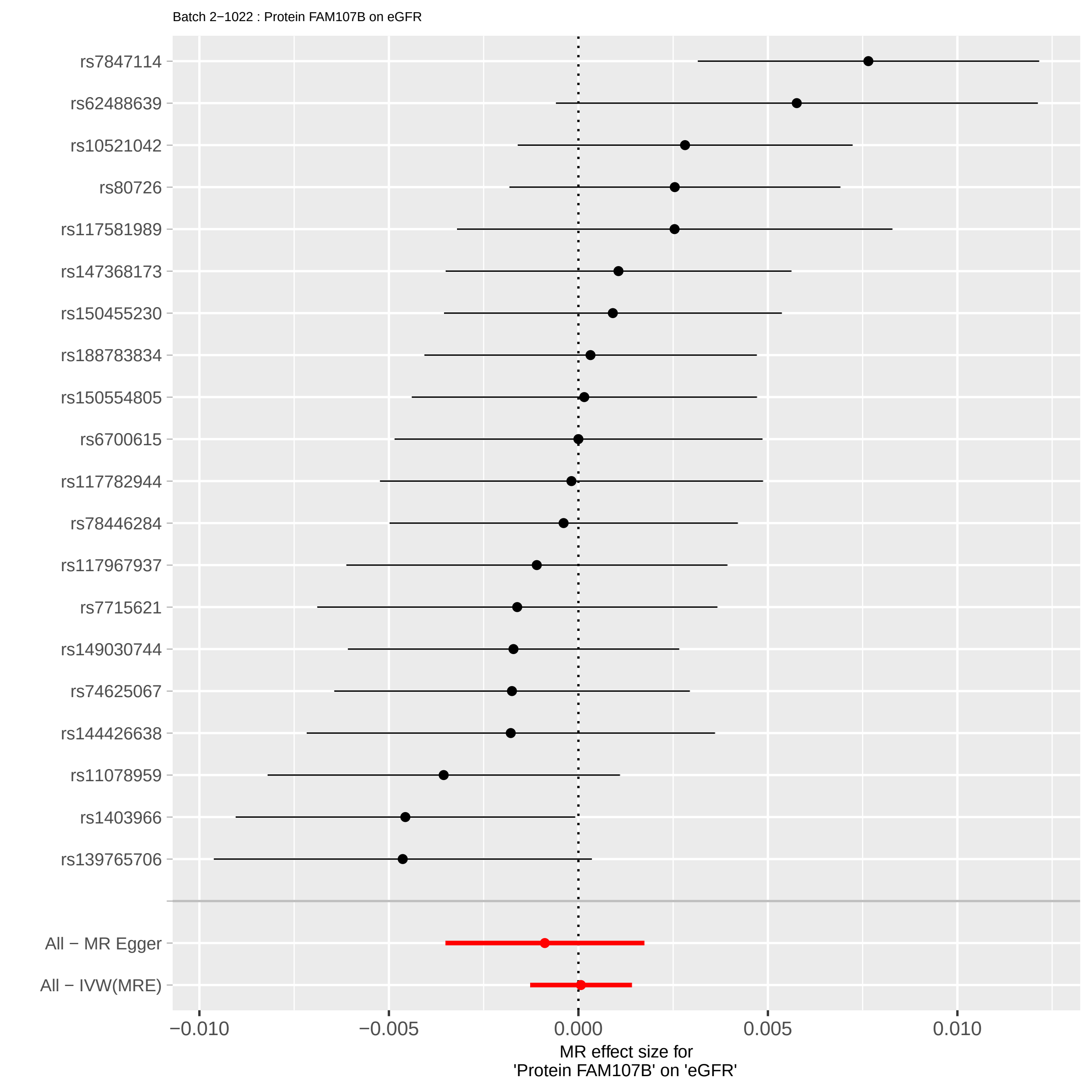

Batch 2−1022 : Protein FAM107B on eGFR
rs7847114
rs62488639
rs10521042
rs80726
rs117581989
rs147368173
rs150455230
rs188783834
rs150554805
rs6700615
rs117782944
rs78446284
rs117967937
rs7715621
rs149030744
rs74625067
rs144426638
rs11078959
rs1403966
rs139765706
All − MR Egger
All − IVW(MRE)
−0.010
−0.005
0.000
0.005
0.010
MR effect size for
'Protein FAM107B' on 'eGFR'

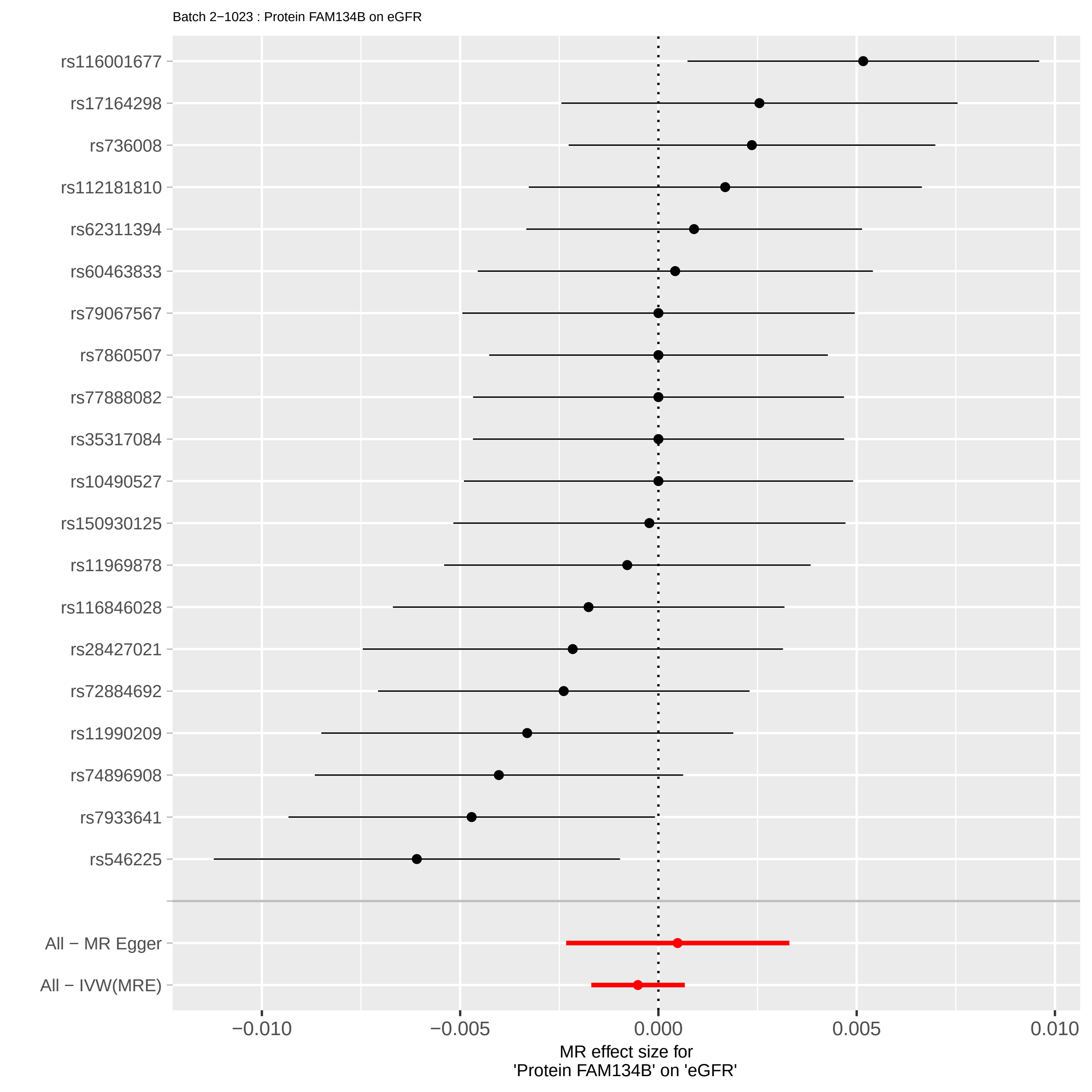

Batch 2−1023 : Protein FAM134B on eGFR
rs116001677
rs17164298
rs736008
rs112181810
rs62311394
rs60463833
rs79067567
rs7860507
rs77888082
rs35317084
rs10490527
rs150930125
rs11969878
rs116846028
rs28427021
rs72884692
rs11990209
rs74896908
rs7933641
rs546225
All − MR Egger
All − IVW(MRE)
−0.010
−0.005
0.000
0.005
0.010
MR effect size for
'Protein FAM134B' on 'eGFR'

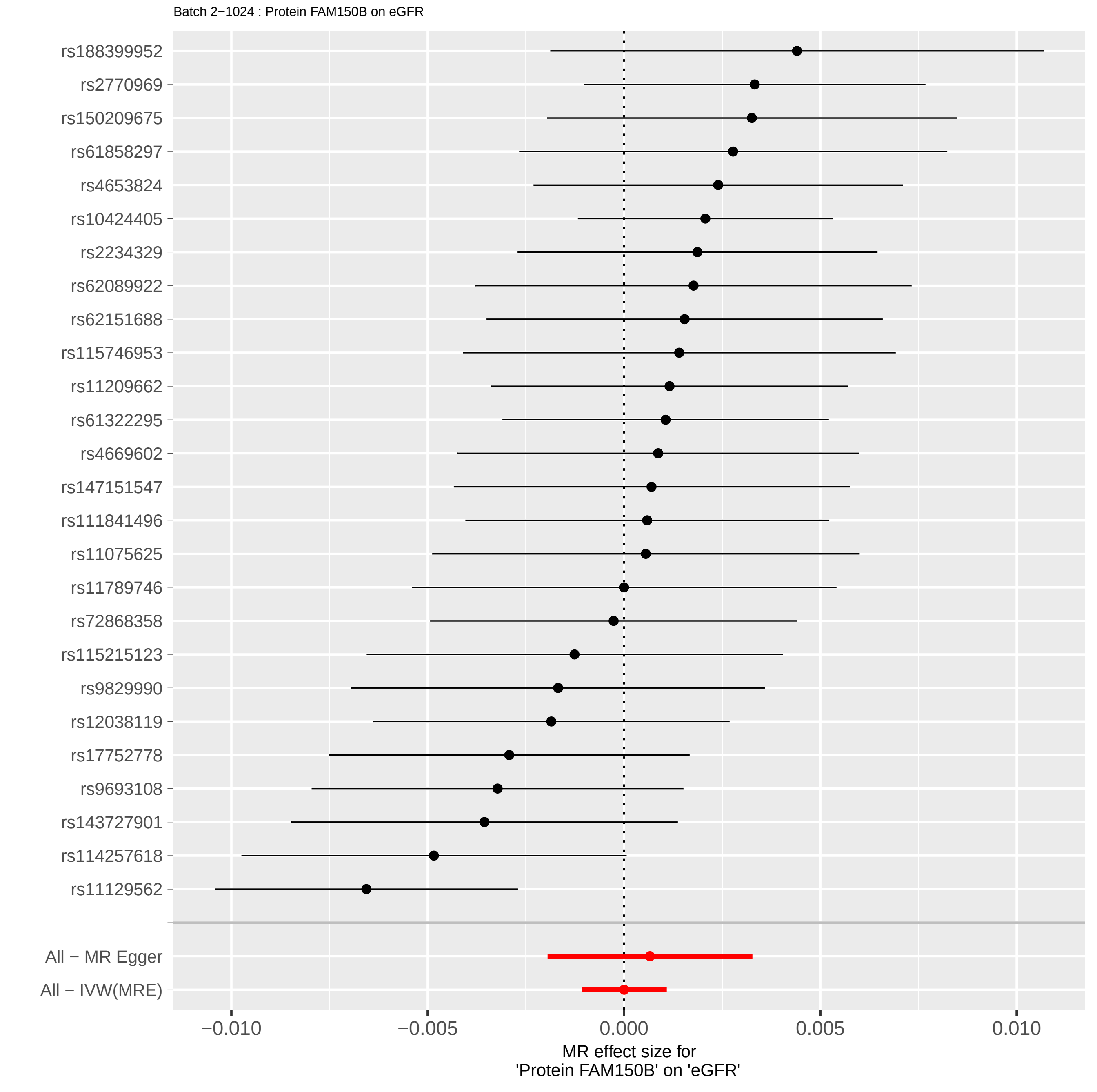

Batch 2−1024 : Protein FAM150B on eGFR
rs188399952
rs2770969
rs150209675
rs61858297
rs4653824
rs10424405
rs2234329
rs62089922
rs62151688
rs115746953
rs11209662
rs61322295
rs4669602
rs147151547
rs111841496
rs11075625
rs11789746
rs72868358
rs115215123
rs9829990
rs12038119
rs17752778
rs9693108
rs143727901
rs114257618
rs11129562
All − MR Egger
All − IVW(MRE)
−0.010
−0.005
0.000
0.005
0.010
MR effect size for
'Protein FAM150B' on 'eGFR'

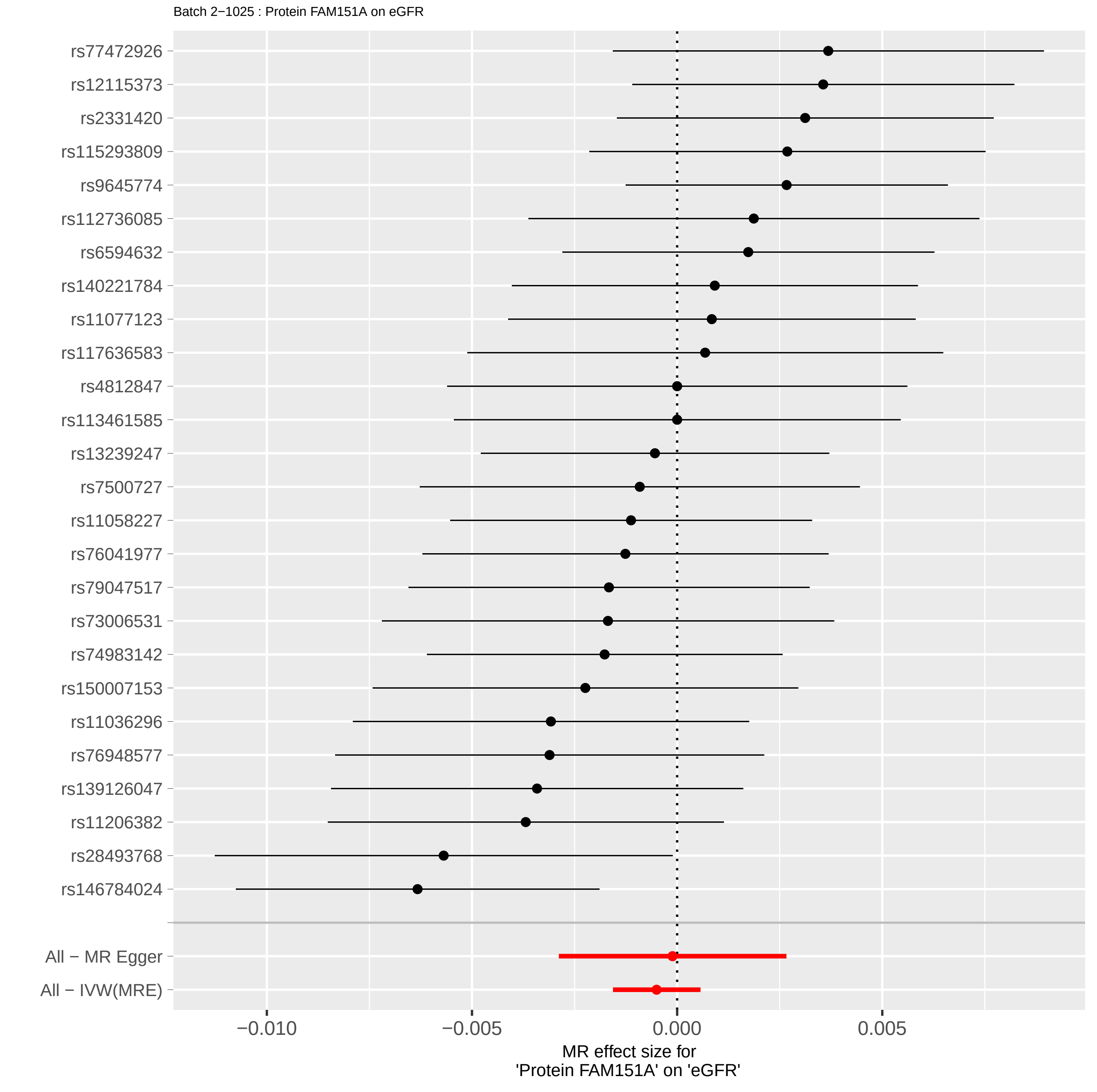
Batch 2−1025 : Protein FAM151A on eGFR
rs77472926
rs12115373
rs2331420
rs115293809
rs9645774
rs112736085
rs6594632
rs140221784
rs11077123
rs117636583
rs4812847
rs113461585
rs13239247
rs7500727
rs11058227
rs76041977
rs79047517
rs73006531
rs74983142
rs150007153
rs11036296
rs76948577
rs139126047
rs11206382
rs28493768
rs146784024
All − MR Egger
All − IVW(MRE)
−0.010
−0.005
0.000
0.005
MR effect size for
'Protein FAM151A' on 'eGFR'

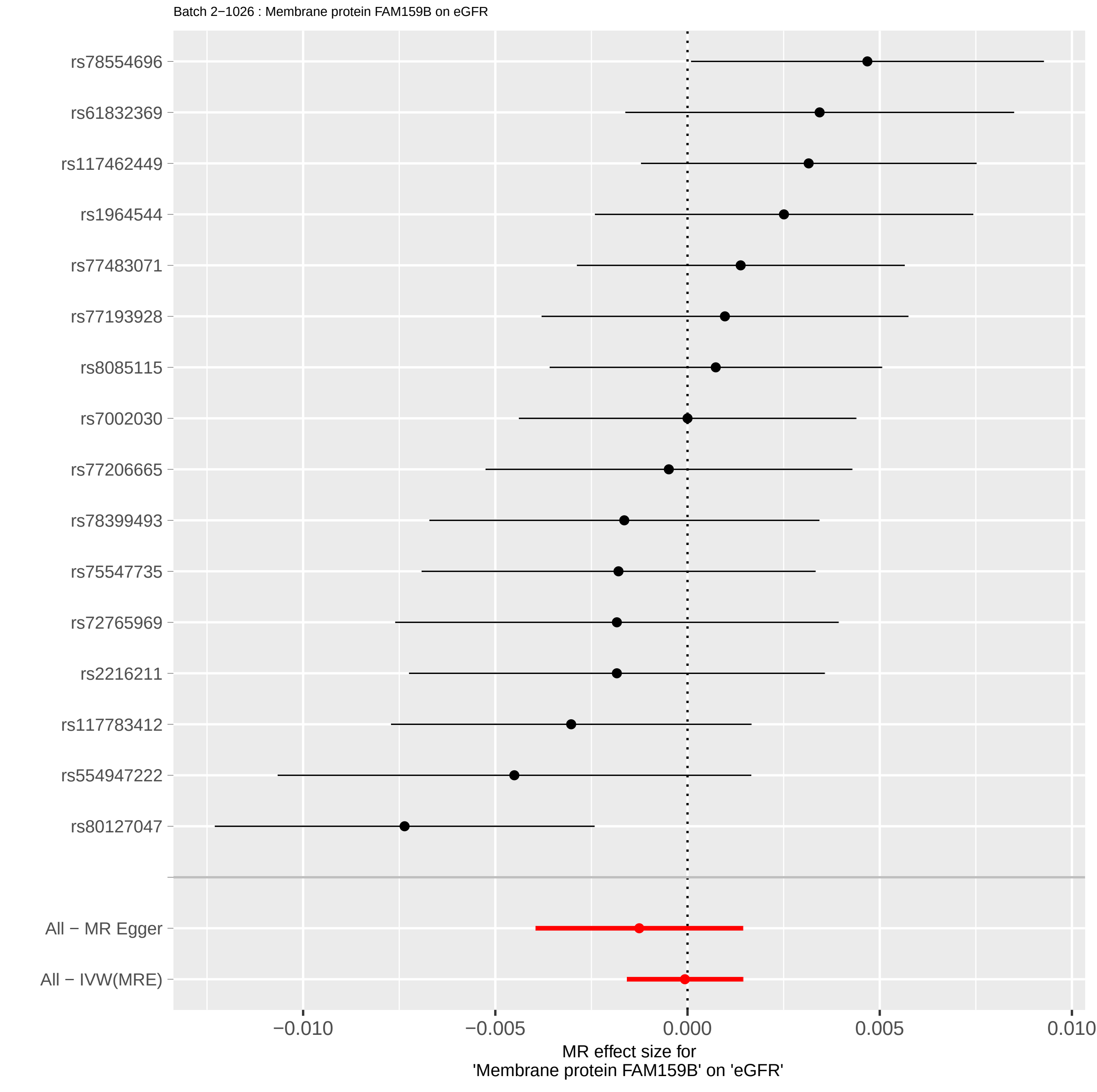

Batch 2−1026 : Membrane protein FAM159B on eGFR
rs78554696
rs61832369
rs117462449
rs1964544
rs77483071
rs77193928
rs8085115
rs7002030
rs77206665
rs78399493
rs75547735
rs72765969
rs2216211
rs117783412
rs554947222
rs80127047
All − MR Egger
All − IVW(MRE)
−0.010
−0.005
0.000
0.005
0.010
MR effect size for
'Membrane protein FAM159B' on 'eGFR'

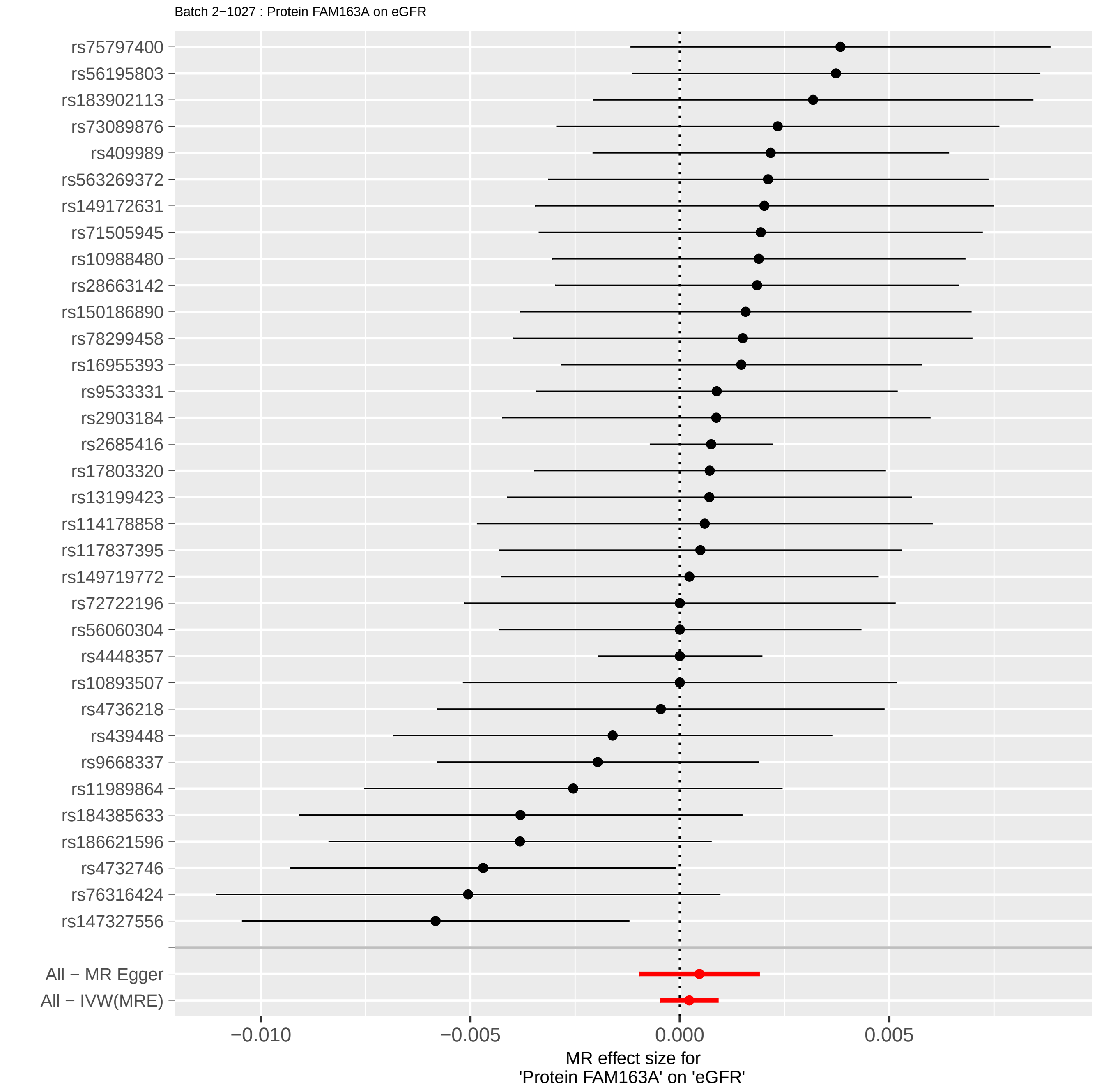

Batch 2−1027 : Protein FAM163A on eGFR
rs75797400
rs56195803
rs183902113
rs73089876
rs409989
rs563269372
rs149172631
rs71505945
rs10988480
rs28663142
rs150186890
rs78299458
rs16955393
rs9533331
rs2903184
rs2685416
rs17803320
rs13199423
rs114178858
rs117837395
rs149719772
rs72722196
rs56060304
rs4448357
rs10893507
rs4736218
rs439448
rs9668337
rs11989864
rs184385633
rs186621596
rs4732746
rs76316424
rs147327556
All − MR Egger
All − IVW(MRE)
−0.010
−0.005
0.000
0.005
MR effect size for
'Protein FAM163A' on 'eGFR'

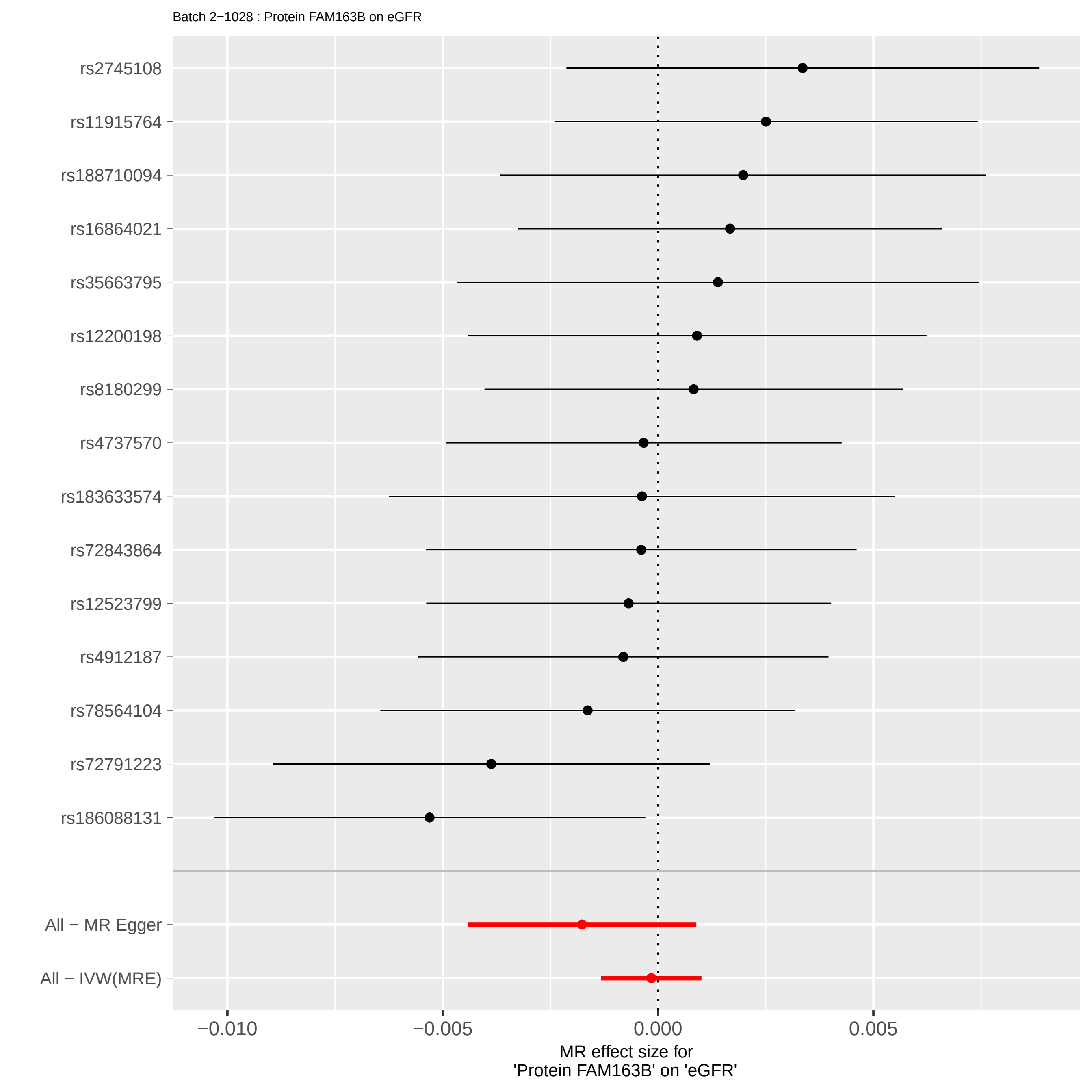
Batch 2−1028 : Protein FAM163B on eGFR
rs2745108
rs11915764
rs188710094
rs16864021
rs35663795
rs12200198
rs8180299
rs4737570
rs183633574
rs72843864
rs12523799
rs4912187
rs78564104
rs72791223
rs186088131
All − MR Egger
All − IVW(MRE)
−0.010
−0.005
0.000
0.005
MR effect size for
'Protein FAM163B' on 'eGFR'

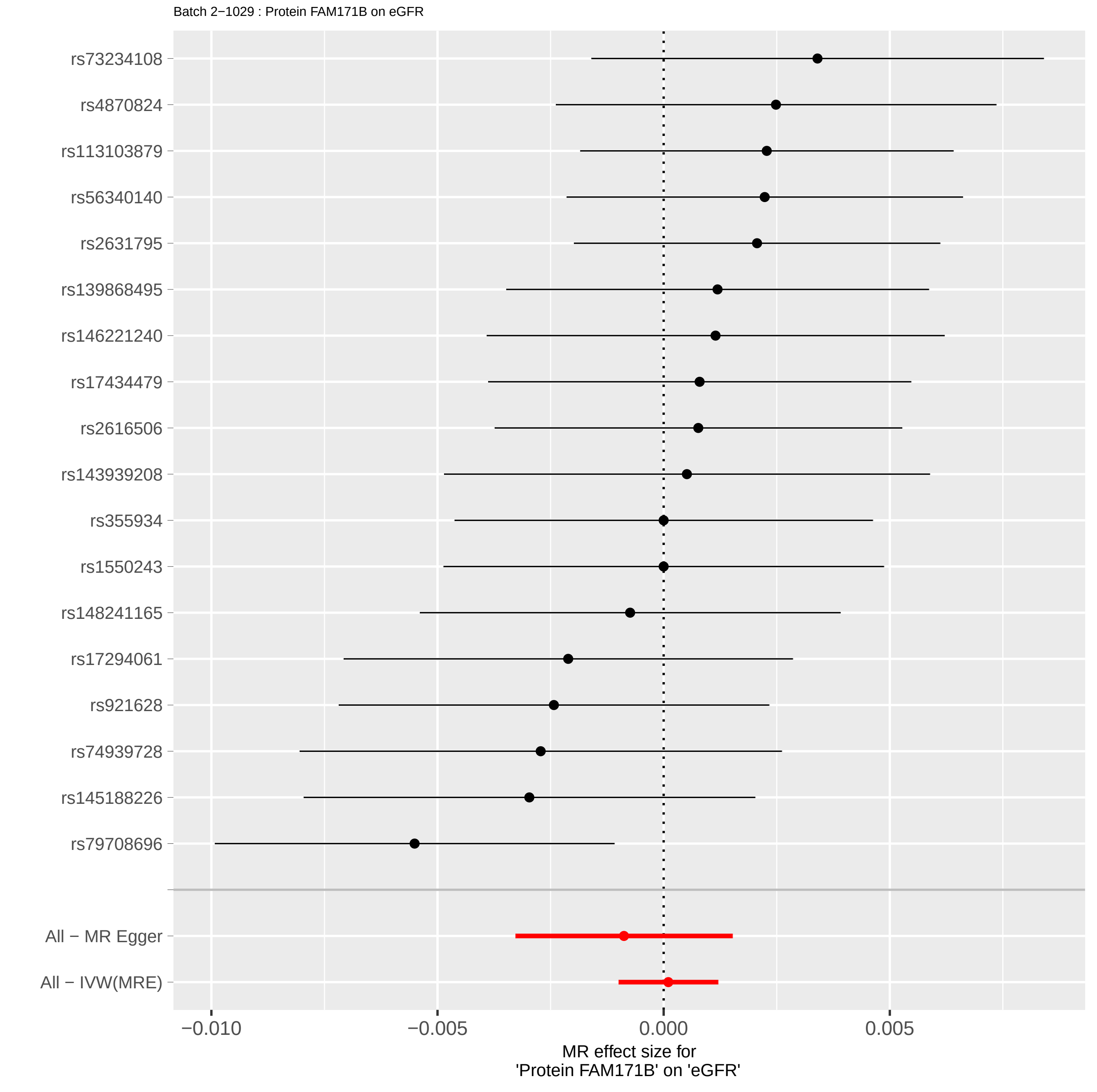

Batch 2−1029 : Protein FAM171B on eGFR
rs73234108
rs4870824
rs113103879
rs56340140
rs2631795
rs139868495
rs146221240
rs17434479
rs2616506
rs143939208
rs355934
rs1550243
rs148241165
rs17294061
rs921628
rs74939728
rs145188226
rs79708696
All − MR Egger
All − IVW(MRE)
−0.010
−0.005
0.000
0.005
MR effect size for
'Protein FAM171B' on 'eGFR'

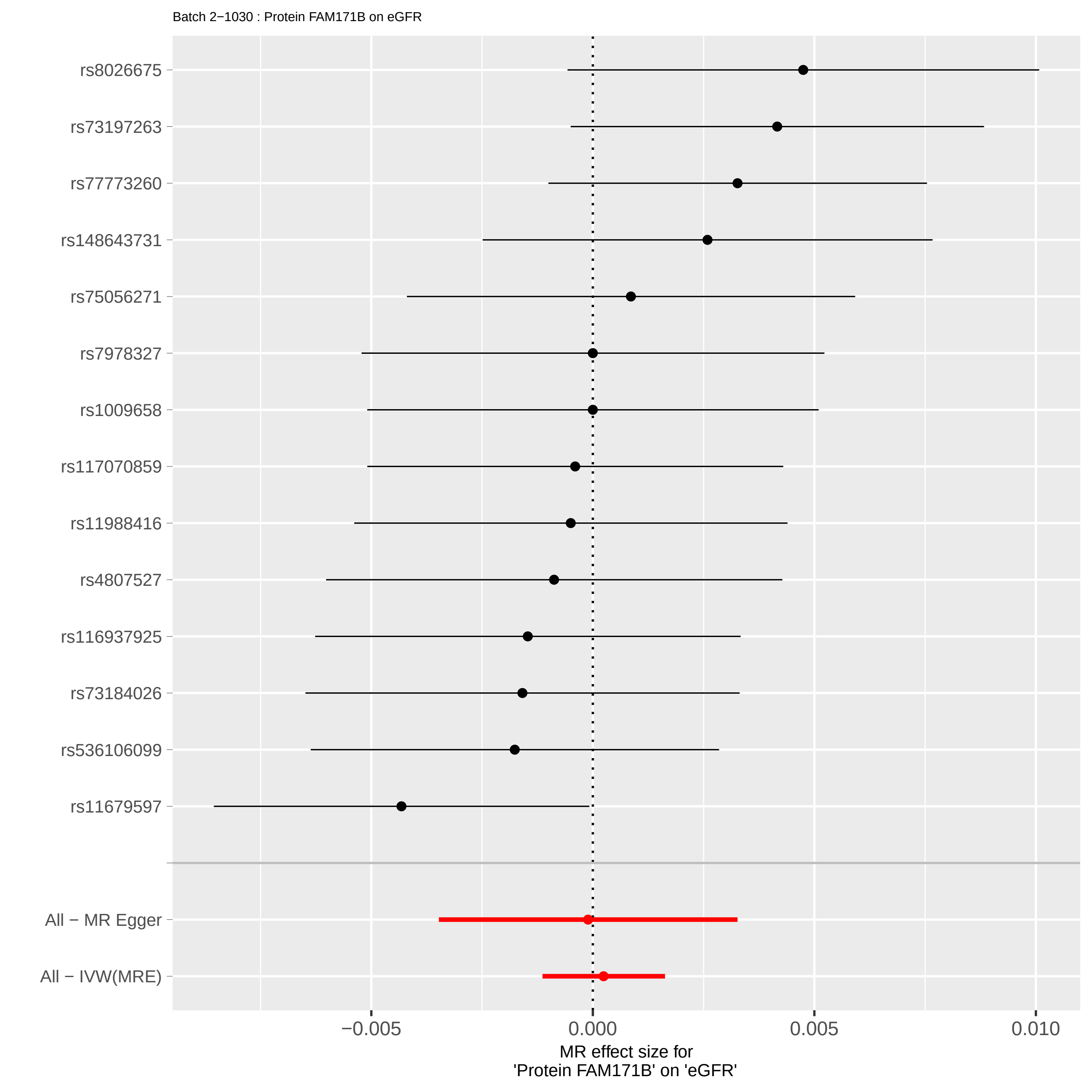
Batch 2−1030 : Protein FAM171B on eGFR
rs8026675
rs73197263
rs77773260
rs148643731
rs75056271
rs7978327
rs1009658
rs117070859
rs11988416
rs4807527
rs116937925
rs73184026
rs536106099
rs11679597
All − MR Egger
All − IVW(MRE)
−0.005
0.000
0.005
0.010
MR effect size for
'Protein FAM171B' on 'eGFR'

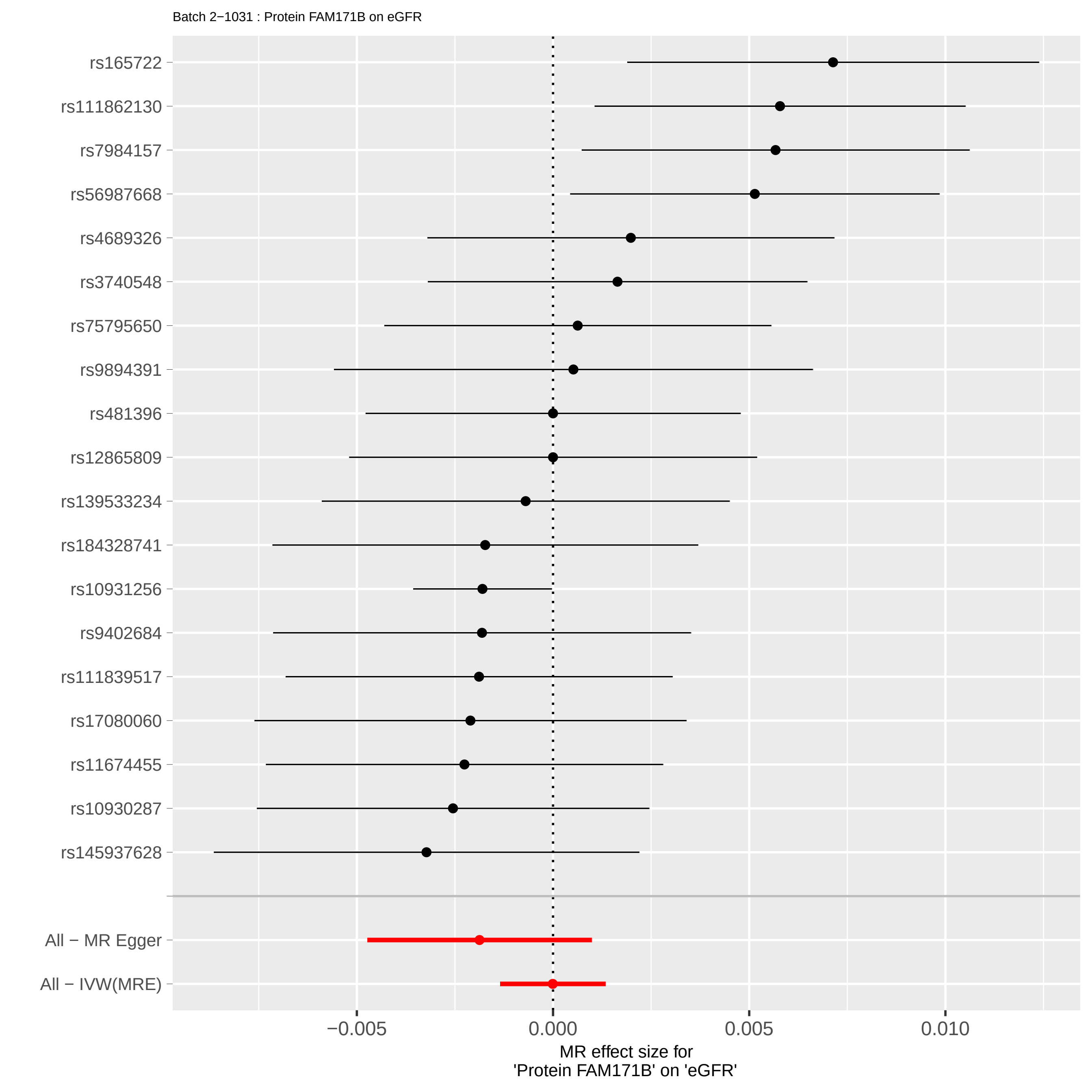

Batch 2−1031 : Protein FAM171B on eGFR
rs165722
rs111862130
rs7984157
rs56987668
rs4689326
rs3740548
rs75795650
rs9894391
rs481396
rs12865809
rs139533234
rs184328741
rs10931256
rs9402684
rs111839517
rs17080060
rs11674455
rs10930287
rs145937628
All − MR Egger
All − IVW(MRE)
−0.005
0.000
0.005
0.010
MR effect size for
'Protein FAM171B' on 'eGFR'

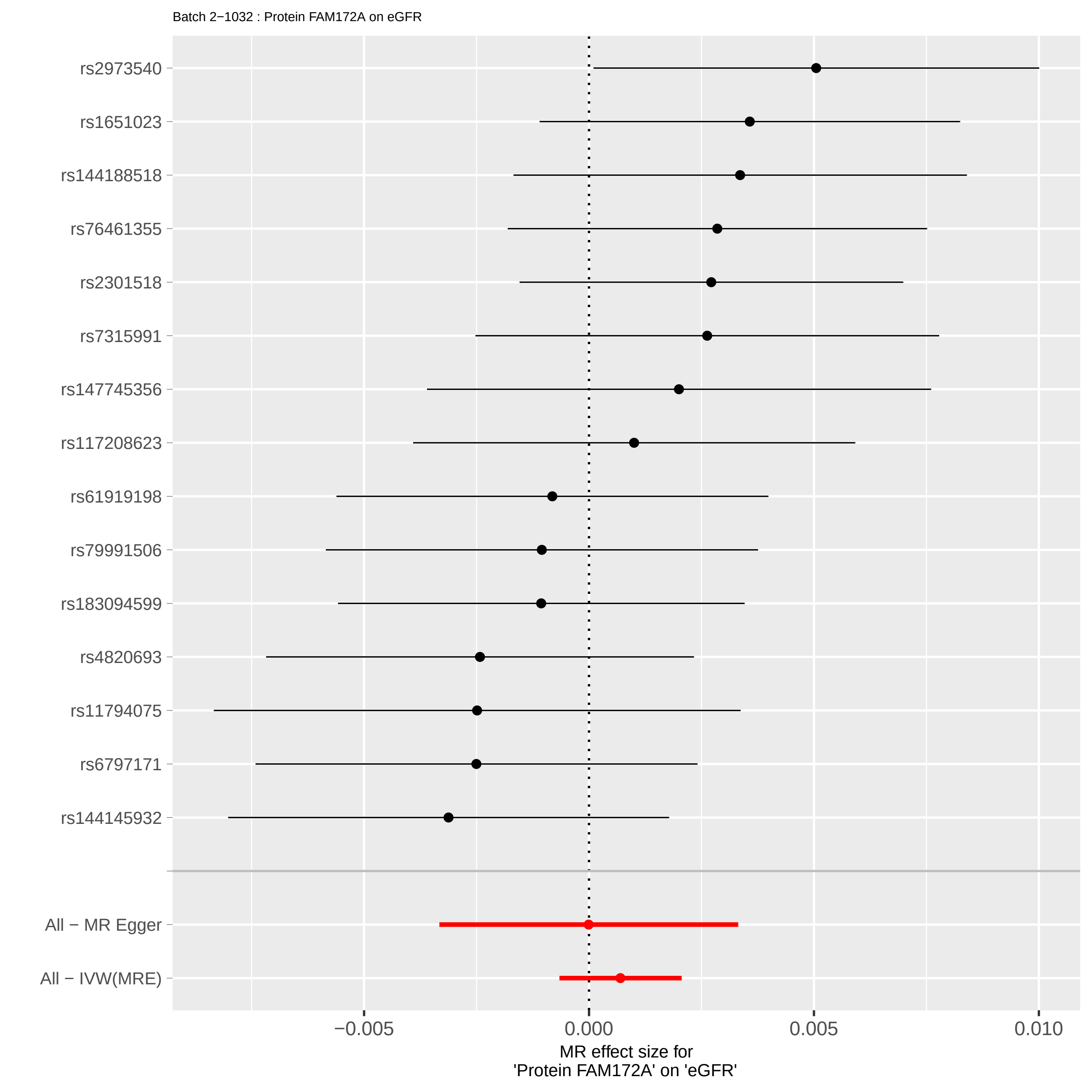

Batch 2−1032 : Protein FAM172A on eGFR
rs2973540
rs1651023
rs144188518
rs76461355
rs2301518
rs7315991
rs147745356
rs117208623
rs61919198
rs79991506
rs183094599
rs4820693
rs11794075
rs6797171
rs144145932
All − MR Egger
All − IVW(MRE)
−0.005
0.000
0.005
0.010
MR effect size for
'Protein FAM172A' on 'eGFR'

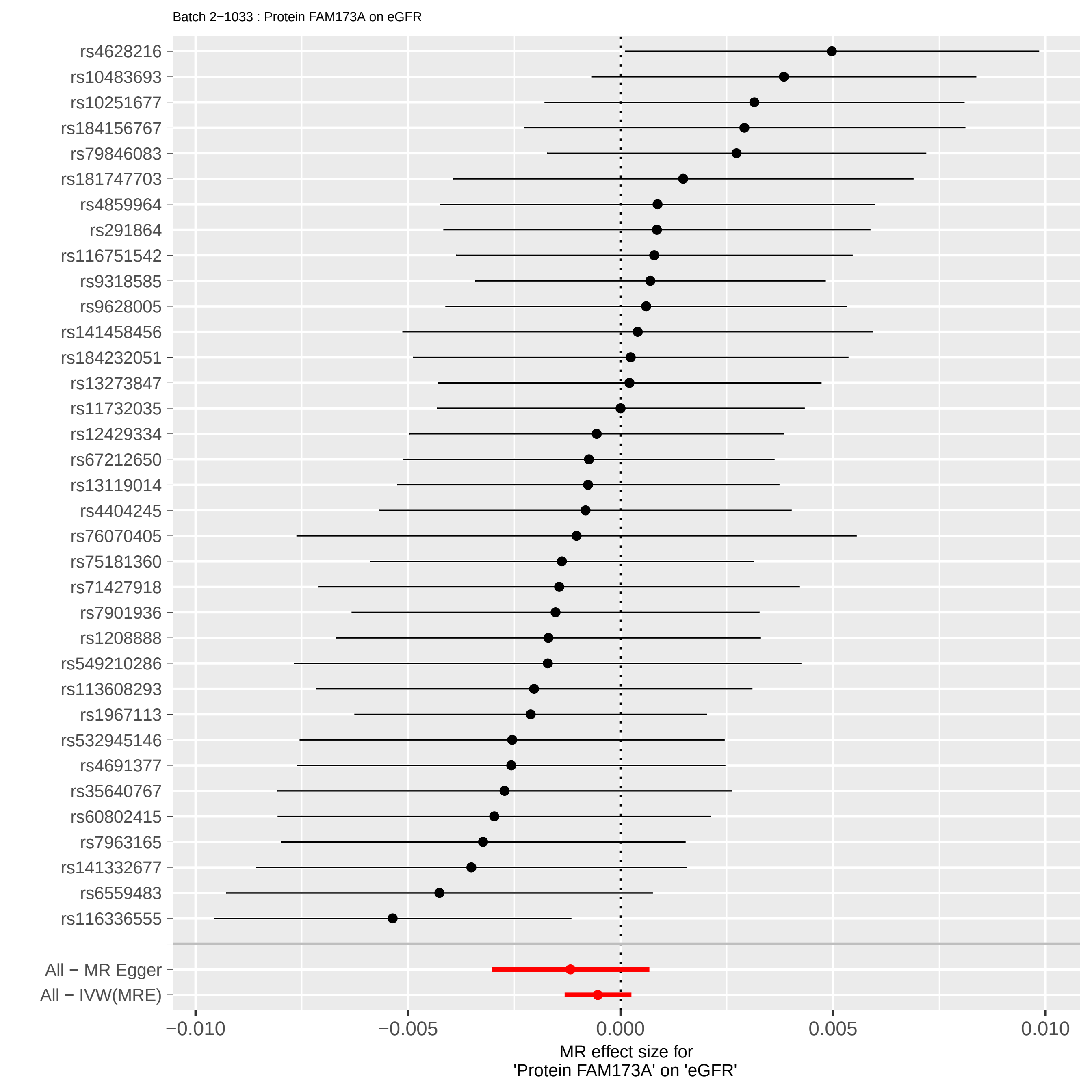
Batch 2−1033 : Protein FAM173A on eGFR
rs4628216
rs10483693
rs10251677
rs184156767
rs79846083
rs181747703
rs4859964
rs291864
rs116751542
rs9318585
rs9628005
rs141458456
rs184232051
rs13273847
rs11732035
rs12429334
rs67212650
rs13119014
rs4404245
rs76070405
rs75181360
rs71427918
rs7901936
rs1208888
rs549210286
rs113608293
rs1967113
rs532945146
rs4691377
rs35640767
rs60802415
rs7963165
rs141332677
rs6559483
rs116336555
All − MR Egger
All − IVW(MRE)
−0.010
−0.005
0.000
0.005
0.010
MR effect size for
'Protein FAM173A' on 'eGFR'

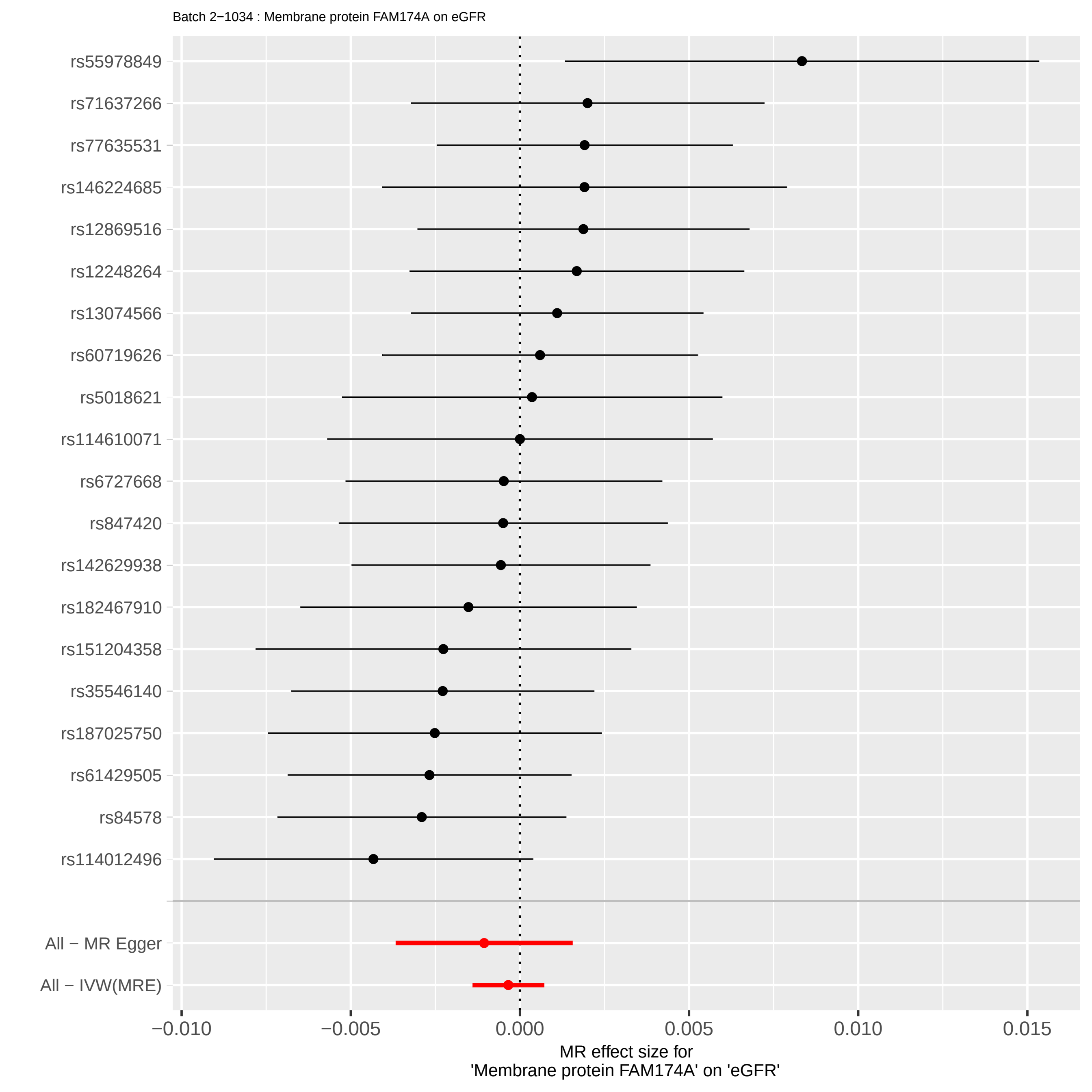

Batch 2−1034 : Membrane protein FAM174A on eGFR
rs55978849
rs71637266
rs77635531
rs146224685
rs12869516
rs12248264
rs13074566
rs60719626
rs5018621
rs114610071
rs6727668
rs847420
rs142629938
rs182467910
rs151204358
rs35546140
rs187025750
rs61429505
rs84578
rs114012496
All − MR Egger
All − IVW(MRE)
−0.010
−0.005
0.000
0.005
0.010
0.015
MR effect size for
'Membrane protein FAM174A' on 'eGFR'

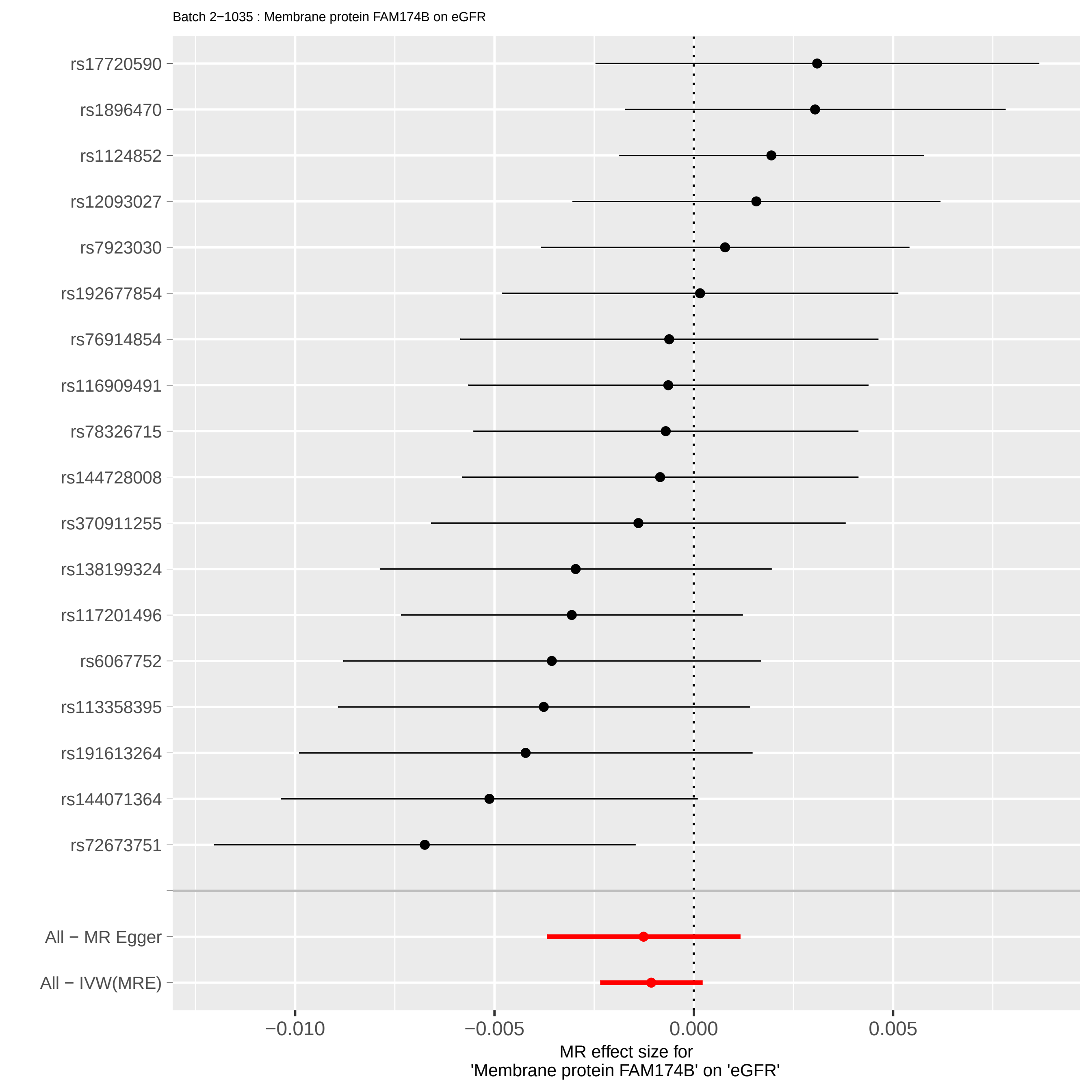

Batch 2−1035 : Membrane protein FAM174B on eGFR
rs17720590
rs1896470
rs1124852
rs12093027
rs7923030
rs192677854
rs76914854
rs116909491
rs78326715
rs144728008
rs370911255
rs138199324
rs117201496
rs6067752
rs113358395
rs191613264
rs144071364
rs72673751
All − MR Egger
All − IVW(MRE)
−0.010
−0.005
0.000
0.005
MR effect size for
'Membrane protein FAM174B' on 'eGFR'

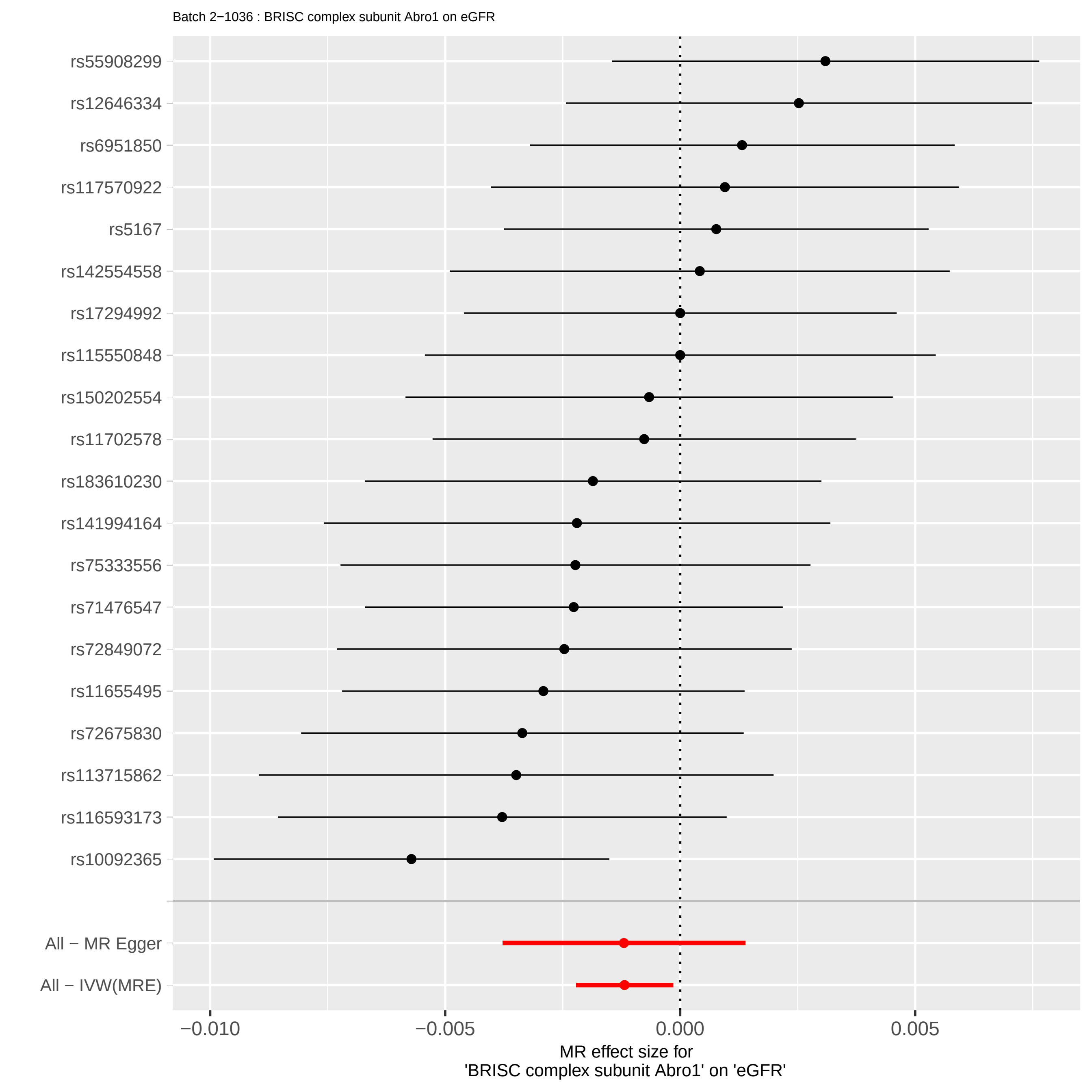

Batch 2−1036 : BRISC complex subunit Abro1 on eGFR
rs55908299
rs12646334
rs6951850
rs117570922
rs5167
rs142554558
rs17294992
rs115550848
rs150202554
rs11702578
rs183610230
rs141994164
rs75333556
rs71476547
rs72849072
rs11655495
rs72675830
rs113715862
rs116593173
rs10092365
All − MR Egger
All − IVW(MRE)
−0.010
−0.005
0.000
0.005
MR effect size for
'BRISC complex subunit Abro1' on 'eGFR'

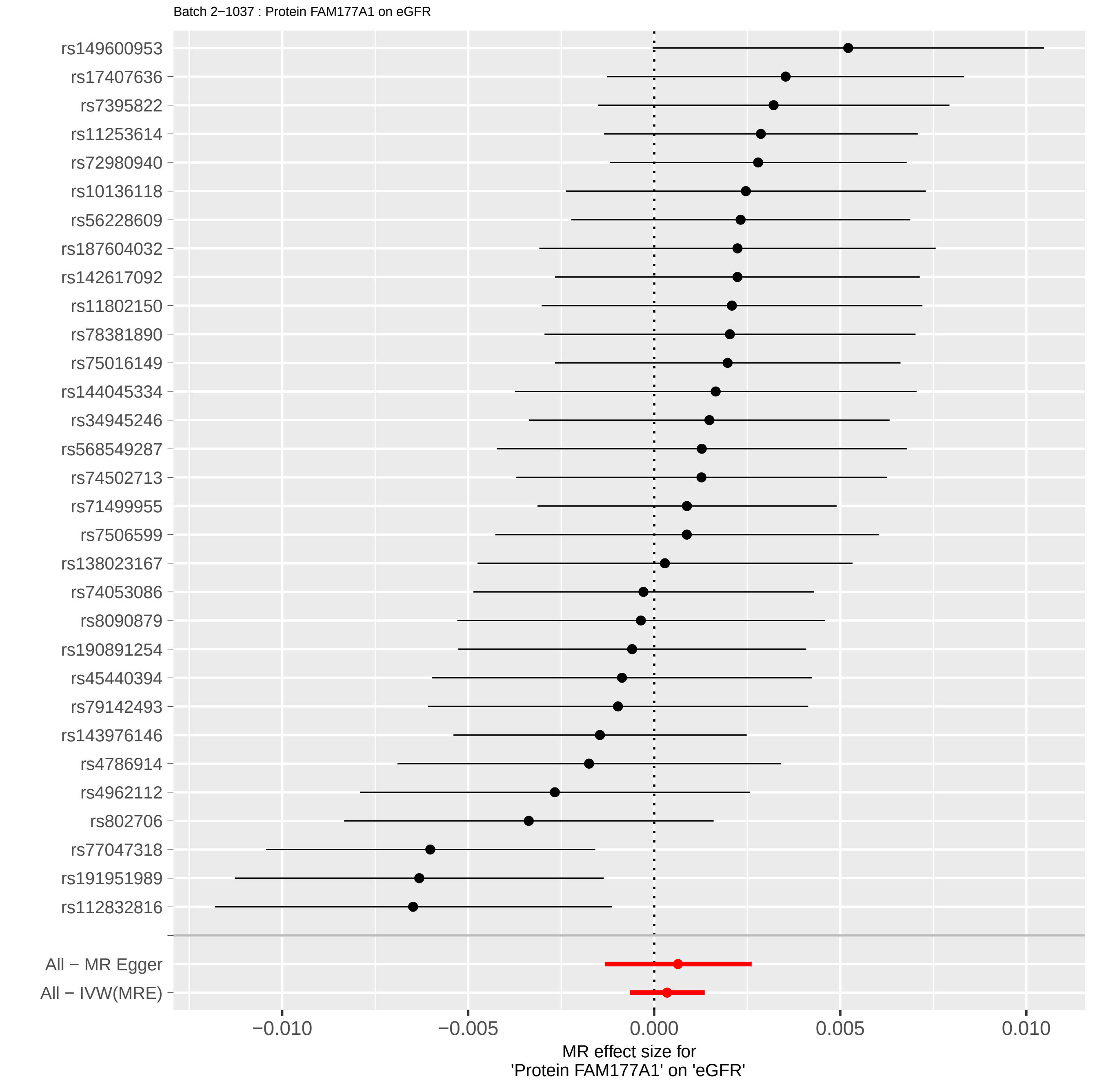

Batch 2−1037 : Protein FAM177A1 on eGFR
rs149600953
rs17407636
rs7395822
rs11253614
rs72980940
rs10136118
rs56228609
rs187604032
rs142617092
rs11802150
rs78381890
rs75016149
rs144045334
rs34945246
rs568549287
rs74502713
rs71499955
rs7506599
rs138023167
rs74053086
rs8090879
rs190891254
rs45440394
rs79142493
rs143976146
rs4786914
rs4962112
rs802706
rs77047318
rs191951989
rs112832816
All − MR Egger
All − IVW(MRE)
−0.010
−0.005
0.000
0.005
0.010
MR effect size for
'Protein FAM177A1' on 'eGFR'

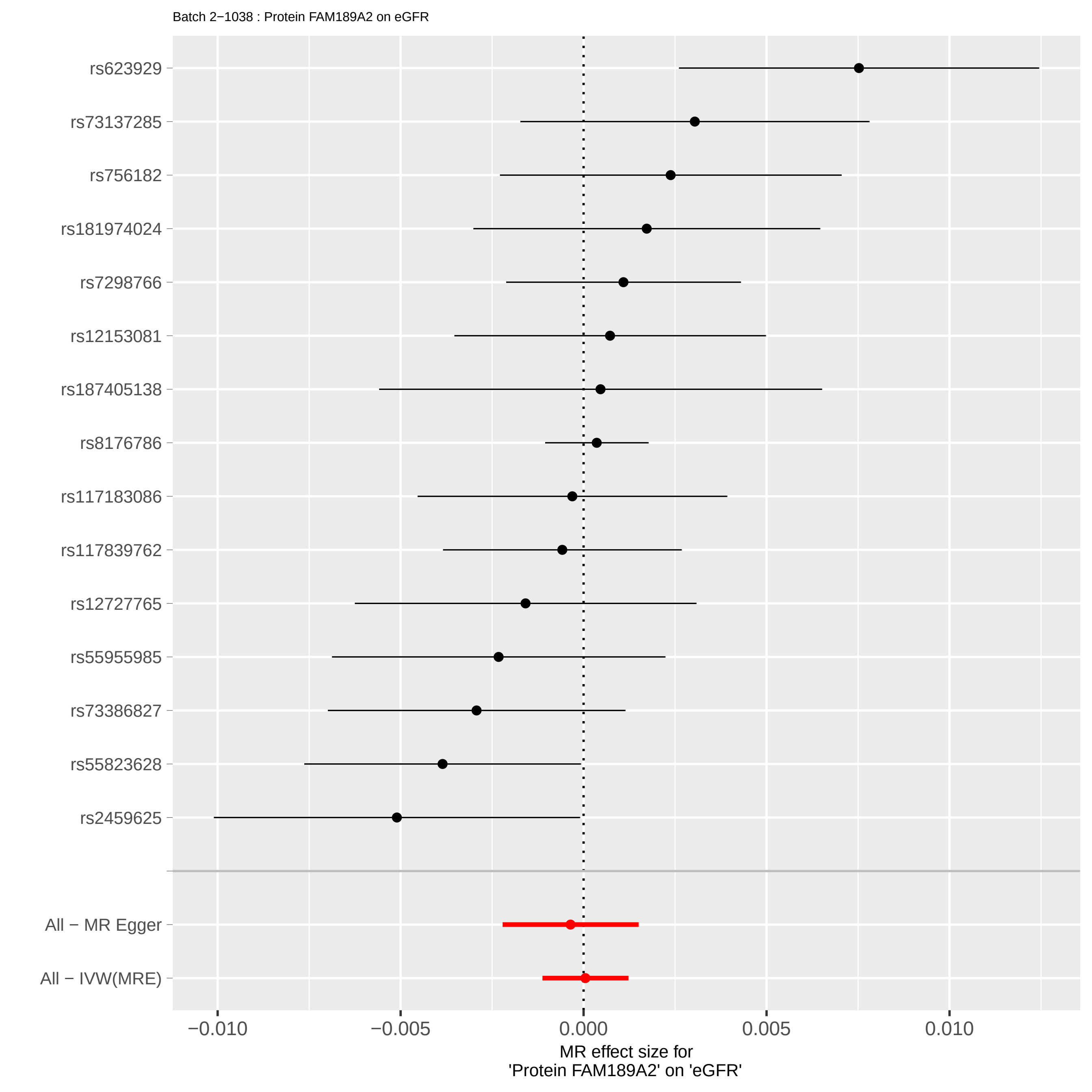
Batch 2−1038 : Protein FAM189A2 on eGFR
rs623929
rs73137285
rs756182
rs181974024
rs7298766
rs12153081
rs187405138
rs8176786
rs117183086
rs117839762
rs12727765
rs55955985
rs73386827
rs55823628
rs2459625
All − MR Egger
All − IVW(MRE)
−0.010
−0.005
0.000
0.005
0.010
MR effect size for
'Protein FAM189A2' on 'eGFR'

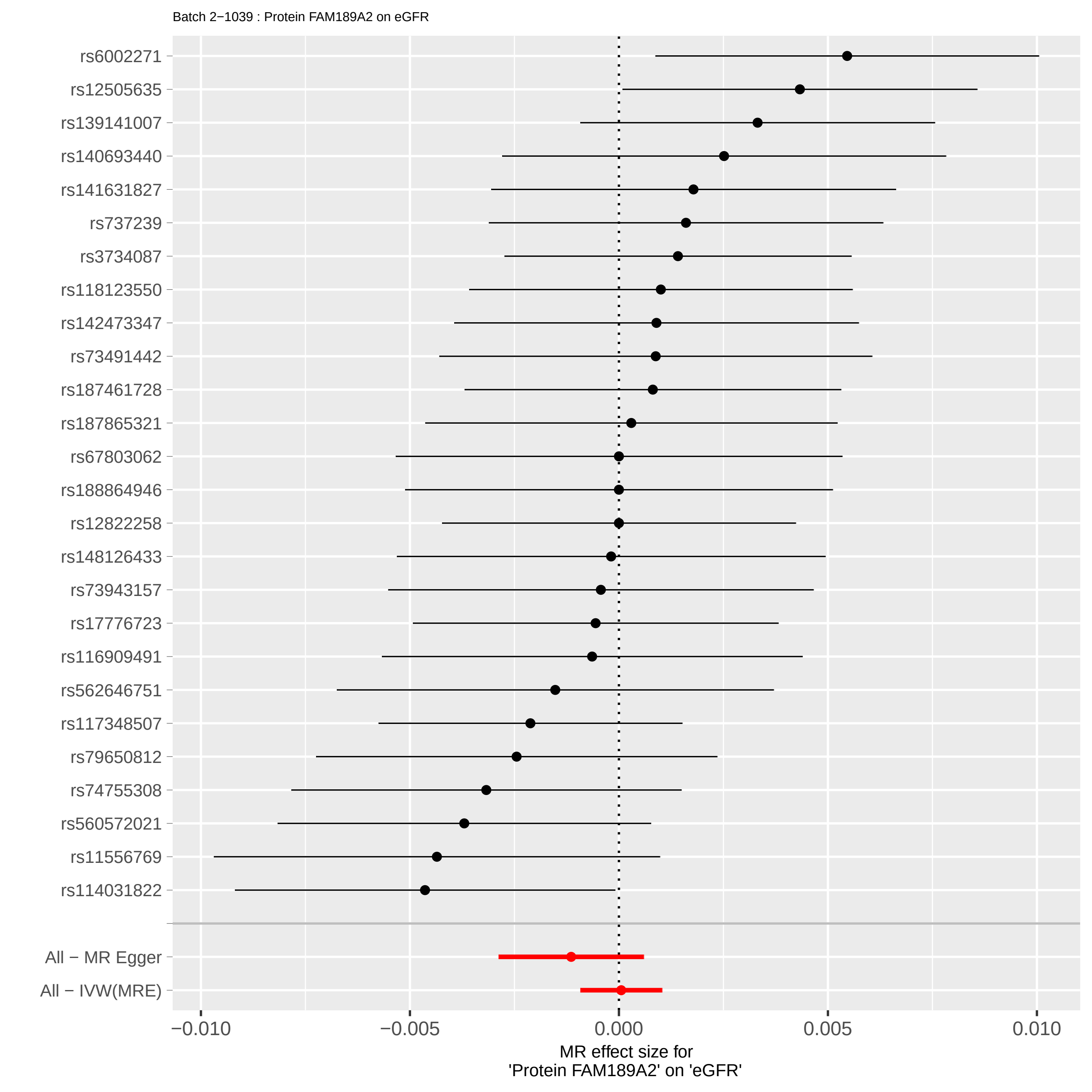
Batch 2−1039 : Protein FAM189A2 on eGFR
rs6002271
rs12505635
rs139141007
rs140693440
rs141631827
rs737239
rs3734087
rs118123550
rs142473347
rs73491442
rs187461728
rs187865321
rs67803062
rs188864946
rs12822258
rs148126433
rs73943157
rs17776723
rs116909491
rs562646751
rs117348507
rs79650812
rs74755308
rs560572021
rs11556769
rs114031822
All − MR Egger
All − IVW(MRE)
−0.010
−0.005
0.000
0.005
0.010
MR effect size for
'Protein FAM189A2' on 'eGFR'

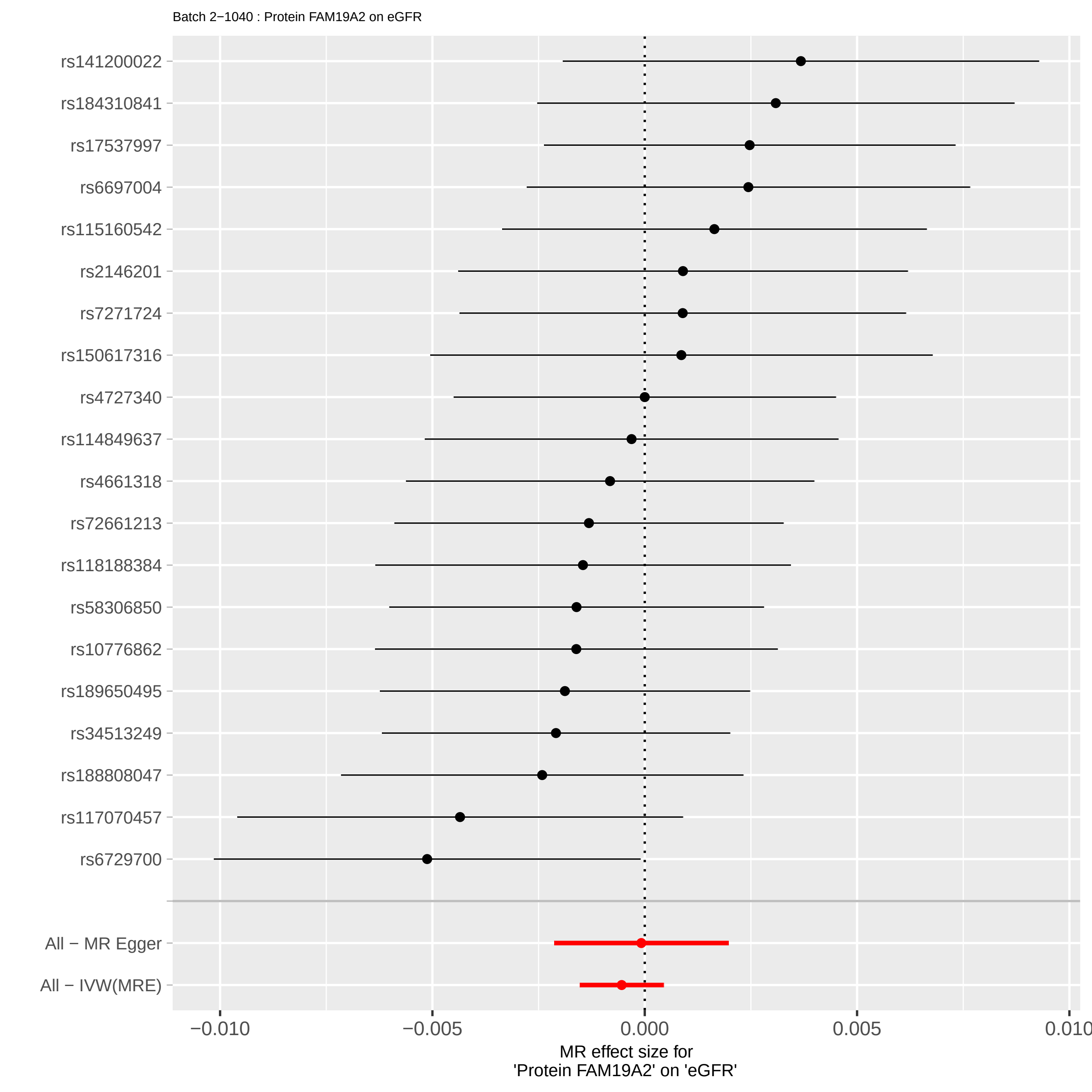

Batch 2−1040 : Protein FAM19A2 on eGFR
rs141200022
rs184310841
rs17537997
rs6697004
rs115160542
rs2146201
rs7271724
rs150617316
rs4727340
rs114849637
rs4661318
rs72661213
rs118188384
rs58306850
rs10776862
rs189650495
rs34513249
rs188808047
rs117070457
rs6729700
All − MR Egger
All − IVW(MRE)
−0.010
−0.005
0.000
0.005
0.010
MR effect size for
'Protein FAM19A2' on 'eGFR'

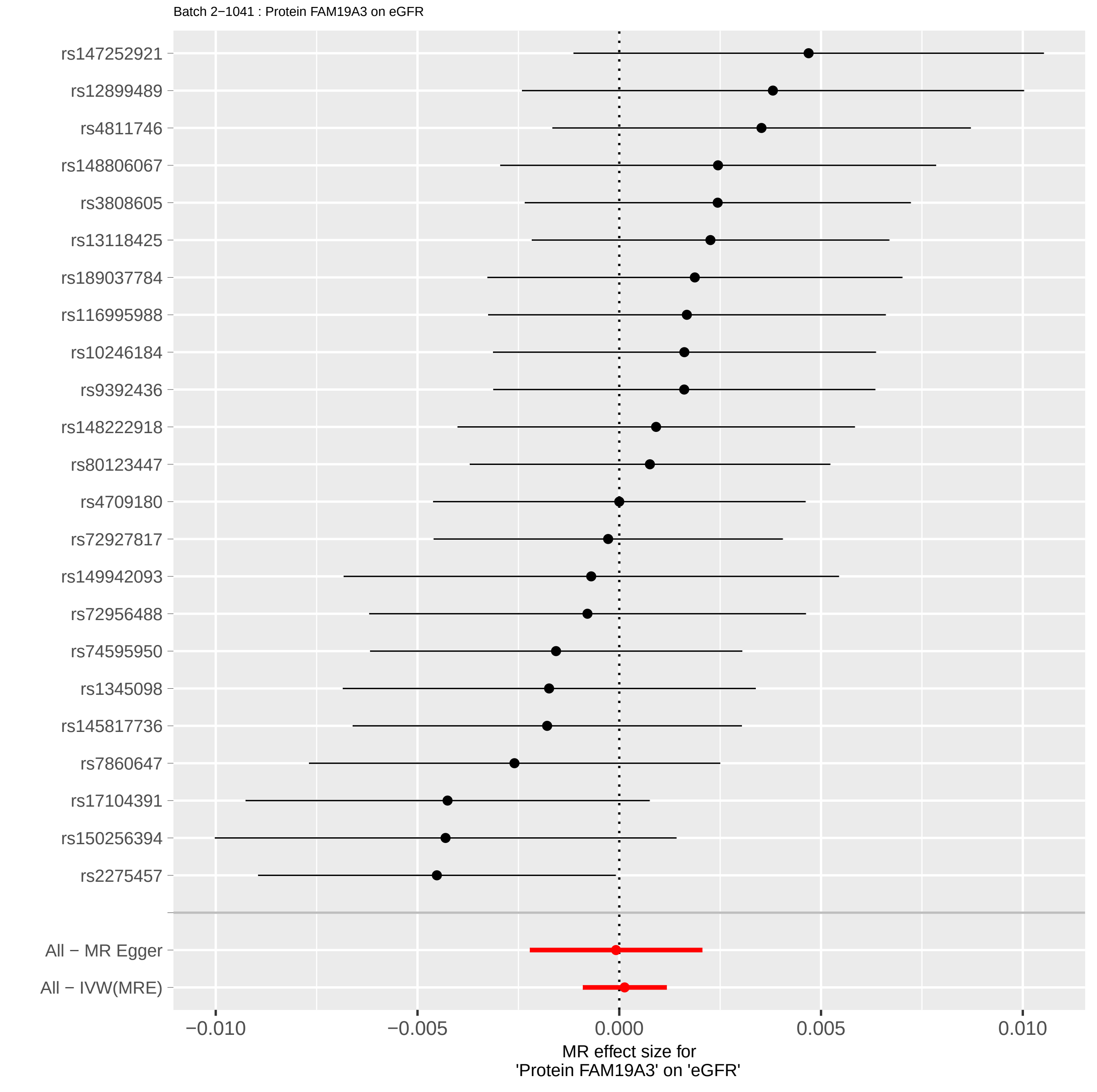

Batch 2−1041 : Protein FAM19A3 on eGFR
rs147252921
rs12899489
rs4811746
rs148806067
rs3808605
rs13118425
rs189037784
rs116995988
rs10246184
rs9392436
rs148222918
rs80123447
rs4709180
rs72927817
rs149942093
rs72956488
rs74595950
rs1345098
rs145817736
rs7860647
rs17104391
rs150256394
rs2275457
All − MR Egger
All − IVW(MRE)
−0.010
−0.005
0.000
0.005
0.010
MR effect size for
'Protein FAM19A3' on 'eGFR'

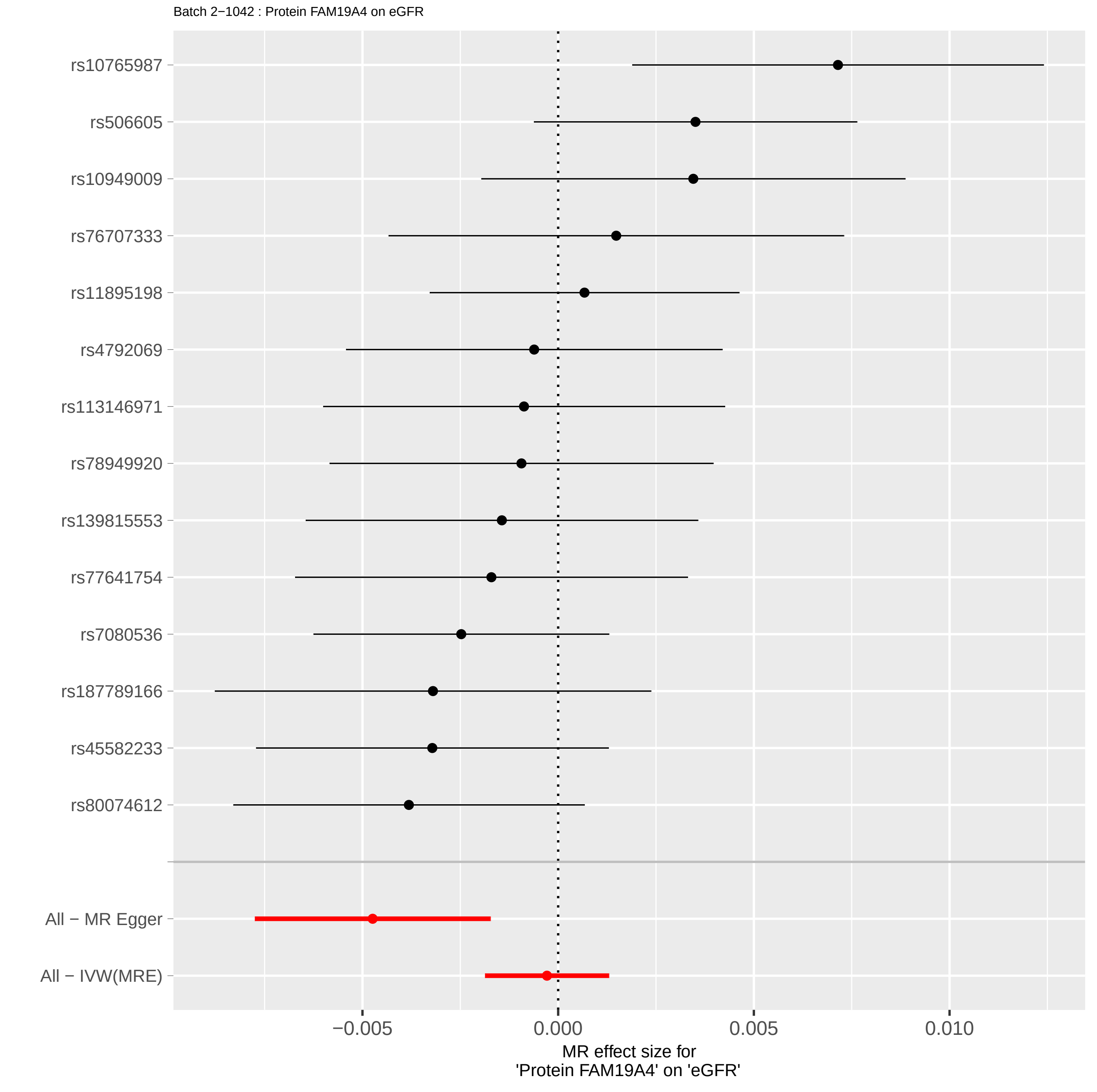

Batch 2−1042 : Protein FAM19A4 on eGFR
rs10765987
rs506605
rs10949009
rs76707333
rs11895198
rs4792069
rs113146971
rs78949920
rs139815553
rs77641754
rs7080536
rs187789166
rs45582233
rs80074612
All − MR Egger
All − IVW(MRE)
−0.005
0.000
0.005
0.010
MR effect size for
'Protein FAM19A4' on 'eGFR'

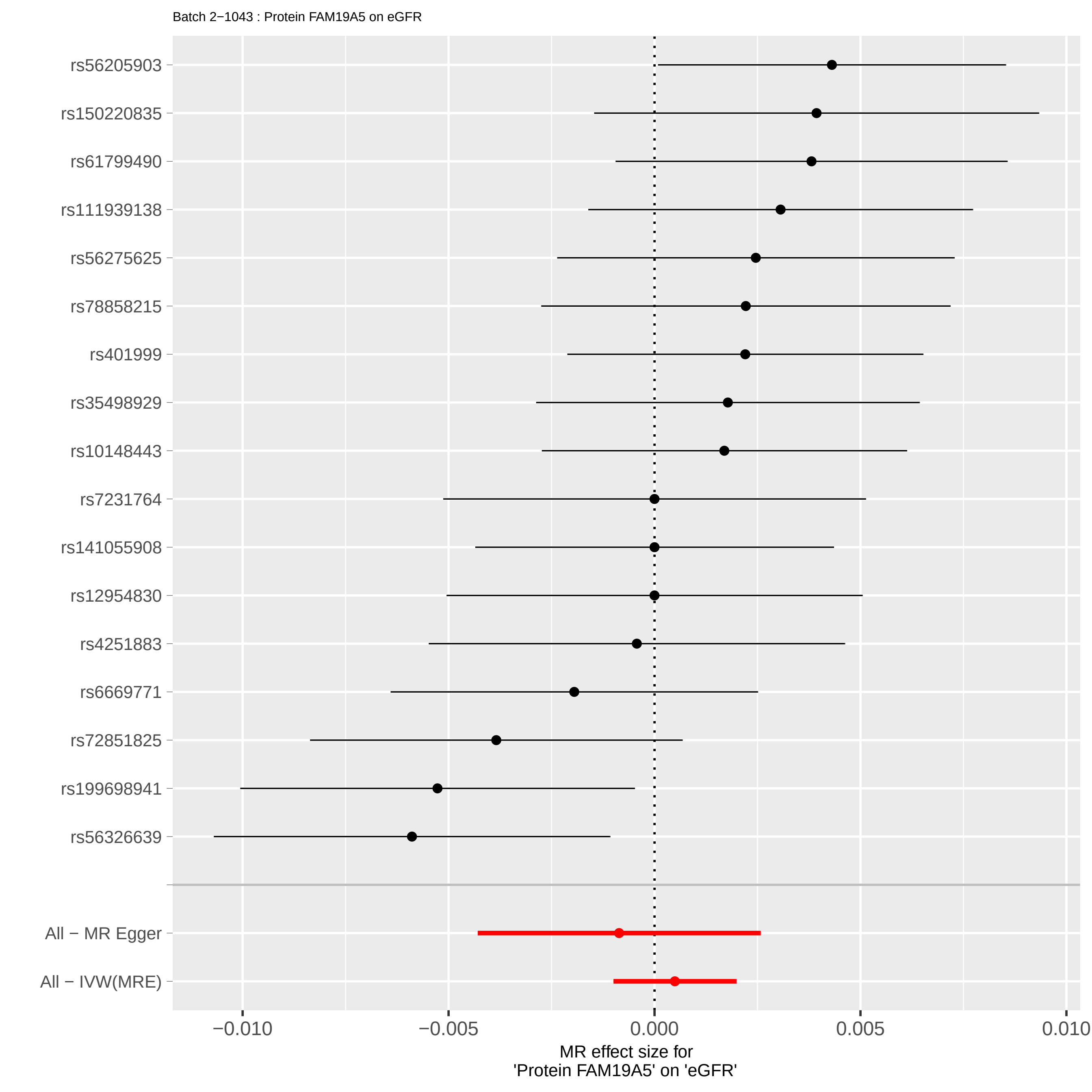

Batch 2−1043 : Protein FAM19A5 on eGFR
rs56205903
rs150220835
rs61799490
rs111939138
rs56275625
rs78858215
rs401999
rs35498929
rs10148443
rs7231764
rs141055908
rs12954830
rs4251883
rs6669771
rs72851825
rs199698941
rs56326639
All − MR Egger
All − IVW(MRE)
−0.010
−0.005
0.000
0.005
0.010
MR effect size for
'Protein FAM19A5' on 'eGFR'

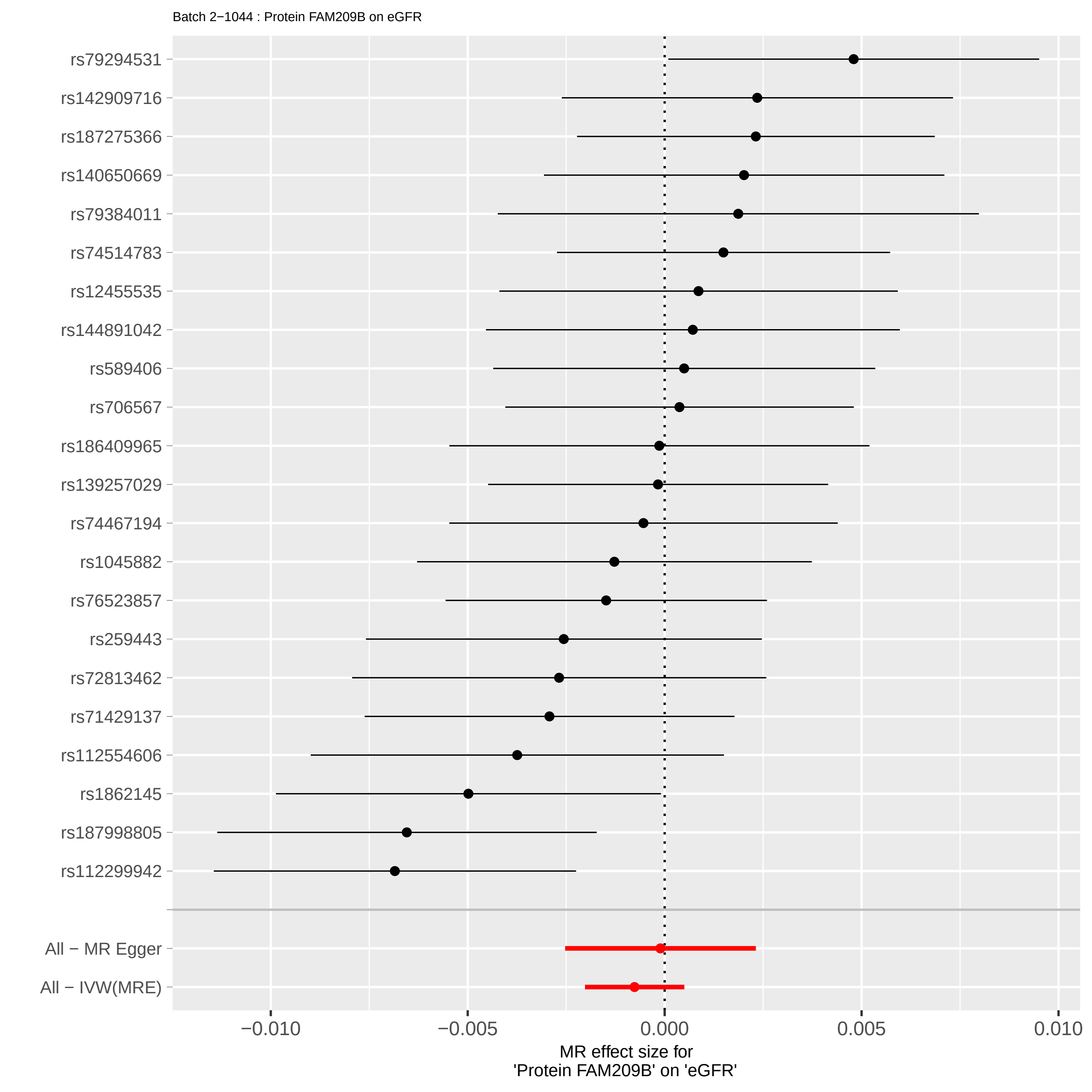
Batch 2−1044 : Protein FAM209B on eGFR
rs79294531
rs142909716
rs187275366
rs140650669
rs79384011
rs74514783
rs12455535
rs144891042
rs589406
rs706567
rs186409965
rs139257029
rs74467194
rs1045882
rs76523857
rs259443
rs72813462
rs71429137
rs112554606
rs1862145
rs187998805
rs112299942
All − MR Egger
All − IVW(MRE)
−0.010
−0.005
0.000
0.005
0.010
MR effect size for
'Protein FAM209B' on 'eGFR'

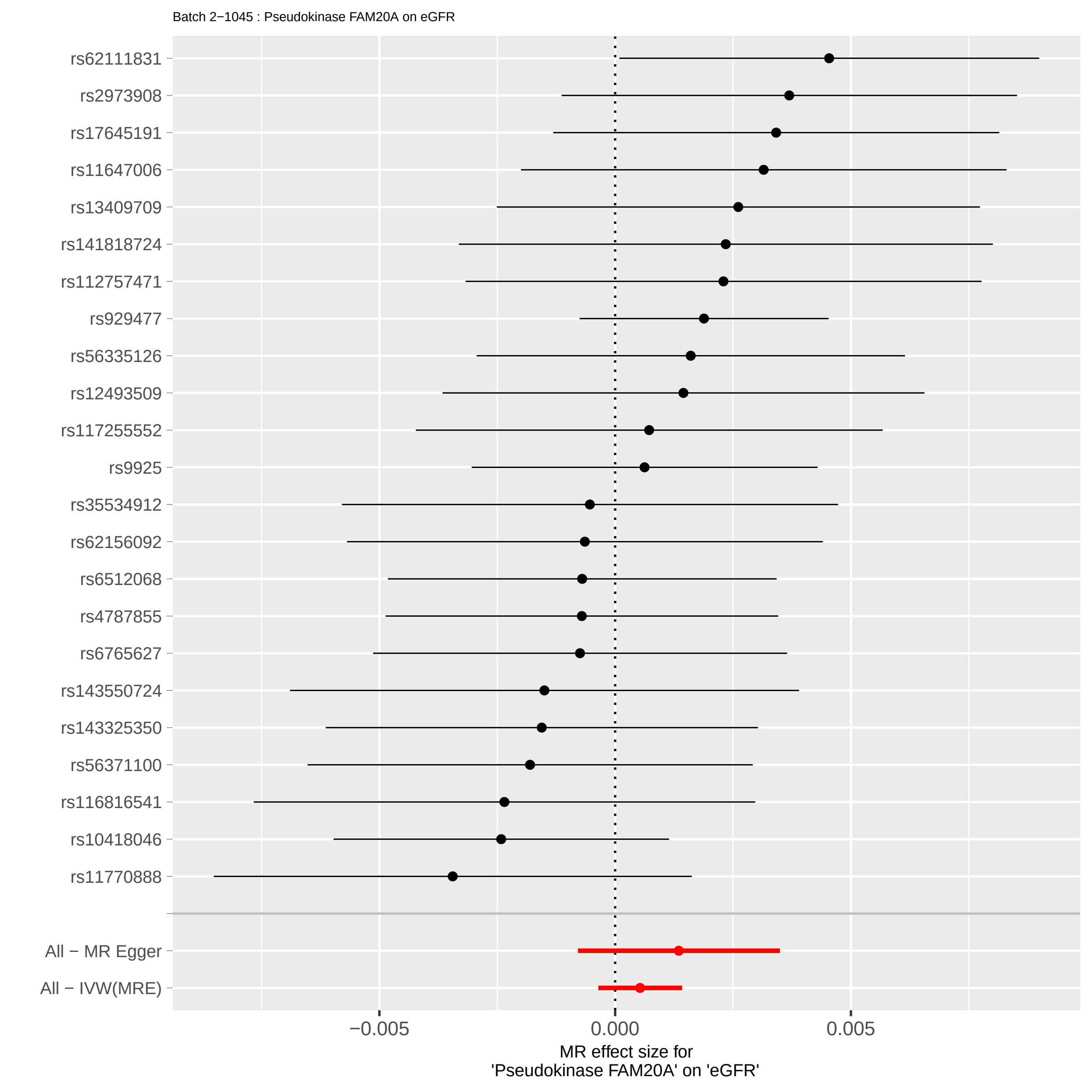

Batch 2−1045 : Pseudokinase FAM20A on eGFR
rs62111831
rs2973908
rs17645191
rs11647006
rs13409709
rs141818724
rs112757471
rs929477
rs56335126
rs12493509
rs117255552
rs9925
rs35534912
rs62156092
rs6512068
rs4787855
rs6765627
rs143550724
rs143325350
rs56371100
rs116816541
rs10418046
rs11770888
All − MR Egger
All − IVW(MRE)
−0.005
0.000
0.005
MR effect size for
'Pseudokinase FAM20A' on 'eGFR'

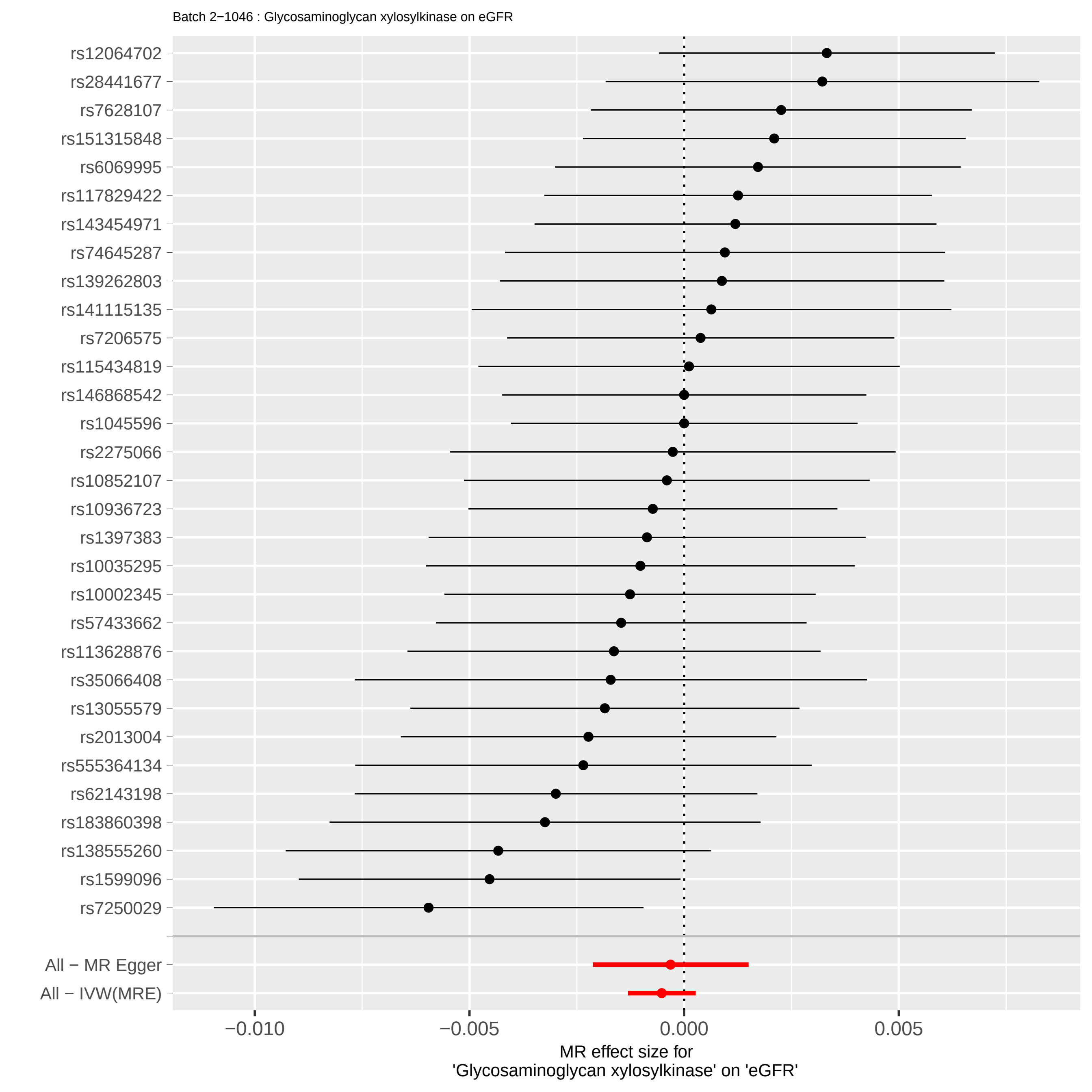
Batch 2−1046 : Glycosaminoglycan xylosylkinase on eGFR
rs12064702
rs28441677
rs7628107
rs151315848
rs6069995
rs117829422
rs143454971
rs74645287
rs139262803
rs141115135
rs7206575
rs115434819
rs146868542
rs1045596
rs2275066
rs10852107
rs10936723
rs1397383
rs10035295
rs10002345
rs57433662
rs113628876
rs35066408
rs13055579
rs2013004
rs555364134
rs62143198
rs183860398
rs138555260
rs1599096
rs7250029
All − MR Egger
All − IVW(MRE)
−0.010
−0.005
0.000
0.005
MR effect size for
'Glycosaminoglycan xylosylkinase' on 'eGFR'

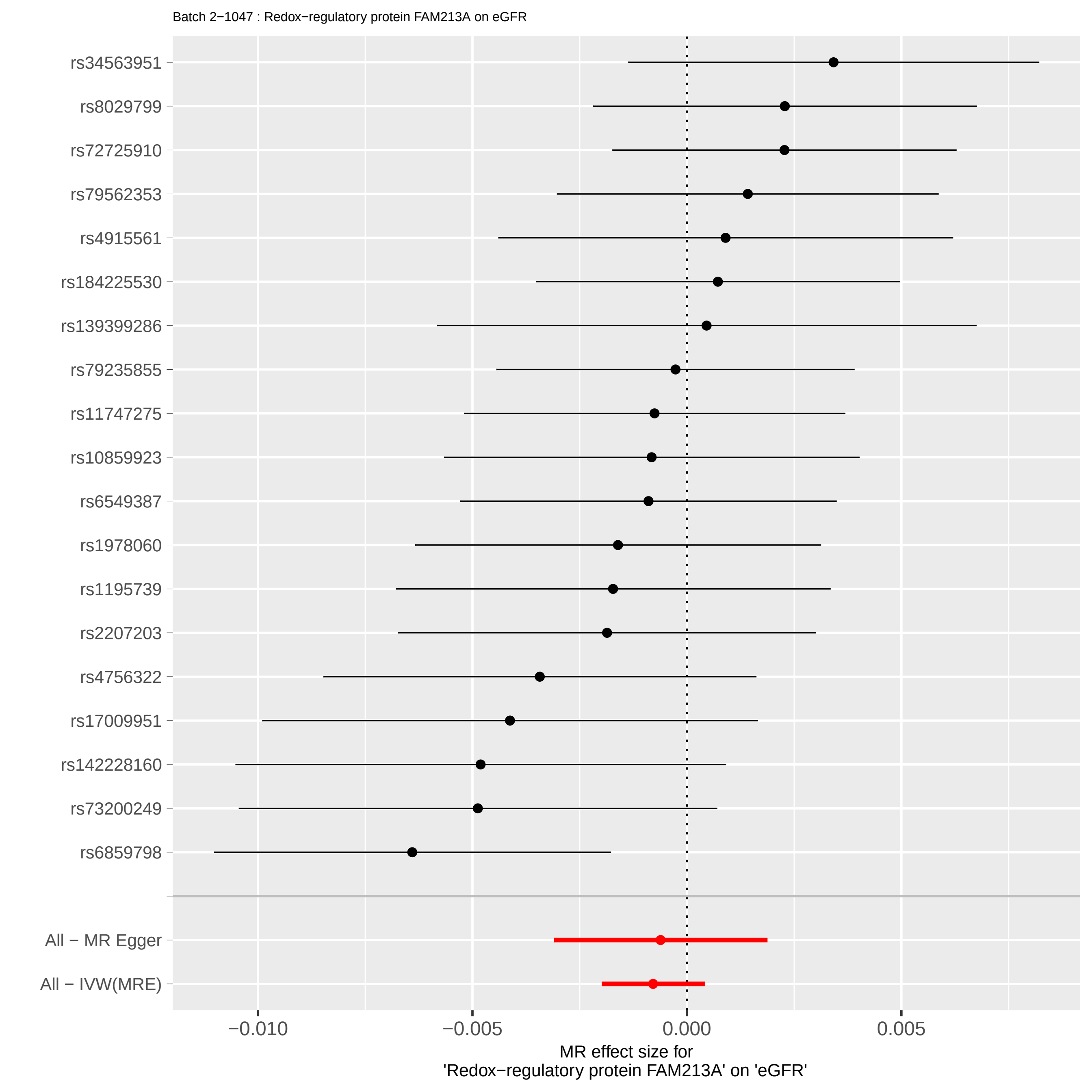

Batch 2−1047 : Redox−regulatory protein FAM213A on eGFR
rs34563951
rs8029799
rs72725910
rs79562353
rs4915561
rs184225530
rs139399286
rs79235855
rs11747275
rs10859923
rs6549387
rs1978060
rs1195739
rs2207203
rs4756322
rs17009951
rs142228160
rs73200249
rs6859798
All − MR Egger
All − IVW(MRE)
−0.010
−0.005
0.000
0.005
MR effect size for
'Redox−regulatory protein FAM213A' on 'eGFR'

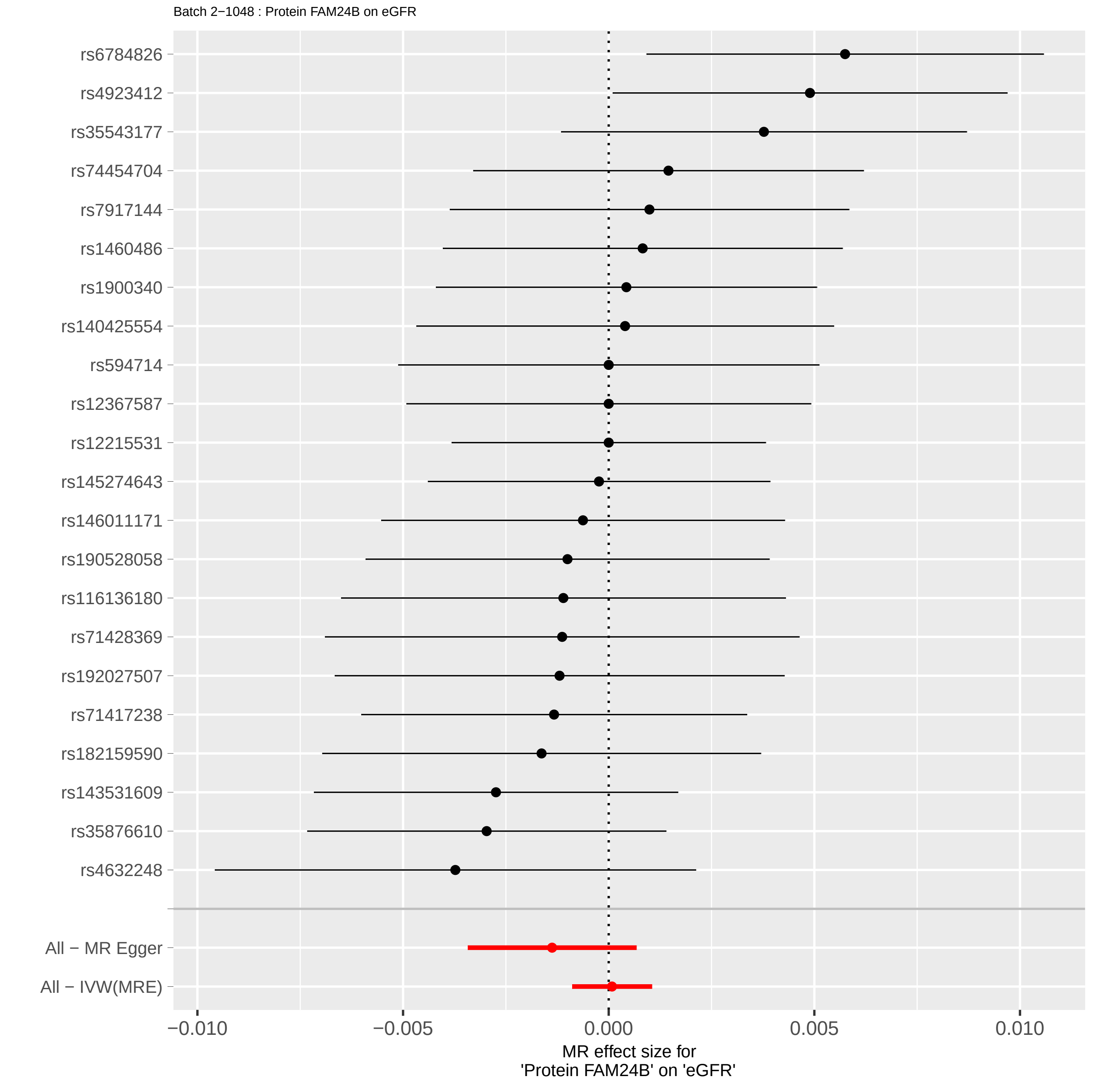

Batch 2−1048 : Protein FAM24B on eGFR
rs6784826
rs4923412
rs35543177
rs74454704
rs7917144
rs1460486
rs1900340
rs140425554
rs594714
rs12367587
rs12215531
rs145274643
rs146011171
rs190528058
rs116136180
rs71428369
rs192027507
rs71417238
rs182159590
rs143531609
rs35876610
rs4632248
All − MR Egger
All − IVW(MRE)
−0.010
−0.005
0.000
0.005
0.010
MR effect size for
'Protein FAM24B' on 'eGFR'

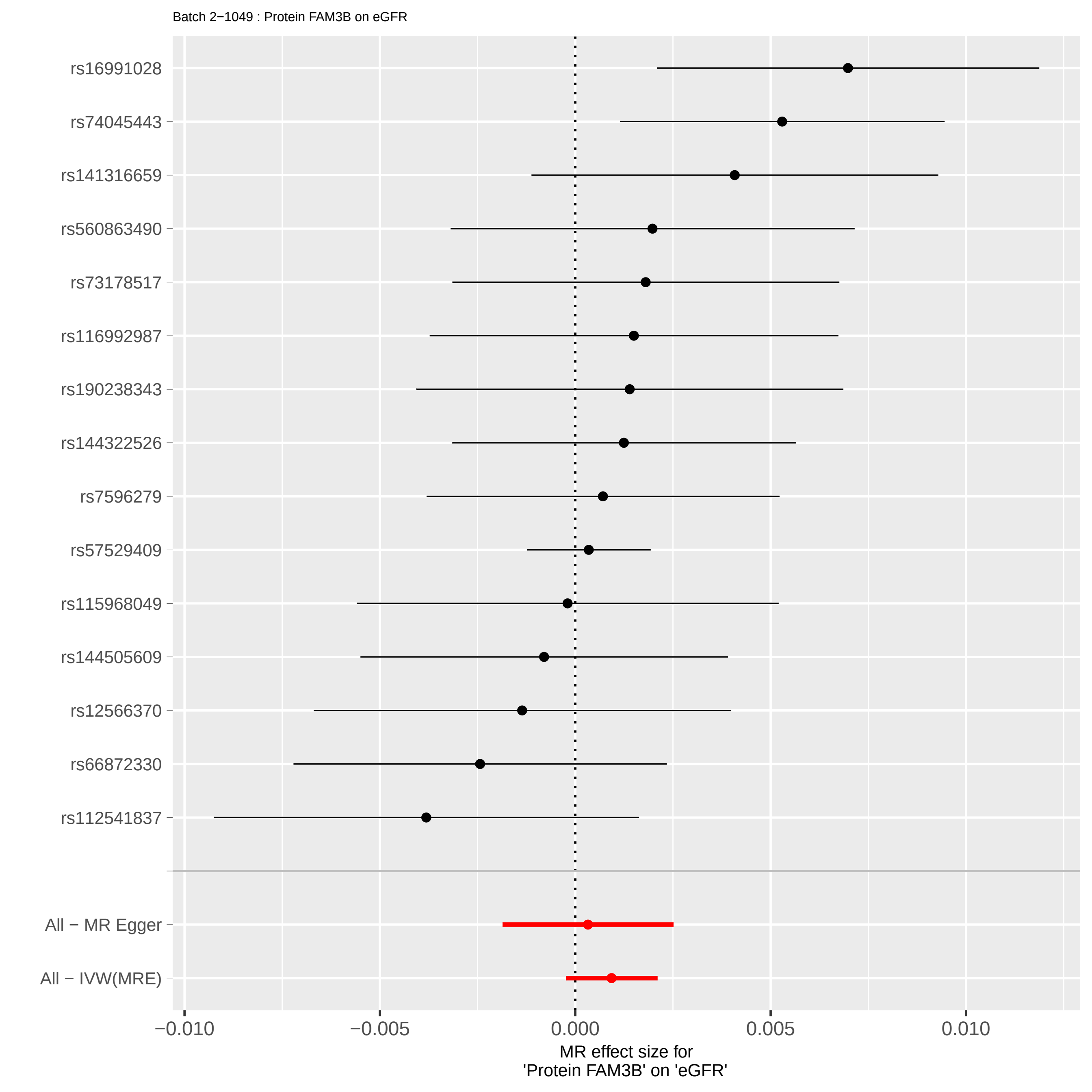

Batch 2−1049 : Protein FAM3B on eGFR
rs16991028
rs74045443
rs141316659
rs560863490
rs73178517
rs116992987
rs190238343
rs144322526
rs7596279
rs57529409
rs115968049
rs144505609
rs12566370
rs66872330
rs112541837
All − MR Egger
All − IVW(MRE)
−0.010
−0.005
0.000
0.005
0.010
MR effect size for
'Protein FAM3B' on 'eGFR'

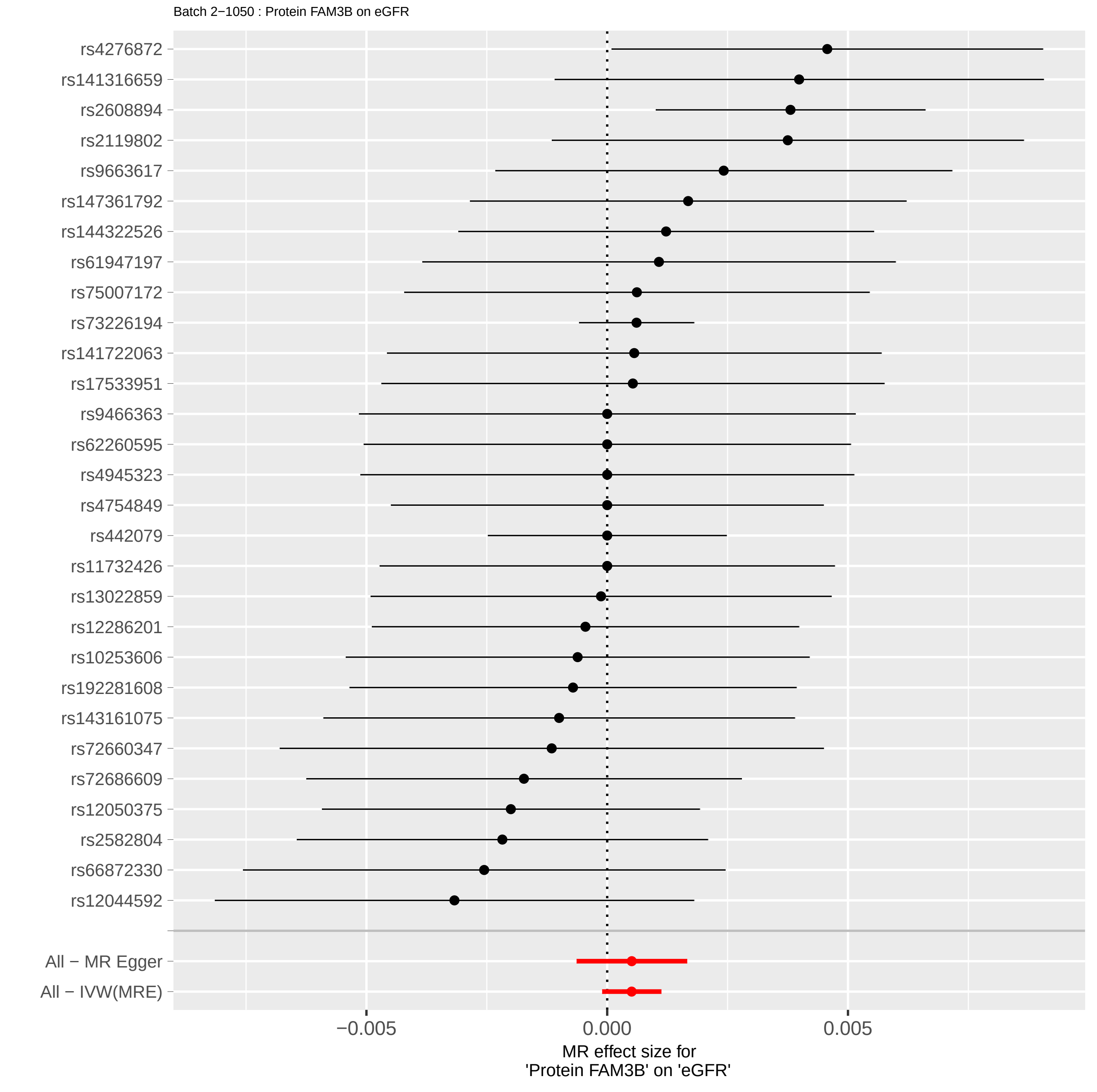

Batch 2−1050 : Protein FAM3B on eGFR
rs4276872
rs141316659
rs2608894
rs2119802
rs9663617
rs147361792
rs144322526
rs61947197
rs75007172
rs73226194
rs141722063
rs17533951
rs9466363
rs62260595
rs4945323
rs4754849
rs442079
rs11732426
rs13022859
rs12286201
rs10253606
rs192281608
rs143161075
rs72660347
rs72686609
rs12050375
rs2582804
rs66872330
rs12044592
All − MR Egger
All − IVW(MRE)
−0.005
0.000
0.005
MR effect size for
'Protein FAM3B' on 'eGFR'

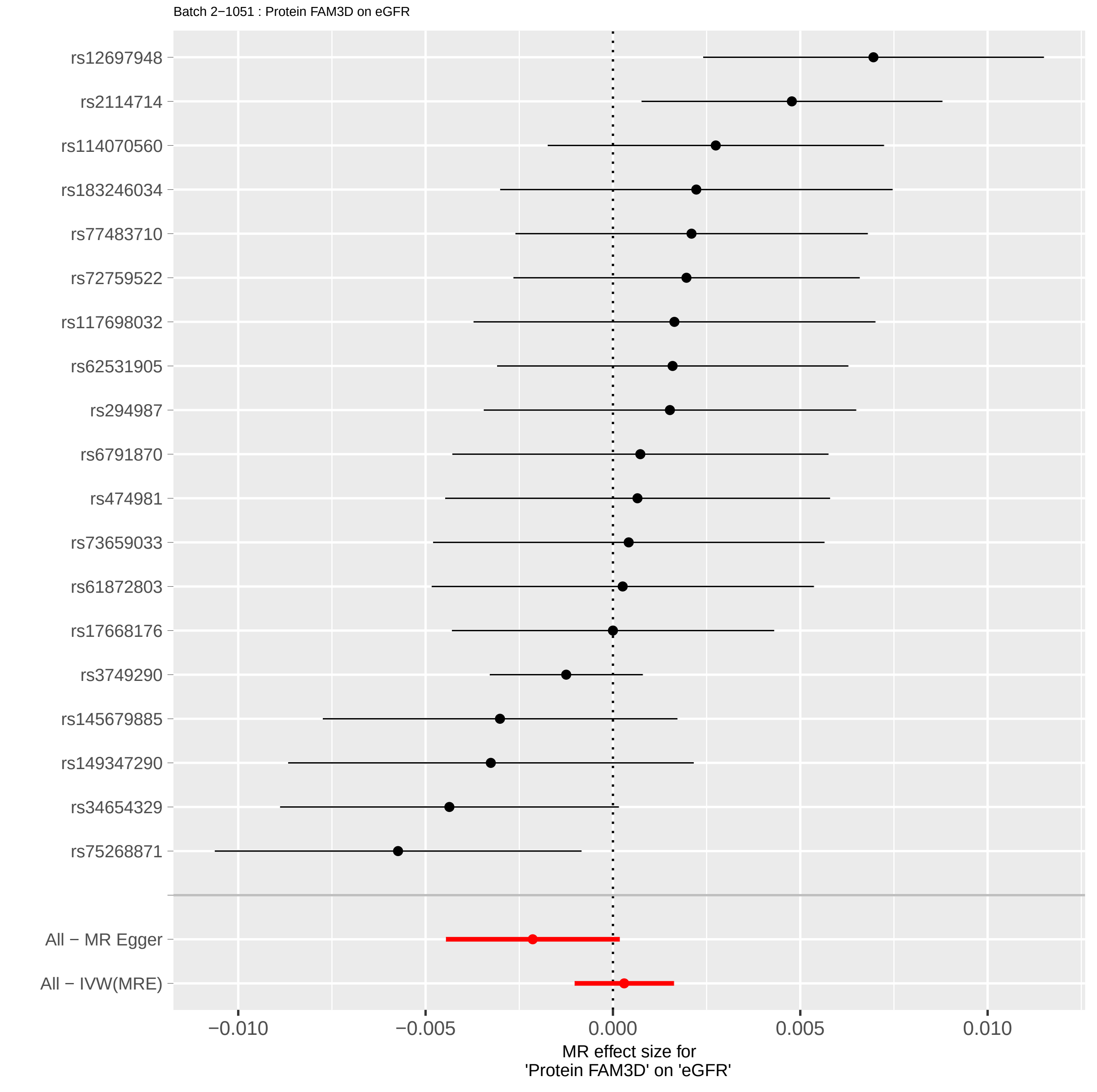
Batch 2−1051 : Protein FAM3D on eGFR
rs12697948
rs2114714
rs114070560
rs183246034
rs77483710
rs72759522
rs117698032
rs62531905
rs294987
rs6791870
rs474981
rs73659033
rs61872803
rs17668176
rs3749290
rs145679885
rs149347290
rs34654329
rs75268871
All − MR Egger
All − IVW(MRE)
−0.010
−0.005
0.000
0.005
0.010
MR effect size for
'Protein FAM3D' on 'eGFR'

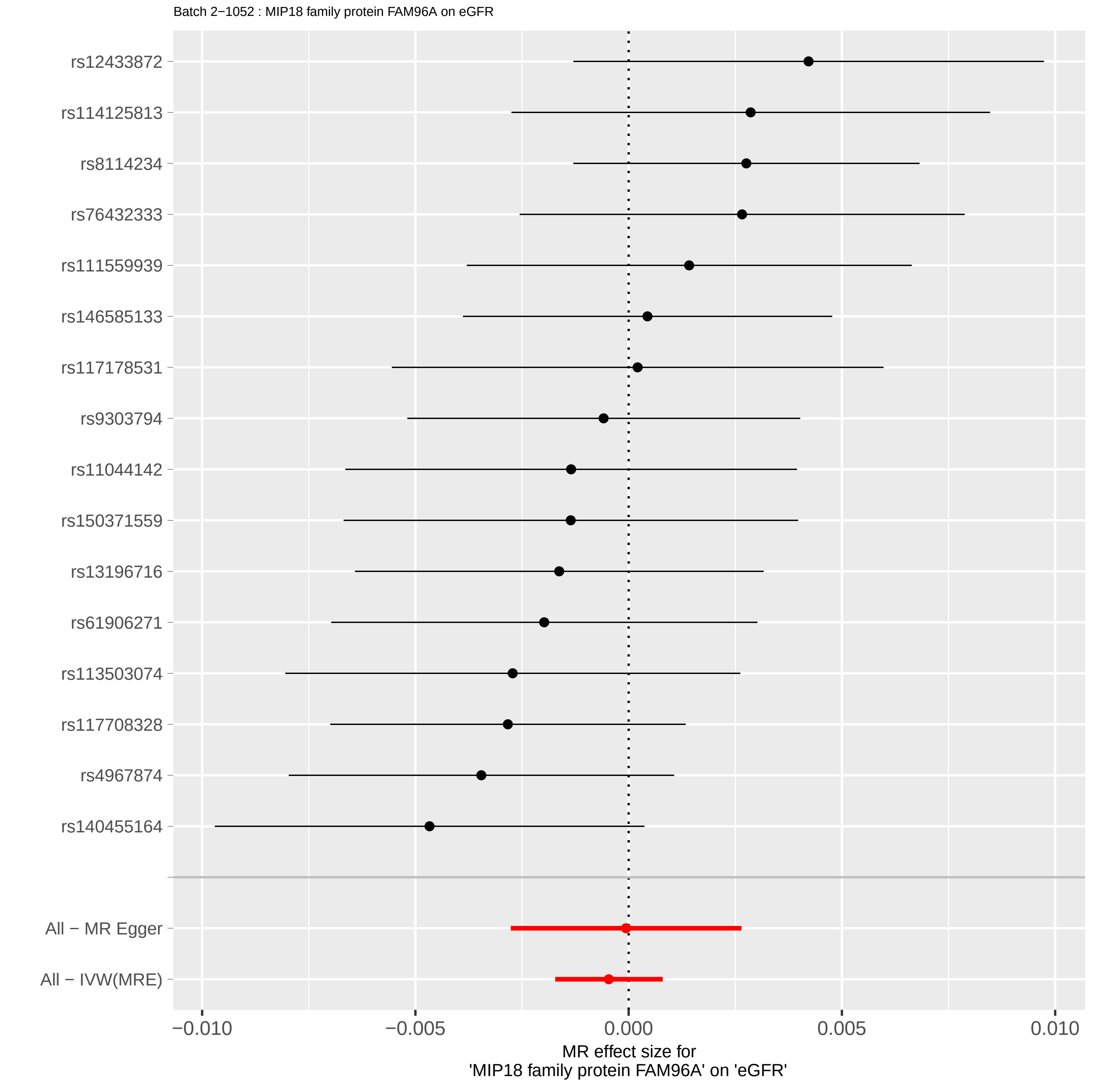

Batch 2−1052 : MIP18 family protein FAM96A on eGFR
rs12433872
rs114125813
rs8114234
rs76432333
rs111559939
rs146585133
rs117178531
rs9303794
rs11044142
rs150371559
rs13196716
rs61906271
rs113503074
rs117708328
rs4967874
rs140455164
All − MR Egger
All − IVW(MRE)
−0.010
−0.005
0.000
0.005
0.010
MR effect size for
'MIP18 family protein FAM96A' on 'eGFR'

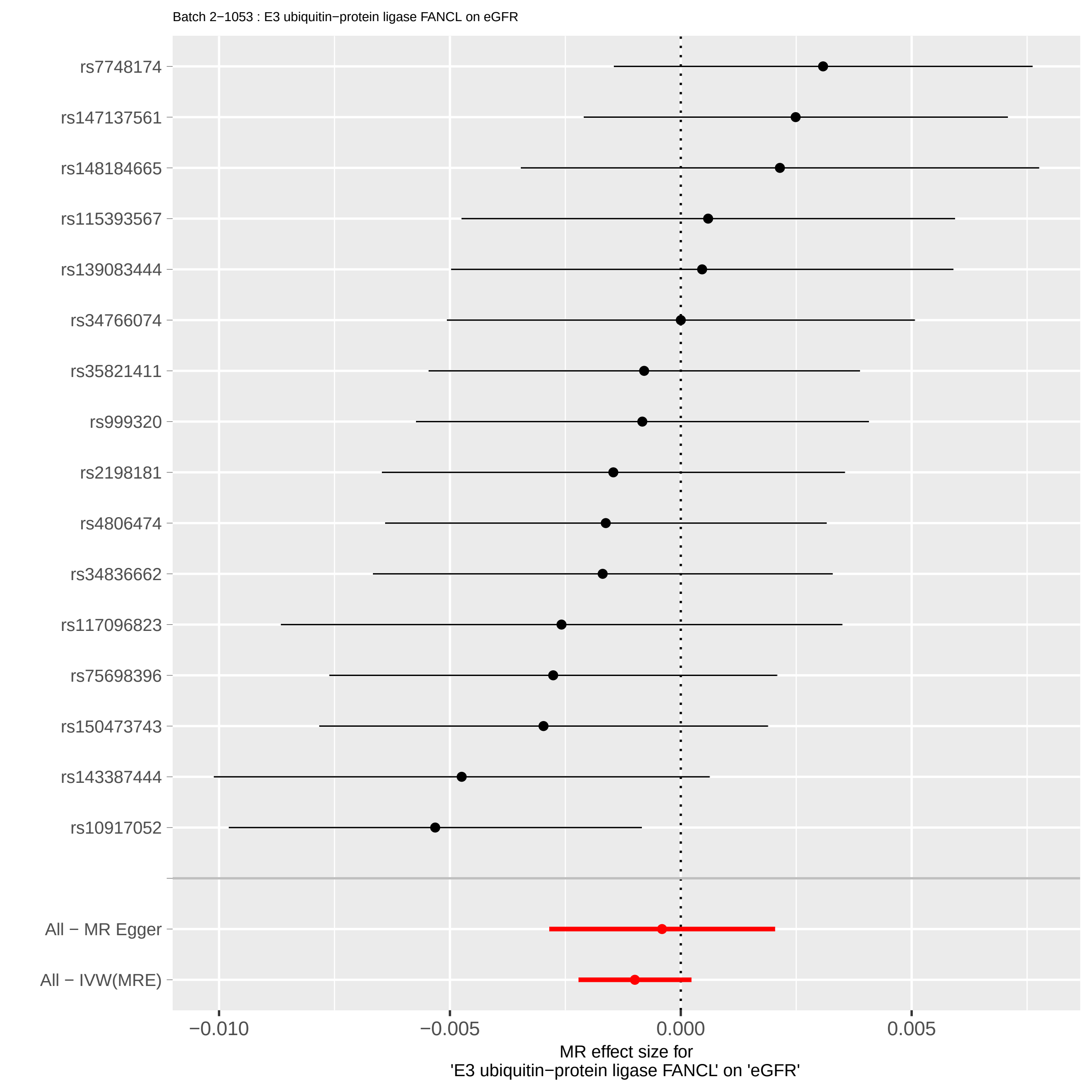

Batch 2−1053 : E3 ubiquitin−protein ligase FANCL on eGFR
rs7748174
rs147137561
rs148184665
rs115393567
rs139083444
rs34766074
rs35821411
rs999320
rs2198181
rs4806474
rs34836662
rs117096823
rs75698396
rs150473743
rs143387444
rs10917052
All − MR Egger
All − IVW(MRE)
−0.010
−0.005
0.000
0.005
MR effect size for
'E3 ubiquitin−protein ligase FANCL' on 'eGFR'

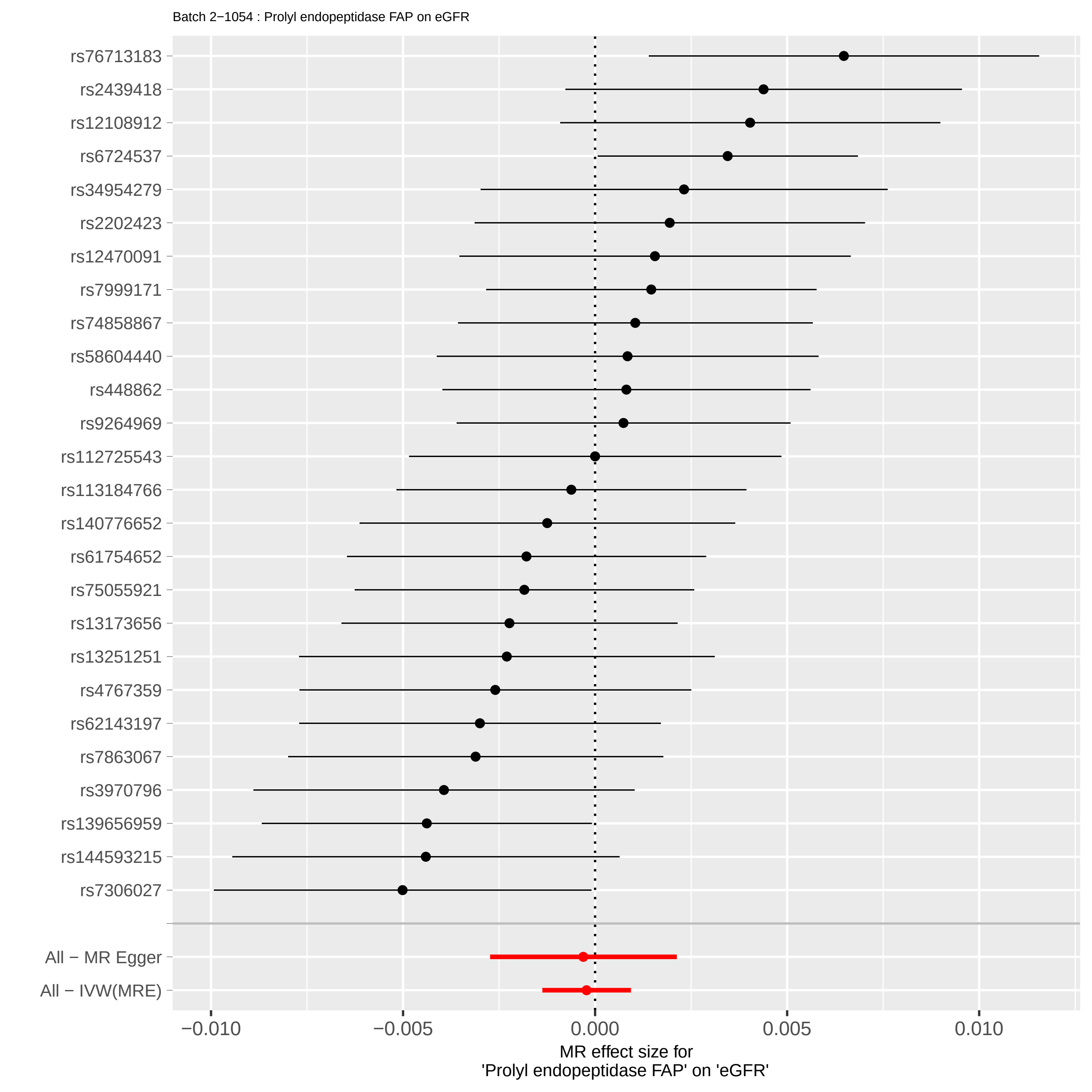

Batch 2−1054 : Prolyl endopeptidase FAP on eGFR
rs76713183
rs2439418
rs12108912
rs6724537
rs34954279
rs2202423
rs12470091
rs7999171
rs74858867
rs58604440
rs448862
rs9264969
rs112725543
rs113184766
rs140776652
rs61754652
rs75055921
rs13173656
rs13251251
rs4767359
rs62143197
rs7863067
rs3970796
rs139656959
rs144593215
rs7306027
All − MR Egger
All − IVW(MRE)
−0.010
−0.005
0.000
0.005
0.010
MR effect size for
'Prolyl endopeptidase FAP' on 'eGFR'

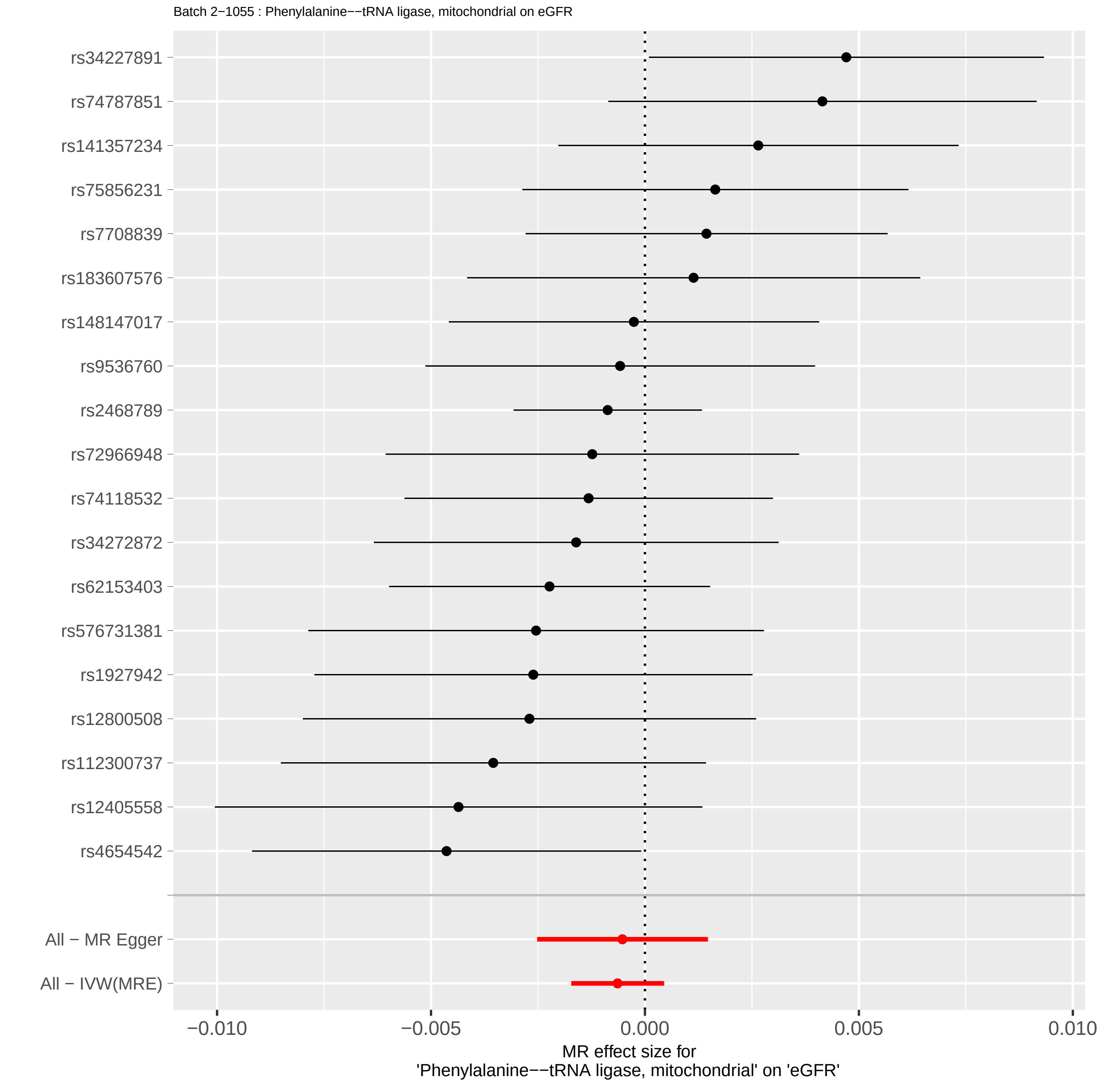

Batch 2−1055 : Phenylalanine−−tRNA ligase, mitochondrial on eGFR
rs34227891
rs74787851
rs141357234
rs75856231
rs7708839
rs183607576
rs148147017
rs9536760
rs2468789
rs72966948
rs74118532
rs34272872
rs62153403
rs576731381
rs1927942
rs12800508
rs112300737
rs12405558
rs4654542
All − MR Egger
All − IVW(MRE)
−0.010
−0.005
0.000
0.005
0.010
MR effect size for
'Phenylalanine−−tRNA ligase, mitochondrial' on 'eGFR'

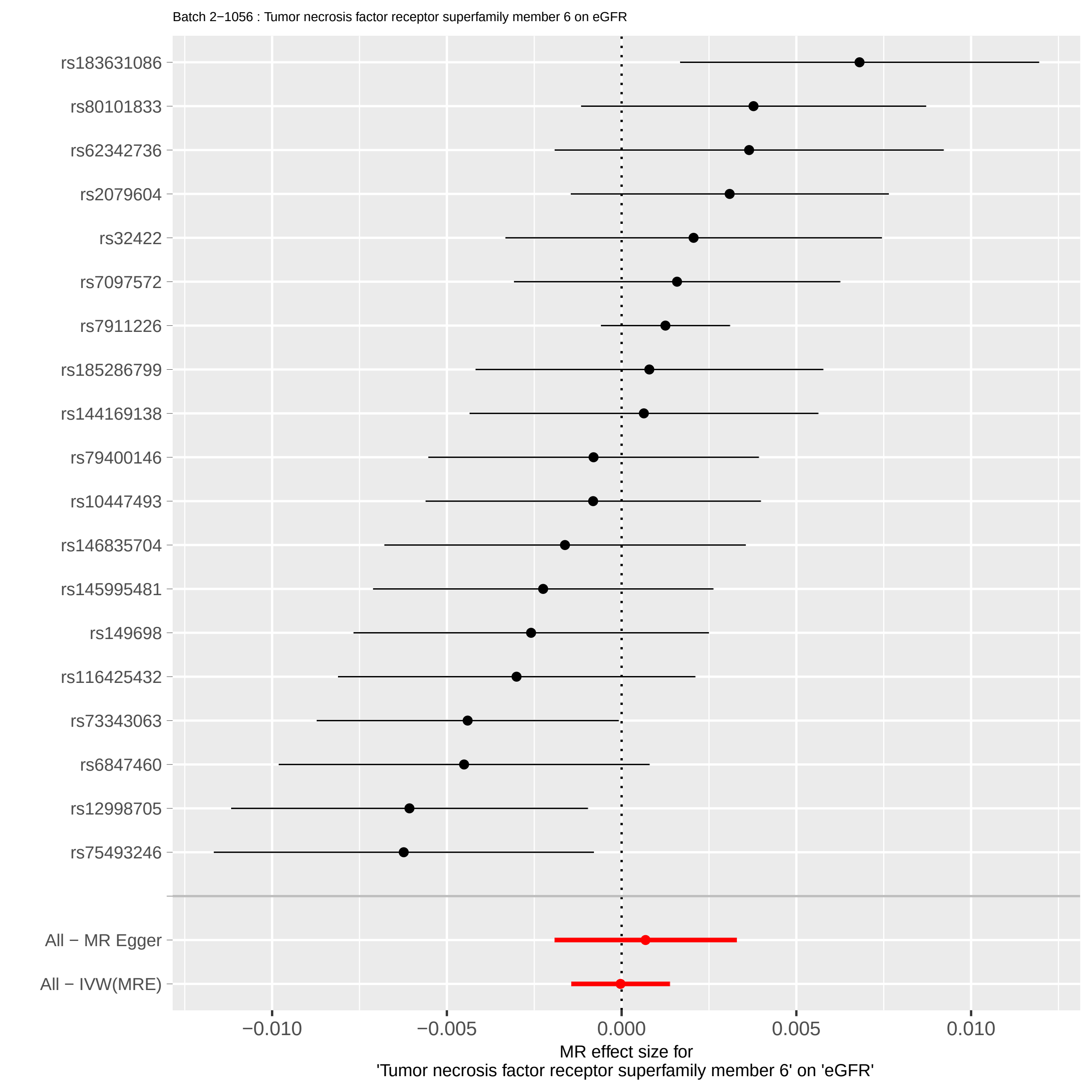

Batch 2−1056 : Tumor necrosis factor receptor superfamily member 6 on eGFR
rs183631086
rs80101833
rs62342736
rs2079604
rs32422
rs7097572
rs7911226
rs185286799
rs144169138
rs79400146
rs10447493
rs146835704
rs145995481
rs149698
rs116425432
rs73343063
rs6847460
rs12998705
rs75493246
All − MR Egger
All − IVW(MRE)
−0.010
−0.005
0.000
0.005
0.010
MR effect size for
'Tumor necrosis factor receptor superfamily member 6' on 'eGFR'

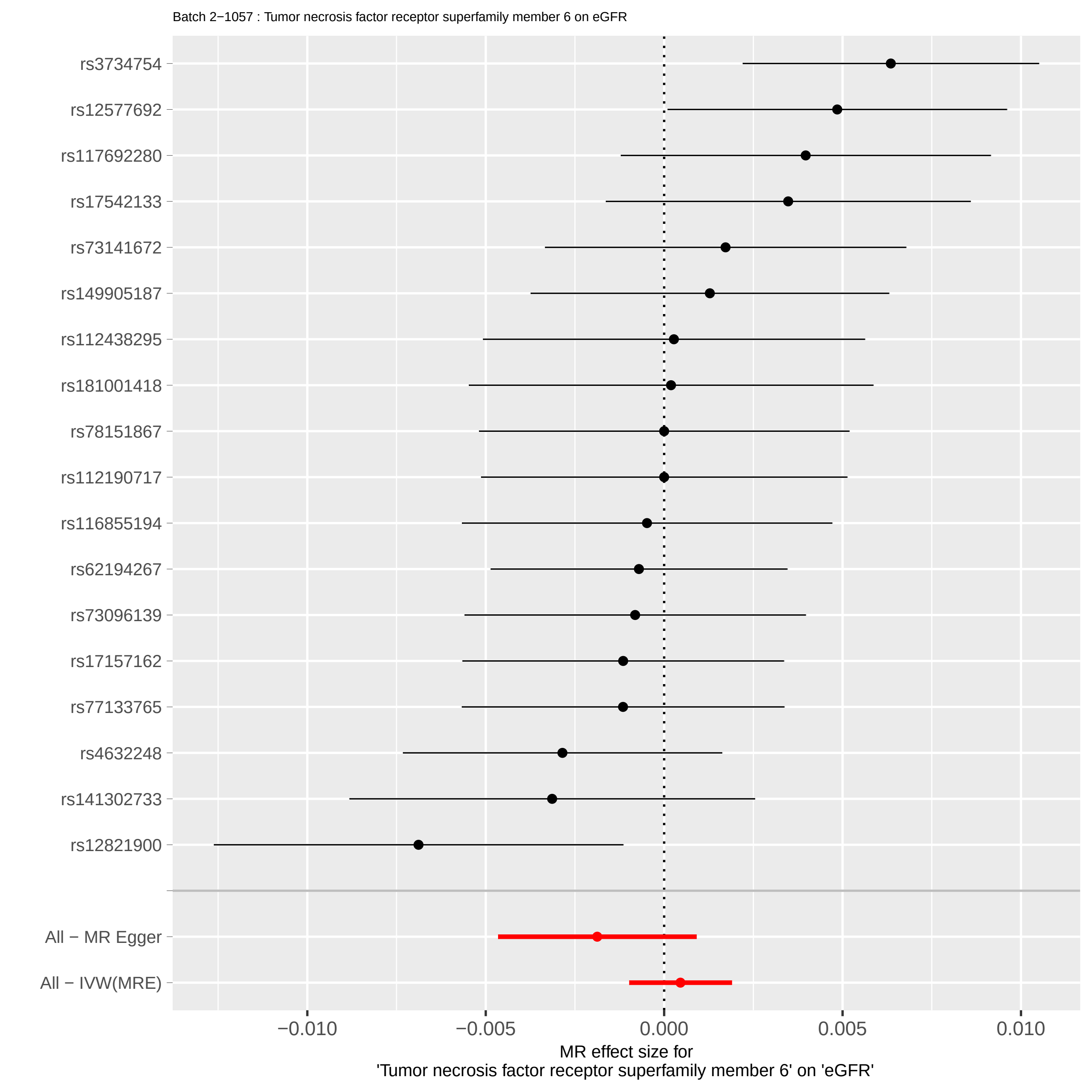
Batch 2−1057 : Tumor necrosis factor receptor superfamily member 6 on eGFR
rs3734754
rs12577692
rs117692280
rs17542133
rs73141672
rs149905187
rs112438295
rs181001418
rs78151867
rs112190717
rs116855194
rs62194267
rs73096139
rs17157162
rs77133765
rs4632248
rs141302733
rs12821900
All − MR Egger
All − IVW(MRE)
−0.010
−0.005
0.000
0.005
0.010
MR effect size for
'Tumor necrosis factor receptor superfamily member 6' on 'eGFR'

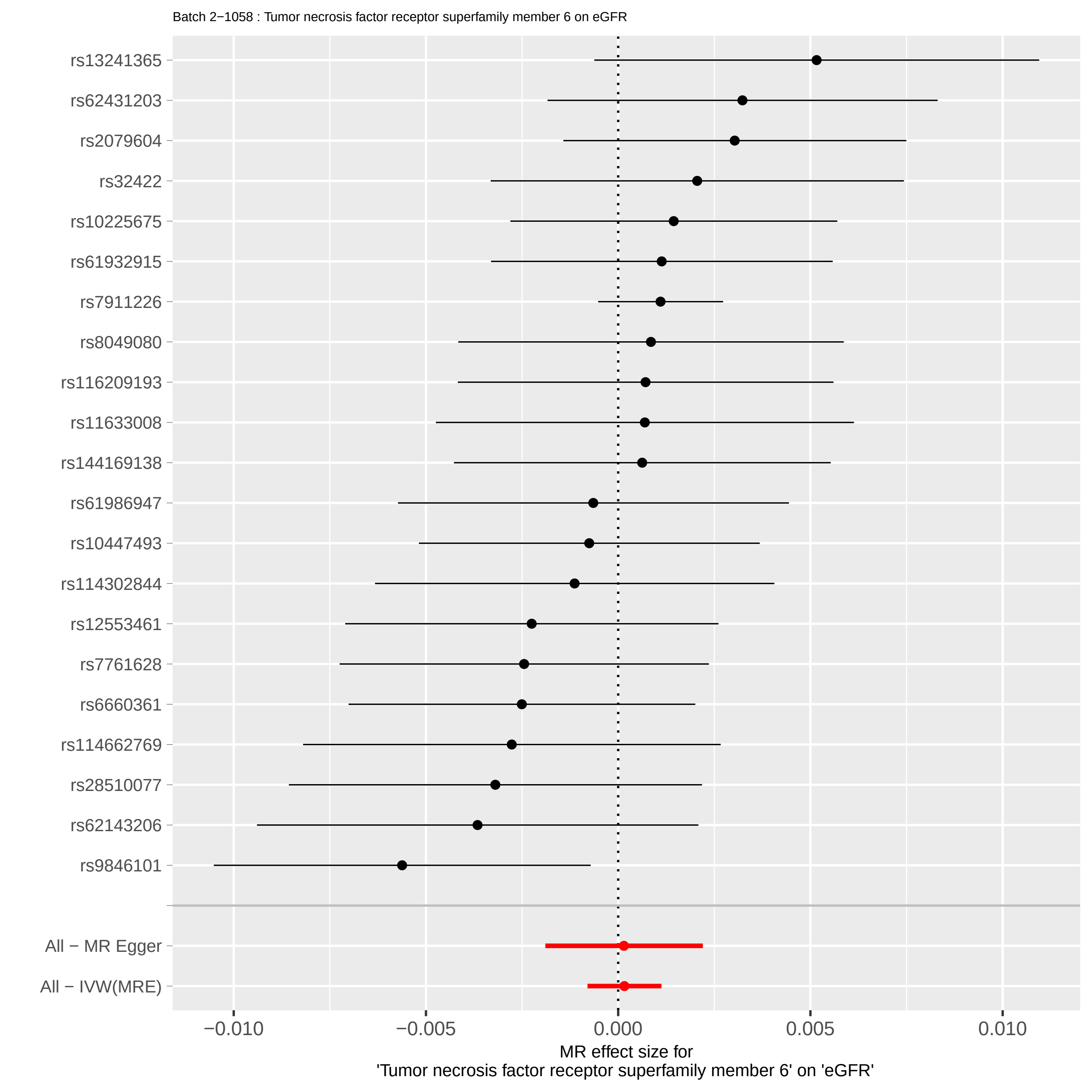
Batch 2−1058 : Tumor necrosis factor receptor superfamily member 6 on eGFR
rs13241365
rs62431203
rs2079604
rs32422
rs10225675
rs61932915
rs7911226
rs8049080
rs116209193
rs11633008
rs144169138
rs61986947
rs10447493
rs114302844
rs12553461
rs7761628
rs6660361
rs114662769
rs28510077
rs62143206
rs9846101
All − MR Egger
All − IVW(MRE)
−0.010
−0.005
0.000
0.005
0.010
MR effect size for
'Tumor necrosis factor receptor superfamily member 6' on 'eGFR'

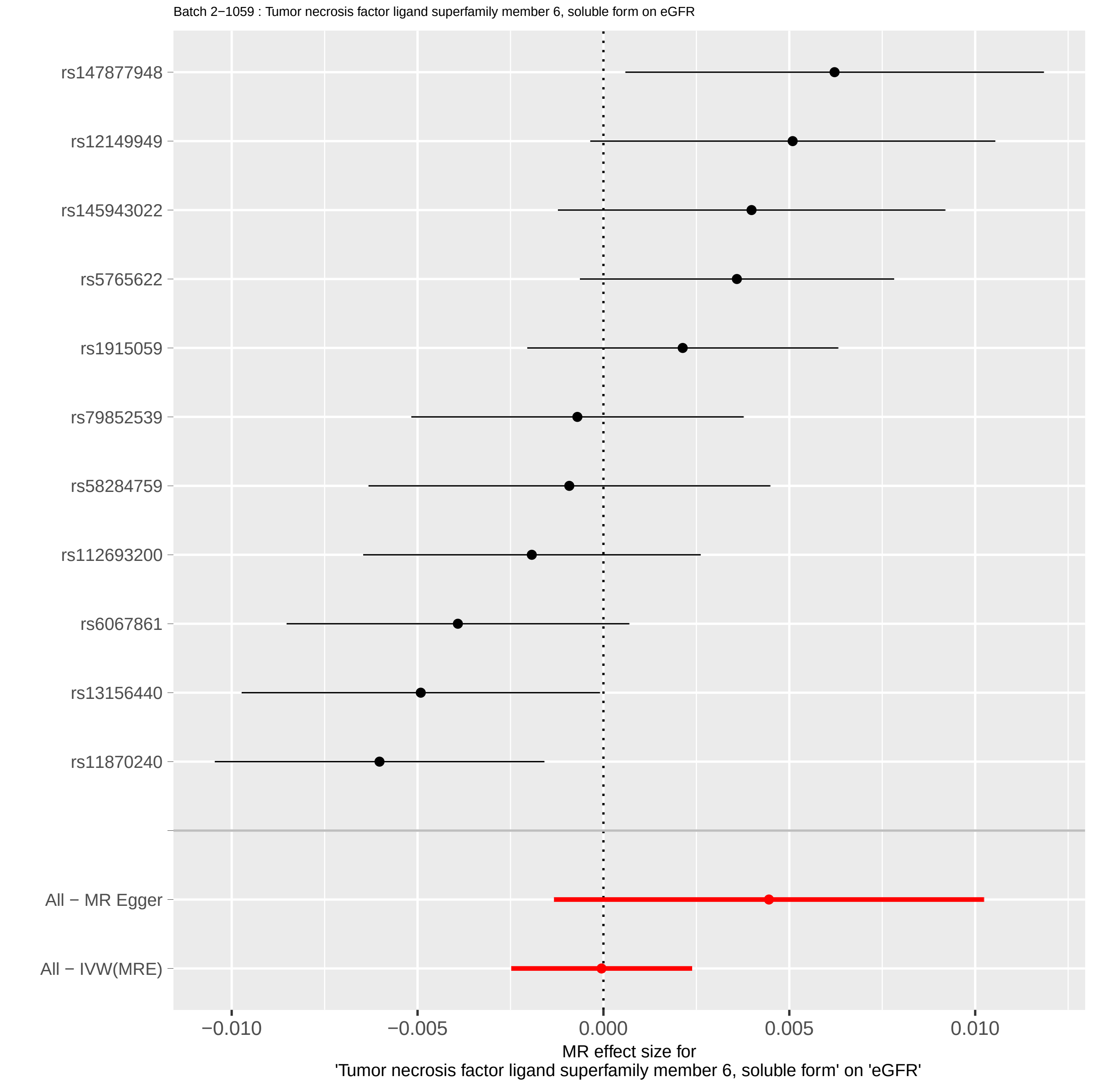

Batch 2−1059 : Tumor necrosis factor ligand superfamily member 6, soluble form on eGFR
rs147877948
rs12149949
rs145943022
rs5765622
rs1915059
rs79852539
rs58284759
rs112693200
rs6067861
rs13156440
rs11870240
All − MR Egger
All − IVW(MRE)
−0.010
−0.005
0.000
0.005
0.010
MR effect size for
'Tumor necrosis factor ligand superfamily member 6, soluble form' on 'eGFR'

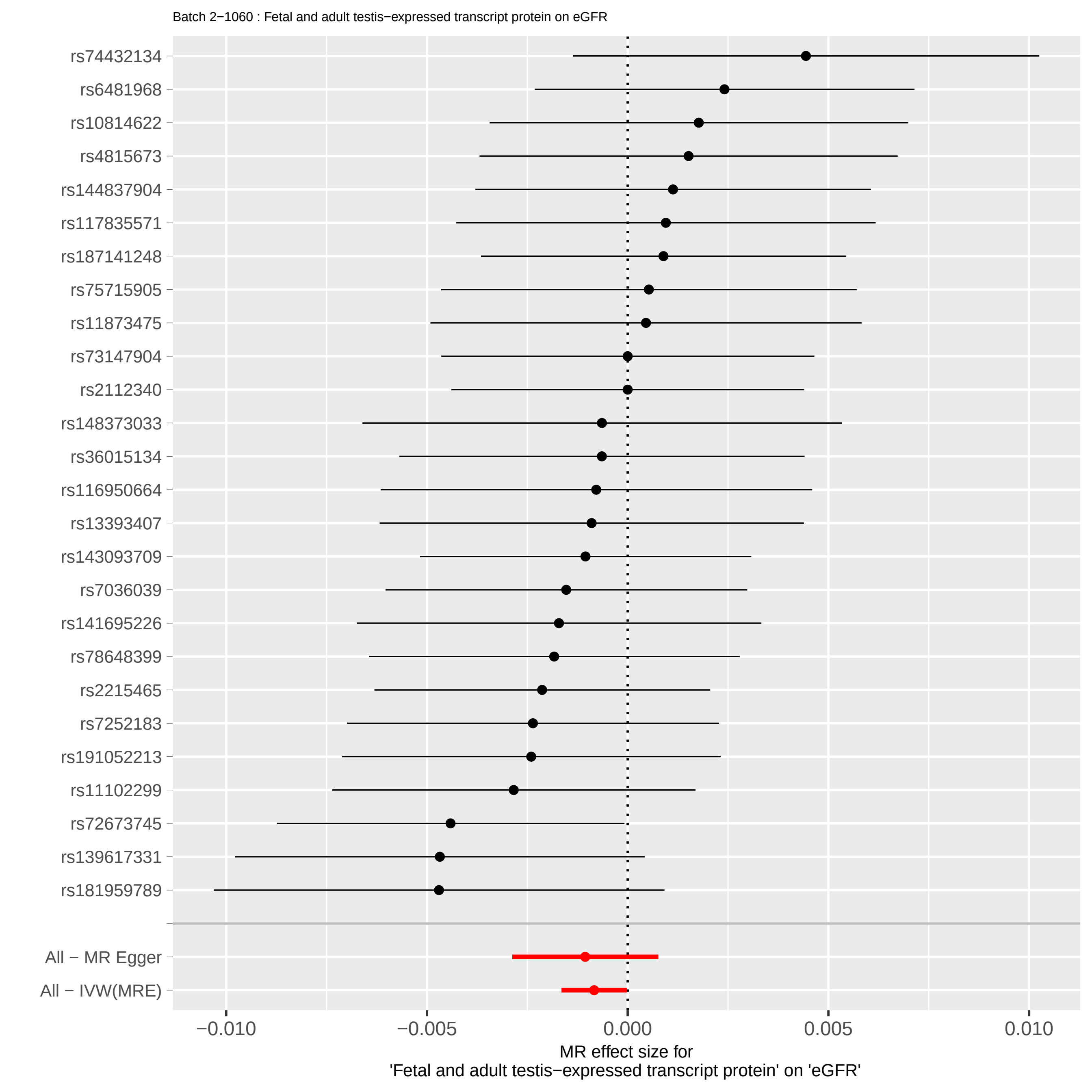

Batch 2−1060 : Fetal and adult testis−expressed transcript protein on eGFR
rs74432134
rs6481968
rs10814622
rs4815673
rs144837904
rs117835571
rs187141248
rs75715905
rs11873475
rs73147904
rs2112340
rs148373033
rs36015134
rs116950664
rs13393407
rs143093709
rs7036039
rs141695226
rs78648399
rs2215465
rs7252183
rs191052213
rs11102299
rs72673745
rs139617331
rs181959789
All − MR Egger
All − IVW(MRE)
−0.010
−0.005
0.000
0.005
0.010
MR effect size for
'Fetal and adult testis−expressed transcript protein' on 'eGFR'

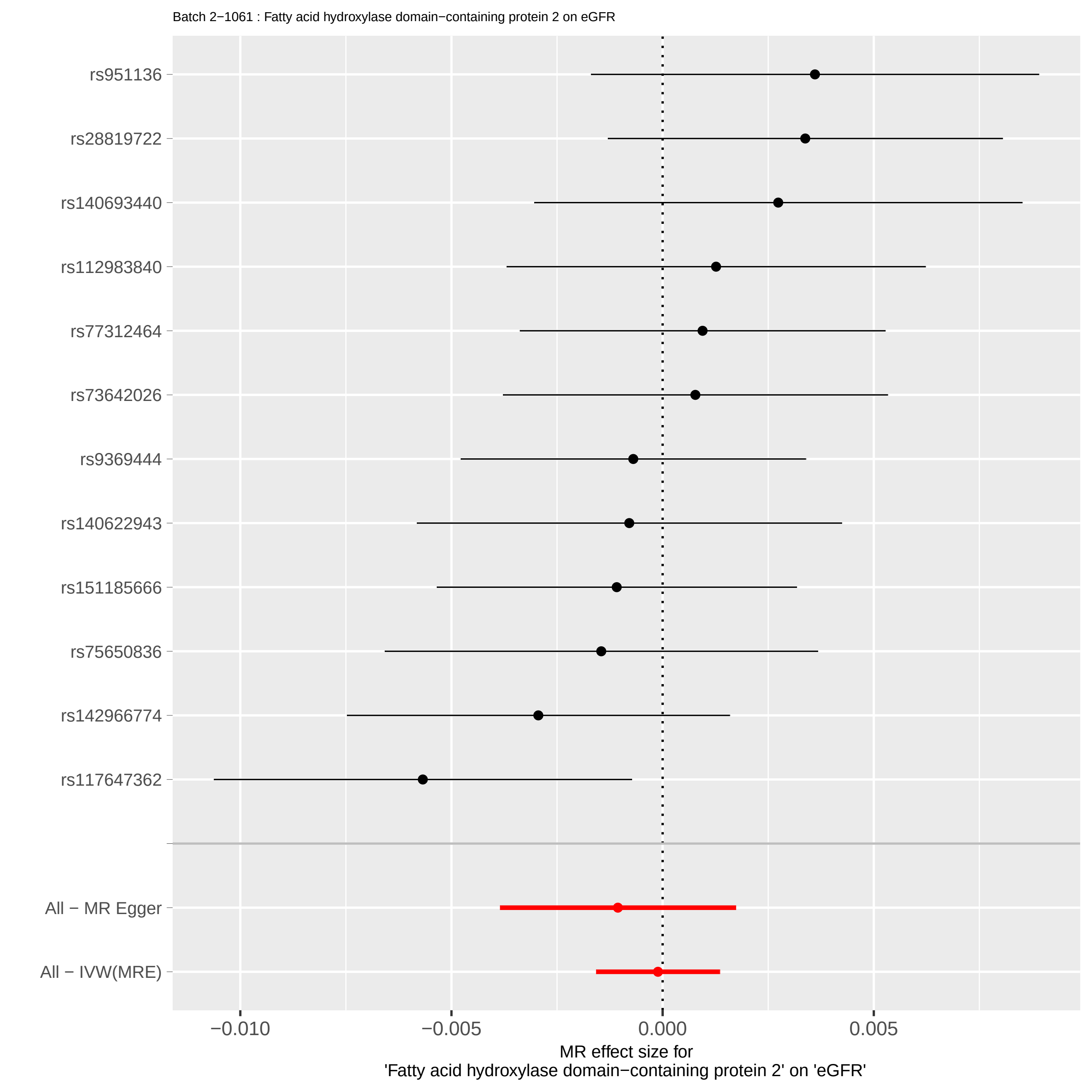

Batch 2−1061 : Fatty acid hydroxylase domain−containing protein 2 on eGFR
rs951136
rs28819722
rs140693440
rs112983840
rs77312464
rs73642026
rs9369444
rs140622943
rs151185666
rs75650836
rs142966774
rs117647362
All − MR Egger
All − IVW(MRE)
−0.010
−0.005
0.000
0.005
MR effect size for
'Fatty acid hydroxylase domain−containing protein 2' on 'eGFR'

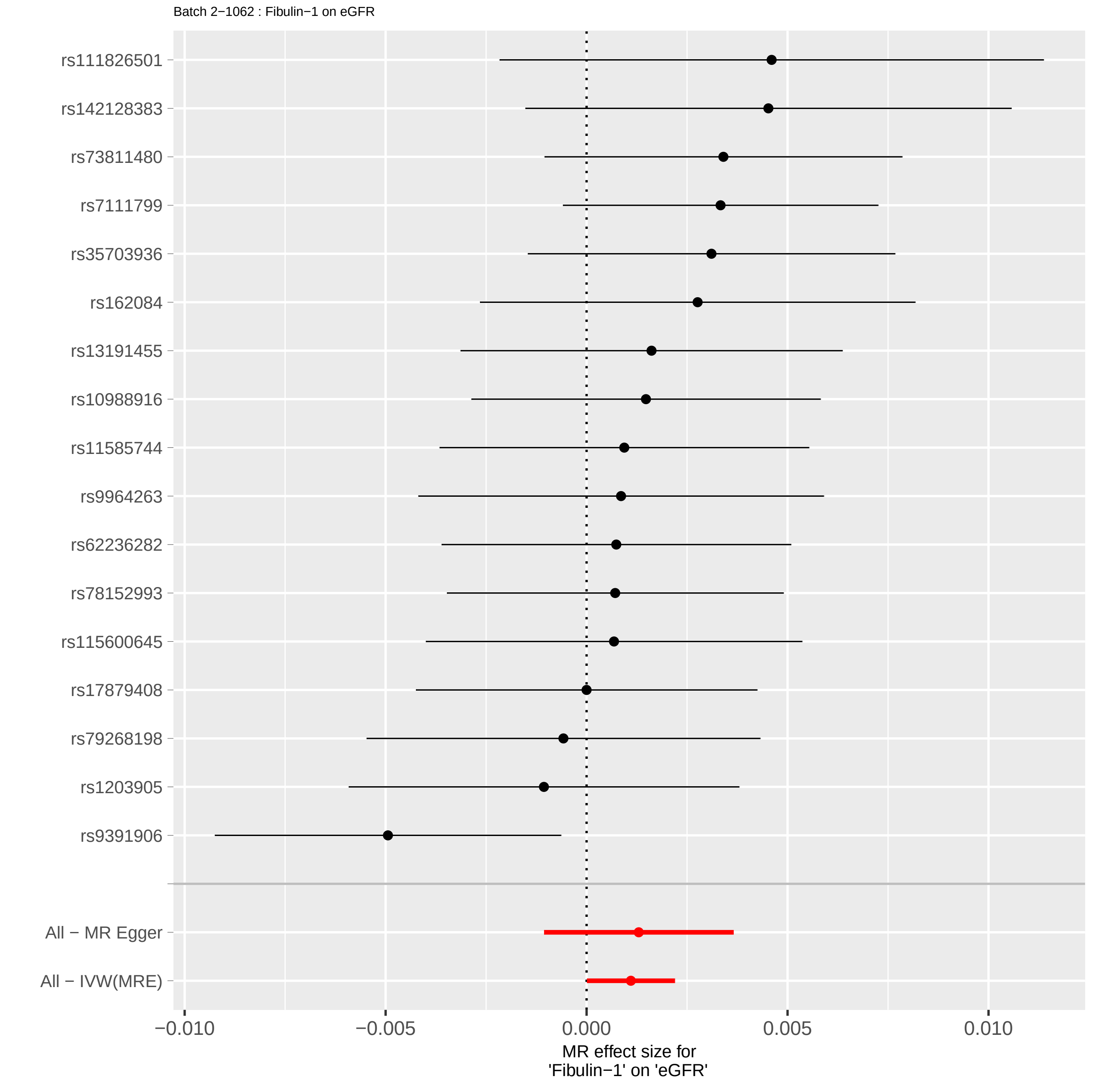
Batch 2−1062 : Fibulin−1 on eGFR
rs111826501
rs142128383
rs73811480
rs7111799
rs35703936
rs162084
rs13191455
rs10988916
rs11585744
rs9964263
rs62236282
rs78152993
rs115600645
rs17879408
rs79268198
rs1203905
rs9391906
All − MR Egger
All − IVW(MRE)
−0.010
−0.005
0.000
0.005
0.010
MR effect size for
'Fibulin−1' on 'eGFR'

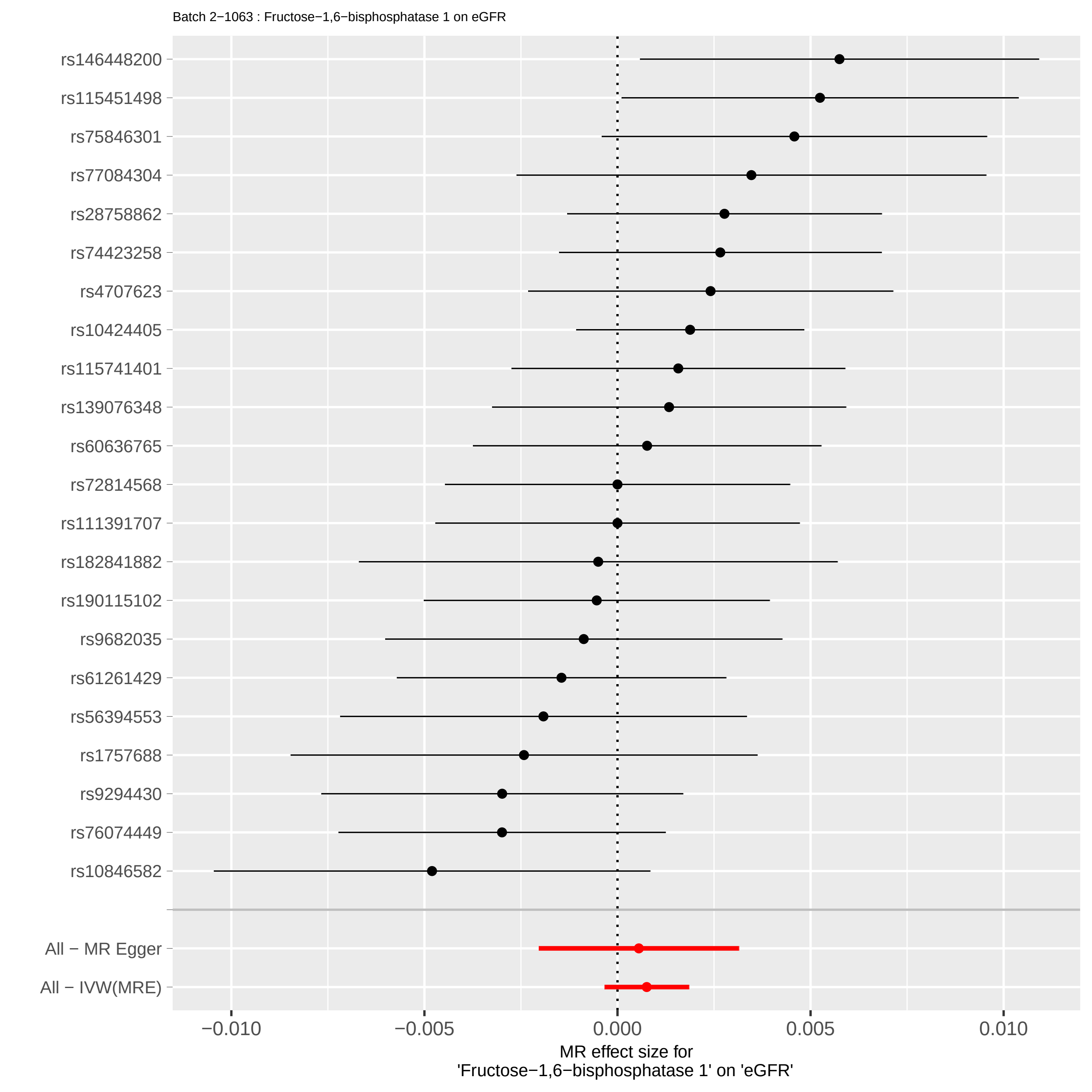

Batch 2−1063 : Fructose−1,6−bisphosphatase 1 on eGFR
rs146448200
rs115451498
rs75846301
rs77084304
rs28758862
rs74423258
rs4707623
rs10424405
rs115741401
rs139076348
rs60636765
rs72814568
rs111391707
rs182841882
rs190115102
rs9682035
rs61261429
rs56394553
rs1757688
rs9294430
rs76074449
rs10846582
All − MR Egger
All − IVW(MRE)
−0.010
−0.005
0.000
0.005
0.010
MR effect size for
'Fructose−1,6−bisphosphatase 1' on 'eGFR'

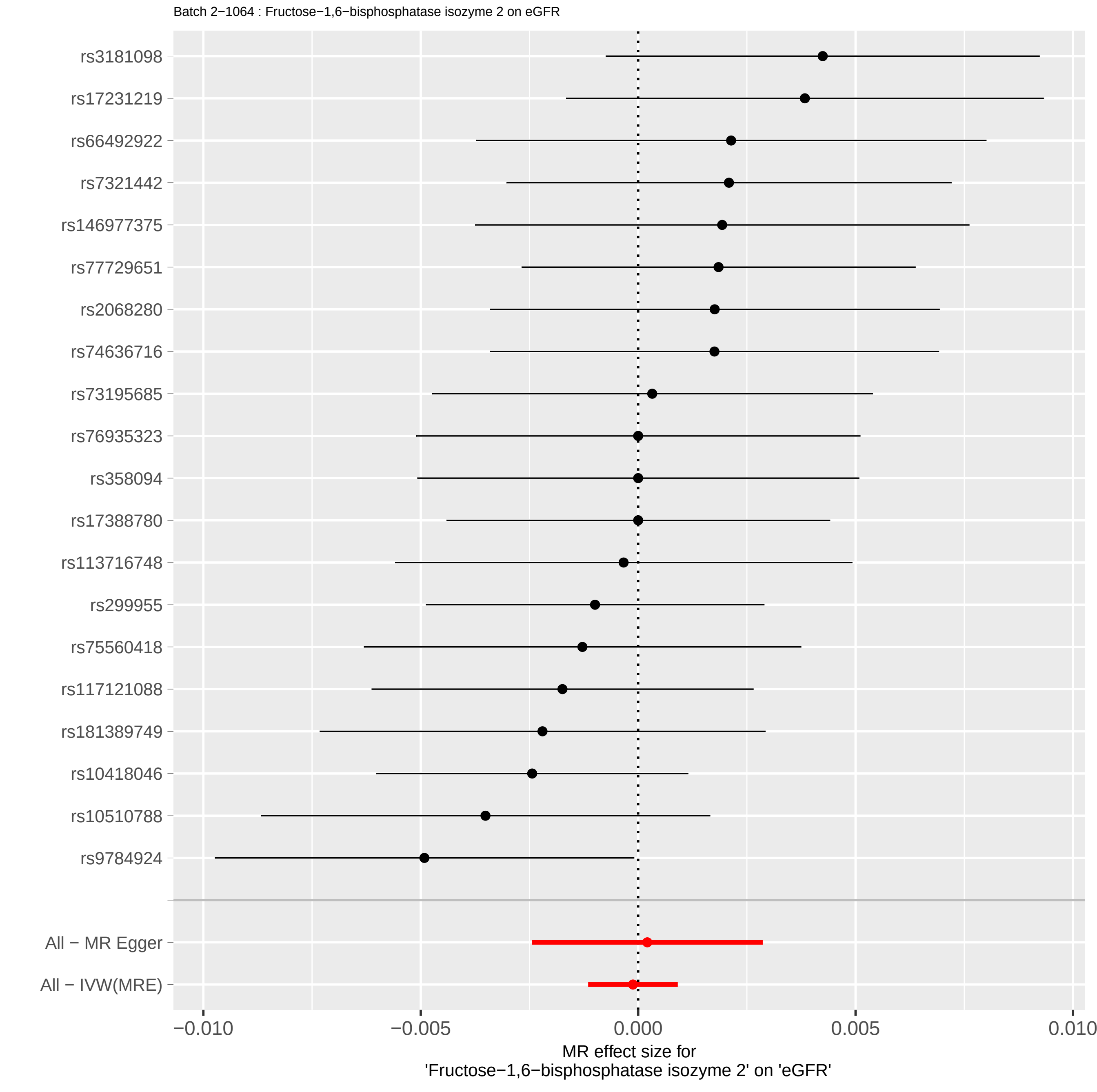
Batch 2−1064 : Fructose−1,6−bisphosphatase isozyme 2 on eGFR
rs3181098
rs17231219
rs66492922
rs7321442
rs146977375
rs77729651
rs2068280
rs74636716
rs73195685
rs76935323
rs358094
rs17388780
rs113716748
rs299955
rs75560418
rs117121088
rs181389749
rs10418046
rs10510788
rs9784924
All − MR Egger
All − IVW(MRE)
−0.010
−0.005
0.000
0.005
0.010
MR effect size for
'Fructose−1,6−bisphosphatase isozyme 2' on 'eGFR'

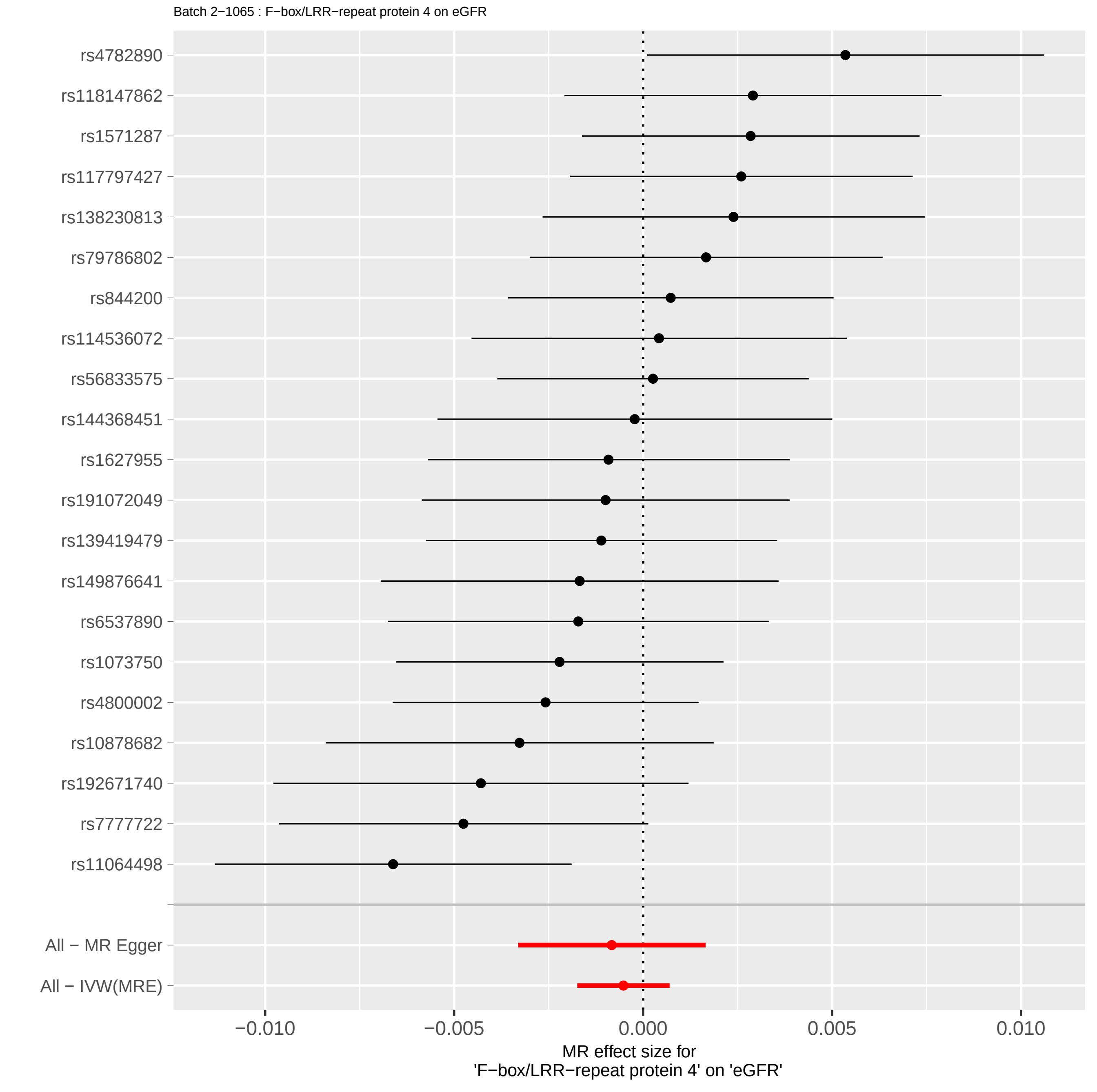

Batch 2−1065 : F−box/LRR−repeat protein 4 on eGFR
rs4782890
rs118147862
rs1571287
rs117797427
rs138230813
rs79786802
rs844200
rs114536072
rs56833575
rs144368451
rs1627955
rs191072049
rs139419479
rs149876641
rs6537890
rs1073750
rs4800002
rs10878682
rs192671740
rs7777722
rs11064498
All − MR Egger
All − IVW(MRE)
−0.010
−0.005
0.000
0.005
0.010
MR effect size for
'F−box/LRR−repeat protein 4' on 'eGFR'

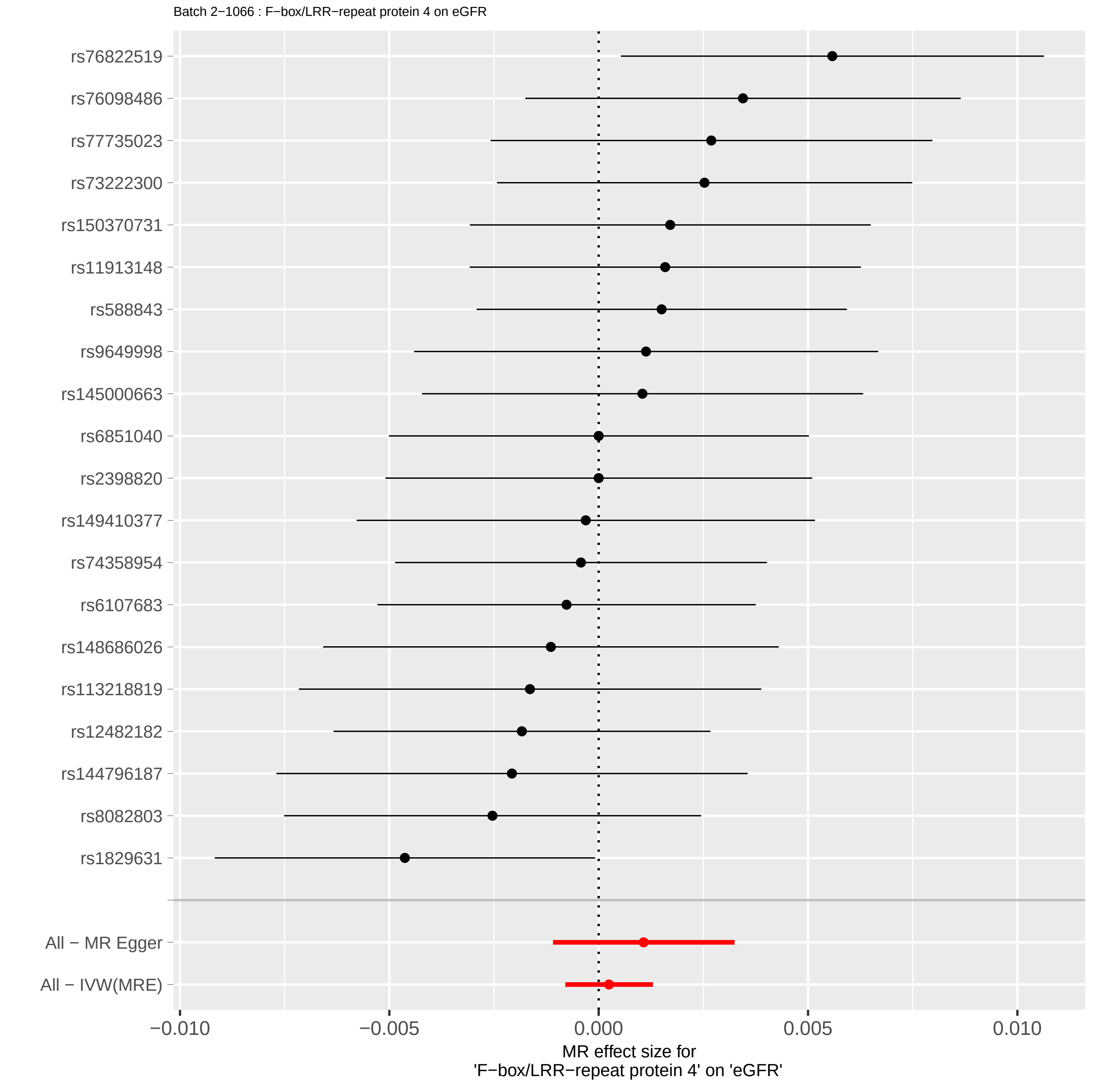

Batch 2−1066 : F−box/LRR−repeat protein 4 on eGFR
rs76822519
rs76098486
rs77735023
rs73222300
rs150370731
rs11913148
rs588843
rs9649998
rs145000663
rs6851040
rs2398820
rs149410377
rs74358954
rs6107683
rs148686026
rs113218819
rs12482182
rs144796187
rs8082803
rs1829631
All − MR Egger
All − IVW(MRE)
−0.010
−0.005
0.000
0.005
0.010
MR effect size for
'F−box/LRR−repeat protein 4' on 'eGFR'

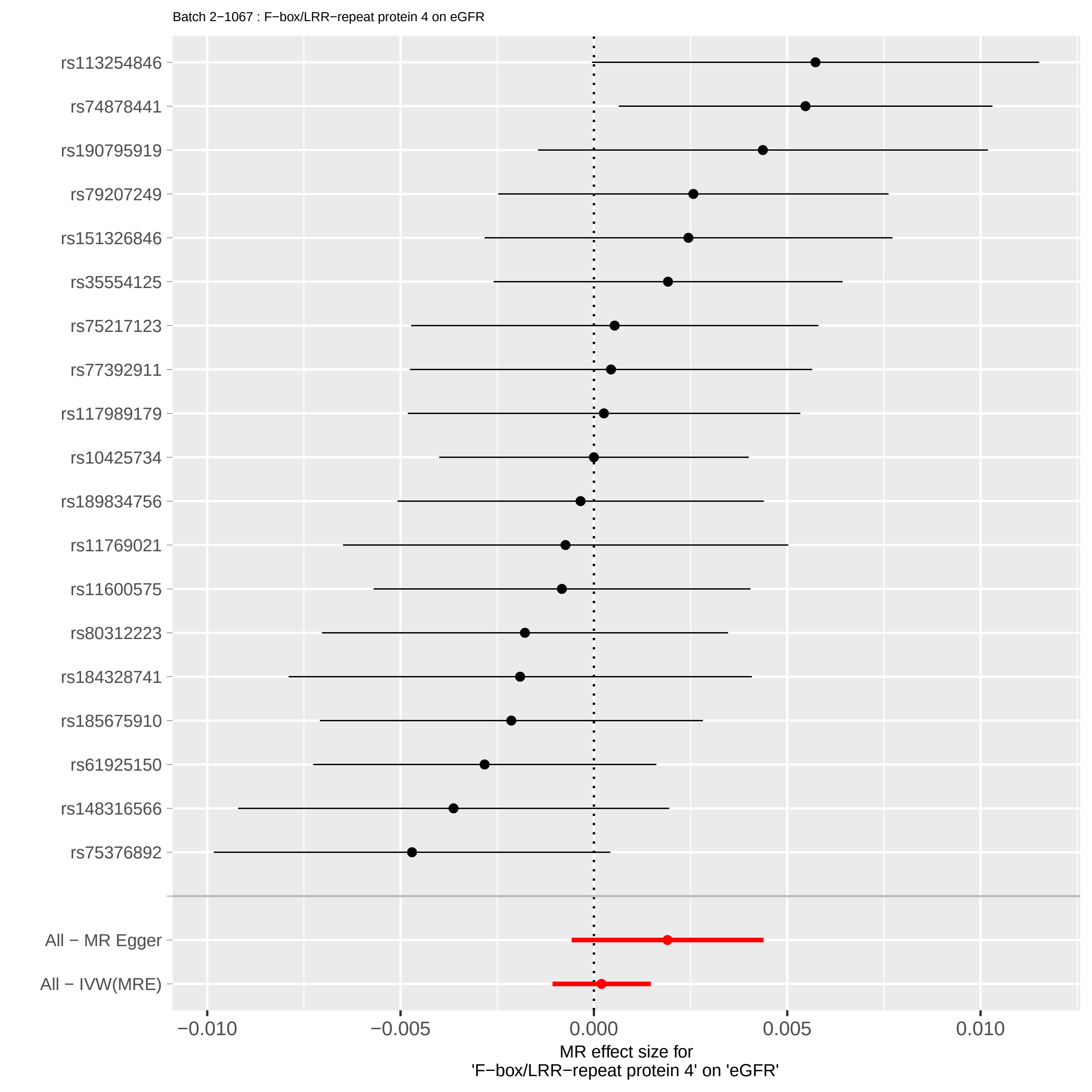
Batch 2−1067 : F−box/LRR−repeat protein 4 on eGFR
rs113254846
rs74878441
rs190795919
rs79207249
rs151326846
rs35554125
rs75217123
rs77392911
rs117989179
rs10425734
rs189834756
rs11769021
rs11600575
rs80312223
rs184328741
rs185675910
rs61925150
rs148316566
rs75376892
All − MR Egger
All − IVW(MRE)
−0.010
−0.005
0.000
0.005
0.010
MR effect size for
'F−box/LRR−repeat protein 4' on 'eGFR'

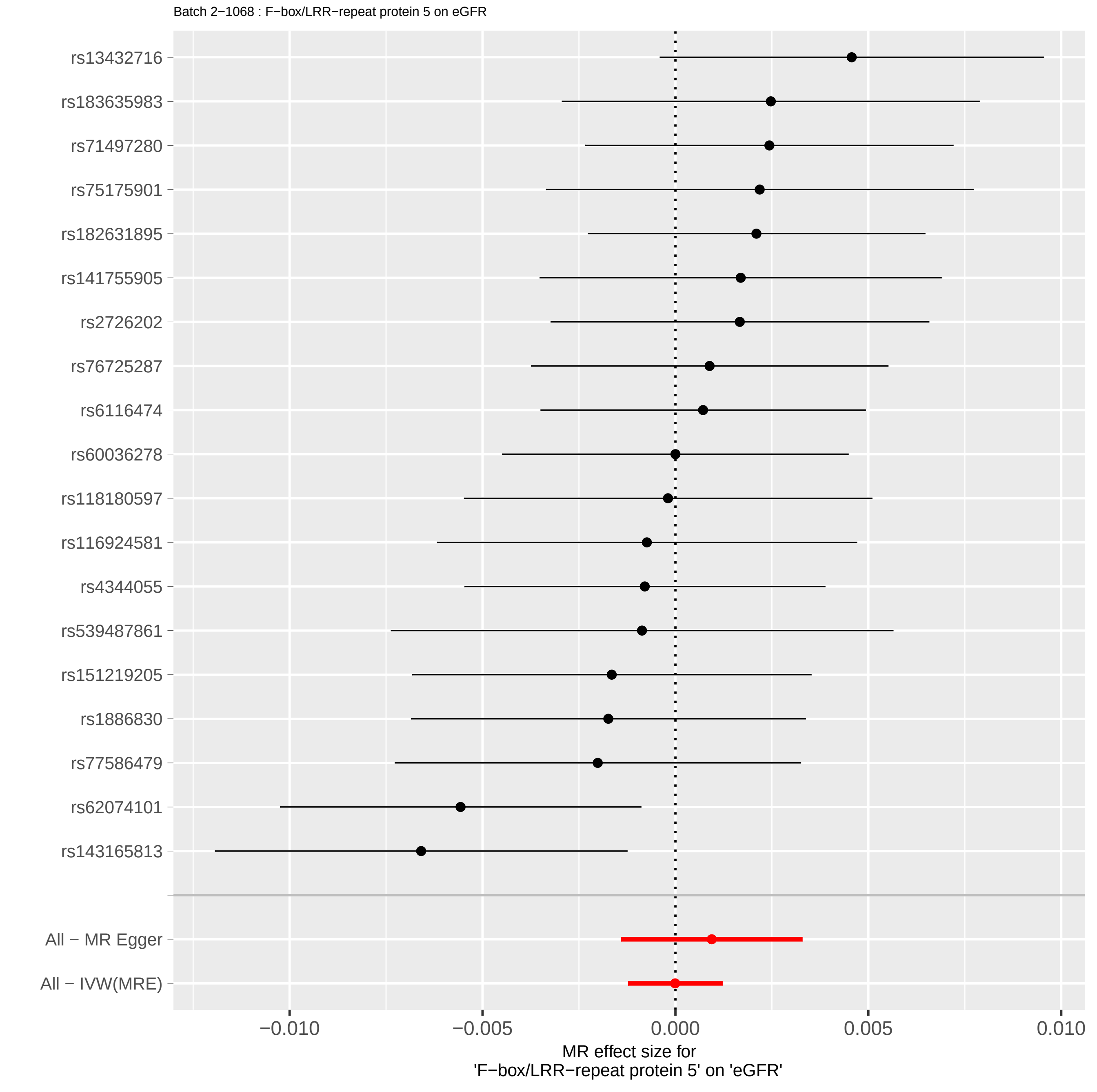

Batch 2−1068 : F−box/LRR−repeat protein 5 on eGFR
rs13432716
rs183635983
rs71497280
rs75175901
rs182631895
rs141755905
rs2726202
rs76725287
rs6116474
rs60036278
rs118180597
rs116924581
rs4344055
rs539487861
rs151219205
rs1886830
rs77586479
rs62074101
rs143165813
All − MR Egger
All − IVW(MRE)
−0.010
−0.005
0.000
0.005
0.010
MR effect size for
'F−box/LRR−repeat protein 5' on 'eGFR'

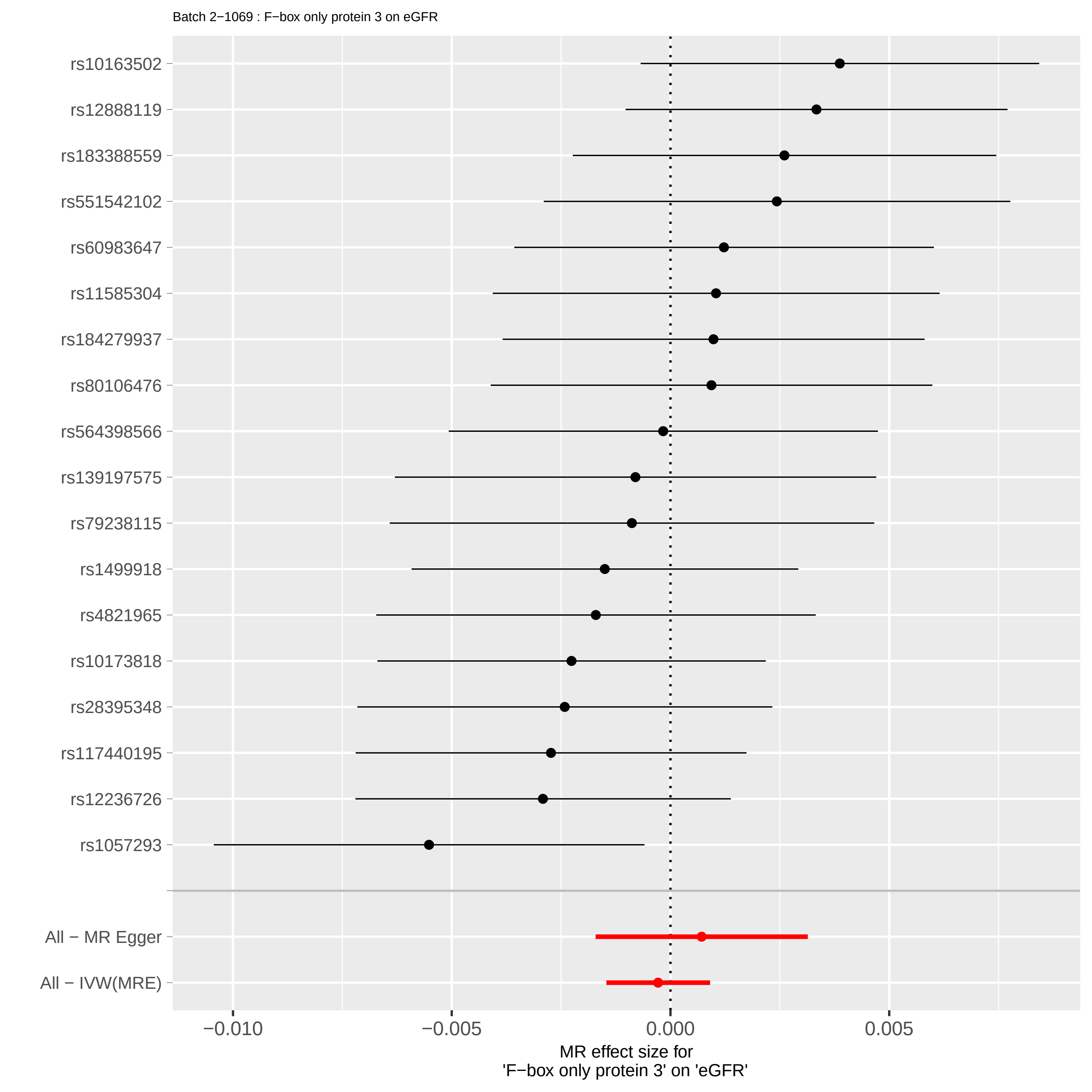

Batch 2−1069 : F−box only protein 3 on eGFR
rs10163502
rs12888119
rs183388559
rs551542102
rs60983647
rs11585304
rs184279937
rs80106476
rs564398566
rs139197575
rs79238115
rs1499918
rs4821965
rs10173818
rs28395348
rs117440195
rs12236726
rs1057293
All − MR Egger
All − IVW(MRE)
−0.010
−0.005
0.000
0.005
MR effect size for
'F−box only protein 3' on 'eGFR'

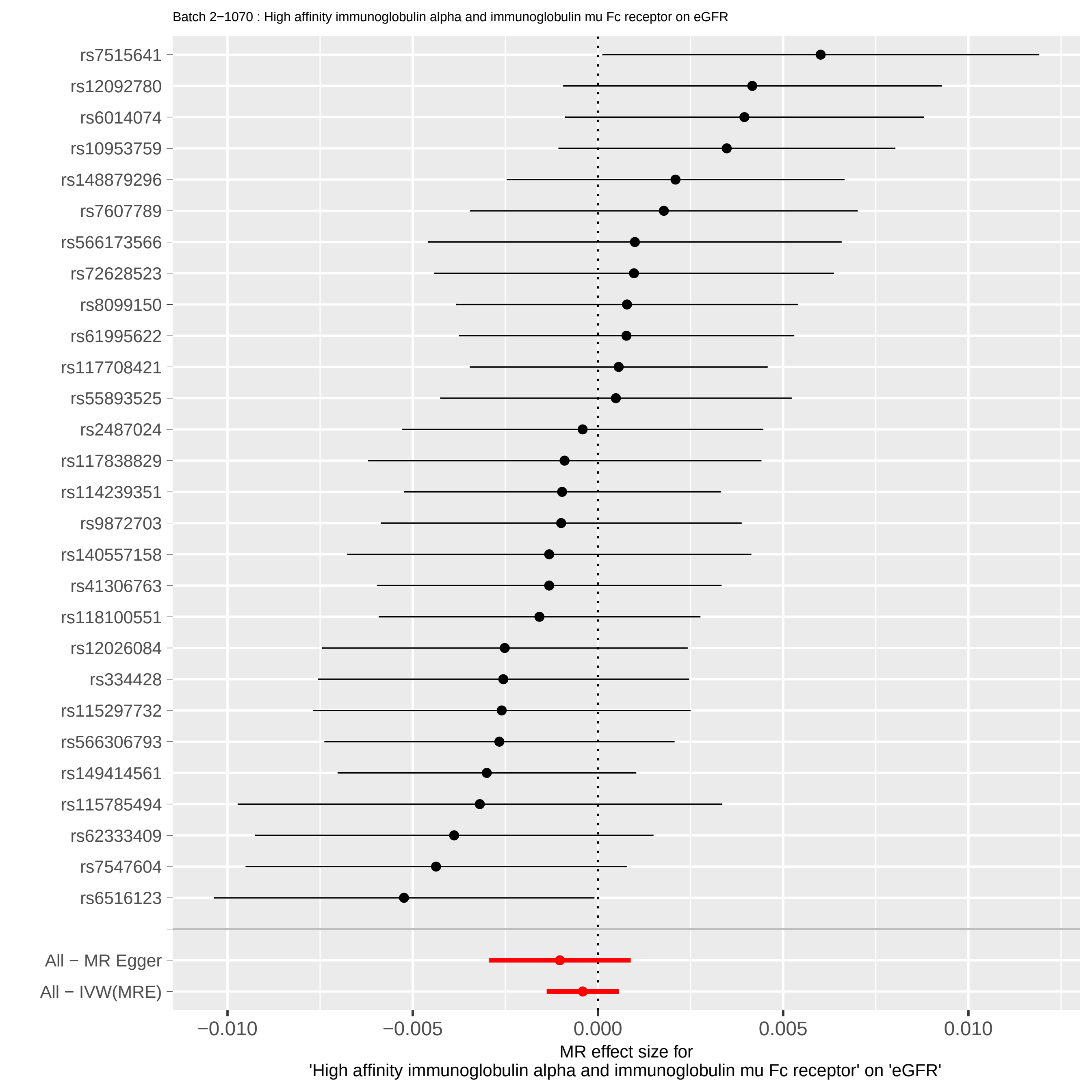

Batch 2−1070 : High affinity immunoglobulin alpha and immunoglobulin mu Fc receptor on eGFR
rs7515641
rs12092780
rs6014074
rs10953759
rs148879296
rs7607789
rs566173566
rs72628523
rs8099150
rs61995622
rs117708421
rs55893525
rs2487024
rs117838829
rs114239351
rs9872703
rs140557158
rs41306763
rs118100551
rs12026084
rs334428
rs115297732
rs566306793
rs149414561
rs115785494
rs62333409
rs7547604
rs6516123
All − MR Egger
All − IVW(MRE)
−0.010
−0.005
0.000
0.005
0.010
MR effect size for
'High affinity immunoglobulin alpha and immunoglobulin mu Fc receptor' on 'eGFR'

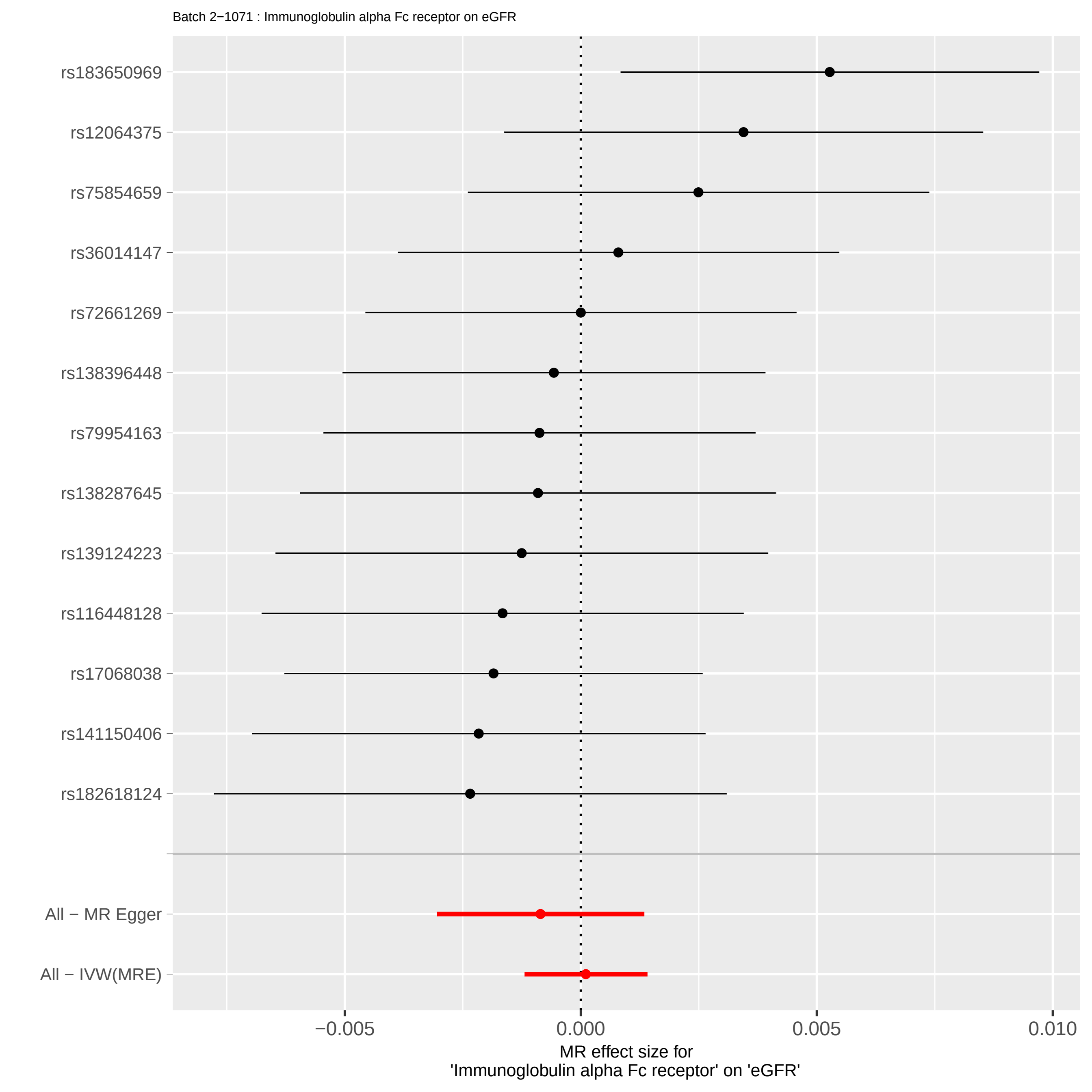
Batch 2−1071 : Immunoglobulin alpha Fc receptor on eGFR
rs183650969
rs12064375
rs75854659
rs36014147
rs72661269
rs138396448
rs79954163
rs138287645
rs139124223
rs116448128
rs17068038
rs141150406
rs182618124
All − MR Egger
All − IVW(MRE)
−0.005
0.000
0.005
0.010
MR effect size for
'Immunoglobulin alpha Fc receptor' on 'eGFR'

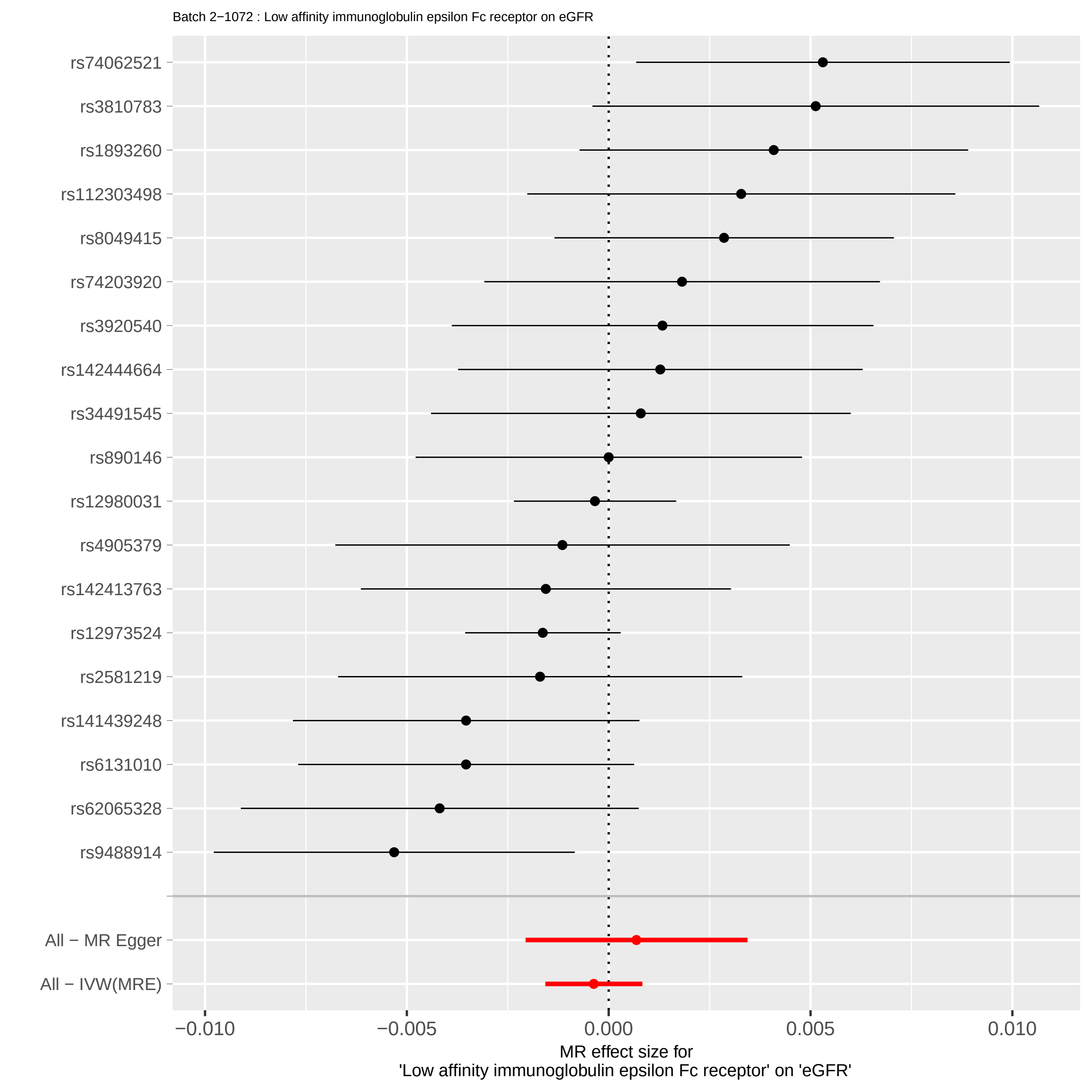

Batch 2−1072 : Low affinity immunoglobulin epsilon Fc receptor on eGFR
rs74062521
rs3810783
rs1893260
rs112303498
rs8049415
rs74203920
rs3920540
rs142444664
rs34491545
rs890146
rs12980031
rs4905379
rs142413763
rs12973524
rs2581219
rs141439248
rs6131010
rs62065328
rs9488914
All − MR Egger
All − IVW(MRE)
−0.010
−0.005
0.000
0.005
0.010
MR effect size for
'Low affinity immunoglobulin epsilon Fc receptor' on 'eGFR'

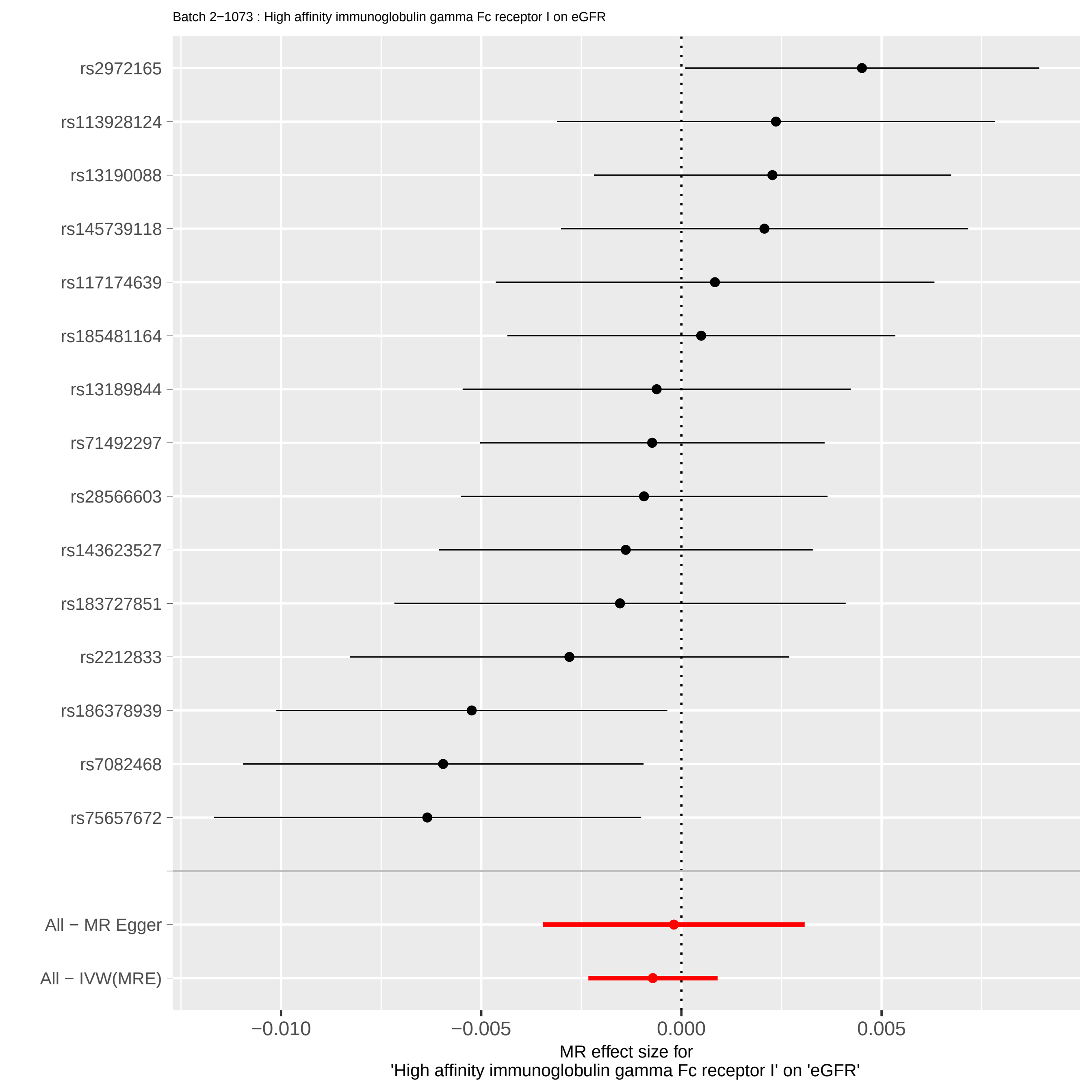

Batch 2−1073 : High affinity immunoglobulin gamma Fc receptor I on eGFR
rs2972165
rs113928124
rs13190088
rs145739118
rs117174639
rs185481164
rs13189844
rs71492297
rs28566603
rs143623527
rs183727851
rs2212833
rs186378939
rs7082468
rs75657672
All − MR Egger
All − IVW(MRE)
−0.010
−0.005
0.000
0.005
MR effect size for
'High affinity immunoglobulin gamma Fc receptor I' on 'eGFR'

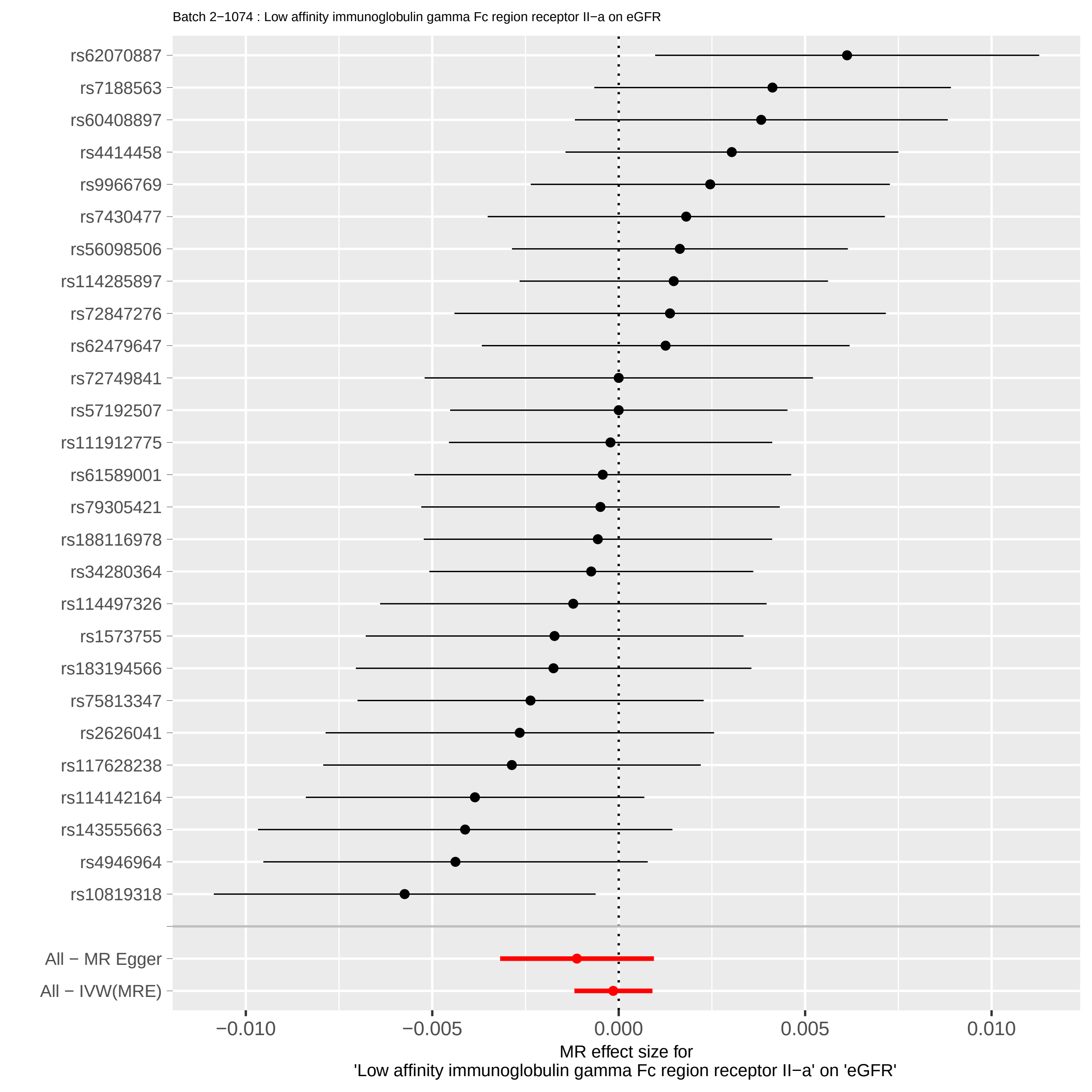

Batch 2−1074 : Low affinity immunoglobulin gamma Fc region receptor II−a on eGFR
rs62070887
rs7188563
rs60408897
rs4414458
rs9966769
rs7430477
rs56098506
rs114285897
rs72847276
rs62479647
rs72749841
rs57192507
rs111912775
rs61589001
rs79305421
rs188116978
rs34280364
rs114497326
rs1573755
rs183194566
rs75813347
rs2626041
rs117628238
rs114142164
rs143555663
rs4946964
rs10819318
All − MR Egger
All − IVW(MRE)
−0.010
−0.005
0.000
0.005
0.010
MR effect size for
'Low affinity immunoglobulin gamma Fc region receptor II−a' on 'eGFR'

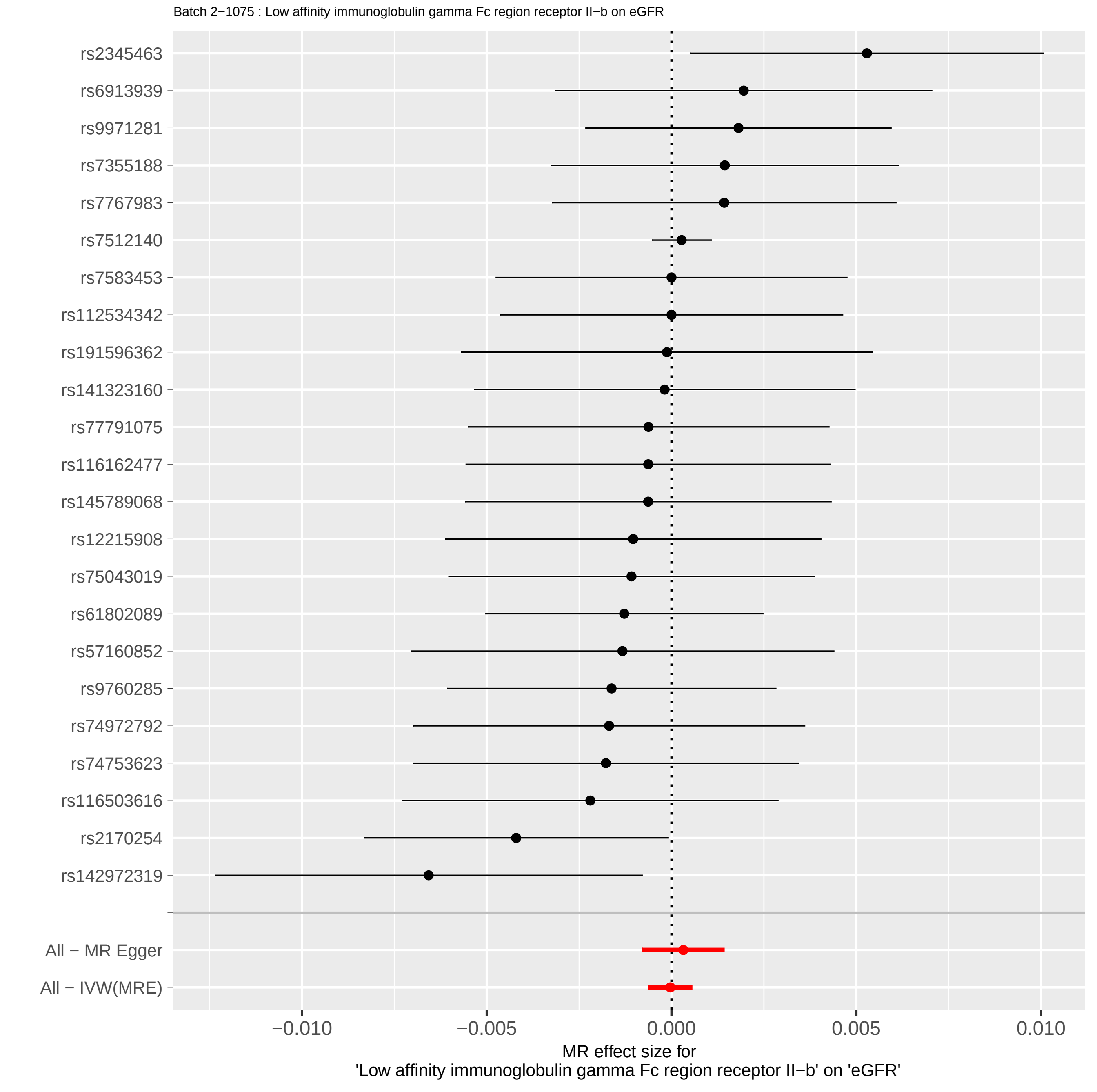

Batch 2−1075 : Low affinity immunoglobulin gamma Fc region receptor II−b on eGFR
rs2345463
rs6913939
rs9971281
rs7355188
rs7767983
rs7512140
rs7583453
rs112534342
rs191596362
rs141323160
rs77791075
rs116162477
rs145789068
rs12215908
rs75043019
rs61802089
rs57160852
rs9760285
rs74972792
rs74753623
rs116503616
rs2170254
rs142972319
All − MR Egger
All − IVW(MRE)
−0.010
−0.005
0.000
0.005
0.010
MR effect size for
'Low affinity immunoglobulin gamma Fc region receptor II−b' on 'eGFR'

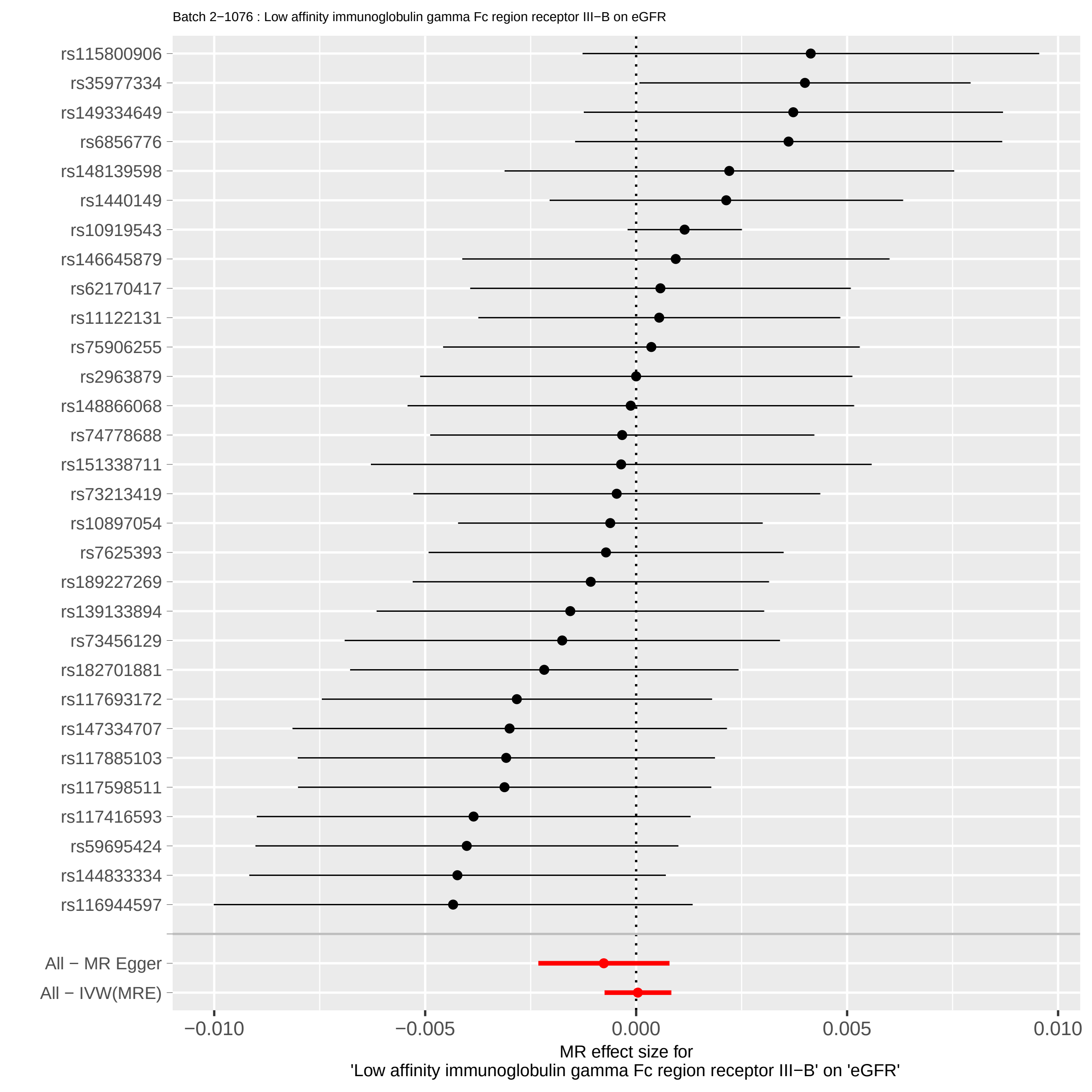

Batch 2−1076 : Low affinity immunoglobulin gamma Fc region receptor III−B on eGFR
rs115800906
rs35977334
rs149334649
rs6856776
rs148139598
rs1440149
rs10919543
rs146645879
rs62170417
rs11122131
rs75906255
rs2963879
rs148866068
rs74778688
rs151338711
rs73213419
rs10897054
rs7625393
rs189227269
rs139133894
rs73456129
rs182701881
rs117693172
rs147334707
rs117885103
rs117598511
rs117416593
rs59695424
rs144833334
rs116944597
All − MR Egger
All − IVW(MRE)
−0.010
−0.005
0.000
0.005
0.010
MR effect size for
'Low affinity immunoglobulin gamma Fc region receptor III−B' on 'eGFR'

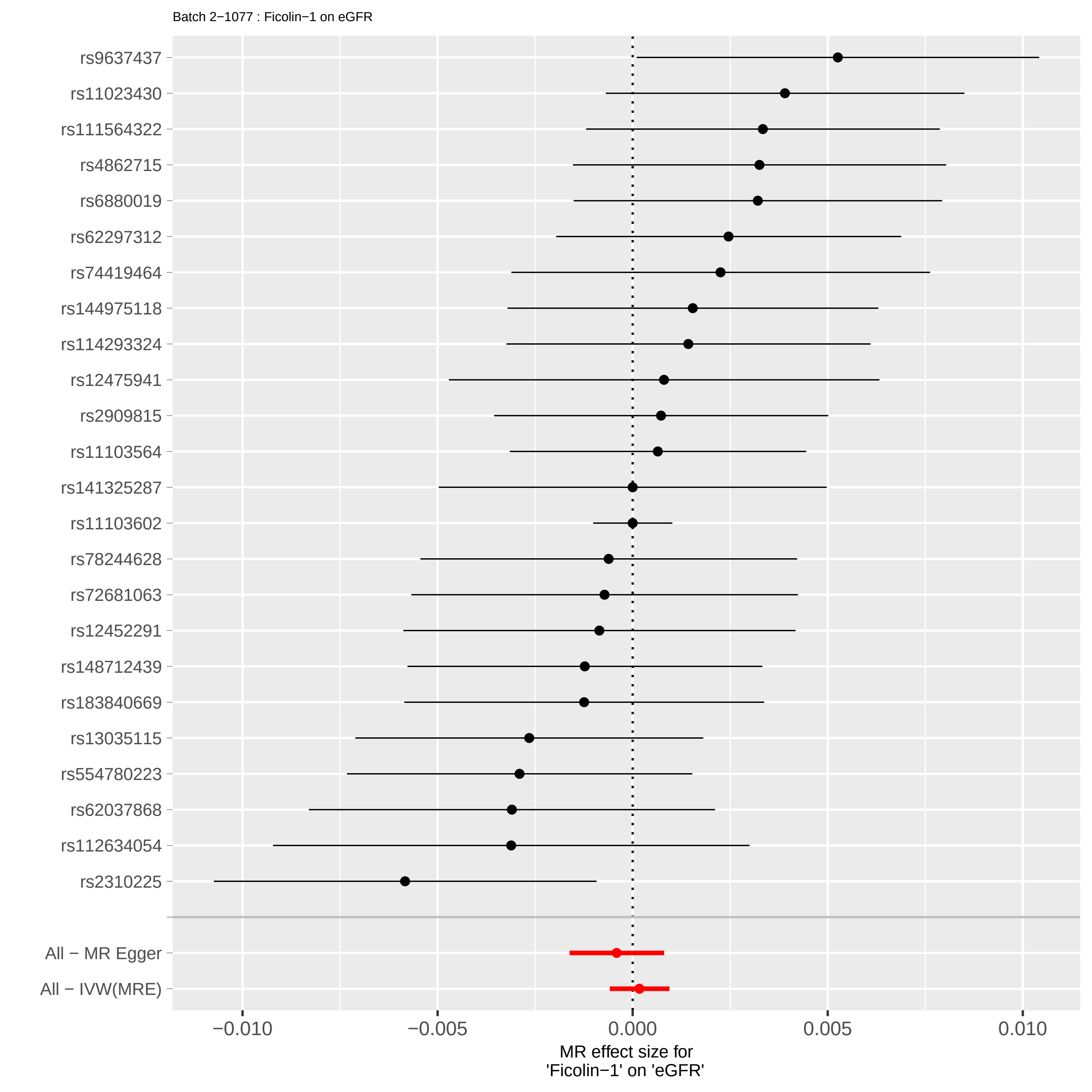
Batch 2−1077 : Ficolin−1 on eGFR
rs9637437
rs11023430
rs111564322
rs4862715
rs6880019
rs62297312
rs74419464
rs144975118
rs114293324
rs12475941
rs2909815
rs11103564
rs141325287
rs11103602
rs78244628
rs72681063
rs12452291
rs148712439
rs183840669
rs13035115
rs554780223
rs62037868
rs112634054
rs2310225
All − MR Egger
All − IVW(MRE)
−0.010
−0.005
0.000
0.005
0.010
MR effect size for
'Ficolin−1' on 'eGFR'

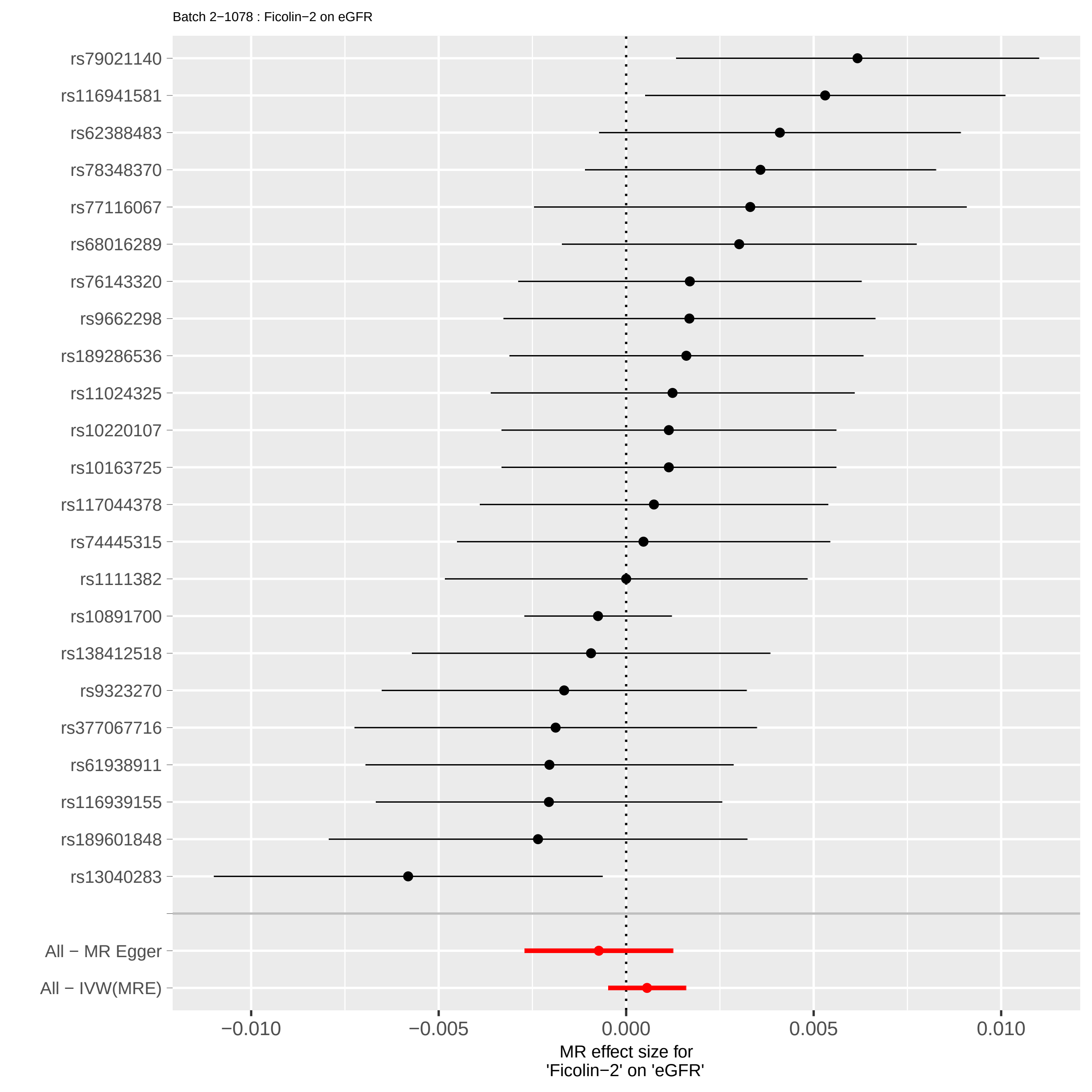
Batch 2−1078 : Ficolin−2 on eGFR
rs79021140
rs116941581
rs62388483
rs78348370
rs77116067
rs68016289
rs76143320
rs9662298
rs189286536
rs11024325
rs10220107
rs10163725
rs117044378
rs74445315
rs1111382
rs10891700
rs138412518
rs9323270
rs377067716
rs61938911
rs116939155
rs189601848
rs13040283
All − MR Egger
All − IVW(MRE)
−0.010
−0.005
0.000
0.005
0.010
MR effect size for
'Ficolin−2' on 'eGFR'

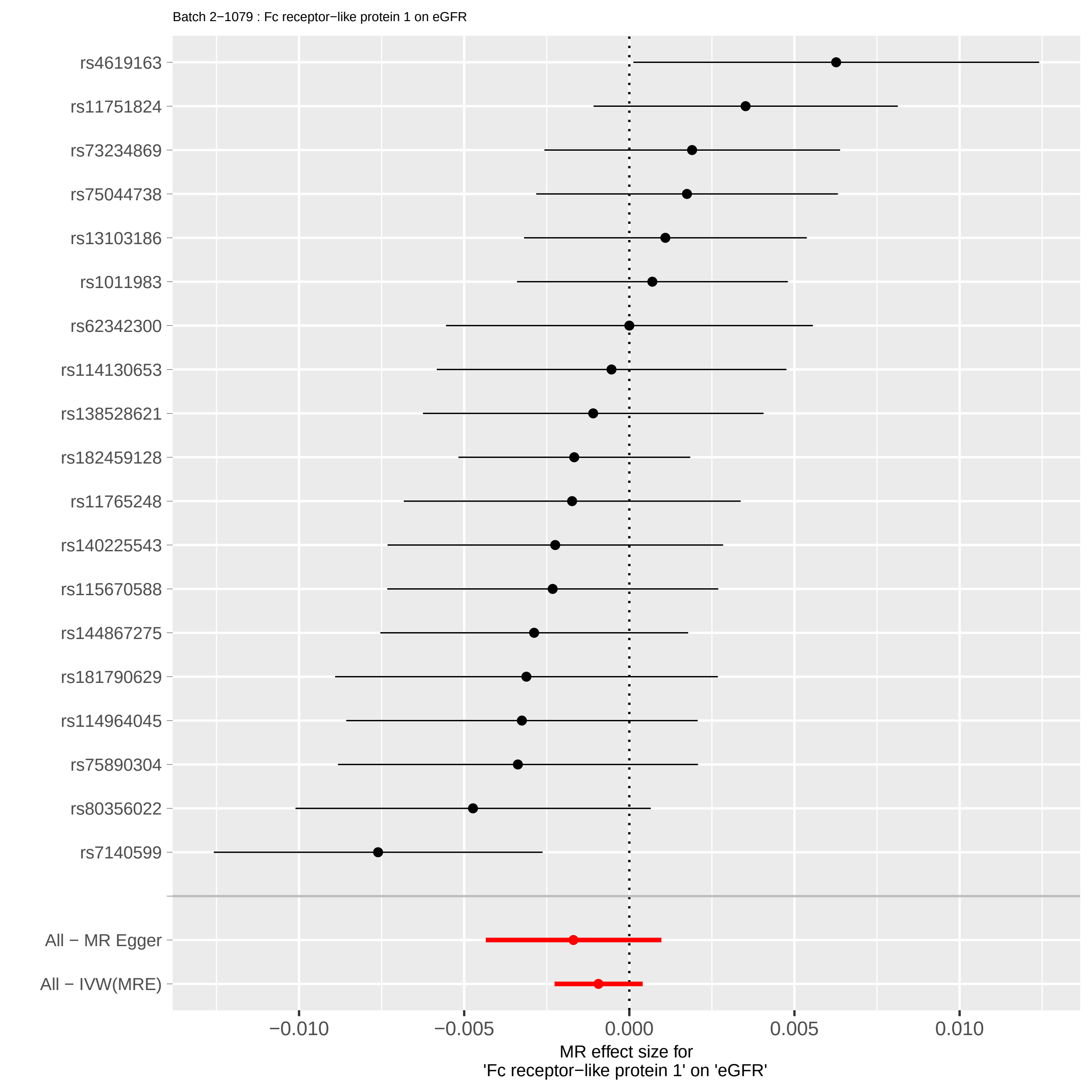

Batch 2−1079 : Fc receptor−like protein 1 on eGFR
rs4619163
rs11751824
rs73234869
rs75044738
rs13103186
rs1011983
rs62342300
rs114130653
rs138528621
rs182459128
rs11765248
rs140225543
rs115670588
rs144867275
rs181790629
rs114964045
rs75890304
rs80356022
rs7140599
All − MR Egger
All − IVW(MRE)
−0.010
−0.005
0.000
0.005
0.010
MR effect size for
'Fc receptor−like protein 1' on 'eGFR'

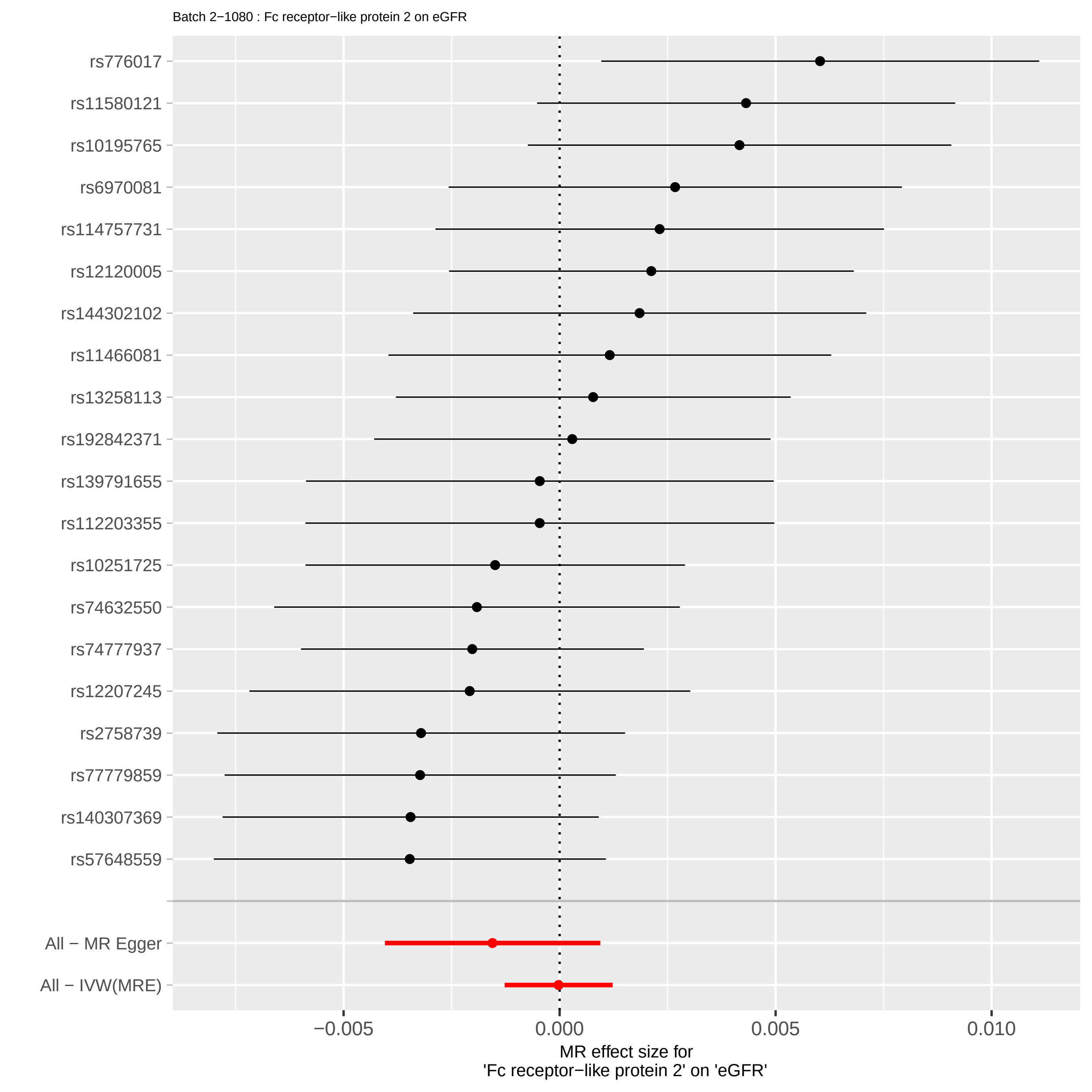
Batch 2−1080 : Fc receptor−like protein 2 on eGFR
rs776017
rs11580121
rs10195765
rs6970081
rs114757731
rs12120005
rs144302102
rs11466081
rs13258113
rs192842371
rs139791655
rs112203355
rs10251725
rs74632550
rs74777937
rs12207245
rs2758739
rs77779859
rs140307369
rs57648559
All − MR Egger
All − IVW(MRE)
−0.005
0.000
0.005
0.010
MR effect size for
'Fc receptor−like protein 2' on 'eGFR'

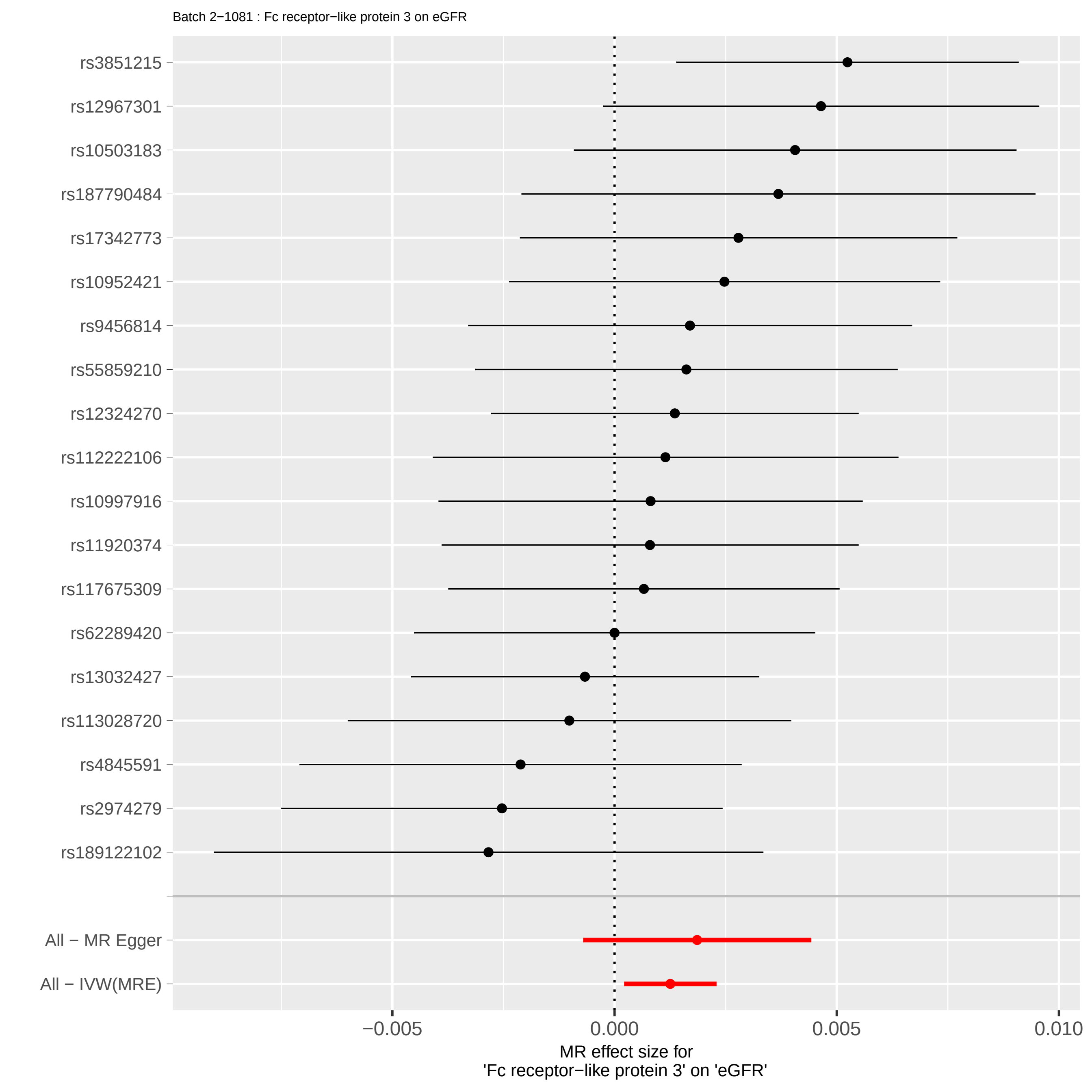

Batch 2−1081 : Fc receptor−like protein 3 on eGFR
rs3851215
rs12967301
rs10503183
rs187790484
rs17342773
rs10952421
rs9456814
rs55859210
rs12324270
rs112222106
rs10997916
rs11920374
rs117675309
rs62289420
rs13032427
rs113028720
rs4845591
rs2974279
rs189122102
All − MR Egger
All − IVW(MRE)
−0.005
0.000
0.005
0.010
MR effect size for
'Fc receptor−like protein 3' on 'eGFR'

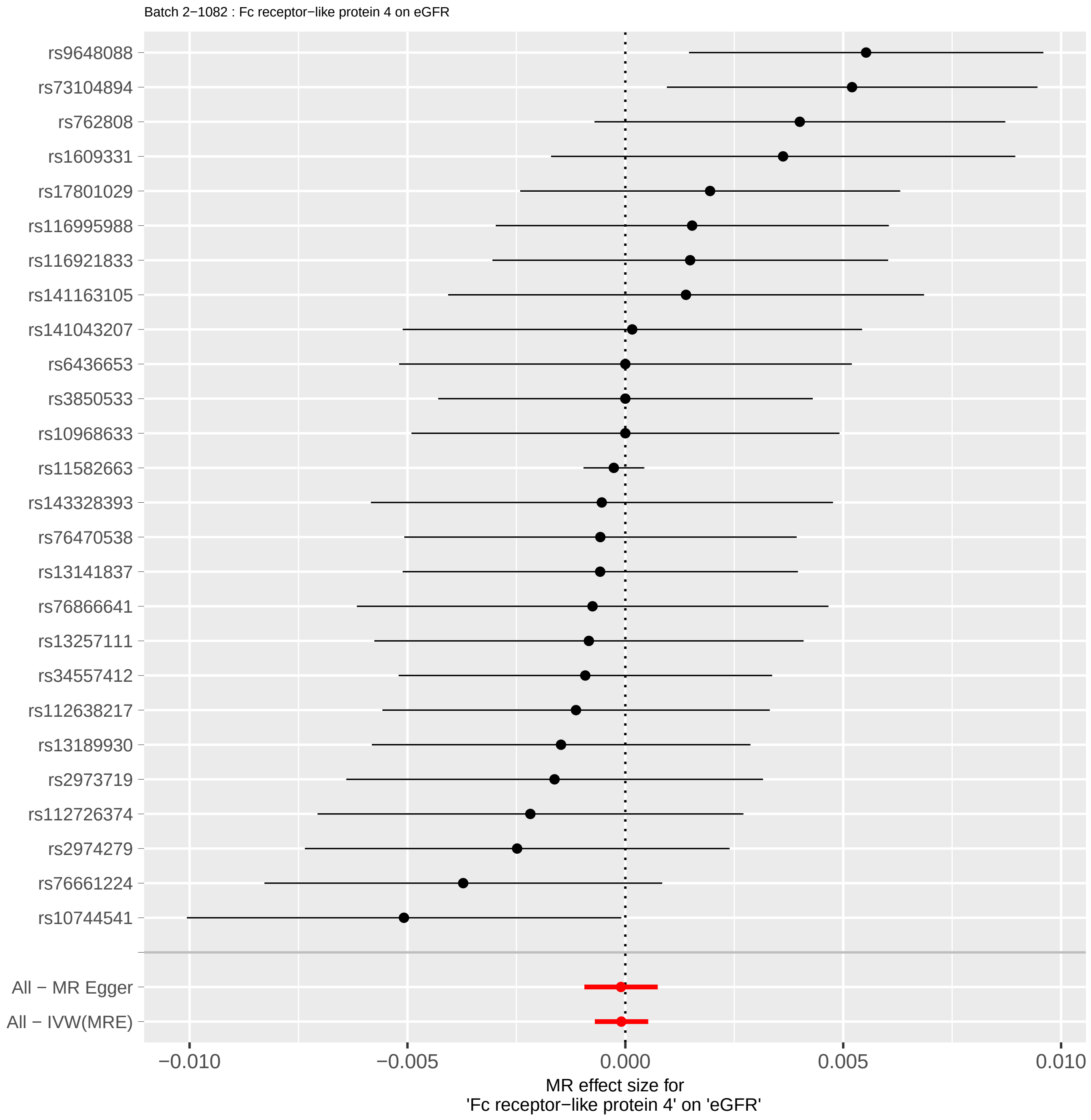

Batch 2−1082 : Fc receptor−like protein 4 on eGFR
rs9648088
rs73104894
rs762808
rs1609331
rs17801029
rs116995988
rs116921833
rs141163105
rs141043207
rs6436653
rs3850533
rs10968633
rs11582663
rs143328393
rs76470538
rs13141837
rs76866641
rs13257111
rs34557412
rs112638217
rs13189930
rs2973719
rs112726374
rs2974279
rs76661224
rs10744541
All − MR Egger
All − IVW(MRE)
−0.010
−0.005
0.000
0.005
0.010
MR effect size for
'Fc receptor−like protein 4' on 'eGFR'

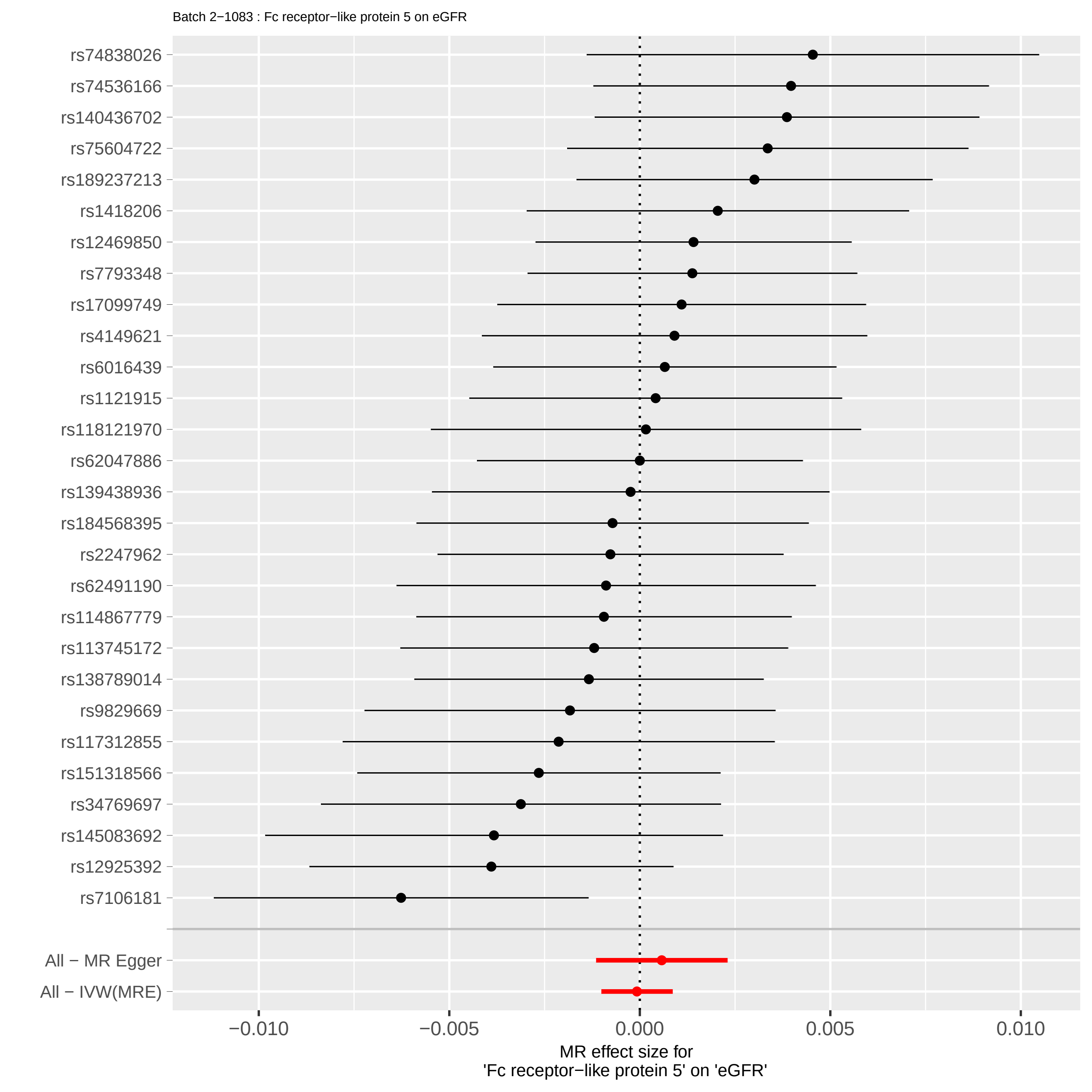

Batch 2−1083 : Fc receptor−like protein 5 on eGFR
rs74838026
rs74536166
rs140436702
rs75604722
rs189237213
rs1418206
rs12469850
rs7793348
rs17099749
rs4149621
rs6016439
rs1121915
rs118121970
rs62047886
rs139438936
rs184568395
rs2247962
rs62491190
rs114867779
rs113745172
rs138789014
rs9829669
rs117312855
rs151318566
rs34769697
rs145083692
rs12925392
rs7106181
All − MR Egger
All − IVW(MRE)
−0.010
−0.005
0.000
0.005
0.010
MR effect size for
'Fc receptor−like protein 5' on 'eGFR'

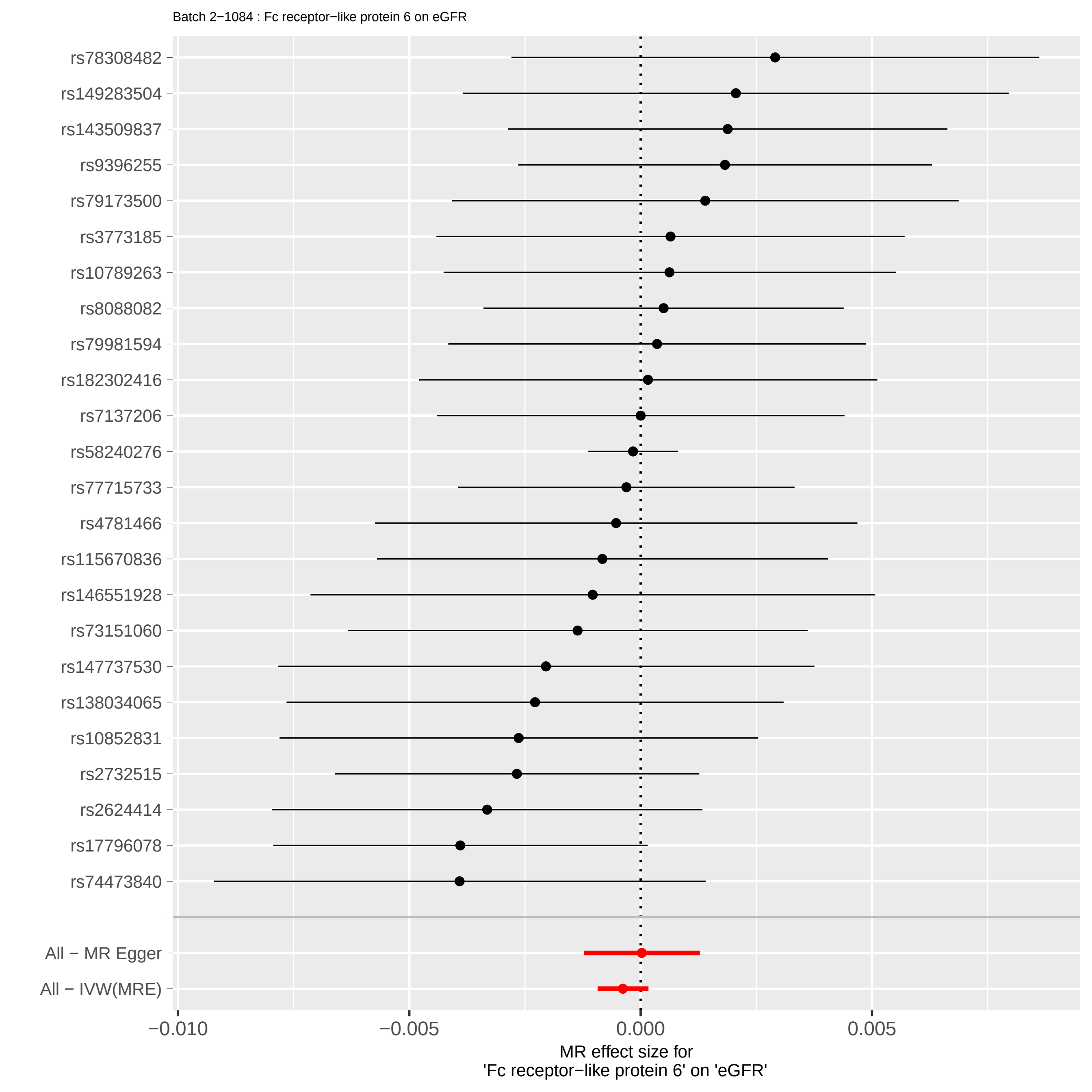

Batch 2−1084 : Fc receptor−like protein 6 on eGFR
rs78308482
rs149283504
rs143509837
rs9396255
rs79173500
rs3773185
rs10789263
rs8088082
rs79981594
rs182302416
rs7137206
rs58240276
rs77715733
rs4781466
rs115670836
rs146551928
rs73151060
rs147737530
rs138034065
rs10852831
rs2732515
rs2624414
rs17796078
rs74473840
All − MR Egger
All − IVW(MRE)
−0.010
−0.005
0.000
0.005
MR effect size for
'Fc receptor−like protein 6' on 'eGFR'

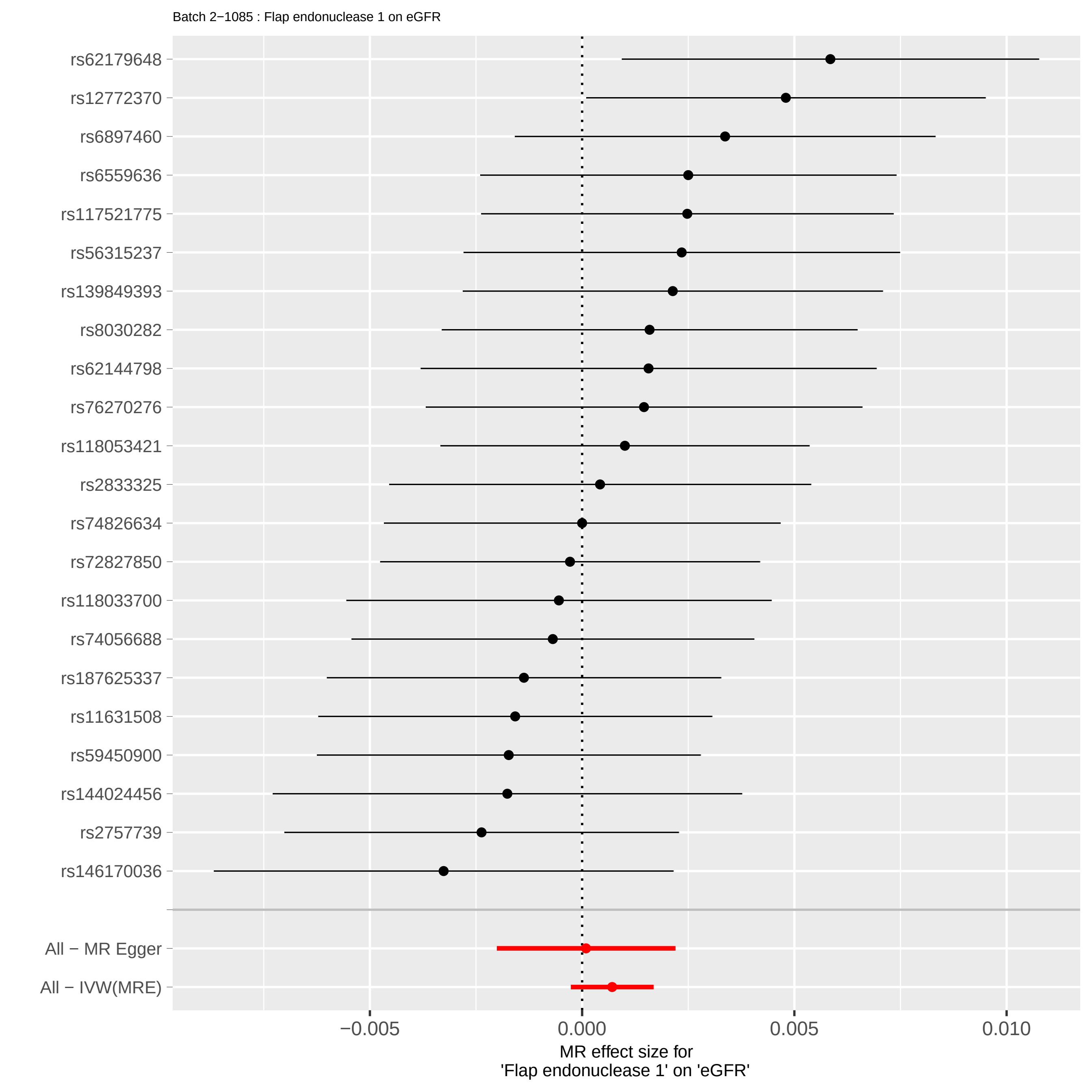

Batch 2−1085 : Flap endonuclease 1 on eGFR
rs62179648
rs12772370
rs6897460
rs6559636
rs117521775
rs56315237
rs139849393
rs8030282
rs62144798
rs76270276
rs118053421
rs2833325
rs74826634
rs72827850
rs118033700
rs74056688
rs187625337
rs11631508
rs59450900
rs144024456
rs2757739
rs146170036
All − MR Egger
All − IVW(MRE)
−0.005
0.000
0.005
0.010
MR effect size for
'Flap endonuclease 1' on 'eGFR'

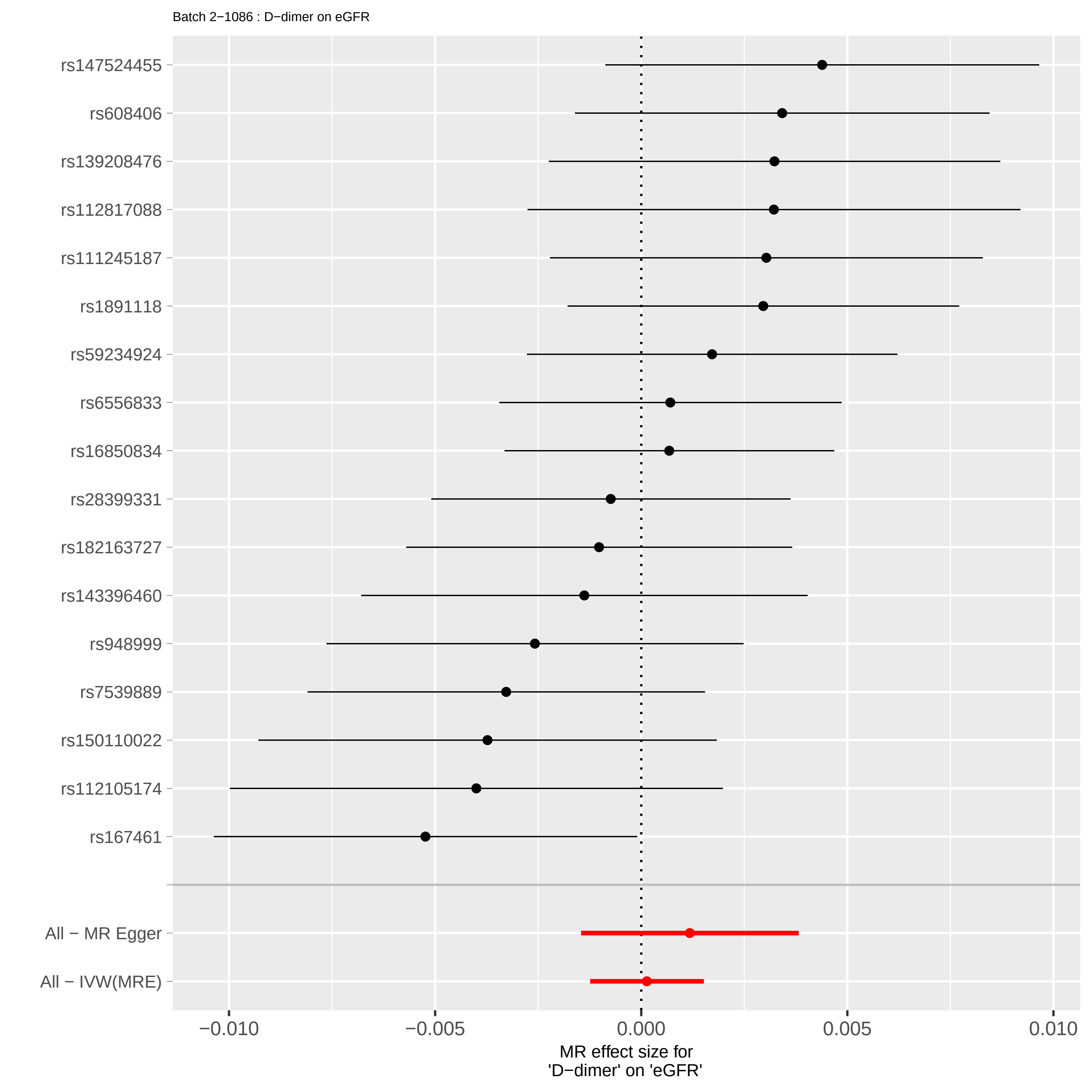
Batch 2−1086 : D−dimer on eGFR
rs147524455
rs608406
rs139208476
rs112817088
rs111245187
rs1891118
rs59234924
rs6556833
rs16850834
rs28399331
rs182163727
rs143396460
rs948999
rs7539889
rs150110022
rs112105174
rs167461
All − MR Egger
All − IVW(MRE)
−0.010
−0.005
0.000
0.005
0.010
MR effect size for
'D−dimer' on 'eGFR'

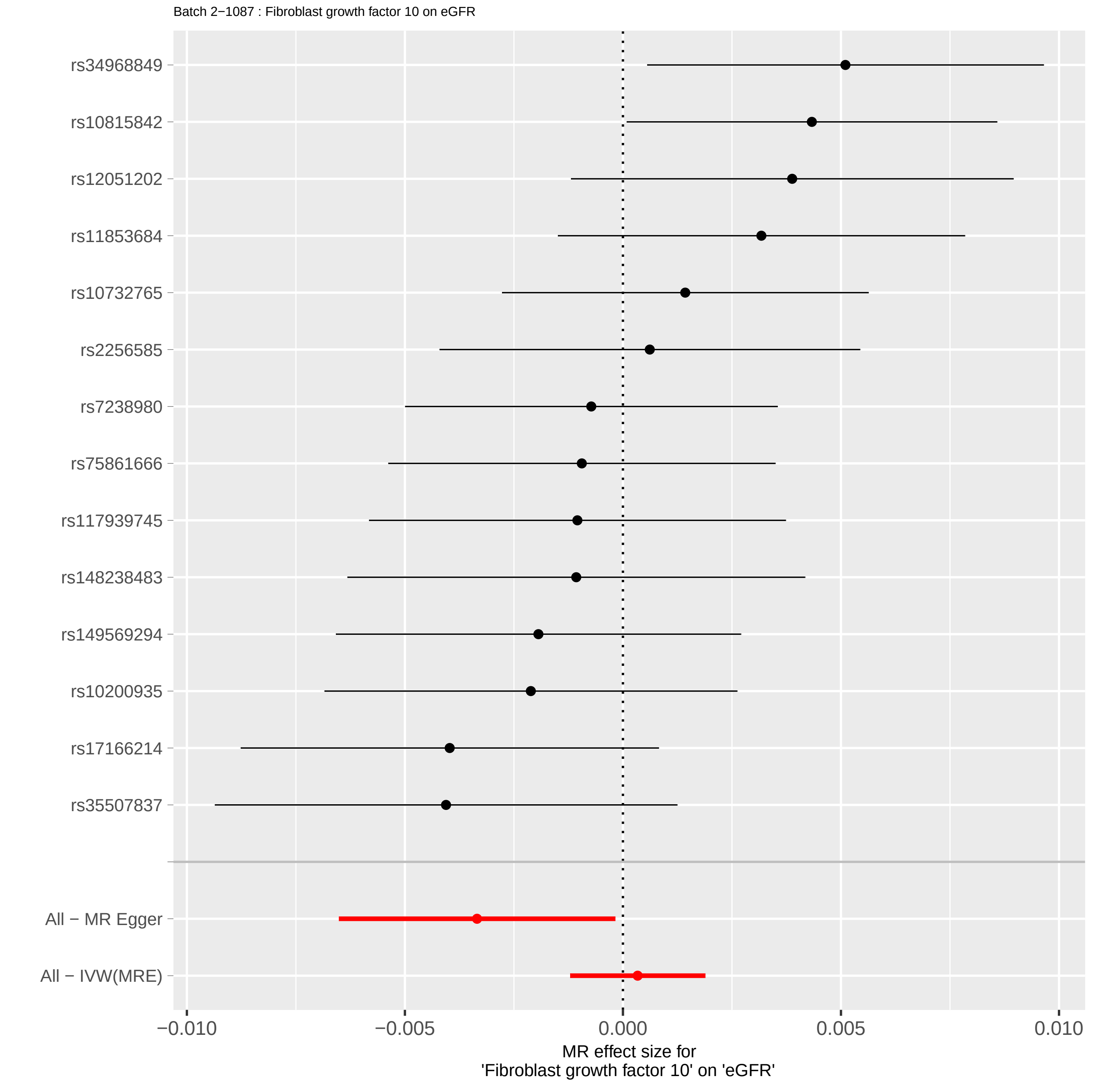

Batch 2−1087 : Fibroblast growth factor 10 on eGFR
rs34968849
rs10815842
rs12051202
rs11853684
rs10732765
rs2256585
rs7238980
rs75861666
rs117939745
rs148238483
rs149569294
rs10200935
rs17166214
rs35507837
All − MR Egger
All − IVW(MRE)
−0.010
−0.005
0.000
0.005
0.010
MR effect size for
'Fibroblast growth factor 10' on 'eGFR'

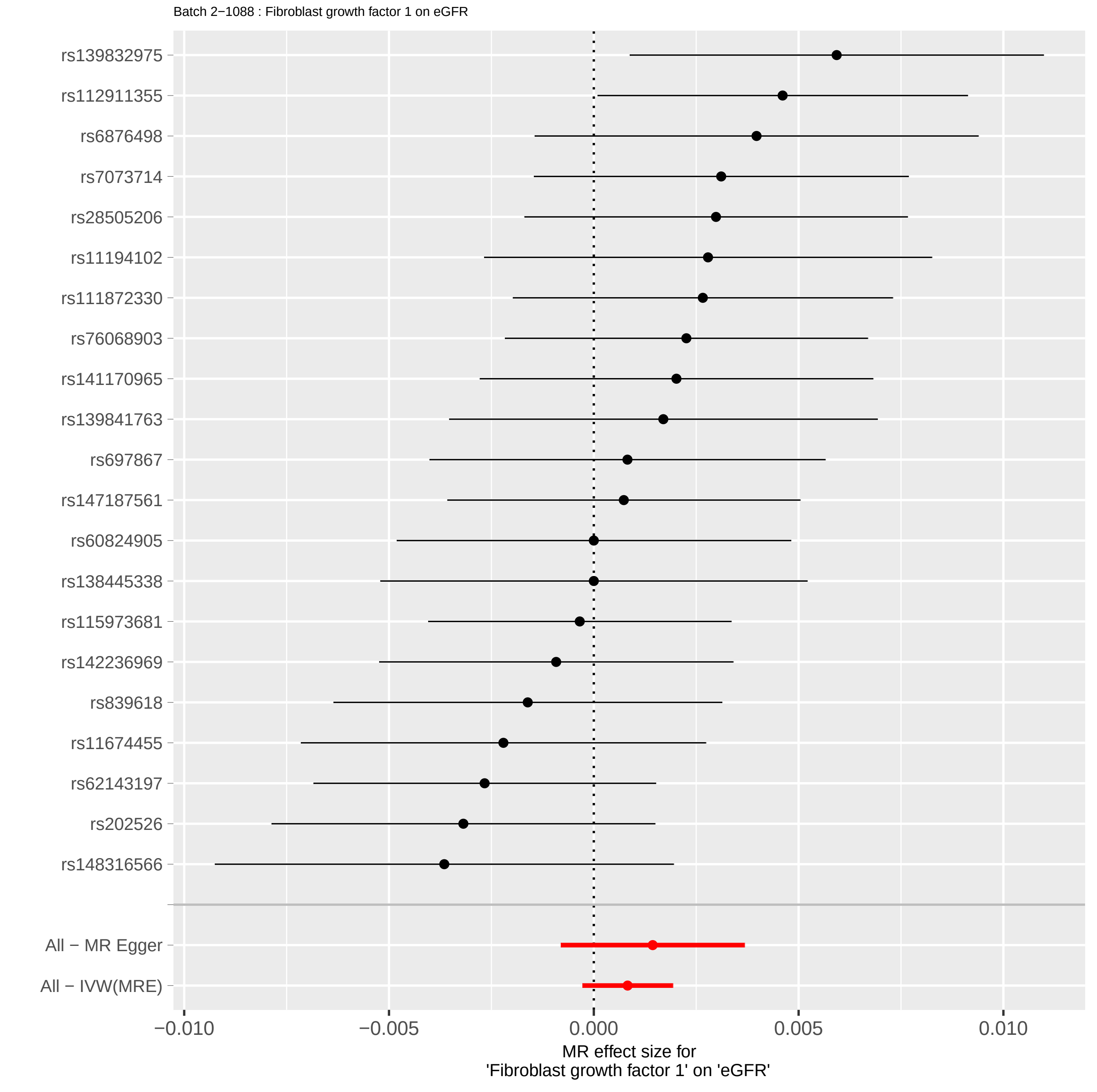
Batch 2−1088 : Fibroblast growth factor 1 on eGFR
rs139832975
rs112911355
rs6876498
rs7073714
rs28505206
rs11194102
rs111872330
rs76068903
rs141170965
rs139841763
rs697867
rs147187561
rs60824905
rs138445338
rs115973681
rs142236969
rs839618
rs11674455
rs62143197
rs202526
rs148316566
All − MR Egger
All − IVW(MRE)
−0.010
−0.005
0.000
0.005
0.010
MR effect size for
'Fibroblast growth factor 1' on 'eGFR'

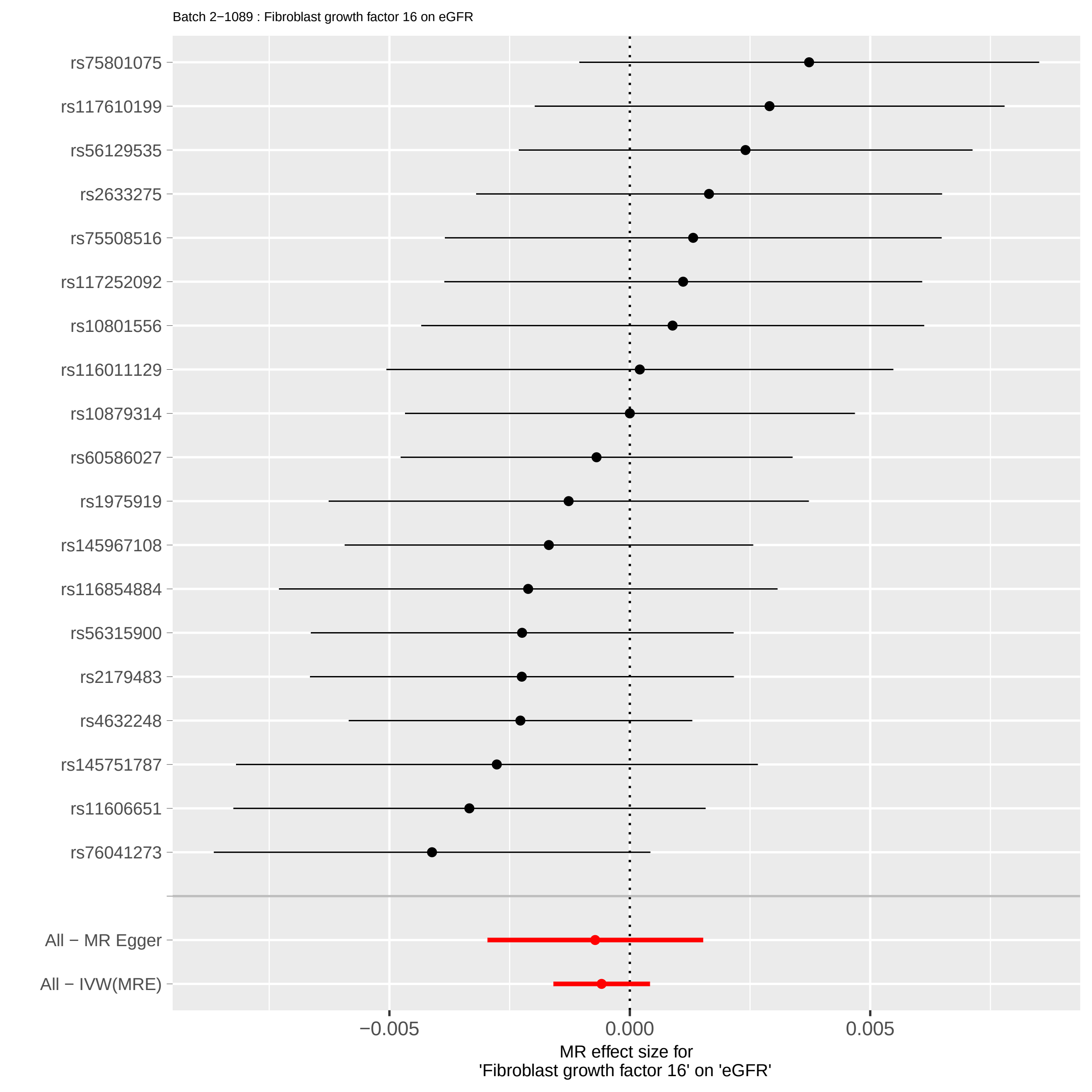
Batch 2−1089 : Fibroblast growth factor 16 on eGFR
rs75801075
rs117610199
rs56129535
rs2633275
rs75508516
rs117252092
rs10801556
rs116011129
rs10879314
rs60586027
rs1975919
rs145967108
rs116854884
rs56315900
rs2179483
rs4632248
rs145751787
rs11606651
rs76041273
All − MR Egger
All − IVW(MRE)
−0.005
0.000
0.005
MR effect size for
'Fibroblast growth factor 16' on 'eGFR'

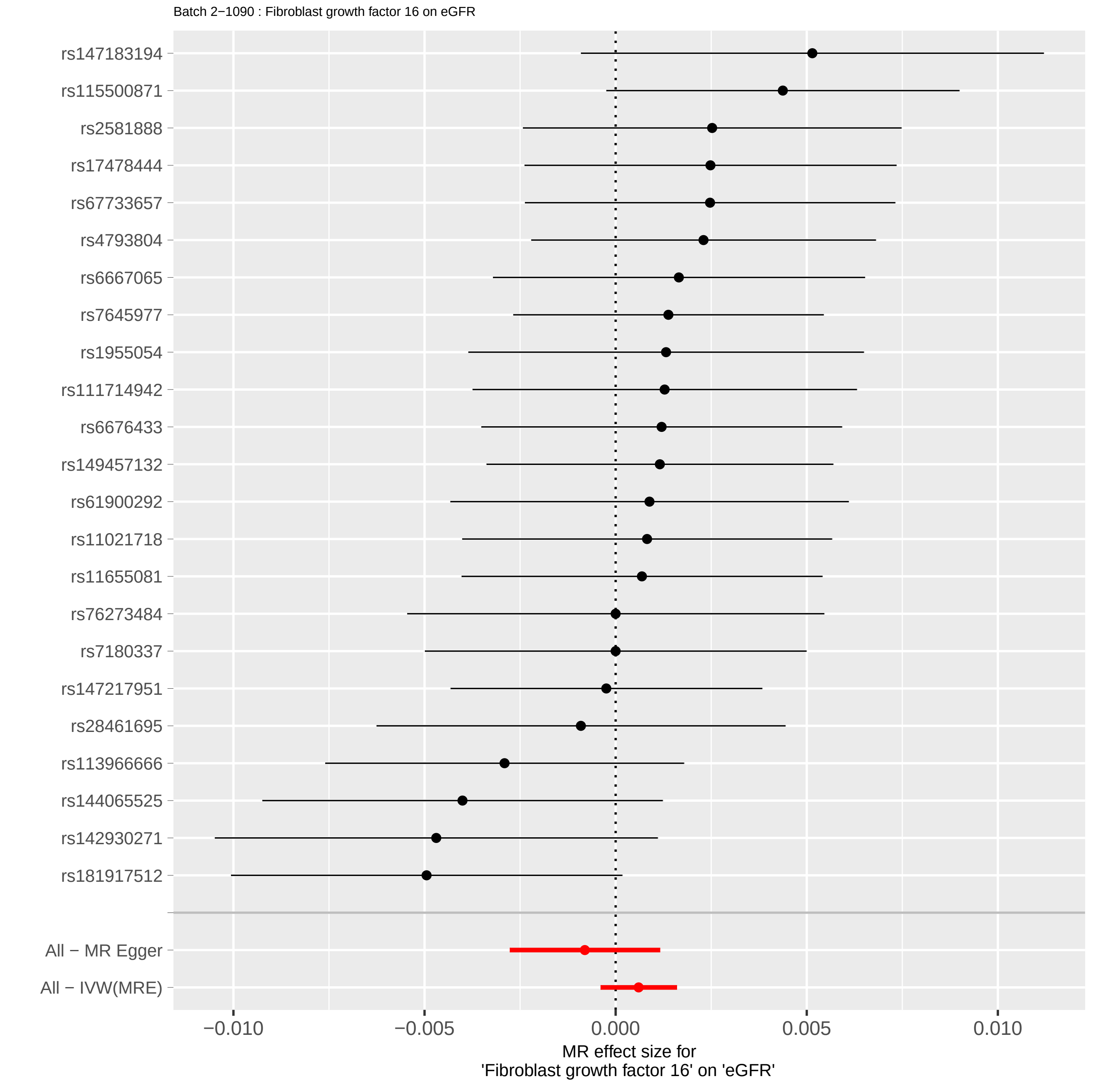

Batch 2−1090 : Fibroblast growth factor 16 on eGFR
rs147183194
rs115500871
rs2581888
rs17478444
rs67733657
rs4793804
rs6667065
rs7645977
rs1955054
rs111714942
rs6676433
rs149457132
rs61900292
rs11021718
rs11655081
rs76273484
rs7180337
rs147217951
rs28461695
rs113966666
rs144065525
rs142930271
rs181917512
All − MR Egger
All − IVW(MRE)
−0.010
−0.005
0.000
0.005
0.010
MR effect size for
'Fibroblast growth factor 16' on 'eGFR'

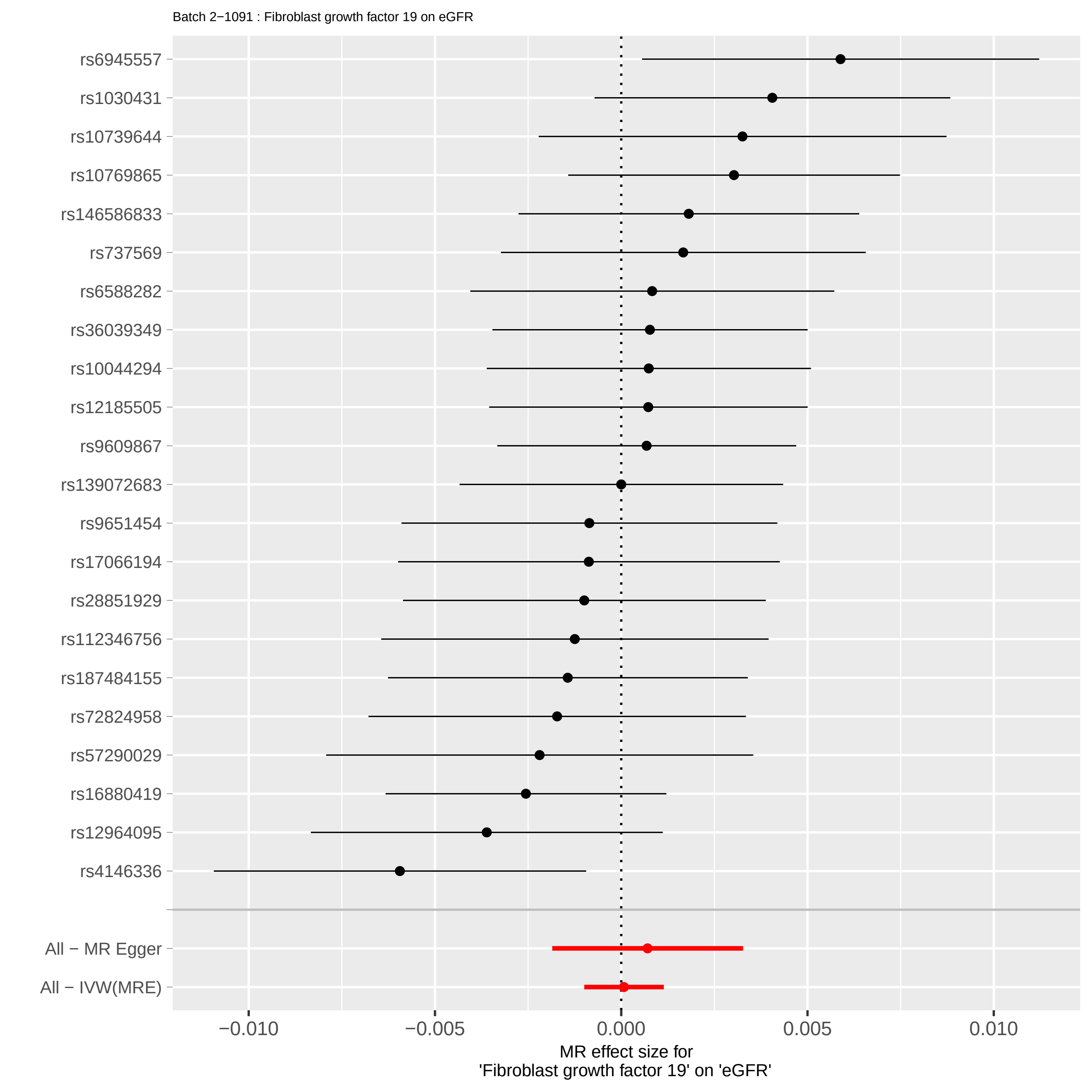

Batch 2−1091 : Fibroblast growth factor 19 on eGFR
rs6945557
rs1030431
rs10739644
rs10769865
rs146586833
rs737569
rs6588282
rs36039349
rs10044294
rs12185505
rs9609867
rs139072683
rs9651454
rs17066194
rs28851929
rs112346756
rs187484155
rs72824958
rs57290029
rs16880419
rs12964095
rs4146336
All − MR Egger
All − IVW(MRE)
−0.010
−0.005
0.000
0.005
0.010
MR effect size for
'Fibroblast growth factor 19' on 'eGFR'

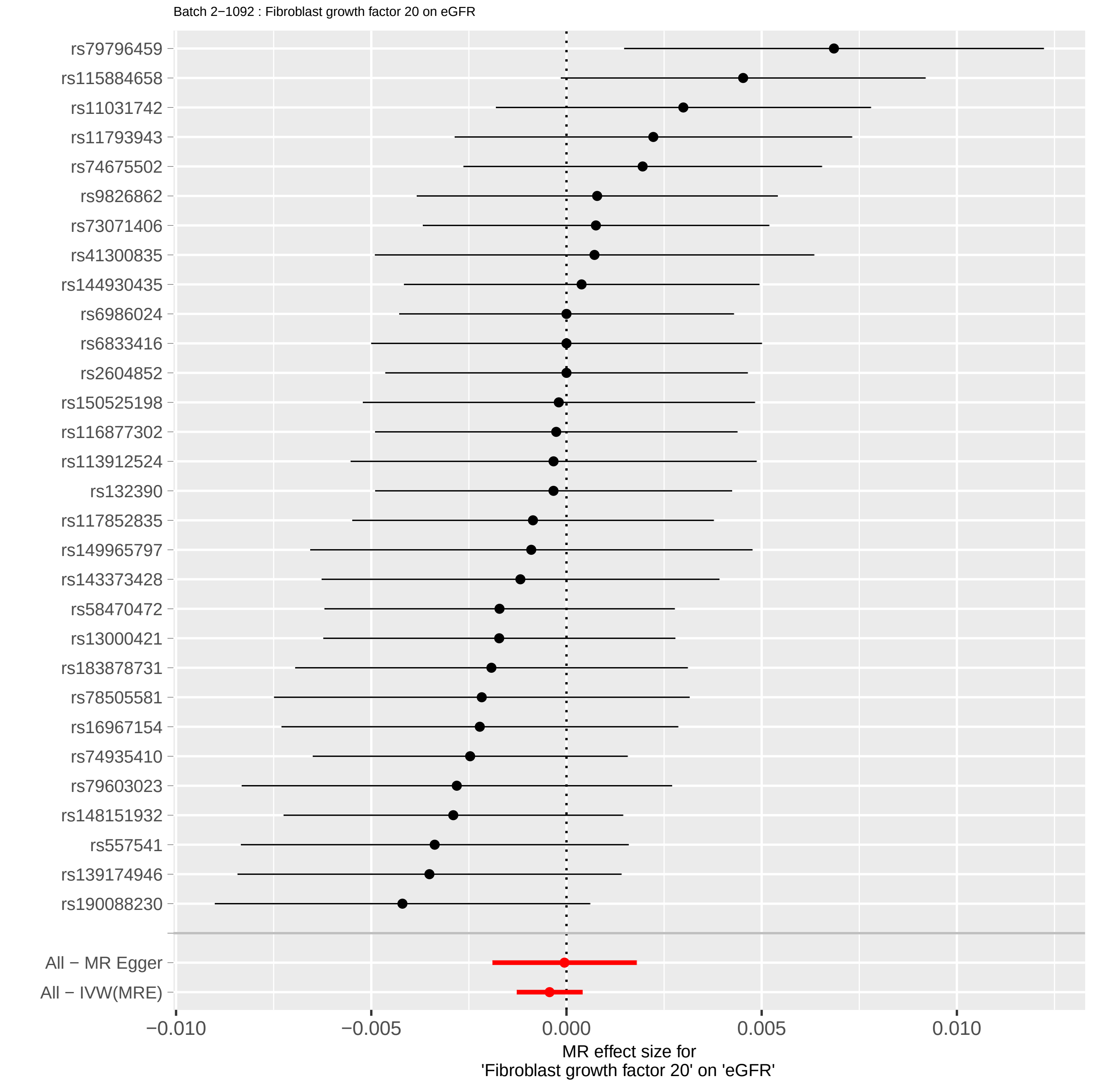
Batch 2−1092 : Fibroblast growth factor 20 on eGFR
rs79796459
rs115884658
rs11031742
rs11793943
rs74675502
rs9826862
rs73071406
rs41300835
rs144930435
rs6986024
rs6833416
rs2604852
rs150525198
rs116877302
rs113912524
rs132390
rs117852835
rs149965797
rs143373428
rs58470472
rs13000421
rs183878731
rs78505581
rs16967154
rs74935410
rs79603023
rs148151932
rs557541
rs139174946
rs190088230
All − MR Egger
All − IVW(MRE)
−0.010
−0.005
0.000
0.005
0.010
MR effect size for
'Fibroblast growth factor 20' on 'eGFR'

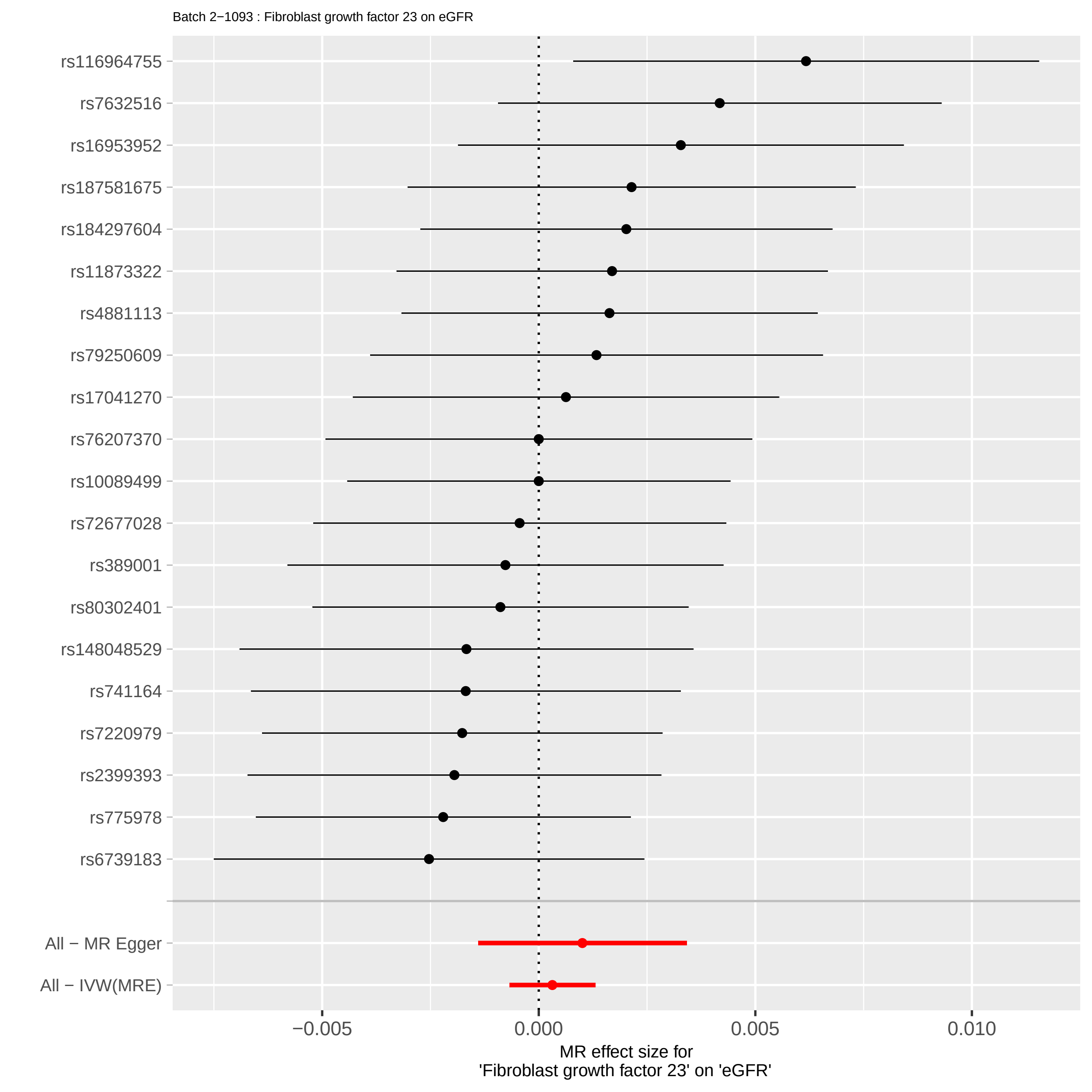

Batch 2−1093 : Fibroblast growth factor 23 on eGFR
rs116964755
rs7632516
rs16953952
rs187581675
rs184297604
rs11873322
rs4881113
rs79250609
rs17041270
rs76207370
rs10089499
rs72677028
rs389001
rs80302401
rs148048529
rs741164
rs7220979
rs2399393
rs775978
rs6739183
All − MR Egger
All − IVW(MRE)
−0.005
0.000
0.005
0.010
MR effect size for
'Fibroblast growth factor 23' on 'eGFR'

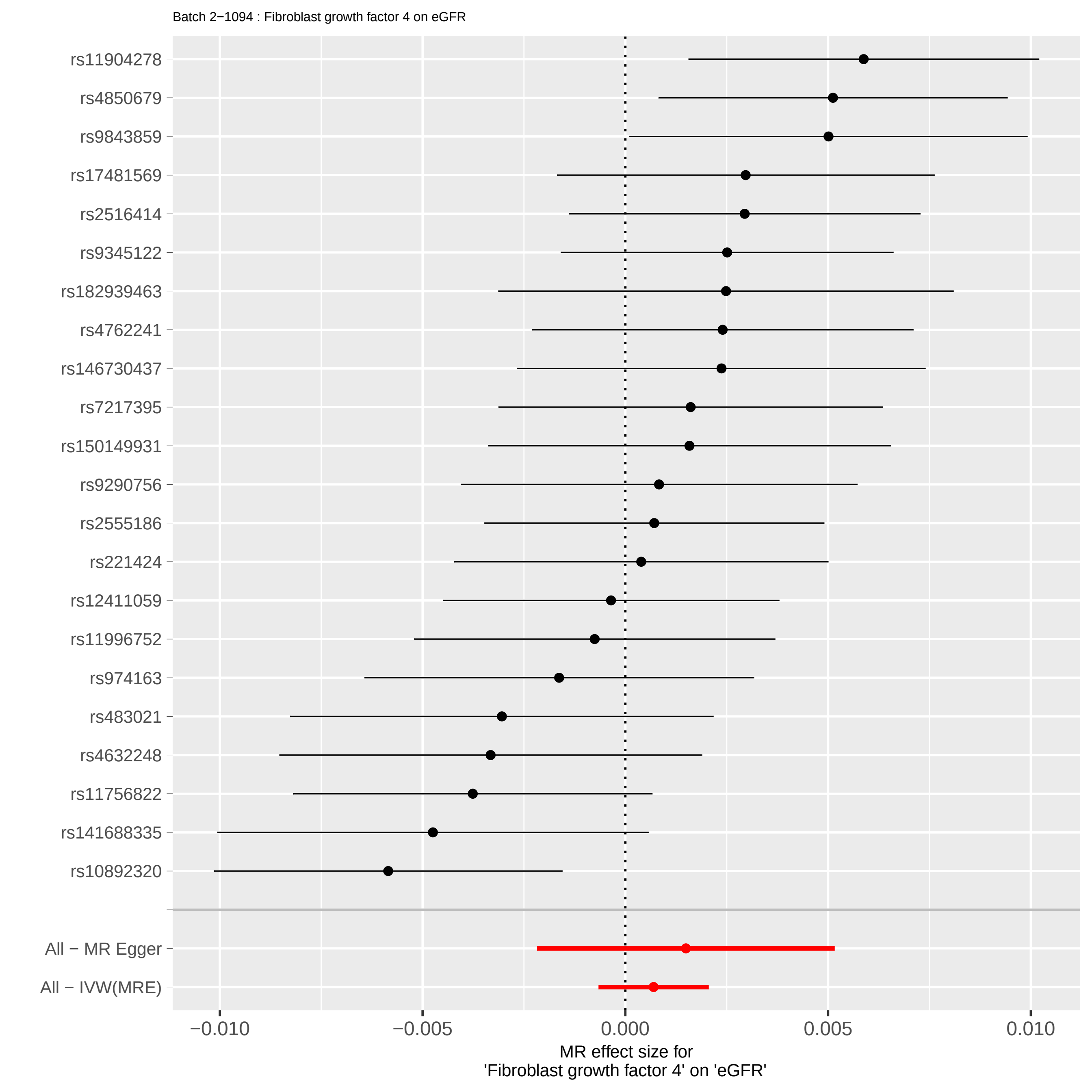
Batch 2−1094 : Fibroblast growth factor 4 on eGFR
rs11904278
rs4850679
rs9843859
rs17481569
rs2516414
rs9345122
rs182939463
rs4762241
rs146730437
rs7217395
rs150149931
rs9290756
rs2555186
rs221424
rs12411059
rs11996752
rs974163
rs483021
rs4632248
rs11756822
rs141688335
rs10892320
All − MR Egger
All − IVW(MRE)
−0.010
−0.005
0.000
0.005
0.010
MR effect size for
'Fibroblast growth factor 4' on 'eGFR'

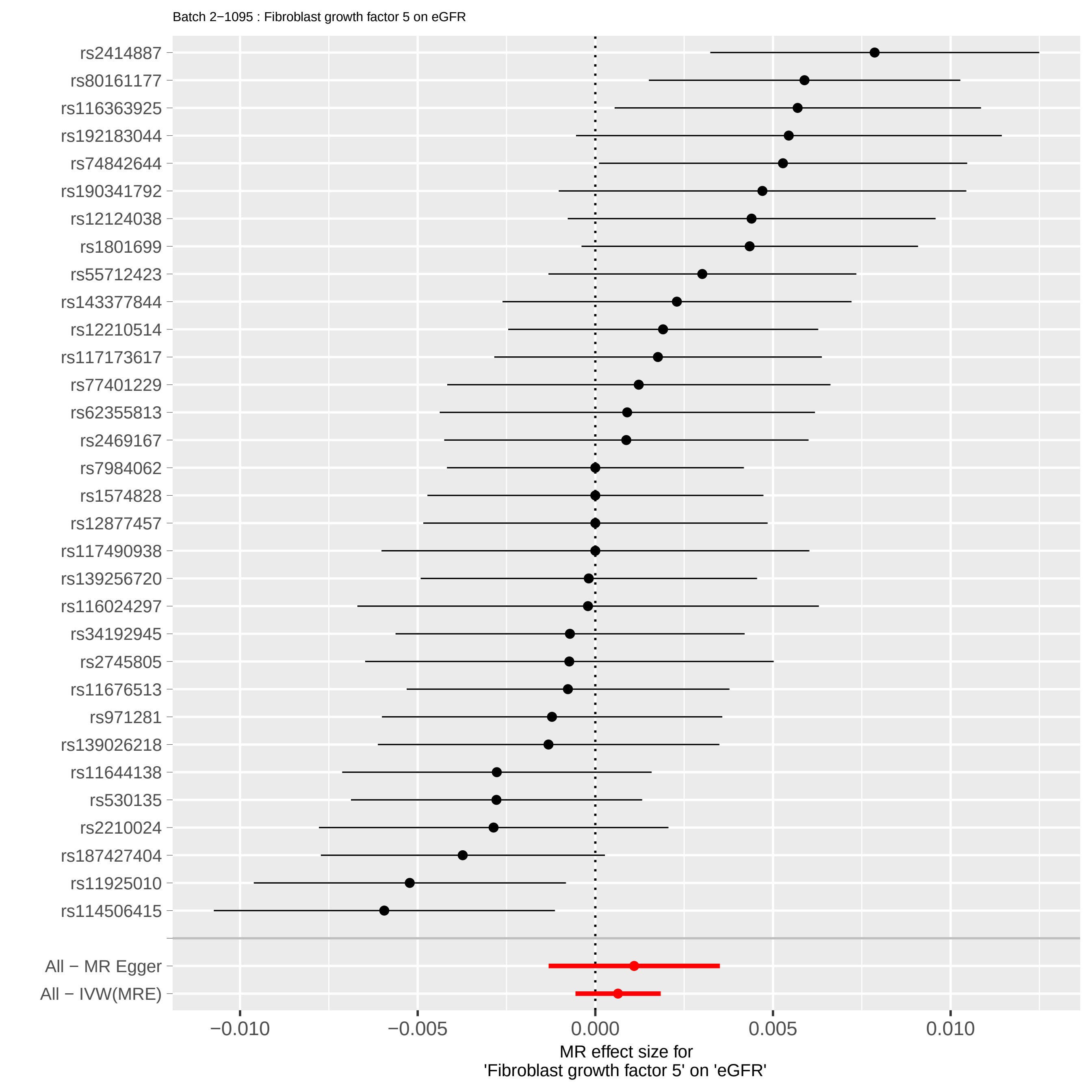

Batch 2−1095 : Fibroblast growth factor 5 on eGFR
rs2414887
rs80161177
rs116363925
rs192183044
rs74842644
rs190341792
rs12124038
rs1801699
rs55712423
rs143377844
rs12210514
rs117173617
rs77401229
rs62355813
rs2469167
rs7984062
rs1574828
rs12877457
rs117490938
rs139256720
rs116024297
rs34192945
rs2745805
rs11676513
rs971281
rs139026218
rs11644138
rs530135
rs2210024
rs187427404
rs11925010
rs114506415
All − MR Egger
All − IVW(MRE)
−0.010
−0.005
0.000
0.005
0.010
MR effect size for
'Fibroblast growth factor 5' on 'eGFR'

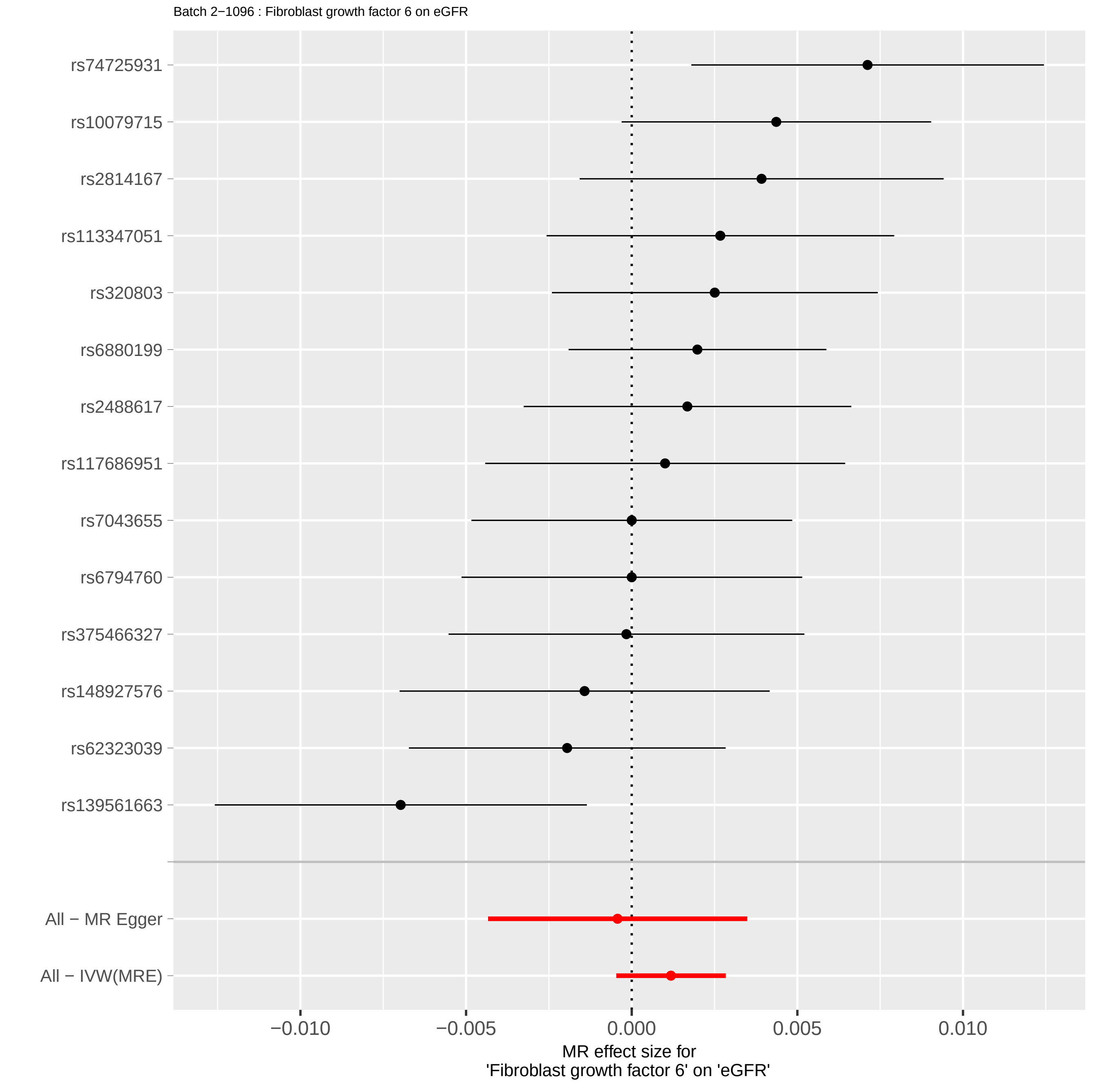
Batch 2−1096 : Fibroblast growth factor 6 on eGFR
rs74725931
rs10079715
rs2814167
rs113347051
rs320803
rs6880199
rs2488617
rs117686951
rs7043655
rs6794760
rs375466327
rs148927576
rs62323039
rs139561663
All − MR Egger
All − IVW(MRE)
−0.010
−0.005
0.000
0.005
0.010
MR effect size for
'Fibroblast growth factor 6' on 'eGFR'

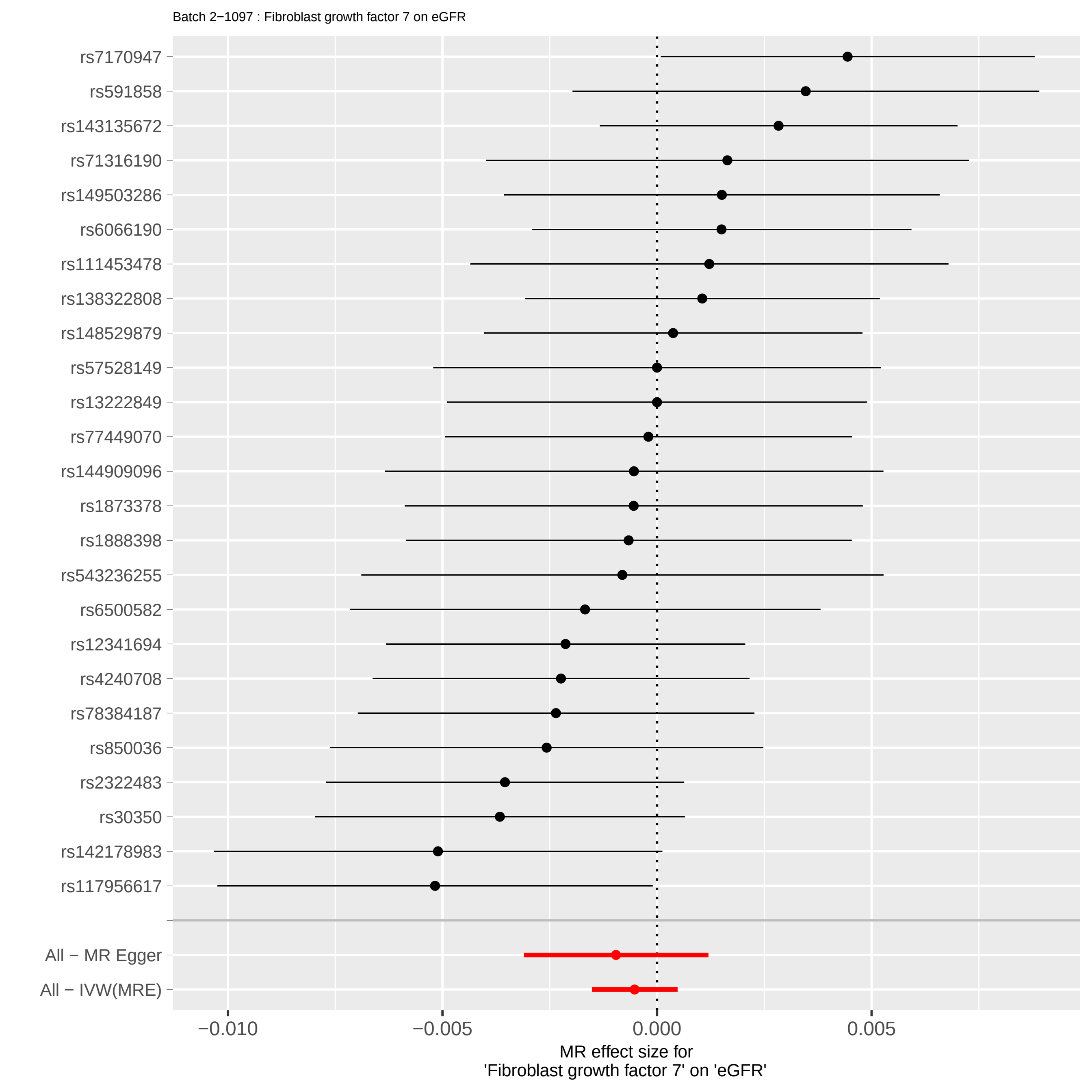
Batch 2−1097 : Fibroblast growth factor 7 on eGFR
rs7170947
rs591858
rs143135672
rs71316190
rs149503286
rs6066190
rs111453478
rs138322808
rs148529879
rs57528149
rs13222849
rs77449070
rs144909096
rs1873378
rs1888398
rs543236255
rs6500582
rs12341694
rs4240708
rs78384187
rs850036
rs2322483
rs30350
rs142178983
rs117956617
All − MR Egger
All − IVW(MRE)
−0.010
−0.005
0.000
0.005
MR effect size for
'Fibroblast growth factor 7' on 'eGFR'

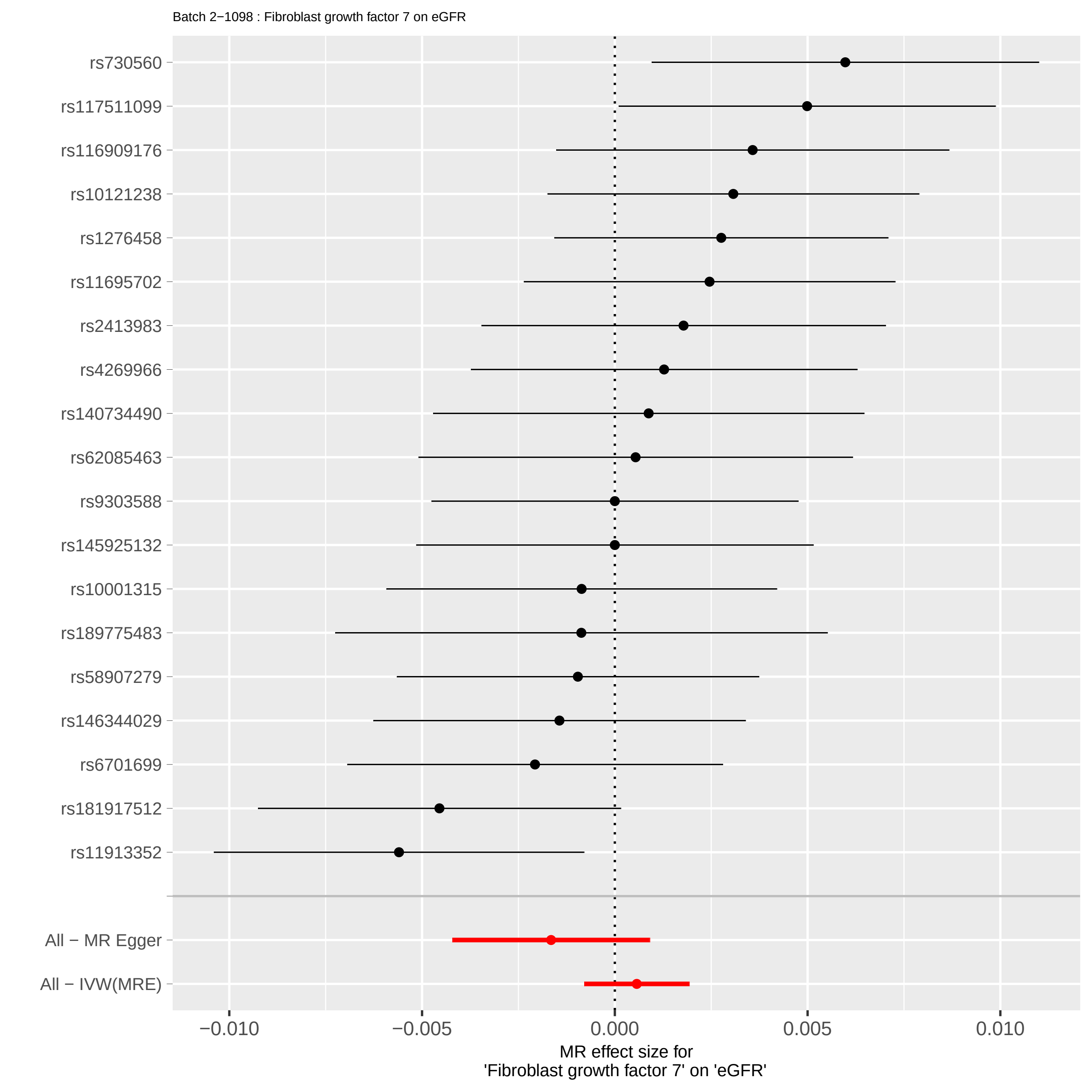
Batch 2−1098 : Fibroblast growth factor 7 on eGFR
rs730560
rs117511099
rs116909176
rs10121238
rs1276458
rs11695702
rs2413983
rs4269966
rs140734490
rs62085463
rs9303588
rs145925132
rs10001315
rs189775483
rs58907279
rs146344029
rs6701699
rs181917512
rs11913352
All − MR Egger
All − IVW(MRE)
−0.010
−0.005
0.000
0.005
0.010
MR effect size for
'Fibroblast growth factor 7' on 'eGFR'

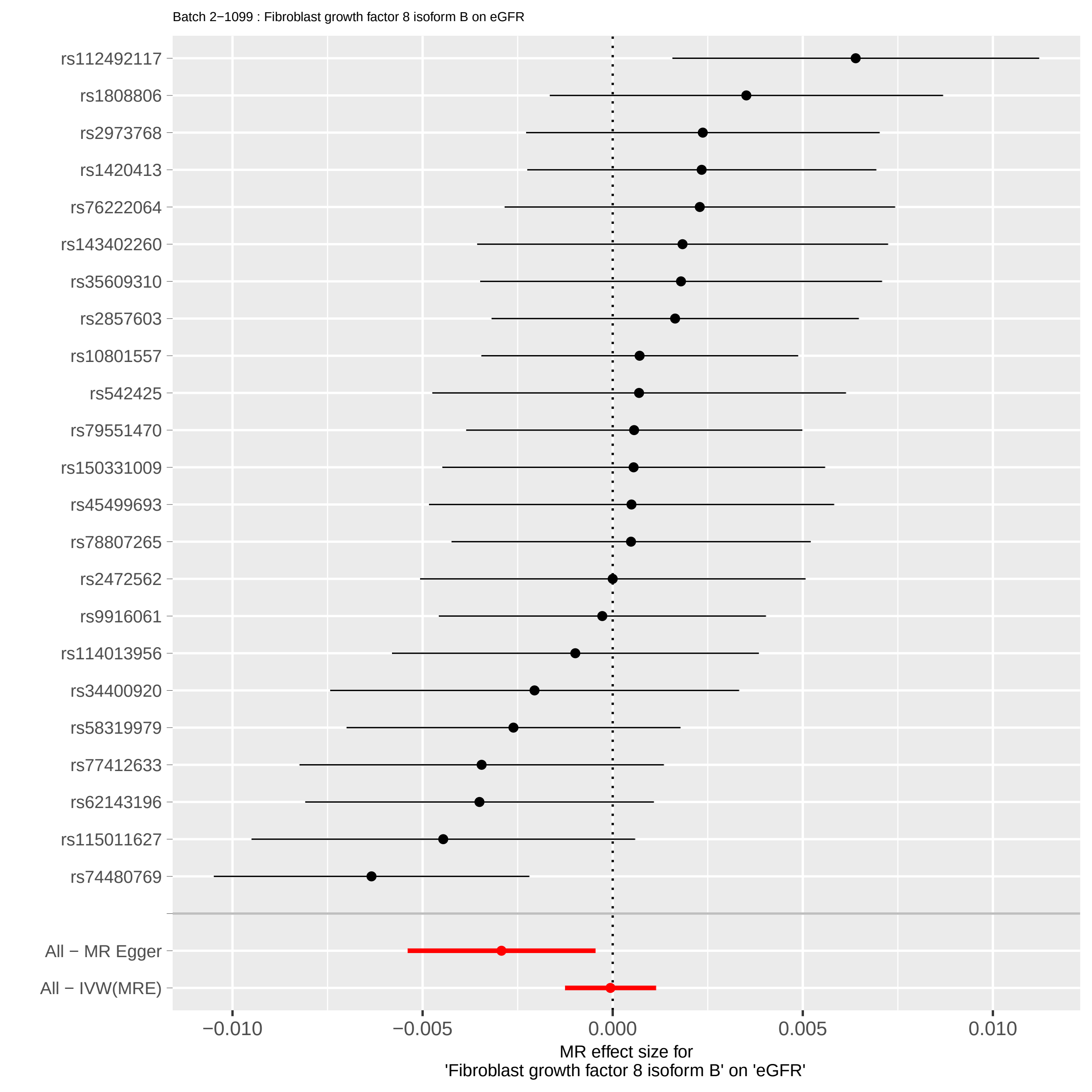
Batch 2−1099 : Fibroblast growth factor 8 isoform B on eGFR
rs112492117
rs1808806
rs2973768
rs1420413
rs76222064
rs143402260
rs35609310
rs2857603
rs10801557
rs542425
rs79551470
rs150331009
rs45499693
rs78807265
rs2472562
rs9916061
rs114013956
rs34400920
rs58319979
rs77412633
rs62143196
rs115011627
rs74480769
All − MR Egger
All − IVW(MRE)
−0.010
−0.005
0.000
0.005
0.010
MR effect size for
'Fibroblast growth factor 8 isoform B' on 'eGFR'

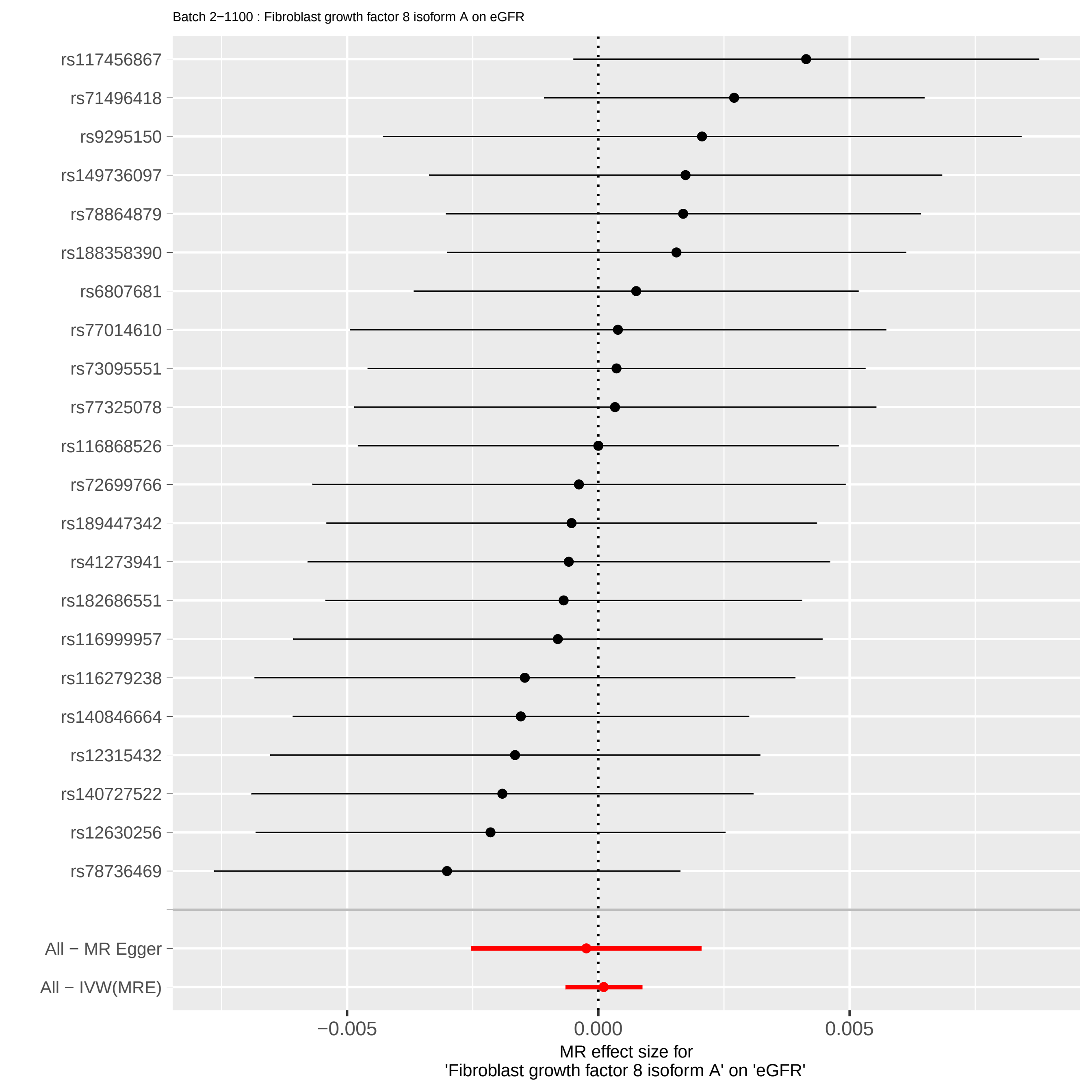

Batch 2−1100 : Fibroblast growth factor 8 isoform A on eGFR
rs117456867
rs71496418
rs9295150
rs149736097
rs78864879
rs188358390
rs6807681
rs77014610
rs73095551
rs77325078
rs116868526
rs72699766
rs189447342
rs41273941
rs182686551
rs116999957
rs116279238
rs140846664
rs12315432
rs140727522
rs12630256
rs78736469
All − MR Egger
All − IVW(MRE)
−0.005
0.000
0.005
MR effect size for
'Fibroblast growth factor 8 isoform A' on 'eGFR'

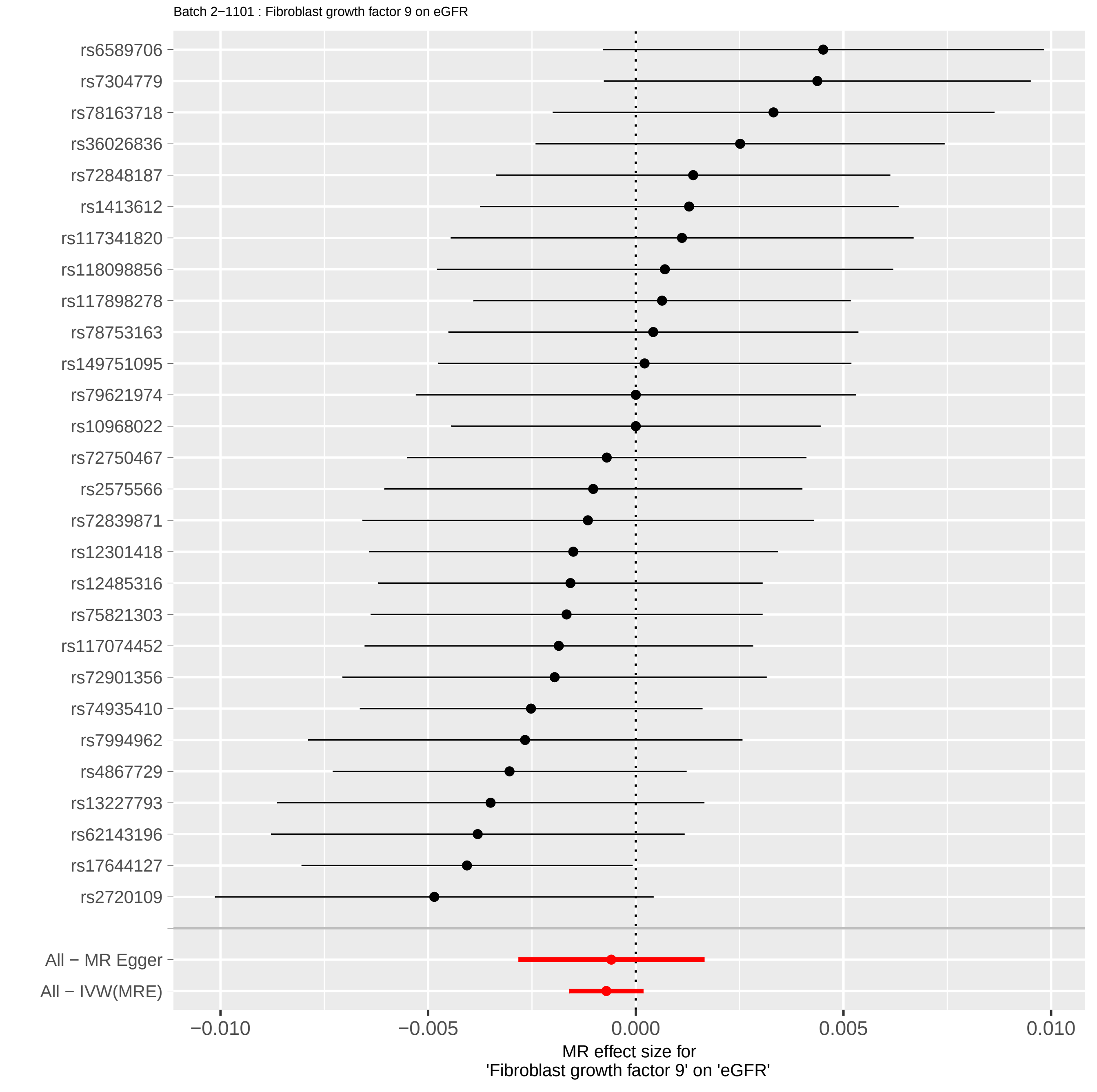

Batch 2−1101 : Fibroblast growth factor 9 on eGFR
rs6589706
rs7304779
rs78163718
rs36026836
rs72848187
rs1413612
rs117341820
rs118098856
rs117898278
rs78753163
rs149751095
rs79621974
rs10968022
rs72750467
rs2575566
rs72839871
rs12301418
rs12485316
rs75821303
rs117074452
rs72901356
rs74935410
rs7994962
rs4867729
rs13227793
rs62143196
rs17644127
rs2720109
All − MR Egger
All − IVW(MRE)
−0.010
−0.005
0.000
0.005
0.010
MR effect size for
'Fibroblast growth factor 9' on 'eGFR'

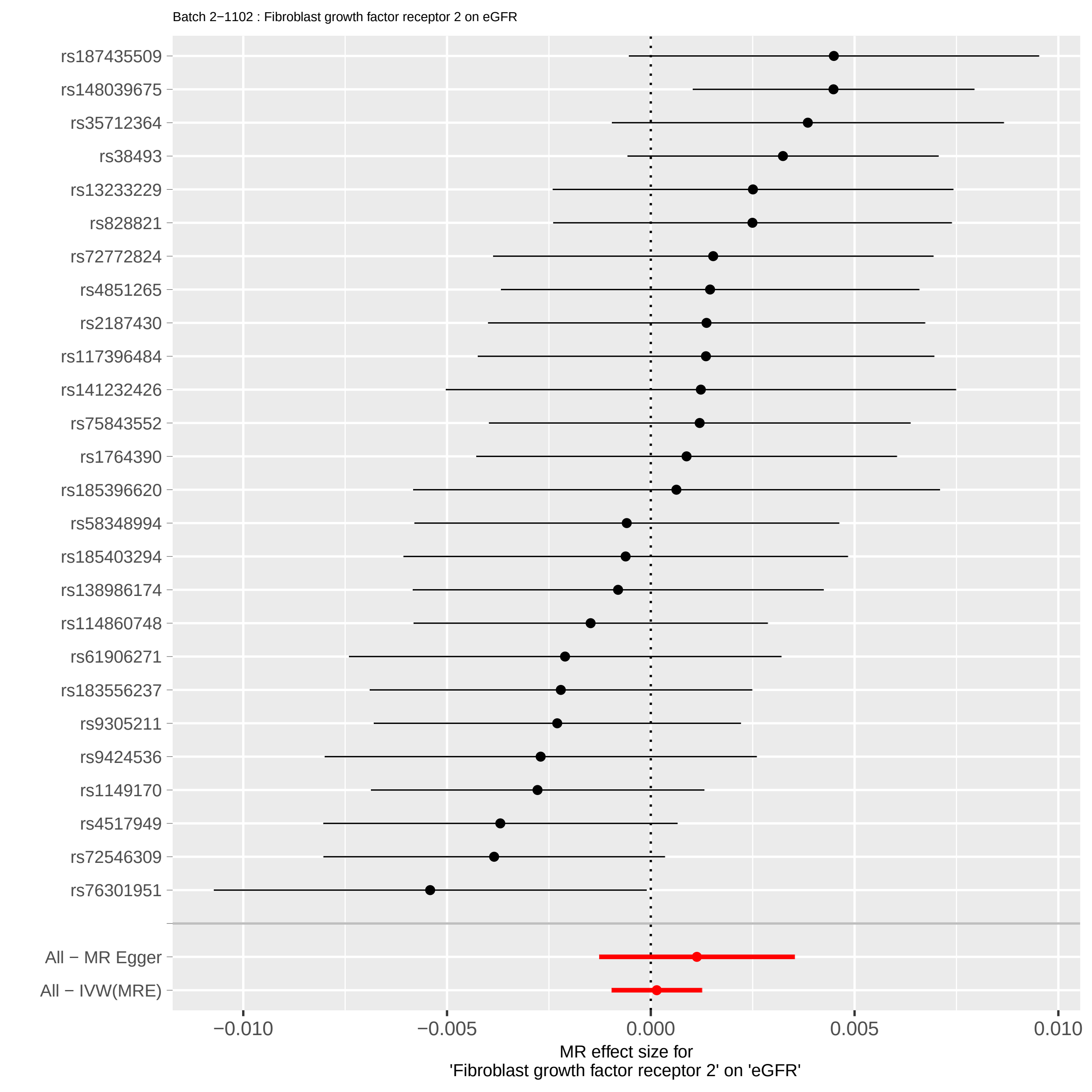
Batch 2−1102 : Fibroblast growth factor receptor 2 on eGFR
rs187435509
rs148039675
rs35712364
rs38493
rs13233229
rs828821
rs72772824
rs4851265
rs2187430
rs117396484
rs141232426
rs75843552
rs1764390
rs185396620
rs58348994
rs185403294
rs138986174
rs114860748
rs61906271
rs183556237
rs9305211
rs9424536
rs1149170
rs4517949
rs72546309
rs76301951
All − MR Egger
All − IVW(MRE)
−0.010
−0.005
0.000
0.005
0.010
MR effect size for
'Fibroblast growth factor receptor 2' on 'eGFR'

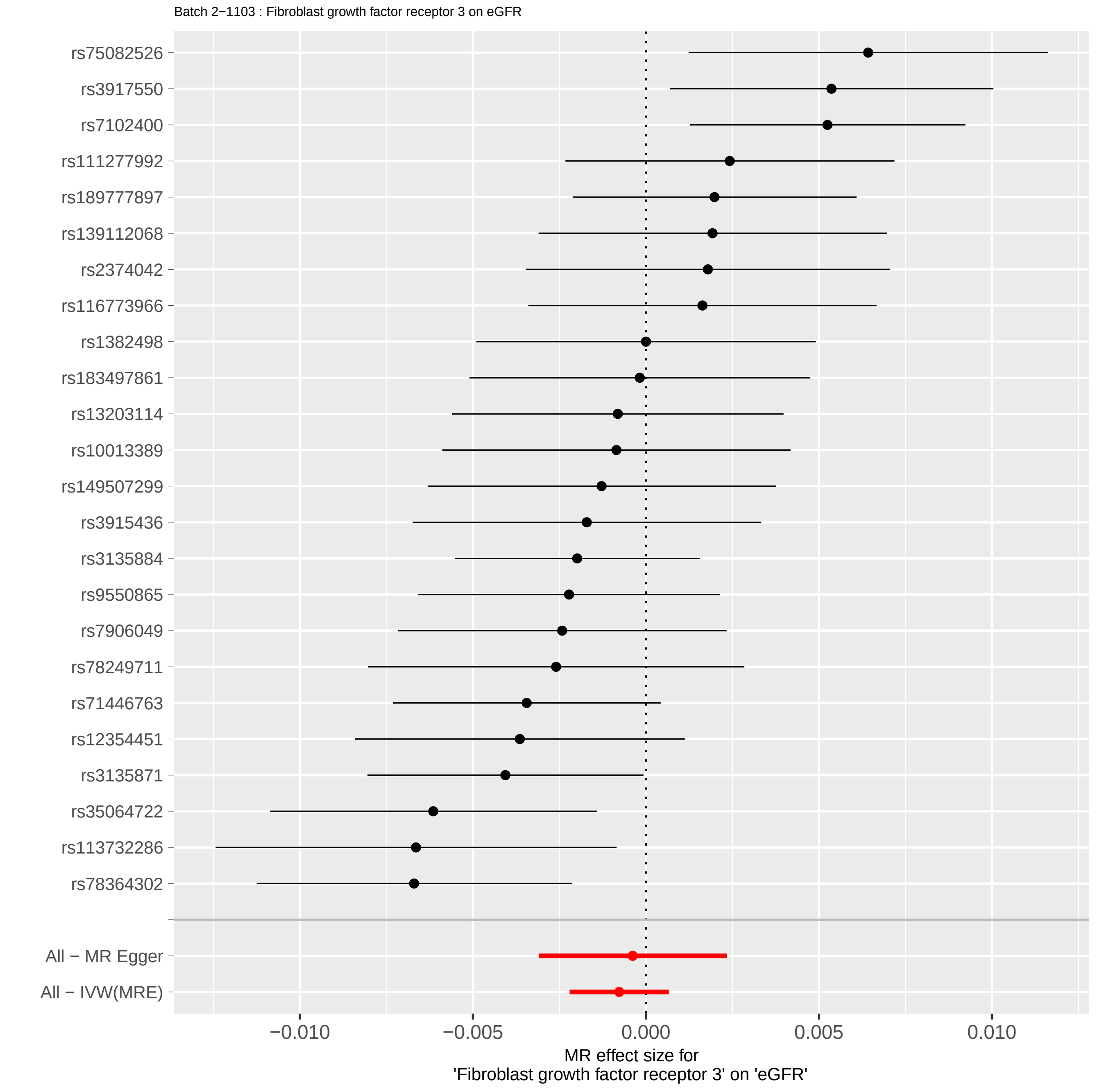
Batch 2−1103 : Fibroblast growth factor receptor 3 on eGFR
rs75082526
rs3917550
rs7102400
rs111277992
rs189777897
rs139112068
rs2374042
rs116773966
rs1382498
rs183497861
rs13203114
rs10013389
rs149507299
rs3915436
rs3135884
rs9550865
rs7906049
rs78249711
rs71446763
rs12354451
rs3135871
rs35064722
rs113732286
rs78364302
All − MR Egger
All − IVW(MRE)
−0.010
−0.005
0.000
0.005
0.010
MR effect size for
'Fibroblast growth factor receptor 3' on 'eGFR'

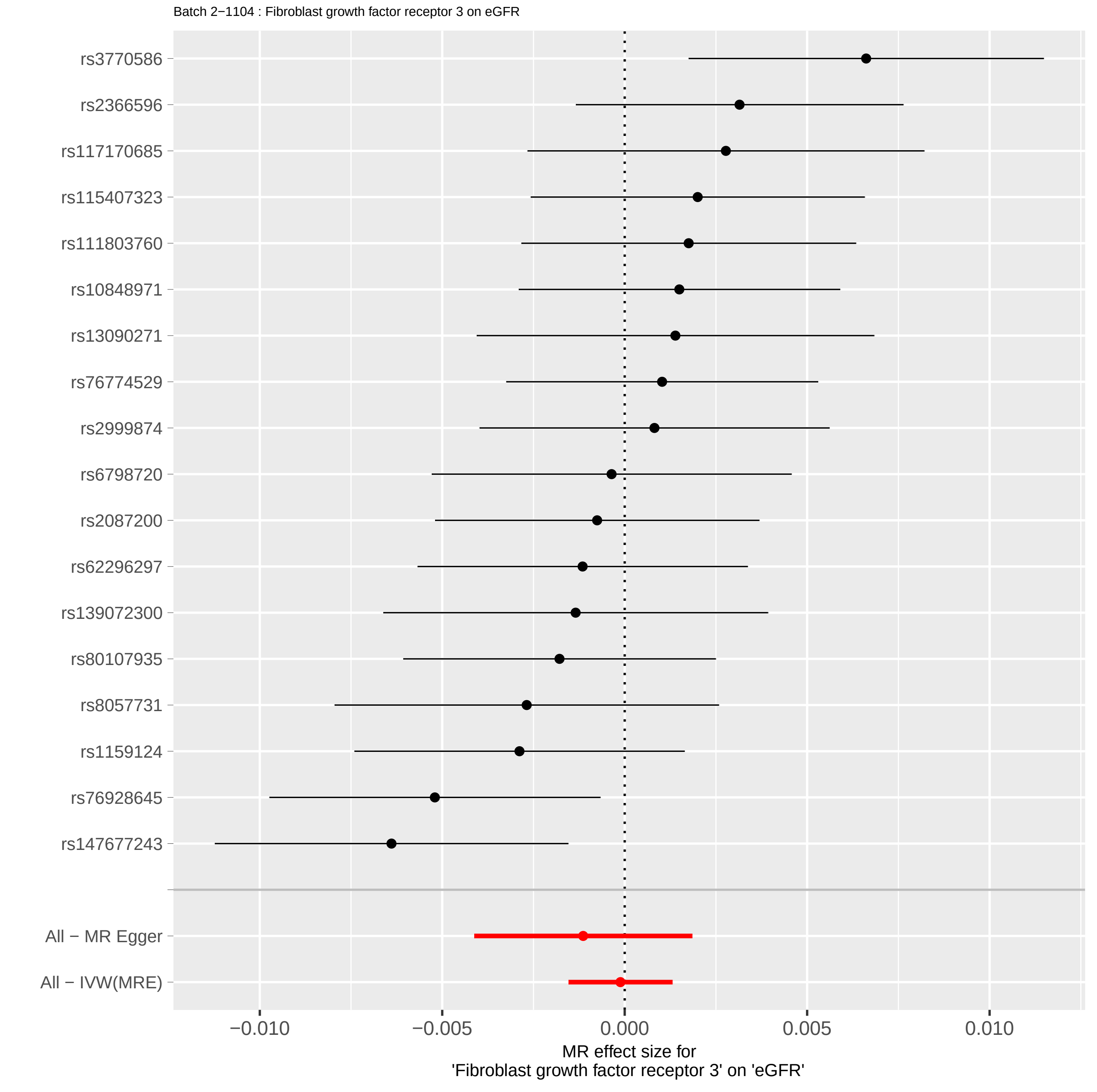

Batch 2−1104 : Fibroblast growth factor receptor 3 on eGFR
rs3770586
rs2366596
rs117170685
rs115407323
rs111803760
rs10848971
rs13090271
rs76774529
rs2999874
rs6798720
rs2087200
rs62296297
rs139072300
rs80107935
rs8057731
rs1159124
rs76928645
rs147677243
All − MR Egger
All − IVW(MRE)
−0.010
−0.005
0.000
0.005
0.010
MR effect size for
'Fibroblast growth factor receptor 3' on 'eGFR'

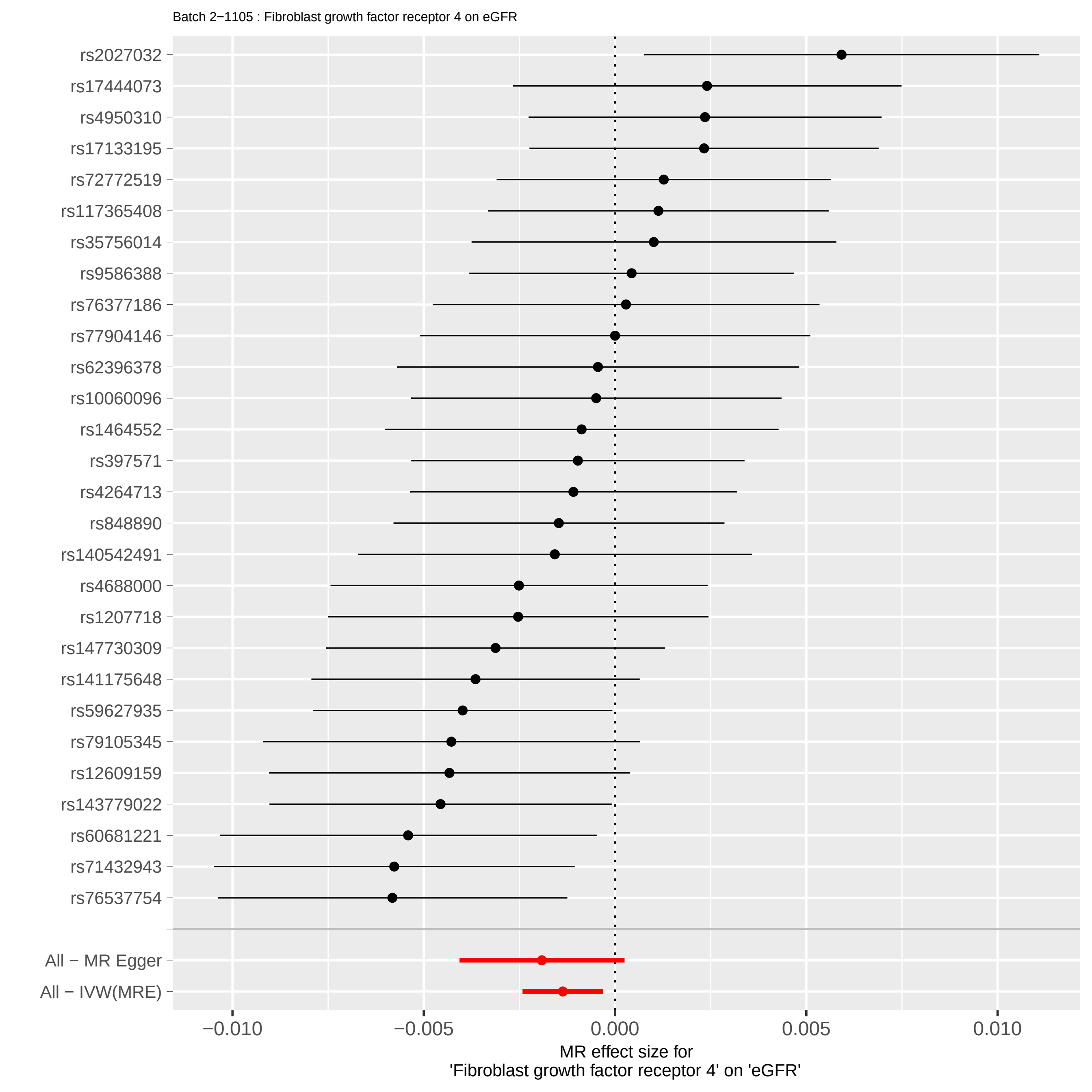
Batch 2−1105 : Fibroblast growth factor receptor 4 on eGFR
rs2027032
rs17444073
rs4950310
rs17133195
rs72772519
rs117365408
rs35756014
rs9586388
rs76377186
rs77904146
rs62396378
rs10060096
rs1464552
rs397571
rs4264713
rs848890
rs140542491
rs4688000
rs1207718
rs147730309
rs141175648
rs59627935
rs79105345
rs12609159
rs143779022
rs60681221
rs71432943
rs76537754
All − MR Egger
All − IVW(MRE)
−0.010
−0.005
0.000
0.005
0.010
MR effect size for
'Fibroblast growth factor receptor 4' on 'eGFR'

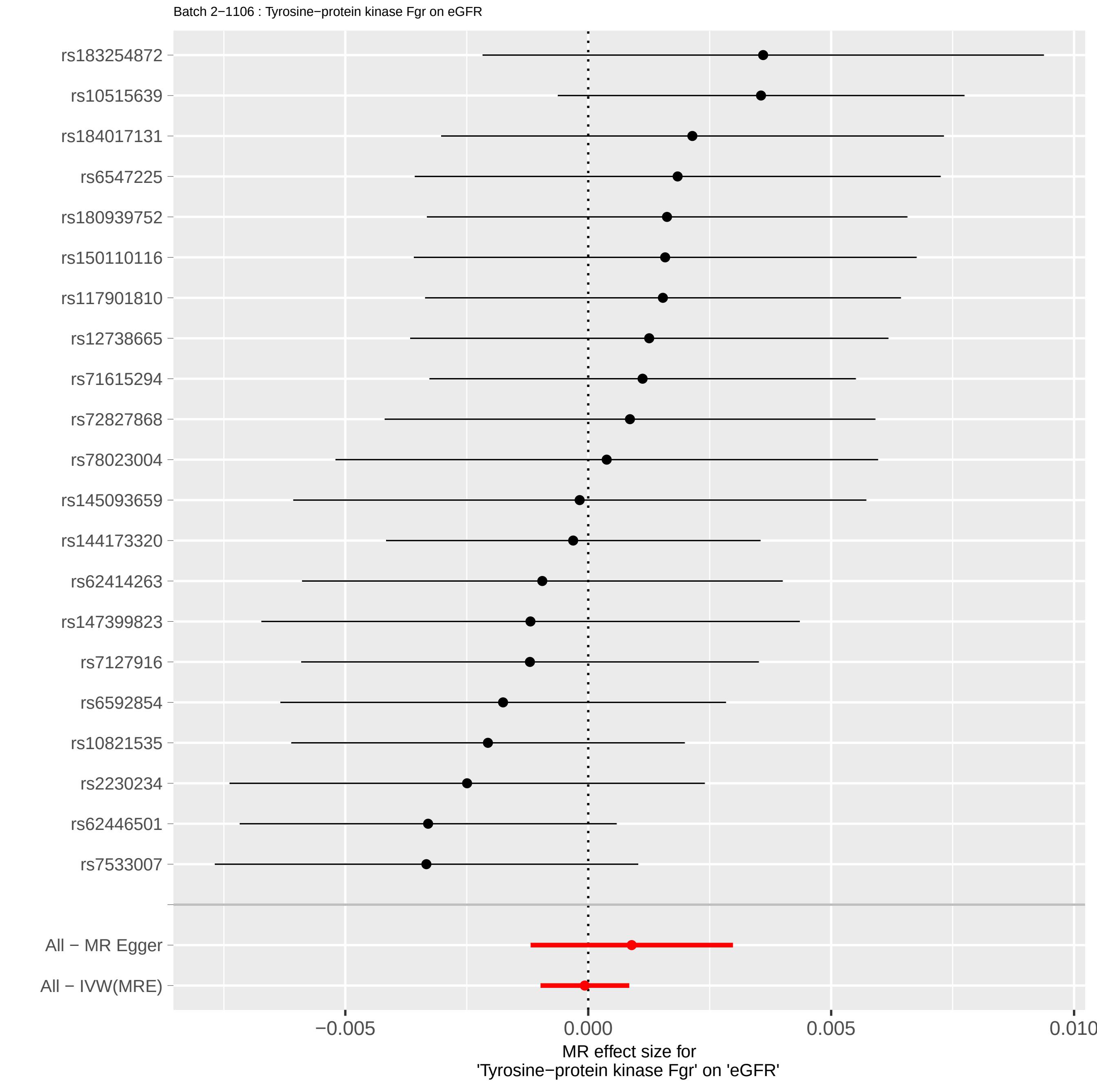

Batch 2−1106 : Tyrosine−protein kinase Fgr on eGFR
rs183254872
rs10515639
rs184017131
rs6547225
rs180939752
rs150110116
rs117901810
rs12738665
rs71615294
rs72827868
rs78023004
rs145093659
rs144173320
rs62414263
rs147399823
rs7127916
rs6592854
rs10821535
rs2230234
rs62446501
rs7533007
All − MR Egger
All − IVW(MRE)
−0.005
0.000
0.005
0.010
MR effect size for
'Tyrosine−protein kinase Fgr' on 'eGFR'

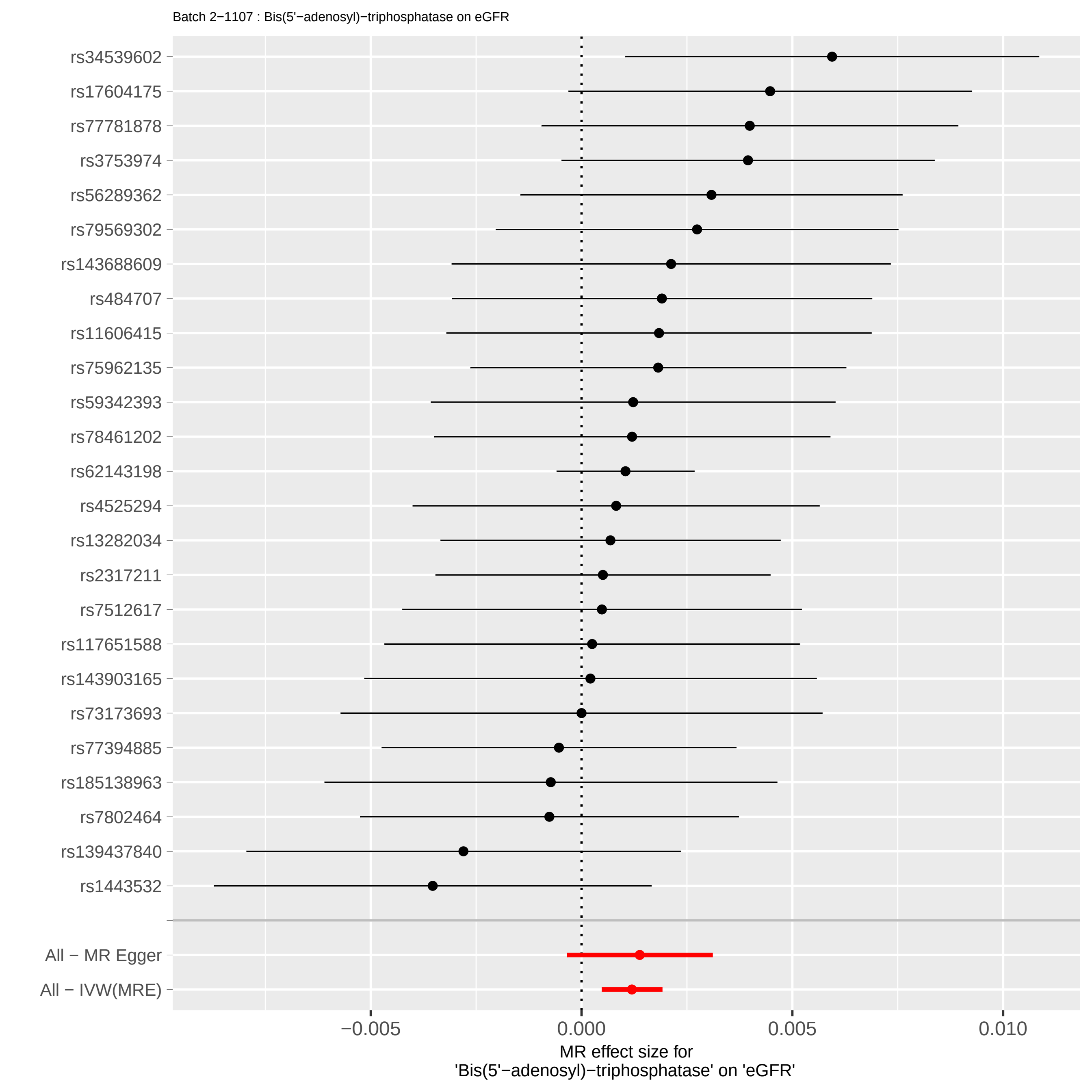

Batch 2−1107 : Bis(5'−adenosyl)−triphosphatase on eGFR
rs34539602
rs17604175
rs77781878
rs3753974
rs56289362
rs79569302
rs143688609
rs484707
rs11606415
rs75962135
rs59342393
rs78461202
rs62143198
rs4525294
rs13282034
rs2317211
rs7512617
rs117651588
rs143903165
rs73173693
rs77394885
rs185138963
rs7802464
rs139437840
rs1443532
All − MR Egger
All − IVW(MRE)
−0.005
0.000
0.005
0.010
MR effect size for
'Bis(5'−adenosyl)−triphosphatase' on 'eGFR'

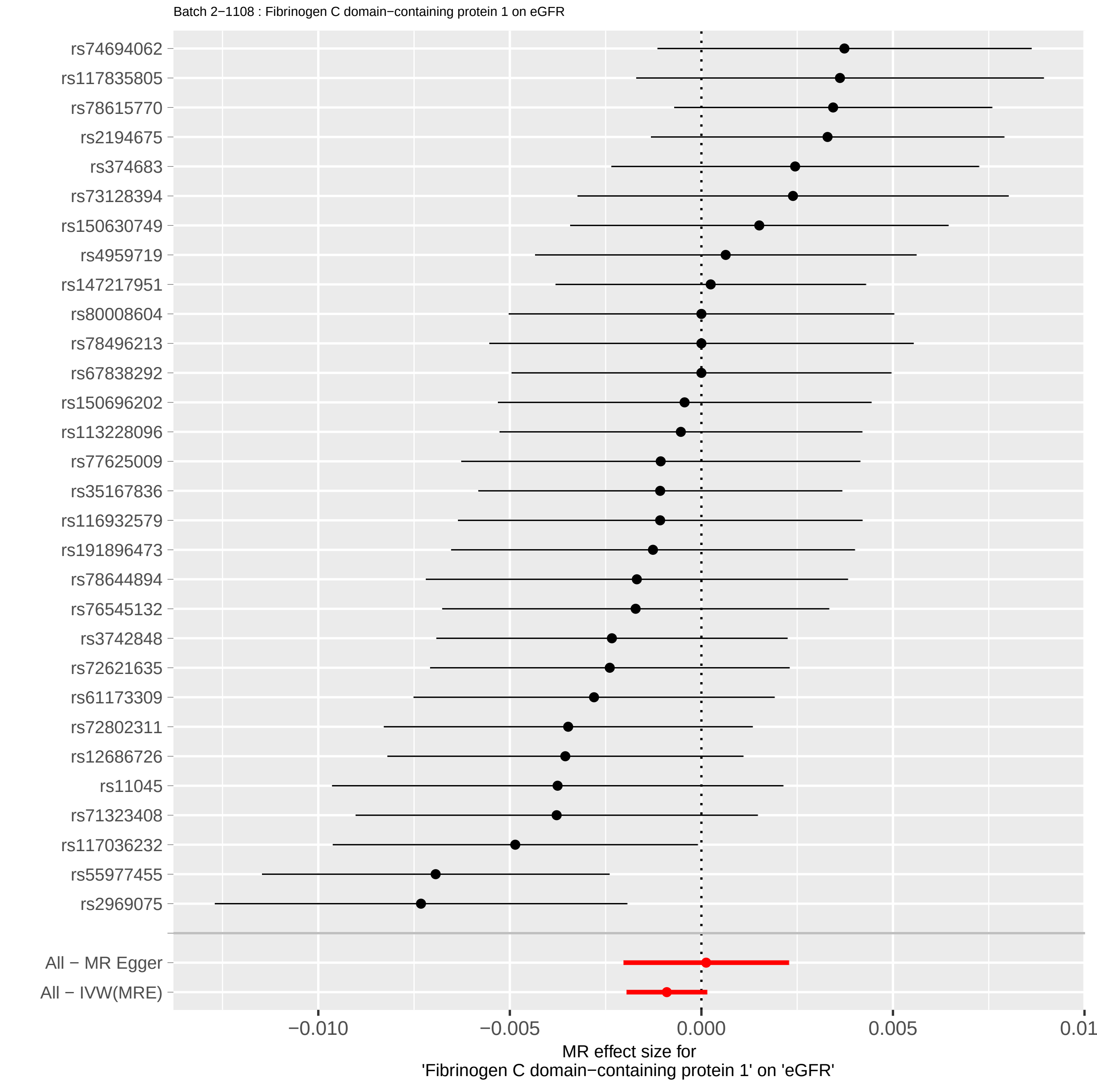

Batch 2−1108 : Fibrinogen C domain−containing protein 1 on eGFR
rs74694062
rs117835805
rs78615770
rs2194675
rs374683
rs73128394
rs150630749
rs4959719
rs147217951
rs80008604
rs78496213
rs67838292
rs150696202
rs113228096
rs77625009
rs35167836
rs116932579
rs191896473
rs78644894
rs76545132
rs3742848
rs72621635
rs61173309
rs72802311
rs12686726
rs11045
rs71323408
rs117036232
rs55977455
rs2969075
All − MR Egger
All − IVW(MRE)
−0.010
−0.005
0.000
0.005
0.01
MR effect size for
'Fibrinogen C domain−containing protein 1' on 'eGFR'

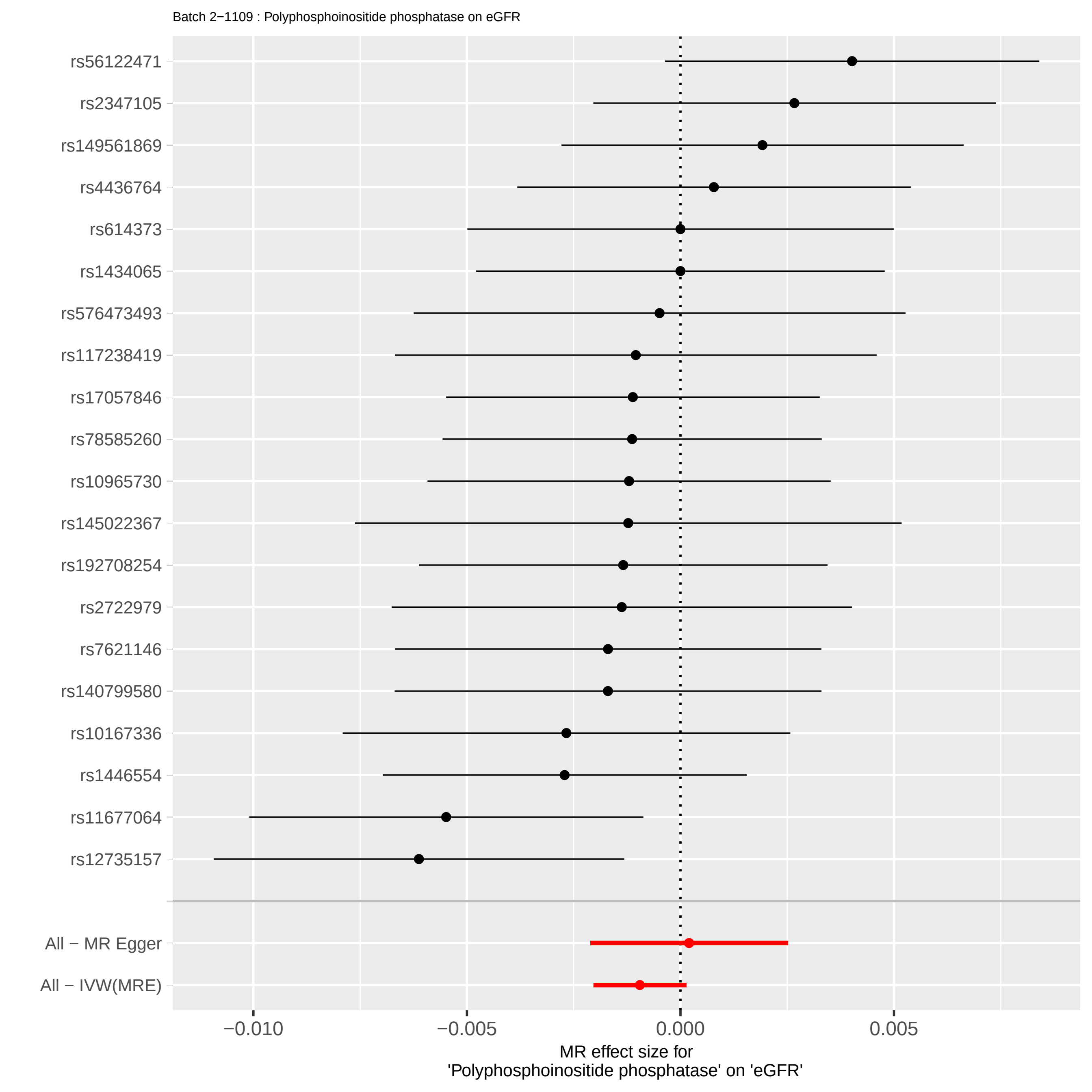

Batch 2−1109 : Polyphosphoinositide phosphatase on eGFR
rs56122471
rs2347105
rs149561869
rs4436764
rs614373
rs1434065
rs576473493
rs117238419
rs17057846
rs78585260
rs10965730
rs145022367
rs192708254
rs2722979
rs7621146
rs140799580
rs10167336
rs1446554
rs11677064
rs12735157
All − MR Egger
All − IVW(MRE)
−0.010
−0.005
0.000
0.005
MR effect size for
'Polyphosphoinositide phosphatase' on 'eGFR'

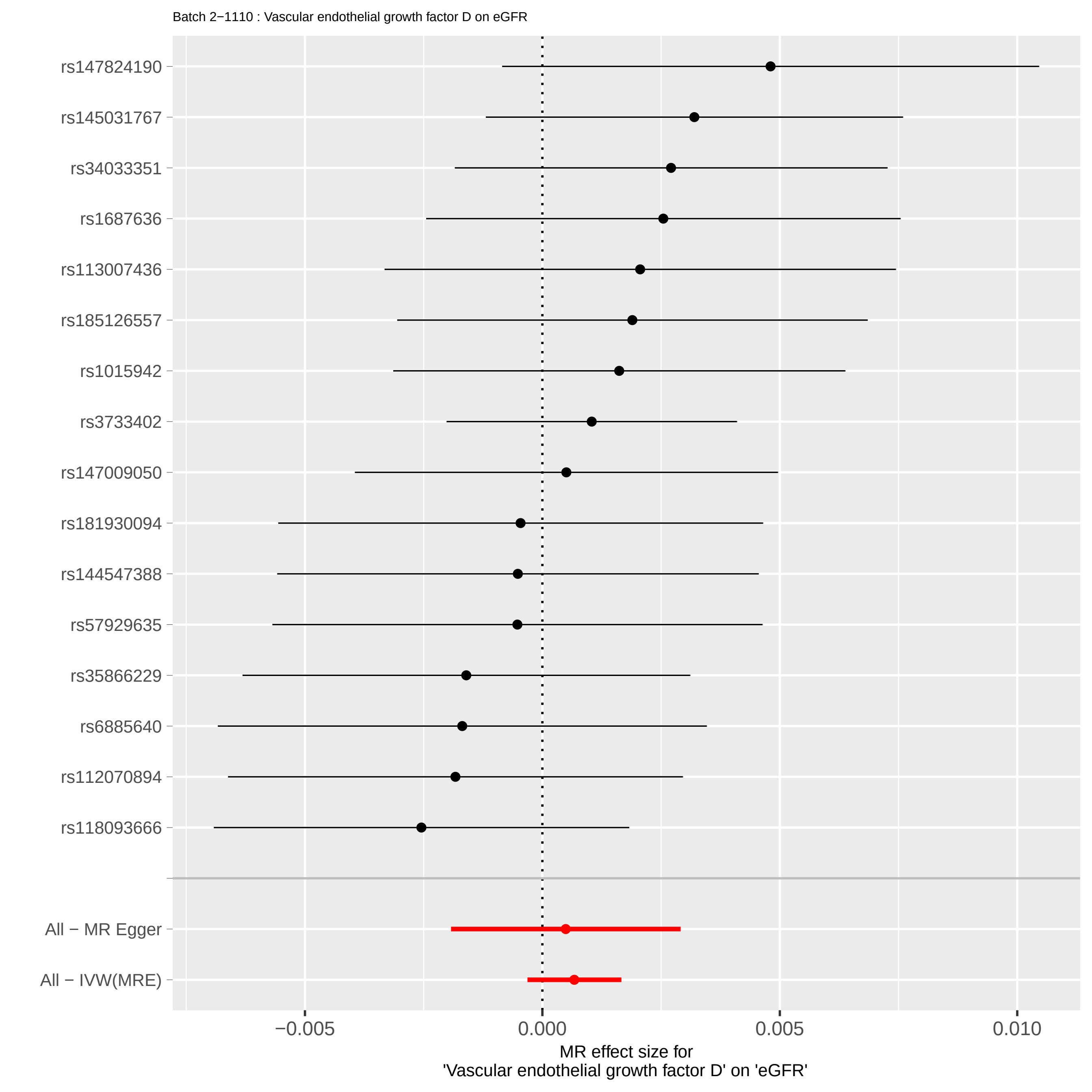

Batch 2−1110 : Vascular endothelial growth factor D on eGFR
rs147824190
rs145031767
rs34033351
rs1687636
rs113007436
rs185126557
rs1015942
rs3733402
rs147009050
rs181930094
rs144547388
rs57929635
rs35866229
rs6885640
rs112070894
rs118093666
All − MR Egger
All − IVW(MRE)
−0.005
0.000
0.005
0.010
MR effect size for
'Vascular endothelial growth factor D' on 'eGFR'

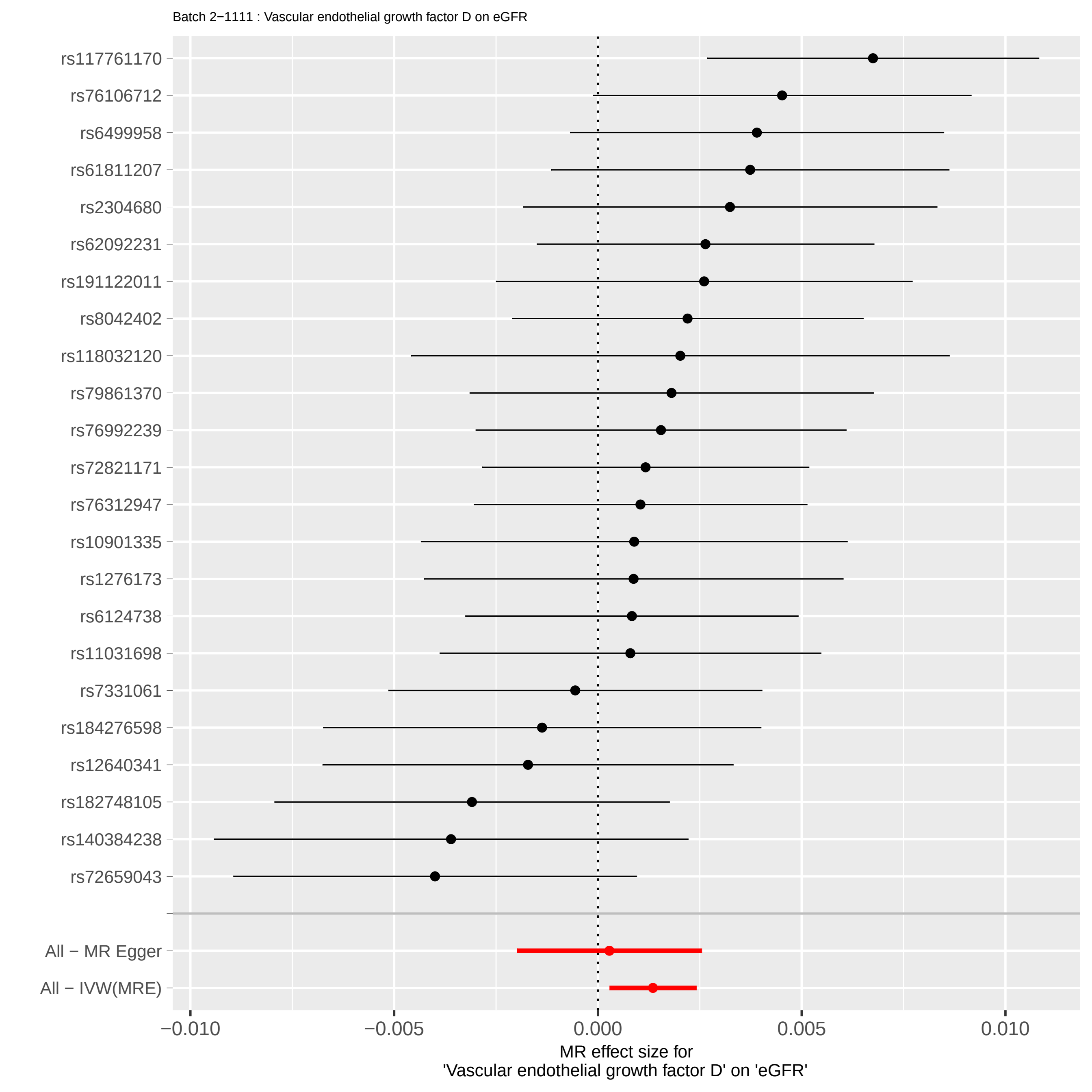
Batch 2−1111 : Vascular endothelial growth factor D on eGFR
rs117761170
rs76106712
rs6499958
rs61811207
rs2304680
rs62092231
rs191122011
rs8042402
rs118032120
rs79861370
rs76992239
rs72821171
rs76312947
rs10901335
rs1276173
rs6124738
rs11031698
rs7331061
rs184276598
rs12640341
rs182748105
rs140384238
rs72659043
All − MR Egger
All − IVW(MRE)
−0.010
−0.005
0.000
0.005
0.010
MR effect size for
'Vascular endothelial growth factor D' on 'eGFR'

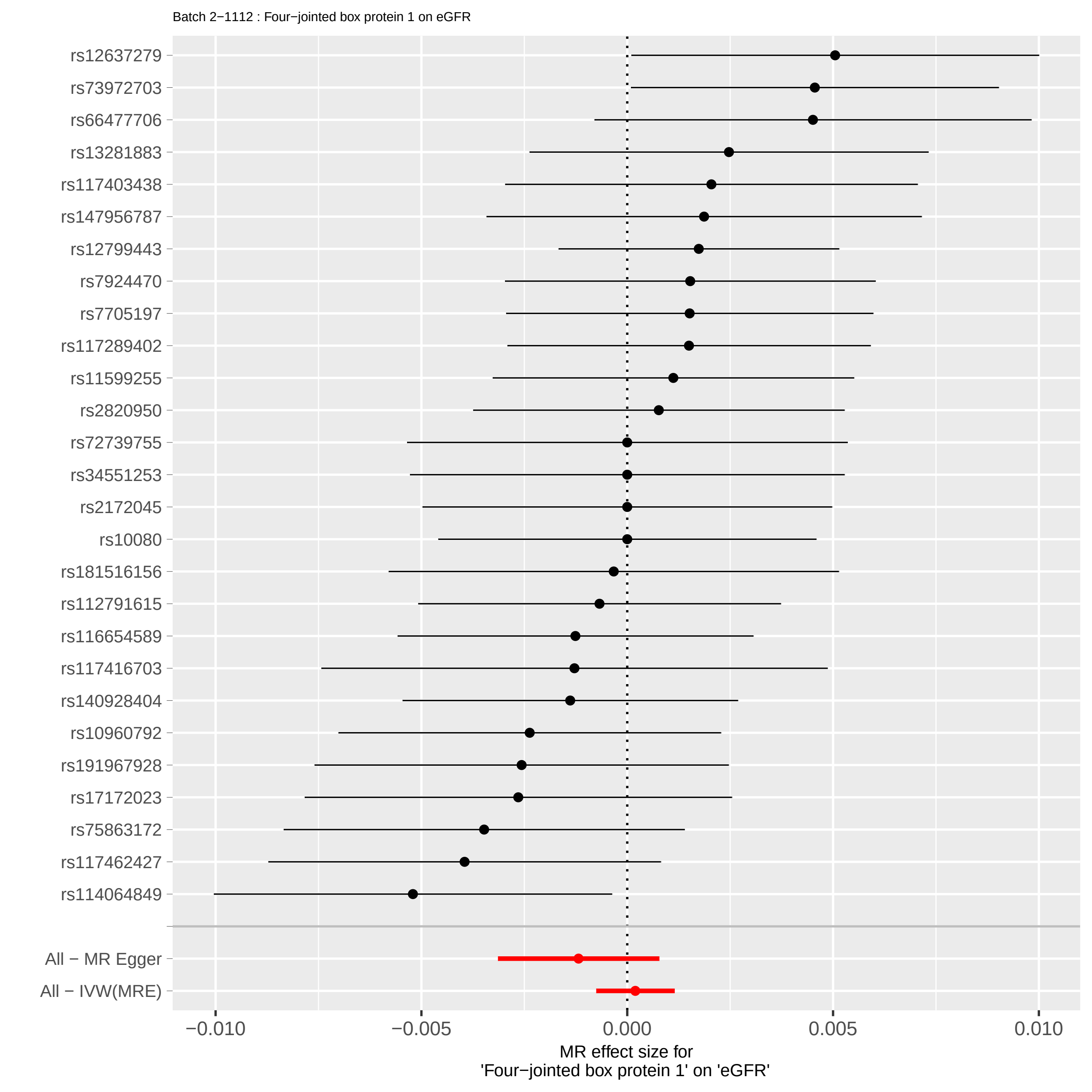

Batch 2−1112 : Four−jointed box protein 1 on eGFR
rs12637279
rs73972703
rs66477706
rs13281883
rs117403438
rs147956787
rs12799443
rs7924470
rs7705197
rs117289402
rs11599255
rs2820950
rs72739755
rs34551253
rs2172045
rs10080
rs181516156
rs112791615
rs116654589
rs117416703
rs140928404
rs10960792
rs191967928
rs17172023
rs75863172
rs117462427
rs114064849
All − MR Egger
All − IVW(MRE)
−0.010
−0.005
0.000
0.005
0.010
MR effect size for
'Four−jointed box protein 1' on 'eGFR'

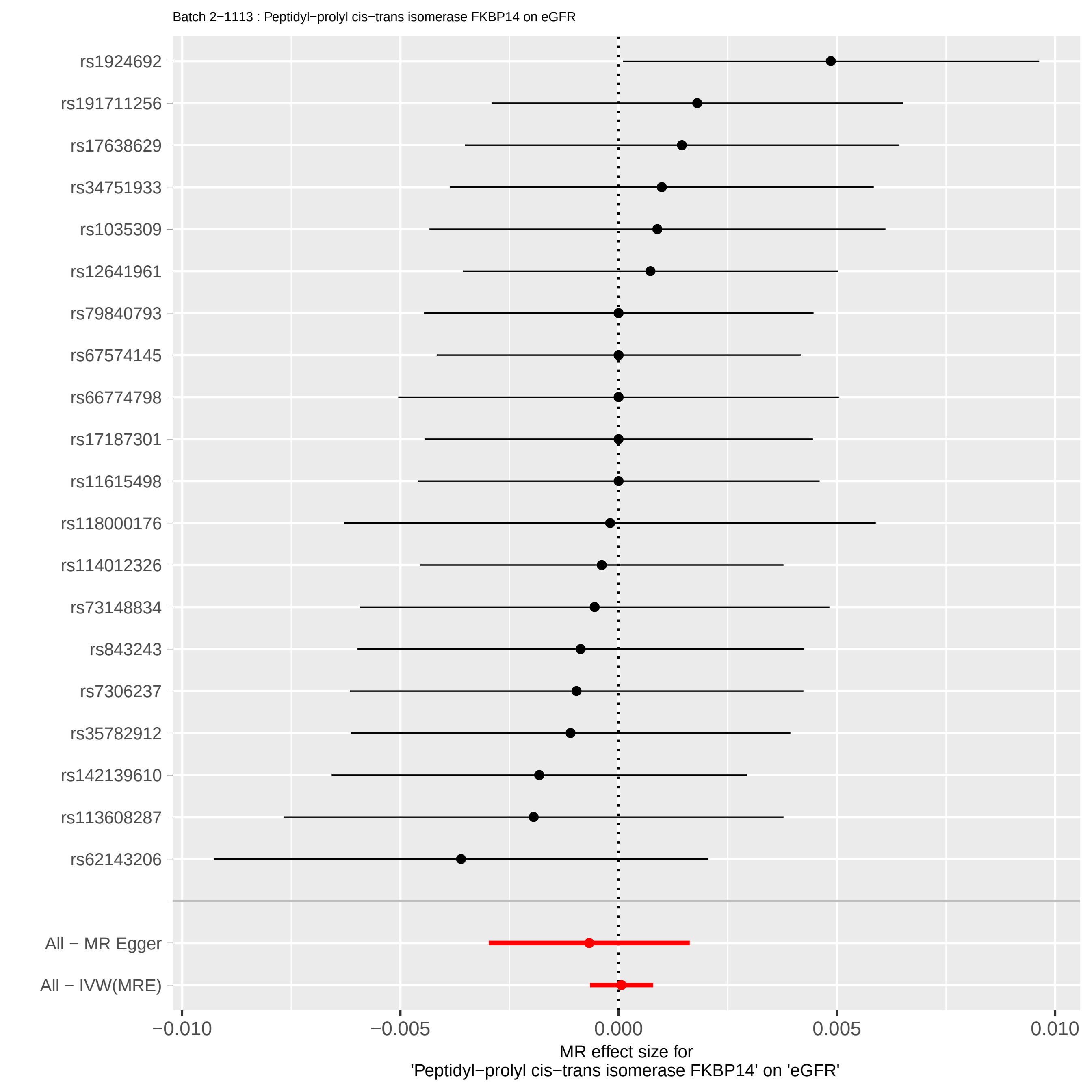

Batch 2−1113 : Peptidyl−prolyl cis−trans isomerase FKBP14 on eGFR
rs1924692
rs191711256
rs17638629
rs34751933
rs1035309
rs12641961
rs79840793
rs67574145
rs66774798
rs17187301
rs11615498
rs118000176
rs114012326
rs73148834
rs843243
rs7306237
rs35782912
rs142139610
rs113608287
rs62143206
All − MR Egger
All − IVW(MRE)
−0.010
−0.005
0.000
0.005
0.010
MR effect size for
'Peptidyl−prolyl cis−trans isomerase FKBP14' on 'eGFR'

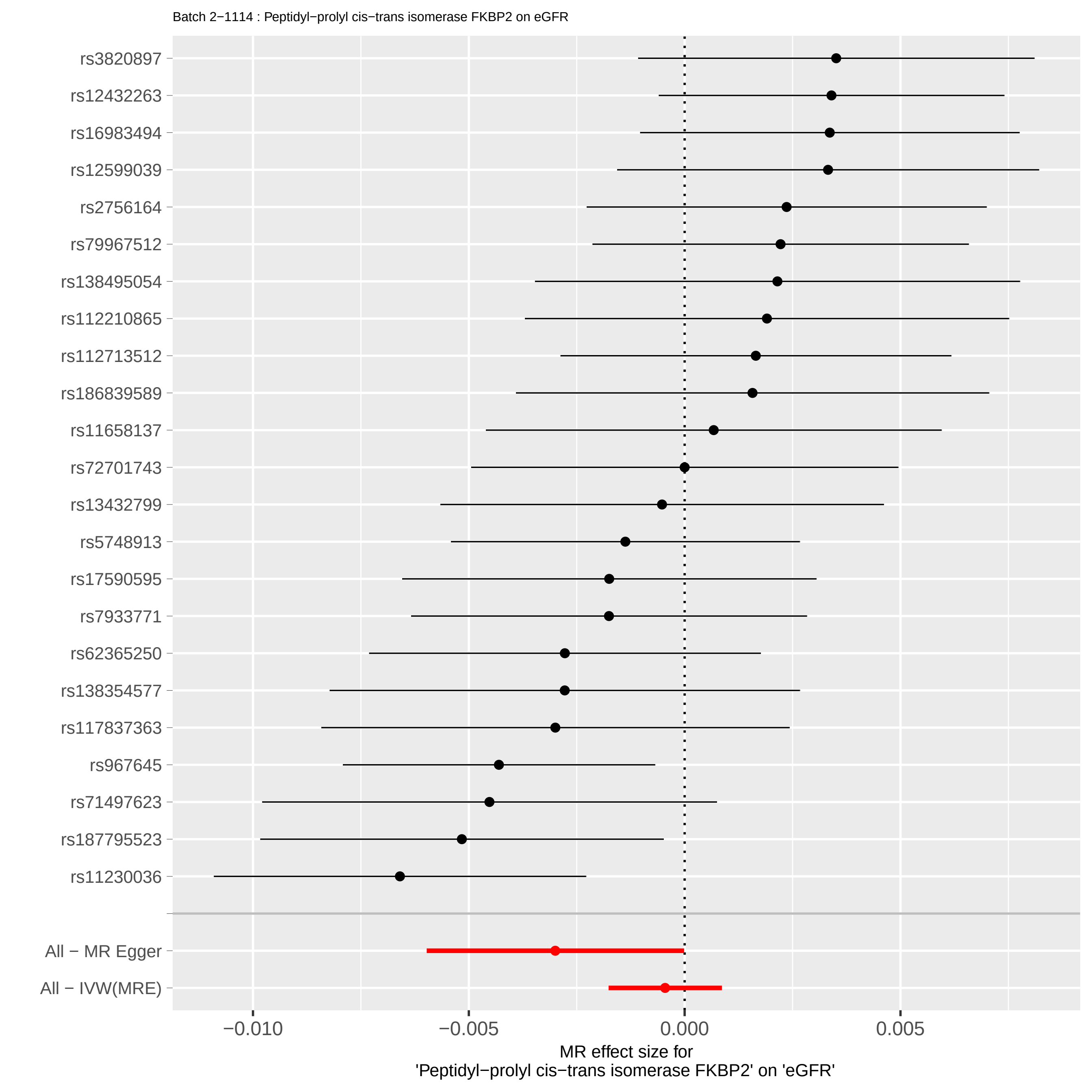

Batch 2−1114 : Peptidyl−prolyl cis−trans isomerase FKBP2 on eGFR
rs3820897
rs12432263
rs16983494
rs12599039
rs2756164
rs79967512
rs138495054
rs112210865
rs112713512
rs186839589
rs11658137
rs72701743
rs13432799
rs5748913
rs17590595
rs7933771
rs62365250
rs138354577
rs117837363
rs967645
rs71497623
rs187795523
rs11230036
All − MR Egger
All − IVW(MRE)
−0.010
−0.005
0.000
0.005
MR effect size for
'Peptidyl−prolyl cis−trans isomerase FKBP2' on 'eGFR'

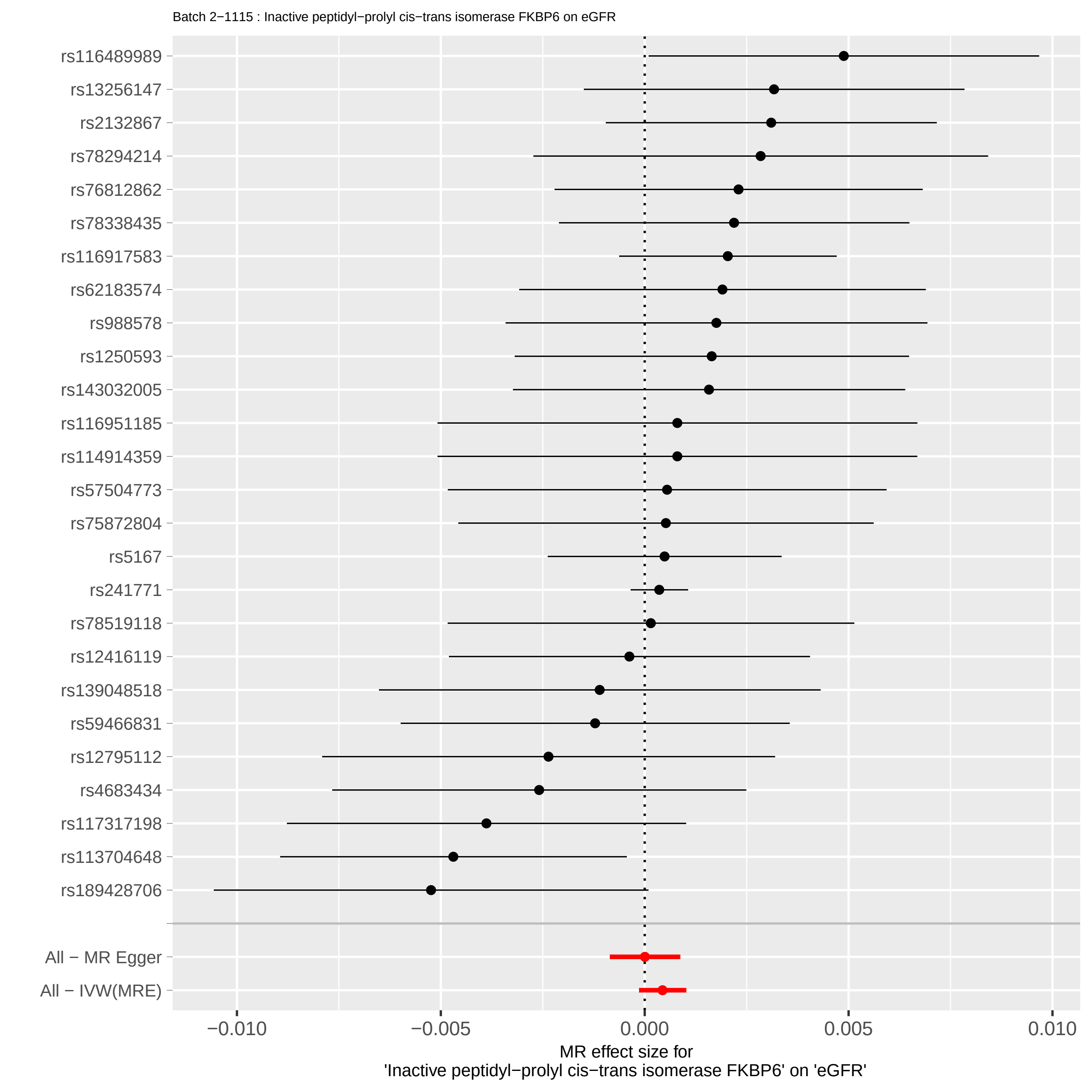

Batch 2−1115 : Inactive peptidyl−prolyl cis−trans isomerase FKBP6 on eGFR
rs116489989
rs13256147
rs2132867
rs78294214
rs76812862
rs78338435
rs116917583
rs62183574
rs988578
rs1250593
rs143032005
rs116951185
rs114914359
rs57504773
rs75872804
rs5167
rs241771
rs78519118
rs12416119
rs139048518
rs59466831
rs12795112
rs4683434
rs117317198
rs113704648
rs189428706
All − MR Egger
All − IVW(MRE)
−0.010
−0.005
0.000
0.005
0.010
MR effect size for
'Inactive peptidyl−prolyl cis−trans isomerase FKBP6' on 'eGFR'

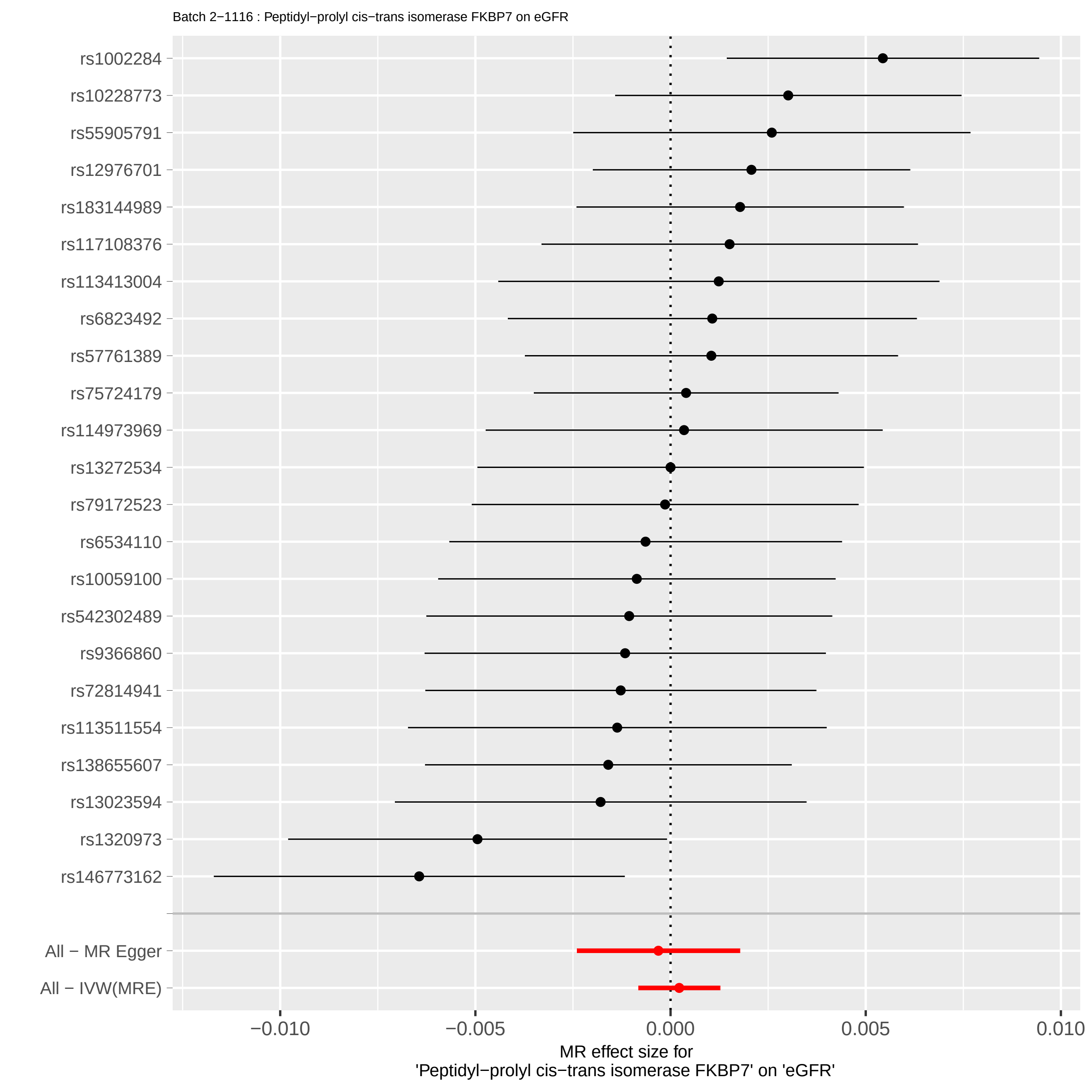

Batch 2−1116 : Peptidyl−prolyl cis−trans isomerase FKBP7 on eGFR
rs1002284
rs10228773
rs55905791
rs12976701
rs183144989
rs117108376
rs113413004
rs6823492
rs57761389
rs75724179
rs114973969
rs13272534
rs79172523
rs6534110
rs10059100
rs542302489
rs9366860
rs72814941
rs113511554
rs138655607
rs13023594
rs1320973
rs146773162
All − MR Egger
All − IVW(MRE)
−0.010
−0.005
0.000
0.005
0.010
MR effect size for
'Peptidyl−prolyl cis−trans isomerase FKBP7' on 'eGFR'

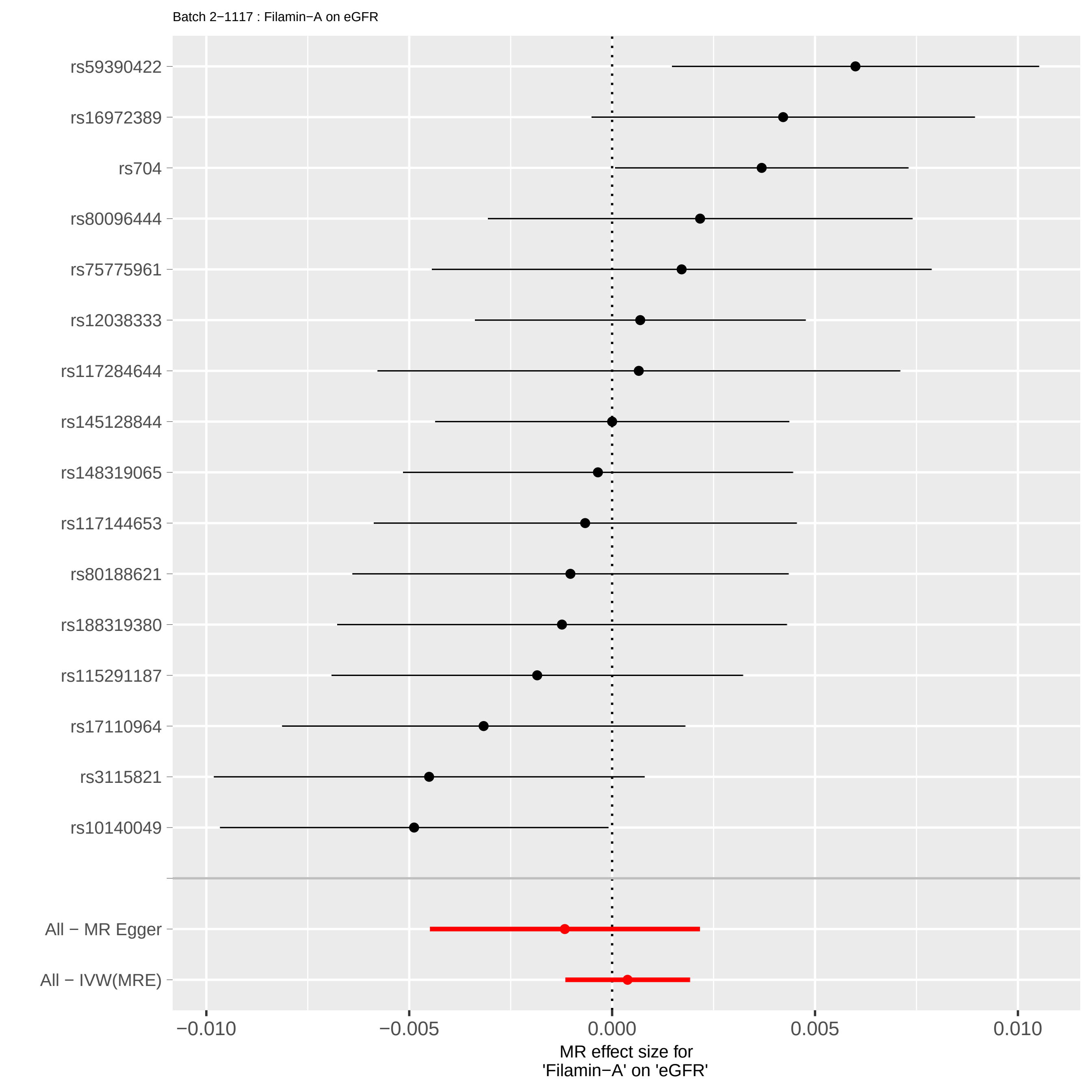
Batch 2−1117 : Filamin−A on eGFR
rs59390422
rs16972389
rs704
rs80096444
rs75775961
rs12038333
rs117284644
rs145128844
rs148319065
rs117144653
rs80188621
rs188319380
rs115291187
rs17110964
rs3115821
rs10140049
All − MR Egger
All − IVW(MRE)
−0.010
−0.005
0.000
0.005
0.010
MR effect size for
'Filamin−A' on 'eGFR'

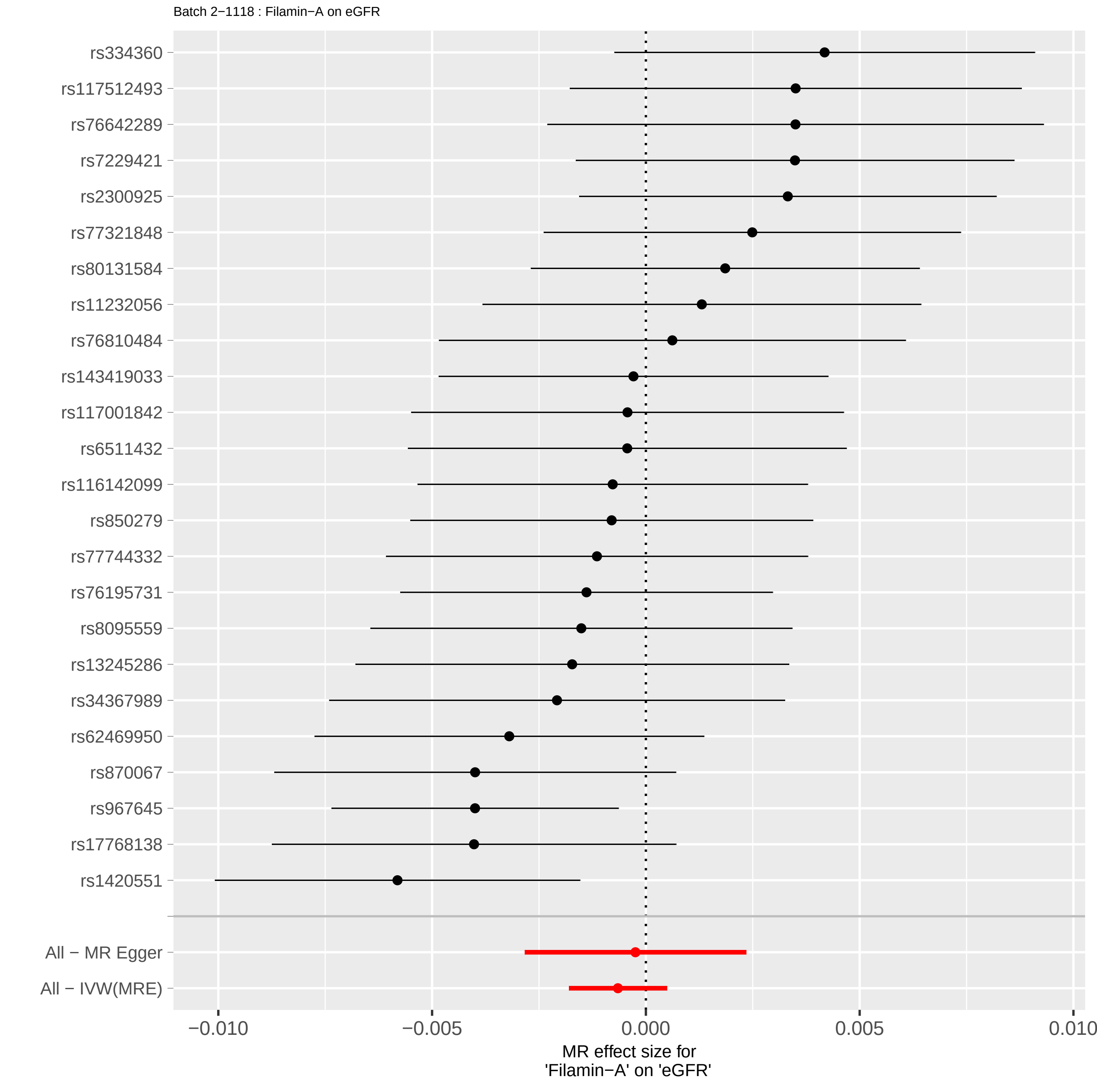

Batch 2−1118 : Filamin−A on eGFR
rs334360
rs117512493
rs76642289
rs7229421
rs2300925
rs77321848
rs80131584
rs11232056
rs76810484
rs143419033
rs117001842
rs6511432
rs116142099
rs850279
rs77744332
rs76195731
rs8095559
rs13245286
rs34367989
rs62469950
rs870067
rs967645
rs17768138
rs1420551
All − MR Egger
All − IVW(MRE)
−0.010
−0.005
0.000
0.005
0.010
MR effect size for
'Filamin−A' on 'eGFR'

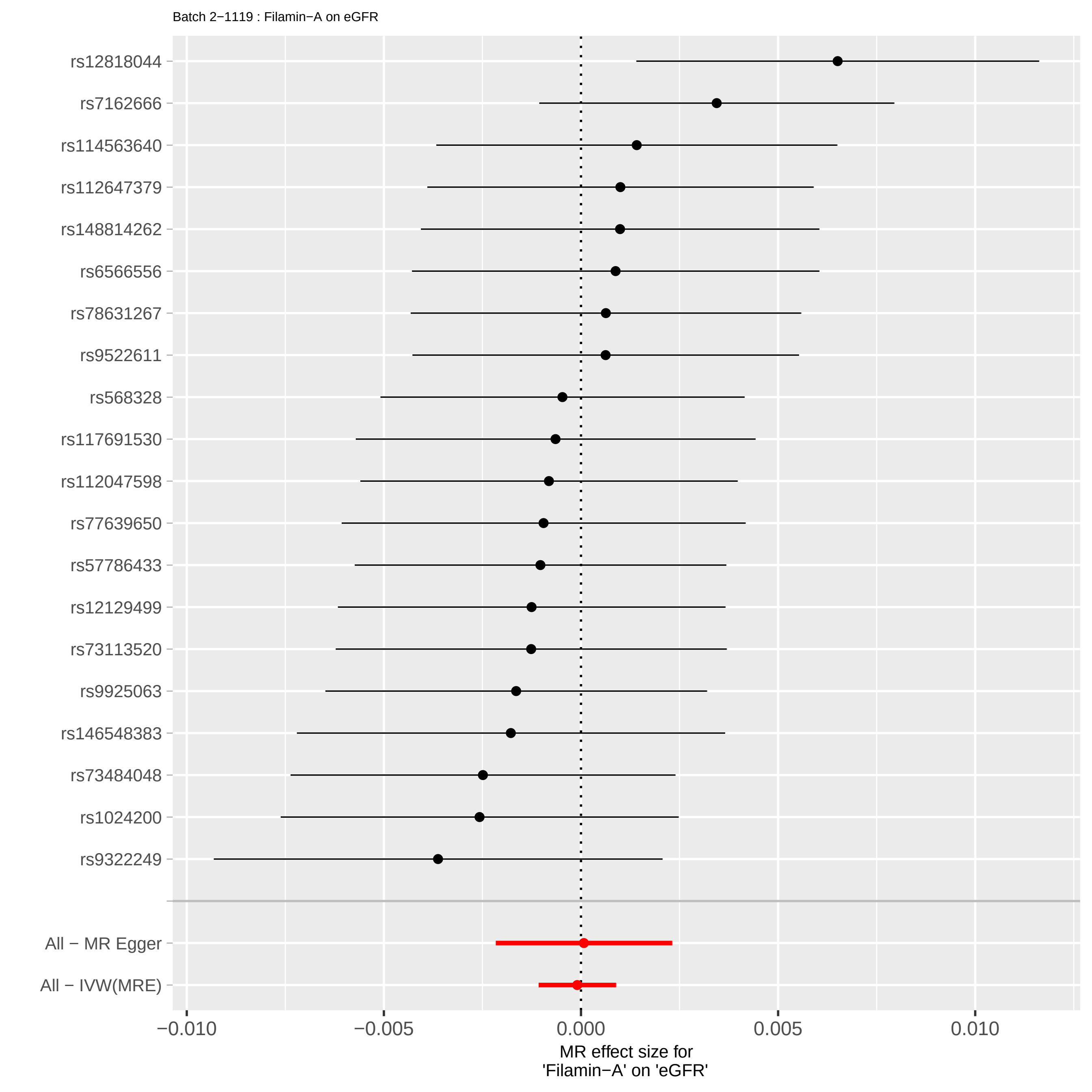

Batch 2−1119 : Filamin−A on eGFR
rs12818044
rs7162666
rs114563640
rs112647379
rs148814262
rs6566556
rs78631267
rs9522611
rs568328
rs117691530
rs112047598
rs77639650
rs57786433
rs12129499
rs73113520
rs9925063
rs146548383
rs73484048
rs1024200
rs9322249
All − MR Egger
All − IVW(MRE)
−0.010
−0.005
0.000
0.005
0.010
MR effect size for
'Filamin−A' on 'eGFR'

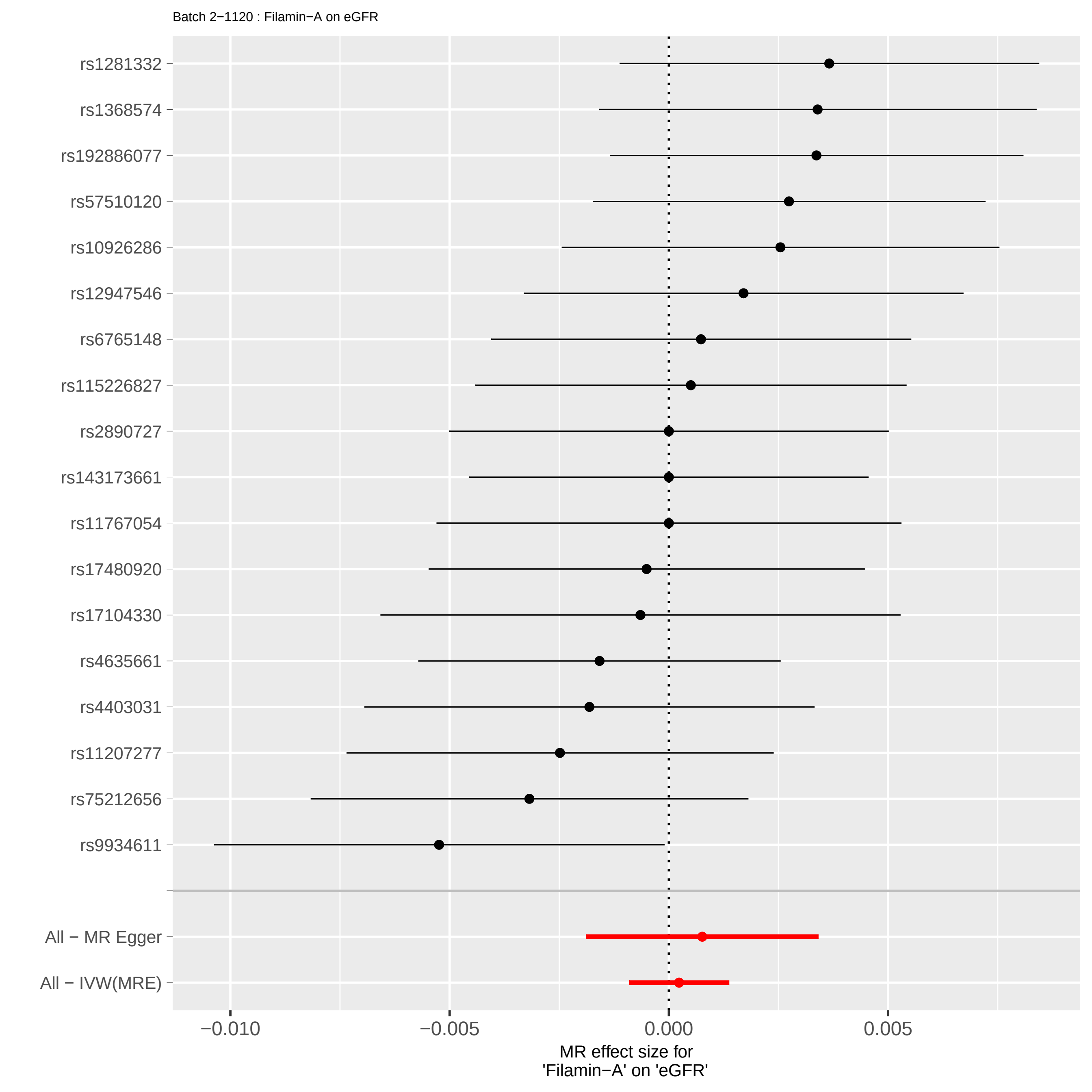

Batch 2−1120 : Filamin−A on eGFR
rs1281332
rs1368574
rs192886077
rs57510120
rs10926286
rs12947546
rs6765148
rs115226827
rs2890727
rs143173661
rs11767054
rs17480920
rs17104330
rs4635661
rs4403031
rs11207277
rs75212656
rs9934611
All − MR Egger
All − IVW(MRE)
−0.010
−0.005
0.000
0.005
MR effect size for
'Filamin−A' on 'eGFR'

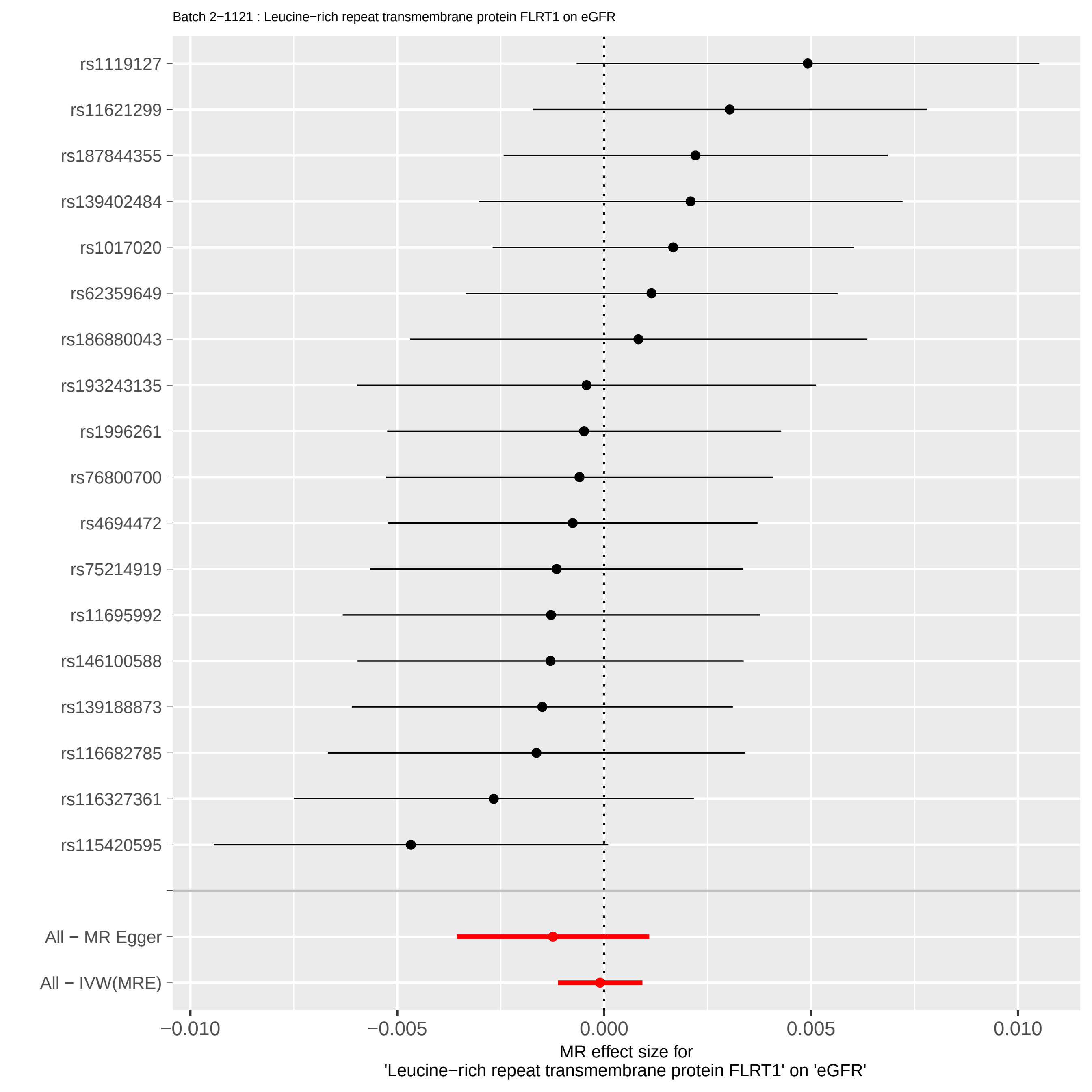

Batch 2−1121 : Leucine−rich repeat transmembrane protein FLRT1 on eGFR
rs1119127
rs11621299
rs187844355
rs139402484
rs1017020
rs62359649
rs186880043
rs193243135
rs1996261
rs76800700
rs4694472
rs75214919
rs11695992
rs146100588
rs139188873
rs116682785
rs116327361
rs115420595
All − MR Egger
All − IVW(MRE)
−0.010
−0.005
0.000
0.005
0.010
MR effect size for
'Leucine−rich repeat transmembrane protein FLRT1' on 'eGFR'

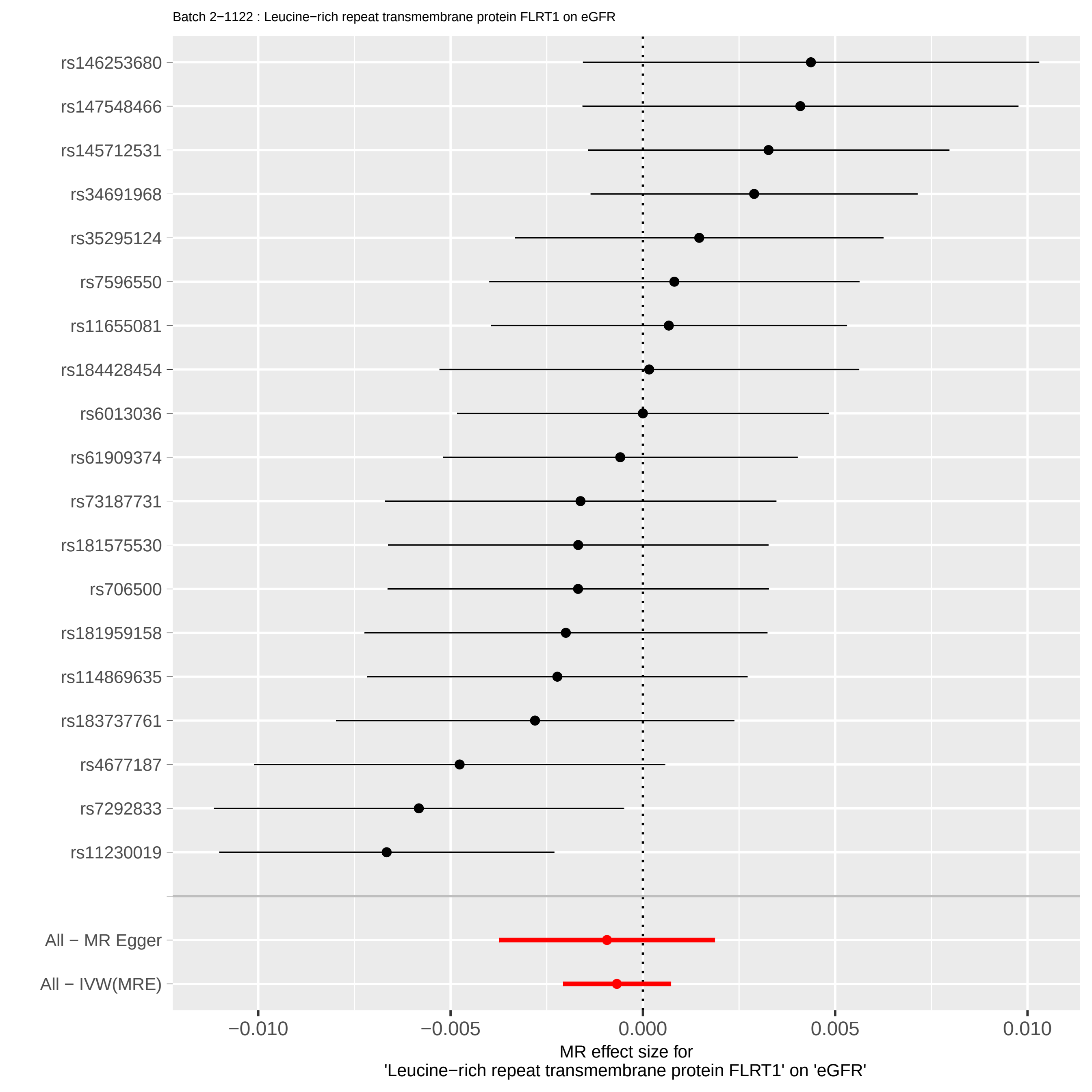

Batch 2−1122 : Leucine−rich repeat transmembrane protein FLRT1 on eGFR
rs146253680
rs147548466
rs145712531
rs34691968
rs35295124
rs7596550
rs11655081
rs184428454
rs6013036
rs61909374
rs73187731
rs181575530
rs706500
rs181959158
rs114869635
rs183737761
rs4677187
rs7292833
rs11230019
All − MR Egger
All − IVW(MRE)
−0.010
−0.005
0.000
0.005
0.010
MR effect size for
'Leucine−rich repeat transmembrane protein FLRT1' on 'eGFR'

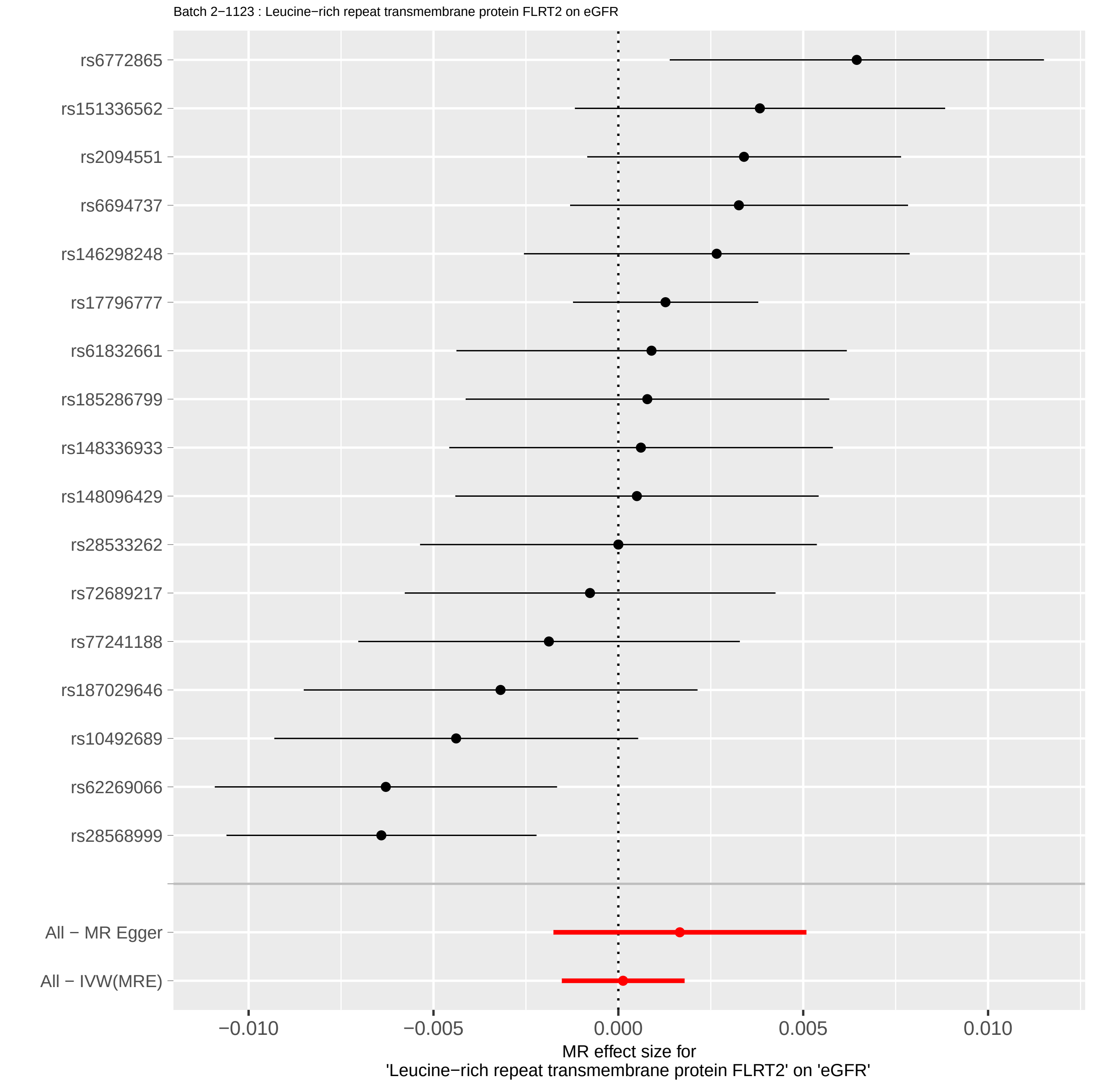

Batch 2−1123 : Leucine−rich repeat transmembrane protein FLRT2 on eGFR
rs6772865
rs151336562
rs2094551
rs6694737
rs146298248
rs17796777
rs61832661
rs185286799
rs148336933
rs148096429
rs28533262
rs72689217
rs77241188
rs187029646
rs10492689
rs62269066
rs28568999
All − MR Egger
All − IVW(MRE)
−0.010
−0.005
0.000
0.005
0.010
MR effect size for
'Leucine−rich repeat transmembrane protein FLRT2' on 'eGFR'

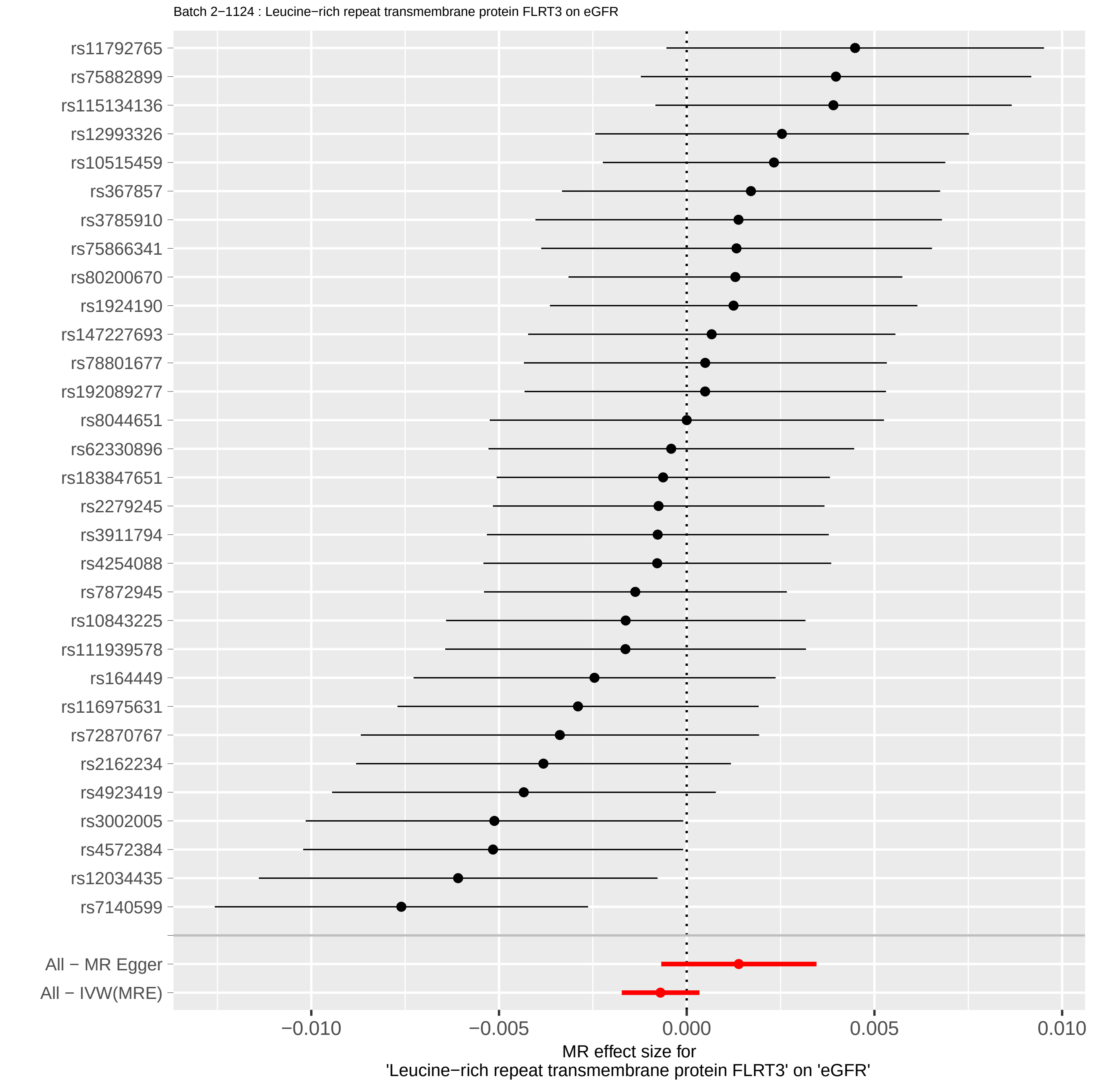
Batch 2−1124 : Leucine−rich repeat transmembrane protein FLRT3 on eGFR
rs11792765
rs75882899
rs115134136
rs12993326
rs10515459
rs367857
rs3785910
rs75866341
rs80200670
rs1924190
rs147227693
rs78801677
rs192089277
rs8044651
rs62330896
rs183847651
rs2279245
rs3911794
rs4254088
rs7872945
rs10843225
rs111939578
rs164449
rs116975631
rs72870767
rs2162234
rs4923419
rs3002005
rs4572384
rs12034435
rs7140599
All − MR Egger
All − IVW(MRE)
−0.010
−0.005
0.000
0.005
0.010
MR effect size for
'Leucine−rich repeat transmembrane protein FLRT3' on 'eGFR'

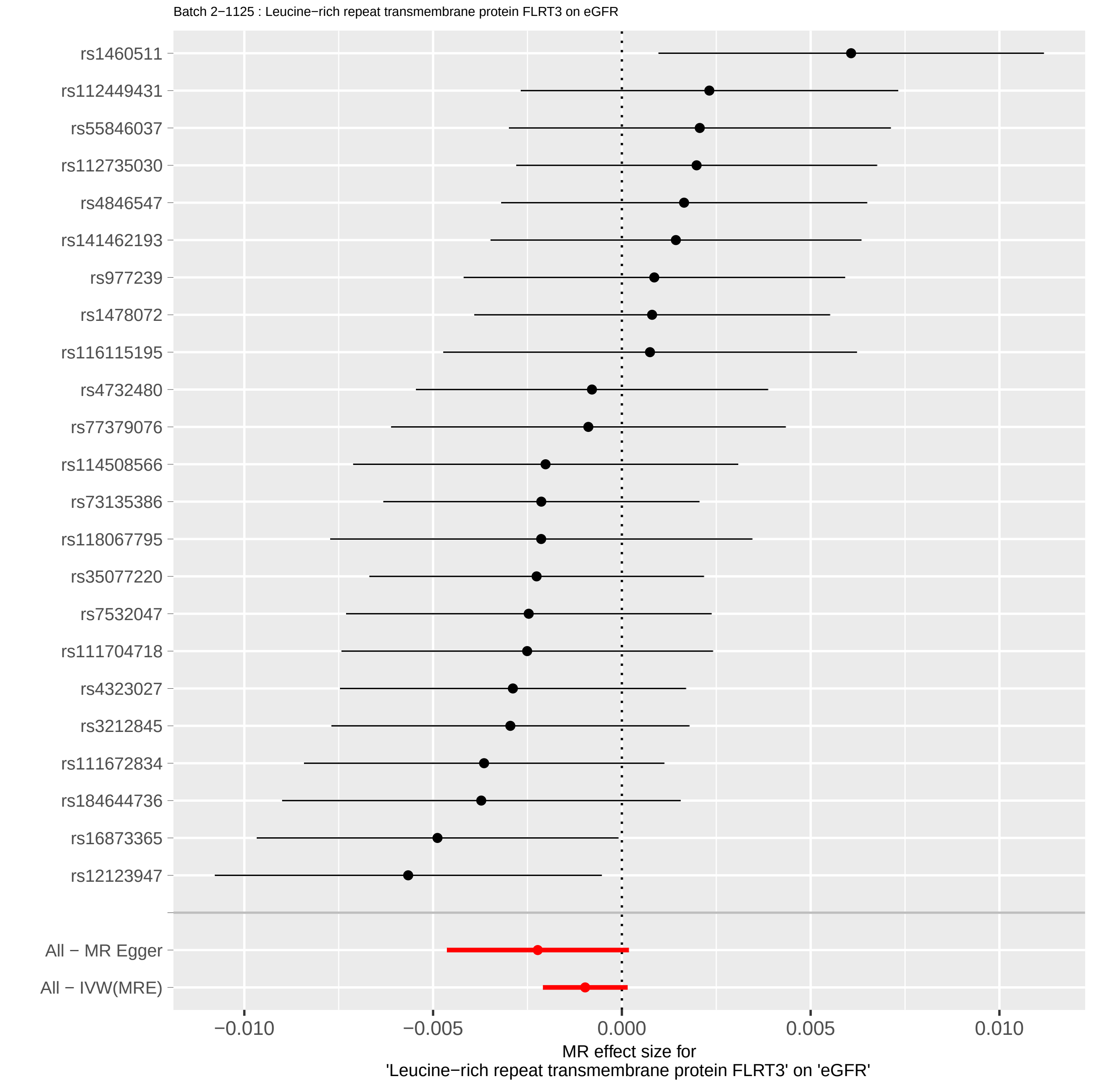
Batch 2−1125 : Leucine−rich repeat transmembrane protein FLRT3 on eGFR
rs1460511
rs112449431
rs55846037
rs112735030
rs4846547
rs141462193
rs977239
rs1478072
rs116115195
rs4732480
rs77379076
rs114508566
rs73135386
rs118067795
rs35077220
rs7532047
rs111704718
rs4323027
rs3212845
rs111672834
rs184644736
rs16873365
rs12123947
All − MR Egger
All − IVW(MRE)
−0.010
−0.005
0.000
0.005
0.010
MR effect size for
'Leucine−rich repeat transmembrane protein FLRT3' on 'eGFR'

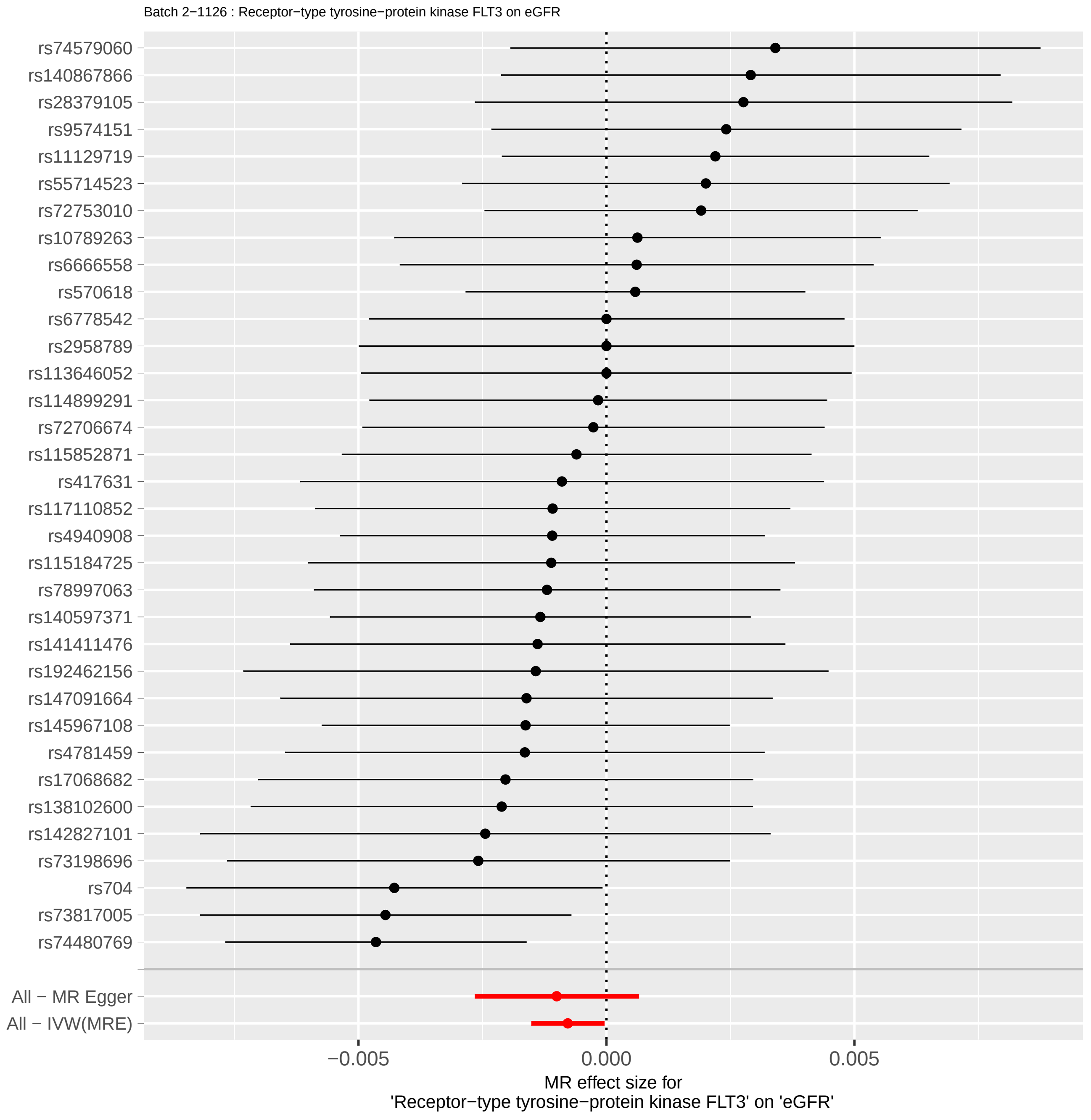

Batch 2−1126 : Receptor−type tyrosine−protein kinase FLT3 on eGFR
rs74579060
rs140867866
rs28379105
rs9574151
rs11129719
rs55714523
rs72753010
rs10789263
rs6666558
rs570618
rs6778542
rs2958789
rs113646052
rs114899291
rs72706674
rs115852871
rs417631
rs117110852
rs4940908
rs115184725
rs78997063
rs140597371
rs141411476
rs192462156
rs147091664
rs145967108
rs4781459
rs17068682
rs138102600
rs142827101
rs73198696
rs704
rs73817005
rs74480769
All − MR Egger
All − IVW(MRE)
−0.005
0.000
0.005
MR effect size for
'Receptor−type tyrosine−protein kinase FLT3' on 'eGFR'

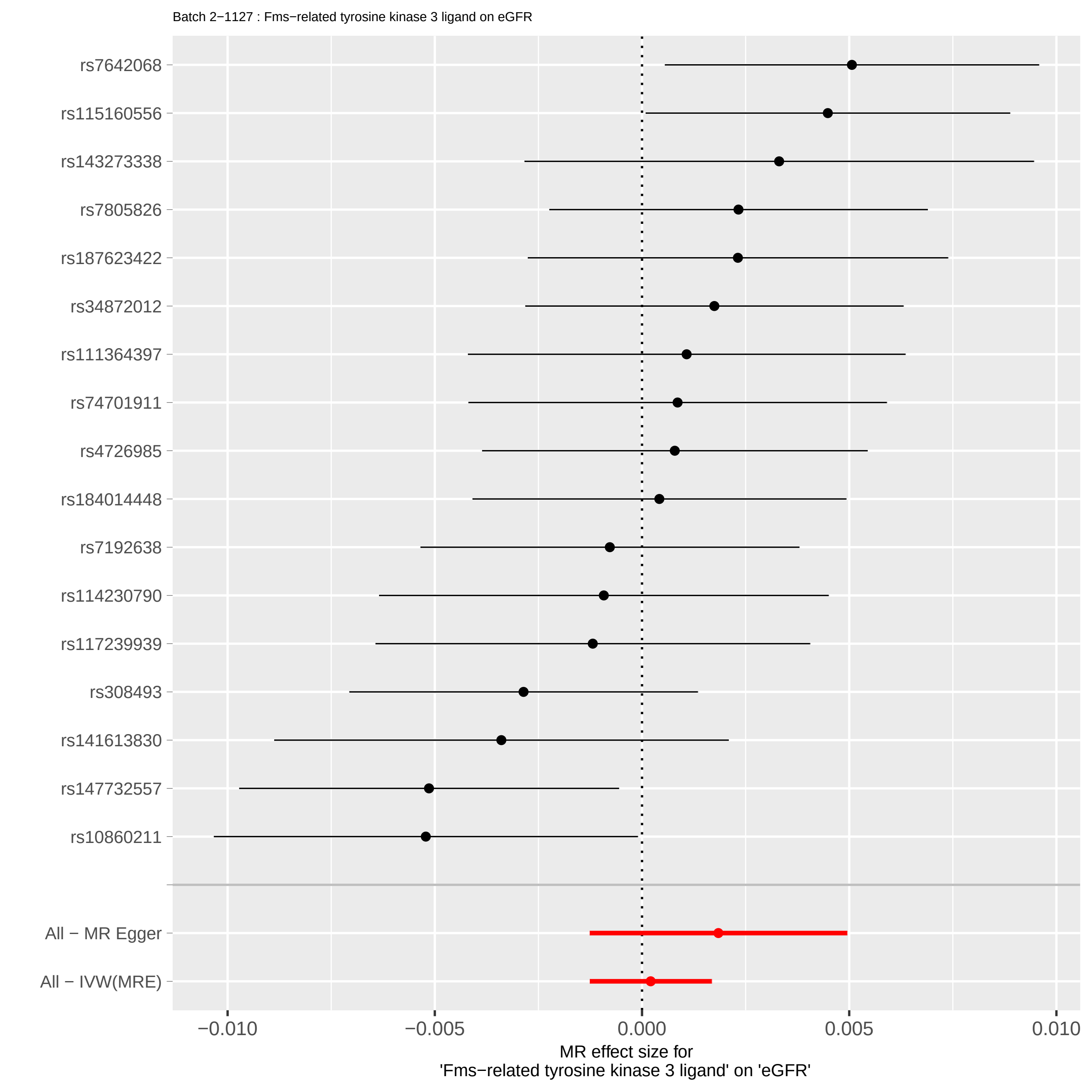

Batch 2−1127 : Fms−related tyrosine kinase 3 ligand on eGFR
rs7642068
rs115160556
rs143273338
rs7805826
rs187623422
rs34872012
rs111364397
rs74701911
rs4726985
rs184014448
rs7192638
rs114230790
rs117239939
rs308493
rs141613830
rs147732557
rs10860211
All − MR Egger
All − IVW(MRE)
−0.010
−0.005
0.000
0.005
0.010
MR effect size for
'Fms−related tyrosine kinase 3 ligand' on 'eGFR'

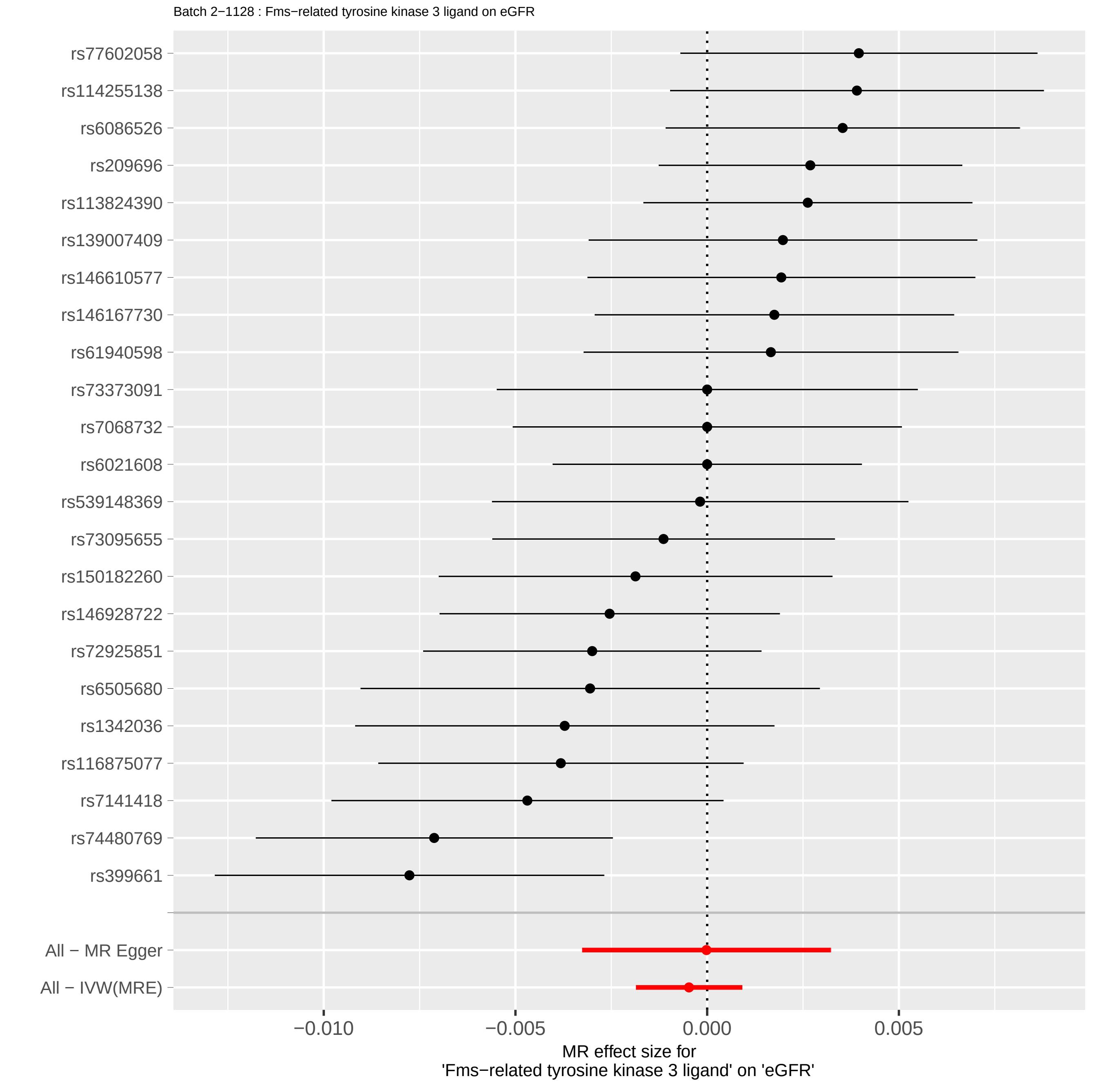

Batch 2−1128 : Fms−related tyrosine kinase 3 ligand on eGFR
rs77602058
rs114255138
rs6086526
rs209696
rs113824390
rs139007409
rs146610577
rs146167730
rs61940598
rs73373091
rs7068732
rs6021608
rs539148369
rs73095655
rs150182260
rs146928722
rs72925851
rs6505680
rs1342036
rs116875077
rs7141418
rs74480769
rs399661
All − MR Egger
All − IVW(MRE)
−0.010
−0.005
0.000
0.005
MR effect size for
'Fms−related tyrosine kinase 3 ligand' on 'eGFR'

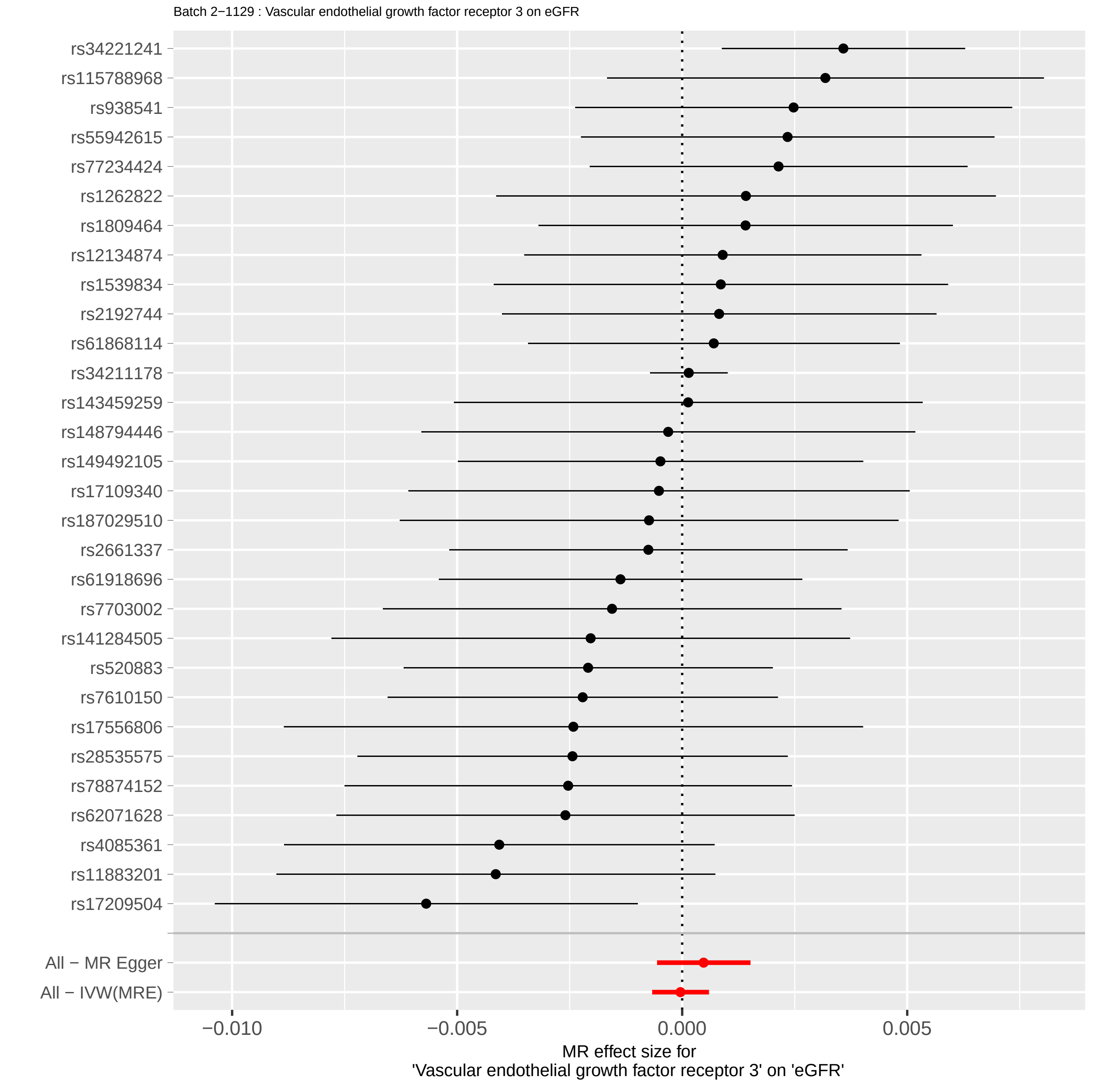

Batch 2−1129 : Vascular endothelial growth factor receptor 3 on eGFR
rs34221241
rs115788968
rs938541
rs55942615
rs77234424
rs1262822
rs1809464
rs12134874
rs1539834
rs2192744
rs61868114
rs34211178
rs143459259
rs148794446
rs149492105
rs17109340
rs187029510
rs2661337
rs61918696
rs7703002
rs141284505
rs520883
rs7610150
rs17556806
rs28535575
rs78874152
rs62071628
rs4085361
rs11883201
rs17209504
All − MR Egger
All − IVW(MRE)
−0.010
−0.005
0.000
0.005
MR effect size for
'Vascular endothelial growth factor receptor 3' on 'eGFR'

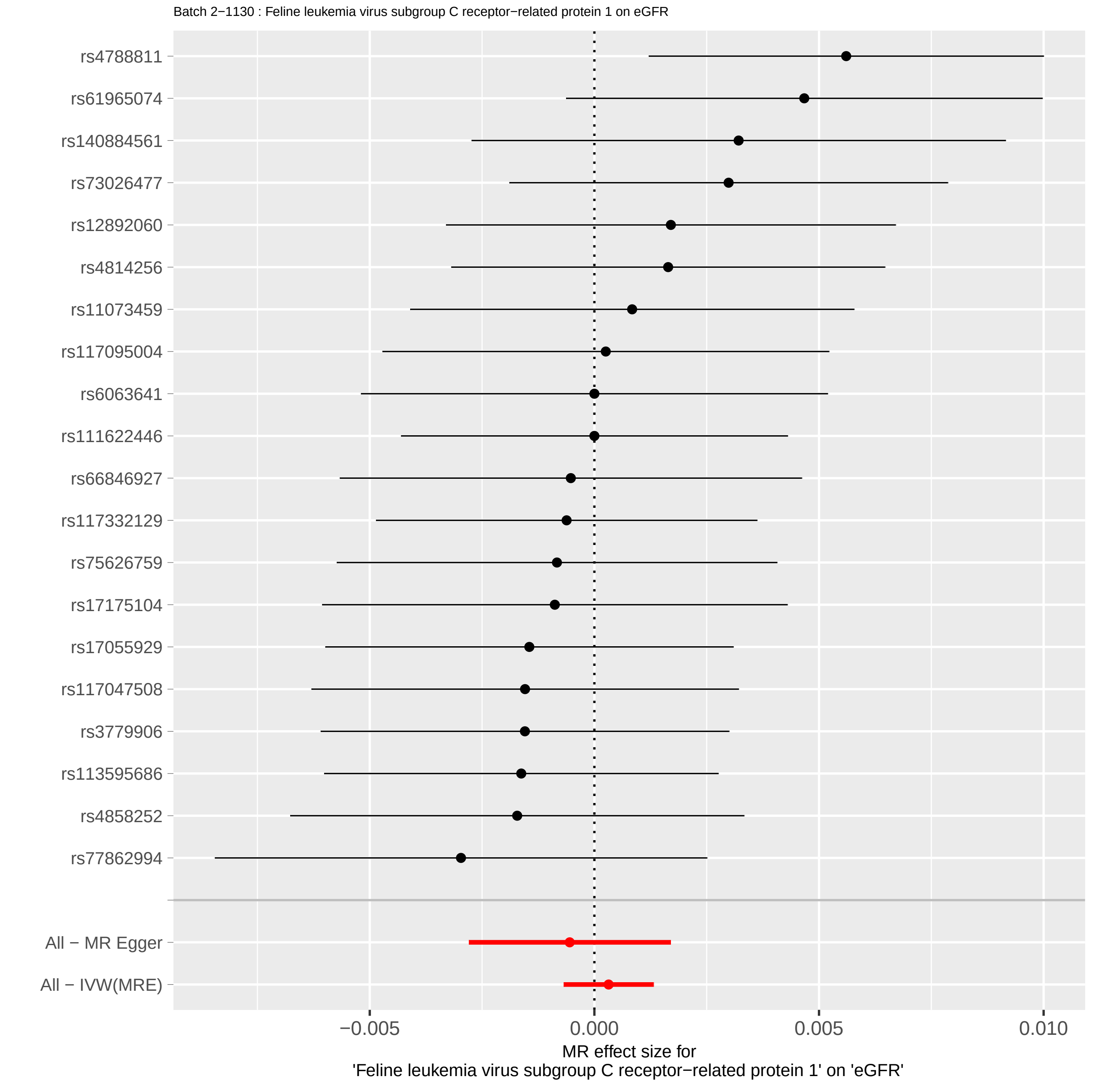

Batch 2−1130 : Feline leukemia virus subgroup C receptor−related protein 1 on eGFR
rs4788811
rs61965074
rs140884561
rs73026477
rs12892060
rs4814256
rs11073459
rs117095004
rs6063641
rs111622446
rs66846927
rs117332129
rs75626759
rs17175104
rs17055929
rs117047508
rs3779906
rs113595686
rs4858252
rs77862994
All − MR Egger
All − IVW(MRE)
−0.005
0.000
0.005
0.010
MR effect size for
'Feline leukemia virus subgroup C receptor−related protein 1' on 'eGFR'

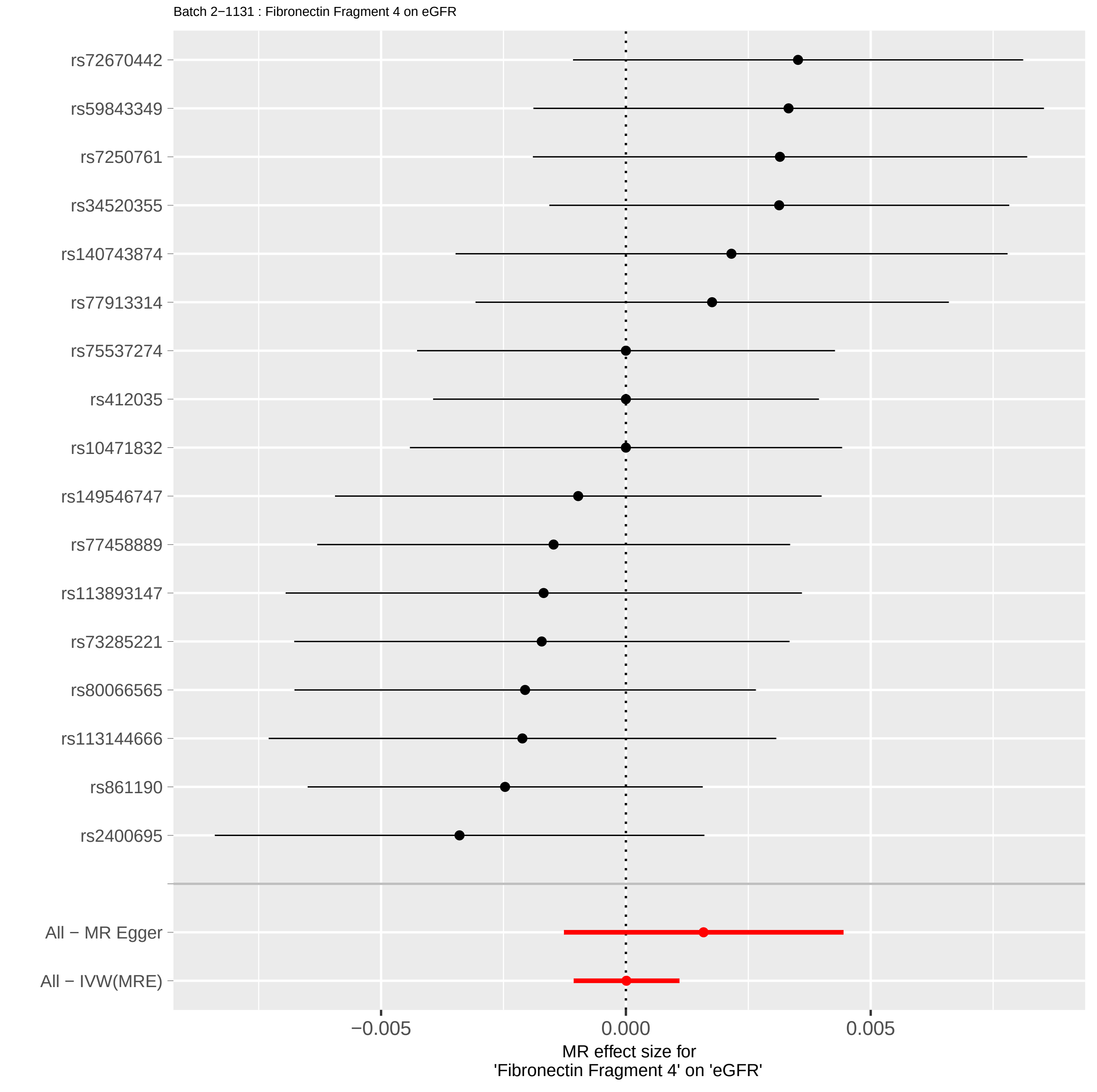

Batch 2−1131 : Fibronectin Fragment 4 on eGFR
rs72670442
rs59843349
rs7250761
rs34520355
rs140743874
rs77913314
rs75537274
rs412035
rs10471832
rs149546747
rs77458889
rs113893147
rs73285221
rs80066565
rs113144666
rs861190
rs2400695
All − MR Egger
All − IVW(MRE)
−0.005
0.000
0.005
MR effect size for
'Fibronectin Fragment 4' on 'eGFR'

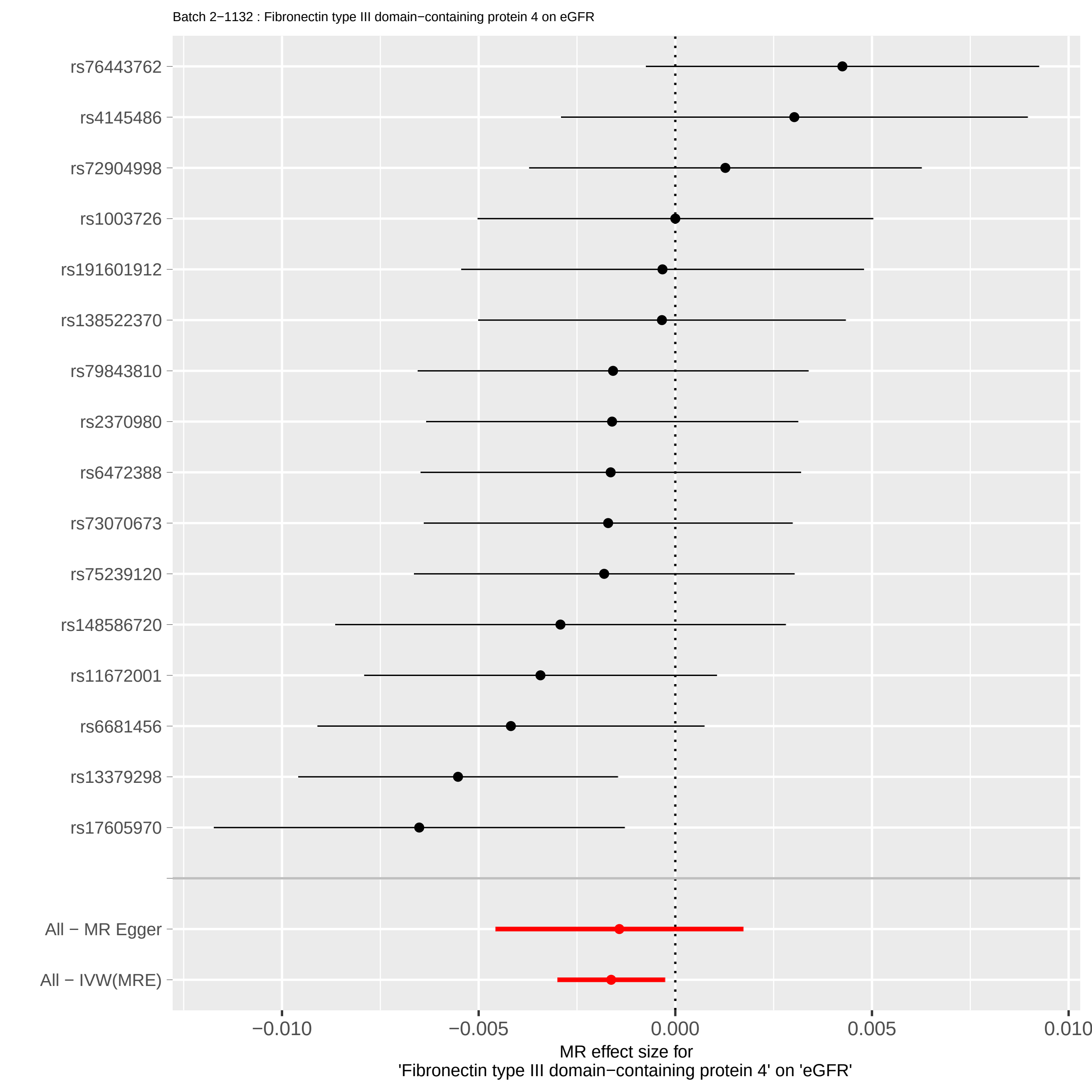

Batch 2−1132 : Fibronectin type III domain−containing protein 4 on eGFR
rs76443762
rs4145486
rs72904998
rs1003726
rs191601912
rs138522370
rs79843810
rs2370980
rs6472388
rs73070673
rs75239120
rs148586720
rs11672001
rs6681456
rs13379298
rs17605970
All − MR Egger
All − IVW(MRE)
−0.010
−0.005
0.000
0.005
0.010
MR effect size for
'Fibronectin type III domain−containing protein 4' on 'eGFR'

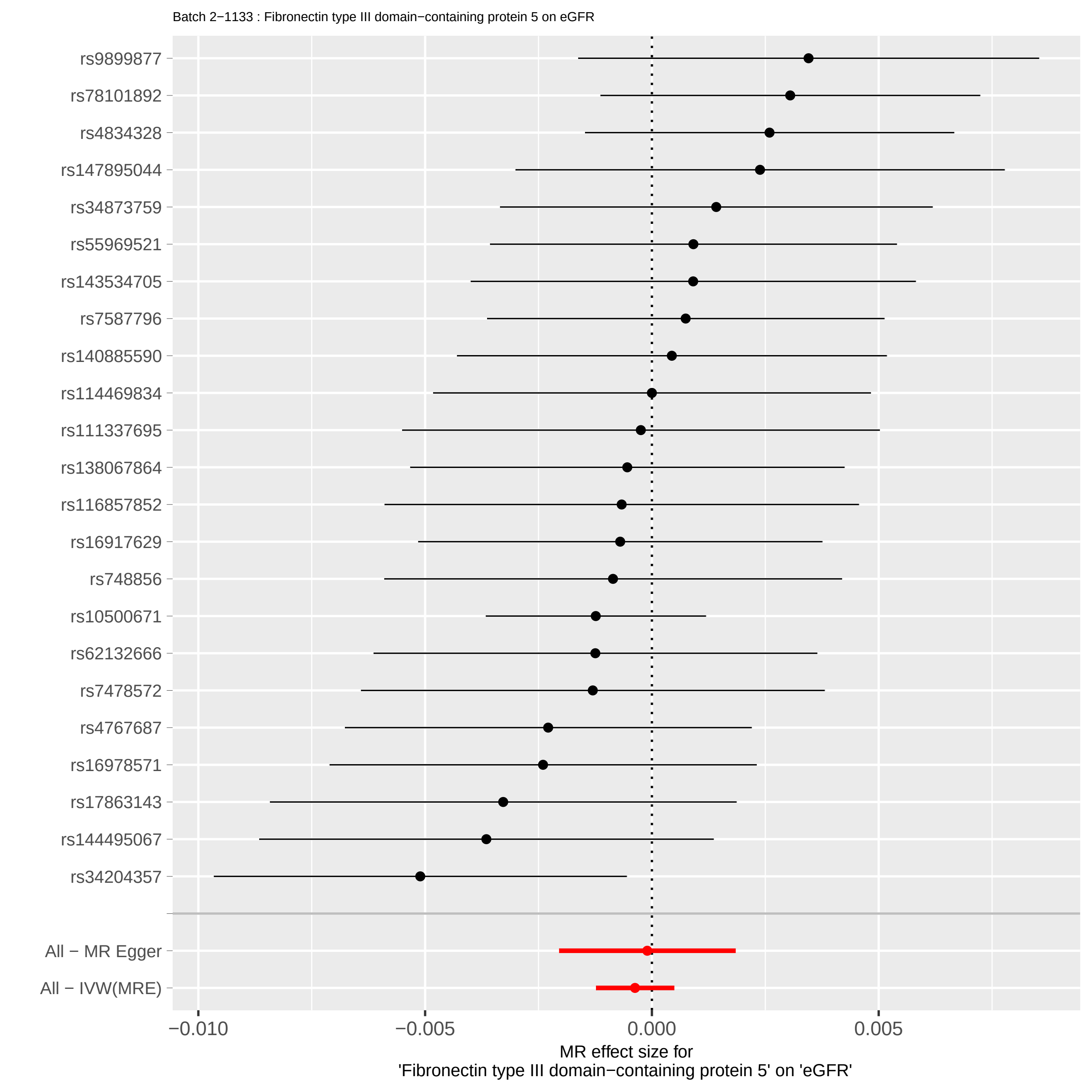
Batch 2−1133 : Fibronectin type III domain−containing protein 5 on eGFR
rs9899877
rs78101892
rs4834328
rs147895044
rs34873759
rs55969521
rs143534705
rs7587796
rs140885590
rs114469834
rs111337695
rs138067864
rs116857852
rs16917629
rs748856
rs10500671
rs62132666
rs7478572
rs4767687
rs16978571
rs17863143
rs144495067
rs34204357
All − MR Egger
All − IVW(MRE)
−0.010
−0.005
0.000
0.005
MR effect size for
'Fibronectin type III domain−containing protein 5' on 'eGFR'

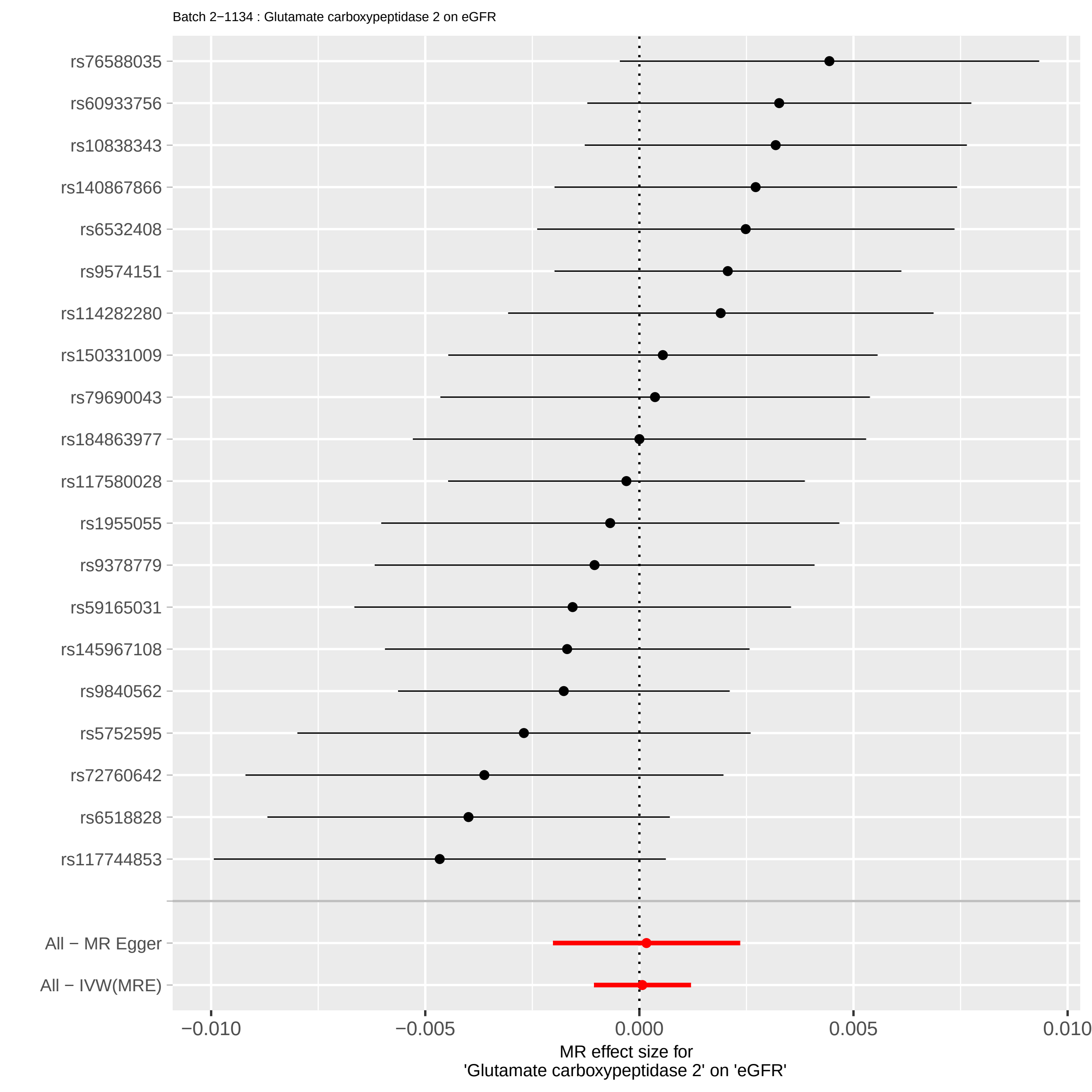
Batch 2−1134 : Glutamate carboxypeptidase 2 on eGFR
rs76588035
rs60933756
rs10838343
rs140867866
rs6532408
rs9574151
rs114282280
rs150331009
rs79690043
rs184863977
rs117580028
rs1955055
rs9378779
rs59165031
rs145967108
rs9840562
rs5752595
rs72760642
rs6518828
rs117744853
All − MR Egger
All − IVW(MRE)
−0.010
−0.005
0.000
0.005
0.010
MR effect size for
'Glutamate carboxypeptidase 2' on 'eGFR'

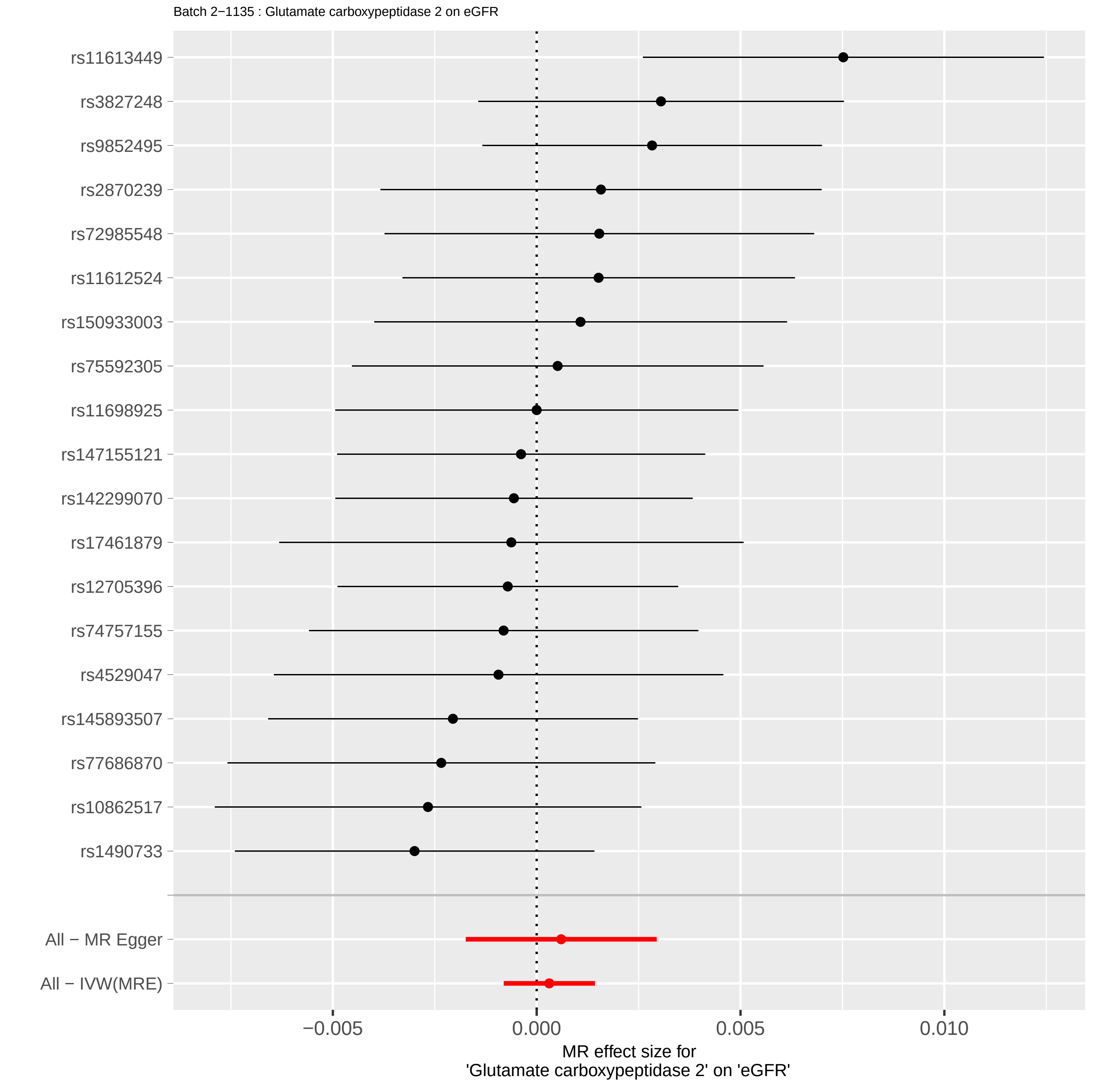

Batch 2−1135 : Glutamate carboxypeptidase 2 on eGFR
rs11613449
rs3827248
rs9852495
rs2870239
rs72985548
rs11612524
rs150933003
rs75592305
rs11698925
rs147155121
rs142299070
rs17461879
rs12705396
rs74757155
rs4529047
rs145893507
rs77686870
rs10862517
rs1490733
All − MR Egger
All − IVW(MRE)
−0.005
0.000
0.005
0.010
MR effect size for
'Glutamate carboxypeptidase 2' on 'eGFR'

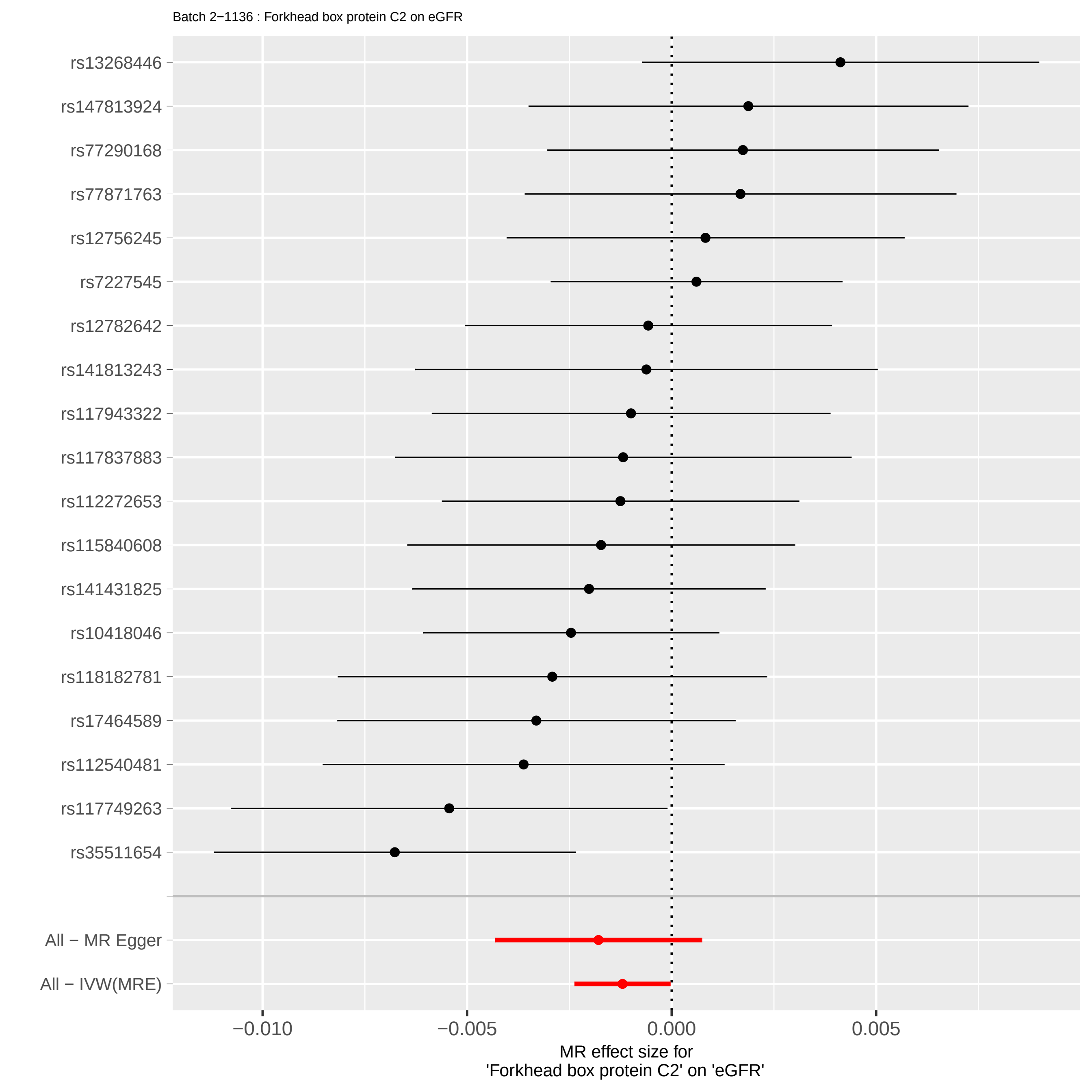

Batch 2−1136 : Forkhead box protein C2 on eGFR
rs13268446
rs147813924
rs77290168
rs77871763
rs12756245
rs7227545
rs12782642
rs141813243
rs117943322
rs117837883
rs112272653
rs115840608
rs141431825
rs10418046
rs118182781
rs17464589
rs112540481
rs117749263
rs35511654
All − MR Egger
All − IVW(MRE)
−0.010
−0.005
0.000
0.005
MR effect size for
'Forkhead box protein C2' on 'eGFR'

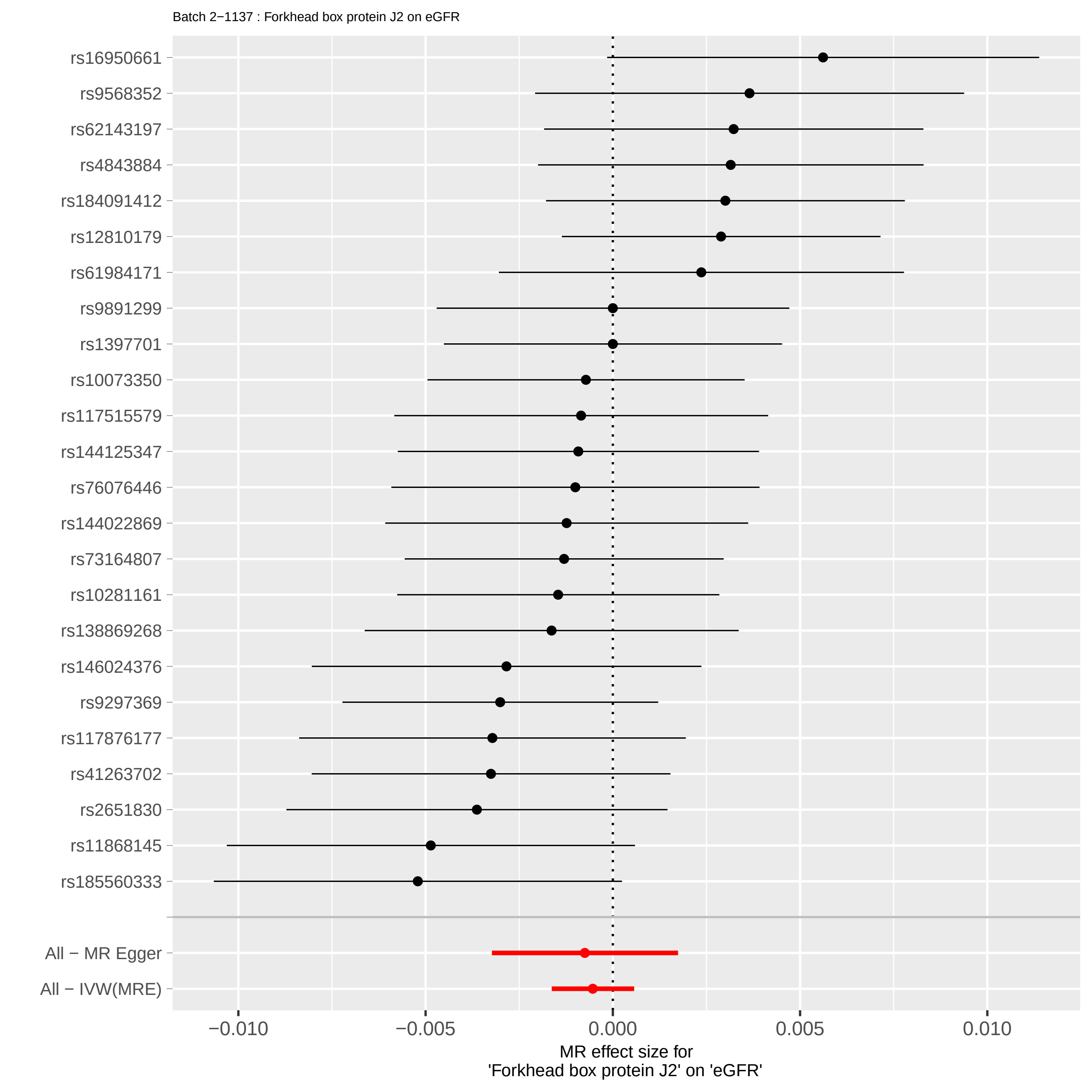
Batch 2−1137 : Forkhead box protein J2 on eGFR
rs16950661
rs9568352
rs62143197
rs4843884
rs184091412
rs12810179
rs61984171
rs9891299
rs1397701
rs10073350
rs117515579
rs144125347
rs76076446
rs144022869
rs73164807
rs10281161
rs138869268
rs146024376
rs9297369
rs117876177
rs41263702
rs2651830
rs11868145
rs185560333
All − MR Egger
All − IVW(MRE)
−0.010
−0.005
0.000
0.005
0.010
MR effect size for
'Forkhead box protein J2' on 'eGFR'

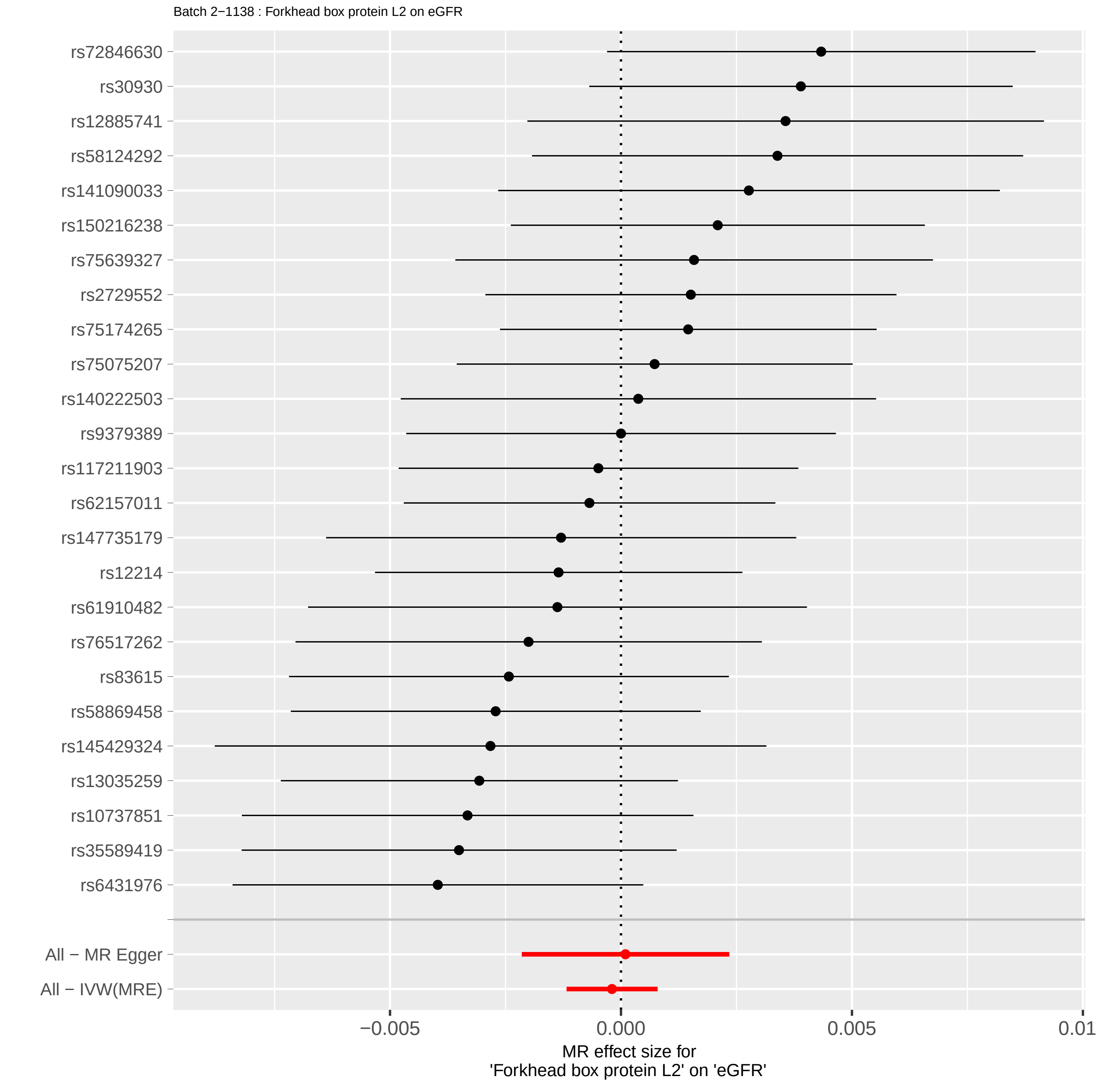

Batch 2−1138 : Forkhead box protein L2 on eGFR
rs72846630
rs30930
rs12885741
rs58124292
rs141090033
rs150216238
rs75639327
rs2729552
rs75174265
rs75075207
rs140222503
rs9379389
rs117211903
rs62157011
rs147735179
rs12214
rs61910482
rs76517262
rs83615
rs58869458
rs145429324
rs13035259
rs10737851
rs35589419
rs6431976
All − MR Egger
All − IVW(MRE)
−0.005
0.000
0.005
0.01
MR effect size for
'Forkhead box protein L2' on 'eGFR'

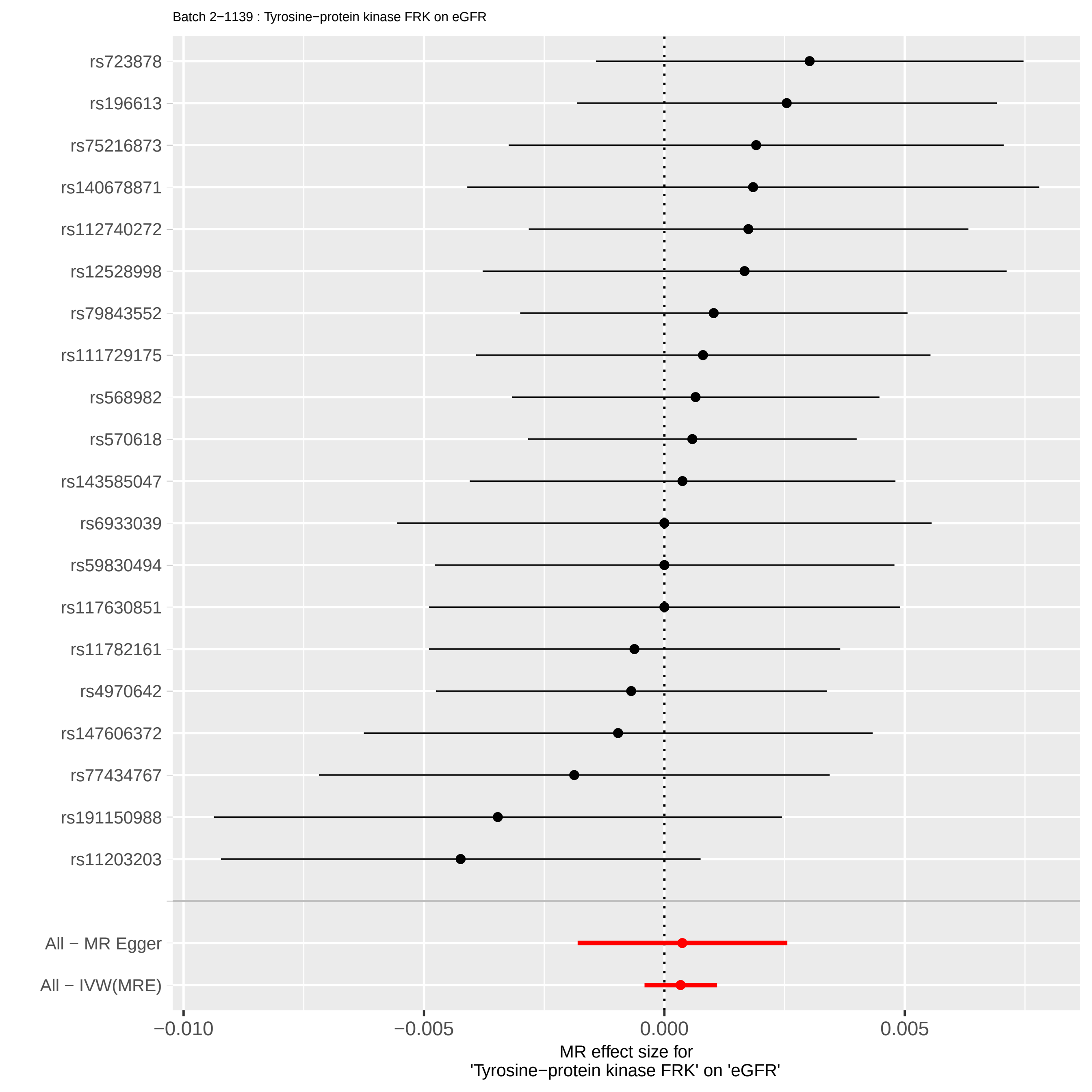
Batch 2−1139 : Tyrosine−protein kinase FRK on eGFR
rs723878
rs196613
rs75216873
rs140678871
rs112740272
rs12528998
rs79843552
rs111729175
rs568982
rs570618
rs143585047
rs6933039
rs59830494
rs117630851
rs11782161
rs4970642
rs147606372
rs77434767
rs191150988
rs11203203
All − MR Egger
All − IVW(MRE)
−0.010
−0.005
0.000
0.005
MR effect size for
'Tyrosine−protein kinase FRK' on 'eGFR'

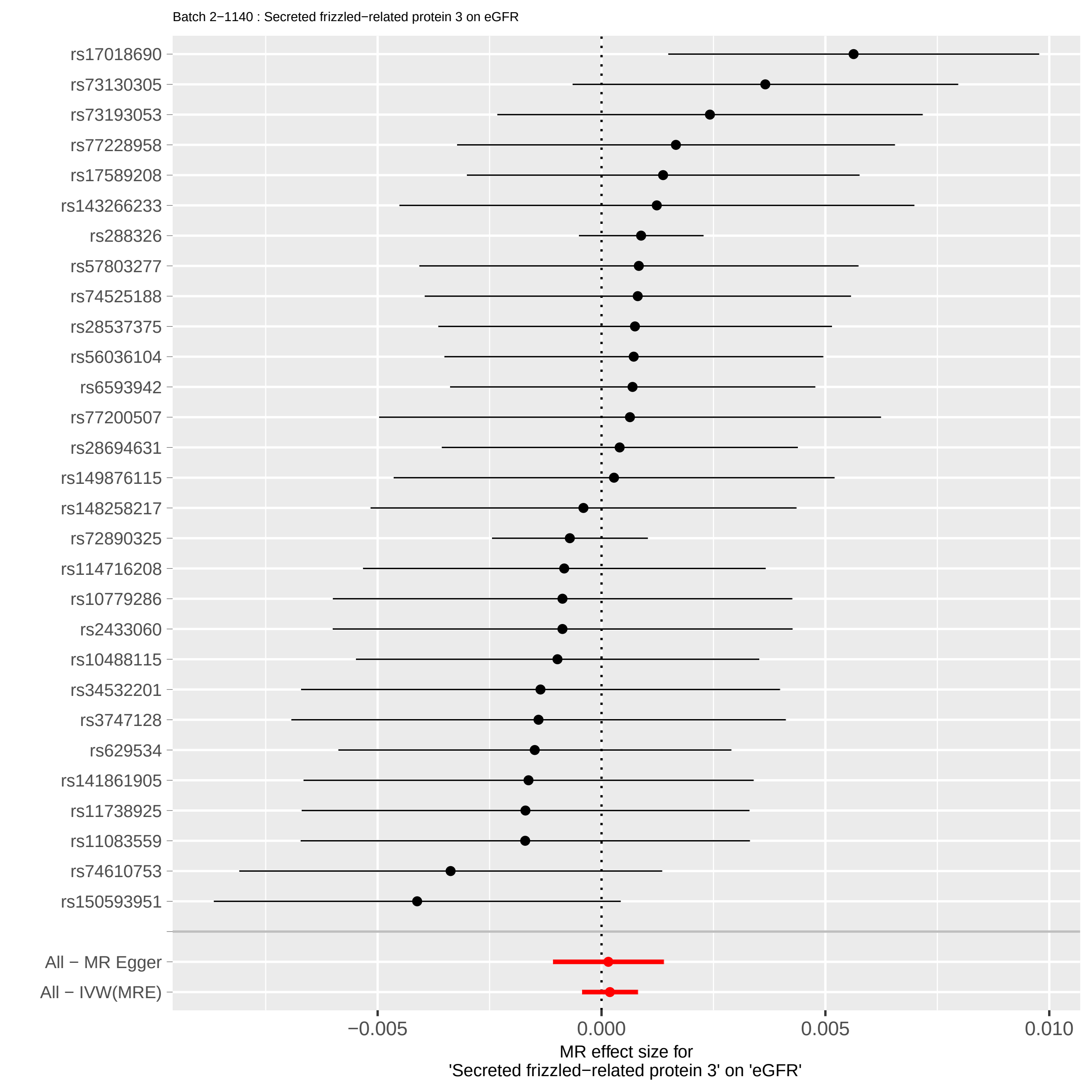

Batch 2−1140 : Secreted frizzled−related protein 3 on eGFR
rs17018690
rs73130305
rs73193053
rs77228958
rs17589208
rs143266233
rs288326
rs57803277
rs74525188
rs28537375
rs56036104
rs6593942
rs77200507
rs28694631
rs149876115
rs148258217
rs72890325
rs114716208
rs10779286
rs2433060
rs10488115
rs34532201
rs3747128
rs629534
rs141861905
rs11738925
rs11083559
rs74610753
rs150593951
All − MR Egger
All − IVW(MRE)
−0.005
0.000
0.005
0.010
MR effect size for
'Secreted frizzled−related protein 3' on 'eGFR'

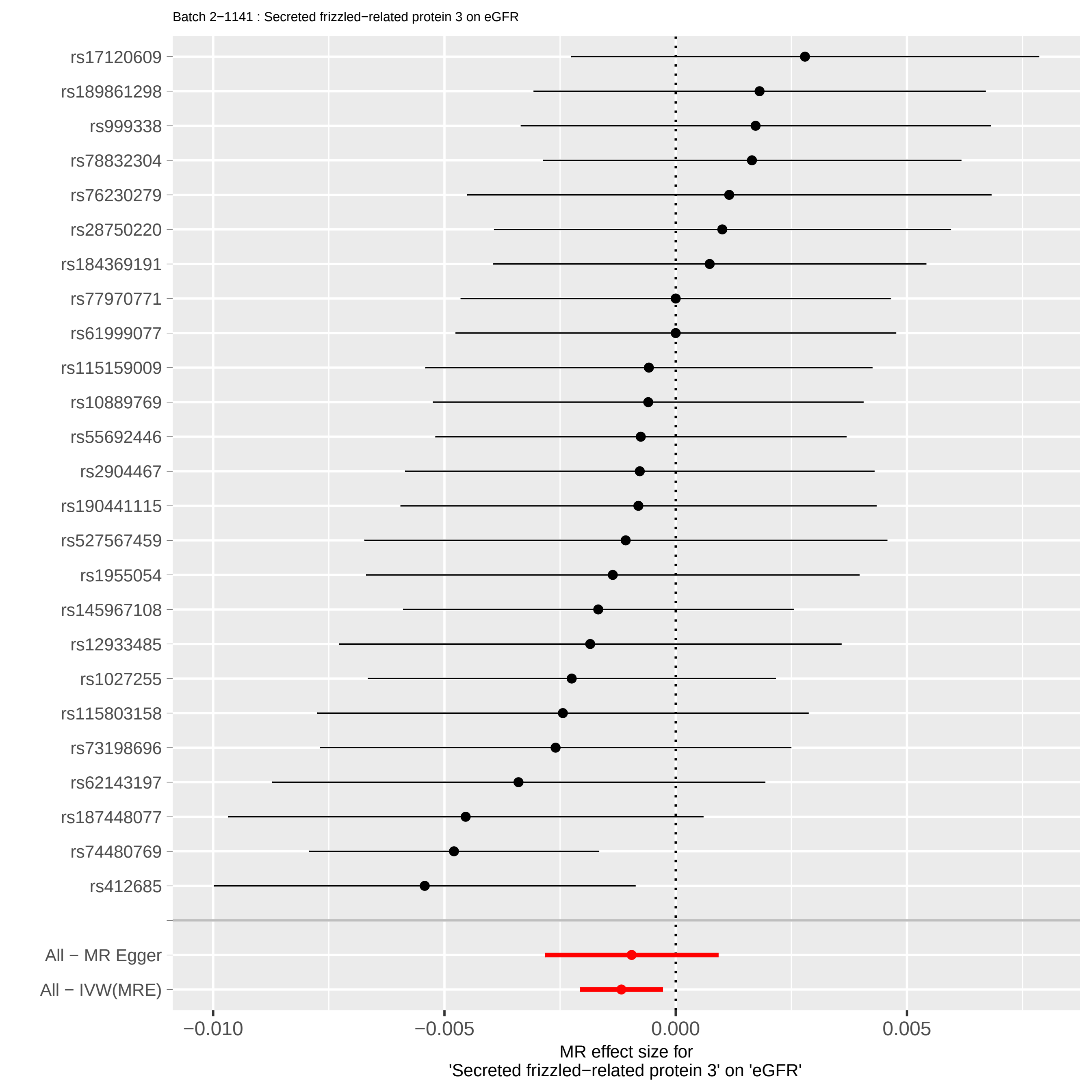

Batch 2−1141 : Secreted frizzled−related protein 3 on eGFR
rs17120609
rs189861298
rs999338
rs78832304
rs76230279
rs28750220
rs184369191
rs77970771
rs61999077
rs115159009
rs10889769
rs55692446
rs2904467
rs190441115
rs527567459
rs1955054
rs145967108
rs12933485
rs1027255
rs115803158
rs73198696
rs62143197
rs187448077
rs74480769
rs412685
All − MR Egger
All − IVW(MRE)
−0.010
−0.005
0.000
0.005
MR effect size for
'Secreted frizzled−related protein 3' on 'eGFR'

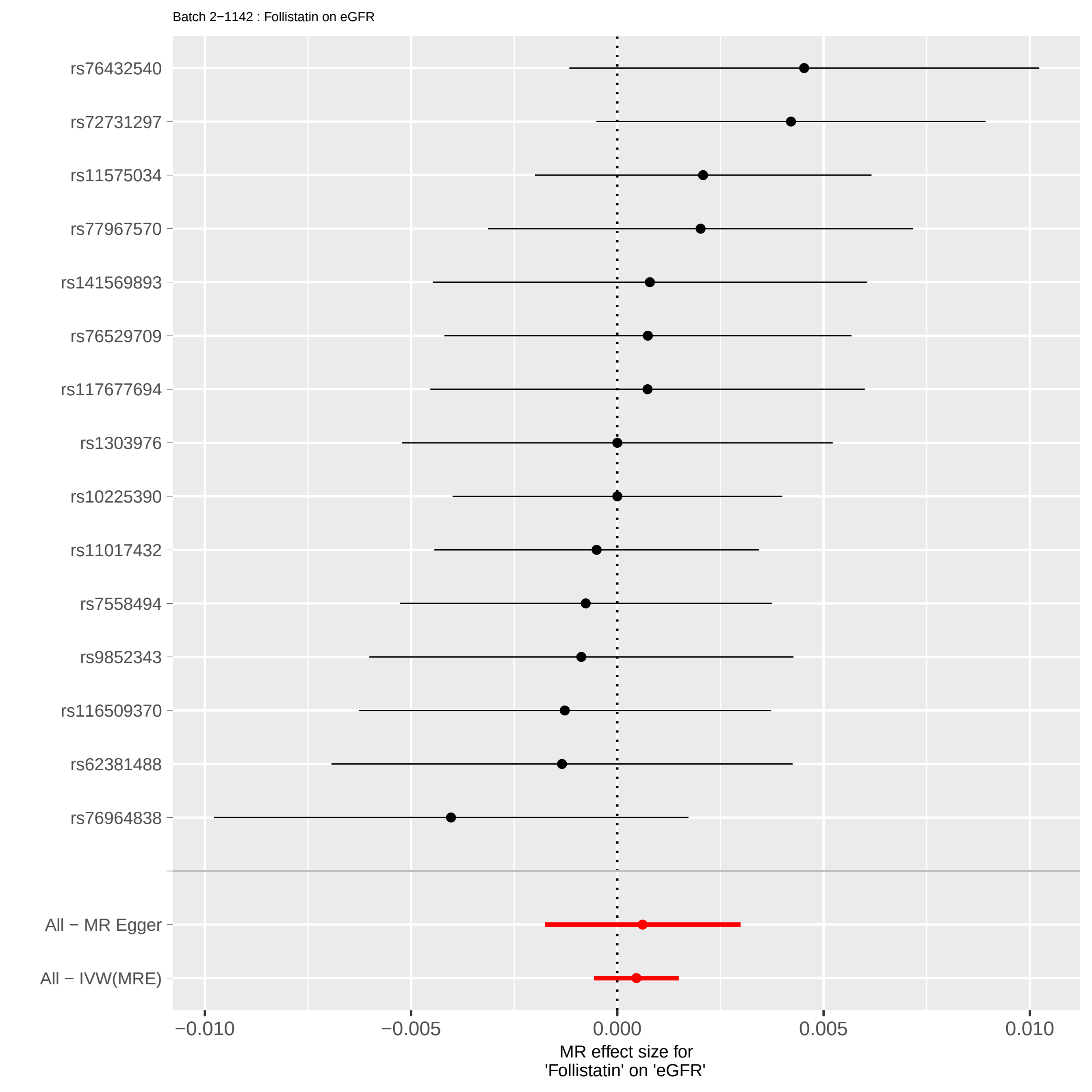

Batch 2−1142 : Follistatin on eGFR
rs76432540
rs72731297
rs11575034
rs77967570
rs141569893
rs76529709
rs117677694
rs1303976
rs10225390
rs11017432
rs7558494
rs9852343
rs116509370
rs62381488
rs76964838
All − MR Egger
All − IVW(MRE)
−0.010
−0.005
0.000
0.005
0.010
MR effect size for
'Follistatin' on 'eGFR'

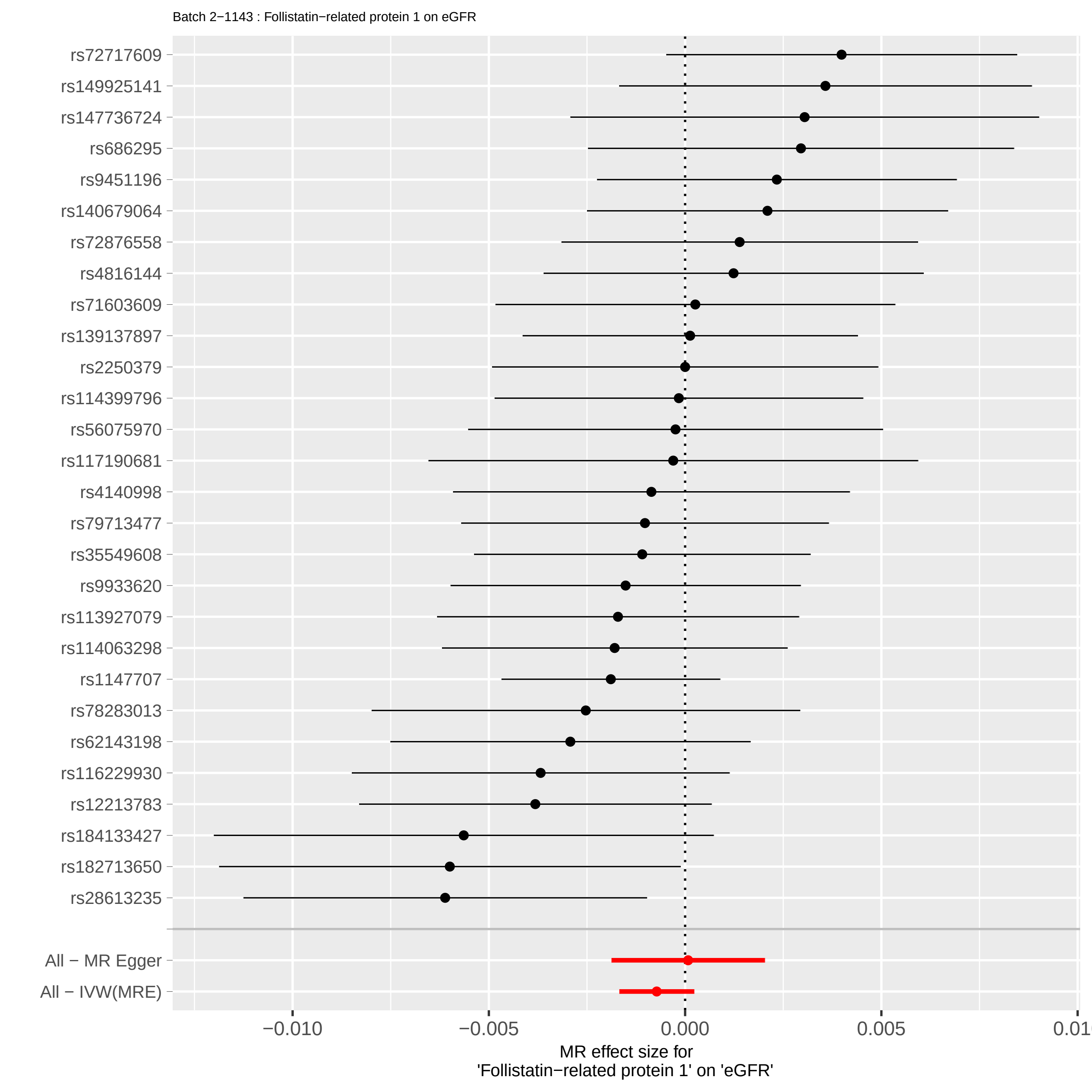
Batch 2−1143 : Follistatin−related protein 1 on eGFR
rs72717609
rs149925141
rs147736724
rs686295
rs9451196
rs140679064
rs72876558
rs4816144
rs71603609
rs139137897
rs2250379
rs114399796
rs56075970
rs117190681
rs4140998
rs79713477
rs35549608
rs9933620
rs113927079
rs114063298
rs1147707
rs78283013
rs62143198
rs116229930
rs12213783
rs184133427
rs182713650
rs28613235
All − MR Egger
All − IVW(MRE)
−0.010
−0.005
0.000
0.005
0.01
MR effect size for
'Follistatin−related protein 1' on 'eGFR'

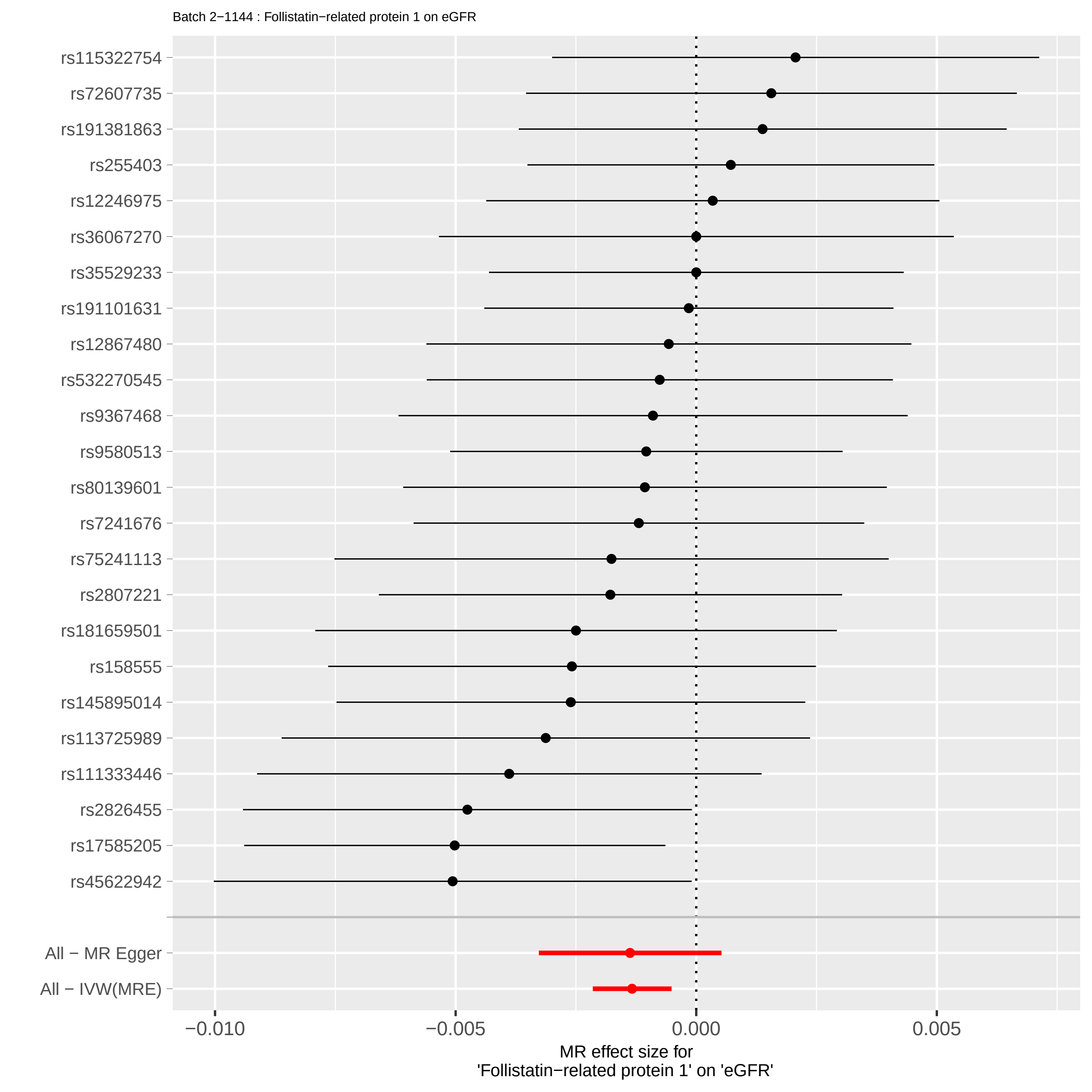

Batch 2−1144 : Follistatin−related protein 1 on eGFR
rs115322754
rs72607735
rs191381863
rs255403
rs12246975
rs36067270
rs35529233
rs191101631
rs12867480
rs532270545
rs9367468
rs9580513
rs80139601
rs7241676
rs75241113
rs2807221
rs181659501
rs158555
rs145895014
rs113725989
rs111333446
rs2826455
rs17585205
rs45622942
All − MR Egger
All − IVW(MRE)
−0.010
−0.005
0.000
0.005
MR effect size for
'Follistatin−related protein 1' on 'eGFR'

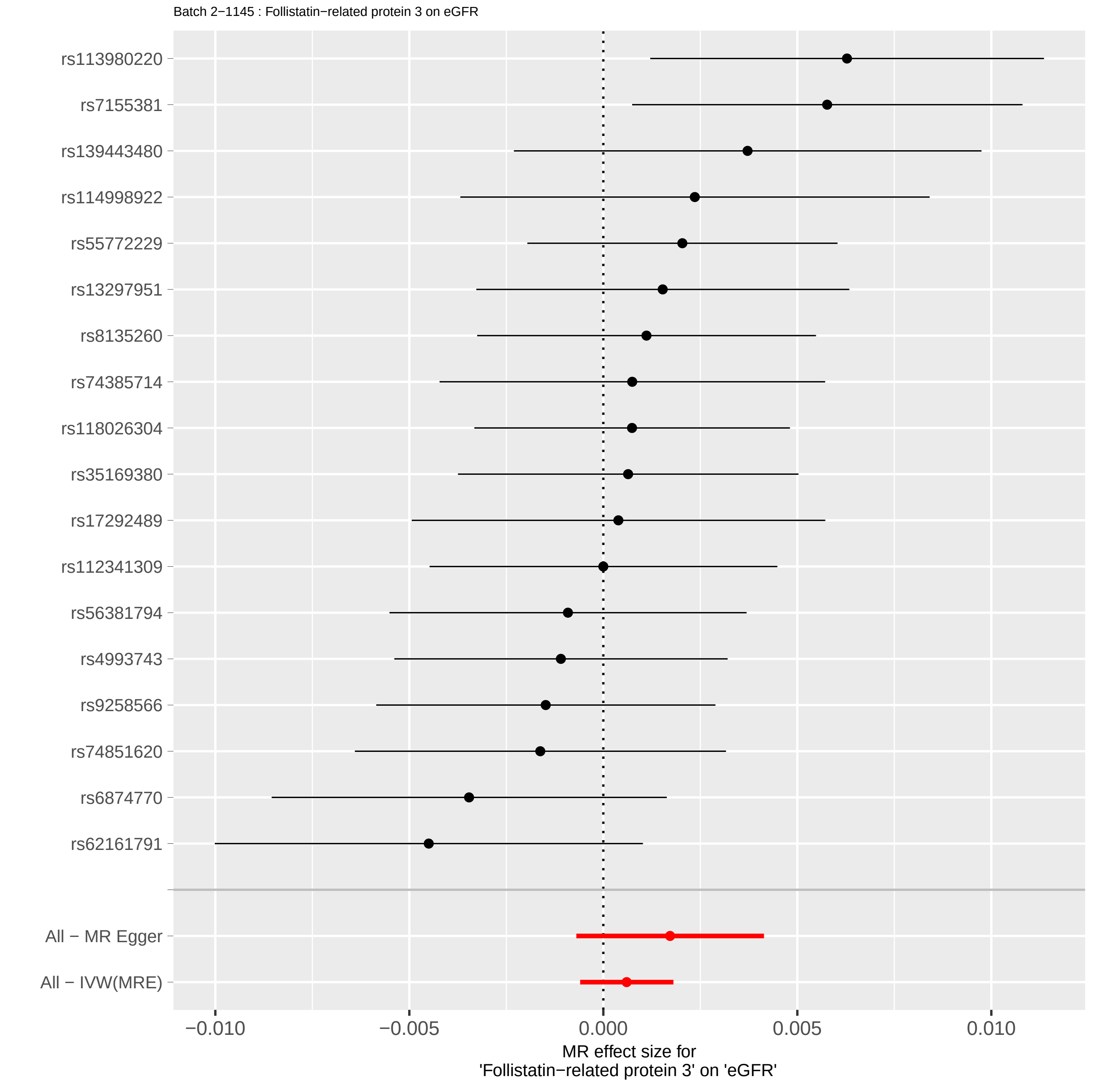

Batch 2−1145 : Follistatin−related protein 3 on eGFR
rs113980220
rs7155381
rs139443480
rs114998922
rs55772229
rs13297951
rs8135260
rs74385714
rs118026304
rs35169380
rs17292489
rs112341309
rs56381794
rs4993743
rs9258566
rs74851620
rs6874770
rs62161791
All − MR Egger
All − IVW(MRE)
−0.010
−0.005
0.000
0.005
0.010
MR effect size for
'Follistatin−related protein 3' on 'eGFR'

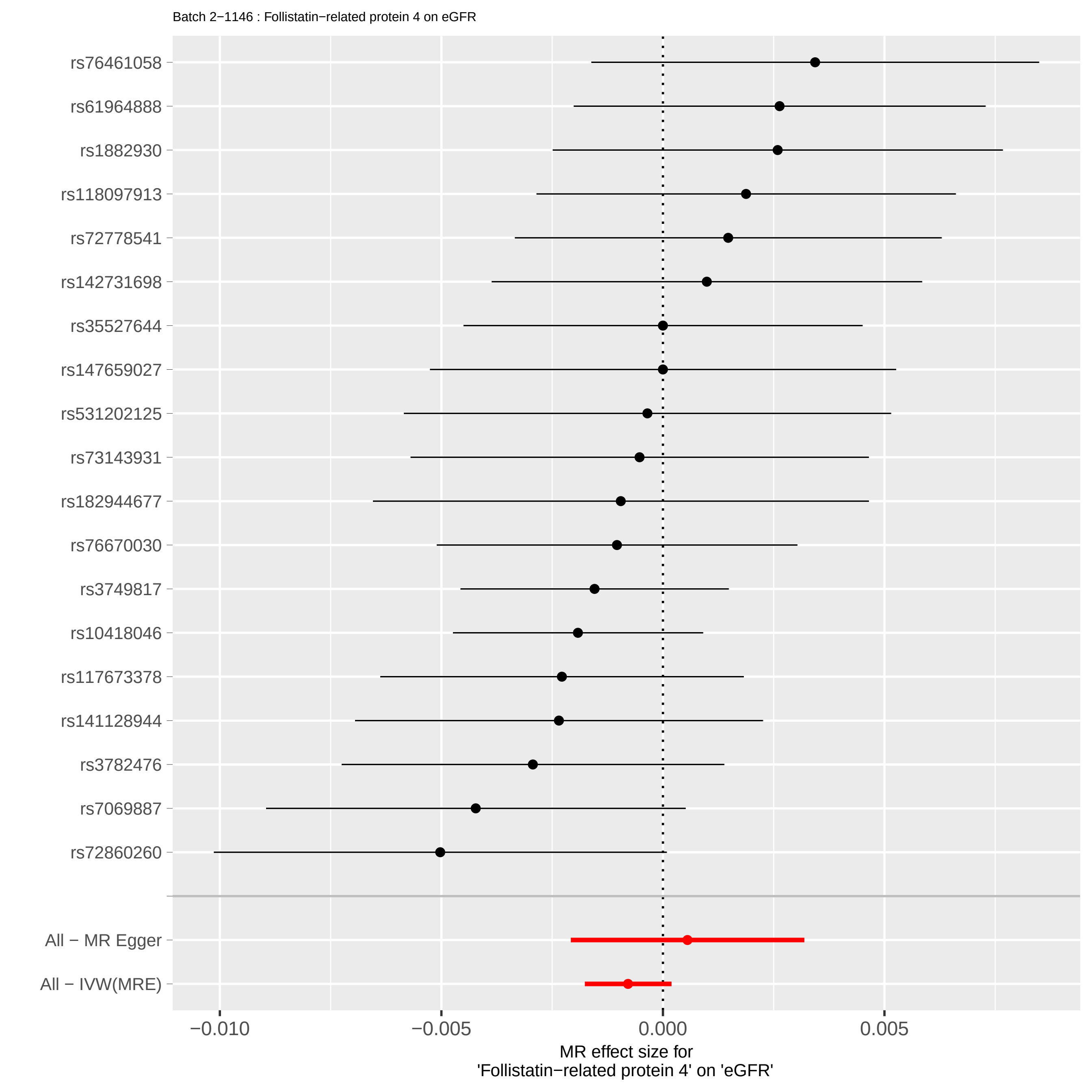

Batch 2−1146 : Follistatin−related protein 4 on eGFR
rs76461058
rs61964888
rs1882930
rs118097913
rs72778541
rs142731698
rs35527644
rs147659027
rs531202125
rs73143931
rs182944677
rs76670030
rs3749817
rs10418046
rs117673378
rs141128944
rs3782476
rs7069887
rs72860260
All − MR Egger
All − IVW(MRE)
−0.010
−0.005
0.000
0.005
MR effect size for
'Follistatin−related protein 4' on 'eGFR'

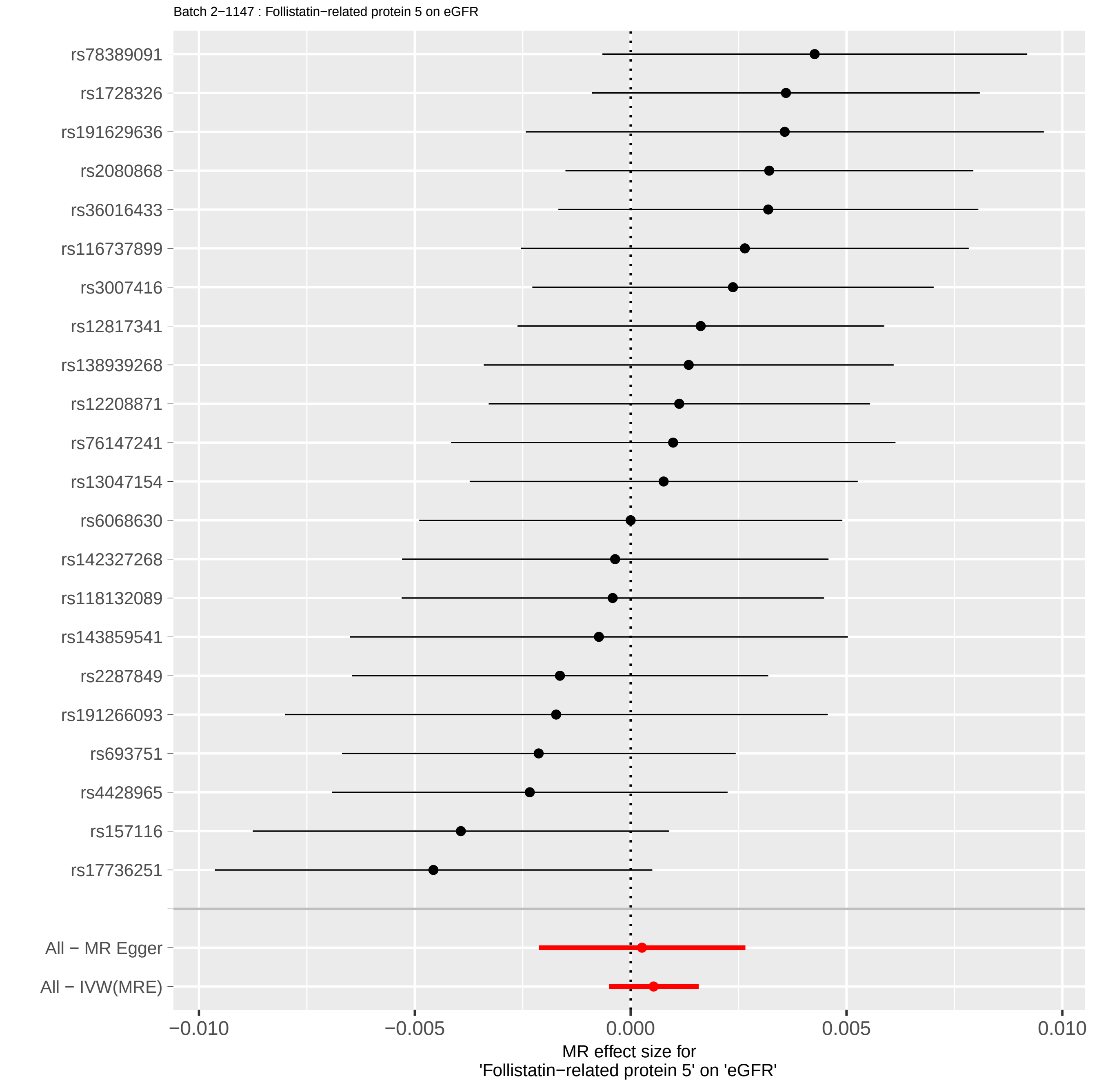

Batch 2−1147 : Follistatin−related protein 5 on eGFR
rs78389091
rs1728326
rs191629636
rs2080868
rs36016433
rs116737899
rs3007416
rs12817341
rs138939268
rs12208871
rs76147241
rs13047154
rs6068630
rs142327268
rs118132089
rs143859541
rs2287849
rs191266093
rs693751
rs4428965
rs157116
rs17736251
All − MR Egger
All − IVW(MRE)
−0.010
−0.005
0.000
0.005
0.010
MR effect size for
'Follistatin−related protein 5' on 'eGFR'

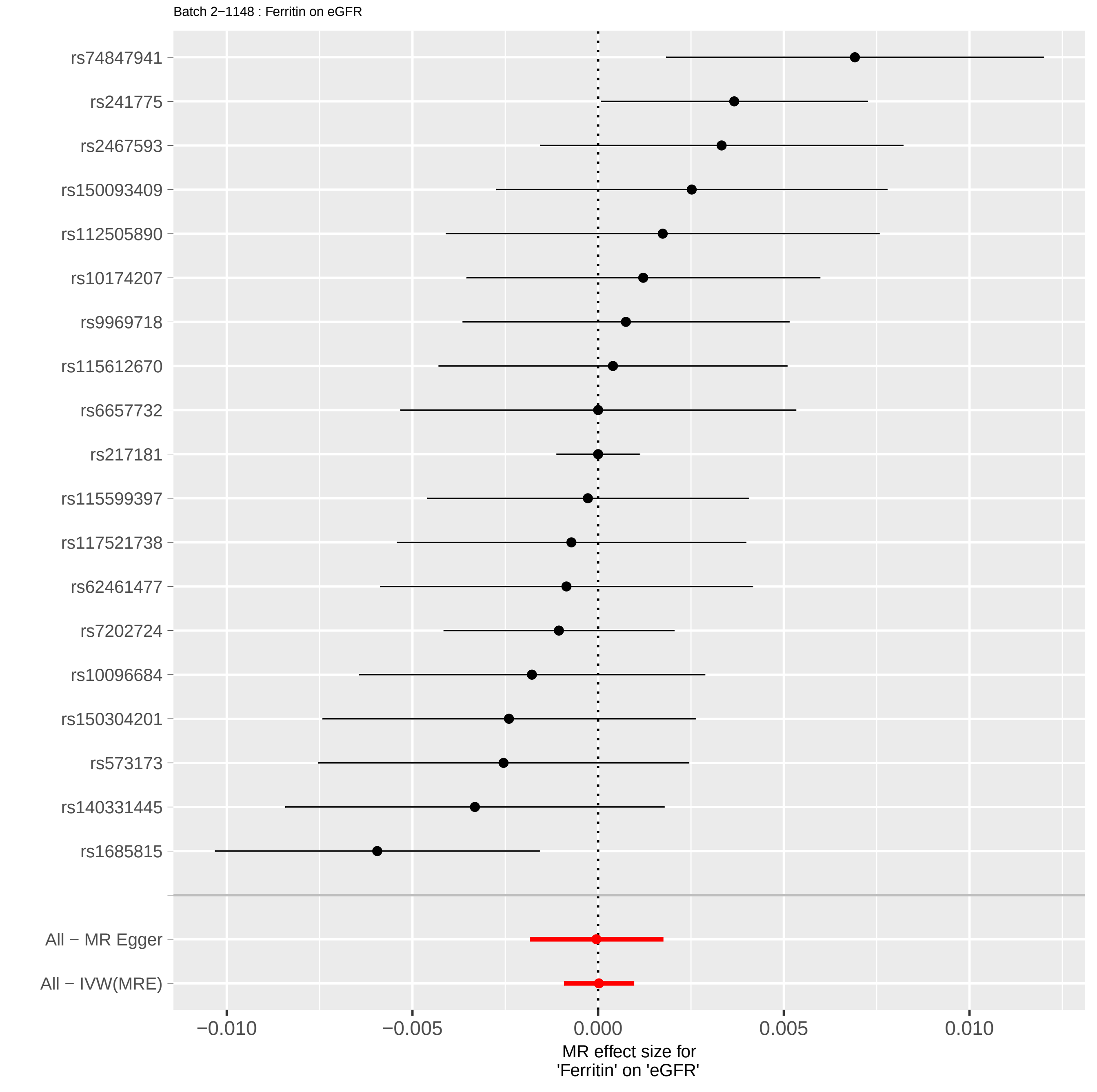
Batch 2−1148 : Ferritin on eGFR
rs74847941
rs241775
rs2467593
rs150093409
rs112505890
rs10174207
rs9969718
rs115612670
rs6657732
rs217181
rs115599397
rs117521738
rs62461477
rs7202724
rs10096684
rs150304201
rs573173
rs140331445
rs1685815
All − MR Egger
All − IVW(MRE)
−0.010
−0.005
0.000
0.005
0.010
MR effect size for
'Ferritin' on 'eGFR'

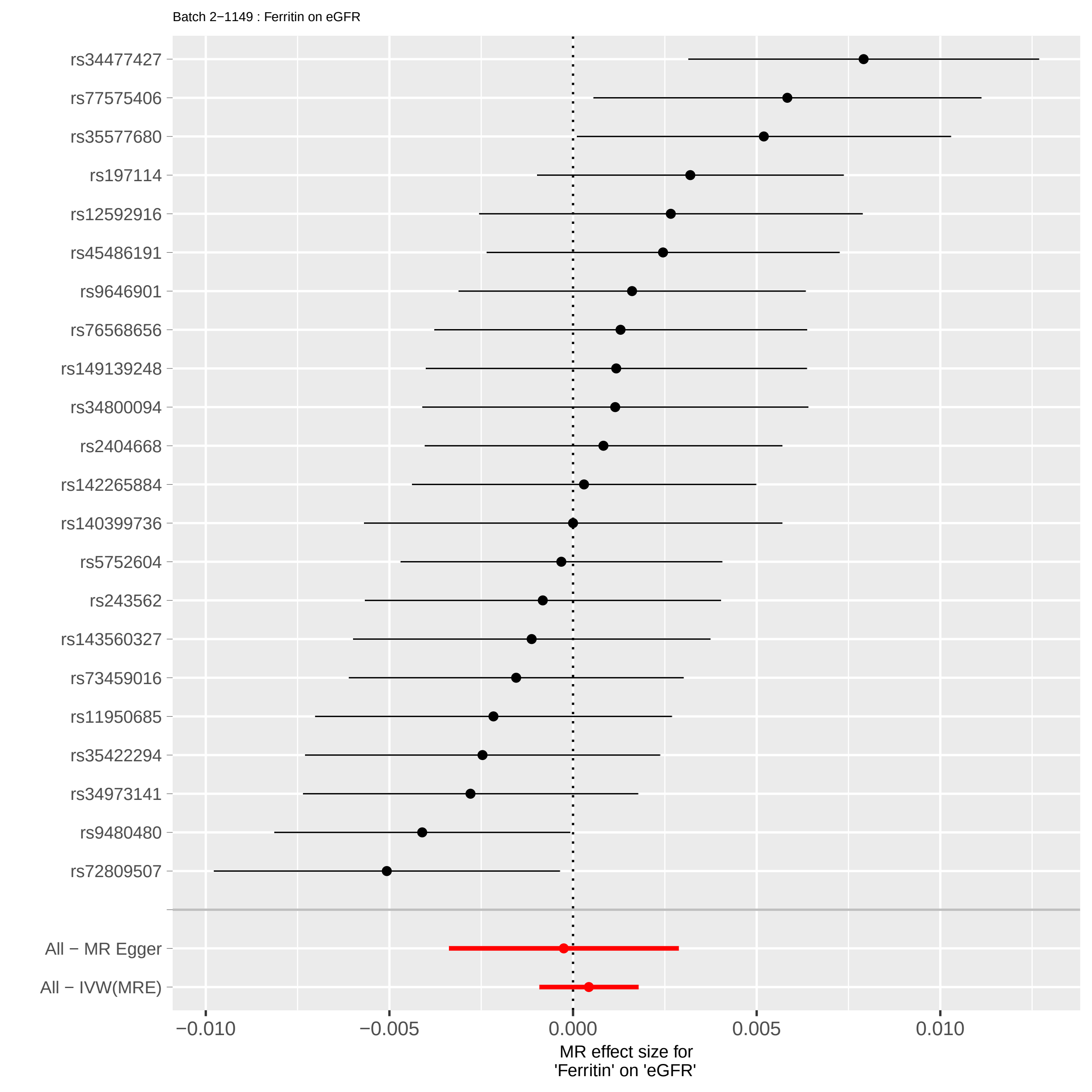
Batch 2−1149 : Ferritin on eGFR
rs34477427
rs77575406
rs35577680
rs197114
rs12592916
rs45486191
rs9646901
rs76568656
rs149139248
rs34800094
rs2404668
rs142265884
rs140399736
rs5752604
rs243562
rs143560327
rs73459016
rs11950685
rs35422294
rs34973141
rs9480480
rs72809507
All − MR Egger
All − IVW(MRE)
−0.010
−0.005
0.000
0.005
0.010
MR effect size for
'Ferritin' on 'eGFR'

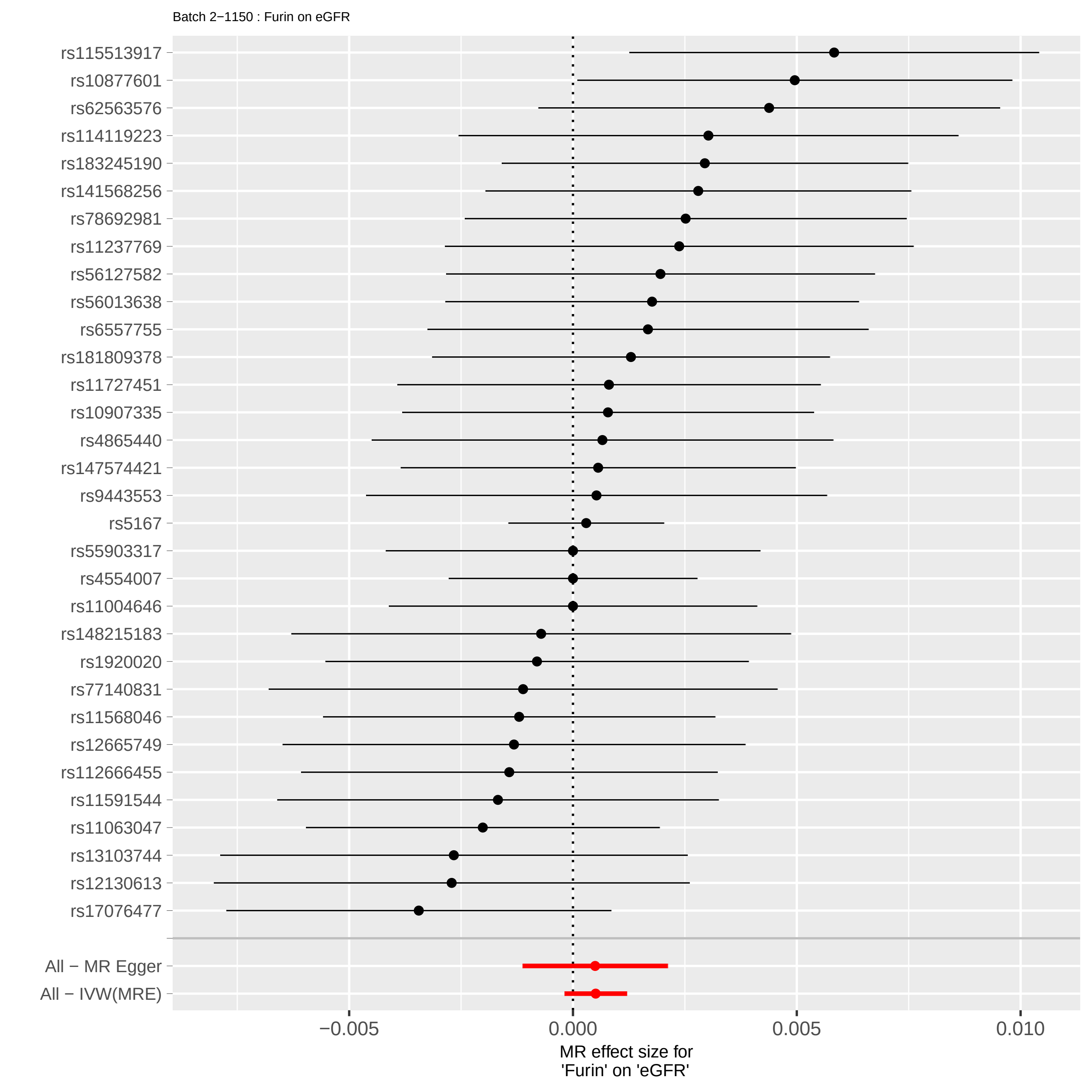
Batch 2−1150 : Furin on eGFR
rs115513917
rs10877601
rs62563576
rs114119223
rs183245190
rs141568256
rs78692981
rs11237769
rs56127582
rs56013638
rs6557755
rs181809378
rs11727451
rs10907335
rs4865440
rs147574421
rs9443553
rs5167
rs55903317
rs4554007
rs11004646
rs148215183
rs1920020
rs77140831
rs11568046
rs12665749
rs112666455
rs11591544
rs11063047
rs13103744
rs12130613
rs17076477
All − MR Egger
All − IVW(MRE)
−0.005
0.000
0.005
0.010
MR effect size for
'Furin' on 'eGFR'

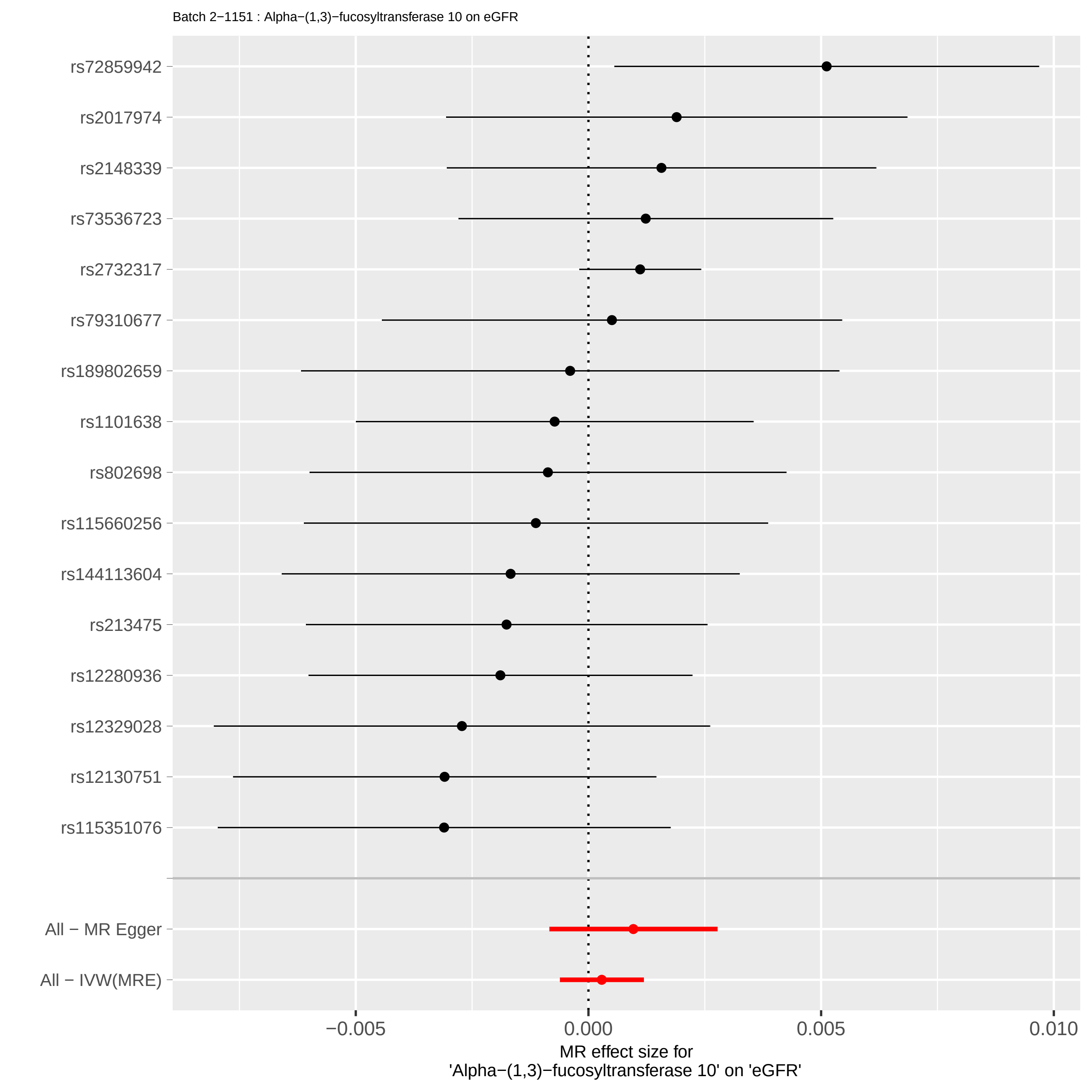

Batch 2−1151 : Alpha−(1,3)−fucosyltransferase 10 on eGFR
rs72859942
rs2017974
rs2148339
rs73536723
rs2732317
rs79310677
rs189802659
rs1101638
rs802698
rs115660256
rs144113604
rs213475
rs12280936
rs12329028
rs12130751
rs115351076
All − MR Egger
All − IVW(MRE)
−0.005
0.000
0.005
0.010
MR effect size for
'Alpha−(1,3)−fucosyltransferase 10' on 'eGFR'

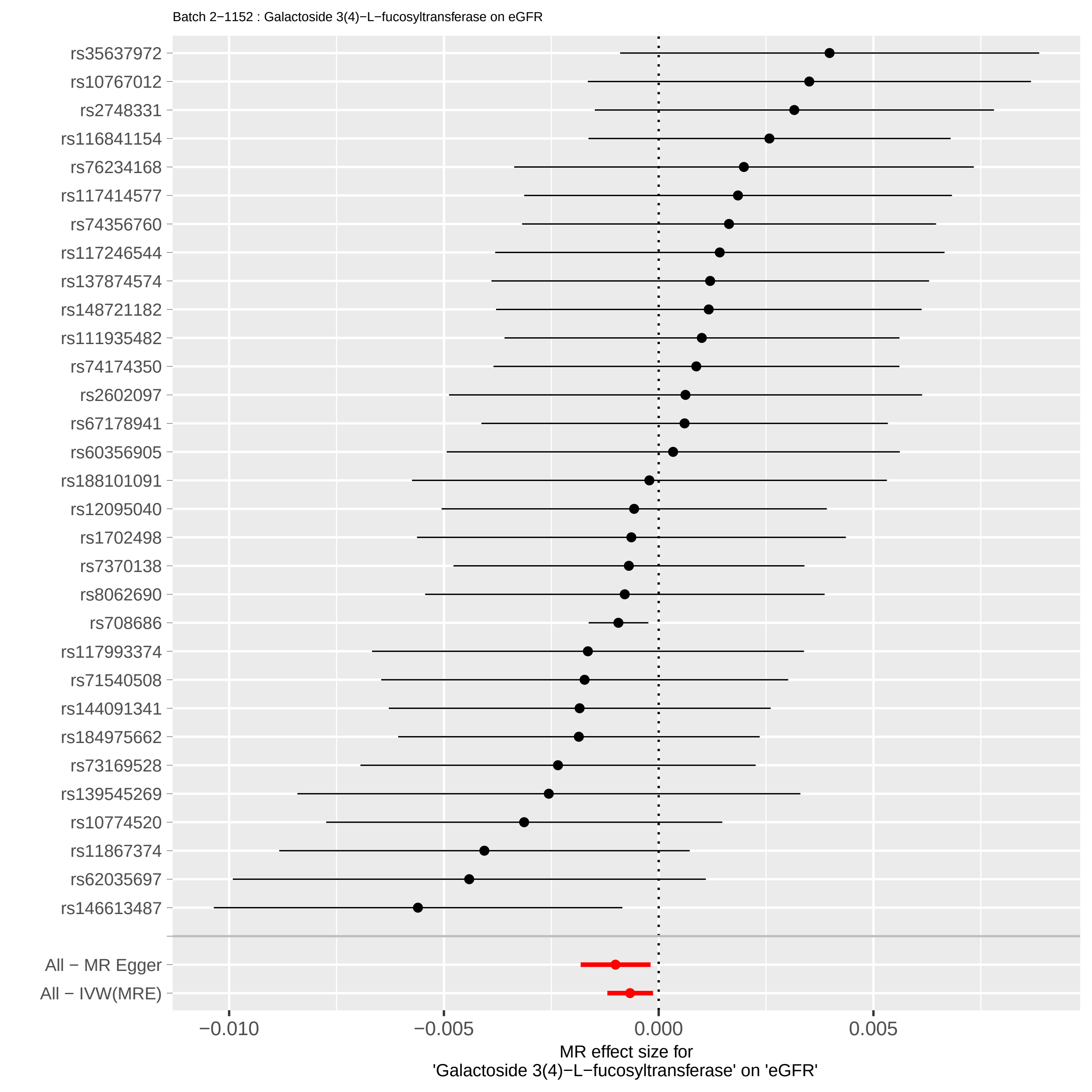

Batch 2−1152 : Galactoside 3(4)−L−fucosyltransferase on eGFR
rs35637972
rs10767012
rs2748331
rs116841154
rs76234168
rs117414577
rs74356760
rs117246544
rs137874574
rs148721182
rs111935482
rs74174350
rs2602097
rs67178941
rs60356905
rs188101091
rs12095040
rs1702498
rs7370138
rs8062690
rs708686
rs117993374
rs71540508
rs144091341
rs184975662
rs73169528
rs139545269
rs10774520
rs11867374
rs62035697
rs146613487
All − MR Egger
All − IVW(MRE)
−0.010
−0.005
0.000
0.005
MR effect size for
'Galactoside 3(4)−L−fucosyltransferase' on 'eGFR'

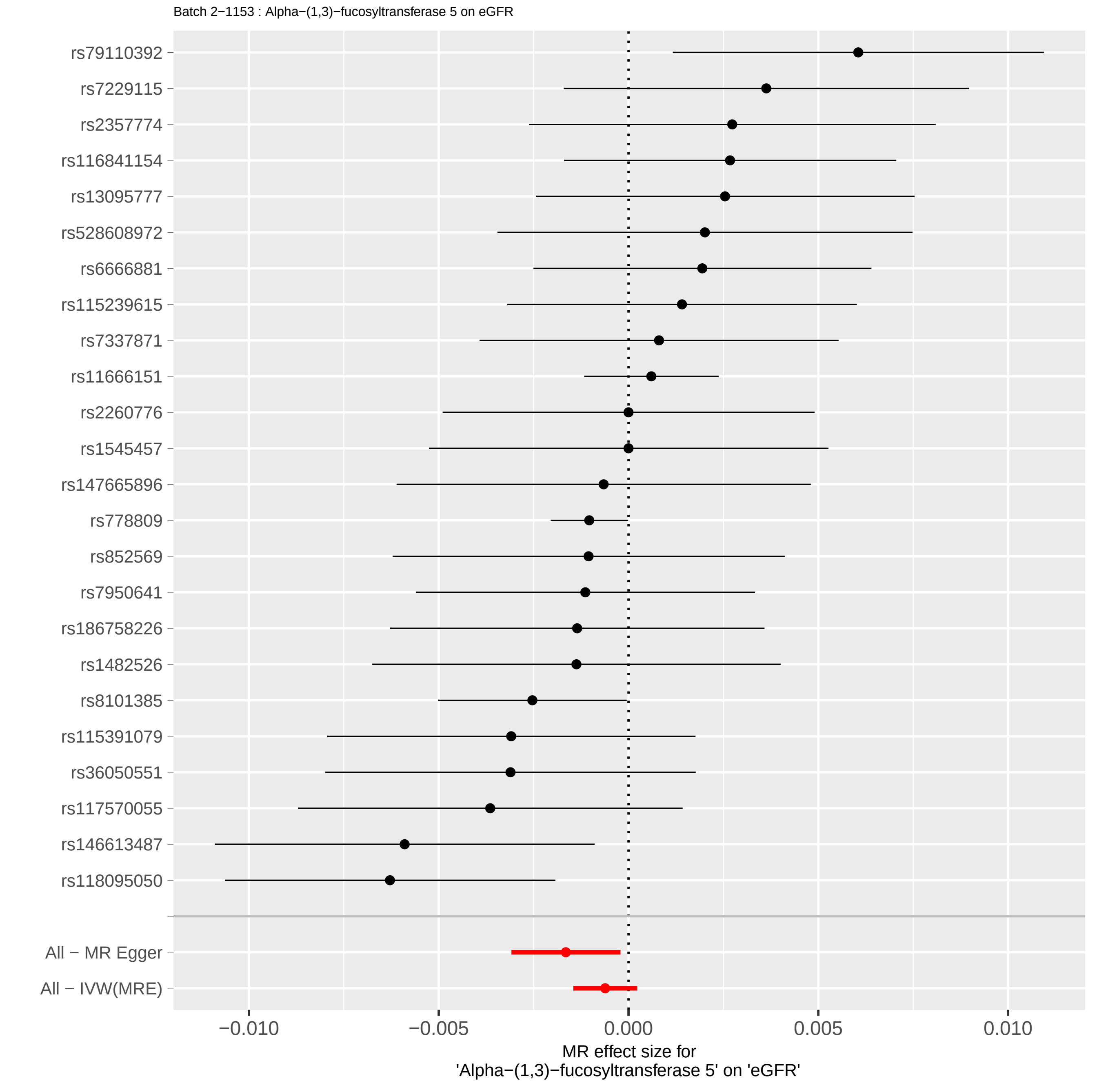

Batch 2−1153 : Alpha−(1,3)−fucosyltransferase 5 on eGFR
rs79110392
rs7229115
rs2357774
rs116841154
rs13095777
rs528608972
rs6666881
rs115239615
rs7337871
rs11666151
rs2260776
rs1545457
rs147665896
rs778809
rs852569
rs7950641
rs186758226
rs1482526
rs8101385
rs115391079
rs36050551
rs117570055
rs146613487
rs118095050
All − MR Egger
All − IVW(MRE)
−0.010
−0.005
0.000
0.005
0.010
MR effect size for
'Alpha−(1,3)−fucosyltransferase 5' on 'eGFR'

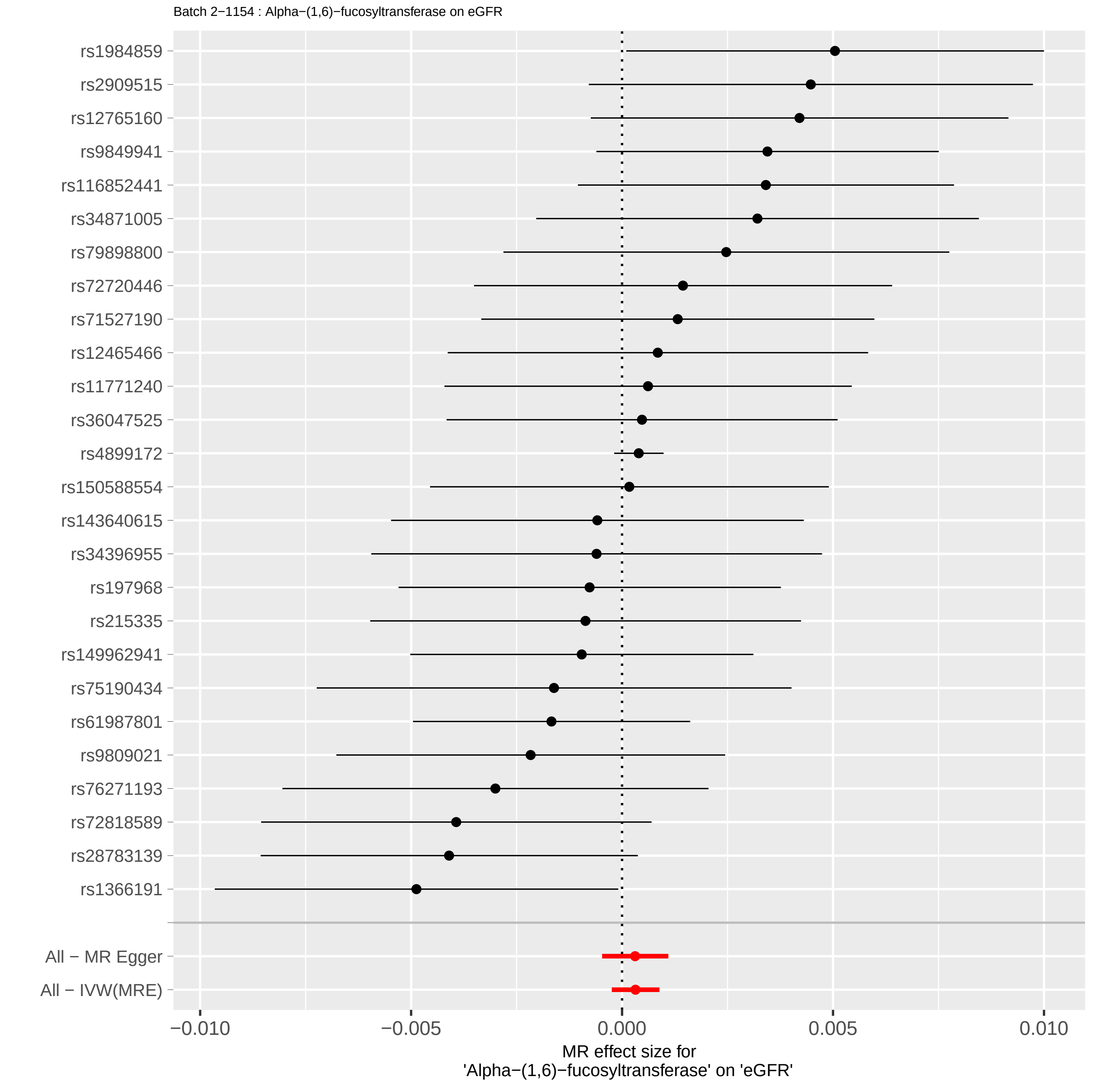
Batch 2−1154 : Alpha−(1,6)−fucosyltransferase on eGFR
rs1984859
rs2909515
rs12765160
rs9849941
rs116852441
rs34871005
rs79898800
rs72720446
rs71527190
rs12465466
rs11771240
rs36047525
rs4899172
rs150588554
rs143640615
rs34396955
rs197968
rs215335
rs149962941
rs75190434
rs61987801
rs9809021
rs76271193
rs72818589
rs28783139
rs1366191
All − MR Egger
All − IVW(MRE)
−0.010
−0.005
0.000
0.005
0.010
MR effect size for
'Alpha−(1,6)−fucosyltransferase' on 'eGFR'

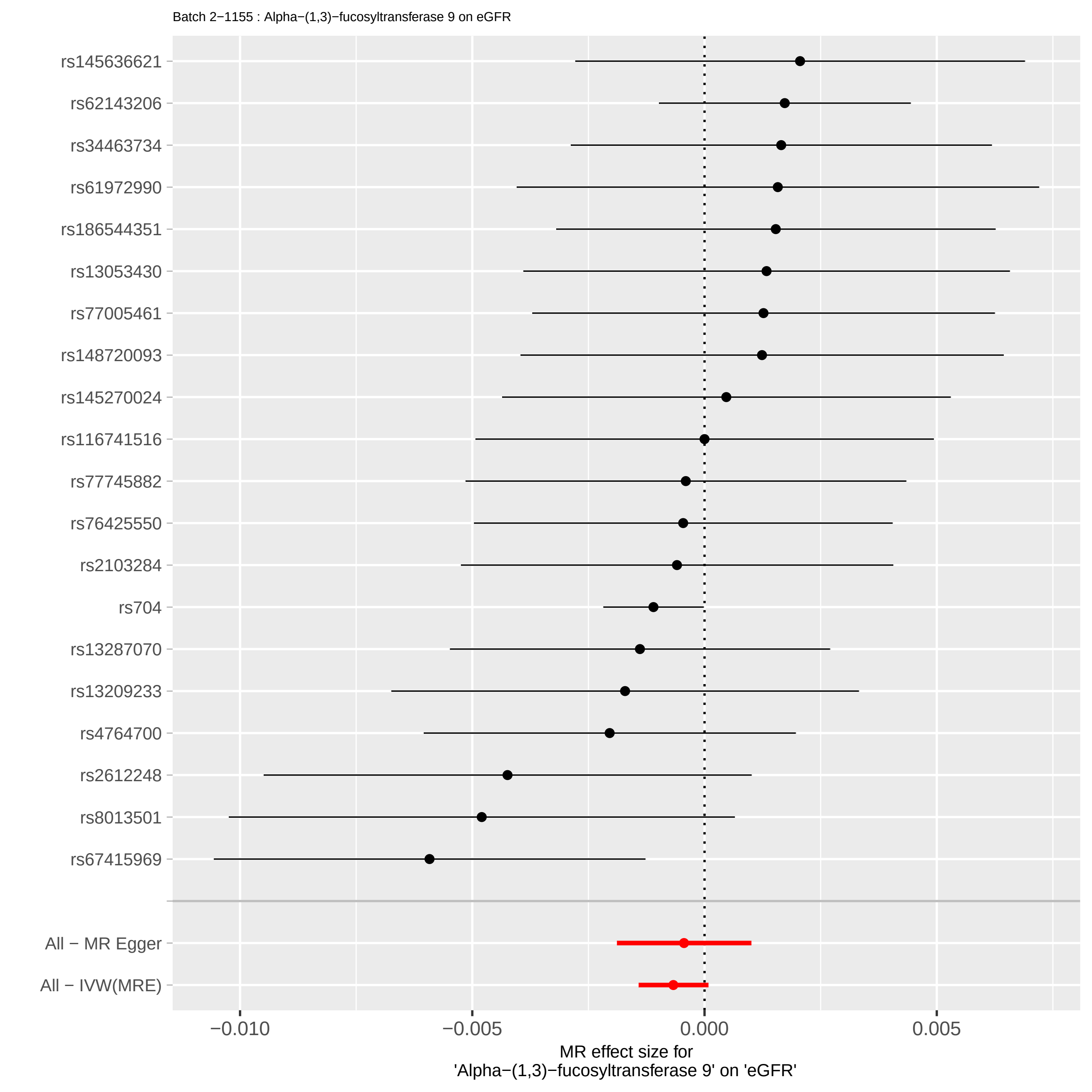

Batch 2−1155 : Alpha−(1,3)−fucosyltransferase 9 on eGFR
rs145636621
rs62143206
rs34463734
rs61972990
rs186544351
rs13053430
rs77005461
rs148720093
rs145270024
rs116741516
rs77745882
rs76425550
rs2103284
rs704
rs13287070
rs13209233
rs4764700
rs2612248
rs8013501
rs67415969
All − MR Egger
All − IVW(MRE)
−0.010
−0.005
0.000
0.005
MR effect size for
'Alpha−(1,3)−fucosyltransferase 9' on 'eGFR'

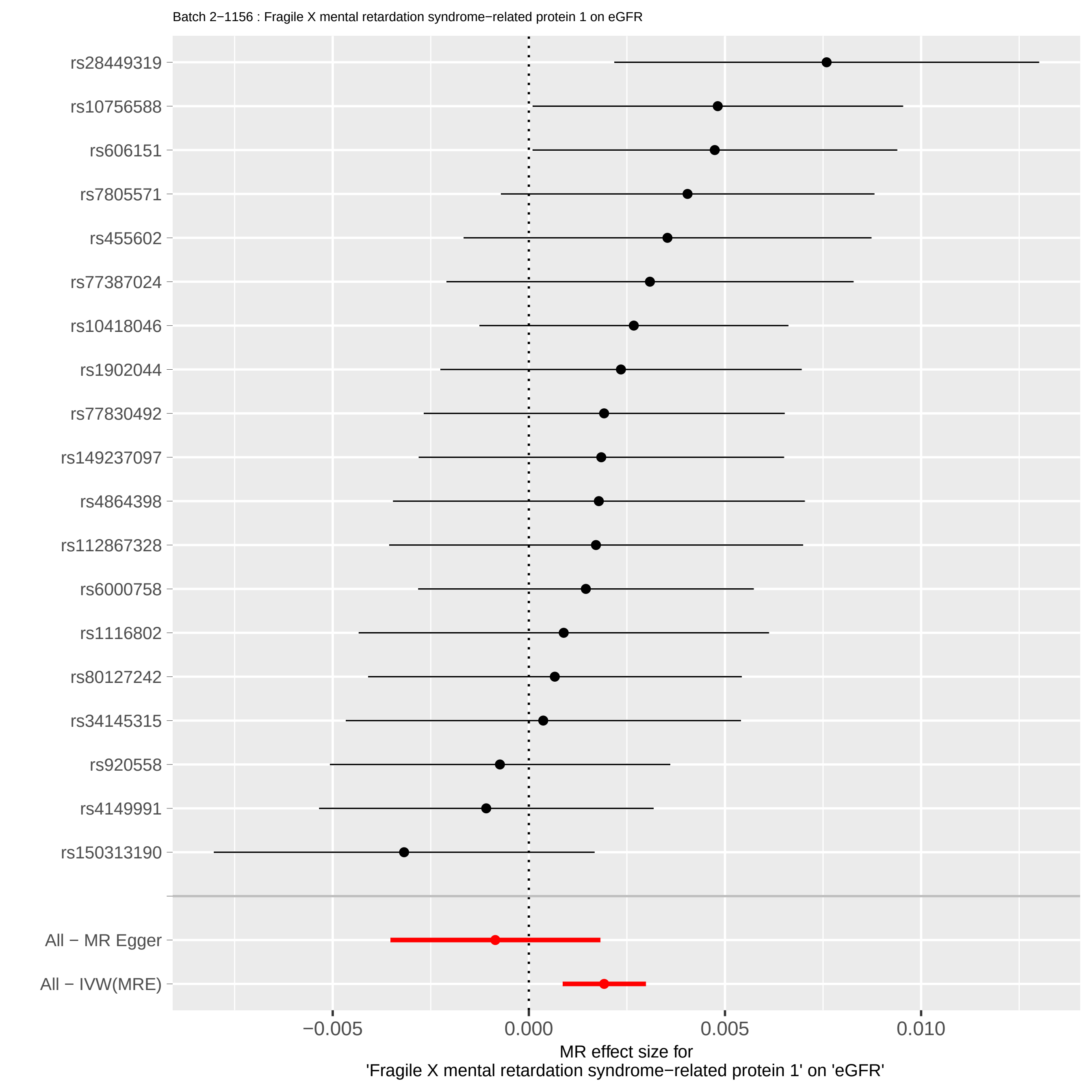
Batch 2−1156 : Fragile X mental retardation syndrome−related protein 1 on eGFR
rs28449319
rs10756588
rs606151
rs7805571
rs455602
rs77387024
rs10418046
rs1902044
rs77830492
rs149237097
rs4864398
rs112867328
rs6000758
rs1116802
rs80127242
rs34145315
rs920558
rs4149991
rs150313190
All − MR Egger
All − IVW(MRE)
−0.005
0.000
0.005
0.010
MR effect size for
'Fragile X mental retardation syndrome−related protein 1' on 'eGFR'

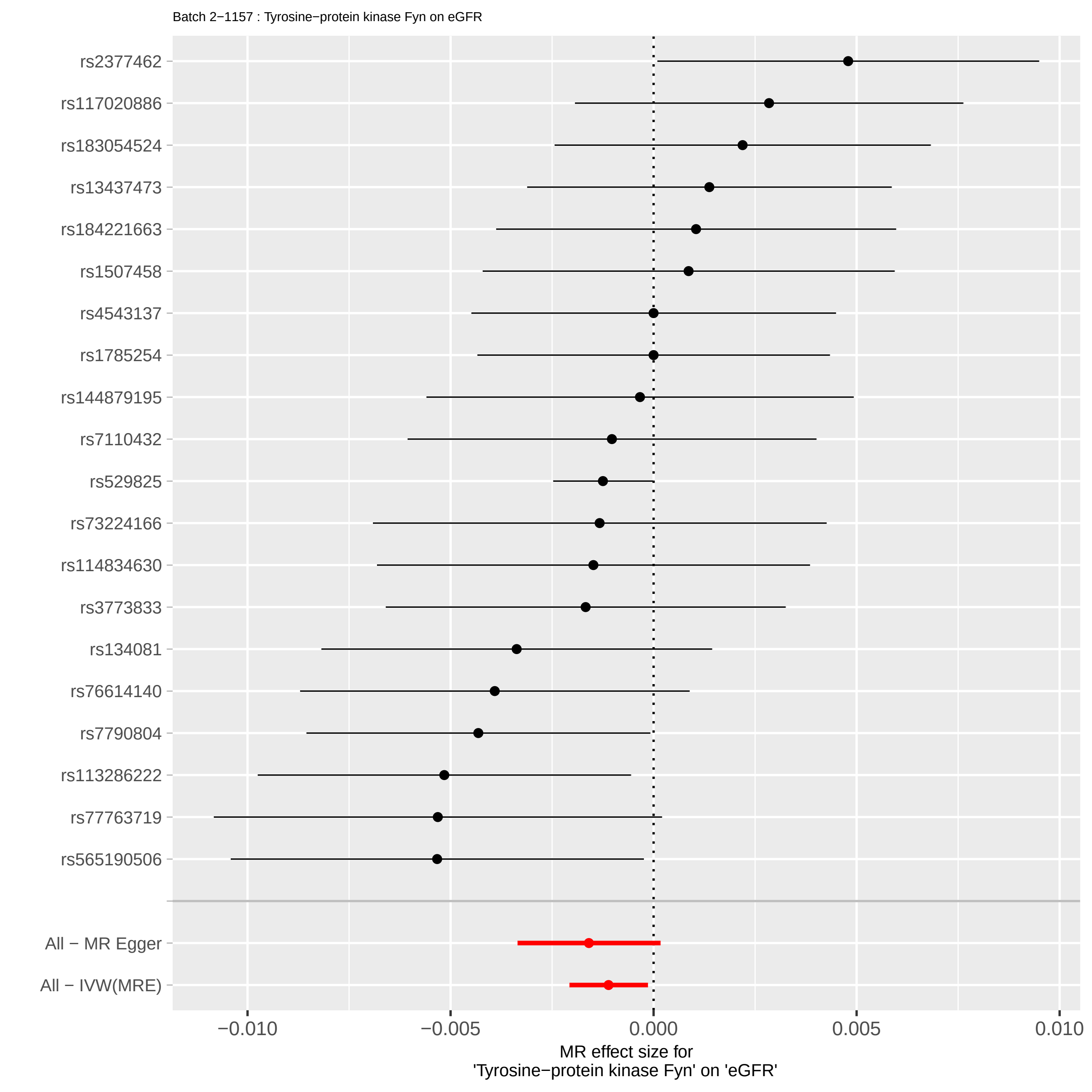
Batch 2−1157 : Tyrosine−protein kinase Fyn on eGFR
rs2377462
rs117020886
rs183054524
rs13437473
rs184221663
rs1507458
rs4543137
rs1785254
rs144879195
rs7110432
rs529825
rs73224166
rs114834630
rs3773833
rs134081
rs76614140
rs7790804
rs113286222
rs77763719
rs565190506
All − MR Egger
All − IVW(MRE)
−0.010
−0.005
0.000
0.005
0.010
MR effect size for
'Tyrosine−protein kinase Fyn' on 'eGFR'

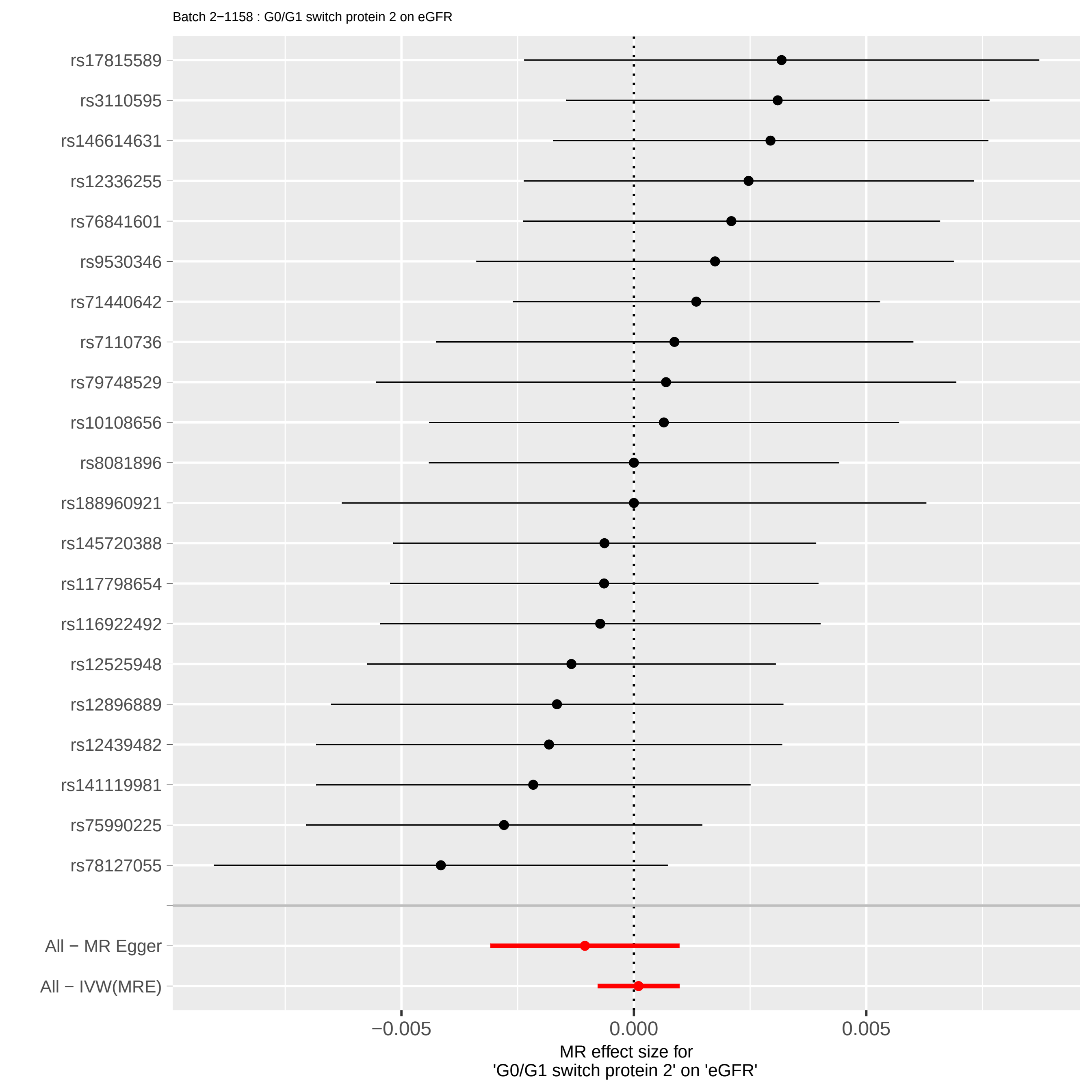
Batch 2−1158 : G0/G1 switch protein 2 on eGFR
rs17815589
rs3110595
rs146614631
rs12336255
rs76841601
rs9530346
rs71440642
rs7110736
rs79748529
rs10108656
rs8081896
rs188960921
rs145720388
rs117798654
rs116922492
rs12525948
rs12896889
rs12439482
rs141119981
rs75990225
rs78127055
All − MR Egger
All − IVW(MRE)
−0.005
0.000
0.005
MR effect size for
'G0/G1 switch protein 2' on 'eGFR'

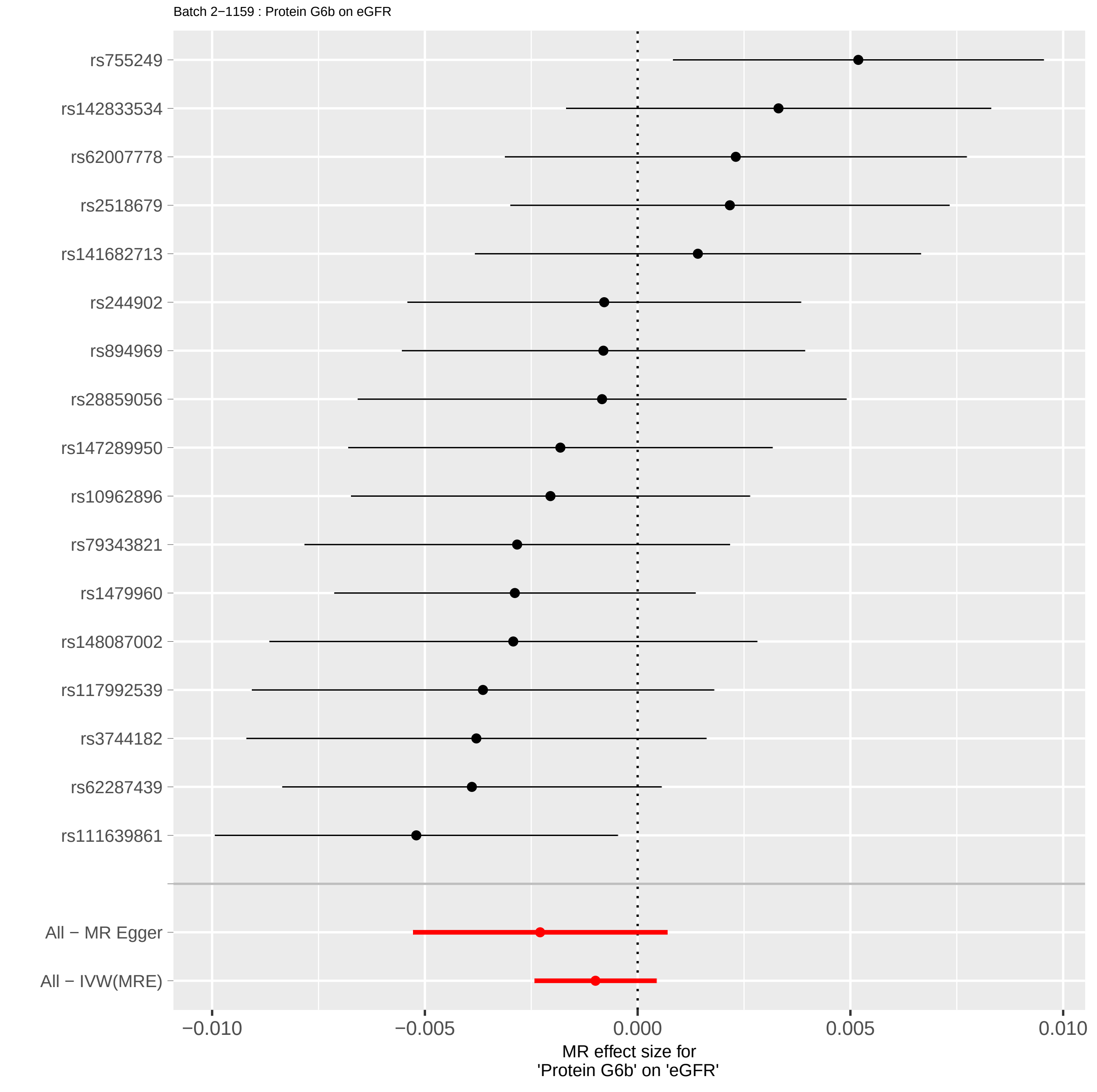

Batch 2−1159 : Protein G6b on eGFR
rs755249
rs142833534
rs62007778
rs2518679
rs141682713
rs244902
rs894969
rs28859056
rs147289950
rs10962896
rs79343821
rs1479960
rs148087002
rs117992539
rs3744182
rs62287439
rs111639861
All − MR Egger
All − IVW(MRE)
−0.010
−0.005
0.000
0.005
0.010
MR effect size for
'Protein G6b' on 'eGFR'

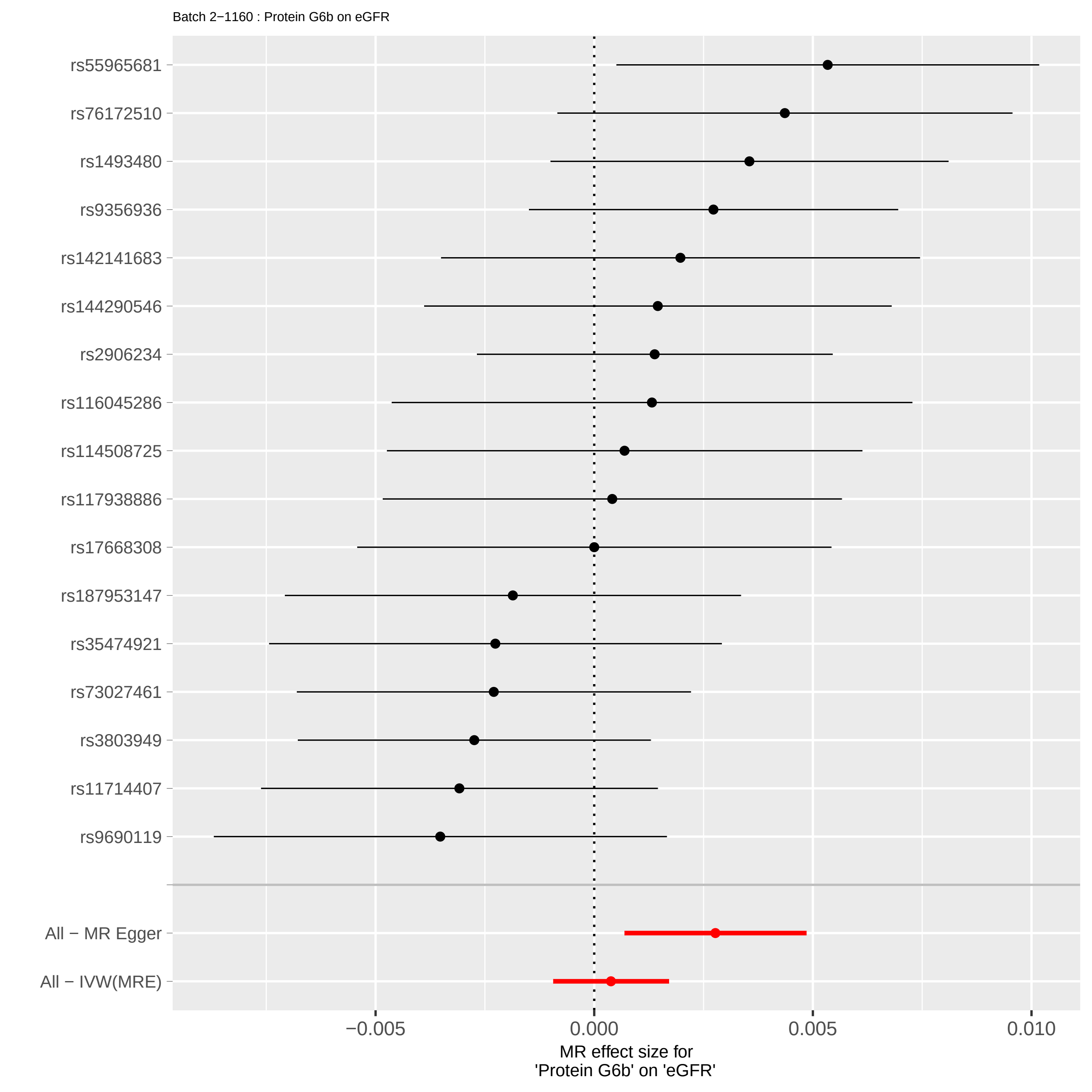

Batch 2−1160 : Protein G6b on eGFR
rs55965681
rs76172510
rs1493480
rs9356936
rs142141683
rs144290546
rs2906234
rs116045286
rs114508725
rs117938886
rs17668308
rs187953147
rs35474921
rs73027461
rs3803949
rs11714407
rs9690119
All − MR Egger
All − IVW(MRE)
−0.005
0.000
0.005
0.010
MR effect size for
'Protein G6b' on 'eGFR'

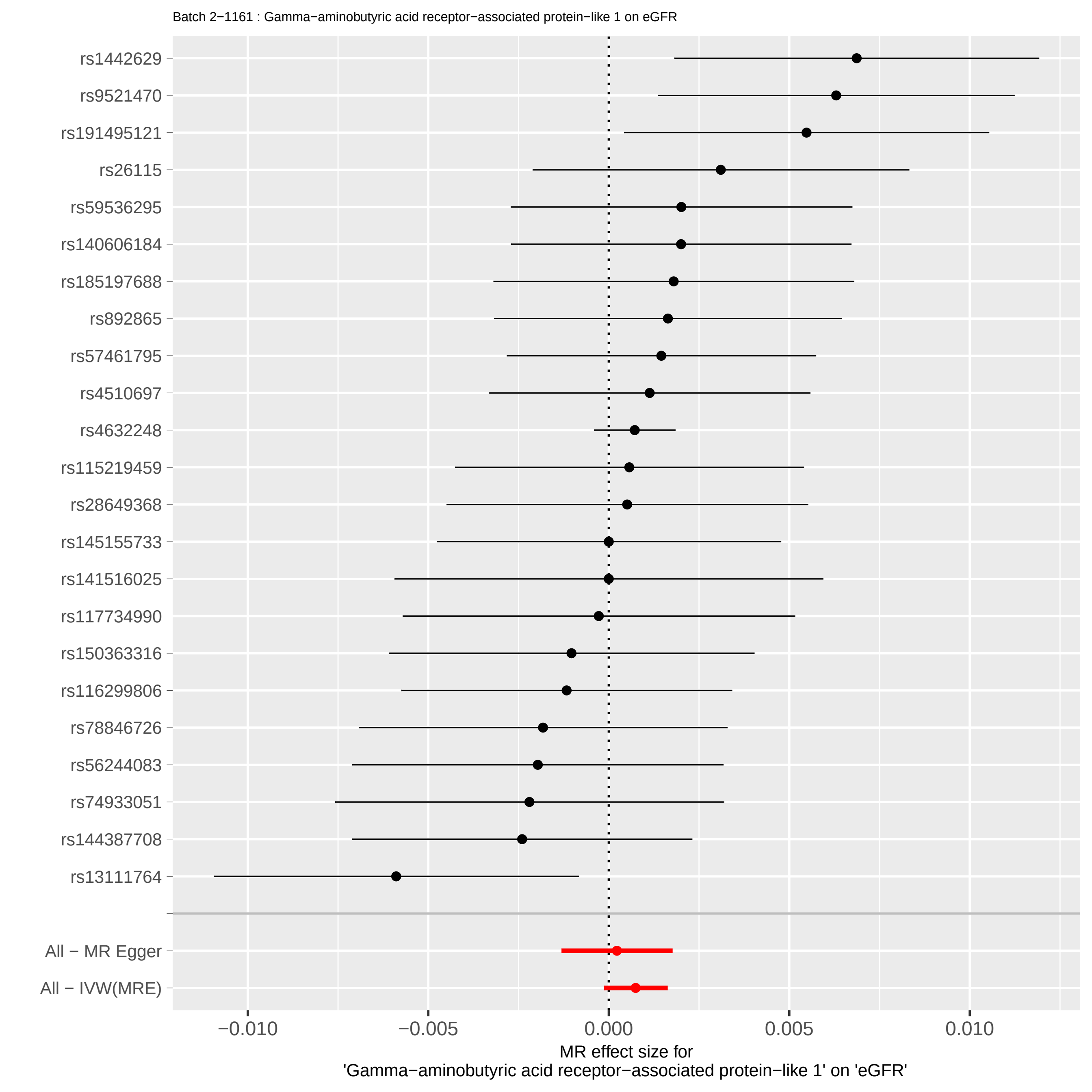

Batch 2−1161 : Gamma−aminobutyric acid receptor−associated protein−like 1 on eGFR
rs1442629
rs9521470
rs191495121
rs26115
rs59536295
rs140606184
rs185197688
rs892865
rs57461795
rs4510697
rs4632248
rs115219459
rs28649368
rs145155733
rs141516025
rs117734990
rs150363316
rs116299806
rs78846726
rs56244083
rs74933051
rs144387708
rs13111764
All − MR Egger
All − IVW(MRE)
−0.010
−0.005
0.000
0.005
0.010
MR effect size for
'Gamma−aminobutyric acid receptor−associated protein−like 1' on 'eGFR'

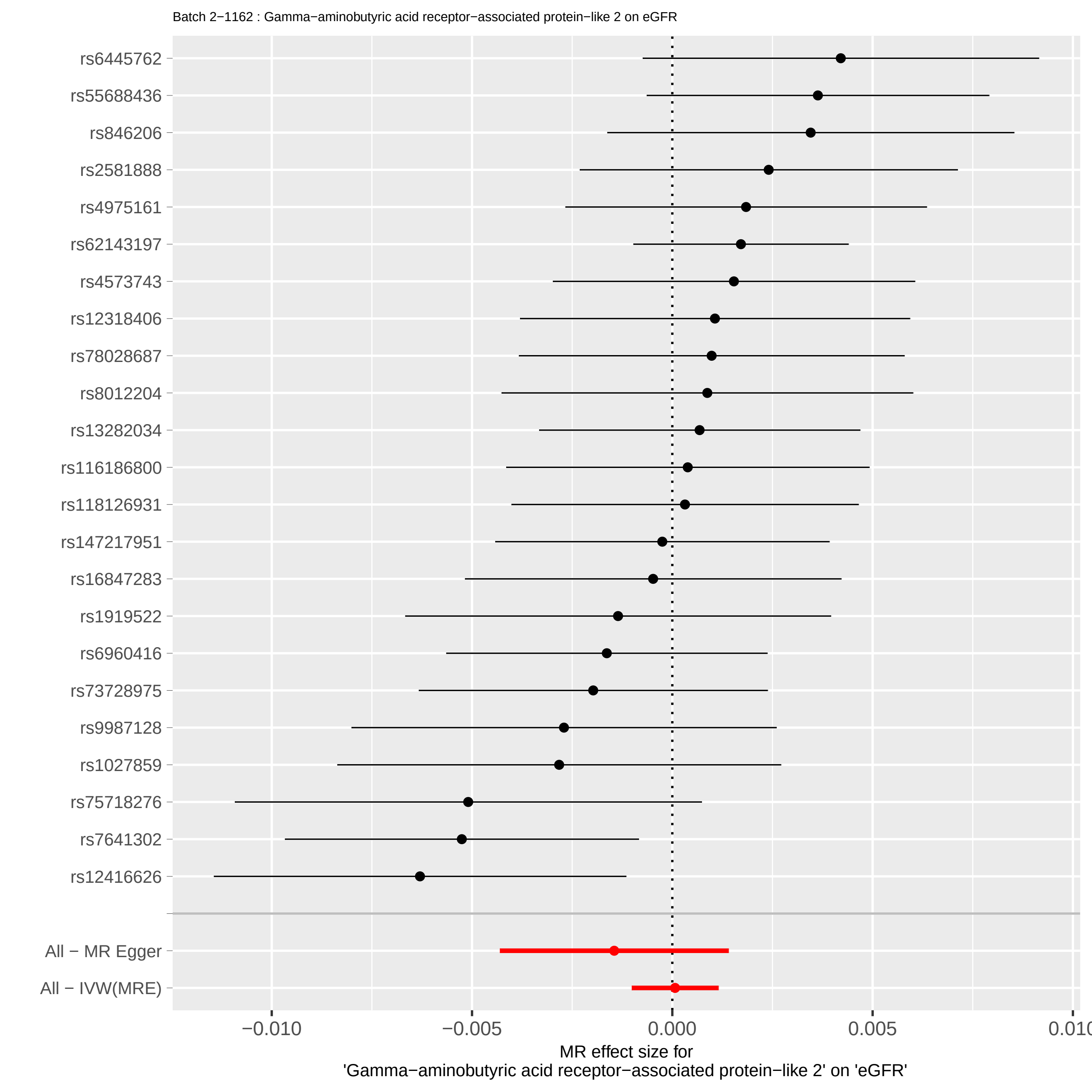
Batch 2−1162 : Gamma−aminobutyric acid receptor−associated protein−like 2 on eGFR
rs6445762
rs55688436
rs846206
rs2581888
rs4975161
rs62143197
rs4573743
rs12318406
rs78028687
rs8012204
rs13282034
rs116186800
rs118126931
rs147217951
rs16847283
rs1919522
rs6960416
rs73728975
rs9987128
rs1027859
rs75718276
rs7641302
rs12416626
All − MR Egger
All − IVW(MRE)
−0.010
−0.005
0.000
0.005
0.010
MR effect size for
'Gamma−aminobutyric acid receptor−associated protein−like 2' on 'eGFR'

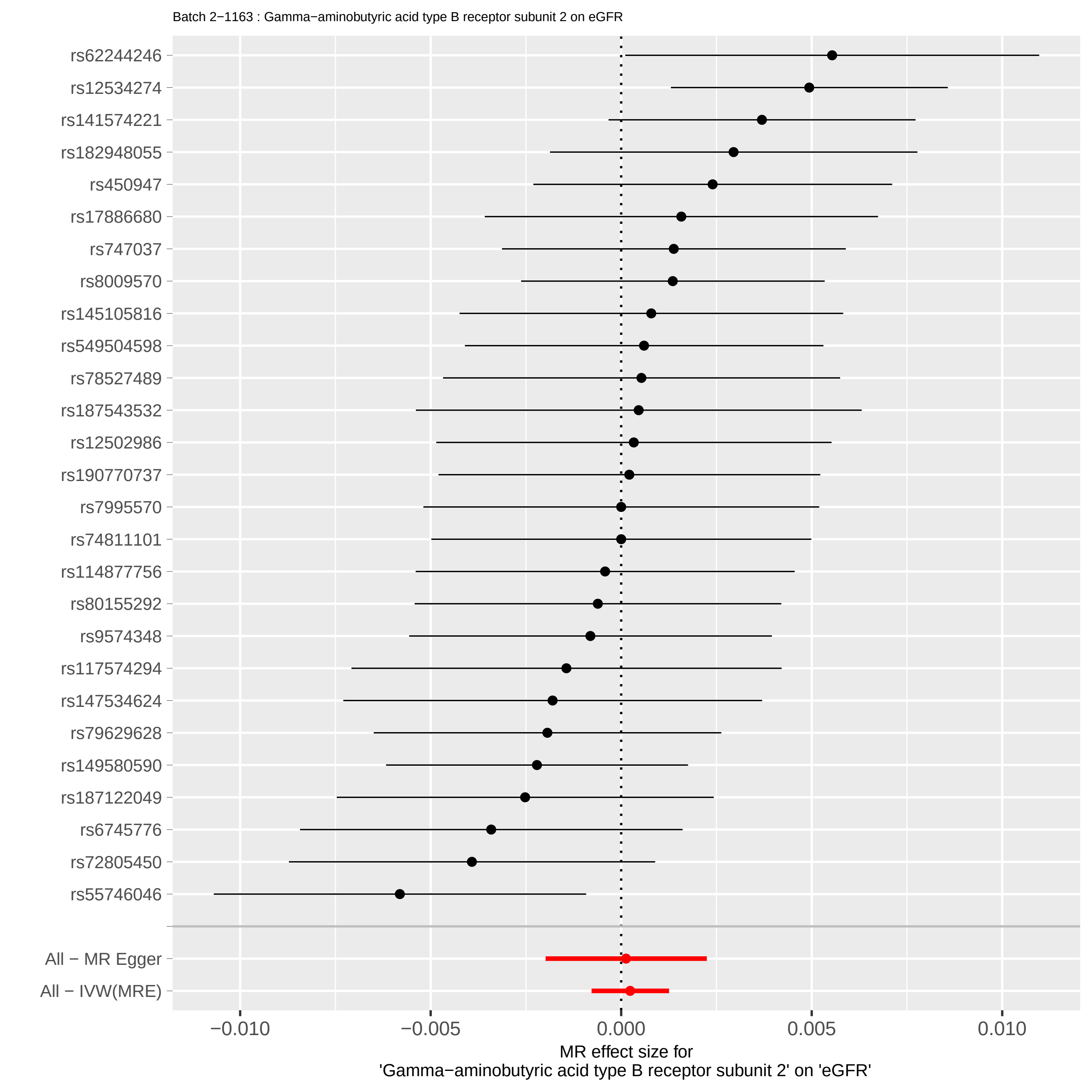
Batch 2−1163 : Gamma−aminobutyric acid type B receptor subunit 2 on eGFR
rs62244246
rs12534274
rs141574221
rs182948055
rs450947
rs17886680
rs747037
rs8009570
rs145105816
rs549504598
rs78527489
rs187543532
rs12502986
rs190770737
rs7995570
rs74811101
rs114877756
rs80155292
rs9574348
rs117574294
rs147534624
rs79629628
rs149580590
rs187122049
rs6745776
rs72805450
rs55746046
All − MR Egger
All − IVW(MRE)
−0.010
−0.005
0.000
0.005
0.010
MR effect size for
'Gamma−aminobutyric acid type B receptor subunit 2' on 'eGFR'

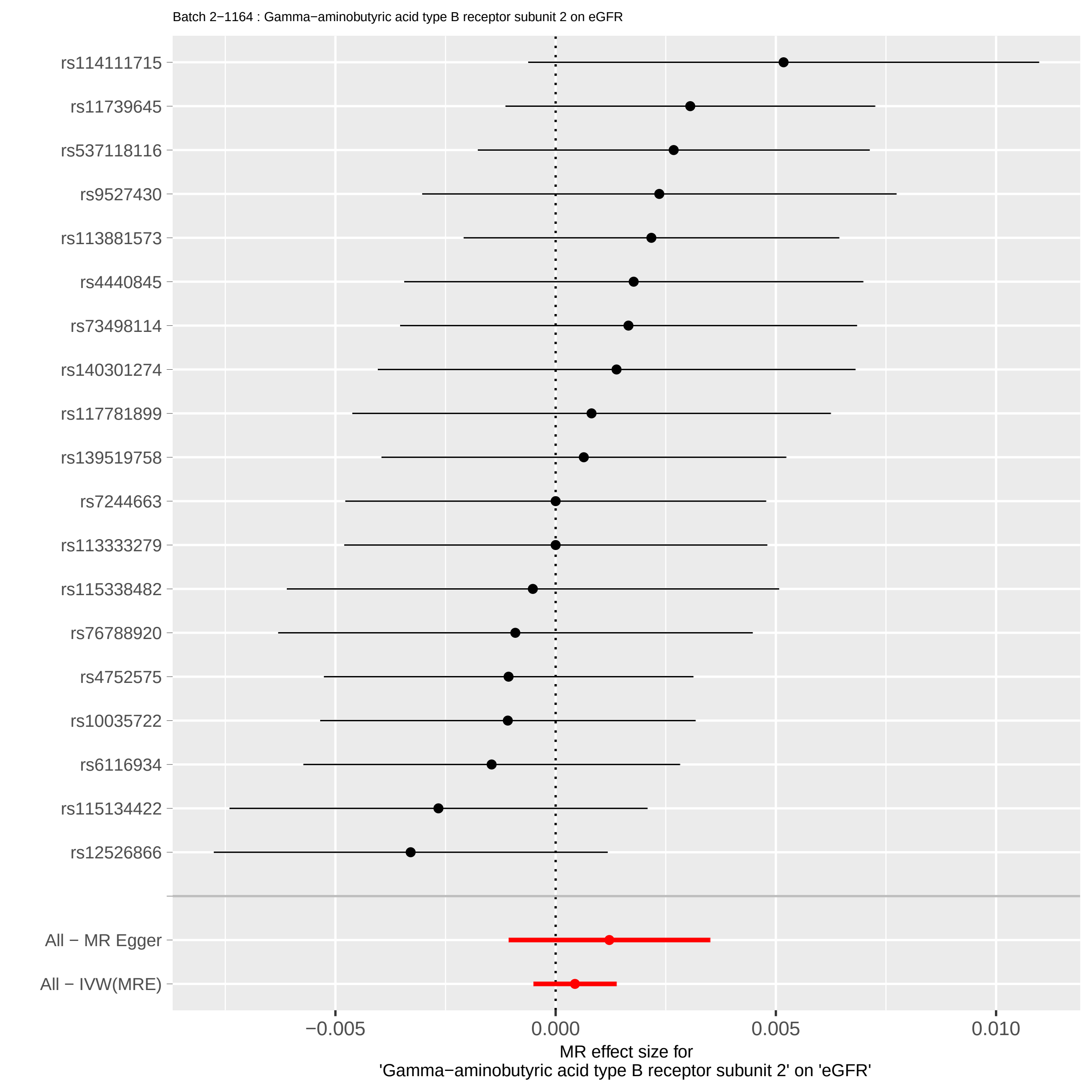
Batch 2−1164 : Gamma−aminobutyric acid type B receptor subunit 2 on eGFR
rs114111715
rs11739645
rs537118116
rs9527430
rs113881573
rs4440845
rs73498114
rs140301274
rs117781899
rs139519758
rs7244663
rs113333279
rs115338482
rs76788920
rs4752575
rs10035722
rs6116934
rs115134422
rs12526866
All − MR Egger
All − IVW(MRE)
−0.005
0.000
0.005
0.010
MR effect size for
'Gamma−aminobutyric acid type B receptor subunit 2' on 'eGFR'

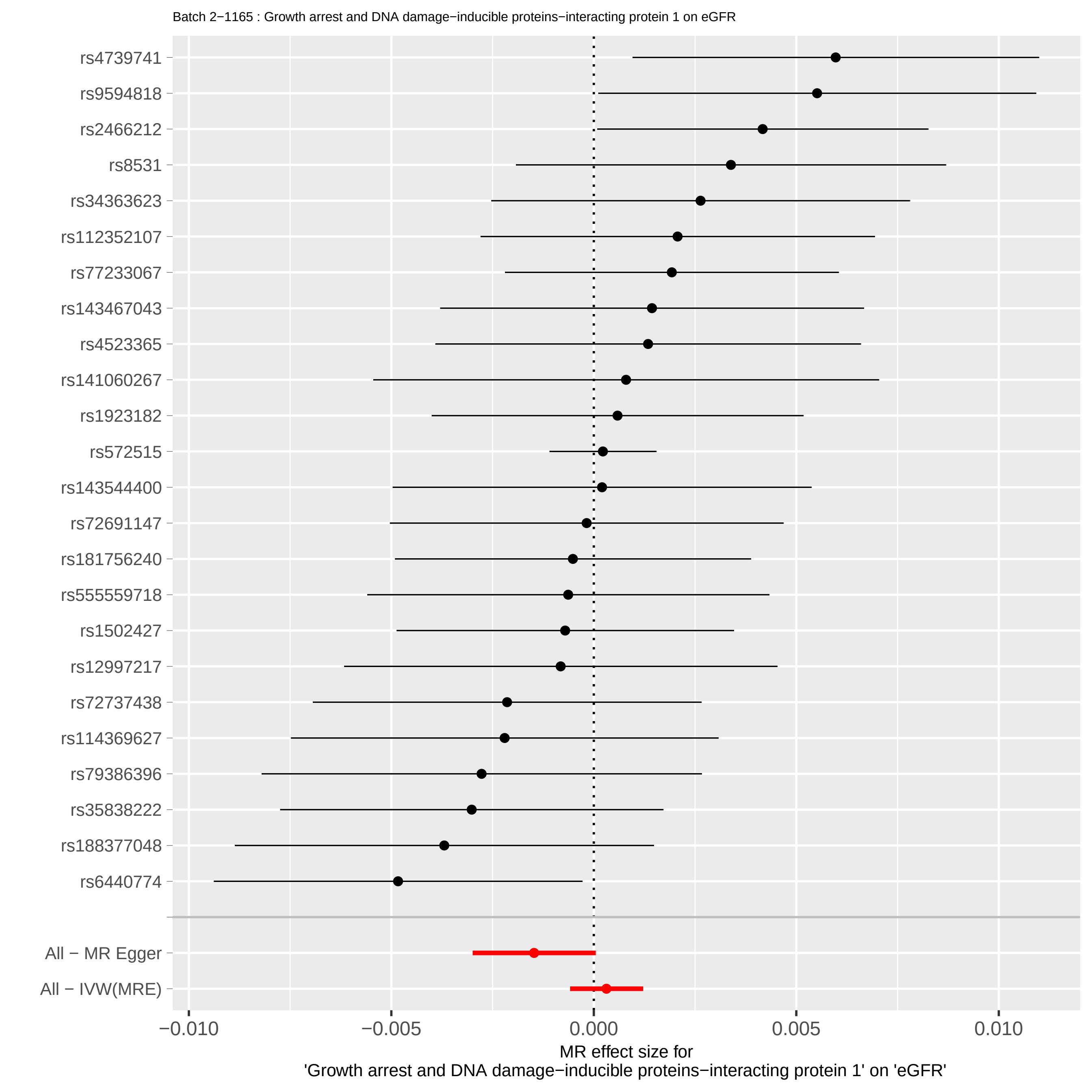

Batch 2−1165 : Growth arrest and DNA damage−inducible proteins−interacting protein 1 on eGFR
rs4739741
rs9594818
rs2466212
rs8531
rs34363623
rs112352107
rs77233067
rs143467043
rs4523365
rs141060267
rs1923182
rs572515
rs143544400
rs72691147
rs181756240
rs555559718
rs1502427
rs12997217
rs72737438
rs114369627
rs79386396
rs35838222
rs188377048
rs6440774
All − MR Egger
All − IVW(MRE)
−0.010
−0.005
0.000
0.005
0.010
MR effect size for
'Growth arrest and DNA damage−inducible proteins−interacting protein 1' on 'eGFR'

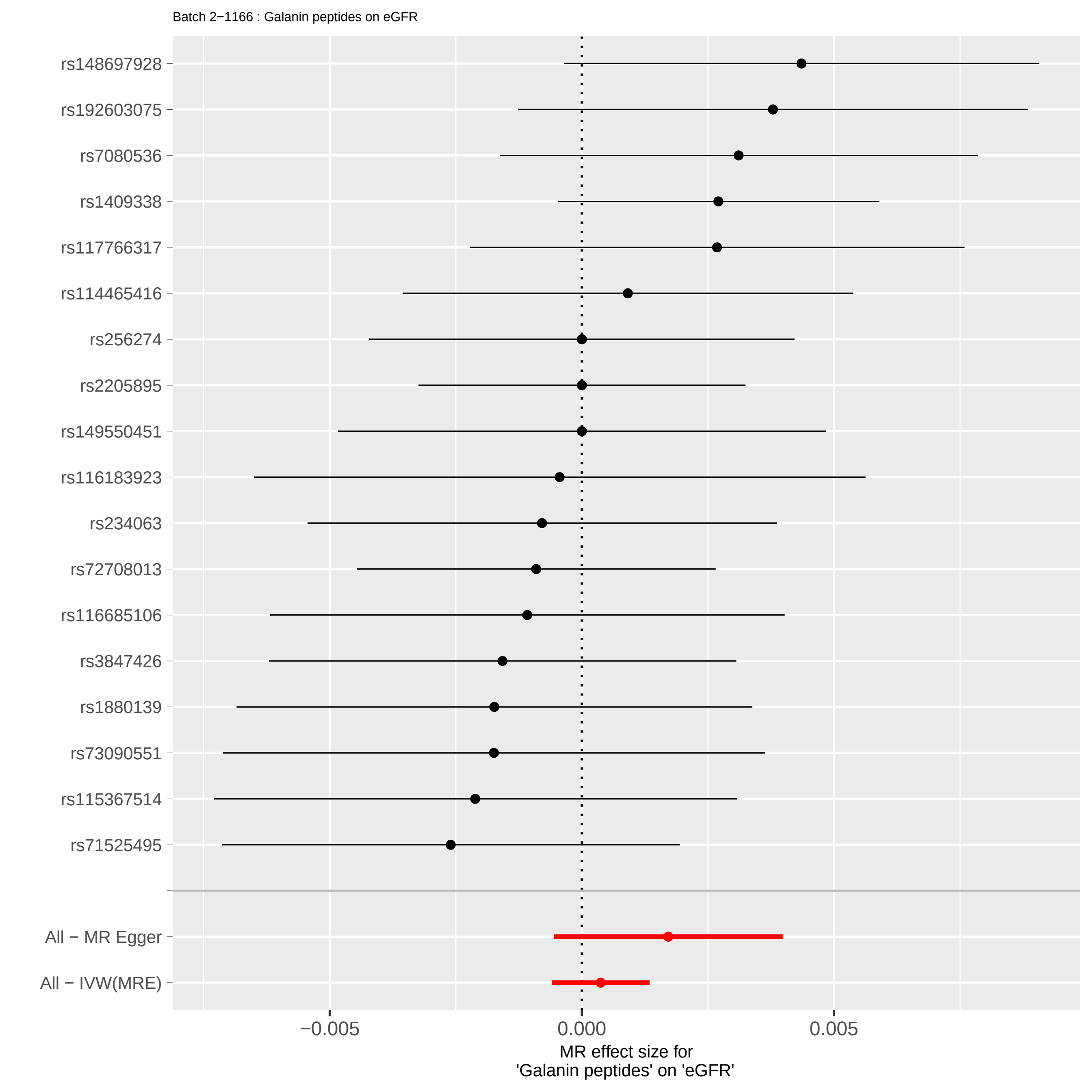

Batch 2−1166 : Galanin peptides on eGFR
rs148697928
rs192603075
rs7080536
rs1409338
rs117766317
rs114465416
rs256274
rs2205895
rs149550451
rs116183923
rs234063
rs72708013
rs116685106
rs3847426
rs1880139
rs73090551
rs115367514
rs71525495
All − MR Egger
All − IVW(MRE)
−0.005
0.000
0.005
MR effect size for
'Galanin peptides' on 'eGFR'

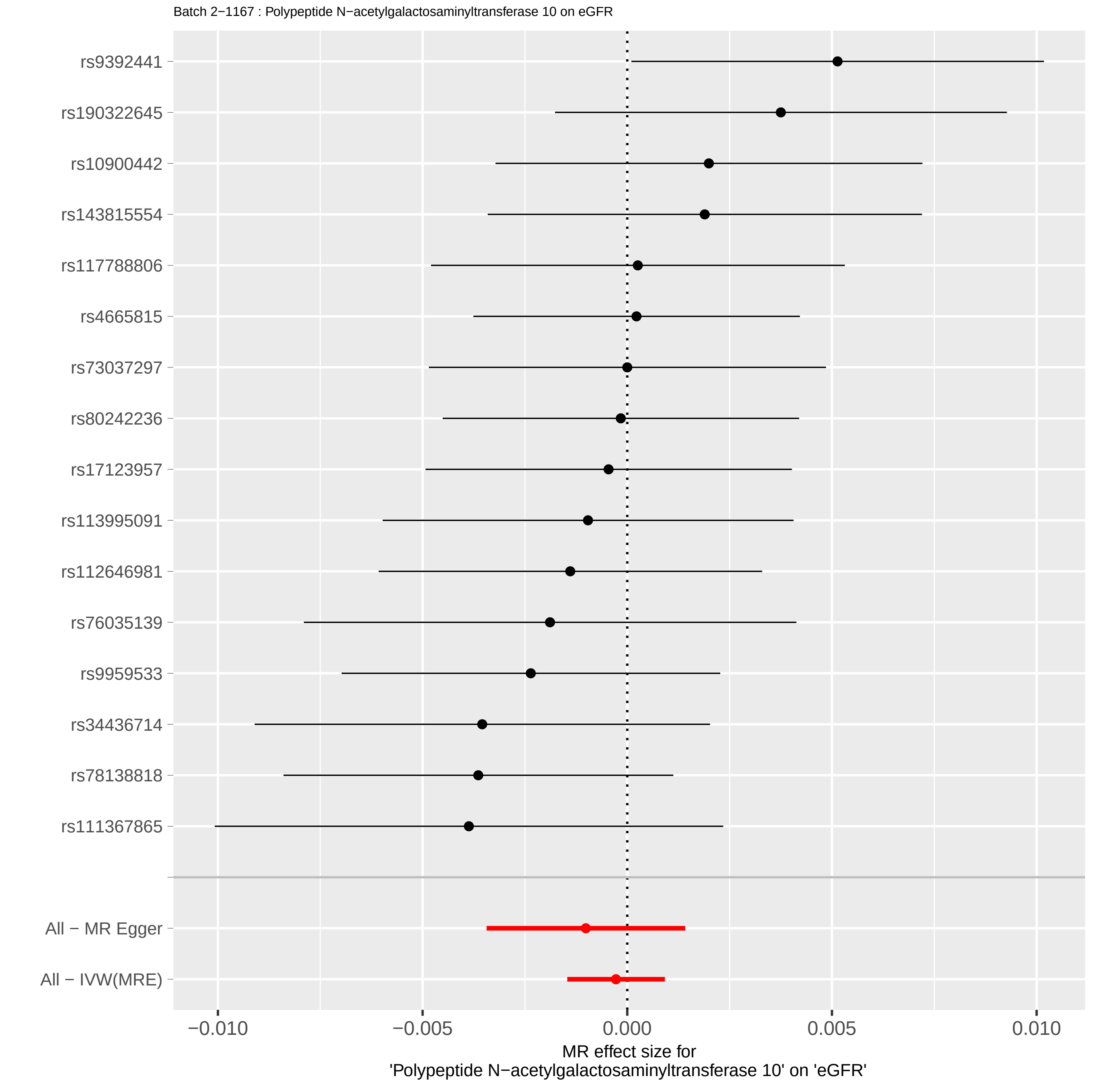

Batch 2−1167 : Polypeptide N−acetylgalactosaminyltransferase 10 on eGFR
rs9392441
rs190322645
rs10900442
rs143815554
rs117788806
rs4665815
rs73037297
rs80242236
rs17123957
rs113995091
rs112646981
rs76035139
rs9959533
rs34436714
rs78138818
rs111367865
All − MR Egger
All − IVW(MRE)
−0.010
−0.005
0.000
0.005
0.010
MR effect size for
'Polypeptide N−acetylgalactosaminyltransferase 10' on 'eGFR'

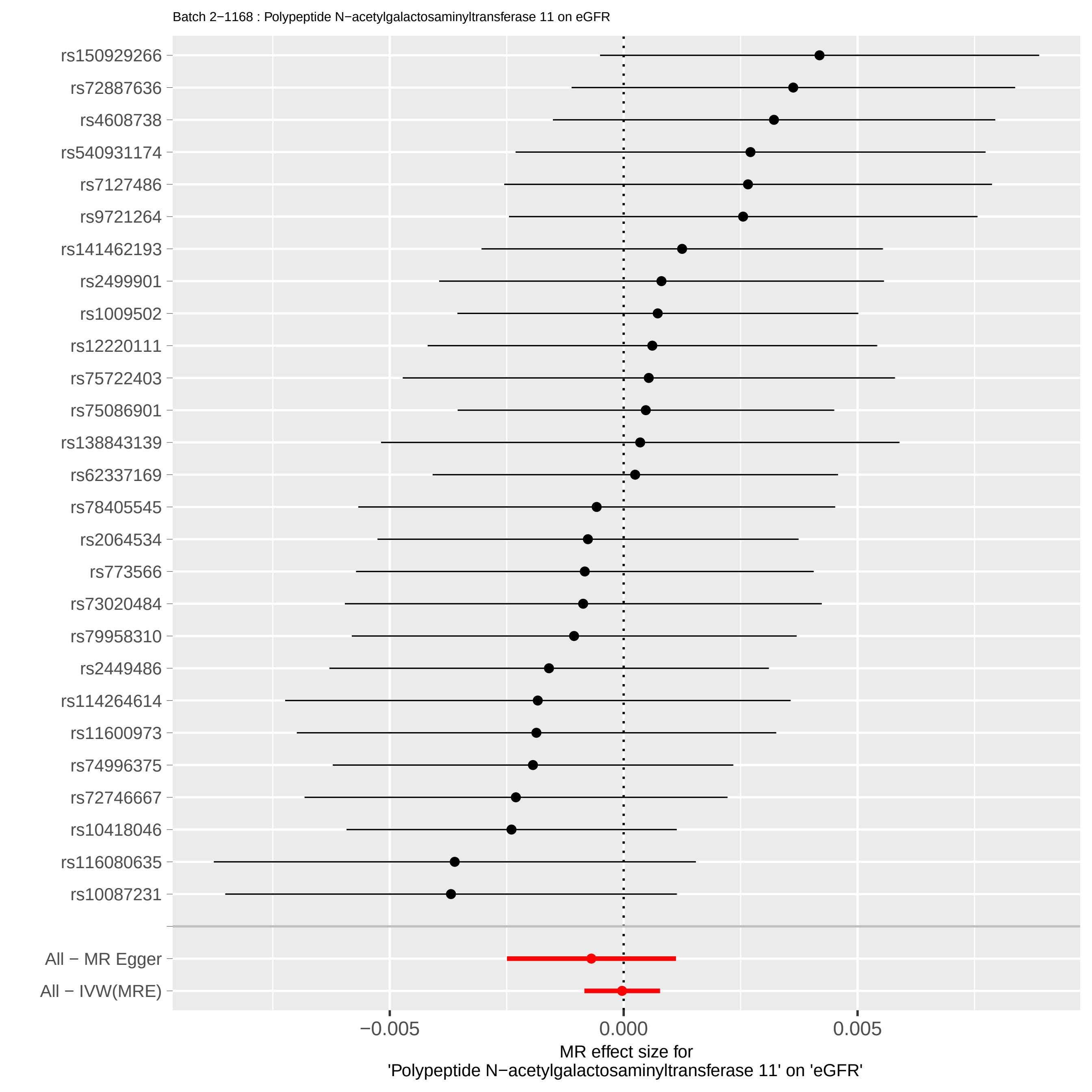

Batch 2−1168 : Polypeptide N−acetylgalactosaminyltransferase 11 on eGFR
rs150929266
rs72887636
rs4608738
rs540931174
rs7127486
rs9721264
rs141462193
rs2499901
rs1009502
rs12220111
rs75722403
rs75086901
rs138843139
rs62337169
rs78405545
rs2064534
rs773566
rs73020484
rs79958310
rs2449486
rs114264614
rs11600973
rs74996375
rs72746667
rs10418046
rs116080635
rs10087231
All − MR Egger
All − IVW(MRE)
−0.005
0.000
0.005
MR effect size for
'Polypeptide N−acetylgalactosaminyltransferase 11' on 'eGFR'

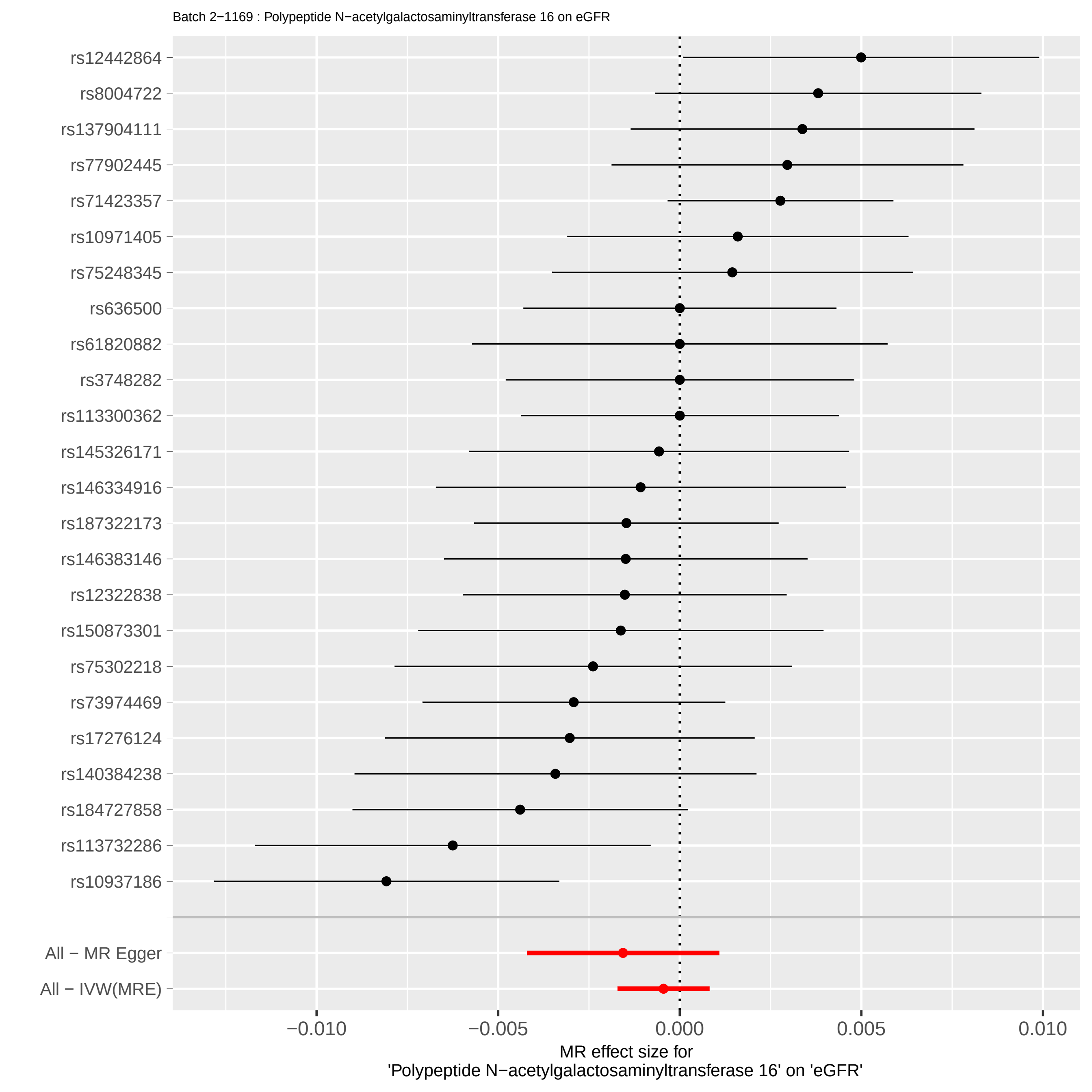

Batch 2−1169 : Polypeptide N−acetylgalactosaminyltransferase 16 on eGFR
rs12442864
rs8004722
rs137904111
rs77902445
rs71423357
rs10971405
rs75248345
rs636500
rs61820882
rs3748282
rs113300362
rs145326171
rs146334916
rs187322173
rs146383146
rs12322838
rs150873301
rs75302218
rs73974469
rs17276124
rs140384238
rs184727858
rs113732286
rs10937186
All − MR Egger
All − IVW(MRE)
−0.010
−0.005
0.000
0.005
0.010
MR effect size for
'Polypeptide N−acetylgalactosaminyltransferase 16' on 'eGFR'

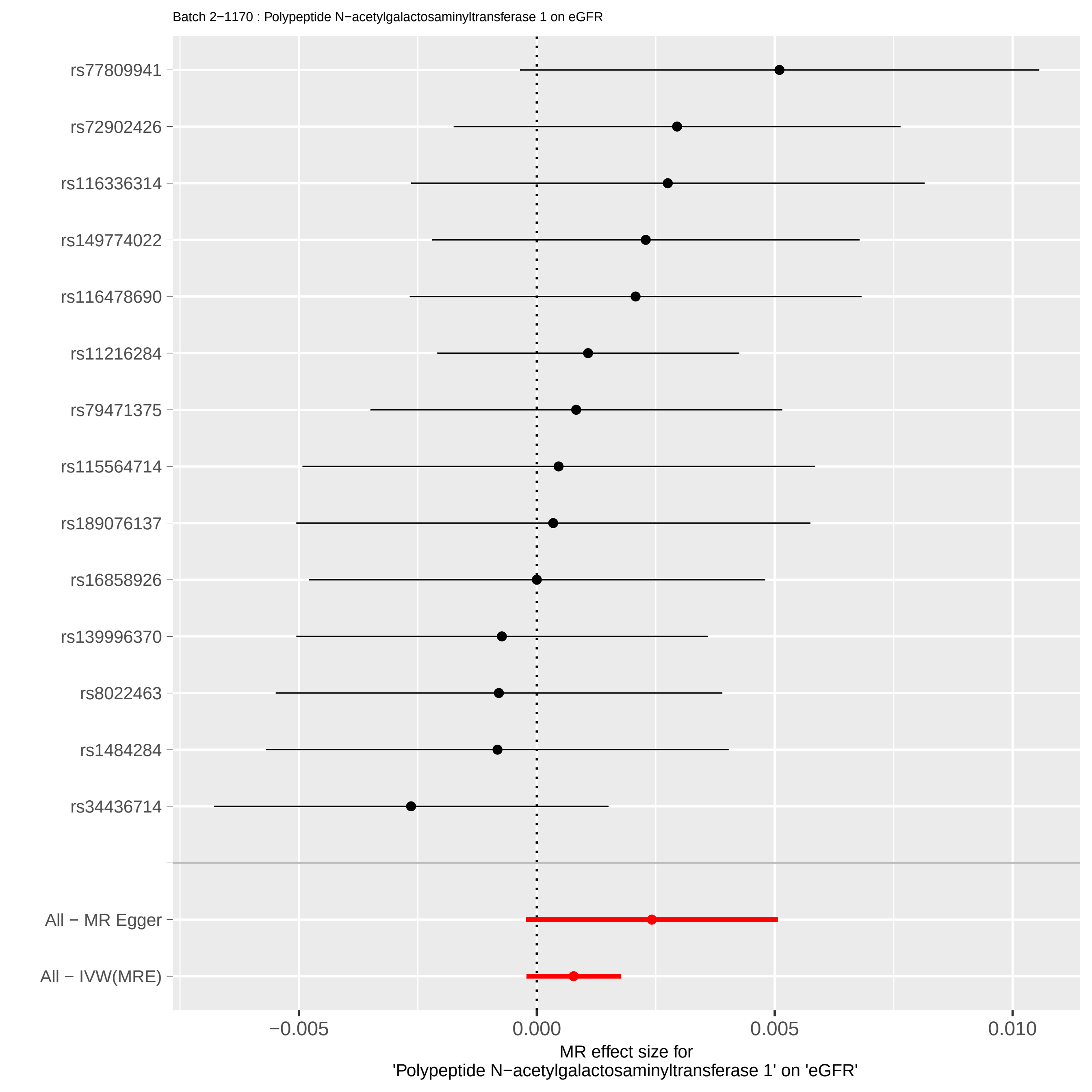
Batch 2−1170 : Polypeptide N−acetylgalactosaminyltransferase 1 on eGFR
rs77809941
rs72902426
rs116336314
rs149774022
rs116478690
rs11216284
rs79471375
rs115564714
rs189076137
rs16858926
rs139996370
rs8022463
rs1484284
rs34436714
All − MR Egger
All − IVW(MRE)
−0.005
0.000
0.005
0.010
MR effect size for
'Polypeptide N−acetylgalactosaminyltransferase 1' on 'eGFR'

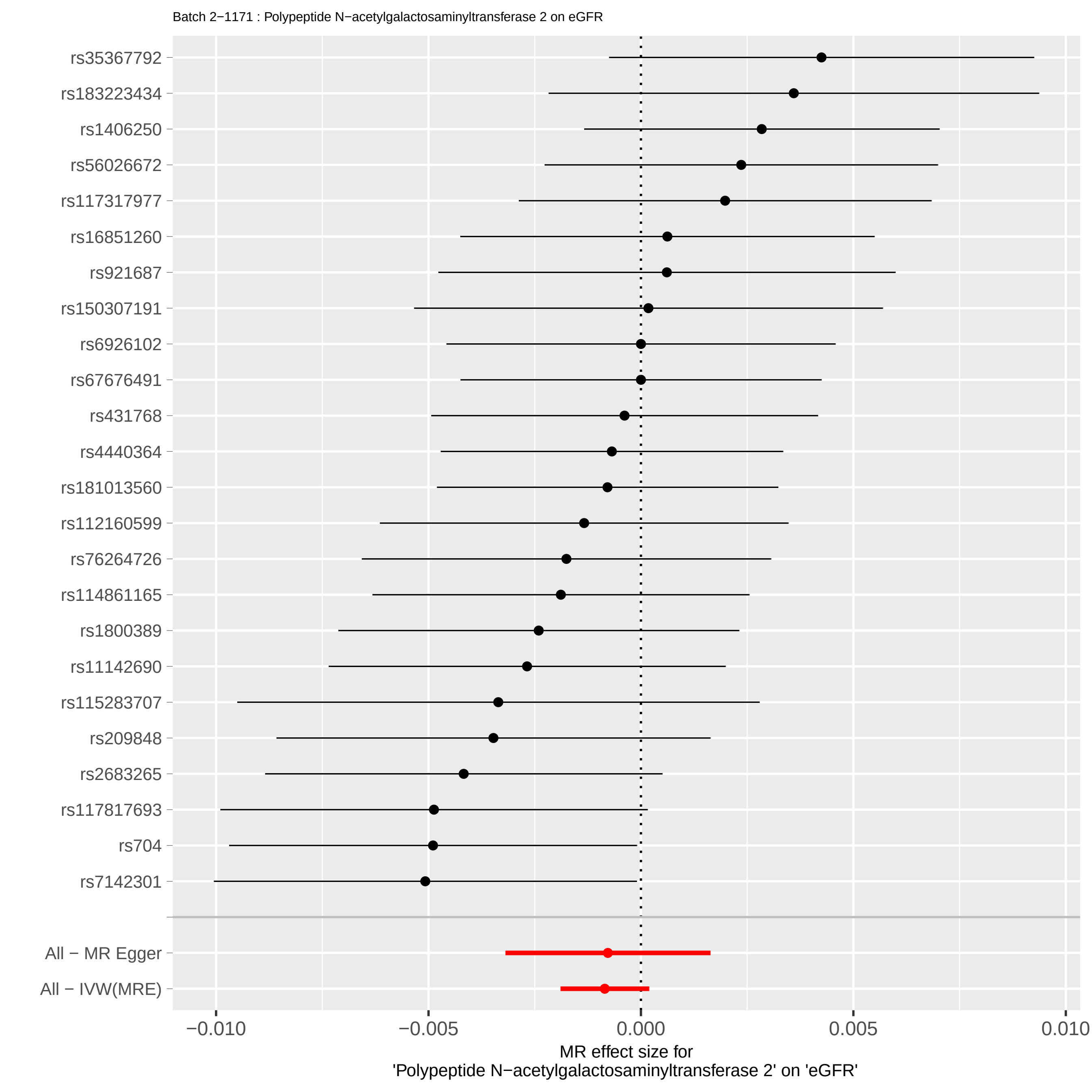

Batch 2−1171 : Polypeptide N−acetylgalactosaminyltransferase 2 on eGFR
rs35367792
rs183223434
rs1406250
rs56026672
rs117317977
rs16851260
rs921687
rs150307191
rs6926102
rs67676491
rs431768
rs4440364
rs181013560
rs112160599
rs76264726
rs114861165
rs1800389
rs11142690
rs115283707
rs209848
rs2683265
rs117817693
rs704
rs7142301
All − MR Egger
All − IVW(MRE)
−0.010
−0.005
0.000
0.005
0.010
MR effect size for
'Polypeptide N−acetylgalactosaminyltransferase 2' on 'eGFR'

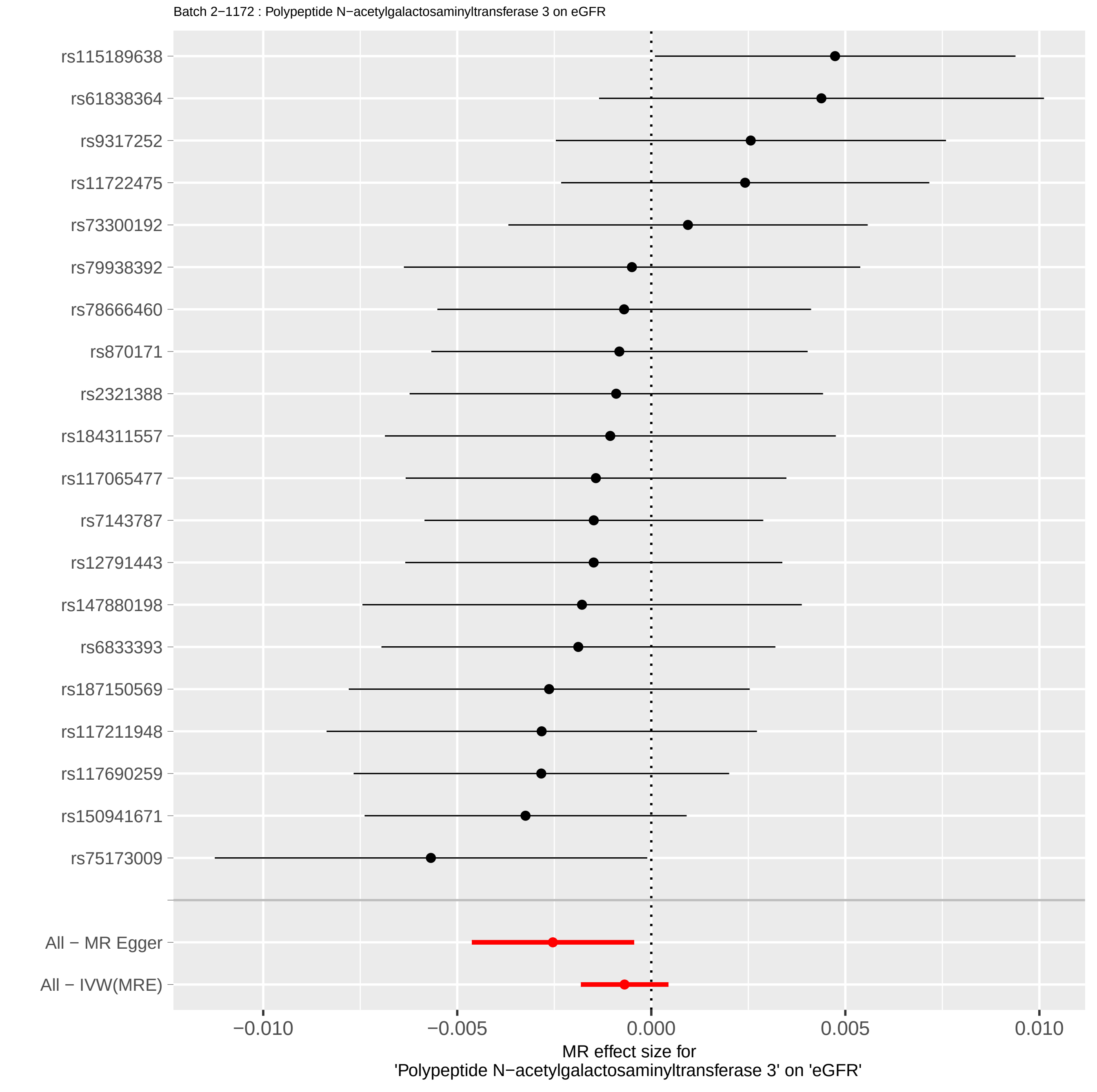

Batch 2−1172 : Polypeptide N−acetylgalactosaminyltransferase 3 on eGFR
rs115189638
rs61838364
rs9317252
rs11722475
rs73300192
rs79938392
rs78666460
rs870171
rs2321388
rs184311557
rs117065477
rs7143787
rs12791443
rs147880198
rs6833393
rs187150569
rs117211948
rs117690259
rs150941671
rs75173009
All − MR Egger
All − IVW(MRE)
−0.010
−0.005
0.000
0.005
0.010
MR effect size for
'Polypeptide N−acetylgalactosaminyltransferase 3' on 'eGFR'

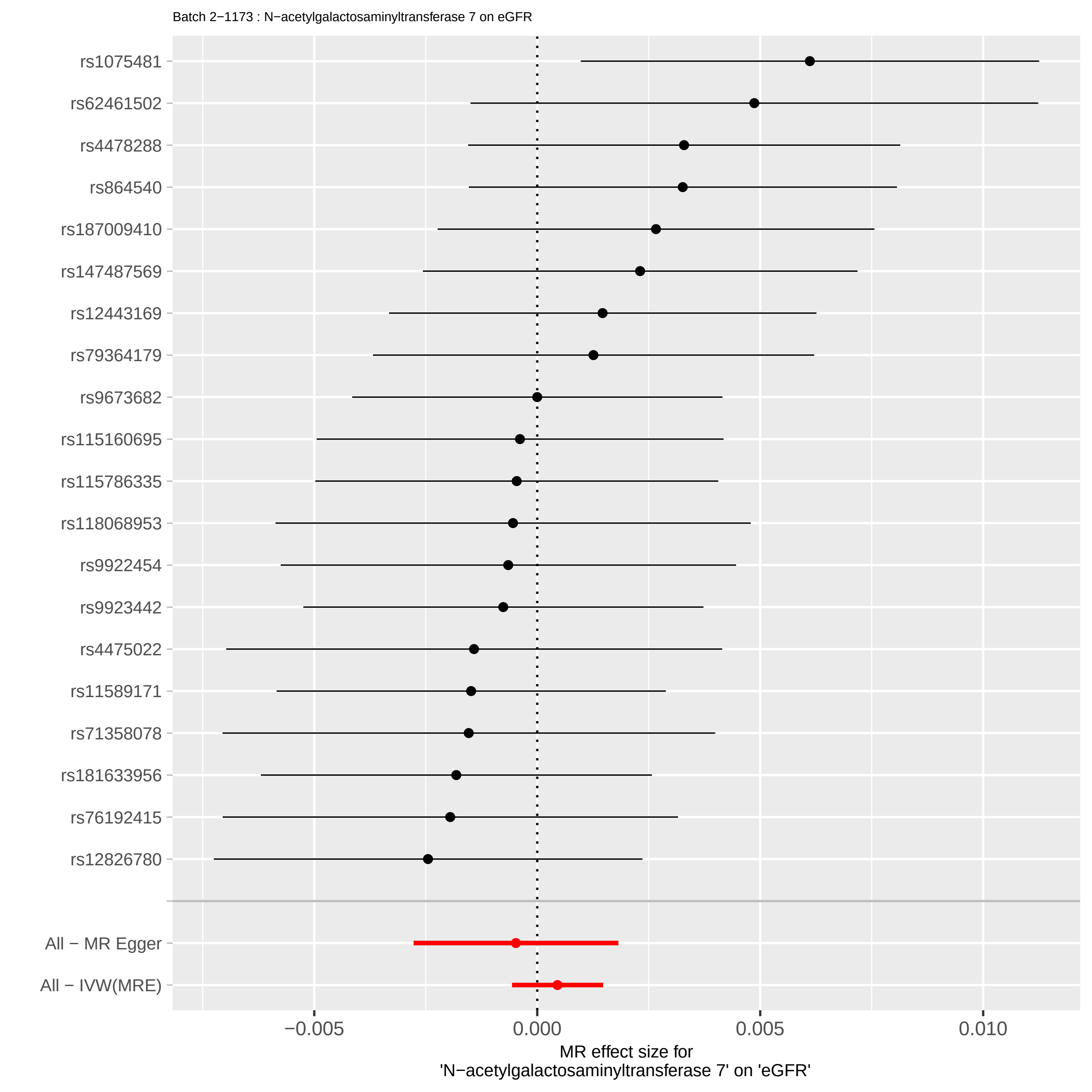

Batch 2−1173 : N−acetylgalactosaminyltransferase 7 on eGFR
rs1075481
rs62461502
rs4478288
rs864540
rs187009410
rs147487569
rs12443169
rs79364179
rs9673682
rs115160695
rs115786335
rs118068953
rs9922454
rs9923442
rs4475022
rs11589171
rs71358078
rs181633956
rs76192415
rs12826780
All − MR Egger
All − IVW(MRE)
−0.005
0.000
0.005
0.010
MR effect size for
'N−acetylgalactosaminyltransferase 7' on 'eGFR'

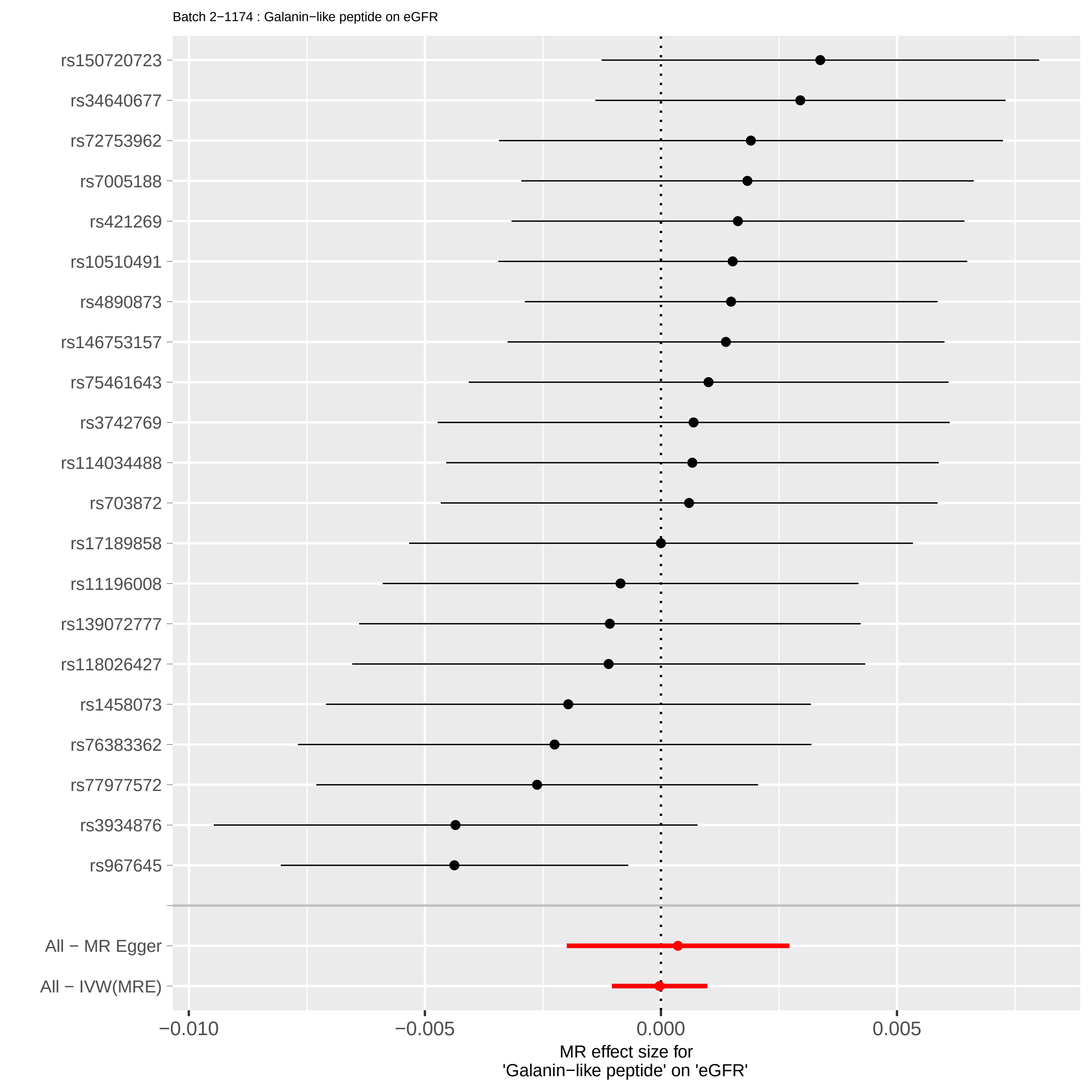
Batch 2−1174 : Galanin−like peptide on eGFR
rs150720723
rs34640677
rs72753962
rs7005188
rs421269
rs10510491
rs4890873
rs146753157
rs75461643
rs3742769
rs114034488
rs703872
rs17189858
rs11196008
rs139072777
rs118026427
rs1458073
rs76383362
rs77977572
rs3934876
rs967645
All − MR Egger
All − IVW(MRE)
−0.010
−0.005
0.000
0.005
MR effect size for
'Galanin−like peptide' on 'eGFR'

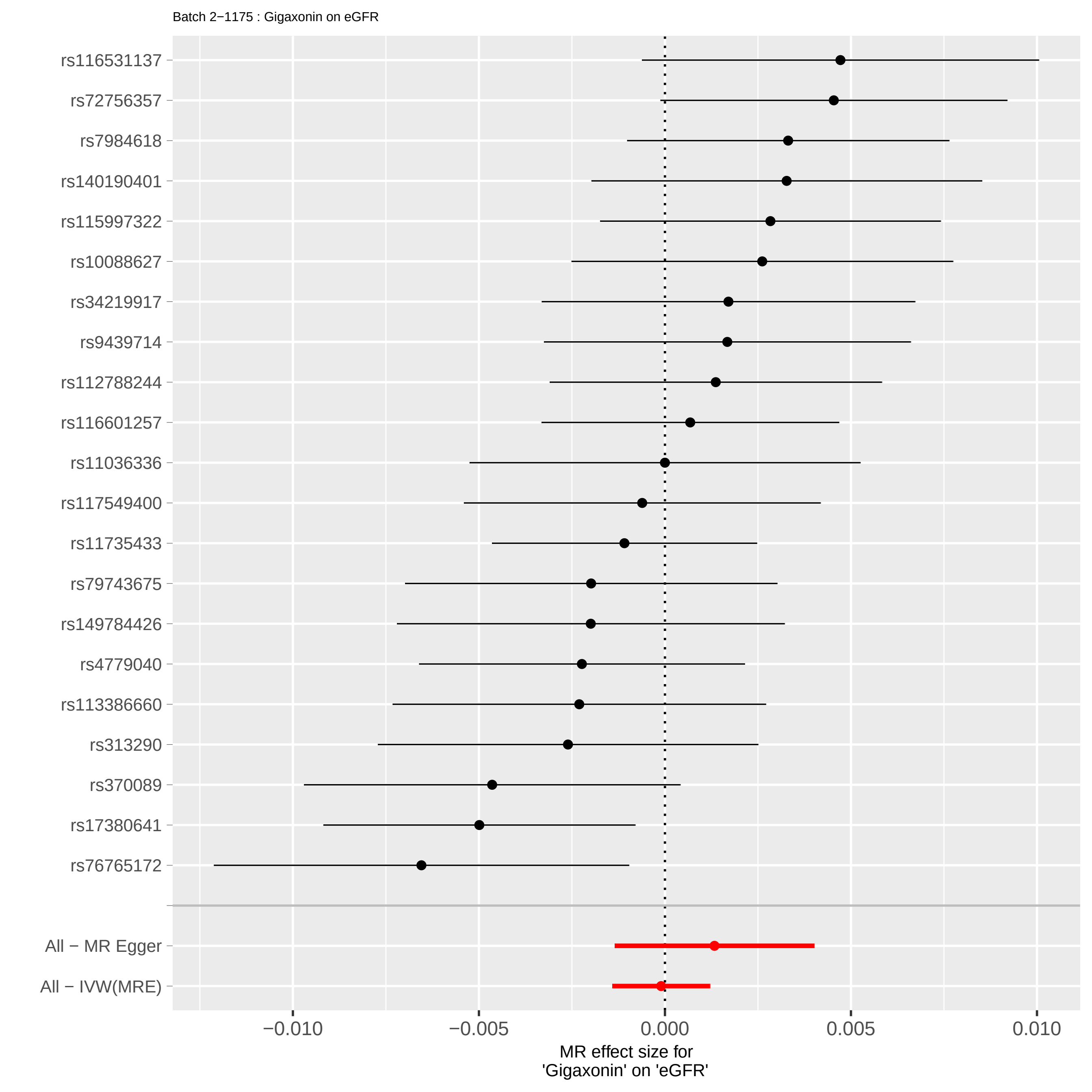

Batch 2−1175 : Gigaxonin on eGFR
rs116531137
rs72756357
rs7984618
rs140190401
rs115997322
rs10088627
rs34219917
rs9439714
rs112788244
rs116601257
rs11036336
rs117549400
rs11735433
rs79743675
rs149784426
rs4779040
rs113386660
rs313290
rs370089
rs17380641
rs76765172
All − MR Egger
All − IVW(MRE)
−0.010
−0.005
0.000
0.005
0.010
MR effect size for
'Gigaxonin' on 'eGFR'

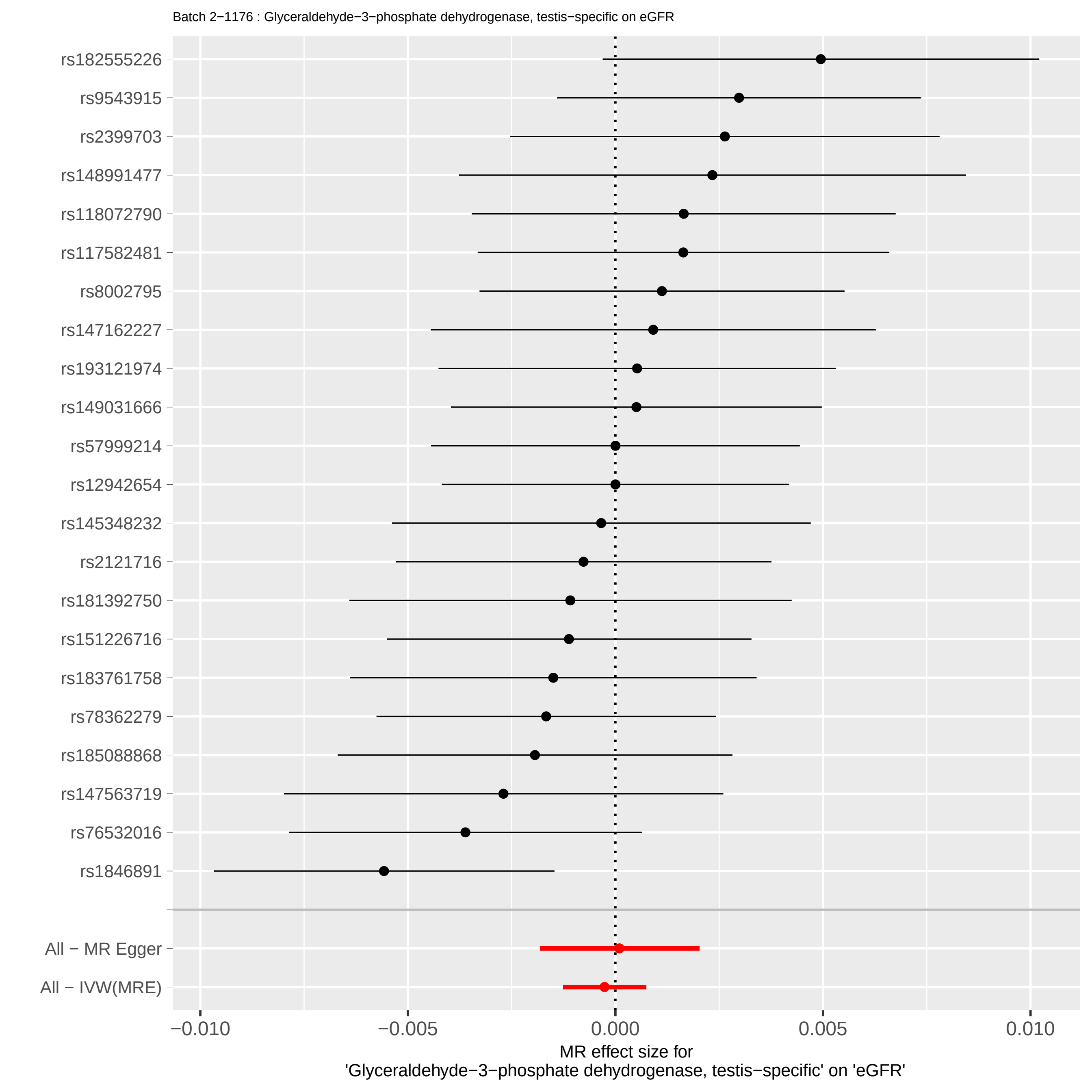

Batch 2−1176 : Glyceraldehyde−3−phosphate dehydrogenase, testis−specific on eGFR
rs182555226
rs9543915
rs2399703
rs148991477
rs118072790
rs117582481
rs8002795
rs147162227
rs193121974
rs149031666
rs57999214
rs12942654
rs145348232
rs2121716
rs181392750
rs151226716
rs183761758
rs78362279
rs185088868
rs147563719
rs76532016
rs1846891
All − MR Egger
All − IVW(MRE)
−0.010
−0.005
0.000
0.005
0.010
MR effect size for
'Glyceraldehyde−3−phosphate dehydrogenase, testis−specific' on 'eGFR'

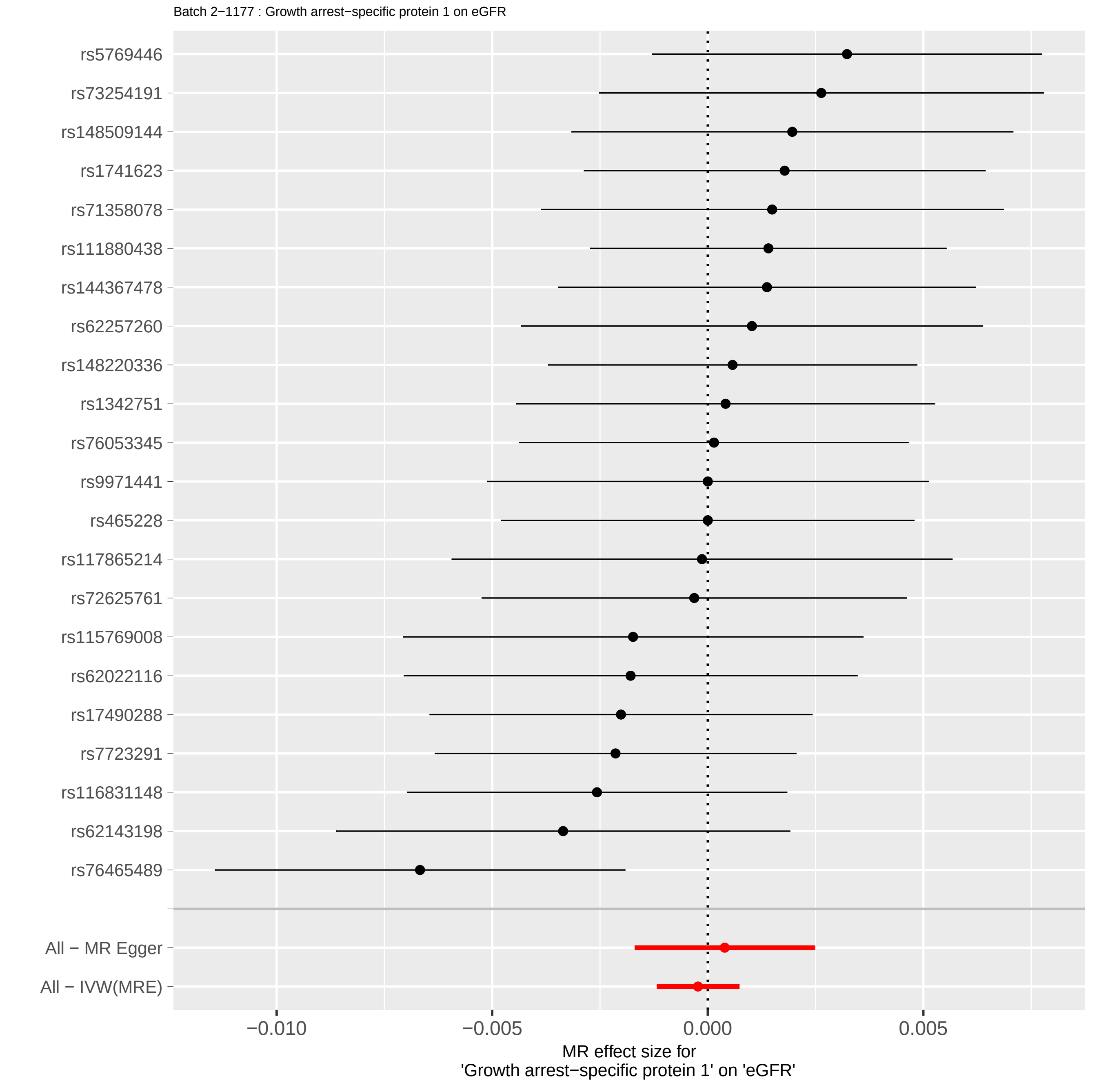

Batch 2−1177 : Growth arrest−specific protein 1 on eGFR
rs5769446
rs73254191
rs148509144
rs1741623
rs71358078
rs111880438
rs144367478
rs62257260
rs148220336
rs1342751
rs76053345
rs9971441
rs465228
rs117865214
rs72625761
rs115769008
rs62022116
rs17490288
rs7723291
rs116831148
rs62143198
rs76465489
All − MR Egger
All − IVW(MRE)
−0.010
−0.005
0.000
0.005
MR effect size for
'Growth arrest−specific protein 1' on 'eGFR'

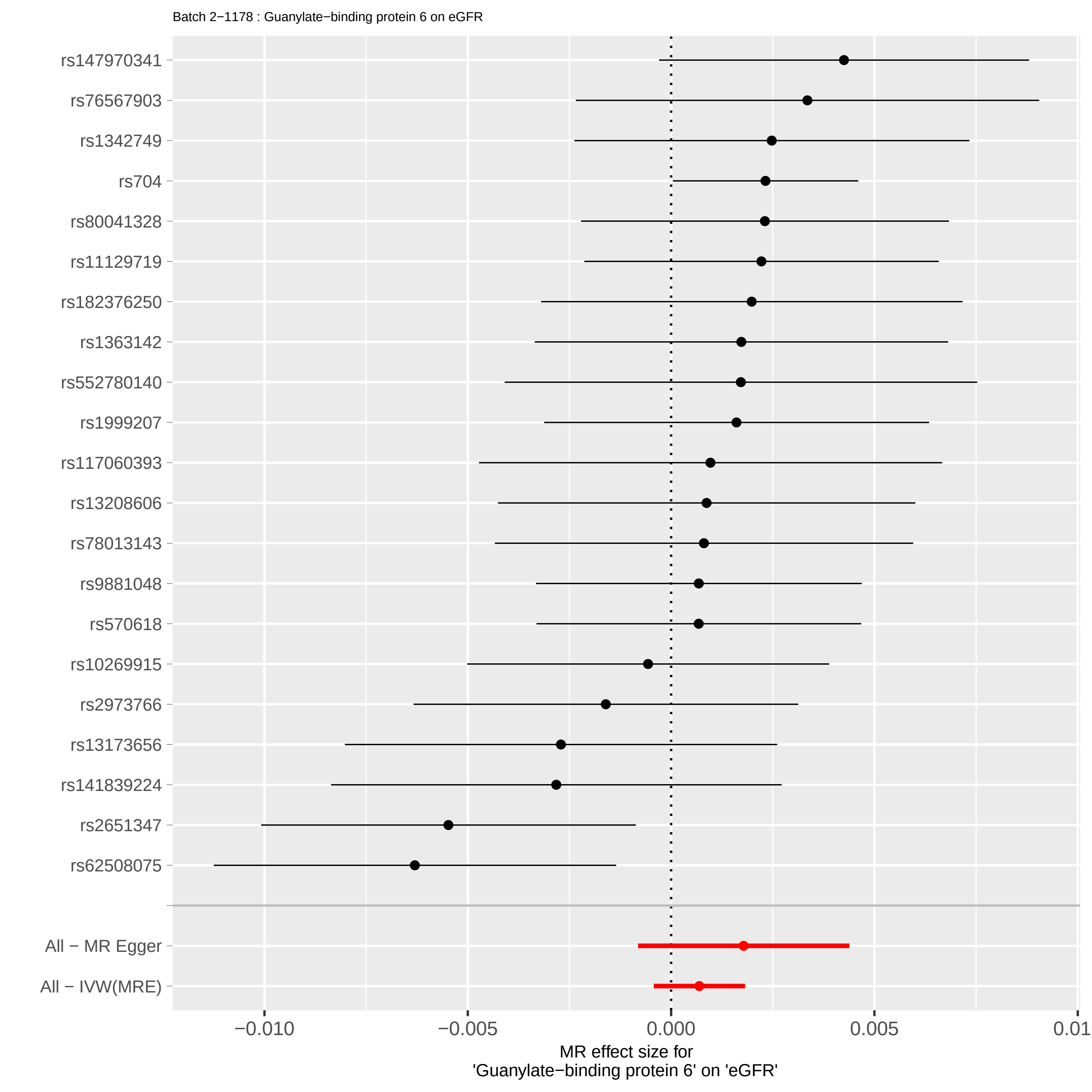

Batch 2−1178 : Guanylate−binding protein 6 on eGFR
rs147970341
rs76567903
rs1342749
rs704
rs80041328
rs11129719
rs182376250
rs1363142
rs552780140
rs1999207
rs117060393
rs13208606
rs78013143
rs9881048
rs570618
rs10269915
rs2973766
rs13173656
rs141839224
rs2651347
rs62508075
All − MR Egger
All − IVW(MRE)
−0.010
−0.005
0.000
0.005
0.01
MR effect size for
'Guanylate−binding protein 6' on 'eGFR'

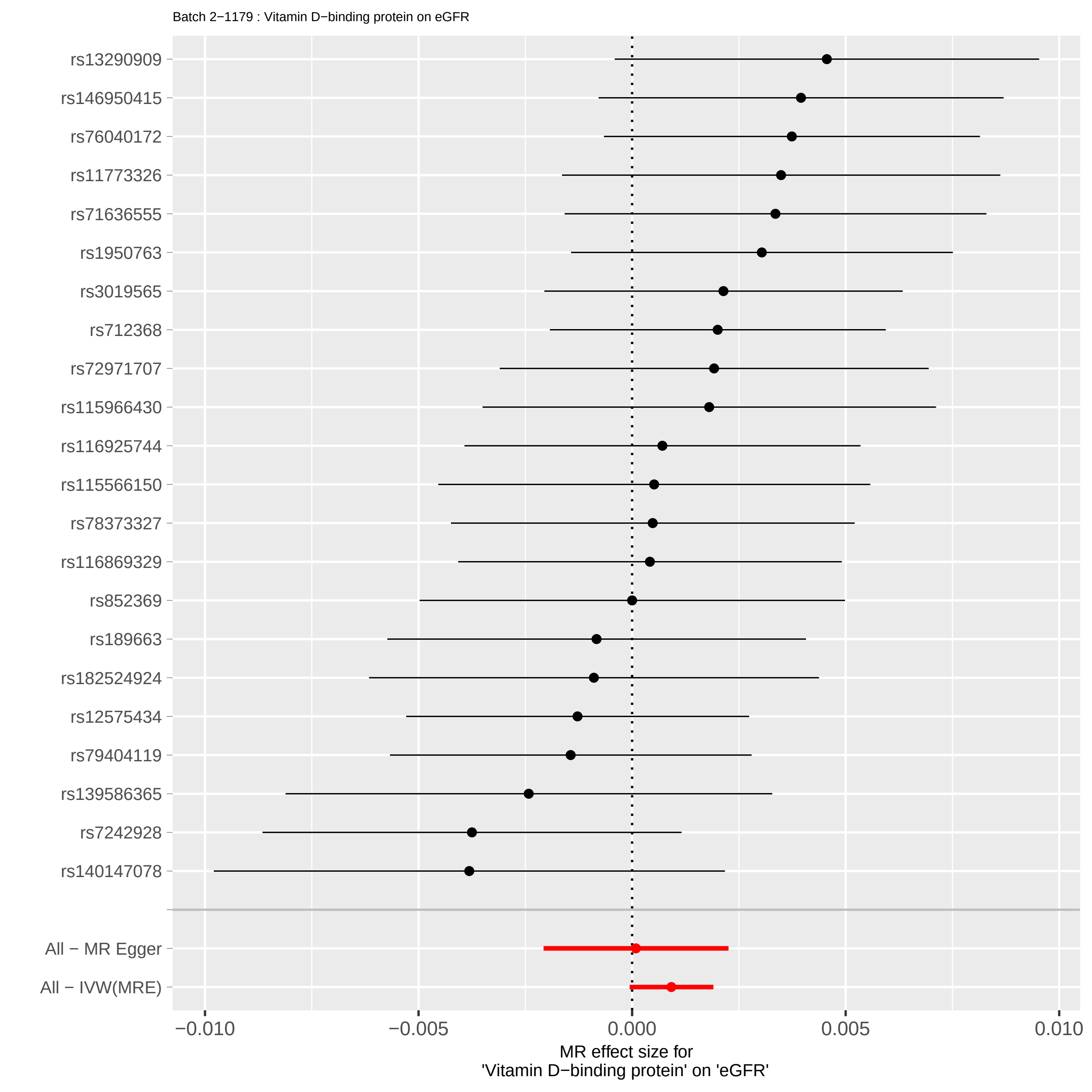

Batch 2−1179 : Vitamin D−binding protein on eGFR
rs13290909
rs146950415
rs76040172
rs11773326
rs71636555
rs1950763
rs3019565
rs712368
rs72971707
rs115966430
rs116925744
rs115566150
rs78373327
rs116869329
rs852369
rs189663
rs182524924
rs12575434
rs79404119
rs139586365
rs7242928
rs140147078
All − MR Egger
All − IVW(MRE)
−0.010
−0.005
0.000
0.005
0.010
MR effect size for
'Vitamin D−binding protein' on 'eGFR'

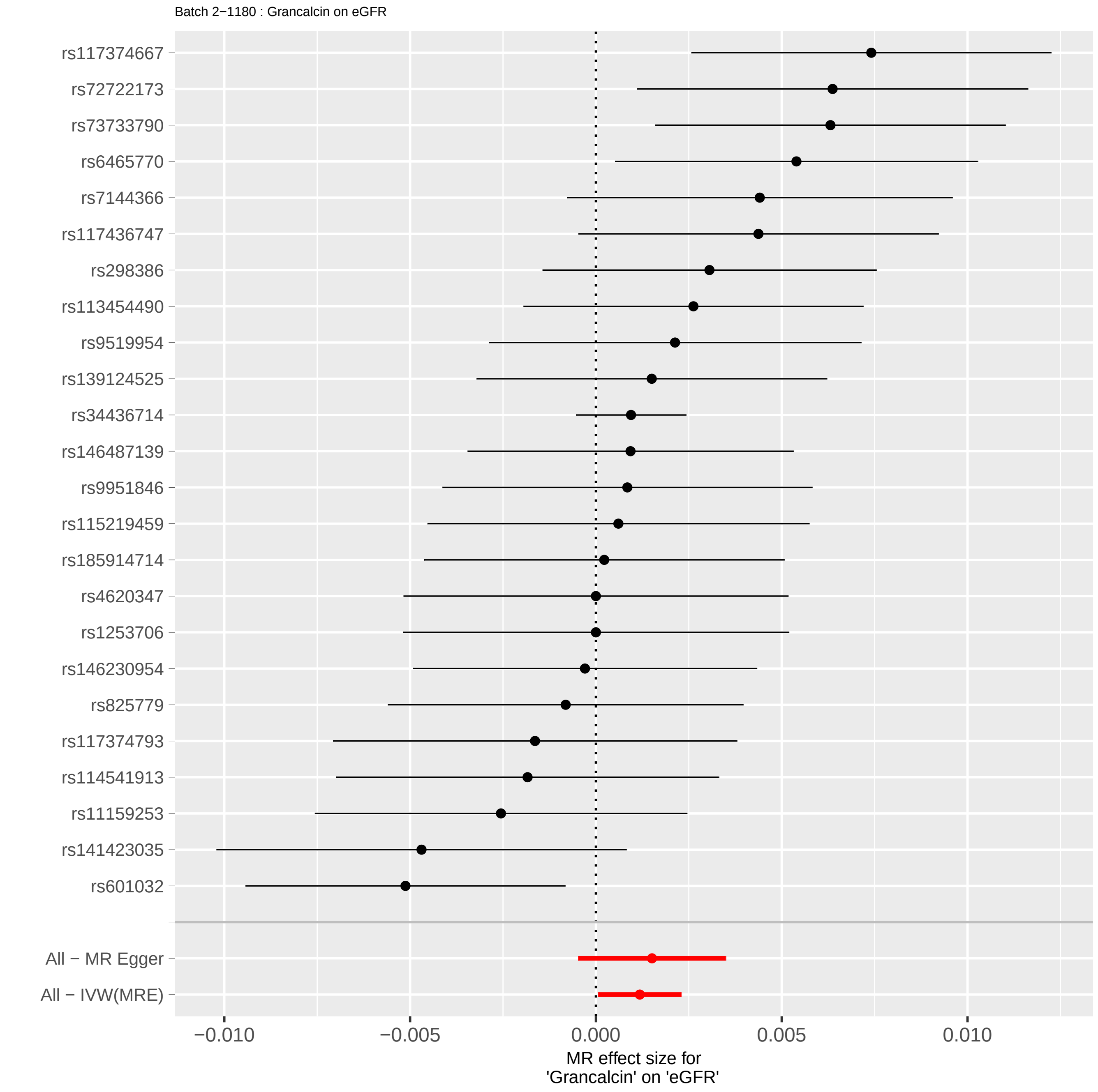

Batch 2−1180 : Grancalcin on eGFR
rs117374667
rs72722173
rs73733790
rs6465770
rs7144366
rs117436747
rs298386
rs113454490
rs9519954
rs139124525
rs34436714
rs146487139
rs9951846
rs115219459
rs185914714
rs4620347
rs1253706
rs146230954
rs825779
rs117374793
rs114541913
rs11159253
rs141423035
rs601032
All − MR Egger
All − IVW(MRE)
−0.010
−0.005
0.000
0.005
0.010
MR effect size for
'Grancalcin' on 'eGFR'

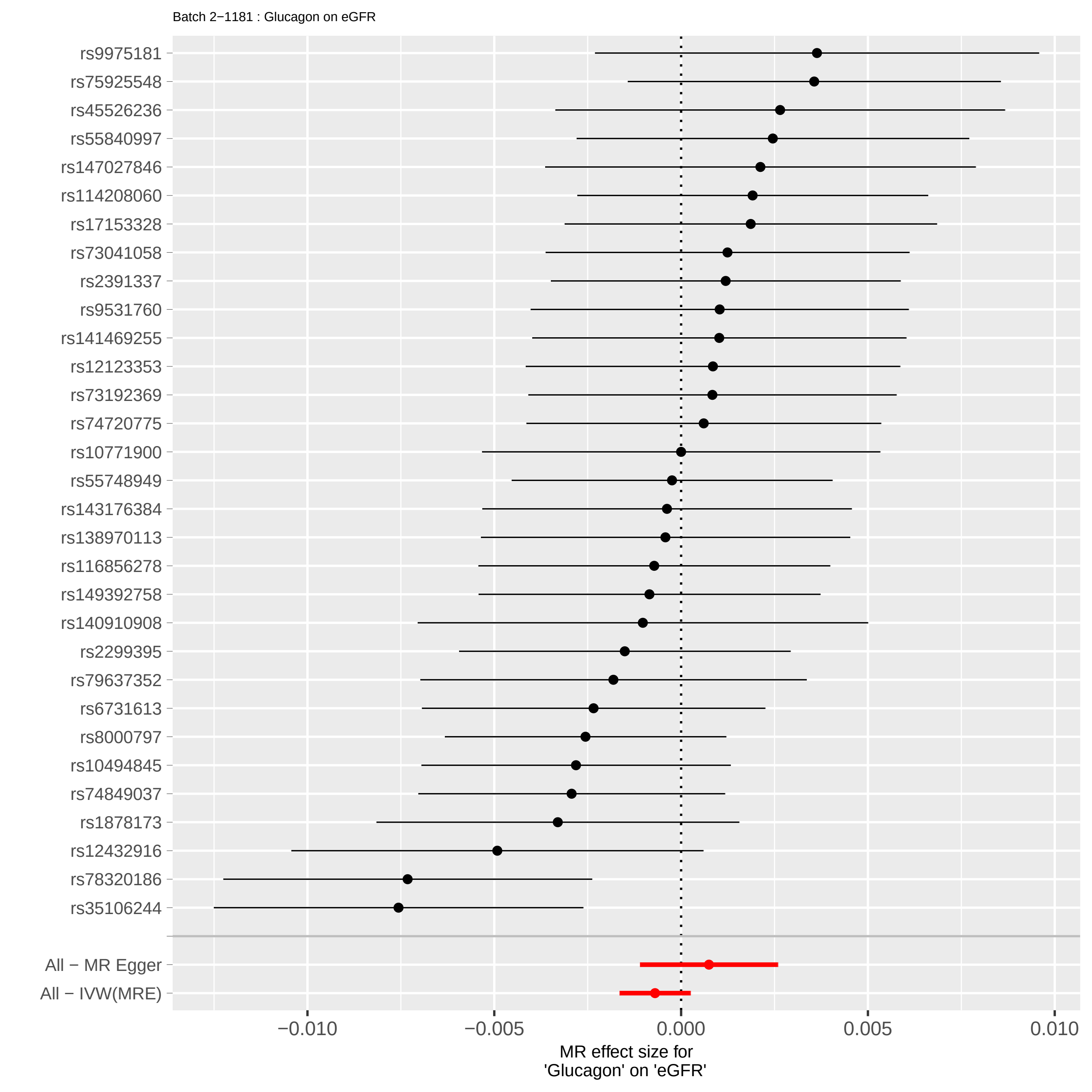

Batch 2−1181 : Glucagon on eGFR
rs9975181
rs75925548
rs45526236
rs55840997
rs147027846
rs114208060
rs17153328
rs73041058
rs2391337
rs9531760
rs141469255
rs12123353
rs73192369
rs74720775
rs10771900
rs55748949
rs143176384
rs138970113
rs116856278
rs149392758
rs140910908
rs2299395
rs79637352
rs6731613
rs8000797
rs10494845
rs74849037
rs1878173
rs12432916
rs78320186
rs35106244
All − MR Egger
All − IVW(MRE)
−0.010
−0.005
0.000
0.005
0.010
MR effect size for
'Glucagon' on 'eGFR'

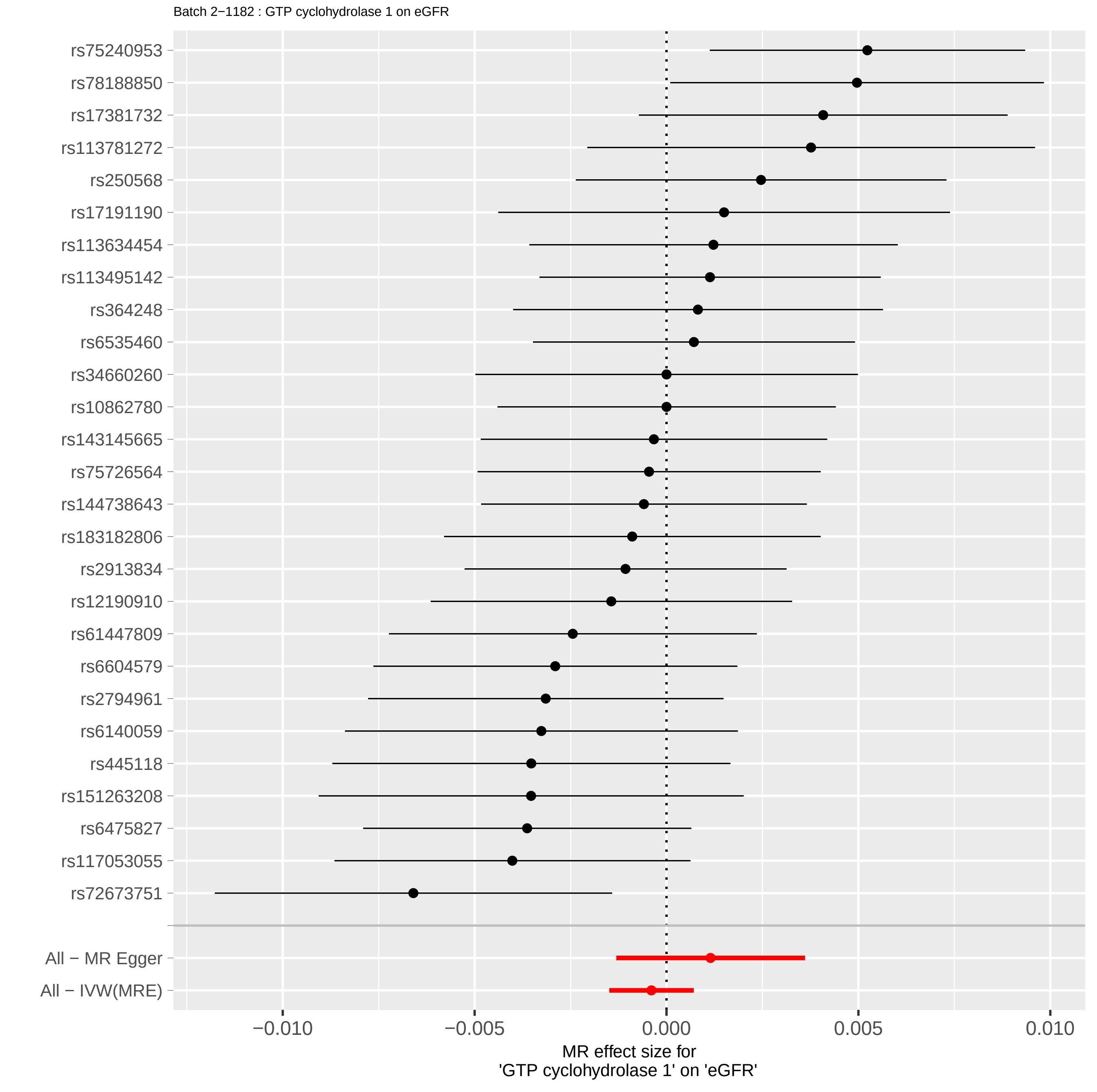

Batch 2−1182 : GTP cyclohydrolase 1 on eGFR
rs75240953
rs78188850
rs17381732
rs113781272
rs250568
rs17191190
rs113634454
rs113495142
rs364248
rs6535460
rs34660260
rs10862780
rs143145665
rs75726564
rs144738643
rs183182806
rs2913834
rs12190910
rs61447809
rs6604579
rs2794961
rs6140059
rs445118
rs151263208
rs6475827
rs117053055
rs72673751
All − MR Egger
All − IVW(MRE)
−0.010
−0.005
0.000
0.005
0.010
MR effect size for
'GTP cyclohydrolase 1' on 'eGFR'

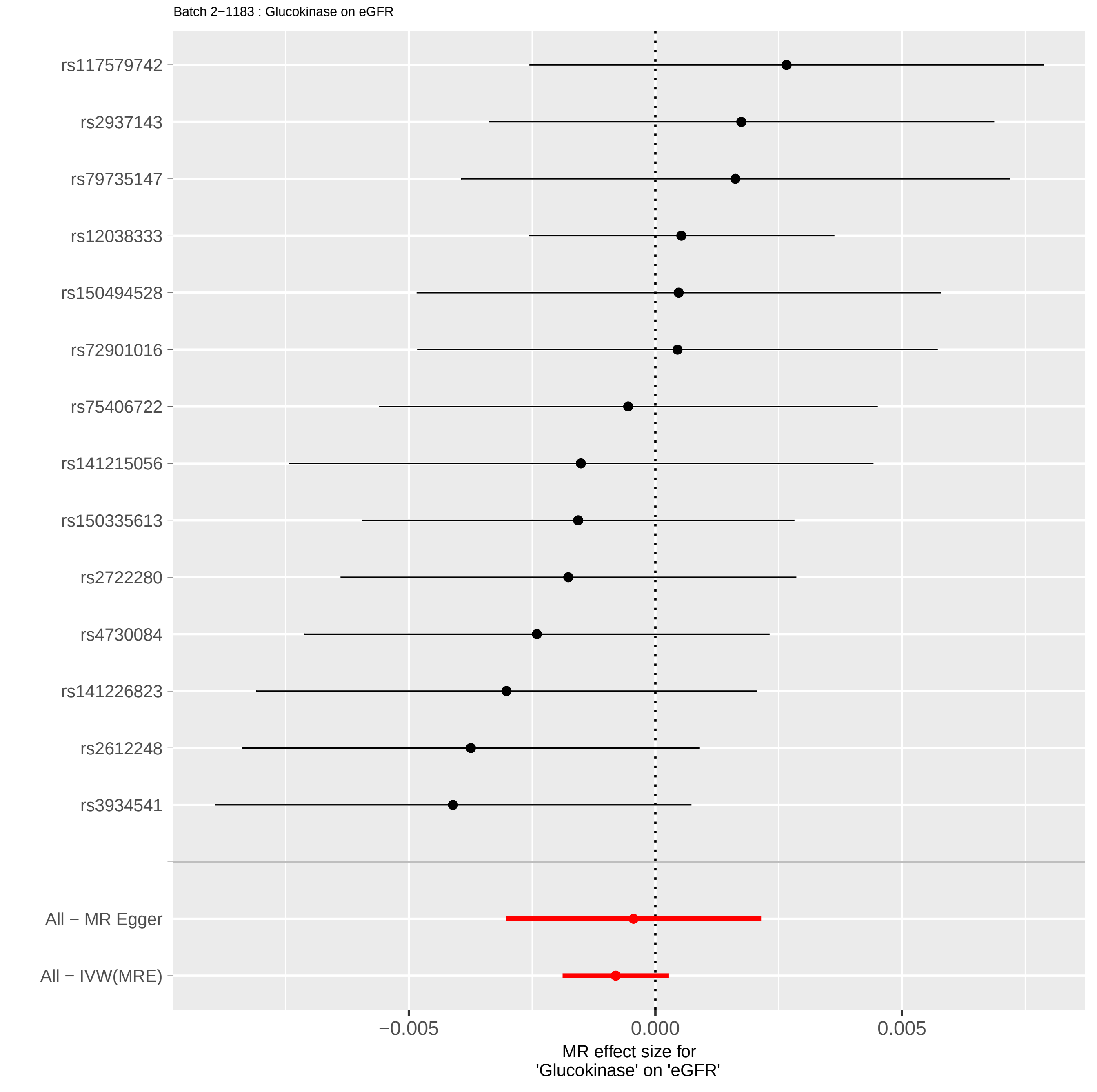

Batch 2−1183 : Glucokinase on eGFR
rs117579742
rs2937143
rs79735147
rs12038333
rs150494528
rs72901016
rs75406722
rs141215056
rs150335613
rs2722280
rs4730084
rs141226823
rs2612248
rs3934541
All − MR Egger
All − IVW(MRE)
−0.005
0.000
0.005
MR effect size for
'Glucokinase' on 'eGFR'

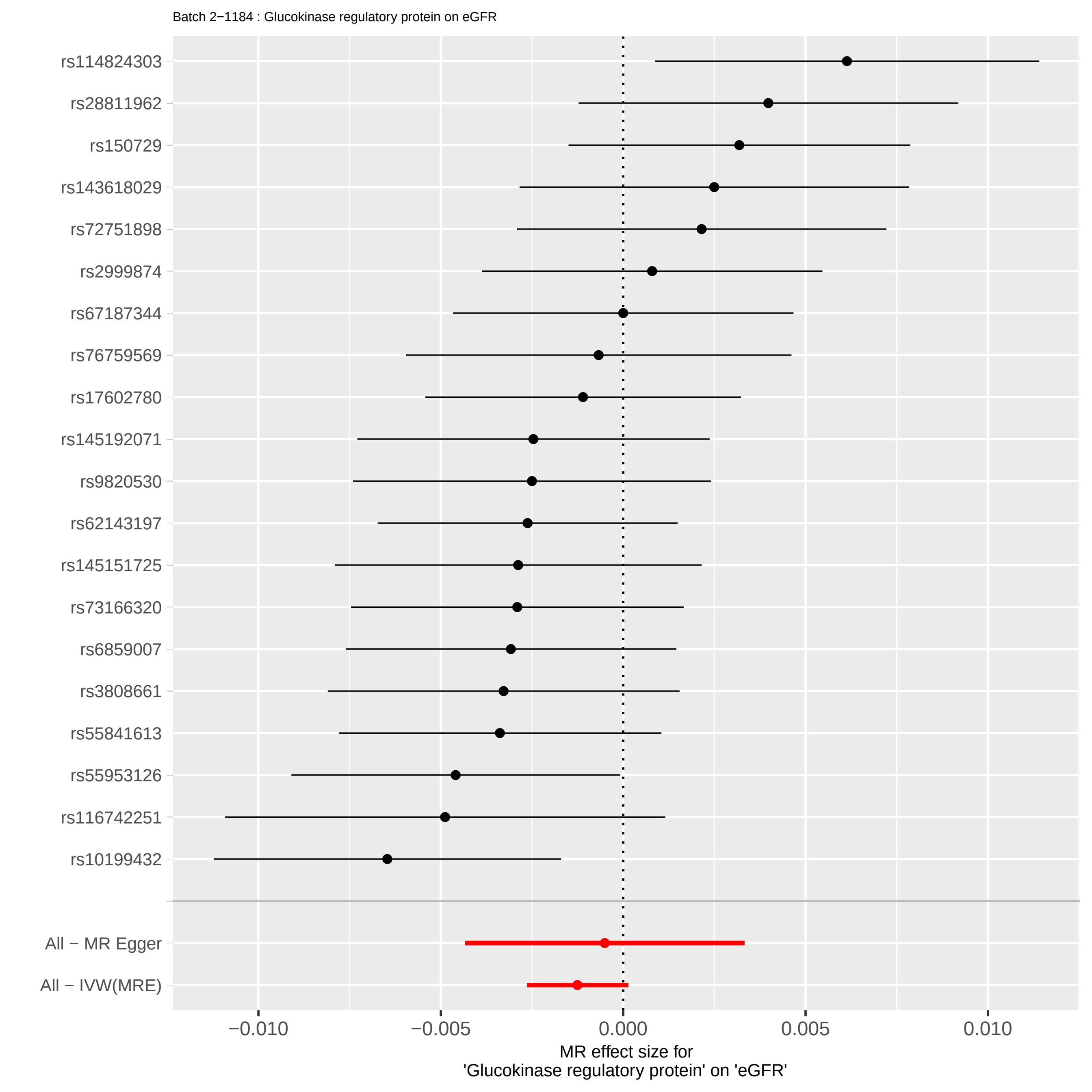

Batch 2−1184 : Glucokinase regulatory protein on eGFR
rs114824303
rs28811962
rs150729
rs143618029
rs72751898
rs2999874
rs67187344
rs76759569
rs17602780
rs145192071
rs9820530
rs62143197
rs145151725
rs73166320
rs6859007
rs3808661
rs55841613
rs55953126
rs116742251
rs10199432
All − MR Egger
All − IVW(MRE)
−0.010
−0.005
0.000
0.005
0.010
MR effect size for
'Glucokinase regulatory protein' on 'eGFR'

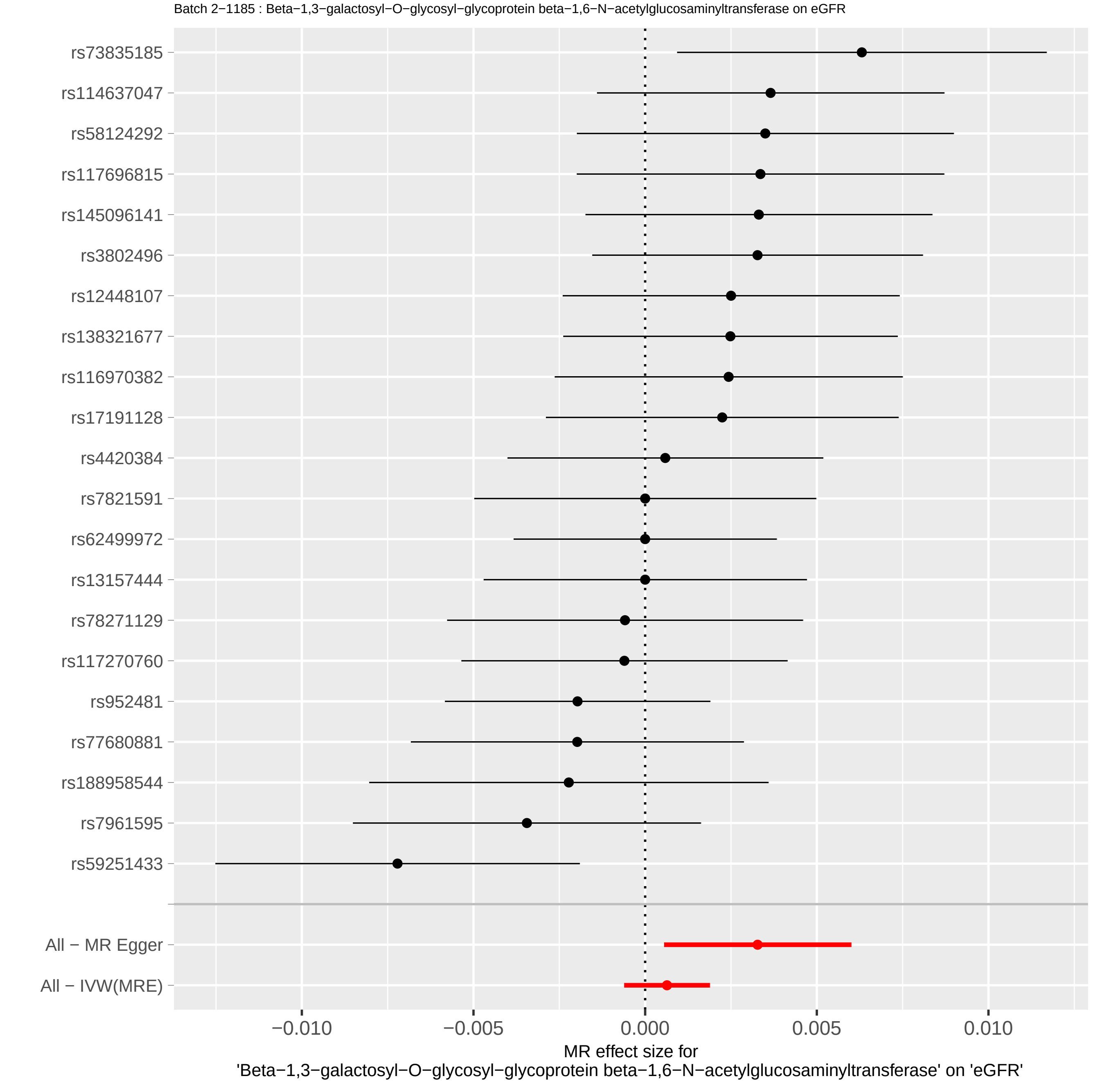

Batch 2−1185 : Beta−1,3−galactosyl−O−glycosyl−glycoprotein beta−1,6−N−acetylglucosaminyltransferase on eGFR
rs73835185
rs114637047
rs58124292
rs117696815
rs145096141
rs3802496
rs12448107
rs138321677
rs116970382
rs17191128
rs4420384
rs7821591
rs62499972
rs13157444
rs78271129
rs117270760
rs952481
rs77680881
rs188958544
rs7961595
rs59251433
All − MR Egger
All − IVW(MRE)
−0.010
−0.005
0.000
0.005
0.010
MR effect size for
'Beta−1,3−galactosyl−O−glycosyl−glycoprotein beta−1,6−N−acetylglucosaminyltransferase' on 'eGFR'

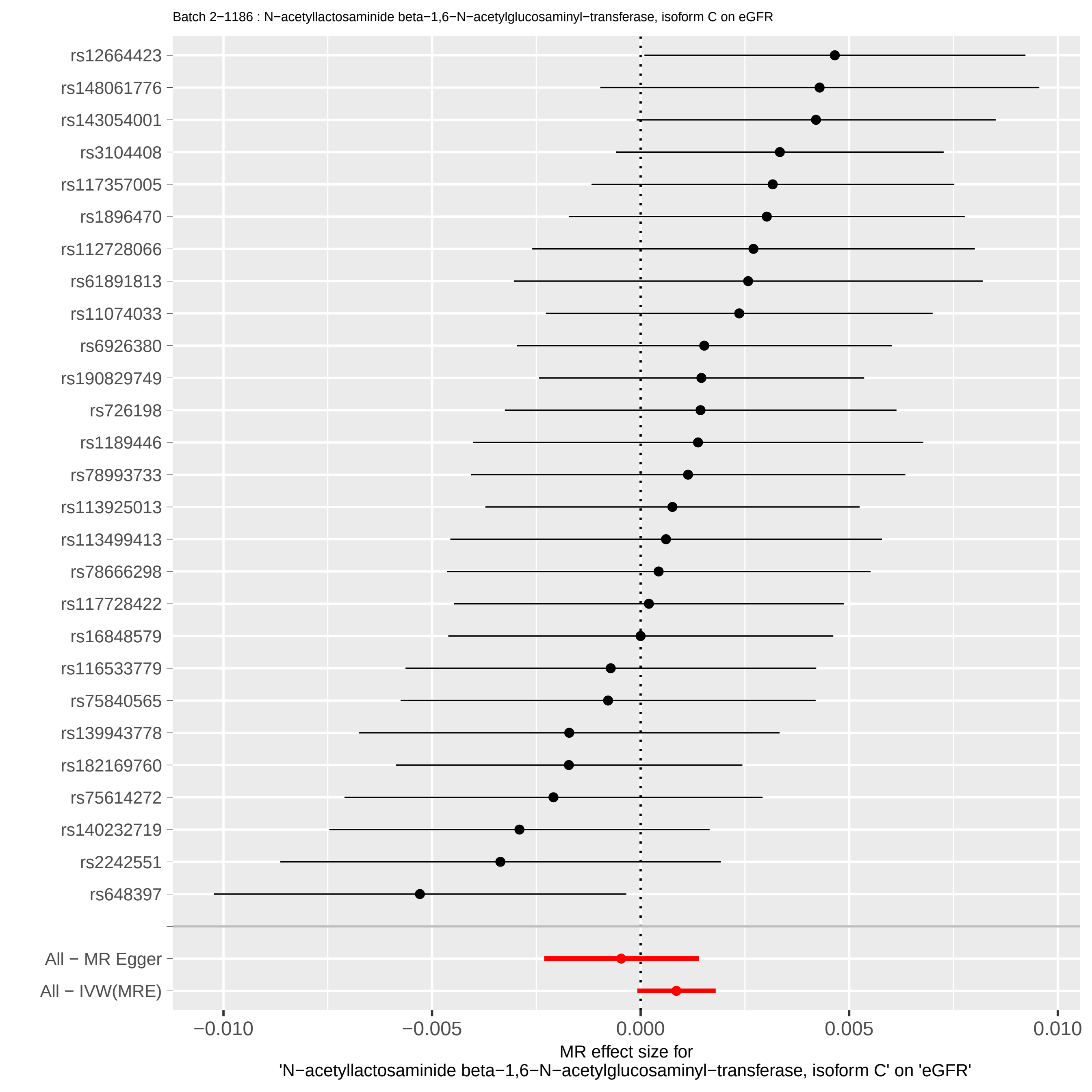

Batch 2−1186 : N−acetyllactosaminide beta−1,6−N−acetylglucosaminyl−transferase, isoform C on eGFR
rs12664423
rs148061776
rs143054001
rs3104408
rs117357005
rs1896470
rs112728066
rs61891813
rs11074033
rs6926380
rs190829749
rs726198
rs1189446
rs78993733
rs113925013
rs113499413
rs78666298
rs117728422
rs16848579
rs116533779
rs75840565
rs139943778
rs182169760
rs75614272
rs140232719
rs2242551
rs648397
All − MR Egger
All − IVW(MRE)
−0.010
−0.005
0.000
0.005
0.010
MR effect size for
'N−acetyllactosaminide beta−1,6−N−acetylglucosaminyl−transferase, isoform C' on 'eGFR'

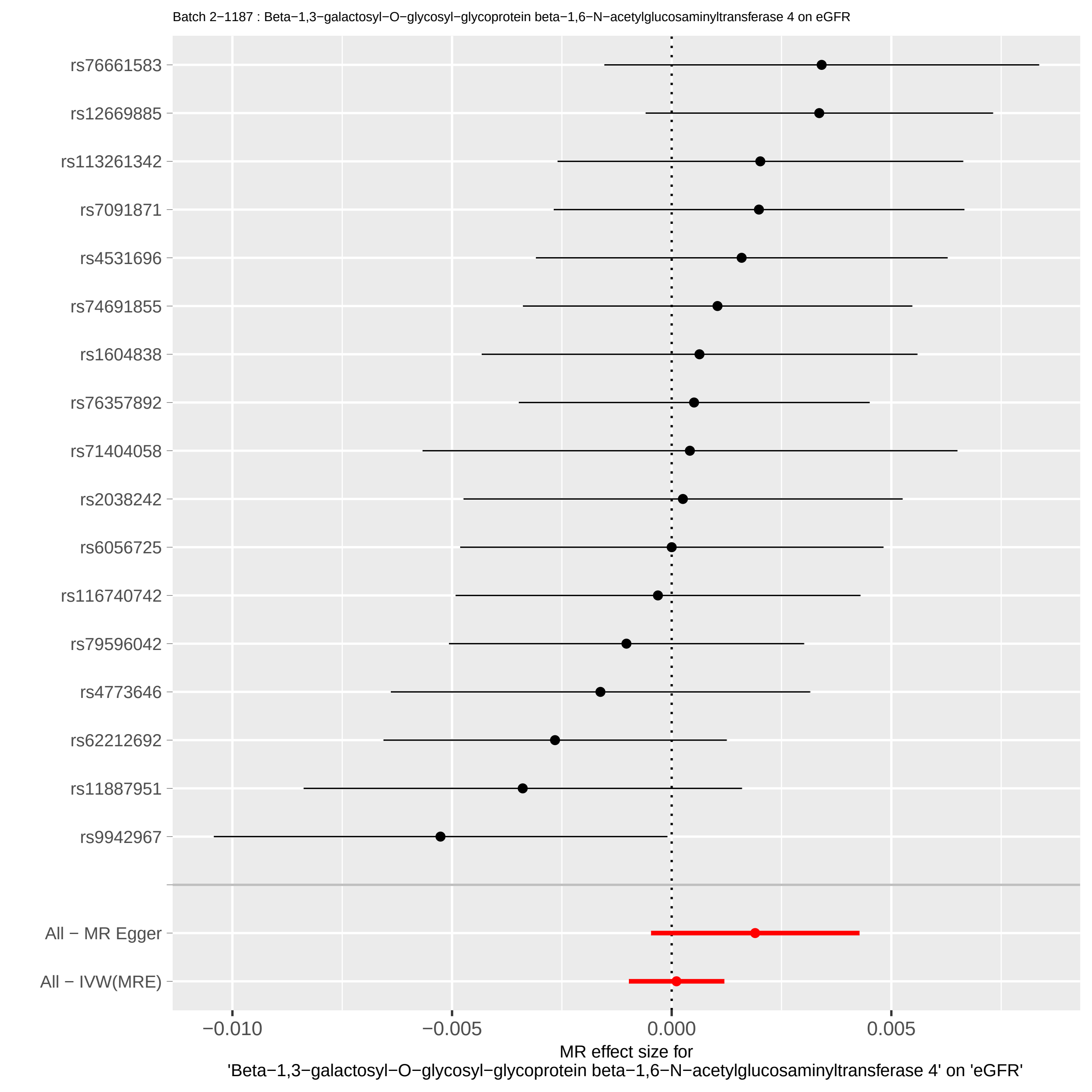

Batch 2−1187 : Beta−1,3−galactosyl−O−glycosyl−glycoprotein beta−1,6−N−acetylglucosaminyltransferase 4 on eGFR
rs76661583
rs12669885
rs113261342
rs7091871
rs4531696
rs74691855
rs1604838
rs76357892
rs71404058
rs2038242
rs6056725
rs116740742
rs79596042
rs4773646
rs62212692
rs11887951
rs9942967
All − MR Egger
All − IVW(MRE)
−0.010
−0.005
0.000
0.005
MR effect size for
'Beta−1,3−galactosyl−O−glycosyl−glycoprotein beta−1,6−N−acetylglucosaminyltransferase 4' on 'eGFR'

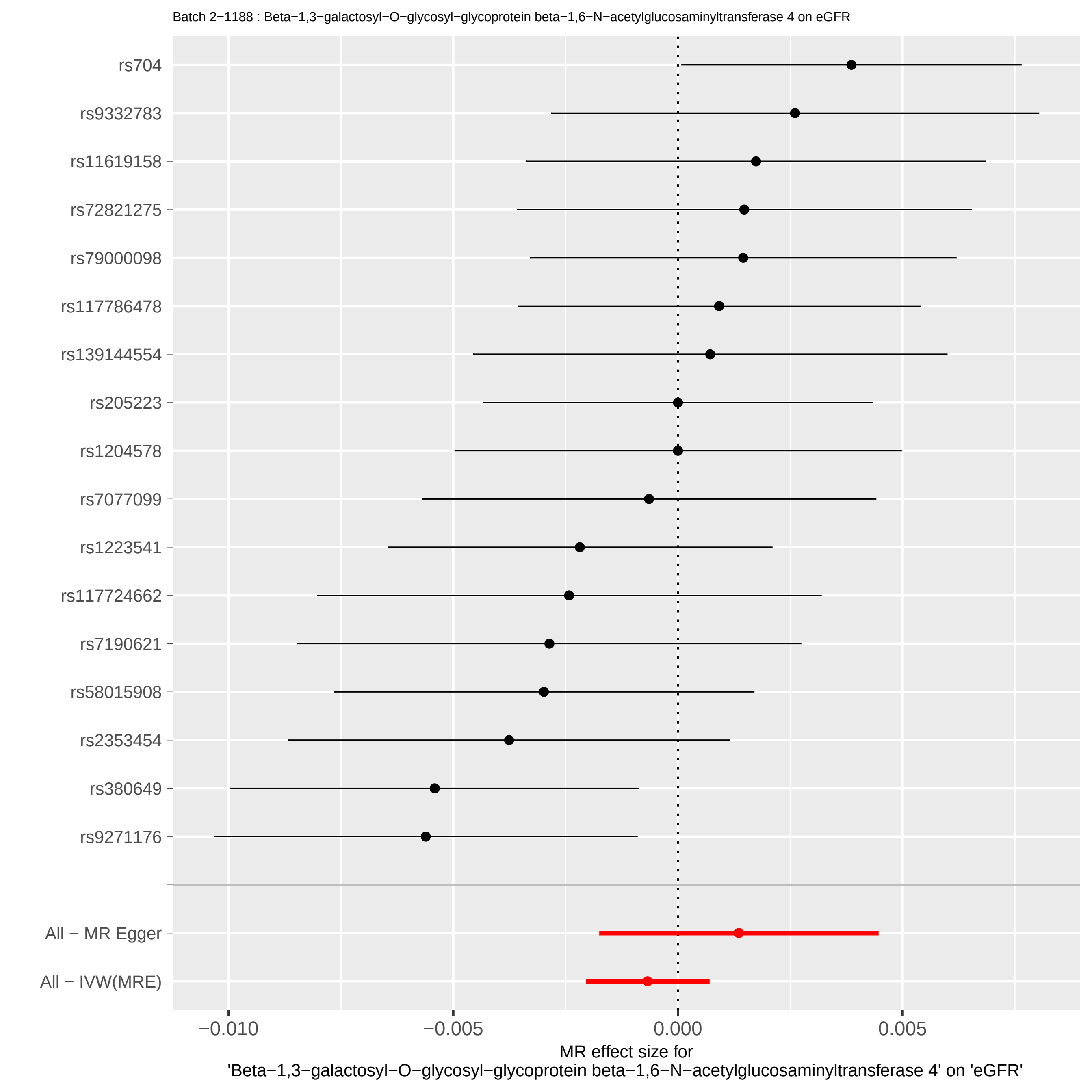

Batch 2−1188 : Beta−1,3−galactosyl−O−glycosyl−glycoprotein beta−1,6−N−acetylglucosaminyltransferase 4 on eGFR
rs704
rs9332783
rs11619158
rs72821275
rs79000098
rs117786478
rs139144554
rs205223
rs1204578
rs7077099
rs1223541
rs117724662
rs7190621
rs58015908
rs2353454
rs380649
rs9271176
All − MR Egger
All − IVW(MRE)
−0.010
−0.005
0.000
0.005
MR effect size for
'Beta−1,3−galactosyl−O−glycosyl−glycoprotein beta−1,6−N−acetylglucosaminyltransferase 4' on 'eGFR'

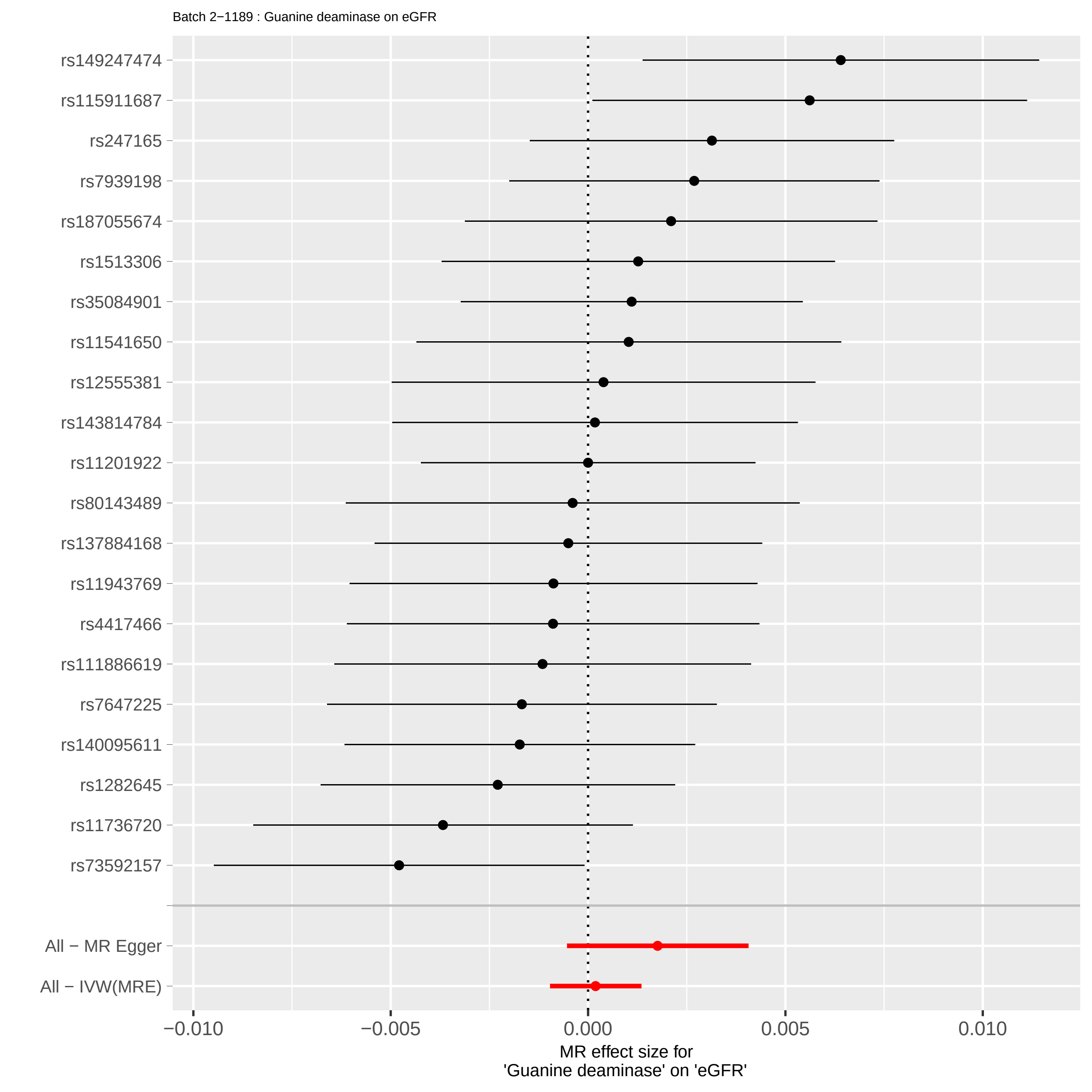

Batch 2−1189 : Guanine deaminase on eGFR
rs149247474
rs115911687
rs247165
rs7939198
rs187055674
rs1513306
rs35084901
rs11541650
rs12555381
rs143814784
rs11201922
rs80143489
rs137884168
rs11943769
rs4417466
rs111886619
rs7647225
rs140095611
rs1282645
rs11736720
rs73592157
All − MR Egger
All − IVW(MRE)
−0.010
−0.005
0.000
0.005
0.010
MR effect size for
'Guanine deaminase' on 'eGFR'

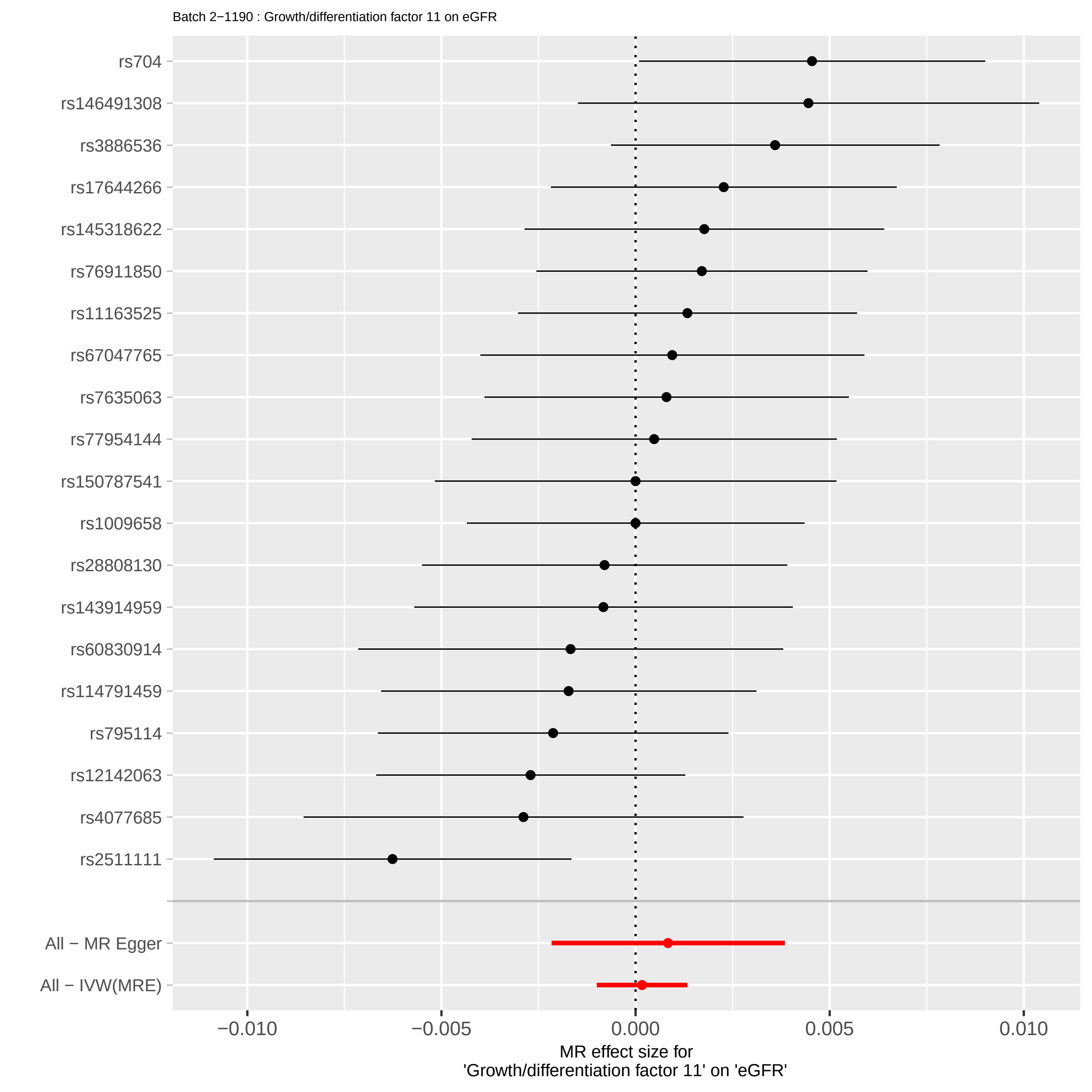
Batch 2−1190 : Growth/differentiation factor 11 on eGFR
rs704
rs146491308
rs3886536
rs17644266
rs145318622
rs76911850
rs11163525
rs67047765
rs7635063
rs77954144
rs150787541
rs1009658
rs28808130
rs143914959
rs60830914
rs114791459
rs795114
rs12142063
rs4077685
rs2511111
All − MR Egger
All − IVW(MRE)
−0.010
−0.005
0.000
0.005
0.010
MR effect size for
'Growth/differentiation factor 11' on 'eGFR'

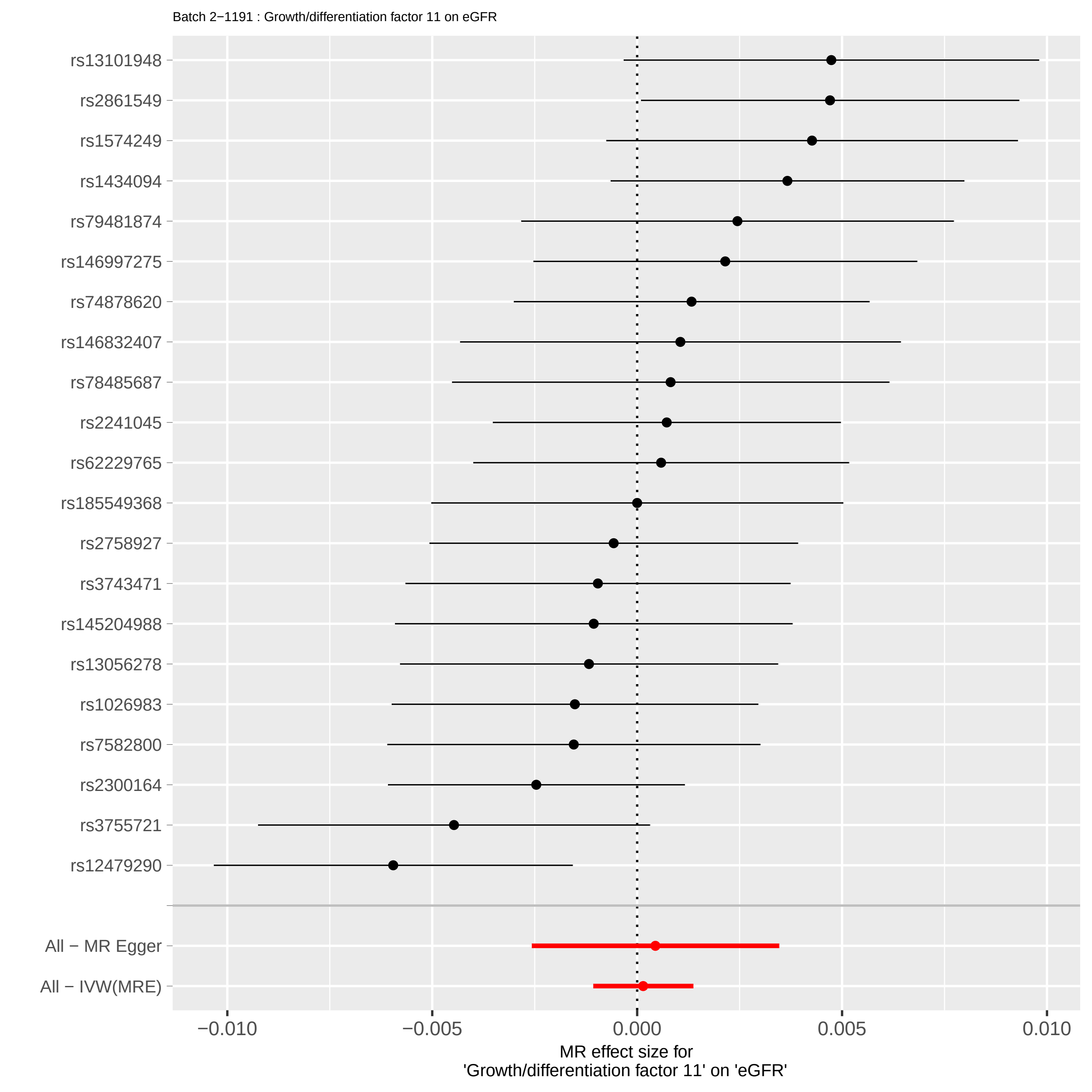

Batch 2−1191 : Growth/differentiation factor 11 on eGFR
rs13101948
rs2861549
rs1574249
rs1434094
rs79481874
rs146997275
rs74878620
rs146832407
rs78485687
rs2241045
rs62229765
rs185549368
rs2758927
rs3743471
rs145204988
rs13056278
rs1026983
rs7582800
rs2300164
rs3755721
rs12479290
All − MR Egger
All − IVW(MRE)
−0.010
−0.005
0.000
0.005
0.010
MR effect size for
'Growth/differentiation factor 11' on 'eGFR'

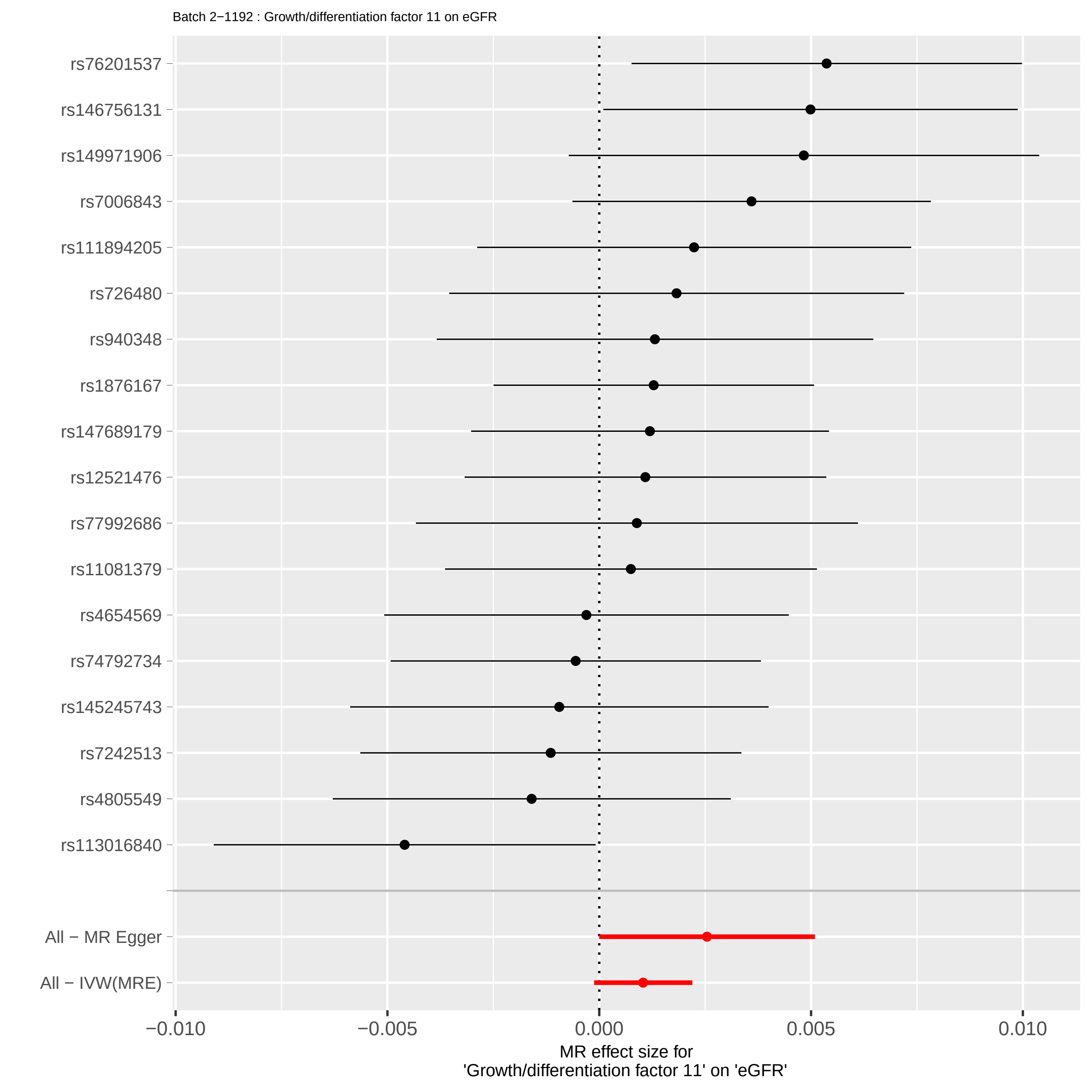

Batch 2−1192 : Growth/differentiation factor 11 on eGFR
rs76201537
rs146756131
rs149971906
rs7006843
rs111894205
rs726480
rs940348
rs1876167
rs147689179
rs12521476
rs77992686
rs11081379
rs4654569
rs74792734
rs145245743
rs7242513
rs4805549
rs113016840
All − MR Egger
All − IVW(MRE)
−0.010
−0.005
0.000
0.005
0.010
MR effect size for
'Growth/differentiation factor 11' on 'eGFR'

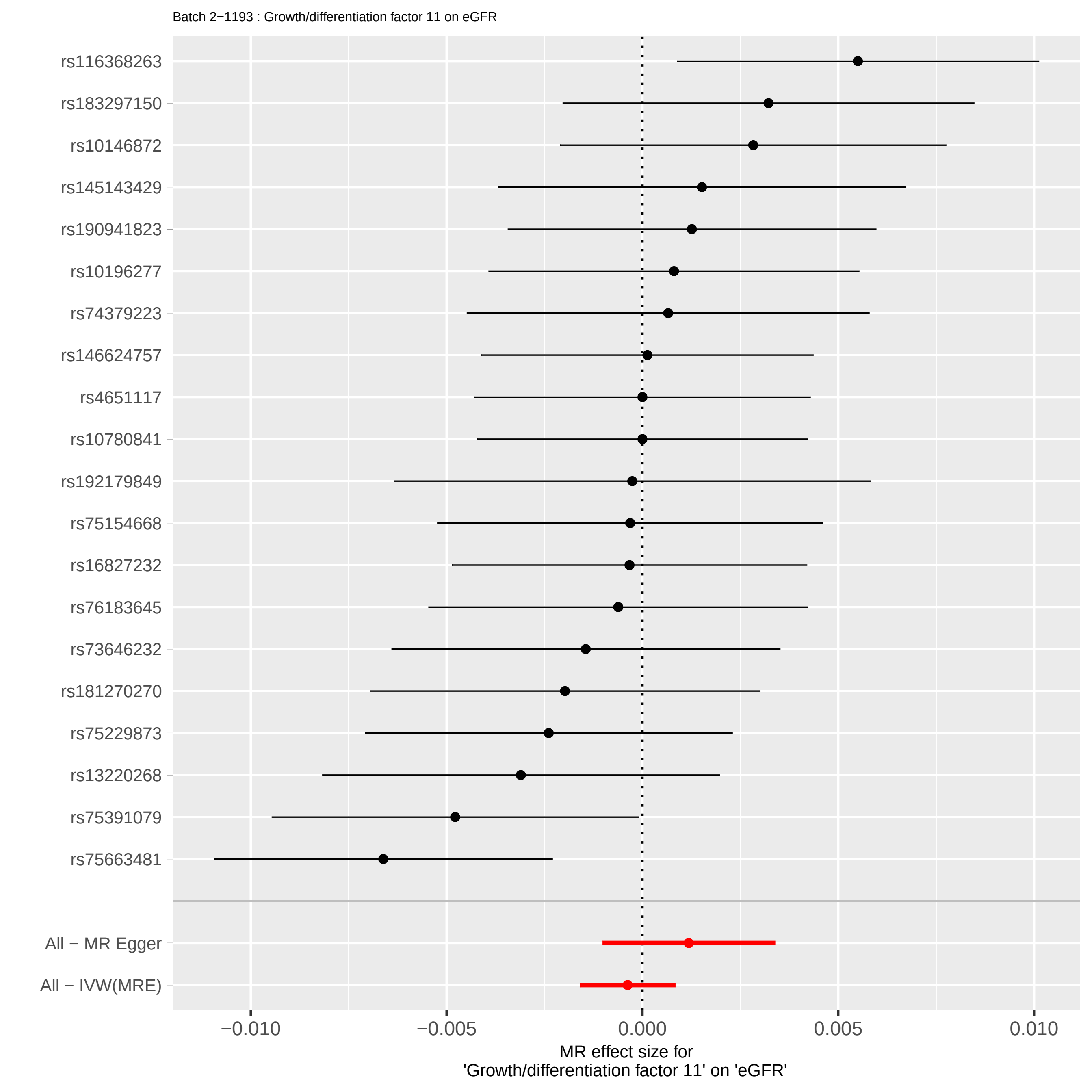

Batch 2−1193 : Growth/differentiation factor 11 on eGFR
rs116368263
rs183297150
rs10146872
rs145143429
rs190941823
rs10196277
rs74379223
rs146624757
rs4651117
rs10780841
rs192179849
rs75154668
rs16827232
rs76183645
rs73646232
rs181270270
rs75229873
rs13220268
rs75391079
rs75663481
All − MR Egger
All − IVW(MRE)
−0.010
−0.005
0.000
0.005
0.010
MR effect size for
'Growth/differentiation factor 11' on 'eGFR'

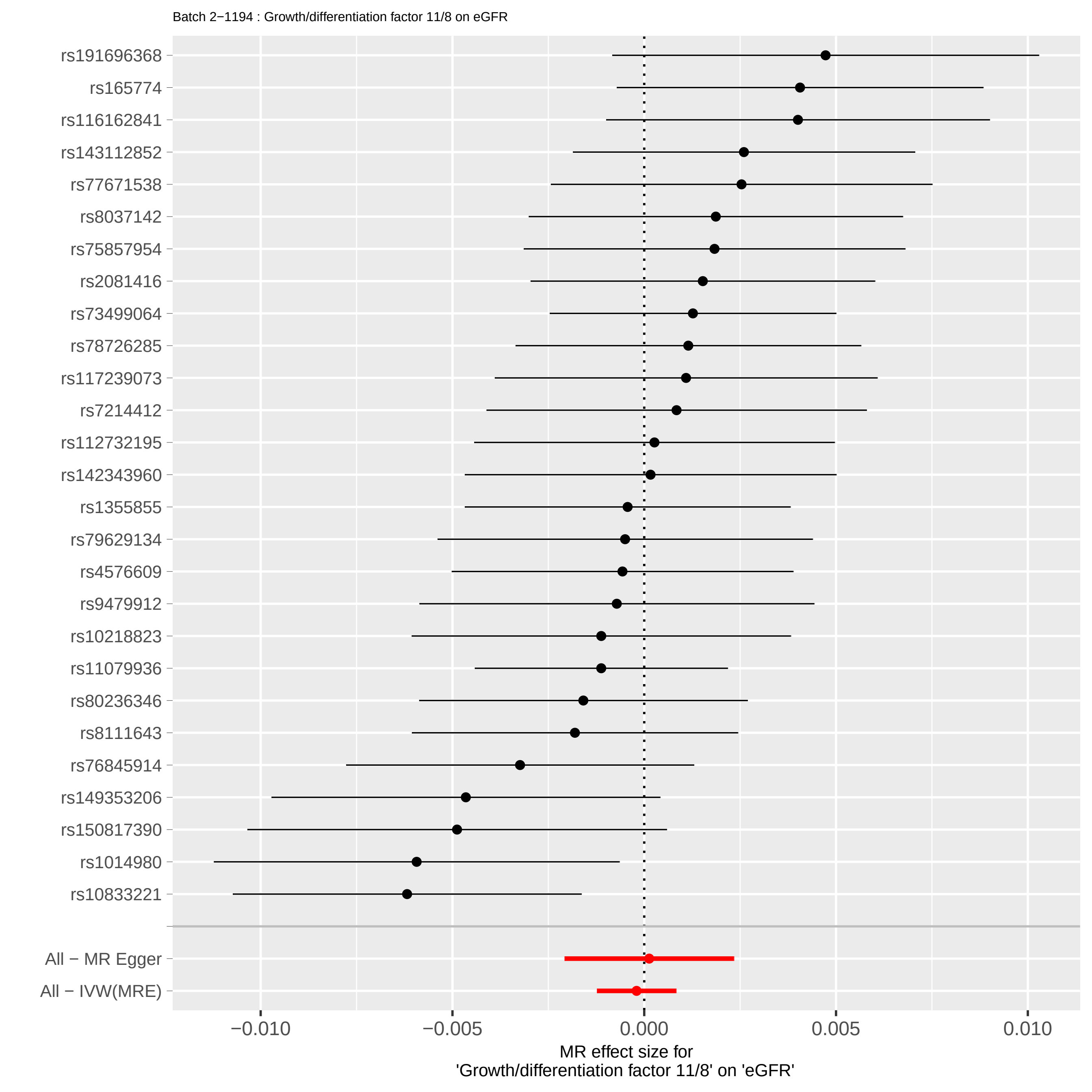

Batch 2−1194 : Growth/differentiation factor 11/8 on eGFR
rs191696368
rs165774
rs116162841
rs143112852
rs77671538
rs8037142
rs75857954
rs2081416
rs73499064
rs78726285
rs117239073
rs7214412
rs112732195
rs142343960
rs1355855
rs79629134
rs4576609
rs9479912
rs10218823
rs11079936
rs80236346
rs8111643
rs76845914
rs149353206
rs150817390
rs1014980
rs10833221
All − MR Egger
All − IVW(MRE)
−0.010
−0.005
0.000
0.005
0.010
MR effect size for
'Growth/differentiation factor 11/8' on 'eGFR'

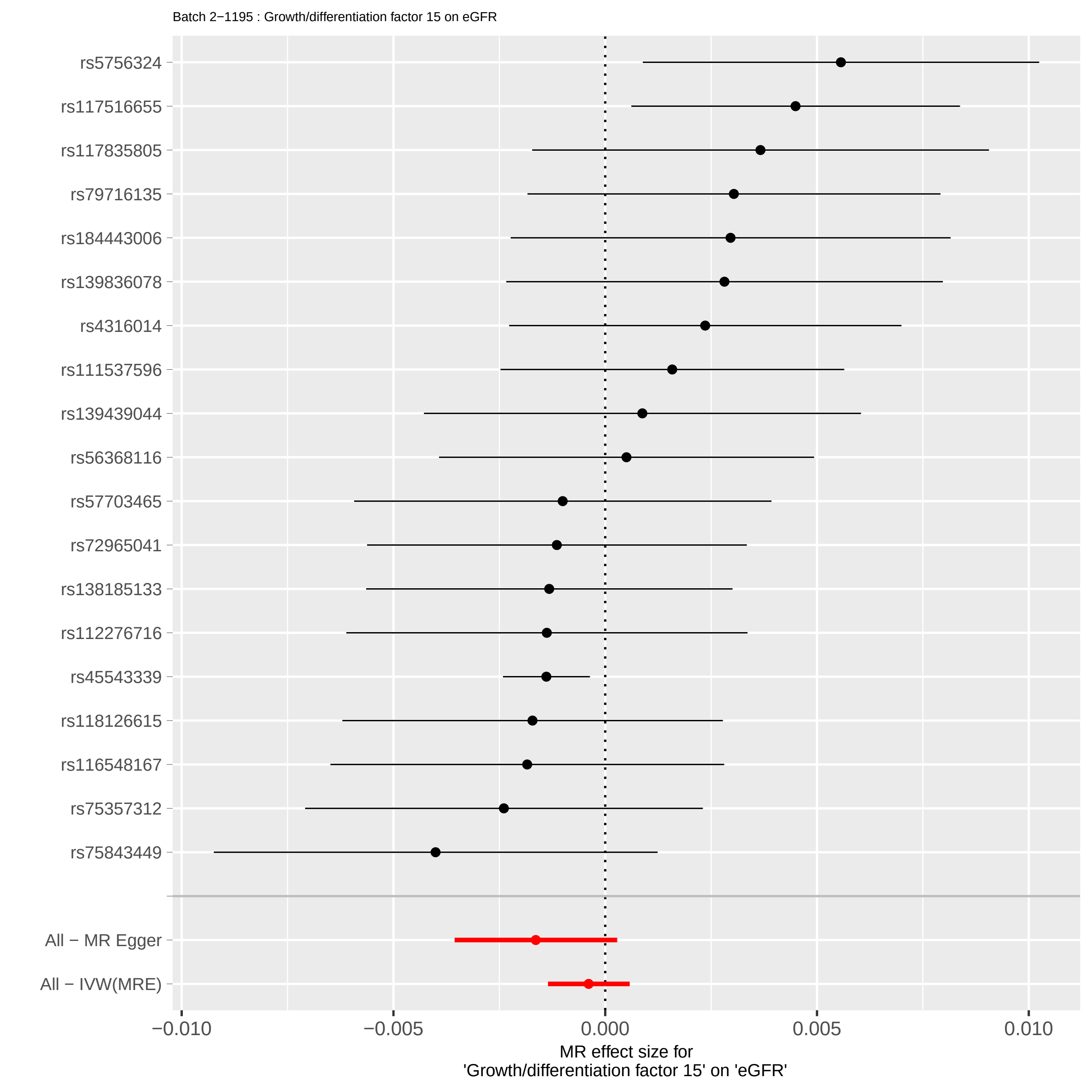
Batch 2−1195 : Growth/differentiation factor 15 on eGFR
rs5756324
rs117516655
rs117835805
rs79716135
rs184443006
rs139836078
rs4316014
rs111537596
rs139439044
rs56368116
rs57703465
rs72965041
rs138185133
rs112276716
rs45543339
rs118126615
rs116548167
rs75357312
rs75843449
All − MR Egger
All − IVW(MRE)
−0.010
−0.005
0.000
0.005
0.010
MR effect size for
'Growth/differentiation factor 15' on 'eGFR'

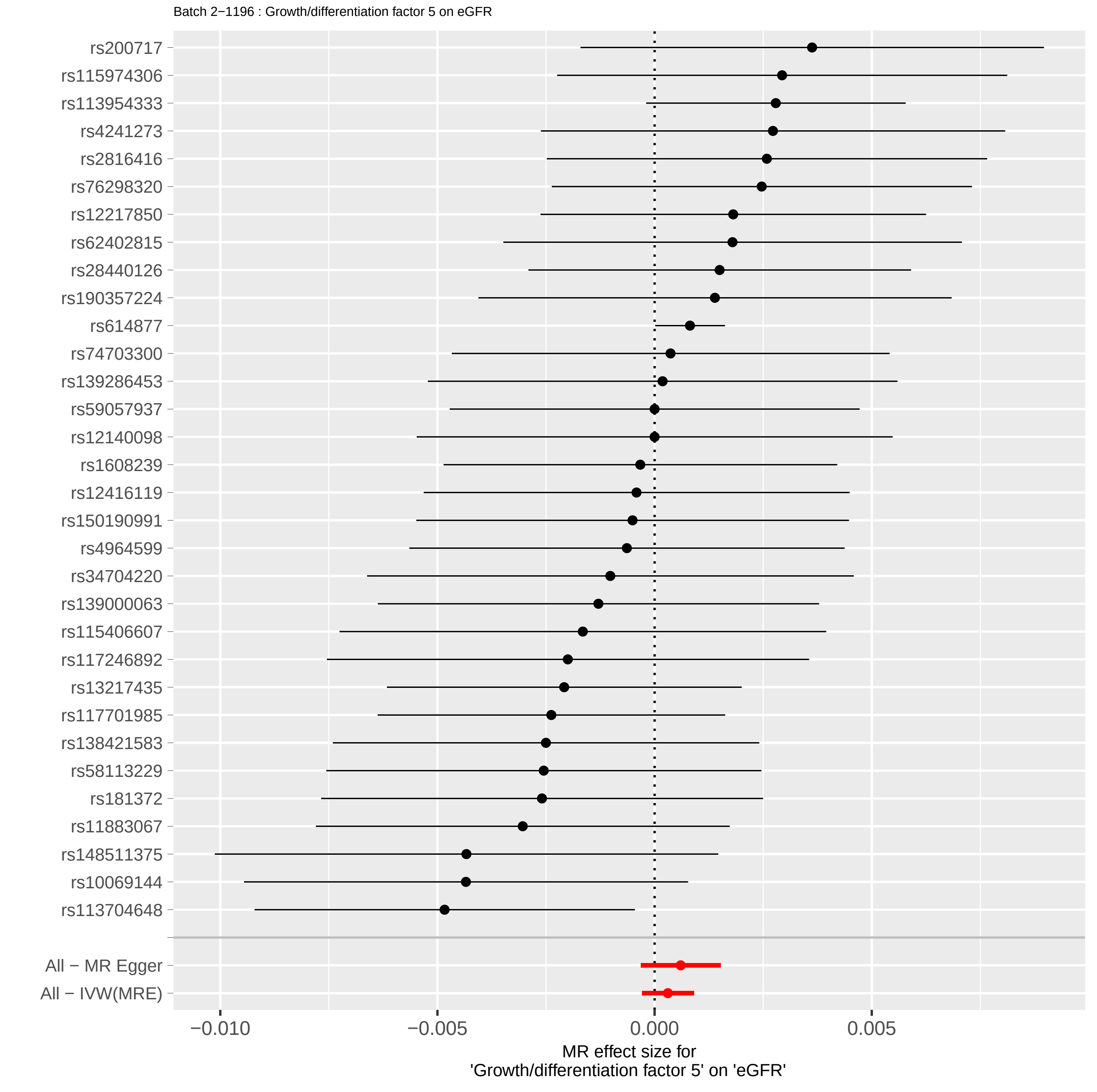

Batch 2−1196 : Growth/differentiation factor 5 on eGFR
rs200717
rs115974306
rs113954333
rs4241273
rs2816416
rs76298320
rs12217850
rs62402815
rs28440126
rs190357224
rs614877
rs74703300
rs139286453
rs59057937
rs12140098
rs1608239
rs12416119
rs150190991
rs4964599
rs34704220
rs139000063
rs115406607
rs117246892
rs13217435
rs117701985
rs138421583
rs58113229
rs181372
rs11883067
rs148511375
rs10069144
rs113704648
All − MR Egger
All − IVW(MRE)
−0.010
−0.005
0.000
0.005
MR effect size for
'Growth/differentiation factor 5' on 'eGFR'

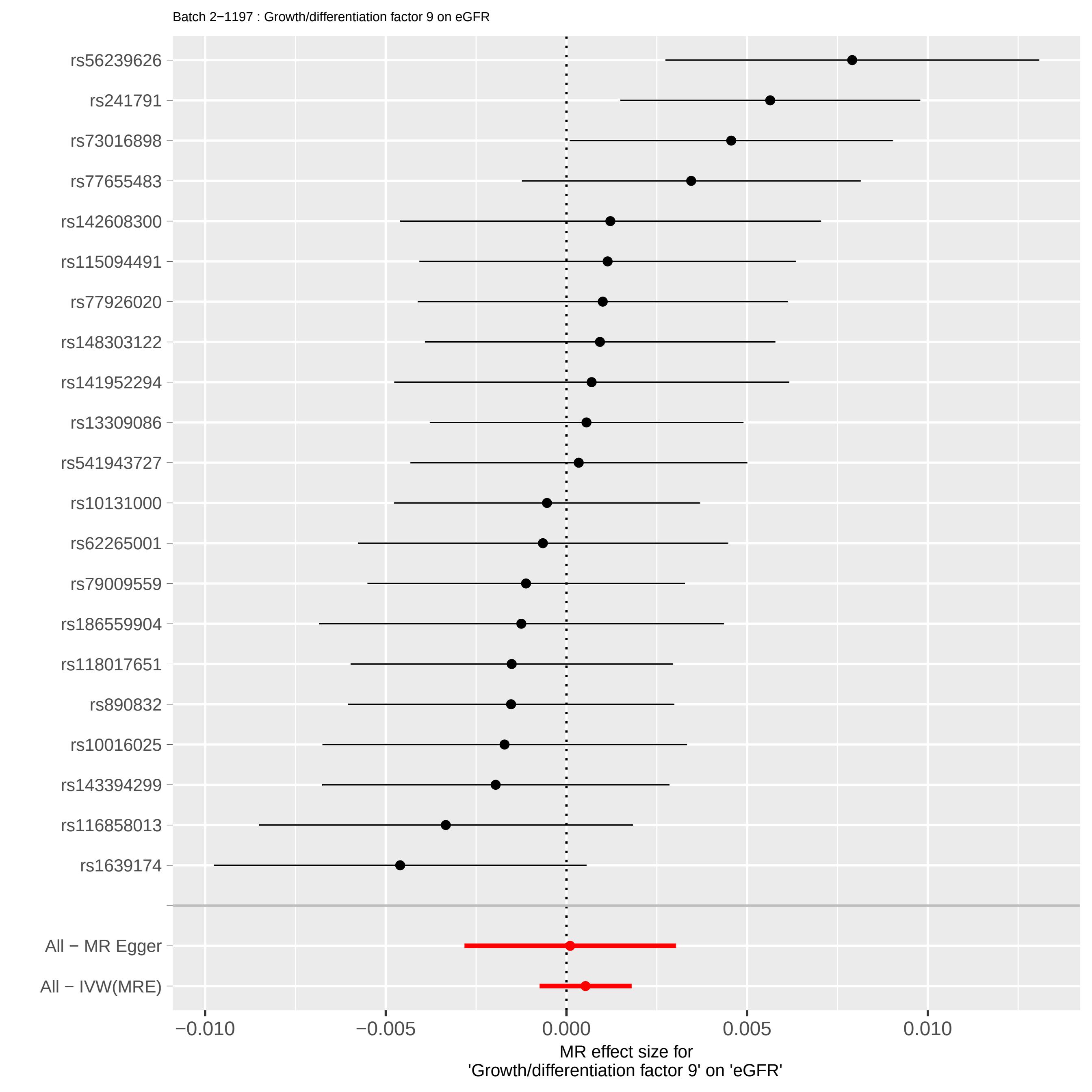

Batch 2−1197 : Growth/differentiation factor 9 on eGFR
rs56239626
rs241791
rs73016898
rs77655483
rs142608300
rs115094491
rs77926020
rs148303122
rs141952294
rs13309086
rs541943727
rs10131000
rs62265001
rs79009559
rs186559904
rs118017651
rs890832
rs10016025
rs143394299
rs116858013
rs1639174
All − MR Egger
All − IVW(MRE)
−0.010
−0.005
0.000
0.005
0.010
MR effect size for
'Growth/differentiation factor 9' on 'eGFR'

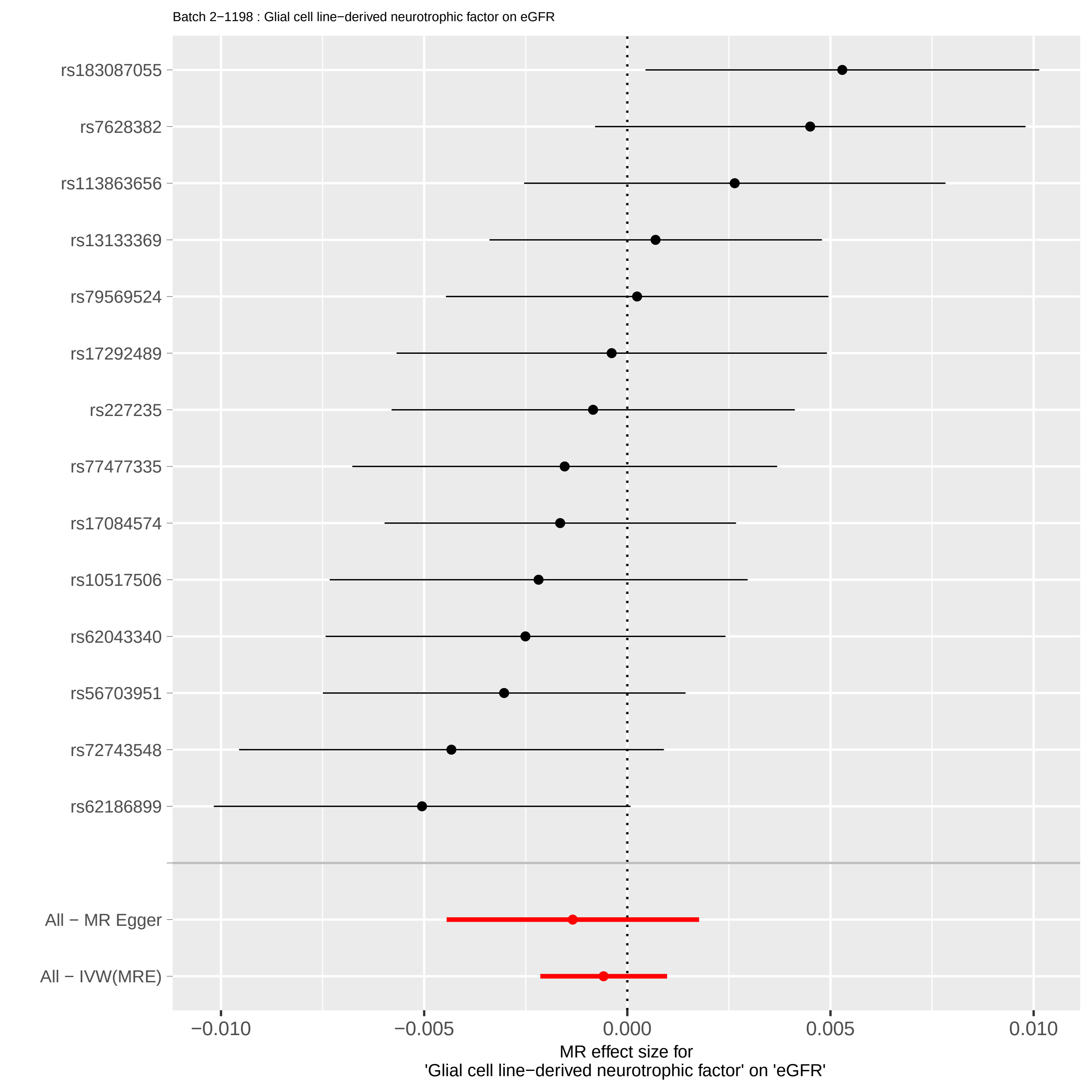
Batch 2−1198 : Glial cell line−derived neurotrophic factor on eGFR
rs183087055
rs7628382
rs113863656
rs13133369
rs79569524
rs17292489
rs227235
rs77477335
rs17084574
rs10517506
rs62043340
rs56703951
rs72743548
rs62186899
All − MR Egger
All − IVW(MRE)
−0.010
−0.005
0.000
0.005
0.010
MR effect size for
'Glial cell line−derived neurotrophic factor' on 'eGFR'

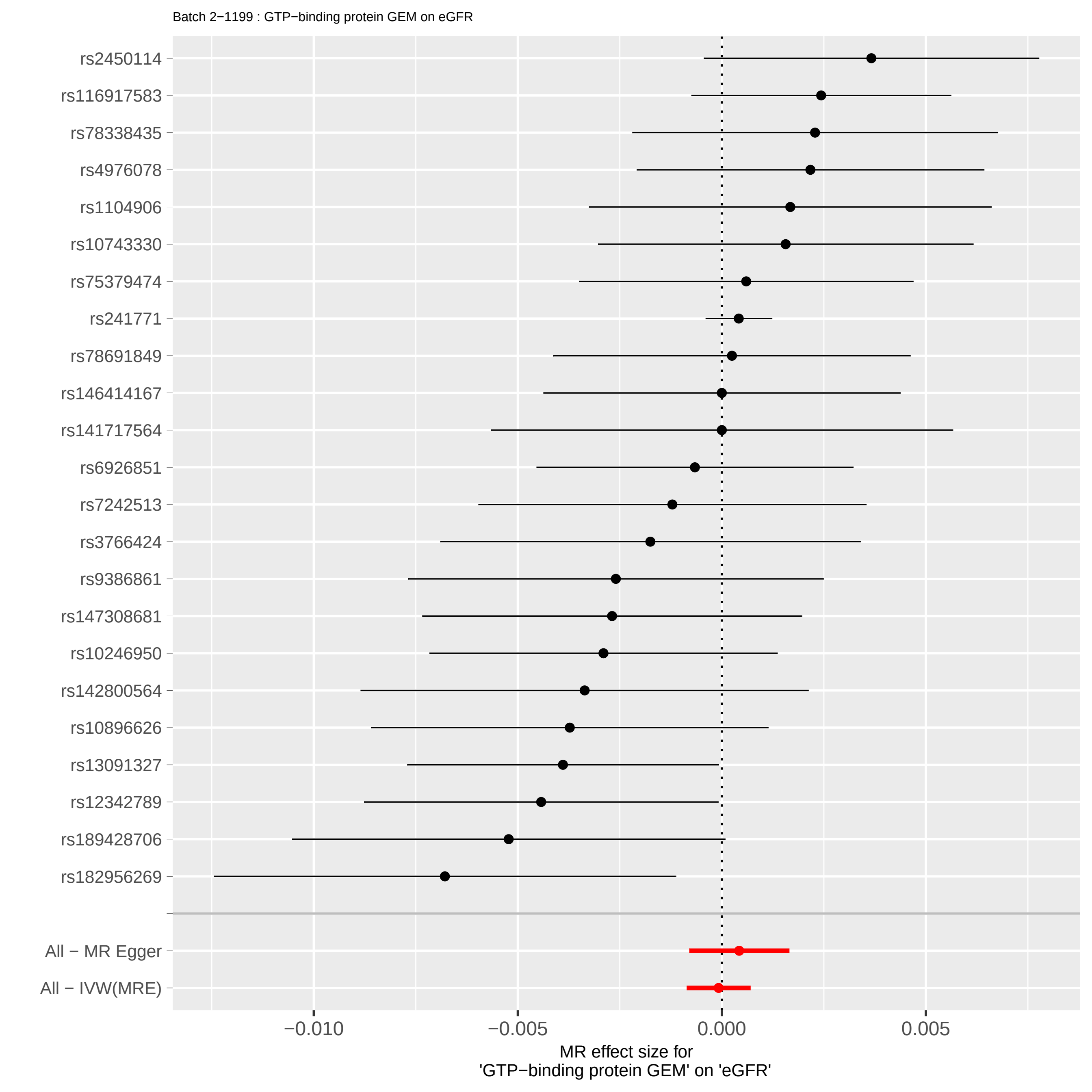

Batch 2−1199 : GTP−binding protein GEM on eGFR
rs2450114
rs116917583
rs78338435
rs4976078
rs1104906
rs10743330
rs75379474
rs241771
rs78691849
rs146414167
rs141717564
rs6926851
rs7242513
rs3766424
rs9386861
rs147308681
rs10246950
rs142800564
rs10896626
rs13091327
rs12342789
rs189428706
rs182956269
All − MR Egger
All − IVW(MRE)
−0.010
−0.005
0.000
0.005
MR effect size for
'GTP−binding protein GEM' on 'eGFR'

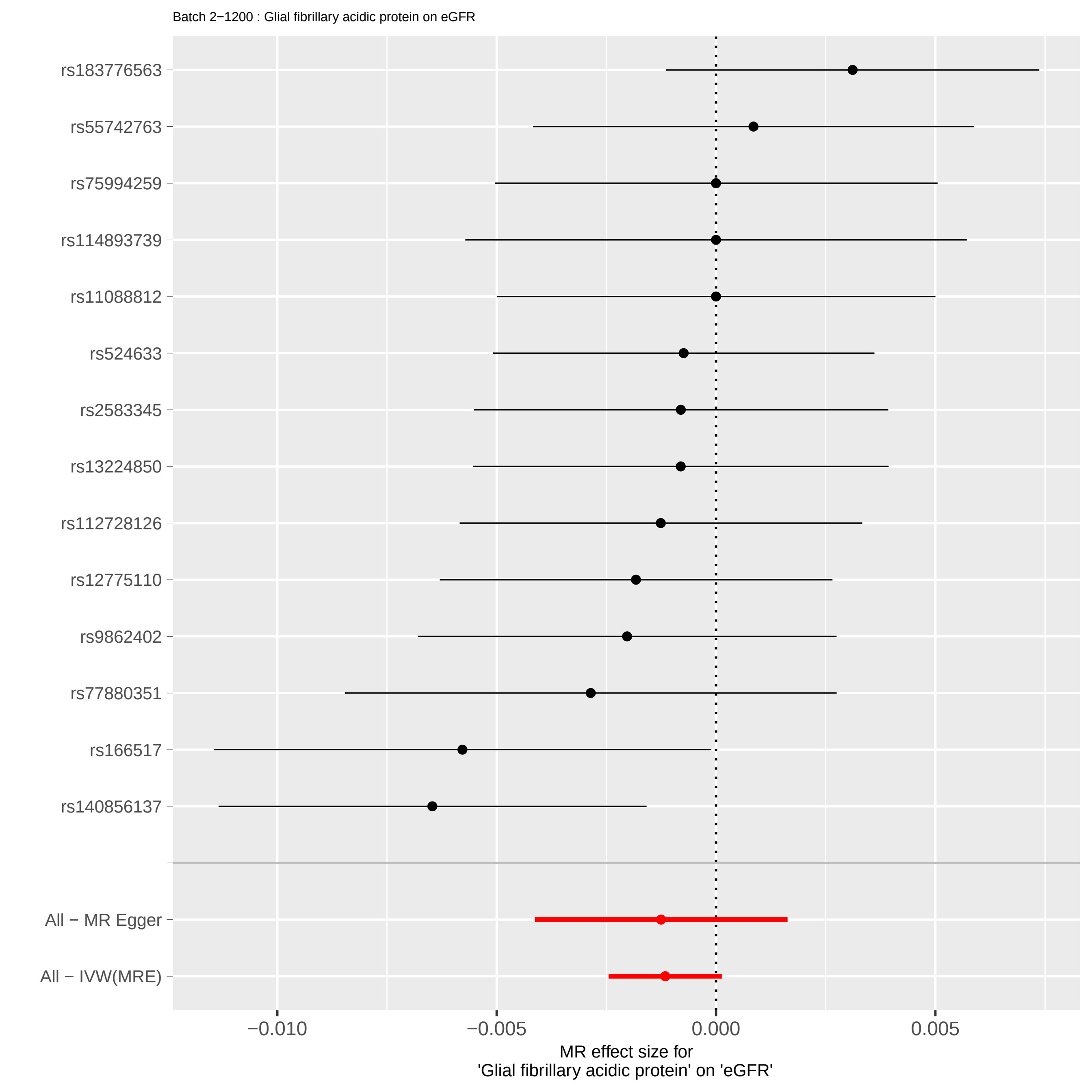
Batch 2−1200 : Glial fibrillary acidic protein on eGFR
rs183776563
rs55742763
rs75994259
rs114893739
rs11088812
rs524633
rs2583345
rs13224850
rs112728126
rs12775110
rs9862402
rs77880351
rs166517
rs140856137
All − MR Egger
All − IVW(MRE)
−0.010
−0.005
0.000
0.005
MR effect size for
'Glial fibrillary acidic protein' on 'eGFR'

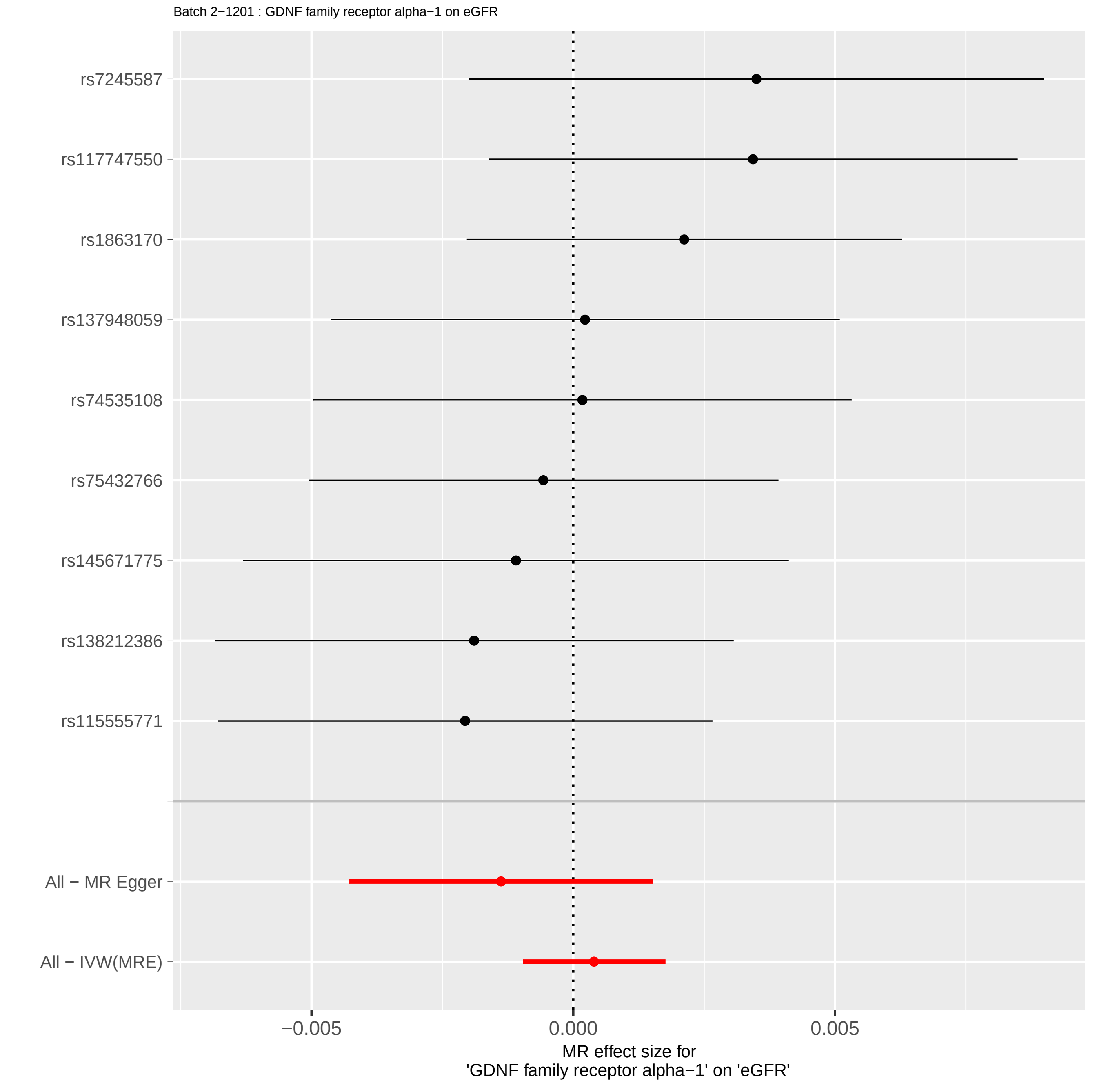

Batch 2−1201 : GDNF family receptor alpha−1 on eGFR
rs7245587
rs117747550
rs1863170
rs137948059
rs74535108
rs75432766
rs145671775
rs138212386
rs115555771
All − MR Egger
All − IVW(MRE)
−0.005
0.000
0.005
MR effect size for
'GDNF family receptor alpha−1' on 'eGFR'

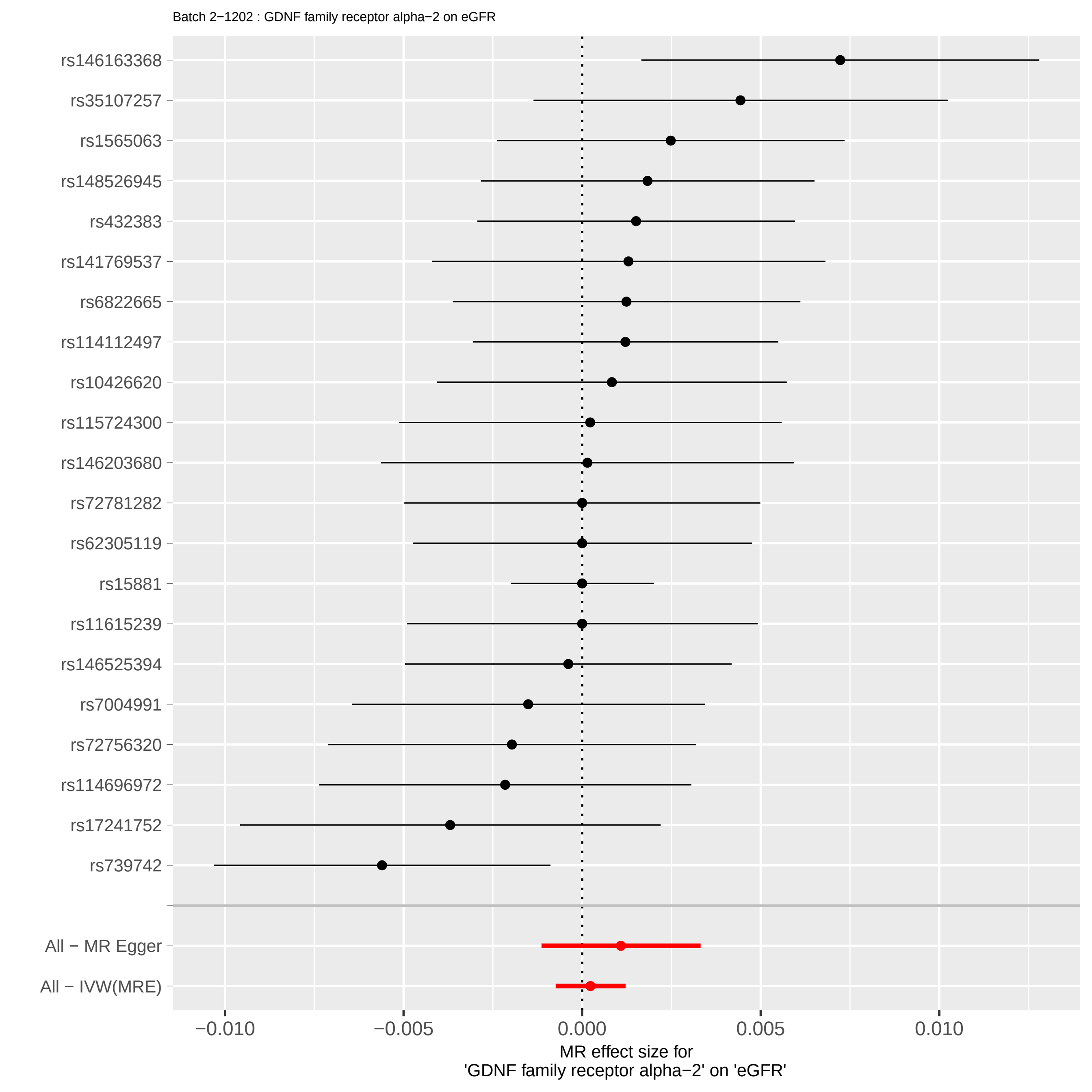

Batch 2−1202 : GDNF family receptor alpha−2 on eGFR
rs146163368
rs35107257
rs1565063
rs148526945
rs432383
rs141769537
rs6822665
rs114112497
rs10426620
rs115724300
rs146203680
rs72781282
rs62305119
rs15881
rs11615239
rs146525394
rs7004991
rs72756320
rs114696972
rs17241752
rs739742
All − MR Egger
All − IVW(MRE)
−0.010
−0.005
0.000
0.005
0.010
MR effect size for
'GDNF family receptor alpha−2' on 'eGFR'

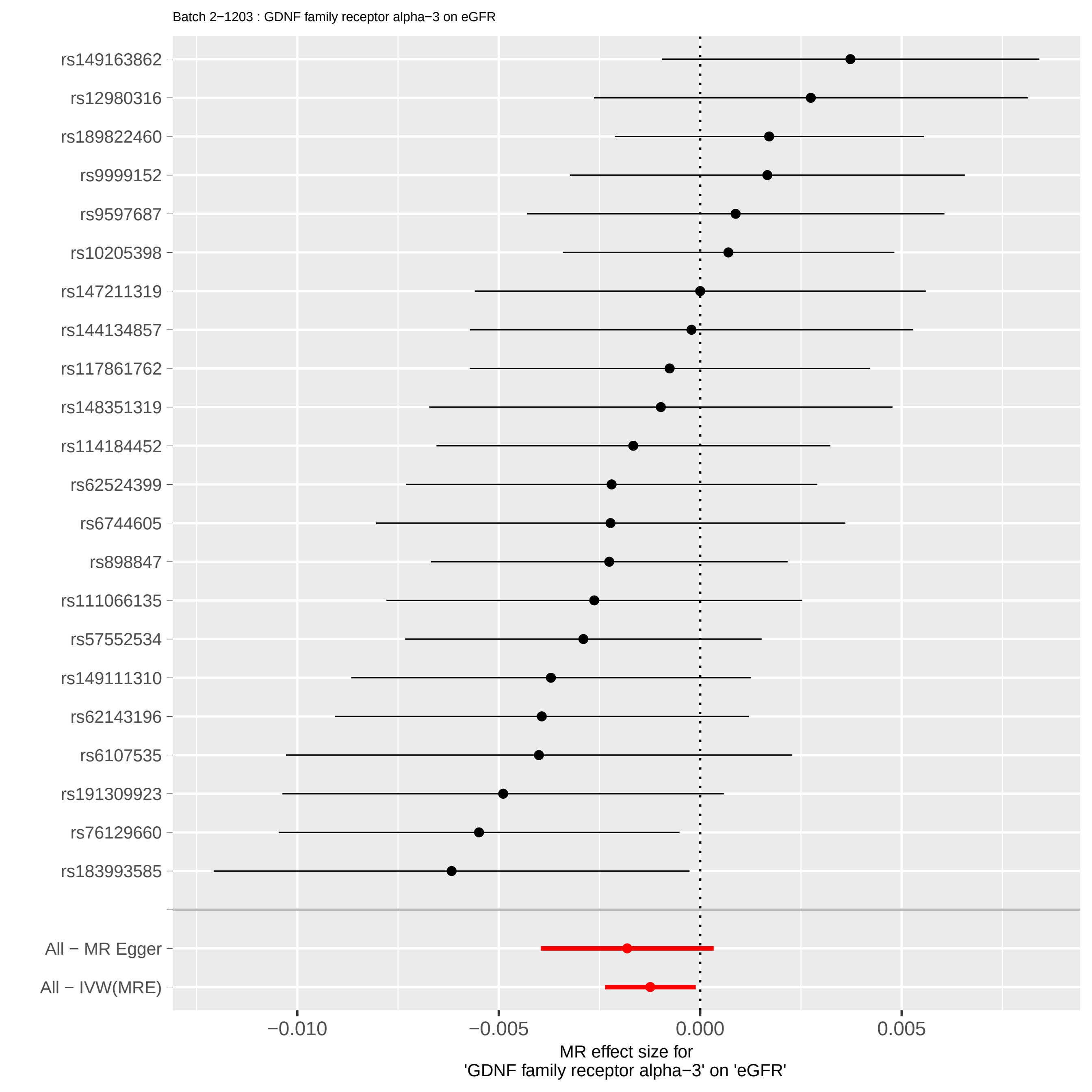

Batch 2−1203 : GDNF family receptor alpha−3 on eGFR
rs149163862
rs12980316
rs189822460
rs9999152
rs9597687
rs10205398
rs147211319
rs144134857
rs117861762
rs148351319
rs114184452
rs62524399
rs6744605
rs898847
rs111066135
rs57552534
rs149111310
rs62143196
rs6107535
rs191309923
rs76129660
rs183993585
All − MR Egger
All − IVW(MRE)
−0.010
−0.005
0.000
0.005
MR effect size for
'GDNF family receptor alpha−3' on 'eGFR'

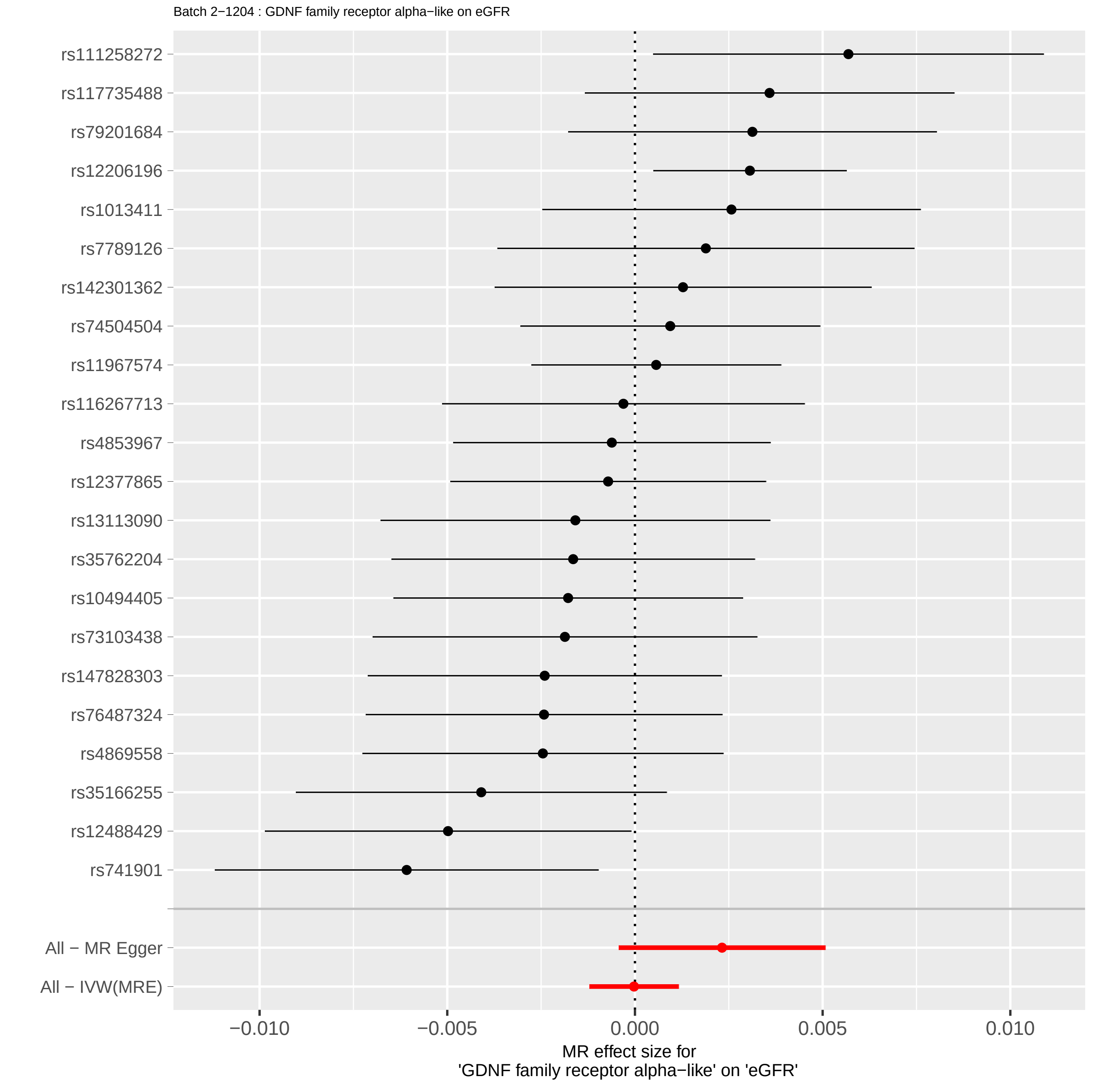

Batch 2−1204 : GDNF family receptor alpha−like on eGFR
rs111258272
rs117735488
rs79201684
rs12206196
rs1013411
rs7789126
rs142301362
rs74504504
rs11967574
rs116267713
rs4853967
rs12377865
rs13113090
rs35762204
rs10494405
rs73103438
rs147828303
rs76487324
rs4869558
rs35166255
rs12488429
rs741901
All − MR Egger
All − IVW(MRE)
−0.010
−0.005
0.000
0.005
0.010
MR effect size for
'GDNF family receptor alpha−like' on 'eGFR'

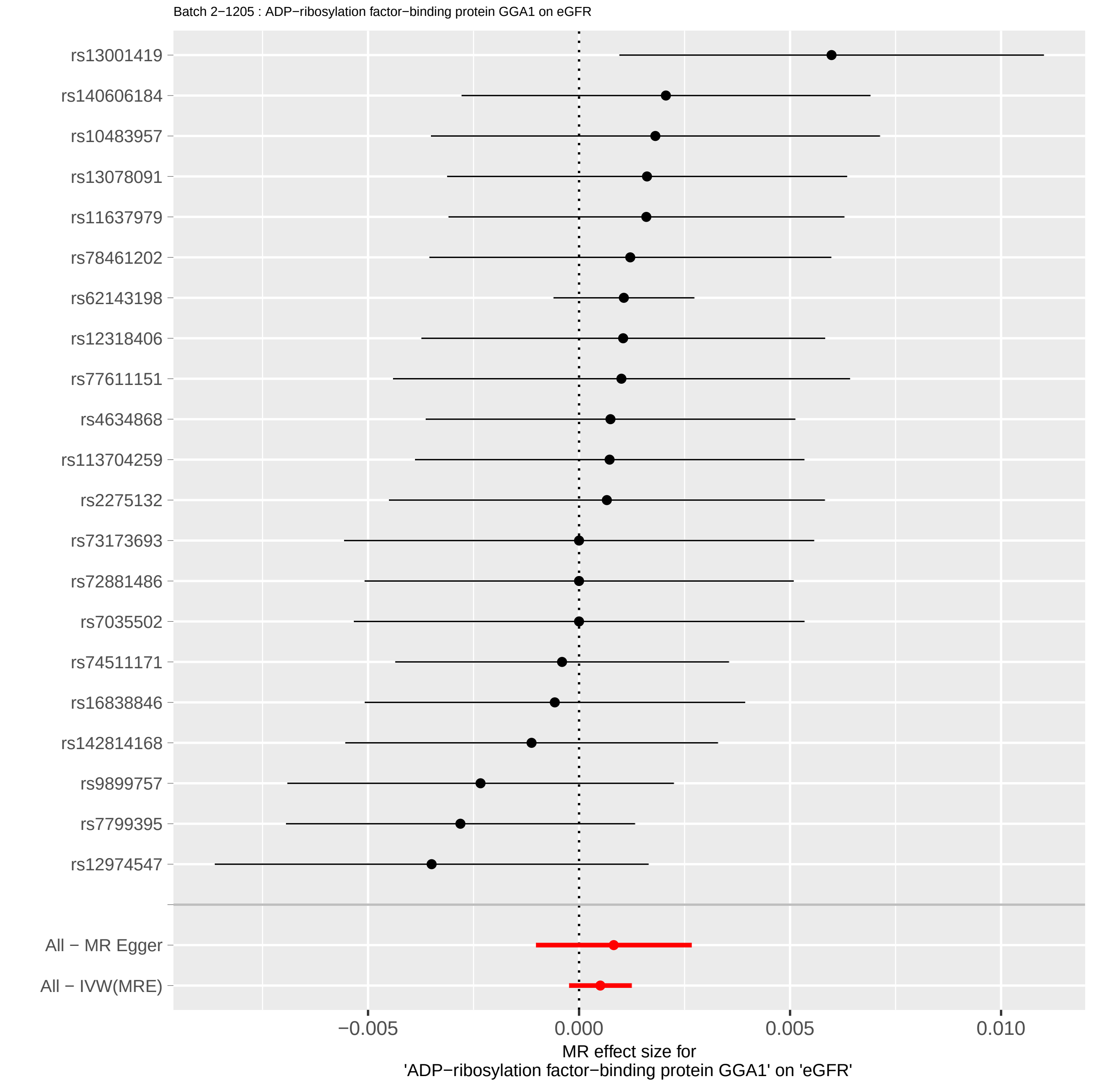

Batch 2−1205 : ADP−ribosylation factor−binding protein GGA1 on eGFR
rs13001419
rs140606184
rs10483957
rs13078091
rs11637979
rs78461202
rs62143198
rs12318406
rs77611151
rs4634868
rs113704259
rs2275132
rs73173693
rs72881486
rs7035502
rs74511171
rs16838846
rs142814168
rs9899757
rs7799395
rs12974547
All − MR Egger
All − IVW(MRE)
−0.005
0.000
0.005
0.010
MR effect size for
'ADP−ribosylation factor−binding protein GGA1' on 'eGFR'

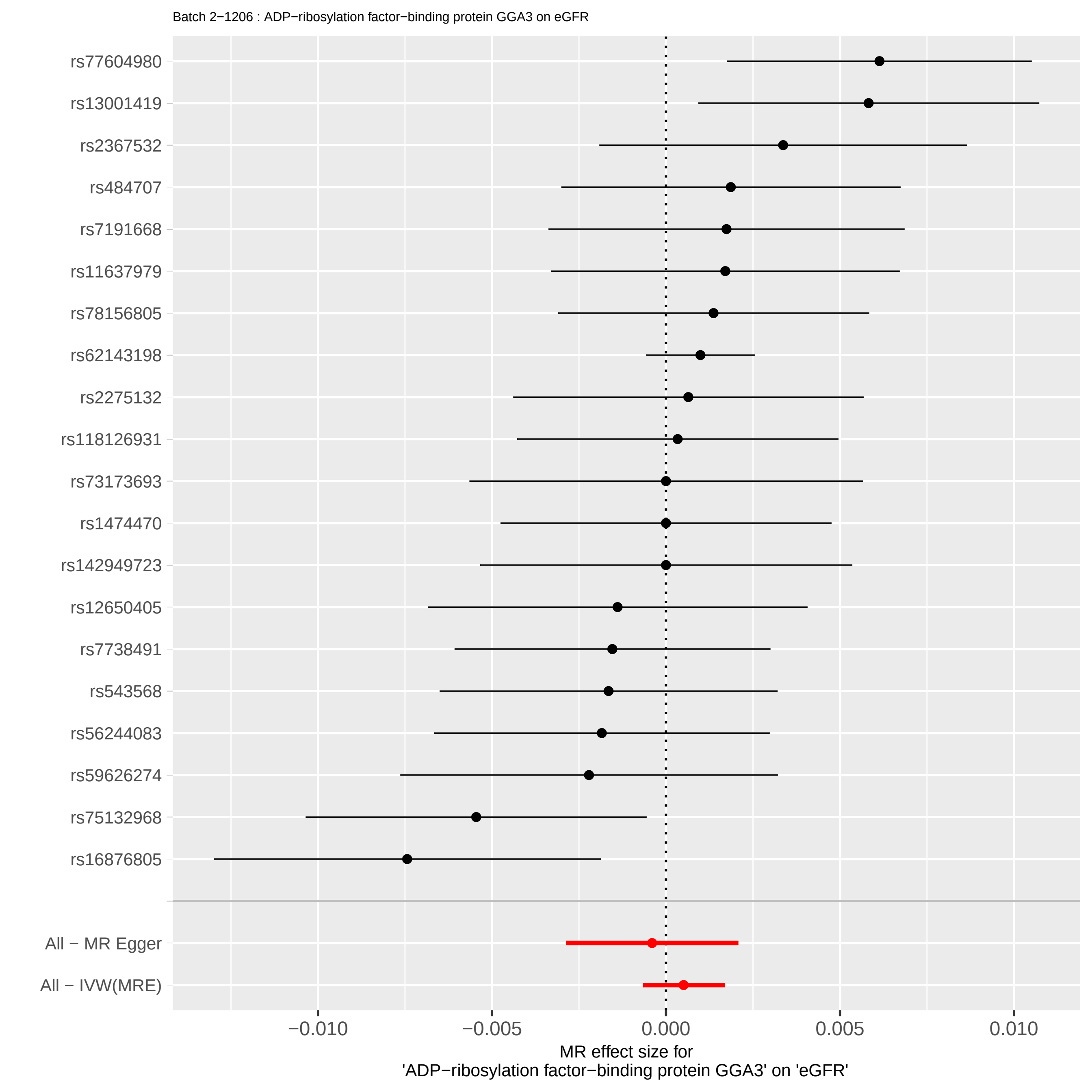

Batch 2−1206 : ADP−ribosylation factor−binding protein GGA3 on eGFR
rs77604980
rs13001419
rs2367532
rs484707
rs7191668
rs11637979
rs78156805
rs62143198
rs2275132
rs118126931
rs73173693
rs1474470
rs142949723
rs12650405
rs7738491
rs543568
rs56244083
rs59626274
rs75132968
rs16876805
All − MR Egger
All − IVW(MRE)
−0.010
−0.005
0.000
0.005
0.010
MR effect size for
'ADP−ribosylation factor−binding protein GGA3' on 'eGFR'

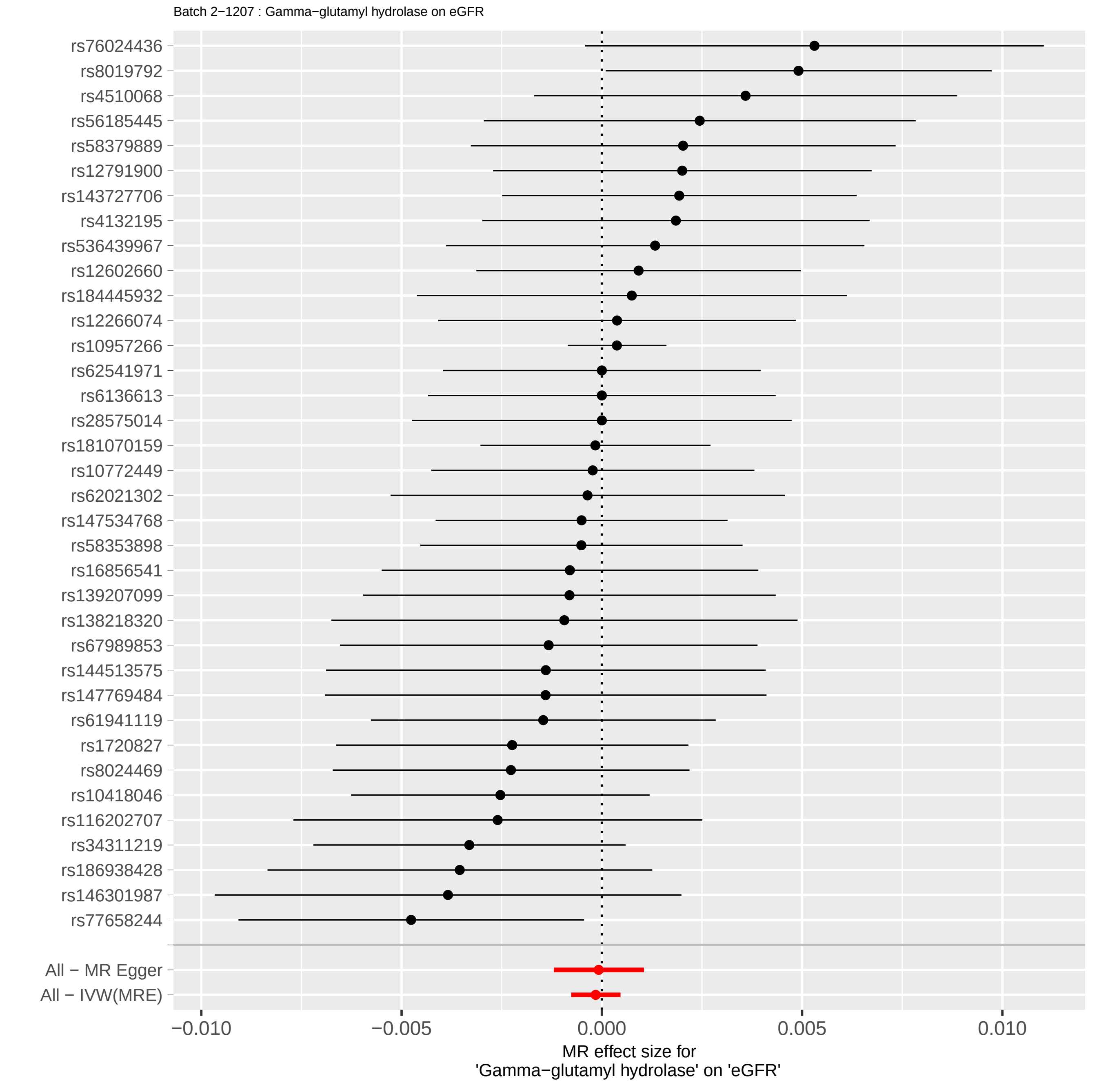

Batch 2−1207 : Gamma−glutamyl hydrolase on eGFR
rs76024436
rs8019792
rs4510068
rs56185445
rs58379889
rs12791900
rs143727706
rs4132195
rs536439967
rs12602660
rs184445932
rs12266074
rs10957266
rs62541971
rs6136613
rs28575014
rs181070159
rs10772449
rs62021302
rs147534768
rs58353898
rs16856541
rs139207099
rs138218320
rs67989853
rs144513575
rs147769484
rs61941119
rs1720827
rs8024469
rs10418046
rs116202707
rs34311219
rs186938428
rs146301987
rs77658244
All − MR Egger
All − IVW(MRE)
−0.010
−0.005
0.000
0.005
0.010
MR effect size for
'Gamma−glutamyl hydrolase' on 'eGFR'

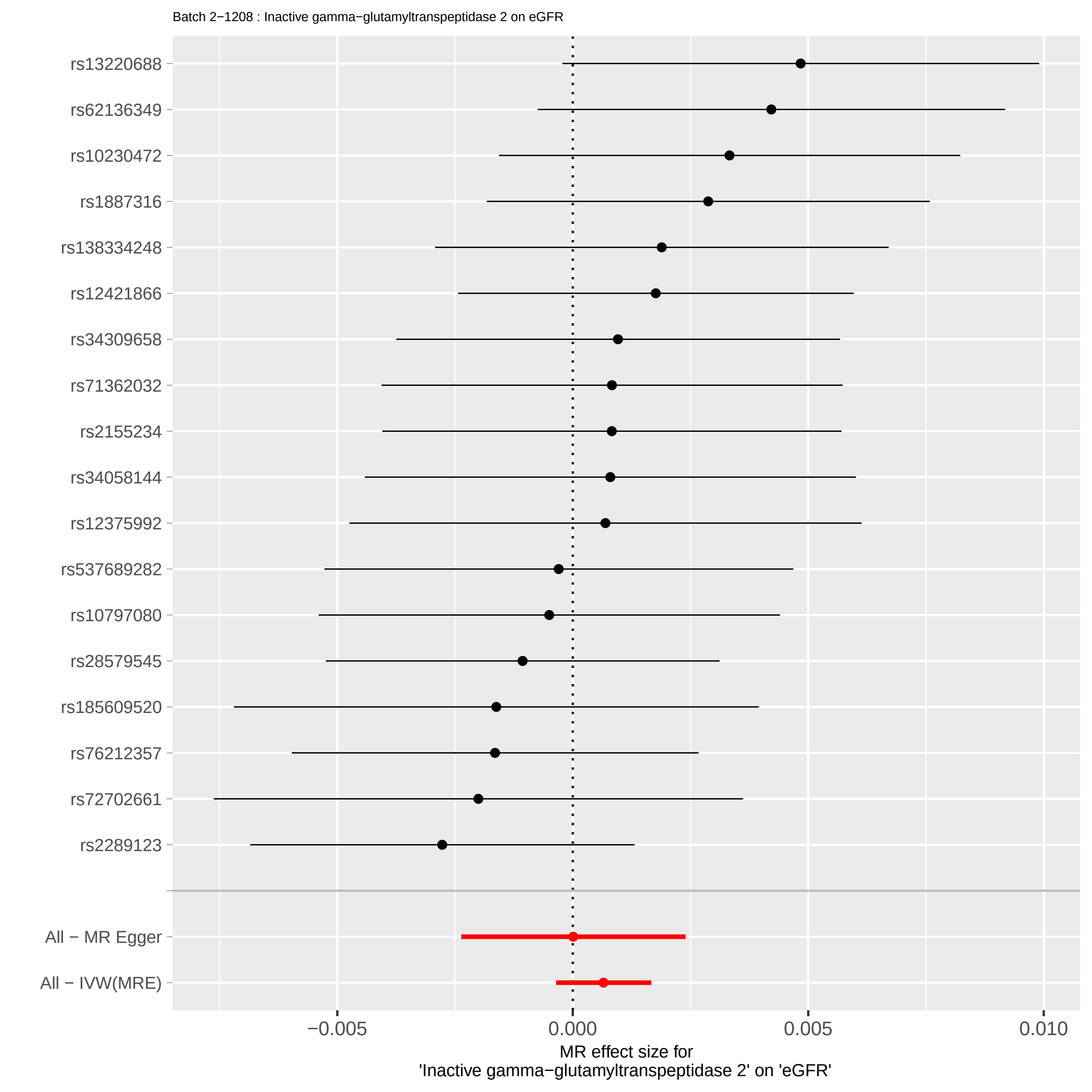

Batch 2−1208 : Inactive gamma−glutamyltranspeptidase 2 on eGFR
rs13220688
rs62136349
rs10230472
rs1887316
rs138334248
rs12421866
rs34309658
rs71362032
rs2155234
rs34058144
rs12375992
rs537689282
rs10797080
rs28579545
rs185609520
rs76212357
rs72702661
rs2289123
All − MR Egger
All − IVW(MRE)
−0.005
0.000
0.005
0.010
MR effect size for
'Inactive gamma−glutamyltranspeptidase 2' on 'eGFR'

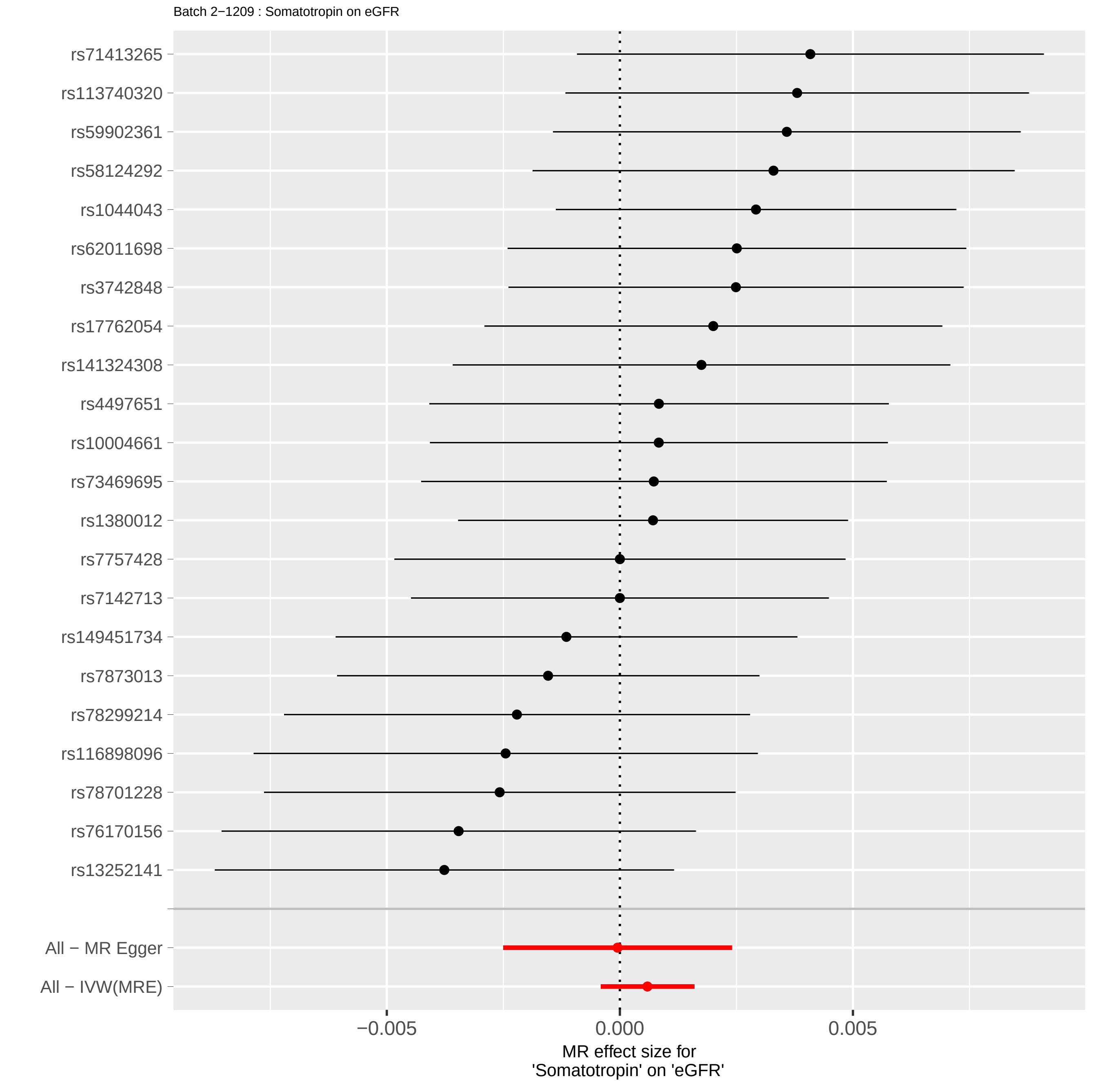

Batch 2−1209 : Somatotropin on eGFR
rs71413265
rs113740320
rs59902361
rs58124292
rs1044043
rs62011698
rs3742848
rs17762054
rs141324308
rs4497651
rs10004661
rs73469695
rs1380012
rs7757428
rs7142713
rs149451734
rs7873013
rs78299214
rs116898096
rs78701228
rs76170156
rs13252141
All − MR Egger
All − IVW(MRE)
−0.005
0.000
0.005
MR effect size for
'Somatotropin' on 'eGFR'

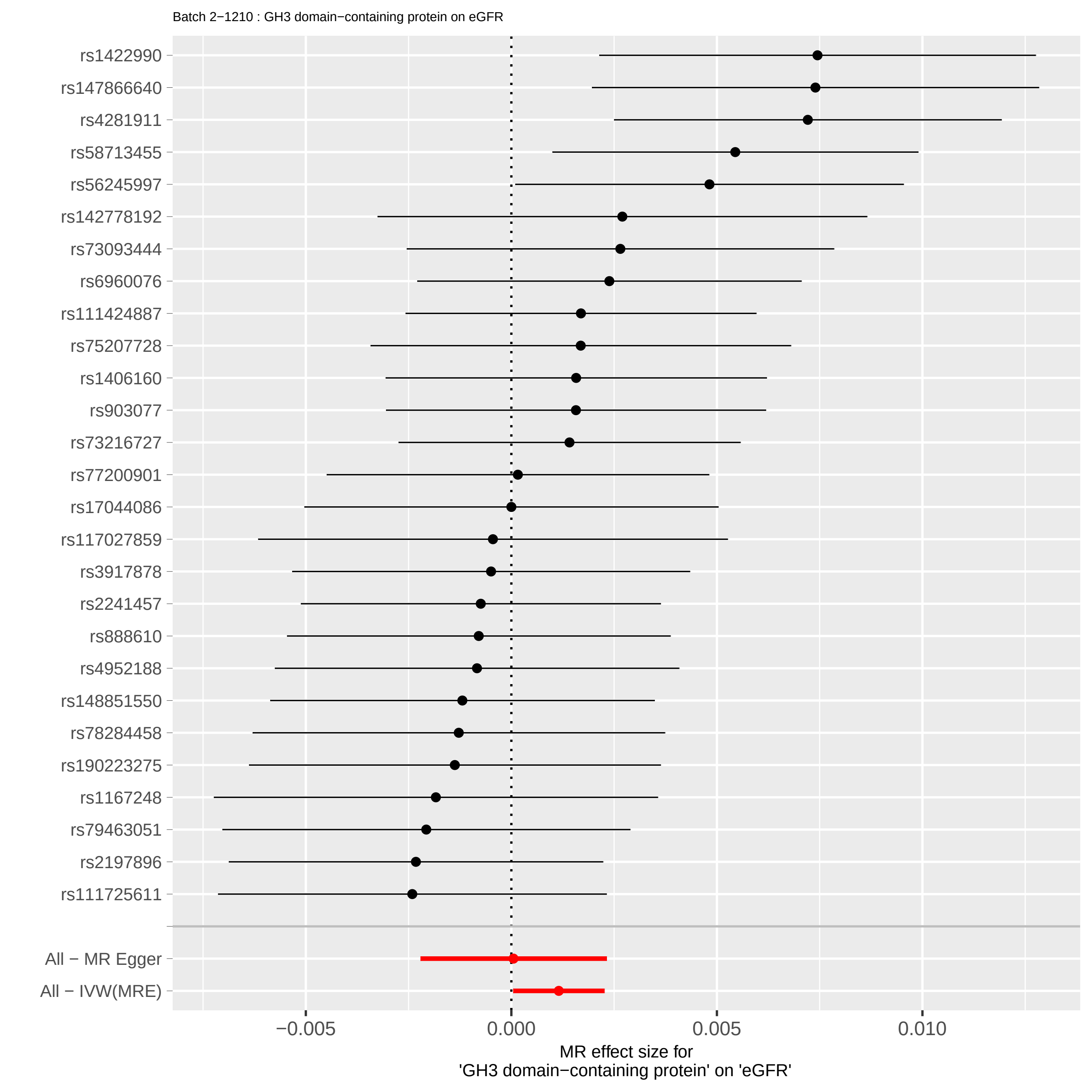

Batch 2−1210 : GH3 domain−containing protein on eGFR
rs1422990
rs147866640
rs4281911
rs58713455
rs56245997
rs142778192
rs73093444
rs6960076
rs111424887
rs75207728
rs1406160
rs903077
rs73216727
rs77200901
rs17044086
rs117027859
rs3917878
rs2241457
rs888610
rs4952188
rs148851550
rs78284458
rs190223275
rs1167248
rs79463051
rs2197896
rs111725611
All − MR Egger
All − IVW(MRE)
−0.005
0.000
0.005
0.010
MR effect size for
'GH3 domain−containing protein' on 'eGFR'

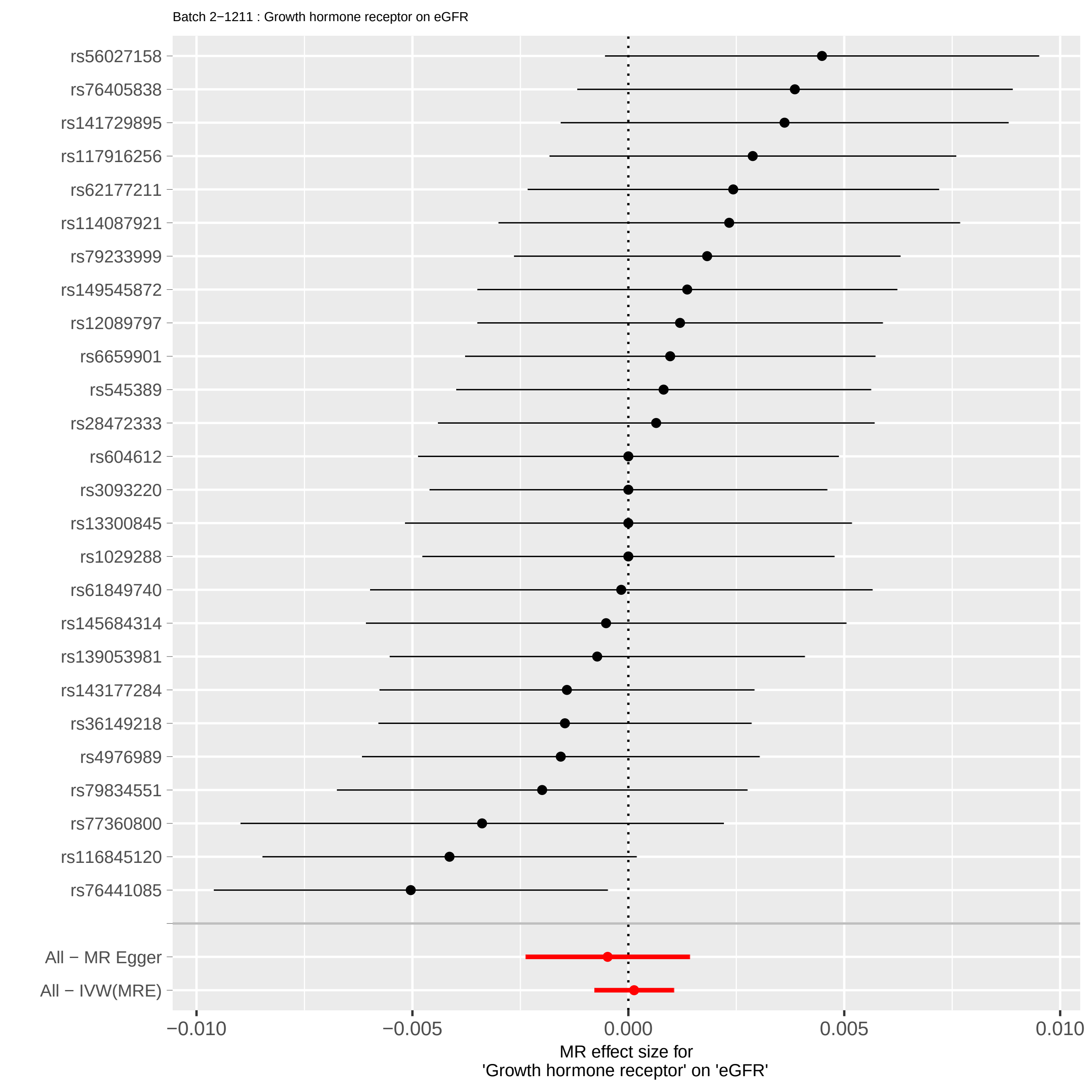
Batch 2−1211 : Growth hormone receptor on eGFR
rs56027158
rs76405838
rs141729895
rs117916256
rs62177211
rs114087921
rs79233999
rs149545872
rs12089797
rs6659901
rs545389
rs28472333
rs604612
rs3093220
rs13300845
rs1029288
rs61849740
rs145684314
rs139053981
rs143177284
rs36149218
rs4976989
rs79834551
rs77360800
rs116845120
rs76441085
All − MR Egger
All − IVW(MRE)
−0.010
−0.005
0.000
0.005
0.010
MR effect size for
'Growth hormone receptor' on 'eGFR'

Batch 2−1212 : Appetite−regulating hormone on eGFR

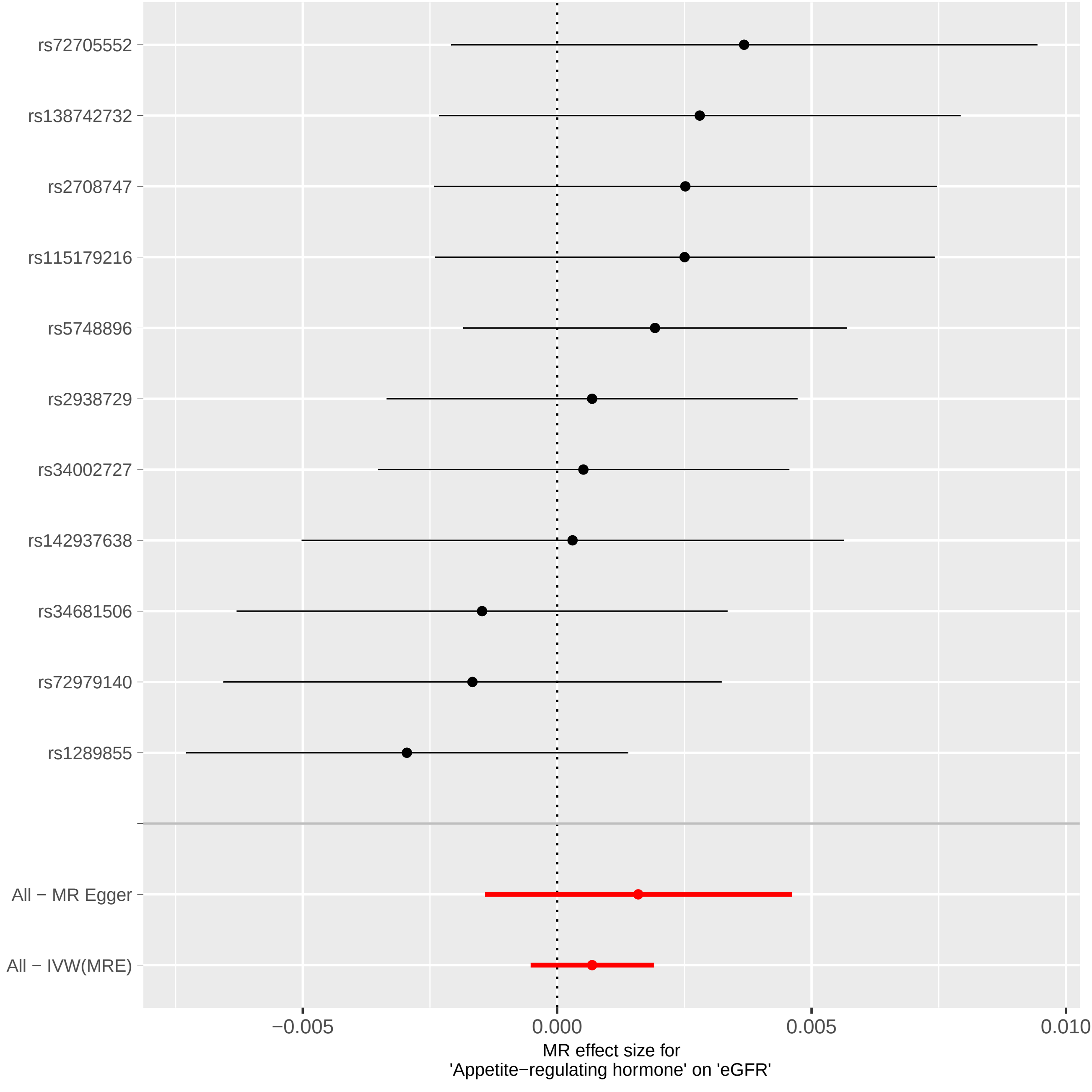

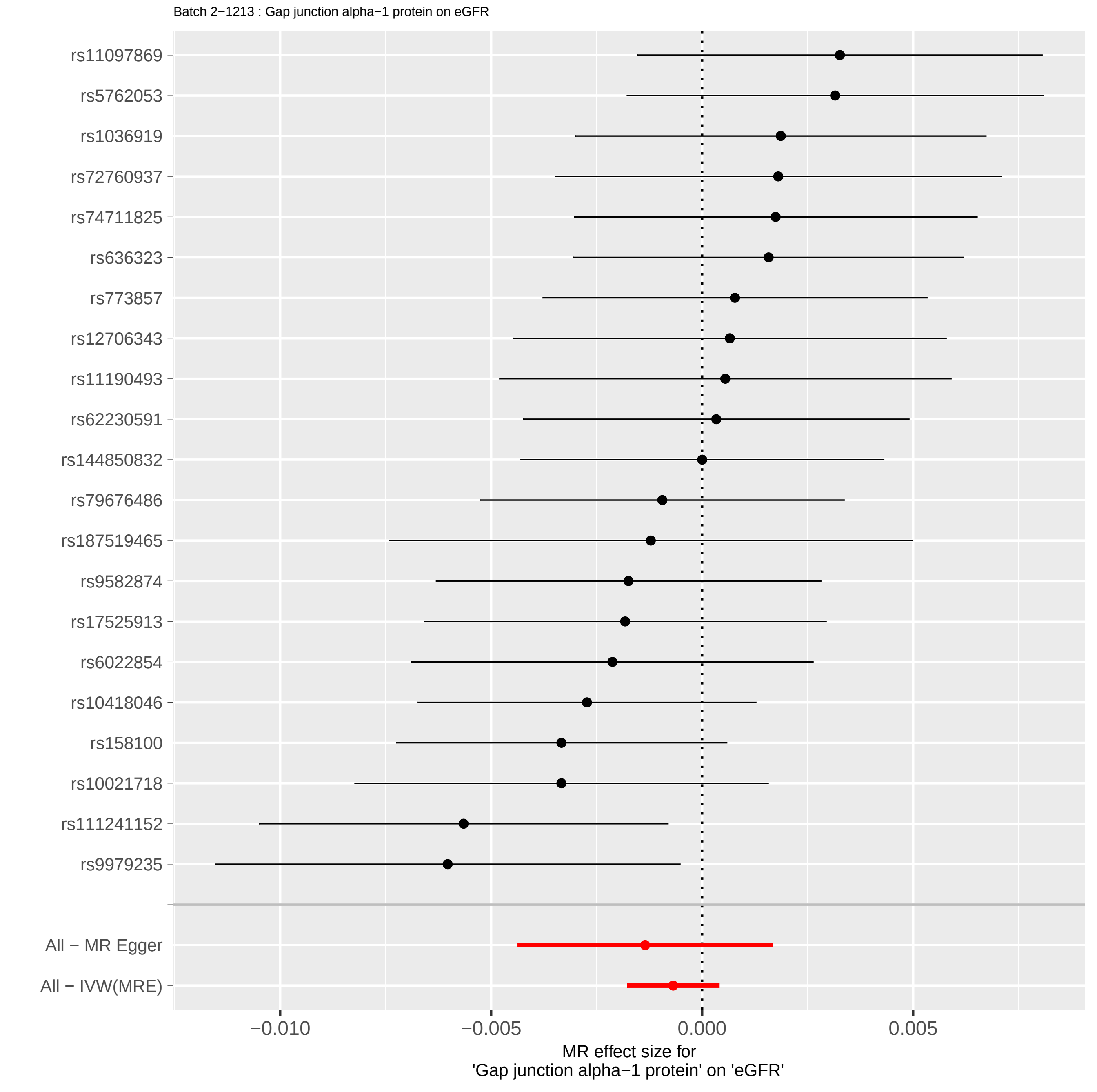
Batch 2−1213 : Gap junction alpha−1 protein on eGFR
rs11097869
rs5762053
rs1036919
rs72760937
rs74711825
rs636323
rs773857
rs12706343
rs11190493
rs62230591
rs144850832
rs79676486
rs187519465
rs9582874
rs17525913
rs6022854
rs10418046
rs158100
rs10021718
rs111241152
rs9979235
All − MR Egger
All − IVW(MRE)
−0.010
−0.005
0.000
0.005
MR effect size for
'Gap junction alpha−1 protein' on 'eGFR'

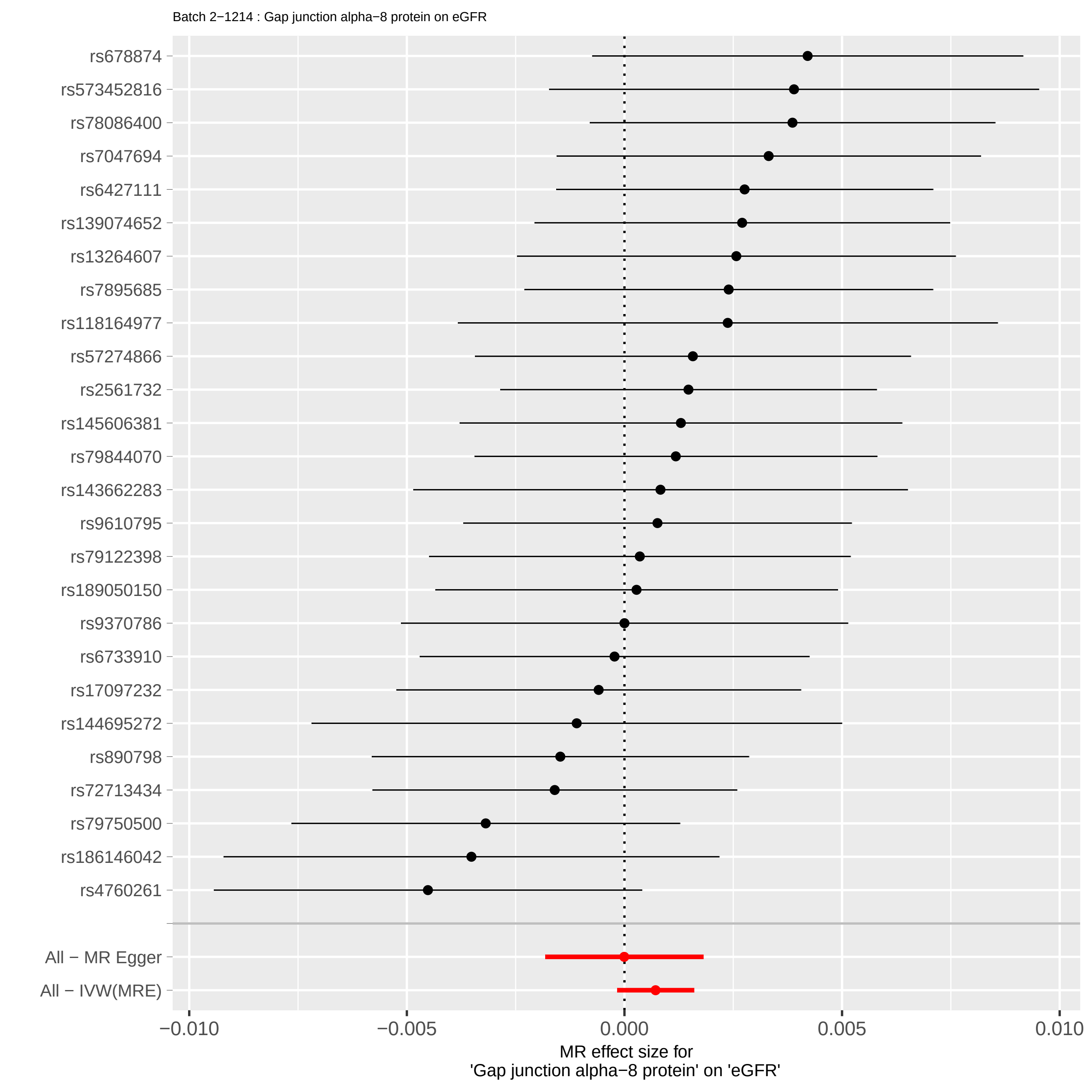

Batch 2−1214 : Gap junction alpha−8 protein on eGFR
rs678874
rs573452816
rs78086400
rs7047694
rs6427111
rs139074652
rs13264607
rs7895685
rs118164977
rs57274866
rs2561732
rs145606381
rs79844070
rs143662283
rs9610795
rs79122398
rs189050150
rs9370786
rs6733910
rs17097232
rs144695272
rs890798
rs72713434
rs79750500
rs186146042
rs4760261
All − MR Egger
All − IVW(MRE)
−0.010
−0.005
0.000
0.005
0.010
MR effect size for
'Gap junction alpha−8 protein' on 'eGFR'

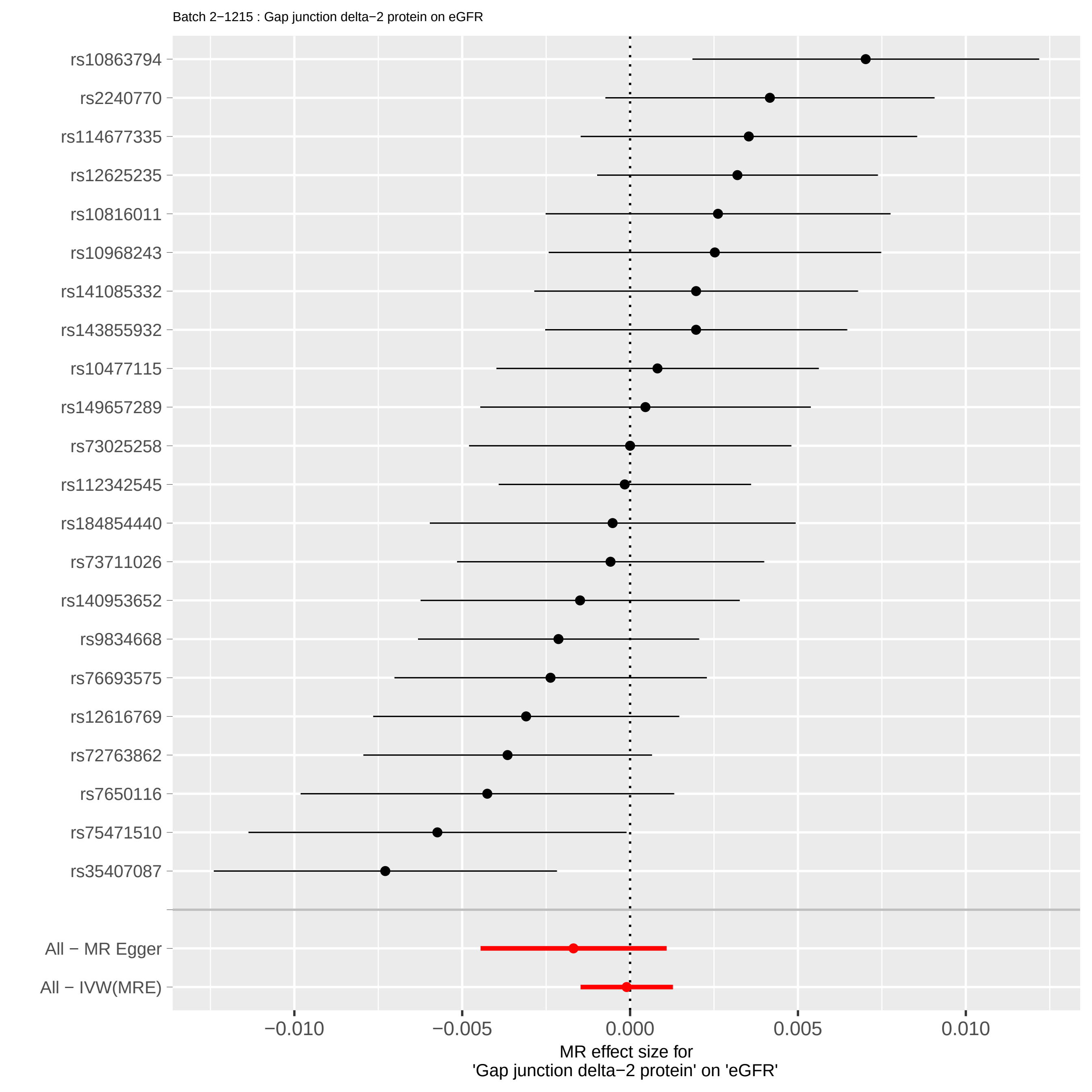

Batch 2−1215 : Gap junction delta−2 protein on eGFR
rs10863794
rs2240770
rs114677335
rs12625235
rs10816011
rs10968243
rs141085332
rs143855932
rs10477115
rs149657289
rs73025258
rs112342545
rs184854440
rs73711026
rs140953652
rs9834668
rs76693575
rs12616769
rs72763862
rs7650116
rs75471510
rs35407087
All − MR Egger
All − IVW(MRE)
−0.010
−0.005
0.000
0.005
0.010
MR effect size for
'Gap junction delta−2 protein' on 'eGFR'

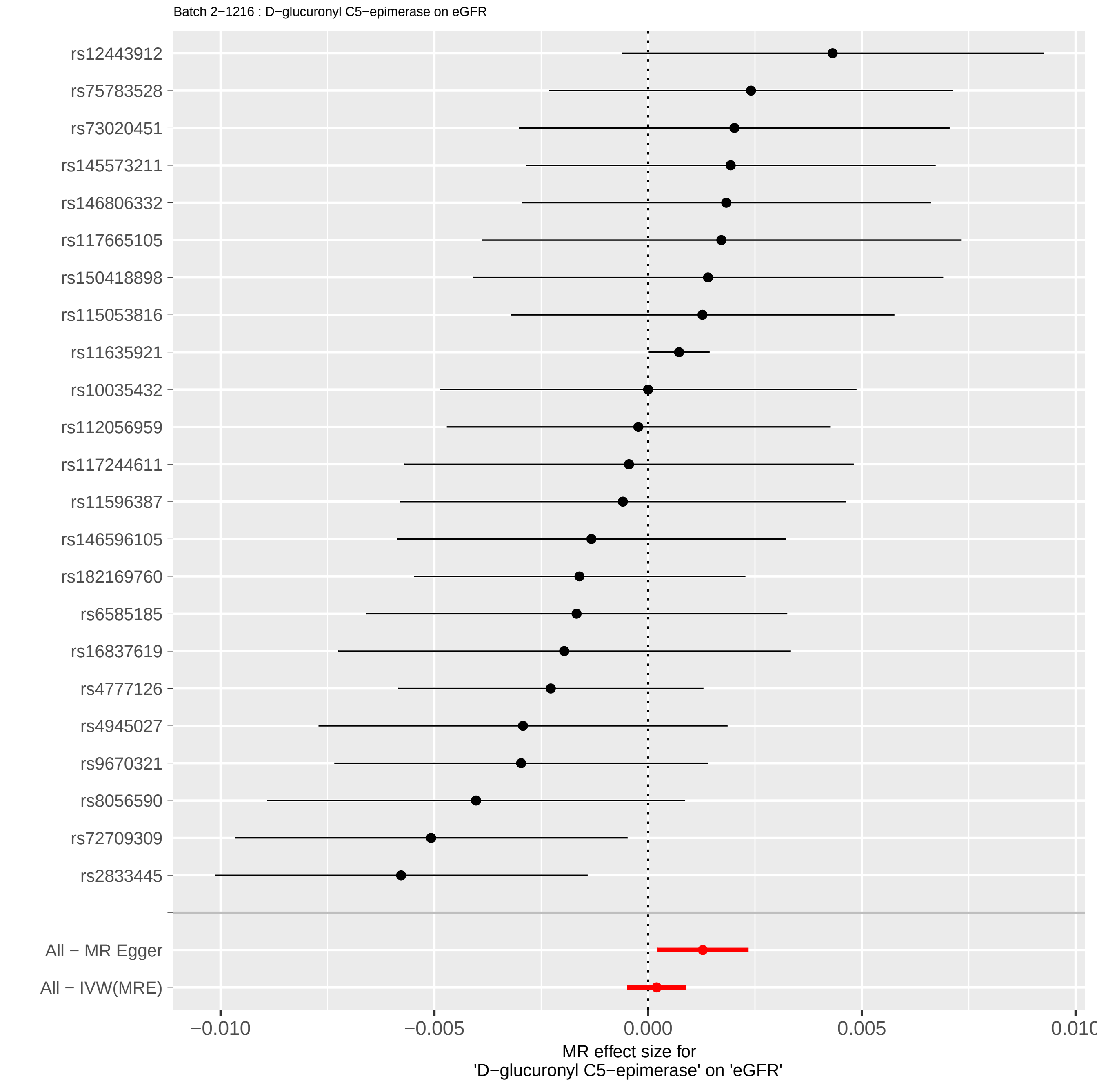

Batch 2−1216 : D−glucuronyl C5−epimerase on eGFR
rs12443912
rs75783528
rs73020451
rs145573211
rs146806332
rs117665105
rs150418898
rs115053816
rs11635921
rs10035432
rs112056959
rs117244611
rs11596387
rs146596105
rs182169760
rs6585185
rs16837619
rs4777126
rs4945027
rs9670321
rs8056590
rs72709309
rs2833445
All − MR Egger
All − IVW(MRE)
−0.010
−0.005
0.000
0.005
0.010
MR effect size for
'D−glucuronyl C5−epimerase' on 'eGFR'

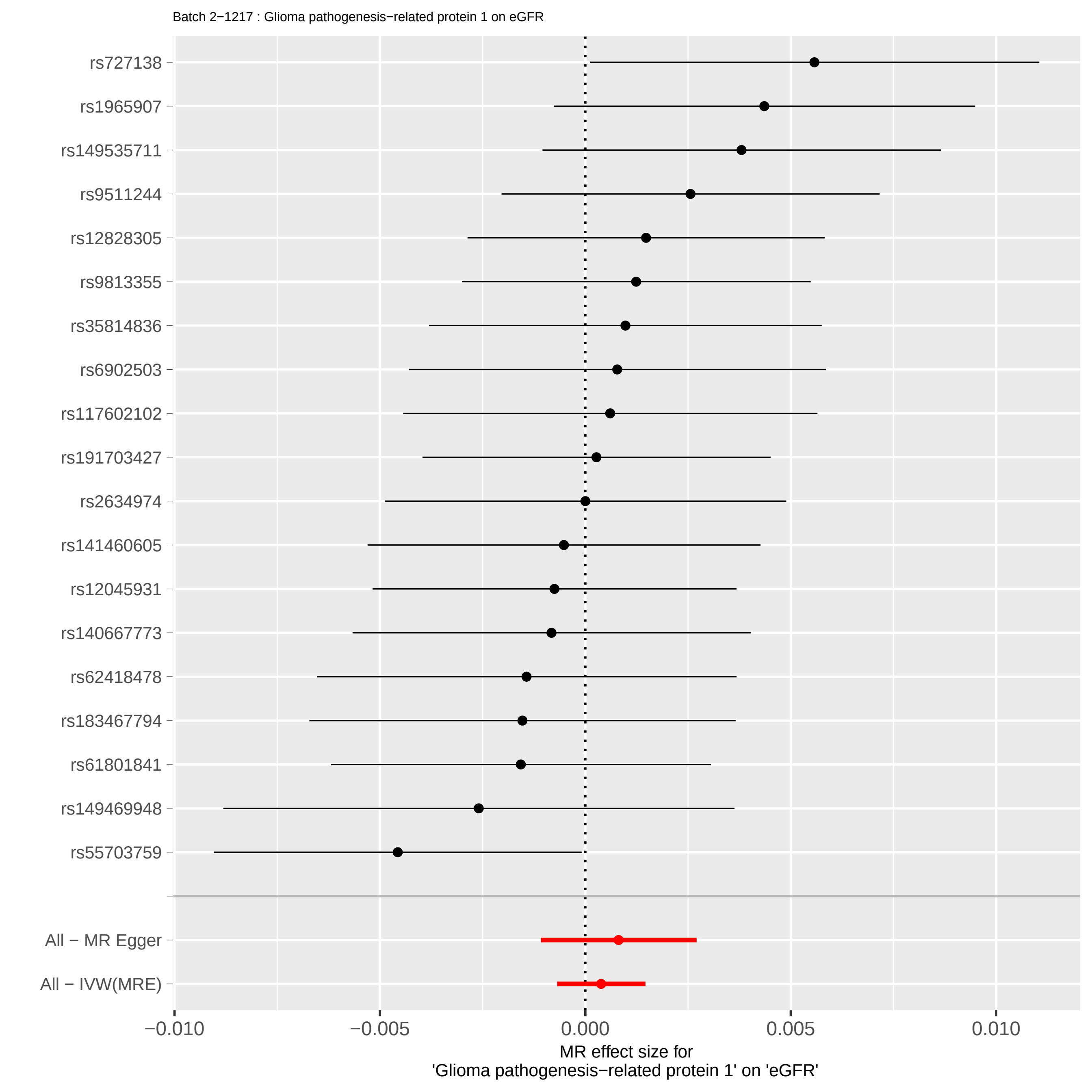
Batch 2−1217 : Glioma pathogenesis−related protein 1 on eGFR
rs727138
rs1965907
rs149535711
rs9511244
rs12828305
rs9813355
rs35814836
rs6902503
rs117602102
rs191703427
rs2634974
rs141460605
rs12045931
rs140667773
rs62418478
rs183467794
rs61801841
rs149469948
rs55703759
All − MR Egger
All − IVW(MRE)
−0.010
−0.005
0.000
0.005
0.010
MR effect size for
'Glioma pathogenesis−related protein 1' on 'eGFR'

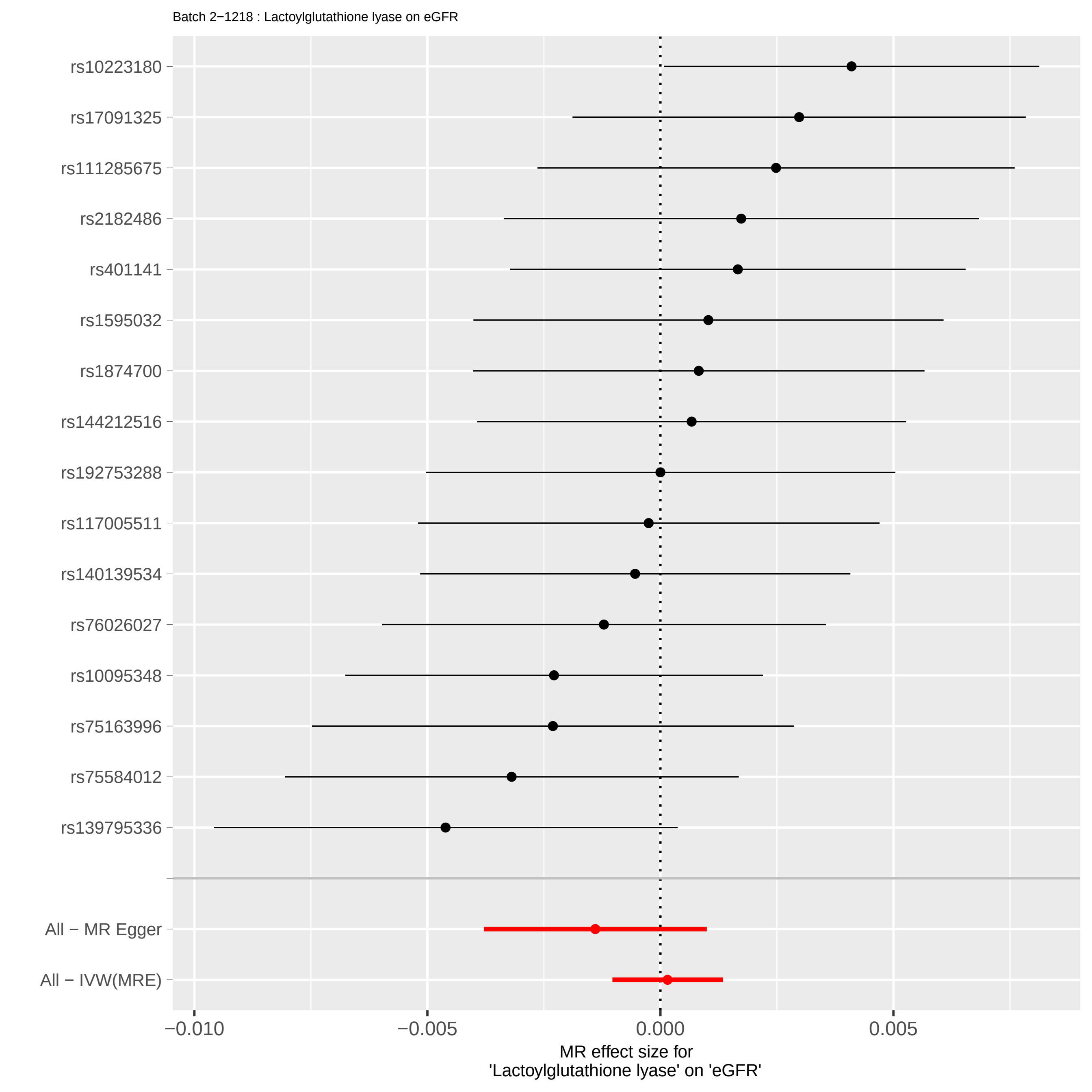
Batch 2−1218 : Lactoylglutathione lyase on eGFR
rs10223180
rs17091325
rs111285675
rs2182486
rs401141
rs1595032
rs1874700
rs144212516
rs192753288
rs117005511
rs140139534
rs76026027
rs10095348
rs75163996
rs75584012
rs139795336
All − MR Egger
All − IVW(MRE)
−0.010
−0.005
0.000
0.005
MR effect size for
'Lactoylglutathione lyase' on 'eGFR'

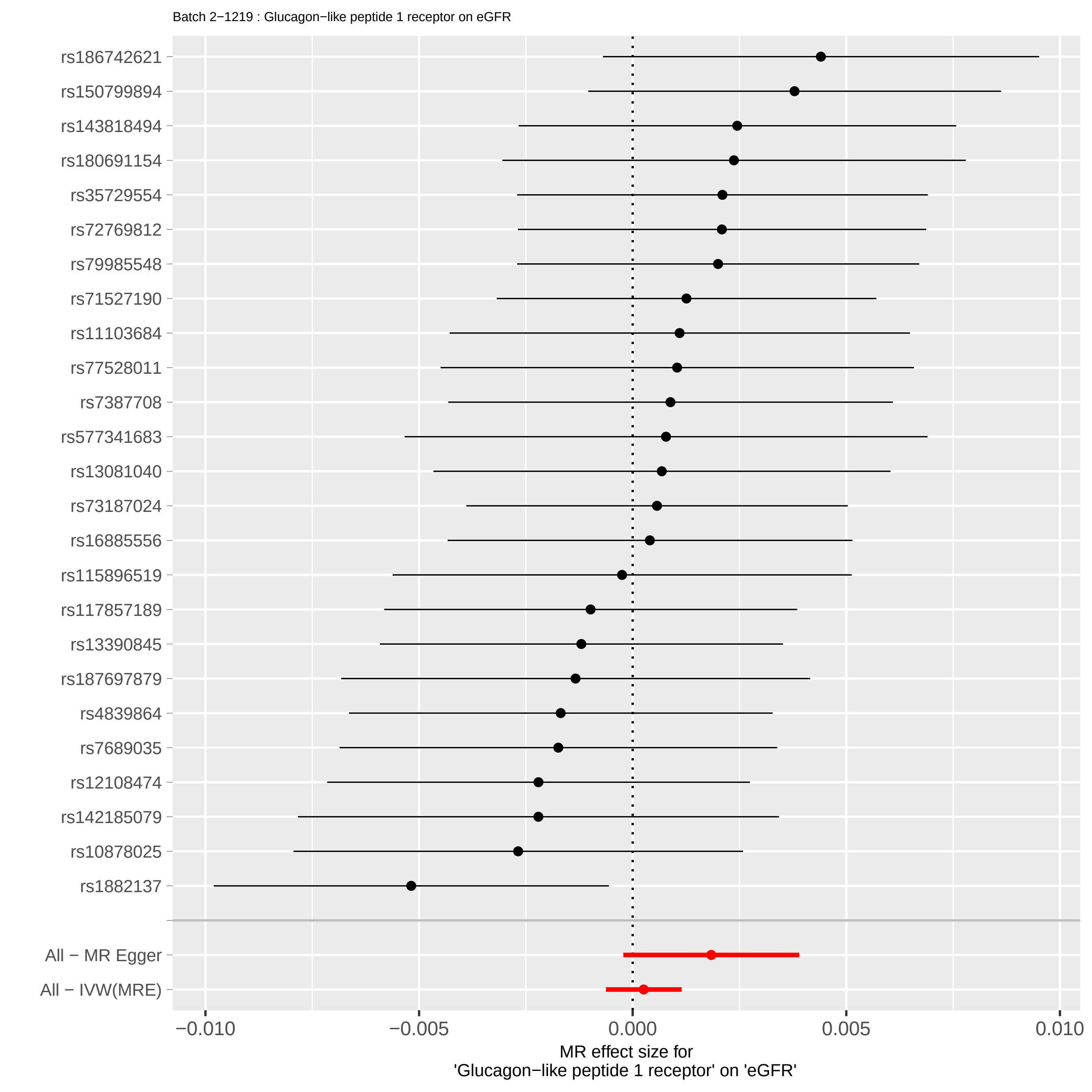
Batch 2−1219 : Glucagon−like peptide 1 receptor on eGFR
rs186742621
rs150799894
rs143818494
rs180691154
rs35729554
rs72769812
rs79985548
rs71527190
rs11103684
rs77528011
rs7387708
rs577341683
rs13081040
rs73187024
rs16885556
rs115896519
rs117857189
rs13390845
rs187697879
rs4839864
rs7689035
rs12108474
rs142185079
rs10878025
rs1882137
All − MR Egger
All − IVW(MRE)
−0.010
−0.005
0.000
0.005
0.010
MR effect size for
'Glucagon−like peptide 1 receptor' on 'eGFR'

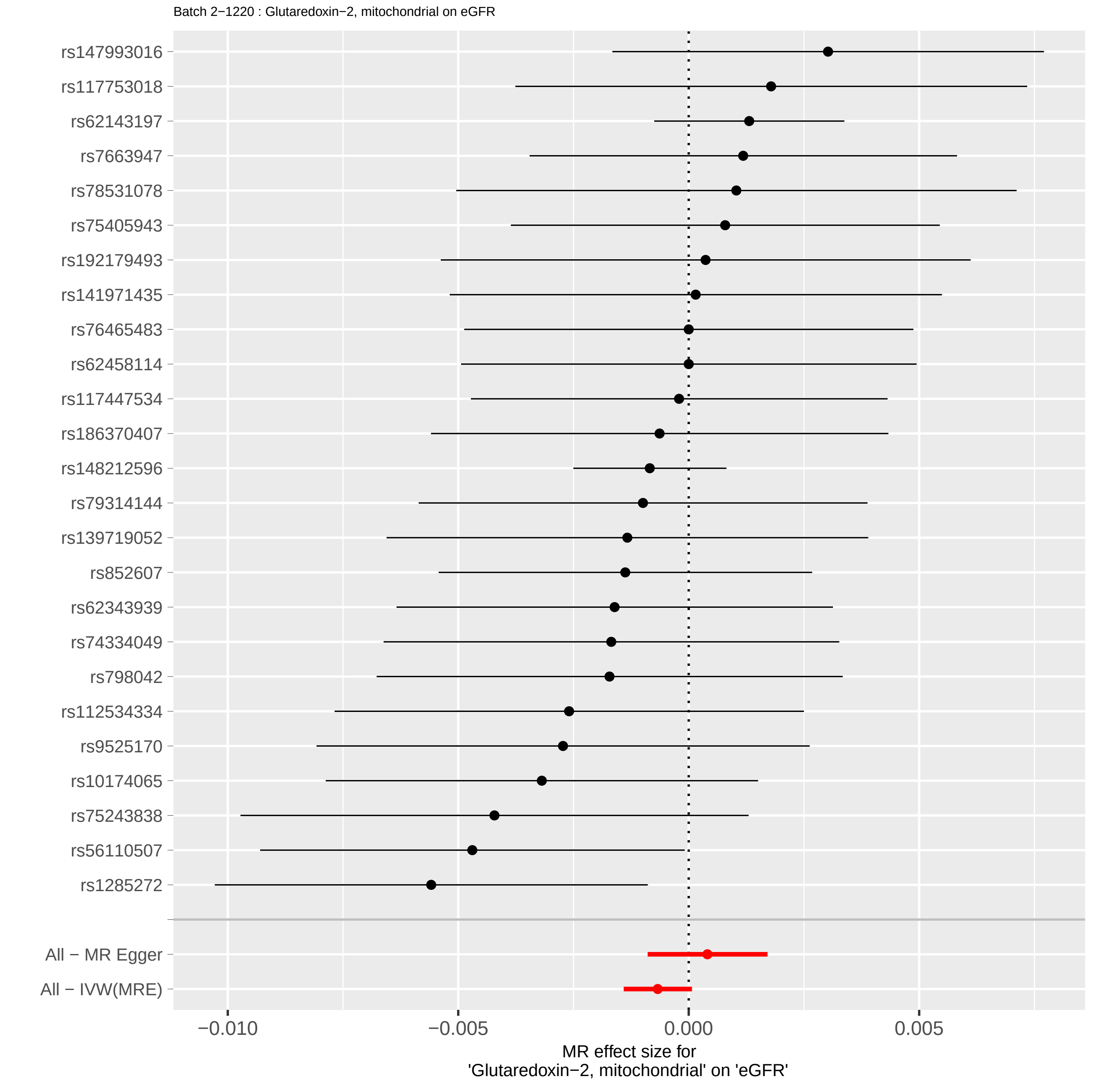
Batch 2−1220 : Glutaredoxin−2, mitochondrial on eGFR
rs147993016
rs117753018
rs62143197
rs7663947
rs78531078
rs75405943
rs192179493
rs141971435
rs76465483
rs62458114
rs117447534
rs186370407
rs148212596
rs79314144
rs139719052
rs852607
rs62343939
rs74334049
rs798042
rs112534334
rs9525170
rs10174065
rs75243838
rs56110507
rs1285272
All − MR Egger
All − IVW(MRE)
−0.010
−0.005
0.000
0.005
MR effect size for
'Glutaredoxin−2, mitochondrial' on 'eGFR'

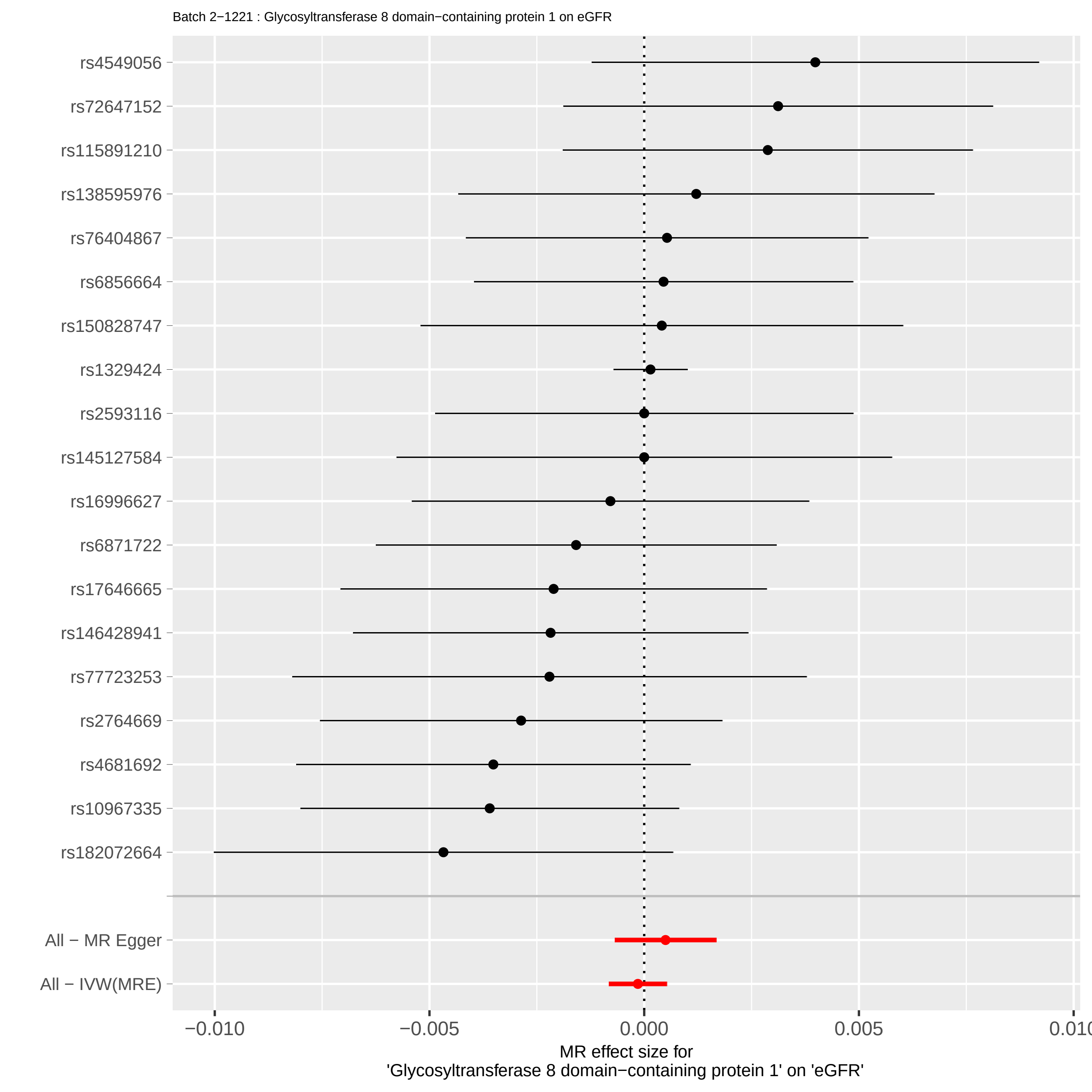
Batch 2−1221 : Glycosyltransferase 8 domain−containing protein 1 on eGFR
rs4549056
rs72647152
rs115891210
rs138595976
rs76404867
rs6856664
rs150828747
rs1329424
rs2593116
rs145127584
rs16996627
rs6871722
rs17646665
rs146428941
rs77723253
rs2764669
rs4681692
rs10967335
rs182072664
All − MR Egger
All − IVW(MRE)
−0.010
−0.005
0.000
0.005
0.010
MR effect size for
'Glycosyltransferase 8 domain−containing protein 1' on 'eGFR'

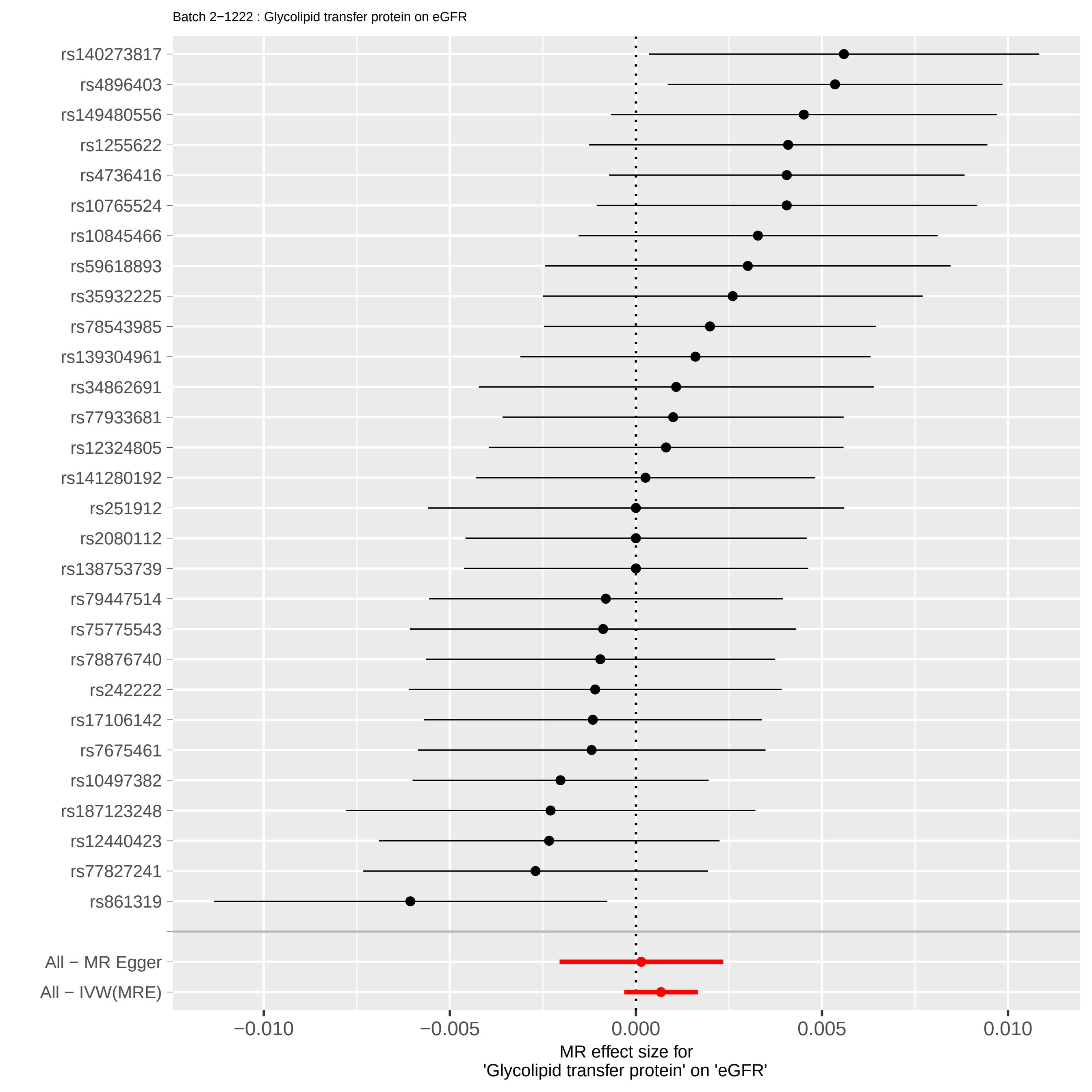
Batch 2−1222 : Glycolipid transfer protein on eGFR
rs140273817
rs4896403
rs149480556
rs1255622
rs4736416
rs10765524
rs10845466
rs59618893
rs35932225
rs78543985
rs139304961
rs34862691
rs77933681
rs12324805
rs141280192
rs251912
rs2080112
rs138753739
rs79447514
rs75775543
rs78876740
rs242222
rs17106142
rs7675461
rs10497382
rs187123248
rs12440423
rs77827241
rs861319
All − MR Egger
All − IVW(MRE)
−0.010
−0.005
0.000
0.005
0.010
MR effect size for
'Glycolipid transfer protein' on 'eGFR'

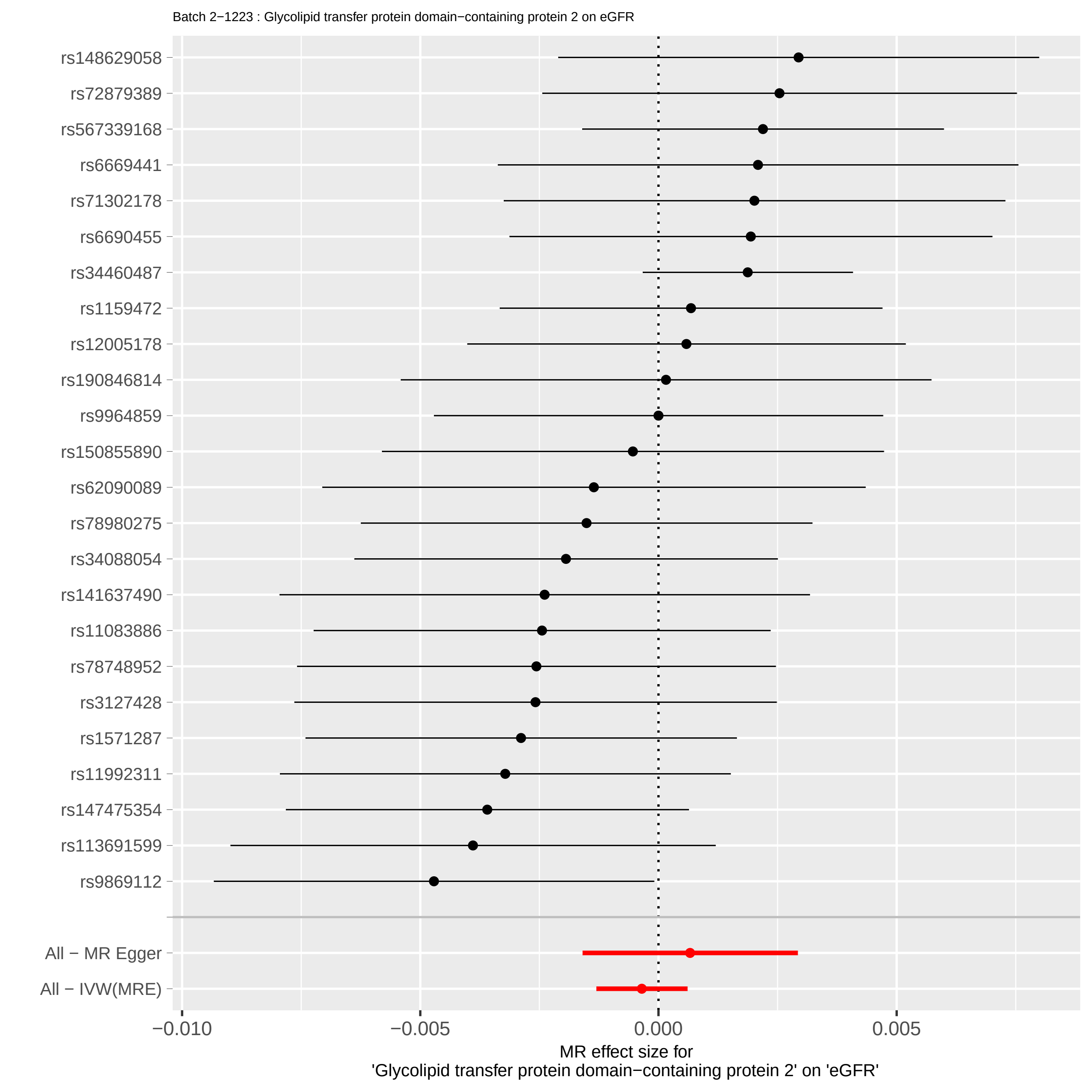
Batch 2−1223 : Glycolipid transfer protein domain−containing protein 2 on eGFR
rs148629058
rs72879389
rs567339168
rs6669441
rs71302178
rs6690455
rs34460487
rs1159472
rs12005178
rs190846814
rs9964859
rs150855890
rs62090089
rs78980275
rs34088054
rs141637490
rs11083886
rs78748952
rs3127428
rs1571287
rs11992311
rs147475354
rs113691599
rs9869112
All − MR Egger
All − IVW(MRE)
−0.010
−0.005
0.000
0.005
MR effect size for
'Glycolipid transfer protein domain−containing protein 2' on 'eGFR'

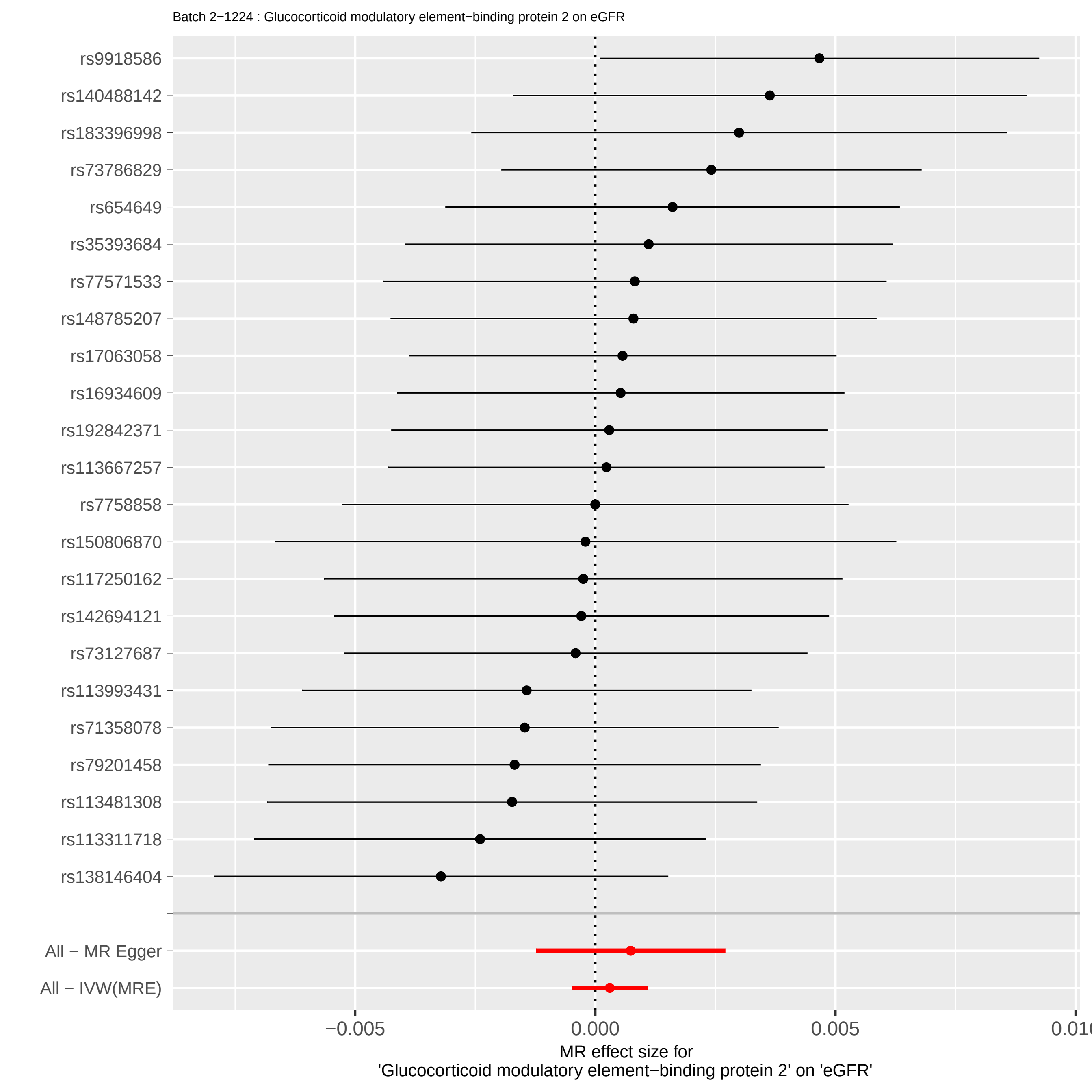
Batch 2−1224 : Glucocorticoid modulatory element−binding protein 2 on eGFR
rs9918586
rs140488142
rs183396998
rs73786829
rs654649
rs35393684
rs77571533
rs148785207
rs17063058
rs16934609
rs192842371
rs113667257
rs7758858
rs150806870
rs117250162
rs142694121
rs73127687
rs113993431
rs71358078
rs79201458
rs113481308
rs113311718
rs138146404
All − MR Egger
All − IVW(MRE)
−0.005
0.000
0.005
0.010
MR effect size for
'Glucocorticoid modulatory element−binding protein 2' on 'eGFR'

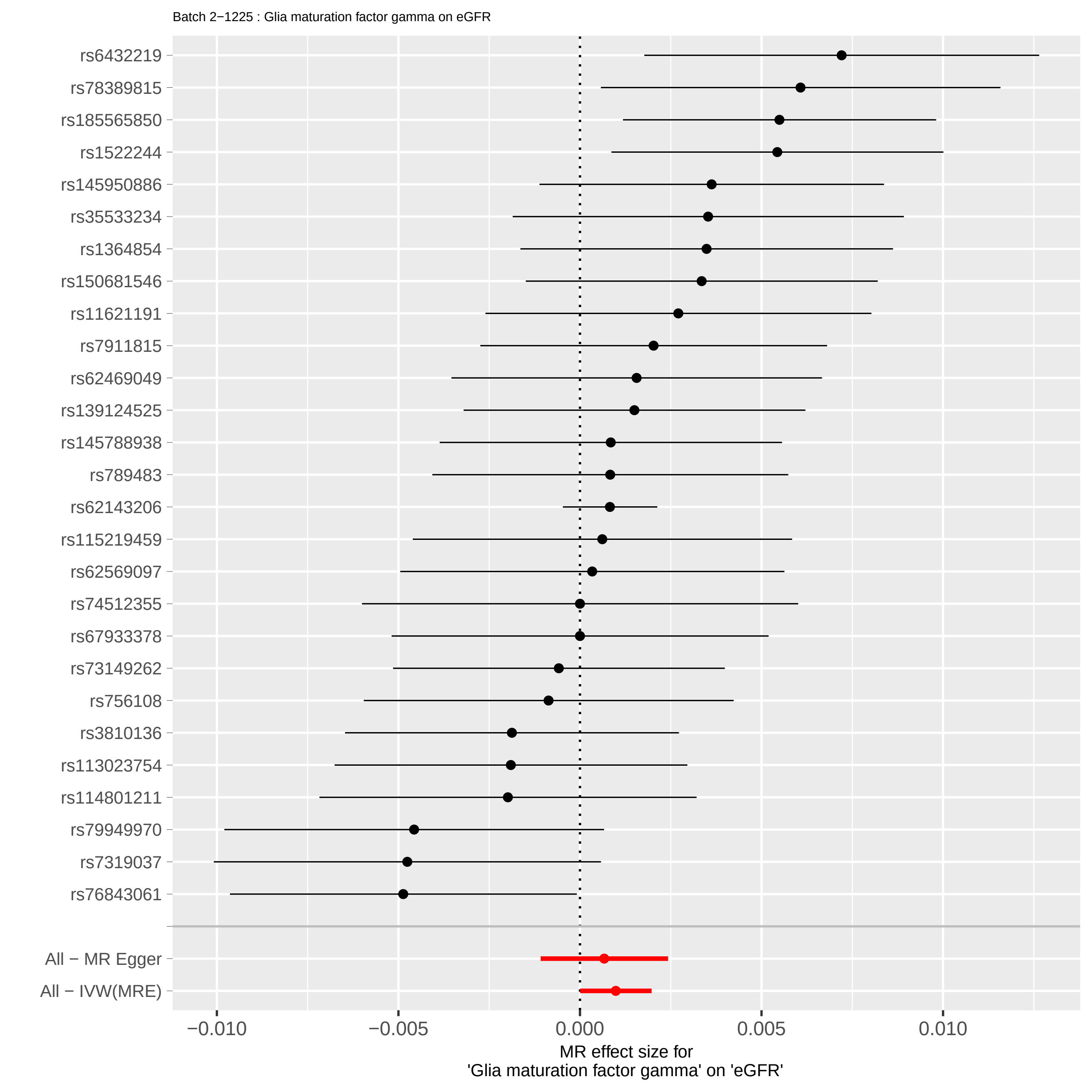

Batch 2−1225 : Glia maturation factor gamma on eGFR
rs6432219
rs78389815
rs185565850
rs1522244
rs145950886
rs35533234
rs1364854
rs150681546
rs11621191
rs7911815
rs62469049
rs139124525
rs145788938
rs789483
rs62143206
rs115219459
rs62569097
rs74512355
rs67933378
rs73149262
rs756108
rs3810136
rs113023754
rs114801211
rs79949970
rs7319037
rs76843061
All − MR Egger
All − IVW(MRE)
−0.010
−0.005
0.000
0.005
0.010
MR effect size for
'Glia maturation factor gamma' on 'eGFR'

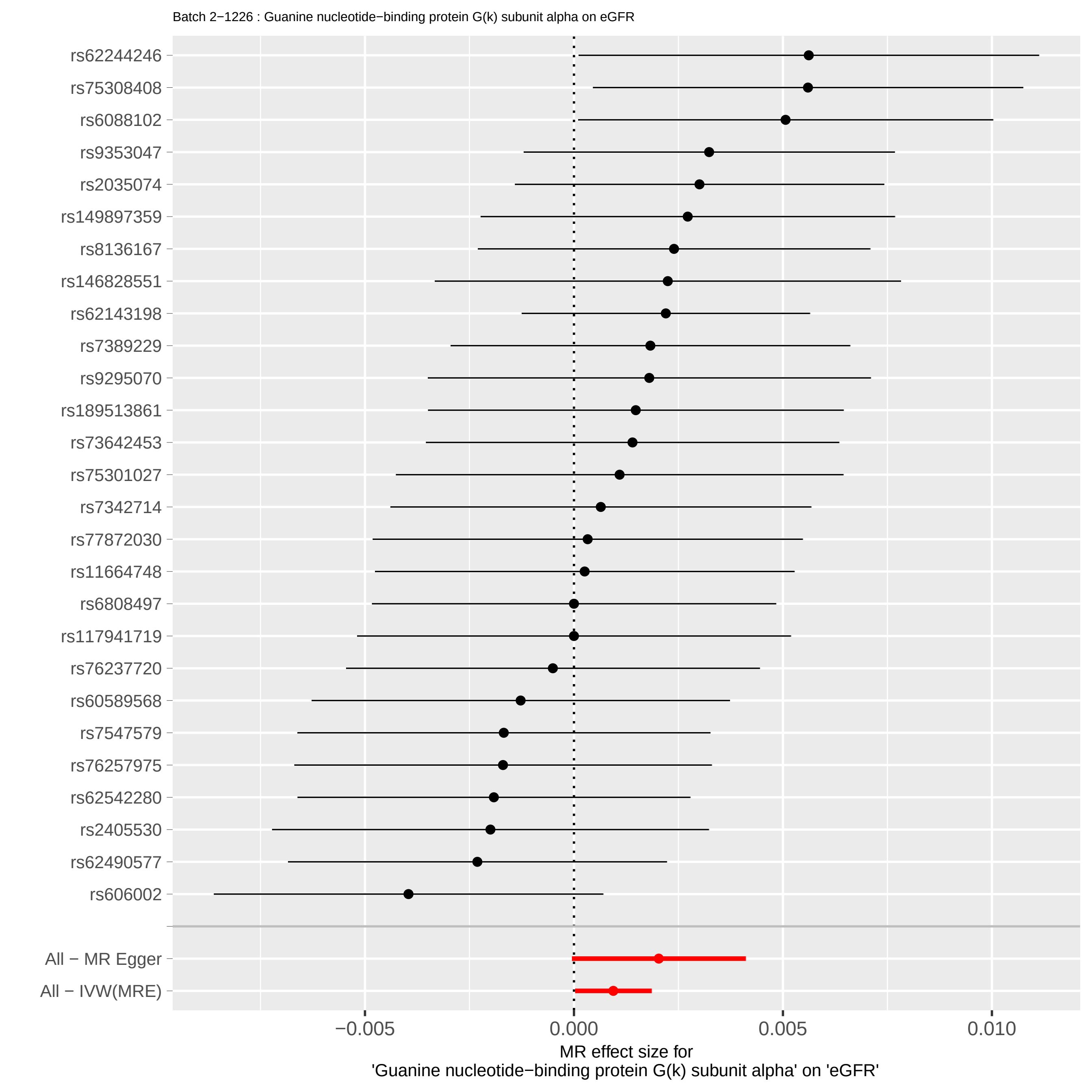

Batch 2−1226 : Guanine nucleotide−binding protein G(k) subunit alpha on eGFR
rs62244246
rs75308408
rs6088102
rs9353047
rs2035074
rs149897359
rs8136167
rs146828551
rs62143198
rs7389229
rs9295070
rs189513861
rs73642453
rs75301027
rs7342714
rs77872030
rs11664748
rs6808497
rs117941719
rs76237720
rs60589568
rs7547579
rs76257975
rs62542280
rs2405530
rs62490577
rs606002
All − MR Egger
All − IVW(MRE)
−0.005
0.000
0.005
0.010
MR effect size for
'Guanine nucleotide−binding protein G(k) subunit alpha' on 'eGFR'

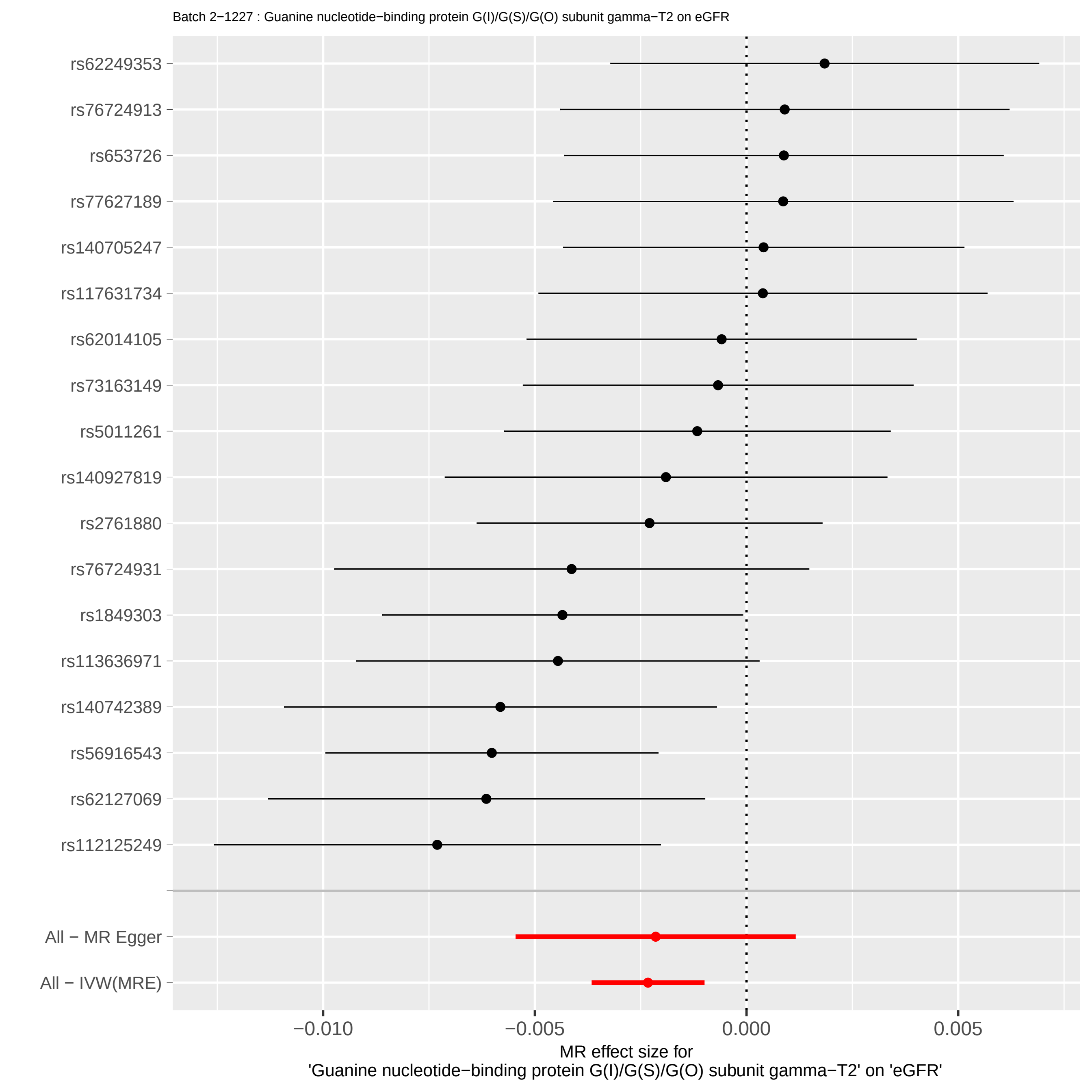

Batch 2−1227 : Guanine nucleotide−binding protein G(I)/G(S)/G(O) subunit gamma−T2 on eGFR
rs62249353
rs76724913
rs653726
rs77627189
rs140705247
rs117631734
rs62014105
rs73163149
rs5011261
rs140927819
rs2761880
rs76724931
rs1849303
rs113636971
rs140742389
rs56916543
rs62127069
rs112125249
All − MR Egger
All − IVW(MRE)
−0.010
−0.005
0.000
0.005
MR effect size for
'Guanine nucleotide−binding protein G(I)/G(S)/G(O) subunit gamma−T2' on 'eGFR'

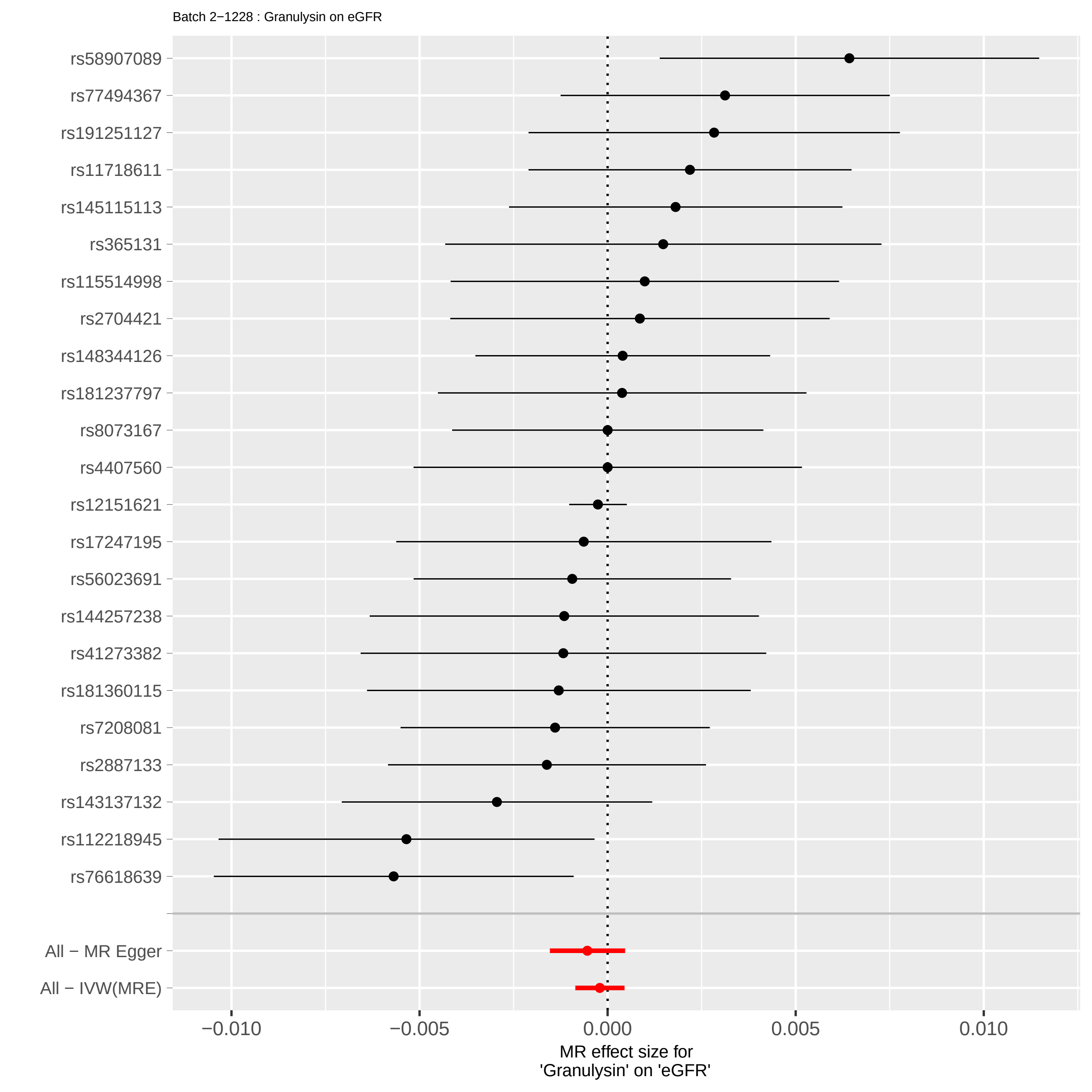

Batch 2−1228 : Granulysin on eGFR
rs58907089
rs77494367
rs191251127
rs11718611
rs145115113
rs365131
rs115514998
rs2704421
rs148344126
rs181237797
rs8073167
rs4407560
rs12151621
rs17247195
rs56023691
rs144257238
rs41273382
rs181360115
rs7208081
rs2887133
rs143137132
rs112218945
rs76618639
All − MR Egger
All − IVW(MRE)
−0.010
−0.005
0.000
0.005
0.010
MR effect size for
'Granulysin' on 'eGFR'

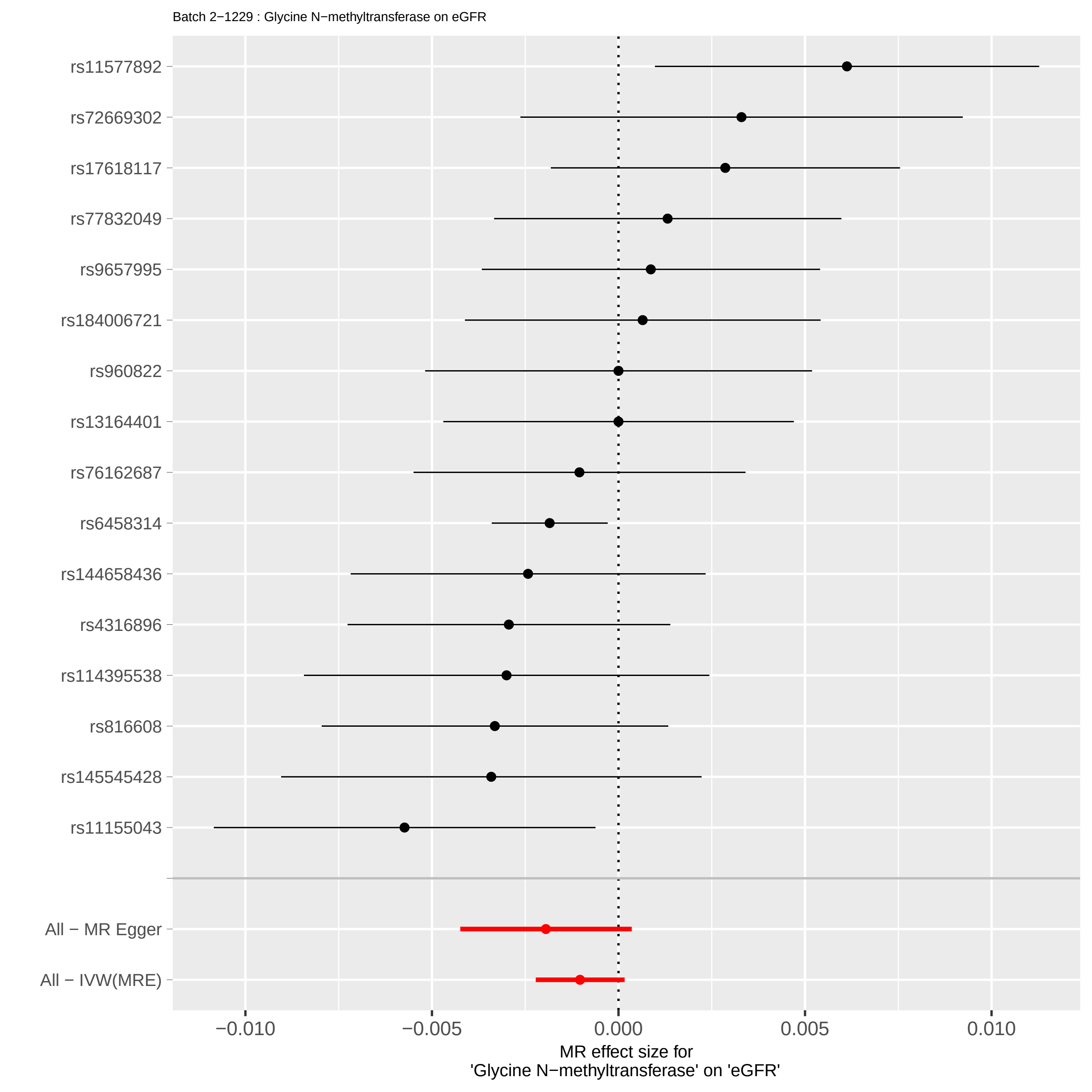
Batch 2−1229 : Glycine N−methyltransferase on eGFR
rs11577892
rs72669302
rs17618117
rs77832049
rs9657995
rs184006721
rs960822
rs13164401
rs76162687
rs6458314
rs144658436
rs4316896
rs114395538
rs816608
rs145545428
rs11155043
All − MR Egger
All − IVW(MRE)
−0.010
−0.005
0.000
0.005
0.010
MR effect size for
'Glycine N−methyltransferase' on 'eGFR'

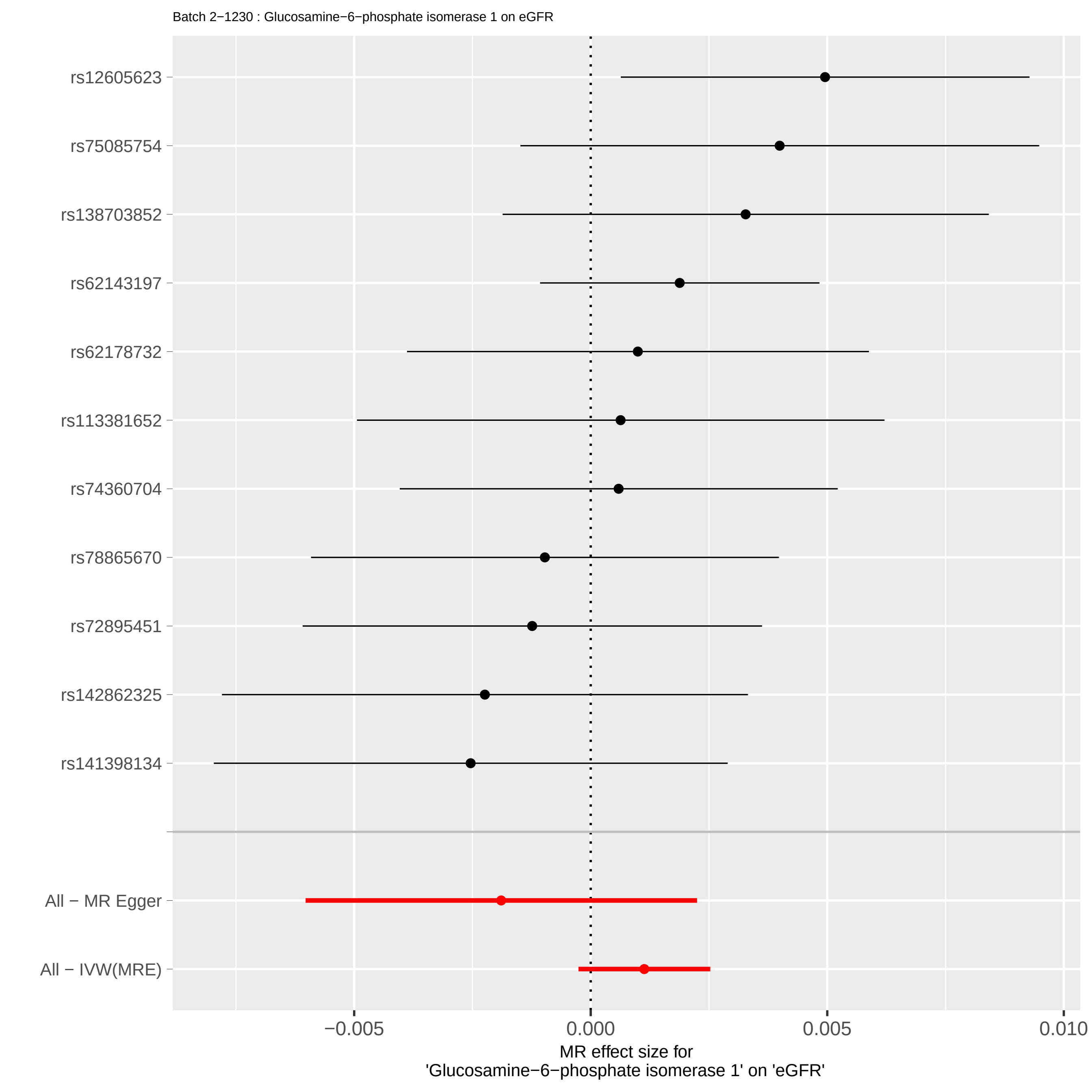

Batch 2−1230 : Glucosamine−6−phosphate isomerase 1 on eGFR
rs12605623
rs75085754
rs138703852
rs62143197
rs62178732
rs113381652
rs74360704
rs78865670
rs72895451
rs142862325
rs141398134
All − MR Egger
All − IVW(MRE)
−0.005
0.000
0.005
0.010
MR effect size for
'Glucosamine−6−phosphate isomerase 1' on 'eGFR'

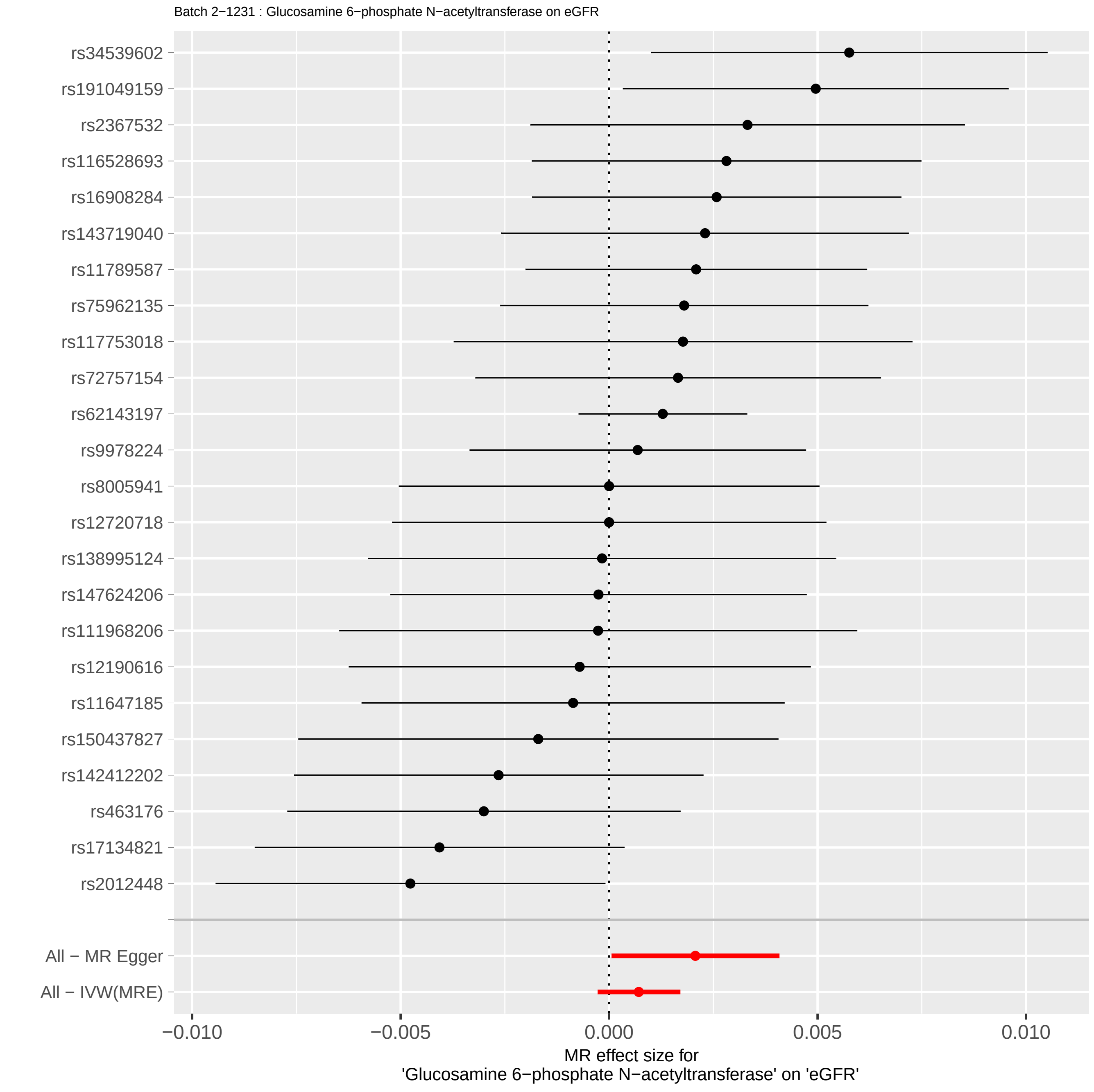

Batch 2−1231 : Glucosamine 6−phosphate N−acetyltransferase on eGFR
rs34539602
rs191049159
rs2367532
rs116528693
rs16908284
rs143719040
rs11789587
rs75962135
rs117753018
rs72757154
rs62143197
rs9978224
rs8005941
rs12720718
rs138995124
rs147624206
rs111968206
rs12190616
rs11647185
rs150437827
rs142412202
rs463176
rs17134821
rs2012448
All − MR Egger
All − IVW(MRE)
−0.010
−0.005
0.000
0.005
0.010
MR effect size for
'Glucosamine 6−phosphate N−acetyltransferase' on 'eGFR'

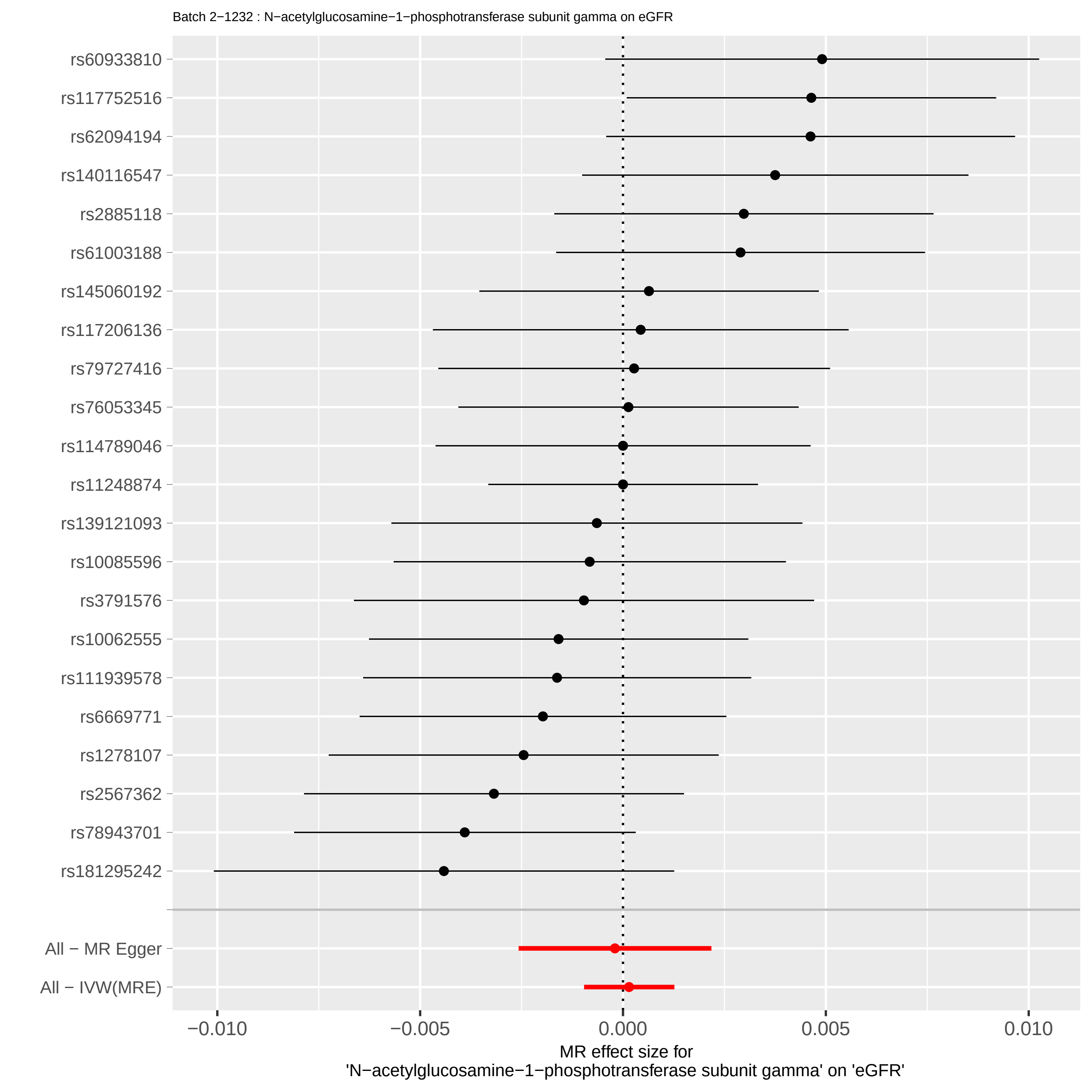

Batch 2−1232 : N−acetylglucosamine−1−phosphotransferase subunit gamma on eGFR
rs60933810
rs117752516
rs62094194
rs140116547
rs2885118
rs61003188
rs145060192
rs117206136
rs79727416
rs76053345
rs114789046
rs11248874
rs139121093
rs10085596
rs3791576
rs10062555
rs111939578
rs6669771
rs1278107
rs2567362
rs78943701
rs181295242
All − MR Egger
All − IVW(MRE)
−0.010
−0.005
0.000
0.005
0.010
MR effect size for
'N−acetylglucosamine−1−phosphotransferase subunit gamma' on 'eGFR'

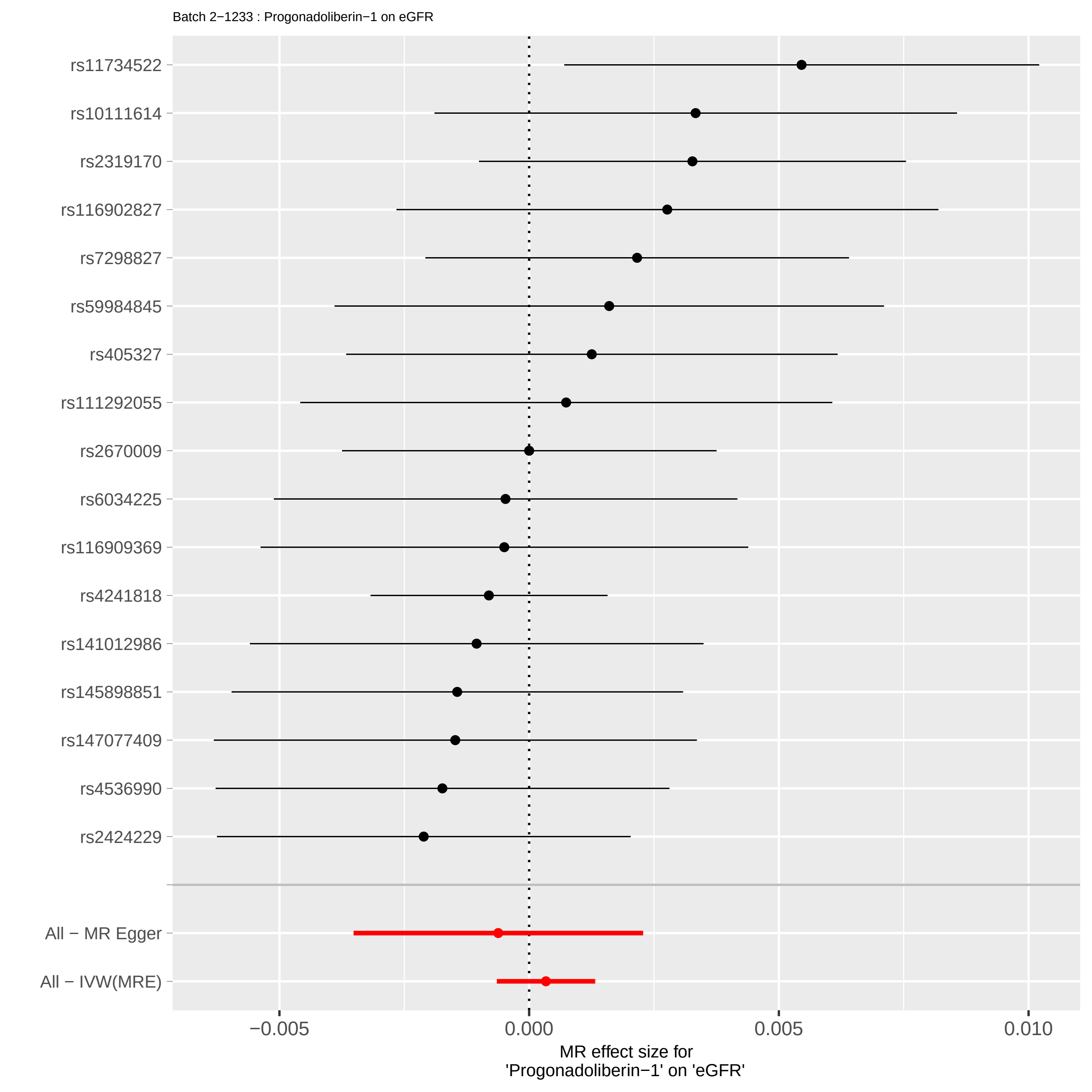

Batch 2−1233 : Progonadoliberin−1 on eGFR
rs11734522
rs10111614
rs2319170
rs116902827
rs7298827
rs59984845
rs405327
rs111292055
rs2670009
rs6034225
rs116909369
rs4241818
rs141012986
rs145898851
rs147077409
rs4536990
rs2424229
All − MR Egger
All − IVW(MRE)
−0.005
0.000
0.005
0.010
MR effect size for
'Progonadoliberin−1' on 'eGFR'

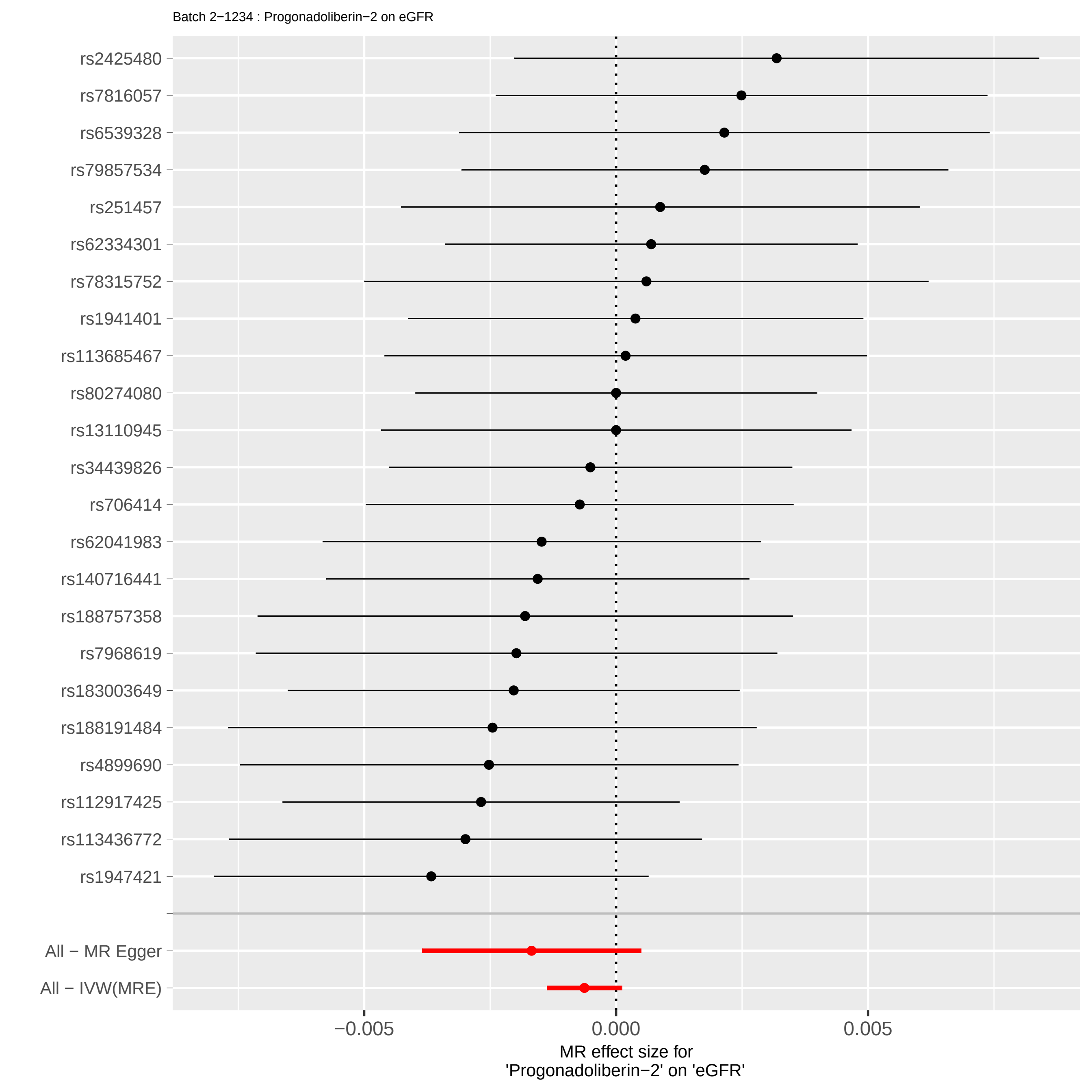
Batch 2−1234 : Progonadoliberin−2 on eGFR
rs2425480
rs7816057
rs6539328
rs79857534
rs251457
rs62334301
rs78315752
rs1941401
rs113685467
rs80274080
rs13110945
rs34439826
rs706414
rs62041983
rs140716441
rs188757358
rs7968619
rs183003649
rs188191484
rs4899690
rs112917425
rs113436772
rs1947421
All − MR Egger
All − IVW(MRE)
−0.005
0.000
0.005
MR effect size for
'Progonadoliberin−2' on 'eGFR'

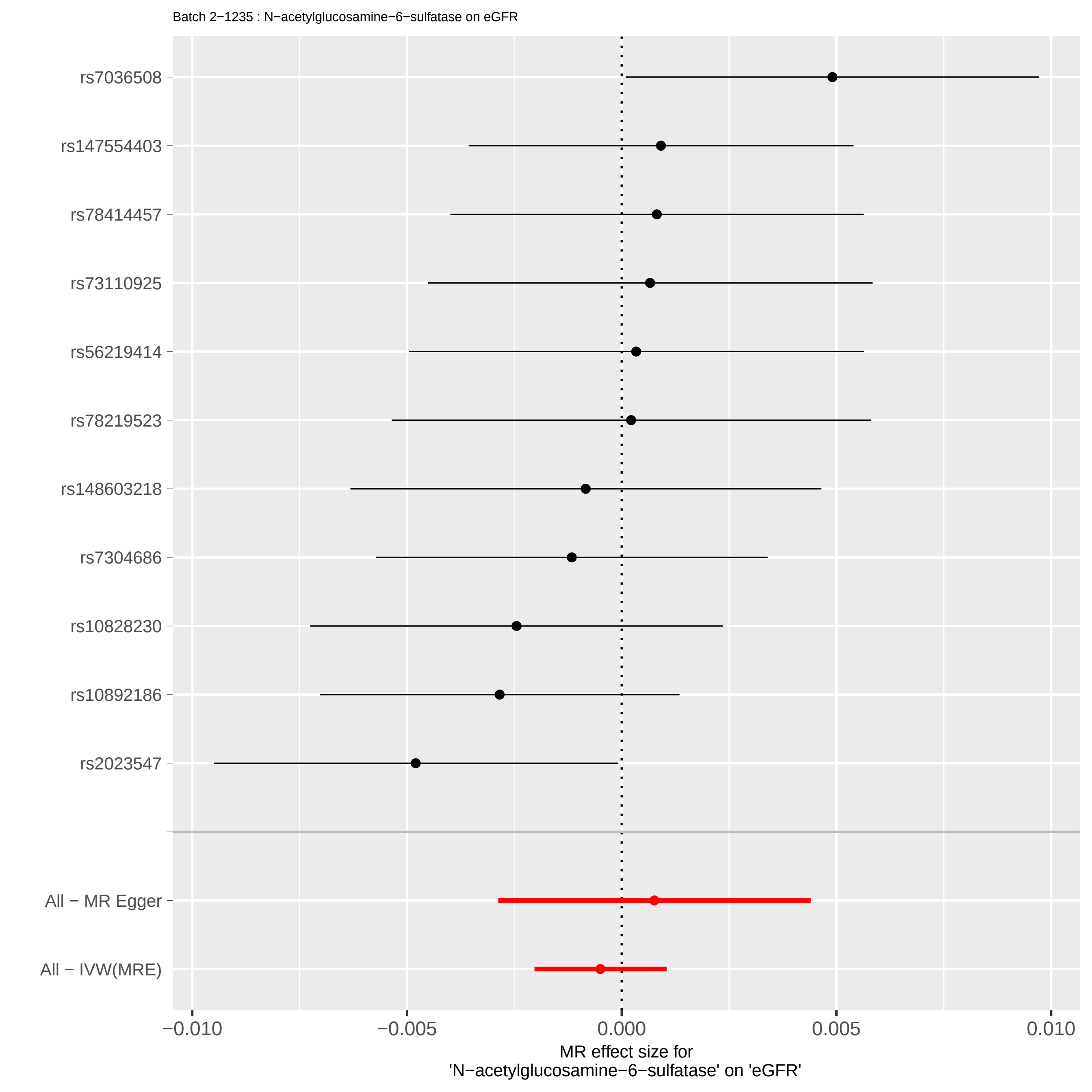

Batch 2−1235 : N−acetylglucosamine−6−sulfatase on eGFR
rs7036508
rs147554403
rs78414457
rs73110925
rs56219414
rs78219523
rs148603218
rs7304686
rs10828230
rs10892186
rs2023547
All − MR Egger
All − IVW(MRE)
−0.010
−0.005
0.000
0.005
0.010
MR effect size for
'N−acetylglucosamine−6−sulfatase' on 'eGFR'

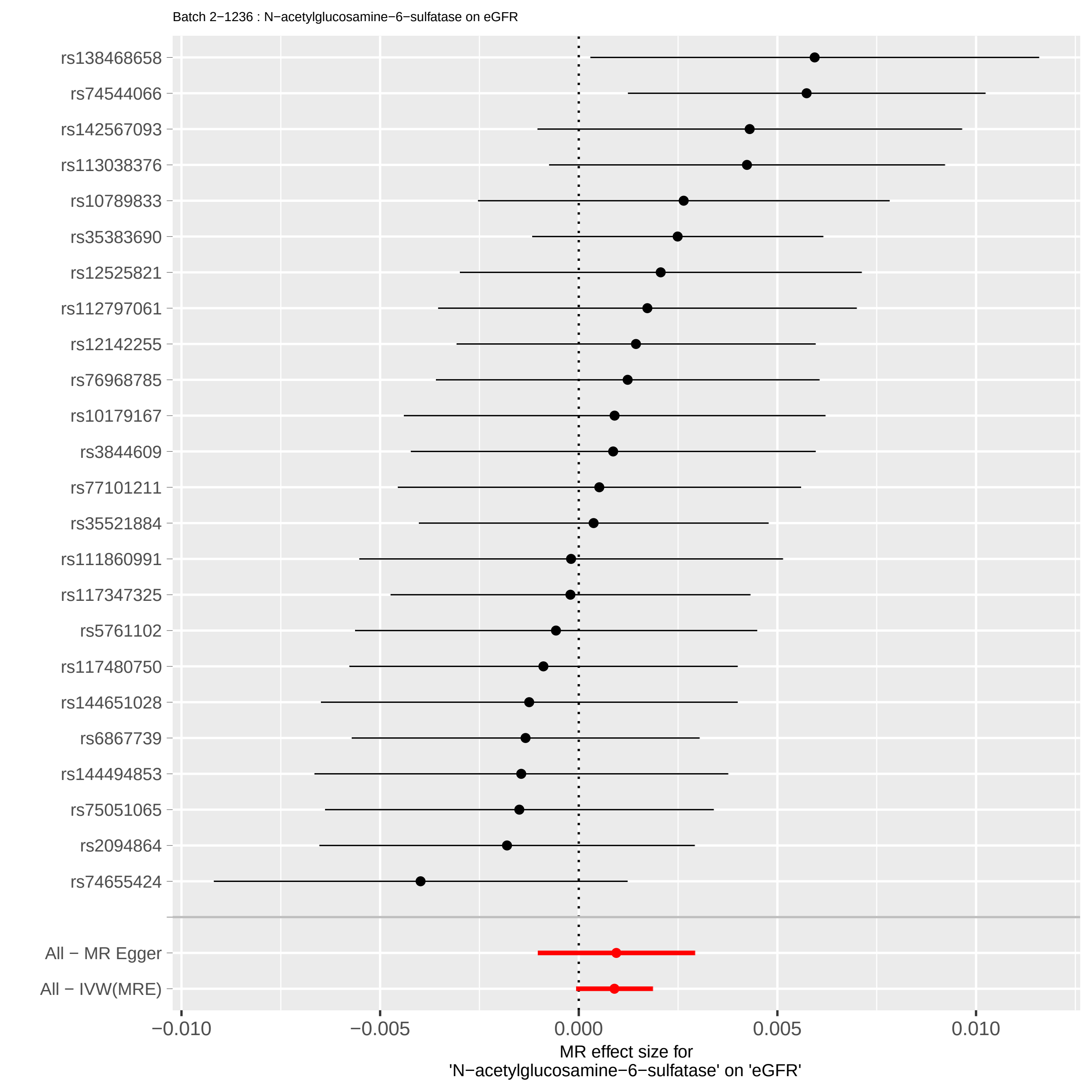
Batch 2−1236 : N−acetylglucosamine−6−sulfatase on eGFR
rs138468658
rs74544066
rs142567093
rs113038376
rs10789833
rs35383690
rs12525821
rs112797061
rs12142255
rs76968785
rs10179167
rs3844609
rs77101211
rs35521884
rs111860991
rs117347325
rs5761102
rs117480750
rs144651028
rs6867739
rs144494853
rs75051065
rs2094864
rs74655424
All − MR Egger
All − IVW(MRE)
−0.010
−0.005
0.000
0.005
0.010
MR effect size for
'N−acetylglucosamine−6−sulfatase' on 'eGFR'

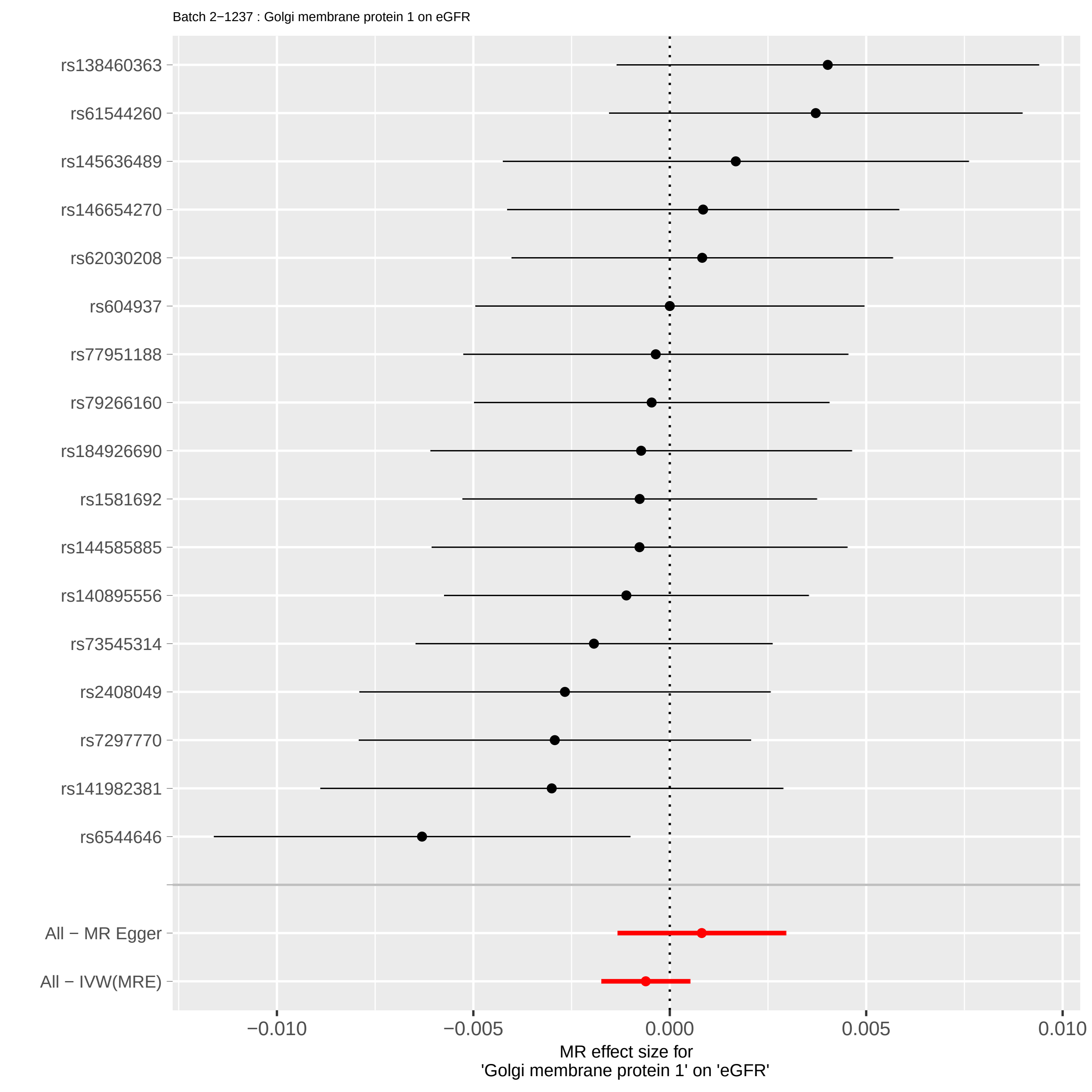

Batch 2−1237 : Golgi membrane protein 1 on eGFR
rs138460363
rs61544260
rs145636489
rs146654270
rs62030208
rs604937
rs77951188
rs79266160
rs184926690
rs1581692
rs144585885
rs140895556
rs73545314
rs2408049
rs7297770
rs141982381
rs6544646
All − MR Egger
All − IVW(MRE)
−0.010
−0.005
0.000
0.005
0.010
MR effect size for
'Golgi membrane protein 1' on 'eGFR'

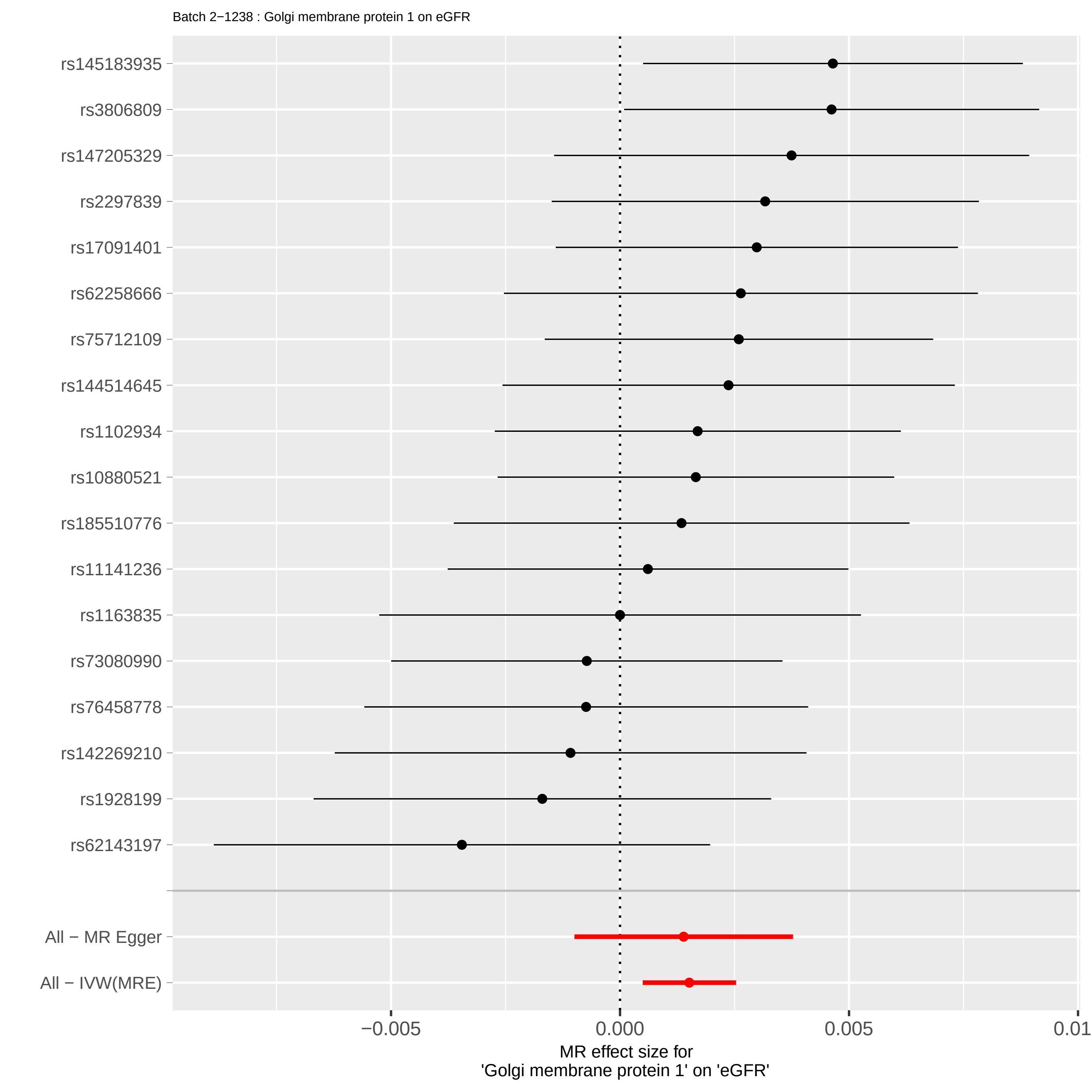

Batch 2−1238 : Golgi membrane protein 1 on eGFR
rs145183935
rs3806809
rs147205329
rs2297839
rs17091401
rs62258666
rs75712109
rs144514645
rs1102934
rs10880521
rs185510776
rs11141236
rs1163835
rs73080990
rs76458778
rs142269210
rs1928199
rs62143197
All − MR Egger
All − IVW(MRE)
−0.005
0.000
0.005
0.01
MR effect size for
'Golgi membrane protein 1' on 'eGFR'

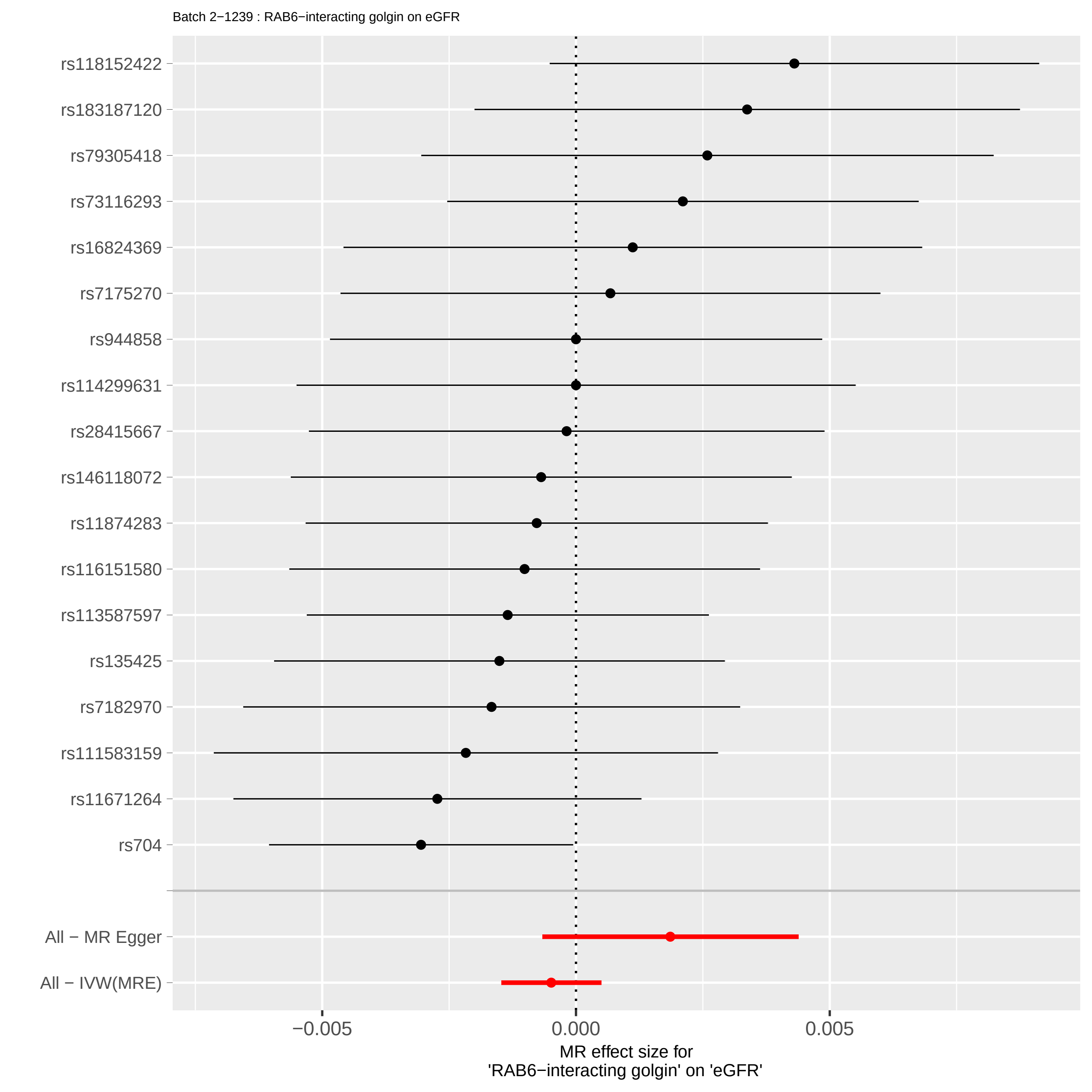

Batch 2−1239 : RAB6−interacting golgin on eGFR
rs118152422
rs183187120
rs79305418
rs73116293
rs16824369
rs7175270
rs944858
rs114299631
rs28415667
rs146118072
rs11874283
rs116151580
rs113587597
rs135425
rs7182970
rs111583159
rs11671264
rs704
All − MR Egger
All − IVW(MRE)
−0.005
0.000
0.005
MR effect size for
'RAB6−interacting golgin' on 'eGFR'

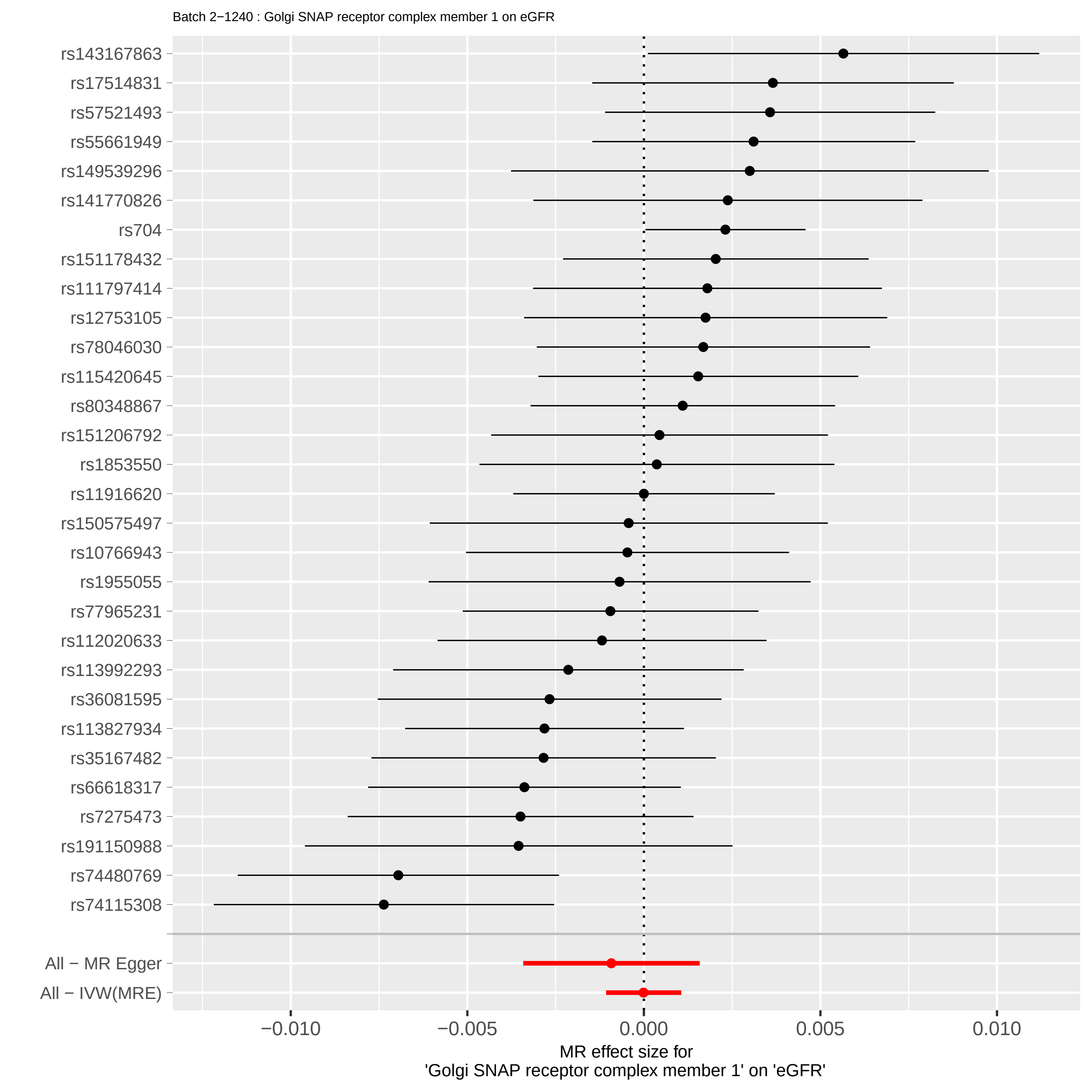

Batch 2−1240 : Golgi SNAP receptor complex member 1 on eGFR
rs143167863
rs17514831
rs57521493
rs55661949
rs149539296
rs141770826
rs704
rs151178432
rs111797414
rs12753105
rs78046030
rs115420645
rs80348867
rs151206792
rs1853550
rs11916620
rs150575497
rs10766943
rs1955055
rs77965231
rs112020633
rs113992293
rs36081595
rs113827934
rs35167482
rs66618317
rs7275473
rs191150988
rs74480769
rs74115308
All − MR Egger
All − IVW(MRE)
−0.010
−0.005
0.000
0.005
0.010
MR effect size for
'Golgi SNAP receptor complex member 1' on 'eGFR'

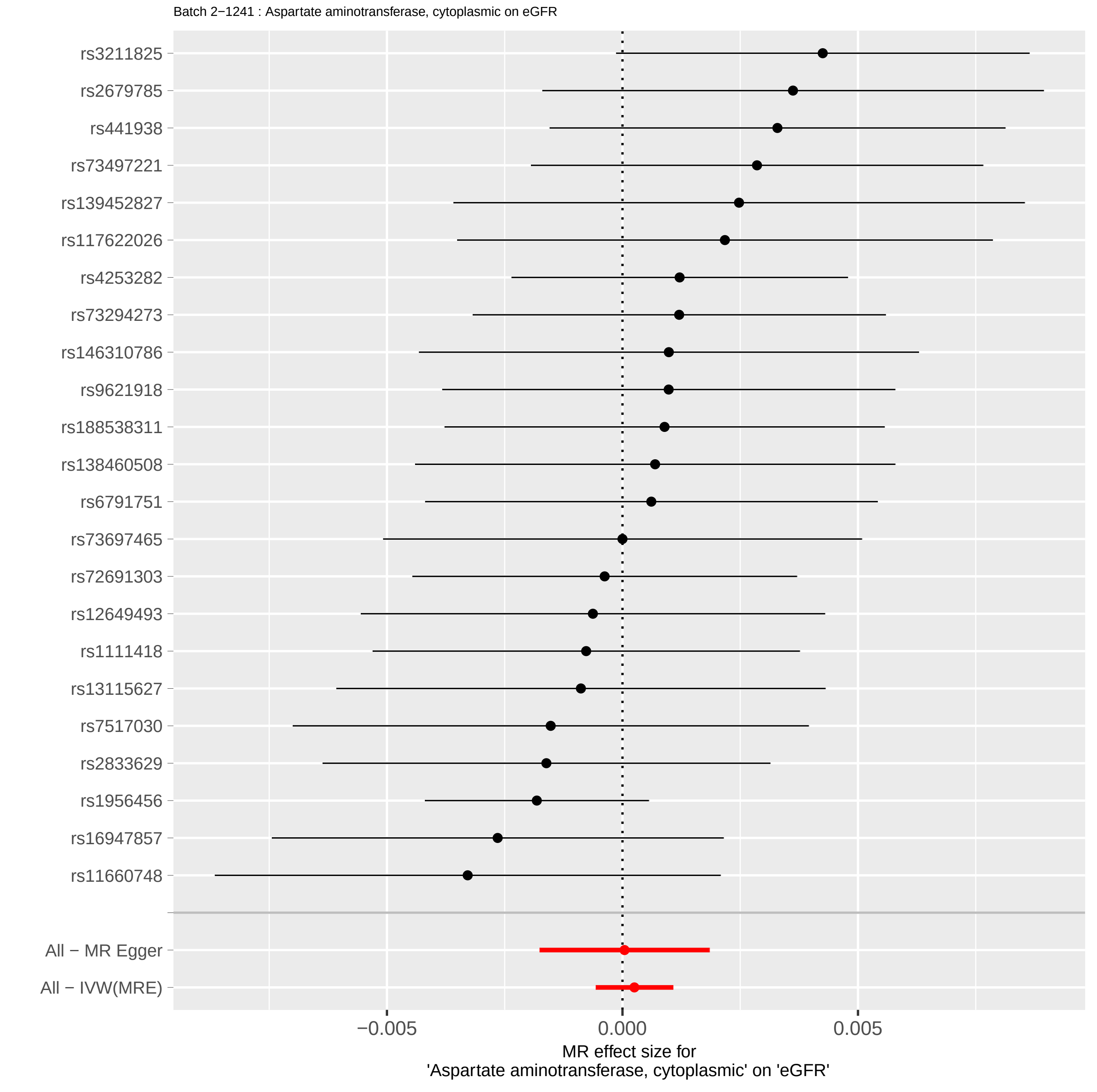

Batch 2−1241 : Aspartate aminotransferase, cytoplasmic on eGFR
rs3211825
rs2679785
rs441938
rs73497221
rs139452827
rs117622026
rs4253282
rs73294273
rs146310786
rs9621918
rs188538311
rs138460508
rs6791751
rs73697465
rs72691303
rs12649493
rs1111418
rs13115627
rs7517030
rs2833629
rs1956456
rs16947857
rs11660748
All − MR Egger
All − IVW(MRE)
−0.005
0.000
0.005
MR effect size for
'Aspartate aminotransferase, cytoplasmic' on 'eGFR'

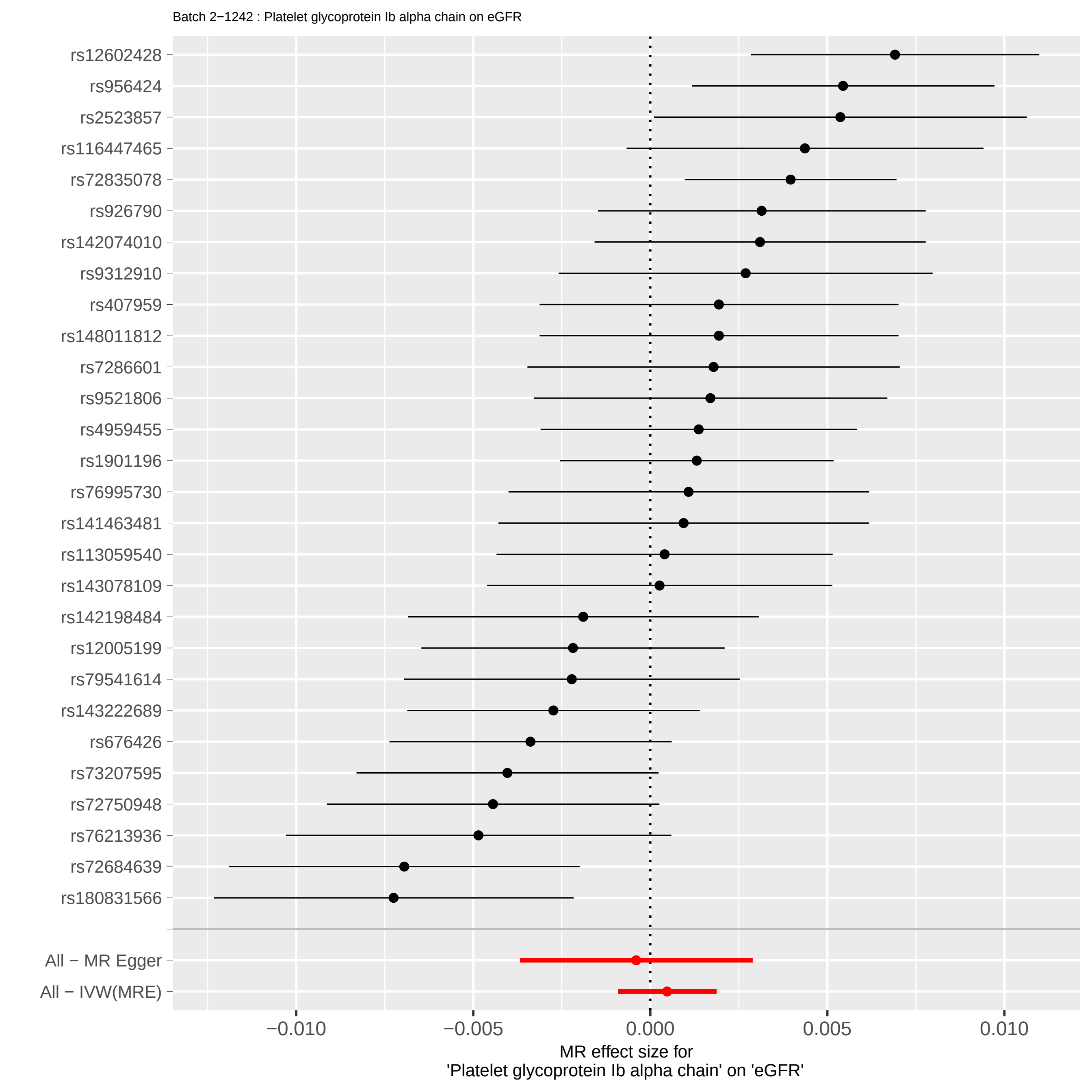

Batch 2−1242 : Platelet glycoprotein Ib alpha chain on eGFR
rs12602428
rs956424
rs2523857
rs116447465
rs72835078
rs926790
rs142074010
rs9312910
rs407959
rs148011812
rs7286601
rs9521806
rs4959455
rs1901196
rs76995730
rs141463481
rs113059540
rs143078109
rs142198484
rs12005199
rs79541614
rs143222689
rs676426
rs73207595
rs72750948
rs76213936
rs72684639
rs180831566
All − MR Egger
All − IVW(MRE)
−0.010
−0.005
0.000
0.005
0.010
MR effect size for
'Platelet glycoprotein Ib alpha chain' on 'eGFR'

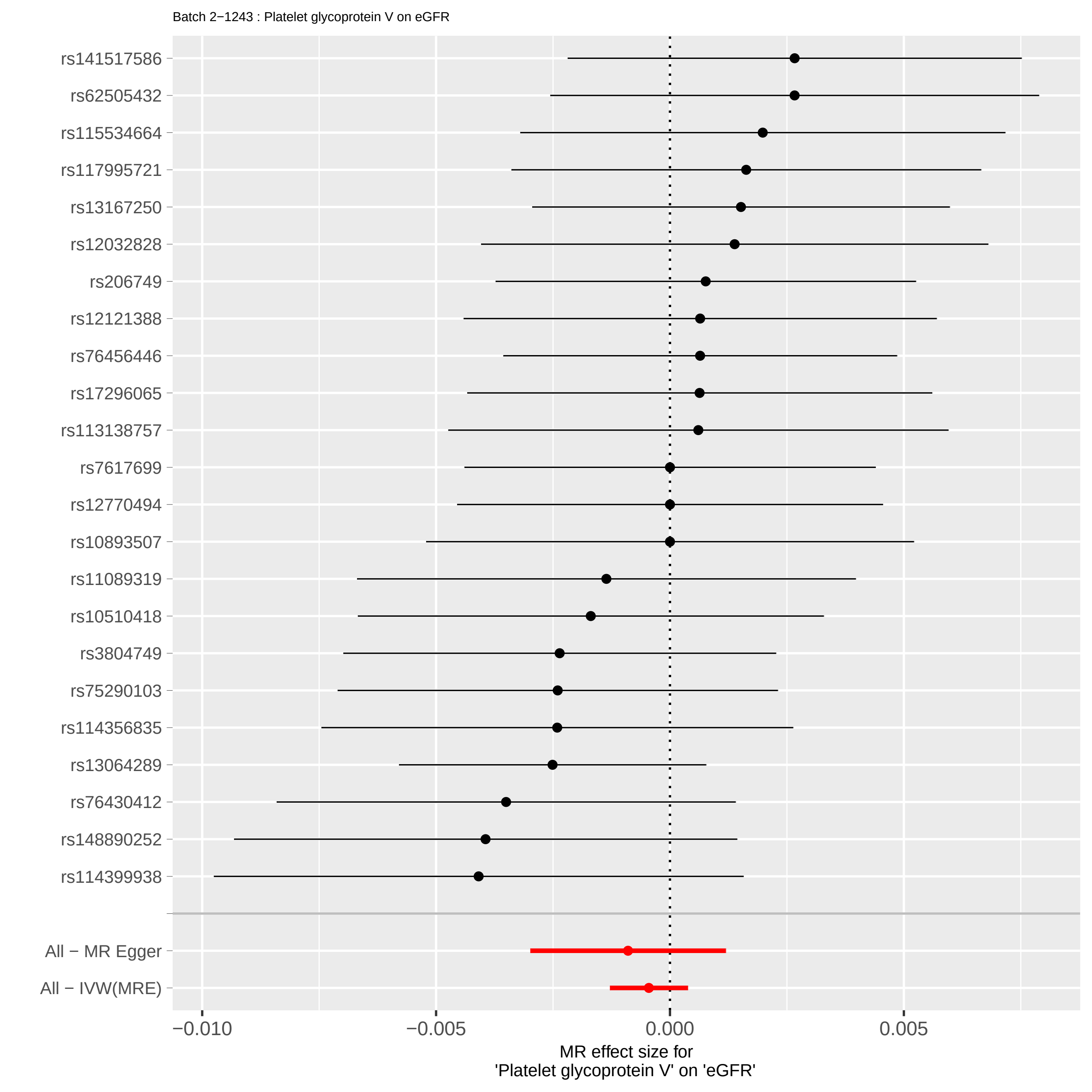
Batch 2−1243 : Platelet glycoprotein V on eGFR
rs141517586
rs62505432
rs115534664
rs117995721
rs13167250
rs12032828
rs206749
rs12121388
rs76456446
rs17296065
rs113138757
rs7617699
rs12770494
rs10893507
rs11089319
rs10510418
rs3804749
rs75290103
rs114356835
rs13064289
rs76430412
rs148890252
rs114399938
All − MR Egger
All − IVW(MRE)
−0.010
−0.005
0.000
0.005
MR effect size for
'Platelet glycoprotein V' on 'eGFR'

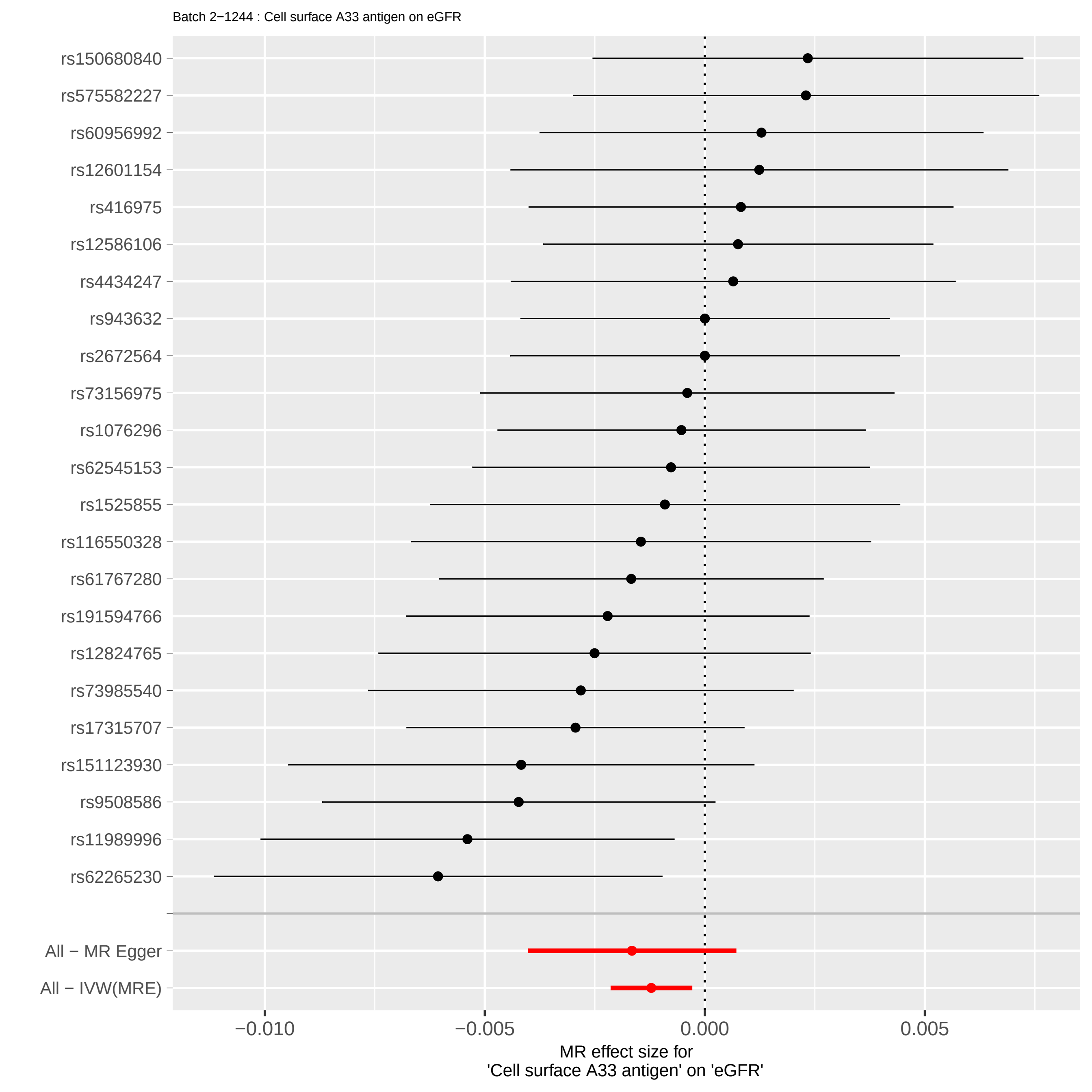

Batch 2−1244 : Cell surface A33 antigen on eGFR
rs150680840
rs575582227
rs60956992
rs12601154
rs416975
rs12586106
rs4434247
rs943632
rs2672564
rs73156975
rs1076296
rs62545153
rs1525855
rs116550328
rs61767280
rs191594766
rs12824765
rs73985540
rs17315707
rs151123930
rs9508586
rs11989996
rs62265230
All − MR Egger
All − IVW(MRE)
−0.010
−0.005
0.000
0.005
MR effect size for
'Cell surface A33 antigen' on 'eGFR'

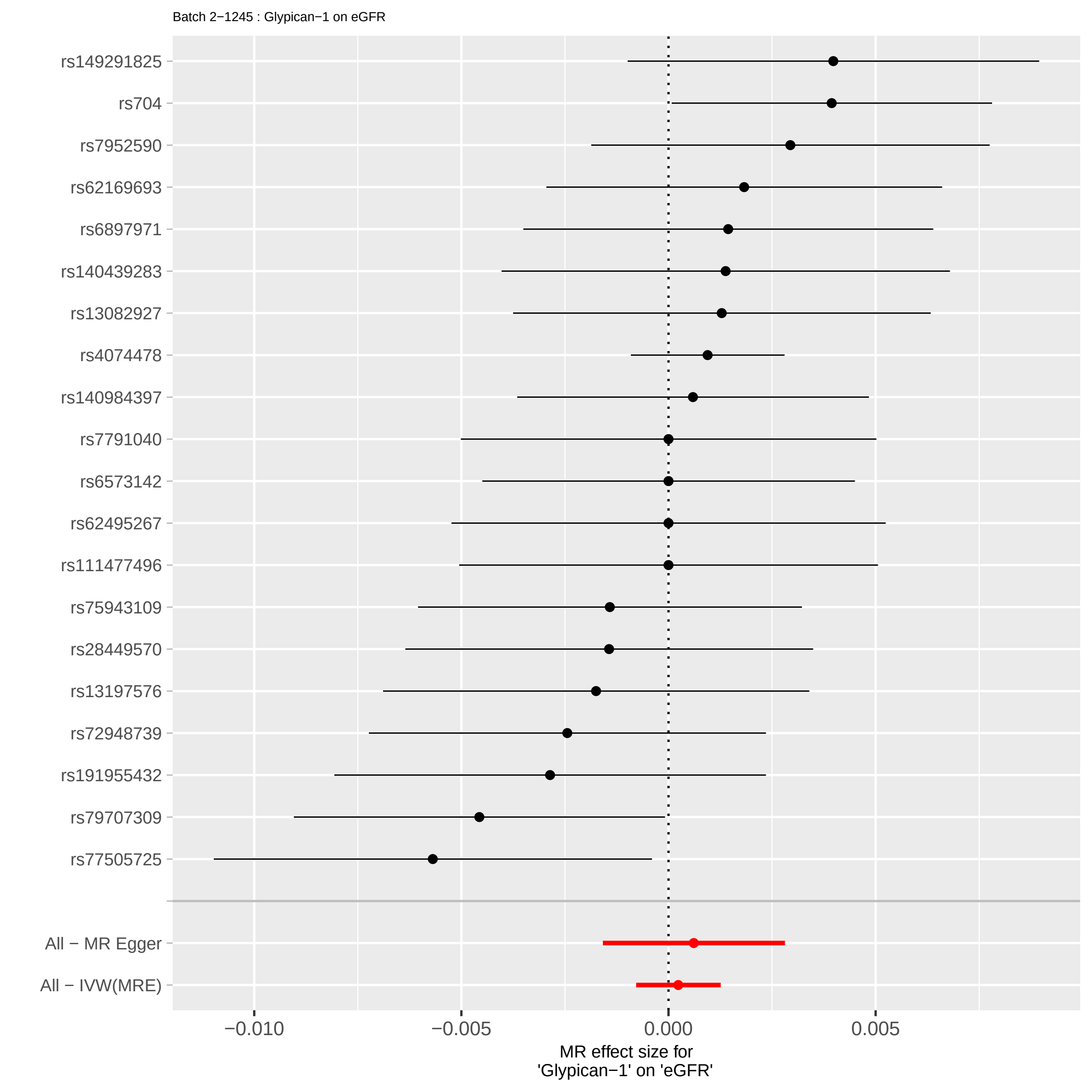

Batch 2−1245 : Glypican−1 on eGFR
rs149291825
rs704
rs7952590
rs62169693
rs6897971
rs140439283
rs13082927
rs4074478
rs140984397
rs7791040
rs6573142
rs62495267
rs111477496
rs75943109
rs28449570
rs13197576
rs72948739
rs191955432
rs79707309
rs77505725
All − MR Egger
All − IVW(MRE)
−0.010
−0.005
0.000
0.005
MR effect size for
'Glypican−1' on 'eGFR'

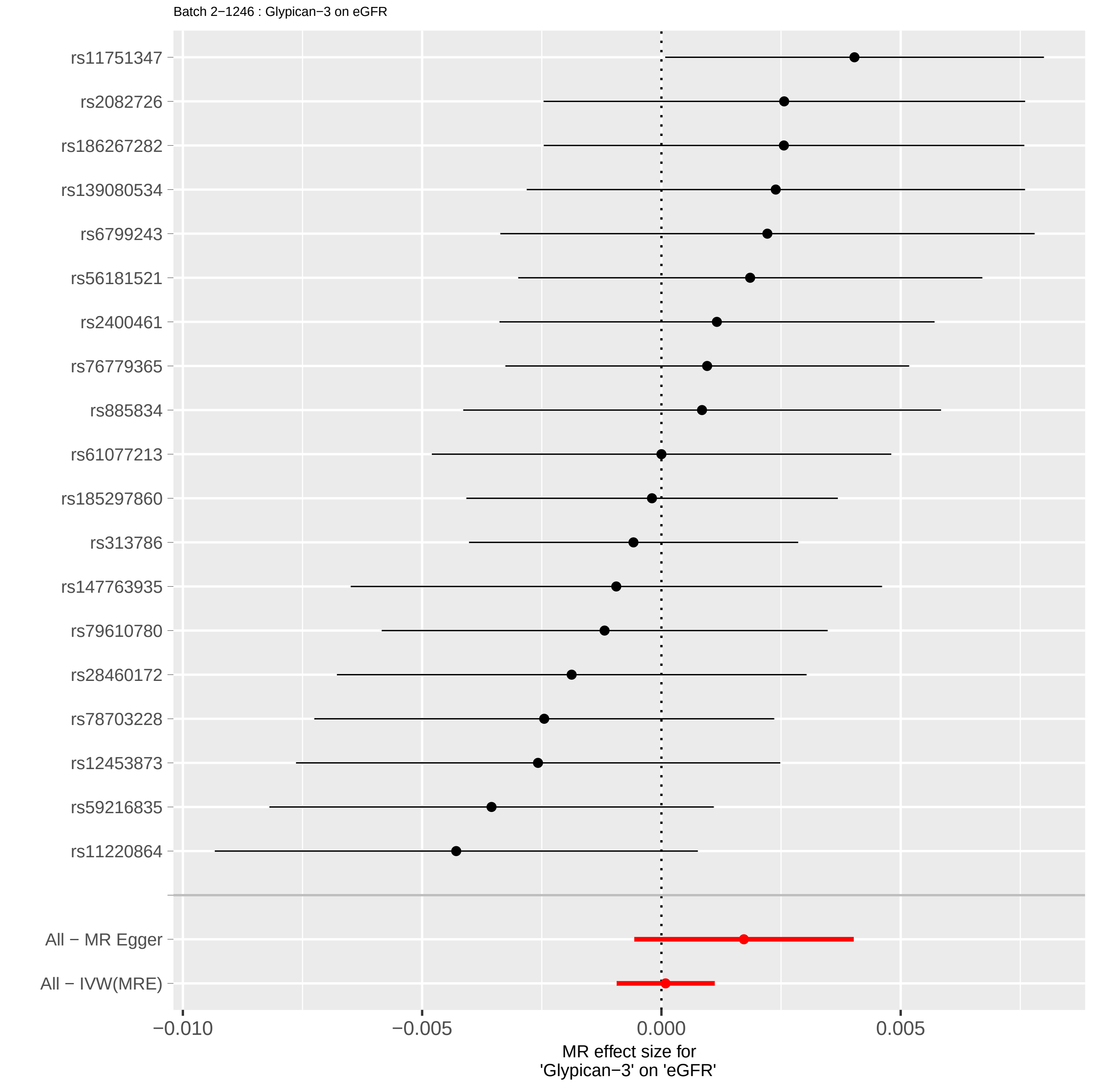

Batch 2−1246 : Glypican−3 on eGFR
rs11751347
rs2082726
rs186267282
rs139080534
rs6799243
rs56181521
rs2400461
rs76779365
rs885834
rs61077213
rs185297860
rs313786
rs147763935
rs79610780
rs28460172
rs78703228
rs12453873
rs59216835
rs11220864
All − MR Egger
All − IVW(MRE)
−0.010
−0.005
0.000
0.005
MR effect size for
'Glypican−3' on 'eGFR'

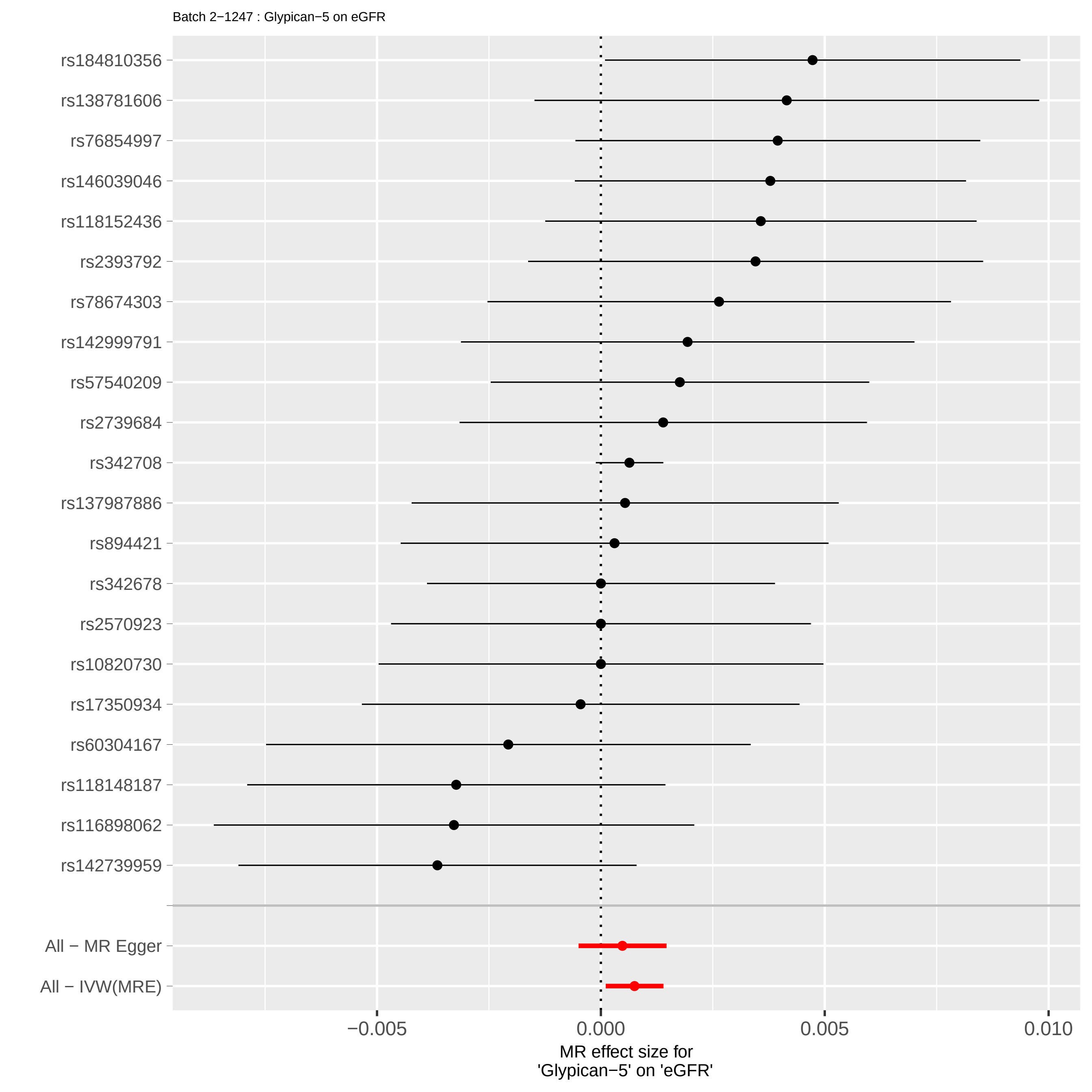

Batch 2−1247 : Glypican−5 on eGFR
rs184810356
rs138781606
rs76854997
rs146039046
rs118152436
rs2393792
rs78674303
rs142999791
rs57540209
rs2739684
rs342708
rs137987886
rs894421
rs342678
rs2570923
rs10820730
rs17350934
rs60304167
rs118148187
rs116898062
rs142739959
All − MR Egger
All − IVW(MRE)
−0.005
0.000
0.005
0.010
MR effect size for
'Glypican−5' on 'eGFR'

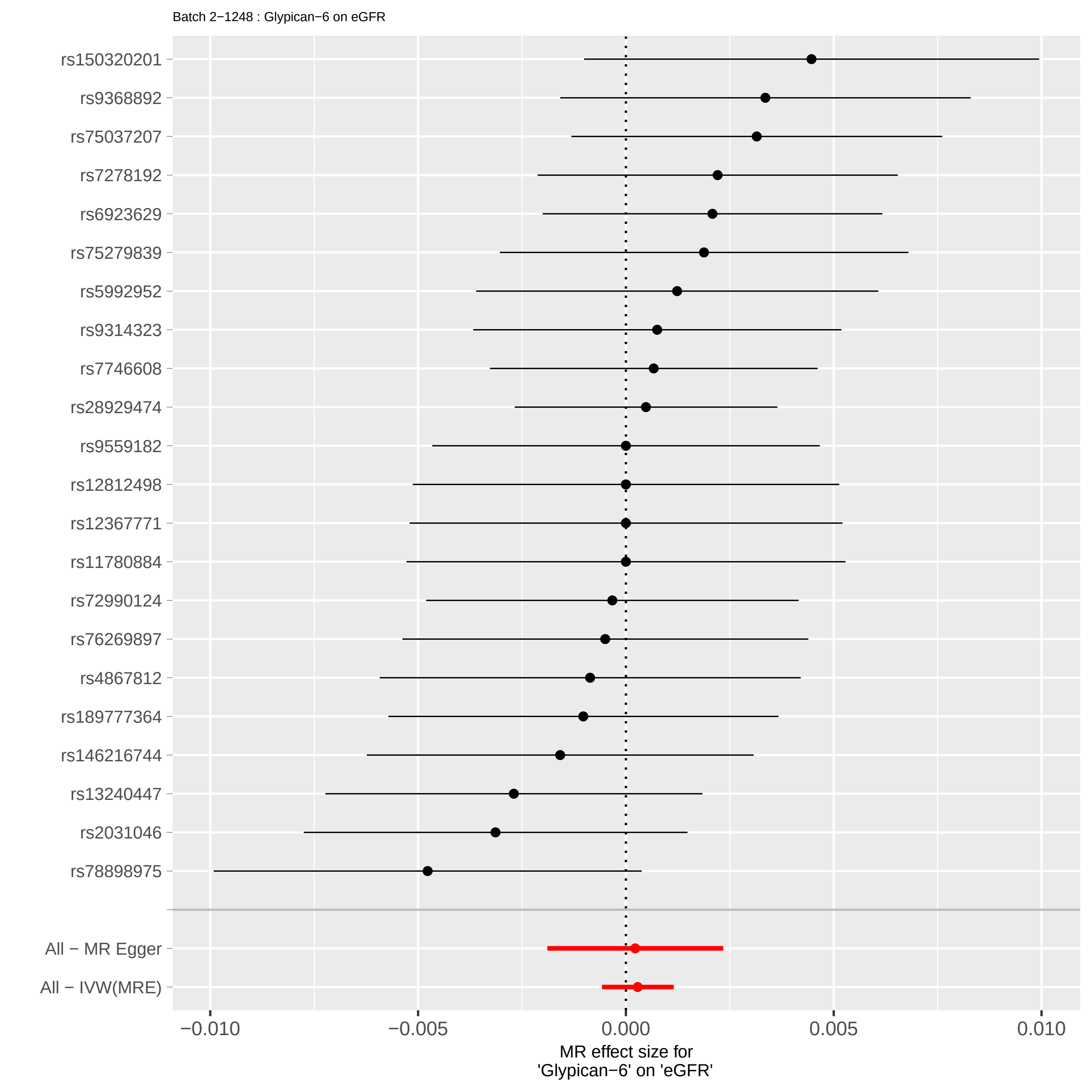

Batch 2−1248 : Glypican−6 on eGFR
rs150320201
rs9368892
rs75037207
rs7278192
rs6923629
rs75279839
rs5992952
rs9314323
rs7746608
rs28929474
rs9559182
rs12812498
rs12367771
rs11780884
rs72990124
rs76269897
rs4867812
rs189777364
rs146216744
rs13240447
rs2031046
rs78898975
All − MR Egger
All − IVW(MRE)
−0.010
−0.005
0.000
0.005
0.010
MR effect size for
'Glypican−6' on 'eGFR'

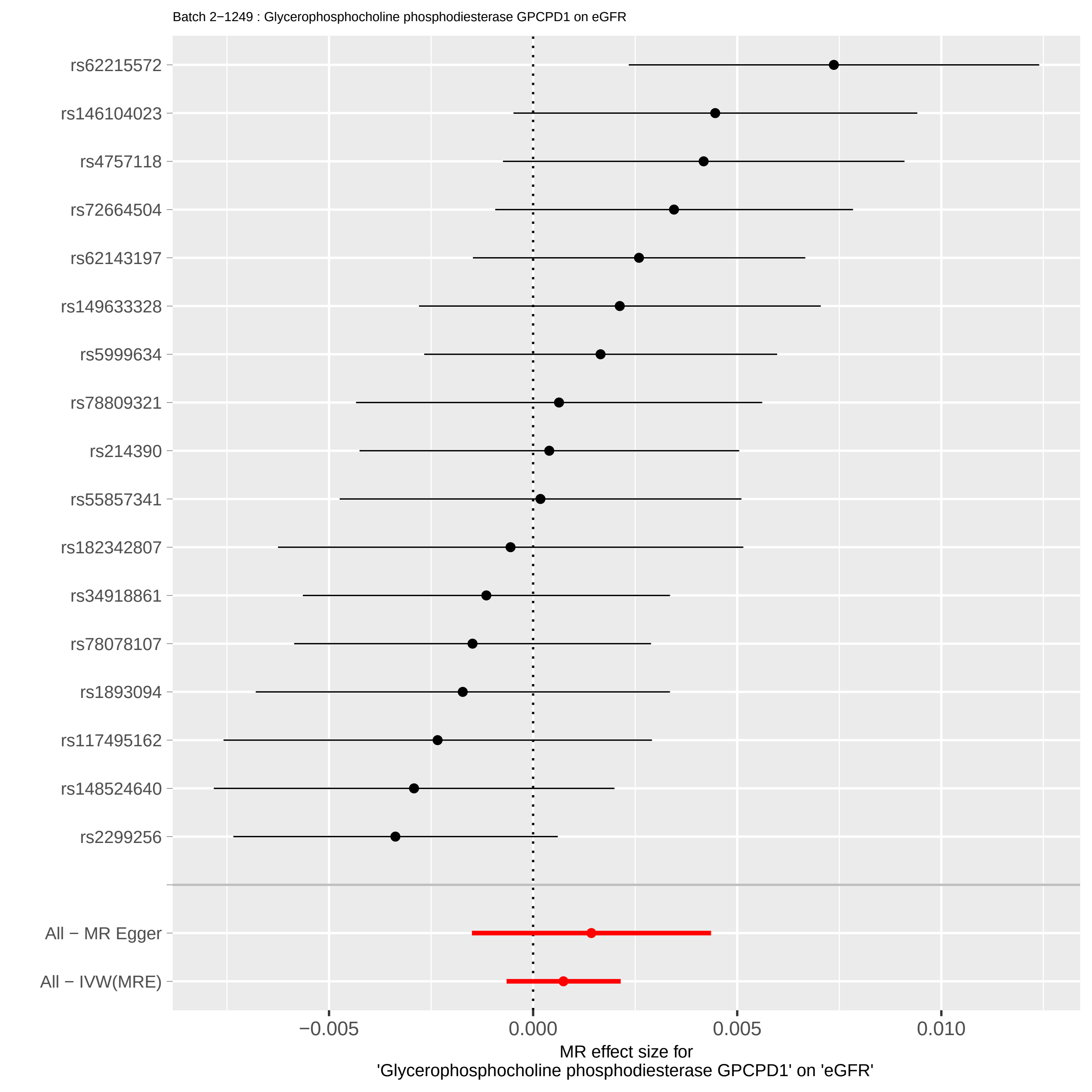

Batch 2−1249 : Glycerophosphocholine phosphodiesterase GPCPD1 on eGFR
rs62215572
rs146104023
rs4757118
rs72664504
rs62143197
rs149633328
rs5999634
rs78809321
rs214390
rs55857341
rs182342807
rs34918861
rs78078107
rs1893094
rs117495162
rs148524640
rs2299256
All − MR Egger
All − IVW(MRE)
−0.005
0.000
0.005
0.010
MR effect size for
'Glycerophosphocholine phosphodiesterase GPCPD1' on 'eGFR'

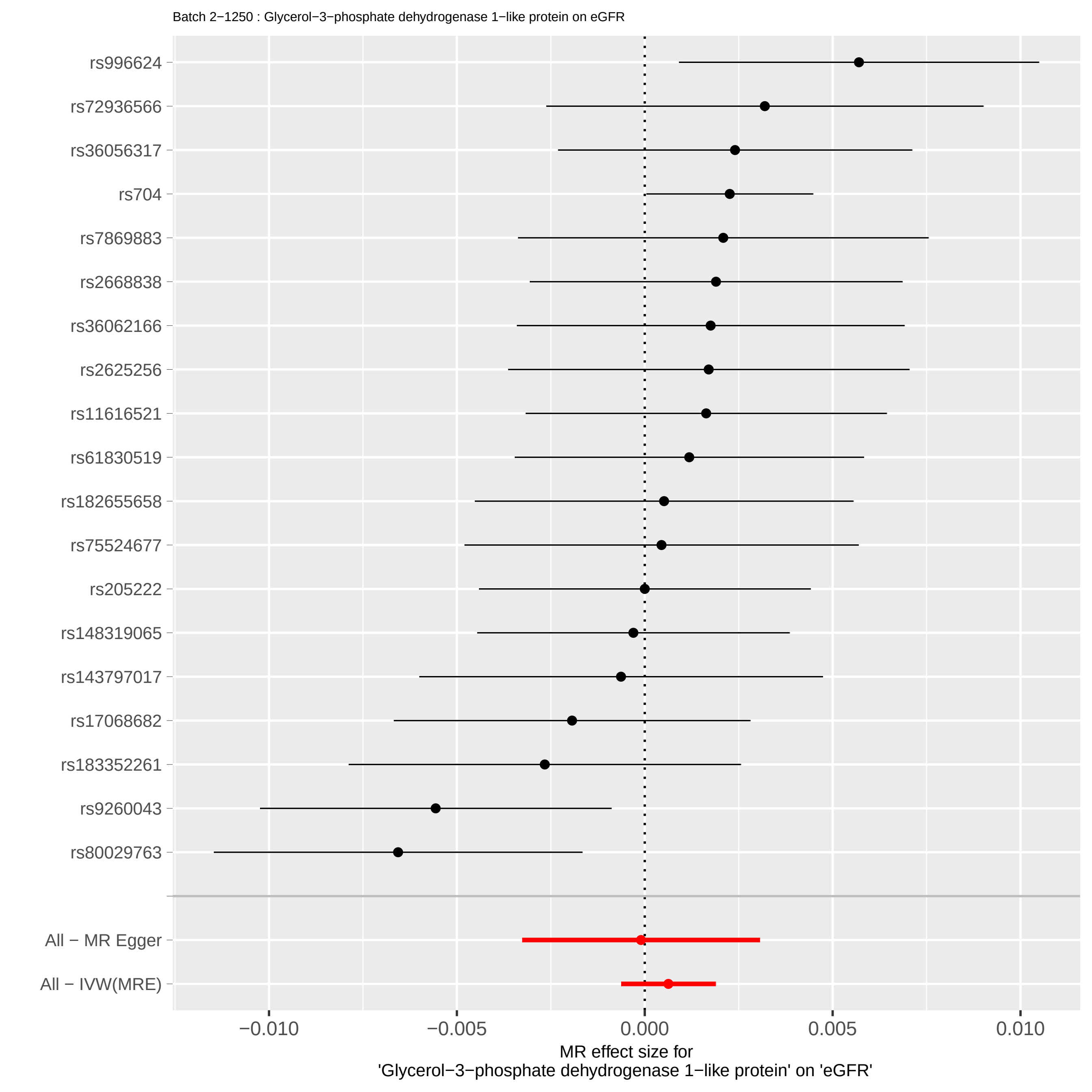

Batch 2−1250 : Glycerol−3−phosphate dehydrogenase 1−like protein on eGFR
rs996624
rs72936566
rs36056317
rs704
rs7869883
rs2668838
rs36062166
rs2625256
rs11616521
rs61830519
rs182655658
rs75524677
rs205222
rs148319065
rs143797017
rs17068682
rs183352261
rs9260043
rs80029763
All − MR Egger
All − IVW(MRE)
−0.010
−0.005
0.000
0.005
0.010
MR effect size for
'Glycerol−3−phosphate dehydrogenase 1−like protein' on 'eGFR'

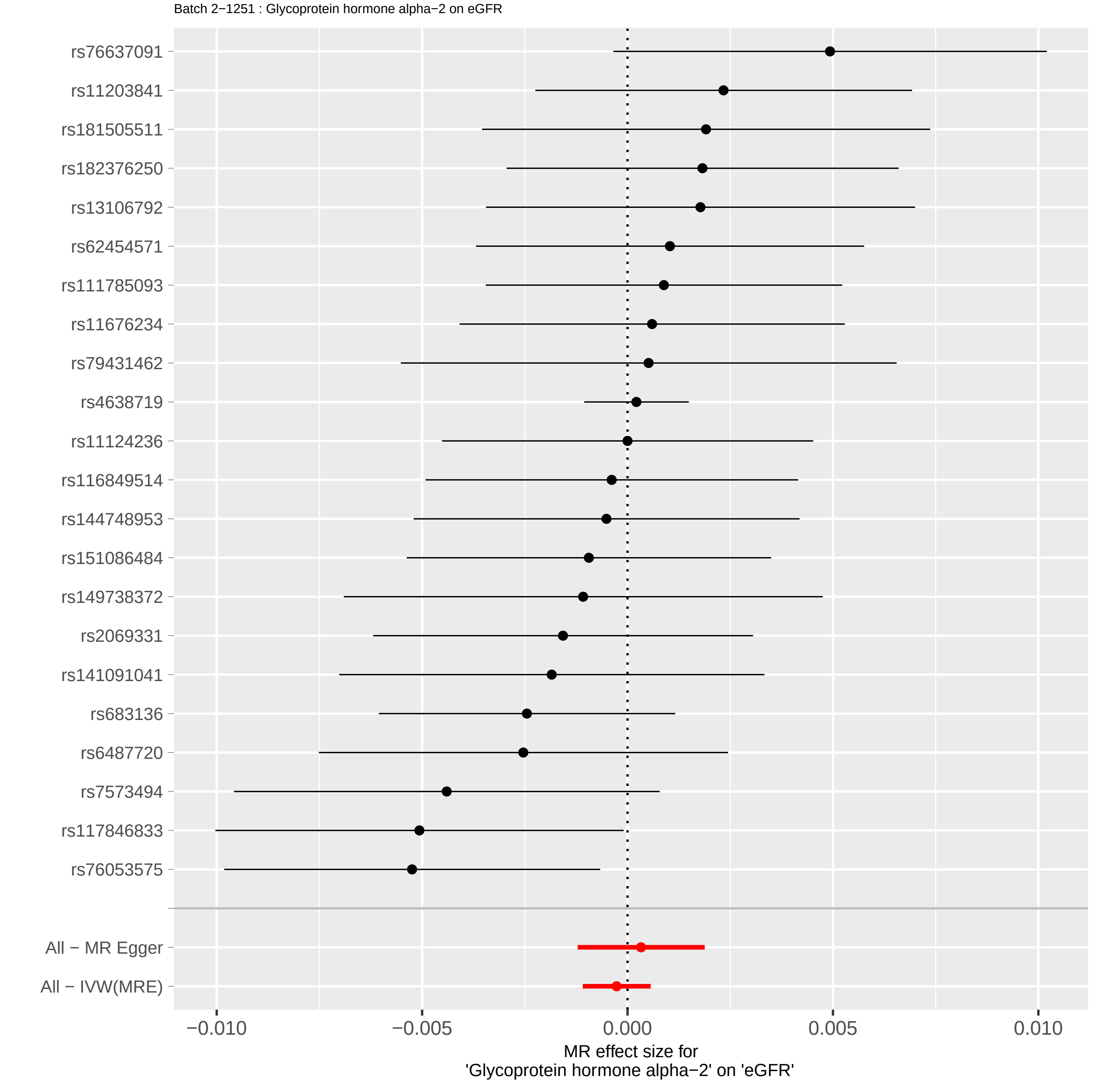
Batch 2−1251 : Glycoprotein hormone alpha−2 on eGFR
rs76637091
rs11203841
rs181505511
rs182376250
rs13106792
rs62454571
rs111785093
rs11676234
rs79431462
rs4638719
rs11124236
rs116849514
rs144748953
rs151086484
rs149738372
rs2069331
rs141091041
rs683136
rs6487720
rs7573494
rs117846833
rs76053575
All − MR Egger
All − IVW(MRE)
−0.010
−0.005
0.000
0.005
0.010
MR effect size for
'Glycoprotein hormone alpha−2' on 'eGFR'

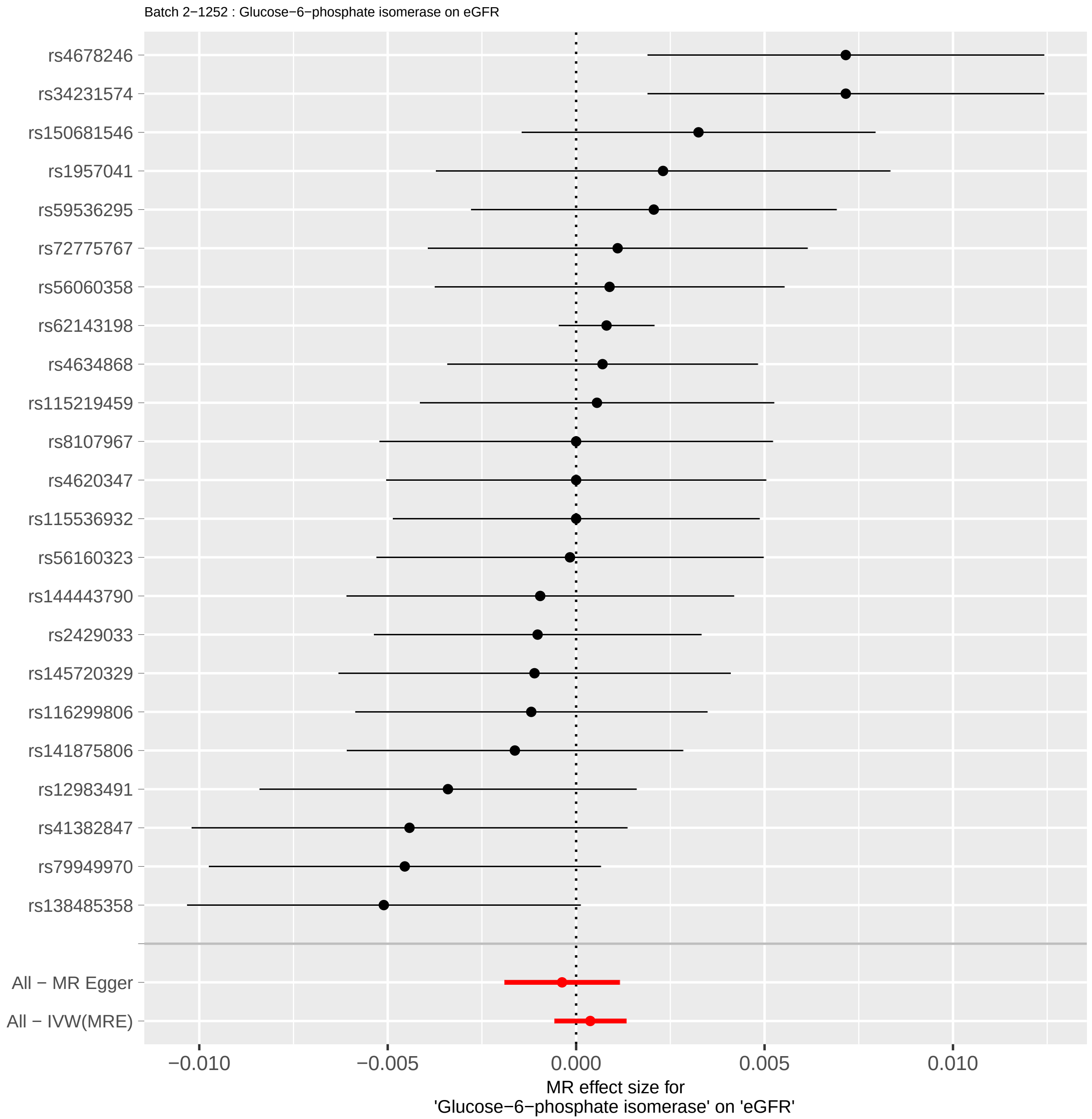

Batch 2−1252 : Glucose−6−phosphate isomerase on eGFR
rs4678246
rs34231574
rs150681546
rs1957041
rs59536295
rs72775767
rs56060358
rs62143198
rs4634868
rs115219459
rs8107967
rs4620347
rs115536932
rs56160323
rs144443790
rs2429033
rs145720329
rs116299806
rs141875806
rs12983491
rs41382847
rs79949970
rs138485358
All − MR Egger
All − IVW(MRE)
−0.010
−0.005
0.000
0.005
0.010
MR effect size for
'Glucose−6−phosphate isomerase' on 'eGFR'

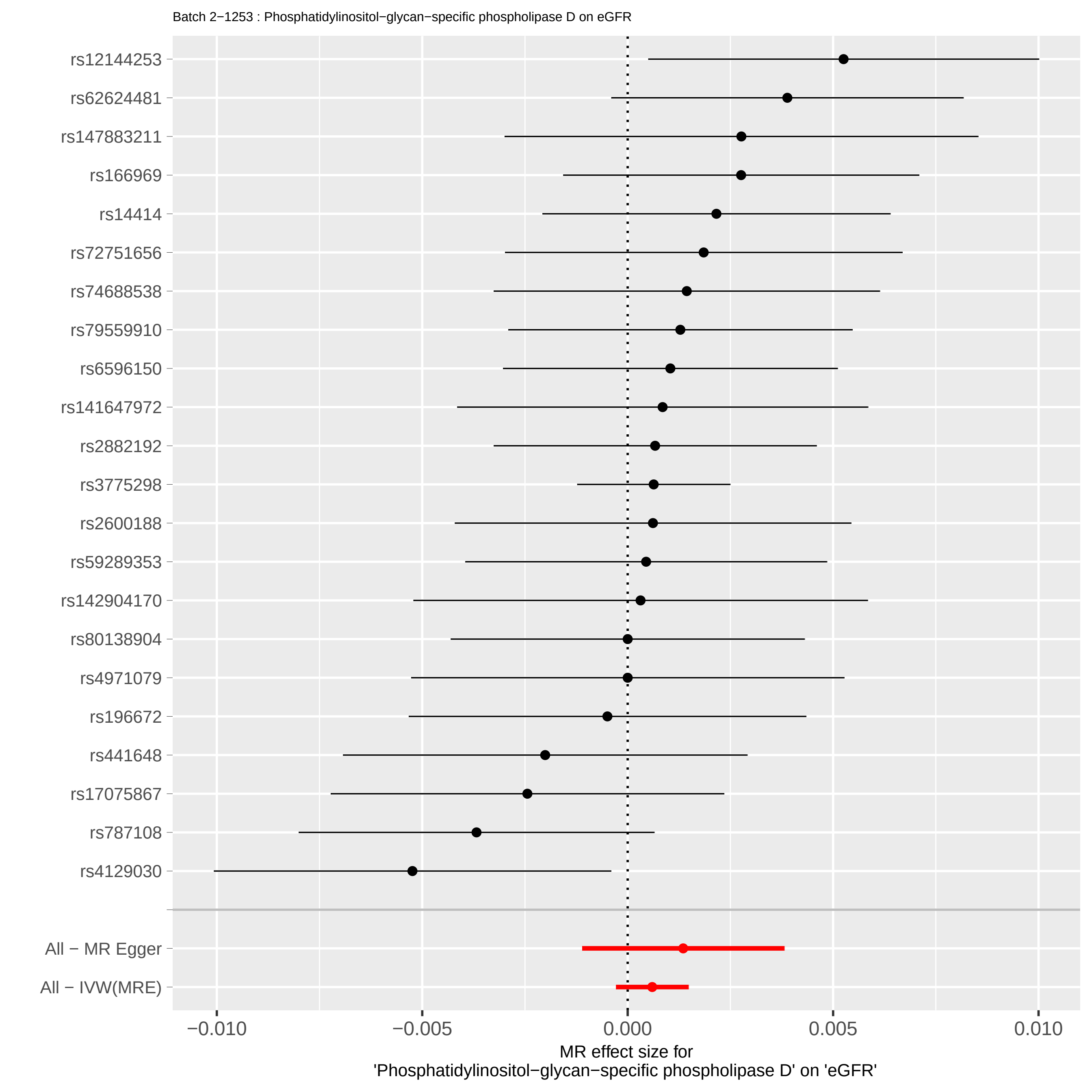

Batch 2−1253 : Phosphatidylinositol−glycan−specific phospholipase D on eGFR
rs12144253
rs62624481
rs147883211
rs166969
rs14414
rs72751656
rs74688538
rs79559910
rs6596150
rs141647972
rs2882192
rs3775298
rs2600188
rs59289353
rs142904170
rs80138904
rs4971079
rs196672
rs441648
rs17075867
rs787108
rs4129030
All − MR Egger
All − IVW(MRE)
−0.010
−0.005
0.000
0.005
0.010
MR effect size for
'Phosphatidylinositol−glycan−specific phospholipase D' on 'eGFR'

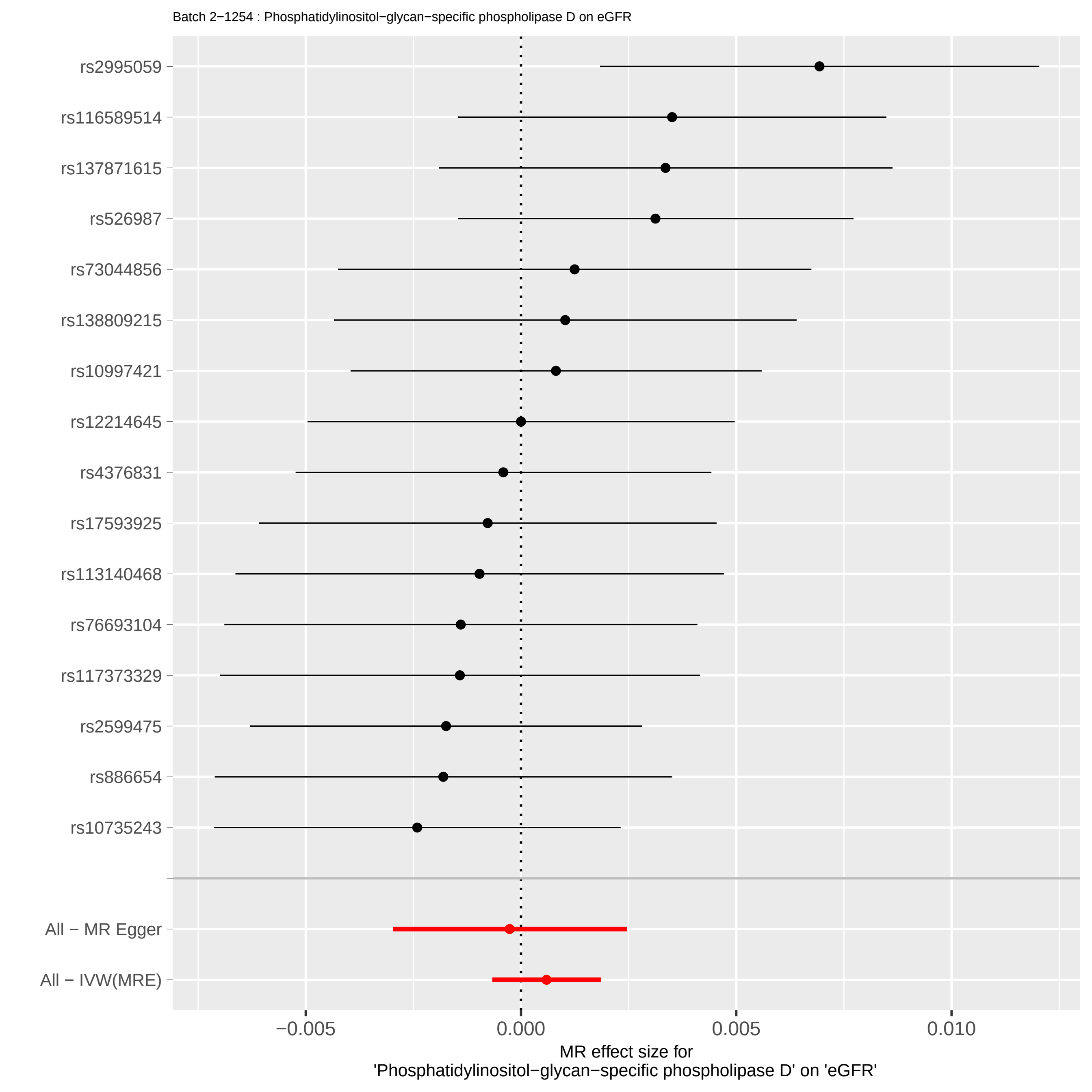

Batch 2−1254 : Phosphatidylinositol−glycan−specific phospholipase D on eGFR
rs2995059
rs116589514
rs137871615
rs526987
rs73044856
rs138809215
rs10997421
rs12214645
rs4376831
rs17593925
rs113140468
rs76693104
rs117373329
rs2599475
rs886654
rs10735243
All − MR Egger
All − IVW(MRE)
−0.005
0.000
0.005
0.010
MR effect size for
'Phosphatidylinositol−glycan−specific phospholipase D' on 'eGFR'

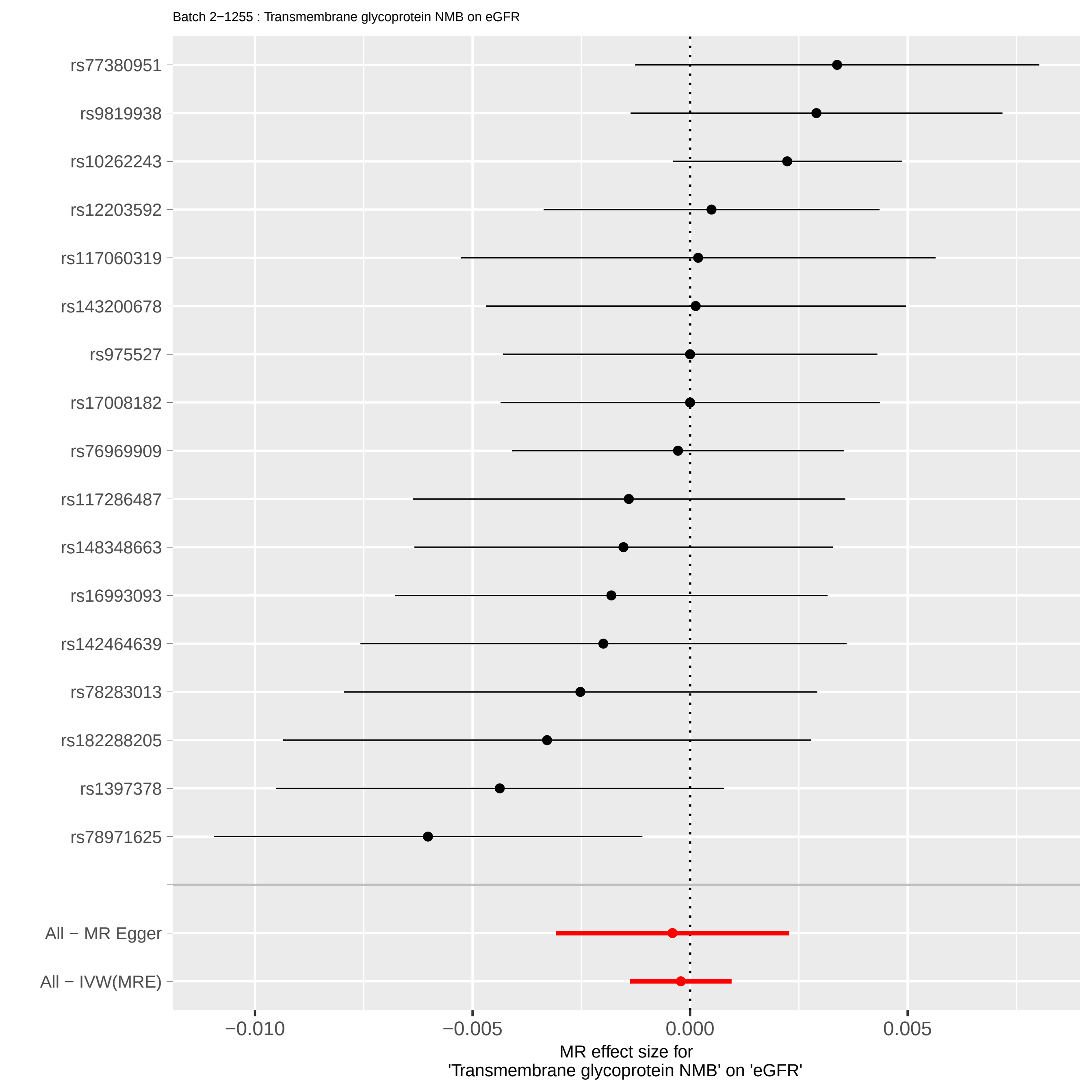

Batch 2−1255 : Transmembrane glycoprotein NMB on eGFR
rs77380951
rs9819938
rs10262243
rs12203592
rs117060319
rs143200678
rs975527
rs17008182
rs76969909
rs117286487
rs148348663
rs16993093
rs142464639
rs78283013
rs182288205
rs1397378
rs78971625
All − MR Egger
All − IVW(MRE)
−0.010
−0.005
0.000
0.005
MR effect size for
'Transmembrane glycoprotein NMB' on 'eGFR'

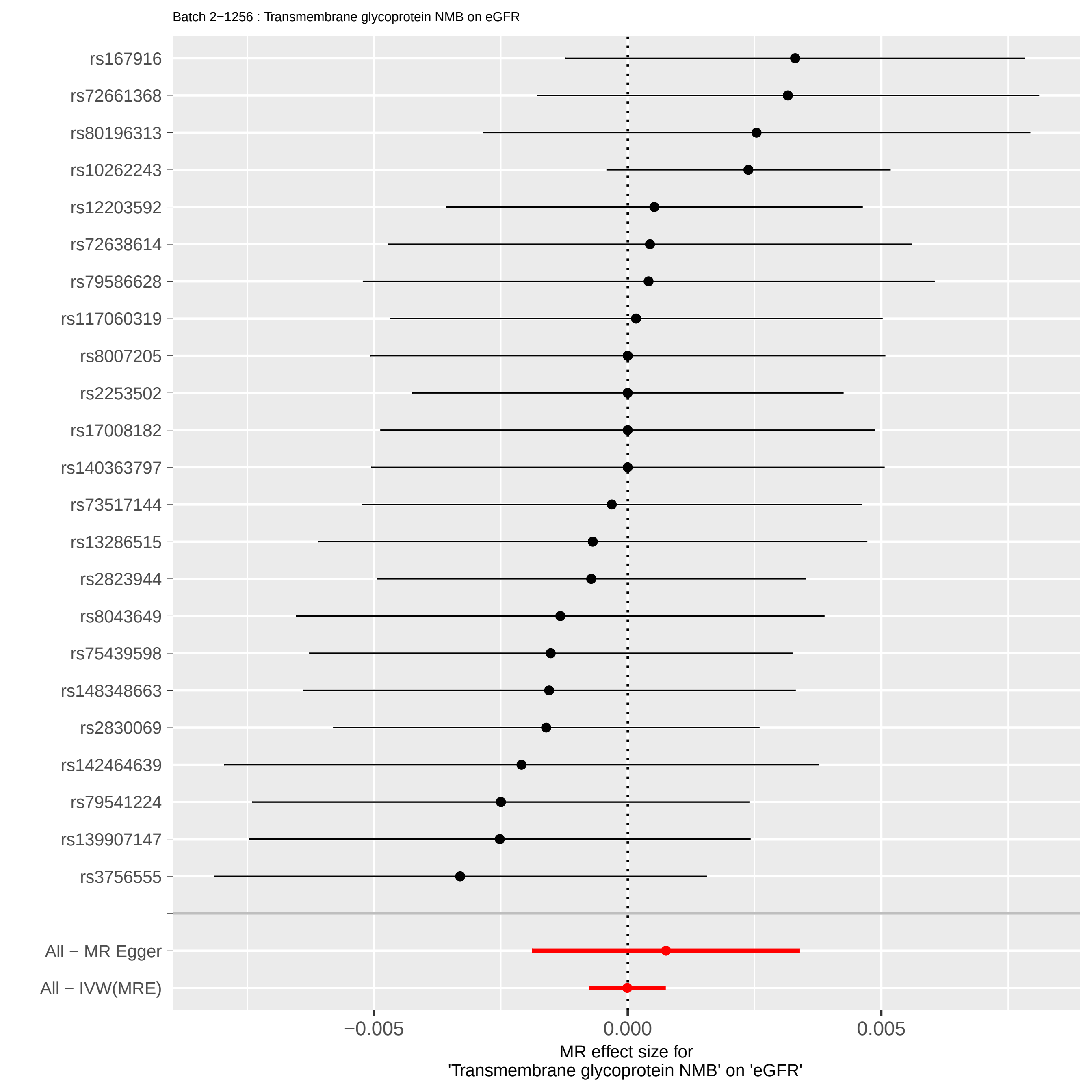
Batch 2−1256 : Transmembrane glycoprotein NMB on eGFR
rs167916
rs72661368
rs80196313
rs10262243
rs12203592
rs72638614
rs79586628
rs117060319
rs8007205
rs2253502
rs17008182
rs140363797
rs73517144
rs13286515
rs2823944
rs8043649
rs75439598
rs148348663
rs2830069
rs142464639
rs79541224
rs139907147
rs3756555
All − MR Egger
All − IVW(MRE)
−0.005
0.000
0.005
MR effect size for
'Transmembrane glycoprotein NMB' on 'eGFR'

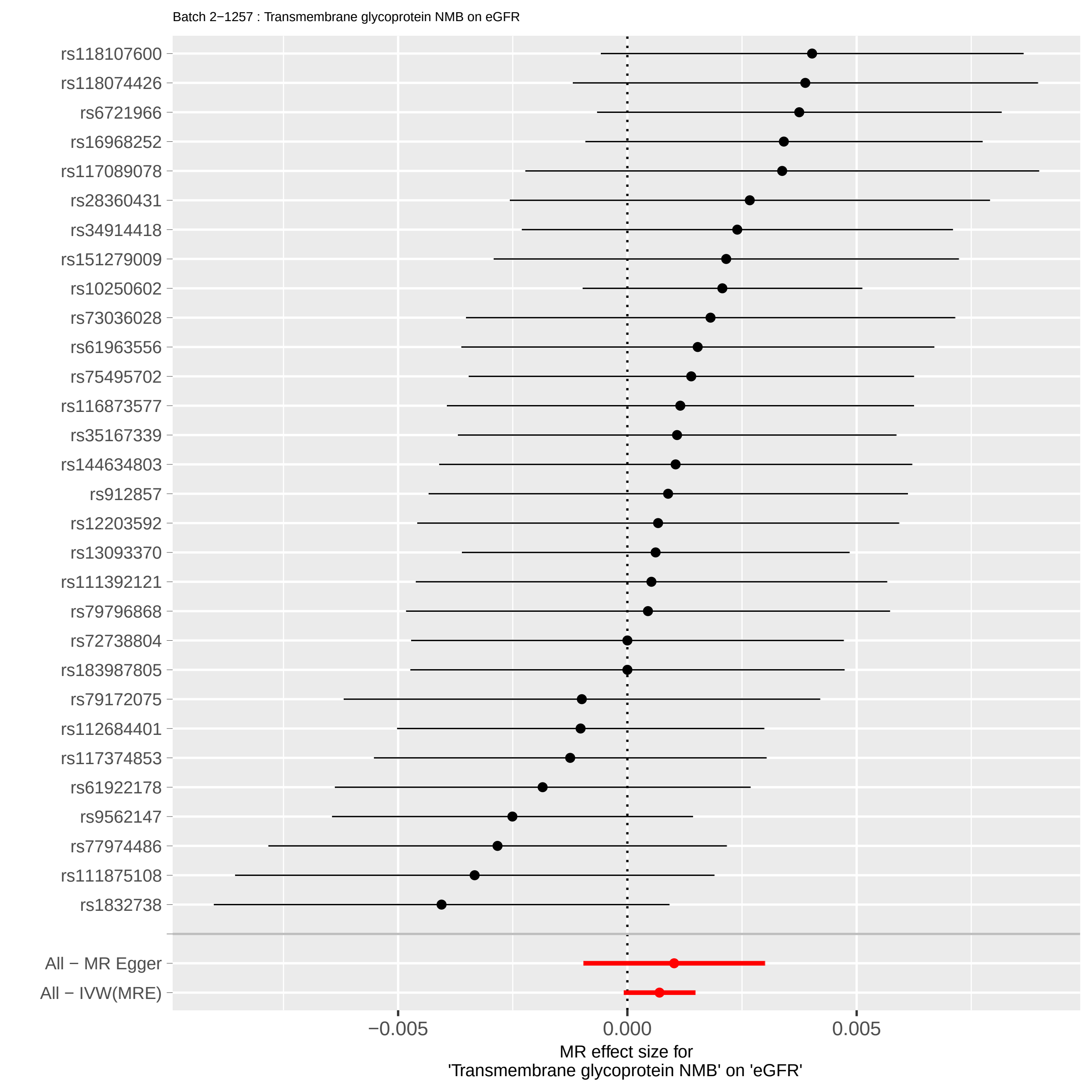

Batch 2−1257 : Transmembrane glycoprotein NMB on eGFR
rs118107600
rs118074426
rs6721966
rs16968252
rs117089078
rs28360431
rs34914418
rs151279009
rs10250602
rs73036028
rs61963556
rs75495702
rs116873577
rs35167339
rs144634803
rs912857
rs12203592
rs13093370
rs111392121
rs79796868
rs72738804
rs183987805
rs79172075
rs112684401
rs117374853
rs61922178
rs9562147
rs77974486
rs111875108
rs1832738
All − MR Egger
All − IVW(MRE)
−0.005
0.000
0.005
MR effect size for
'Transmembrane glycoprotein NMB' on 'eGFR'

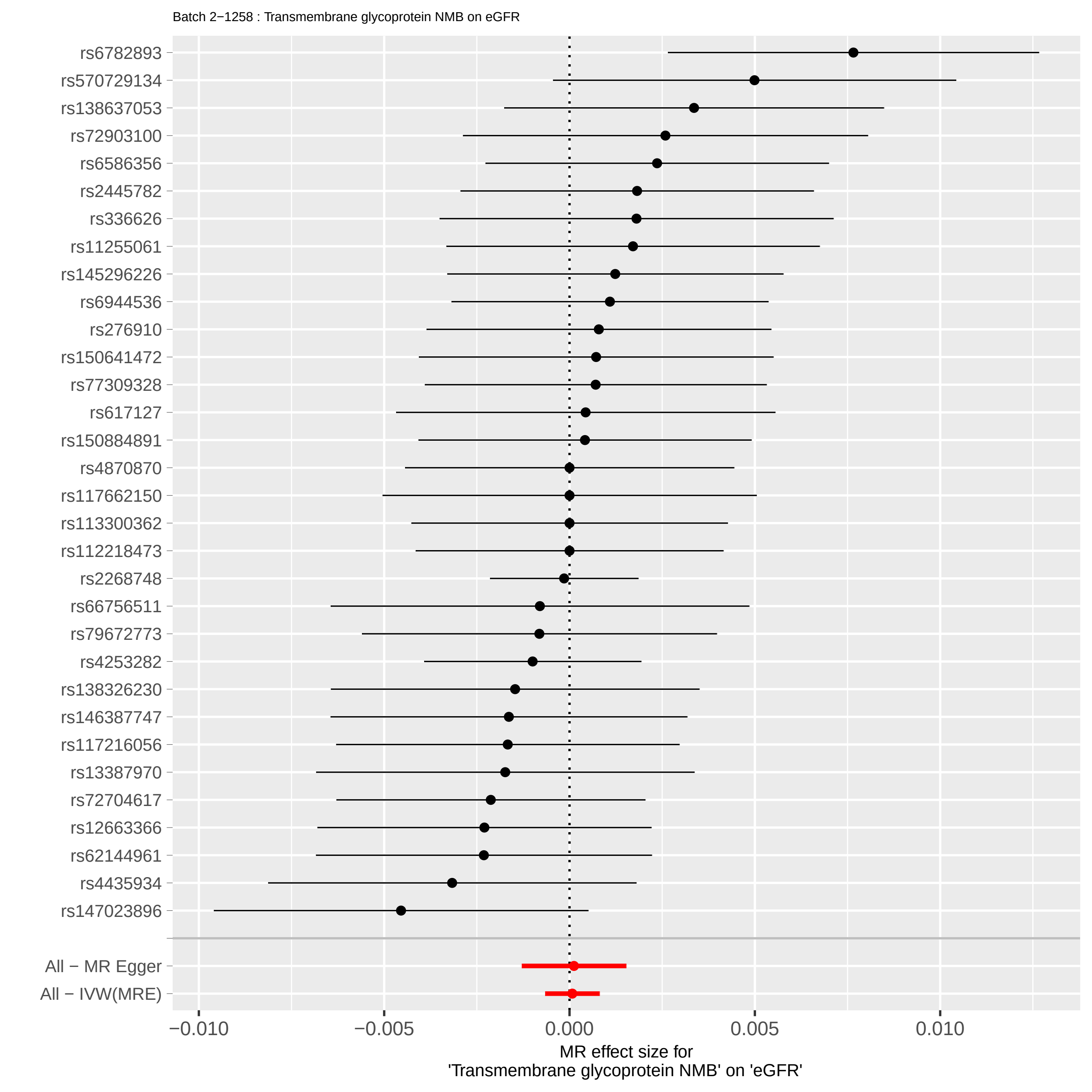
Batch 2−1258 : Transmembrane glycoprotein NMB on eGFR
rs6782893
rs570729134
rs138637053
rs72903100
rs6586356
rs2445782
rs336626
rs11255061
rs145296226
rs6944536
rs276910
rs150641472
rs77309328
rs617127
rs150884891
rs4870870
rs117662150
rs113300362
rs112218473
rs2268748
rs66756511
rs79672773
rs4253282
rs138326230
rs146387747
rs117216056
rs13387970
rs72704617
rs12663366
rs62144961
rs4435934
rs147023896
All − MR Egger
All − IVW(MRE)
−0.010
−0.005
0.000
0.005
0.010
MR effect size for
'Transmembrane glycoprotein NMB' on 'eGFR'

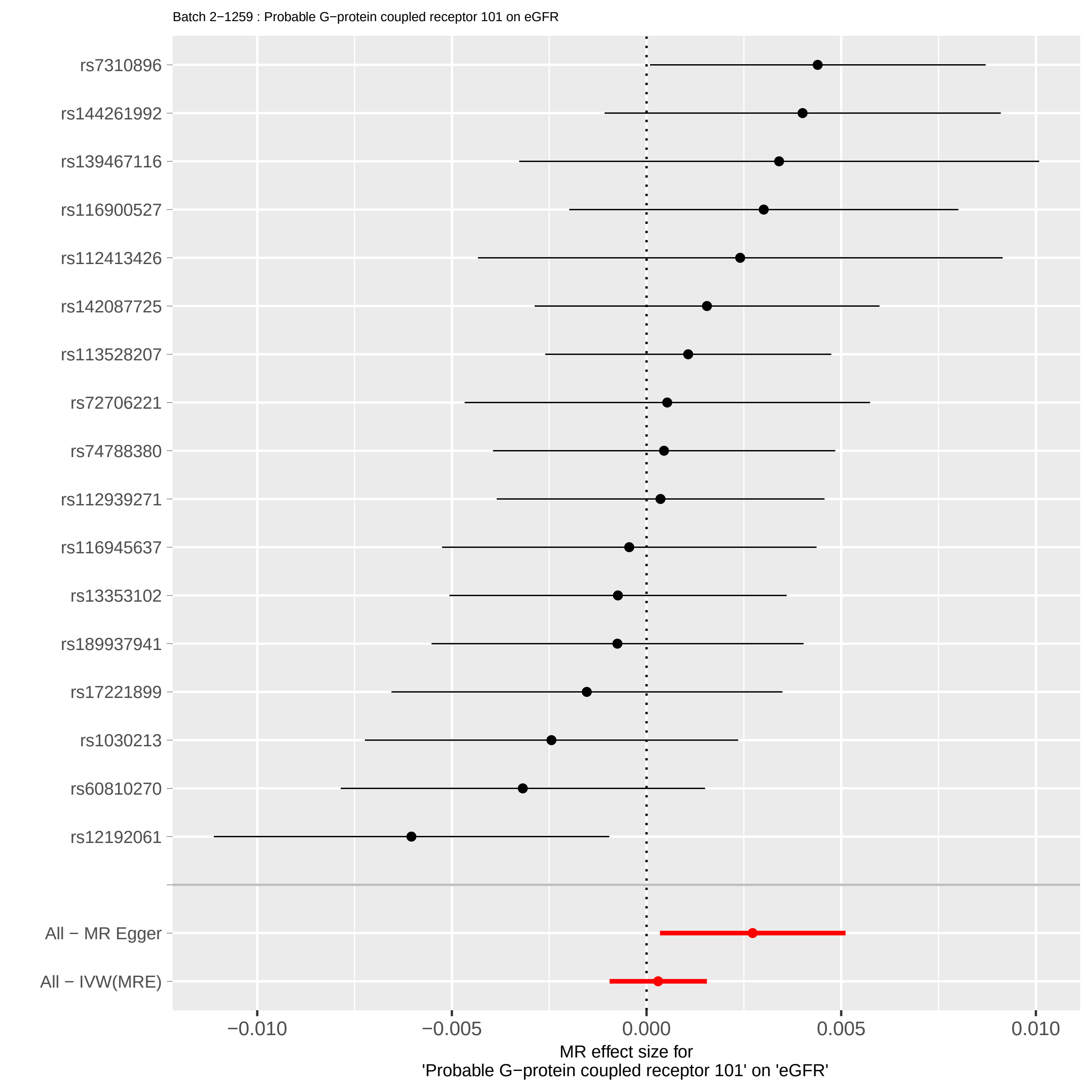
Batch 2−1259 : Probable G−protein coupled receptor 101 on eGFR
rs7310896
rs144261992
rs139467116
rs116900527
rs112413426
rs142087725
rs113528207
rs72706221
rs74788380
rs112939271
rs116945637
rs13353102
rs189937941
rs17221899
rs1030213
rs60810270
rs12192061
All − MR Egger
All − IVW(MRE)
−0.010
−0.005
0.000
0.005
0.010
MR effect size for
'Probable G−protein coupled receptor 101' on 'eGFR'

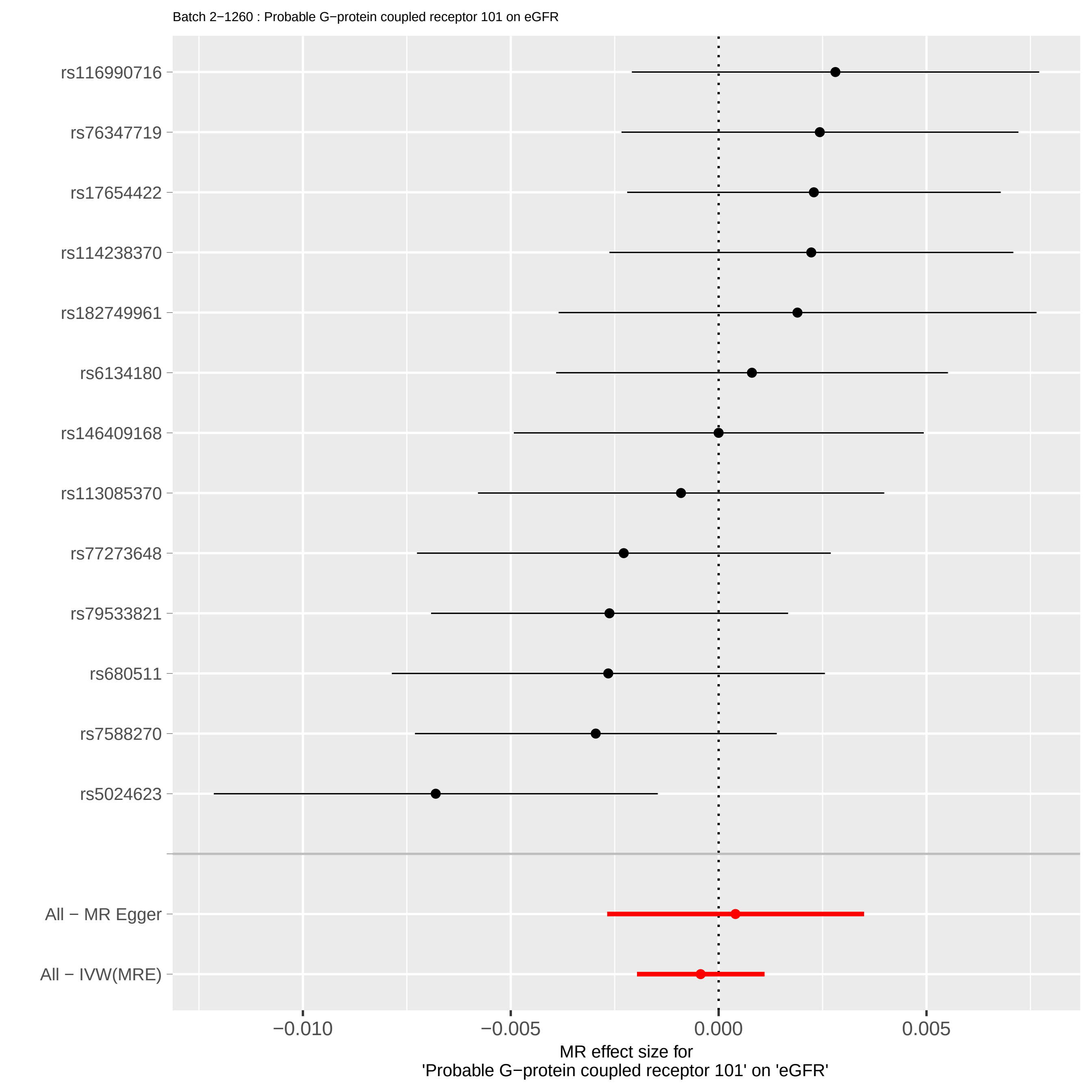

Batch 2−1260 : Probable G−protein coupled receptor 101 on eGFR
rs116990716
rs76347719
rs17654422
rs114238370
rs182749961
rs6134180
rs146409168
rs113085370
rs77273648
rs79533821
rs680511
rs7588270
rs5024623
All − MR Egger
All − IVW(MRE)
−0.010
−0.005
0.000
0.005
MR effect size for
'Probable G−protein coupled receptor 101' on 'eGFR'

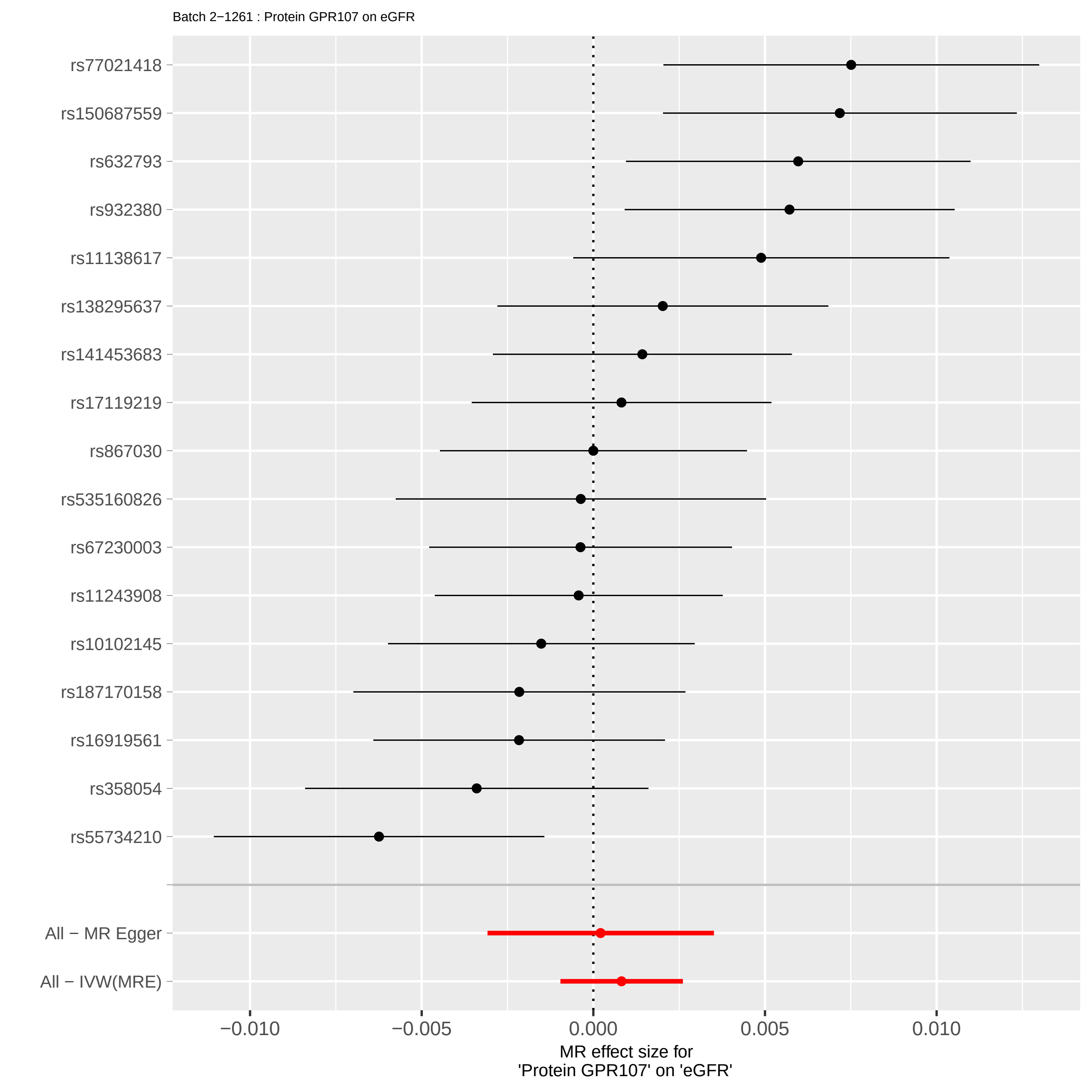

Batch 2−1261 : Protein GPR107 on eGFR
rs77021418
rs150687559
rs632793
rs932380
rs11138617
rs138295637
rs141453683
rs17119219
rs867030
rs535160826
rs67230003
rs11243908
rs10102145
rs187170158
rs16919561
rs358054
rs55734210
All − MR Egger
All − IVW(MRE)
−0.010
−0.005
0.000
0.005
0.010
MR effect size for
'Protein GPR107' on 'eGFR'

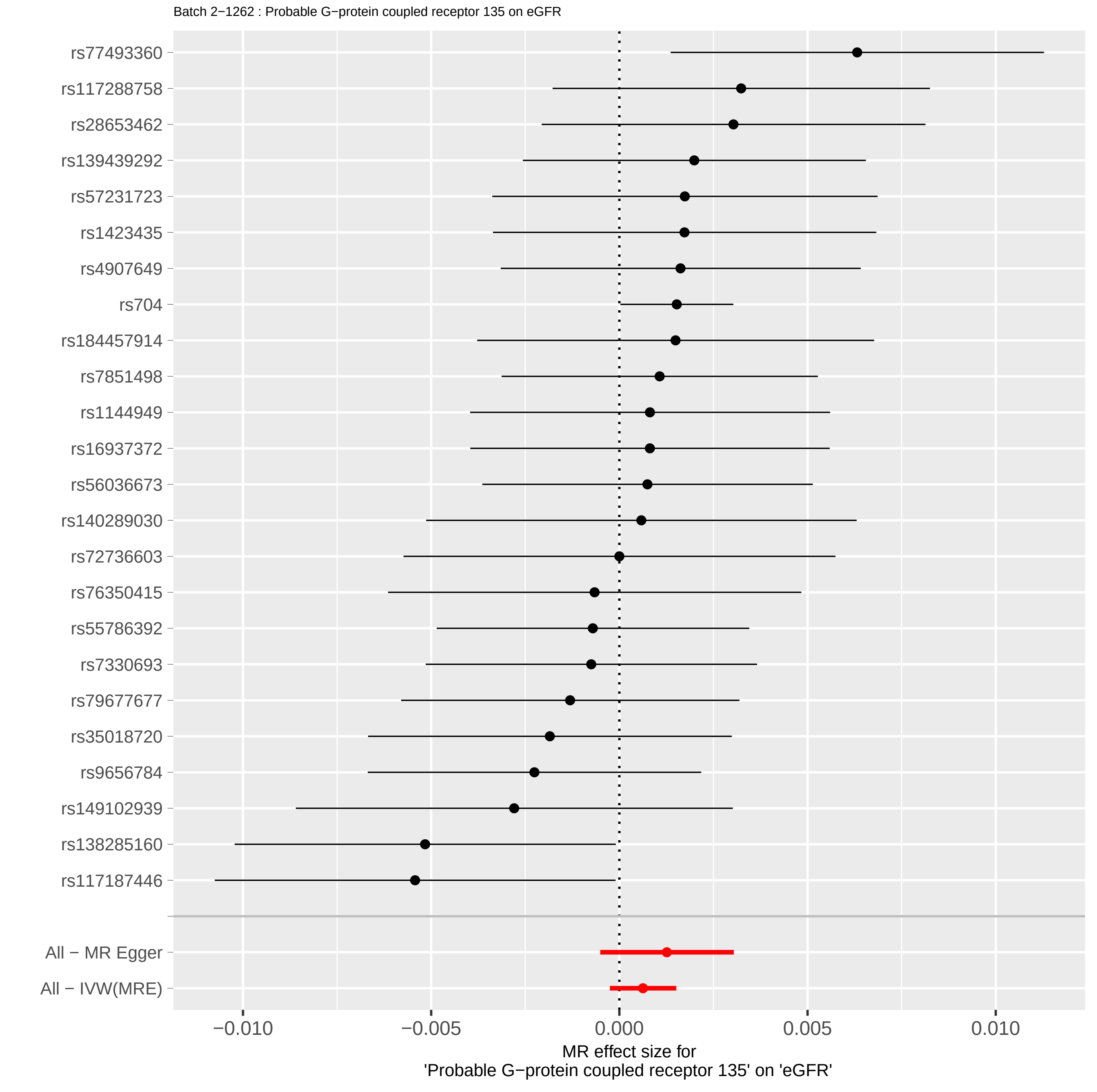

Batch 2−1262 : Probable G−protein coupled receptor 135 on eGFR
rs77493360
rs117288758
rs28653462
rs139439292
rs57231723
rs1423435
rs4907649
rs704
rs184457914
rs7851498
rs1144949
rs16937372
rs56036673
rs140289030
rs72736603
rs76350415
rs55786392
rs7330693
rs79677677
rs35018720
rs9656784
rs149102939
rs138285160
rs117187446
All − MR Egger
All − IVW(MRE)
−0.010
−0.005
0.000
0.005
0.010
MR effect size for
'Probable G−protein coupled receptor 135' on 'eGFR'

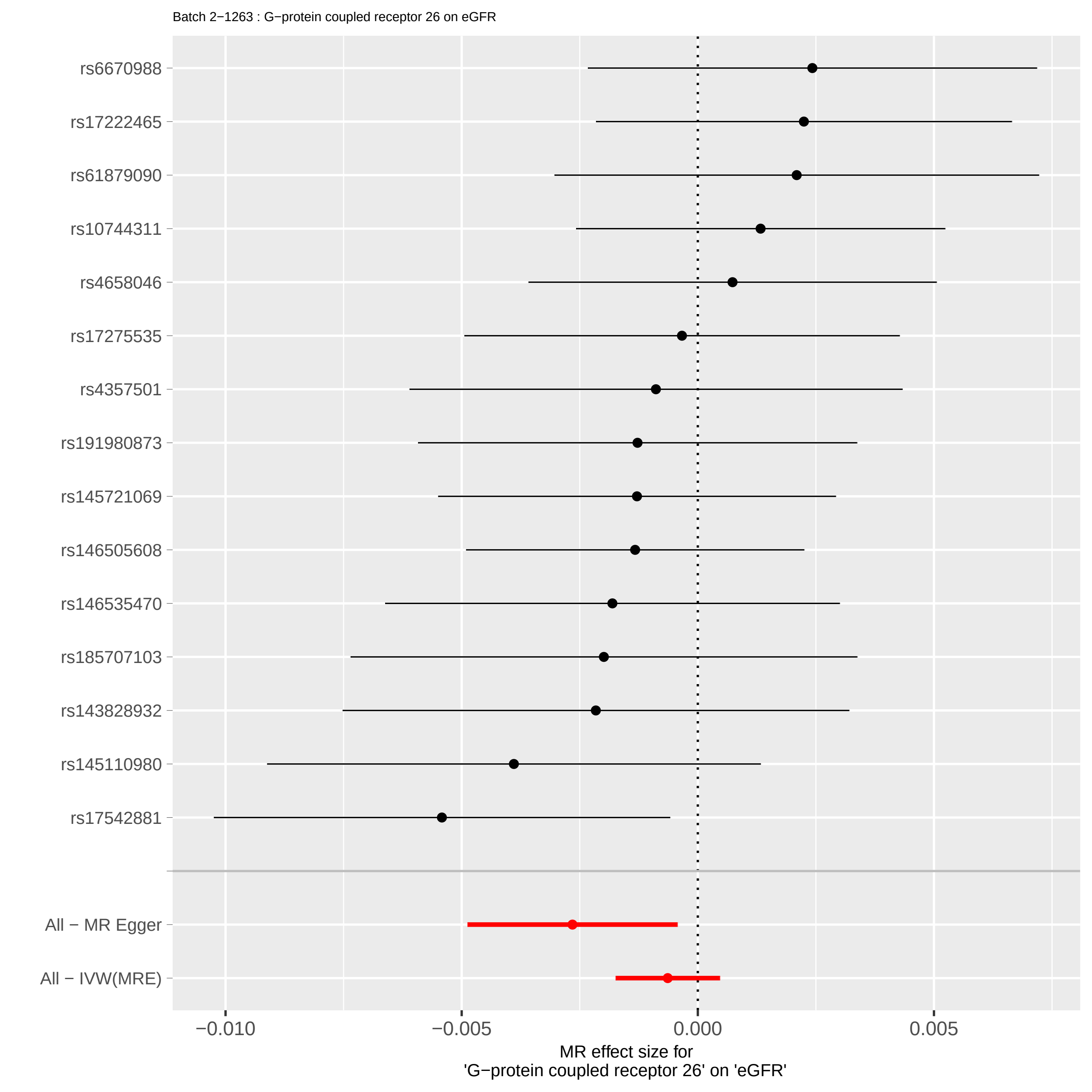
Batch 2−1263 : G−protein coupled receptor 26 on eGFR
rs6670988
rs17222465
rs61879090
rs10744311
rs4658046
rs17275535
rs4357501
rs191980873
rs145721069
rs146505608
rs146535470
rs185707103
rs143828932
rs145110980
rs17542881
All − MR Egger
All − IVW(MRE)
−0.010
−0.005
0.000
0.005
MR effect size for
'G−protein coupled receptor 26' on 'eGFR'

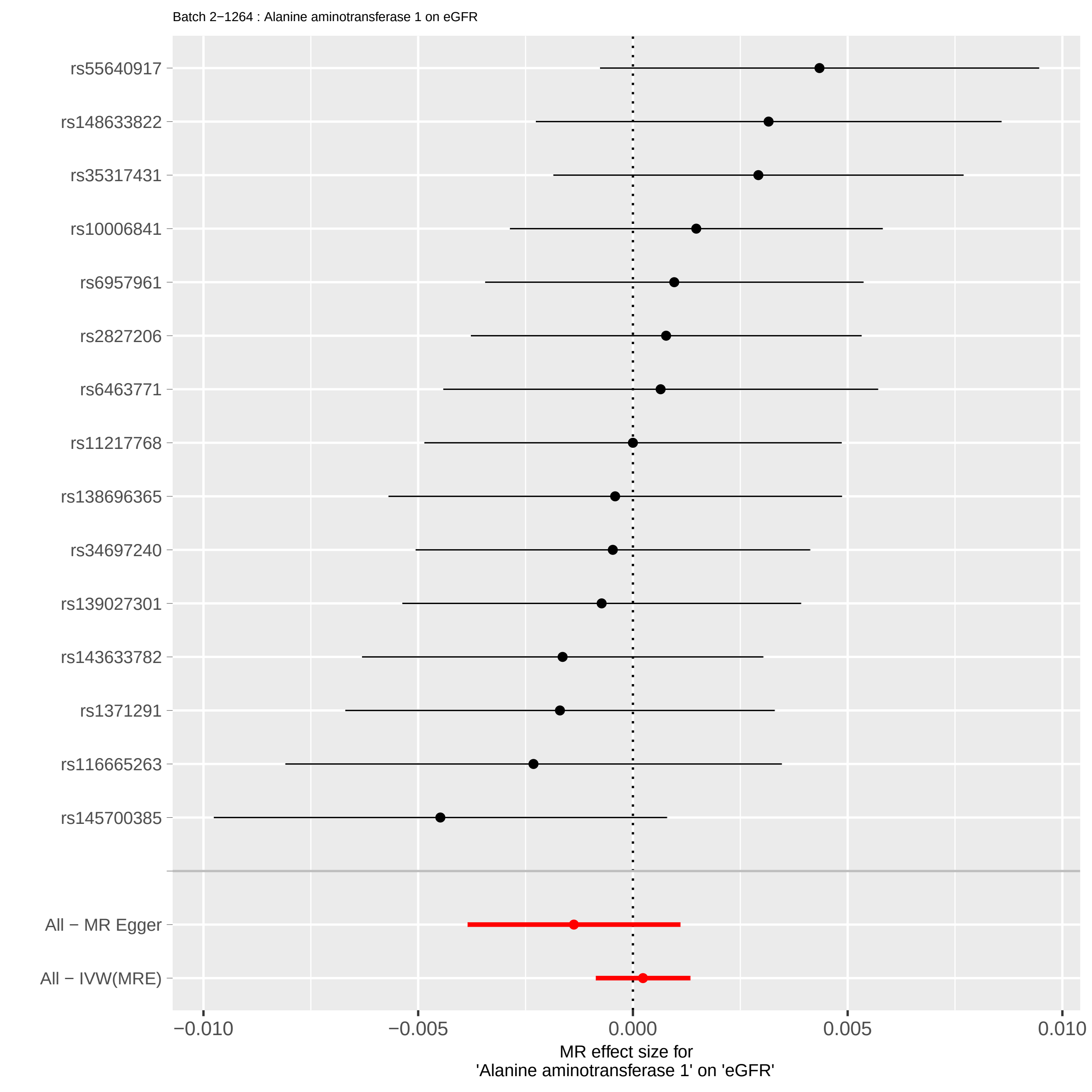

Batch 2−1264 : Alanine aminotransferase 1 on eGFR
rs55640917
rs148633822
rs35317431
rs10006841
rs6957961
rs2827206
rs6463771
rs11217768
rs138696365
rs34697240
rs139027301
rs143633782
rs1371291
rs116665263
rs145700385
All − MR Egger
All − IVW(MRE)
−0.010
−0.005
0.000
0.005
0.010
MR effect size for
'Alanine aminotransferase 1' on 'eGFR'

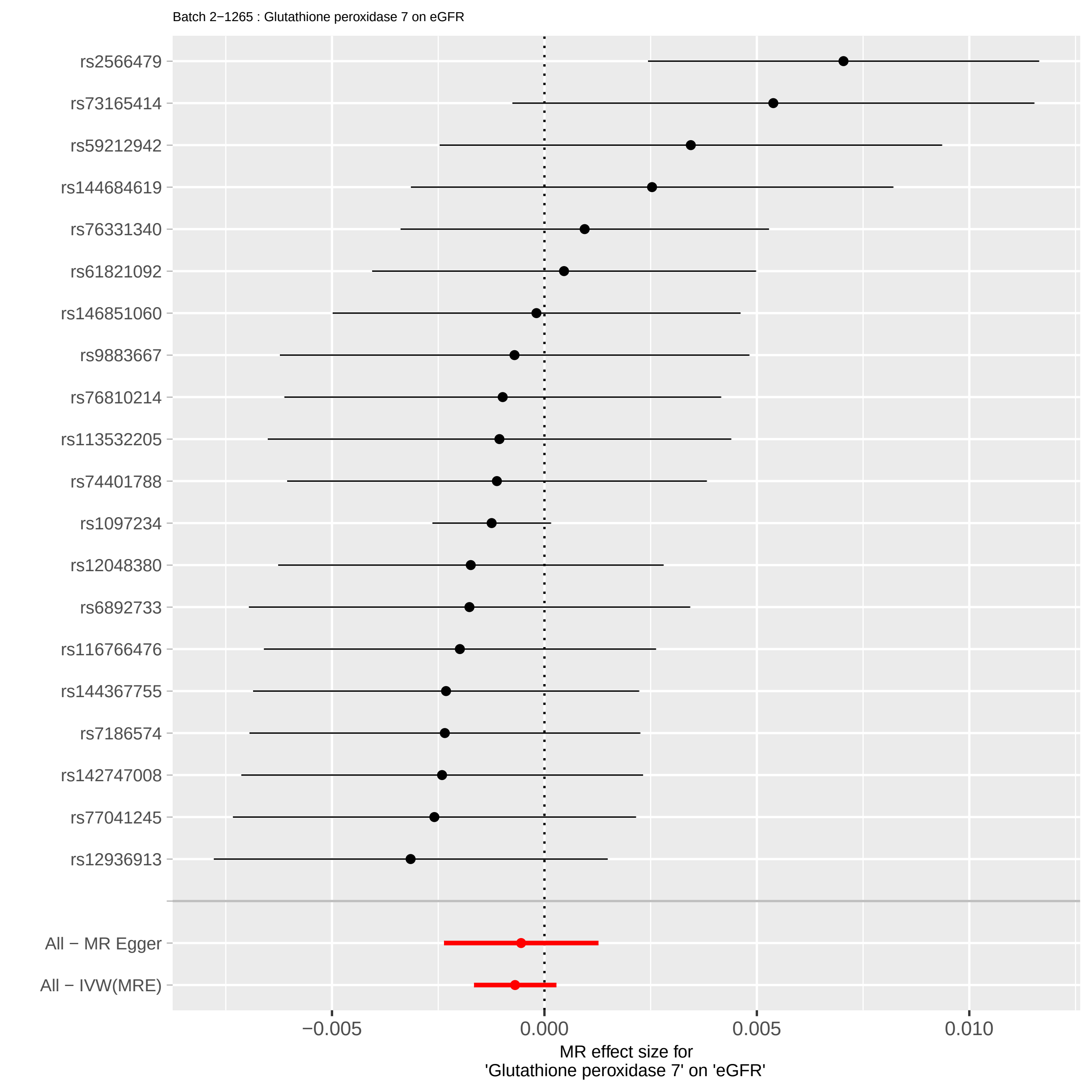
Batch 2−1265 : Glutathione peroxidase 7 on eGFR
rs2566479
rs73165414
rs59212942
rs144684619
rs76331340
rs61821092
rs146851060
rs9883667
rs76810214
rs113532205
rs74401788
rs1097234
rs12048380
rs6892733
rs116766476
rs144367755
rs7186574
rs142747008
rs77041245
rs12936913
All − MR Egger
All − IVW(MRE)
−0.005
0.000
0.005
0.010
MR effect size for
'Glutathione peroxidase 7' on 'eGFR'

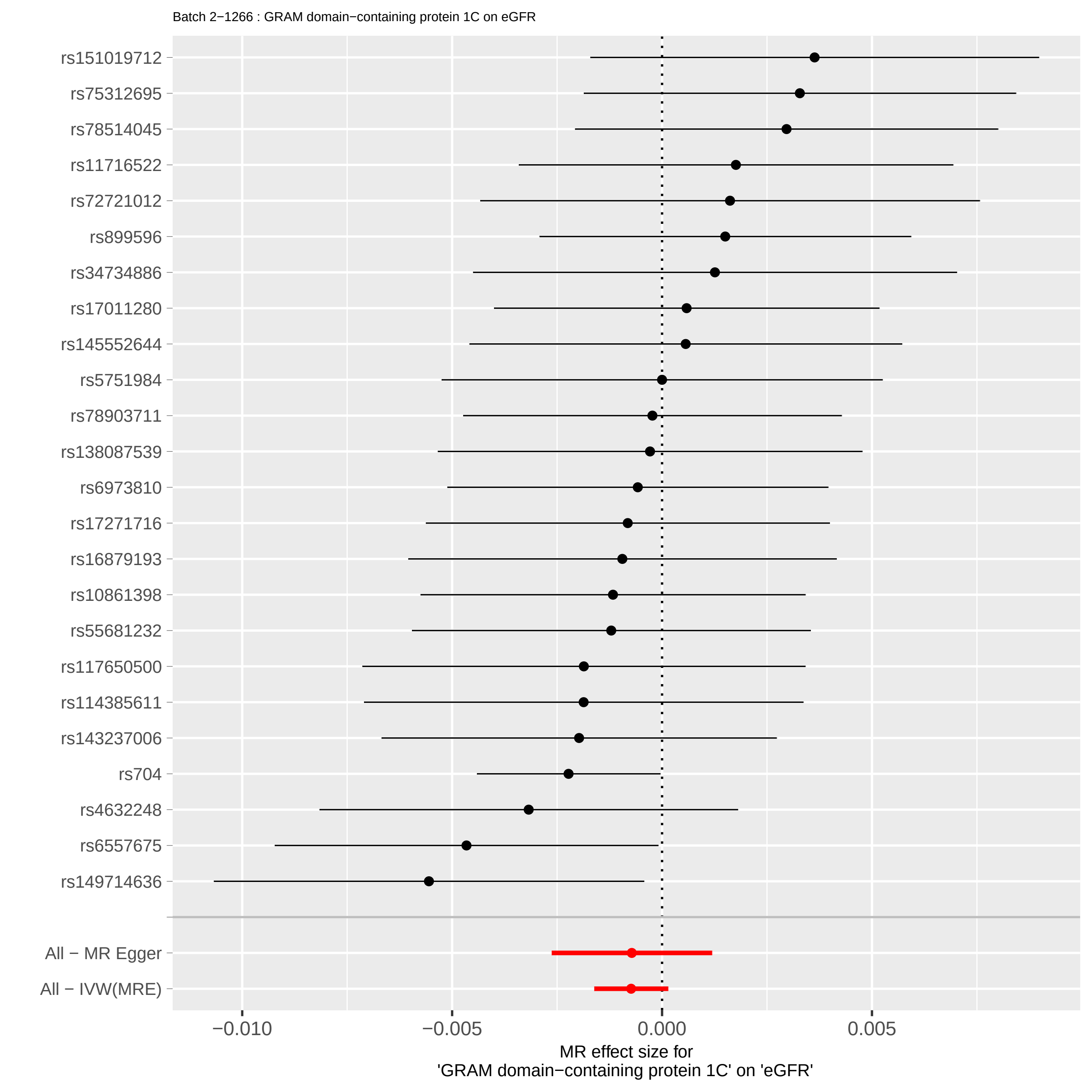
Batch 2−1266 : GRAM domain−containing protein 1C on eGFR
rs151019712
rs75312695
rs78514045
rs11716522
rs72721012
rs899596
rs34734886
rs17011280
rs145552644
rs5751984
rs78903711
rs138087539
rs6973810
rs17271716
rs16879193
rs10861398
rs55681232
rs117650500
rs114385611
rs143237006
rs704
rs4632248
rs6557675
rs149714636
All − MR Egger
All − IVW(MRE)
−0.010
−0.005
0.000
0.005
MR effect size for
'GRAM domain−containing protein 1C' on 'eGFR'

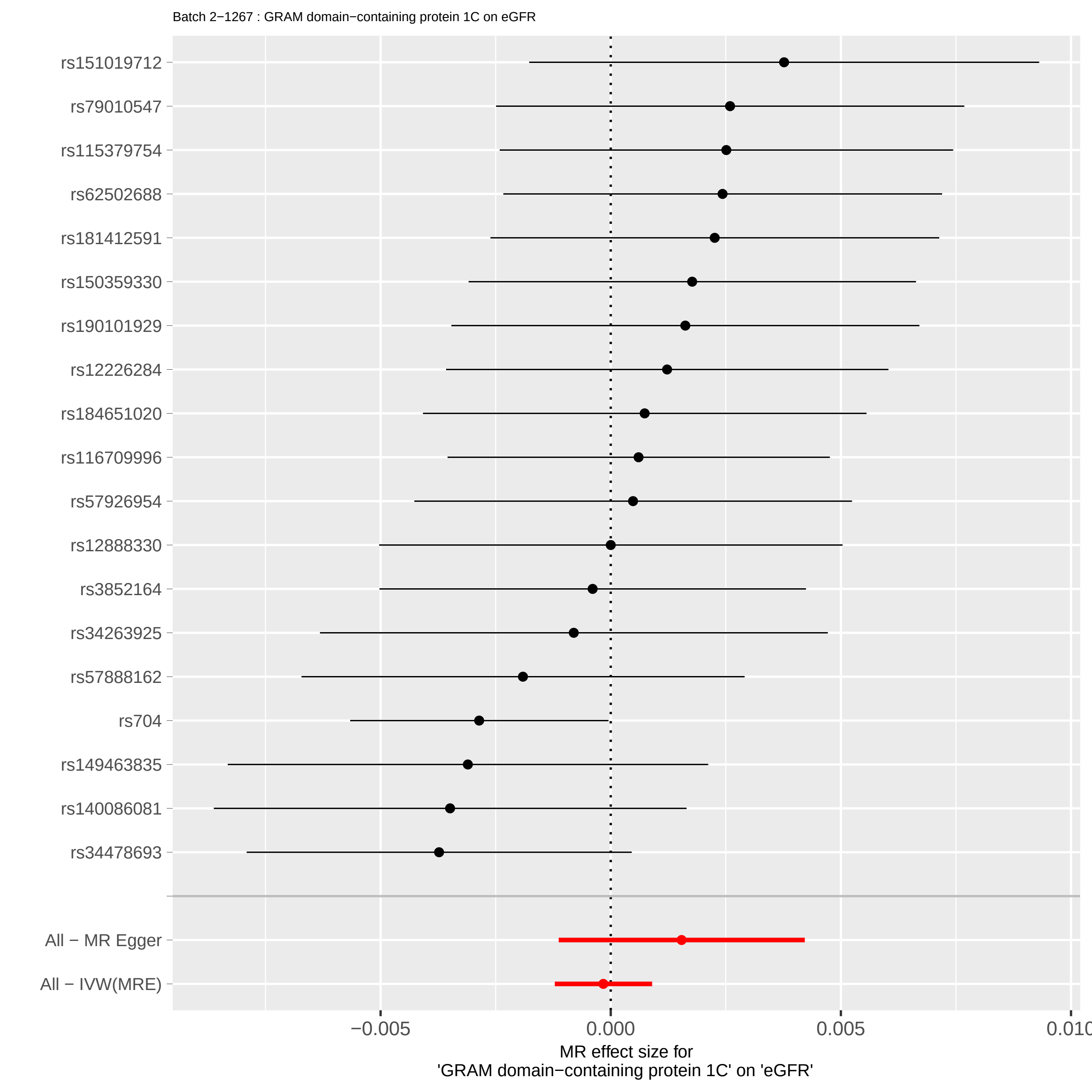

Batch 2−1267 : GRAM domain−containing protein 1C on eGFR
rs151019712
rs79010547
rs115379754
rs62502688
rs181412591
rs150359330
rs190101929
rs12226284
rs184651020
rs116709996
rs57926954
rs12888330
rs3852164
rs34263925
rs57888162
rs704
rs149463835
rs140086081
rs34478693
All − MR Egger
All − IVW(MRE)
−0.005
0.000
0.005
0.010
MR effect size for
'GRAM domain−containing protein 1C' on 'eGFR'

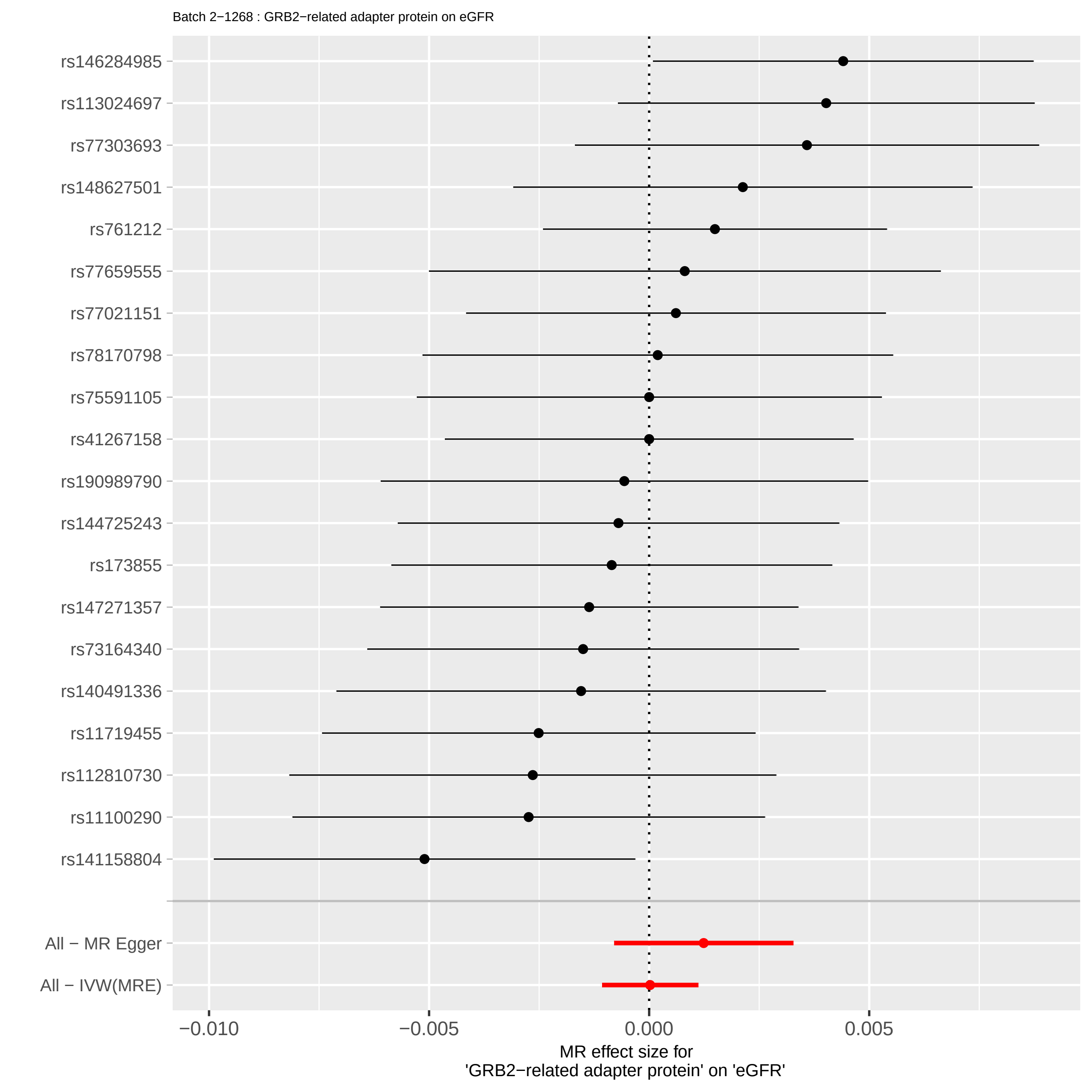

Batch 2−1268 : GRB2−related adapter protein on eGFR
rs146284985
rs113024697
rs77303693
rs148627501
rs761212
rs77659555
rs77021151
rs78170798
rs75591105
rs41267158
rs190989790
rs144725243
rs173855
rs147271357
rs73164340
rs140491336
rs11719455
rs112810730
rs11100290
rs141158804
All − MR Egger
All − IVW(MRE)
−0.010
−0.005
0.000
0.005
MR effect size for
'GRB2−related adapter protein' on 'eGFR'

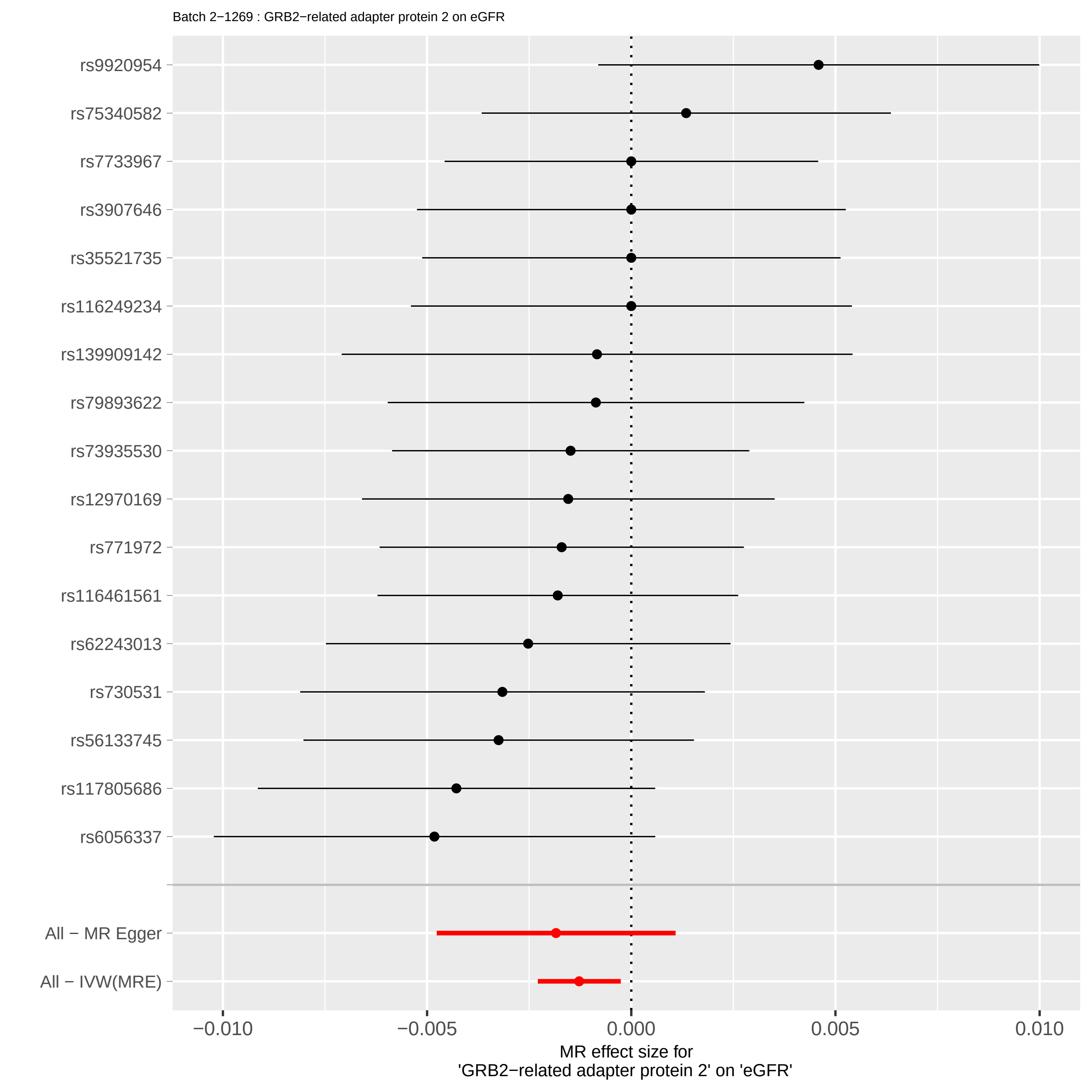

Batch 2−1269 : GRB2−related adapter protein 2 on eGFR
rs9920954
rs75340582
rs7733967
rs3907646
rs35521735
rs116249234
rs139909142
rs79893622
rs73935530
rs12970169
rs771972
rs116461561
rs62243013
rs730531
rs56133745
rs117805686
rs6056337
All − MR Egger
All − IVW(MRE)
−0.010
−0.005
0.000
0.005
0.010
MR effect size for
'GRB2−related adapter protein 2' on 'eGFR'

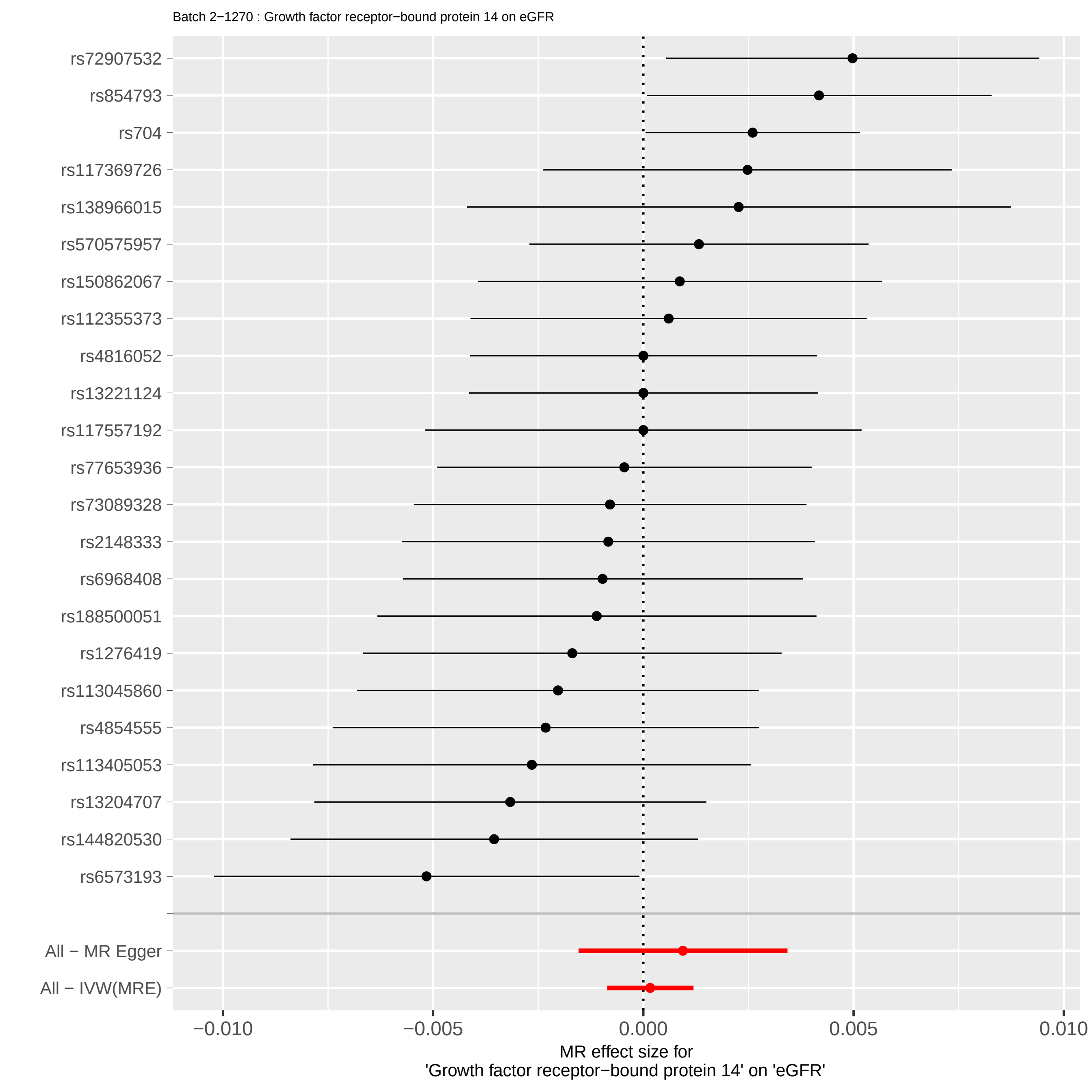

Batch 2−1270 : Growth factor receptor−bound protein 14 on eGFR
rs72907532
rs854793
rs704
rs117369726
rs138966015
rs570575957
rs150862067
rs112355373
rs4816052
rs13221124
rs117557192
rs77653936
rs73089328
rs2148333
rs6968408
rs188500051
rs1276419
rs113045860
rs4854555
rs113405053
rs13204707
rs144820530
rs6573193
All − MR Egger
All − IVW(MRE)
−0.010
−0.005
0.000
0.005
0.010
MR effect size for
'Growth factor receptor−bound protein 14' on 'eGFR'

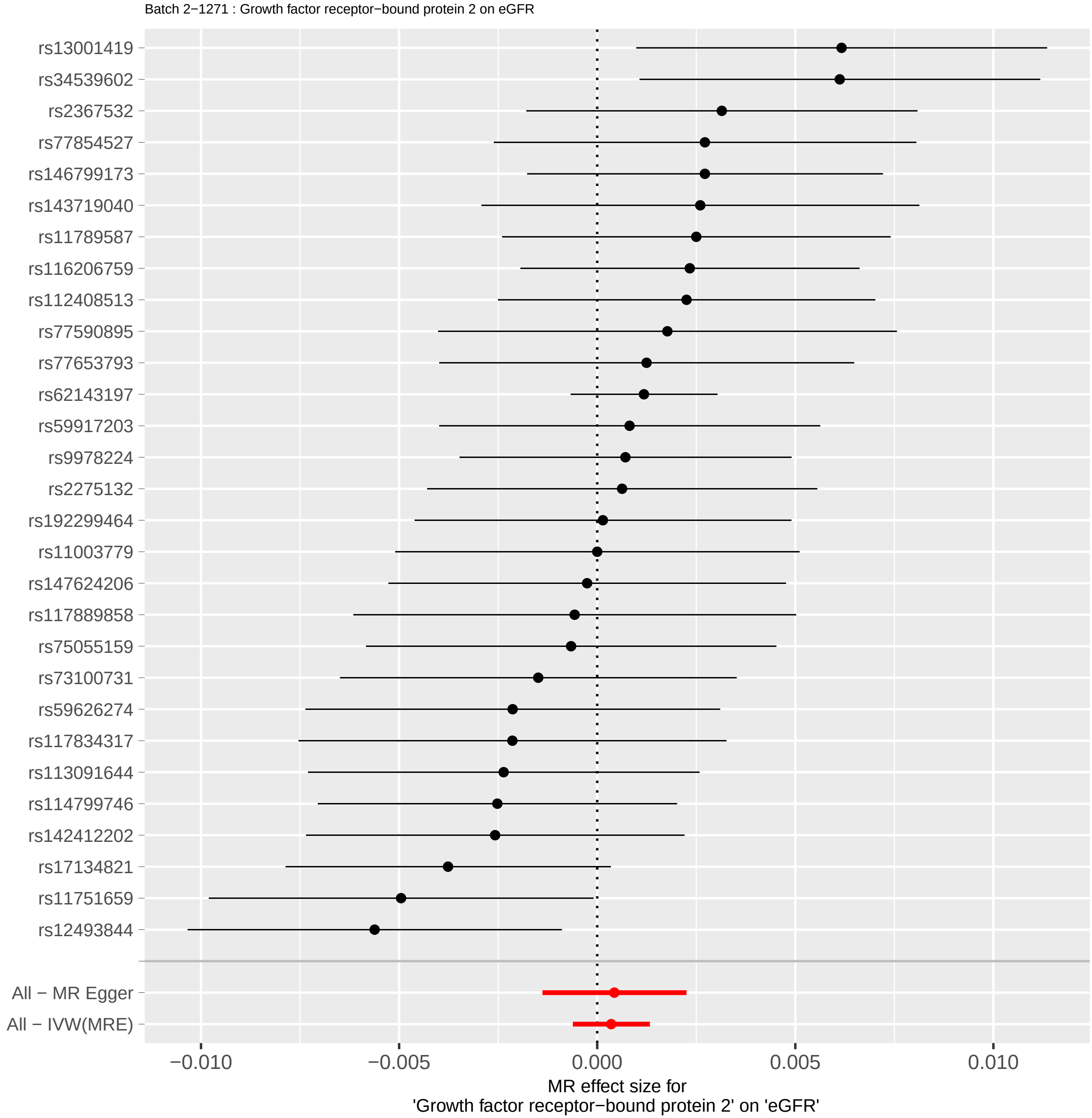

Batch 2−1271 : Growth factor receptor−bound protein 2 on eGFR
rs13001419
rs34539602
rs2367532
rs77854527
rs146799173
rs143719040
rs11789587
rs116206759
rs112408513
rs77590895
rs77653793
rs62143197
rs59917203
rs9978224
rs2275132
rs192299464
rs11003779
rs147624206
rs117889858
rs75055159
rs73100731
rs59626274
rs117834317
rs113091644
rs114799746
rs142412202
rs17134821
rs11751659
rs12493844
All − MR Egger
All − IVW(MRE)
−0.010
−0.005
0.000
0.005
0.010
MR effect size for
'Growth factor receptor−bound protein 2' on 'eGFR'

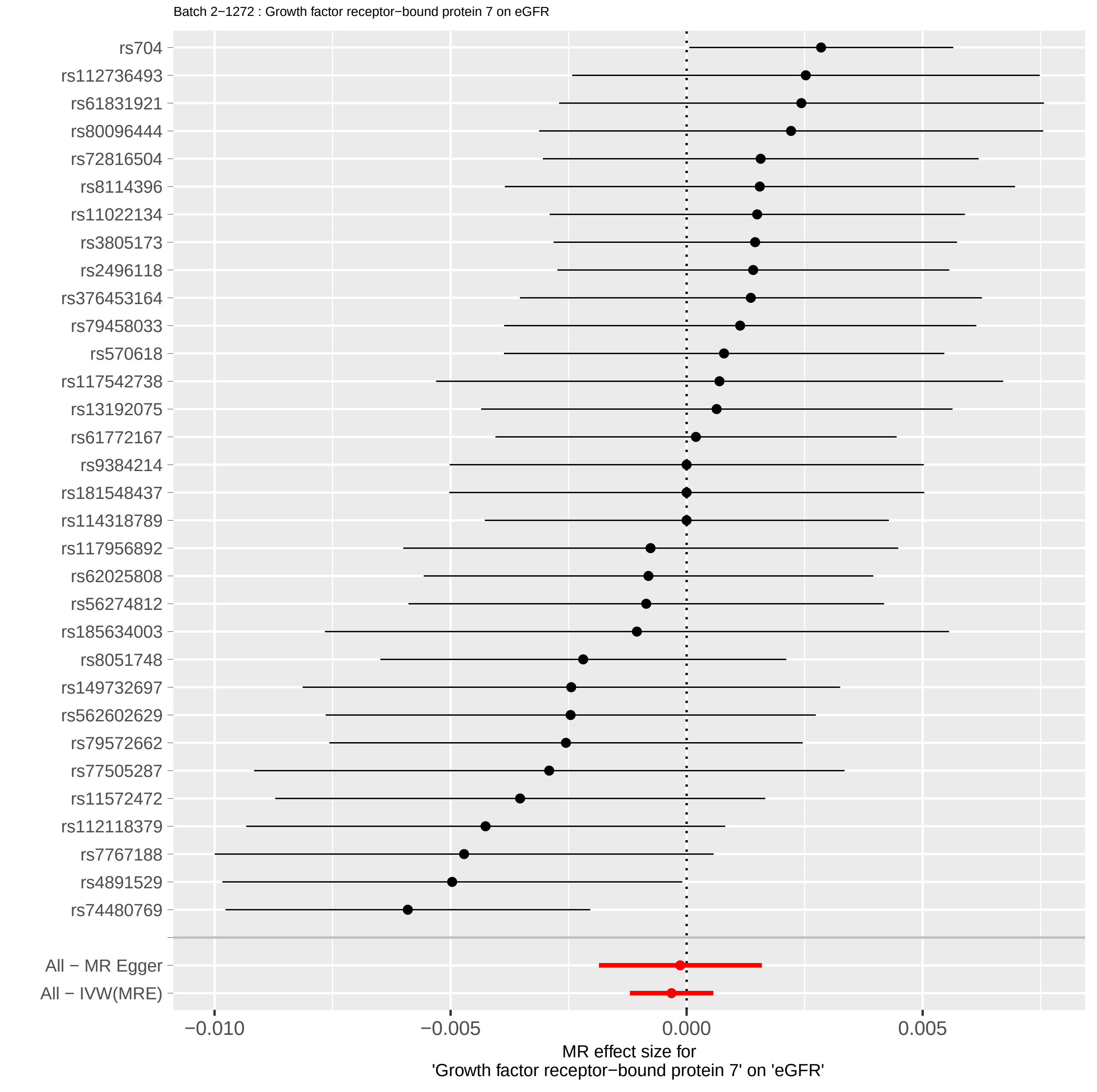

Batch 2−1272 : Growth factor receptor−bound protein 7 on eGFR
rs704
rs112736493
rs61831921
rs80096444
rs72816504
rs8114396
rs11022134
rs3805173
rs2496118
rs376453164
rs79458033
rs570618
rs117542738
rs13192075
rs61772167
rs9384214
rs181548437
rs114318789
rs117956892
rs62025808
rs56274812
rs185634003
rs8051748
rs149732697
rs562602629
rs79572662
rs77505287
rs11572472
rs112118379
rs7767188
rs4891529
rs74480769
All − MR Egger
All − IVW(MRE)
−0.010
−0.005
0.000
0.005
MR effect size for
'Growth factor receptor−bound protein 7' on 'eGFR'

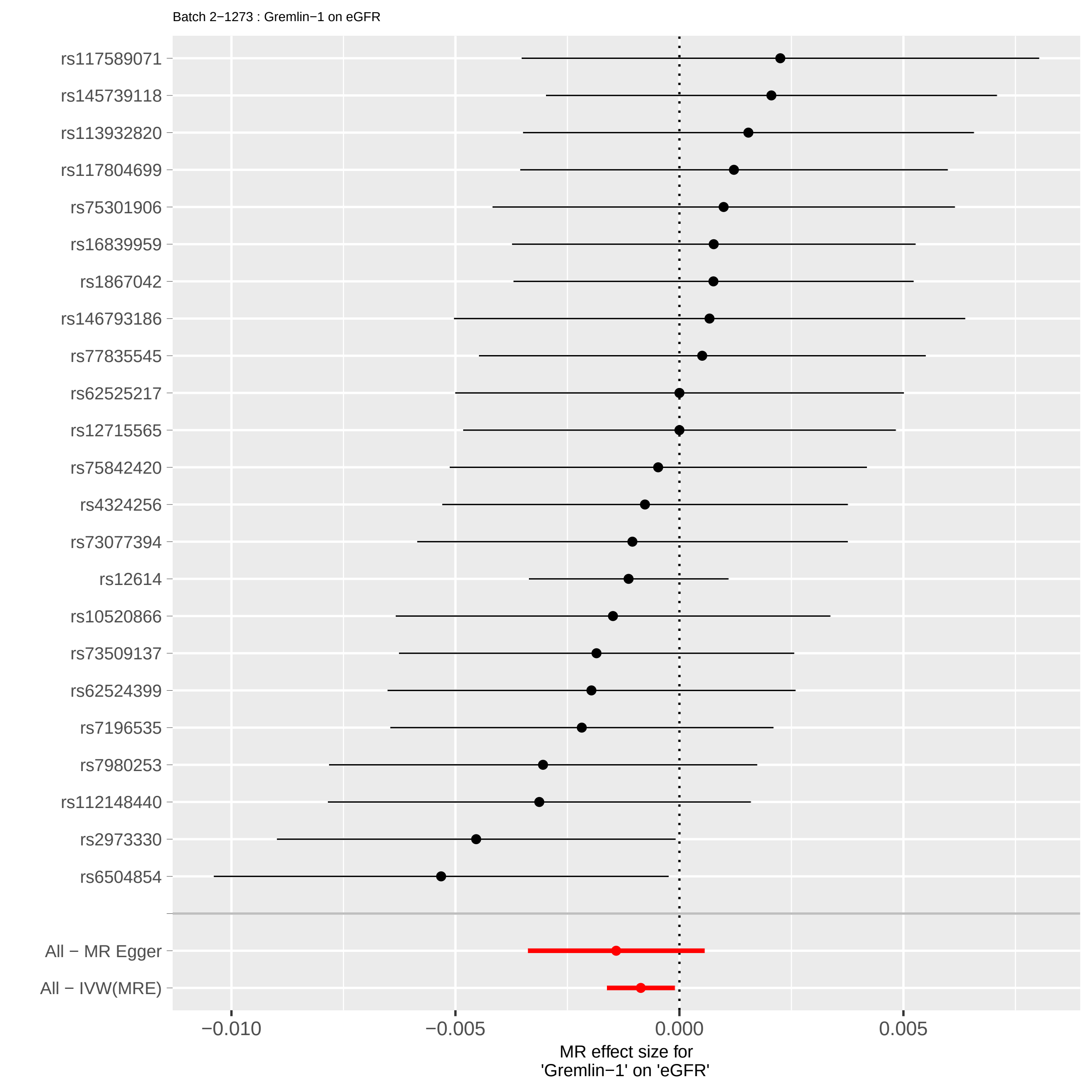

Batch 2−1273 : Gremlin−1 on eGFR
rs117589071
rs145739118
rs113932820
rs117804699
rs75301906
rs16839959
rs1867042
rs146793186
rs77835545
rs62525217
rs12715565
rs75842420
rs4324256
rs73077394
rs12614
rs10520866
rs73509137
rs62524399
rs7196535
rs7980253
rs112148440
rs2973330
rs6504854
All − MR Egger
All − IVW(MRE)
−0.010
−0.005
0.000
0.005
MR effect size for
'Gremlin−1' on 'eGFR'

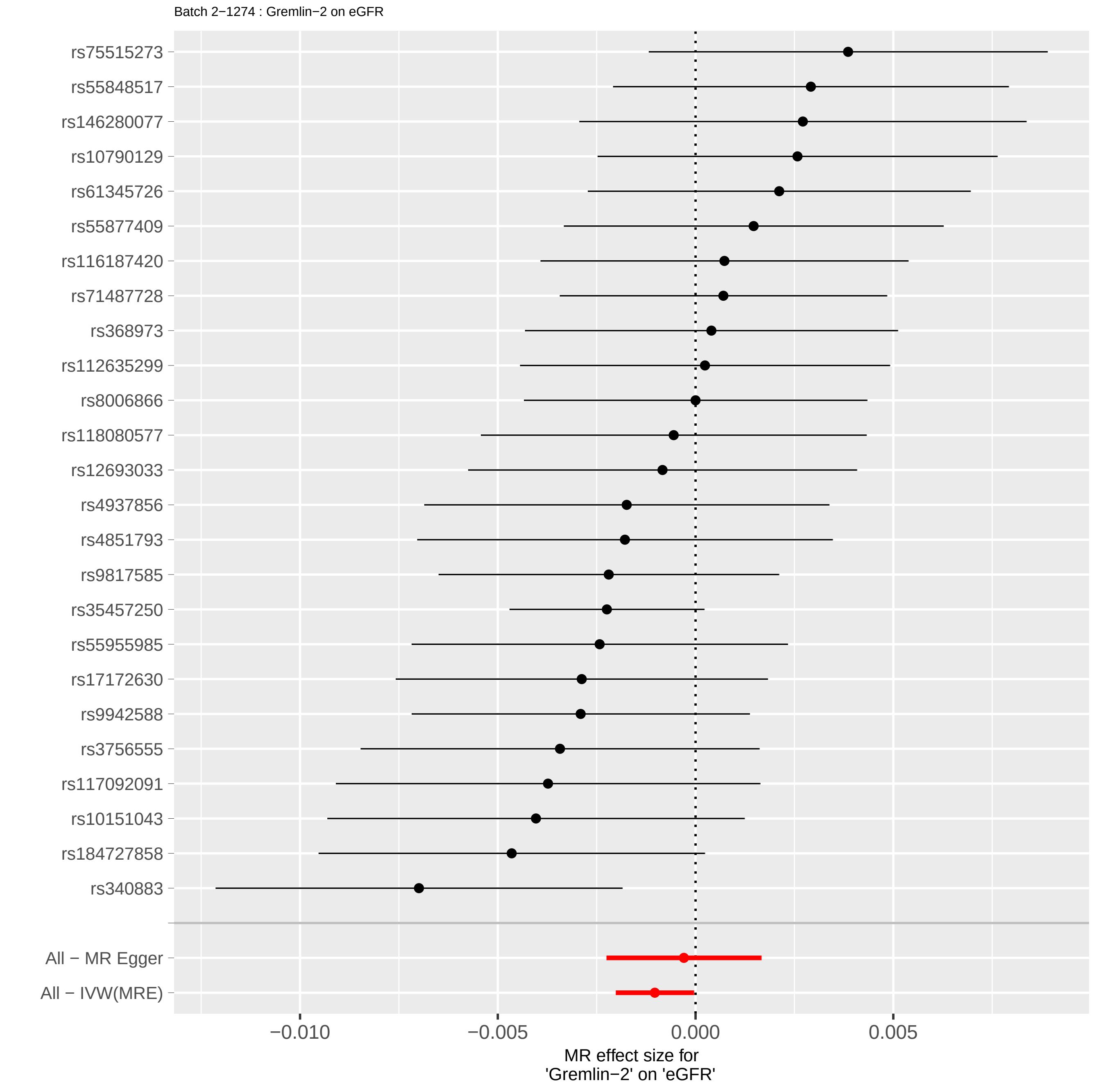

Batch 2−1274 : Gremlin−2 on eGFR
rs75515273
rs55848517
rs146280077
rs10790129
rs61345726
rs55877409
rs116187420
rs71487728
rs368973
rs112635299
rs8006866
rs118080577
rs12693033
rs4937856
rs4851793
rs9817585
rs35457250
rs55955985
rs17172630
rs9942588
rs3756555
rs117092091
rs10151043
rs184727858
rs340883
All − MR Egger
All − IVW(MRE)
−0.010
−0.005
0.000
0.005
MR effect size for
'Gremlin−2' on 'eGFR'

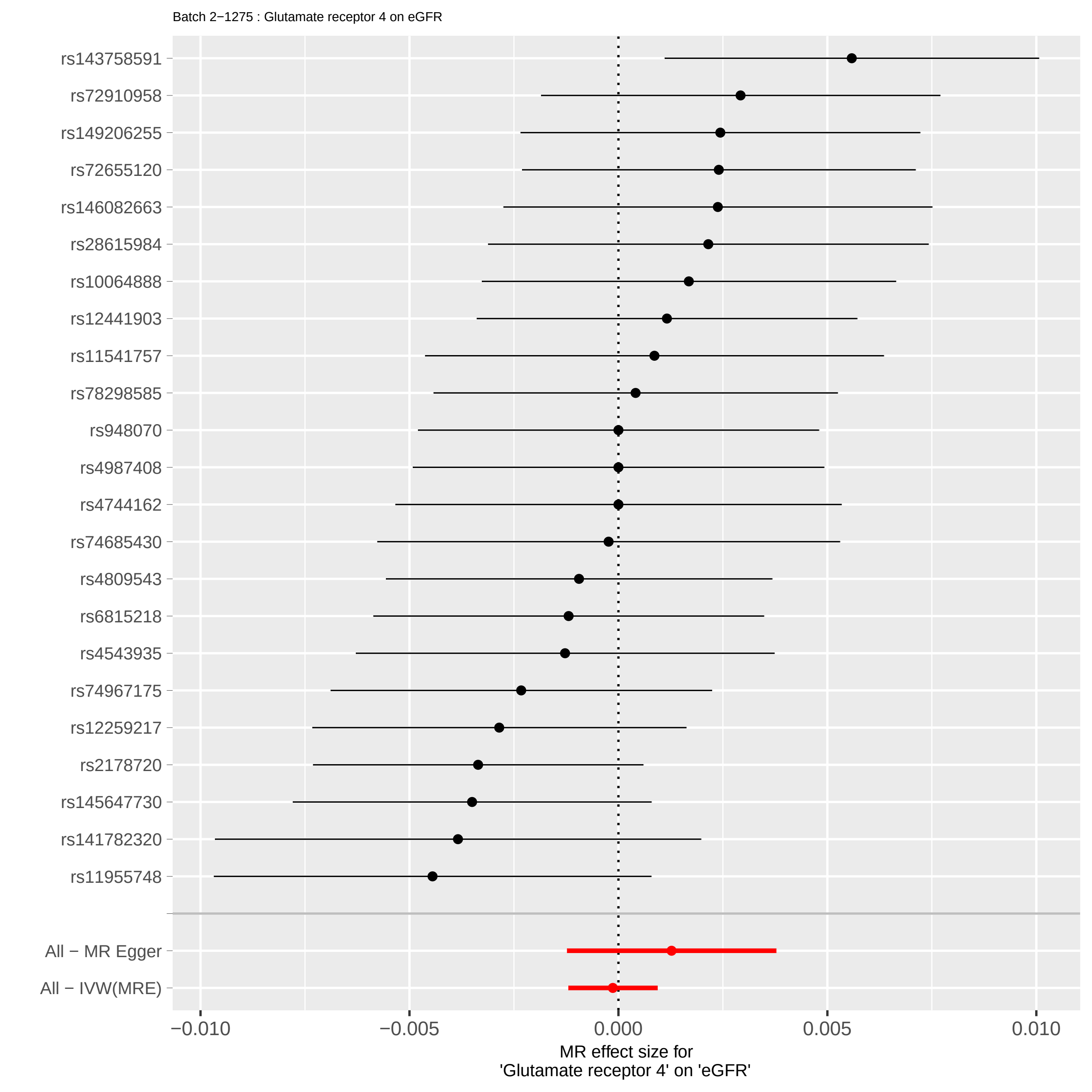
Batch 2−1275 : Glutamate receptor 4 on eGFR
rs143758591
rs72910958
rs149206255
rs72655120
rs146082663
rs28615984
rs10064888
rs12441903
rs11541757
rs78298585
rs948070
rs4987408
rs4744162
rs74685430
rs4809543
rs6815218
rs4543935
rs74967175
rs12259217
rs2178720
rs145647730
rs141782320
rs11955748
All − MR Egger
All − IVW(MRE)
−0.010
−0.005
0.000
0.005
0.010
MR effect size for
'Glutamate receptor 4' on 'eGFR'

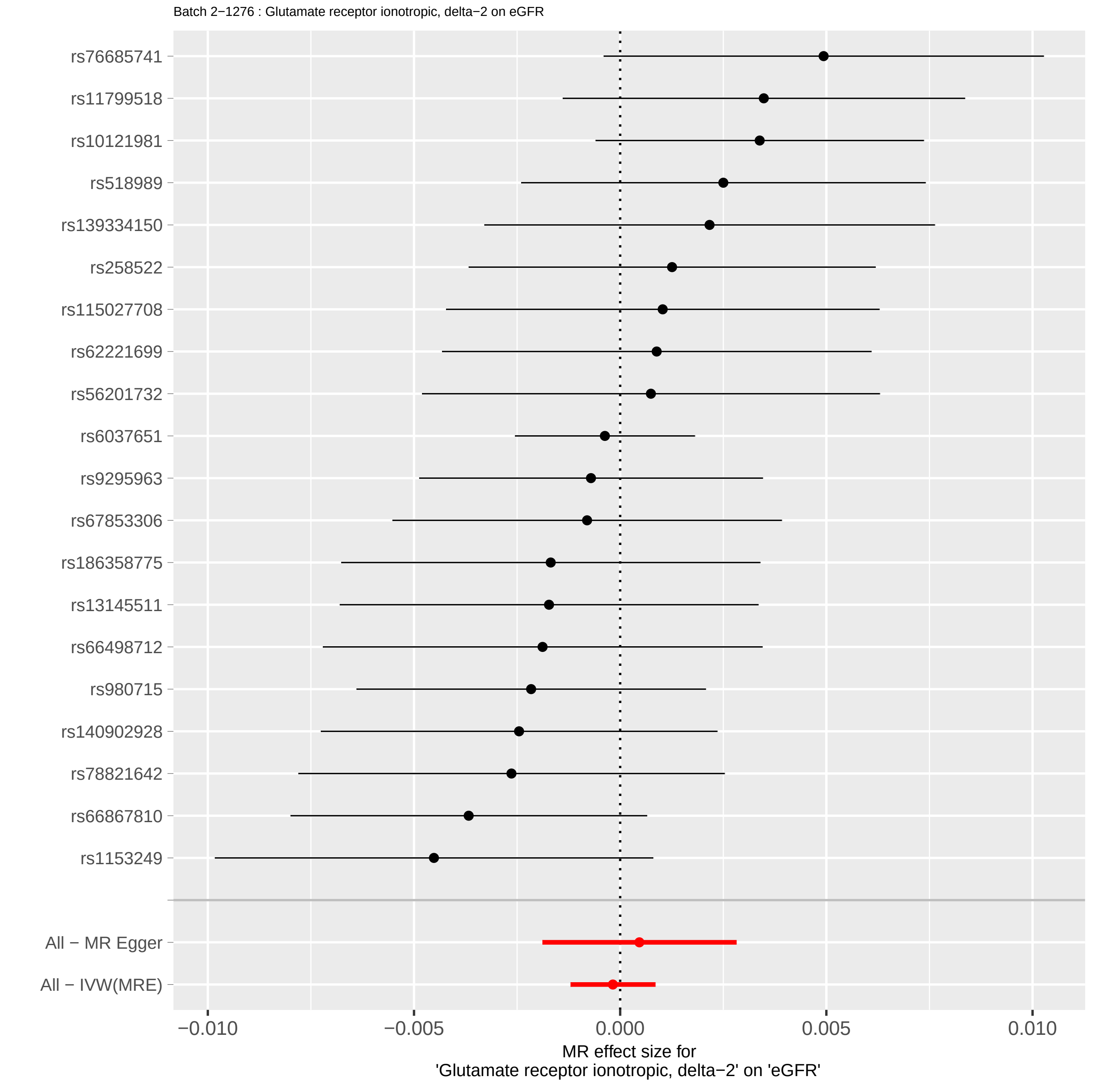

Batch 2−1276 : Glutamate receptor ionotropic, delta−2 on eGFR
rs76685741
rs11799518
rs10121981
rs518989
rs139334150
rs258522
rs115027708
rs62221699
rs56201732
rs6037651
rs9295963
rs67853306
rs186358775
rs13145511
rs66498712
rs980715
rs140902928
rs78821642
rs66867810
rs1153249
All − MR Egger
All − IVW(MRE)
−0.010
−0.005
0.000
0.005
0.010
MR effect size for
'Glutamate receptor ionotropic, delta−2' on 'eGFR'

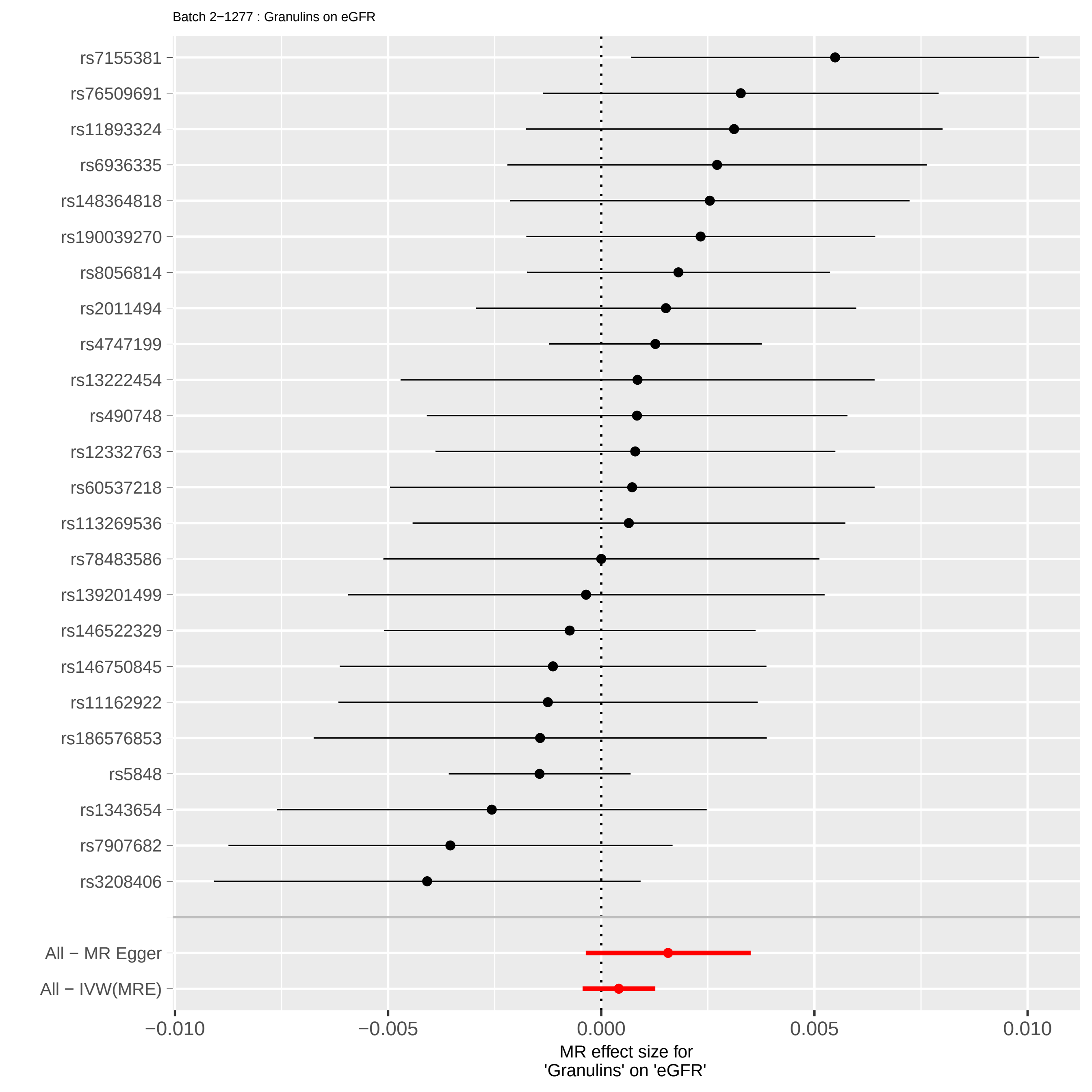

Batch 2−1277 : Granulins on eGFR
rs7155381
rs76509691
rs11893324
rs6936335
rs148364818
rs190039270
rs8056814
rs2011494
rs4747199
rs13222454
rs490748
rs12332763
rs60537218
rs113269536
rs78483586
rs139201499
rs146522329
rs146750845
rs11162922
rs186576853
rs5848
rs1343654
rs7907682
rs3208406
All − MR Egger
All − IVW(MRE)
−0.010
−0.005
0.000
0.005
0.010
MR effect size for
'Granulins' on 'eGFR'

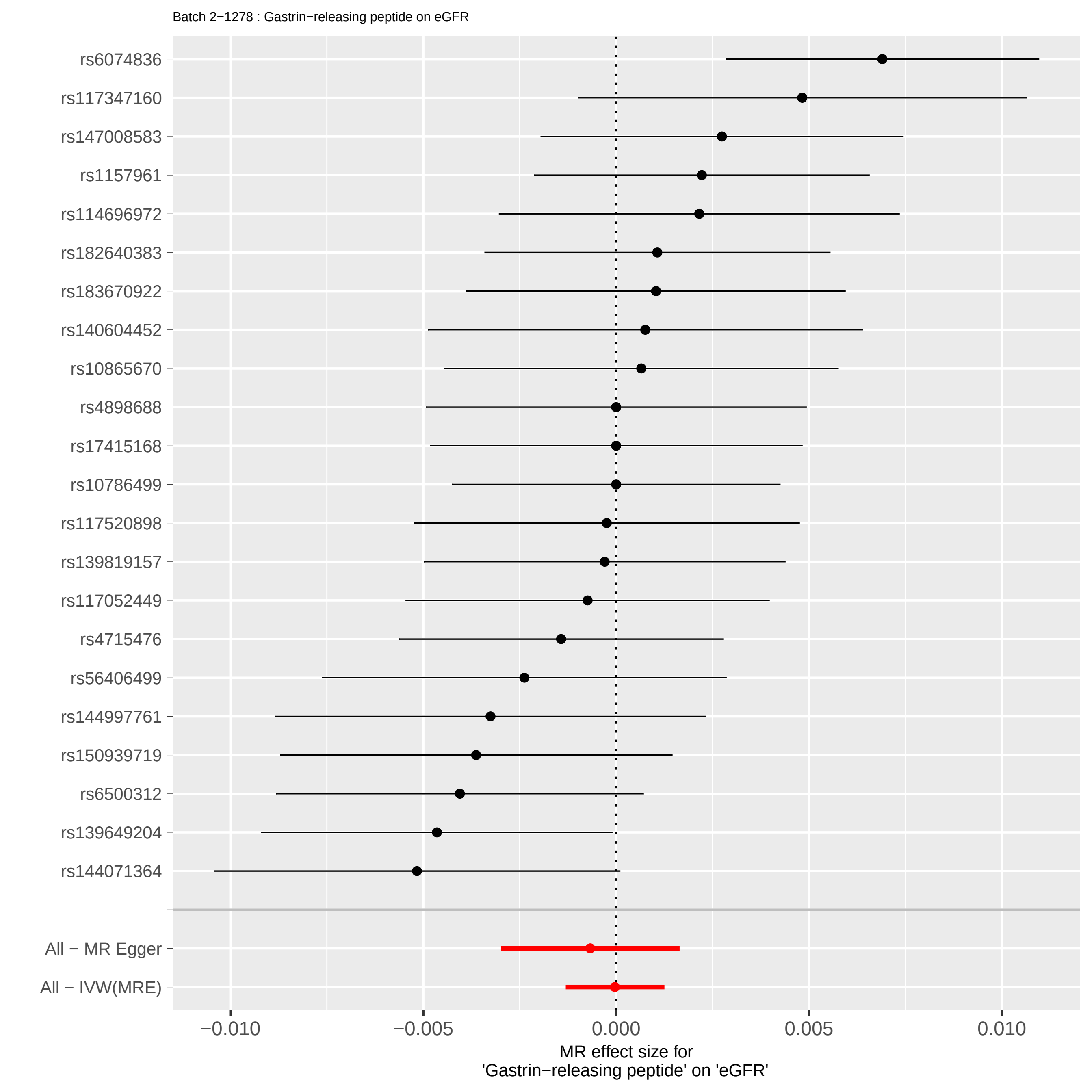

Batch 2−1278 : Gastrin−releasing peptide on eGFR
rs6074836
rs117347160
rs147008583
rs1157961
rs114696972
rs182640383
rs183670922
rs140604452
rs10865670
rs4898688
rs17415168
rs10786499
rs117520898
rs139819157
rs117052449
rs4715476
rs56406499
rs144997761
rs150939719
rs6500312
rs139649204
rs144071364
All − MR Egger
All − IVW(MRE)
−0.010
−0.005
0.000
0.005
0.010
MR effect size for
'Gastrin−releasing peptide' on 'eGFR'

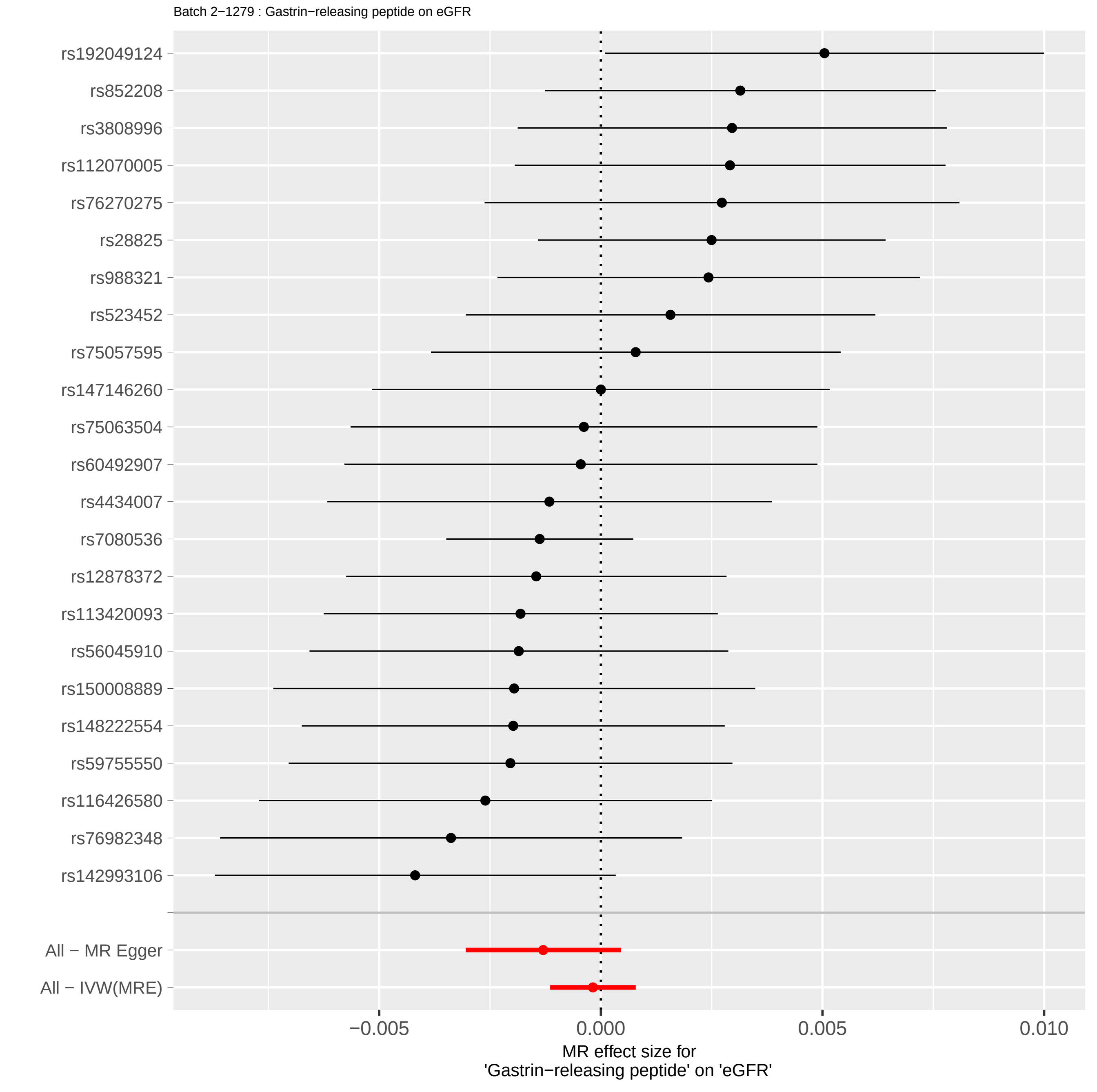

Batch 2−1279 : Gastrin−releasing peptide on eGFR
rs192049124
rs852208
rs3808996
rs112070005
rs76270275
rs28825
rs988321
rs523452
rs75057595
rs147146260
rs75063504
rs60492907
rs4434007
rs7080536
rs12878372
rs113420093
rs56045910
rs150008889
rs148222554
rs59755550
rs116426580
rs76982348
rs142993106
All − MR Egger
All − IVW(MRE)
−0.005
0.000
0.005
0.010
MR effect size for
'Gastrin−releasing peptide' on 'eGFR'

Batch 2−1280 : Gastrin−releasing peptide on eGFR

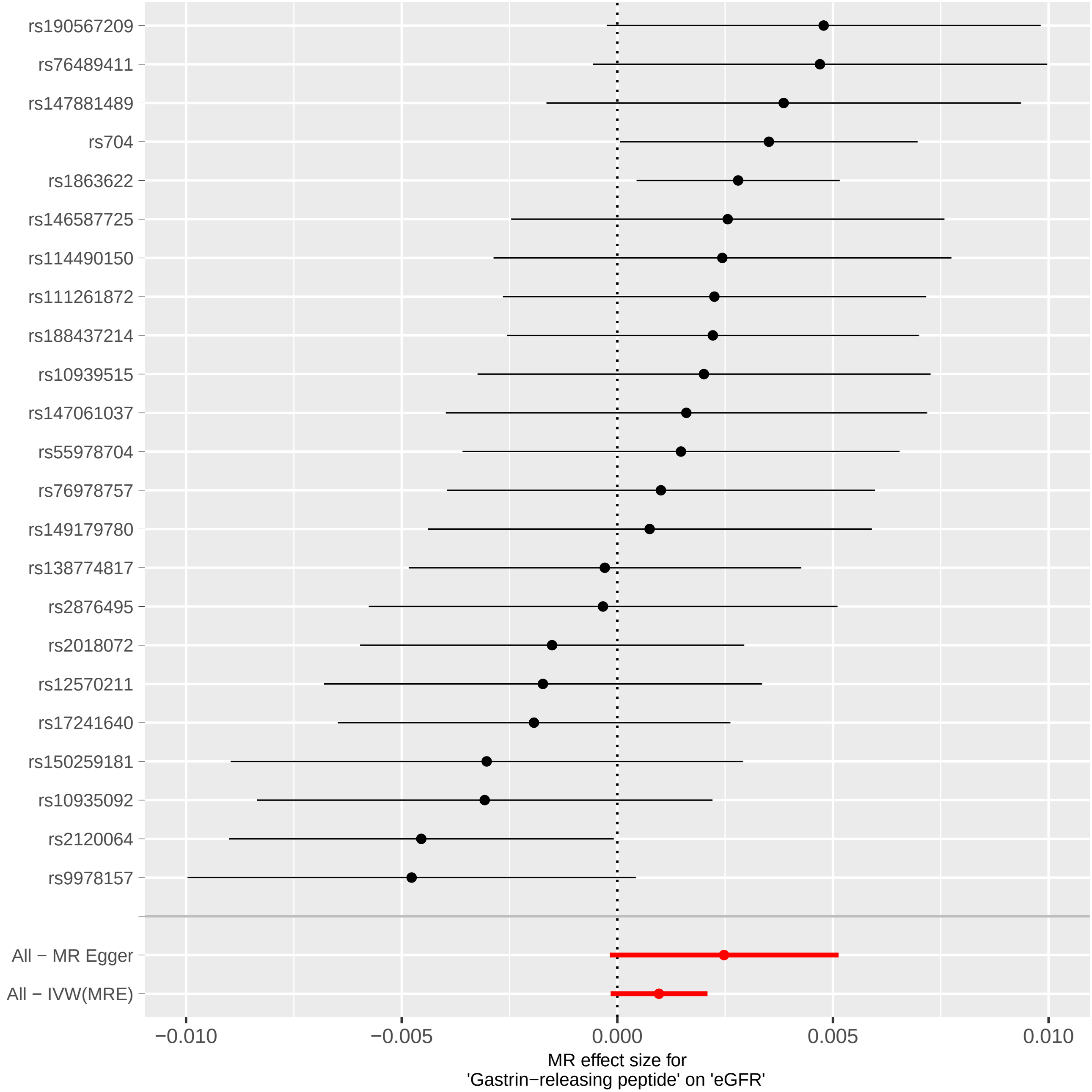

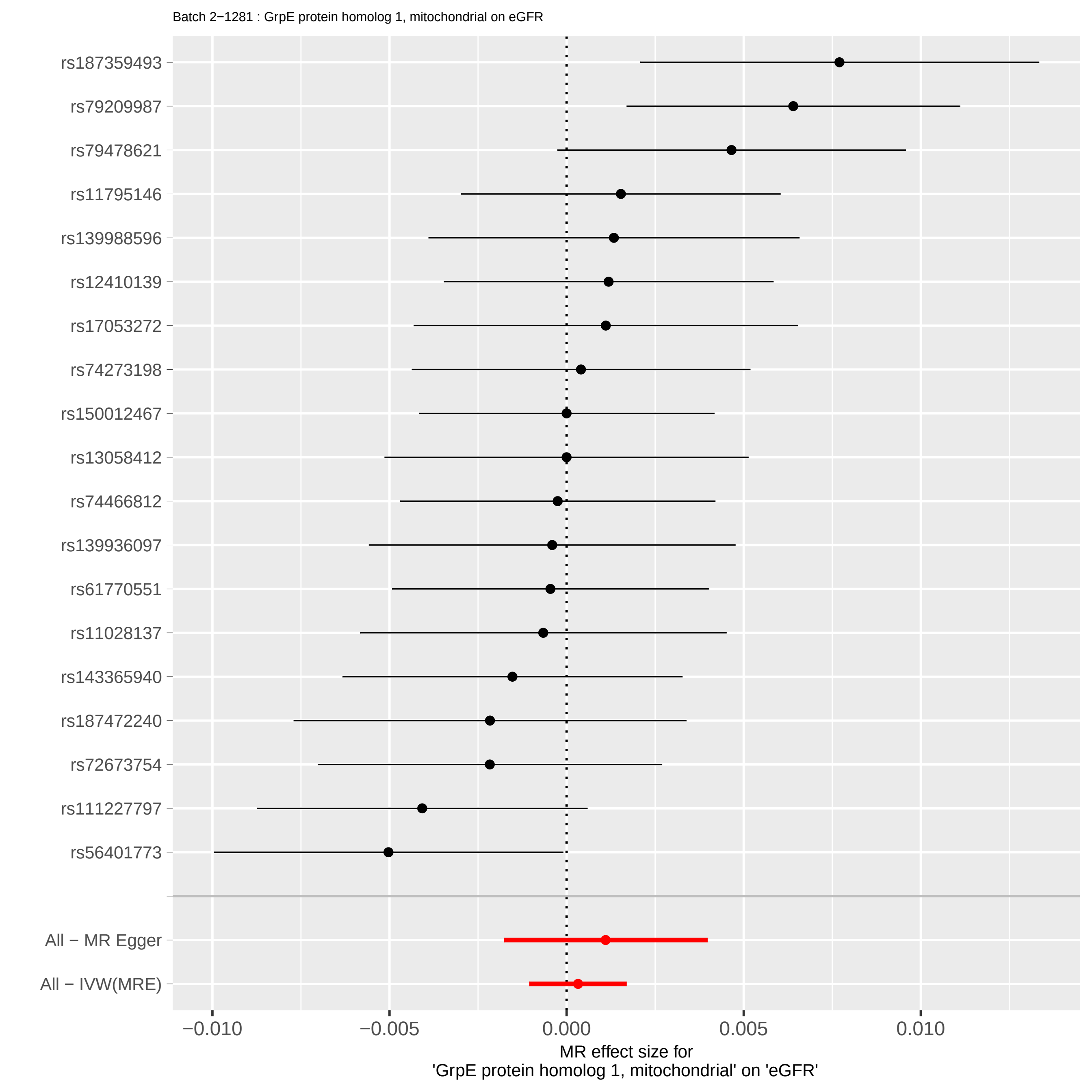
Batch 2−1281 : GrpE protein homolog 1, mitochondrial on eGFR
rs187359493
rs79209987
rs79478621
rs11795146
rs139988596
rs12410139
rs17053272
rs74273198
rs150012467
rs13058412
rs74466812
rs139936097
rs61770551
rs11028137
rs143365940
rs187472240
rs72673754
rs111227797
rs56401773
All − MR Egger
All − IVW(MRE)
−0.010
−0.005
0.000
0.005
0.010
MR effect size for
'GrpE protein homolog 1, mitochondrial' on 'eGFR'

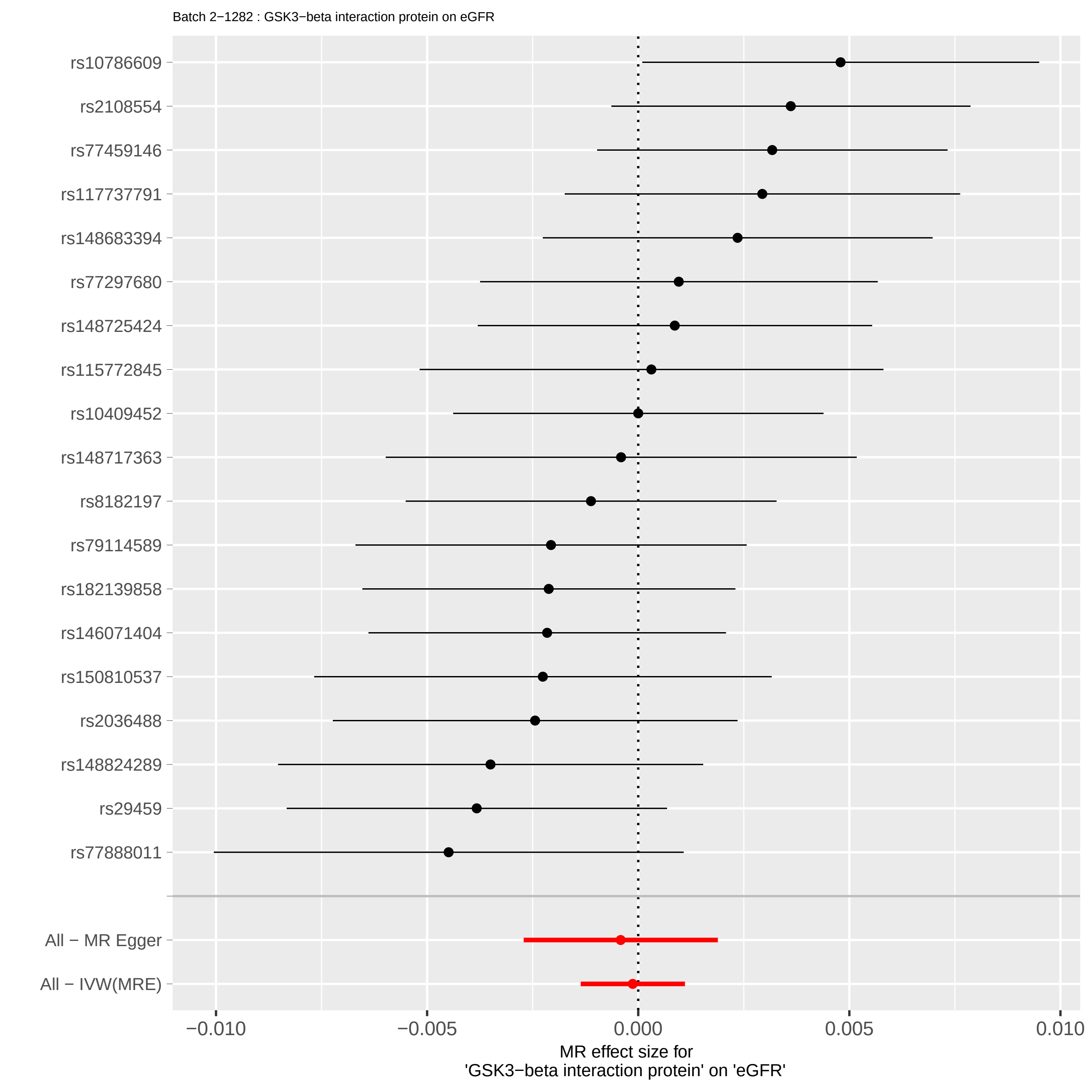

Batch 2−1282 : GSK3−beta interaction protein on eGFR
rs10786609
rs2108554
rs77459146
rs117737791
rs148683394
rs77297680
rs148725424
rs115772845
rs10409452
rs148717363
rs8182197
rs79114589
rs182139858
rs146071404
rs150810537
rs2036488
rs148824289
rs29459
rs77888011
All − MR Egger
All − IVW(MRE)
−0.010
−0.005
0.000
0.005
0.010
MR effect size for
'GSK3−beta interaction protein' on 'eGFR'

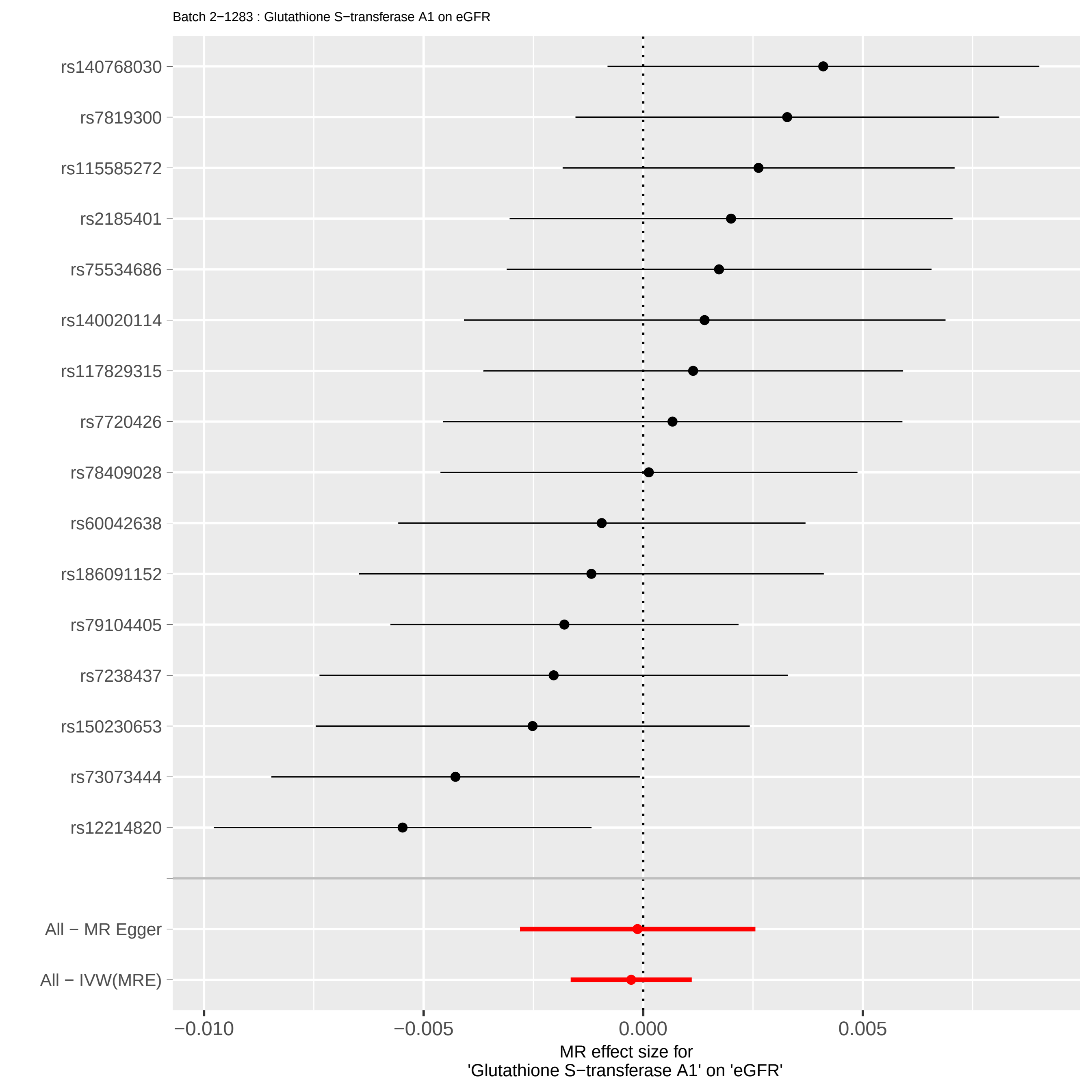

Batch 2−1283 : Glutathione S−transferase A1 on eGFR
rs140768030
rs7819300
rs115585272
rs2185401
rs75534686
rs140020114
rs117829315
rs7720426
rs78409028
rs60042638
rs186091152
rs79104405
rs7238437
rs150230653
rs73073444
rs12214820
All − MR Egger
All − IVW(MRE)
−0.010
−0.005
0.000
0.005
MR effect size for
'Glutathione S−transferase A1' on 'eGFR'

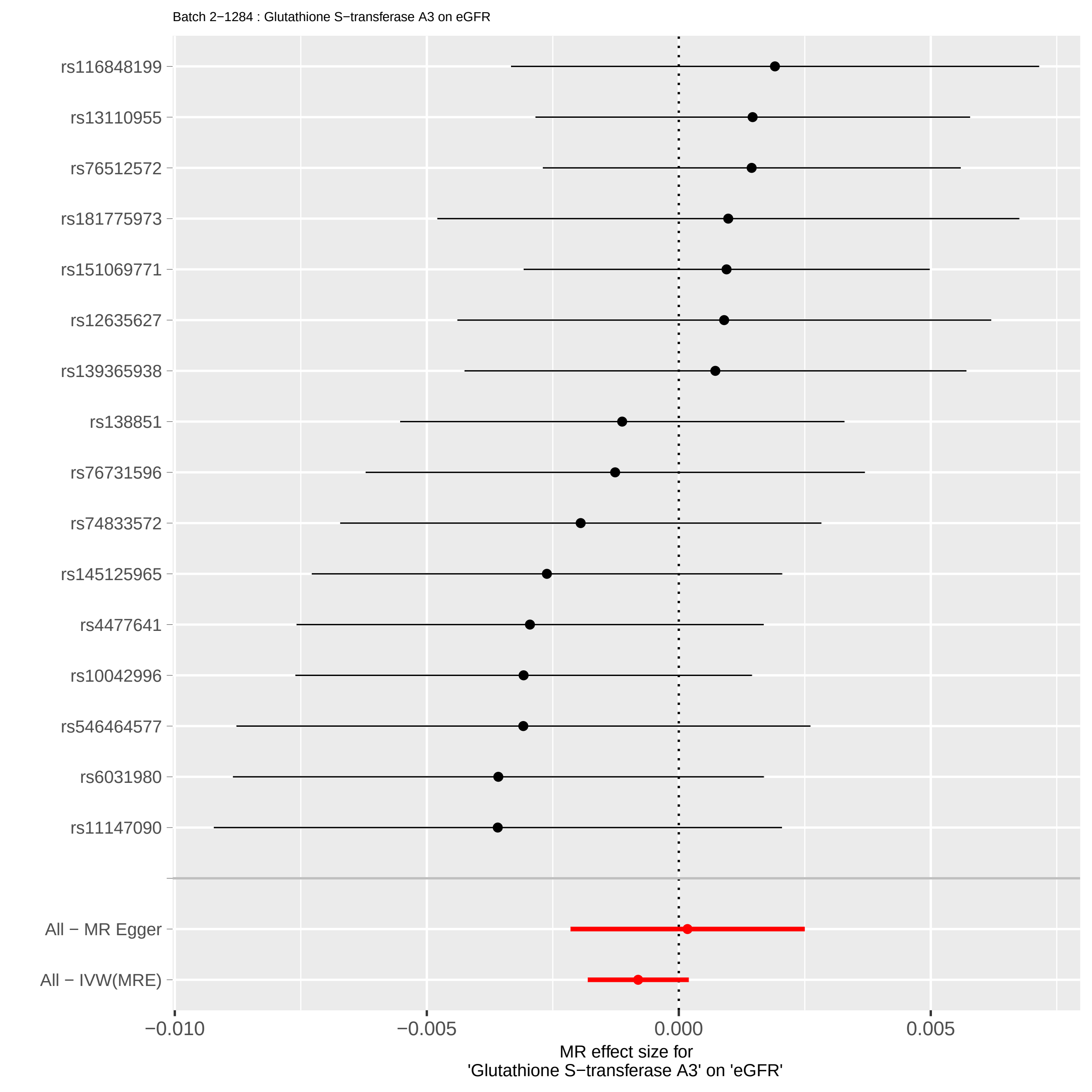

Batch 2−1284 : Glutathione S−transferase A3 on eGFR
rs116848199
rs13110955
rs76512572
rs181775973
rs151069771
rs12635627
rs139365938
rs138851
rs76731596
rs74833572
rs145125965
rs4477641
rs10042996
rs546464577
rs6031980
rs11147090
All − MR Egger
All − IVW(MRE)
−0.010
−0.005
0.000
0.005
MR effect size for
'Glutathione S−transferase A3' on 'eGFR'

Batch 2−1285 : Glutathione S−transferase A4 on eGFR

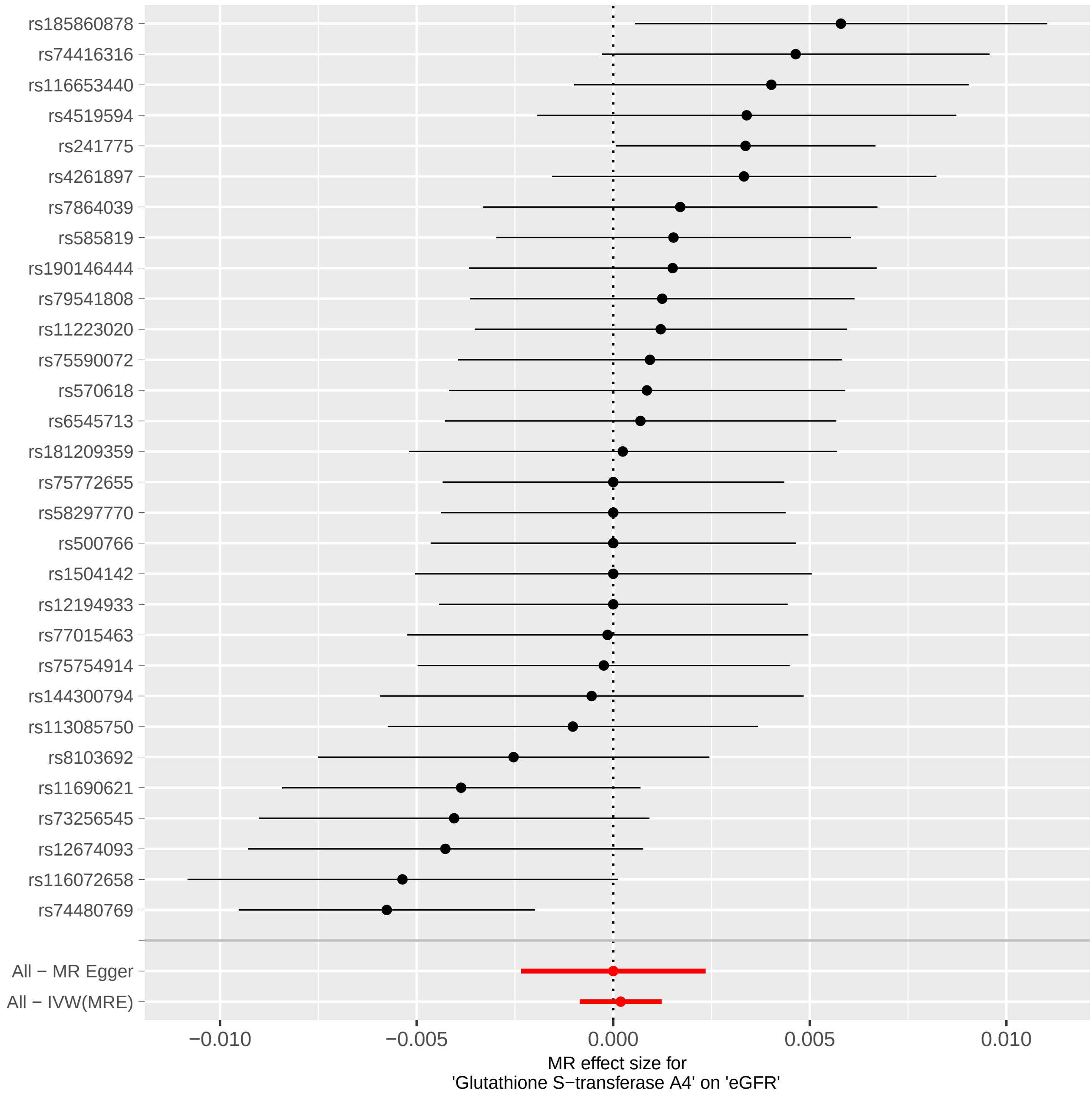

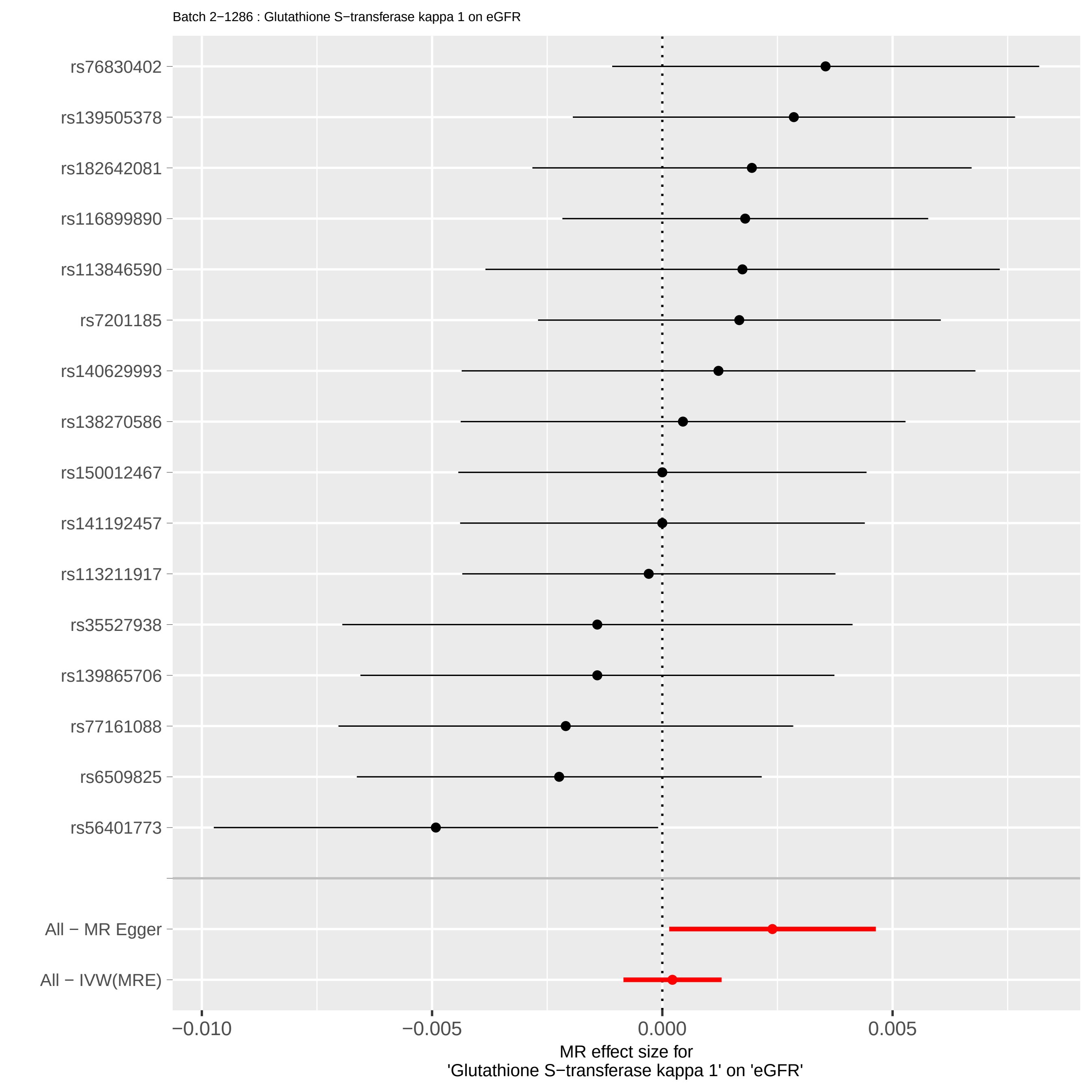

Batch 2−1286 : Glutathione S−transferase kappa 1 on eGFR
rs76830402
rs139505378
rs182642081
rs116899890
rs113846590
rs7201185
rs140629993
rs138270586
rs150012467
rs141192457
rs113211917
rs35527938
rs139865706
rs77161088
rs6509825
rs56401773
All − MR Egger
All − IVW(MRE)
−0.010
−0.005
0.000
0.005
MR effect size for
'Glutathione S−transferase kappa 1' on 'eGFR'

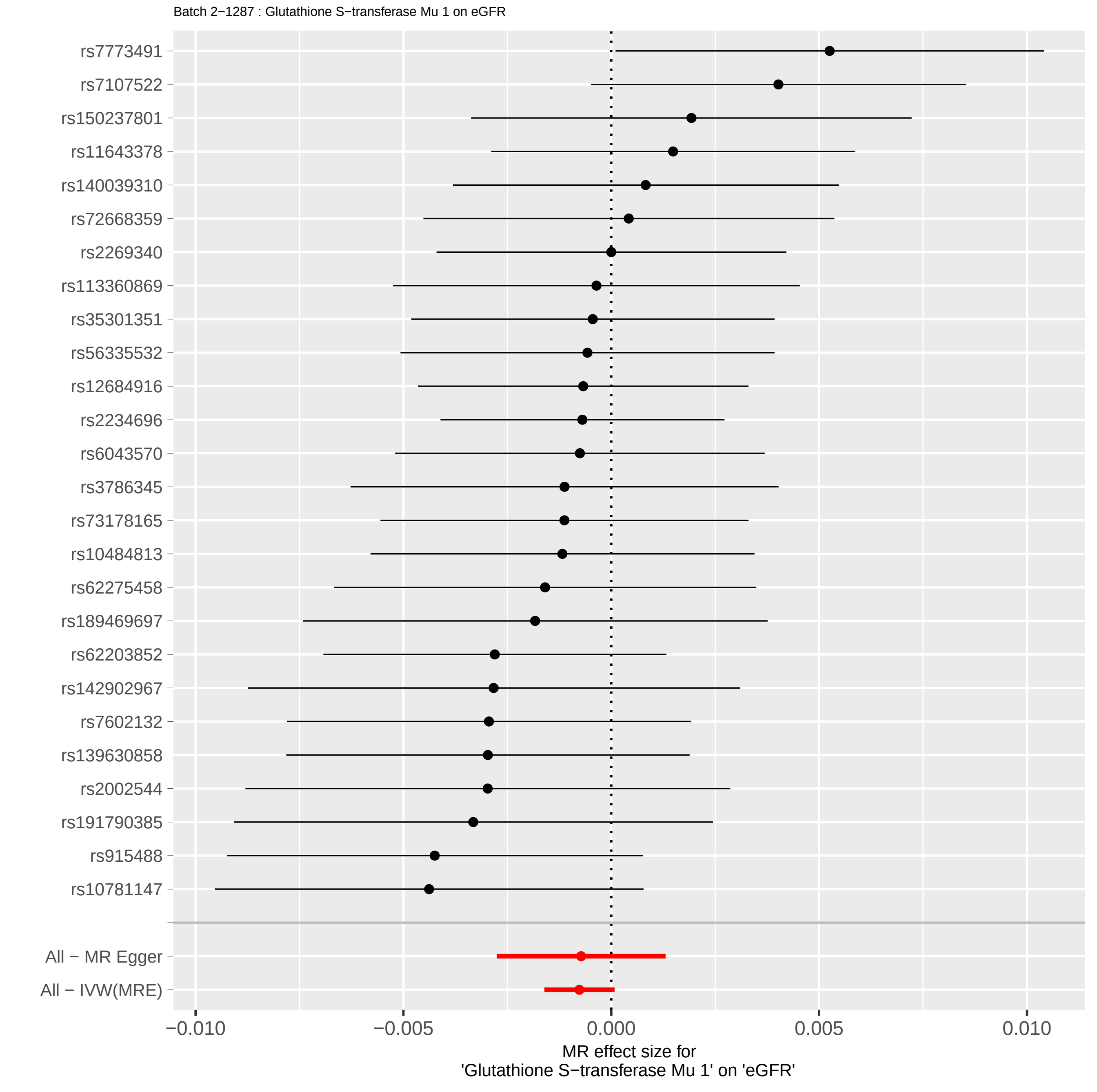

Batch 2−1287 : Glutathione S−transferase Mu 1 on eGFR
rs7773491
rs7107522
rs150237801
rs11643378
rs140039310
rs72668359
rs2269340
rs113360869
rs35301351
rs56335532
rs12684916
rs2234696
rs6043570
rs3786345
rs73178165
rs10484813
rs62275458
rs189469697
rs62203852
rs142902967
rs7602132
rs139630858
rs2002544
rs191790385
rs915488
rs10781147
All − MR Egger
All − IVW(MRE)
−0.010
−0.005
0.000
0.005
0.010
MR effect size for
'Glutathione S−transferase Mu 1' on 'eGFR'

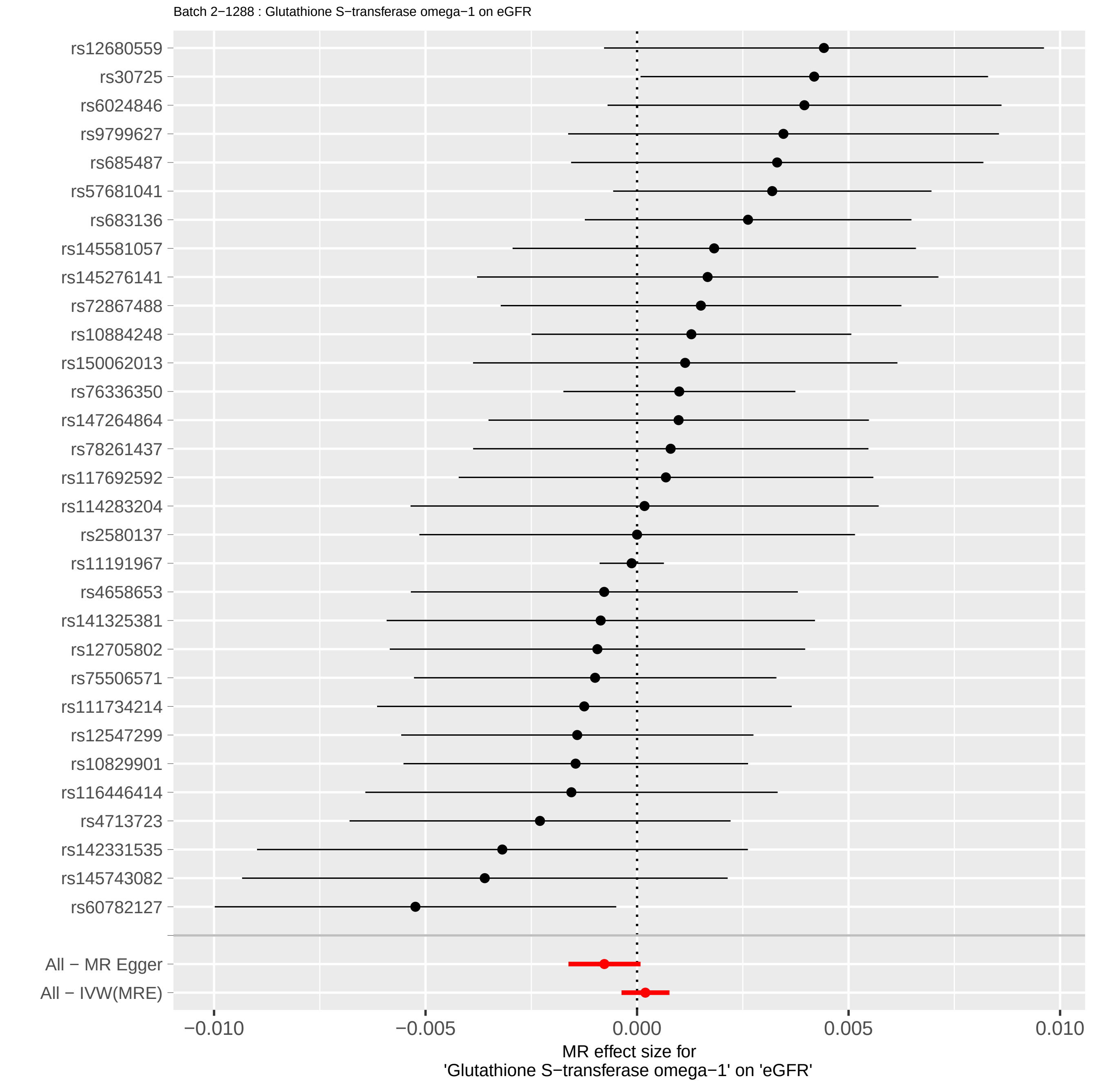

Batch 2−1288 : Glutathione S−transferase omega−1 on eGFR
rs12680559
rs30725
rs6024846
rs9799627
rs685487
rs57681041
rs683136
rs145581057
rs145276141
rs72867488
rs10884248
rs150062013
rs76336350
rs147264864
rs78261437
rs117692592
rs114283204
rs2580137
rs11191967
rs4658653
rs141325381
rs12705802
rs75506571
rs111734214
rs12547299
rs10829901
rs116446414
rs4713723
rs142331535
rs145743082
rs60782127
All − MR Egger
All − IVW(MRE)
−0.010
−0.005
0.000
0.005
0.010
MR effect size for
'Glutathione S−transferase omega−1' on 'eGFR'

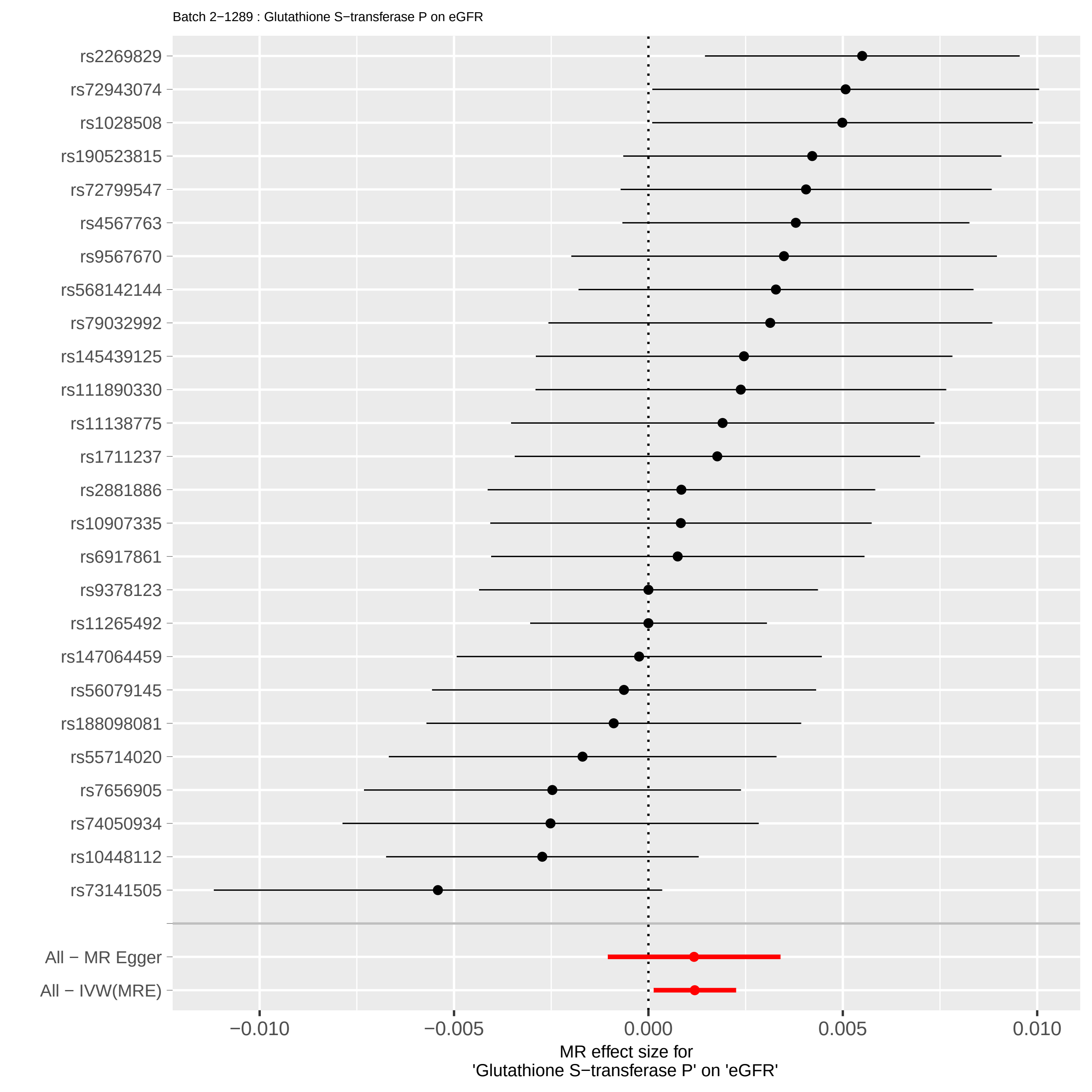

Batch 2−1289 : Glutathione S−transferase P on eGFR
rs2269829
rs72943074
rs1028508
rs190523815
rs72799547
rs4567763
rs9567670
rs568142144
rs79032992
rs145439125
rs111890330
rs11138775
rs1711237
rs2881886
rs10907335
rs6917861
rs9378123
rs11265492
rs147064459
rs56079145
rs188098081
rs55714020
rs7656905
rs74050934
rs10448112
rs73141505
All − MR Egger
All − IVW(MRE)
−0.010
−0.005
0.000
0.005
0.010
MR effect size for
'Glutathione S−transferase P' on 'eGFR'

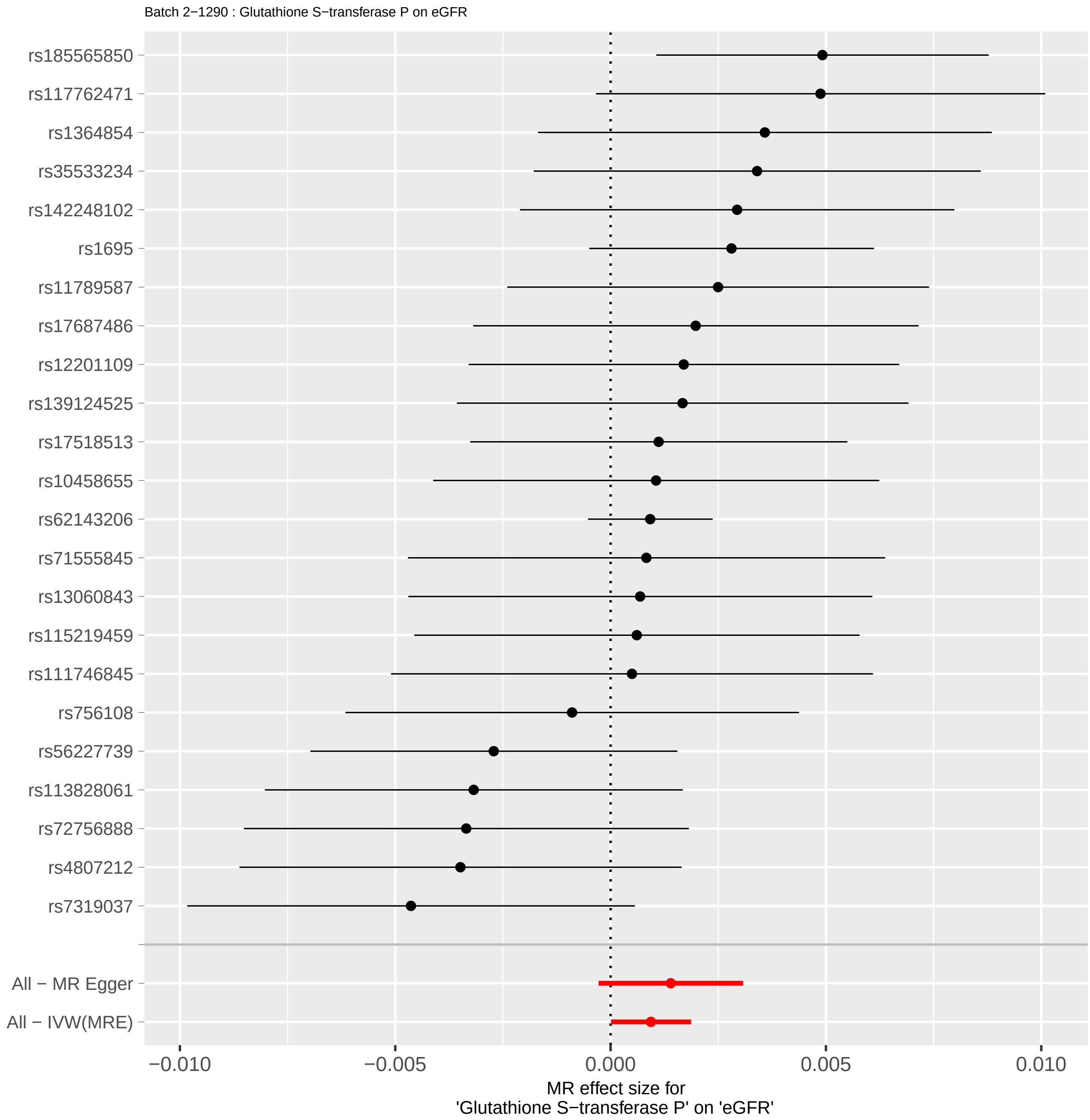

Batch 2−1290 : Glutathione S−transferase P on eGFR
rs185565850
rs117762471
rs1364854
rs35533234
rs142248102
rs1695
rs11789587
rs17687486
rs12201109
rs139124525
rs17518513
rs10458655
rs62143206
rs71555845
rs13060843
rs115219459
rs111746845
rs756108
rs56227739
rs113828061
rs72756888
rs4807212
rs7319037
All − MR Egger
All − IVW(MRE)
−0.010
−0.005
0.000
0.005
0.010
MR effect size for
'Glutathione S−transferase P' on 'eGFR'

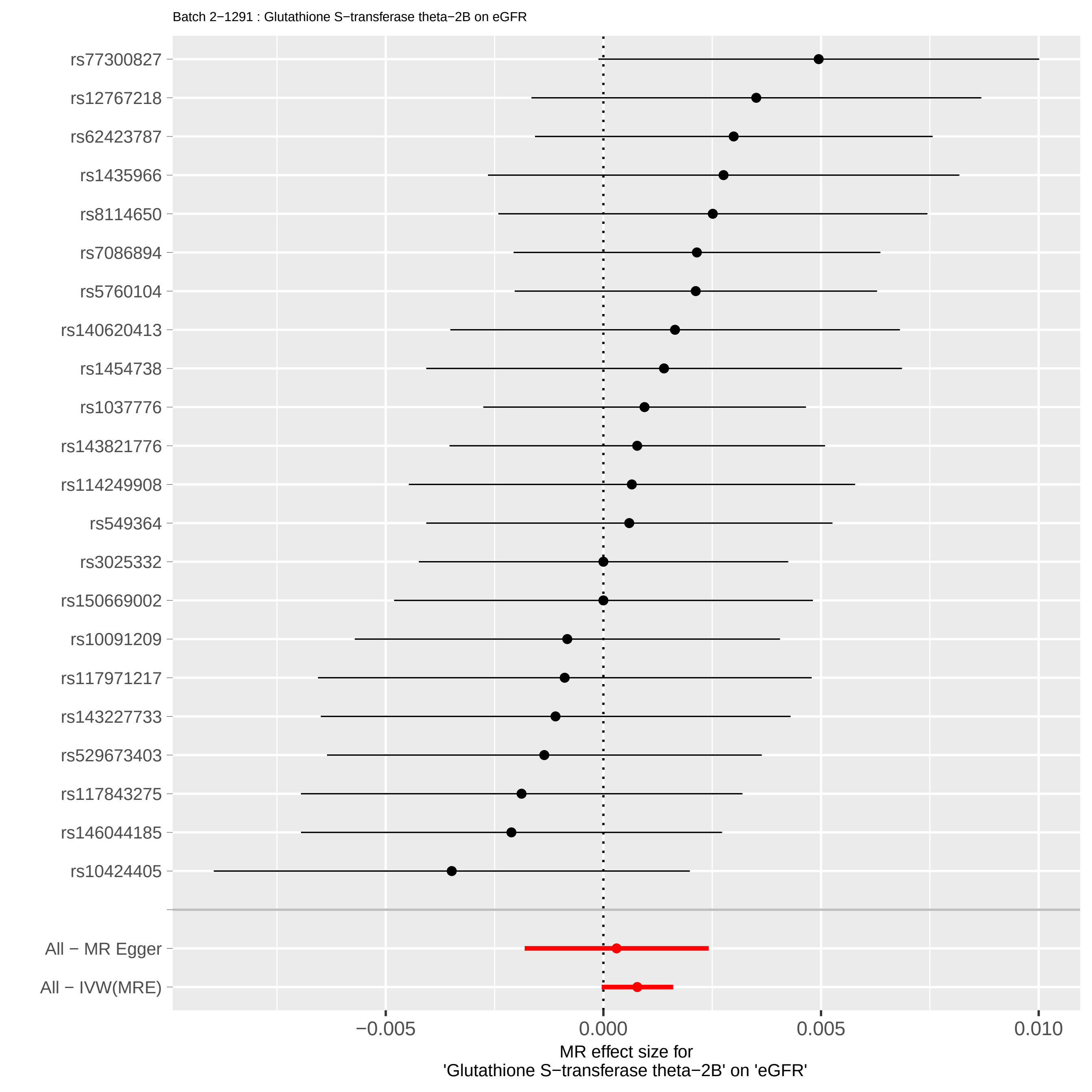

Batch 2−1291 : Glutathione S−transferase theta−2B on eGFR
rs77300827
rs12767218
rs62423787
rs1435966
rs8114650
rs7086894
rs5760104
rs140620413
rs1454738
rs1037776
rs143821776
rs114249908
rs549364
rs3025332
rs150669002
rs10091209
rs117971217
rs143227733
rs529673403
rs117843275
rs146044185
rs10424405
All − MR Egger
All − IVW(MRE)
−0.005
0.000
0.005
0.010
MR effect size for
'Glutathione S−transferase theta−2B' on 'eGFR'

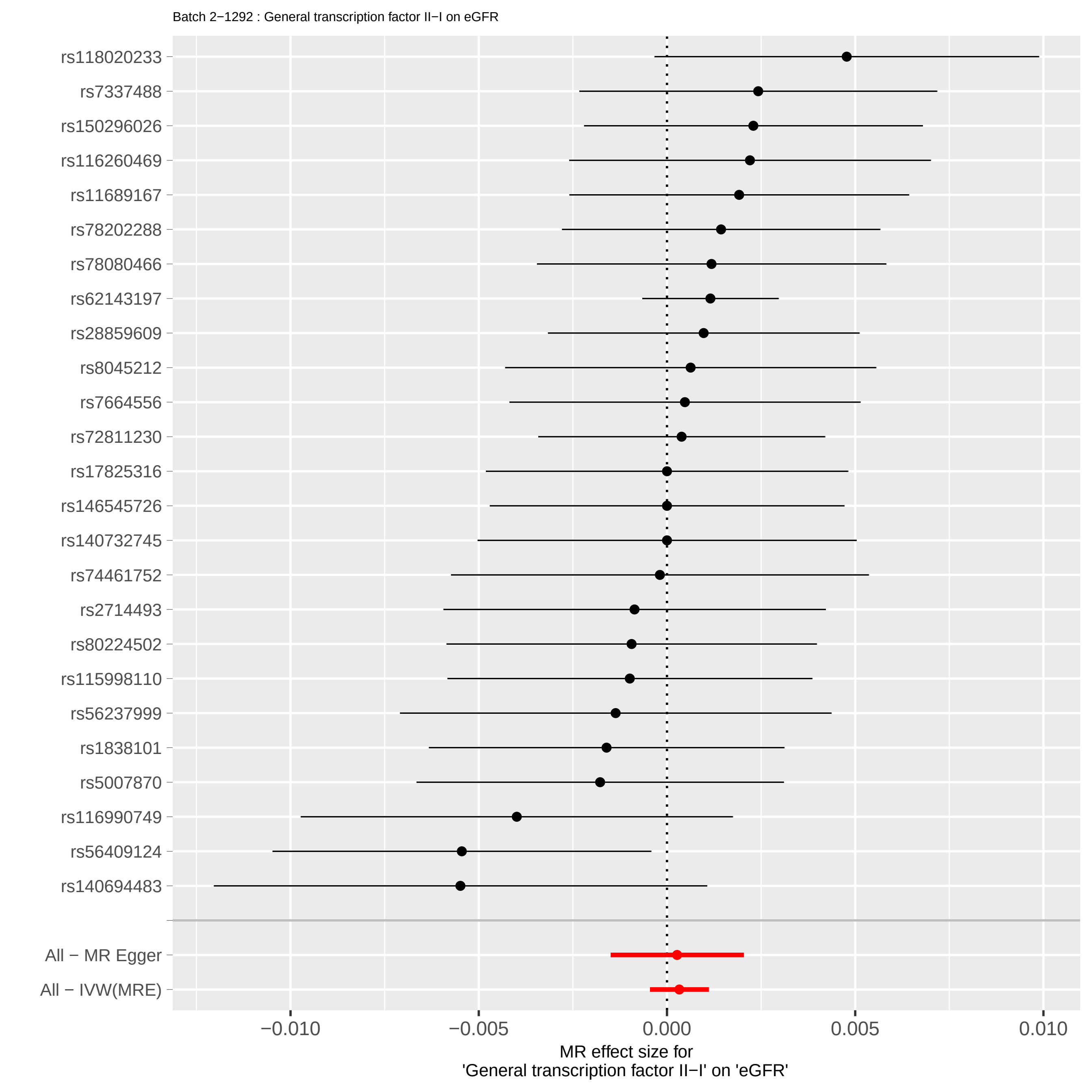
Batch 2−1292 : General transcription factor II−I on eGFR
rs118020233
rs7337488
rs150296026
rs116260469
rs11689167
rs78202288
rs78080466
rs62143197
rs28859609
rs8045212
rs7664556
rs72811230
rs17825316
rs146545726
rs140732745
rs74461752
rs2714493
rs80224502
rs115998110
rs56237999
rs1838101
rs5007870
rs116990749
rs56409124
rs140694483
All − MR Egger
All − IVW(MRE)
−0.010
−0.005
0.000
0.005
0.010
MR effect size for
'General transcription factor II−I' on 'eGFR'

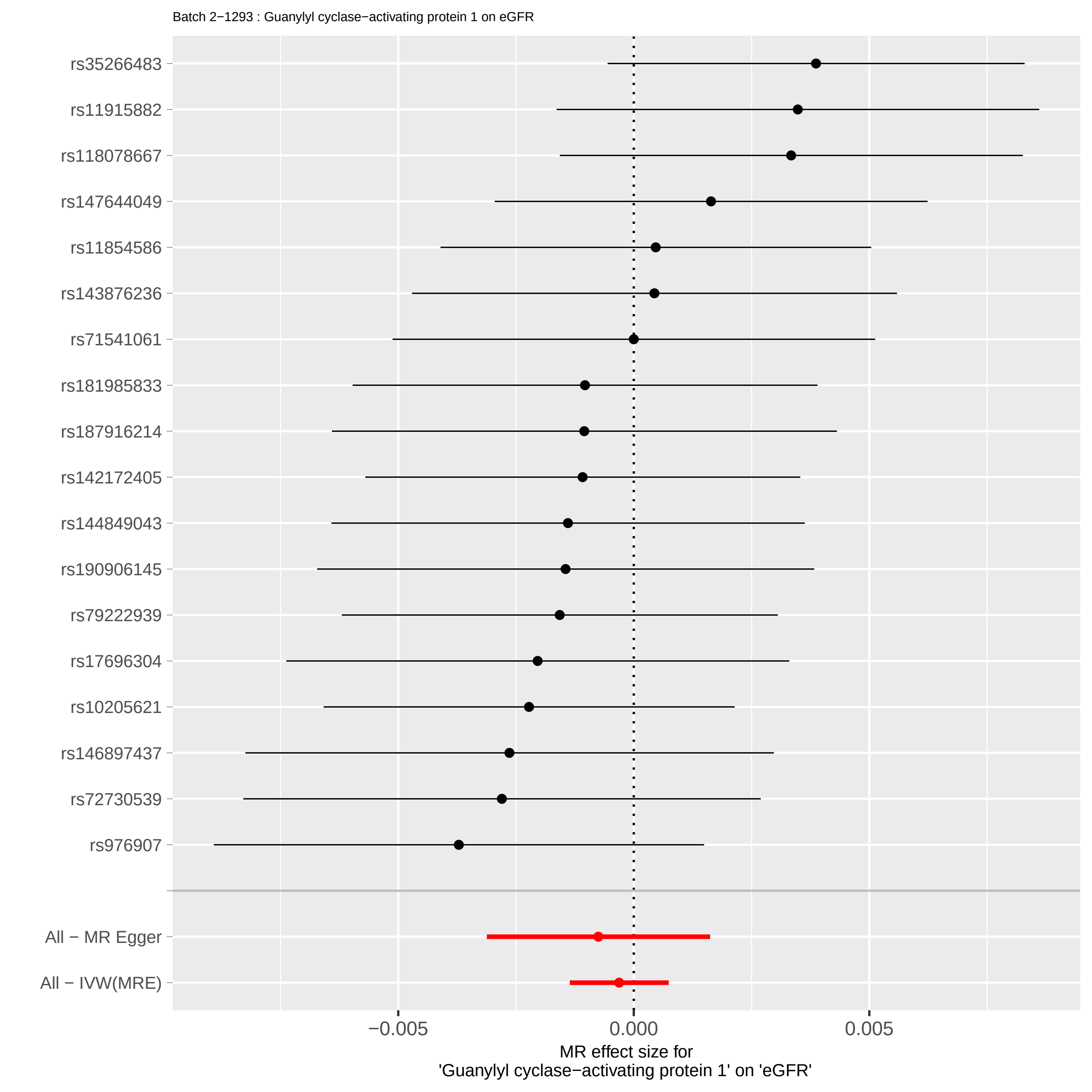

Batch 2−1293 : Guanylyl cyclase−activating protein 1 on eGFR
rs35266483
rs11915882
rs118078667
rs147644049
rs11854586
rs143876236
rs71541061
rs181985833
rs187916214
rs142172405
rs144849043
rs190906145
rs79222939
rs17696304
rs10205621
rs146897437
rs72730539
rs976907
All − MR Egger
All − IVW(MRE)
−0.005
0.000
0.005
MR effect size for
'Guanylyl cyclase−activating protein 1' on 'eGFR'

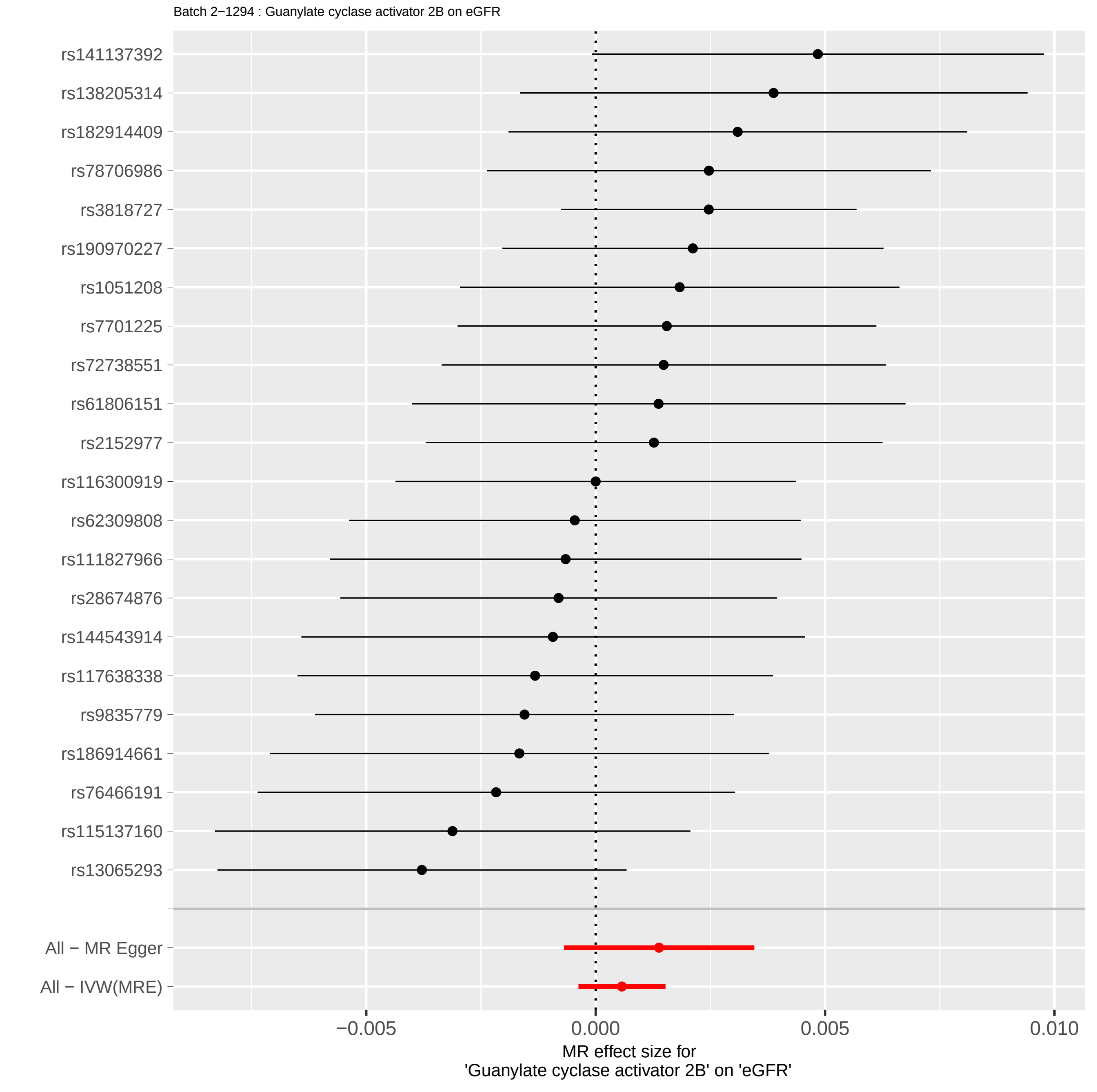
Batch 2−1294 : Guanylate cyclase activator 2B on eGFR
rs141137392
rs138205314
rs182914409
rs78706986
rs3818727
rs190970227
rs1051208
rs7701225
rs72738551
rs61806151
rs2152977
rs116300919
rs62309808
rs111827966
rs28674876
rs144543914
rs117638338
rs9835779
rs186914661
rs76466191
rs115137160
rs13065293
All − MR Egger
All − IVW(MRE)
−0.005
0.000
0.005
0.010
MR effect size for
'Guanylate cyclase activator 2B' on 'eGFR'

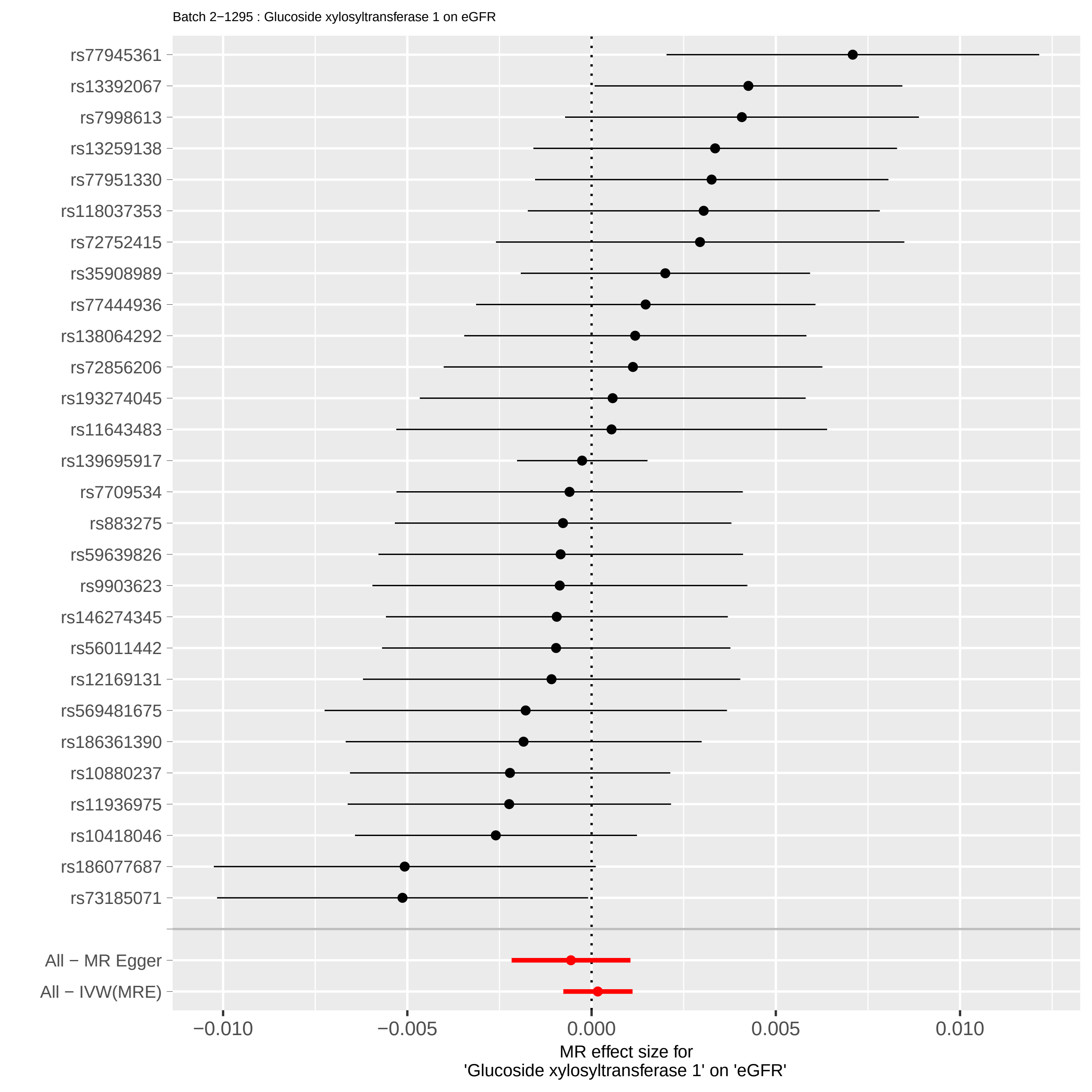
Batch 2−1295 : Glucoside xylosyltransferase 1 on eGFR
rs77945361
rs13392067
rs7998613
rs13259138
rs77951330
rs118037353
rs72752415
rs35908989
rs77444936
rs138064292
rs72856206
rs193274045
rs11643483
rs139695917
rs7709534
rs883275
rs59639826
rs9903623
rs146274345
rs56011442
rs12169131
rs569481675
rs186361390
rs10880237
rs11936975
rs10418046
rs186077687
rs73185071
All − MR Egger
All − IVW(MRE)
−0.010
−0.005
0.000
0.005
0.010
MR effect size for
'Glucoside xylosyltransferase 1' on 'eGFR'

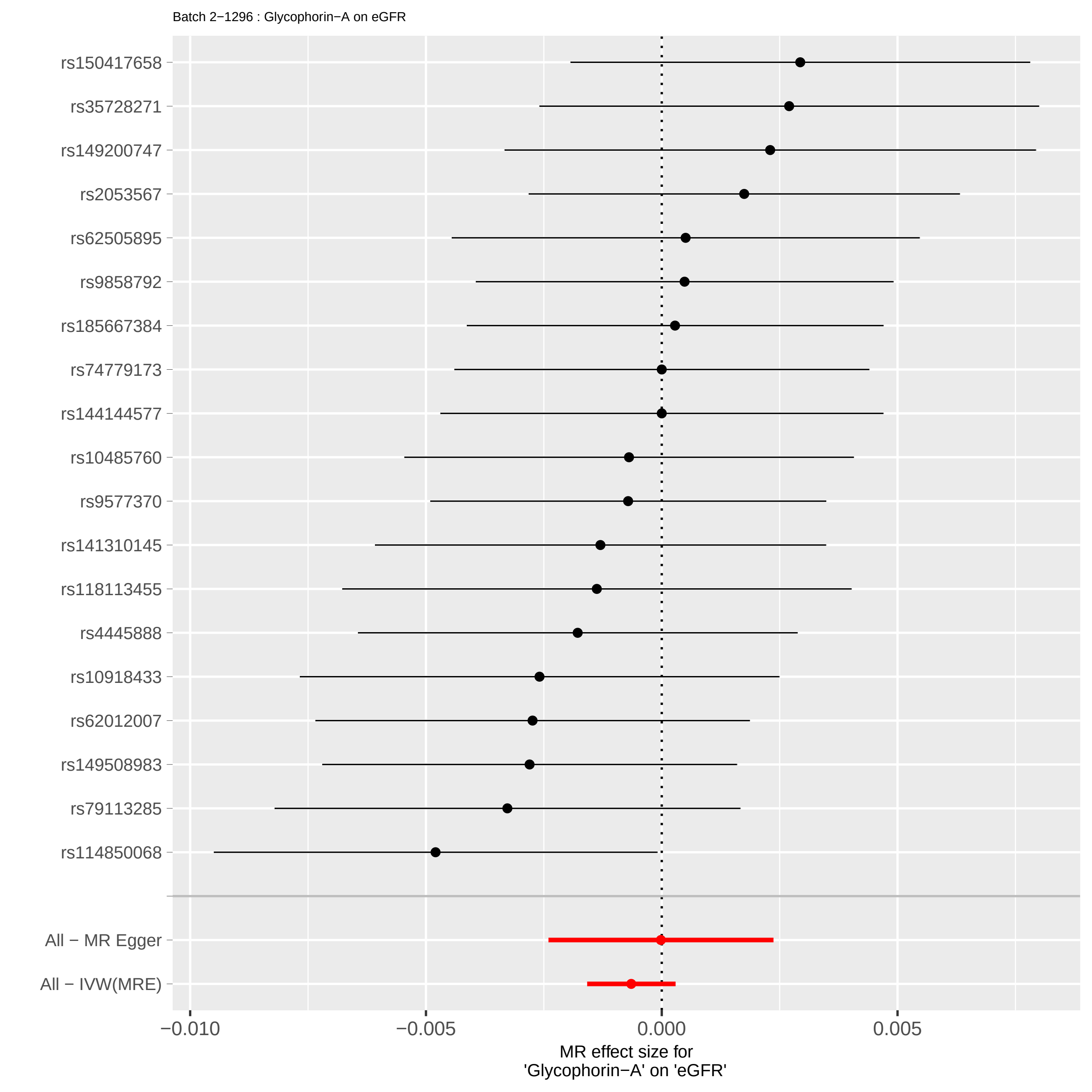

Batch 2−1296 : Glycophorin−A on eGFR
rs150417658
rs35728271
rs149200747
rs2053567
rs62505895
rs9858792
rs185667384
rs74779173
rs144144577
rs10485760
rs9577370
rs141310145
rs118113455
rs4445888
rs10918433
rs62012007
rs149508983
rs79113285
rs114850068
All − MR Egger
All − IVW(MRE)
−0.010
−0.005
0.000
0.005
MR effect size for
'Glycophorin−A' on 'eGFR'

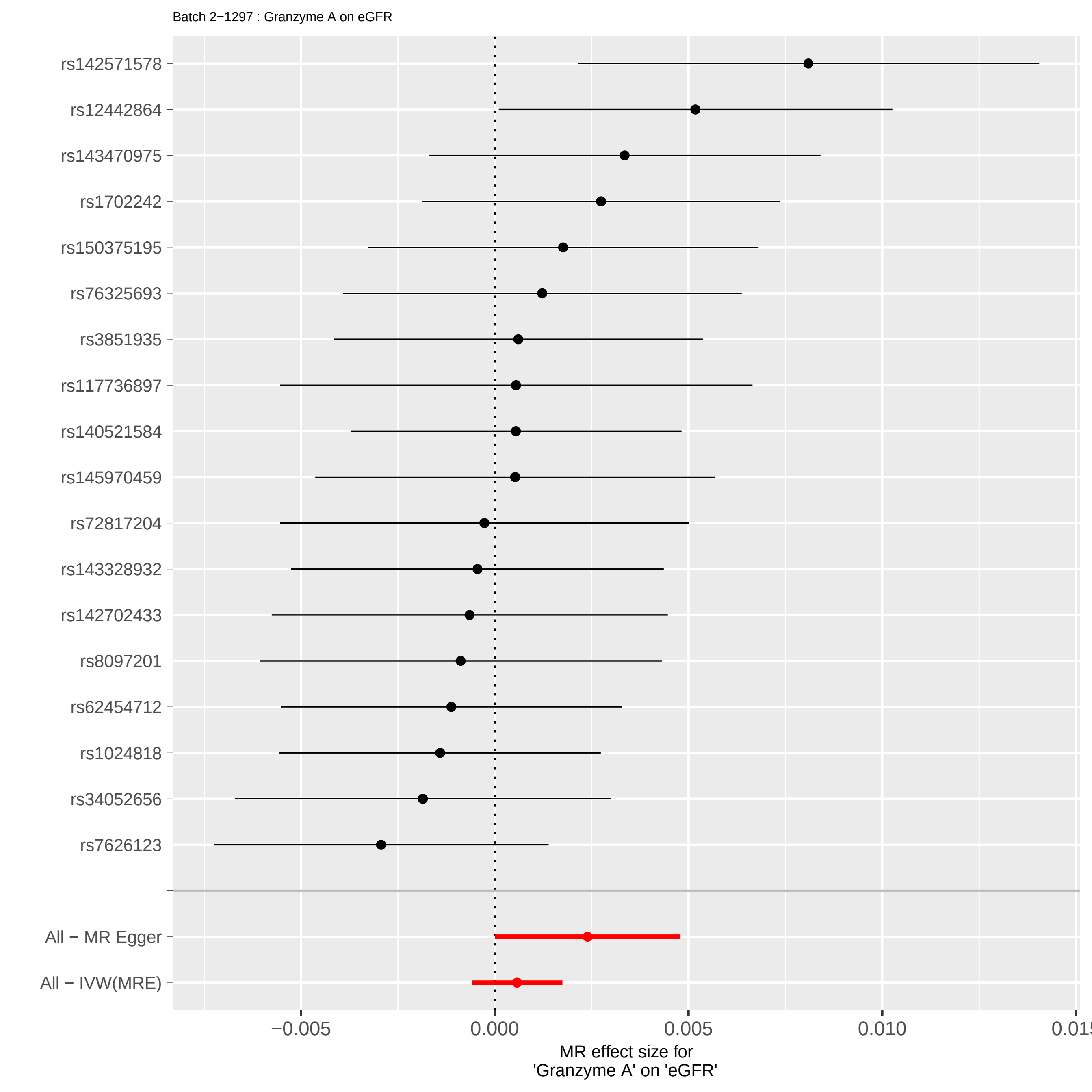

Batch 2−1297 : Granzyme A on eGFR
rs142571578
rs12442864
rs143470975
rs1702242
rs150375195
rs76325693
rs3851935
rs117736897
rs140521584
rs145970459
rs72817204
rs143328932
rs142702433
rs8097201
rs62454712
rs1024818
rs34052656
rs7626123
All − MR Egger
All − IVW(MRE)
−0.005
0.000
0.005
0.010
MR effect size for
'Granzyme A' on 'eGFR'

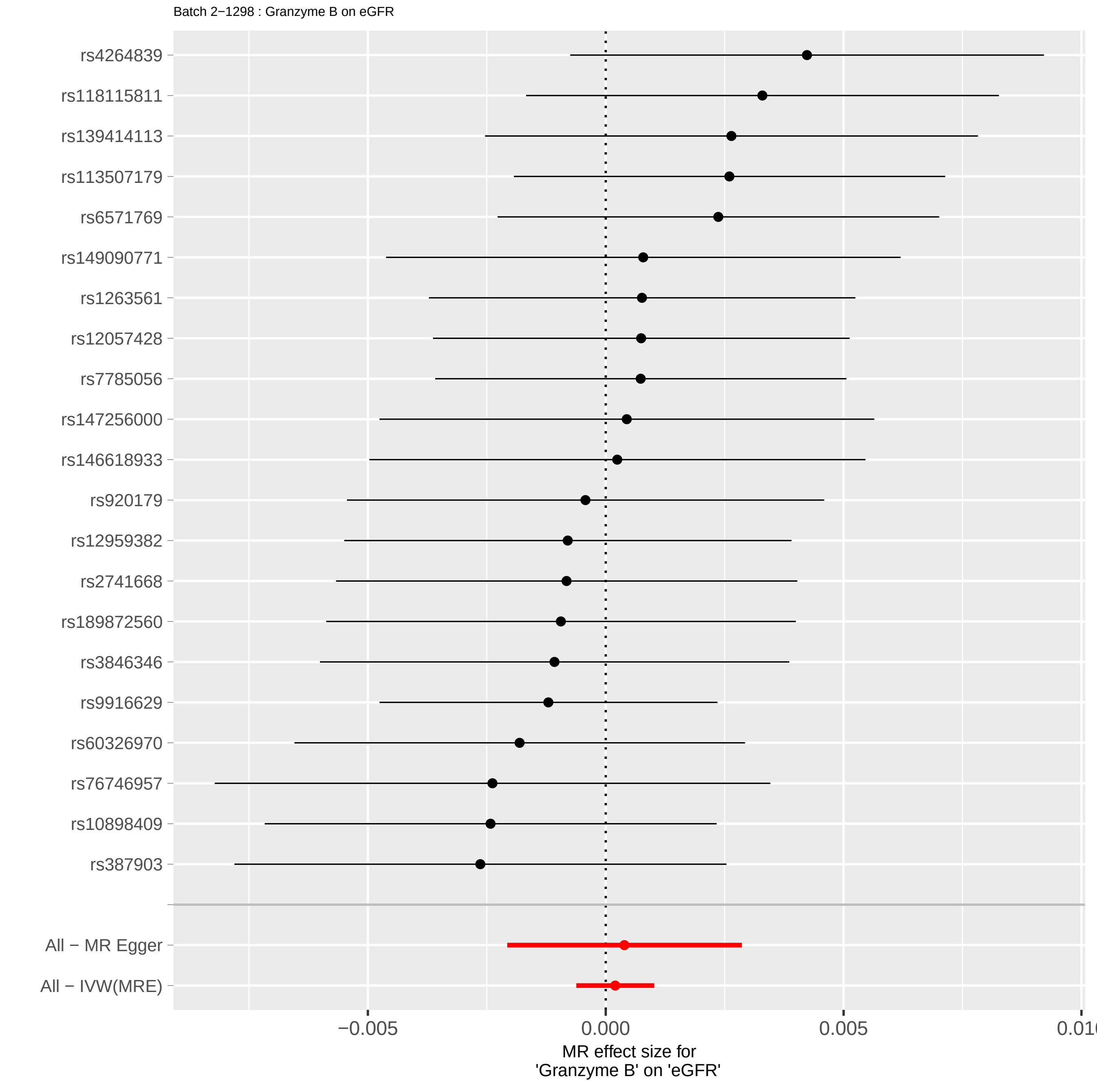
Batch 2−1298 : Granzyme B on eGFR
rs4264839
rs118115811
rs139414113
rs113507179
rs6571769
rs149090771
rs1263561
rs12057428
rs7785056
rs147256000
rs146618933
rs920179
rs12959382
rs2741668
rs189872560
rs3846346
rs9916629
rs60326970
rs76746957
rs10898409
rs387903
All − MR Egger
All − IVW(MRE)
−0.005
0.000
0.005
MR effect size for
'Granzyme B' on 'eGFR'

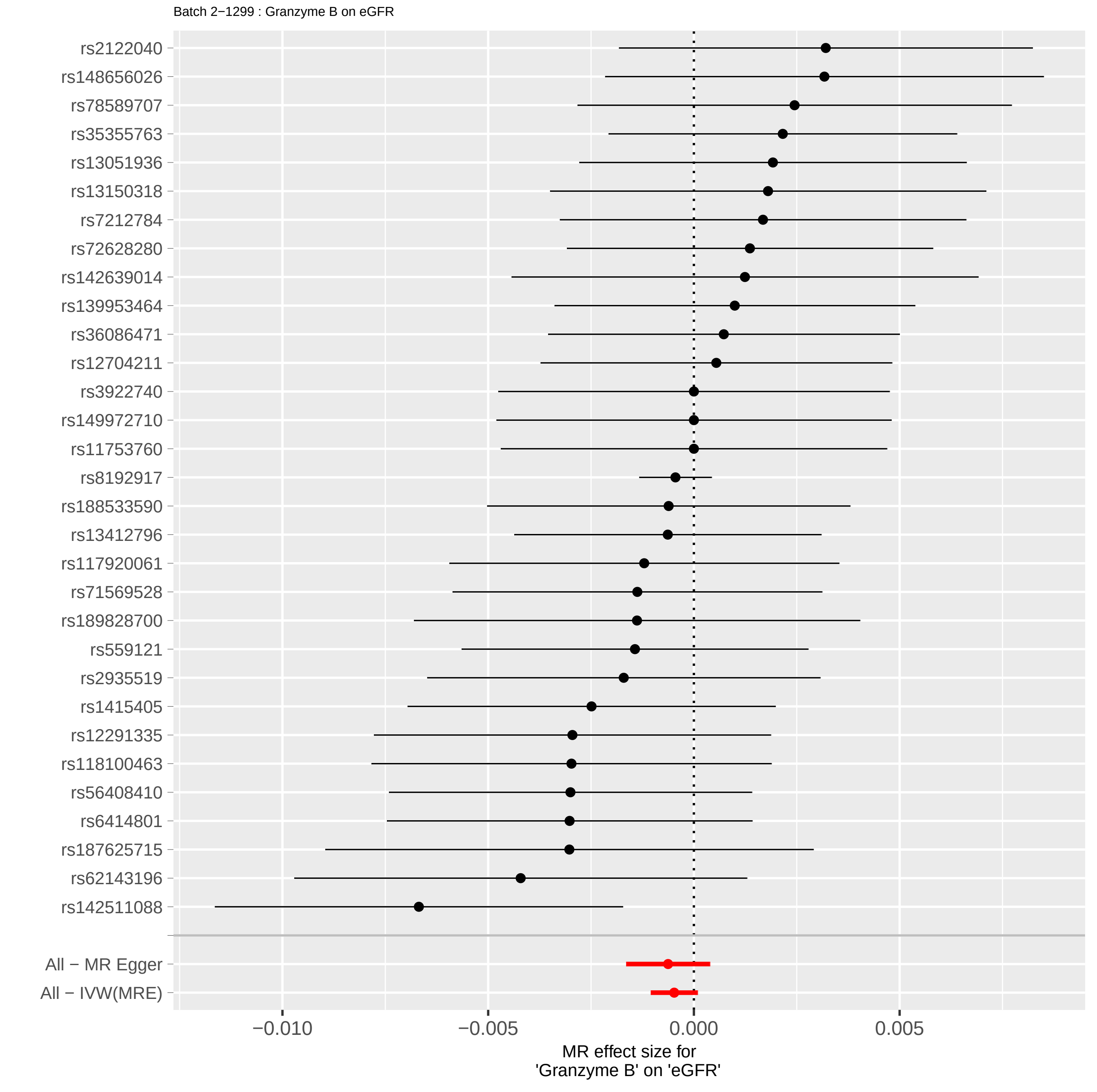

Batch 2−1299 : Granzyme B on eGFR
rs2122040
rs148656026
rs78589707
rs35355763
rs13051936
rs13150318
rs7212784
rs72628280
rs142639014
rs139953464
rs36086471
rs12704211
rs3922740
rs149972710
rs11753760
rs8192917
rs188533590
rs13412796
rs117920061
rs71569528
rs189828700
rs559121
rs2935519
rs1415405
rs12291335
rs118100463
rs56408410
rs6414801
rs187625715
rs62143196
rs142511088
All − MR Egger
All − IVW(MRE)
−0.010
−0.005
0.000
0.005
MR effect size for
'Granzyme B' on 'eGFR'

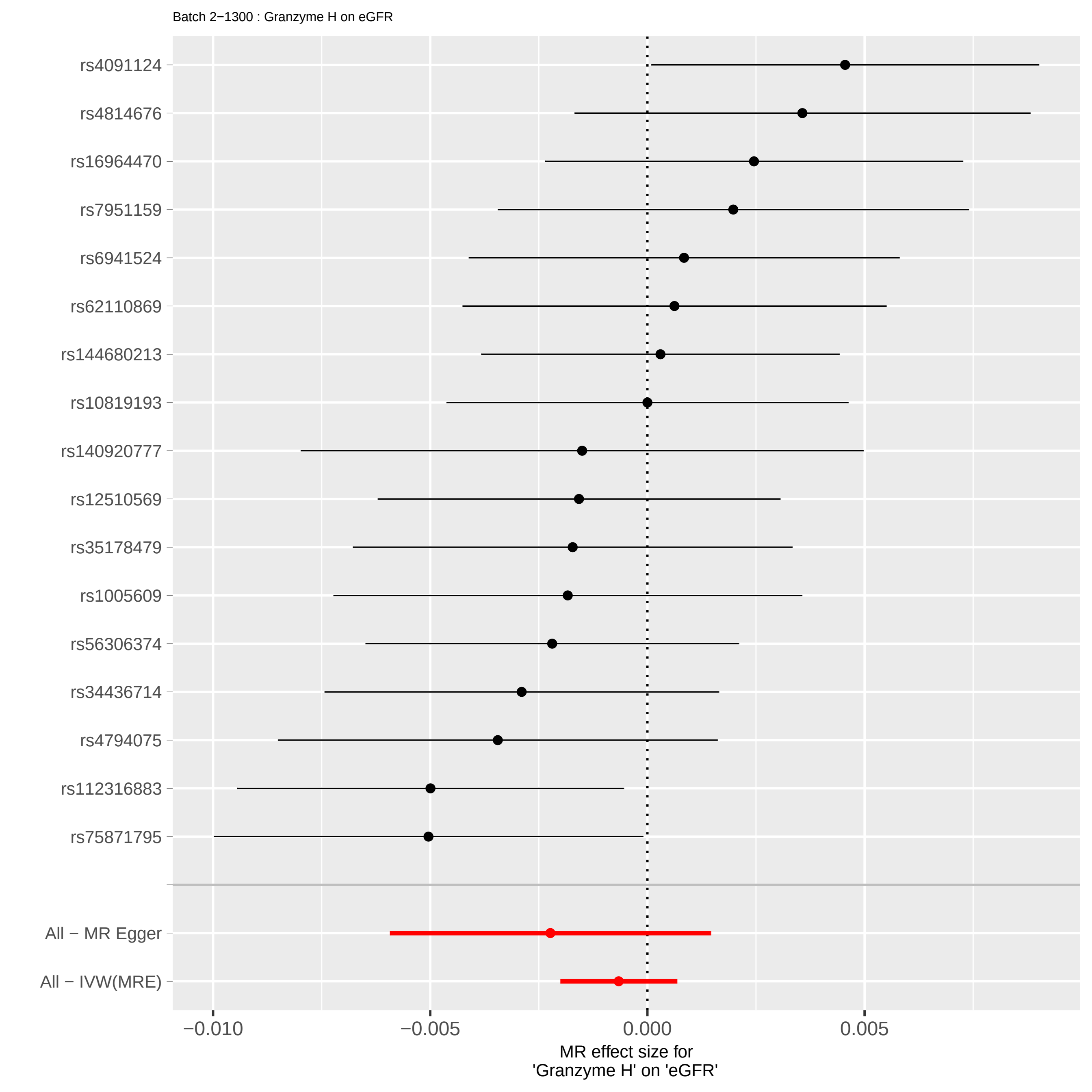
Batch 2−1300 : Granzyme H on eGFR
rs4091124
rs4814676
rs16964470
rs7951159
rs6941524
rs62110869
rs144680213
rs10819193
rs140920777
rs12510569
rs35178479
rs1005609
rs56306374
rs34436714
rs4794075
rs112316883
rs75871795
All − MR Egger
All − IVW(MRE)
−0.010
−0.005
0.000
0.005
MR effect size for
'Granzyme H' on 'eGFR'

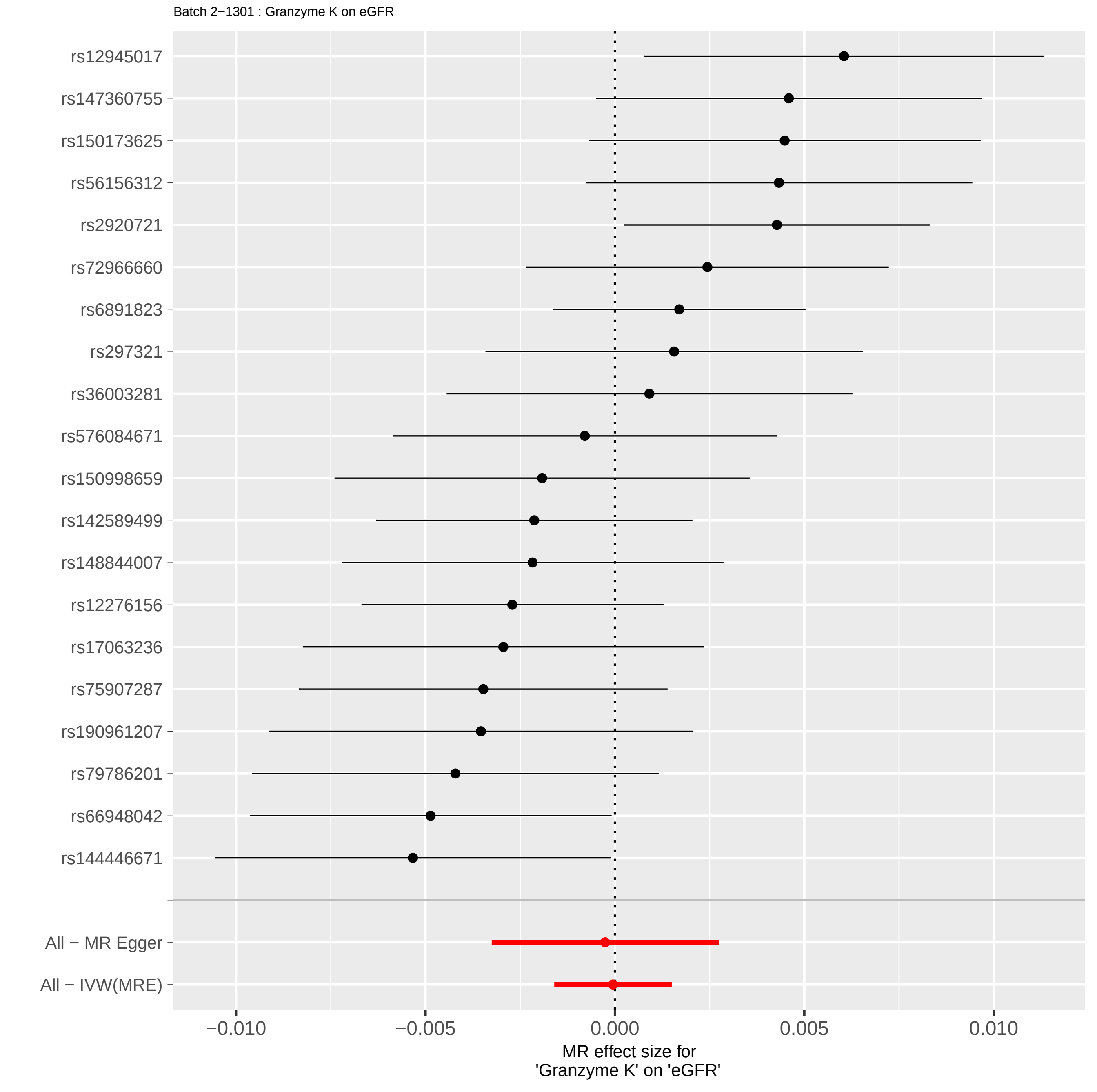

Batch 2−1301 : Granzyme K on eGFR
rs12945017
rs147360755
rs150173625
rs56156312
rs2920721
rs72966660
rs6891823
rs297321
rs36003281
rs576084671
rs150998659
rs142589499
rs148844007
rs12276156
rs17063236
rs75907287
rs190961207
rs79786201
rs66948042
rs144446671
All − MR Egger
All − IVW(MRE)
−0.010
−0.005
0.000
0.005
0.010
MR effect size for
'Granzyme K' on 'eGFR'

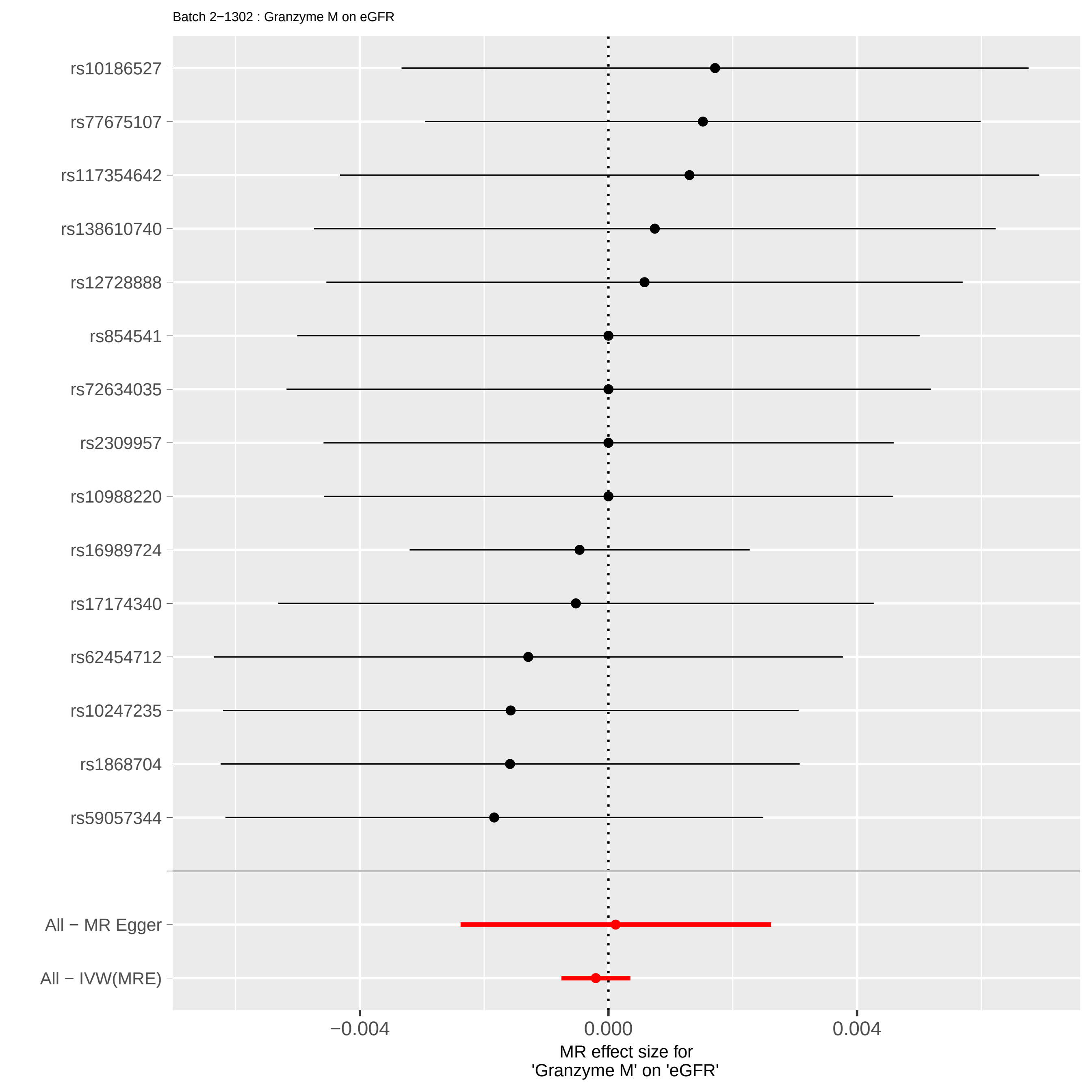

Batch 2−1302 : Granzyme M on eGFR
rs10186527
rs77675107
rs117354642
rs138610740
rs12728888
rs854541
rs72634035
rs2309957
rs10988220
rs16989724
rs17174340
rs62454712
rs10247235
rs1868704
rs59057344
All − MR Egger
All − IVW(MRE)
−0.004
0.000
0.004
MR effect size for
'Granzyme M' on 'eGFR'

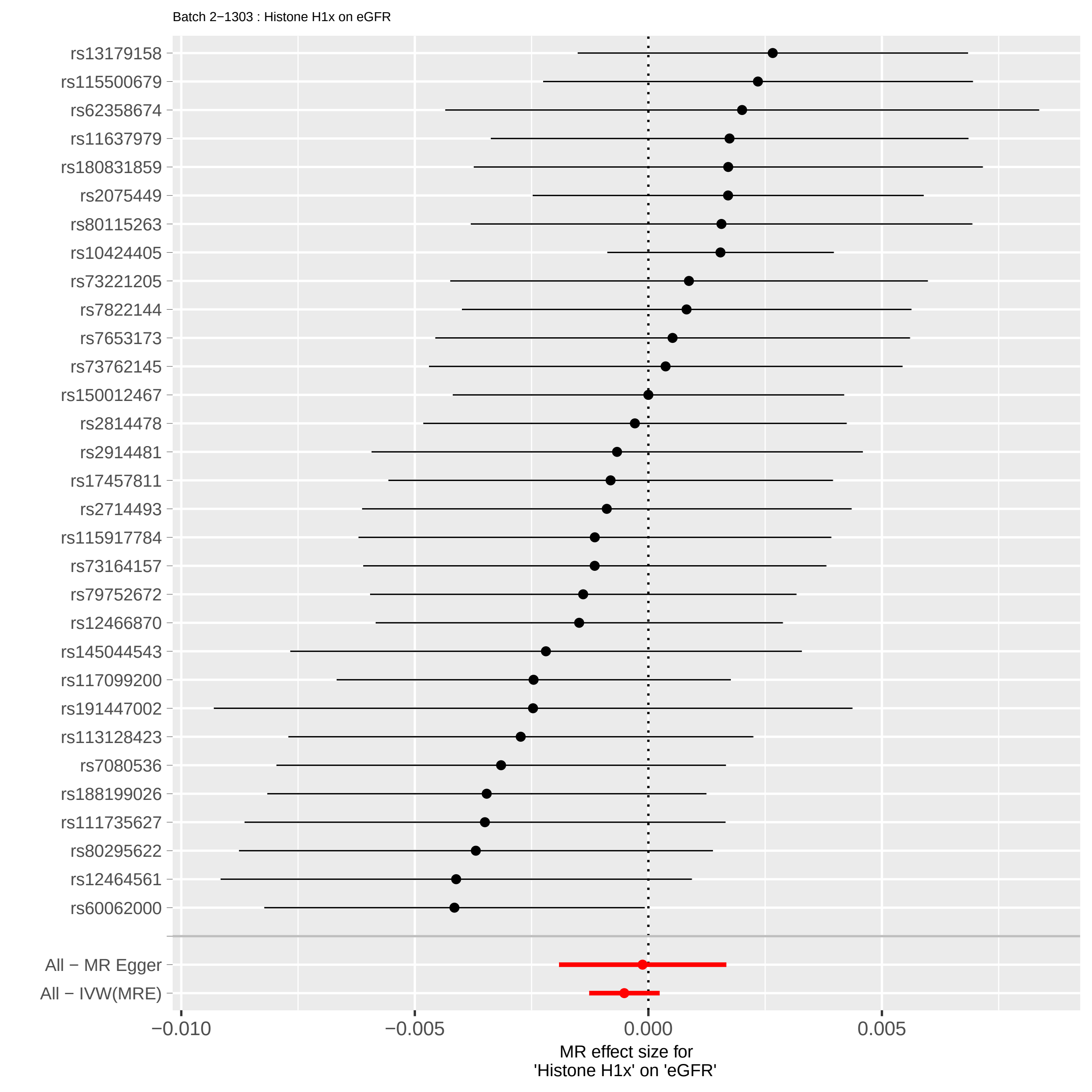

Batch 2−1303 : Histone H1x on eGFR
rs13179158
rs115500679
rs62358674
rs11637979
rs180831859
rs2075449
rs80115263
rs10424405
rs73221205
rs7822144
rs7653173
rs73762145
rs150012467
rs2814478
rs2914481
rs17457811
rs2714493
rs115917784
rs73164157
rs79752672
rs12466870
rs145044543
rs117099200
rs191447002
rs113128423
rs7080536
rs188199026
rs111735627
rs80295622
rs12464561
rs60062000
All − MR Egger
All − IVW(MRE)
−0.010
−0.005
0.000
0.005
MR effect size for
'Histone H1x' on 'eGFR'

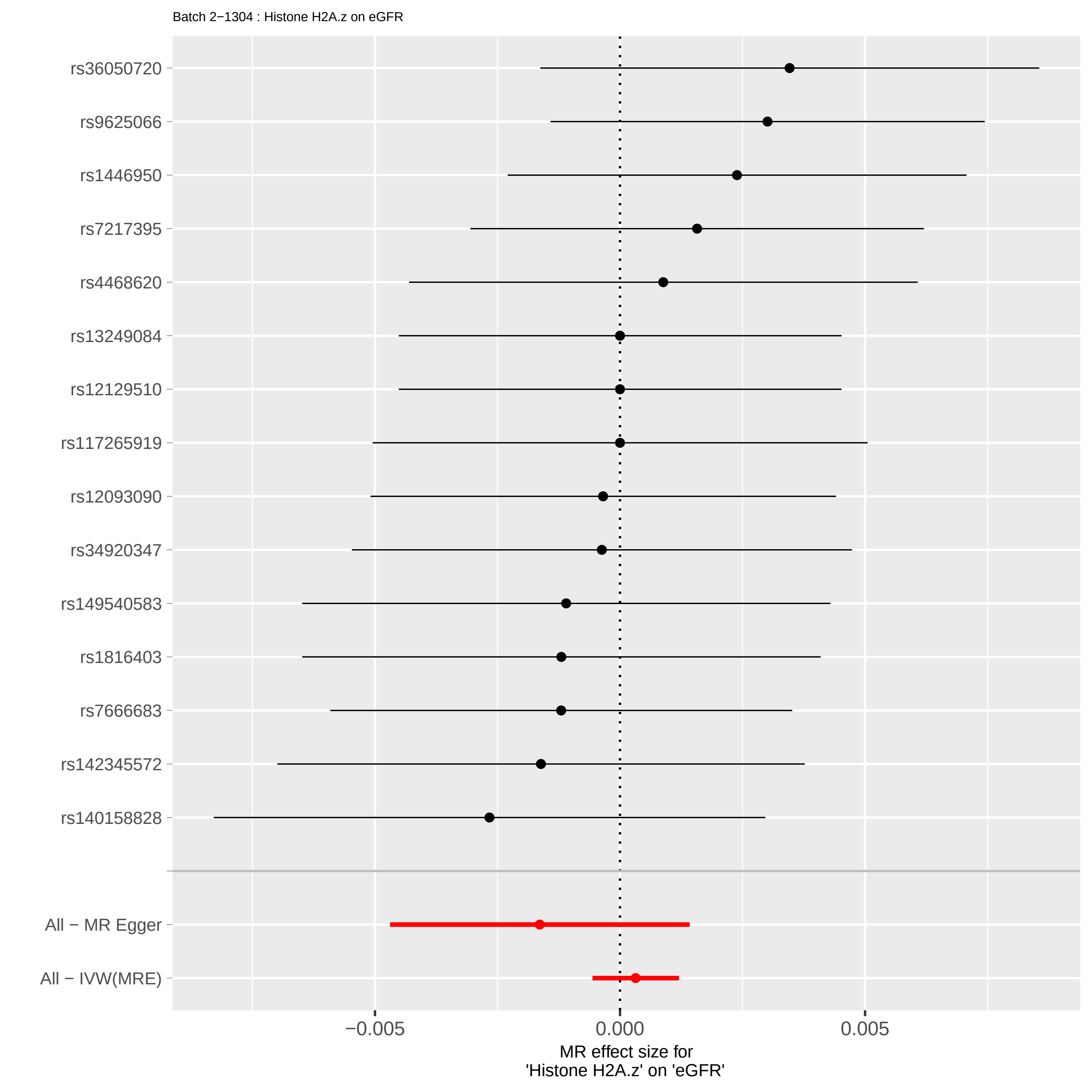

Batch 2−1304 : Histone H2A.z on eGFR
rs36050720
rs9625066
rs1446950
rs7217395
rs4468620
rs13249084
rs12129510
rs117265919
rs12093090
rs34920347
rs149540583
rs1816403
rs7666683
rs142345572
rs140158828
All − MR Egger
All − IVW(MRE)
−0.005
0.000
0.005
MR effect size for
'Histone H2A.z' on 'eGFR'

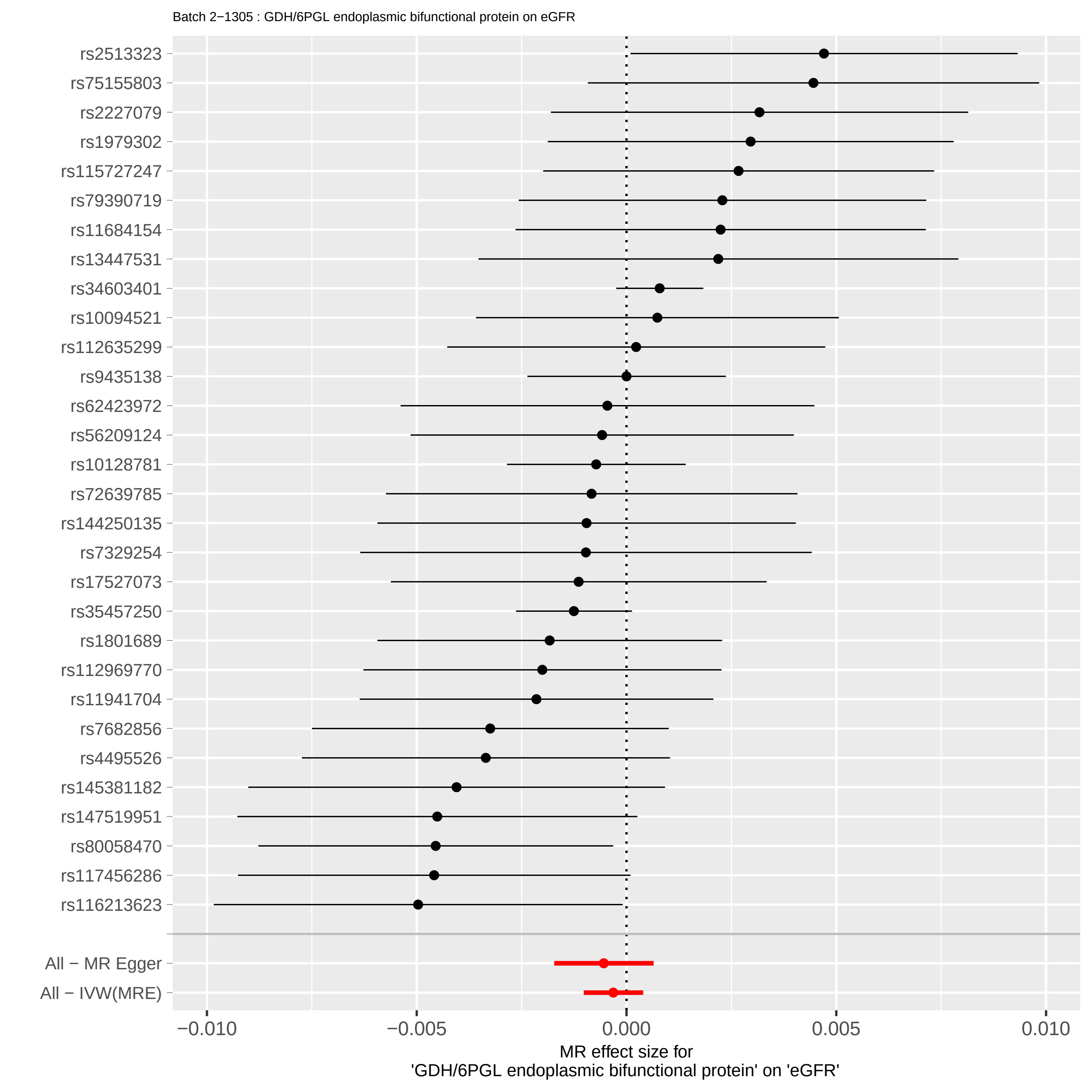
Batch 2−1305 : GDH/6PGL endoplasmic bifunctional protein on eGFR
rs2513323
rs75155803
rs2227079
rs1979302
rs115727247
rs79390719
rs11684154
rs13447531
rs34603401
rs10094521
rs112635299
rs9435138
rs62423972
rs56209124
rs10128781
rs72639785
rs144250135
rs7329254
rs17527073
rs35457250
rs1801689
rs112969770
rs11941704
rs7682856
rs4495526
rs145381182
rs147519951
rs80058470
rs117456286
rs116213623
All − MR Egger
All − IVW(MRE)
−0.010
−0.005
0.000
0.005
0.010
MR effect size for
'GDH/6PGL endoplasmic bifunctional protein' on 'eGFR'

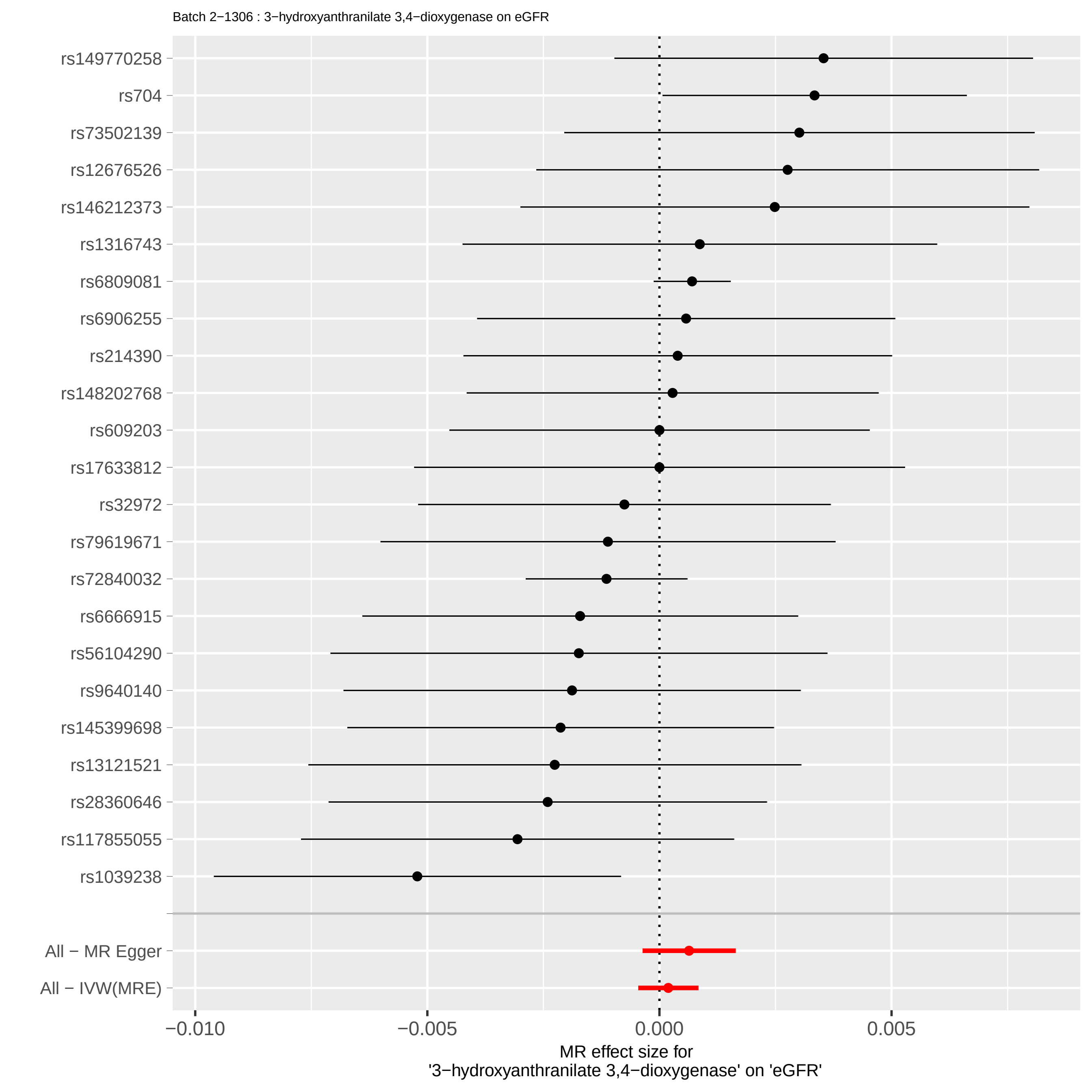
Batch 2−1306 : 3−hydroxyanthranilate 3,4−dioxygenase on eGFR
rs149770258
rs704
rs73502139
rs12676526
rs146212373
rs1316743
rs6809081
rs6906255
rs214390
rs148202768
rs609203
rs17633812
rs32972
rs79619671
rs72840032
rs6666915
rs56104290
rs9640140
rs145399698
rs13121521
rs28360646
rs117855055
rs1039238
All − MR Egger
All − IVW(MRE)
−0.010
−0.005
0.000
0.005
MR effect size for
'3−hydroxyanthranilate 3,4−dioxygenase' on 'eGFR'

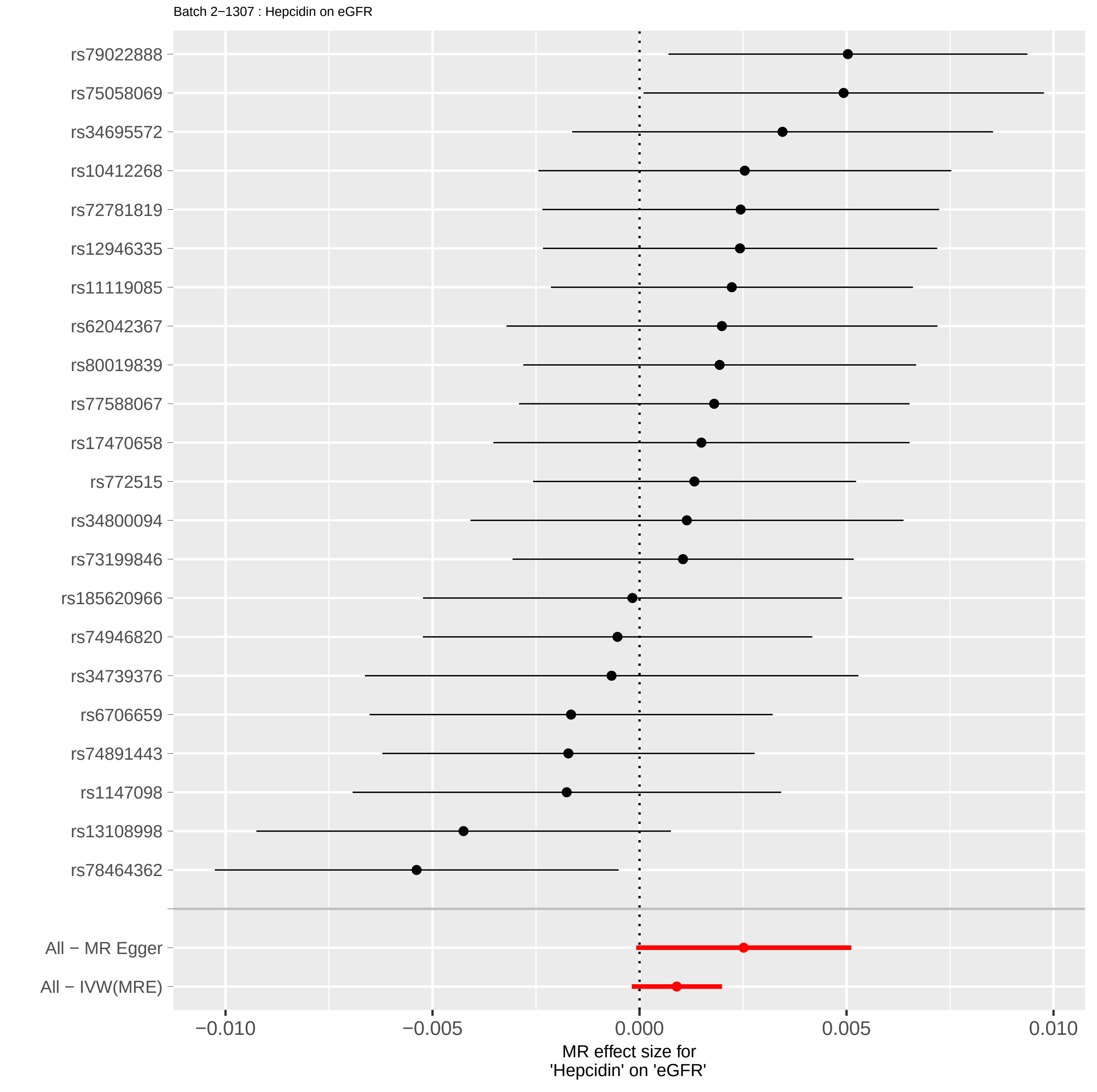
Batch 2−1307 : Hepcidin on eGFR
rs79022888
rs75058069
rs34695572
rs10412268
rs72781819
rs12946335
rs11119085
rs62042367
rs80019839
rs77588067
rs17470658
rs772515
rs34800094
rs73199846
rs185620966
rs74946820
rs34739376
rs6706659
rs74891443
rs1147098
rs13108998
rs78464362
All − MR Egger
All − IVW(MRE)
−0.010
−0.005
0.000
0.005
0.010
MR effect size for
'Hepcidin' on 'eGFR'

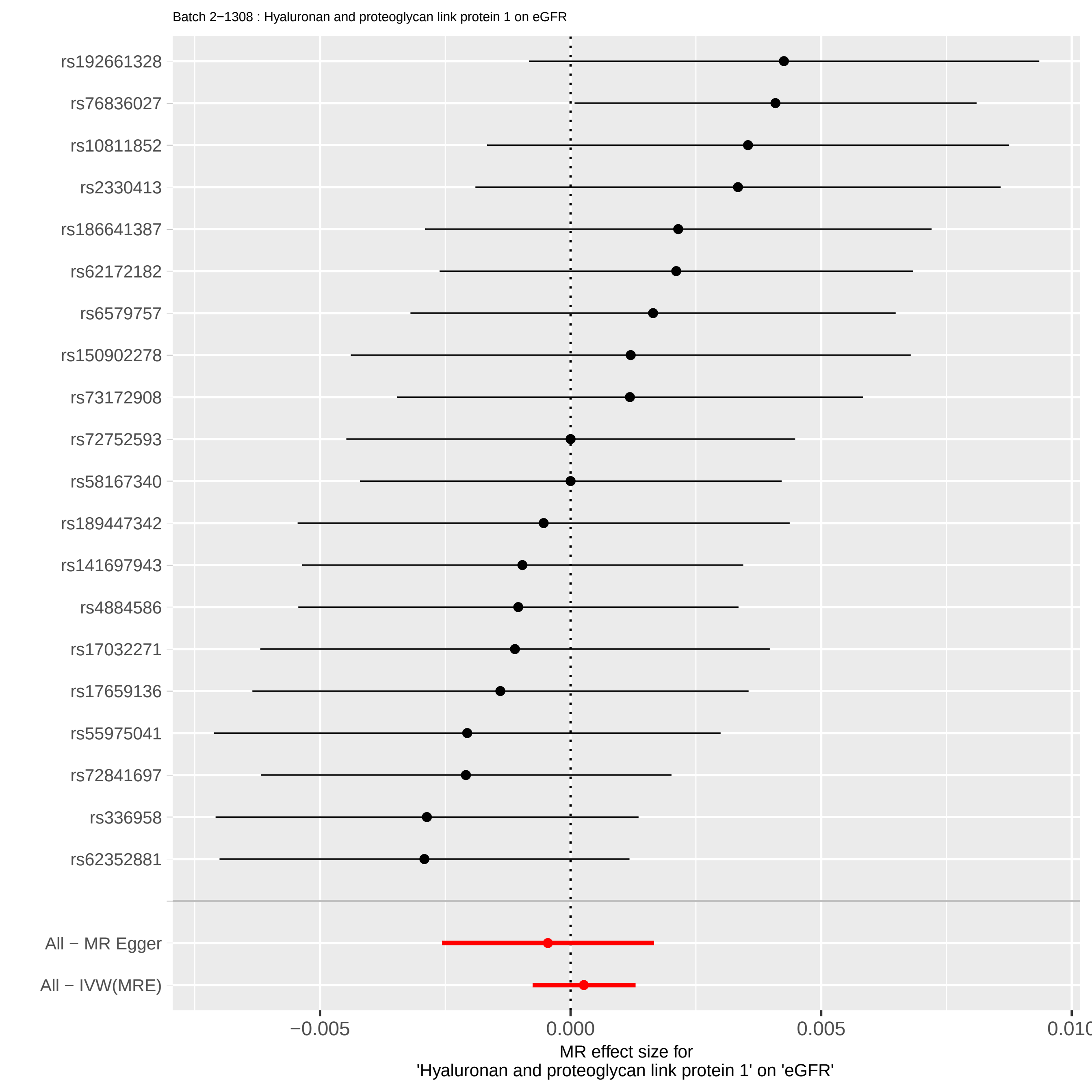

Batch 2−1308 : Hyaluronan and proteoglycan link protein 1 on eGFR
rs192661328
rs76836027
rs10811852
rs2330413
rs186641387
rs62172182
rs6579757
rs150902278
rs73172908
rs72752593
rs58167340
rs189447342
rs141697943
rs4884586
rs17032271
rs17659136
rs55975041
rs72841697
rs336958
rs62352881
All − MR Egger
All − IVW(MRE)
−0.005
0.000
0.005
0.010
MR effect size for
'Hyaluronan and proteoglycan link protein 1' on 'eGFR'

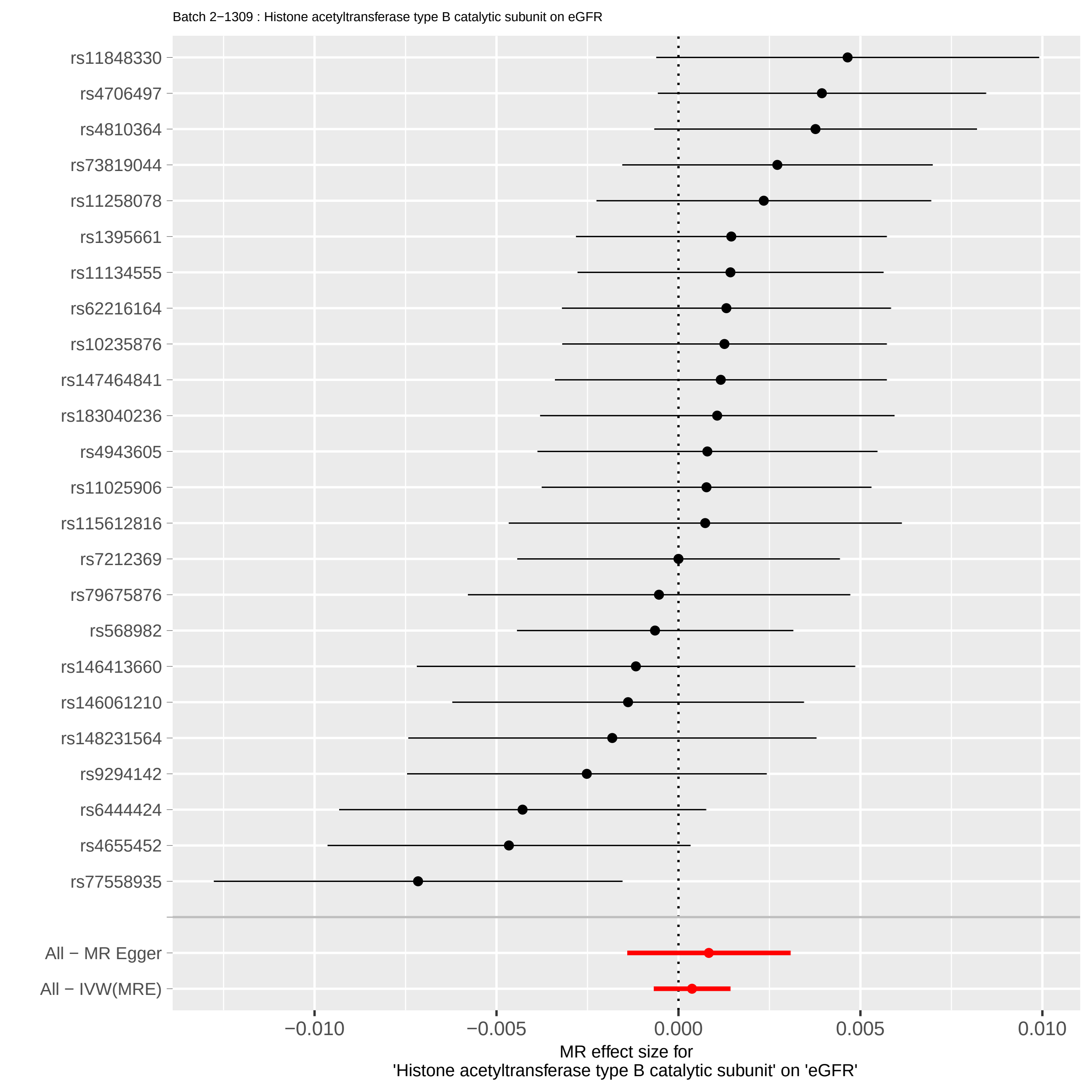

Batch 2−1309 : Histone acetyltransferase type B catalytic subunit on eGFR
rs11848330
rs4706497
rs4810364
rs73819044
rs11258078
rs1395661
rs11134555
rs62216164
rs10235876
rs147464841
rs183040236
rs4943605
rs11025906
rs115612816
rs7212369
rs79675876
rs568982
rs146413660
rs146061210
rs148231564
rs9294142
rs6444424
rs4655452
rs77558935
All − MR Egger
All − IVW(MRE)
−0.010
−0.005
0.000
0.005
0.010
MR effect size for
'Histone acetyltransferase type B catalytic subunit' on 'eGFR'

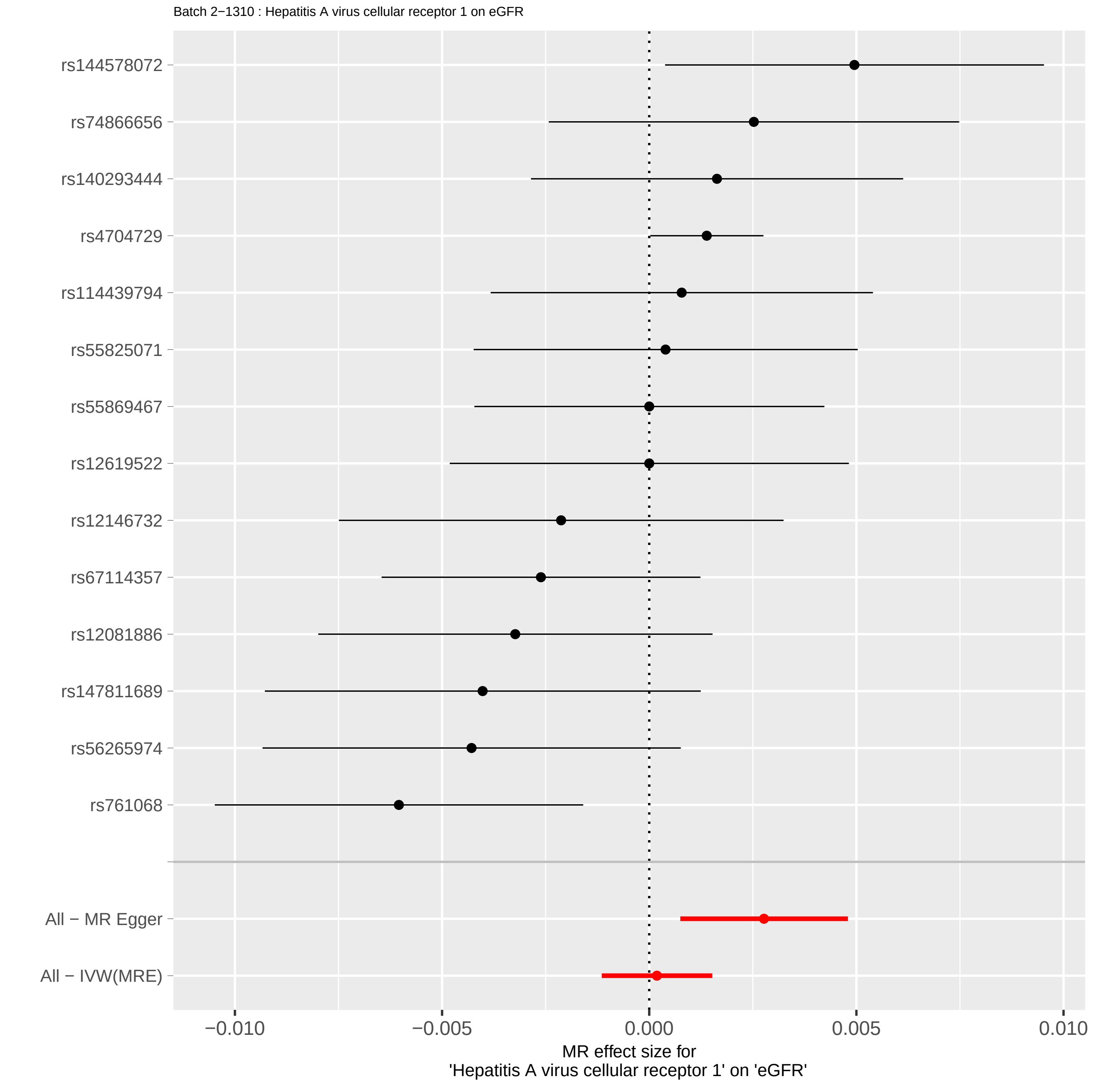
Batch 2−1310 : Hepatitis A virus cellular receptor 1 on eGFR
rs144578072
rs74866656
rs140293444
rs4704729
rs114439794
rs55825071
rs55869467
rs12619522
rs12146732
rs67114357
rs12081886
rs147811689
rs56265974
rs761068
All − MR Egger
All − IVW(MRE)
−0.010
−0.005
0.000
0.005
0.010
MR effect size for
'Hepatitis A virus cellular receptor 1' on 'eGFR'

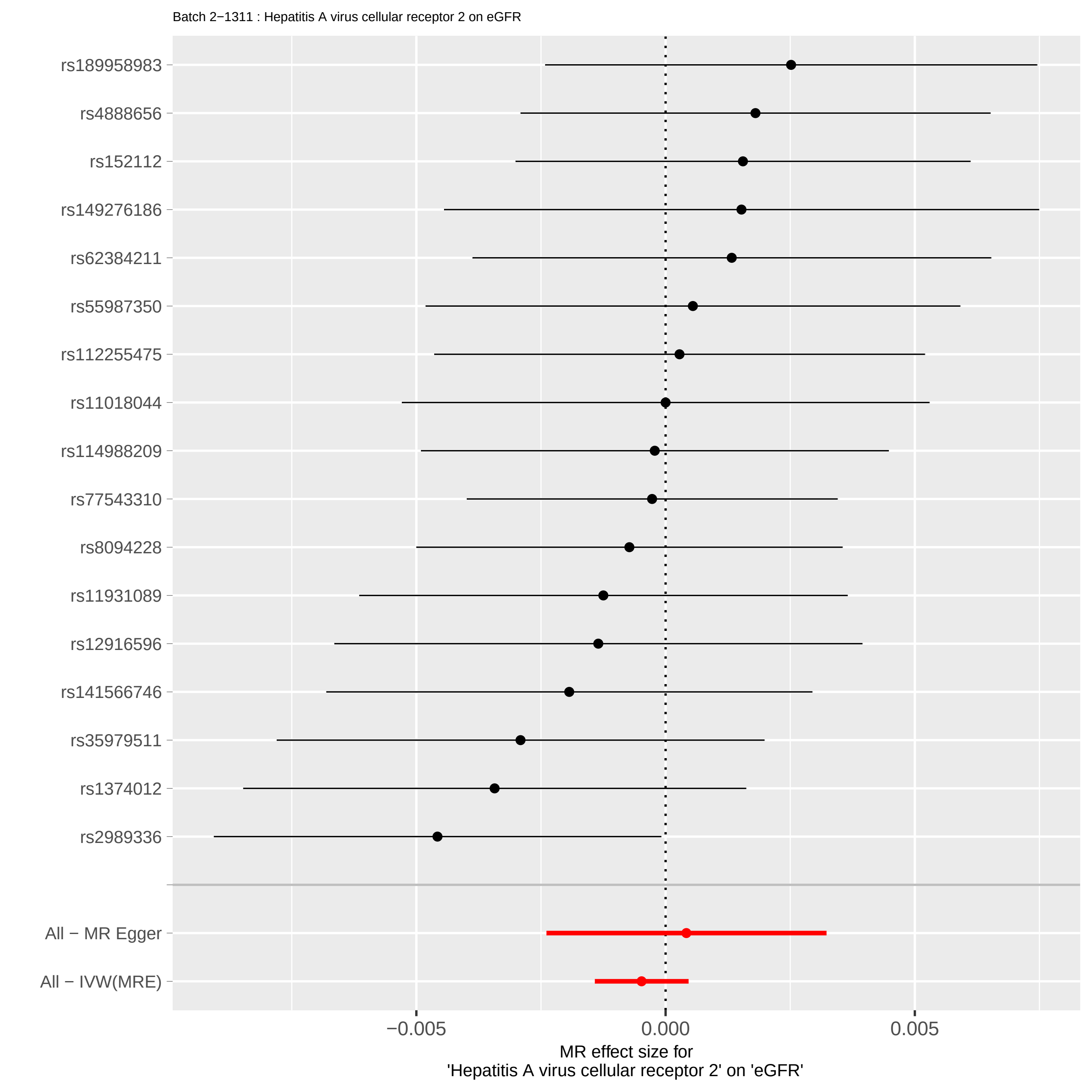
Batch 2−1311 : Hepatitis A virus cellular receptor 2 on eGFR
rs189958983
rs4888656
rs152112
rs149276186
rs62384211
rs55987350
rs112255475
rs11018044
rs114988209
rs77543310
rs8094228
rs11931089
rs12916596
rs141566746
rs35979511
rs1374012
rs2989336
All − MR Egger
All − IVW(MRE)
−0.005
0.000
0.005
MR effect size for
'Hepatitis A virus cellular receptor 2' on 'eGFR'

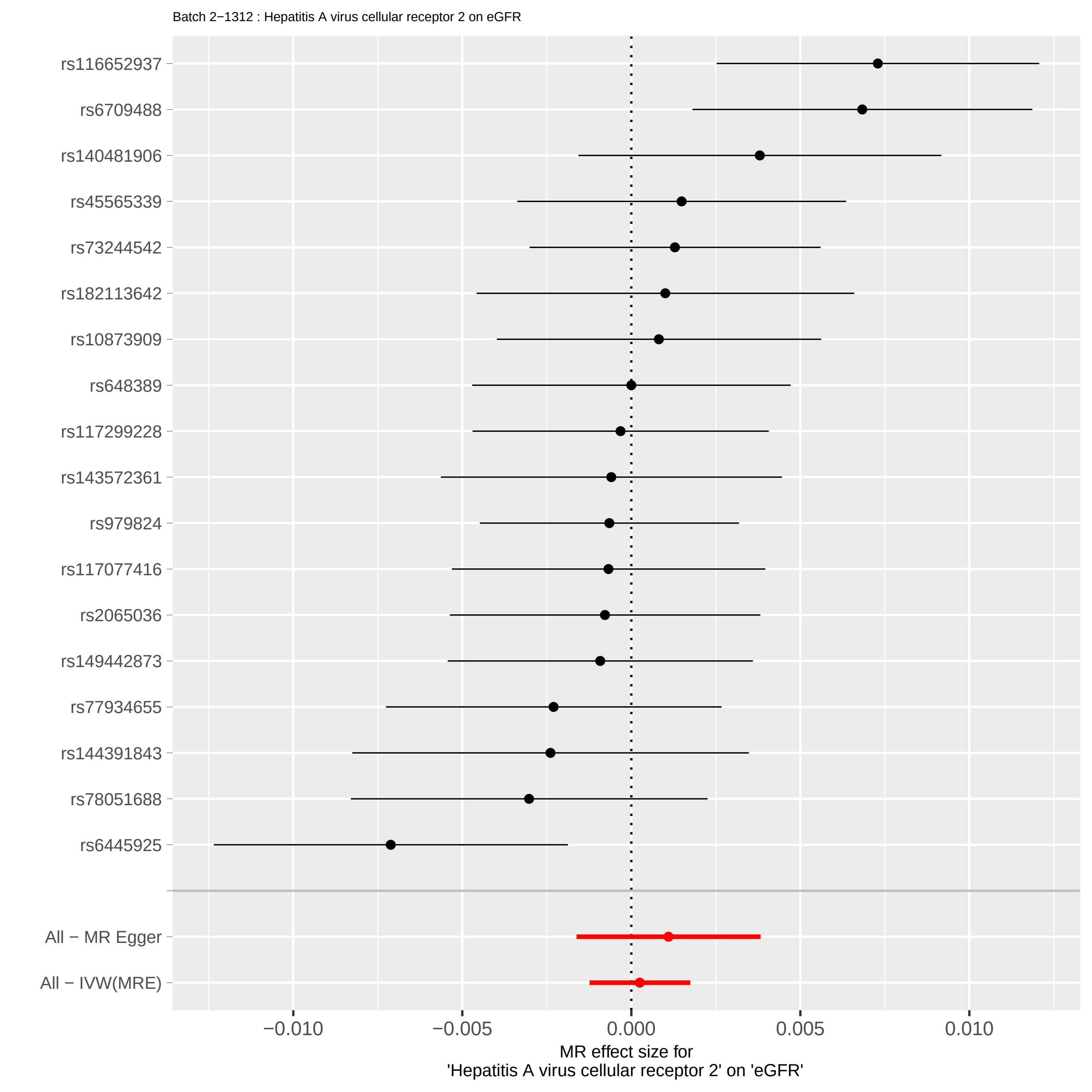

Batch 2−1312 : Hepatitis A virus cellular receptor 2 on eGFR
rs116652937
rs6709488
rs140481906
rs45565339
rs73244542
rs182113642
rs10873909
rs648389
rs117299228
rs143572361
rs979824
rs117077416
rs2065036
rs149442873
rs77934655
rs144391843
rs78051688
rs6445925
All − MR Egger
All − IVW(MRE)
−0.010
−0.005
0.000
0.005
0.010
MR effect size for
'Hepatitis A virus cellular receptor 2' on 'eGFR'

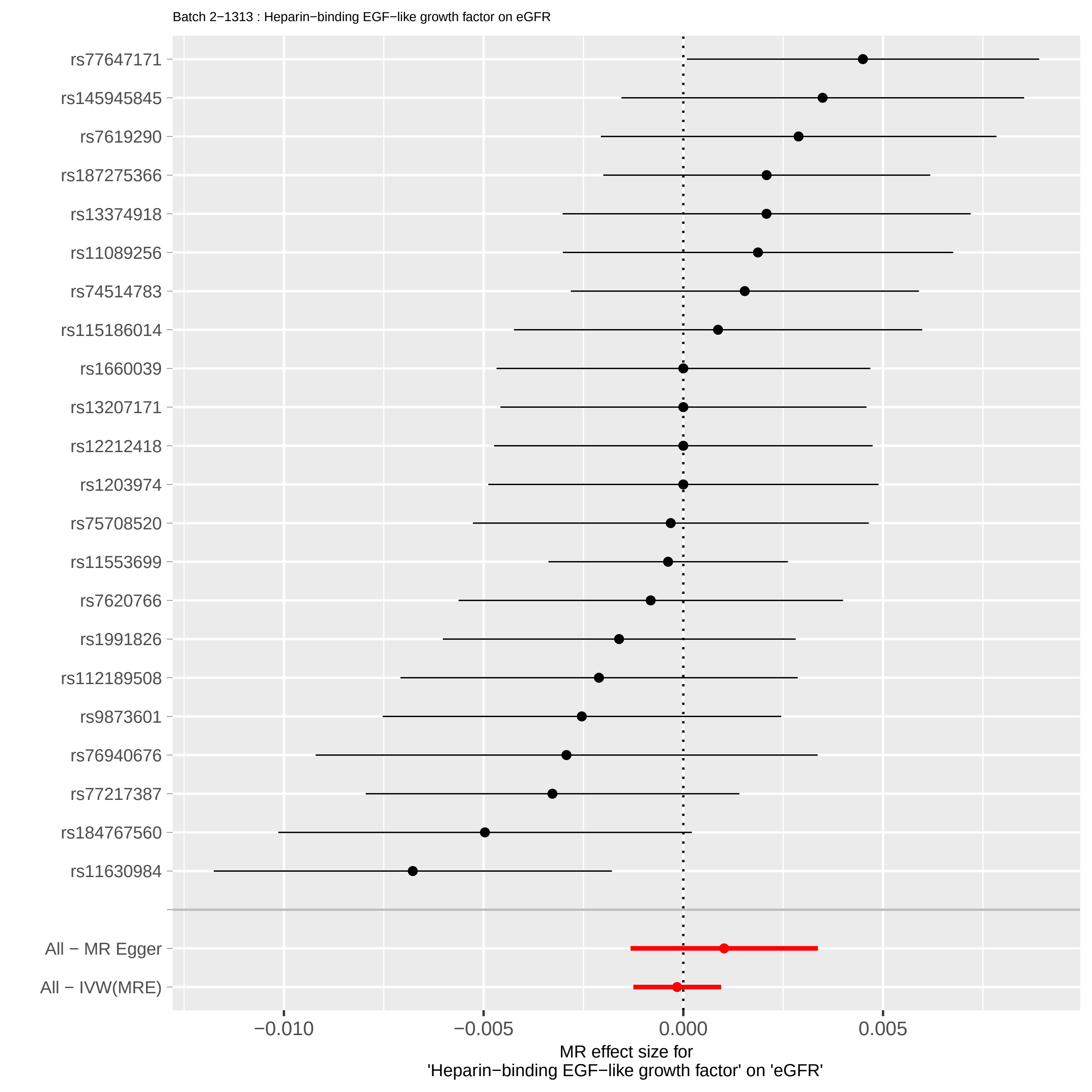

Batch 2−1313 : Heparin−binding EGF−like growth factor on eGFR
rs77647171
rs145945845
rs7619290
rs187275366
rs13374918
rs11089256
rs74514783
rs115186014
rs1660039
rs13207171
rs12212418
rs1203974
rs75708520
rs11553699
rs7620766
rs1991826
rs112189508
rs9873601
rs76940676
rs77217387
rs184767560
rs11630984
All − MR Egger
All − IVW(MRE)
−0.010
−0.005
0.000
0.005
MR effect size for
'Heparin−binding EGF−like growth factor' on 'eGFR'

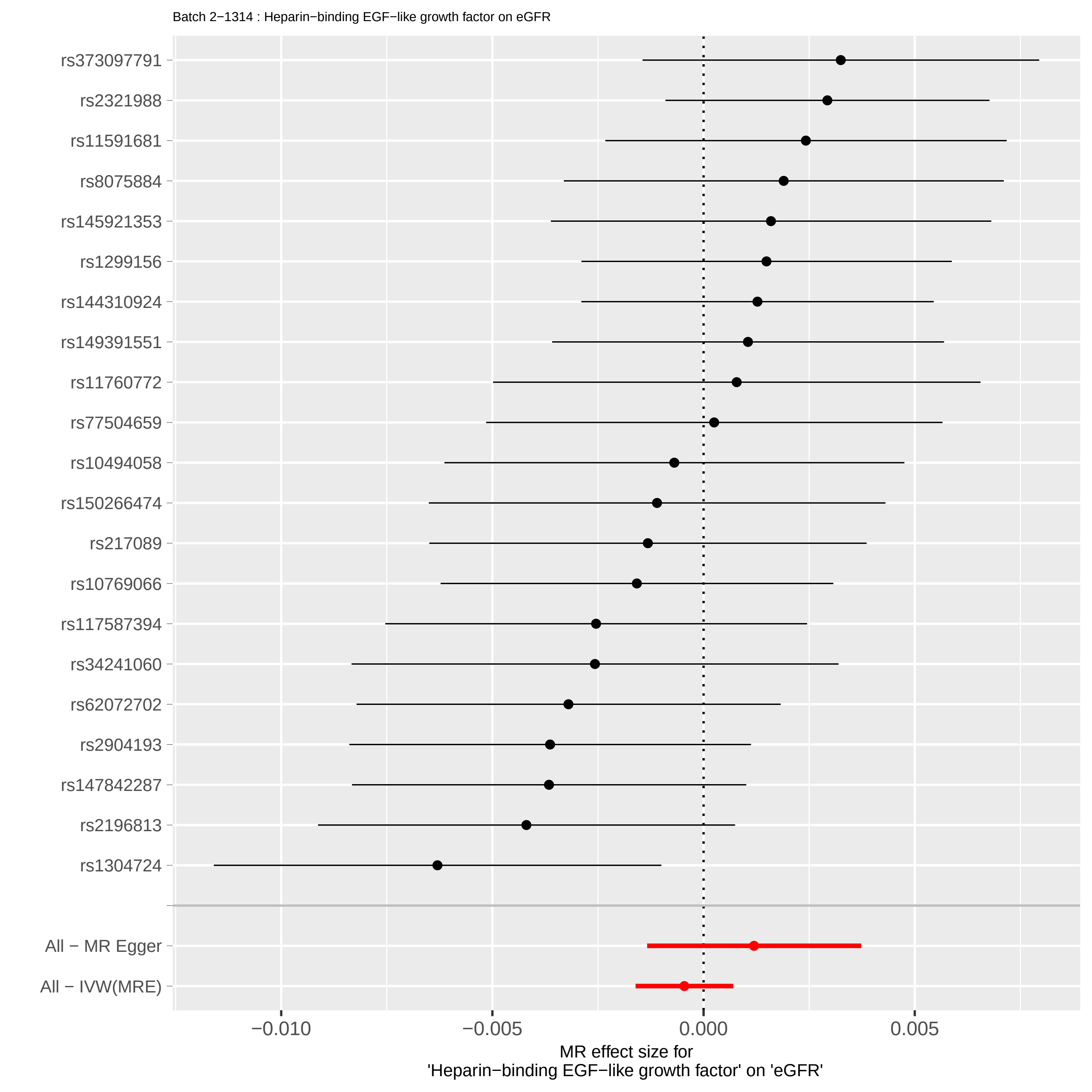

Batch 2−1314 : Heparin−binding EGF−like growth factor on eGFR
rs373097791
rs2321988
rs11591681
rs8075884
rs145921353
rs1299156
rs144310924
rs149391551
rs11760772
rs77504659
rs10494058
rs150266474
rs217089
rs10769066
rs117587394
rs34241060
rs62072702
rs2904193
rs147842287
rs2196813
rs1304724
All − MR Egger
All − IVW(MRE)
−0.010
−0.005
0.000
0.005
MR effect size for
'Heparin−binding EGF−like growth factor' on 'eGFR'

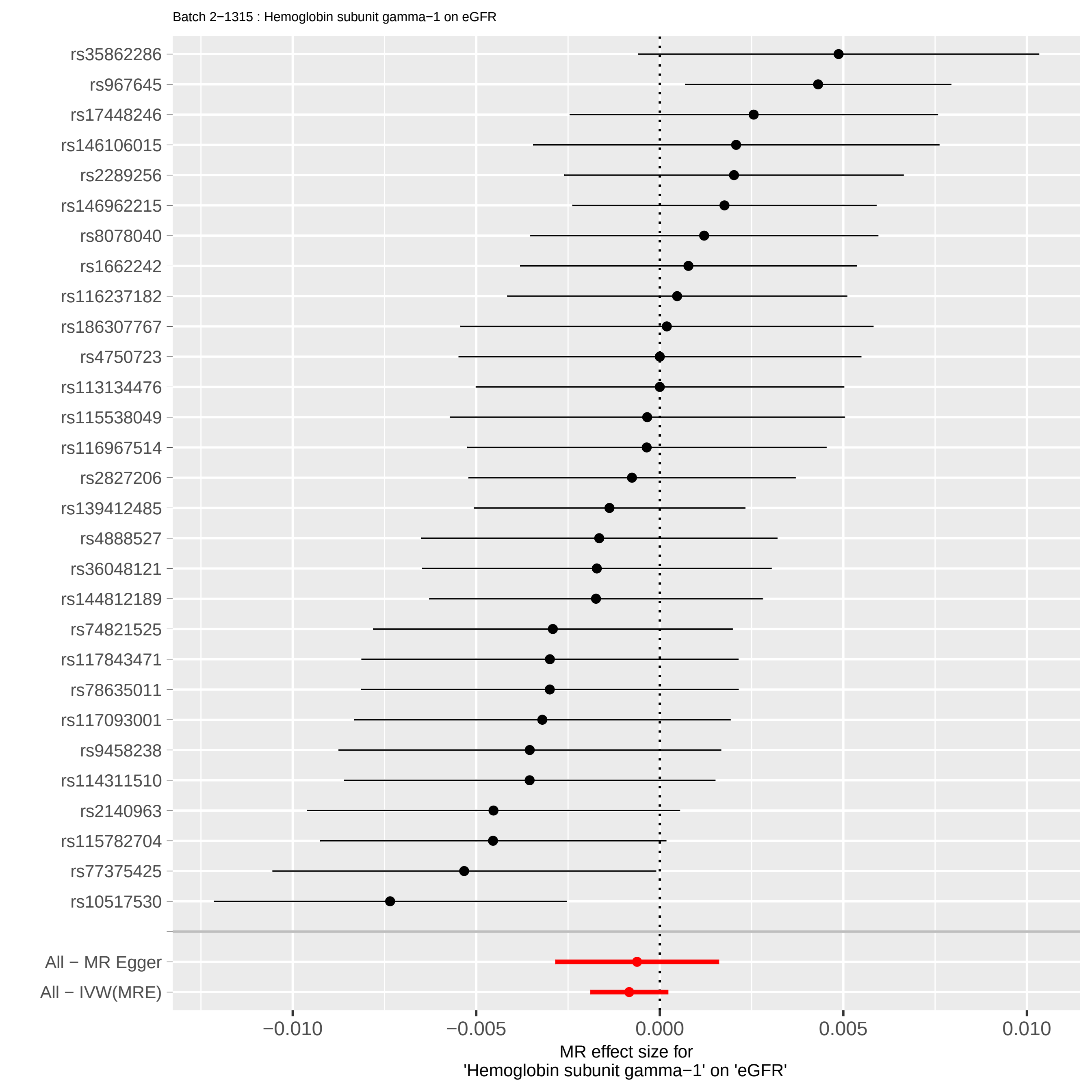

Batch 2−1315 : Hemoglobin subunit gamma−1 on eGFR
rs35862286
rs967645
rs17448246
rs146106015
rs2289256
rs146962215
rs8078040
rs1662242
rs116237182
rs186307767
rs4750723
rs113134476
rs115538049
rs116967514
rs2827206
rs139412485
rs4888527
rs36048121
rs144812189
rs74821525
rs117843471
rs78635011
rs117093001
rs9458238
rs114311510
rs2140963
rs115782704
rs77375425
rs10517530
All − MR Egger
All − IVW(MRE)
−0.010
−0.005
0.000
0.005
0.010
MR effect size for
'Hemoglobin subunit gamma−1' on 'eGFR'

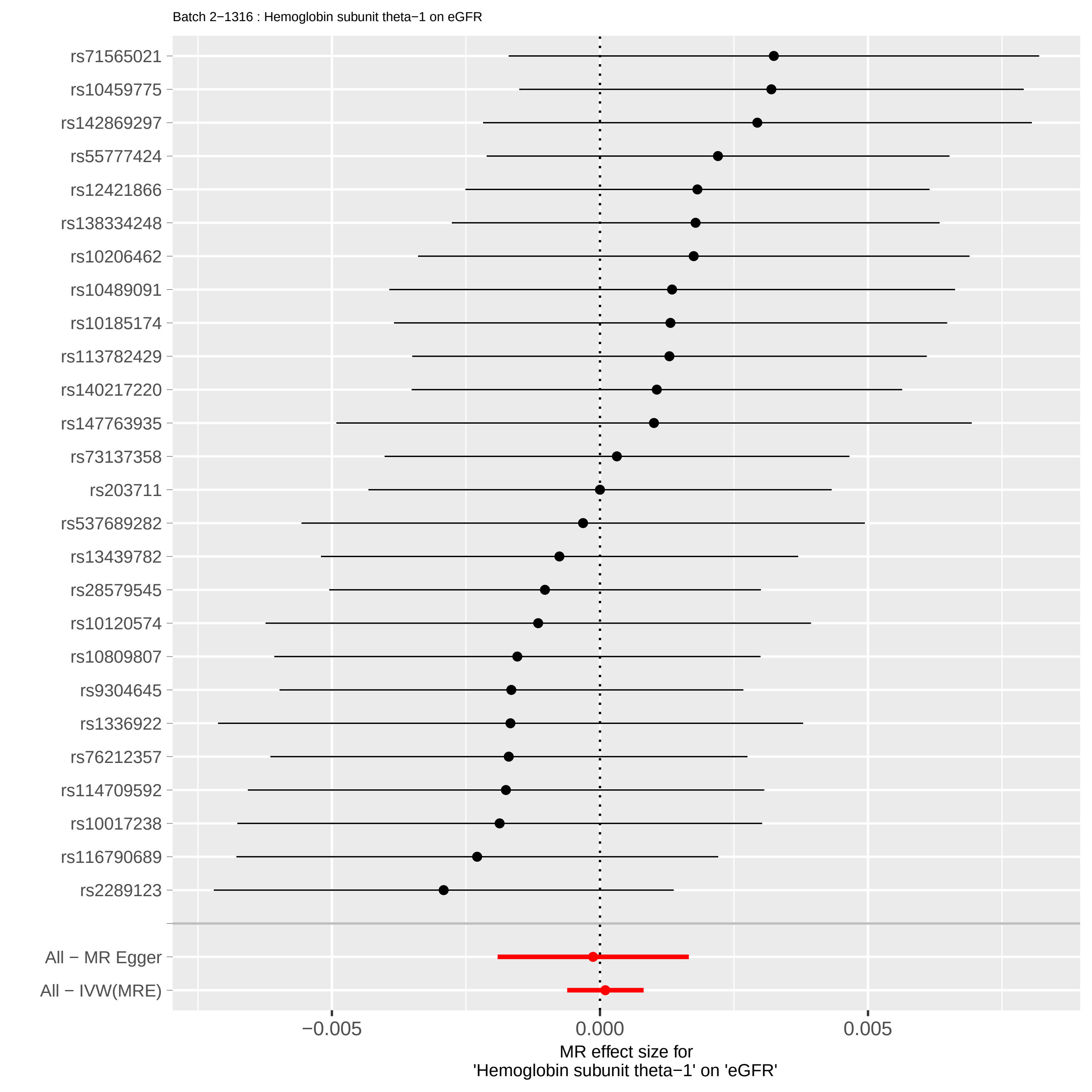

Batch 2−1316 : Hemoglobin subunit theta−1 on eGFR
rs71565021
rs10459775
rs142869297
rs55777424
rs12421866
rs138334248
rs10206462
rs10489091
rs10185174
rs113782429
rs140217220
rs147763935
rs73137358
rs203711
rs537689282
rs13439782
rs28579545
rs10120574
rs10809807
rs9304645
rs1336922
rs76212357
rs114709592
rs10017238
rs116790689
rs2289123
All − MR Egger
All − IVW(MRE)
−0.005
0.000
0.005
MR effect size for
'Hemoglobin subunit theta−1' on 'eGFR'

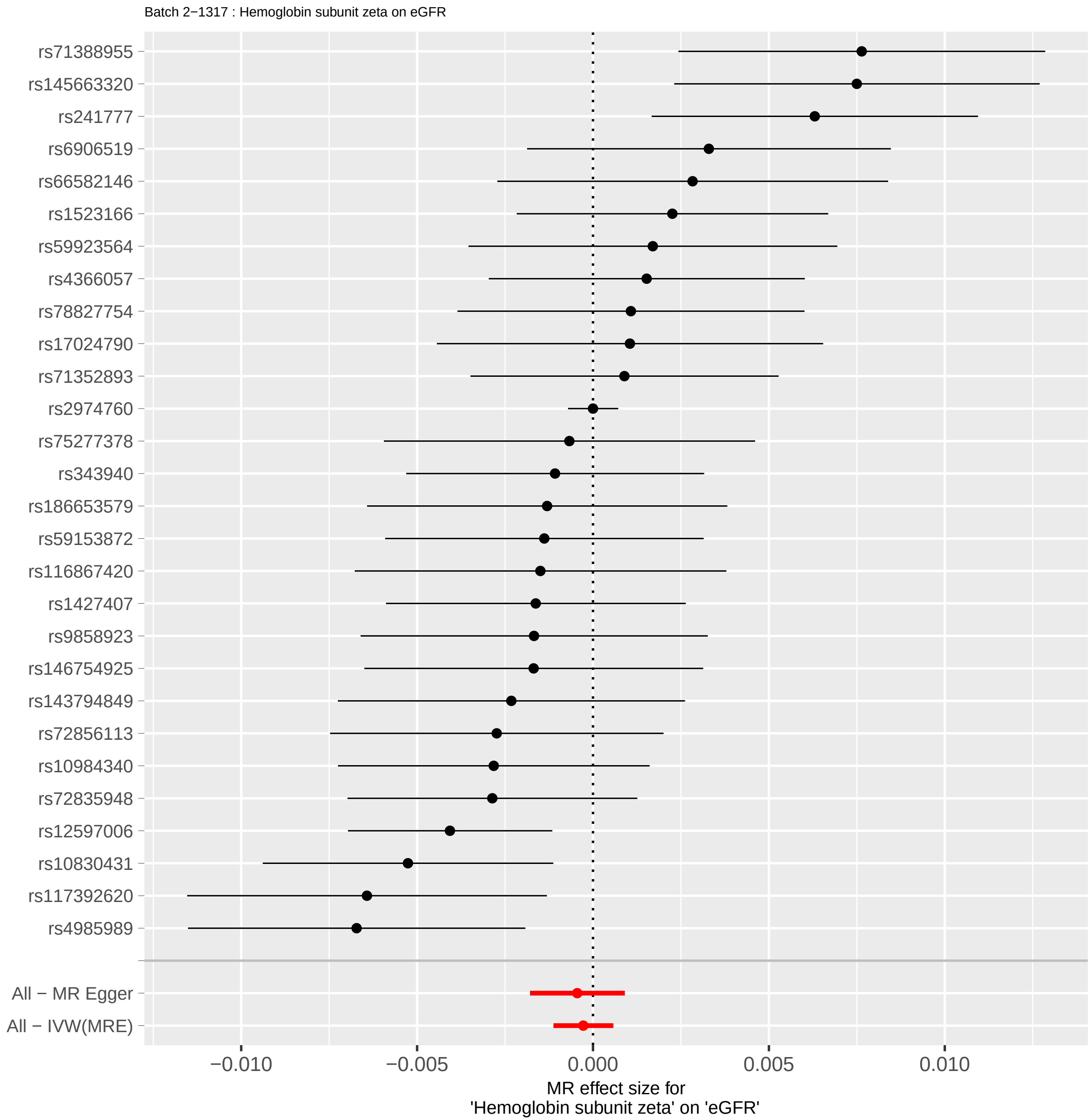

Batch 2−1317 : Hemoglobin subunit zeta on eGFR
rs71388955
rs145663320
rs241777
rs6906519
rs66582146
rs1523166
rs59923564
rs4366057
rs78827754
rs17024790
rs71352893
rs2974760
rs75277378
rs343940
rs186653579
rs59153872
rs116867420
rs1427407
rs9858923
rs146754925
rs143794849
rs72856113
rs10984340
rs72835948
rs12597006
rs10830431
rs117392620
rs4985989
All − MR Egger
All − IVW(MRE)
−0.010
−0.005
0.000
0.005
0.010
MR effect size for
'Hemoglobin subunit zeta' on 'eGFR'

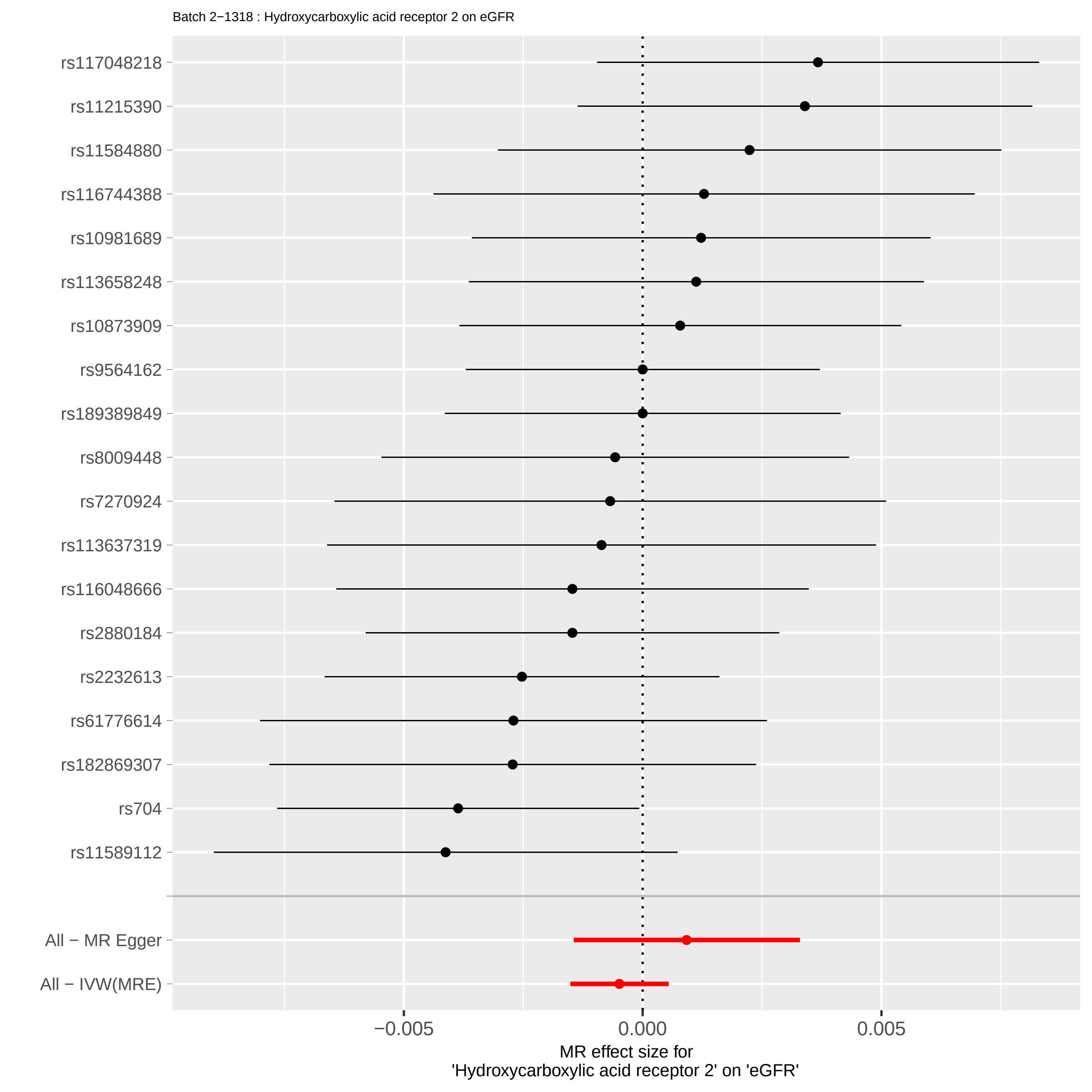

Batch 2−1318 : Hydroxycarboxylic acid receptor 2 on eGFR
rs117048218
rs11215390
rs11584880
rs116744388
rs10981689
rs113658248
rs10873909
rs9564162
rs189389849
rs8009448
rs7270924
rs113637319
rs116048666
rs2880184
rs2232613
rs61776614
rs182869307
rs704
rs11589112
All − MR Egger
All − IVW(MRE)
−0.005
0.000
0.005
MR effect size for
'Hydroxycarboxylic acid receptor 2' on 'eGFR'

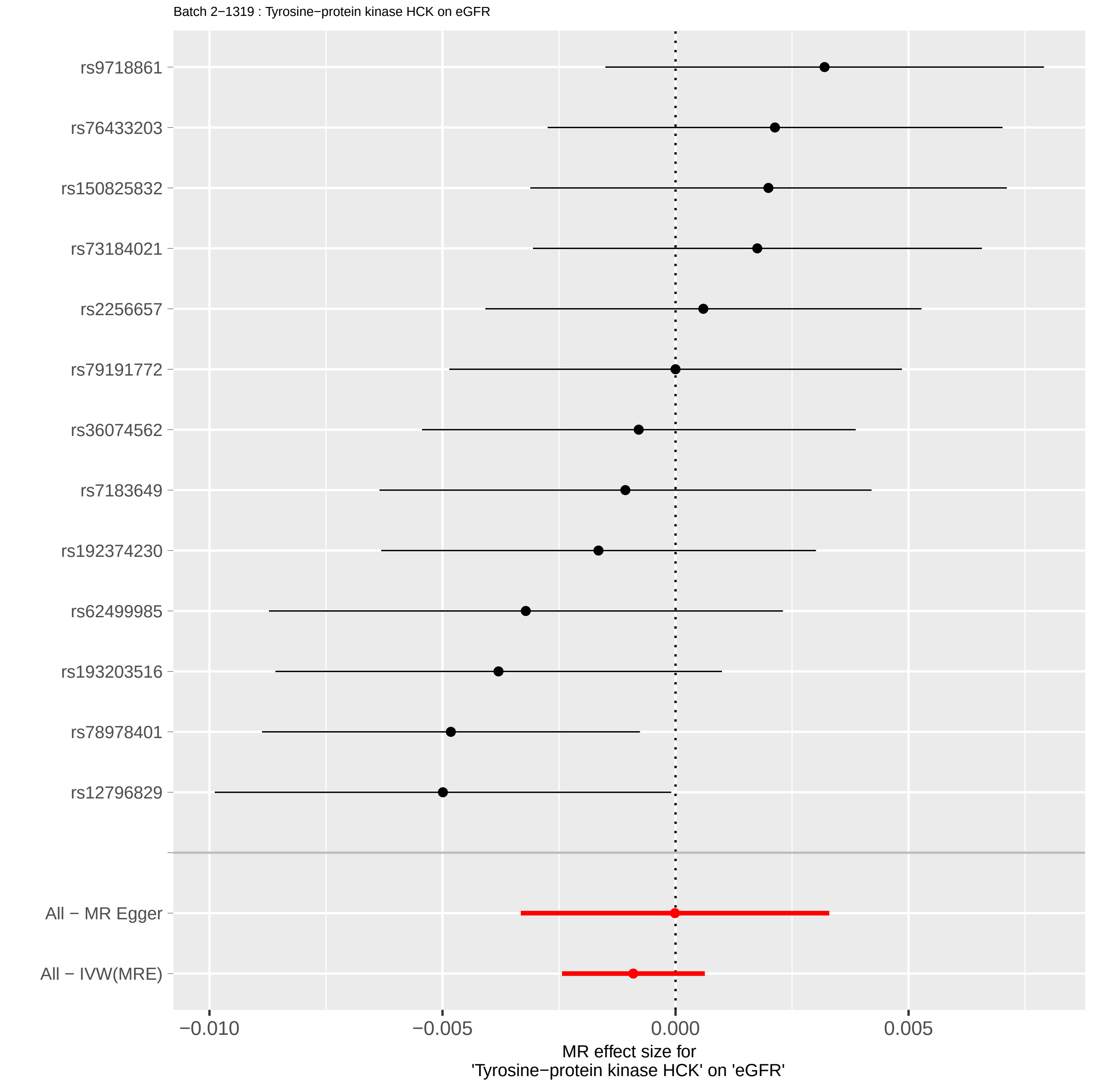

Batch 2−1319 : Tyrosine−protein kinase HCK on eGFR
rs9718861
rs76433203
rs150825832
rs73184021
rs2256657
rs79191772
rs36074562
rs7183649
rs192374230
rs62499985
rs193203516
rs78978401
rs12796829
All − MR Egger
All − IVW(MRE)
−0.010
−0.005
0.000
0.005
MR effect size for
'Tyrosine−protein kinase HCK' on 'eGFR'

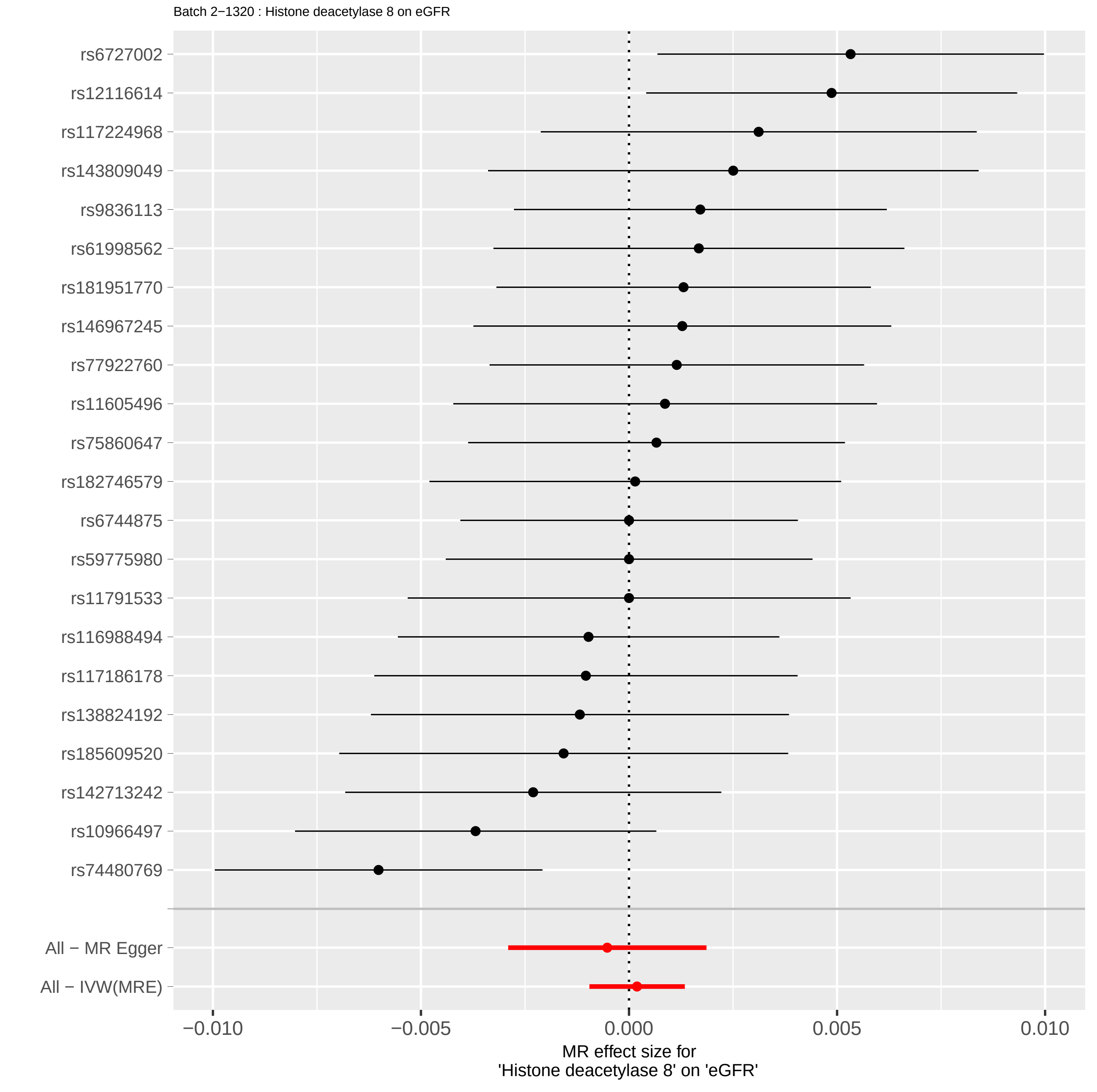

Batch 2−1320 : Histone deacetylase 8 on eGFR
rs6727002
rs12116614
rs117224968
rs143809049
rs9836113
rs61998562
rs181951770
rs146967245
rs77922760
rs11605496
rs75860647
rs182746579
rs6744875
rs59775980
rs11791533
rs116988494
rs117186178
rs138824192
rs185609520
rs142713242
rs10966497
rs74480769
All − MR Egger
All − IVW(MRE)
−0.010
−0.005
0.000
0.005
0.010
MR effect size for
'Histone deacetylase 8' on 'eGFR'

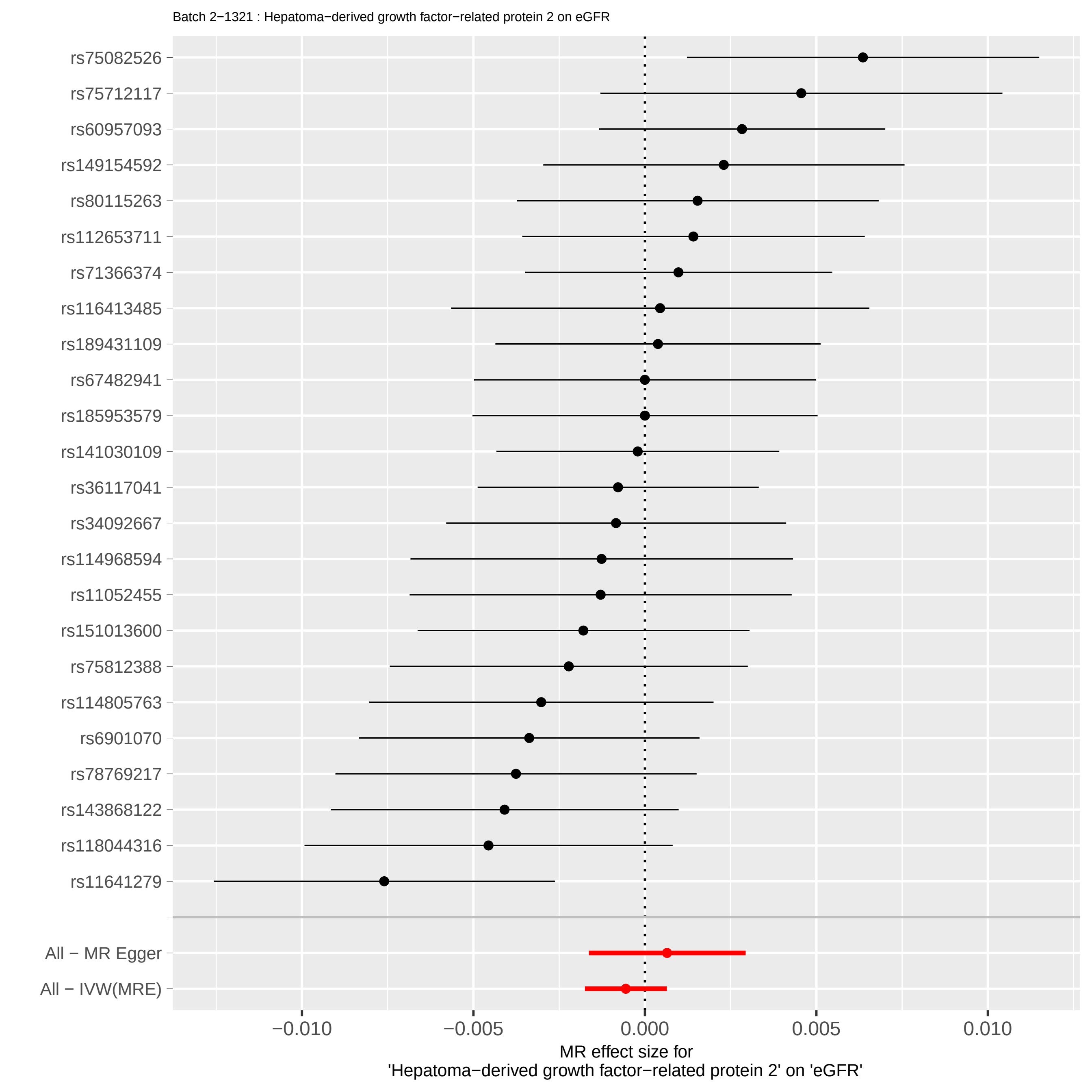

Batch 2−1321 : Hepatoma−derived growth factor−related protein 2 on eGFR
rs75082526
rs75712117
rs60957093
rs149154592
rs80115263
rs112653711
rs71366374
rs116413485
rs189431109
rs67482941
rs185953579
rs141030109
rs36117041
rs34092667
rs114968594
rs11052455
rs151013600
rs75812388
rs114805763
rs6901070
rs78769217
rs143868122
rs118044316
rs11641279
All − MR Egger
All − IVW(MRE)
−0.010
−0.005
0.000
0.005
0.010
MR effect size for
'Hepatoma−derived growth factor−related protein 2' on 'eGFR'

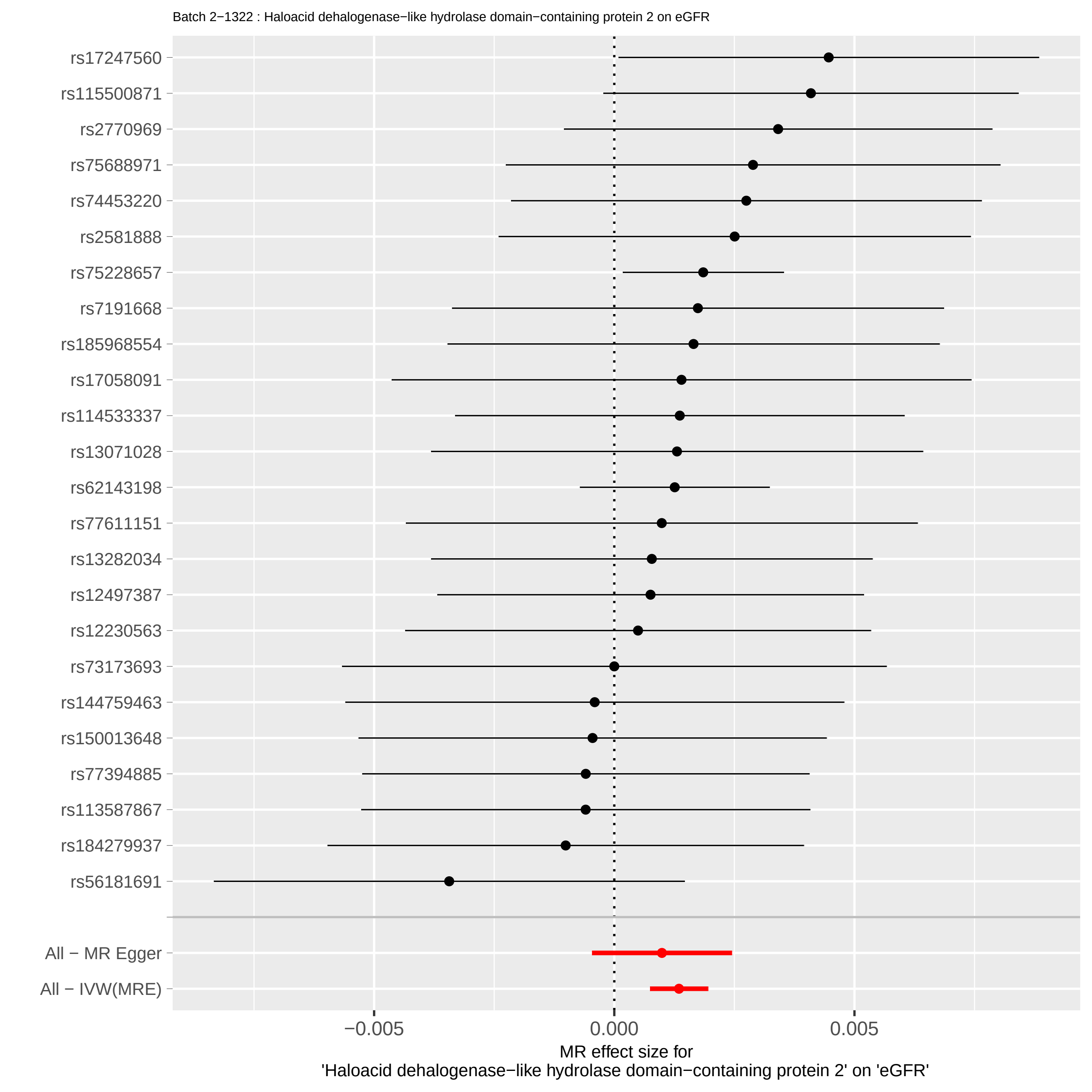
Batch 2−1322 : Haloacid dehalogenase−like hydrolase domain−containing protein 2 on eGFR
rs17247560
rs115500871
rs2770969
rs75688971
rs74453220
rs2581888
rs75228657
rs7191668
rs185968554
rs17058091
rs114533337
rs13071028
rs62143198
rs77611151
rs13282034
rs12497387
rs12230563
rs73173693
rs144759463
rs150013648
rs77394885
rs113587867
rs184279937
rs56181691
All − MR Egger
All − IVW(MRE)
−0.005
0.000
0.005
MR effect size for
'Haloacid dehalogenase−like hydrolase domain−containing protein 2' on 'eGFR'

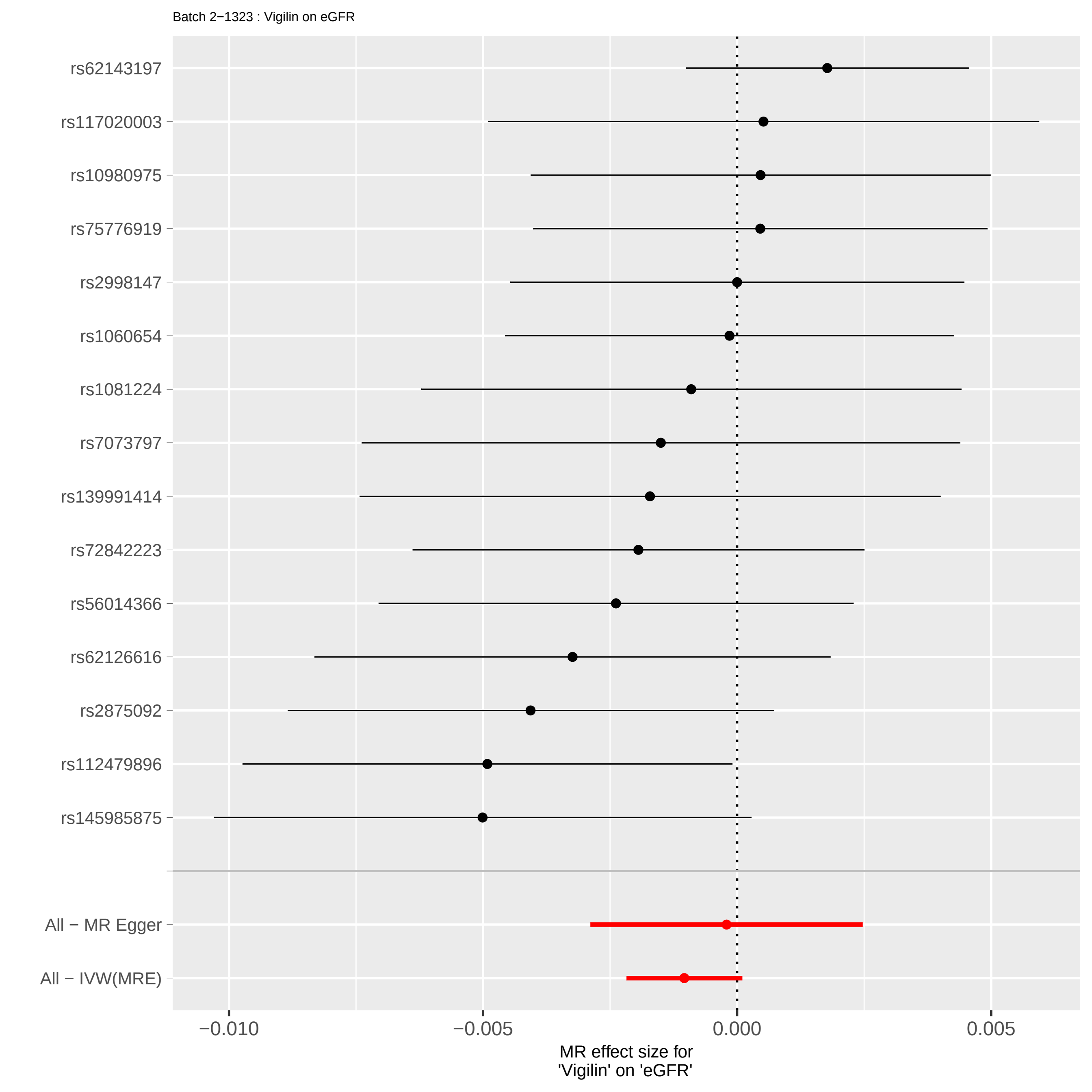

Batch 2−1323 : Vigilin on eGFR
rs62143197
rs117020003
rs10980975
rs75776919
rs2998147
rs1060654
rs1081224
rs7073797
rs139991414
rs72842223
rs56014366
rs62126616
rs2875092
rs112479896
rs145985875
All − MR Egger
All − IVW(MRE)
−0.010
−0.005
0.000
0.005
MR effect size for
'Vigilin' on 'eGFR'

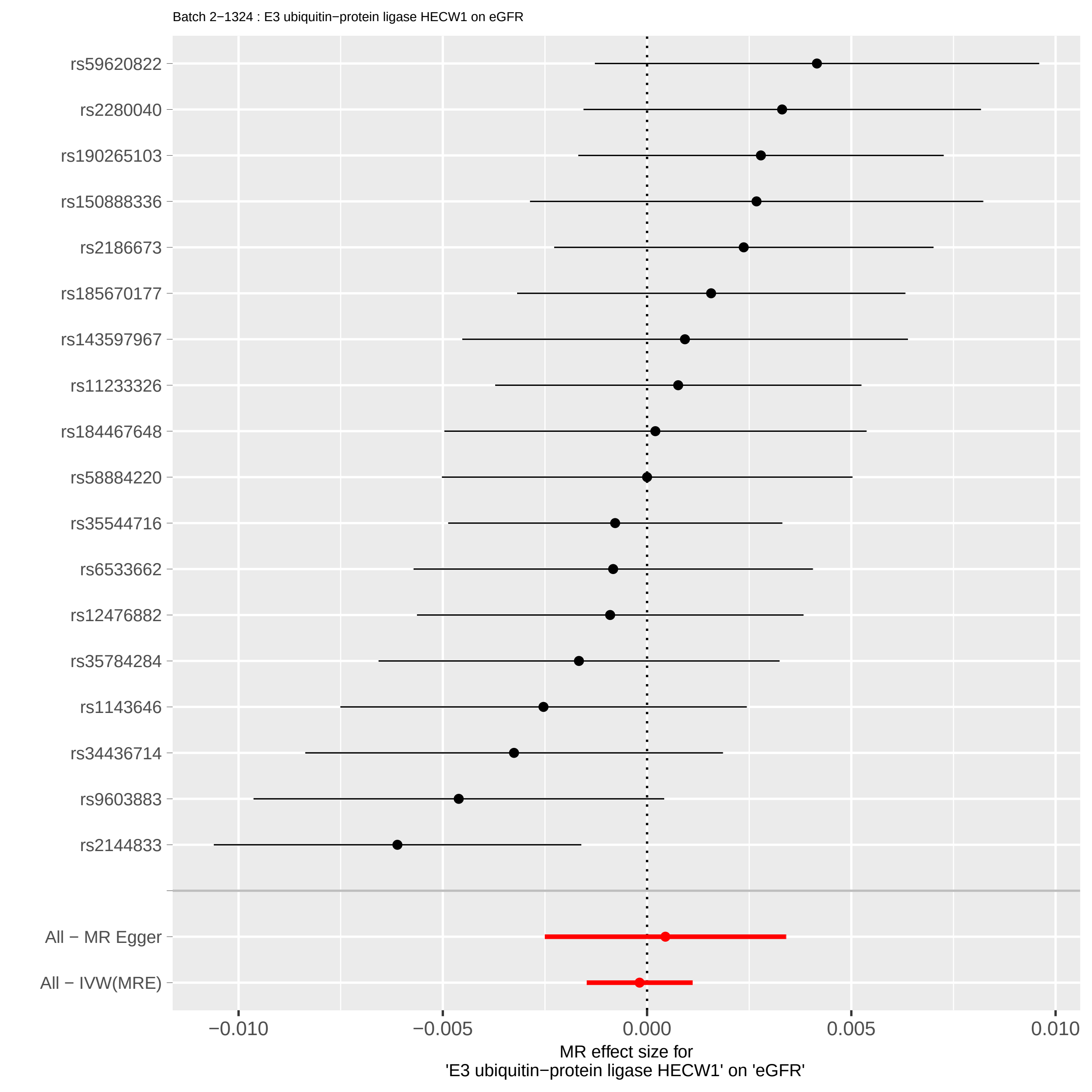

Batch 2−1324 : E3 ubiquitin−protein ligase HECW1 on eGFR
rs59620822
rs2280040
rs190265103
rs150888336
rs2186673
rs185670177
rs143597967
rs11233326
rs184467648
rs58884220
rs35544716
rs6533662
rs12476882
rs35784284
rs1143646
rs34436714
rs9603883
rs2144833
All − MR Egger
All − IVW(MRE)
−0.010
−0.005
0.000
0.005
0.010
MR effect size for
'E3 ubiquitin−protein ligase HECW1' on 'eGFR'

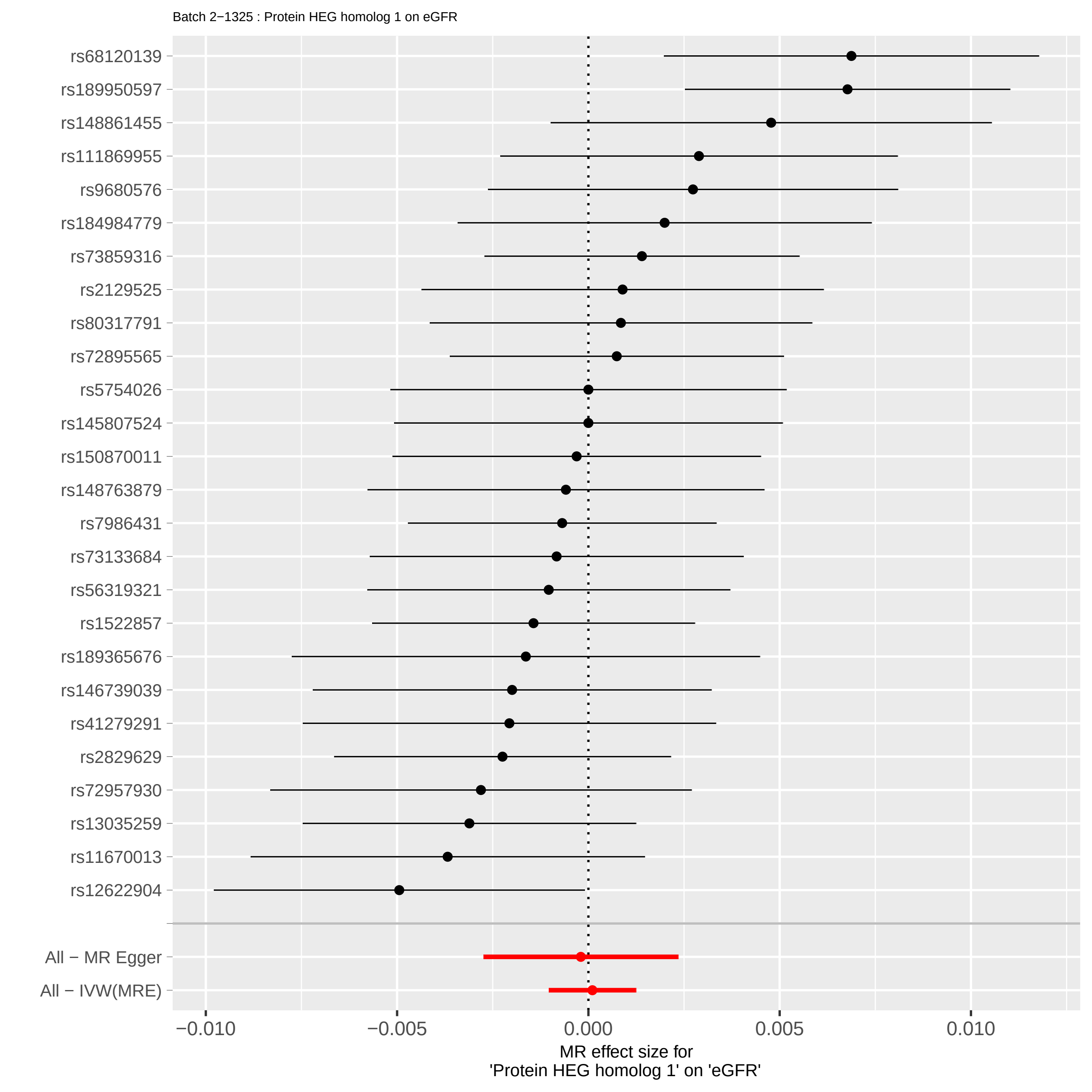

Batch 2−1325 : Protein HEG homolog 1 on eGFR
rs68120139
rs189950597
rs148861455
rs111869955
rs9680576
rs184984779
rs73859316
rs2129525
rs80317791
rs72895565
rs5754026
rs145807524
rs150870011
rs148763879
rs7986431
rs73133684
rs56319321
rs1522857
rs189365676
rs146739039
rs41279291
rs2829629
rs72957930
rs13035259
rs11670013
rs12622904
All − MR Egger
All − IVW(MRE)
−0.010
−0.005
0.000
0.005
0.010
MR effect size for
'Protein HEG homolog 1' on 'eGFR'

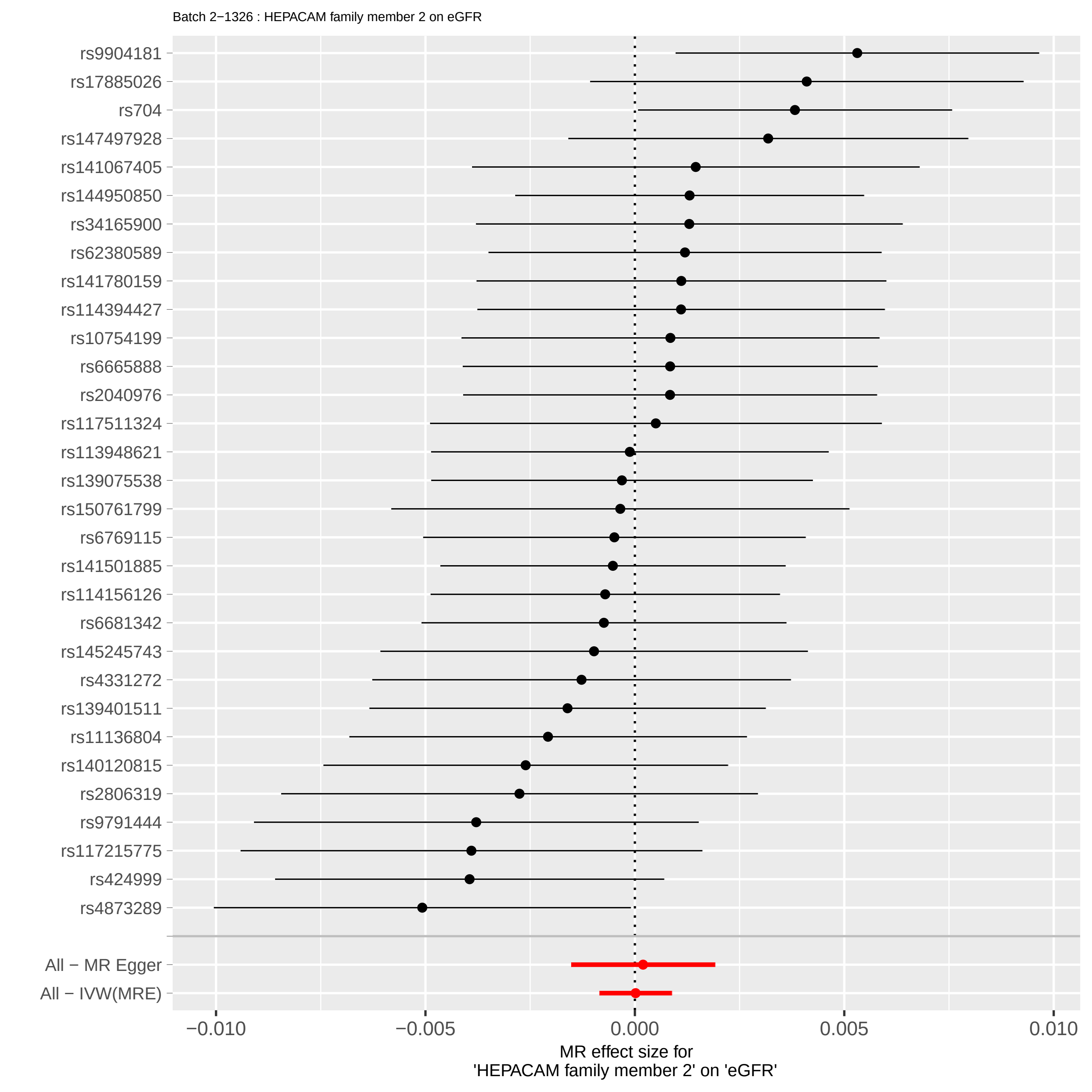

Batch 2−1326 : HEPACAM family member 2 on eGFR
rs9904181
rs17885026
rs704
rs147497928
rs141067405
rs144950850
rs34165900
rs62380589
rs141780159
rs114394427
rs10754199
rs6665888
rs2040976
rs117511324
rs113948621
rs139075538
rs150761799
rs6769115
rs141501885
rs114156126
rs6681342
rs145245743
rs4331272
rs139401511
rs11136804
rs140120815
rs2806319
rs9791444
rs117215775
rs424999
rs4873289
All − MR Egger
All − IVW(MRE)
−0.010
−0.005
0.000
0.005
0.010
MR effect size for
'HEPACAM family member 2' on 'eGFR'

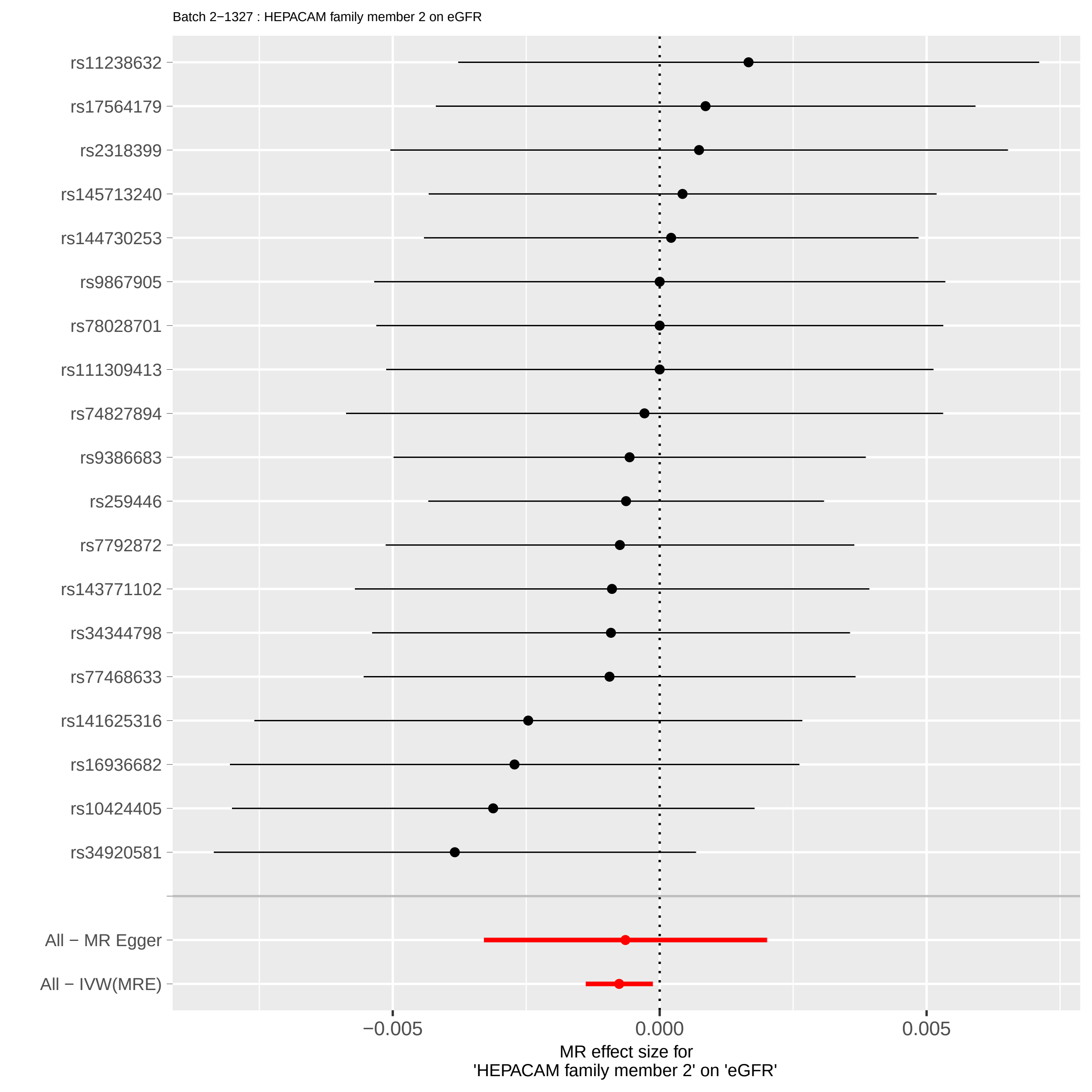
Batch 2−1327 : HEPACAM family member 2 on eGFR
rs11238632
rs17564179
rs2318399
rs145713240
rs144730253
rs9867905
rs78028701
rs111309413
rs74827894
rs9386683
rs259446
rs7792872
rs143771102
rs34344798
rs77468633
rs141625316
rs16936682
rs10424405
rs34920581
All − MR Egger
All − IVW(MRE)
−0.005
0.000
0.005
MR effect size for
'HEPACAM family member 2' on 'eGFR'

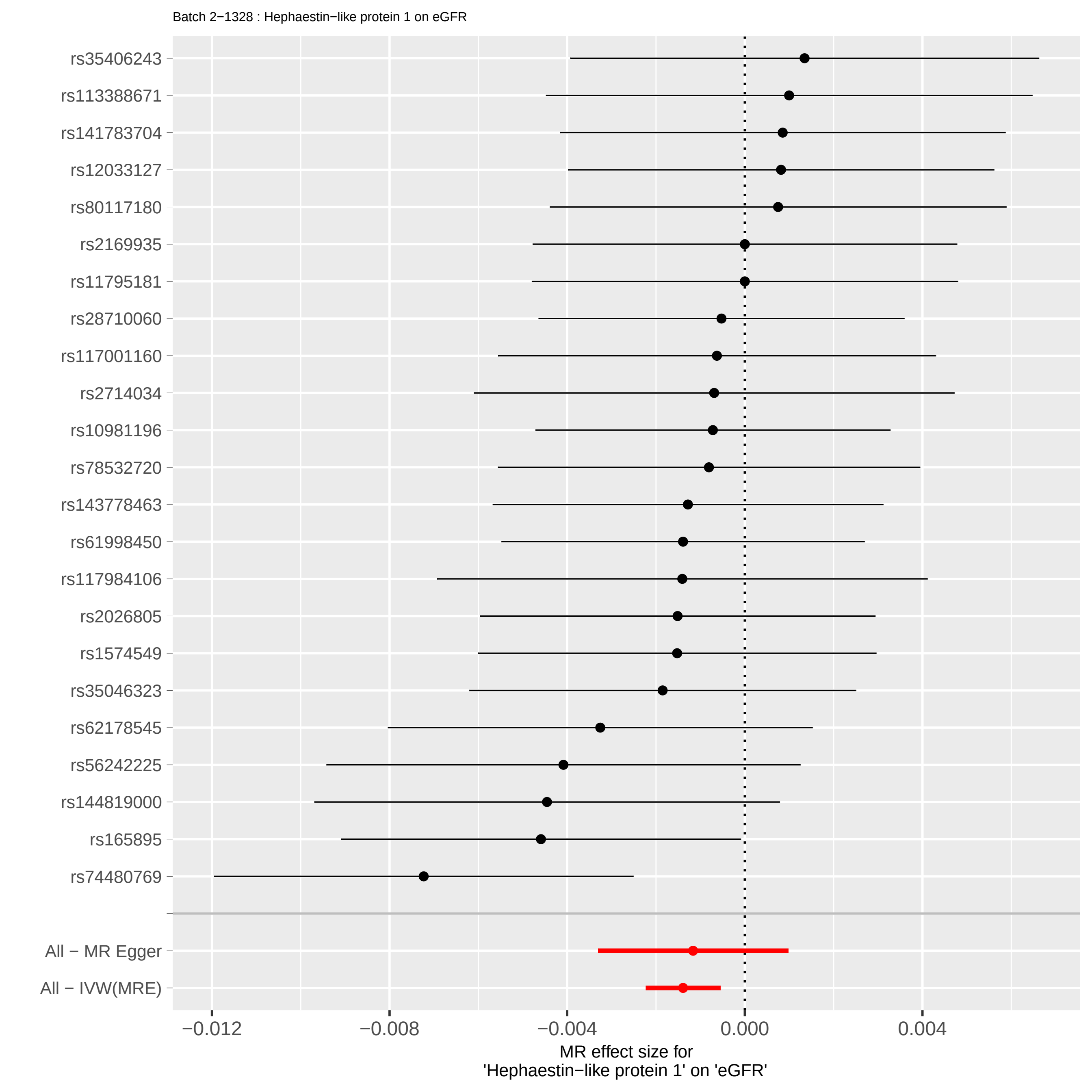

Batch 2−1328 : Hephaestin−like protein 1 on eGFR
rs35406243
rs113388671
rs141783704
rs12033127
rs80117180
rs2169935
rs11795181
rs28710060
rs117001160
rs2714034
rs10981196
rs78532720
rs143778463
rs61998450
rs117984106
rs2026805
rs1574549
rs35046323
rs62178545
rs56242225
rs144819000
rs165895
rs74480769
All − MR Egger
All − IVW(MRE)
−0.012
−0.008
−0.004
0.000
0.004
MR effect size for
'Hephaestin−like protein 1' on 'eGFR'

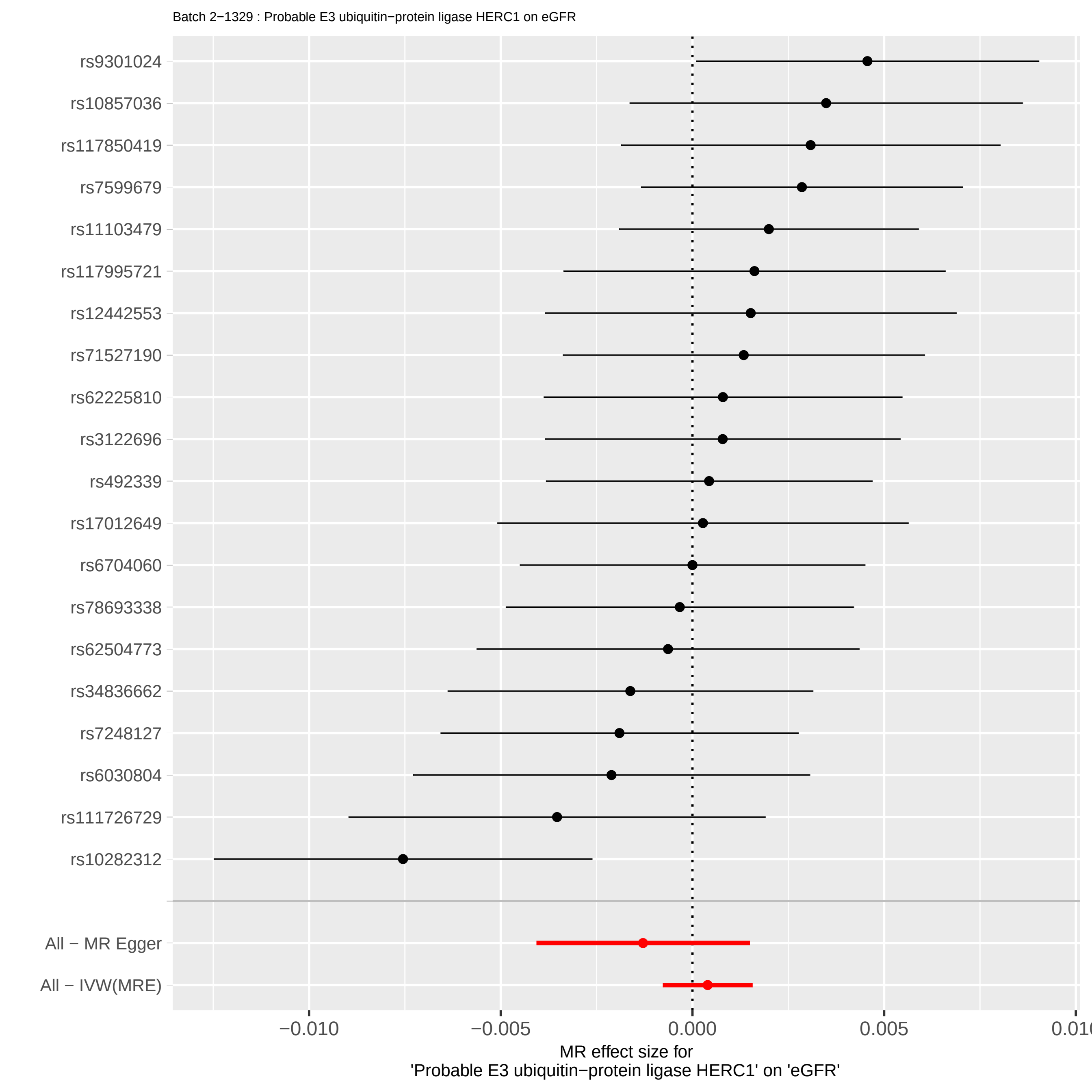

Batch 2−1329 : Probable E3 ubiquitin−protein ligase HERC1 on eGFR
rs9301024
rs10857036
rs117850419
rs7599679
rs11103479
rs117995721
rs12442553
rs71527190
rs62225810
rs3122696
rs492339
rs17012649
rs6704060
rs78693338
rs62504773
rs34836662
rs7248127
rs6030804
rs111726729
rs10282312
All − MR Egger
All − IVW(MRE)
−0.010
−0.005
0.000
0.005
0.010
MR effect size for
'Probable E3 ubiquitin−protein ligase HERC1' on 'eGFR'

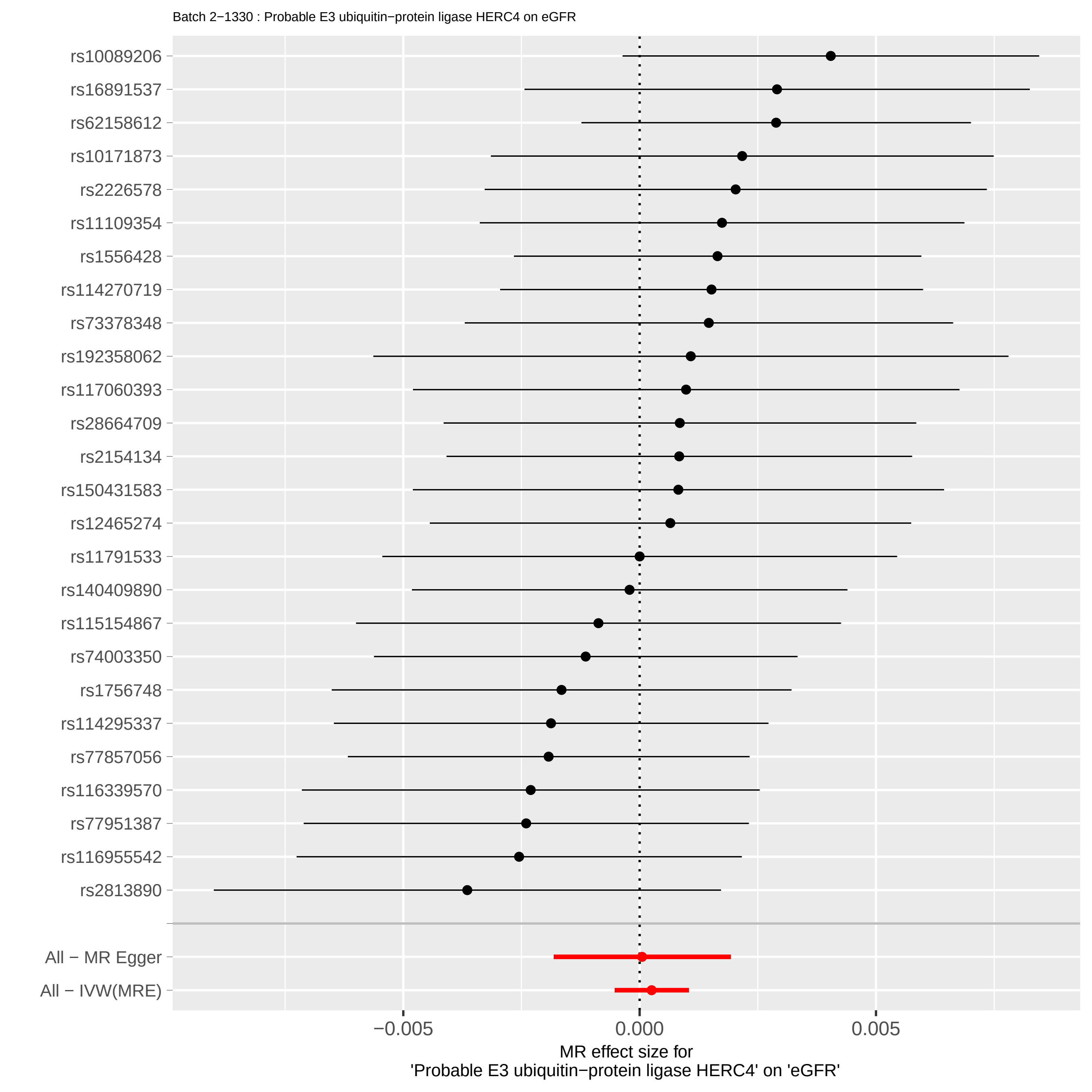

Batch 2−1330 : Probable E3 ubiquitin−protein ligase HERC4 on eGFR
rs10089206
rs16891537
rs62158612
rs10171873
rs2226578
rs11109354
rs1556428
rs114270719
rs73378348
rs192358062
rs117060393
rs28664709
rs2154134
rs150431583
rs12465274
rs11791533
rs140409890
rs115154867
rs74003350
rs1756748
rs114295337
rs77857056
rs116339570
rs77951387
rs116955542
rs2813890
All − MR Egger
All − IVW(MRE)
−0.005
0.000
0.005
MR effect size for
'Probable E3 ubiquitin−protein ligase HERC4' on 'eGFR'

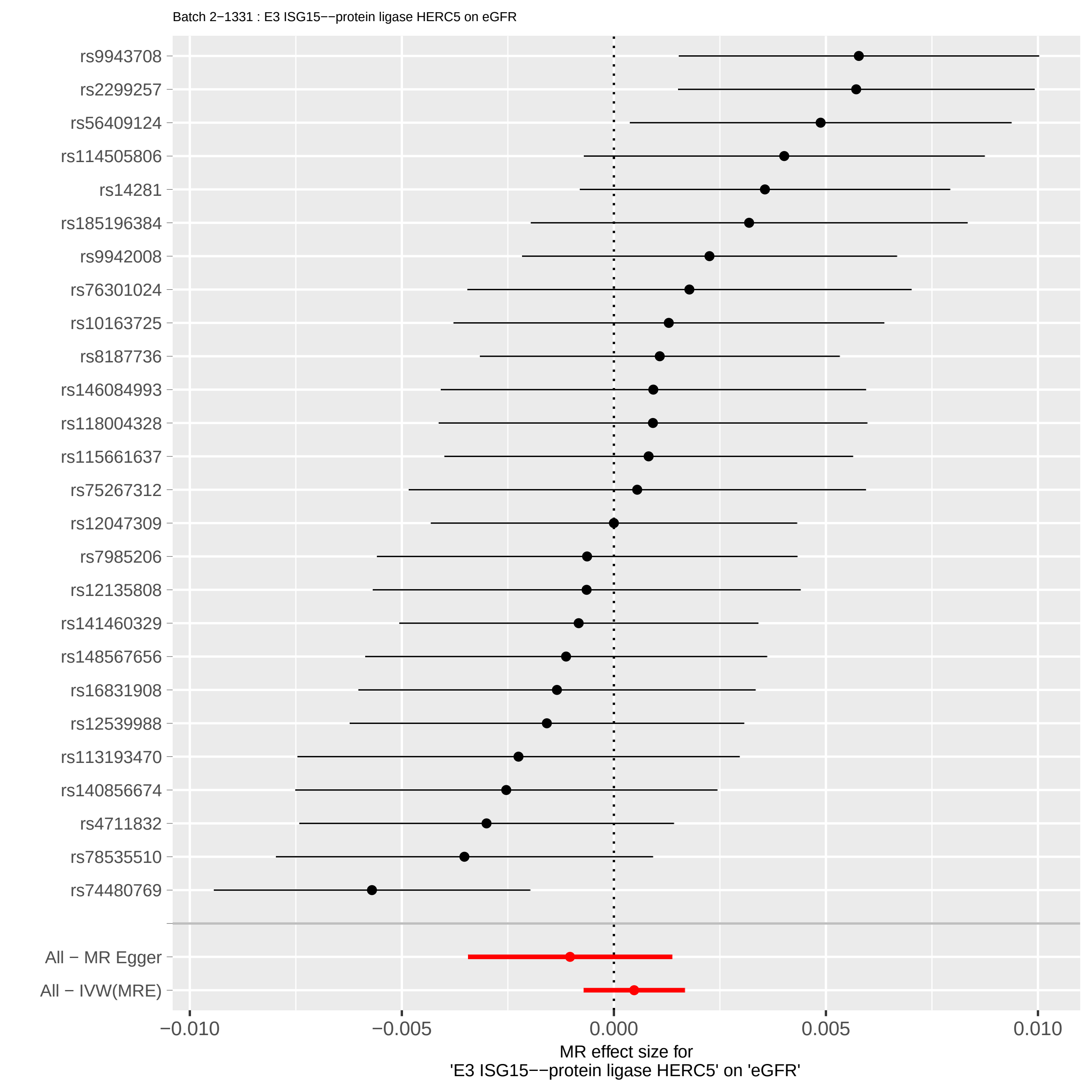

Batch 2−1331 : E3 ISG15−−protein ligase HERC5 on eGFR
rs9943708
rs2299257
rs56409124
rs114505806
rs14281
rs185196384
rs9942008
rs76301024
rs10163725
rs8187736
rs146084993
rs118004328
rs115661637
rs75267312
rs12047309
rs7985206
rs12135808
rs141460329
rs148567656
rs16831908
rs12539988
rs113193470
rs140856674
rs4711832
rs78535510
rs74480769
All − MR Egger
All − IVW(MRE)
−0.010
−0.005
0.000
0.005
0.010
MR effect size for
'E3 ISG15−−protein ligase HERC5' on 'eGFR'

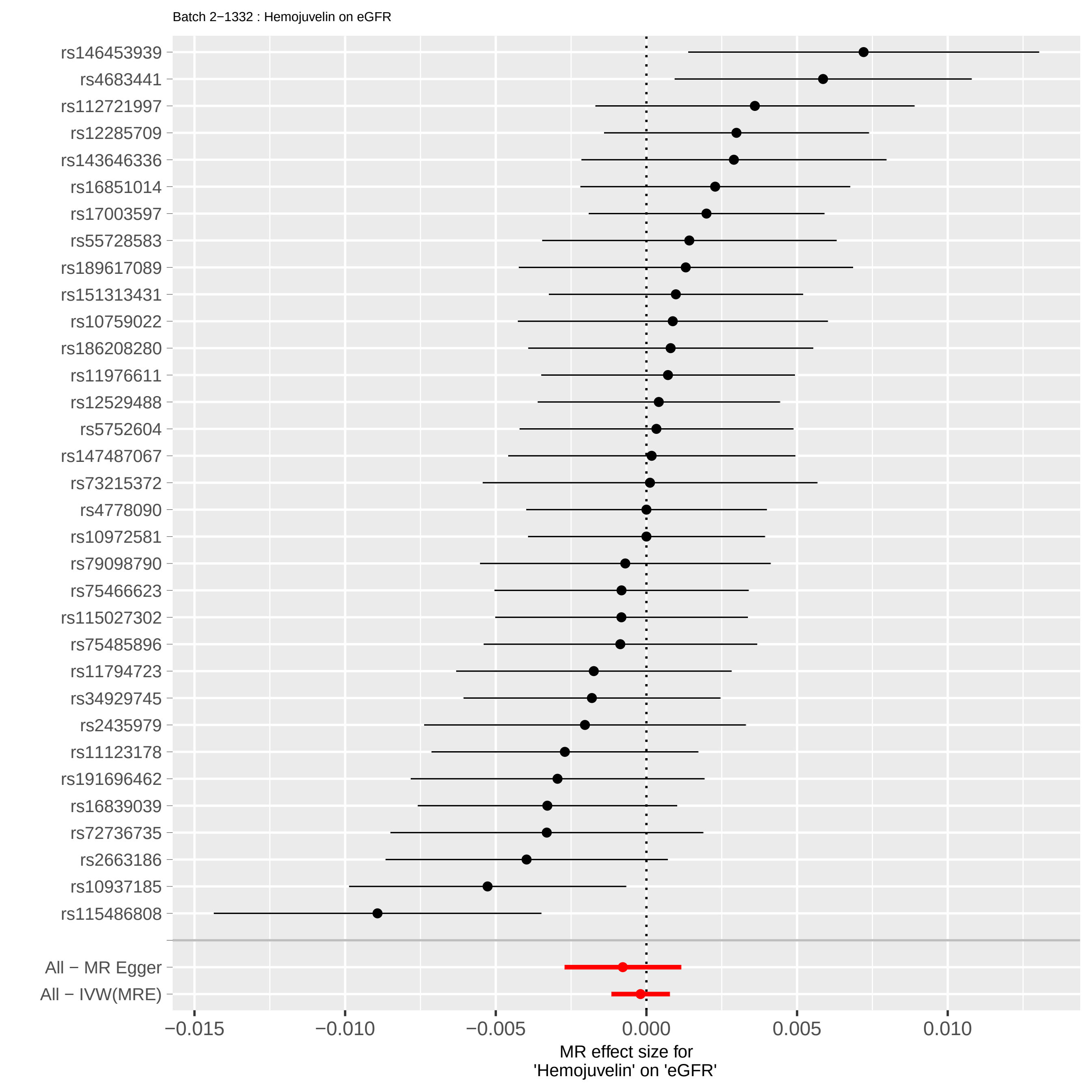

Batch 2−1332 : Hemojuvelin on eGFR
rs146453939
rs4683441
rs112721997
rs12285709
rs143646336
rs16851014
rs17003597
rs55728583
rs189617089
rs151313431
rs10759022
rs186208280
rs11976611
rs12529488
rs5752604
rs147487067
rs73215372
rs4778090
rs10972581
rs79098790
rs75466623
rs115027302
rs75485896
rs11794723
rs34929745
rs2435979
rs11123178
rs191696462
rs16839039
rs72736735
rs2663186
rs10937185
rs115486808
All − MR Egger
All − IVW(MRE)
−0.015
−0.010
−0.005
0.000
0.005
0.010
MR effect size for
'Hemojuvelin' on 'eGFR'

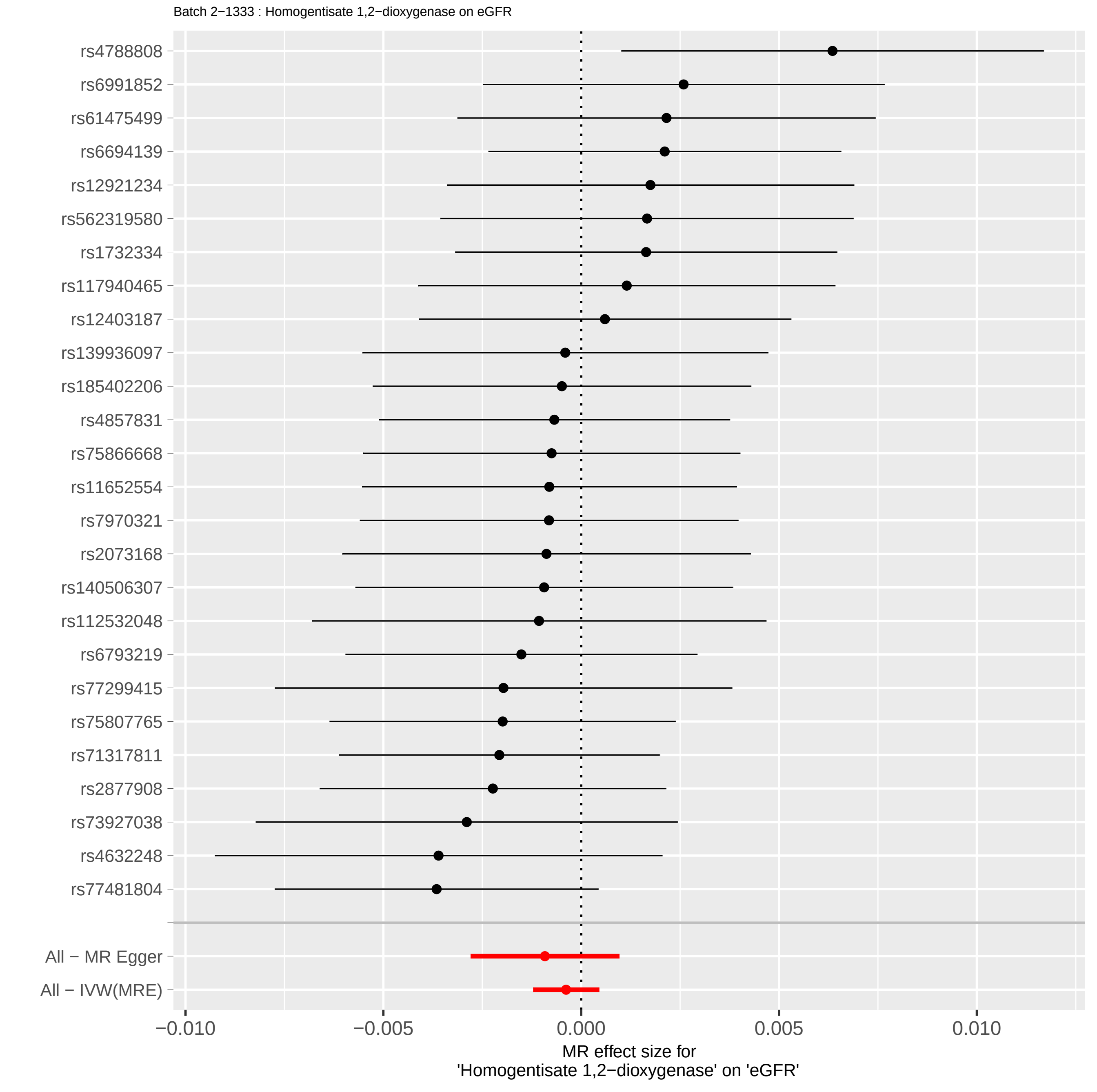

Batch 2−1333 : Homogentisate 1,2−dioxygenase on eGFR
rs4788808
rs6991852
rs61475499
rs6694139
rs12921234
rs562319580
rs1732334
rs117940465
rs12403187
rs139936097
rs185402206
rs4857831
rs75866668
rs11652554
rs7970321
rs2073168
rs140506307
rs112532048
rs6793219
rs77299415
rs75807765
rs71317811
rs2877908
rs73927038
rs4632248
rs77481804
All − MR Egger
All − IVW(MRE)
−0.010
−0.005
0.000
0.005
0.010
MR effect size for
'Homogentisate 1,2−dioxygenase' on 'eGFR'

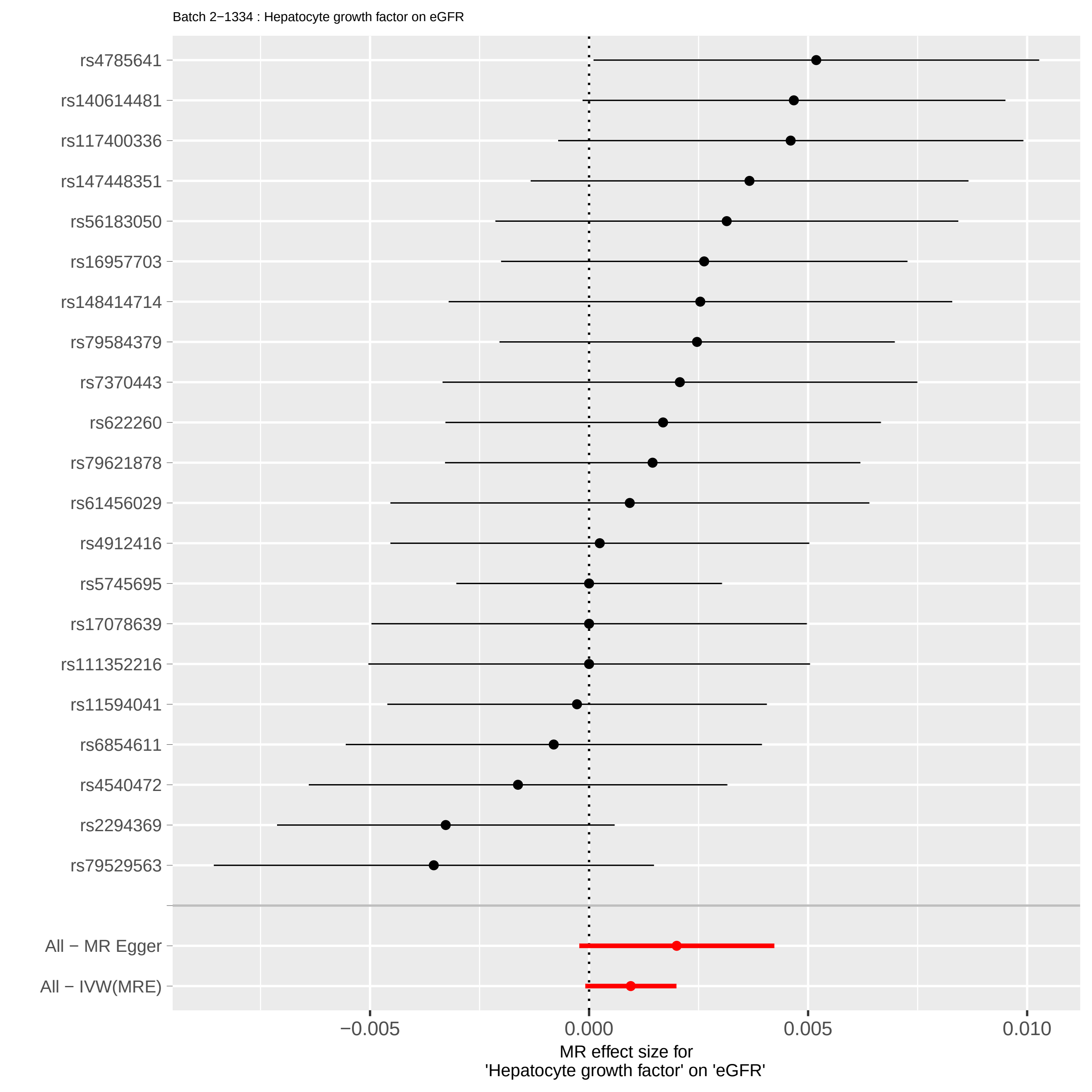

Batch 2−1334 : Hepatocyte growth factor on eGFR
rs4785641
rs140614481
rs117400336
rs147448351
rs56183050
rs16957703
rs148414714
rs79584379
rs7370443
rs622260
rs79621878
rs61456029
rs4912416
rs5745695
rs17078639
rs111352216
rs11594041
rs6854611
rs4540472
rs2294369
rs79529563
All − MR Egger
All − IVW(MRE)
−0.005
0.000
0.005
0.010
MR effect size for
'Hepatocyte growth factor' on 'eGFR'

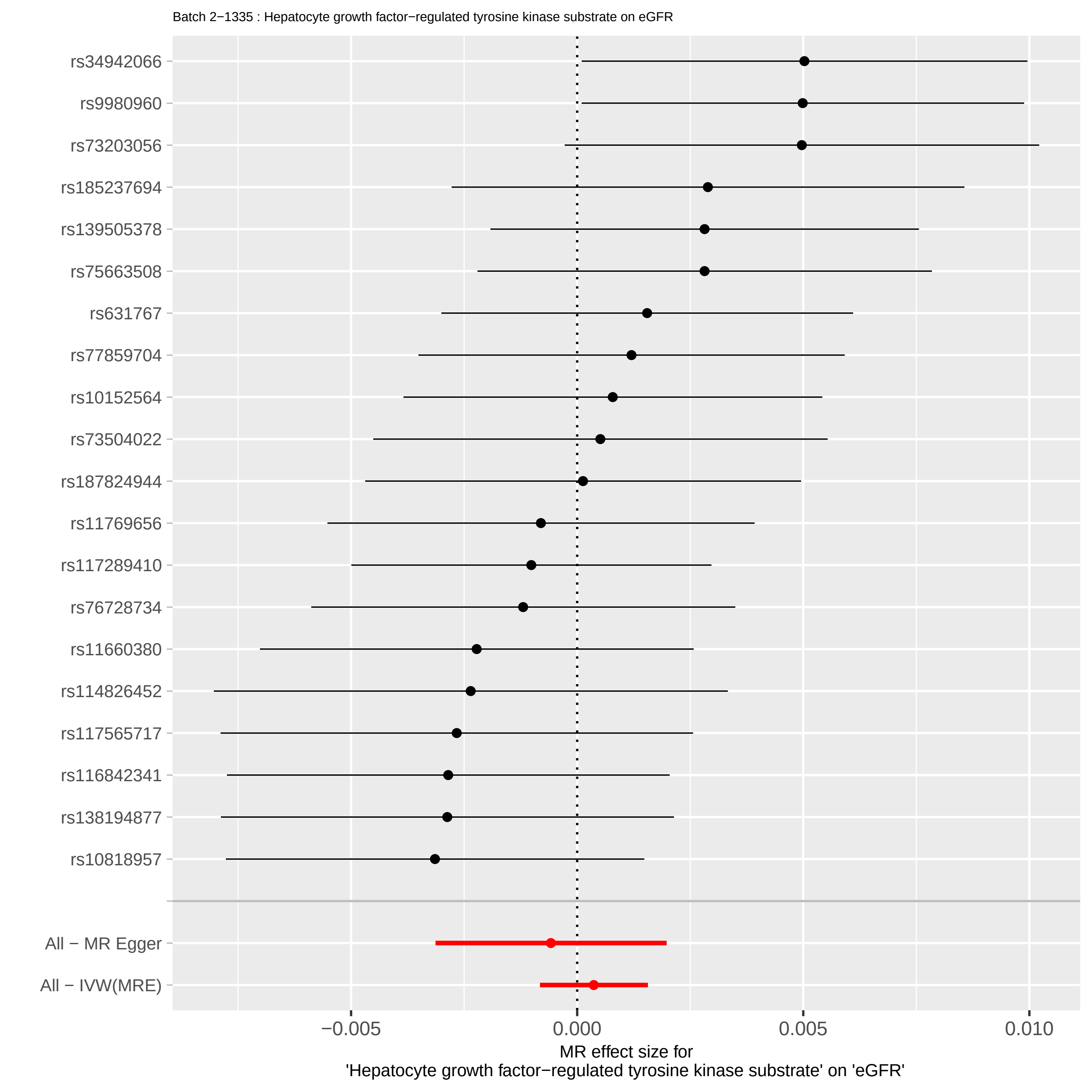

Batch 2−1335 : Hepatocyte growth factor−regulated tyrosine kinase substrate on eGFR
rs34942066
rs9980960
rs73203056
rs185237694
rs139505378
rs75663508
rs631767
rs77859704
rs10152564
rs73504022
rs187824944
rs11769656
rs117289410
rs76728734
rs11660380
rs114826452
rs117565717
rs116842341
rs138194877
rs10818957
All − MR Egger
All − IVW(MRE)
−0.005
0.000
0.005
0.010
MR effect size for
'Hepatocyte growth factor−regulated tyrosine kinase substrate' on 'eGFR'

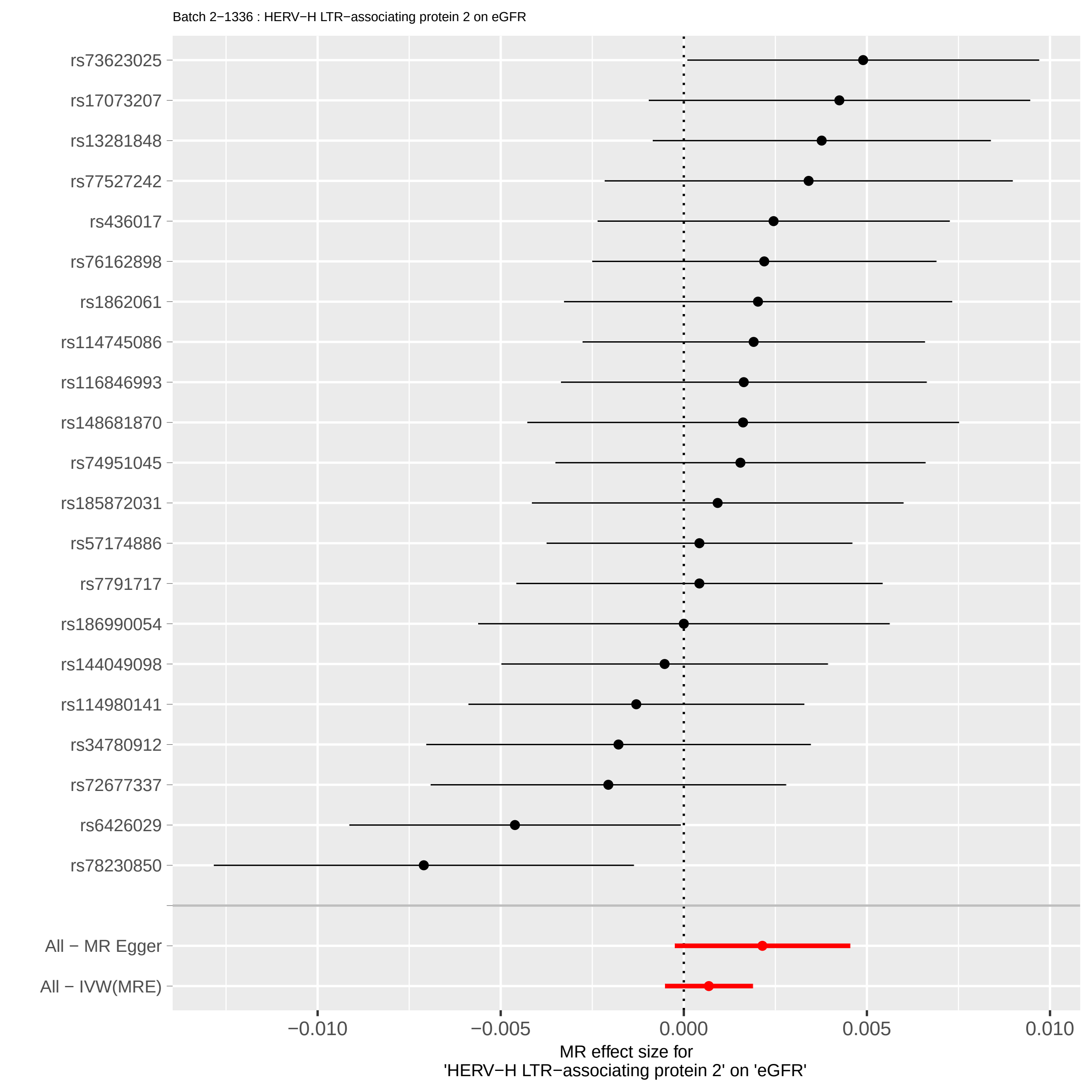

Batch 2−1336 : HERV−H LTR−associating protein 2 on eGFR
rs73623025
rs17073207
rs13281848
rs77527242
rs436017
rs76162898
rs1862061
rs114745086
rs116846993
rs148681870
rs74951045
rs185872031
rs57174886
rs7791717
rs186990054
rs144049098
rs114980141
rs34780912
rs72677337
rs6426029
rs78230850
All − MR Egger
All − IVW(MRE)
−0.010
−0.005
0.000
0.005
0.010
MR effect size for
'HERV−H LTR−associating protein 2' on 'eGFR'

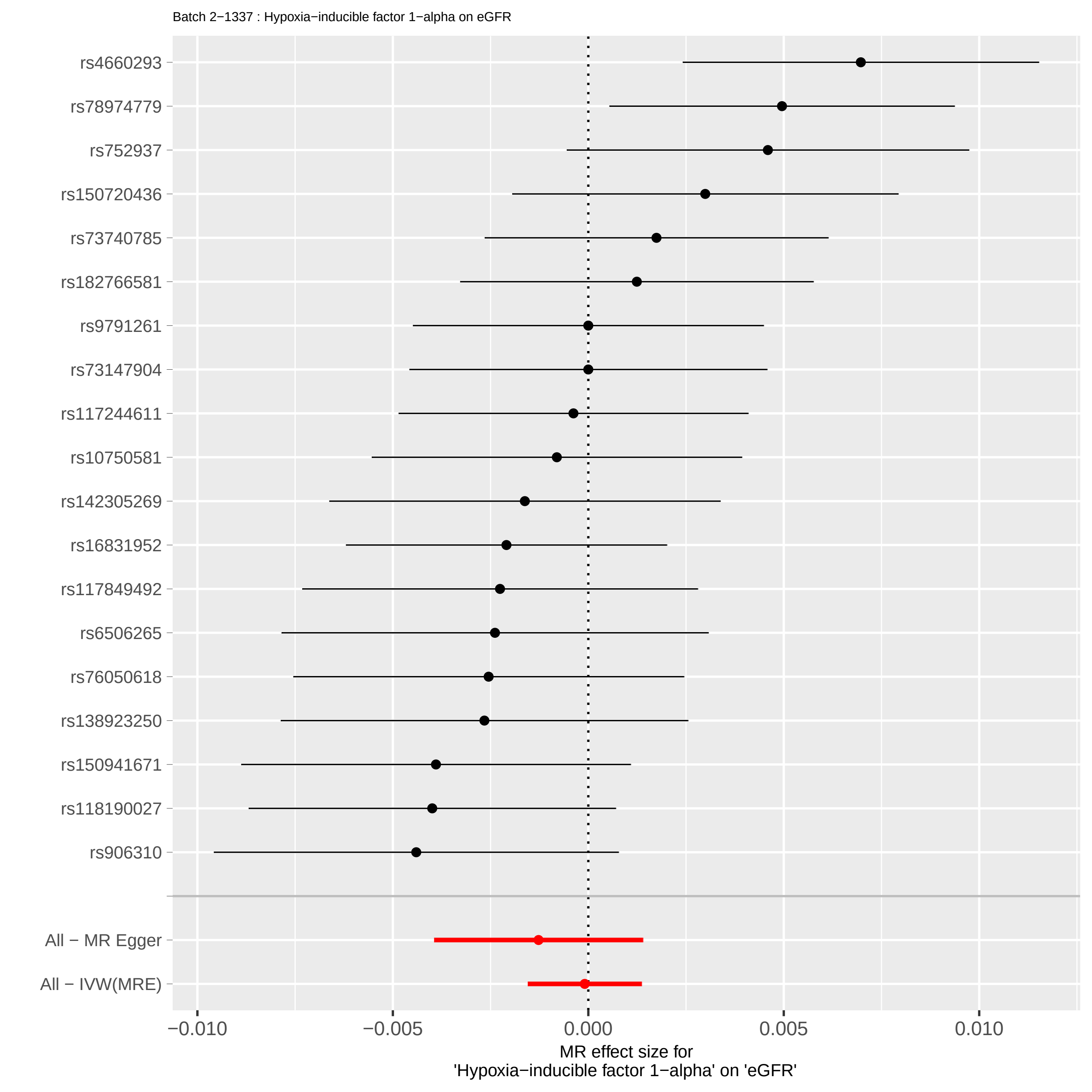

Batch 2−1337 : Hypoxia−inducible factor 1−alpha on eGFR
rs4660293
rs78974779
rs752937
rs150720436
rs73740785
rs182766581
rs9791261
rs73147904
rs117244611
rs10750581
rs142305269
rs16831952
rs117849492
rs6506265
rs76050618
rs138923250
rs150941671
rs118190027
rs906310
All − MR Egger
All − IVW(MRE)
−0.010
−0.005
0.000
0.005
0.010
MR effect size for
'Hypoxia−inducible factor 1−alpha' on 'eGFR'

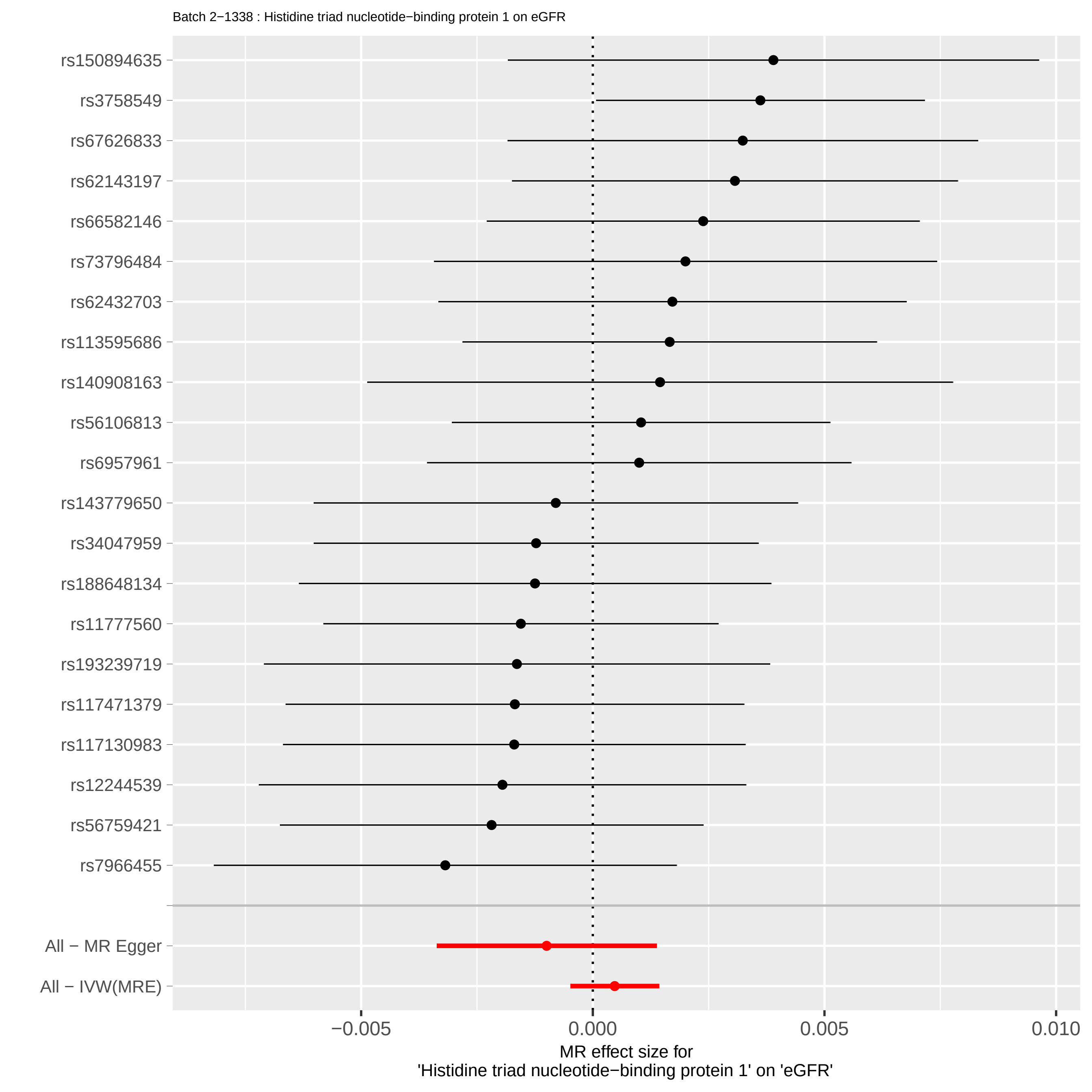
Batch 2−1338 : Histidine triad nucleotide−binding protein 1 on eGFR
rs150894635
rs3758549
rs67626833
rs62143197
rs66582146
rs73796484
rs62432703
rs113595686
rs140908163
rs56106813
rs6957961
rs143779650
rs34047959
rs188648134
rs11777560
rs193239719
rs117471379
rs117130983
rs12244539
rs56759421
rs7966455
All − MR Egger
All − IVW(MRE)
−0.005
0.000
0.005
0.010
MR effect size for
'Histidine triad nucleotide−binding protein 1' on 'eGFR'

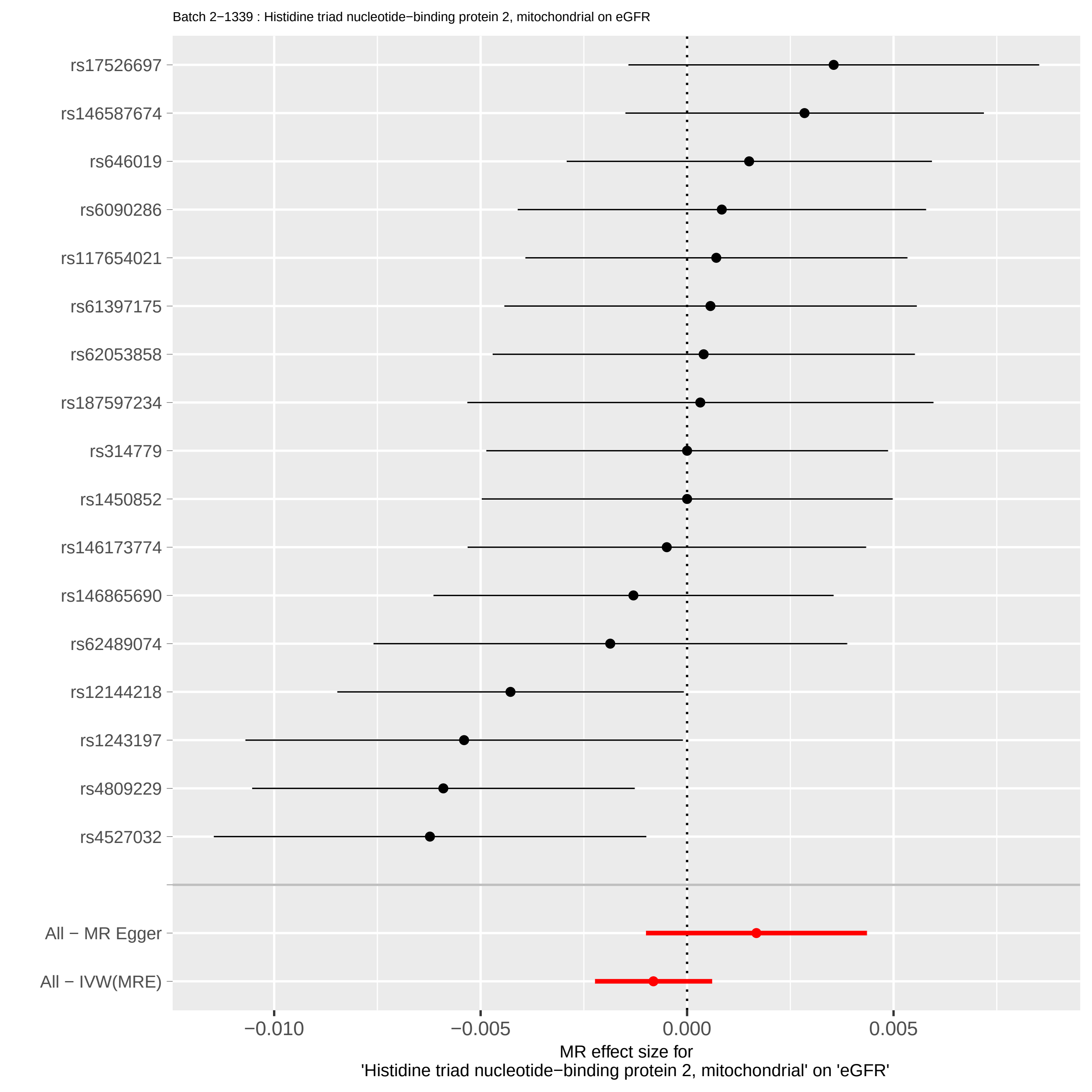
Batch 2−1339 : Histidine triad nucleotide−binding protein 2, mitochondrial on eGFR
rs17526697
rs146587674
rs646019
rs6090286
rs117654021
rs61397175
rs62053858
rs187597234
rs314779
rs1450852
rs146173774
rs146865690
rs62489074
rs12144218
rs1243197
rs4809229
rs4527032
All − MR Egger
All − IVW(MRE)
−0.010
−0.005
0.000
0.005
MR effect size for
'Histidine triad nucleotide−binding protein 2, mitochondrial' on 'eGFR'

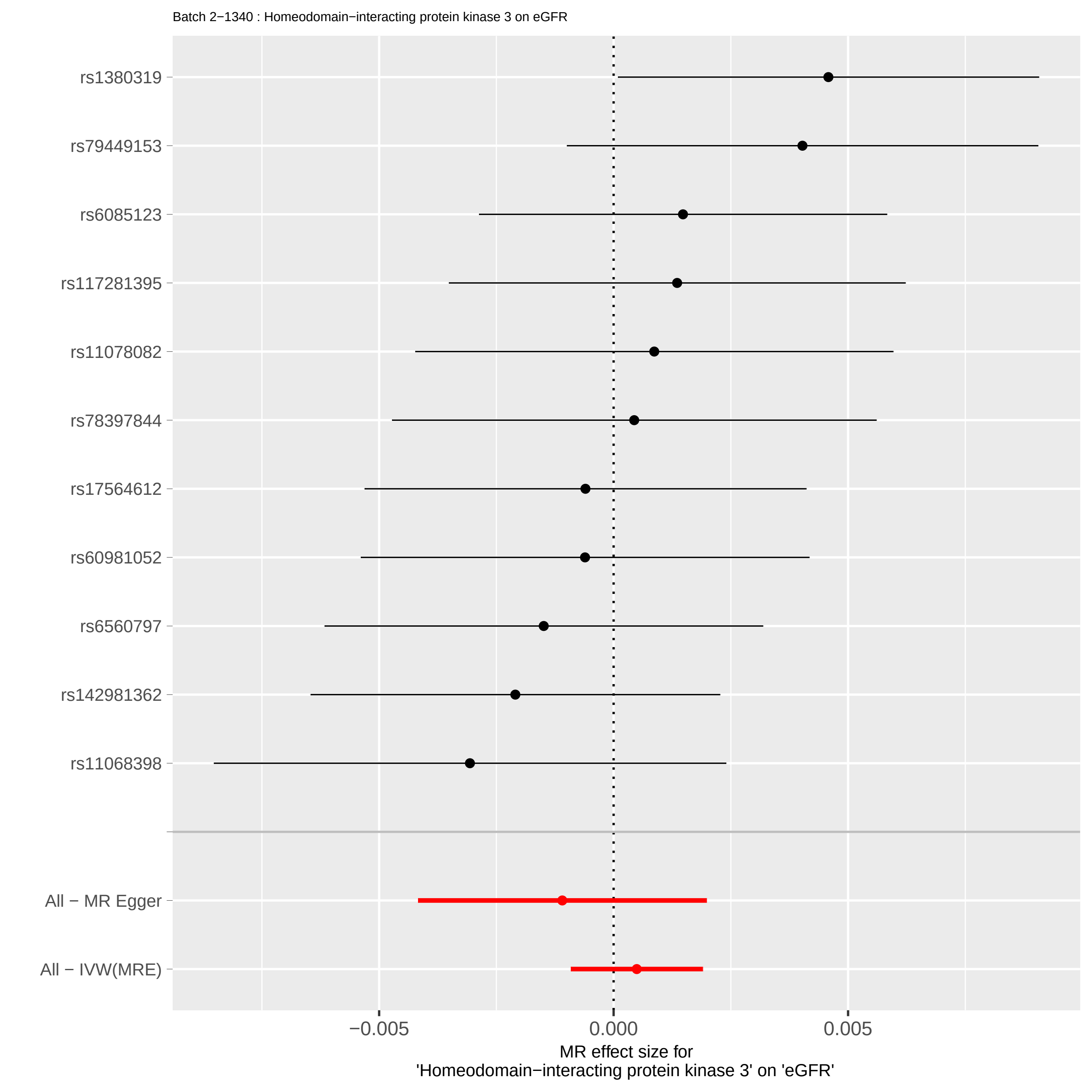

Batch 2−1340 : Homeodomain−interacting protein kinase 3 on eGFR
rs1380319
rs79449153
rs6085123
rs117281395
rs11078082
rs78397844
rs17564612
rs60981052
rs6560797
rs142981362
rs11068398
All − MR Egger
All − IVW(MRE)
−0.005
0.000
0.005
MR effect size for
'Homeodomain−interacting protein kinase 3' on 'eGFR'

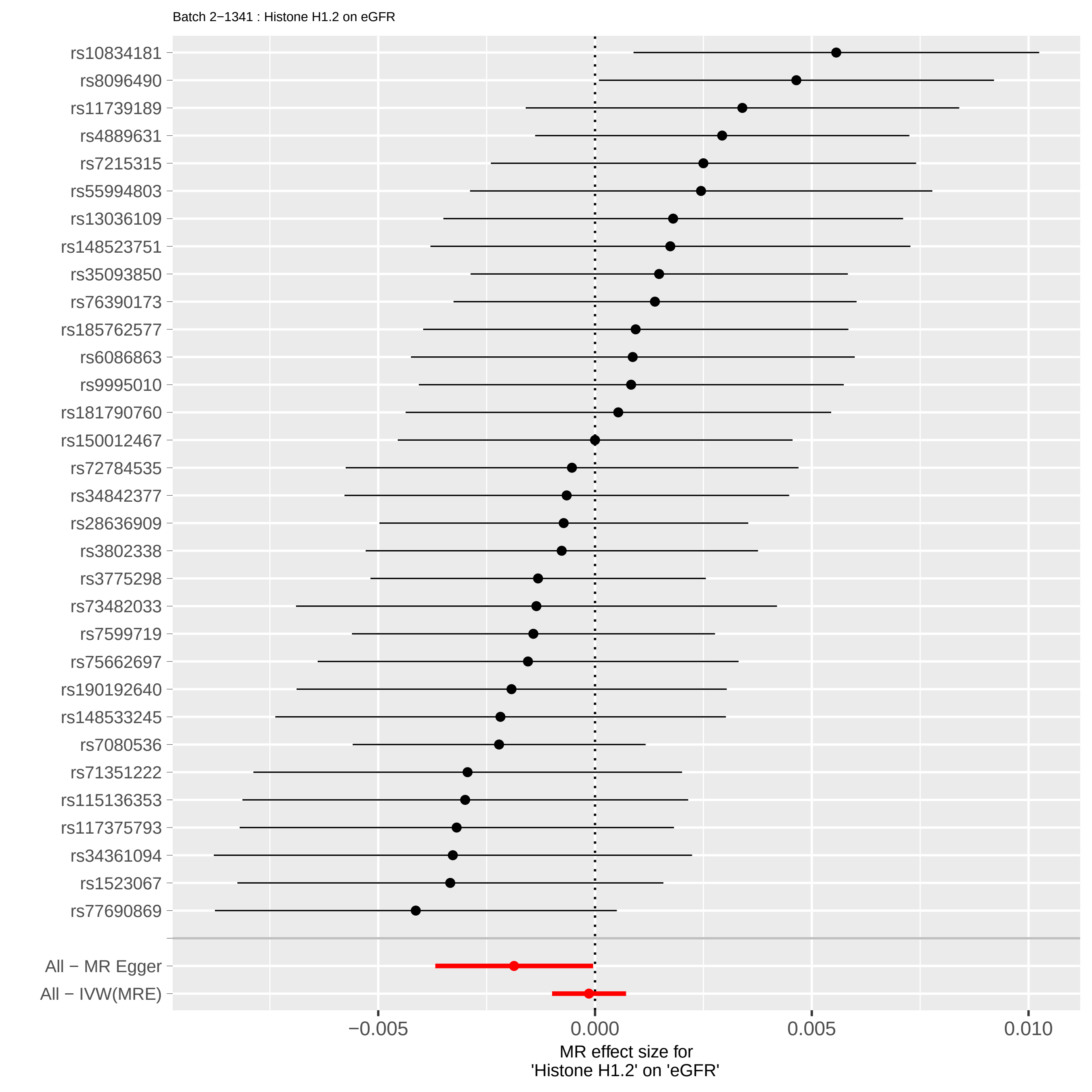
Batch 2−1341 : Histone H1.2 on eGFR
rs10834181
rs8096490
rs11739189
rs4889631
rs7215315
rs55994803
rs13036109
rs148523751
rs35093850
rs76390173
rs185762577
rs6086863
rs9995010
rs181790760
rs150012467
rs72784535
rs34842377
rs28636909
rs3802338
rs3775298
rs73482033
rs7599719
rs75662697
rs190192640
rs148533245
rs7080536
rs71351222
rs115136353
rs117375793
rs34361094
rs1523067
rs77690869
All − MR Egger
All − IVW(MRE)
−0.005
0.000
0.005
0.010
MR effect size for
'Histone H1.2' on 'eGFR'

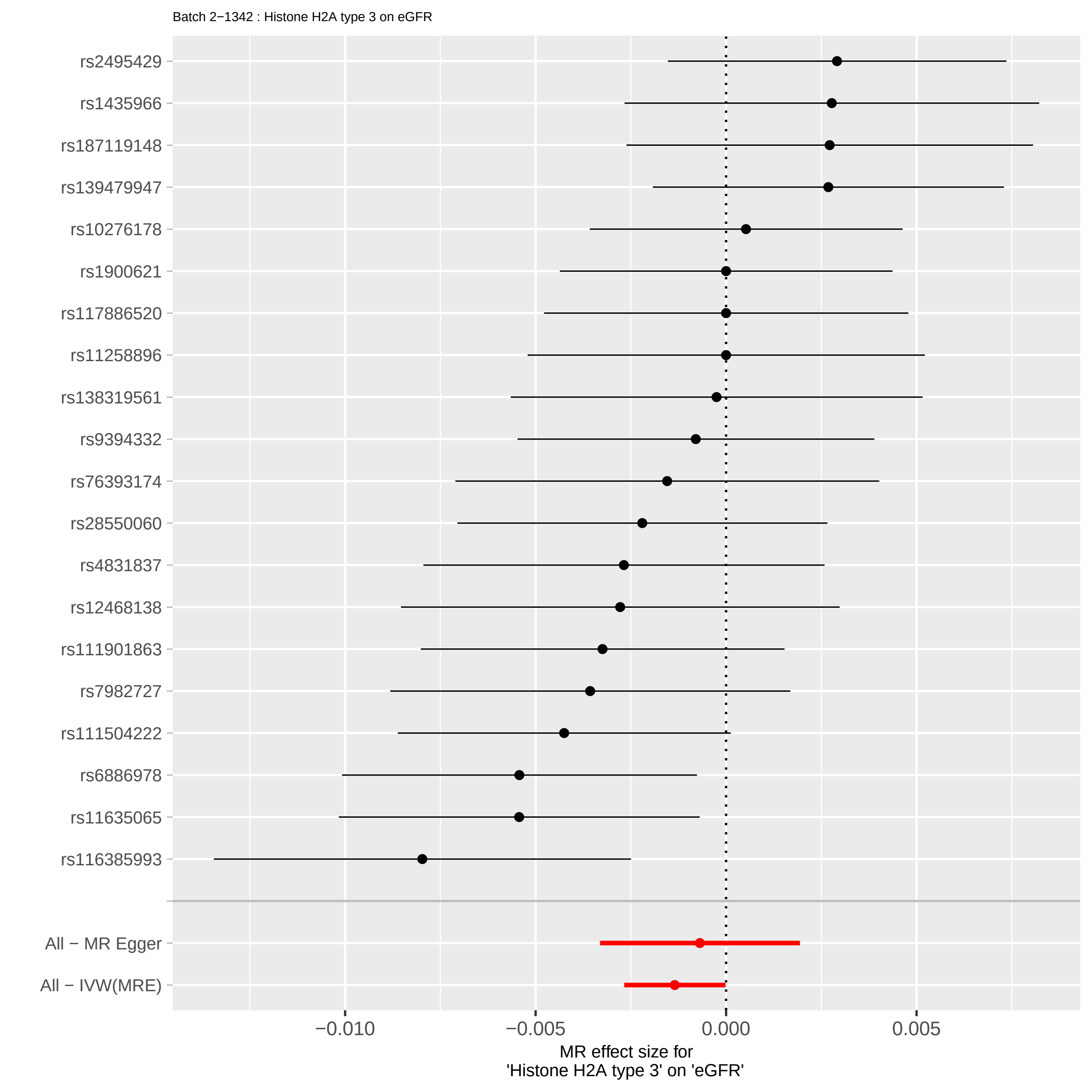

Batch 2−1342 : Histone H2A type 3 on eGFR
rs2495429
rs1435966
rs187119148
rs139479947
rs10276178
rs1900621
rs117886520
rs11258896
rs138319561
rs9394332
rs76393174
rs28550060
rs4831837
rs12468138
rs111901863
rs7982727
rs111504222
rs6886978
rs11635065
rs116385993
All − MR Egger
All − IVW(MRE)
−0.010
−0.005
0.000
0.005
MR effect size for
'Histone H2A type 3' on 'eGFR'

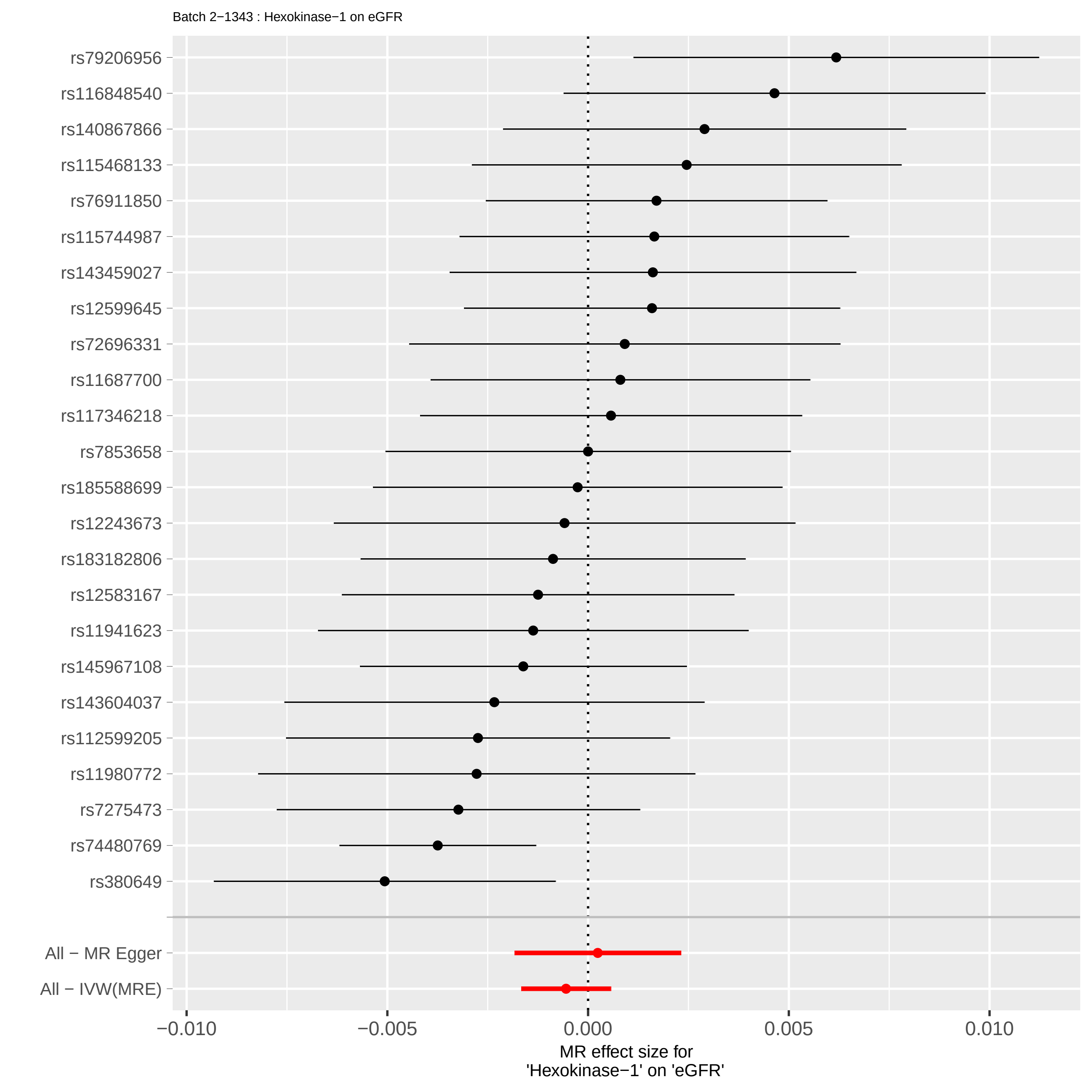

Batch 2−1343 : Hexokinase−1 on eGFR
rs79206956
rs116848540
rs140867866
rs115468133
rs76911850
rs115744987
rs143459027
rs12599645
rs72696331
rs11687700
rs117346218
rs7853658
rs185588699
rs12243673
rs183182806
rs12583167
rs11941623
rs145967108
rs143604037
rs112599205
rs11980772
rs7275473
rs74480769
rs380649
All − MR Egger
All − IVW(MRE)
−0.010
−0.005
0.000
0.005
0.010
MR effect size for
'Hexokinase−1' on 'eGFR'

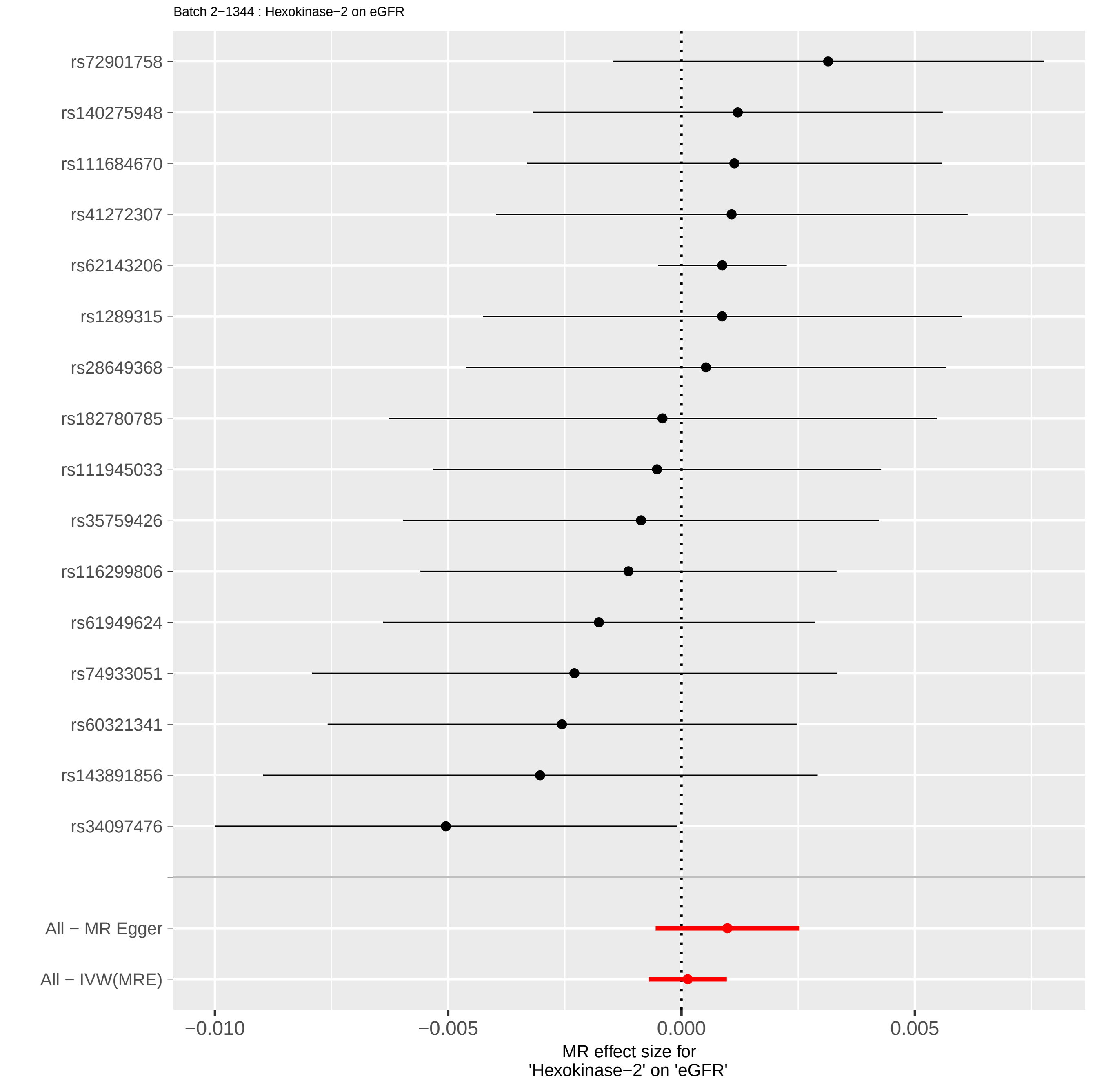

Batch 2−1344 : Hexokinase−2 on eGFR
rs72901758
rs140275948
rs111684670
rs41272307
rs62143206
rs1289315
rs28649368
rs182780785
rs111945033
rs35759426
rs116299806
rs61949624
rs74933051
rs60321341
rs143891856
rs34097476
All − MR Egger
All − IVW(MRE)
−0.010
−0.005
0.000
0.005
MR effect size for
'Hexokinase−2' on 'eGFR'

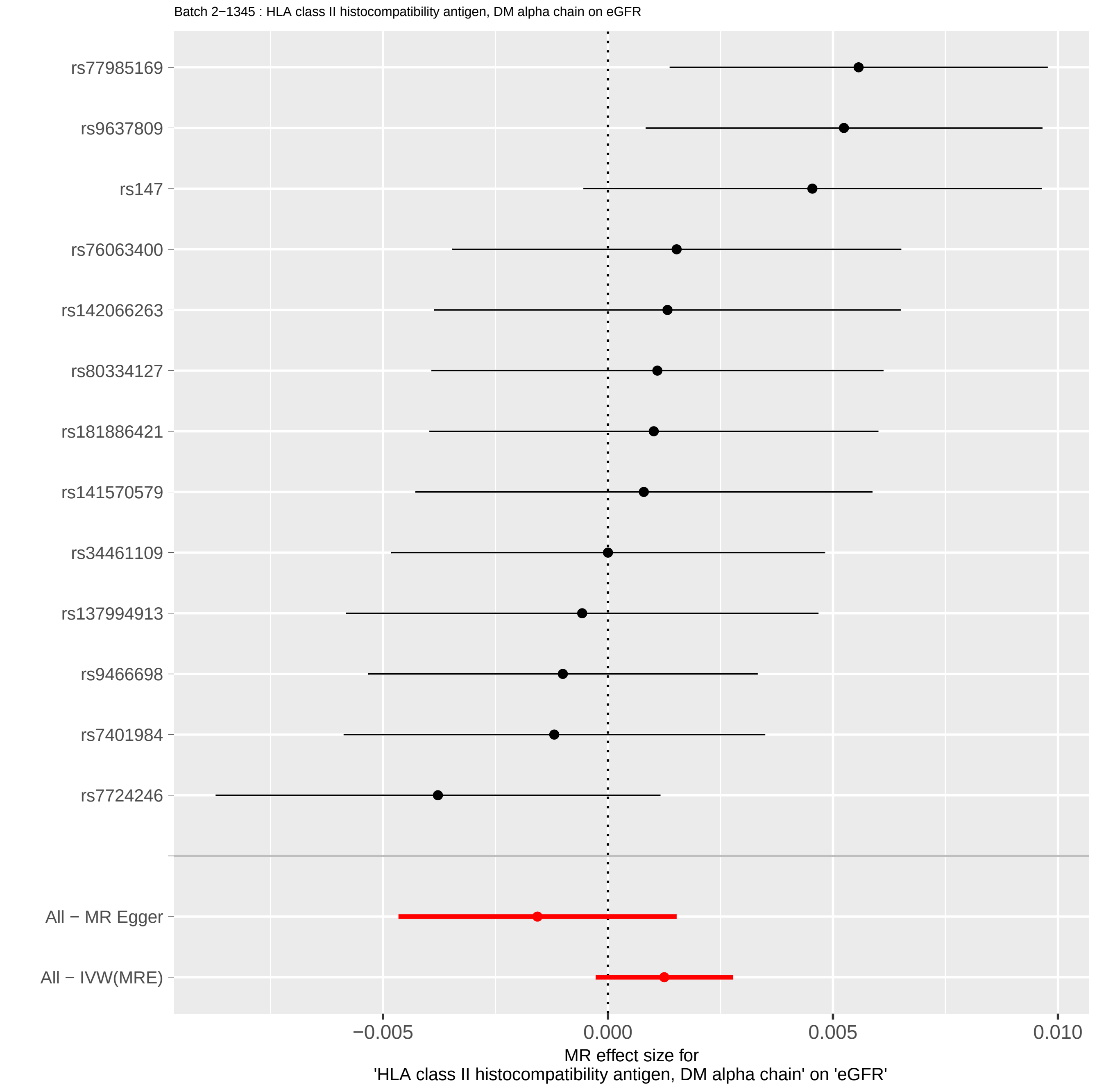

Batch 2−1345 : HLA class II histocompatibility antigen, DM alpha chain on eGFR
rs77985169
rs9637809
rs147
rs76063400
rs142066263
rs80334127
rs181886421
rs141570579
rs34461109
rs137994913
rs9466698
rs7401984
rs7724246
All − MR Egger
All − IVW(MRE)
−0.005
0.000
0.005
0.010
MR effect size for
'HLA class II histocompatibility antigen, DM alpha chain' on 'eGFR'

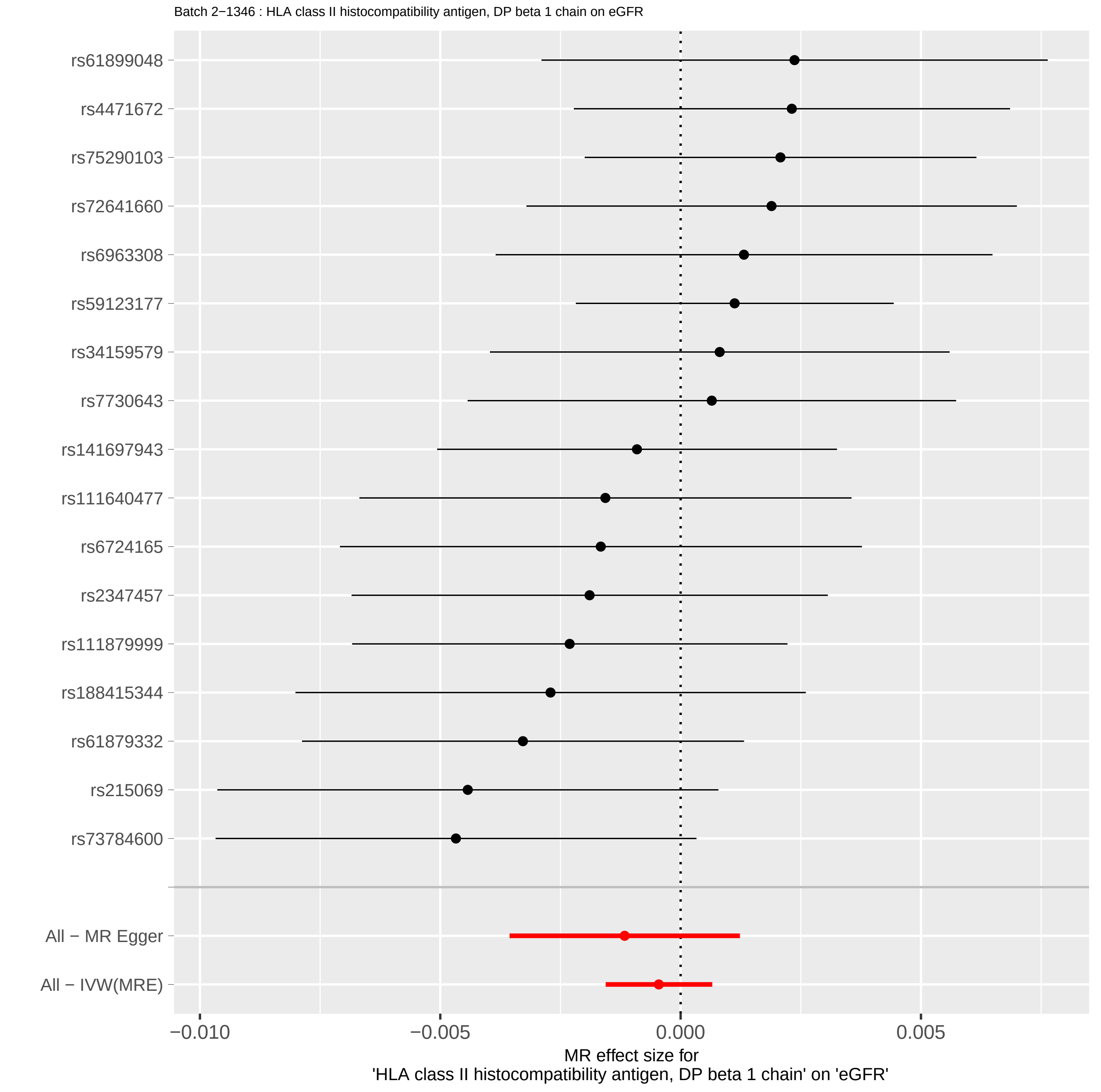

Batch 2−1346 : HLA class II histocompatibility antigen, DP beta 1 chain on eGFR
rs61899048
rs4471672
rs75290103
rs72641660
rs6963308
rs59123177
rs34159579
rs7730643
rs141697943
rs111640477
rs6724165
rs2347457
rs111879999
rs188415344
rs61879332
rs215069
rs73784600
All − MR Egger
All − IVW(MRE)
−0.010
−0.005
0.000
0.005
MR effect size for
'HLA class II histocompatibility antigen, DP beta 1 chain' on 'eGFR'

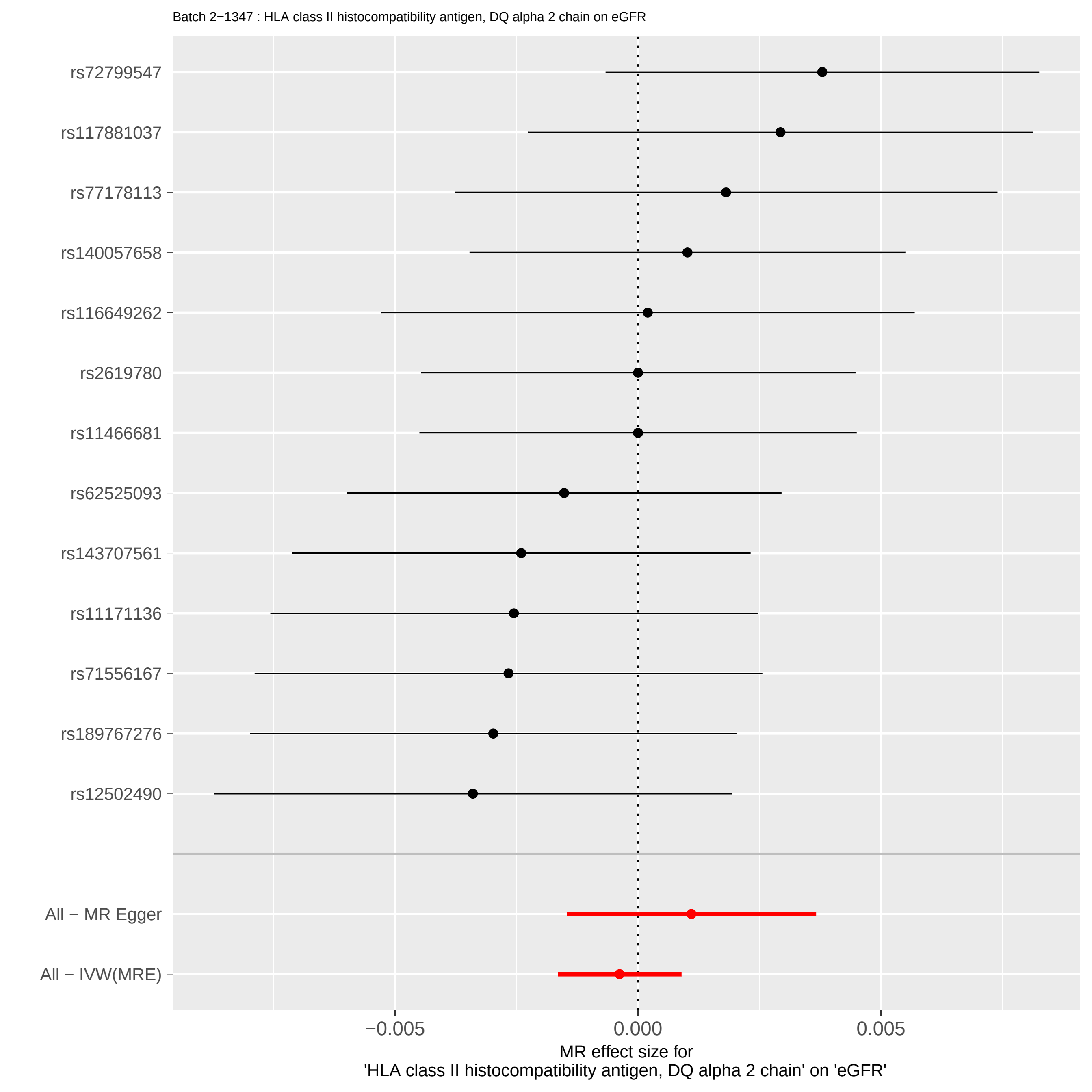
Batch 2−1347 : HLA class II histocompatibility antigen, DQ alpha 2 chain on eGFR
rs72799547
rs117881037
rs77178113
rs140057658
rs116649262
rs2619780
rs11466681
rs62525093
rs143707561
rs11171136
rs71556167
rs189767276
rs12502490
All − MR Egger
All − IVW(MRE)
−0.005
0.000
0.005
MR effect size for
'HLA class II histocompatibility antigen, DQ alpha 2 chain' on 'eGFR'

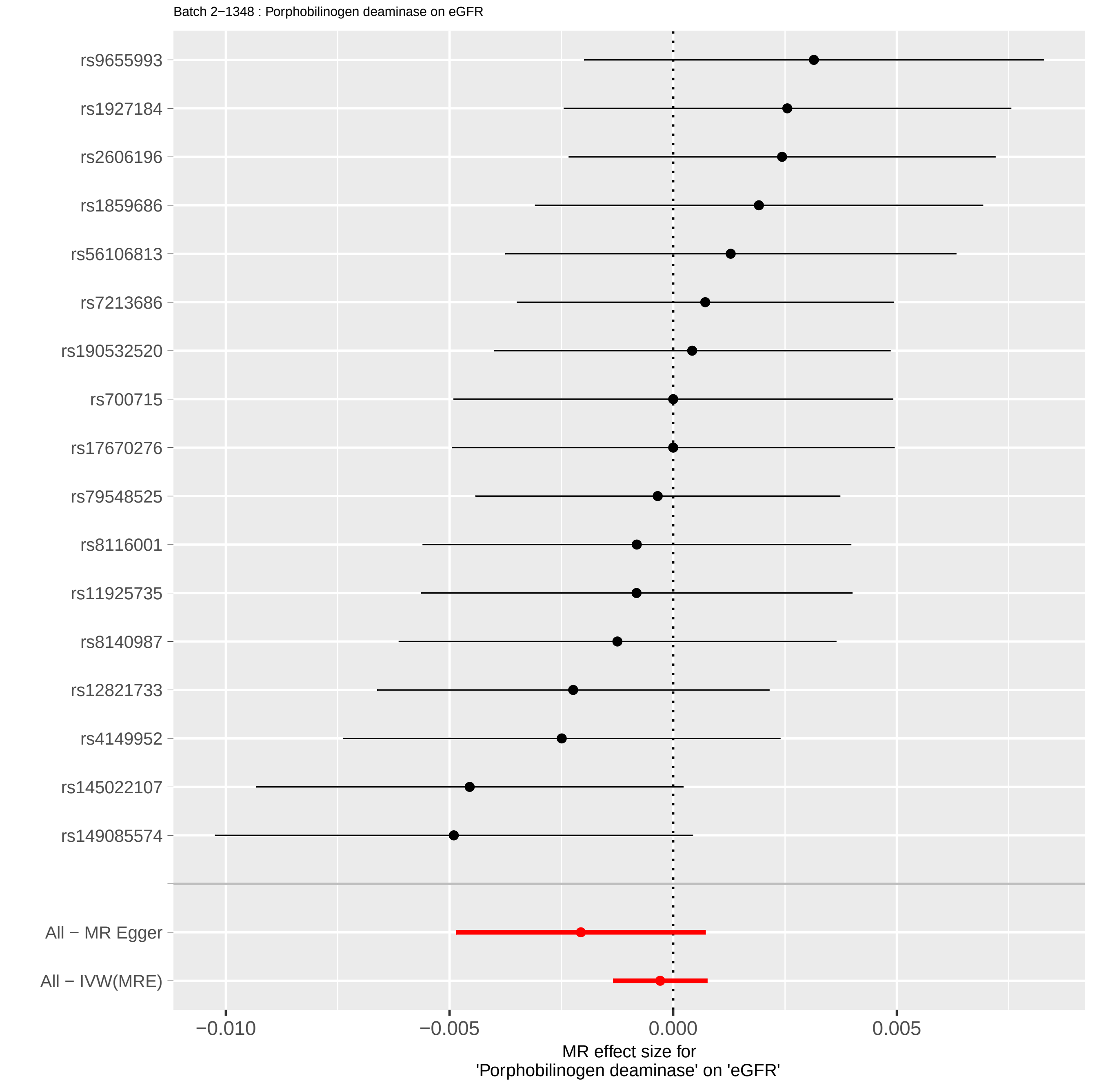

Batch 2−1348 : Porphobilinogen deaminase on eGFR
rs9655993
rs1927184
rs2606196
rs1859686
rs56106813
rs7213686
rs190532520
rs700715
rs17670276
rs79548525
rs8116001
rs11925735
rs8140987
rs12821733
rs4149952
rs145022107
rs149085574
All − MR Egger
All − IVW(MRE)
−0.010
−0.005
0.000
0.005
MR effect size for
'Porphobilinogen deaminase' on 'eGFR'

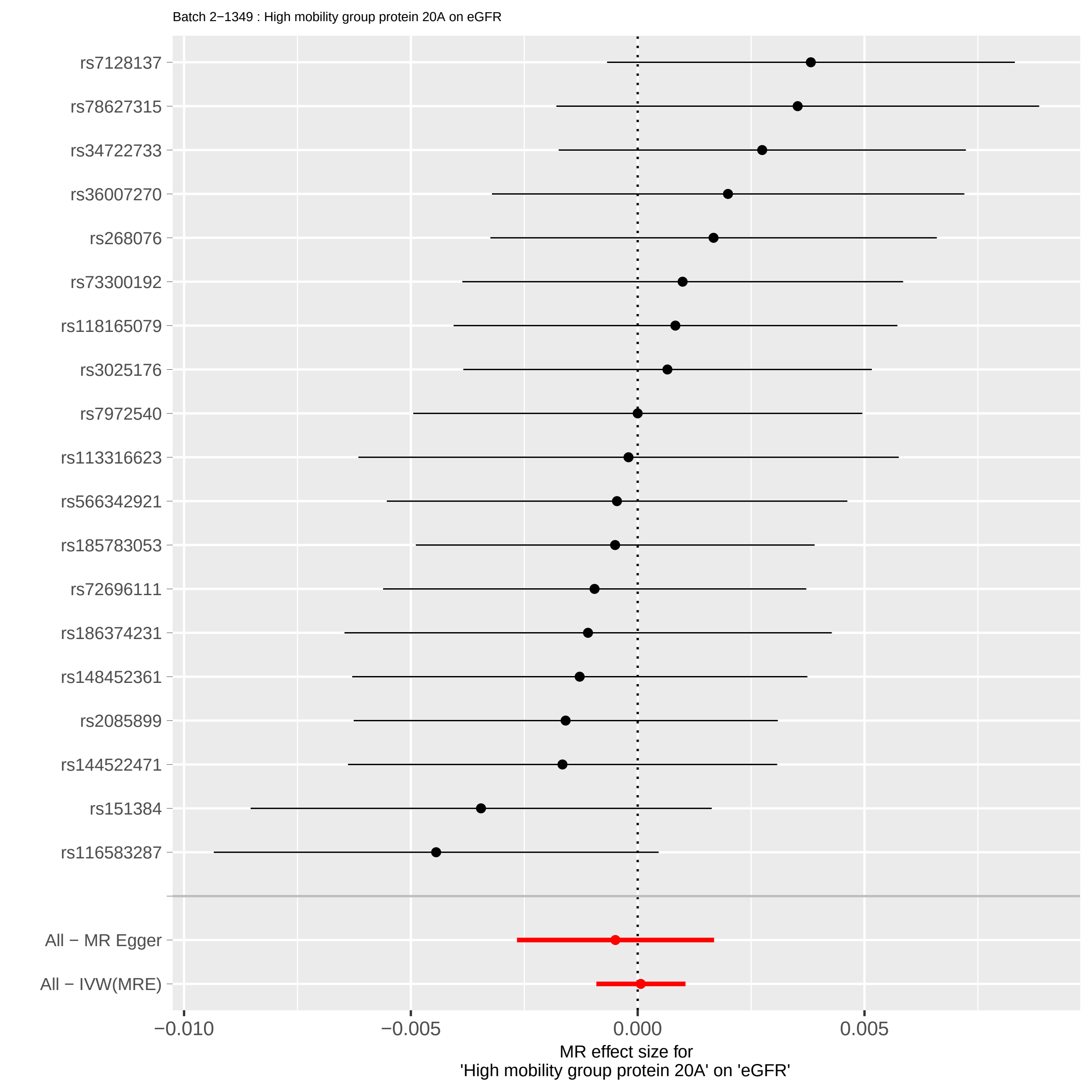

Batch 2−1349 : High mobility group protein 20A on eGFR
rs7128137
rs78627315
rs34722733
rs36007270
rs268076
rs73300192
rs118165079
rs3025176
rs7972540
rs113316623
rs566342921
rs185783053
rs72696111
rs186374231
rs148452361
rs2085899
rs144522471
rs151384
rs116583287
All − MR Egger
All − IVW(MRE)
−0.010
−0.005
0.000
0.005
MR effect size for
'High mobility group protein 20A' on 'eGFR'

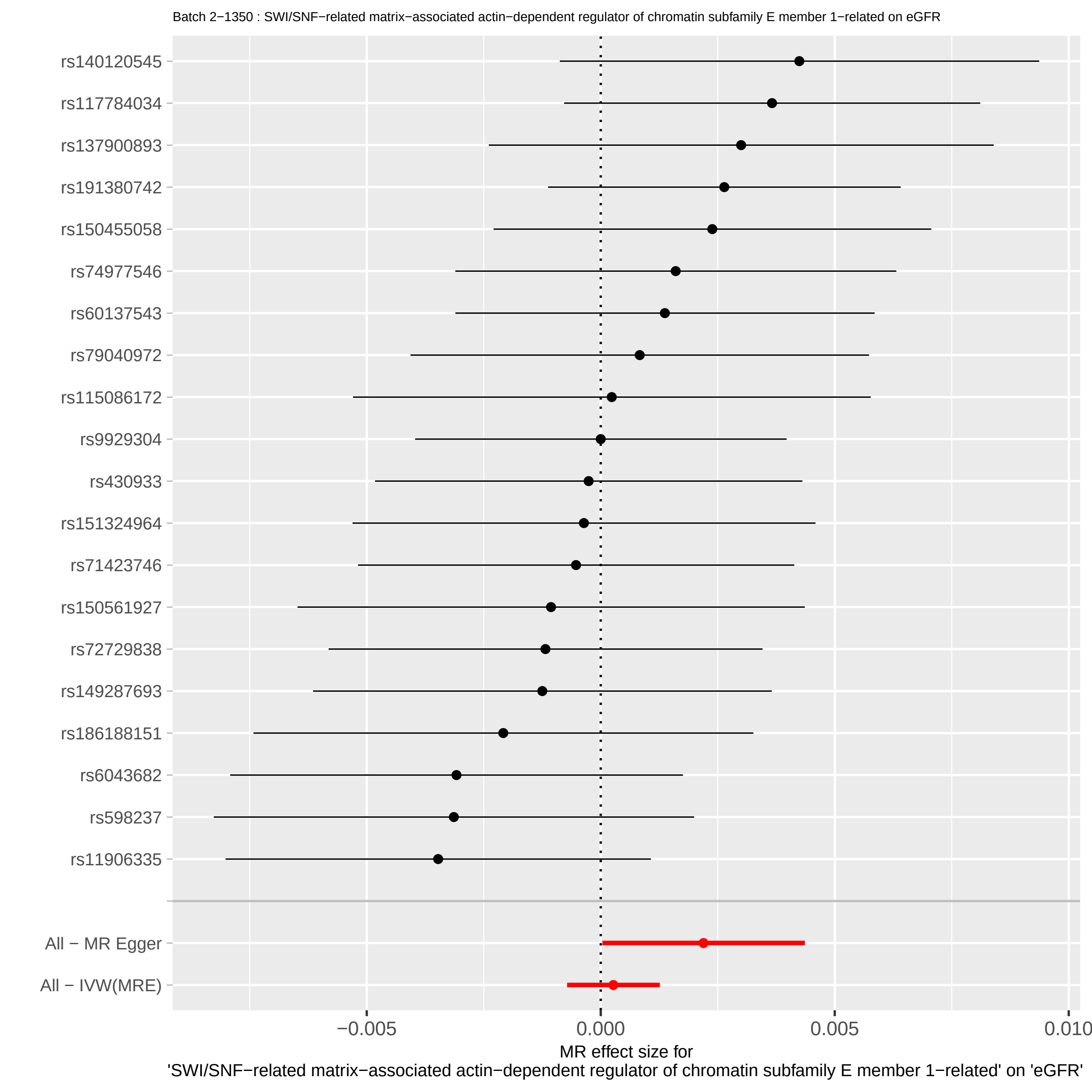
Batch 2−1350 : SWI/SNF−related matrix−associated actin−dependent regulator of chromatin subfamily E member 1−related on eGFR
rs140120545
rs117784034
rs137900893
rs191380742
rs150455058
rs74977546
rs60137543
rs79040972
rs115086172
rs9929304
rs430933
rs151324964
rs71423746
rs150561927
rs72729838
rs149287693
rs186188151
rs6043682
rs598237
rs11906335
All − MR Egger
All − IVW(MRE)
−0.005
0.000
0.005
0.010
MR effect size for
'SWI/SNF−related matrix−associated actin−dependent regulator of chromatin subfamily E member 1−related' on 'eGFR'

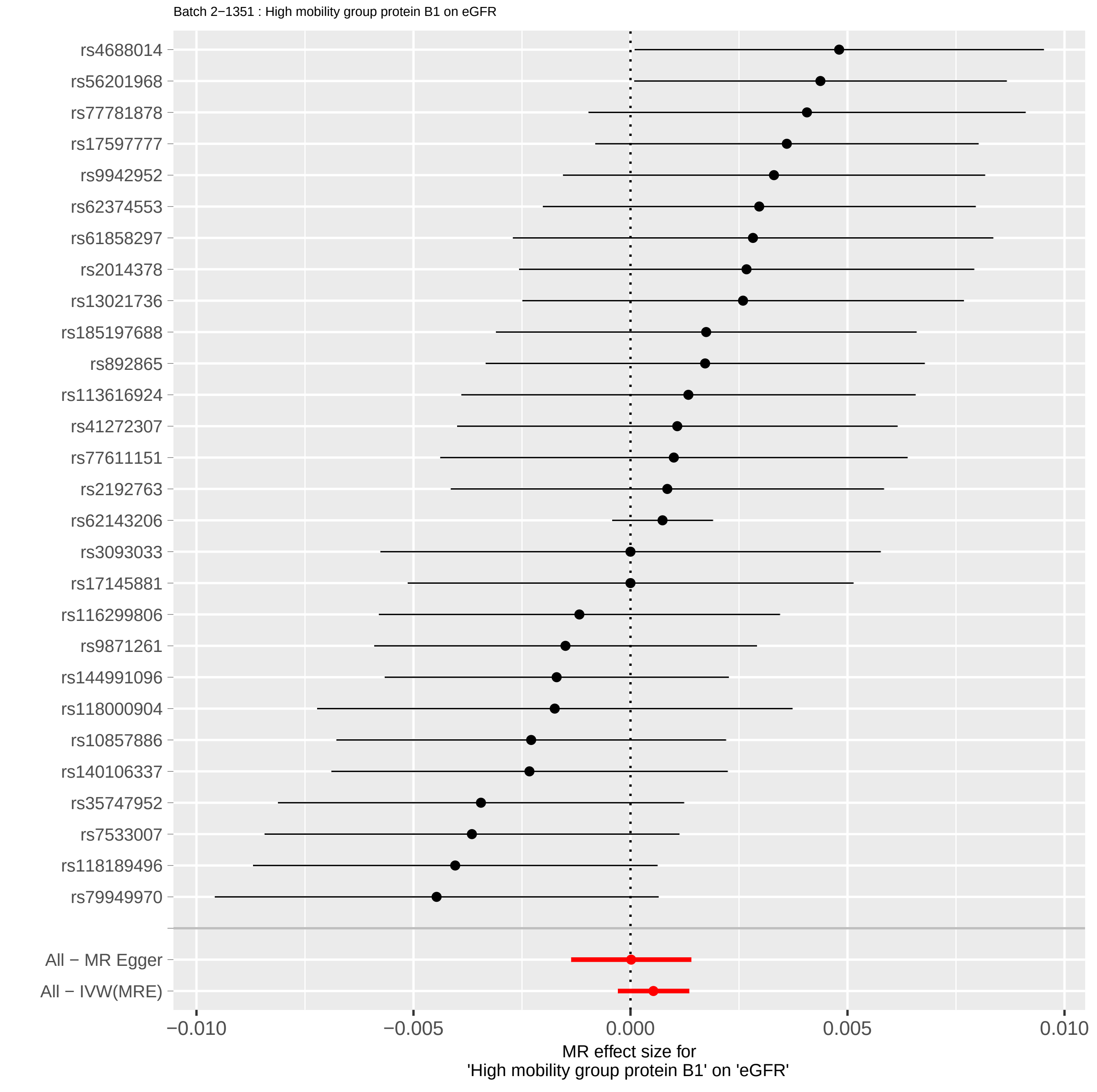

Batch 2−1351 : High mobility group protein B1 on eGFR
rs4688014
rs56201968
rs77781878
rs17597777
rs9942952
rs62374553
rs61858297
rs2014378
rs13021736
rs185197688
rs892865
rs113616924
rs41272307
rs77611151
rs2192763
rs62143206
rs3093033
rs17145881
rs116299806
rs9871261
rs144991096
rs118000904
rs10857886
rs140106337
rs35747952
rs7533007
rs118189496
rs79949970
All − MR Egger
All − IVW(MRE)
−0.010
−0.005
0.000
0.005
0.010
MR effect size for
'High mobility group protein B1' on 'eGFR'

Batch 2−1352 : High mobility group protein B2 on eGFR

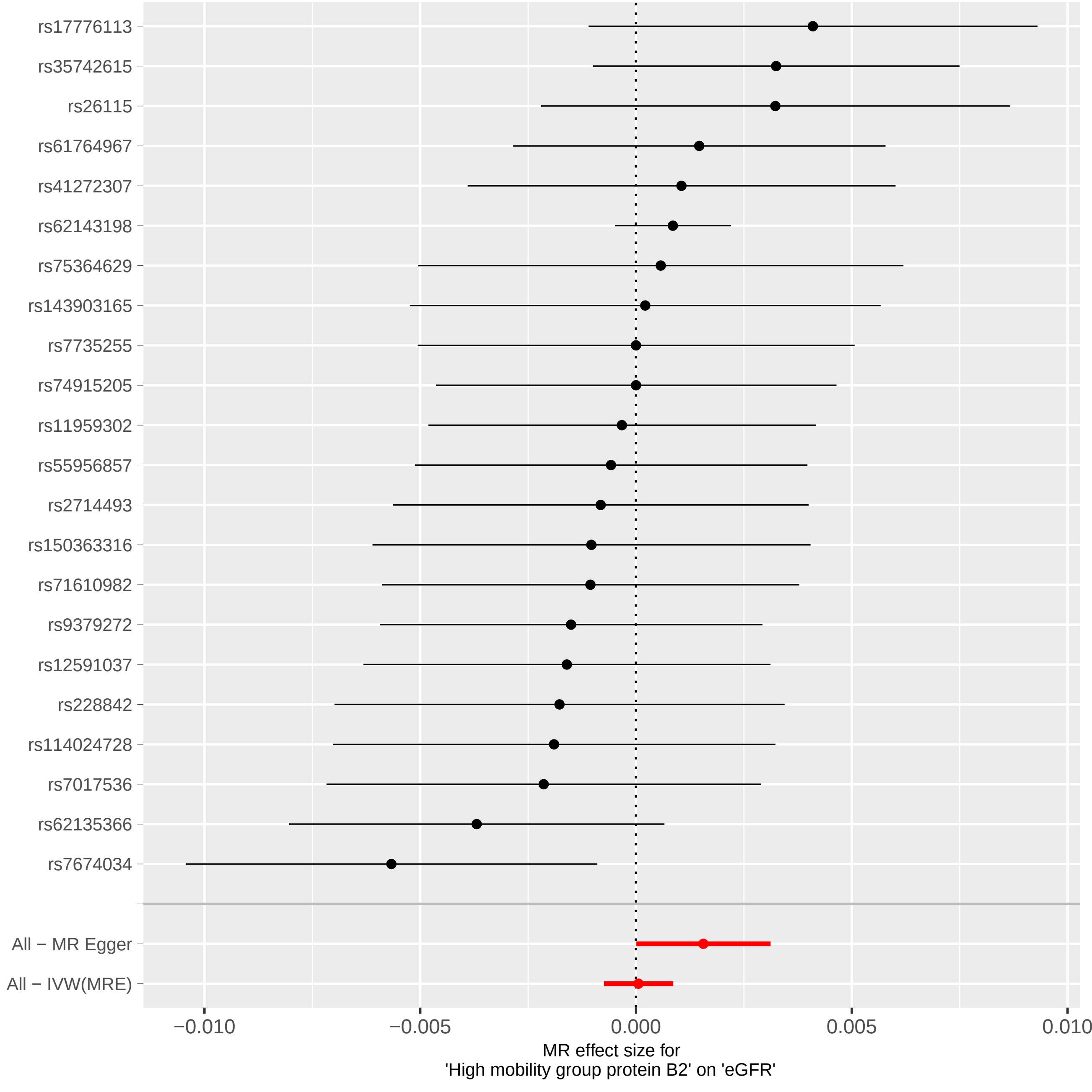

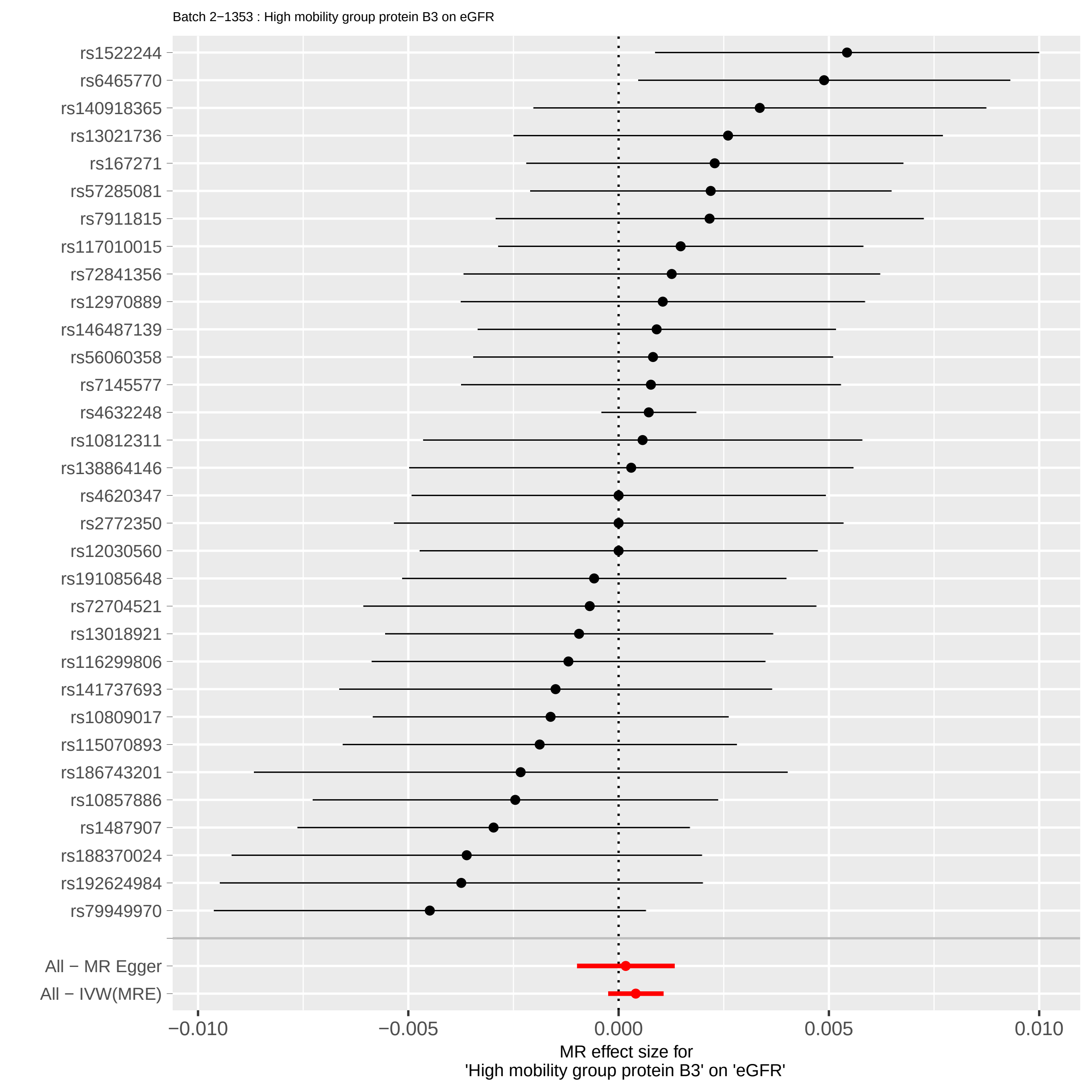

Batch 2−1353 : High mobility group protein B3 on eGFR
rs1522244
rs6465770
rs140918365
rs13021736
rs167271
rs57285081
rs7911815
rs117010015
rs72841356
rs12970889
rs146487139
rs56060358
rs7145577
rs4632248
rs10812311
rs138864146
rs4620347
rs2772350
rs12030560
rs191085648
rs72704521
rs13018921
rs116299806
rs141737693
rs10809017
rs115070893
rs186743201
rs10857886
rs1487907
rs188370024
rs192624984
rs79949970
All − MR Egger
All − IVW(MRE)
−0.010
−0.005
0.000
0.005
0.010
MR effect size for
'High mobility group protein B3' on 'eGFR'

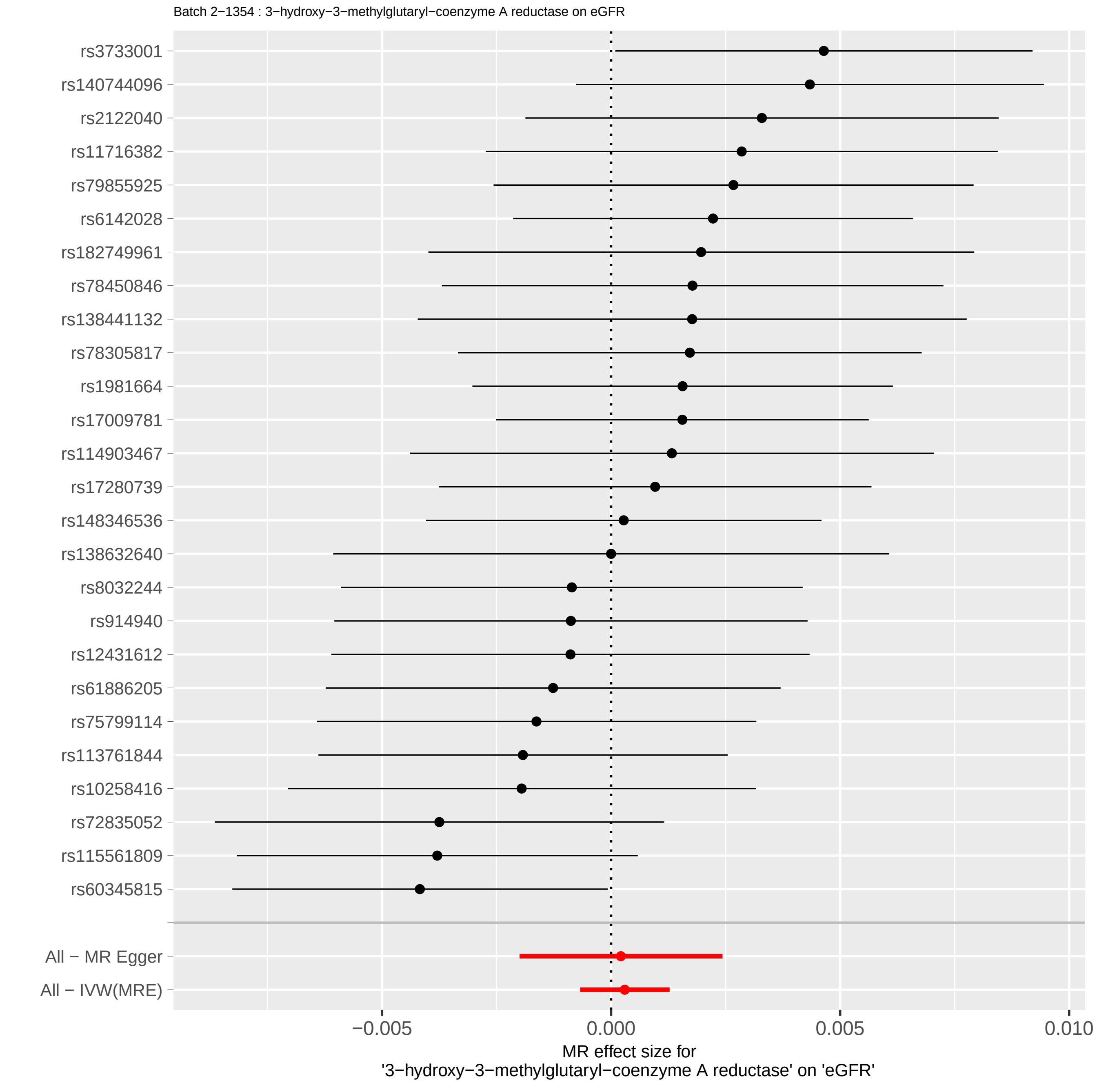

Batch 2−1354 : 3−hydroxy−3−methylglutaryl−coenzyme A reductase on eGFR
rs3733001
rs140744096
rs2122040
rs11716382
rs79855925
rs6142028
rs182749961
rs78450846
rs138441132
rs78305817
rs1981664
rs17009781
rs114903467
rs17280739
rs148346536
rs138632640
rs8032244
rs914940
rs12431612
rs61886205
rs75799114
rs113761844
rs10258416
rs72835052
rs115561809
rs60345815
All − MR Egger
All − IVW(MRE)
−0.005
0.000
0.005
0.010
MR effect size for
'3−hydroxy−3−methylglutaryl−coenzyme A reductase' on 'eGFR'

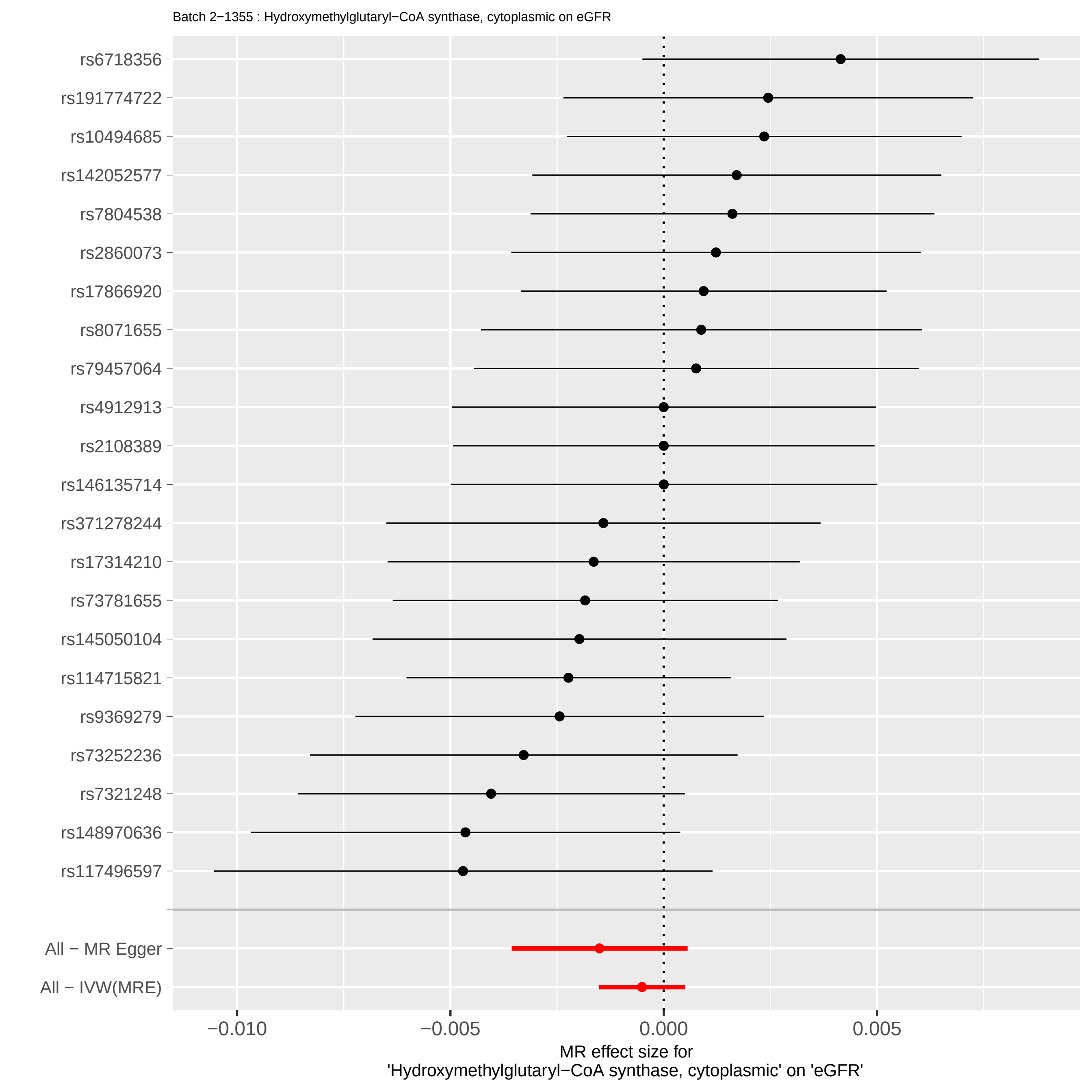

Batch 2−1355 : Hydroxymethylglutaryl−CoA synthase, cytoplasmic on eGFR
rs6718356
rs191774722
rs10494685
rs142052577
rs7804538
rs2860073
rs17866920
rs8071655
rs79457064
rs4912913
rs2108389
rs146135714
rs371278244
rs17314210
rs73781655
rs145050104
rs114715821
rs9369279
rs73252236
rs7321248
rs148970636
rs117496597
All − MR Egger
All − IVW(MRE)
−0.010
−0.005
0.000
0.005
MR effect size for
'Hydroxymethylglutaryl−CoA synthase, cytoplasmic' on 'eGFR'

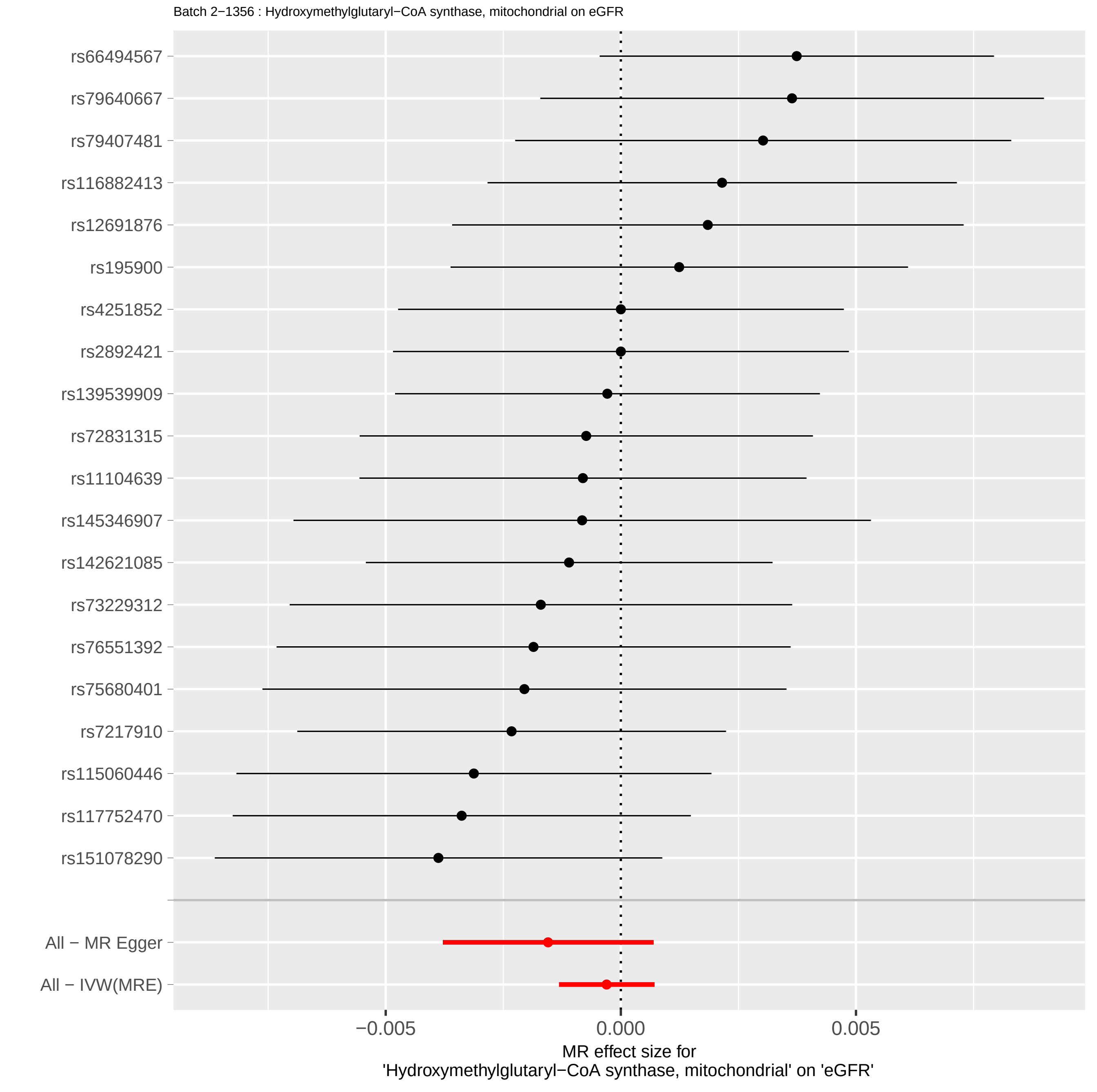

Batch 2−1356 : Hydroxymethylglutaryl−CoA synthase, mitochondrial on eGFR
rs66494567
rs79640667
rs79407481
rs116882413
rs12691876
rs195900
rs4251852
rs2892421
rs139539909
rs72831315
rs11104639
rs145346907
rs142621085
rs73229312
rs76551392
rs75680401
rs7217910
rs115060446
rs117752470
rs151078290
All − MR Egger
All − IVW(MRE)
−0.005
0.000
0.005
MR effect size for
'Hydroxymethylglutaryl−CoA synthase, mitochondrial' on 'eGFR'

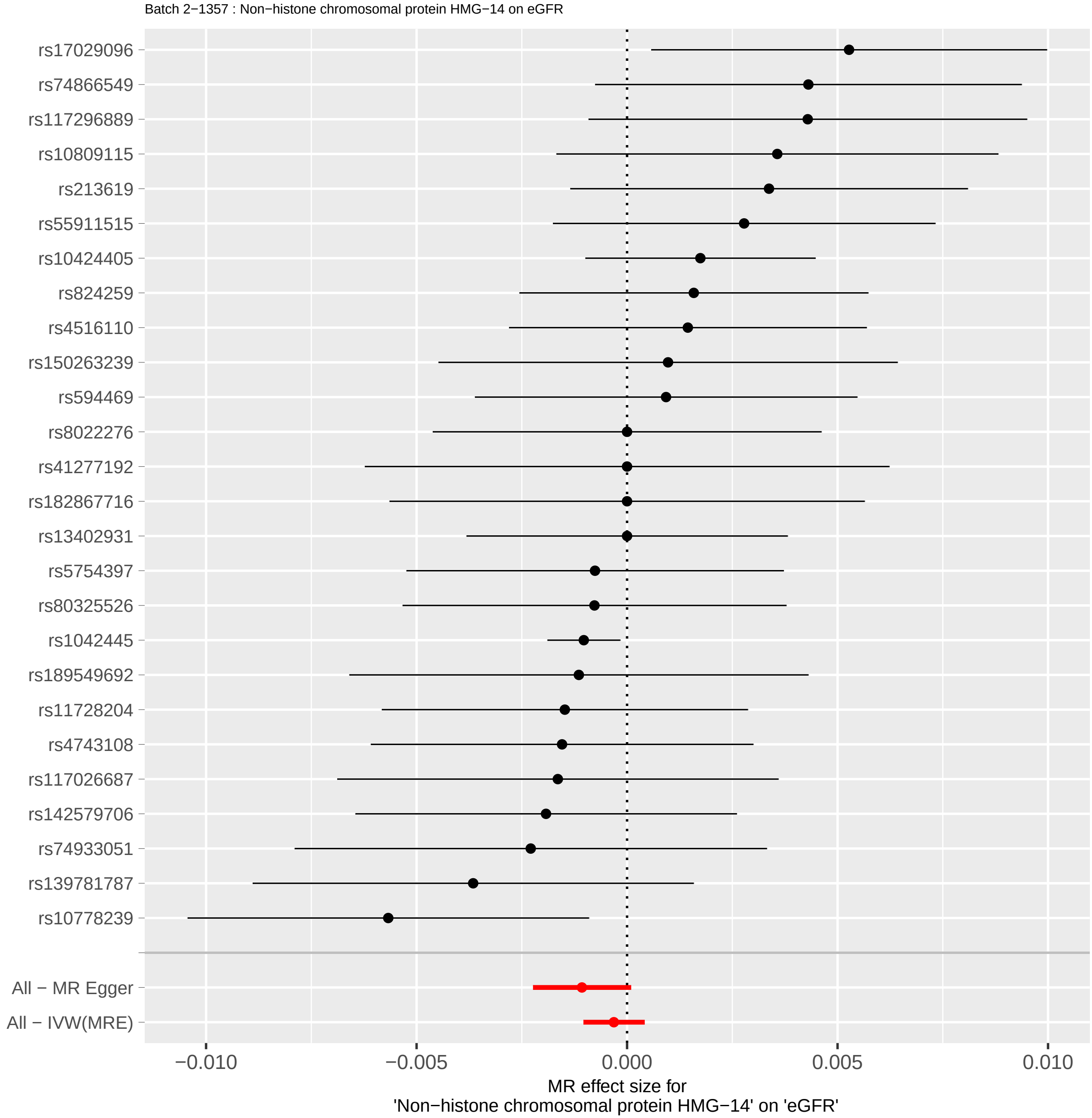
Batch 2−1357 : Non−histone chromosomal protein HMG−14 on eGFR
rs17029096
rs74866549
rs117296889
rs10809115
rs213619
rs55911515
rs10424405
rs824259
rs4516110
rs150263239
rs594469
rs8022276
rs41277192
rs182867716
rs13402931
rs5754397
rs80325526
rs1042445
rs189549692
rs11728204
rs4743108
rs117026687
rs142579706
rs74933051
rs139781787
rs10778239
All − MR Egger
All − IVW(MRE)
−0.010
−0.005
0.000
0.005
0.010
MR effect size for
'Non−histone chromosomal protein HMG−14' on 'eGFR'

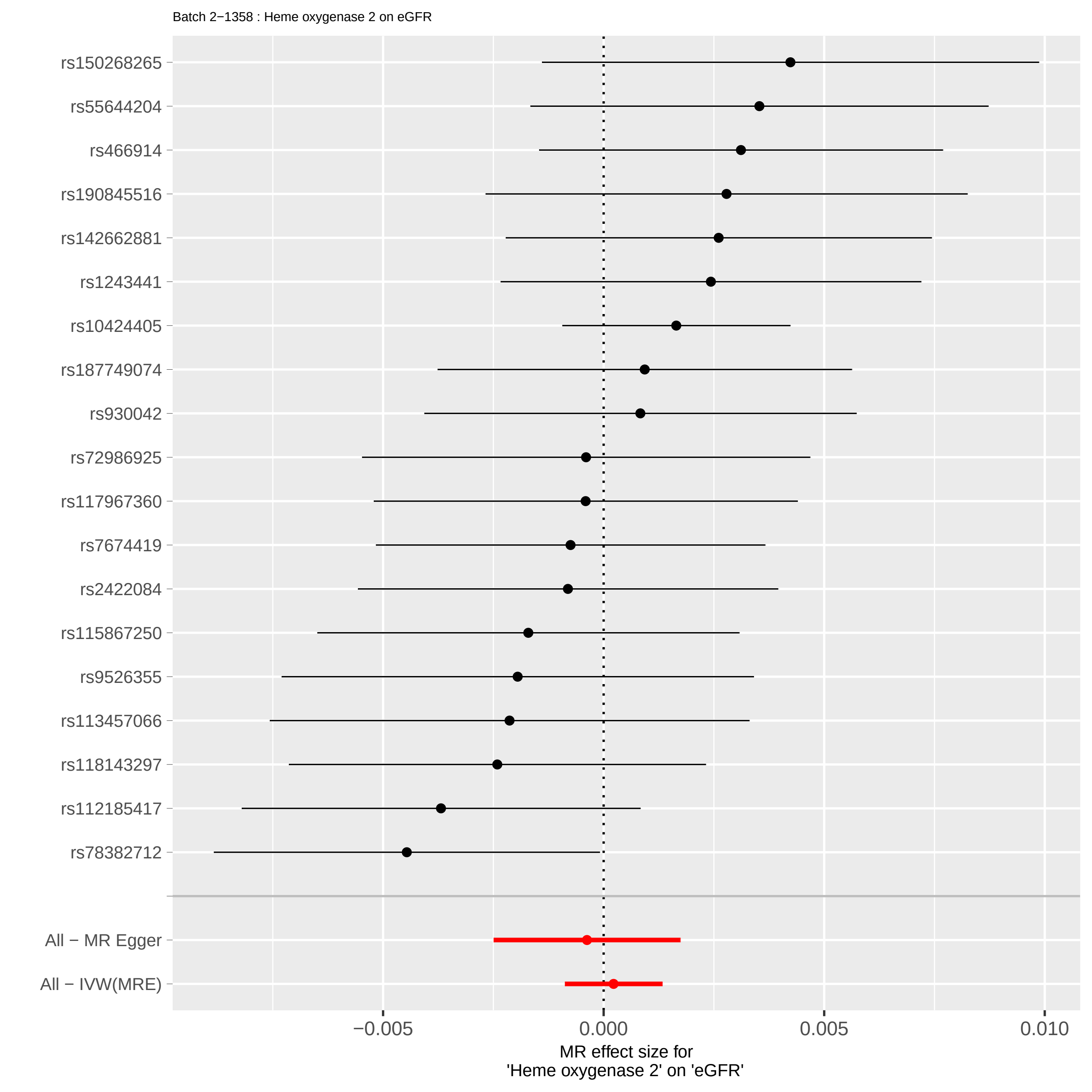

Batch 2−1358 : Heme oxygenase 2 on eGFR
rs150268265
rs55644204
rs466914
rs190845516
rs142662881
rs1243441
rs10424405
rs187749074
rs930042
rs72986925
rs117967360
rs7674419
rs2422084
rs115867250
rs9526355
rs113457066
rs118143297
rs112185417
rs78382712
All − MR Egger
All − IVW(MRE)
−0.005
0.000
0.005
0.010
MR effect size for
'Heme oxygenase 2' on 'eGFR'

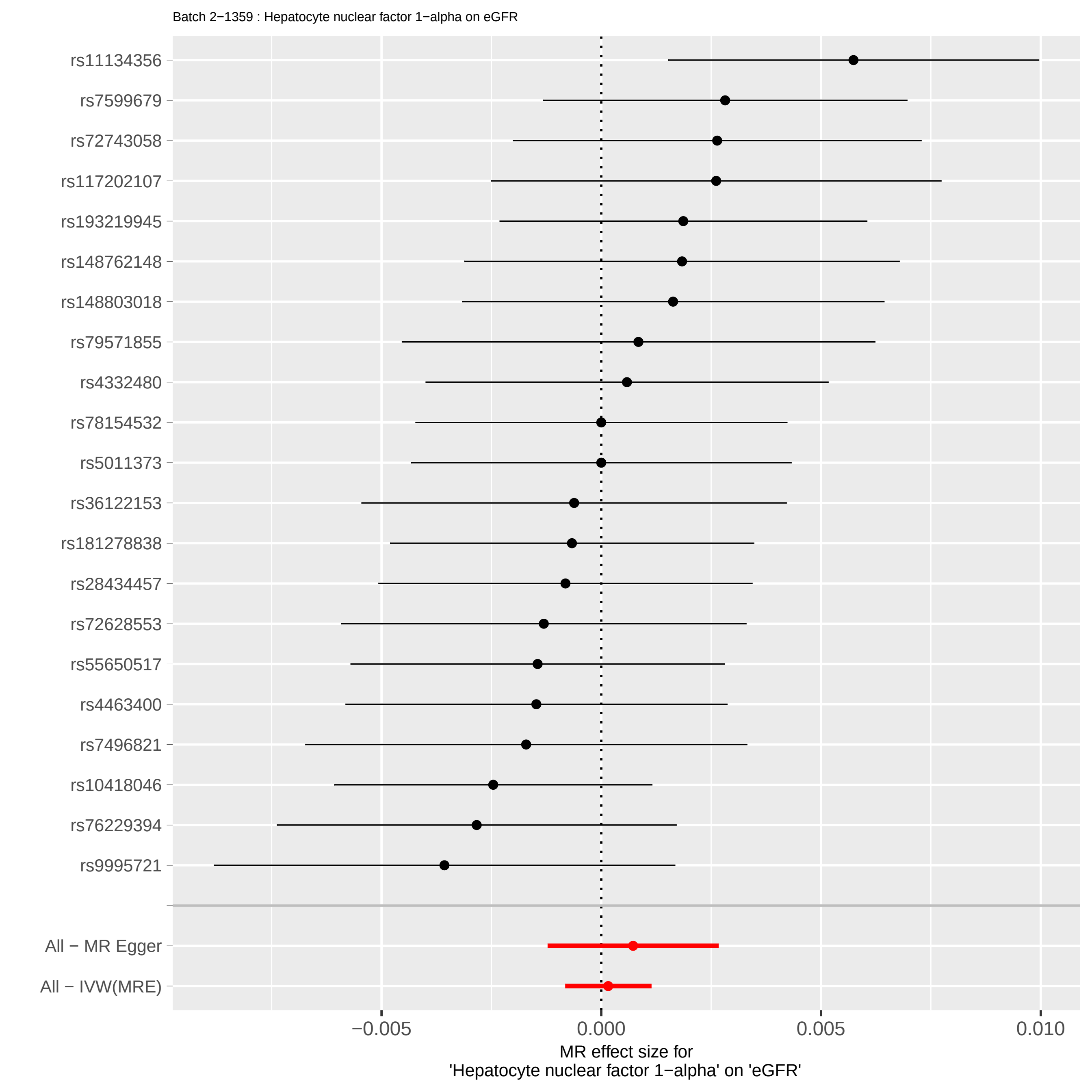
Batch 2−1359 : Hepatocyte nuclear factor 1−alpha on eGFR
rs11134356
rs7599679
rs72743058
rs117202107
rs193219945
rs148762148
rs148803018
rs79571855
rs4332480
rs78154532
rs5011373
rs36122153
rs181278838
rs28434457
rs72628553
rs55650517
rs4463400
rs7496821
rs10418046
rs76229394
rs9995721
All − MR Egger
All − IVW(MRE)
−0.005
0.000
0.005
0.010
MR effect size for
'Hepatocyte nuclear factor 1−alpha' on 'eGFR'

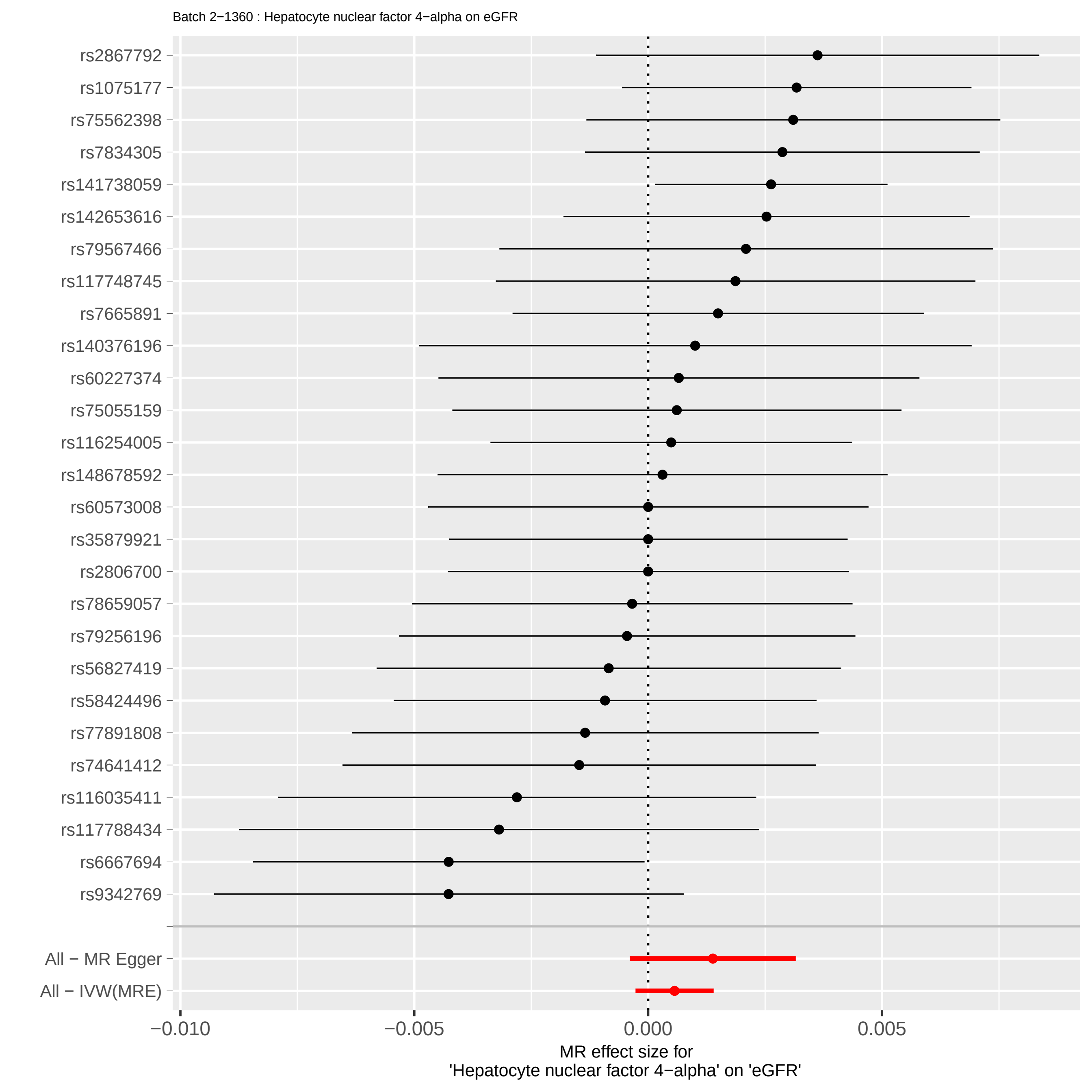

Batch 2−1360 : Hepatocyte nuclear factor 4−alpha on eGFR
rs2867792
rs1075177
rs75562398
rs7834305
rs141738059
rs142653616
rs79567466
rs117748745
rs7665891
rs140376196
rs60227374
rs75055159
rs116254005
rs148678592
rs60573008
rs35879921
rs2806700
rs78659057
rs79256196
rs56827419
rs58424496
rs77891808
rs74641412
rs116035411
rs117788434
rs6667694
rs9342769
All − MR Egger
All − IVW(MRE)
−0.010
−0.005
0.000
0.005
MR effect size for
'Hepatocyte nuclear factor 4−alpha' on 'eGFR'

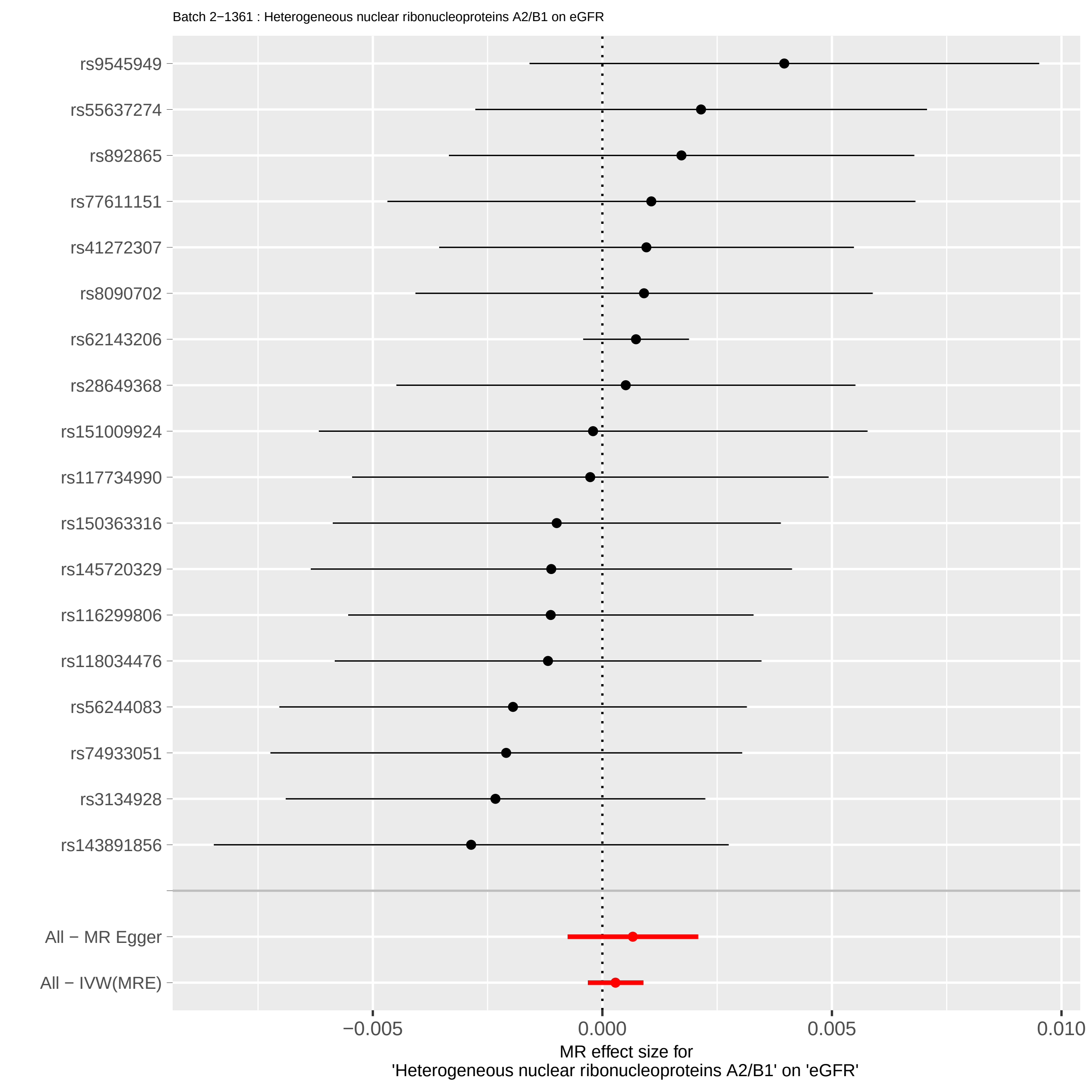

Batch 2−1361 : Heterogeneous nuclear ribonucleoproteins A2/B1 on eGFR
rs9545949
rs55637274
rs892865
rs77611151
rs41272307
rs8090702
rs62143206
rs28649368
rs151009924
rs117734990
rs150363316
rs145720329
rs116299806
rs118034476
rs56244083
rs74933051
rs3134928
rs143891856
All − MR Egger
All − IVW(MRE)
−0.005
0.000
0.005
0.010
MR effect size for
'Heterogeneous nuclear ribonucleoproteins A2/B1' on 'eGFR'

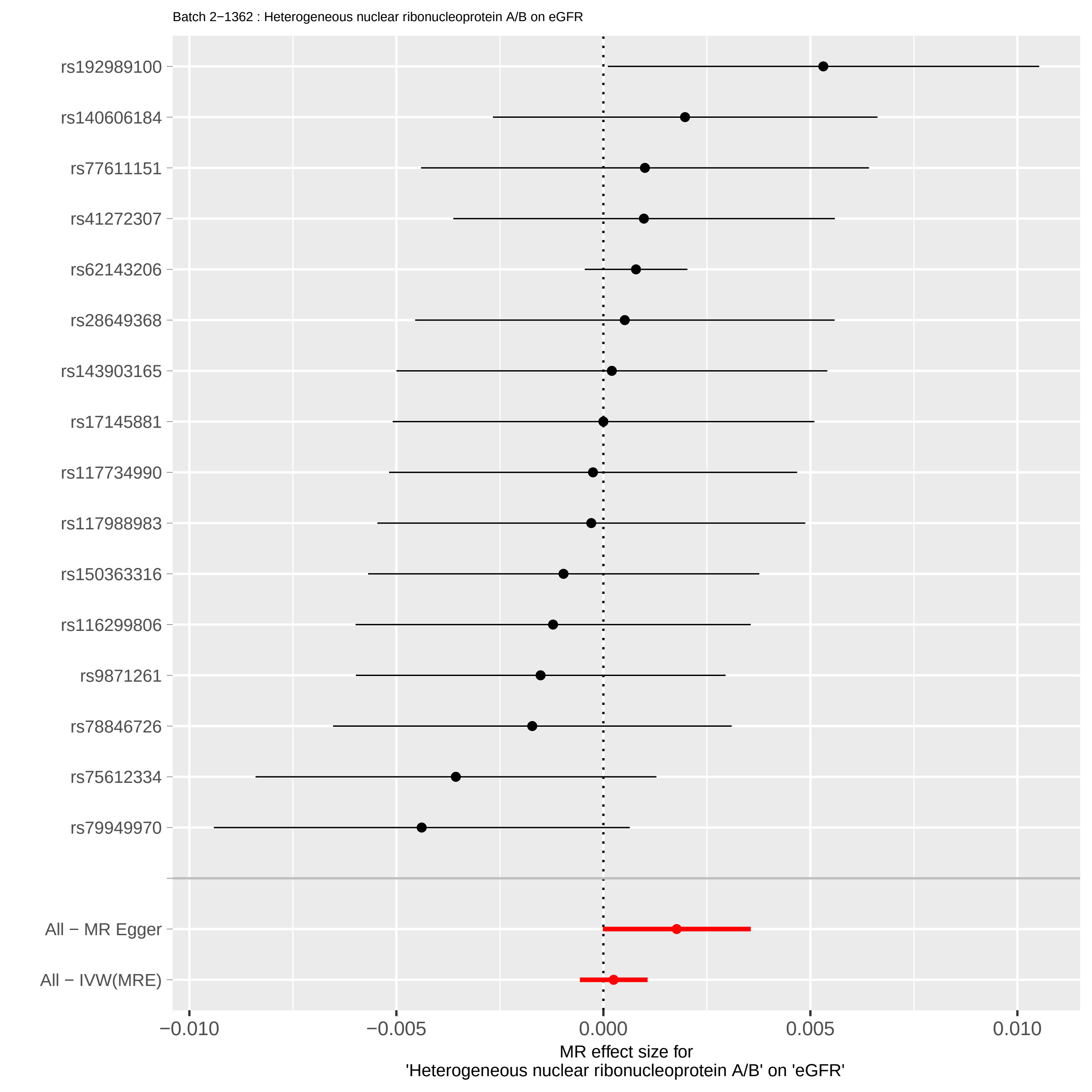

Batch 2−1362 : Heterogeneous nuclear ribonucleoprotein A/B on eGFR
rs192989100
rs140606184
rs77611151
rs41272307
rs62143206
rs28649368
rs143903165
rs17145881
rs117734990
rs117988983
rs150363316
rs116299806
rs9871261
rs78846726
rs75612334
rs79949970
All − MR Egger
All − IVW(MRE)
−0.010
−0.005
0.000
0.005
0.010
MR effect size for
'Heterogeneous nuclear ribonucleoprotein A/B' on 'eGFR'

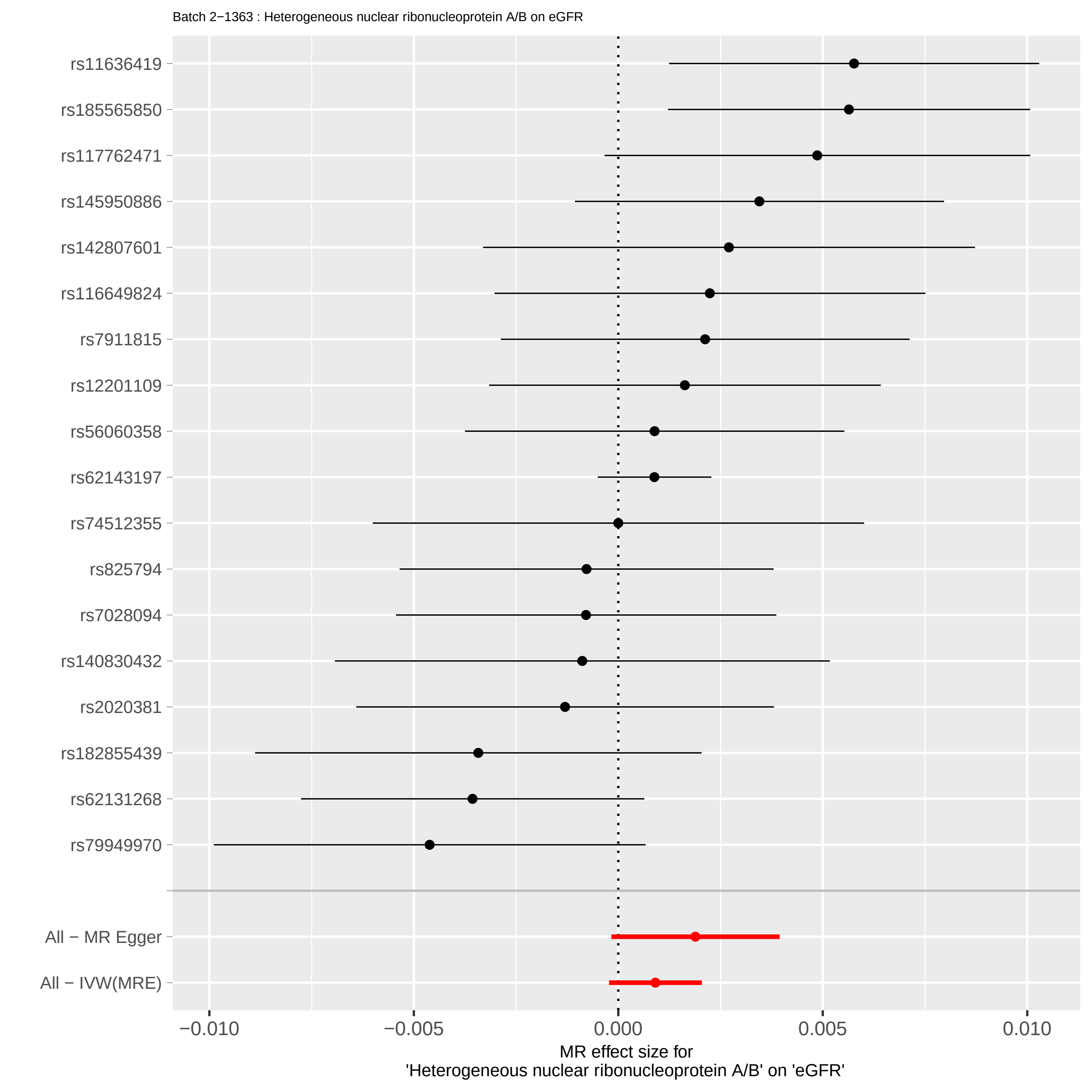
Batch 2−1363 : Heterogeneous nuclear ribonucleoprotein A/B on eGFR
rs11636419
rs185565850
rs117762471
rs145950886
rs142807601
rs116649824
rs7911815
rs12201109
rs56060358
rs62143197
rs74512355
rs825794
rs7028094
rs140830432
rs2020381
rs182855439
rs62131268
rs79949970
All − MR Egger
All − IVW(MRE)
−0.010
−0.005
0.000
0.005
0.010
MR effect size for
'Heterogeneous nuclear ribonucleoprotein A/B' on 'eGFR'

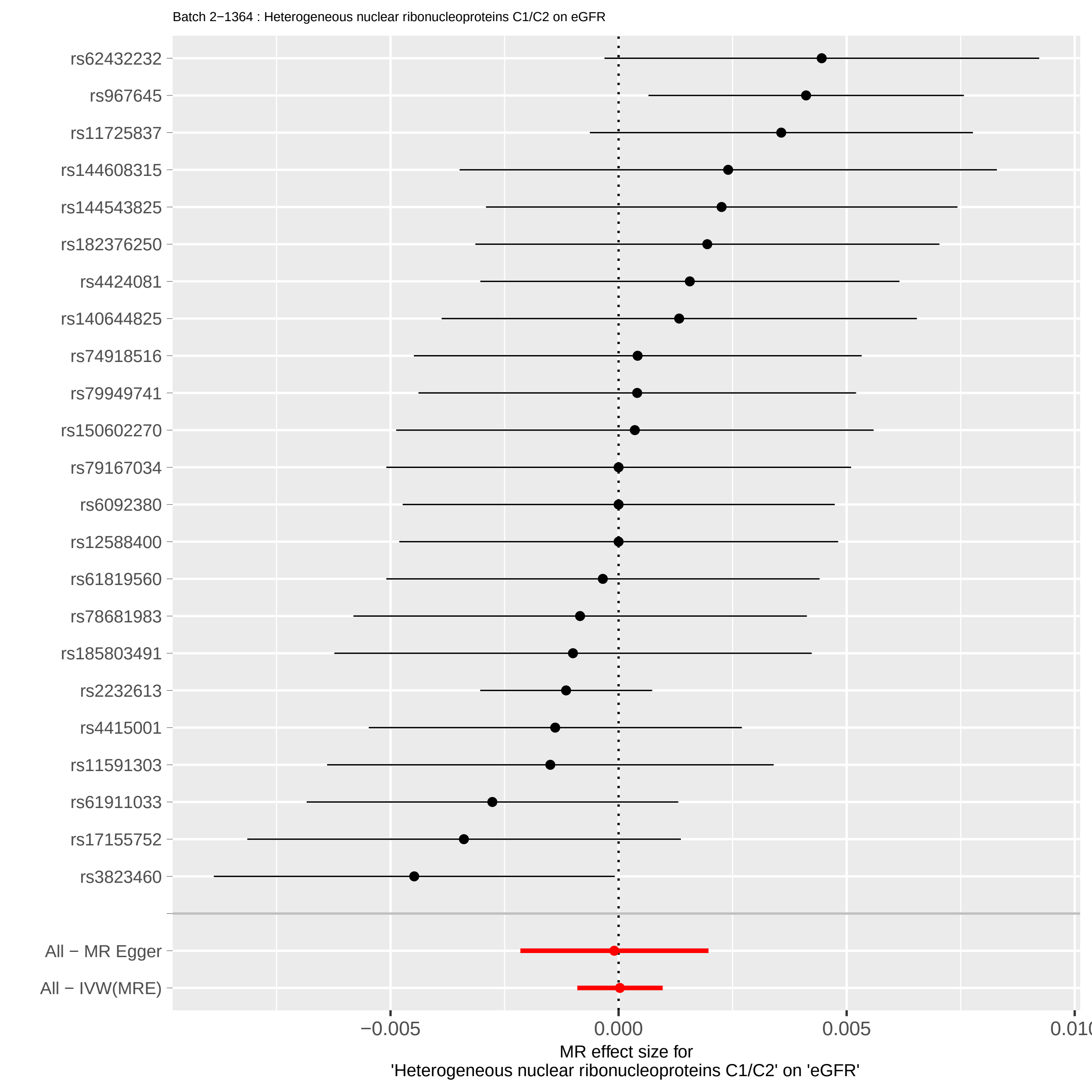

Batch 2−1364 : Heterogeneous nuclear ribonucleoproteins C1/C2 on eGFR
rs62432232
rs967645
rs11725837
rs144608315
rs144543825
rs182376250
rs4424081
rs140644825
rs74918516
rs79949741
rs150602270
rs79167034
rs6092380
rs12588400
rs61819560
rs78681983
rs185803491
rs2232613
rs4415001
rs11591303
rs61911033
rs17155752
rs3823460
All − MR Egger
All − IVW(MRE)
−0.005
0.000
0.005
0.010
MR effect size for
'Heterogeneous nuclear ribonucleoproteins C1/C2' on 'eGFR'

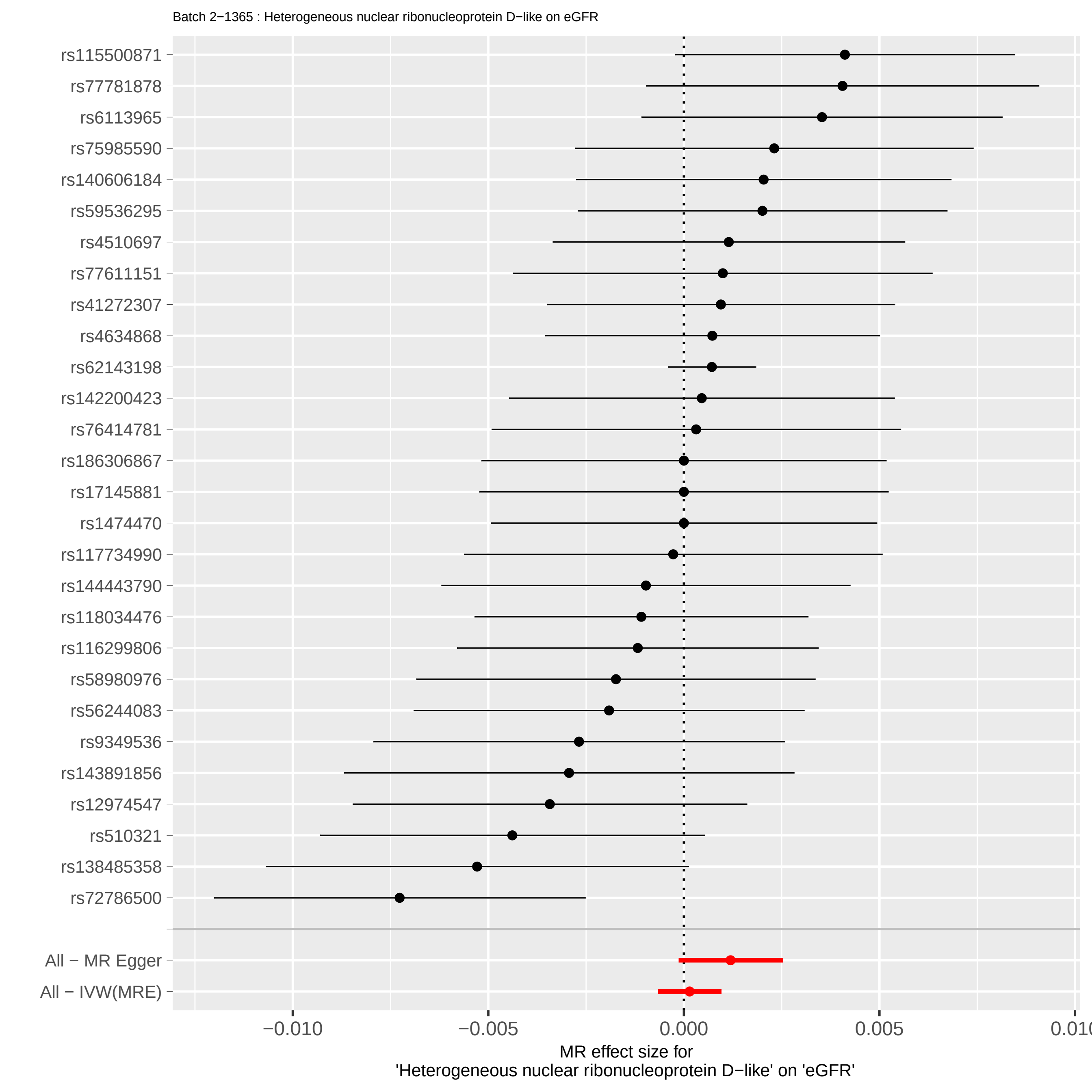

Batch 2−1365 : Heterogeneous nuclear ribonucleoprotein D−like on eGFR
rs115500871
rs77781878
rs6113965
rs75985590
rs140606184
rs59536295
rs4510697
rs77611151
rs41272307
rs4634868
rs62143198
rs142200423
rs76414781
rs186306867
rs17145881
rs1474470
rs117734990
rs144443790
rs118034476
rs116299806
rs58980976
rs56244083
rs9349536
rs143891856
rs12974547
rs510321
rs138485358
rs72786500
All − MR Egger
All − IVW(MRE)
−0.010
−0.005
0.000
0.005
0.010
MR effect size for
'Heterogeneous nuclear ribonucleoprotein D−like' on 'eGFR'

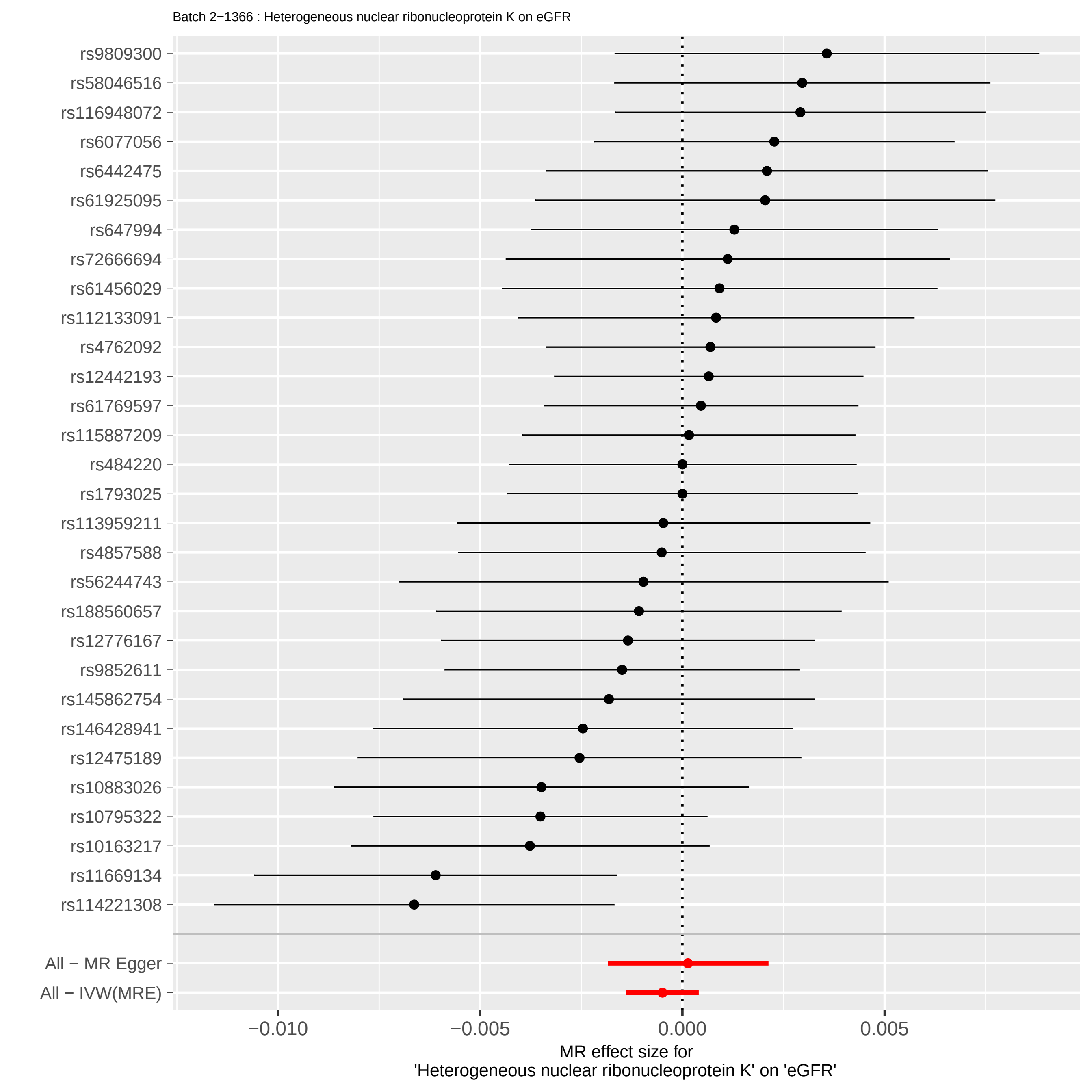

Batch 2−1366 : Heterogeneous nuclear ribonucleoprotein K on eGFR
rs9809300
rs58046516
rs116948072
rs6077056
rs6442475
rs61925095
rs647994
rs72666694
rs61456029
rs112133091
rs4762092
rs12442193
rs61769597
rs115887209
rs484220
rs1793025
rs113959211
rs4857588
rs56244743
rs188560657
rs12776167
rs9852611
rs145862754
rs146428941
rs12475189
rs10883026
rs10795322
rs10163217
rs11669134
rs114221308
All − MR Egger
All − IVW(MRE)
−0.010
−0.005
0.000
0.005
MR effect size for
'Heterogeneous nuclear ribonucleoprotein K' on 'eGFR'

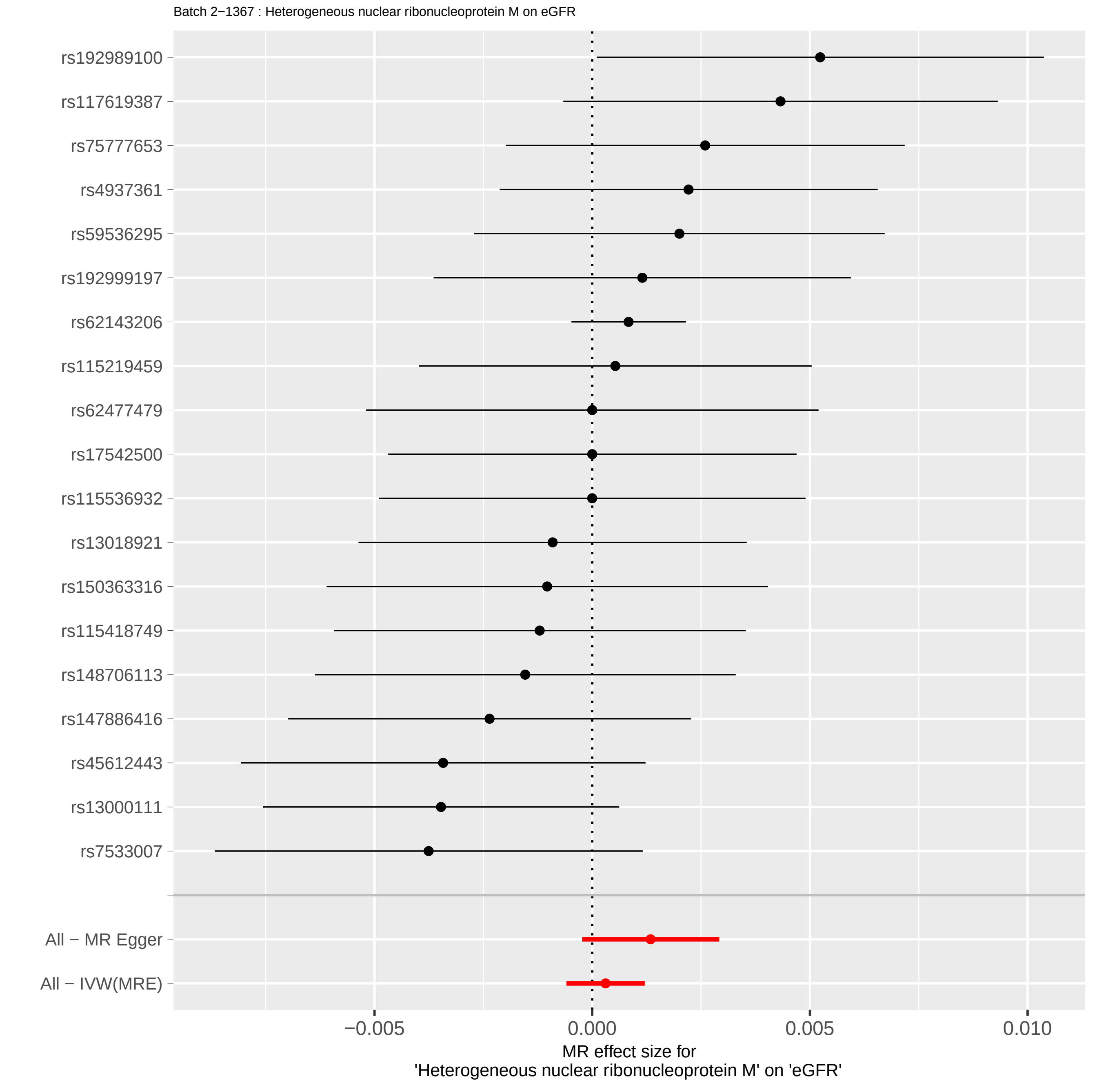
Batch 2−1367 : Heterogeneous nuclear ribonucleoprotein M on eGFR
rs192989100
rs117619387
rs75777653
rs4937361
rs59536295
rs192999197
rs62143206
rs115219459
rs62477479
rs17542500
rs115536932
rs13018921
rs150363316
rs115418749
rs148706113
rs147886416
rs45612443
rs13000111
rs7533007
All − MR Egger
All − IVW(MRE)
−0.005
0.000
0.005
0.010
MR effect size for
'Heterogeneous nuclear ribonucleoprotein M' on 'eGFR'

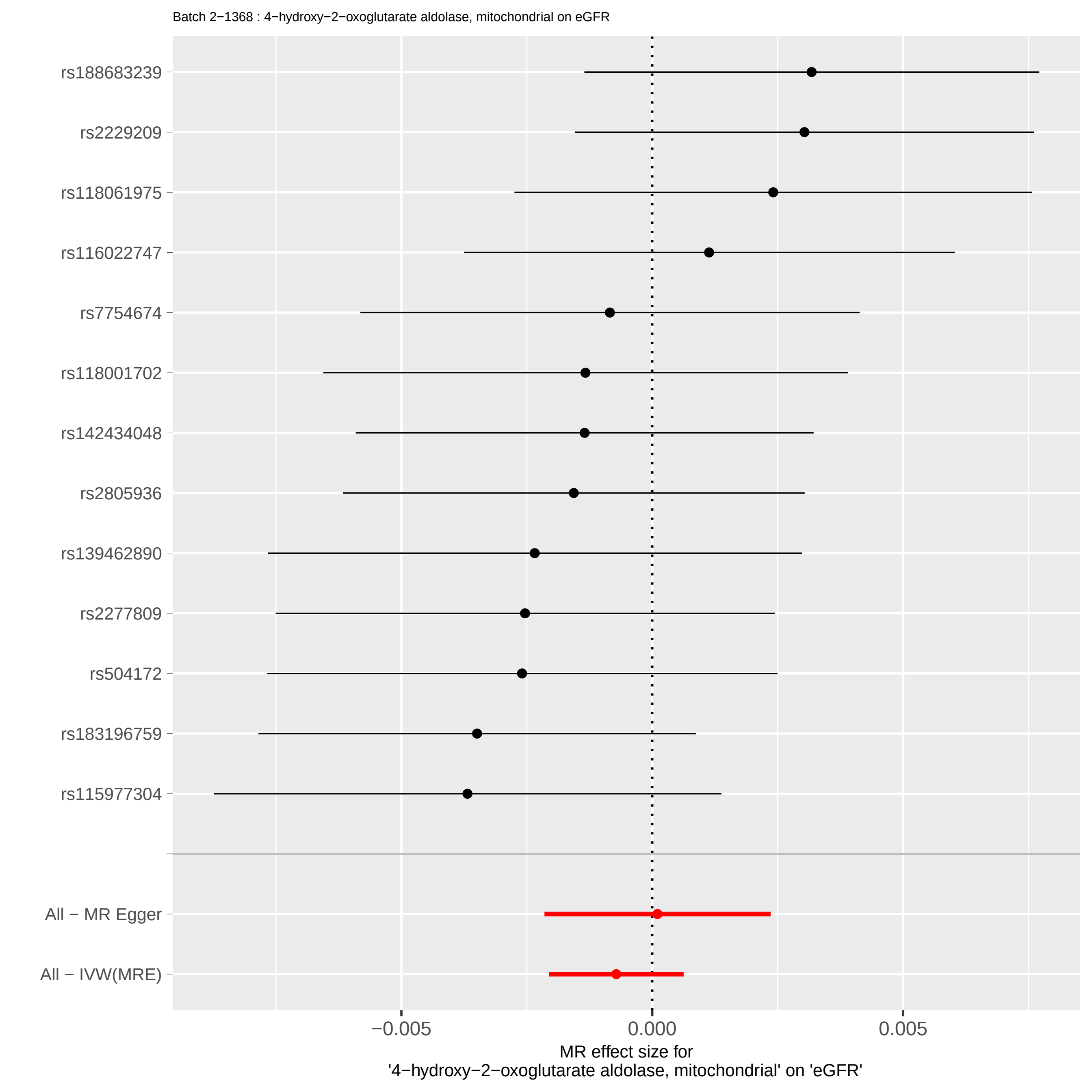

Batch 2−1368 : 4−hydroxy−2−oxoglutarate aldolase, mitochondrial on eGFR
rs188683239
rs2229209
rs118061975
rs116022747
rs7754674
rs118001702
rs142434048
rs2805936
rs139462890
rs2277809
rs504172
rs183196759
rs115977304
All − MR Egger
All − IVW(MRE)
−0.005
0.000
0.005
MR effect size for
'4−hydroxy−2−oxoglutarate aldolase, mitochondrial' on 'eGFR'

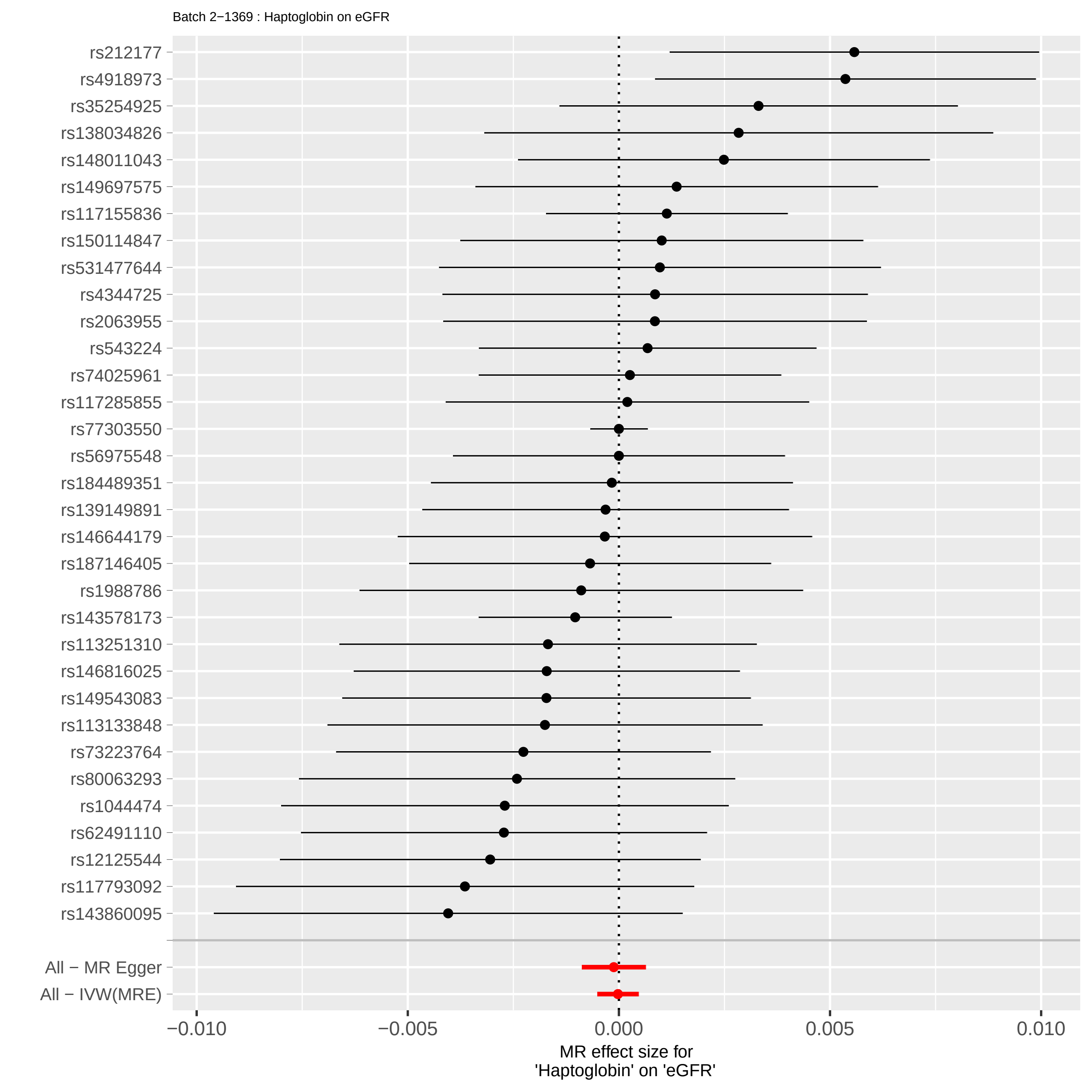
Batch 2−1369 : Haptoglobin on eGFR
rs212177
rs4918973
rs35254925
rs138034826
rs148011043
rs149697575
rs117155836
rs150114847
rs531477644
rs4344725
rs2063955
rs543224
rs74025961
rs117285855
rs77303550
rs56975548
rs184489351
rs139149891
rs146644179
rs187146405
rs1988786
rs143578173
rs113251310
rs146816025
rs149543083
rs113133848
rs73223764
rs80063293
rs1044474
rs62491110
rs12125544
rs117793092
rs143860095
All − MR Egger
All − IVW(MRE)
−0.010
−0.005
0.000
0.005
0.010
MR effect size for
'Haptoglobin' on 'eGFR'

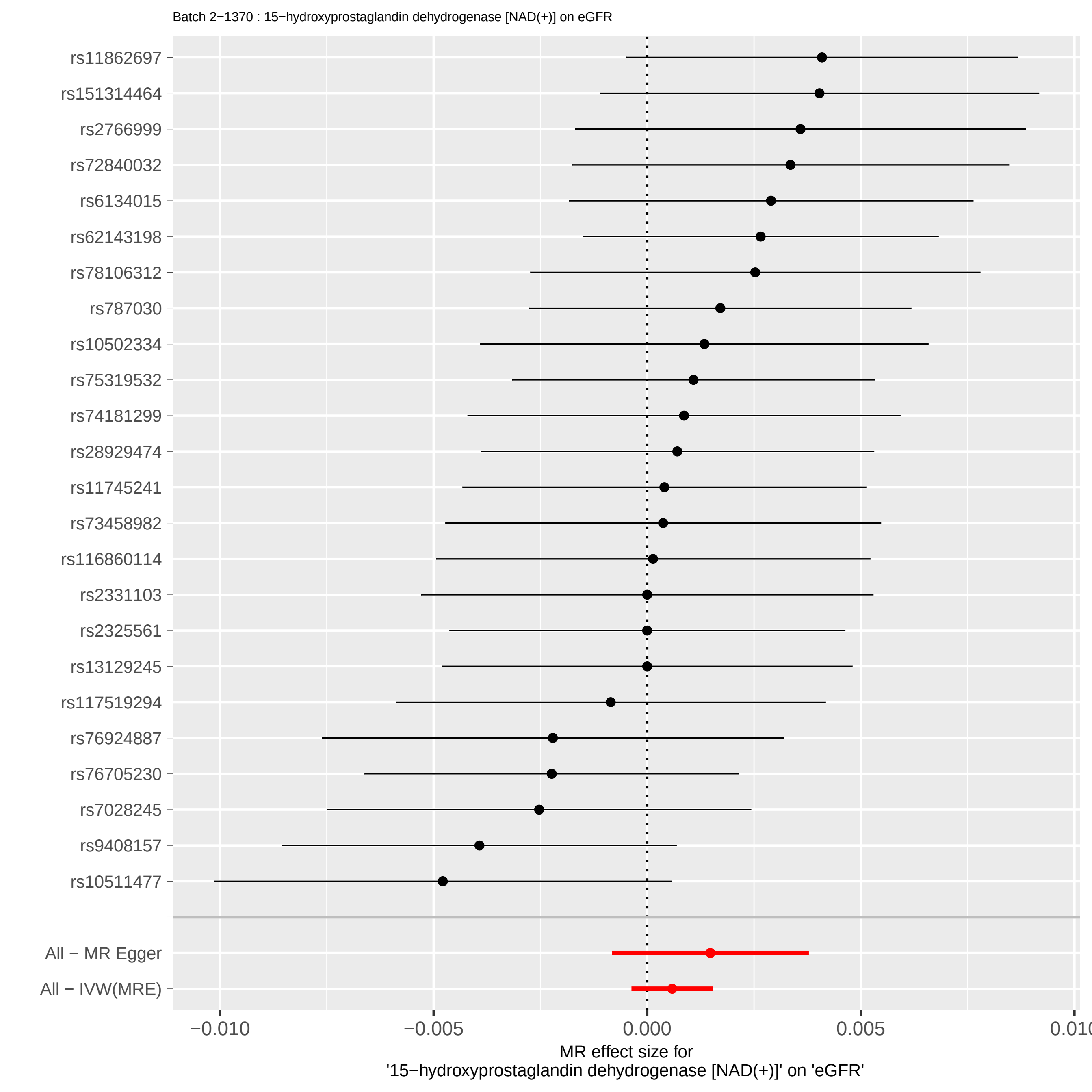

Batch 2−1370 : 15−hydroxyprostaglandin dehydrogenase [NAD(+)] on eGFR
rs11862697
rs151314464
rs2766999
rs72840032
rs6134015
rs62143198
rs78106312
rs787030
rs10502334
rs75319532
rs74181299
rs28929474
rs11745241
rs73458982
rs116860114
rs2331103
rs2325561
rs13129245
rs117519294
rs76924887
rs76705230
rs7028245
rs9408157
rs10511477
All − MR Egger
All − IVW(MRE)
−0.010
−0.005
0.000
0.005
0.010
MR effect size for
'15−hydroxyprostaglandin dehydrogenase [NAD(+)]' on 'eGFR'

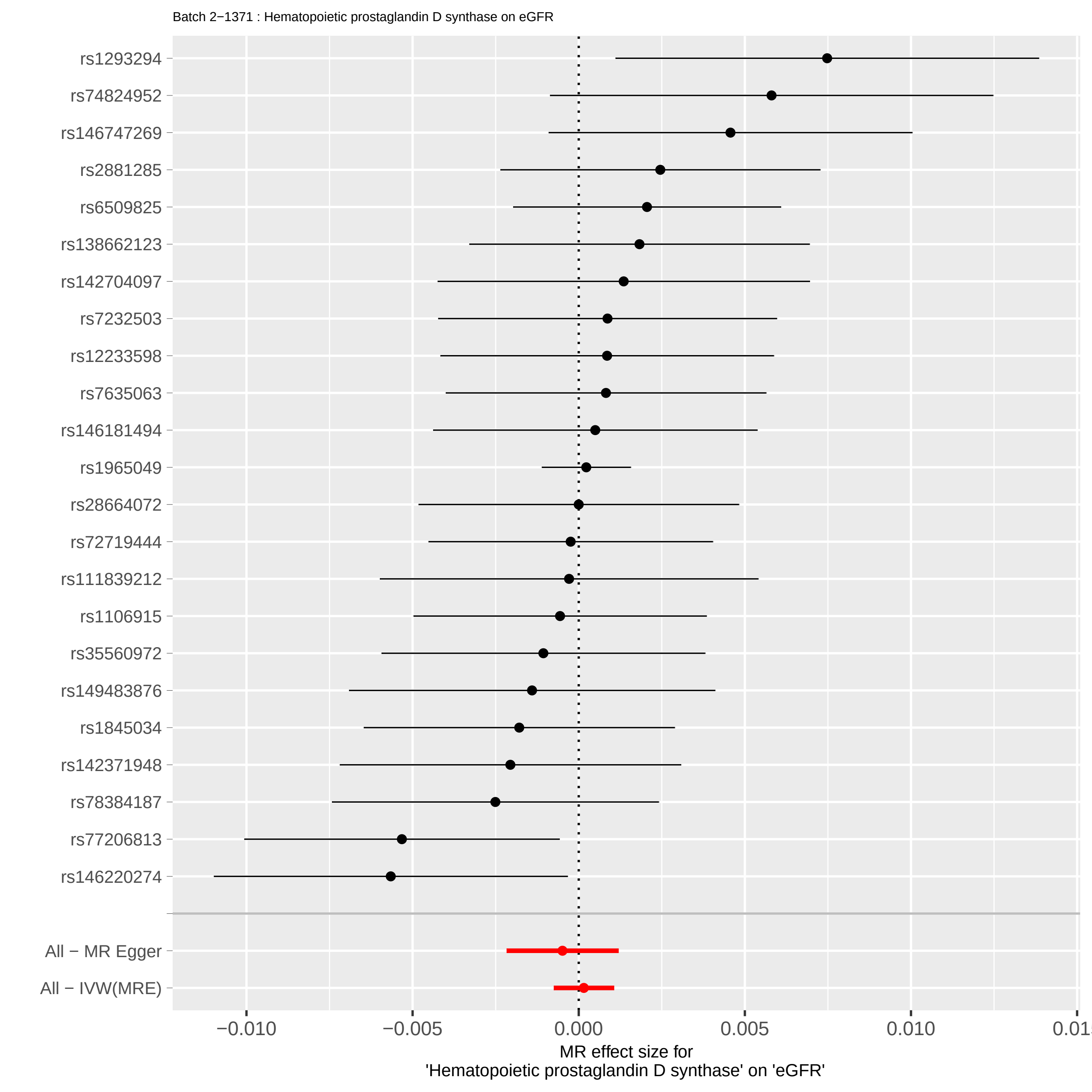

Batch 2−1371 : Hematopoietic prostaglandin D synthase on eGFR
rs1293294
rs74824952
rs146747269
rs2881285
rs6509825
rs138662123
rs142704097
rs7232503
rs12233598
rs7635063
rs146181494
rs1965049
rs28664072
rs72719444
rs111839212
rs1106915
rs35560972
rs149483876
rs1845034
rs142371948
rs78384187
rs77206813
rs146220274
All − MR Egger
All − IVW(MRE)
−0.010
−0.005
0.000
0.005
0.010
MR effect size for
'Hematopoietic prostaglandin D synthase' on 'eGFR'

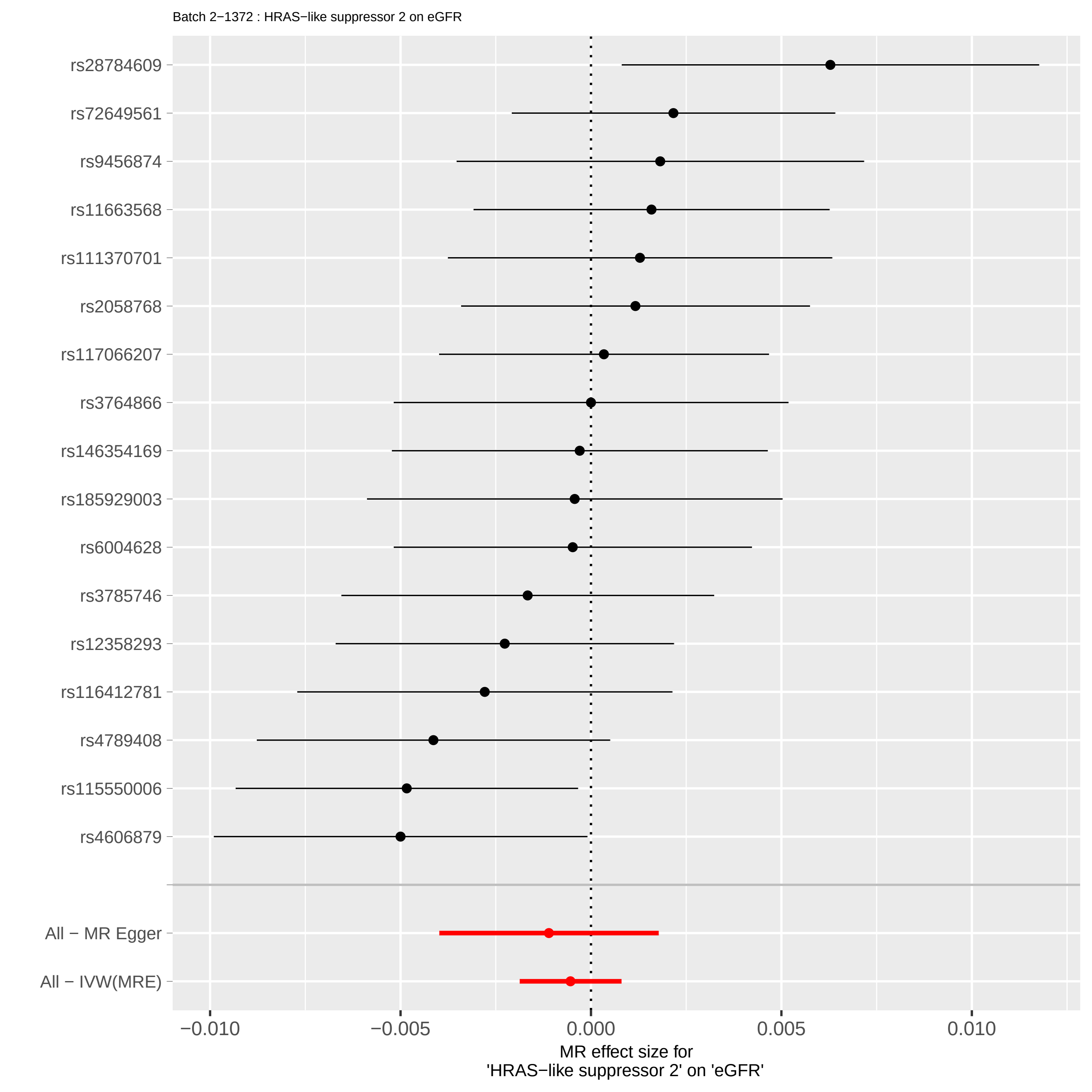

Batch 2−1372 : HRAS−like suppressor 2 on eGFR
rs28784609
rs72649561
rs9456874
rs11663568
rs111370701
rs2058768
rs117066207
rs3764866
rs146354169
rs185929003
rs6004628
rs3785746
rs12358293
rs116412781
rs4789408
rs115550006
rs4606879
All − MR Egger
All − IVW(MRE)
−0.010
−0.005
0.000
0.005
0.010
MR effect size for
'HRAS−like suppressor 2' on 'eGFR'

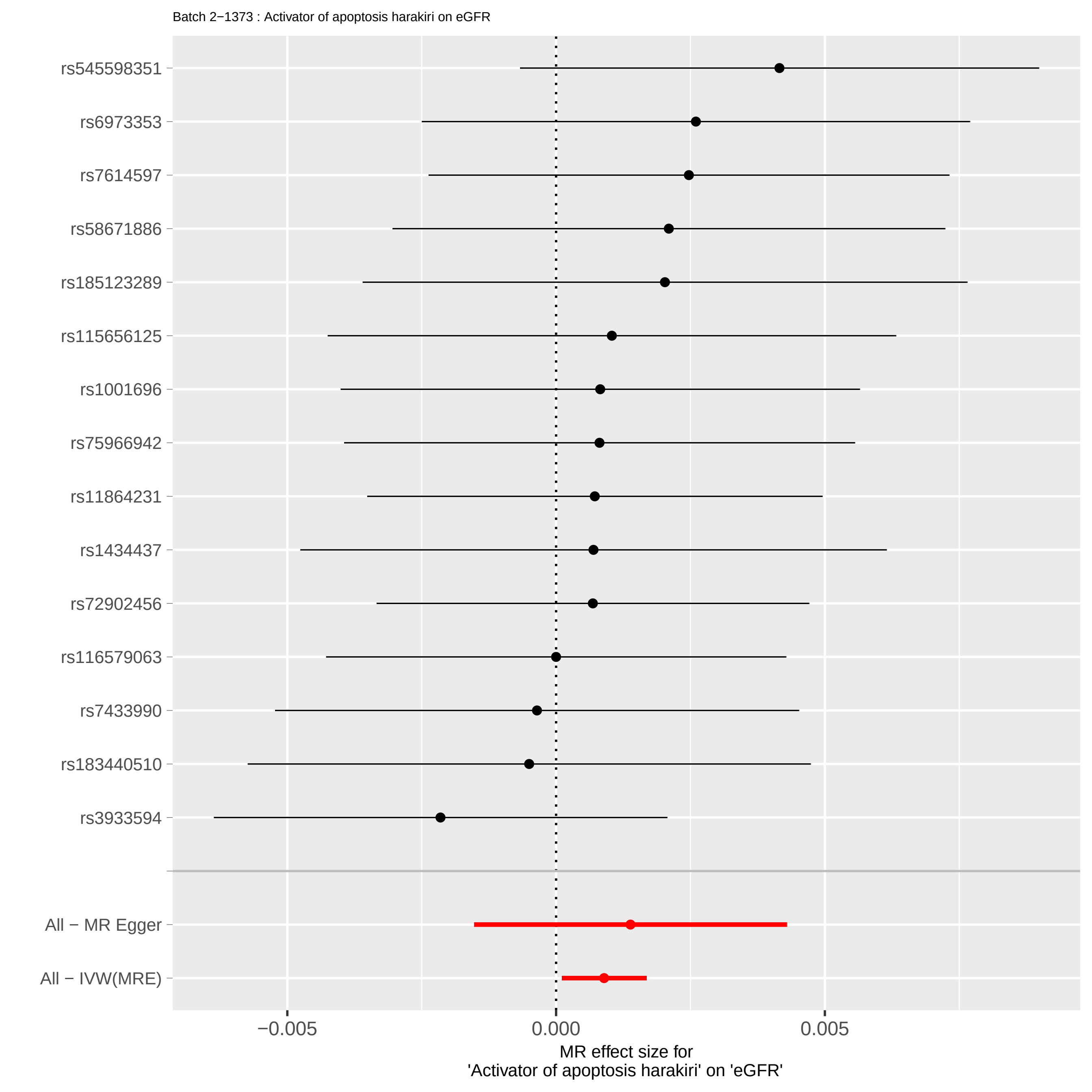

Batch 2−1373 : Activator of apoptosis harakiri on eGFR
rs545598351
rs6973353
rs7614597
rs58671886
rs185123289
rs115656125
rs1001696
rs75966942
rs11864231
rs1434437
rs72902456
rs116579063
rs7433990
rs183440510
rs3933594
All − MR Egger
All − IVW(MRE)
−0.005
0.000
0.005
MR effect size for
'Activator of apoptosis harakiri' on 'eGFR'

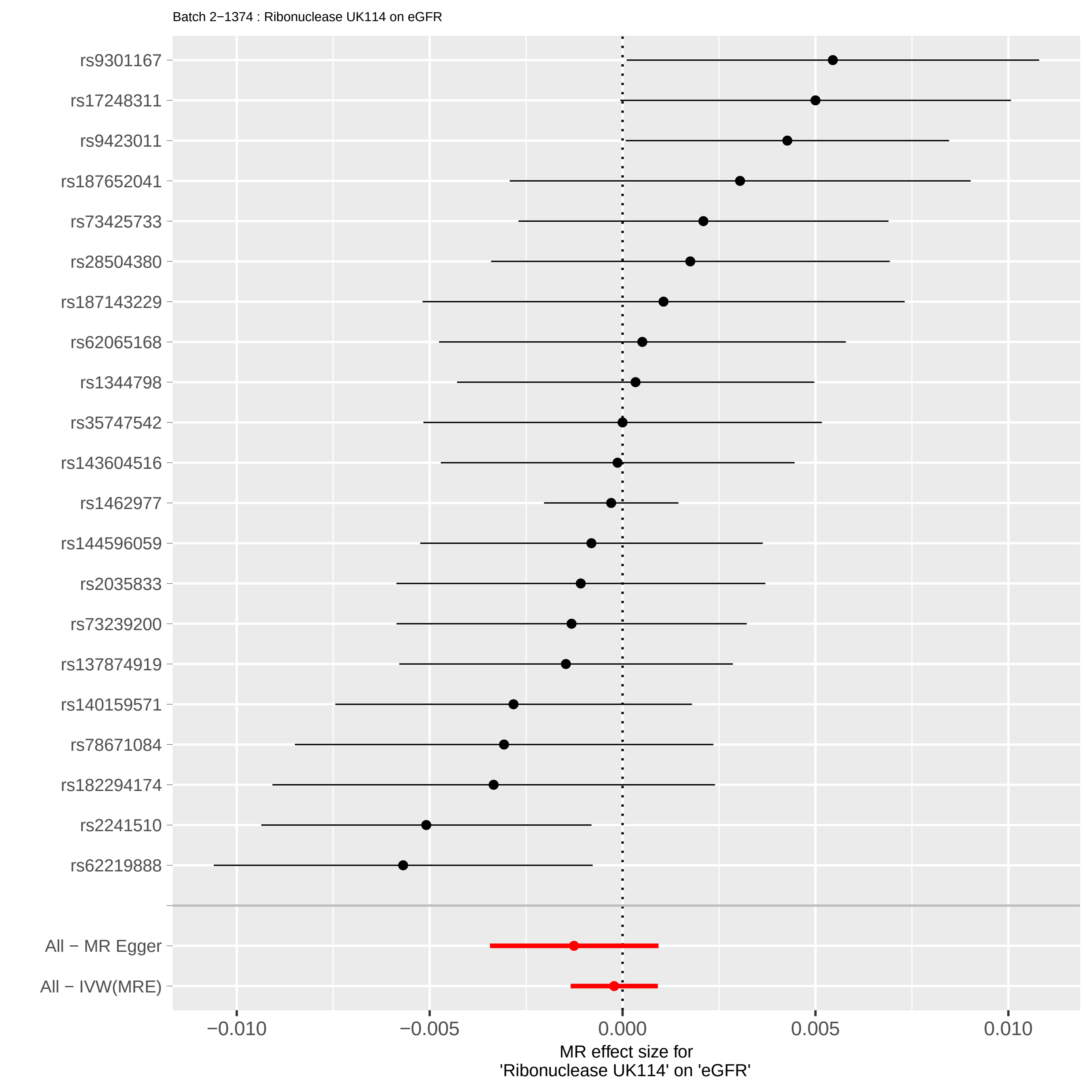

Batch 2−1374 : Ribonuclease UK114 on eGFR
rs9301167
rs17248311
rs9423011
rs187652041
rs73425733
rs28504380
rs187143229
rs62065168
rs1344798
rs35747542
rs143604516
rs1462977
rs144596059
rs2035833
rs73239200
rs137874919
rs140159571
rs78671084
rs182294174
rs2241510
rs62219888
All − MR Egger
All − IVW(MRE)
−0.010
−0.005
0.000
0.005
0.010
MR effect size for
'Ribonuclease UK114' on 'eGFR'

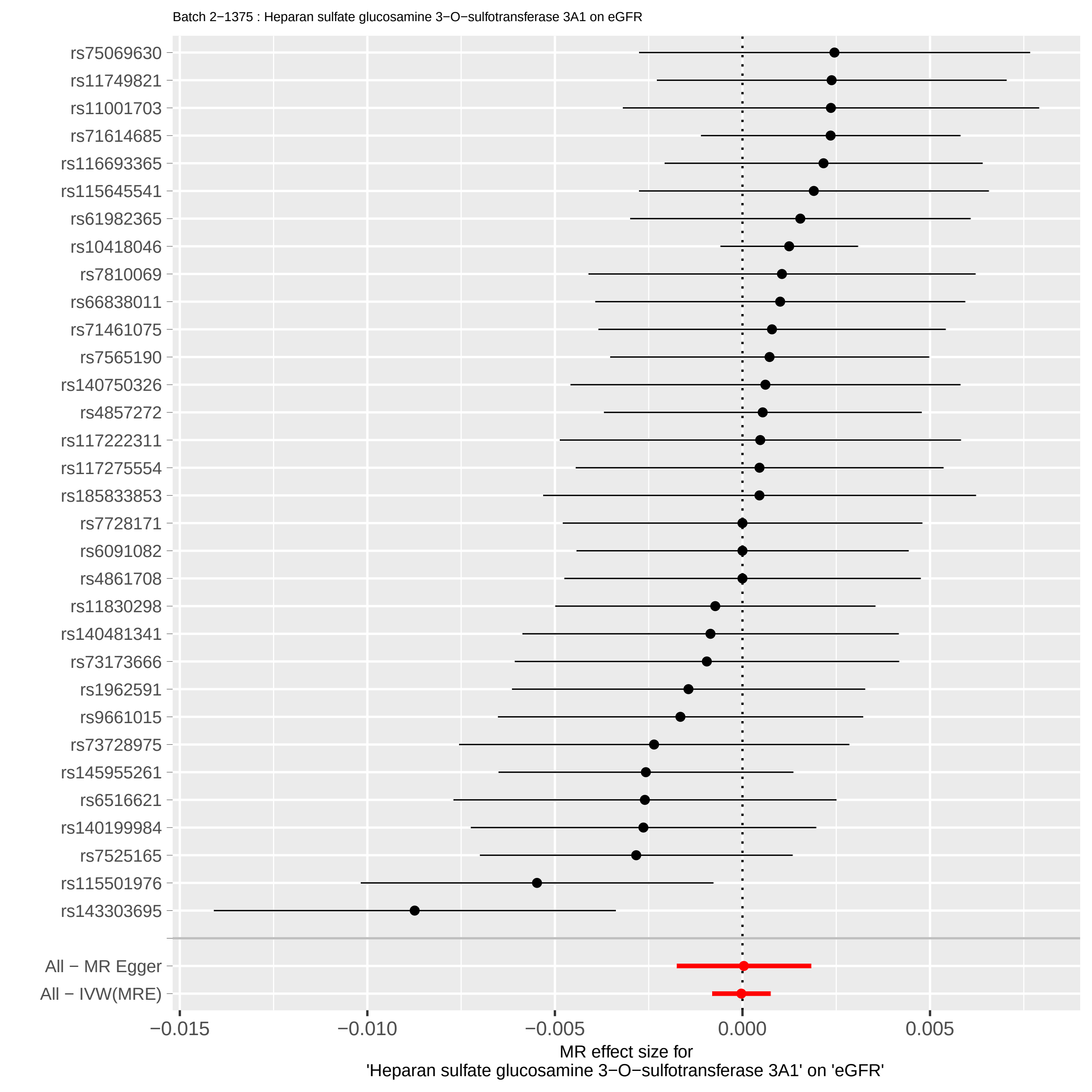
Batch 2−1375 : Heparan sulfate glucosamine 3−O−sulfotransferase 3A1 on eGFR
rs75069630
rs11749821
rs11001703
rs71614685
rs116693365
rs115645541
rs61982365
rs10418046
rs7810069
rs66838011
rs71461075
rs7565190
rs140750326
rs4857272
rs117222311
rs117275554
rs185833853
rs7728171
rs6091082
rs4861708
rs11830298
rs140481341
rs73173666
rs1962591
rs9661015
rs73728975
rs145955261
rs6516621
rs140199984
rs7525165
rs115501976
rs143303695
All − MR Egger
All − IVW(MRE)
−0.015
−0.010
−0.005
0.000
0.005
MR effect size for
'Heparan sulfate glucosamine 3−O−sulfotransferase 3A1' on 'eGFR'

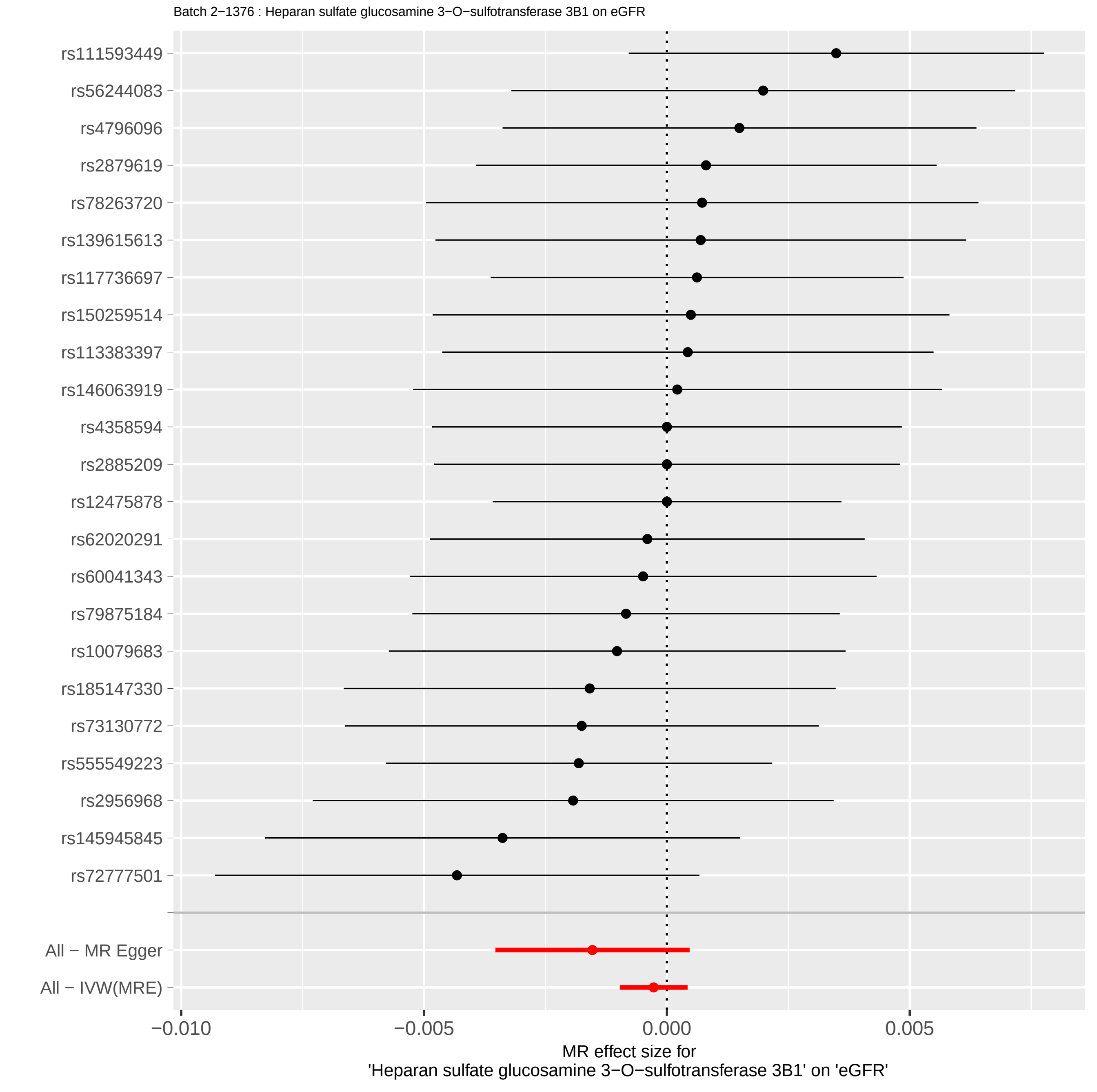

Batch 2−1376 : Heparan sulfate glucosamine 3−O−sulfotransferase 3B1 on eGFR
rs111593449
rs56244083
rs4796096
rs2879619
rs78263720
rs139615613
rs117736697
rs150259514
rs113383397
rs146063919
rs4358594
rs2885209
rs12475878
rs62020291
rs60041343
rs79875184
rs10079683
rs185147330
rs73130772
rs555549223
rs2956968
rs145945845
rs72777501
All − MR Egger
All − IVW(MRE)
−0.010
−0.005
0.000
0.005
MR effect size for
'Heparan sulfate glucosamine 3−O−sulfotransferase 3B1' on 'eGFR'

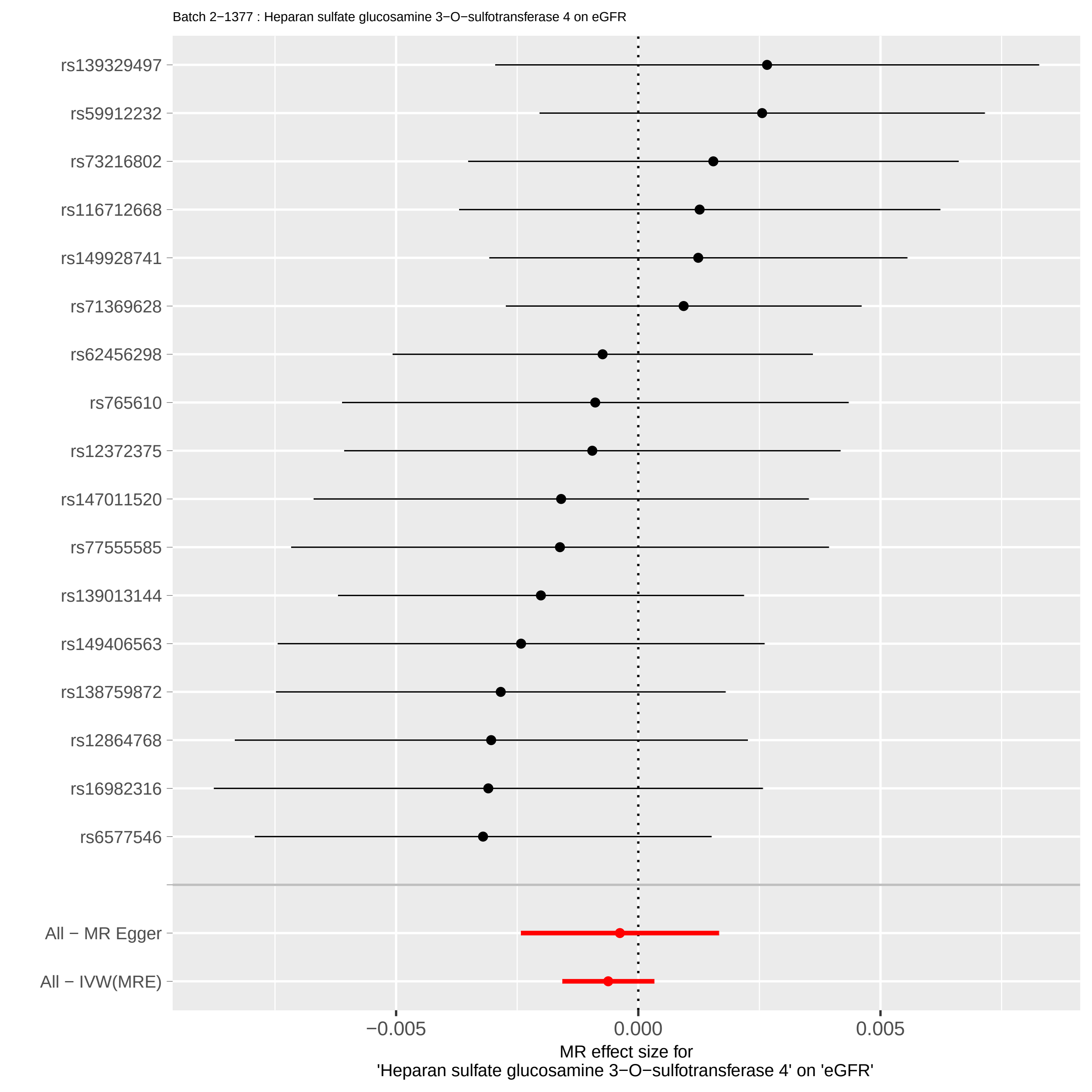

Batch 2−1377 : Heparan sulfate glucosamine 3−O−sulfotransferase 4 on eGFR
rs139329497
rs59912232
rs73216802
rs116712668
rs149928741
rs71369628
rs62456298
rs765610
rs12372375
rs147011520
rs77555585
rs139013144
rs149406563
rs138759872
rs12864768
rs16982316
rs6577546
All − MR Egger
All − IVW(MRE)
−0.005
0.000
0.005
MR effect size for
'Heparan sulfate glucosamine 3−O−sulfotransferase 4' on 'eGFR'

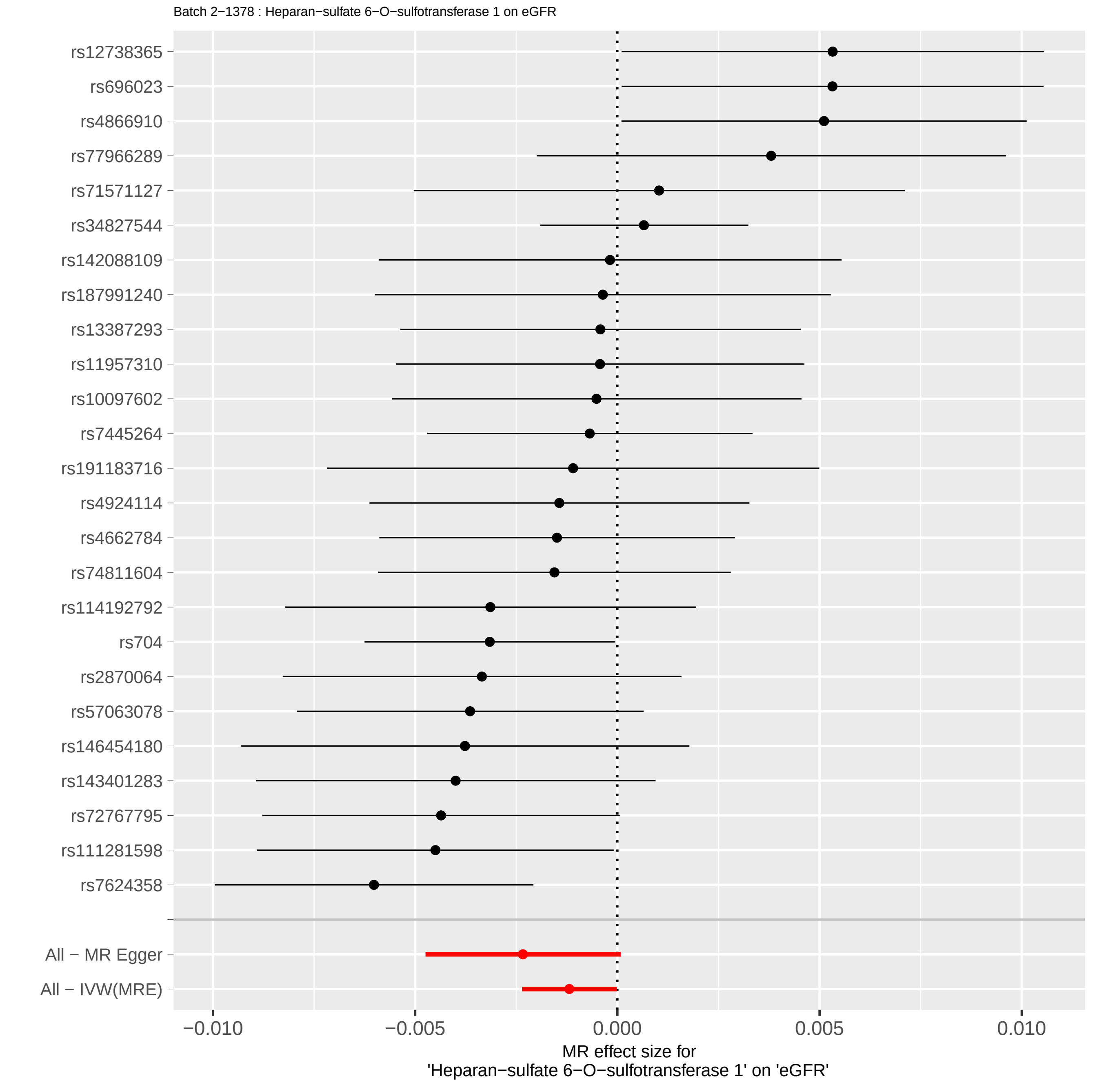

Batch 2−1378 : Heparan−sulfate 6−O−sulfotransferase 1 on eGFR
rs12738365
rs696023
rs4866910
rs77966289
rs71571127
rs34827544
rs142088109
rs187991240
rs13387293
rs11957310
rs10097602
rs7445264
rs191183716
rs4924114
rs4662784
rs74811604
rs114192792
rs704
rs2870064
rs57063078
rs146454180
rs143401283
rs72767795
rs111281598
rs7624358
All − MR Egger
All − IVW(MRE)
−0.010
−0.005
0.000
0.005
0.010
MR effect size for
'Heparan−sulfate 6−O−sulfotransferase 1' on 'eGFR'

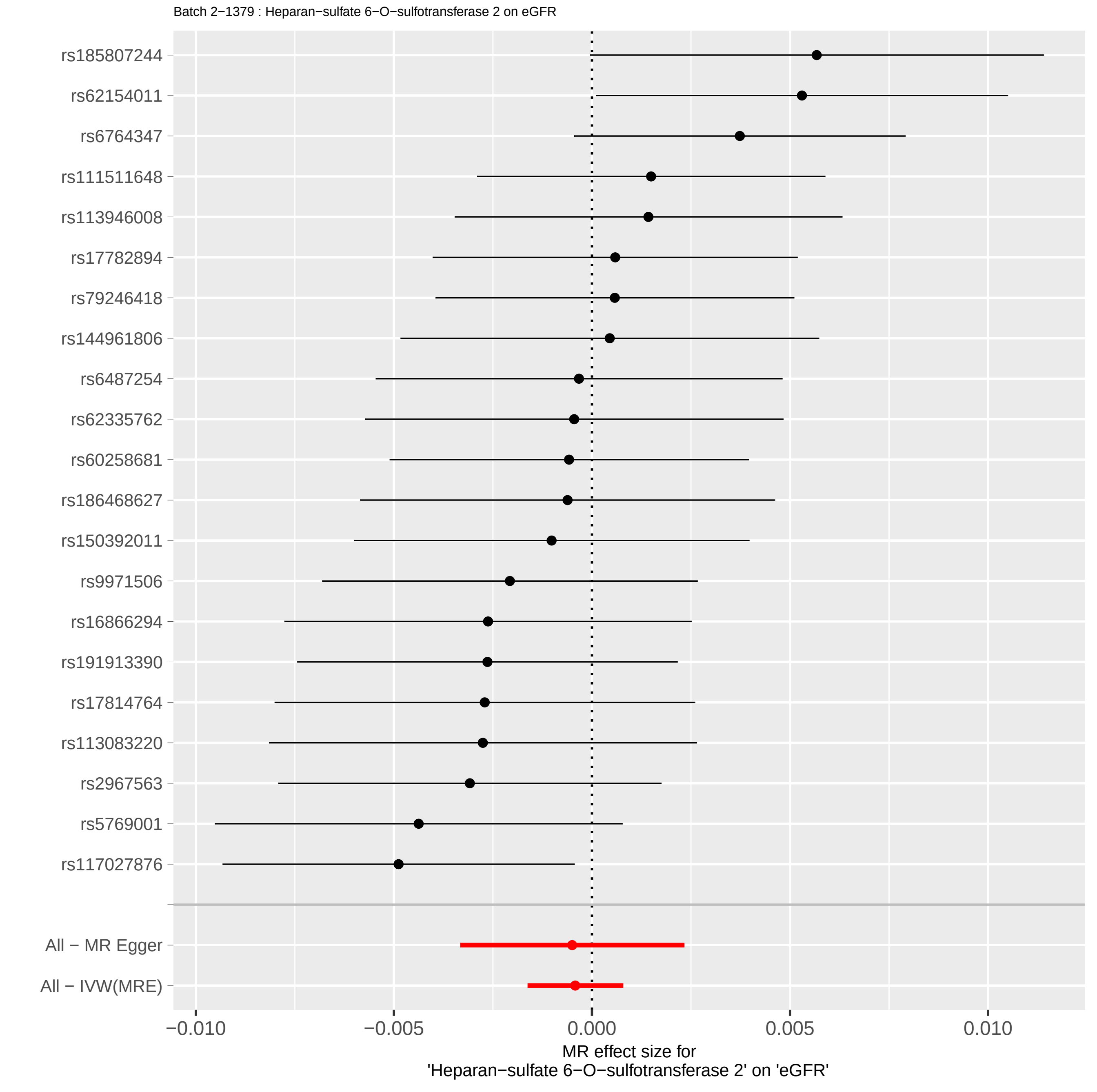
Batch 2−1379 : Heparan−sulfate 6−O−sulfotransferase 2 on eGFR
rs185807244
rs62154011
rs6764347
rs111511648
rs113946008
rs17782894
rs79246418
rs144961806
rs6487254
rs62335762
rs60258681
rs186468627
rs150392011
rs9971506
rs16866294
rs191913390
rs17814764
rs113083220
rs2967563
rs5769001
rs117027876
All − MR Egger
All − IVW(MRE)
−0.010
−0.005
0.000
0.005
0.010
MR effect size for
'Heparan−sulfate 6−O−sulfotransferase 2' on 'eGFR'

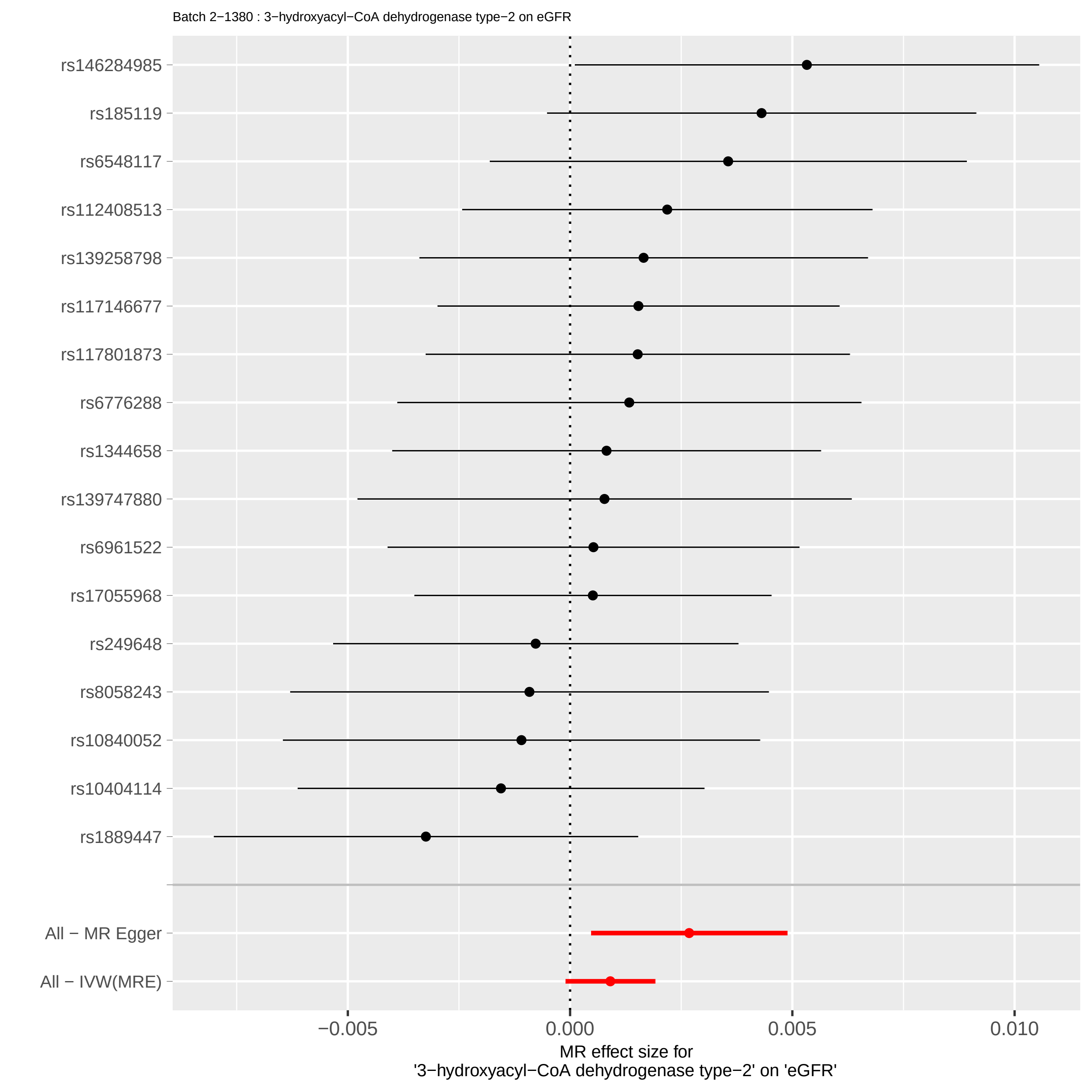

Batch 2−1380 : 3−hydroxyacyl−CoA dehydrogenase type−2 on eGFR
rs146284985
rs185119
rs6548117
rs112408513
rs139258798
rs117146677
rs117801873
rs6776288
rs1344658
rs139747880
rs6961522
rs17055968
rs249648
rs8058243
rs10840052
rs10404114
rs1889447
All − MR Egger
All − IVW(MRE)
−0.005
0.000
0.005
0.010
MR effect size for
'3−hydroxyacyl−CoA dehydrogenase type−2' on 'eGFR'

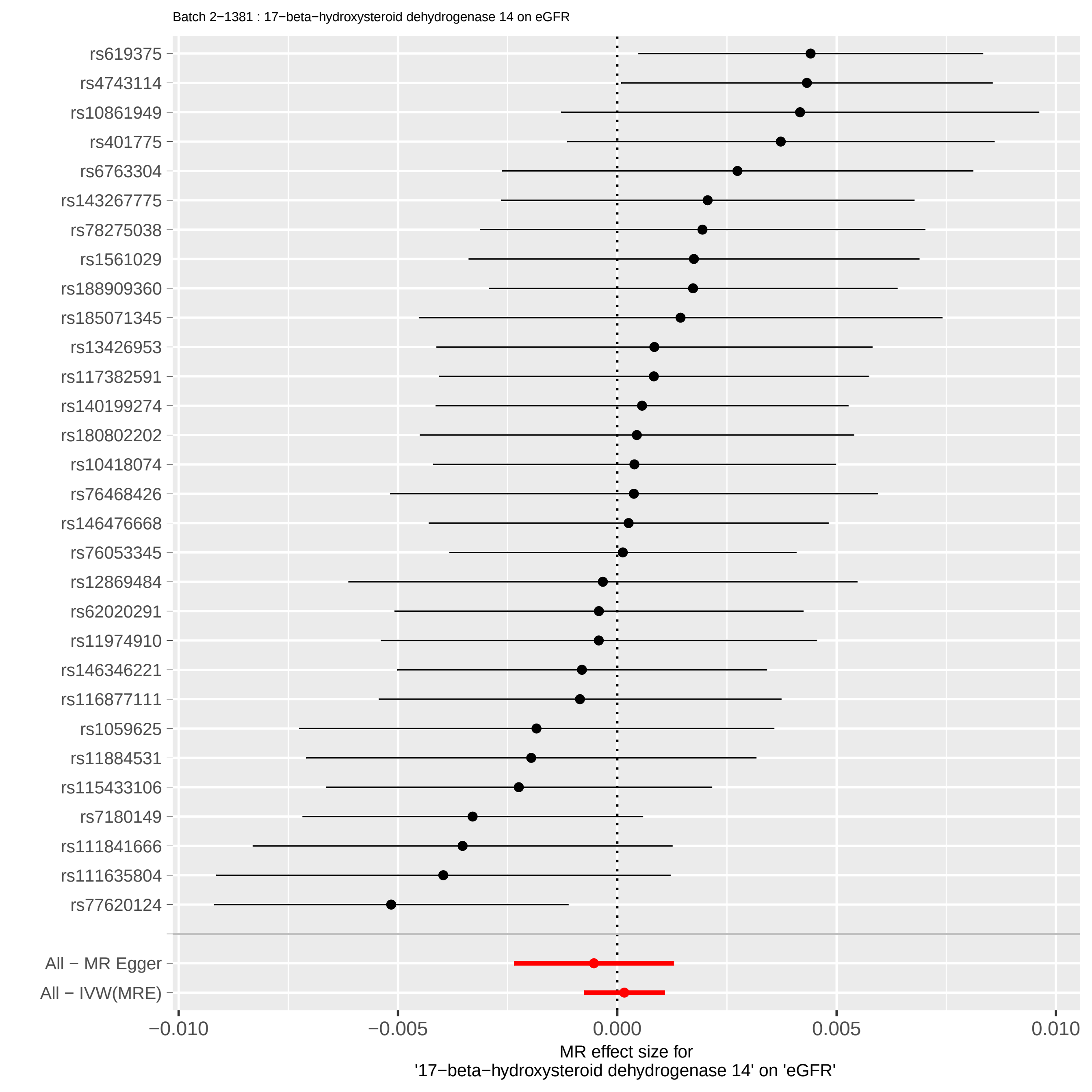

Batch 2−1381 : 17−beta−hydroxysteroid dehydrogenase 14 on eGFR
rs619375
rs4743114
rs10861949
rs401775
rs6763304
rs143267775
rs78275038
rs1561029
rs188909360
rs185071345
rs13426953
rs117382591
rs140199274
rs180802202
rs10418074
rs76468426
rs146476668
rs76053345
rs12869484
rs62020291
rs11974910
rs146346221
rs116877111
rs1059625
rs11884531
rs115433106
rs7180149
rs111841666
rs111635804
rs77620124
All − MR Egger
All − IVW(MRE)
−0.010
−0.005
0.000
0.005
0.010
MR effect size for
'17−beta−hydroxysteroid dehydrogenase 14' on 'eGFR'

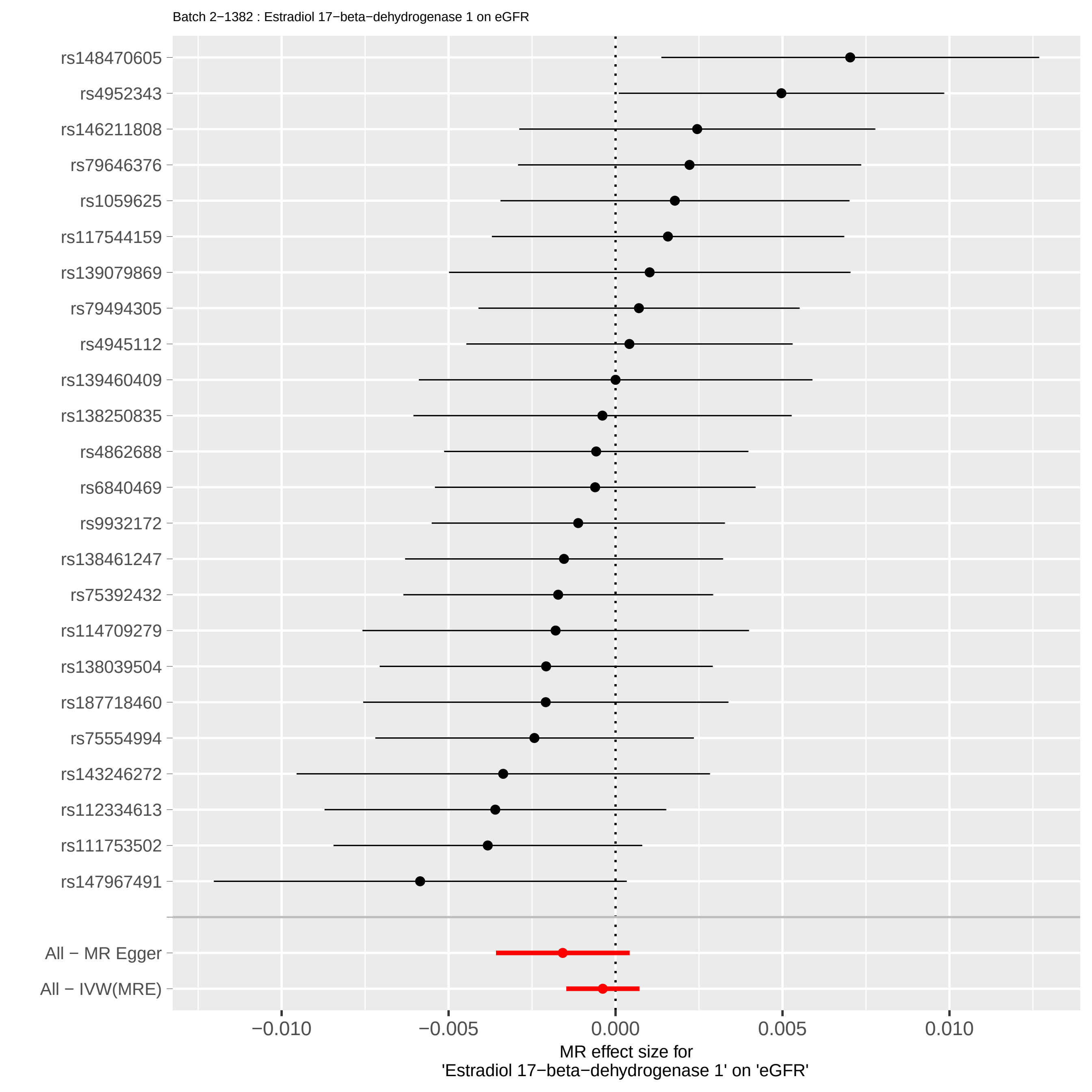

Batch 2−1382 : Estradiol 17−beta−dehydrogenase 1 on eGFR
rs148470605
rs4952343
rs146211808
rs79646376
rs1059625
rs117544159
rs139079869
rs79494305
rs4945112
rs139460409
rs138250835
rs4862688
rs6840469
rs9932172
rs138461247
rs75392432
rs114709279
rs138039504
rs187718460
rs75554994
rs143246272
rs112334613
rs111753502
rs147967491
All − MR Egger
All − IVW(MRE)
−0.010
−0.005
0.000
0.005
0.010
MR effect size for
'Estradiol 17−beta−dehydrogenase 1' on 'eGFR'

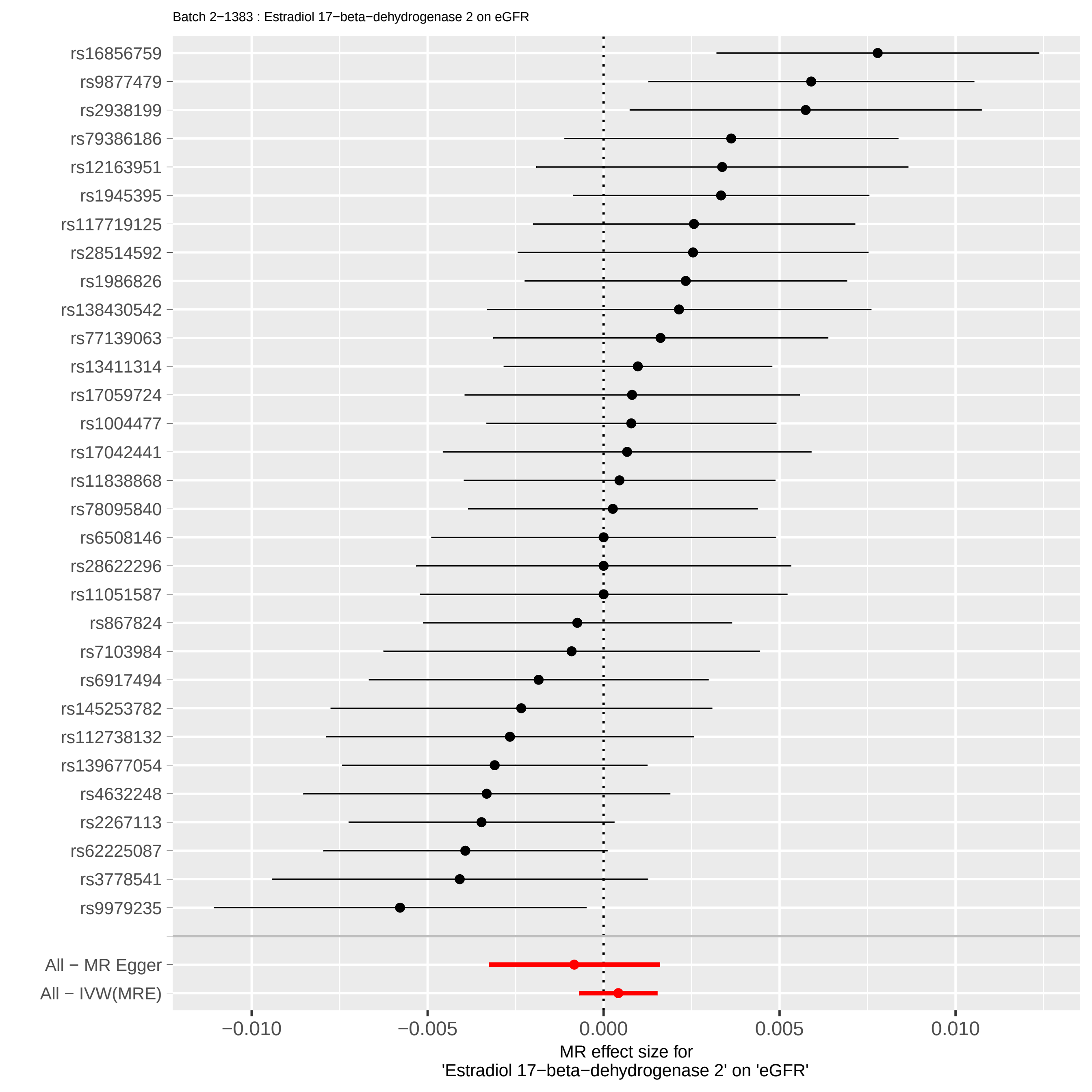

Batch 2−1383 : Estradiol 17−beta−dehydrogenase 2 on eGFR
rs16856759
rs9877479
rs2938199
rs79386186
rs12163951
rs1945395
rs117719125
rs28514592
rs1986826
rs138430542
rs77139063
rs13411314
rs17059724
rs1004477
rs17042441
rs11838868
rs78095840
rs6508146
rs28622296
rs11051587
rs867824
rs7103984
rs6917494
rs145253782
rs112738132
rs139677054
rs4632248
rs2267113
rs62225087
rs3778541
rs9979235
All − MR Egger
All − IVW(MRE)
−0.010
−0.005
0.000
0.005
0.010
MR effect size for
'Estradiol 17−beta−dehydrogenase 2' on 'eGFR'

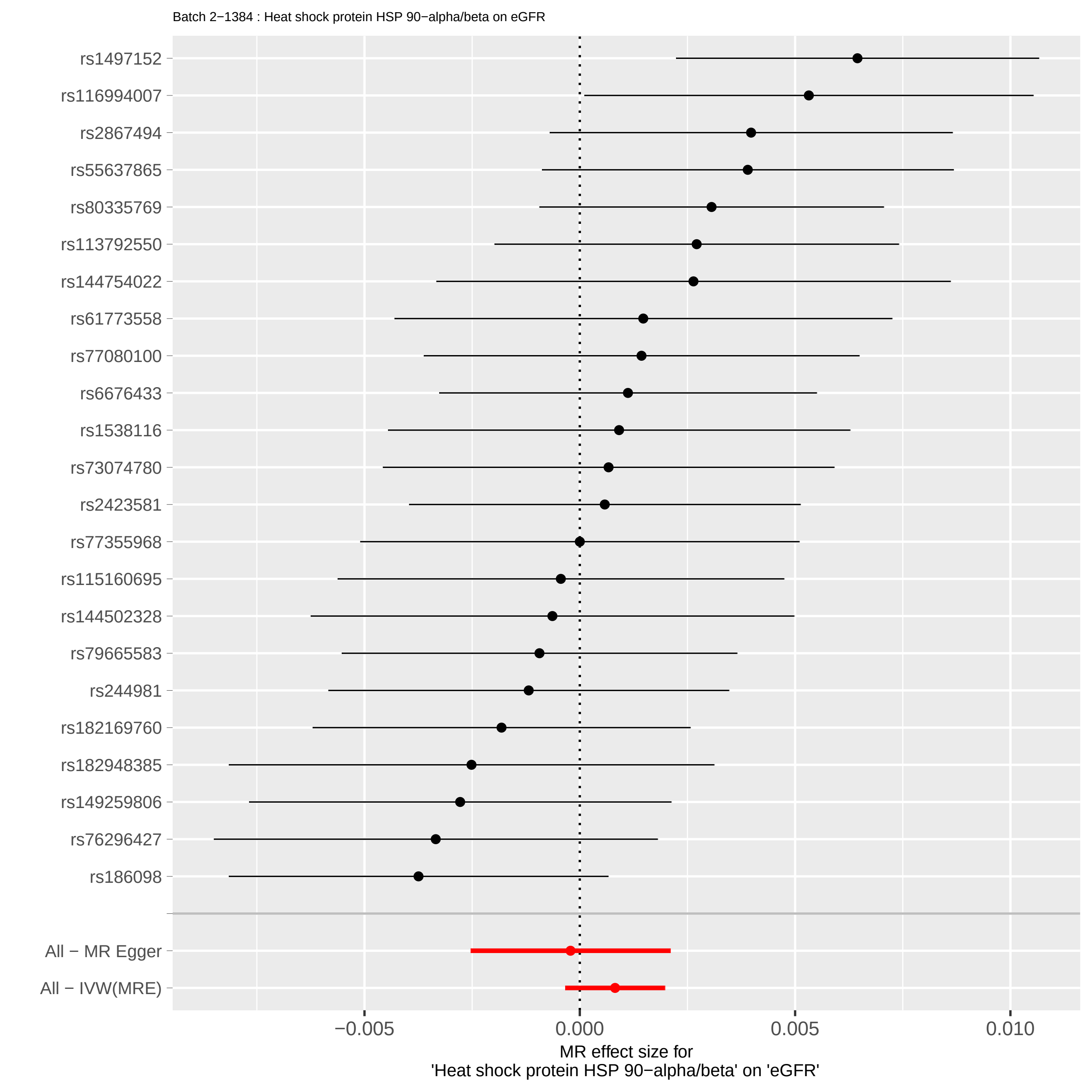
Batch 2−1384 : Heat shock protein HSP 90−alpha/beta on eGFR
rs1497152
rs116994007
rs2867494
rs55637865
rs80335769
rs113792550
rs144754022
rs61773558
rs77080100
rs6676433
rs1538116
rs73074780
rs2423581
rs77355968
rs115160695
rs144502328
rs79665583
rs244981
rs182169760
rs182948385
rs149259806
rs76296427
rs186098
All − MR Egger
All − IVW(MRE)
−0.005
0.000
0.005
0.010
MR effect size for
'Heat shock protein HSP 90−alpha/beta' on 'eGFR'

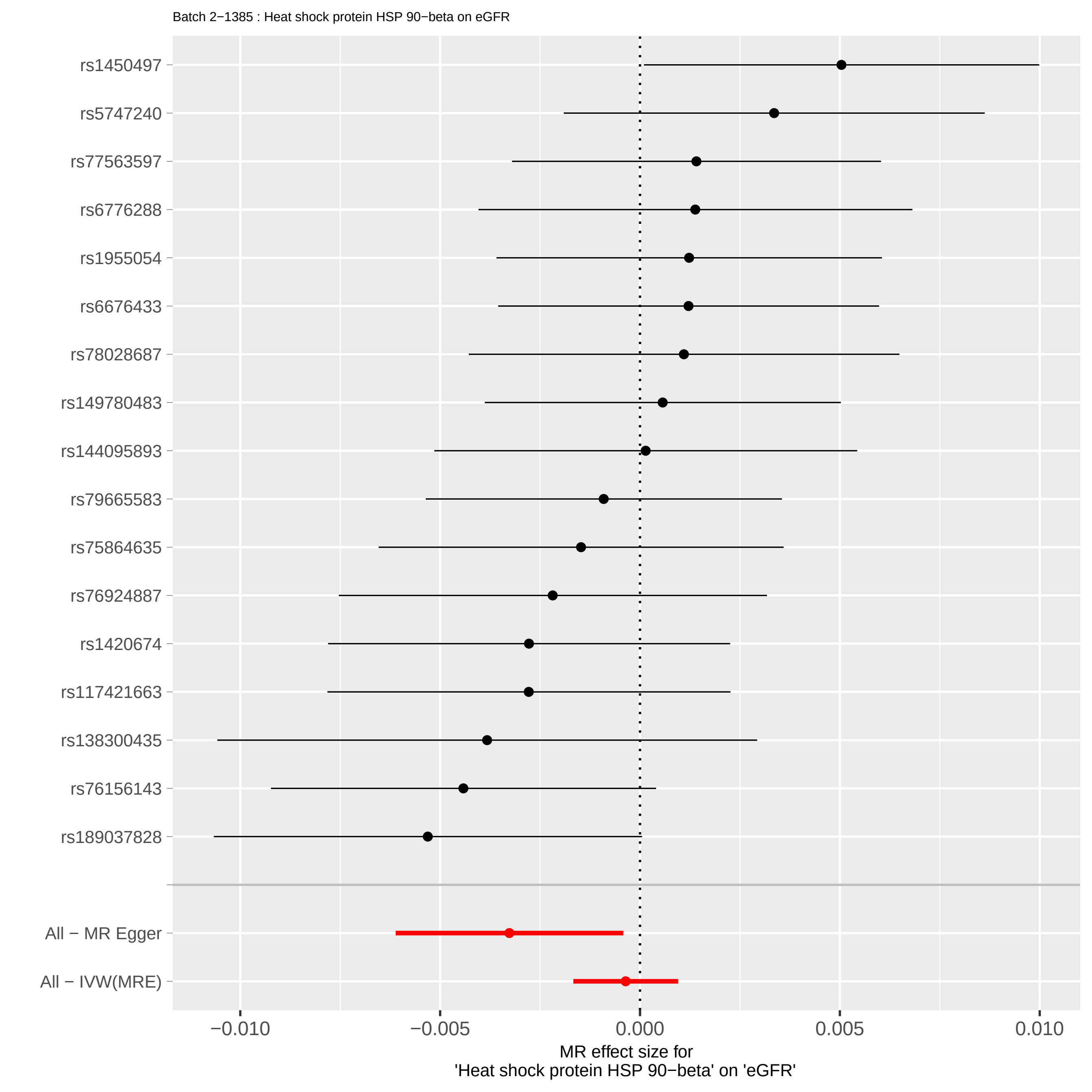
Batch 2−1385 : Heat shock protein HSP 90−beta on eGFR
rs1450497
rs5747240
rs77563597
rs6776288
rs1955054
rs6676433
rs78028687
rs149780483
rs144095893
rs79665583
rs75864635
rs76924887
rs1420674
rs117421663
rs138300435
rs76156143
rs189037828
All − MR Egger
All − IVW(MRE)
−0.010
−0.005
0.000
0.005
0.010
MR effect size for
'Heat shock protein HSP 90−beta' on 'eGFR'

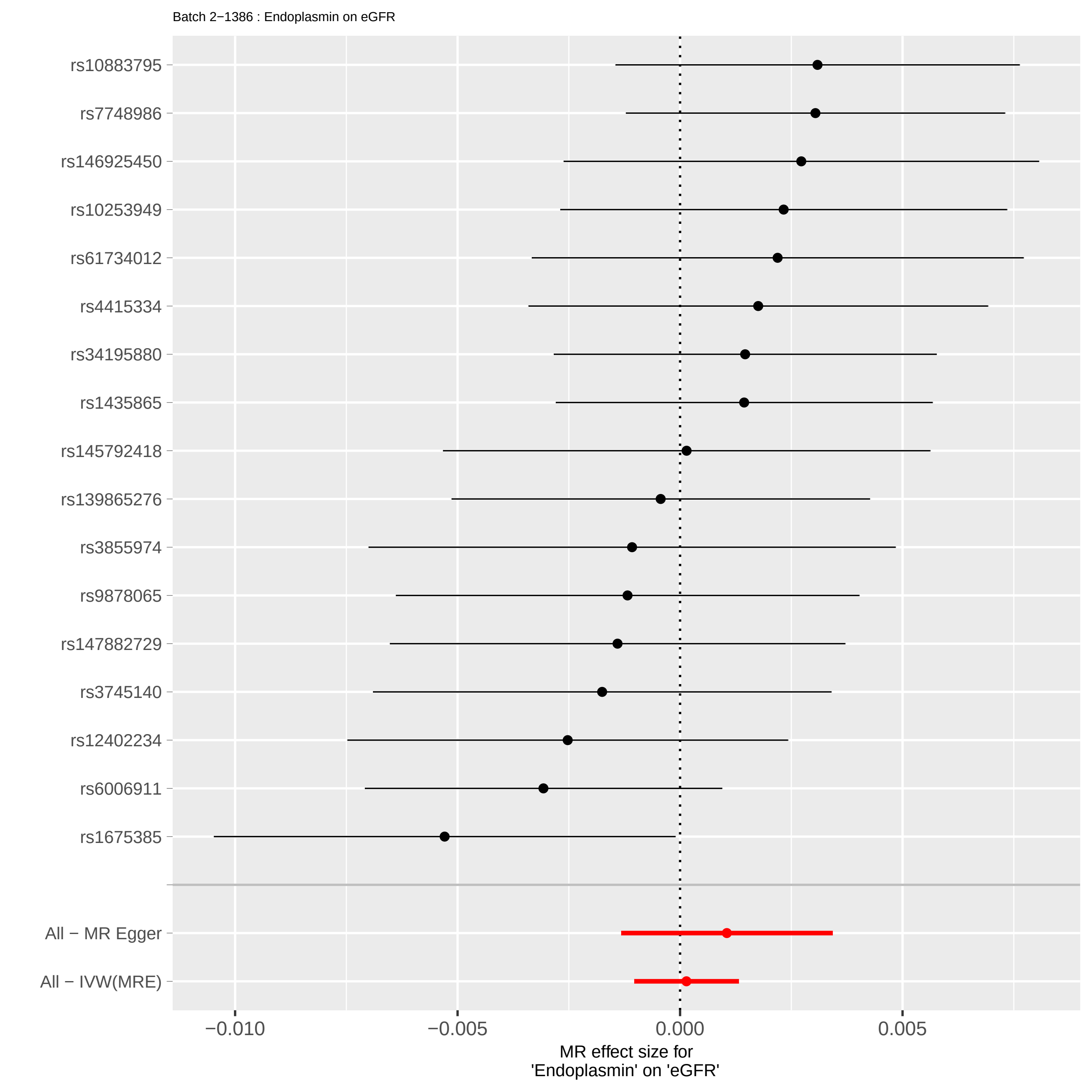

Batch 2−1386 : Endoplasmin on eGFR
rs10883795
rs7748986
rs146925450
rs10253949
rs61734012
rs4415334
rs34195880
rs1435865
rs145792418
rs139865276
rs3855974
rs9878065
rs147882729
rs3745140
rs12402234
rs6006911
rs1675385
All − MR Egger
All − IVW(MRE)
−0.010
−0.005
0.000
0.005
MR effect size for
'Endoplasmin' on 'eGFR'

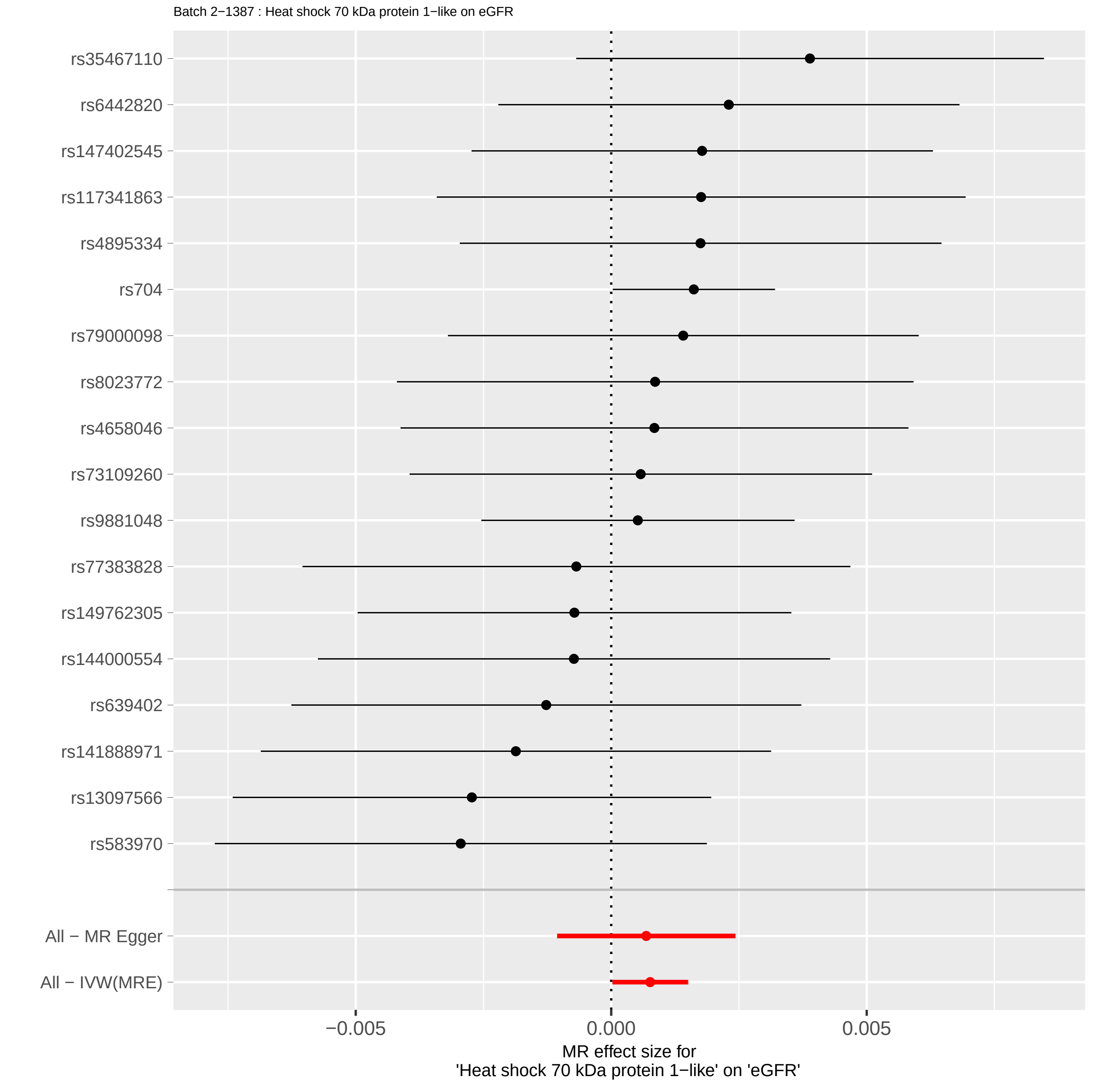
Batch 2−1387 : Heat shock 70 kDa protein 1−like on eGFR
rs35467110
rs6442820
rs147402545
rs117341863
rs4895334
rs704
rs79000098
rs8023772
rs4658046
rs73109260
rs9881048
rs77383828
rs149762305
rs144000554
rs639402
rs141888971
rs13097566
rs583970
All − MR Egger
All − IVW(MRE)
−0.005
0.000
0.005
MR effect size for
'Heat shock 70 kDa protein 1−like' on 'eGFR'

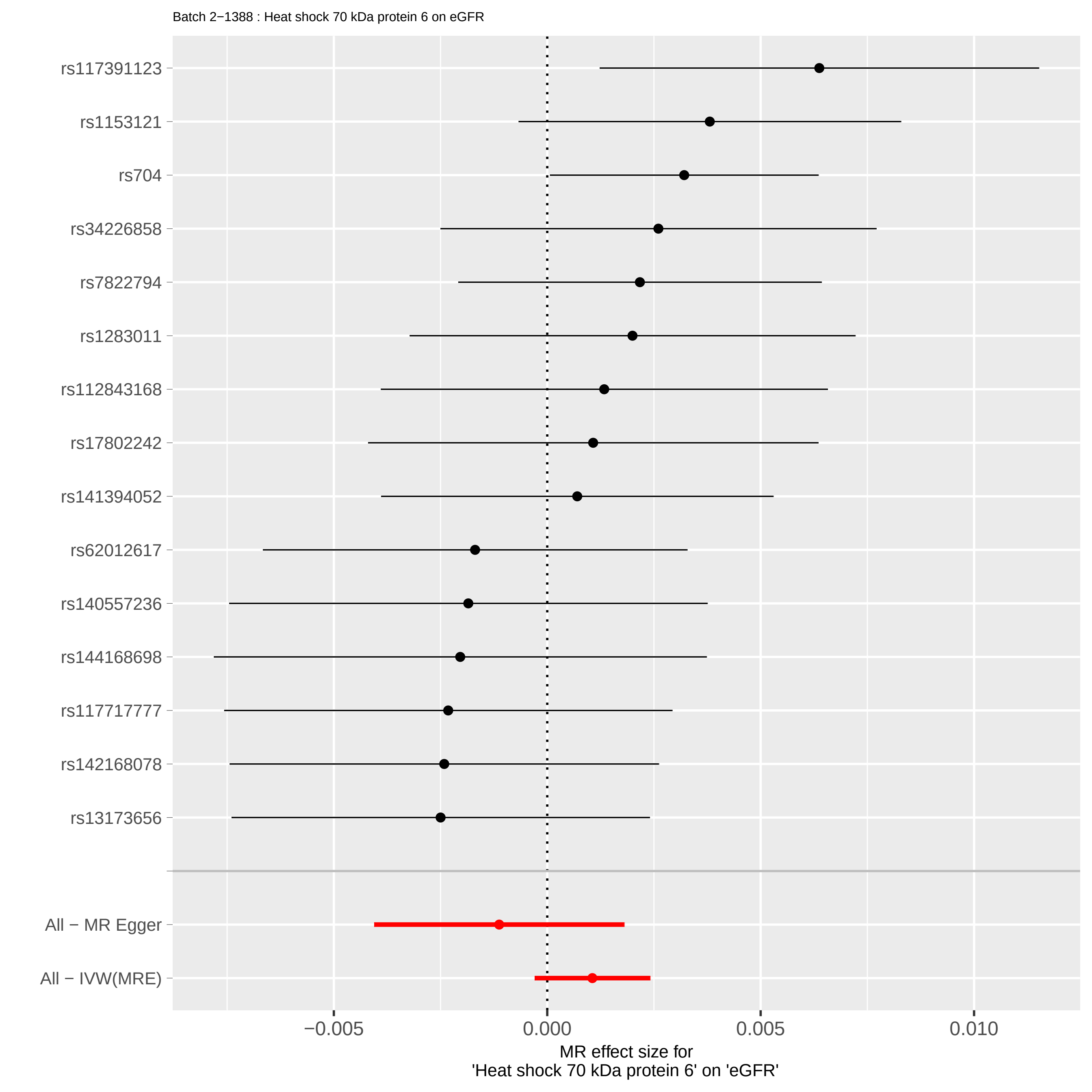

Batch 2−1388 : Heat shock 70 kDa protein 6 on eGFR
rs117391123
rs1153121
rs704
rs34226858
rs7822794
rs1283011
rs112843168
rs17802242
rs141394052
rs62012617
rs140557236
rs144168698
rs117717777
rs142168078
rs13173656
All − MR Egger
All − IVW(MRE)
−0.005
0.000
0.005
0.010
MR effect size for
'Heat shock 70 kDa protein 6' on 'eGFR'

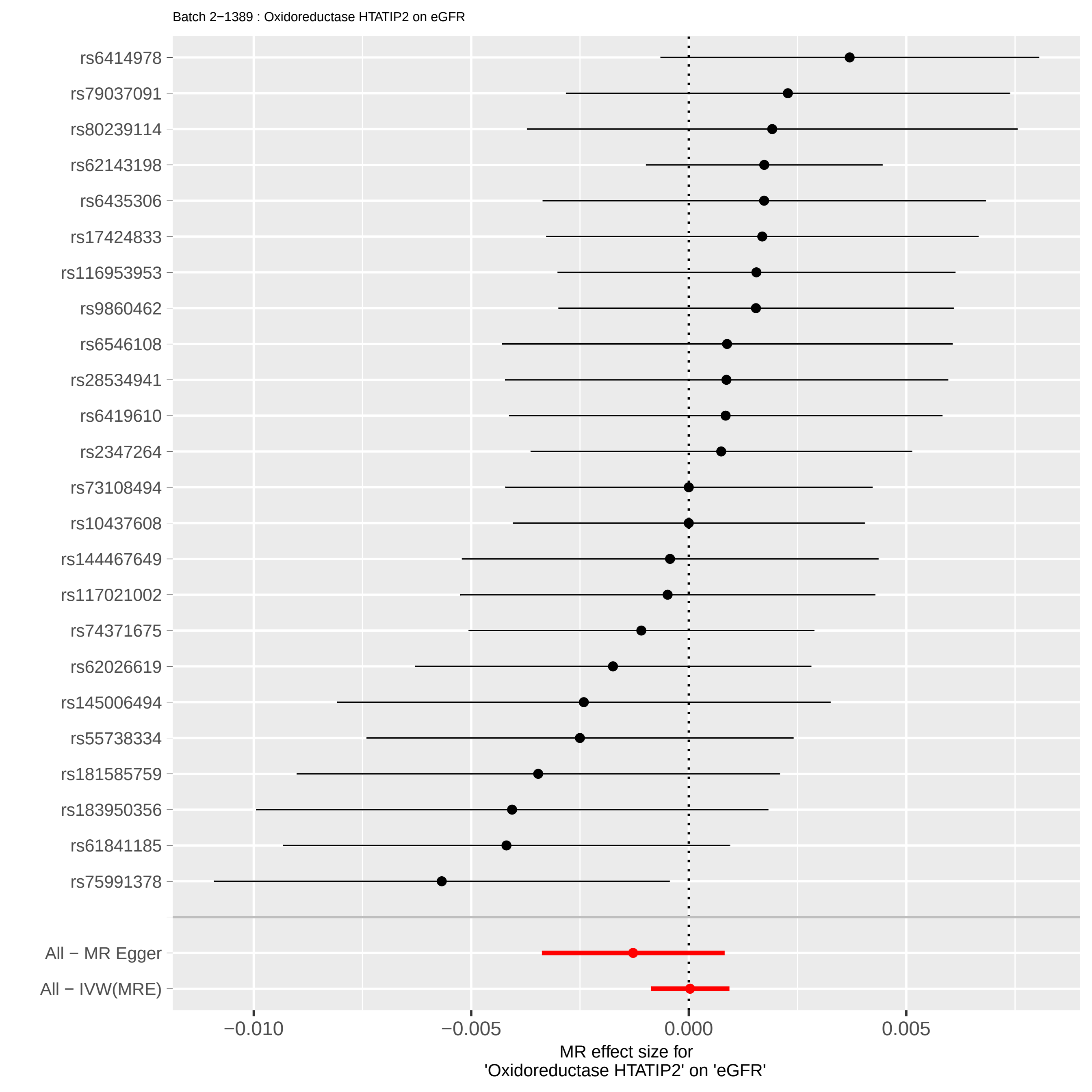

Batch 2−1389 : Oxidoreductase HTATIP2 on eGFR
rs6414978
rs79037091
rs80239114
rs62143198
rs6435306
rs17424833
rs116953953
rs9860462
rs6546108
rs28534941
rs6419610
rs2347264
rs73108494
rs10437608
rs144467649
rs117021002
rs74371675
rs62026619
rs145006494
rs55738334
rs181585759
rs183950356
rs61841185
rs75991378
All − MR Egger
All − IVW(MRE)
−0.010
−0.005
0.000
0.005
MR effect size for
'Oxidoreductase HTATIP2' on 'eGFR'

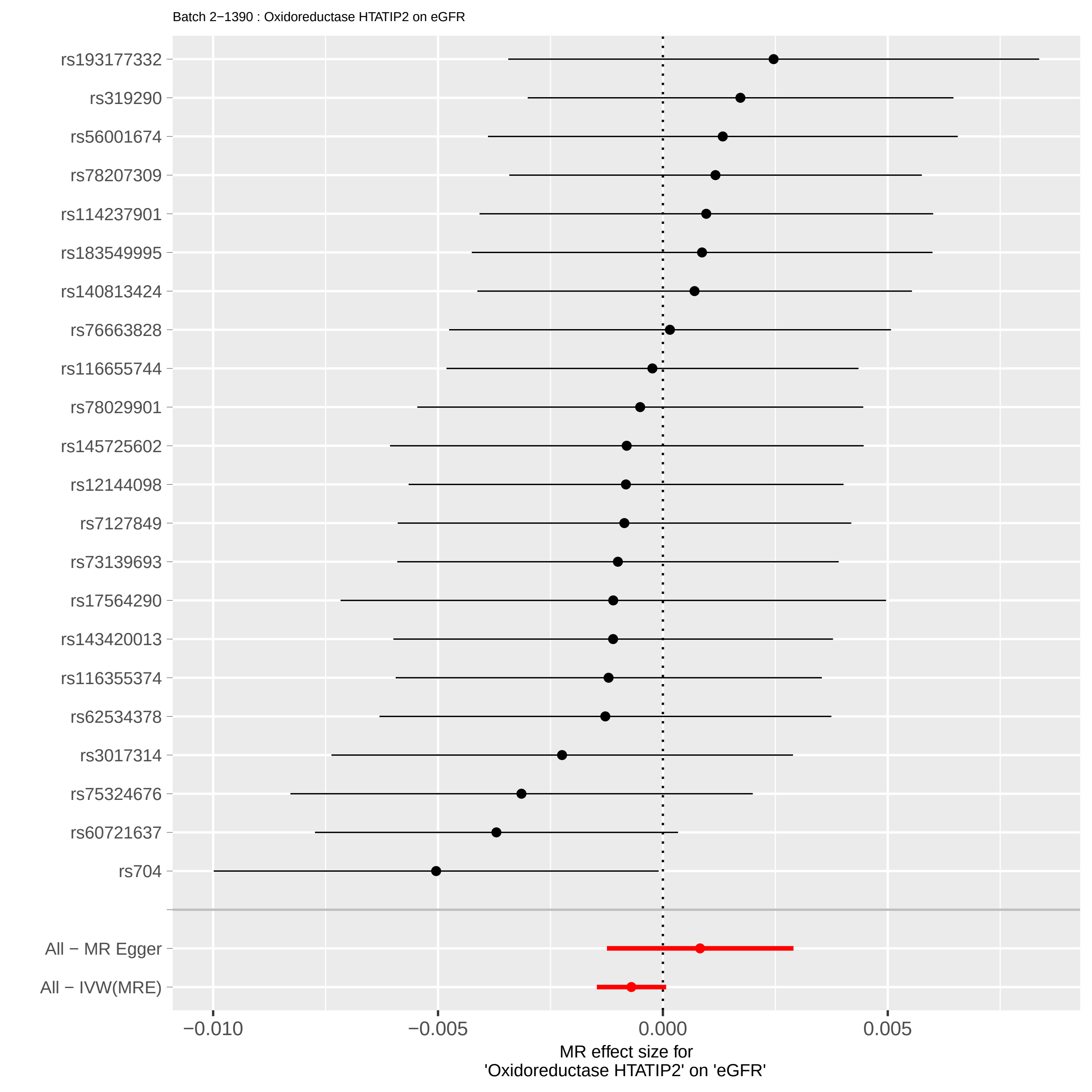
Batch 2−1390 : Oxidoreductase HTATIP2 on eGFR
rs193177332
rs319290
rs56001674
rs78207309
rs114237901
rs183549995
rs140813424
rs76663828
rs116655744
rs78029901
rs145725602
rs12144098
rs7127849
rs73139693
rs17564290
rs143420013
rs116355374
rs62534378
rs3017314
rs75324676
rs60721637
rs704
All − MR Egger
All − IVW(MRE)
−0.010
−0.005
0.000
0.005
MR effect size for
'Oxidoreductase HTATIP2' on 'eGFR'

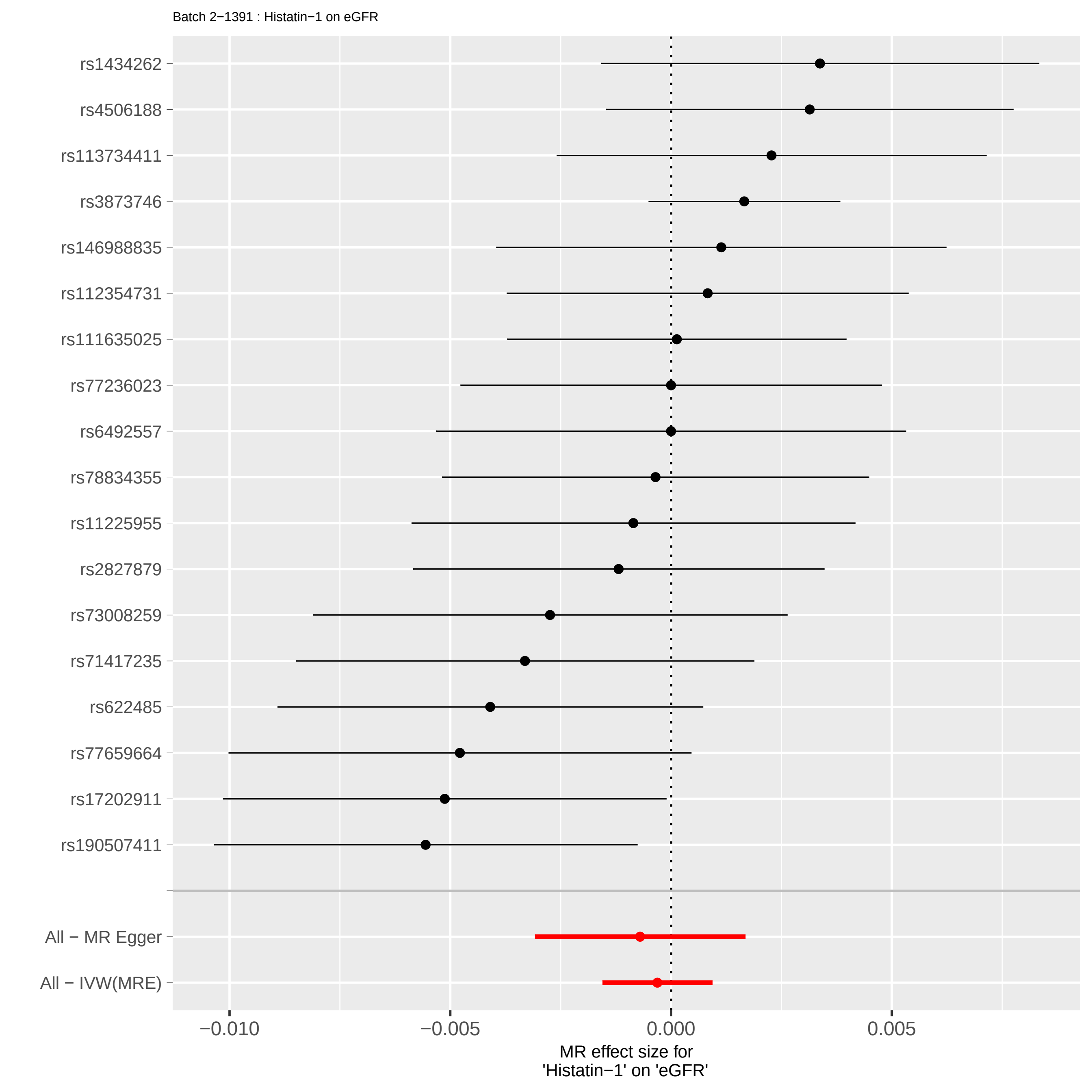
Batch 2−1391 : Histatin−1 on eGFR
rs1434262
rs4506188
rs113734411
rs3873746
rs146988835
rs112354731
rs111635025
rs77236023
rs6492557
rs78834355
rs11225955
rs2827879
rs73008259
rs71417235
rs622485
rs77659664
rs17202911
rs190507411
All − MR Egger
All − IVW(MRE)
−0.010
−0.005
0.000
0.005
MR effect size for
'Histatin−1' on 'eGFR'

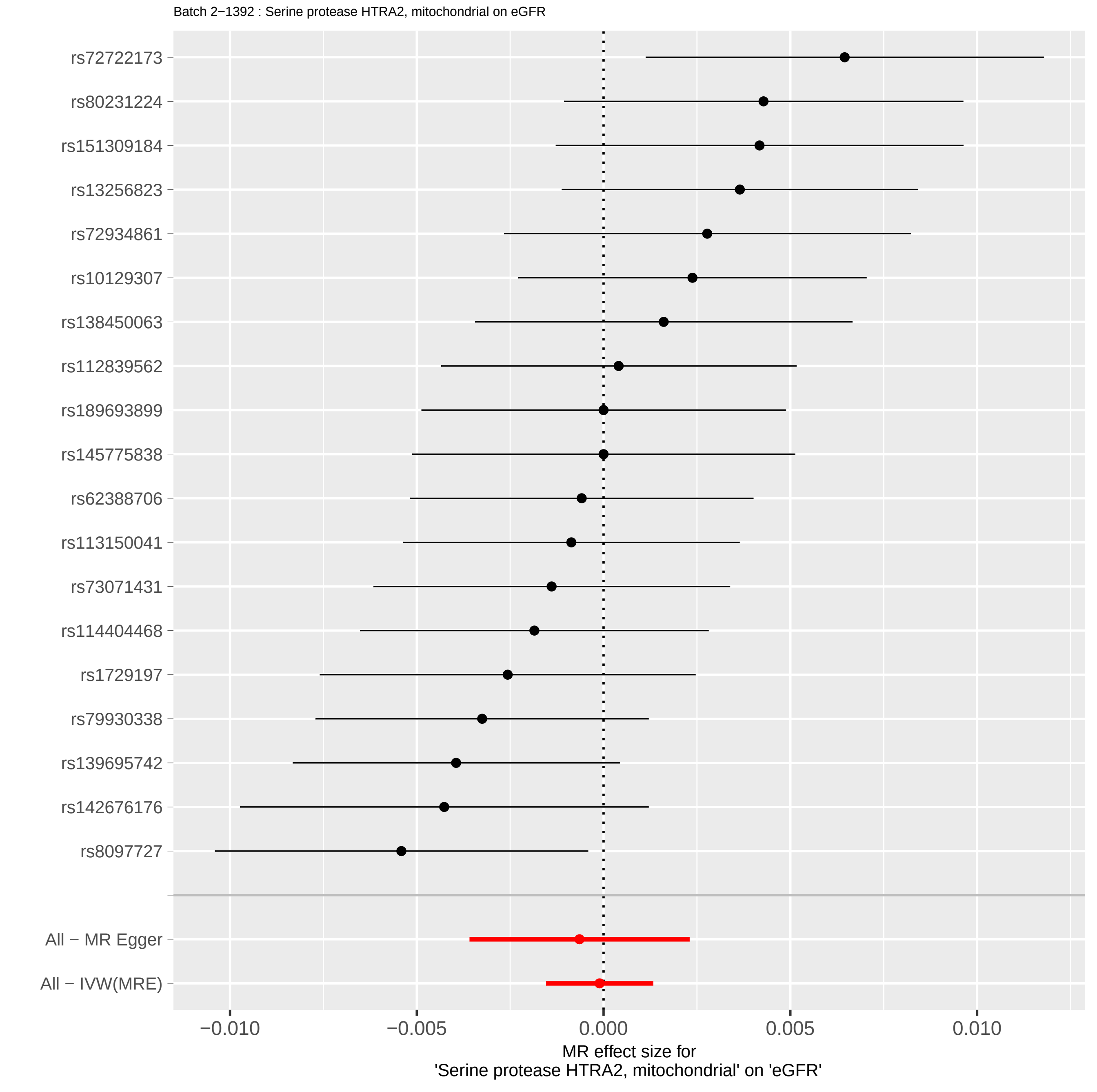

Batch 2−1392 : Serine protease HTRA2, mitochondrial on eGFR
rs72722173
rs80231224
rs151309184
rs13256823
rs72934861
rs10129307
rs138450063
rs112839562
rs189693899
rs145775838
rs62388706
rs113150041
rs73071431
rs114404468
rs1729197
rs79930338
rs139695742
rs142676176
rs8097727
All − MR Egger
All − IVW(MRE)
−0.010
−0.005
0.000
0.005
0.010
MR effect size for
'Serine protease HTRA2, mitochondrial' on 'eGFR'

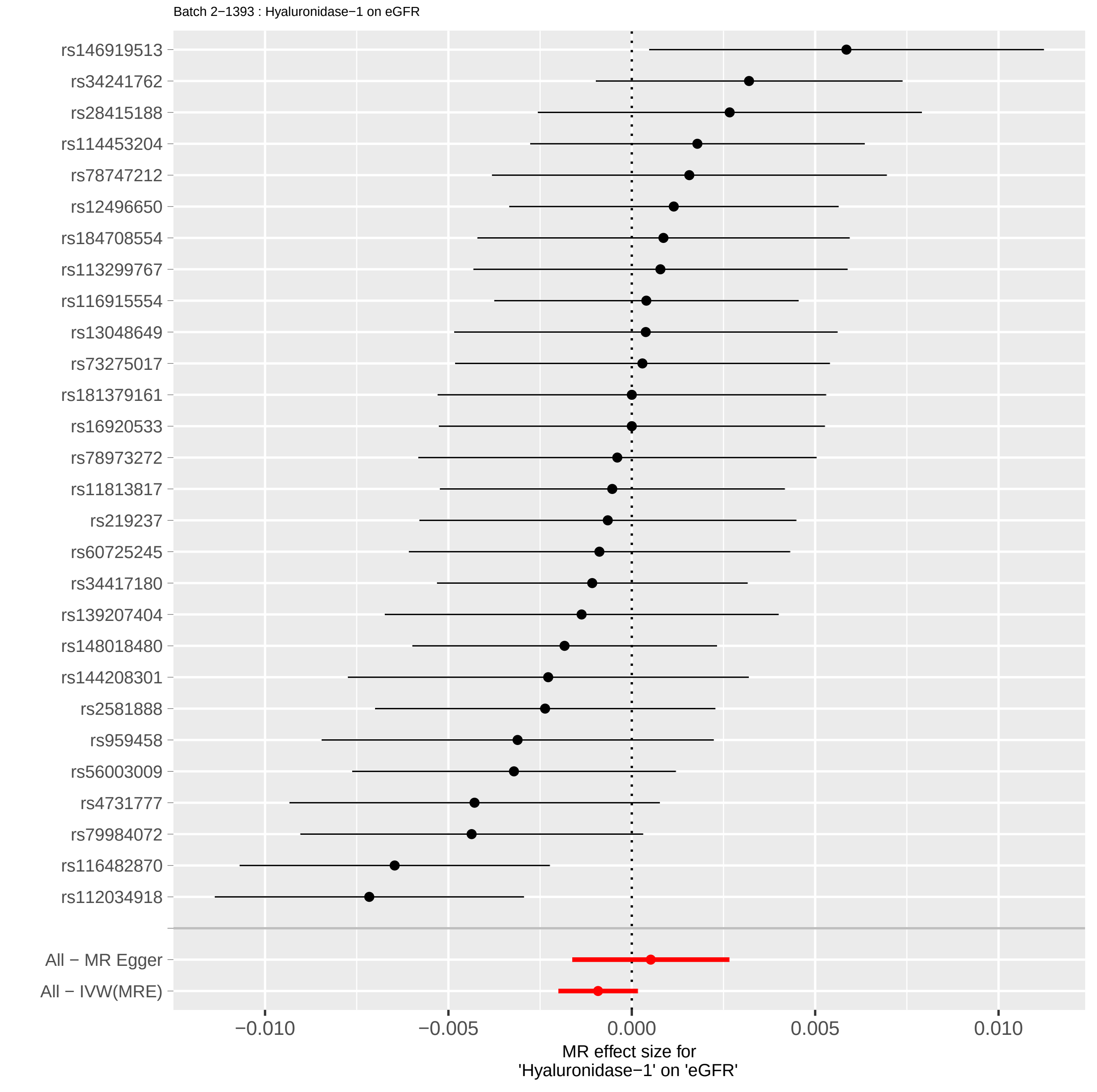

Batch 2−1393 : Hyaluronidase−1 on eGFR
rs146919513
rs34241762
rs28415188
rs114453204
rs78747212
rs12496650
rs184708554
rs113299767
rs116915554
rs13048649
rs73275017
rs181379161
rs16920533
rs78973272
rs11813817
rs219237
rs60725245
rs34417180
rs139207404
rs148018480
rs144208301
rs2581888
rs959458
rs56003009
rs4731777
rs79984072
rs116482870
rs112034918
All − MR Egger
All − IVW(MRE)
−0.010
−0.005
0.000
0.005
0.010
MR effect size for
'Hyaluronidase−1' on 'eGFR'

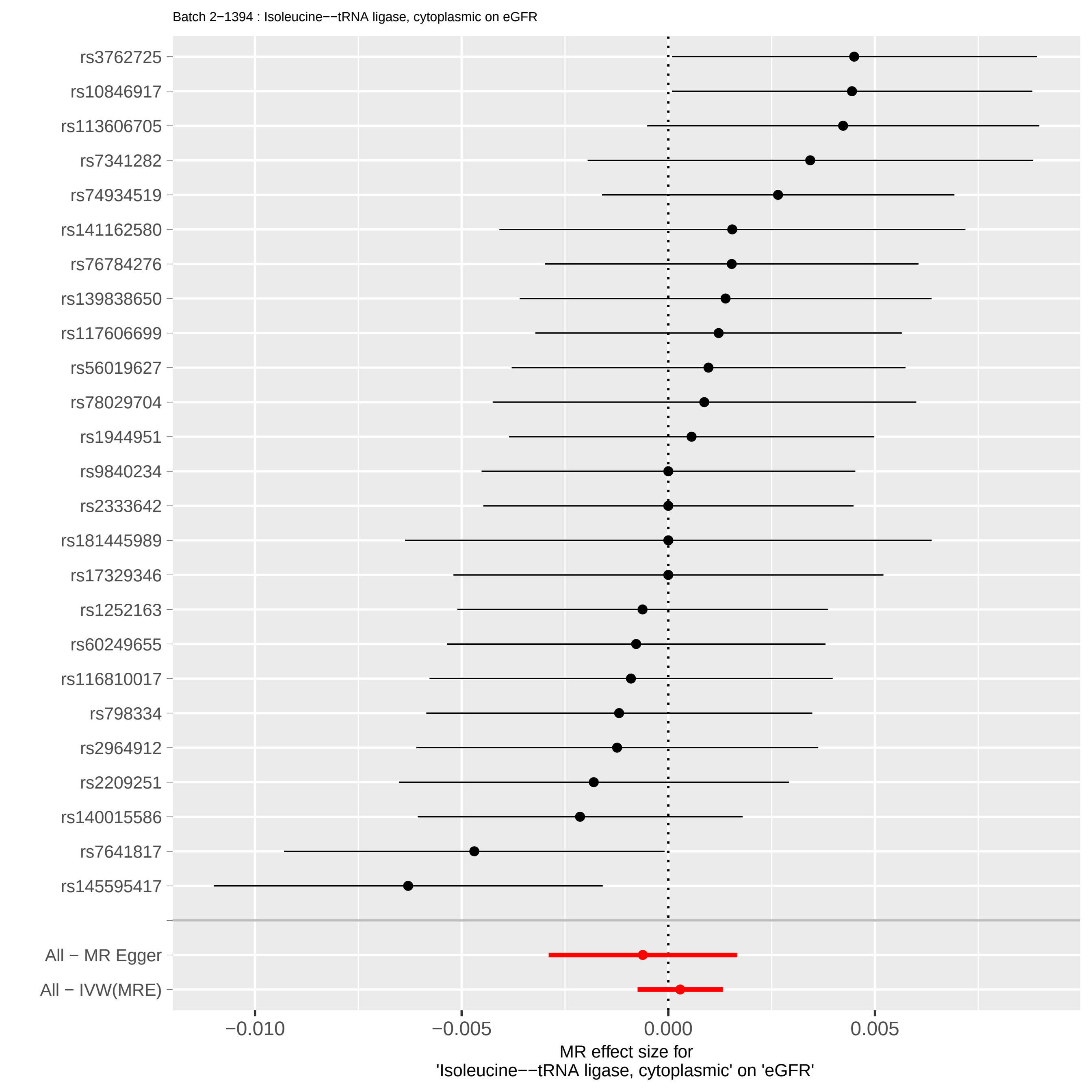

Batch 2−1394 : Isoleucine−−tRNA ligase, cytoplasmic on eGFR
rs3762725
rs10846917
rs113606705
rs7341282
rs74934519
rs141162580
rs76784276
rs139838650
rs117606699
rs56019627
rs78029704
rs1944951
rs9840234
rs2333642
rs181445989
rs17329346
rs1252163
rs60249655
rs116810017
rs798334
rs2964912
rs2209251
rs140015586
rs7641817
rs145595417
All − MR Egger
All − IVW(MRE)
−0.010
−0.005
0.000
0.005
MR effect size for
'Isoleucine−−tRNA ligase, cytoplasmic' on 'eGFR'

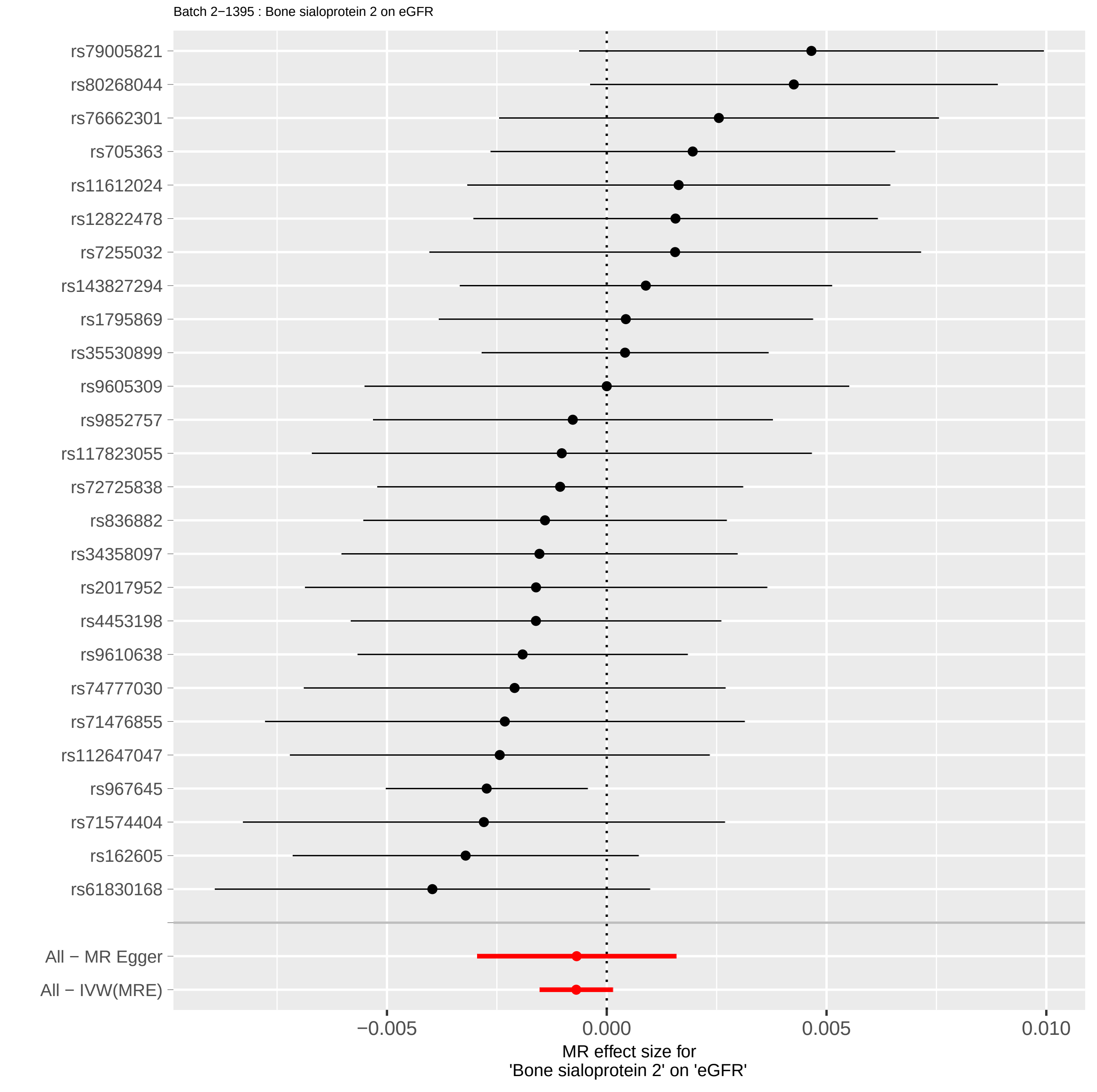

Batch 2−1395 : Bone sialoprotein 2 on eGFR
rs79005821
rs80268044
rs76662301
rs705363
rs11612024
rs12822478
rs7255032
rs143827294
rs1795869
rs35530899
rs9605309
rs9852757
rs117823055
rs72725838
rs836882
rs34358097
rs2017952
rs4453198
rs9610638
rs74777030
rs71476855
rs112647047
rs967645
rs71574404
rs162605
rs61830168
All − MR Egger
All − IVW(MRE)
−0.005
0.000
0.005
0.010
MR effect size for
'Bone sialoprotein 2' on 'eGFR'

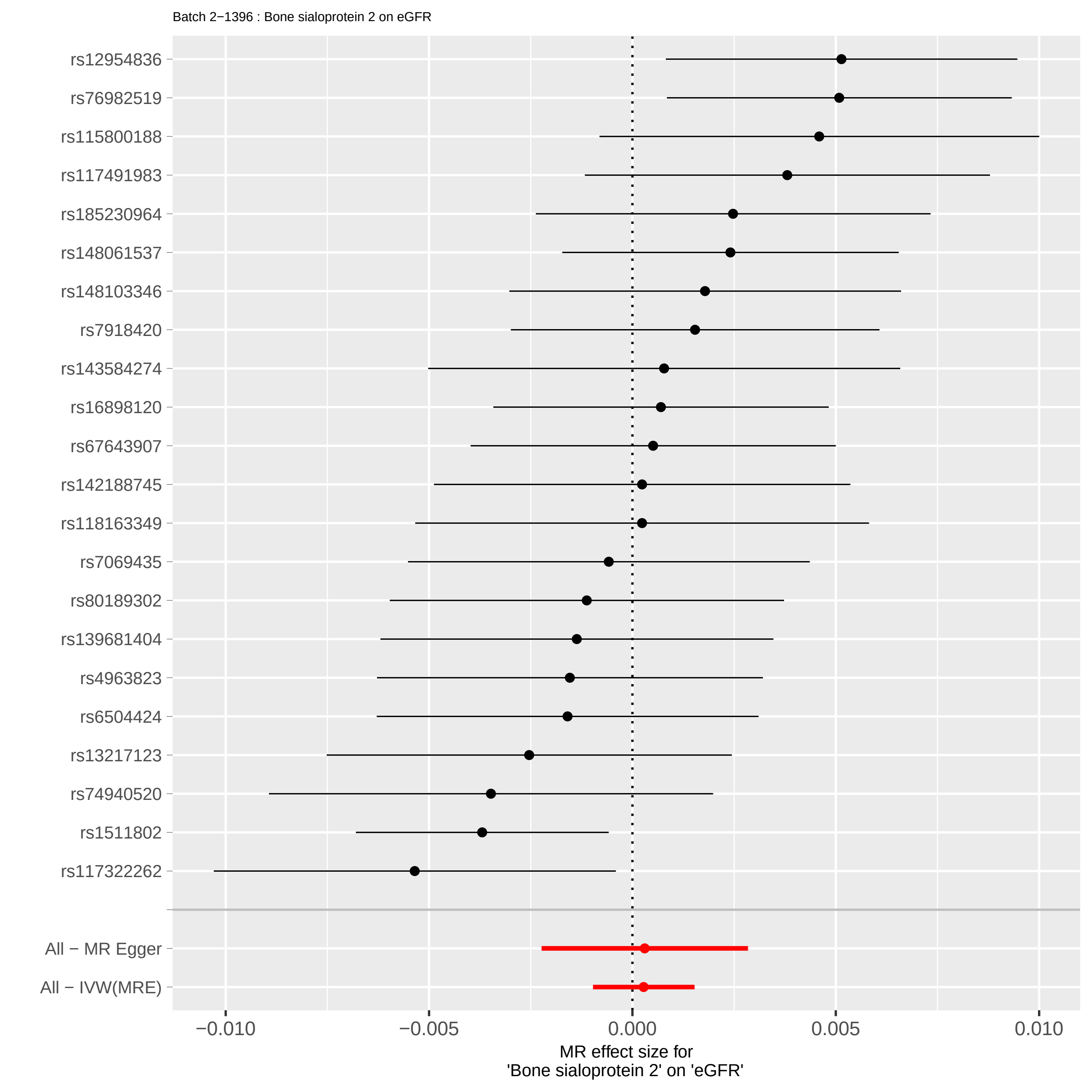

Batch 2−1396 : Bone sialoprotein 2 on eGFR
rs12954836
rs76982519
rs115800188
rs117491983
rs185230964
rs148061537
rs148103346
rs7918420
rs143584274
rs16898120
rs67643907
rs142188745
rs118163349
rs7069435
rs80189302
rs139681404
rs4963823
rs6504424
rs13217123
rs74940520
rs1511802
rs117322262
All − MR Egger
All − IVW(MRE)
−0.010
−0.005
0.000
0.005
0.010
MR effect size for
'Bone sialoprotein 2' on 'eGFR'

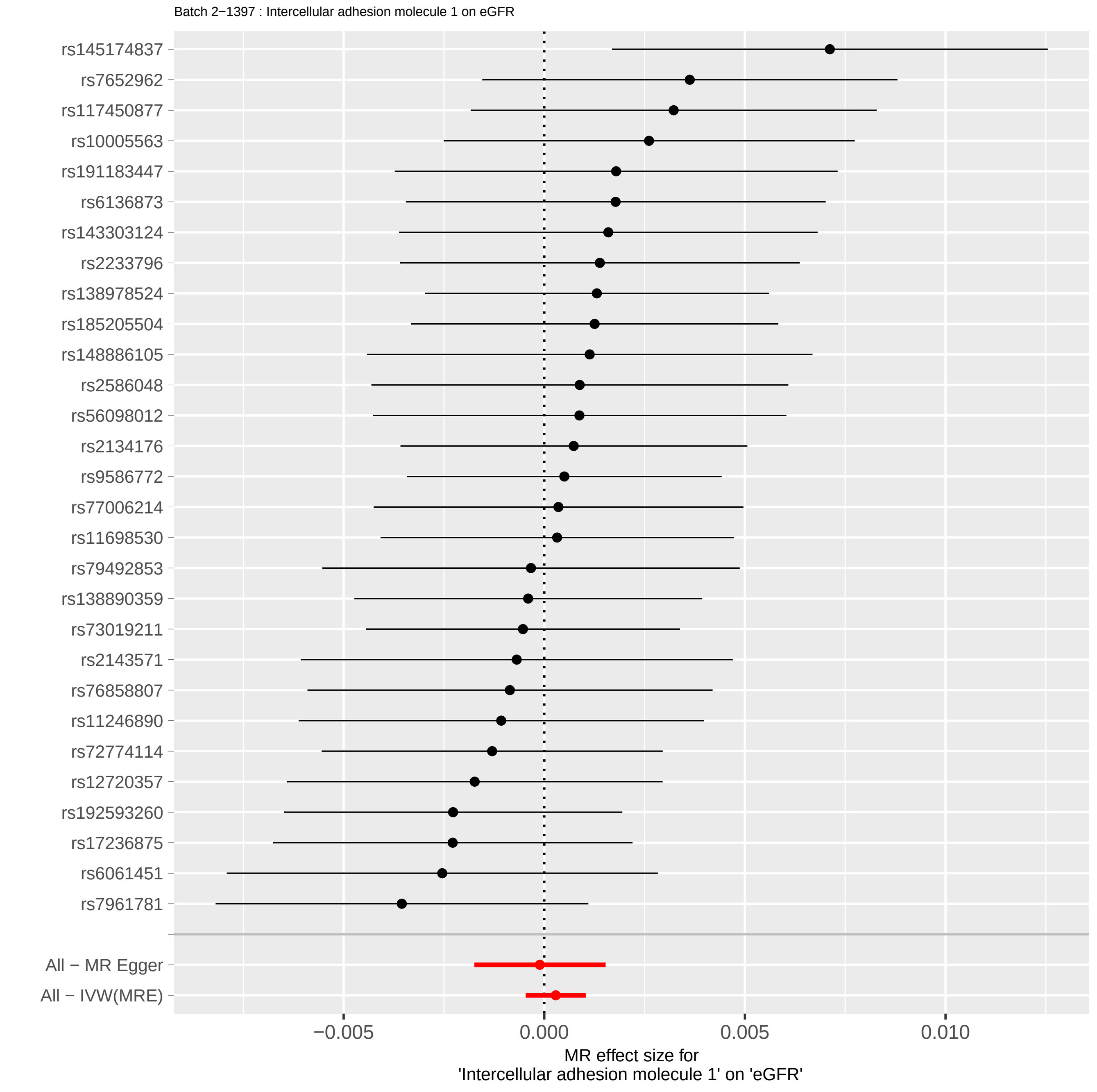
Batch 2−1397 : Intercellular adhesion molecule 1 on eGFR
rs145174837
rs7652962
rs117450877
rs10005563
rs191183447
rs6136873
rs143303124
rs2233796
rs138978524
rs185205504
rs148886105
rs2586048
rs56098012
rs2134176
rs9586772
rs77006214
rs11698530
rs79492853
rs138890359
rs73019211
rs2143571
rs76858807
rs11246890
rs72774114
rs12720357
rs192593260
rs17236875
rs6061451
rs7961781
All − MR Egger
All − IVW(MRE)
−0.005
0.000
0.005
0.010
MR effect size for
'Intercellular adhesion molecule 1' on 'eGFR'

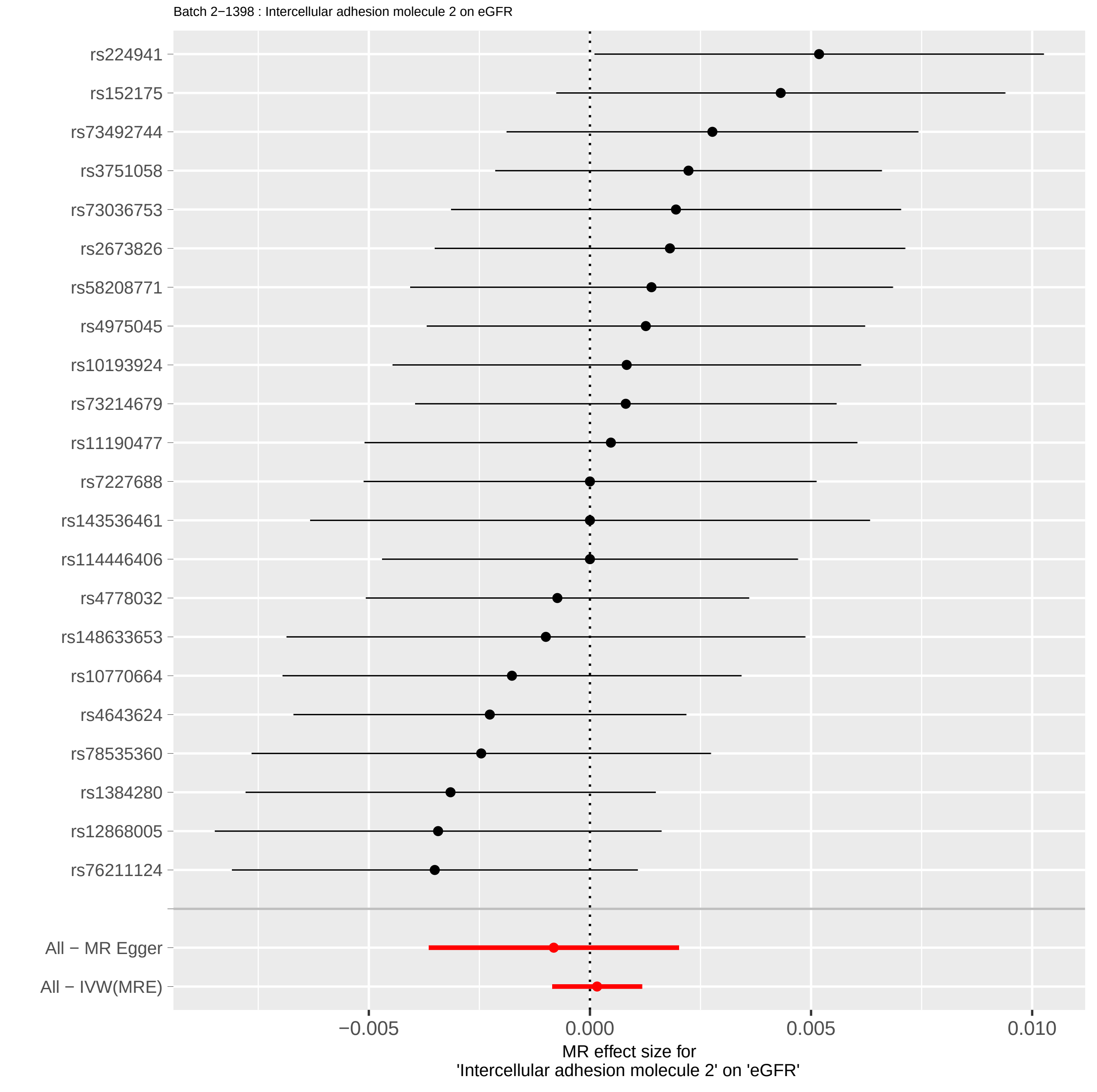
Batch 2−1398 : Intercellular adhesion molecule 2 on eGFR
rs224941
rs152175
rs73492744
rs3751058
rs73036753
rs2673826
rs58208771
rs4975045
rs10193924
rs73214679
rs11190477
rs7227688
rs143536461
rs114446406
rs4778032
rs148633653
rs10770664
rs4643624
rs78535360
rs1384280
rs12868005
rs76211124
All − MR Egger
All − IVW(MRE)
−0.005
0.000
0.005
0.010
MR effect size for
'Intercellular adhesion molecule 2' on 'eGFR'

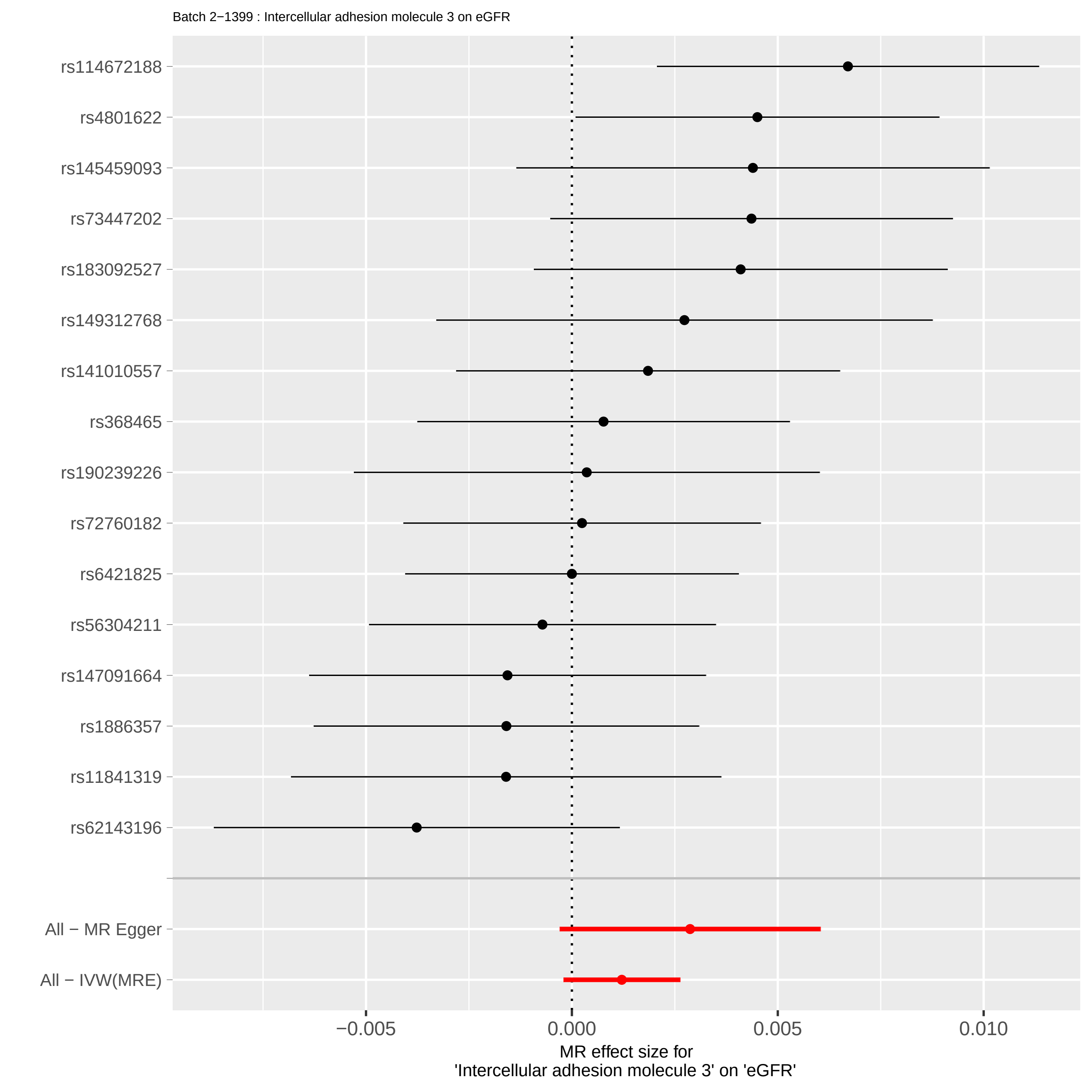

Batch 2−1399 : Intercellular adhesion molecule 3 on eGFR
rs114672188
rs4801622
rs145459093
rs73447202
rs183092527
rs149312768
rs141010557
rs368465
rs190239226
rs72760182
rs6421825
rs56304211
rs147091664
rs1886357
rs11841319
rs62143196
All − MR Egger
All − IVW(MRE)
−0.005
0.000
0.005
0.010
MR effect size for
'Intercellular adhesion molecule 3' on 'eGFR'

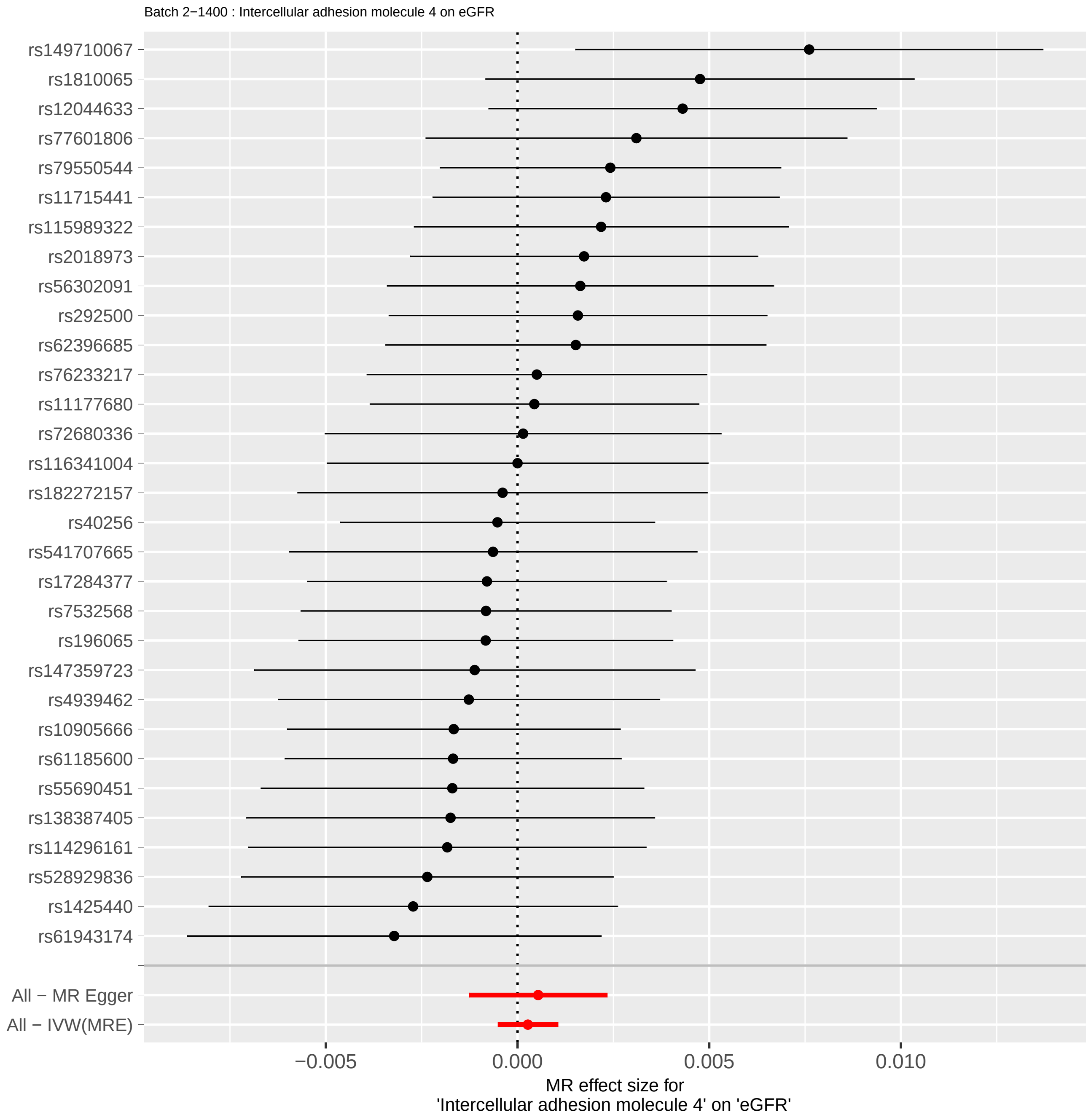
Batch 2−1400 : Intercellular adhesion molecule 4 on eGFR
rs149710067
rs1810065
rs12044633
rs77601806
rs79550544
rs11715441
rs115989322
rs2018973
rs56302091
rs292500
rs62396685
rs76233217
rs11177680
rs72680336
rs116341004
rs182272157
rs40256
rs541707665
rs17284377
rs7532568
rs196065
rs147359723
rs4939462
rs10905666
rs61185600
rs55690451
rs138387405
rs114296161
rs528929836
rs1425440
rs61943174
All − MR Egger
All − IVW(MRE)
−0.005
0.000
0.005
0.010
MR effect size for
'Intercellular adhesion molecule 4' on 'eGFR'

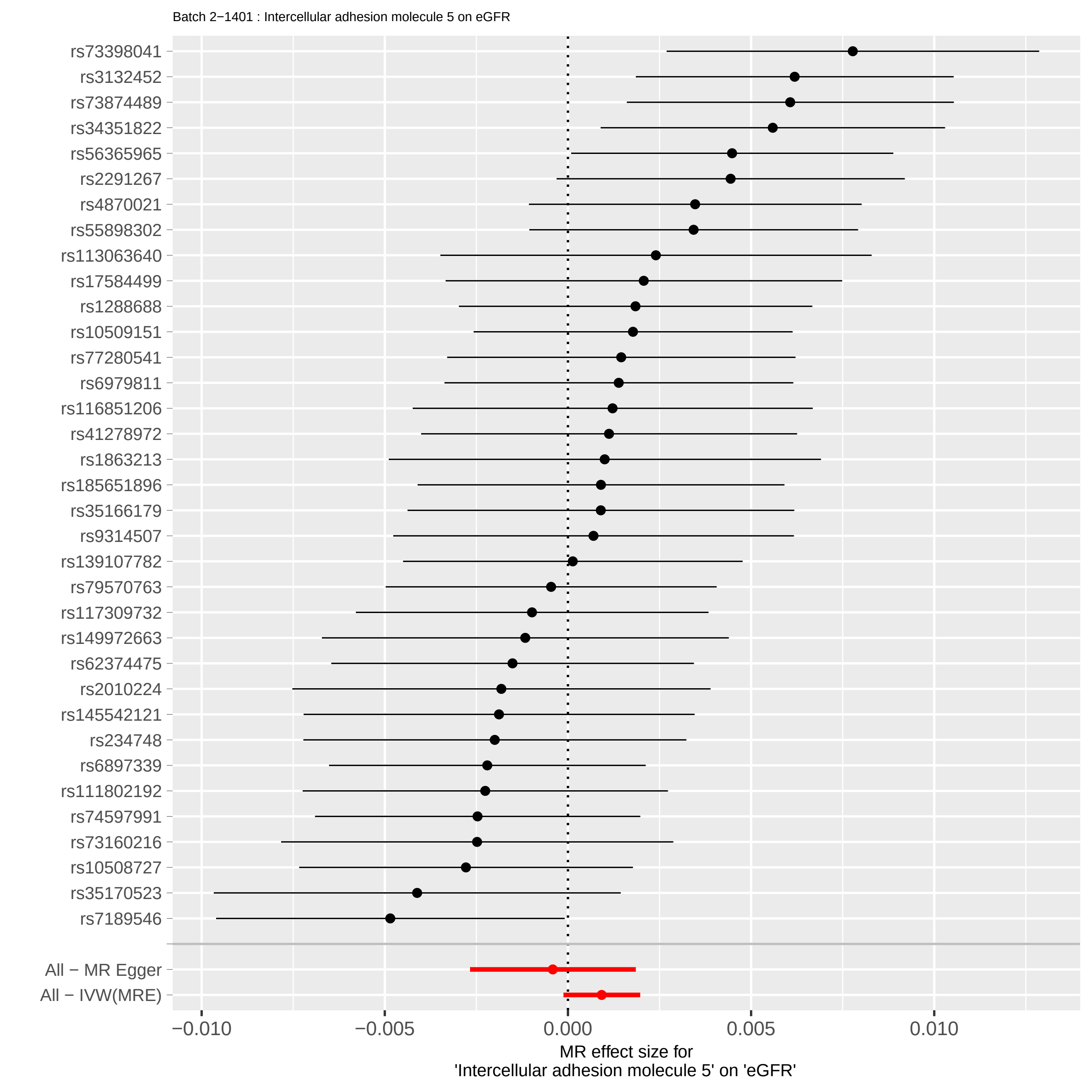

Batch 2−1401 : Intercellular adhesion molecule 5 on eGFR
rs73398041
rs3132452
rs73874489
rs34351822
rs56365965
rs2291267
rs4870021
rs55898302
rs113063640
rs17584499
rs1288688
rs10509151
rs77280541
rs6979811
rs116851206
rs41278972
rs1863213
rs185651896
rs35166179
rs9314507
rs139107782
rs79570763
rs117309732
rs149972663
rs62374475
rs2010224
rs145542121
rs234748
rs6897339
rs111802192
rs74597991
rs73160216
rs10508727
rs35170523
rs7189546
All − MR Egger
All − IVW(MRE)
−0.010
−0.005
0.000
0.005
0.010
MR effect size for
'Intercellular adhesion molecule 5' on 'eGFR'

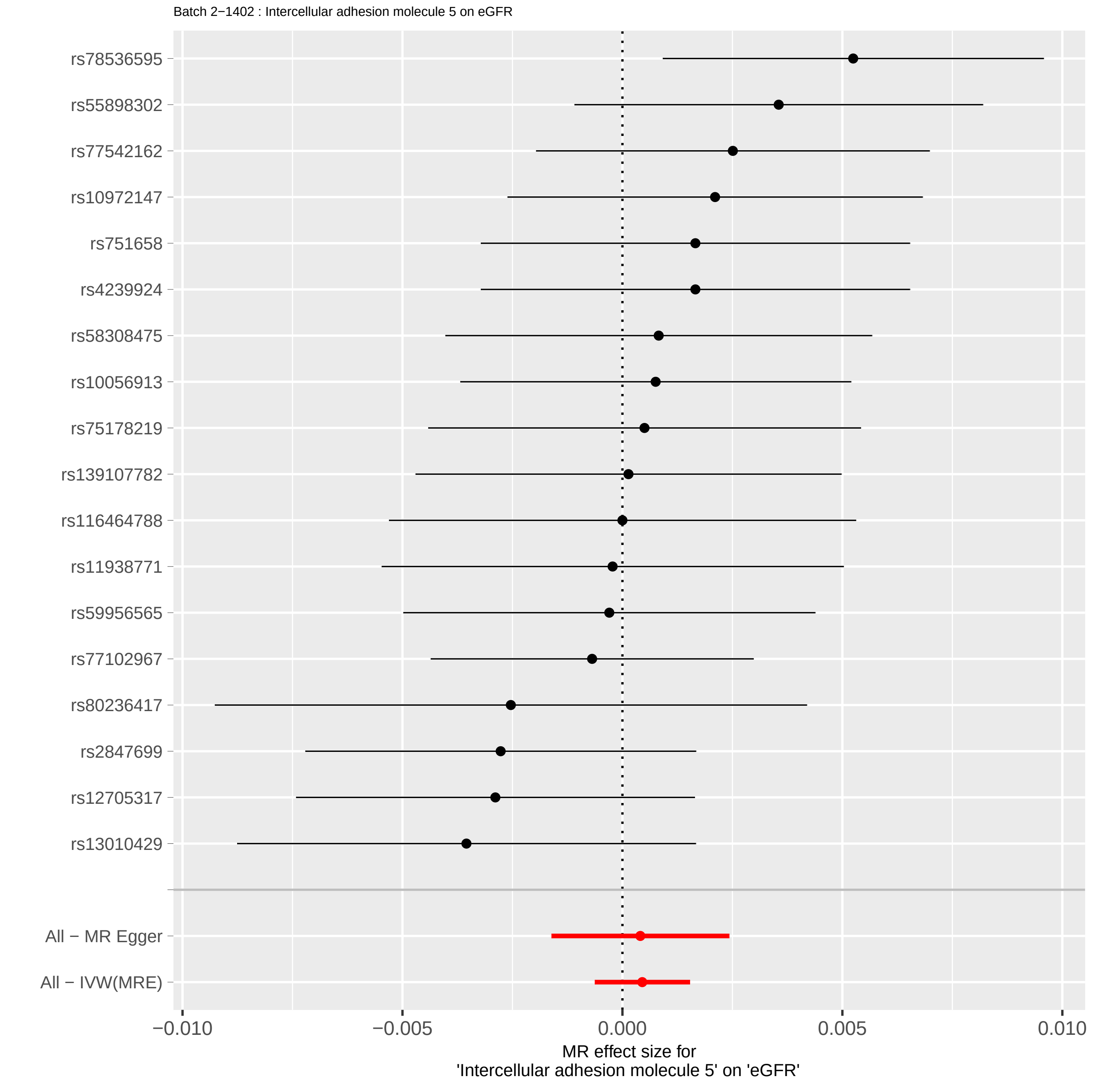
Batch 2−1402 : Intercellular adhesion molecule 5 on eGFR
rs78536595
rs55898302
rs77542162
rs10972147
rs751658
rs4239924
rs58308475
rs10056913
rs75178219
rs139107782
rs116464788
rs11938771
rs59956565
rs77102967
rs80236417
rs2847699
rs12705317
rs13010429
All − MR Egger
All − IVW(MRE)
−0.010
−0.005
0.000
0.005
0.010
MR effect size for
'Intercellular adhesion molecule 5' on 'eGFR'

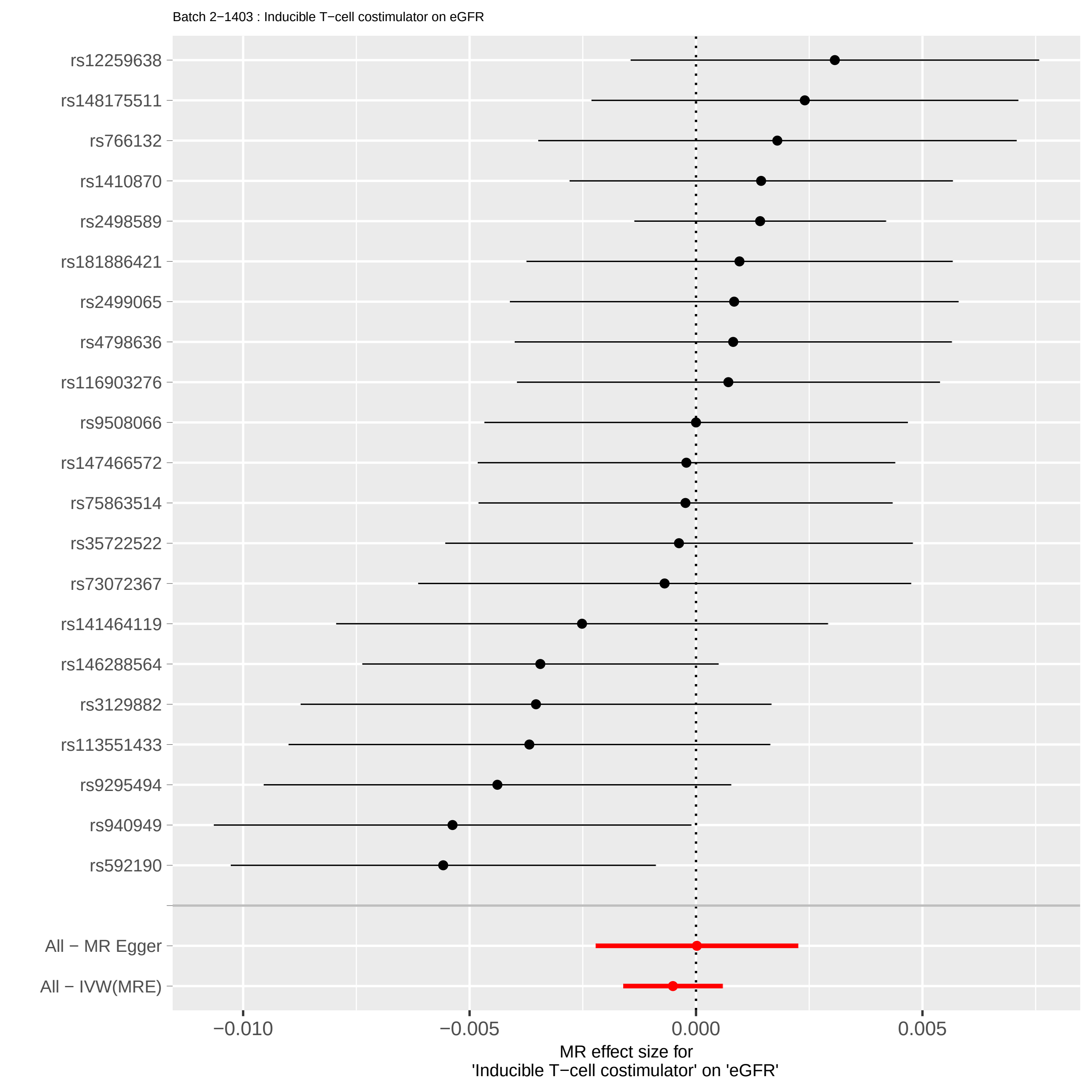

Batch 2−1403 : Inducible T−cell costimulator on eGFR
rs12259638
rs148175511
rs766132
rs1410870
rs2498589
rs181886421
rs2499065
rs4798636
rs116903276
rs9508066
rs147466572
rs75863514
rs35722522
rs73072367
rs141464119
rs146288564
rs3129882
rs113551433
rs9295494
rs940949
rs592190
All − MR Egger
All − IVW(MRE)
−0.010
−0.005
0.000
0.005
MR effect size for
'Inducible T−cell costimulator' on 'eGFR'

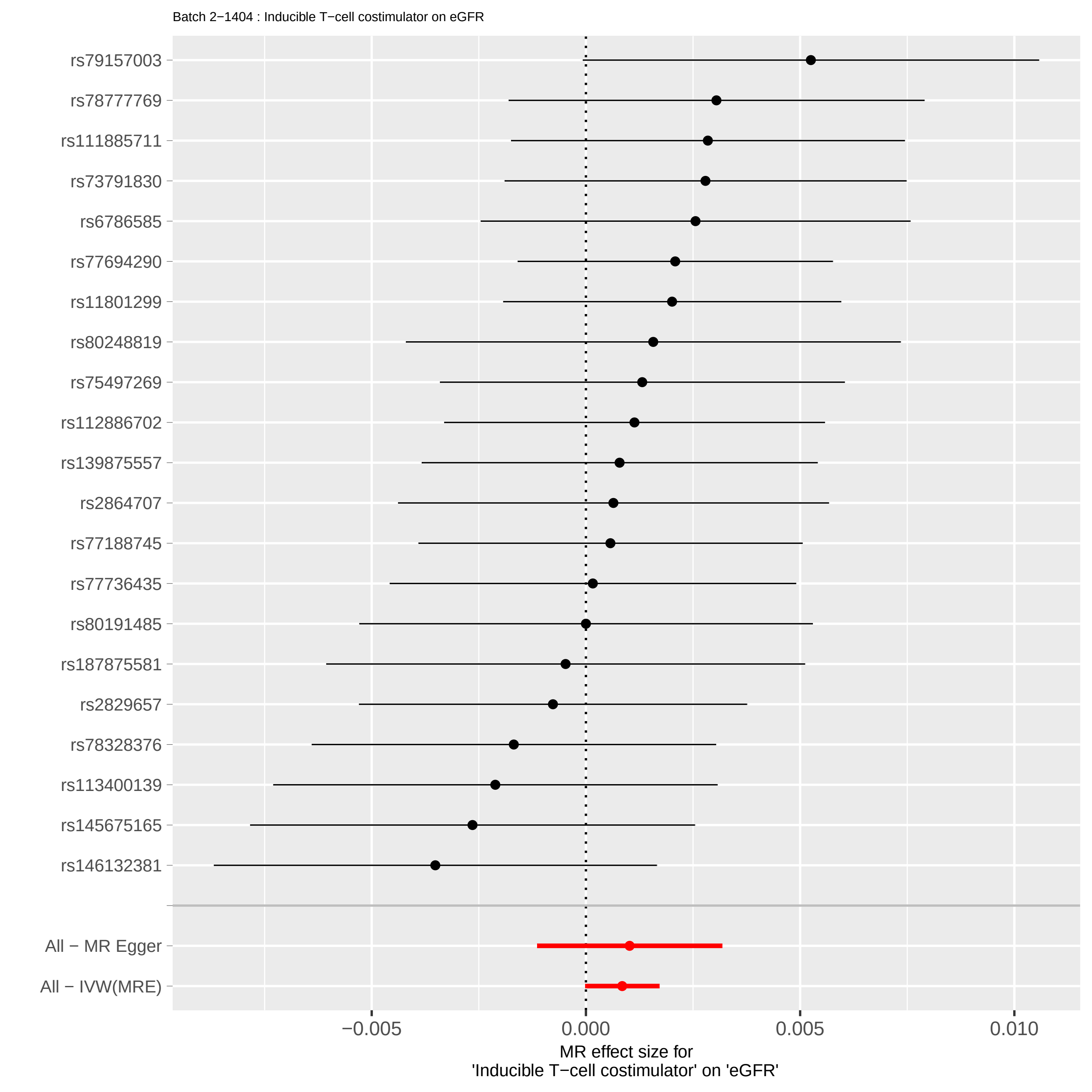
Batch 2−1404 : Inducible T−cell costimulator on eGFR
rs79157003
rs78777769
rs111885711
rs73791830
rs6786585
rs77694290
rs11801299
rs80248819
rs75497269
rs112886702
rs139875557
rs2864707
rs77188745
rs77736435
rs80191485
rs187875581
rs2829657
rs78328376
rs113400139
rs145675165
rs146132381
All − MR Egger
All − IVW(MRE)
−0.005
0.000
0.005
0.010
MR effect size for
'Inducible T−cell costimulator' on 'eGFR'

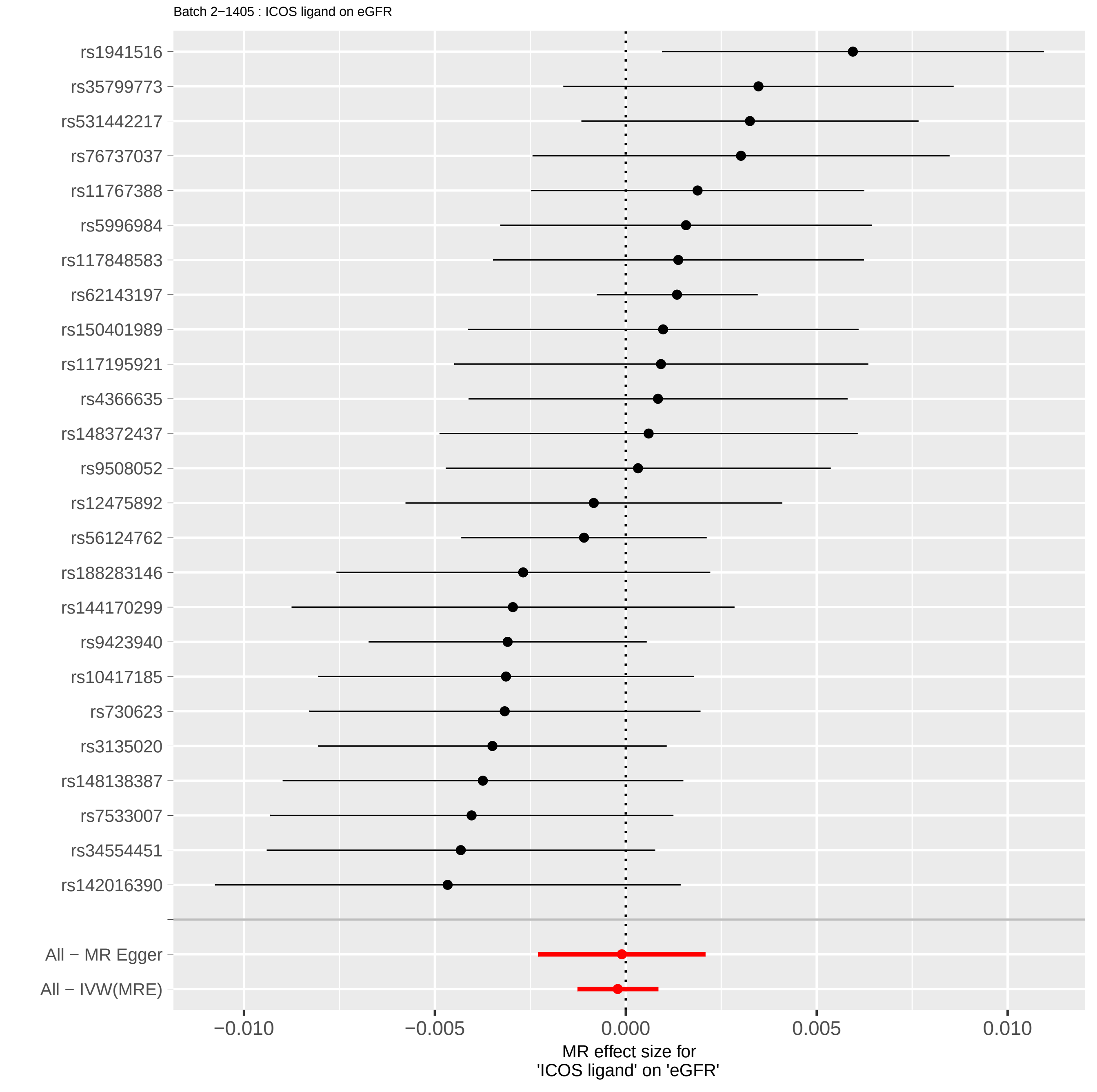

Batch 2−1405 : ICOS ligand on eGFR
rs1941516
rs35799773
rs531442217
rs76737037
rs11767388
rs5996984
rs117848583
rs62143197
rs150401989
rs117195921
rs4366635
rs148372437
rs9508052
rs12475892
rs56124762
rs188283146
rs144170299
rs9423940
rs10417185
rs730623
rs3135020
rs148138387
rs7533007
rs34554451
rs142016390
All − MR Egger
All − IVW(MRE)
−0.010
−0.005
0.000
0.005
0.010
MR effect size for
'ICOS ligand' on 'eGFR'

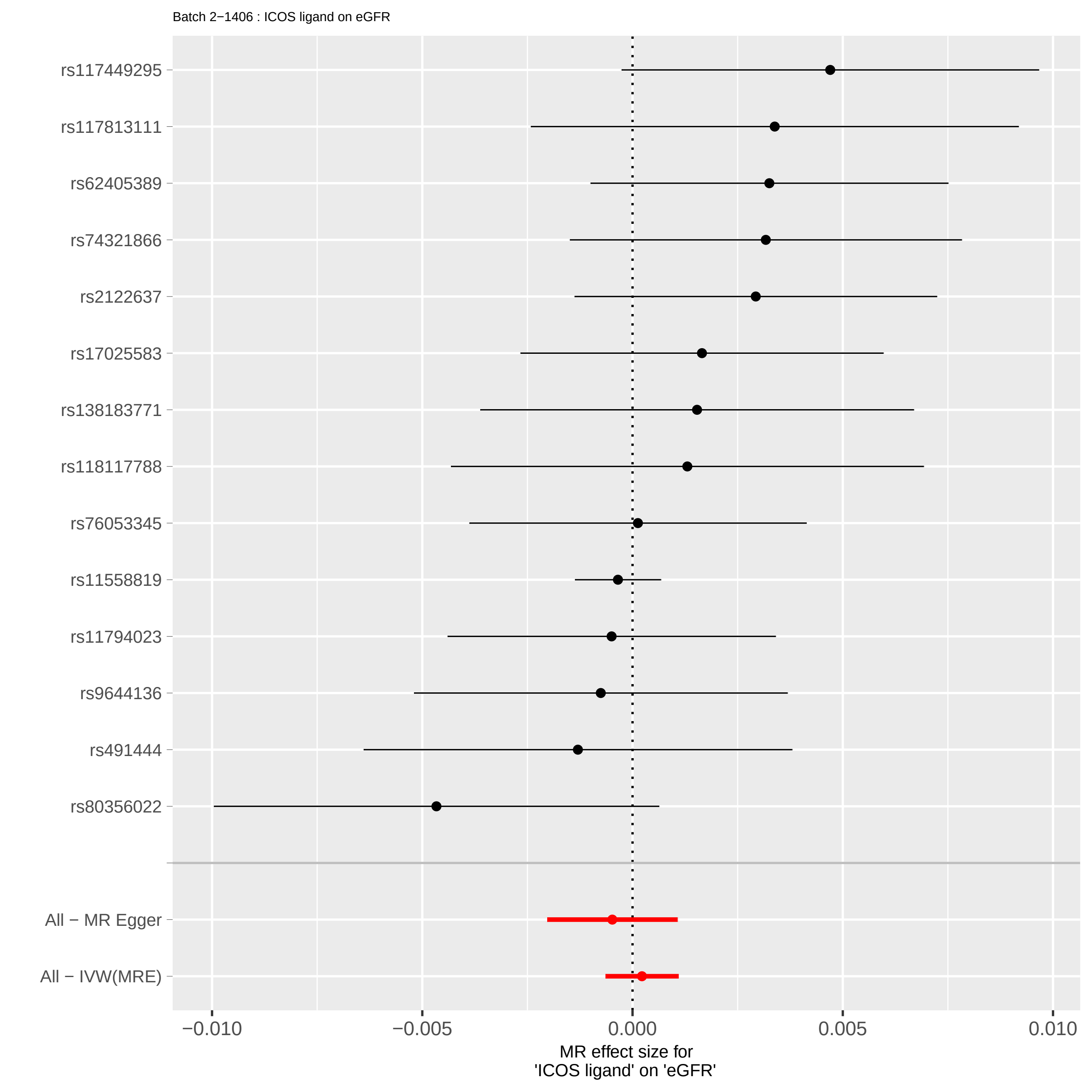

Batch 2−1406 : ICOS ligand on eGFR
rs117449295
rs117813111
rs62405389
rs74321866
rs2122637
rs17025583
rs138183771
rs118117788
rs76053345
rs11558819
rs11794023
rs9644136
rs491444
rs80356022
All − MR Egger
All − IVW(MRE)
−0.010
−0.005
0.000
0.005
0.010
MR effect size for
'ICOS ligand' on 'eGFR'

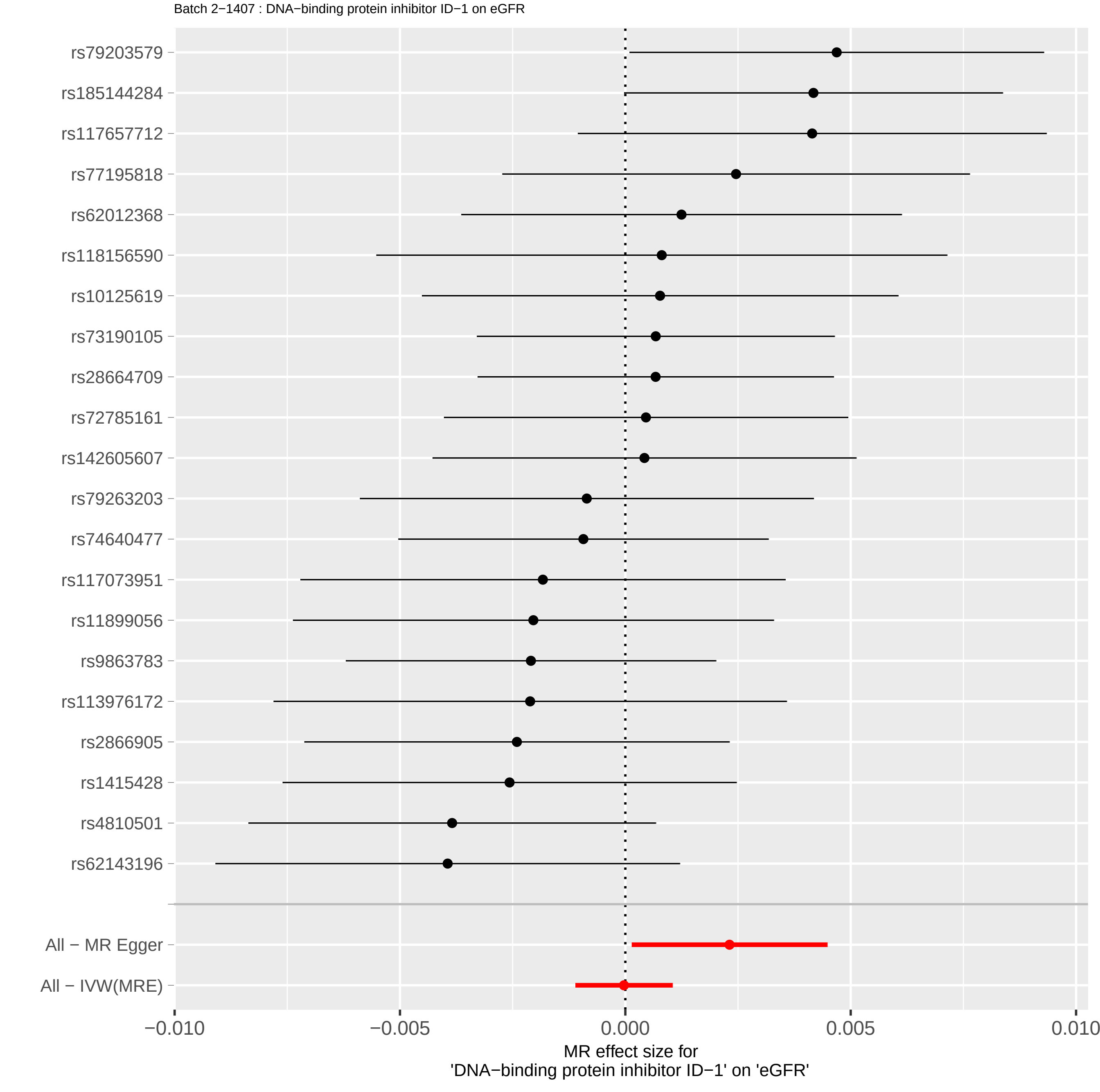

Batch 2−1407 : DNA−binding protein inhibitor ID−1 on eGFR
rs79203579
rs185144284
rs117657712
rs77195818
rs62012368
rs118156590
rs10125619
rs73190105
rs28664709
rs72785161
rs142605607
rs79263203
rs74640477
rs117073951
rs11899056
rs9863783
rs113976172
rs2866905
rs1415428
rs4810501
rs62143196
All − MR Egger
All − IVW(MRE)
−0.010
−0.005
0.000
0.005
0.010
MR effect size for
'DNA−binding protein inhibitor ID−1' on 'eGFR'

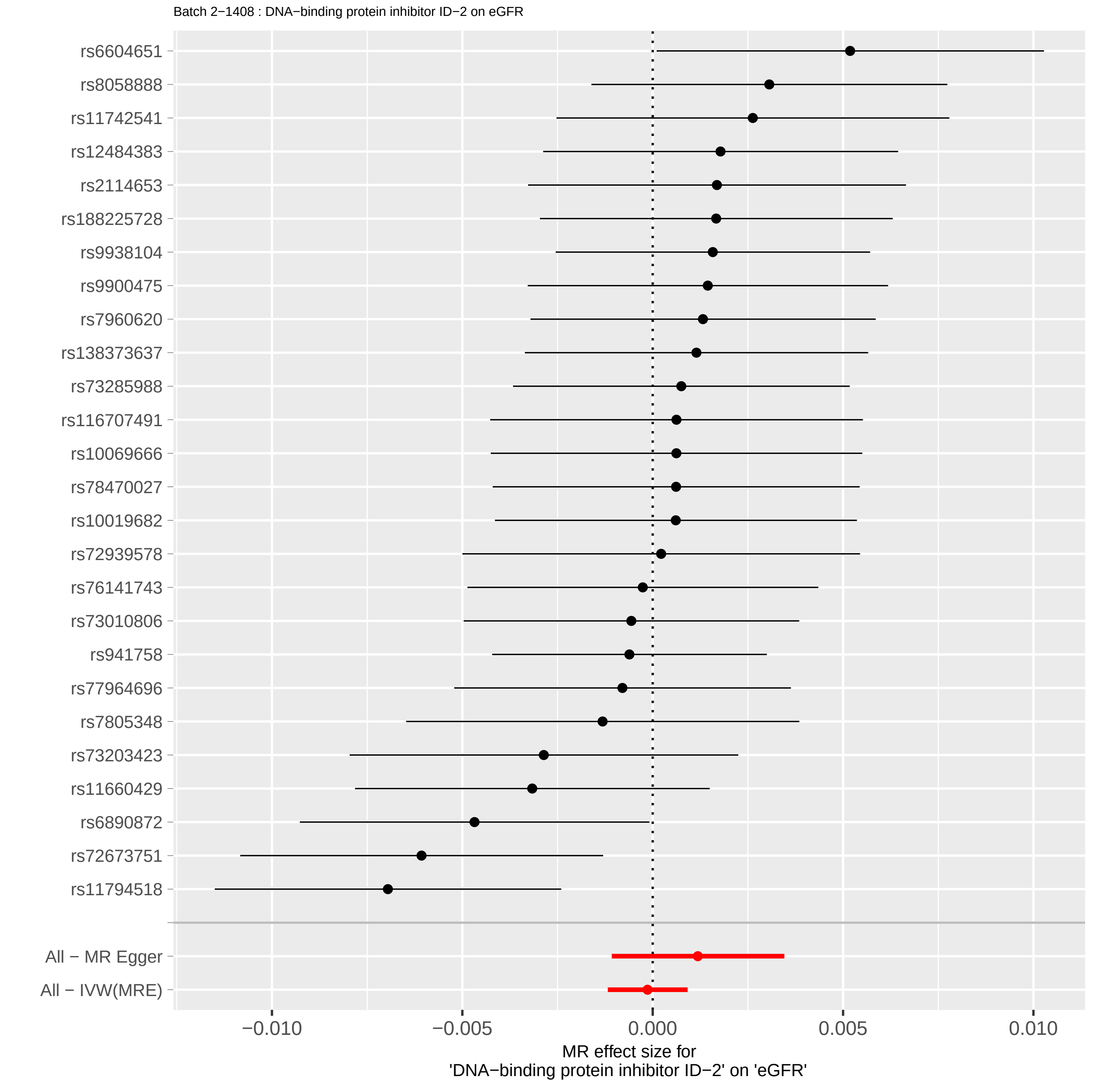

Batch 2−1408 : DNA−binding protein inhibitor ID−2 on eGFR
rs6604651
rs8058888
rs11742541
rs12484383
rs2114653
rs188225728
rs9938104
rs9900475
rs7960620
rs138373637
rs73285988
rs116707491
rs10069666
rs78470027
rs10019682
rs72939578
rs76141743
rs73010806
rs941758
rs77964696
rs7805348
rs73203423
rs11660429
rs6890872
rs72673751
rs11794518
All − MR Egger
All − IVW(MRE)
−0.010
−0.005
0.000
0.005
0.010
MR effect size for
'DNA−binding protein inhibitor ID−2' on 'eGFR'

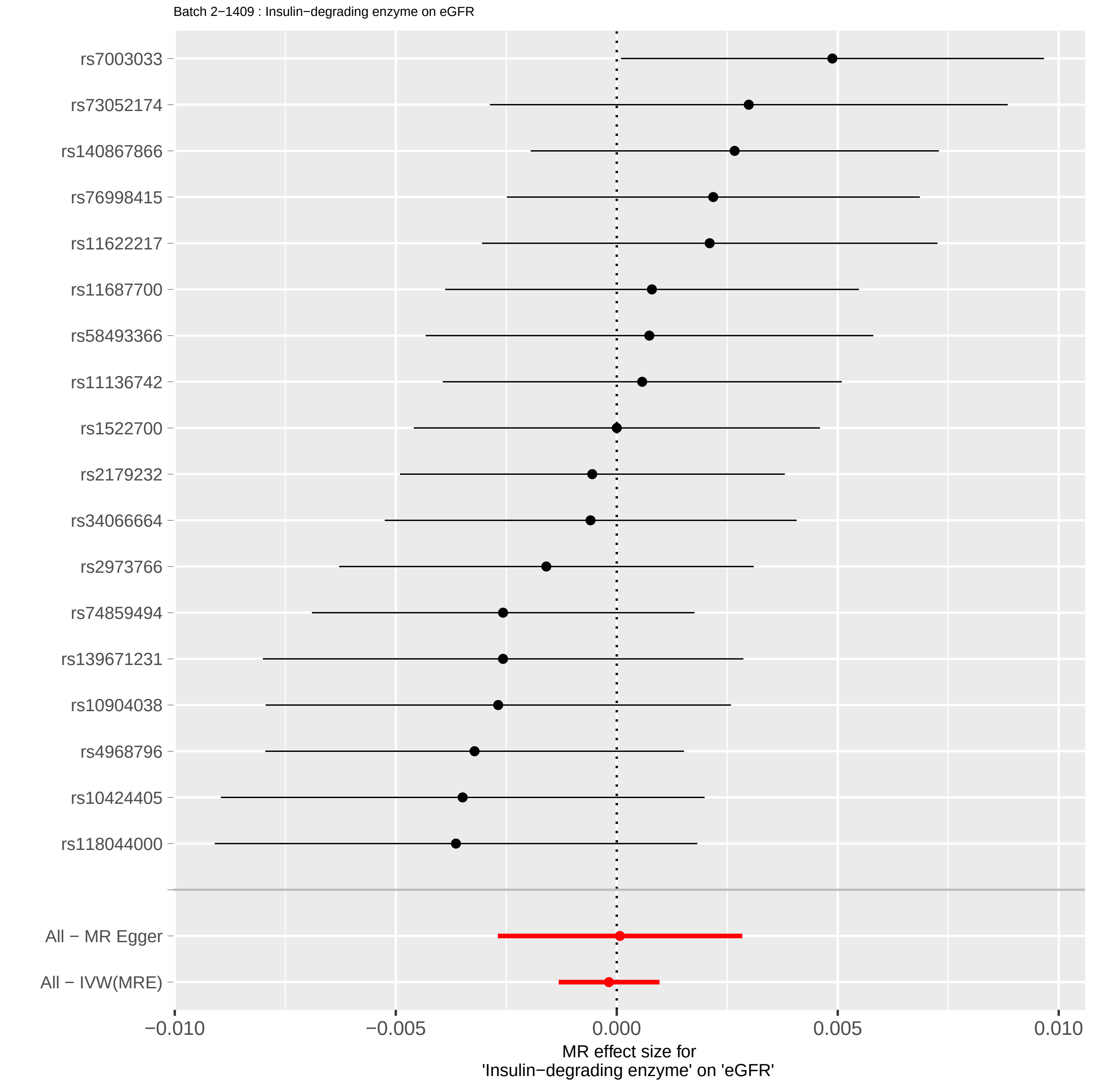

Batch 2−1409 : Insulin−degrading enzyme on eGFR
rs7003033
rs73052174
rs140867866
rs76998415
rs11622217
rs11687700
rs58493366
rs11136742
rs1522700
rs2179232
rs34066664
rs2973766
rs74859494
rs139671231
rs10904038
rs4968796
rs10424405
rs118044000
All − MR Egger
All − IVW(MRE)
−0.010
−0.005
0.000
0.005
0.010
MR effect size for
'Insulin−degrading enzyme' on 'eGFR'

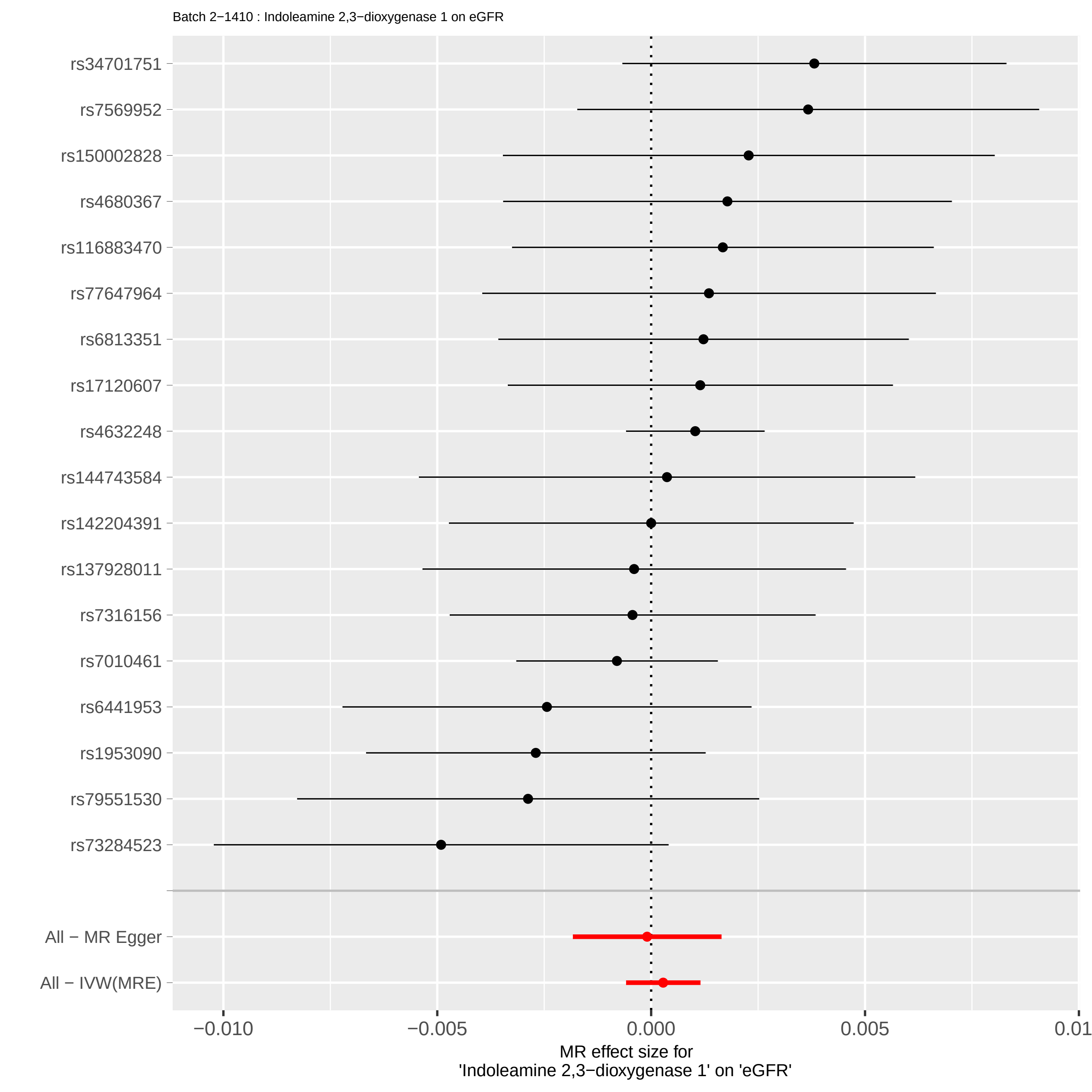
Batch 2−1410 : Indoleamine 2,3−dioxygenase 1 on eGFR
rs34701751
rs7569952
rs150002828
rs4680367
rs116883470
rs77647964
rs6813351
rs17120607
rs4632248
rs144743584
rs142204391
rs137928011
rs7316156
rs7010461
rs6441953
rs1953090
rs79551530
rs73284523
All − MR Egger
All − IVW(MRE)
−0.010
−0.005
0.000
0.005
0.01
MR effect size for
'Indoleamine 2,3−dioxygenase 1' on 'eGFR'

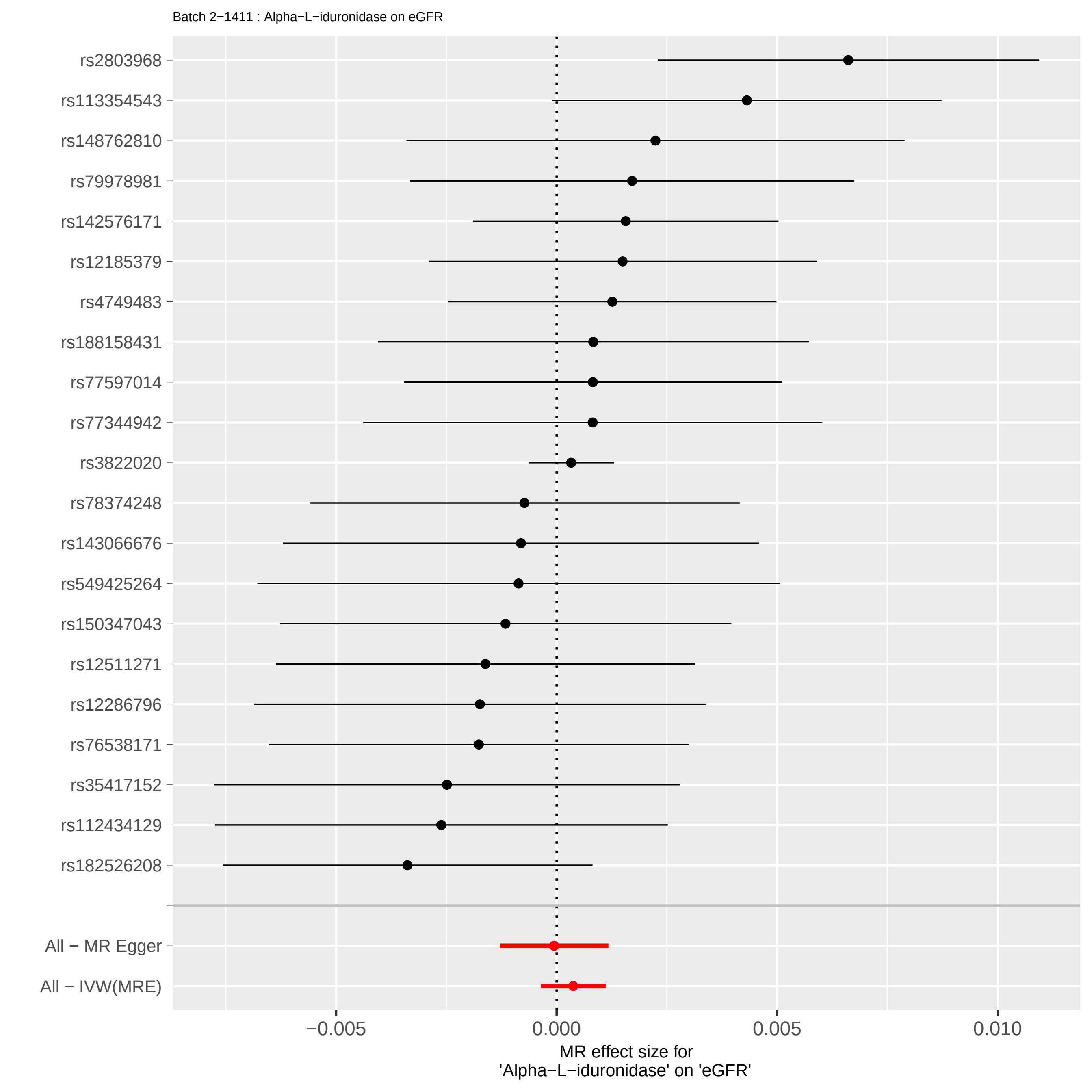

Batch 2−1411 : Alpha−L−iduronidase on eGFR
rs2803968
rs113354543
rs148762810
rs79978981
rs142576171
rs12185379
rs4749483
rs188158431
rs77597014
rs77344942
rs3822020
rs78374248
rs143066676
rs549425264
rs150347043
rs12511271
rs12286796
rs76538171
rs35417152
rs112434129
rs182526208
All − MR Egger
All − IVW(MRE)
−0.005
0.000
0.005
0.010
MR effect size for
'Alpha−L−iduronidase' on 'eGFR'

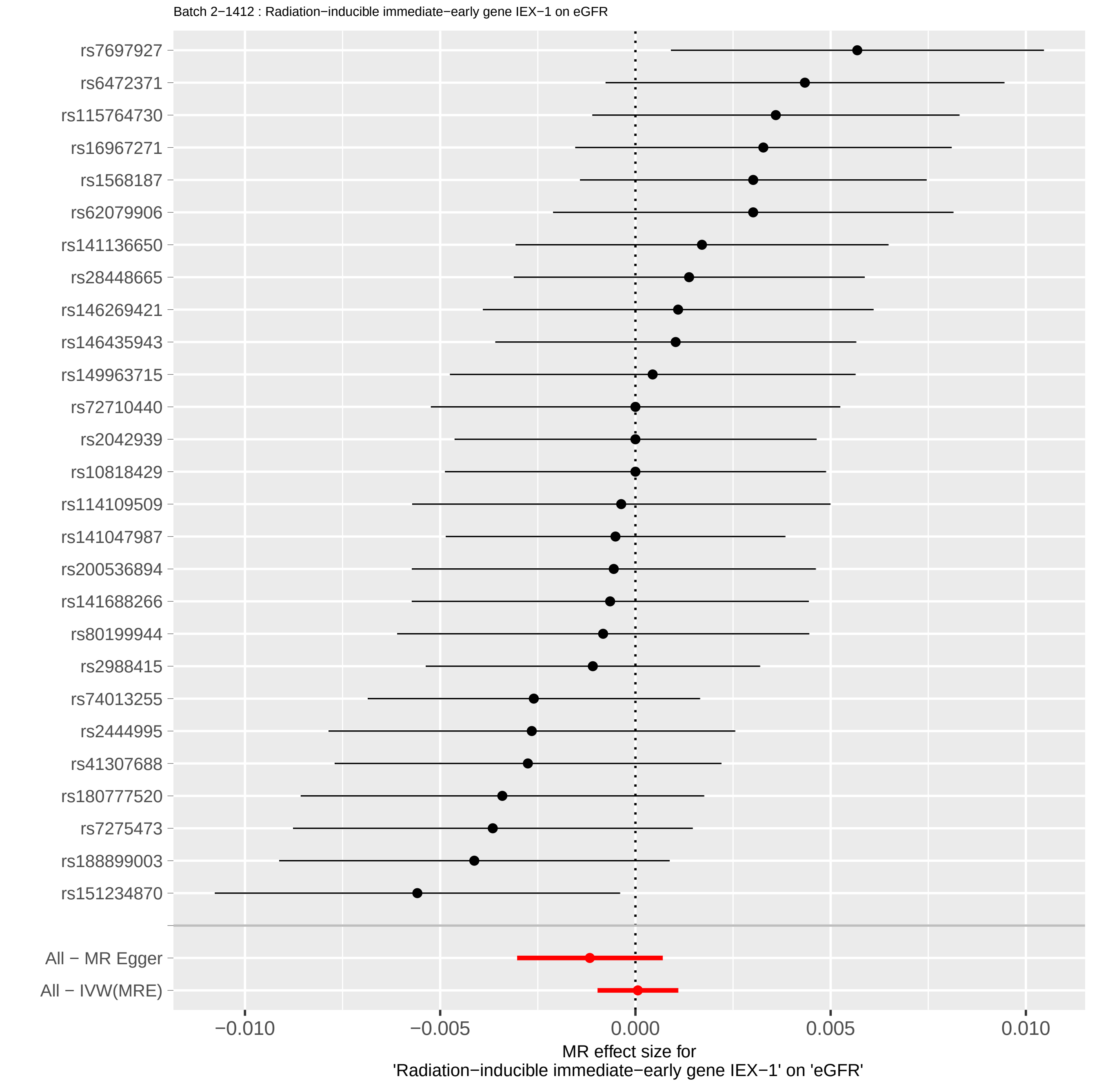

Batch 2−1412 : Radiation−inducible immediate−early gene IEX−1 on eGFR
rs7697927
rs6472371
rs115764730
rs16967271
rs1568187
rs62079906
rs141136650
rs28448665
rs146269421
rs146435943
rs149963715
rs72710440
rs2042939
rs10818429
rs114109509
rs141047987
rs200536894
rs141688266
rs80199944
rs2988415
rs74013255
rs2444995
rs41307688
rs180777520
rs7275473
rs188899003
rs151234870
All − MR Egger
All − IVW(MRE)
−0.010
−0.005
0.000
0.005
0.010
MR effect size for
'Radiation−inducible immediate−early gene IEX−1' on 'eGFR'

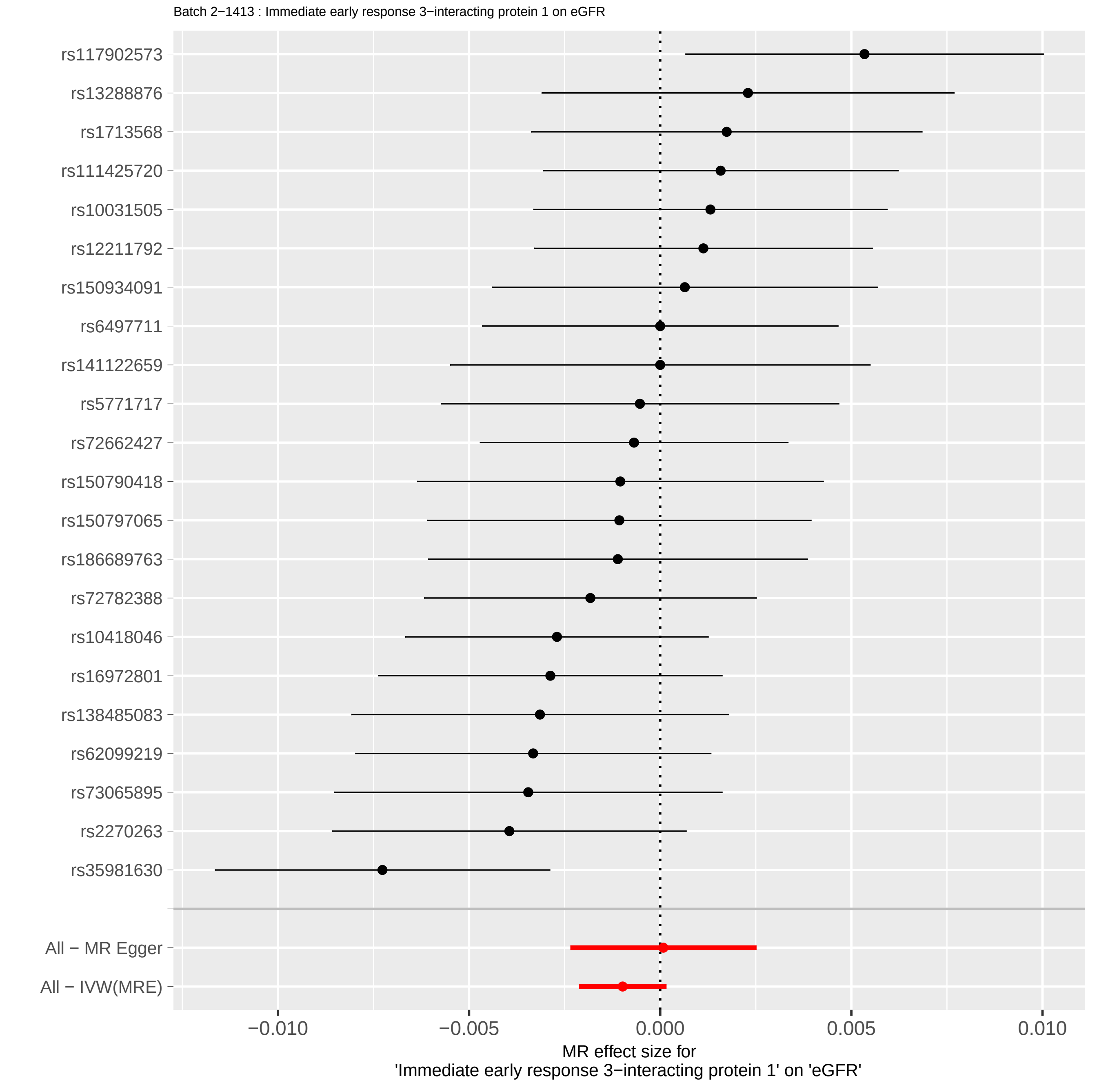

Batch 2−1413 : Immediate early response 3−interacting protein 1 on eGFR
rs117902573
rs13288876
rs1713568
rs111425720
rs10031505
rs12211792
rs150934091
rs6497711
rs141122659
rs5771717
rs72662427
rs150790418
rs150797065
rs186689763
rs72782388
rs10418046
rs16972801
rs138485083
rs62099219
rs73065895
rs2270263
rs35981630
All − MR Egger
All − IVW(MRE)
−0.010
−0.005
0.000
0.005
0.010
MR effect size for
'Immediate early response 3−interacting protein 1' on 'eGFR'

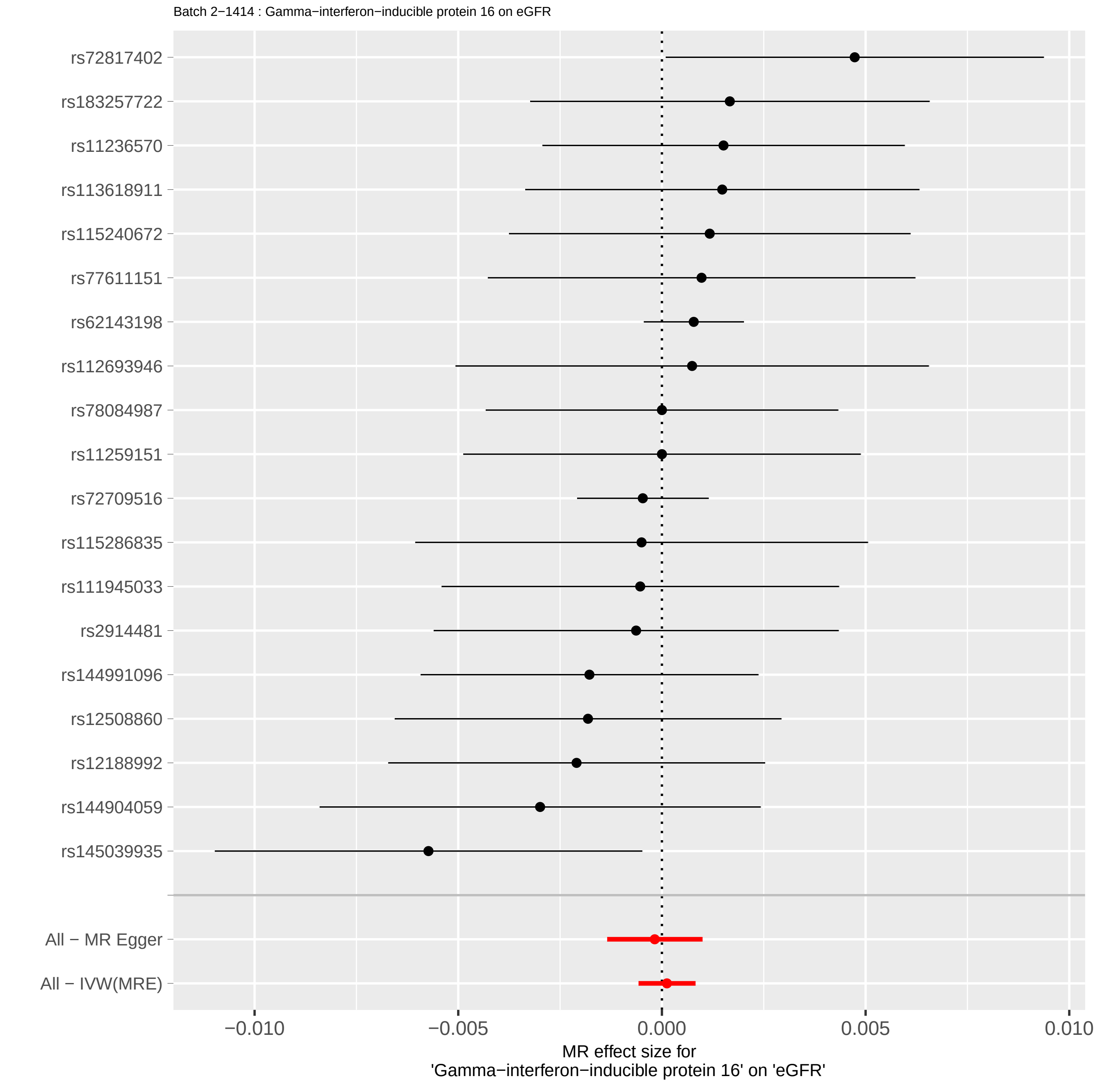

Batch 2−1414 : Gamma−interferon−inducible protein 16 on eGFR
rs72817402
rs183257722
rs11236570
rs113618911
rs115240672
rs77611151
rs62143198
rs112693946
rs78084987
rs11259151
rs72709516
rs115286835
rs111945033
rs2914481
rs144991096
rs12508860
rs12188992
rs144904059
rs145039935
All − MR Egger
All − IVW(MRE)
−0.010
−0.005
0.000
0.005
0.010
MR effect size for
'Gamma−interferon−inducible protein 16' on 'eGFR'

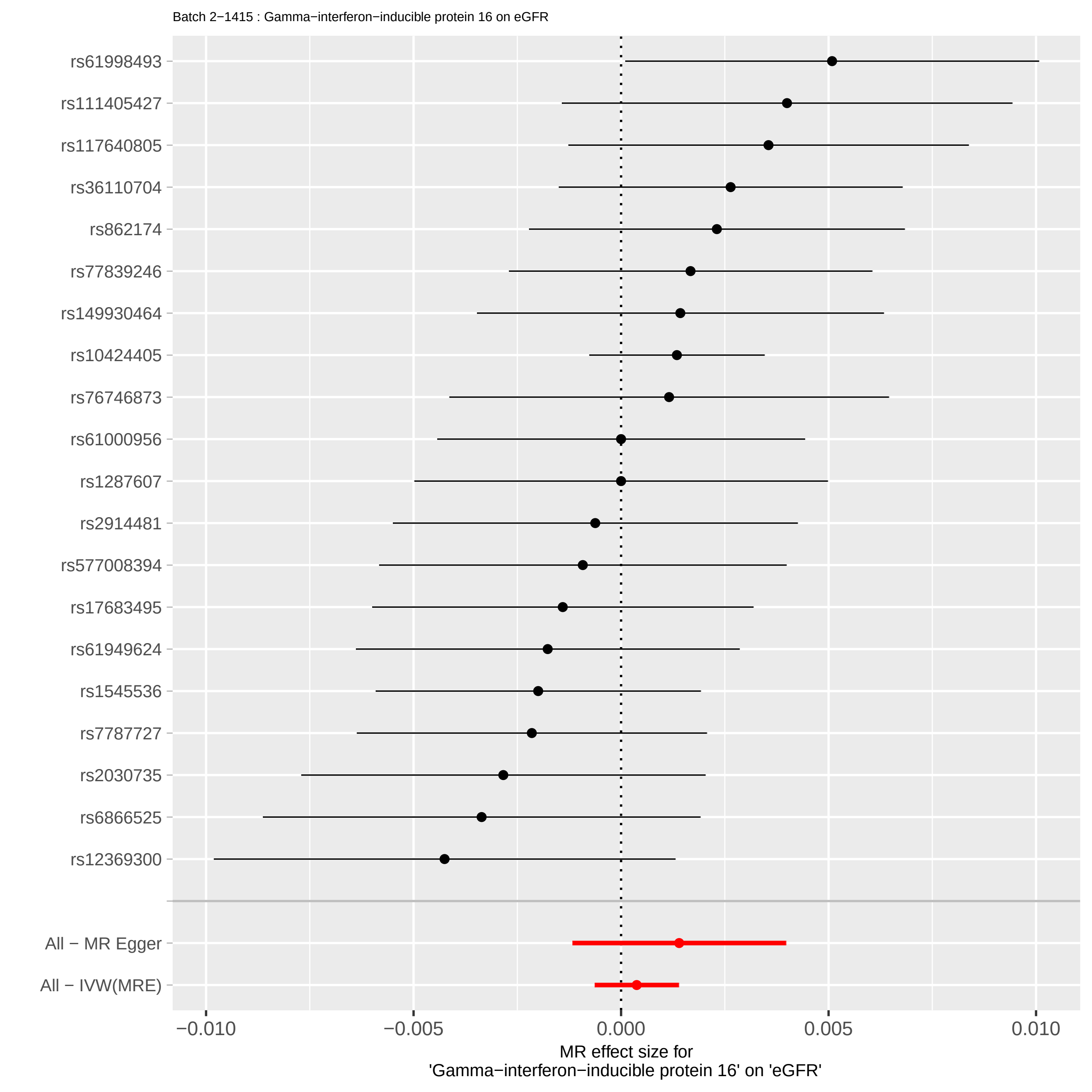

Batch 2−1415 : Gamma−interferon−inducible protein 16 on eGFR
rs61998493
rs111405427
rs117640805
rs36110704
rs862174
rs77839246
rs149930464
rs10424405
rs76746873
rs61000956
rs1287607
rs2914481
rs577008394
rs17683495
rs61949624
rs1545536
rs7787727
rs2030735
rs6866525
rs12369300
All − MR Egger
All − IVW(MRE)
−0.010
−0.005
0.000
0.005
0.010
MR effect size for
'Gamma−interferon−inducible protein 16' on 'eGFR'

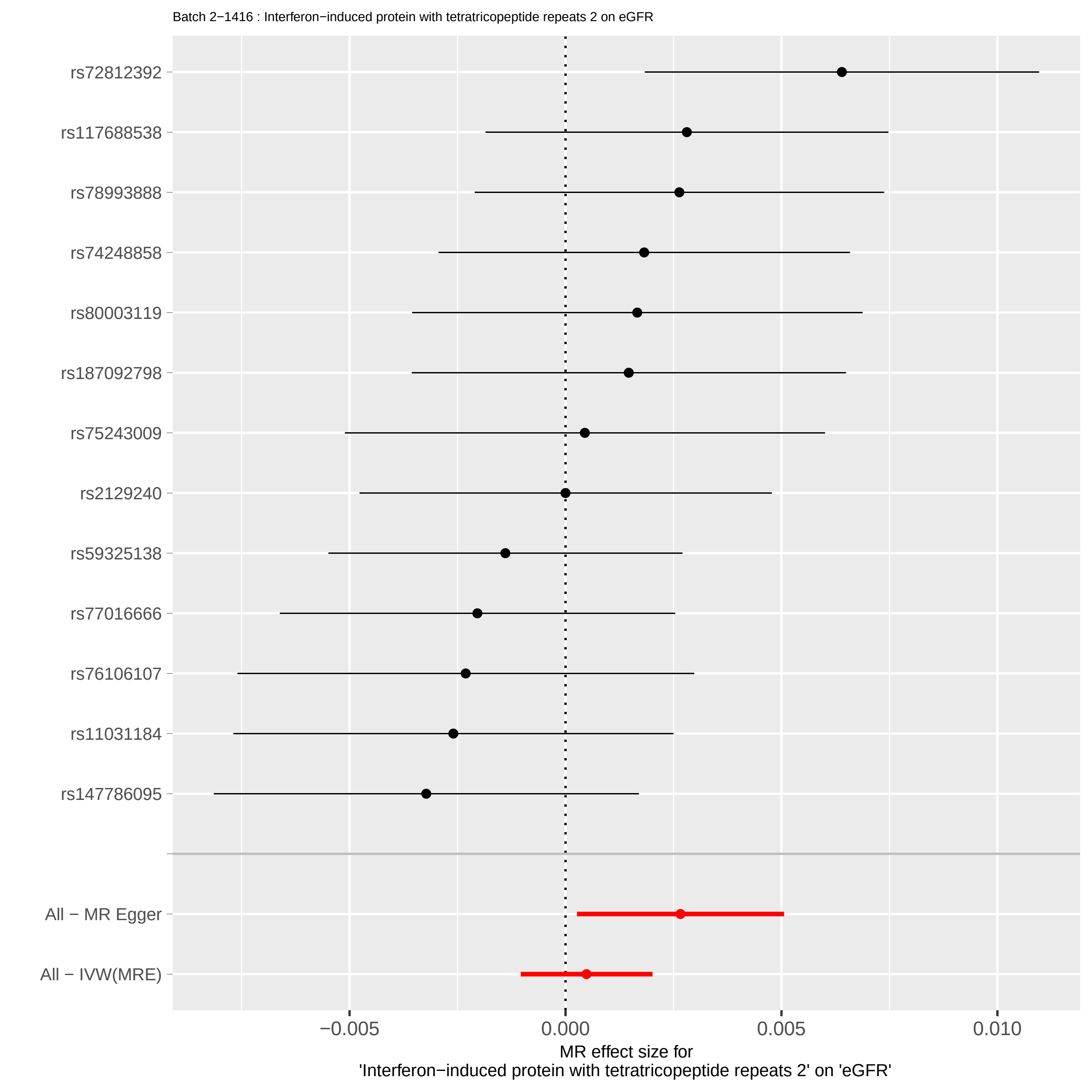
Batch 2−1416 : Interferon−induced protein with tetratricopeptide repeats 2 on eGFR
rs72812392
rs117688538
rs78993888
rs74248858
rs80003119
rs187092798
rs75243009
rs2129240
rs59325138
rs77016666
rs76106107
rs11031184
rs147786095
All − MR Egger
All − IVW(MRE)
−0.005
0.000
0.005
0.010
MR effect size for
'Interferon−induced protein with tetratricopeptide repeats 2' on 'eGFR'

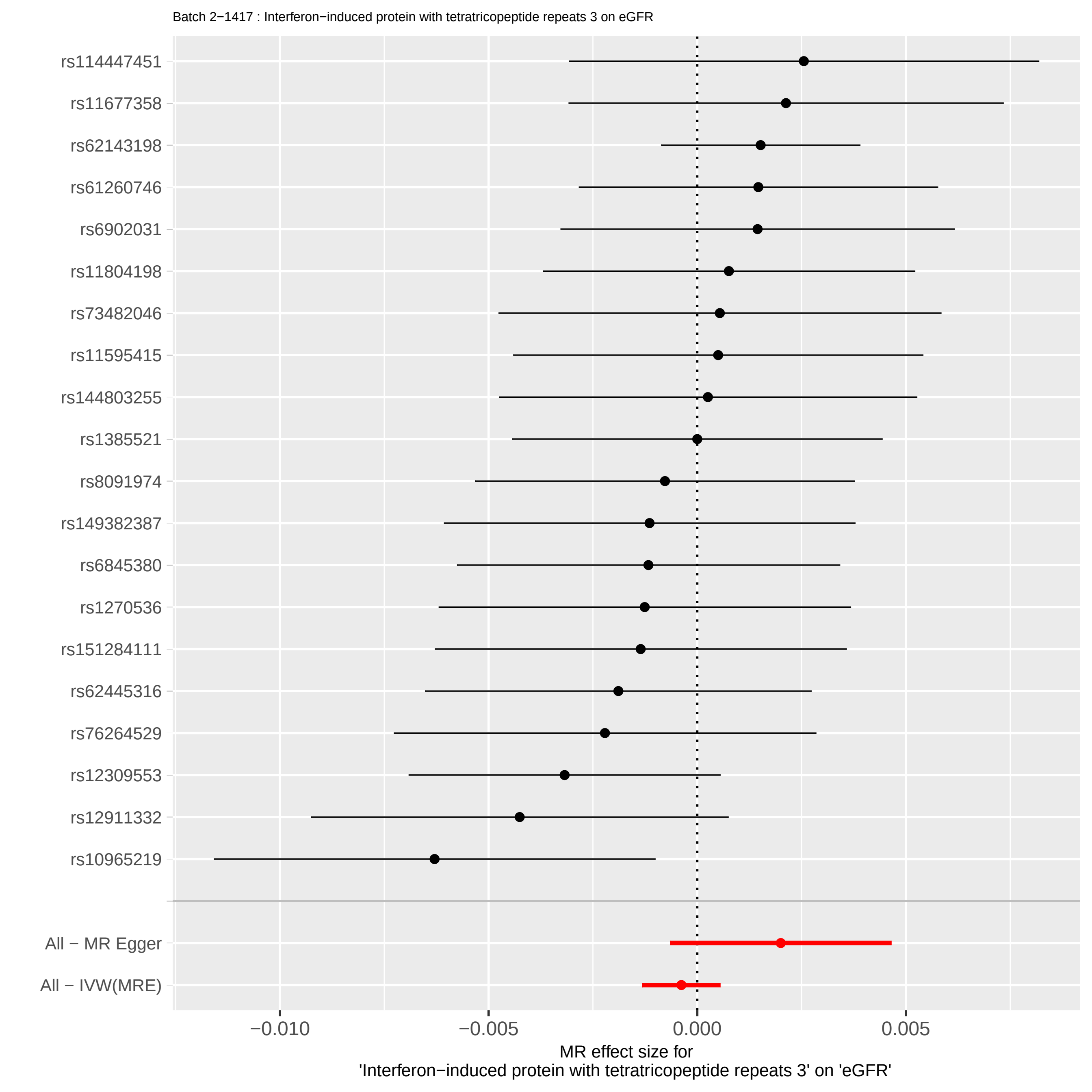
Batch 2−1417 : Interferon−induced protein with tetratricopeptide repeats 3 on eGFR
rs114447451
rs11677358
rs62143198
rs61260746
rs6902031
rs11804198
rs73482046
rs11595415
rs144803255
rs1385521
rs8091974
rs149382387
rs6845380
rs1270536
rs151284111
rs62445316
rs76264529
rs12309553
rs12911332
rs10965219
All − MR Egger
All − IVW(MRE)
−0.010
−0.005
0.000
0.005
MR effect size for
'Interferon−induced protein with tetratricopeptide repeats 3' on 'eGFR'

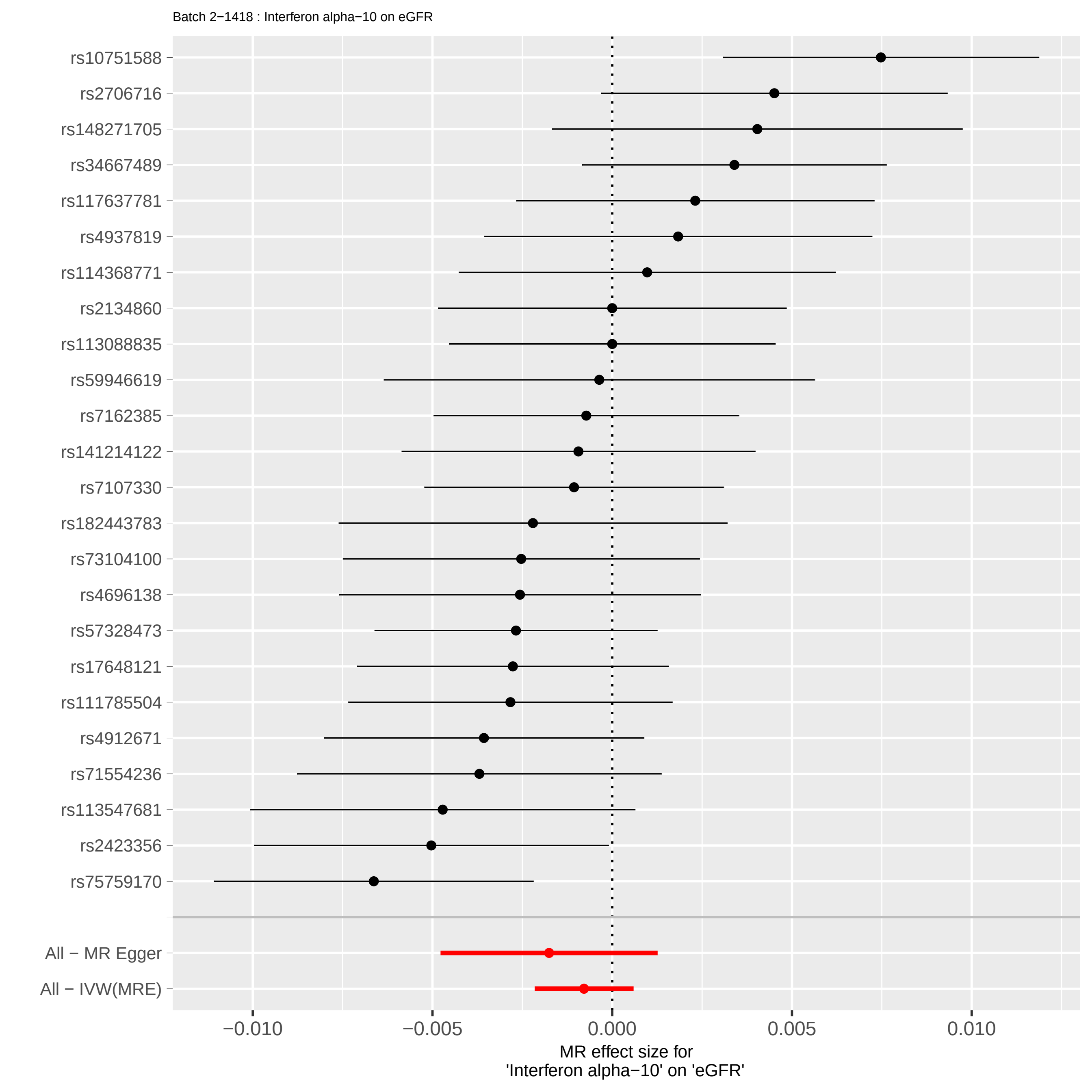
Batch 2−1418 : Interferon alpha−10 on eGFR
rs10751588
rs2706716
rs148271705
rs34667489
rs117637781
rs4937819
rs114368771
rs2134860
rs113088835
rs59946619
rs7162385
rs141214122
rs7107330
rs182443783
rs73104100
rs4696138
rs57328473
rs17648121
rs111785504
rs4912671
rs71554236
rs113547681
rs2423356
rs75759170
All − MR Egger
All − IVW(MRE)
−0.010
−0.005
0.000
0.005
0.010
MR effect size for
'Interferon alpha−10' on 'eGFR'

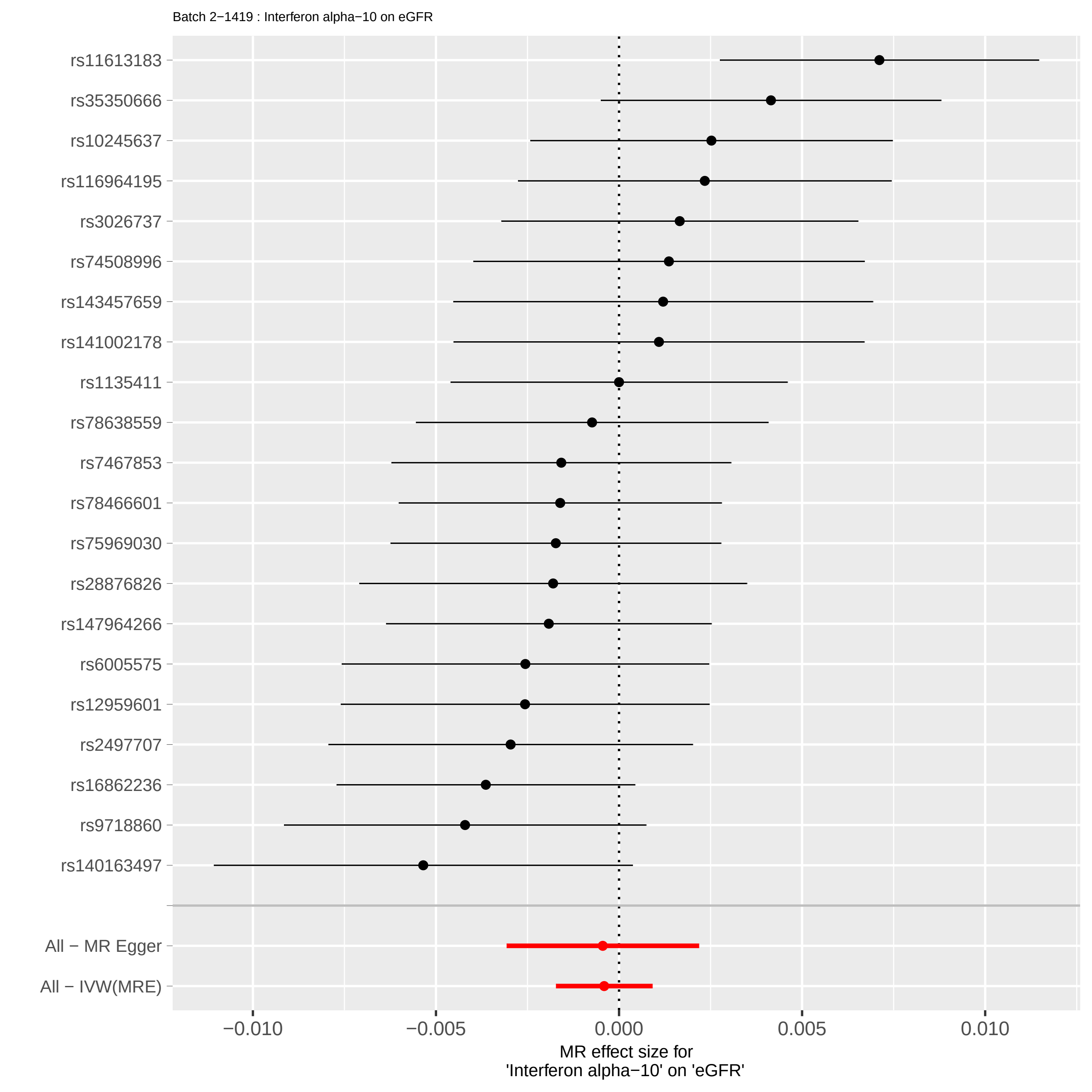

Batch 2−1419 : Interferon alpha−10 on eGFR
rs11613183
rs35350666
rs10245637
rs116964195
rs3026737
rs74508996
rs143457659
rs141002178
rs1135411
rs78638559
rs7467853
rs78466601
rs75969030
rs28876826
rs147964266
rs6005575
rs12959601
rs2497707
rs16862236
rs9718860
rs140163497
All − MR Egger
All − IVW(MRE)
−0.010
−0.005
0.000
0.005
0.010
MR effect size for
'Interferon alpha−10' on 'eGFR'

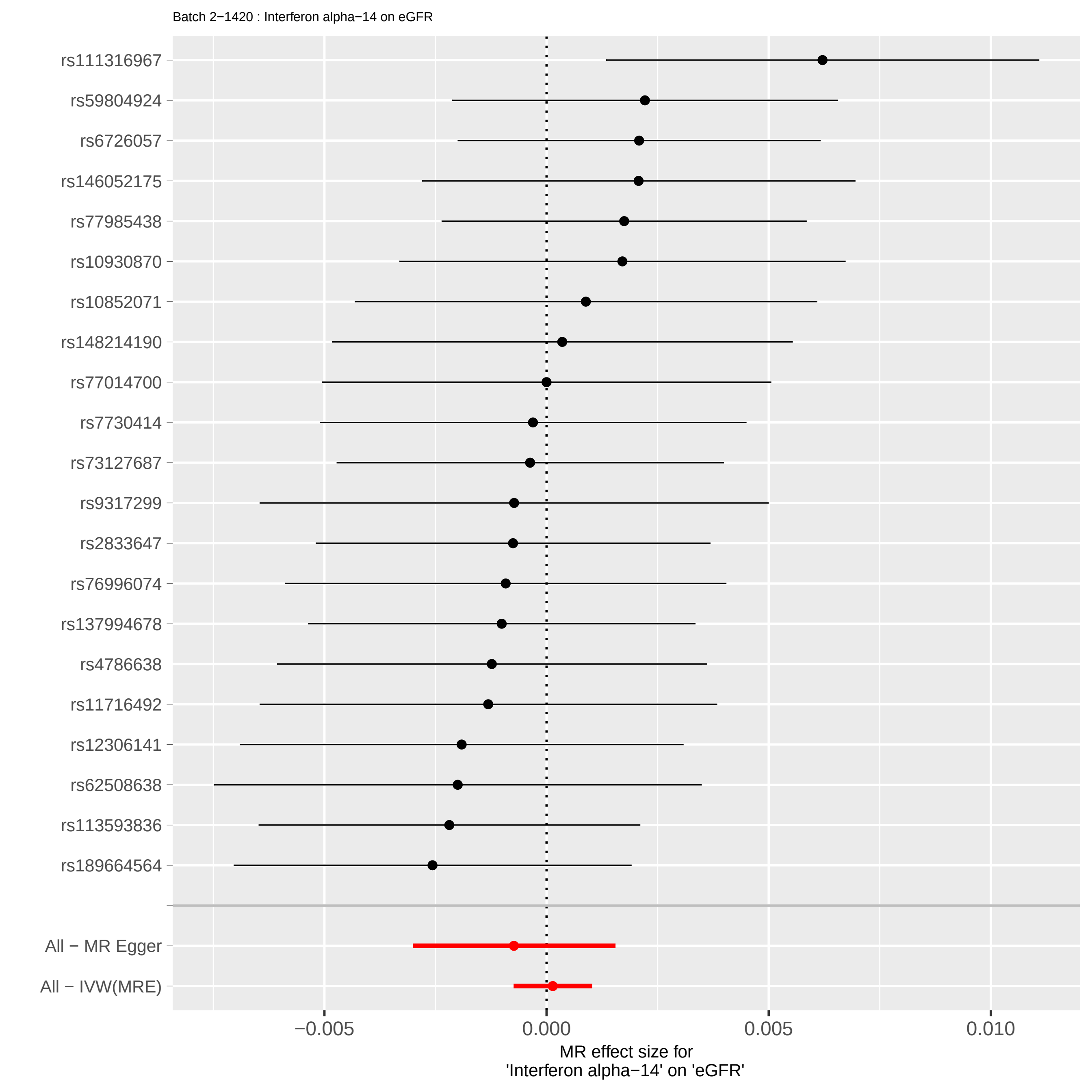

Batch 2−1420 : Interferon alpha−14 on eGFR
rs111316967
rs59804924
rs6726057
rs146052175
rs77985438
rs10930870
rs10852071
rs148214190
rs77014700
rs7730414
rs73127687
rs9317299
rs2833647
rs76996074
rs137994678
rs4786638
rs11716492
rs12306141
rs62508638
rs113593836
rs189664564
All − MR Egger
All − IVW(MRE)
−0.005
0.000
0.005
0.010
MR effect size for
'Interferon alpha−14' on 'eGFR'

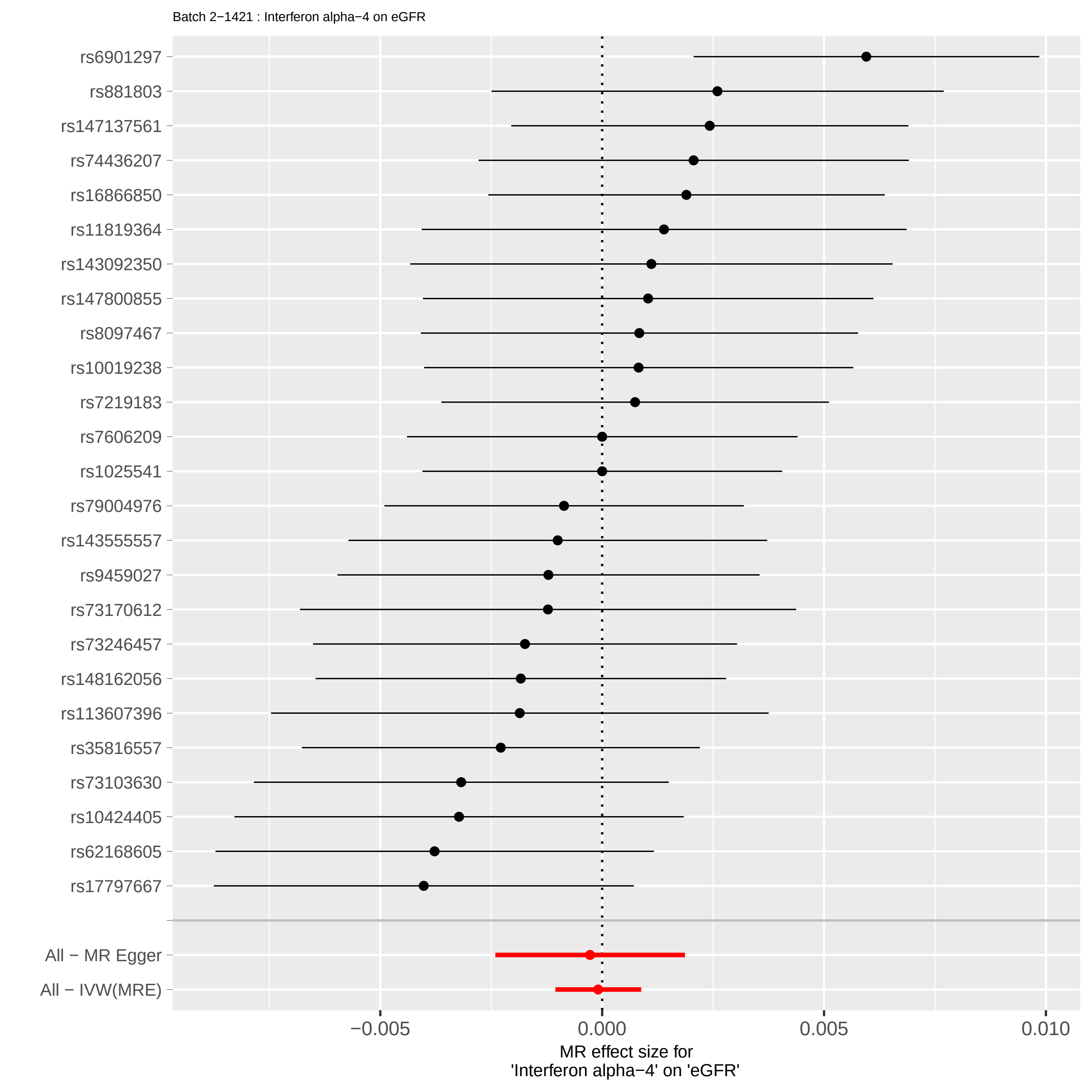

Batch 2−1421 : Interferon alpha−4 on eGFR
rs6901297
rs881803
rs147137561
rs74436207
rs16866850
rs11819364
rs143092350
rs147800855
rs8097467
rs10019238
rs7219183
rs7606209
rs1025541
rs79004976
rs143555557
rs9459027
rs73170612
rs73246457
rs148162056
rs113607396
rs35816557
rs73103630
rs10424405
rs62168605
rs17797667
All − MR Egger
All − IVW(MRE)
−0.005
0.000
0.005
0.010
MR effect size for
'Interferon alpha−4' on 'eGFR'

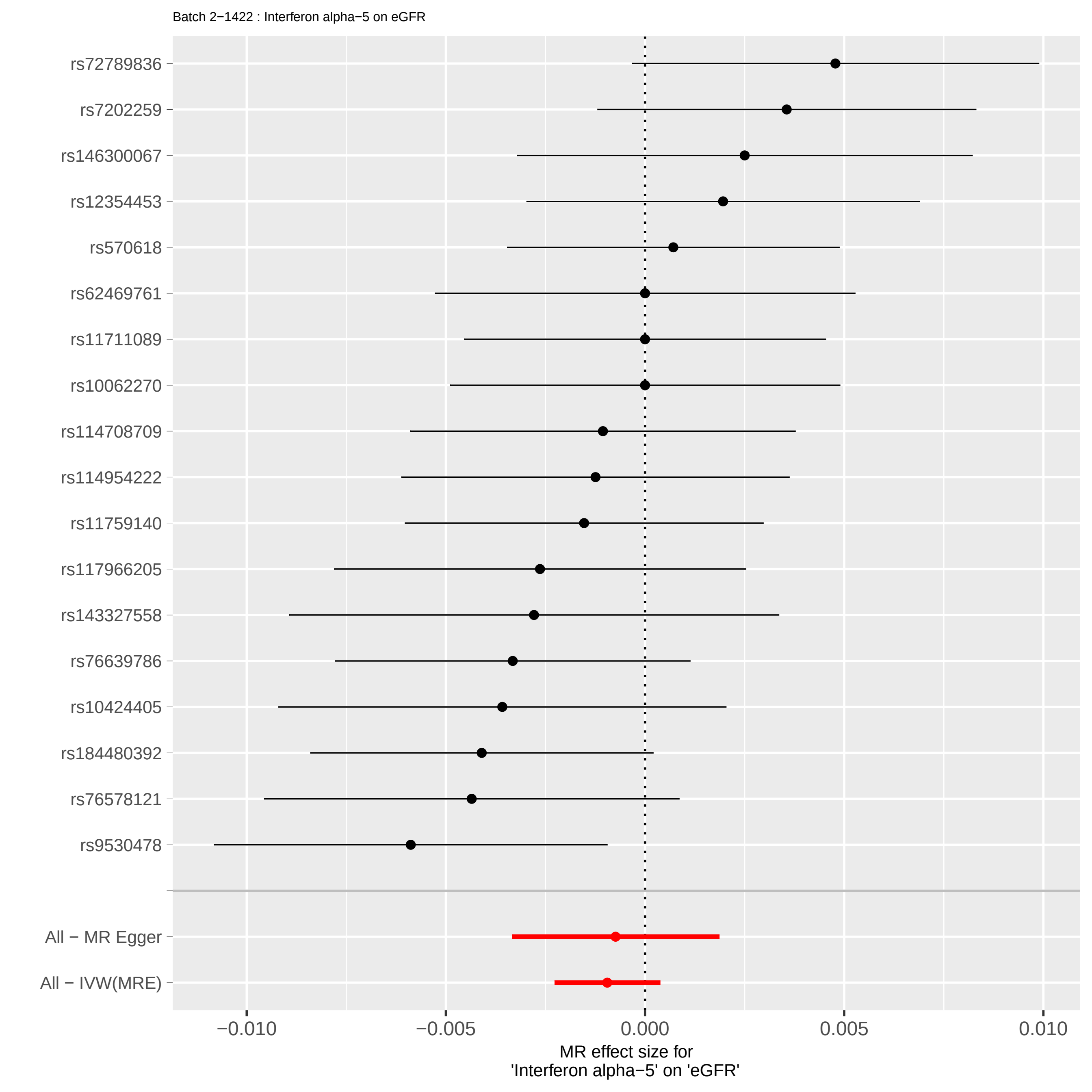

Batch 2−1422 : Interferon alpha−5 on eGFR
rs72789836
rs7202259
rs146300067
rs12354453
rs570618
rs62469761
rs11711089
rs10062270
rs114708709
rs114954222
rs11759140
rs117966205
rs143327558
rs76639786
rs10424405
rs184480392
rs76578121
rs9530478
All − MR Egger
All − IVW(MRE)
−0.010
−0.005
0.000
0.005
0.010
MR effect size for
'Interferon alpha−5' on 'eGFR'

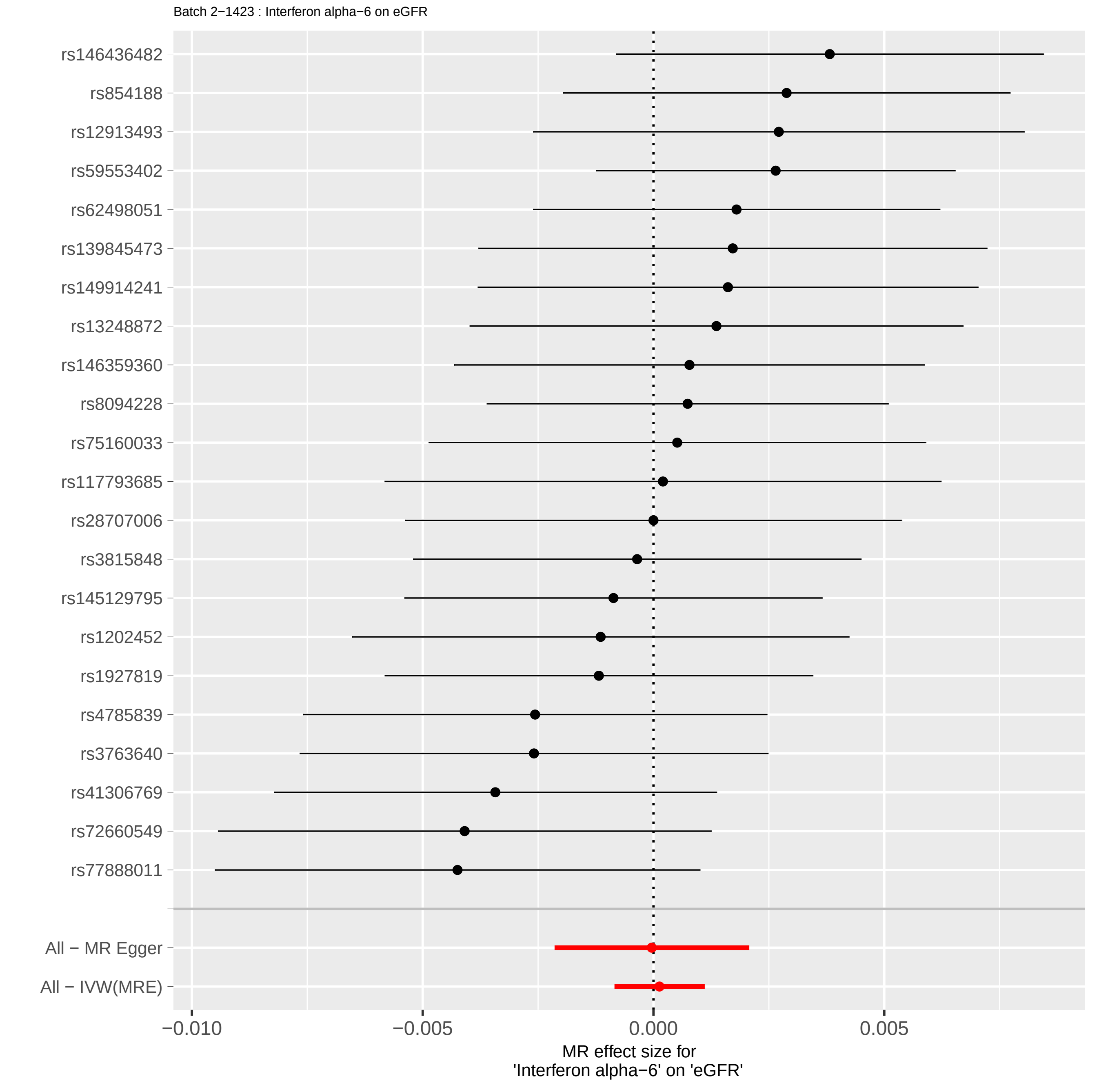

Batch 2−1423 : Interferon alpha−6 on eGFR
rs146436482
rs854188
rs12913493
rs59553402
rs62498051
rs139845473
rs149914241
rs13248872
rs146359360
rs8094228
rs75160033
rs117793685
rs28707006
rs3815848
rs145129795
rs1202452
rs1927819
rs4785839
rs3763640
rs41306769
rs72660549
rs77888011
All − MR Egger
All − IVW(MRE)
−0.010
−0.005
0.000
0.005
MR effect size for
'Interferon alpha−6' on 'eGFR'

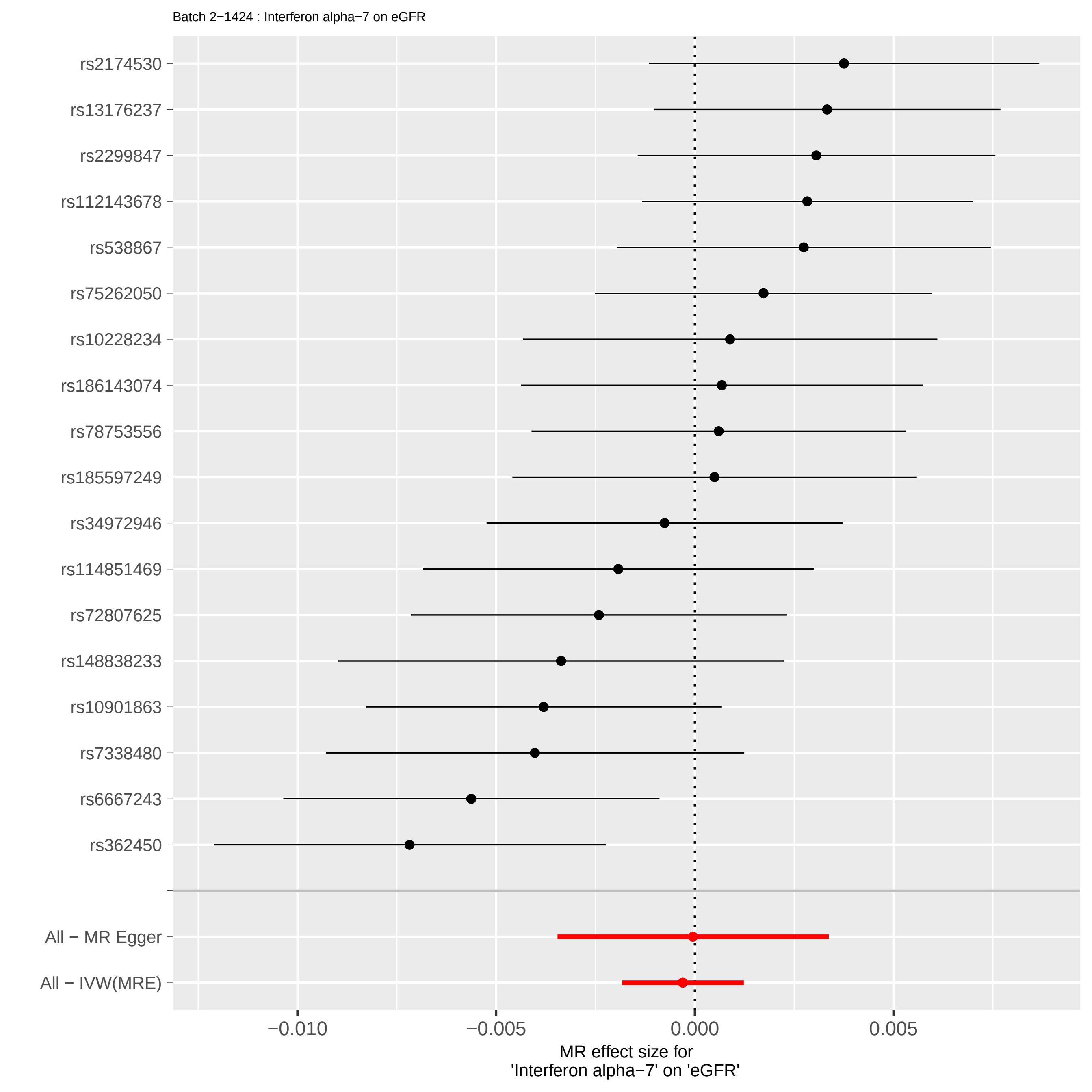
Batch 2−1424 : Interferon alpha−7 on eGFR
rs2174530
rs13176237
rs2299847
rs112143678
rs538867
rs75262050
rs10228234
rs186143074
rs78753556
rs185597249
rs34972946
rs114851469
rs72807625
rs148838233
rs10901863
rs7338480
rs6667243
rs362450
All − MR Egger
All − IVW(MRE)
−0.010
−0.005
0.000
0.005
MR effect size for
'Interferon alpha−7' on 'eGFR'

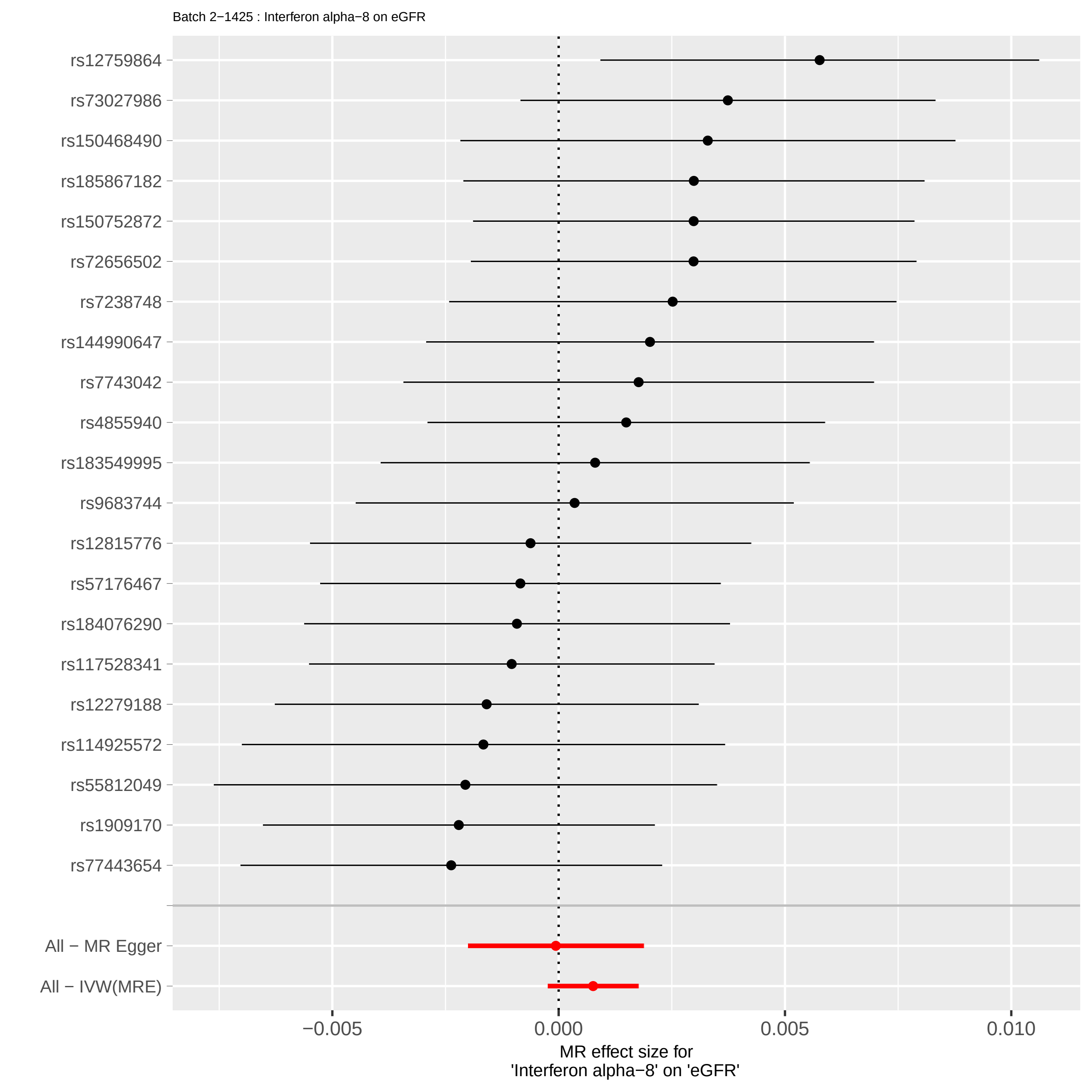
Batch 2−1425 : Interferon alpha−8 on eGFR
rs12759864
rs73027986
rs150468490
rs185867182
rs150752872
rs72656502
rs7238748
rs144990647
rs7743042
rs4855940
rs183549995
rs9683744
rs12815776
rs57176467
rs184076290
rs117528341
rs12279188
rs114925572
rs55812049
rs1909170
rs77443654
All − MR Egger
All − IVW(MRE)
−0.005
0.000
0.005
0.010
MR effect size for
'Interferon alpha−8' on 'eGFR'

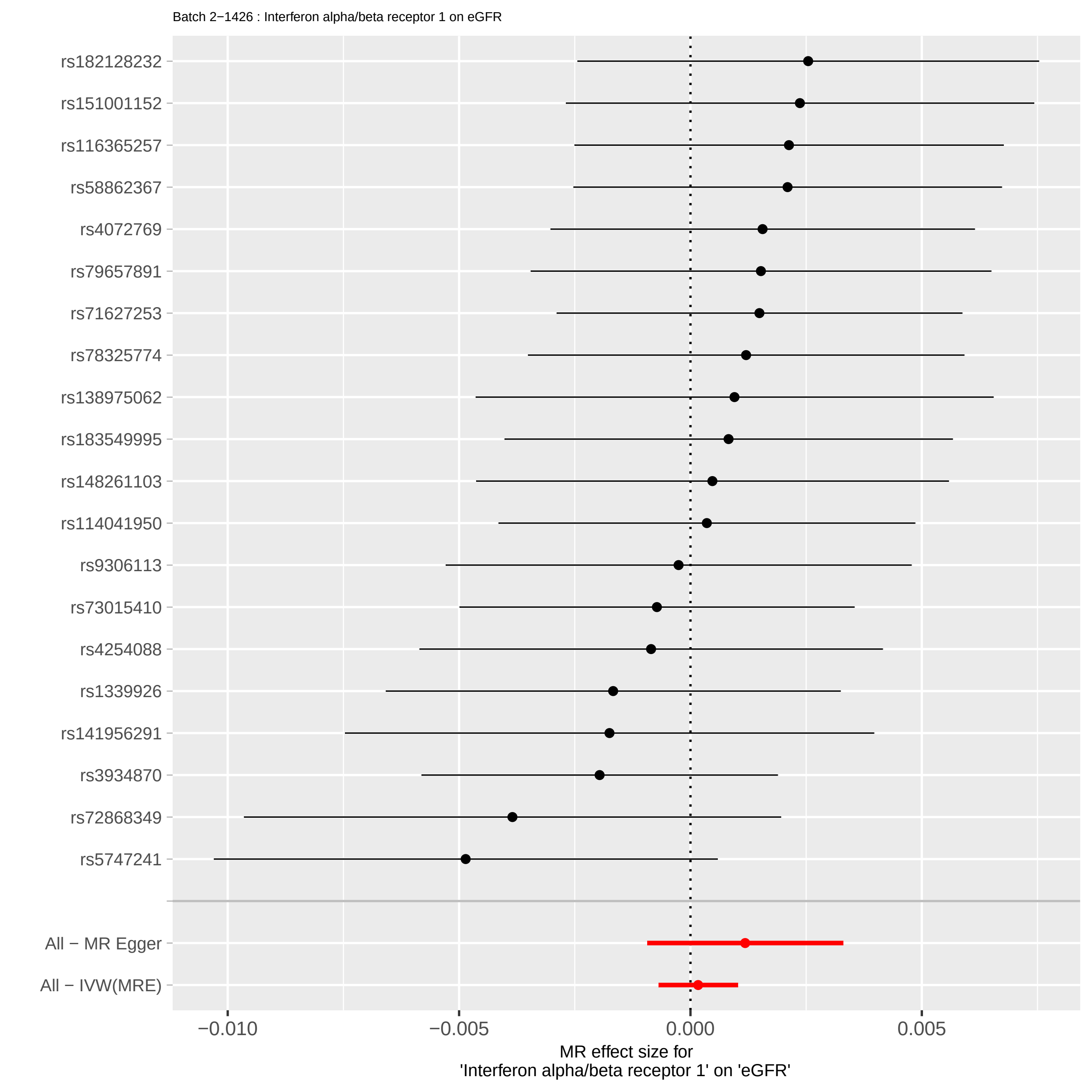

Batch 2−1426 : Interferon alpha/beta receptor 1 on eGFR
rs182128232
rs151001152
rs116365257
rs58862367
rs4072769
rs79657891
rs71627253
rs78325774
rs138975062
rs183549995
rs148261103
rs114041950
rs9306113
rs73015410
rs4254088
rs1339926
rs141956291
rs3934870
rs72868349
rs5747241
All − MR Egger
All − IVW(MRE)
−0.010
−0.005
0.000
0.005
MR effect size for
'Interferon alpha/beta receptor 1' on 'eGFR'

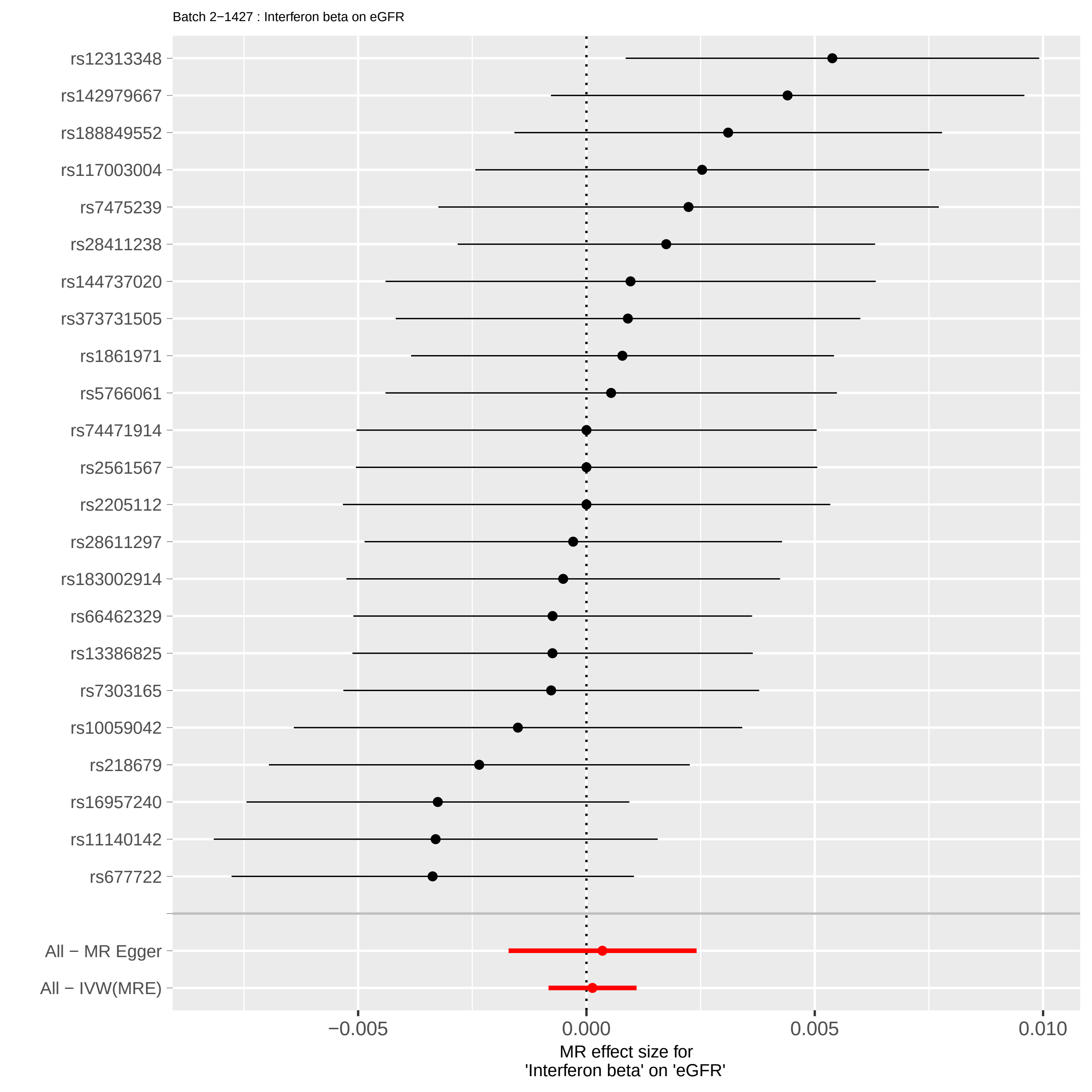

Batch 2−1427 : Interferon beta on eGFR
rs12313348
rs142979667
rs188849552
rs117003004
rs7475239
rs28411238
rs144737020
rs373731505
rs1861971
rs5766061
rs74471914
rs2561567
rs2205112
rs28611297
rs183002914
rs66462329
rs13386825
rs7303165
rs10059042
rs218679
rs16957240
rs11140142
rs677722
All − MR Egger
All − IVW(MRE)
−0.005
0.000
0.005
0.010
MR effect size for
'Interferon beta' on 'eGFR'

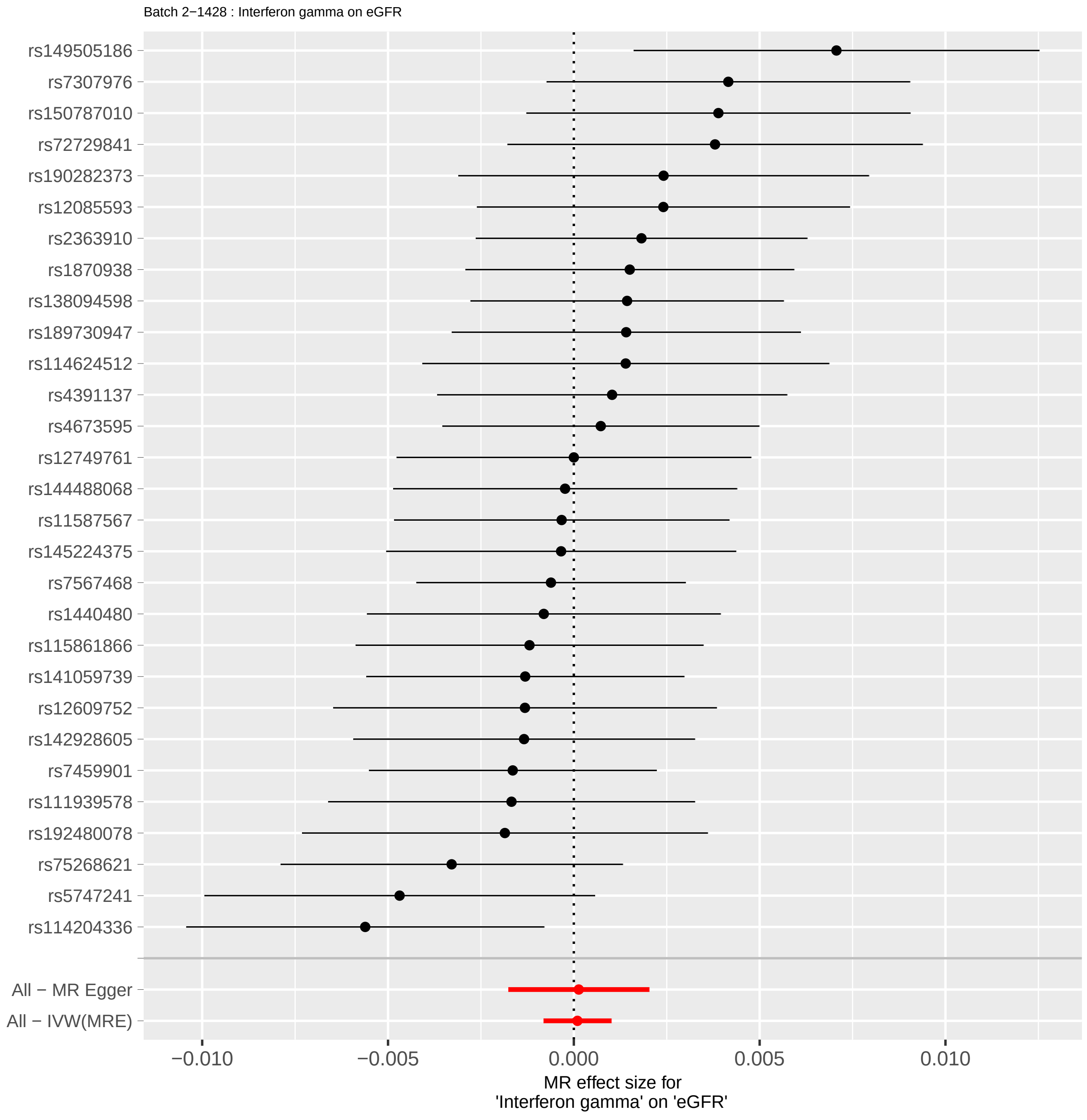

Batch 2−1428 : Interferon gamma on eGFR
rs149505186
rs7307976
rs150787010
rs72729841
rs190282373
rs12085593
rs2363910
rs1870938
rs138094598
rs189730947
rs114624512
rs4391137
rs4673595
rs12749761
rs144488068
rs11587567
rs145224375
rs7567468
rs1440480
rs115861866
rs141059739
rs12609752
rs142928605
rs7459901
rs111939578
rs192480078
rs75268621
rs5747241
rs114204336
All − MR Egger
All − IVW(MRE)
−0.010
−0.005
0.000
0.005
0.010
MR effect size for
'Interferon gamma' on 'eGFR'

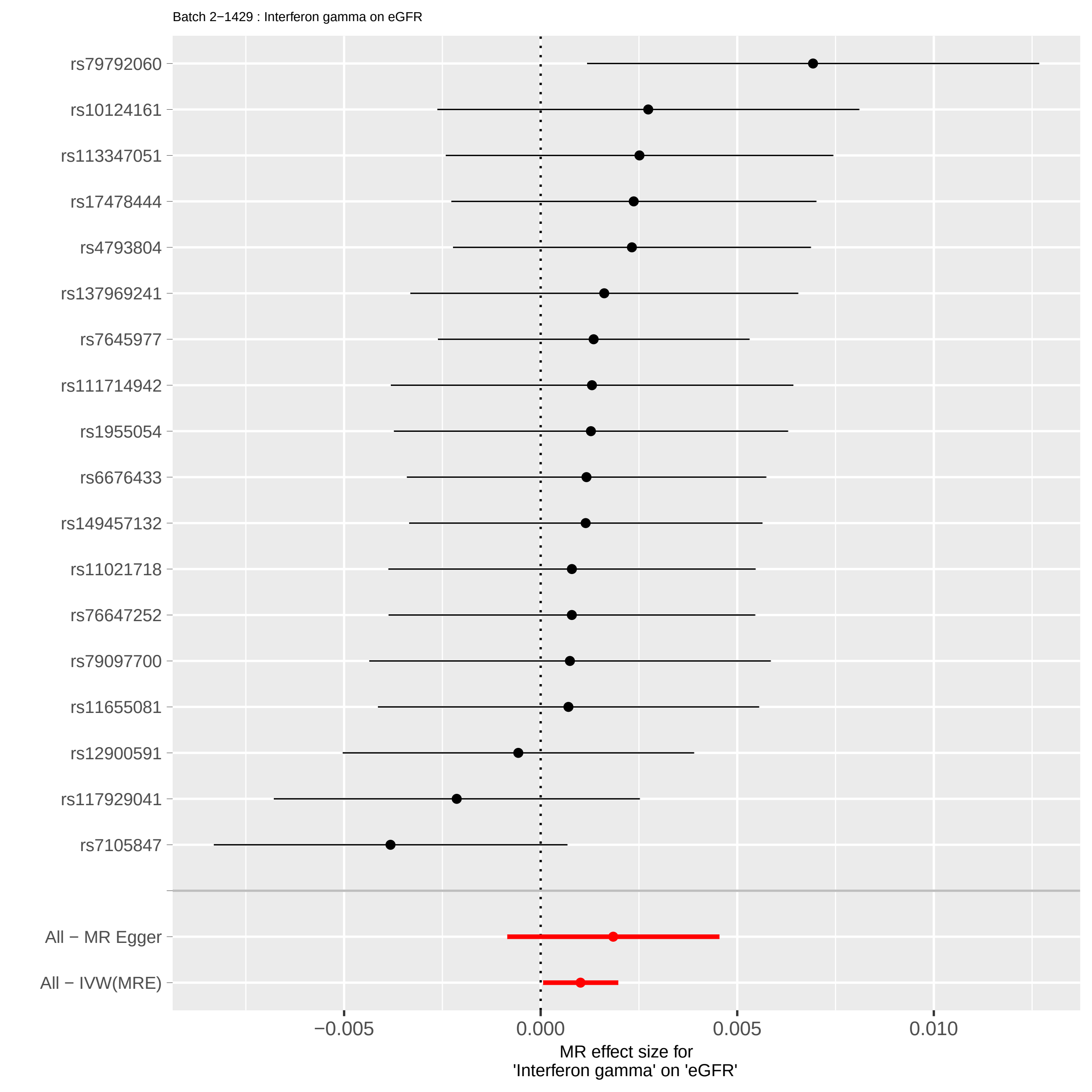

Batch 2−1429 : Interferon gamma on eGFR
rs79792060
rs10124161
rs113347051
rs17478444
rs4793804
rs137969241
rs7645977
rs111714942
rs1955054
rs6676433
rs149457132
rs11021718
rs76647252
rs79097700
rs11655081
rs12900591
rs117929041
rs7105847
All − MR Egger
All − IVW(MRE)
−0.005
0.000
0.005
0.010
MR effect size for
'Interferon gamma' on 'eGFR'

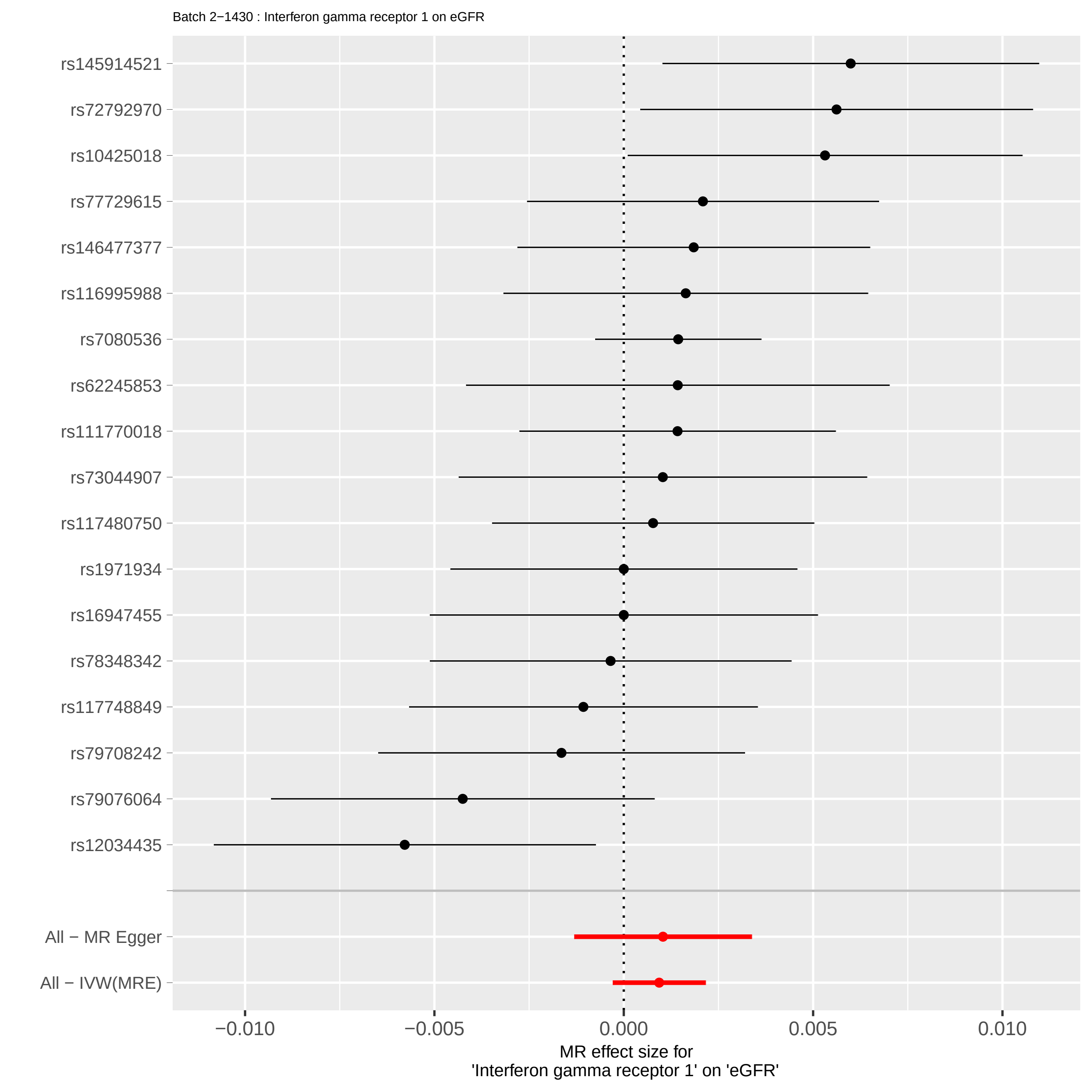
Batch 2−1430 : Interferon gamma receptor 1 on eGFR
rs145914521
rs72792970
rs10425018
rs77729615
rs146477377
rs116995988
rs7080536
rs62245853
rs111770018
rs73044907
rs117480750
rs1971934
rs16947455
rs78348342
rs117748849
rs79708242
rs79076064
rs12034435
All − MR Egger
All − IVW(MRE)
−0.010
−0.005
0.000
0.005
0.010
MR effect size for
'Interferon gamma receptor 1' on 'eGFR'

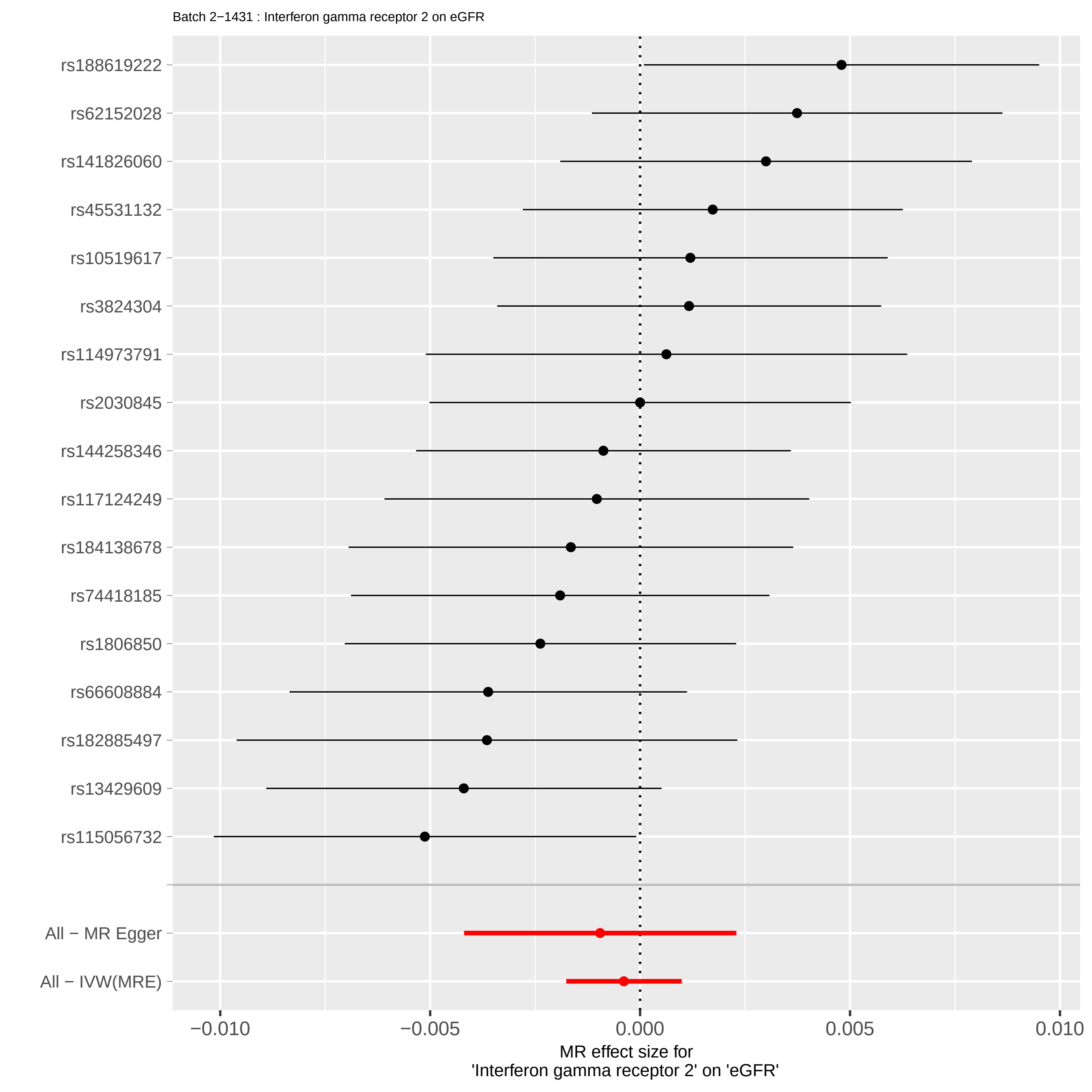
Batch 2−1431 : Interferon gamma receptor 2 on eGFR
rs188619222
rs62152028
rs141826060
rs45531132
rs10519617
rs3824304
rs114973791
rs2030845
rs144258346
rs117124249
rs184138678
rs74418185
rs1806850
rs66608884
rs182885497
rs13429609
rs115056732
All − MR Egger
All − IVW(MRE)
−0.010
−0.005
0.000
0.005
0.010
MR effect size for
'Interferon gamma receptor 2' on 'eGFR'

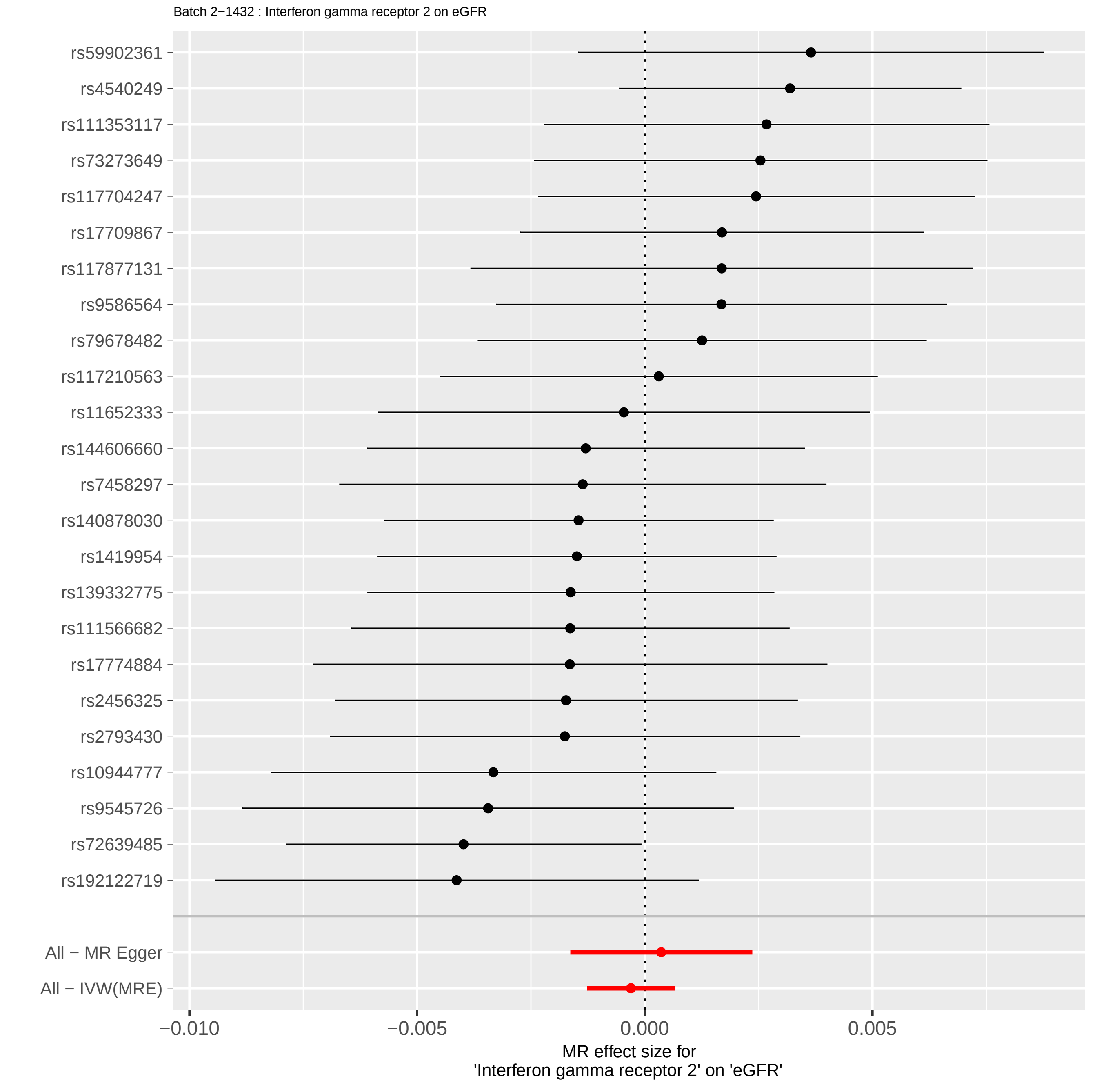

Batch 2−1432 : Interferon gamma receptor 2 on eGFR
rs59902361
rs4540249
rs111353117
rs73273649
rs117704247
rs17709867
rs117877131
rs9586564
rs79678482
rs117210563
rs11652333
rs144606660
rs7458297
rs140878030
rs1419954
rs139332775
rs111566682
rs17774884
rs2456325
rs2793430
rs10944777
rs9545726
rs72639485
rs192122719
All − MR Egger
All − IVW(MRE)
−0.010
−0.005
0.000
0.005
MR effect size for
'Interferon gamma receptor 2' on 'eGFR'

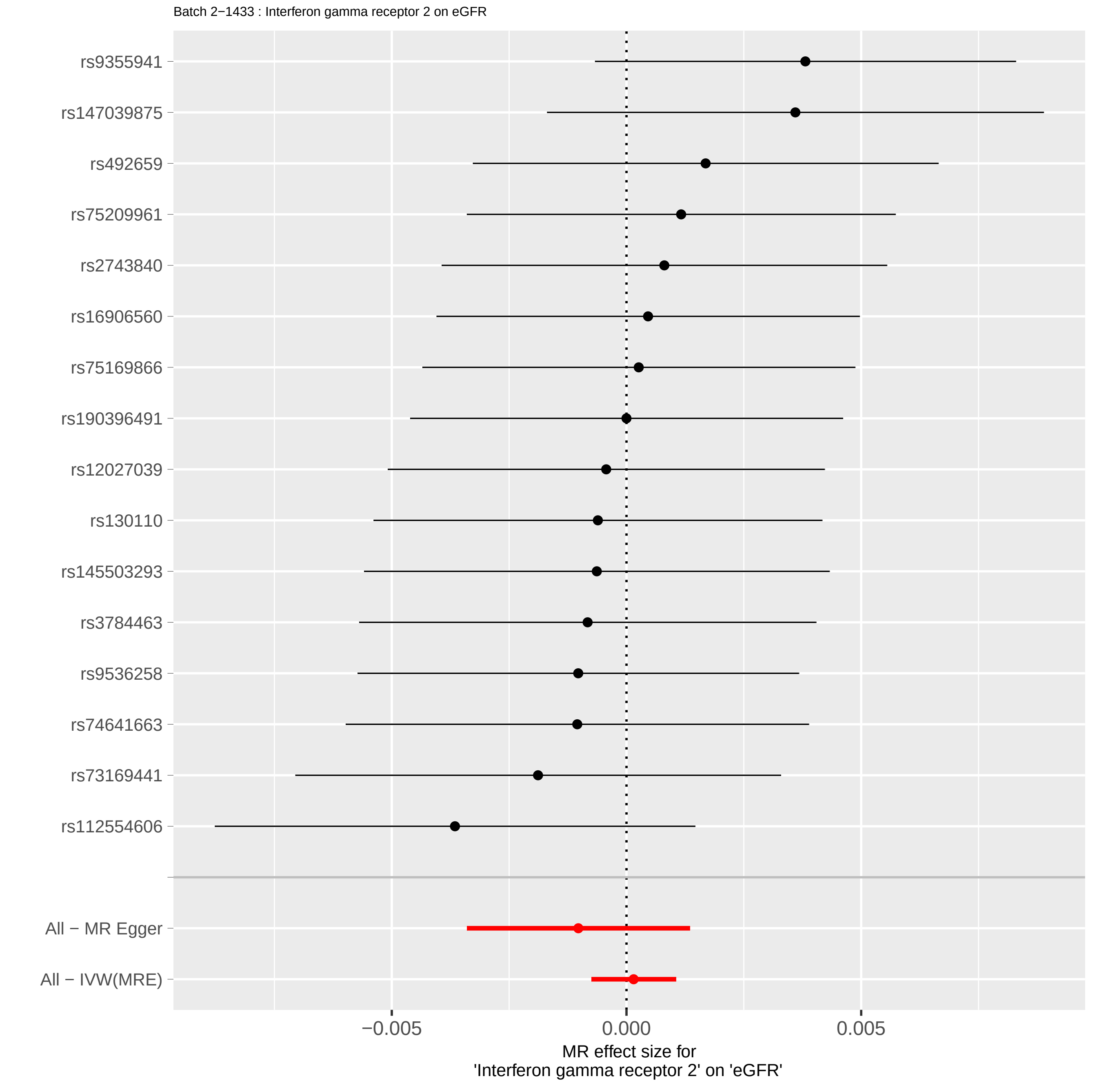

Batch 2−1433 : Interferon gamma receptor 2 on eGFR
rs9355941
rs147039875
rs492659
rs75209961
rs2743840
rs16906560
rs75169866
rs190396491
rs12027039
rs130110
rs145503293
rs3784463
rs9536258
rs74641663
rs73169441
rs112554606
All − MR Egger
All − IVW(MRE)
−0.005
0.000
0.005
MR effect size for
'Interferon gamma receptor 2' on 'eGFR'

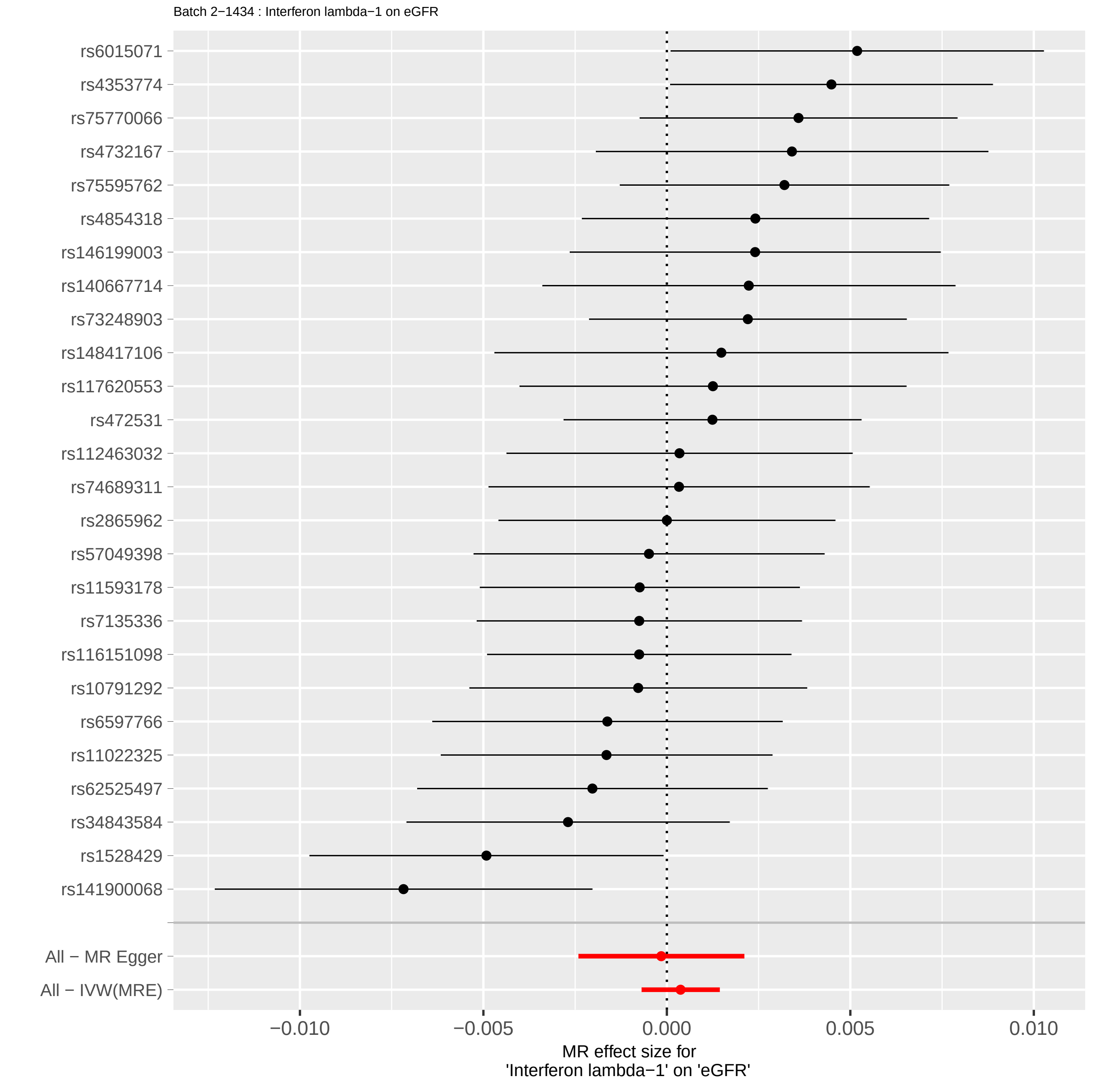

Batch 2−1434 : Interferon lambda−1 on eGFR
rs6015071
rs4353774
rs75770066
rs4732167
rs75595762
rs4854318
rs146199003
rs140667714
rs73248903
rs148417106
rs117620553
rs472531
rs112463032
rs74689311
rs2865962
rs57049398
rs11593178
rs7135336
rs116151098
rs10791292
rs6597766
rs11022325
rs62525497
rs34843584
rs1528429
rs141900068
All − MR Egger
All − IVW(MRE)
−0.010
−0.005
0.000
0.005
0.010
MR effect size for
'Interferon lambda−1' on 'eGFR'

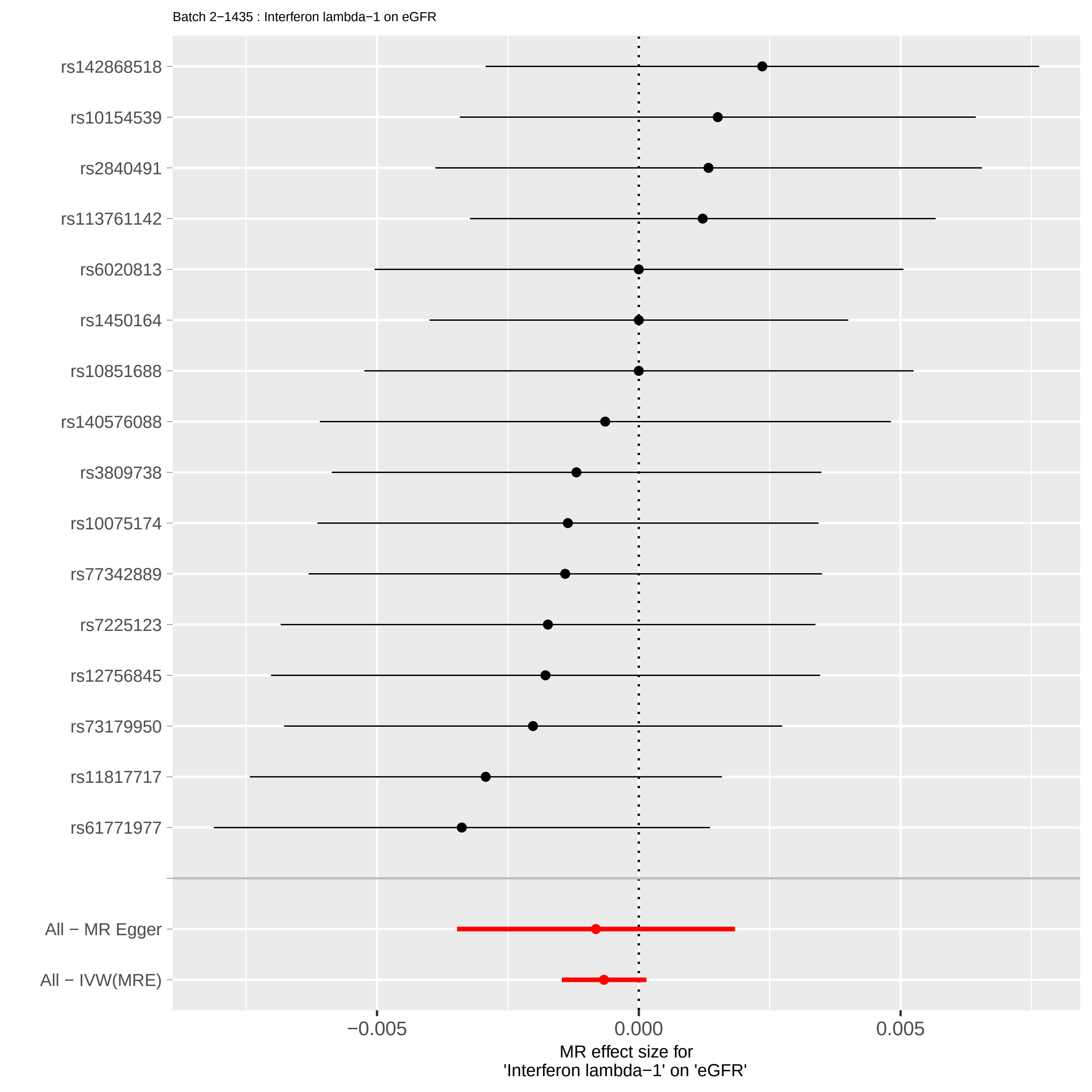

Batch 2−1435 : Interferon lambda−1 on eGFR
rs142868518
rs10154539
rs2840491
rs113761142
rs6020813
rs1450164
rs10851688
rs140576088
rs3809738
rs10075174
rs77342889
rs7225123
rs12756845
rs73179950
rs11817717
rs61771977
All − MR Egger
All − IVW(MRE)
−0.005
0.000
0.005
MR effect size for
'Interferon lambda−1' on 'eGFR'

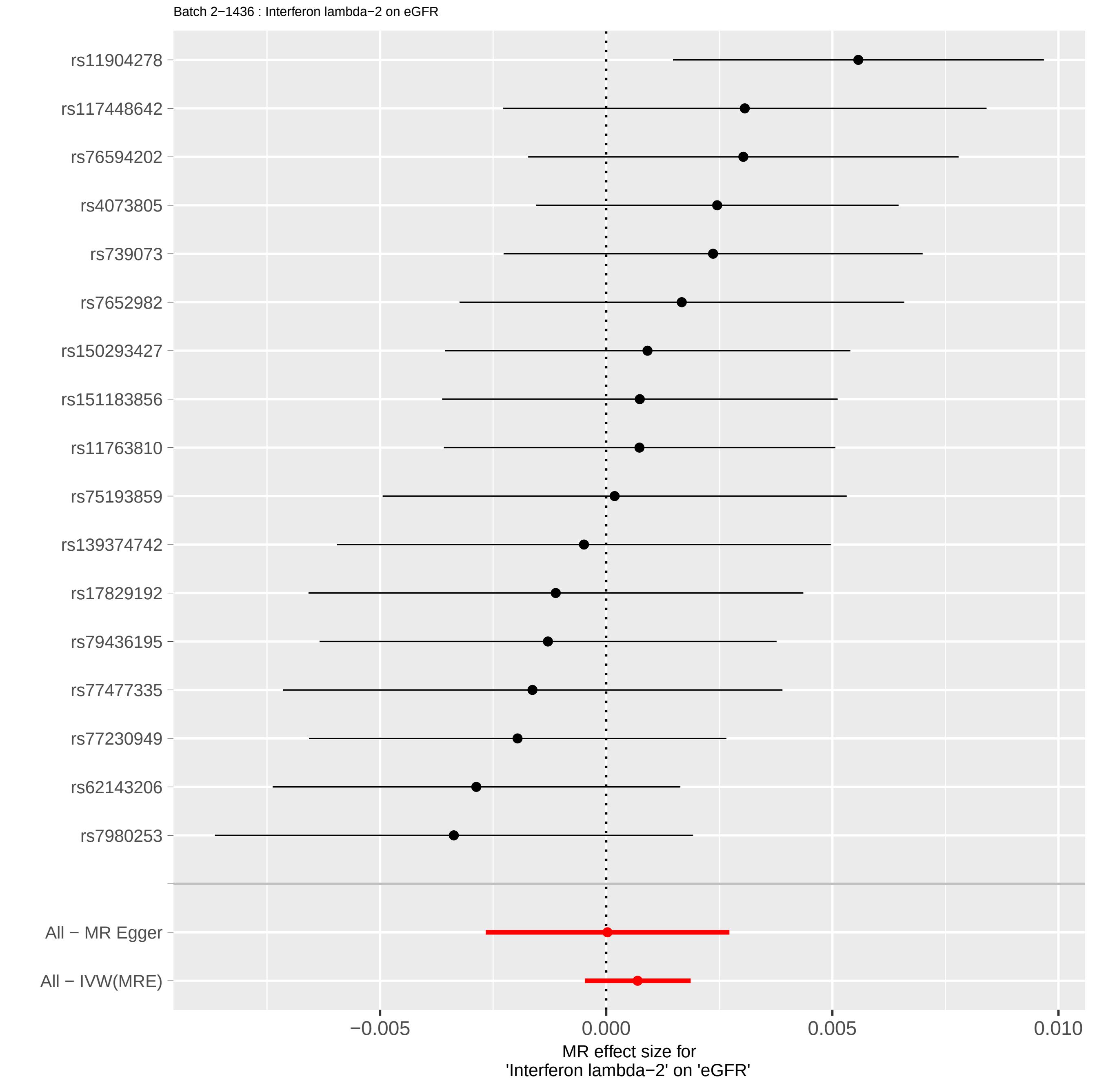
Batch 2−1436 : Interferon lambda−2 on eGFR
rs11904278
rs117448642
rs76594202
rs4073805
rs739073
rs7652982
rs150293427
rs151183856
rs11763810
rs75193859
rs139374742
rs17829192
rs79436195
rs77477335
rs77230949
rs62143206
rs7980253
All − MR Egger
All − IVW(MRE)
−0.005
0.000
0.005
0.010
MR effect size for
'Interferon lambda−2' on 'eGFR'

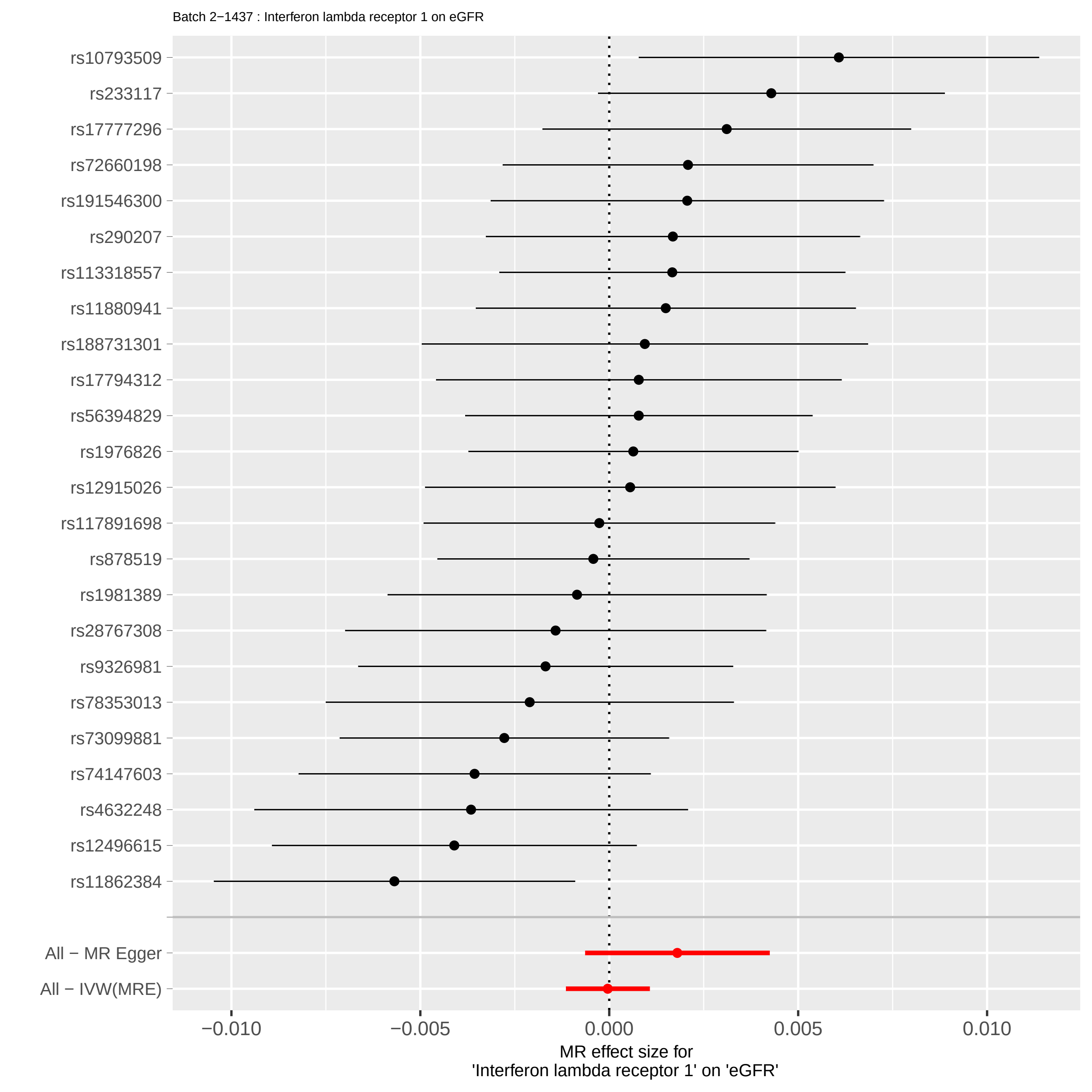

Batch 2−1437 : Interferon lambda receptor 1 on eGFR
rs10793509
rs233117
rs17777296
rs72660198
rs191546300
rs290207
rs113318557
rs11880941
rs188731301
rs17794312
rs56394829
rs1976826
rs12915026
rs117891698
rs878519
rs1981389
rs28767308
rs9326981
rs78353013
rs73099881
rs74147603
rs4632248
rs12496615
rs11862384
All − MR Egger
All − IVW(MRE)
−0.010
−0.005
0.000
0.005
0.010
MR effect size for
'Interferon lambda receptor 1' on 'eGFR'

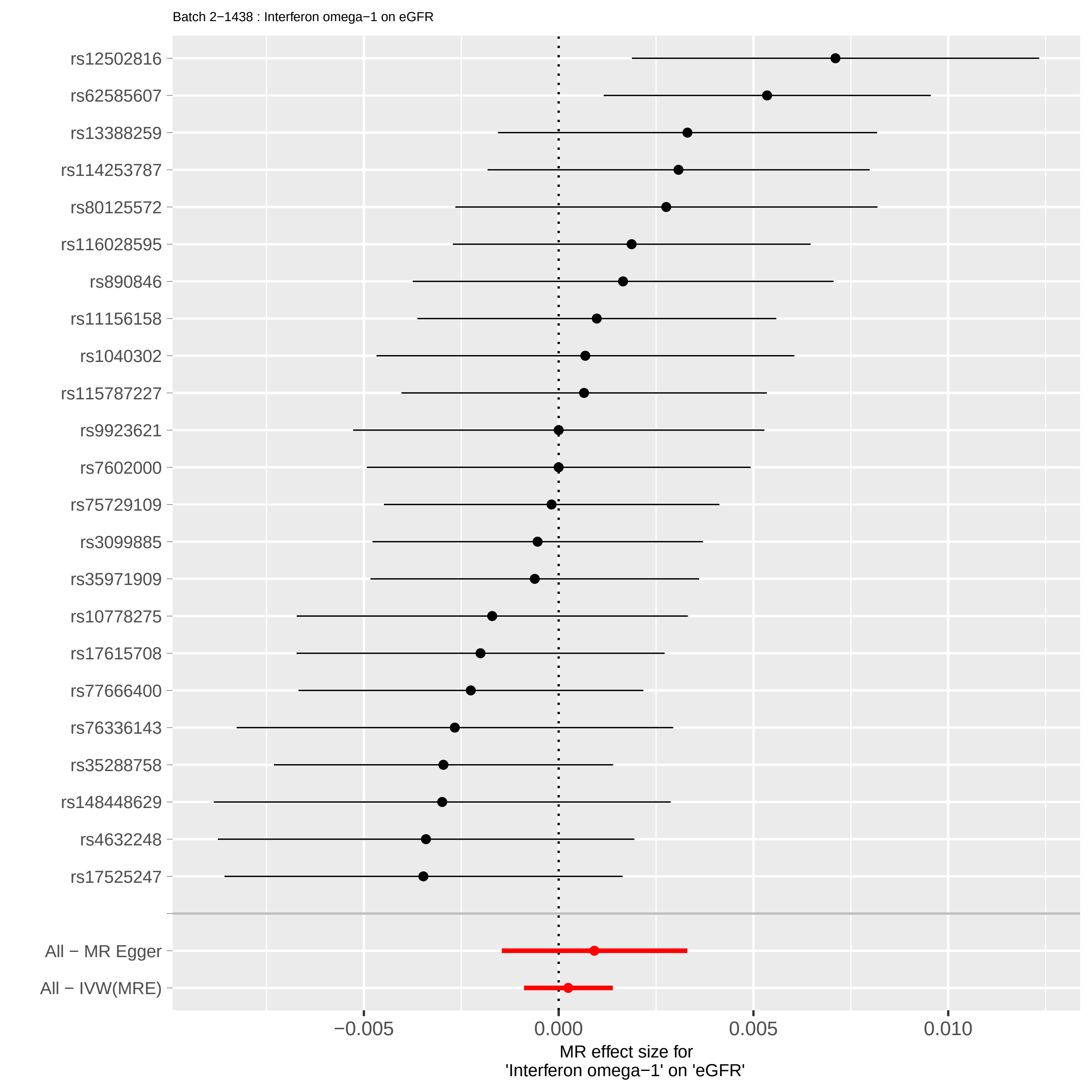

Batch 2−1438 : Interferon omega−1 on eGFR
rs12502816
rs62585607
rs13388259
rs114253787
rs80125572
rs116028595
rs890846
rs11156158
rs1040302
rs115787227
rs9923621
rs7602000
rs75729109
rs3099885
rs35971909
rs10778275
rs17615708
rs77666400
rs76336143
rs35288758
rs148448629
rs4632248
rs17525247
All − MR Egger
All − IVW(MRE)
−0.005
0.000
0.005
0.010
MR effect size for
'Interferon omega−1' on 'eGFR'

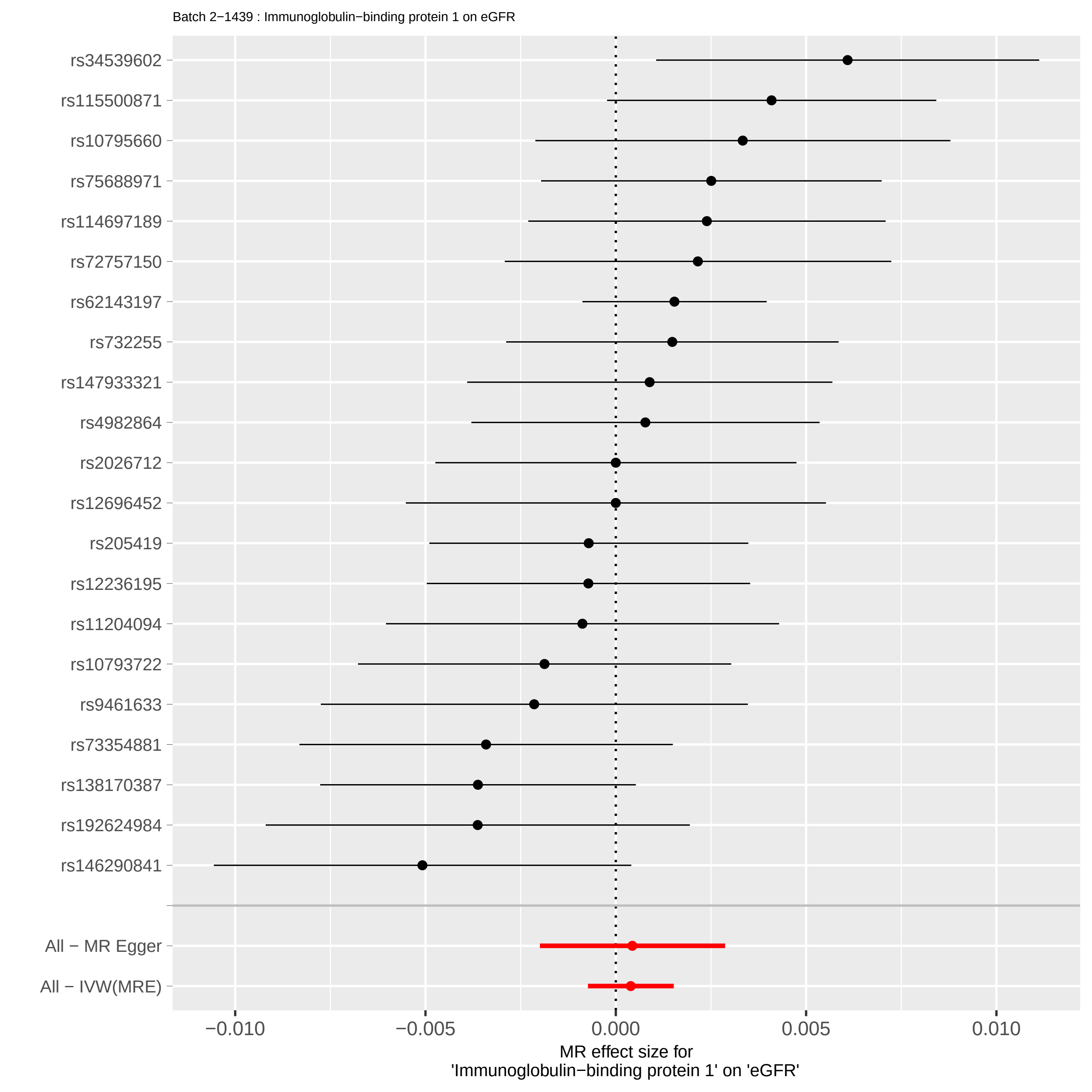

Batch 2−1439 : Immunoglobulin−binding protein 1 on eGFR
rs34539602
rs115500871
rs10795660
rs75688971
rs114697189
rs72757150
rs62143197
rs732255
rs147933321
rs4982864
rs2026712
rs12696452
rs205419
rs12236195
rs11204094
rs10793722
rs9461633
rs73354881
rs138170387
rs192624984
rs146290841
All − MR Egger
All − IVW(MRE)
−0.010
−0.005
0.000
0.005
0.010
MR effect size for
'Immunoglobulin−binding protein 1' on 'eGFR'

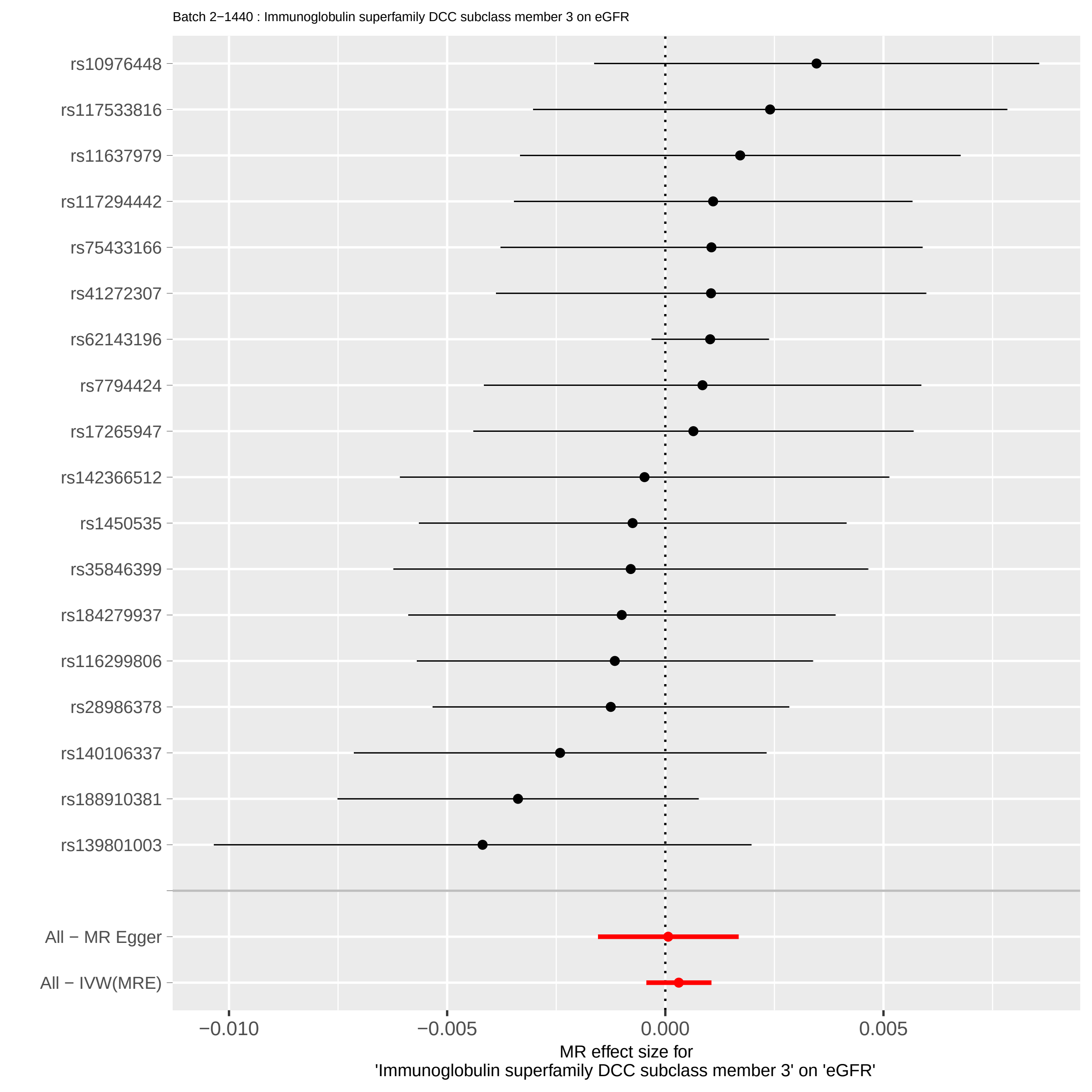

Batch 2−1440 : Immunoglobulin superfamily DCC subclass member 3 on eGFR
rs10976448
rs117533816
rs11637979
rs117294442
rs75433166
rs41272307
rs62143196
rs7794424
rs17265947
rs142366512
rs1450535
rs35846399
rs184279937
rs116299806
rs28986378
rs140106337
rs188910381
rs139801003
All − MR Egger
All − IVW(MRE)
−0.010
−0.005
0.000
0.005
MR effect size for
'Immunoglobulin superfamily DCC subclass member 3' on 'eGFR'

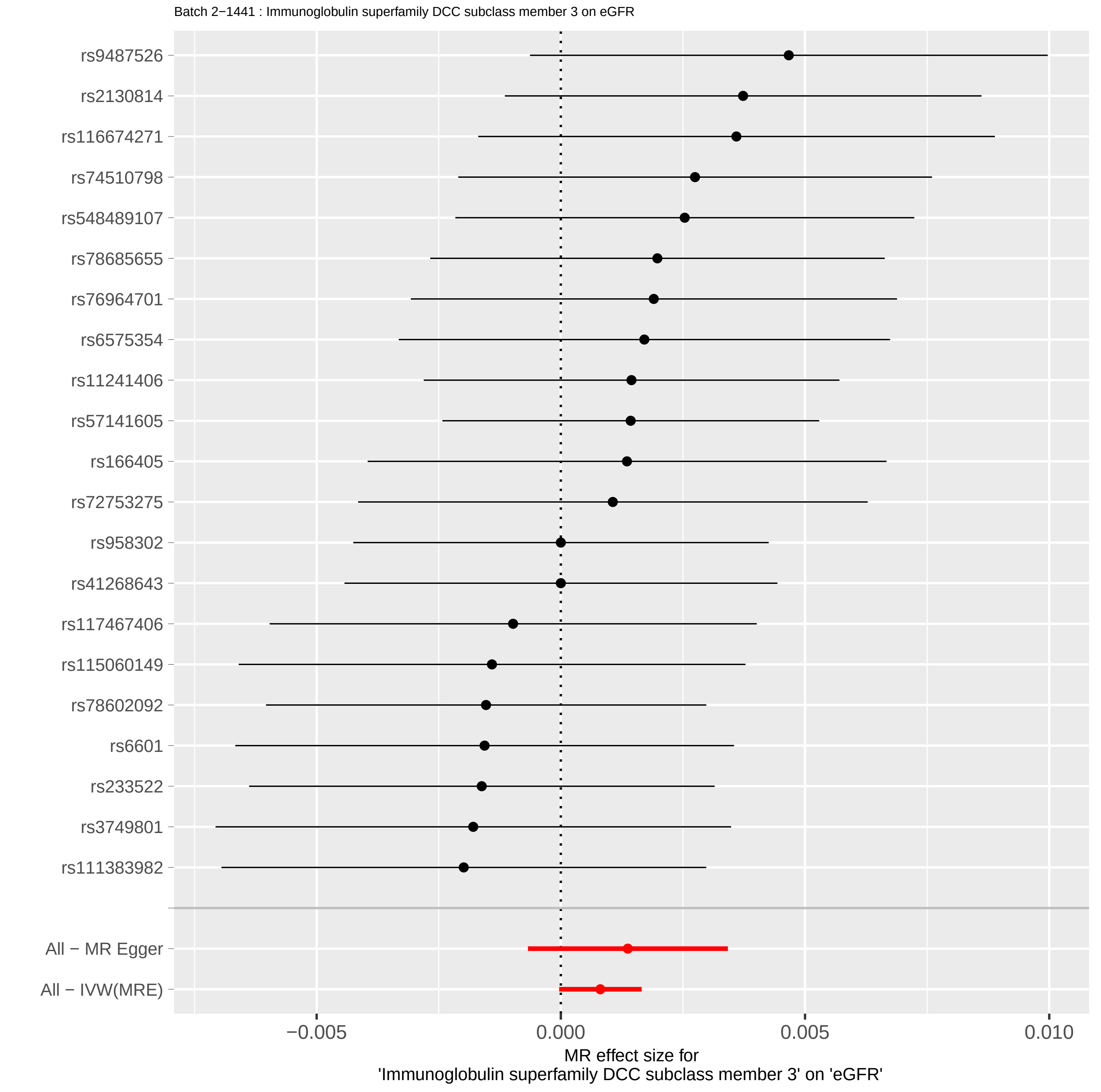

Batch 2−1441 : Immunoglobulin superfamily DCC subclass member 3 on eGFR
rs9487526
rs2130814
rs116674271
rs74510798
rs548489107
rs78685655
rs76964701
rs6575354
rs11241406
rs57141605
rs166405
rs72753275
rs958302
rs41268643
rs117467406
rs115060149
rs78602092
rs6601
rs233522
rs3749801
rs111383982
All − MR Egger
All − IVW(MRE)
−0.005
0.000
0.005
0.010
MR effect size for
'Immunoglobulin superfamily DCC subclass member 3' on 'eGFR'

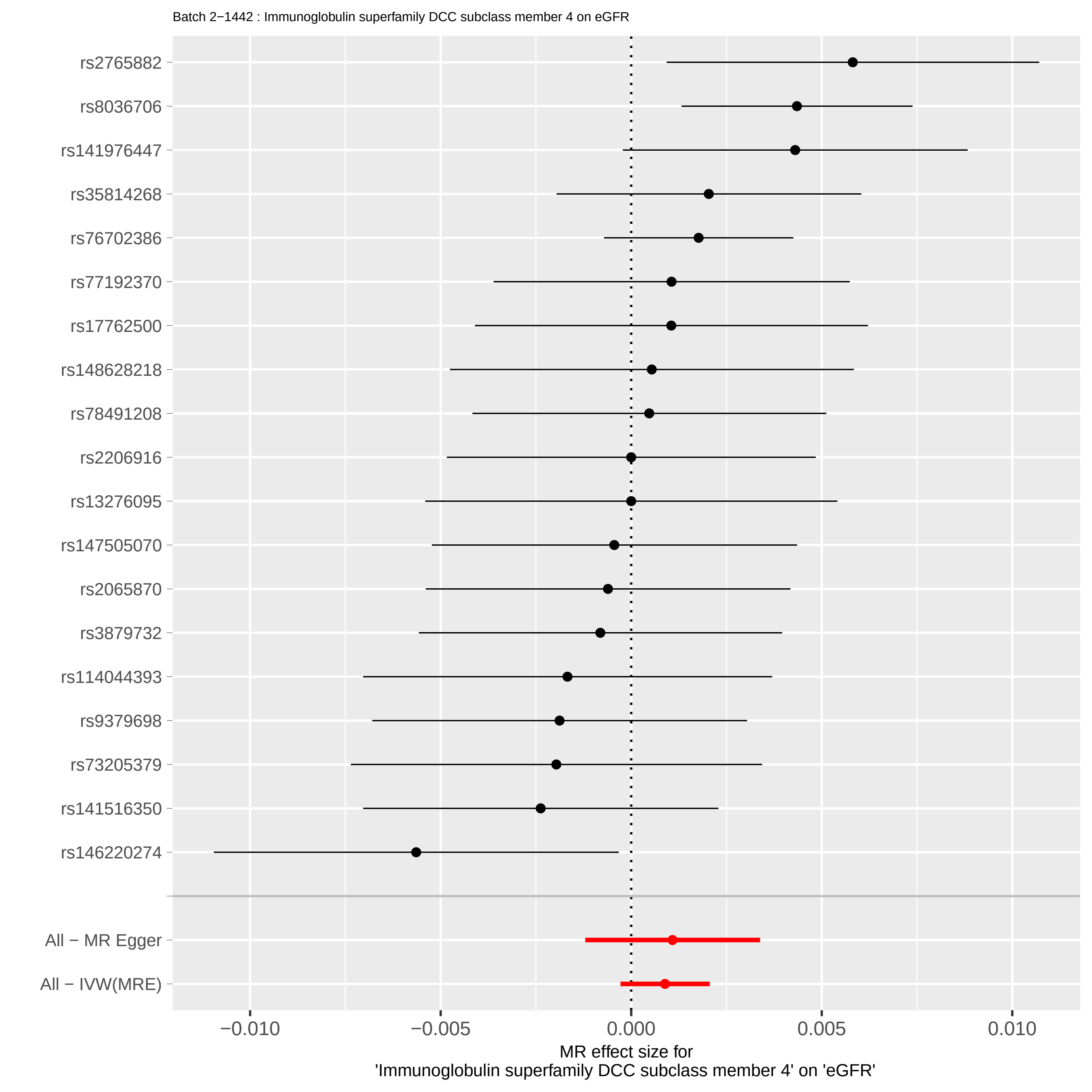

Batch 2−1442 : Immunoglobulin superfamily DCC subclass member 4 on eGFR
rs2765882
rs8036706
rs141976447
rs35814268
rs76702386
rs77192370
rs17762500
rs148628218
rs78491208
rs2206916
rs13276095
rs147505070
rs2065870
rs3879732
rs114044393
rs9379698
rs73205379
rs141516350
rs146220274
All − MR Egger
All − IVW(MRE)
−0.010
−0.005
0.000
0.005
0.010
MR effect size for
'Immunoglobulin superfamily DCC subclass member 4' on 'eGFR'

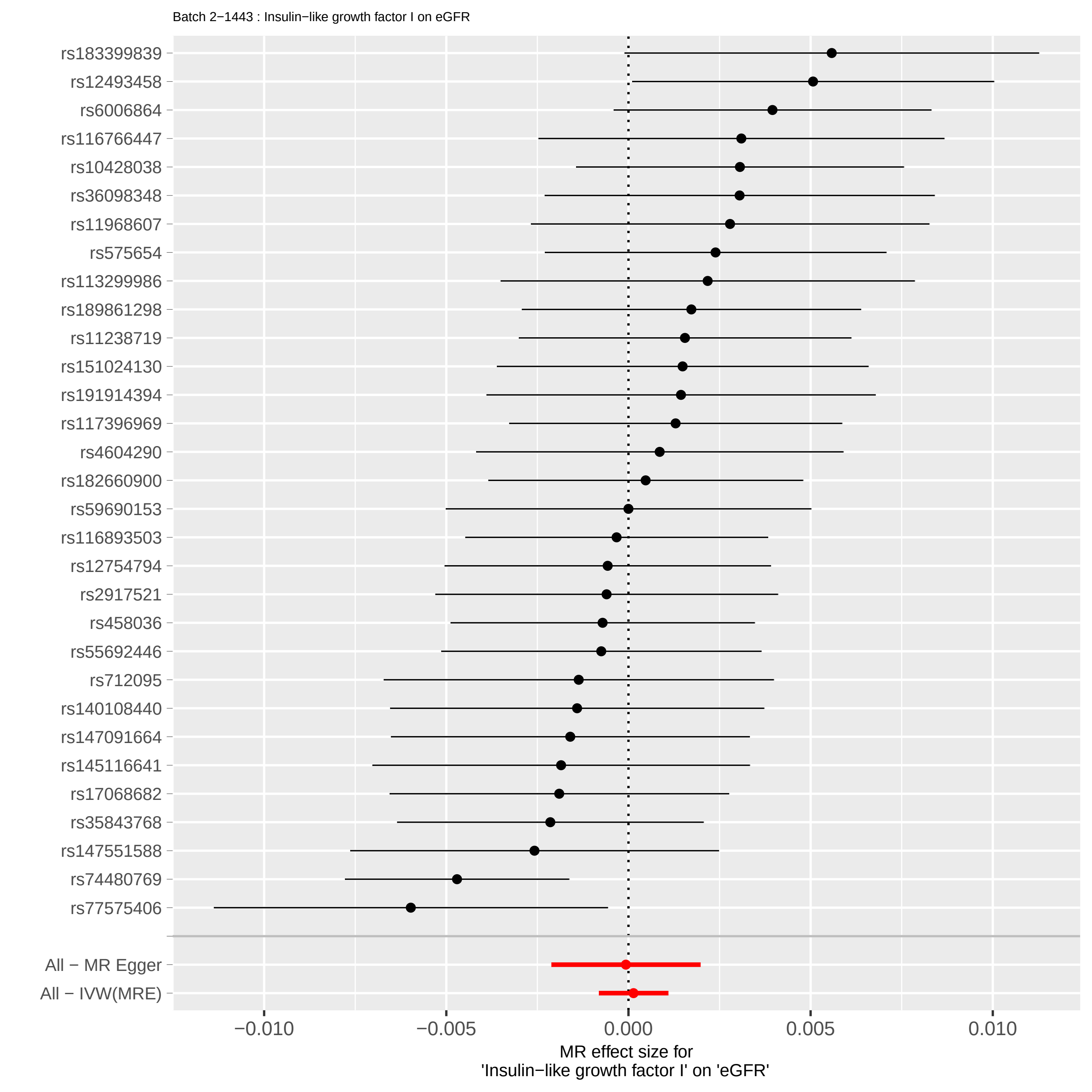

Batch 2−1443 : Insulin−like growth factor I on eGFR
rs183399839
rs12493458
rs6006864
rs116766447
rs10428038
rs36098348
rs11968607
rs575654
rs113299986
rs189861298
rs11238719
rs151024130
rs191914394
rs117396969
rs4604290
rs182660900
rs59690153
rs116893503
rs12754794
rs2917521
rs458036
rs55692446
rs712095
rs140108440
rs147091664
rs145116641
rs17068682
rs35843768
rs147551588
rs74480769
rs77575406
All − MR Egger
All − IVW(MRE)
−0.010
−0.005
0.000
0.005
0.010
MR effect size for
'Insulin−like growth factor I' on 'eGFR'

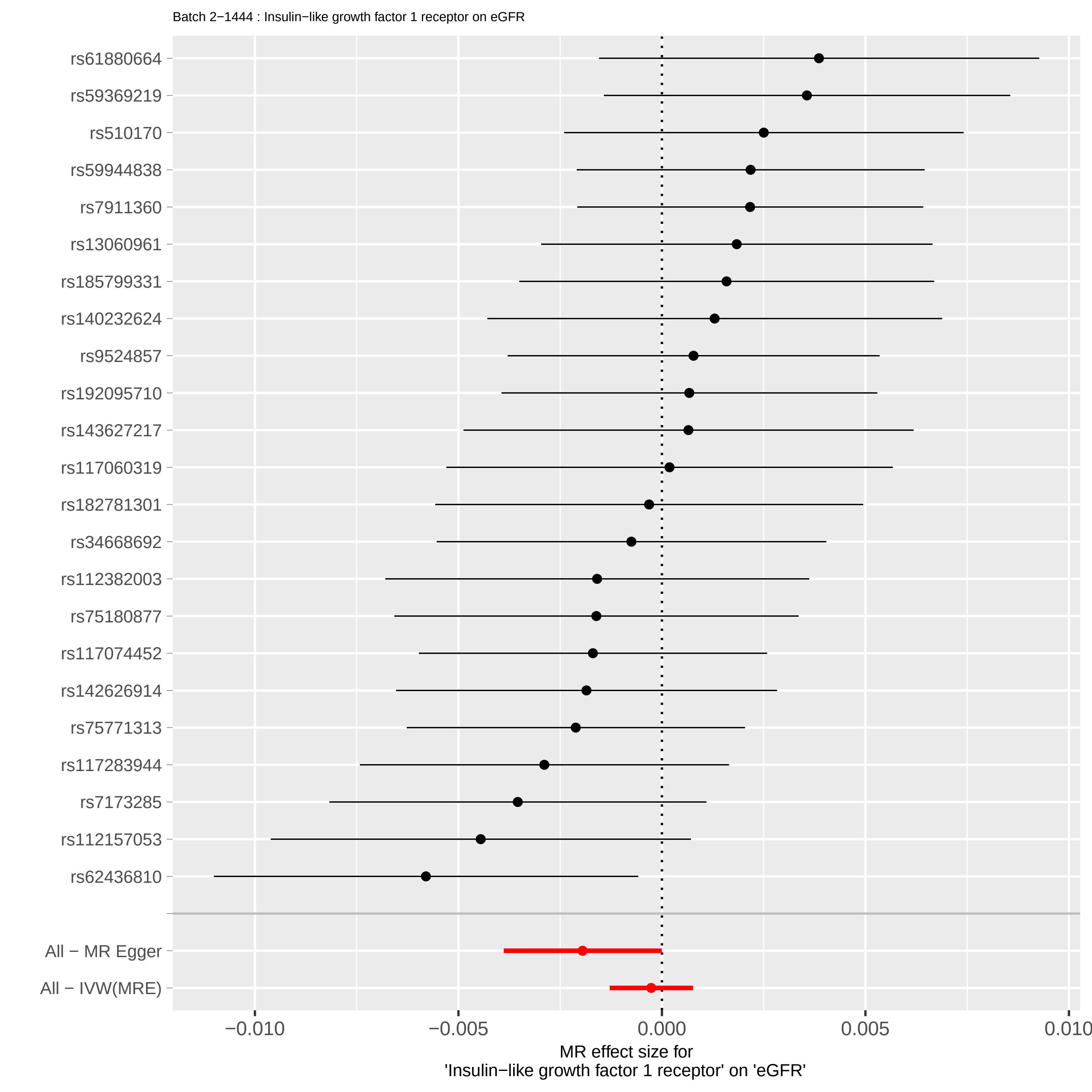

Batch 2−1444 : Insulin−like growth factor 1 receptor on eGFR
rs61880664
rs59369219
rs510170
rs59944838
rs7911360
rs13060961
rs185799331
rs140232624
rs9524857
rs192095710
rs143627217
rs117060319
rs182781301
rs34668692
rs112382003
rs75180877
rs117074452
rs142626914
rs75771313
rs117283944
rs7173285
rs112157053
rs62436810
All − MR Egger
All − IVW(MRE)
−0.010
−0.005
0.000
0.005
0.010
MR effect size for
'Insulin−like growth factor 1 receptor' on 'eGFR'

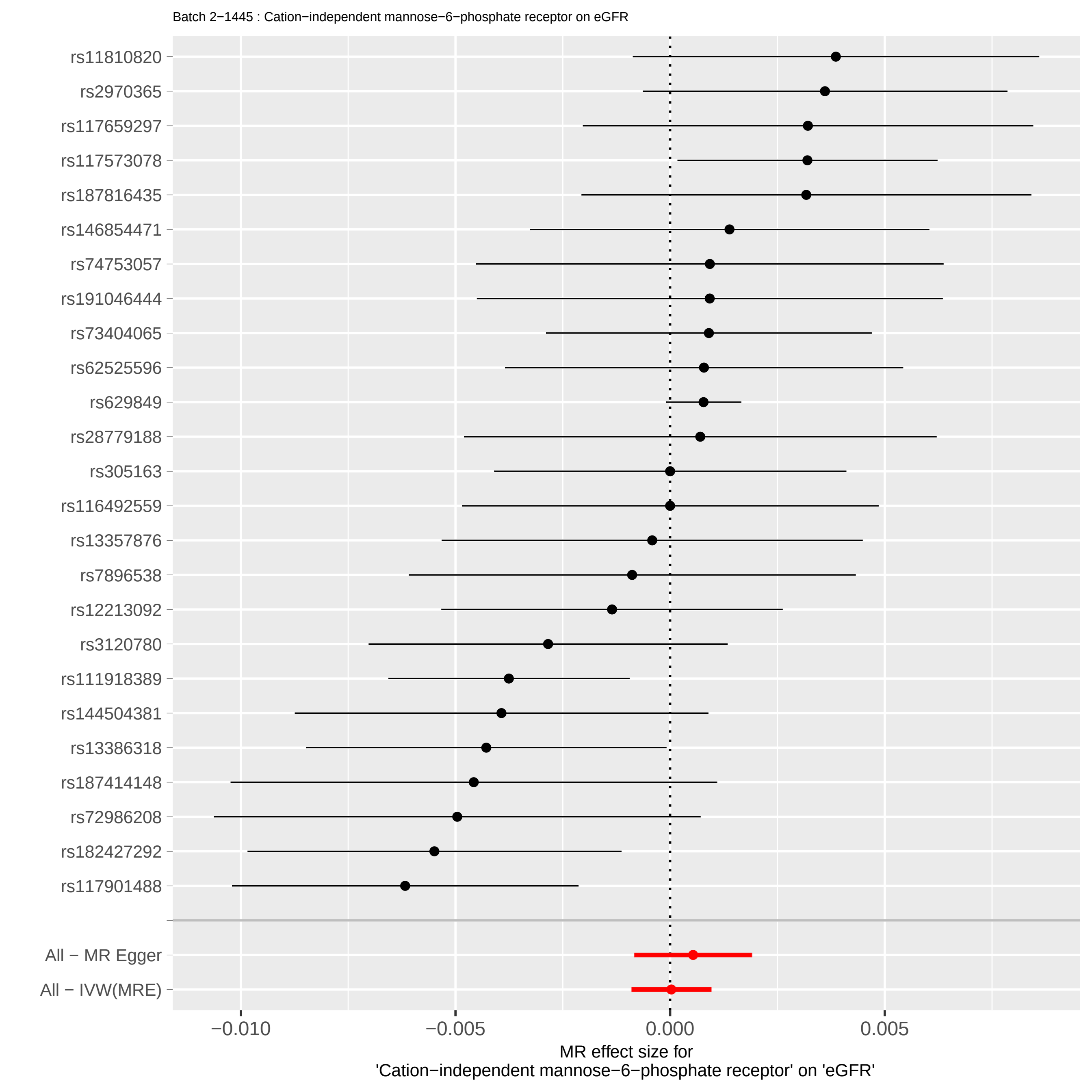

Batch 2−1445 : Cation−independent mannose−6−phosphate receptor on eGFR
rs11810820
rs2970365
rs117659297
rs117573078
rs187816435
rs146854471
rs74753057
rs191046444
rs73404065
rs62525596
rs629849
rs28779188
rs305163
rs116492559
rs13357876
rs7896538
rs12213092
rs3120780
rs111918389
rs144504381
rs13386318
rs187414148
rs72986208
rs182427292
rs117901488
All − MR Egger
All − IVW(MRE)
−0.010
−0.005
0.000
0.005
MR effect size for
'Cation−independent mannose−6−phosphate receptor' on 'eGFR'

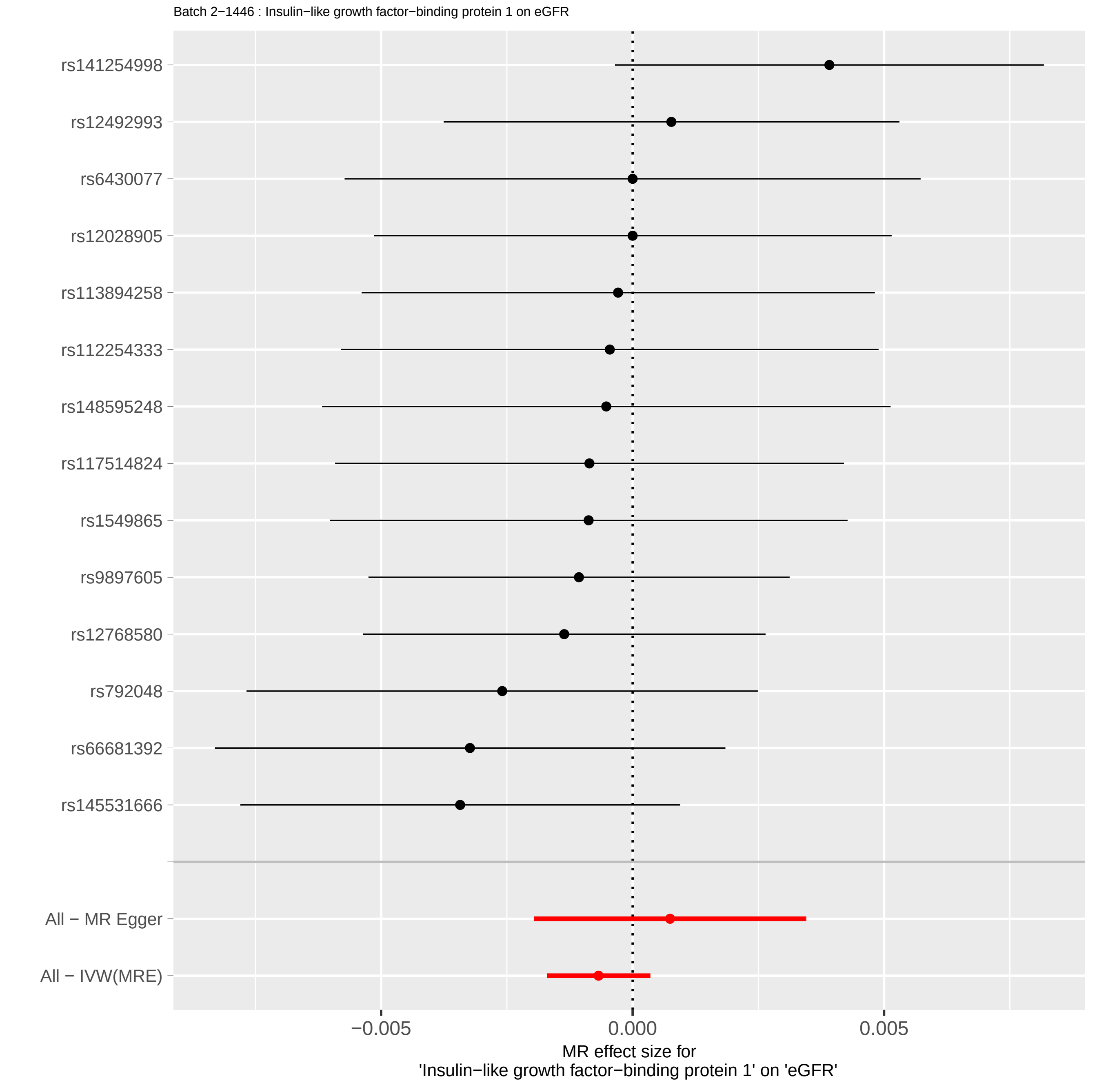

Batch 2−1446 : Insulin−like growth factor−binding protein 1 on eGFR
rs141254998
rs12492993
rs6430077
rs12028905
rs113894258
rs112254333
rs148595248
rs117514824
rs1549865
rs9897605
rs12768580
rs792048
rs66681392
rs145531666
All − MR Egger
All − IVW(MRE)
−0.005
0.000
0.005
MR effect size for
'Insulin−like growth factor−binding protein 1' on 'eGFR'

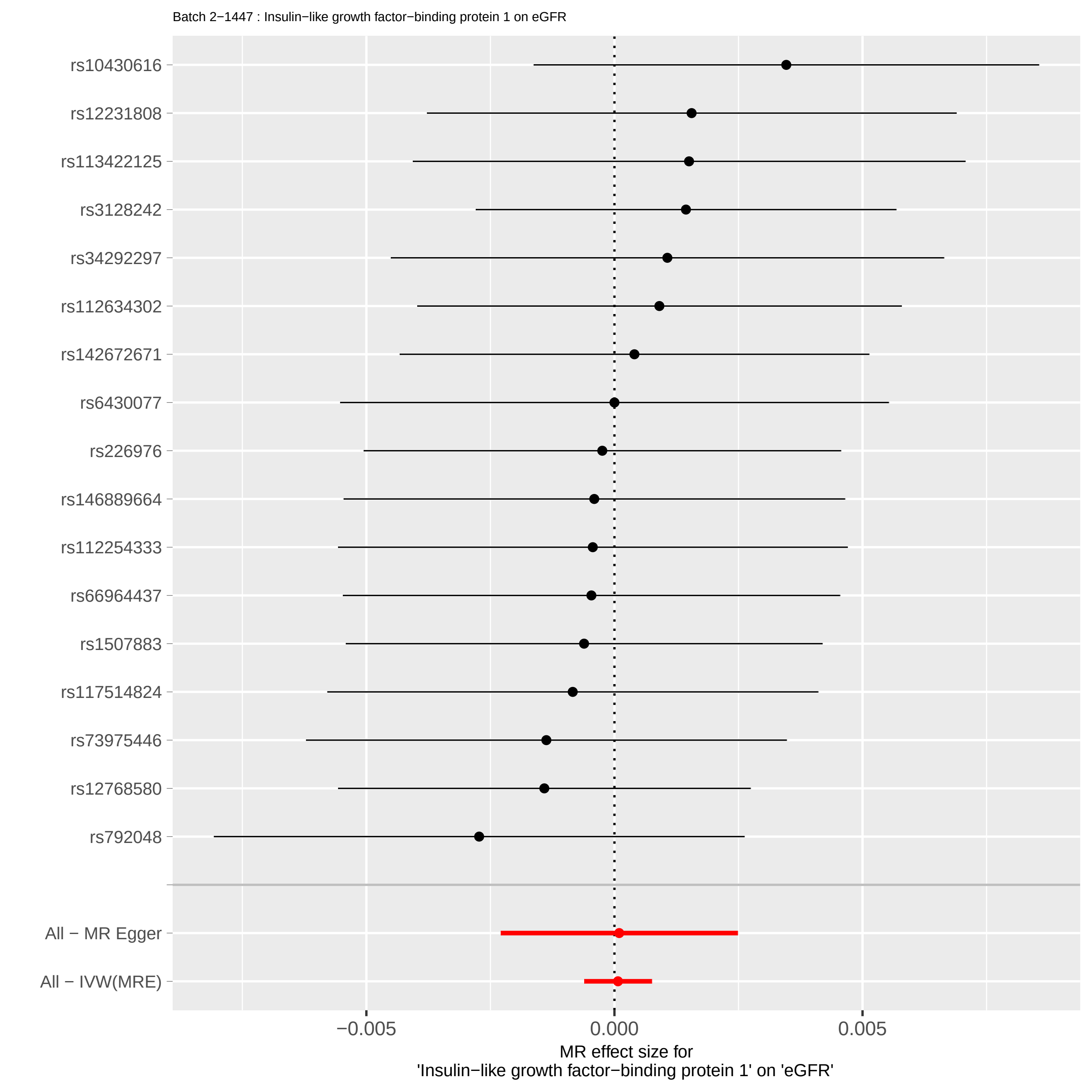

Batch 2−1447 : Insulin−like growth factor−binding protein 1 on eGFR
rs10430616
rs12231808
rs113422125
rs3128242
rs34292297
rs112634302
rs142672671
rs6430077
rs226976
rs146889664
rs112254333
rs66964437
rs1507883
rs117514824
rs73975446
rs12768580
rs792048
All − MR Egger
All − IVW(MRE)
−0.005
0.000
0.005
MR effect size for
'Insulin−like growth factor−binding protein 1' on 'eGFR'

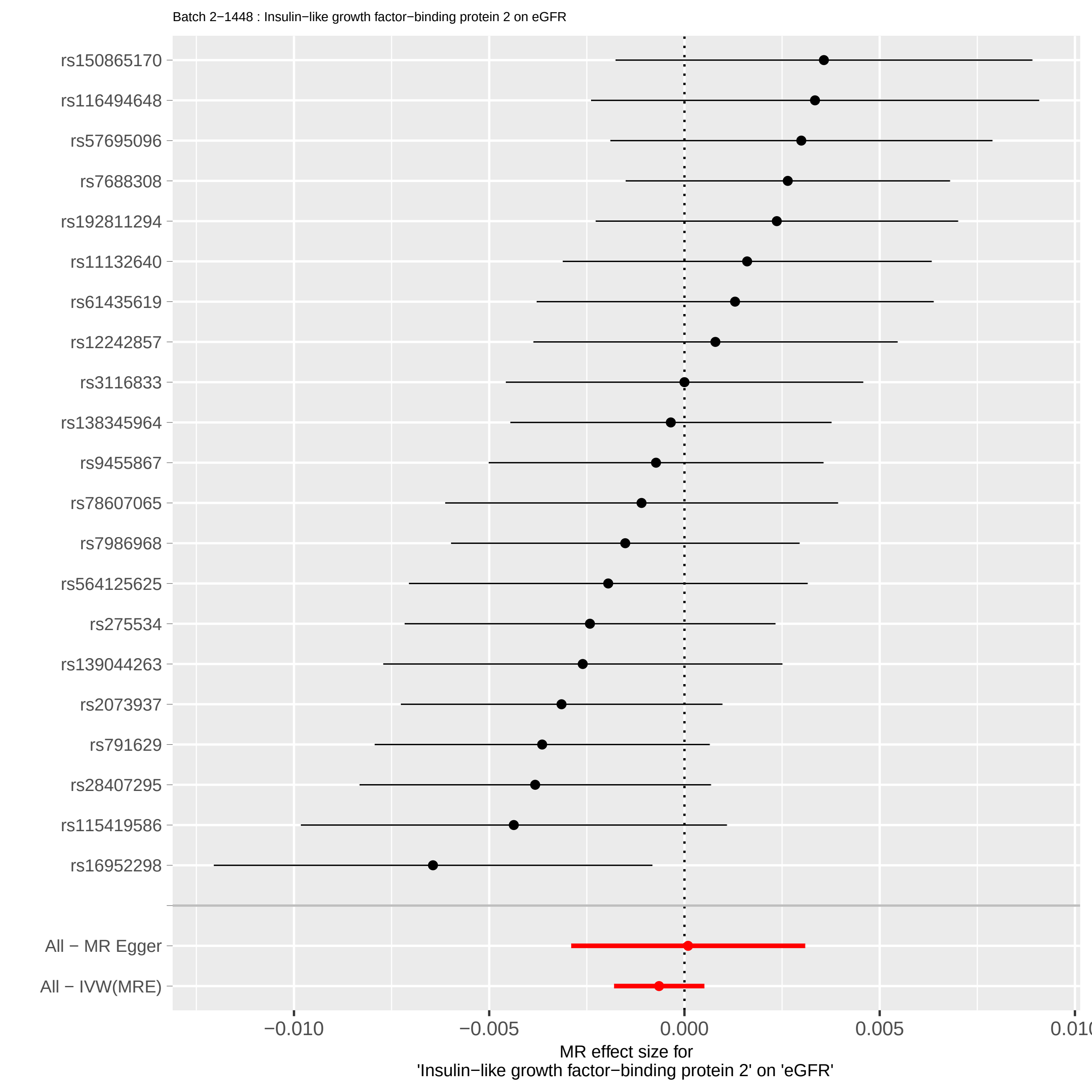

Batch 2−1448 : Insulin−like growth factor−binding protein 2 on eGFR
rs150865170
rs116494648
rs57695096
rs7688308
rs192811294
rs11132640
rs61435619
rs12242857
rs3116833
rs138345964
rs9455867
rs78607065
rs7986968
rs564125625
rs275534
rs139044263
rs2073937
rs791629
rs28407295
rs115419586
rs16952298
All − MR Egger
All − IVW(MRE)
−0.010
−0.005
0.000
0.005
MR effect size for
'Insulin−like growth factor−binding protein 2' on 'eGFR'

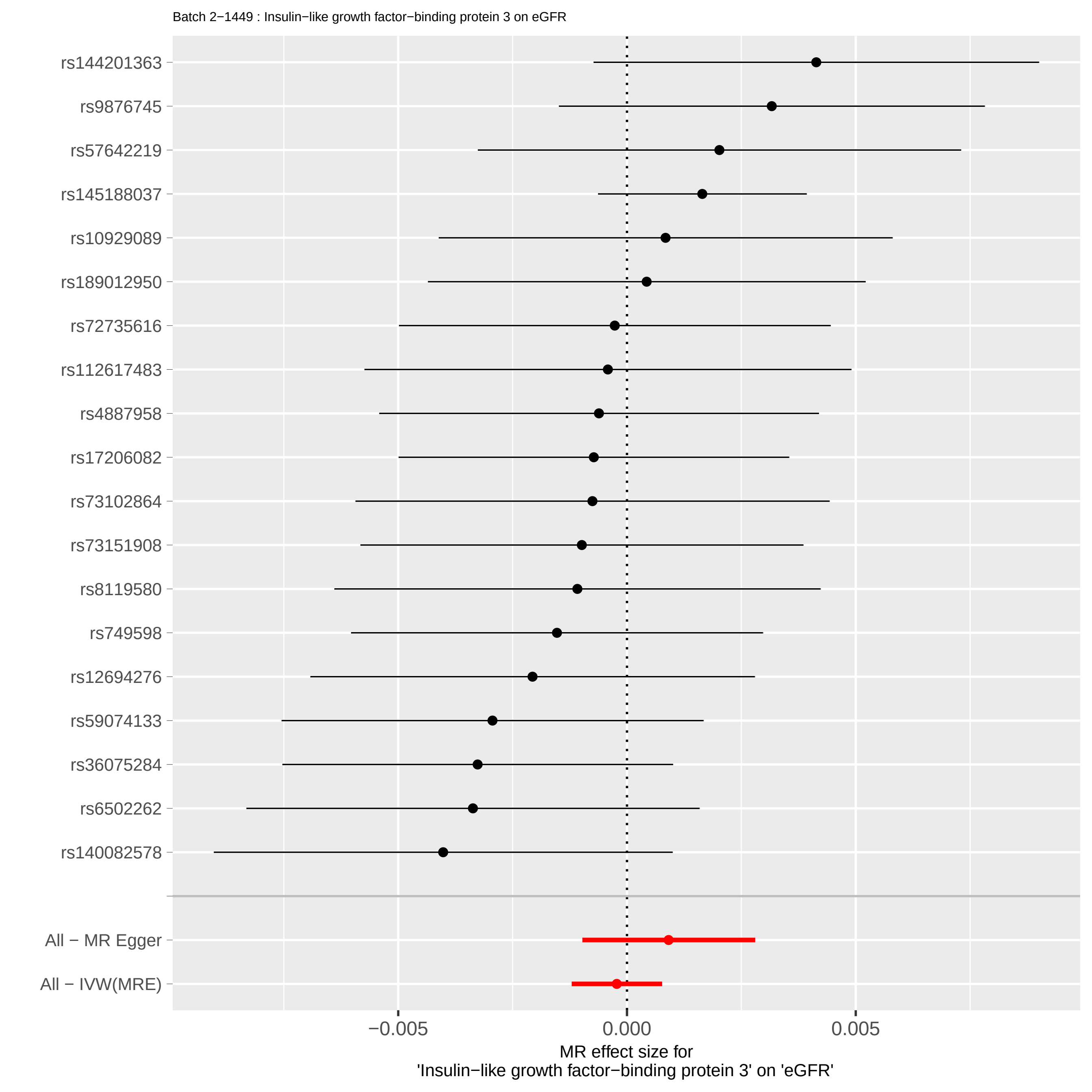

Batch 2−1449 : Insulin−like growth factor−binding protein 3 on eGFR
rs144201363
rs9876745
rs57642219
rs145188037
rs10929089
rs189012950
rs72735616
rs112617483
rs4887958
rs17206082
rs73102864
rs73151908
rs8119580
rs749598
rs12694276
rs59074133
rs36075284
rs6502262
rs140082578
All − MR Egger
All − IVW(MRE)
−0.005
0.000
0.005
MR effect size for
'Insulin−like growth factor−binding protein 3' on 'eGFR'

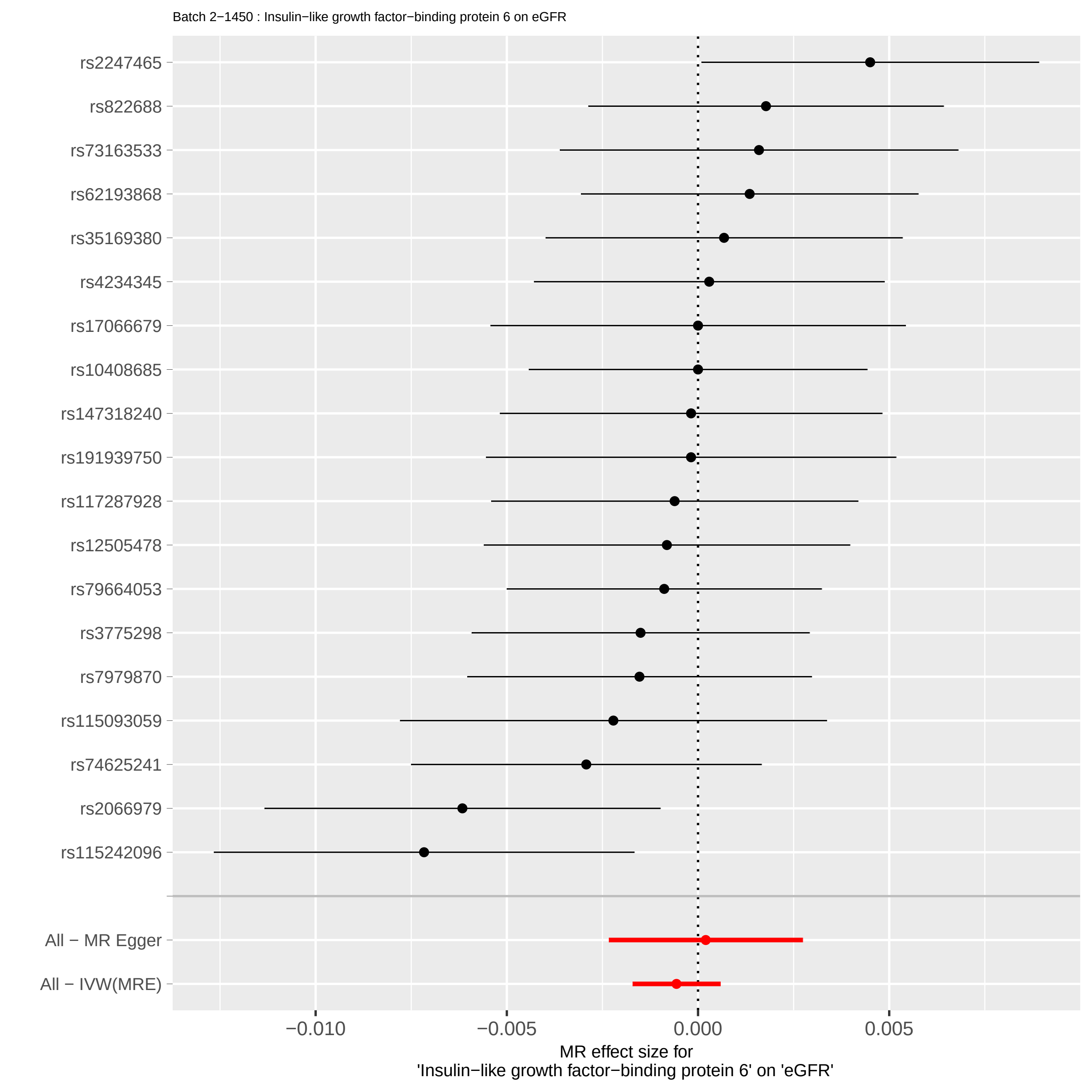

Batch 2−1450 : Insulin−like growth factor−binding protein 6 on eGFR
rs2247465
rs822688
rs73163533
rs62193868
rs35169380
rs4234345
rs17066679
rs10408685
rs147318240
rs191939750
rs117287928
rs12505478
rs79664053
rs3775298
rs7979870
rs115093059
rs74625241
rs2066979
rs115242096
All − MR Egger
All − IVW(MRE)
−0.010
−0.005
0.000
0.005
MR effect size for
'Insulin−like growth factor−binding protein 6' on 'eGFR'

Batch 2−1451 : Insulin−like growth factor−binding protein 7 on eGFR

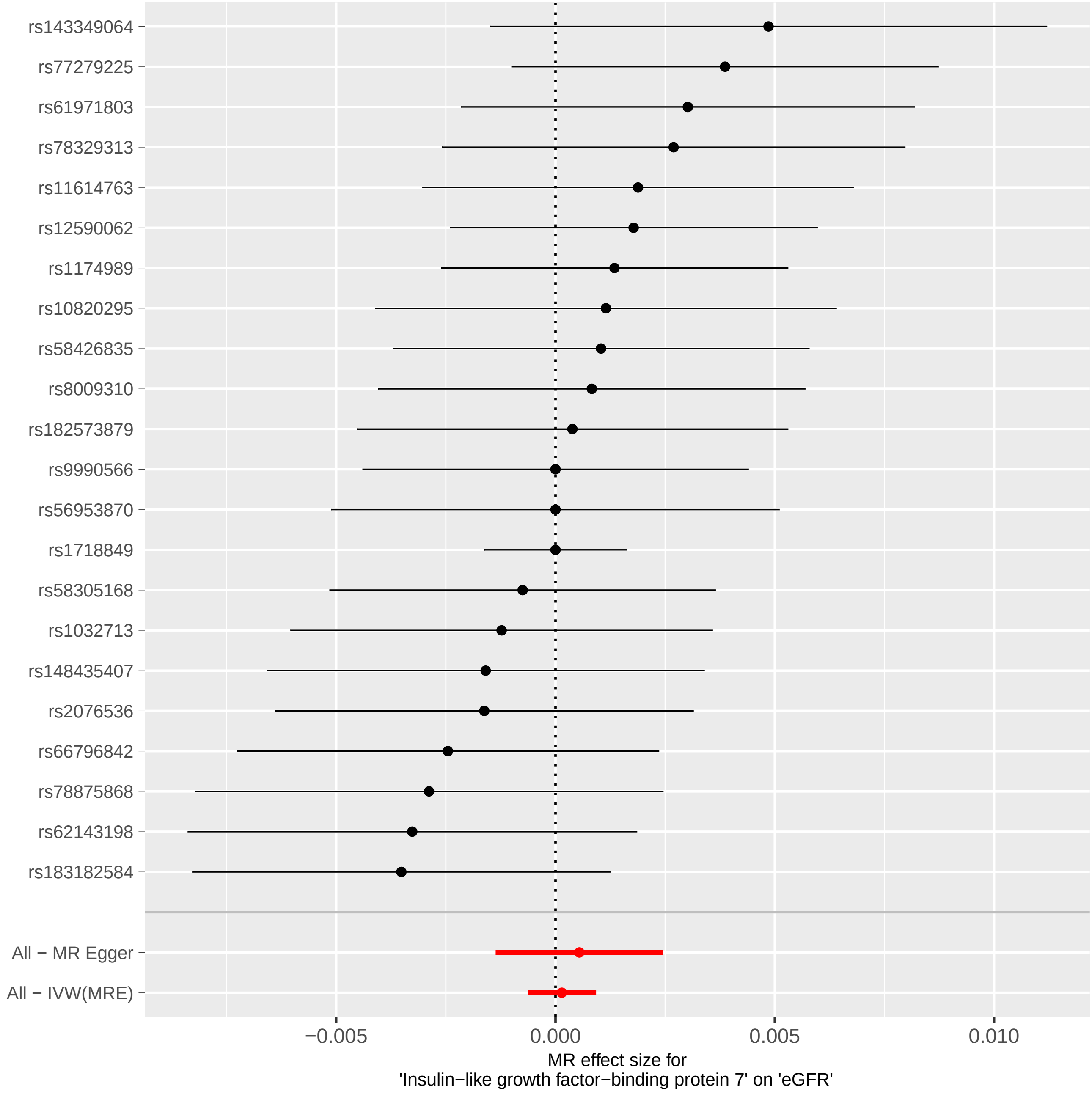

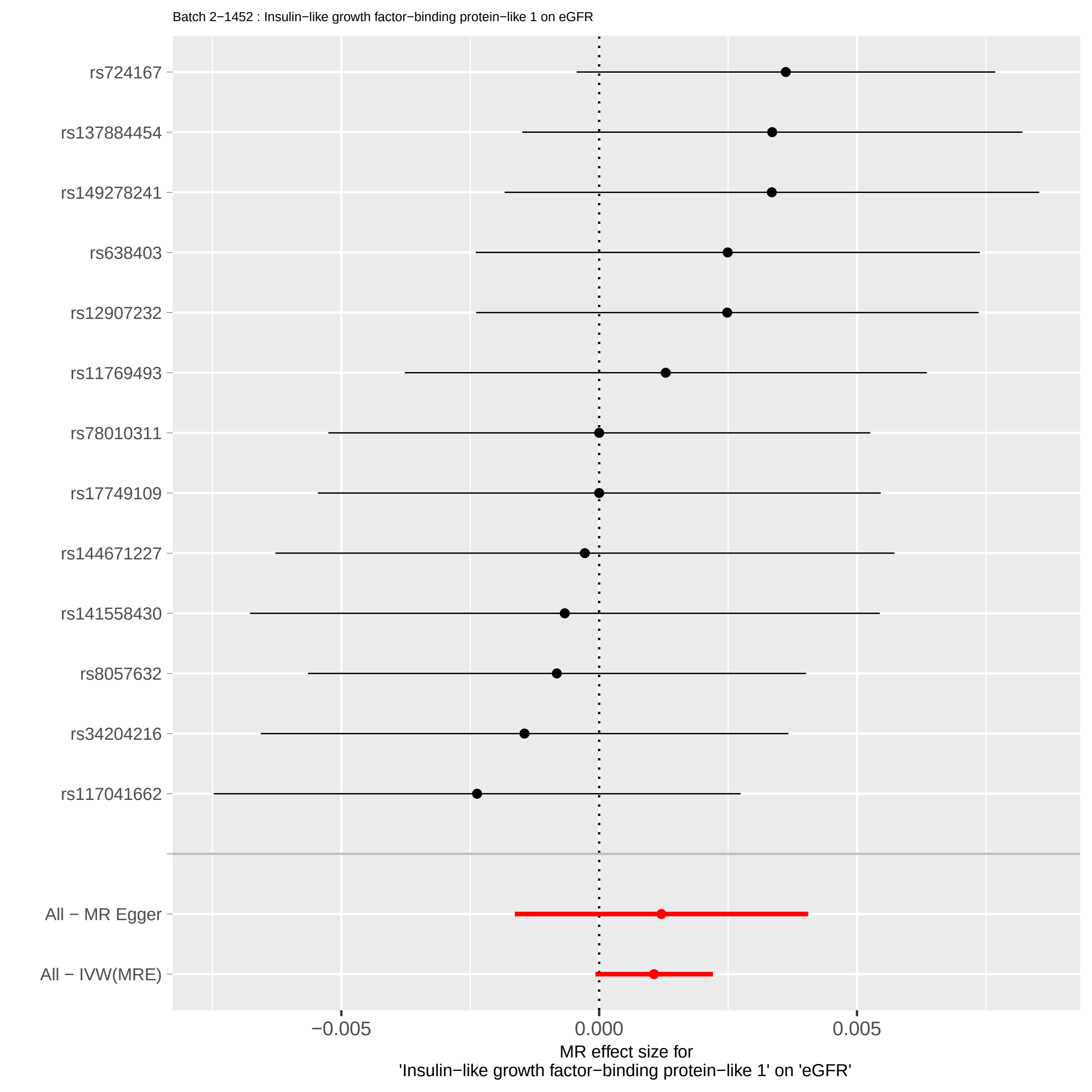
Batch 2−1452 : Insulin−like growth factor−binding protein−like 1 on eGFR
rs724167
rs137884454
rs149278241
rs638403
rs12907232
rs11769493
rs78010311
rs17749109
rs144671227
rs141558430
rs8057632
rs34204216
rs117041662
All − MR Egger
All − IVW(MRE)
−0.005
0.000
0.005
MR effect size for
'Insulin−like growth factor−binding protein−like 1' on 'eGFR'

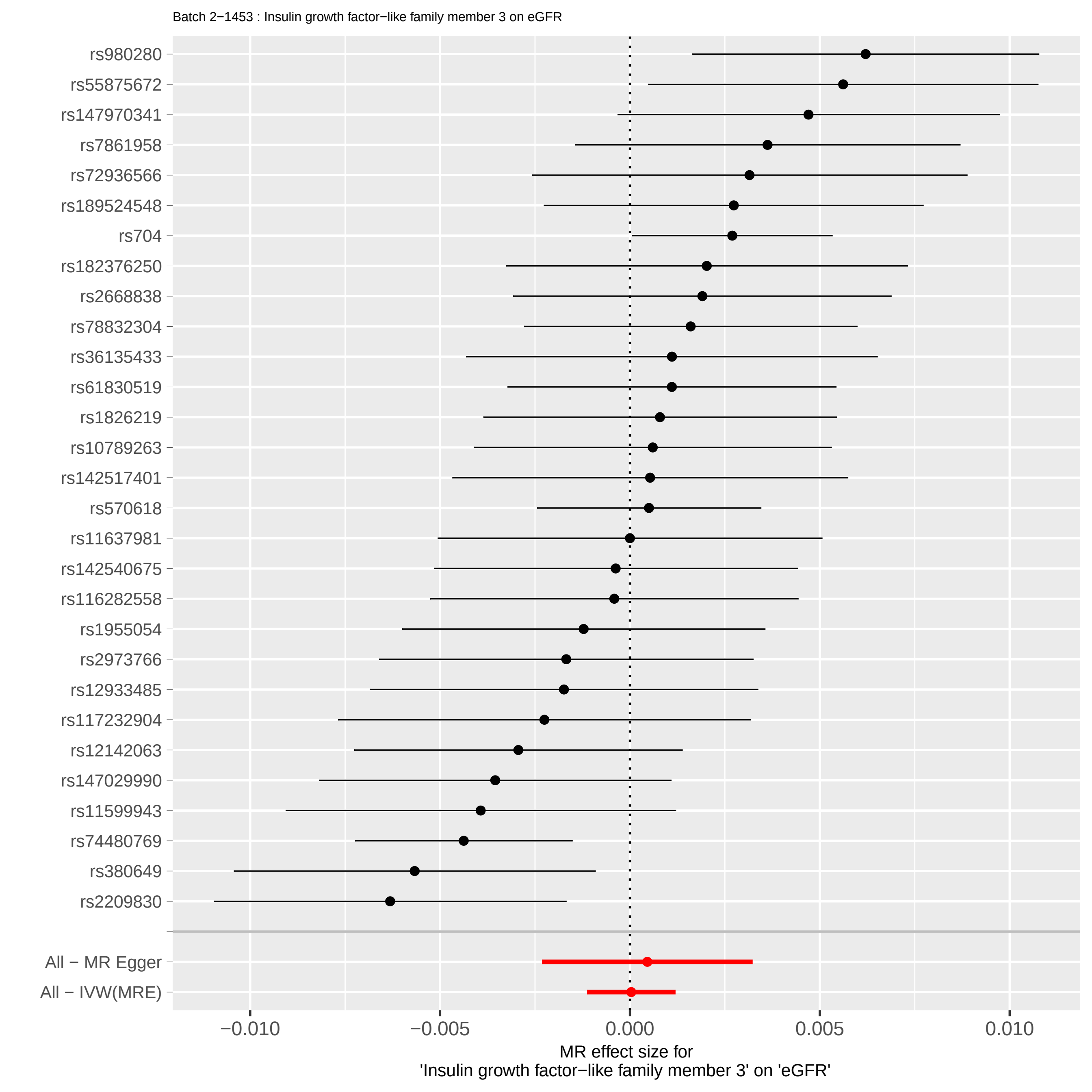
Batch 2−1453 : Insulin growth factor−like family member 3 on eGFR
rs980280
rs55875672
rs147970341
rs7861958
rs72936566
rs189524548
rs704
rs182376250
rs2668838
rs78832304
rs36135433
rs61830519
rs1826219
rs10789263
rs142517401
rs570618
rs11637981
rs142540675
rs116282558
rs1955054
rs2973766
rs12933485
rs117232904
rs12142063
rs147029990
rs11599943
rs74480769
rs380649
rs2209830
All − MR Egger
All − IVW(MRE)
−0.010
−0.005
0.000
0.005
0.010
MR effect size for
'Insulin growth factor−like family member 3' on 'eGFR'

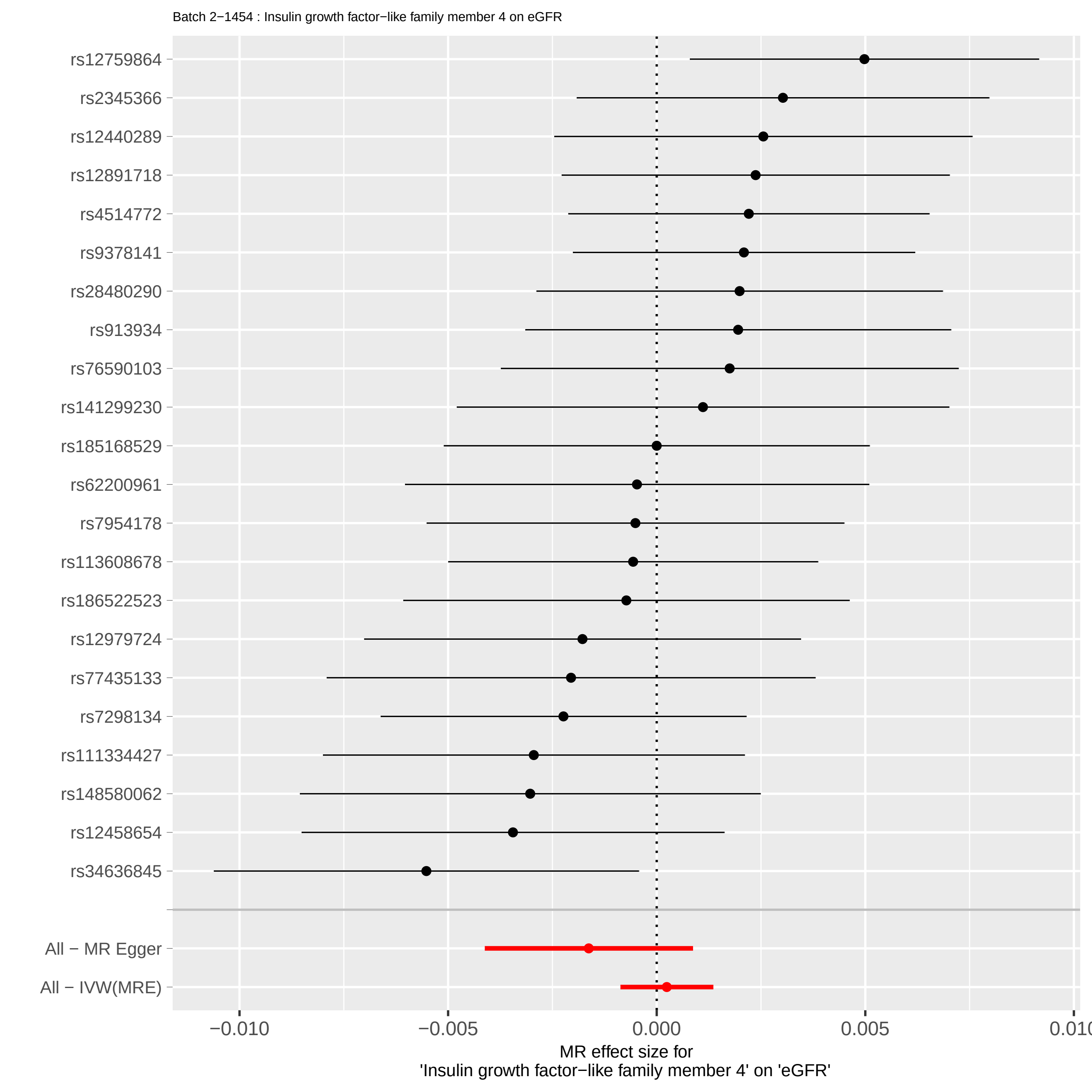
Batch 2−1454 : Insulin growth factor−like family member 4 on eGFR
rs12759864
rs2345366
rs12440289
rs12891718
rs4514772
rs9378141
rs28480290
rs913934
rs76590103
rs141299230
rs185168529
rs62200961
rs7954178
rs113608678
rs186522523
rs12979724
rs77435133
rs7298134
rs111334427
rs148580062
rs12458654
rs34636845
All − MR Egger
All − IVW(MRE)
−0.010
−0.005
0.000
0.005
0.010
MR effect size for
'Insulin growth factor−like family member 4' on 'eGFR'

Batch 2−1455 : IGF−like family receptor 1 on eGFR

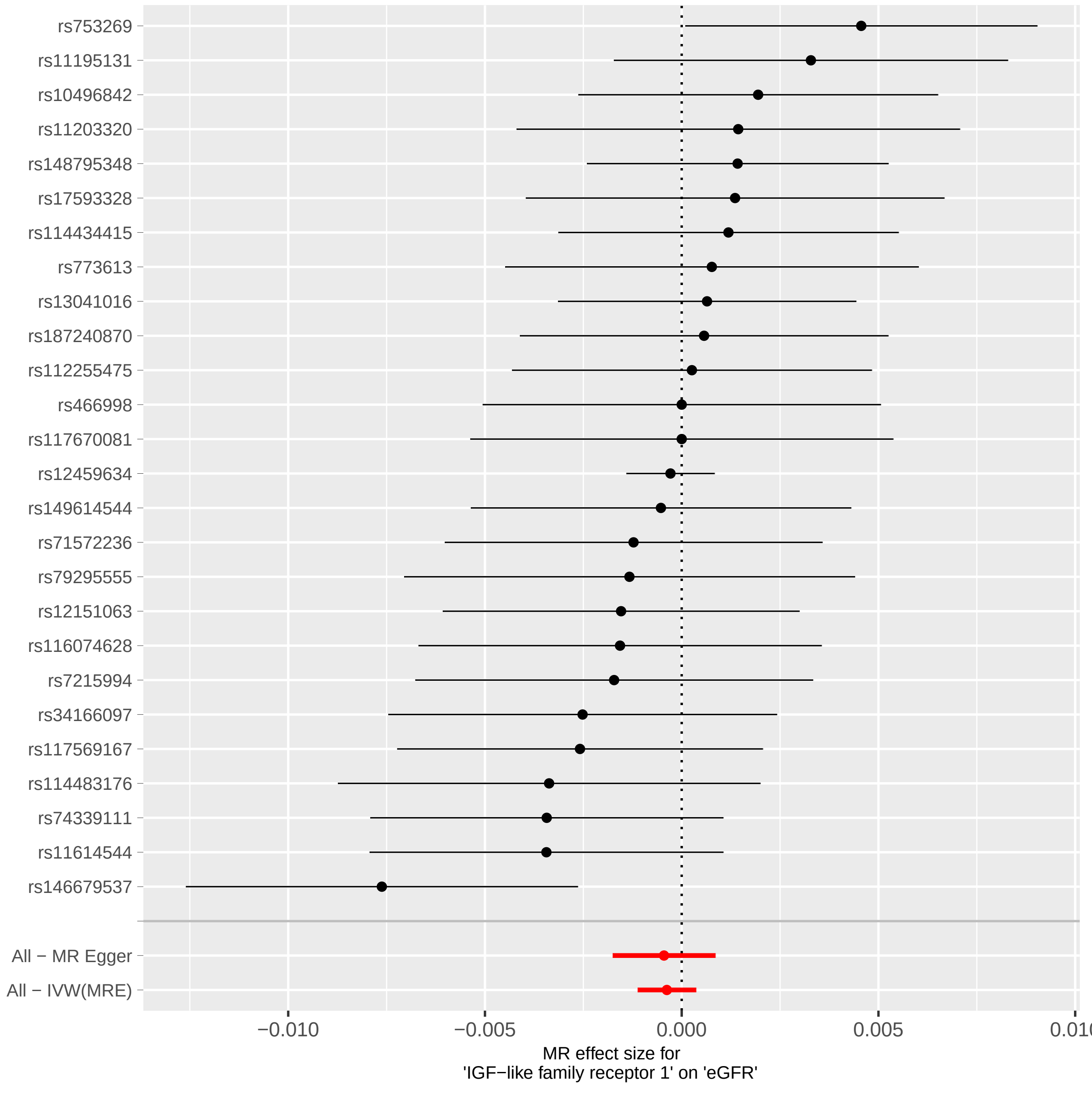

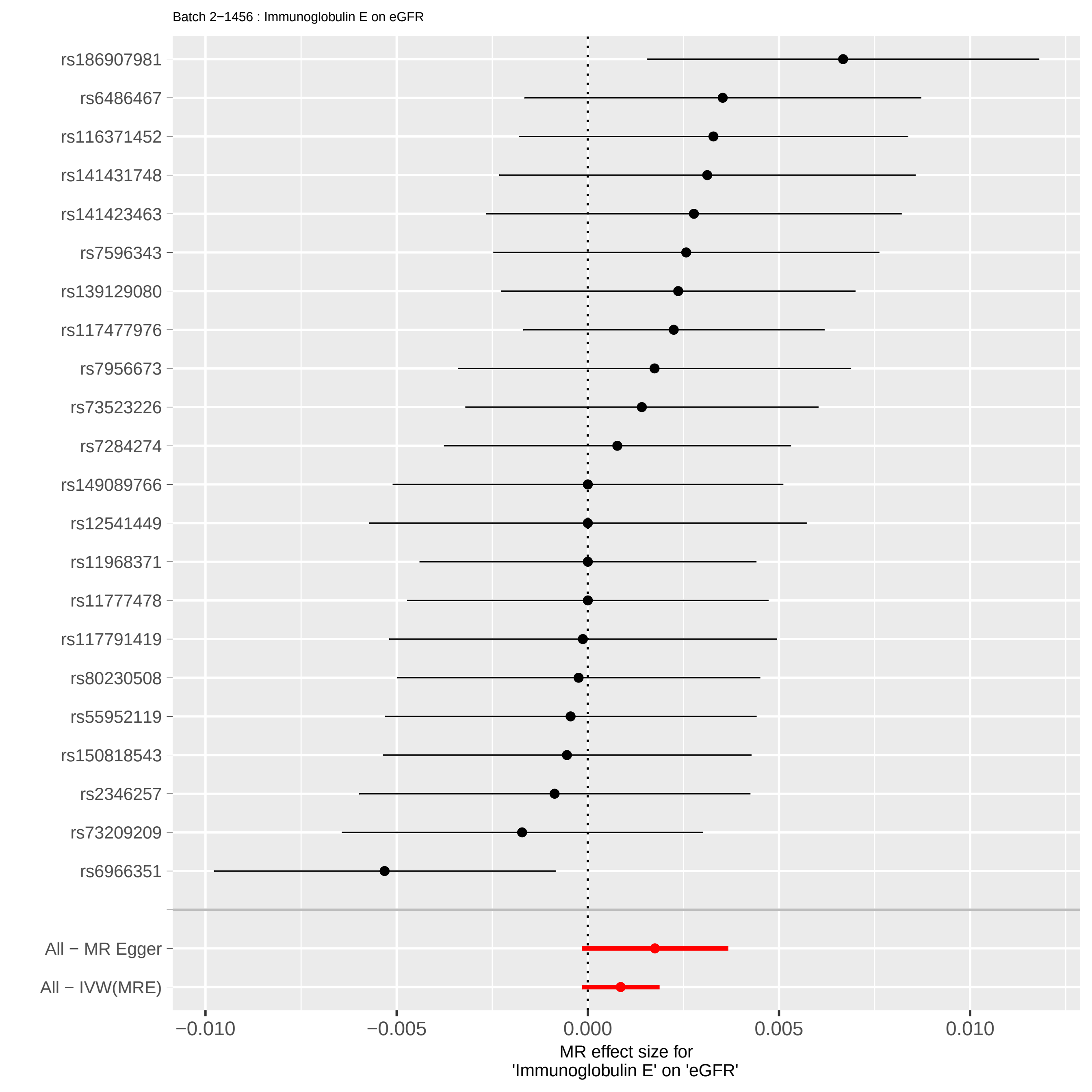
Batch 2−1456 : Immunoglobulin E on eGFR
rs186907981
rs6486467
rs116371452
rs141431748
rs141423463
rs7596343
rs139129080
rs117477976
rs7956673
rs73523226
rs7284274
rs149089766
rs12541449
rs11968371
rs11777478
rs117791419
rs80230508
rs55952119
rs150818543
rs2346257
rs73209209
rs6966351
All − MR Egger
All − IVW(MRE)
−0.010
−0.005
0.000
0.005
0.010
MR effect size for
'Immunoglobulin E' on 'eGFR'

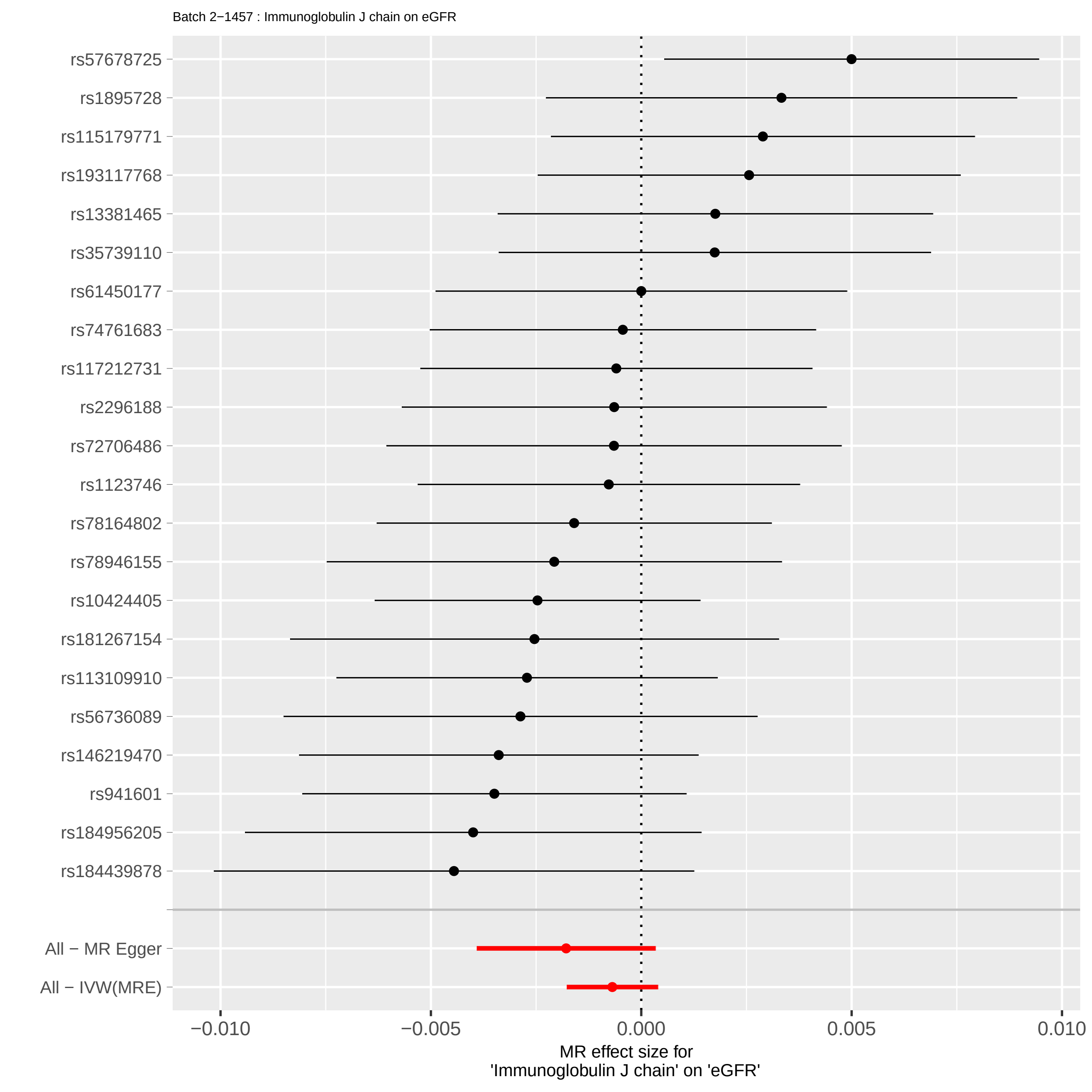

Batch 2−1457 : Immunoglobulin J chain on eGFR
rs57678725
rs1895728
rs115179771
rs193117768
rs13381465
rs35739110
rs61450177
rs74761683
rs117212731
rs2296188
rs72706486
rs1123746
rs78164802
rs78946155
rs10424405
rs181267154
rs113109910
rs56736089
rs146219470
rs941601
rs184956205
rs184439878
All − MR Egger
All − IVW(MRE)
−0.010
−0.005
0.000
0.005
0.010
MR effect size for
'Immunoglobulin J chain' on 'eGFR'

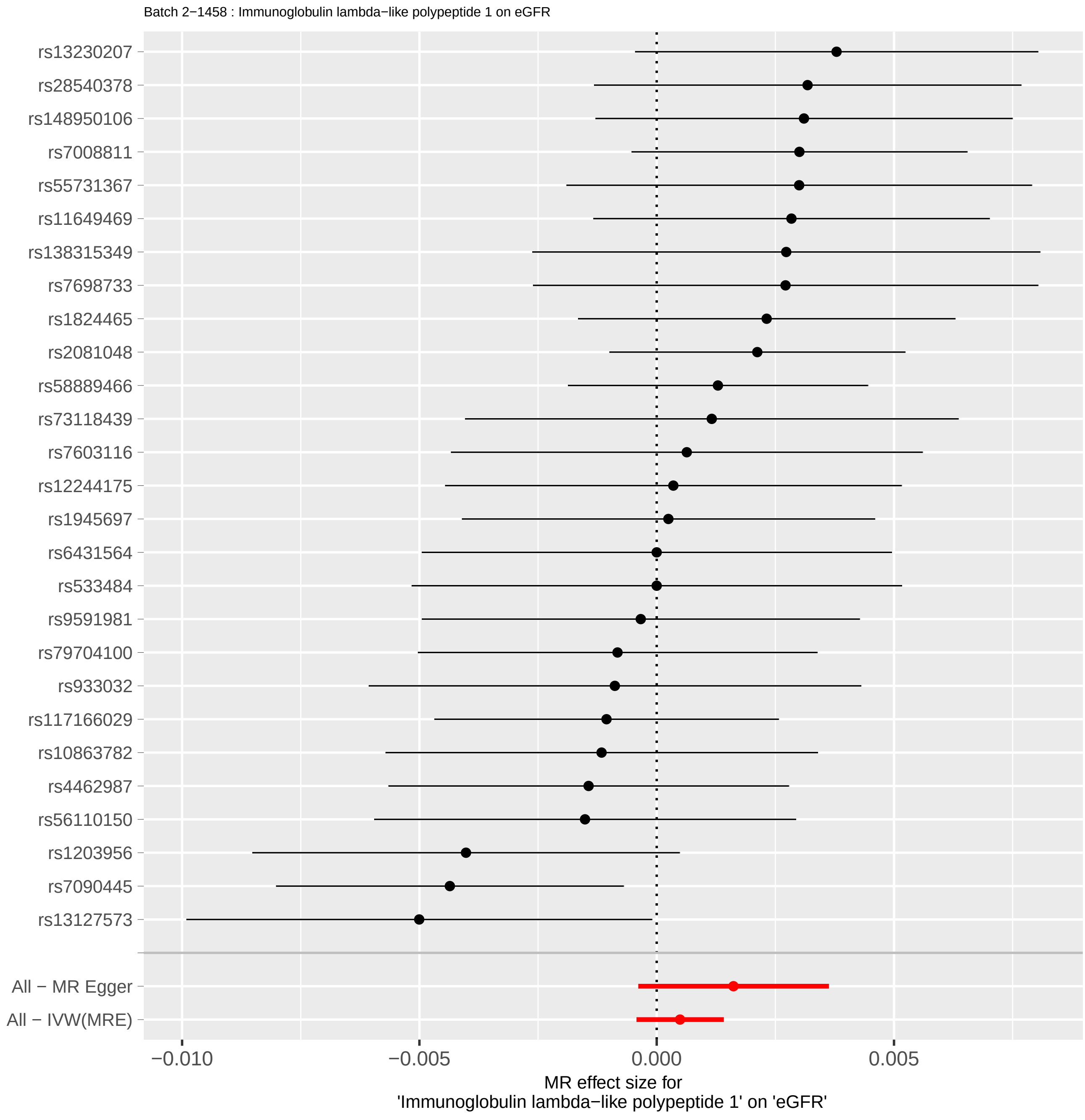
Batch 2−1458 : Immunoglobulin lambda−like polypeptide 1 on eGFR
rs13230207
rs28540378
rs148950106
rs7008811
rs55731367
rs11649469
rs138315349
rs7698733
rs1824465
rs2081048
rs58889466
rs73118439
rs7603116
rs12244175
rs1945697
rs6431564
rs533484
rs9591981
rs79704100
rs933032
rs117166029
rs10863782
rs4462987
rs56110150
rs1203956
rs7090445
rs13127573
All − MR Egger
All − IVW(MRE)
−0.010
−0.005
0.000
0.005
MR effect size for
'Immunoglobulin lambda−like polypeptide 1' on 'eGFR'

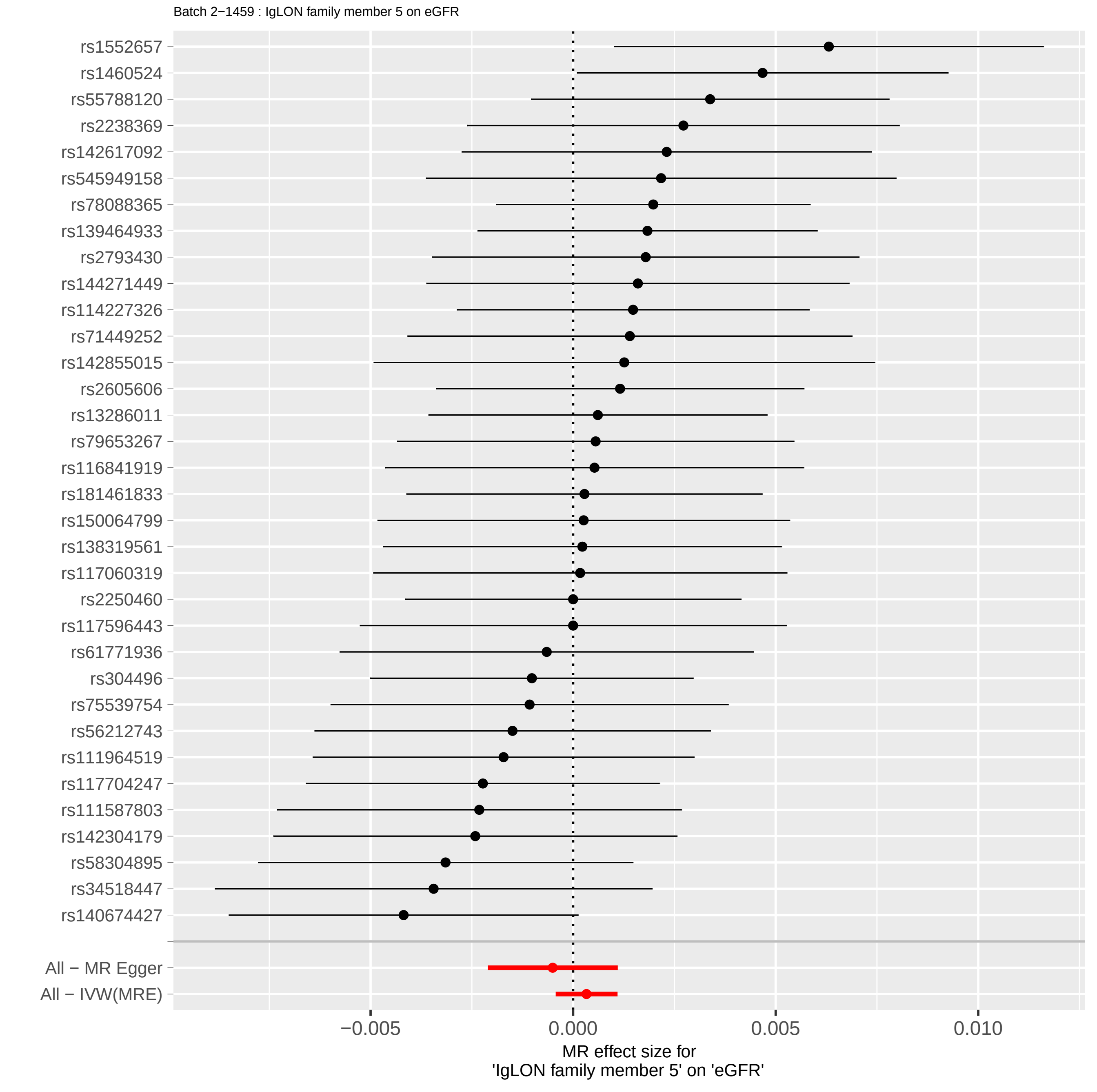

Batch 2−1459 : IgLON family member 5 on eGFR
rs1552657
rs1460524
rs55788120
rs2238369
rs142617092
rs545949158
rs78088365
rs139464933
rs2793430
rs144271449
rs114227326
rs71449252
rs142855015
rs2605606
rs13286011
rs79653267
rs116841919
rs181461833
rs150064799
rs138319561
rs117060319
rs2250460
rs117596443
rs61771936
rs304496
rs75539754
rs56212743
rs111964519
rs117704247
rs111587803
rs142304179
rs58304895
rs34518447
rs140674427
All − MR Egger
All − IVW(MRE)
−0.005
0.000
0.005
0.010
MR effect size for
'IgLON family member 5' on 'eGFR'

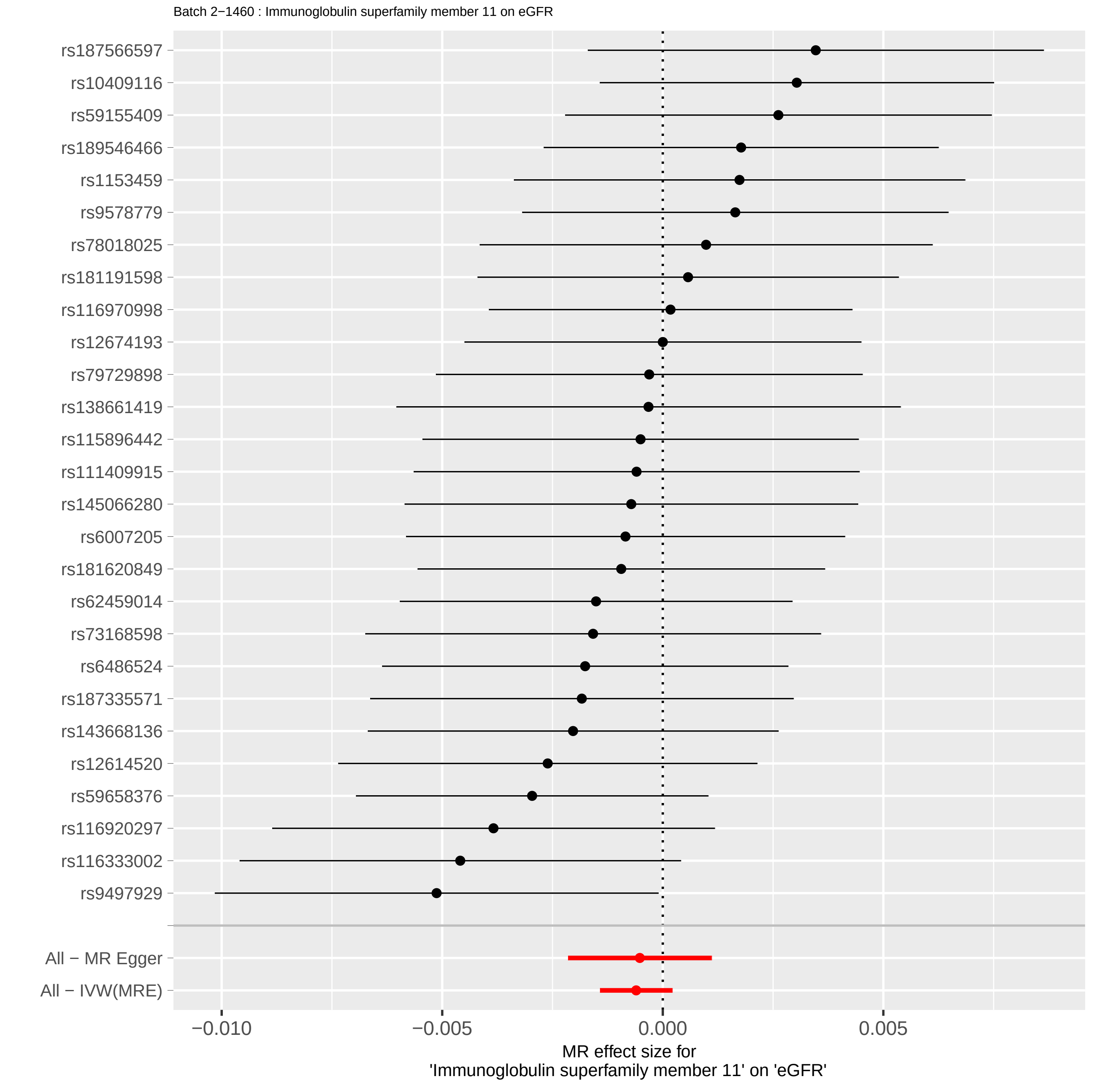
Batch 2−1460 : Immunoglobulin superfamily member 11 on eGFR
rs187566597
rs10409116
rs59155409
rs189546466
rs1153459
rs9578779
rs78018025
rs181191598
rs116970998
rs12674193
rs79729898
rs138661419
rs115896442
rs111409915
rs145066280
rs6007205
rs181620849
rs62459014
rs73168598
rs6486524
rs187335571
rs143668136
rs12614520
rs59658376
rs116920297
rs116333002
rs9497929
All − MR Egger
All − IVW(MRE)
−0.010
−0.005
0.000
0.005
MR effect size for
'Immunoglobulin superfamily member 11' on 'eGFR'

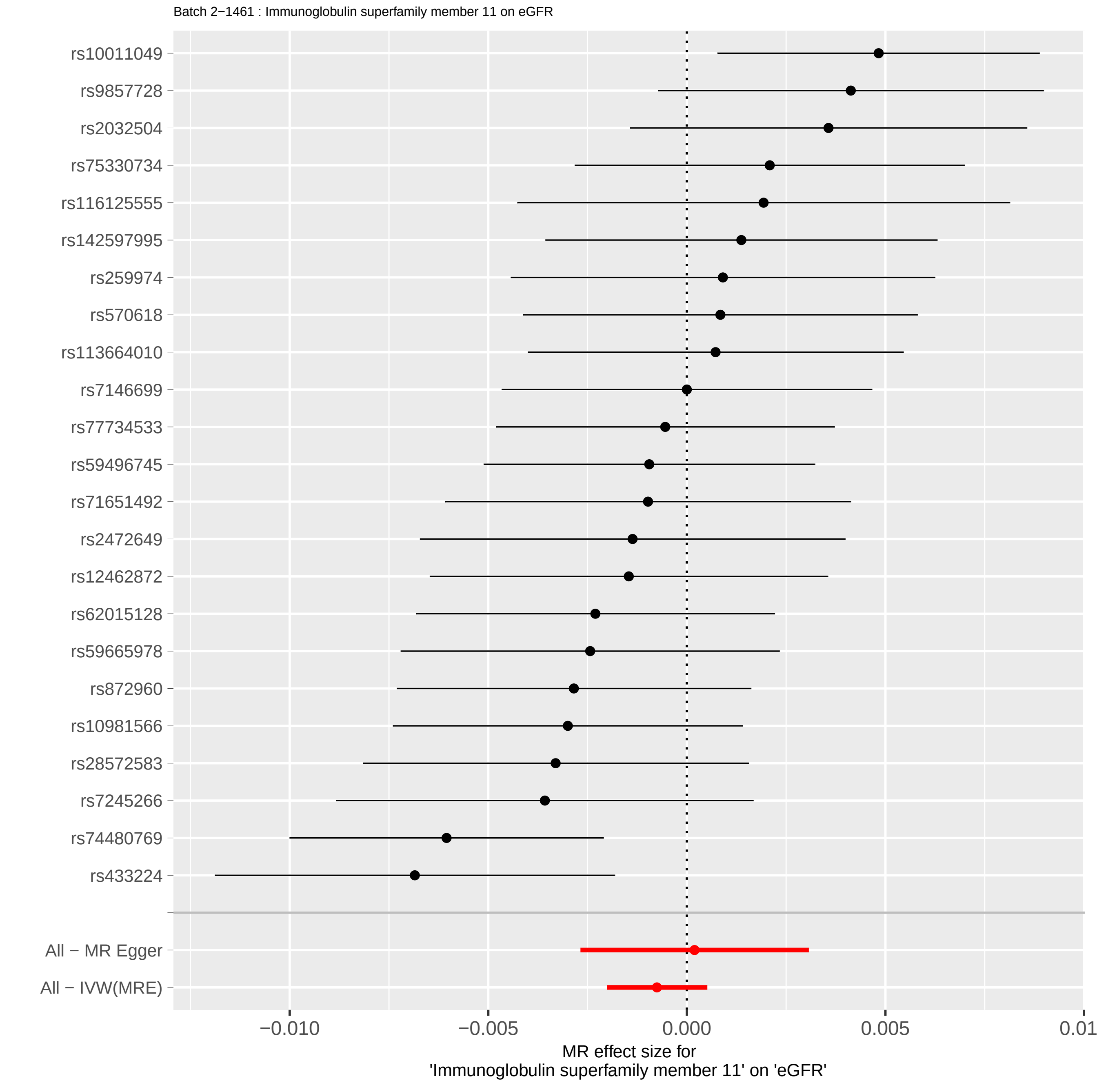

Batch 2−1461 : Immunoglobulin superfamily member 11 on eGFR
rs10011049
rs9857728
rs2032504
rs75330734
rs116125555
rs142597995
rs259974
rs570618
rs113664010
rs7146699
rs77734533
rs59496745
rs71651492
rs2472649
rs12462872
rs62015128
rs59665978
rs872960
rs10981566
rs28572583
rs7245266
rs74480769
rs433224
All − MR Egger
All − IVW(MRE)
−0.010
−0.005
0.000
0.005
0.01
MR effect size for
'Immunoglobulin superfamily member 11' on 'eGFR'

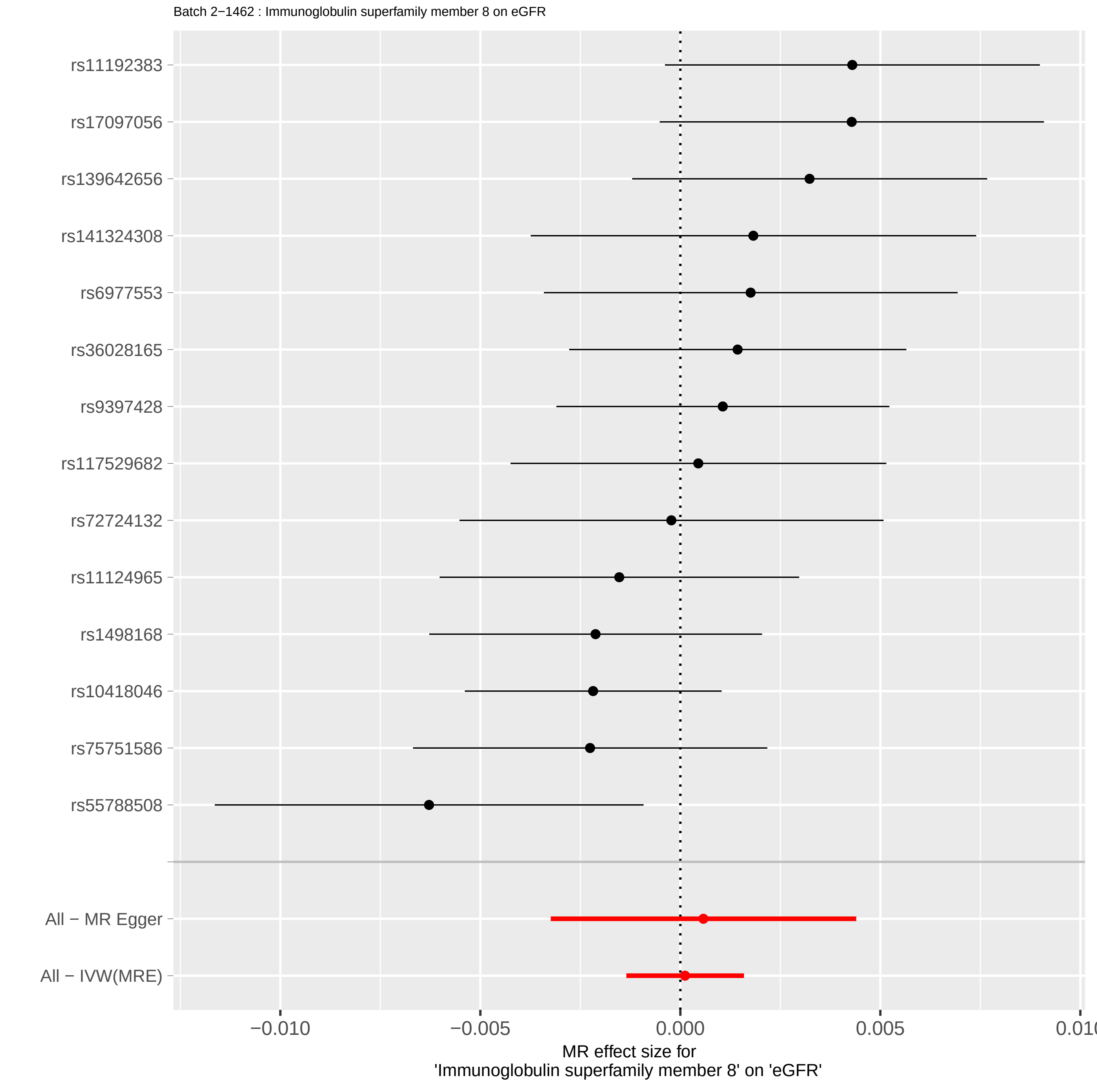

Batch 2−1462 : Immunoglobulin superfamily member 8 on eGFR
rs11192383
rs17097056
rs139642656
rs141324308
rs6977553
rs36028165
rs9397428
rs117529682
rs72724132
rs11124965
rs1498168
rs10418046
rs75751586
rs55788508
All − MR Egger
All − IVW(MRE)
−0.010
−0.005
0.000
0.005
MR effect size for
'Immunoglobulin superfamily member 8' on 'eGFR'

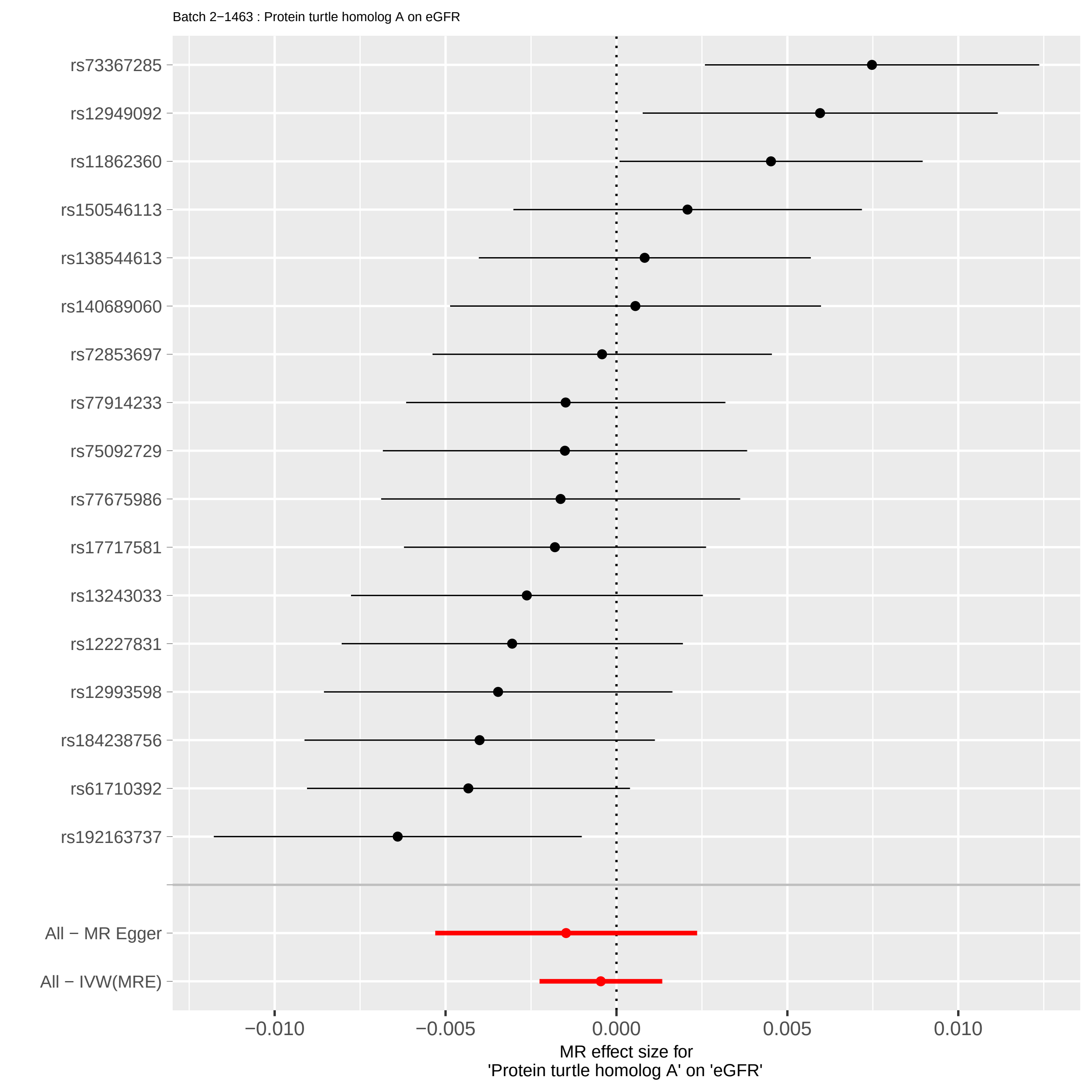
Batch 2−1463 : Protein turtle homolog A on eGFR
rs73367285
rs12949092
rs11862360
rs150546113
rs138544613
rs140689060
rs72853697
rs77914233
rs75092729
rs77675986
rs17717581
rs13243033
rs12227831
rs12993598
rs184238756
rs61710392
rs192163737
All − MR Egger
All − IVW(MRE)
−0.010
−0.005
0.000
0.005
0.010
MR effect size for
'Protein turtle homolog A' on 'eGFR'

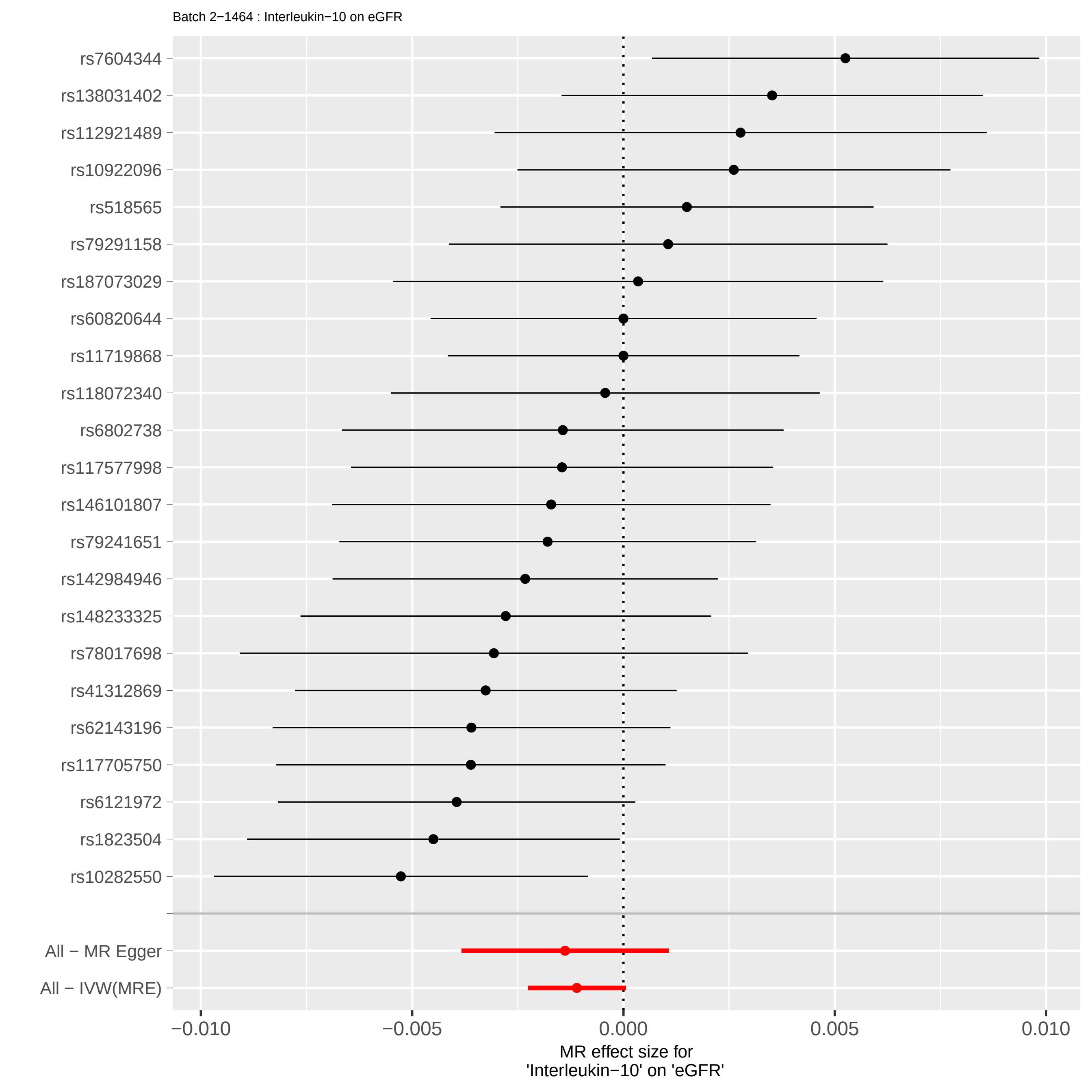

Batch 2−1464 : Interleukin−10 on eGFR
rs7604344
rs138031402
rs112921489
rs10922096
rs518565
rs79291158
rs187073029
rs60820644
rs11719868
rs118072340
rs6802738
rs117577998
rs146101807
rs79241651
rs142984946
rs148233325
rs78017698
rs41312869
rs62143196
rs117705750
rs6121972
rs1823504
rs10282550
All − MR Egger
All − IVW(MRE)
−0.010
−0.005
0.000
0.005
0.010
MR effect size for
'Interleukin−10' on 'eGFR'

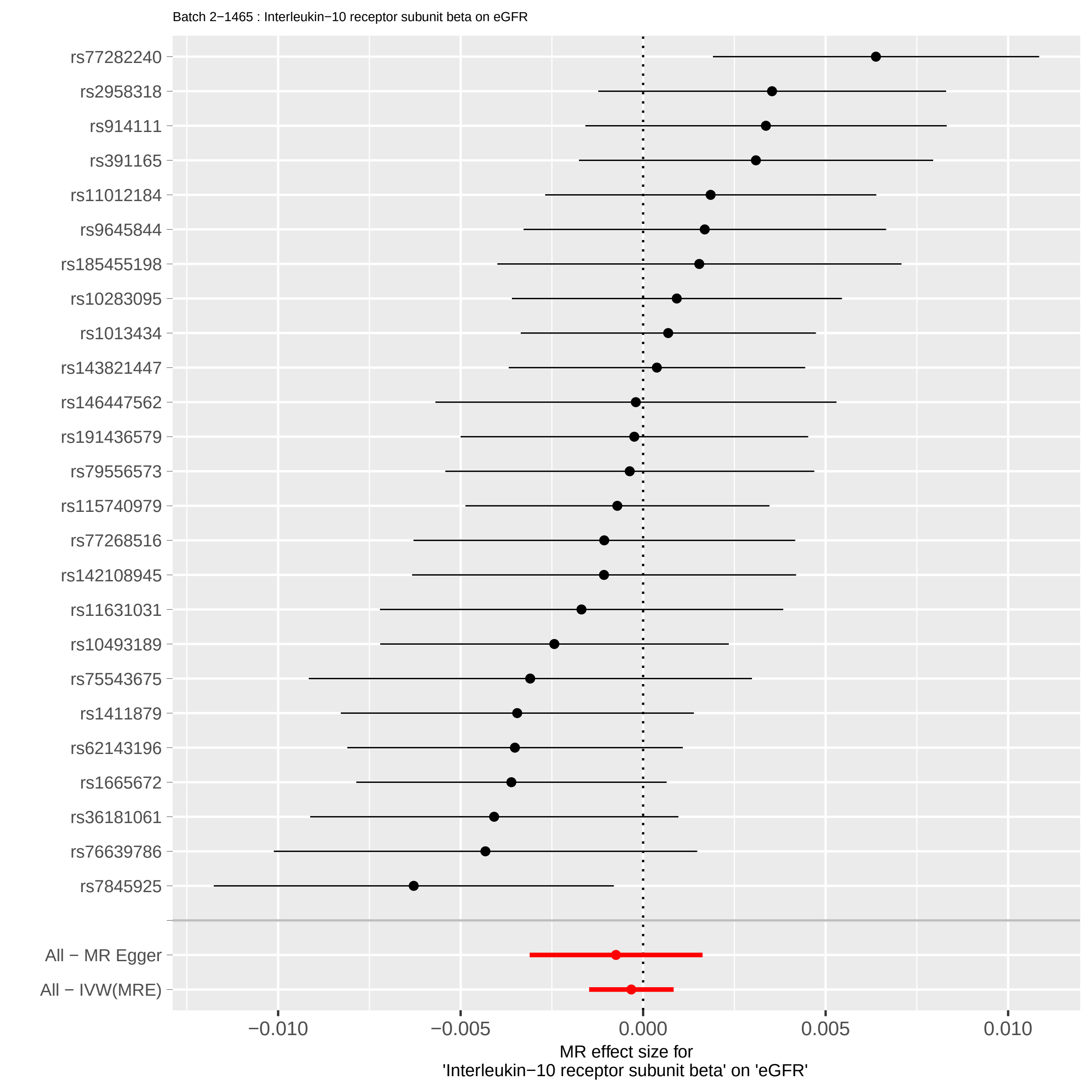

Batch 2−1465 : Interleukin−10 receptor subunit beta on eGFR
rs77282240
rs2958318
rs914111
rs391165
rs11012184
rs9645844
rs185455198
rs10283095
rs1013434
rs143821447
rs146447562
rs191436579
rs79556573
rs115740979
rs77268516
rs142108945
rs11631031
rs10493189
rs75543675
rs1411879
rs62143196
rs1665672
rs36181061
rs76639786
rs7845925
All − MR Egger
All − IVW(MRE)
−0.010
−0.005
0.000
0.005
0.010
MR effect size for
'Interleukin−10 receptor subunit beta' on 'eGFR'

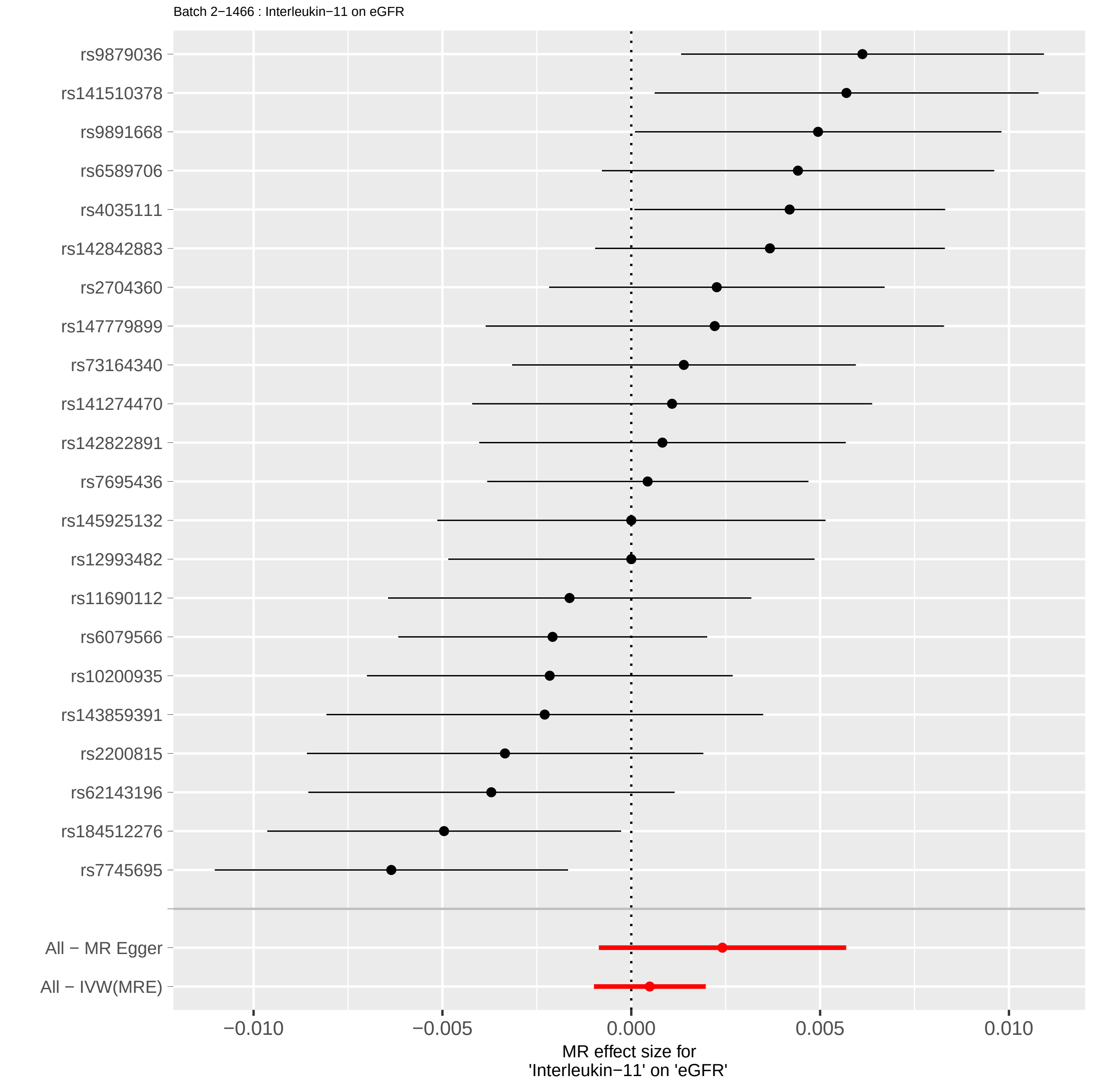

Batch 2−1466 : Interleukin−11 on eGFR
rs9879036
rs141510378
rs9891668
rs6589706
rs4035111
rs142842883
rs2704360
rs147779899
rs73164340
rs141274470
rs142822891
rs7695436
rs145925132
rs12993482
rs11690112
rs6079566
rs10200935
rs143859391
rs2200815
rs62143196
rs184512276
rs7745695
All − MR Egger
All − IVW(MRE)
−0.010
−0.005
0.000
0.005
0.010
MR effect size for
'Interleukin−11' on 'eGFR'

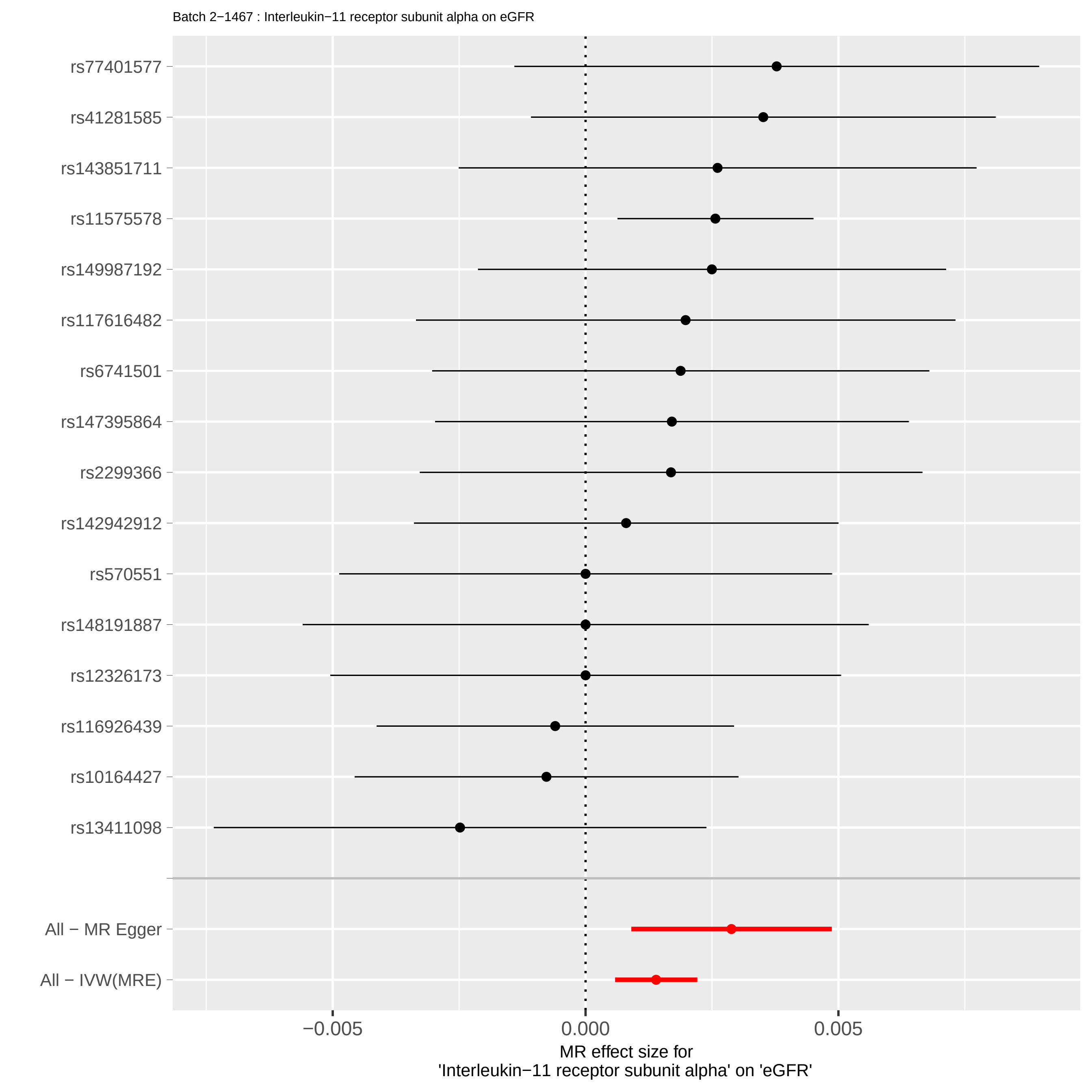
Batch 2−1467 : Interleukin−11 receptor subunit alpha on eGFR
rs77401577
rs41281585
rs143851711
rs11575578
rs149987192
rs117616482
rs6741501
rs147395864
rs2299366
rs142942912
rs570551
rs148191887
rs12326173
rs116926439
rs10164427
rs13411098
All − MR Egger
All − IVW(MRE)
−0.005
0.000
0.005
MR effect size for
'Interleukin−11 receptor subunit alpha' on 'eGFR'

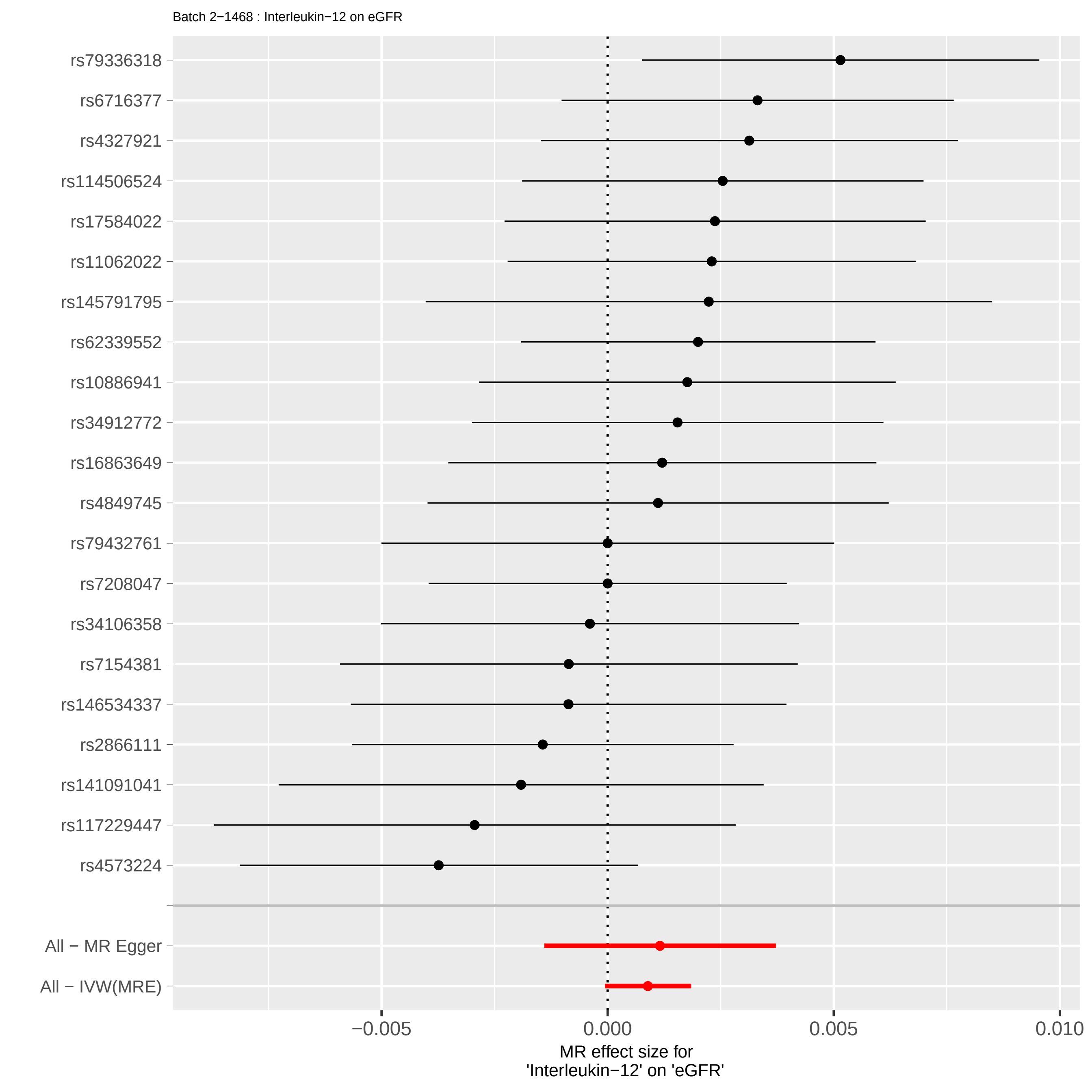
Batch 2−1468 : Interleukin−12 on eGFR
rs79336318
rs6716377
rs4327921
rs114506524
rs17584022
rs11062022
rs145791795
rs62339552
rs10886941
rs34912772
rs16863649
rs4849745
rs79432761
rs7208047
rs34106358
rs7154381
rs146534337
rs2866111
rs141091041
rs117229447
rs4573224
All − MR Egger
All − IVW(MRE)
−0.005
0.000
0.005
0.010
MR effect size for
'Interleukin−12' on 'eGFR'

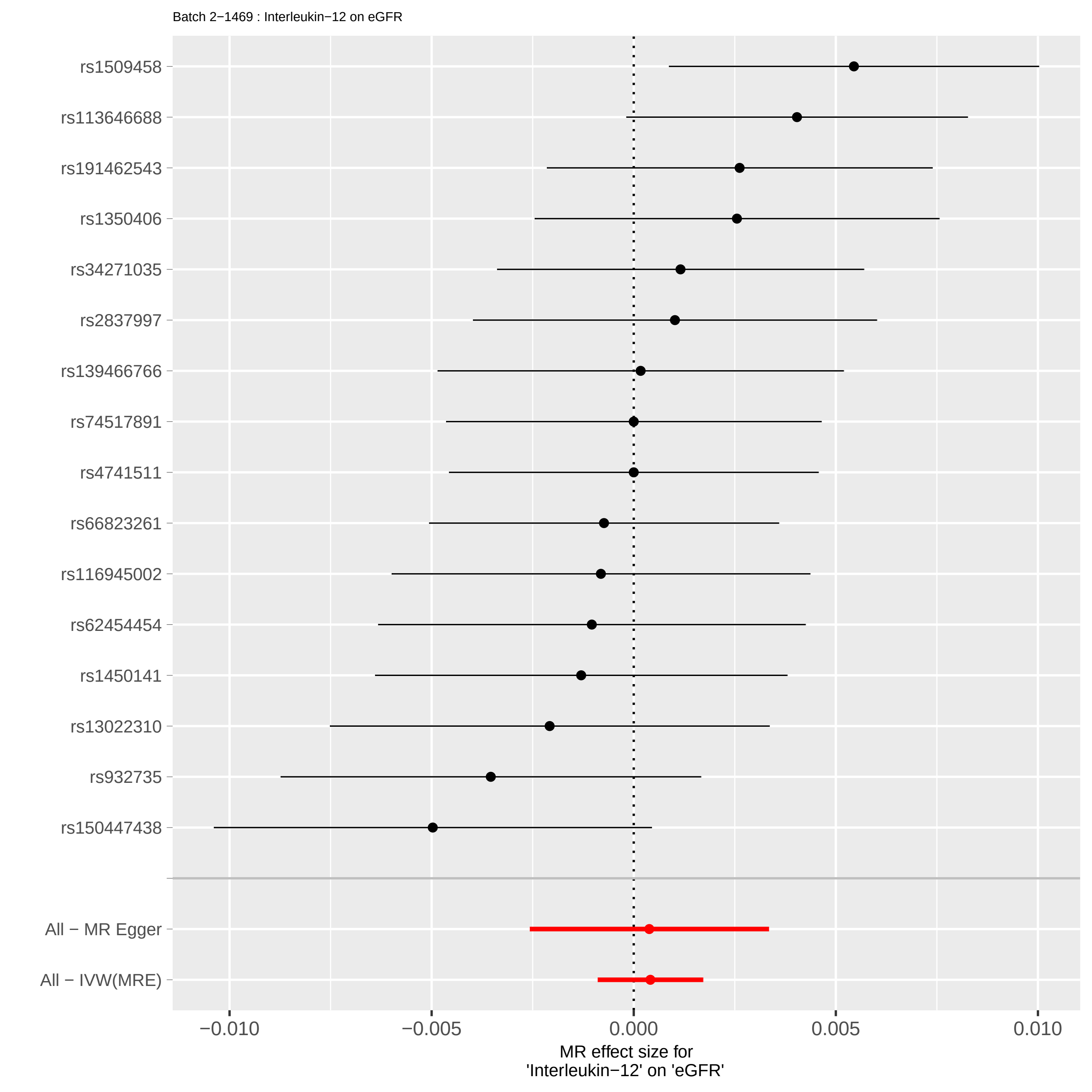

Batch 2−1469 : Interleukin−12 on eGFR
rs1509458
rs113646688
rs191462543
rs1350406
rs34271035
rs2837997
rs139466766
rs74517891
rs4741511
rs66823261
rs116945002
rs62454454
rs1450141
rs13022310
rs932735
rs150447438
All − MR Egger
All − IVW(MRE)
−0.010
−0.005
0.000
0.005
0.010
MR effect size for
'Interleukin−12' on 'eGFR'

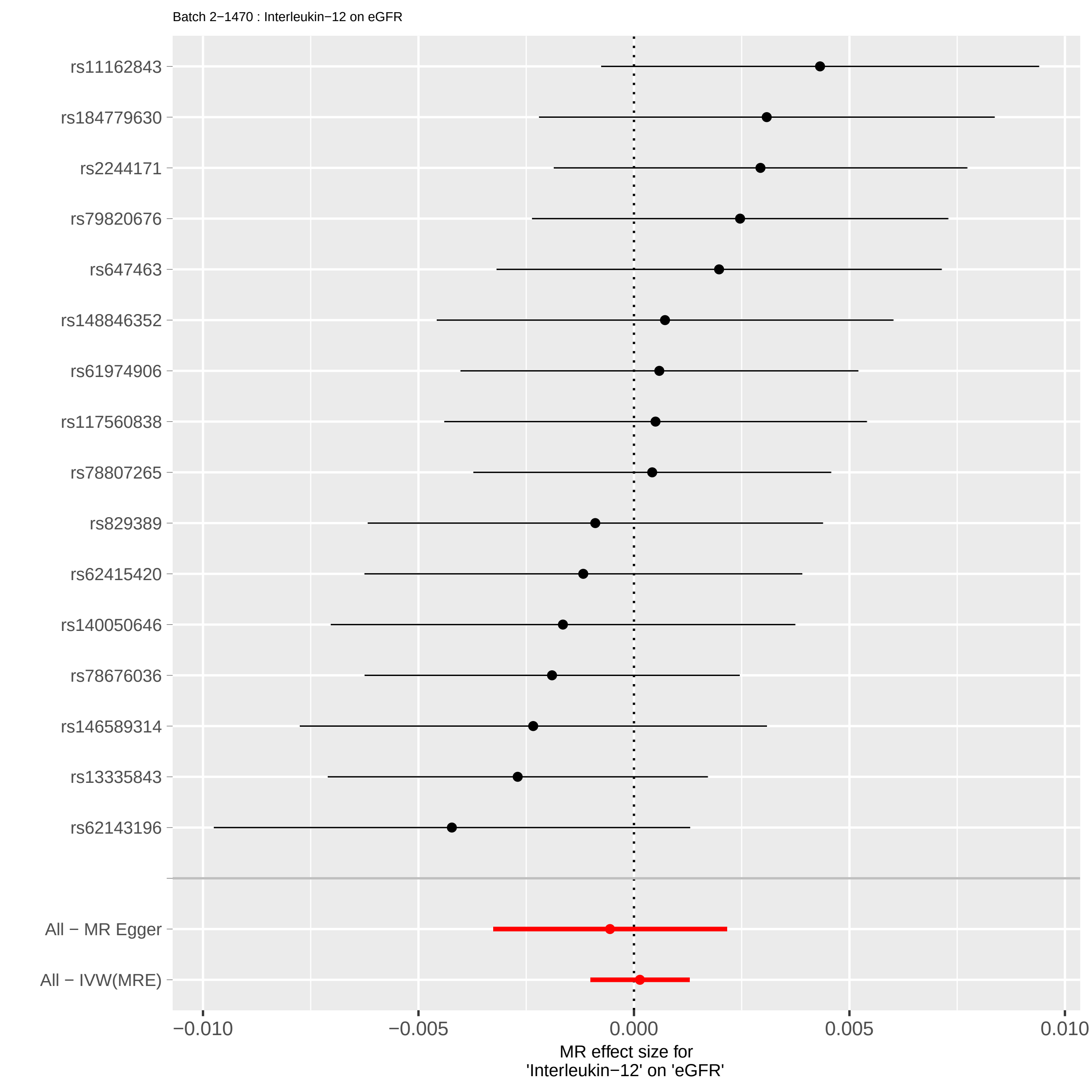

Batch 2−1470 : Interleukin−12 on eGFR
rs11162843
rs184779630
rs2244171
rs79820676
rs647463
rs148846352
rs61974906
rs117560838
rs78807265
rs829389
rs62415420
rs140050646
rs78676036
rs146589314
rs13335843
rs62143196
All − MR Egger
All − IVW(MRE)
−0.010
−0.005
0.000
0.005
0.010
MR effect size for
'Interleukin−12' on 'eGFR'

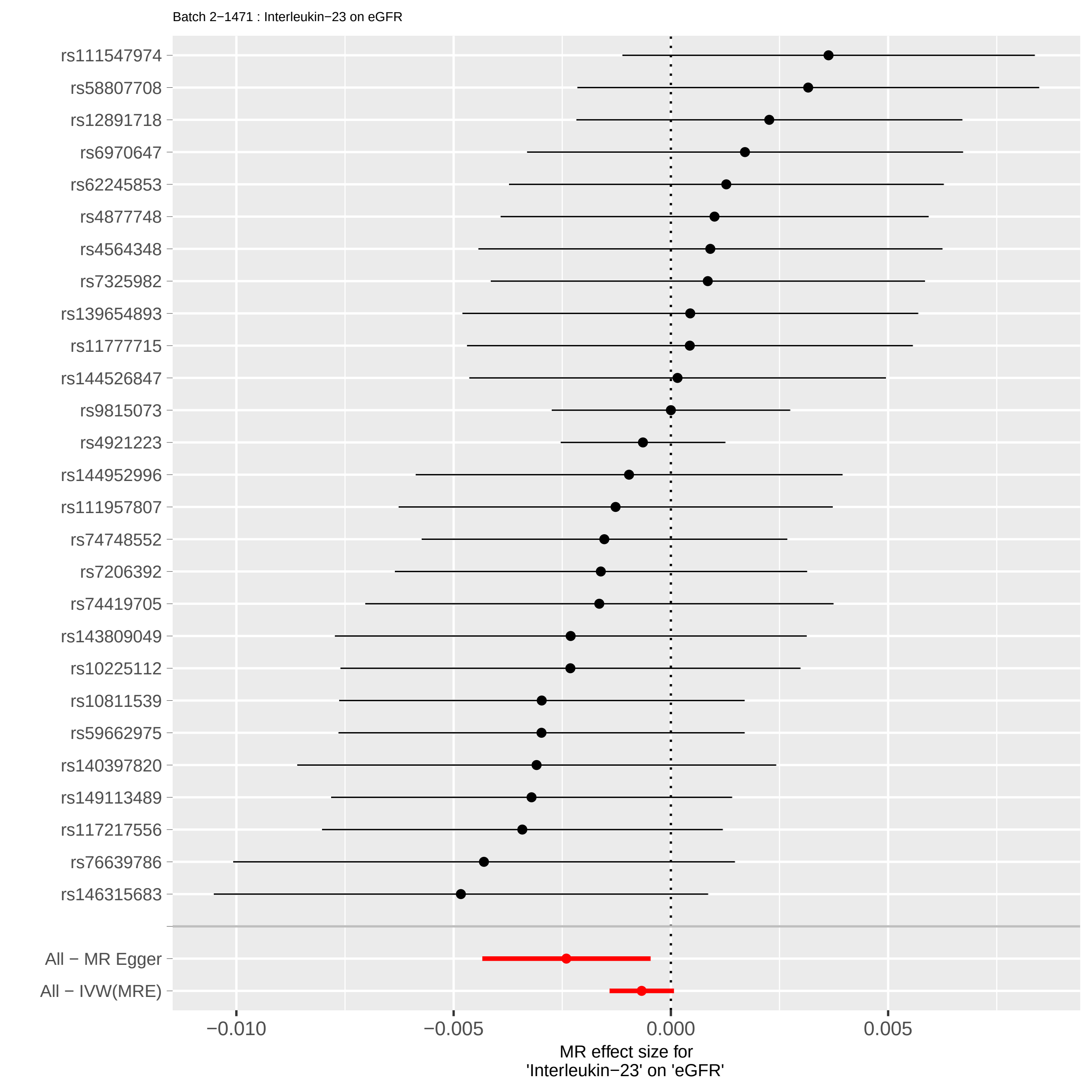

Batch 2−1471 : Interleukin−23 on eGFR
rs111547974
rs58807708
rs12891718
rs6970647
rs62245853
rs4877748
rs4564348
rs7325982
rs139654893
rs11777715
rs144526847
rs9815073
rs4921223
rs144952996
rs111957807
rs74748552
rs7206392
rs74419705
rs143809049
rs10225112
rs10811539
rs59662975
rs140397820
rs149113489
rs117217556
rs76639786
rs146315683
All − MR Egger
All − IVW(MRE)
−0.010
−0.005
0.000
0.005
MR effect size for
'Interleukin−23' on 'eGFR'

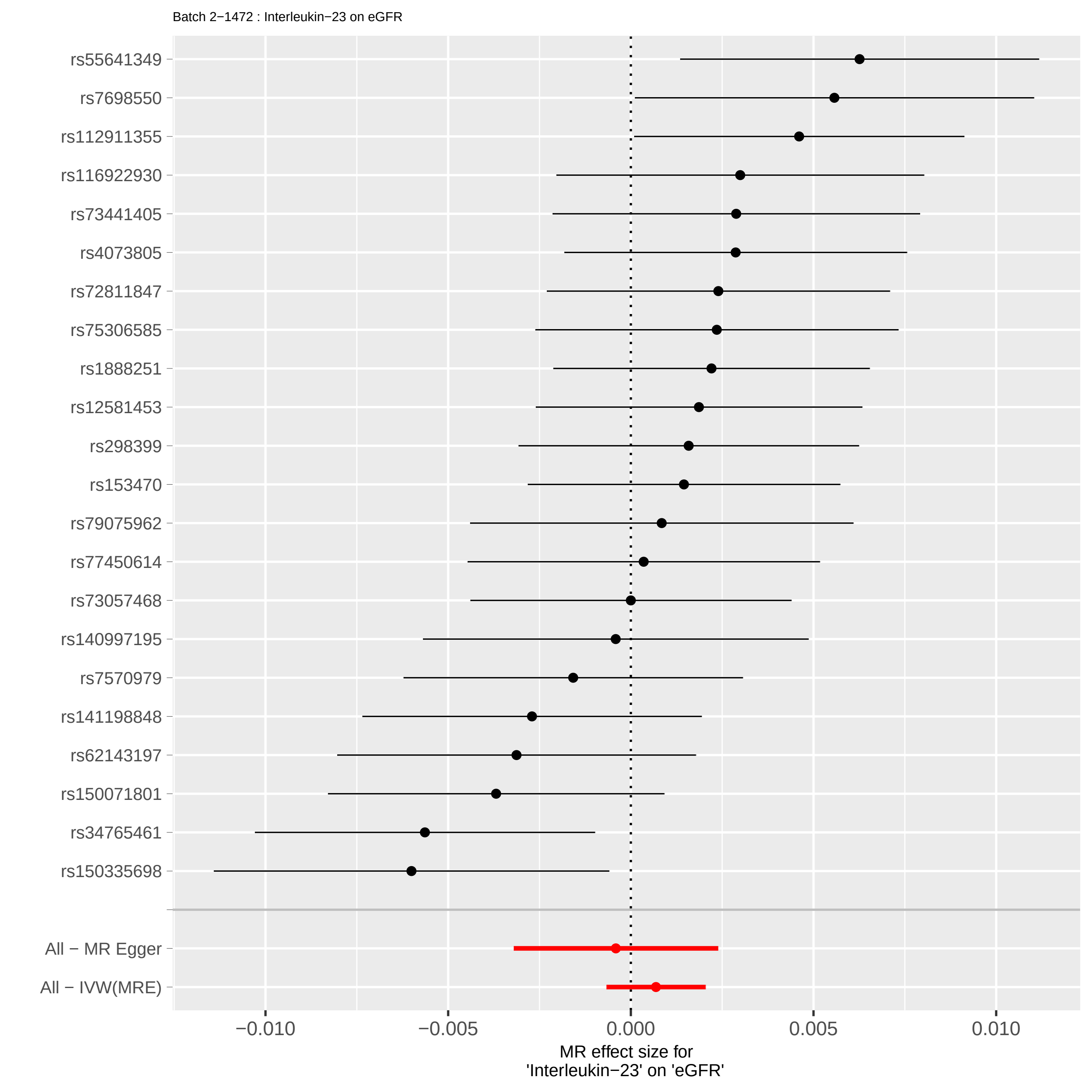
Batch 2−1472 : Interleukin−23 on eGFR
rs55641349
rs7698550
rs112911355
rs116922930
rs73441405
rs4073805
rs72811847
rs75306585
rs1888251
rs12581453
rs298399
rs153470
rs79075962
rs77450614
rs73057468
rs140997195
rs7570979
rs141198848
rs62143197
rs150071801
rs34765461
rs150335698
All − MR Egger
All − IVW(MRE)
−0.010
−0.005
0.000
0.005
0.010
MR effect size for
'Interleukin−23' on 'eGFR'

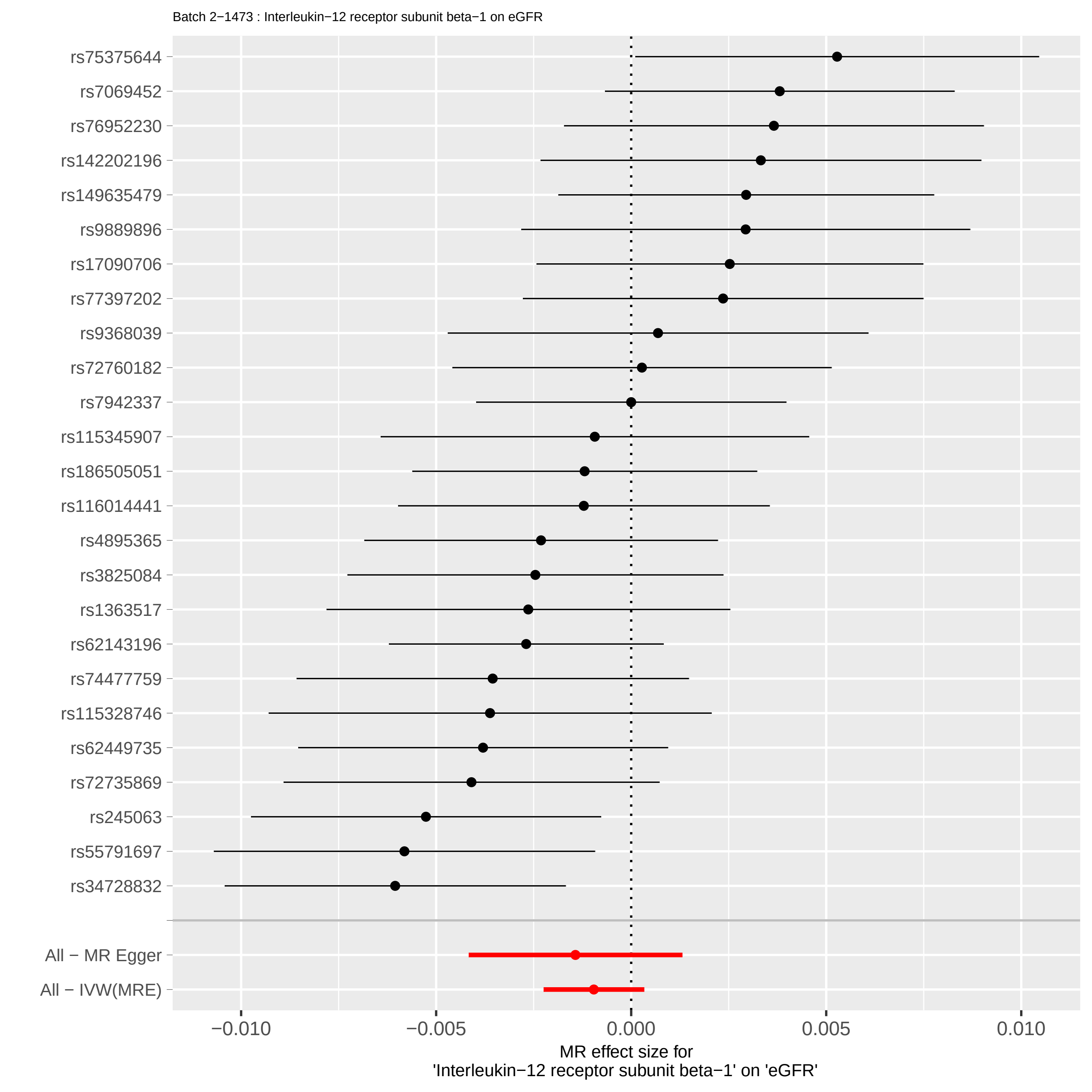

Batch 2−1473 : Interleukin−12 receptor subunit beta−1 on eGFR
rs75375644
rs7069452
rs76952230
rs142202196
rs149635479
rs9889896
rs17090706
rs77397202
rs9368039
rs72760182
rs7942337
rs115345907
rs186505051
rs116014441
rs4895365
rs3825084
rs1363517
rs62143196
rs74477759
rs115328746
rs62449735
rs72735869
rs245063
rs55791697
rs34728832
All − MR Egger
All − IVW(MRE)
−0.010
−0.005
0.000
0.005
0.010
MR effect size for
'Interleukin−12 receptor subunit beta−1' on 'eGFR'

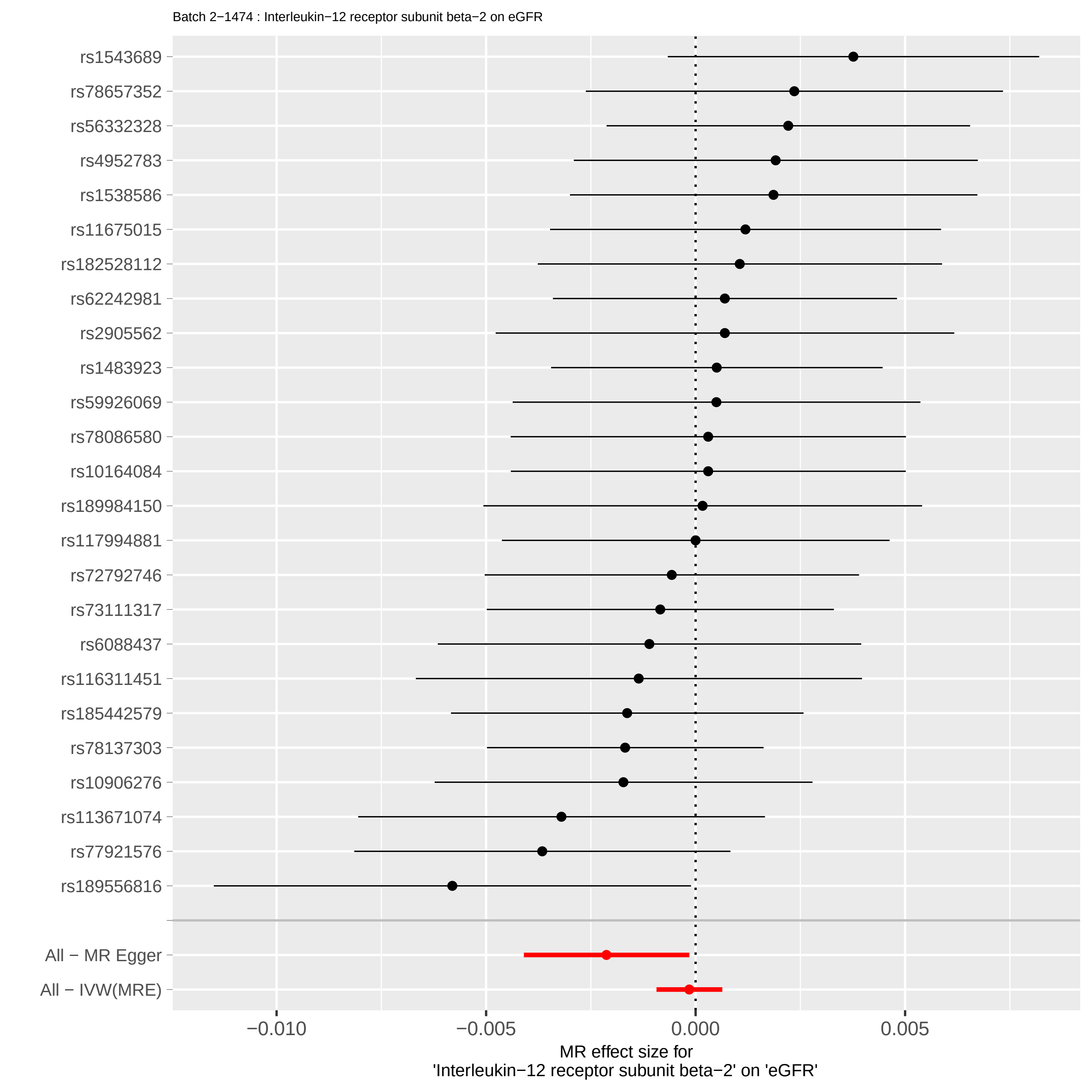

Batch 2−1474 : Interleukin−12 receptor subunit beta−2 on eGFR
rs1543689
rs78657352
rs56332328
rs4952783
rs1538586
rs11675015
rs182528112
rs62242981
rs2905562
rs1483923
rs59926069
rs78086580
rs10164084
rs189984150
rs117994881
rs72792746
rs73111317
rs6088437
rs116311451
rs185442579
rs78137303
rs10906276
rs113671074
rs77921576
rs189556816
All − MR Egger
All − IVW(MRE)
−0.010
−0.005
0.000
0.005
MR effect size for
'Interleukin−12 receptor subunit beta−2' on 'eGFR'

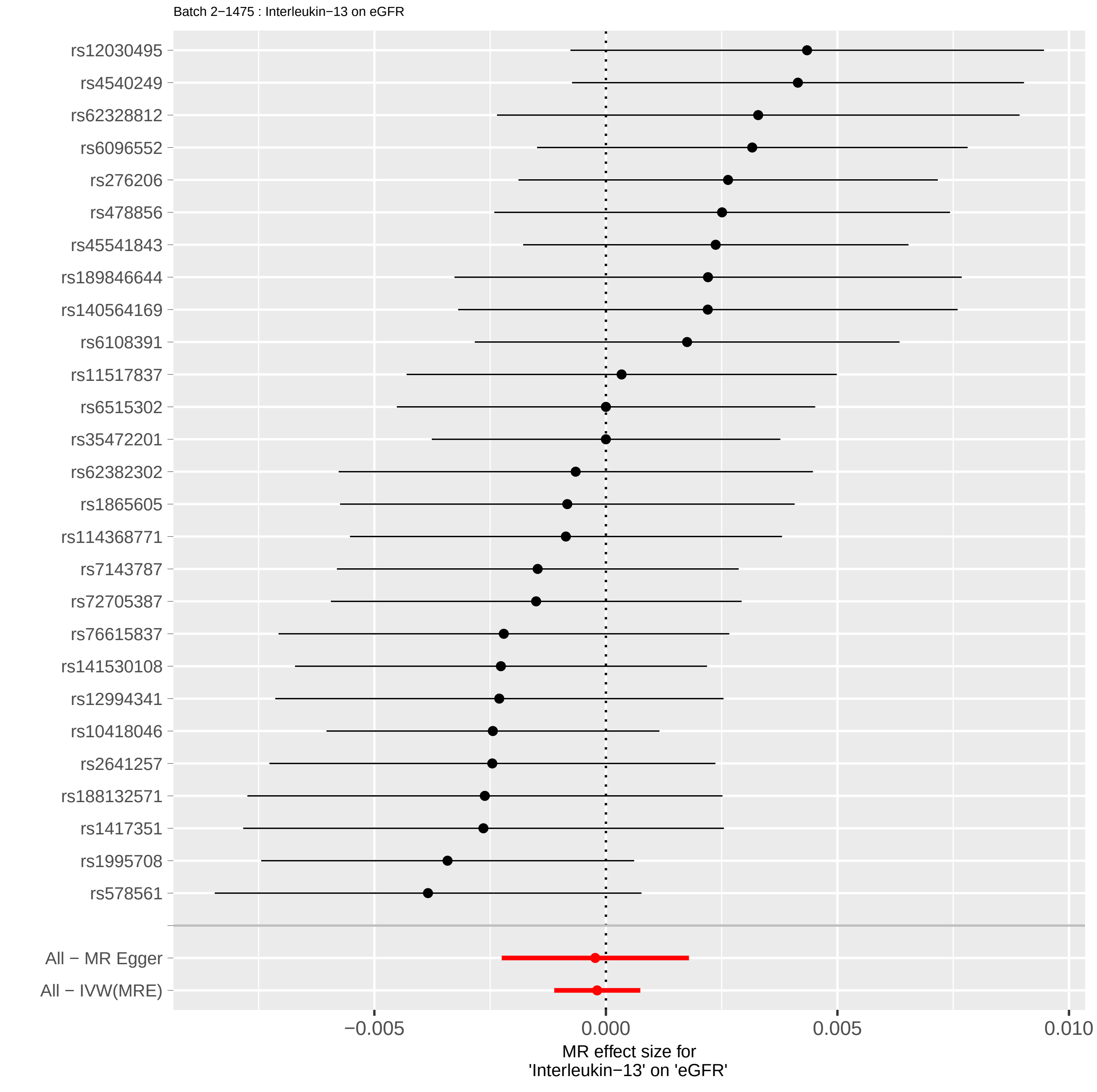

Batch 2−1475 : Interleukin−13 on eGFR
rs12030495
rs4540249
rs62328812
rs6096552
rs276206
rs478856
rs45541843
rs189846644
rs140564169
rs6108391
rs11517837
rs6515302
rs35472201
rs62382302
rs1865605
rs114368771
rs7143787
rs72705387
rs76615837
rs141530108
rs12994341
rs10418046
rs2641257
rs188132571
rs1417351
rs1995708
rs578561
All − MR Egger
All − IVW(MRE)
−0.005
0.000
0.005
0.010
MR effect size for
'Interleukin−13' on 'eGFR'

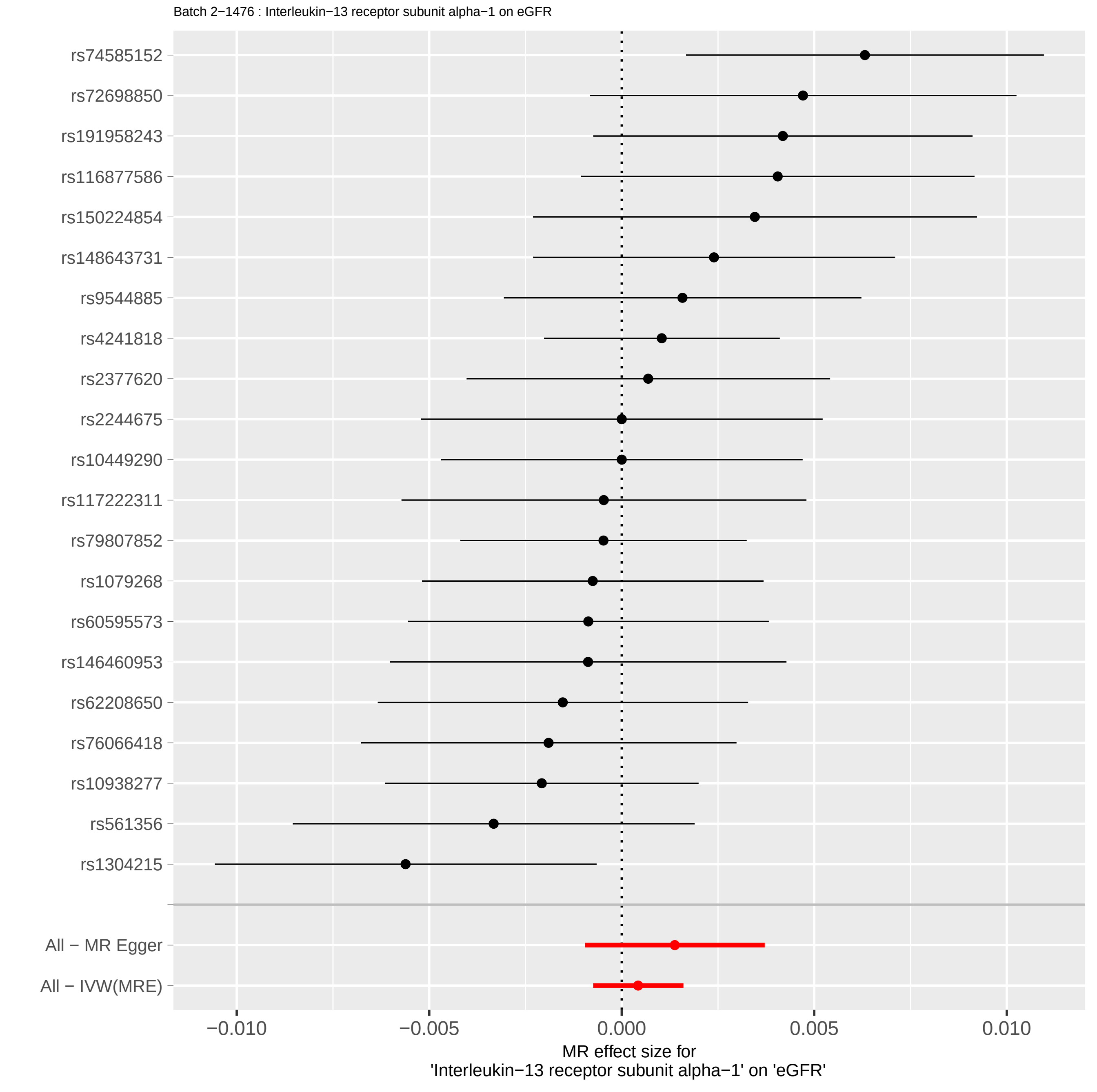

Batch 2−1476 : Interleukin−13 receptor subunit alpha−1 on eGFR
rs74585152
rs72698850
rs191958243
rs116877586
rs150224854
rs148643731
rs9544885
rs4241818
rs2377620
rs2244675
rs10449290
rs117222311
rs79807852
rs1079268
rs60595573
rs146460953
rs62208650
rs76066418
rs10938277
rs561356
rs1304215
All − MR Egger
All − IVW(MRE)
−0.010
−0.005
0.000
0.005
0.010
MR effect size for
'Interleukin−13 receptor subunit alpha−1' on 'eGFR'

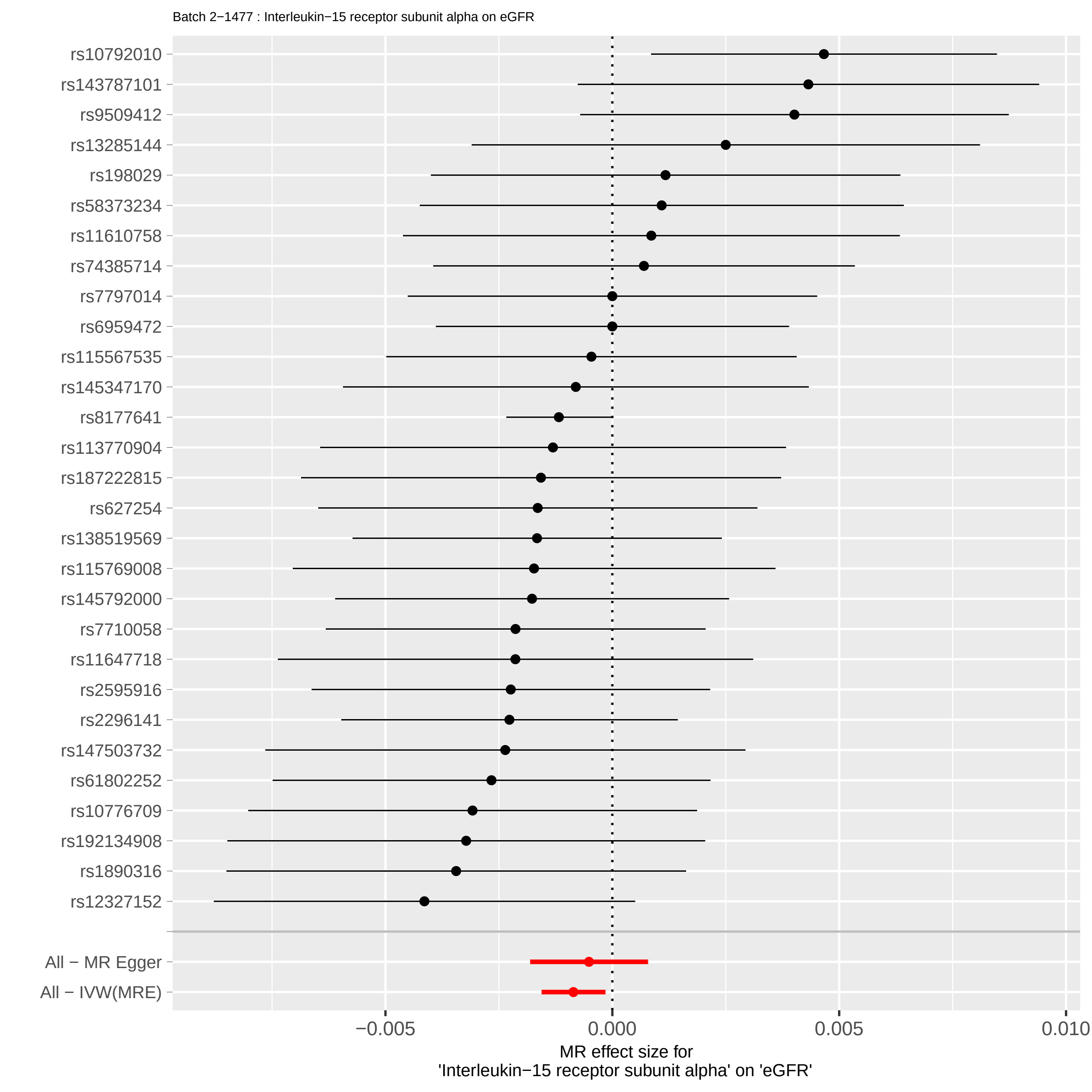

Batch 2−1477 : Interleukin−15 receptor subunit alpha on eGFR
rs10792010
rs143787101
rs9509412
rs13285144
rs198029
rs58373234
rs11610758
rs74385714
rs7797014
rs6959472
rs115567535
rs145347170
rs8177641
rs113770904
rs187222815
rs627254
rs138519569
rs115769008
rs145792000
rs7710058
rs11647718
rs2595916
rs2296141
rs147503732
rs61802252
rs10776709
rs192134908
rs1890316
rs12327152
All − MR Egger
All − IVW(MRE)
−0.005
0.000
0.005
0.010
MR effect size for
'Interleukin−15 receptor subunit alpha' on 'eGFR'

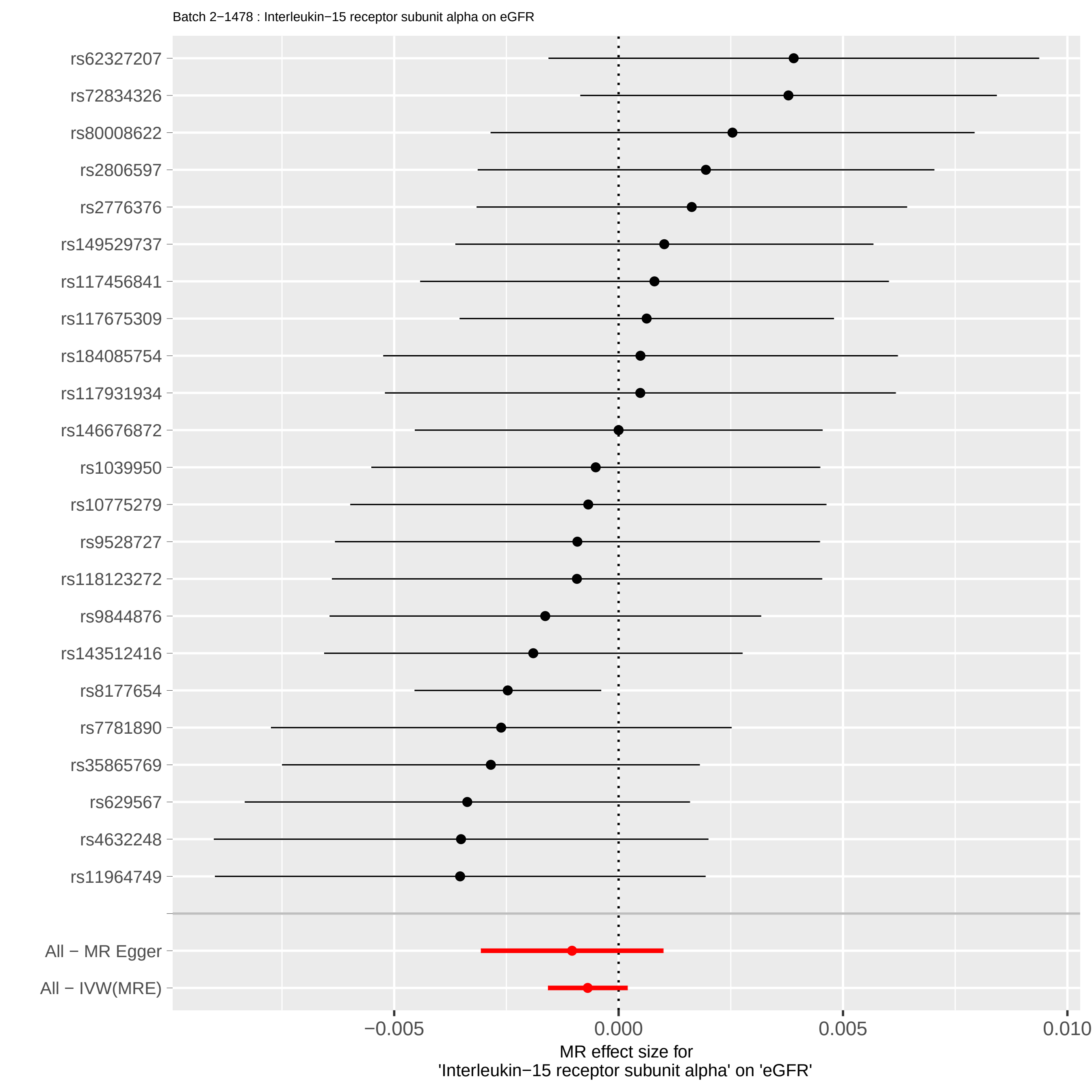
Batch 2−1478 : Interleukin−15 receptor subunit alpha on eGFR
rs62327207
rs72834326
rs80008622
rs2806597
rs2776376
rs149529737
rs117456841
rs117675309
rs184085754
rs117931934
rs146676872
rs1039950
rs10775279
rs9528727
rs118123272
rs9844876
rs143512416
rs8177654
rs7781890
rs35865769
rs629567
rs4632248
rs11964749
All − MR Egger
All − IVW(MRE)
−0.005
0.000
0.005
0.010
MR effect size for
'Interleukin−15 receptor subunit alpha' on 'eGFR'

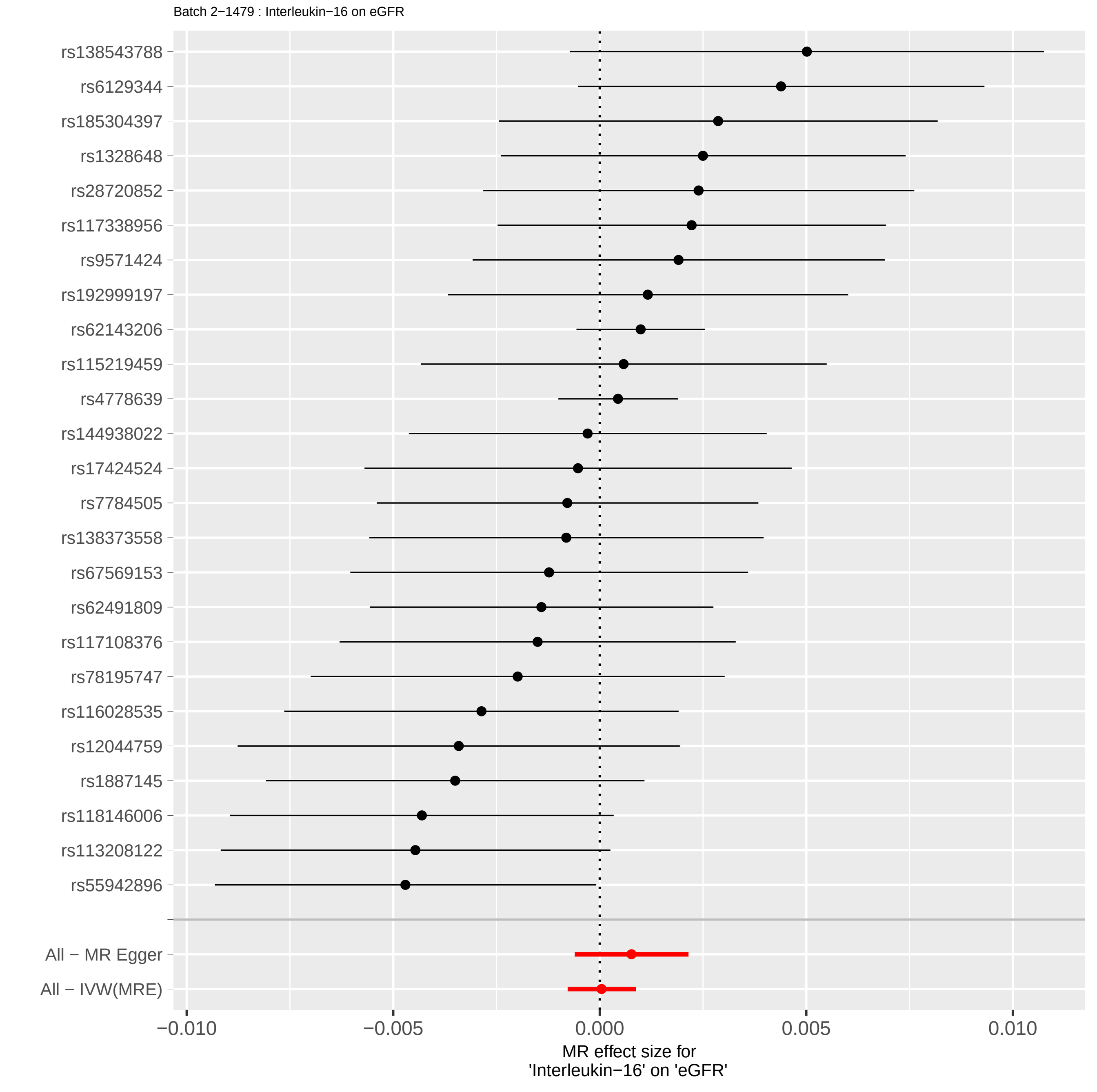
Batch 2−1479 : Interleukin−16 on eGFR
rs138543788
rs6129344
rs185304397
rs1328648
rs28720852
rs117338956
rs9571424
rs192999197
rs62143206
rs115219459
rs4778639
rs144938022
rs17424524
rs7784505
rs138373558
rs67569153
rs62491809
rs117108376
rs78195747
rs116028535
rs12044759
rs1887145
rs118146006
rs113208122
rs55942896
All − MR Egger
All − IVW(MRE)
−0.010
−0.005
0.000
0.005
0.010
MR effect size for
'Interleukin−16' on 'eGFR'

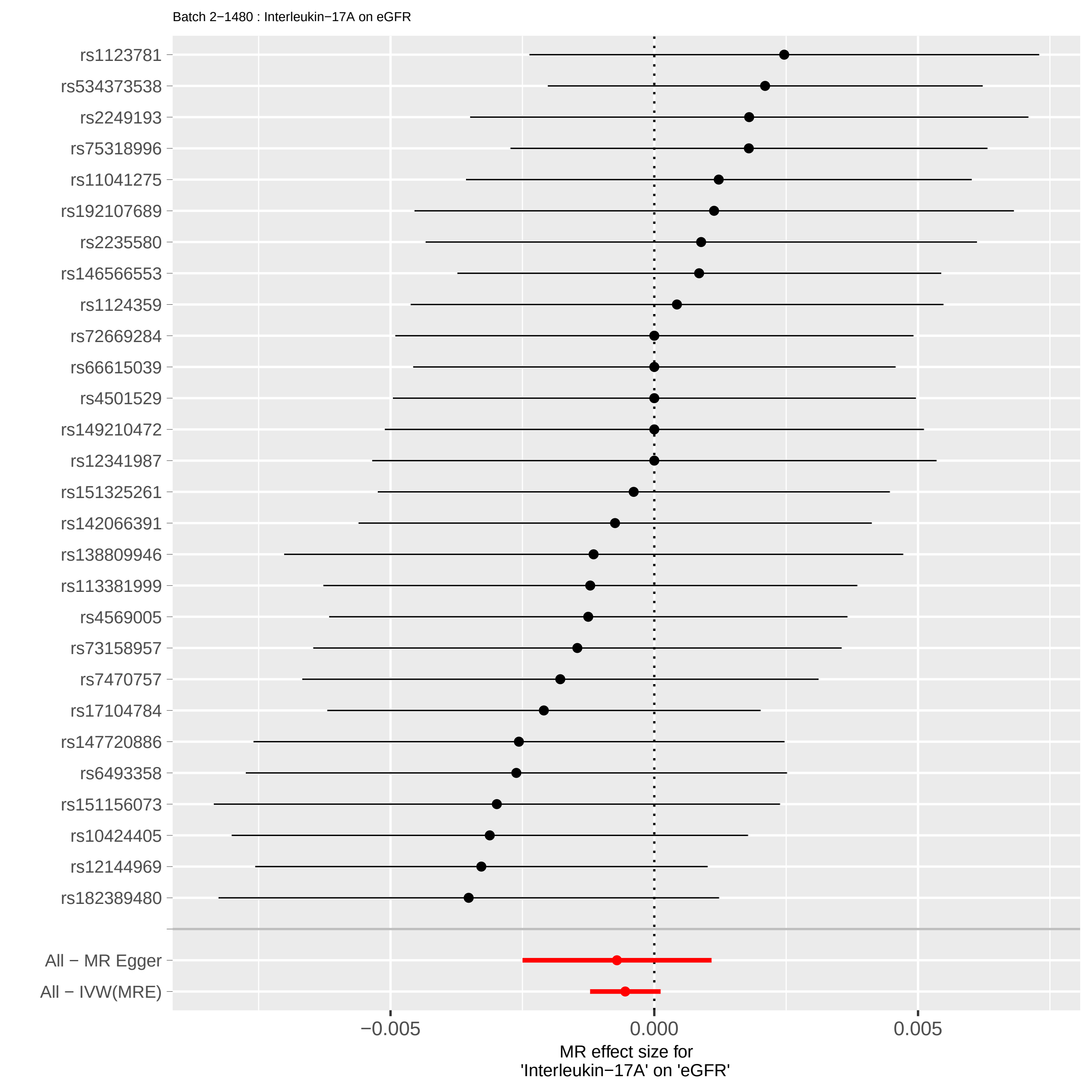

Batch 2−1480 : Interleukin−17A on eGFR
rs1123781
rs534373538
rs2249193
rs75318996
rs11041275
rs192107689
rs2235580
rs146566553
rs1124359
rs72669284
rs66615039
rs4501529
rs149210472
rs12341987
rs151325261
rs142066391
rs138809946
rs113381999
rs4569005
rs73158957
rs7470757
rs17104784
rs147720886
rs6493358
rs151156073
rs10424405
rs12144969
rs182389480
All − MR Egger
All − IVW(MRE)
−0.005
0.000
0.005
MR effect size for
'Interleukin−17A' on 'eGFR'

Batch 2−1481 : Interleukin−17A on eGFR

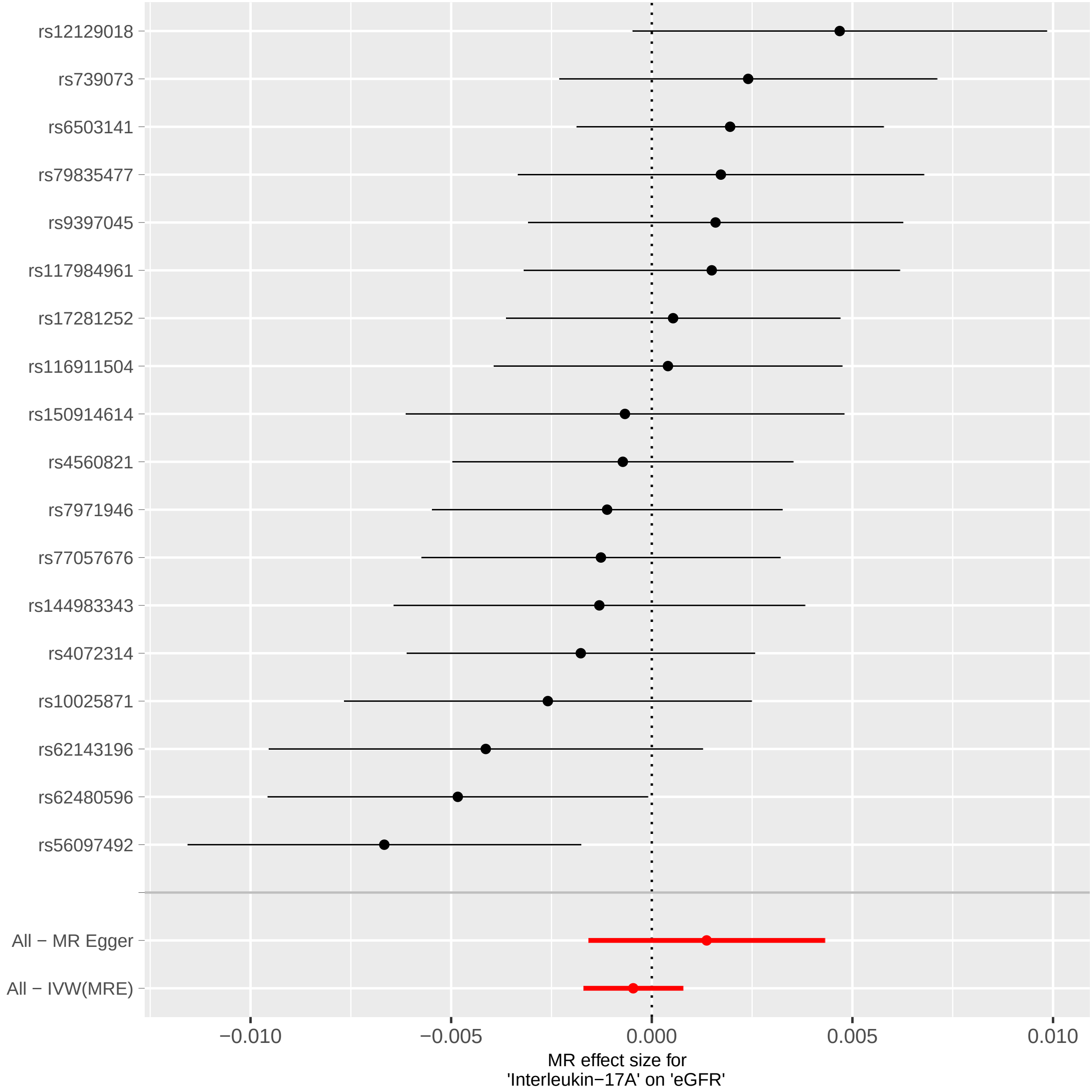

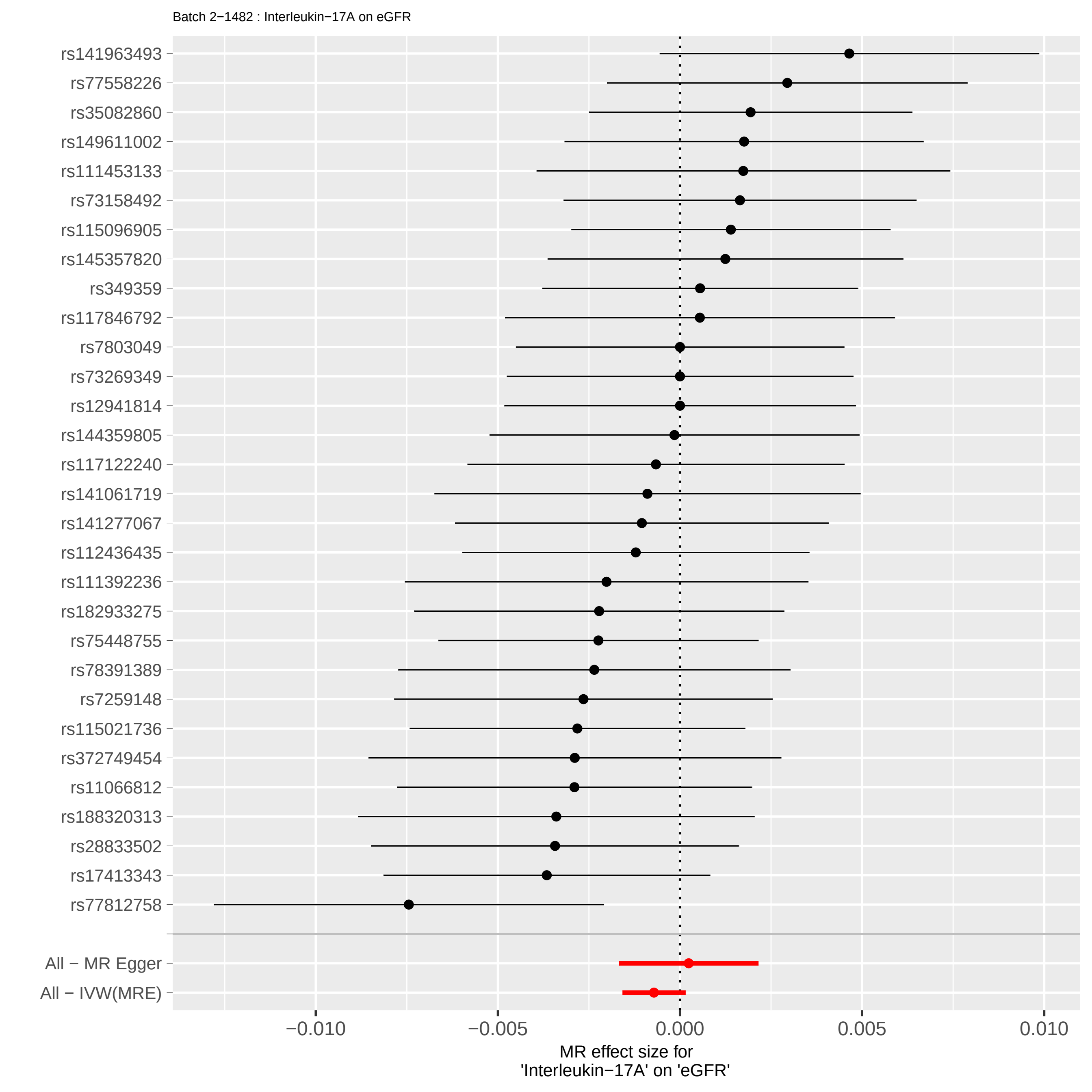

Batch 2−1482 : Interleukin−17A on eGFR
rs141963493
rs77558226
rs35082860
rs149611002
rs111453133
rs73158492
rs115096905
rs145357820
rs349359
rs117846792
rs7803049
rs73269349
rs12941814
rs144359805
rs117122240
rs141061719
rs141277067
rs112436435
rs111392236
rs182933275
rs75448755
rs78391389
rs7259148
rs115021736
rs372749454
rs11066812
rs188320313
rs28833502
rs17413343
rs77812758
All − MR Egger
All − IVW(MRE)
−0.010
−0.005
0.000
0.005
0.010
MR effect size for
'Interleukin−17A' on 'eGFR'

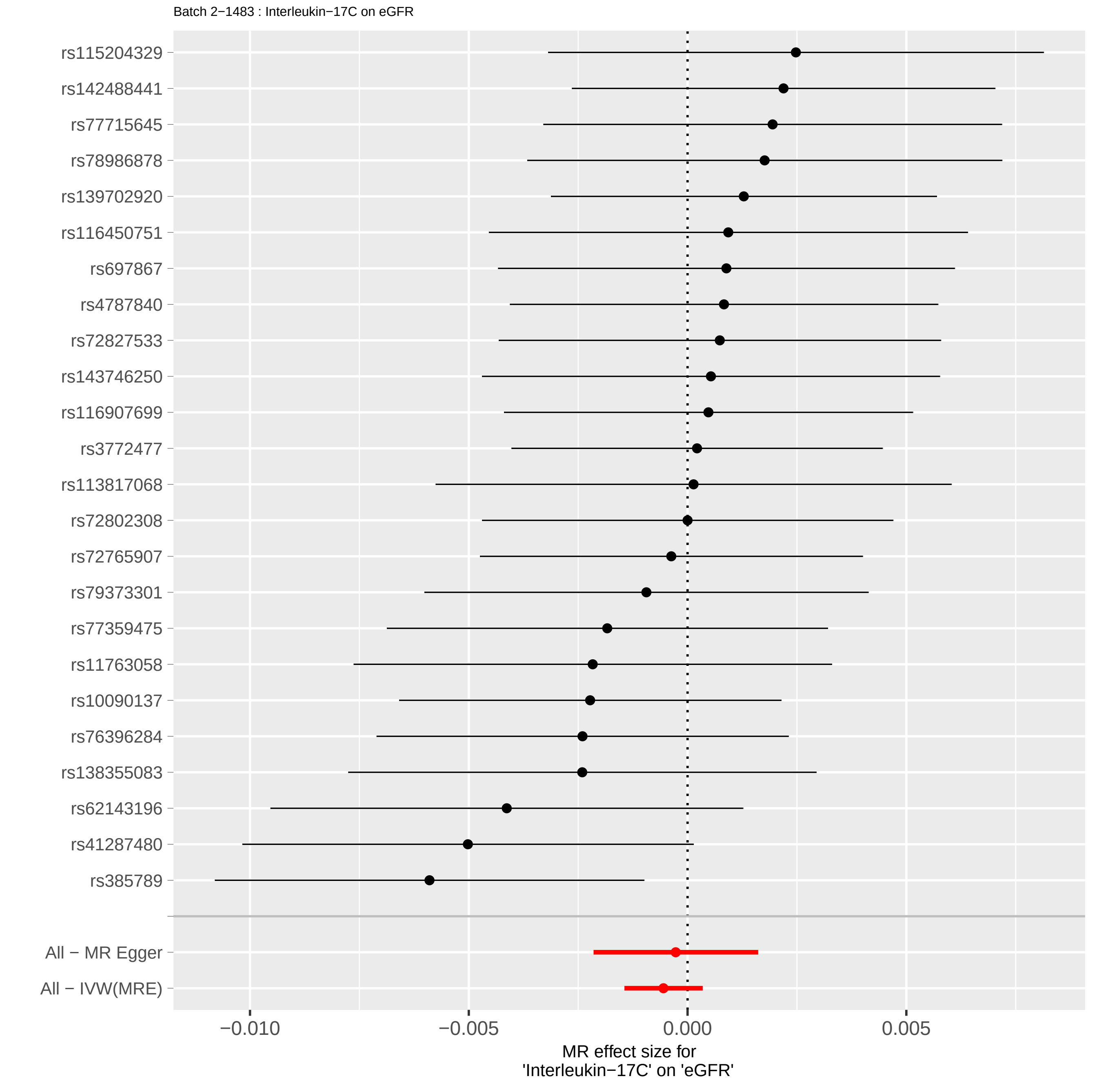

Batch 2−1483 : Interleukin−17C on eGFR
rs115204329
rs142488441
rs77715645
rs78986878
rs139702920
rs116450751
rs697867
rs4787840
rs72827533
rs143746250
rs116907699
rs3772477
rs113817068
rs72802308
rs72765907
rs79373301
rs77359475
rs11763058
rs10090137
rs76396284
rs138355083
rs62143196
rs41287480
rs385789
All − MR Egger
All − IVW(MRE)
−0.010
−0.005
0.000
0.005
MR effect size for
'Interleukin−17C' on 'eGFR'

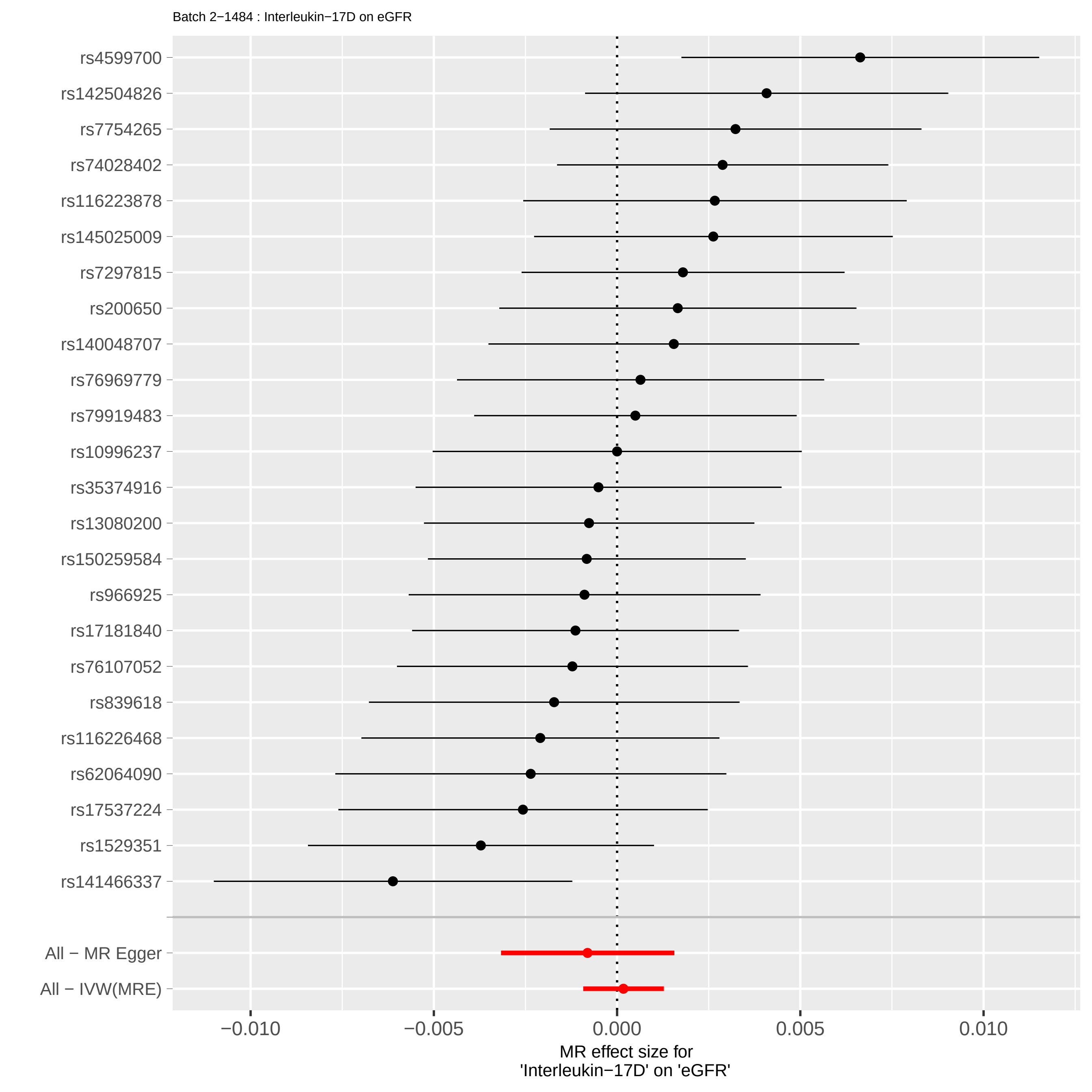

Batch 2−1484 : Interleukin−17D on eGFR
rs4599700
rs142504826
rs7754265
rs74028402
rs116223878
rs145025009
rs7297815
rs200650
rs140048707
rs76969779
rs79919483
rs10996237
rs35374916
rs13080200
rs150259584
rs966925
rs17181840
rs76107052
rs839618
rs116226468
rs62064090
rs17537224
rs1529351
rs141466337
All − MR Egger
All − IVW(MRE)
−0.010
−0.005
0.000
0.005
0.010
MR effect size for
'Interleukin−17D' on 'eGFR'

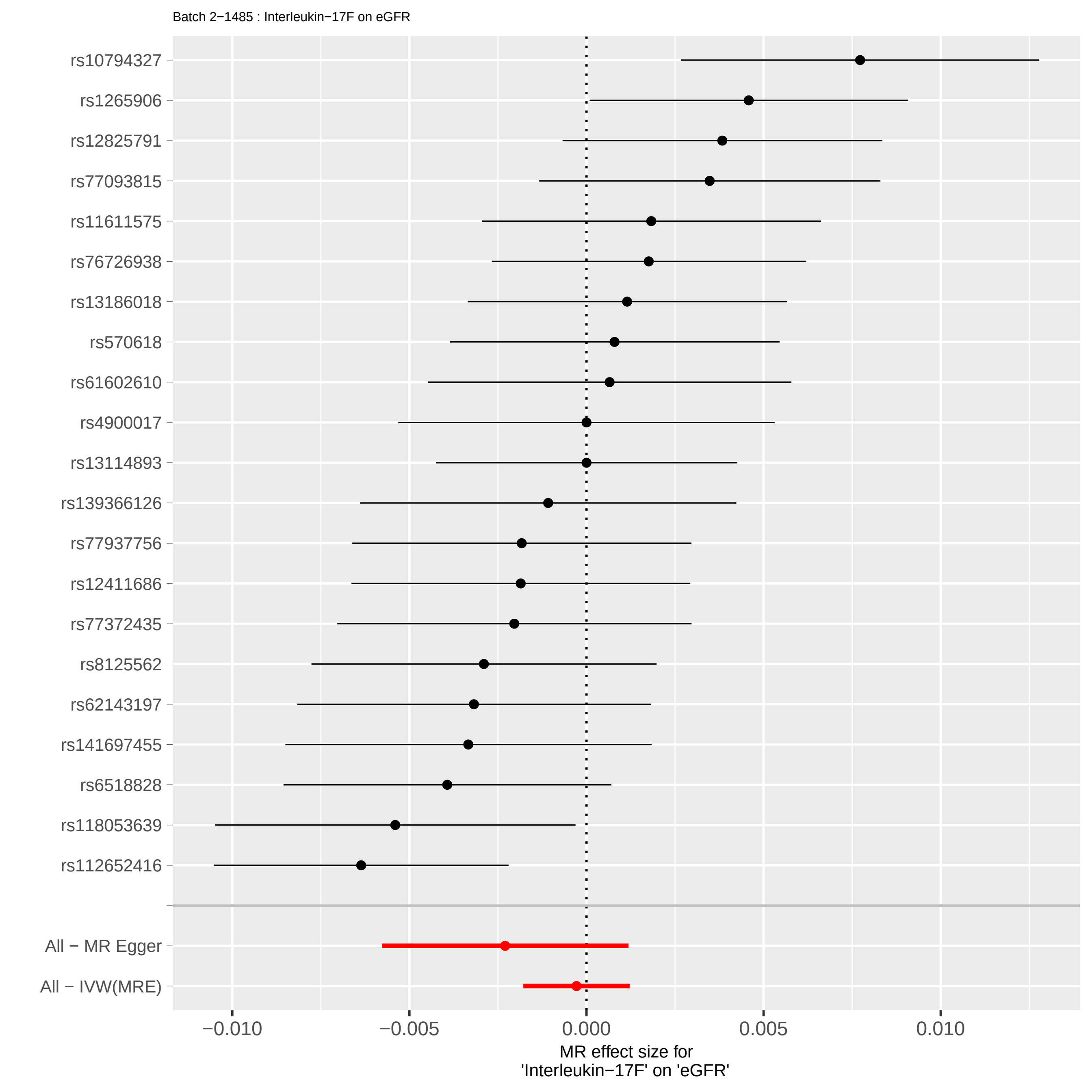
Batch 2−1485 : Interleukin−17F on eGFR
rs10794327
rs1265906
rs12825791
rs77093815
rs11611575
rs76726938
rs13186018
rs570618
rs61602610
rs4900017
rs13114893
rs139366126
rs77937756
rs12411686
rs77372435
rs8125562
rs62143197
rs141697455
rs6518828
rs118053639
rs112652416
All − MR Egger
All − IVW(MRE)
−0.010
−0.005
0.000
0.005
0.010
MR effect size for
'Interleukin−17F' on 'eGFR'

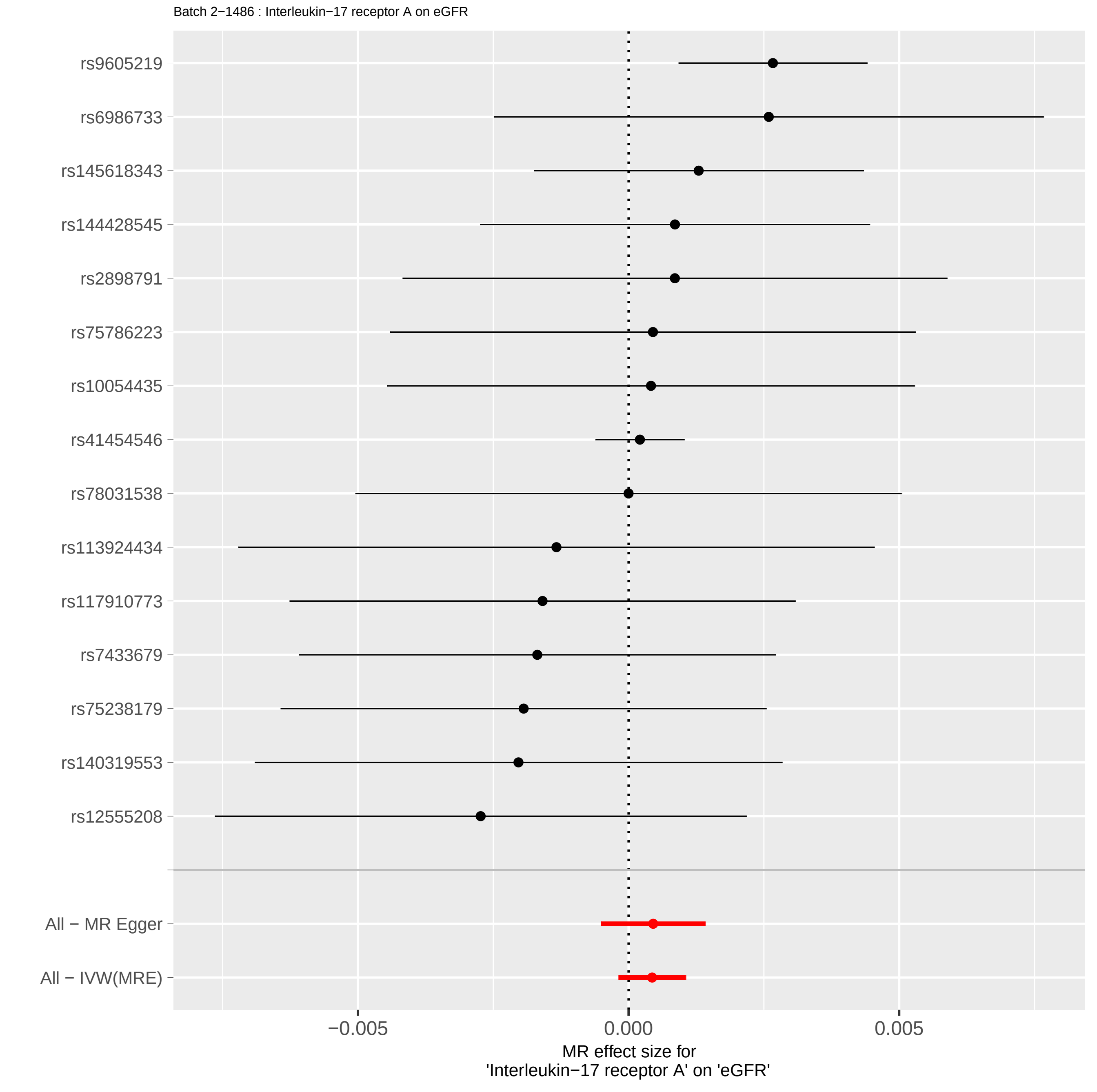
Batch 2−1486 : Interleukin−17 receptor A on eGFR
rs9605219
rs6986733
rs145618343
rs144428545
rs2898791
rs75786223
rs10054435
rs41454546
rs78031538
rs113924434
rs117910773
rs7433679
rs75238179
rs140319553
rs12555208
All − MR Egger
All − IVW(MRE)
−0.005
0.000
0.005
MR effect size for
'Interleukin−17 receptor A' on 'eGFR'

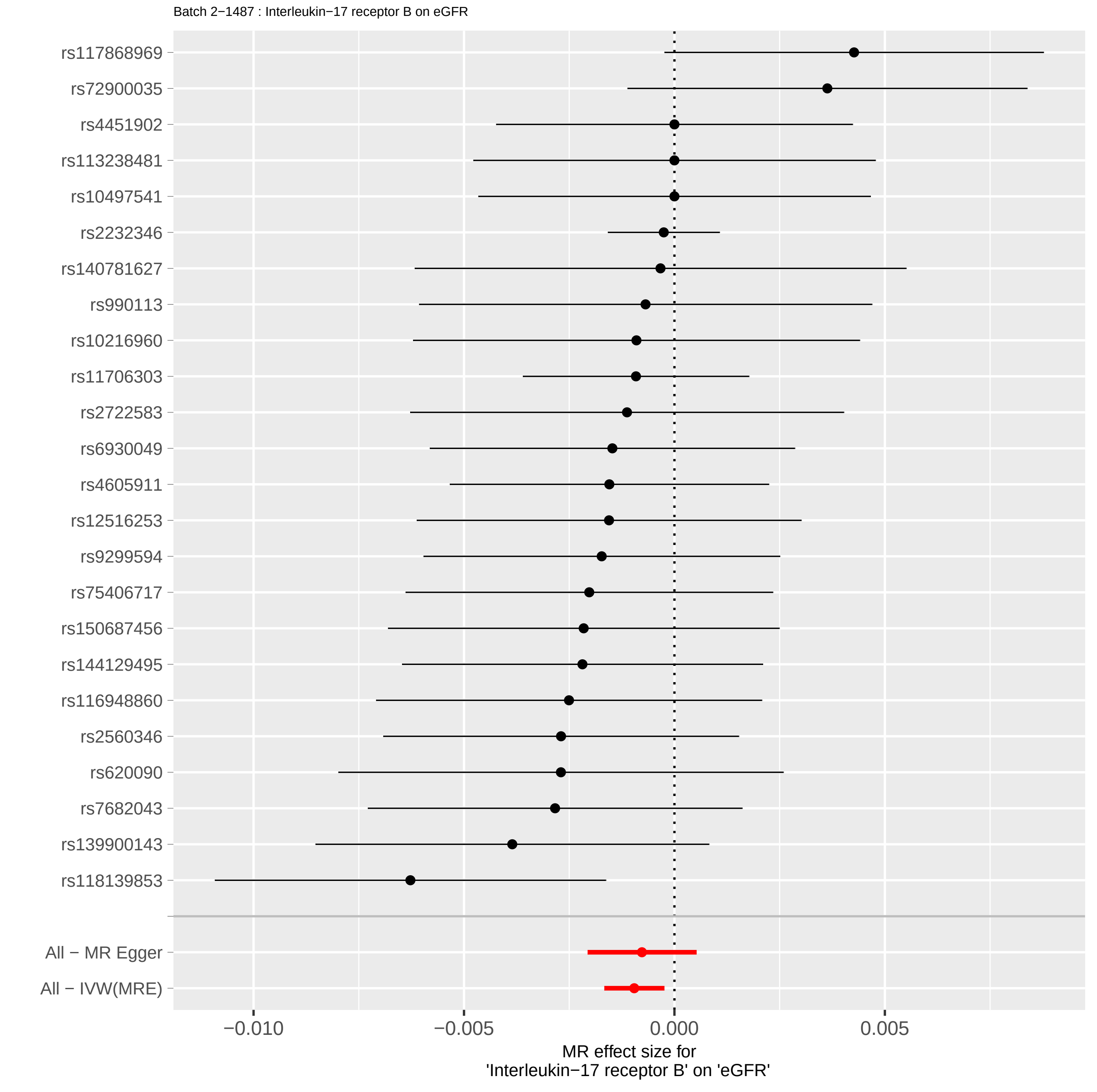
Batch 2−1487 : Interleukin−17 receptor B on eGFR
rs117868969
rs72900035
rs4451902
rs113238481
rs10497541
rs2232346
rs140781627
rs990113
rs10216960
rs11706303
rs2722583
rs6930049
rs4605911
rs12516253
rs9299594
rs75406717
rs150687456
rs144129495
rs116948860
rs2560346
rs620090
rs7682043
rs139900143
rs118139853
All − MR Egger
All − IVW(MRE)
−0.010
−0.005
0.000
0.005
MR effect size for
'Interleukin−17 receptor B' on 'eGFR'

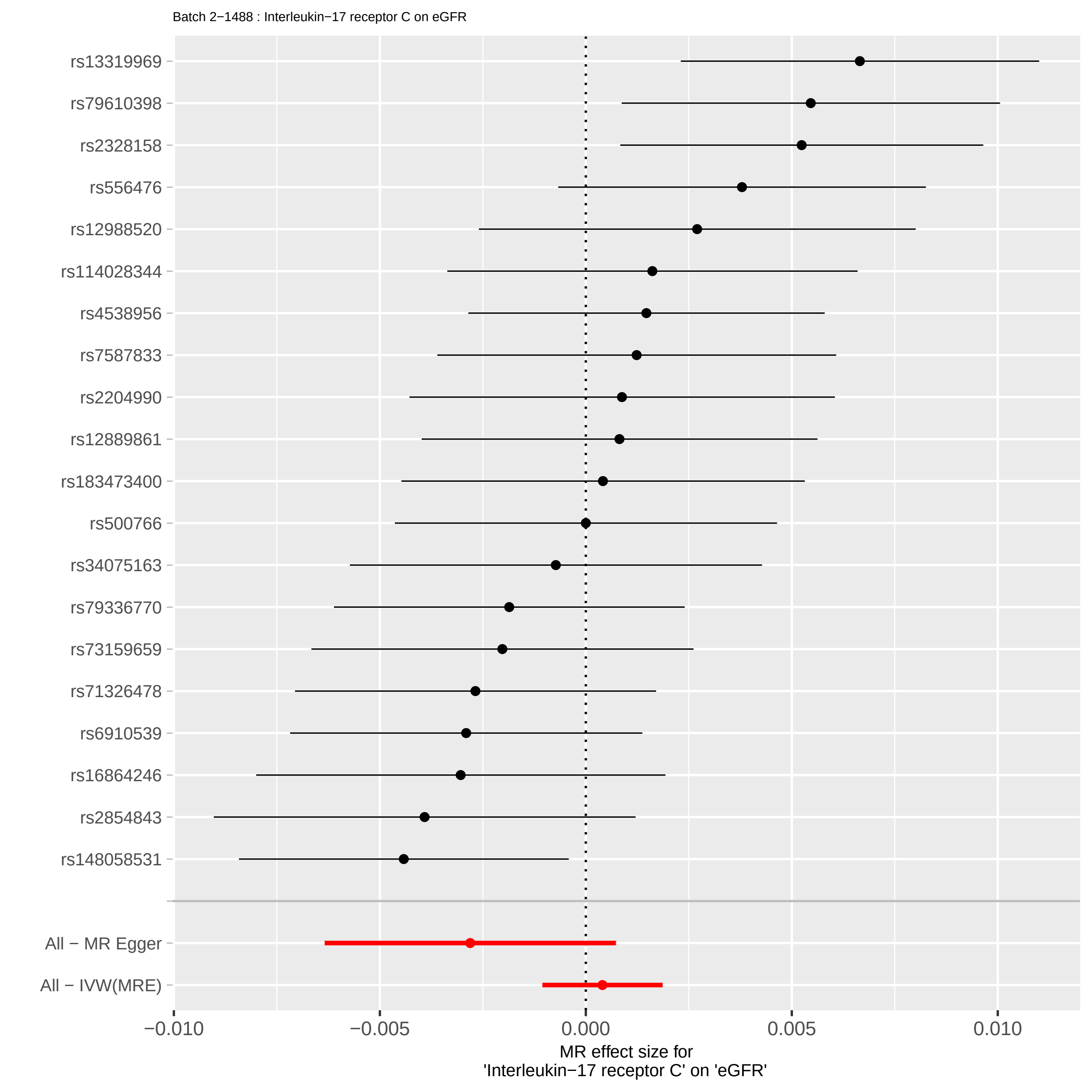

Batch 2−1488 : Interleukin−17 receptor C on eGFR
rs13319969
rs79610398
rs2328158
rs556476
rs12988520
rs114028344
rs4538956
rs7587833
rs2204990
rs12889861
rs183473400
rs500766
rs34075163
rs79336770
rs73159659
rs71326478
rs6910539
rs16864246
rs2854843
rs148058531
All − MR Egger
All − IVW(MRE)
−0.010
−0.005
0.000
0.005
0.010
MR effect size for
'Interleukin−17 receptor C' on 'eGFR'

Batch 2−1489 : Interleukin−17 receptor D on eGFR

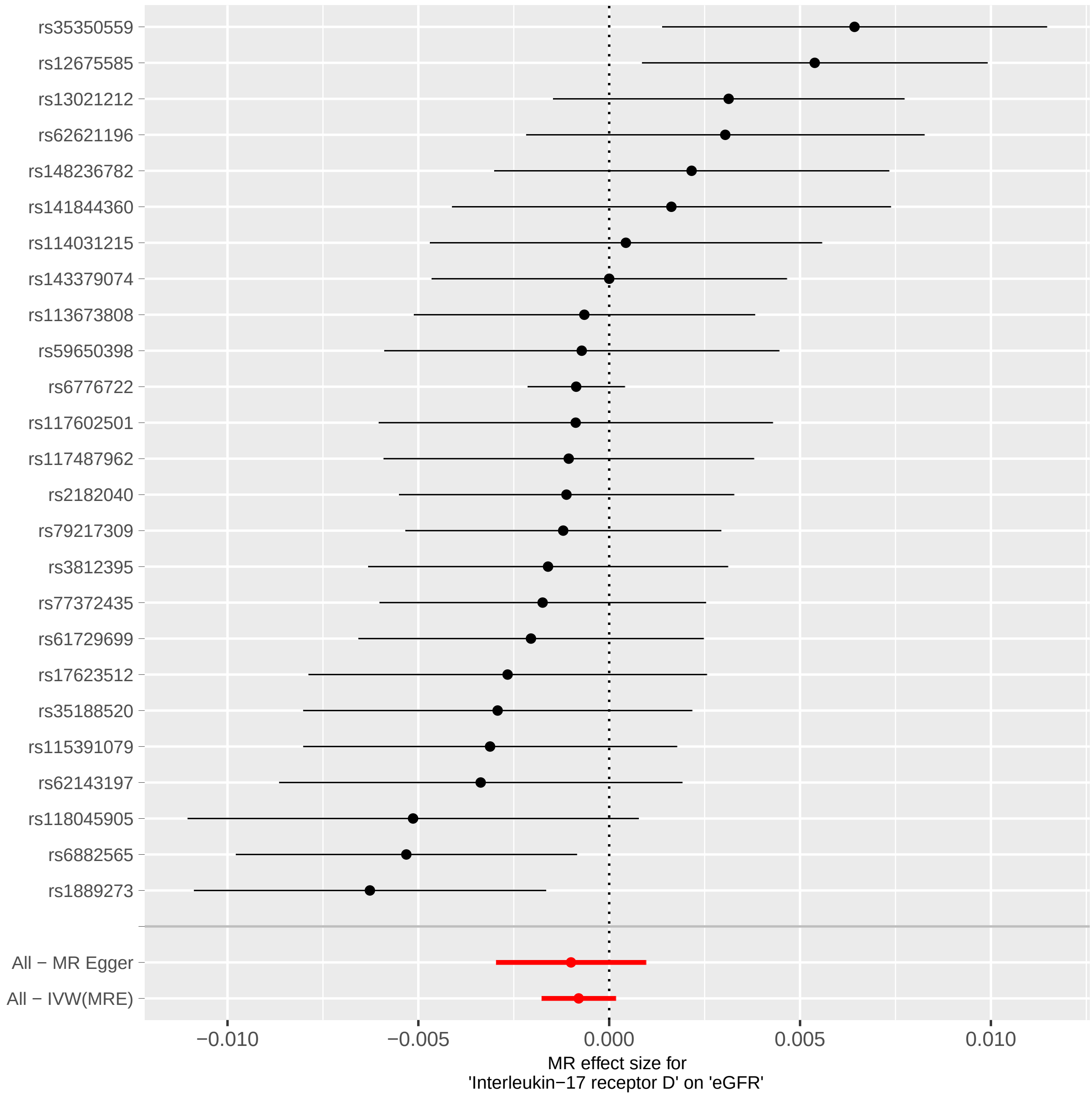

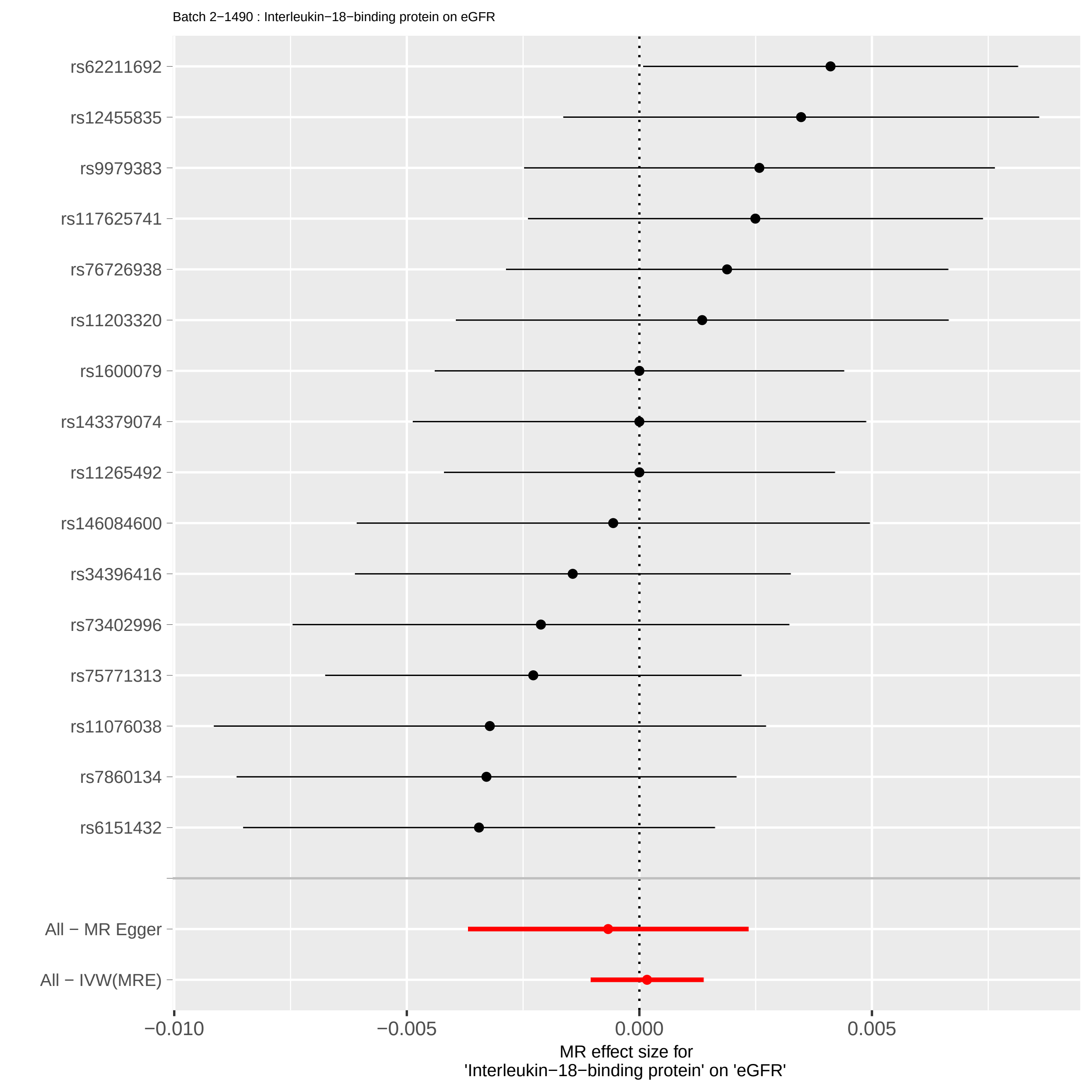

Batch 2−1490 : Interleukin−18−binding protein on eGFR
rs62211692
rs12455835
rs9979383
rs117625741
rs76726938
rs11203320
rs1600079
rs143379074
rs11265492
rs146084600
rs34396416
rs73402996
rs75771313
rs11076038
rs7860134
rs6151432
All − MR Egger
All − IVW(MRE)
−0.010
−0.005
0.000
0.005
MR effect size for
'Interleukin−18−binding protein' on 'eGFR'

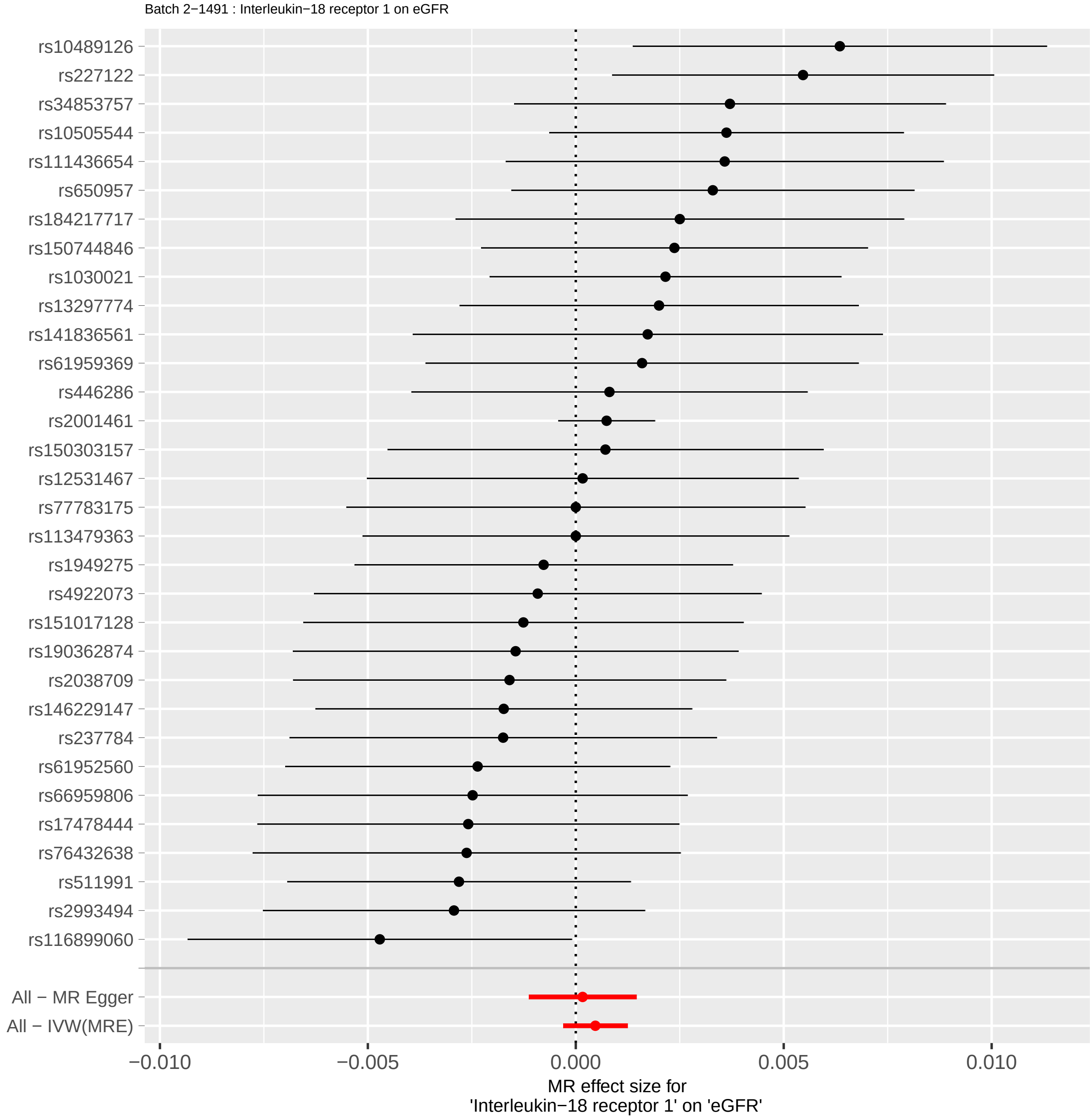

Batch 2−1491 : Interleukin−18 receptor 1 on eGFR
rs10489126
rs227122
rs34853757
rs10505544
rs111436654
rs650957
rs184217717
rs150744846
rs1030021
rs13297774
rs141836561
rs61959369
rs446286
rs2001461
rs150303157
rs12531467
rs77783175
rs113479363
rs1949275
rs4922073
rs151017128
rs190362874
rs2038709
rs146229147
rs237784
rs61952560
rs66959806
rs17478444
rs76432638
rs511991
rs2993494
rs116899060
All − MR Egger
All − IVW(MRE)
−0.010
−0.005
0.000
0.005
0.010
MR effect size for
'Interleukin−18 receptor 1' on 'eGFR'

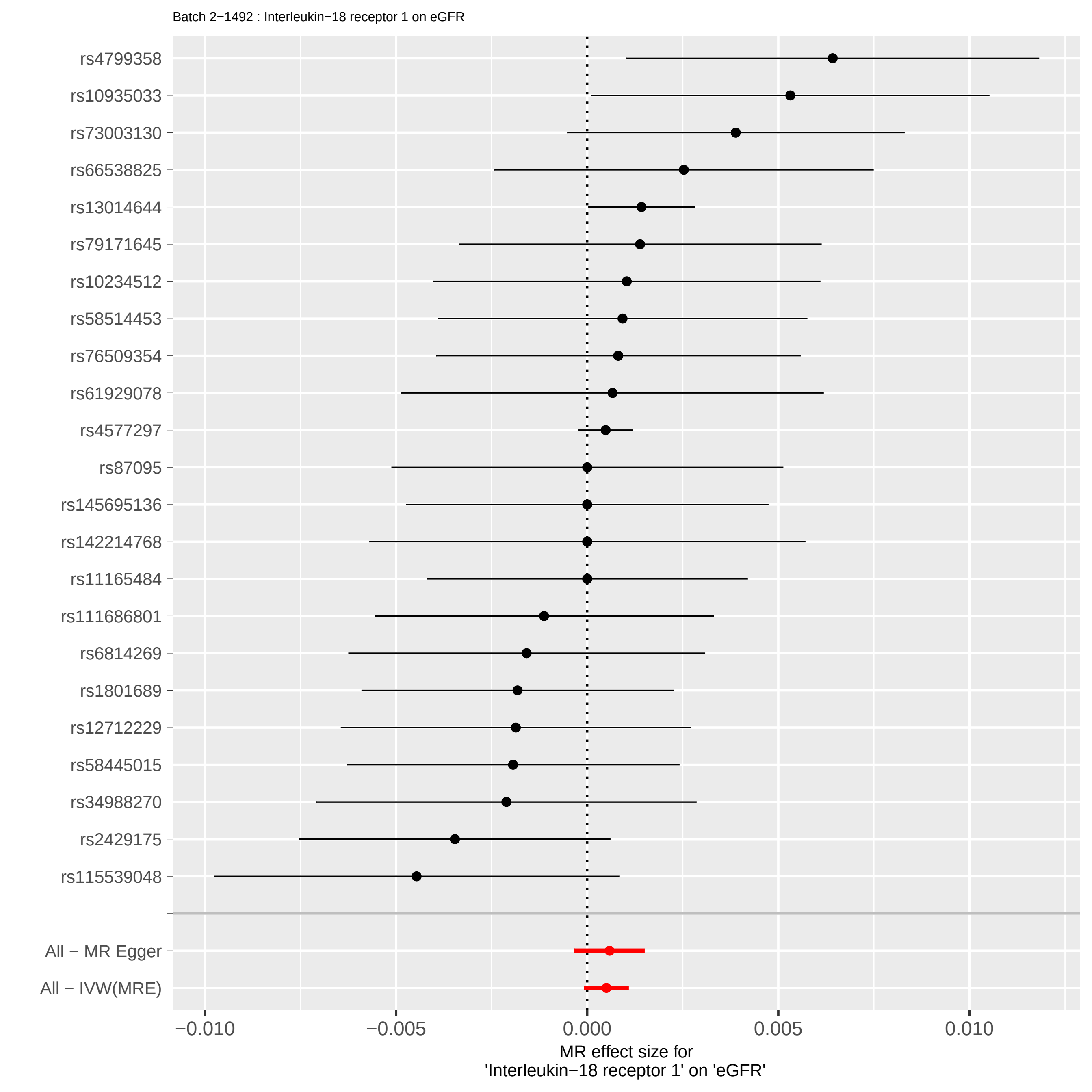

Batch 2−1492 : Interleukin−18 receptor 1 on eGFR
rs4799358
rs10935033
rs73003130
rs66538825
rs13014644
rs79171645
rs10234512
rs58514453
rs76509354
rs61929078
rs4577297
rs87095
rs145695136
rs142214768
rs11165484
rs111686801
rs6814269
rs1801689
rs12712229
rs58445015
rs34988270
rs2429175
rs115539048
All − MR Egger
All − IVW(MRE)
−0.010
−0.005
0.000
0.005
0.010
MR effect size for
'Interleukin−18 receptor 1' on 'eGFR'

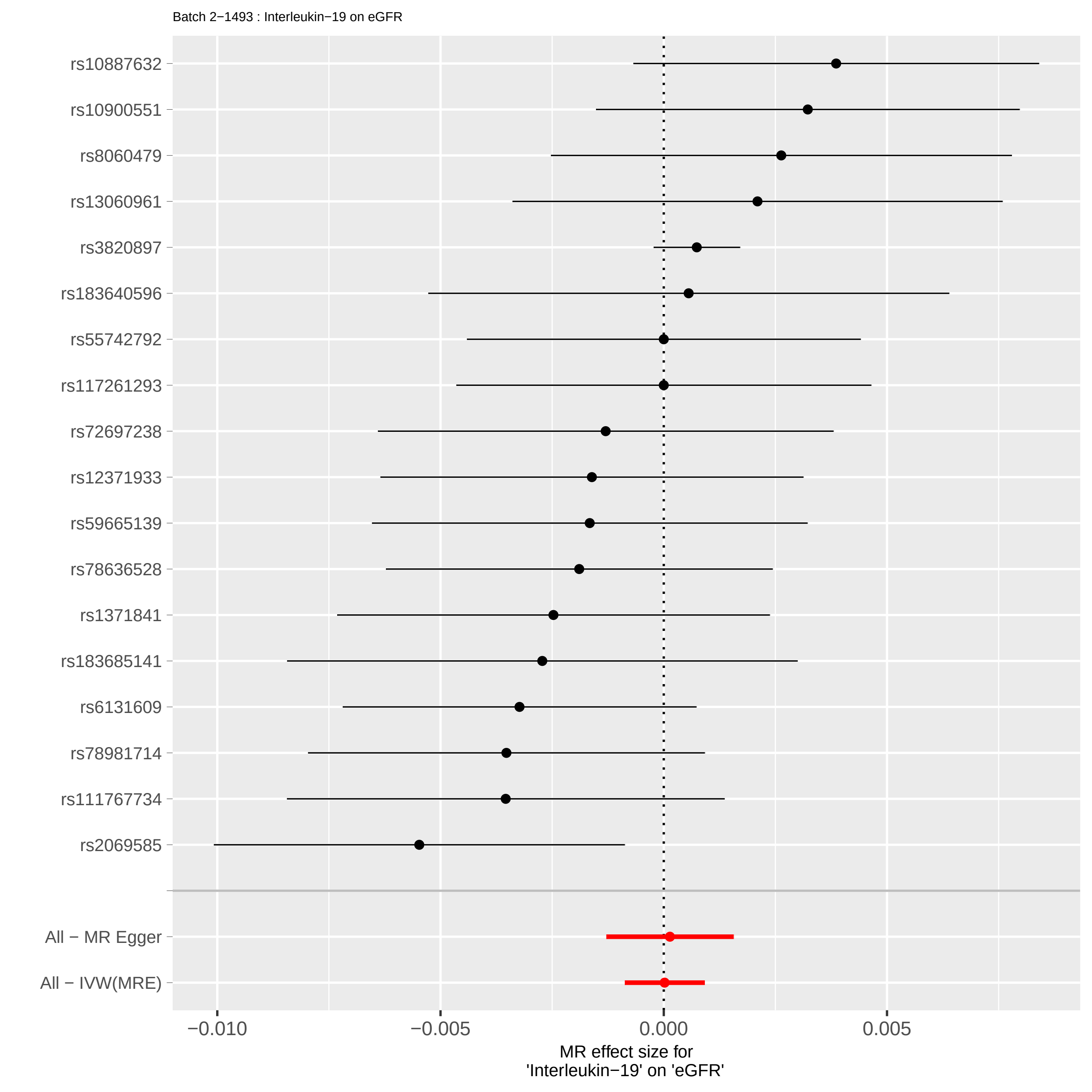

Batch 2−1493 : Interleukin−19 on eGFR
rs10887632
rs10900551
rs8060479
rs13060961
rs3820897
rs183640596
rs55742792
rs117261293
rs72697238
rs12371933
rs59665139
rs78636528
rs1371841
rs183685141
rs6131609
rs78981714
rs111767734
rs2069585
All − MR Egger
All − IVW(MRE)
−0.010
−0.005
0.000
0.005
MR effect size for
'Interleukin−19' on 'eGFR'

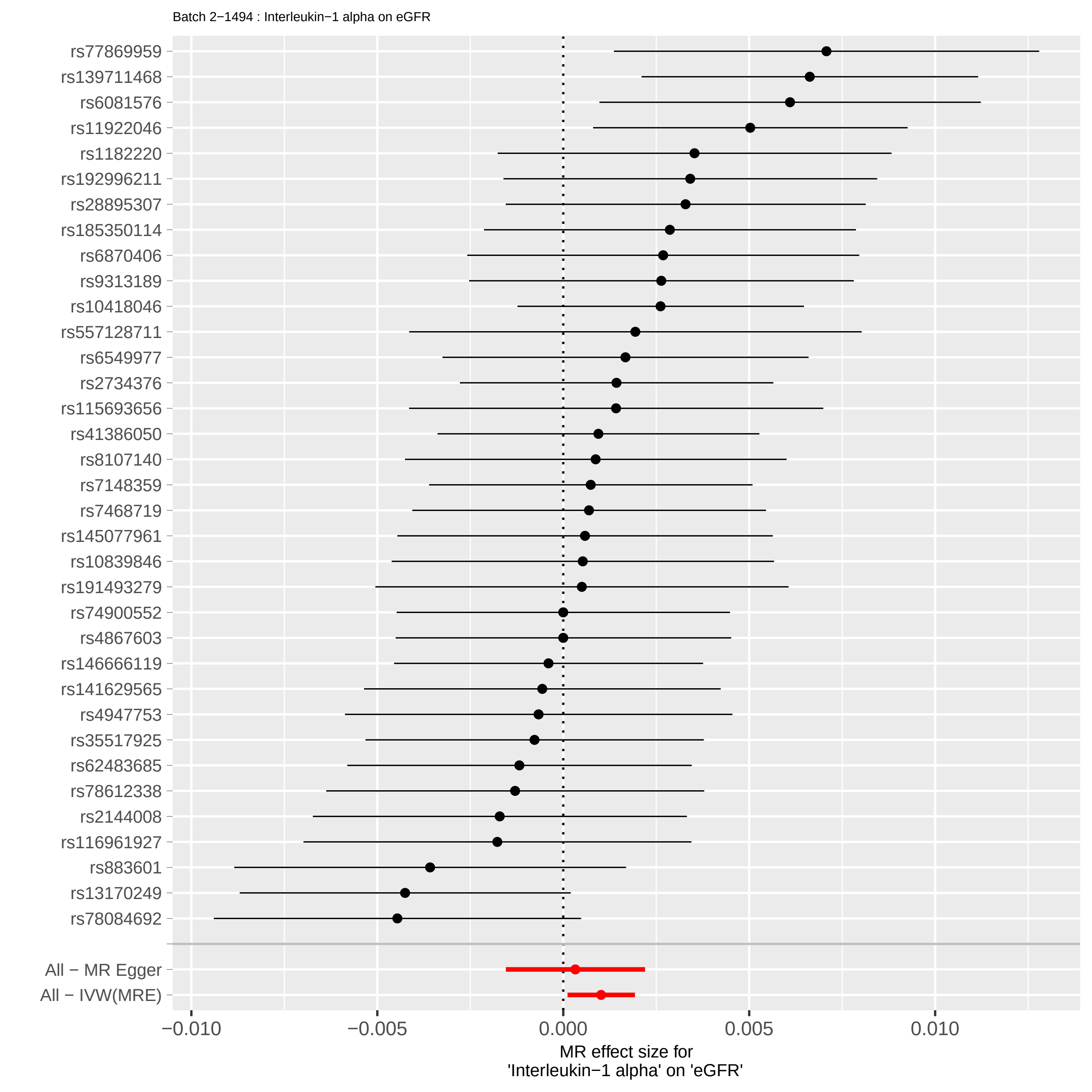

Batch 2−1494 : Interleukin−1 alpha on eGFR
rs77869959
rs139711468
rs6081576
rs11922046
rs1182220
rs192996211
rs28895307
rs185350114
rs6870406
rs9313189
rs10418046
rs557128711
rs6549977
rs2734376
rs115693656
rs41386050
rs8107140
rs7148359
rs7468719
rs145077961
rs10839846
rs191493279
rs74900552
rs4867603
rs146666119
rs141629565
rs4947753
rs35517925
rs62483685
rs78612338
rs2144008
rs116961927
rs883601
rs13170249
rs78084692
All − MR Egger
All − IVW(MRE)
−0.010
−0.005
0.000
0.005
0.010
MR effect size for
'Interleukin−1 alpha' on 'eGFR'

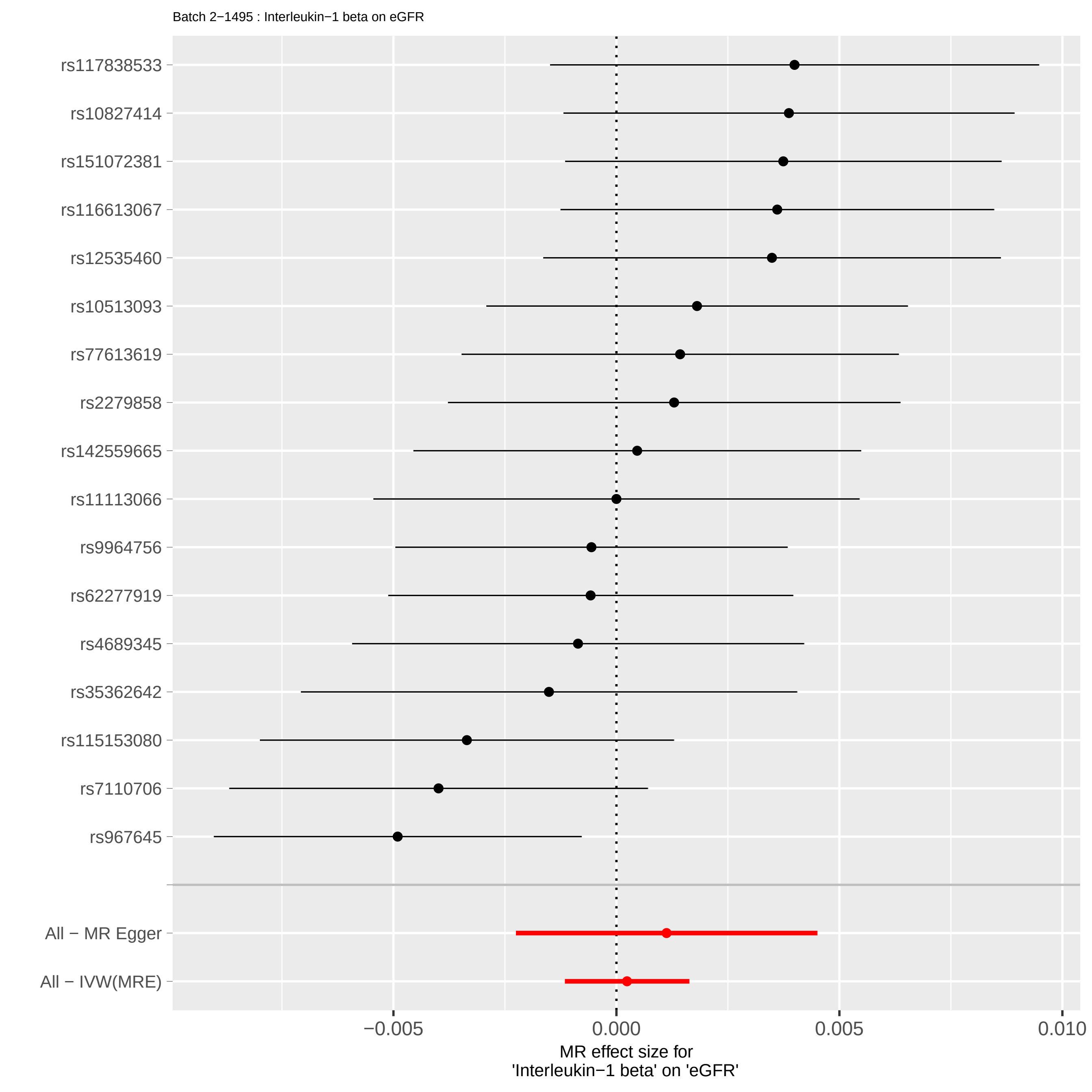
Batch 2−1495 : Interleukin−1 beta on eGFR
rs117838533
rs10827414
rs151072381
rs116613067
rs12535460
rs10513093
rs77613619
rs2279858
rs142559665
rs11113066
rs9964756
rs62277919
rs4689345
rs35362642
rs115153080
rs7110706
rs967645
All − MR Egger
All − IVW(MRE)
−0.005
0.000
0.005
0.010
MR effect size for
'Interleukin−1 beta' on 'eGFR'

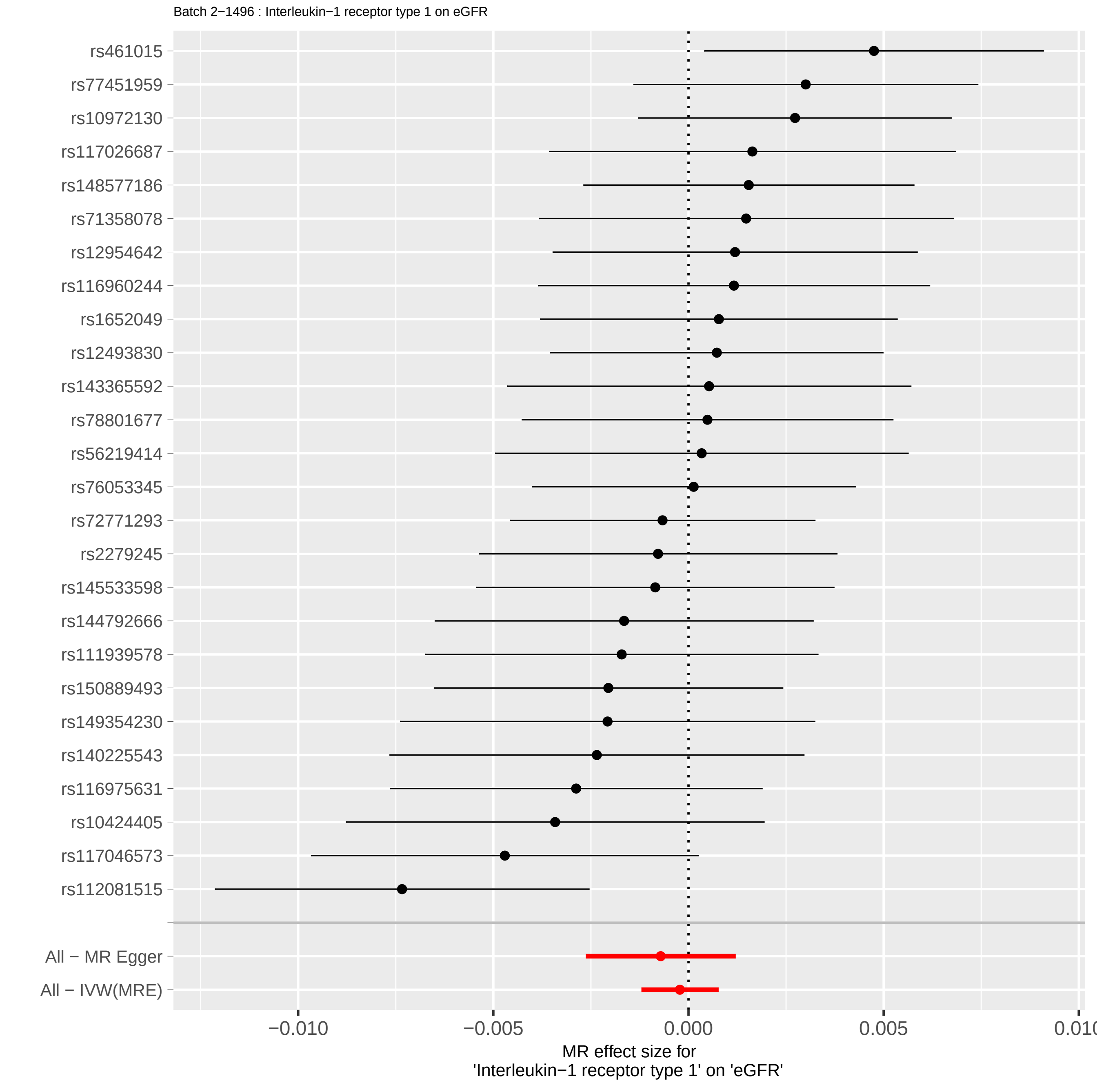

Batch 2−1496 : Interleukin−1 receptor type 1 on eGFR
rs461015
rs77451959
rs10972130
rs117026687
rs148577186
rs71358078
rs12954642
rs116960244
rs1652049
rs12493830
rs143365592
rs78801677
rs56219414
rs76053345
rs72771293
rs2279245
rs145533598
rs144792666
rs111939578
rs150889493
rs149354230
rs140225543
rs116975631
rs10424405
rs117046573
rs112081515
All − MR Egger
All − IVW(MRE)
−0.010
−0.005
0.000
0.005
0.010
MR effect size for
'Interleukin−1 receptor type 1' on 'eGFR'

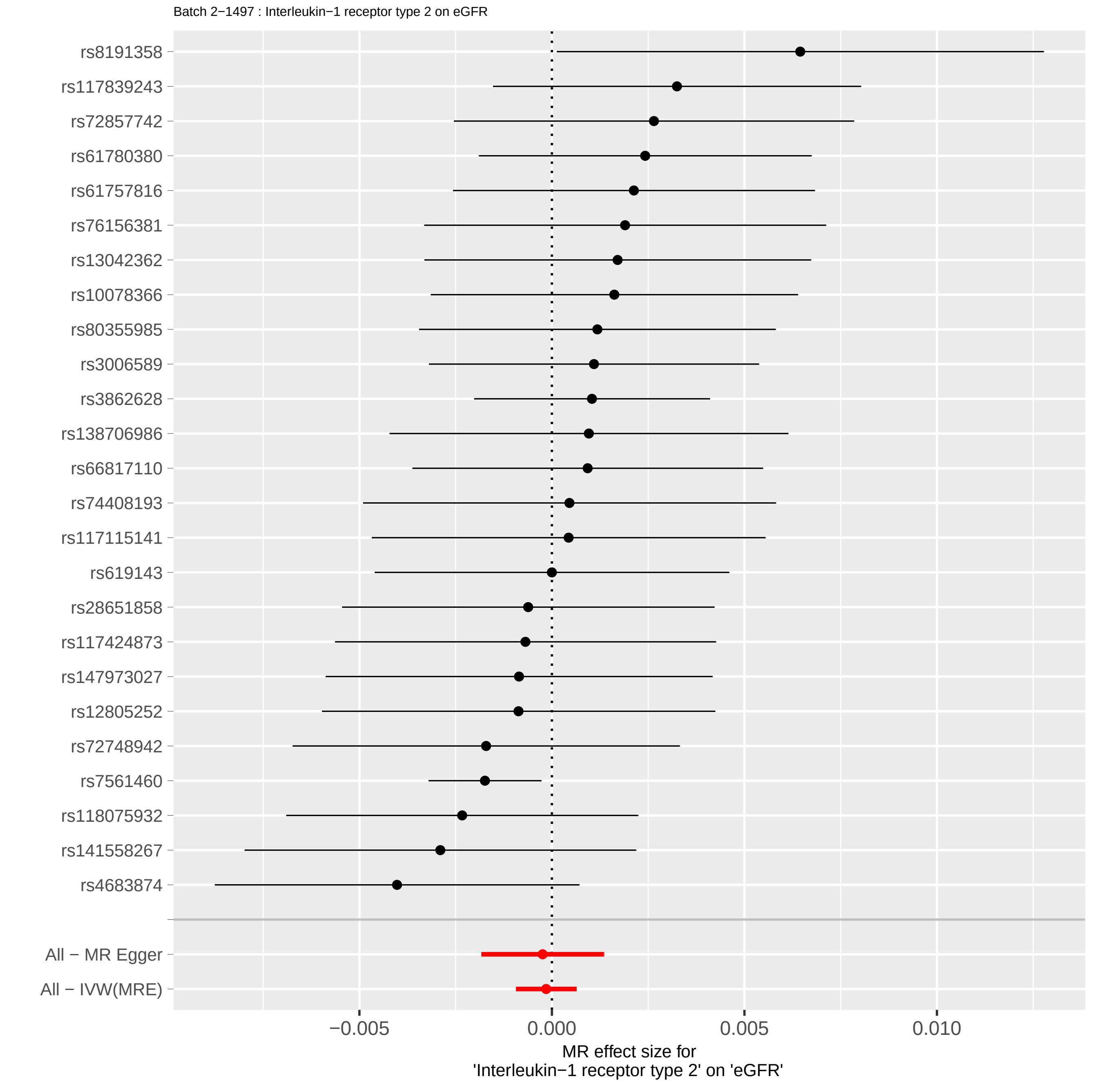
Batch 2−1497 : Interleukin−1 receptor type 2 on eGFR
rs8191358
rs117839243
rs72857742
rs61780380
rs61757816
rs76156381
rs13042362
rs10078366
rs80355985
rs3006589
rs3862628
rs138706986
rs66817110
rs74408193
rs117115141
rs619143
rs28651858
rs117424873
rs147973027
rs12805252
rs72748942
rs7561460
rs118075932
rs141558267
rs4683874
All − MR Egger
All − IVW(MRE)
−0.005
0.000
0.005
0.010
MR effect size for
'Interleukin−1 receptor type 2' on 'eGFR'

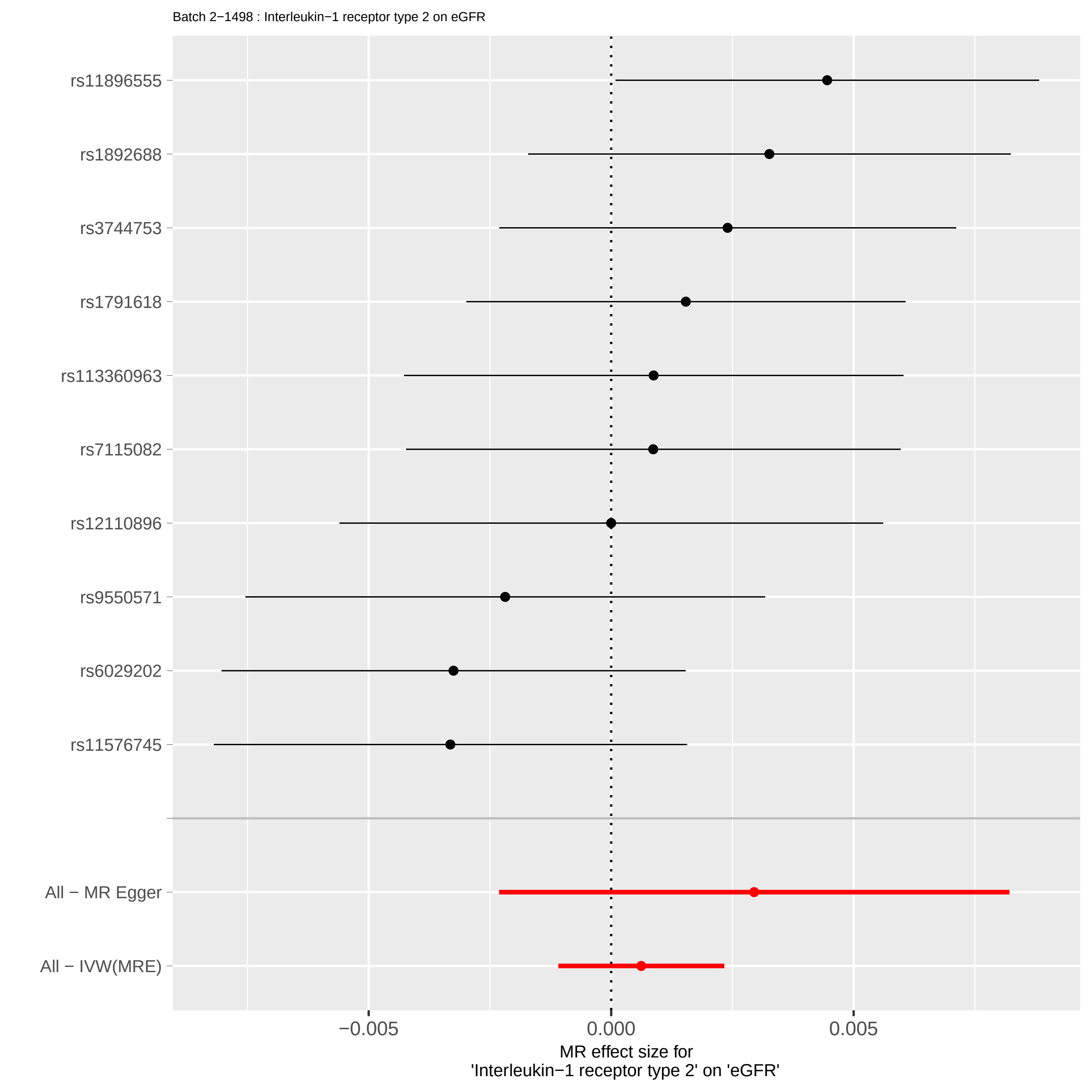
Batch 2−1498 : Interleukin−1 receptor type 2 on eGFR
rs11896555
rs1892688
rs3744753
rs1791618
rs113360963
rs7115082
rs12110896
rs9550571
rs6029202
rs11576745
All − MR Egger
All − IVW(MRE)
−0.005
0.000
0.005
MR effect size for
'Interleukin−1 receptor type 2' on 'eGFR'

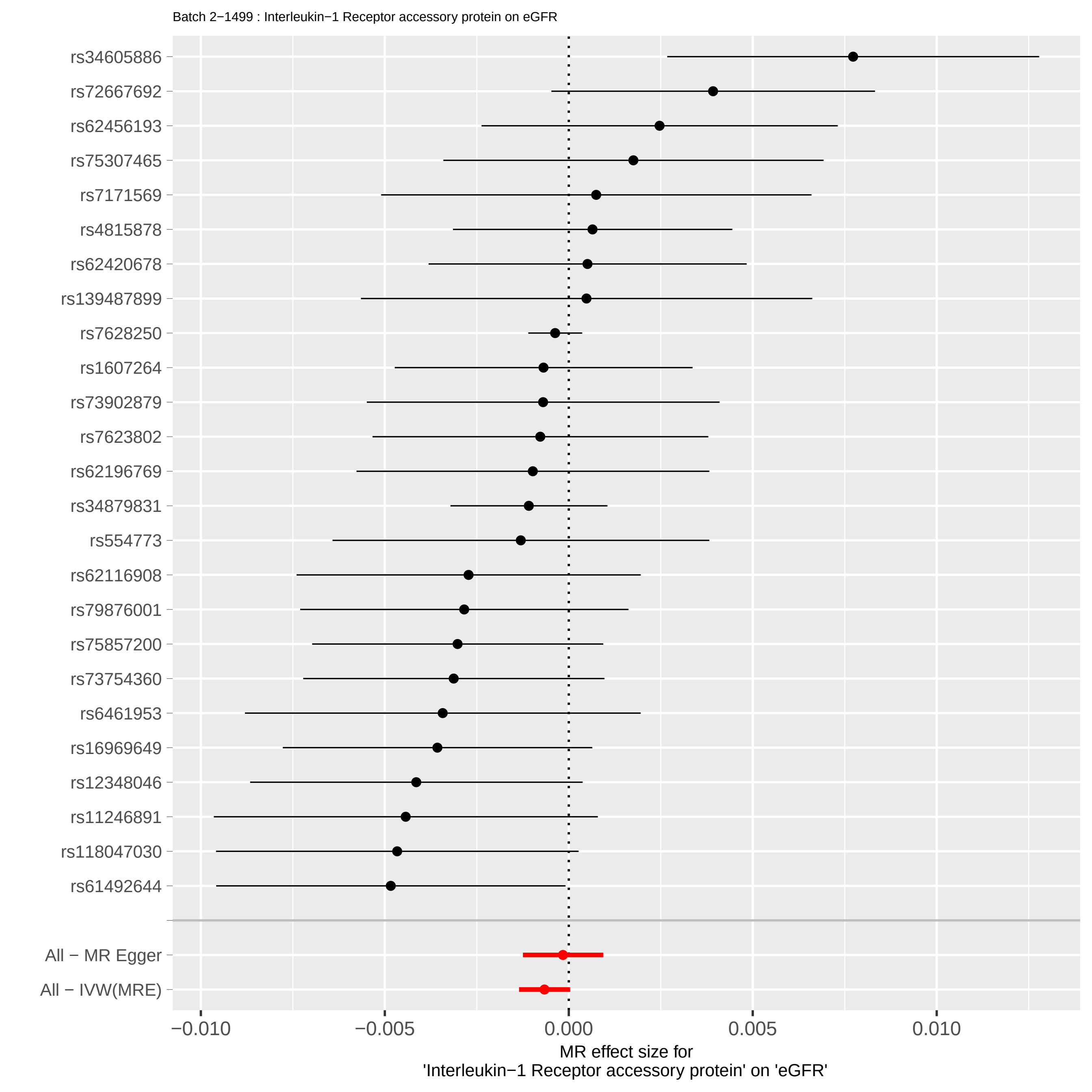

Batch 2−1499 : Interleukin−1 Receptor accessory protein on eGFR
rs34605886
rs72667692
rs62456193
rs75307465
rs7171569
rs4815878
rs62420678
rs139487899
rs7628250
rs1607264
rs73902879
rs7623802
rs62196769
rs34879831
rs554773
rs62116908
rs79876001
rs75857200
rs73754360
rs6461953
rs16969649
rs12348046
rs11246891
rs118047030
rs61492644
All − MR Egger
All − IVW(MRE)
−0.010
−0.005
0.000
0.005
0.010
MR effect size for
'Interleukin−1 Receptor accessory protein' on 'eGFR'

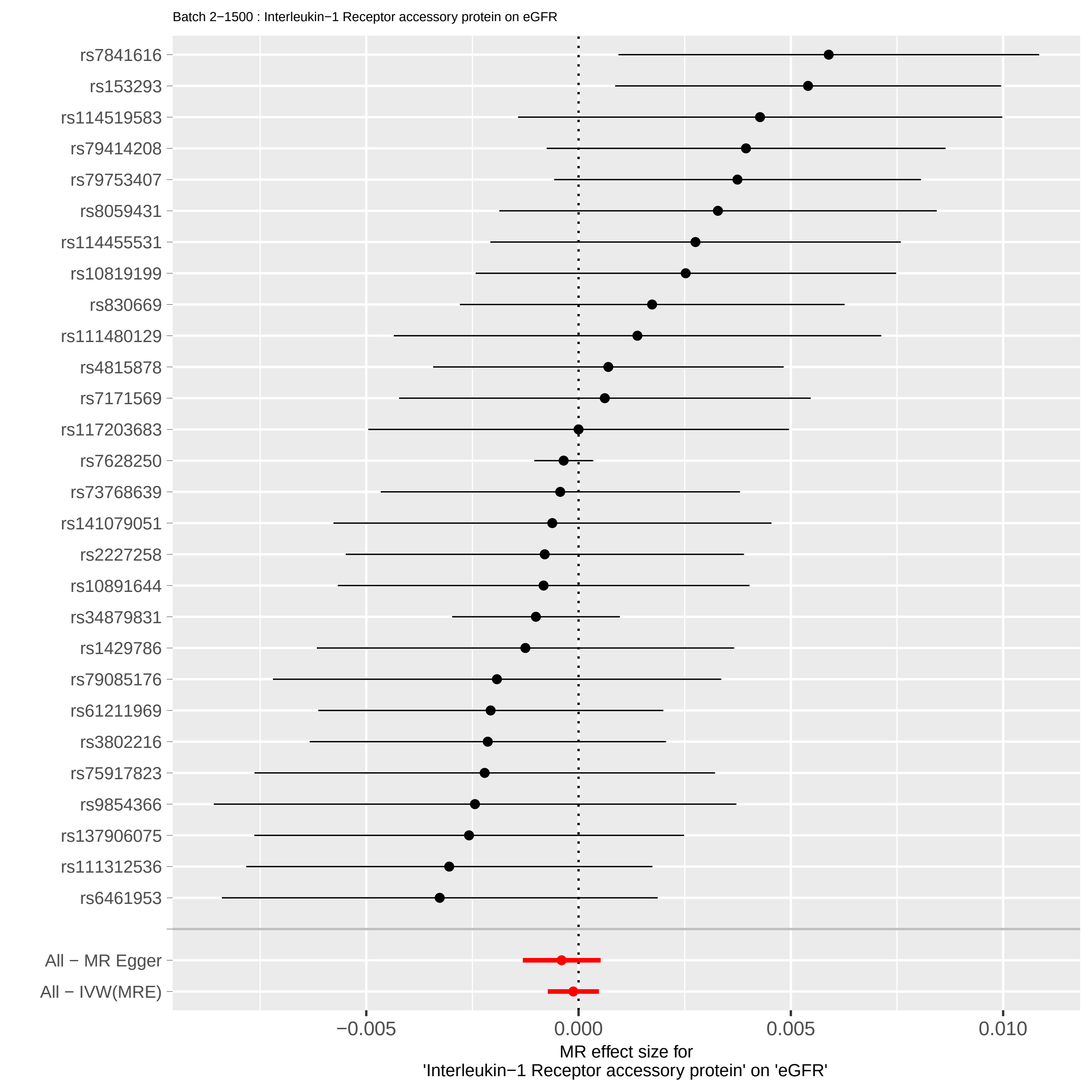

Batch 2−1500 : Interleukin−1 Receptor accessory protein on eGFR
rs7841616
rs153293
rs114519583
rs79414208
rs79753407
rs8059431
rs114455531
rs10819199
rs830669
rs111480129
rs4815878
rs7171569
rs117203683
rs7628250
rs73768639
rs141079051
rs2227258
rs10891644
rs34879831
rs1429786
rs79085176
rs61211969
rs3802216
rs75917823
rs9854366
rs137906075
rs111312536
rs6461953
All − MR Egger
All − IVW(MRE)
−0.005
0.000
0.005
0.010
MR effect size for
'Interleukin−1 Receptor accessory protein' on 'eGFR'

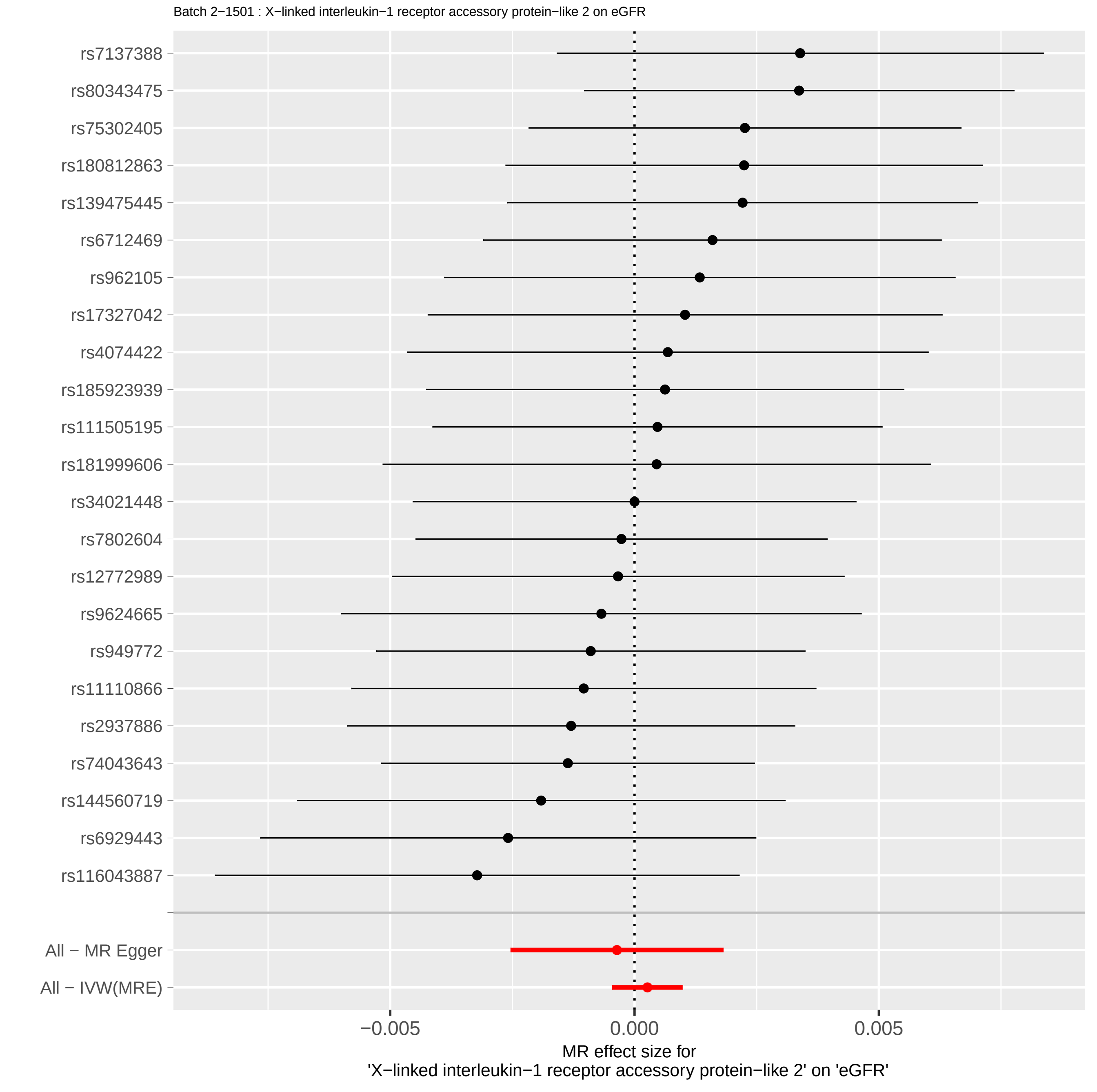

Batch 2−1501 : X−linked interleukin−1 receptor accessory protein−like 2 on eGFR
rs7137388
rs80343475
rs75302405
rs180812863
rs139475445
rs6712469
rs962105
rs17327042
rs4074422
rs185923939
rs111505195
rs181999606
rs34021448
rs7802604
rs12772989
rs9624665
rs949772
rs11110866
rs2937886
rs74043643
rs144560719
rs6929443
rs116043887
All − MR Egger
All − IVW(MRE)
−0.005
0.000
0.005
MR effect size for
'X−linked interleukin−1 receptor accessory protein−like 2' on 'eGFR'

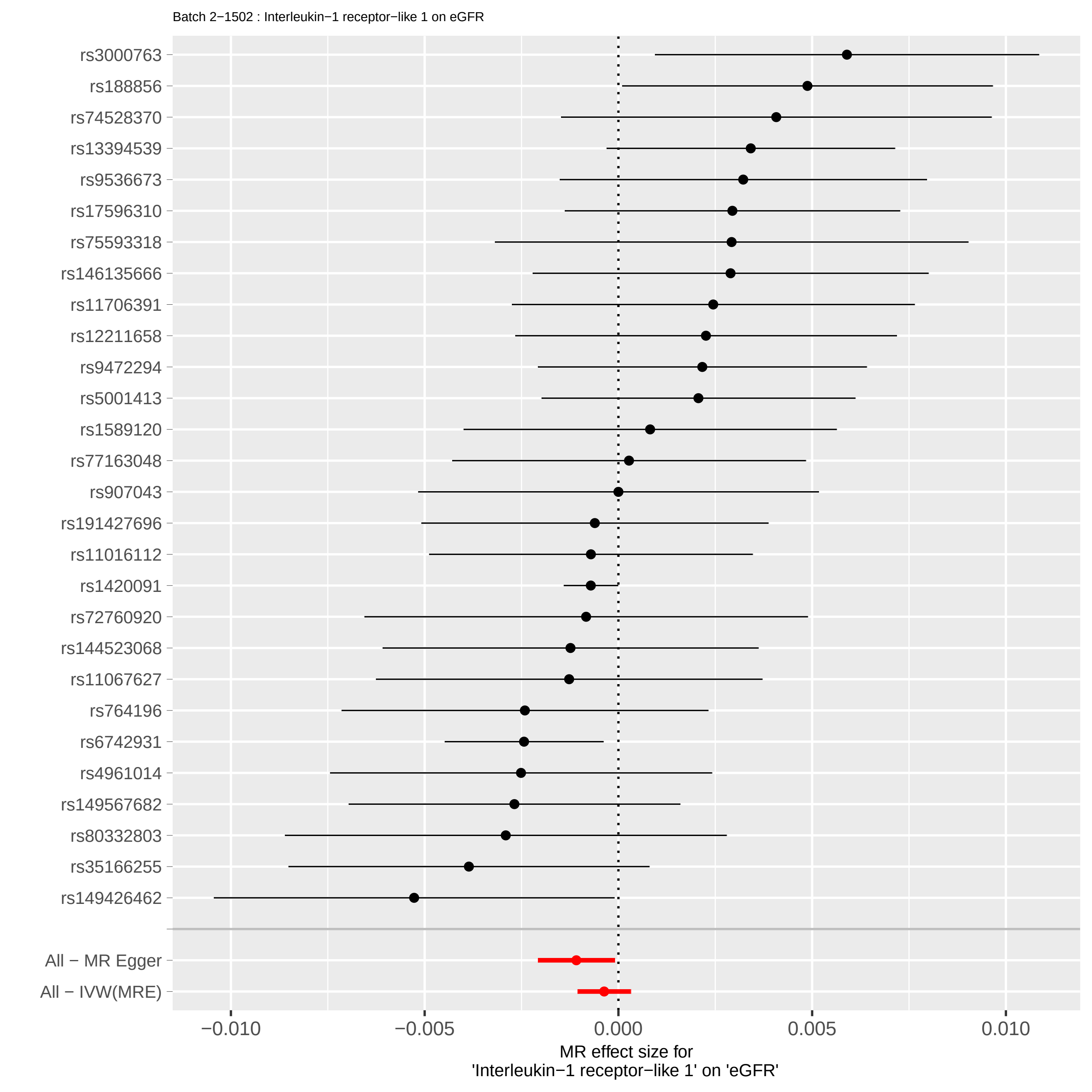
Batch 2−1502 : Interleukin−1 receptor−like 1 on eGFR
rs3000763
rs188856
rs74528370
rs13394539
rs9536673
rs17596310
rs75593318
rs146135666
rs11706391
rs12211658
rs9472294
rs5001413
rs1589120
rs77163048
rs907043
rs191427696
rs11016112
rs1420091
rs72760920
rs144523068
rs11067627
rs764196
rs6742931
rs4961014
rs149567682
rs80332803
rs35166255
rs149426462
All − MR Egger
All − IVW(MRE)
−0.010
−0.005
0.000
0.005
0.010
MR effect size for
'Interleukin−1 receptor−like 1' on 'eGFR'

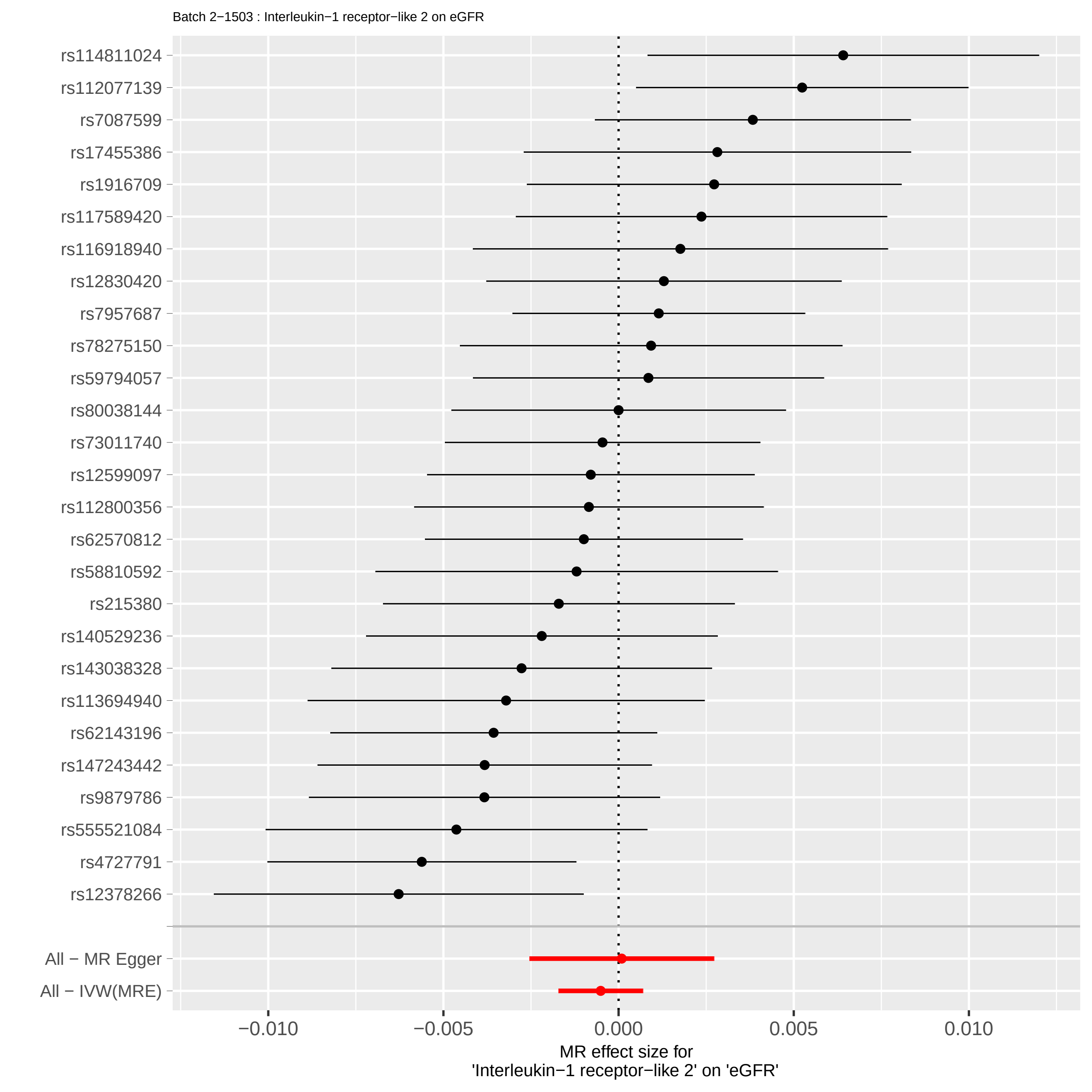

Batch 2−1503 : Interleukin−1 receptor−like 2 on eGFR
rs114811024
rs112077139
rs7087599
rs17455386
rs1916709
rs117589420
rs116918940
rs12830420
rs7957687
rs78275150
rs59794057
rs80038144
rs73011740
rs12599097
rs112800356
rs62570812
rs58810592
rs215380
rs140529236
rs143038328
rs113694940
rs62143196
rs147243442
rs9879786
rs555521084
rs4727791
rs12378266
All − MR Egger
All − IVW(MRE)
−0.010
−0.005
0.000
0.005
0.010
MR effect size for
'Interleukin−1 receptor−like 2' on 'eGFR'

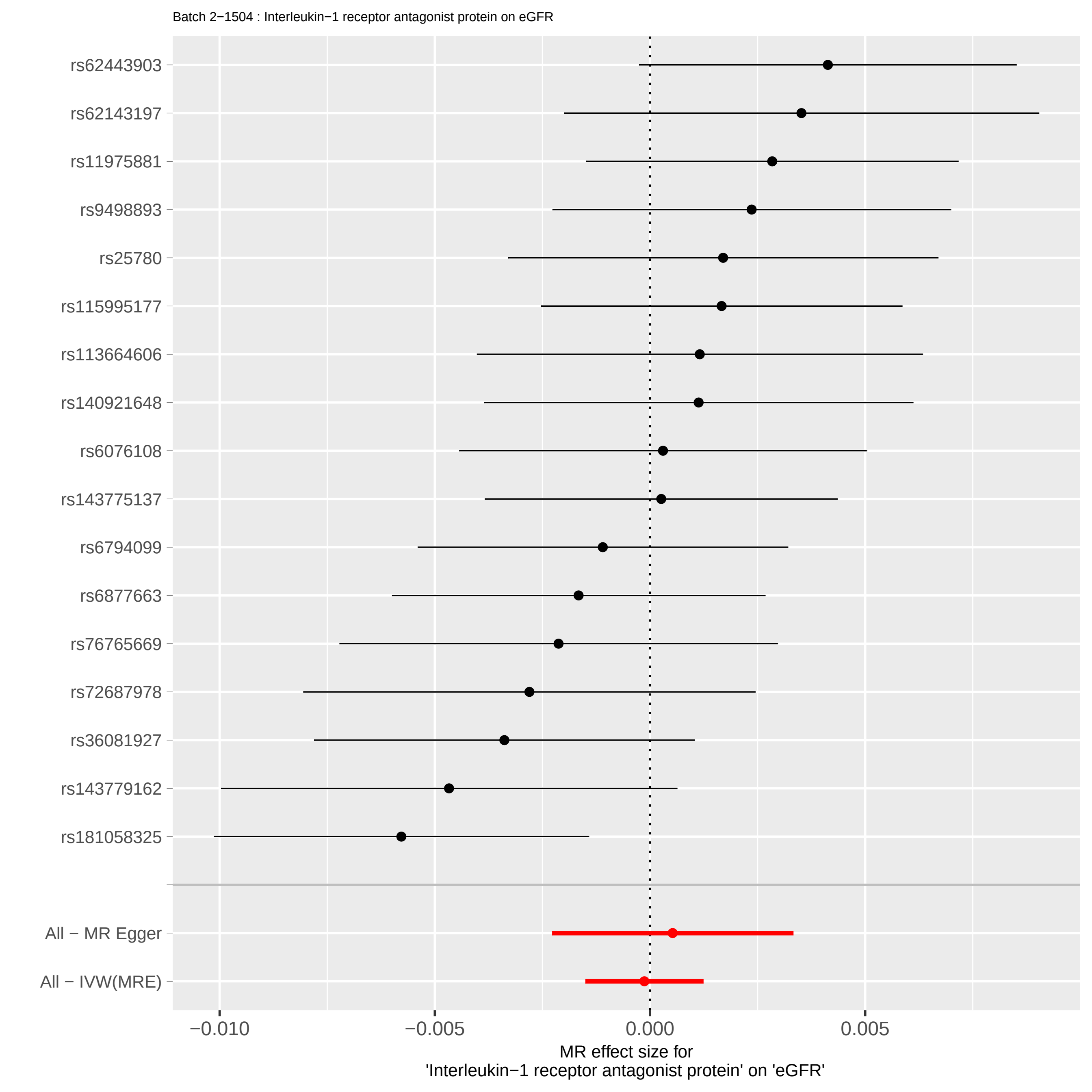
Batch 2−1504 : Interleukin−1 receptor antagonist protein on eGFR
rs62443903
rs62143197
rs11975881
rs9498893
rs25780
rs115995177
rs113664606
rs140921648
rs6076108
rs143775137
rs6794099
rs6877663
rs76765669
rs72687978
rs36081927
rs143779162
rs181058325
All − MR Egger
All − IVW(MRE)
−0.010
−0.005
0.000
0.005
MR effect size for
'Interleukin−1 receptor antagonist protein' on 'eGFR'

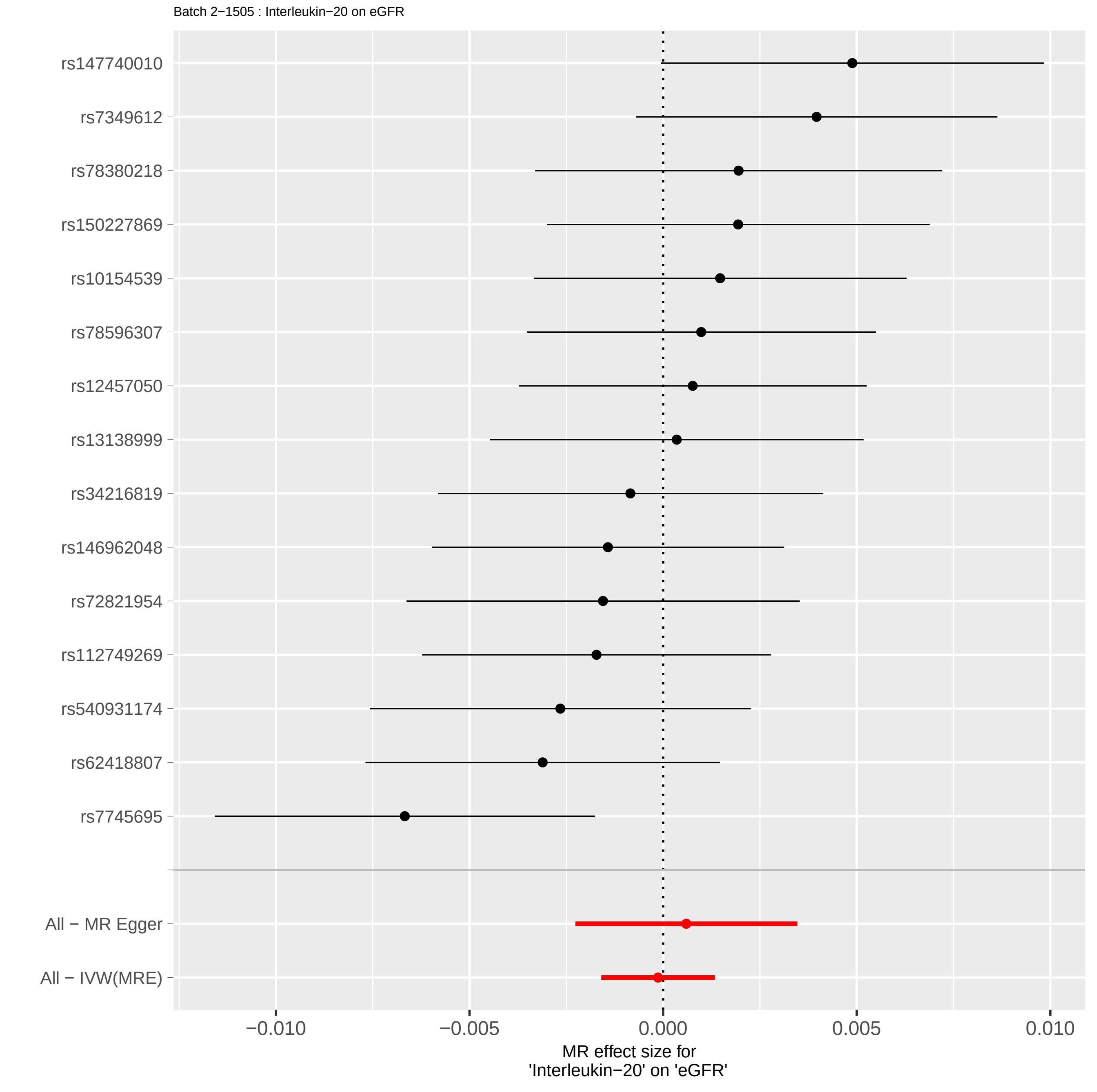

Batch 2−1505 : Interleukin−20 on eGFR
rs147740010
rs7349612
rs78380218
rs150227869
rs10154539
rs78596307
rs12457050
rs13138999
rs34216819
rs146962048
rs72821954
rs112749269
rs540931174
rs62418807
rs7745695
All − MR Egger
All − IVW(MRE)
−0.010
−0.005
0.000
0.005
0.010
MR effect size for
'Interleukin−20' on 'eGFR'

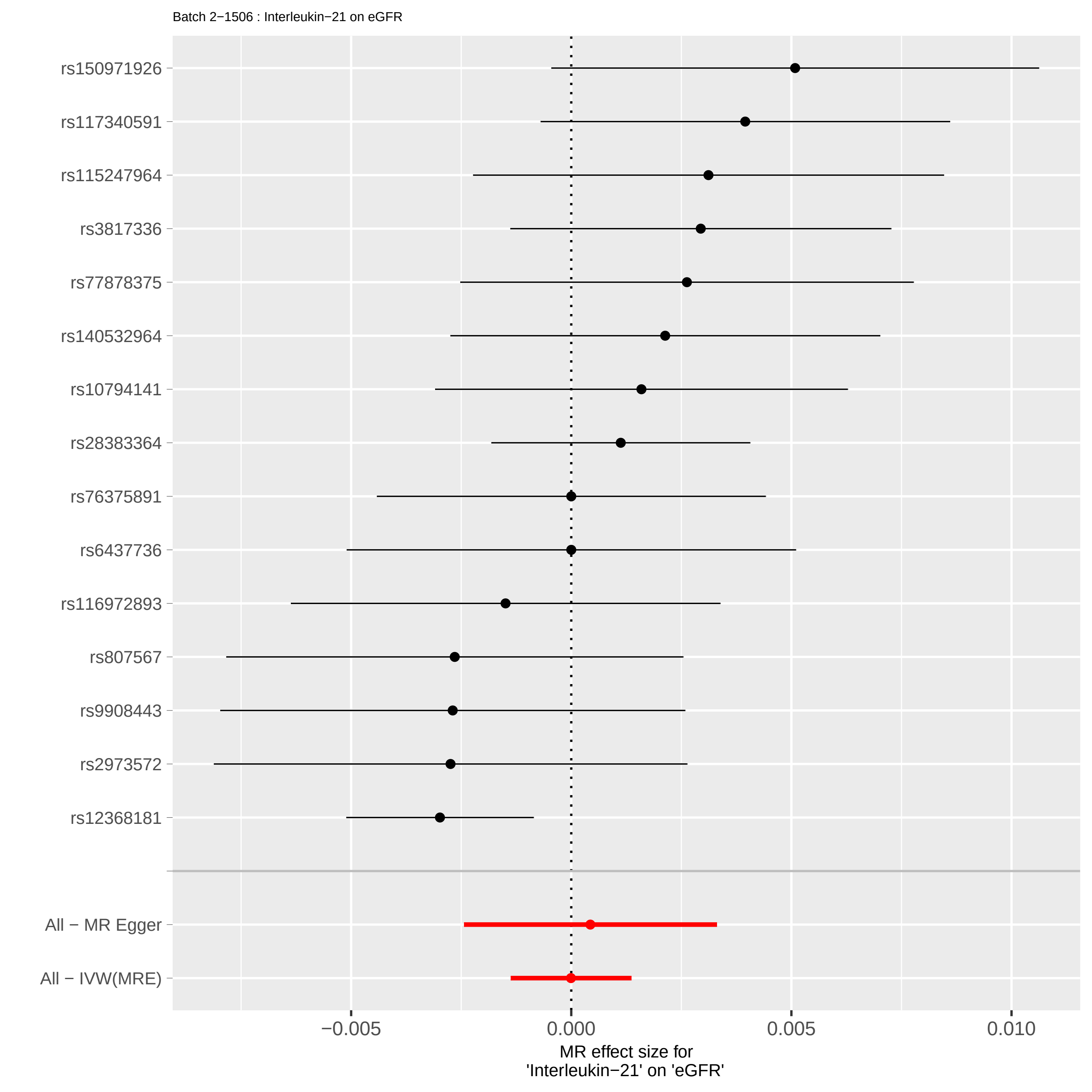
Batch 2−1506 : Interleukin−21 on eGFR
rs150971926
rs117340591
rs115247964
rs3817336
rs77878375
rs140532964
rs10794141
rs28383364
rs76375891
rs6437736
rs116972893
rs807567
rs9908443
rs2973572
rs12368181
All − MR Egger
All − IVW(MRE)
−0.005
0.000
0.005
0.010
MR effect size for
'Interleukin−21' on 'eGFR'

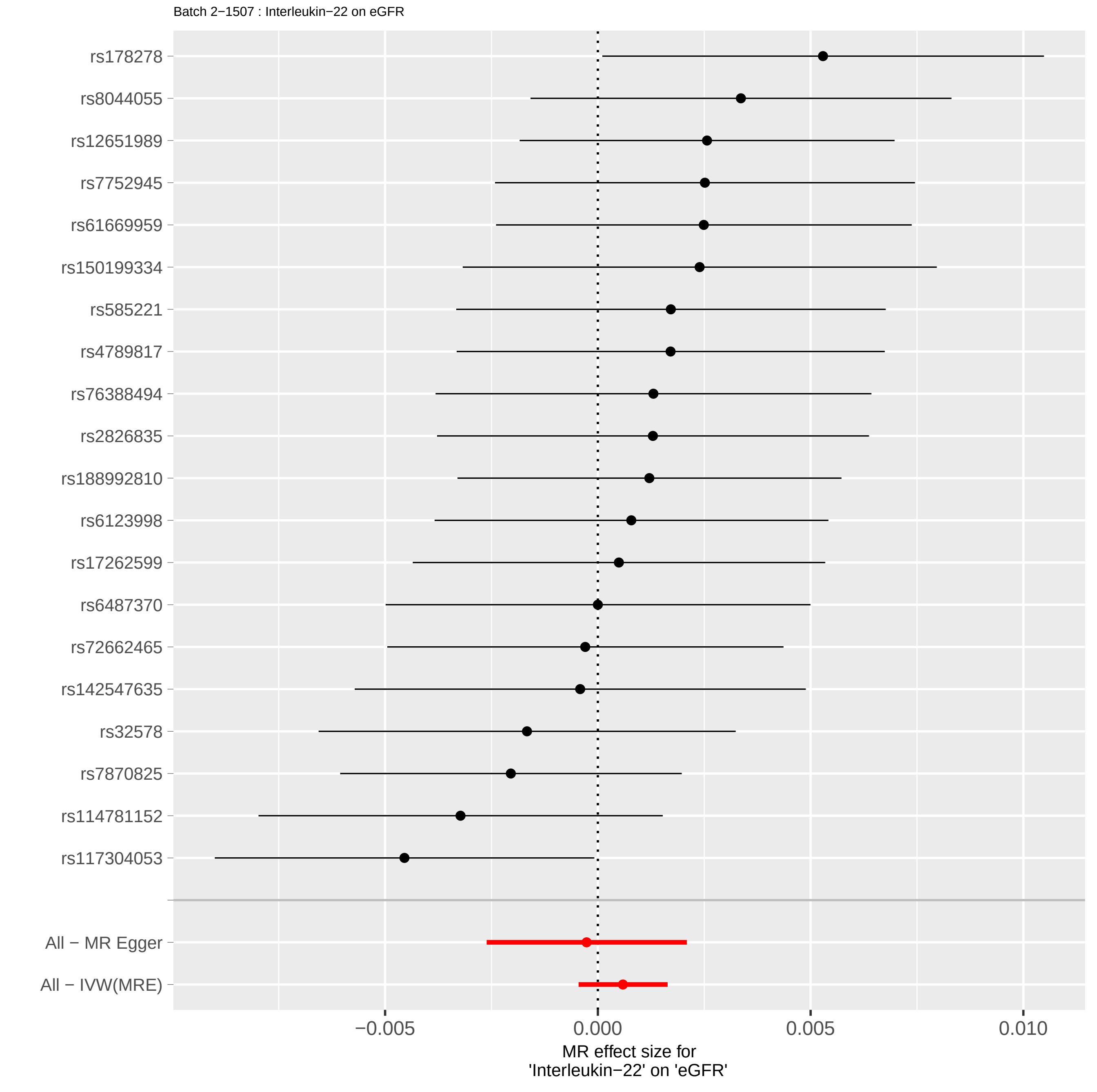
Batch 2−1507 : Interleukin−22 on eGFR
rs178278
rs8044055
rs12651989
rs7752945
rs61669959
rs150199334
rs585221
rs4789817
rs76388494
rs2826835
rs188992810
rs6123998
rs17262599
rs6487370
rs72662465
rs142547635
rs32578
rs7870825
rs114781152
rs117304053
All − MR Egger
All − IVW(MRE)
−0.005
0.000
0.005
0.010
MR effect size for
'Interleukin−22' on 'eGFR'

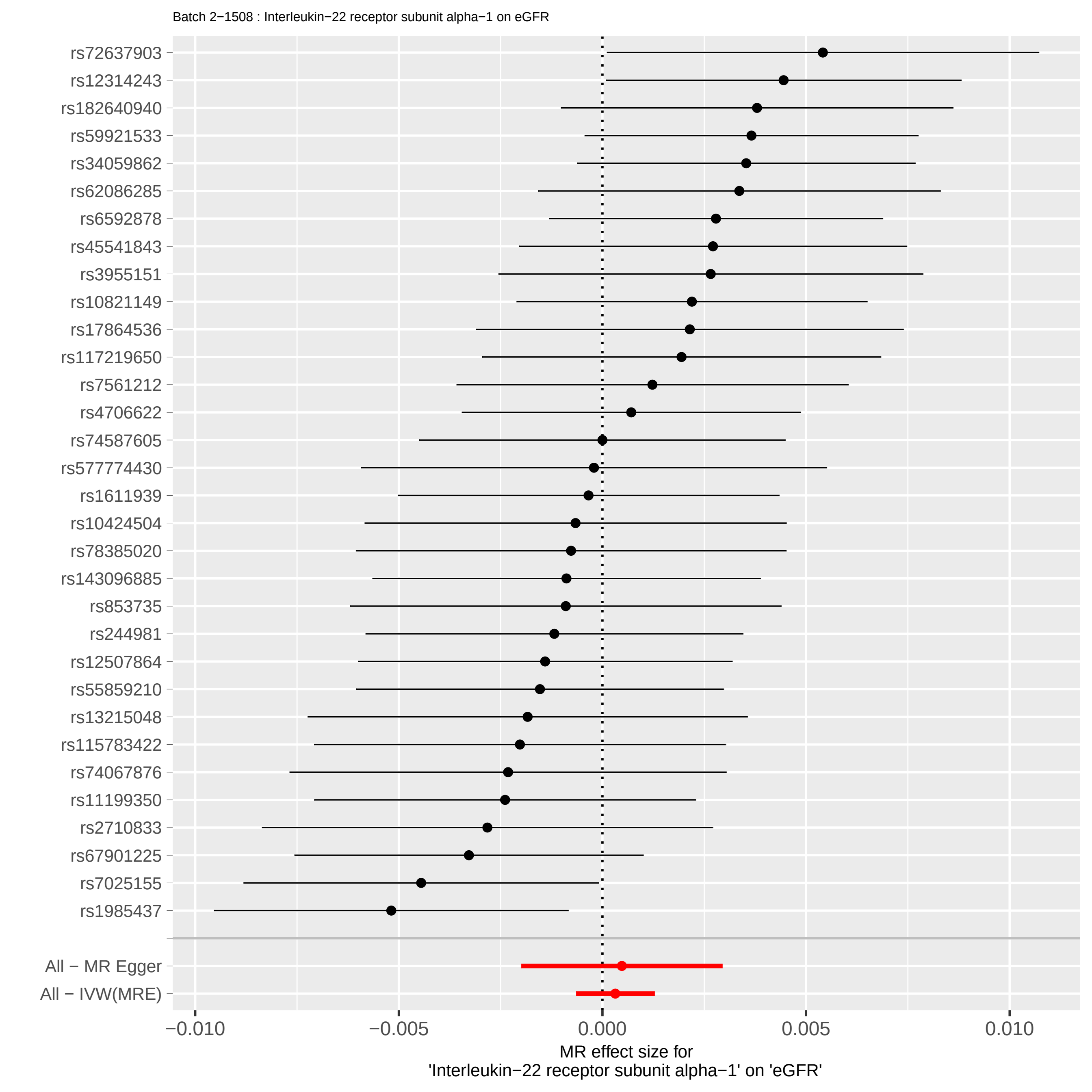
Batch 2−1508 : Interleukin−22 receptor subunit alpha−1 on eGFR
rs72637903
rs12314243
rs182640940
rs59921533
rs34059862
rs62086285
rs6592878
rs45541843
rs3955151
rs10821149
rs17864536
rs117219650
rs7561212
rs4706622
rs74587605
rs577774430
rs1611939
rs10424504
rs78385020
rs143096885
rs853735
rs244981
rs12507864
rs55859210
rs13215048
rs115783422
rs74067876
rs11199350
rs2710833
rs67901225
rs7025155
rs1985437
All − MR Egger
All − IVW(MRE)
−0.010
−0.005
0.000
0.005
0.010
MR effect size for
'Interleukin−22 receptor subunit alpha−1' on 'eGFR'

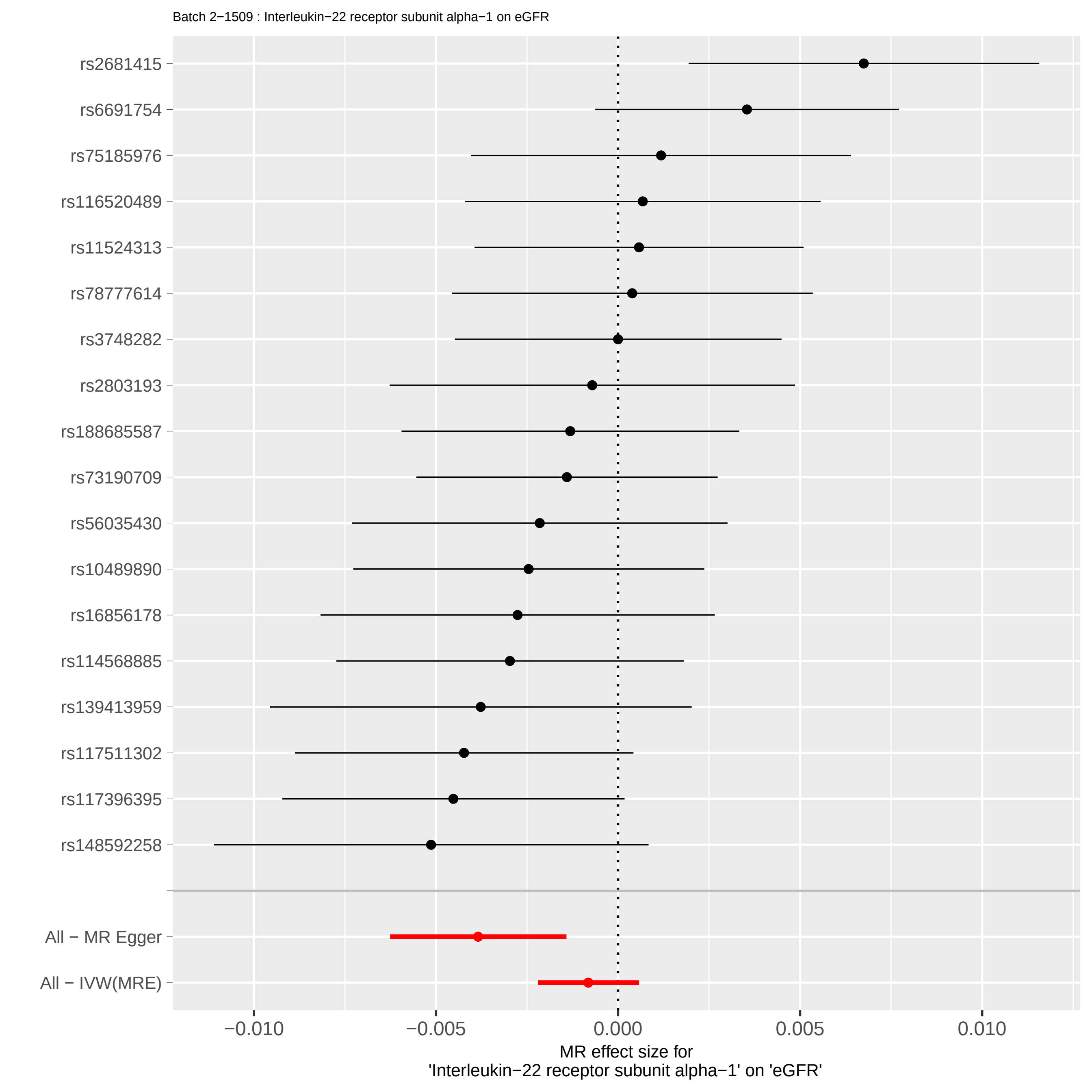
Batch 2−1509 : Interleukin−22 receptor subunit alpha−1 on eGFR
rs2681415
rs6691754
rs75185976
rs116520489
rs11524313
rs78777614
rs3748282
rs2803193
rs188685587
rs73190709
rs56035430
rs10489890
rs16856178
rs114568885
rs139413959
rs117511302
rs117396395
rs148592258
All − MR Egger
All − IVW(MRE)
−0.010
−0.005
0.000
0.005
0.010
MR effect size for
'Interleukin−22 receptor subunit alpha−1' on 'eGFR'

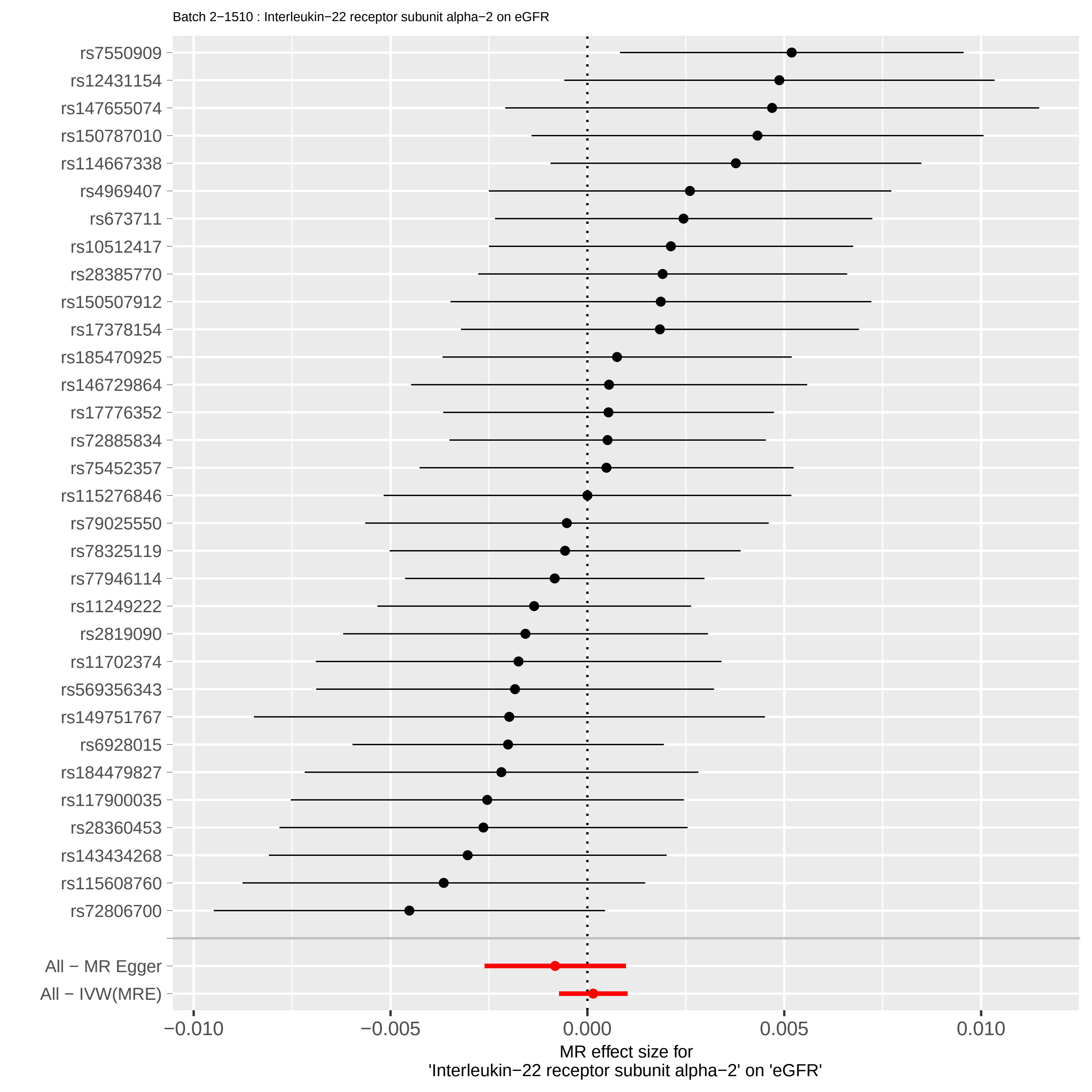
Batch 2−1510 : Interleukin−22 receptor subunit alpha−2 on eGFR
rs7550909
rs12431154
rs147655074
rs150787010
rs114667338
rs4969407
rs673711
rs10512417
rs28385770
rs150507912
rs17378154
rs185470925
rs146729864
rs17776352
rs72885834
rs75452357
rs115276846
rs79025550
rs78325119
rs77946114
rs11249222
rs2819090
rs11702374
rs569356343
rs149751767
rs6928015
rs184479827
rs117900035
rs28360453
rs143434268
rs115608760
rs72806700
All − MR Egger
All − IVW(MRE)
−0.010
−0.005
0.000
0.005
0.010
MR effect size for
'Interleukin−22 receptor subunit alpha−2' on 'eGFR'

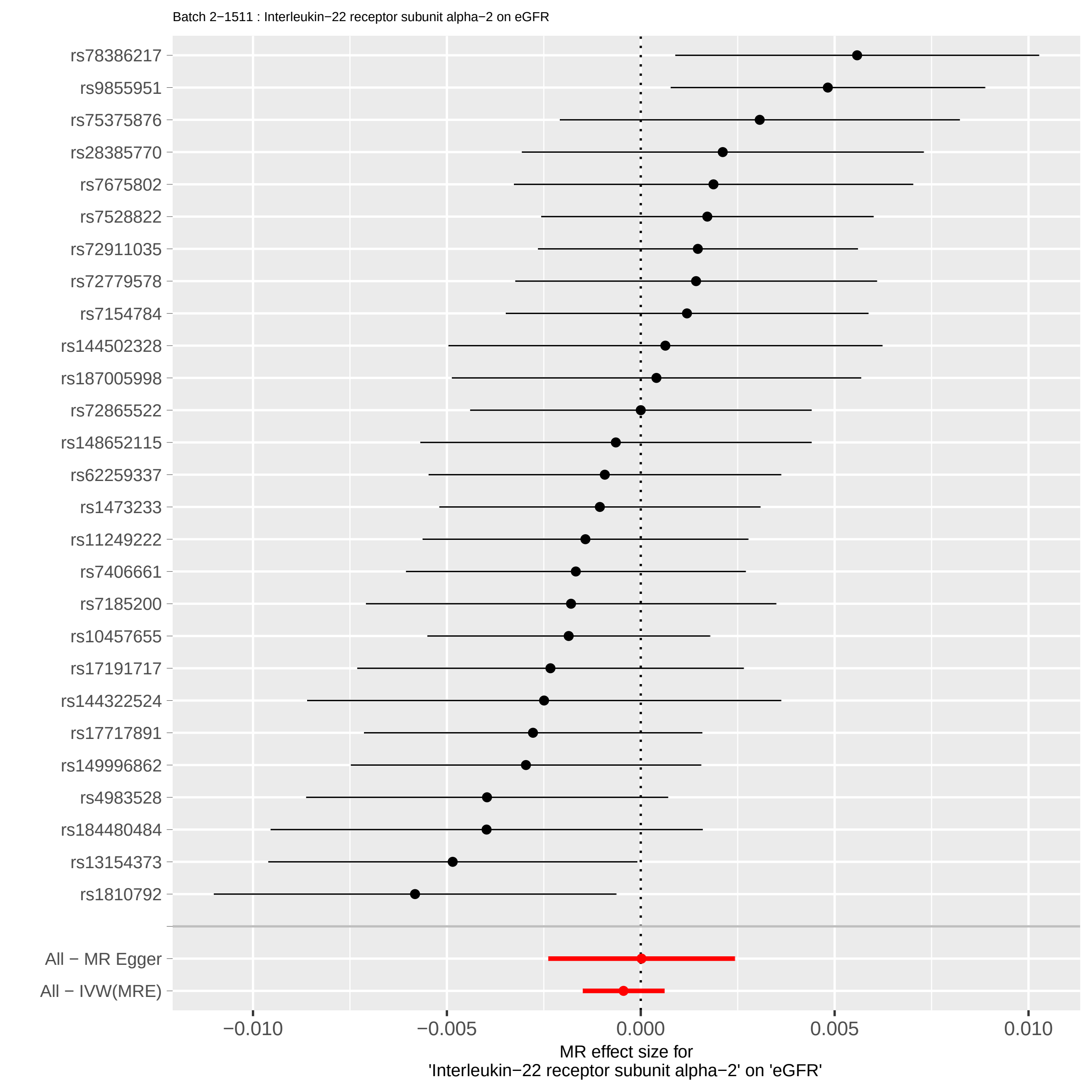
Batch 2−1511 : Interleukin−22 receptor subunit alpha−2 on eGFR
rs78386217
rs9855951
rs75375876
rs28385770
rs7675802
rs7528822
rs72911035
rs72779578
rs7154784
rs144502328
rs187005998
rs72865522
rs148652115
rs62259337
rs1473233
rs11249222
rs7406661
rs7185200
rs10457655
rs17191717
rs144322524
rs17717891
rs149996862
rs4983528
rs184480484
rs13154373
rs1810792
All − MR Egger
All − IVW(MRE)
−0.010
−0.005
0.000
0.005
0.010
MR effect size for
'Interleukin−22 receptor subunit alpha−2' on 'eGFR'

Batch 2−1512 : Interleukin−2 on eGFR

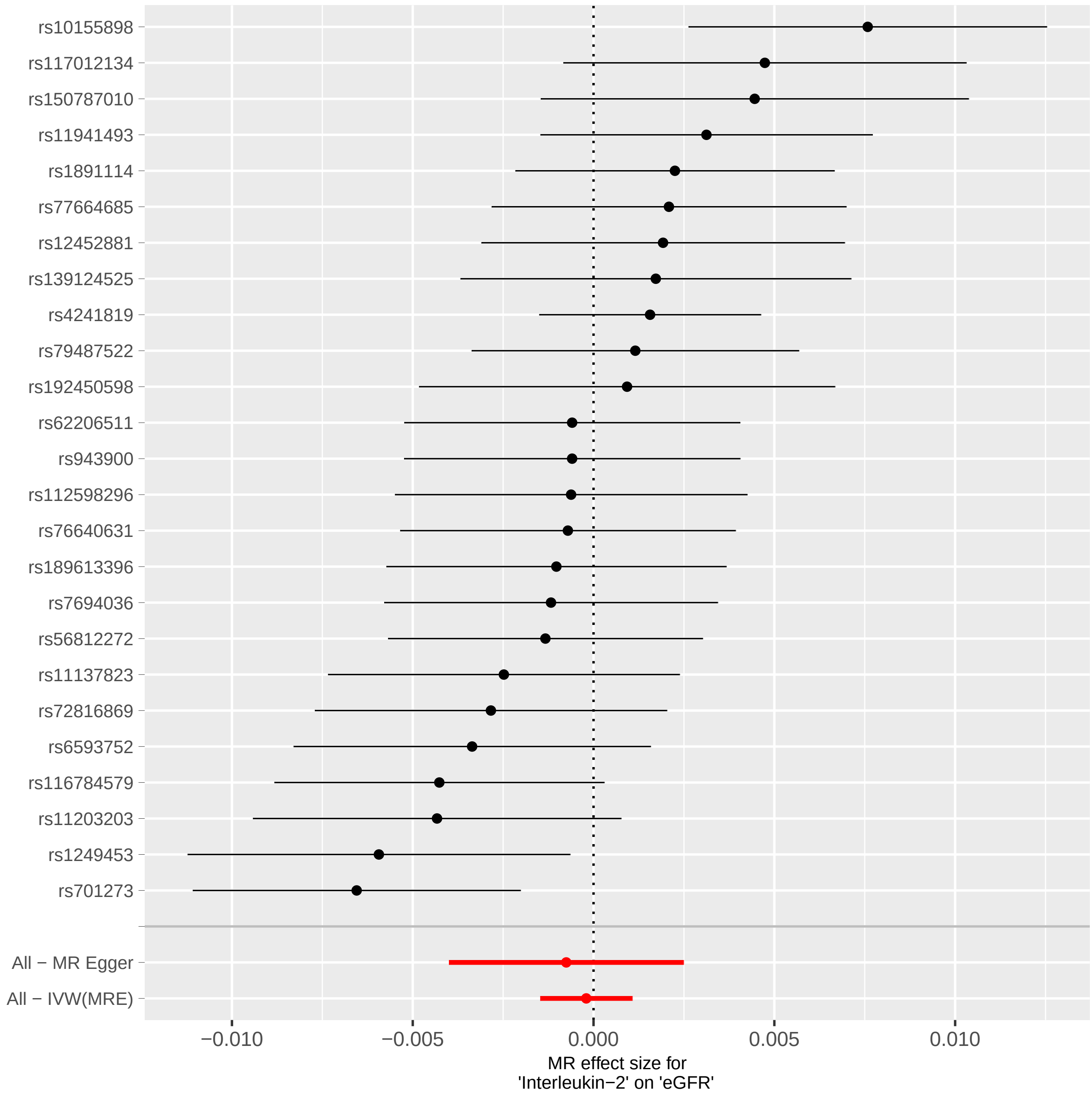

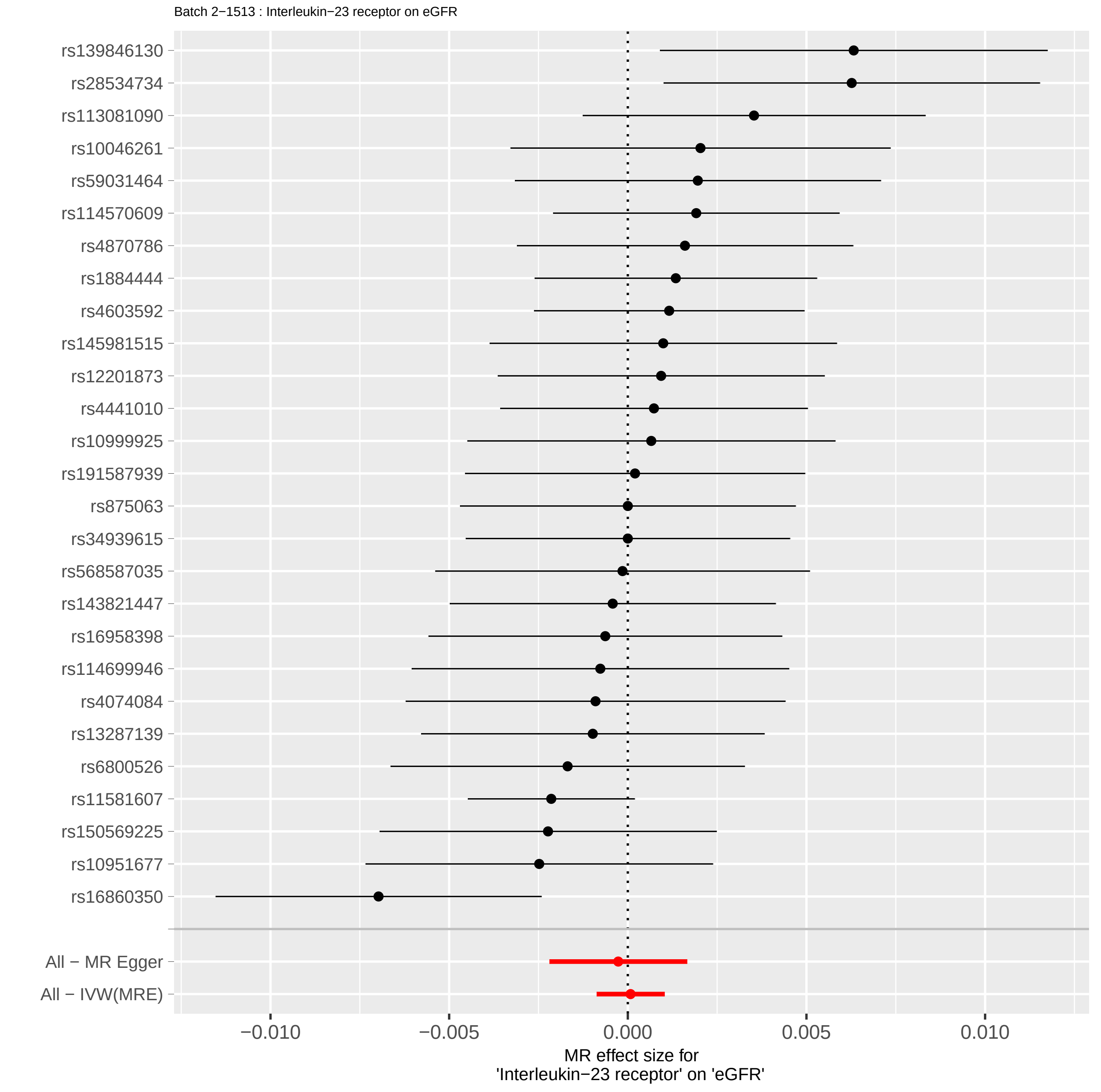
Batch 2−1513 : Interleukin−23 receptor on eGFR
rs139846130
rs28534734
rs113081090
rs10046261
rs59031464
rs114570609
rs4870786
rs1884444
rs4603592
rs145981515
rs12201873
rs4441010
rs10999925
rs191587939
rs875063
rs34939615
rs568587035
rs143821447
rs16958398
rs114699946
rs4074084
rs13287139
rs6800526
rs11581607
rs150569225
rs10951677
rs16860350
All − MR Egger
All − IVW(MRE)
−0.010
−0.005
0.000
0.005
0.010
MR effect size for
'Interleukin−23 receptor' on 'eGFR'

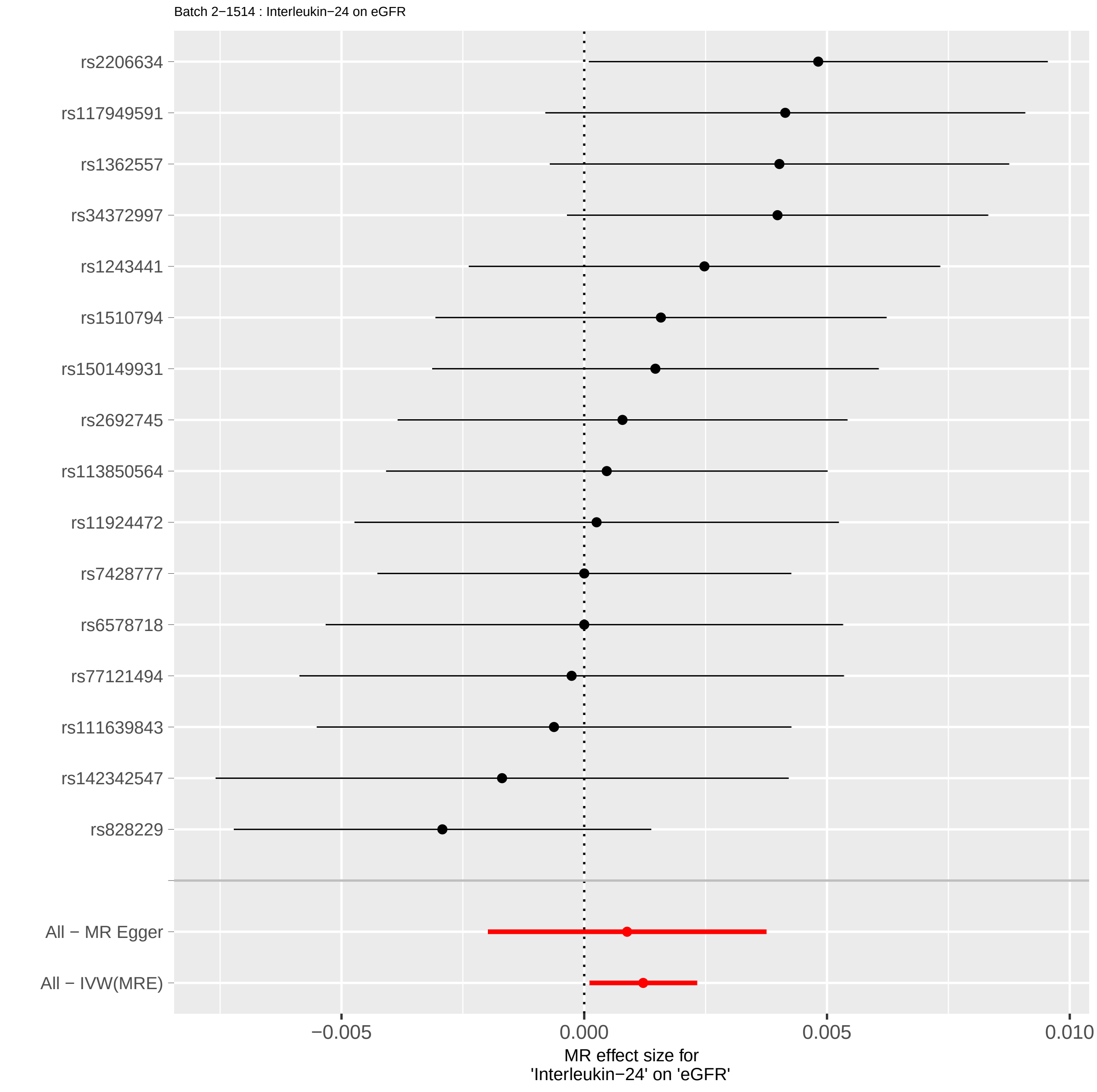

Batch 2−1514 : Interleukin−24 on eGFR
rs2206634
rs117949591
rs1362557
rs34372997
rs1243441
rs1510794
rs150149931
rs2692745
rs113850564
rs11924472
rs7428777
rs6578718
rs77121494
rs111639843
rs142342547
rs828229
All − MR Egger
All − IVW(MRE)
−0.005
0.000
0.005
0.010
MR effect size for
'Interleukin−24' on 'eGFR'

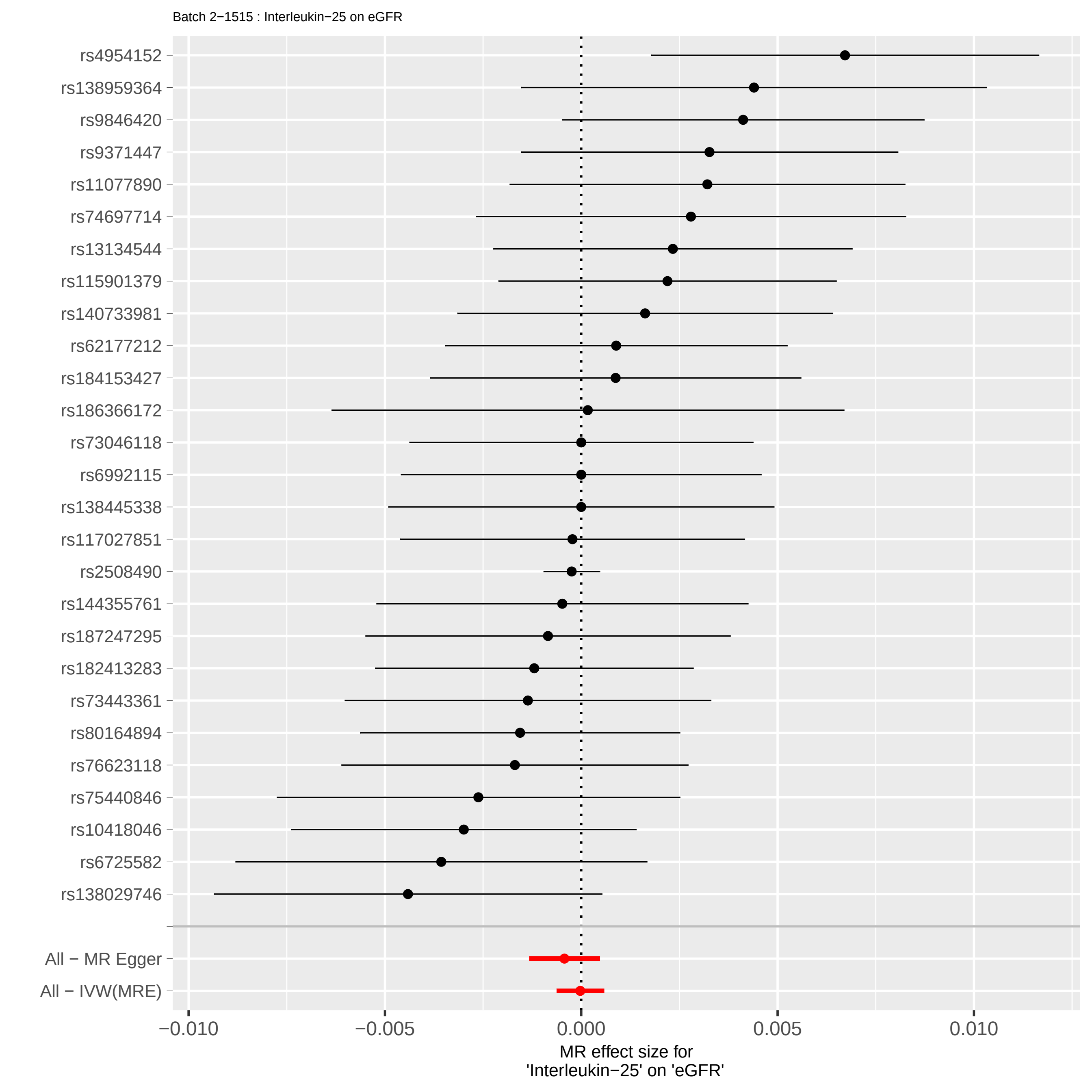

Batch 2−1515 : Interleukin−25 on eGFR
rs4954152
rs138959364
rs9846420
rs9371447
rs11077890
rs74697714
rs13134544
rs115901379
rs140733981
rs62177212
rs184153427
rs186366172
rs73046118
rs6992115
rs138445338
rs117027851
rs2508490
rs144355761
rs187247295
rs182413283
rs73443361
rs80164894
rs76623118
rs75440846
rs10418046
rs6725582
rs138029746
All − MR Egger
All − IVW(MRE)
−0.010
−0.005
0.000
0.005
0.010
MR effect size for
'Interleukin−25' on 'eGFR'

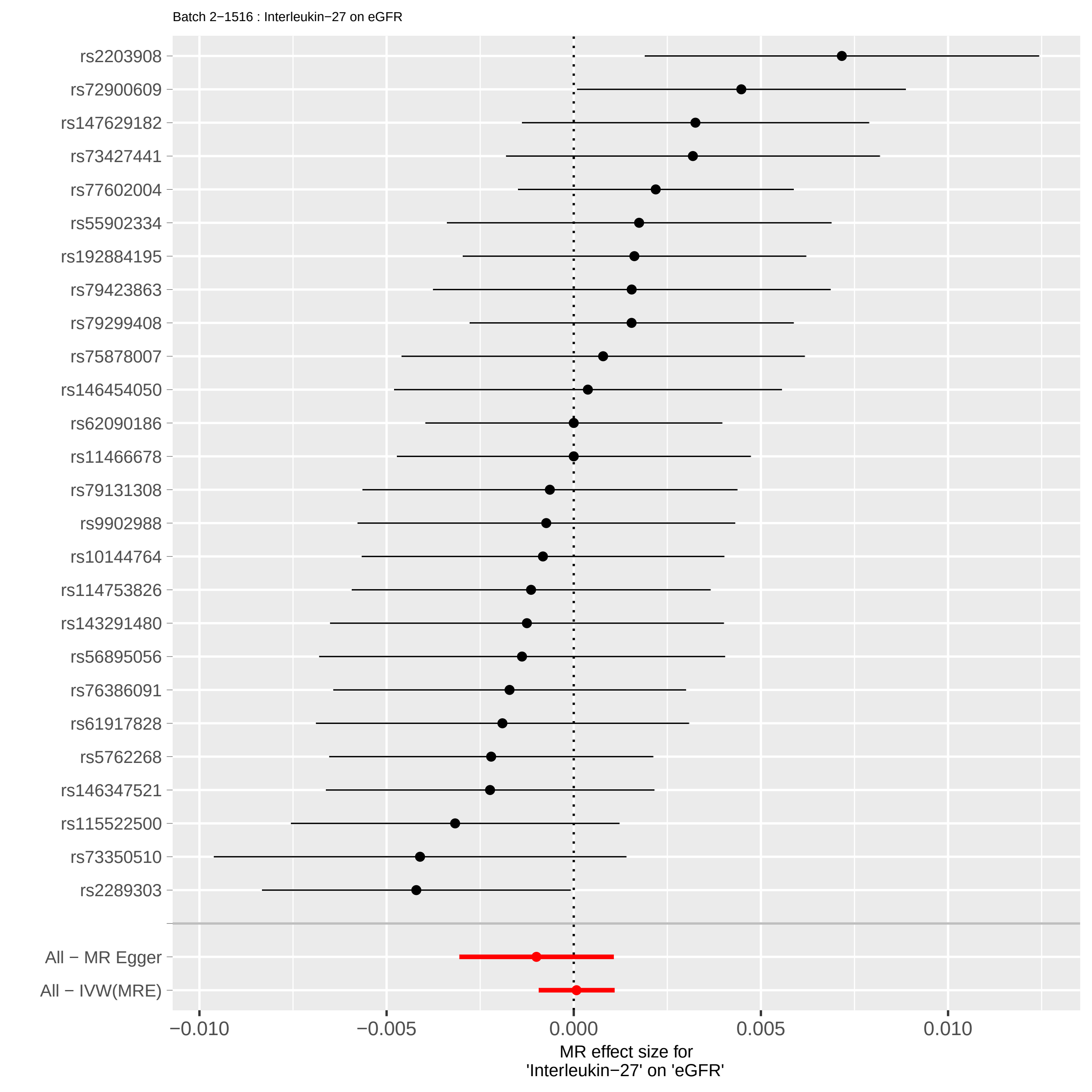

Batch 2−1516 : Interleukin−27 on eGFR
rs2203908
rs72900609
rs147629182
rs73427441
rs77602004
rs55902334
rs192884195
rs79423863
rs79299408
rs75878007
rs146454050
rs62090186
rs11466678
rs79131308
rs9902988
rs10144764
rs114753826
rs143291480
rs56895056
rs76386091
rs61917828
rs5762268
rs146347521
rs115522500
rs73350510
rs2289303
All − MR Egger
All − IVW(MRE)
−0.010
−0.005
0.000
0.005
0.010
MR effect size for
'Interleukin−27' on 'eGFR'

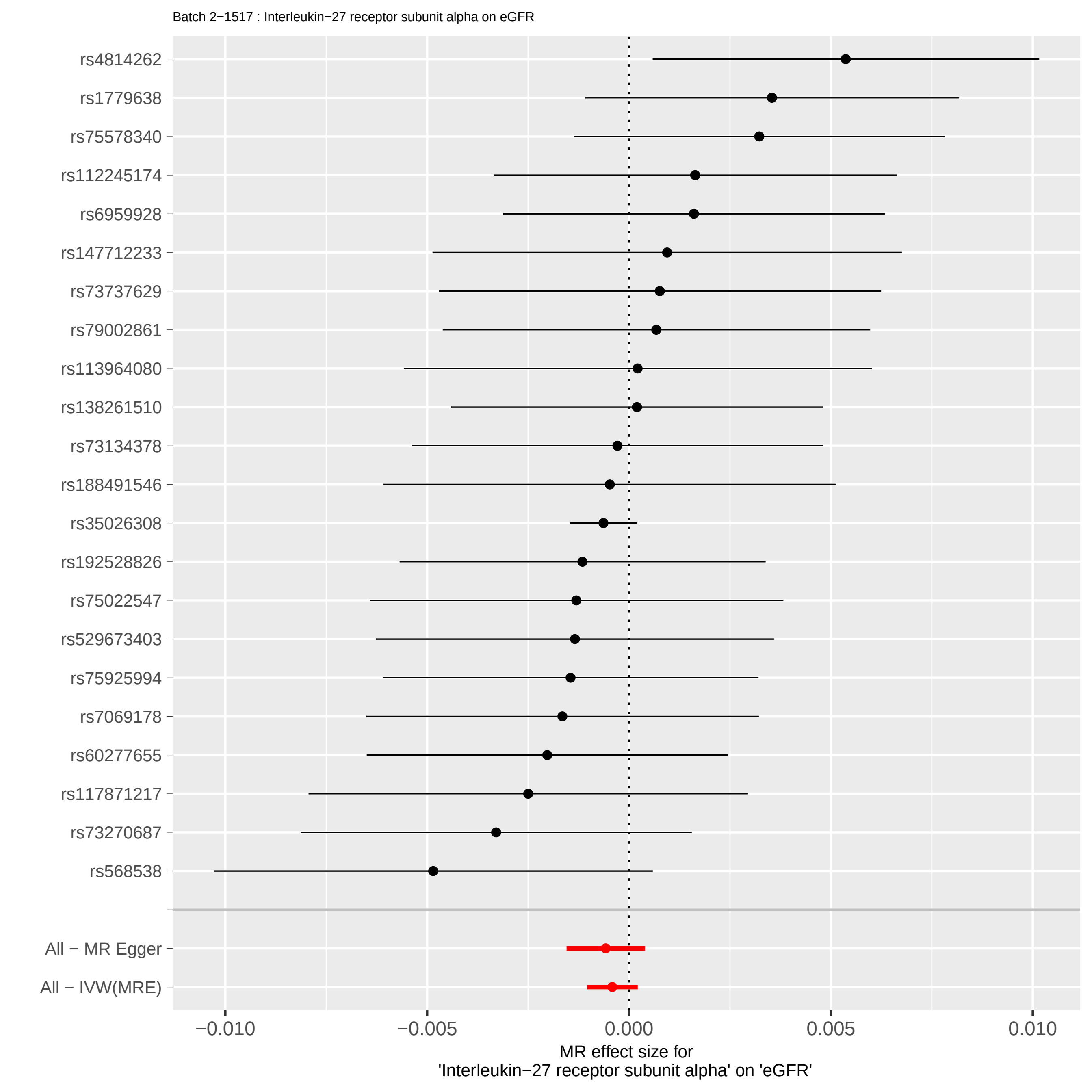

Batch 2−1517 : Interleukin−27 receptor subunit alpha on eGFR
rs4814262
rs1779638
rs75578340
rs112245174
rs6959928
rs147712233
rs73737629
rs79002861
rs113964080
rs138261510
rs73134378
rs188491546
rs35026308
rs192528826
rs75022547
rs529673403
rs75925994
rs7069178
rs60277655
rs117871217
rs73270687
rs568538
All − MR Egger
All − IVW(MRE)
−0.010
−0.005
0.000
0.005
0.010
MR effect size for
'Interleukin−27 receptor subunit alpha' on 'eGFR'

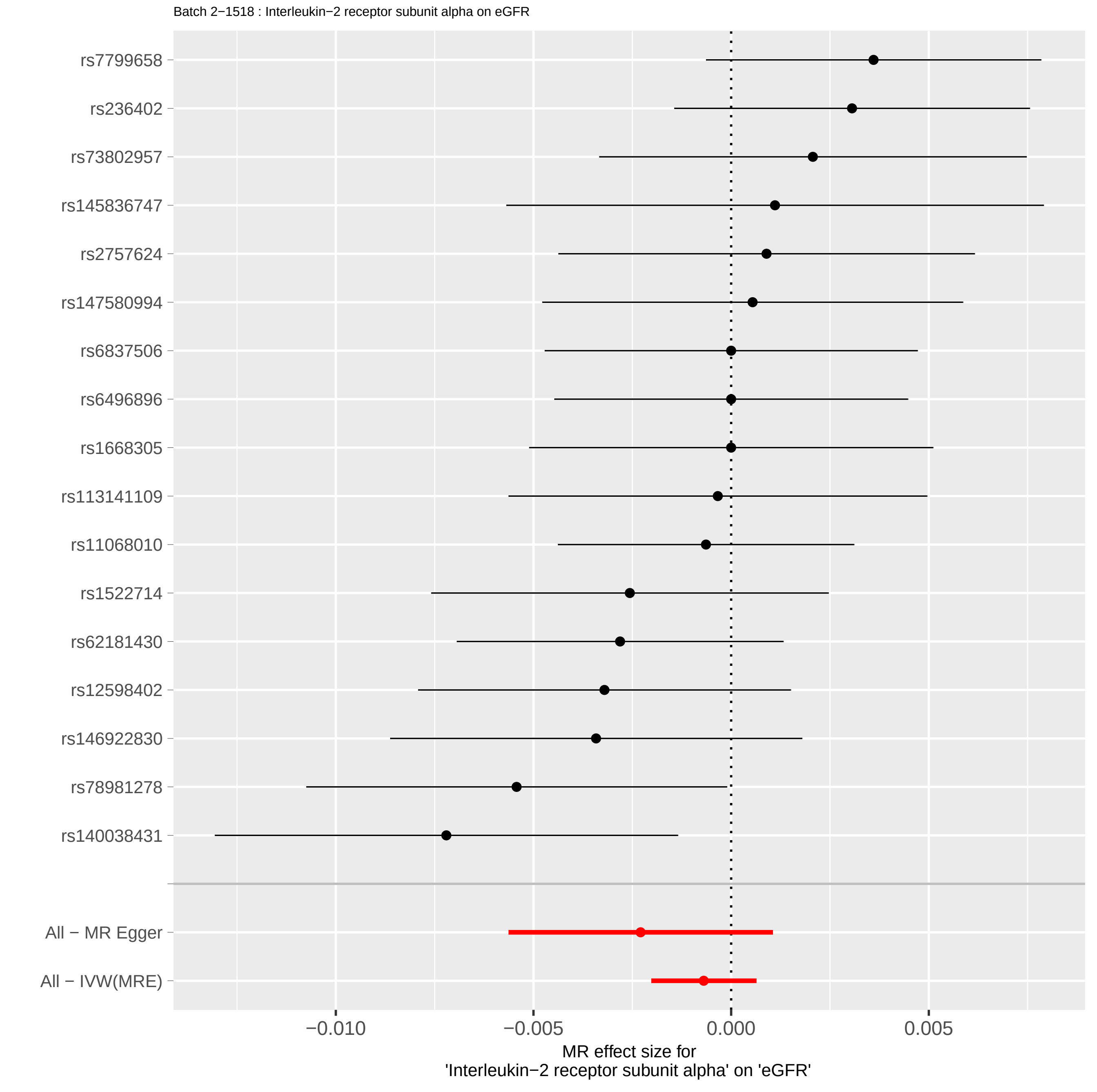

Batch 2−1518 : Interleukin−2 receptor subunit alpha on eGFR
rs7799658
rs236402
rs73802957
rs145836747
rs2757624
rs147580994
rs6837506
rs6496896
rs1668305
rs113141109
rs11068010
rs1522714
rs62181430
rs12598402
rs146922830
rs78981278
rs140038431
All − MR Egger
All − IVW(MRE)
−0.010
−0.005
0.000
0.005
MR effect size for
'Interleukin−2 receptor subunit alpha' on 'eGFR'

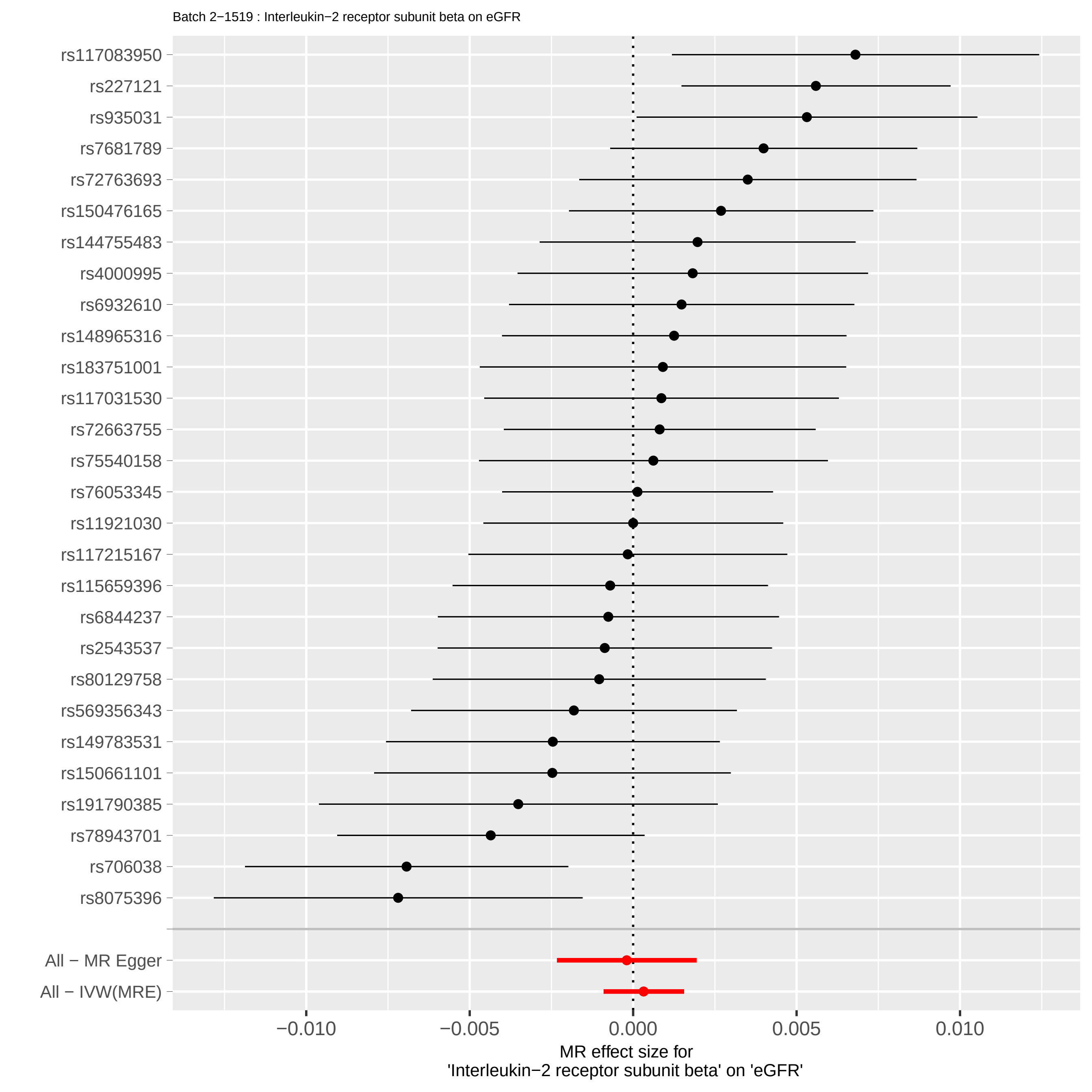

Batch 2−1519 : Interleukin−2 receptor subunit beta on eGFR
rs117083950
rs227121
rs935031
rs7681789
rs72763693
rs150476165
rs144755483
rs4000995
rs6932610
rs148965316
rs183751001
rs117031530
rs72663755
rs75540158
rs76053345
rs11921030
rs117215167
rs115659396
rs6844237
rs2543537
rs80129758
rs569356343
rs149783531
rs150661101
rs191790385
rs78943701
rs706038
rs8075396
All − MR Egger
All − IVW(MRE)
−0.010
−0.005
0.000
0.005
0.010
MR effect size for
'Interleukin−2 receptor subunit beta' on 'eGFR'

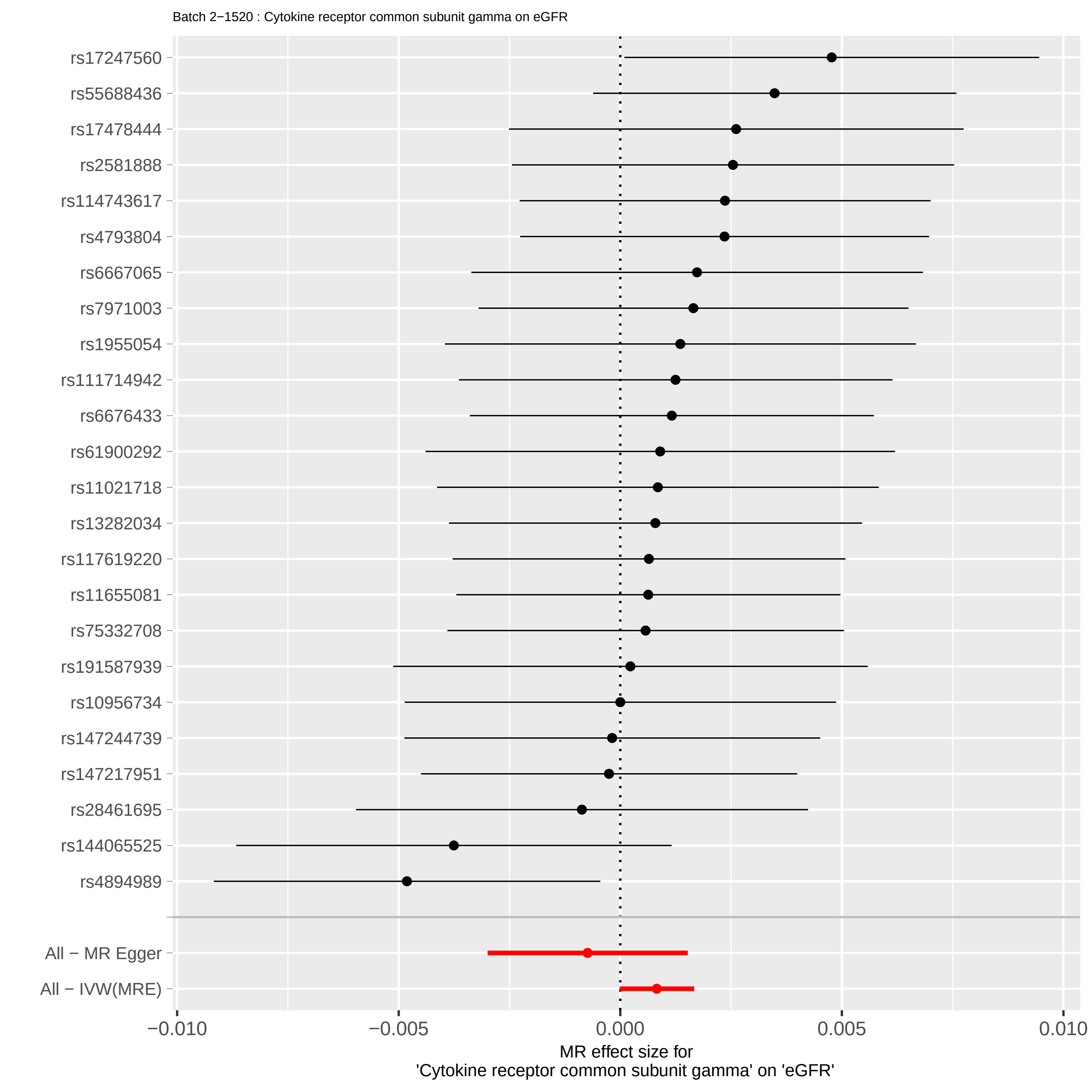

Batch 2−1520 : Cytokine receptor common subunit gamma on eGFR
rs17247560
rs55688436
rs17478444
rs2581888
rs114743617
rs4793804
rs6667065
rs7971003
rs1955054
rs111714942
rs6676433
rs61900292
rs11021718
rs13282034
rs117619220
rs11655081
rs75332708
rs191587939
rs10956734
rs147244739
rs147217951
rs28461695
rs144065525
rs4894989
All − MR Egger
All − IVW(MRE)
−0.010
−0.005
0.000
0.005
0.010
MR effect size for
'Cytokine receptor common subunit gamma' on 'eGFR'

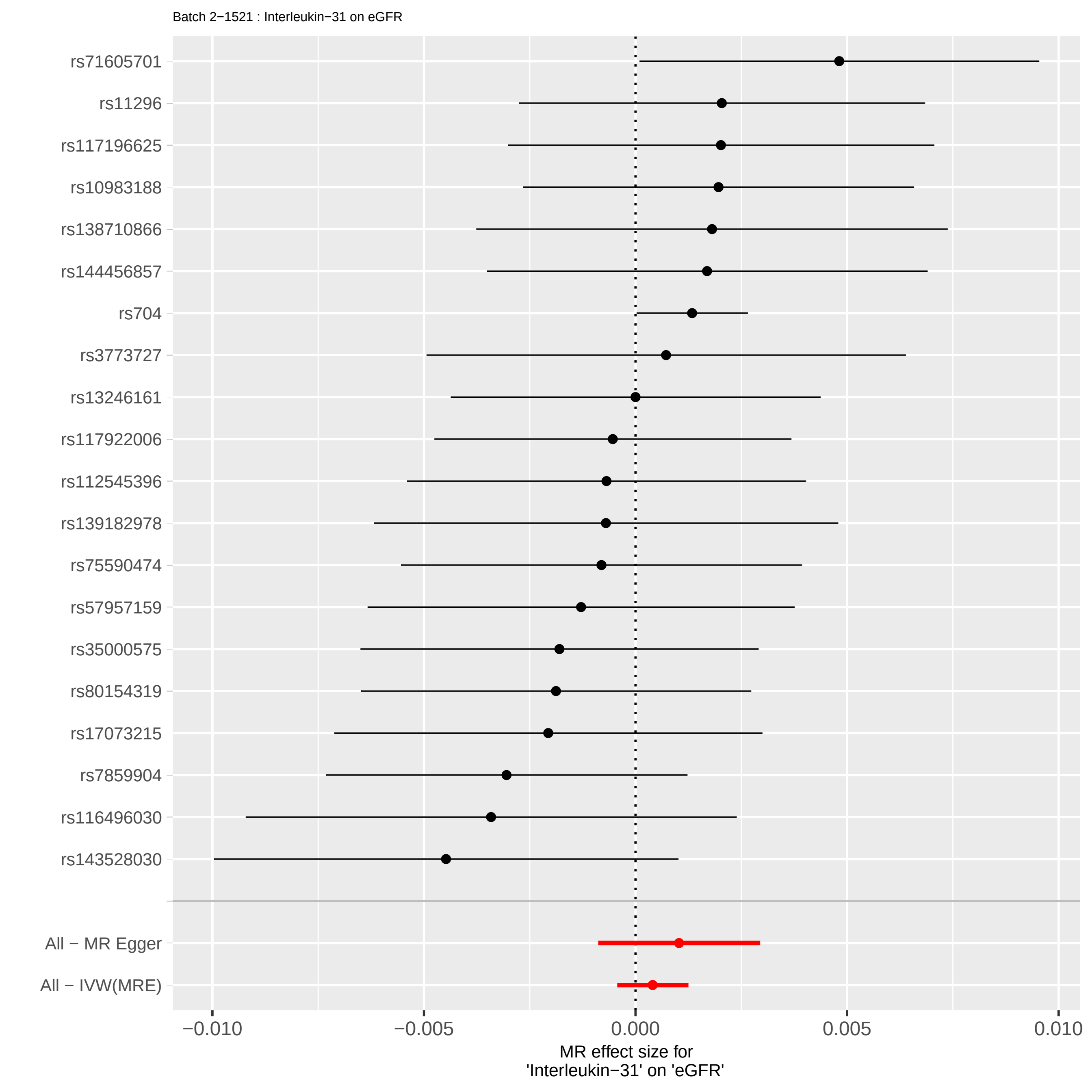

Batch 2−1521 : Interleukin−31 on eGFR
rs71605701
rs11296
rs117196625
rs10983188
rs138710866
rs144456857
rs704
rs3773727
rs13246161
rs117922006
rs112545396
rs139182978
rs75590474
rs57957159
rs35000575
rs80154319
rs17073215
rs7859904
rs116496030
rs143528030
All − MR Egger
All − IVW(MRE)
−0.010
−0.005
0.000
0.005
0.010
MR effect size for
'Interleukin−31' on 'eGFR'

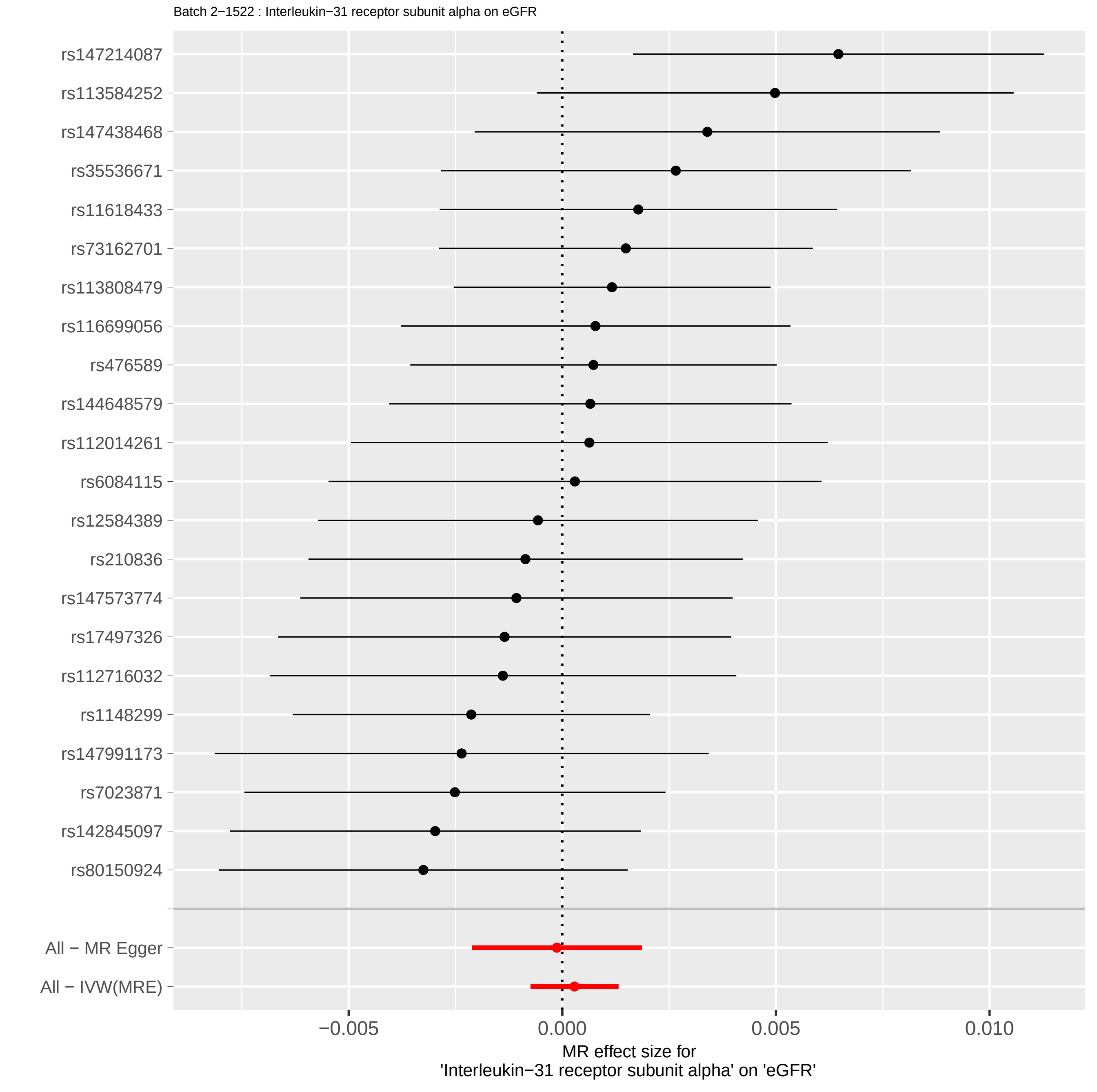

Batch 2−1522 : Interleukin−31 receptor subunit alpha on eGFR
rs147214087
rs113584252
rs147438468
rs35536671
rs11618433
rs73162701
rs113808479
rs116699056
rs476589
rs144648579
rs112014261
rs6084115
rs12584389
rs210836
rs147573774
rs17497326
rs112716032
rs1148299
rs147991173
rs7023871
rs142845097
rs80150924
All − MR Egger
All − IVW(MRE)
−0.005
0.000
0.005
0.010
MR effect size for
'Interleukin−31 receptor subunit alpha' on 'eGFR'

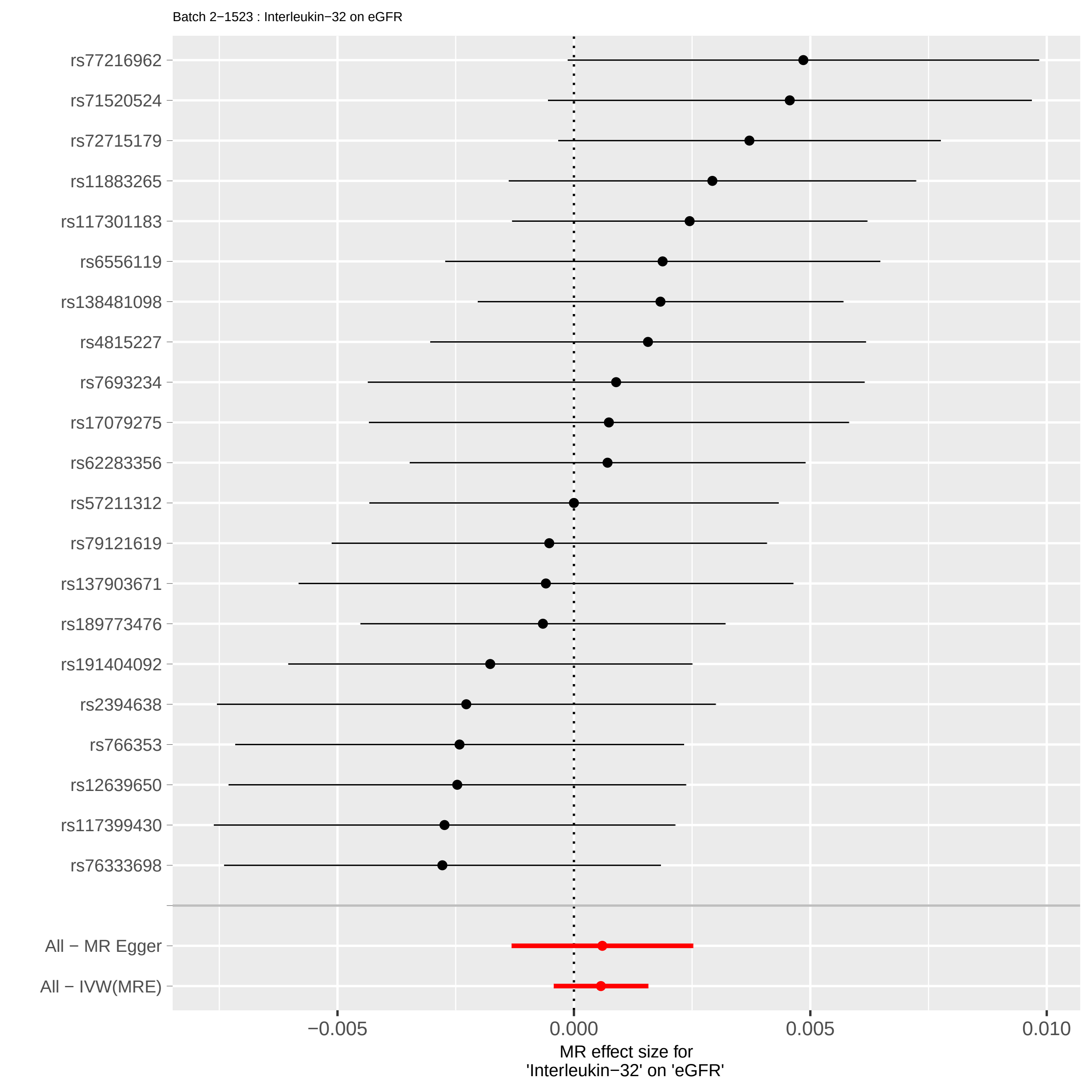

Batch 2−1523 : Interleukin−32 on eGFR
rs77216962
rs71520524
rs72715179
rs11883265
rs117301183
rs6556119
rs138481098
rs4815227
rs7693234
rs17079275
rs62283356
rs57211312
rs79121619
rs137903671
rs189773476
rs191404092
rs2394638
rs766353
rs12639650
rs117399430
rs76333698
All − MR Egger
All − IVW(MRE)
−0.005
0.000
0.005
0.010
MR effect size for
'Interleukin−32' on 'eGFR'

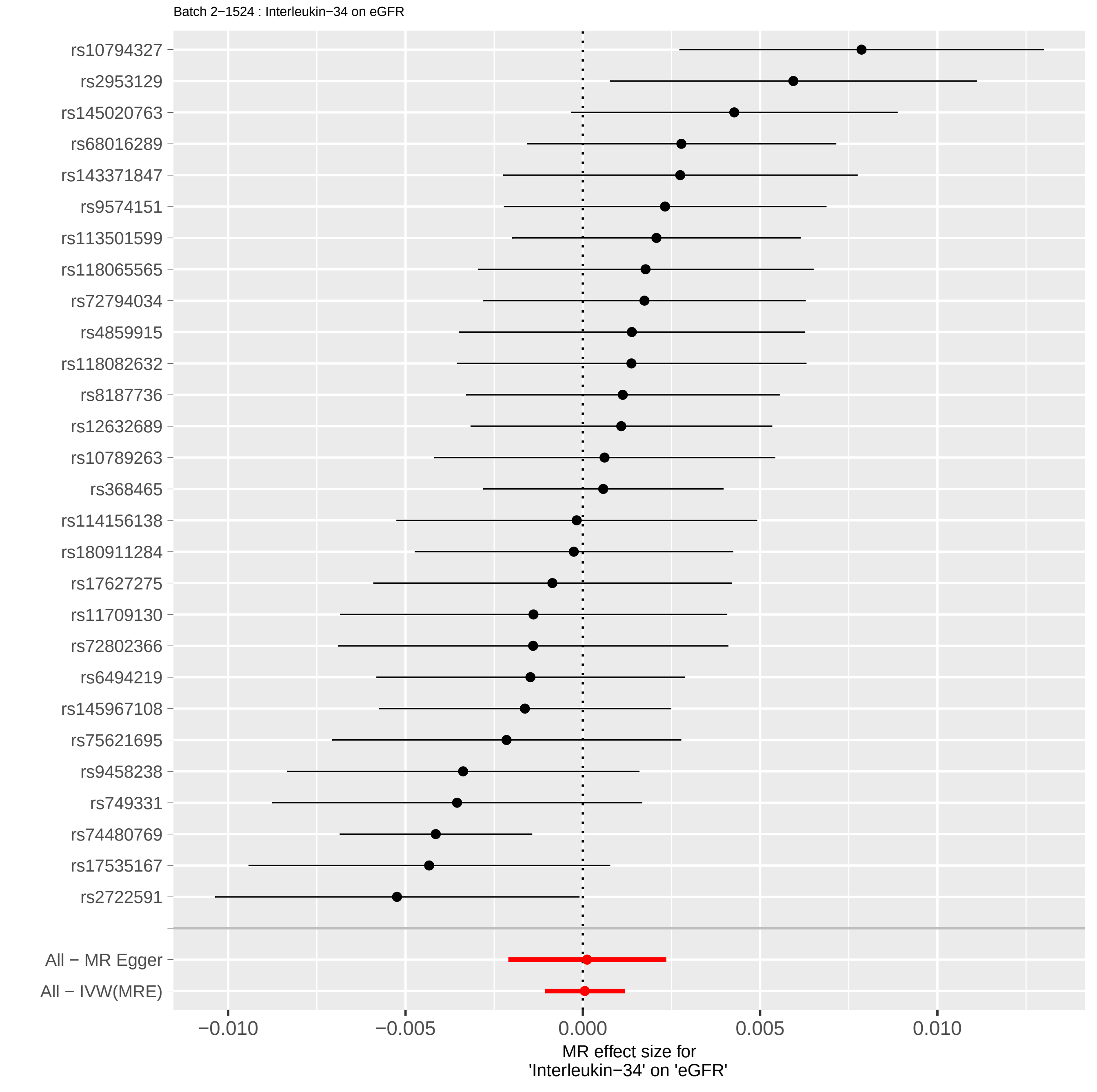
Batch 2−1524 : Interleukin−34 on eGFR
rs10794327
rs2953129
rs145020763
rs68016289
rs143371847
rs9574151
rs113501599
rs118065565
rs72794034
rs4859915
rs118082632
rs8187736
rs12632689
rs10789263
rs368465
rs114156138
rs180911284
rs17627275
rs11709130
rs72802366
rs6494219
rs145967108
rs75621695
rs9458238
rs749331
rs74480769
rs17535167
rs2722591
All − MR Egger
All − IVW(MRE)
−0.010
−0.005
0.000
0.005
0.010
MR effect size for
'Interleukin−34' on 'eGFR'

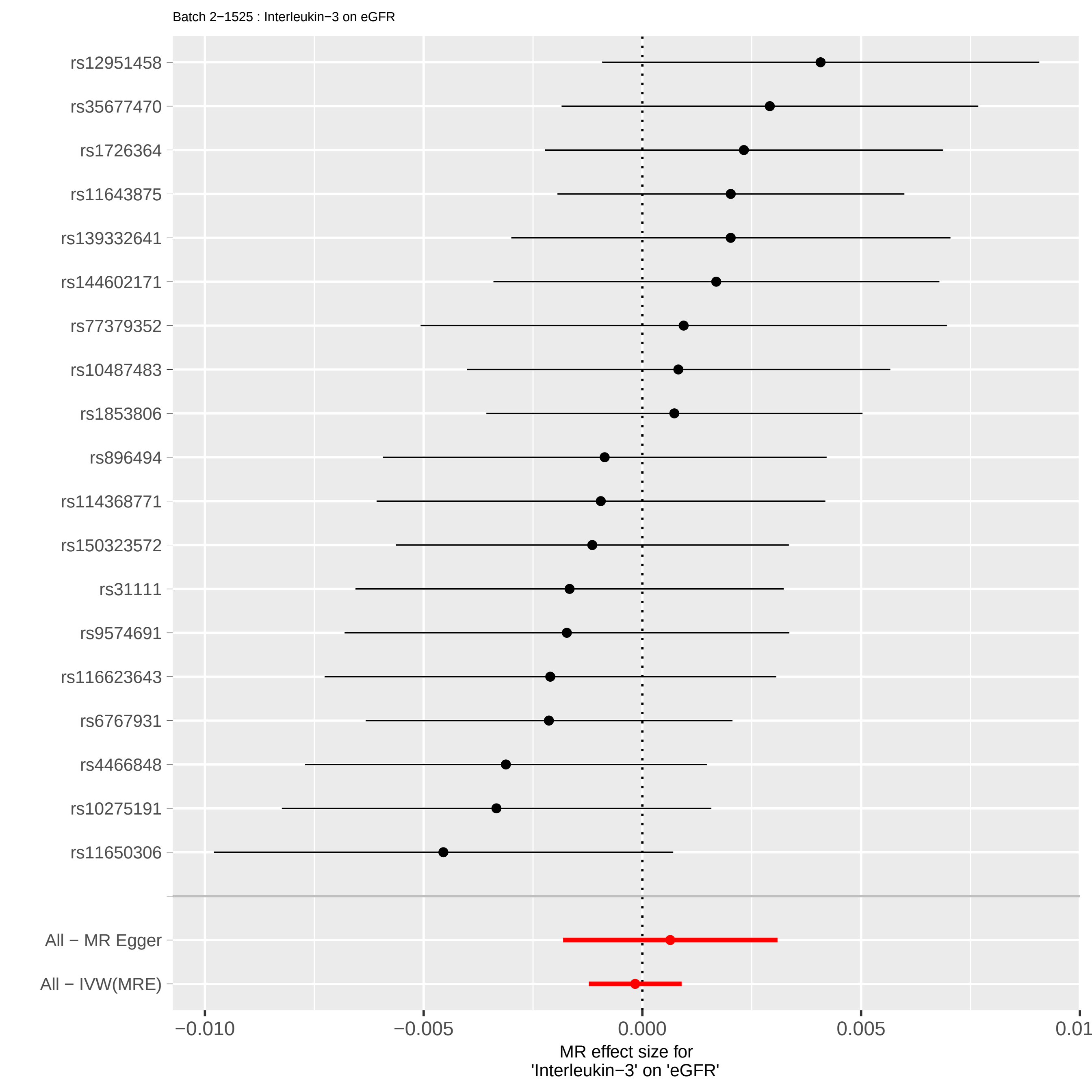

Batch 2−1525 : Interleukin−3 on eGFR
rs12951458
rs35677470
rs1726364
rs11643875
rs139332641
rs144602171
rs77379352
rs10487483
rs1853806
rs896494
rs114368771
rs150323572
rs31111
rs9574691
rs116623643
rs6767931
rs4466848
rs10275191
rs11650306
All − MR Egger
All − IVW(MRE)
−0.010
−0.005
0.000
0.005
0.01
MR effect size for
'Interleukin−3' on 'eGFR'

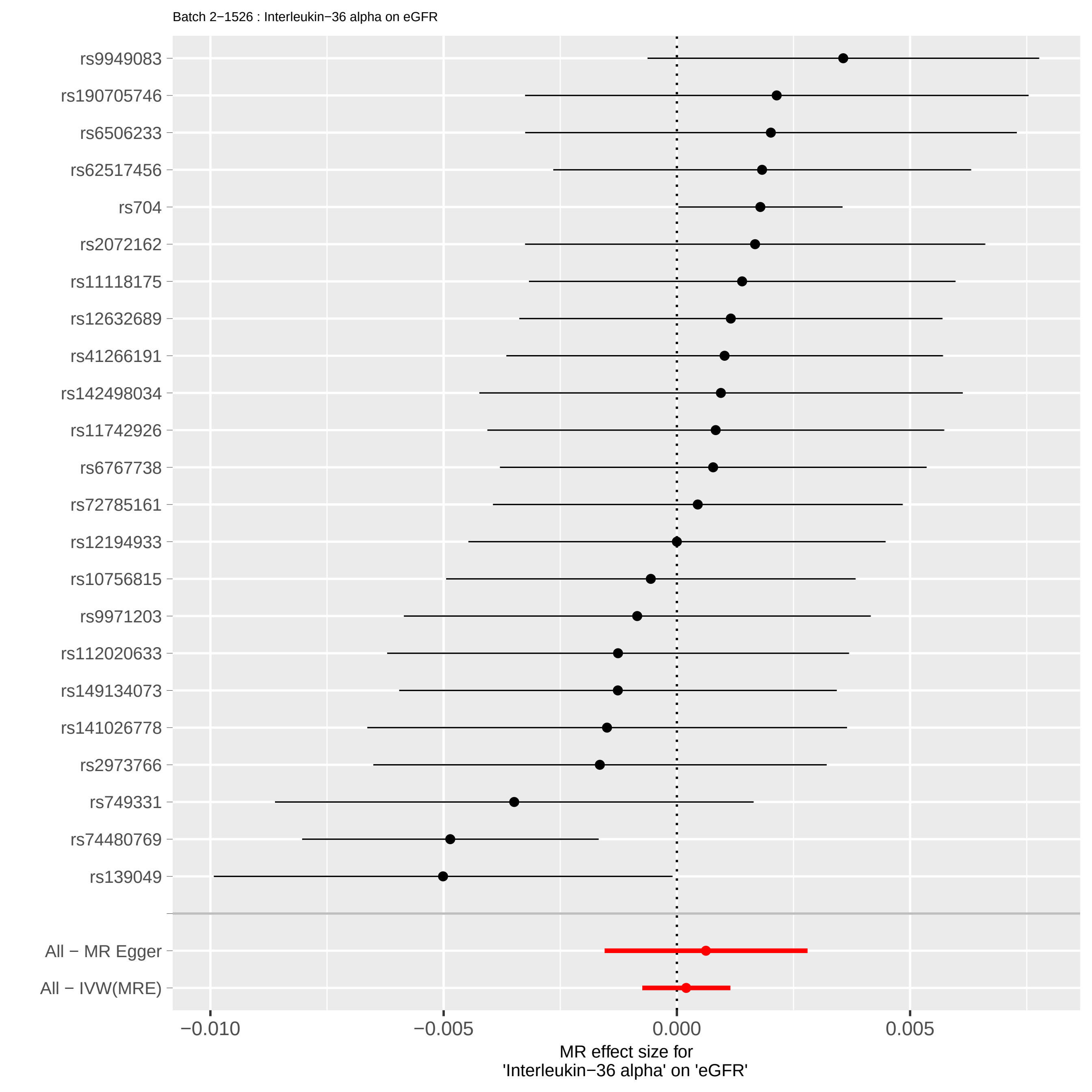
Batch 2−1526 : Interleukin−36 alpha on eGFR
rs9949083
rs190705746
rs6506233
rs62517456
rs704
rs2072162
rs11118175
rs12632689
rs41266191
rs142498034
rs11742926
rs6767738
rs72785161
rs12194933
rs10756815
rs9971203
rs112020633
rs149134073
rs141026778
rs2973766
rs749331
rs74480769
rs139049
All − MR Egger
All − IVW(MRE)
−0.010
−0.005
0.000
0.005
MR effect size for
'Interleukin−36 alpha' on 'eGFR'

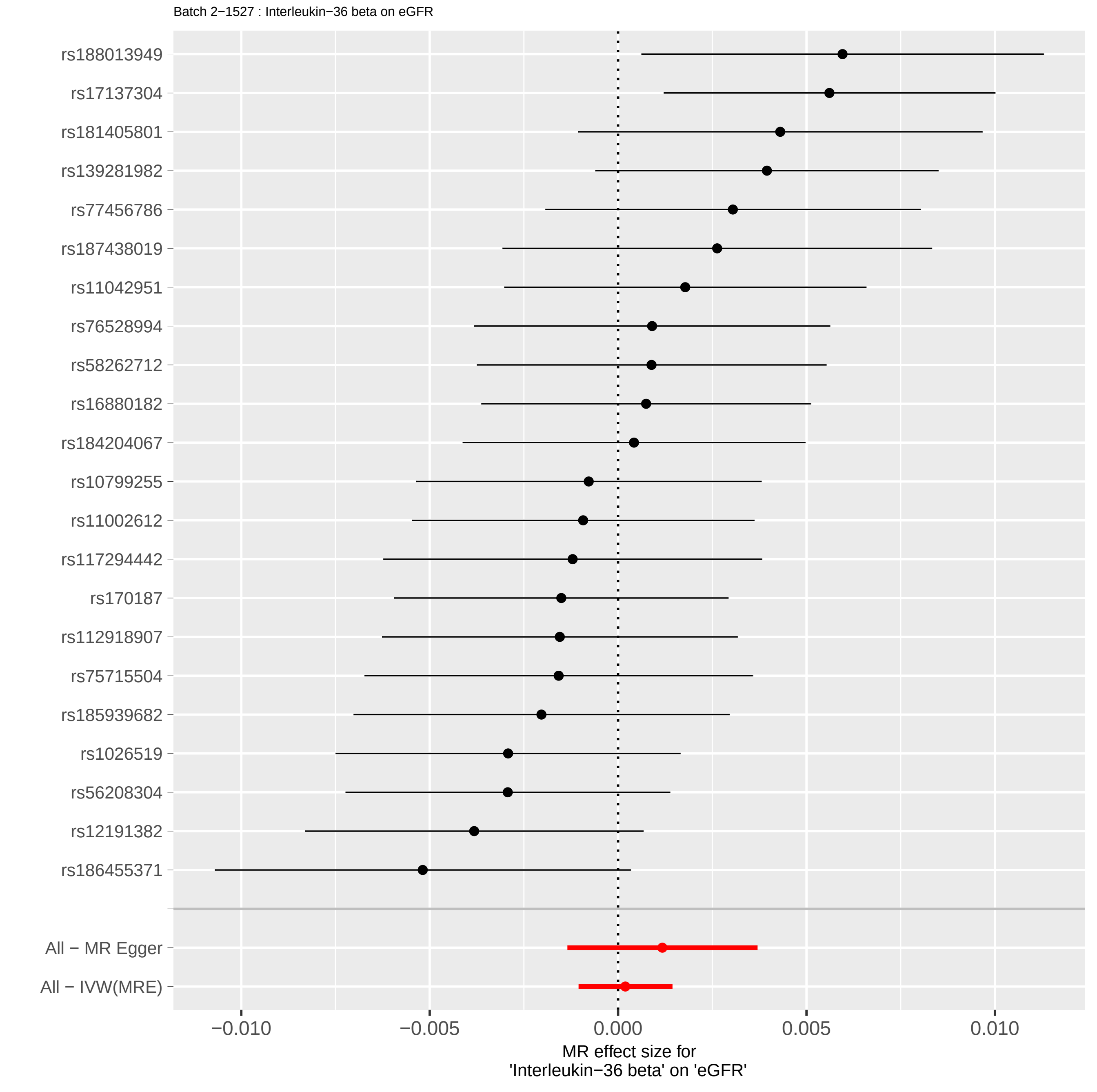

Batch 2−1527 : Interleukin−36 beta on eGFR
rs188013949
rs17137304
rs181405801
rs139281982
rs77456786
rs187438019
rs11042951
rs76528994
rs58262712
rs16880182
rs184204067
rs10799255
rs11002612
rs117294442
rs170187
rs112918907
rs75715504
rs185939682
rs1026519
rs56208304
rs12191382
rs186455371
All − MR Egger
All − IVW(MRE)
−0.010
−0.005
0.000
0.005
0.010
MR effect size for
'Interleukin−36 beta' on 'eGFR'

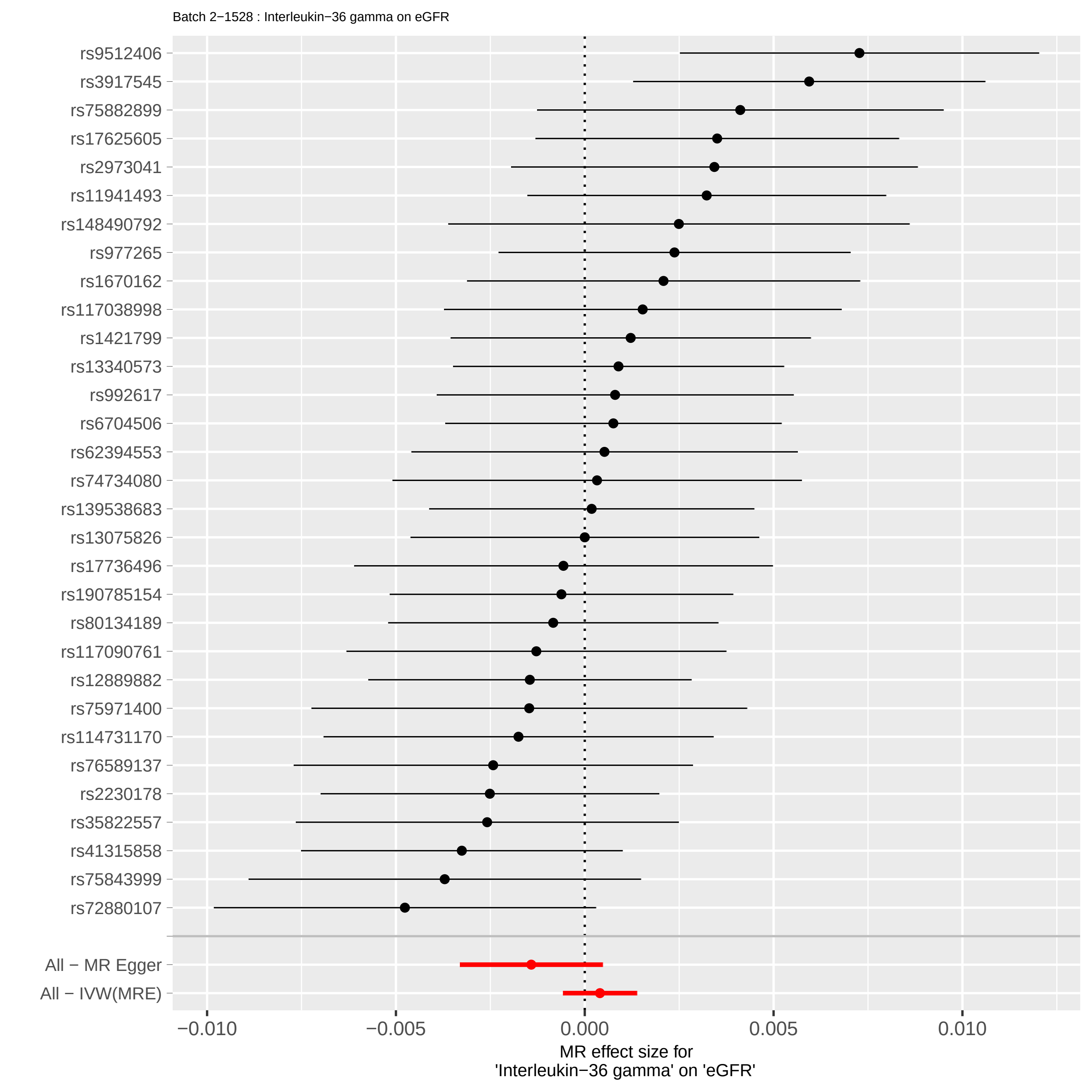
Batch 2−1528 : Interleukin−36 gamma on eGFR
rs9512406
rs3917545
rs75882899
rs17625605
rs2973041
rs11941493
rs148490792
rs977265
rs1670162
rs117038998
rs1421799
rs13340573
rs992617
rs6704506
rs62394553
rs74734080
rs139538683
rs13075826
rs17736496
rs190785154
rs80134189
rs117090761
rs12889882
rs75971400
rs114731170
rs76589137
rs2230178
rs35822557
rs41315858
rs75843999
rs72880107
All − MR Egger
All − IVW(MRE)
−0.010
−0.005
0.000
0.005
0.010
MR effect size for
'Interleukin−36 gamma' on 'eGFR'

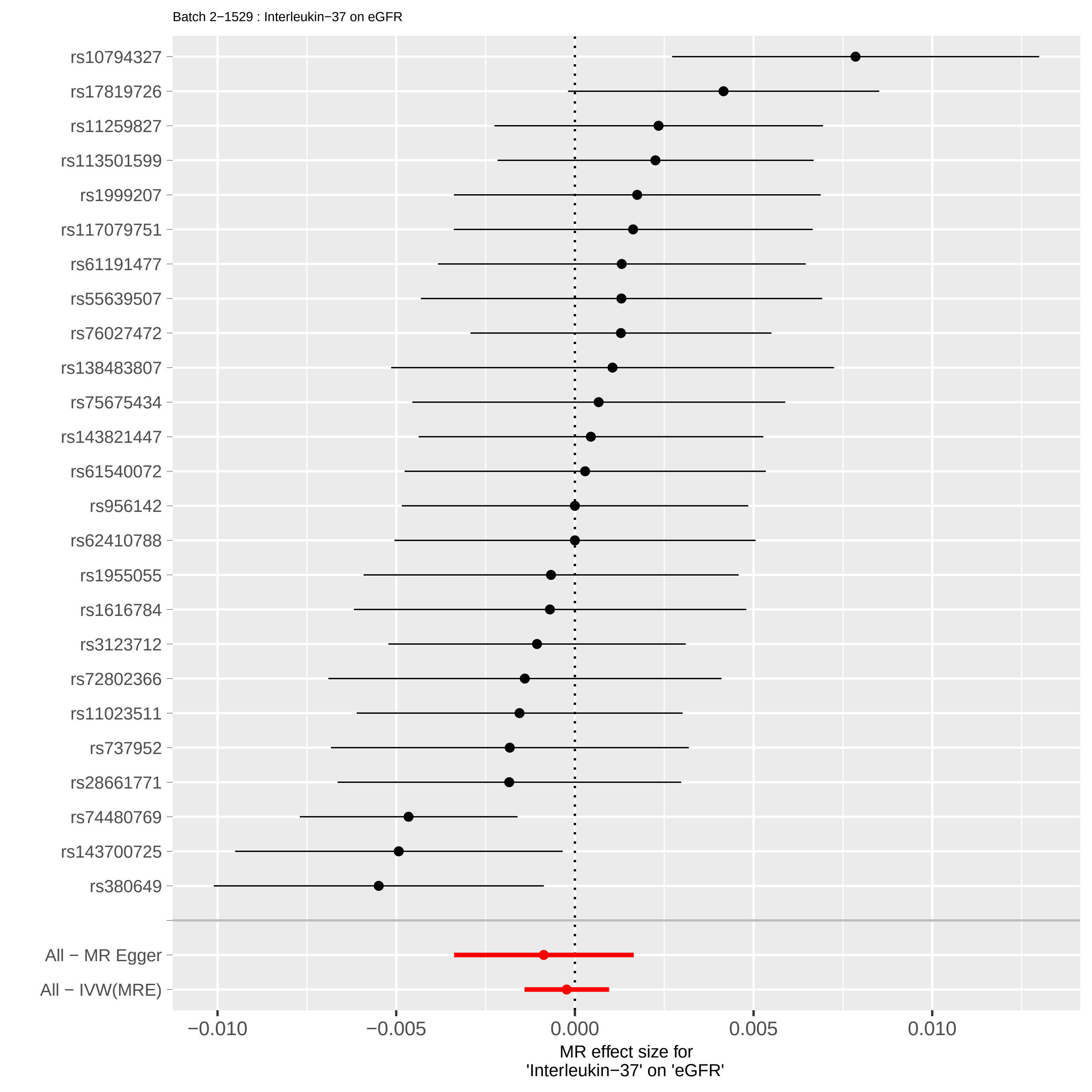

Batch 2−1529 : Interleukin−37 on eGFR
rs10794327
rs17819726
rs11259827
rs113501599
rs1999207
rs117079751
rs61191477
rs55639507
rs76027472
rs138483807
rs75675434
rs143821447
rs61540072
rs956142
rs62410788
rs1955055
rs1616784
rs3123712
rs72802366
rs11023511
rs737952
rs28661771
rs74480769
rs143700725
rs380649
All − MR Egger
All − IVW(MRE)
−0.010
−0.005
0.000
0.005
0.010
MR effect size for
'Interleukin−37' on 'eGFR'

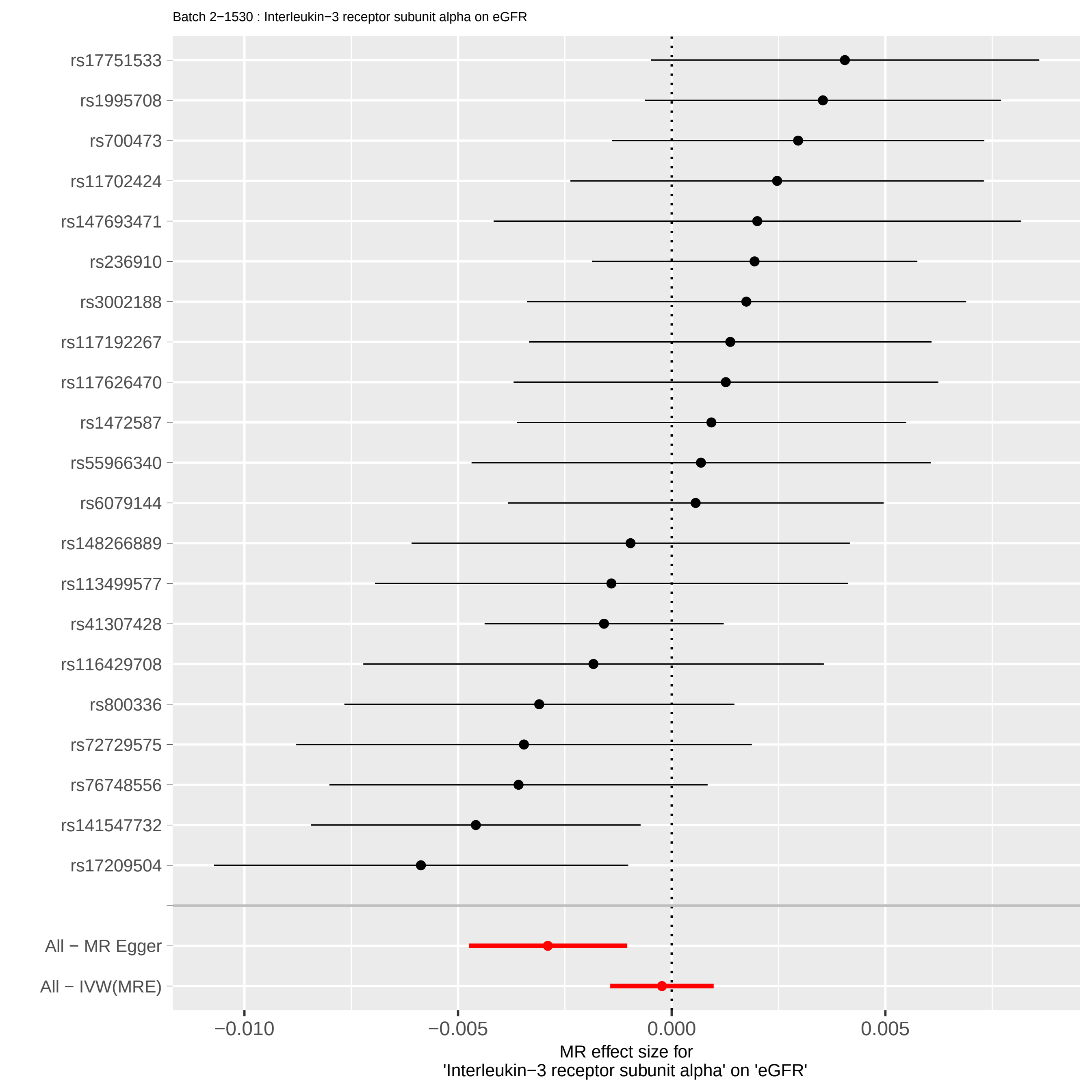
Batch 2−1530 : Interleukin−3 receptor subunit alpha on eGFR
rs17751533
rs1995708
rs700473
rs11702424
rs147693471
rs236910
rs3002188
rs117192267
rs117626470
rs1472587
rs55966340
rs6079144
rs148266889
rs113499577
rs41307428
rs116429708
rs800336
rs72729575
rs76748556
rs141547732
rs17209504
All − MR Egger
All − IVW(MRE)
−0.010
−0.005
0.000
0.005
MR effect size for
'Interleukin−3 receptor subunit alpha' on 'eGFR'

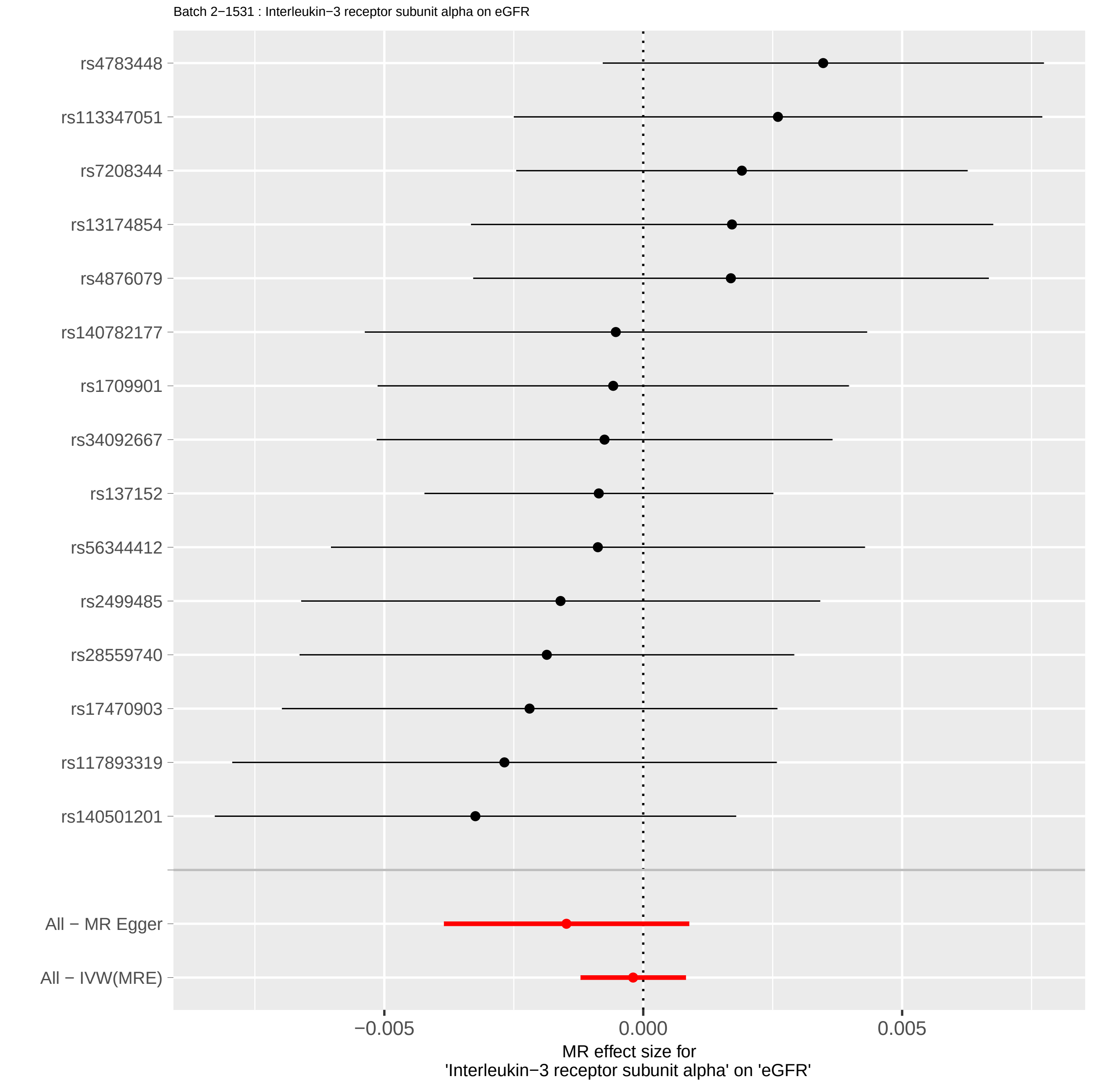

Batch 2−1531 : Interleukin−3 receptor subunit alpha on eGFR
rs4783448
rs113347051
rs7208344
rs13174854
rs4876079
rs140782177
rs1709901
rs34092667
rs137152
rs56344412
rs2499485
rs28559740
rs17470903
rs117893319
rs140501201
All − MR Egger
All − IVW(MRE)
−0.005
0.000
0.005
MR effect size for
'Interleukin−3 receptor subunit alpha' on 'eGFR'

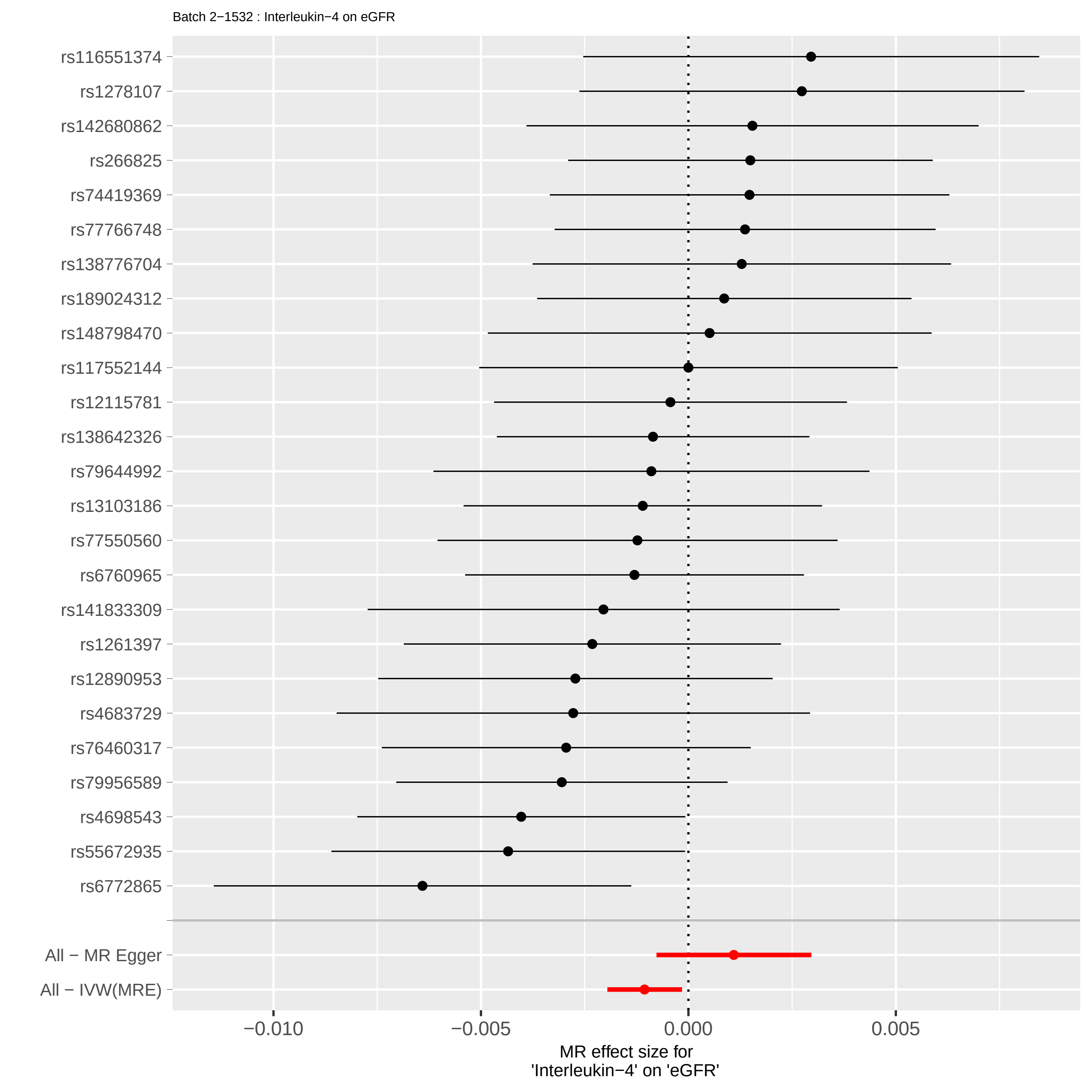

Batch 2−1532 : Interleukin−4 on eGFR
rs116551374
rs1278107
rs142680862
rs266825
rs74419369
rs77766748
rs138776704
rs189024312
rs148798470
rs117552144
rs12115781
rs138642326
rs79644992
rs13103186
rs77550560
rs6760965
rs141833309
rs1261397
rs12890953
rs4683729
rs76460317
rs79956589
rs4698543
rs55672935
rs6772865
All − MR Egger
All − IVW(MRE)
−0.010
−0.005
0.000
0.005
MR effect size for
'Interleukin−4' on 'eGFR'

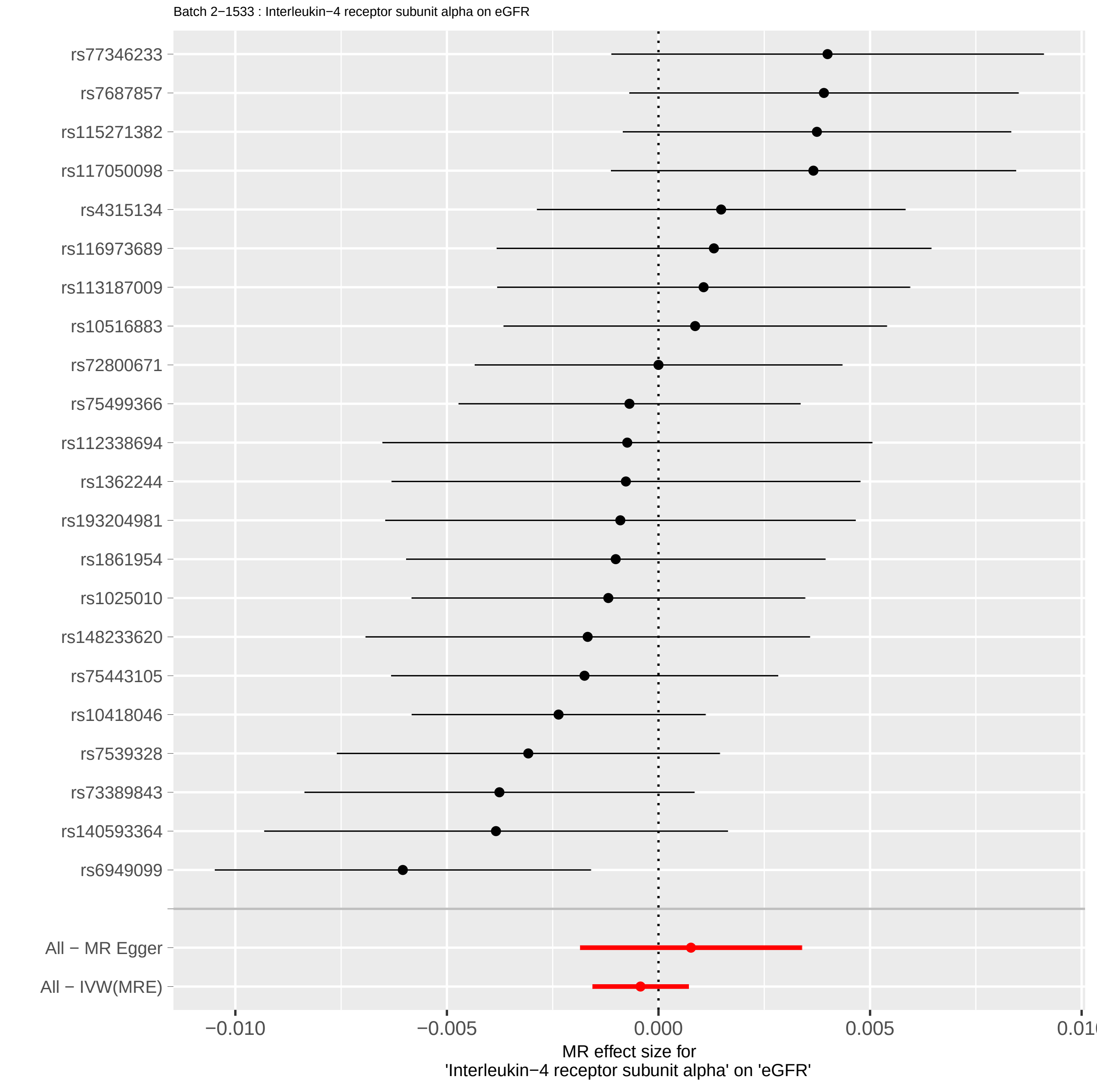
Batch 2−1533 : Interleukin−4 receptor subunit alpha on eGFR
rs77346233
rs7687857
rs115271382
rs117050098
rs4315134
rs116973689
rs113187009
rs10516883
rs72800671
rs75499366
rs112338694
rs1362244
rs193204981
rs1861954
rs1025010
rs148233620
rs75443105
rs10418046
rs7539328
rs73389843
rs140593364
rs6949099
All − MR Egger
All − IVW(MRE)
−0.010
−0.005
0.000
0.005
0.01
MR effect size for
'Interleukin−4 receptor subunit alpha' on 'eGFR'

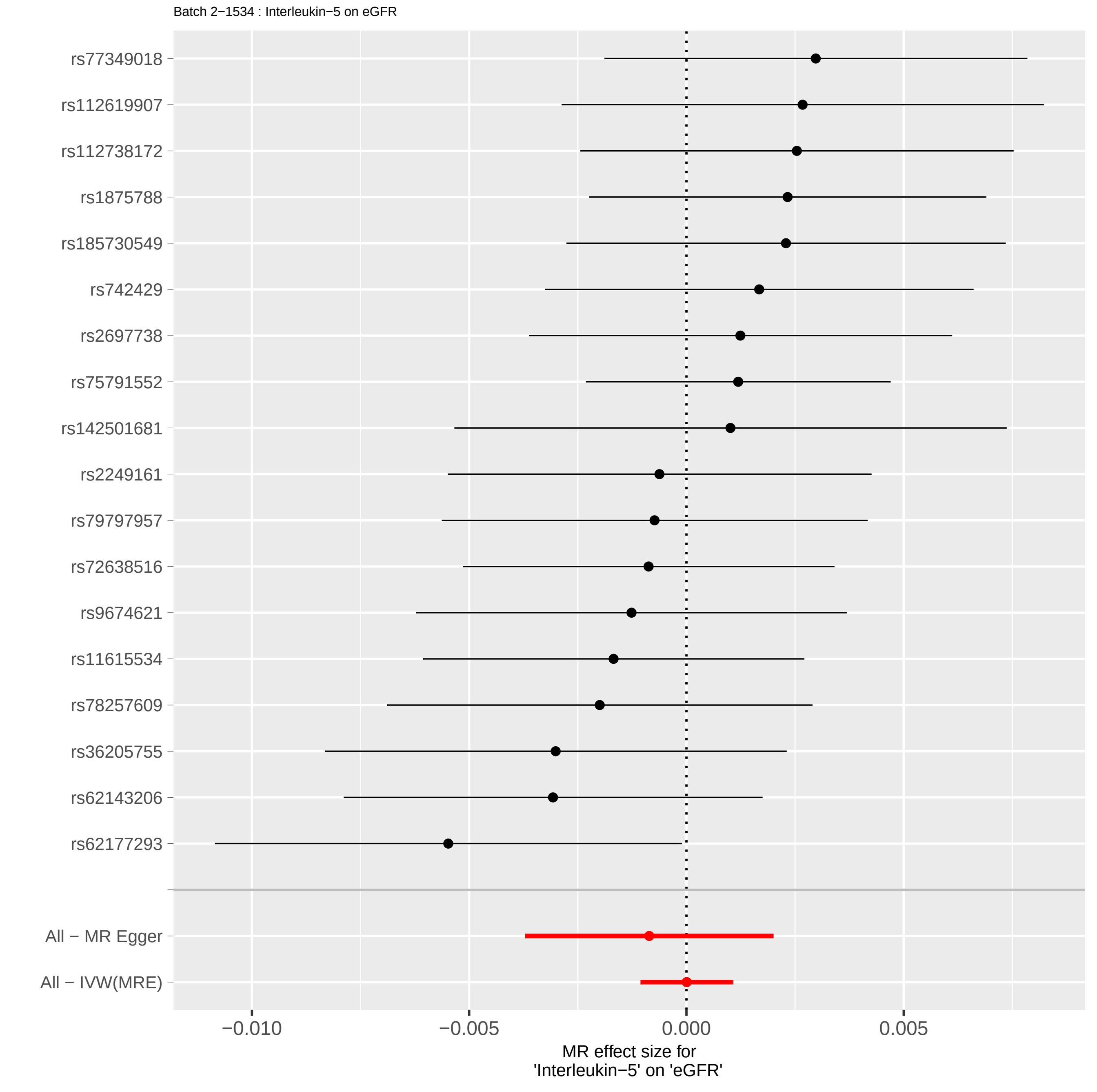

Batch 2−1534 : Interleukin−5 on eGFR
rs77349018
rs112619907
rs112738172
rs1875788
rs185730549
rs742429
rs2697738
rs75791552
rs142501681
rs2249161
rs79797957
rs72638516
rs9674621
rs11615534
rs78257609
rs36205755
rs62143206
rs62177293
All − MR Egger
All − IVW(MRE)
−0.010
−0.005
0.000
0.005
MR effect size for
'Interleukin−5' on 'eGFR'

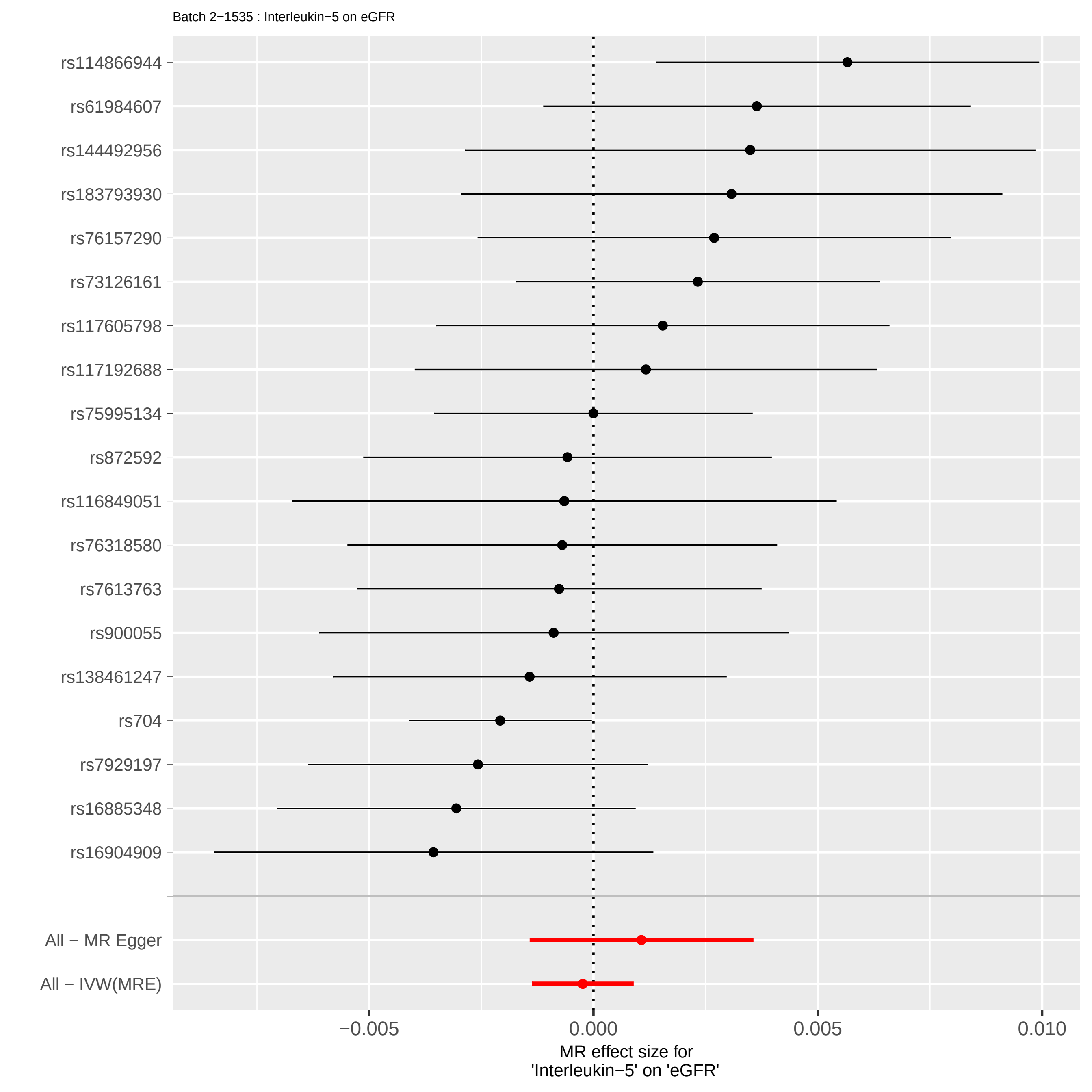
Batch 2−1535 : Interleukin−5 on eGFR
rs114866944
rs61984607
rs144492956
rs183793930
rs76157290
rs73126161
rs117605798
rs117192688
rs75995134
rs872592
rs116849051
rs76318580
rs7613763
rs900055
rs138461247
rs704
rs7929197
rs16885348
rs16904909
All − MR Egger
All − IVW(MRE)
−0.005
0.000
0.005
0.010
MR effect size for
'Interleukin−5' on 'eGFR'

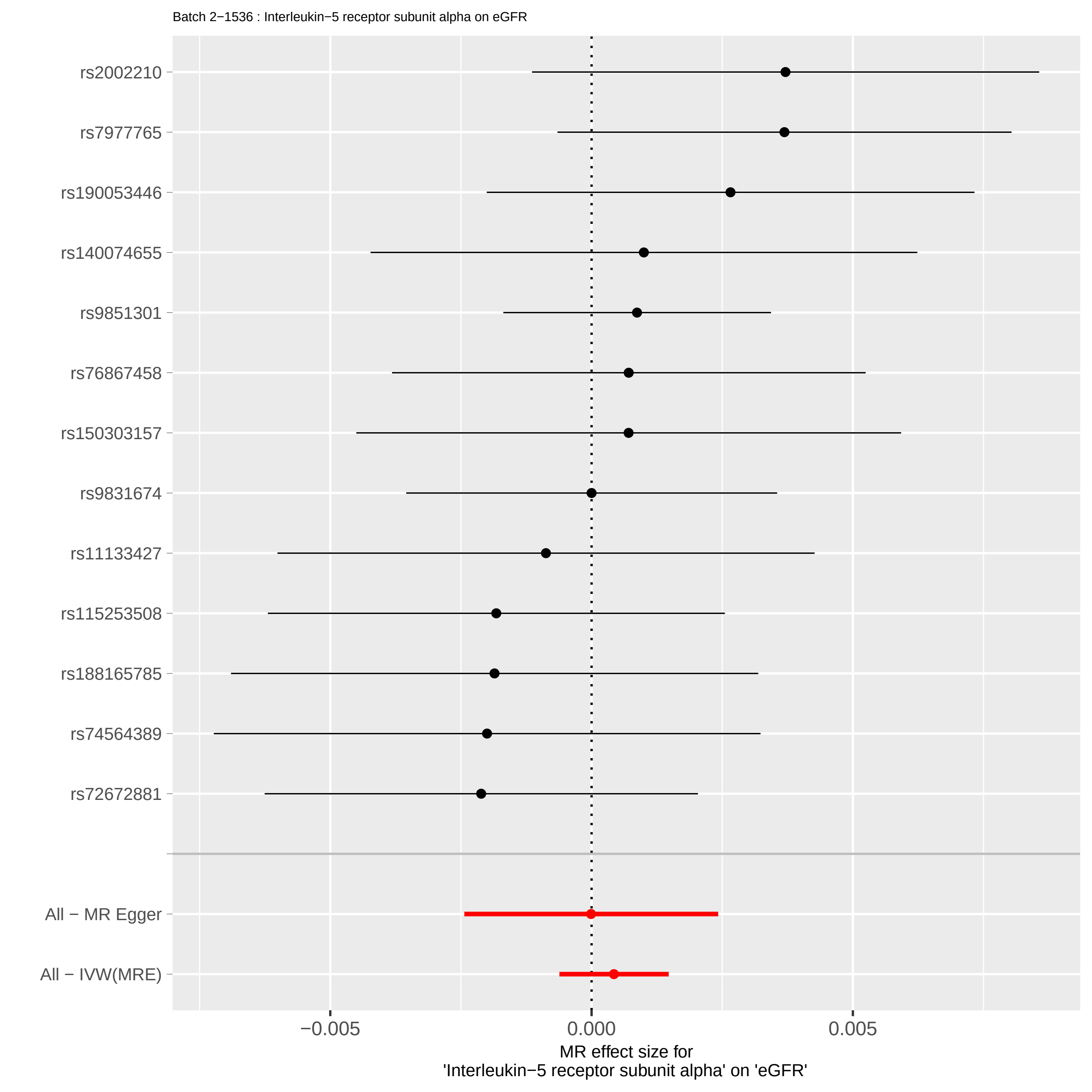

Batch 2−1536 : Interleukin−5 receptor subunit alpha on eGFR
rs2002210
rs7977765
rs190053446
rs140074655
rs9851301
rs76867458
rs150303157
rs9831674
rs11133427
rs115253508
rs188165785
rs74564389
rs72672881
All − MR Egger
All − IVW(MRE)
−0.005
0.000
0.005
MR effect size for
'Interleukin−5 receptor subunit alpha' on 'eGFR'

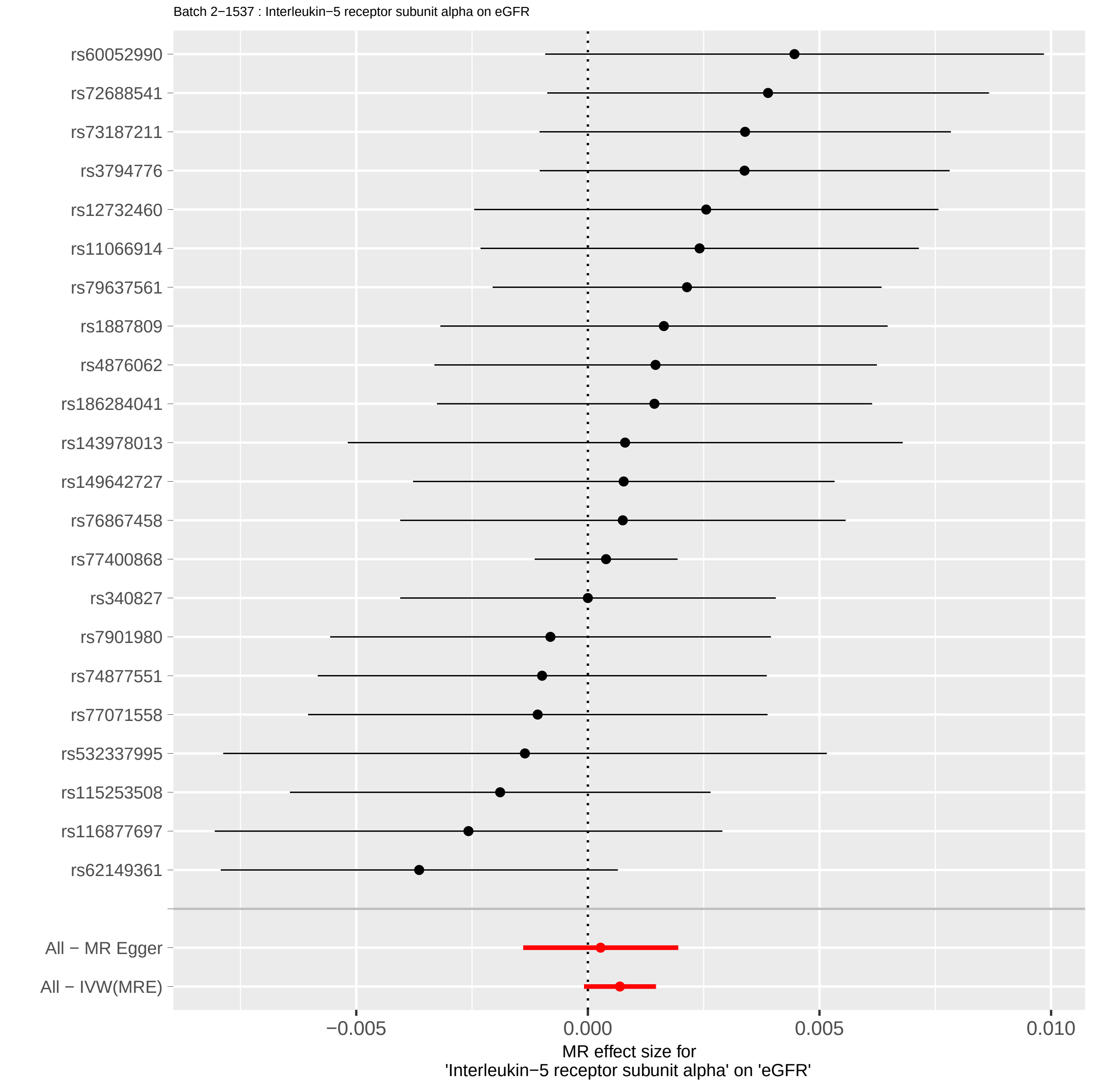

Batch 2−1537 : Interleukin−5 receptor subunit alpha on eGFR
rs60052990
rs72688541
rs73187211
rs3794776
rs12732460
rs11066914
rs79637561
rs1887809
rs4876062
rs186284041
rs143978013
rs149642727
rs76867458
rs77400868
rs340827
rs7901980
rs74877551
rs77071558
rs532337995
rs115253508
rs116877697
rs62149361
All − MR Egger
All − IVW(MRE)
−0.005
0.000
0.005
0.010
MR effect size for
'Interleukin−5 receptor subunit alpha' on 'eGFR'

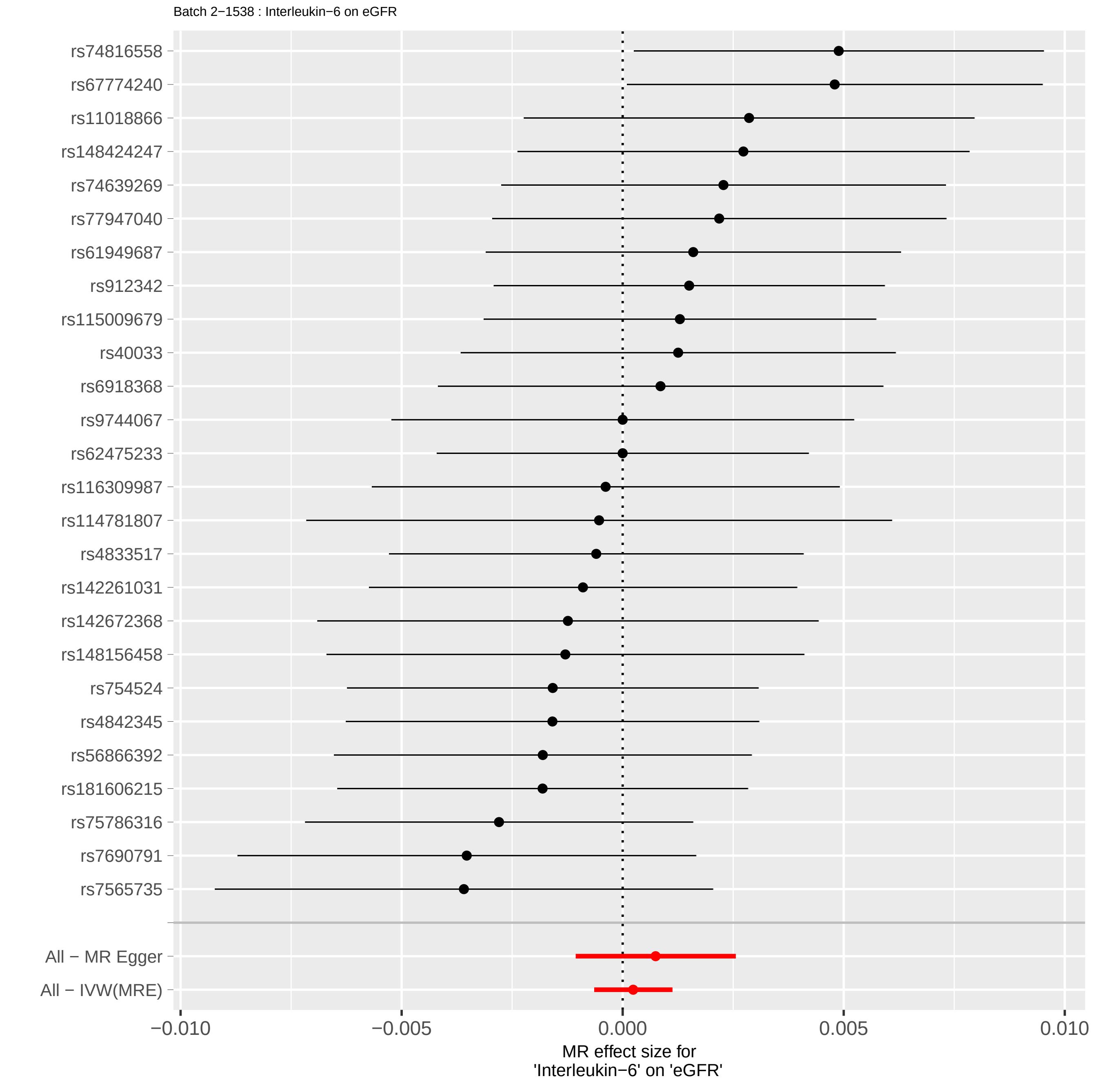
Batch 2−1538 : Interleukin−6 on eGFR
rs74816558
rs67774240
rs11018866
rs148424247
rs74639269
rs77947040
rs61949687
rs912342
rs115009679
rs40033
rs6918368
rs9744067
rs62475233
rs116309987
rs114781807
rs4833517
rs142261031
rs142672368
rs148156458
rs754524
rs4842345
rs56866392
rs181606215
rs75786316
rs7690791
rs7565735
All − MR Egger
All − IVW(MRE)
−0.010
−0.005
0.000
0.005
0.010
MR effect size for
'Interleukin−6' on 'eGFR'

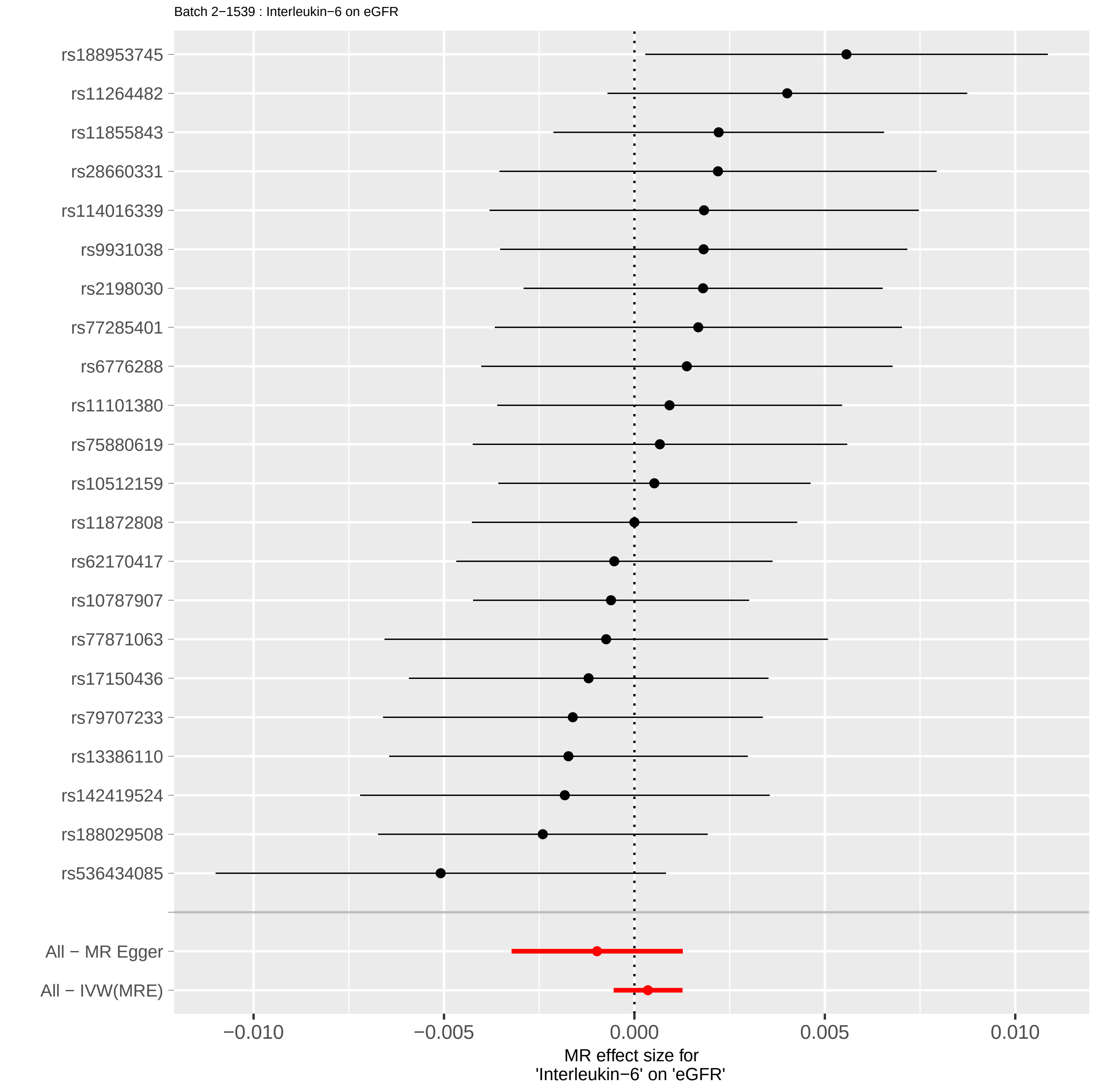

Batch 2−1539 : Interleukin−6 on eGFR
rs188953745
rs11264482
rs11855843
rs28660331
rs114016339
rs9931038
rs2198030
rs77285401
rs6776288
rs11101380
rs75880619
rs10512159
rs11872808
rs62170417
rs10787907
rs77871063
rs17150436
rs79707233
rs13386110
rs142419524
rs188029508
rs536434085
All − MR Egger
All − IVW(MRE)
−0.010
−0.005
0.000
0.005
0.010
MR effect size for
'Interleukin−6' on 'eGFR'

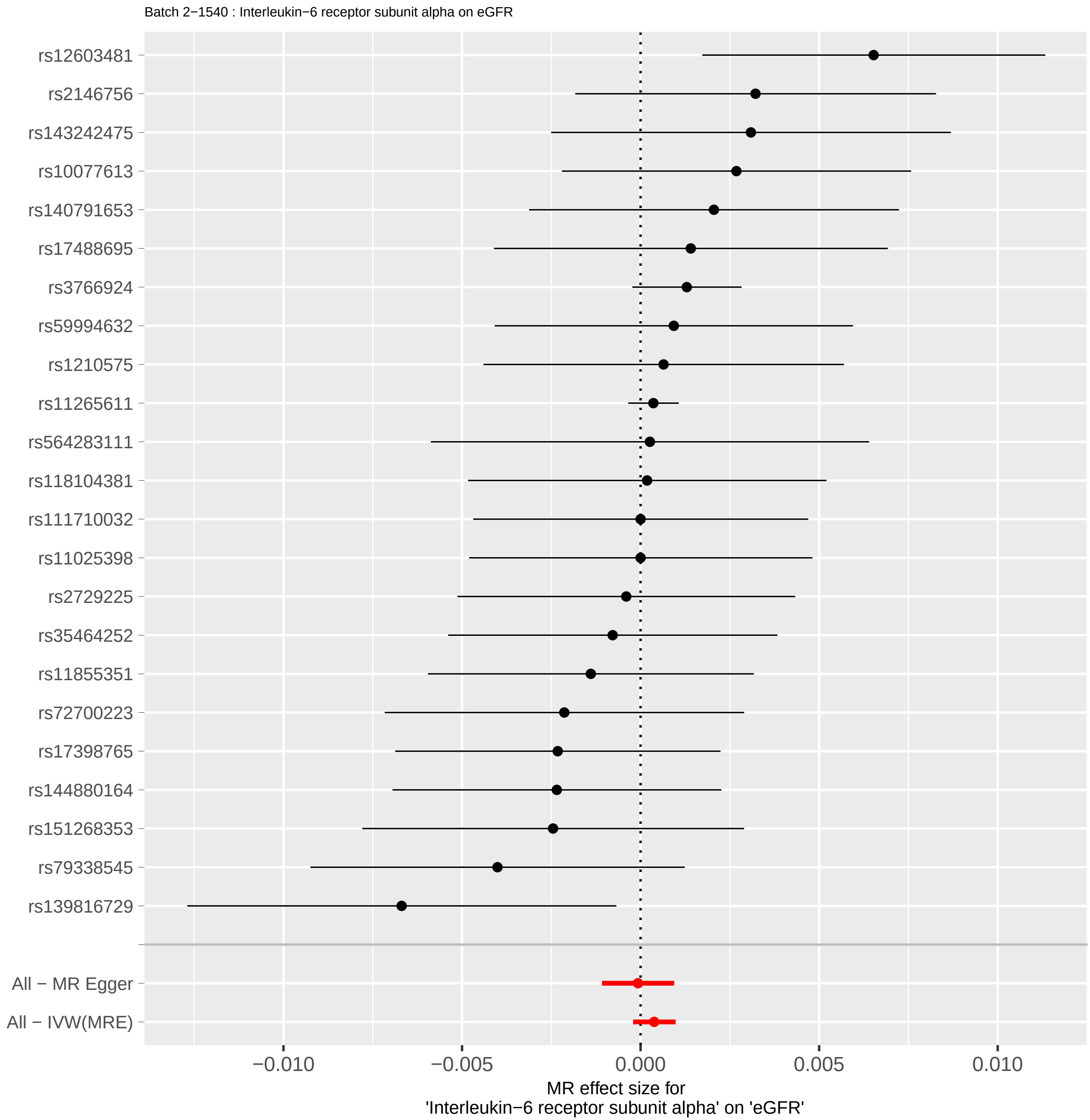

Batch 2−1540 : Interleukin−6 receptor subunit alpha on eGFR
rs12603481
rs2146756
rs143242475
rs10077613
rs140791653
rs17488695
rs3766924
rs59994632
rs1210575
rs11265611
rs564283111
rs118104381
rs111710032
rs11025398
rs2729225
rs35464252
rs11855351
rs72700223
rs17398765
rs144880164
rs151268353
rs79338545
rs139816729
All − MR Egger
All − IVW(MRE)
−0.010
−0.005
0.000
0.005
0.010
MR effect size for
'Interleukin−6 receptor subunit alpha' on 'eGFR'

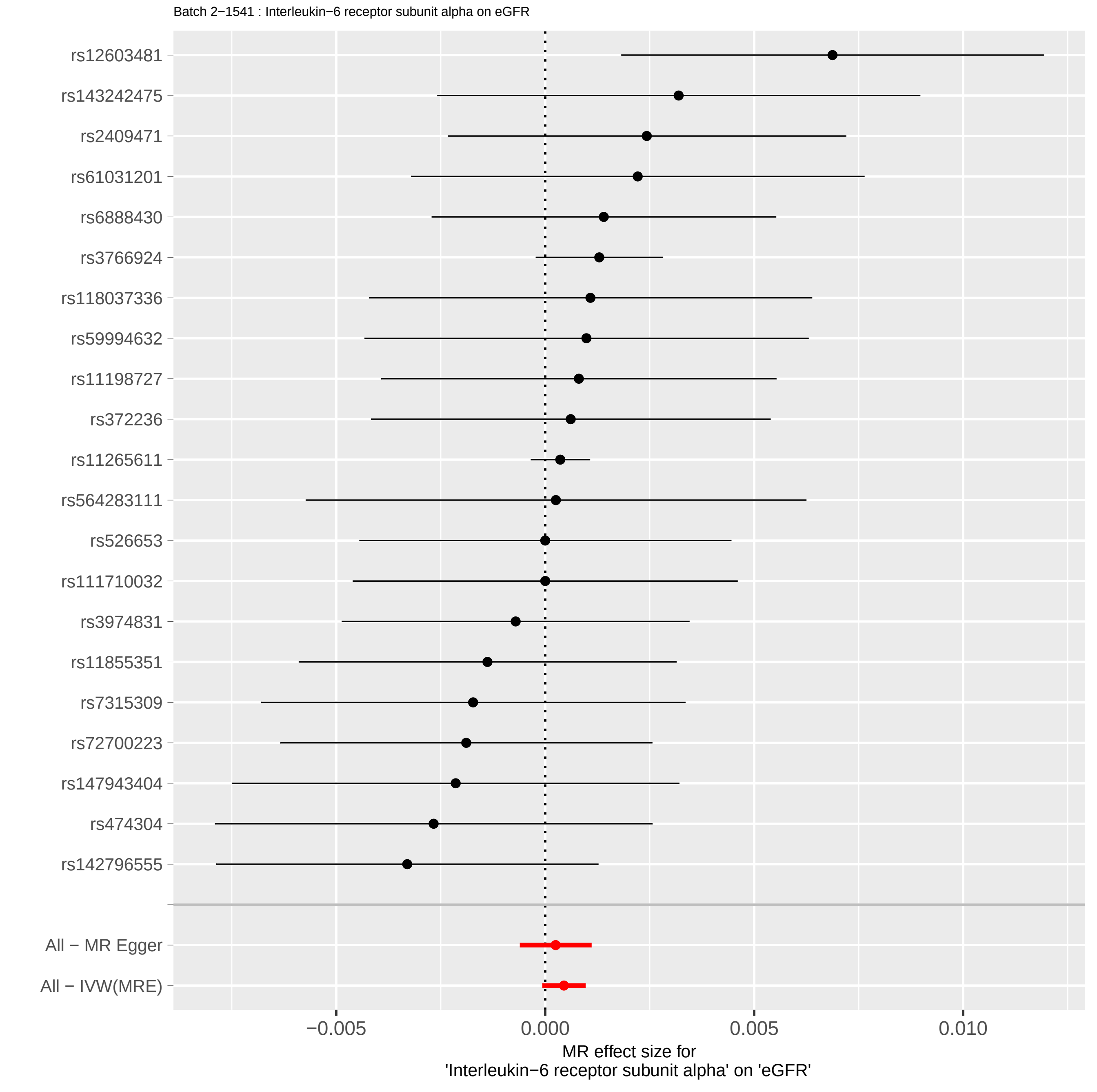

Batch 2−1541 : Interleukin−6 receptor subunit alpha on eGFR
rs12603481
rs143242475
rs2409471
rs61031201
rs6888430
rs3766924
rs118037336
rs59994632
rs11198727
rs372236
rs11265611
rs564283111
rs526653
rs111710032
rs3974831
rs11855351
rs7315309
rs72700223
rs147943404
rs474304
rs142796555
All − MR Egger
All − IVW(MRE)
−0.005
0.000
0.005
0.010
MR effect size for
'Interleukin−6 receptor subunit alpha' on 'eGFR'

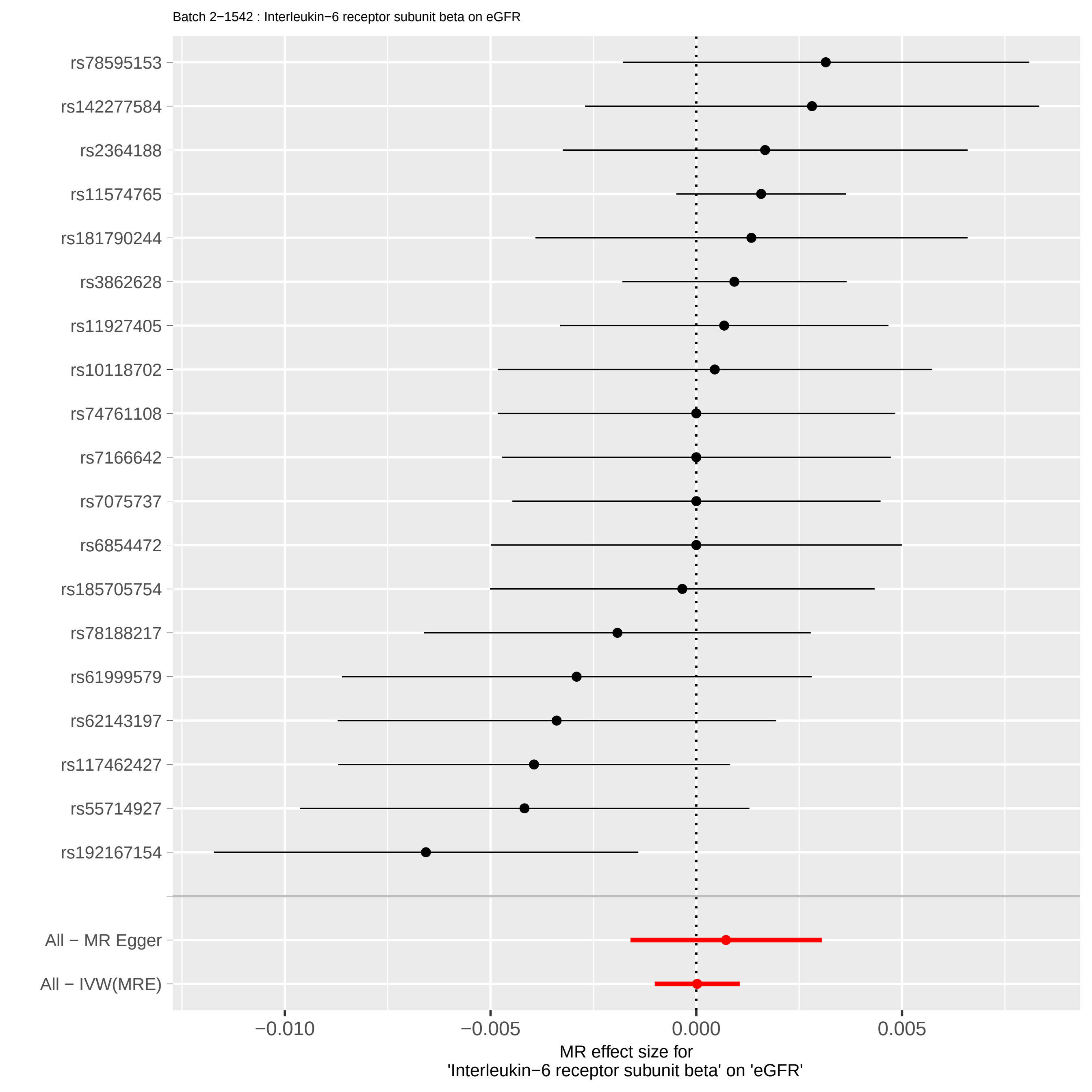
Batch 2−1542 : Interleukin−6 receptor subunit beta on eGFR
rs78595153
rs142277584
rs2364188
rs11574765
rs181790244
rs3862628
rs11927405
rs10118702
rs74761108
rs7166642
rs7075737
rs6854472
rs185705754
rs78188217
rs61999579
rs62143197
rs117462427
rs55714927
rs192167154
All − MR Egger
All − IVW(MRE)
−0.010
−0.005
0.000
0.005
MR effect size for
'Interleukin−6 receptor subunit beta' on 'eGFR'

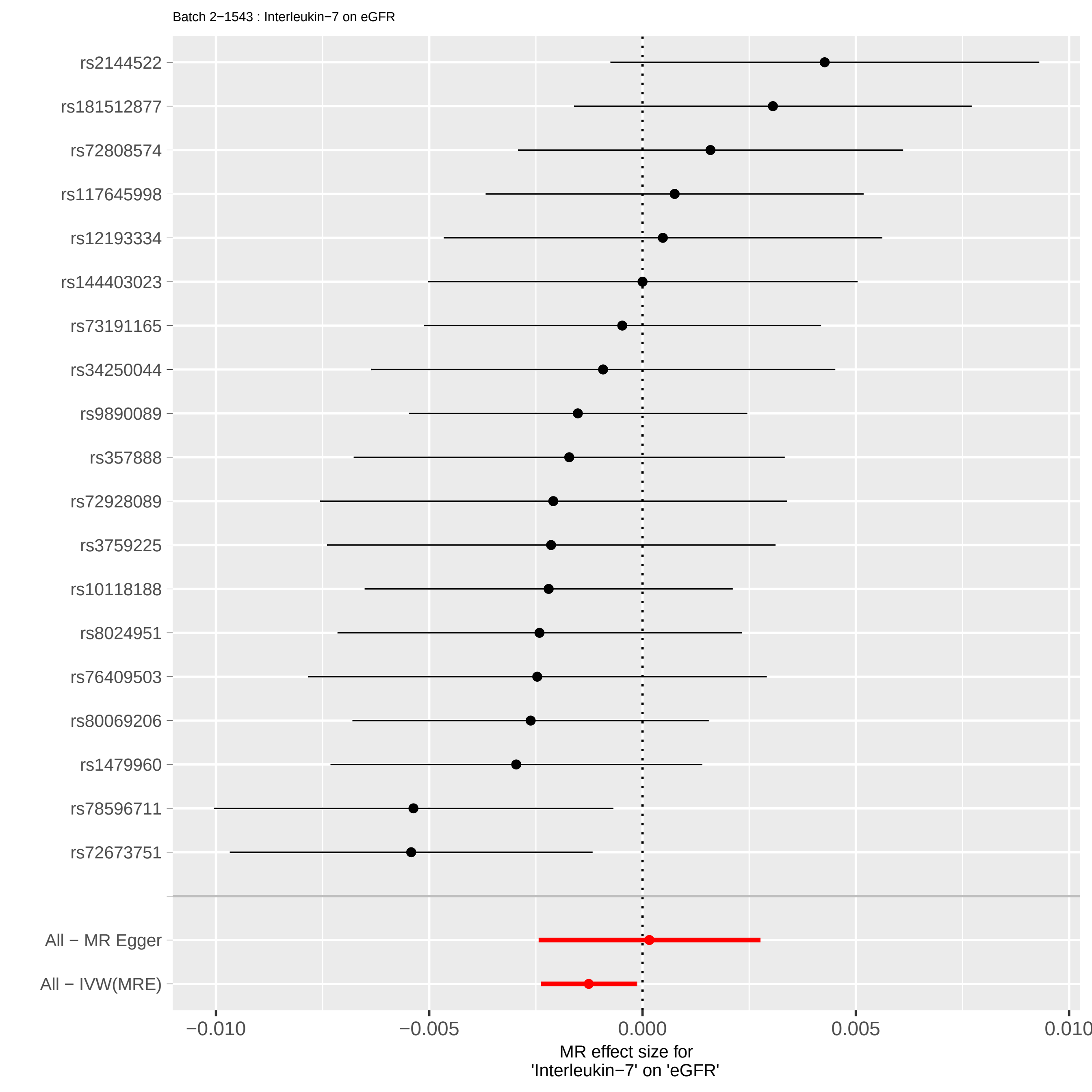

Batch 2−1543 : Interleukin−7 on eGFR
rs2144522
rs181512877
rs72808574
rs117645998
rs12193334
rs144403023
rs73191165
rs34250044
rs9890089
rs357888
rs72928089
rs3759225
rs10118188
rs8024951
rs76409503
rs80069206
rs1479960
rs78596711
rs72673751
All − MR Egger
All − IVW(MRE)
−0.010
−0.005
0.000
0.005
0.010
MR effect size for
'Interleukin−7' on 'eGFR'

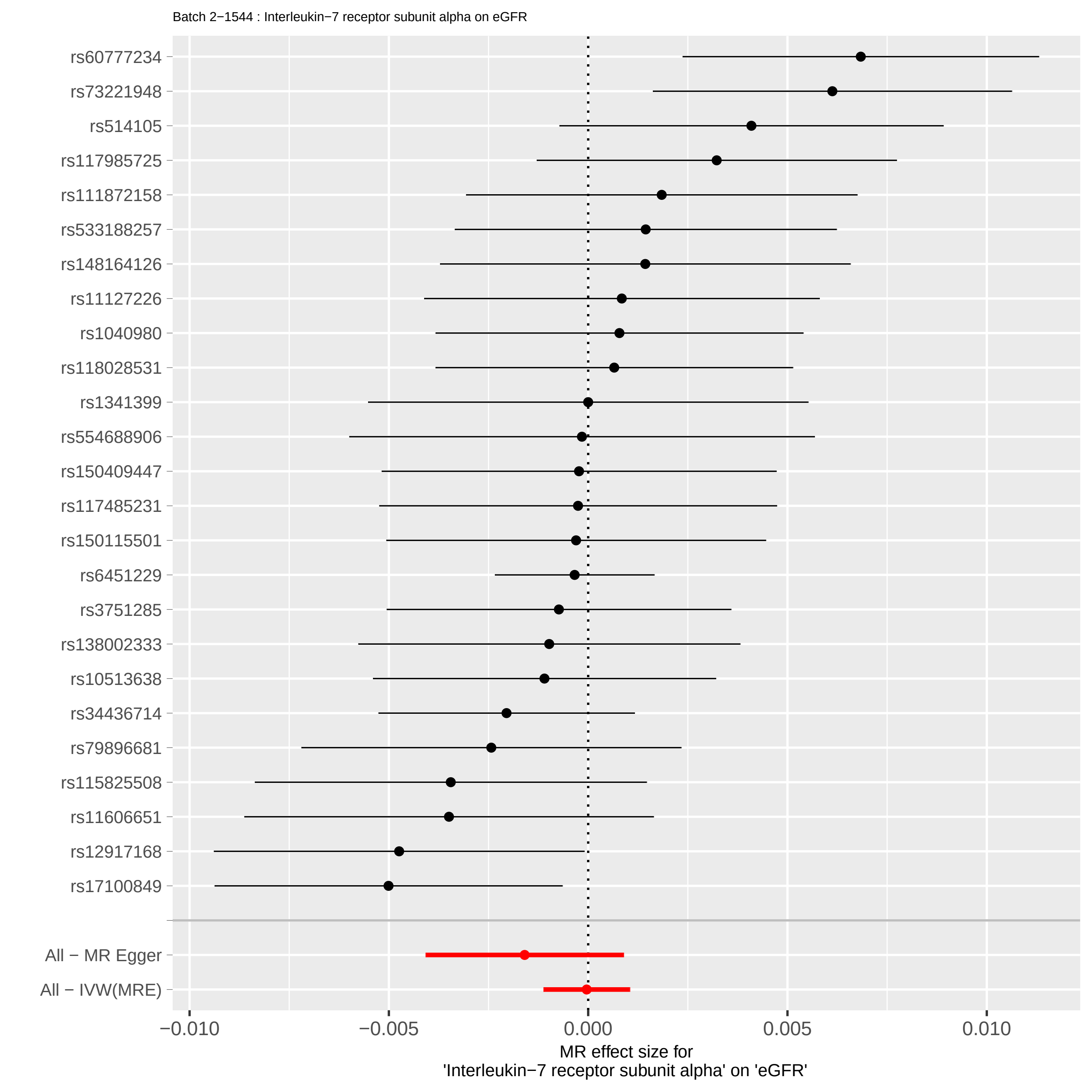

Batch 2−1544 : Interleukin−7 receptor subunit alpha on eGFR
rs60777234
rs73221948
rs514105
rs117985725
rs111872158
rs533188257
rs148164126
rs11127226
rs1040980
rs118028531
rs1341399
rs554688906
rs150409447
rs117485231
rs150115501
rs6451229
rs3751285
rs138002333
rs10513638
rs34436714
rs79896681
rs115825508
rs11606651
rs12917168
rs17100849
All − MR Egger
All − IVW(MRE)
−0.010
−0.005
0.000
0.005
0.010
MR effect size for
'Interleukin−7 receptor subunit alpha' on 'eGFR'

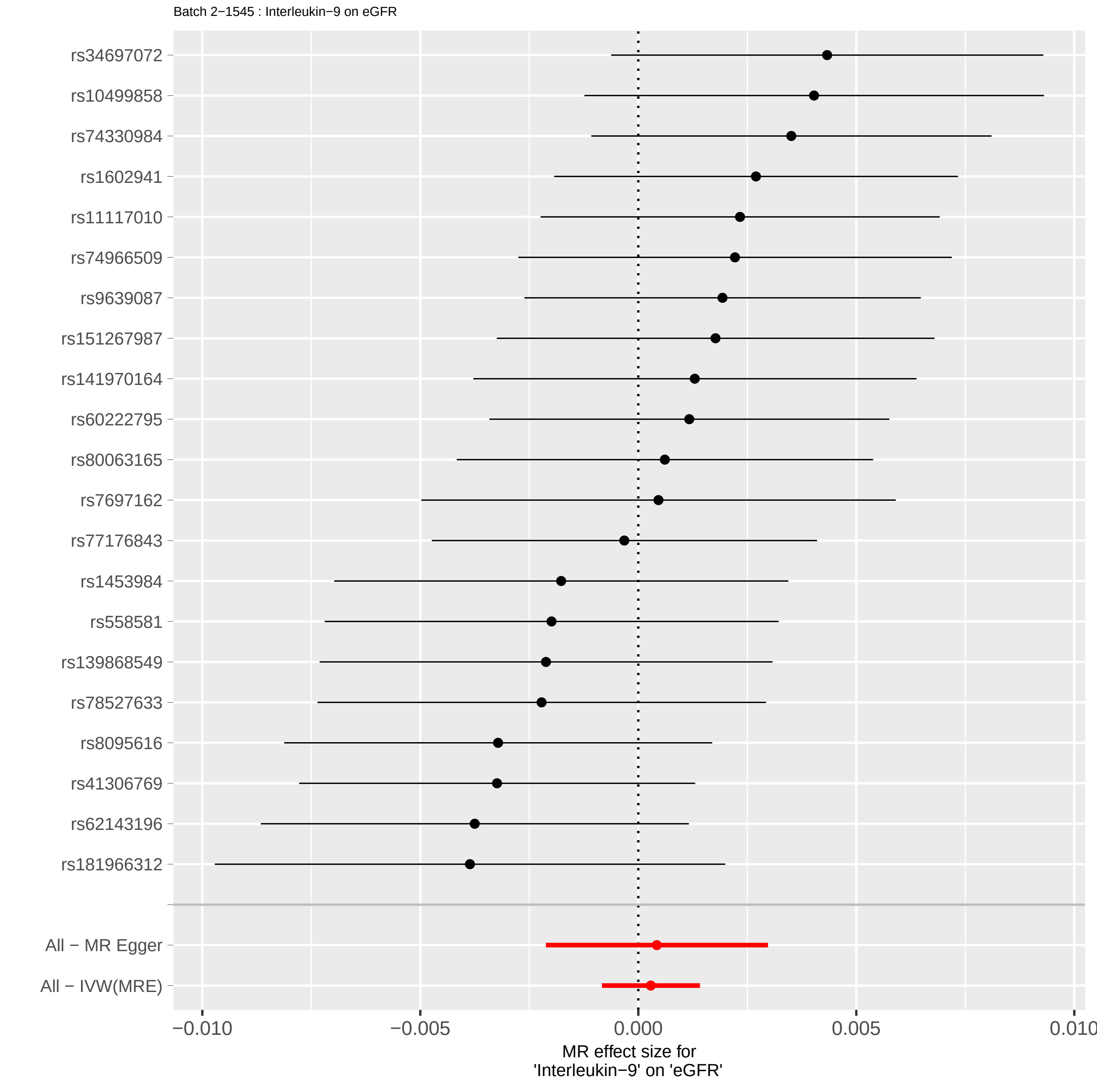

Batch 2−1545 : Interleukin−9 on eGFR
rs34697072
rs10499858
rs74330984
rs1602941
rs11117010
rs74966509
rs9639087
rs151267987
rs141970164
rs60222795
rs80063165
rs7697162
rs77176843
rs1453984
rs558581
rs139868549
rs78527633
rs8095616
rs41306769
rs62143196
rs181966312
All − MR Egger
All − IVW(MRE)
−0.010
−0.005
0.000
0.005
0.010
MR effect size for
'Interleukin−9' on 'eGFR'

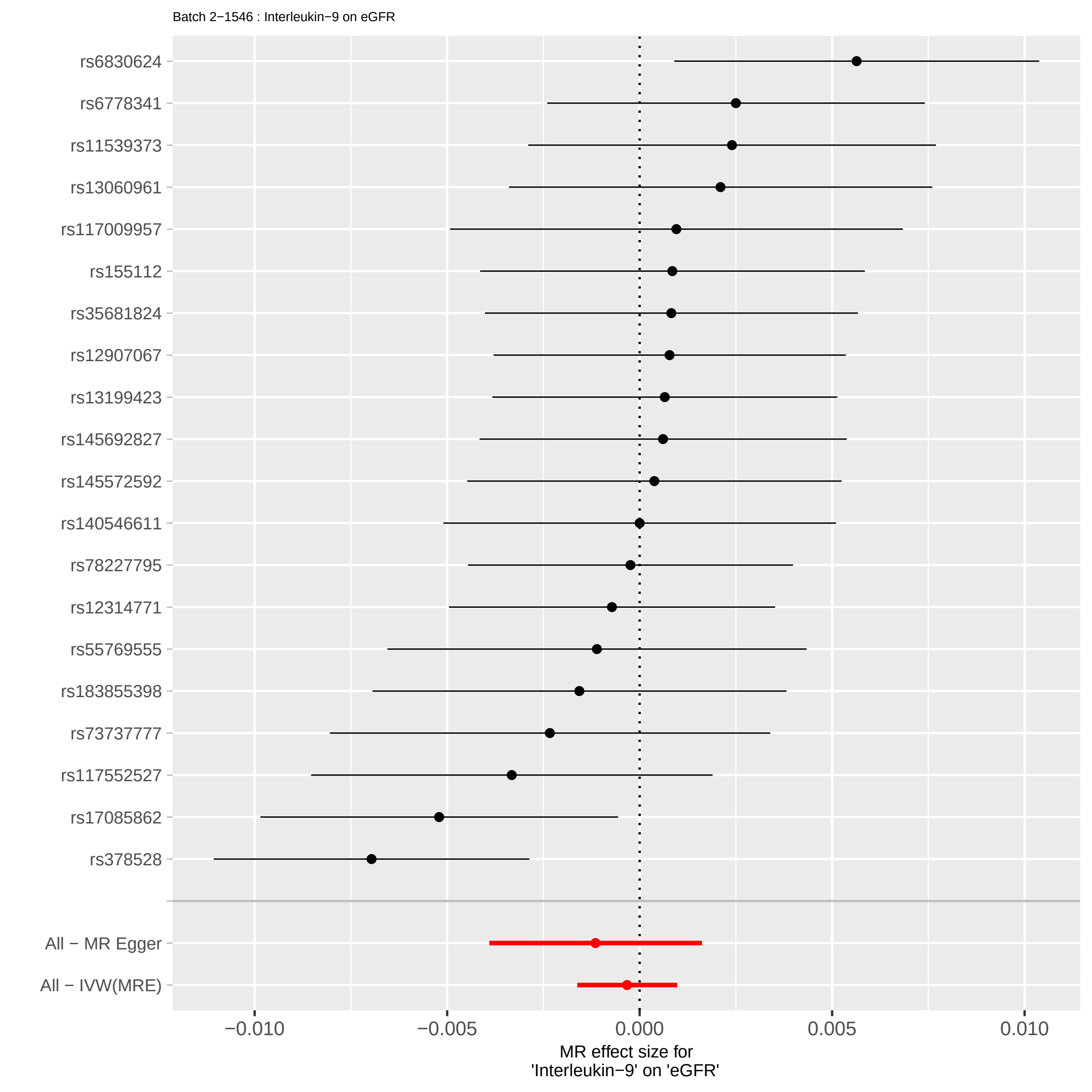

Batch 2−1546 : Interleukin−9 on eGFR
rs6830624
rs6778341
rs11539373
rs13060961
rs117009957
rs155112
rs35681824
rs12907067
rs13199423
rs145692827
rs145572592
rs140546611
rs78227795
rs12314771
rs55769555
rs183855398
rs73737777
rs117552527
rs17085862
rs378528
All − MR Egger
All − IVW(MRE)
−0.010
−0.005
0.000
0.005
0.010
MR effect size for
'Interleukin−9' on 'eGFR'

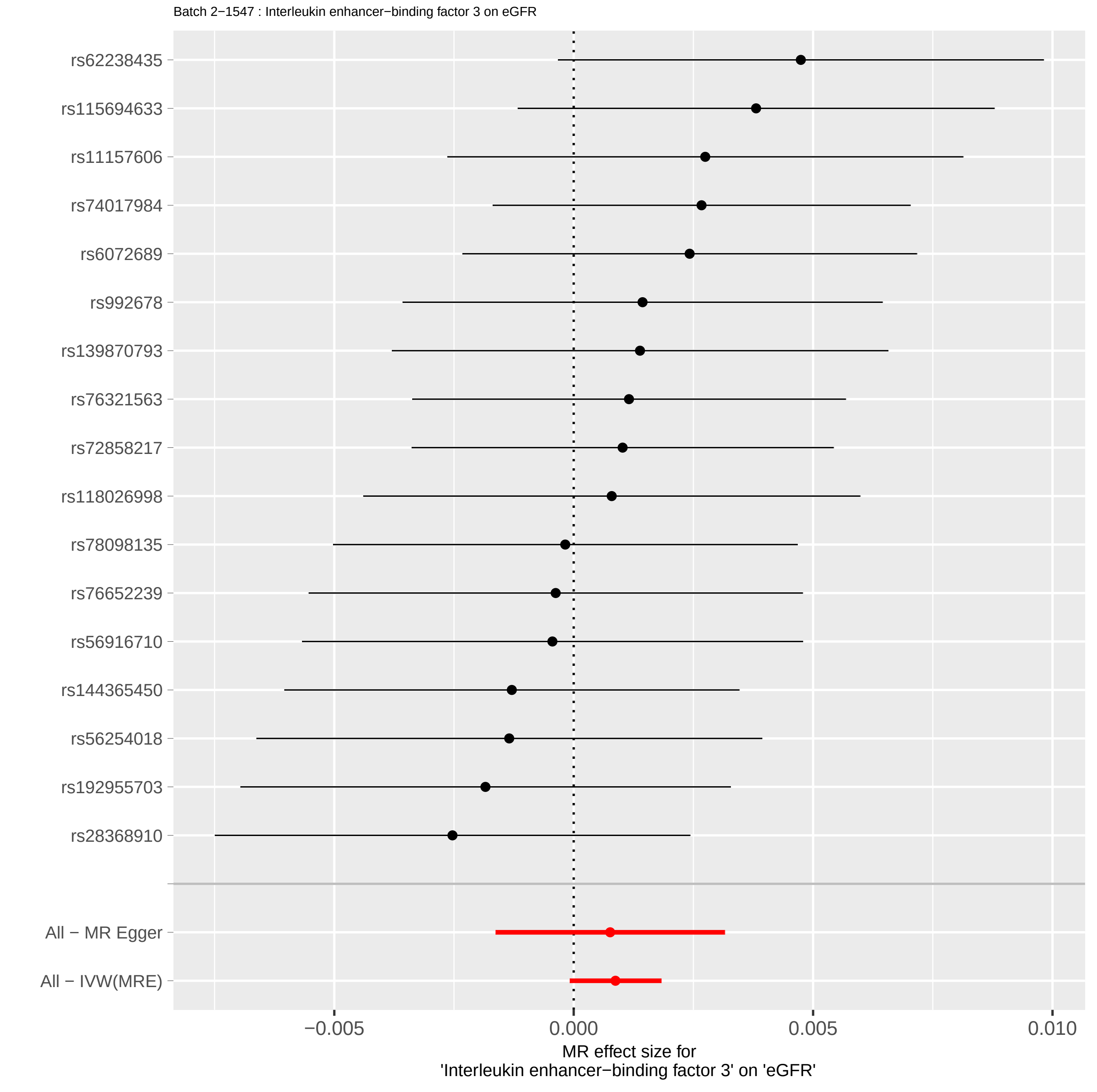
Batch 2−1547 : Interleukin enhancer−binding factor 3 on eGFR
rs62238435
rs115694633
rs11157606
rs74017984
rs6072689
rs992678
rs139870793
rs76321563
rs72858217
rs118026998
rs78098135
rs76652239
rs56916710
rs144365450
rs56254018
rs192955703
rs28368910
All − MR Egger
All − IVW(MRE)
−0.005
0.000
0.005
0.010
MR effect size for
'Interleukin enhancer−binding factor 3' on 'eGFR'

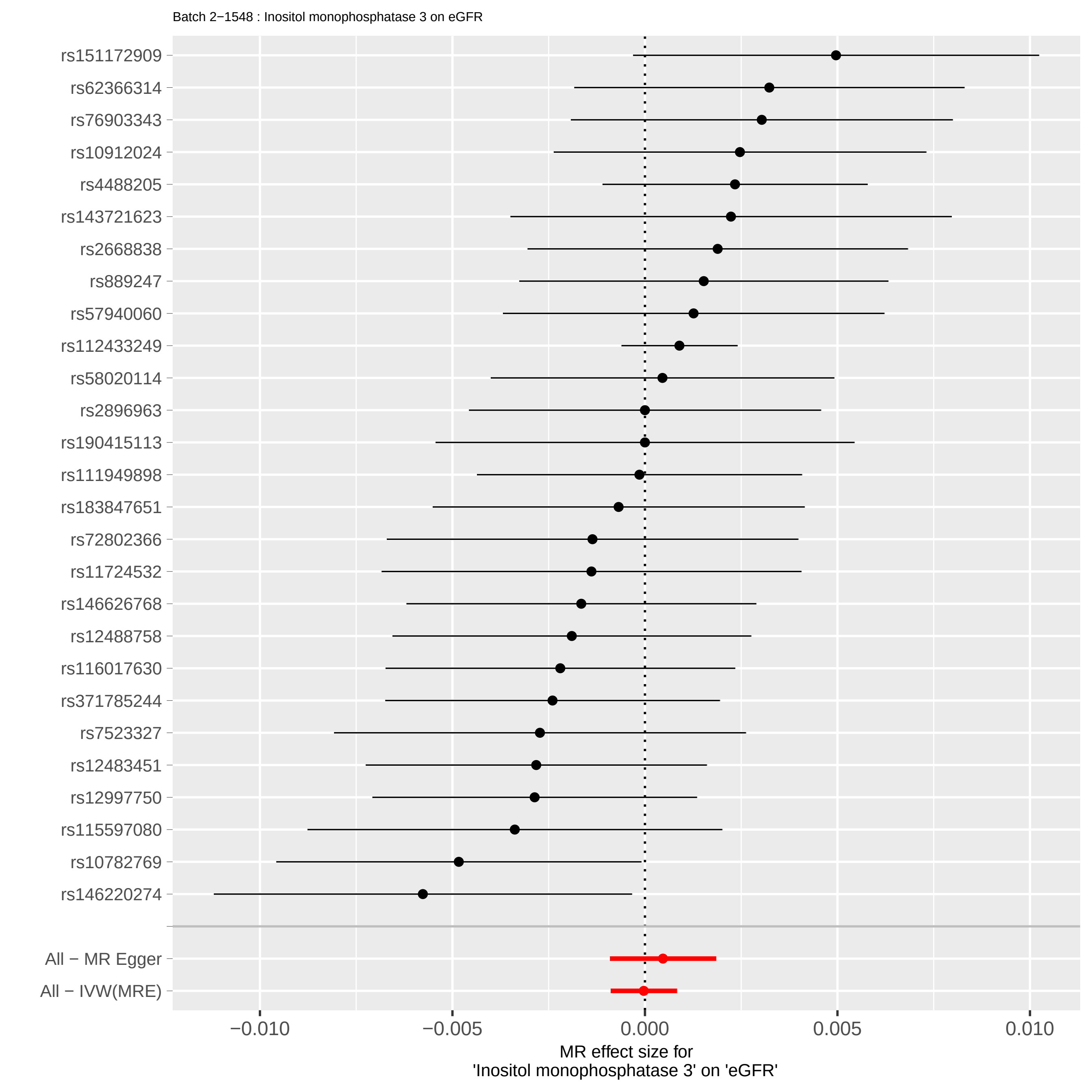

Batch 2−1548 : Inositol monophosphatase 3 on eGFR
rs151172909
rs62366314
rs76903343
rs10912024
rs4488205
rs143721623
rs2668838
rs889247
rs57940060
rs112433249
rs58020114
rs2896963
rs190415113
rs111949898
rs183847651
rs72802366
rs11724532
rs146626768
rs12488758
rs116017630
rs371785244
rs7523327
rs12483451
rs12997750
rs115597080
rs10782769
rs146220274
All − MR Egger
All − IVW(MRE)
−0.010
−0.005
0.000
0.005
0.010
MR effect size for
'Inositol monophosphatase 3' on 'eGFR'

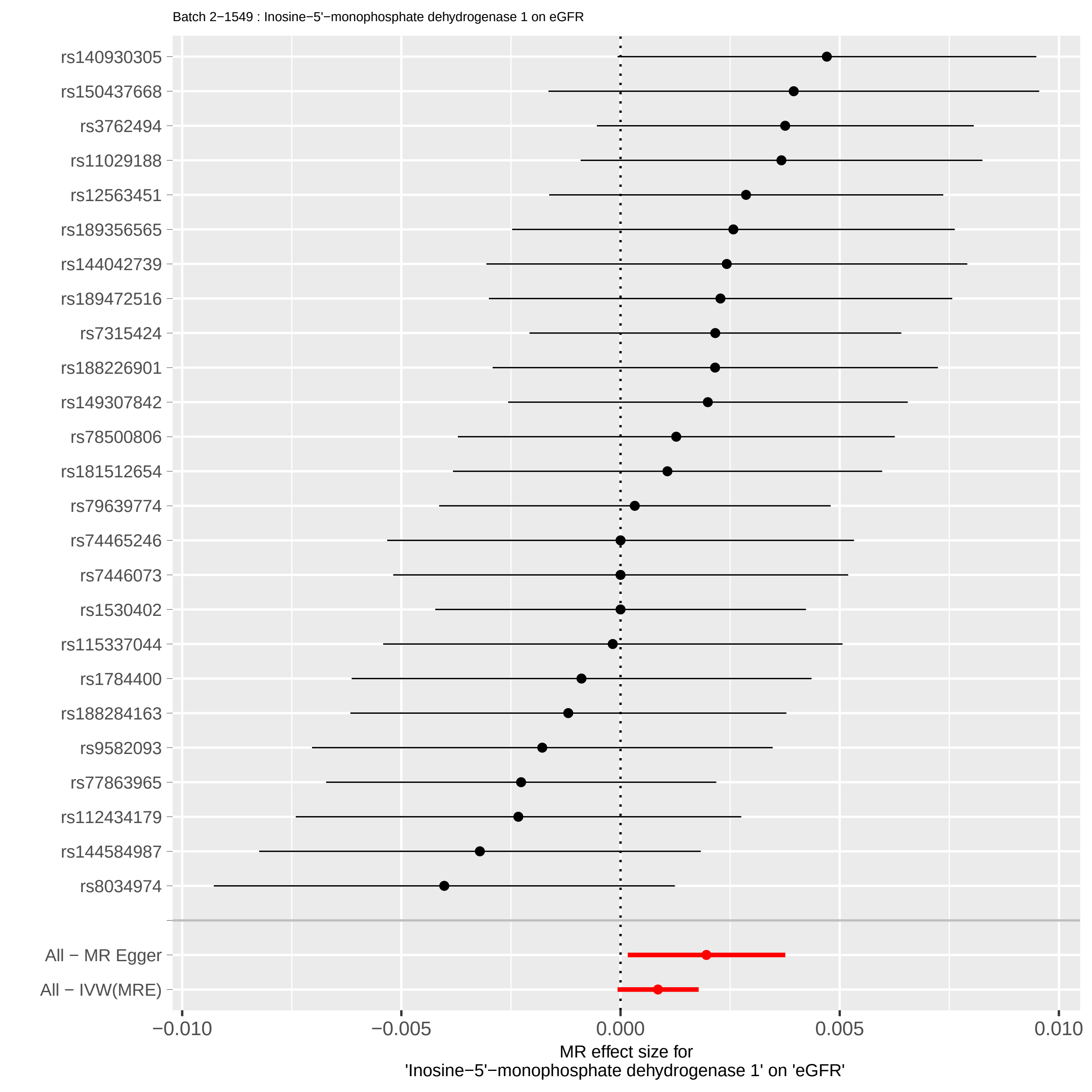

Batch 2−1549 : Inosine−5'−monophosphate dehydrogenase 1 on eGFR
rs140930305
rs150437668
rs3762494
rs11029188
rs12563451
rs189356565
rs144042739
rs189472516
rs7315424
rs188226901
rs149307842
rs78500806
rs181512654
rs79639774
rs74465246
rs7446073
rs1530402
rs115337044
rs1784400
rs188284163
rs9582093
rs77863965
rs112434179
rs144584987
rs8034974
All − MR Egger
All − IVW(MRE)
−0.010
−0.005
0.000
0.005
0.010
MR effect size for
'Inosine−5'−monophosphate dehydrogenase 1' on 'eGFR'

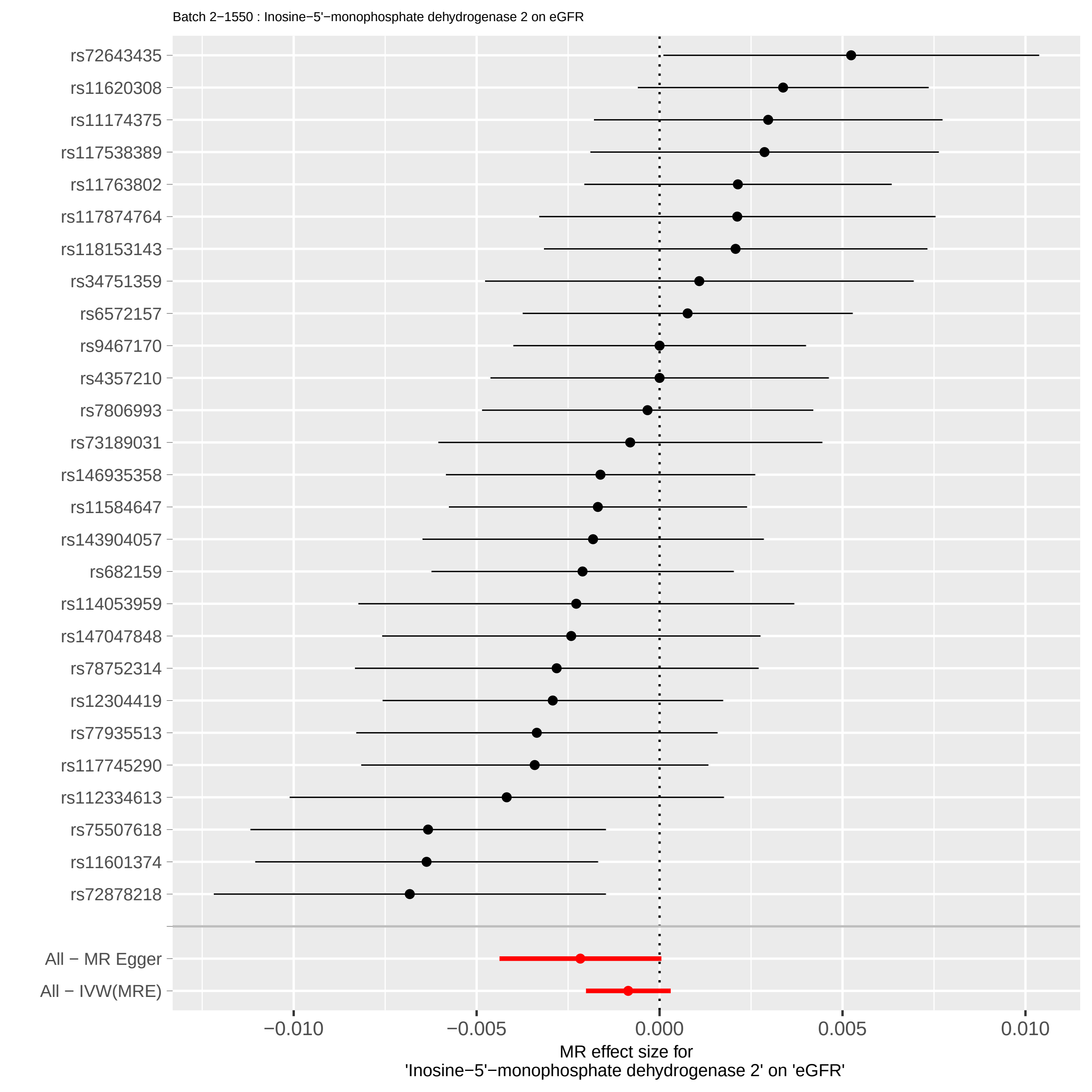
Batch 2−1550 : Inosine−5'−monophosphate dehydrogenase 2 on eGFR
rs72643435
rs11620308
rs11174375
rs117538389
rs11763802
rs117874764
rs118153143
rs34751359
rs6572157
rs9467170
rs4357210
rs7806993
rs73189031
rs146935358
rs11584647
rs143904057
rs682159
rs114053959
rs147047848
rs78752314
rs12304419
rs77935513
rs117745290
rs112334613
rs75507618
rs11601374
rs72878218
All − MR Egger
All − IVW(MRE)
−0.010
−0.005
0.000
0.005
0.010
MR effect size for
'Inosine−5'−monophosphate dehydrogenase 2' on 'eGFR'

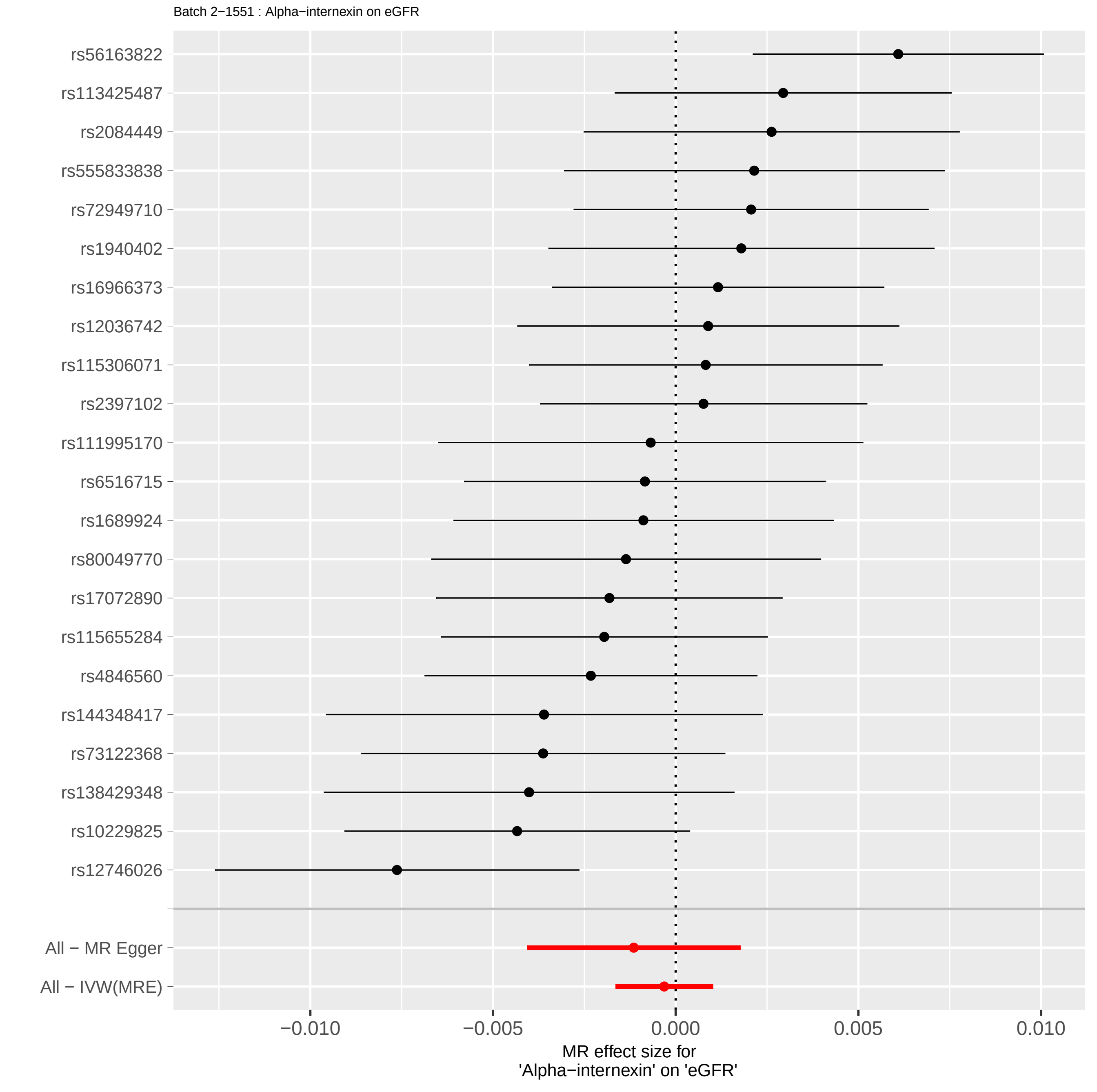

Batch 2−1551 : Alpha−internexin on eGFR
rs56163822
rs113425487
rs2084449
rs555833838
rs72949710
rs1940402
rs16966373
rs12036742
rs115306071
rs2397102
rs111995170
rs6516715
rs1689924
rs80049770
rs17072890
rs115655284
rs4846560
rs144348417
rs73122368
rs138429348
rs10229825
rs12746026
All − MR Egger
All − IVW(MRE)
−0.010
−0.005
0.000
0.005
0.010
MR effect size for
'Alpha−internexin' on 'eGFR'

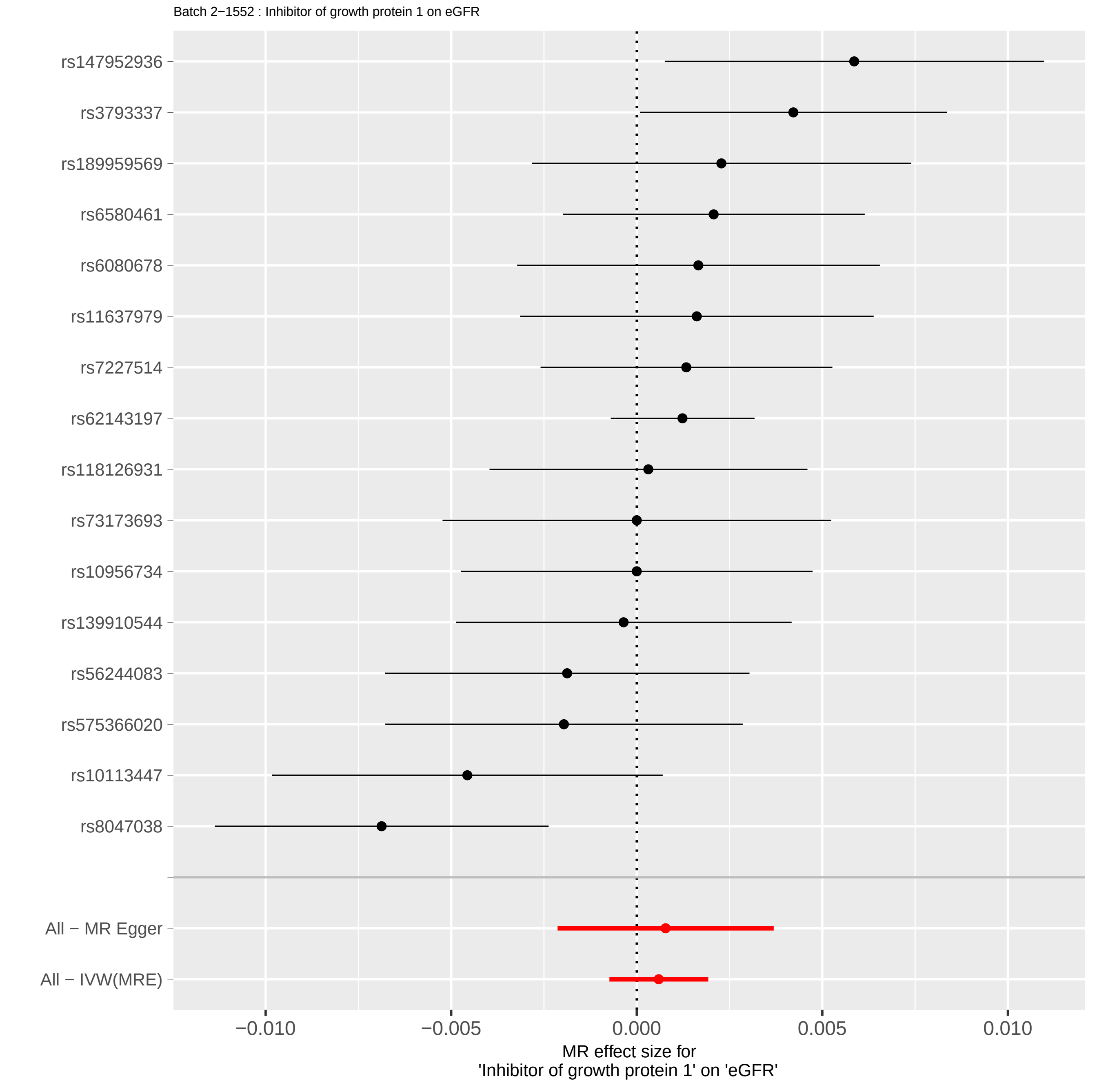

Batch 2−1552 : Inhibitor of growth protein 1 on eGFR
rs147952936
rs3793337
rs189959569
rs6580461
rs6080678
rs11637979
rs7227514
rs62143197
rs118126931
rs73173693
rs10956734
rs139910544
rs56244083
rs575366020
rs10113447
rs8047038
All − MR Egger
All − IVW(MRE)
−0.010
−0.005
0.000
0.005
0.010
MR effect size for
'Inhibitor of growth protein 1' on 'eGFR'

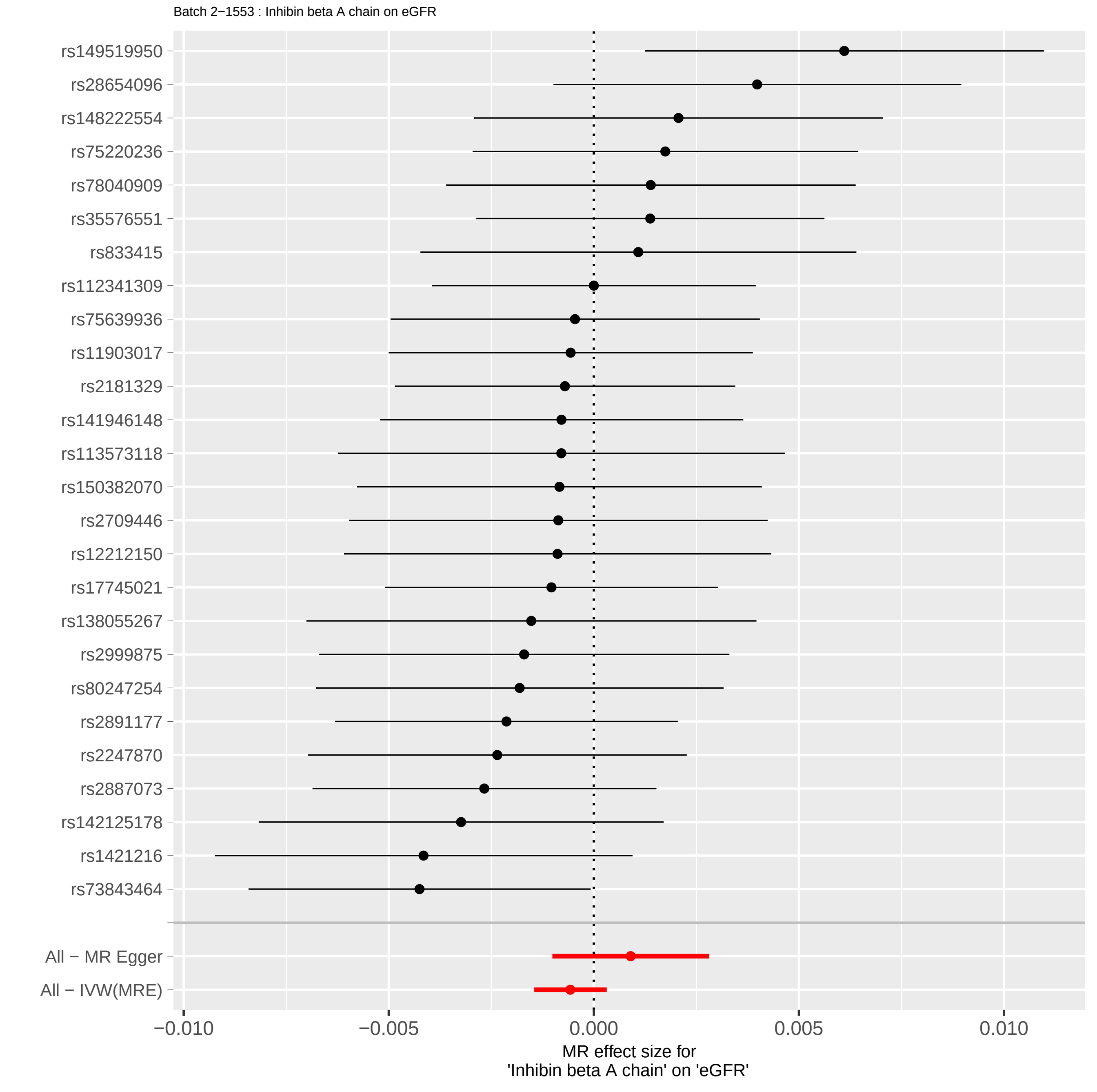

Batch 2−1553 : Inhibin beta A chain on eGFR
rs149519950
rs28654096
rs148222554
rs75220236
rs78040909
rs35576551
rs833415
rs112341309
rs75639936
rs11903017
rs2181329
rs141946148
rs113573118
rs150382070
rs2709446
rs12212150
rs17745021
rs138055267
rs2999875
rs80247254
rs2891177
rs2247870
rs2887073
rs142125178
rs1421216
rs73843464
All − MR Egger
All − IVW(MRE)
−0.010
−0.005
0.000
0.005
0.010
MR effect size for
'Inhibin beta A chain' on 'eGFR'

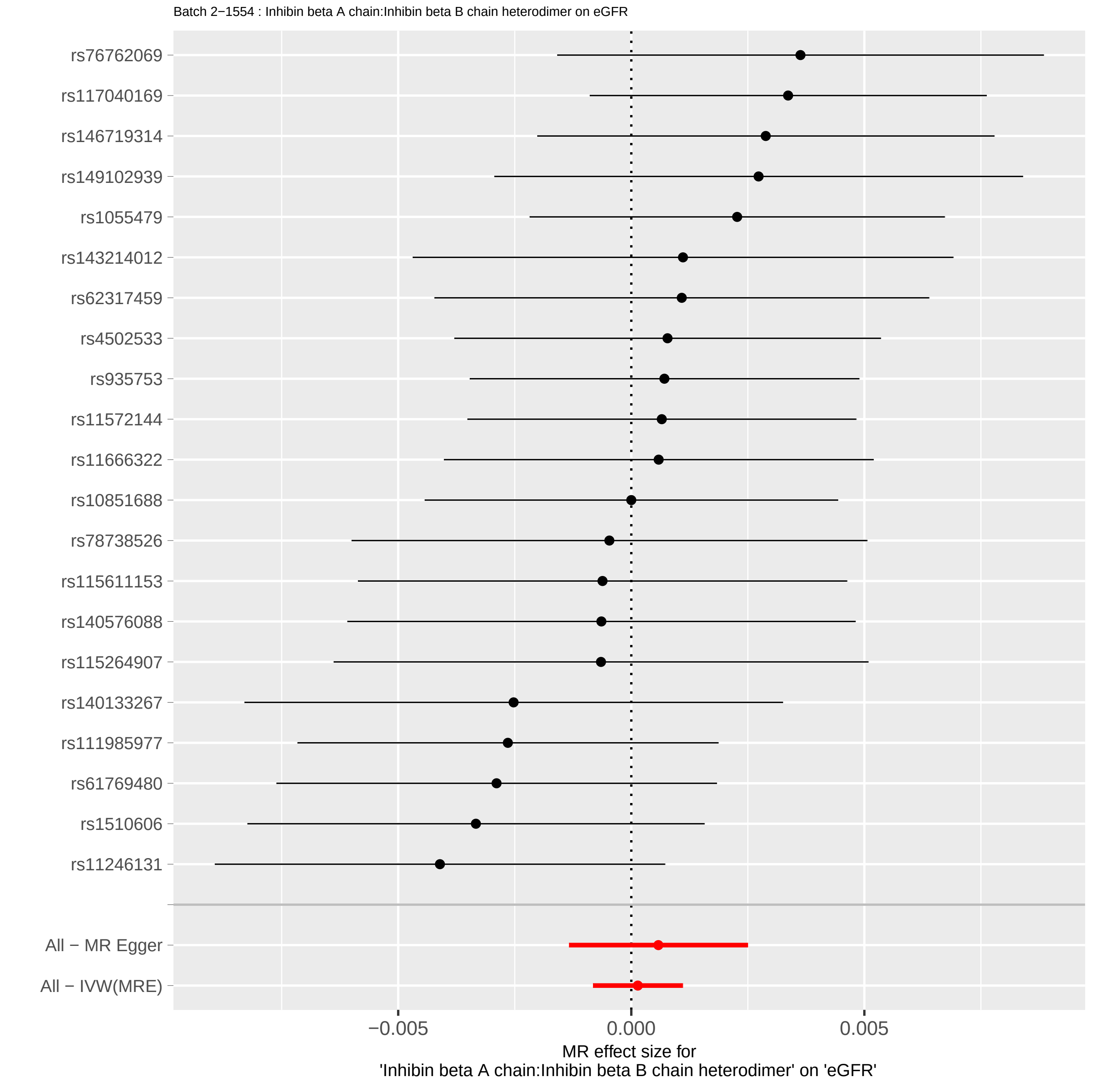

Batch 2−1554 : Inhibin beta A chain:Inhibin beta B chain heterodimer on eGFR
rs76762069
rs117040169
rs146719314
rs149102939
rs1055479
rs143214012
rs62317459
rs4502533
rs935753
rs11572144
rs11666322
rs10851688
rs78738526
rs115611153
rs140576088
rs115264907
rs140133267
rs111985977
rs61769480
rs1510606
rs11246131
All − MR Egger
All − IVW(MRE)
−0.005
0.000
0.005
MR effect size for
'Inhibin beta A chain:Inhibin beta B chain heterodimer' on 'eGFR'

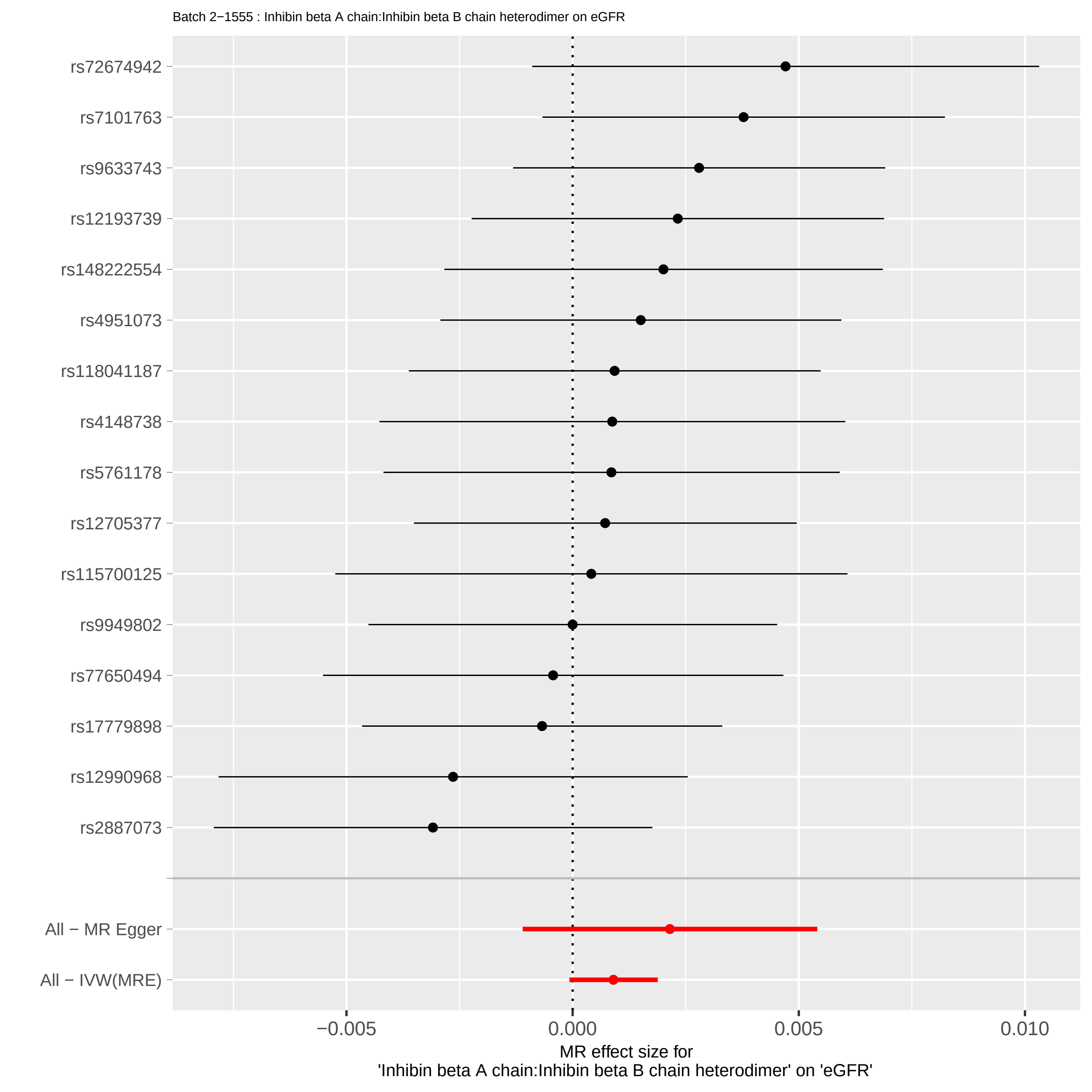

Batch 2−1555 : Inhibin beta A chain:Inhibin beta B chain heterodimer on eGFR
rs72674942
rs7101763
rs9633743
rs12193739
rs148222554
rs4951073
rs118041187
rs4148738
rs5761178
rs12705377
rs115700125
rs9949802
rs77650494
rs17779898
rs12990968
rs2887073
All − MR Egger
All − IVW(MRE)
−0.005
0.000
0.005
0.010
MR effect size for
'Inhibin beta A chain:Inhibin beta B chain heterodimer' on 'eGFR'

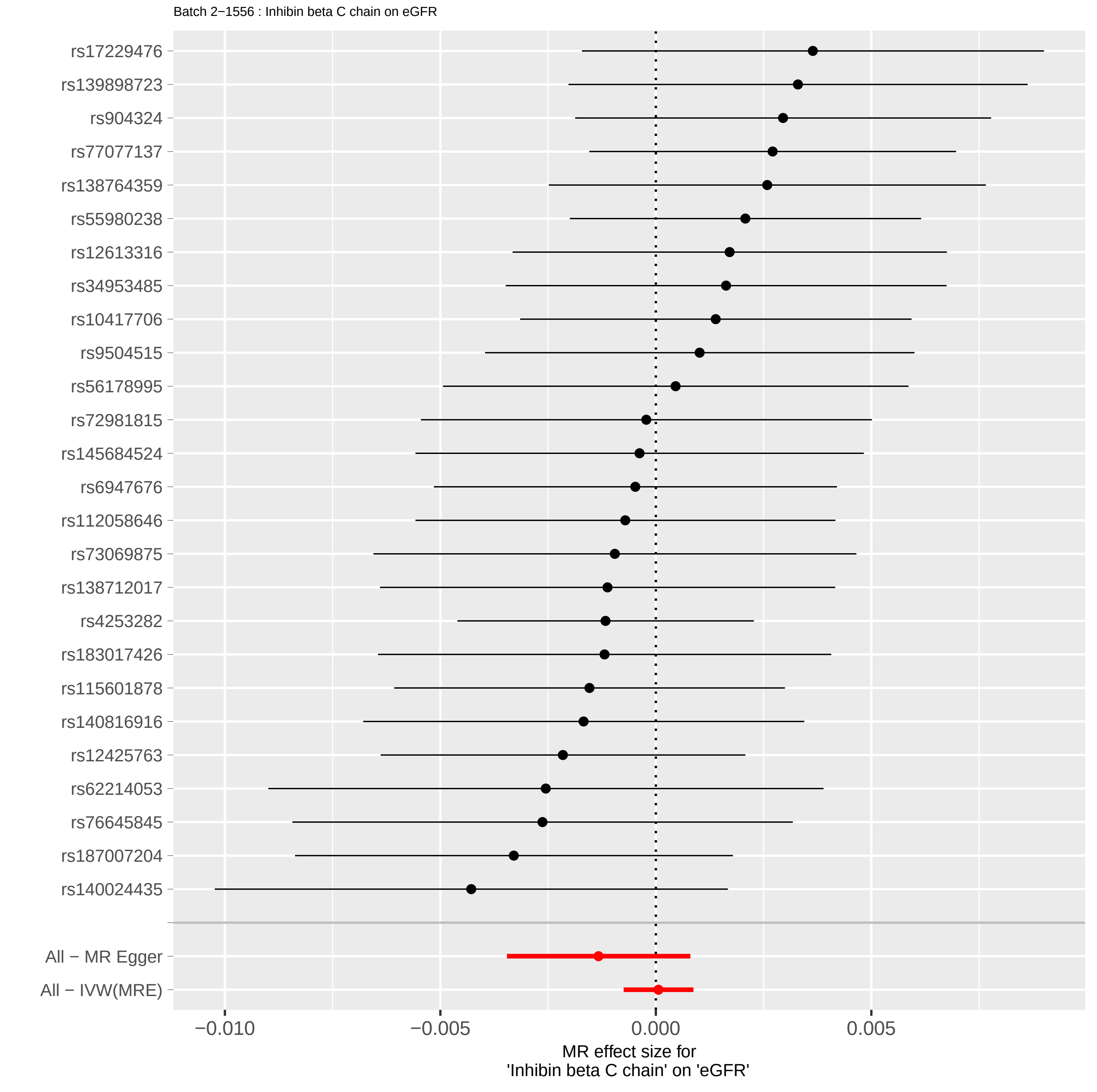

Batch 2−1556 : Inhibin beta C chain on eGFR
rs17229476
rs139898723
rs904324
rs77077137
rs138764359
rs55980238
rs12613316
rs34953485
rs10417706
rs9504515
rs56178995
rs72981815
rs145684524
rs6947676
rs112058646
rs73069875
rs138712017
rs4253282
rs183017426
rs115601878
rs140816916
rs12425763
rs62214053
rs76645845
rs187007204
rs140024435
All − MR Egger
All − IVW(MRE)
−0.010
−0.005
0.000
0.005
MR effect size for
'Inhibin beta C chain' on 'eGFR'

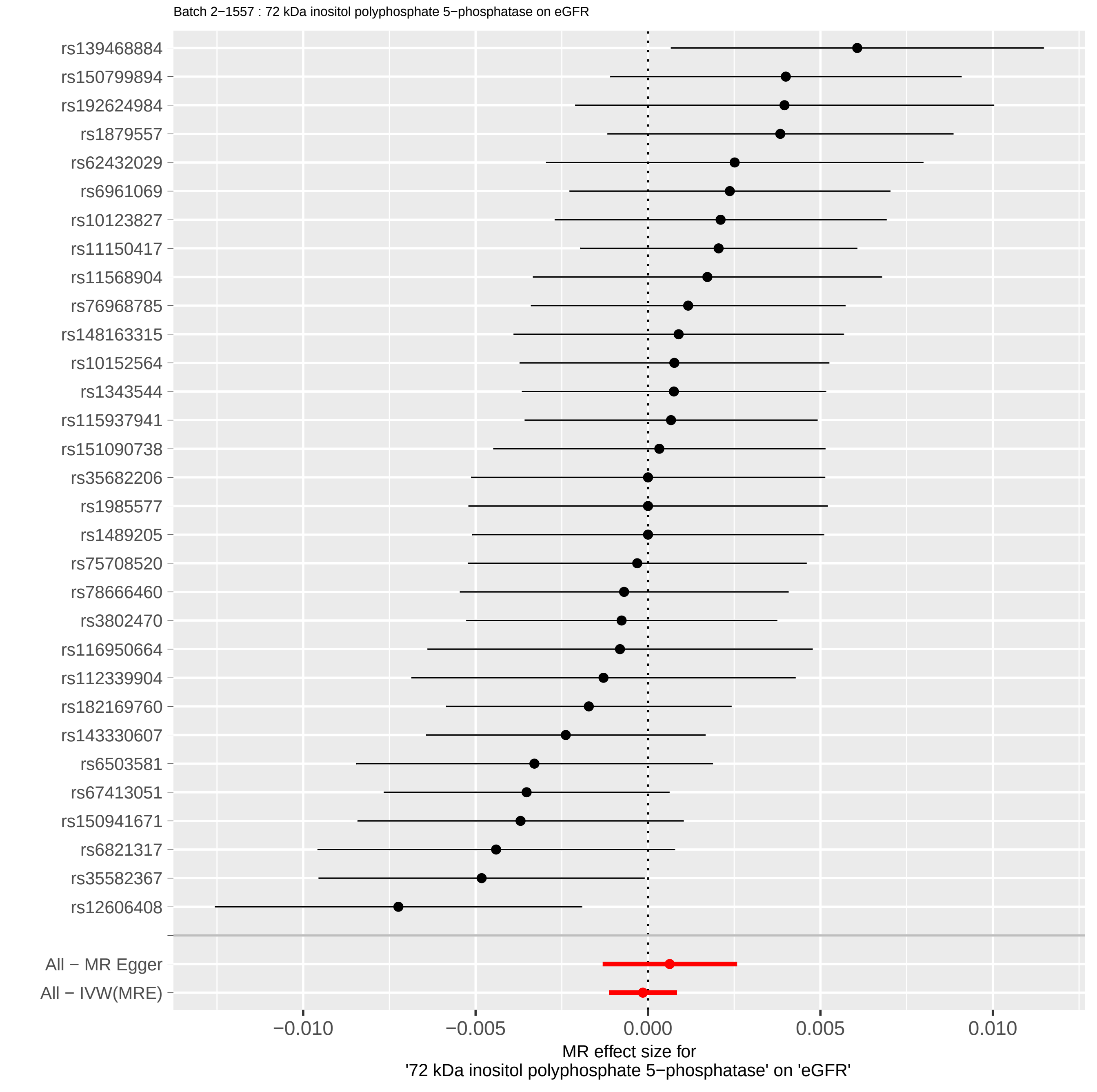
Batch 2−1557 : 72 kDa inositol polyphosphate 5−phosphatase on eGFR
rs139468884
rs150799894
rs192624984
rs1879557
rs62432029
rs6961069
rs10123827
rs11150417
rs11568904
rs76968785
rs148163315
rs10152564
rs1343544
rs115937941
rs151090738
rs35682206
rs1985577
rs1489205
rs75708520
rs78666460
rs3802470
rs116950664
rs112339904
rs182169760
rs143330607
rs6503581
rs67413051
rs150941671
rs6821317
rs35582367
rs12606408
All − MR Egger
All − IVW(MRE)
−0.010
−0.005
0.000
0.005
0.010
MR effect size for
'72 kDa inositol polyphosphate 5−phosphatase' on 'eGFR'

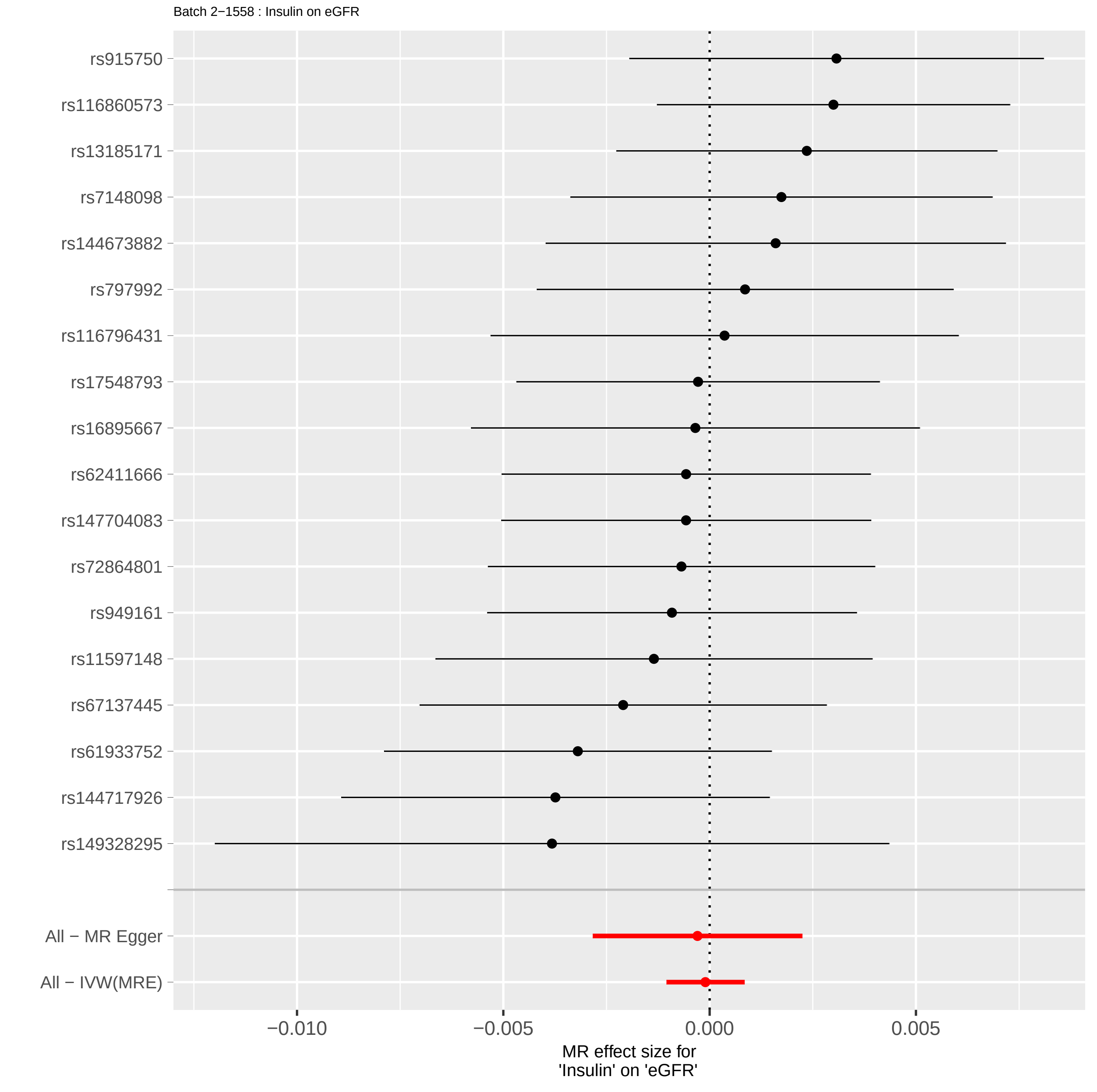
Batch 2−1558 : Insulin on eGFR
rs915750
rs116860573
rs13185171
rs7148098
rs144673882
rs797992
rs116796431
rs17548793
rs16895667
rs62411666
rs147704083
rs72864801
rs949161
rs11597148
rs67137445
rs61933752
rs144717926
rs149328295
All − MR Egger
All − IVW(MRE)
−0.010
−0.005
0.000
0.005
MR effect size for
'Insulin' on 'eGFR'

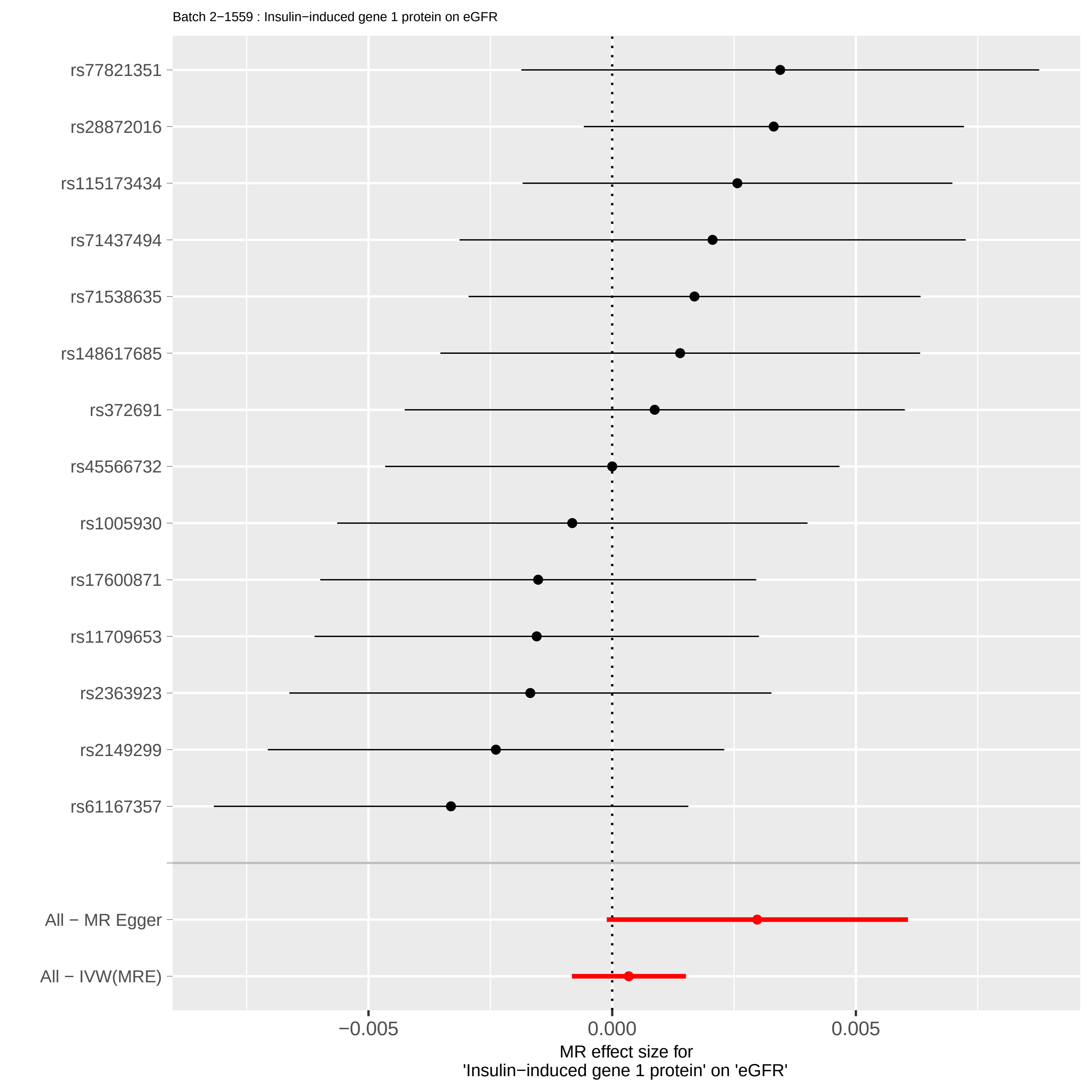

Batch 2−1559 : Insulin−induced gene 1 protein on eGFR
rs77821351
rs28872016
rs115173434
rs71437494
rs71538635
rs148617685
rs372691
rs45566732
rs1005930
rs17600871
rs11709653
rs2363923
rs2149299
rs61167357
All − MR Egger
All − IVW(MRE)
−0.005
0.000
0.005
MR effect size for
'Insulin−induced gene 1 protein' on 'eGFR'

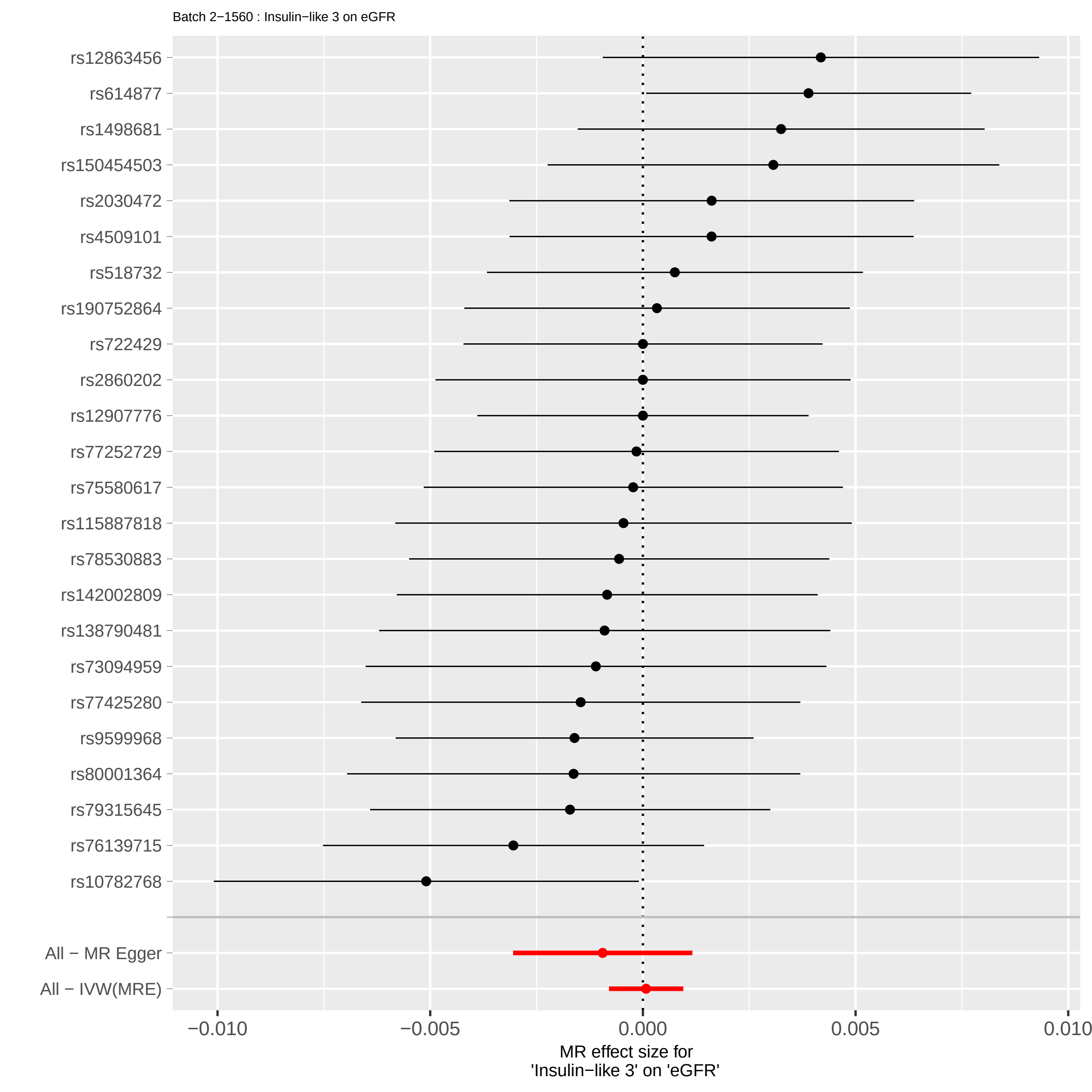
Batch 2−1560 : Insulin−like 3 on eGFR
rs12863456
rs614877
rs1498681
rs150454503
rs2030472
rs4509101
rs518732
rs190752864
rs722429
rs2860202
rs12907776
rs77252729
rs75580617
rs115887818
rs78530883
rs142002809
rs138790481
rs73094959
rs77425280
rs9599968
rs80001364
rs79315645
rs76139715
rs10782768
All − MR Egger
All − IVW(MRE)
−0.010
−0.005
0.000
0.005
0.010
MR effect size for
'Insulin−like 3' on 'eGFR'

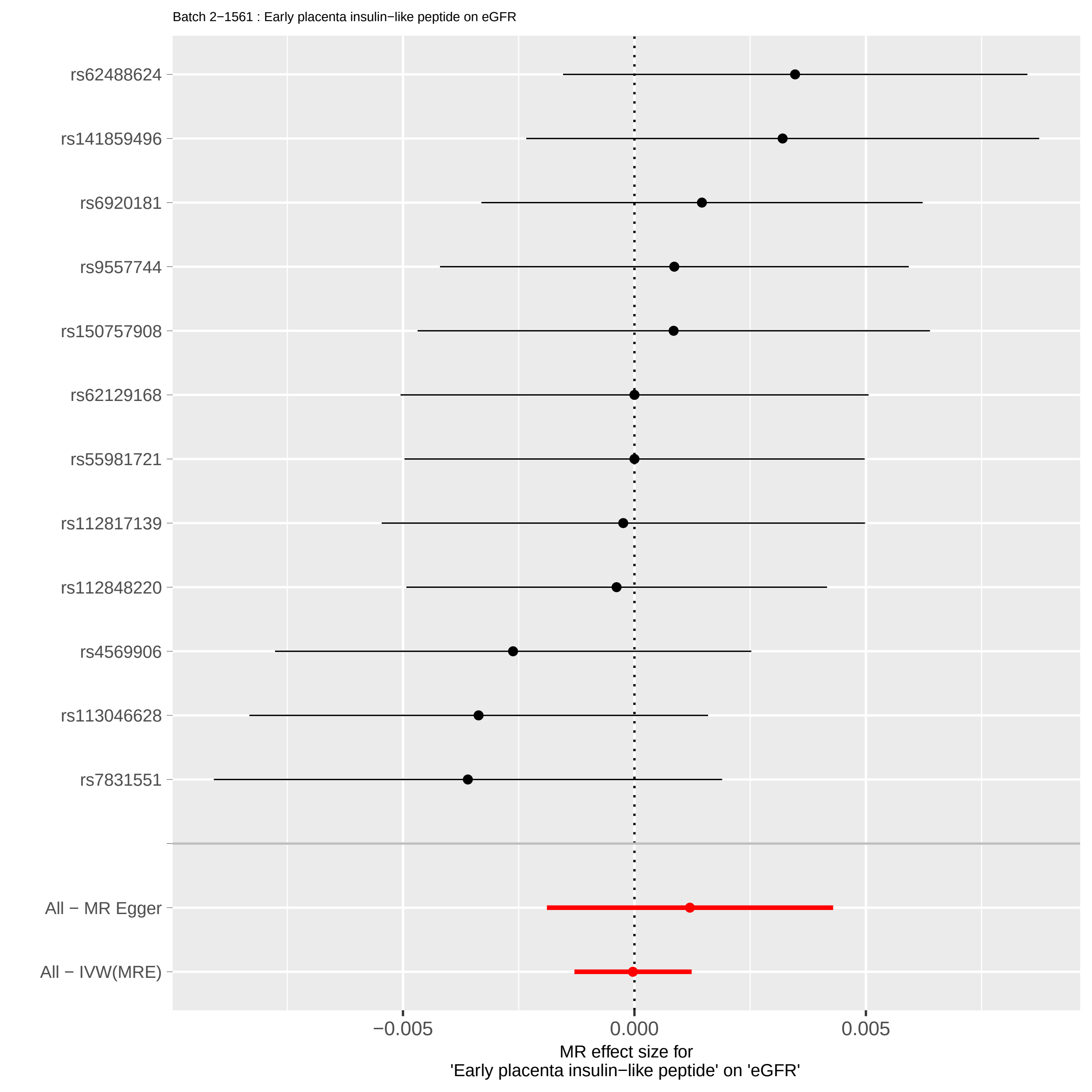
Batch 2−1561 : Early placenta insulin−like peptide on eGFR
rs62488624
rs141859496
rs6920181
rs9557744
rs150757908
rs62129168
rs55981721
rs112817139
rs112848220
rs4569906
rs113046628
rs7831551
All − MR Egger
All − IVW(MRE)
−0.005
0.000
0.005
MR effect size for
'Early placenta insulin−like peptide' on 'eGFR'

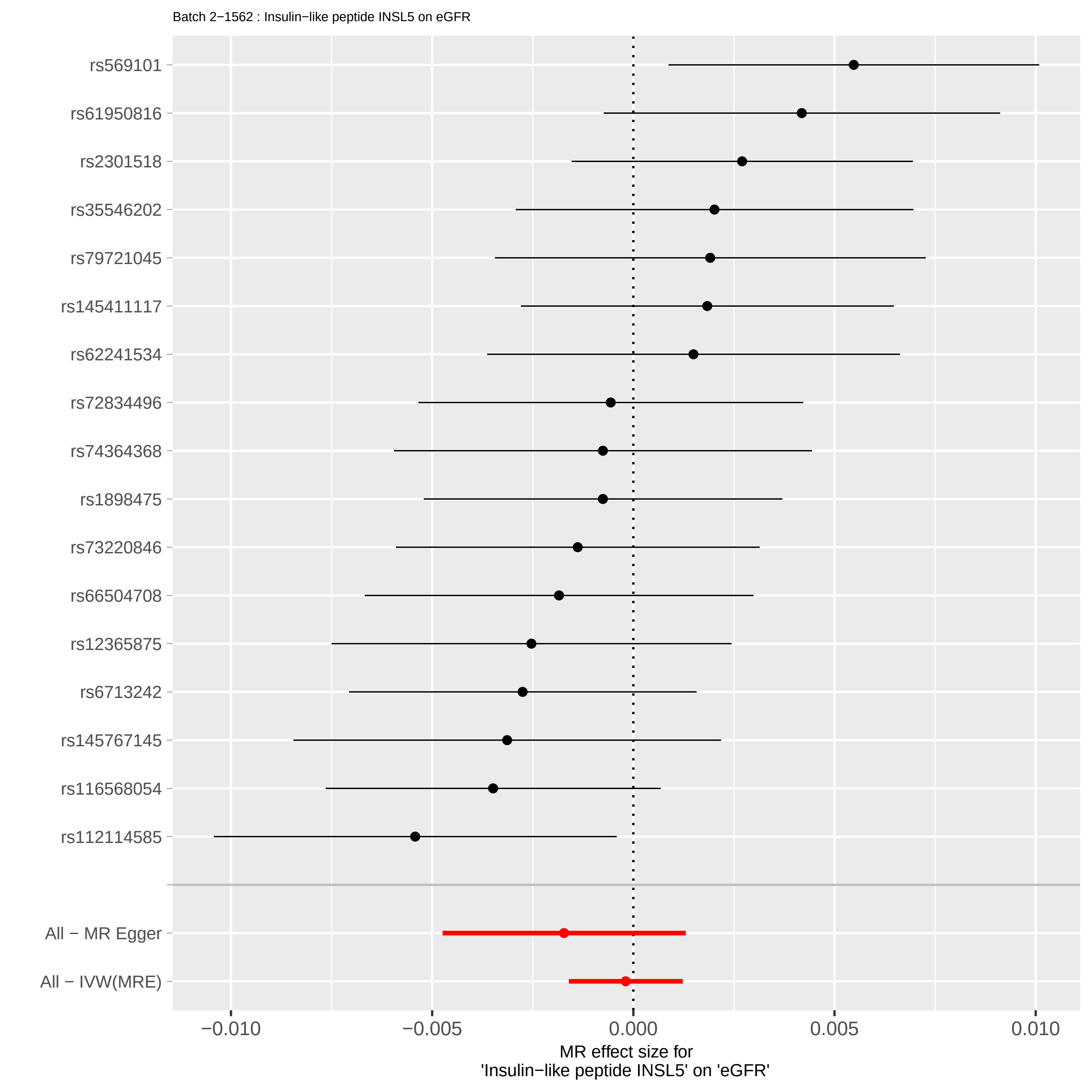

Batch 2−1562 : Insulin−like peptide INSL5 on eGFR
rs569101
rs61950816
rs2301518
rs35546202
rs79721045
rs145411117
rs62241534
rs72834496
rs74364368
rs1898475
rs73220846
rs66504708
rs12365875
rs6713242
rs145767145
rs116568054
rs112114585
All − MR Egger
All − IVW(MRE)
−0.010
−0.005
0.000
0.005
0.010
MR effect size for
'Insulin−like peptide INSL5' on 'eGFR'

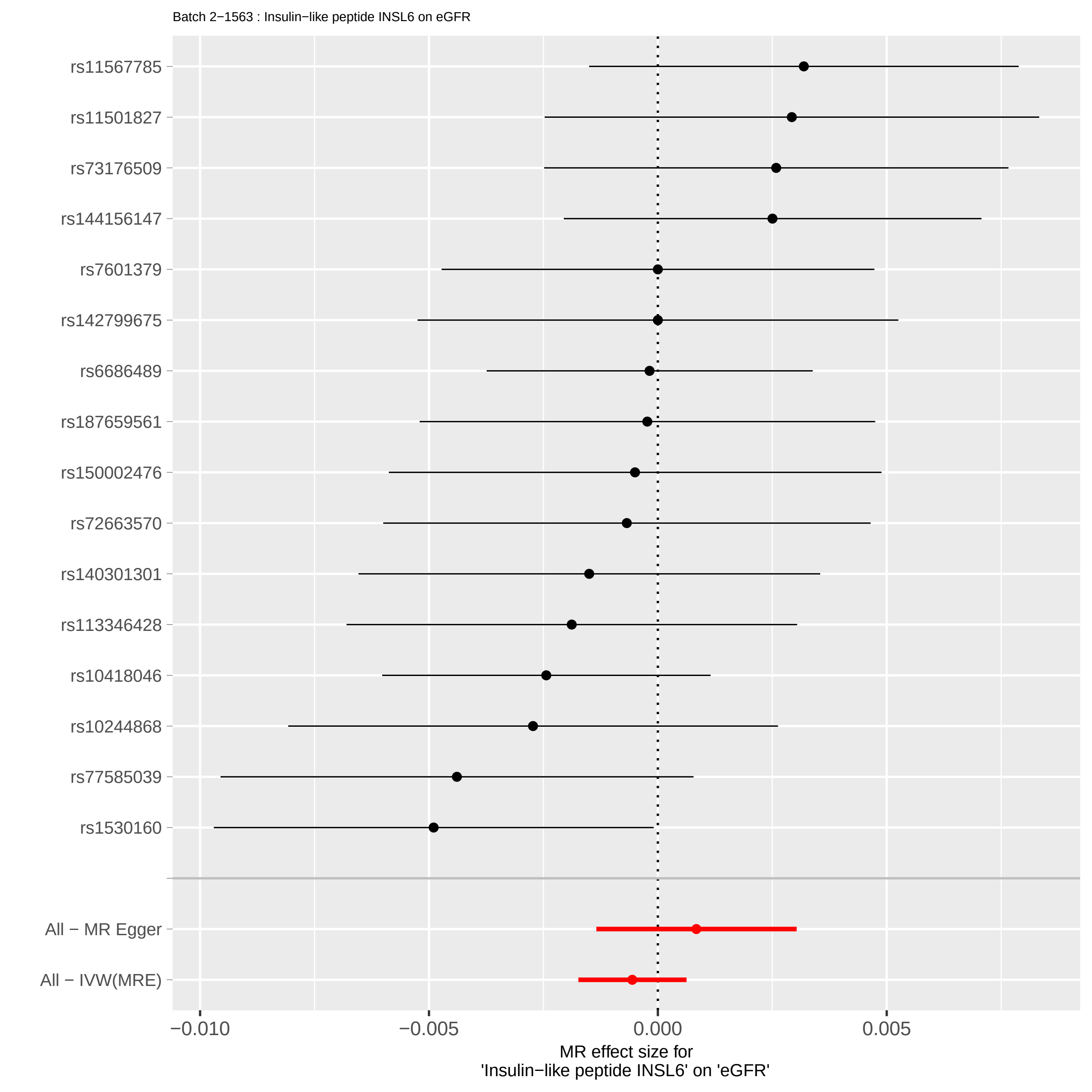

Batch 2−1563 : Insulin−like peptide INSL6 on eGFR
rs11567785
rs11501827
rs73176509
rs144156147
rs7601379
rs142799675
rs6686489
rs187659561
rs150002476
rs72663570
rs140301301
rs113346428
rs10418046
rs10244868
rs77585039
rs1530160
All − MR Egger
All − IVW(MRE)
−0.010
−0.005
0.000
0.005
MR effect size for
'Insulin−like peptide INSL6' on 'eGFR'

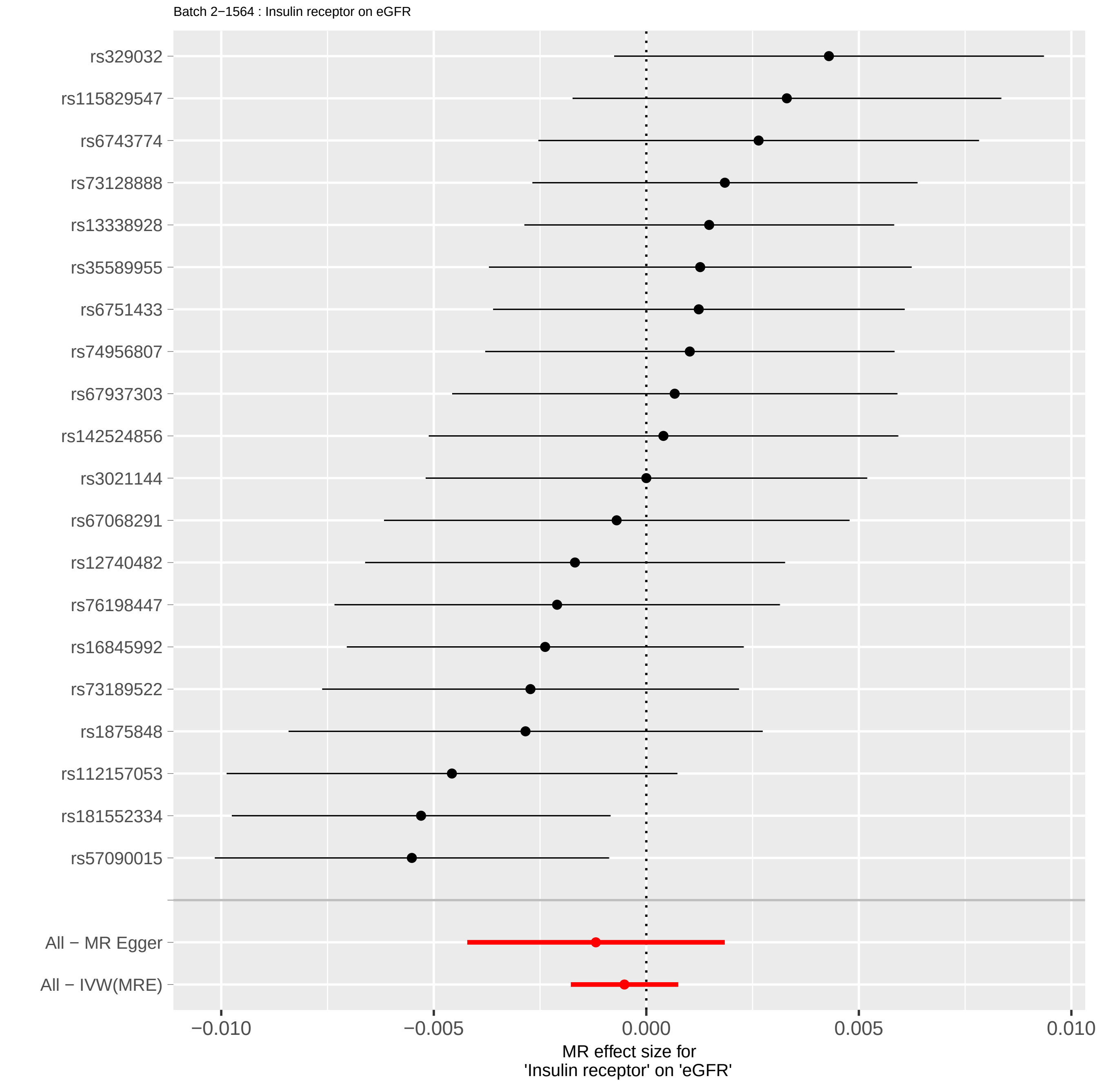
Batch 2−1564 : Insulin receptor on eGFR
rs329032
rs115829547
rs6743774
rs73128888
rs13338928
rs35589955
rs6751433
rs74956807
rs67937303
rs142524856
rs3021144
rs67068291
rs12740482
rs76198447
rs16845992
rs73189522
rs1875848
rs112157053
rs181552334
rs57090015
All − MR Egger
All − IVW(MRE)
−0.010
−0.005
0.000
0.005
0.010
MR effect size for
'Insulin receptor' on 'eGFR'

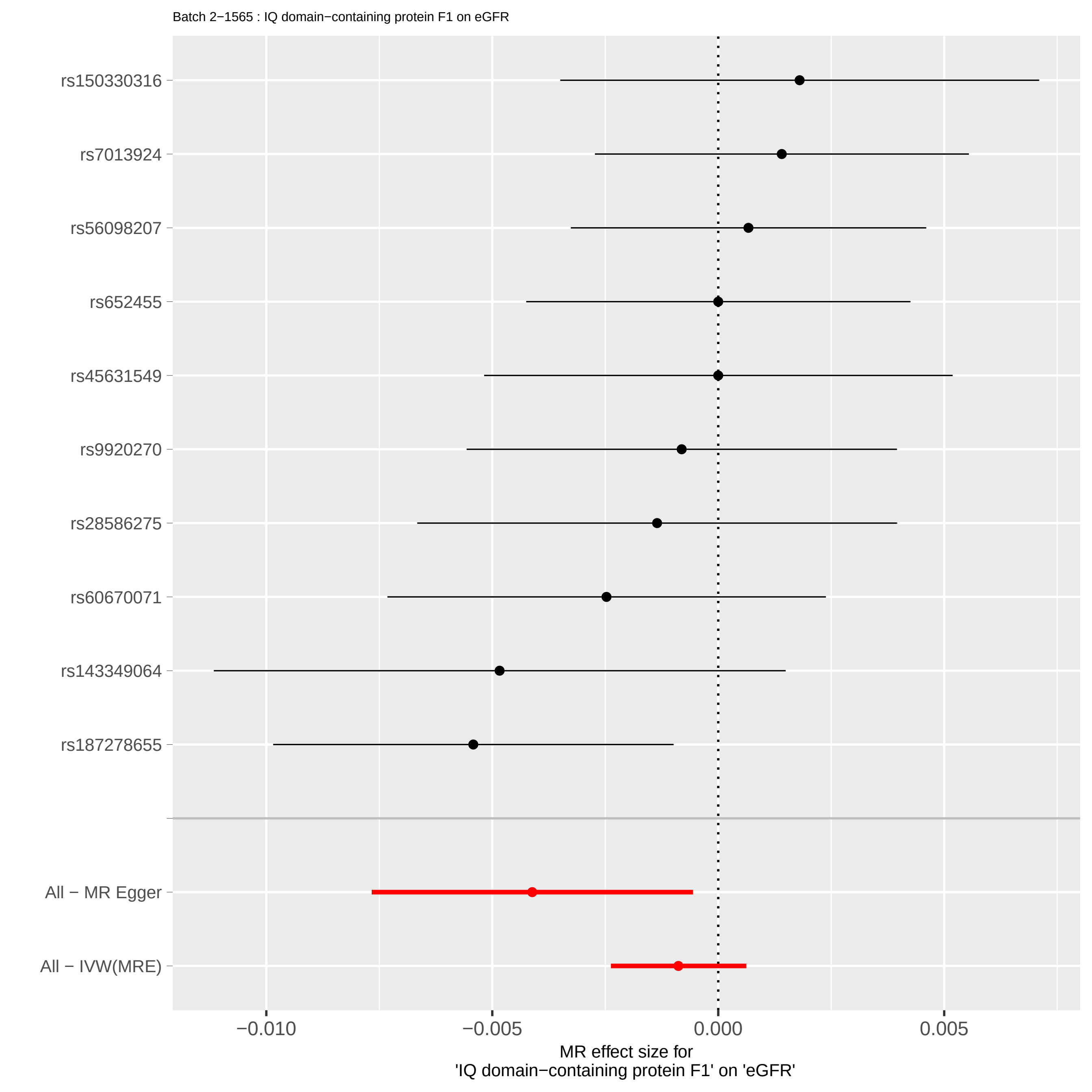
Batch 2−1565 : IQ domain−containing protein F1 on eGFR
rs150330316
rs7013924
rs56098207
rs652455
rs45631549
rs9920270
rs28586275
rs60670071
rs143349064
rs187278655
All − MR Egger
All − IVW(MRE)
−0.010
−0.005
0.000
0.005
MR effect size for
'IQ domain−containing protein F1' on 'eGFR'

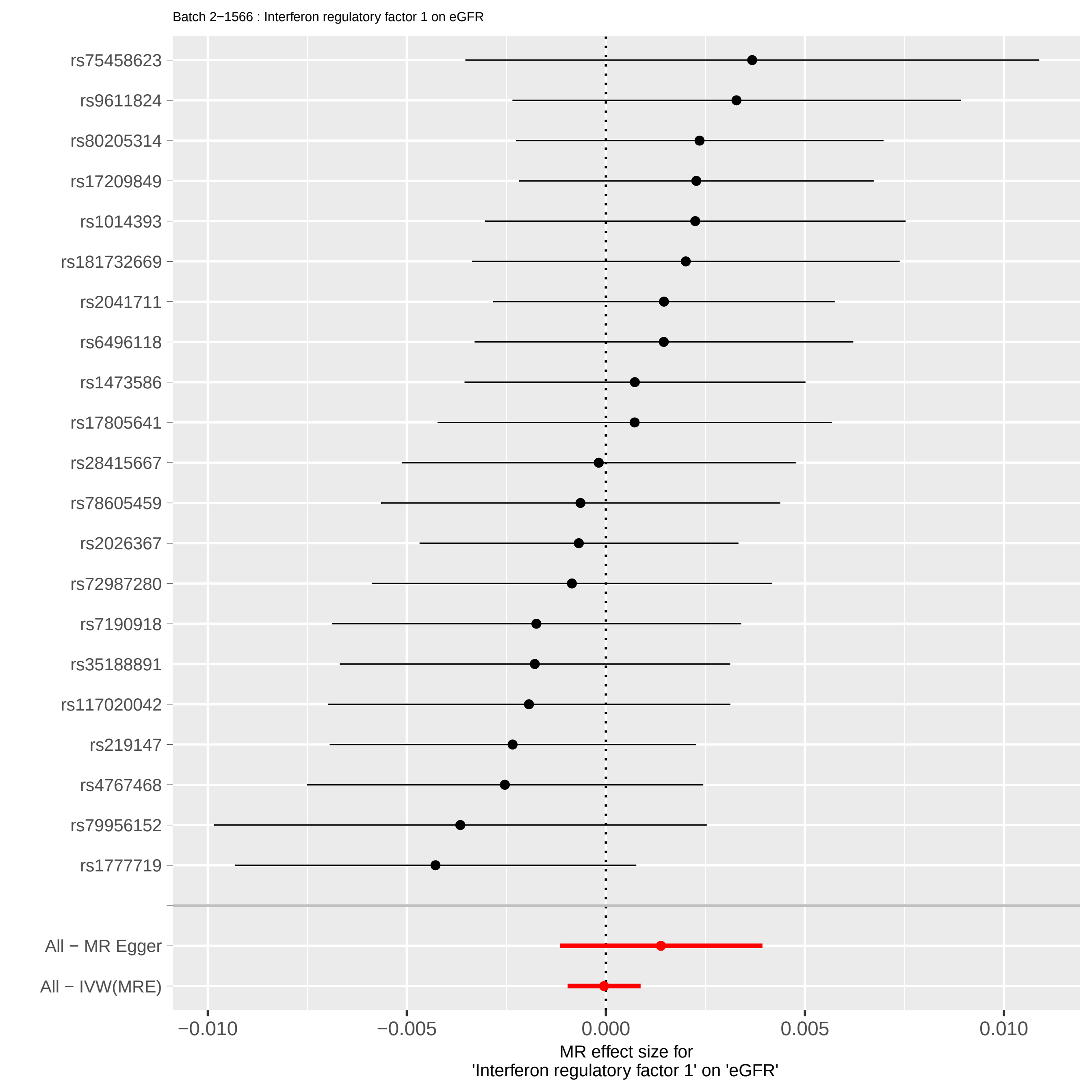

Batch 2−1566 : Interferon regulatory factor 1 on eGFR
rs75458623
rs9611824
rs80205314
rs17209849
rs1014393
rs181732669
rs2041711
rs6496118
rs1473586
rs17805641
rs28415667
rs78605459
rs2026367
rs72987280
rs7190918
rs35188891
rs117020042
rs219147
rs4767468
rs79956152
rs1777719
All − MR Egger
All − IVW(MRE)
−0.010
−0.005
0.000
0.005
0.010
MR effect size for
'Interferon regulatory factor 1' on 'eGFR'

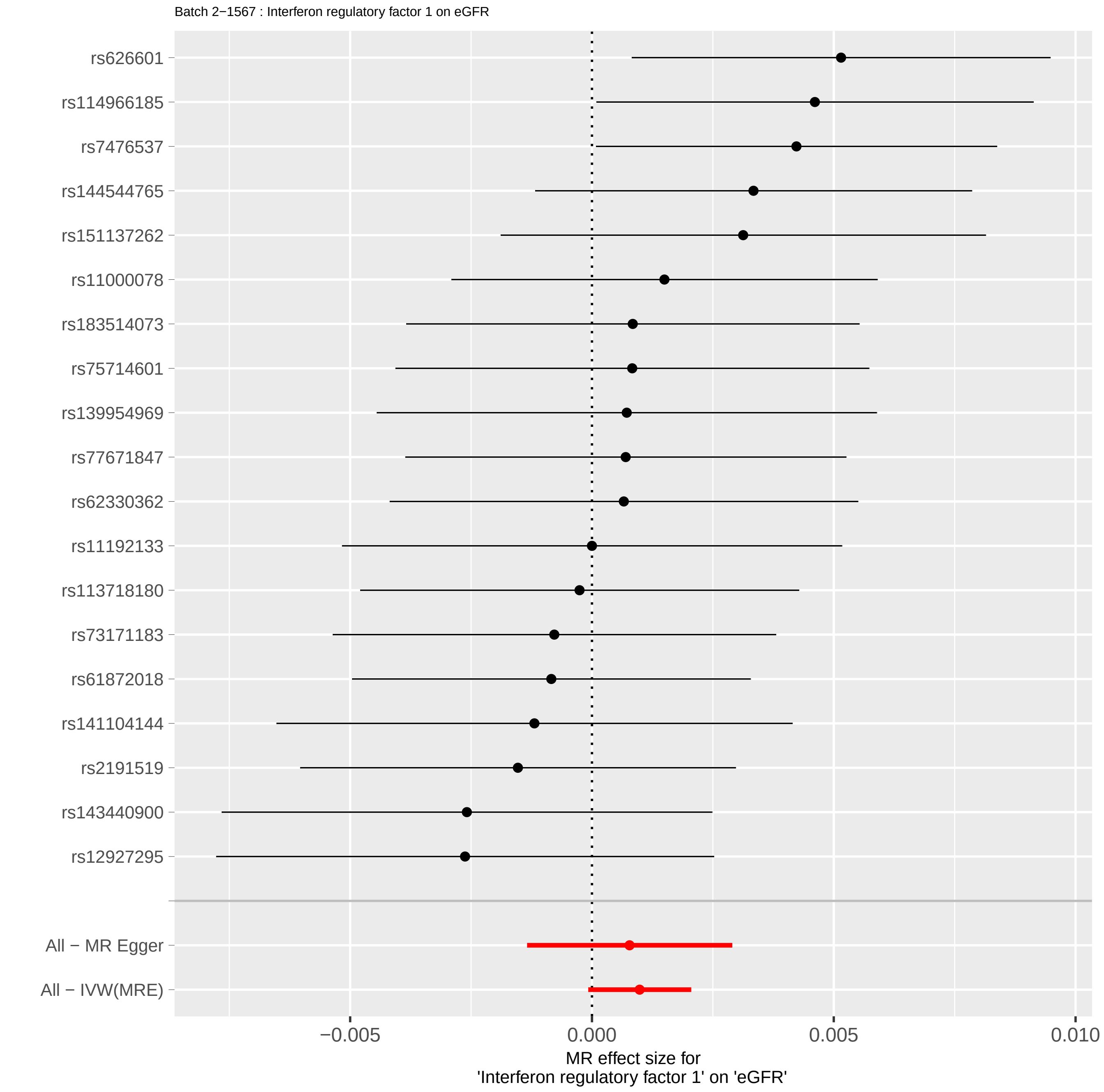

Batch 2−1567 : Interferon regulatory factor 1 on eGFR
rs626601
rs114966185
rs7476537
rs144544765
rs151137262
rs11000078
rs183514073
rs75714601
rs139954969
rs77671847
rs62330362
rs11192133
rs113718180
rs73171183
rs61872018
rs141104144
rs2191519
rs143440900
rs12927295
All − MR Egger
All − IVW(MRE)
−0.005
0.000
0.005
0.010
MR effect size for
'Interferon regulatory factor 1' on 'eGFR'

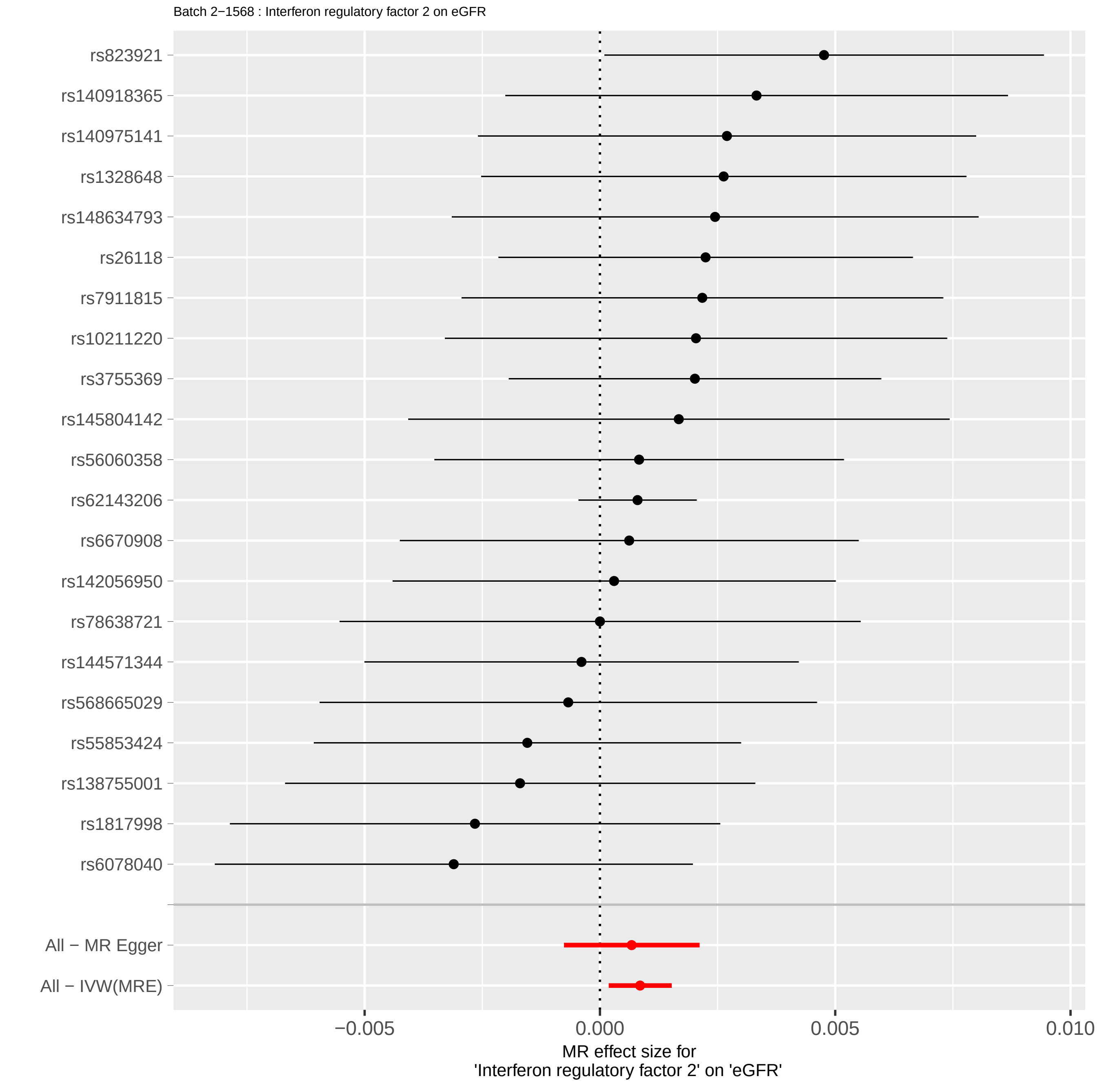

Batch 2−1568 : Interferon regulatory factor 2 on eGFR
rs823921
rs140918365
rs140975141
rs1328648
rs148634793
rs26118
rs7911815
rs10211220
rs3755369
rs145804142
rs56060358
rs62143206
rs6670908
rs142056950
rs78638721
rs144571344
rs568665029
rs55853424
rs138755001
rs1817998
rs6078040
All − MR Egger
All − IVW(MRE)
−0.005
0.000
0.005
0.010
MR effect size for
'Interferon regulatory factor 2' on 'eGFR'

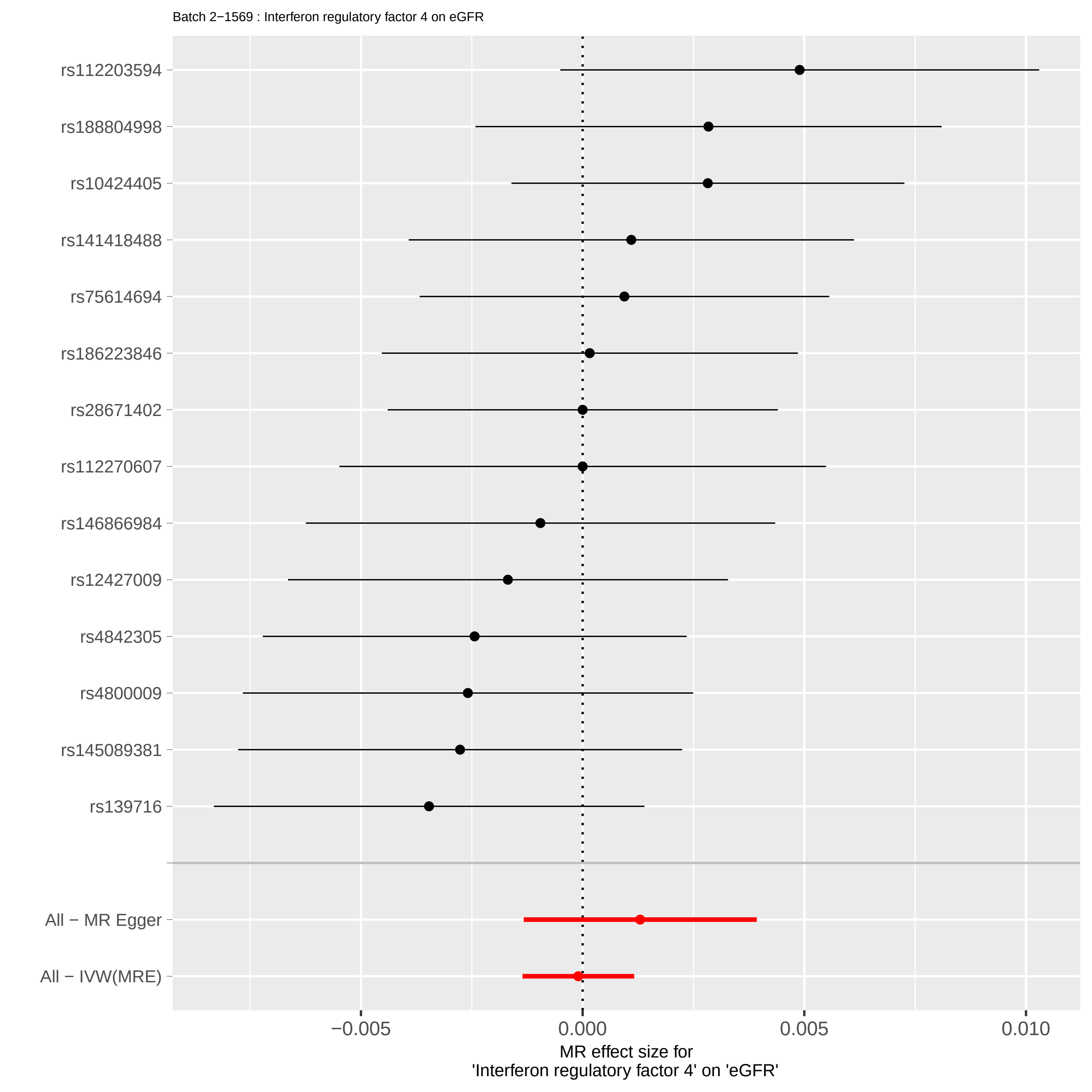

Batch 2−1569 : Interferon regulatory factor 4 on eGFR
rs112203594
rs188804998
rs10424405
rs141418488
rs75614694
rs186223846
rs28671402
rs112270607
rs146866984
rs12427009
rs4842305
rs4800009
rs145089381
rs139716
All − MR Egger
All − IVW(MRE)
−0.005
0.000
0.005
0.010
MR effect size for
'Interferon regulatory factor 4' on 'eGFR'

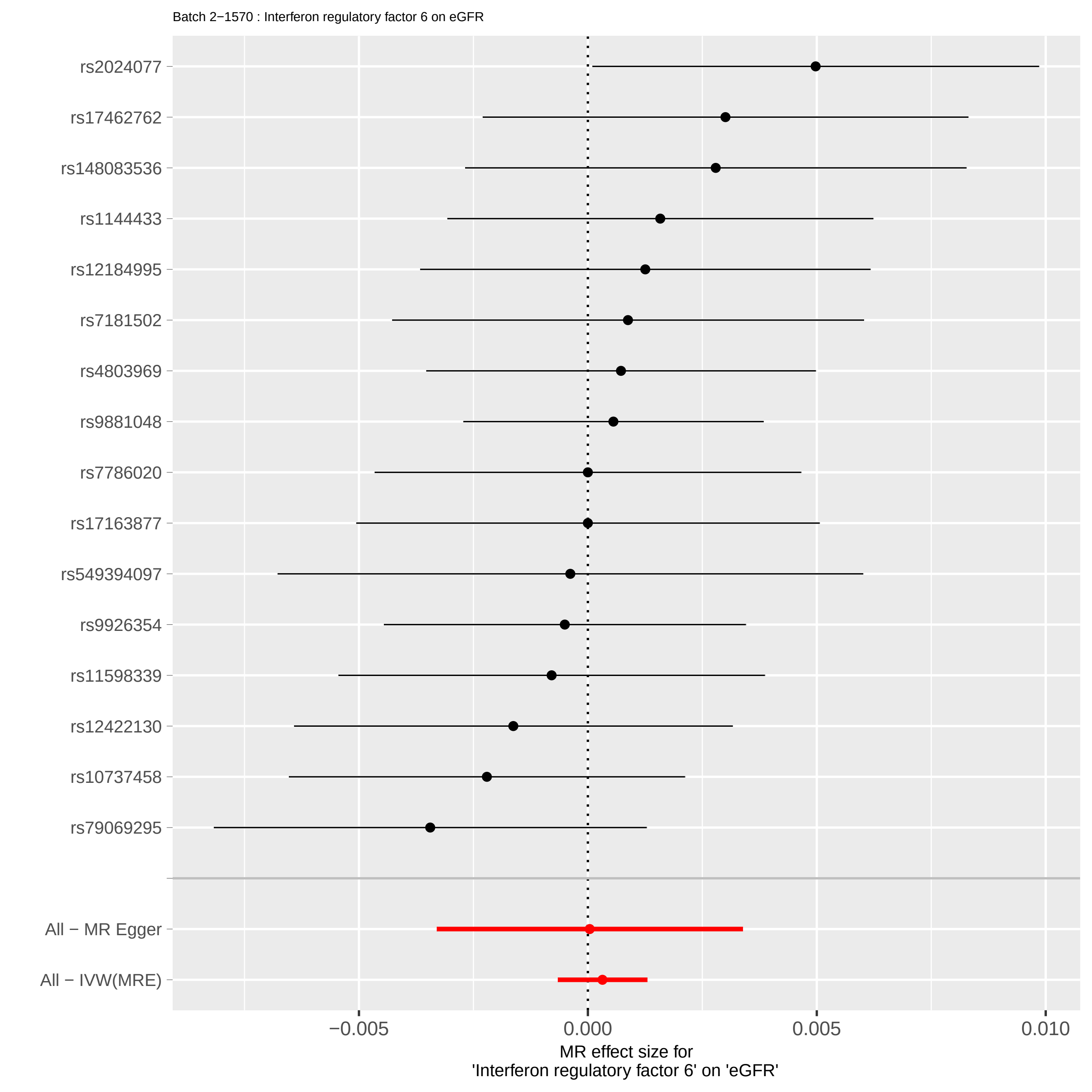
Batch 2−1570 : Interferon regulatory factor 6 on eGFR
rs2024077
rs17462762
rs148083536
rs1144433
rs12184995
rs7181502
rs4803969
rs9881048
rs7786020
rs17163877
rs549394097
rs9926354
rs11598339
rs12422130
rs10737458
rs79069295
All − MR Egger
All − IVW(MRE)
−0.005
0.000
0.005
0.010
MR effect size for
'Interferon regulatory factor 6' on 'eGFR'

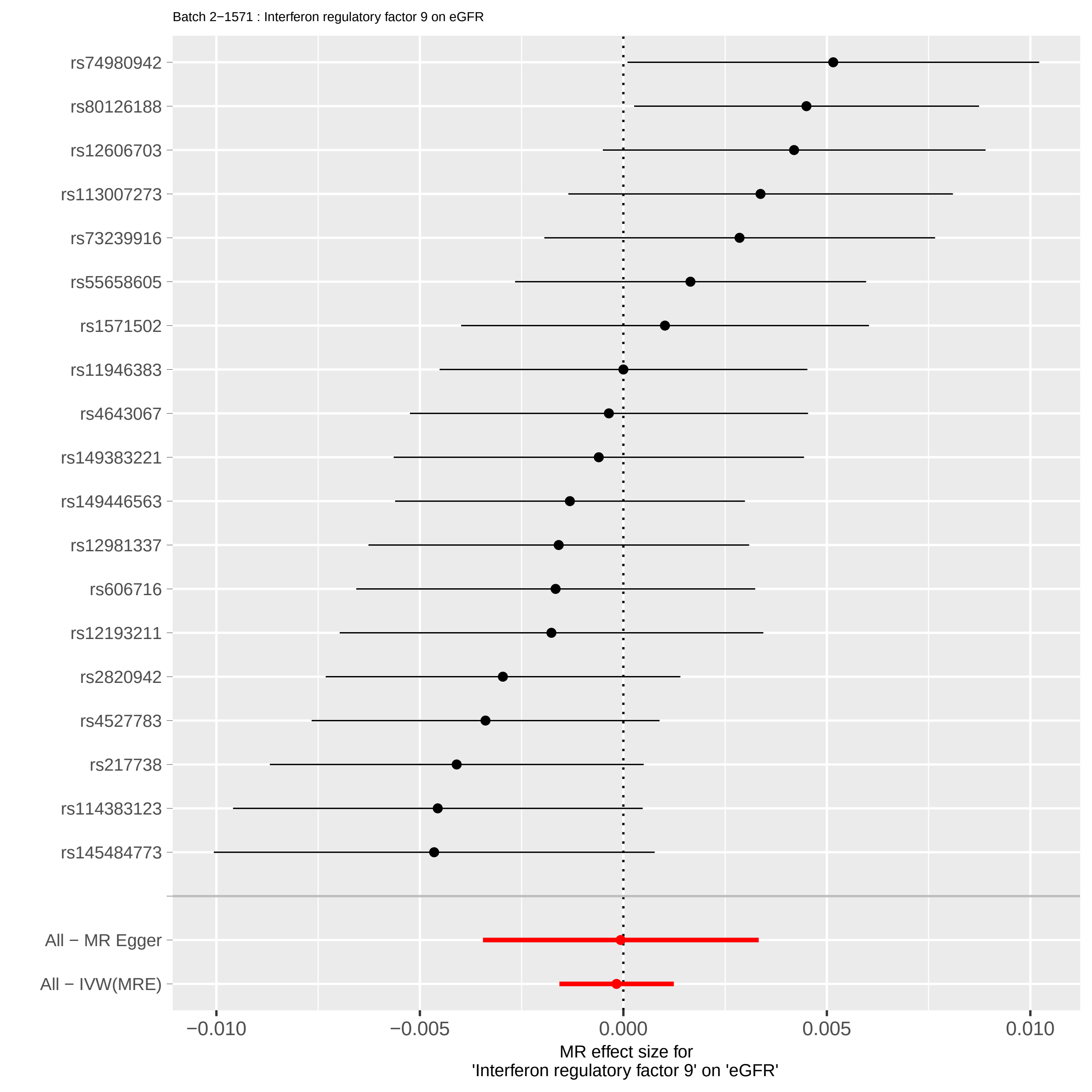
Batch 2−1571 : Interferon regulatory factor 9 on eGFR
rs74980942
rs80126188
rs12606703
rs113007273
rs73239916
rs55658605
rs1571502
rs11946383
rs4643067
rs149383221
rs149446563
rs12981337
rs606716
rs12193211
rs2820942
rs4527783
rs217738
rs114383123
rs145484773
All − MR Egger
All − IVW(MRE)
−0.010
−0.005
0.000
0.005
0.010
MR effect size for
'Interferon regulatory factor 9' on 'eGFR'

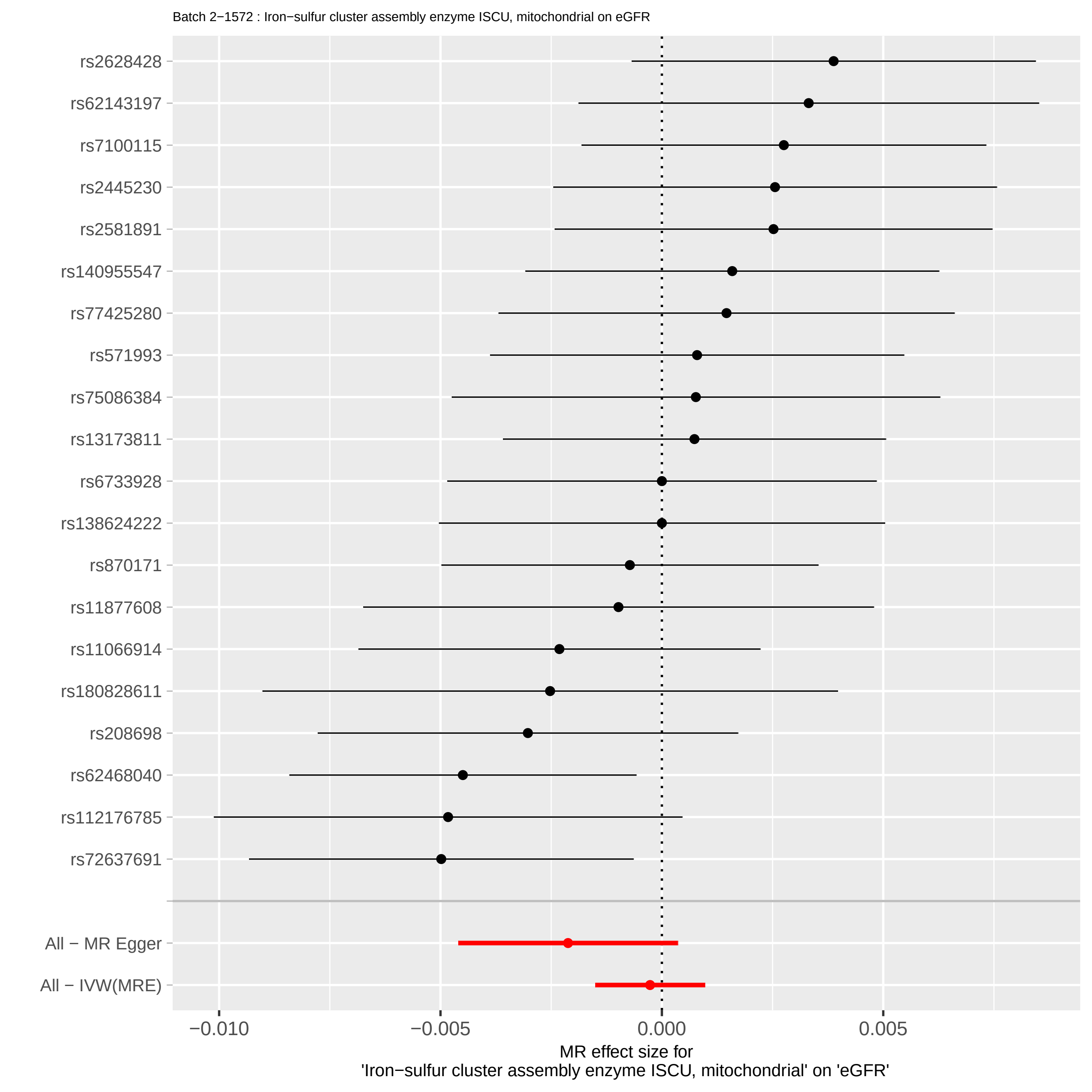

Batch 2−1572 : Iron−sulfur cluster assembly enzyme ISCU, mitochondrial on eGFR
rs2628428
rs62143197
rs7100115
rs2445230
rs2581891
rs140955547
rs77425280
rs571993
rs75086384
rs13173811
rs6733928
rs138624222
rs870171
rs11877608
rs11066914
rs180828611
rs208698
rs62468040
rs112176785
rs72637691
All − MR Egger
All − IVW(MRE)
−0.010
−0.005
0.000
0.005
MR effect size for
'Iron−sulfur cluster assembly enzyme ISCU, mitochondrial' on 'eGFR'

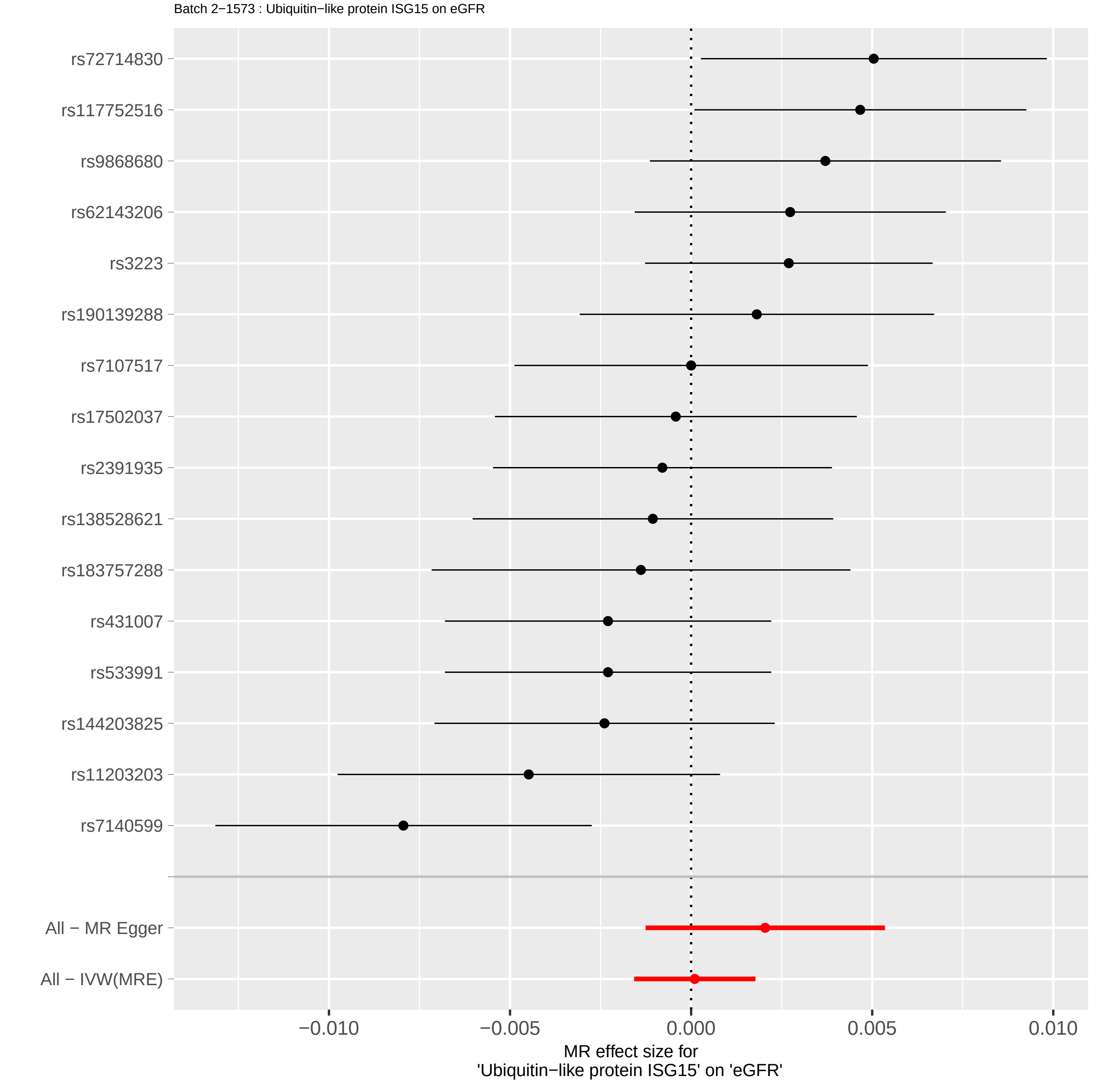

Batch 2−1573 : Ubiquitin−like protein ISG15 on eGFR
rs72714830
rs117752516
rs9868680
rs62143206
rs3223
rs190139288
rs7107517
rs17502037
rs2391935
rs138528621
rs183757288
rs431007
rs533991
rs144203825
rs11203203
rs7140599
All − MR Egger
All − IVW(MRE)
−0.010
−0.005
0.000
0.005
0.010
MR effect size for
'Ubiquitin−like protein ISG15' on 'eGFR'

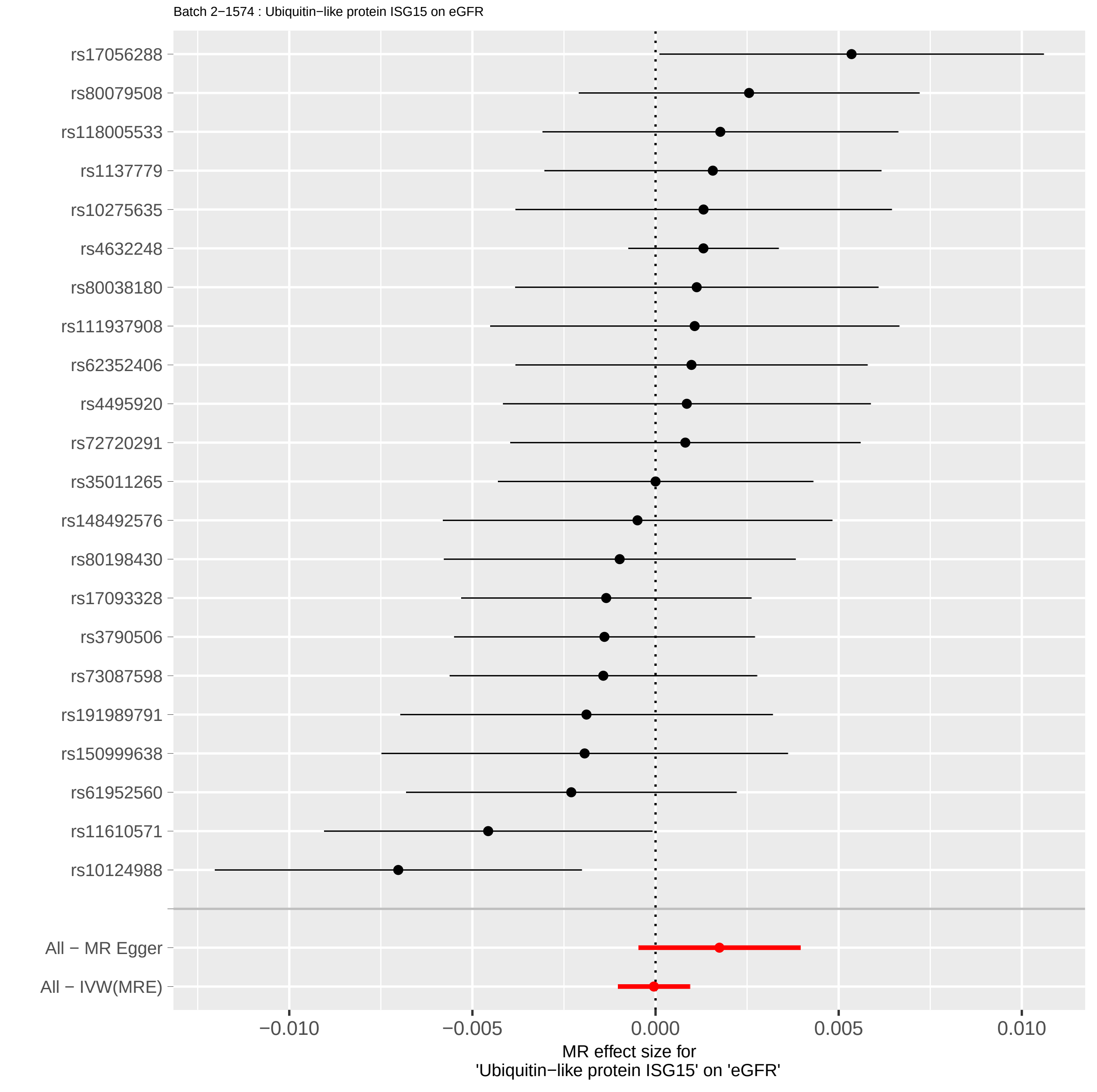
Batch 2−1574 : Ubiquitin−like protein ISG15 on eGFR
rs17056288
rs80079508
rs118005533
rs1137779
rs10275635
rs4632248
rs80038180
rs111937908
rs62352406
rs4495920
rs72720291
rs35011265
rs148492576
rs80198430
rs17093328
rs3790506
rs73087598
rs191989791
rs150999638
rs61952560
rs11610571
rs10124988
All − MR Egger
All − IVW(MRE)
−0.010
−0.005
0.000
0.005
0.010
MR effect size for
'Ubiquitin−like protein ISG15' on 'eGFR'

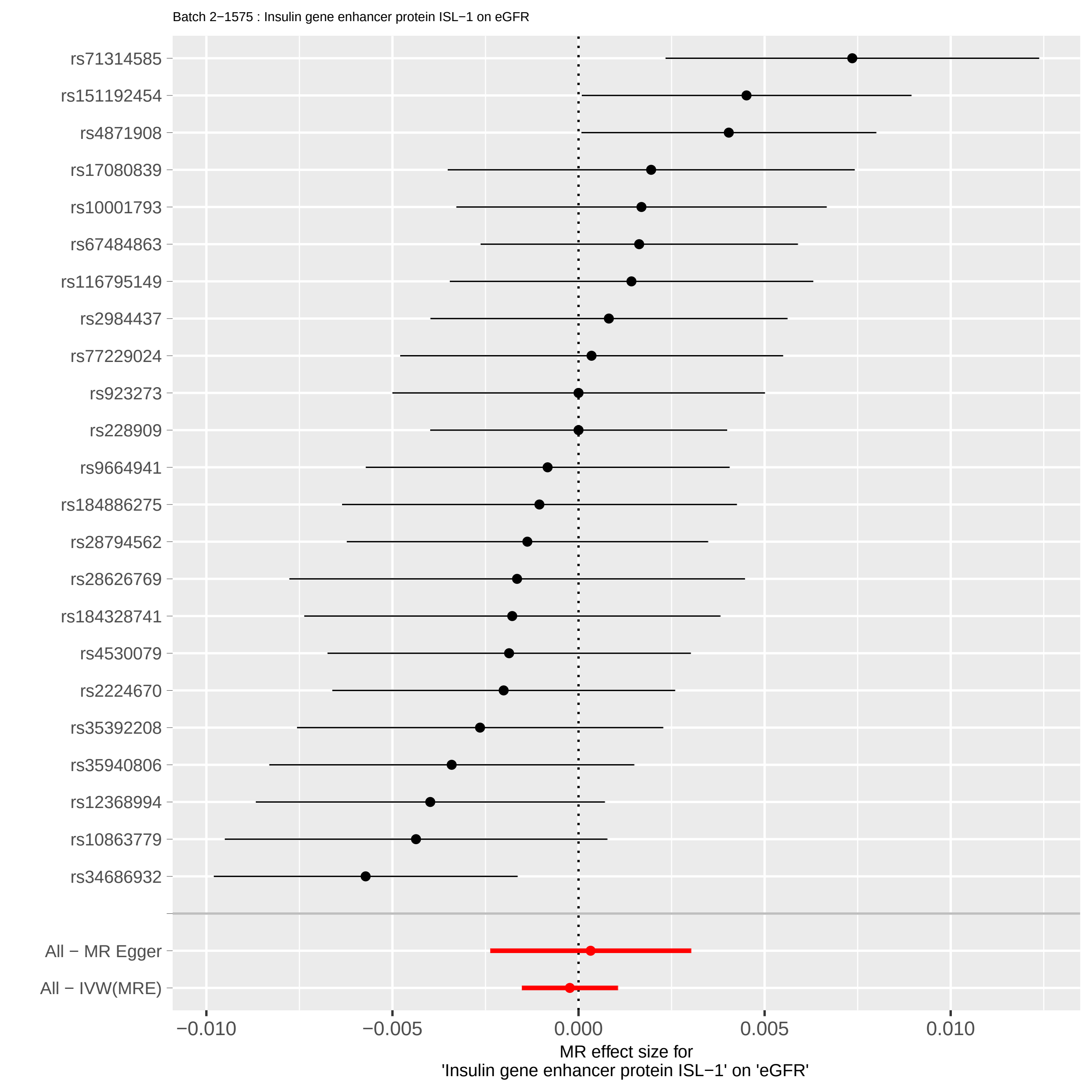
Batch 2−1575 : Insulin gene enhancer protein ISL−1 on eGFR
rs71314585
rs151192454
rs4871908
rs17080839
rs10001793
rs67484863
rs116795149
rs2984437
rs77229024
rs923273
rs228909
rs9664941
rs184886275
rs28794562
rs28626769
rs184328741
rs4530079
rs2224670
rs35392208
rs35940806
rs12368994
rs10863779
rs34686932
All − MR Egger
All − IVW(MRE)
−0.010
−0.005
0.000
0.005
0.010
MR effect size for
'Insulin gene enhancer protein ISL−1' on 'eGFR'

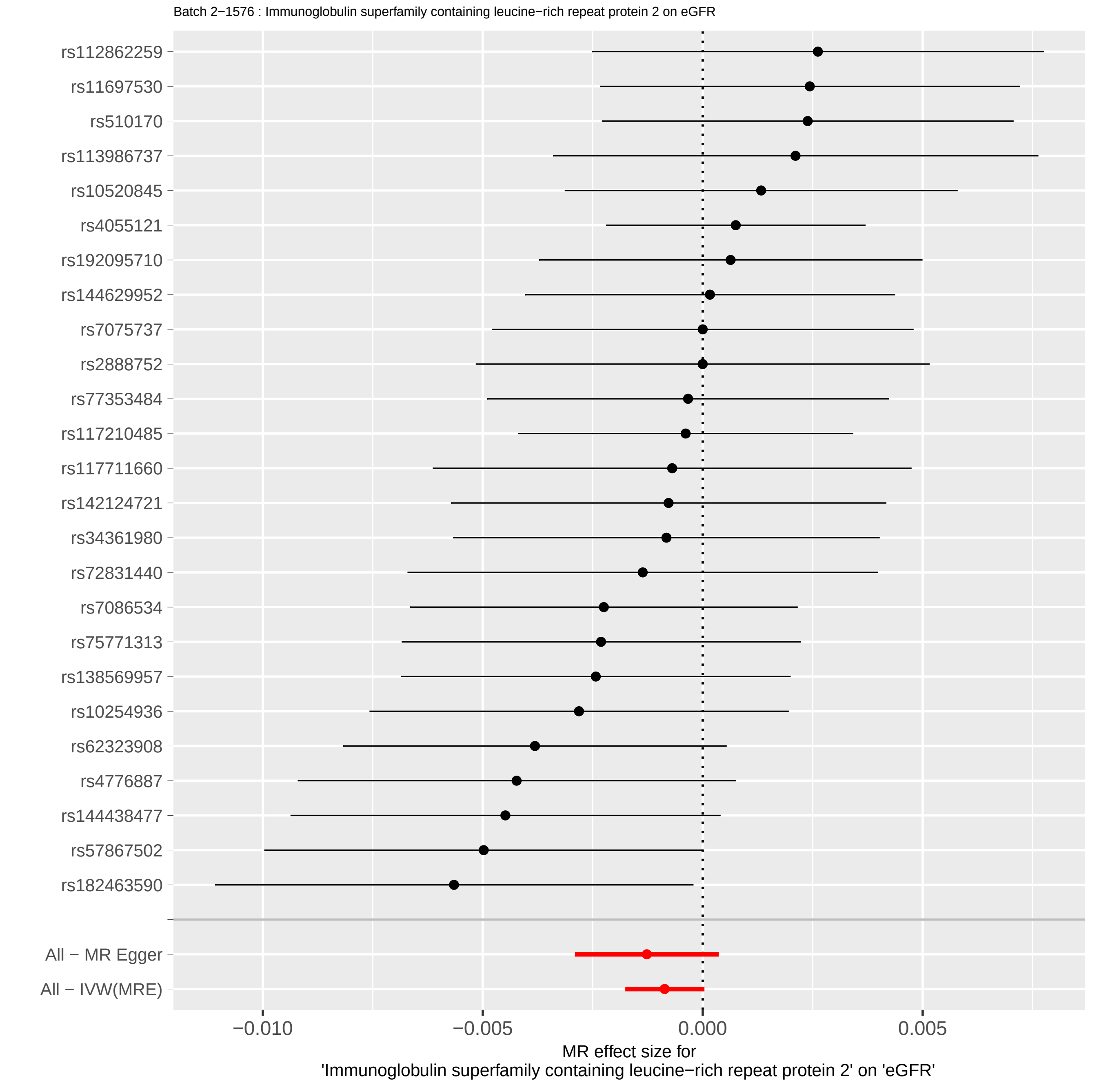
Batch 2−1576 : Immunoglobulin superfamily containing leucine−rich repeat protein 2 on eGFR
rs112862259
rs11697530
rs510170
rs113986737
rs10520845
rs4055121
rs192095710
rs144629952
rs7075737
rs2888752
rs77353484
rs117210485
rs117711660
rs142124721
rs34361980
rs72831440
rs7086534
rs75771313
rs138569957
rs10254936
rs62323908
rs4776887
rs144438477
rs57867502
rs182463590
All − MR Egger
All − IVW(MRE)
−0.010
−0.005
0.000
0.005
MR effect size for
'Immunoglobulin superfamily containing leucine−rich repeat protein 2' on 'eGFR'

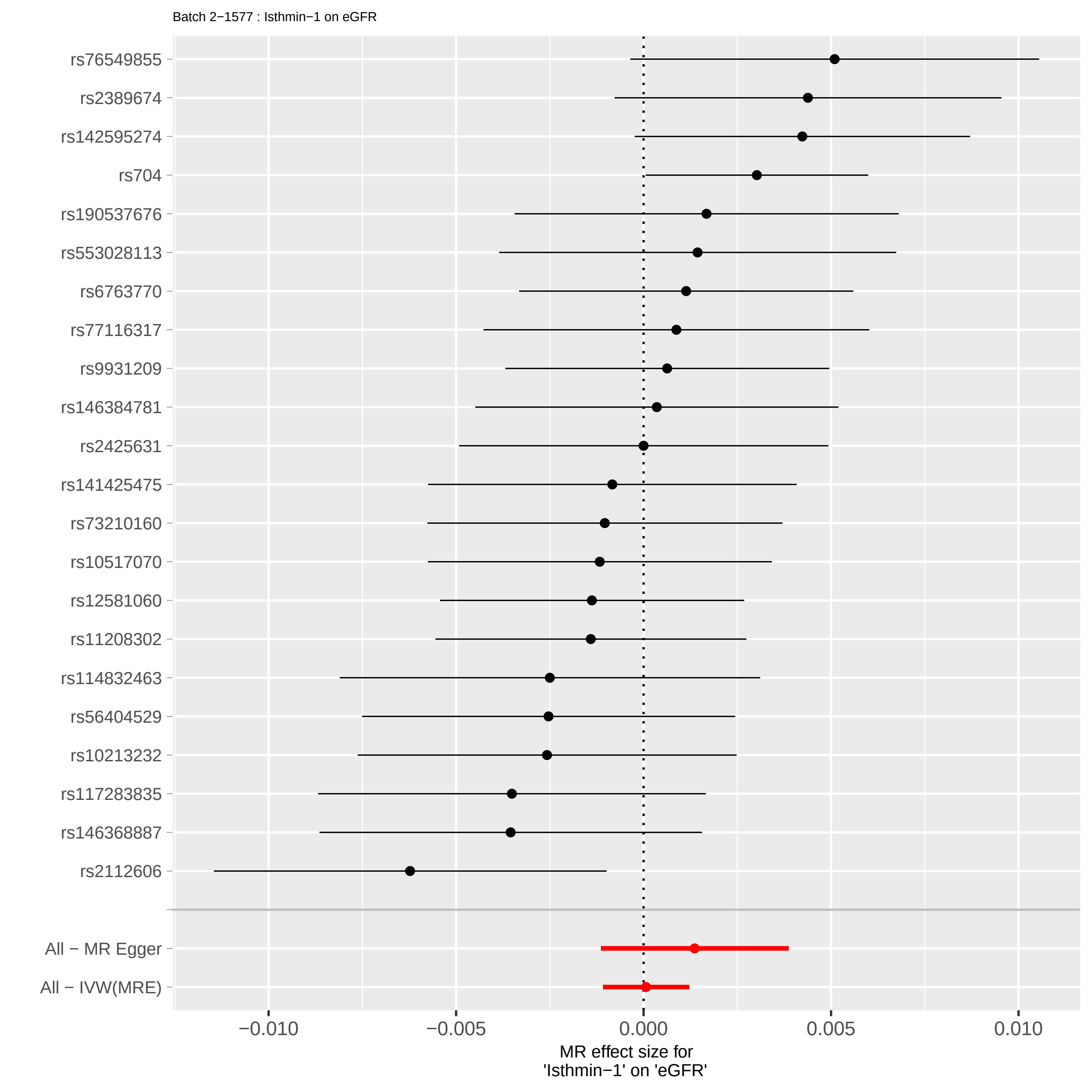

Batch 2−1577 : Isthmin−1 on eGFR
rs76549855
rs2389674
rs142595274
rs704
rs190537676
rs553028113
rs6763770
rs77116317
rs9931209
rs146384781
rs2425631
rs141425475
rs73210160
rs10517070
rs12581060
rs11208302
rs114832463
rs56404529
rs10213232
rs117283835
rs146368887
rs2112606
All − MR Egger
All − IVW(MRE)
−0.010
−0.005
0.000
0.005
0.010
MR effect size for
'Isthmin−1' on 'eGFR'

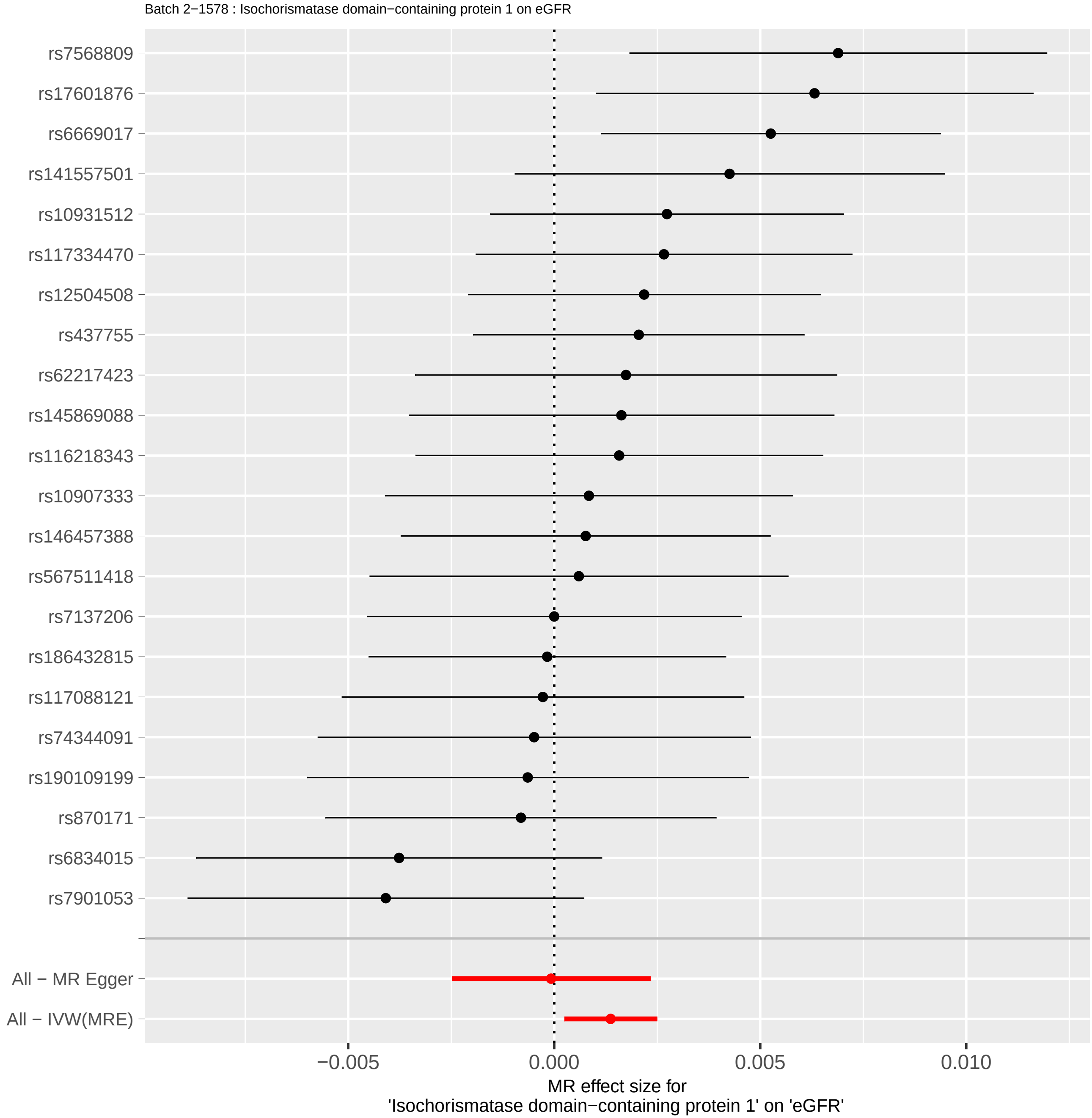

Batch 2−1578 : Isochorismatase domain−containing protein 1 on eGFR
rs7568809
rs17601876
rs6669017
rs141557501
rs10931512
rs117334470
rs12504508
rs437755
rs62217423
rs145869088
rs116218343
rs10907333
rs146457388
rs567511418
rs7137206
rs186432815
rs117088121
rs74344091
rs190109199
rs870171
rs6834015
rs7901053
All − MR Egger
All − IVW(MRE)
−0.005
0.000
0.005
0.010
MR effect size for
'Isochorismatase domain−containing protein 1' on 'eGFR'

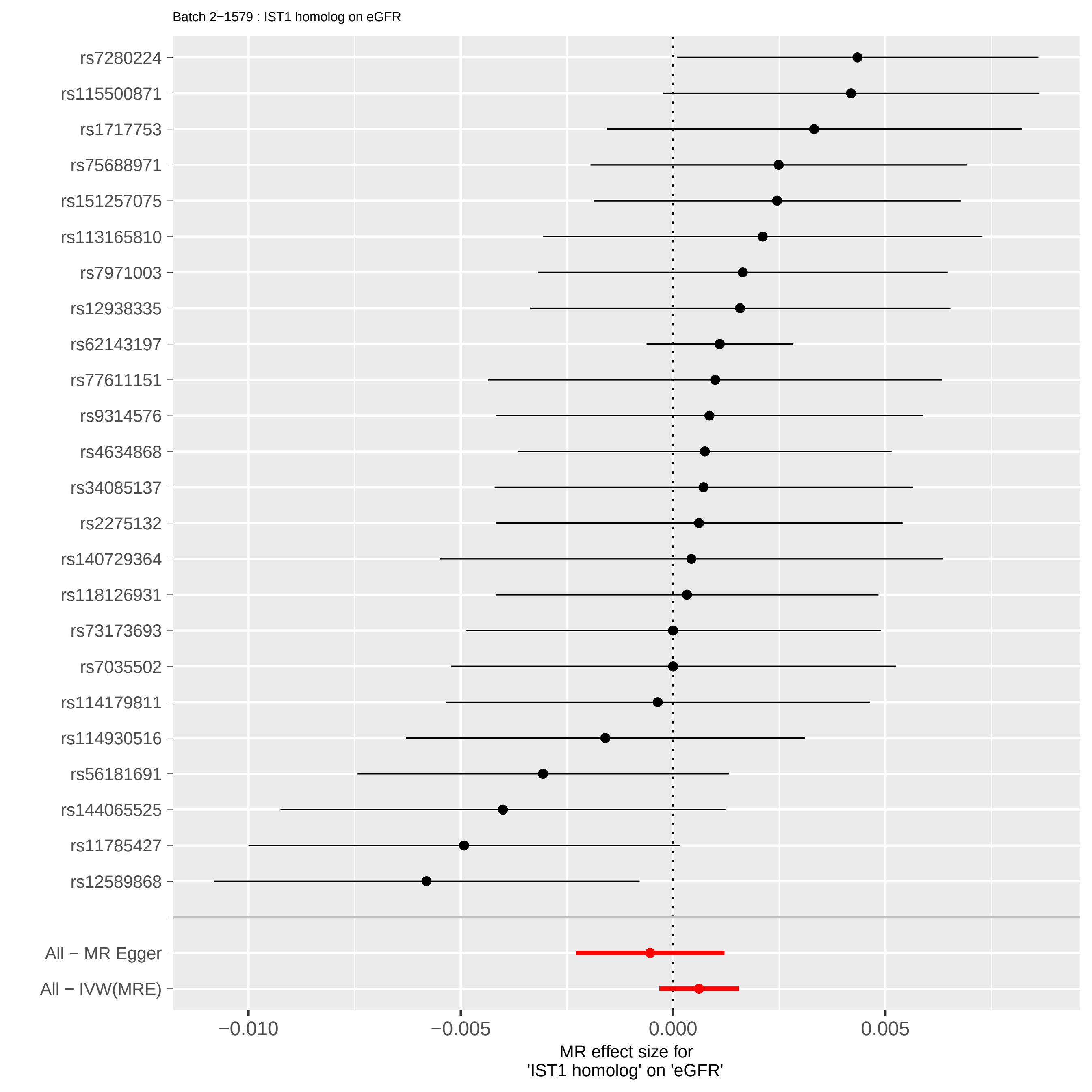
Batch 2−1579 : IST1 homolog on eGFR
rs7280224
rs115500871
rs1717753
rs75688971
rs151257075
rs113165810
rs7971003
rs12938335
rs62143197
rs77611151
rs9314576
rs4634868
rs34085137
rs2275132
rs140729364
rs118126931
rs73173693
rs7035502
rs114179811
rs114930516
rs56181691
rs144065525
rs11785427
rs12589868
All − MR Egger
All − IVW(MRE)
−0.010
−0.005
0.000
0.005
MR effect size for
'IST1 homolog' on 'eGFR'

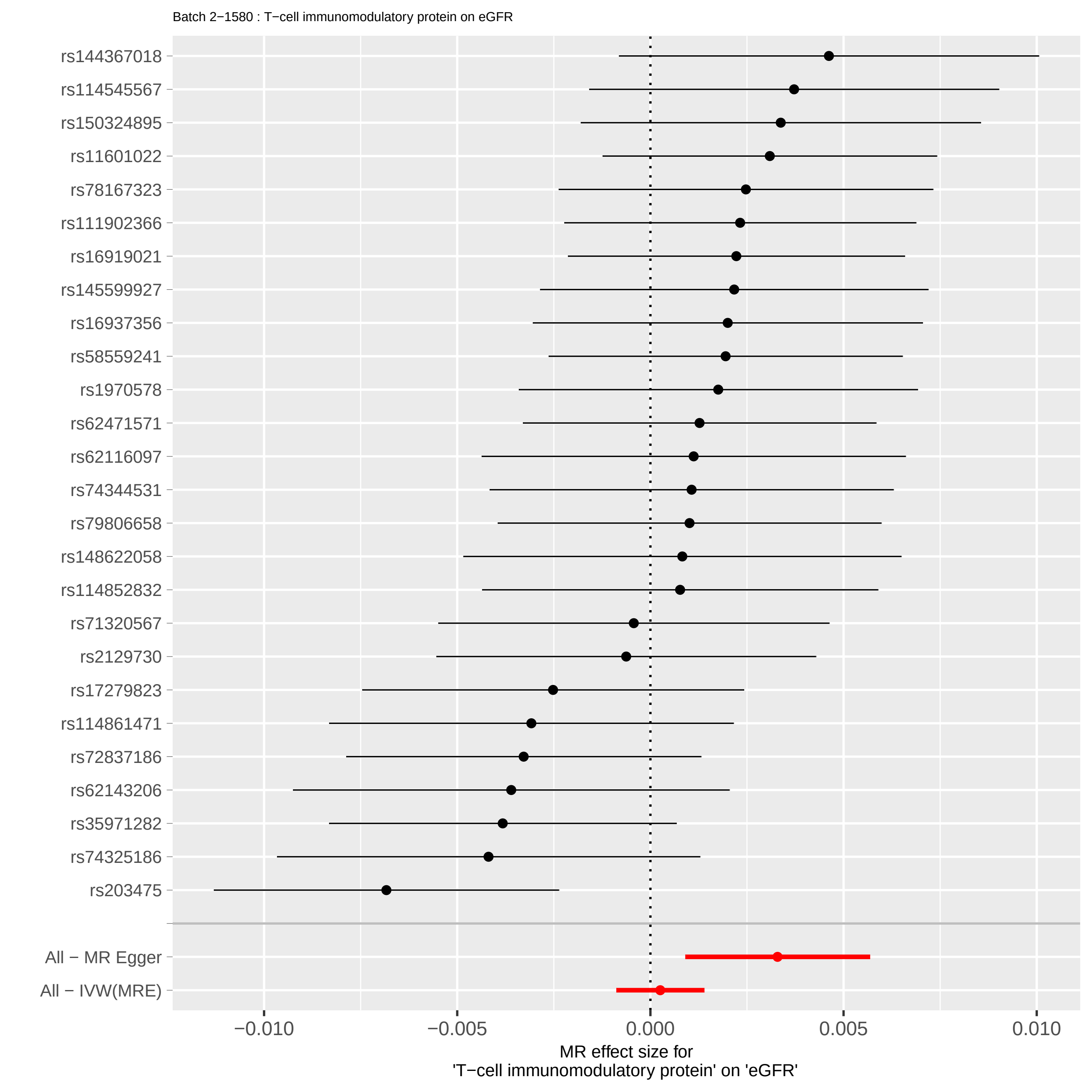

Batch 2−1580 : T−cell immunomodulatory protein on eGFR
rs144367018
rs114545567
rs150324895
rs11601022
rs78167323
rs111902366
rs16919021
rs145599927
rs16937356
rs58559241
rs1970578
rs62471571
rs62116097
rs74344531
rs79806658
rs148622058
rs114852832
rs71320567
rs2129730
rs17279823
rs114861471
rs72837186
rs62143206
rs35971282
rs74325186
rs203475
All − MR Egger
All − IVW(MRE)
−0.010
−0.005
0.000
0.005
0.010
MR effect size for
'T−cell immunomodulatory protein' on 'eGFR'

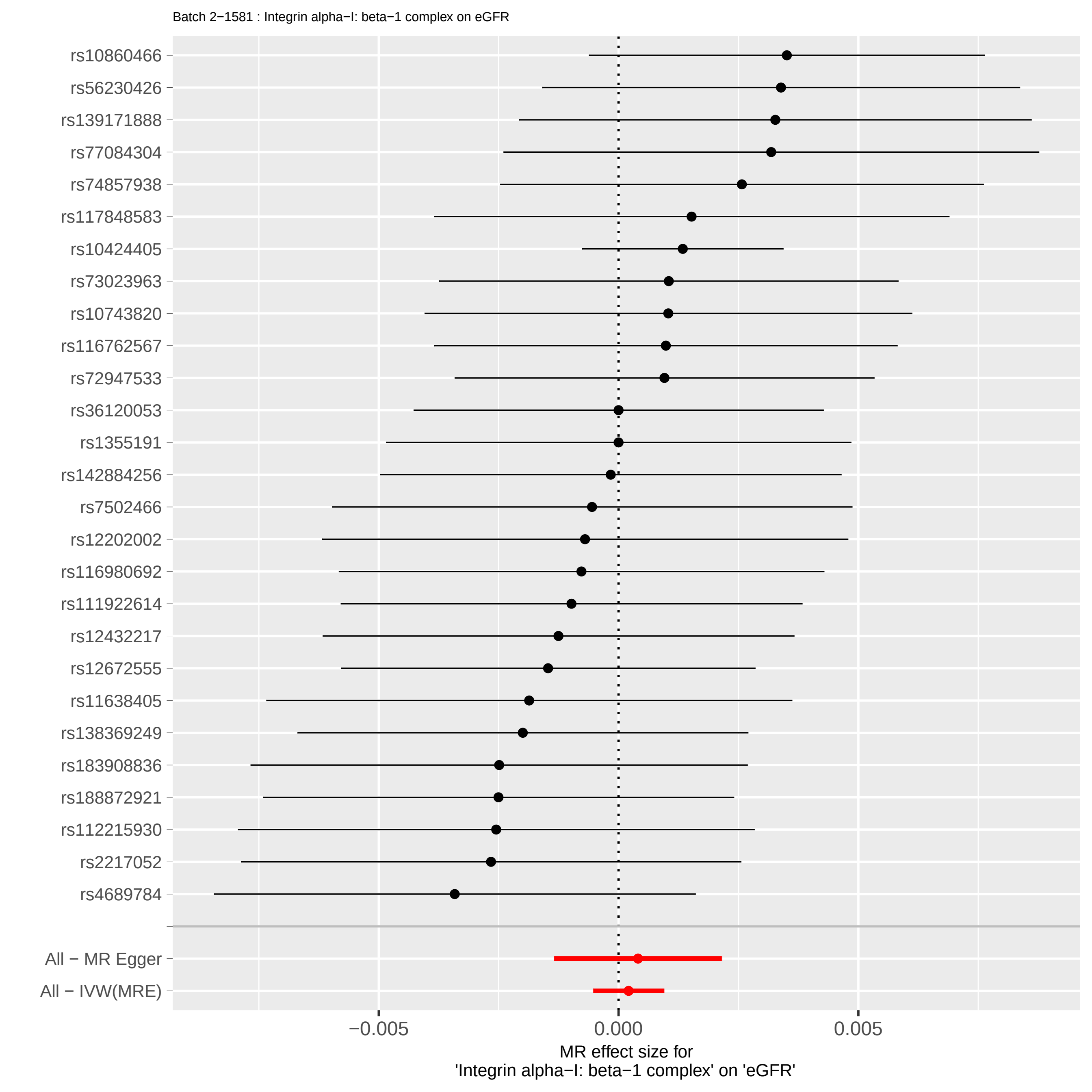
Batch 2−1581 : Integrin alpha−I: beta−1 complex on eGFR
rs10860466
rs56230426
rs139171888
rs77084304
rs74857938
rs117848583
rs10424405
rs73023963
rs10743820
rs116762567
rs72947533
rs36120053
rs1355191
rs142884256
rs7502466
rs12202002
rs116980692
rs111922614
rs12432217
rs12672555
rs11638405
rs138369249
rs183908836
rs188872921
rs112215930
rs2217052
rs4689784
All − MR Egger
All − IVW(MRE)
−0.005
0.000
0.005
MR effect size for
'Integrin alpha−I: beta−1 complex' on 'eGFR'

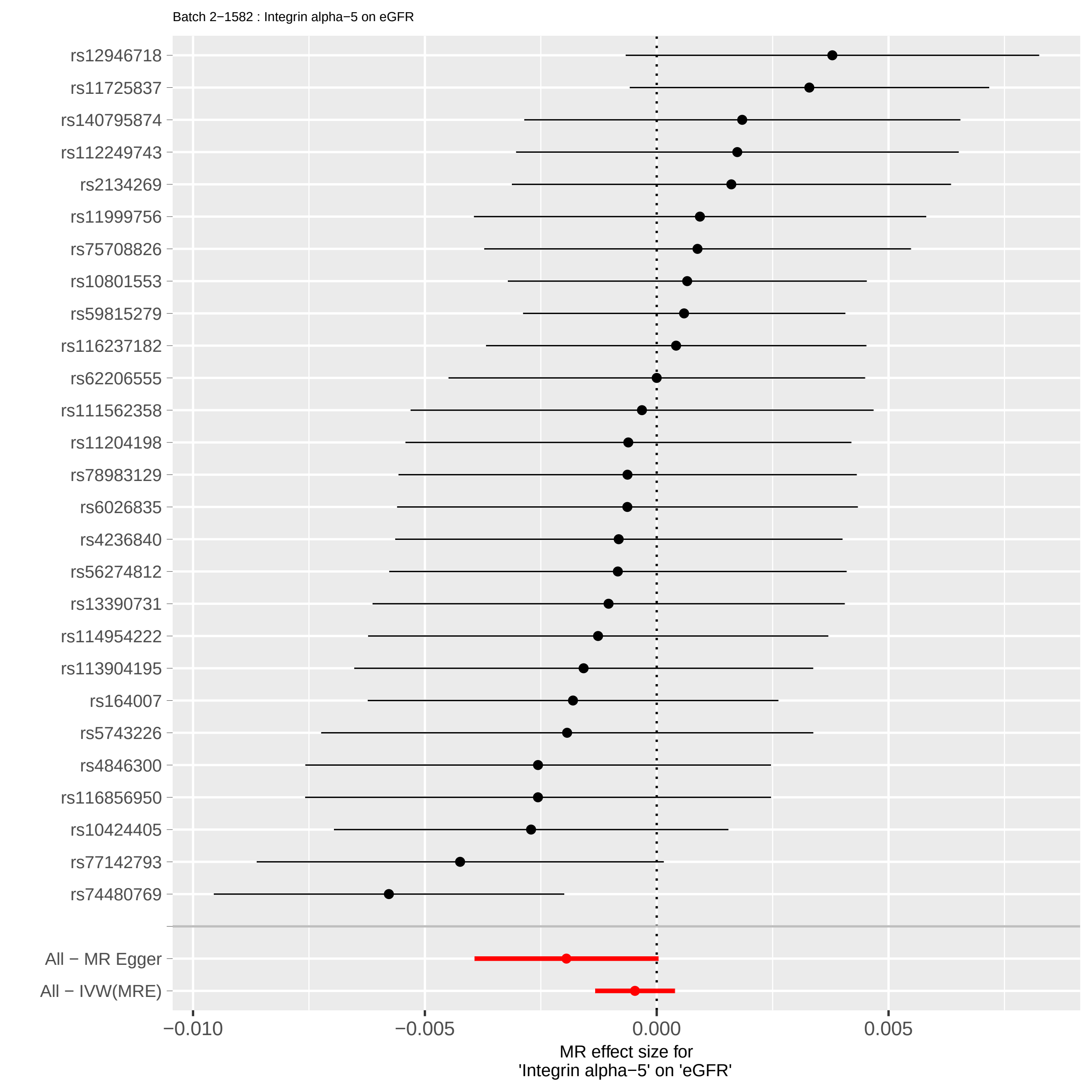
Batch 2−1582 : Integrin alpha−5 on eGFR
rs12946718
rs11725837
rs140795874
rs112249743
rs2134269
rs11999756
rs75708826
rs10801553
rs59815279
rs116237182
rs62206555
rs111562358
rs11204198
rs78983129
rs6026835
rs4236840
rs56274812
rs13390731
rs114954222
rs113904195
rs164007
rs5743226
rs4846300
rs116856950
rs10424405
rs77142793
rs74480769
All − MR Egger
All − IVW(MRE)
−0.010
−0.005
0.000
0.005
MR effect size for
'Integrin alpha−5' on 'eGFR'

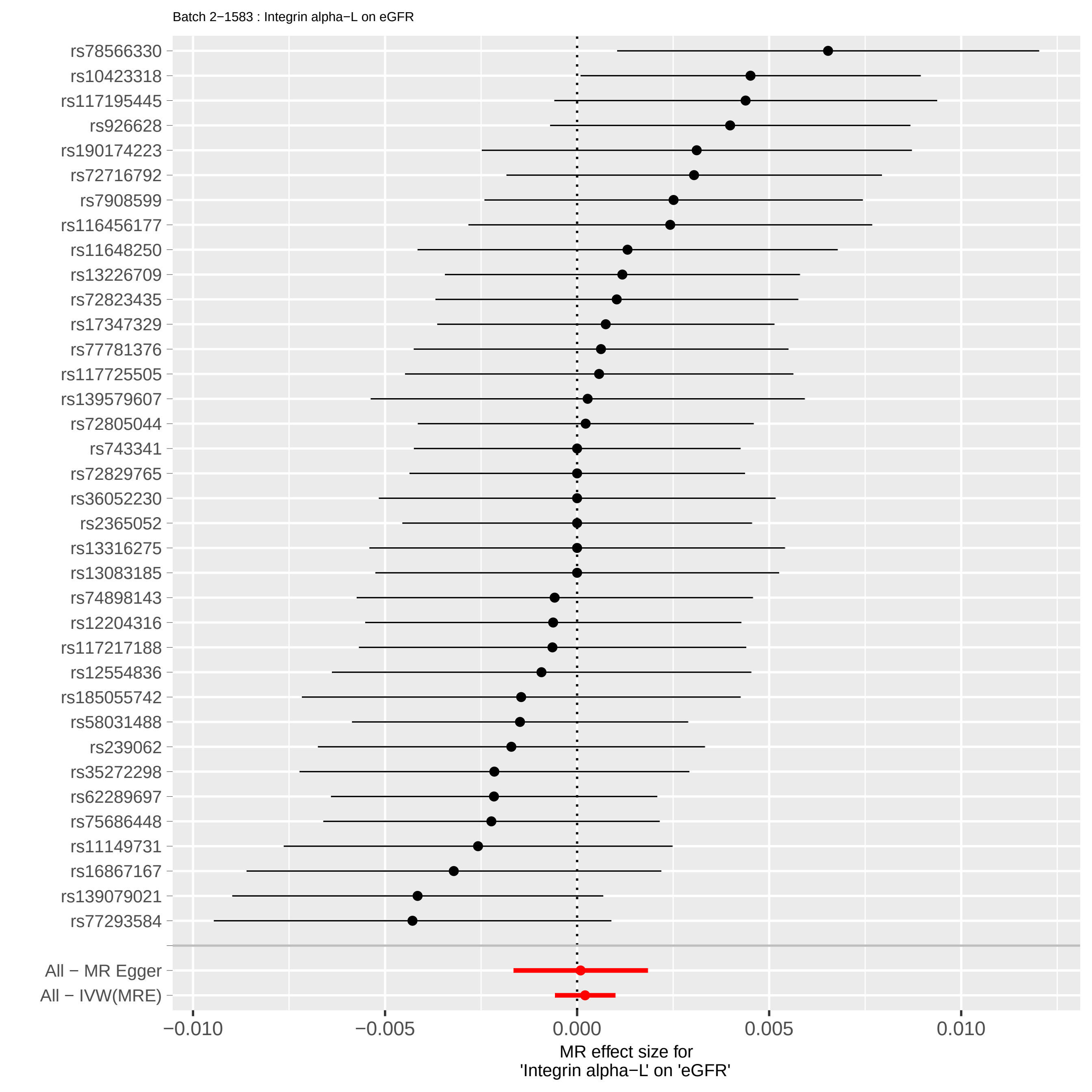

Batch 2−1583 : Integrin alpha−L on eGFR
rs78566330
rs10423318
rs117195445
rs926628
rs190174223
rs72716792
rs7908599
rs116456177
rs11648250
rs13226709
rs72823435
rs17347329
rs77781376
rs117725505
rs139579607
rs72805044
rs743341
rs72829765
rs36052230
rs2365052
rs13316275
rs13083185
rs74898143
rs12204316
rs117217188
rs12554836
rs185055742
rs58031488
rs239062
rs35272298
rs62289697
rs75686448
rs11149731
rs16867167
rs139079021
rs77293584
All − MR Egger
All − IVW(MRE)
−0.010
−0.005
0.000
0.005
0.010
MR effect size for
'Integrin alpha−L' on 'eGFR'

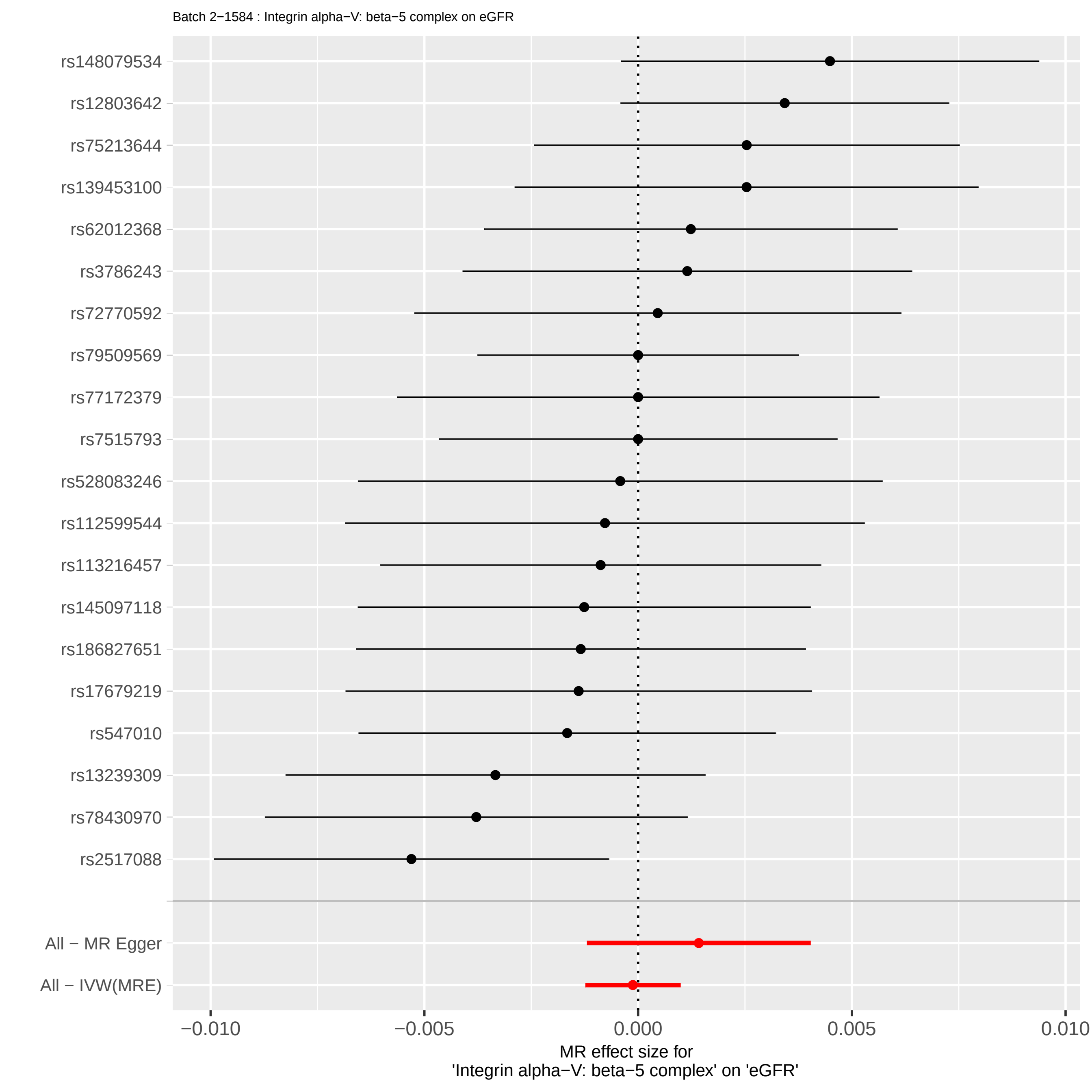
Batch 2−1584 : Integrin alpha−V: beta−5 complex on eGFR
rs148079534
rs12803642
rs75213644
rs139453100
rs62012368
rs3786243
rs72770592
rs79509569
rs77172379
rs7515793
rs528083246
rs112599544
rs113216457
rs145097118
rs186827651
rs17679219
rs547010
rs13239309
rs78430970
rs2517088
All − MR Egger
All − IVW(MRE)
−0.010
−0.005
0.000
0.005
0.010
MR effect size for
'Integrin alpha−V: beta−5 complex' on 'eGFR'

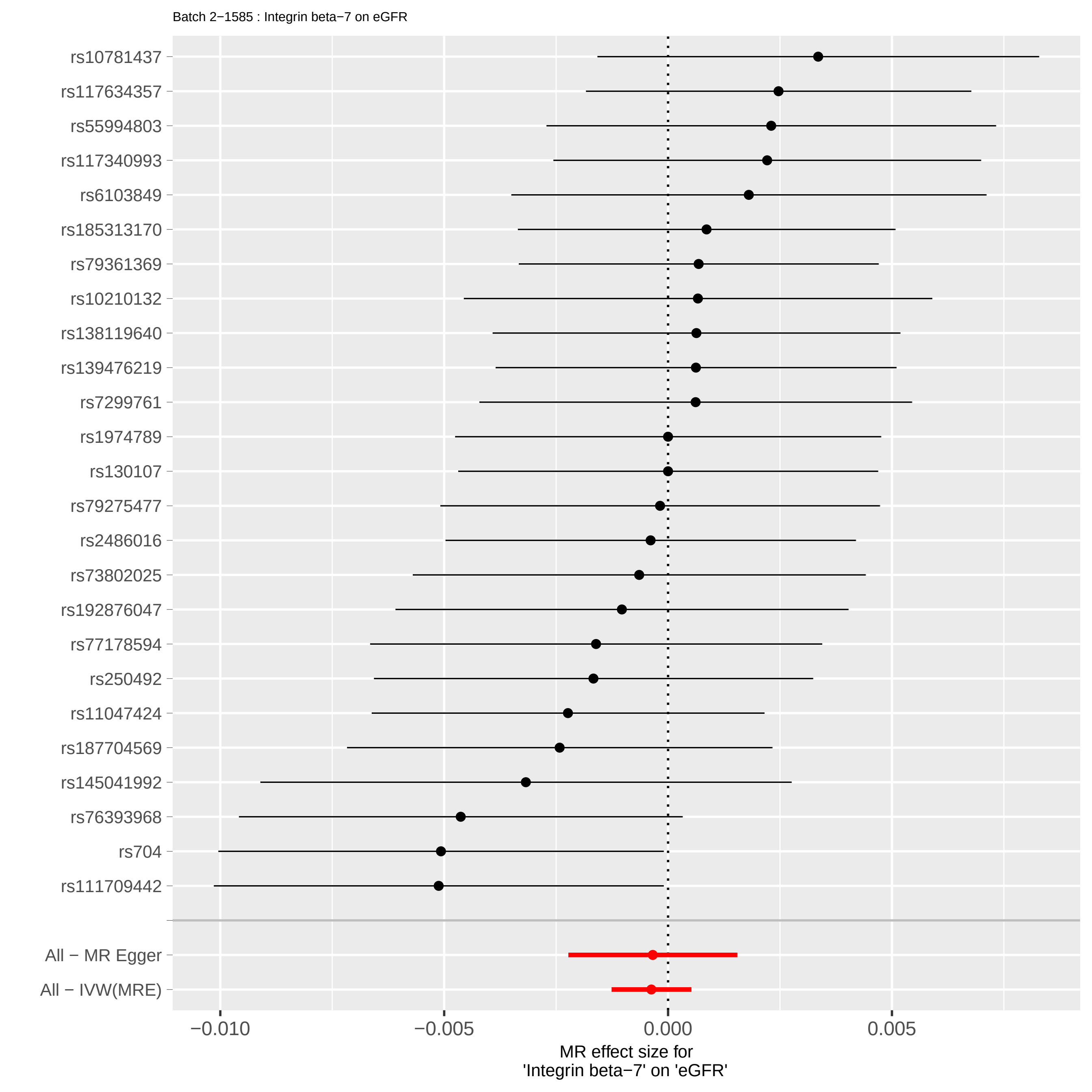
Batch 2−1585 : Integrin beta−7 on eGFR
rs10781437
rs117634357
rs55994803
rs117340993
rs6103849
rs185313170
rs79361369
rs10210132
rs138119640
rs139476219
rs7299761
rs1974789
rs130107
rs79275477
rs2486016
rs73802025
rs192876047
rs77178594
rs250492
rs11047424
rs187704569
rs145041992
rs76393968
rs704
rs111709442
All − MR Egger
All − IVW(MRE)
−0.010
−0.005
0.000
0.005
MR effect size for
'Integrin beta−7' on 'eGFR'

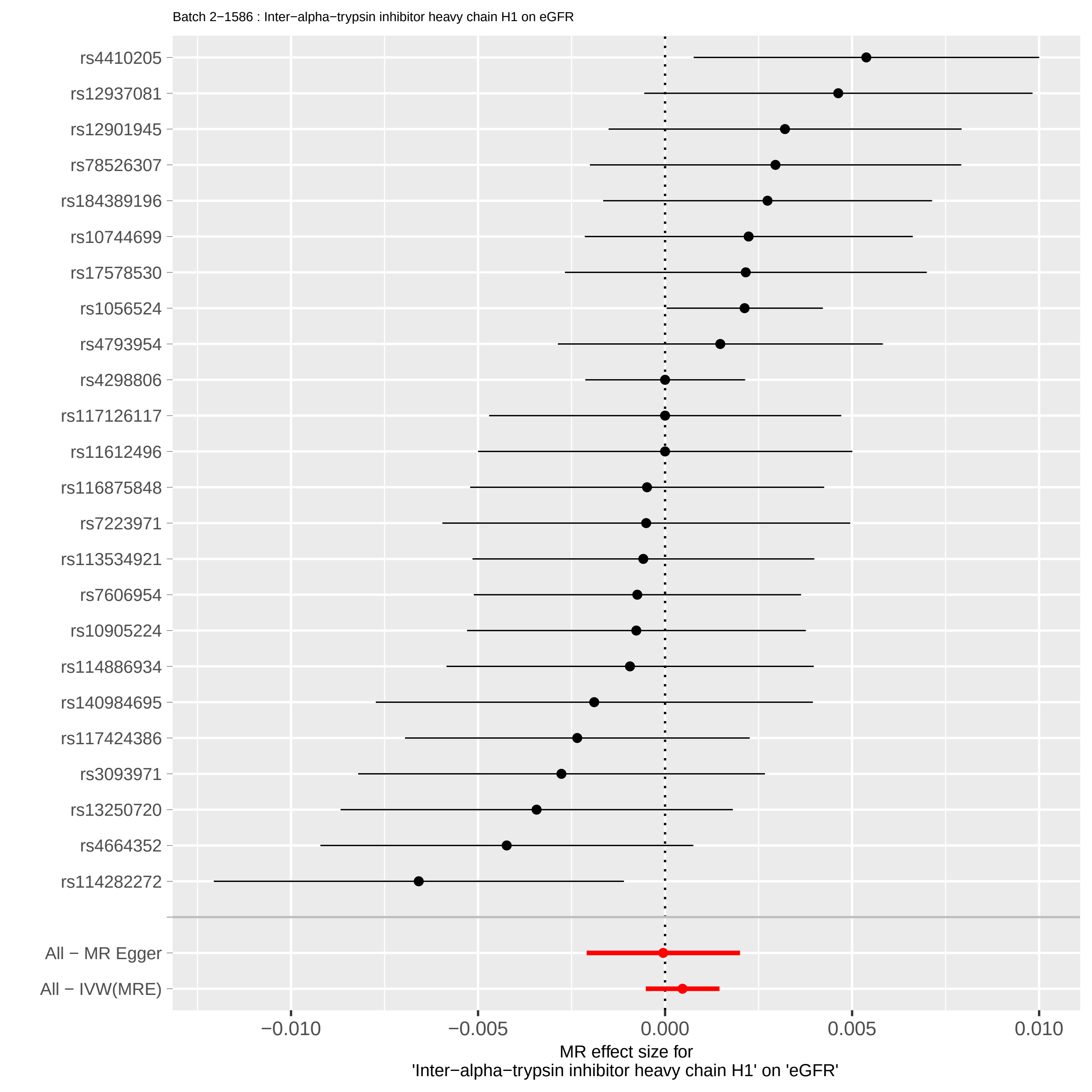
Batch 2−1586 : Inter−alpha−trypsin inhibitor heavy chain H1 on eGFR
rs4410205
rs12937081
rs12901945
rs78526307
rs184389196
rs10744699
rs17578530
rs1056524
rs4793954
rs4298806
rs117126117
rs11612496
rs116875848
rs7223971
rs113534921
rs7606954
rs10905224
rs114886934
rs140984695
rs117424386
rs3093971
rs13250720
rs4664352
rs114282272
All − MR Egger
All − IVW(MRE)
−0.010
−0.005
0.000
0.005
0.010
MR effect size for
'Inter−alpha−trypsin inhibitor heavy chain H1' on 'eGFR'

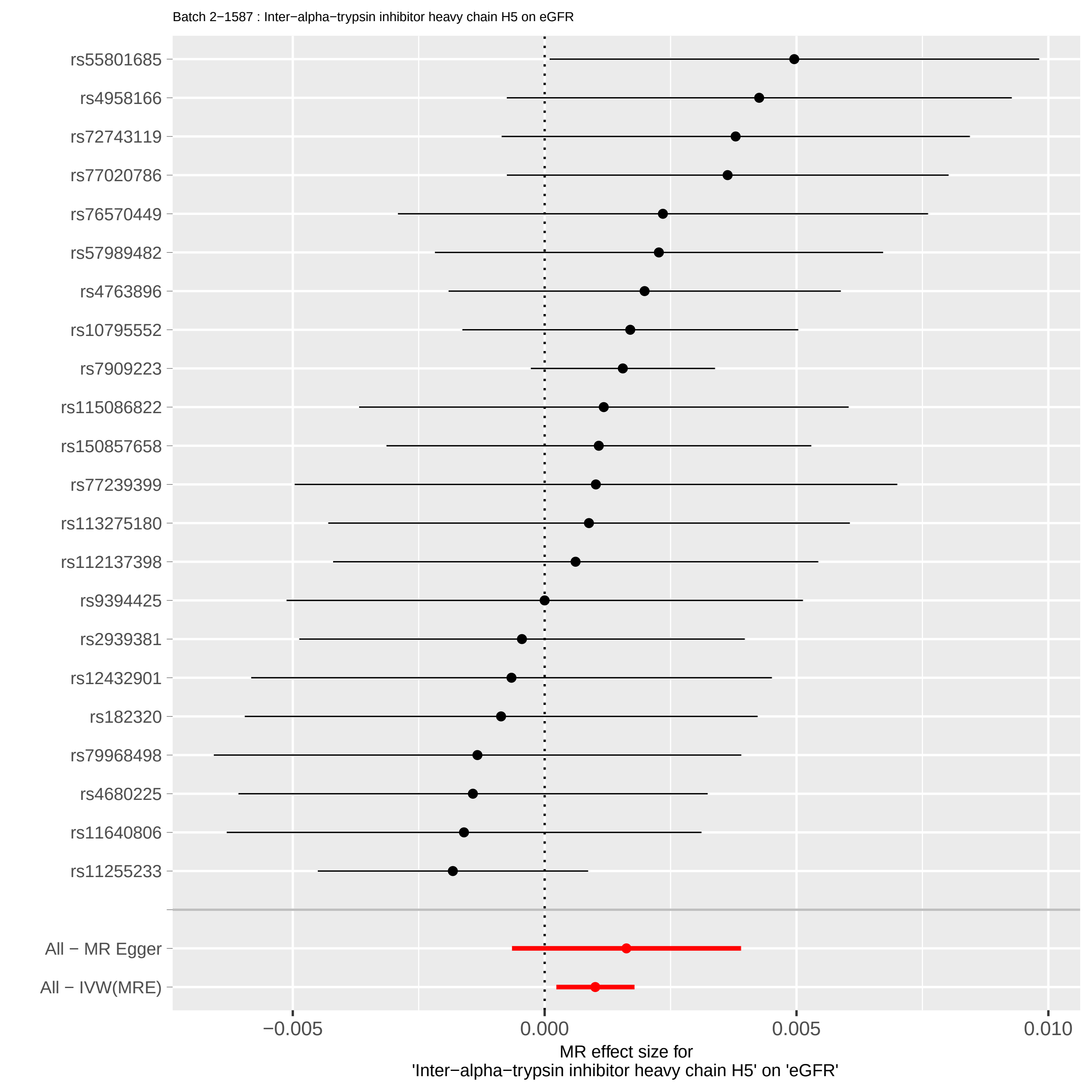

Batch 2−1587 : Inter−alpha−trypsin inhibitor heavy chain H5 on eGFR
rs55801685
rs4958166
rs72743119
rs77020786
rs76570449
rs57989482
rs4763896
rs10795552
rs7909223
rs115086822
rs150857658
rs77239399
rs113275180
rs112137398
rs9394425
rs2939381
rs12432901
rs182320
rs79968498
rs4680225
rs11640806
rs11255233
All − MR Egger
All − IVW(MRE)
−0.005
0.000
0.005
0.010
MR effect size for
'Inter−alpha−trypsin inhibitor heavy chain H5' on 'eGFR'

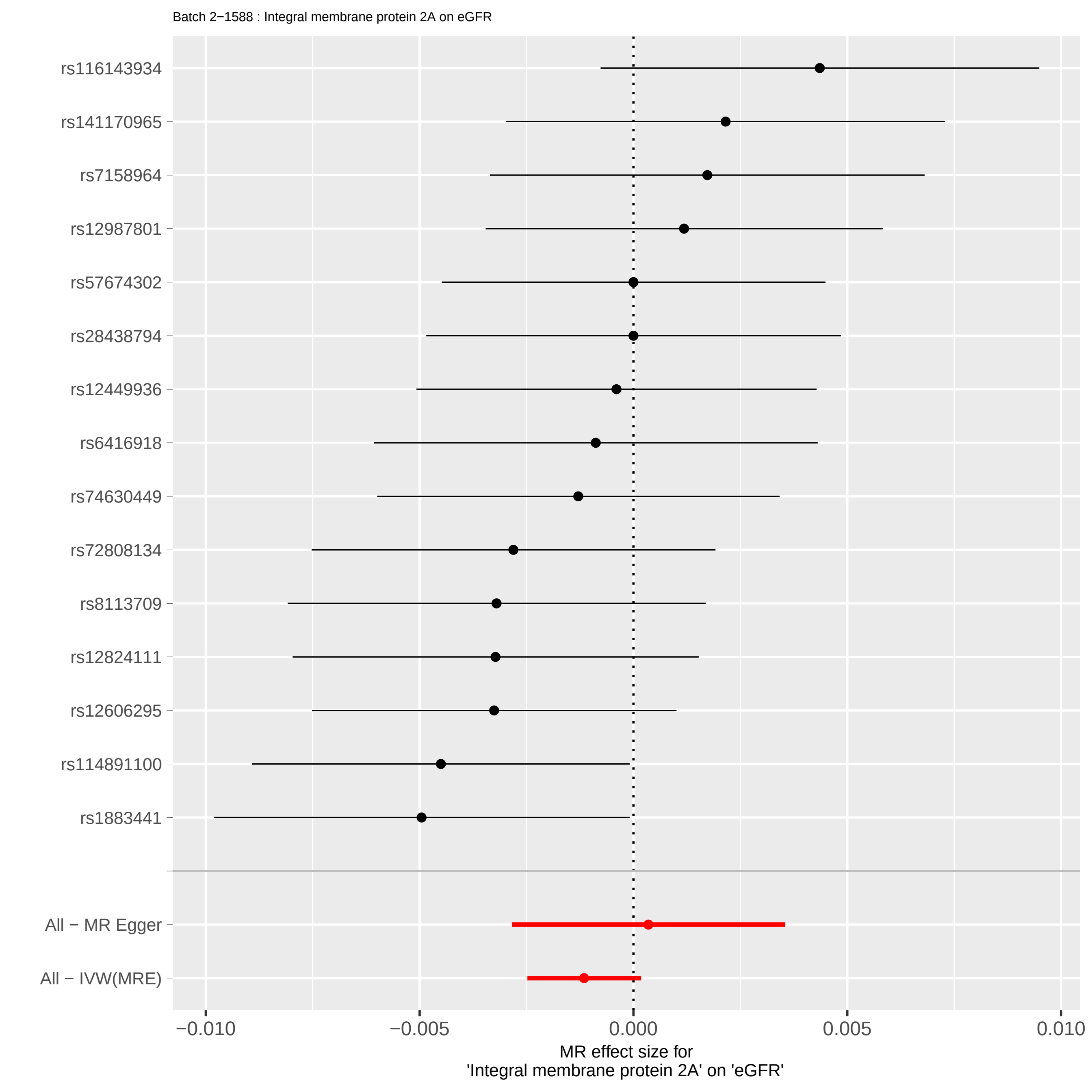

Batch 2−1588 : Integral membrane protein 2A on eGFR
rs116143934
rs141170965
rs7158964
rs12987801
rs57674302
rs28438794
rs12449936
rs6416918
rs74630449
rs72808134
rs8113709
rs12824111
rs12606295
rs114891100
rs1883441
All − MR Egger
All − IVW(MRE)
−0.010
−0.005
0.000
0.005
0.010
MR effect size for
'Integral membrane protein 2A' on 'eGFR'

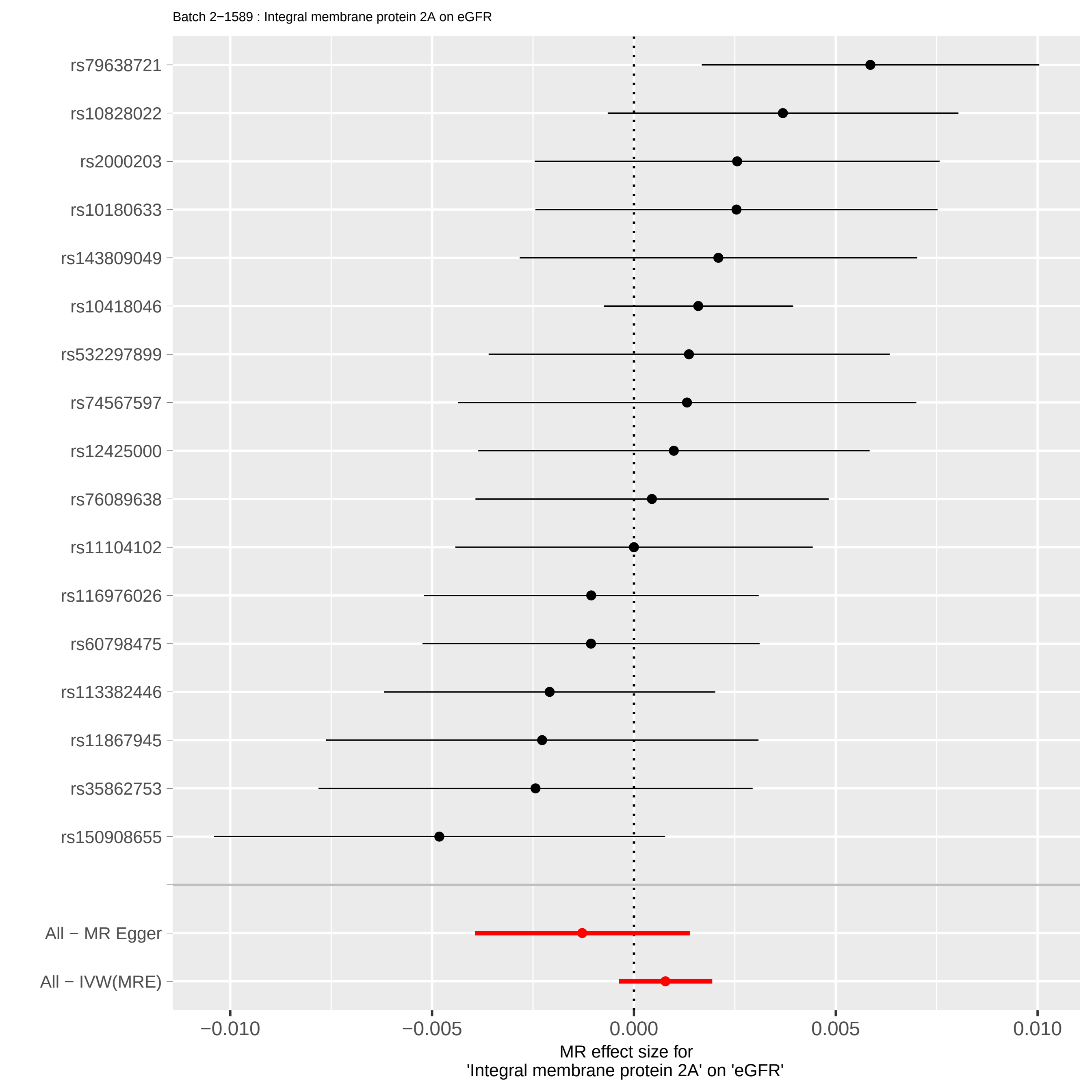
Batch 2−1589 : Integral membrane protein 2A on eGFR
rs79638721
rs10828022
rs2000203
rs10180633
rs143809049
rs10418046
rs532297899
rs74567597
rs12425000
rs76089638
rs11104102
rs116976026
rs60798475
rs113382446
rs11867945
rs35862753
rs150908655
All − MR Egger
All − IVW(MRE)
−0.010
−0.005
0.000
0.005
0.010
MR effect size for
'Integral membrane protein 2A' on 'eGFR'

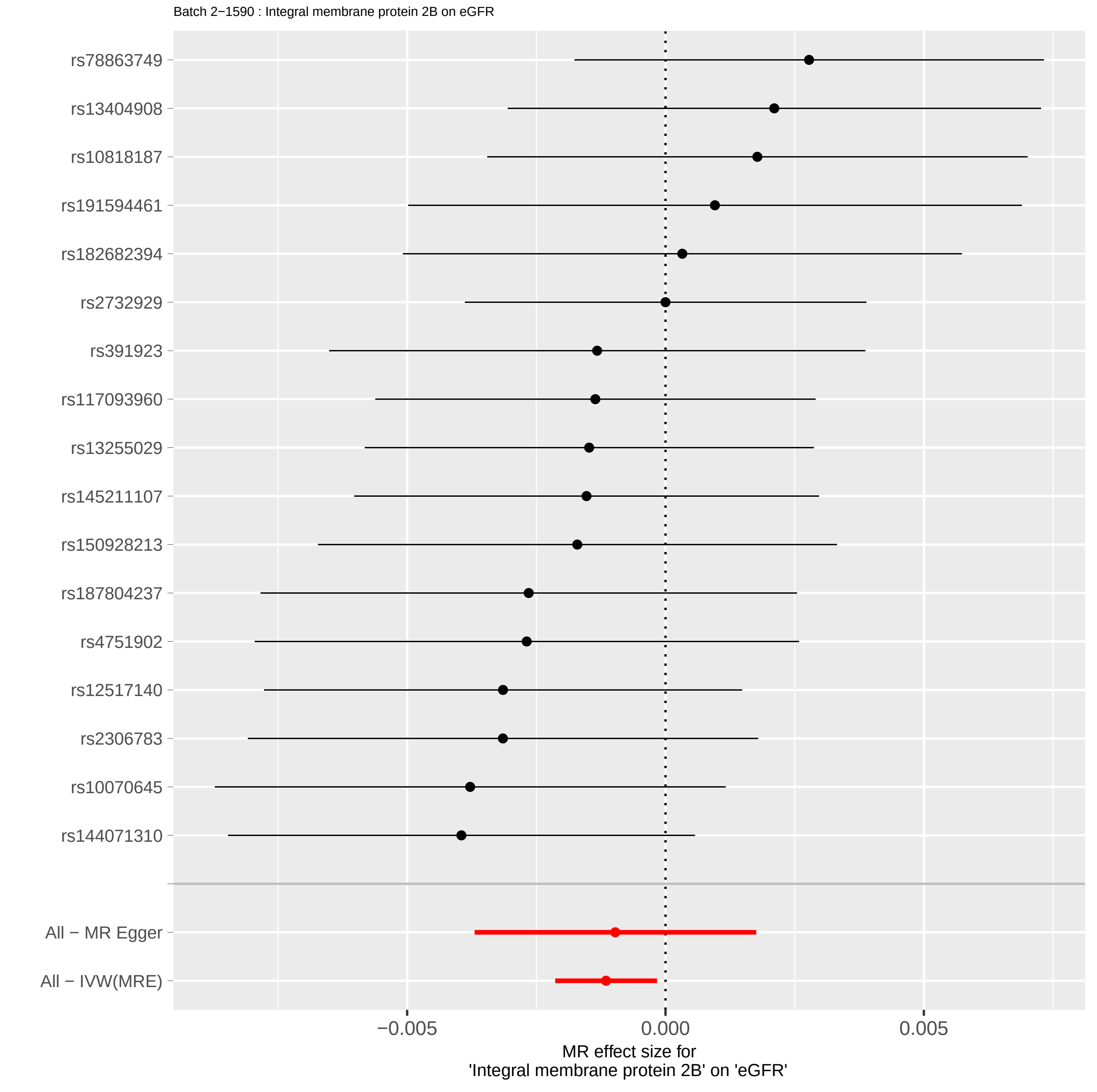
Batch 2−1590 : Integral membrane protein 2B on eGFR
rs78863749
rs13404908
rs10818187
rs191594461
rs182682394
rs2732929
rs391923
rs117093960
rs13255029
rs145211107
rs150928213
rs187804237
rs4751902
rs12517140
rs2306783
rs10070645
rs144071310
All − MR Egger
All − IVW(MRE)
−0.005
0.000
0.005
MR effect size for
'Integral membrane protein 2B' on 'eGFR'

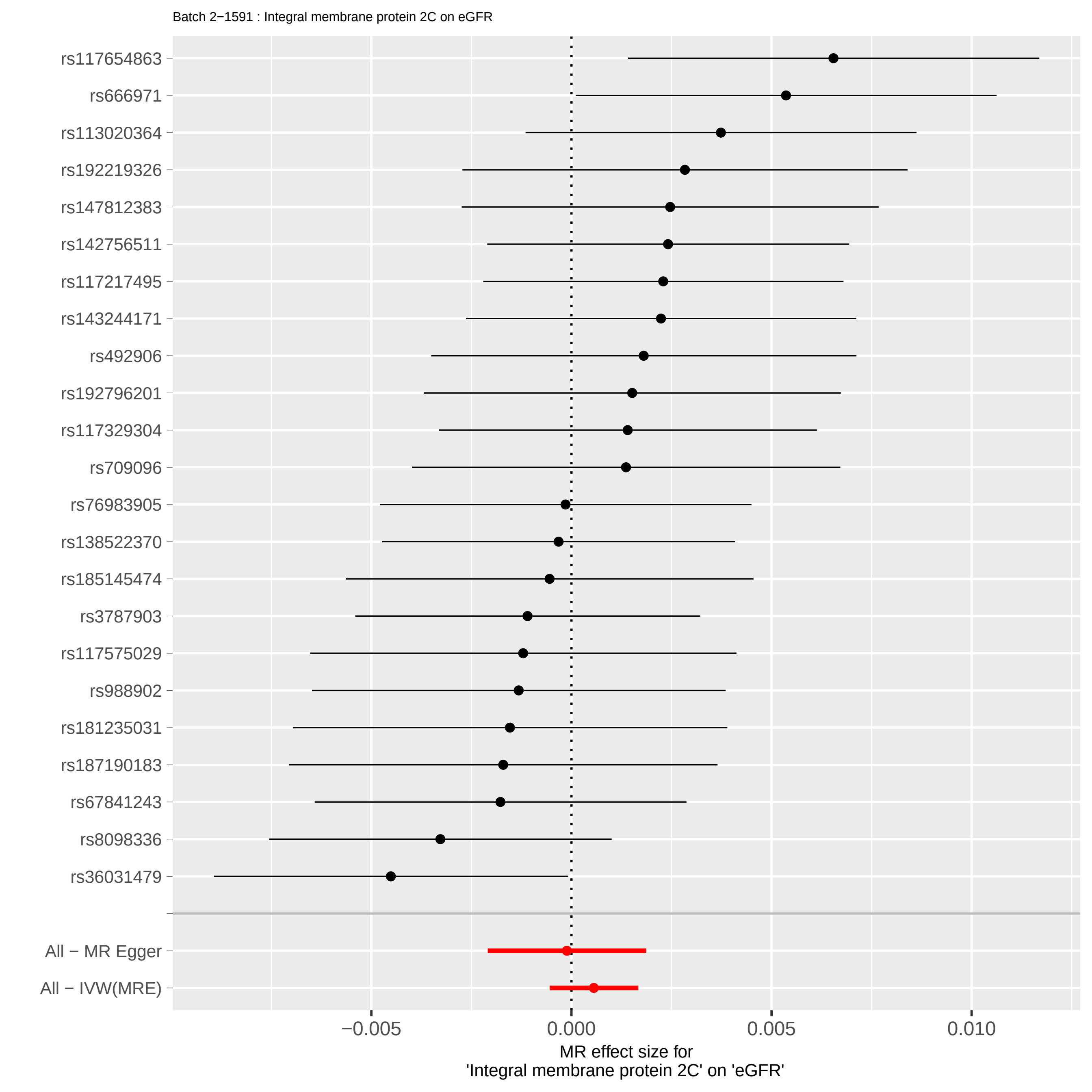

Batch 2−1591 : Integral membrane protein 2C on eGFR
rs117654863
rs666971
rs113020364
rs192219326
rs147812383
rs142756511
rs117217495
rs143244171
rs492906
rs192796201
rs117329304
rs709096
rs76983905
rs138522370
rs185145474
rs3787903
rs117575029
rs988902
rs181235031
rs187190183
rs67841243
rs8098336
rs36031479
All − MR Egger
All − IVW(MRE)
−0.005
0.000
0.005
0.010
MR effect size for
'Integral membrane protein 2C' on 'eGFR'

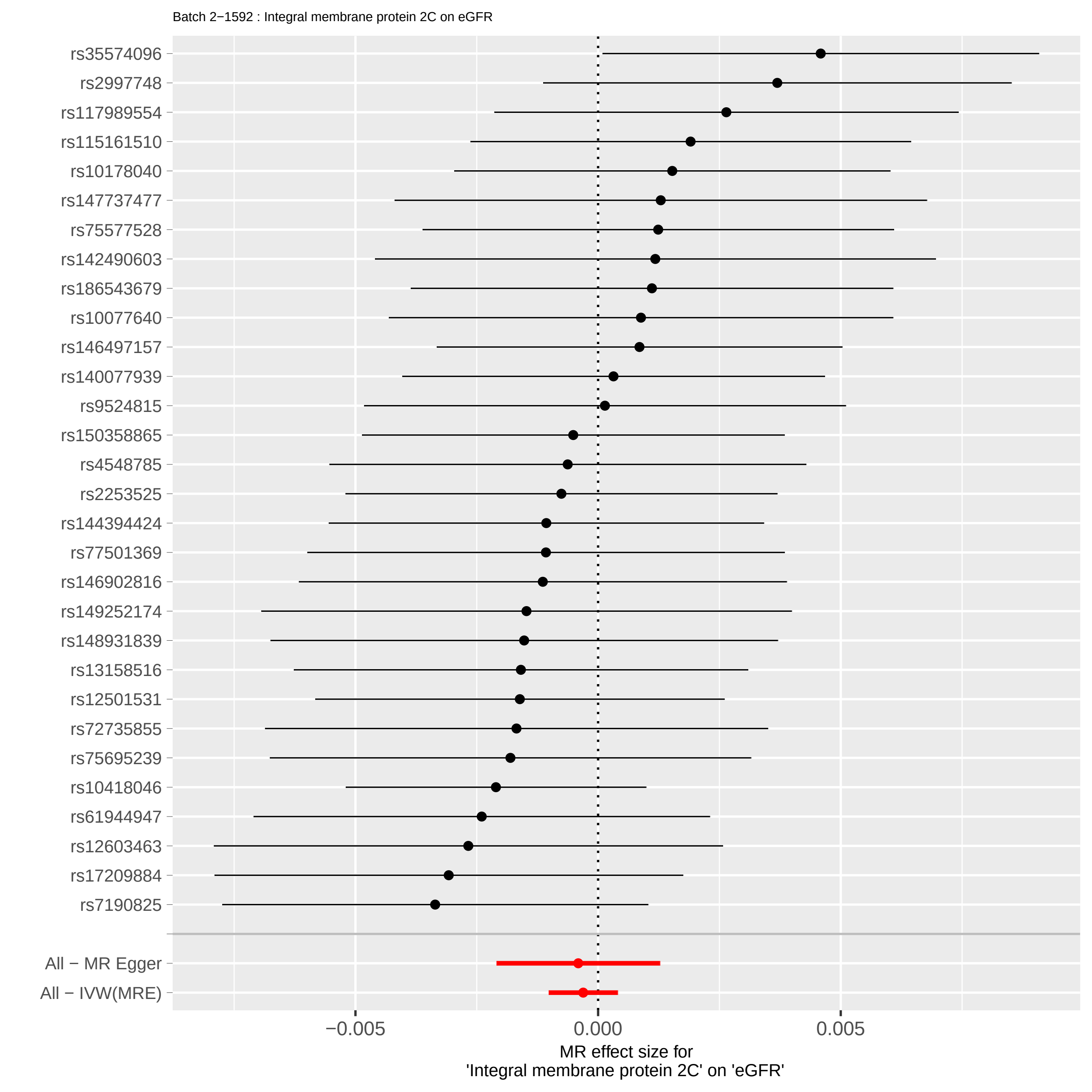

Batch 2−1592 : Integral membrane protein 2C on eGFR
rs35574096
rs2997748
rs117989554
rs115161510
rs10178040
rs147737477
rs75577528
rs142490603
rs186543679
rs10077640
rs146497157
rs140077939
rs9524815
rs150358865
rs4548785
rs2253525
rs144394424
rs77501369
rs146902816
rs149252174
rs148931839
rs13158516
rs12501531
rs72735855
rs75695239
rs10418046
rs61944947
rs12603463
rs17209884
rs7190825
All − MR Egger
All − IVW(MRE)
−0.005
0.000
0.005
MR effect size for
'Integral membrane protein 2C' on 'eGFR'

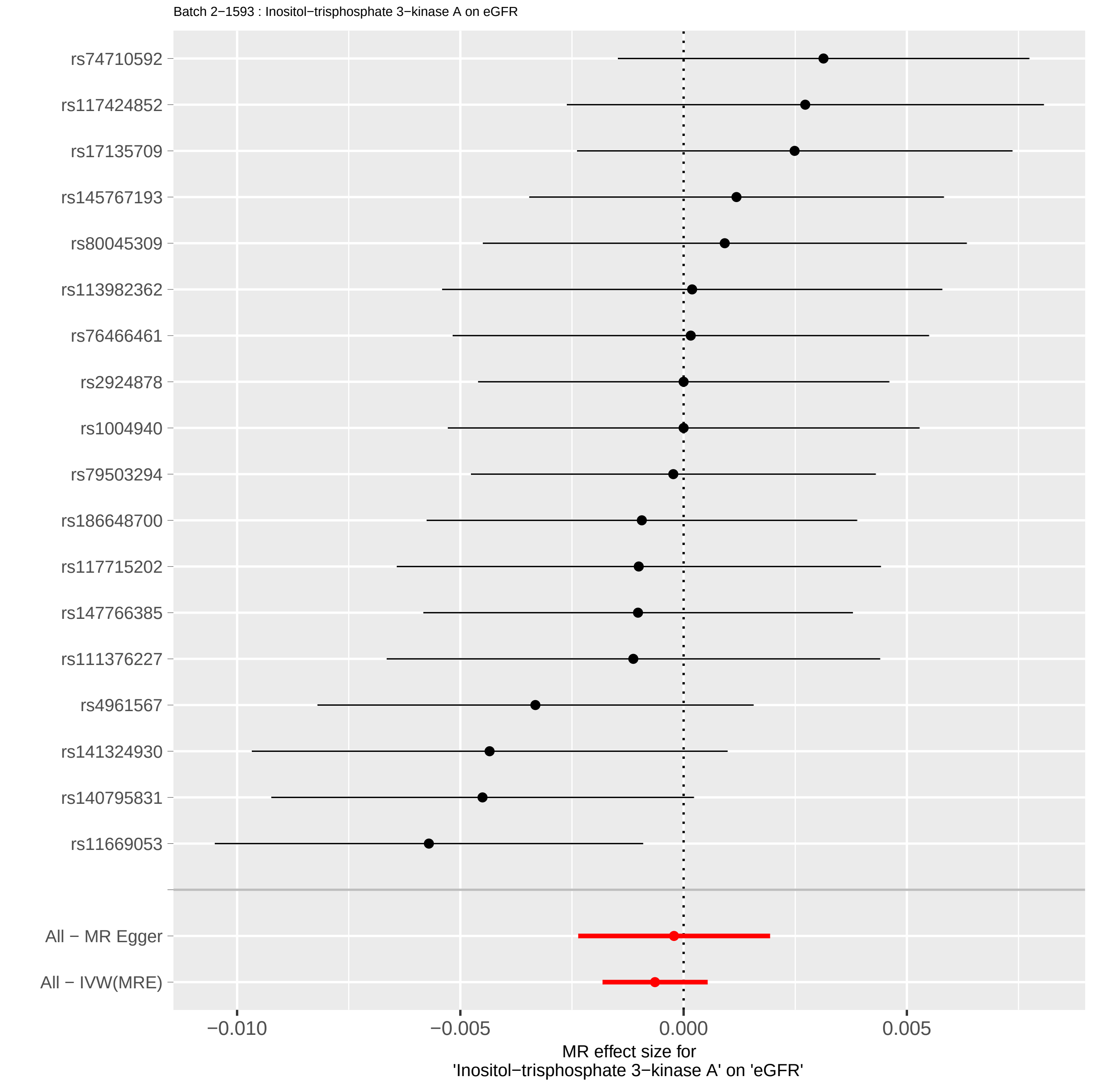

Batch 2−1593 : Inositol−trisphosphate 3−kinase A on eGFR
rs74710592
rs117424852
rs17135709
rs145767193
rs80045309
rs113982362
rs76466461
rs2924878
rs1004940
rs79503294
rs186648700
rs117715202
rs147766385
rs111376227
rs4961567
rs141324930
rs140795831
rs11669053
All − MR Egger
All − IVW(MRE)
−0.010
−0.005
0.000
0.005
MR effect size for
'Inositol−trisphosphate 3−kinase A' on 'eGFR'

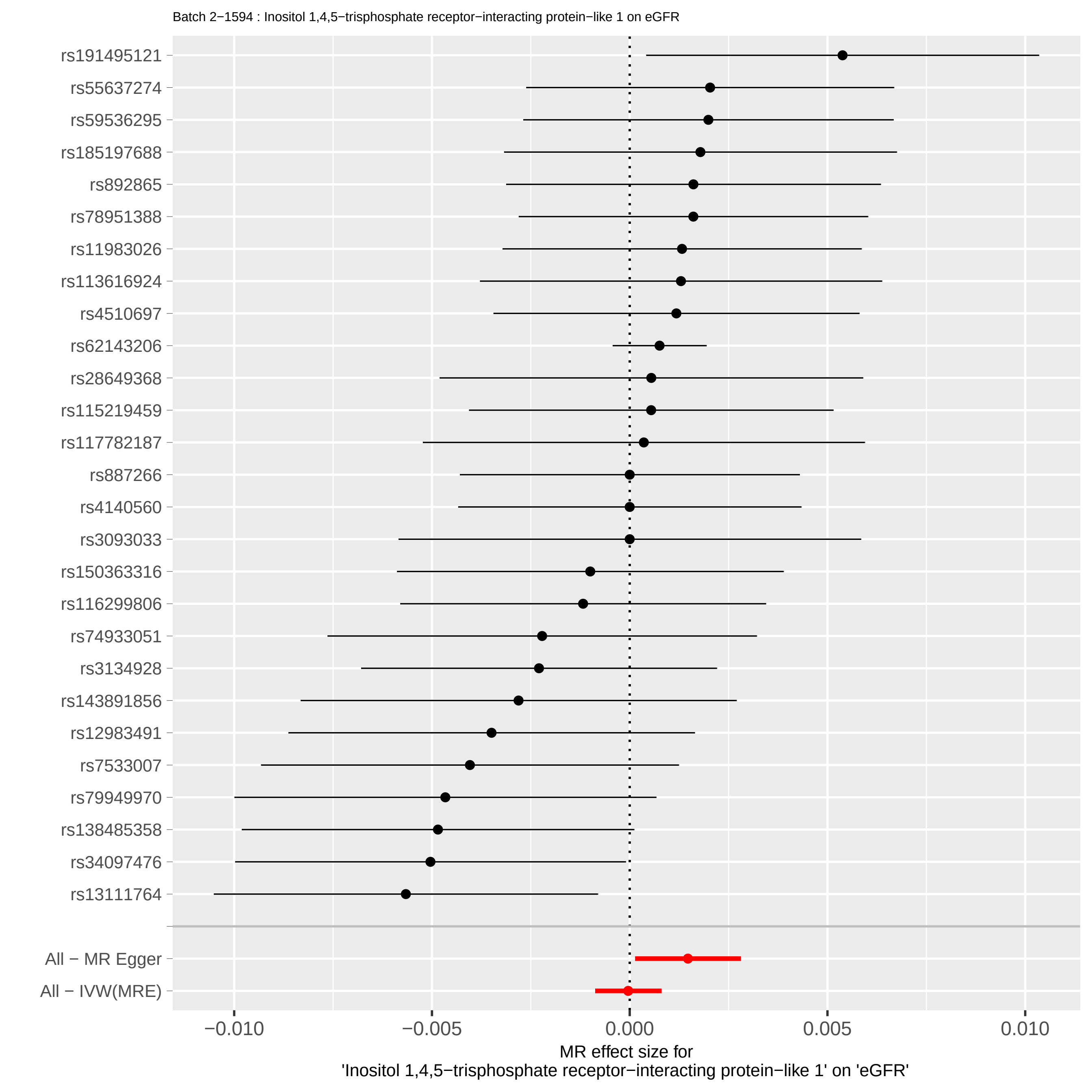
Batch 2−1594 : Inositol 1,4,5−trisphosphate receptor−interacting protein−like 1 on eGFR
rs191495121
rs55637274
rs59536295
rs185197688
rs892865
rs78951388
rs11983026
rs113616924
rs4510697
rs62143206
rs28649368
rs115219459
rs117782187
rs887266
rs4140560
rs3093033
rs150363316
rs116299806
rs74933051
rs3134928
rs143891856
rs12983491
rs7533007
rs79949970
rs138485358
rs34097476
rs13111764
All − MR Egger
All − IVW(MRE)
−0.010
−0.005
0.000
0.005
0.010
MR effect size for
'Inositol 1,4,5−trisphosphate receptor−interacting protein−like 1' on 'eGFR'

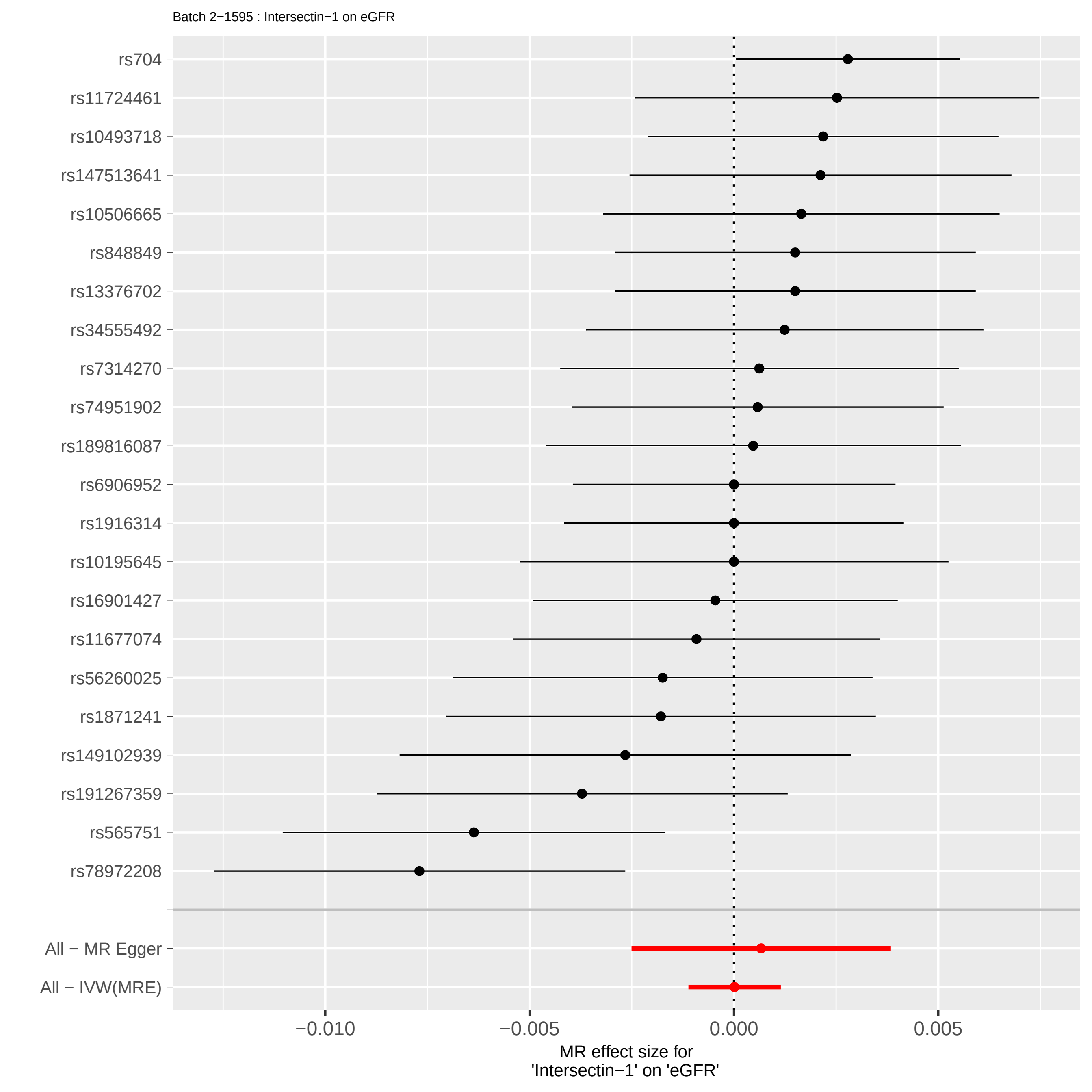

Batch 2−1595 : Intersectin−1 on eGFR
rs704
rs11724461
rs10493718
rs147513641
rs10506665
rs848849
rs13376702
rs34555492
rs7314270
rs74951902
rs189816087
rs6906952
rs1916314
rs10195645
rs16901427
rs11677074
rs56260025
rs1871241
rs149102939
rs191267359
rs565751
rs78972208
All − MR Egger
All − IVW(MRE)
−0.010
−0.005
0.000
0.005
MR effect size for
'Intersectin−1' on 'eGFR'

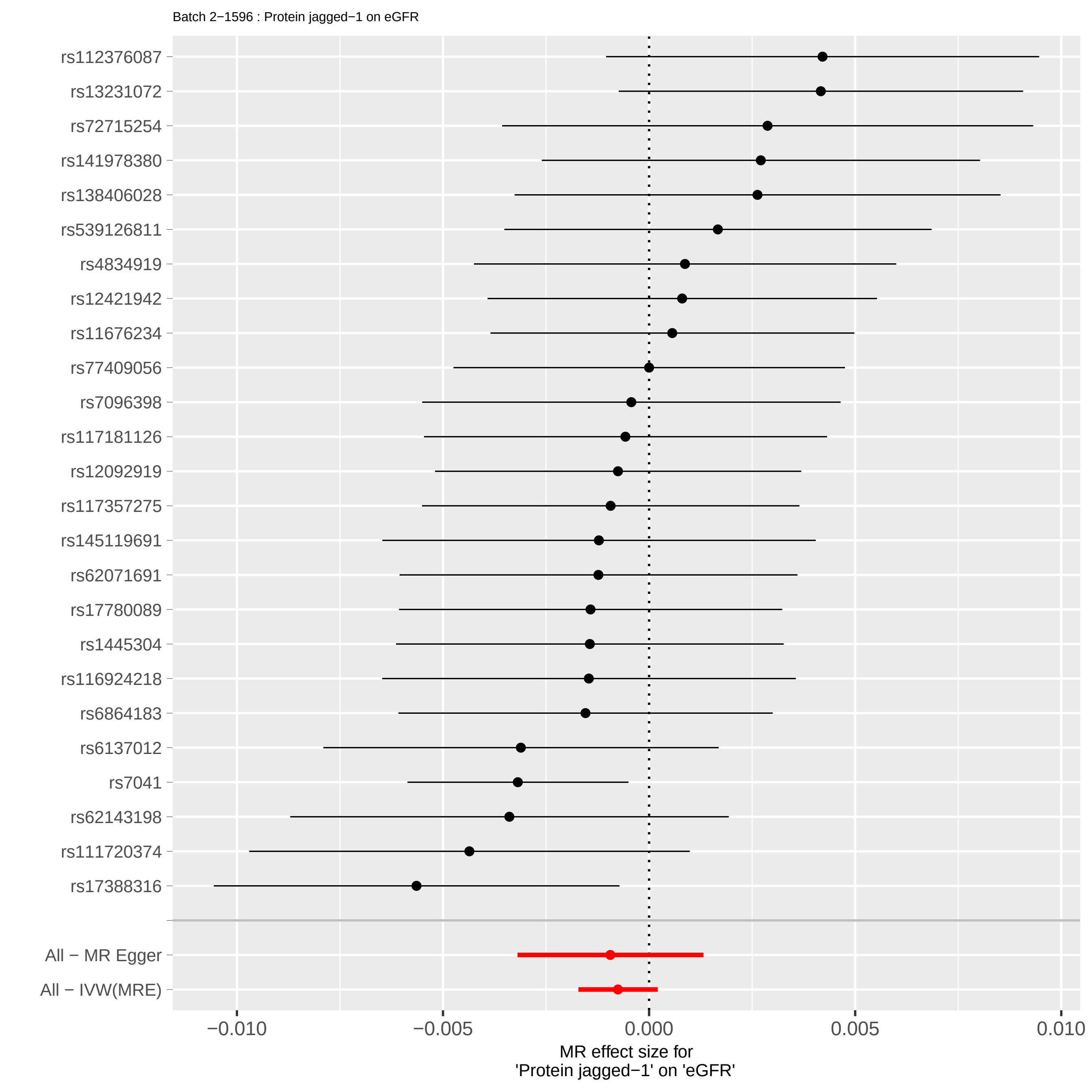

Batch 2−1596 : Protein jagged−1 on eGFR
rs112376087
rs13231072
rs72715254
rs141978380
rs138406028
rs539126811
rs4834919
rs12421942
rs11676234
rs77409056
rs7096398
rs117181126
rs12092919
rs117357275
rs145119691
rs62071691
rs17780089
rs1445304
rs116924218
rs6864183
rs6137012
rs7041
rs62143198
rs111720374
rs17388316
All − MR Egger
All − IVW(MRE)
−0.010
−0.005
0.000
0.005
0.010
MR effect size for
'Protein jagged−1' on 'eGFR'

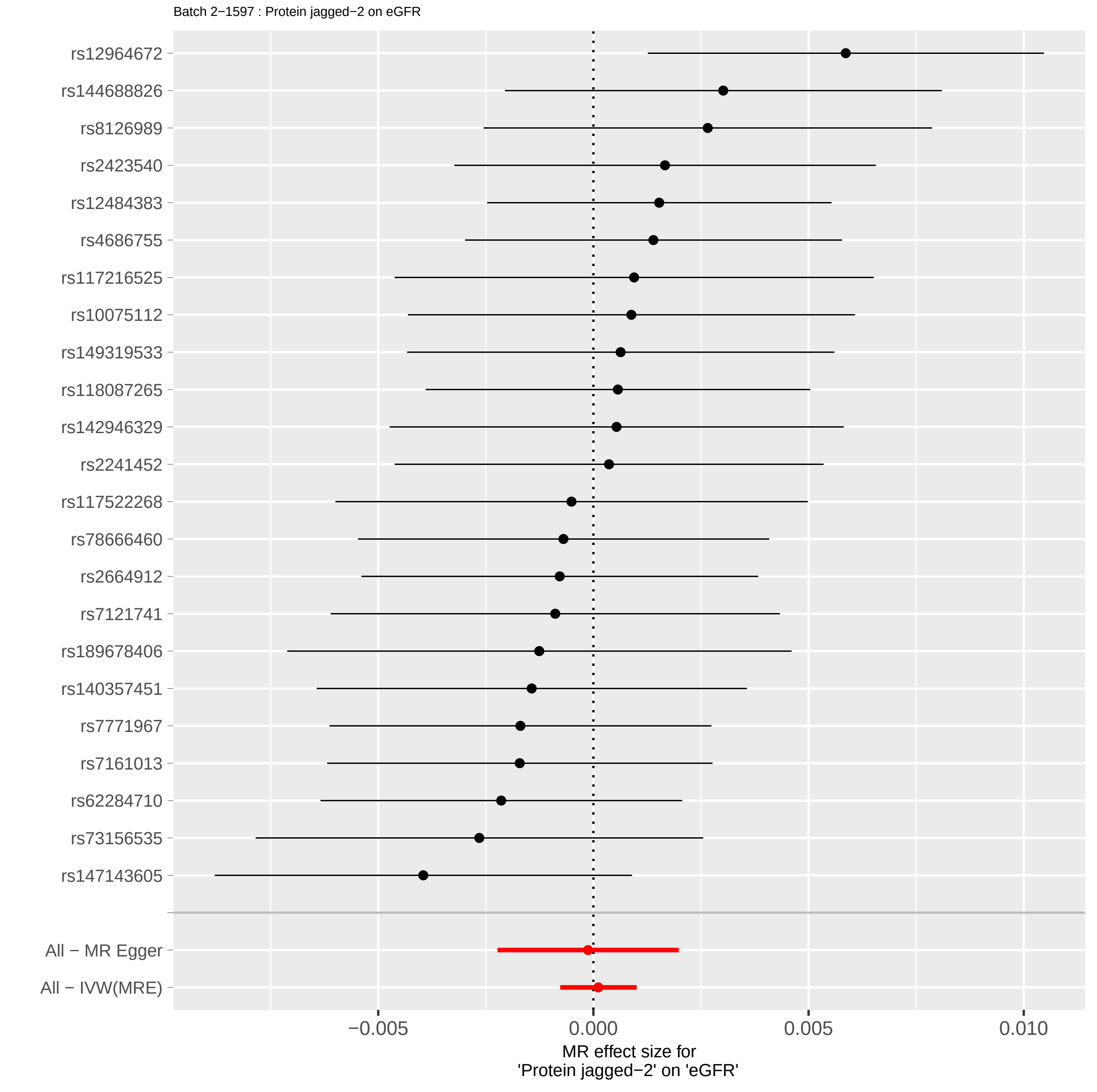

Batch 2−1597 : Protein jagged−2 on eGFR
rs12964672
rs144688826
rs8126989
rs2423540
rs12484383
rs4686755
rs117216525
rs10075112
rs149319533
rs118087265
rs142946329
rs2241452
rs117522268
rs78666460
rs2664912
rs7121741
rs189678406
rs140357451
rs7771967
rs7161013
rs62284710
rs73156535
rs147143605
All − MR Egger
All − IVW(MRE)
−0.005
0.000
0.005
0.010
MR effect size for
'Protein jagged−2' on 'eGFR'

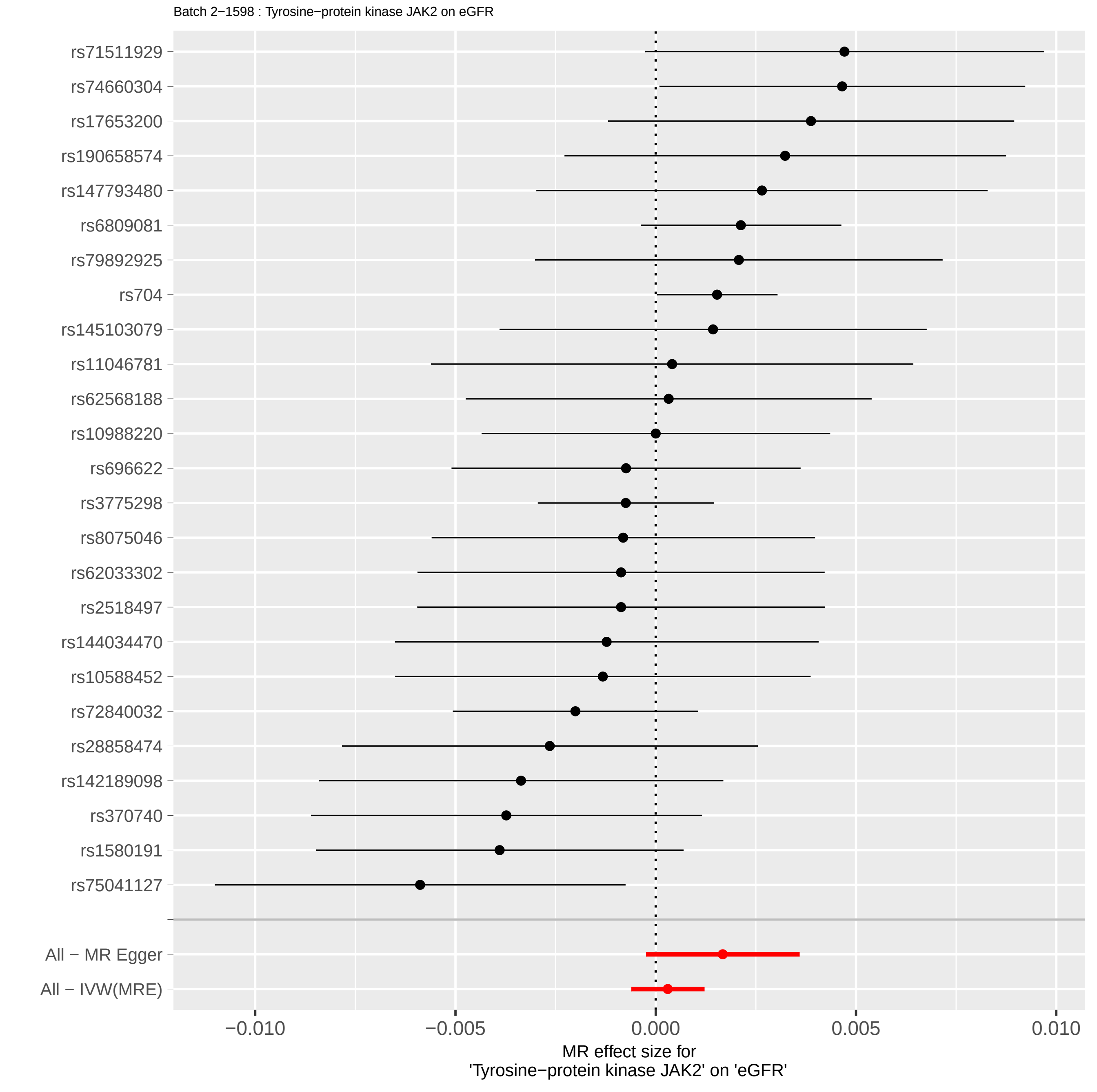

Batch 2−1598 : Tyrosine−protein kinase JAK2 on eGFR
rs71511929
rs74660304
rs17653200
rs190658574
rs147793480
rs6809081
rs79892925
rs704
rs145103079
rs11046781
rs62568188
rs10988220
rs696622
rs3775298
rs8075046
rs62033302
rs2518497
rs144034470
rs10588452
rs72840032
rs28858474
rs142189098
rs370740
rs1580191
rs75041127
All − MR Egger
All − IVW(MRE)
−0.010
−0.005
0.000
0.005
0.010
MR effect size for
'Tyrosine−protein kinase JAK2' on 'eGFR'

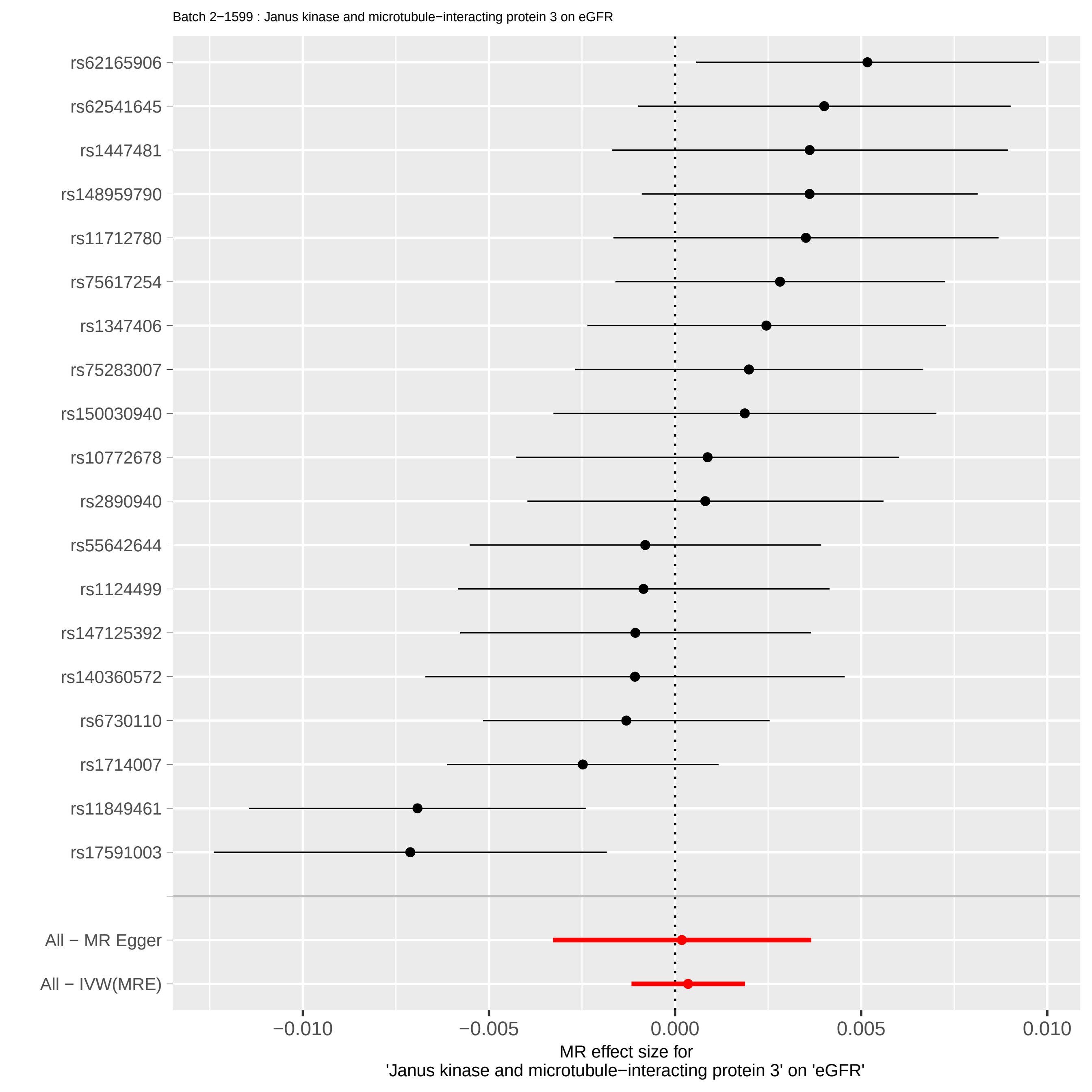
Batch 2−1599 : Janus kinase and microtubule−interacting protein 3 on eGFR
rs62165906
rs62541645
rs1447481
rs148959790
rs11712780
rs75617254
rs1347406
rs75283007
rs150030940
rs10772678
rs2890940
rs55642644
rs1124499
rs147125392
rs140360572
rs6730110
rs1714007
rs11849461
rs17591003
All − MR Egger
All − IVW(MRE)
−0.010
−0.005
0.000
0.005
0.010
MR effect size for
'Janus kinase and microtubule−interacting protein 3' on 'eGFR'

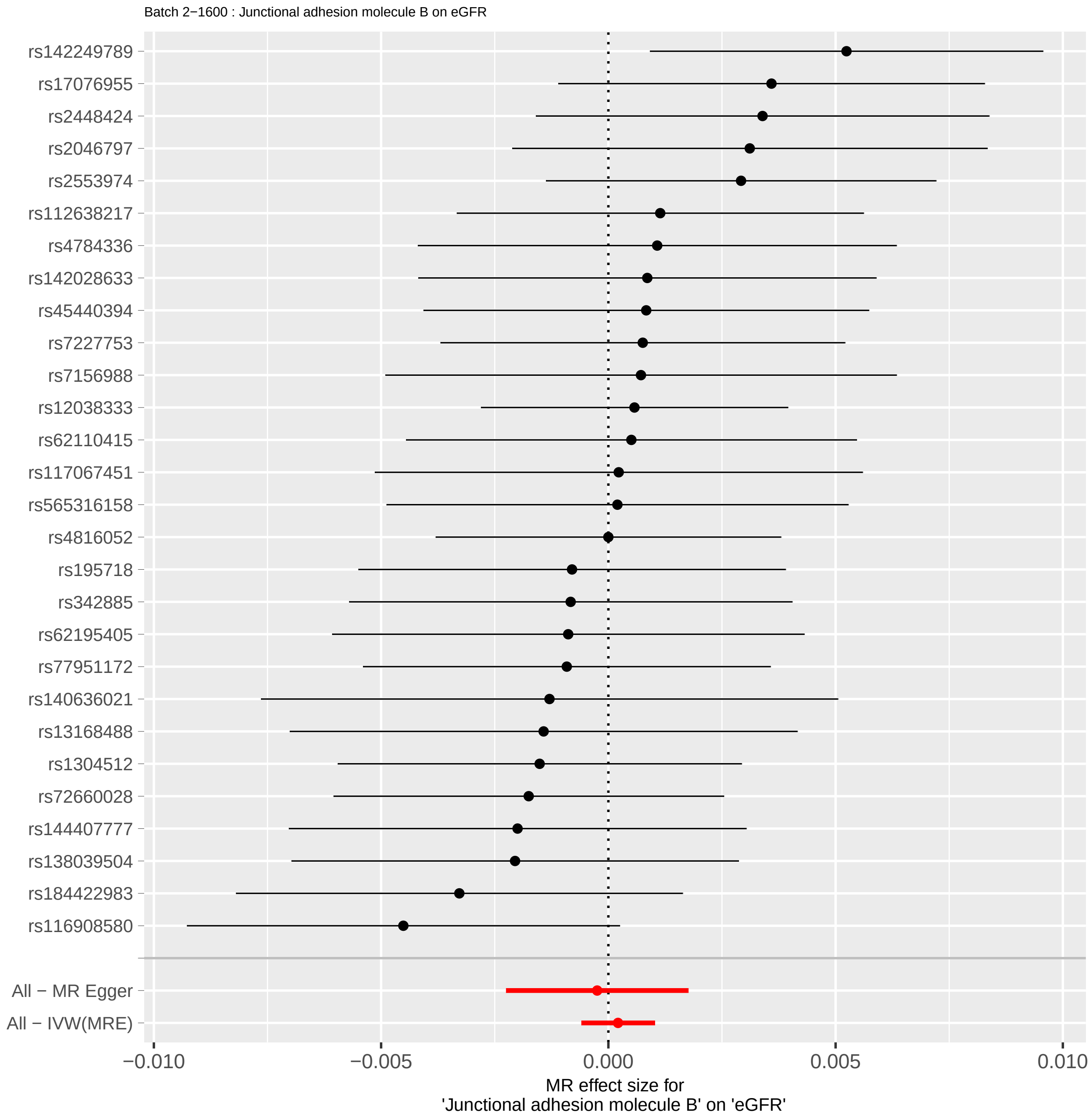

Batch 2−1600 : Junctional adhesion molecule B on eGFR
rs142249789
rs17076955
rs2448424
rs2046797
rs2553974
rs112638217
rs4784336
rs142028633
rs45440394
rs7227753
rs7156988
rs12038333
rs62110415
rs117067451
rs565316158
rs4816052
rs195718
rs342885
rs62195405
rs77951172
rs140636021
rs13168488
rs1304512
rs72660028
rs144407777
rs138039504
rs184422983
rs116908580
All − MR Egger
All − IVW(MRE)
−0.010
−0.005
0.000
0.005
0.010
MR effect size for
'Junctional adhesion molecule B' on 'eGFR'

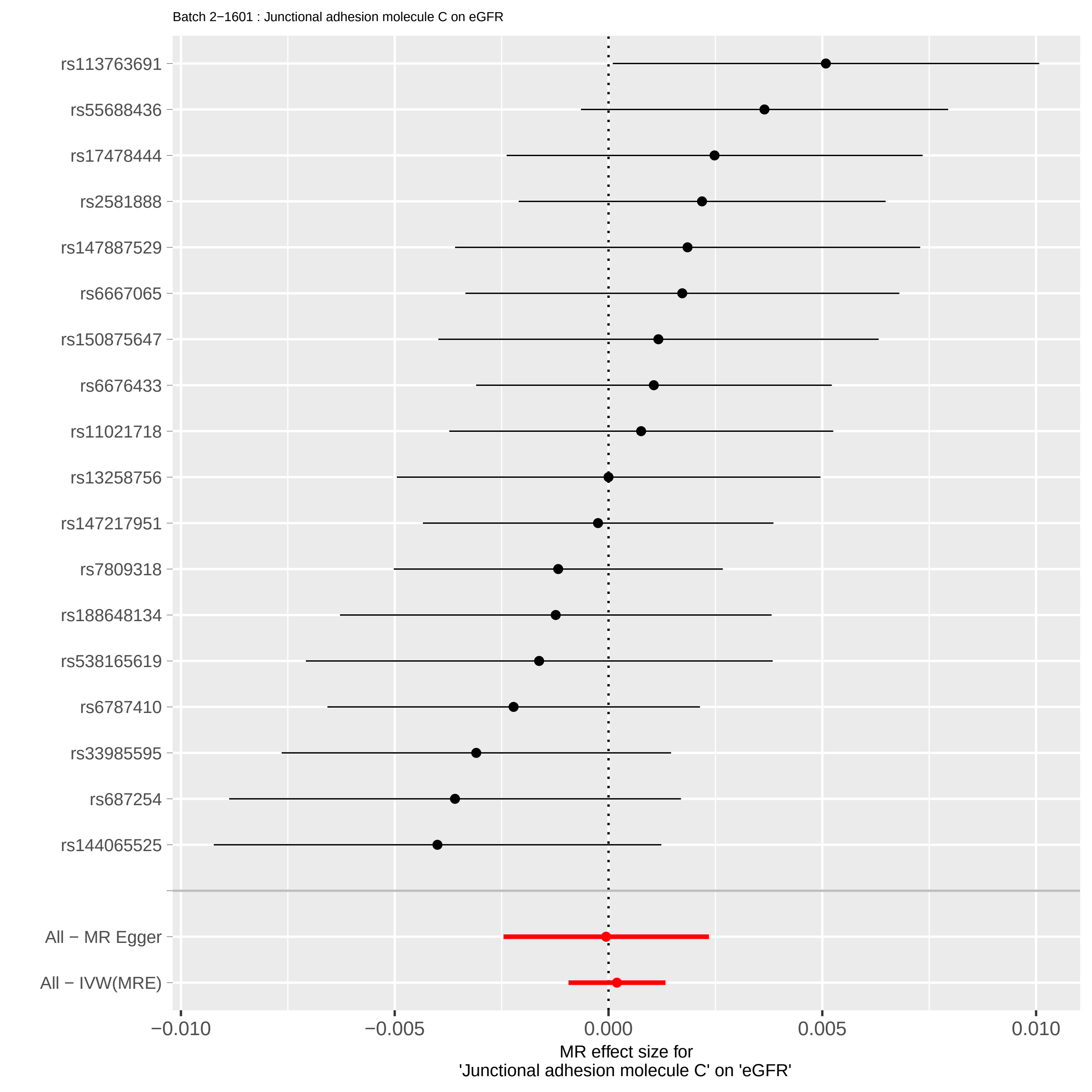

Batch 2−1601 : Junctional adhesion molecule C on eGFR
rs113763691
rs55688436
rs17478444
rs2581888
rs147887529
rs6667065
rs150875647
rs6676433
rs11021718
rs13258756
rs147217951
rs7809318
rs188648134
rs538165619
rs6787410
rs33985595
rs687254
rs144065525
All − MR Egger
All − IVW(MRE)
−0.010
−0.005
0.000
0.005
0.010
MR effect size for
'Junctional adhesion molecule C' on 'eGFR'

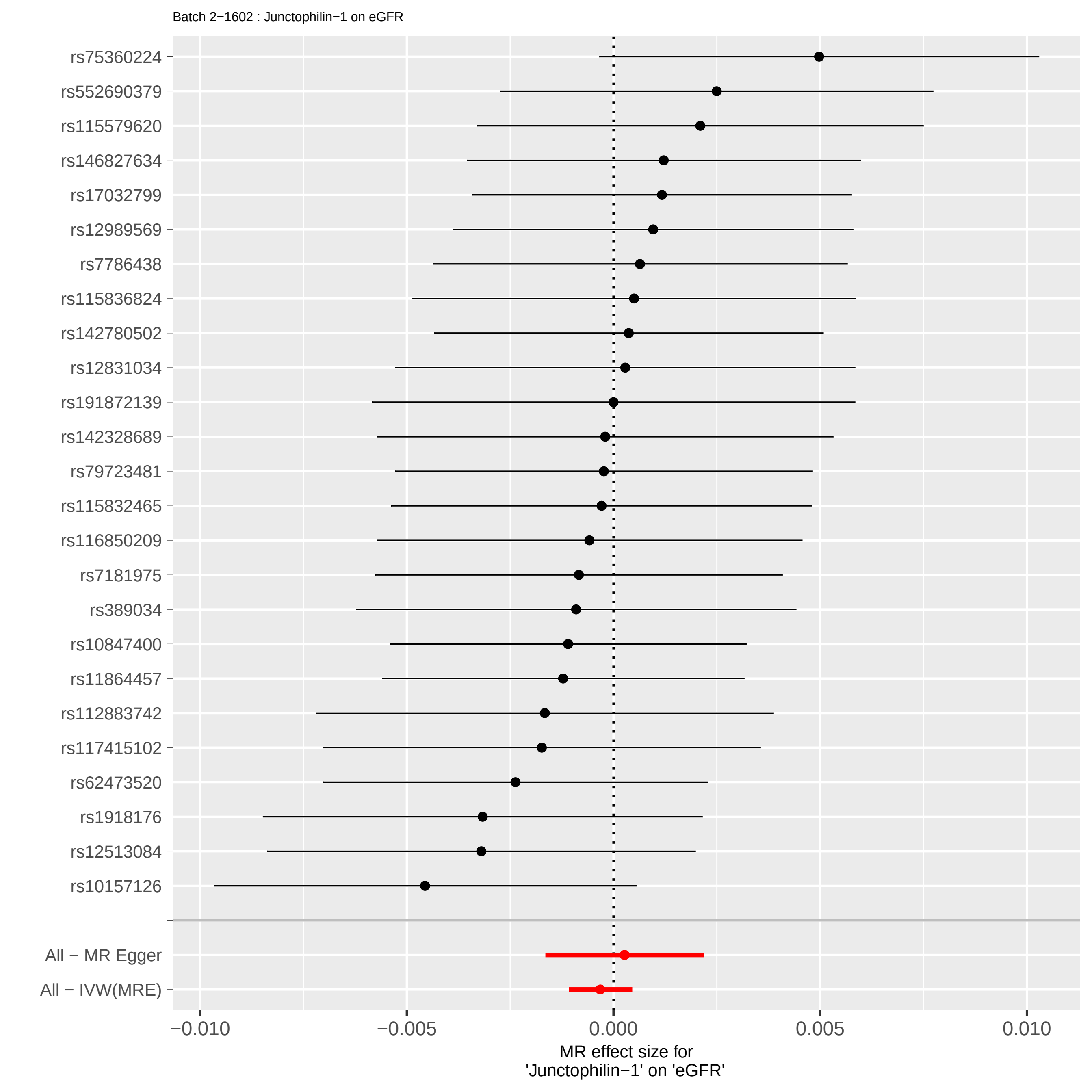

Batch 2−1602 : Junctophilin−1 on eGFR
rs75360224
rs552690379
rs115579620
rs146827634
rs17032799
rs12989569
rs7786438
rs115836824
rs142780502
rs12831034
rs191872139
rs142328689
rs79723481
rs115832465
rs116850209
rs7181975
rs389034
rs10847400
rs11864457
rs112883742
rs117415102
rs62473520
rs1918176
rs12513084
rs10157126
All − MR Egger
All − IVW(MRE)
−0.010
−0.005
0.000
0.005
0.010
MR effect size for
'Junctophilin−1' on 'eGFR'

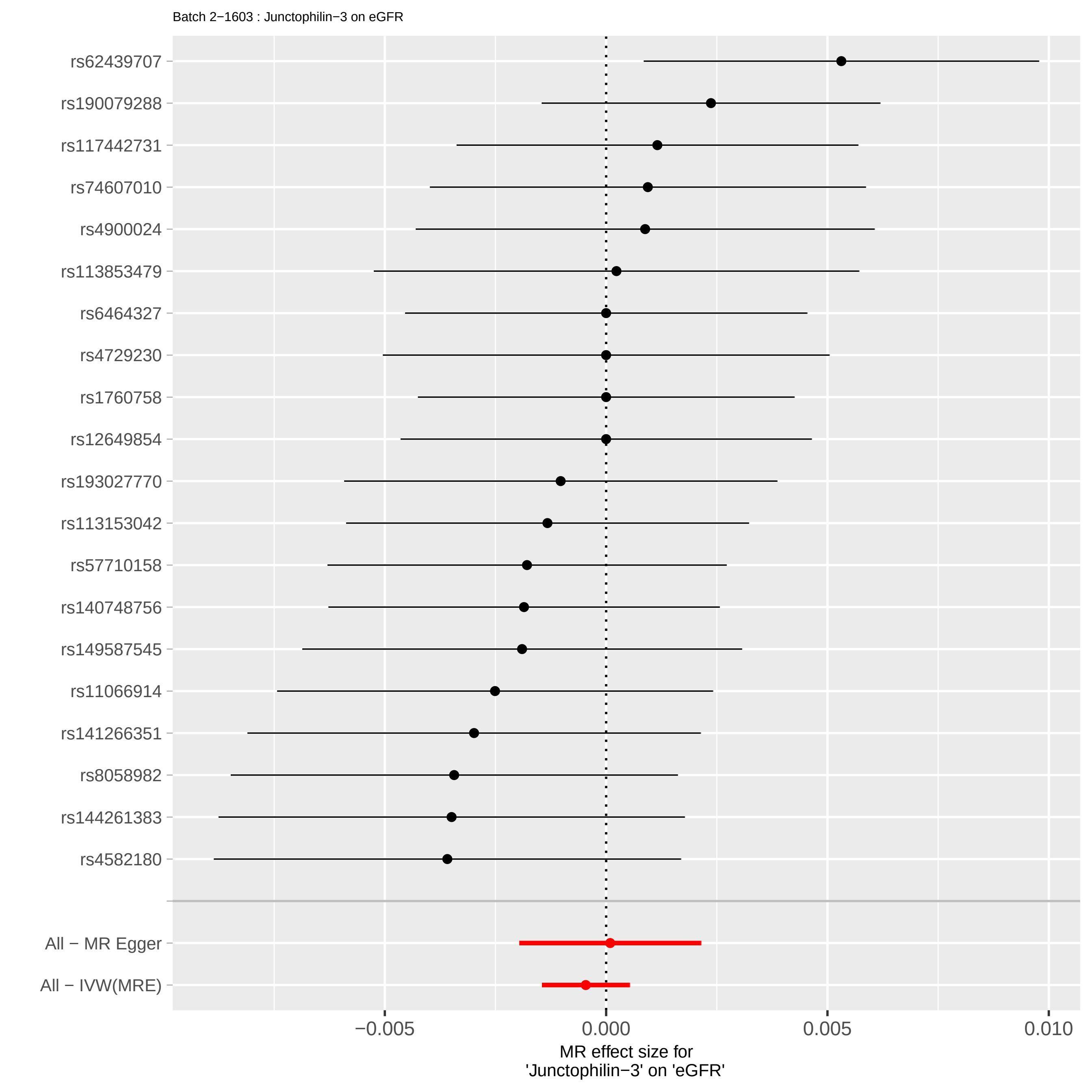
Batch 2−1603 : Junctophilin−3 on eGFR
rs62439707
rs190079288
rs117442731
rs74607010
rs4900024
rs113853479
rs6464327
rs4729230
rs1760758
rs12649854
rs193027770
rs113153042
rs57710158
rs140748756
rs149587545
rs11066914
rs141266351
rs8058982
rs144261383
rs4582180
All − MR Egger
All − IVW(MRE)
−0.005
0.000
0.005
0.010
MR effect size for
'Junctophilin−3' on 'eGFR'

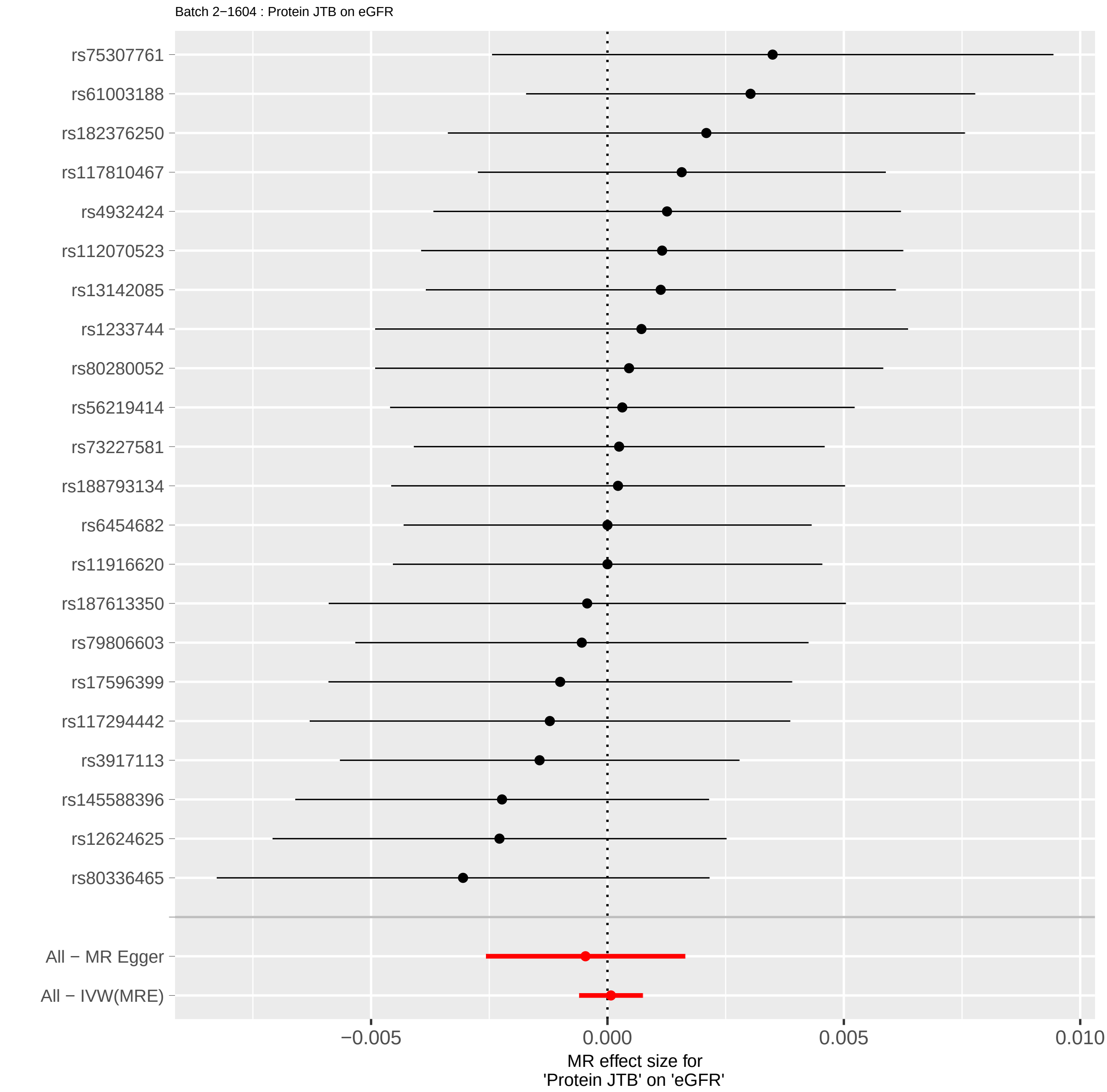

Batch 2−1604 : Protein JTB on eGFR
rs75307761
rs61003188
rs182376250
rs117810467
rs4932424
rs112070523
rs13142085
rs1233744
rs80280052
rs56219414
rs73227581
rs188793134
rs6454682
rs11916620
rs187613350
rs79806603
rs17596399
rs117294442
rs3917113
rs145588396
rs12624625
rs80336465
All − MR Egger
All − IVW(MRE)
−0.005
0.000
0.005
0.010
MR effect size for
'Protein JTB' on 'eGFR'

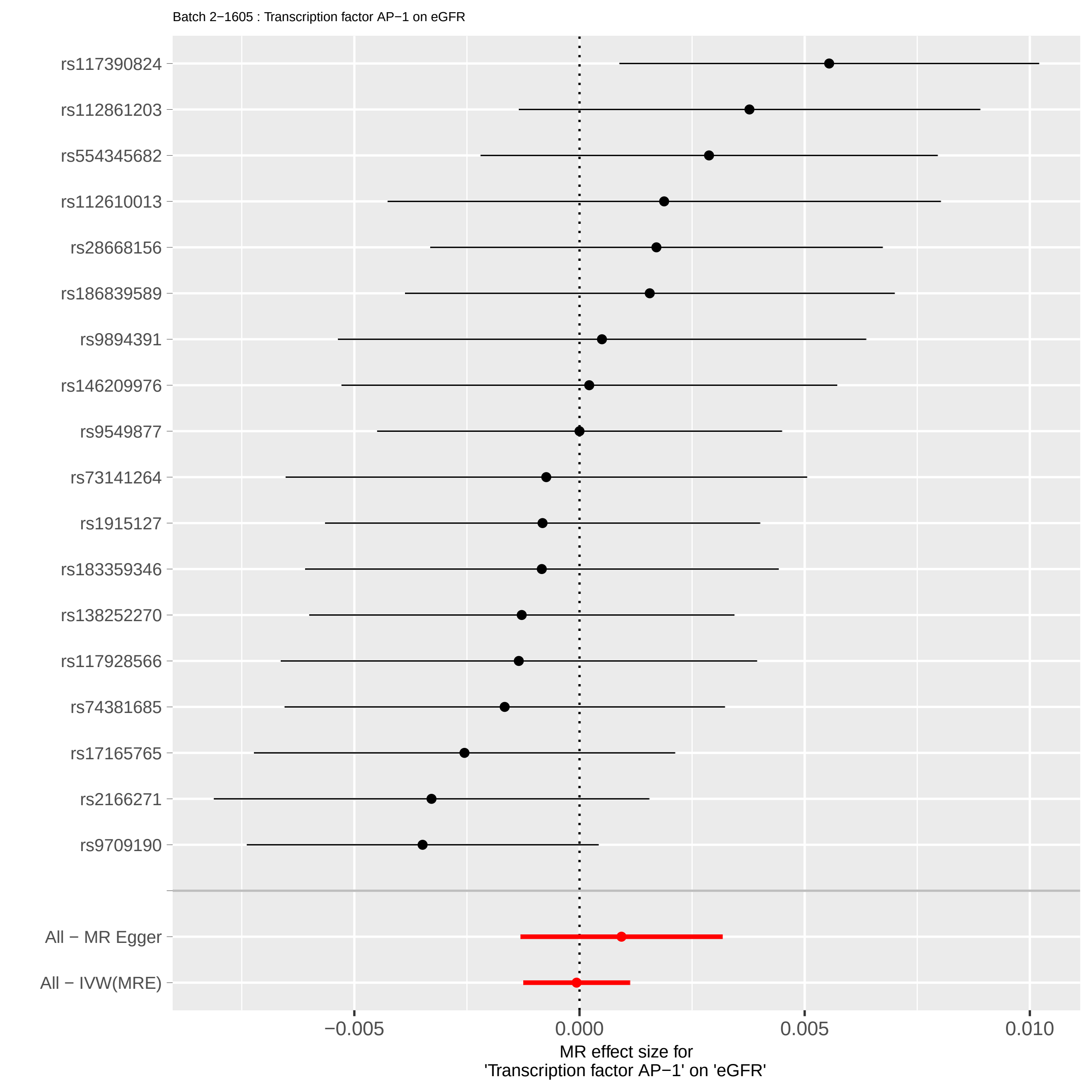

Batch 2−1605 : Transcription factor AP−1 on eGFR
rs117390824
rs112861203
rs554345682
rs112610013
rs28668156
rs186839589
rs9894391
rs146209976
rs9549877
rs73141264
rs1915127
rs183359346
rs138252270
rs117928566
rs74381685
rs17165765
rs2166271
rs9709190
All − MR Egger
All − IVW(MRE)
−0.005
0.000
0.005
0.010
MR effect size for
'Transcription factor AP−1' on 'eGFR'

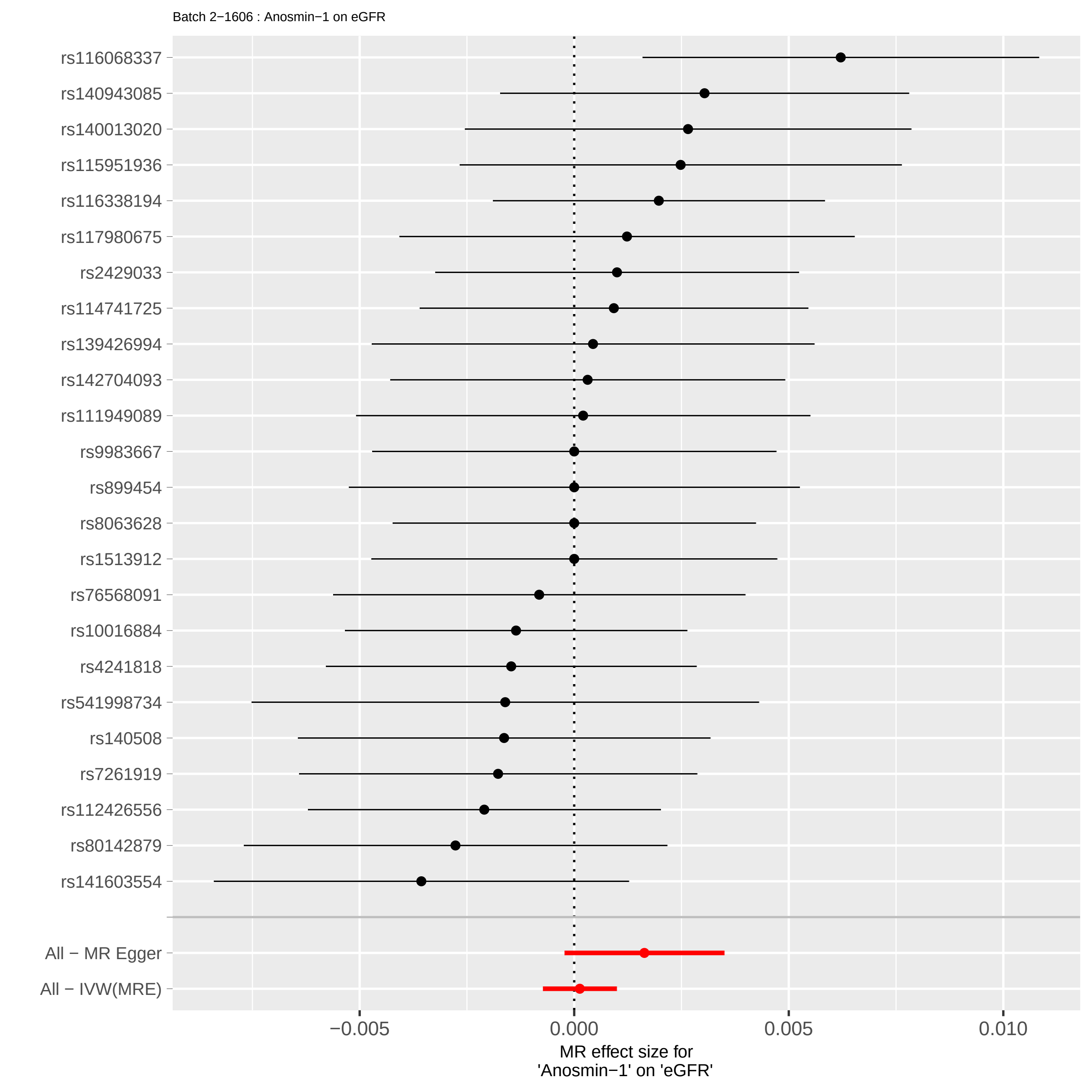

Batch 2−1606 : Anosmin−1 on eGFR
rs116068337
rs140943085
rs140013020
rs115951936
rs116338194
rs117980675
rs2429033
rs114741725
rs139426994
rs142704093
rs111949089
rs9983667
rs899454
rs8063628
rs1513912
rs76568091
rs10016884
rs4241818
rs541998734
rs140508
rs7261919
rs112426556
rs80142879
rs141603554
All − MR Egger
All − IVW(MRE)
−0.005
0.000
0.005
0.010
MR effect size for
'Anosmin−1' on 'eGFR'

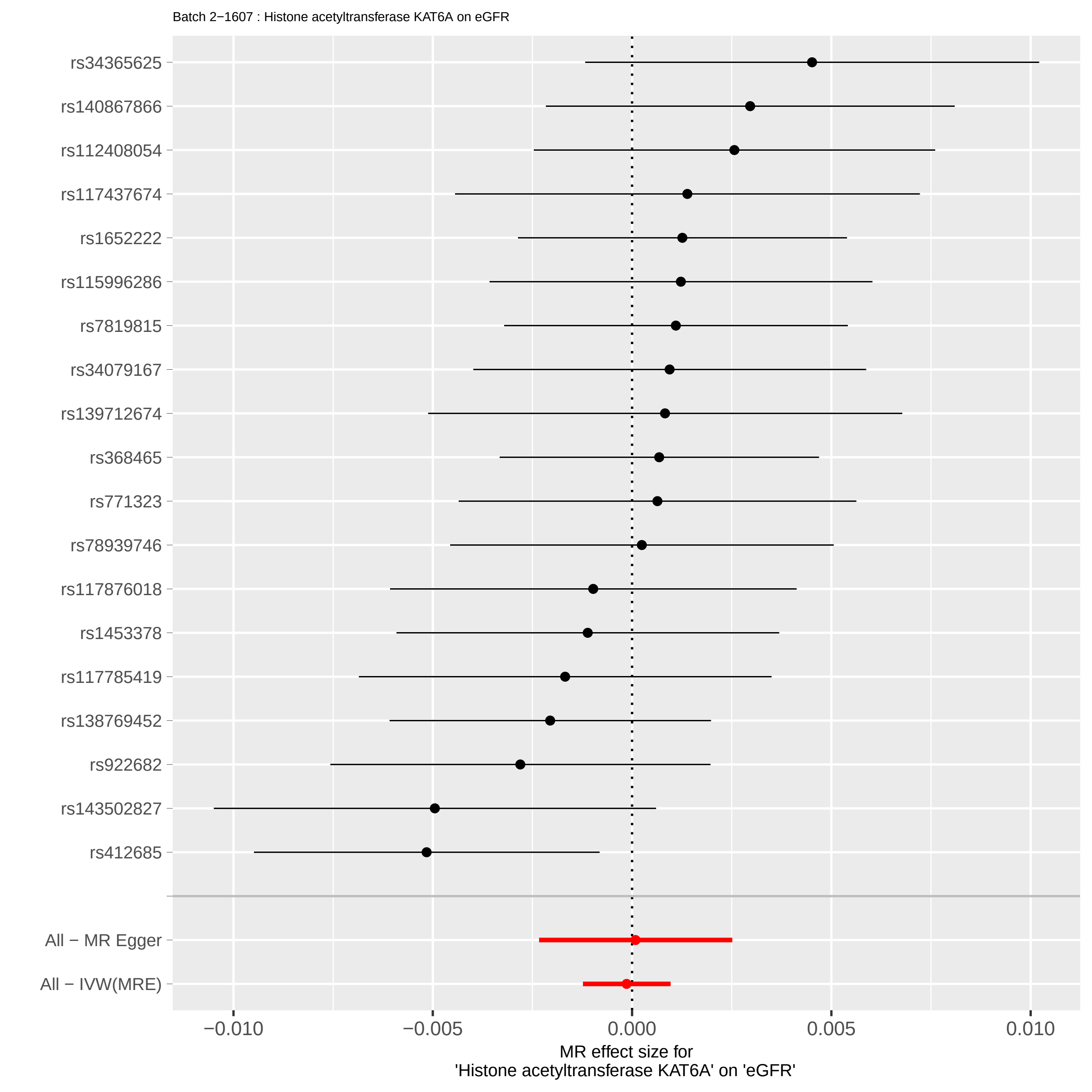

Batch 2−1607 : Histone acetyltransferase KAT6A on eGFR
rs34365625
rs140867866
rs112408054
rs117437674
rs1652222
rs115996286
rs7819815
rs34079167
rs139712674
rs368465
rs771323
rs78939746
rs117876018
rs1453378
rs117785419
rs138769452
rs922682
rs143502827
rs412685
All − MR Egger
All − IVW(MRE)
−0.010
−0.005
0.000
0.005
0.010
MR effect size for
'Histone acetyltransferase KAT6A' on 'eGFR'

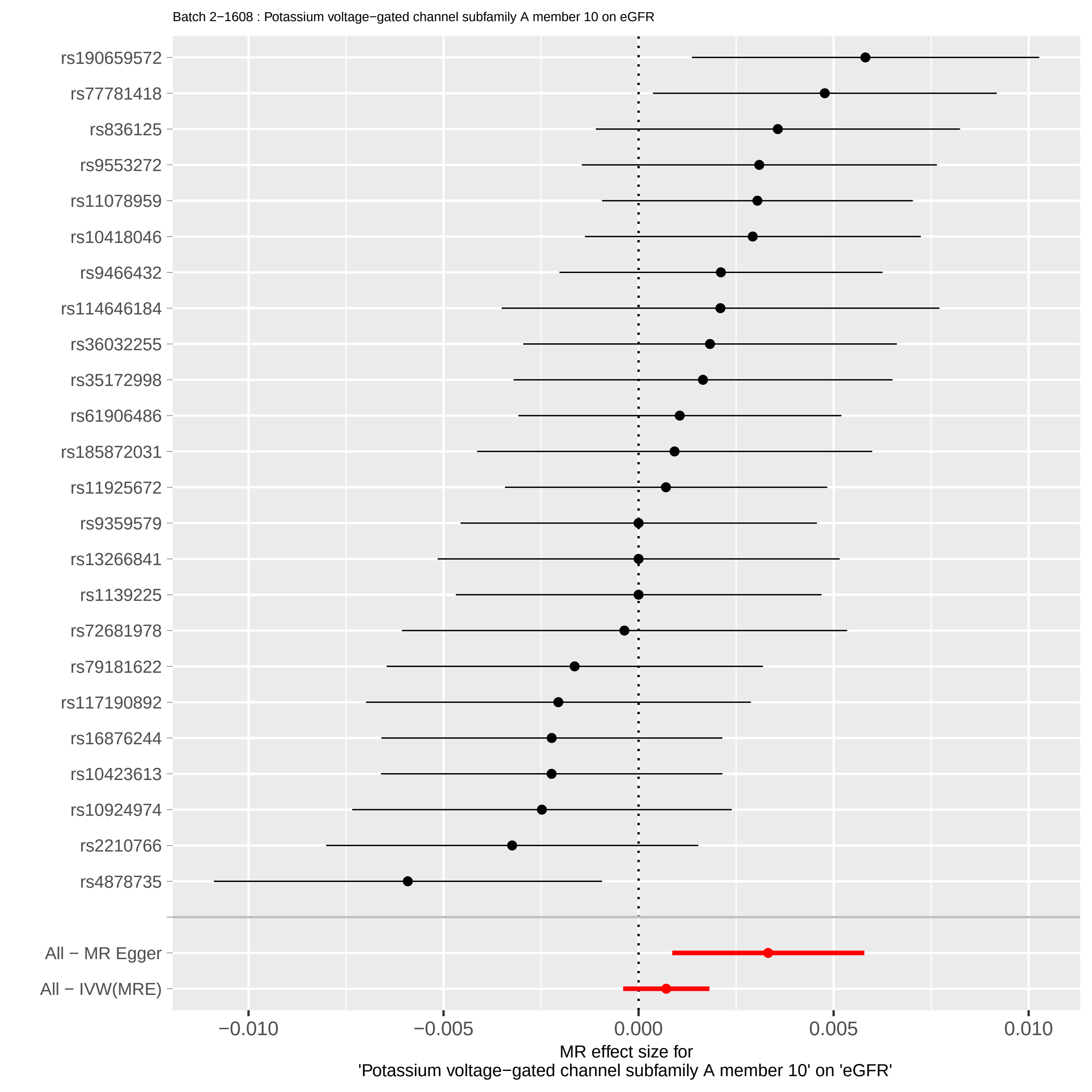

Batch 2−1608 : Potassium voltage−gated channel subfamily A member 10 on eGFR
rs190659572
rs77781418
rs836125
rs9553272
rs11078959
rs10418046
rs9466432
rs114646184
rs36032255
rs35172998
rs61906486
rs185872031
rs11925672
rs9359579
rs13266841
rs1139225
rs72681978
rs79181622
rs117190892
rs16876244
rs10423613
rs10924974
rs2210766
rs4878735
All − MR Egger
All − IVW(MRE)
−0.010
−0.005
0.000
0.005
0.010
MR effect size for
'Potassium voltage−gated channel subfamily A member 10' on 'eGFR'

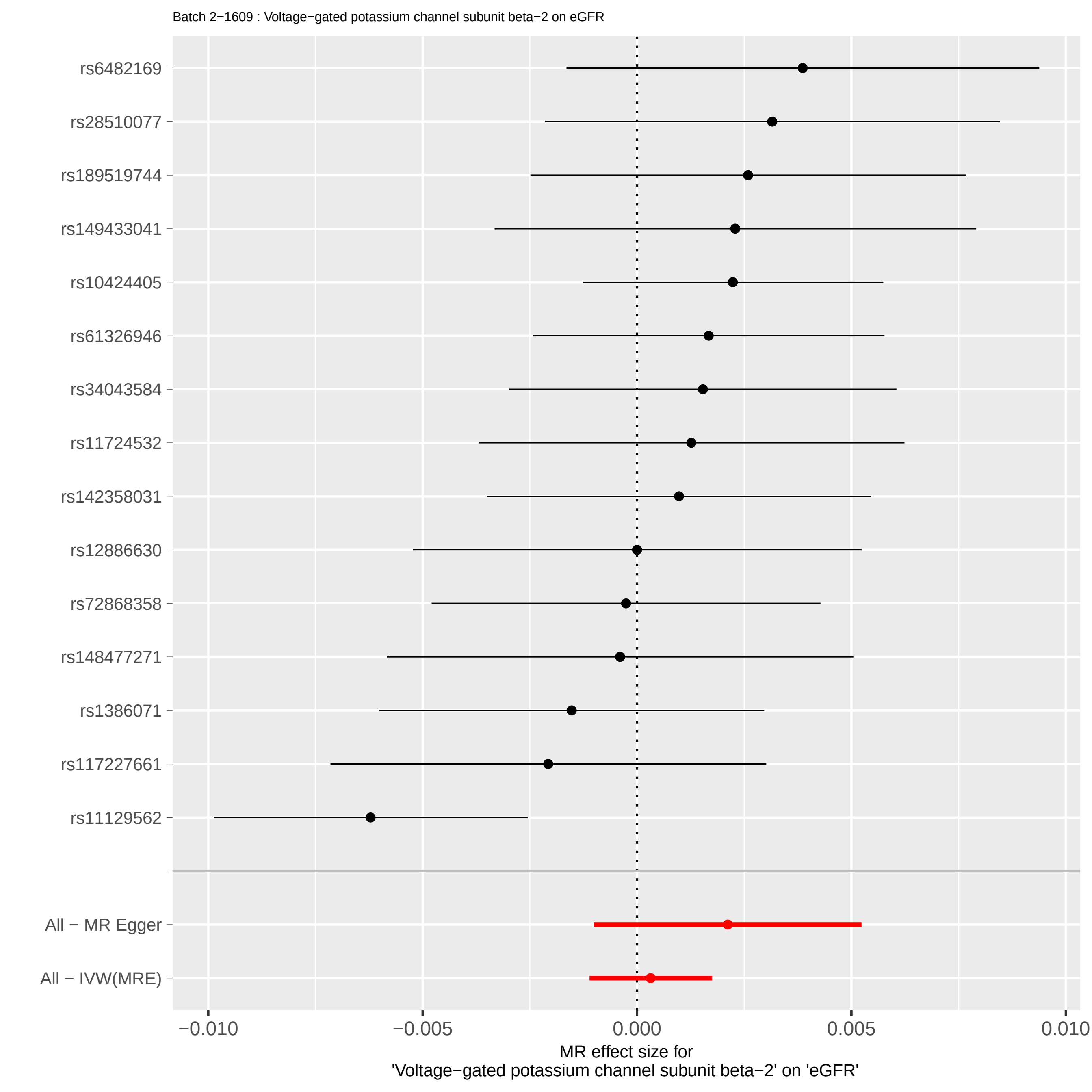
Batch 2−1609 : Voltage−gated potassium channel subunit beta−2 on eGFR
rs6482169
rs28510077
rs189519744
rs149433041
rs10424405
rs61326946
rs34043584
rs11724532
rs142358031
rs12886630
rs72868358
rs148477271
rs1386071
rs117227661
rs11129562
All − MR Egger
All − IVW(MRE)
−0.010
−0.005
0.000
0.005
0.010
MR effect size for
'Voltage−gated potassium channel subunit beta−2' on 'eGFR'

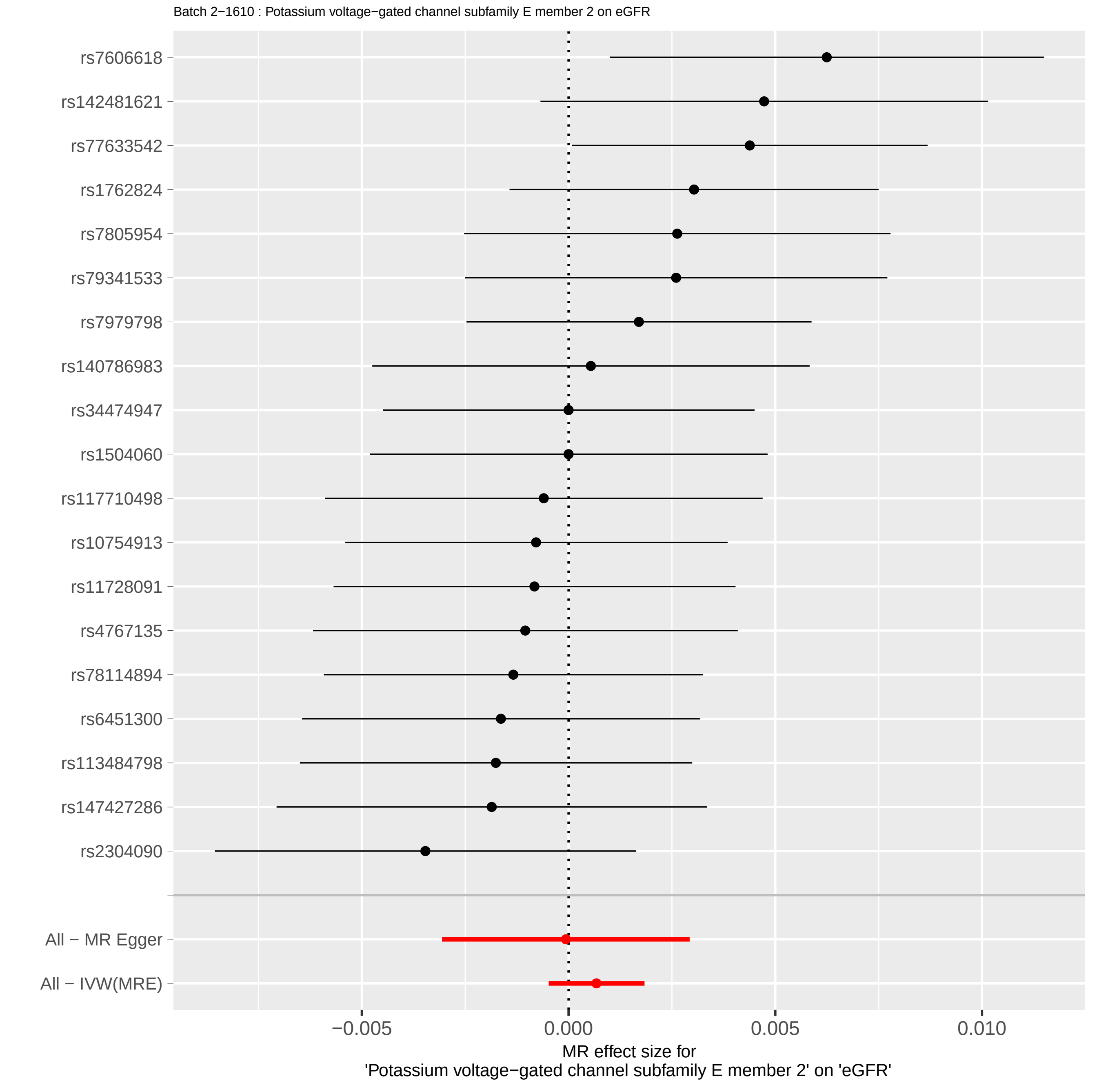

Batch 2−1610 : Potassium voltage−gated channel subfamily E member 2 on eGFR
rs7606618
rs142481621
rs77633542
rs1762824
rs7805954
rs79341533
rs7979798
rs140786983
rs34474947
rs1504060
rs117710498
rs10754913
rs11728091
rs4767135
rs78114894
rs6451300
rs113484798
rs147427286
rs2304090
All − MR Egger
All − IVW(MRE)
−0.005
0.000
0.005
0.010
MR effect size for
'Potassium voltage−gated channel subfamily E member 2' on 'eGFR'

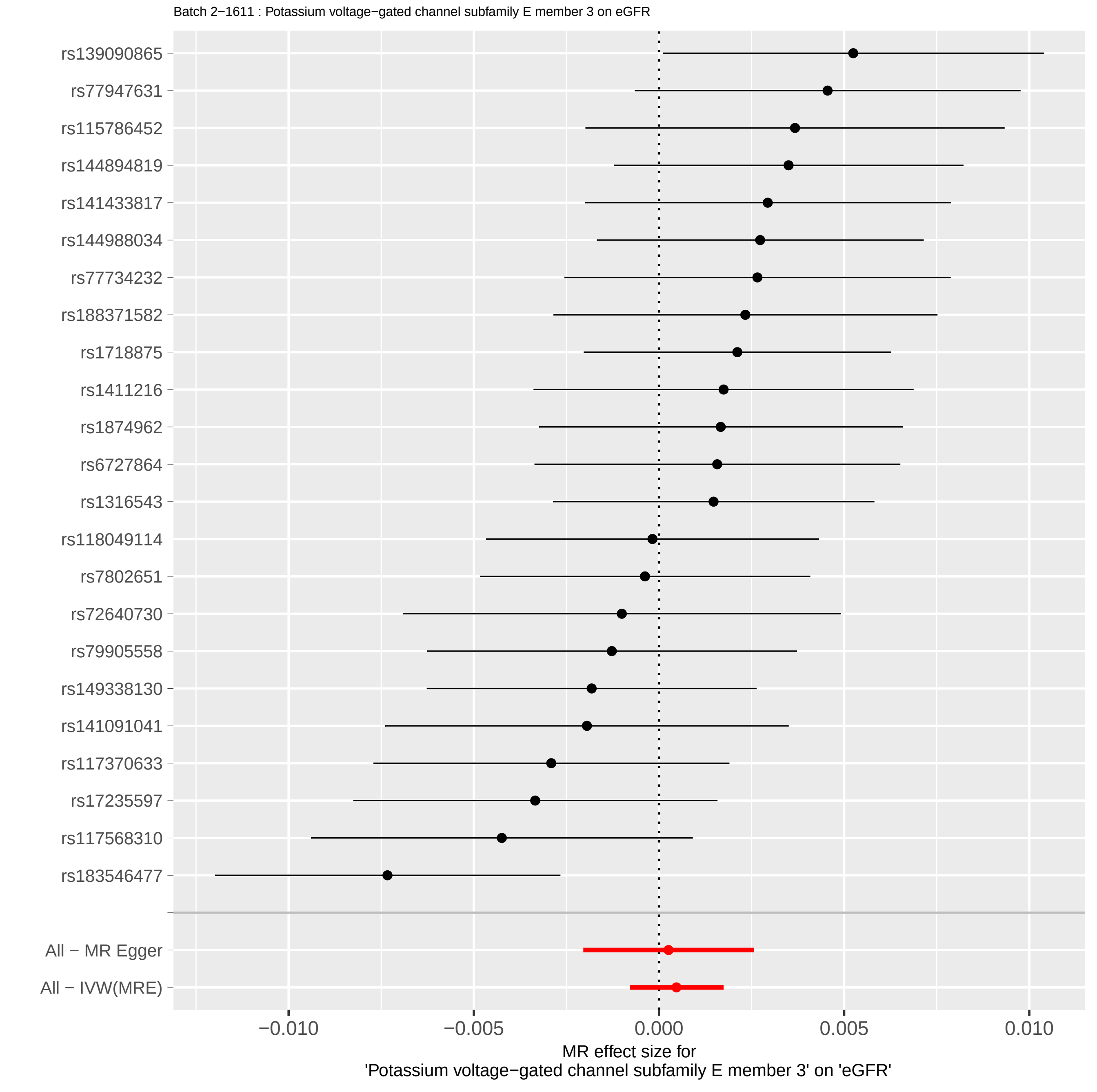

Batch 2−1611 : Potassium voltage−gated channel subfamily E member 3 on eGFR
rs139090865
rs77947631
rs115786452
rs144894819
rs141433817
rs144988034
rs77734232
rs188371582
rs1718875
rs1411216
rs1874962
rs6727864
rs1316543
rs118049114
rs7802651
rs72640730
rs79905558
rs149338130
rs141091041
rs117370633
rs17235597
rs117568310
rs183546477
All − MR Egger
All − IVW(MRE)
−0.010
−0.005
0.000
0.005
0.010
MR effect size for
'Potassium voltage−gated channel subfamily E member 3' on 'eGFR'

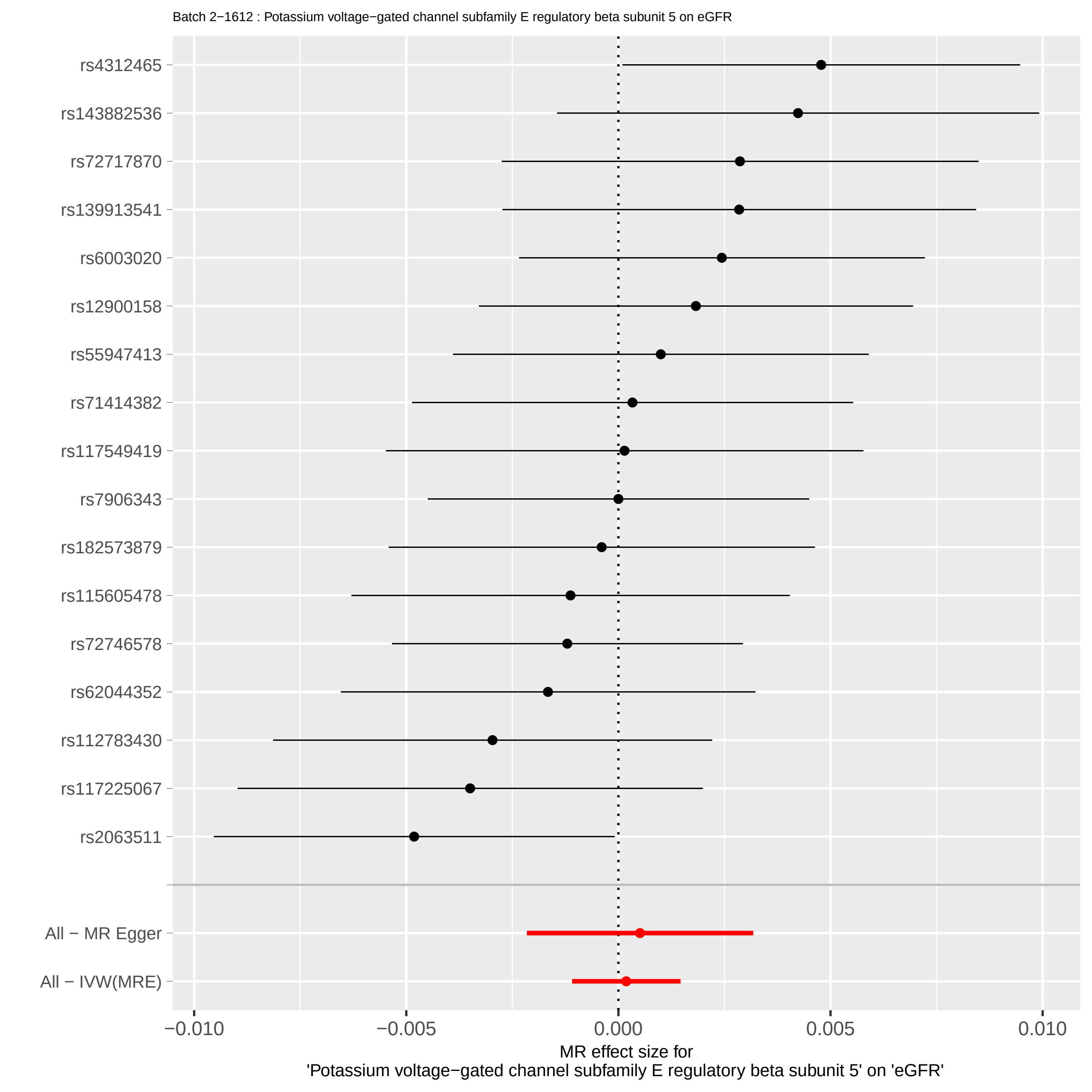

Batch 2−1612 : Potassium voltage−gated channel subfamily E regulatory beta subunit 5 on eGFR
rs4312465
rs143882536
rs72717870
rs139913541
rs6003020
rs12900158
rs55947413
rs71414382
rs117549419
rs7906343
rs182573879
rs115605478
rs72746578
rs62044352
rs112783430
rs117225067
rs2063511
All − MR Egger
All − IVW(MRE)
−0.010
−0.005
0.000
0.005
0.010
MR effect size for
'Potassium voltage−gated channel subfamily E regulatory beta subunit 5' on 'eGFR'

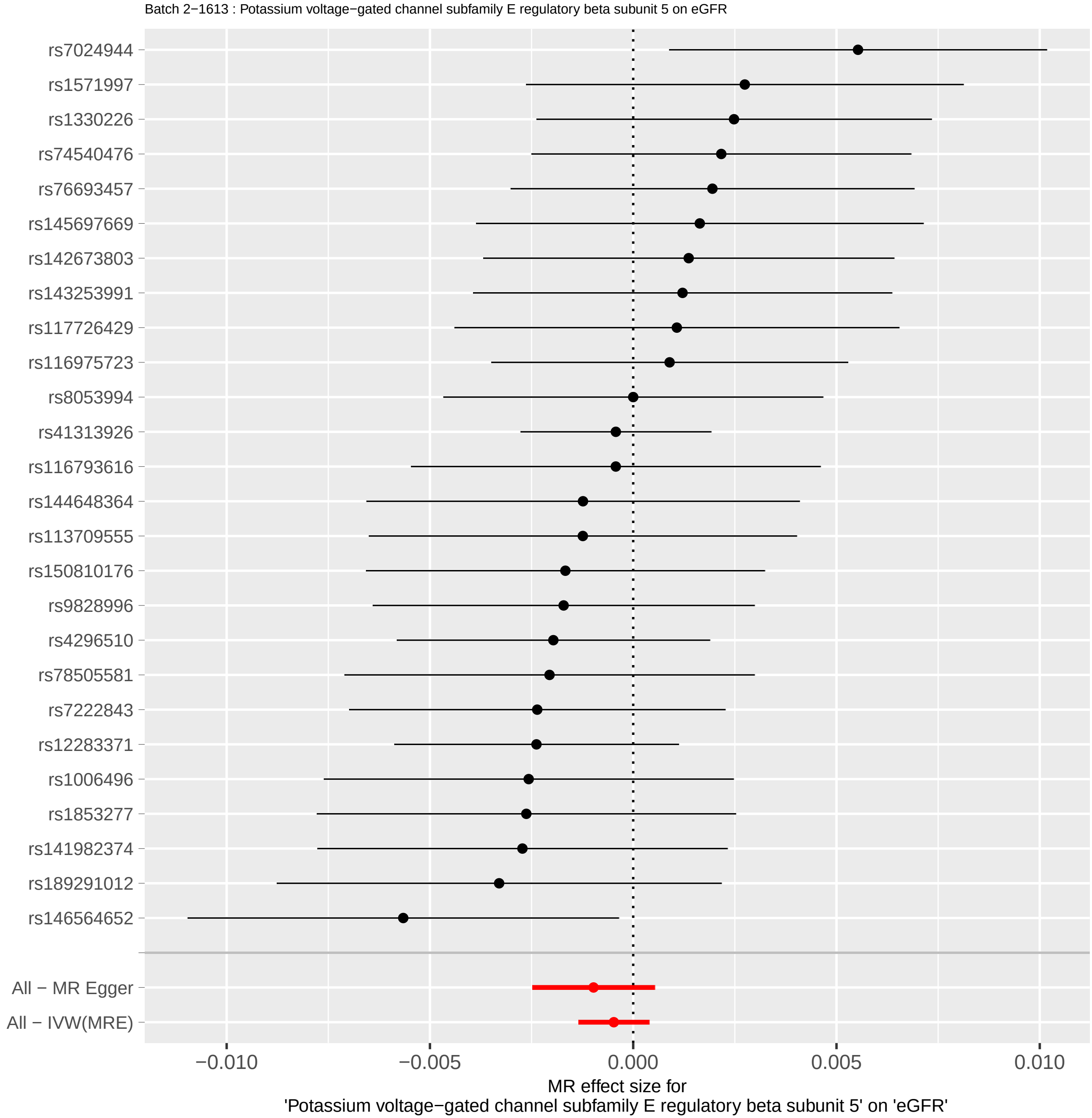
Batch 2−1613 : Potassium voltage−gated channel subfamily E regulatory beta subunit 5 on eGFR
rs7024944
rs1571997
rs1330226
rs74540476
rs76693457
rs145697669
rs142673803
rs143253991
rs117726429
rs116975723
rs8053994
rs41313926
rs116793616
rs144648364
rs113709555
rs150810176
rs9828996
rs4296510
rs78505581
rs7222843
rs12283371
rs1006496
rs1853277
rs141982374
rs189291012
rs146564652
All − MR Egger
All − IVW(MRE)
−0.010
−0.005
0.000
0.005
0.010
MR effect size for
'Potassium voltage−gated channel subfamily E regulatory beta subunit 5' on 'eGFR'

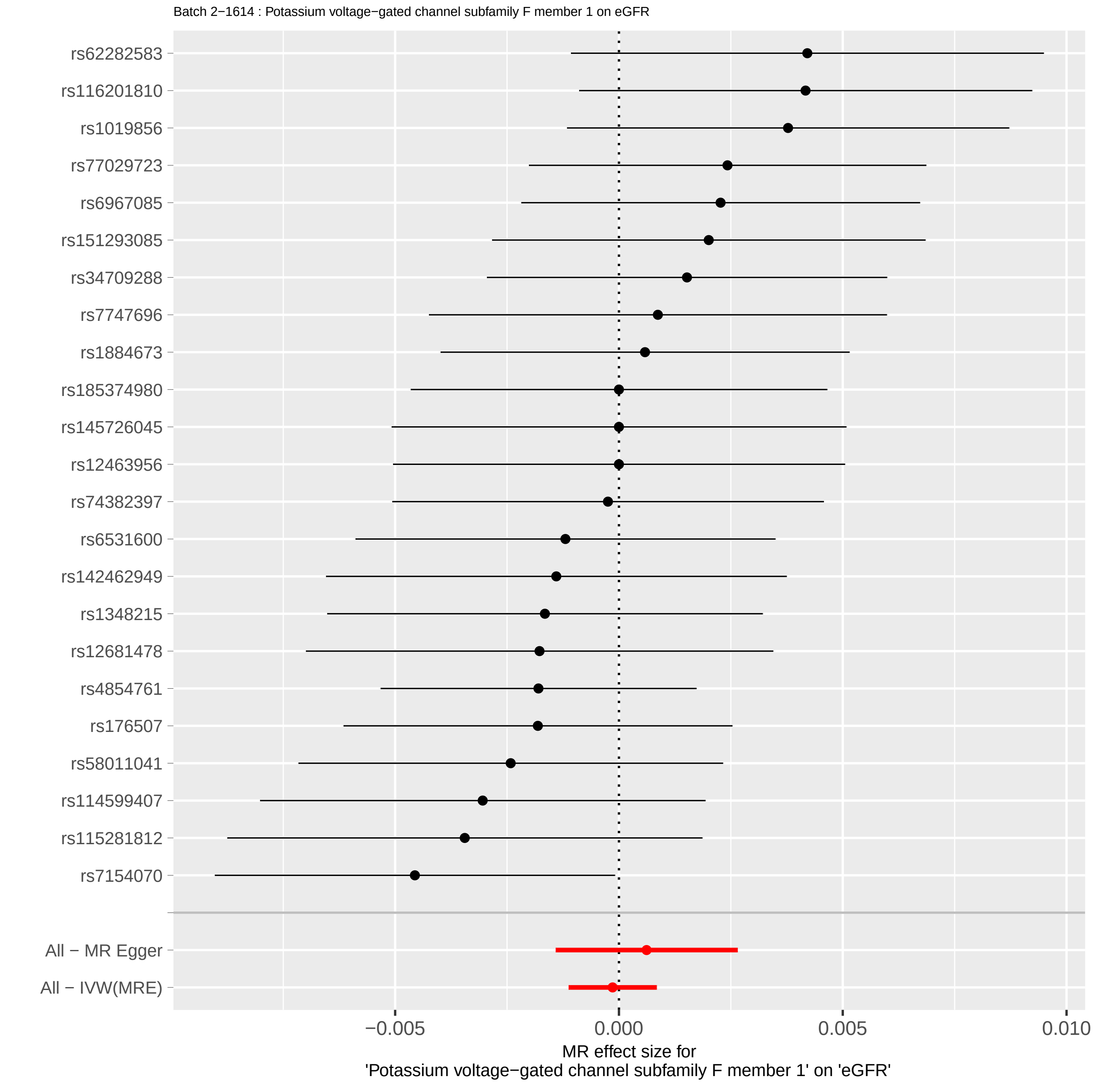

Batch 2−1614 : Potassium voltage−gated channel subfamily F member 1 on eGFR
rs62282583
rs116201810
rs1019856
rs77029723
rs6967085
rs151293085
rs34709288
rs7747696
rs1884673
rs185374980
rs145726045
rs12463956
rs74382397
rs6531600
rs142462949
rs1348215
rs12681478
rs4854761
rs176507
rs58011041
rs114599407
rs115281812
rs7154070
All − MR Egger
All − IVW(MRE)
−0.005
0.000
0.005
0.010
MR effect size for
'Potassium voltage−gated channel subfamily F member 1' on 'eGFR'

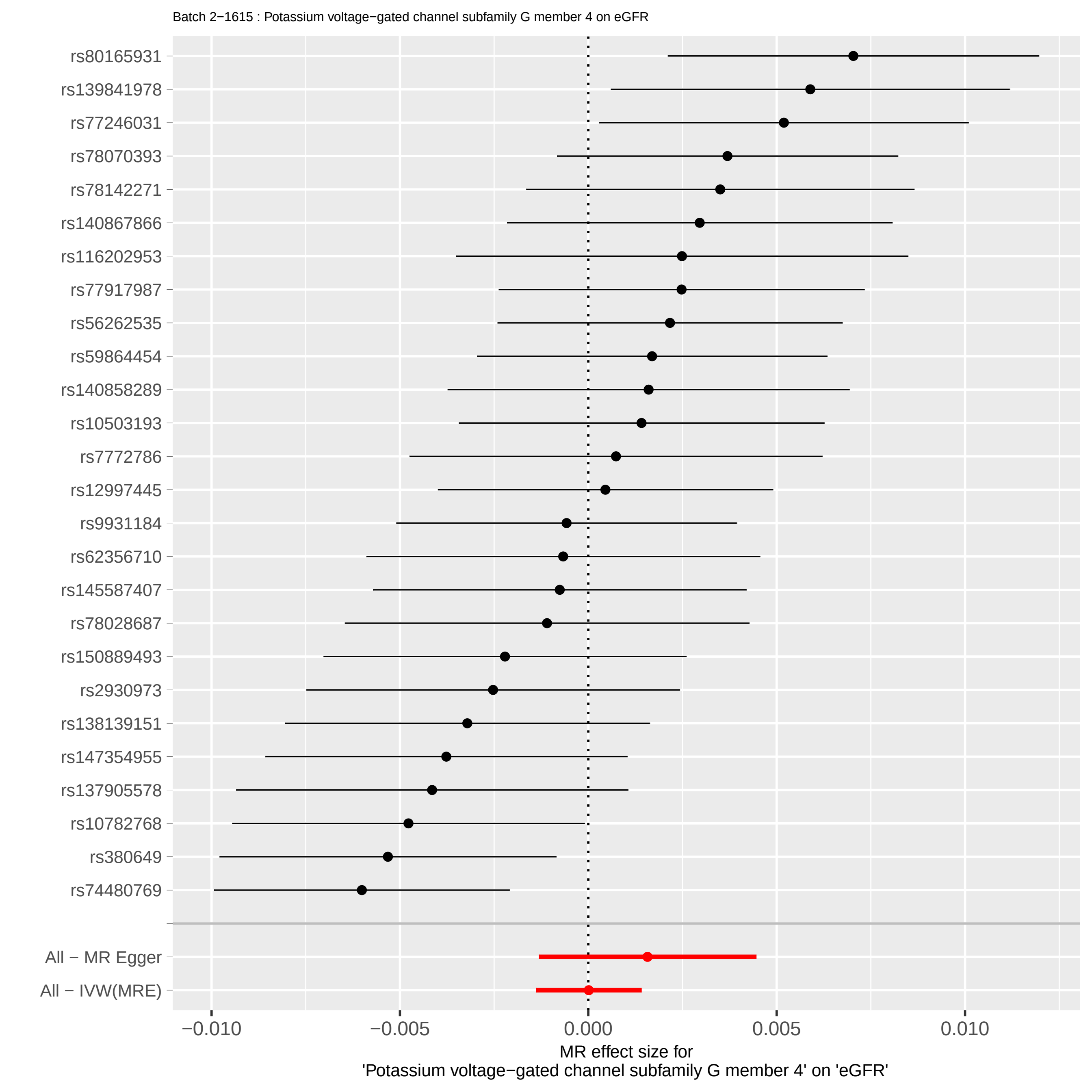
Batch 2−1615 : Potassium voltage−gated channel subfamily G member 4 on eGFR
rs80165931
rs139841978
rs77246031
rs78070393
rs78142271
rs140867866
rs116202953
rs77917987
rs56262535
rs59864454
rs140858289
rs10503193
rs7772786
rs12997445
rs9931184
rs62356710
rs145587407
rs78028687
rs150889493
rs2930973
rs138139151
rs147354955
rs137905578
rs10782768
rs380649
rs74480769
All − MR Egger
All − IVW(MRE)
−0.010
−0.005
0.000
0.005
0.010
MR effect size for
'Potassium voltage−gated channel subfamily G member 4' on 'eGFR'

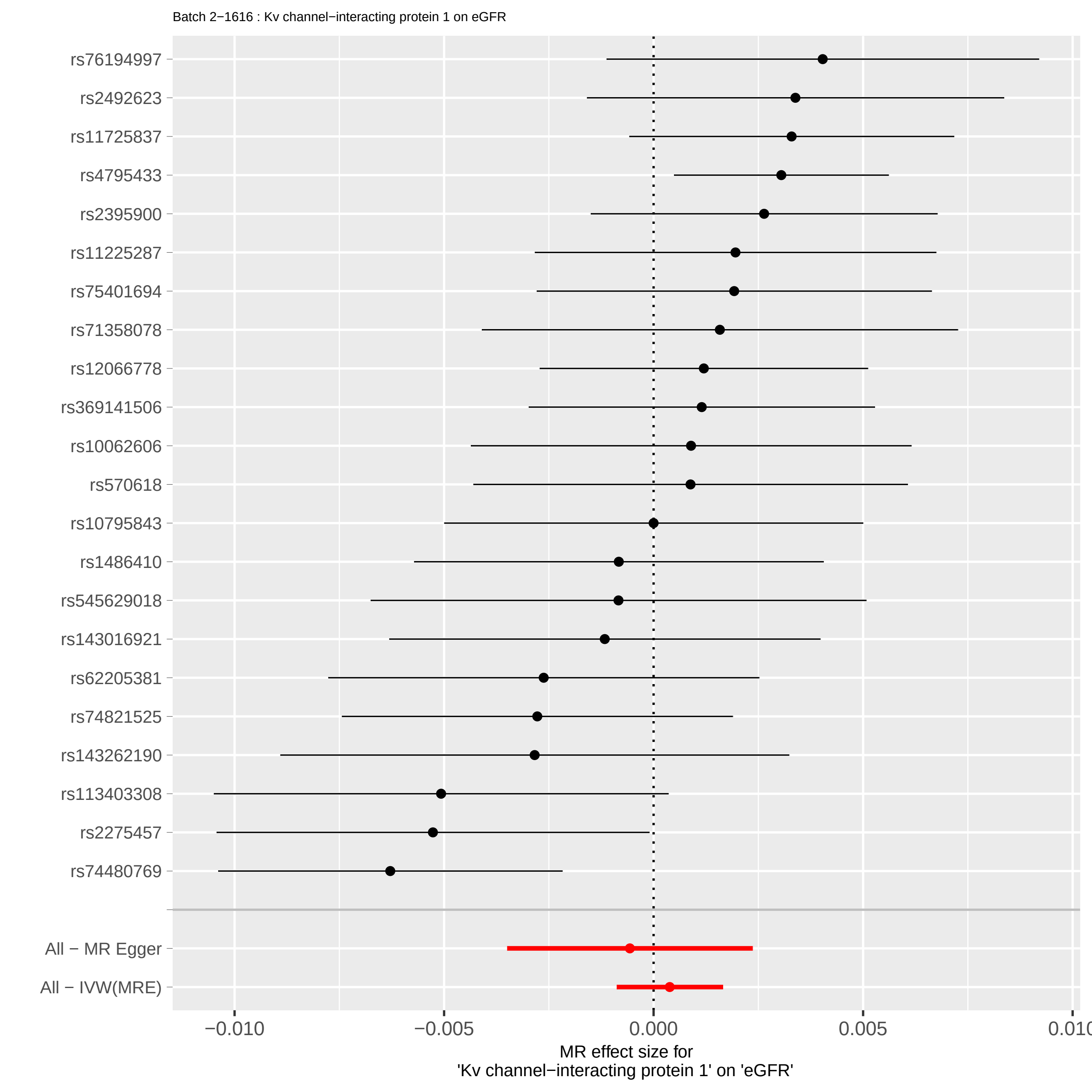
Batch 2−1616 : Kv channel−interacting protein 1 on eGFR
rs76194997
rs2492623
rs11725837
rs4795433
rs2395900
rs11225287
rs75401694
rs71358078
rs12066778
rs369141506
rs10062606
rs570618
rs10795843
rs1486410
rs545629018
rs143016921
rs62205381
rs74821525
rs143262190
rs113403308
rs2275457
rs74480769
All − MR Egger
All − IVW(MRE)
−0.010
−0.005
0.000
0.005
0.010
MR effect size for
'Kv channel−interacting protein 1' on 'eGFR'

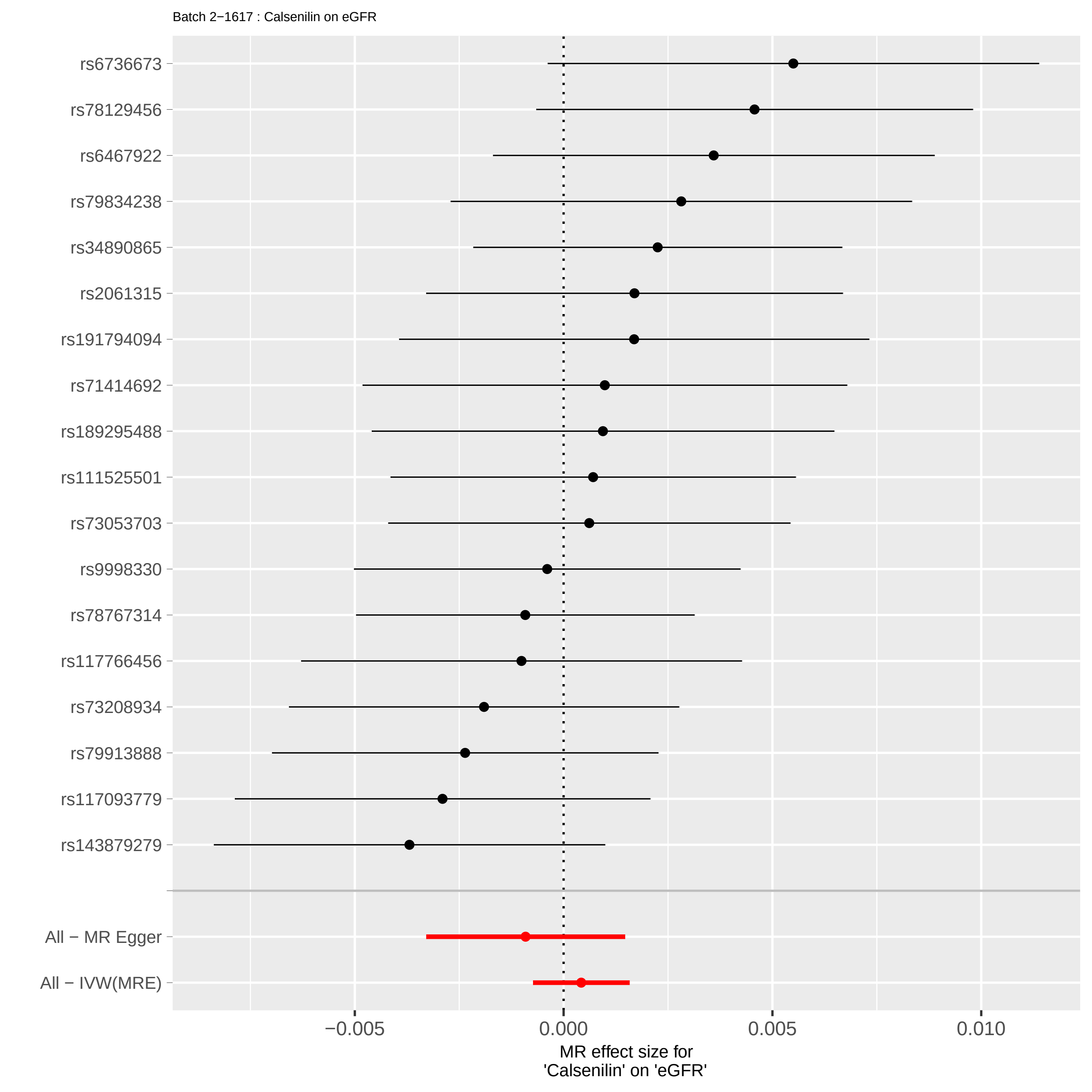
Batch 2−1617 : Calsenilin on eGFR
rs6736673
rs78129456
rs6467922
rs79834238
rs34890865
rs2061315
rs191794094
rs71414692
rs189295488
rs111525501
rs73053703
rs9998330
rs78767314
rs117766456
rs73208934
rs79913888
rs117093779
rs143879279
All − MR Egger
All − IVW(MRE)
−0.005
0.000
0.005
0.010
MR effect size for
'Calsenilin' on 'eGFR'

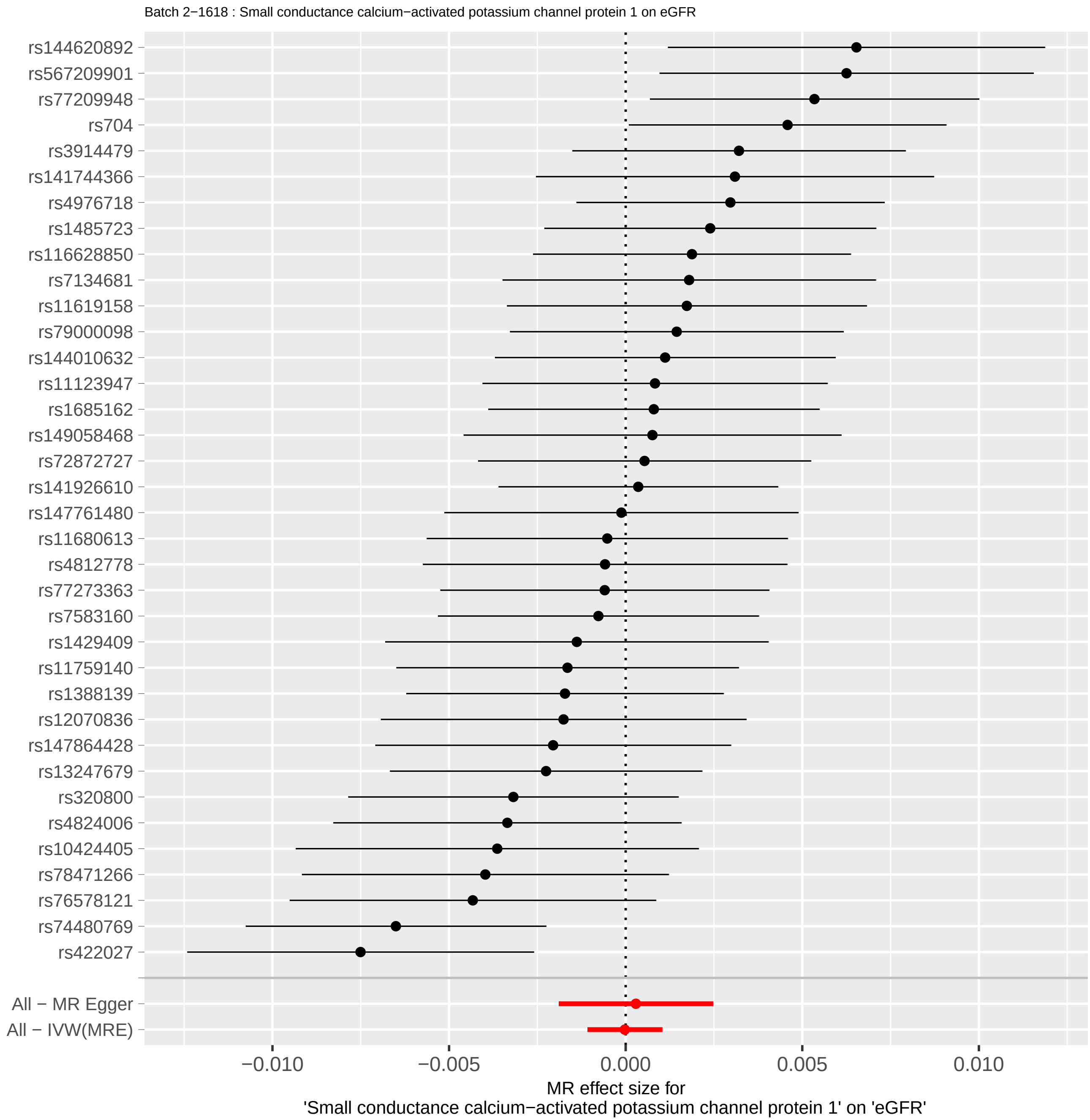

Batch 2−1618 : Small conductance calcium−activated potassium channel protein 1 on eGFR
rs144620892
rs567209901
rs77209948
rs704
rs3914479
rs141744366
rs4976718
rs1485723
rs116628850
rs7134681
rs11619158
rs79000098
rs144010632
rs11123947
rs1685162
rs149058468
rs72872727
rs141926610
rs147761480
rs11680613
rs4812778
rs77273363
rs7583160
rs1429409
rs11759140
rs1388139
rs12070836
rs147864428
rs13247679
rs320800
rs4824006
rs10424405
rs78471266
rs76578121
rs74480769
rs422027
All − MR Egger
All − IVW(MRE)
−0.010
−0.005
0.000
0.005
0.010
MR effect size for
'Small conductance calcium−activated potassium channel protein 1' on 'eGFR'

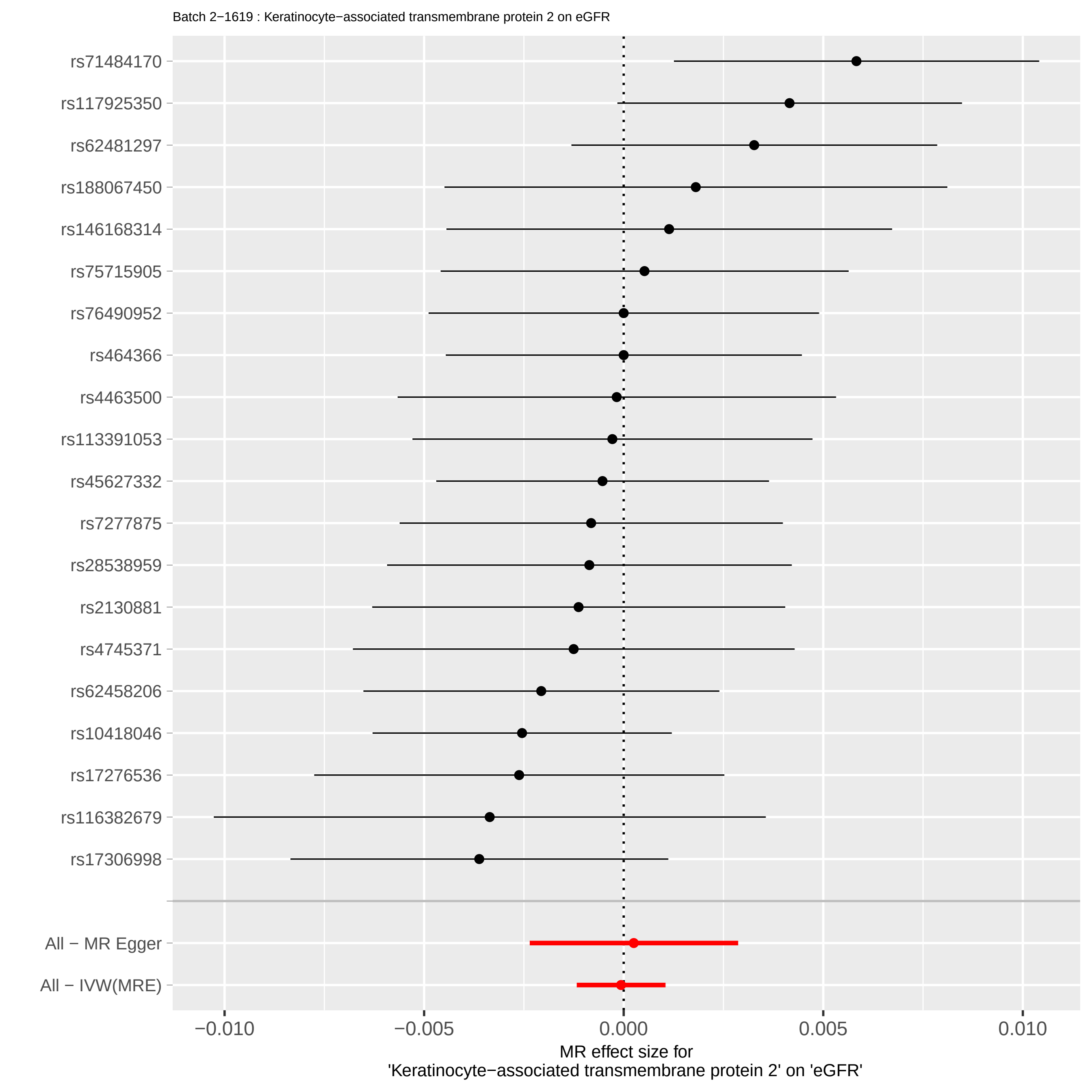
Batch 2−1619 : Keratinocyte−associated transmembrane protein 2 on eGFR
rs71484170
rs117925350
rs62481297
rs188067450
rs146168314
rs75715905
rs76490952
rs464366
rs4463500
rs113391053
rs45627332
rs7277875
rs28538959
rs2130881
rs4745371
rs62458206
rs10418046
rs17276536
rs116382679
rs17306998
All − MR Egger
All − IVW(MRE)
−0.010
−0.005
0.000
0.005
0.010
MR effect size for
'Keratinocyte−associated transmembrane protein 2' on 'eGFR'

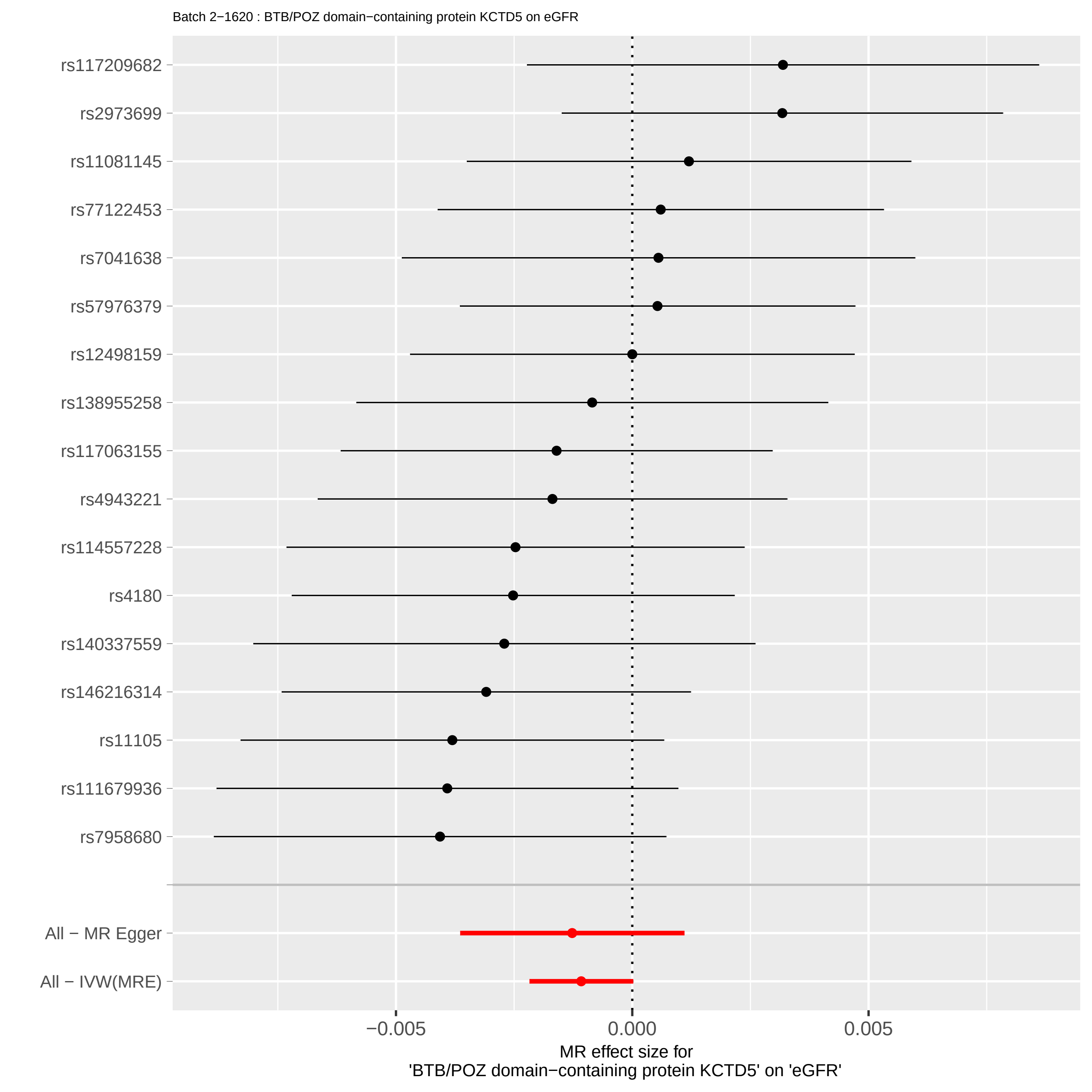

Batch 2−1620 : BTB/POZ domain−containing protein KCTD5 on eGFR
rs117209682
rs2973699
rs11081145
rs77122453
rs7041638
rs57976379
rs12498159
rs138955258
rs117063155
rs4943221
rs114557228
rs4180
rs140337559
rs146216314
rs11105
rs111679936
rs7958680
All − MR Egger
All − IVW(MRE)
−0.005
0.000
0.005
MR effect size for
'BTB/POZ domain−containing protein KCTD5' on 'eGFR'

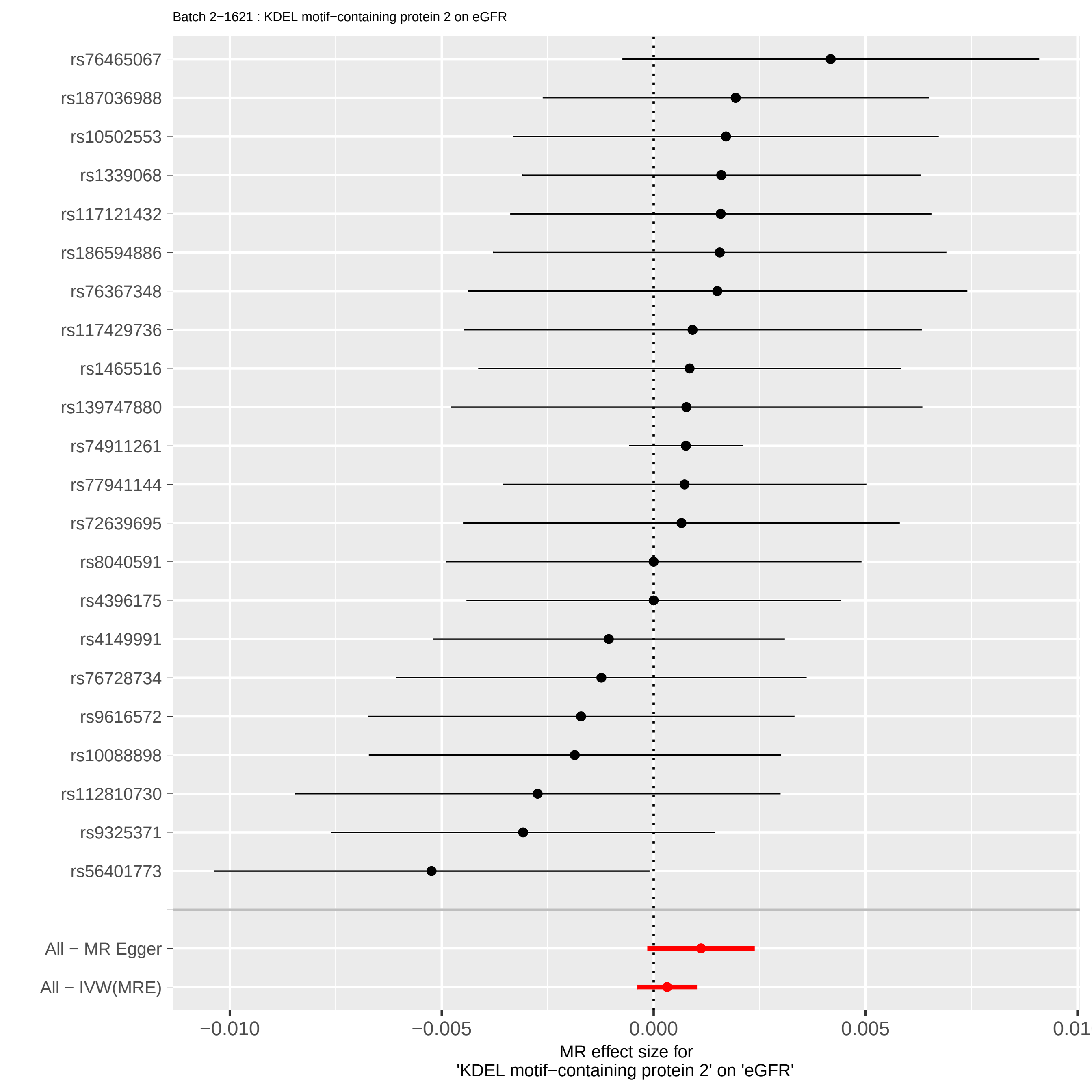

Batch 2−1621 : KDEL motif−containing protein 2 on eGFR
rs76465067
rs187036988
rs10502553
rs1339068
rs117121432
rs186594886
rs76367348
rs117429736
rs1465516
rs139747880
rs74911261
rs77941144
rs72639695
rs8040591
rs4396175
rs4149991
rs76728734
rs9616572
rs10088898
rs112810730
rs9325371
rs56401773
All − MR Egger
All − IVW(MRE)
−0.010
−0.005
0.000
0.005
MR effect size for
'KDEL motif−containing protein 2' on 'eGFR'

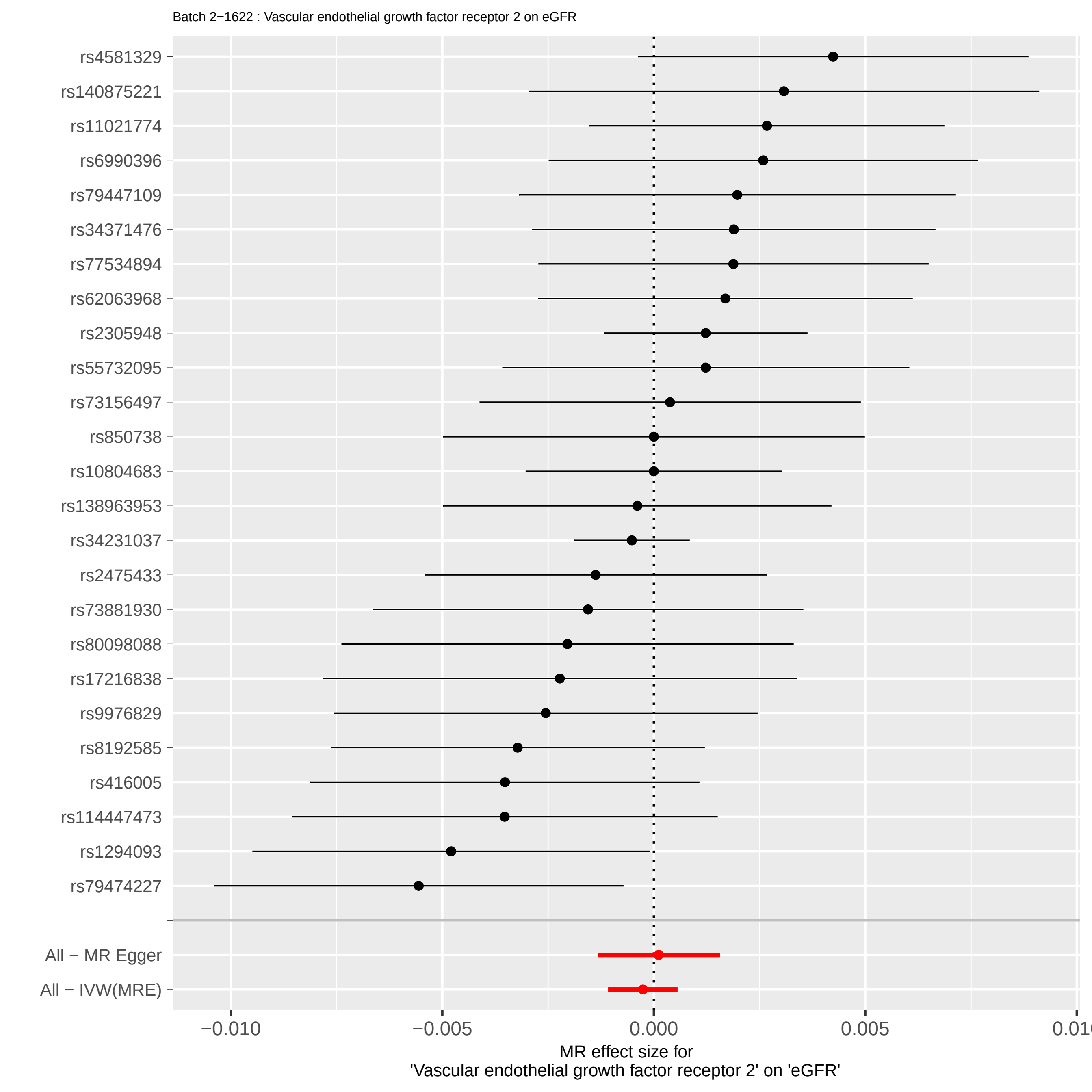

Batch 2−1622 : Vascular endothelial growth factor receptor 2 on eGFR
rs4581329
rs140875221
rs11021774
rs6990396
rs79447109
rs34371476
rs77534894
rs62063968
rs2305948
rs55732095
rs73156497
rs850738
rs10804683
rs138963953
rs34231037
rs2475433
rs73881930
rs80098088
rs17216838
rs9976829
rs8192585
rs416005
rs114447473
rs1294093
rs79474227
All − MR Egger
All − IVW(MRE)
−0.010
−0.005
0.000
0.005
MR effect size for
'Vascular endothelial growth factor receptor 2' on 'eGFR'

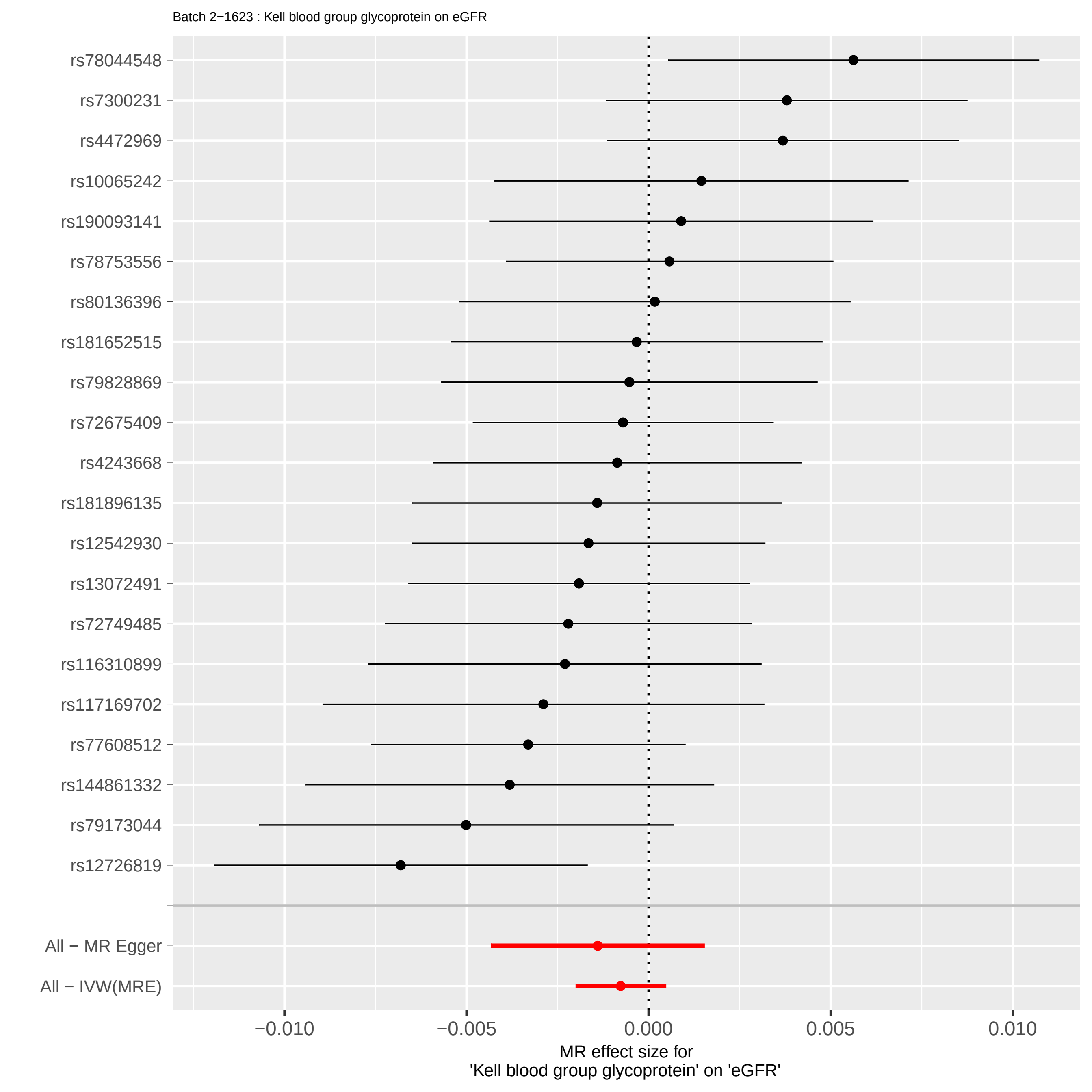

Batch 2−1623 : Kell blood group glycoprotein on eGFR
rs78044548
rs7300231
rs4472969
rs10065242
rs190093141
rs78753556
rs80136396
rs181652515
rs79828869
rs72675409
rs4243668
rs181896135
rs12542930
rs13072491
rs72749485
rs116310899
rs117169702
rs77608512
rs144861332
rs79173044
rs12726819
All − MR Egger
All − IVW(MRE)
−0.010
−0.005
0.000
0.005
0.010
MR effect size for
'Kell blood group glycoprotein' on 'eGFR'

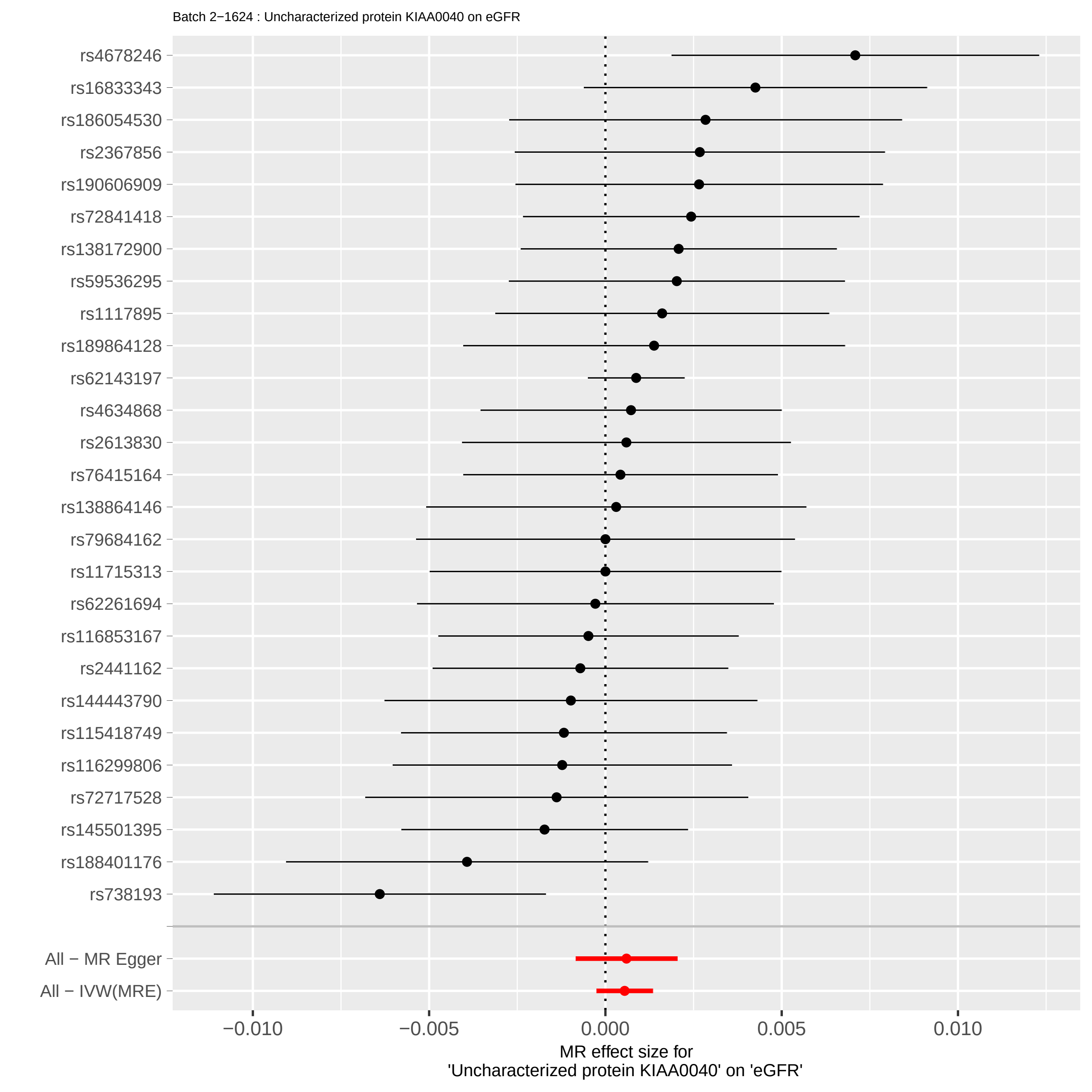
Batch 2−1624 : Uncharacterized protein KIAA0040 on eGFR
rs4678246
rs16833343
rs186054530
rs2367856
rs190606909
rs72841418
rs138172900
rs59536295
rs1117895
rs189864128
rs62143197
rs4634868
rs2613830
rs76415164
rs138864146
rs79684162
rs11715313
rs62261694
rs116853167
rs2441162
rs144443790
rs115418749
rs116299806
rs72717528
rs145501395
rs188401176
rs738193
All − MR Egger
All − IVW(MRE)
−0.010
−0.005
0.000
0.005
0.010
MR effect size for
'Uncharacterized protein KIAA0040' on 'eGFR'

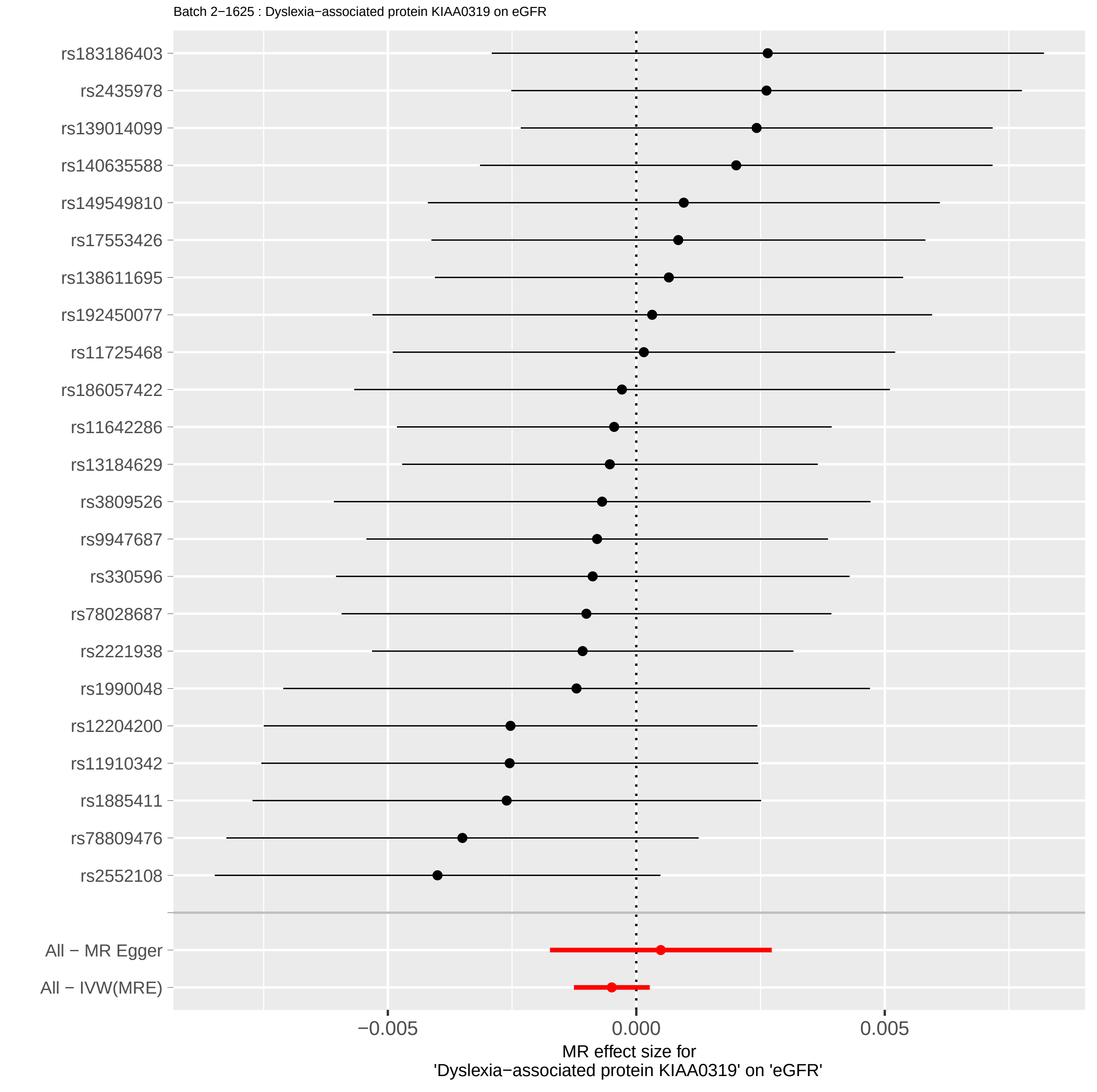

Batch 2−1625 : Dyslexia−associated protein KIAA0319 on eGFR
rs183186403
rs2435978
rs139014099
rs140635588
rs149549810
rs17553426
rs138611695
rs192450077
rs11725468
rs186057422
rs11642286
rs13184629
rs3809526
rs9947687
rs330596
rs78028687
rs2221938
rs1990048
rs12204200
rs11910342
rs1885411
rs78809476
rs2552108
All − MR Egger
All − IVW(MRE)
−0.005
0.000
0.005
MR effect size for
'Dyslexia−associated protein KIAA0319' on 'eGFR'

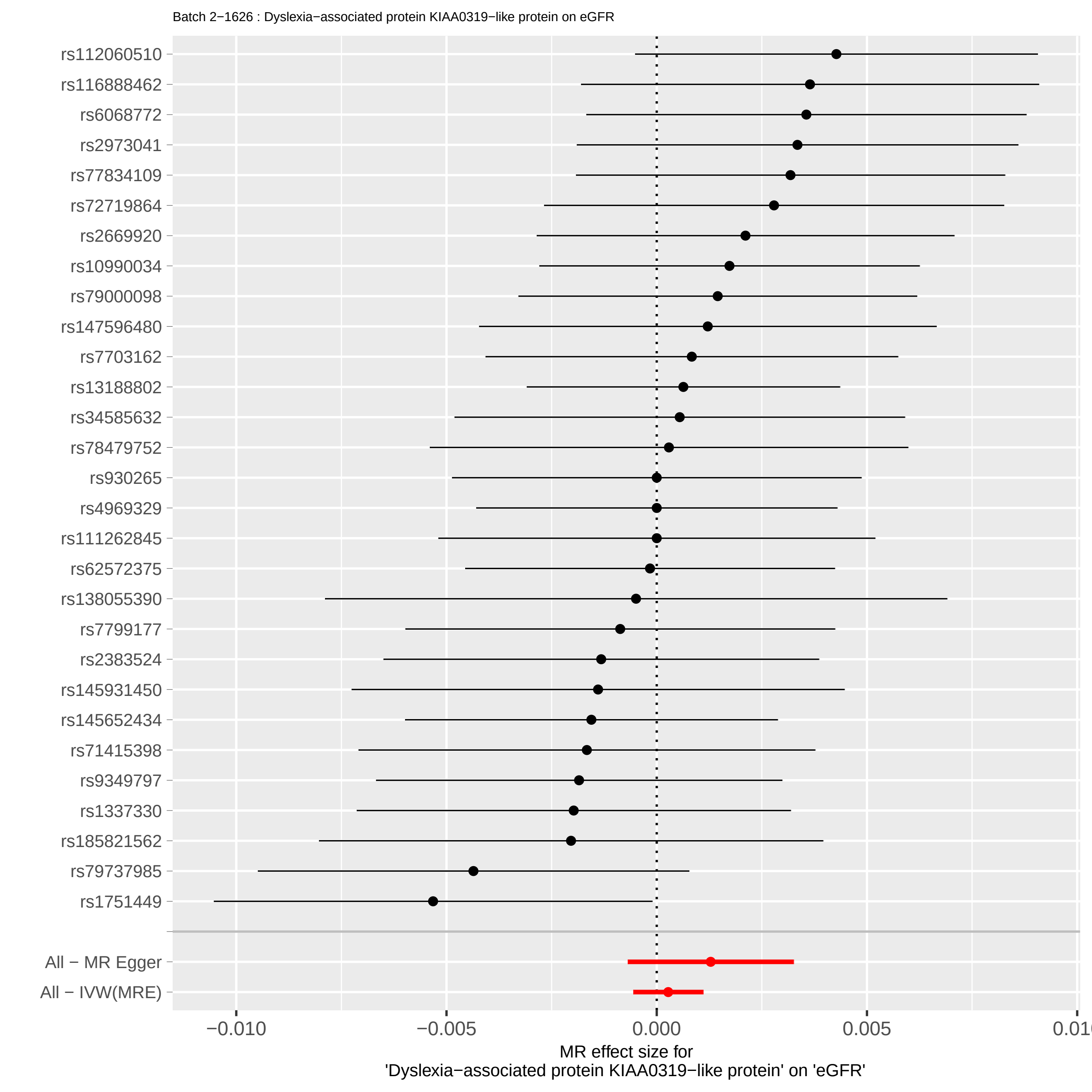

Batch 2−1626 : Dyslexia−associated protein KIAA0319−like protein on eGFR
rs112060510
rs116888462
rs6068772
rs2973041
rs77834109
rs72719864
rs2669920
rs10990034
rs79000098
rs147596480
rs7703162
rs13188802
rs34585632
rs78479752
rs930265
rs4969329
rs111262845
rs62572375
rs138055390
rs7799177
rs2383524
rs145931450
rs145652434
rs71415398
rs9349797
rs1337330
rs185821562
rs79737985
rs1751449
All − MR Egger
All − IVW(MRE)
−0.010
−0.005
0.000
0.005
MR effect size for
'Dyslexia−associated protein KIAA0319−like protein' on 'eGFR'

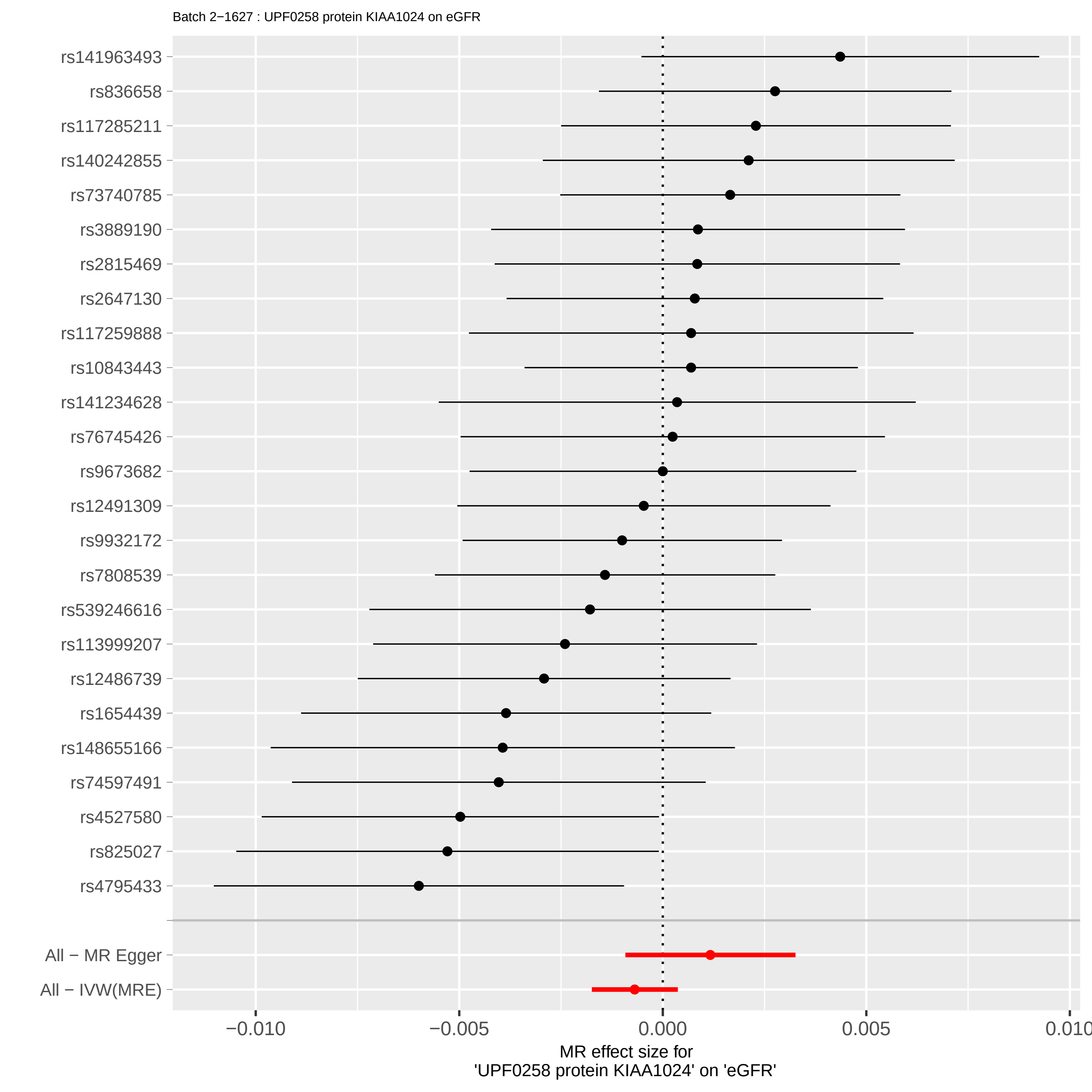

Batch 2−1627 : UPF0258 protein KIAA1024 on eGFR
rs141963493
rs836658
rs117285211
rs140242855
rs73740785
rs3889190
rs2815469
rs2647130
rs117259888
rs10843443
rs141234628
rs76745426
rs9673682
rs12491309
rs9932172
rs7808539
rs539246616
rs113999207
rs12486739
rs1654439
rs148655166
rs74597491
rs4527580
rs825027
rs4795433
All − MR Egger
All − IVW(MRE)
−0.010
−0.005
0.000
0.005
0.010
MR effect size for
'UPF0258 protein KIAA1024' on 'eGFR'

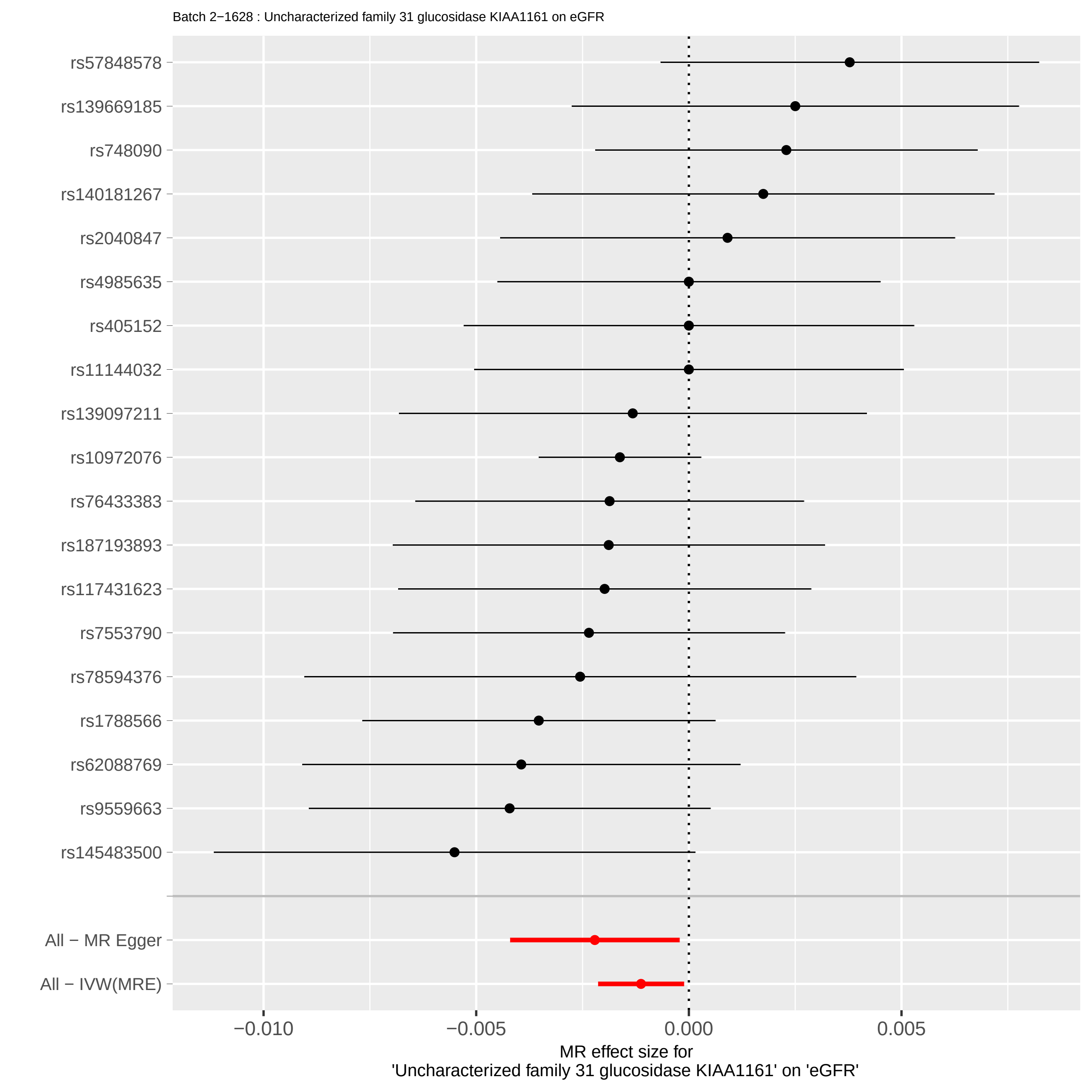
Batch 2−1628 : Uncharacterized family 31 glucosidase KIAA1161 on eGFR
rs57848578
rs139669185
rs748090
rs140181267
rs2040847
rs4985635
rs405152
rs11144032
rs139097211
rs10972076
rs76433383
rs187193893
rs117431623
rs7553790
rs78594376
rs1788566
rs62088769
rs9559663
rs145483500
All − MR Egger
All − IVW(MRE)
−0.010
−0.005
0.000
0.005
MR effect size for
'Uncharacterized family 31 glucosidase KIAA1161' on 'eGFR'

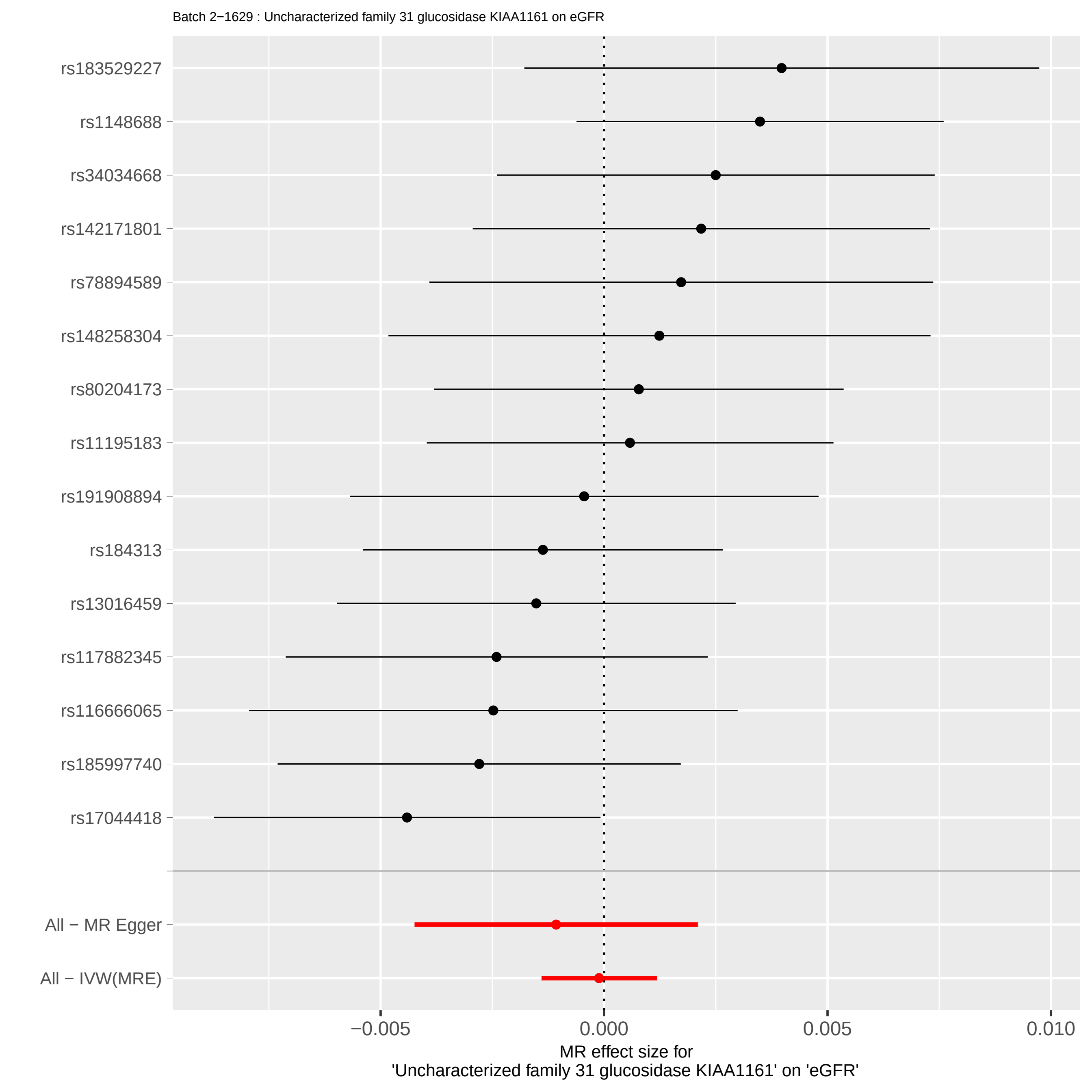

Batch 2−1629 : Uncharacterized family 31 glucosidase KIAA1161 on eGFR
rs183529227
rs1148688
rs34034668
rs142171801
rs78894589
rs148258304
rs80204173
rs11195183
rs191908894
rs184313
rs13016459
rs117882345
rs116666065
rs185997740
rs17044418
All − MR Egger
All − IVW(MRE)
−0.005
0.000
0.005
0.010
MR effect size for
'Uncharacterized family 31 glucosidase KIAA1161' on 'eGFR'

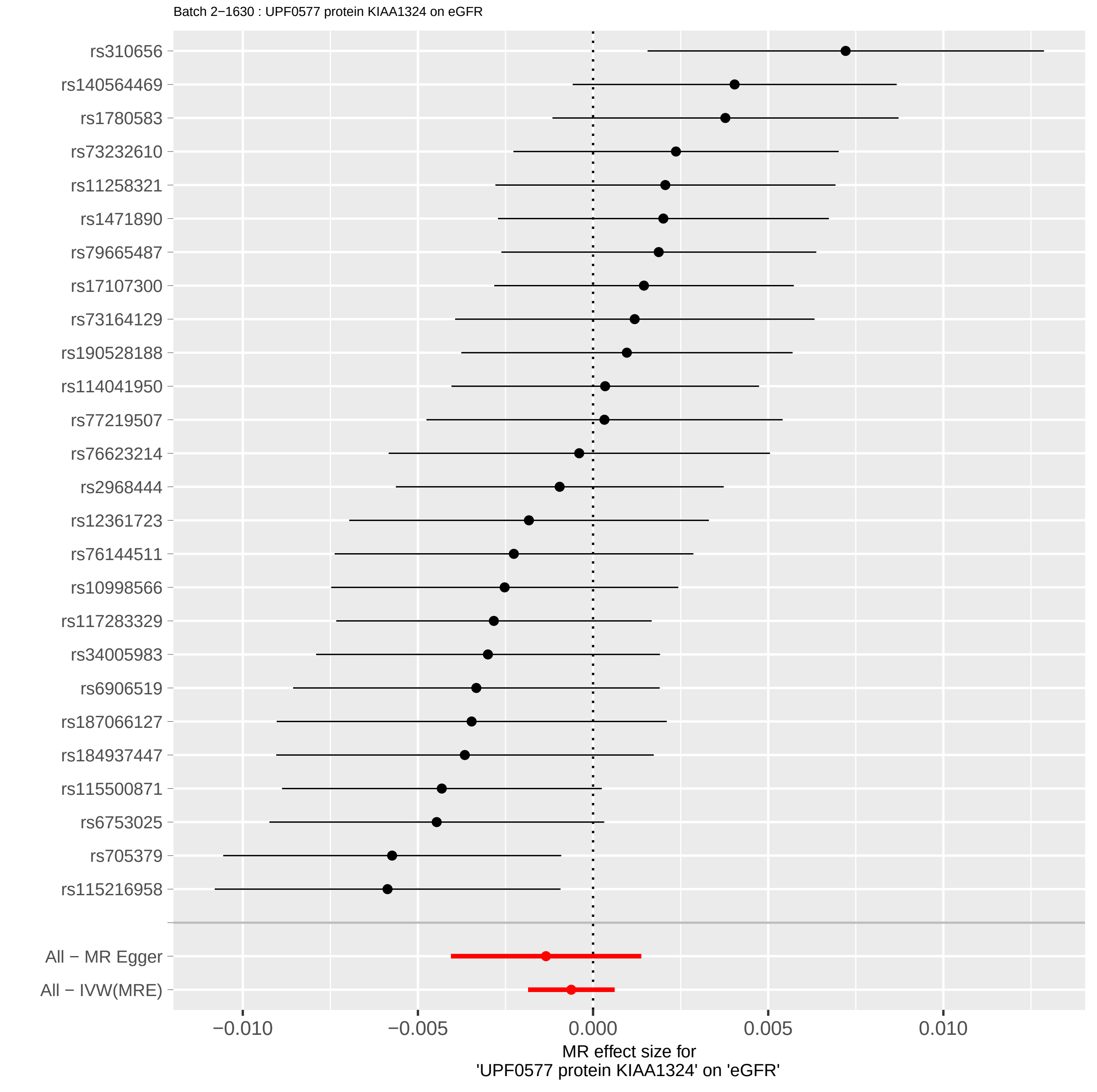

Batch 2−1630 : UPF0577 protein KIAA1324 on eGFR
rs310656
rs140564469
rs1780583
rs73232610
rs11258321
rs1471890
rs79665487
rs17107300
rs73164129
rs190528188
rs114041950
rs77219507
rs76623214
rs2968444
rs12361723
rs76144511
rs10998566
rs117283329
rs34005983
rs6906519
rs187066127
rs184937447
rs115500871
rs6753025
rs705379
rs115216958
All − MR Egger
All − IVW(MRE)
−0.010
−0.005
0.000
0.005
0.010
MR effect size for
'UPF0577 protein KIAA1324' on 'eGFR'

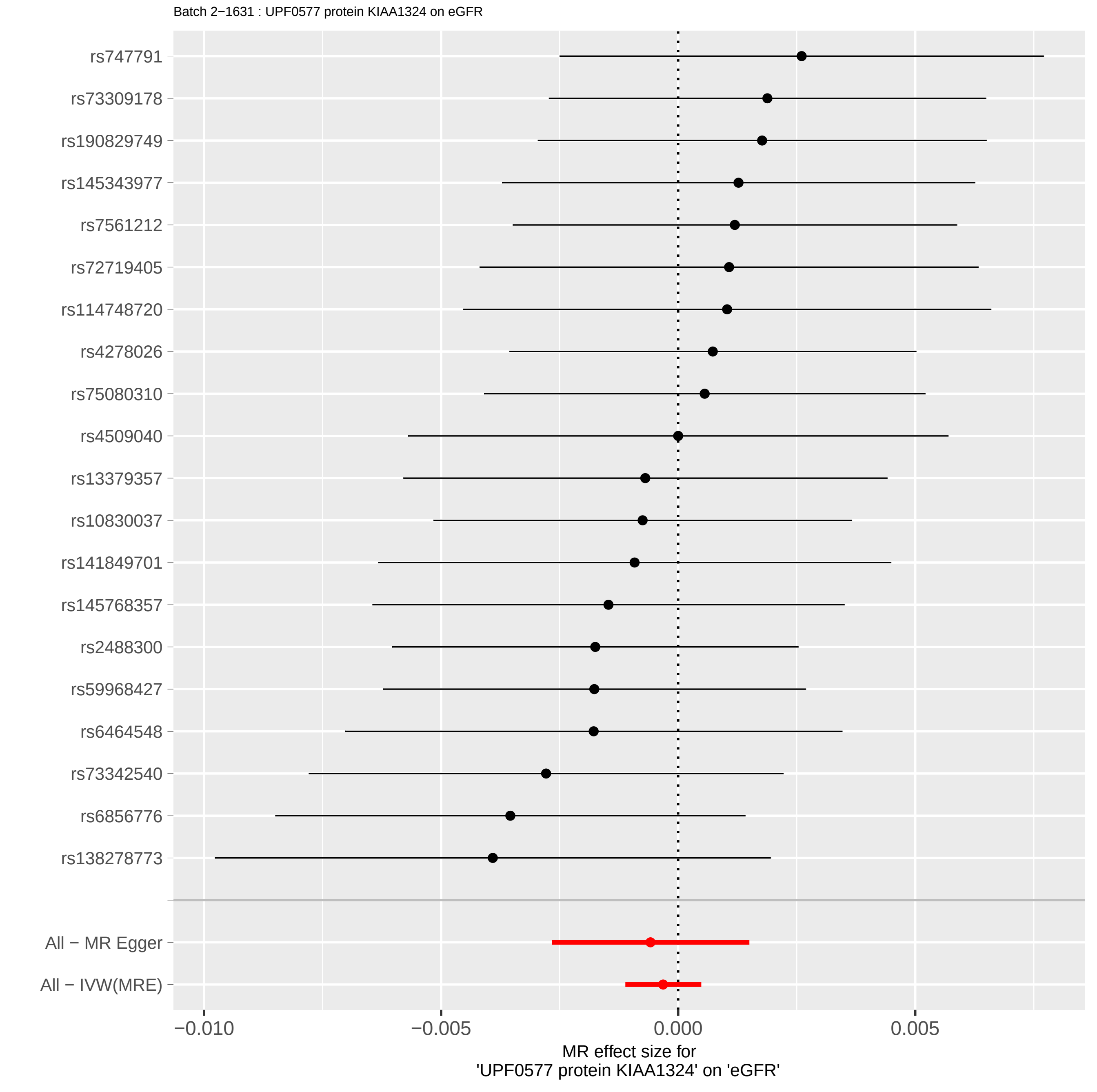

Batch 2−1631 : UPF0577 protein KIAA1324 on eGFR
rs747791
rs73309178
rs190829749
rs145343977
rs7561212
rs72719405
rs114748720
rs4278026
rs75080310
rs4509040
rs13379357
rs10830037
rs141849701
rs145768357
rs2488300
rs59968427
rs6464548
rs73342540
rs6856776
rs138278773
All − MR Egger
All − IVW(MRE)
−0.010
−0.005
0.000
0.005
MR effect size for
'UPF0577 protein KIAA1324' on 'eGFR'

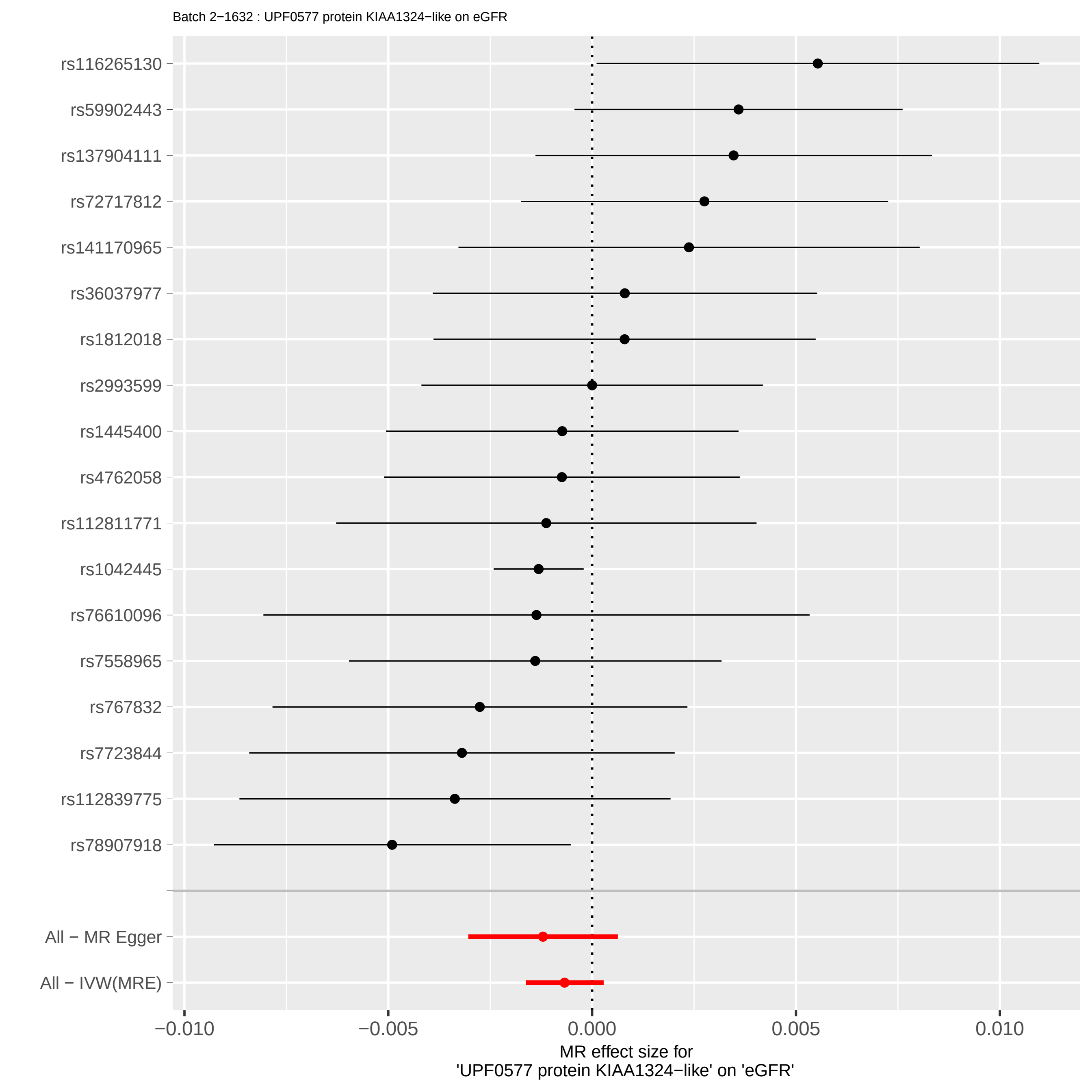
Batch 2−1632 : UPF0577 protein KIAA1324−like on eGFR
rs116265130
rs59902443
rs137904111
rs72717812
rs141170965
rs36037977
rs1812018
rs2993599
rs1445400
rs4762058
rs112811771
rs1042445
rs76610096
rs7558965
rs767832
rs7723844
rs112839775
rs78907918
All − MR Egger
All − IVW(MRE)
−0.010
−0.005
0.000
0.005
0.010
MR effect size for
'UPF0577 protein KIAA1324−like' on 'eGFR'

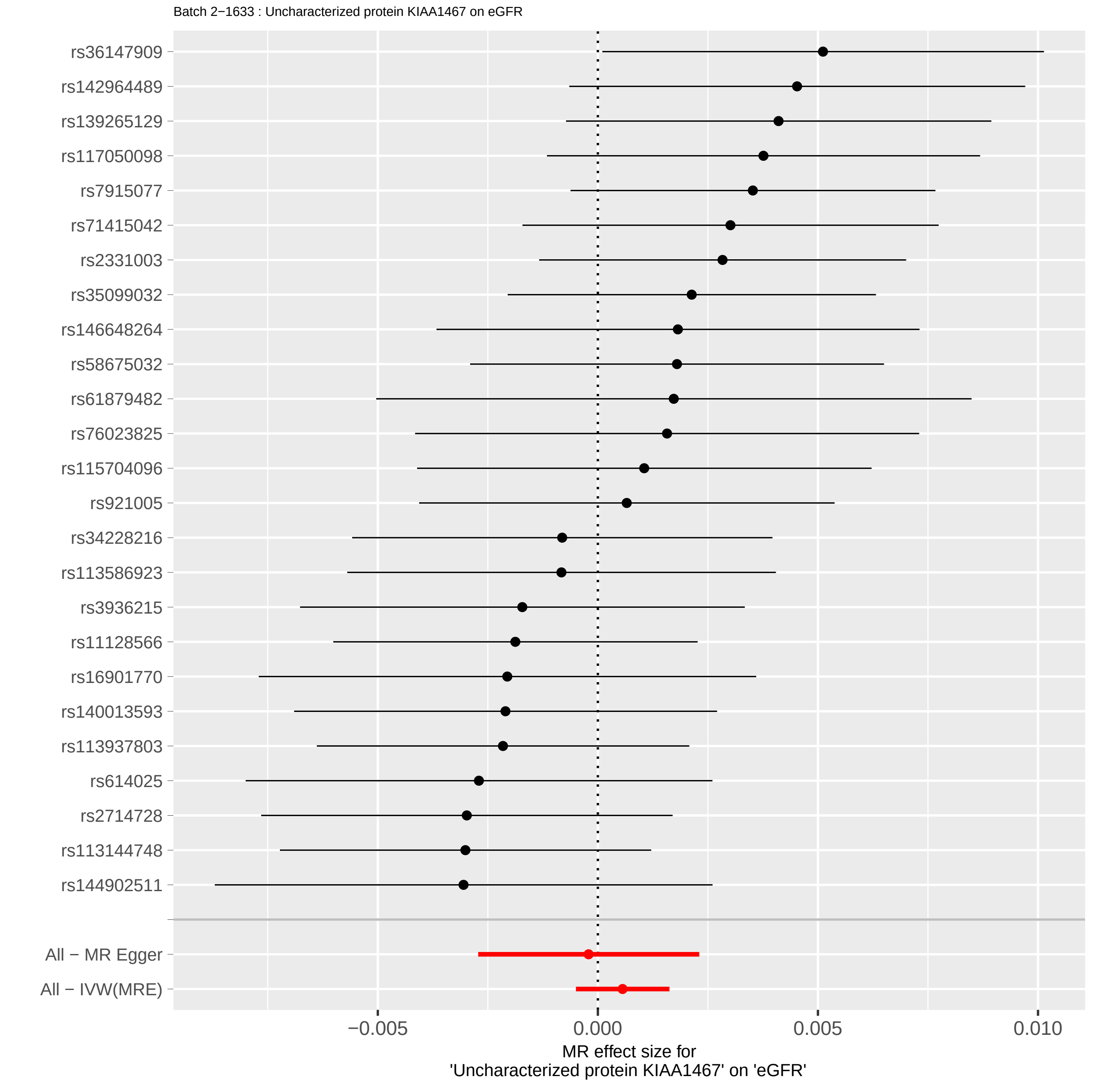

Batch 2−1633 : Uncharacterized protein KIAA1467 on eGFR
rs36147909
rs142964489
rs139265129
rs117050098
rs7915077
rs71415042
rs2331003
rs35099032
rs146648264
rs58675032
rs61879482
rs76023825
rs115704096
rs921005
rs34228216
rs113586923
rs3936215
rs11128566
rs16901770
rs140013593
rs113937803
rs614025
rs2714728
rs113144748
rs144902511
All − MR Egger
All − IVW(MRE)
−0.005
0.000
0.005
0.010
MR effect size for
'Uncharacterized protein KIAA1467' on 'eGFR'

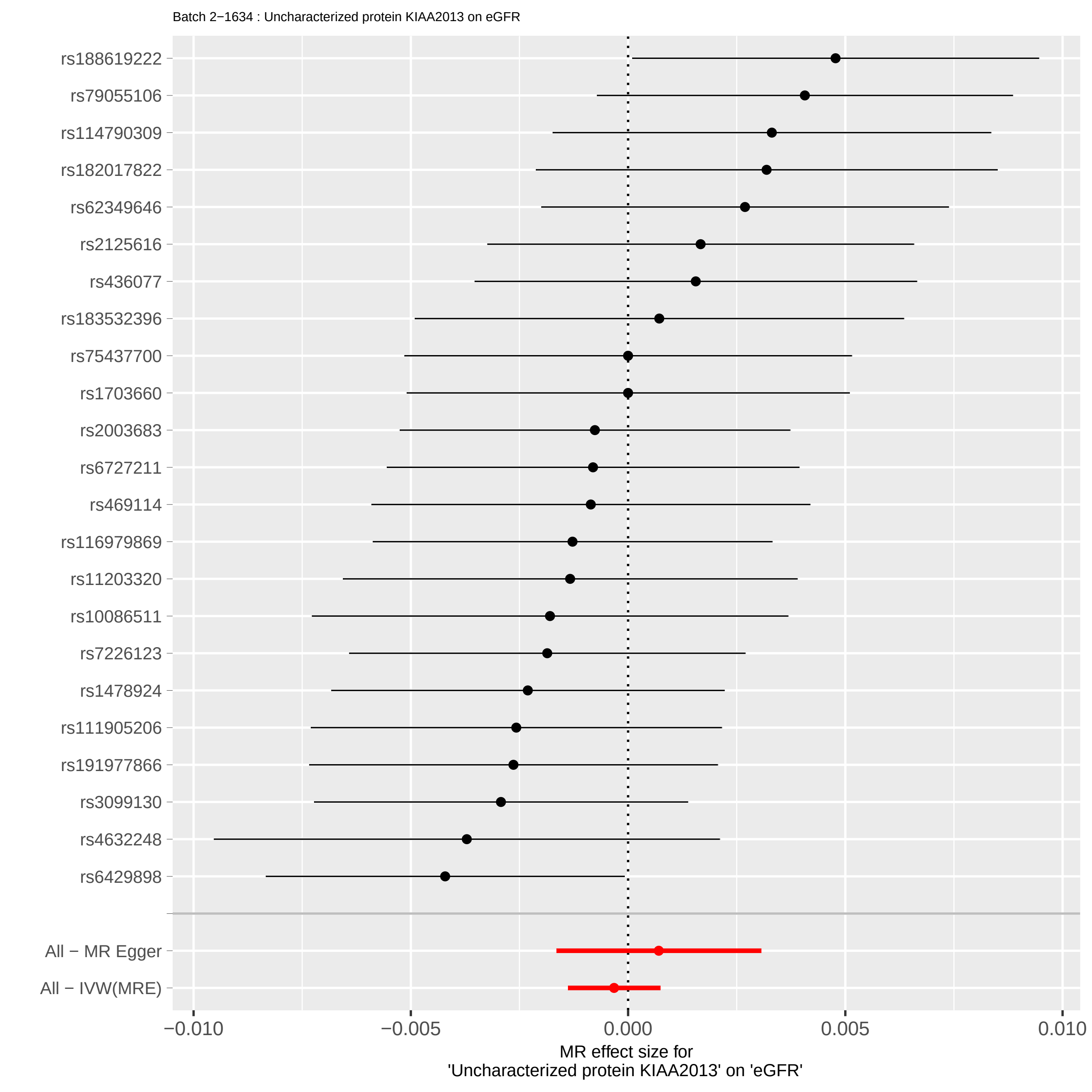

Batch 2−1634 : Uncharacterized protein KIAA2013 on eGFR
rs188619222
rs79055106
rs114790309
rs182017822
rs62349646
rs2125616
rs436077
rs183532396
rs75437700
rs1703660
rs2003683
rs6727211
rs469114
rs116979869
rs11203320
rs10086511
rs7226123
rs1478924
rs111905206
rs191977866
rs3099130
rs4632248
rs6429898
All − MR Egger
All − IVW(MRE)
−0.010
−0.005
0.000
0.005
0.010
MR effect size for
'Uncharacterized protein KIAA2013' on 'eGFR'

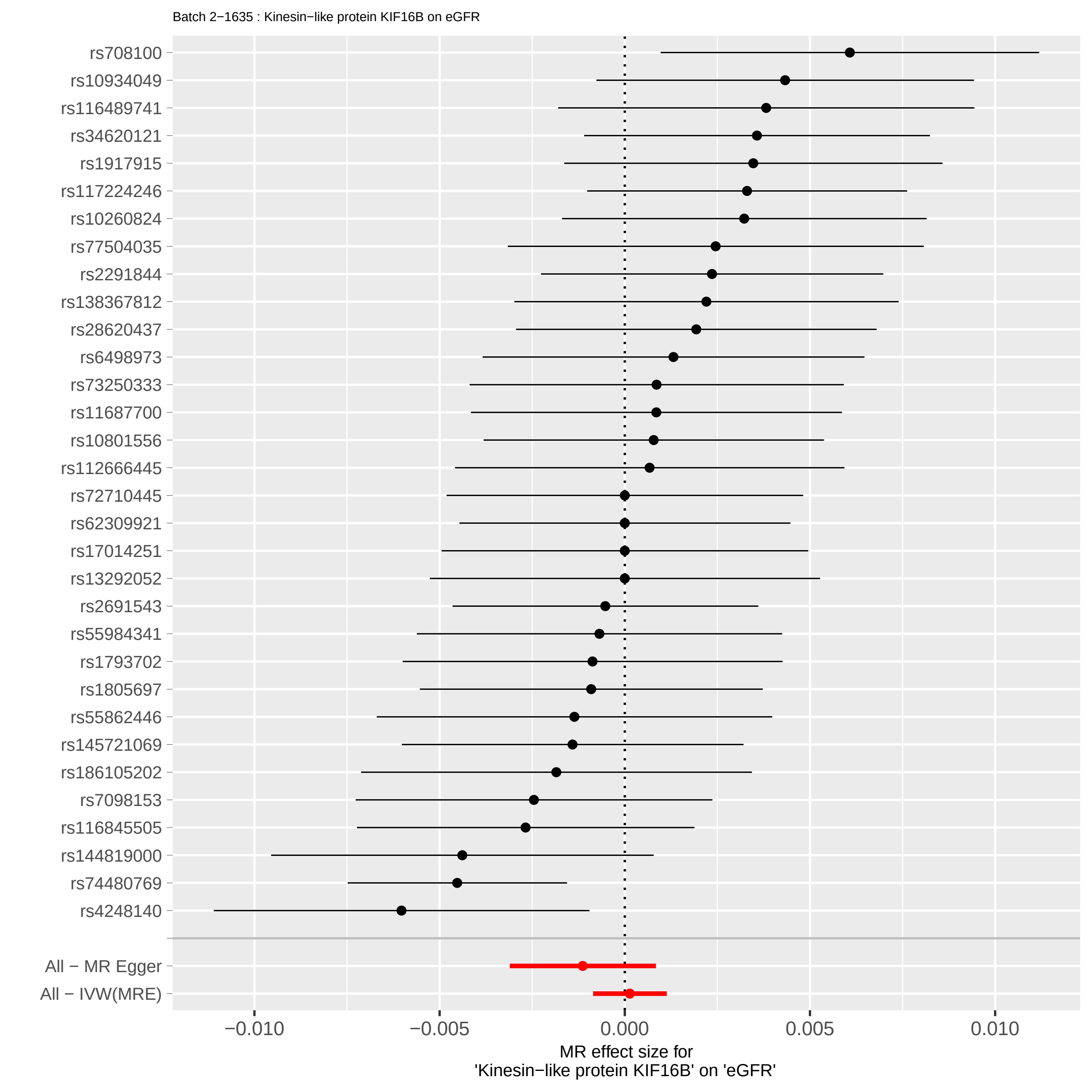

Batch 2−1635 : Kinesin−like protein KIF16B on eGFR
rs708100
rs10934049
rs116489741
rs34620121
rs1917915
rs117224246
rs10260824
rs77504035
rs2291844
rs138367812
rs28620437
rs6498973
rs73250333
rs11687700
rs10801556
rs112666445
rs72710445
rs62309921
rs17014251
rs13292052
rs2691543
rs55984341
rs1793702
rs1805697
rs55862446
rs145721069
rs186105202
rs7098153
rs116845505
rs144819000
rs74480769
rs4248140
All − MR Egger
All − IVW(MRE)
−0.010
−0.005
0.000
0.005
0.010
MR effect size for
'Kinesin−like protein KIF16B' on 'eGFR'

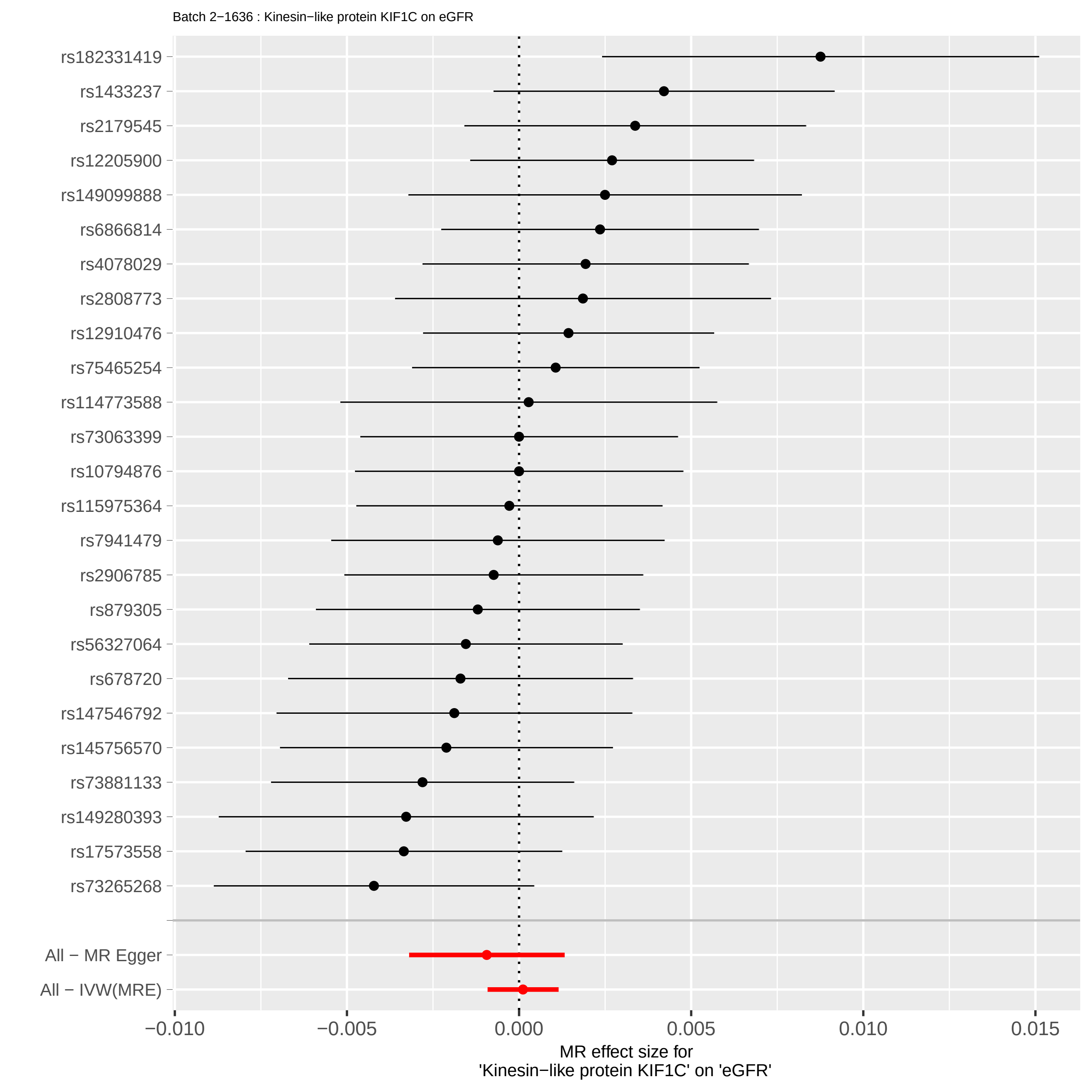

Batch 2−1636 : Kinesin−like protein KIF1C on eGFR
rs182331419
rs1433237
rs2179545
rs12205900
rs149099888
rs6866814
rs4078029
rs2808773
rs12910476
rs75465254
rs114773588
rs73063399
rs10794876
rs115975364
rs7941479
rs2906785
rs879305
rs56327064
rs678720
rs147546792
rs145756570
rs73881133
rs149280393
rs17573558
rs73265268
All − MR Egger
All − IVW(MRE)
−0.010
−0.005
0.000
0.005
0.010
0.015
MR effect size for
'Kinesin−like protein KIF1C' on 'eGFR'

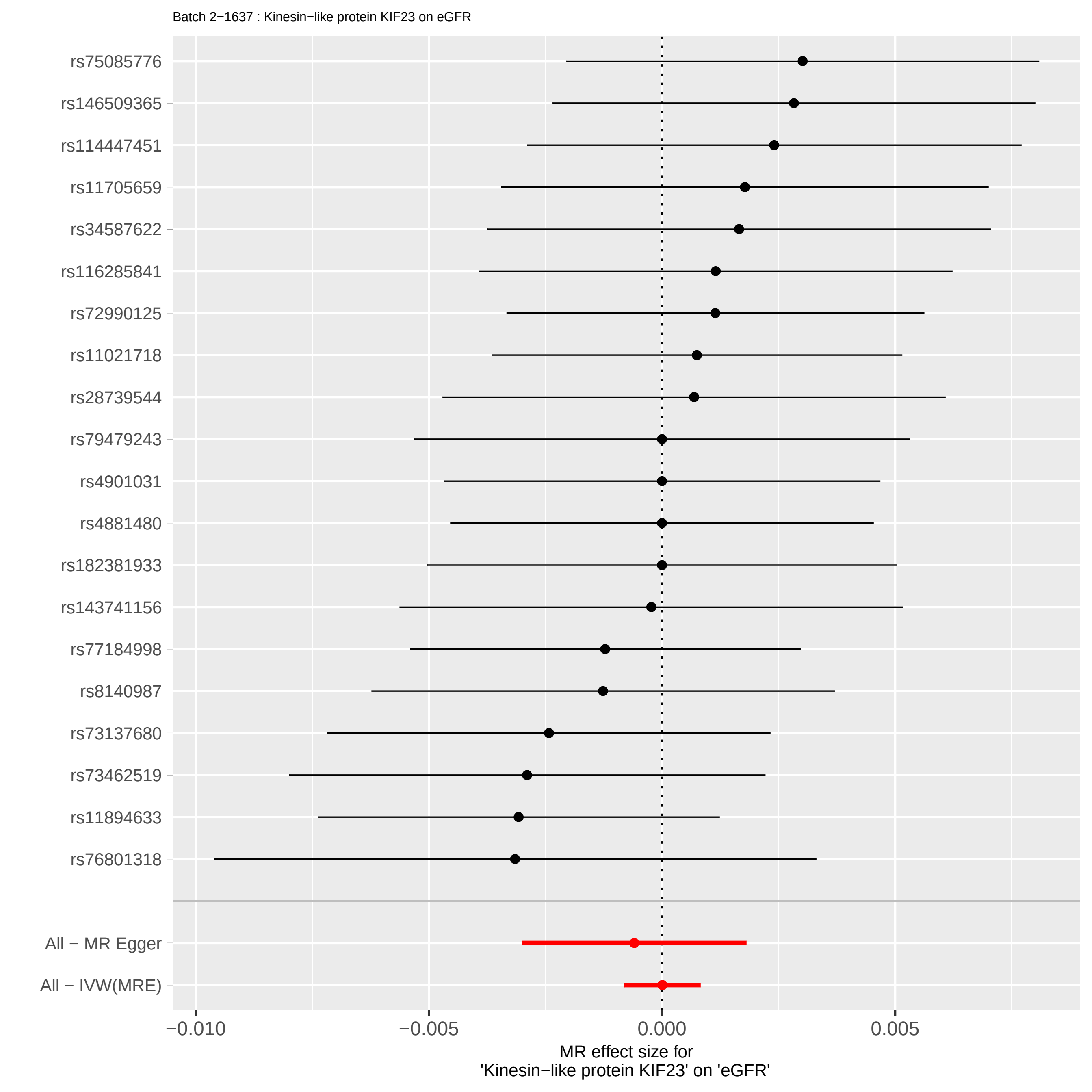

Batch 2−1637 : Kinesin−like protein KIF23 on eGFR
rs75085776
rs146509365
rs114447451
rs11705659
rs34587622
rs116285841
rs72990125
rs11021718
rs28739544
rs79479243
rs4901031
rs4881480
rs182381933
rs143741156
rs77184998
rs8140987
rs73137680
rs73462519
rs11894633
rs76801318
All − MR Egger
All − IVW(MRE)
−0.010
−0.005
0.000
0.005
MR effect size for
'Kinesin−like protein KIF23' on 'eGFR'

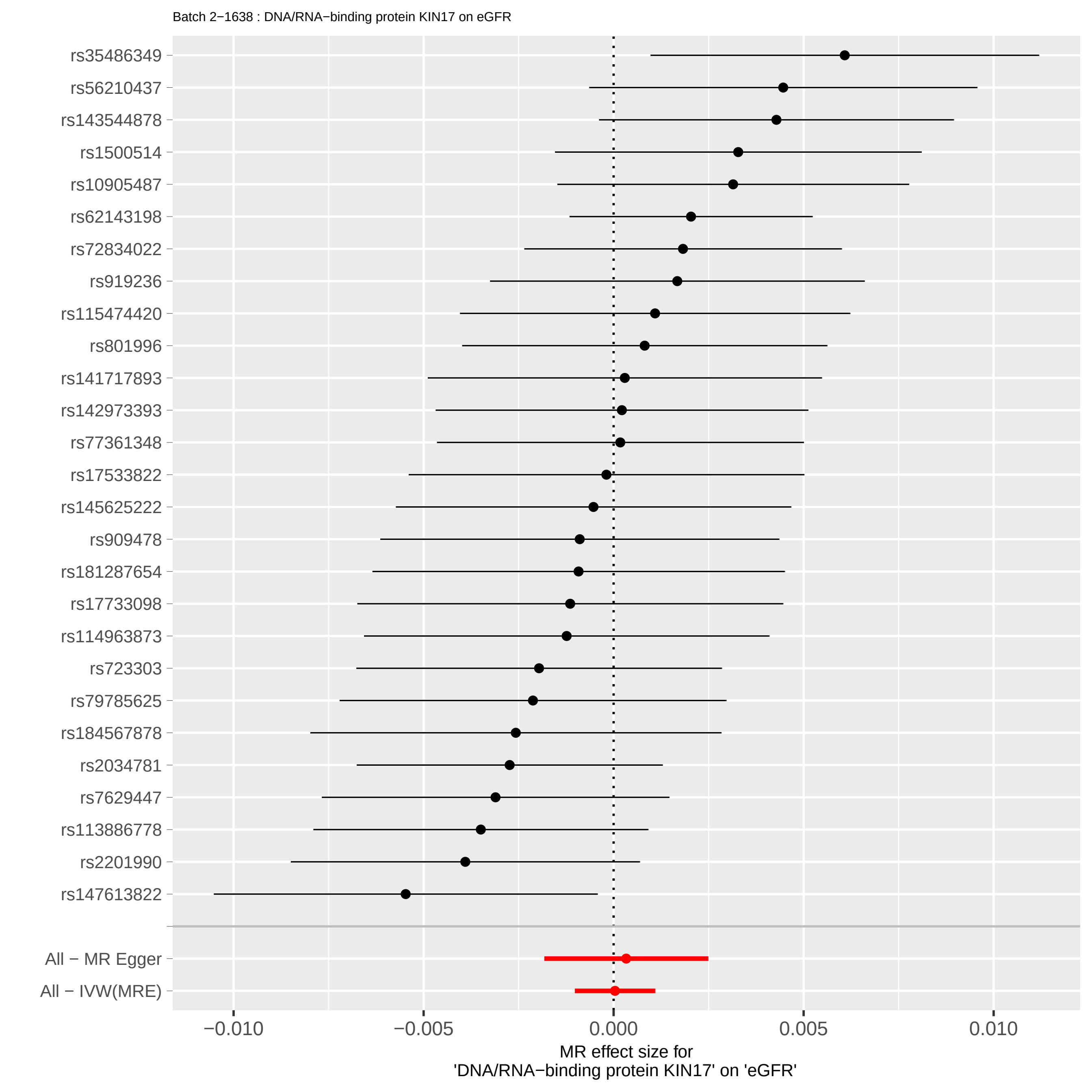

Batch 2−1638 : DNA/RNA−binding protein KIN17 on eGFR
rs35486349
rs56210437
rs143544878
rs1500514
rs10905487
rs62143198
rs72834022
rs919236
rs115474420
rs801996
rs141717893
rs142973393
rs77361348
rs17533822
rs145625222
rs909478
rs181287654
rs17733098
rs114963873
rs723303
rs79785625
rs184567878
rs2034781
rs7629447
rs113886778
rs2201990
rs147613822
All − MR Egger
All − IVW(MRE)
−0.010
−0.005
0.000
0.005
0.010
MR effect size for
'DNA/RNA−binding protein KIN17' on 'eGFR'

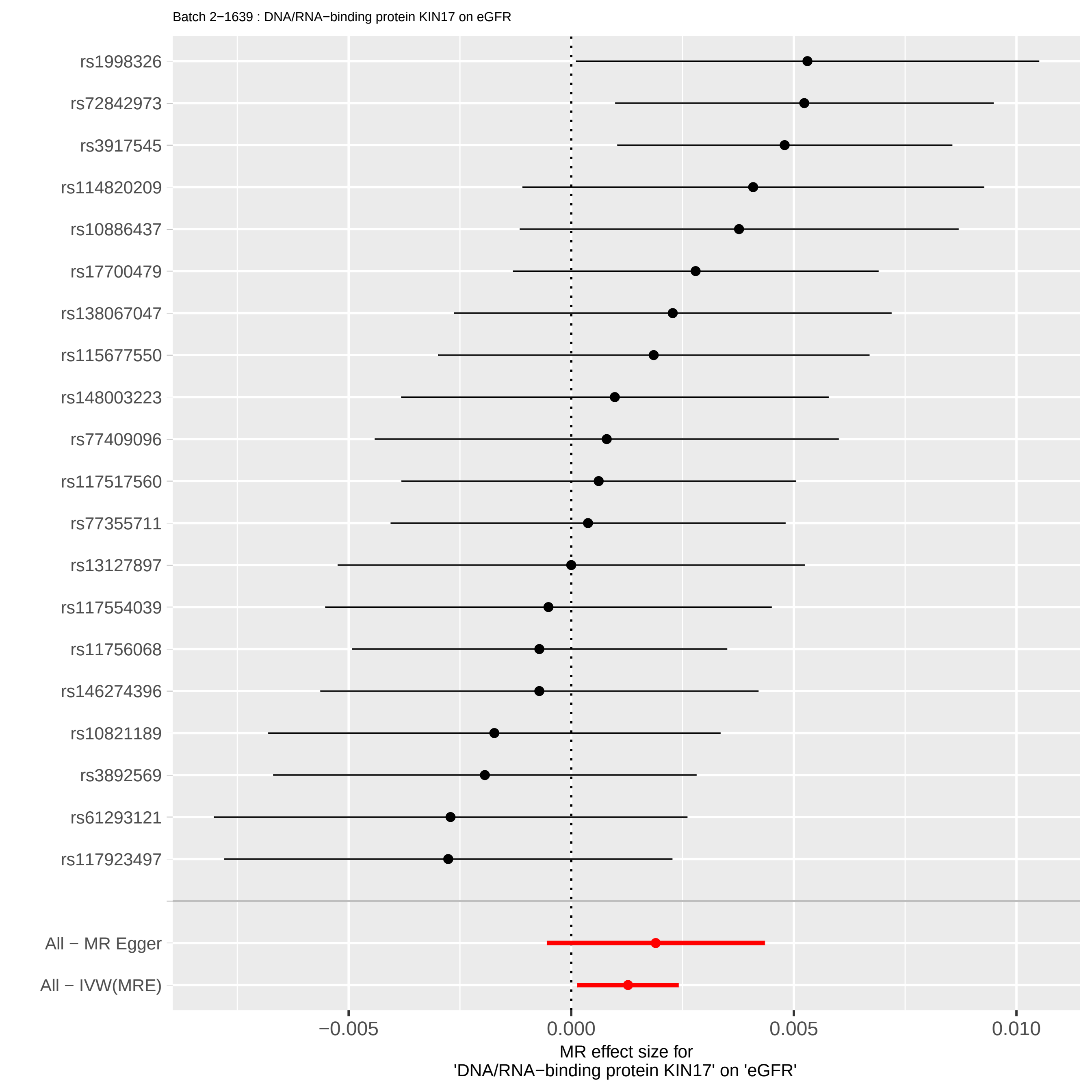

Batch 2−1639 : DNA/RNA−binding protein KIN17 on eGFR
rs1998326
rs72842973
rs3917545
rs114820209
rs10886437
rs17700479
rs138067047
rs115677550
rs148003223
rs77409096
rs117517560
rs77355711
rs13127897
rs117554039
rs11756068
rs146274396
rs10821189
rs3892569
rs61293121
rs117923497
All − MR Egger
All − IVW(MRE)
−0.005
0.000
0.005
0.010
MR effect size for
'DNA/RNA−binding protein KIN17' on 'eGFR'

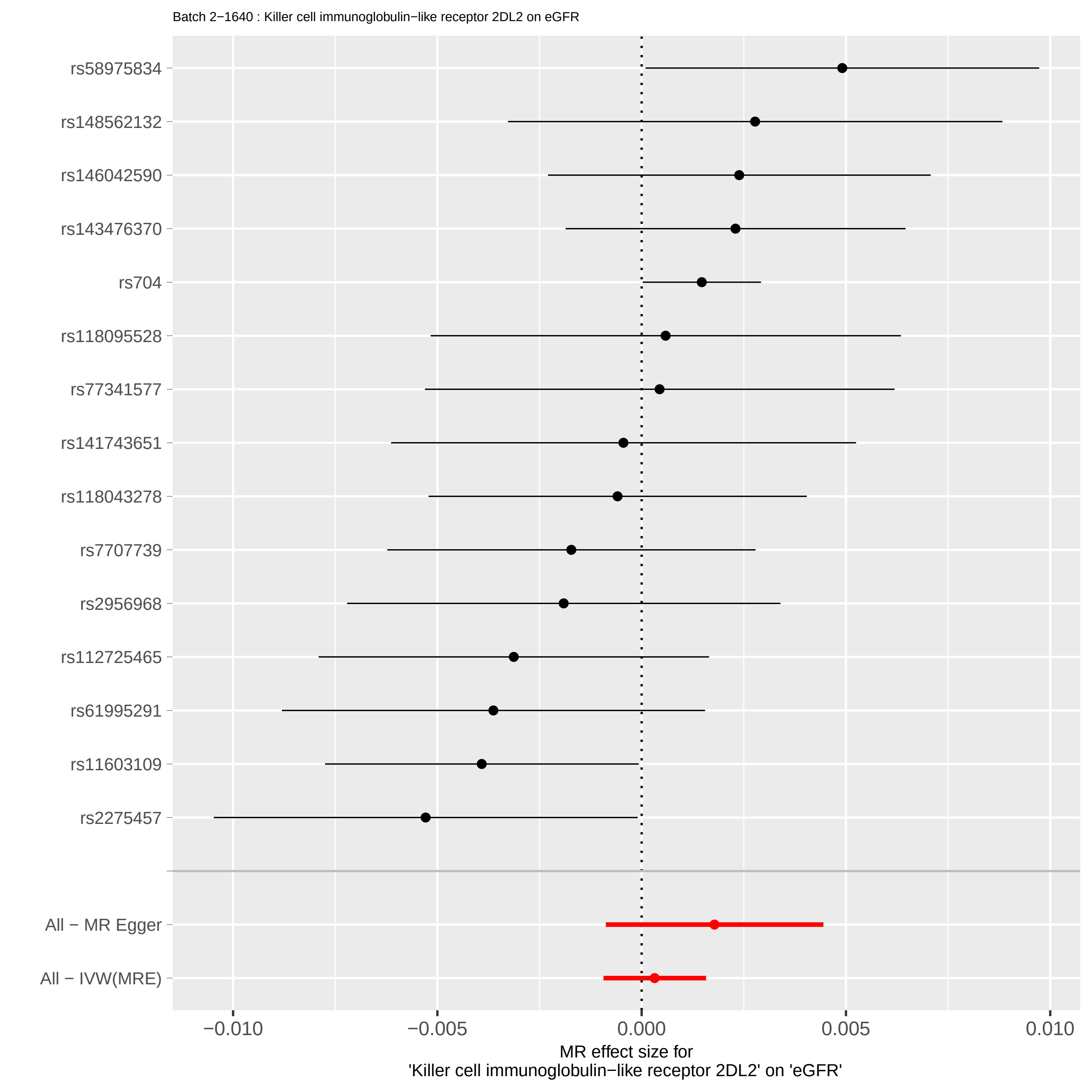
Batch 2−1640 : Killer cell immunoglobulin−like receptor 2DL2 on eGFR
rs58975834
rs148562132
rs146042590
rs143476370
rs704
rs118095528
rs77341577
rs141743651
rs118043278
rs7707739
rs2956968
rs112725465
rs61995291
rs11603109
rs2275457
All − MR Egger
All − IVW(MRE)
−0.010
−0.005
0.000
0.005
0.010
MR effect size for
'Killer cell immunoglobulin−like receptor 2DL2' on 'eGFR'

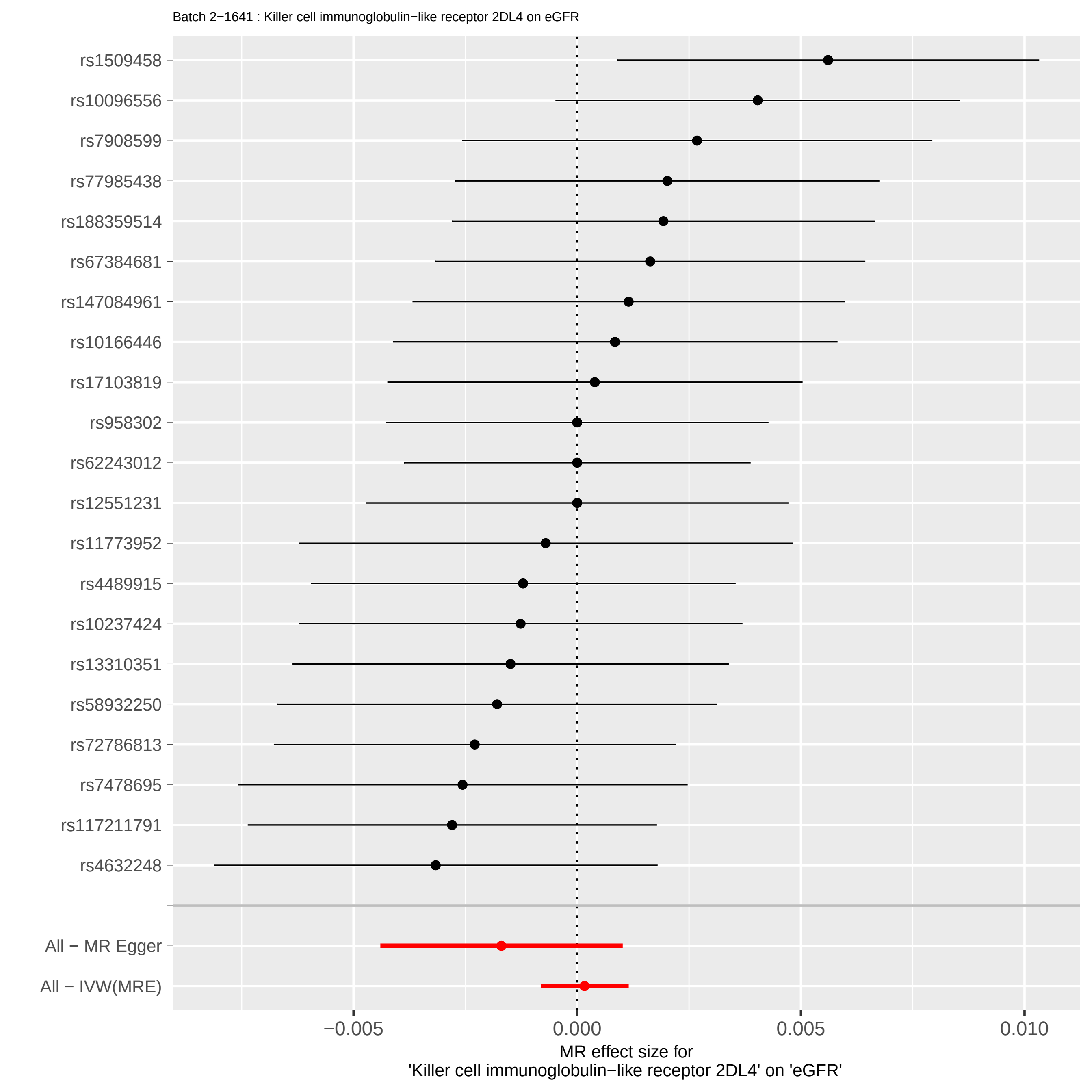
Batch 2−1641 : Killer cell immunoglobulin−like receptor 2DL4 on eGFR
rs1509458
rs10096556
rs7908599
rs77985438
rs188359514
rs67384681
rs147084961
rs10166446
rs17103819
rs958302
rs62243012
rs12551231
rs11773952
rs4489915
rs10237424
rs13310351
rs58932250
rs72786813
rs7478695
rs117211791
rs4632248
All − MR Egger
All − IVW(MRE)
−0.005
0.000
0.005
0.010
MR effect size for
'Killer cell immunoglobulin−like receptor 2DL4' on 'eGFR'

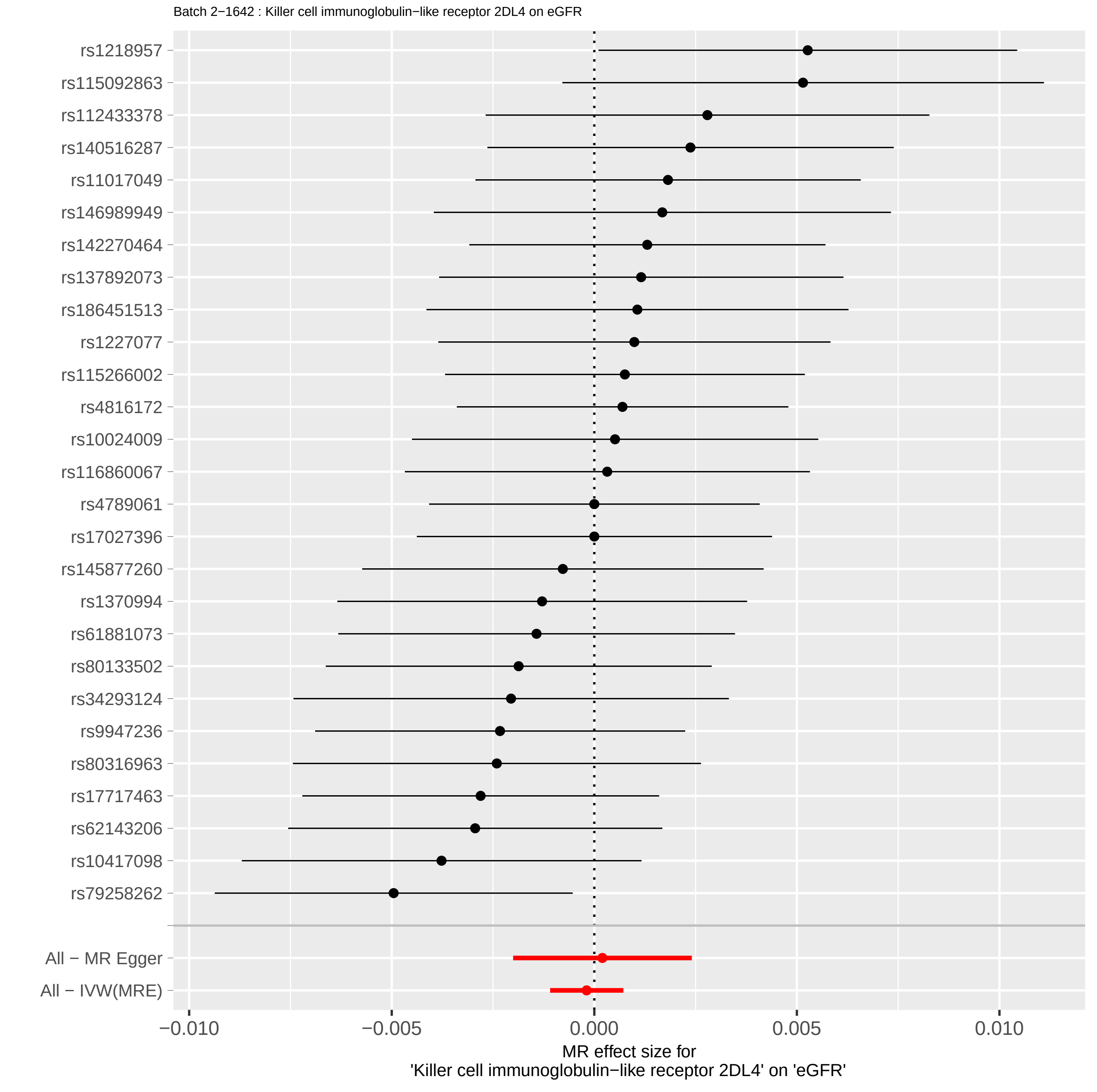

Batch 2−1642 : Killer cell immunoglobulin−like receptor 2DL4 on eGFR
rs1218957
rs115092863
rs112433378
rs140516287
rs11017049
rs146989949
rs142270464
rs137892073
rs186451513
rs1227077
rs115266002
rs4816172
rs10024009
rs116860067
rs4789061
rs17027396
rs145877260
rs1370994
rs61881073
rs80133502
rs34293124
rs9947236
rs80316963
rs17717463
rs62143206
rs10417098
rs79258262
All − MR Egger
All − IVW(MRE)
−0.010
−0.005
0.000
0.005
0.010
MR effect size for
'Killer cell immunoglobulin−like receptor 2DL4' on 'eGFR'

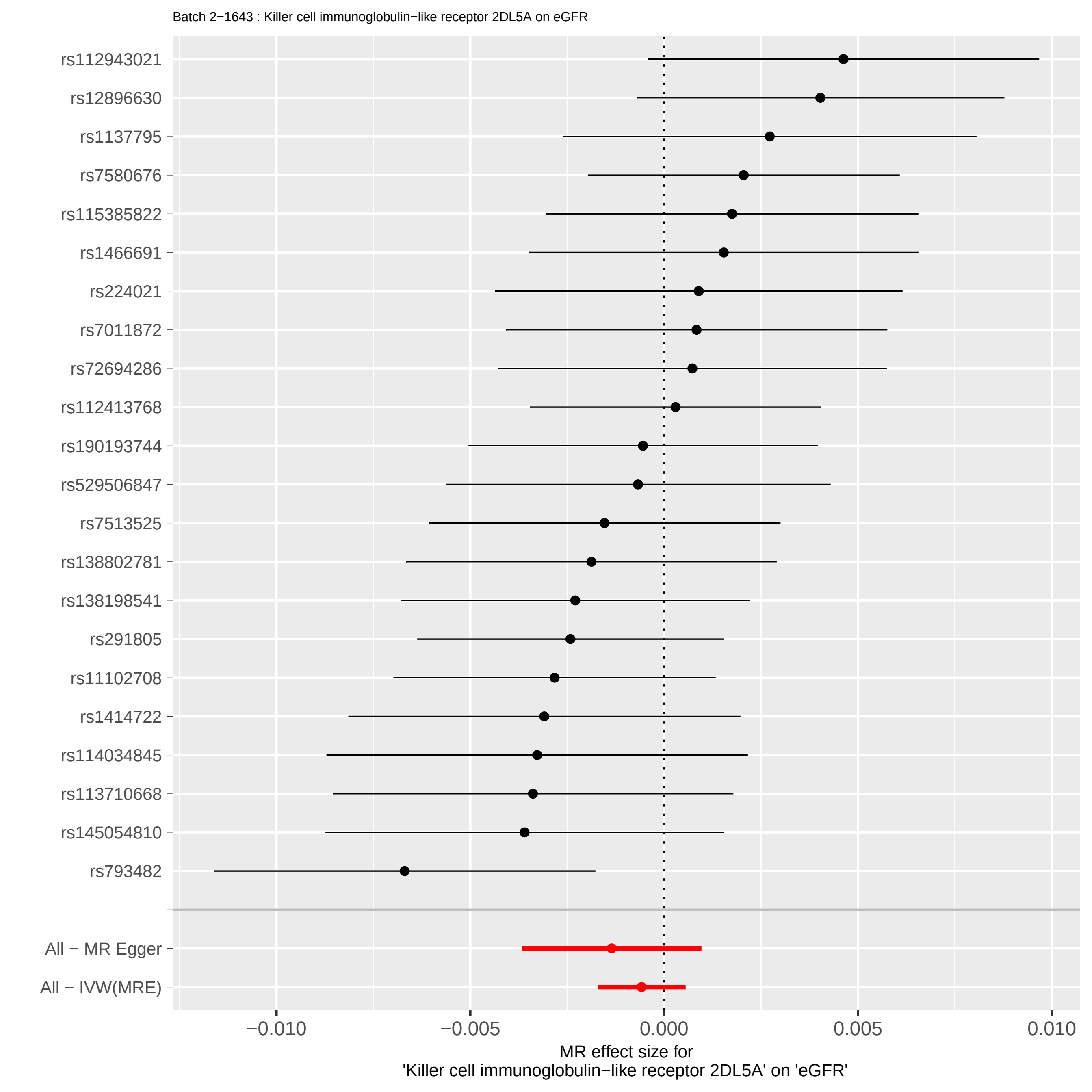

Batch 2−1643 : Killer cell immunoglobulin−like receptor 2DL5A on eGFR
rs112943021
rs12896630
rs1137795
rs7580676
rs115385822
rs1466691
rs224021
rs7011872
rs72694286
rs112413768
rs190193744
rs529506847
rs7513525
rs138802781
rs138198541
rs291805
rs11102708
rs1414722
rs114034845
rs113710668
rs145054810
rs793482
All − MR Egger
All − IVW(MRE)
−0.010
−0.005
0.000
0.005
0.010
MR effect size for
'Killer cell immunoglobulin−like receptor 2DL5A' on 'eGFR'

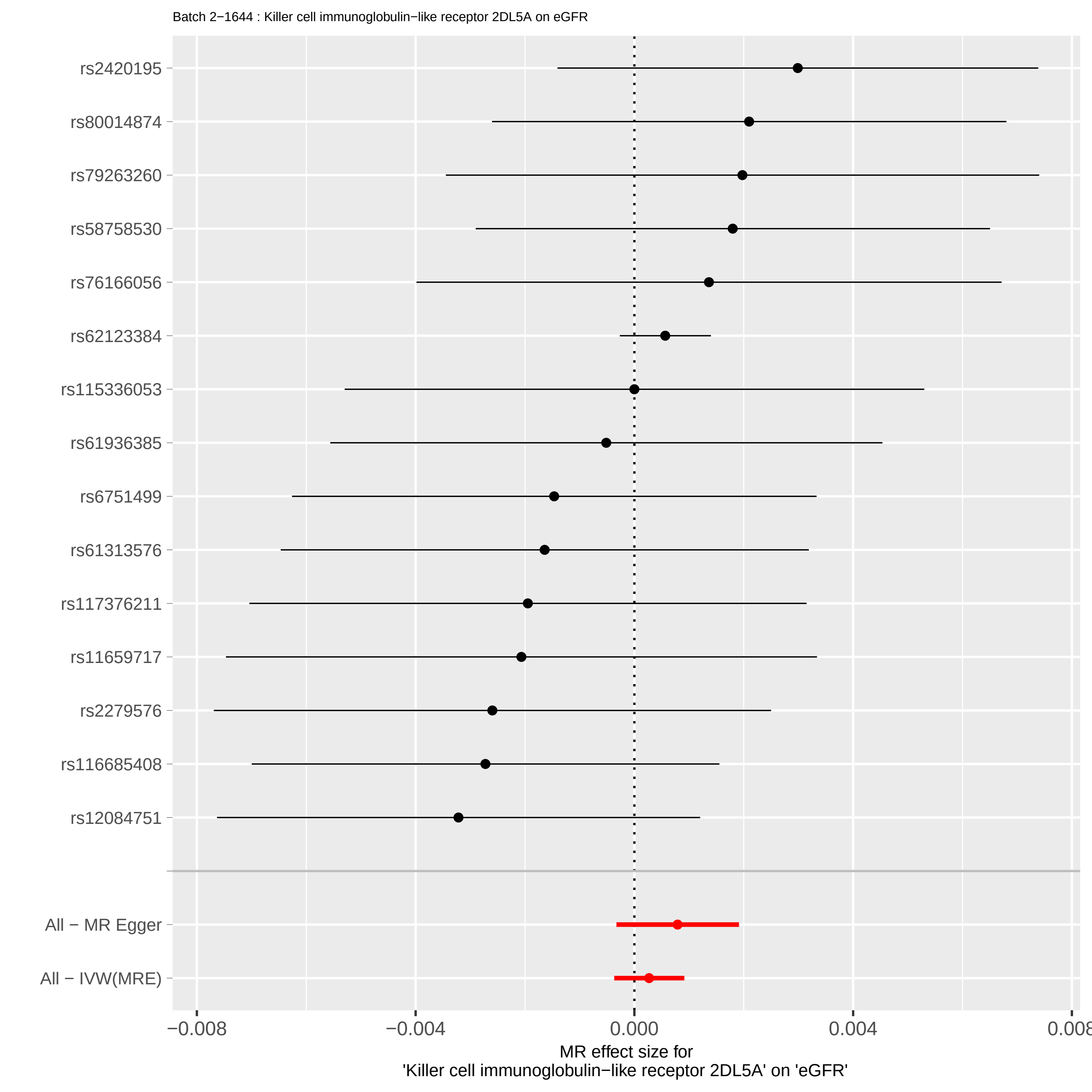

Batch 2−1644 : Killer cell immunoglobulin−like receptor 2DL5A on eGFR
rs2420195
rs80014874
rs79263260
rs58758530
rs76166056
rs62123384
rs115336053
rs61936385
rs6751499
rs61313576
rs117376211
rs11659717
rs2279576
rs116685408
rs12084751
All − MR Egger
All − IVW(MRE)
−0.008
−0.004
0.000
0.004
0.008
MR effect size for
'Killer cell immunoglobulin−like receptor 2DL5A' on 'eGFR'

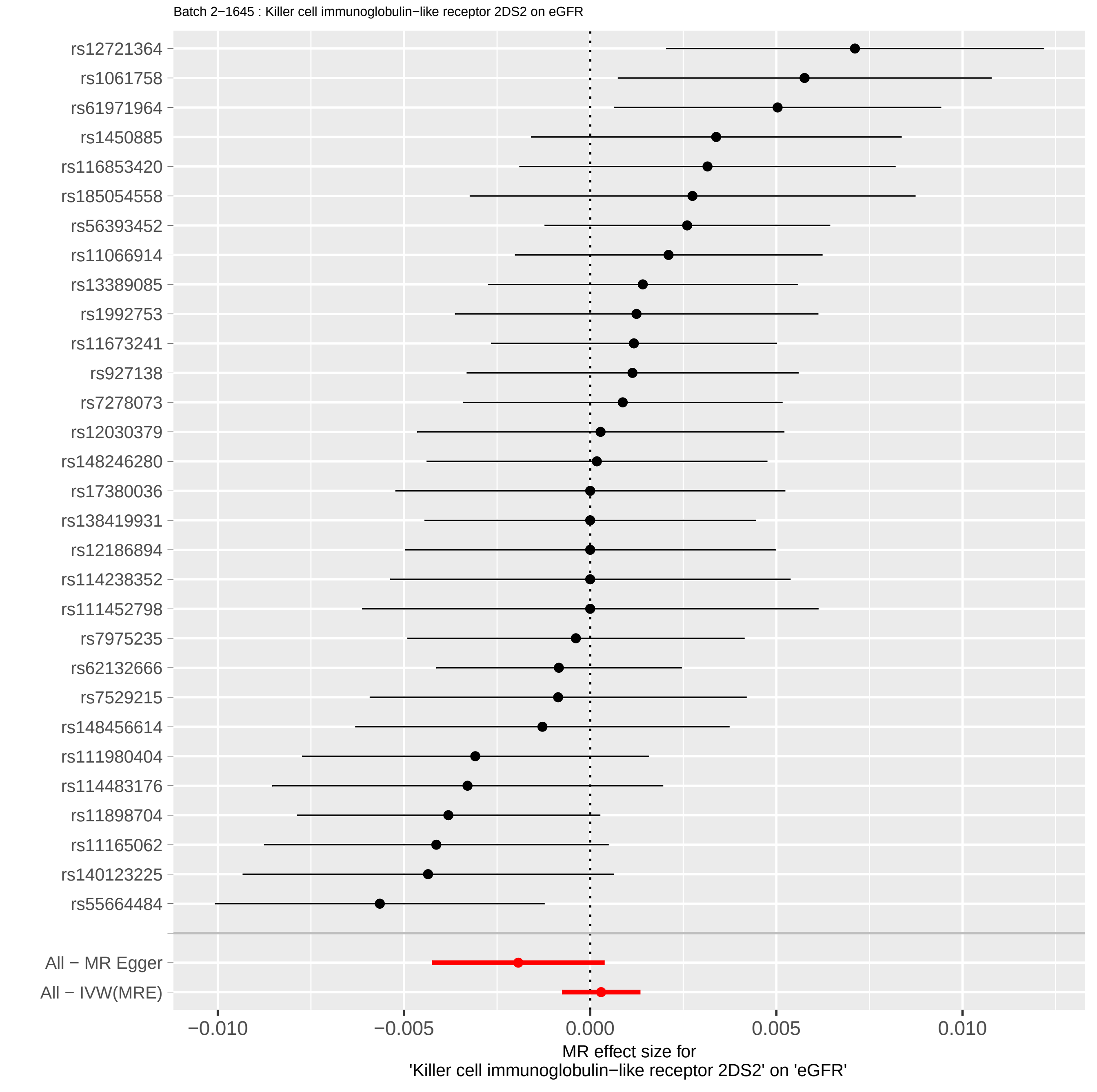

Batch 2−1645 : Killer cell immunoglobulin−like receptor 2DS2 on eGFR
rs12721364
rs1061758
rs61971964
rs1450885
rs116853420
rs185054558
rs56393452
rs11066914
rs13389085
rs1992753
rs11673241
rs927138
rs7278073
rs12030379
rs148246280
rs17380036
rs138419931
rs12186894
rs114238352
rs111452798
rs7975235
rs62132666
rs7529215
rs148456614
rs111980404
rs114483176
rs11898704
rs11165062
rs140123225
rs55664484
All − MR Egger
All − IVW(MRE)
−0.010
−0.005
0.000
0.005
0.010
MR effect size for
'Killer cell immunoglobulin−like receptor 2DS2' on 'eGFR'

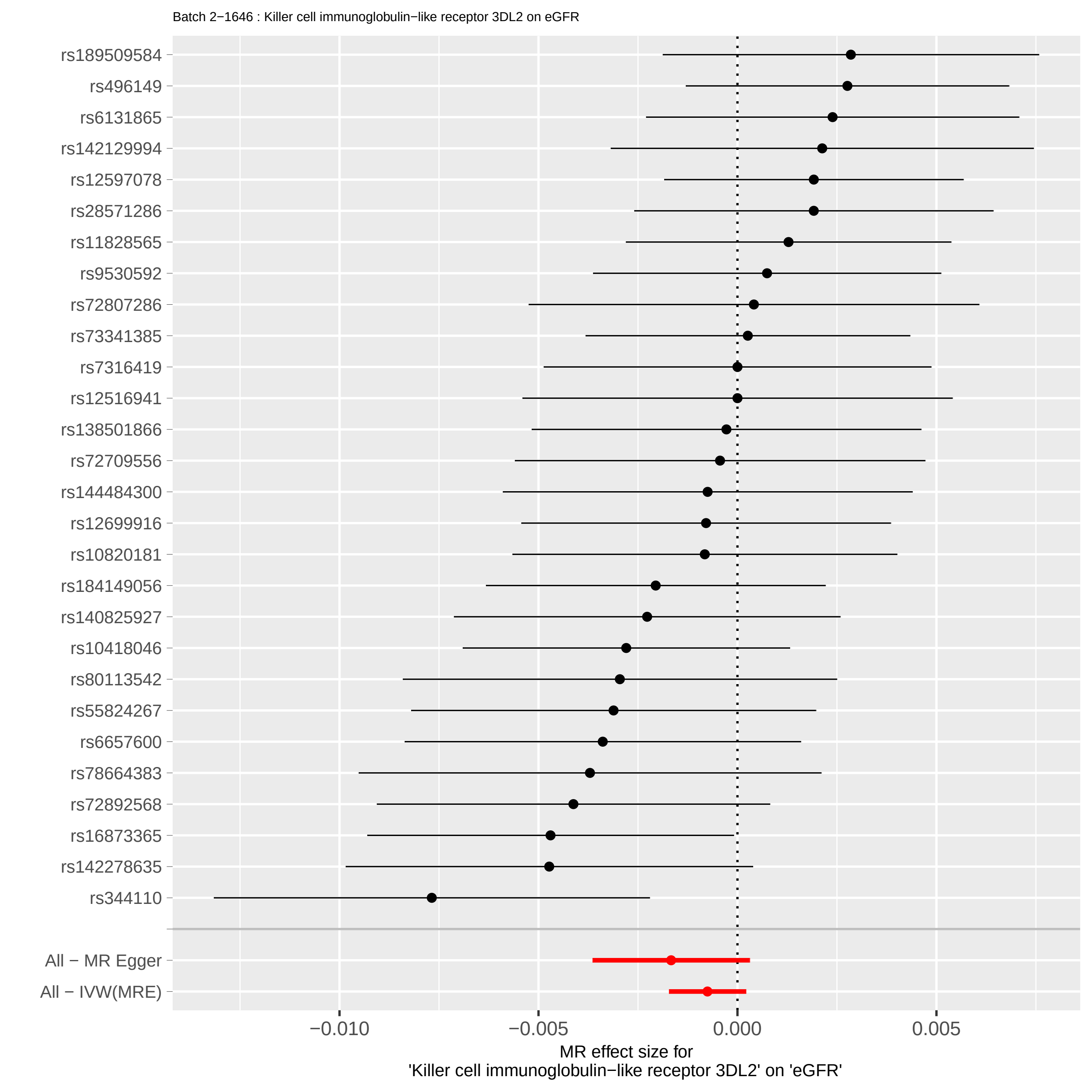

Batch 2−1646 : Killer cell immunoglobulin−like receptor 3DL2 on eGFR
rs189509584
rs496149
rs6131865
rs142129994
rs12597078
rs28571286
rs11828565
rs9530592
rs72807286
rs73341385
rs7316419
rs12516941
rs138501866
rs72709556
rs144484300
rs12699916
rs10820181
rs184149056
rs140825927
rs10418046
rs80113542
rs55824267
rs6657600
rs78664383
rs72892568
rs16873365
rs142278635
rs344110
All − MR Egger
All − IVW(MRE)
−0.010
−0.005
0.000
0.005
MR effect size for
'Killer cell immunoglobulin−like receptor 3DL2' on 'eGFR'

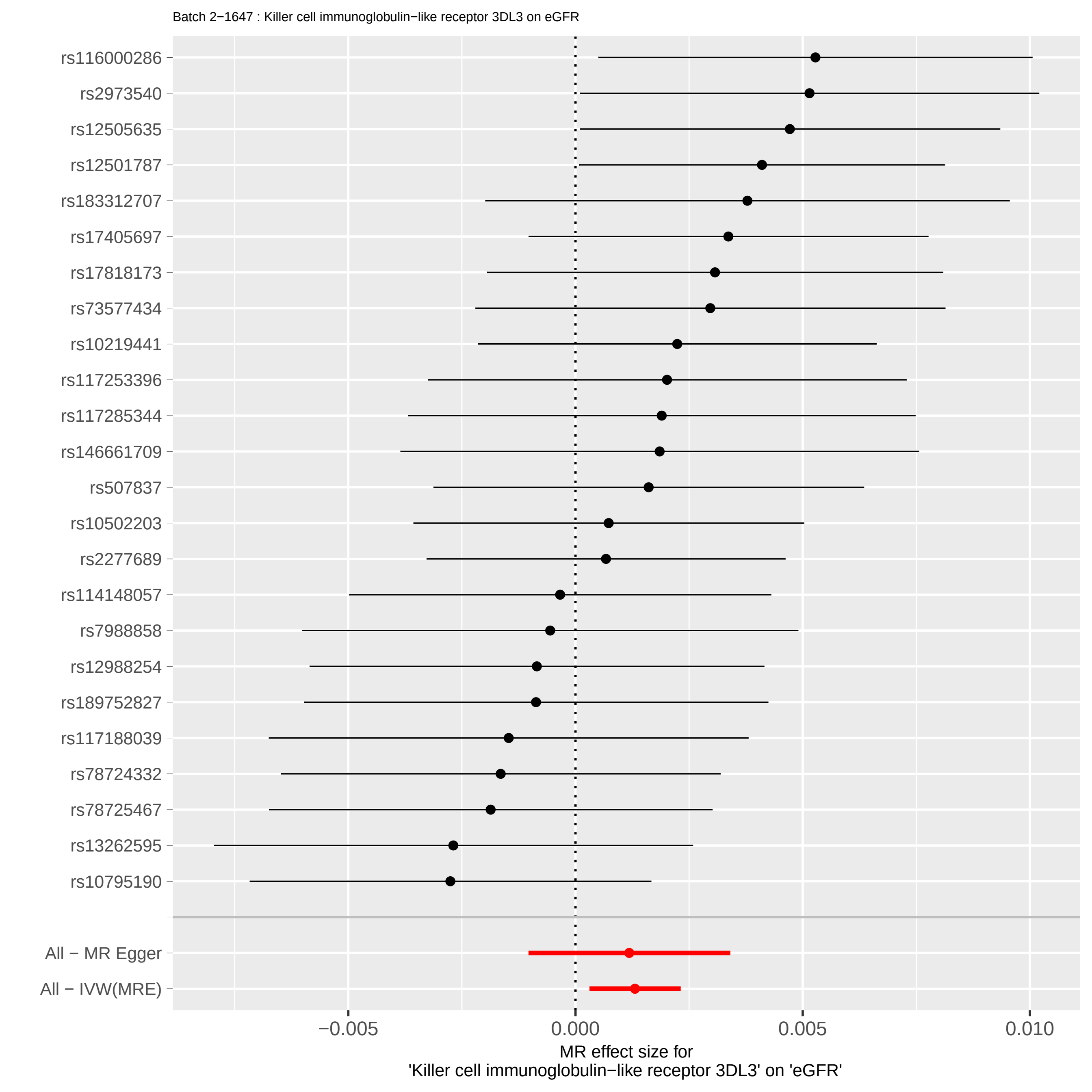

Batch 2−1647 : Killer cell immunoglobulin−like receptor 3DL3 on eGFR
rs116000286
rs2973540
rs12505635
rs12501787
rs183312707
rs17405697
rs17818173
rs73577434
rs10219441
rs117253396
rs117285344
rs146661709
rs507837
rs10502203
rs2277689
rs114148057
rs7988858
rs12988254
rs189752827
rs117188039
rs78724332
rs78725467
rs13262595
rs10795190
All − MR Egger
All − IVW(MRE)
−0.005
0.000
0.005
0.010
MR effect size for
'Killer cell immunoglobulin−like receptor 3DL3' on 'eGFR'

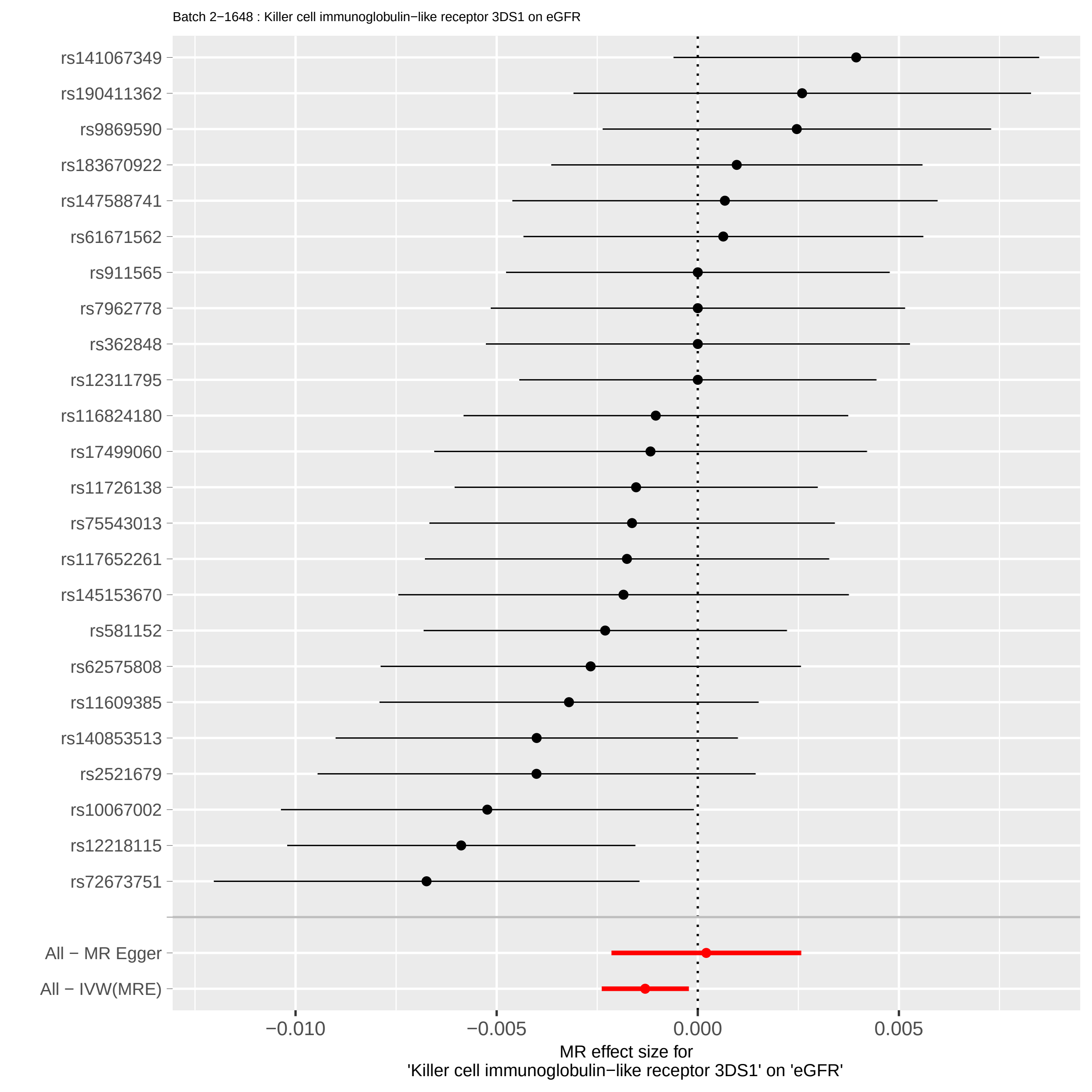
Batch 2−1648 : Killer cell immunoglobulin−like receptor 3DS1 on eGFR
rs141067349
rs190411362
rs9869590
rs183670922
rs147588741
rs61671562
rs911565
rs7962778
rs362848
rs12311795
rs116824180
rs17499060
rs11726138
rs75543013
rs117652261
rs145153670
rs581152
rs62575808
rs11609385
rs140853513
rs2521679
rs10067002
rs12218115
rs72673751
All − MR Egger
All − IVW(MRE)
−0.010
−0.005
0.000
0.005
MR effect size for
'Killer cell immunoglobulin−like receptor 3DS1' on 'eGFR'

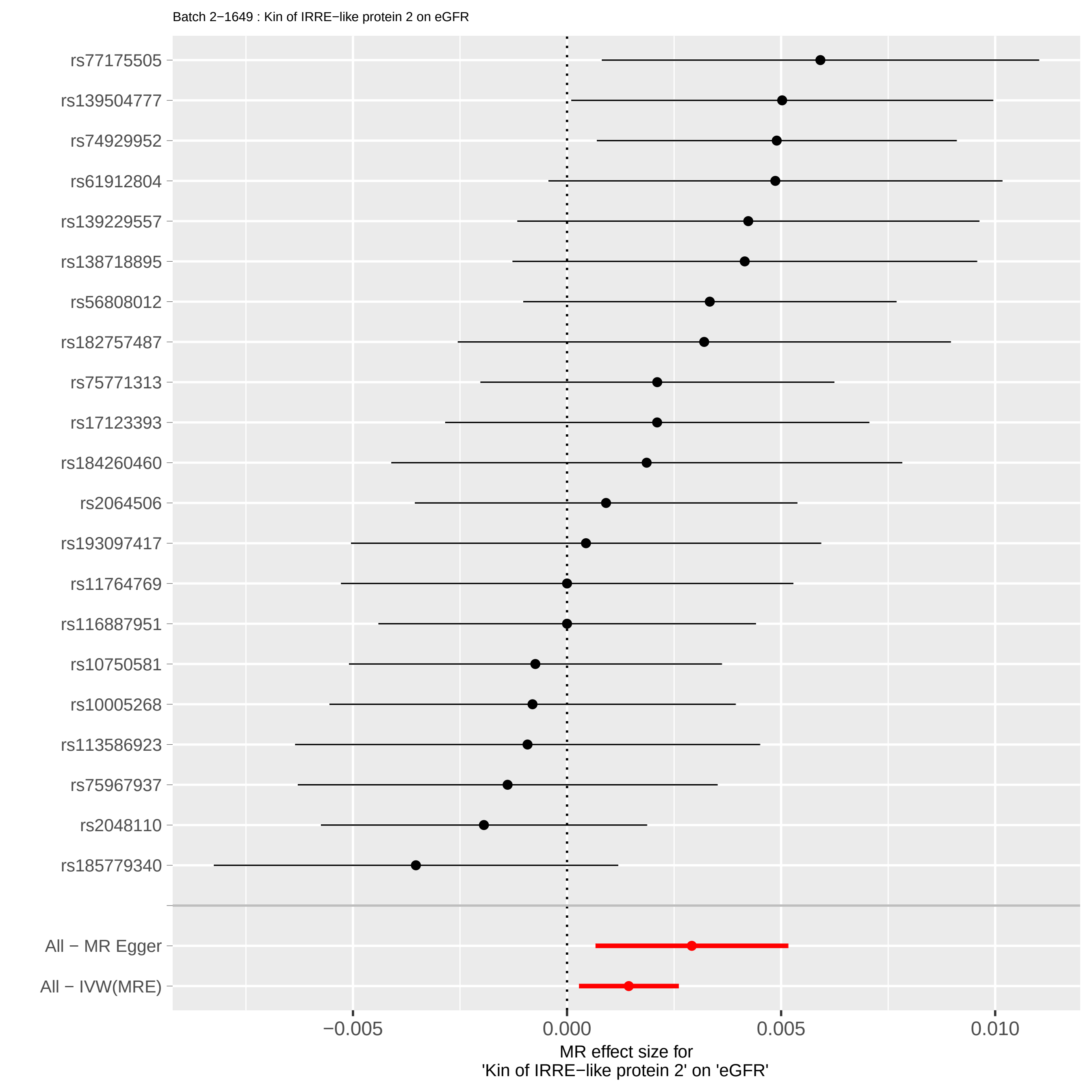

Batch 2−1649 : Kin of IRRE−like protein 2 on eGFR
rs77175505
rs139504777
rs74929952
rs61912804
rs139229557
rs138718895
rs56808012
rs182757487
rs75771313
rs17123393
rs184260460
rs2064506
rs193097417
rs11764769
rs116887951
rs10750581
rs10005268
rs113586923
rs75967937
rs2048110
rs185779340
All − MR Egger
All − IVW(MRE)
−0.005
0.000
0.005
0.010
MR effect size for
'Kin of IRRE−like protein 2' on 'eGFR'

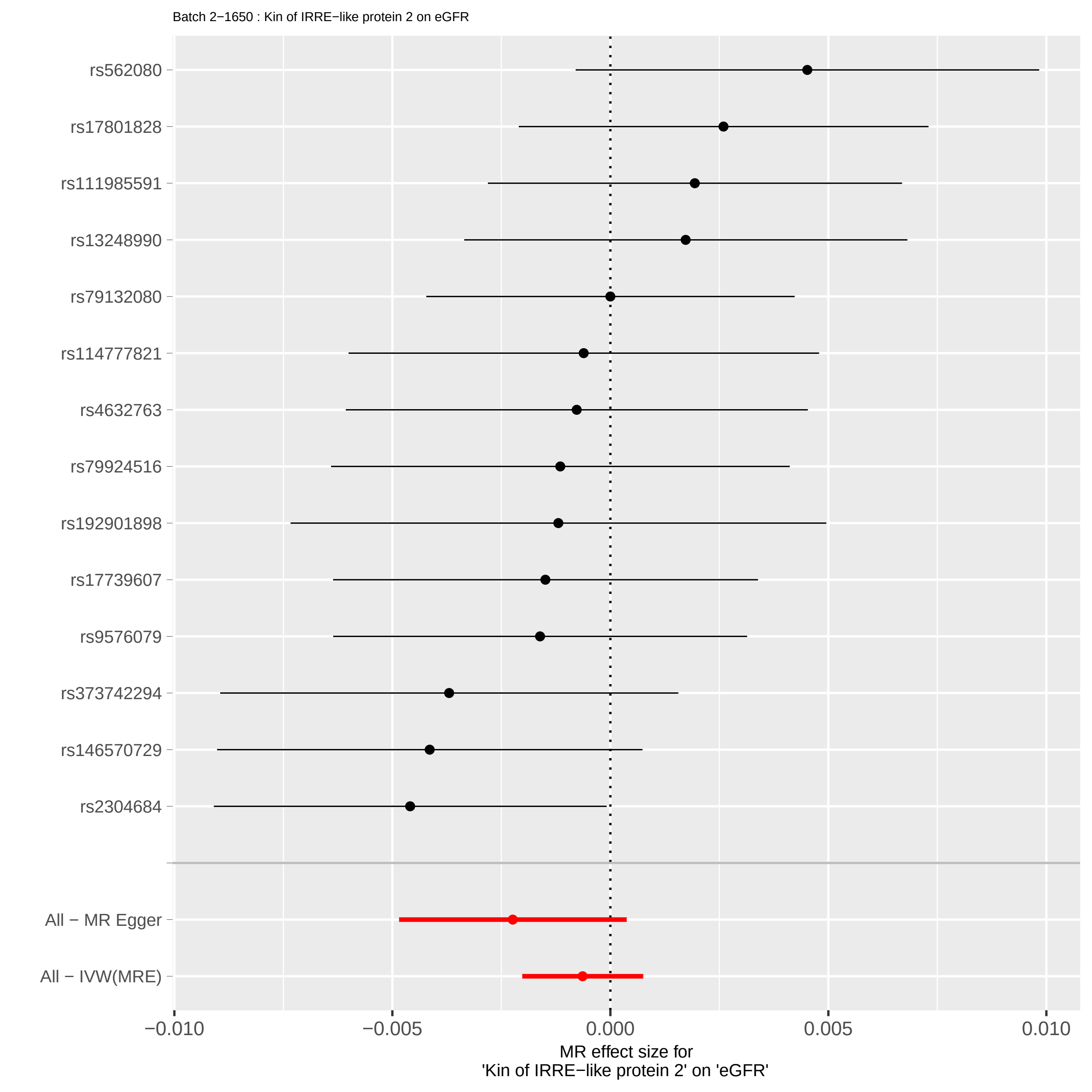

Batch 2−1650 : Kin of IRRE−like protein 2 on eGFR
rs562080
rs17801828
rs111985591
rs13248990
rs79132080
rs114777821
rs4632763
rs79924516
rs192901898
rs17739607
rs9576079
rs373742294
rs146570729
rs2304684
All − MR Egger
All − IVW(MRE)
−0.010
−0.005
0.000
0.005
0.010
MR effect size for
'Kin of IRRE−like protein 2' on 'eGFR'

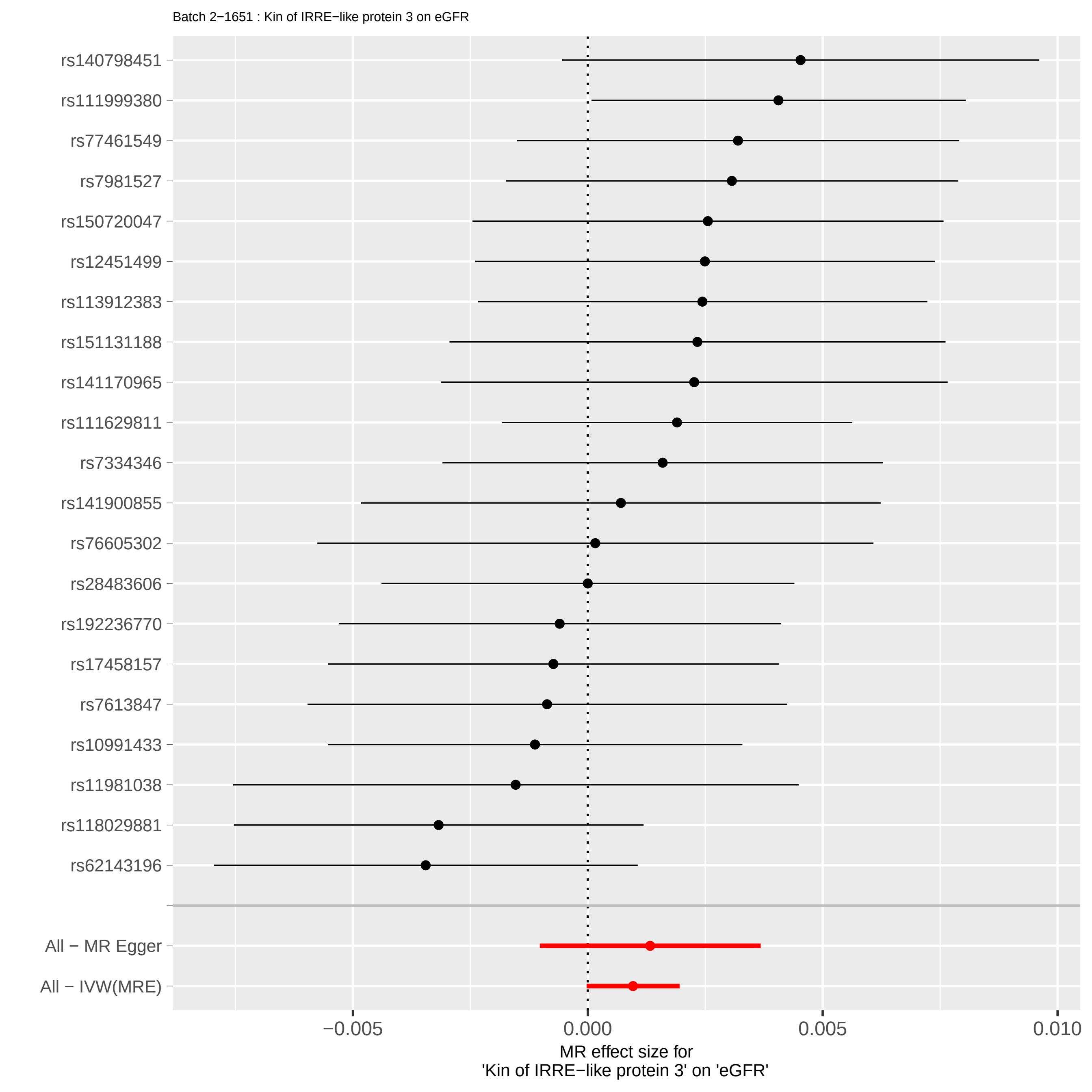

Batch 2−1651 : Kin of IRRE−like protein 3 on eGFR
rs140798451
rs111999380
rs77461549
rs7981527
rs150720047
rs12451499
rs113912383
rs151131188
rs141170965
rs111629811
rs7334346
rs141900855
rs76605302
rs28483606
rs192236770
rs17458157
rs7613847
rs10991433
rs11981038
rs118029881
rs62143196
All − MR Egger
All − IVW(MRE)
−0.005
0.000
0.005
0.010
MR effect size for
'Kin of IRRE−like protein 3' on 'eGFR'

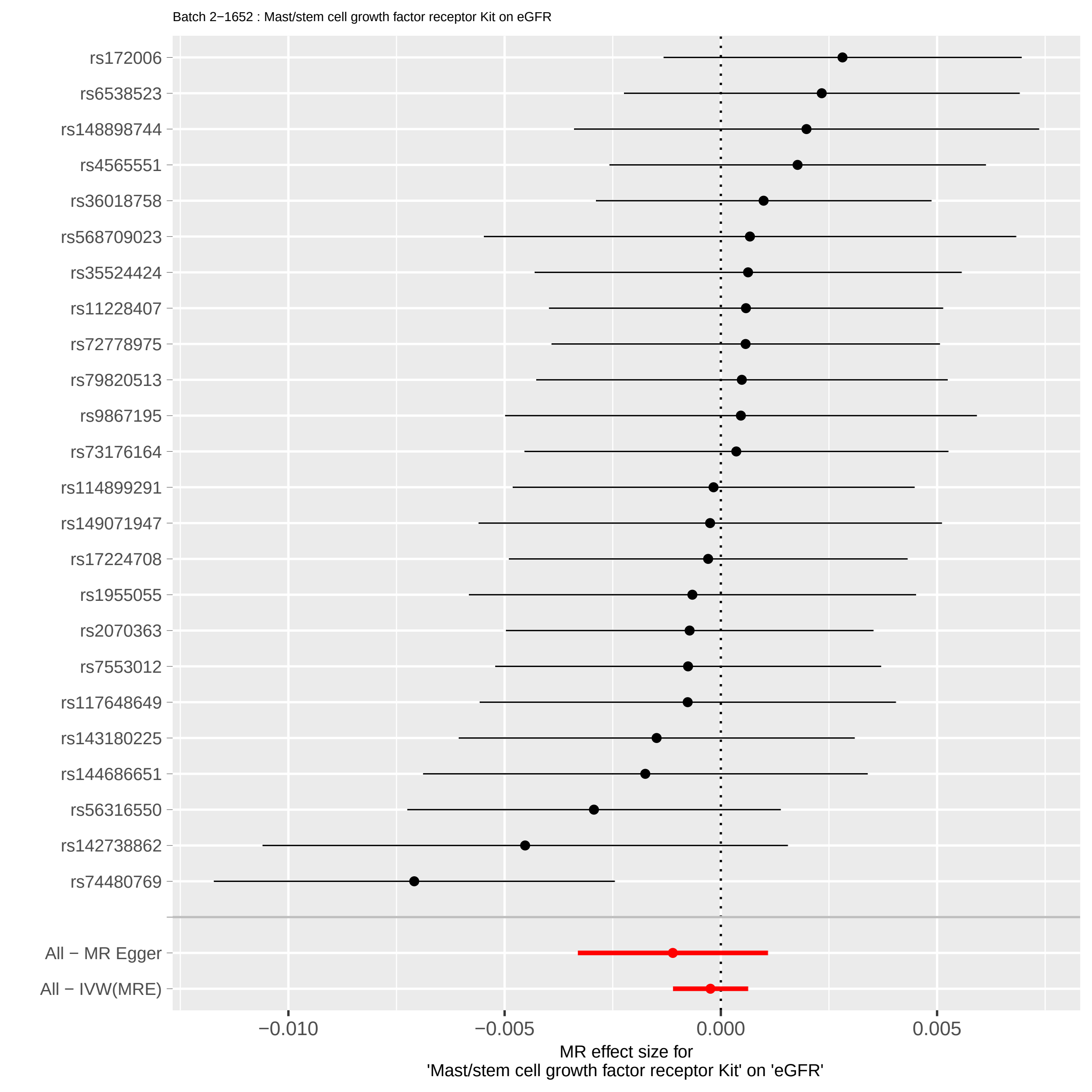
Batch 2−1652 : Mast/stem cell growth factor receptor Kit on eGFR
rs172006
rs6538523
rs148898744
rs4565551
rs36018758
rs568709023
rs35524424
rs11228407
rs72778975
rs79820513
rs9867195
rs73176164
rs114899291
rs149071947
rs17224708
rs1955055
rs2070363
rs7553012
rs117648649
rs143180225
rs144686651
rs56316550
rs142738862
rs74480769
All − MR Egger
All − IVW(MRE)
−0.010
−0.005
0.000
0.005
MR effect size for
'Mast/stem cell growth factor receptor Kit' on 'eGFR'

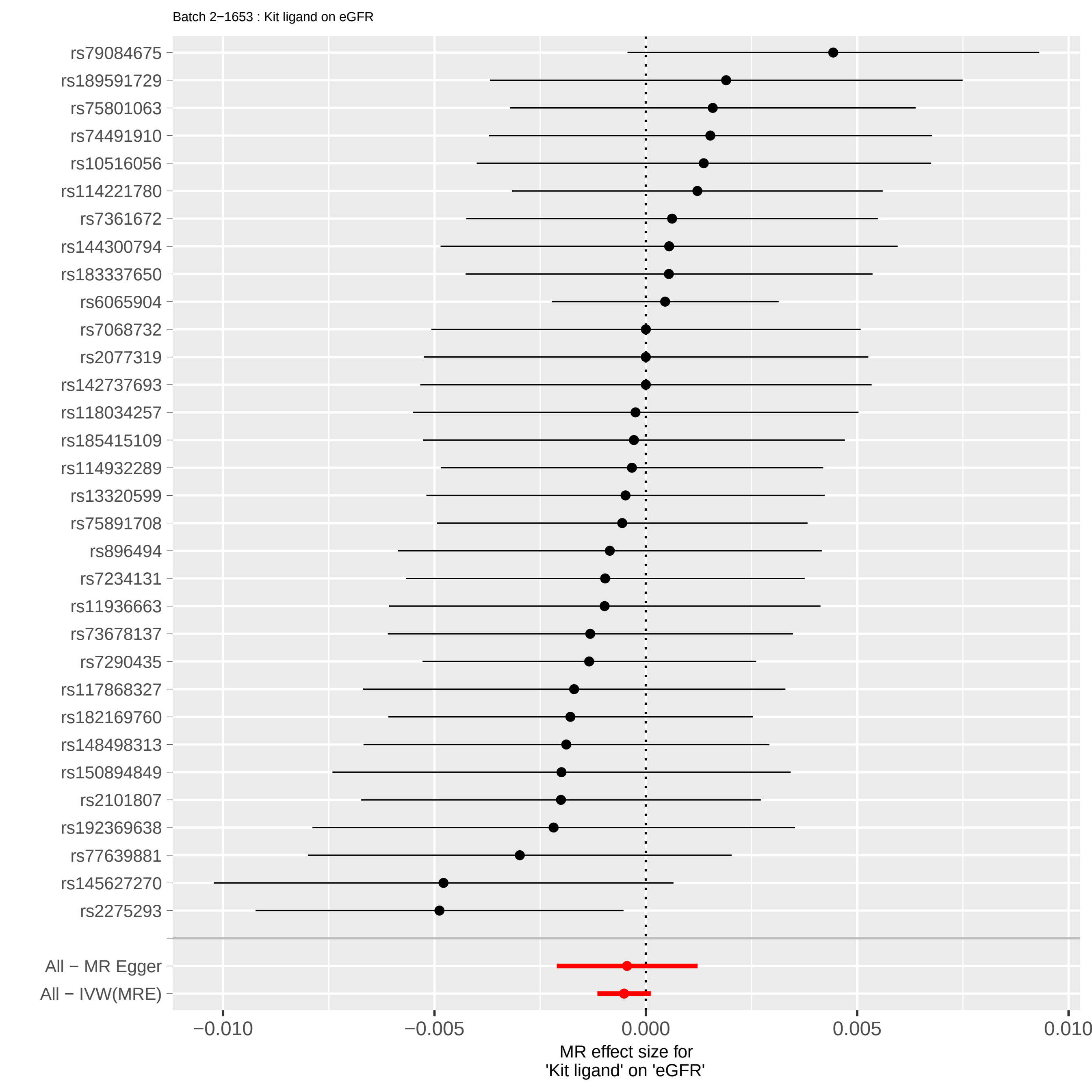

Batch 2−1653 : Kit ligand on eGFR
rs79084675
rs189591729
rs75801063
rs74491910
rs10516056
rs114221780
rs7361672
rs144300794
rs183337650
rs6065904
rs7068732
rs2077319
rs142737693
rs118034257
rs185415109
rs114932289
rs13320599
rs75891708
rs896494
rs7234131
rs11936663
rs73678137
rs7290435
rs117868327
rs182169760
rs148498313
rs150894849
rs2101807
rs192369638
rs77639881
rs145627270
rs2275293
All − MR Egger
All − IVW(MRE)
−0.010
−0.005
0.000
0.005
0.010
MR effect size for
'Kit ligand' on 'eGFR'

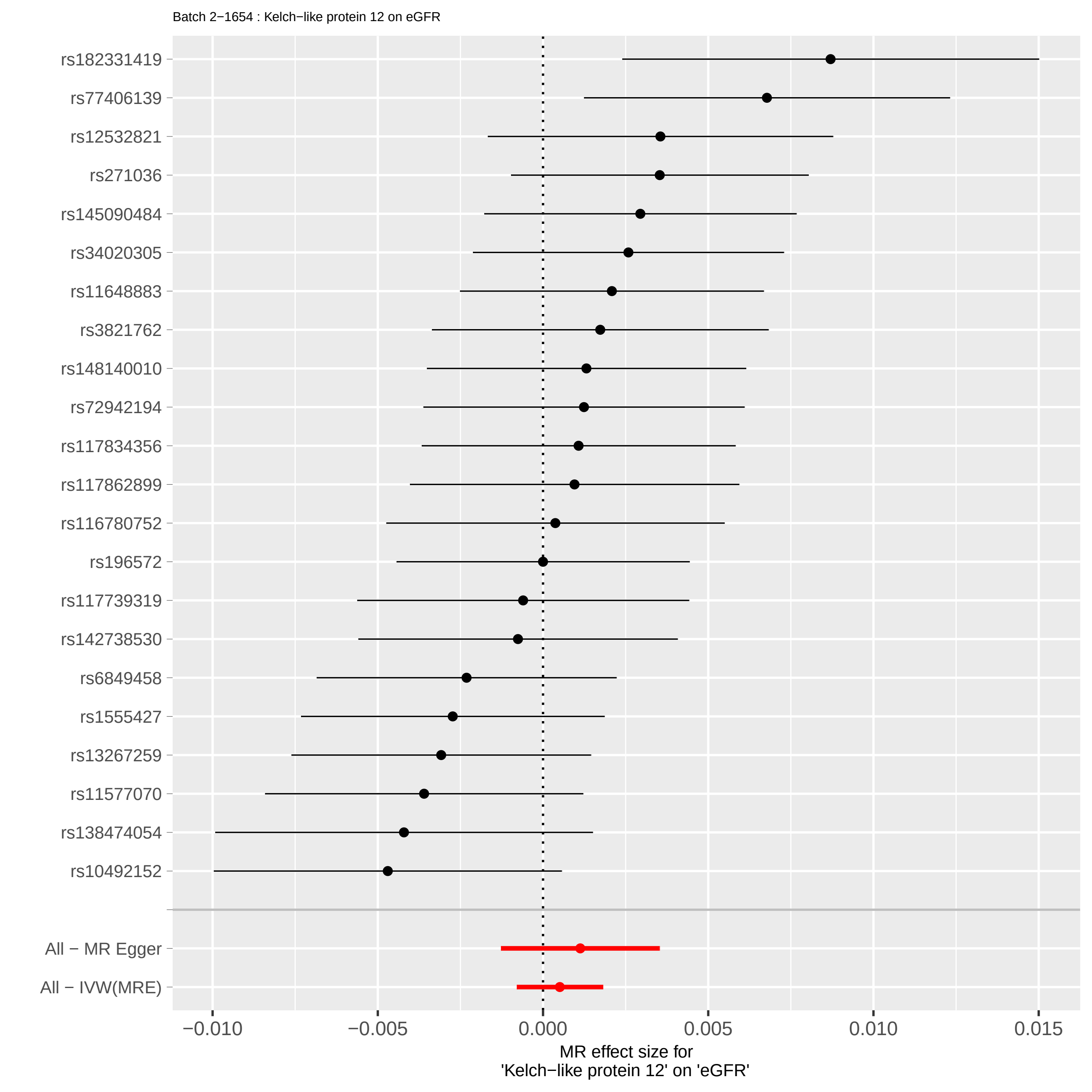

Batch 2−1654 : Kelch−like protein 12 on eGFR
rs182331419
rs77406139
rs12532821
rs271036
rs145090484
rs34020305
rs11648883
rs3821762
rs148140010
rs72942194
rs117834356
rs117862899
rs116780752
rs196572
rs117739319
rs142738530
rs6849458
rs1555427
rs13267259
rs11577070
rs138474054
rs10492152
All − MR Egger
All − IVW(MRE)
−0.010
−0.005
0.000
0.005
0.010
0.015
MR effect size for
'Kelch−like protein 12' on 'eGFR'

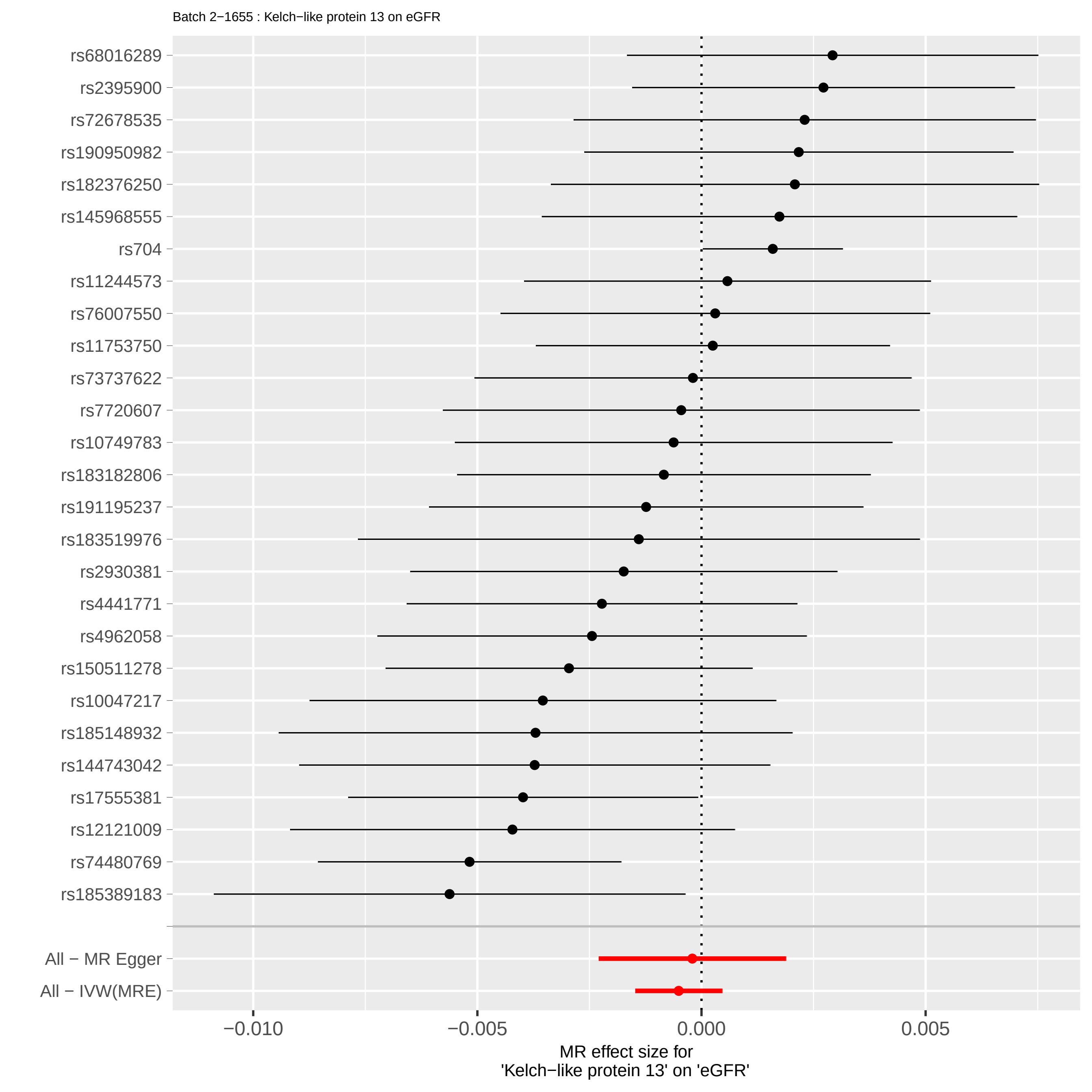

Batch 2−1655 : Kelch−like protein 13 on eGFR
rs68016289
rs2395900
rs72678535
rs190950982
rs182376250
rs145968555
rs704
rs11244573
rs76007550
rs11753750
rs73737622
rs7720607
rs10749783
rs183182806
rs191195237
rs183519976
rs2930381
rs4441771
rs4962058
rs150511278
rs10047217
rs185148932
rs144743042
rs17555381
rs12121009
rs74480769
rs185389183
All − MR Egger
All − IVW(MRE)
−0.010
−0.005
0.000
0.005
MR effect size for
'Kelch−like protein 13' on 'eGFR'

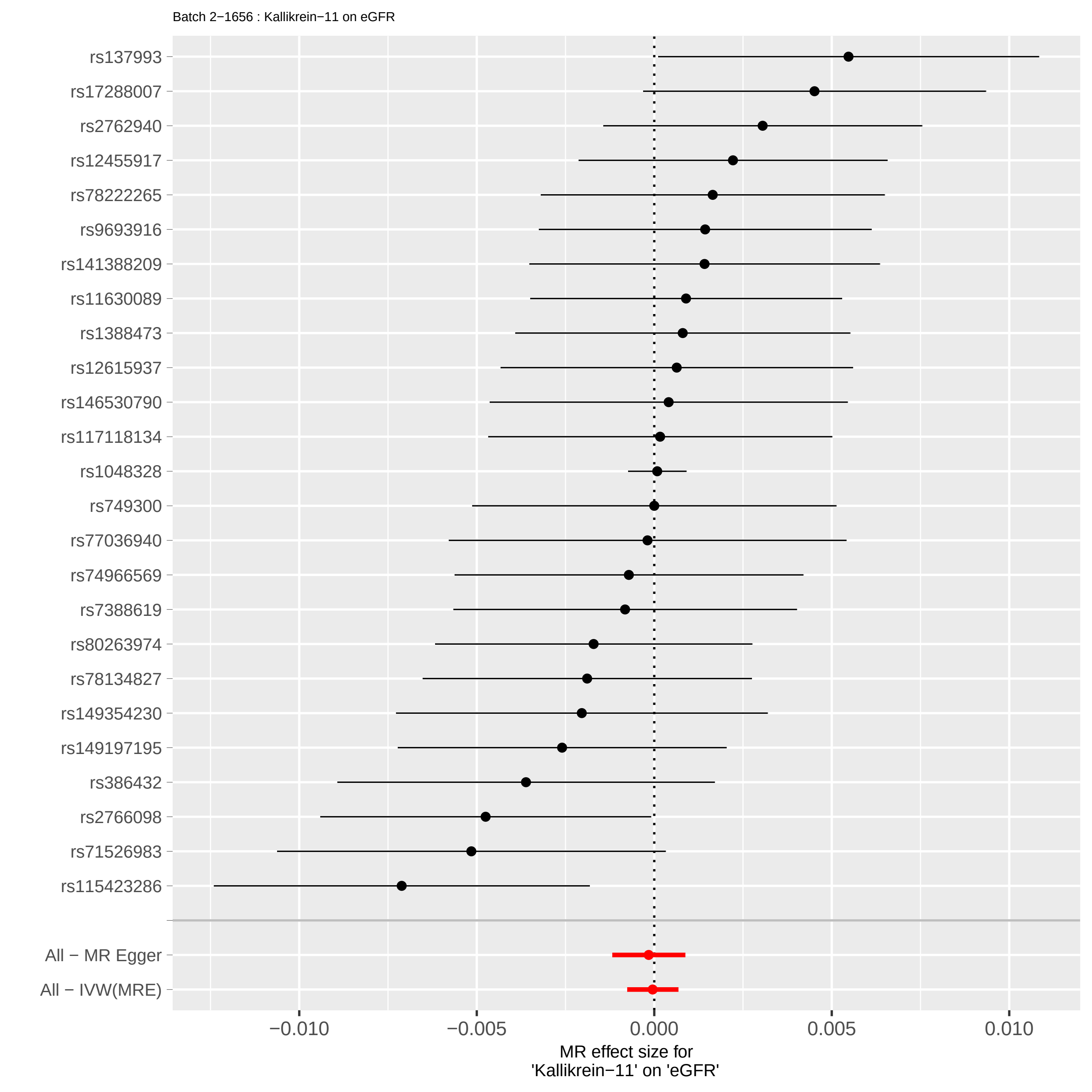
Batch 2−1656 : Kallikrein−11 on eGFR
rs137993
rs17288007
rs2762940
rs12455917
rs78222265
rs9693916
rs141388209
rs11630089
rs1388473
rs12615937
rs146530790
rs117118134
rs1048328
rs749300
rs77036940
rs74966569
rs7388619
rs80263974
rs78134827
rs149354230
rs149197195
rs386432
rs2766098
rs71526983
rs115423286
All − MR Egger
All − IVW(MRE)
−0.010
−0.005
0.000
0.005
0.010
MR effect size for
'Kallikrein−11' on 'eGFR'

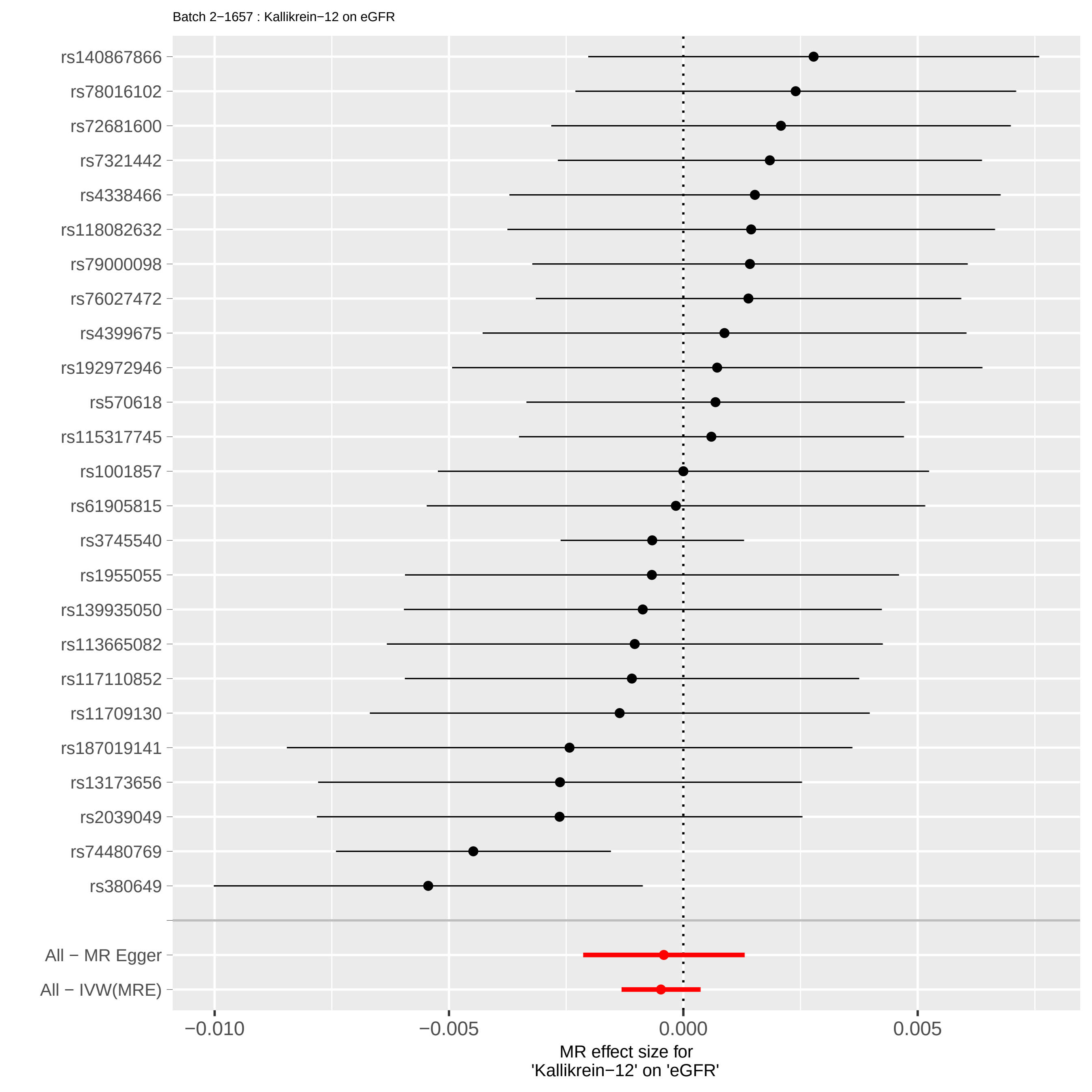

Batch 2−1657 : Kallikrein−12 on eGFR
rs140867866
rs78016102
rs72681600
rs7321442
rs4338466
rs118082632
rs79000098
rs76027472
rs4399675
rs192972946
rs570618
rs115317745
rs1001857
rs61905815
rs3745540
rs1955055
rs139935050
rs113665082
rs117110852
rs11709130
rs187019141
rs13173656
rs2039049
rs74480769
rs380649
All − MR Egger
All − IVW(MRE)
−0.010
−0.005
0.000
0.005
MR effect size for
'Kallikrein−12' on 'eGFR'

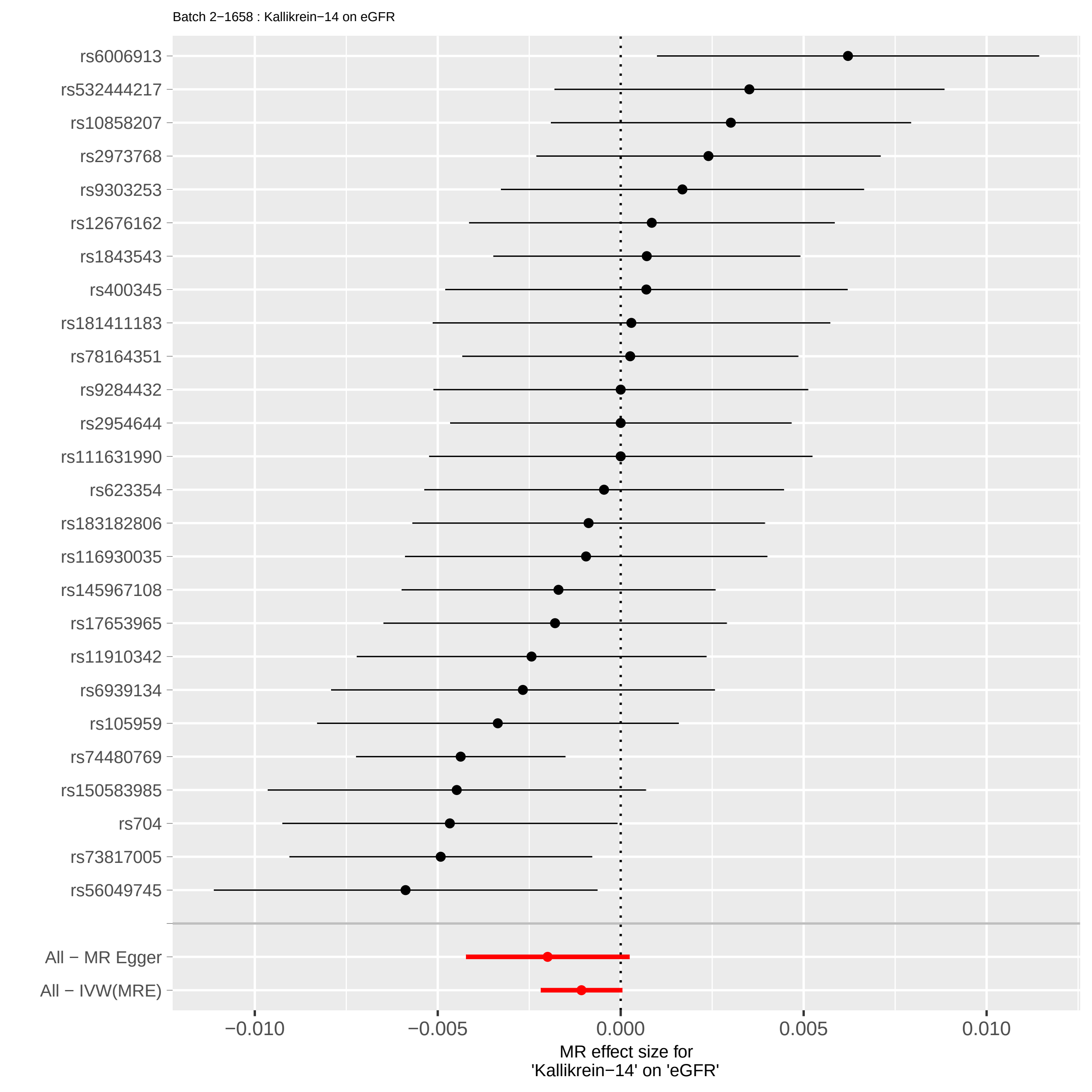

Batch 2−1658 : Kallikrein−14 on eGFR
rs6006913
rs532444217
rs10858207
rs2973768
rs9303253
rs12676162
rs1843543
rs400345
rs181411183
rs78164351
rs9284432
rs2954644
rs111631990
rs623354
rs183182806
rs116930035
rs145967108
rs17653965
rs11910342
rs6939134
rs105959
rs74480769
rs150583985
rs704
rs73817005
rs56049745
All − MR Egger
All − IVW(MRE)
−0.010
−0.005
0.000
0.005
0.010
MR effect size for
'Kallikrein−14' on 'eGFR'

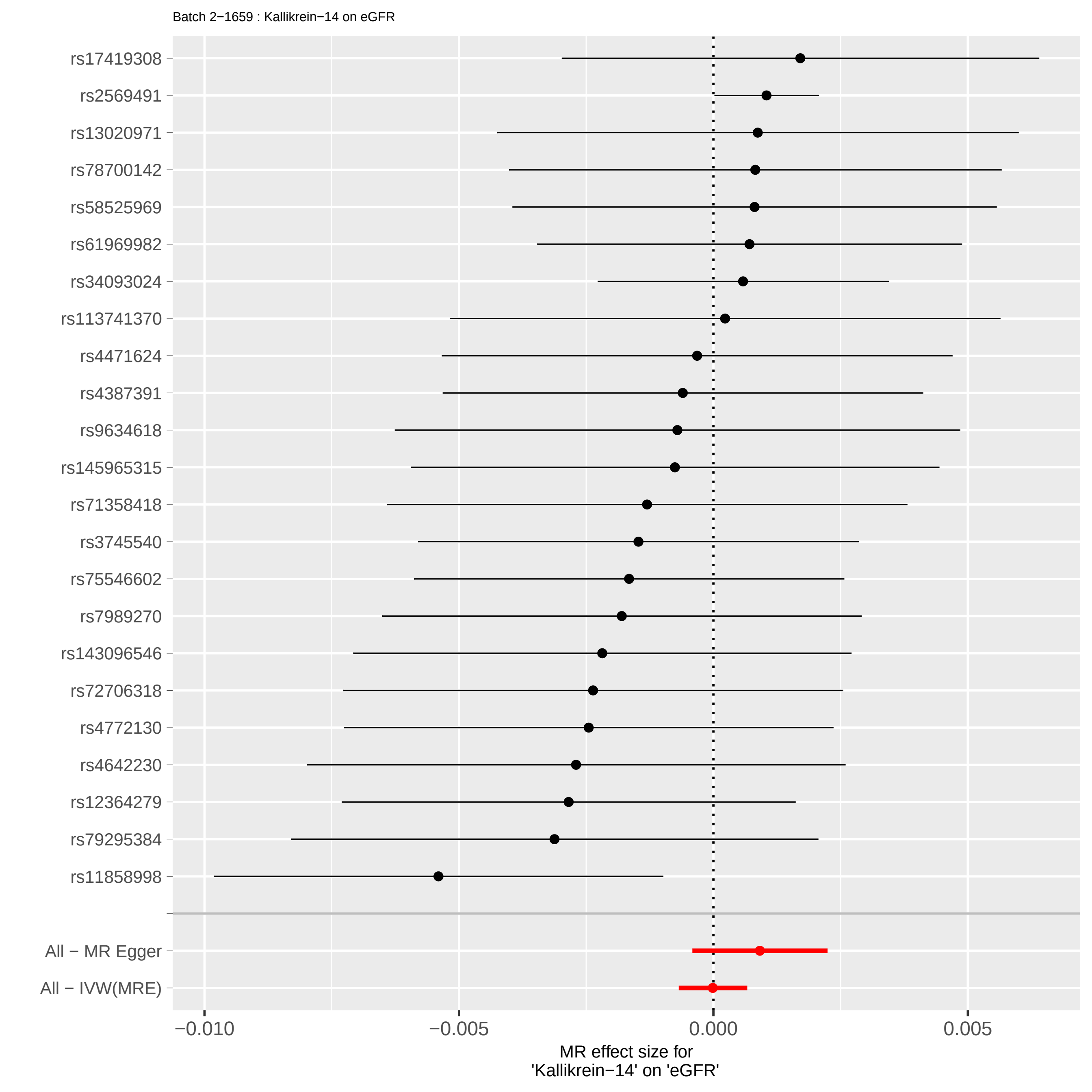

Batch 2−1659 : Kallikrein−14 on eGFR
rs17419308
rs2569491
rs13020971
rs78700142
rs58525969
rs61969982
rs34093024
rs113741370
rs4471624
rs4387391
rs9634618
rs145965315
rs71358418
rs3745540
rs75546602
rs7989270
rs143096546
rs72706318
rs4772130
rs4642230
rs12364279
rs79295384
rs11858998
All − MR Egger
All − IVW(MRE)
−0.010
−0.005
0.000
0.005
MR effect size for
'Kallikrein−14' on 'eGFR'

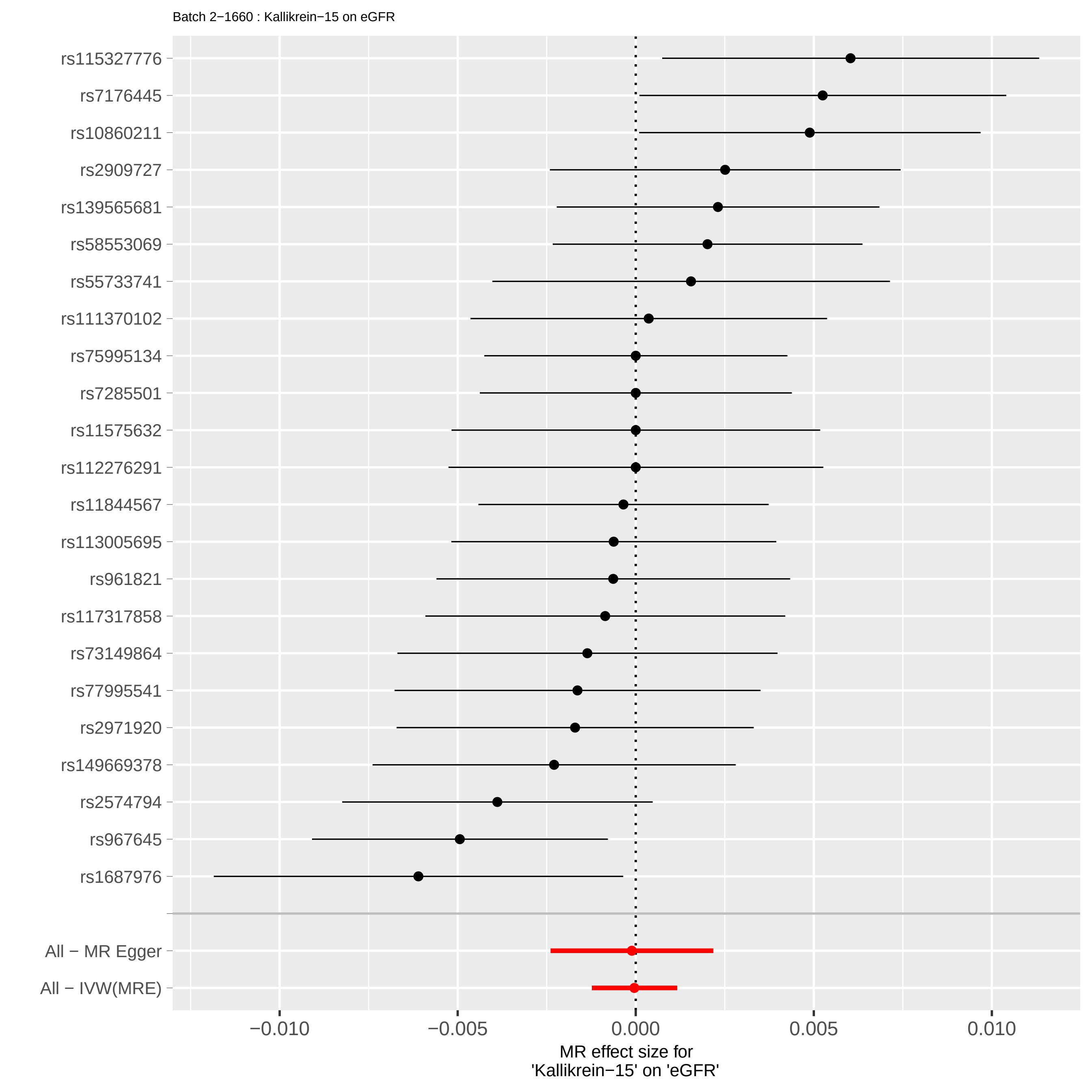

Batch 2−1660 : Kallikrein−15 on eGFR
rs115327776
rs7176445
rs10860211
rs2909727
rs139565681
rs58553069
rs55733741
rs111370102
rs75995134
rs7285501
rs11575632
rs112276291
rs11844567
rs113005695
rs961821
rs117317858
rs73149864
rs77995541
rs2971920
rs149669378
rs2574794
rs967645
rs1687976
All − MR Egger
All − IVW(MRE)
−0.010
−0.005
0.000
0.005
0.010
MR effect size for
'Kallikrein−15' on 'eGFR'

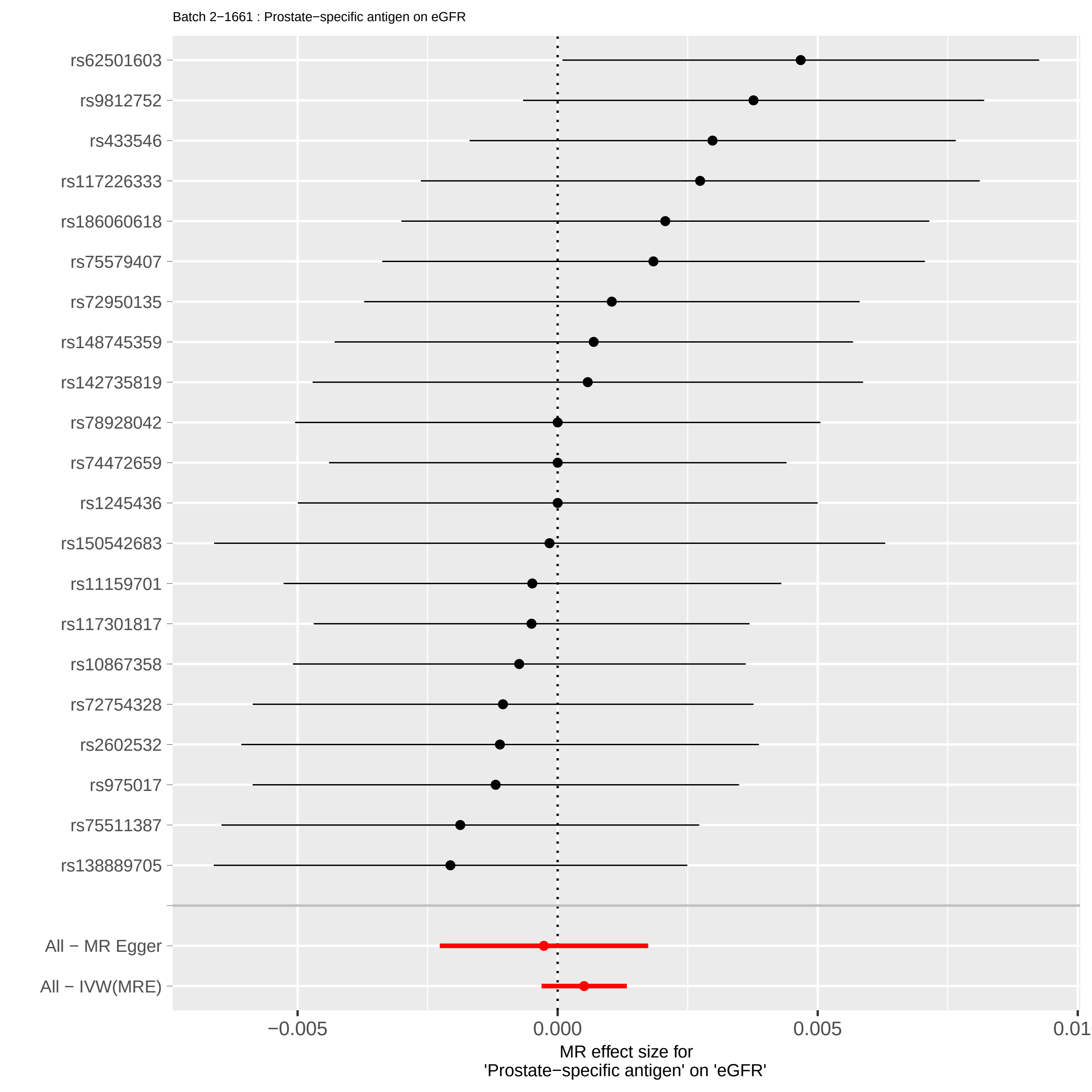
Batch 2−1661 : Prostate−specific antigen on eGFR
rs62501603
rs9812752
rs433546
rs117226333
rs186060618
rs75579407
rs72950135
rs148745359
rs142735819
rs78928042
rs74472659
rs1245436
rs150542683
rs11159701
rs117301817
rs10867358
rs72754328
rs2602532
rs975017
rs75511387
rs138889705
All − MR Egger
All − IVW(MRE)
−0.005
0.000
0.005
0.01
MR effect size for
'Prostate−specific antigen' on 'eGFR'

Batch 2−1662 : Kallikrein−4 on eGFR

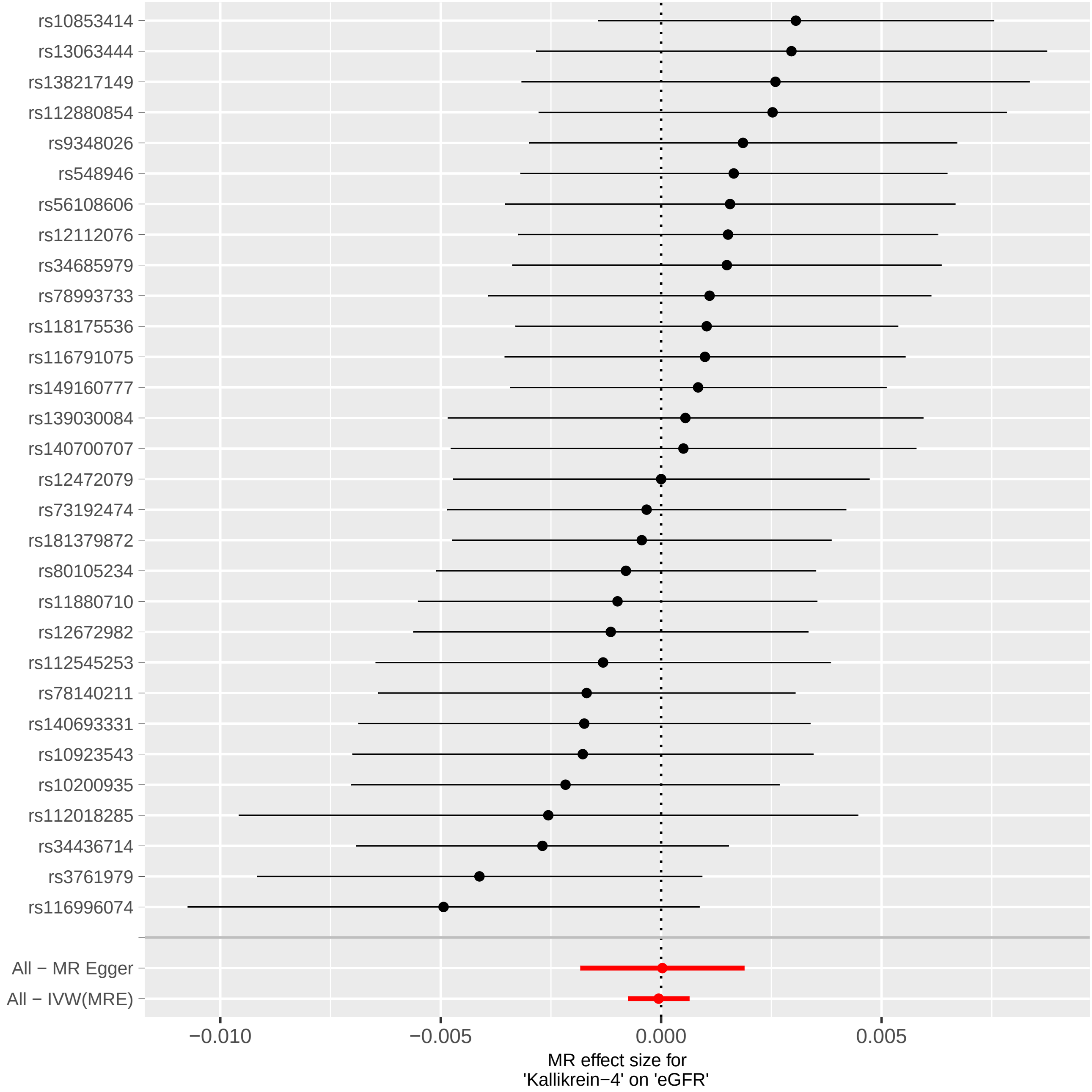

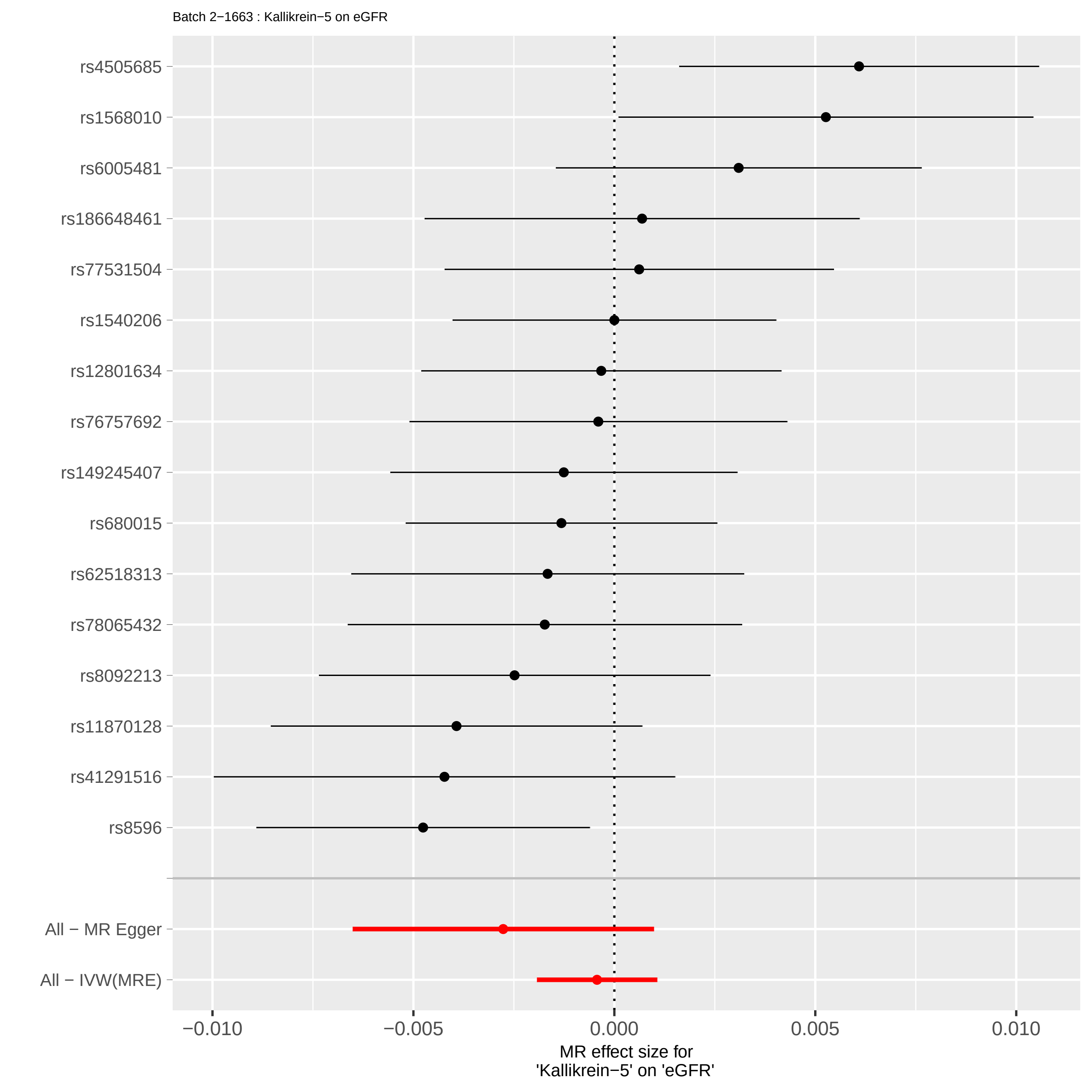

Batch 2−1663 : Kallikrein−5 on eGFR
rs4505685
rs1568010
rs6005481
rs186648461
rs77531504
rs1540206
rs12801634
rs76757692
rs149245407
rs680015
rs62518313
rs78065432
rs8092213
rs11870128
rs41291516
rs8596
All − MR Egger
All − IVW(MRE)
−0.010
−0.005
0.000
0.005
0.010
MR effect size for
'Kallikrein−5' on 'eGFR'

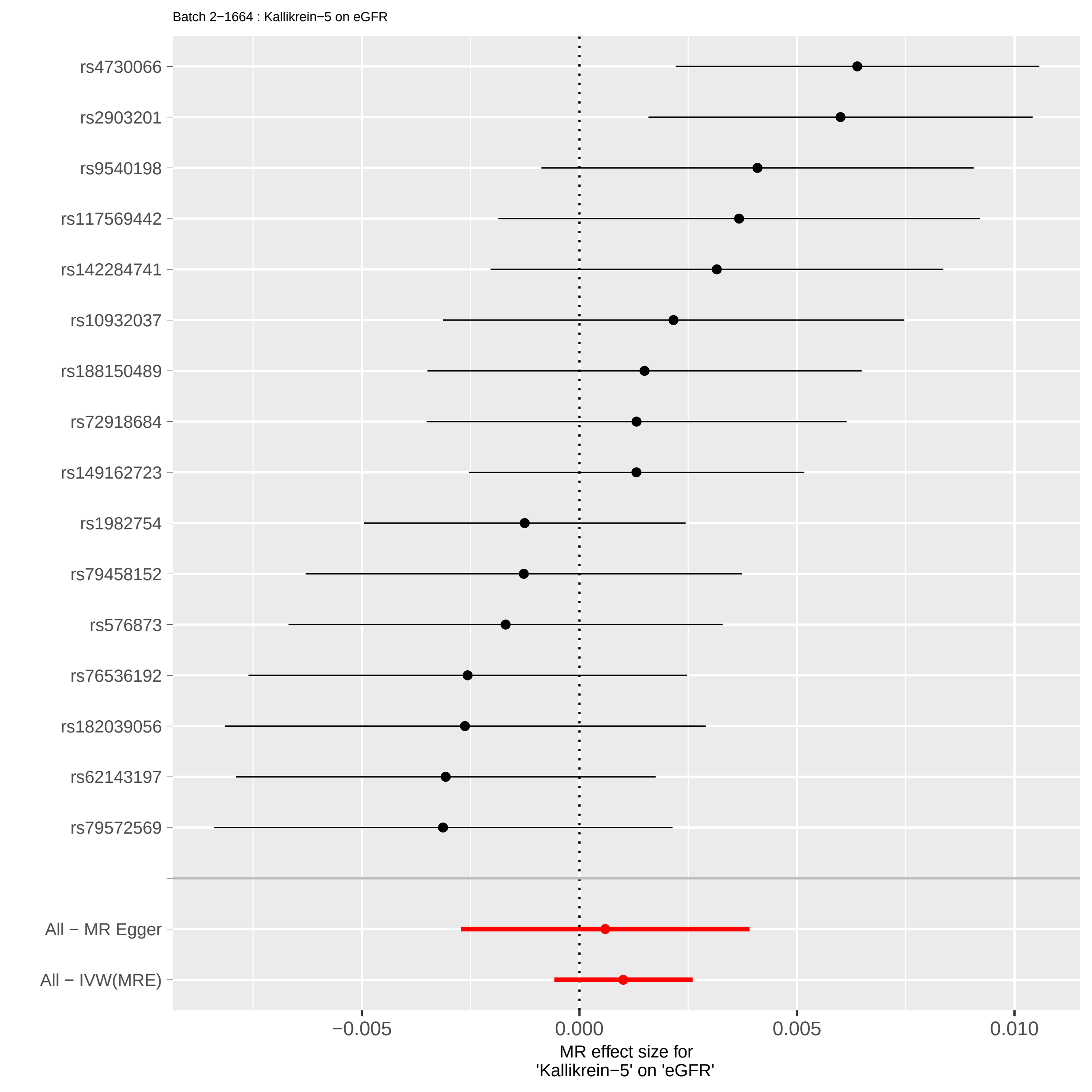

Batch 2−1664 : Kallikrein−5 on eGFR
rs4730066
rs2903201
rs9540198
rs117569442
rs142284741
rs10932037
rs188150489
rs72918684
rs149162723
rs1982754
rs79458152
rs576873
rs76536192
rs182039056
rs62143197
rs79572569
All − MR Egger
All − IVW(MRE)
−0.005
0.000
0.005
0.010
MR effect size for
'Kallikrein−5' on 'eGFR'

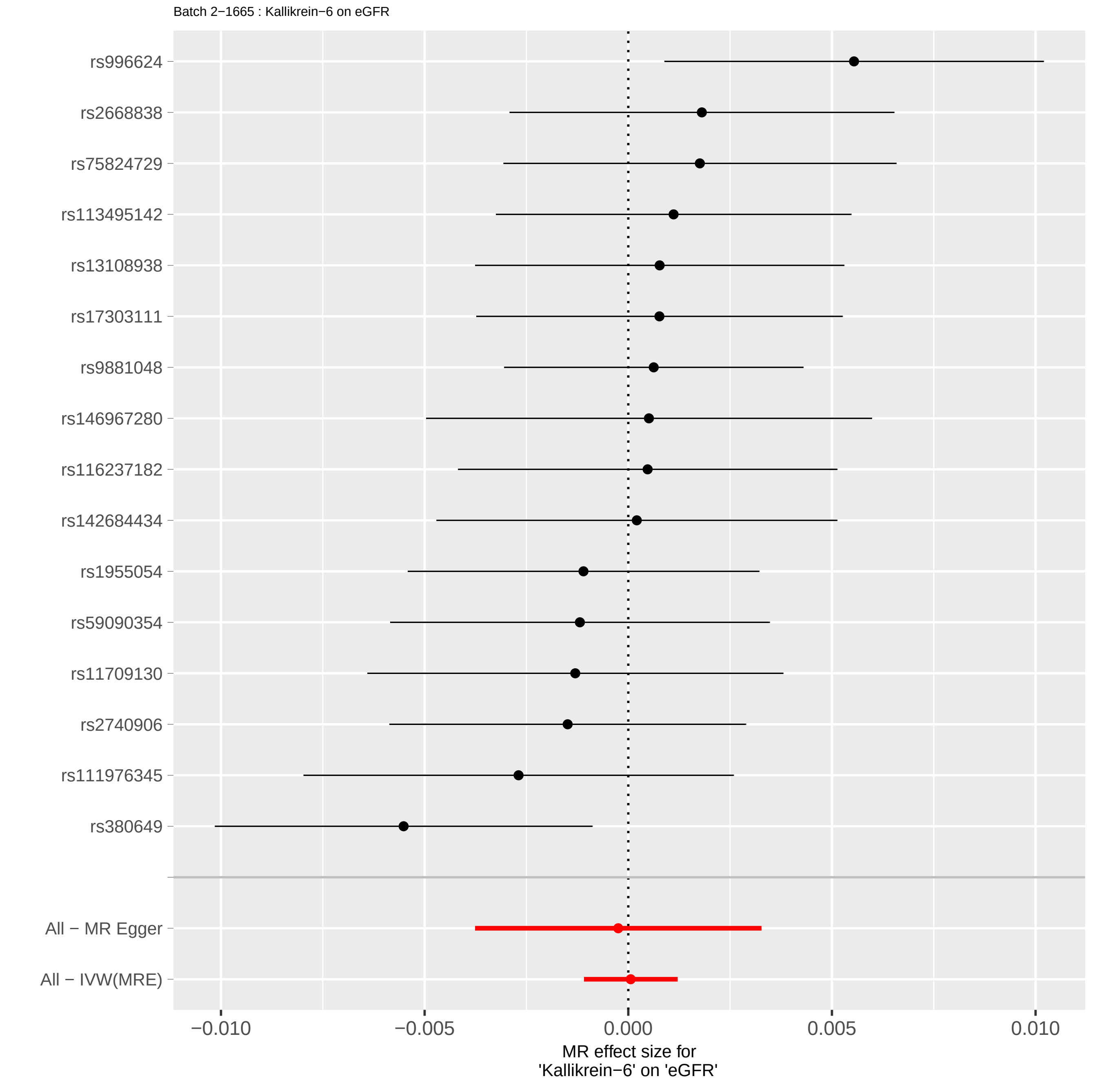

Batch 2−1665 : Kallikrein−6 on eGFR
rs996624
rs2668838
rs75824729
rs113495142
rs13108938
rs17303111
rs9881048
rs146967280
rs116237182
rs142684434
rs1955054
rs59090354
rs11709130
rs2740906
rs111976345
rs380649
All − MR Egger
All − IVW(MRE)
−0.010
−0.005
0.000
0.005
0.010
MR effect size for
'Kallikrein−6' on 'eGFR'

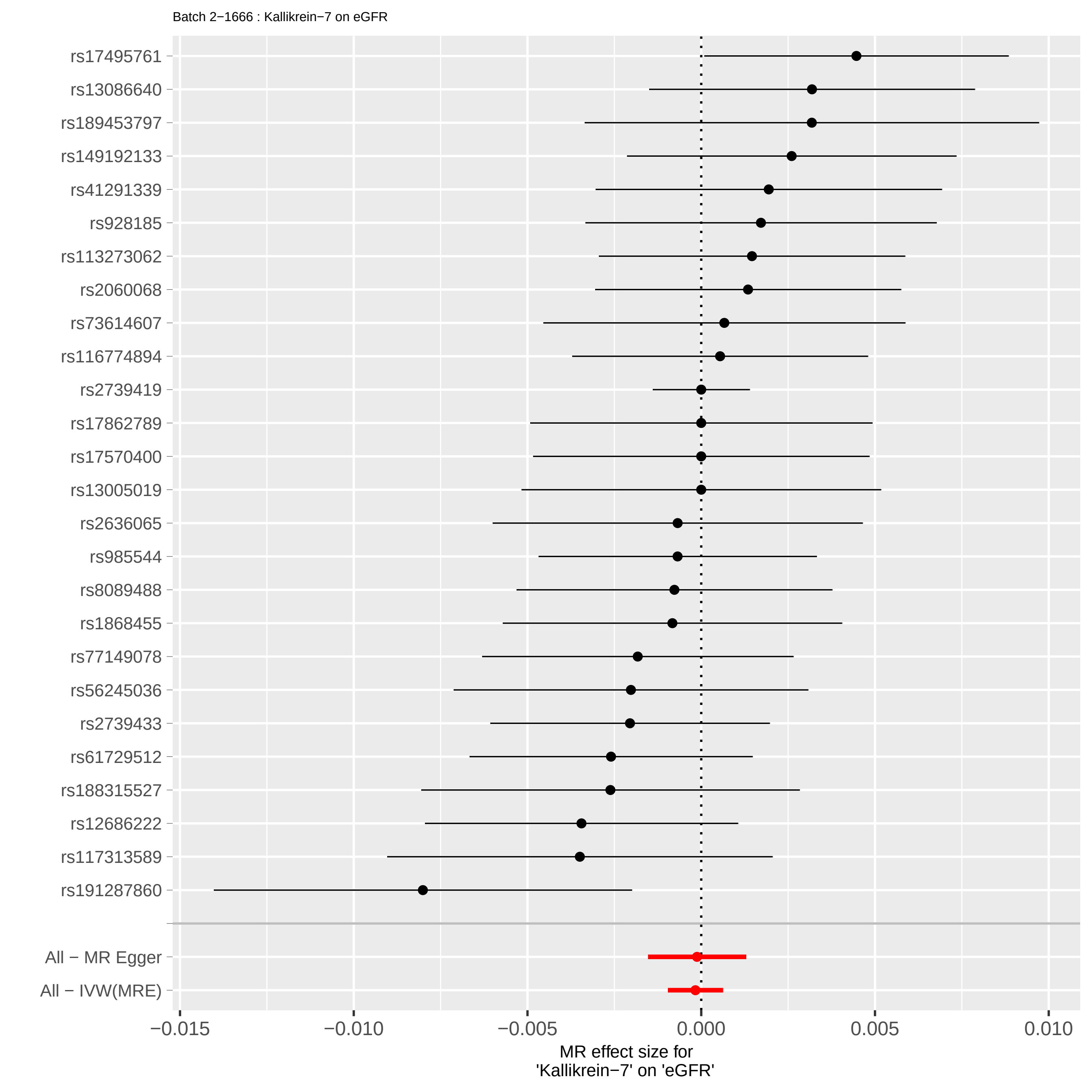

Batch 2−1666 : Kallikrein−7 on eGFR
rs17495761
rs13086640
rs189453797
rs149192133
rs41291339
rs928185
rs113273062
rs2060068
rs73614607
rs116774894
rs2739419
rs17862789
rs17570400
rs13005019
rs2636065
rs985544
rs8089488
rs1868455
rs77149078
rs56245036
rs2739433
rs61729512
rs188315527
rs12686222
rs117313589
rs191287860
All − MR Egger
All − IVW(MRE)
−0.015
−0.010
−0.005
0.000
0.005
0.010
MR effect size for
'Kallikrein−7' on 'eGFR'

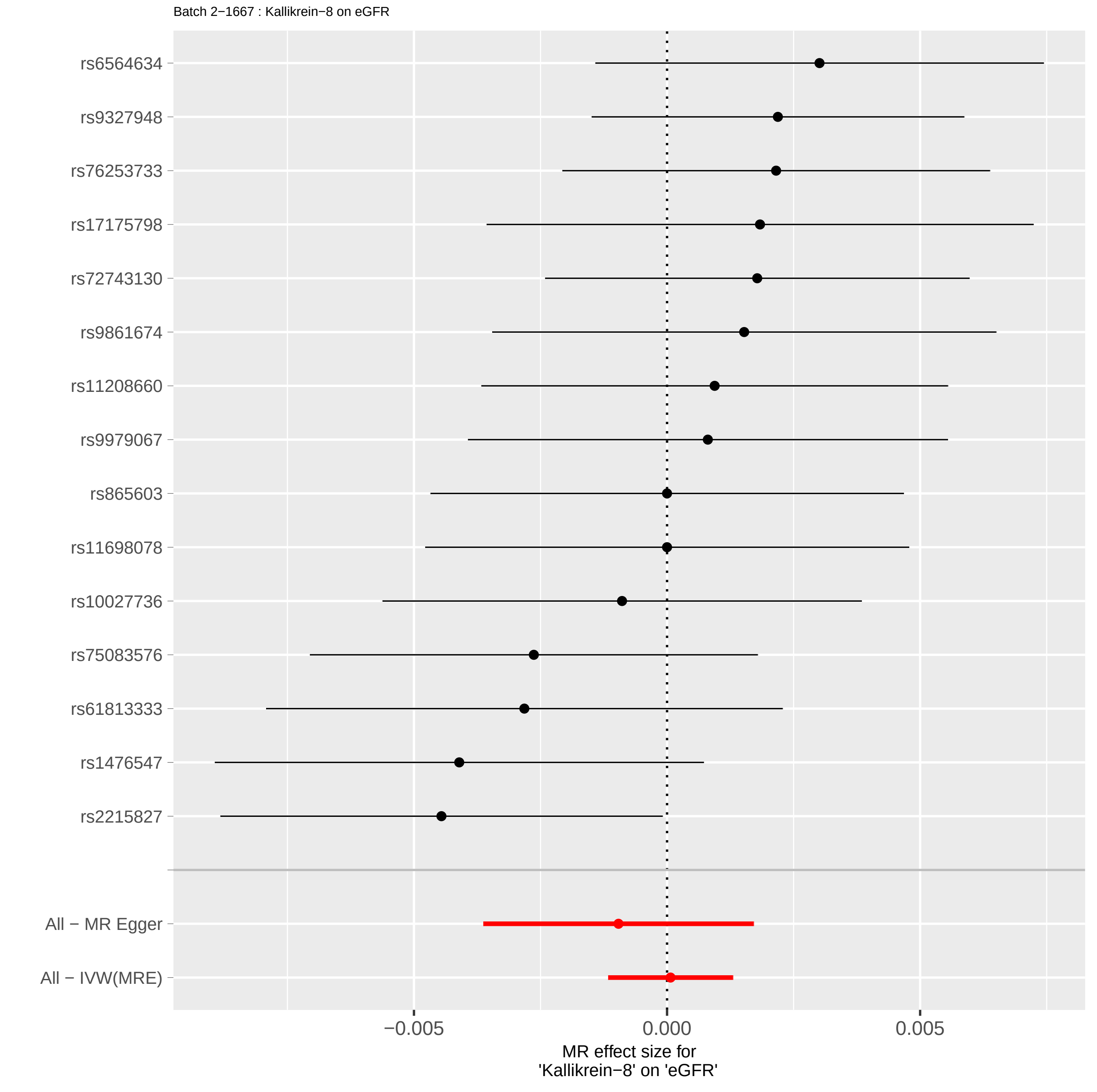

Batch 2−1667 : Kallikrein−8 on eGFR
rs6564634
rs9327948
rs76253733
rs17175798
rs72743130
rs9861674
rs11208660
rs9979067
rs865603
rs11698078
rs10027736
rs75083576
rs61813333
rs1476547
rs2215827
All − MR Egger
All − IVW(MRE)
−0.005
0.000
0.005
MR effect size for
'Kallikrein−8' on 'eGFR'

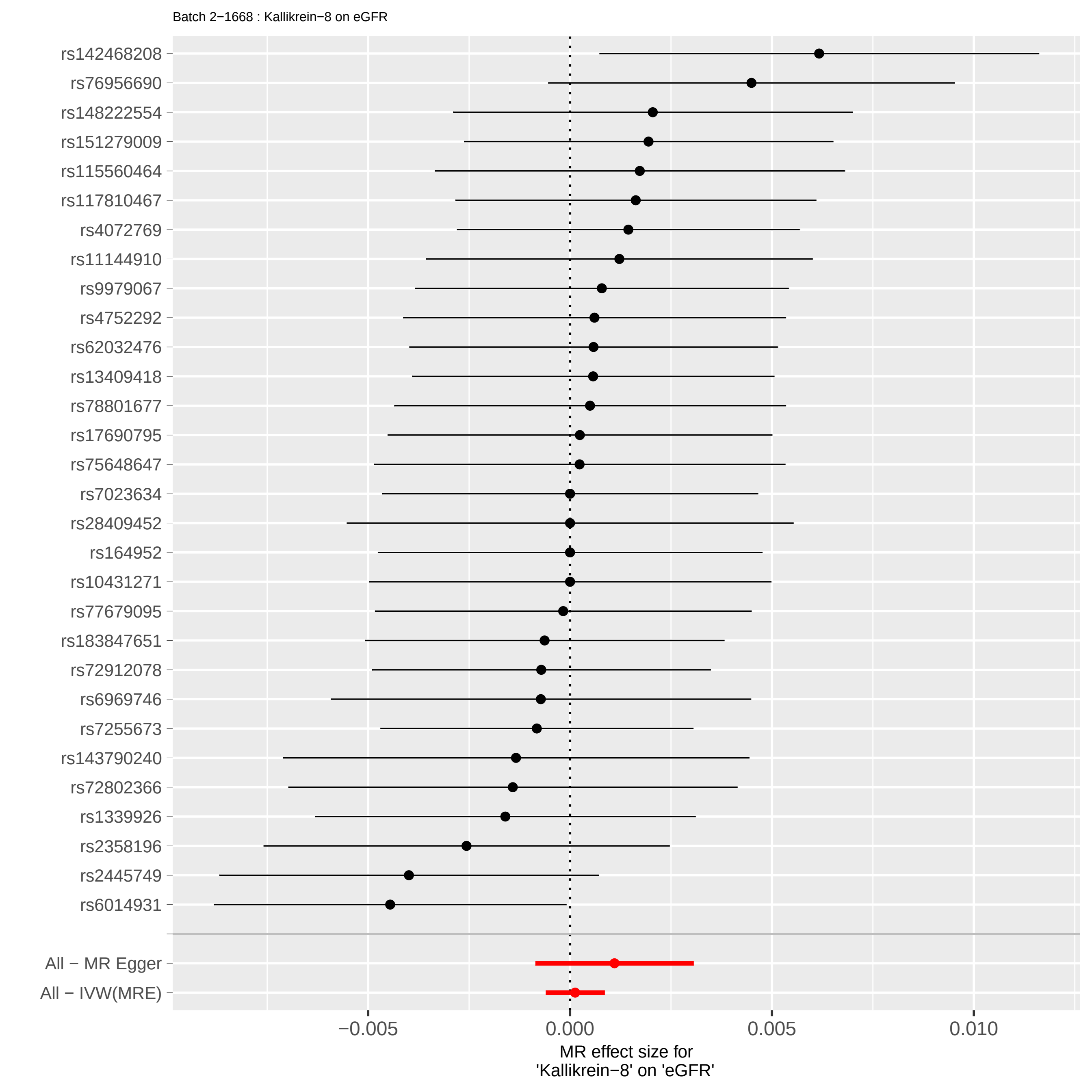

Batch 2−1668 : Kallikrein−8 on eGFR
rs142468208
rs76956690
rs148222554
rs151279009
rs115560464
rs117810467
rs4072769
rs11144910
rs9979067
rs4752292
rs62032476
rs13409418
rs78801677
rs17690795
rs75648647
rs7023634
rs28409452
rs164952
rs10431271
rs77679095
rs183847651
rs72912078
rs6969746
rs7255673
rs143790240
rs72802366
rs1339926
rs2358196
rs2445749
rs6014931
All − MR Egger
All − IVW(MRE)
−0.005
0.000
0.005
0.010
MR effect size for
'Kallikrein−8' on 'eGFR'

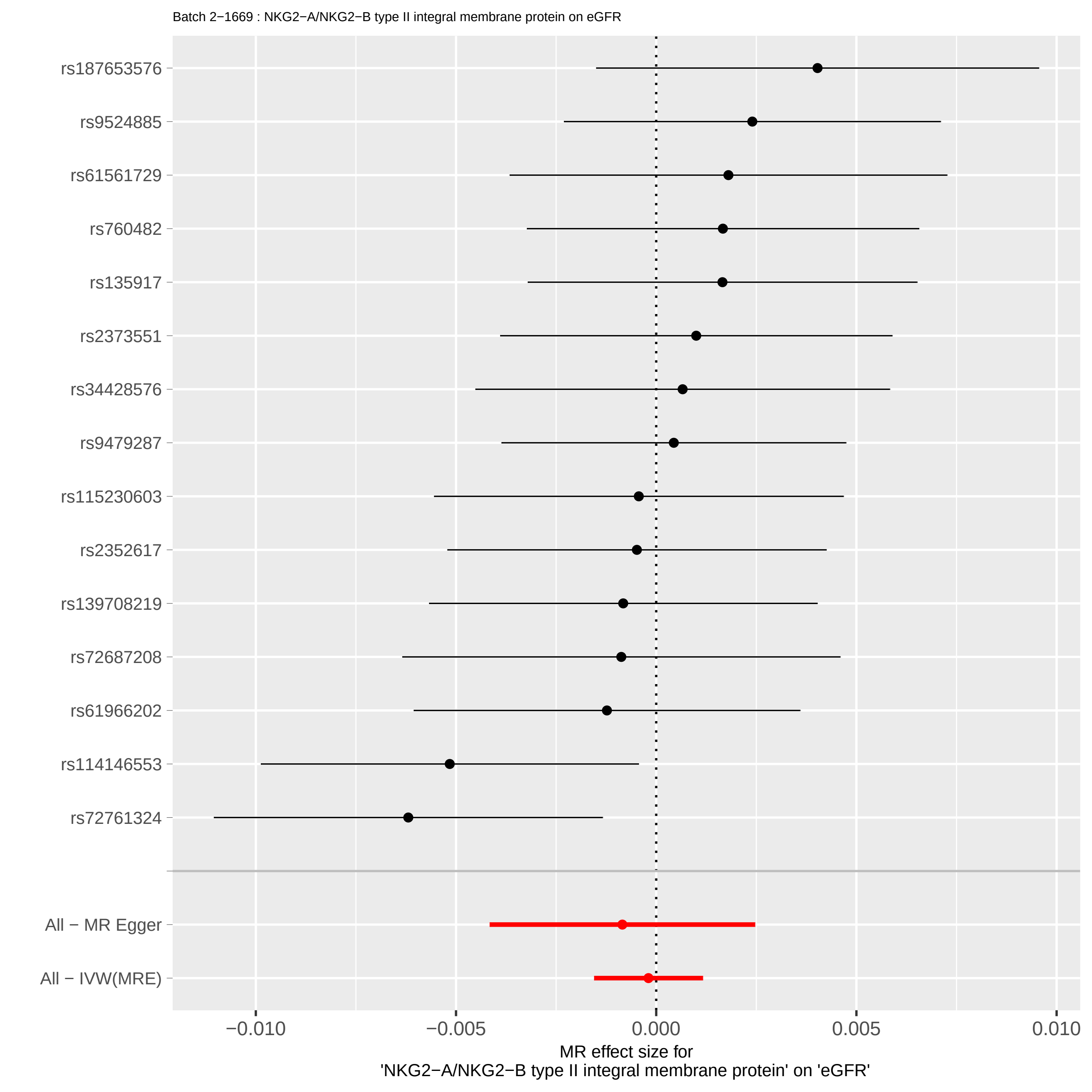

Batch 2−1669 : NKG2−A/NKG2−B type II integral membrane protein on eGFR
rs187653576
rs9524885
rs61561729
rs760482
rs135917
rs2373551
rs34428576
rs9479287
rs115230603
rs2352617
rs139708219
rs72687208
rs61966202
rs114146553
rs72761324
All − MR Egger
All − IVW(MRE)
−0.010
−0.005
0.000
0.005
0.010
MR effect size for
'NKG2−A/NKG2−B type II integral membrane protein' on 'eGFR'

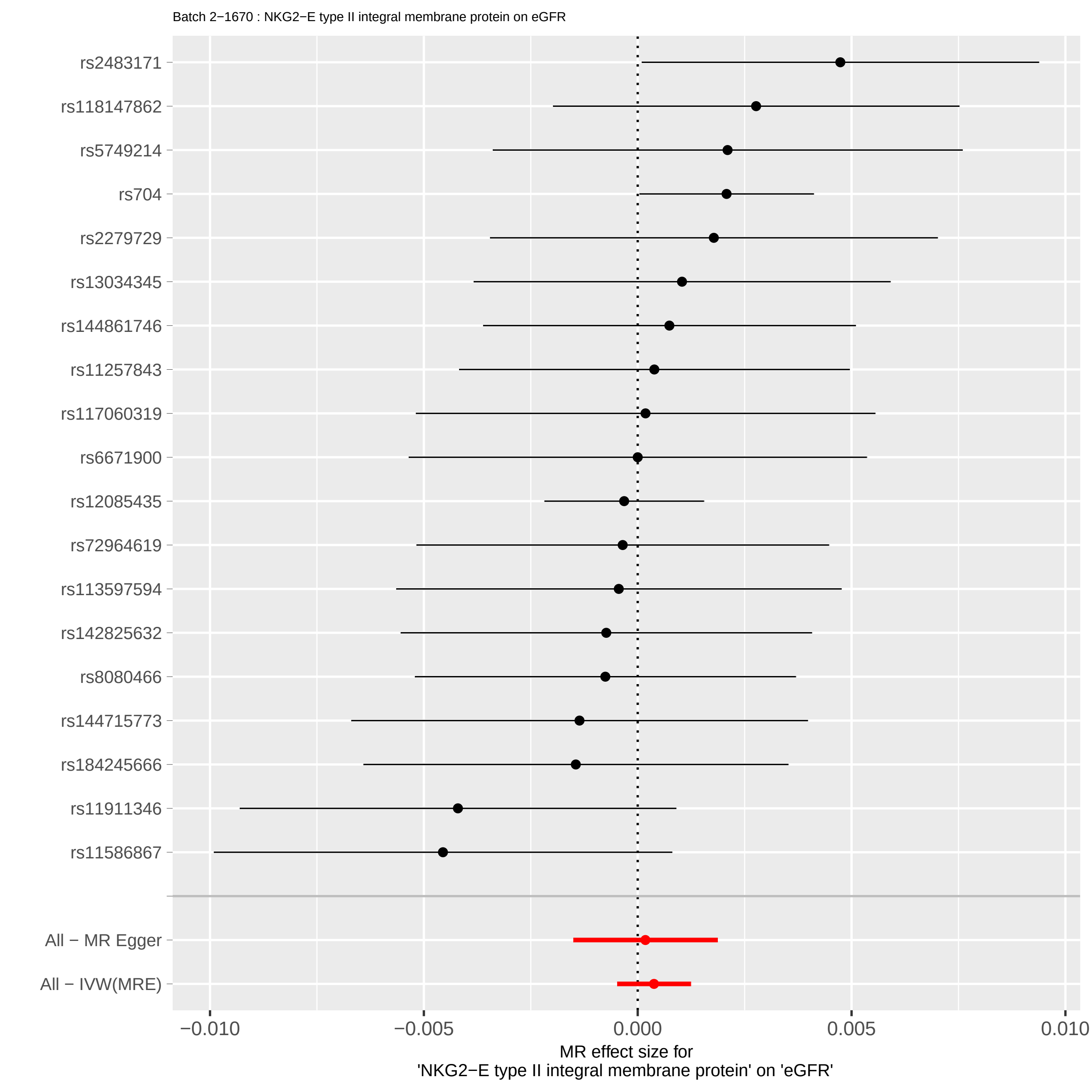

Batch 2−1670 : NKG2−E type II integral membrane protein on eGFR
rs2483171
rs118147862
rs5749214
rs704
rs2279729
rs13034345
rs144861746
rs11257843
rs117060319
rs6671900
rs12085435
rs72964619
rs113597594
rs142825632
rs8080466
rs144715773
rs184245666
rs11911346
rs11586867
All − MR Egger
All − IVW(MRE)
−0.010
−0.005
0.000
0.005
0.010
MR effect size for
'NKG2−E type II integral membrane protein' on 'eGFR'

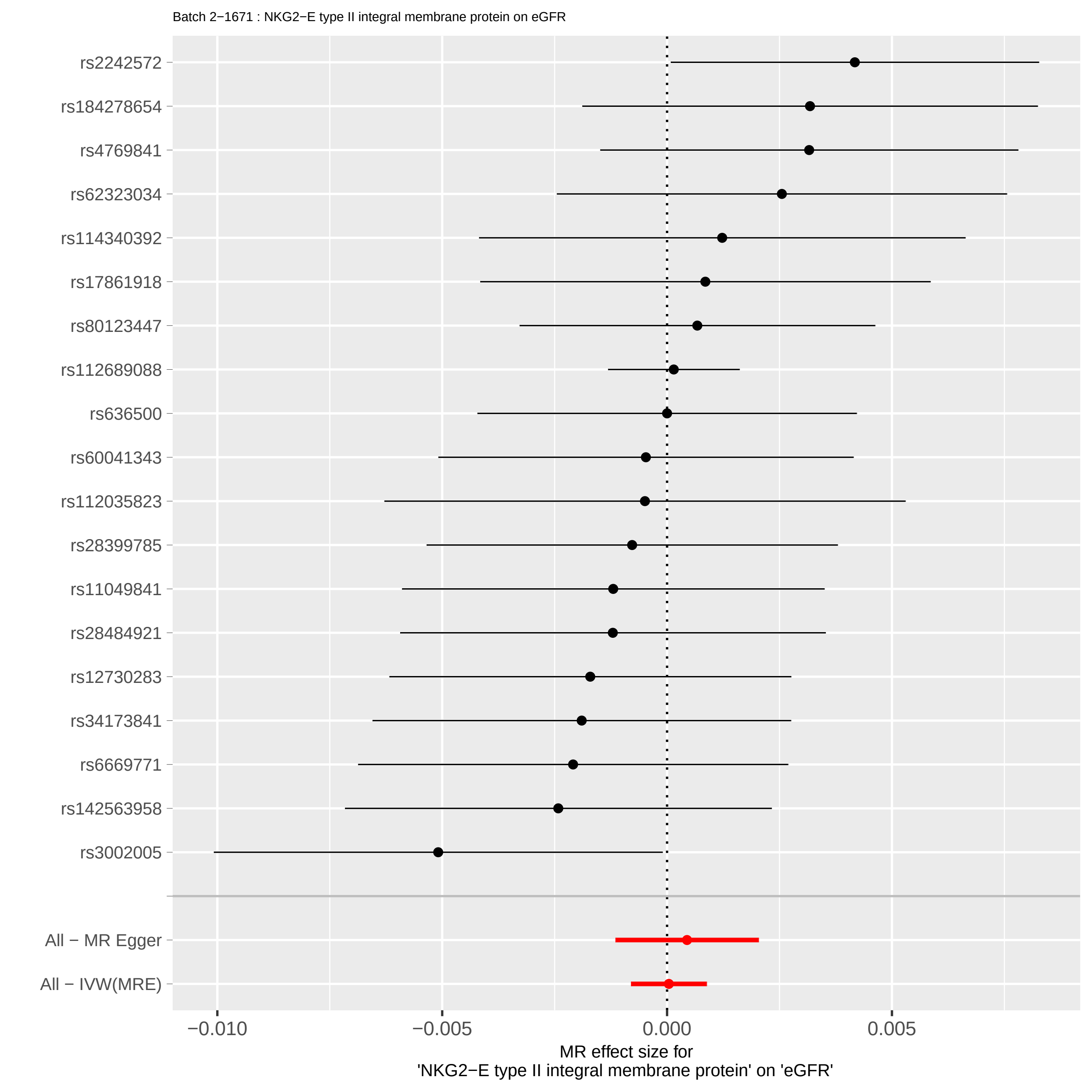
Batch 2−1671 : NKG2−E type II integral membrane protein on eGFR
rs2242572
rs184278654
rs4769841
rs62323034
rs114340392
rs17861918
rs80123447
rs112689088
rs636500
rs60041343
rs112035823
rs28399785
rs11049841
rs28484921
rs12730283
rs34173841
rs6669771
rs142563958
rs3002005
All − MR Egger
All − IVW(MRE)
−0.010
−0.005
0.000
0.005
MR effect size for
'NKG2−E type II integral membrane protein' on 'eGFR'

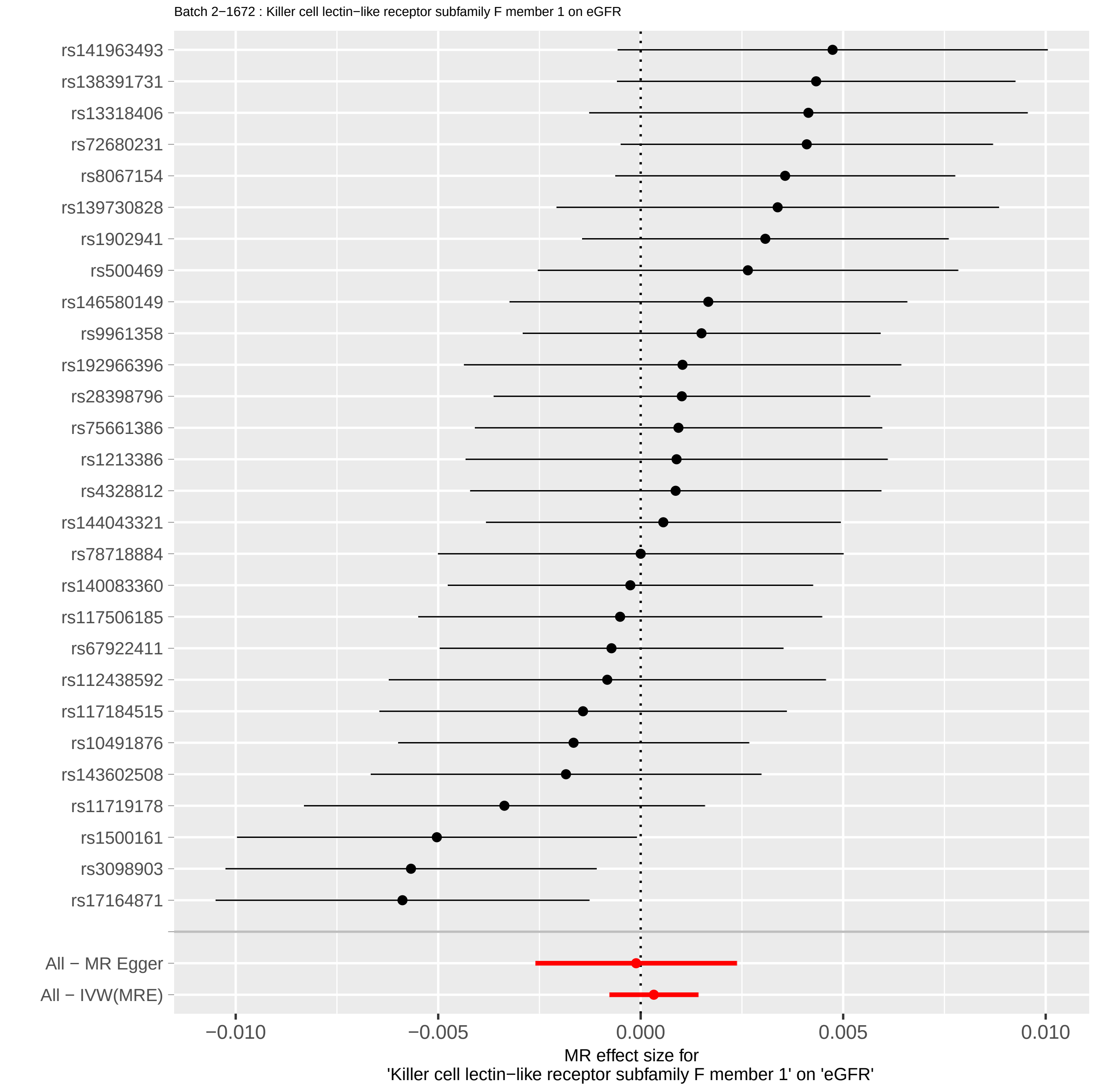

Batch 2−1672 : Killer cell lectin−like receptor subfamily F member 1 on eGFR
rs141963493
rs138391731
rs13318406
rs72680231
rs8067154
rs139730828
rs1902941
rs500469
rs146580149
rs9961358
rs192966396
rs28398796
rs75661386
rs1213386
rs4328812
rs144043321
rs78718884
rs140083360
rs117506185
rs67922411
rs112438592
rs117184515
rs10491876
rs143602508
rs11719178
rs1500161
rs3098903
rs17164871
All − MR Egger
All − IVW(MRE)
−0.010
−0.005
0.000
0.005
0.010
MR effect size for
'Killer cell lectin−like receptor subfamily F member 1' on 'eGFR'

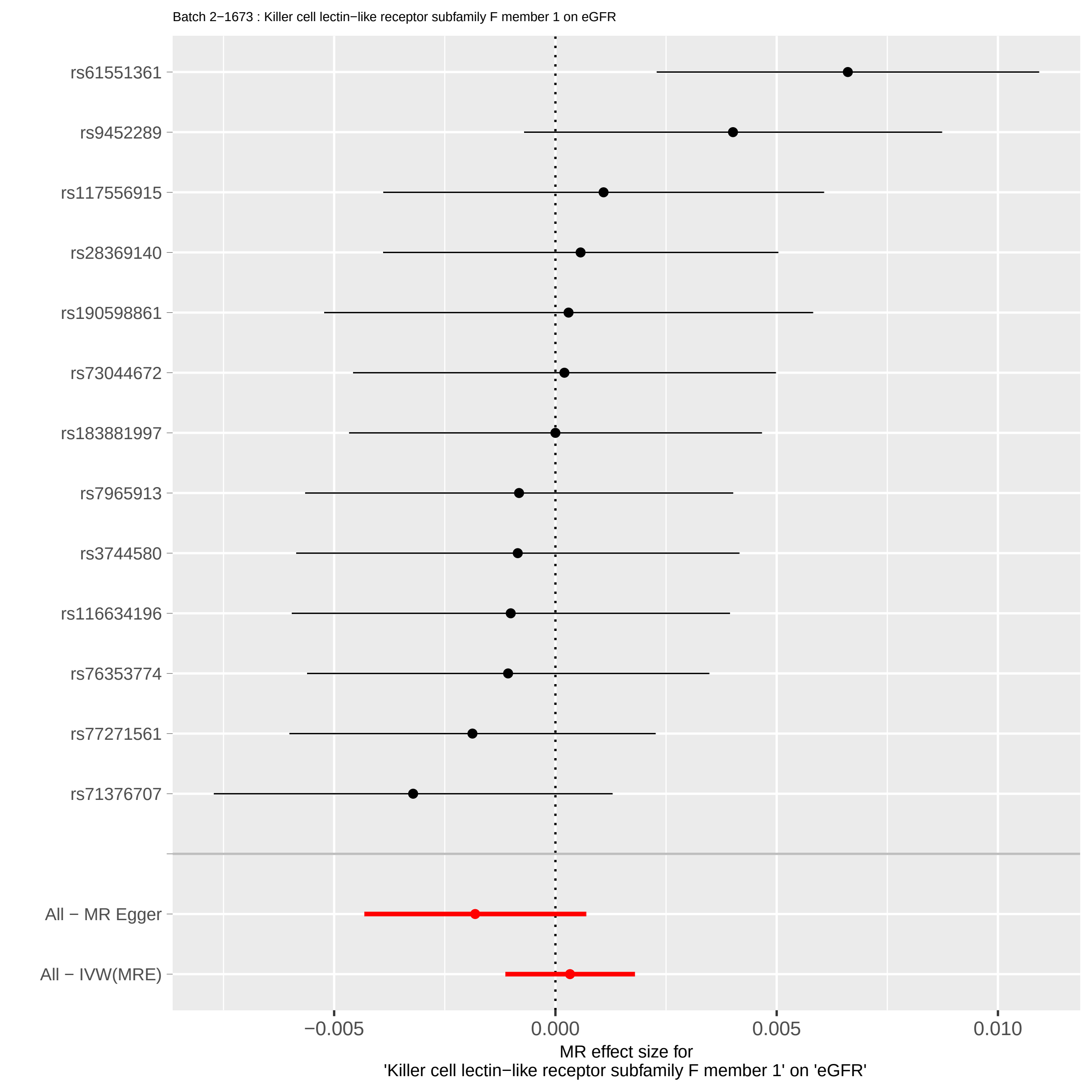

Batch 2−1673 : Killer cell lectin−like receptor subfamily F member 1 on eGFR
rs61551361
rs9452289
rs117556915
rs28369140
rs190598861
rs73044672
rs183881997
rs7965913
rs3744580
rs116634196
rs76353774
rs77271561
rs71376707
All − MR Egger
All − IVW(MRE)
−0.005
0.000
0.005
0.010
MR effect size for
'Killer cell lectin−like receptor subfamily F member 1' on 'eGFR'

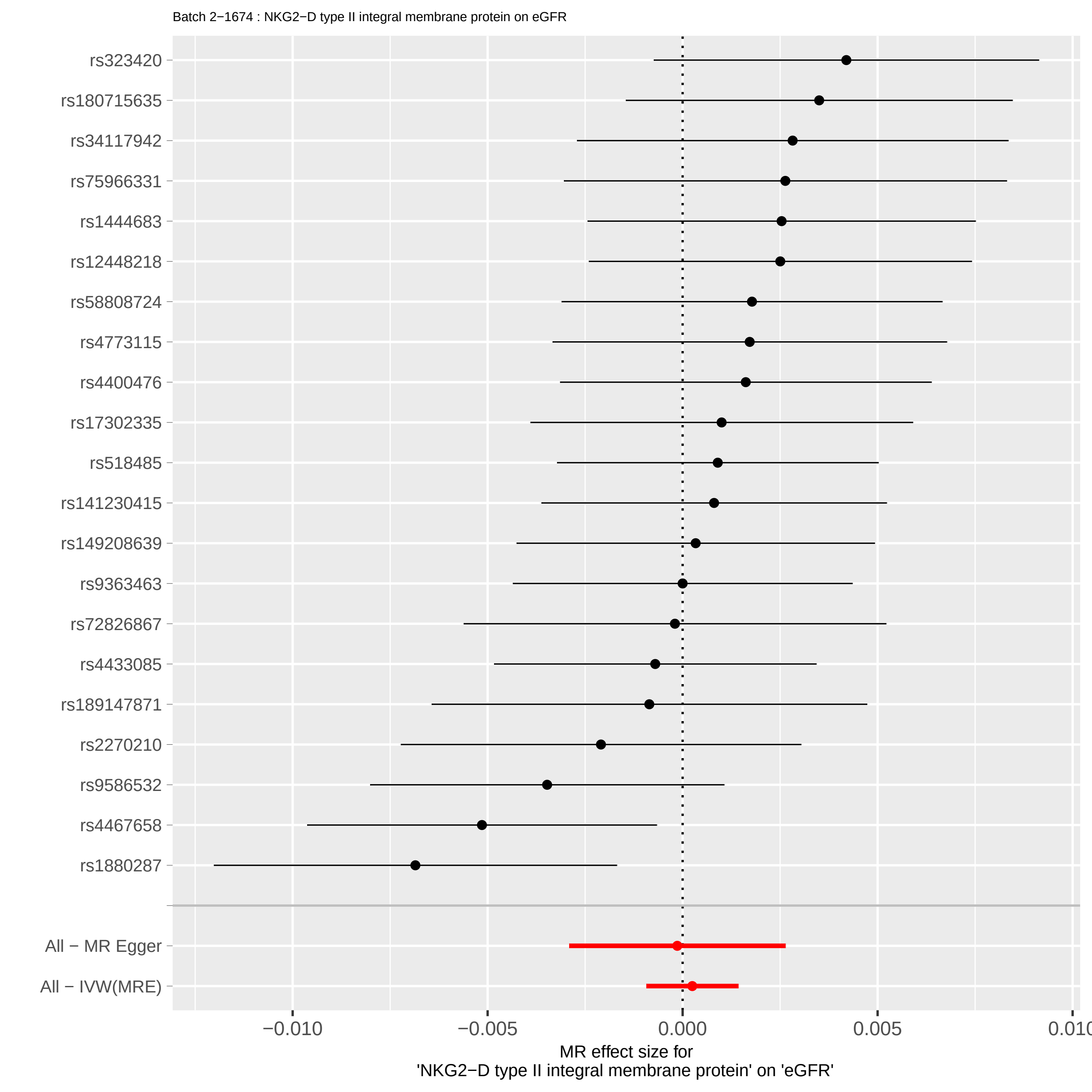

Batch 2−1674 : NKG2−D type II integral membrane protein on eGFR
rs323420
rs180715635
rs34117942
rs75966331
rs1444683
rs12448218
rs58808724
rs4773115
rs4400476
rs17302335
rs518485
rs141230415
rs149208639
rs9363463
rs72826867
rs4433085
rs189147871
rs2270210
rs9586532
rs4467658
rs1880287
All − MR Egger
All − IVW(MRE)
−0.010
−0.005
0.000
0.005
0.010
MR effect size for
'NKG2−D type II integral membrane protein' on 'eGFR'

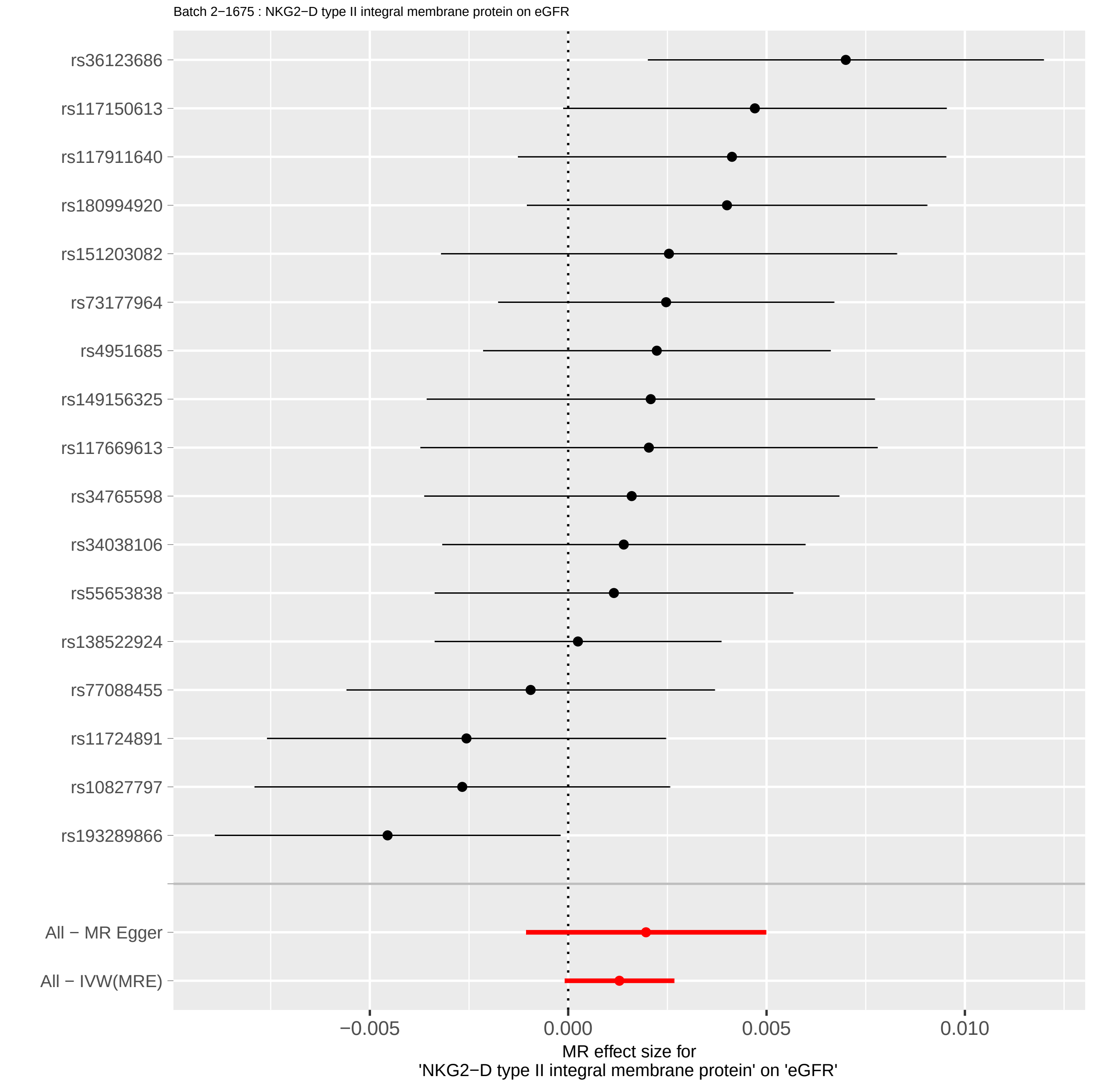

Batch 2−1675 : NKG2−D type II integral membrane protein on eGFR
rs36123686
rs117150613
rs117911640
rs180994920
rs151203082
rs73177964
rs4951685
rs149156325
rs117669613
rs34765598
rs34038106
rs55653838
rs138522924
rs77088455
rs11724891
rs10827797
rs193289866
All − MR Egger
All − IVW(MRE)
−0.005
0.000
0.005
0.010
MR effect size for
'NKG2−D type II integral membrane protein' on 'eGFR'

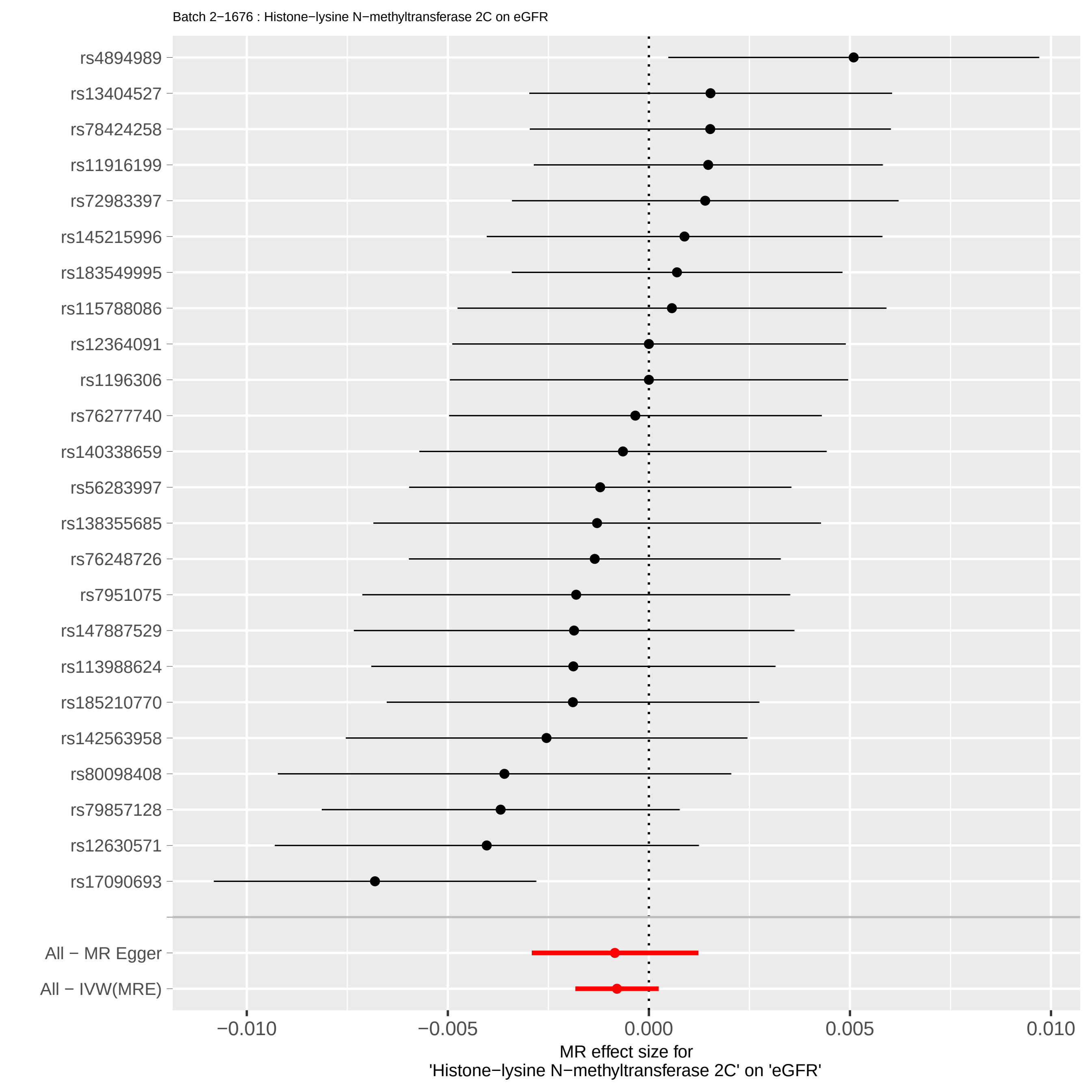
Batch 2−1676 : Histone−lysine N−methyltransferase 2C on eGFR
rs4894989
rs13404527
rs78424258
rs11916199
rs72983397
rs145215996
rs183549995
rs115788086
rs12364091
rs1196306
rs76277740
rs140338659
rs56283997
rs138355685
rs76248726
rs7951075
rs147887529
rs113988624
rs185210770
rs142563958
rs80098408
rs79857128
rs12630571
rs17090693
All − MR Egger
All − IVW(MRE)
−0.010
−0.005
0.000
0.005
0.010
MR effect size for
'Histone−lysine N−methyltransferase 2C' on 'eGFR'

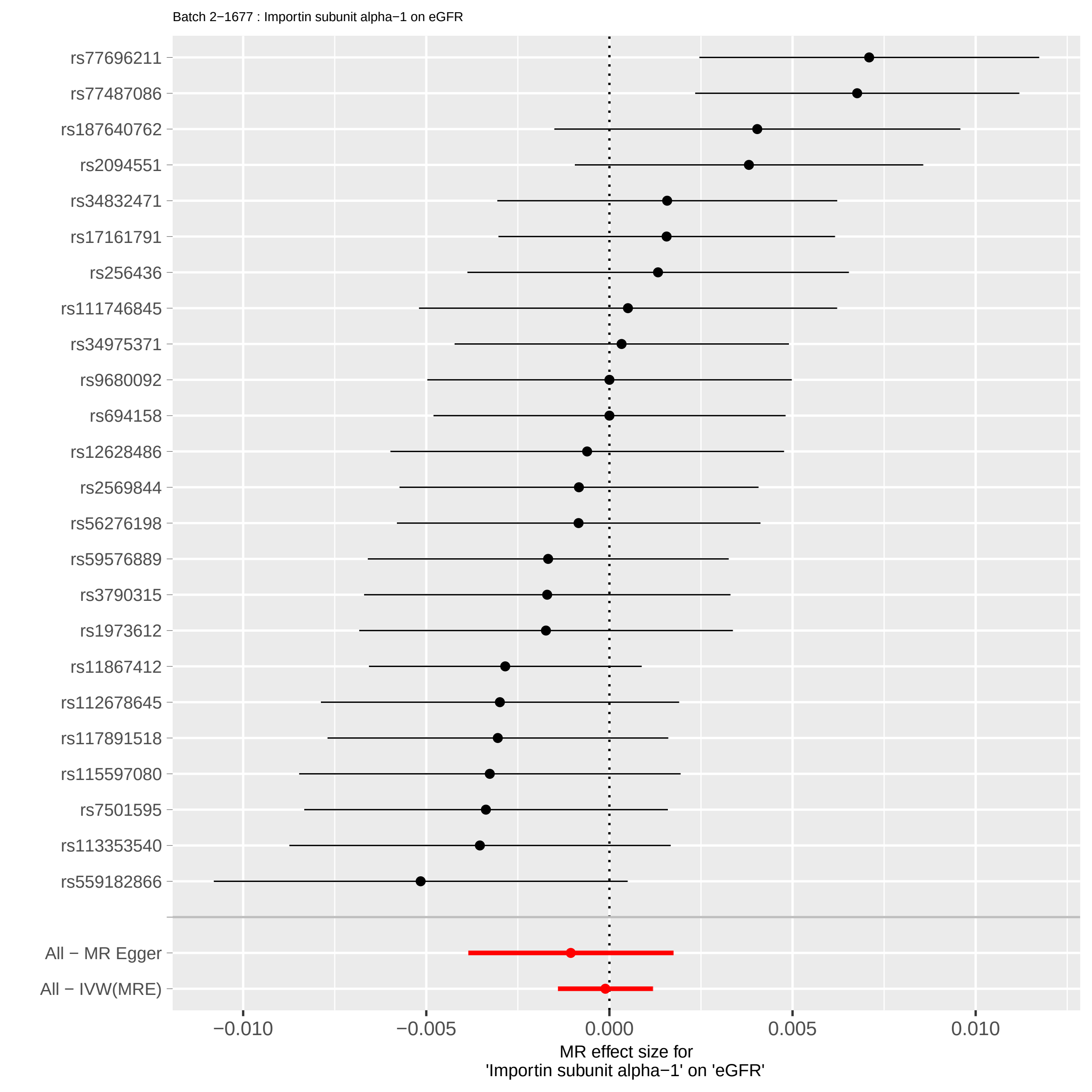

Batch 2−1677 : Importin subunit alpha−1 on eGFR
rs77696211
rs77487086
rs187640762
rs2094551
rs34832471
rs17161791
rs256436
rs111746845
rs34975371
rs9680092
rs694158
rs12628486
rs2569844
rs56276198
rs59576889
rs3790315
rs1973612
rs11867412
rs112678645
rs117891518
rs115597080
rs7501595
rs113353540
rs559182866
All − MR Egger
All − IVW(MRE)
−0.010
−0.005
0.000
0.005
0.010
MR effect size for
'Importin subunit alpha−1' on 'eGFR'

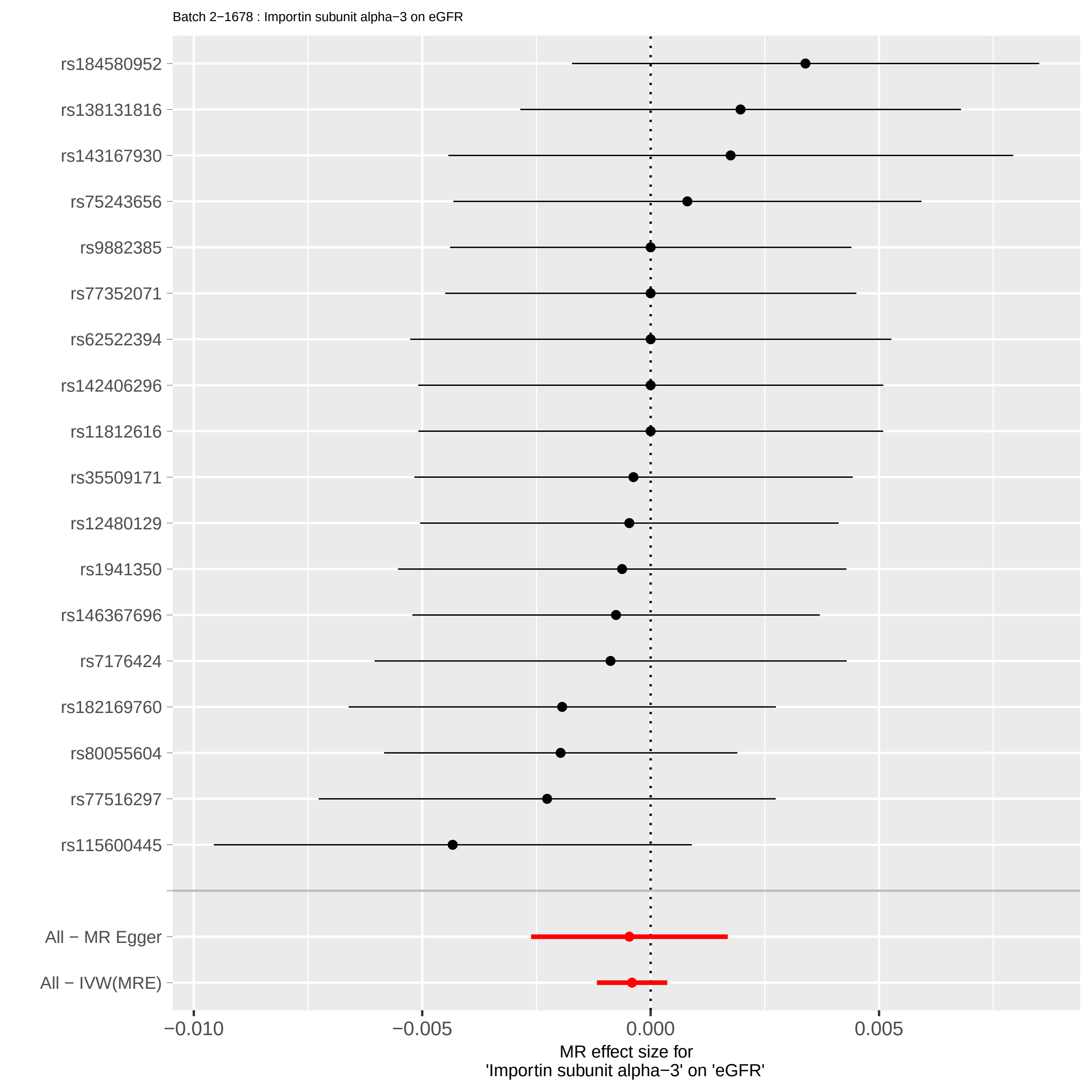
Batch 2−1678 : Importin subunit alpha−3 on eGFR
rs184580952
rs138131816
rs143167930
rs75243656
rs9882385
rs77352071
rs62522394
rs142406296
rs11812616
rs35509171
rs12480129
rs1941350
rs146367696
rs7176424
rs182169760
rs80055604
rs77516297
rs115600445
All − MR Egger
All − IVW(MRE)
−0.010
−0.005
0.000
0.005
MR effect size for
'Importin subunit alpha−3' on 'eGFR'

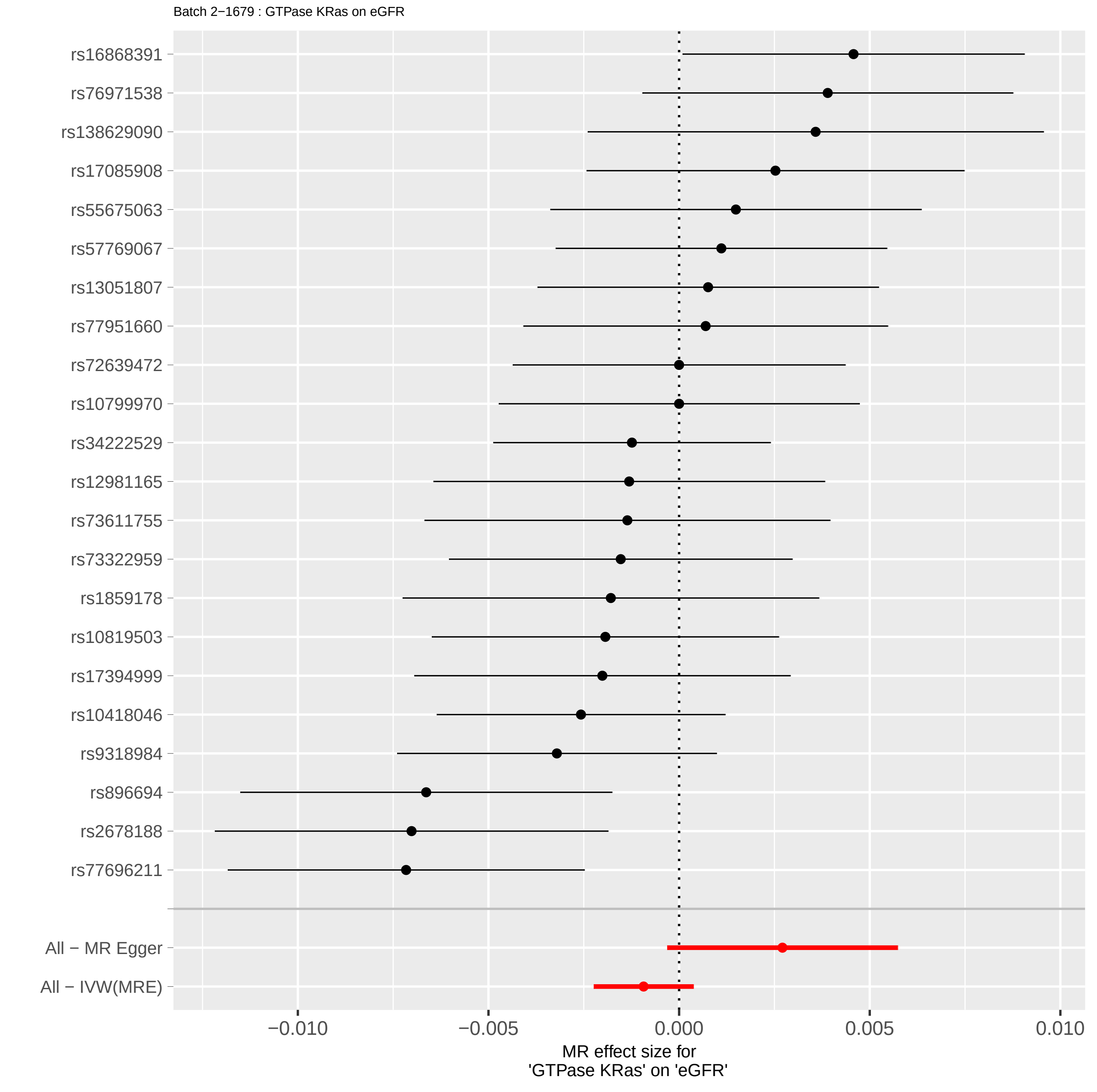

Batch 2−1679 : GTPase KRas on eGFR
rs16868391
rs76971538
rs138629090
rs17085908
rs55675063
rs57769067
rs13051807
rs77951660
rs72639472
rs10799970
rs34222529
rs12981165
rs73611755
rs73322959
rs1859178
rs10819503
rs17394999
rs10418046
rs9318984
rs896694
rs2678188
rs77696211
All − MR Egger
All − IVW(MRE)
−0.010
−0.005
0.000
0.005
0.010
MR effect size for
'GTPase KRas' on 'eGFR'

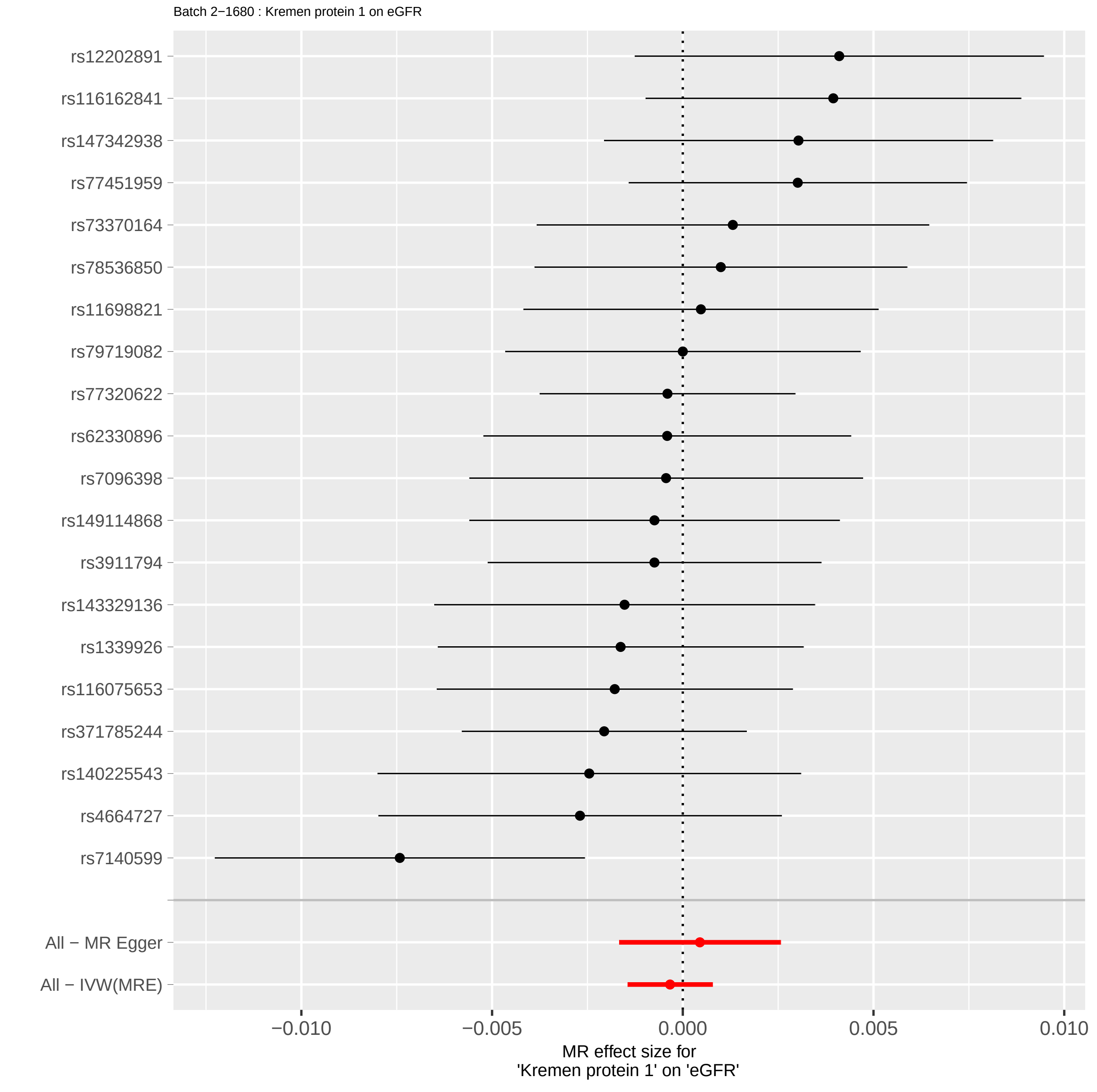

Batch 2−1680 : Kremen protein 1 on eGFR
rs12202891
rs116162841
rs147342938
rs77451959
rs73370164
rs78536850
rs11698821
rs79719082
rs77320622
rs62330896
rs7096398
rs149114868
rs3911794
rs143329136
rs1339926
rs116075653
rs371785244
rs140225543
rs4664727
rs7140599
All − MR Egger
All − IVW(MRE)
−0.010
−0.005
0.000
0.005
0.010
MR effect size for
'Kremen protein 1' on 'eGFR'

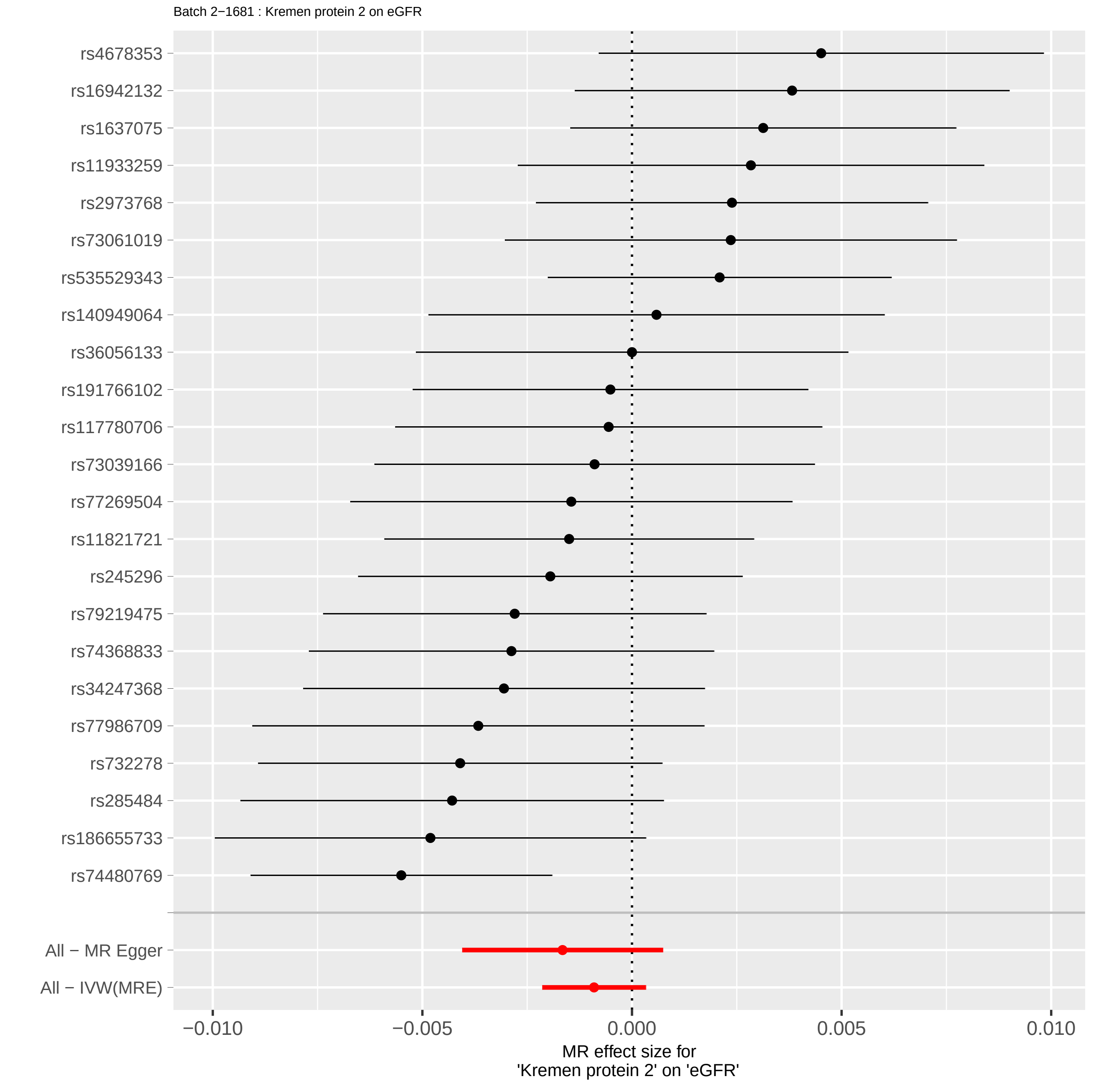

Batch 2−1681 : Kremen protein 2 on eGFR
rs4678353
rs16942132
rs1637075
rs11933259
rs2973768
rs73061019
rs535529343
rs140949064
rs36056133
rs191766102
rs117780706
rs73039166
rs77269504
rs11821721
rs245296
rs79219475
rs74368833
rs34247368
rs77986709
rs732278
rs285484
rs186655733
rs74480769
All − MR Egger
All − IVW(MRE)
−0.010
−0.005
0.000
0.005
0.010
MR effect size for
'Kremen protein 2' on 'eGFR'

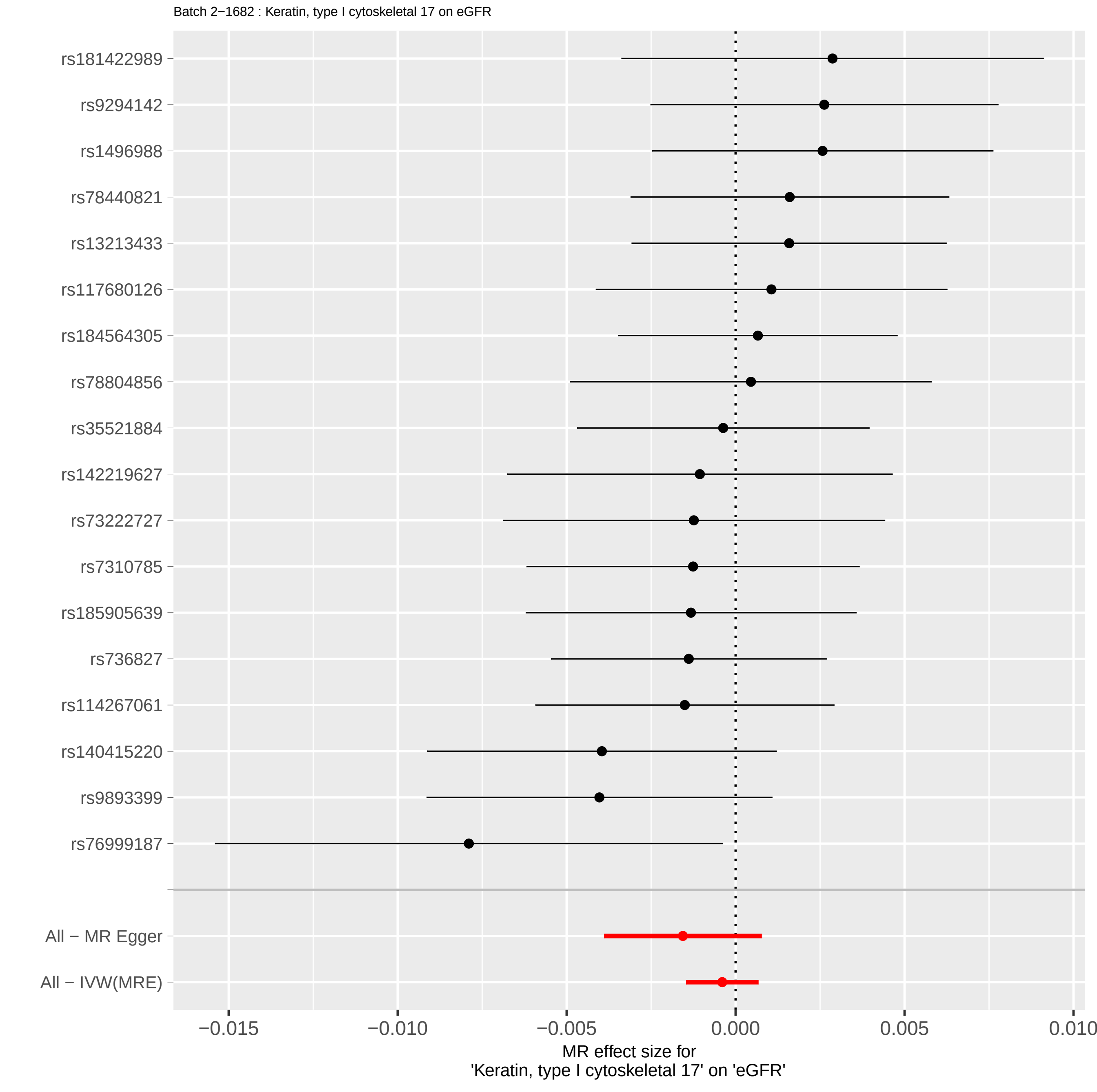
Batch 2−1682 : Keratin, type I cytoskeletal 17 on eGFR
rs181422989
rs9294142
rs1496988
rs78440821
rs13213433
rs117680126
rs184564305
rs78804856
rs35521884
rs142219627
rs73222727
rs7310785
rs185905639
rs736827
rs114267061
rs140415220
rs9893399
rs76999187
All − MR Egger
All − IVW(MRE)
−0.015
−0.010
−0.005
0.000
0.005
0.010
MR effect size for
'Keratin, type I cytoskeletal 17' on 'eGFR'

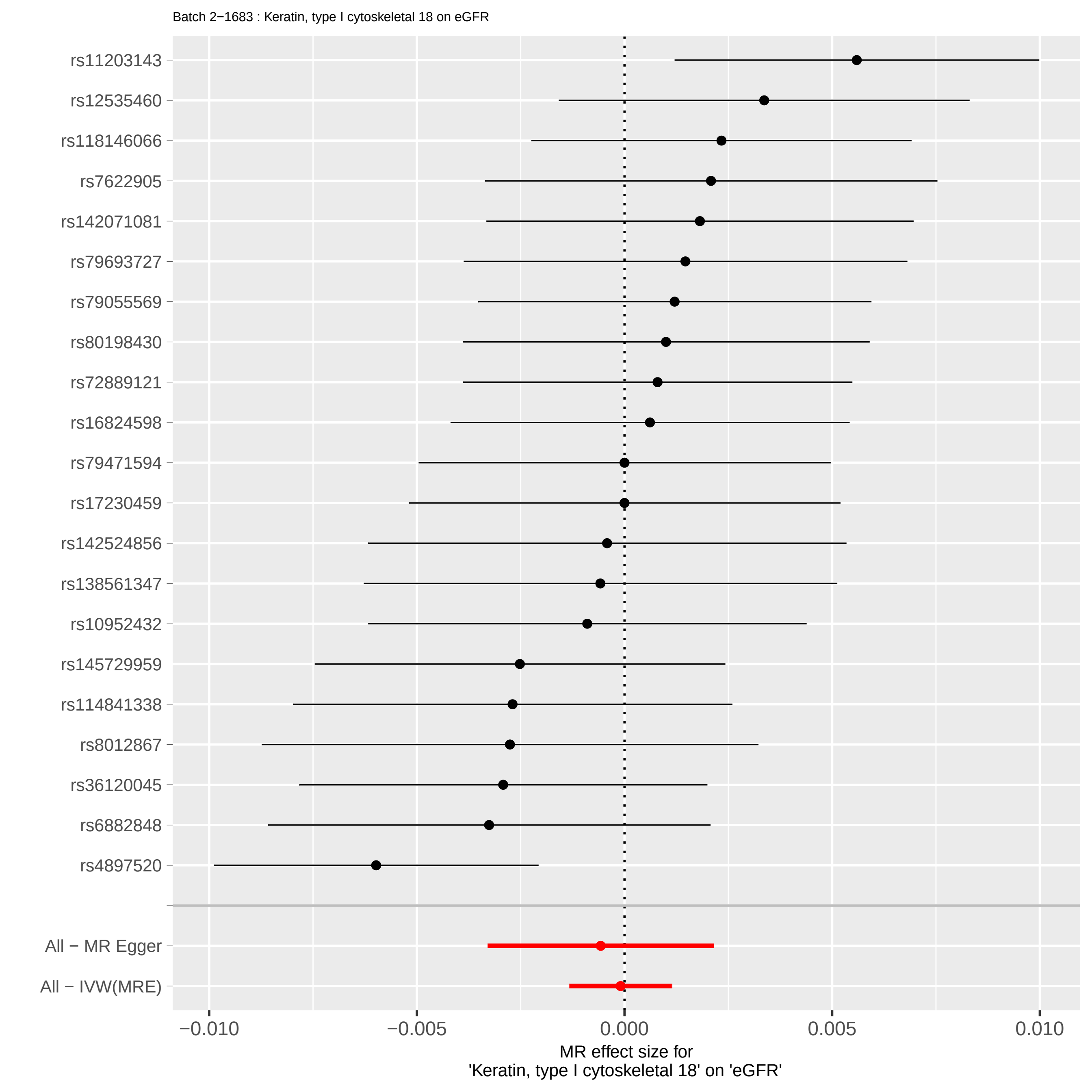
Batch 2−1683 : Keratin, type I cytoskeletal 18 on eGFR
rs11203143
rs12535460
rs118146066
rs7622905
rs142071081
rs79693727
rs79055569
rs80198430
rs72889121
rs16824598
rs79471594
rs17230459
rs142524856
rs138561347
rs10952432
rs145729959
rs114841338
rs8012867
rs36120045
rs6882848
rs4897520
All − MR Egger
All − IVW(MRE)
−0.010
−0.005
0.000
0.005
0.010
MR effect size for
'Keratin, type I cytoskeletal 18' on 'eGFR'

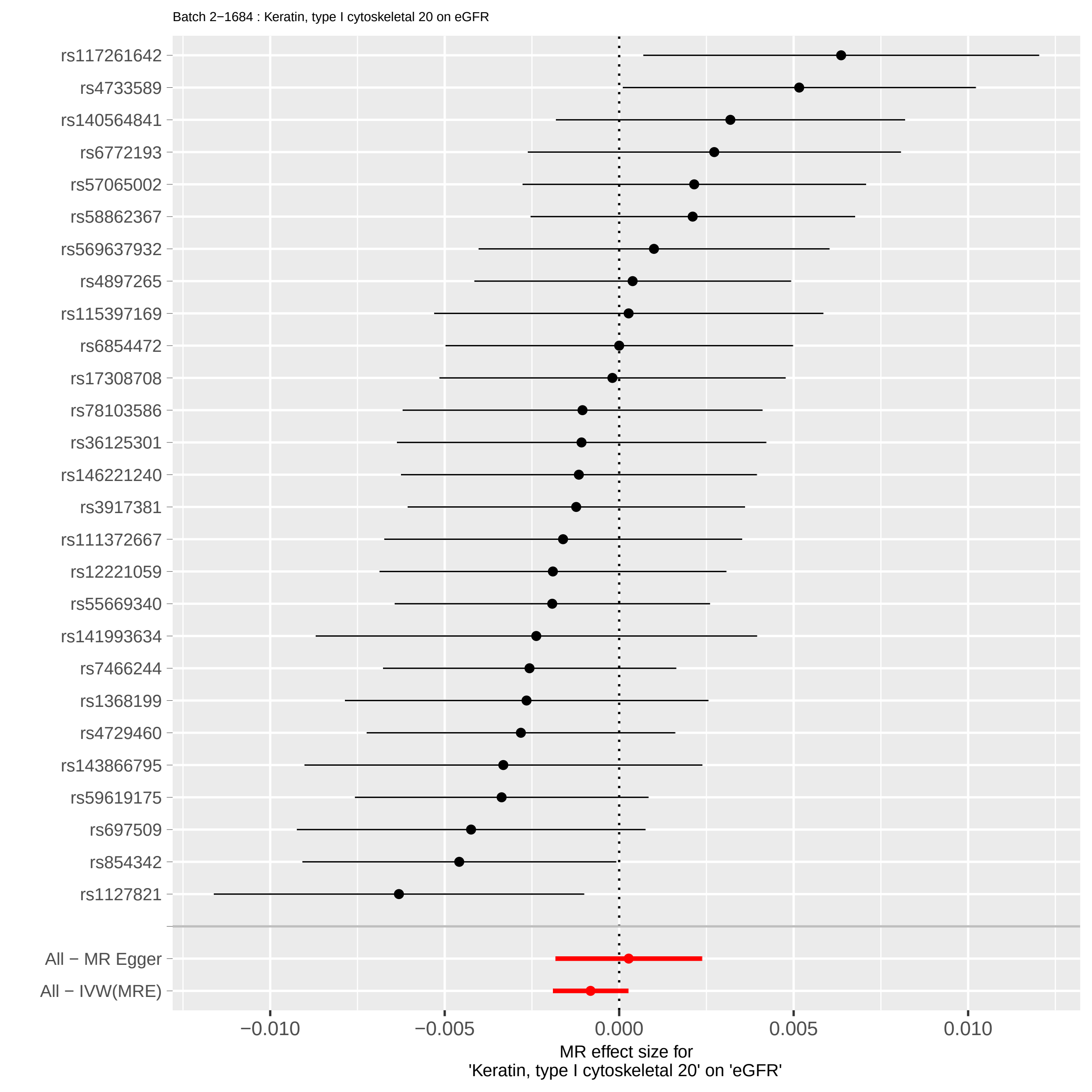
Batch 2−1684 : Keratin, type I cytoskeletal 20 on eGFR
rs117261642
rs4733589
rs140564841
rs6772193
rs57065002
rs58862367
rs569637932
rs4897265
rs115397169
rs6854472
rs17308708
rs78103586
rs36125301
rs146221240
rs3917381
rs111372667
rs12221059
rs55669340
rs141993634
rs7466244
rs1368199
rs4729460
rs143866795
rs59619175
rs697509
rs854342
rs1127821
All − MR Egger
All − IVW(MRE)
−0.010
−0.005
0.000
0.005
0.010
MR effect size for
'Keratin, type I cytoskeletal 20' on 'eGFR'

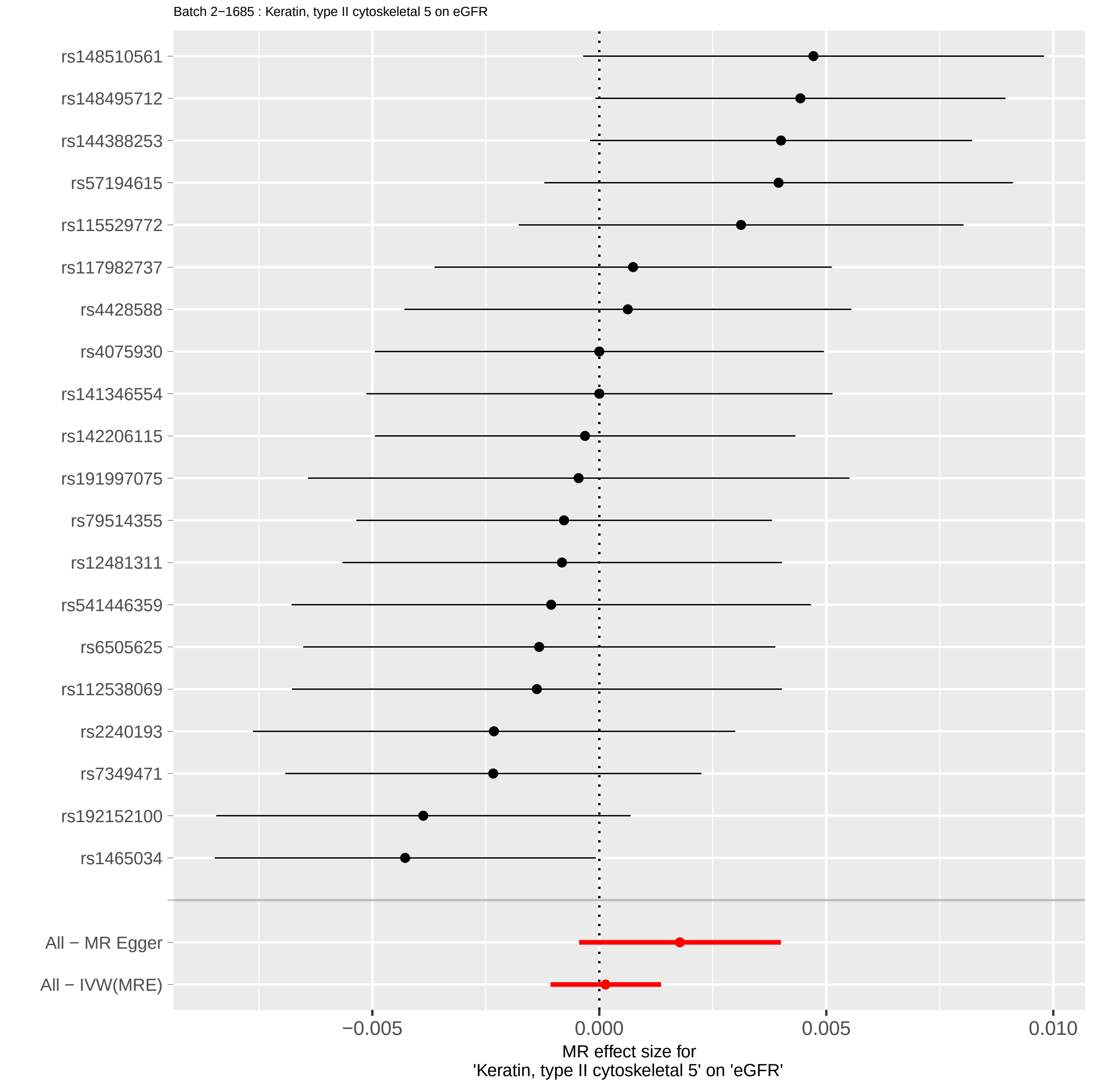

Batch 2−1685 : Keratin, type II cytoskeletal 5 on eGFR
rs148510561
rs148495712
rs144388253
rs57194615
rs115529772
rs117982737
rs4428588
rs4075930
rs141346554
rs142206115
rs191997075
rs79514355
rs12481311
rs541446359
rs6505625
rs112538069
rs2240193
rs7349471
rs192152100
rs1465034
All − MR Egger
All − IVW(MRE)
−0.005
0.000
0.005
0.010
MR effect size for
'Keratin, type II cytoskeletal 5' on 'eGFR'

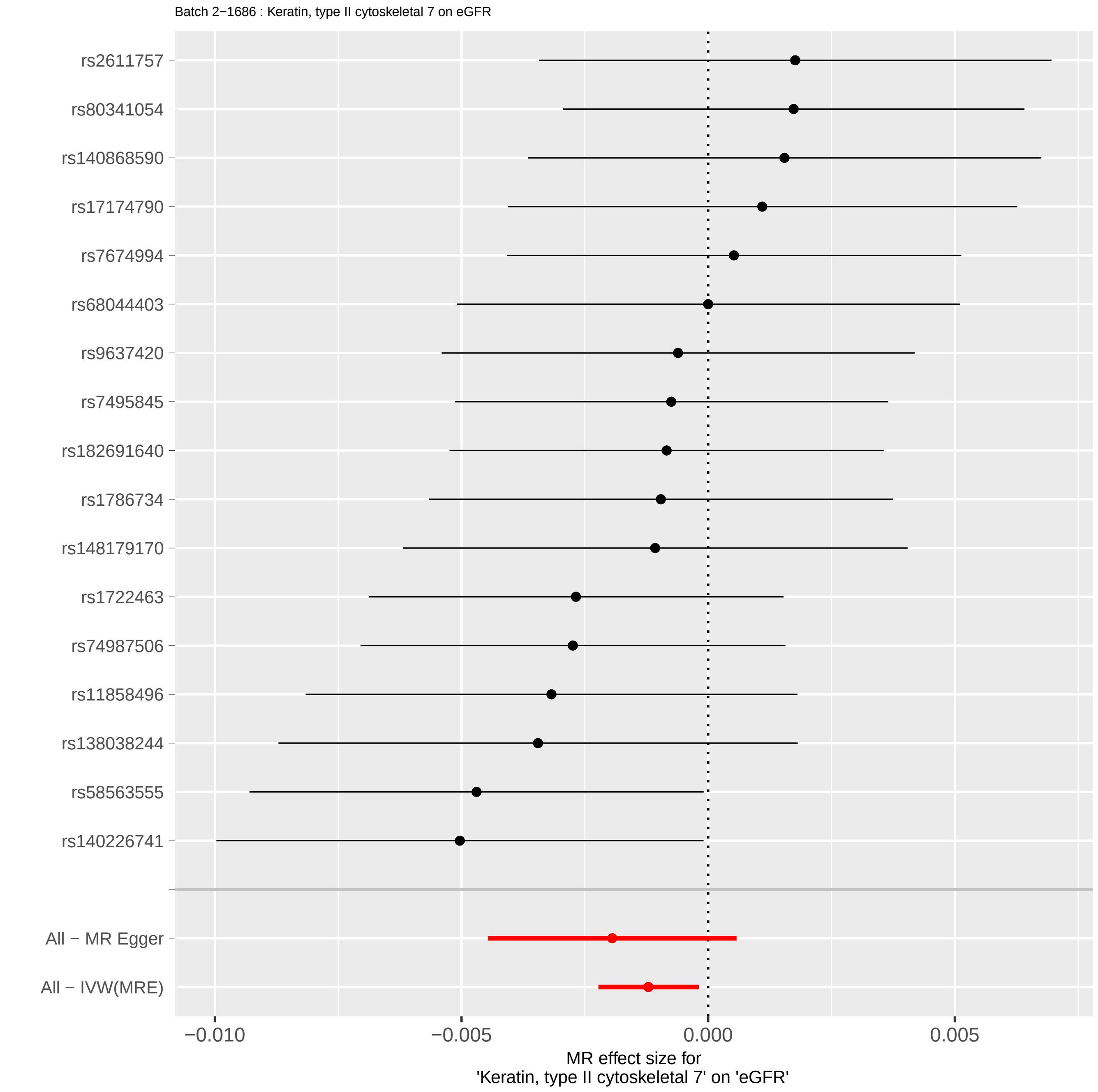

Batch 2−1686 : Keratin, type II cytoskeletal 7 on eGFR
rs2611757
rs80341054
rs140868590
rs17174790
rs7674994
rs68044403
rs9637420
rs7495845
rs182691640
rs1786734
rs148179170
rs1722463
rs74987506
rs11858496
rs138038244
rs58563555
rs140226741
All − MR Egger
All − IVW(MRE)
−0.010
−0.005
0.000
0.005
MR effect size for
'Keratin, type II cytoskeletal 7' on 'eGFR'

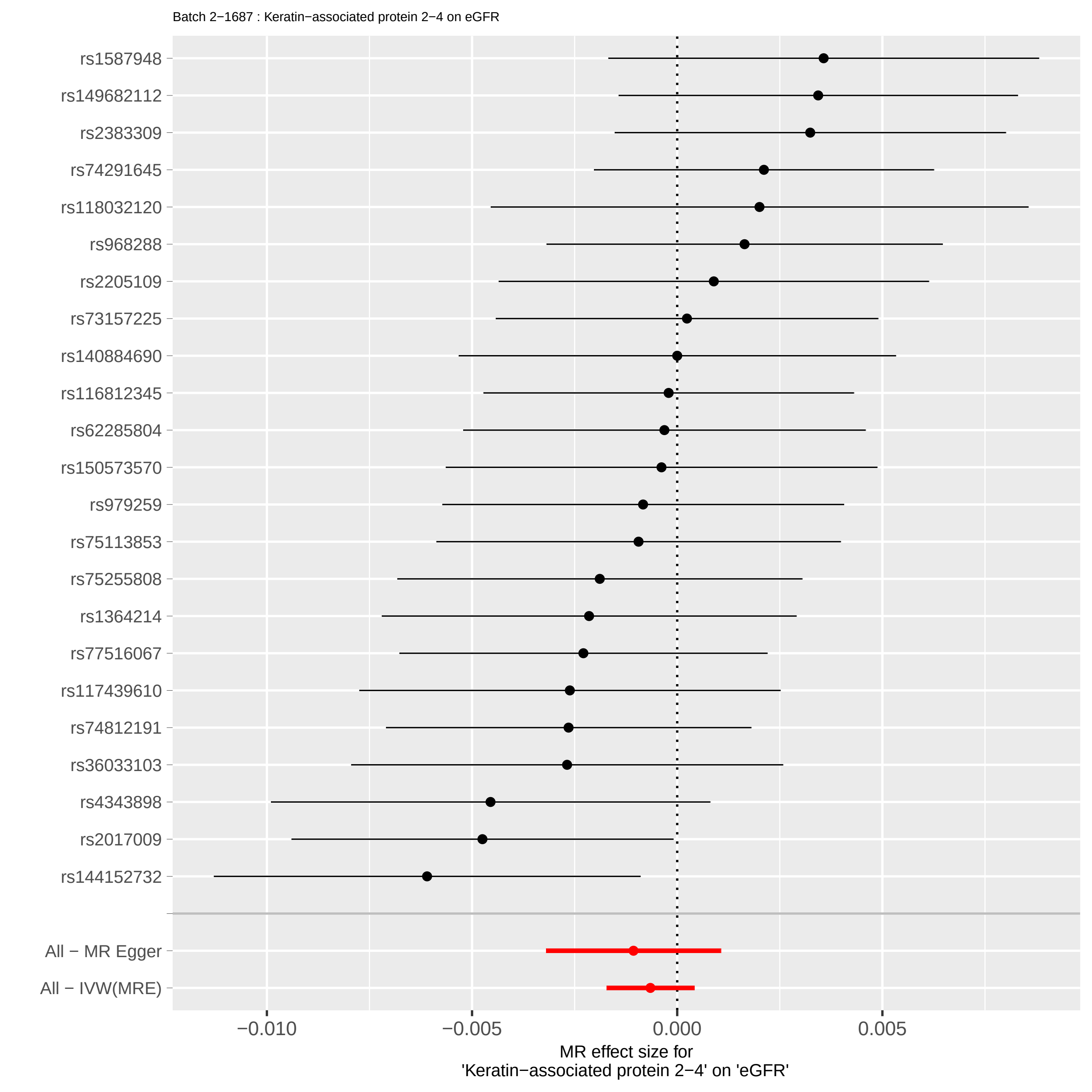
Batch 2−1687 : Keratin−associated protein 2−4 on eGFR
rs1587948
rs149682112
rs2383309
rs74291645
rs118032120
rs968288
rs2205109
rs73157225
rs140884690
rs116812345
rs62285804
rs150573570
rs979259
rs75113853
rs75255808
rs1364214
rs77516067
rs117439610
rs74812191
rs36033103
rs4343898
rs2017009
rs144152732
All − MR Egger
All − IVW(MRE)
−0.010
−0.005
0.000
0.005
MR effect size for
'Keratin−associated protein 2−4' on 'eGFR'

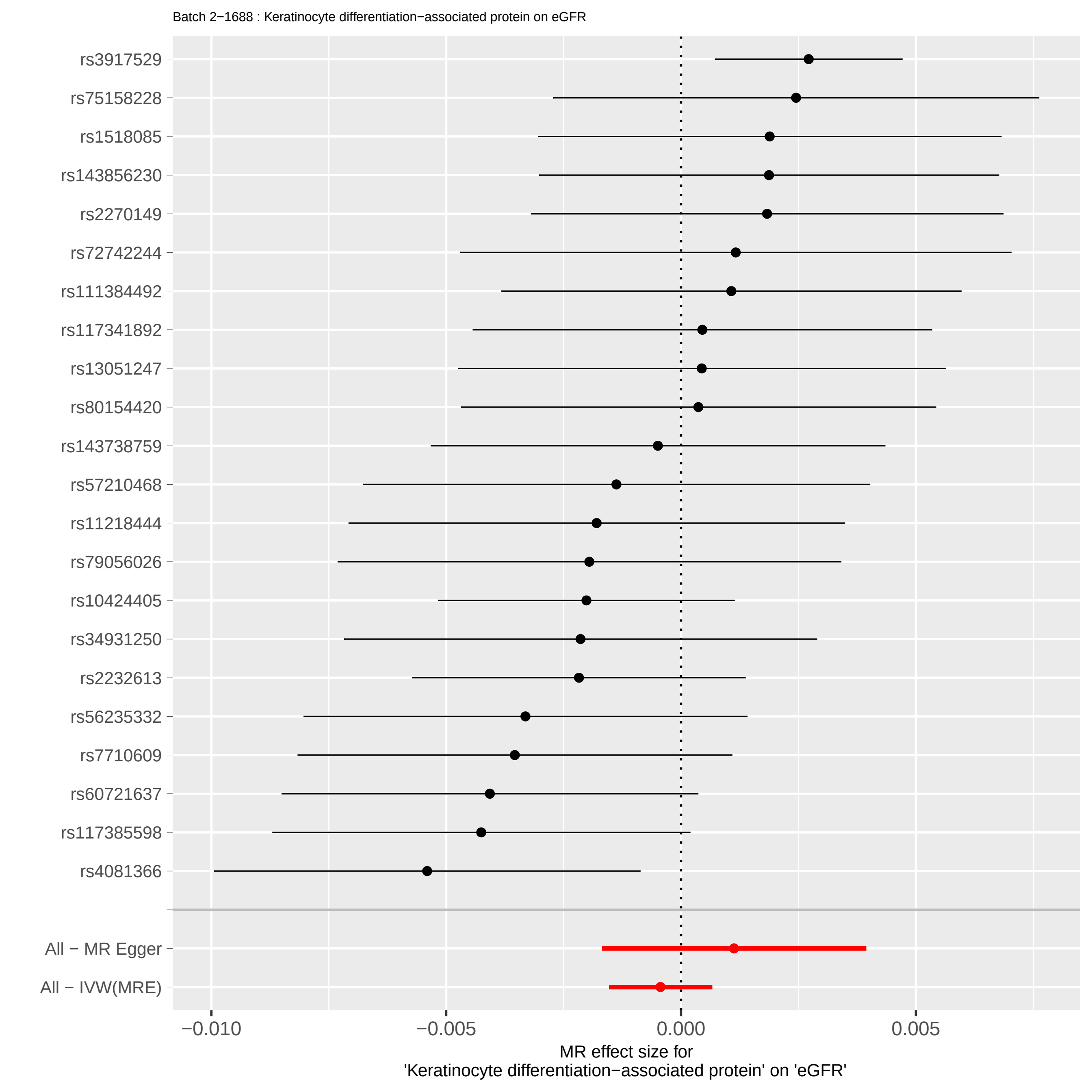
Batch 2−1688 : Keratinocyte differentiation−associated protein on eGFR
rs3917529
rs75158228
rs1518085
rs143856230
rs2270149
rs72742244
rs111384492
rs117341892
rs13051247
rs80154420
rs143738759
rs57210468
rs11218444
rs79056026
rs10424405
rs34931250
rs2232613
rs56235332
rs7710609
rs60721637
rs117385598
rs4081366
All − MR Egger
All − IVW(MRE)
−0.010
−0.005
0.000
0.005
MR effect size for
'Keratinocyte differentiation−associated protein' on 'eGFR'

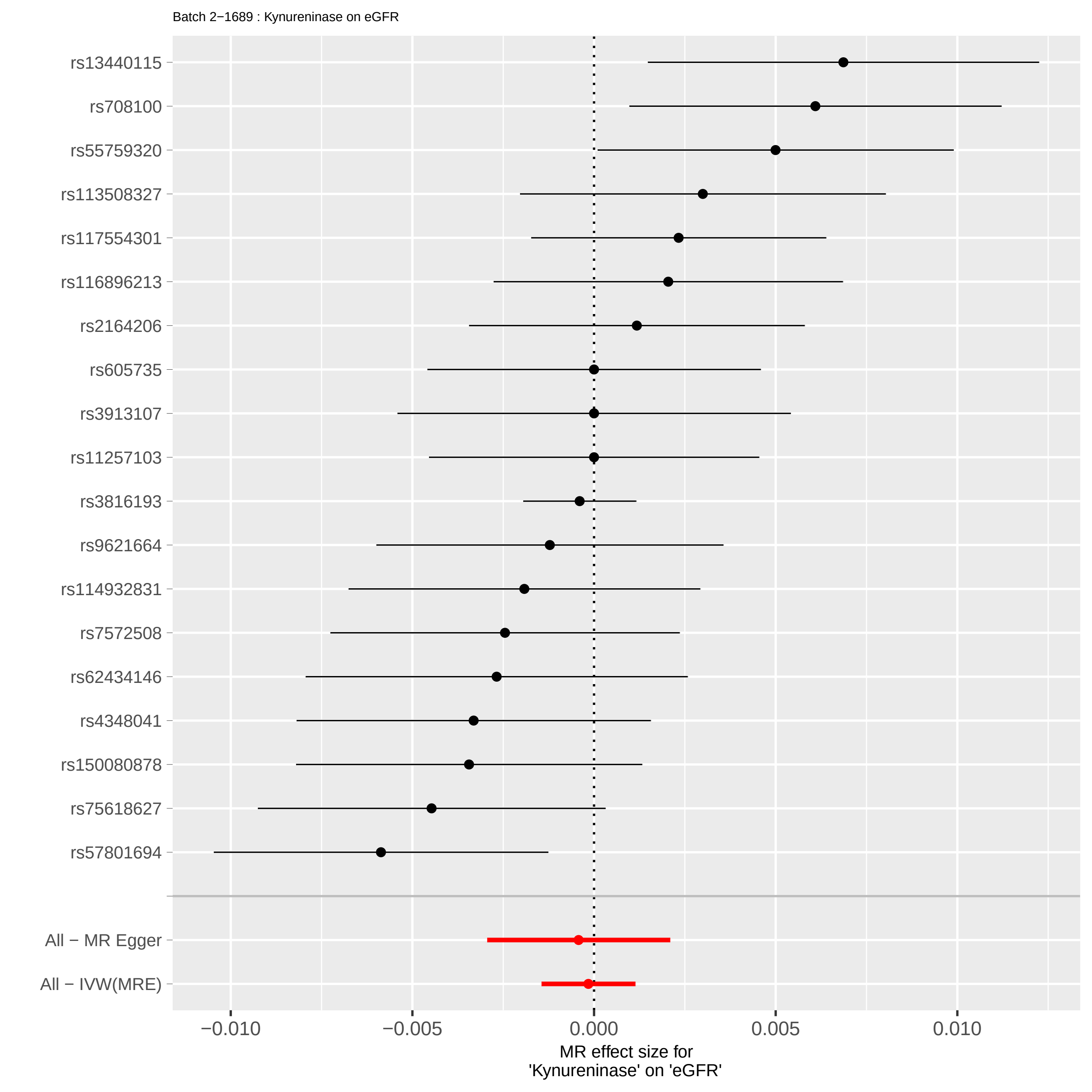
Batch 2−1689 : Kynureninase on eGFR
rs13440115
rs708100
rs55759320
rs113508327
rs117554301
rs116896213
rs2164206
rs605735
rs3913107
rs11257103
rs3816193
rs9621664
rs114932831
rs7572508
rs62434146
rs4348041
rs150080878
rs75618627
rs57801694
All − MR Egger
All − IVW(MRE)
−0.010
−0.005
0.000
0.005
0.010
MR effect size for
'Kynureninase' on 'eGFR'

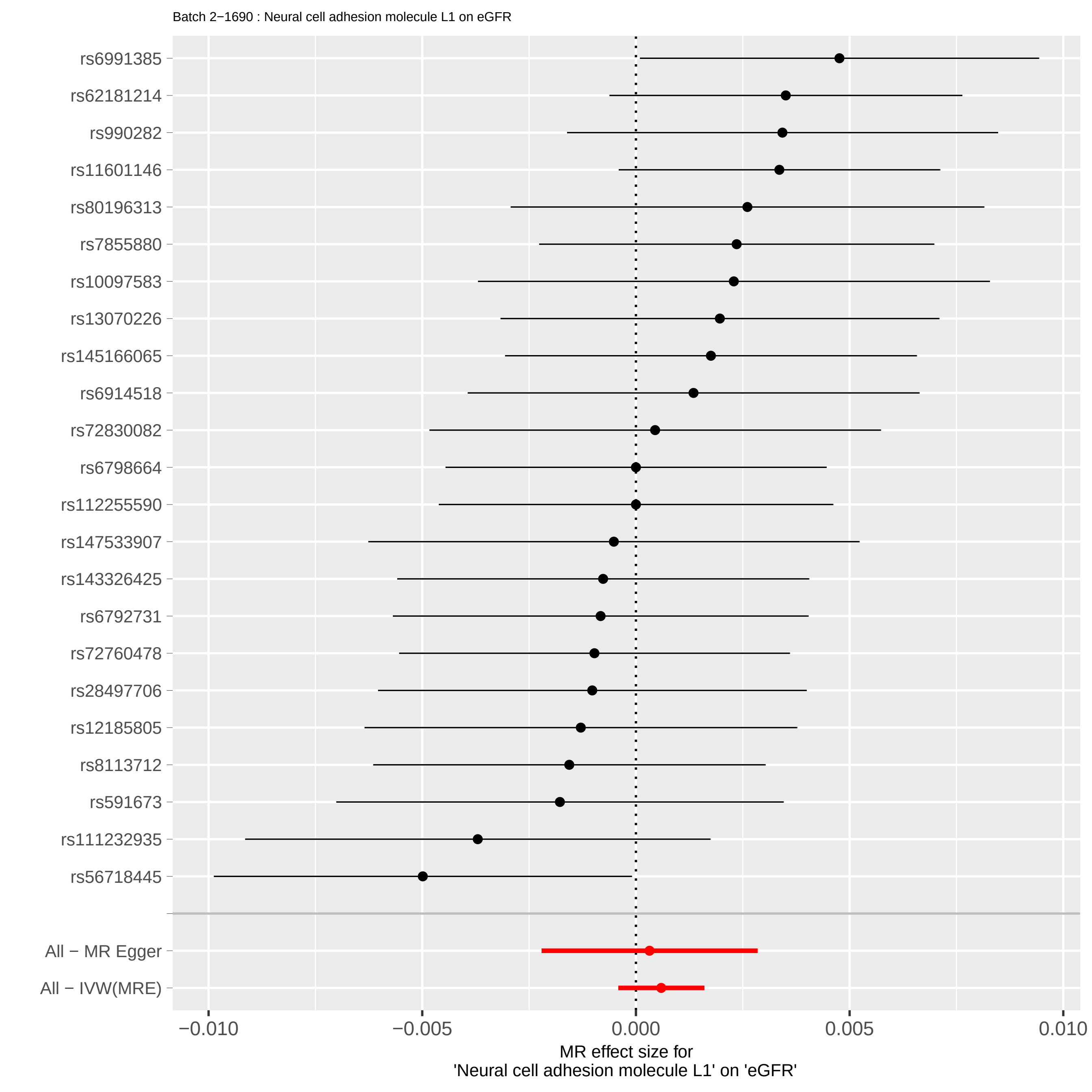

Batch 2−1690 : Neural cell adhesion molecule L1 on eGFR
rs6991385
rs62181214
rs990282
rs11601146
rs80196313
rs7855880
rs10097583
rs13070226
rs145166065
rs6914518
rs72830082
rs6798664
rs112255590
rs147533907
rs143326425
rs6792731
rs72760478
rs28497706
rs12185805
rs8113712
rs591673
rs111232935
rs56718445
All − MR Egger
All − IVW(MRE)
−0.010
−0.005
0.000
0.005
0.010
MR effect size for
'Neural cell adhesion molecule L1' on 'eGFR'

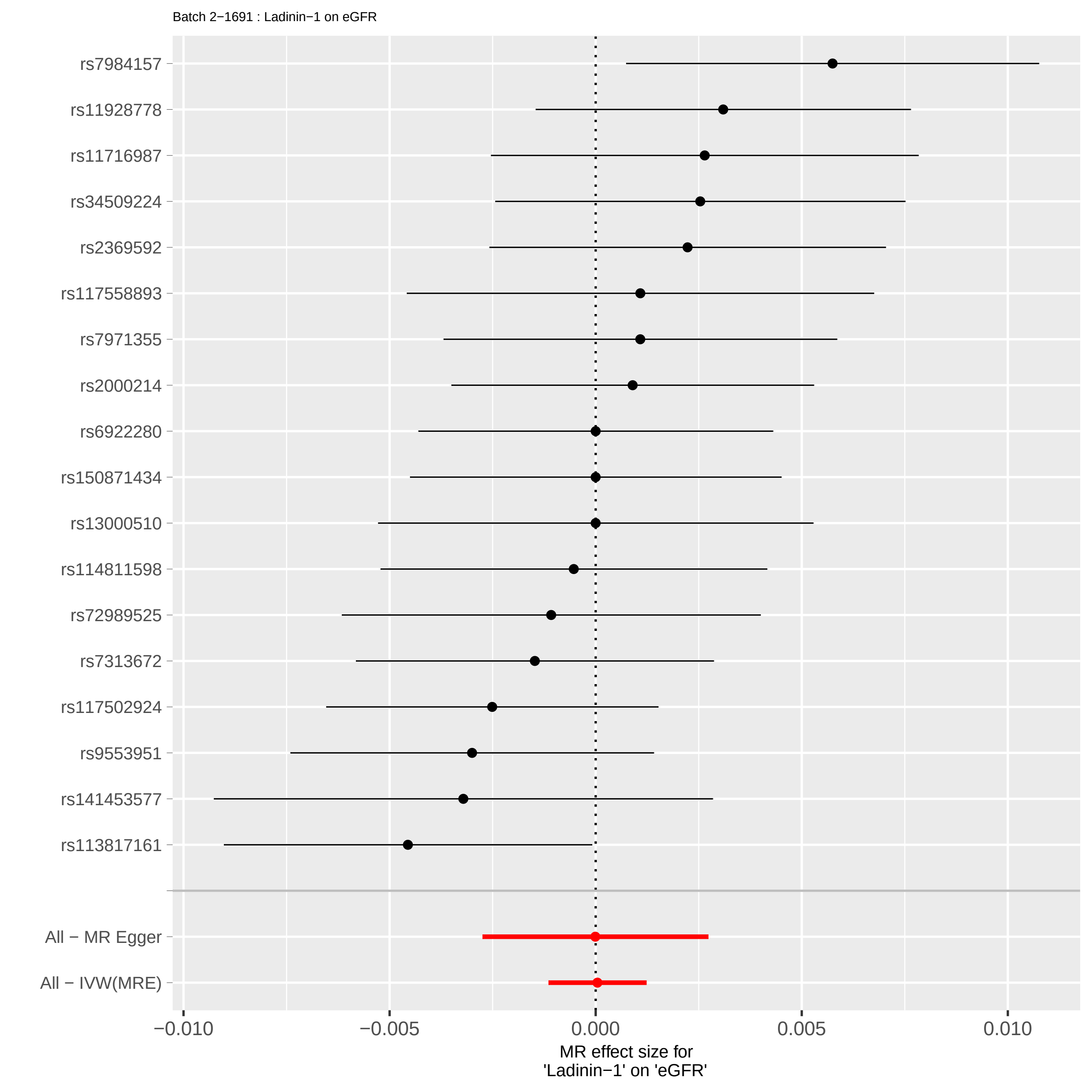
Batch 2−1691 : Ladinin−1 on eGFR
rs7984157
rs11928778
rs11716987
rs34509224
rs2369592
rs117558893
rs7971355
rs2000214
rs6922280
rs150871434
rs13000510
rs114811598
rs72989525
rs7313672
rs117502924
rs9553951
rs141453577
rs113817161
All − MR Egger
All − IVW(MRE)
−0.010
−0.005
0.000
0.005
0.010
MR effect size for
'Ladinin−1' on 'eGFR'

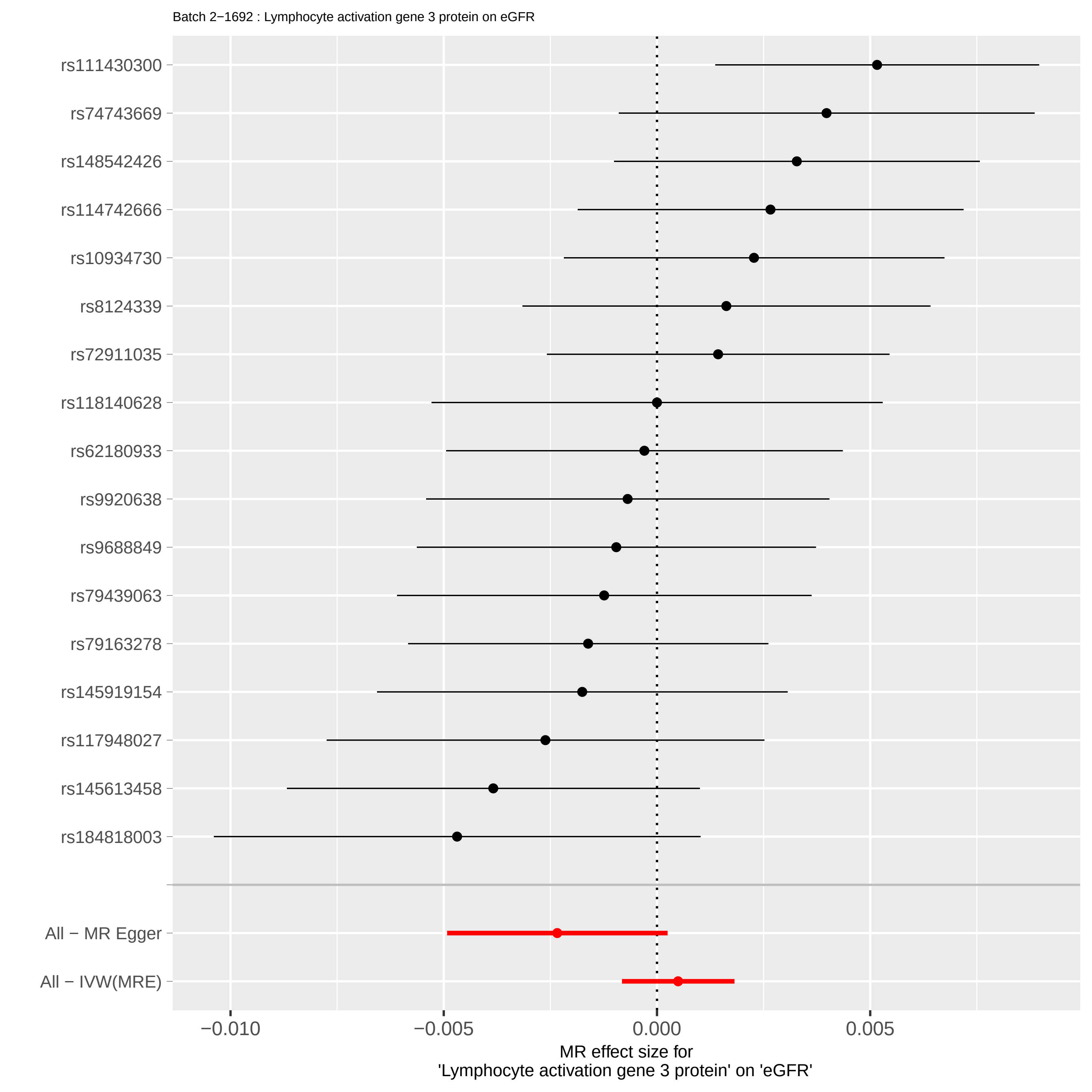

Batch 2−1692 : Lymphocyte activation gene 3 protein on eGFR
rs111430300
rs74743669
rs148542426
rs114742666
rs10934730
rs8124339
rs72911035
rs118140628
rs62180933
rs9920638
rs9688849
rs79439063
rs79163278
rs145919154
rs117948027
rs145613458
rs184818003
All − MR Egger
All − IVW(MRE)
−0.010
−0.005
0.000
0.005
MR effect size for
'Lymphocyte activation gene 3 protein' on 'eGFR'

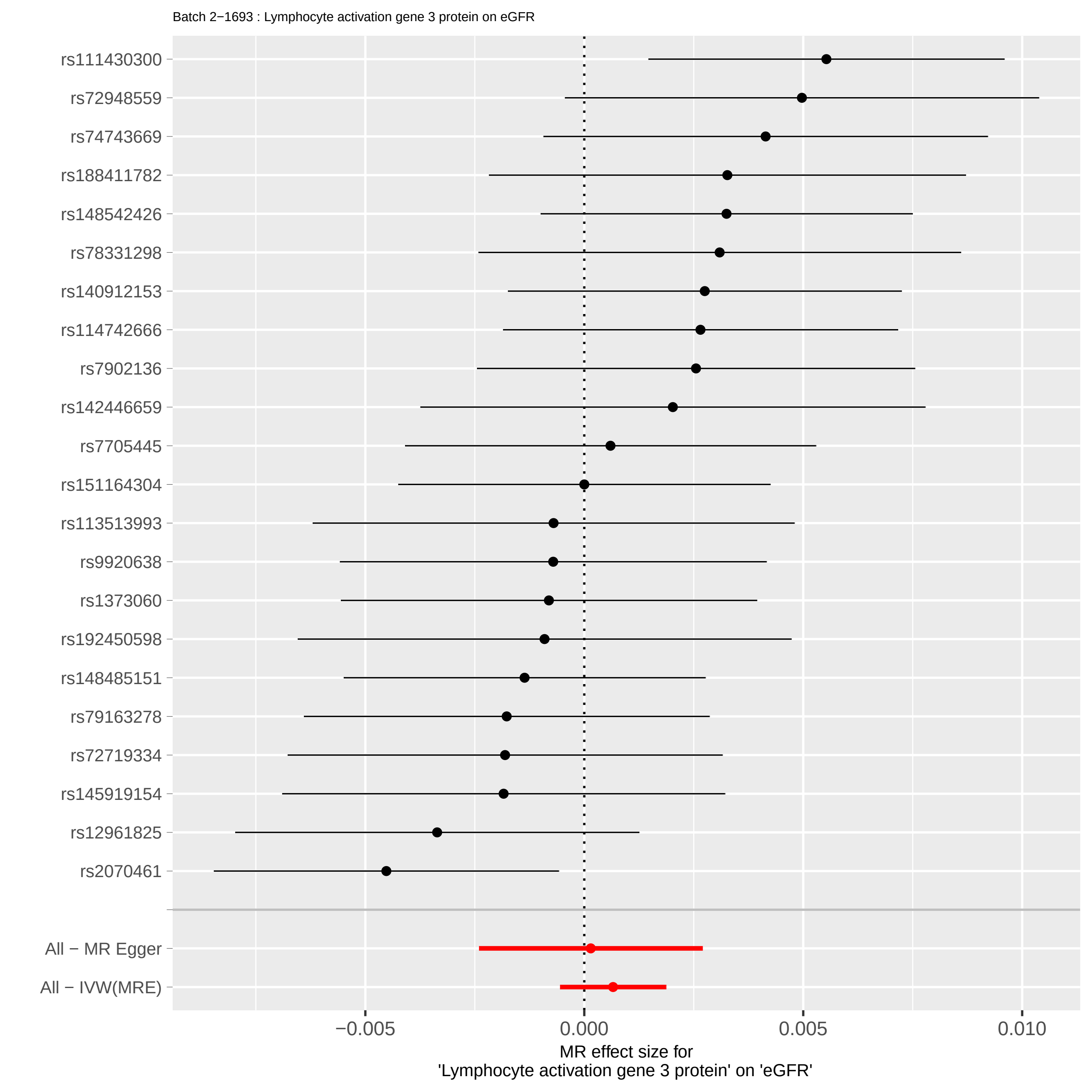

Batch 2−1693 : Lymphocyte activation gene 3 protein on eGFR
rs111430300
rs72948559
rs74743669
rs188411782
rs148542426
rs78331298
rs140912153
rs114742666
rs7902136
rs142446659
rs7705445
rs151164304
rs113513993
rs9920638
rs1373060
rs192450598
rs148485151
rs79163278
rs72719334
rs145919154
rs12961825
rs2070461
All − MR Egger
All − IVW(MRE)
−0.005
0.000
0.005
0.010
MR effect size for
'Lymphocyte activation gene 3 protein' on 'eGFR'

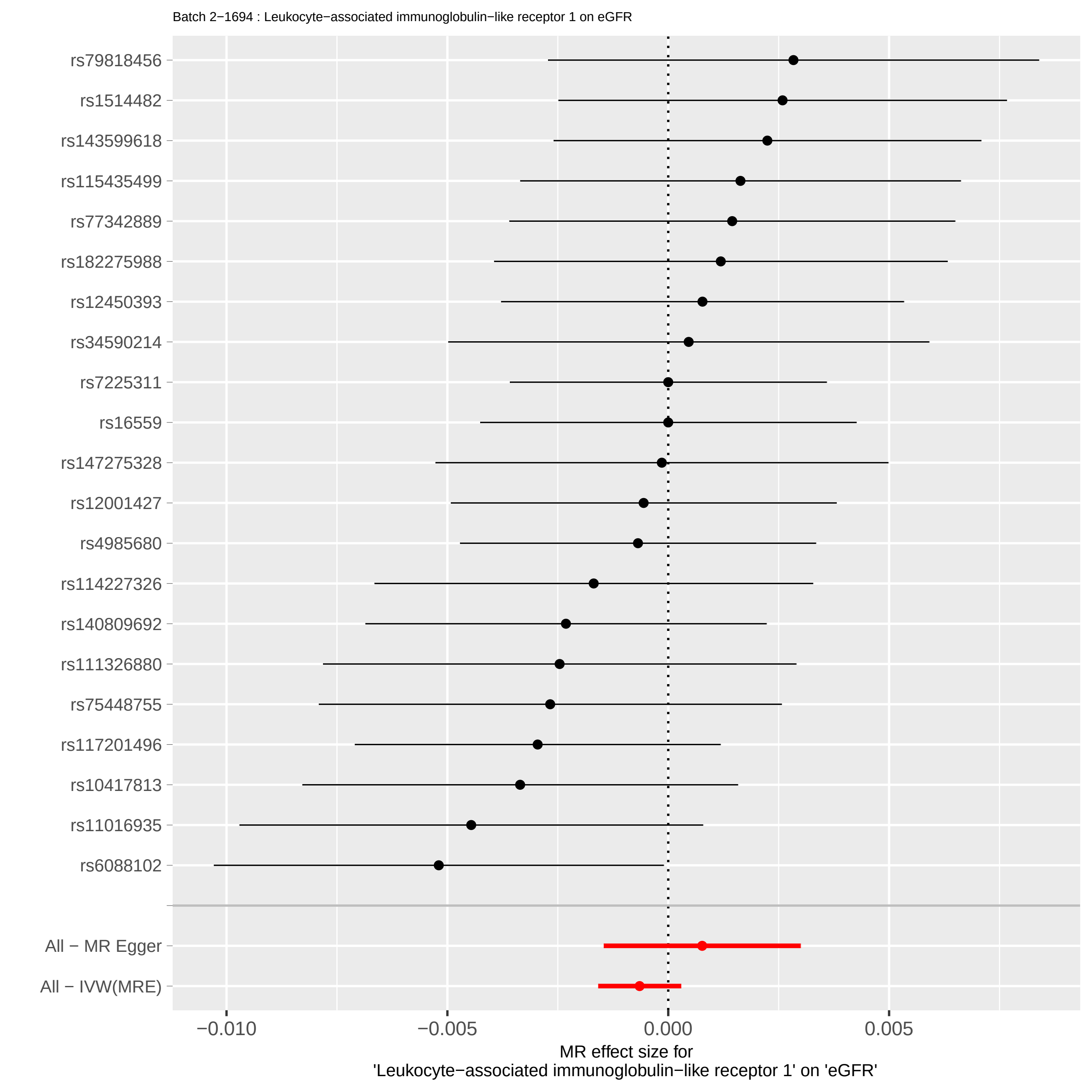

Batch 2−1694 : Leukocyte−associated immunoglobulin−like receptor 1 on eGFR
rs79818456
rs1514482
rs143599618
rs115435499
rs77342889
rs182275988
rs12450393
rs34590214
rs7225311
rs16559
rs147275328
rs12001427
rs4985680
rs114227326
rs140809692
rs111326880
rs75448755
rs117201496
rs10417813
rs11016935
rs6088102
All − MR Egger
All − IVW(MRE)
−0.010
−0.005
0.000
0.005
MR effect size for
'Leukocyte−associated immunoglobulin−like receptor 1' on 'eGFR'

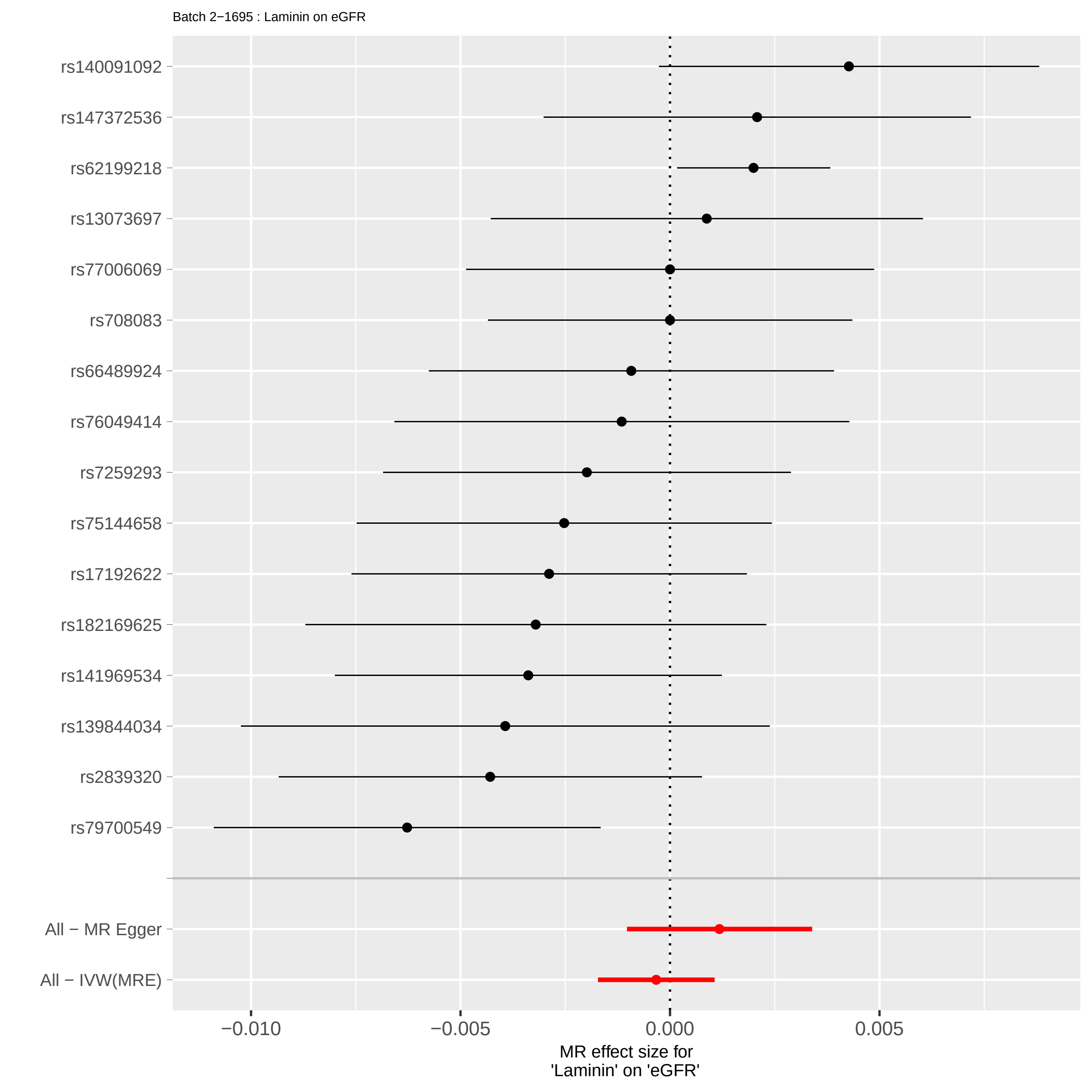

Batch 2−1695 : Laminin on eGFR
rs140091092
rs147372536
rs62199218
rs13073697
rs77006069
rs708083
rs66489924
rs76049414
rs7259293
rs75144658
rs17192622
rs182169625
rs141969534
rs139844034
rs2839320
rs79700549
All − MR Egger
All − IVW(MRE)
−0.010
−0.005
0.000
0.005
MR effect size for
'Laminin' on 'eGFR'

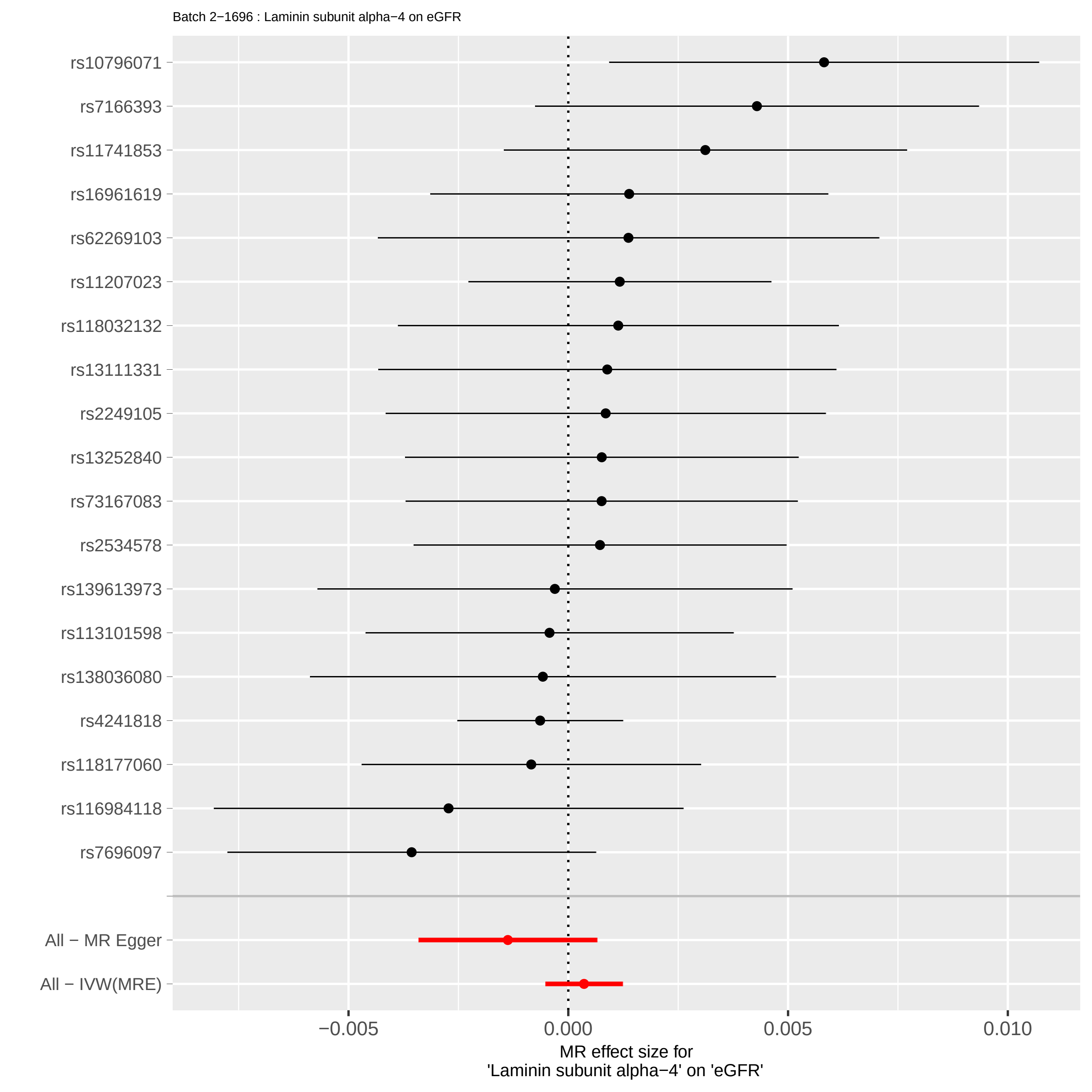
Batch 2−1696 : Laminin subunit alpha−4 on eGFR
rs10796071
rs7166393
rs11741853
rs16961619
rs62269103
rs11207023
rs118032132
rs13111331
rs2249105
rs13252840
rs73167083
rs2534578
rs139613973
rs113101598
rs138036080
rs4241818
rs118177060
rs116984118
rs7696097
All − MR Egger
All − IVW(MRE)
−0.005
0.000
0.005
0.010
MR effect size for
'Laminin subunit alpha−4' on 'eGFR'

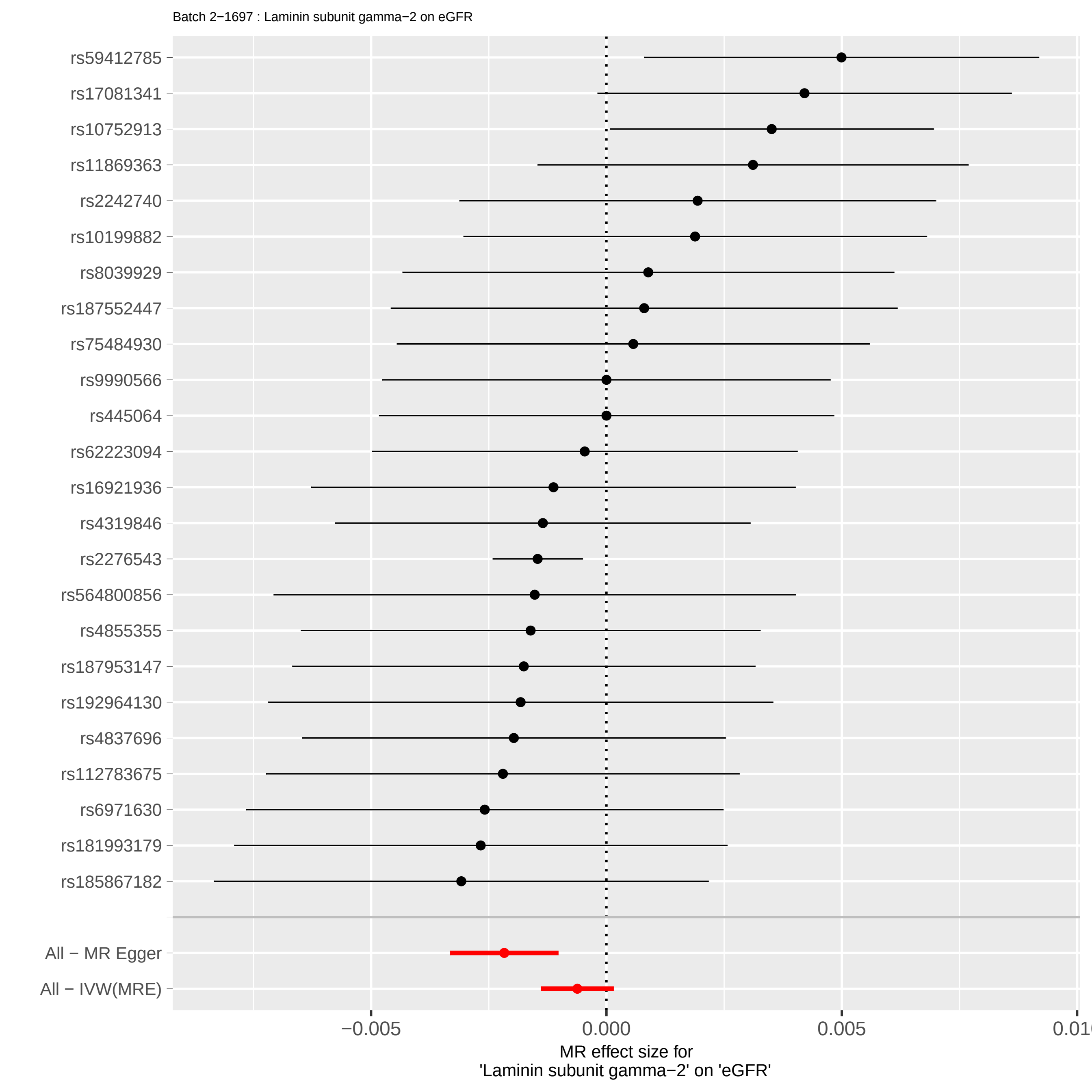

Batch 2−1697 : Laminin subunit gamma−2 on eGFR
rs59412785
rs17081341
rs10752913
rs11869363
rs2242740
rs10199882
rs8039929
rs187552447
rs75484930
rs9990566
rs445064
rs62223094
rs16921936
rs4319846
rs2276543
rs564800856
rs4855355
rs187953147
rs192964130
rs4837696
rs112783675
rs6971630
rs181993179
rs185867182
All − MR Egger
All − IVW(MRE)
−0.005
0.000
0.005
MR effect size for
'Laminin subunit gamma−2' on 'eGFR'

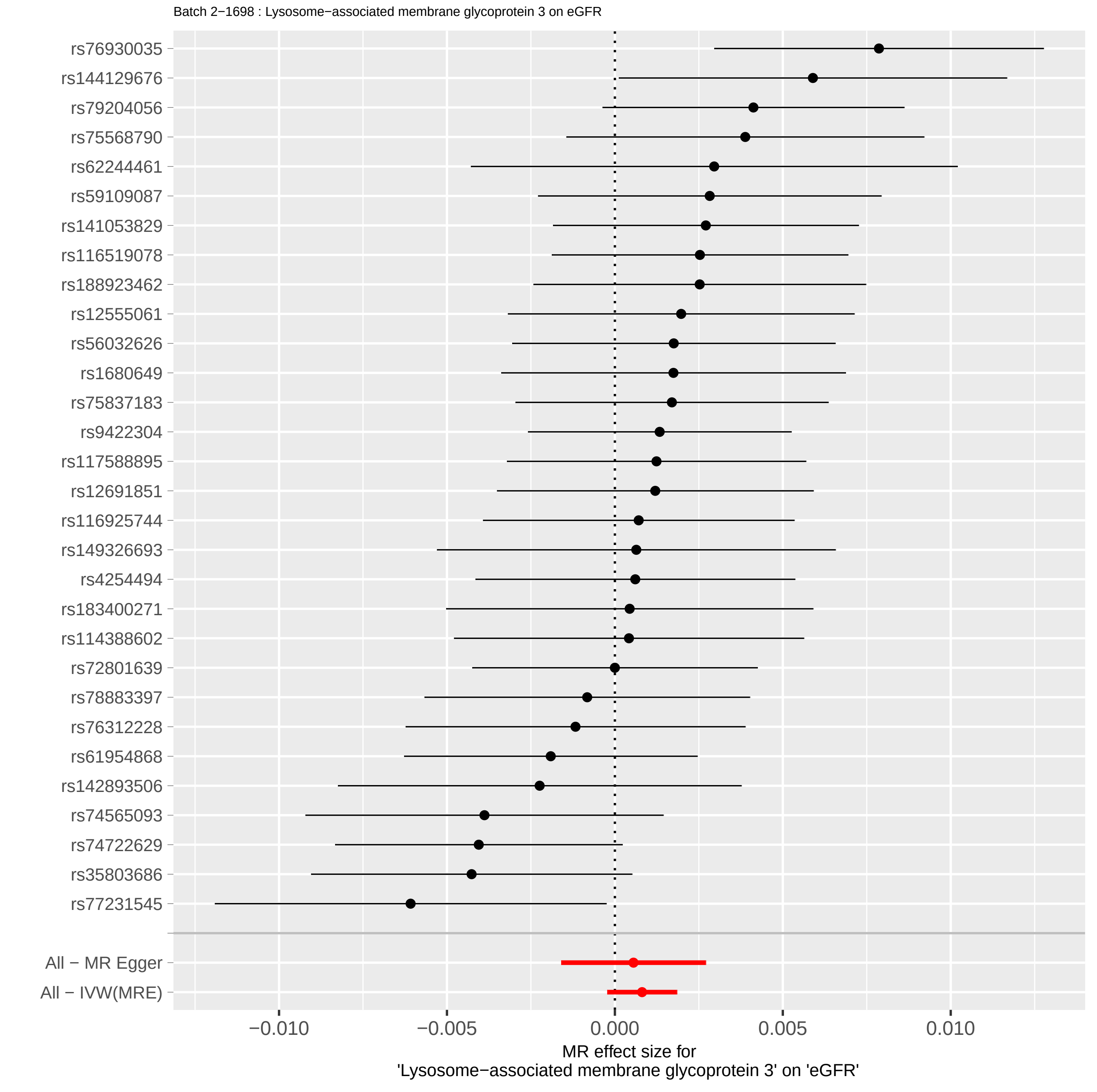

Batch 2−1698 : Lysosome−associated membrane glycoprotein 3 on eGFR
rs76930035
rs144129676
rs79204056
rs75568790
rs62244461
rs59109087
rs141053829
rs116519078
rs188923462
rs12555061
rs56032626
rs1680649
rs75837183
rs9422304
rs117588895
rs12691851
rs116925744
rs149326693
rs4254494
rs183400271
rs114388602
rs72801639
rs78883397
rs76312228
rs61954868
rs142893506
rs74565093
rs74722629
rs35803686
rs77231545
All − MR Egger
All − IVW(MRE)
−0.010
−0.005
0.000
0.005
0.010
MR effect size for
'Lysosome−associated membrane glycoprotein 3' on 'eGFR'

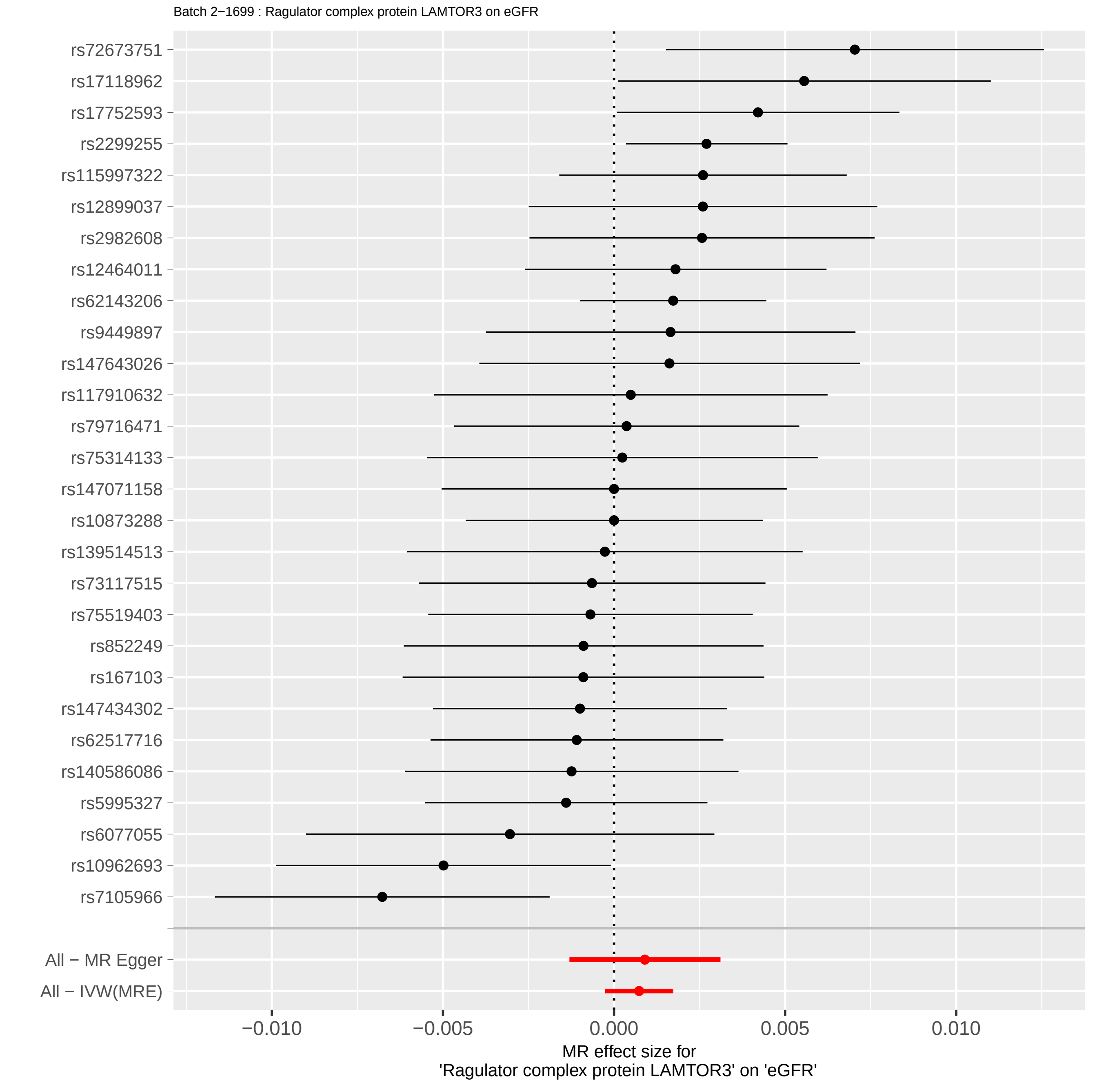

Batch 2−1699 : Ragulator complex protein LAMTOR3 on eGFR
rs72673751
rs17118962
rs17752593
rs2299255
rs115997322
rs12899037
rs2982608
rs12464011
rs62143206
rs9449897
rs147643026
rs117910632
rs79716471
rs75314133
rs147071158
rs10873288
rs139514513
rs73117515
rs75519403
rs852249
rs167103
rs147434302
rs62517716
rs140586086
rs5995327
rs6077055
rs10962693
rs7105966
All − MR Egger
All − IVW(MRE)
−0.010
−0.005
0.000
0.005
0.010
MR effect size for
'Ragulator complex protein LAMTOR3' on 'eGFR'

Batch 2−1700 : Glycosyltransferase−like protein LARGE1 on eGFR

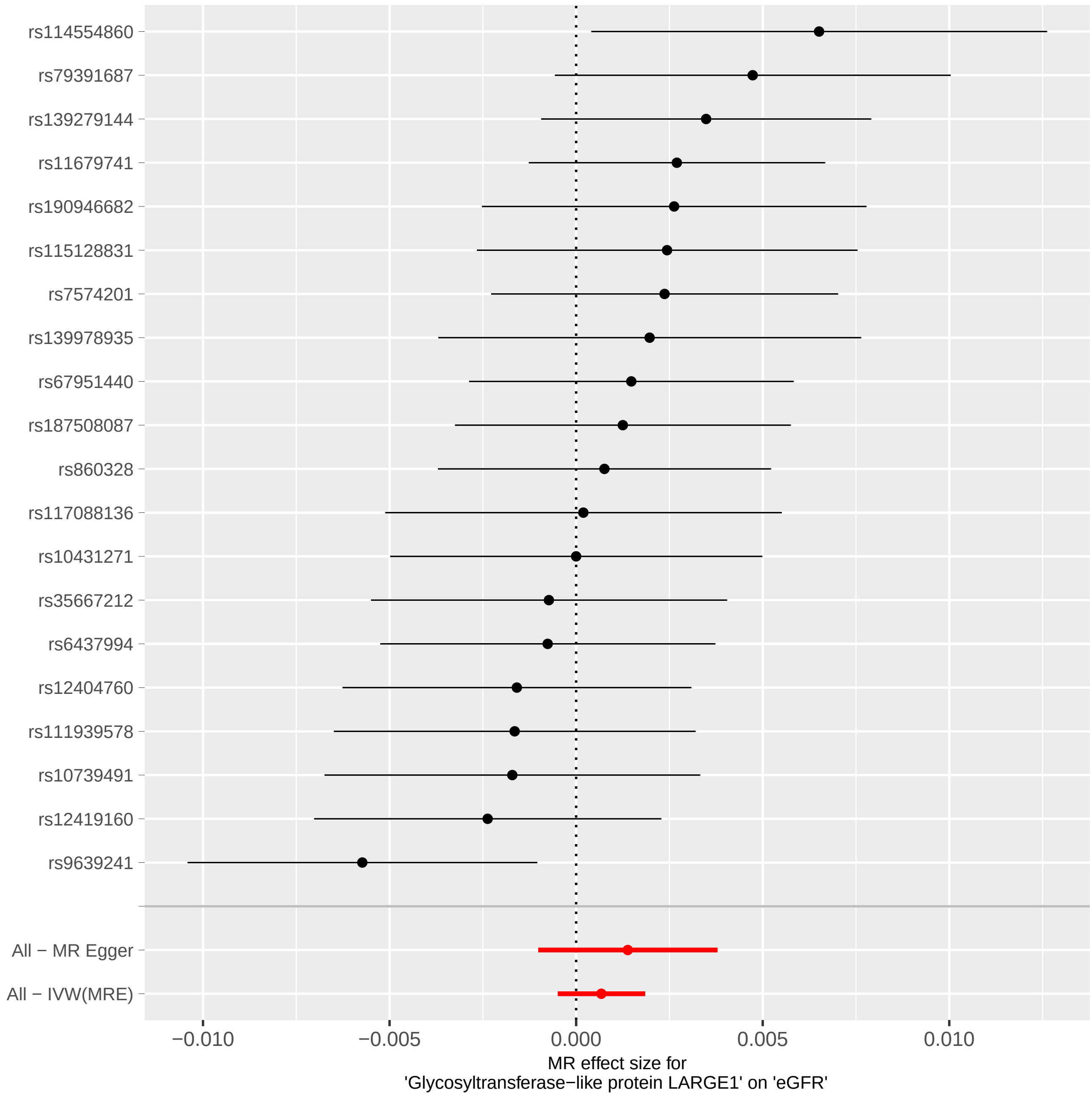

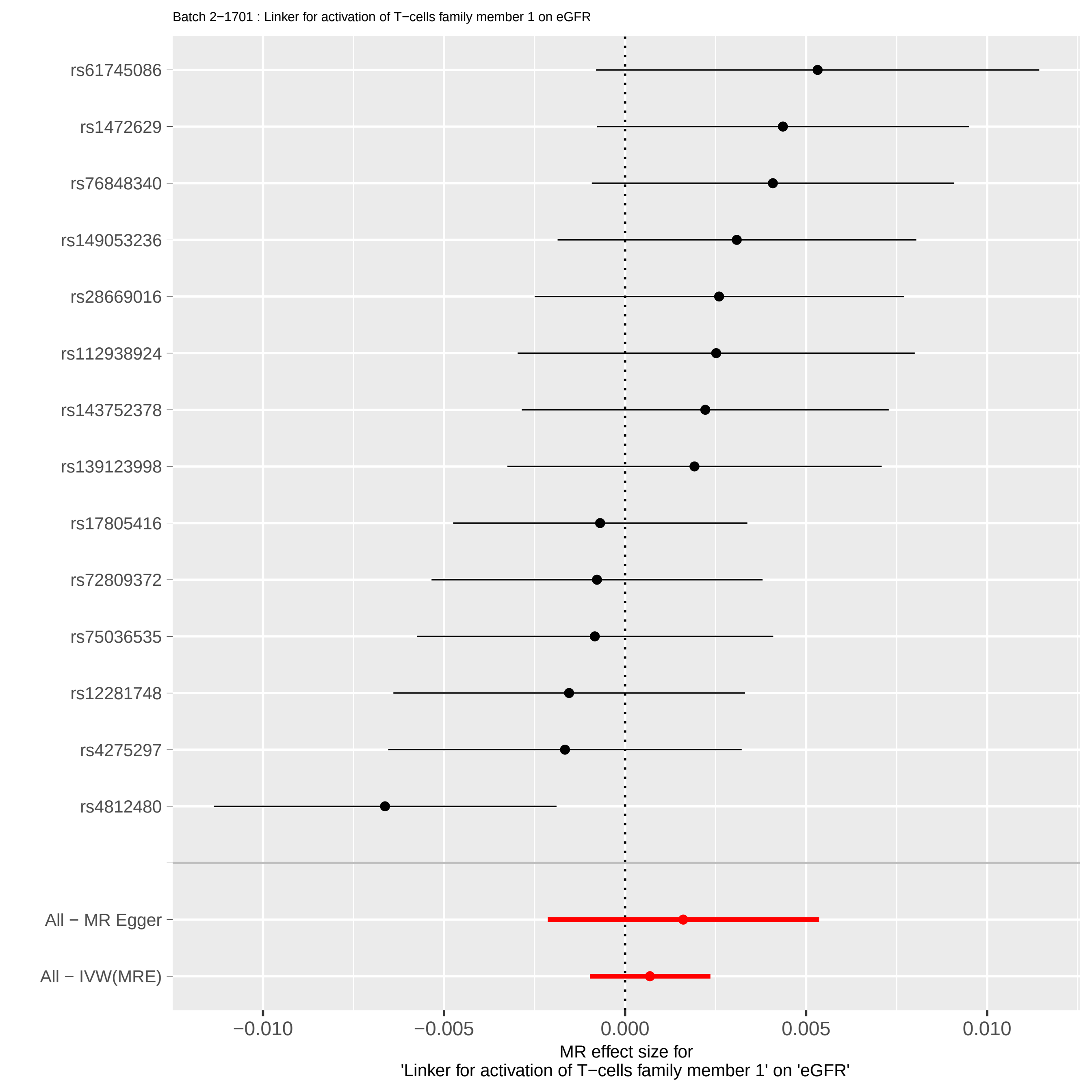
Batch 2−1701 : Linker for activation of T−cells family member 1 on eGFR
rs61745086
rs1472629
rs76848340
rs149053236
rs28669016
rs112938924
rs143752378
rs139123998
rs17805416
rs72809372
rs75036535
rs12281748
rs4275297
rs4812480
All − MR Egger
All − IVW(MRE)
−0.010
−0.005
0.000
0.005
0.010
MR effect size for
'Linker for activation of T−cells family member 1' on 'eGFR'

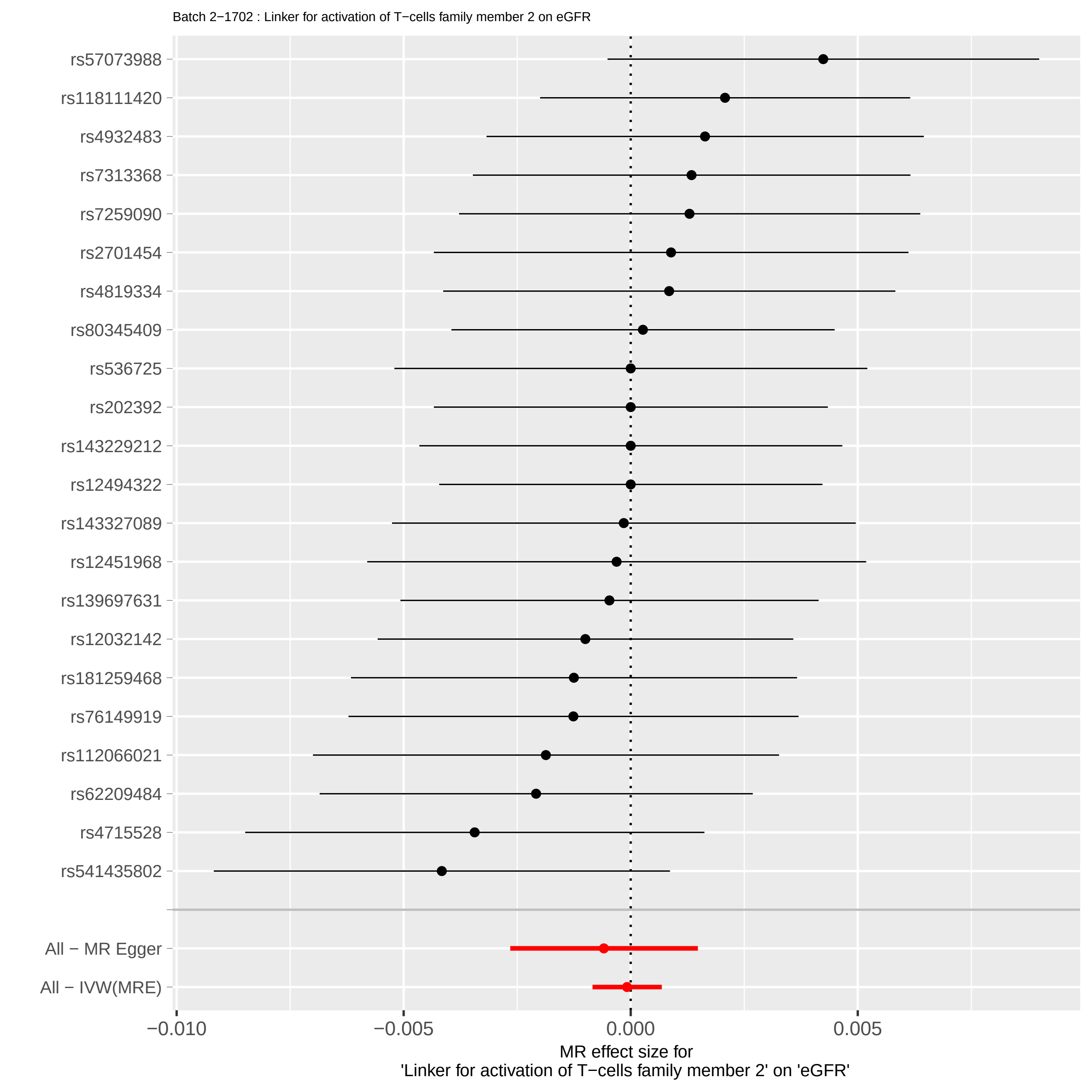
Batch 2−1702 : Linker for activation of T−cells family member 2 on eGFR
rs57073988
rs118111420
rs4932483
rs7313368
rs7259090
rs2701454
rs4819334
rs80345409
rs536725
rs202392
rs143229212
rs12494322
rs143327089
rs12451968
rs139697631
rs12032142
rs181259468
rs76149919
rs112066021
rs62209484
rs4715528
rs541435802
All − MR Egger
All − IVW(MRE)
−0.010
−0.005
0.000
0.005
MR effect size for
'Linker for activation of T−cells family member 2' on 'eGFR'

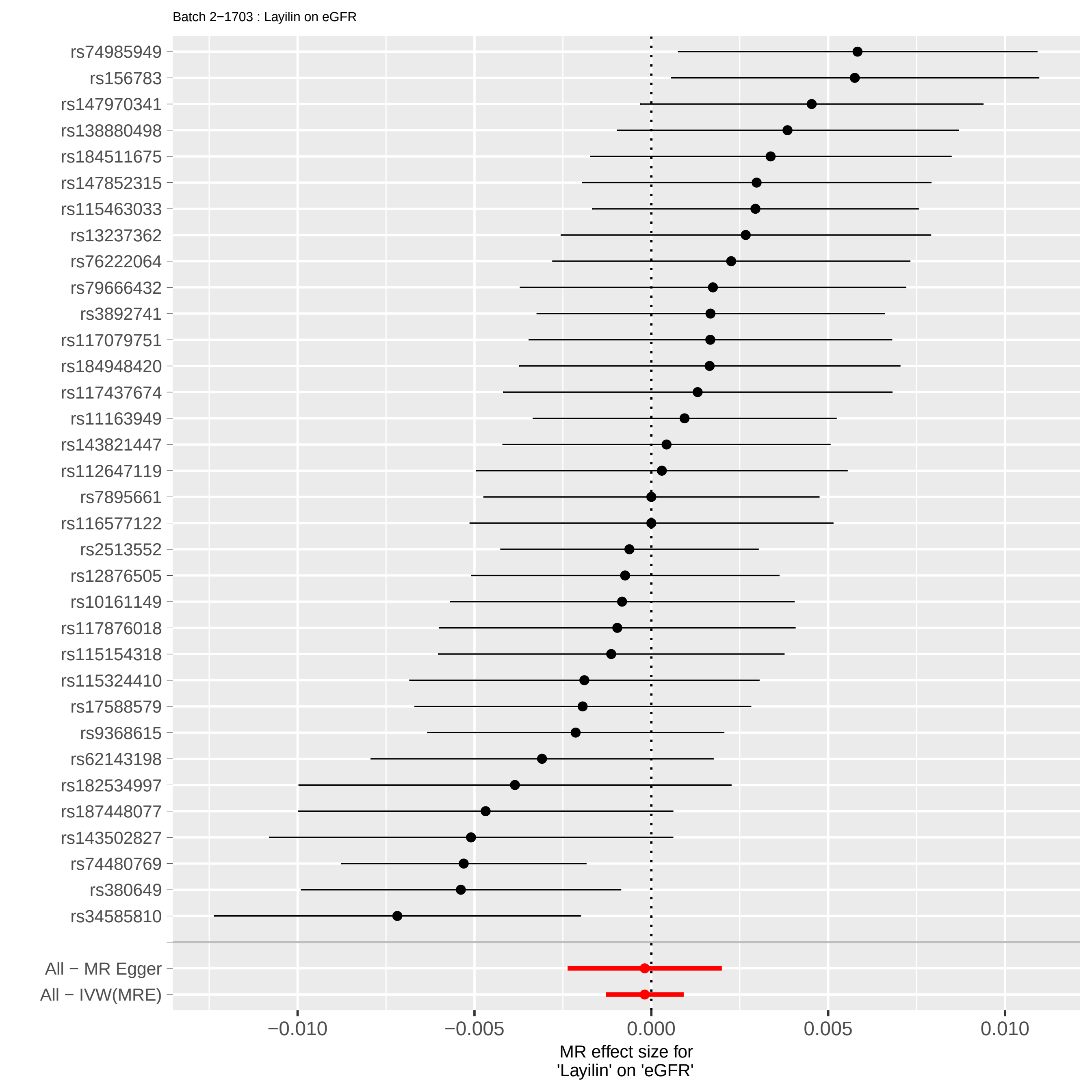
Batch 2−1703 : Layilin on eGFR
rs74985949
rs156783
rs147970341
rs138880498
rs184511675
rs147852315
rs115463033
rs13237362
rs76222064
rs79666432
rs3892741
rs117079751
rs184948420
rs117437674
rs11163949
rs143821447
rs112647119
rs7895661
rs116577122
rs2513552
rs12876505
rs10161149
rs117876018
rs115154318
rs115324410
rs17588579
rs9368615
rs62143198
rs182534997
rs187448077
rs143502827
rs74480769
rs380649
rs34585810
All − MR Egger
All − IVW(MRE)
−0.010
−0.005
0.000
0.005
0.010
MR effect size for
'Layilin' on 'eGFR'

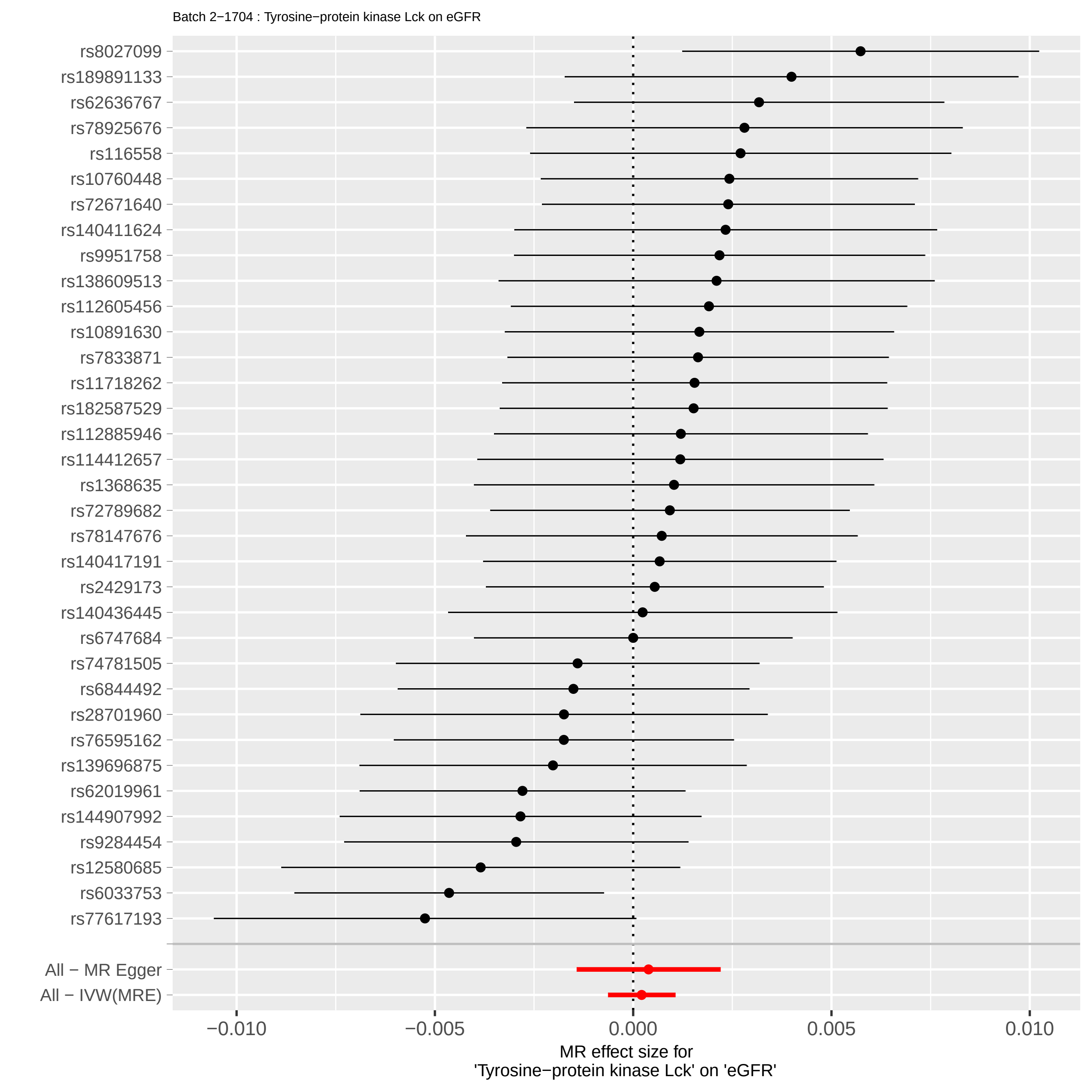

Batch 2−1704 : Tyrosine−protein kinase Lck on eGFR
rs8027099
rs189891133
rs62636767
rs78925676
rs116558
rs10760448
rs72671640
rs140411624
rs9951758
rs138609513
rs112605456
rs10891630
rs7833871
rs11718262
rs182587529
rs112885946
rs114412657
rs1368635
rs72789682
rs78147676
rs140417191
rs2429173
rs140436445
rs6747684
rs74781505
rs6844492
rs28701960
rs76595162
rs139696875
rs62019961
rs144907992
rs9284454
rs12580685
rs6033753
rs77617193
All − MR Egger
All − IVW(MRE)
−0.010
−0.005
0.000
0.005
0.010
MR effect size for
'Tyrosine−protein kinase Lck' on 'eGFR'

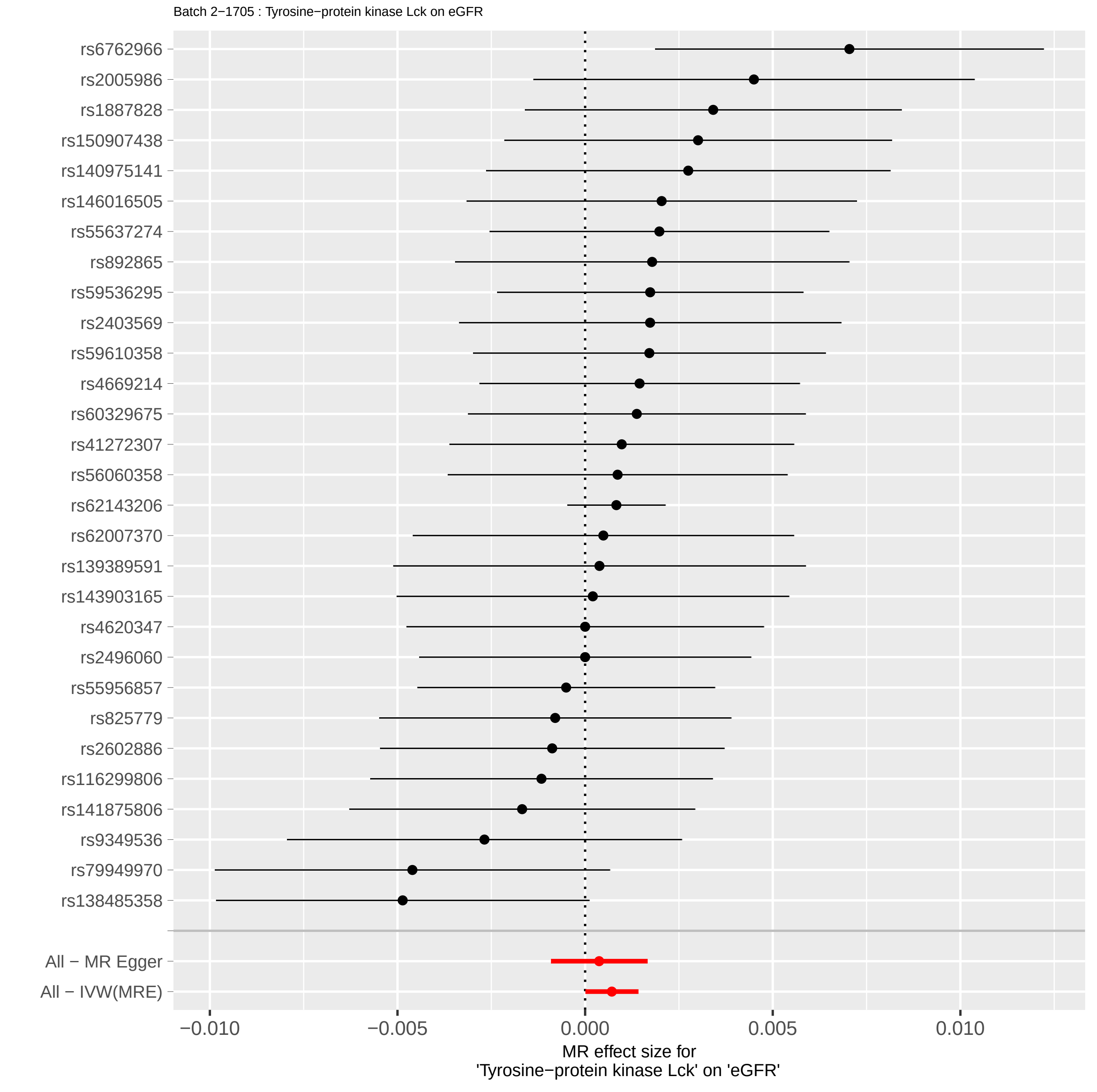

Batch 2−1705 : Tyrosine−protein kinase Lck on eGFR
rs6762966
rs2005986
rs1887828
rs150907438
rs140975141
rs146016505
rs55637274
rs892865
rs59536295
rs2403569
rs59610358
rs4669214
rs60329675
rs41272307
rs56060358
rs62143206
rs62007370
rs139389591
rs143903165
rs4620347
rs2496060
rs55956857
rs825779
rs2602886
rs116299806
rs141875806
rs9349536
rs79949970
rs138485358
All − MR Egger
All − IVW(MRE)
−0.010
−0.005
0.000
0.005
0.010
MR effect size for
'Tyrosine−protein kinase Lck' on 'eGFR'

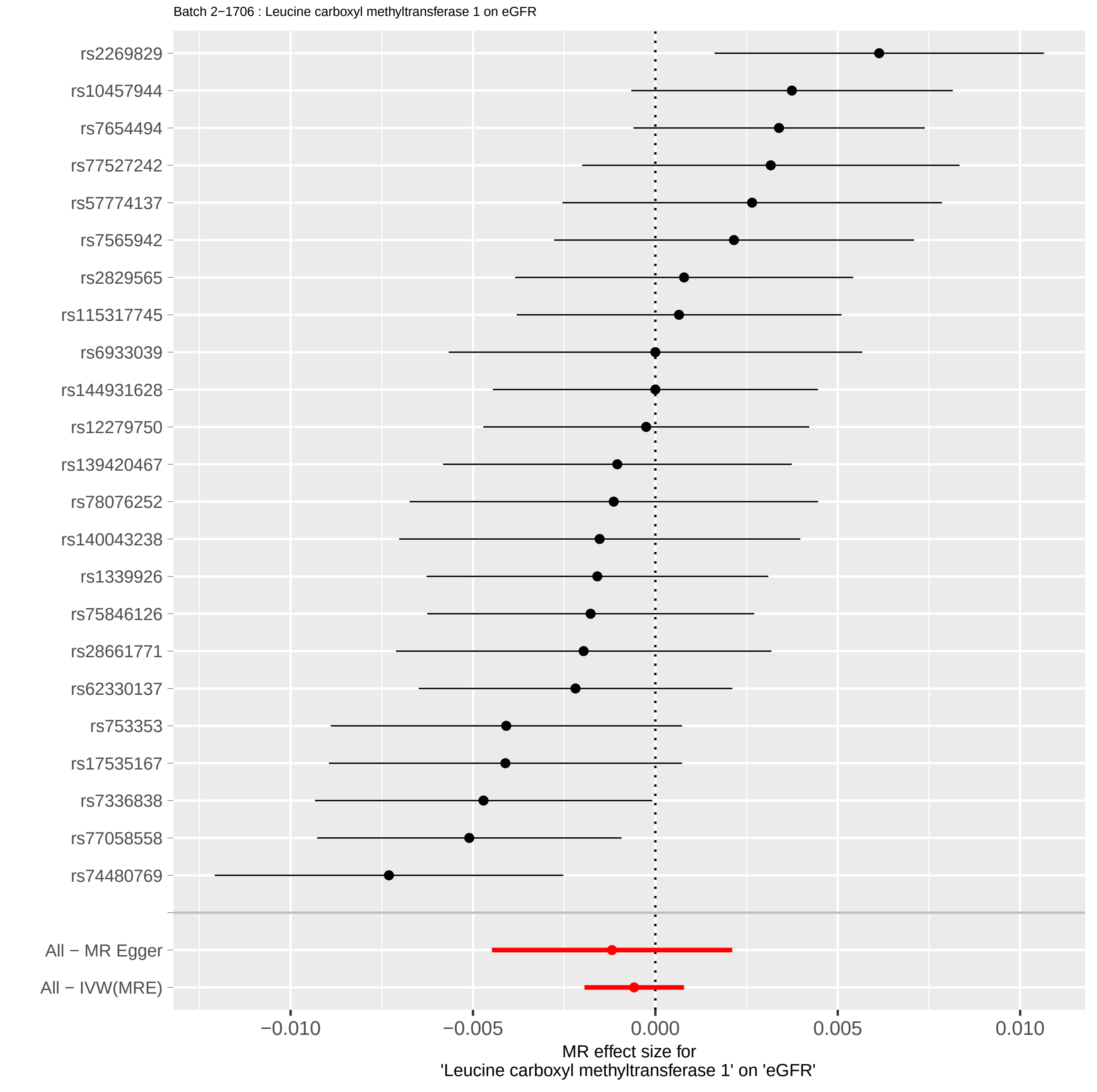

Batch 2−1706 : Leucine carboxyl methyltransferase 1 on eGFR
rs2269829
rs10457944
rs7654494
rs77527242
rs57774137
rs7565942
rs2829565
rs115317745
rs6933039
rs144931628
rs12279750
rs139420467
rs78076252
rs140043238
rs1339926
rs75846126
rs28661771
rs62330137
rs753353
rs17535167
rs7336838
rs77058558
rs74480769
All − MR Egger
All − IVW(MRE)
−0.010
−0.005
0.000
0.005
0.010
MR effect size for
'Leucine carboxyl methyltransferase 1' on 'eGFR'

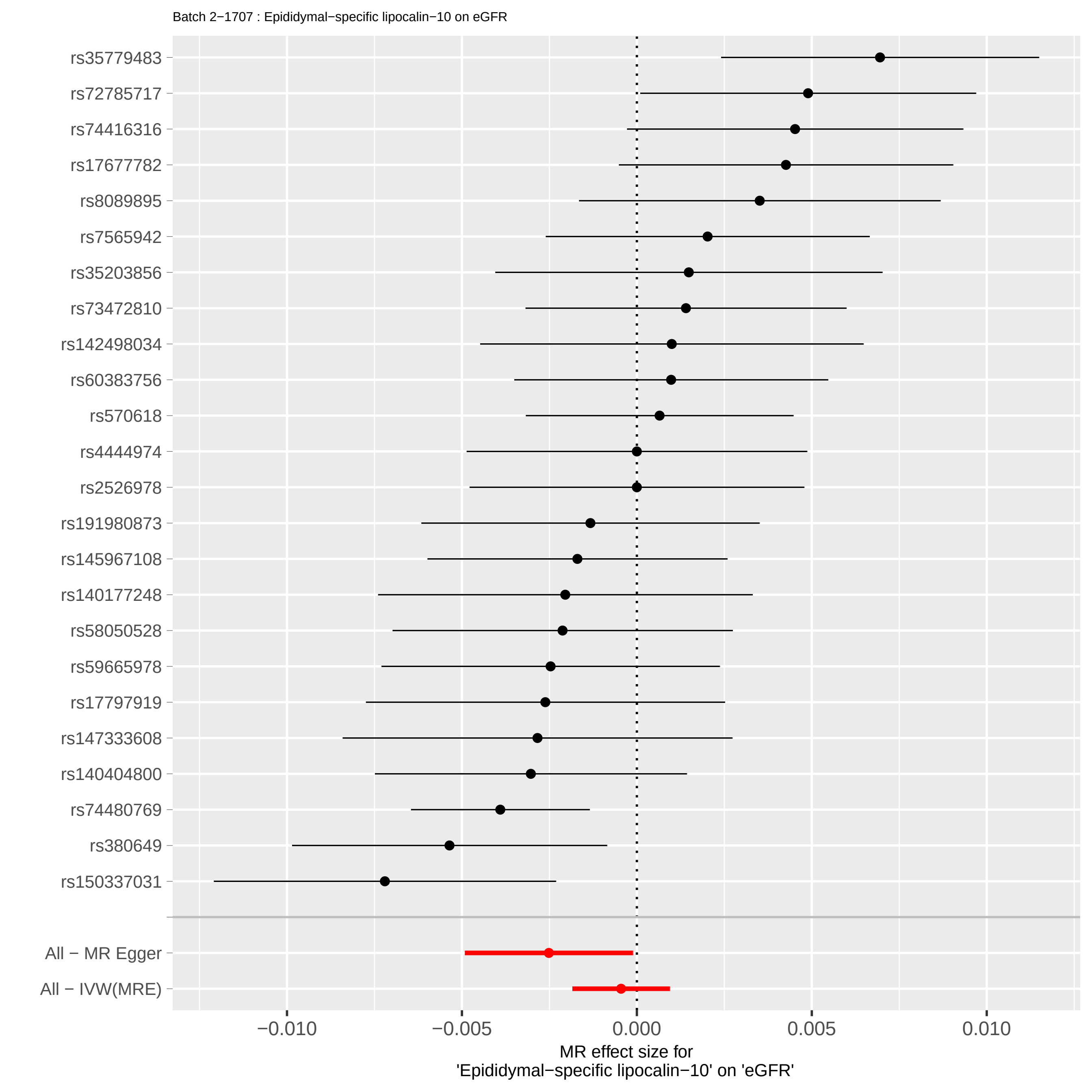
Batch 2−1707 : Epididymal−specific lipocalin−10 on eGFR
rs35779483
rs72785717
rs74416316
rs17677782
rs8089895
rs7565942
rs35203856
rs73472810
rs142498034
rs60383756
rs570618
rs4444974
rs2526978
rs191980873
rs145967108
rs140177248
rs58050528
rs59665978
rs17797919
rs147333608
rs140404800
rs74480769
rs380649
rs150337031
All − MR Egger
All − IVW(MRE)
−0.010
−0.005
0.000
0.005
0.010
MR effect size for
'Epididymal−specific lipocalin−10' on 'eGFR'

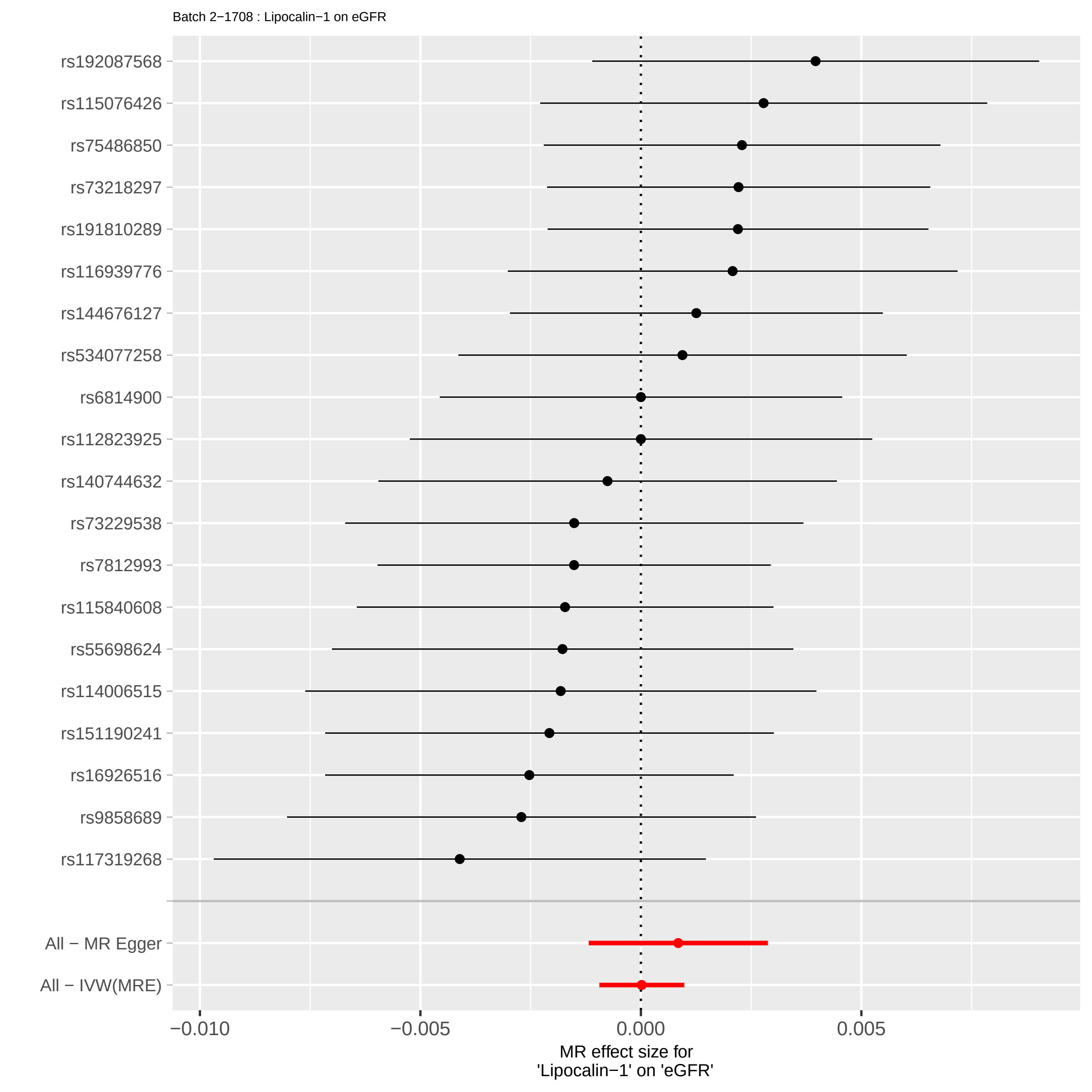
Batch 2−1708 : Lipocalin−1 on eGFR
rs192087568
rs115076426
rs75486850
rs73218297
rs191810289
rs116939776
rs144676127
rs534077258
rs6814900
rs112823925
rs140744632
rs73229538
rs7812993
rs115840608
rs55698624
rs114006515
rs151190241
rs16926516
rs9858689
rs117319268
All − MR Egger
All − IVW(MRE)
−0.010
−0.005
0.000
0.005
MR effect size for
'Lipocalin−1' on 'eGFR'

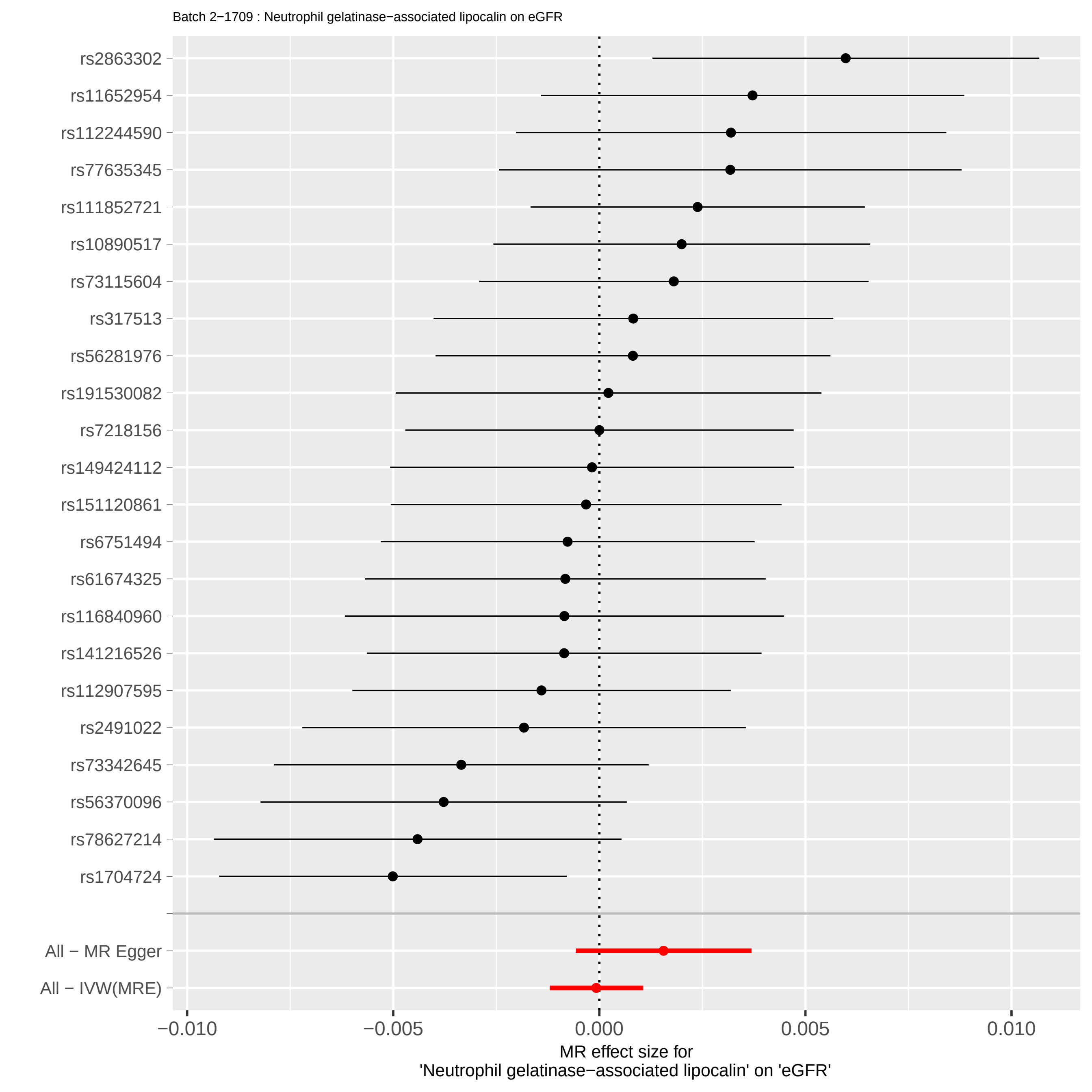

Batch 2−1709 : Neutrophil gelatinase−associated lipocalin on eGFR
rs2863302
rs11652954
rs112244590
rs77635345
rs111852721
rs10890517
rs73115604
rs317513
rs56281976
rs191530082
rs7218156
rs149424112
rs151120861
rs6751494
rs61674325
rs116840960
rs141216526
rs112907595
rs2491022
rs73342645
rs56370096
rs78627214
rs1704724
All − MR Egger
All − IVW(MRE)
−0.010
−0.005
0.000
0.005
0.010
MR effect size for
'Neutrophil gelatinase−associated lipocalin' on 'eGFR'

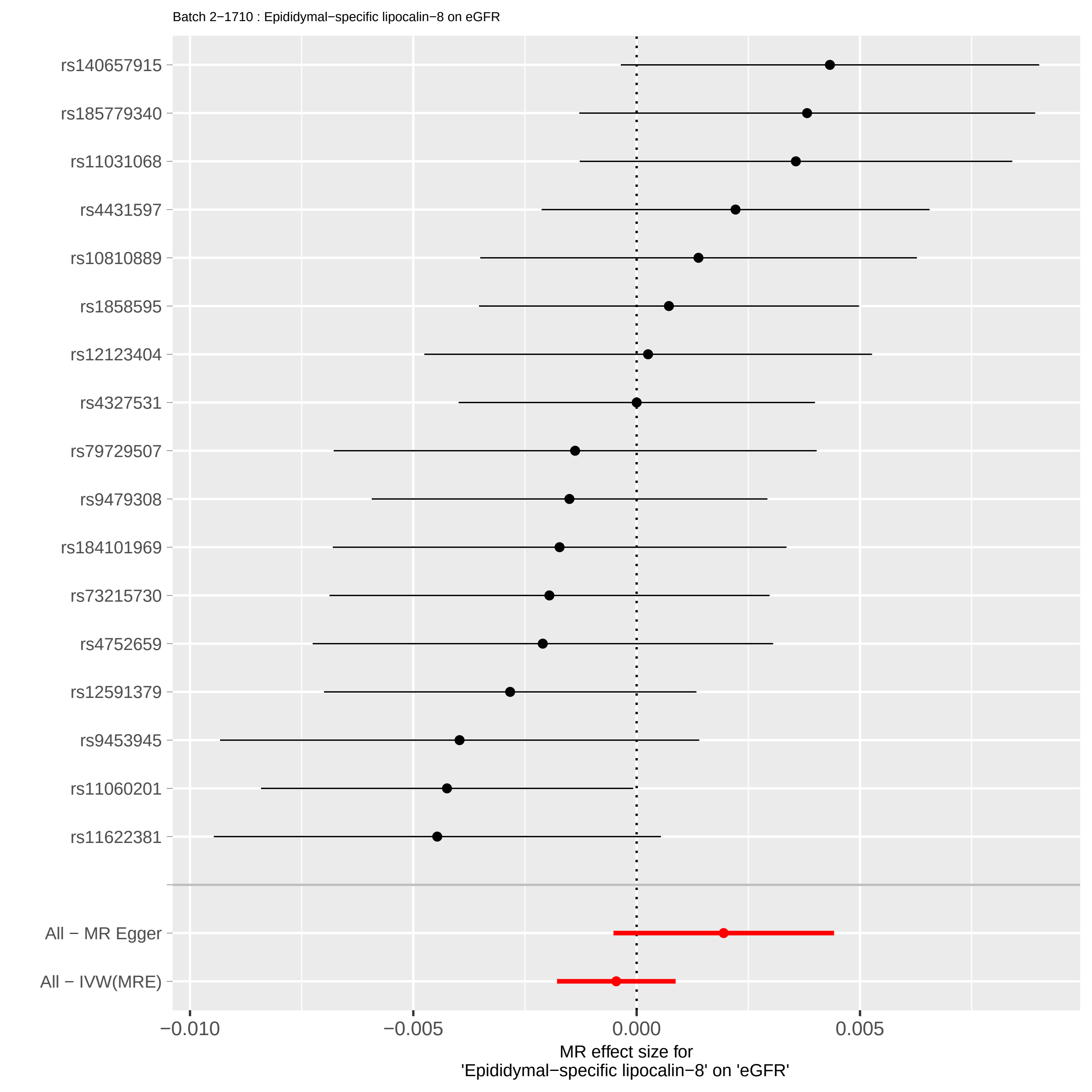

Batch 2−1710 : Epididymal−specific lipocalin−8 on eGFR
rs140657915
rs185779340
rs11031068
rs4431597
rs10810889
rs1858595
rs12123404
rs4327531
rs79729507
rs9479308
rs184101969
rs73215730
rs4752659
rs12591379
rs9453945
rs11060201
rs11622381
All − MR Egger
All − IVW(MRE)
−0.010
−0.005
0.000
0.005
MR effect size for
'Epididymal−specific lipocalin−8' on 'eGFR'

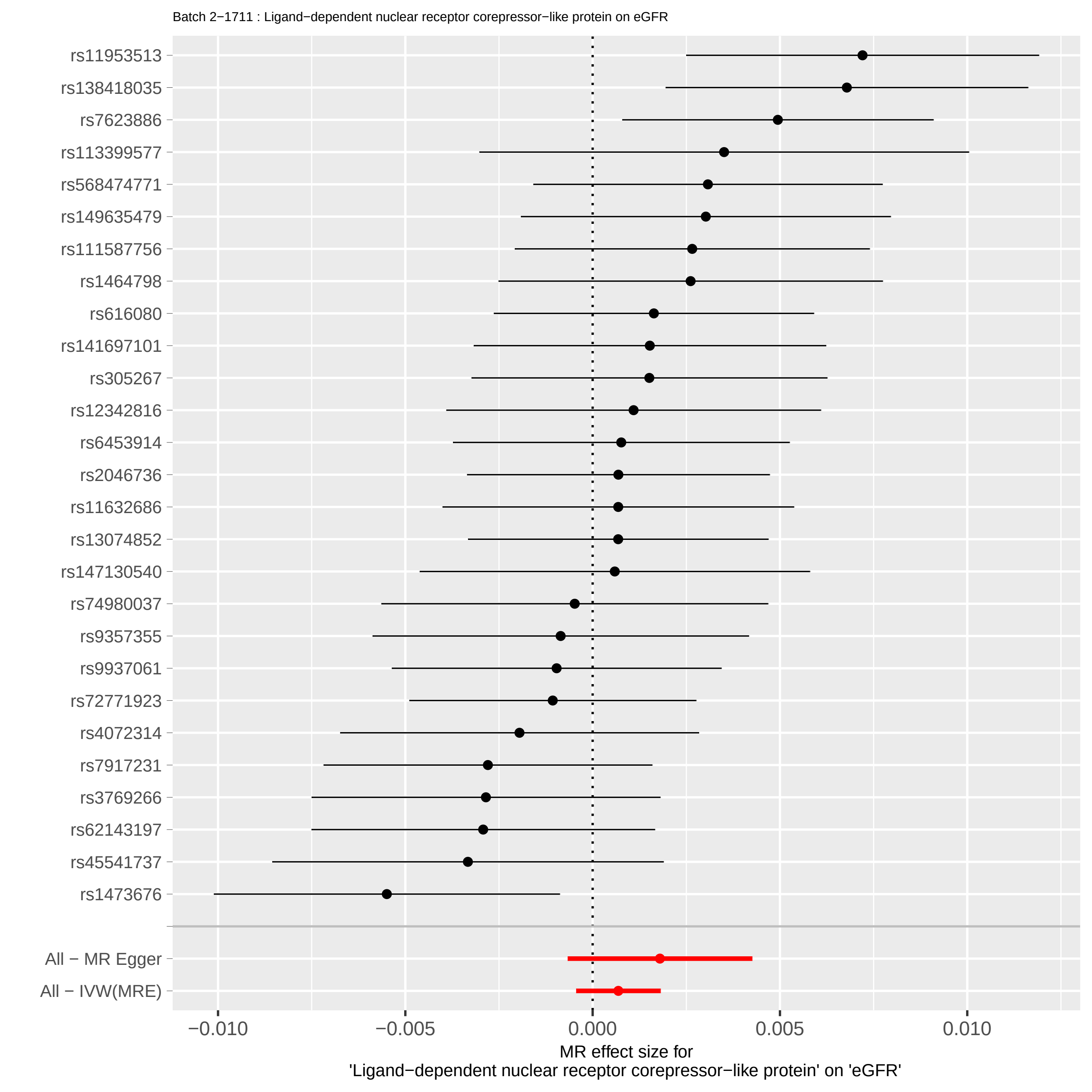
Batch 2−1711 : Ligand−dependent nuclear receptor corepressor−like protein on eGFR
rs11953513
rs138418035
rs7623886
rs113399577
rs568474771
rs149635479
rs111587756
rs1464798
rs616080
rs141697101
rs305267
rs12342816
rs6453914
rs2046736
rs11632686
rs13074852
rs147130540
rs74980037
rs9357355
rs9937061
rs72771923
rs4072314
rs7917231
rs3769266
rs62143197
rs45541737
rs1473676
All − MR Egger
All − IVW(MRE)
−0.010
−0.005
0.000
0.005
0.010
MR effect size for
'Ligand−dependent nuclear receptor corepressor−like protein' on 'eGFR'

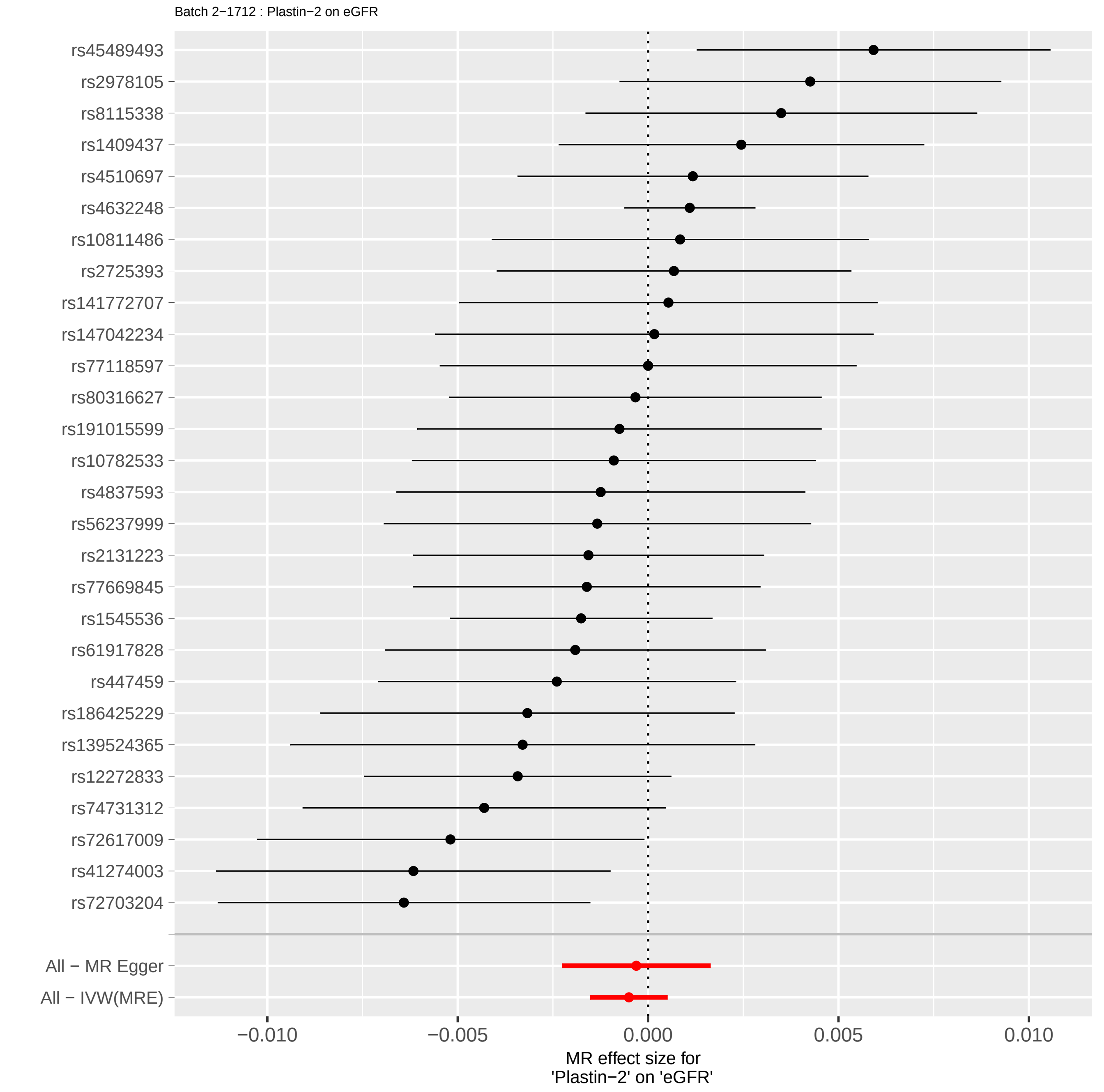

Batch 2−1712 : Plastin−2 on eGFR
rs45489493
rs2978105
rs8115338
rs1409437
rs4510697
rs4632248
rs10811486
rs2725393
rs141772707
rs147042234
rs77118597
rs80316627
rs191015599
rs10782533
rs4837593
rs56237999
rs2131223
rs77669845
rs1545536
rs61917828
rs447459
rs186425229
rs139524365
rs12272833
rs74731312
rs72617009
rs41274003
rs72703204
All − MR Egger
All − IVW(MRE)
−0.010
−0.005
0.000
0.005
0.010
MR effect size for
'Plastin−2' on 'eGFR'

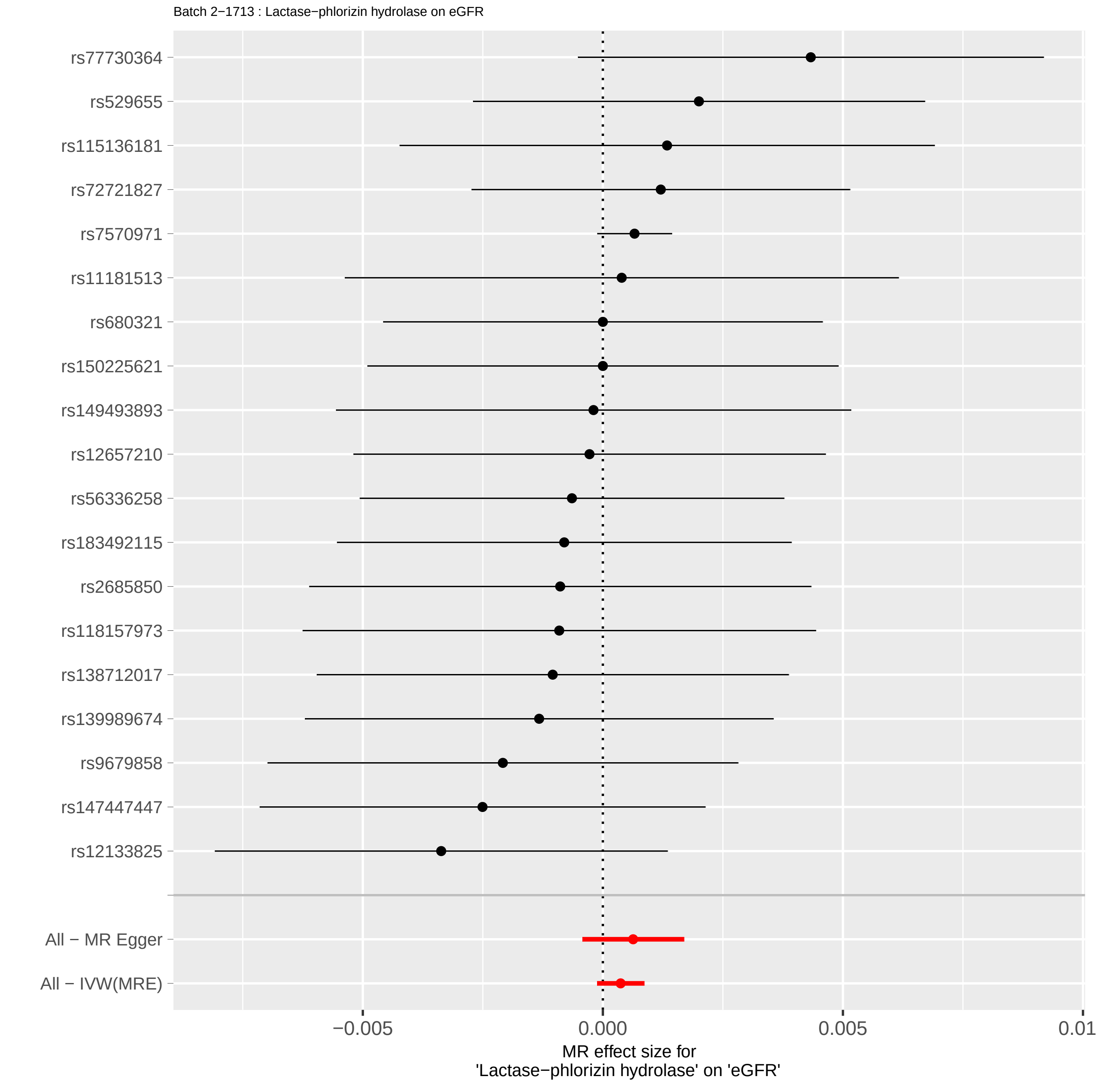

Batch 2−1713 : Lactase−phlorizin hydrolase on eGFR
rs77730364
rs529655
rs115136181
rs72721827
rs7570971
rs11181513
rs680321
rs150225621
rs149493893
rs12657210
rs56336258
rs183492115
rs2685850
rs118157973
rs138712017
rs139989674
rs9679858
rs147447447
rs12133825
All − MR Egger
All − IVW(MRE)
−0.005
0.000
0.005
0.01
MR effect size for
'Lactase−phlorizin hydrolase' on 'eGFR'

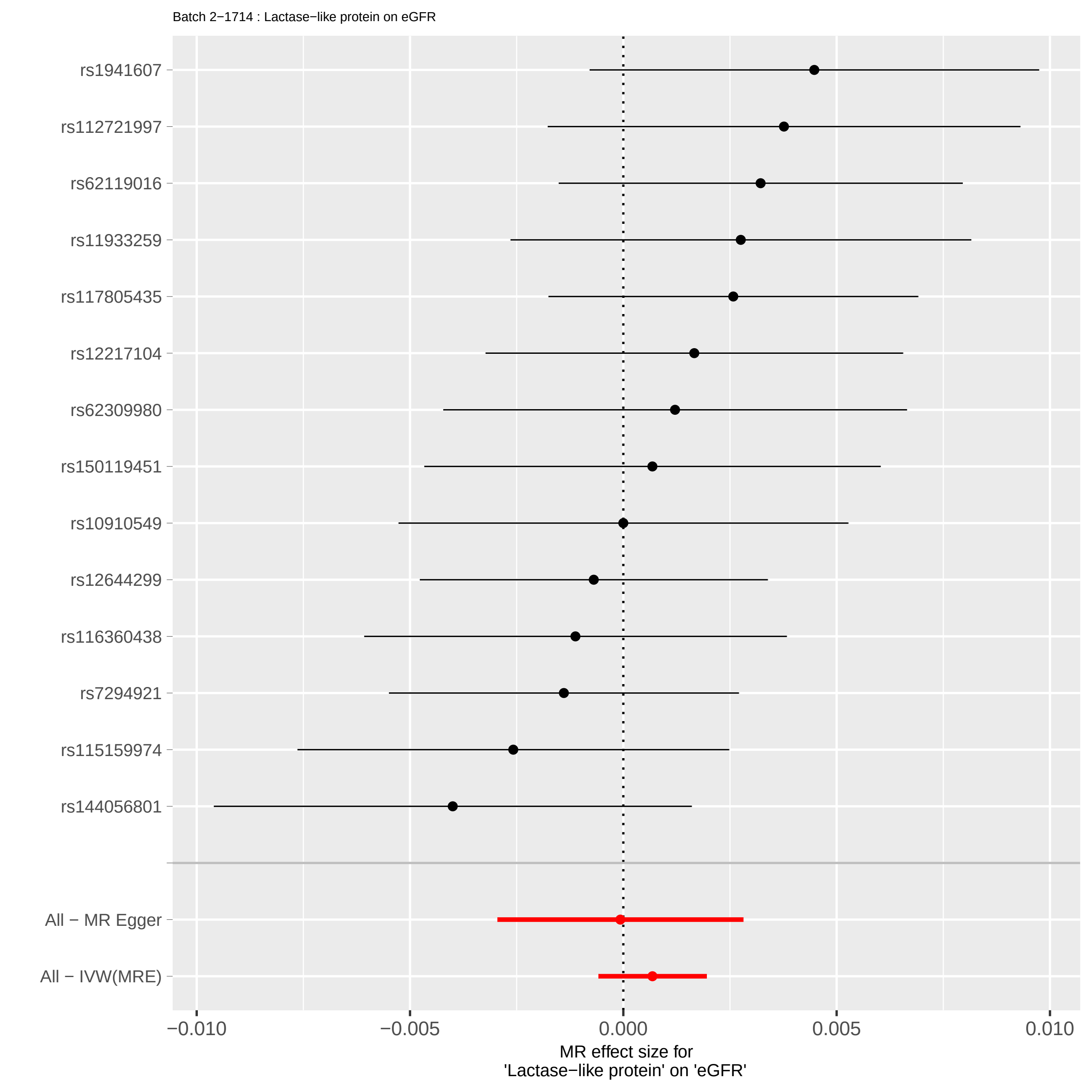

Batch 2−1714 : Lactase−like protein on eGFR
rs1941607
rs112721997
rs62119016
rs11933259
rs117805435
rs12217104
rs62309980
rs150119451
rs10910549
rs12644299
rs116360438
rs7294921
rs115159974
rs144056801
All − MR Egger
All − IVW(MRE)
−0.010
−0.005
0.000
0.005
0.010
MR effect size for
'Lactase−like protein' on 'eGFR'

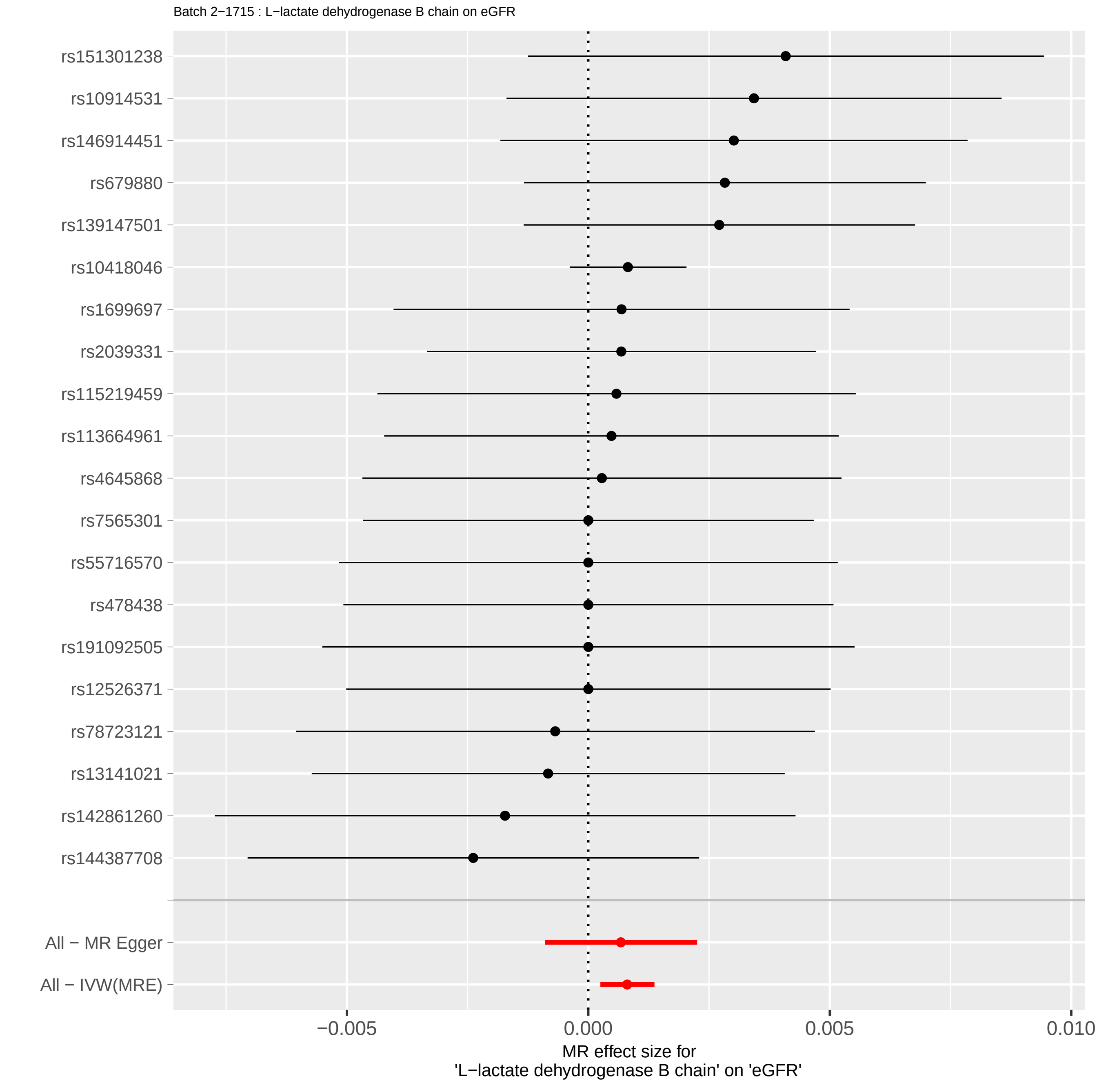

Batch 2−1715 : L−lactate dehydrogenase B chain on eGFR
rs151301238
rs10914531
rs146914451
rs679880
rs139147501
rs10418046
rs1699697
rs2039331
rs115219459
rs113664961
rs4645868
rs7565301
rs55716570
rs478438
rs191092505
rs12526371
rs78723121
rs13141021
rs142861260
rs144387708
All − MR Egger
All − IVW(MRE)
−0.005
0.000
0.005
0.010
MR effect size for
'L−lactate dehydrogenase B chain' on 'eGFR'

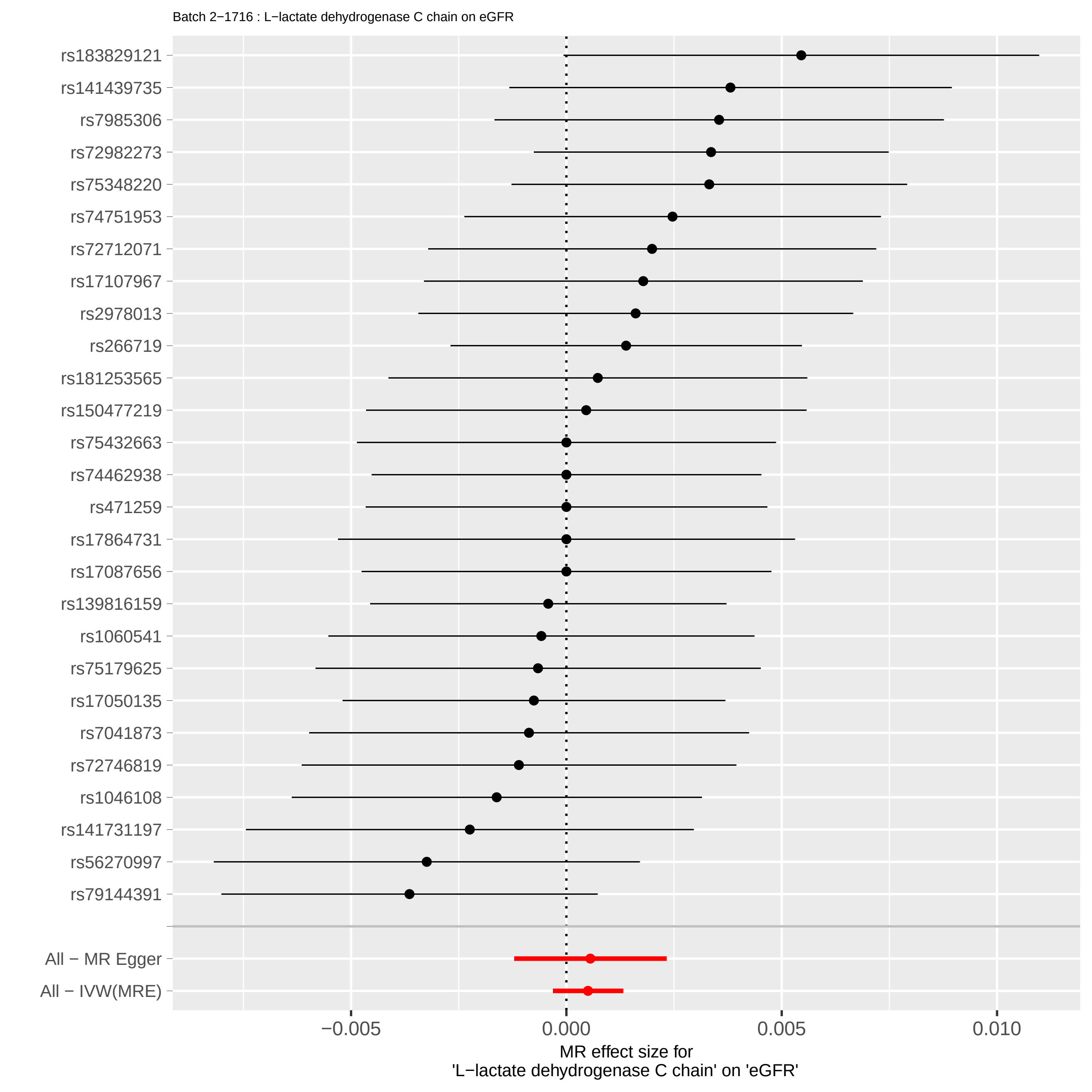
Batch 2−1716 : L−lactate dehydrogenase C chain on eGFR
rs183829121
rs141439735
rs7985306
rs72982273
rs75348220
rs74751953
rs72712071
rs17107967
rs2978013
rs266719
rs181253565
rs150477219
rs75432663
rs74462938
rs471259
rs17864731
rs17087656
rs139816159
rs1060541
rs75179625
rs17050135
rs7041873
rs72746819
rs1046108
rs141731197
rs56270997
rs79144391
All − MR Egger
All − IVW(MRE)
−0.005
0.000
0.005
0.010
MR effect size for
'L−lactate dehydrogenase C chain' on 'eGFR'

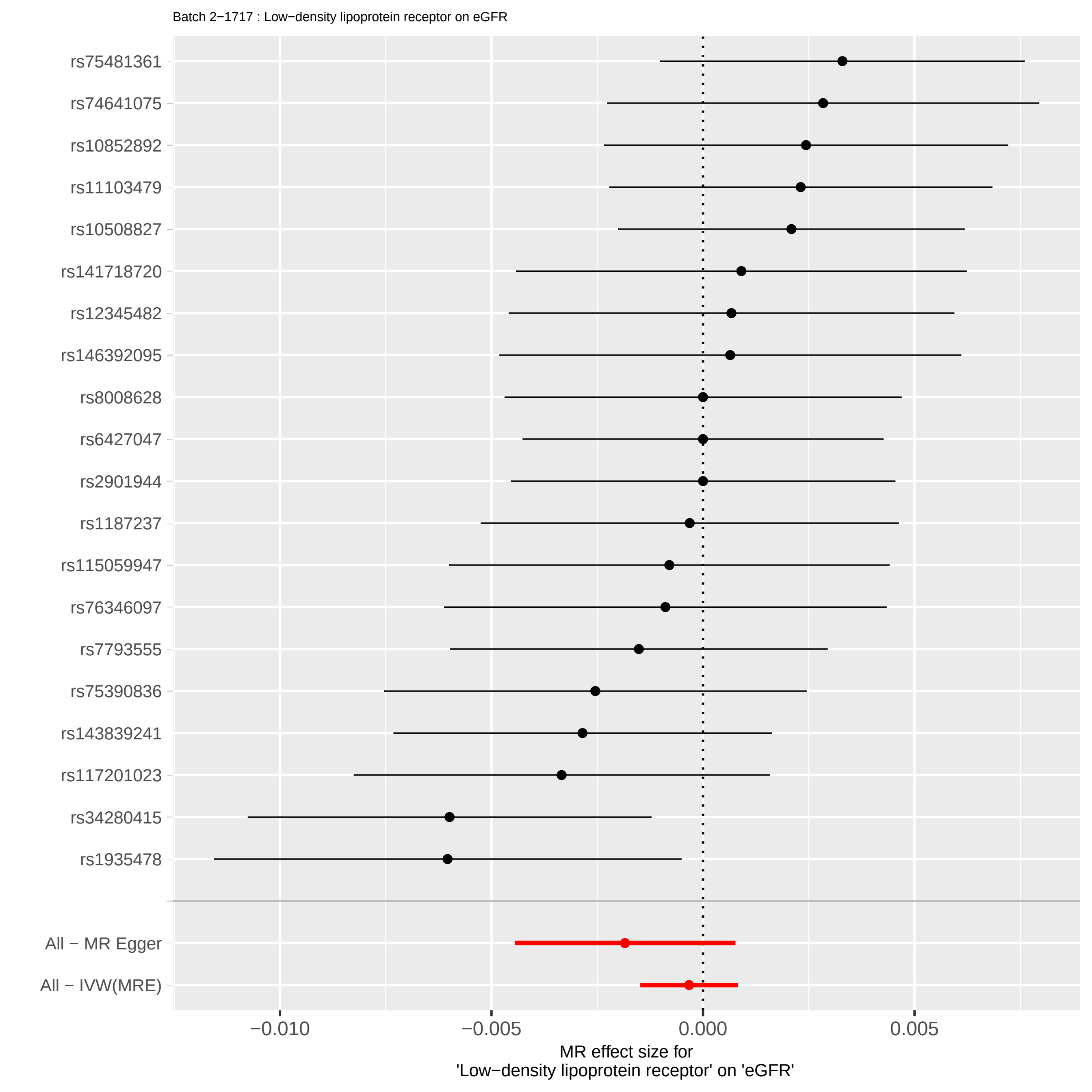
Batch 2−1717 : Low−density lipoprotein receptor on eGFR
rs75481361
rs74641075
rs10852892
rs11103479
rs10508827
rs141718720
rs12345482
rs146392095
rs8008628
rs6427047
rs2901944
rs1187237
rs115059947
rs76346097
rs7793555
rs75390836
rs143839241
rs117201023
rs34280415
rs1935478
All − MR Egger
All − IVW(MRE)
−0.010
−0.005
0.000
0.005
MR effect size for
'Low−density lipoprotein receptor' on 'eGFR'

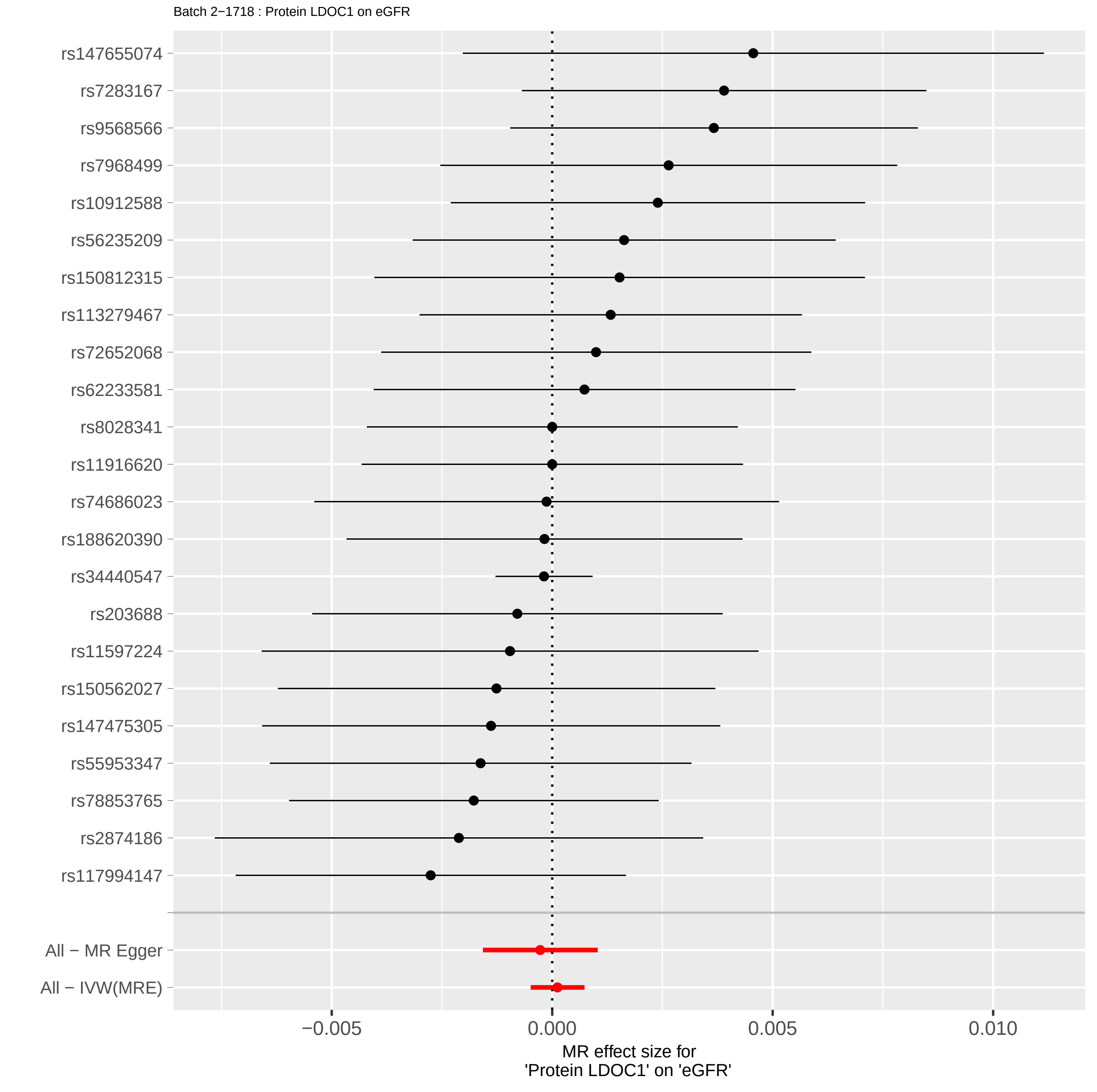

Batch 2−1718 : Protein LDOC1 on eGFR
rs147655074
rs7283167
rs9568566
rs7968499
rs10912588
rs56235209
rs150812315
rs113279467
rs72652068
rs62233581
rs8028341
rs11916620
rs74686023
rs188620390
rs34440547
rs203688
rs11597224
rs150562027
rs147475305
rs55953347
rs78853765
rs2874186
rs117994147
All − MR Egger
All − IVW(MRE)
−0.005
0.000
0.005
0.010
MR effect size for
'Protein LDOC1' on 'eGFR'

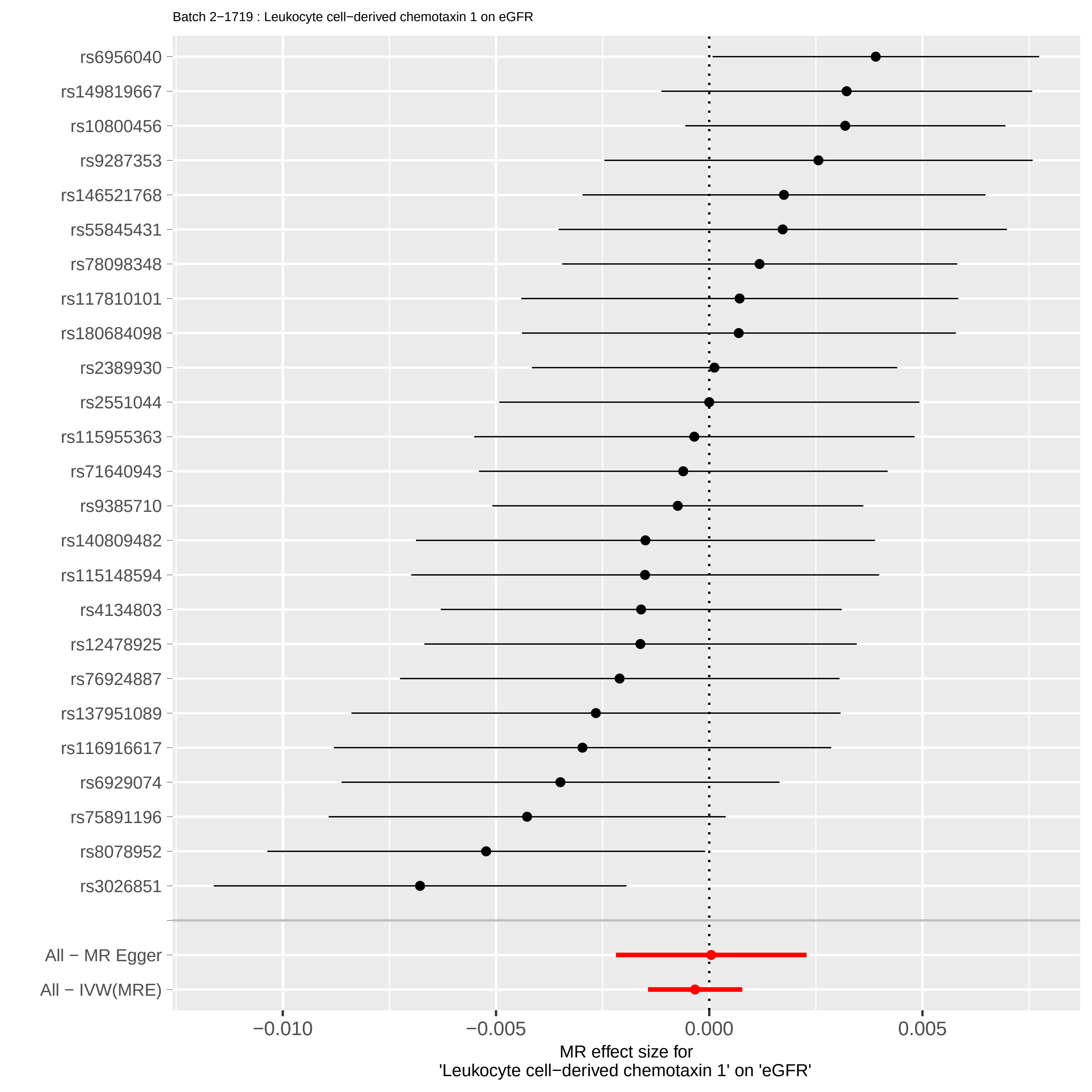

Batch 2−1719 : Leukocyte cell−derived chemotaxin 1 on eGFR
rs6956040
rs149819667
rs10800456
rs9287353
rs146521768
rs55845431
rs78098348
rs117810101
rs180684098
rs2389930
rs2551044
rs115955363
rs71640943
rs9385710
rs140809482
rs115148594
rs4134803
rs12478925
rs76924887
rs137951089
rs116916617
rs6929074
rs75891196
rs8078952
rs3026851
All − MR Egger
All − IVW(MRE)
−0.010
−0.005
0.000
0.005
MR effect size for
'Leukocyte cell−derived chemotaxin 1' on 'eGFR'

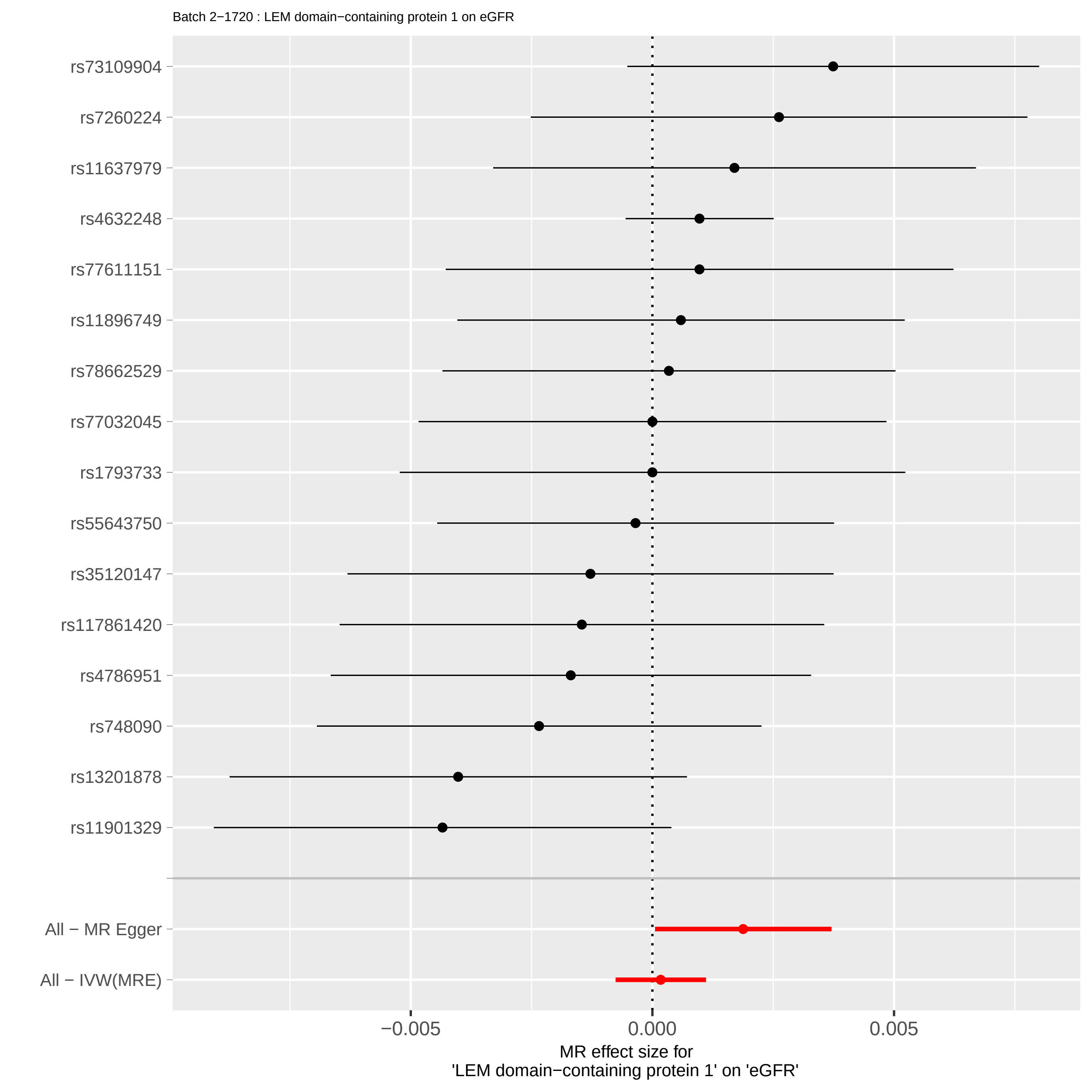
Batch 2−1720 : LEM domain−containing protein 1 on eGFR
rs73109904
rs7260224
rs11637979
rs4632248
rs77611151
rs11896749
rs78662529
rs77032045
rs1793733
rs55643750
rs35120147
rs117861420
rs4786951
rs748090
rs13201878
rs11901329
All − MR Egger
All − IVW(MRE)
−0.005
0.000
0.005
MR effect size for
'LEM domain−containing protein 1' on 'eGFR'

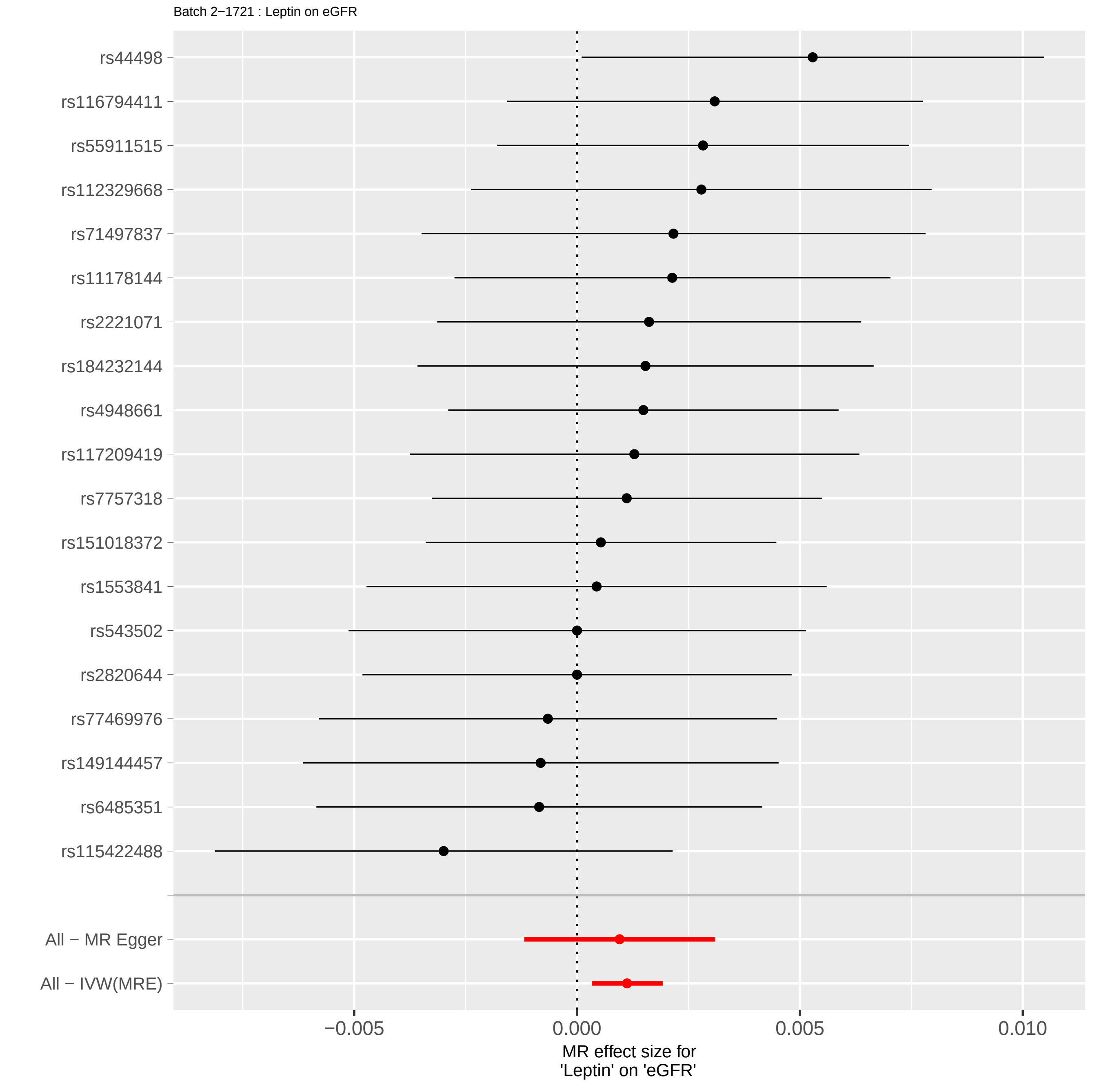
Batch 2−1721 : Leptin on eGFR
rs44498
rs116794411
rs55911515
rs112329668
rs71497837
rs11178144
rs2221071
rs184232144
rs4948661
rs117209419
rs7757318
rs151018372
rs1553841
rs543502
rs2820644
rs77469976
rs149144457
rs6485351
rs115422488
All − MR Egger
All − IVW(MRE)
−0.005
0.000
0.005
0.010
MR effect size for
'Leptin' on 'eGFR'

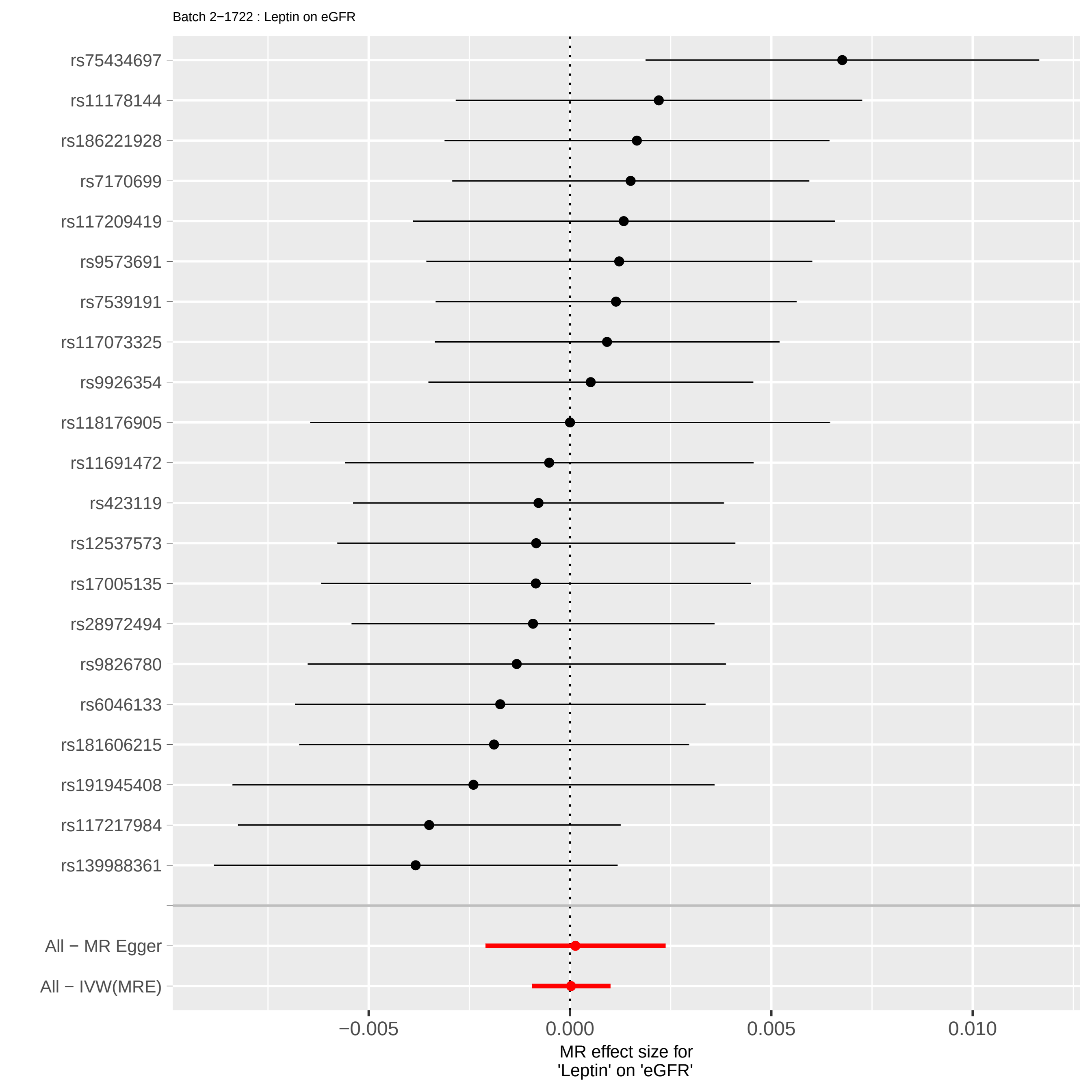

Batch 2−1722 : Leptin on eGFR
rs75434697
rs11178144
rs186221928
rs7170699
rs117209419
rs9573691
rs7539191
rs117073325
rs9926354
rs118176905
rs11691472
rs423119
rs12537573
rs17005135
rs28972494
rs9826780
rs6046133
rs181606215
rs191945408
rs117217984
rs139988361
All − MR Egger
All − IVW(MRE)
−0.005
0.000
0.005
0.010
MR effect size for
'Leptin' on 'eGFR'

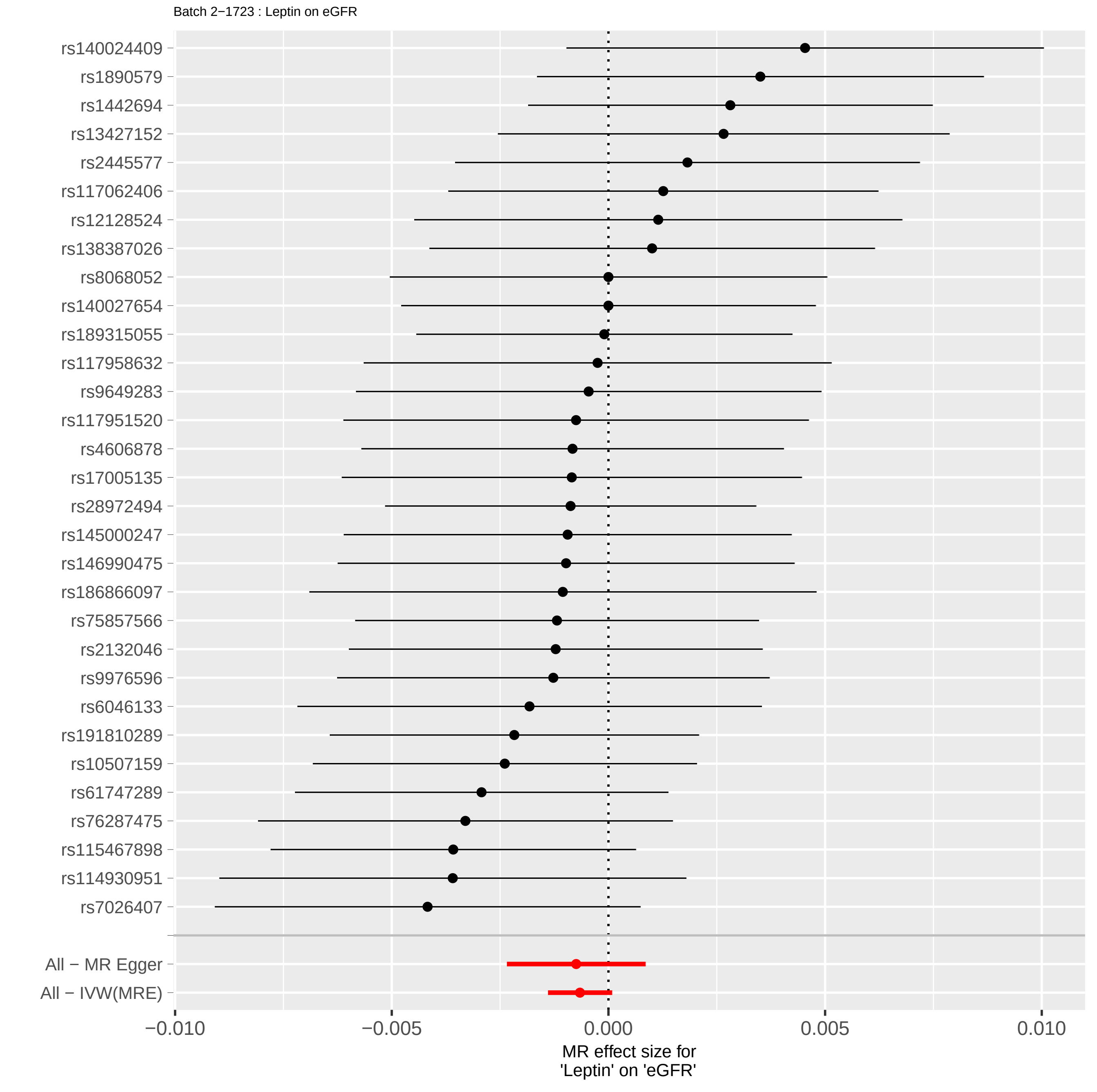
Batch 2−1723 : Leptin on eGFR
rs140024409
rs1890579
rs1442694
rs13427152
rs2445577
rs117062406
rs12128524
rs138387026
rs8068052
rs140027654
rs189315055
rs117958632
rs9649283
rs117951520
rs4606878
rs17005135
rs28972494
rs145000247
rs146990475
rs186866097
rs75857566
rs2132046
rs9976596
rs6046133
rs191810289
rs10507159
rs61747289
rs76287475
rs115467898
rs114930951
rs7026407
All − MR Egger
All − IVW(MRE)
−0.010
−0.005
0.000
0.005
0.010
MR effect size for
'Leptin' on 'eGFR'

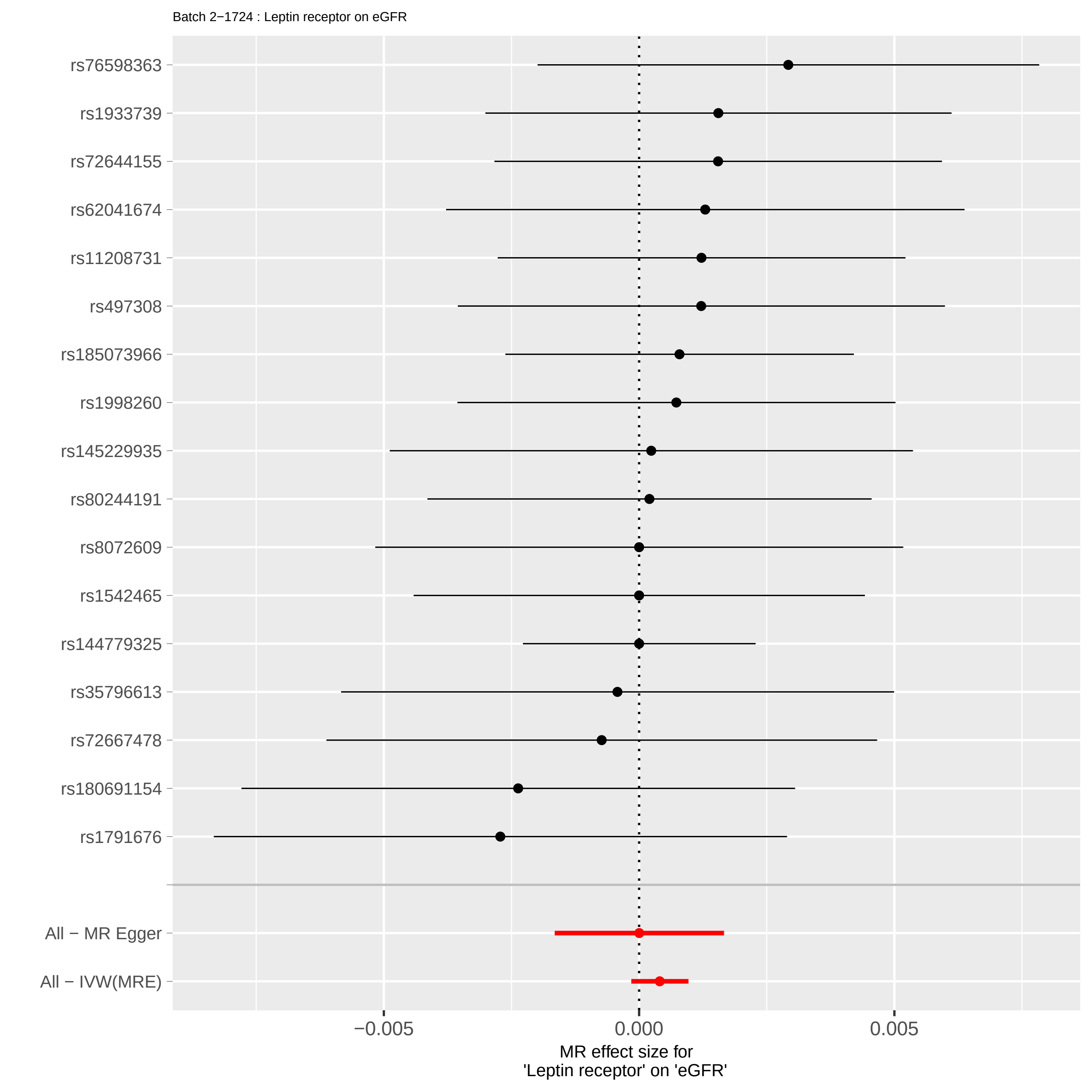

Batch 2−1724 : Leptin receptor on eGFR
rs76598363
rs1933739
rs72644155
rs62041674
rs11208731
rs497308
rs185073966
rs1998260
rs145229935
rs80244191
rs8072609
rs1542465
rs144779325
rs35796613
rs72667478
rs180691154
rs1791676
All − MR Egger
All − IVW(MRE)
−0.005
0.000
0.005
MR effect size for
'Leptin receptor' on 'eGFR'

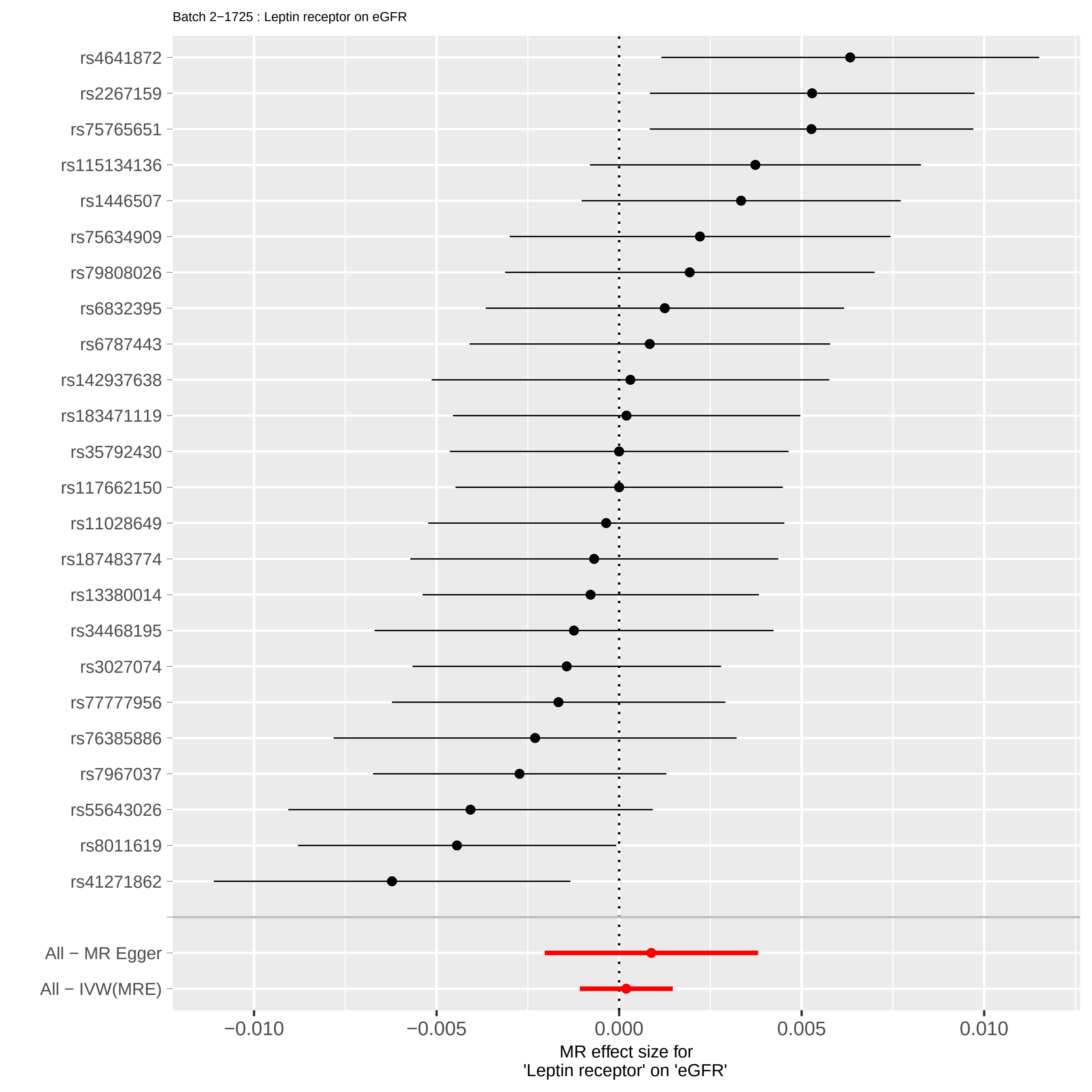

Batch 2−1725 : Leptin receptor on eGFR
rs4641872
rs2267159
rs75765651
rs115134136
rs1446507
rs75634909
rs79808026
rs6832395
rs6787443
rs142937638
rs183471119
rs35792430
rs117662150
rs11028649
rs187483774
rs13380014
rs34468195
rs3027074
rs77777956
rs76385886
rs7967037
rs55643026
rs8011619
rs41271862
All − MR Egger
All − IVW(MRE)
−0.010
−0.005
0.000
0.005
0.010
MR effect size for
'Leptin receptor' on 'eGFR'

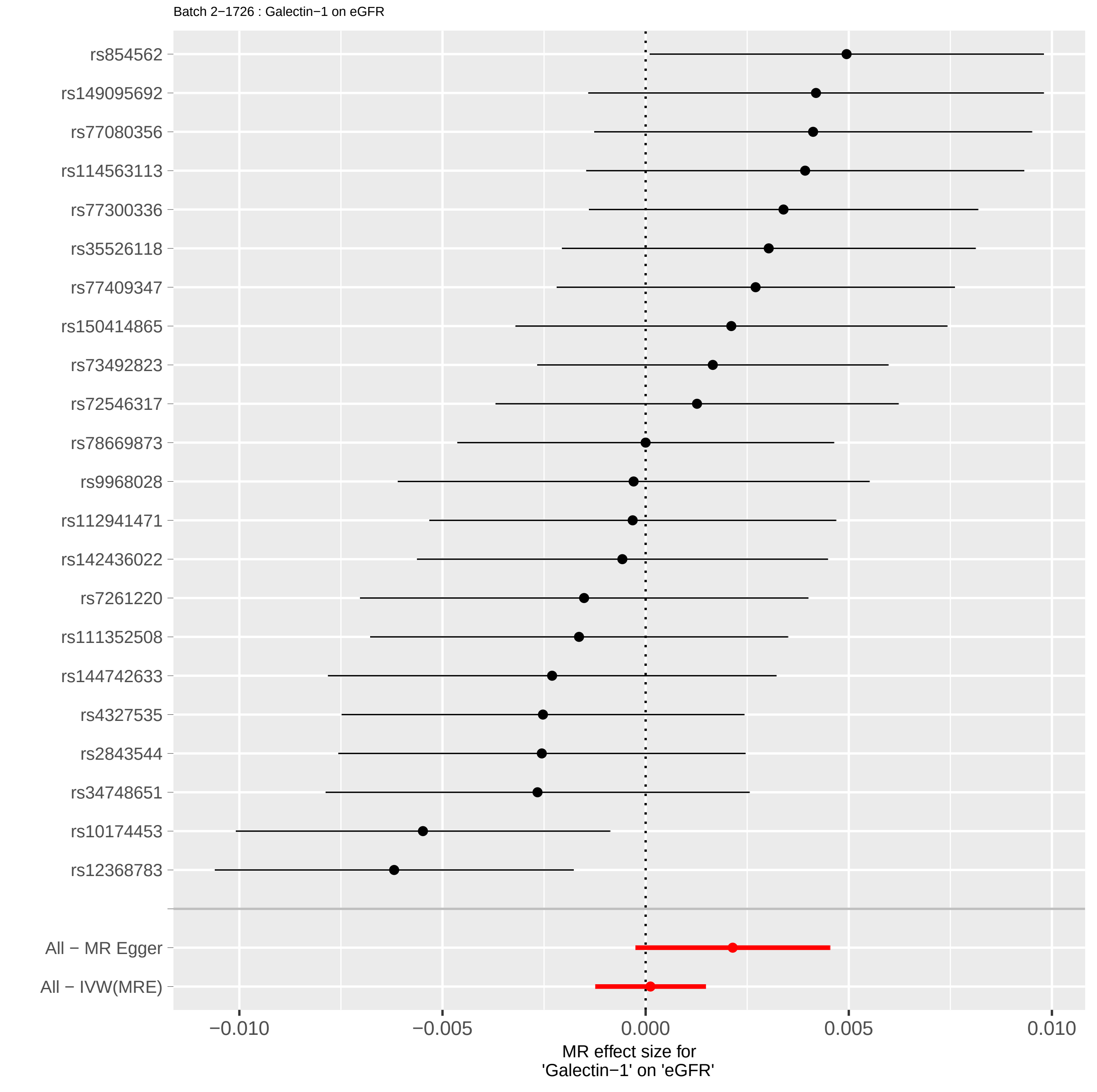

Batch 2−1726 : Galectin−1 on eGFR
rs854562
rs149095692
rs77080356
rs114563113
rs77300336
rs35526118
rs77409347
rs150414865
rs73492823
rs72546317
rs78669873
rs9968028
rs112941471
rs142436022
rs7261220
rs111352508
rs144742633
rs4327535
rs2843544
rs34748651
rs10174453
rs12368783
All − MR Egger
All − IVW(MRE)
−0.010
−0.005
0.000
0.005
0.010
MR effect size for
'Galectin−1' on 'eGFR'

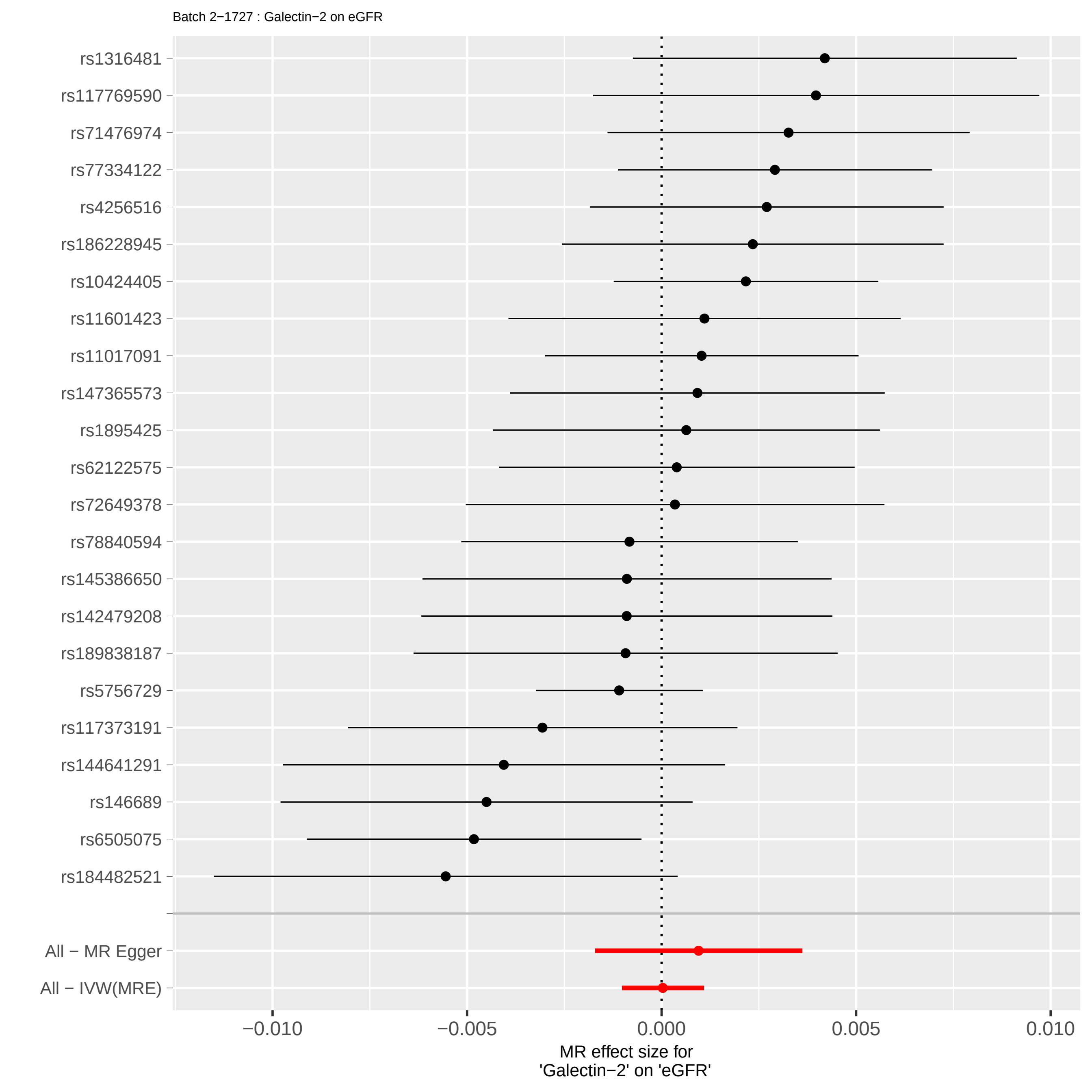

Batch 2−1727 : Galectin−2 on eGFR
rs1316481
rs117769590
rs71476974
rs77334122
rs4256516
rs186228945
rs10424405
rs11601423
rs11017091
rs147365573
rs1895425
rs62122575
rs72649378
rs78840594
rs145386650
rs142479208
rs189838187
rs5756729
rs117373191
rs144641291
rs146689
rs6505075
rs184482521
All − MR Egger
All − IVW(MRE)
−0.010
−0.005
0.000
0.005
0.010
MR effect size for
'Galectin−2' on 'eGFR'

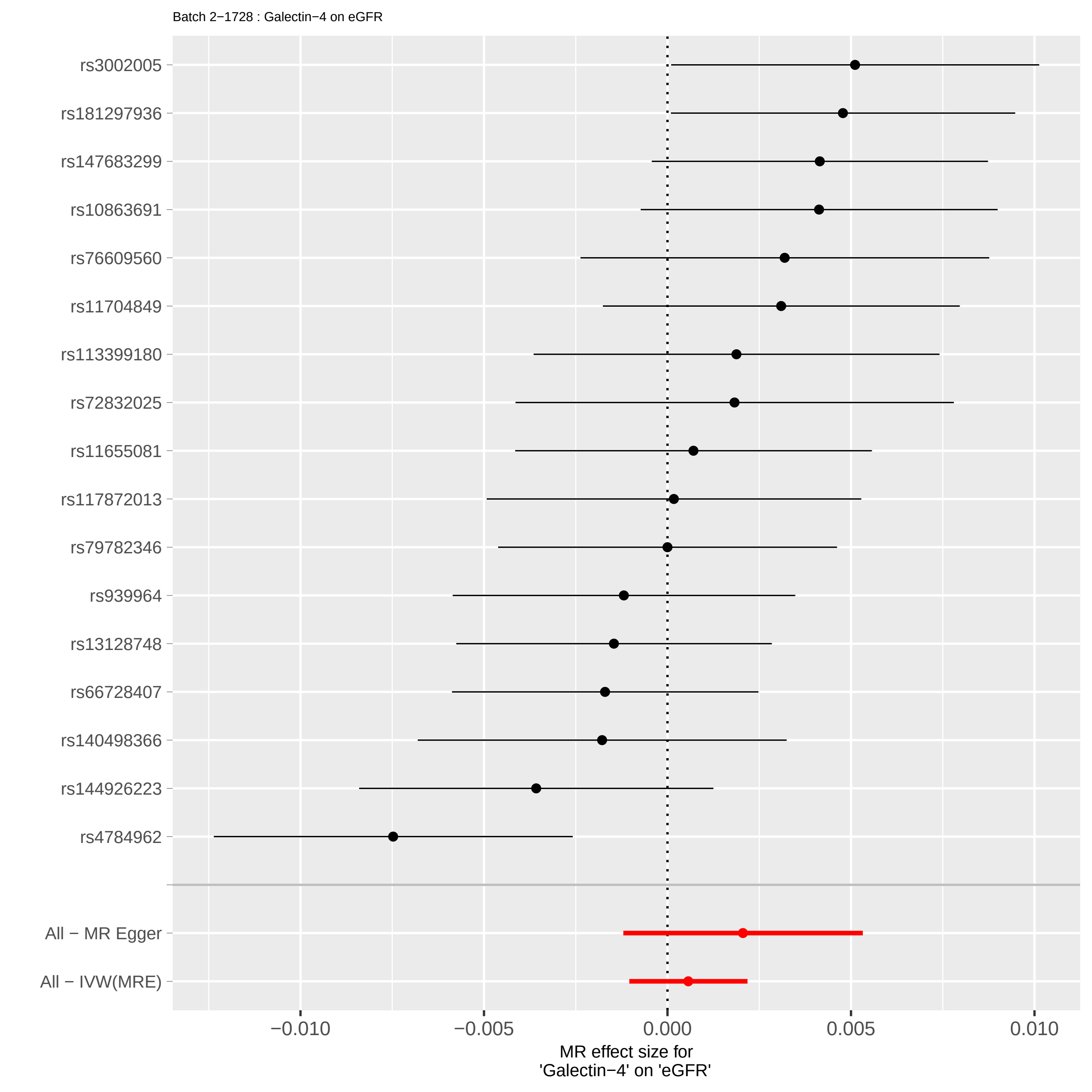

Batch 2−1728 : Galectin−4 on eGFR
rs3002005
rs181297936
rs147683299
rs10863691
rs76609560
rs11704849
rs113399180
rs72832025
rs11655081
rs117872013
rs79782346
rs939964
rs13128748
rs66728407
rs140498366
rs144926223
rs4784962
All − MR Egger
All − IVW(MRE)
−0.010
−0.005
0.000
0.005
0.010
MR effect size for
'Galectin−4' on 'eGFR'

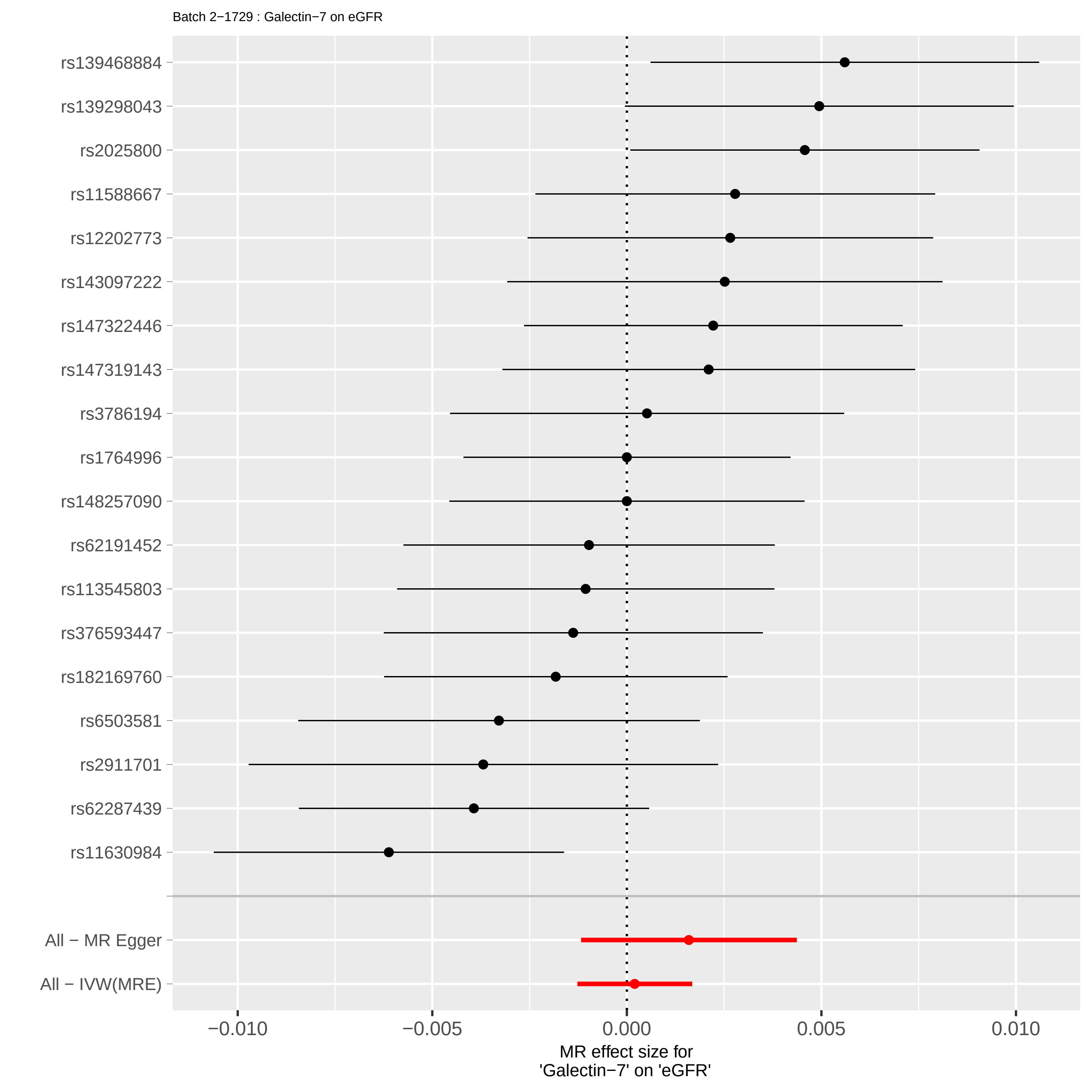
Batch 2−1729 : Galectin−7 on eGFR
rs139468884
rs139298043
rs2025800
rs11588667
rs12202773
rs143097222
rs147322446
rs147319143
rs3786194
rs1764996
rs148257090
rs62191452
rs113545803
rs376593447
rs182169760
rs6503581
rs2911701
rs62287439
rs11630984
All − MR Egger
All − IVW(MRE)
−0.010
−0.005
0.000
0.005
0.010
MR effect size for
'Galectin−7' on 'eGFR'

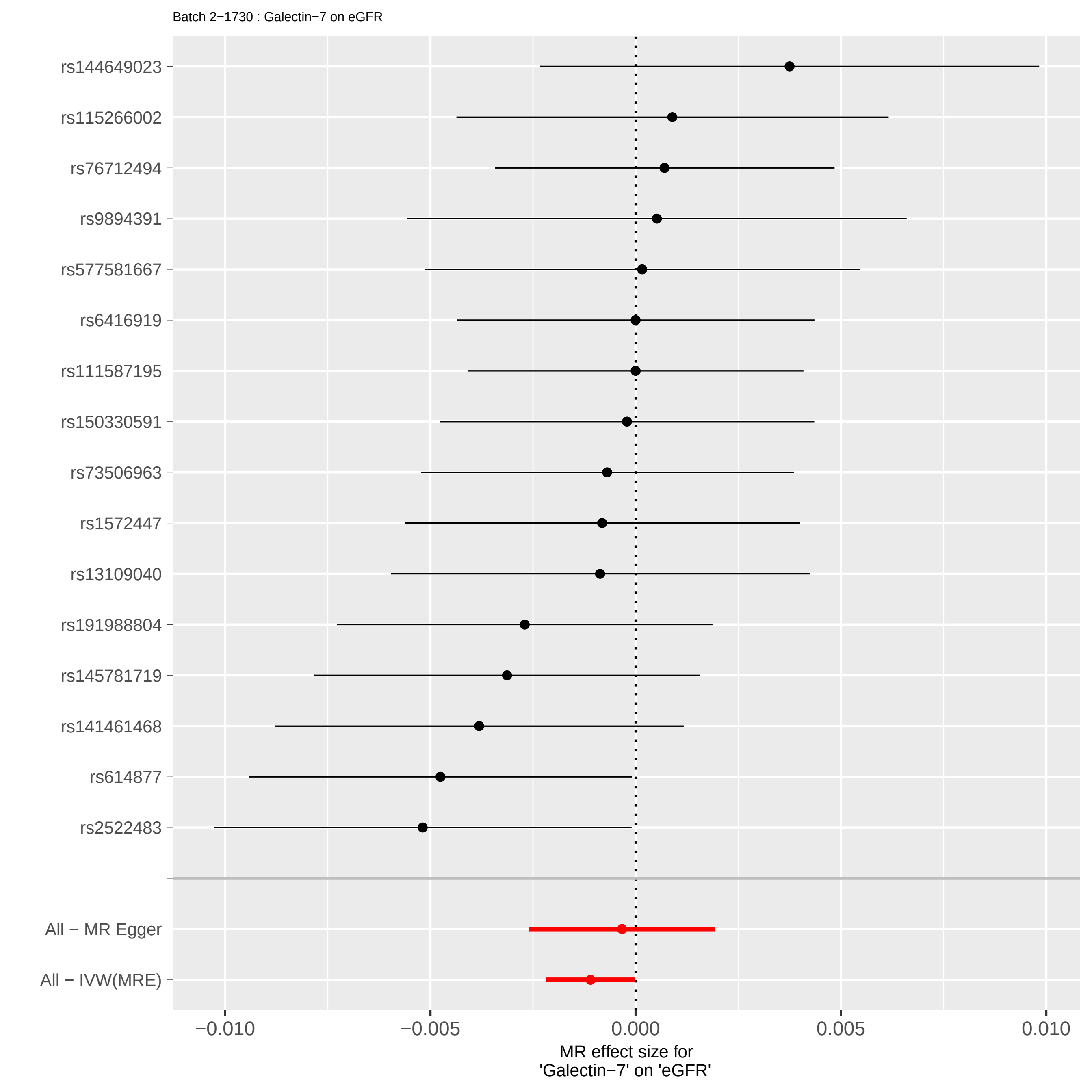

Batch 2−1730 : Galectin−7 on eGFR
rs144649023
rs115266002
rs76712494
rs9894391
rs577581667
rs6416919
rs111587195
rs150330591
rs73506963
rs1572447
rs13109040
rs191988804
rs145781719
rs141461468
rs614877
rs2522483
All − MR Egger
All − IVW(MRE)
−0.010
−0.005
0.000
0.005
0.010
MR effect size for
'Galectin−7' on 'eGFR'

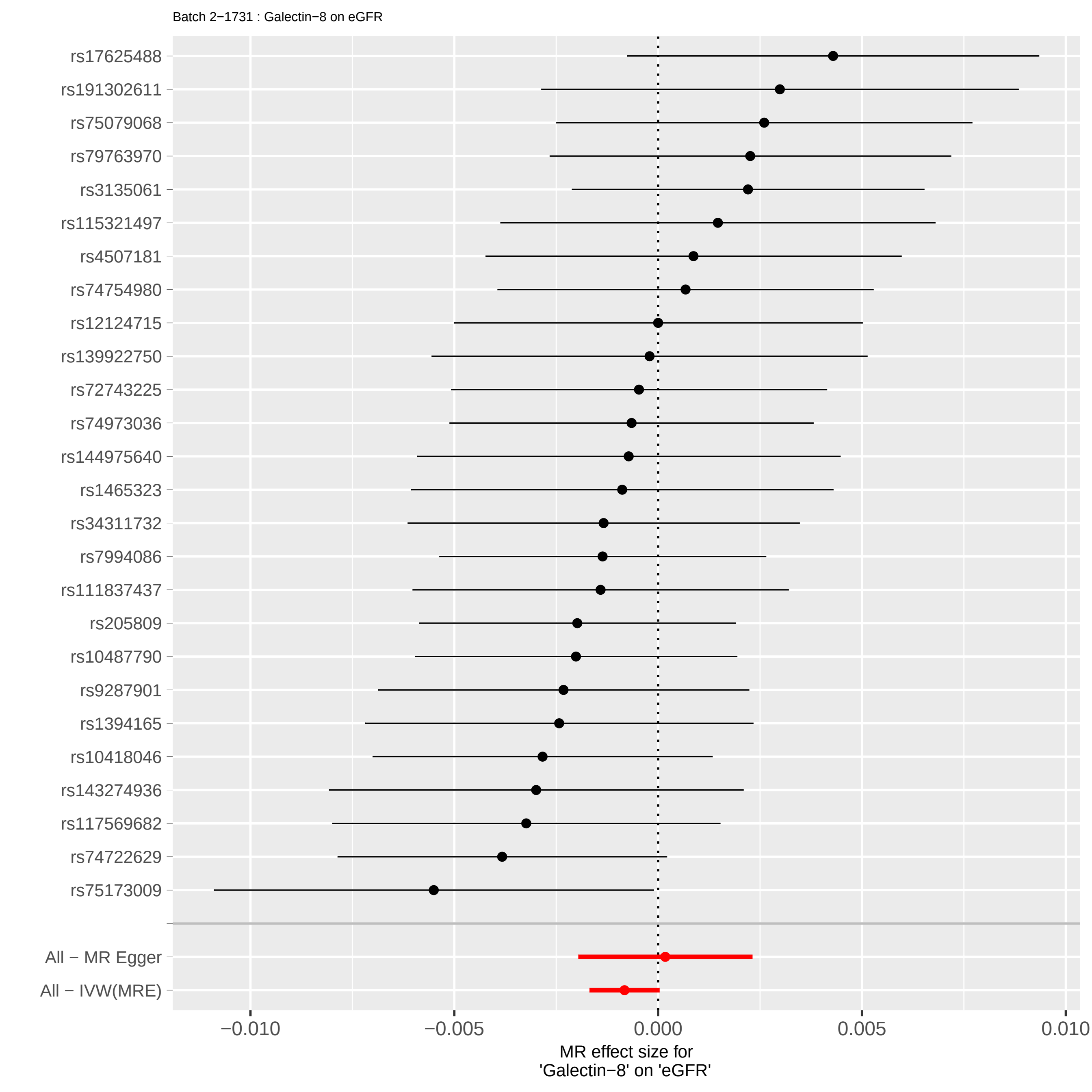

Batch 2−1731 : Galectin−8 on eGFR
rs17625488
rs191302611
rs75079068
rs79763970
rs3135061
rs115321497
rs4507181
rs74754980
rs12124715
rs139922750
rs72743225
rs74973036
rs144975640
rs1465323
rs34311732
rs7994086
rs111837437
rs205809
rs10487790
rs9287901
rs1394165
rs10418046
rs143274936
rs117569682
rs74722629
rs75173009
All − MR Egger
All − IVW(MRE)
−0.010
−0.005
0.000
0.005
0.010
MR effect size for
'Galectin−8' on 'eGFR'

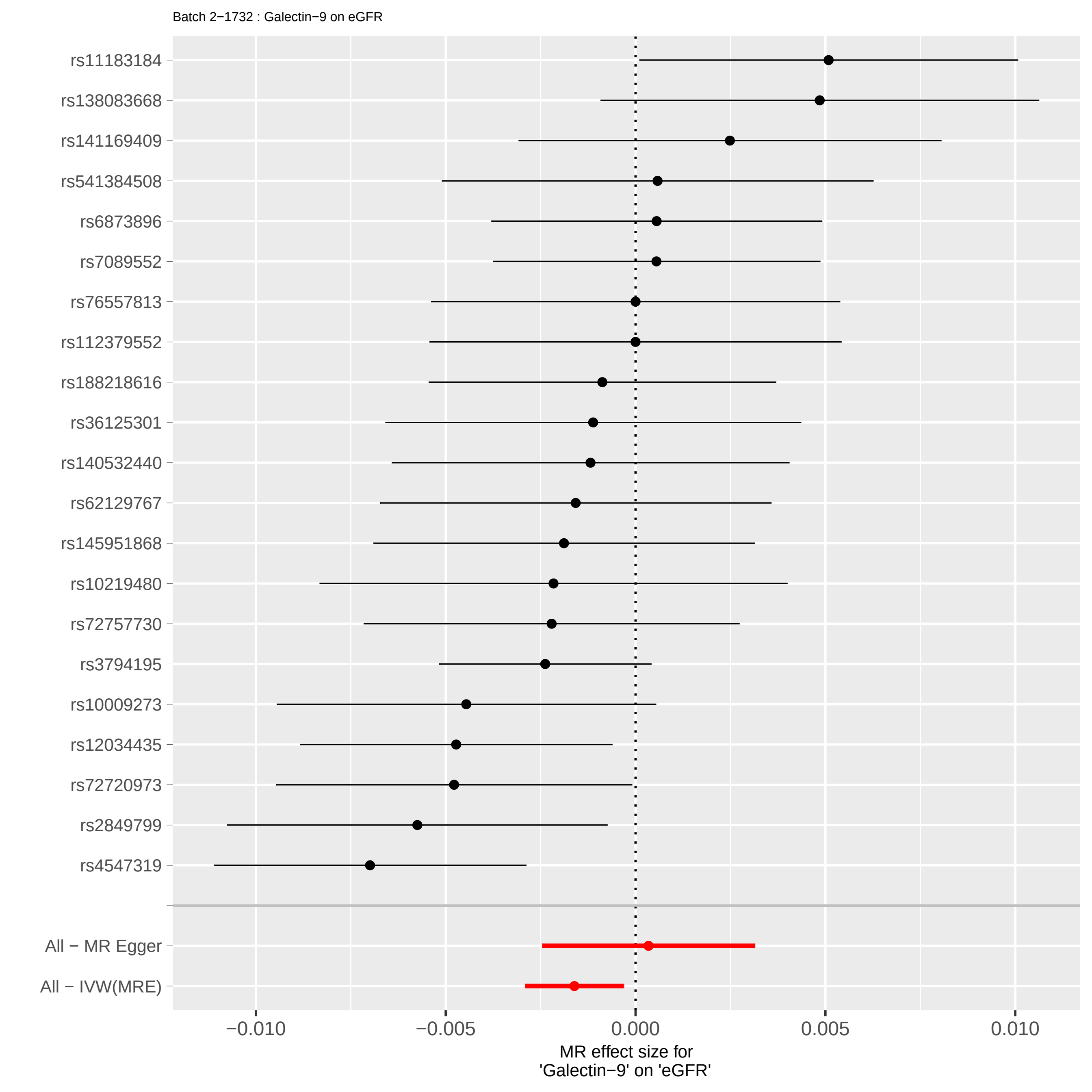

Batch 2−1732 : Galectin−9 on eGFR
rs11183184
rs138083668
rs141169409
rs541384508
rs6873896
rs7089552
rs76557813
rs112379552
rs188218616
rs36125301
rs140532440
rs62129767
rs145951868
rs10219480
rs72757730
rs3794195
rs10009273
rs12034435
rs72720973
rs2849799
rs4547319
All − MR Egger
All − IVW(MRE)
−0.010
−0.005
0.000
0.005
0.010
MR effect size for
'Galectin−9' on 'eGFR'

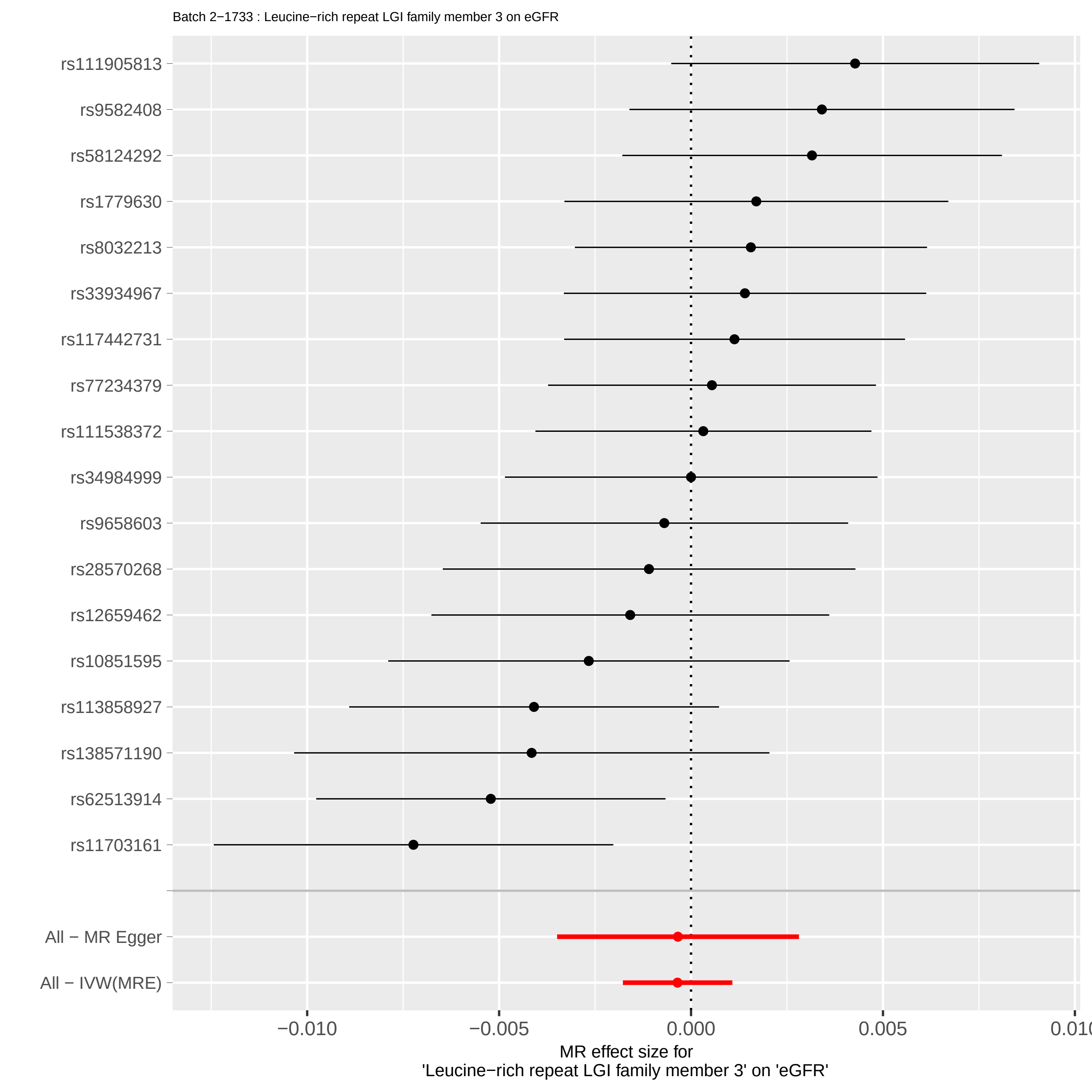

Batch 2−1733 : Leucine−rich repeat LGI family member 3 on eGFR
rs111905813
rs9582408
rs58124292
rs1779630
rs8032213
rs33934967
rs117442731
rs77234379
rs111538372
rs34984999
rs9658603
rs28570268
rs12659462
rs10851595
rs113858927
rs138571190
rs62513914
rs11703161
All − MR Egger
All − IVW(MRE)
−0.010
−0.005
0.000
0.005
MR effect size for
'Leucine−rich repeat LGI family member 3' on 'eGFR'

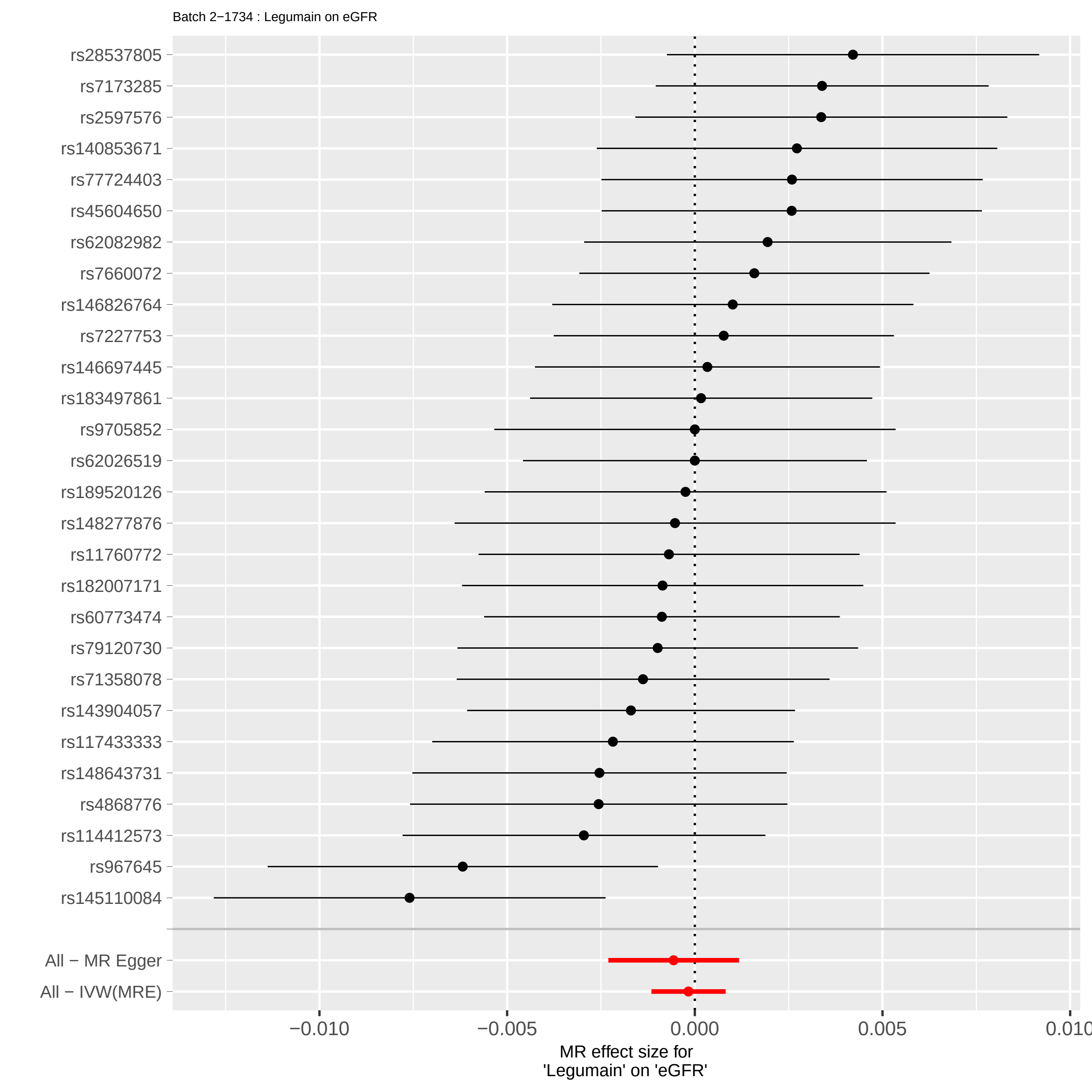
Batch 2−1734 : Legumain on eGFR
rs28537805
rs7173285
rs2597576
rs140853671
rs77724403
rs45604650
rs62082982
rs7660072
rs146826764
rs7227753
rs146697445
rs183497861
rs9705852
rs62026519
rs189520126
rs148277876
rs11760772
rs182007171
rs60773474
rs79120730
rs71358078
rs143904057
rs117433333
rs148643731
rs4868776
rs114412573
rs967645
rs145110084
All − MR Egger
All − IVW(MRE)
−0.010
−0.005
0.000
0.005
0.010
MR effect size for
'Legumain' on 'eGFR'

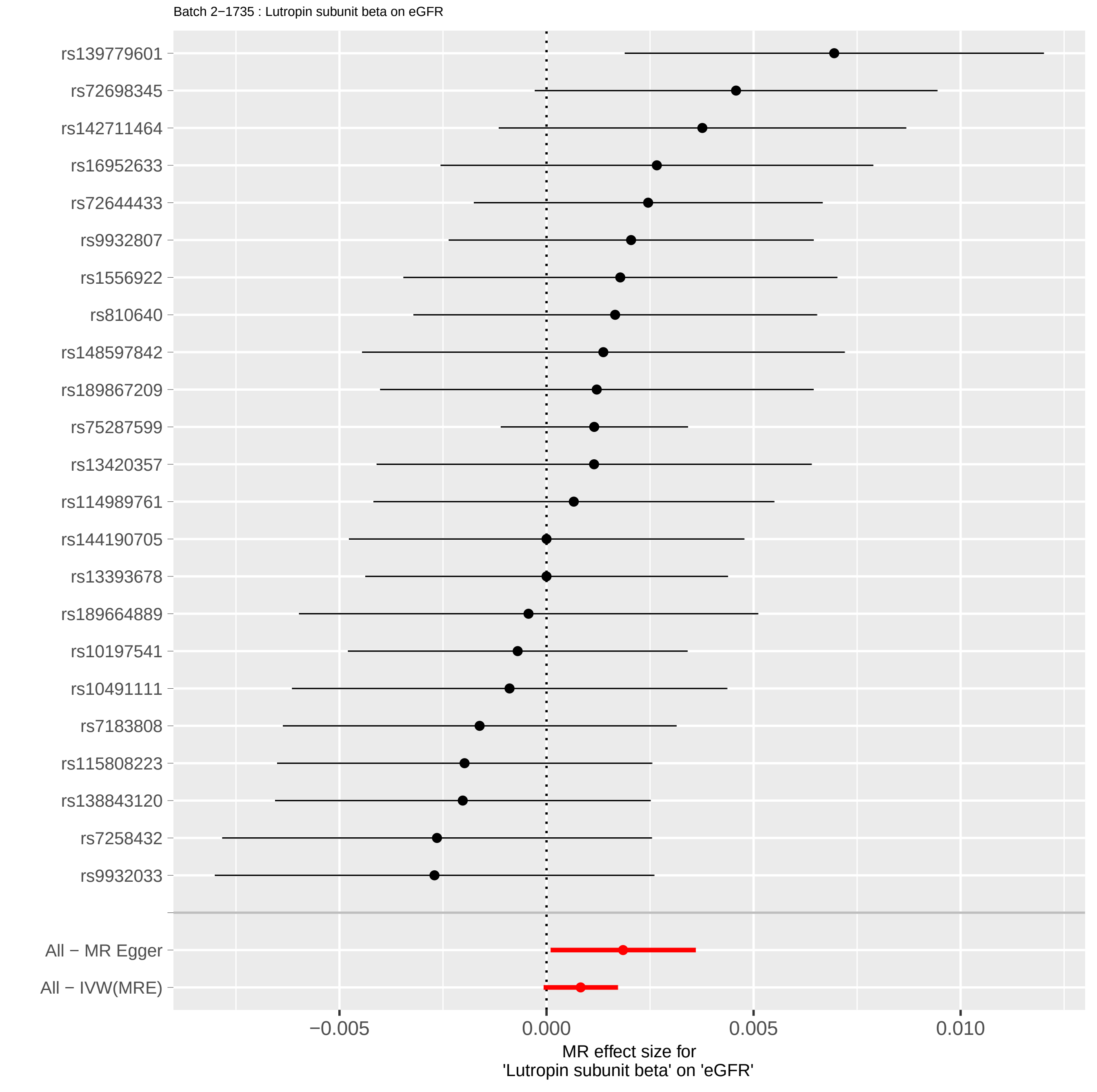
Batch 2−1735 : Lutropin subunit beta on eGFR
rs139779601
rs72698345
rs142711464
rs16952633
rs72644433
rs9932807
rs1556922
rs810640
rs148597842
rs189867209
rs75287599
rs13420357
rs114989761
rs144190705
rs13393678
rs189664889
rs10197541
rs10491111
rs7183808
rs115808223
rs138843120
rs7258432
rs9932033
All − MR Egger
All − IVW(MRE)
−0.005
0.000
0.005
0.010
MR effect size for
'Lutropin subunit beta' on 'eGFR'

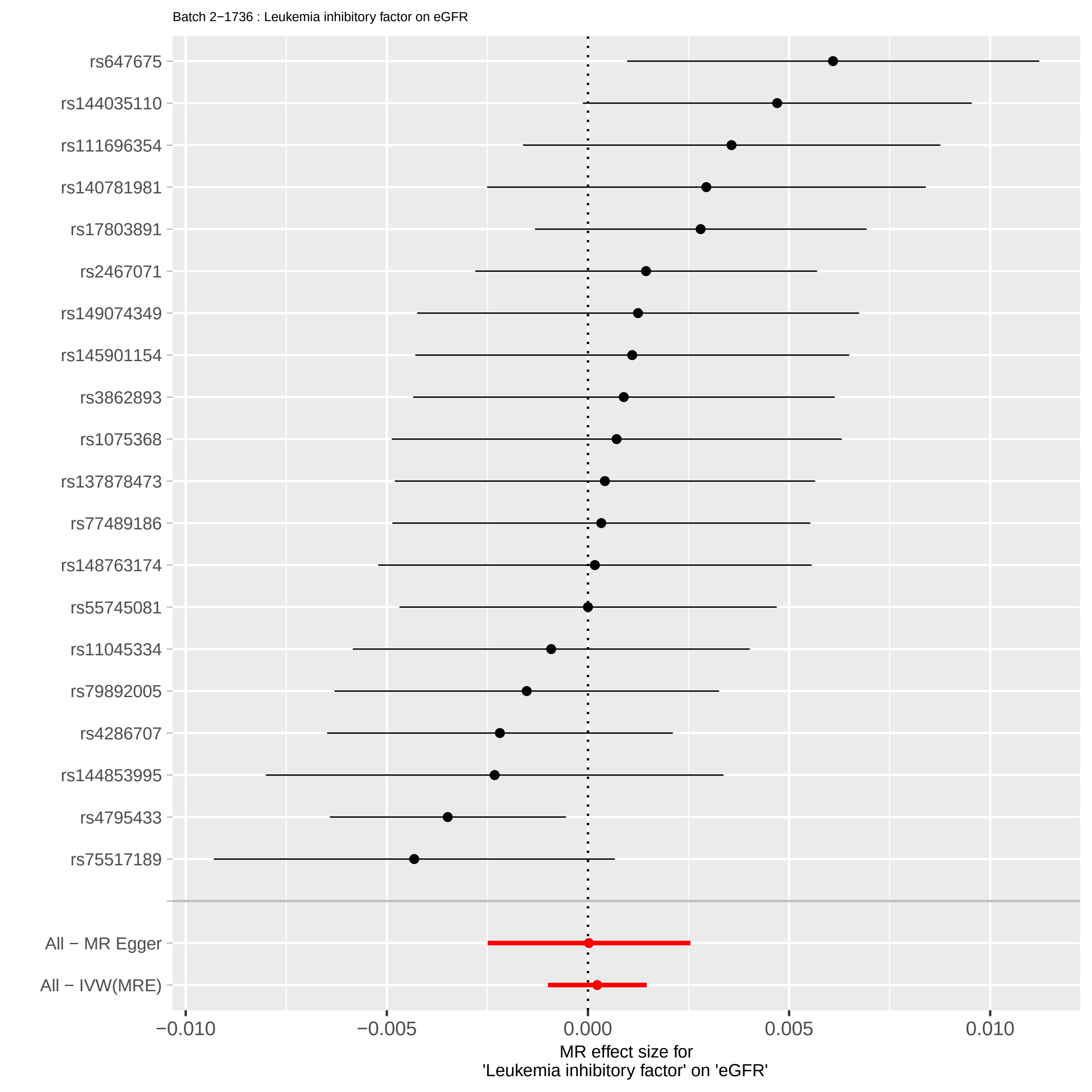

Batch 2−1736 : Leukemia inhibitory factor on eGFR
rs647675
rs144035110
rs111696354
rs140781981
rs17803891
rs2467071
rs149074349
rs145901154
rs3862893
rs1075368
rs137878473
rs77489186
rs148763174
rs55745081
rs11045334
rs79892005
rs4286707
rs144853995
rs4795433
rs75517189
All − MR Egger
All − IVW(MRE)
−0.010
−0.005
0.000
0.005
0.010
MR effect size for
'Leukemia inhibitory factor' on 'eGFR'

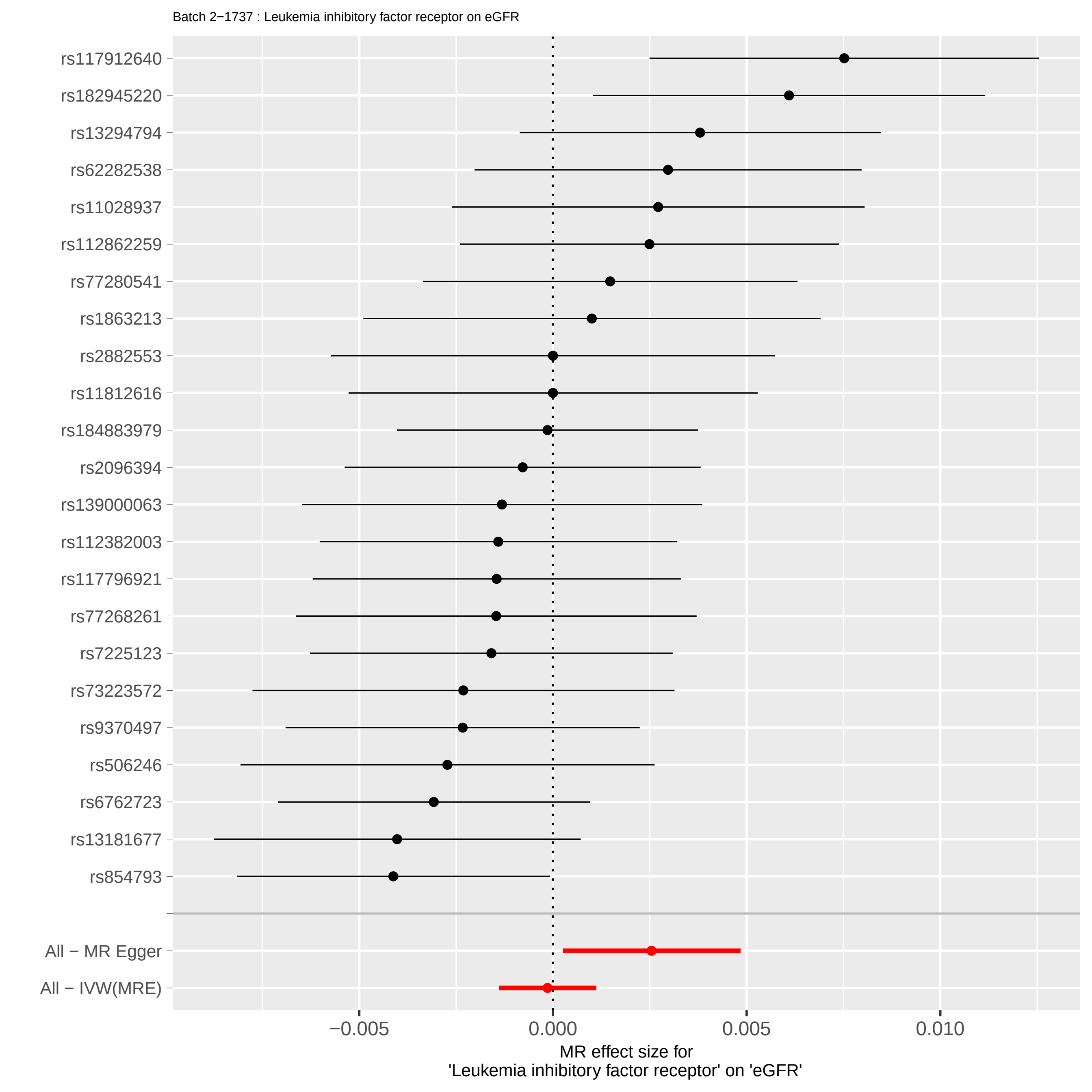

Batch 2−1737 : Leukemia inhibitory factor receptor on eGFR
rs117912640
rs182945220
rs13294794
rs62282538
rs11028937
rs112862259
rs77280541
rs1863213
rs2882553
rs11812616
rs184883979
rs2096394
rs139000063
rs112382003
rs117796921
rs77268261
rs7225123
rs73223572
rs9370497
rs506246
rs6762723
rs13181677
rs854793
All − MR Egger
All − IVW(MRE)
−0.005
0.000
0.005
0.010
MR effect size for
'Leukemia inhibitory factor receptor' on 'eGFR'

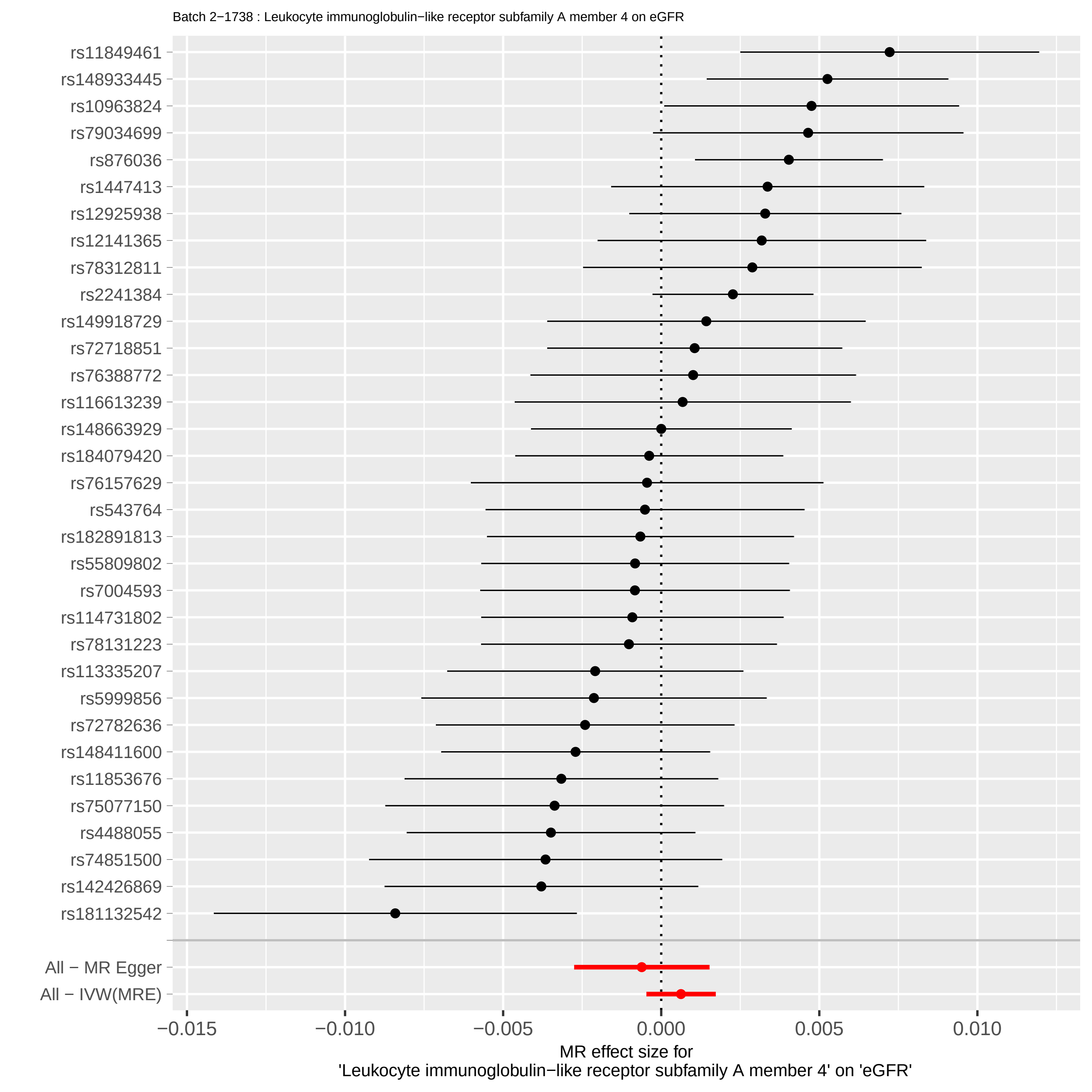
Batch 2−1738 : Leukocyte immunoglobulin−like receptor subfamily A member 4 on eGFR
rs11849461
rs148933445
rs10963824
rs79034699
rs876036
rs1447413
rs12925938
rs12141365
rs78312811
rs2241384
rs149918729
rs72718851
rs76388772
rs116613239
rs148663929
rs184079420
rs76157629
rs543764
rs182891813
rs55809802
rs7004593
rs114731802
rs78131223
rs113335207
rs5999856
rs72782636
rs148411600
rs11853676
rs75077150
rs4488055
rs74851500
rs142426869
rs181132542
All − MR Egger
All − IVW(MRE)
−0.015
−0.010
−0.005
0.000
0.005
0.010
MR effect size for
'Leukocyte immunoglobulin−like receptor subfamily A member 4' on 'eGFR'

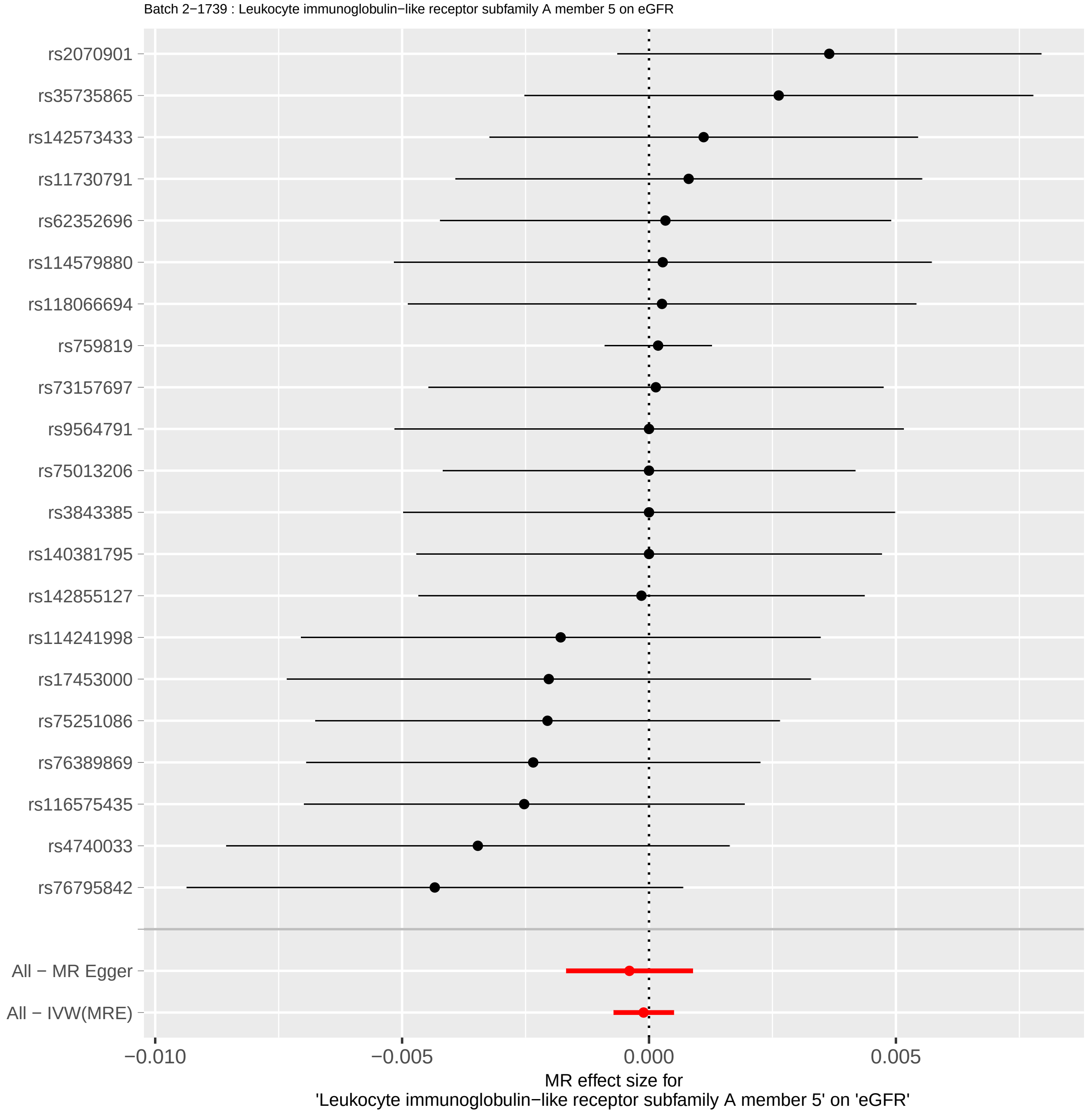

Batch 2−1739 : Leukocyte immunoglobulin−like receptor subfamily A member 5 on eGFR
rs2070901
rs35735865
rs142573433
rs11730791
rs62352696
rs114579880
rs118066694
rs759819
rs73157697
rs9564791
rs75013206
rs3843385
rs140381795
rs142855127
rs114241998
rs17453000
rs75251086
rs76389869
rs116575435
rs4740033
rs76795842
All − MR Egger
All − IVW(MRE)
−0.010
−0.005
0.000
0.005
MR effect size for
'Leukocyte immunoglobulin−like receptor subfamily A member 5' on 'eGFR'

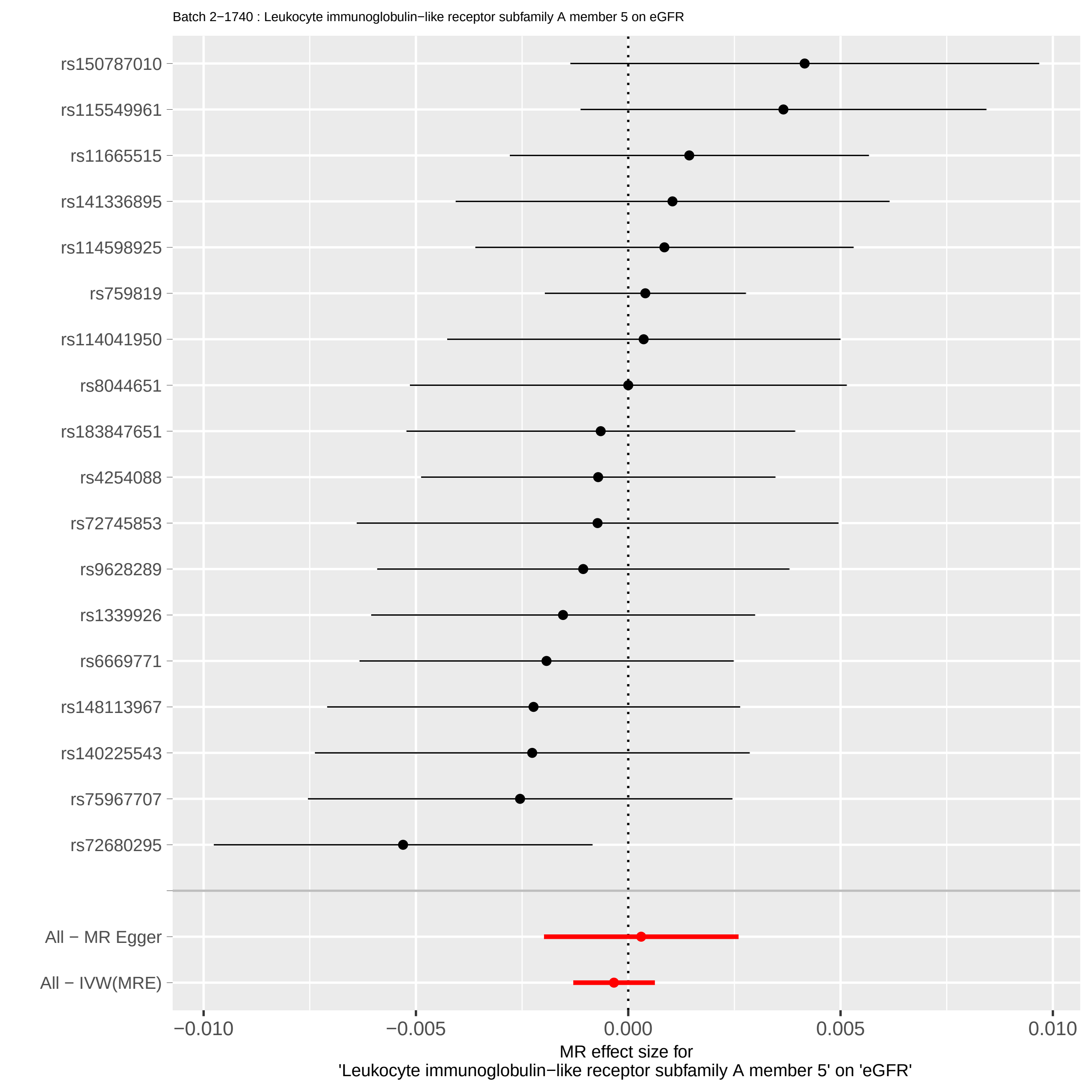
Batch 2−1740 : Leukocyte immunoglobulin−like receptor subfamily A member 5 on eGFR
rs150787010
rs115549961
rs11665515
rs141336895
rs114598925
rs759819
rs114041950
rs8044651
rs183847651
rs4254088
rs72745853
rs9628289
rs1339926
rs6669771
rs148113967
rs140225543
rs75967707
rs72680295
All − MR Egger
All − IVW(MRE)
−0.010
−0.005
0.000
0.005
0.010
MR effect size for
'Leukocyte immunoglobulin−like receptor subfamily A member 5' on 'eGFR'

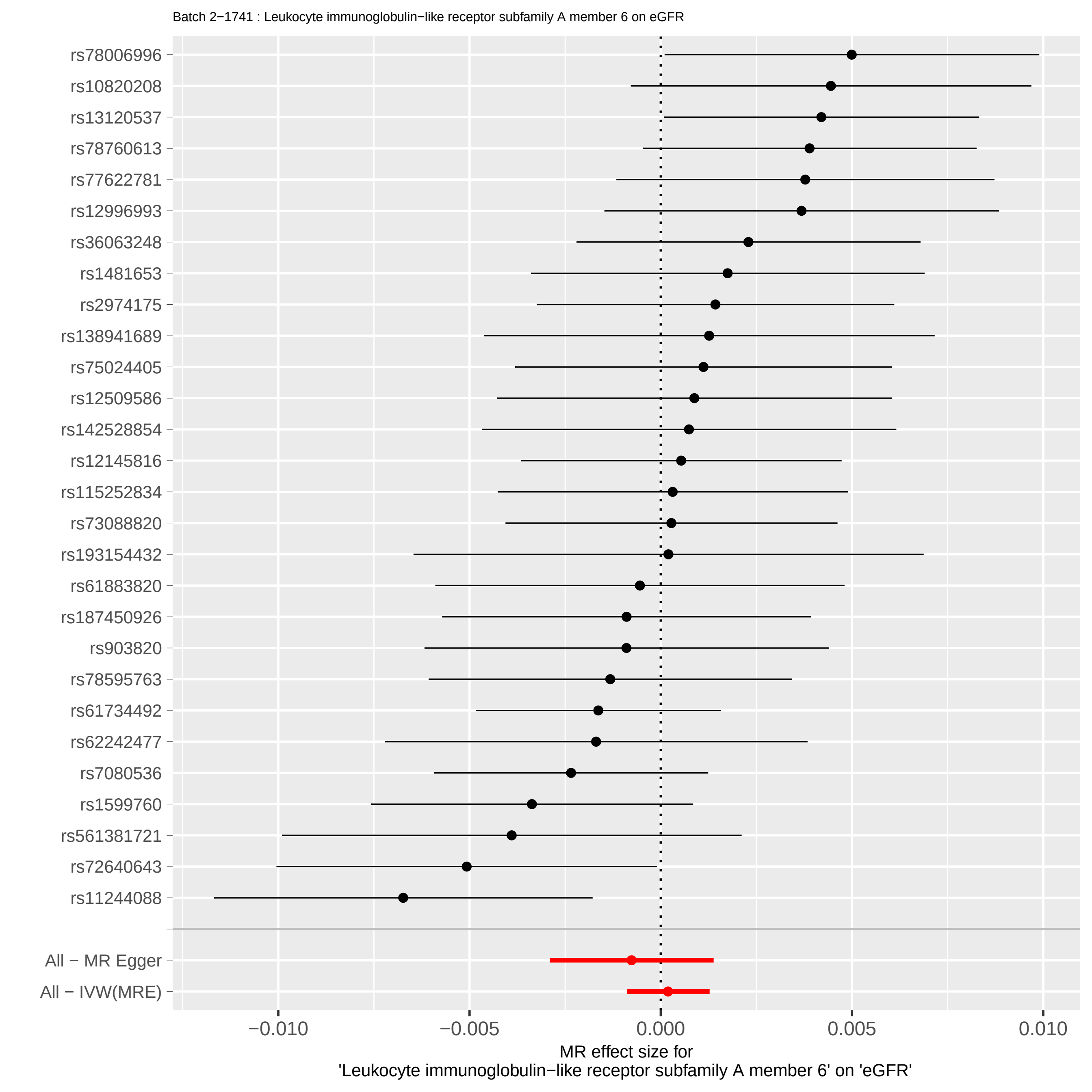
Batch 2−1741 : Leukocyte immunoglobulin−like receptor subfamily A member 6 on eGFR
rs78006996
rs10820208
rs13120537
rs78760613
rs77622781
rs12996993
rs36063248
rs1481653
rs2974175
rs138941689
rs75024405
rs12509586
rs142528854
rs12145816
rs115252834
rs73088820
rs193154432
rs61883820
rs187450926
rs903820
rs78595763
rs61734492
rs62242477
rs7080536
rs1599760
rs561381721
rs72640643
rs11244088
All − MR Egger
All − IVW(MRE)
−0.010
−0.005
0.000
0.005
0.010
MR effect size for
'Leukocyte immunoglobulin−like receptor subfamily A member 6' on 'eGFR'

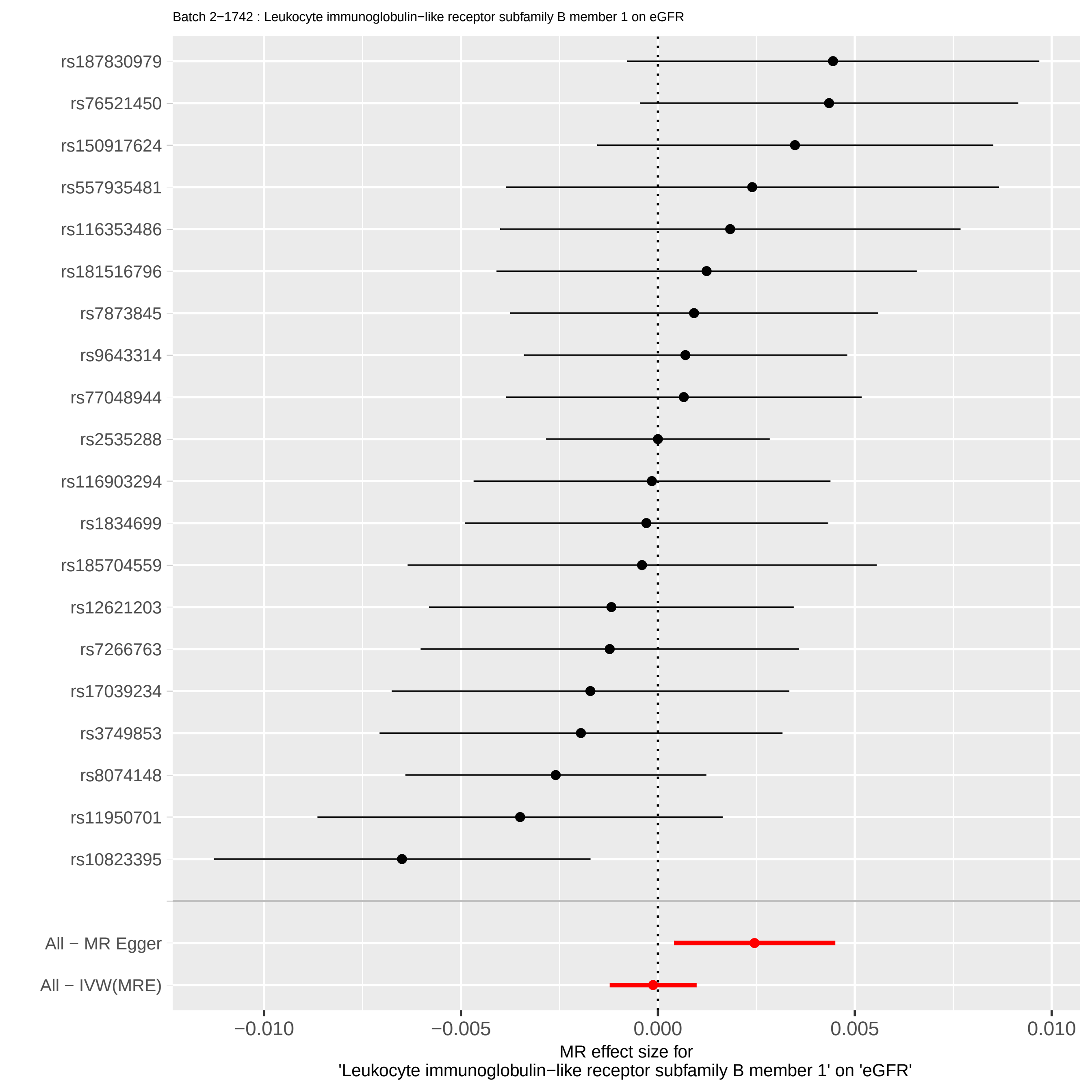
Batch 2−1742 : Leukocyte immunoglobulin−like receptor subfamily B member 1 on eGFR
rs187830979
rs76521450
rs150917624
rs557935481
rs116353486
rs181516796
rs7873845
rs9643314
rs77048944
rs2535288
rs116903294
rs1834699
rs185704559
rs12621203
rs7266763
rs17039234
rs3749853
rs8074148
rs11950701
rs10823395
All − MR Egger
All − IVW(MRE)
−0.010
−0.005
0.000
0.005
0.010
MR effect size for
'Leukocyte immunoglobulin−like receptor subfamily B member 1' on 'eGFR'

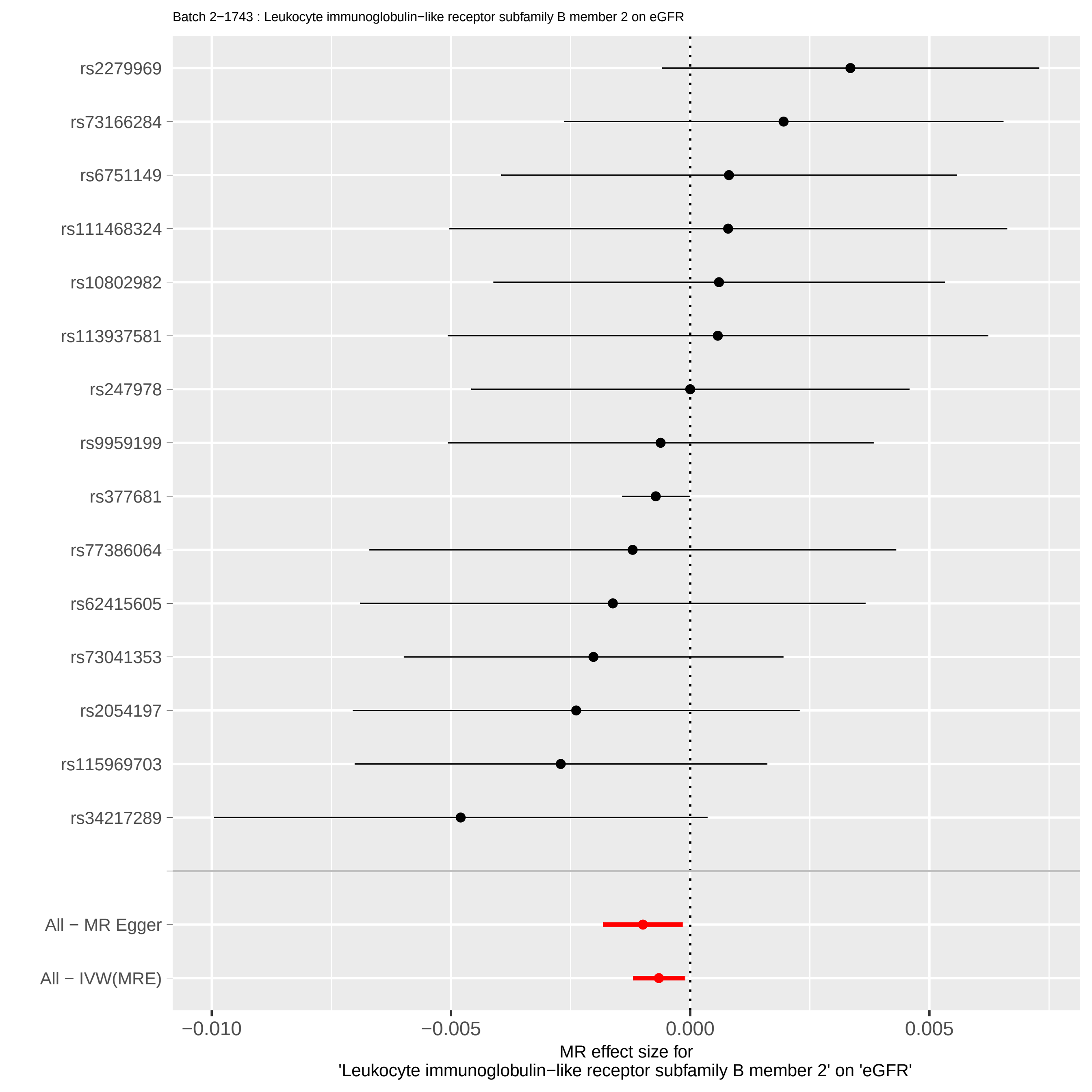

Batch 2−1743 : Leukocyte immunoglobulin−like receptor subfamily B member 2 on eGFR
rs2279969
rs73166284
rs6751149
rs111468324
rs10802982
rs113937581
rs247978
rs9959199
rs377681
rs77386064
rs62415605
rs73041353
rs2054197
rs115969703
rs34217289
All − MR Egger
All − IVW(MRE)
−0.010
−0.005
0.000
0.005
MR effect size for
'Leukocyte immunoglobulin−like receptor subfamily B member 2' on 'eGFR'

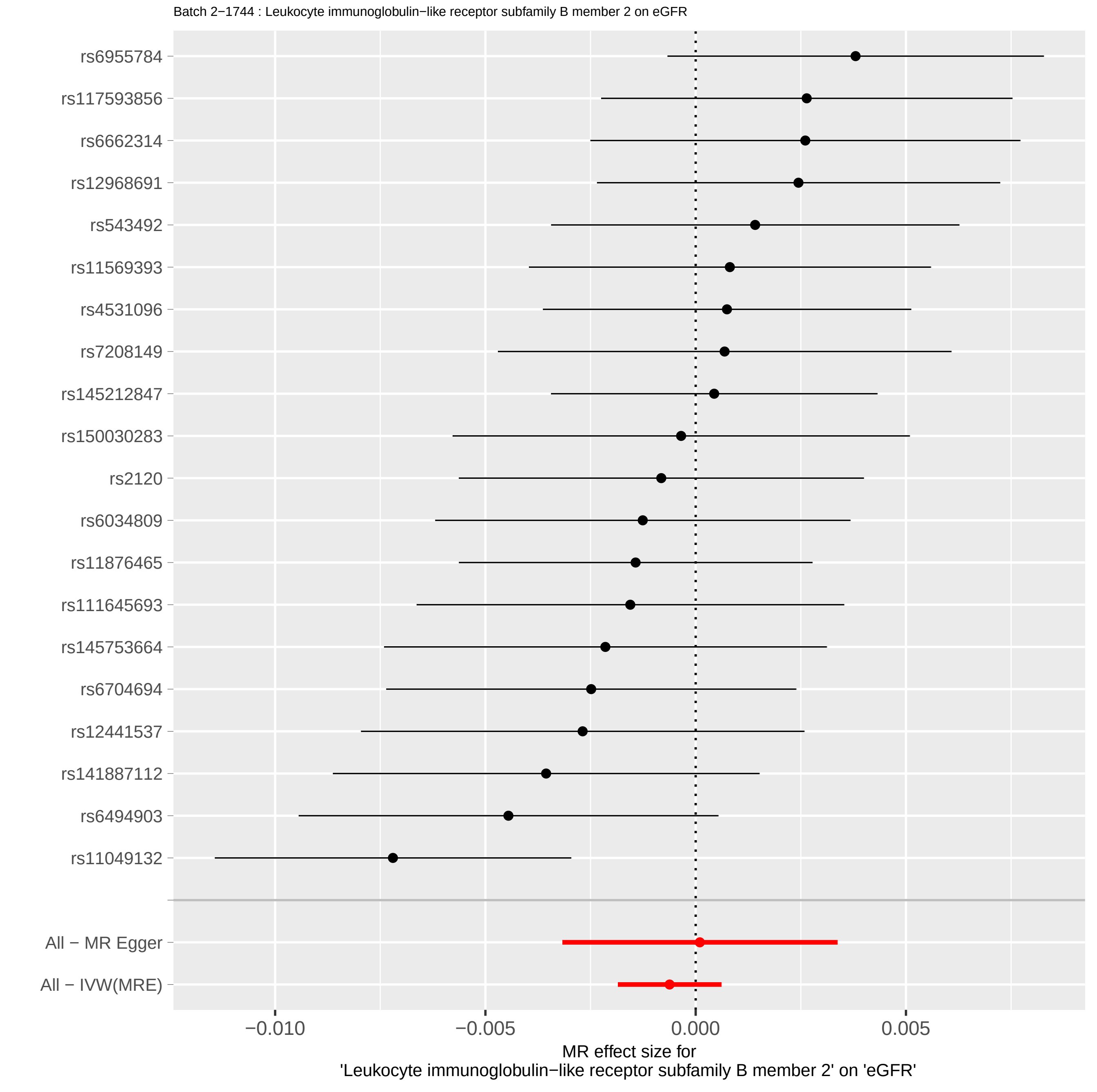

Batch 2−1744 : Leukocyte immunoglobulin−like receptor subfamily B member 2 on eGFR
rs6955784
rs117593856
rs6662314
rs12968691
rs543492
rs11569393
rs4531096
rs7208149
rs145212847
rs150030283
rs2120
rs6034809
rs11876465
rs111645693
rs145753664
rs6704694
rs12441537
rs141887112
rs6494903
rs11049132
All − MR Egger
All − IVW(MRE)
−0.010
−0.005
0.000
0.005
MR effect size for
'Leukocyte immunoglobulin−like receptor subfamily B member 2' on 'eGFR'

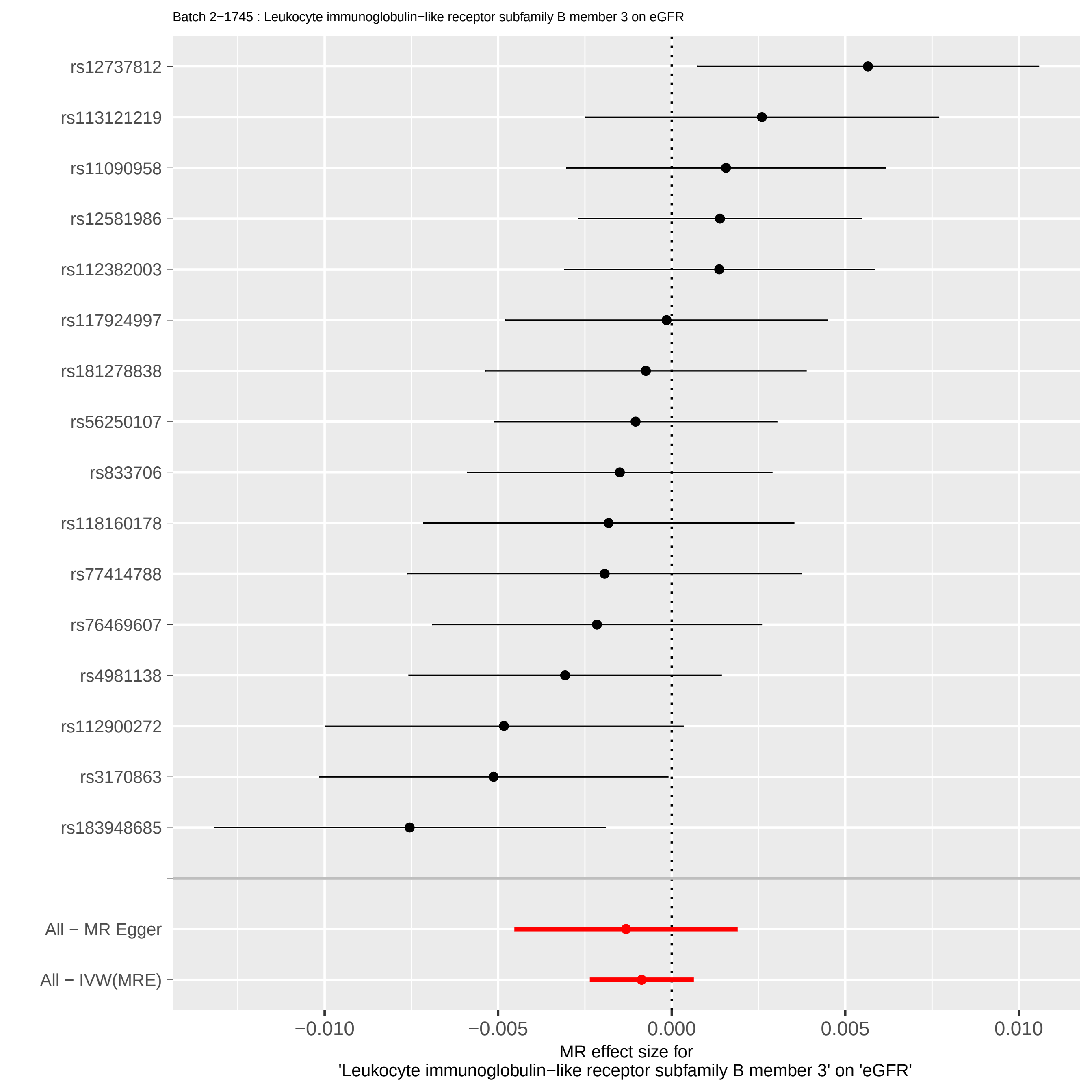

Batch 2−1745 : Leukocyte immunoglobulin−like receptor subfamily B member 3 on eGFR
rs12737812
rs113121219
rs11090958
rs12581986
rs112382003
rs117924997
rs181278838
rs56250107
rs833706
rs118160178
rs77414788
rs76469607
rs4981138
rs112900272
rs3170863
rs183948685
All − MR Egger
All − IVW(MRE)
−0.010
−0.005
0.000
0.005
0.010
MR effect size for
'Leukocyte immunoglobulin−like receptor subfamily B member 3' on 'eGFR'

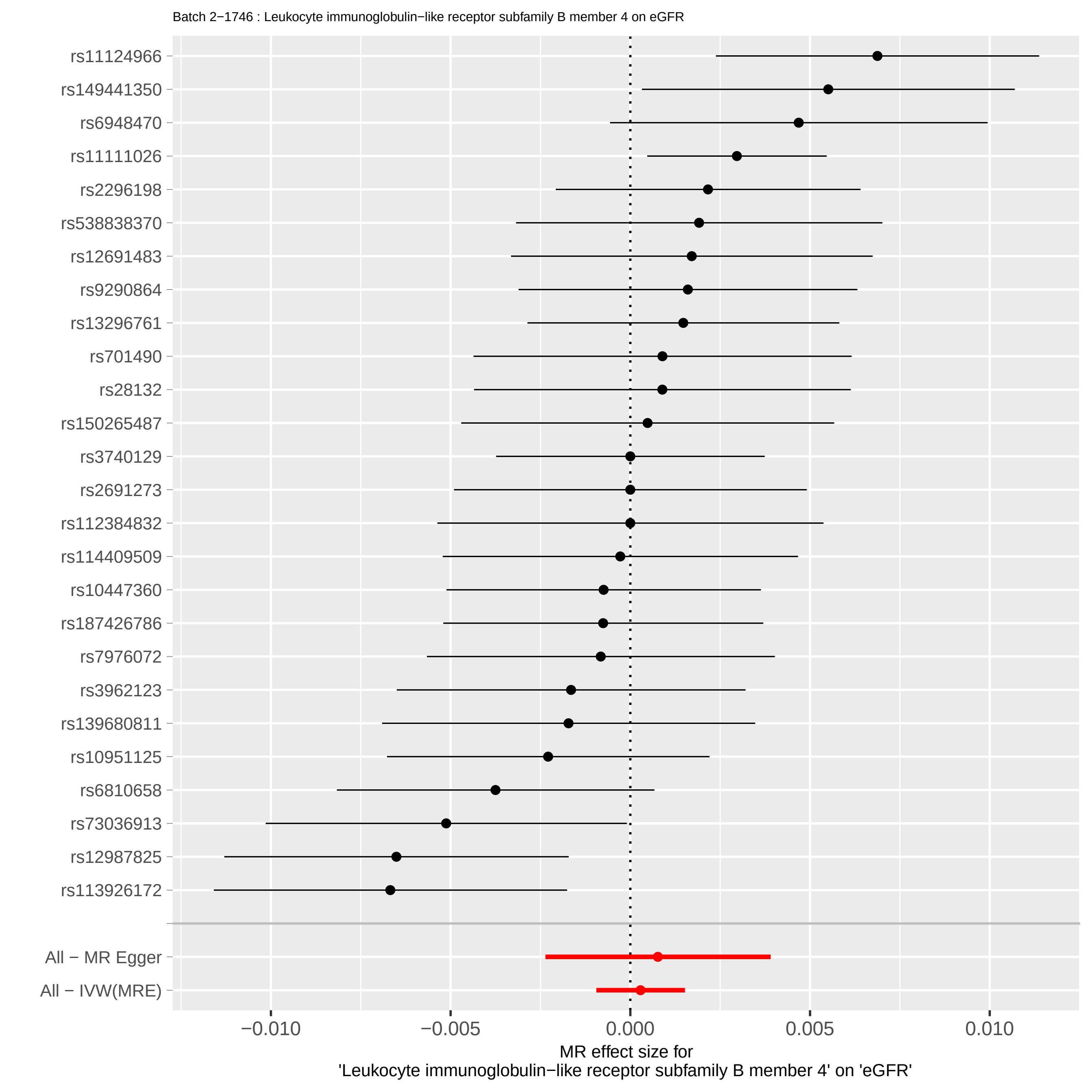

Batch 2−1746 : Leukocyte immunoglobulin−like receptor subfamily B member 4 on eGFR
rs11124966
rs149441350
rs6948470
rs11111026
rs2296198
rs538838370
rs12691483
rs9290864
rs13296761
rs701490
rs28132
rs150265487
rs3740129
rs2691273
rs112384832
rs114409509
rs10447360
rs187426786
rs7976072
rs3962123
rs139680811
rs10951125
rs6810658
rs73036913
rs12987825
rs113926172
All − MR Egger
All − IVW(MRE)
−0.010
−0.005
0.000
0.005
0.010
MR effect size for
'Leukocyte immunoglobulin−like receptor subfamily B member 4' on 'eGFR'

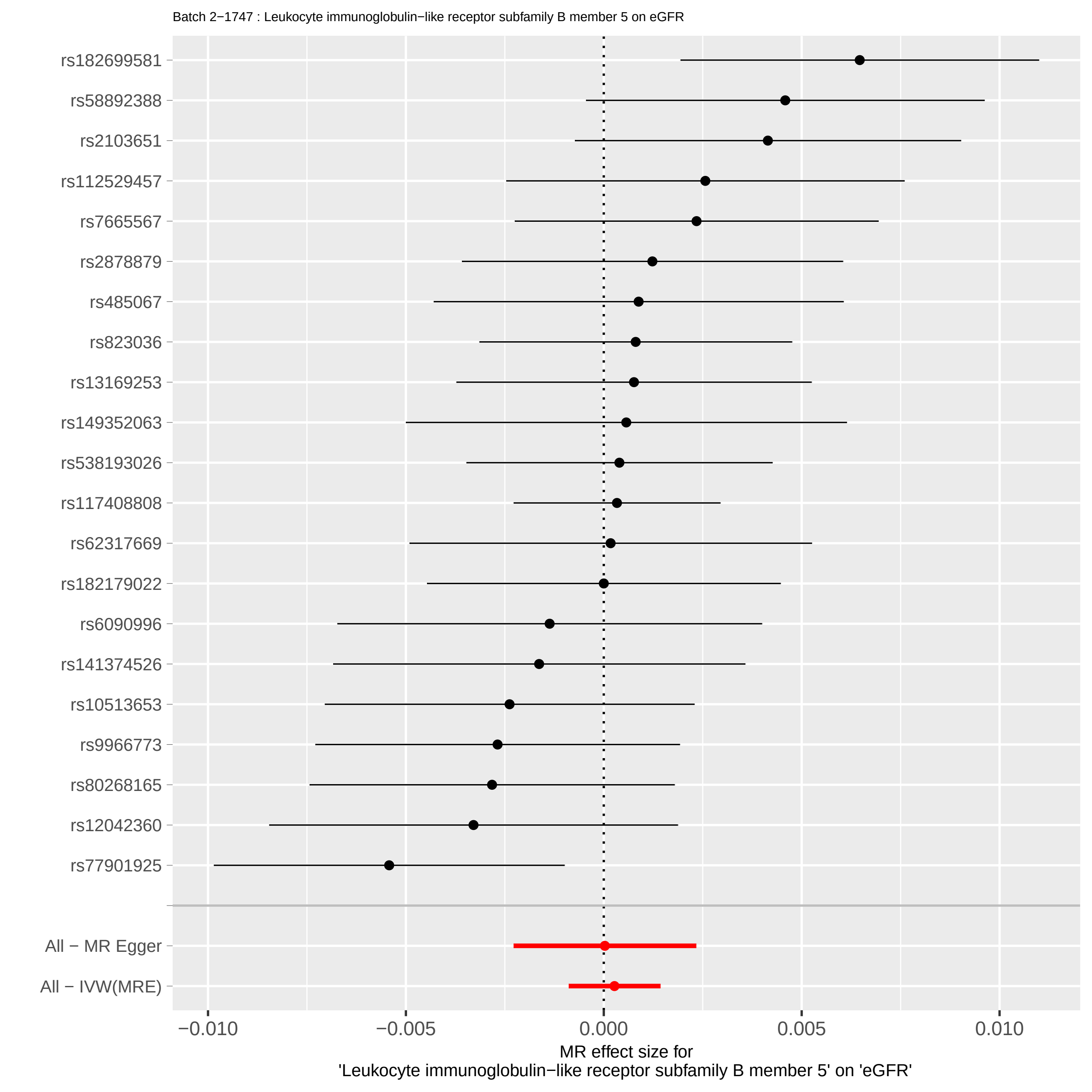

Batch 2−1747 : Leukocyte immunoglobulin−like receptor subfamily B member 5 on eGFR
rs182699581
rs58892388
rs2103651
rs112529457
rs7665567
rs2878879
rs485067
rs823036
rs13169253
rs149352063
rs538193026
rs117408808
rs62317669
rs182179022
rs6090996
rs141374526
rs10513653
rs9966773
rs80268165
rs12042360
rs77901925
All − MR Egger
All − IVW(MRE)
−0.010
−0.005
0.000
0.005
0.010
MR effect size for
'Leukocyte immunoglobulin−like receptor subfamily B member 5' on 'eGFR'

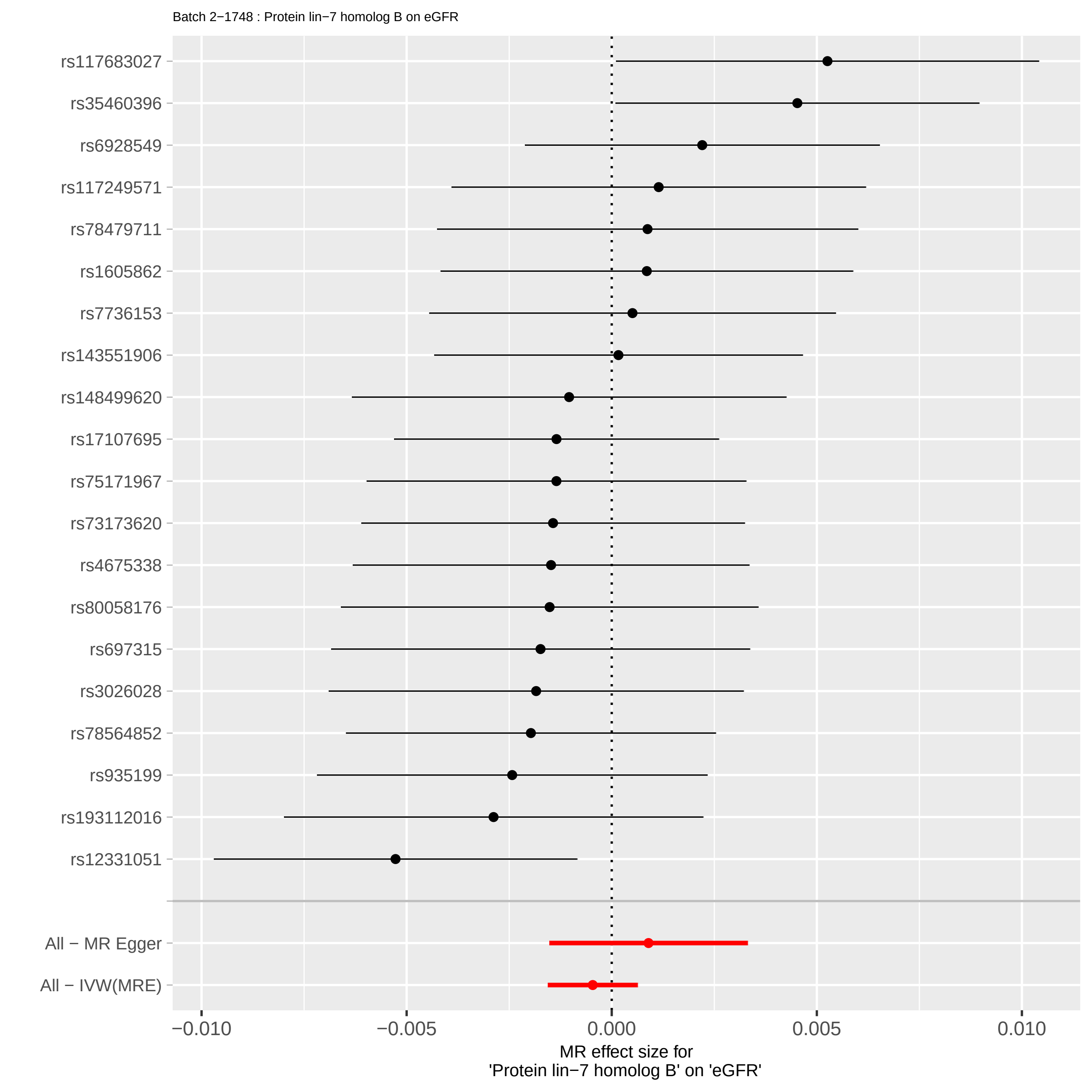
Batch 2−1748 : Protein lin−7 homolog B on eGFR
rs117683027
rs35460396
rs6928549
rs117249571
rs78479711
rs1605862
rs7736153
rs143551906
rs148499620
rs17107695
rs75171967
rs73173620
rs4675338
rs80058176
rs697315
rs3026028
rs78564852
rs935199
rs193112016
rs12331051
All − MR Egger
All − IVW(MRE)
−0.010
−0.005
0.000
0.005
0.010
MR effect size for
'Protein lin−7 homolog B' on 'eGFR'

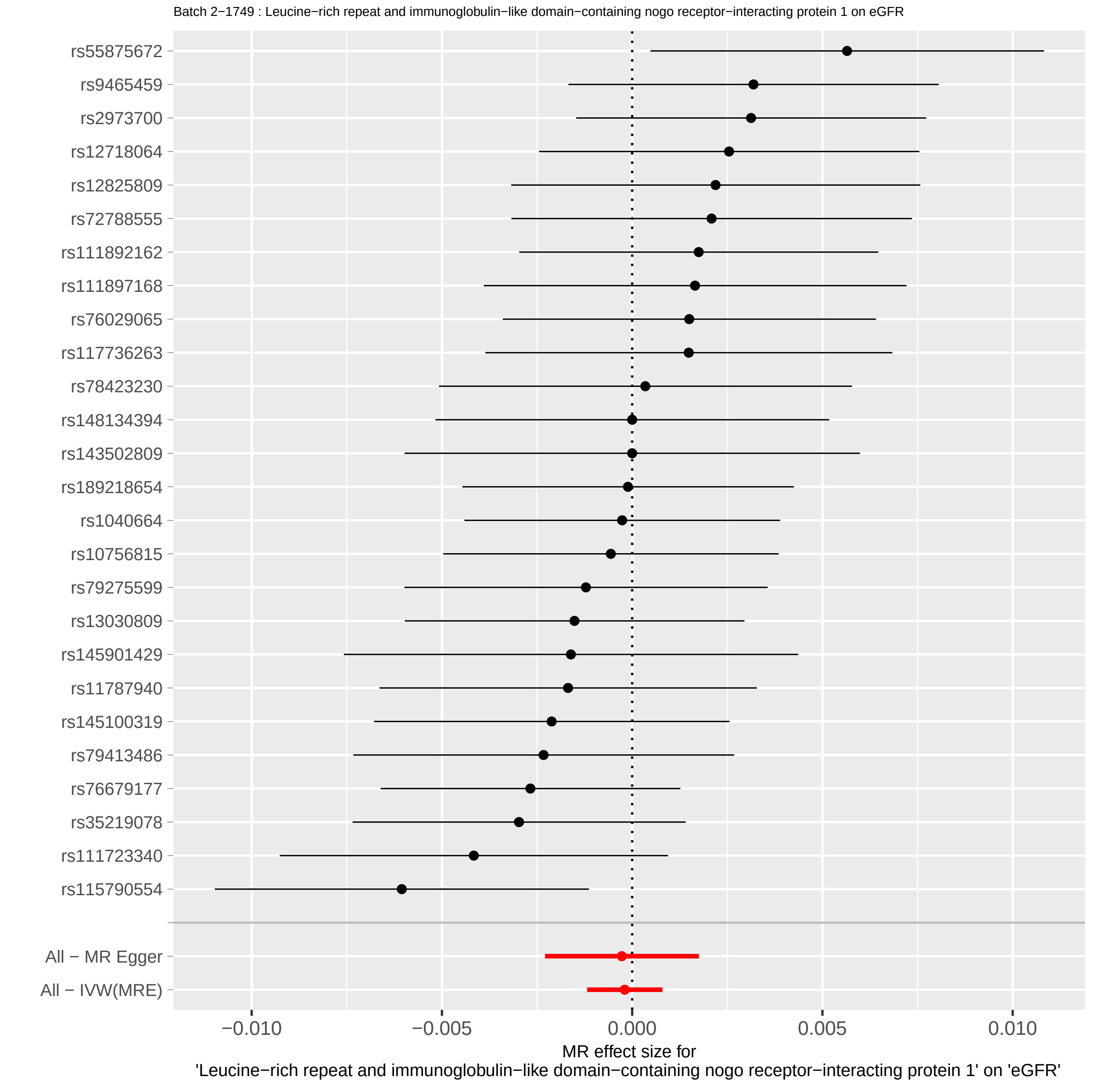

Batch 2−1749 : Leucine−rich repeat and immunoglobulin−like domain−containing nogo receptor−interacting protein 1 on eGFR
rs55875672
rs9465459
rs2973700
rs12718064
rs12825809
rs72788555
rs111892162
rs111897168
rs76029065
rs117736263
rs78423230
rs148134394
rs143502809
rs189218654
rs1040664
rs10756815
rs79275599
rs13030809
rs145901429
rs11787940
rs145100319
rs79413486
rs76679177
rs35219078
rs111723340
rs115790554
All − MR Egger
All − IVW(MRE)
−0.010
−0.005
0.000
0.005
0.010
MR effect size for
'Leucine−rich repeat and immunoglobulin−like domain−containing nogo receptor−interacting protein 1' on 'eGFR'

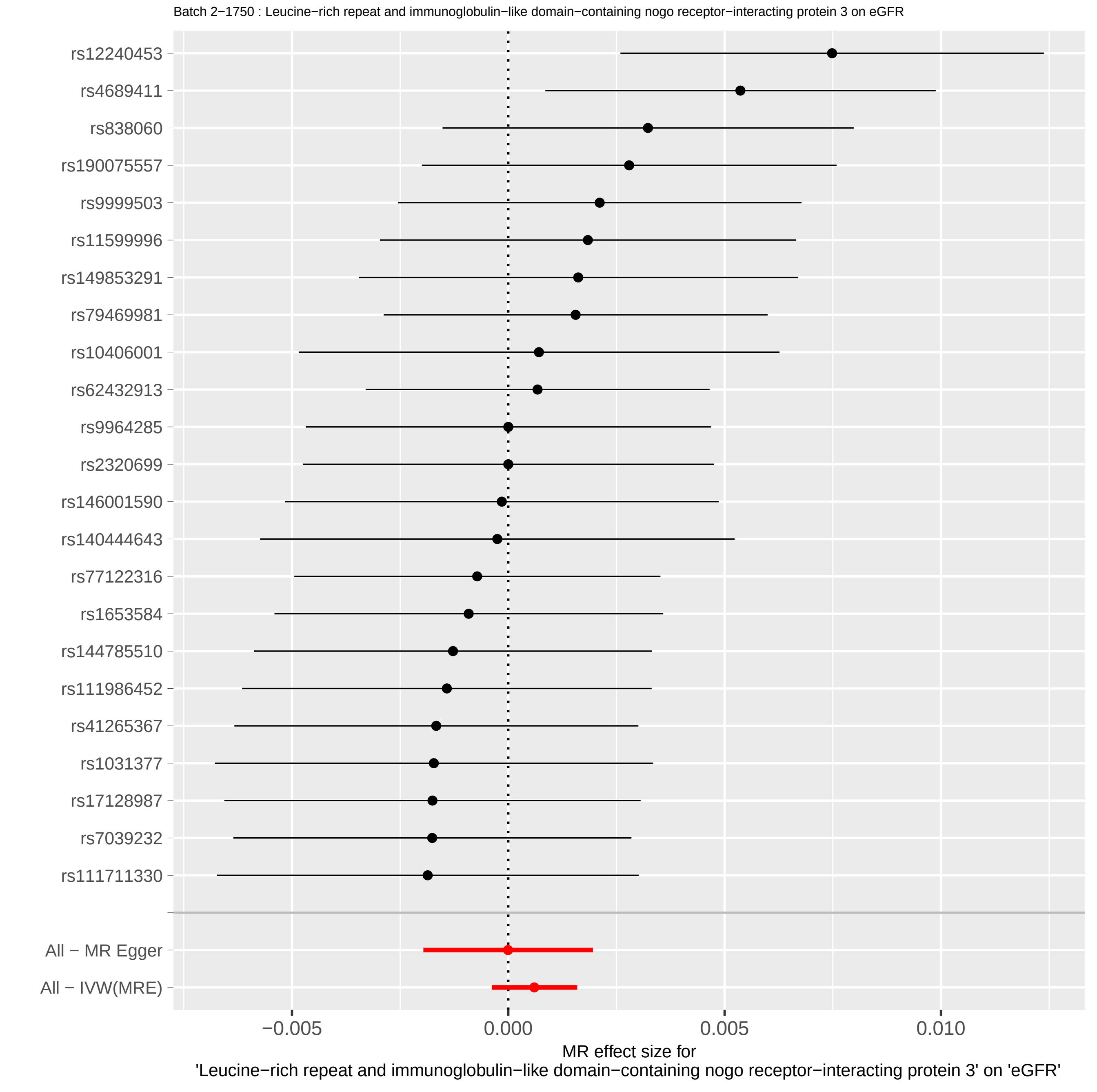

Batch 2−1750 : Leucine−rich repeat and immunoglobulin−like domain−containing nogo receptor−interacting protein 3 on eGFR
rs12240453
rs4689411
rs838060
rs190075557
rs9999503
rs11599996
rs149853291
rs79469981
rs10406001
rs62432913
rs9964285
rs2320699
rs146001590
rs140444643
rs77122316
rs1653584
rs144785510
rs111986452
rs41265367
rs1031377
rs17128987
rs7039232
rs111711330
All − MR Egger
All − IVW(MRE)
−0.005
0.000
0.005
0.010
MR effect size for
'Leucine−rich repeat and immunoglobulin−like domain−containing nogo receptor−interacting protein 3' on 'eGFR'

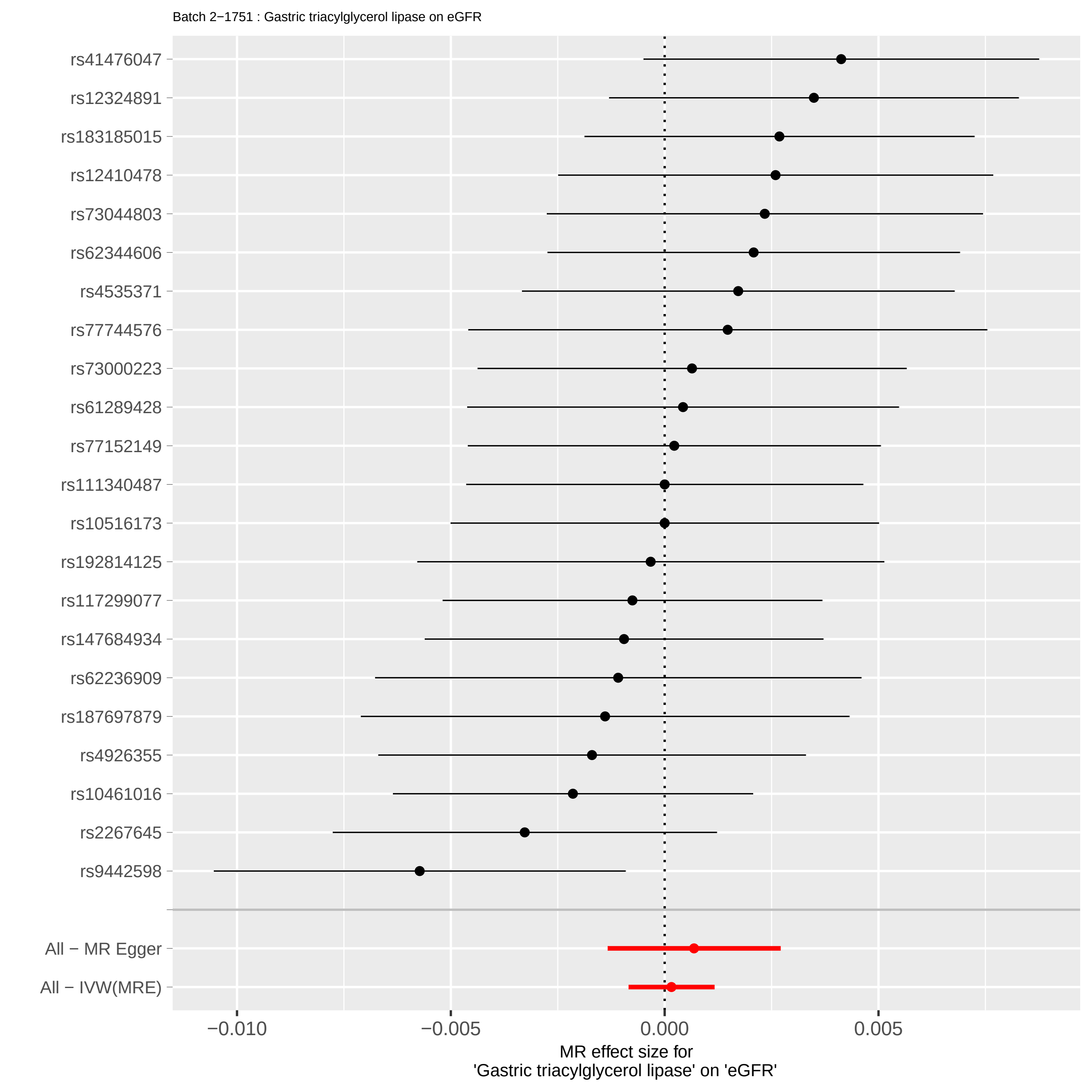

Batch 2−1751 : Gastric triacylglycerol lipase on eGFR
rs41476047
rs12324891
rs183185015
rs12410478
rs73044803
rs62344606
rs4535371
rs77744576
rs73000223
rs61289428
rs77152149
rs111340487
rs10516173
rs192814125
rs117299077
rs147684934
rs62236909
rs187697879
rs4926355
rs10461016
rs2267645
rs9442598
All − MR Egger
All − IVW(MRE)
−0.010
−0.005
0.000
0.005
MR effect size for
'Gastric triacylglycerol lipase' on 'eGFR'

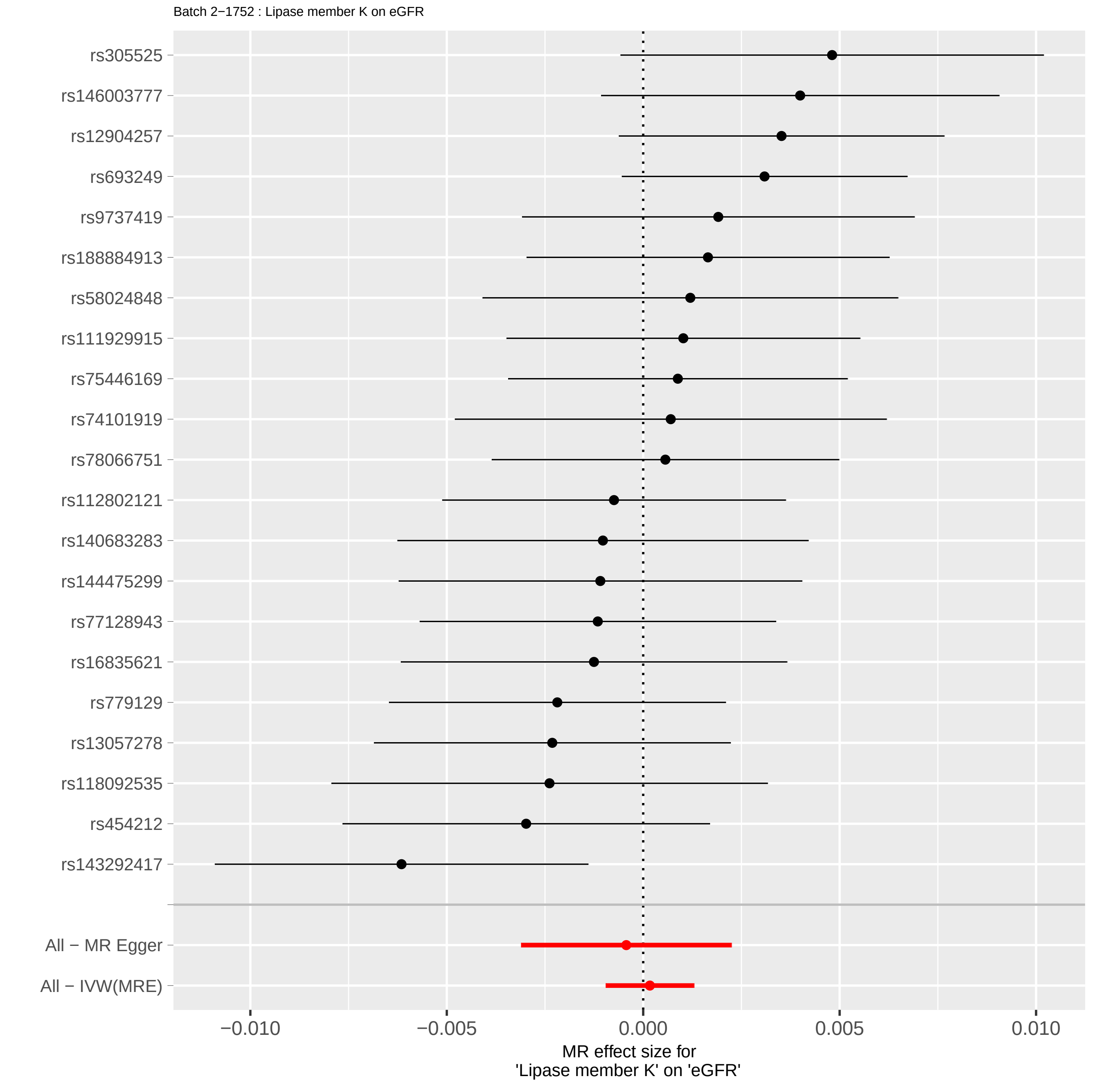

Batch 2−1752 : Lipase member K on eGFR
rs305525
rs146003777
rs12904257
rs693249
rs9737419
rs188884913
rs58024848
rs111929915
rs75446169
rs74101919
rs78066751
rs112802121
rs140683283
rs144475299
rs77128943
rs16835621
rs779129
rs13057278
rs118092535
rs454212
rs143292417
All − MR Egger
All − IVW(MRE)
−0.010
−0.005
0.000
0.005
0.010
MR effect size for
'Lipase member K' on 'eGFR'

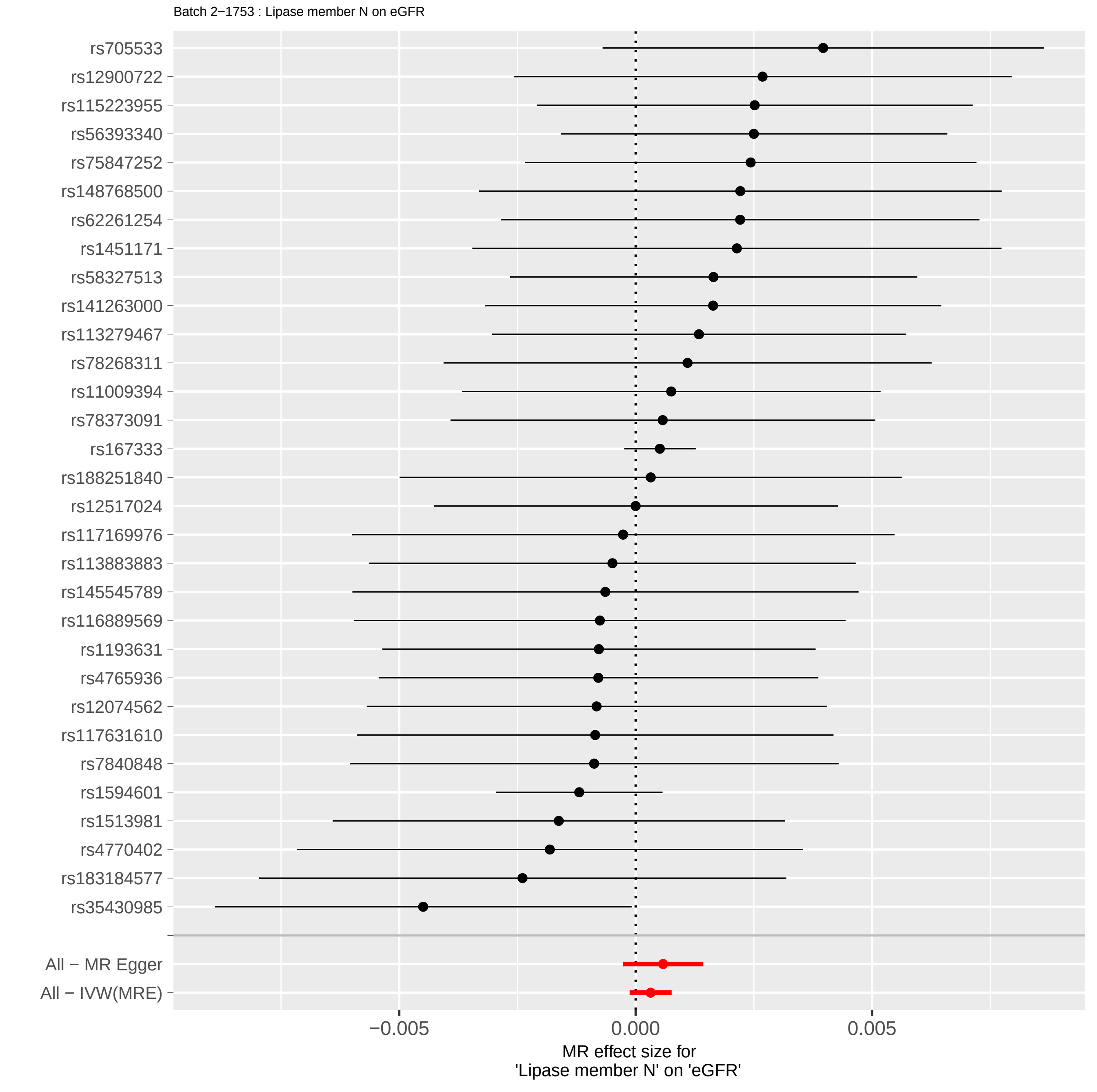

Batch 2−1753 : Lipase member N on eGFR
rs705533
rs12900722
rs115223955
rs56393340
rs75847252
rs148768500
rs62261254
rs1451171
rs58327513
rs141263000
rs113279467
rs78268311
rs11009394
rs78373091
rs167333
rs188251840
rs12517024
rs117169976
rs113883883
rs145545789
rs116889569
rs1193631
rs4765936
rs12074562
rs117631610
rs7840848
rs1594601
rs1513981
rs4770402
rs183184577
rs35430985
All − MR Egger
All − IVW(MRE)
−0.005
0.000
0.005
MR effect size for
'Lipase member N' on 'eGFR'

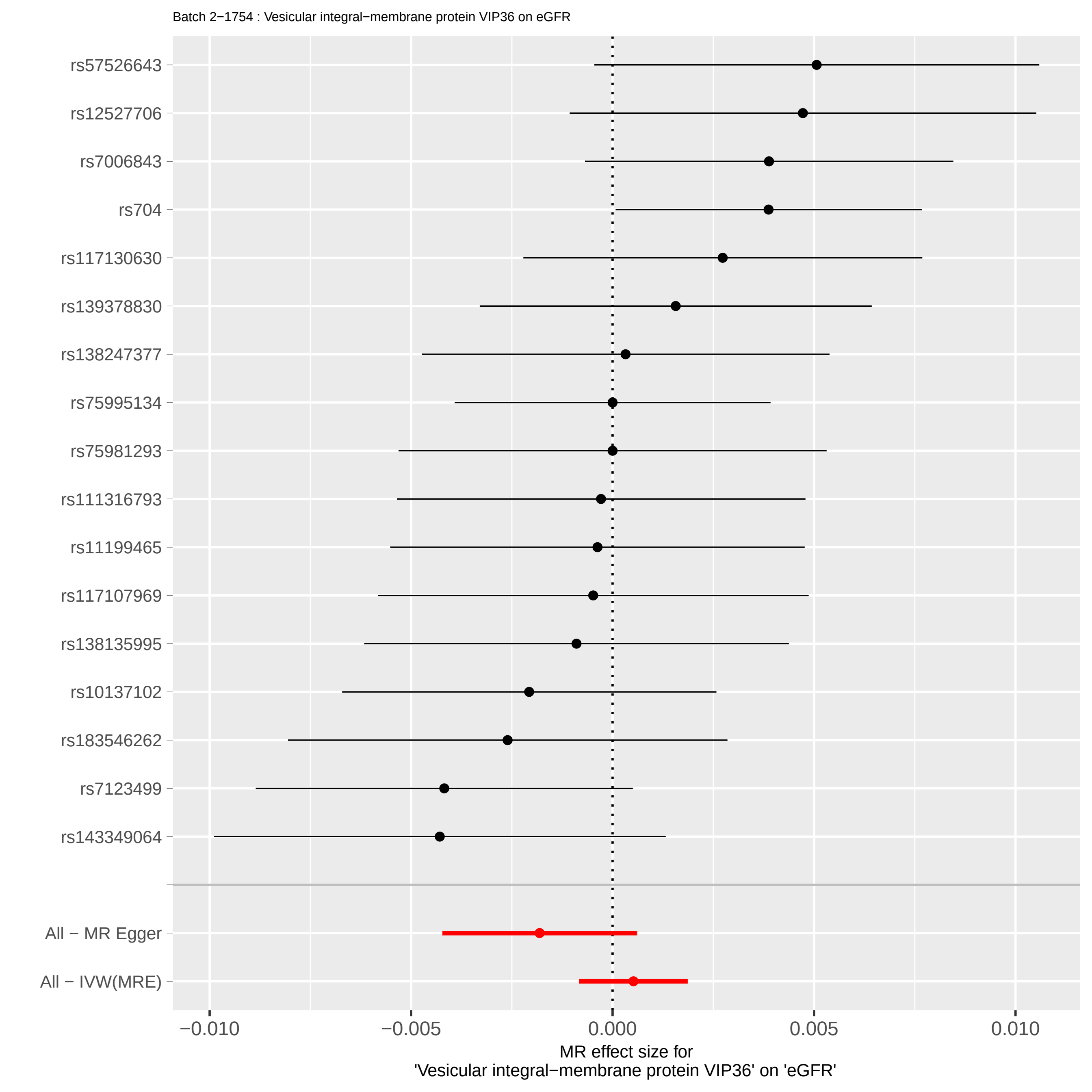

Batch 2−1754 : Vesicular integral−membrane protein VIP36 on eGFR
rs57526643
rs12527706
rs7006843
rs704
rs117130630
rs139378830
rs138247377
rs75995134
rs75981293
rs111316793
rs11199465
rs117107969
rs138135995
rs10137102
rs183546262
rs7123499
rs143349064
All − MR Egger
All − IVW(MRE)
−0.010
−0.005
0.000
0.005
0.010
MR effect size for
'Vesicular integral−membrane protein VIP36' on 'eGFR'

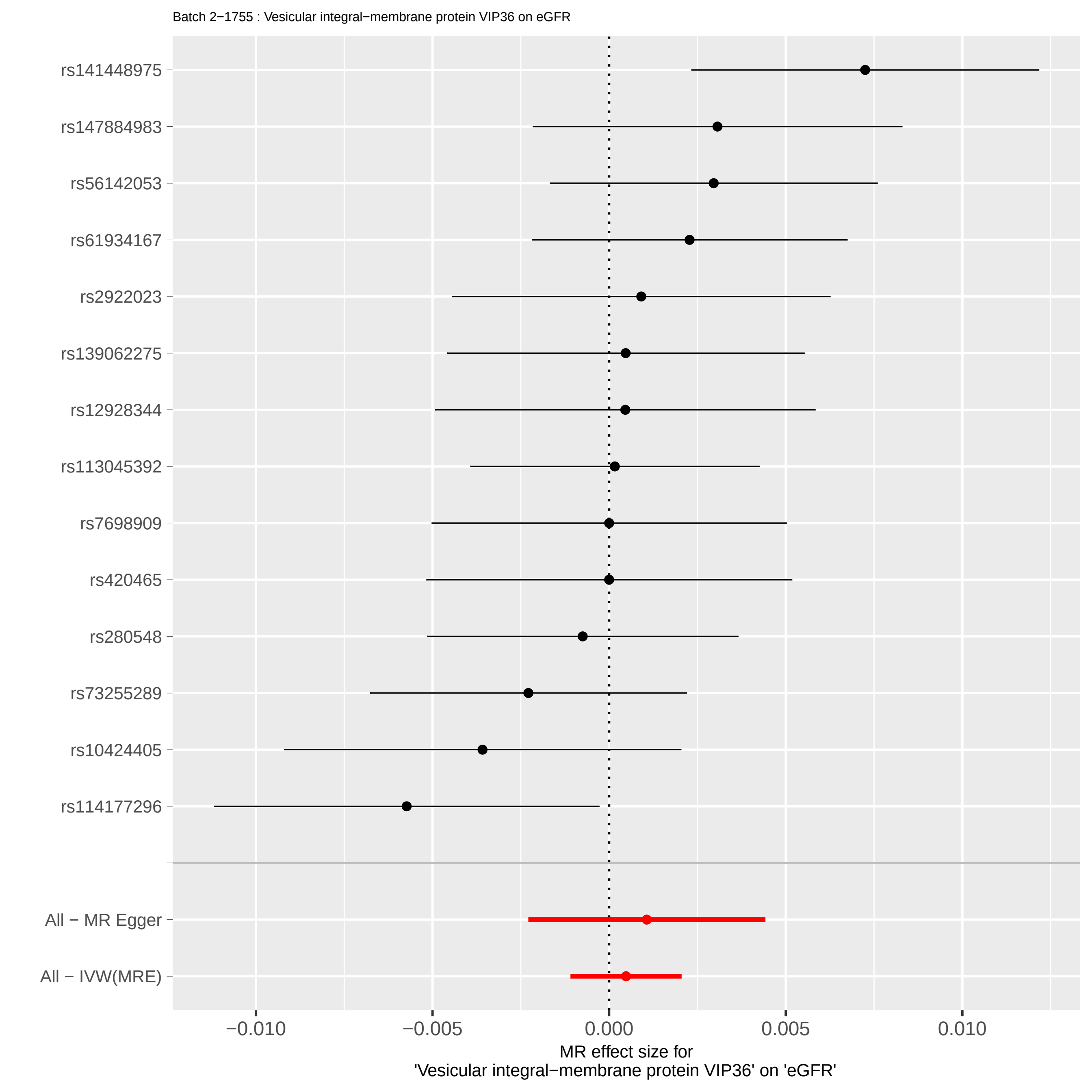

Batch 2−1755 : Vesicular integral−membrane protein VIP36 on eGFR
rs141448975
rs147884983
rs56142053
rs61934167
rs2922023
rs139062275
rs12928344
rs113045392
rs7698909
rs420465
rs280548
rs73255289
rs10424405
rs114177296
All − MR Egger
All − IVW(MRE)
−0.010
−0.005
0.000
0.005
0.010
MR effect size for
'Vesicular integral−membrane protein VIP36' on 'eGFR'

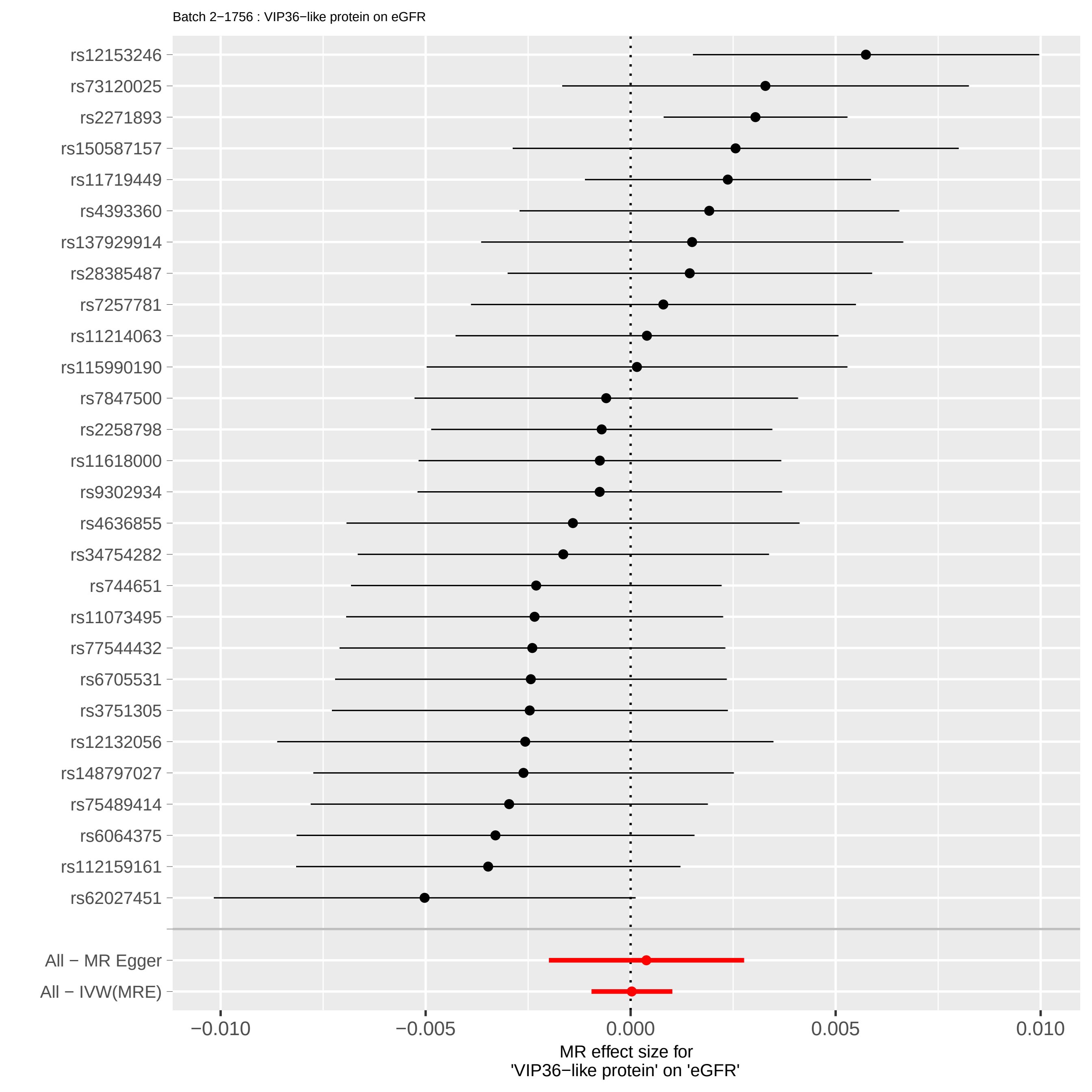

Batch 2−1756 : VIP36−like protein on eGFR
rs12153246
rs73120025
rs2271893
rs150587157
rs11719449
rs4393360
rs137929914
rs28385487
rs7257781
rs11214063
rs115990190
rs7847500
rs2258798
rs11618000
rs9302934
rs4636855
rs34754282
rs744651
rs11073495
rs77544432
rs6705531
rs3751305
rs12132056
rs148797027
rs75489414
rs6064375
rs112159161
rs62027451
All − MR Egger
All − IVW(MRE)
−0.010
−0.005
0.000
0.005
0.010
MR effect size for
'VIP36−like protein' on 'eGFR'

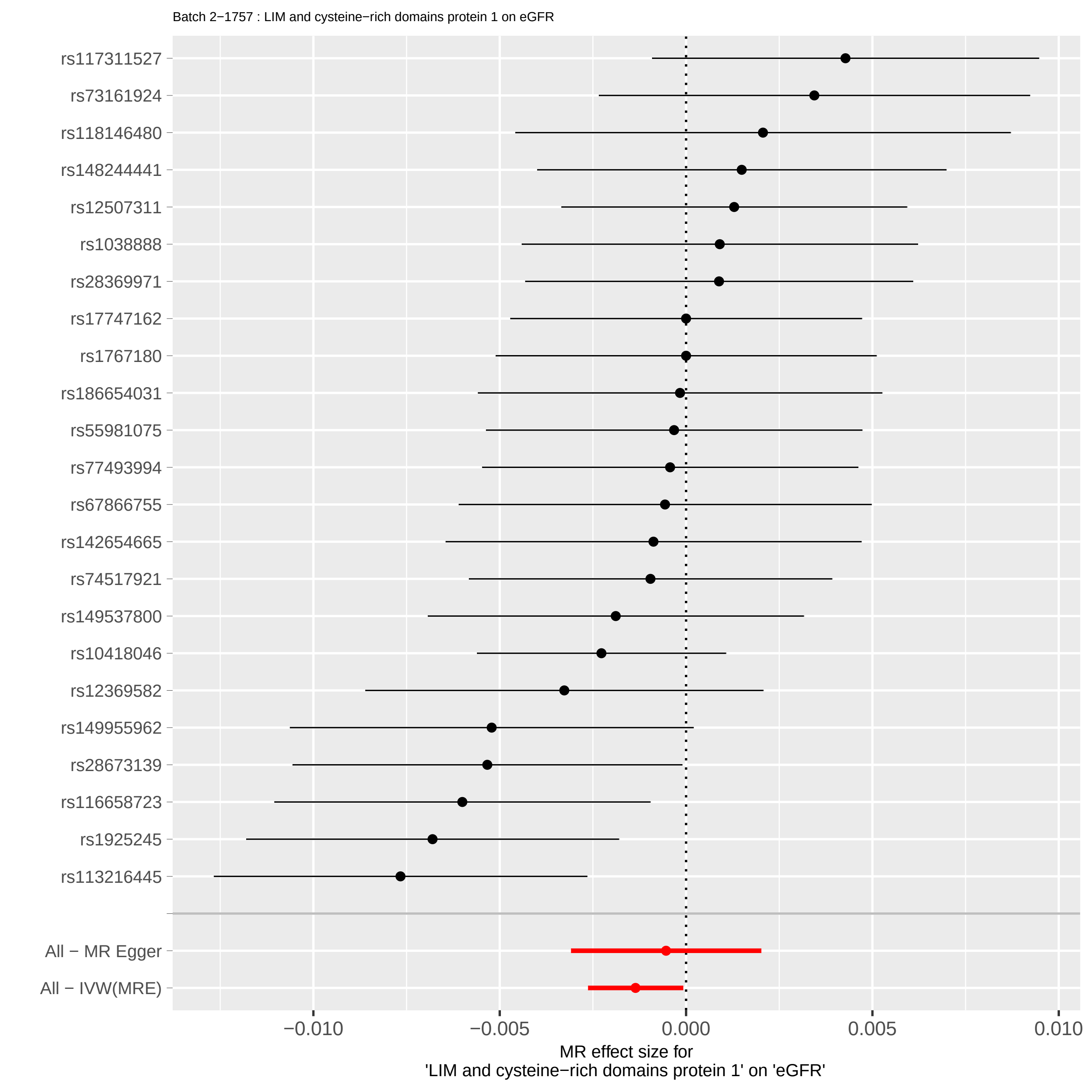

Batch 2−1757 : LIM and cysteine−rich domains protein 1 on eGFR
rs117311527
rs73161924
rs118146480
rs148244441
rs12507311
rs1038888
rs28369971
rs17747162
rs1767180
rs186654031
rs55981075
rs77493994
rs67866755
rs142654665
rs74517921
rs149537800
rs10418046
rs12369582
rs149955962
rs28673139
rs116658723
rs1925245
rs113216445
All − MR Egger
All − IVW(MRE)
−0.010
−0.005
0.000
0.005
0.010
MR effect size for
'LIM and cysteine−rich domains protein 1' on 'eGFR'

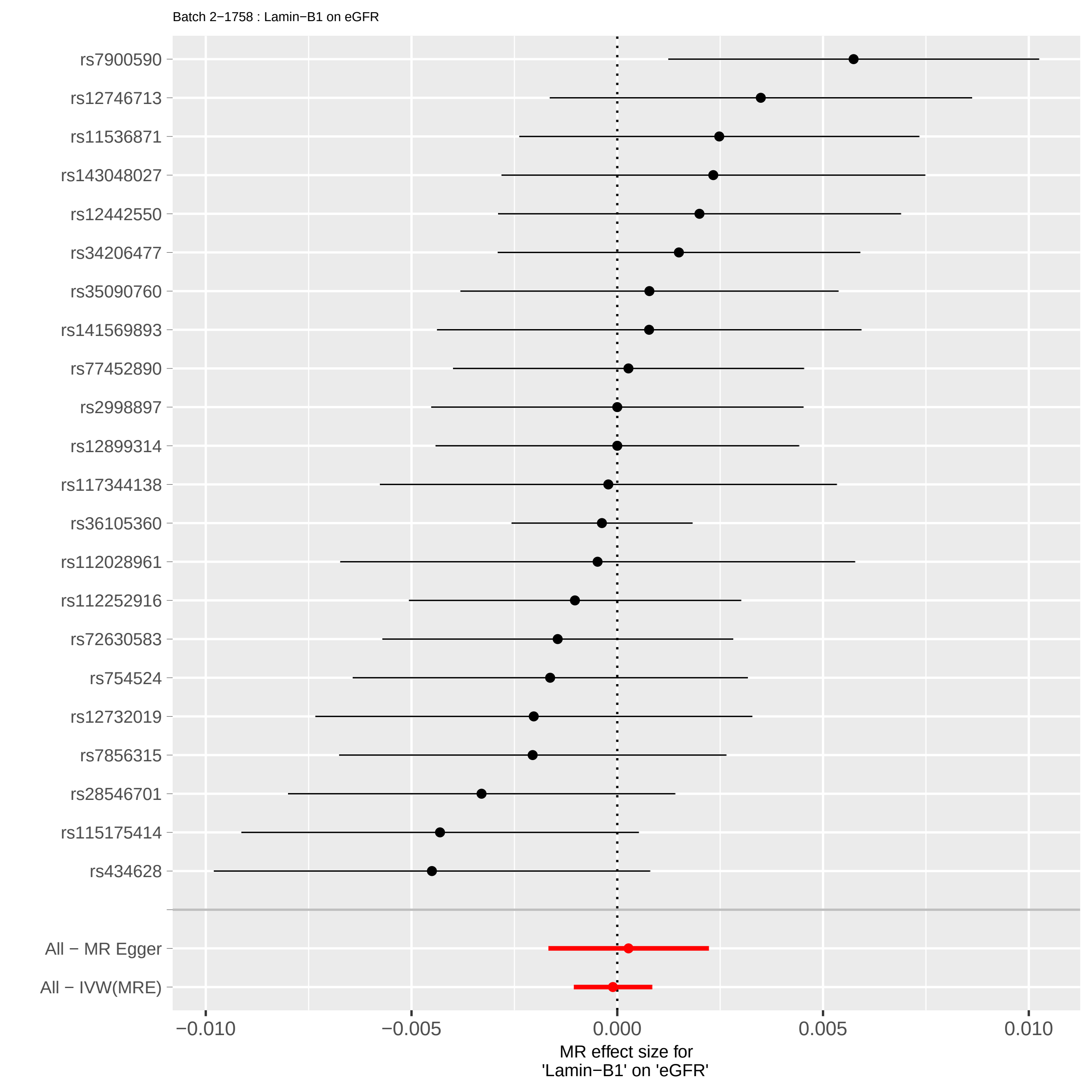
Batch 2−1758 : Lamin−B1 on eGFR
rs7900590
rs12746713
rs11536871
rs143048027
rs12442550
rs34206477
rs35090760
rs141569893
rs77452890
rs2998897
rs12899314
rs117344138
rs36105360
rs112028961
rs112252916
rs72630583
rs754524
rs12732019
rs7856315
rs28546701
rs115175414
rs434628
All − MR Egger
All − IVW(MRE)
−0.010
−0.005
0.000
0.005
0.010
MR effect size for
'Lamin−B1' on 'eGFR'

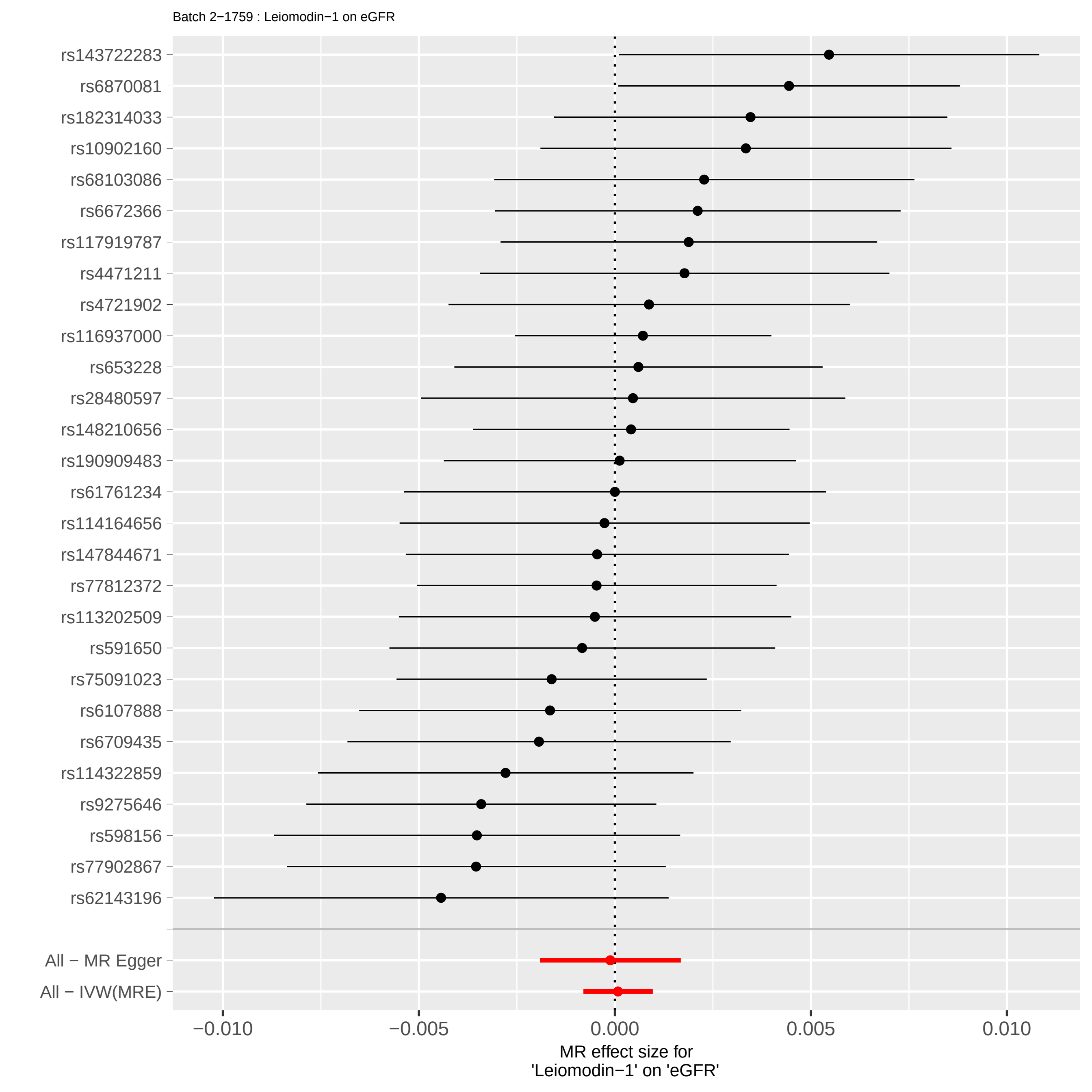

Batch 2−1759 : Leiomodin−1 on eGFR
rs143722283
rs6870081
rs182314033
rs10902160
rs68103086
rs6672366
rs117919787
rs4471211
rs4721902
rs116937000
rs653228
rs28480597
rs148210656
rs190909483
rs61761234
rs114164656
rs147844671
rs77812372
rs113202509
rs591650
rs75091023
rs6107888
rs6709435
rs114322859
rs9275646
rs598156
rs77902867
rs62143196
All − MR Egger
All − IVW(MRE)
−0.010
−0.005
0.000
0.005
0.010
MR effect size for
'Leiomodin−1' on 'eGFR'

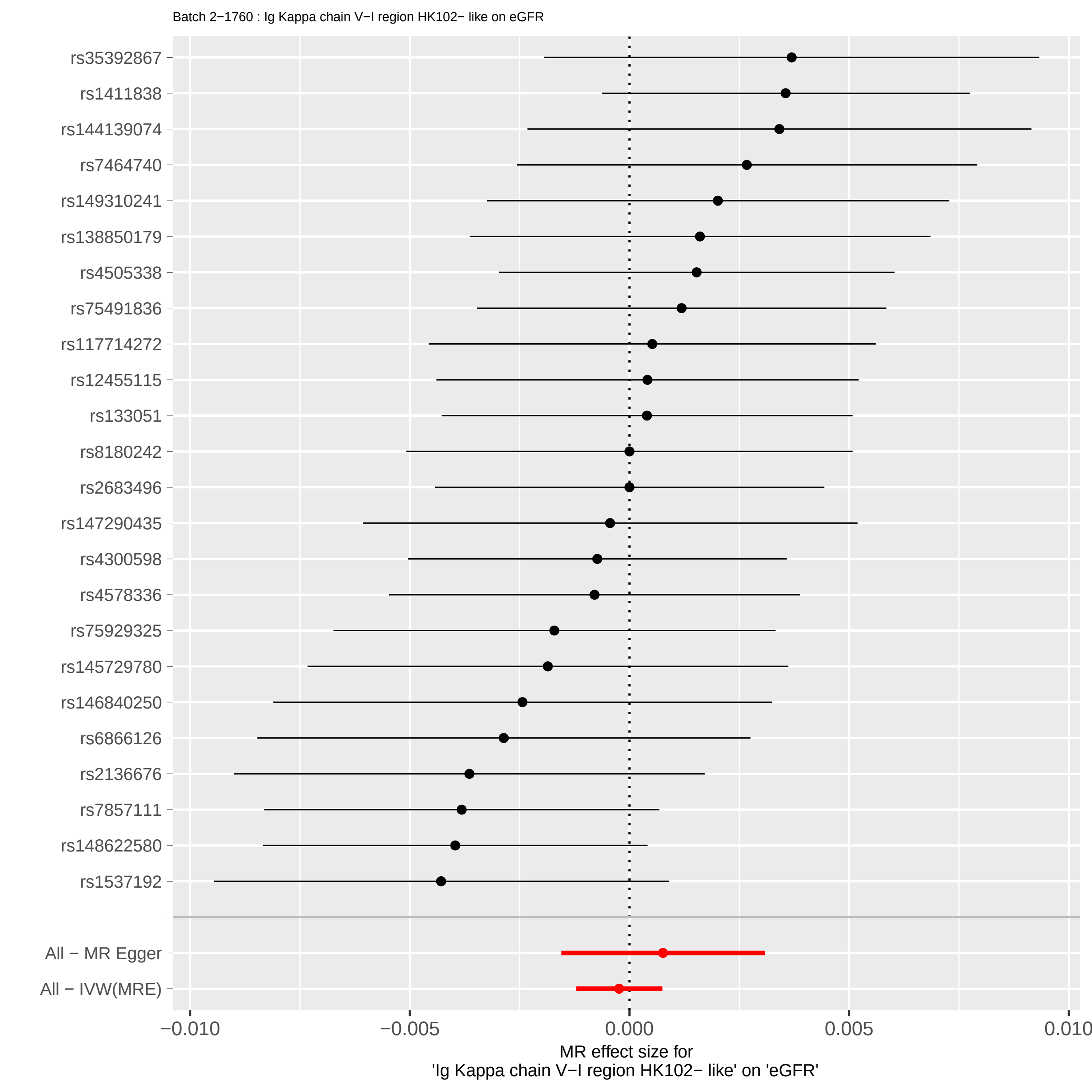

Batch 2−1760 : Ig Kappa chain V−I region HK102− like on eGFR
rs35392867
rs1411838
rs144139074
rs7464740
rs149310241
rs138850179
rs4505338
rs75491836
rs117714272
rs12455115
rs133051
rs8180242
rs2683496
rs147290435
rs4300598
rs4578336
rs75929325
rs145729780
rs146840250
rs6866126
rs2136676
rs7857111
rs148622580
rs1537192
All − MR Egger
All − IVW(MRE)
−0.010
−0.005
0.000
0.005
0.010
MR effect size for
'Ig Kappa chain V−I region HK102− like' on 'eGFR'

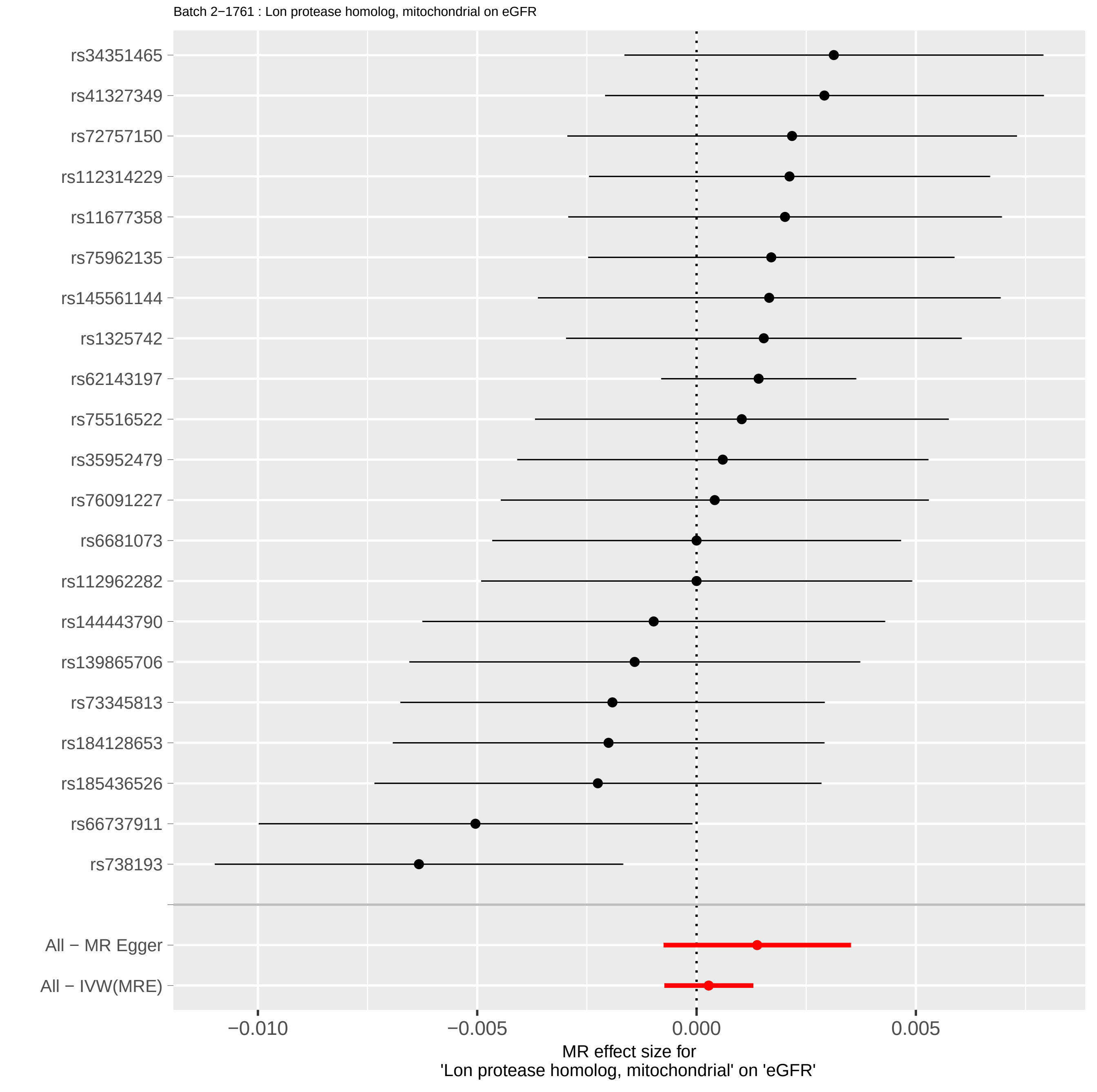

Batch 2−1761 : Lon protease homolog, mitochondrial on eGFR
rs34351465
rs41327349
rs72757150
rs112314229
rs11677358
rs75962135
rs145561144
rs1325742
rs62143197
rs75516522
rs35952479
rs76091227
rs6681073
rs112962282
rs144443790
rs139865706
rs73345813
rs184128653
rs185436526
rs66737911
rs738193
All − MR Egger
All − IVW(MRE)
−0.010
−0.005
0.000
0.005
MR effect size for
'Lon protease homolog, mitochondrial' on 'eGFR'

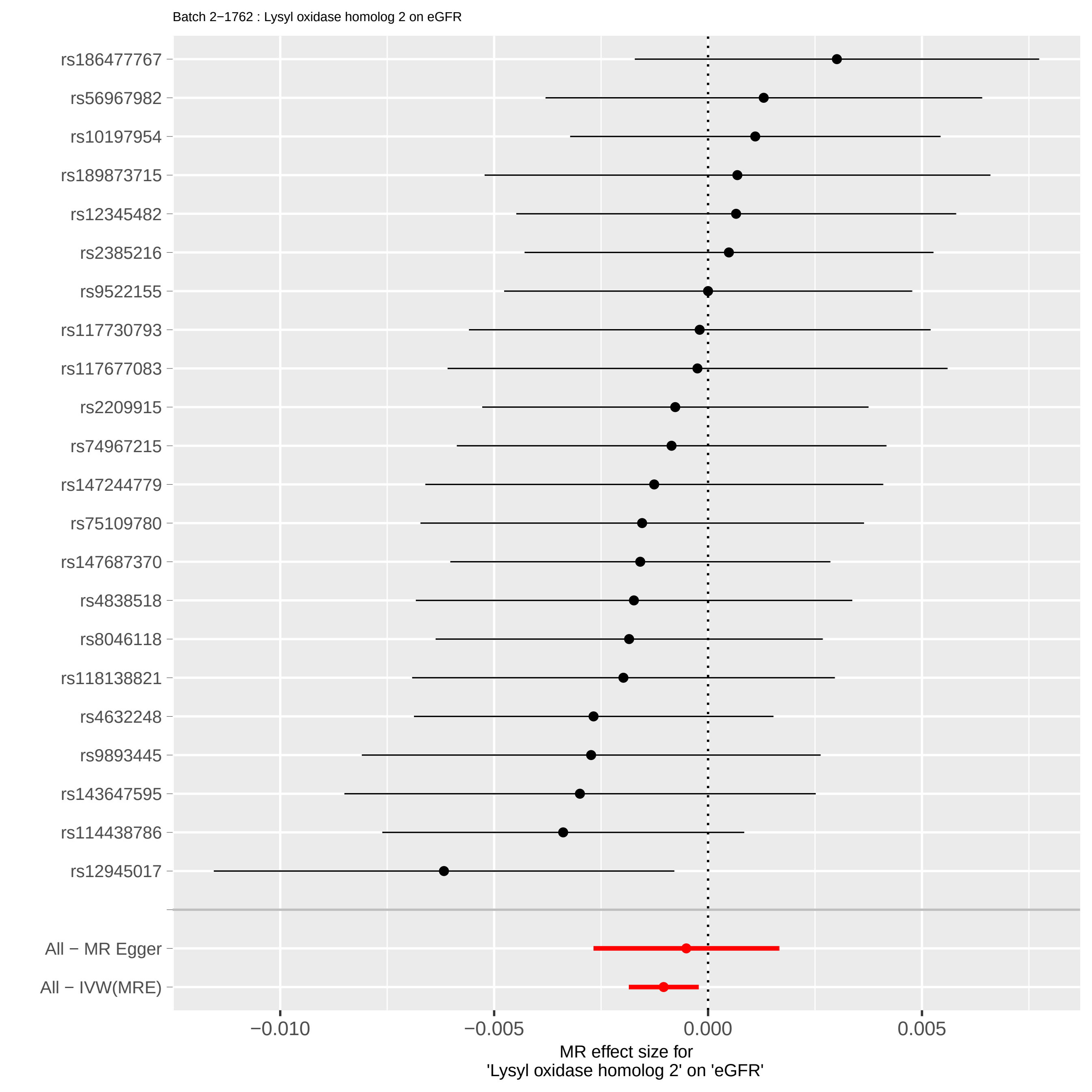

Batch 2−1762 : Lysyl oxidase homolog 2 on eGFR
rs186477767
rs56967982
rs10197954
rs189873715
rs12345482
rs2385216
rs9522155
rs117730793
rs117677083
rs2209915
rs74967215
rs147244779
rs75109780
rs147687370
rs4838518
rs8046118
rs118138821
rs4632248
rs9893445
rs143647595
rs114438786
rs12945017
All − MR Egger
All − IVW(MRE)
−0.010
−0.005
0.000
0.005
MR effect size for
'Lysyl oxidase homolog 2' on 'eGFR'

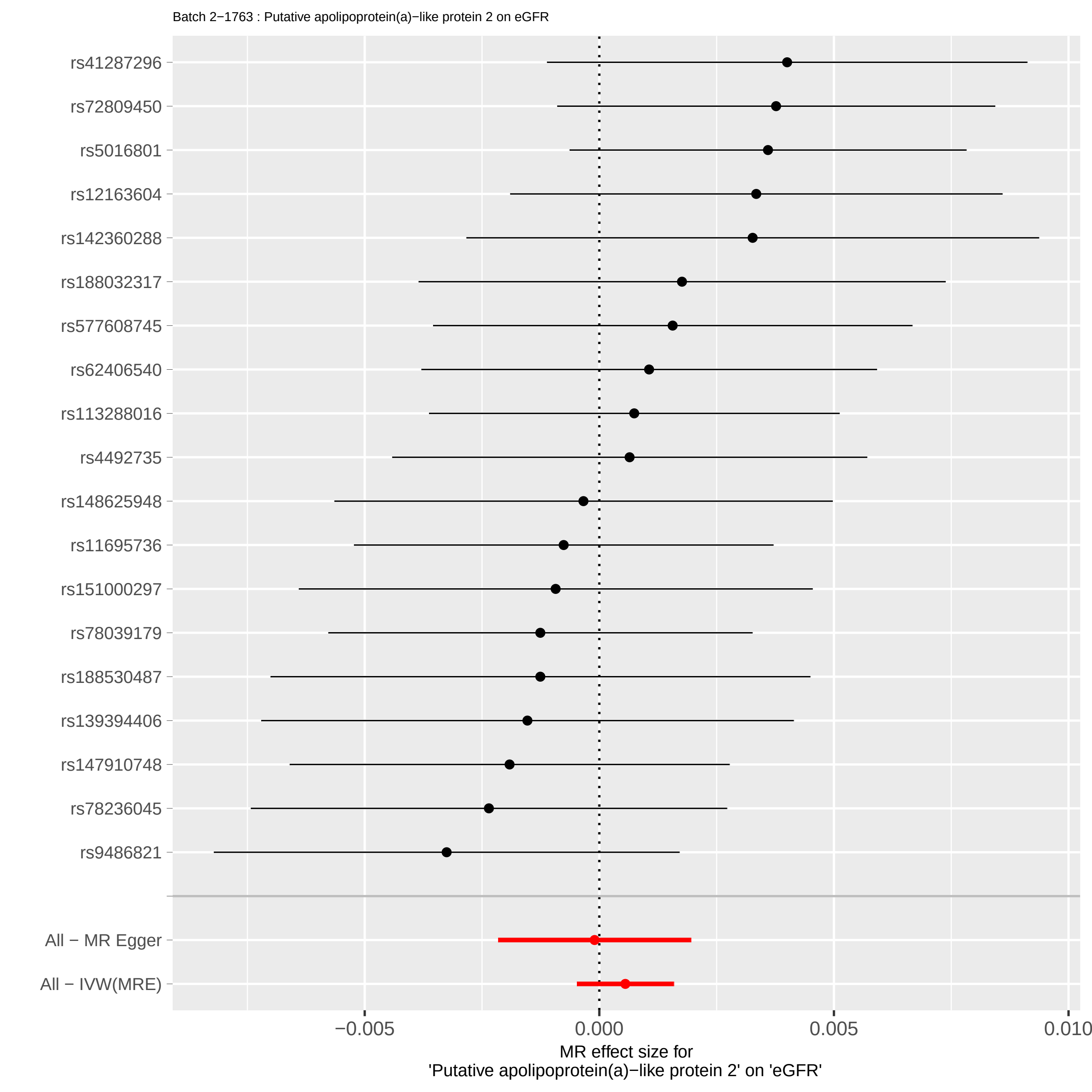

Batch 2−1763 : Putative apolipoprotein(a)−like protein 2 on eGFR
rs41287296
rs72809450
rs5016801
rs12163604
rs142360288
rs188032317
rs577608745
rs62406540
rs113288016
rs4492735
rs148625948
rs11695736
rs151000297
rs78039179
rs188530487
rs139394406
rs147910748
rs78236045
rs9486821
All − MR Egger
All − IVW(MRE)
−0.005
0.000
0.005
0.010
MR effect size for
'Putative apolipoprotein(a)−like protein 2' on 'eGFR'

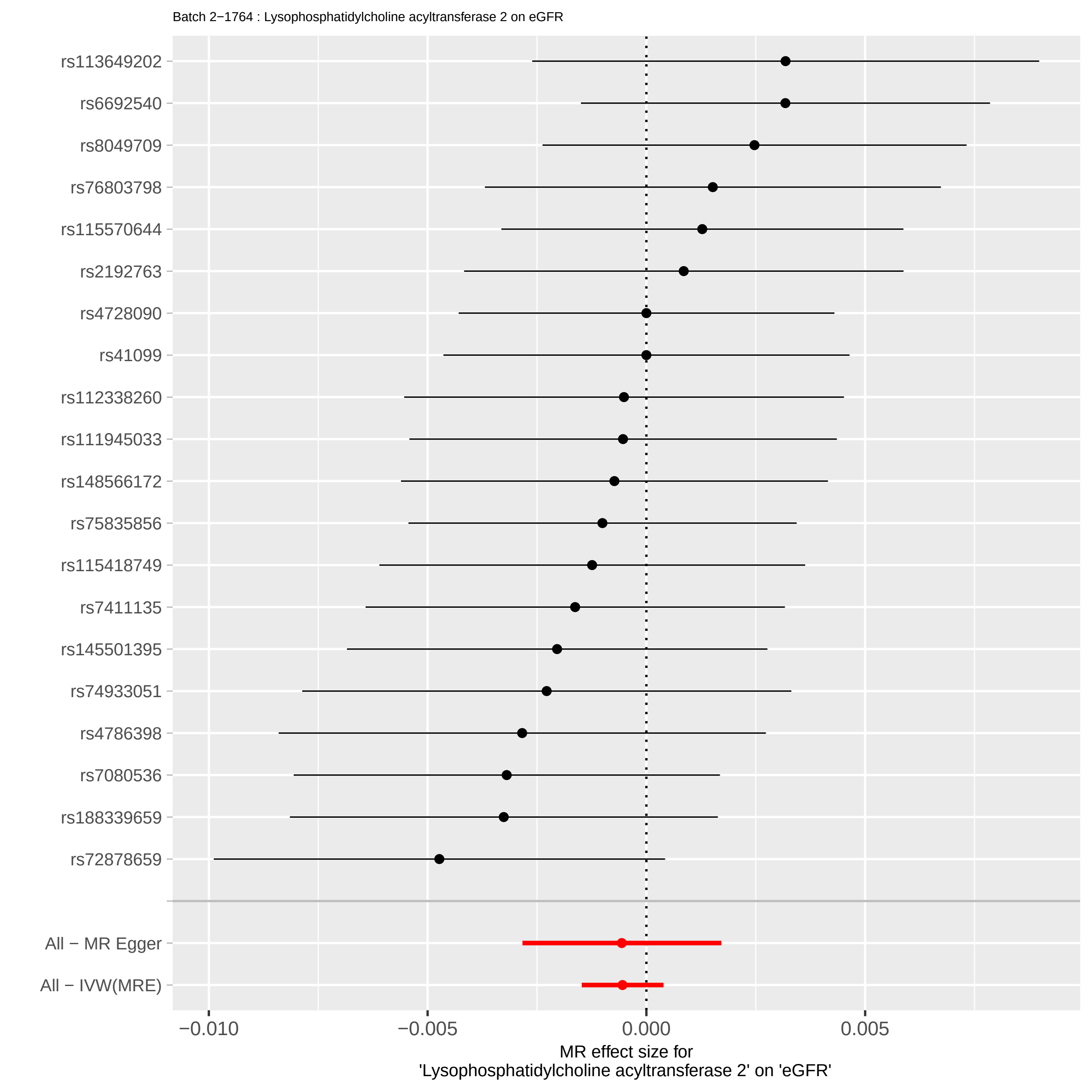
Batch 2−1764 : Lysophosphatidylcholine acyltransferase 2 on eGFR
rs113649202
rs6692540
rs8049709
rs76803798
rs115570644
rs2192763
rs4728090
rs41099
rs112338260
rs111945033
rs148566172
rs75835856
rs115418749
rs7411135
rs145501395
rs74933051
rs4786398
rs7080536
rs188339659
rs72878659
All − MR Egger
All − IVW(MRE)
−0.010
−0.005
0.000
0.005
MR effect size for
'Lysophosphatidylcholine acyltransferase 2' on 'eGFR'

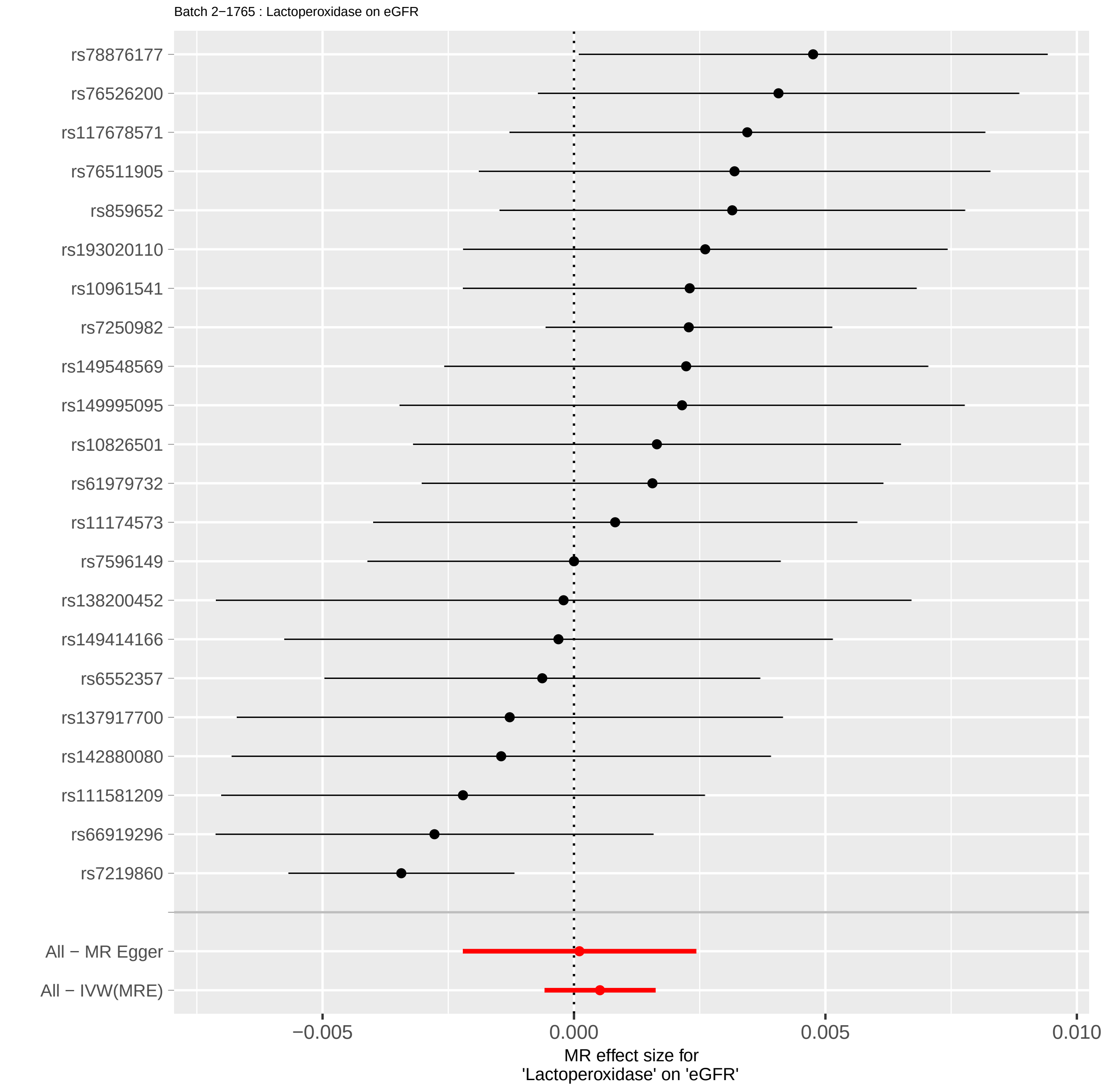
Batch 2−1765 : Lactoperoxidase on eGFR
rs78876177
rs76526200
rs117678571
rs76511905
rs859652
rs193020110
rs10961541
rs7250982
rs149548569
rs149995095
rs10826501
rs61979732
rs11174573
rs7596149
rs138200452
rs149414166
rs6552357
rs137917700
rs142880080
rs111581209
rs66919296
rs7219860
All − MR Egger
All − IVW(MRE)
−0.005
0.000
0.005
0.010
MR effect size for
'Lactoperoxidase' on 'eGFR'

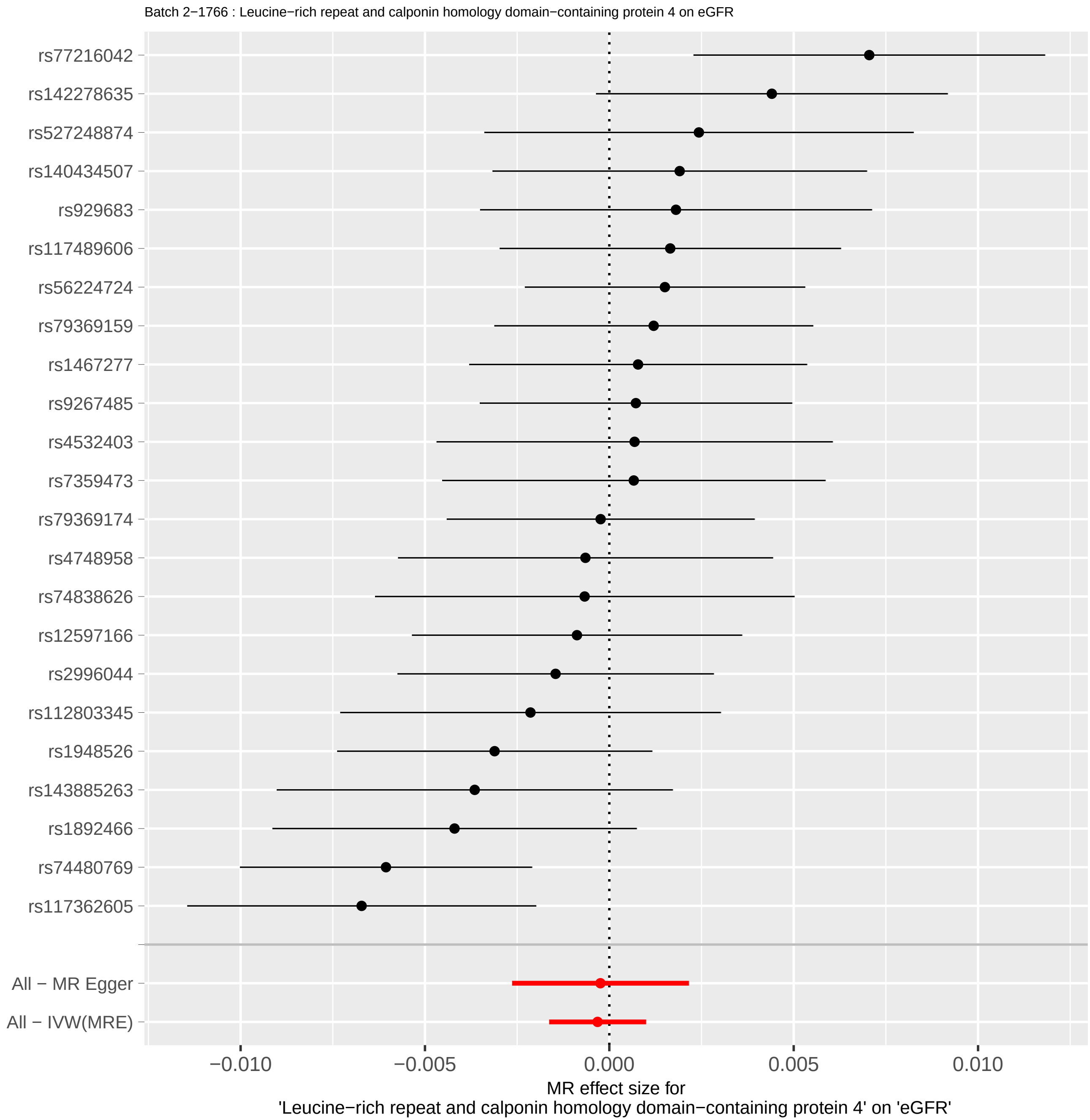

Batch 2−1766 : Leucine−rich repeat and calponin homology domain−containing protein 4 on eGFR
rs77216042
rs142278635
rs527248874
rs140434507
rs929683
rs117489606
rs56224724
rs79369159
rs1467277
rs9267485
rs4532403
rs7359473
rs79369174
rs4748958
rs74838626
rs12597166
rs2996044
rs112803345
rs1948526
rs143885263
rs1892466
rs74480769
rs117362605
All − MR Egger
All − IVW(MRE)
−0.010
−0.005
0.000
0.005
0.010
MR effect size for
'Leucine−rich repeat and calponin homology domain−containing protein 4' on 'eGFR'

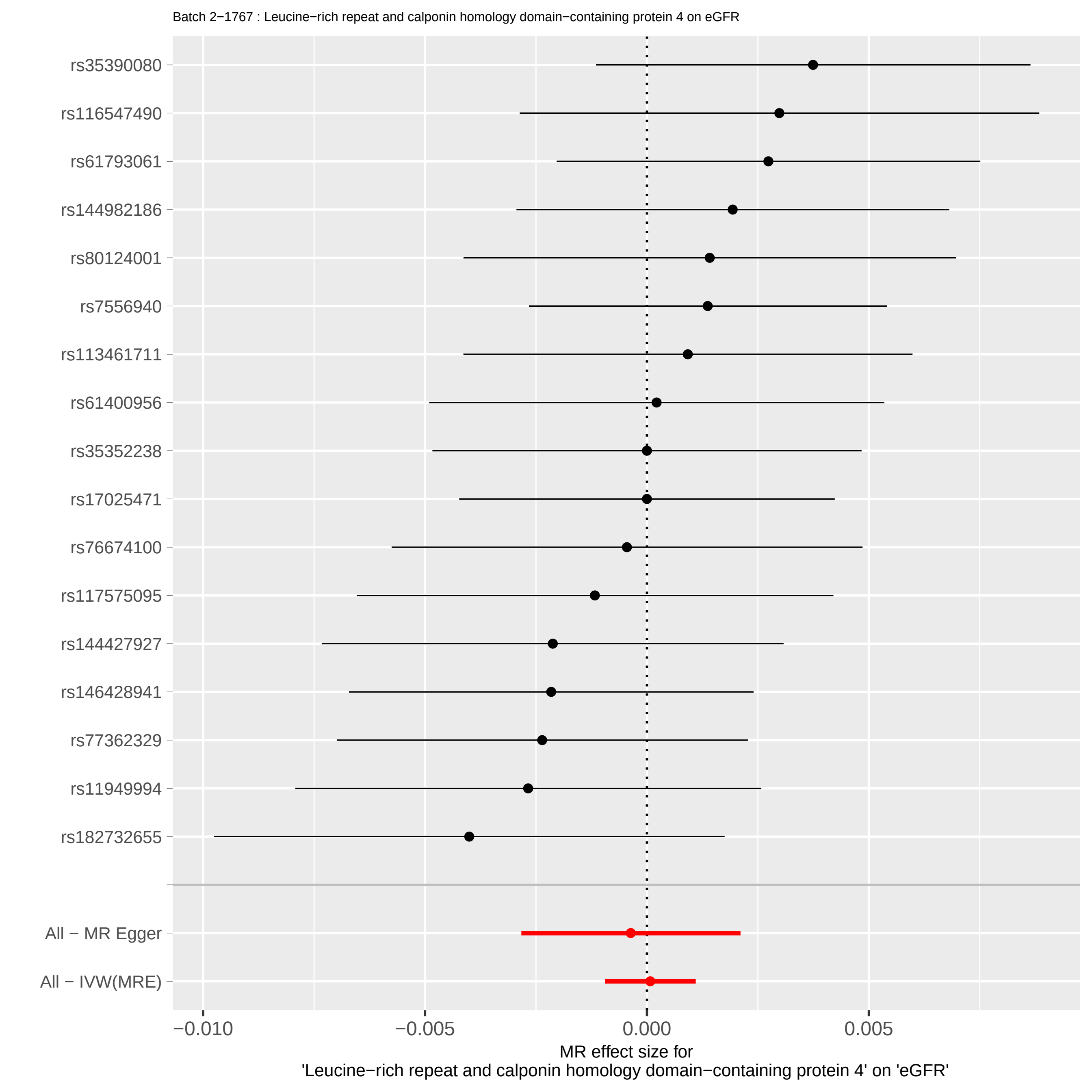
Batch 2−1767 : Leucine−rich repeat and calponin homology domain−containing protein 4 on eGFR
rs35390080
rs116547490
rs61793061
rs144982186
rs80124001
rs7556940
rs113461711
rs61400956
rs35352238
rs17025471
rs76674100
rs117575095
rs144427927
rs146428941
rs77362329
rs11949994
rs182732655
All − MR Egger
All − IVW(MRE)
−0.010
−0.005
0.000
0.005
MR effect size for
'Leucine−rich repeat and calponin homology domain−containing protein 4' on 'eGFR'

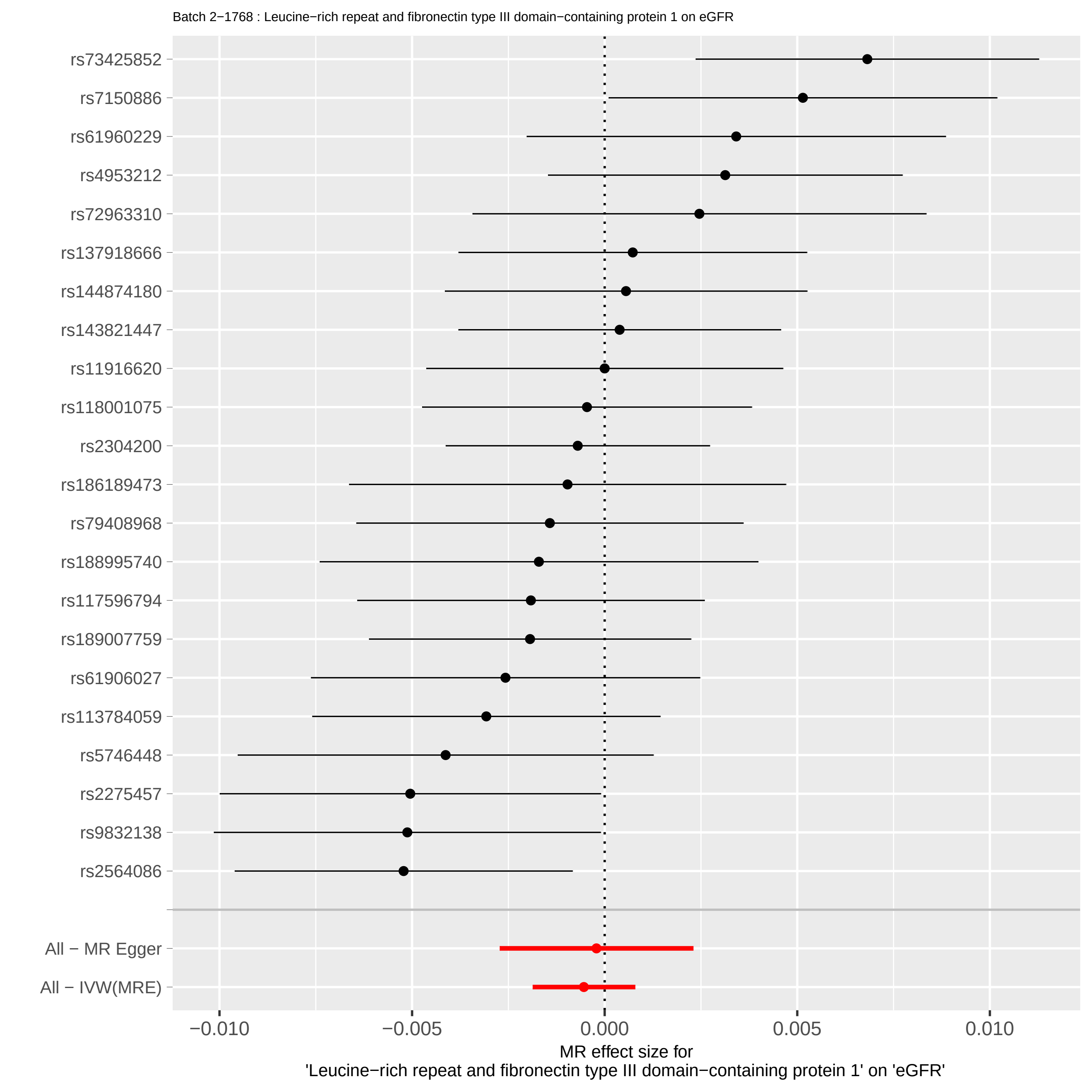

Batch 2−1768 : Leucine−rich repeat and fibronectin type III domain−containing protein 1 on eGFR
rs73425852
rs7150886
rs61960229
rs4953212
rs72963310
rs137918666
rs144874180
rs143821447
rs11916620
rs118001075
rs2304200
rs186189473
rs79408968
rs188995740
rs117596794
rs189007759
rs61906027
rs113784059
rs5746448
rs2275457
rs9832138
rs2564086
All − MR Egger
All − IVW(MRE)
−0.010
−0.005
0.000
0.005
0.010
MR effect size for
'Leucine−rich repeat and fibronectin type III domain−containing protein 1' on 'eGFR'

Batch 2−1769 : Leucine−rich repeat and fibronectin type−III domain−containing protein 2 on eGFR

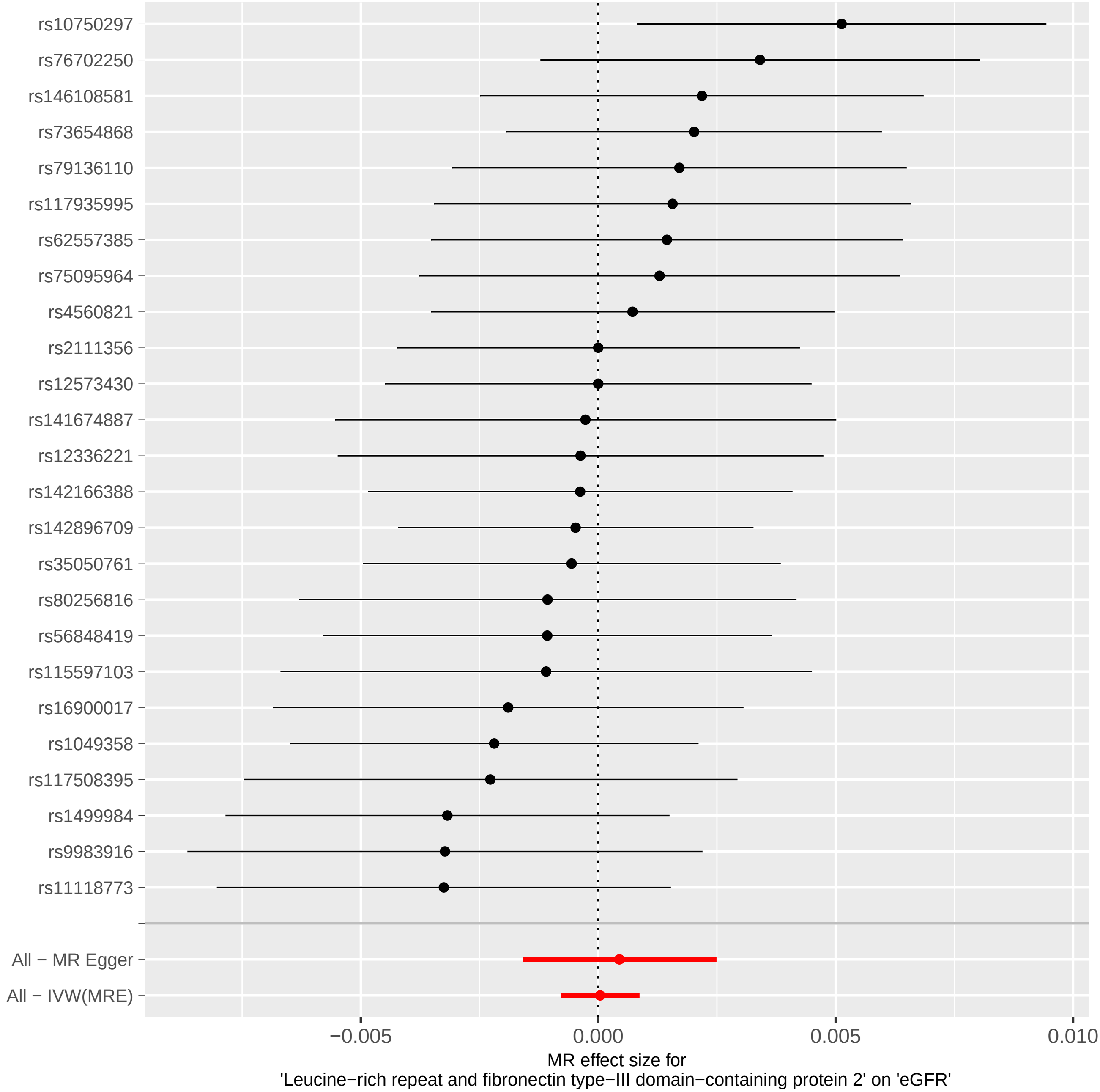

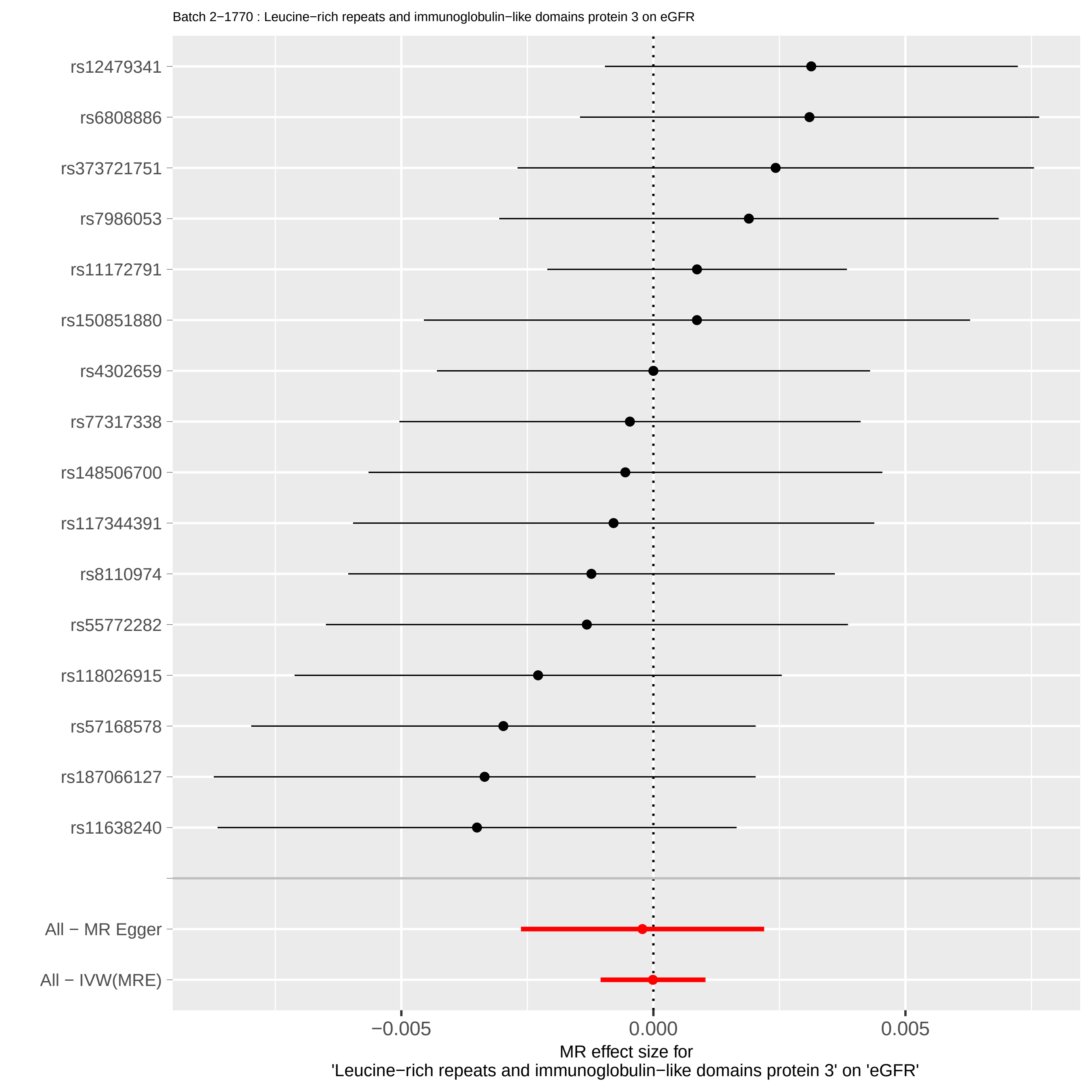

Batch 2−1770 : Leucine−rich repeats and immunoglobulin−like domains protein 3 on eGFR
rs12479341
rs6808886
rs373721751
rs7986053
rs11172791
rs150851880
rs4302659
rs77317338
rs148506700
rs117344391
rs8110974
rs55772282
rs118026915
rs57168578
rs187066127
rs11638240
All − MR Egger
All − IVW(MRE)
−0.005
0.000
0.005
MR effect size for
'Leucine−rich repeats and immunoglobulin−like domains protein 3' on 'eGFR'

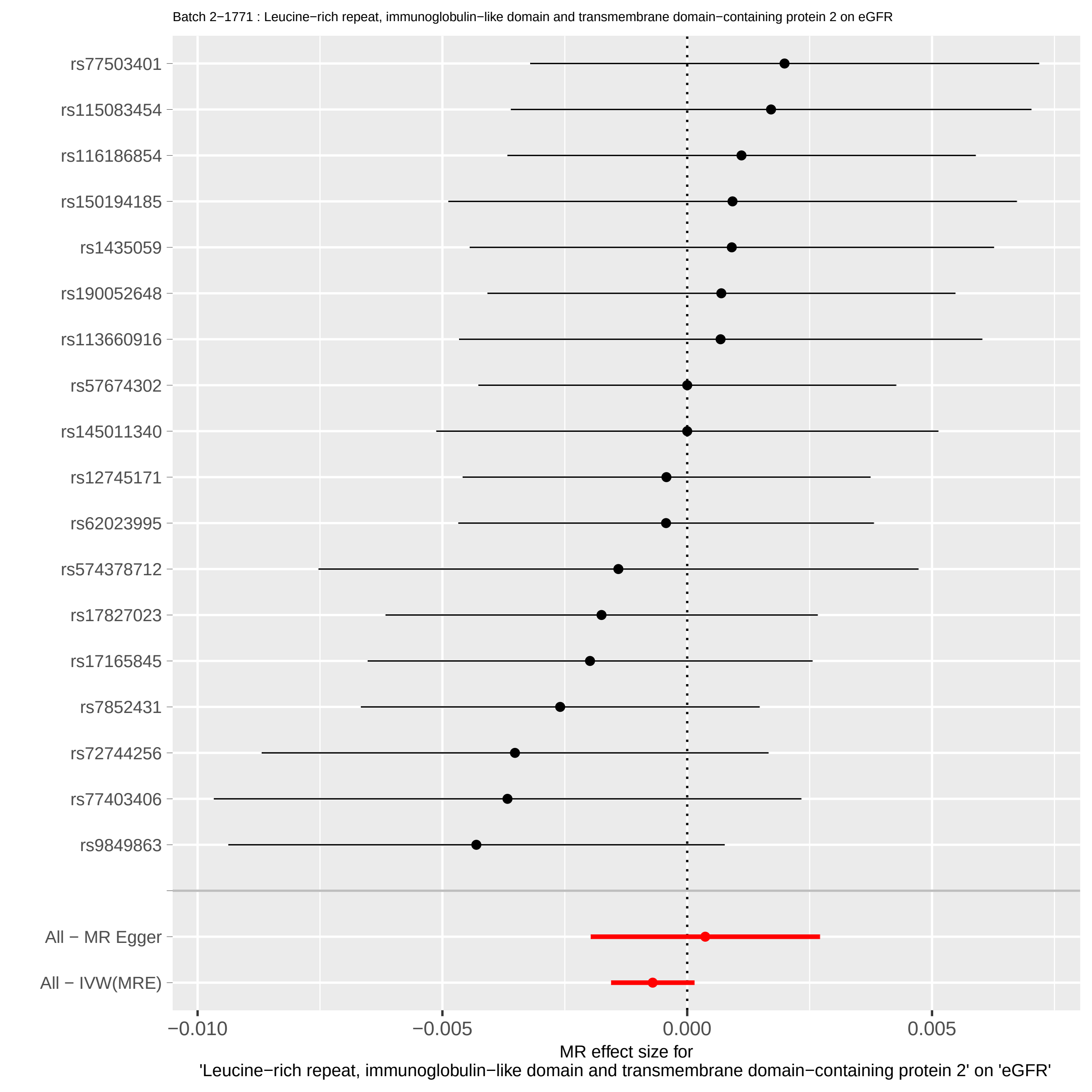

Batch 2−1771 : Leucine−rich repeat, immunoglobulin−like domain and transmembrane domain−containing protein 2 on eGFR
rs77503401
rs115083454
rs116186854
rs150194185
rs1435059
rs190052648
rs113660916
rs57674302
rs145011340
rs12745171
rs62023995
rs574378712
rs17827023
rs17165845
rs7852431
rs72744256
rs77403406
rs9849863
All − MR Egger
All − IVW(MRE)
−0.010
−0.005
0.000
0.005
MR effect size for
'Leucine−rich repeat, immunoglobulin−like domain and transmembrane domain−containing protein 2' on 'eGFR'

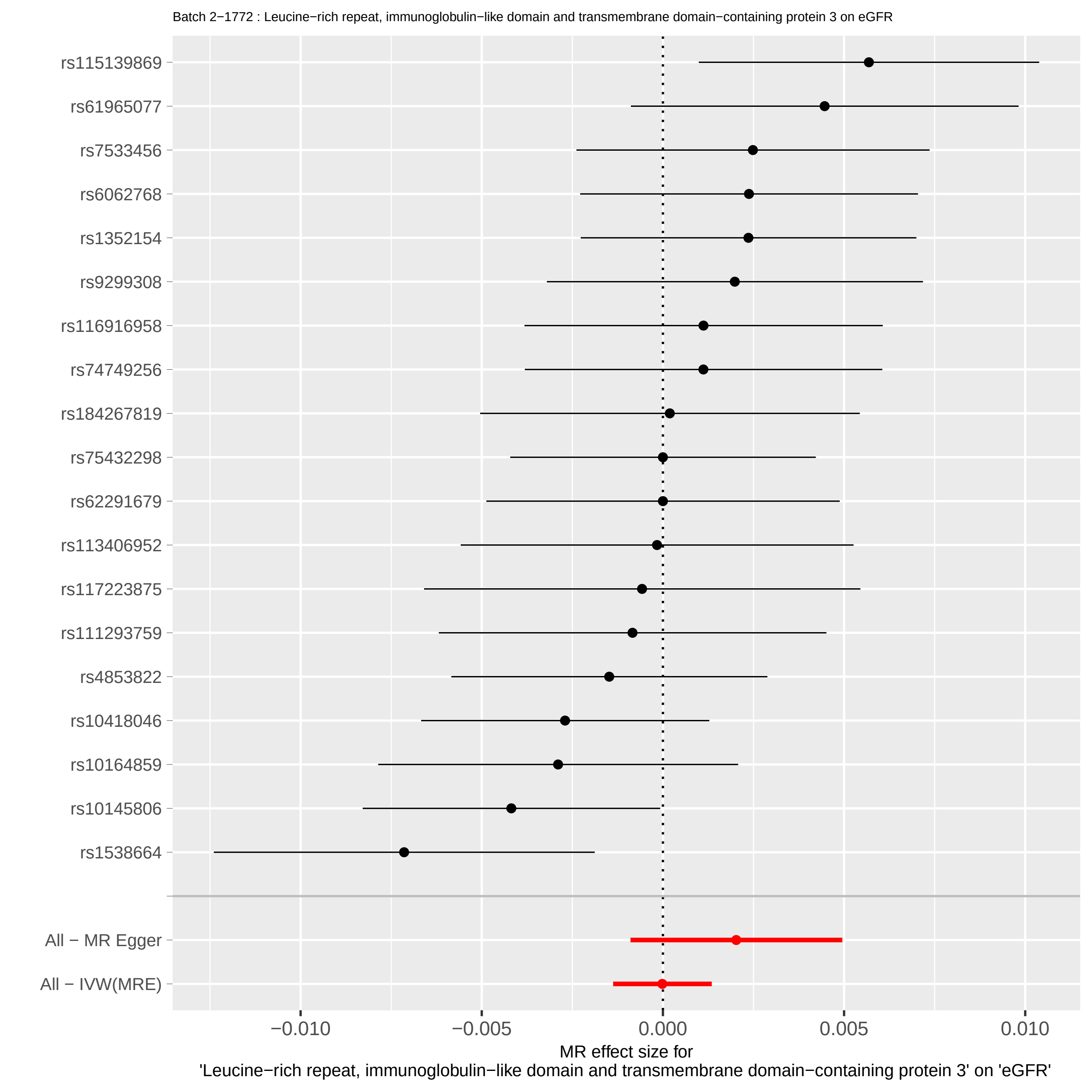

Batch 2−1772 : Leucine−rich repeat, immunoglobulin−like domain and transmembrane domain−containing protein 3 on eGFR
rs115139869
rs61965077
rs7533456
rs6062768
rs1352154
rs9299308
rs116916958
rs74749256
rs184267819
rs75432298
rs62291679
rs113406952
rs117223875
rs111293759
rs4853822
rs10418046
rs10164859
rs10145806
rs1538664
All − MR Egger
All − IVW(MRE)
−0.010
−0.005
0.000
0.005
0.010
MR effect size for
'Leucine−rich repeat, immunoglobulin−like domain and transmembrane domain−containing protein 3' on 'eGFR'

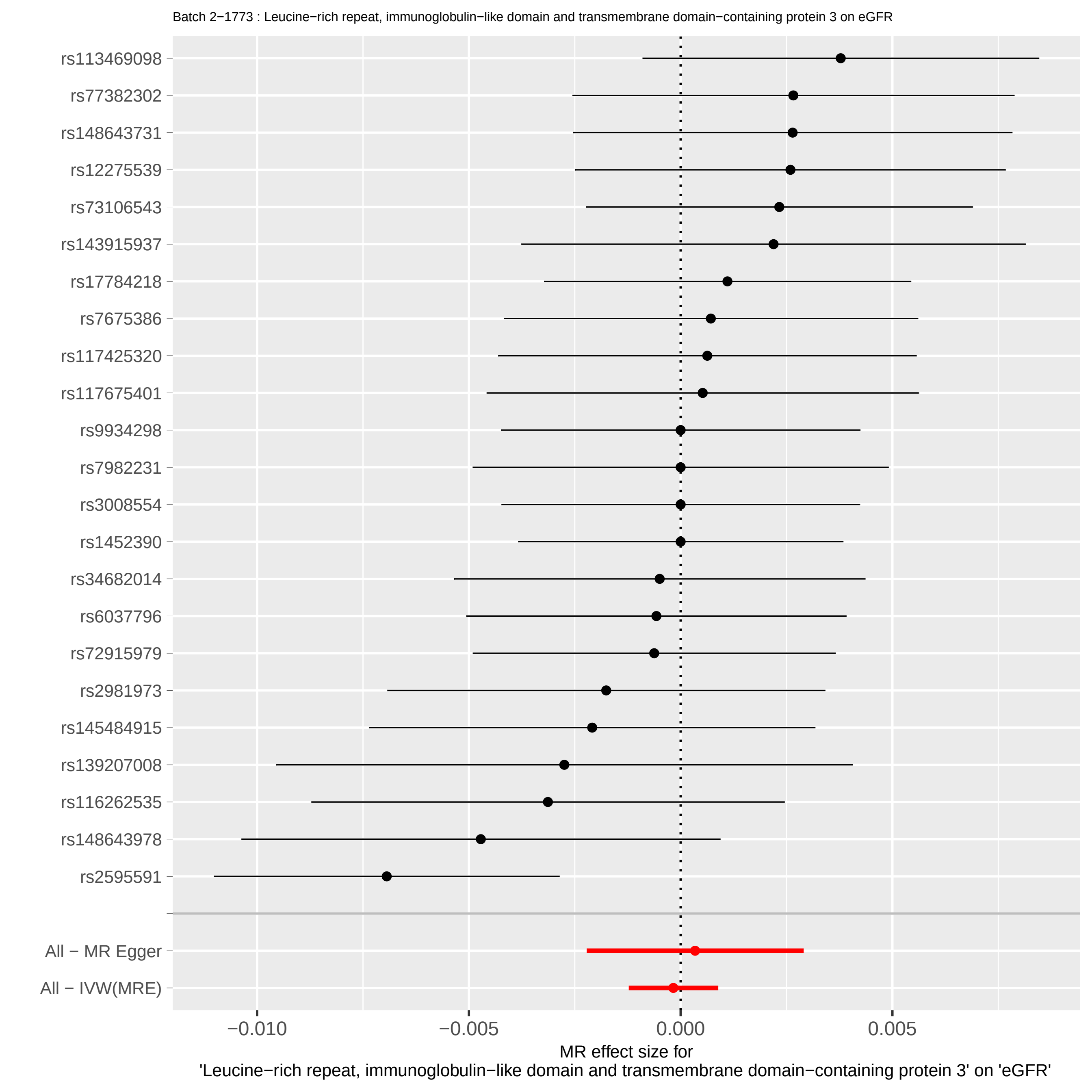

Batch 2−1773 : Leucine−rich repeat, immunoglobulin−like domain and transmembrane domain−containing protein 3 on eGFR
rs113469098
rs77382302
rs148643731
rs12275539
rs73106543
rs143915937
rs17784218
rs7675386
rs117425320
rs117675401
rs9934298
rs7982231
rs3008554
rs1452390
rs34682014
rs6037796
rs72915979
rs2981973
rs145484915
rs139207008
rs116262535
rs148643978
rs2595591
All − MR Egger
All − IVW(MRE)
−0.010
−0.005
0.000
0.005
MR effect size for
'Leucine−rich repeat, immunoglobulin−like domain and transmembrane domain−containing protein 3' on 'eGFR'

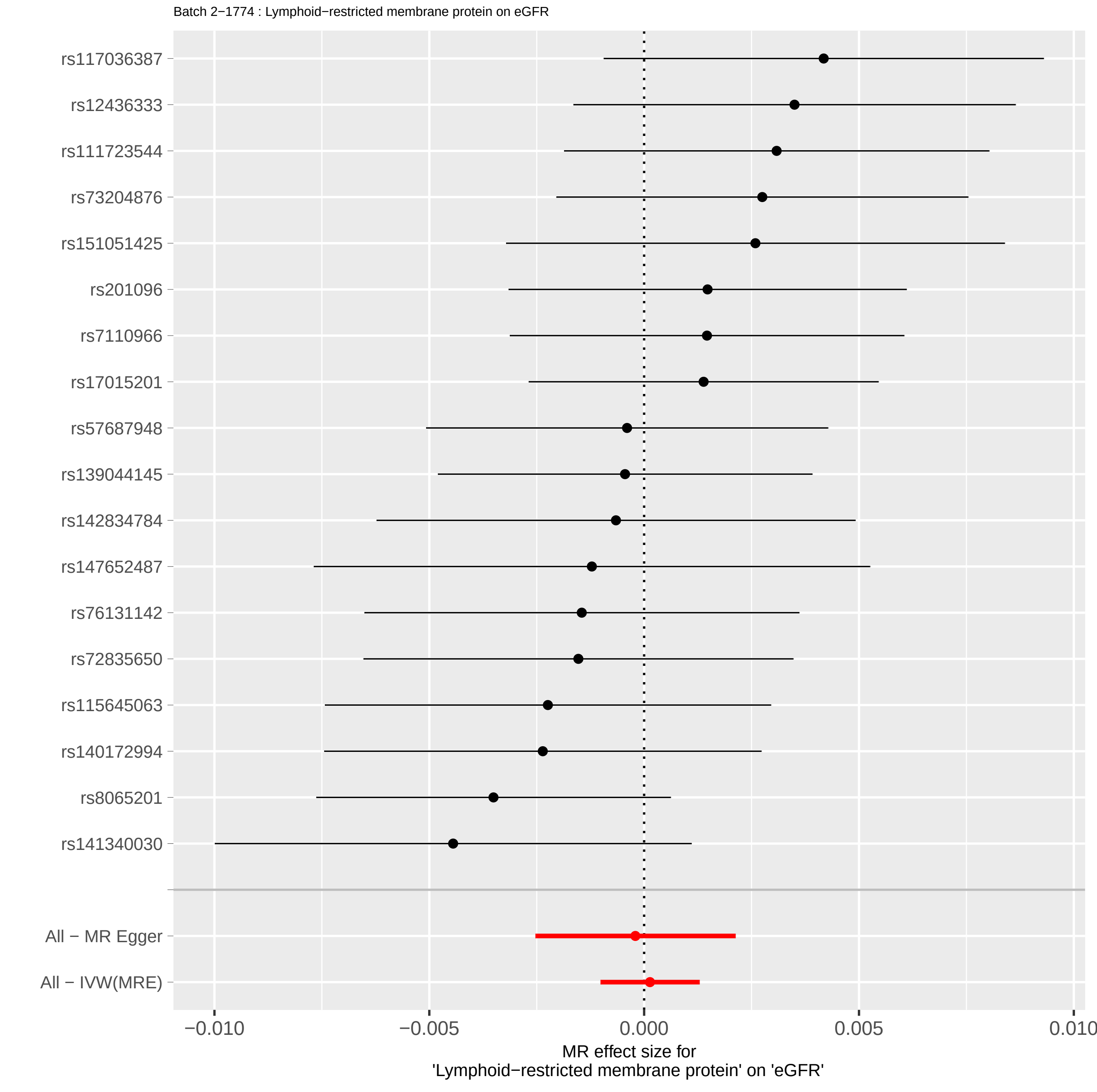

Batch 2−1774 : Lymphoid−restricted membrane protein on eGFR
rs117036387
rs12436333
rs111723544
rs73204876
rs151051425
rs201096
rs7110966
rs17015201
rs57687948
rs139044145
rs142834784
rs147652487
rs76131142
rs72835650
rs115645063
rs140172994
rs8065201
rs141340030
All − MR Egger
All − IVW(MRE)
−0.010
−0.005
0.000
0.005
0.010
MR effect size for
'Lymphoid−restricted membrane protein' on 'eGFR'

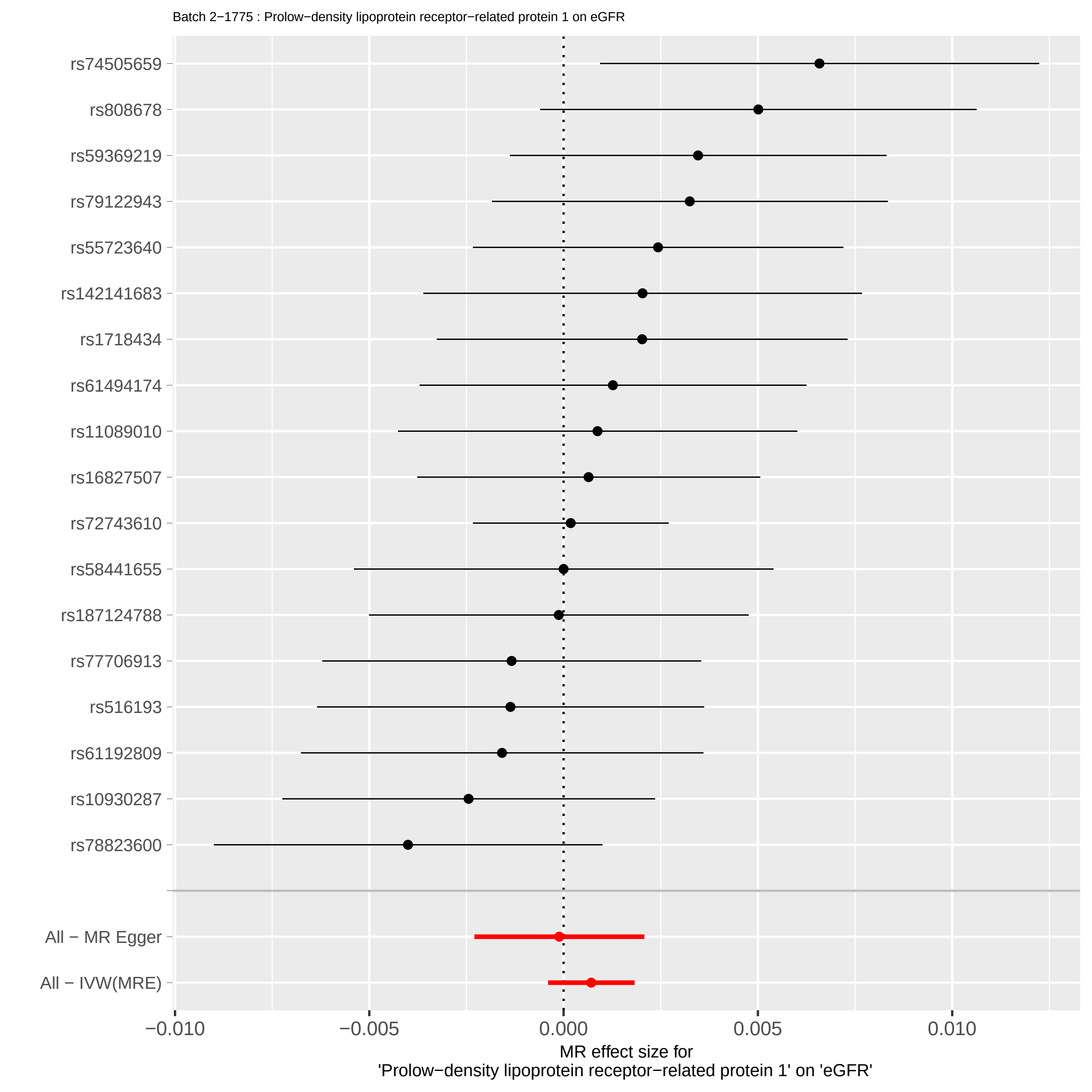

Batch 2−1775 : Prolow−density lipoprotein receptor−related protein 1 on eGFR
rs74505659
rs808678
rs59369219
rs79122943
rs55723640
rs142141683
rs1718434
rs61494174
rs11089010
rs16827507
rs72743610
rs58441655
rs187124788
rs77706913
rs516193
rs61192809
rs10930287
rs78823600
All − MR Egger
All − IVW(MRE)
−0.010
−0.005
0.000
0.005
0.010
MR effect size for
'Prolow−density lipoprotein receptor−related protein 1' on 'eGFR'

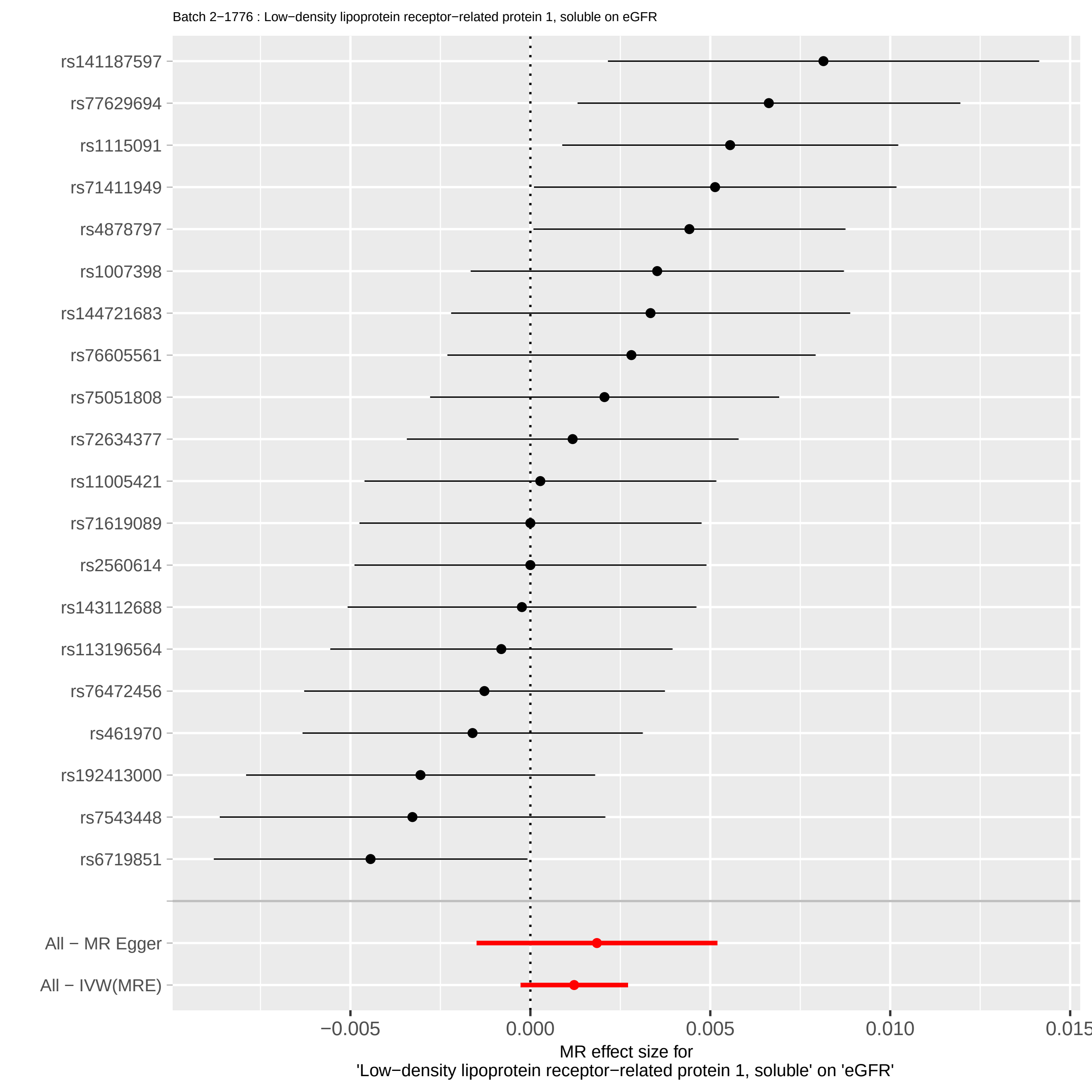
Batch 2−1776 : Low−density lipoprotein receptor−related protein 1, soluble on eGFR
rs141187597
rs77629694
rs1115091
rs71411949
rs4878797
rs1007398
rs144721683
rs76605561
rs75051808
rs72634377
rs11005421
rs71619089
rs2560614
rs143112688
rs113196564
rs76472456
rs461970
rs192413000
rs7543448
rs6719851
All − MR Egger
All − IVW(MRE)
−0.005
0.000
0.005
0.010
0.015
MR effect size for
'Low−density lipoprotein receptor−related protein 1, soluble' on 'eGFR'

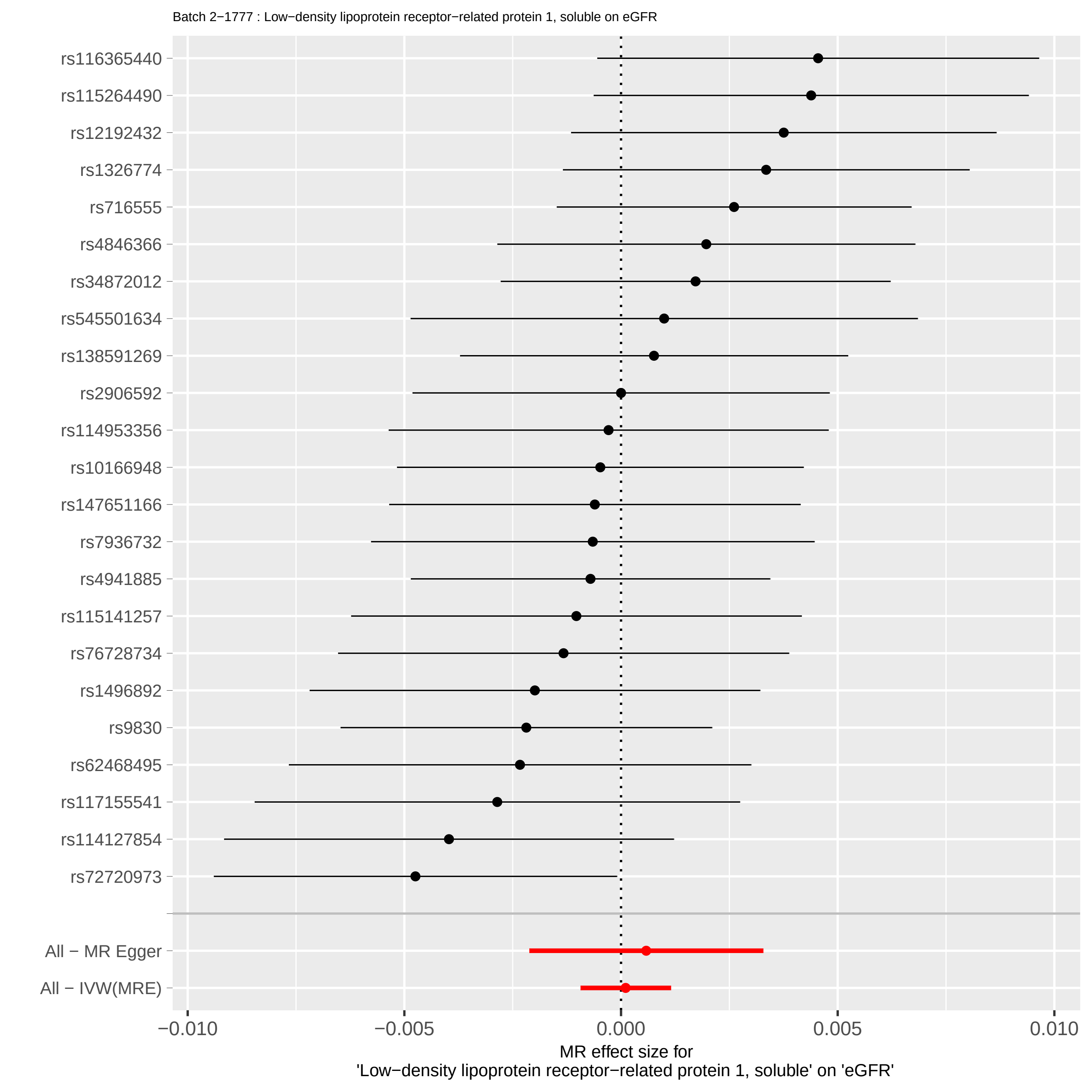

Batch 2−1777 : Low−density lipoprotein receptor−related protein 1, soluble on eGFR
rs116365440
rs115264490
rs12192432
rs1326774
rs716555
rs4846366
rs34872012
rs545501634
rs138591269
rs2906592
rs114953356
rs10166948
rs147651166
rs7936732
rs4941885
rs115141257
rs76728734
rs1496892
rs9830
rs62468495
rs117155541
rs114127854
rs72720973
All − MR Egger
All − IVW(MRE)
−0.010
−0.005
0.000
0.005
0.010
MR effect size for
'Low−density lipoprotein receptor−related protein 1, soluble' on 'eGFR'

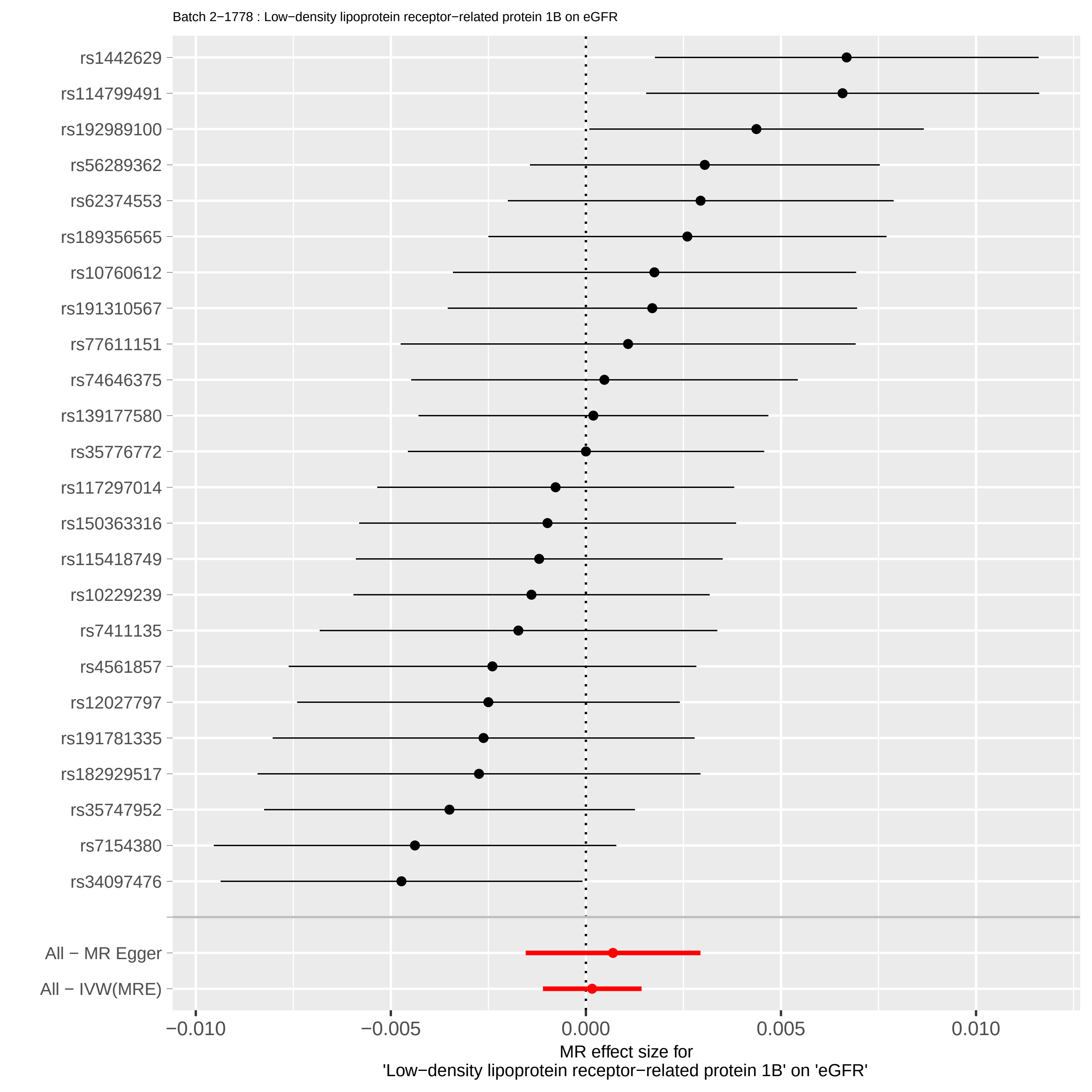

Batch 2−1778 : Low−density lipoprotein receptor−related protein 1B on eGFR
rs1442629
rs114799491
rs192989100
rs56289362
rs62374553
rs189356565
rs10760612
rs191310567
rs77611151
rs74646375
rs139177580
rs35776772
rs117297014
rs150363316
rs115418749
rs10229239
rs7411135
rs4561857
rs12027797
rs191781335
rs182929517
rs35747952
rs7154380
rs34097476
All − MR Egger
All − IVW(MRE)
−0.010
−0.005
0.000
0.005
0.010
MR effect size for
'Low−density lipoprotein receptor−related protein 1B' on 'eGFR'

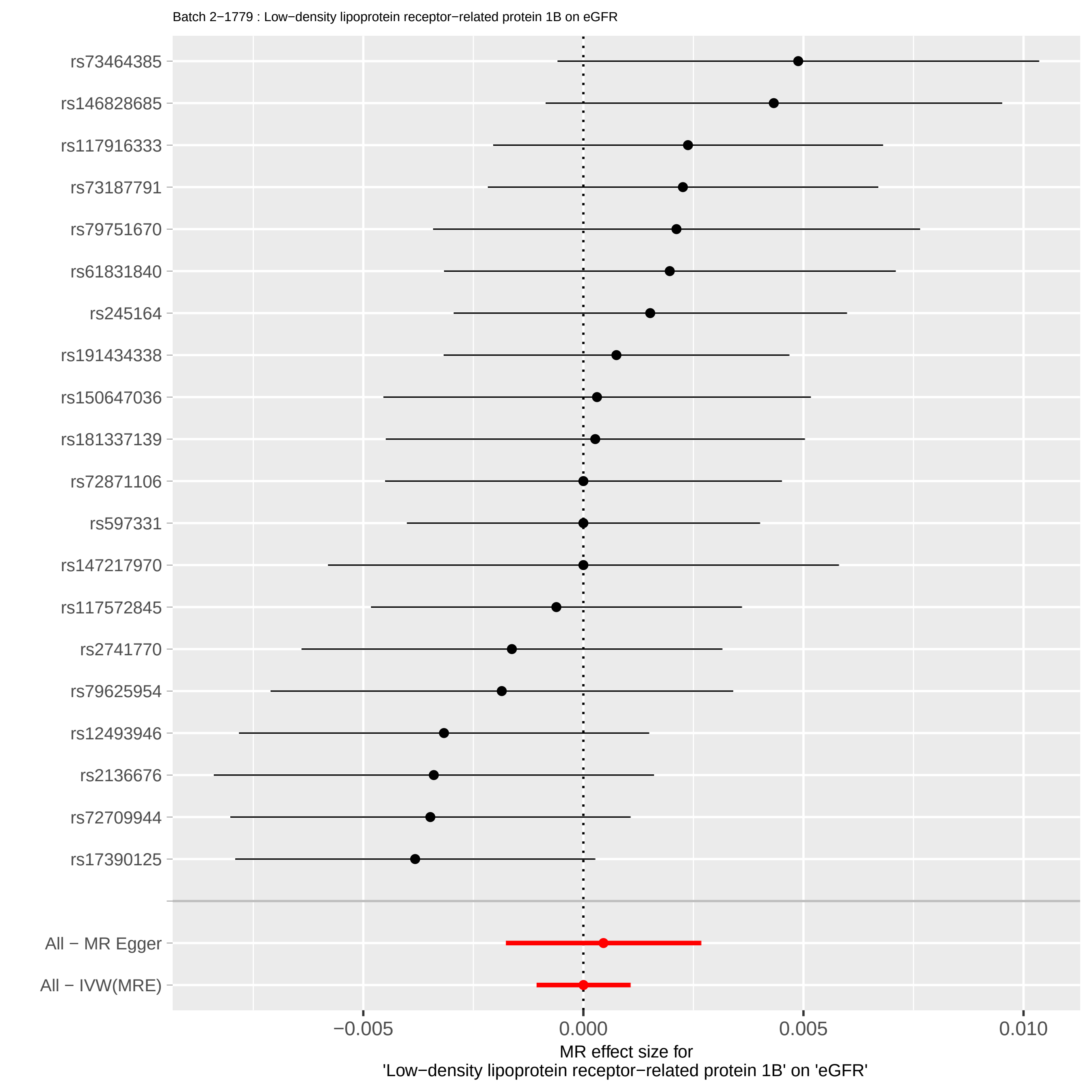

Batch 2−1779 : Low−density lipoprotein receptor−related protein 1B on eGFR
rs73464385
rs146828685
rs117916333
rs73187791
rs79751670
rs61831840
rs245164
rs191434338
rs150647036
rs181337139
rs72871106
rs597331
rs147217970
rs117572845
rs2741770
rs79625954
rs12493946
rs2136676
rs72709944
rs17390125
All − MR Egger
All − IVW(MRE)
−0.005
0.000
0.005
0.010
MR effect size for
'Low−density lipoprotein receptor−related protein 1B' on 'eGFR'

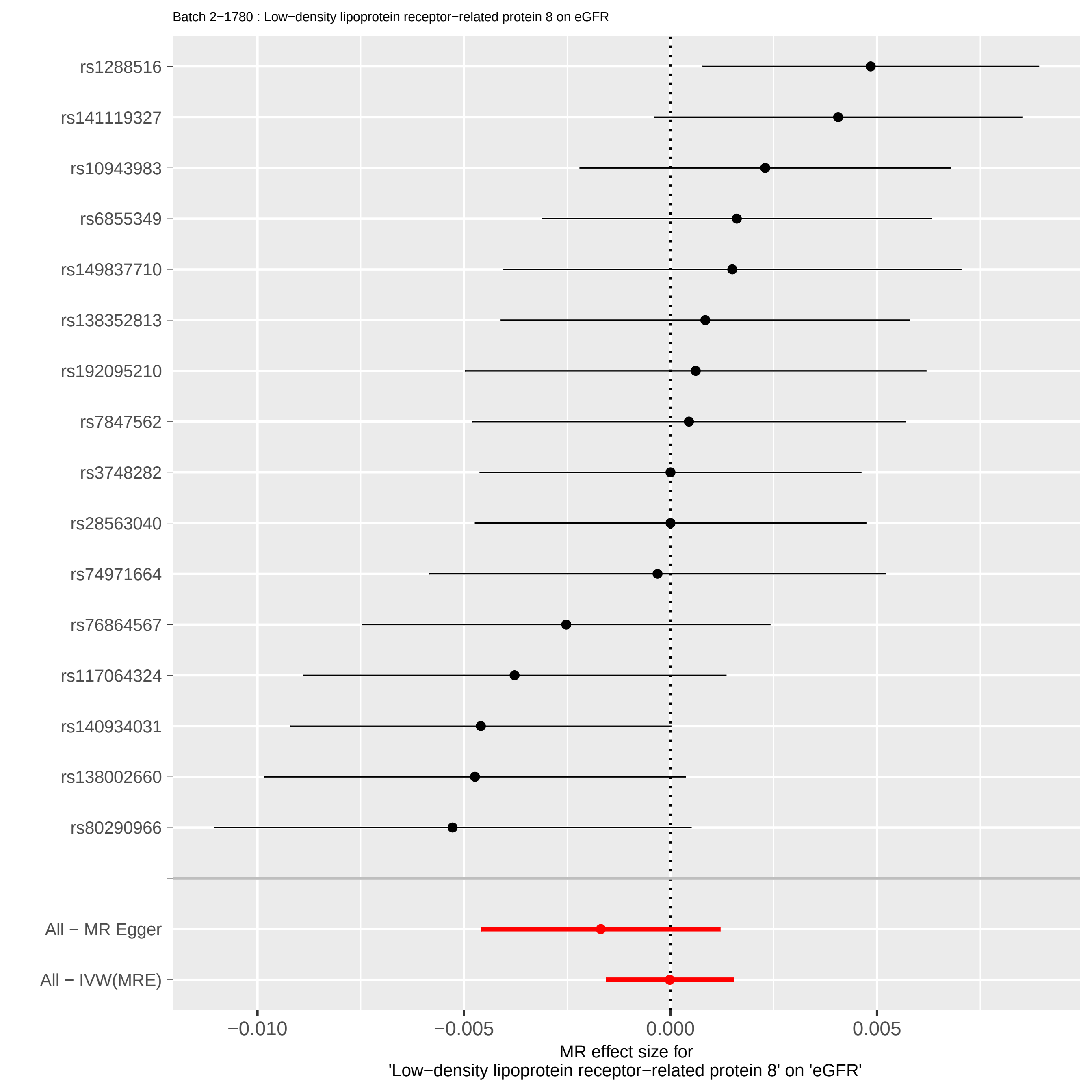

Batch 2−1780 : Low−density lipoprotein receptor−related protein 8 on eGFR
rs1288516
rs141119327
rs10943983
rs6855349
rs149837710
rs138352813
rs192095210
rs7847562
rs3748282
rs28563040
rs74971664
rs76864567
rs117064324
rs140934031
rs138002660
rs80290966
All − MR Egger
All − IVW(MRE)
−0.010
−0.005
0.000
0.005
MR effect size for
'Low−density lipoprotein receptor−related protein 8' on 'eGFR'

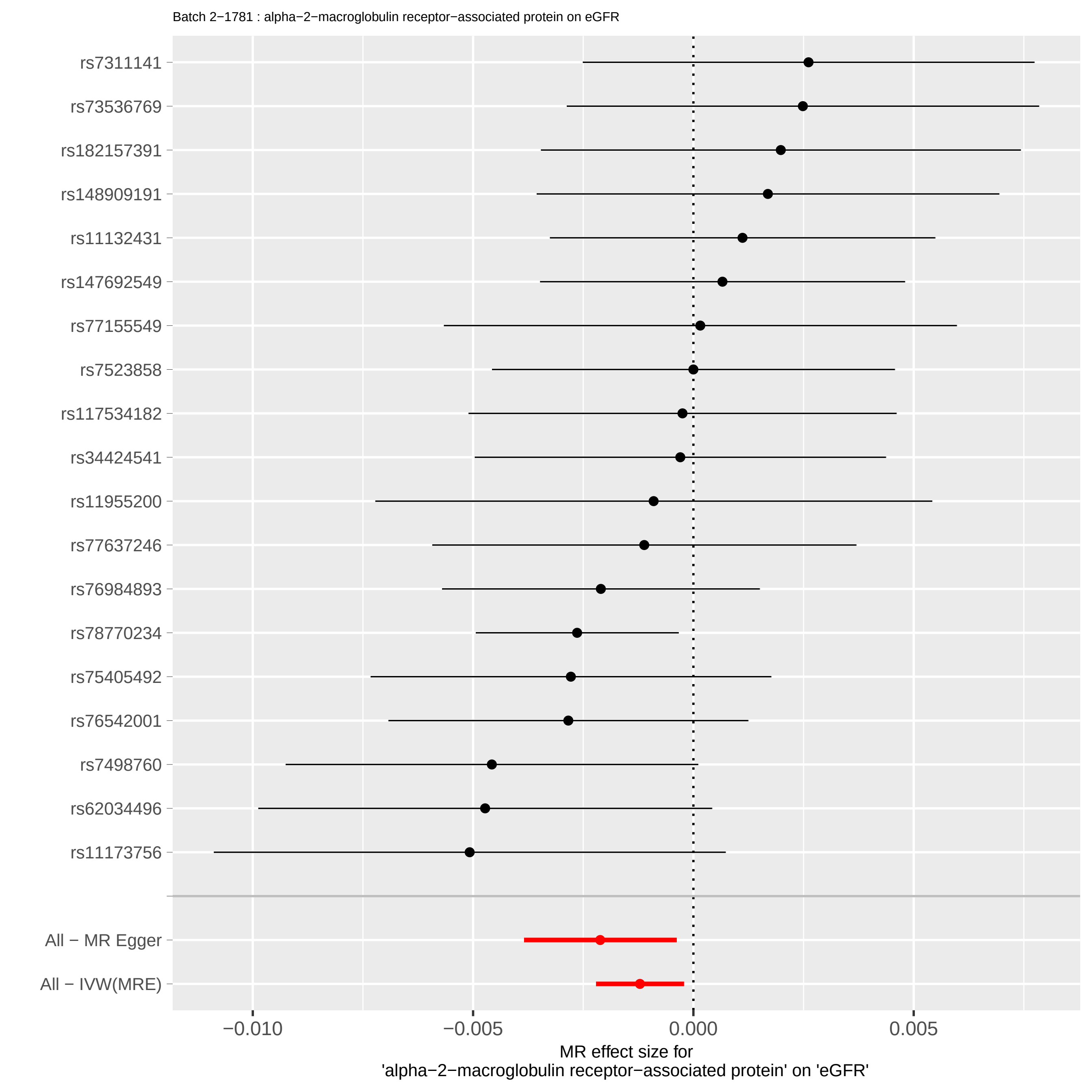
Batch 2−1781 : alpha−2−macroglobulin receptor−associated protein on eGFR
rs7311141
rs73536769
rs182157391
rs148909191
rs11132431
rs147692549
rs77155549
rs7523858
rs117534182
rs34424541
rs11955200
rs77637246
rs76984893
rs78770234
rs75405492
rs76542001
rs7498760
rs62034496
rs11173756
All − MR Egger
All − IVW(MRE)
−0.010
−0.005
0.000
0.005
MR effect size for
'alpha−2−macroglobulin receptor−associated protein' on 'eGFR'

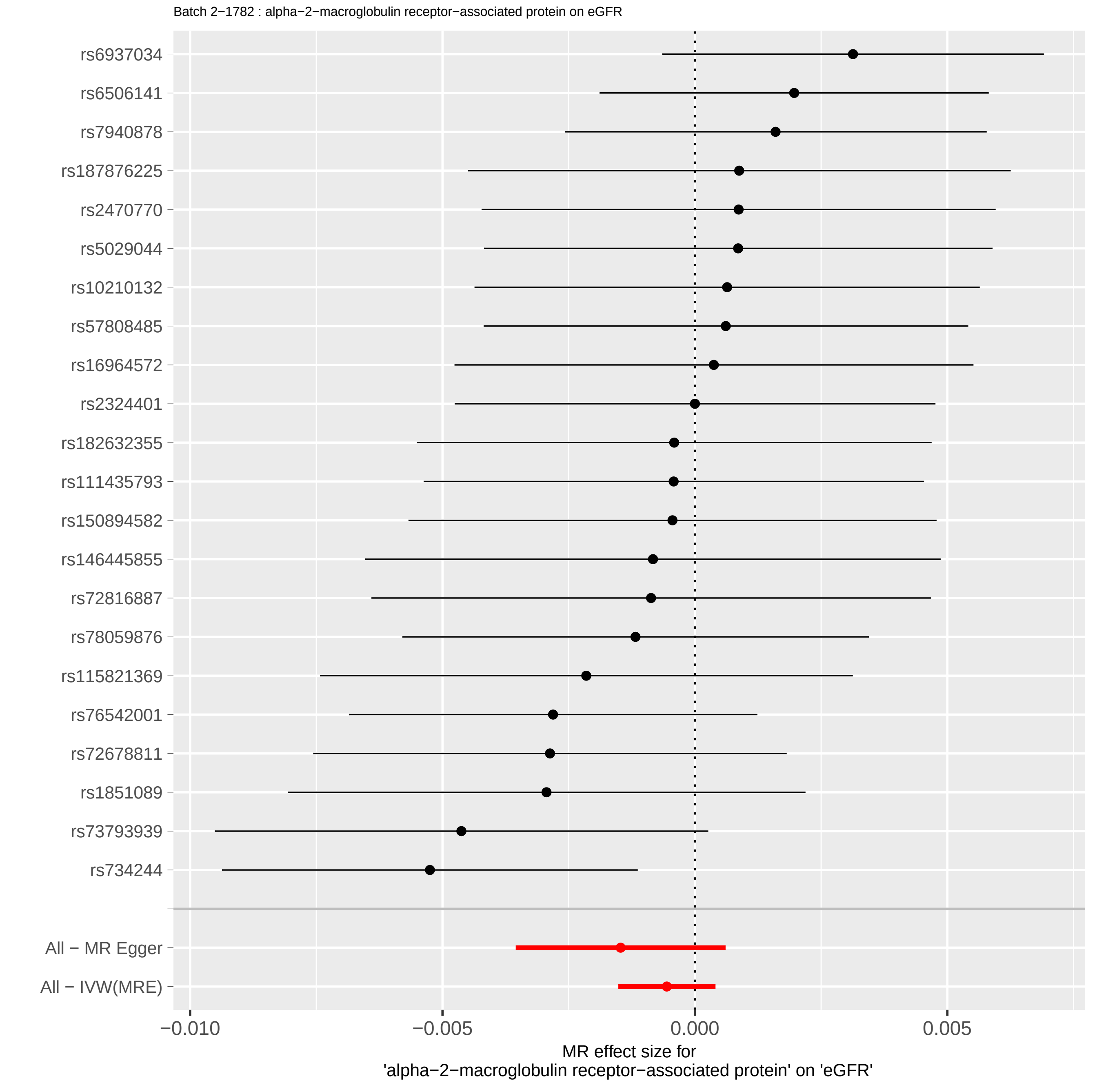

Batch 2−1782 : alpha−2−macroglobulin receptor−associated protein on eGFR
rs6937034
rs6506141
rs7940878
rs187876225
rs2470770
rs5029044
rs10210132
rs57808485
rs16964572
rs2324401
rs182632355
rs111435793
rs150894582
rs146445855
rs72816887
rs78059876
rs115821369
rs76542001
rs72678811
rs1851089
rs73793939
rs734244
All − MR Egger
All − IVW(MRE)
−0.010
−0.005
0.000
0.005
MR effect size for
'alpha−2−macroglobulin receptor−associated protein' on 'eGFR'

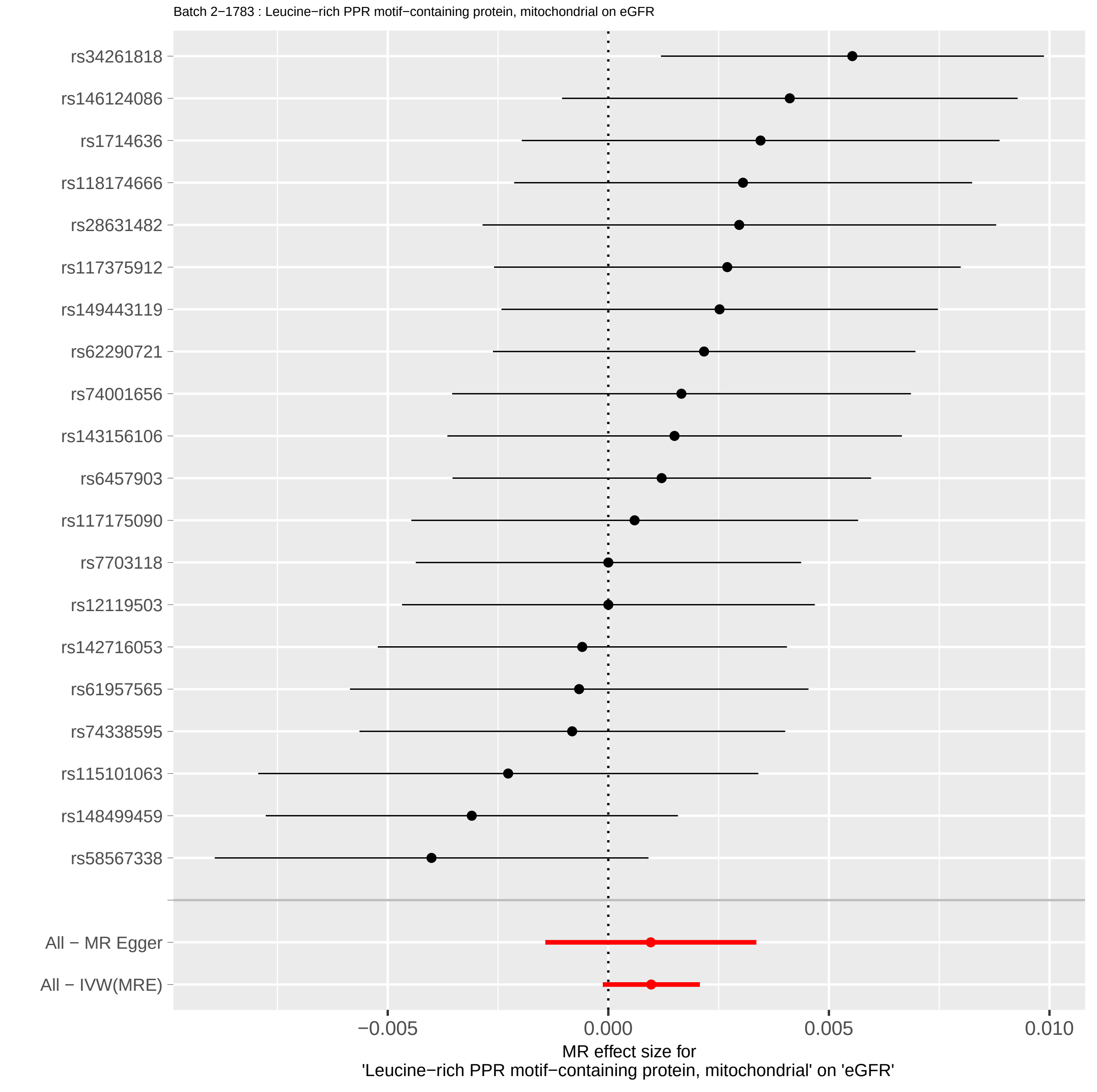

Batch 2−1783 : Leucine−rich PPR motif−containing protein, mitochondrial on eGFR
rs34261818
rs146124086
rs1714636
rs118174666
rs28631482
rs117375912
rs149443119
rs62290721
rs74001656
rs143156106
rs6457903
rs117175090
rs7703118
rs12119503
rs142716053
rs61957565
rs74338595
rs115101063
rs148499459
rs58567338
All − MR Egger
All − IVW(MRE)
−0.005
0.000
0.005
0.010
MR effect size for
'Leucine−rich PPR motif−containing protein, mitochondrial' on 'eGFR'

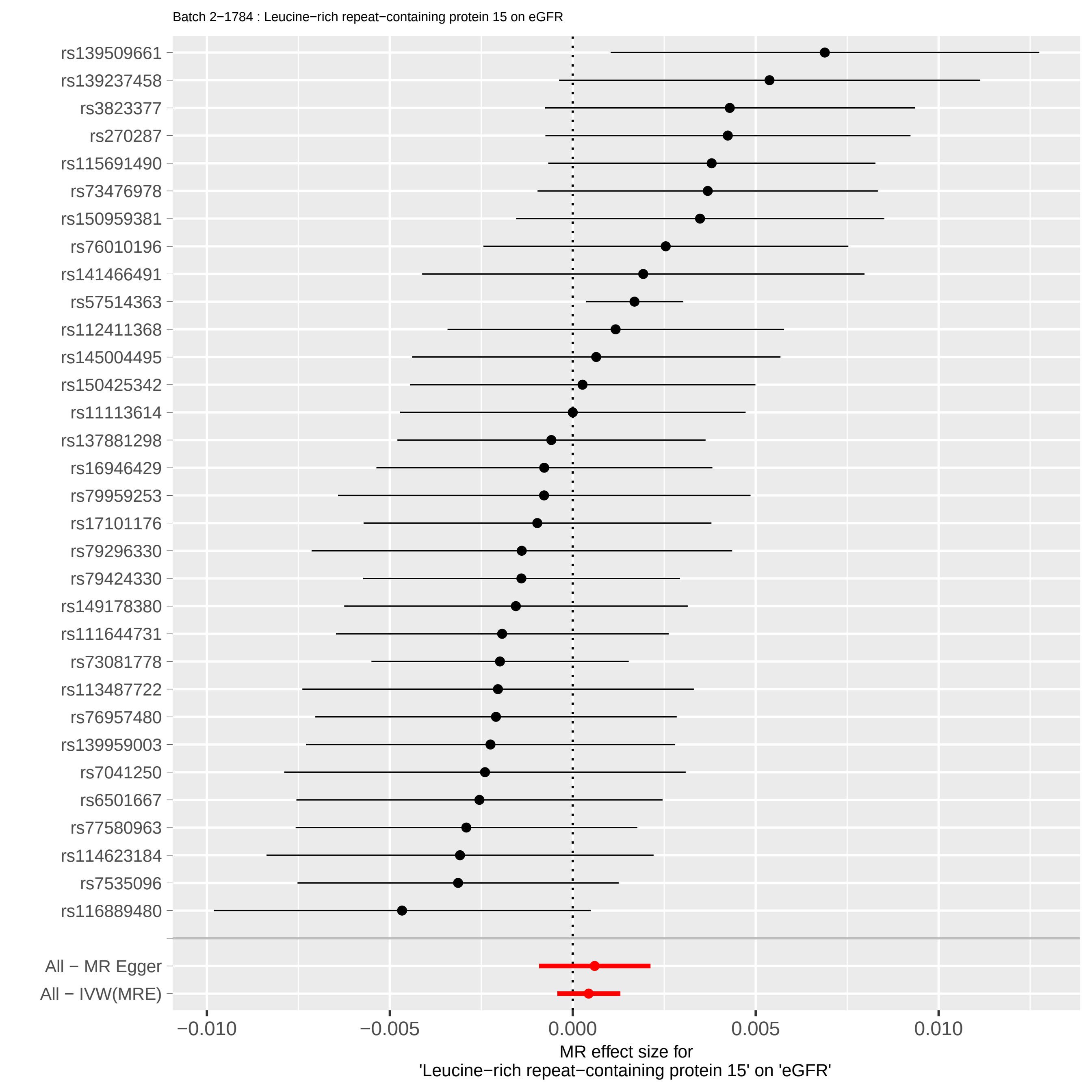

Batch 2−1784 : Leucine−rich repeat−containing protein 15 on eGFR
rs139509661
rs139237458
rs3823377
rs270287
rs115691490
rs73476978
rs150959381
rs76010196
rs141466491
rs57514363
rs112411368
rs145004495
rs150425342
rs11113614
rs137881298
rs16946429
rs79959253
rs17101176
rs79296330
rs79424330
rs149178380
rs111644731
rs73081778
rs113487722
rs76957480
rs139959003
rs7041250
rs6501667
rs77580963
rs114623184
rs7535096
rs116889480
All − MR Egger
All − IVW(MRE)
−0.010
−0.005
0.000
0.005
0.010
MR effect size for
'Leucine−rich repeat−containing protein 15' on 'eGFR'

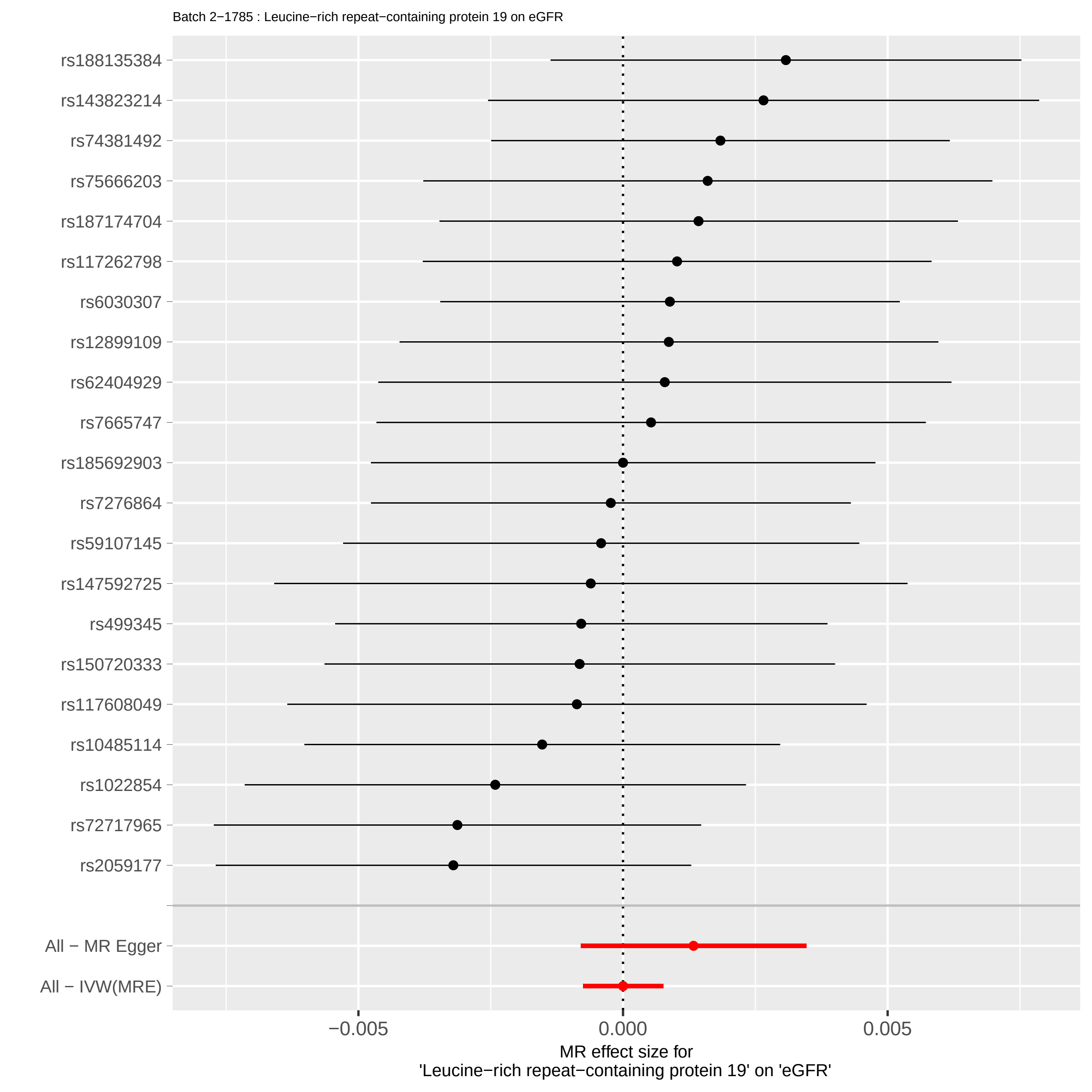

Batch 2−1785 : Leucine−rich repeat−containing protein 19 on eGFR
rs188135384
rs143823214
rs74381492
rs75666203
rs187174704
rs117262798
rs6030307
rs12899109
rs62404929
rs7665747
rs185692903
rs7276864
rs59107145
rs147592725
rs499345
rs150720333
rs117608049
rs10485114
rs1022854
rs72717965
rs2059177
All − MR Egger
All − IVW(MRE)
−0.005
0.000
0.005
MR effect size for
'Leucine−rich repeat−containing protein 19' on 'eGFR'

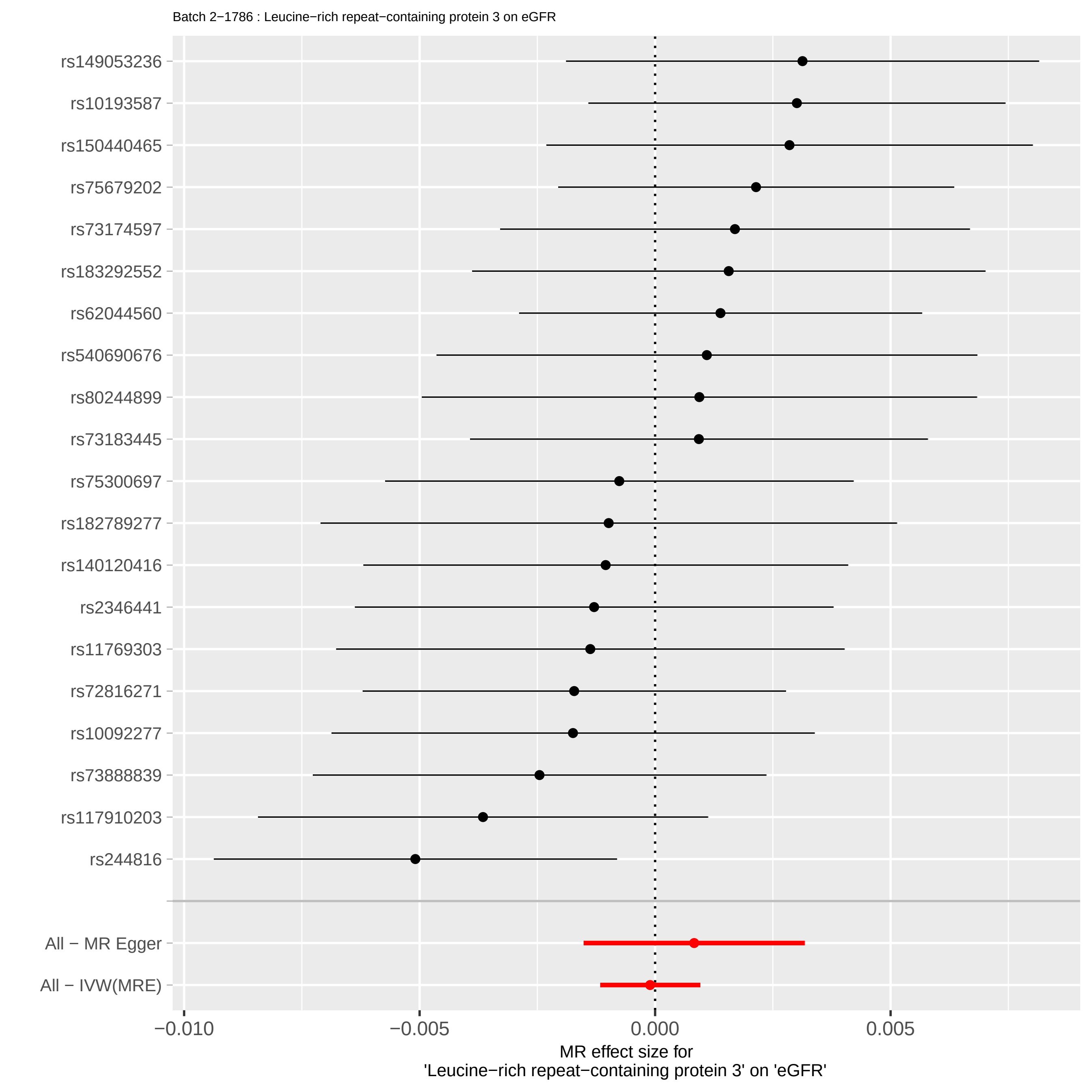

Batch 2−1786 : Leucine−rich repeat−containing protein 3 on eGFR
rs149053236
rs10193587
rs150440465
rs75679202
rs73174597
rs183292552
rs62044560
rs540690676
rs80244899
rs73183445
rs75300697
rs182789277
rs140120416
rs2346441
rs11769303
rs72816271
rs10092277
rs73888839
rs117910203
rs244816
All − MR Egger
All − IVW(MRE)
−0.010
−0.005
0.000
0.005
MR effect size for
'Leucine−rich repeat−containing protein 3' on 'eGFR'

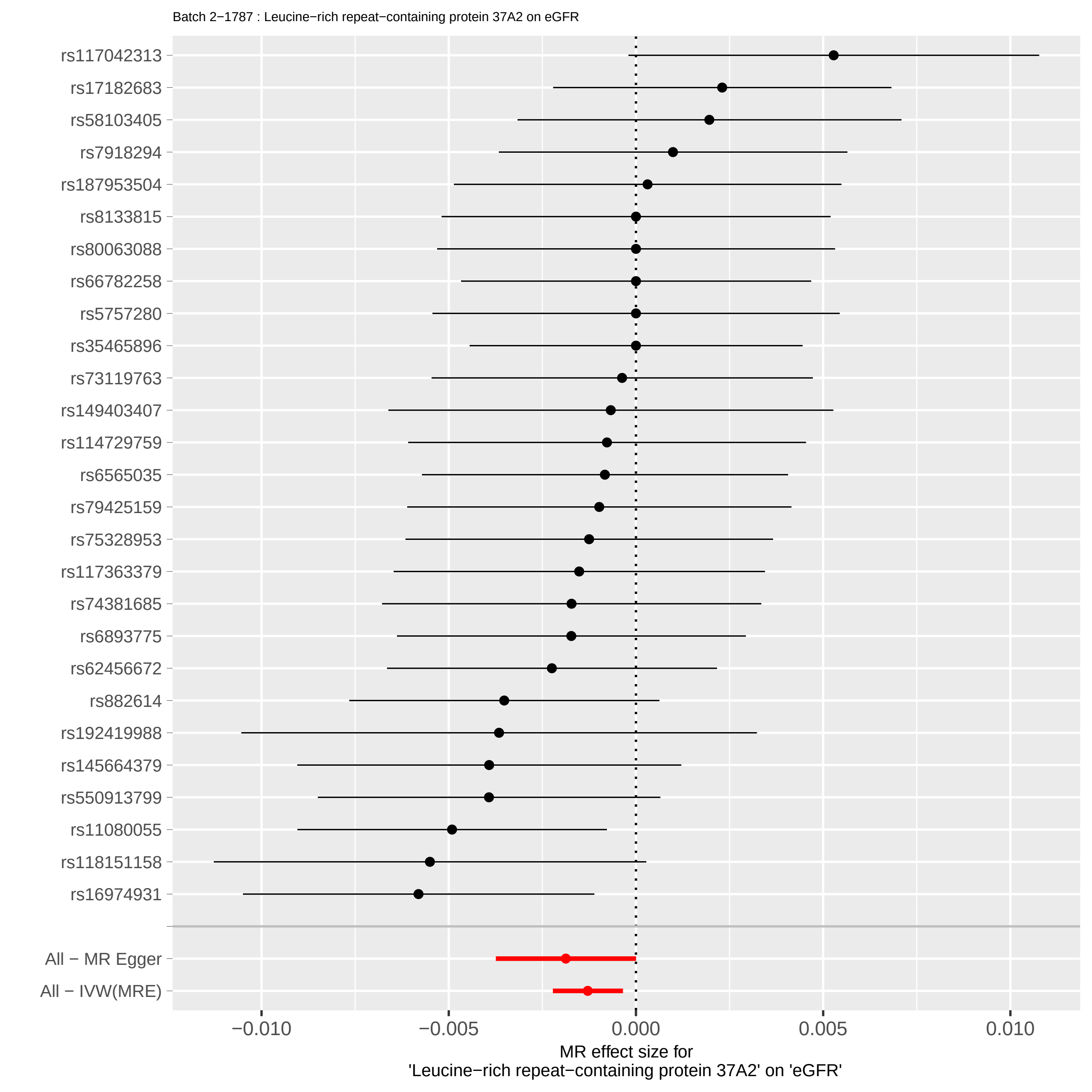

Batch 2−1787 : Leucine−rich repeat−containing protein 37A2 on eGFR
rs117042313
rs17182683
rs58103405
rs7918294
rs187953504
rs8133815
rs80063088
rs66782258
rs5757280
rs35465896
rs73119763
rs149403407
rs114729759
rs6565035
rs79425159
rs75328953
rs117363379
rs74381685
rs6893775
rs62456672
rs882614
rs192419988
rs145664379
rs550913799
rs11080055
rs118151158
rs16974931
All − MR Egger
All − IVW(MRE)
−0.010
−0.005
0.000
0.005
0.010
MR effect size for
'Leucine−rich repeat−containing protein 37A2' on 'eGFR'

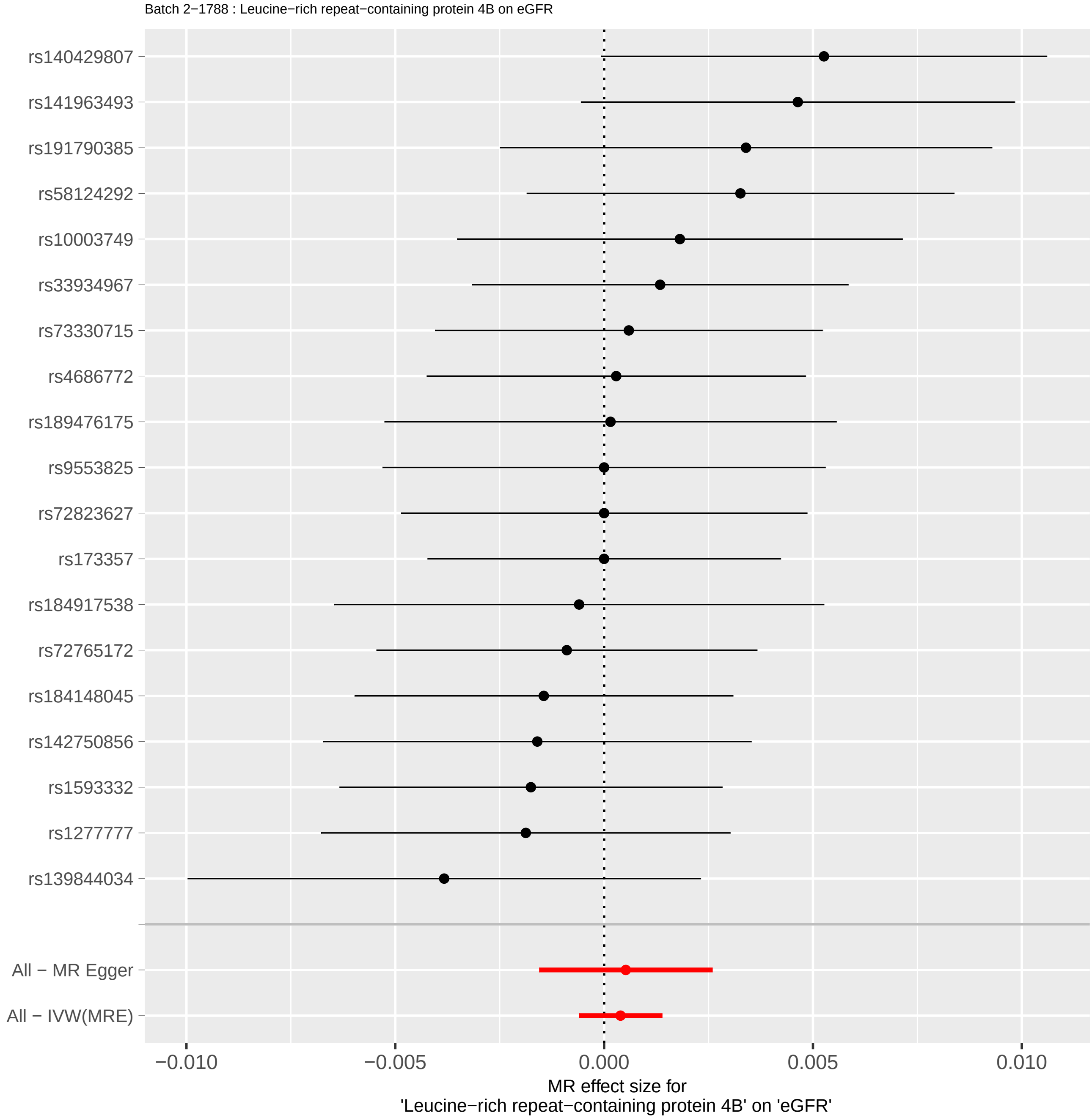
Batch 2−1788 : Leucine−rich repeat−containing protein 4B on eGFR
rs140429807
rs141963493
rs191790385
rs58124292
rs10003749
rs33934967
rs73330715
rs4686772
rs189476175
rs9553825
rs72823627
rs173357
rs184917538
rs72765172
rs184148045
rs142750856
rs1593332
rs1277777
rs139844034
All − MR Egger
All − IVW(MRE)
−0.010
−0.005
0.000
0.005
0.010
MR effect size for
'Leucine−rich repeat−containing protein 4B' on 'eGFR'

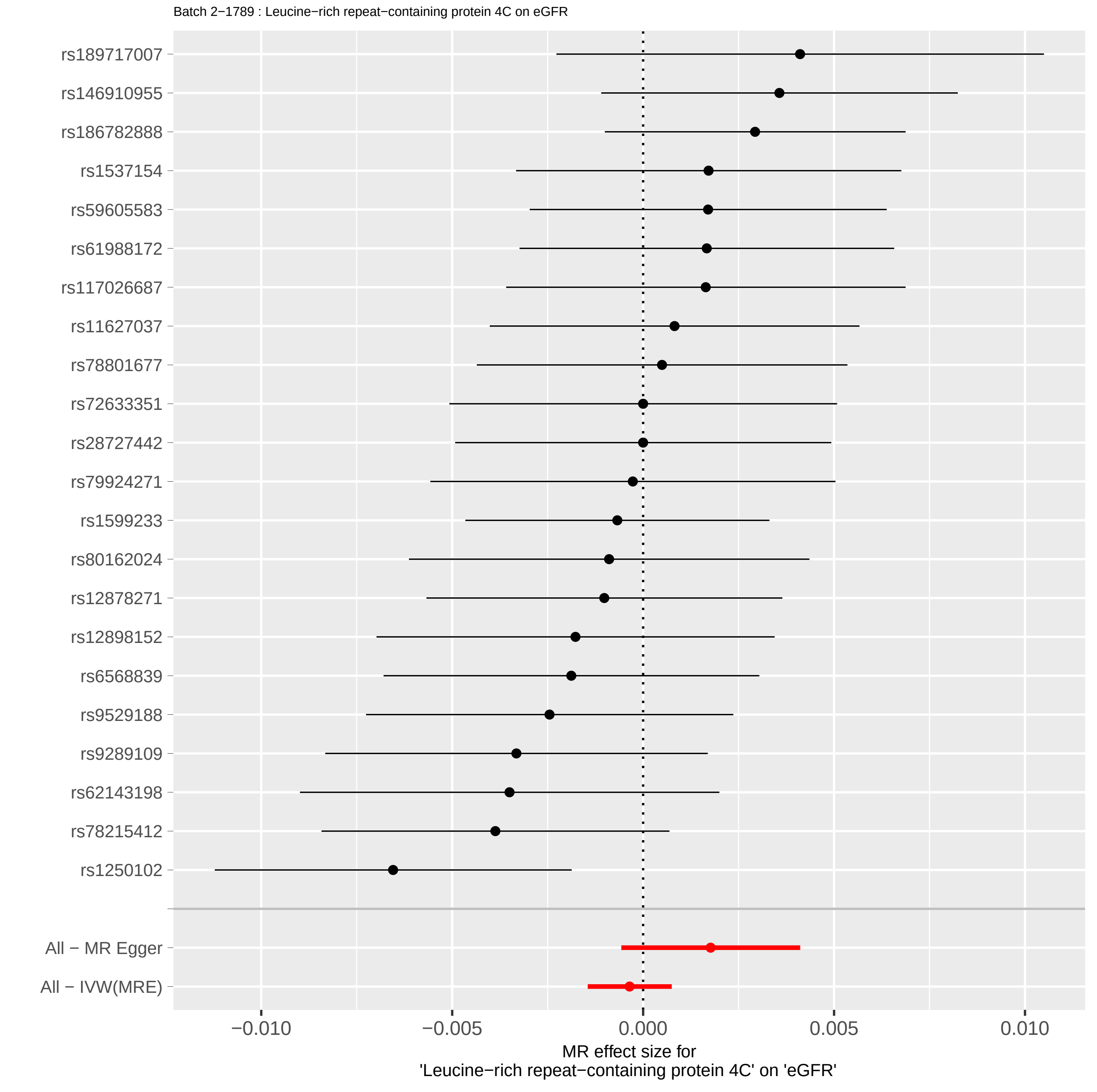

Batch 2−1789 : Leucine−rich repeat−containing protein 4C on eGFR
rs189717007
rs146910955
rs186782888
rs1537154
rs59605583
rs61988172
rs117026687
rs11627037
rs78801677
rs72633351
rs28727442
rs79924271
rs1599233
rs80162024
rs12878271
rs12898152
rs6568839
rs9529188
rs9289109
rs62143198
rs78215412
rs1250102
All − MR Egger
All − IVW(MRE)
−0.010
−0.005
0.000
0.005
0.010
MR effect size for
'Leucine−rich repeat−containing protein 4C' on 'eGFR'

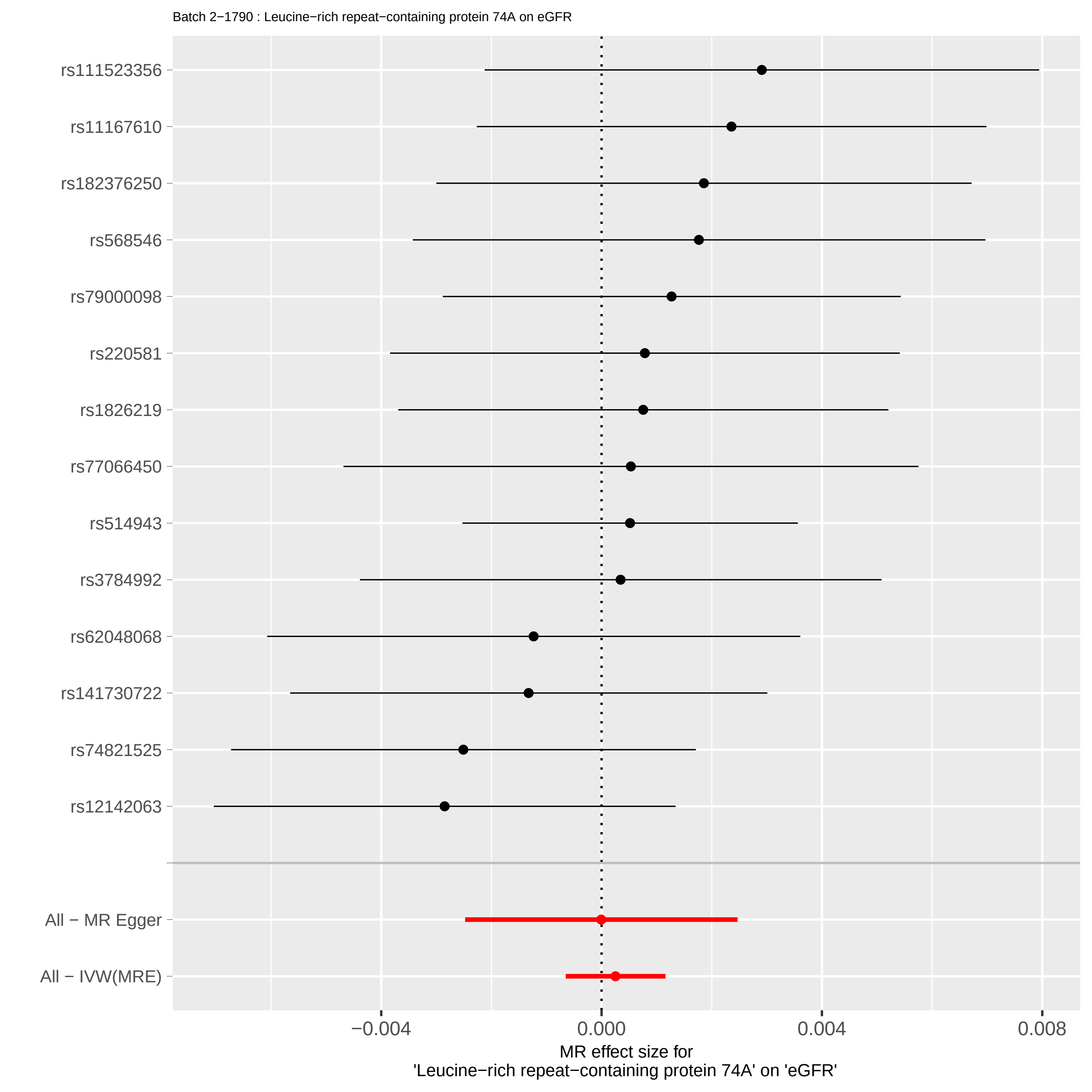

Batch 2−1790 : Leucine−rich repeat−containing protein 74A on eGFR
rs111523356
rs11167610
rs182376250
rs568546
rs79000098
rs220581
rs1826219
rs77066450
rs514943
rs3784992
rs62048068
rs141730722
rs74821525
rs12142063
All − MR Egger
All − IVW(MRE)
−0.004
0.000
0.004
0.008
MR effect size for
'Leucine−rich repeat−containing protein 74A' on 'eGFR'

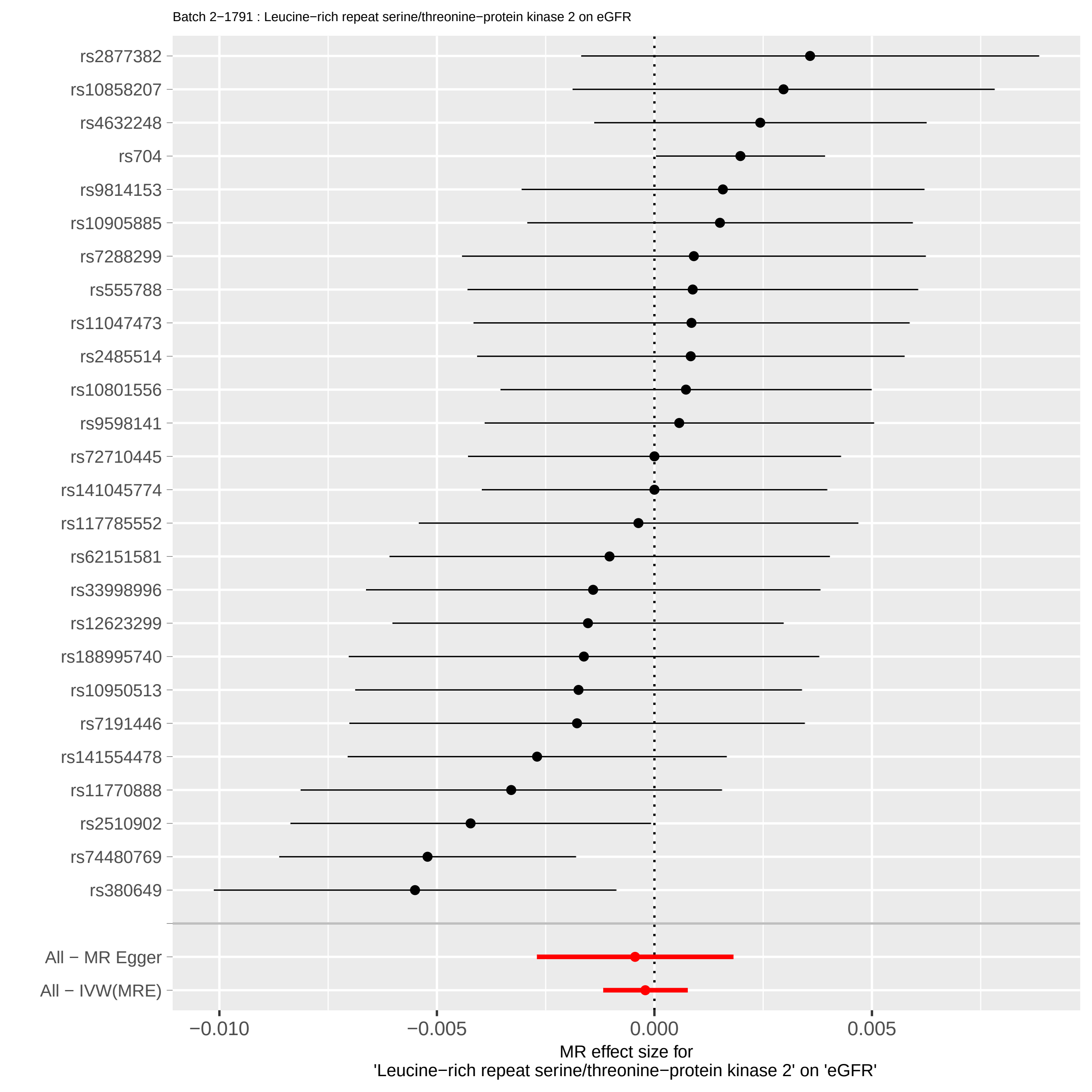

Batch 2−1791 : Leucine−rich repeat serine/threonine−protein kinase 2 on eGFR
rs2877382
rs10858207
rs4632248
rs704
rs9814153
rs10905885
rs7288299
rs555788
rs11047473
rs2485514
rs10801556
rs9598141
rs72710445
rs141045774
rs117785552
rs62151581
rs33998996
rs12623299
rs188995740
rs10950513
rs7191446
rs141554478
rs11770888
rs2510902
rs74480769
rs380649
All − MR Egger
All − IVW(MRE)
−0.010
−0.005
0.000
0.005
MR effect size for
'Leucine−rich repeat serine/threonine−protein kinase 2' on 'eGFR'

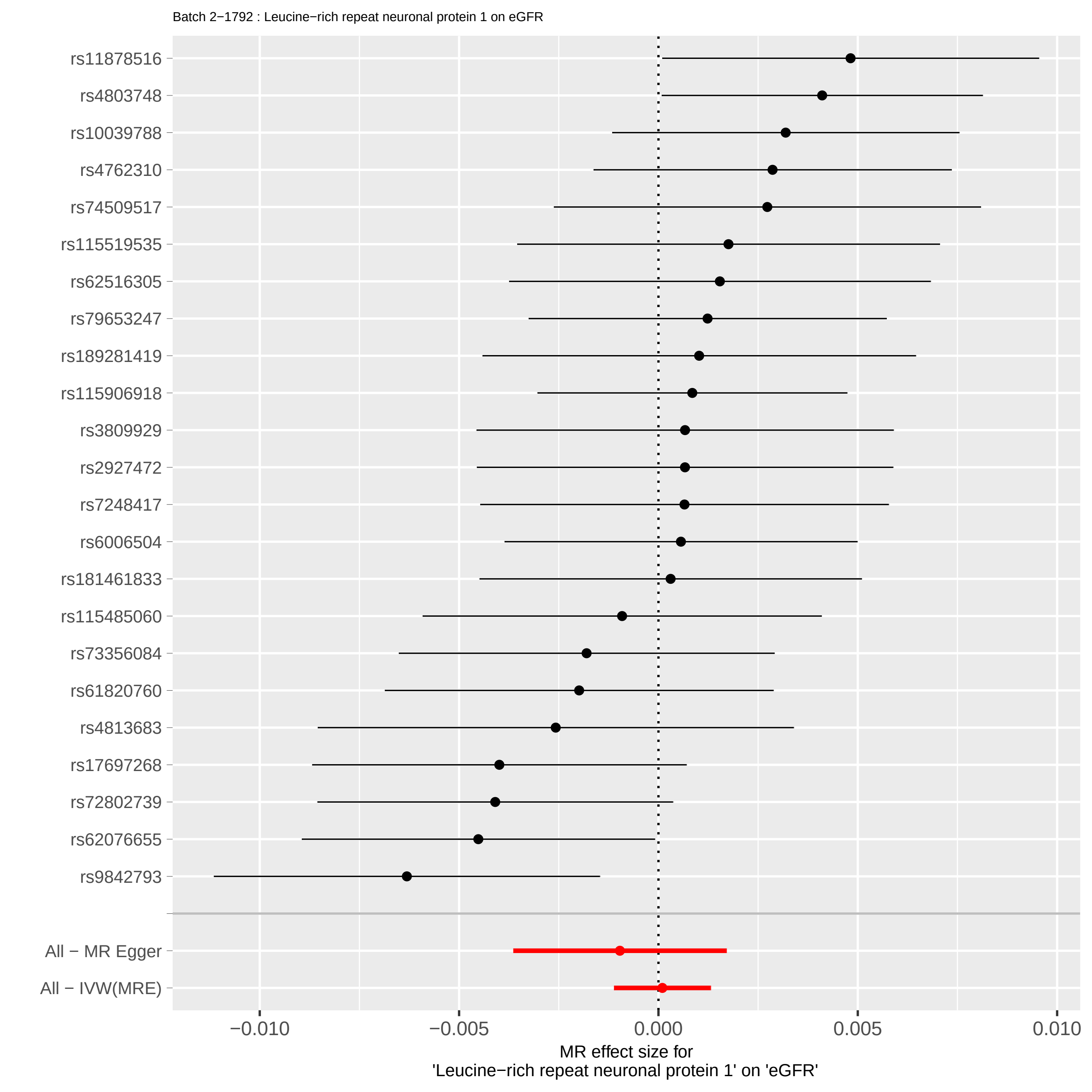

Batch 2−1792 : Leucine−rich repeat neuronal protein 1 on eGFR
rs11878516
rs4803748
rs10039788
rs4762310
rs74509517
rs115519535
rs62516305
rs79653247
rs189281419
rs115906918
rs3809929
rs2927472
rs7248417
rs6006504
rs181461833
rs115485060
rs73356084
rs61820760
rs4813683
rs17697268
rs72802739
rs62076655
rs9842793
All − MR Egger
All − IVW(MRE)
−0.010
−0.005
0.000
0.005
0.010
MR effect size for
'Leucine−rich repeat neuronal protein 1' on 'eGFR'

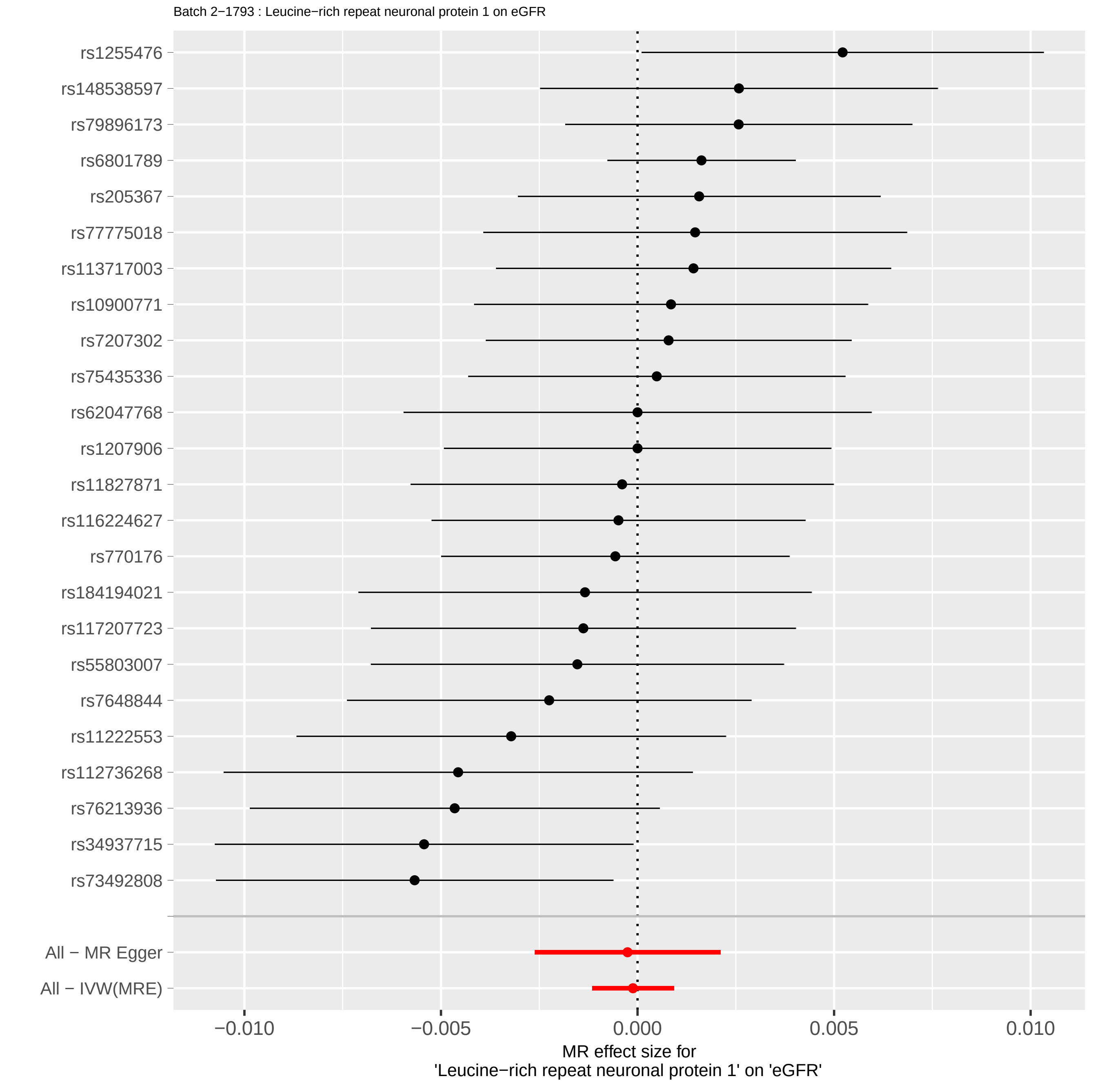

Batch 2−1793 : Leucine−rich repeat neuronal protein 1 on eGFR
rs1255476
rs148538597
rs79896173
rs6801789
rs205367
rs77775018
rs113717003
rs10900771
rs7207302
rs75435336
rs62047768
rs1207906
rs11827871
rs116224627
rs770176
rs184194021
rs117207723
rs55803007
rs7648844
rs11222553
rs112736268
rs76213936
rs34937715
rs73492808
All − MR Egger
All − IVW(MRE)
−0.010
−0.005
0.000
0.005
0.010
MR effect size for
'Leucine−rich repeat neuronal protein 1' on 'eGFR'

Batch 2−1794 : Leucine−rich repeat neuronal protein 3 on eGFR

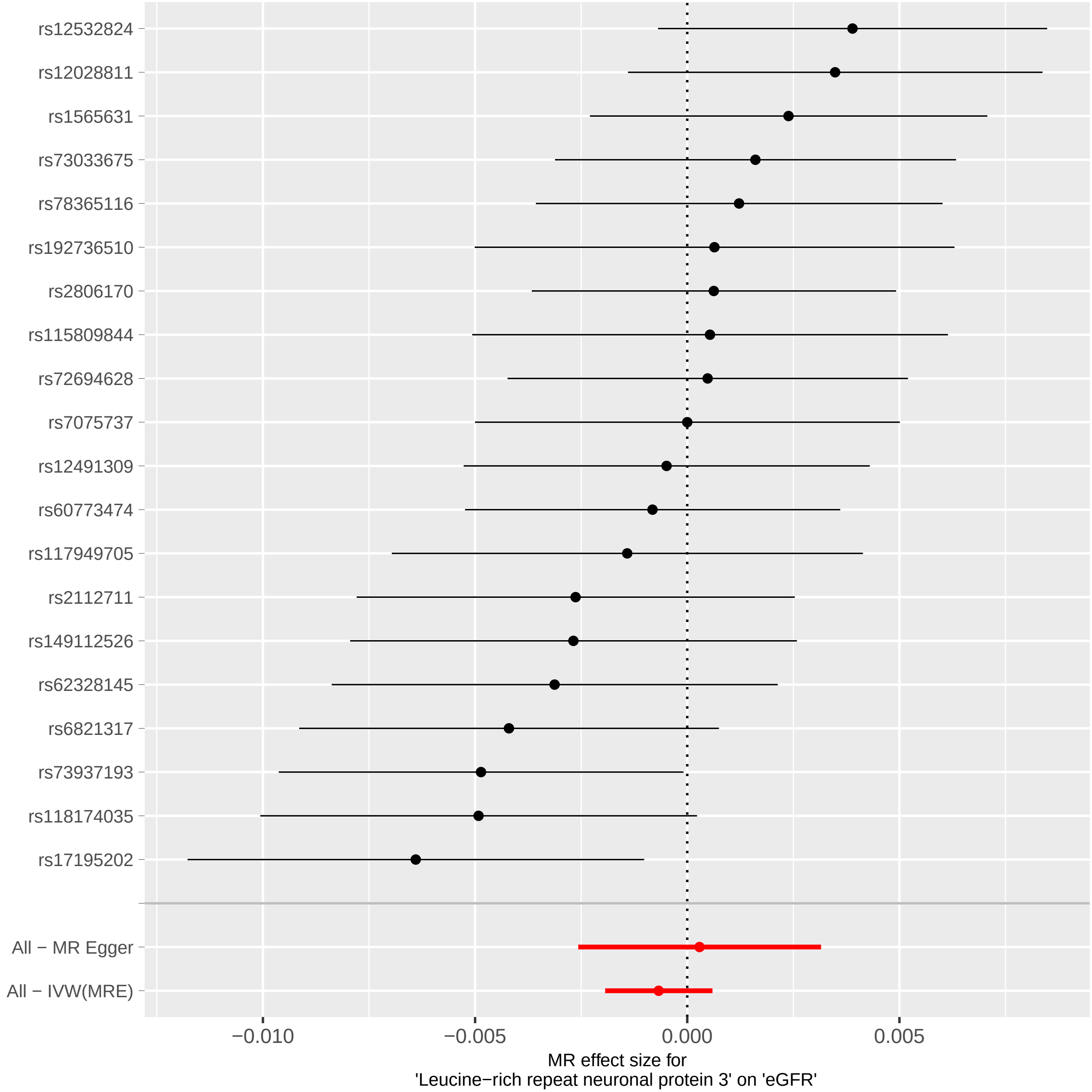

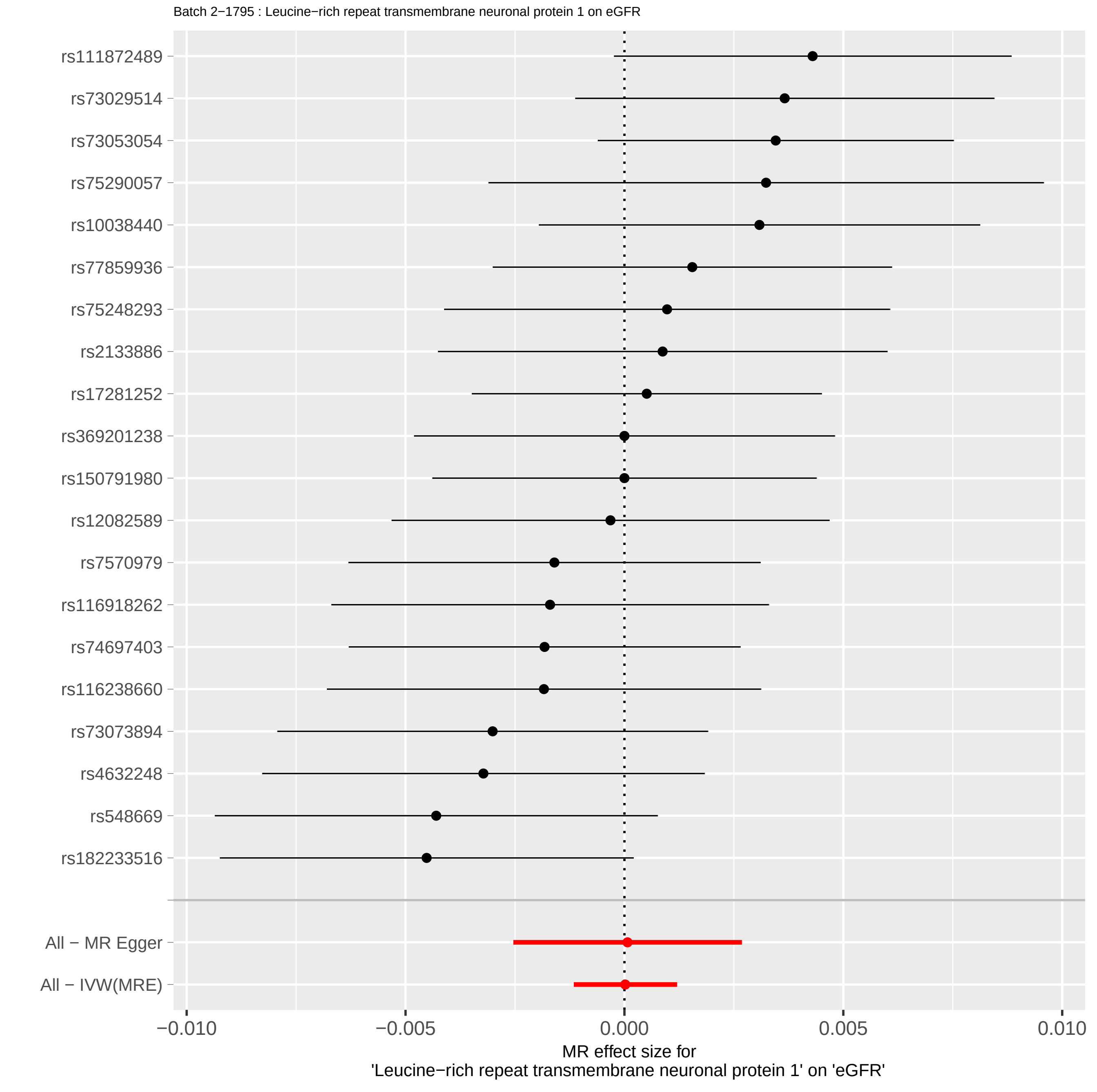

Batch 2−1795 : Leucine−rich repeat transmembrane neuronal protein 1 on eGFR
rs111872489
rs73029514
rs73053054
rs75290057
rs10038440
rs77859936
rs75248293
rs2133886
rs17281252
rs369201238
rs150791980
rs12082589
rs7570979
rs116918262
rs74697403
rs116238660
rs73073894
rs4632248
rs548669
rs182233516
All − MR Egger
All − IVW(MRE)
−0.010
−0.005
0.000
0.005
0.010
MR effect size for
'Leucine−rich repeat transmembrane neuronal protein 1' on 'eGFR'

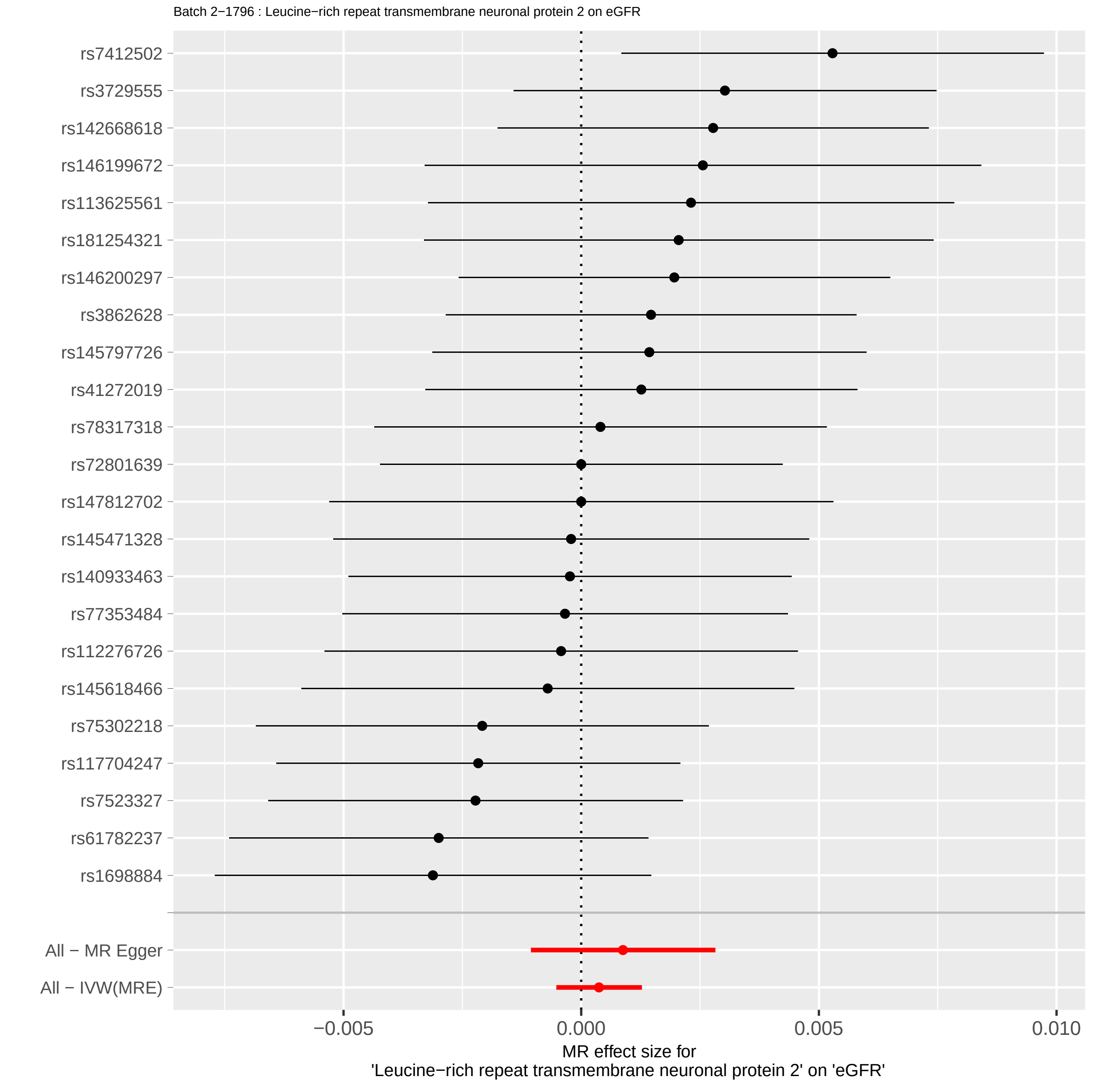
Batch 2−1796 : Leucine−rich repeat transmembrane neuronal protein 2 on eGFR
rs7412502
rs3729555
rs142668618
rs146199672
rs113625561
rs181254321
rs146200297
rs3862628
rs145797726
rs41272019
rs78317318
rs72801639
rs147812702
rs145471328
rs140933463
rs77353484
rs112276726
rs145618466
rs75302218
rs117704247
rs7523327
rs61782237
rs1698884
All − MR Egger
All − IVW(MRE)
−0.005
0.000
0.005
0.010
MR effect size for
'Leucine−rich repeat transmembrane neuronal protein 2' on 'eGFR'

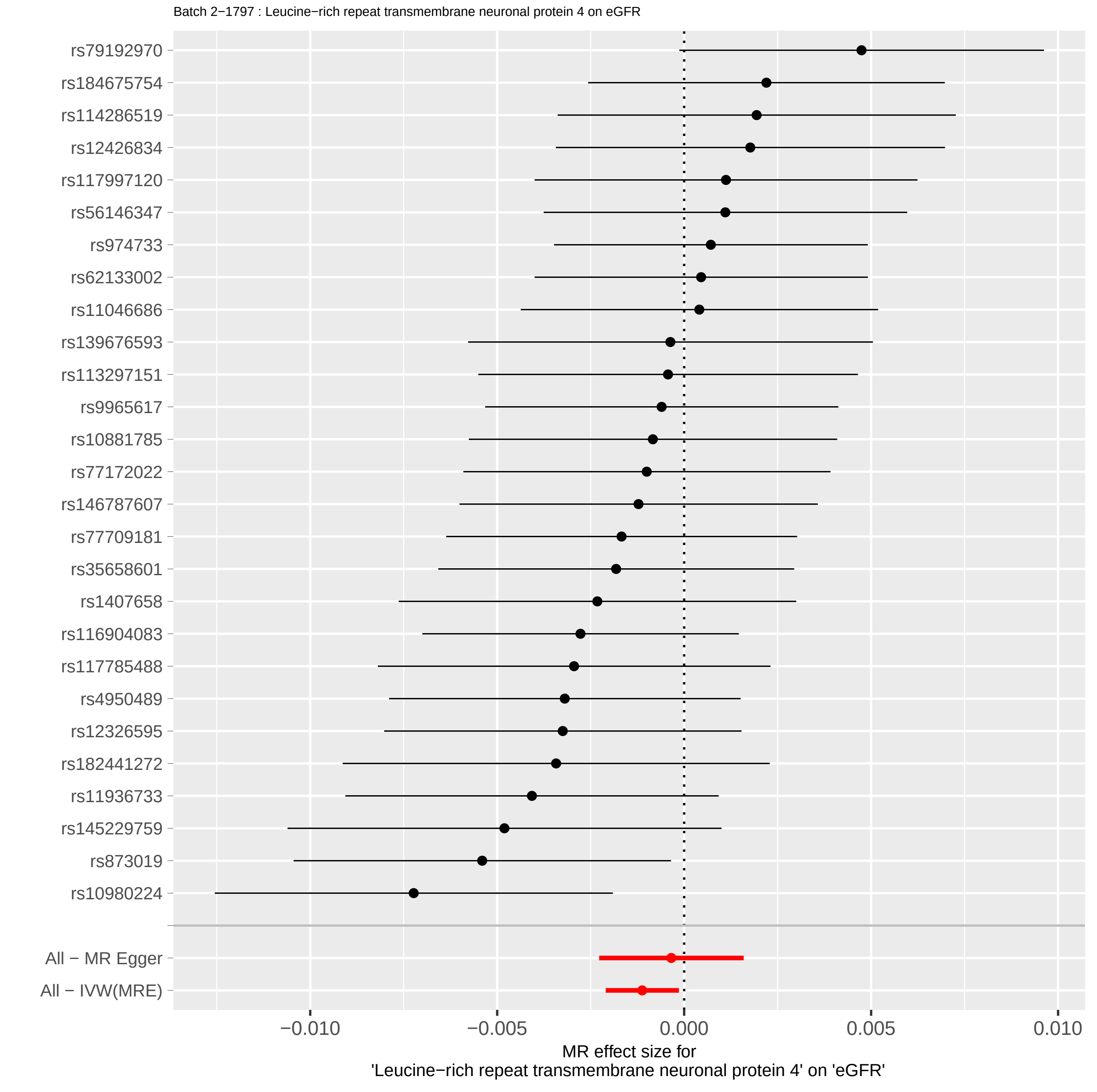

Batch 2−1797 : Leucine−rich repeat transmembrane neuronal protein 4 on eGFR
rs79192970
rs184675754
rs114286519
rs12426834
rs117997120
rs56146347
rs974733
rs62133002
rs11046686
rs139676593
rs113297151
rs9965617
rs10881785
rs77172022
rs146787607
rs77709181
rs35658601
rs1407658
rs116904083
rs117785488
rs4950489
rs12326595
rs182441272
rs11936733
rs145229759
rs873019
rs10980224
All − MR Egger
All − IVW(MRE)
−0.010
−0.005
0.000
0.005
0.010
MR effect size for
'Leucine−rich repeat transmembrane neuronal protein 4' on 'eGFR'

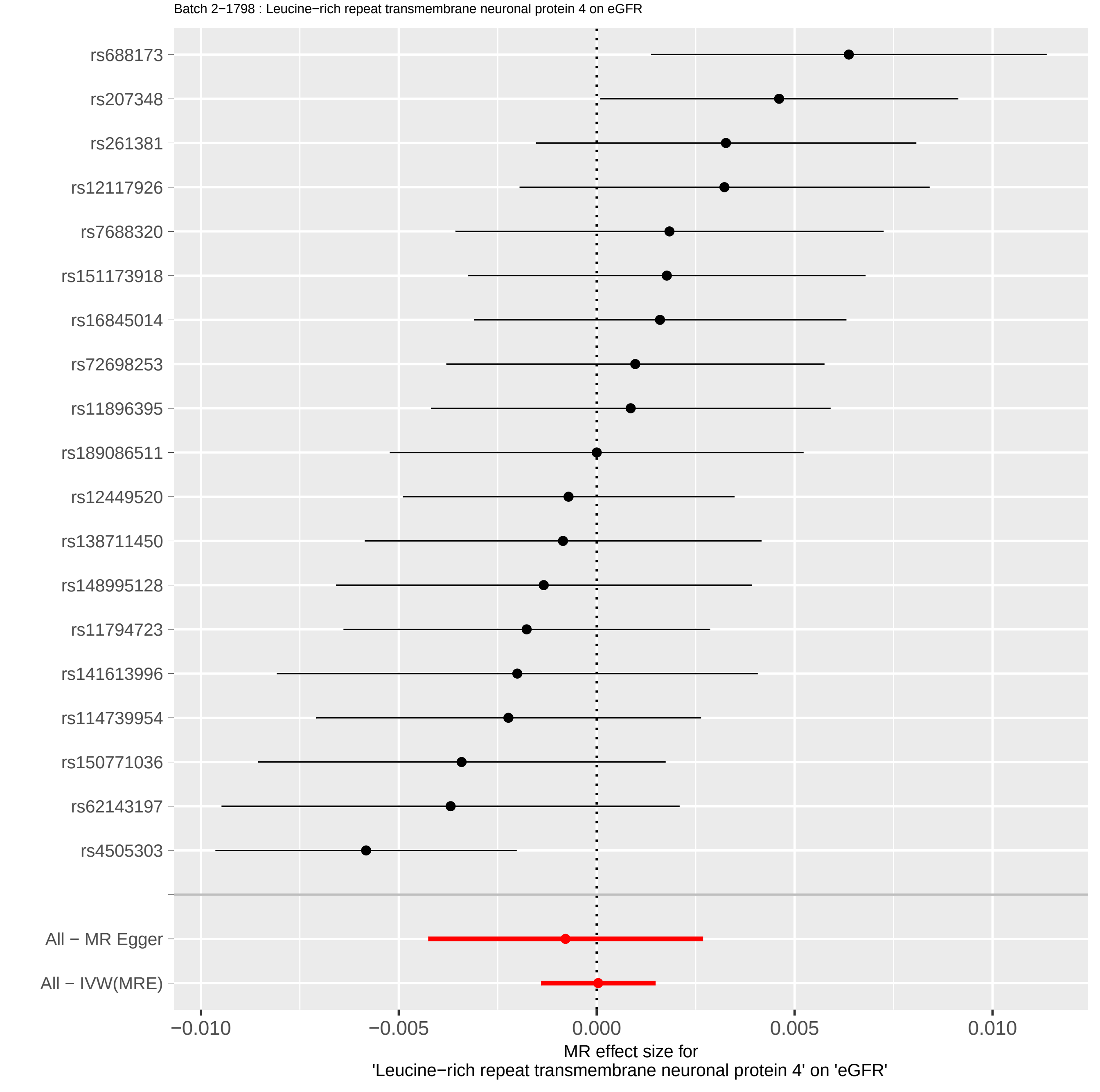
Batch 2−1798 : Leucine−rich repeat transmembrane neuronal protein 4 on eGFR
rs688173
rs207348
rs261381
rs12117926
rs7688320
rs151173918
rs16845014
rs72698253
rs11896395
rs189086511
rs12449520
rs138711450
rs148995128
rs11794723
rs141613996
rs114739954
rs150771036
rs62143197
rs4505303
All − MR Egger
All − IVW(MRE)
−0.010
−0.005
0.000
0.005
0.010
MR effect size for
'Leucine−rich repeat transmembrane neuronal protein 4' on 'eGFR'

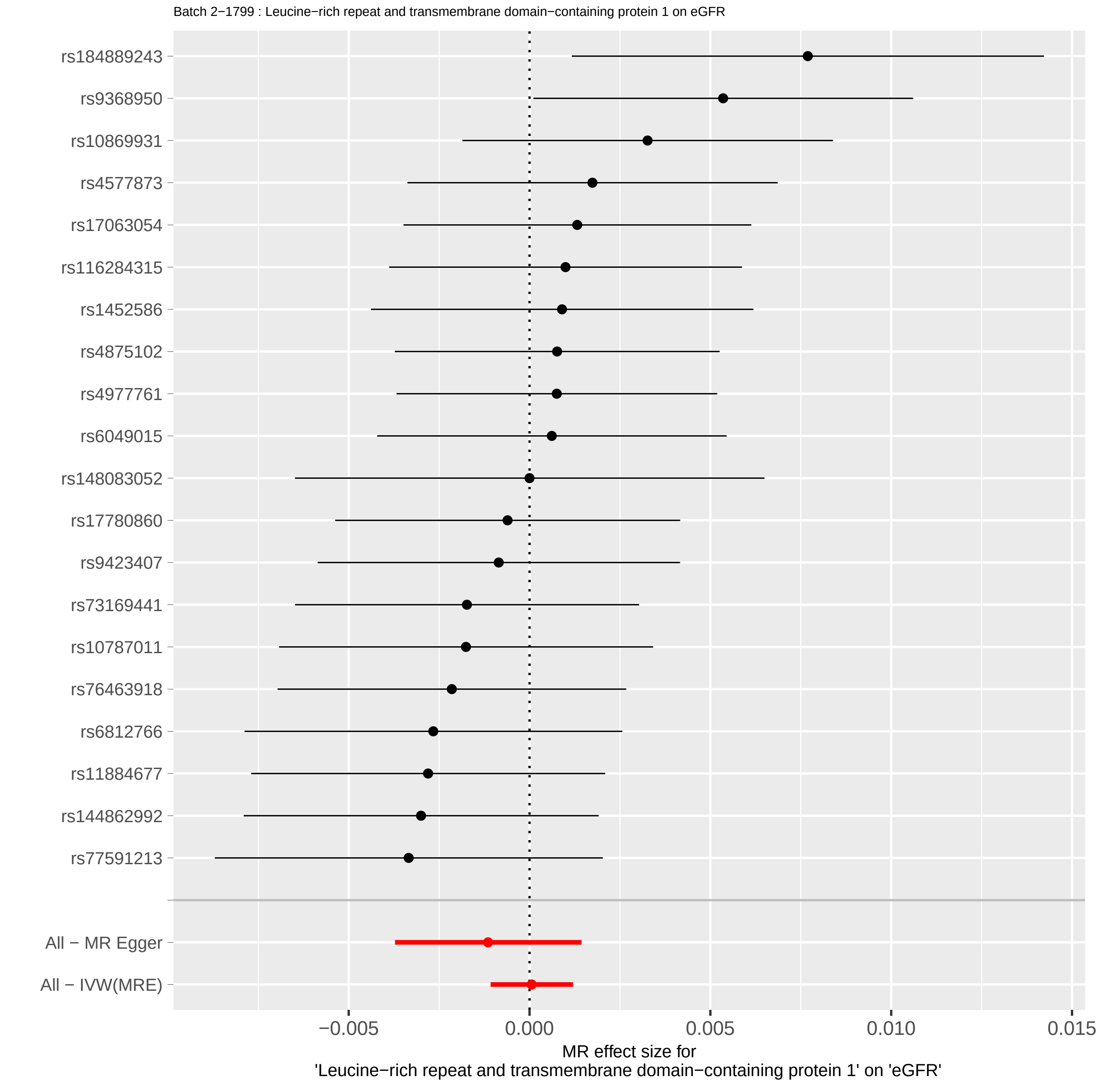

Batch 2−1799 : Leucine−rich repeat and transmembrane domain−containing protein 1 on eGFR
rs184889243
rs9368950
rs10869931
rs4577873
rs17063054
rs116284315
rs1452586
rs4875102
rs4977761
rs6049015
rs148083052
rs17780860
rs9423407
rs73169441
rs10787011
rs76463918
rs6812766
rs11884677
rs144862992
rs77591213
All − MR Egger
All − IVW(MRE)
−0.005
0.000
0.005
0.010
0.015
MR effect size for
'Leucine−rich repeat and transmembrane domain−containing protein 1' on 'eGFR'

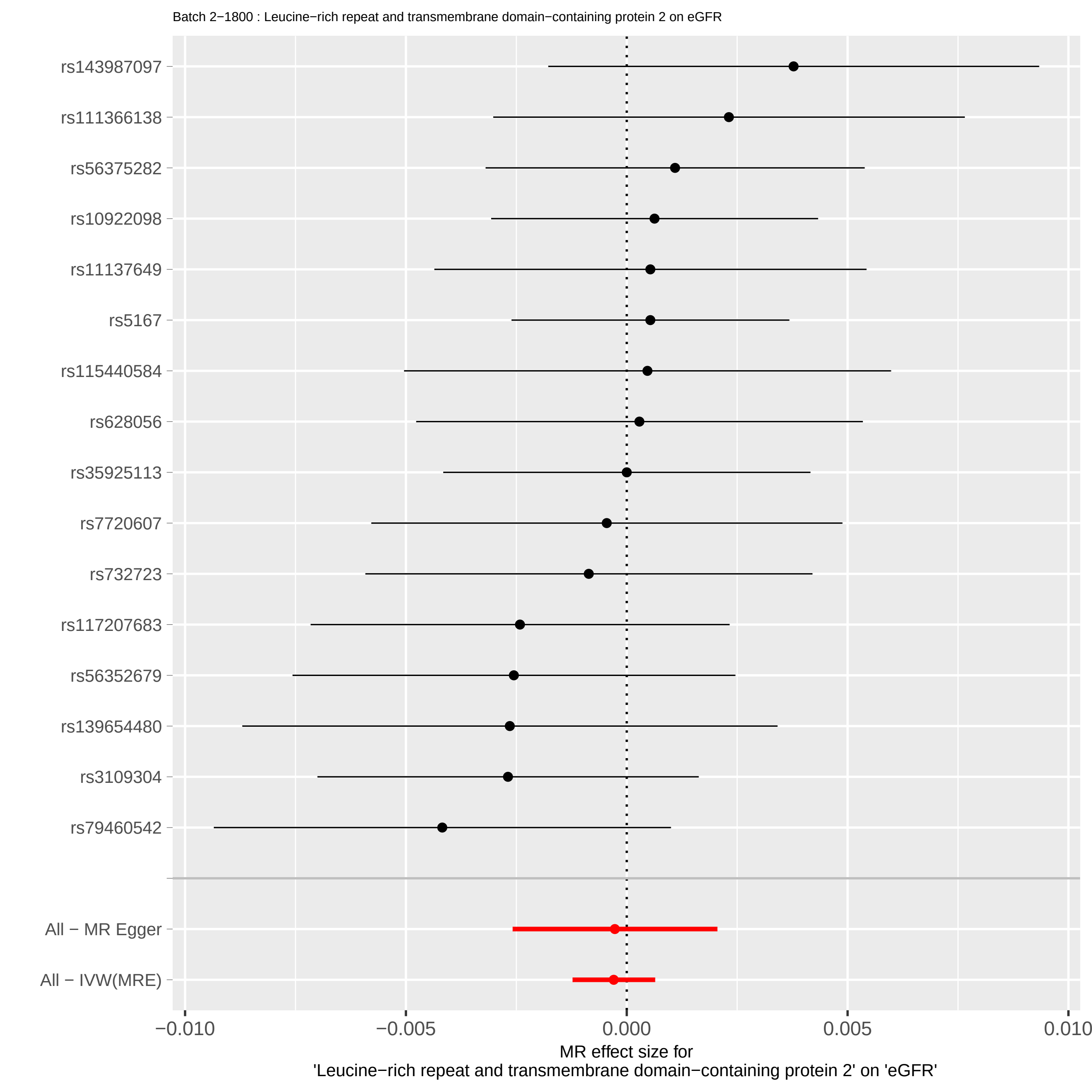

Batch 2−1800 : Leucine−rich repeat and transmembrane domain−containing protein 2 on eGFR
rs143987097
rs111366138
rs56375282
rs10922098
rs11137649
rs5167
rs115440584
rs628056
rs35925113
rs7720607
rs732723
rs117207683
rs56352679
rs139654480
rs3109304
rs79460542
All − MR Egger
All − IVW(MRE)
−0.010
−0.005
0.000
0.005
0.010
MR effect size for
'Leucine−rich repeat and transmembrane domain−containing protein 2' on 'eGFR'

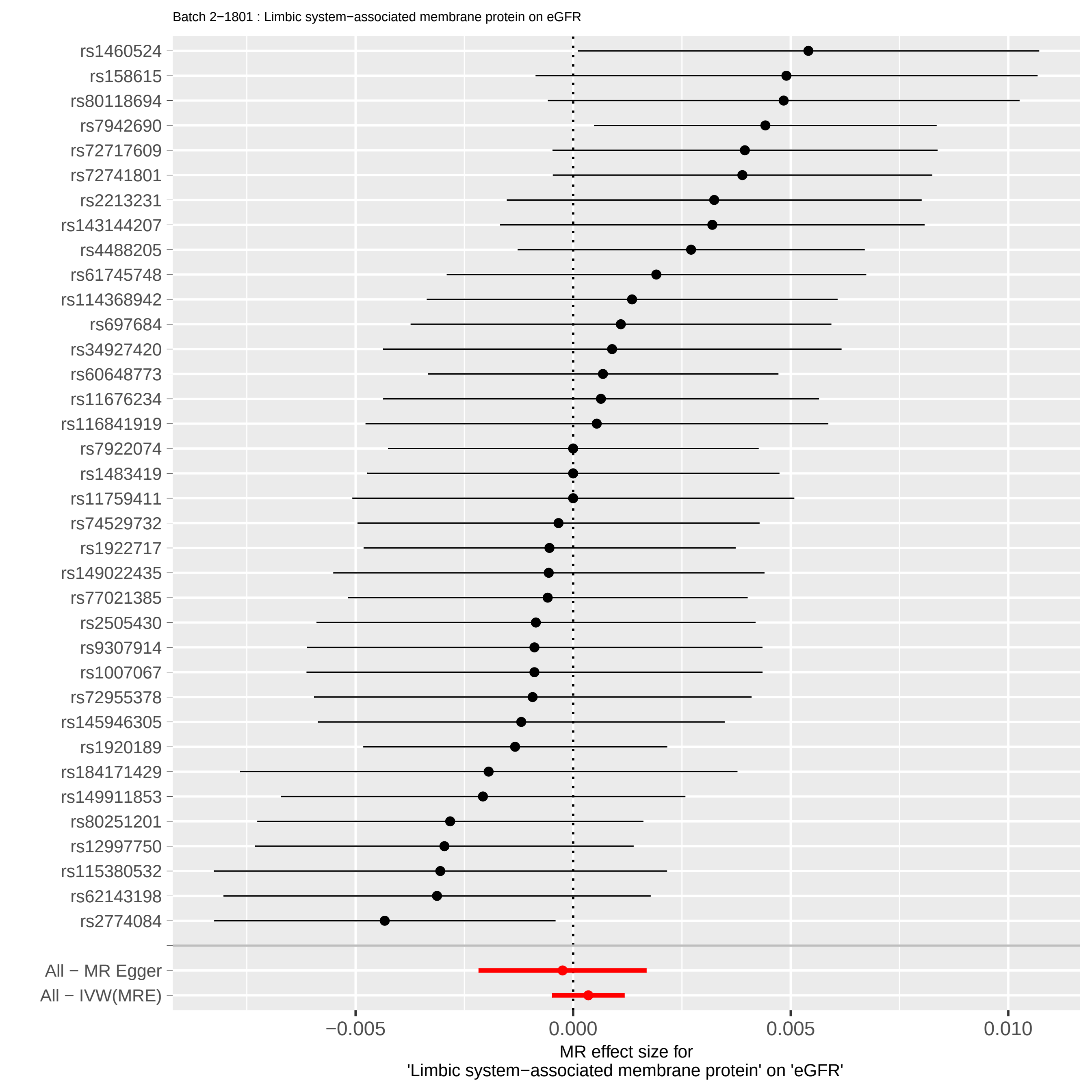

Batch 2−1801 : Limbic system−associated membrane protein on eGFR
rs1460524
rs158615
rs80118694
rs7942690
rs72717609
rs72741801
rs2213231
rs143144207
rs4488205
rs61745748
rs114368942
rs697684
rs34927420
rs60648773
rs11676234
rs116841919
rs7922074
rs1483419
rs11759411
rs74529732
rs1922717
rs149022435
rs77021385
rs2505430
rs9307914
rs1007067
rs72955378
rs145946305
rs1920189
rs184171429
rs149911853
rs80251201
rs12997750
rs115380532
rs62143198
rs2774084
All − MR Egger
All − IVW(MRE)
−0.005
0.000
0.005
0.010
MR effect size for
'Limbic system−associated membrane protein' on 'eGFR'

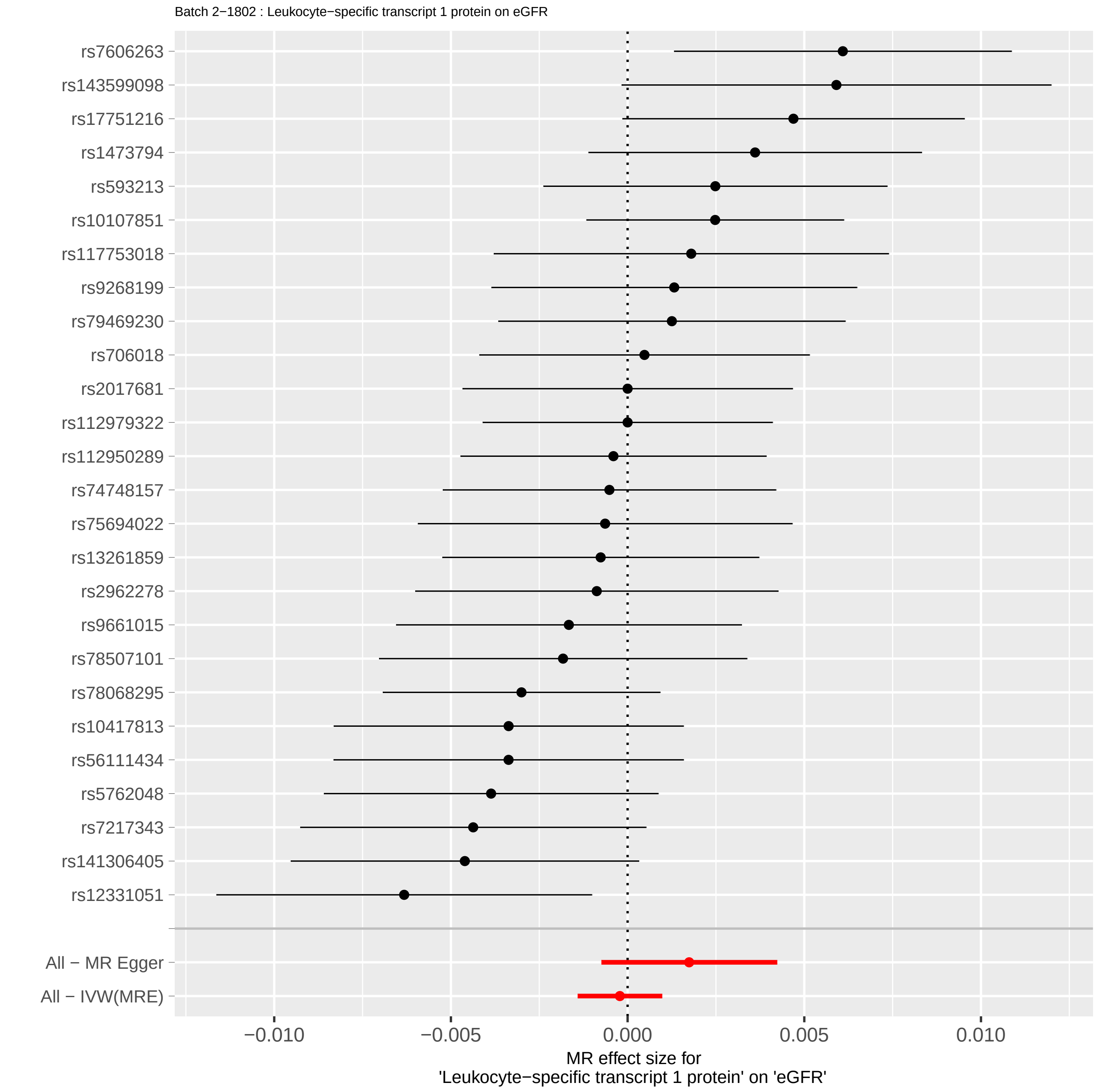

Batch 2−1802 : Leukocyte−specific transcript 1 protein on eGFR
rs7606263
rs143599098
rs17751216
rs1473794
rs593213
rs10107851
rs117753018
rs9268199
rs79469230
rs706018
rs2017681
rs112979322
rs112950289
rs74748157
rs75694022
rs13261859
rs2962278
rs9661015
rs78507101
rs78068295
rs10417813
rs56111434
rs5762048
rs7217343
rs141306405
rs12331051
All − MR Egger
All − IVW(MRE)
−0.010
−0.005
0.000
0.005
0.010
MR effect size for
'Leukocyte−specific transcript 1 protein' on 'eGFR'

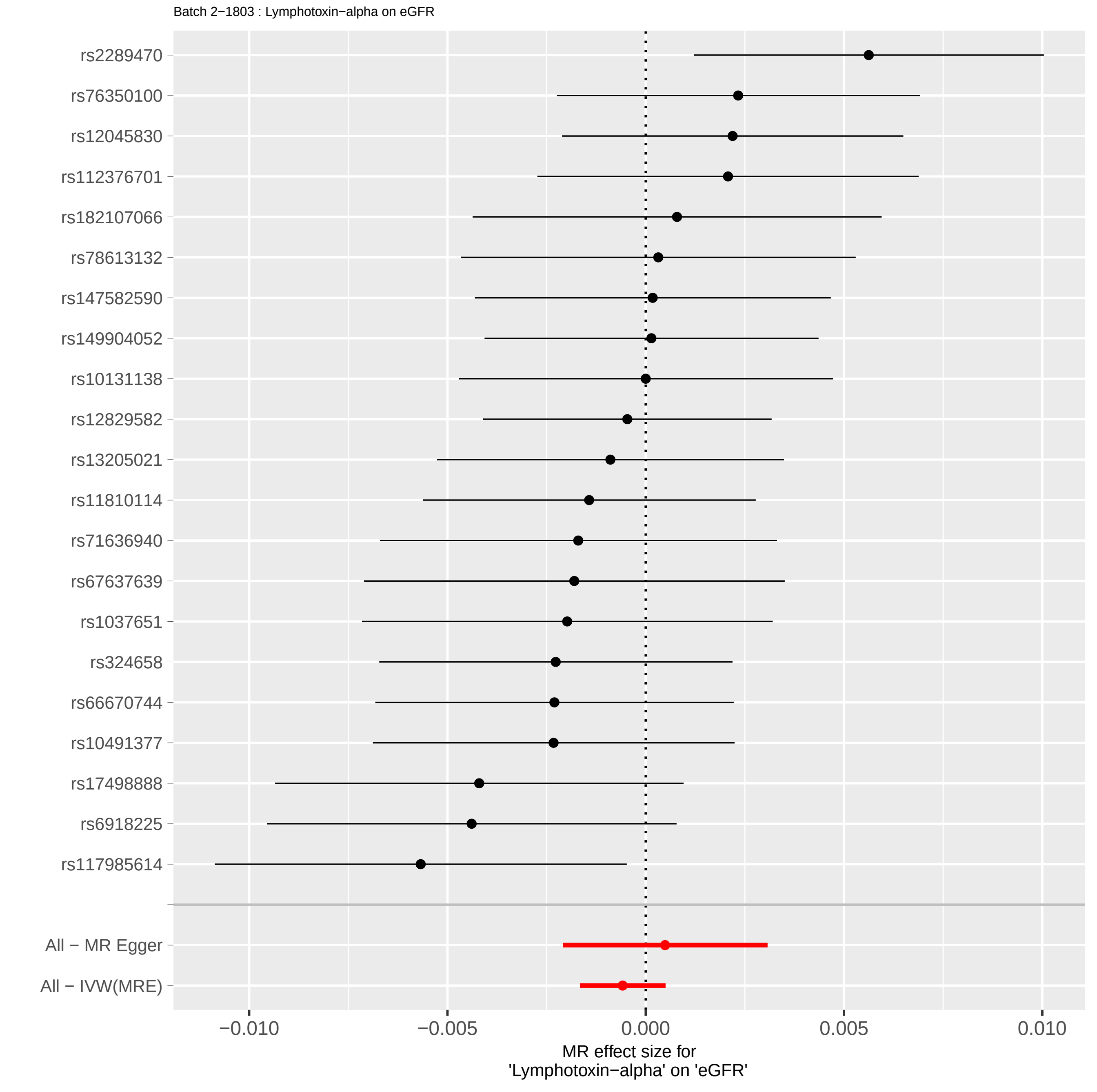

Batch 2−1803 : Lymphotoxin−alpha on eGFR
rs2289470
rs76350100
rs12045830
rs112376701
rs182107066
rs78613132
rs147582590
rs149904052
rs10131138
rs12829582
rs13205021
rs11810114
rs71636940
rs67637639
rs1037651
rs324658
rs66670744
rs10491377
rs17498888
rs6918225
rs117985614
All − MR Egger
All − IVW(MRE)
−0.010
−0.005
0.000
0.005
0.010
MR effect size for
'Lymphotoxin−alpha' on 'eGFR'

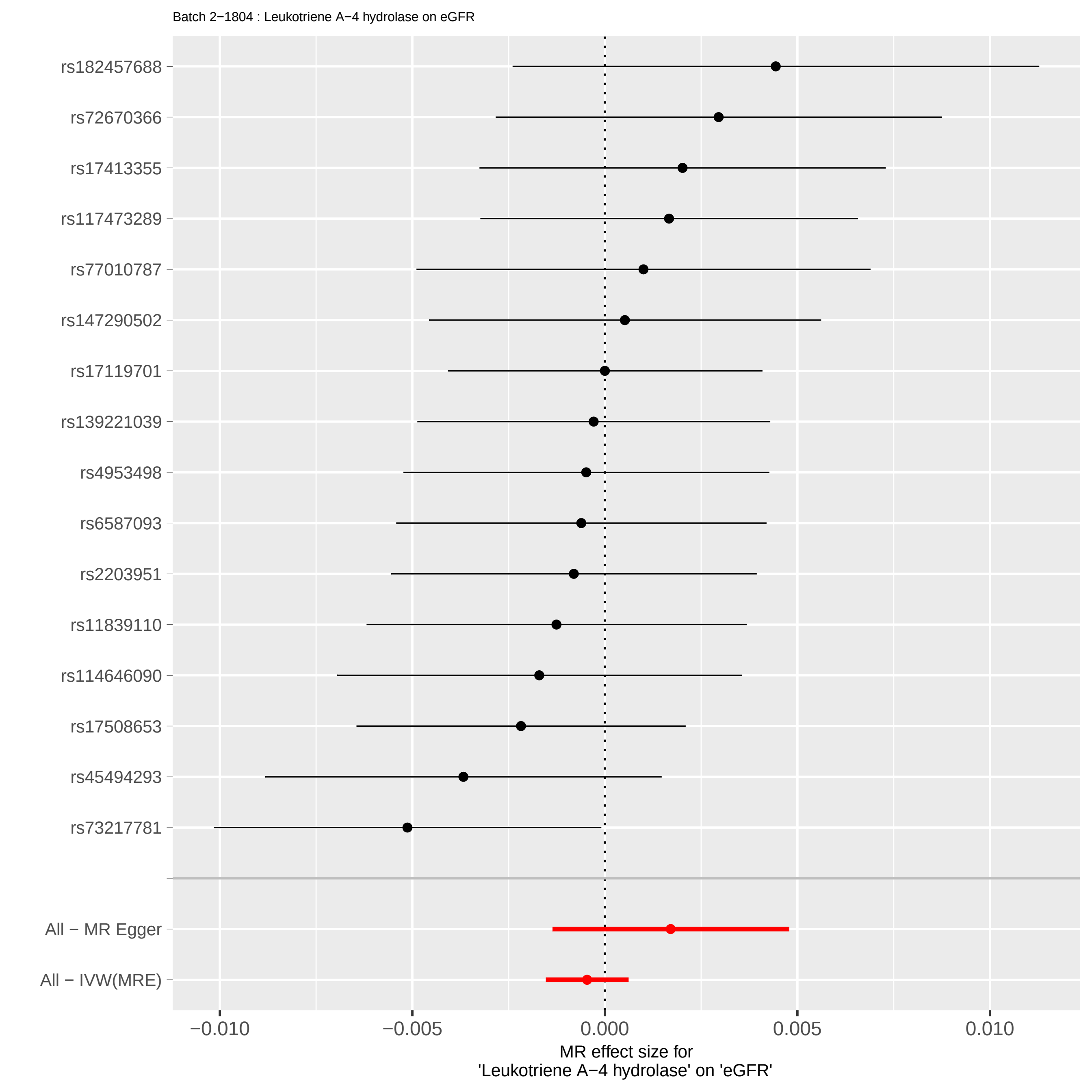
Batch 2−1804 : Leukotriene A−4 hydrolase on eGFR
rs182457688
rs72670366
rs17413355
rs117473289
rs77010787
rs147290502
rs17119701
rs139221039
rs4953498
rs6587093
rs2203951
rs11839110
rs114646090
rs17508653
rs45494293
rs73217781
All − MR Egger
All − IVW(MRE)
−0.010
−0.005
0.000
0.005
0.010
MR effect size for
'Leukotriene A−4 hydrolase' on 'eGFR'

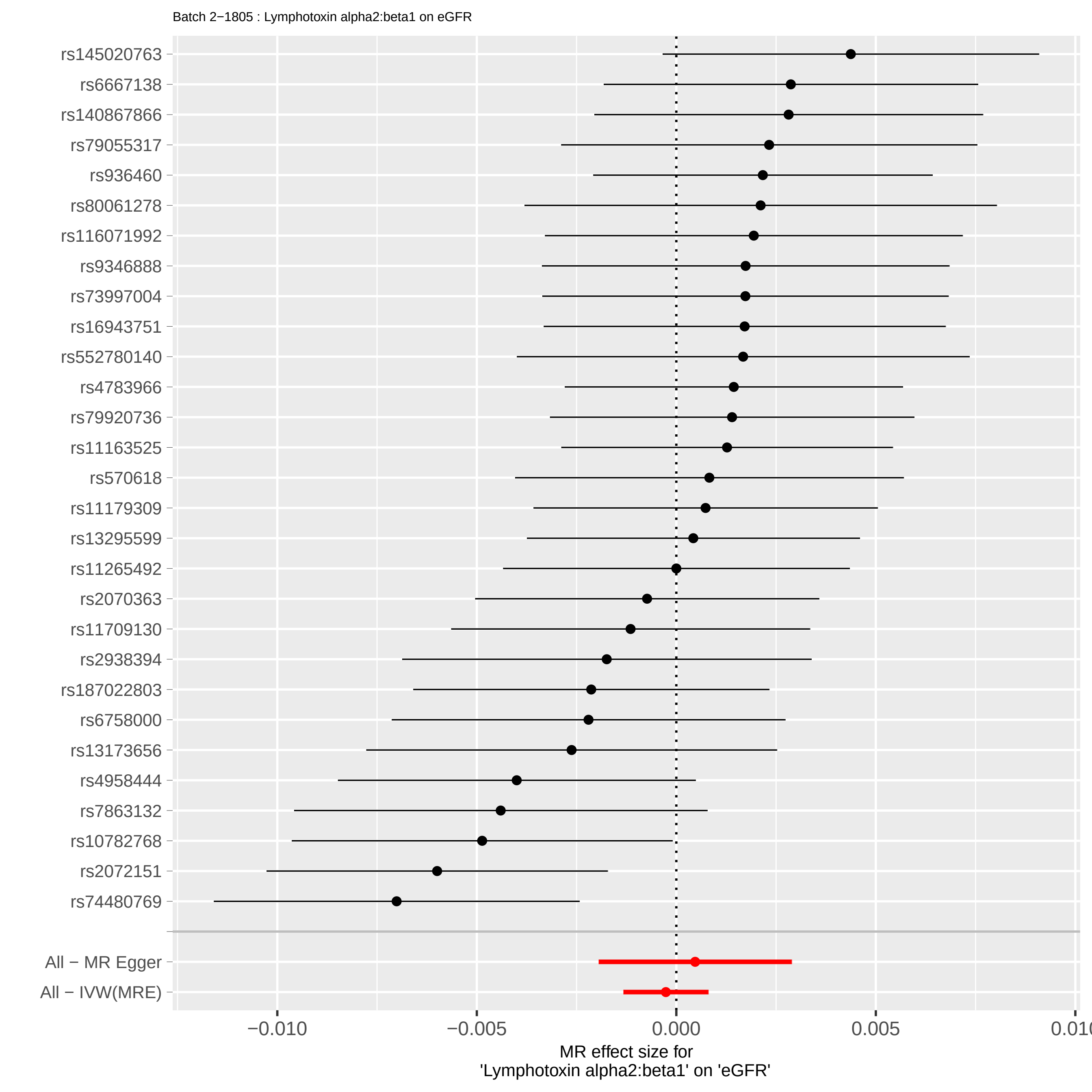

Batch 2−1805 : Lymphotoxin alpha2:beta1 on eGFR
rs145020763
rs6667138
rs140867866
rs79055317
rs936460
rs80061278
rs116071992
rs9346888
rs73997004
rs16943751
rs552780140
rs4783966
rs79920736
rs11163525
rs570618
rs11179309
rs13295599
rs11265492
rs2070363
rs11709130
rs2938394
rs187022803
rs6758000
rs13173656
rs4958444
rs7863132
rs10782768
rs2072151
rs74480769
All − MR Egger
All − IVW(MRE)
−0.010
−0.005
0.000
0.005
0.010
MR effect size for
'Lymphotoxin alpha2:beta1' on 'eGFR'

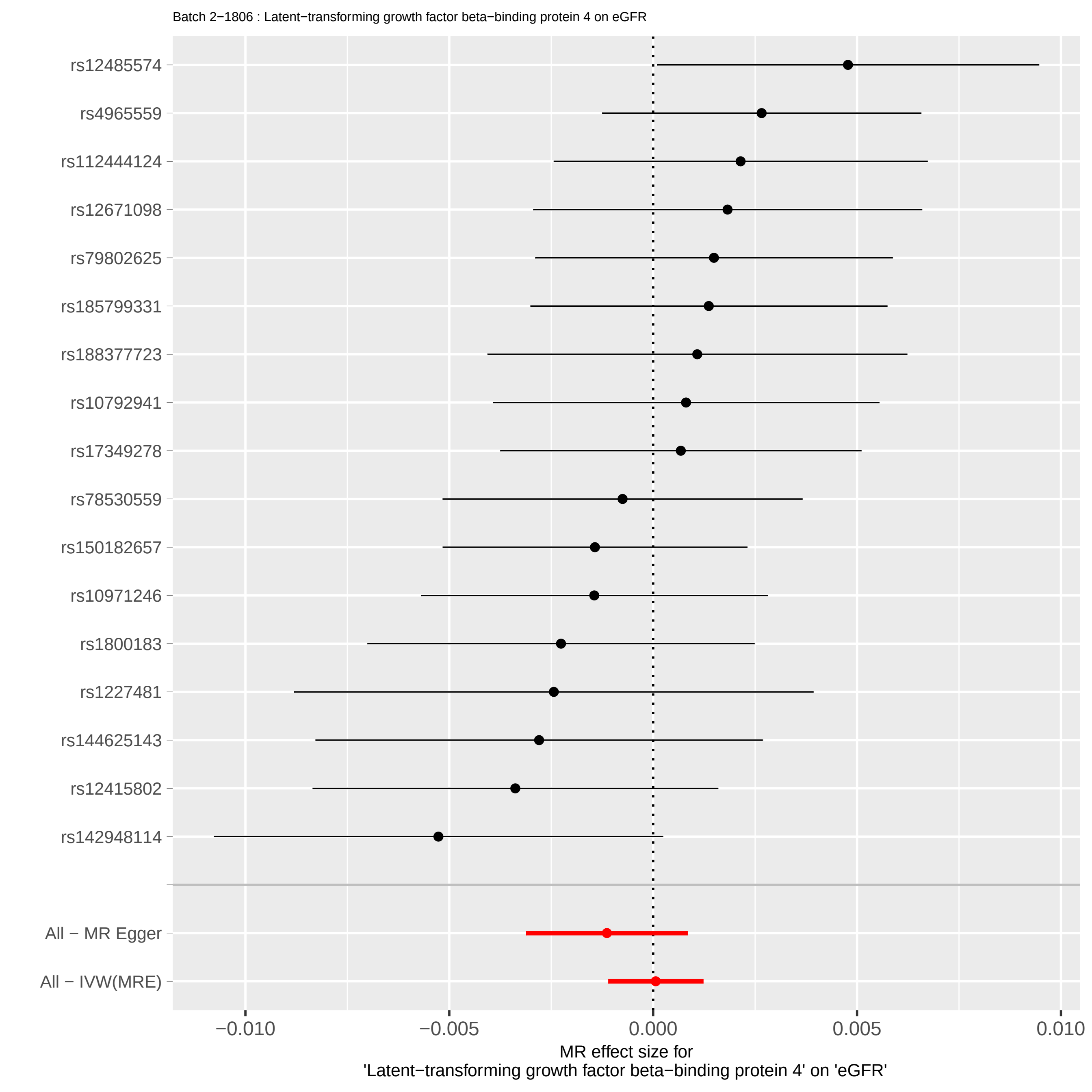
Batch 2−1806 : Latent−transforming growth factor beta−binding protein 4 on eGFR
rs12485574
rs4965559
rs112444124
rs12671098
rs79802625
rs185799331
rs188377723
rs10792941
rs17349278
rs78530559
rs150182657
rs10971246
rs1800183
rs1227481
rs144625143
rs12415802
rs142948114
All − MR Egger
All − IVW(MRE)
−0.010
−0.005
0.000
0.005
0.010
MR effect size for
'Latent−transforming growth factor beta−binding protein 4' on 'eGFR'

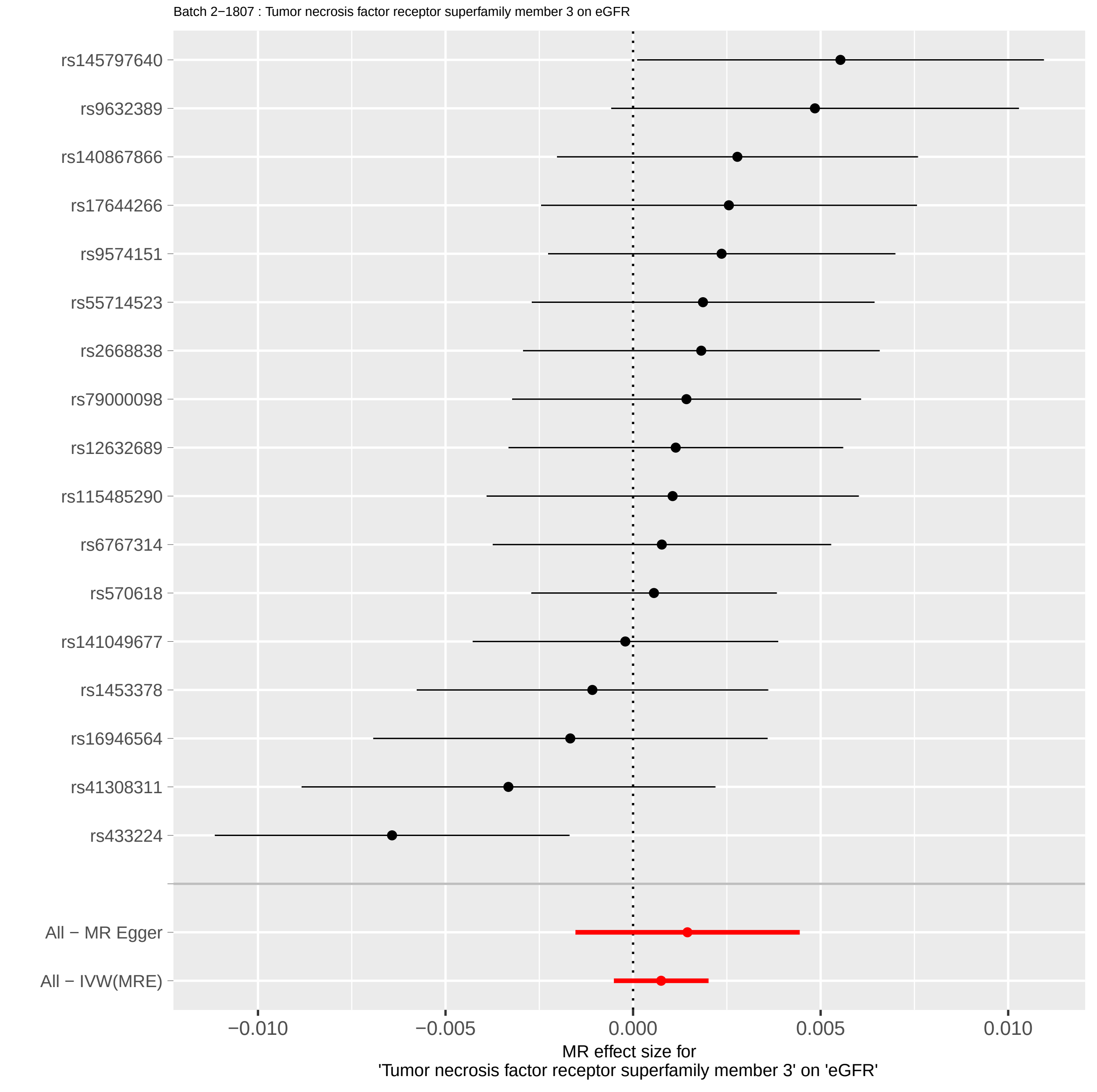
Batch 2−1807 : Tumor necrosis factor receptor superfamily member 3 on eGFR
rs145797640
rs9632389
rs140867866
rs17644266
rs9574151
rs55714523
rs2668838
rs79000098
rs12632689
rs115485290
rs6767314
rs570618
rs141049677
rs1453378
rs16946564
rs41308311
rs433224
All − MR Egger
All − IVW(MRE)
−0.010
−0.005
0.000
0.005
0.010
MR effect size for
'Tumor necrosis factor receptor superfamily member 3' on 'eGFR'

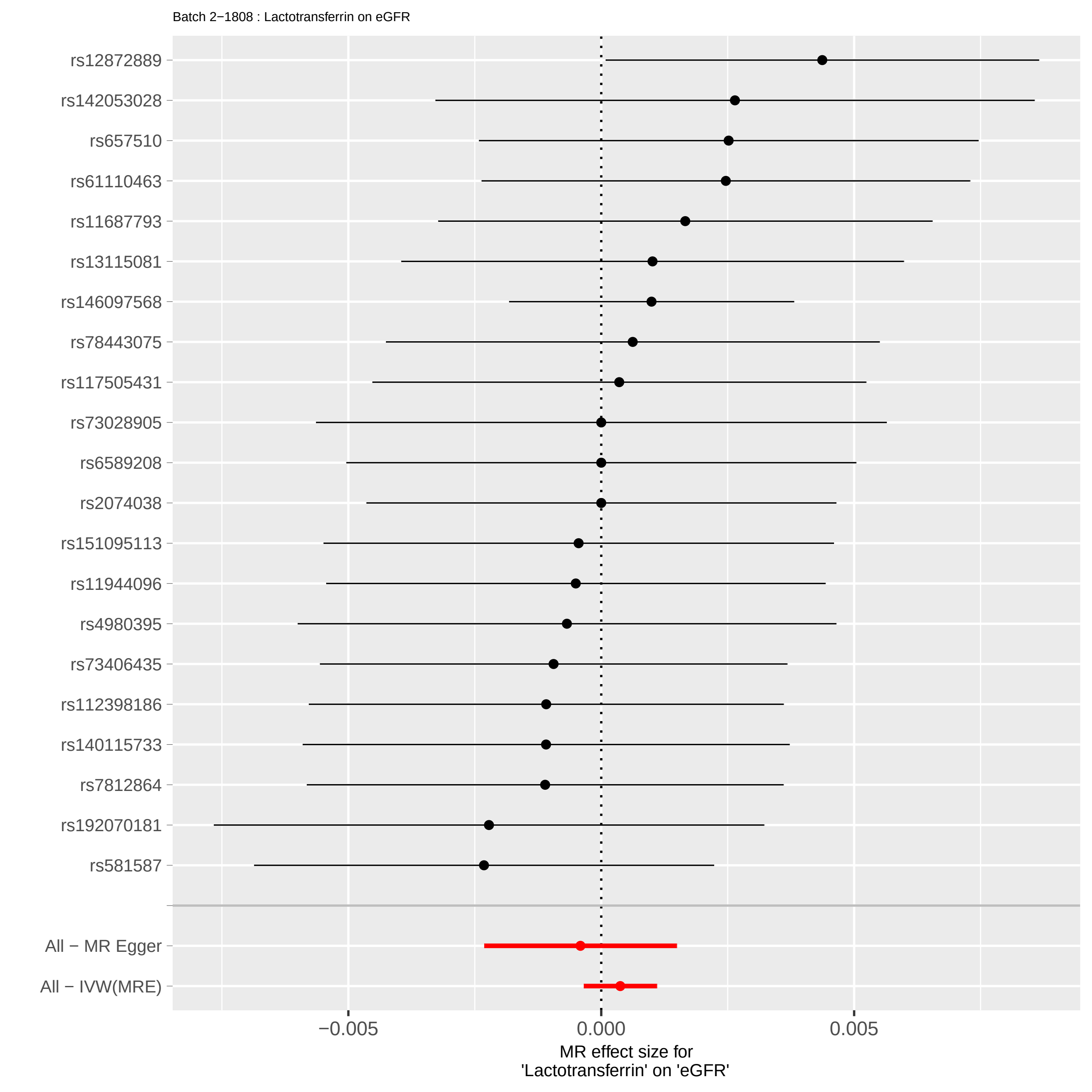
Batch 2−1808 : Lactotransferrin on eGFR
rs12872889
rs142053028
rs657510
rs61110463
rs11687793
rs13115081
rs146097568
rs78443075
rs117505431
rs73028905
rs6589208
rs2074038
rs151095113
rs11944096
rs4980395
rs73406435
rs112398186
rs140115733
rs7812864
rs192070181
rs581587
All − MR Egger
All − IVW(MRE)
−0.005
0.000
0.005
MR effect size for
'Lactotransferrin' on 'eGFR'

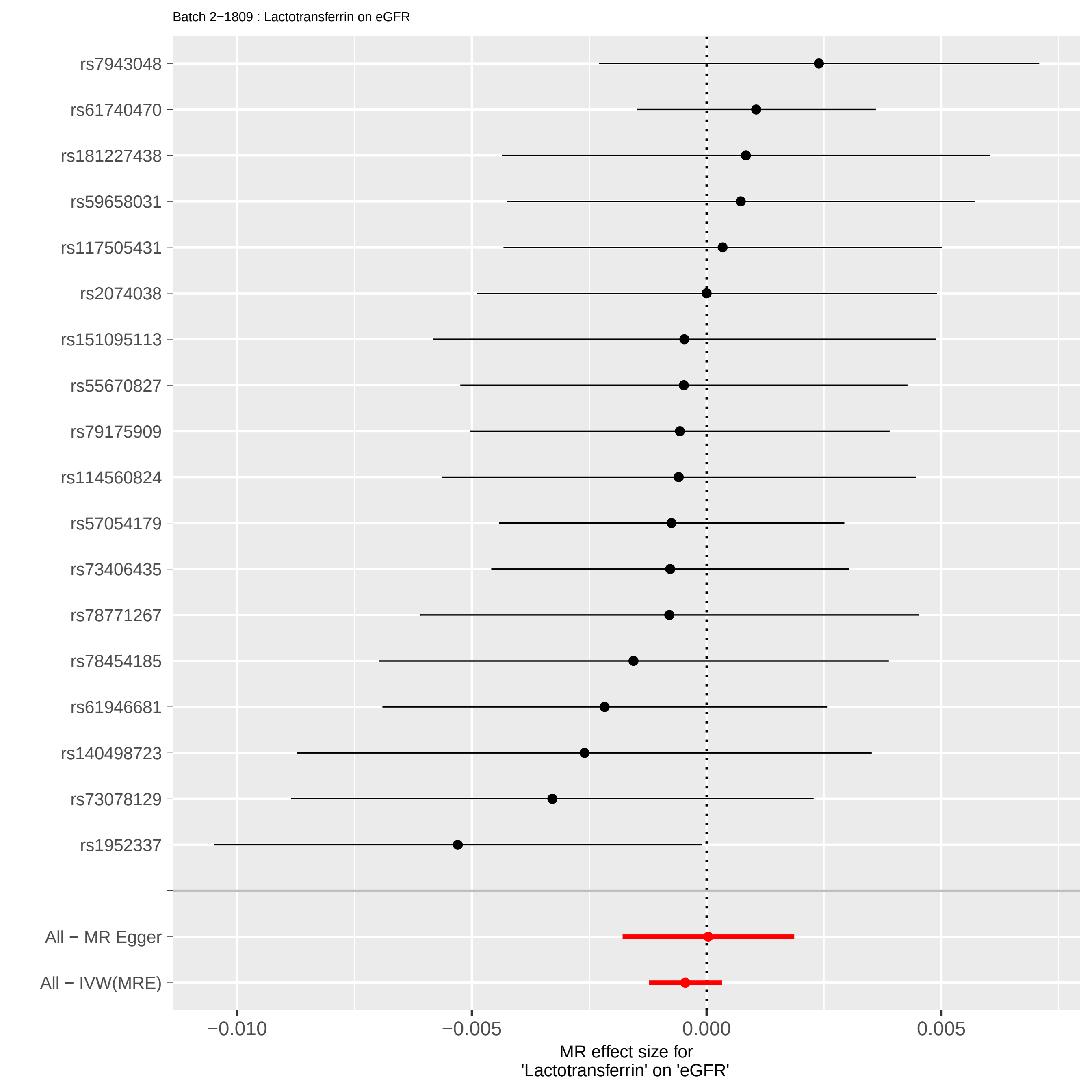
Batch 2−1809 : Lactotransferrin on eGFR
rs7943048
rs61740470
rs181227438
rs59658031
rs117505431
rs2074038
rs151095113
rs55670827
rs79175909
rs114560824
rs57054179
rs73406435
rs78771267
rs78454185
rs61946681
rs140498723
rs73078129
rs1952337
All − MR Egger
All − IVW(MRE)
−0.010
−0.005
0.000
0.005
MR effect size for
'Lactotransferrin' on 'eGFR'

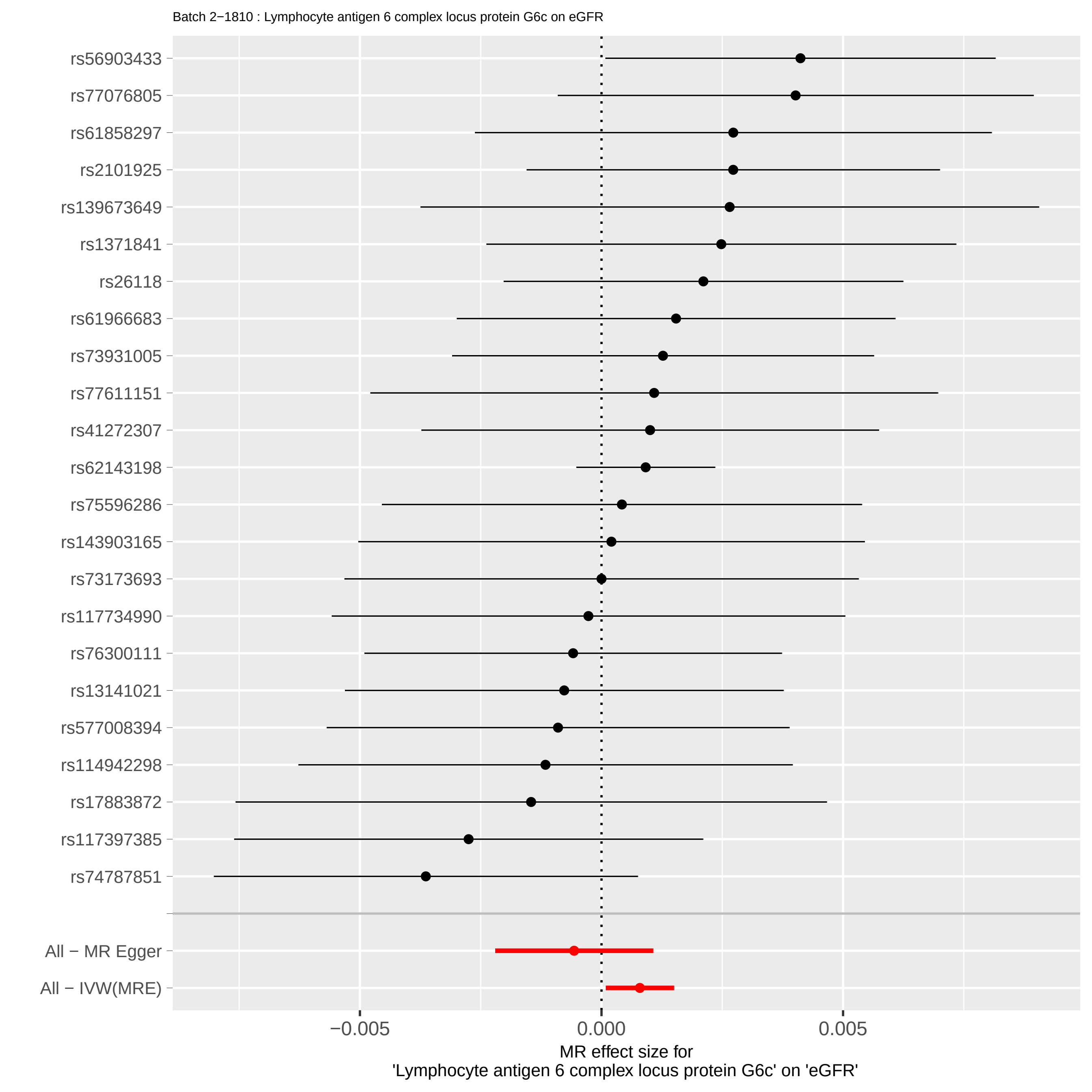

Batch 2−1810 : Lymphocyte antigen 6 complex locus protein G6c on eGFR
rs56903433
rs77076805
rs61858297
rs2101925
rs139673649
rs1371841
rs26118
rs61966683
rs73931005
rs77611151
rs41272307
rs62143198
rs75596286
rs143903165
rs73173693
rs117734990
rs76300111
rs13141021
rs577008394
rs114942298
rs17883872
rs117397385
rs74787851
All − MR Egger
All − IVW(MRE)
−0.005
0.000
0.005
MR effect size for
'Lymphocyte antigen 6 complex locus protein G6c' on 'eGFR'

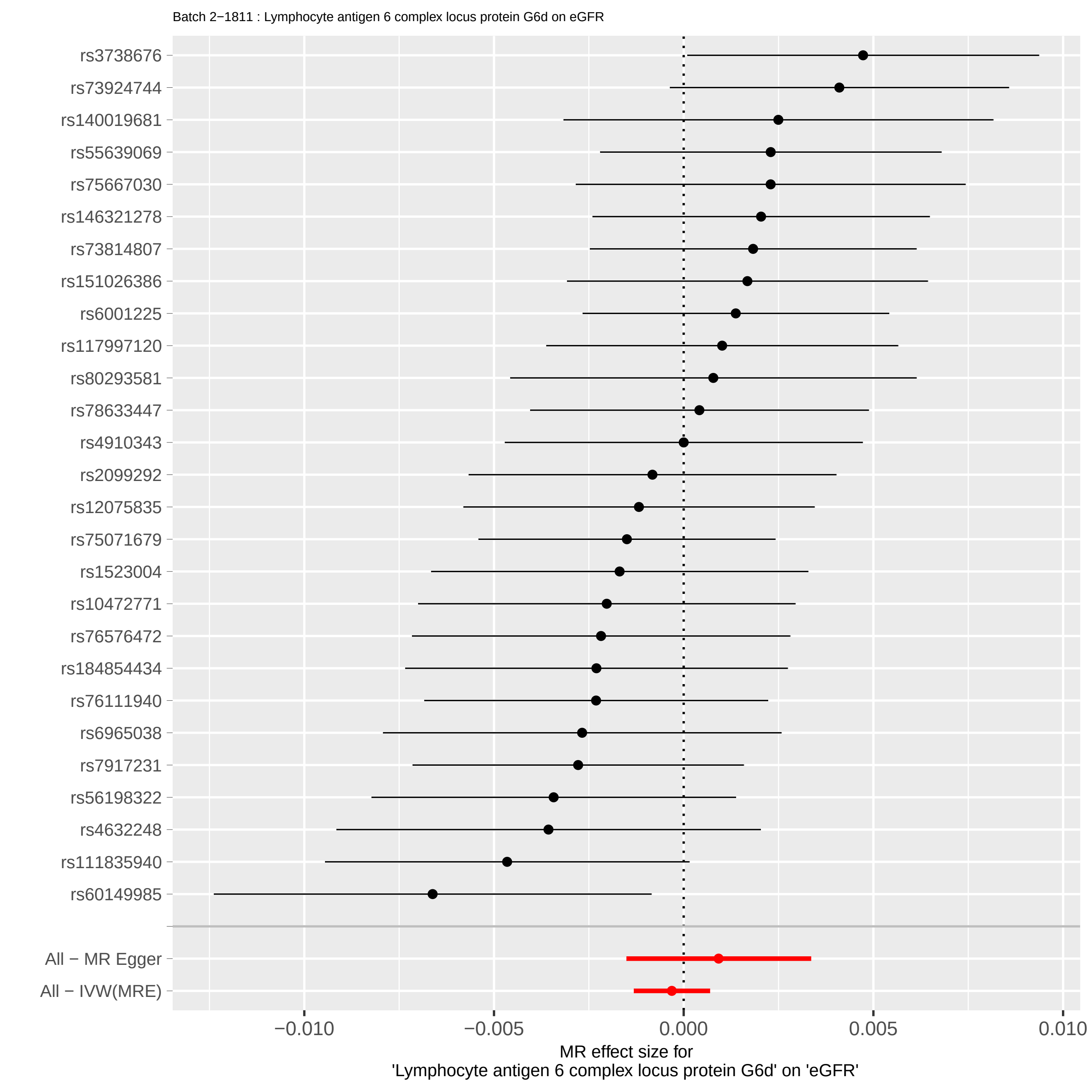
Batch 2−1811 : Lymphocyte antigen 6 complex locus protein G6d on eGFR
rs3738676
rs73924744
rs140019681
rs55639069
rs75667030
rs146321278
rs73814807
rs151026386
rs6001225
rs117997120
rs80293581
rs78633447
rs4910343
rs2099292
rs12075835
rs75071679
rs1523004
rs10472771
rs76576472
rs184854434
rs76111940
rs6965038
rs7917231
rs56198322
rs4632248
rs111835940
rs60149985
All − MR Egger
All − IVW(MRE)
−0.010
−0.005
0.000
0.005
0.010
MR effect size for
'Lymphocyte antigen 6 complex locus protein G6d' on 'eGFR'

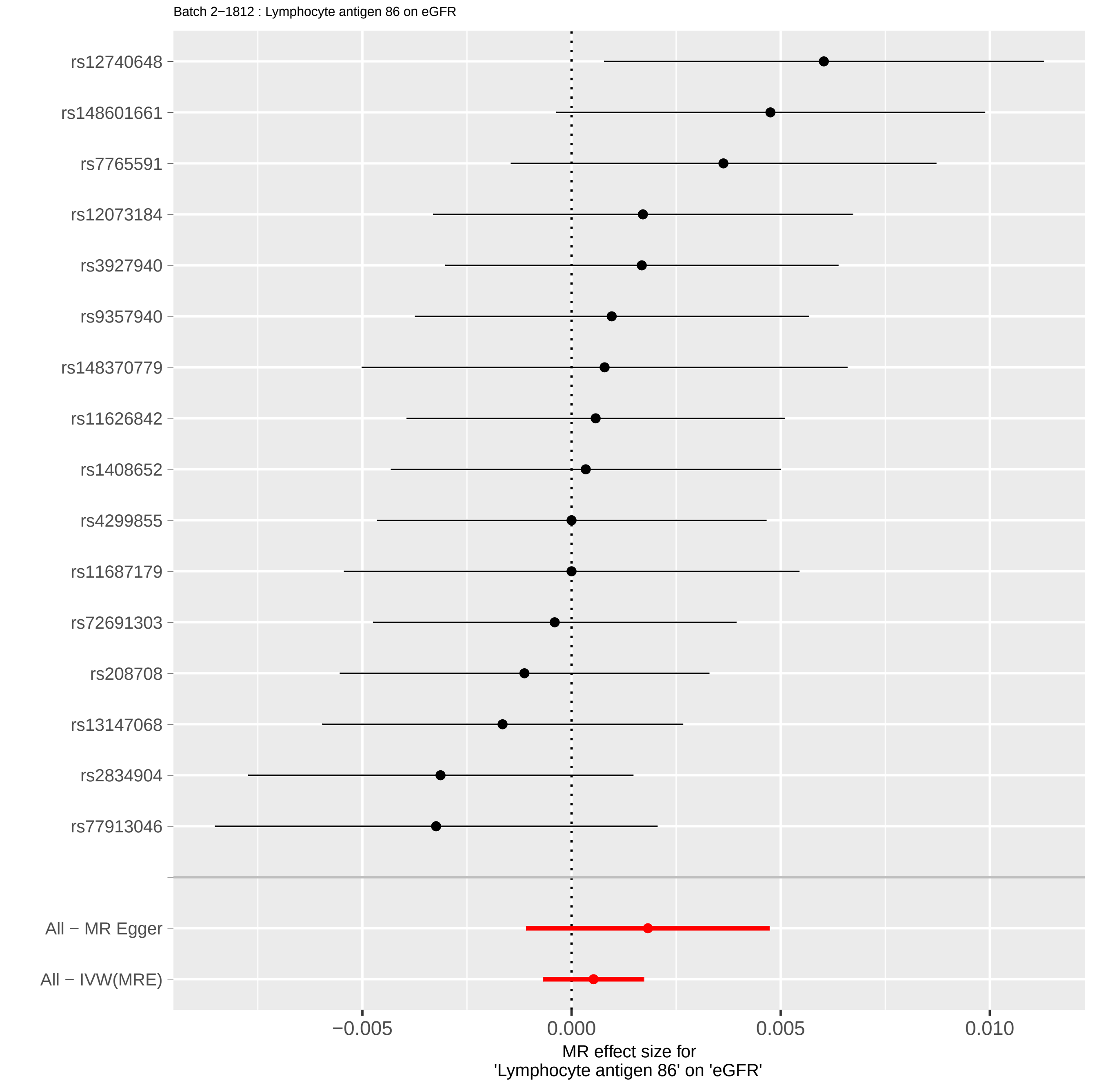

Batch 2−1812 : Lymphocyte antigen 86 on eGFR
rs12740648
rs148601661
rs7765591
rs12073184
rs3927940
rs9357940
rs148370779
rs11626842
rs1408652
rs4299855
rs11687179
rs72691303
rs208708
rs13147068
rs2834904
rs77913046
All − MR Egger
All − IVW(MRE)
−0.005
0.000
0.005
0.010
MR effect size for
'Lymphocyte antigen 86' on 'eGFR'

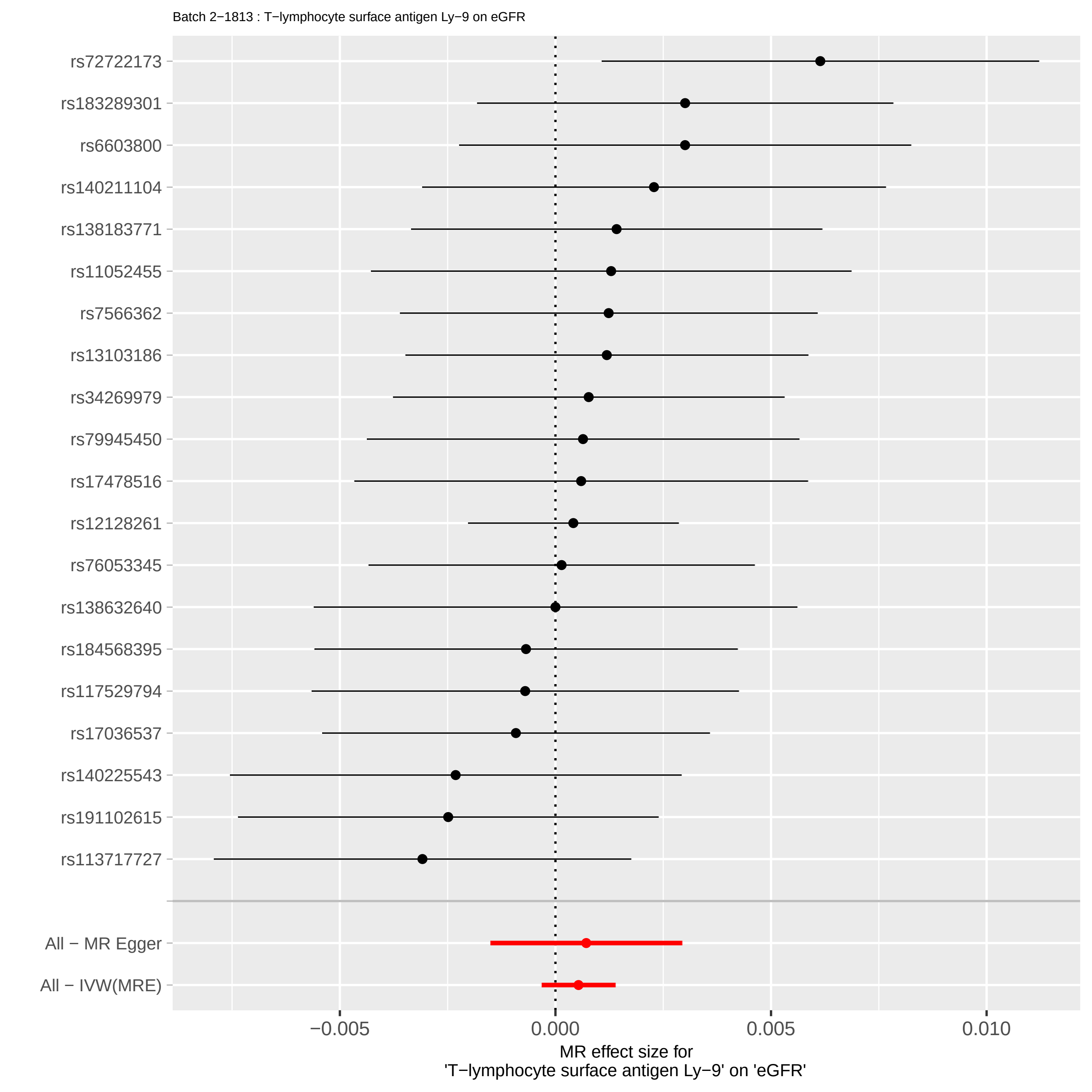

Batch 2−1813 : T−lymphocyte surface antigen Ly−9 on eGFR
rs72722173
rs183289301
rs6603800
rs140211104
rs138183771
rs11052455
rs7566362
rs13103186
rs34269979
rs79945450
rs17478516
rs12128261
rs76053345
rs138632640
rs184568395
rs117529794
rs17036537
rs140225543
rs191102615
rs113717727
All − MR Egger
All − IVW(MRE)
−0.005
0.000
0.005
0.010
MR effect size for
'T−lymphocyte surface antigen Ly−9' on 'eGFR'

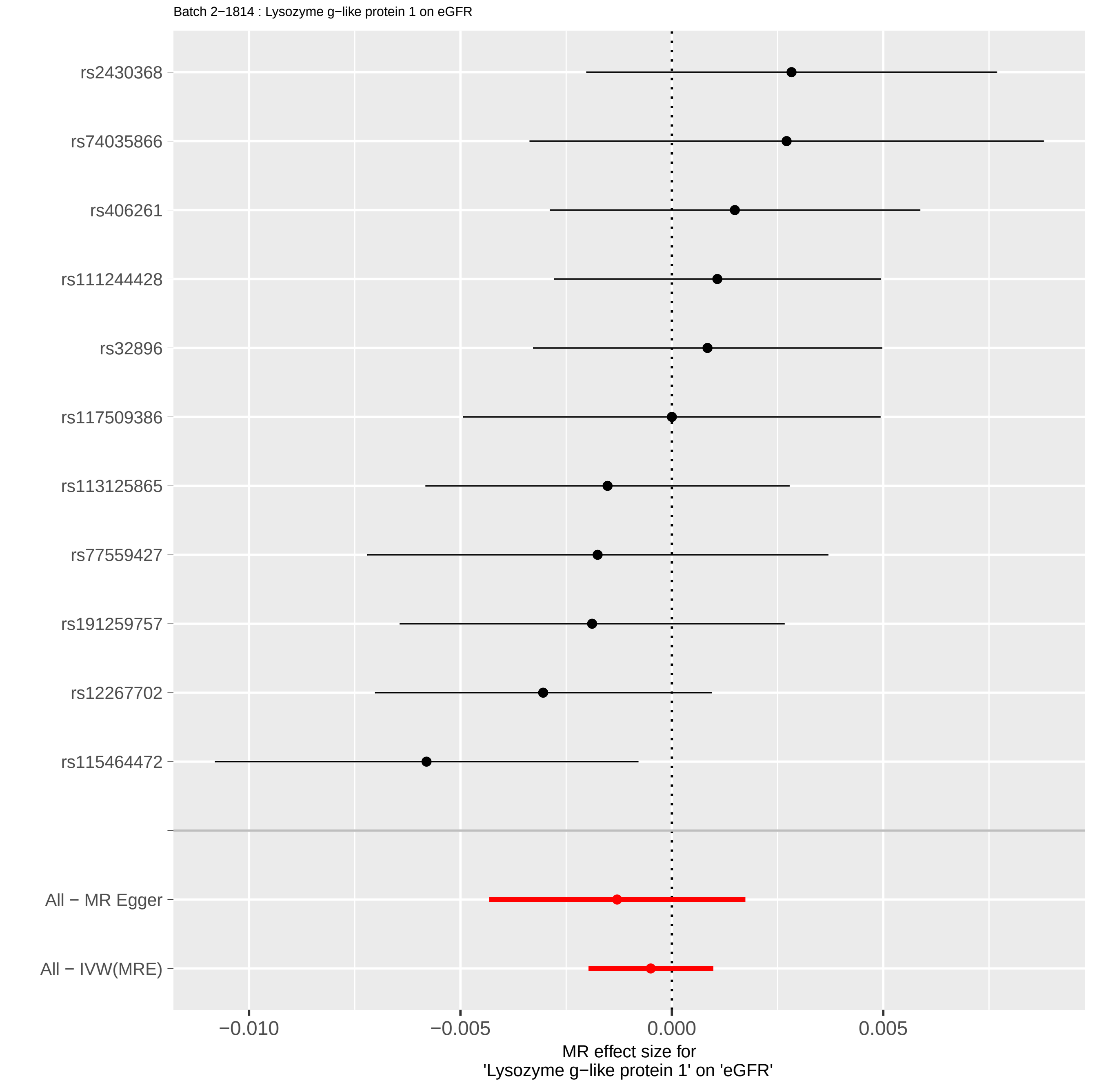

Batch 2−1814 : Lysozyme g−like protein 1 on eGFR
rs2430368
rs74035866
rs406261
rs111244428
rs32896
rs117509386
rs113125865
rs77559427
rs191259757
rs12267702
rs115464472
All − MR Egger
All − IVW(MRE)
−0.010
−0.005
0.000
0.005
MR effect size for
'Lysozyme g−like protein 1' on 'eGFR'

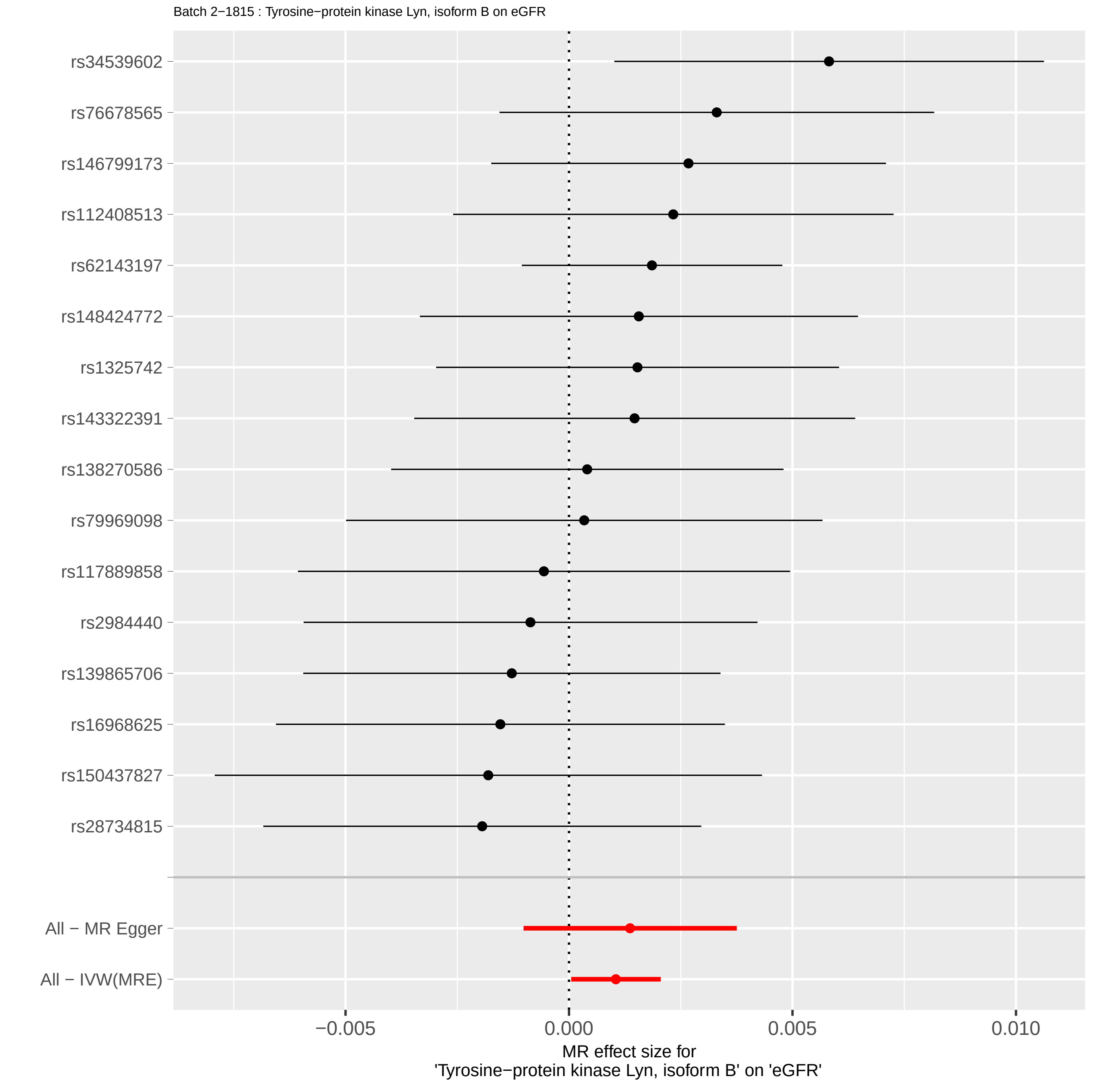

Batch 2−1815 : Tyrosine−protein kinase Lyn, isoform B on eGFR
rs34539602
rs76678565
rs146799173
rs112408513
rs62143197
rs148424772
rs1325742
rs143322391
rs138270586
rs79969098
rs117889858
rs2984440
rs139865706
rs16968625
rs150437827
rs28734815
All − MR Egger
All − IVW(MRE)
−0.005
0.000
0.005
0.010
MR effect size for
'Tyrosine−protein kinase Lyn, isoform B' on 'eGFR'

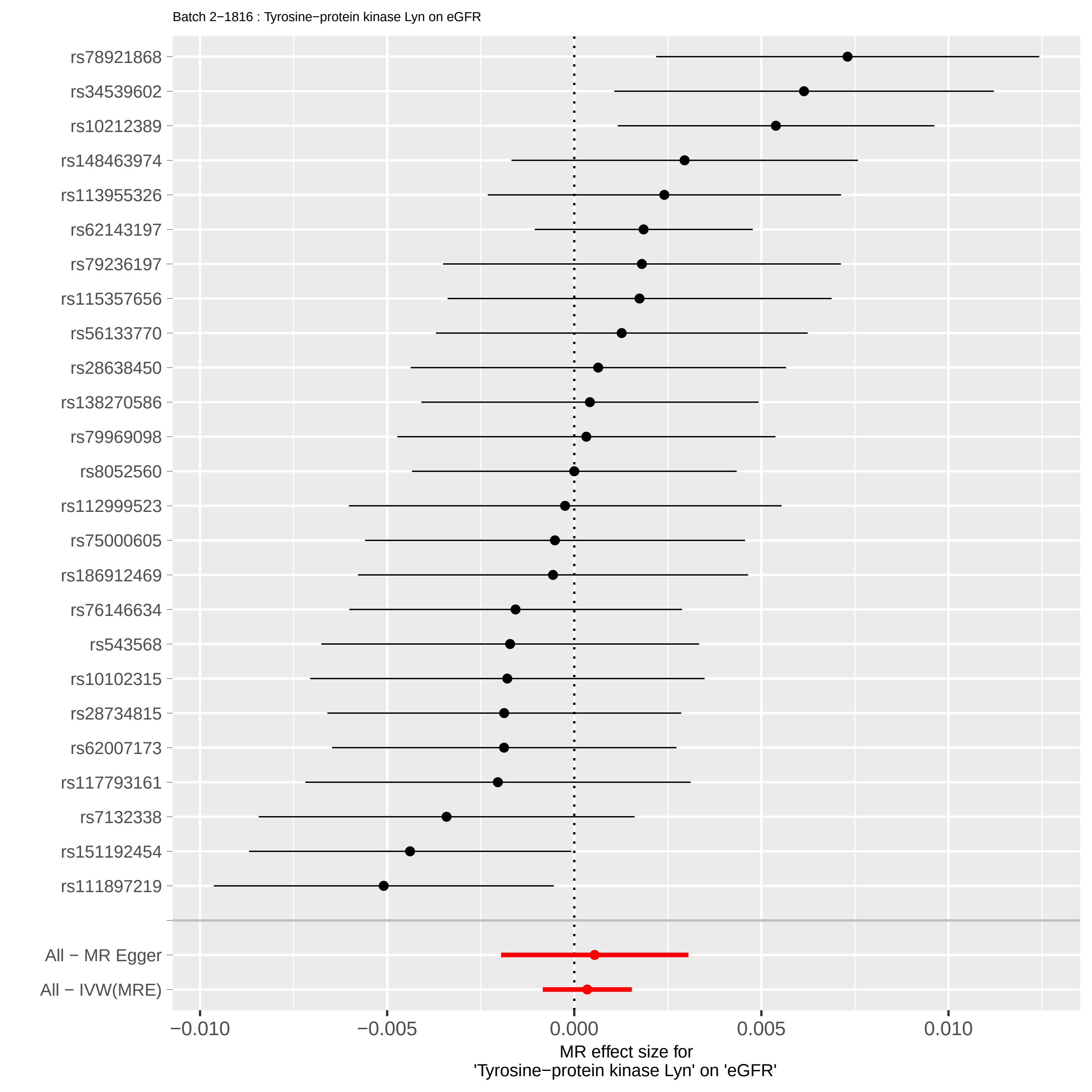
Batch 2−1816 : Tyrosine−protein kinase Lyn on eGFR
rs78921868
rs34539602
rs10212389
rs148463974
rs113955326
rs62143197
rs79236197
rs115357656
rs56133770
rs28638450
rs138270586
rs79969098
rs8052560
rs112999523
rs75000605
rs186912469
rs76146634
rs543568
rs10102315
rs28734815
rs62007173
rs117793161
rs7132338
rs151192454
rs111897219
All − MR Egger
All − IVW(MRE)
−0.010
−0.005
0.000
0.005
0.010
MR effect size for
'Tyrosine−protein kinase Lyn' on 'eGFR'

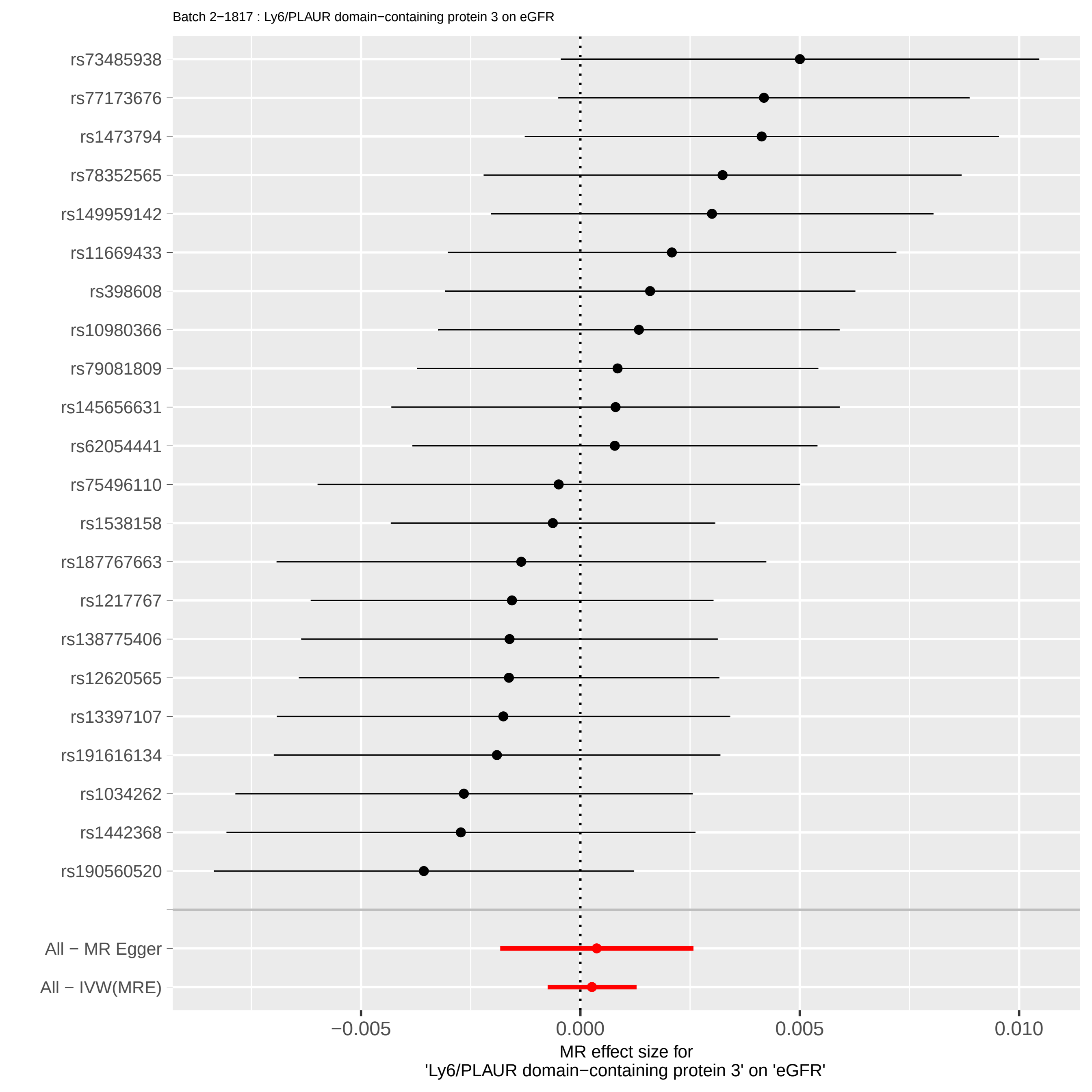

Batch 2−1817 : Ly6/PLAUR domain−containing protein 3 on eGFR
rs73485938
rs77173676
rs1473794
rs78352565
rs149959142
rs11669433
rs398608
rs10980366
rs79081809
rs145656631
rs62054441
rs75496110
rs1538158
rs187767663
rs1217767
rs138775406
rs12620565
rs13397107
rs191616134
rs1034262
rs1442368
rs190560520
All − MR Egger
All − IVW(MRE)
−0.005
0.000
0.005
0.010
MR effect size for
'Ly6/PLAUR domain−containing protein 3' on 'eGFR'

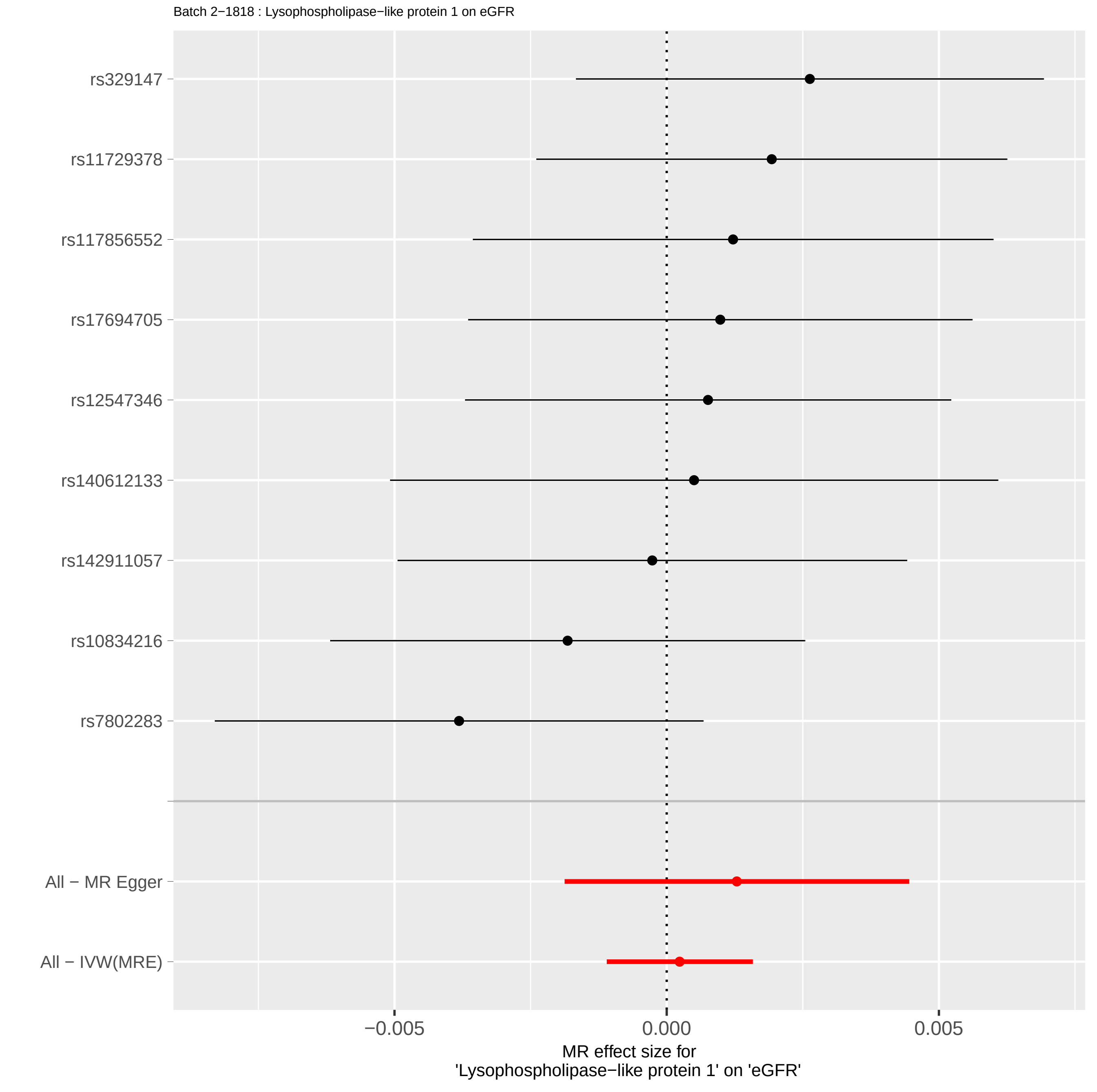
Batch 2−1818 : Lysophospholipase−like protein 1 on eGFR
rs329147
rs11729378
rs117856552
rs17694705
rs12547346
rs140612133
rs142911057
rs10834216
rs7802283
All − MR Egger
All − IVW(MRE)
−0.005
0.000
0.005
MR effect size for
'Lysophospholipase−like protein 1' on 'eGFR'

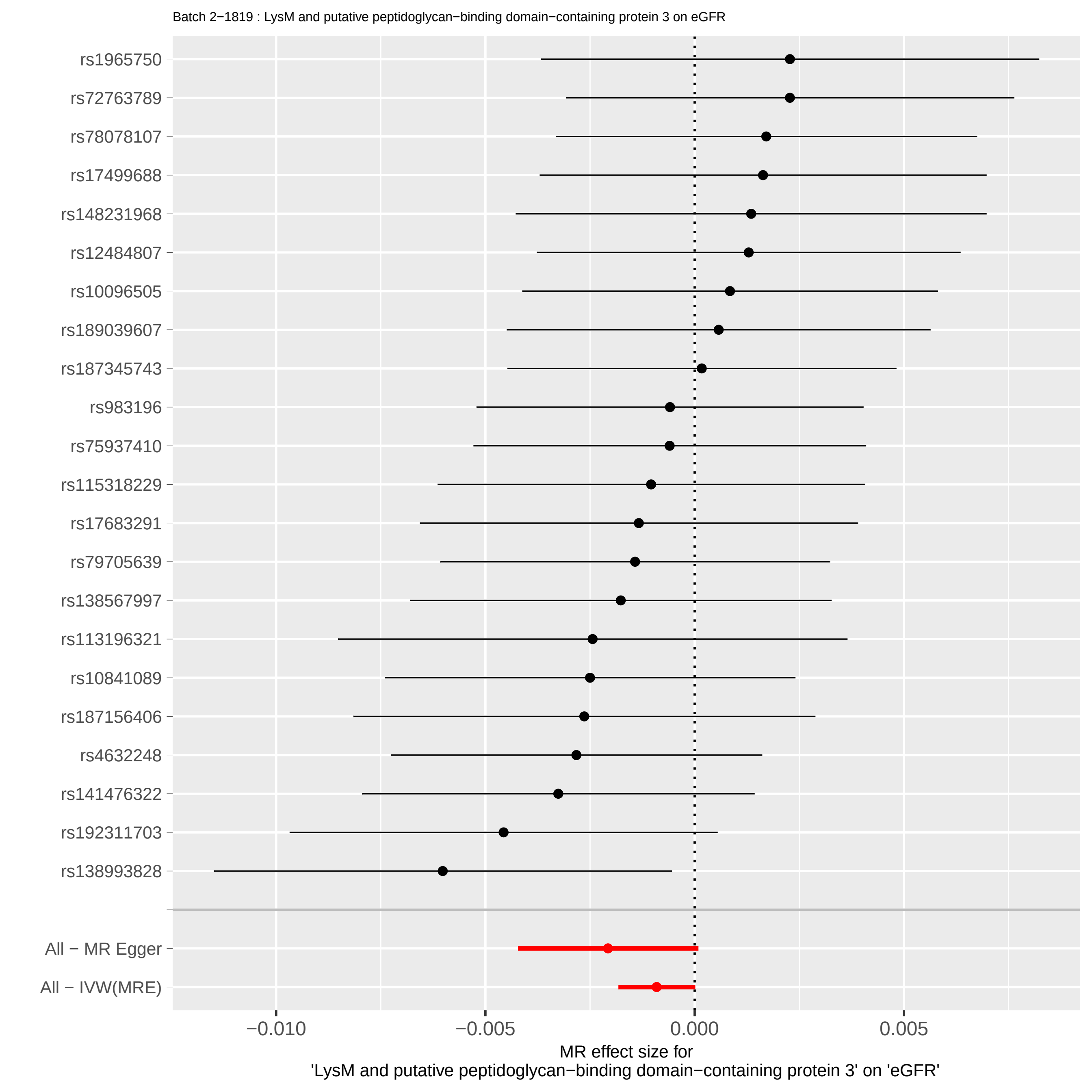

Batch 2−1819 : LysM and putative peptidoglycan−binding domain−containing protein 3 on eGFR
rs1965750
rs72763789
rs78078107
rs17499688
rs148231968
rs12484807
rs10096505
rs189039607
rs187345743
rs983196
rs75937410
rs115318229
rs17683291
rs79705639
rs138567997
rs113196321
rs10841089
rs187156406
rs4632248
rs141476322
rs192311703
rs138993828
All − MR Egger
All − IVW(MRE)
−0.010
−0.005
0.000
0.005
MR effect size for
'LysM and putative peptidoglycan−binding domain−containing protein 3' on 'eGFR'

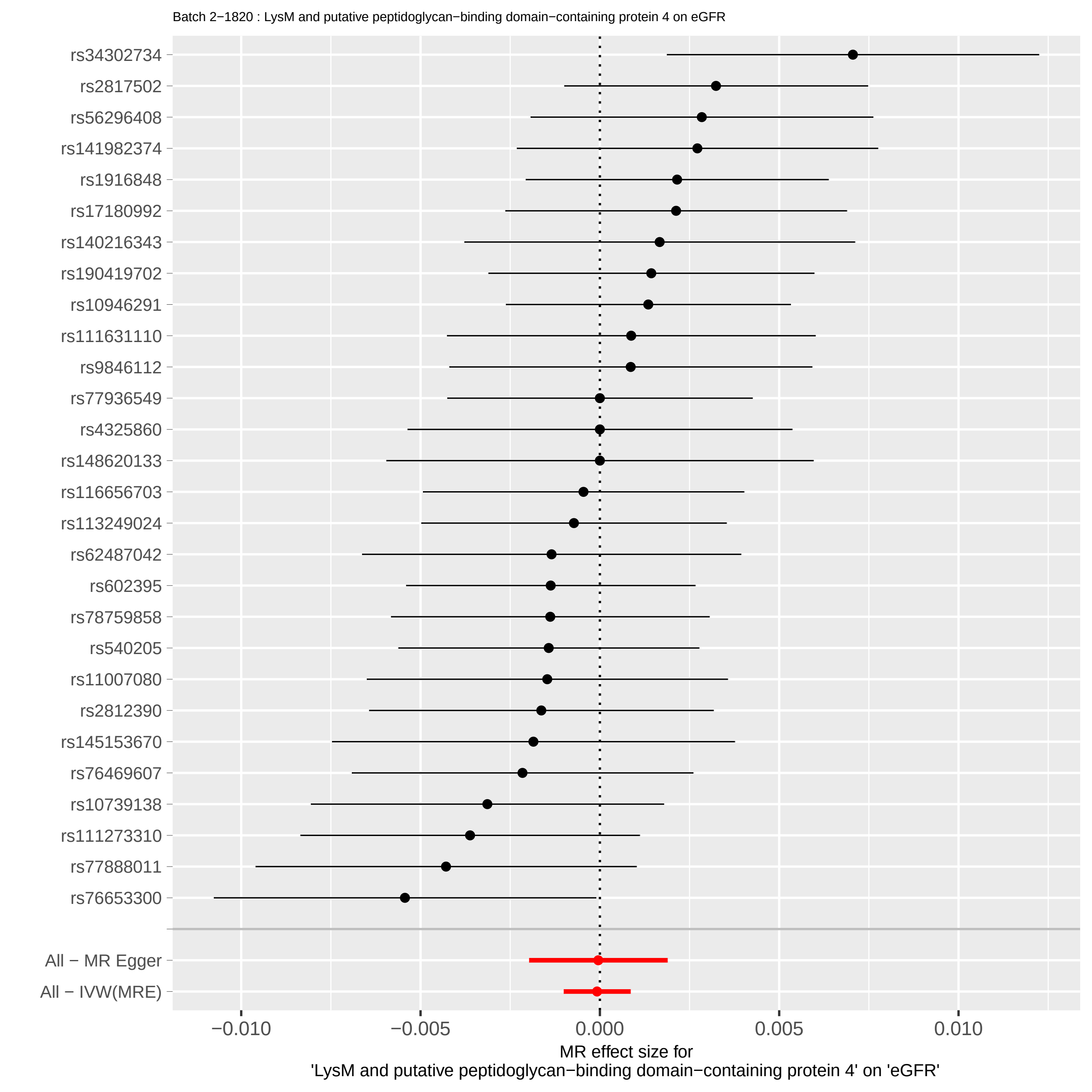

Batch 2−1820 : LysM and putative peptidoglycan−binding domain−containing protein 4 on eGFR
rs34302734
rs2817502
rs56296408
rs141982374
rs1916848
rs17180992
rs140216343
rs190419702
rs10946291
rs111631110
rs9846112
rs77936549
rs4325860
rs148620133
rs116656703
rs113249024
rs62487042
rs602395
rs78759858
rs540205
rs11007080
rs2812390
rs145153670
rs76469607
rs10739138
rs111273310
rs77888011
rs76653300
All − MR Egger
All − IVW(MRE)
−0.010
−0.005
0.000
0.005
0.010
MR effect size for
'LysM and putative peptidoglycan−binding domain−containing protein 4' on 'eGFR'

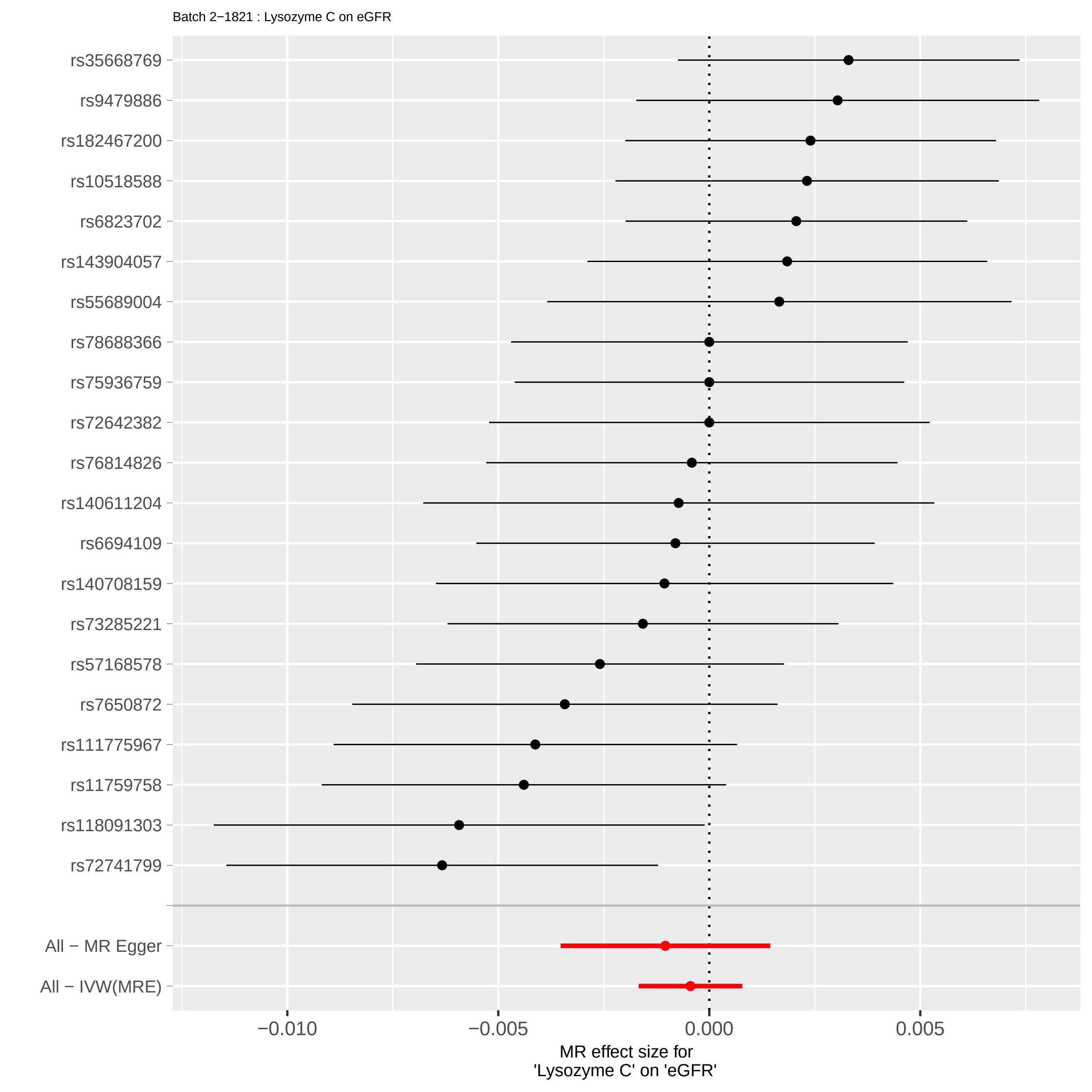

Batch 2−1821 : Lysozyme C on eGFR
rs35668769
rs9479886
rs182467200
rs10518588
rs6823702
rs143904057
rs55689004
rs78688366
rs75936759
rs72642382
rs76814826
rs140611204
rs6694109
rs140708159
rs73285221
rs57168578
rs7650872
rs111775967
rs11759758
rs118091303
rs72741799
All − MR Egger
All − IVW(MRE)
−0.010
−0.005
0.000
0.005
MR effect size for
'Lysozyme C' on 'eGFR'

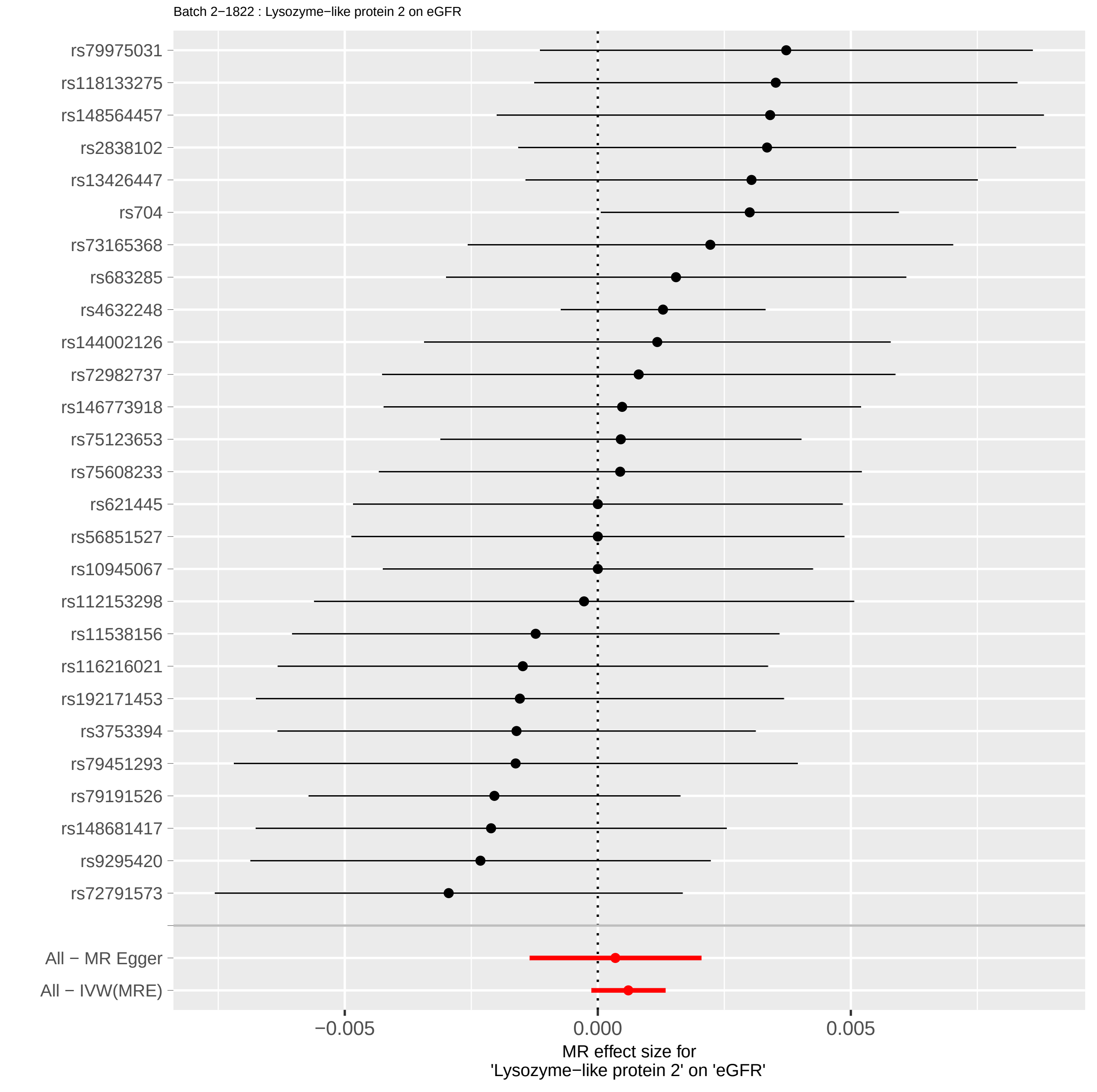

Batch 2−1822 : Lysozyme−like protein 2 on eGFR
rs79975031
rs118133275
rs148564457
rs2838102
rs13426447
rs704
rs73165368
rs683285
rs4632248
rs144002126
rs72982737
rs146773918
rs75123653
rs75608233
rs621445
rs56851527
rs10945067
rs112153298
rs11538156
rs116216021
rs192171453
rs3753394
rs79451293
rs79191526
rs148681417
rs9295420
rs72791573
All − MR Egger
All − IVW(MRE)
−0.005
0.000
0.005
MR effect size for
'Lysozyme−like protein 2' on 'eGFR'

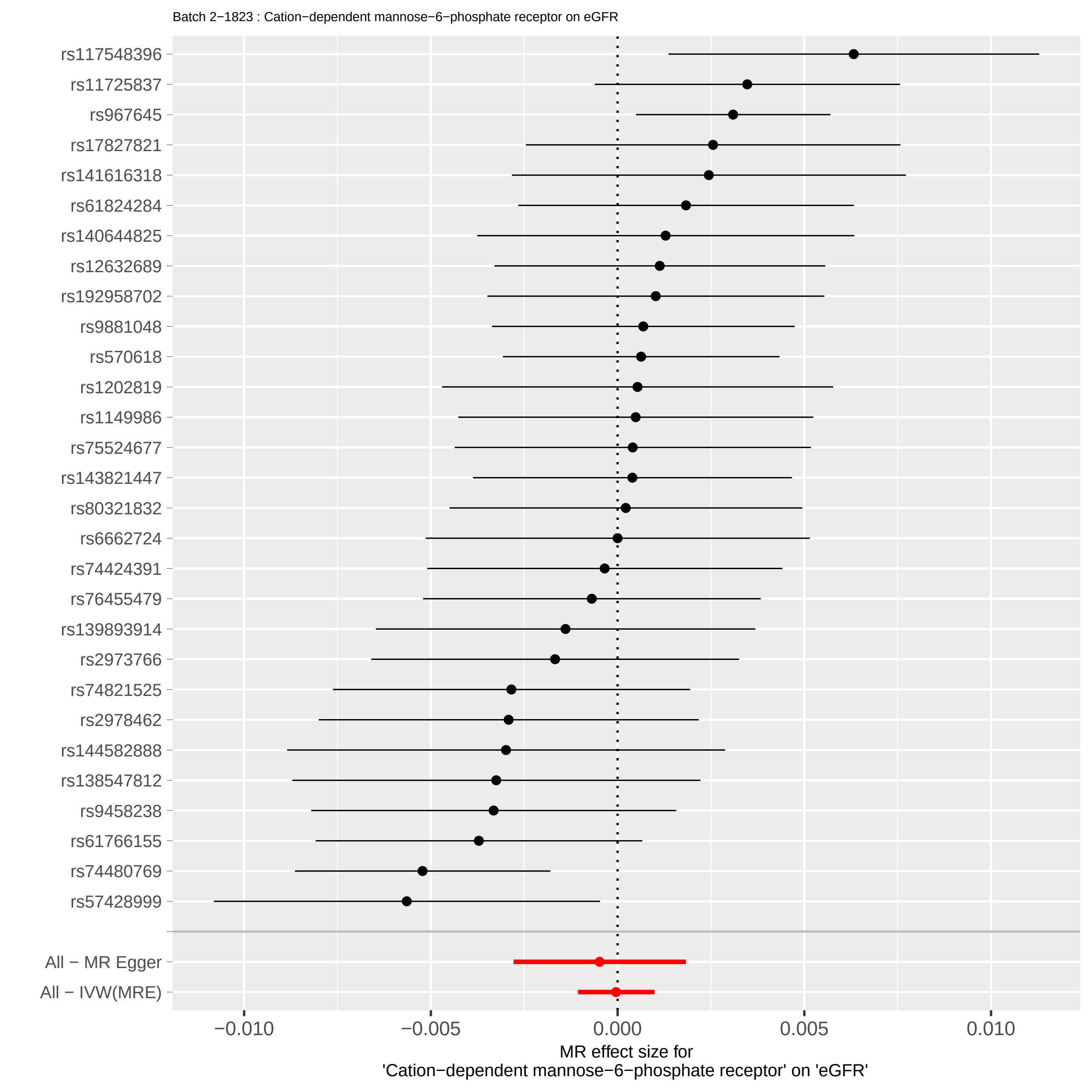
Batch 2−1823 : Cation−dependent mannose−6−phosphate receptor on eGFR
rs117548396
rs11725837
rs967645
rs17827821
rs141616318
rs61824284
rs140644825
rs12632689
rs192958702
rs9881048
rs570618
rs1202819
rs1149986
rs75524677
rs143821447
rs80321832
rs6662724
rs74424391
rs76455479
rs139893914
rs2973766
rs74821525
rs2978462
rs144582888
rs138547812
rs9458238
rs61766155
rs74480769
rs57428999
All − MR Egger
All − IVW(MRE)
−0.010
−0.005
0.000
0.005
0.010
MR effect size for
'Cation−dependent mannose−6−phosphate receptor' on 'eGFR'

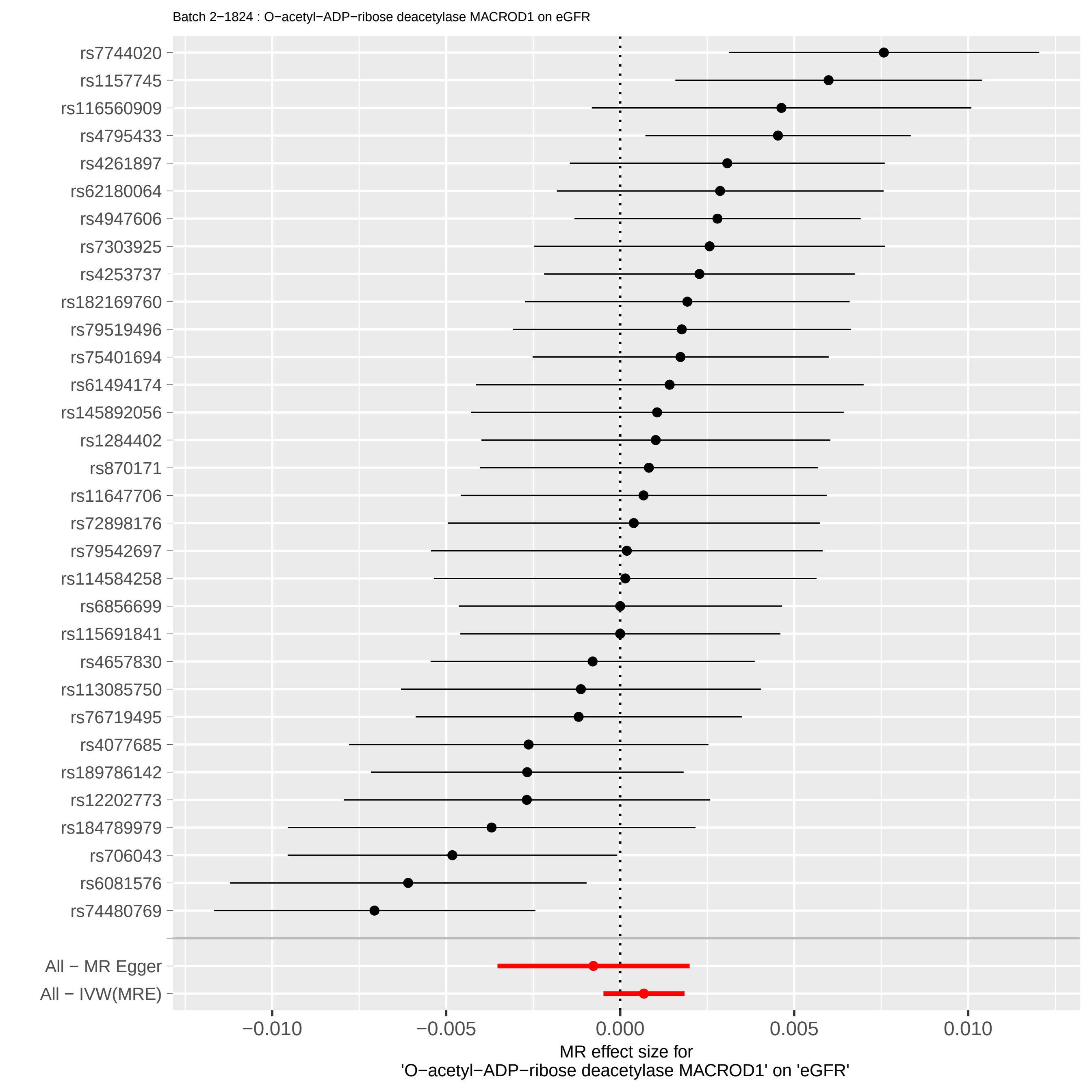

Batch 2−1824 : O−acetyl−ADP−ribose deacetylase MACROD1 on eGFR
rs7744020
rs1157745
rs116560909
rs4795433
rs4261897
rs62180064
rs4947606
rs7303925
rs4253737
rs182169760
rs79519496
rs75401694
rs61494174
rs145892056
rs1284402
rs870171
rs11647706
rs72898176
rs79542697
rs114584258
rs6856699
rs115691841
rs4657830
rs113085750
rs76719495
rs4077685
rs189786142
rs12202773
rs184789979
rs706043
rs6081576
rs74480769
All − MR Egger
All − IVW(MRE)
−0.010
−0.005
0.000
0.005
0.010
MR effect size for
'O−acetyl−ADP−ribose deacetylase MACROD1' on 'eGFR'

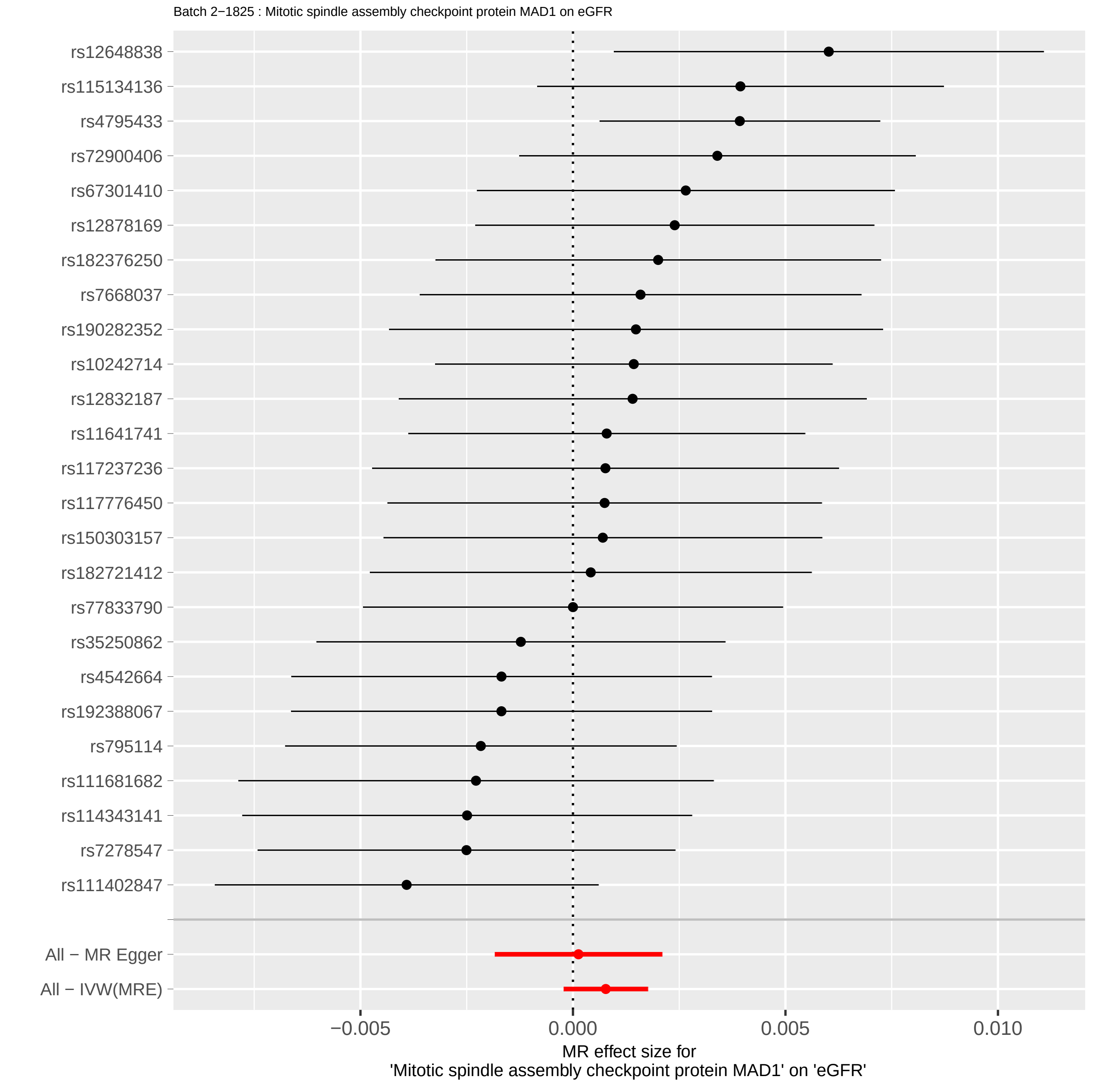

Batch 2−1825 : Mitotic spindle assembly checkpoint protein MAD1 on eGFR
rs12648838
rs115134136
rs4795433
rs72900406
rs67301410
rs12878169
rs182376250
rs7668037
rs190282352
rs10242714
rs12832187
rs11641741
rs117237236
rs117776450
rs150303157
rs182721412
rs77833790
rs35250862
rs4542664
rs192388067
rs795114
rs111681682
rs114343141
rs7278547
rs111402847
All − MR Egger
All − IVW(MRE)
−0.005
0.000
0.005
0.010
MR effect size for
'Mitotic spindle assembly checkpoint protein MAD1' on 'eGFR'

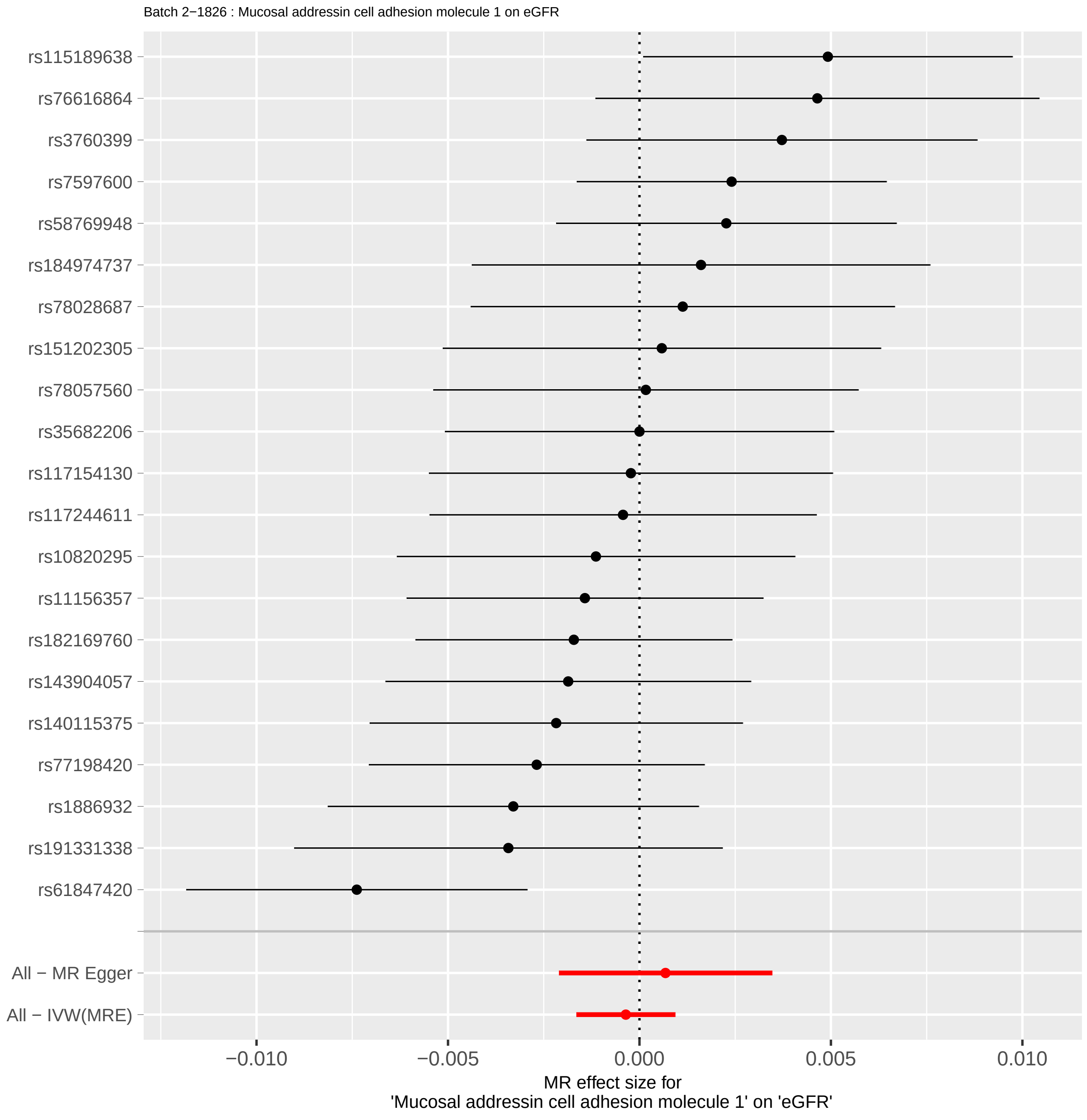

Batch 2−1826 : Mucosal addressin cell adhesion molecule 1 on eGFR
rs115189638
rs76616864
rs3760399
rs7597600
rs58769948
rs184974737
rs78028687
rs151202305
rs78057560
rs35682206
rs117154130
rs117244611
rs10820295
rs11156357
rs182169760
rs143904057
rs140115375
rs77198420
rs1886932
rs191331338
rs61847420
All − MR Egger
All − IVW(MRE)
−0.010
−0.005
0.000
0.005
0.010
MR effect size for
'Mucosal addressin cell adhesion molecule 1' on 'eGFR'

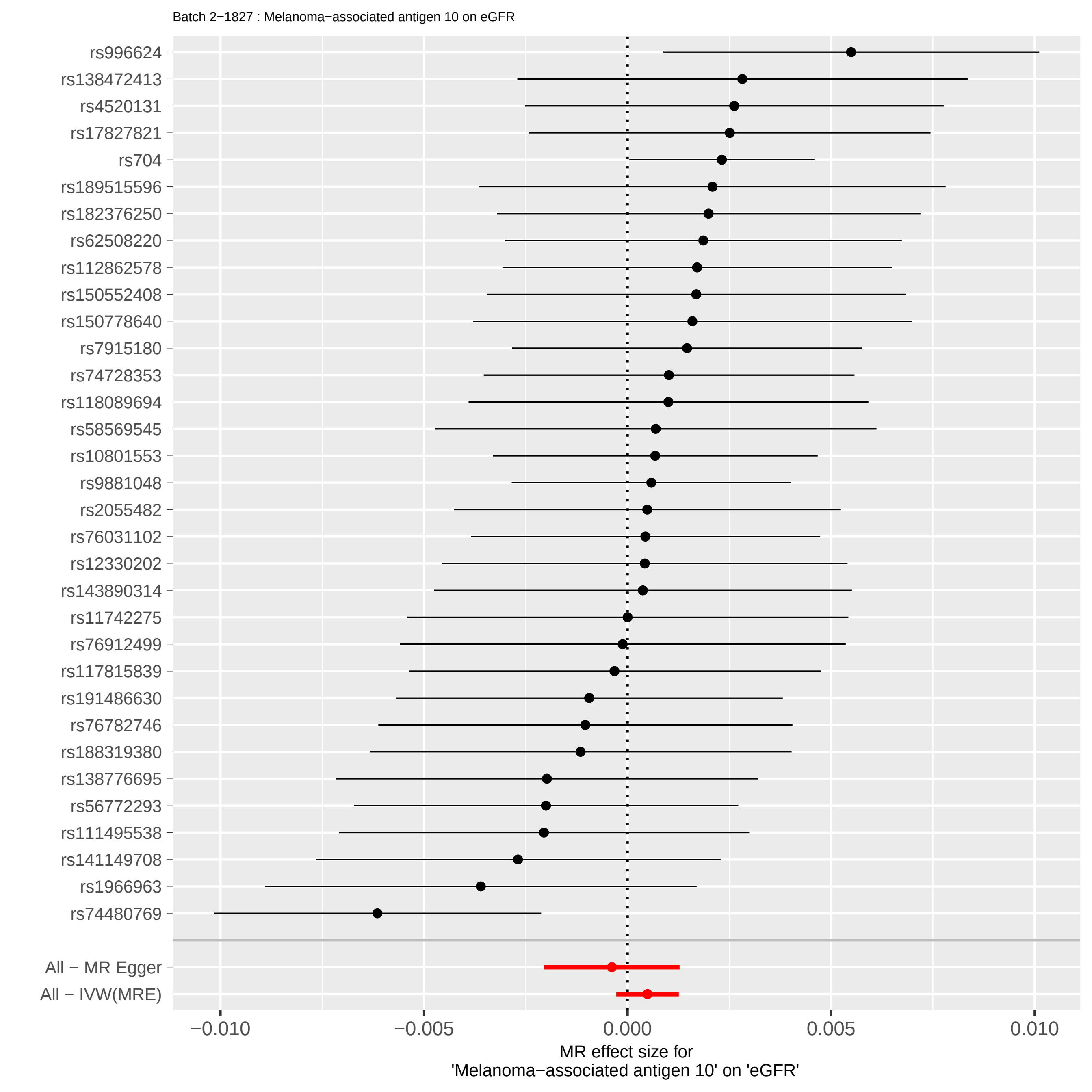
Batch 2−1827 : Melanoma−associated antigen 10 on eGFR
rs996624
rs138472413
rs4520131
rs17827821
rs704
rs189515596
rs182376250
rs62508220
rs112862578
rs150552408
rs150778640
rs7915180
rs74728353
rs118089694
rs58569545
rs10801553
rs9881048
rs2055482
rs76031102
rs12330202
rs143890314
rs11742275
rs76912499
rs117815839
rs191486630
rs76782746
rs188319380
rs138776695
rs56772293
rs111495538
rs141149708
rs1966963
rs74480769
All − MR Egger
All − IVW(MRE)
−0.010
−0.005
0.000
0.005
0.010
MR effect size for
'Melanoma−associated antigen 10' on 'eGFR'

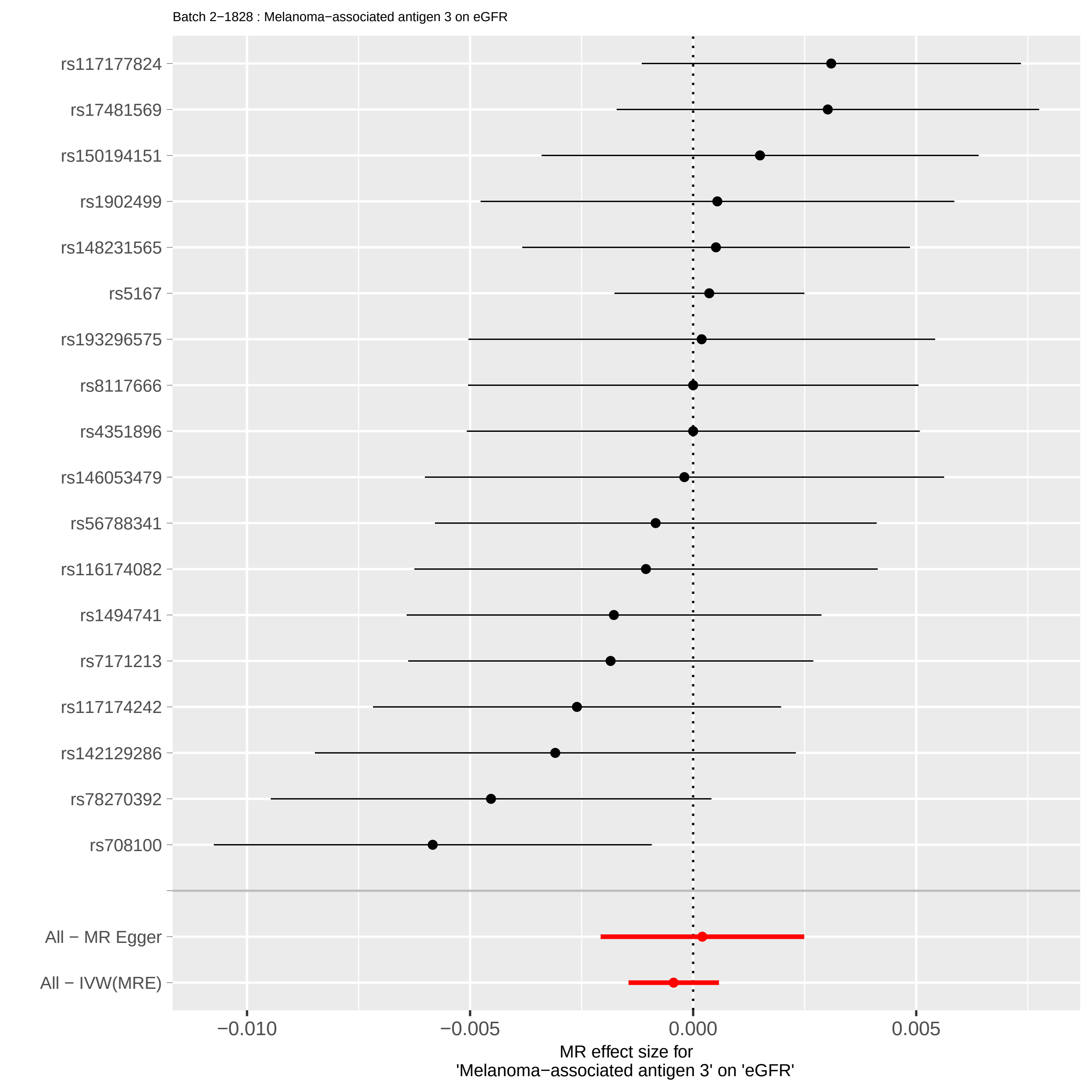
Batch 2−1828 : Melanoma−associated antigen 3 on eGFR
rs117177824
rs17481569
rs150194151
rs1902499
rs148231565
rs5167
rs193296575
rs8117666
rs4351896
rs146053479
rs56788341
rs116174082
rs1494741
rs7171213
rs117174242
rs142129286
rs78270392
rs708100
All − MR Egger
All − IVW(MRE)
−0.010
−0.005
0.000
0.005
MR effect size for
'Melanoma−associated antigen 3' on 'eGFR'

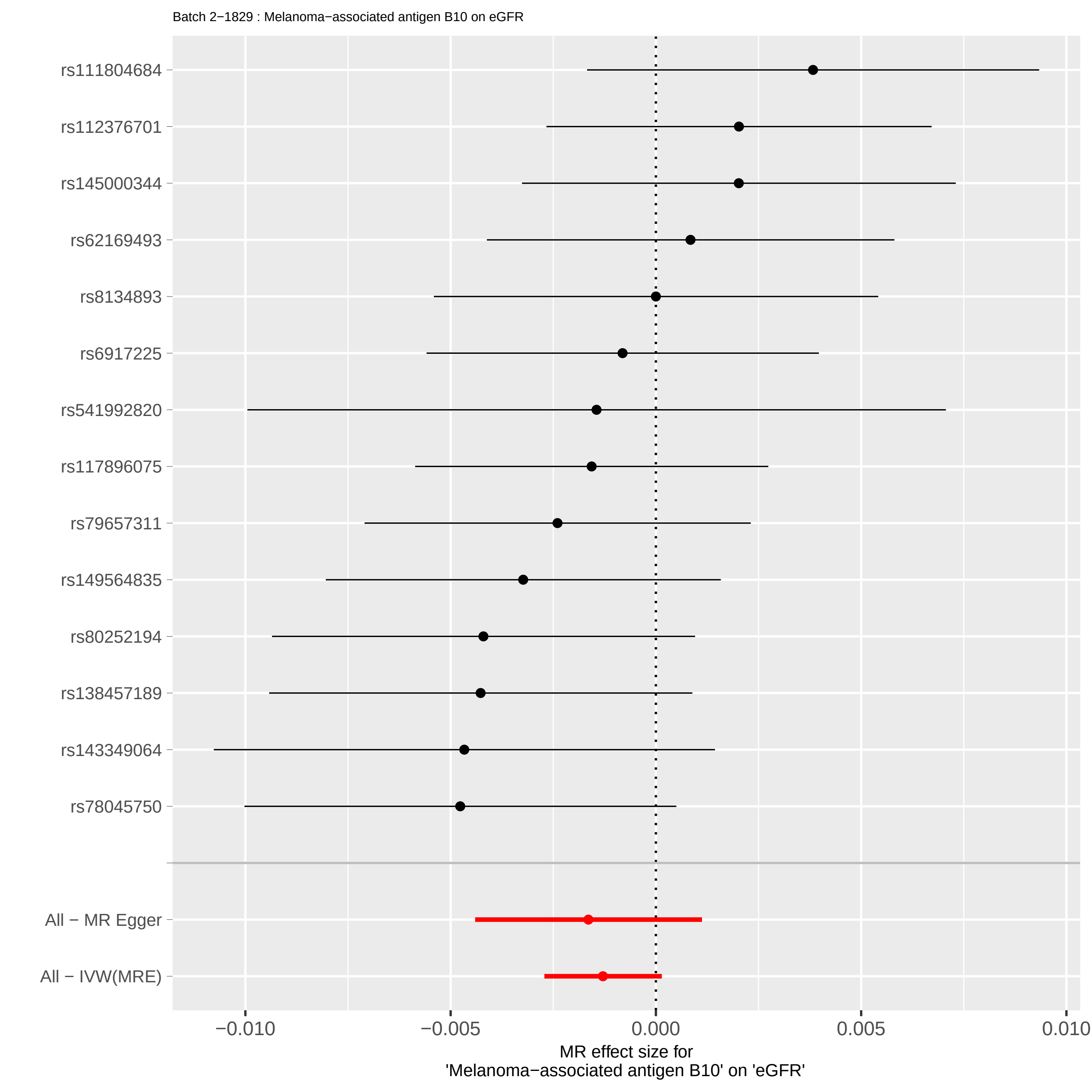

Batch 2−1829 : Melanoma−associated antigen B10 on eGFR
rs111804684
rs112376701
rs145000344
rs62169493
rs8134893
rs6917225
rs541992820
rs117896075
rs79657311
rs149564835
rs80252194
rs138457189
rs143349064
rs78045750
All − MR Egger
All − IVW(MRE)
−0.010
−0.005
0.000
0.005
0.010
MR effect size for
'Melanoma−associated antigen B10' on 'eGFR'

Batch 2−1830 : Membrane−associated guanylate kinase, WW and PDZ domain−containing protein 2 on eGFR

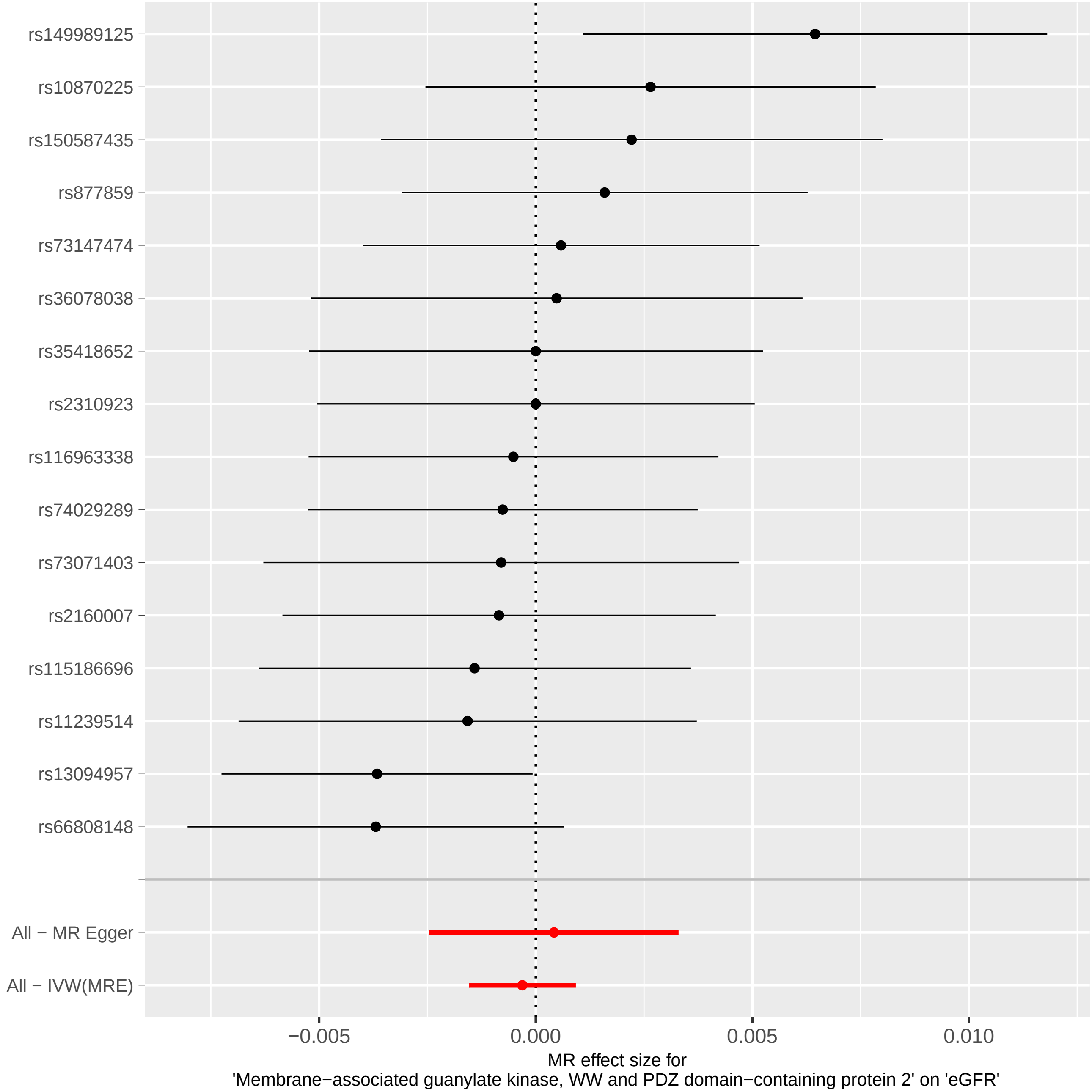

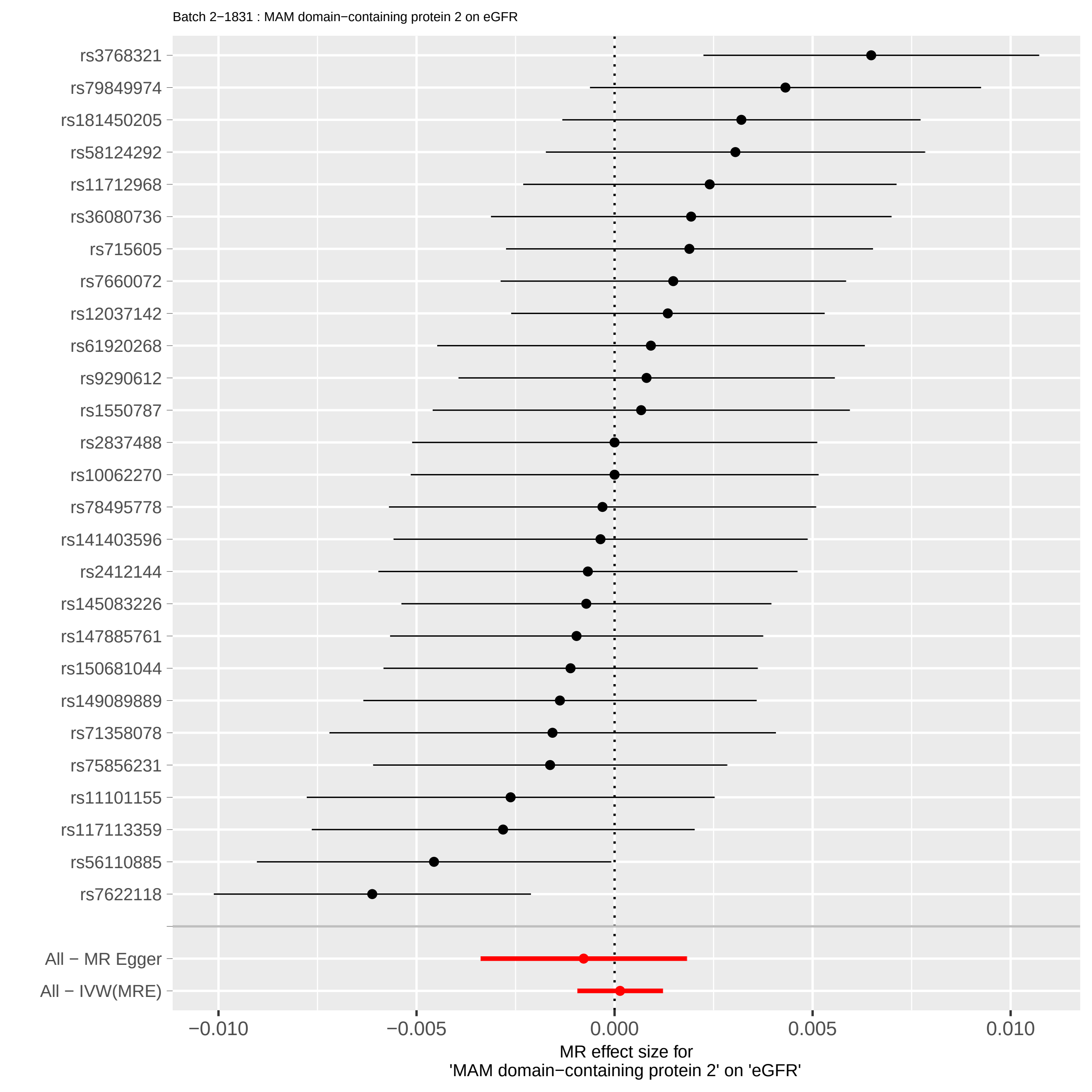

Batch 2−1831 : MAM domain−containing protein 2 on eGFR
rs3768321
rs79849974
rs181450205
rs58124292
rs11712968
rs36080736
rs715605
rs7660072
rs12037142
rs61920268
rs9290612
rs1550787
rs2837488
rs10062270
rs78495778
rs141403596
rs2412144
rs145083226
rs147885761
rs150681044
rs149089889
rs71358078
rs75856231
rs11101155
rs117113359
rs56110885
rs7622118
All − MR Egger
All − IVW(MRE)
−0.010
−0.005
0.000
0.005
0.010
MR effect size for
'MAM domain−containing protein 2' on 'eGFR'

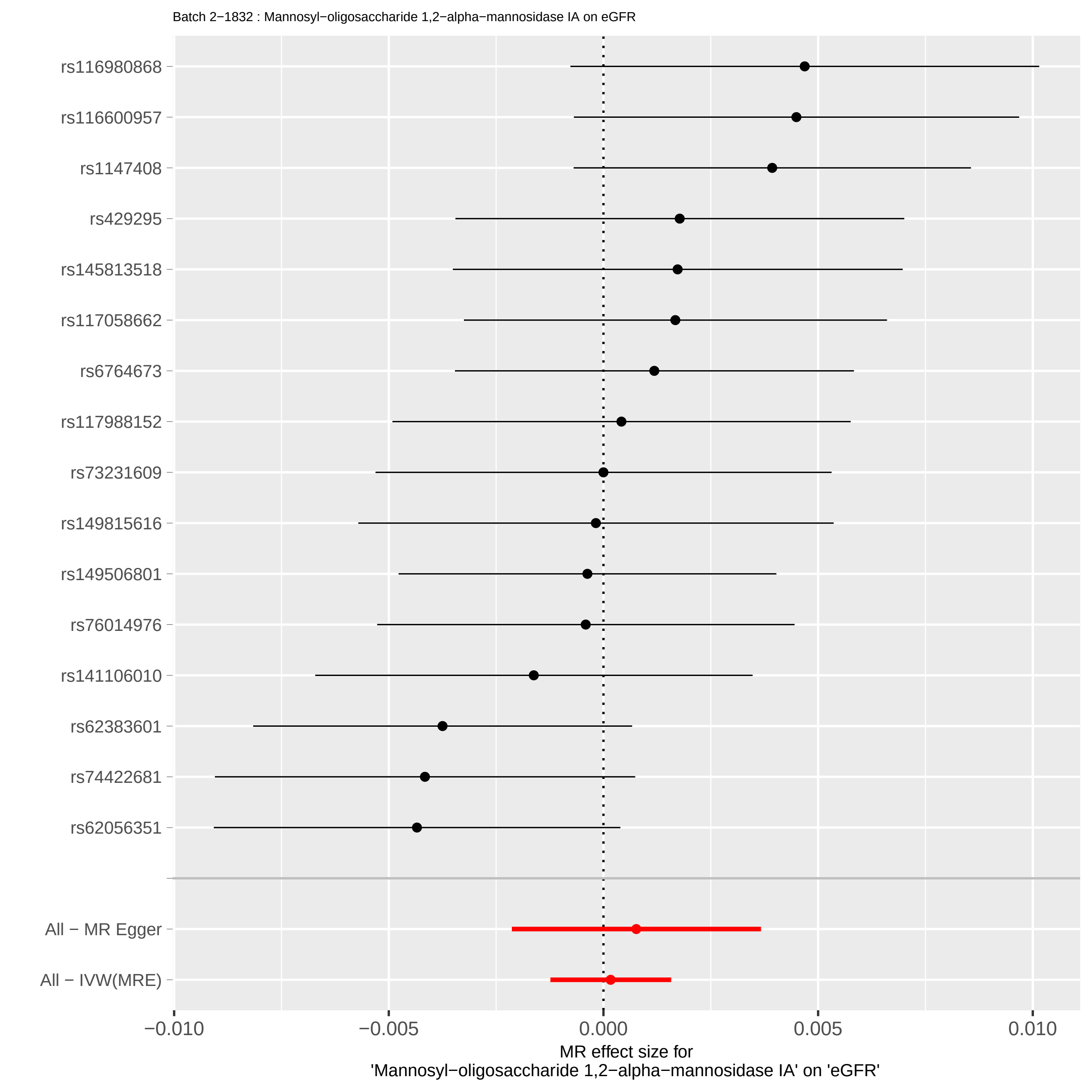

Batch 2−1832 : Mannosyl−oligosaccharide 1,2−alpha−mannosidase IA on eGFR
rs116980868
rs116600957
rs1147408
rs429295
rs145813518
rs117058662
rs6764673
rs117988152
rs73231609
rs149815616
rs149506801
rs76014976
rs141106010
rs62383601
rs74422681
rs62056351
All − MR Egger
All − IVW(MRE)
−0.010
−0.005
0.000
0.005
0.010
MR effect size for
'Mannosyl−oligosaccharide 1,2−alpha−mannosidase IA' on 'eGFR'

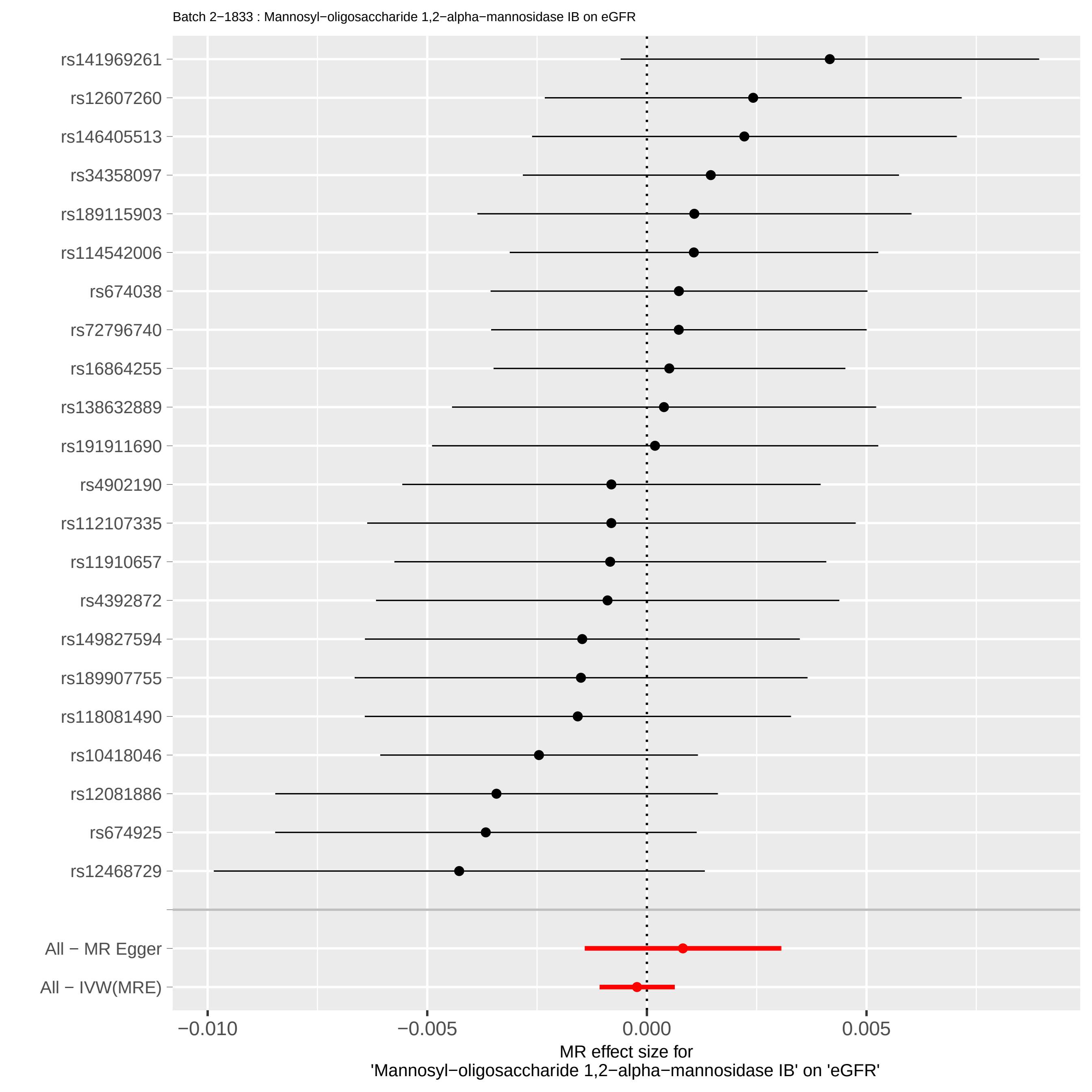
Batch 2−1833 : Mannosyl−oligosaccharide 1,2−alpha−mannosidase IB on eGFR
rs141969261
rs12607260
rs146405513
rs34358097
rs189115903
rs114542006
rs674038
rs72796740
rs16864255
rs138632889
rs191911690
rs4902190
rs112107335
rs11910657
rs4392872
rs149827594
rs189907755
rs118081490
rs10418046
rs12081886
rs674925
rs12468729
All − MR Egger
All − IVW(MRE)
−0.010
−0.005
0.000
0.005
MR effect size for
'Mannosyl−oligosaccharide 1,2−alpha−mannosidase IB' on 'eGFR'

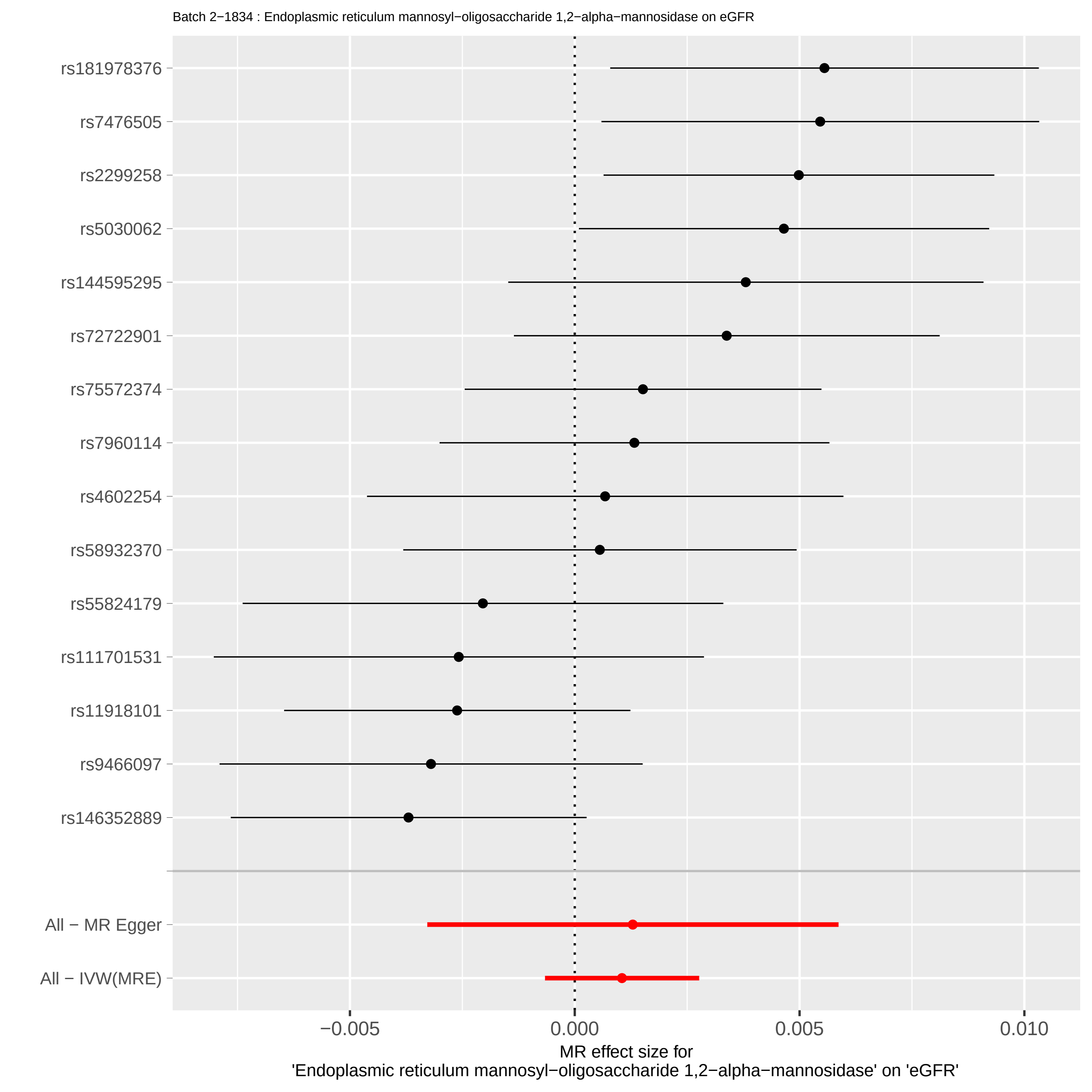

Batch 2−1834 : Endoplasmic reticulum mannosyl−oligosaccharide 1,2−alpha−mannosidase on eGFR
rs181978376
rs7476505
rs2299258
rs5030062
rs144595295
rs72722901
rs75572374
rs7960114
rs4602254
rs58932370
rs55824179
rs111701531
rs11918101
rs9466097
rs146352889
All − MR Egger
All − IVW(MRE)
−0.005
0.000
0.005
0.010
MR effect size for
'Endoplasmic reticulum mannosyl−oligosaccharide 1,2−alpha−mannosidase' on 'eGFR'

Batch 2−1835 : Epididymis−specific alpha−mannosidase on eGFR

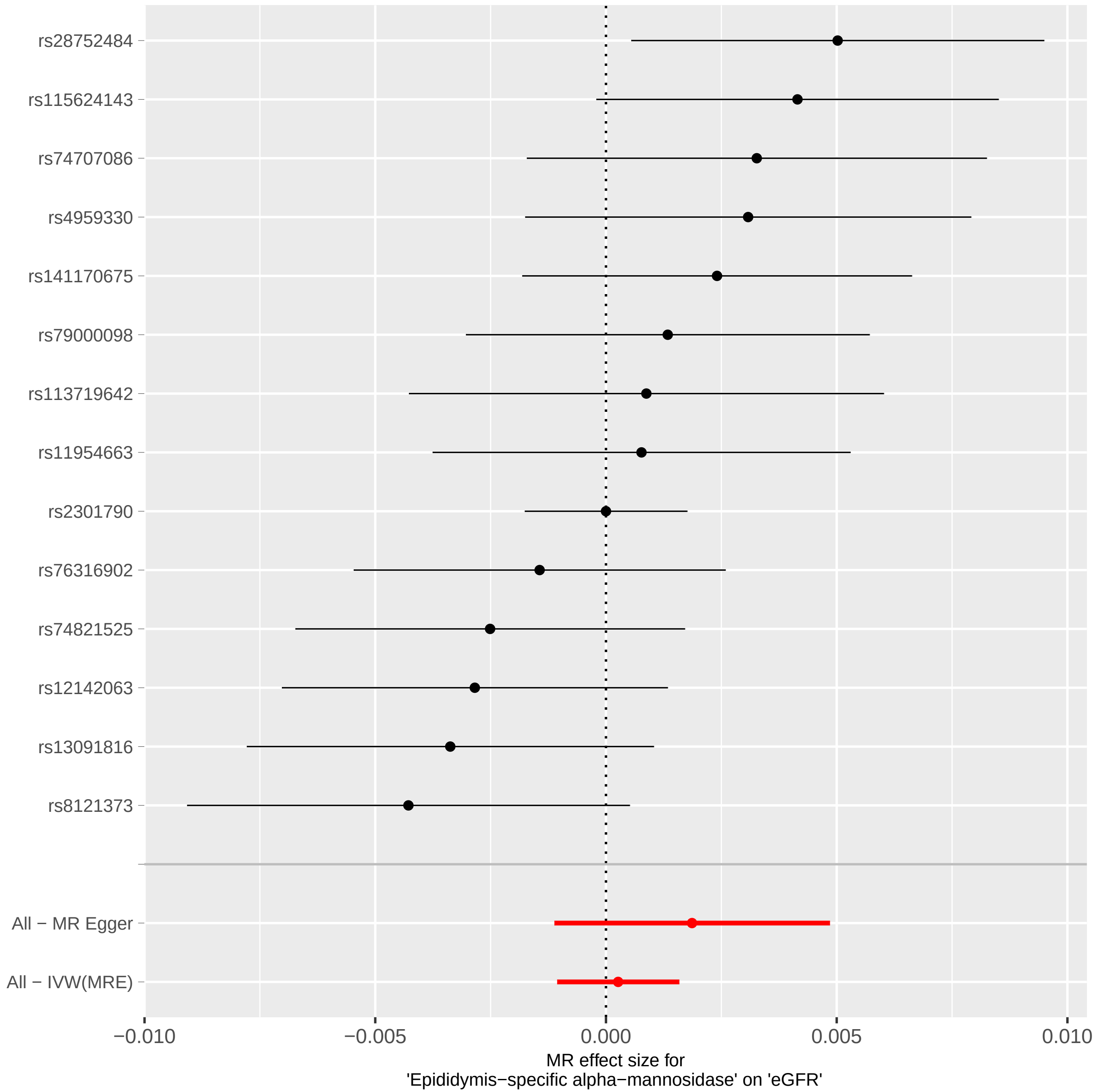

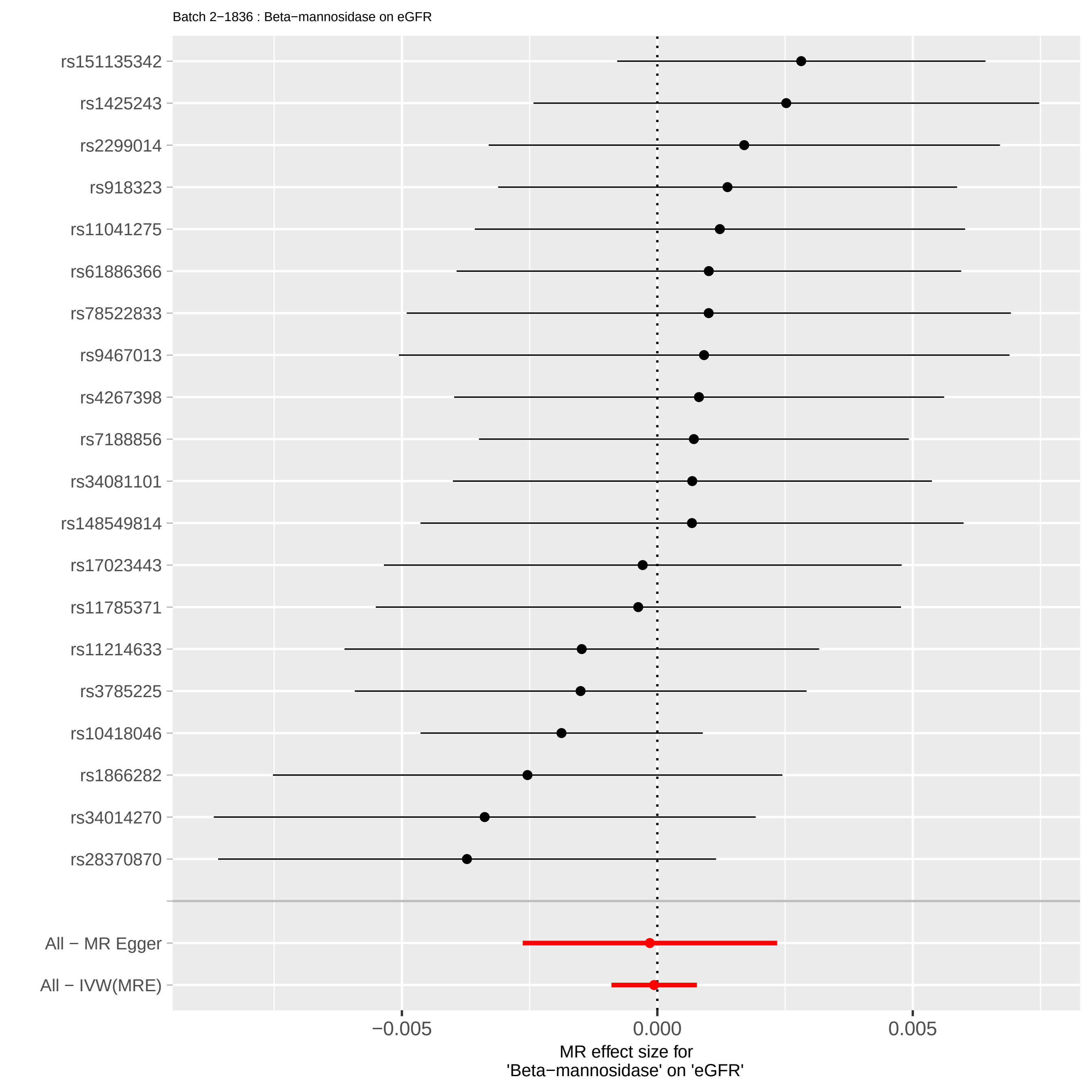

Batch 2−1836 : Beta−mannosidase on eGFR
rs151135342
rs1425243
rs2299014
rs918323
rs11041275
rs61886366
rs78522833
rs9467013
rs4267398
rs7188856
rs34081101
rs148549814
rs17023443
rs11785371
rs11214633
rs3785225
rs10418046
rs1866282
rs34014270
rs28370870
All − MR Egger
All − IVW(MRE)
−0.005
0.000
0.005
MR effect size for
'Beta−mannosidase' on 'eGFR'

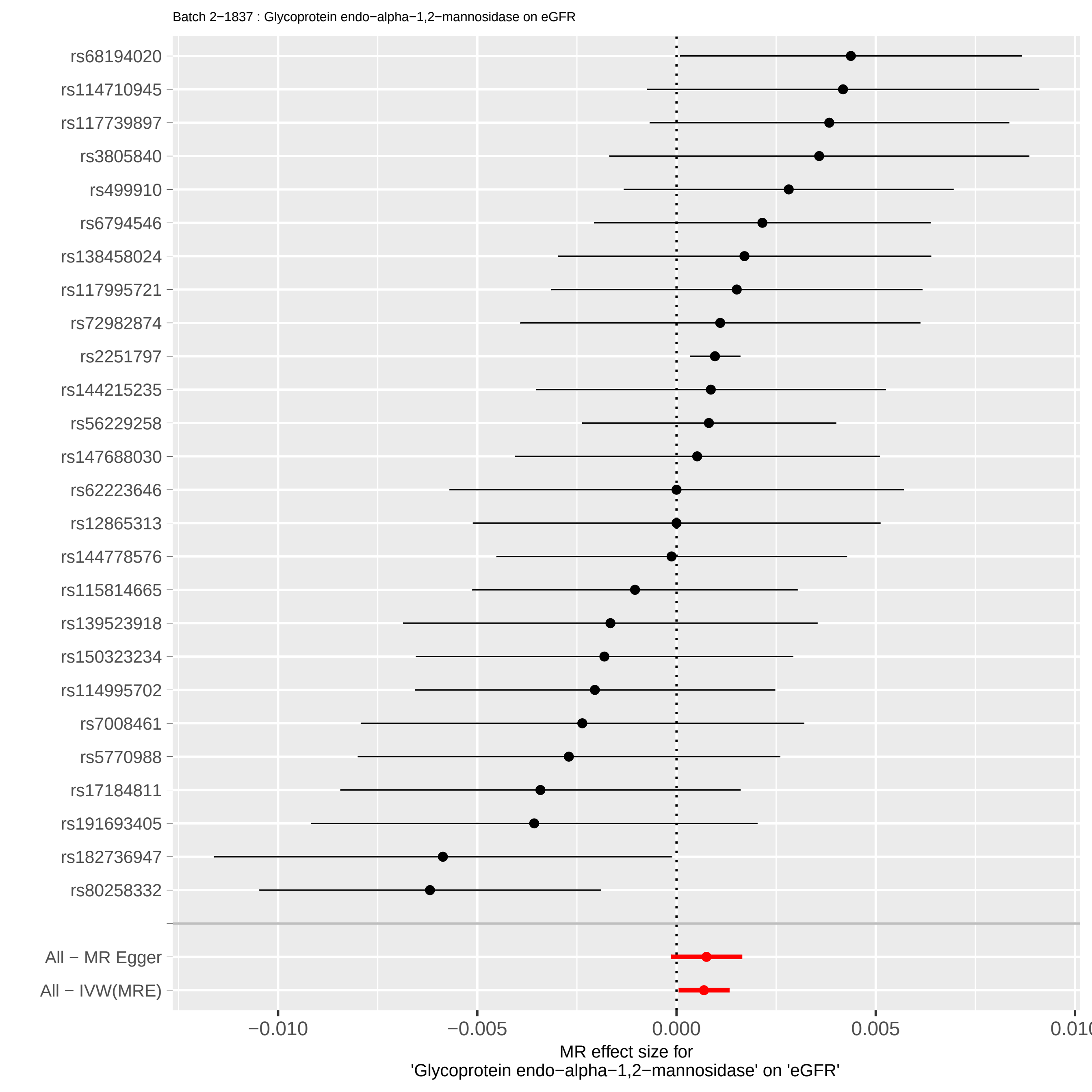

Batch 2−1837 : Glycoprotein endo−alpha−1,2−mannosidase on eGFR
rs68194020
rs114710945
rs117739897
rs3805840
rs499910
rs6794546
rs138458024
rs117995721
rs72982874
rs2251797
rs144215235
rs56229258
rs147688030
rs62223646
rs12865313
rs144778576
rs115814665
rs139523918
rs150323234
rs114995702
rs7008461
rs5770988
rs17184811
rs191693405
rs182736947
rs80258332
All − MR Egger
All − IVW(MRE)
−0.010
−0.005
0.000
0.005
0.010
MR effect size for
'Glycoprotein endo−alpha−1,2−mannosidase' on 'eGFR'

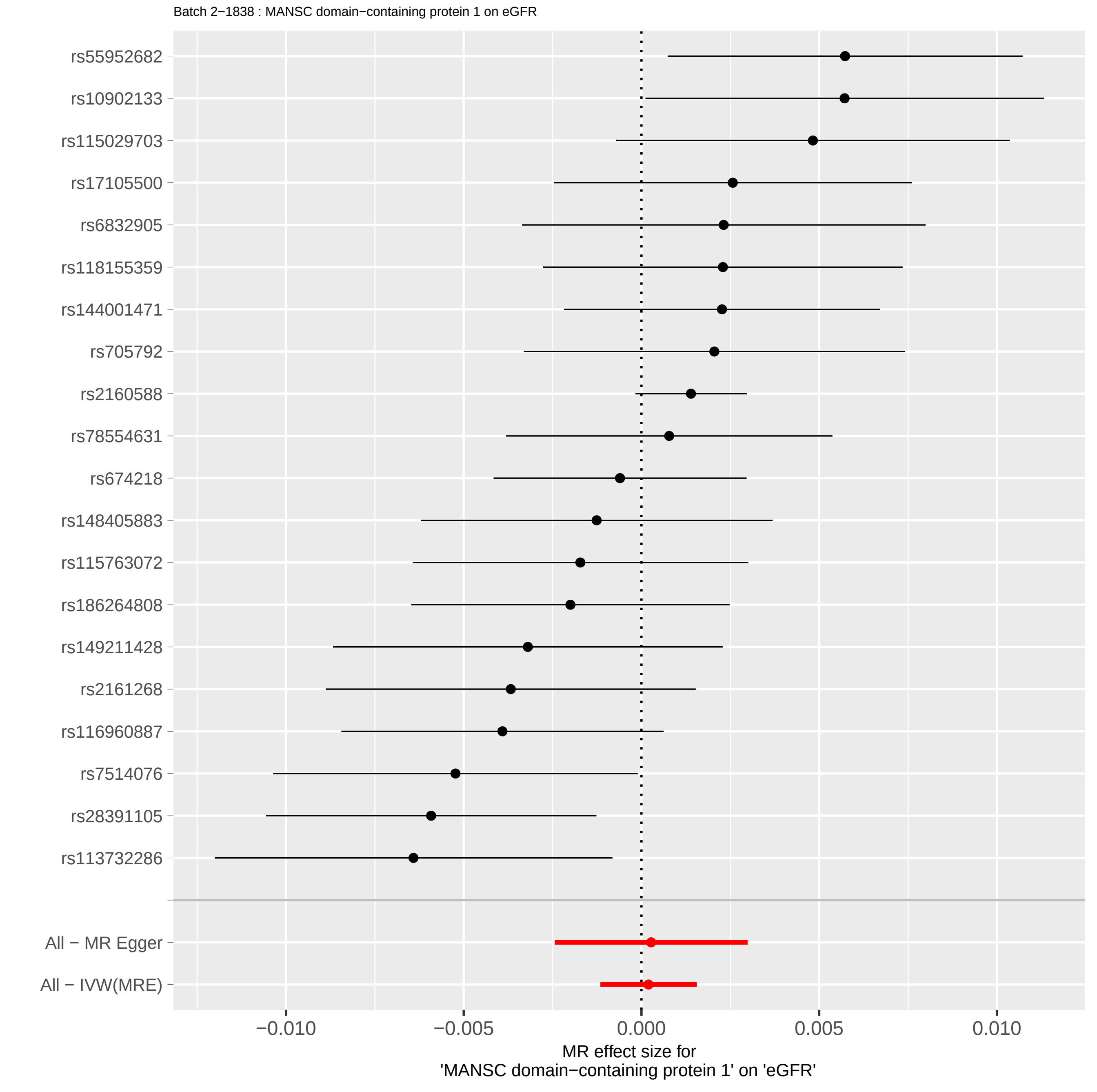

Batch 2−1838 : MANSC domain−containing protein 1 on eGFR
rs55952682
rs10902133
rs115029703
rs17105500
rs6832905
rs118155359
rs144001471
rs705792
rs2160588
rs78554631
rs674218
rs148405883
rs115763072
rs186264808
rs149211428
rs2161268
rs116960887
rs7514076
rs28391105
rs113732286
All − MR Egger
All − IVW(MRE)
−0.010
−0.005
0.000
0.005
0.010
MR effect size for
'MANSC domain−containing protein 1' on 'eGFR'

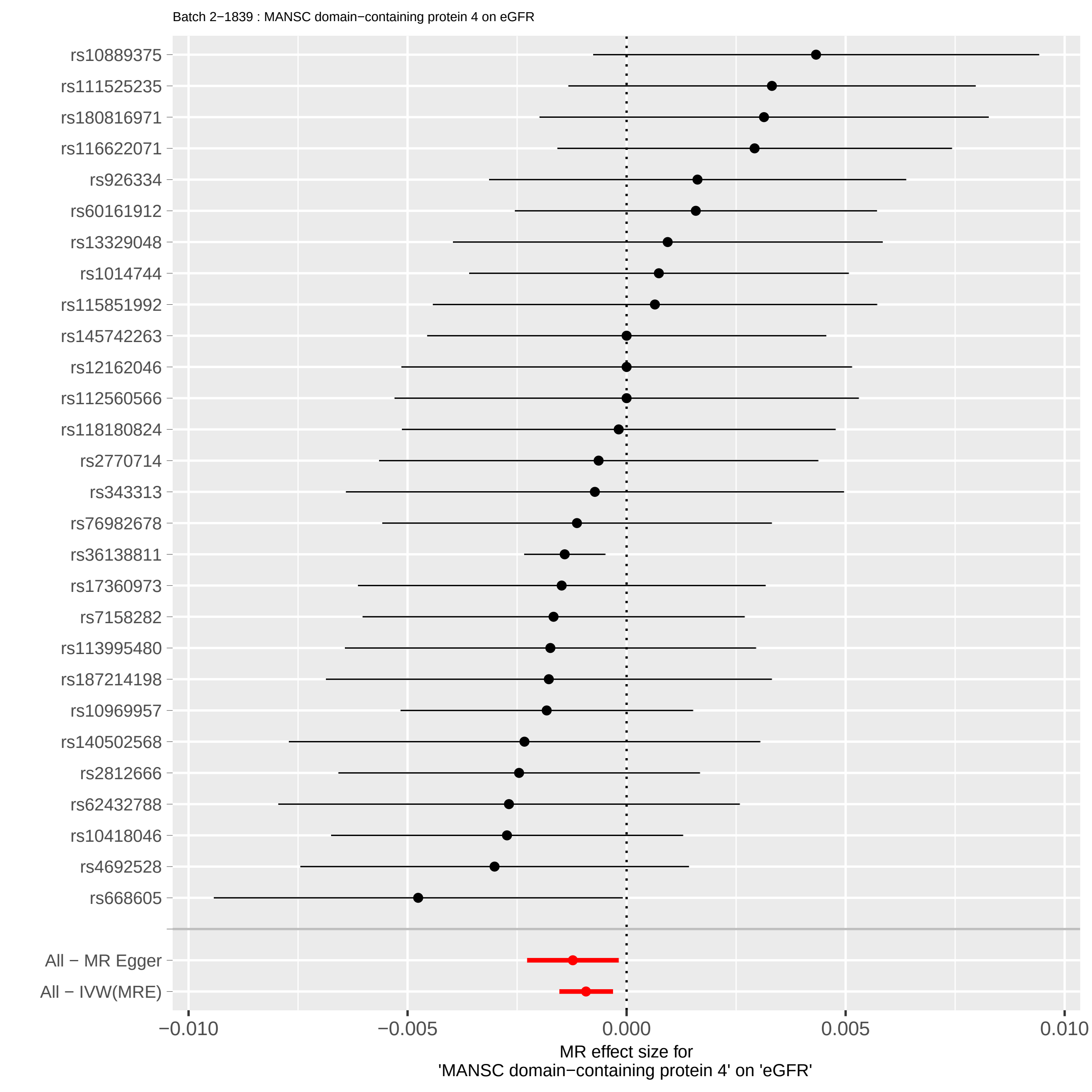

Batch 2−1839 : MANSC domain−containing protein 4 on eGFR
rs10889375
rs111525235
rs180816971
rs116622071
rs926334
rs60161912
rs13329048
rs1014744
rs115851992
rs145742263
rs12162046
rs112560566
rs118180824
rs2770714
rs343313
rs76982678
rs36138811
rs17360973
rs7158282
rs113995480
rs187214198
rs10969957
rs140502568
rs2812666
rs62432788
rs10418046
rs4692528
rs668605
All − MR Egger
All − IVW(MRE)
−0.010
−0.005
0.000
0.005
0.010
MR effect size for
'MANSC domain−containing protein 4' on 'eGFR'

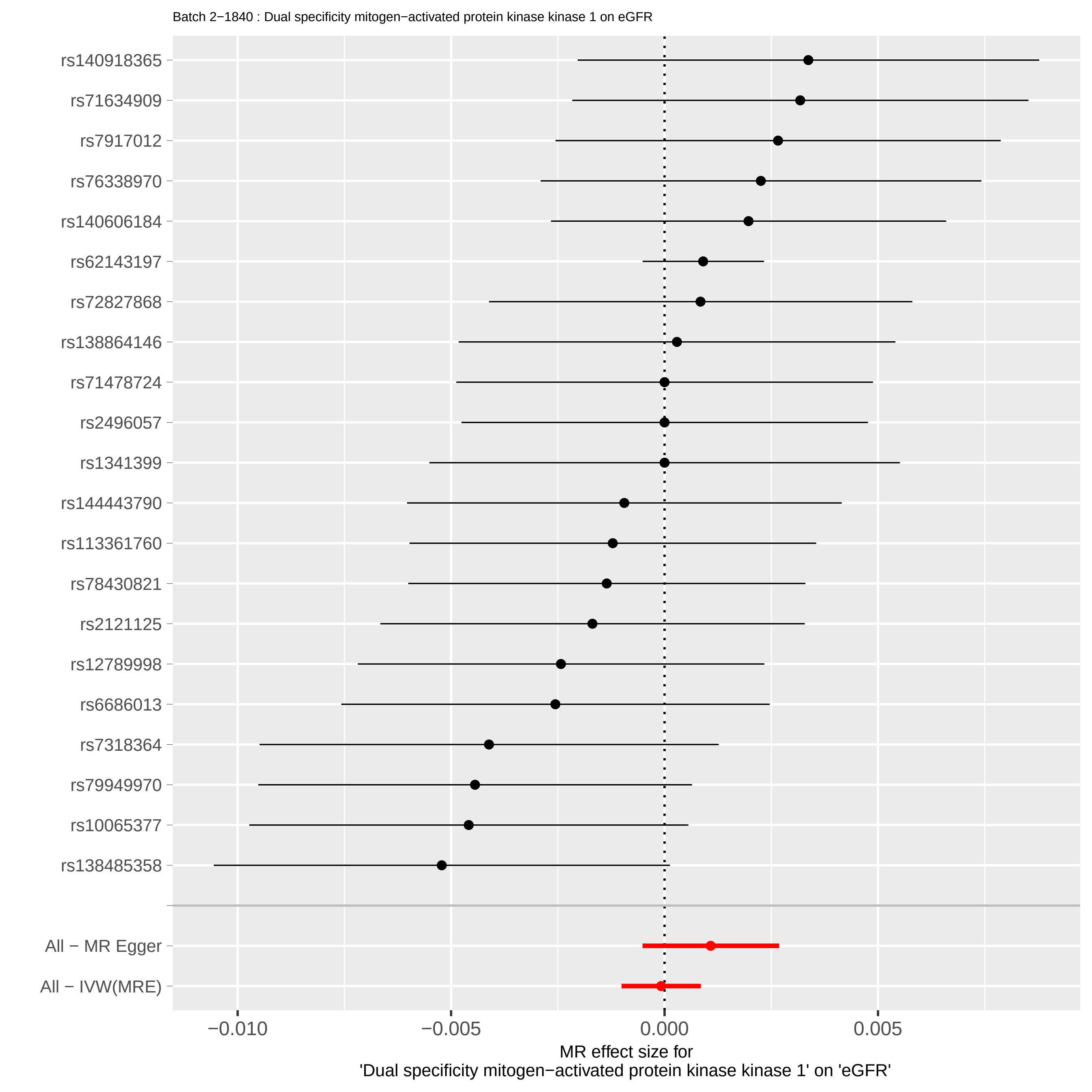

Batch 2−1840 : Dual specificity mitogen−activated protein kinase kinase 1 on eGFR
rs140918365
rs71634909
rs7917012
rs76338970
rs140606184
rs62143197
rs72827868
rs138864146
rs71478724
rs2496057
rs1341399
rs144443790
rs113361760
rs78430821
rs2121125
rs12789998
rs6686013
rs7318364
rs79949970
rs10065377
rs138485358
All − MR Egger
All − IVW(MRE)
−0.010
−0.005
0.000
0.005
MR effect size for
'Dual specificity mitogen−activated protein kinase kinase 1' on 'eGFR'

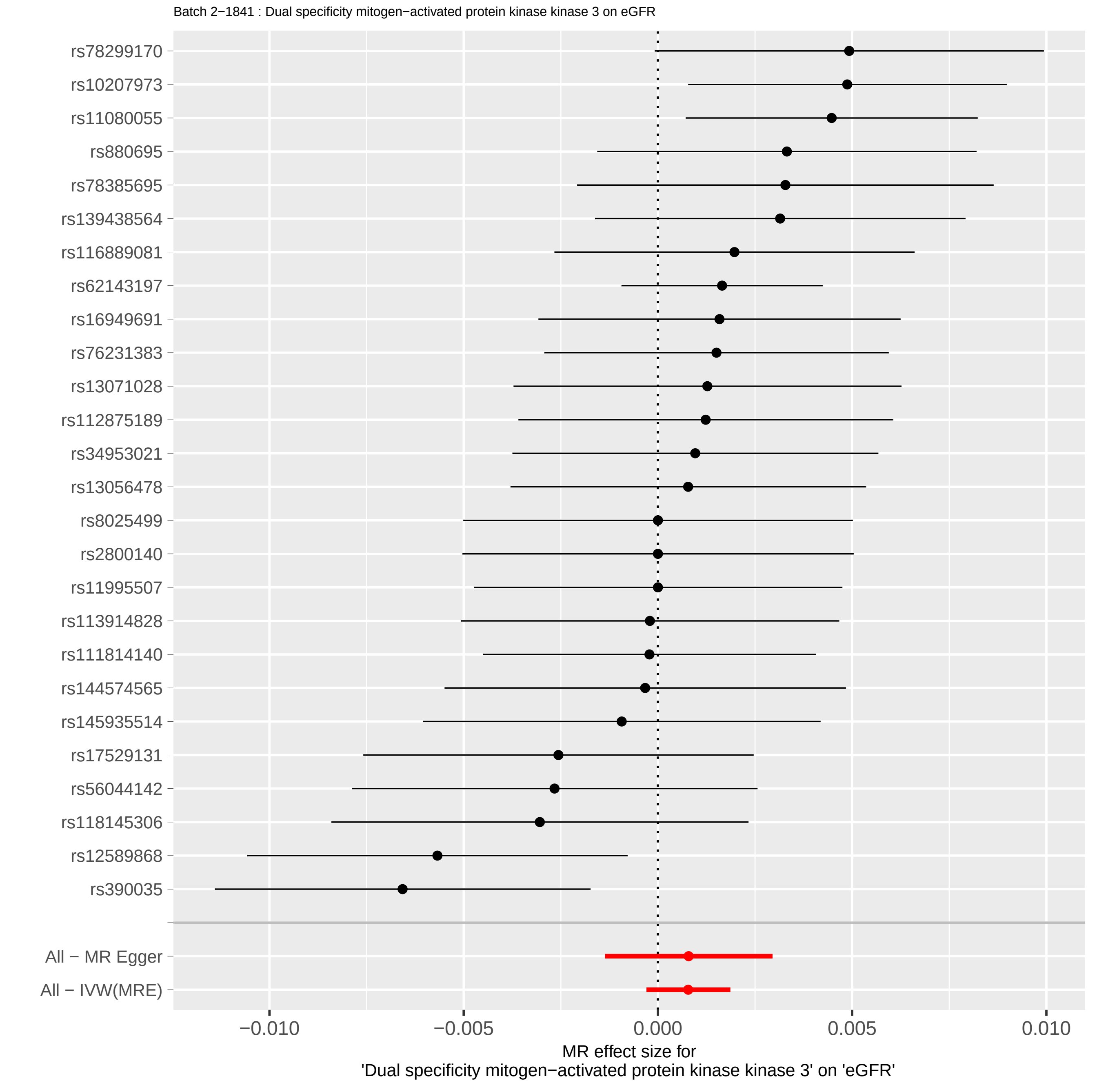

Batch 2−1841 : Dual specificity mitogen−activated protein kinase kinase 3 on eGFR
rs78299170
rs10207973
rs11080055
rs880695
rs78385695
rs139438564
rs116889081
rs62143197
rs16949691
rs76231383
rs13071028
rs112875189
rs34953021
rs13056478
rs8025499
rs2800140
rs11995507
rs113914828
rs111814140
rs144574565
rs145935514
rs17529131
rs56044142
rs118145306
rs12589868
rs390035
All − MR Egger
All − IVW(MRE)
−0.010
−0.005
0.000
0.005
0.010
MR effect size for
'Dual specificity mitogen−activated protein kinase kinase 3' on 'eGFR'

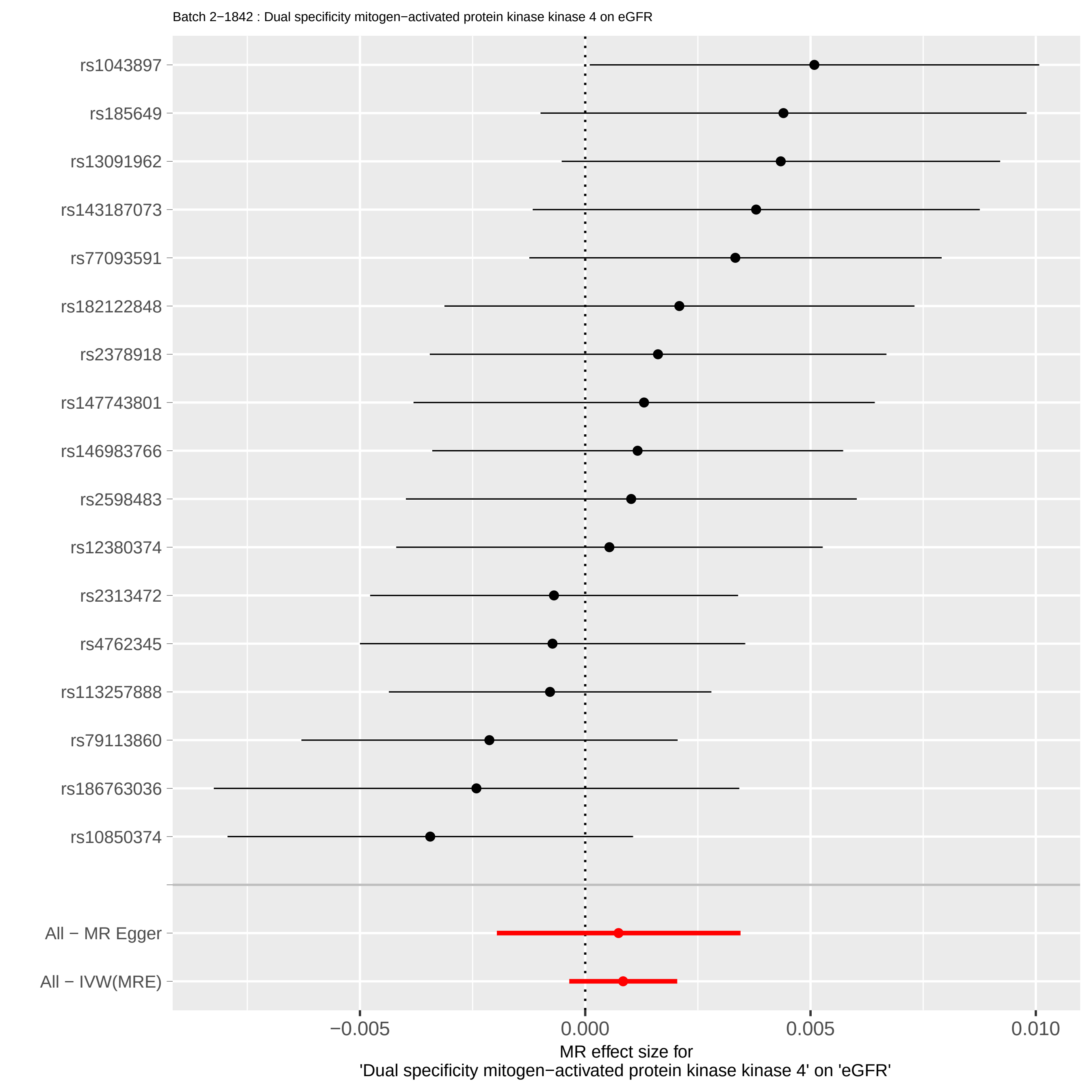

Batch 2−1842 : Dual specificity mitogen−activated protein kinase kinase 4 on eGFR
rs1043897
rs185649
rs13091962
rs143187073
rs77093591
rs182122848
rs2378918
rs147743801
rs146983766
rs2598483
rs12380374
rs2313472
rs4762345
rs113257888
rs79113860
rs186763036
rs10850374
All − MR Egger
All − IVW(MRE)
−0.005
0.000
0.005
0.010
MR effect size for
'Dual specificity mitogen−activated protein kinase kinase 4' on 'eGFR'

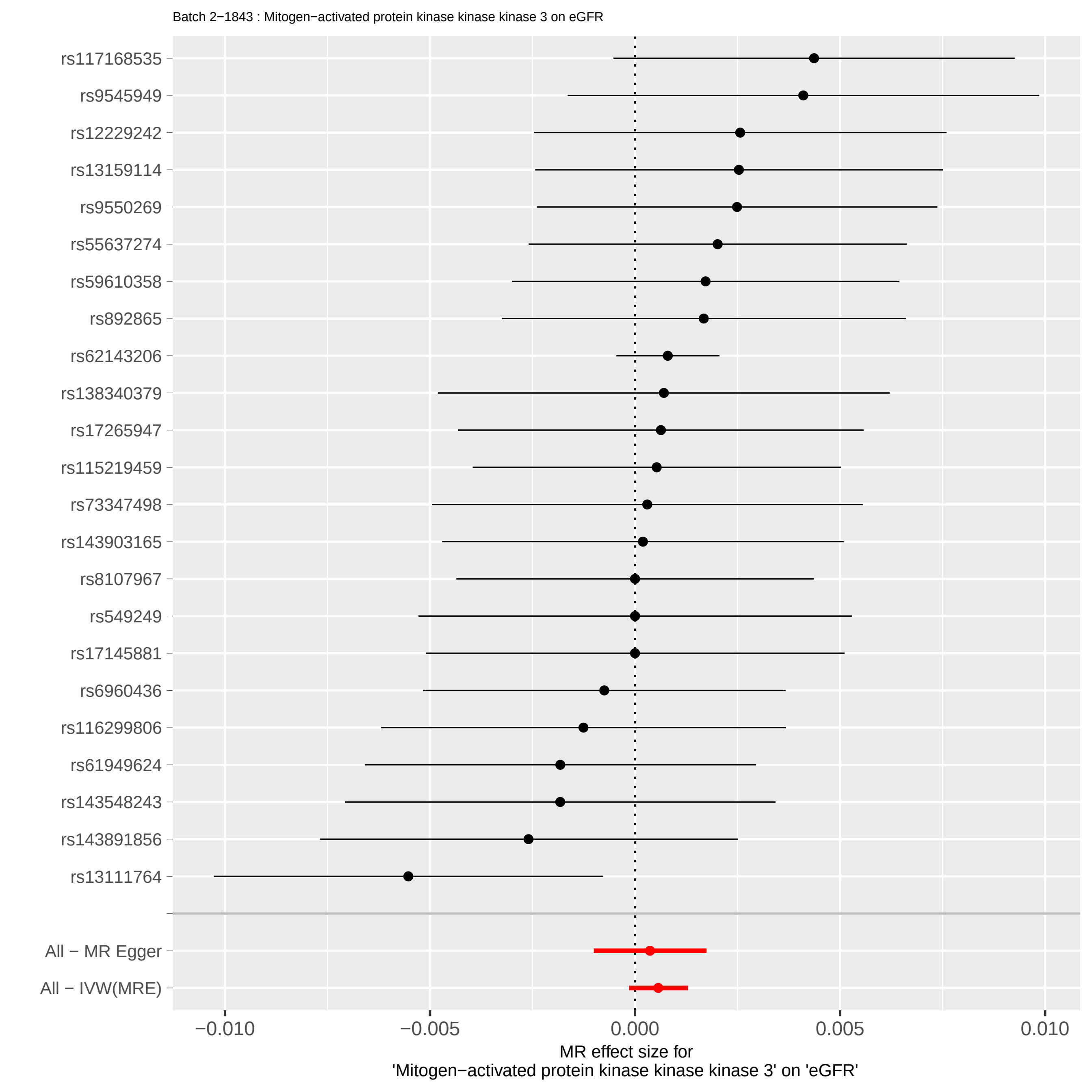

Batch 2−1843 : Mitogen−activated protein kinase kinase kinase 3 on eGFR
rs117168535
rs9545949
rs12229242
rs13159114
rs9550269
rs55637274
rs59610358
rs892865
rs62143206
rs138340379
rs17265947
rs115219459
rs73347498
rs143903165
rs8107967
rs549249
rs17145881
rs6960436
rs116299806
rs61949624
rs143548243
rs143891856
rs13111764
All − MR Egger
All − IVW(MRE)
−0.010
−0.005
0.000
0.005
0.010
MR effect size for
'Mitogen−activated protein kinase kinase kinase 3' on 'eGFR'

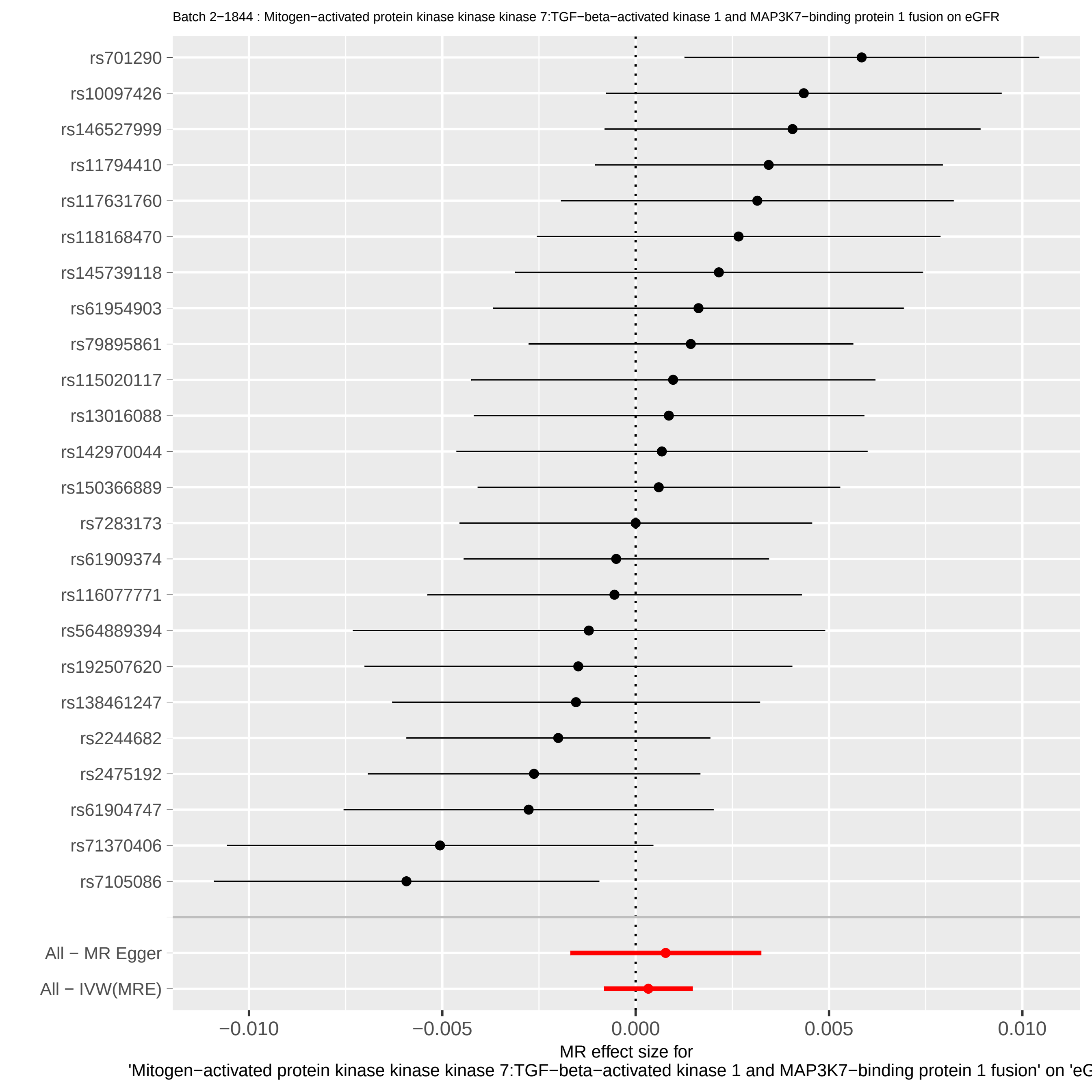

Batch 2−1844 : Mitogen−activated protein kinase kinase kinase 7:TGF−beta−activated kinase 1 and MAP3K7−binding protein 1 fusion on eGFR
rs701290
rs10097426
rs146527999
rs11794410
rs117631760
rs118168470
rs145739118
rs61954903
rs79895861
rs115020117
rs13016088
rs142970044
rs150366889
rs7283173
rs61909374
rs116077771
rs564889394
rs192507620
rs138461247
rs2244682
rs2475192
rs61904747
rs71370406
rs7105086
All − MR Egger
All − IVW(MRE)
−0.010
−0.005
0.000
0.005
0.010
MR effect size for
'Mitogen−activated protein kinase kinase kinase 7:TGF−beta−activated kinase 1 and MAP3K7−binding protein 1 fusion' on 'eG

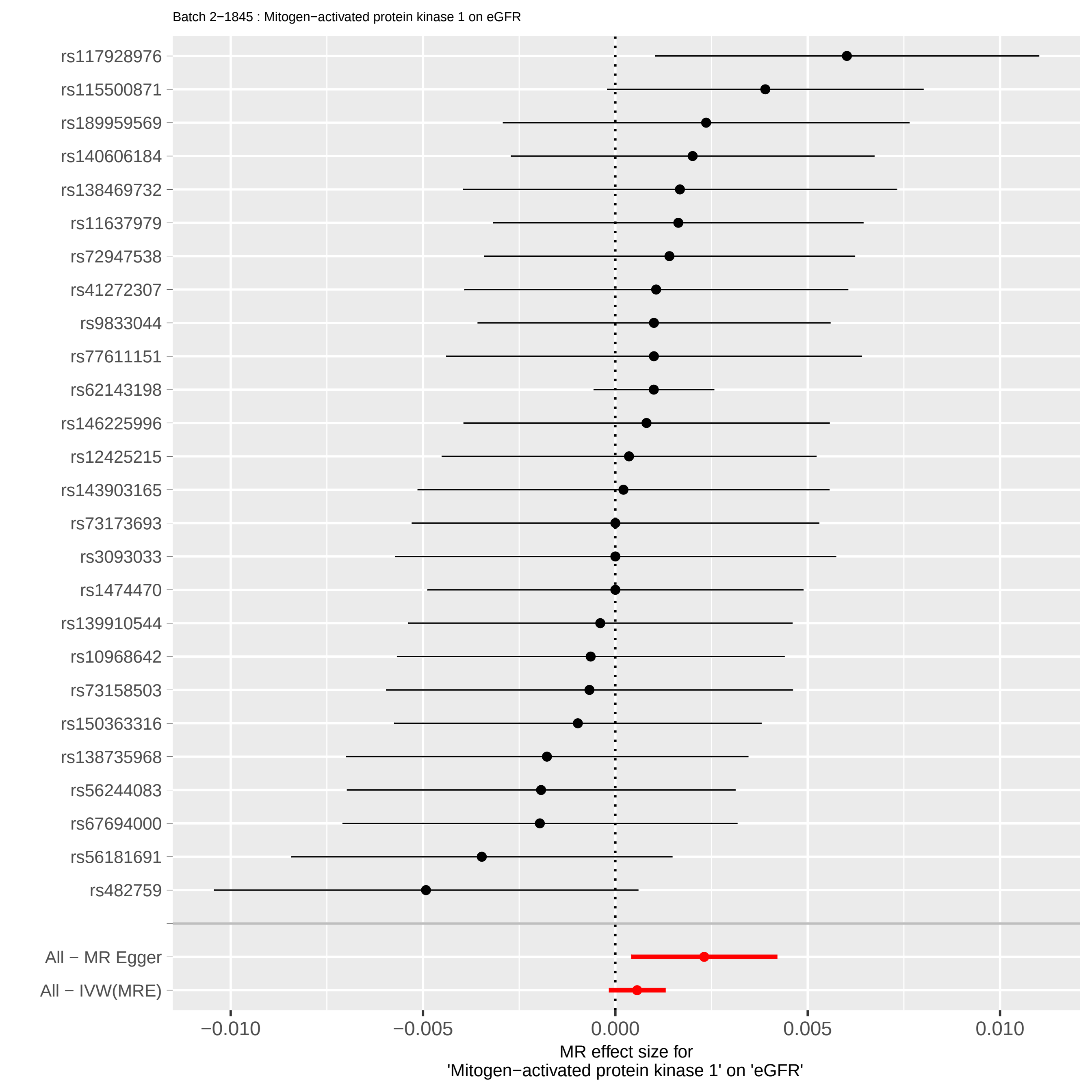

Batch 2−1845 : Mitogen−activated protein kinase 1 on eGFR
rs117928976
rs115500871
rs189959569
rs140606184
rs138469732
rs11637979
rs72947538
rs41272307
rs9833044
rs77611151
rs62143198
rs146225996
rs12425215
rs143903165
rs73173693
rs3093033
rs1474470
rs139910544
rs10968642
rs73158503
rs150363316
rs138735968
rs56244083
rs67694000
rs56181691
rs482759
All − MR Egger
All − IVW(MRE)
−0.010
−0.005
0.000
0.005
0.010
MR effect size for
'Mitogen−activated protein kinase 1' on 'eGFR'

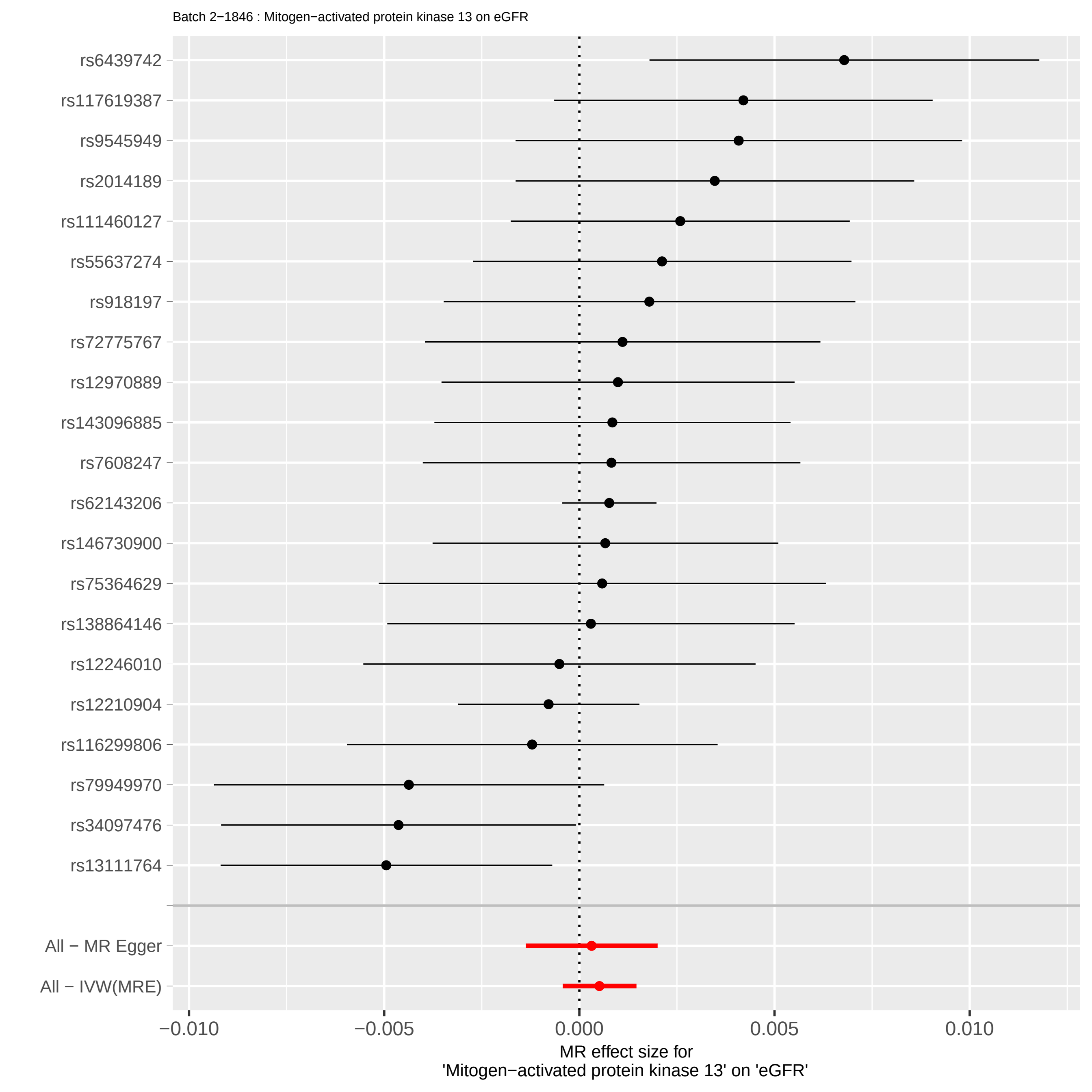

Batch 2−1846 : Mitogen−activated protein kinase 13 on eGFR
rs6439742
rs117619387
rs9545949
rs2014189
rs111460127
rs55637274
rs918197
rs72775767
rs12970889
rs143096885
rs7608247
rs62143206
rs146730900
rs75364629
rs138864146
rs12246010
rs12210904
rs116299806
rs79949970
rs34097476
rs13111764
All − MR Egger
All − IVW(MRE)
−0.010
−0.005
0.000
0.005
0.010
MR effect size for
'Mitogen−activated protein kinase 13' on 'eGFR'

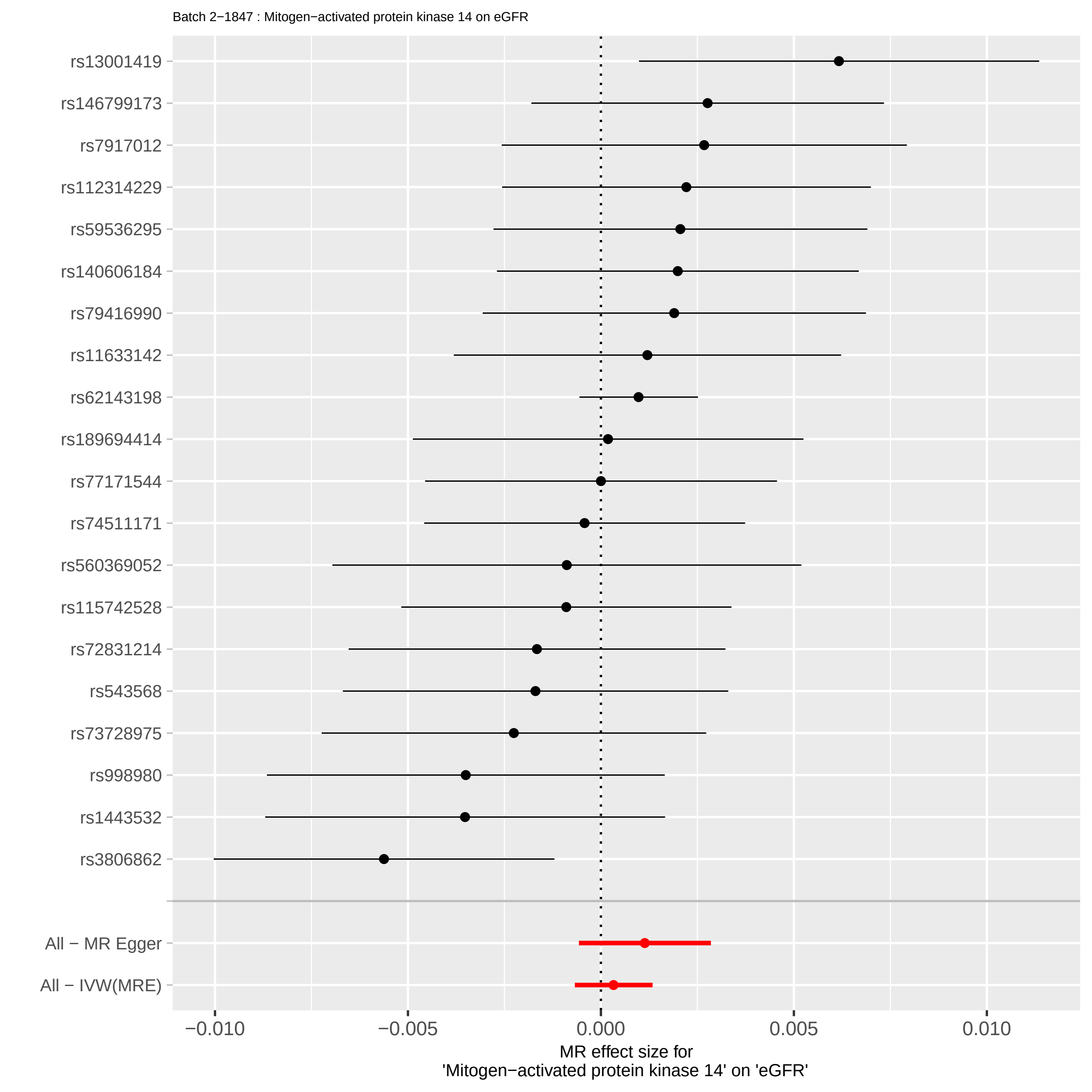

Batch 2−1847 : Mitogen−activated protein kinase 14 on eGFR
rs13001419
rs146799173
rs7917012
rs112314229
rs59536295
rs140606184
rs79416990
rs11633142
rs62143198
rs189694414
rs77171544
rs74511171
rs560369052
rs115742528
rs72831214
rs543568
rs73728975
rs998980
rs1443532
rs3806862
All − MR Egger
All − IVW(MRE)
−0.010
−0.005
0.000
0.005
0.010
MR effect size for
'Mitogen−activated protein kinase 14' on 'eGFR'

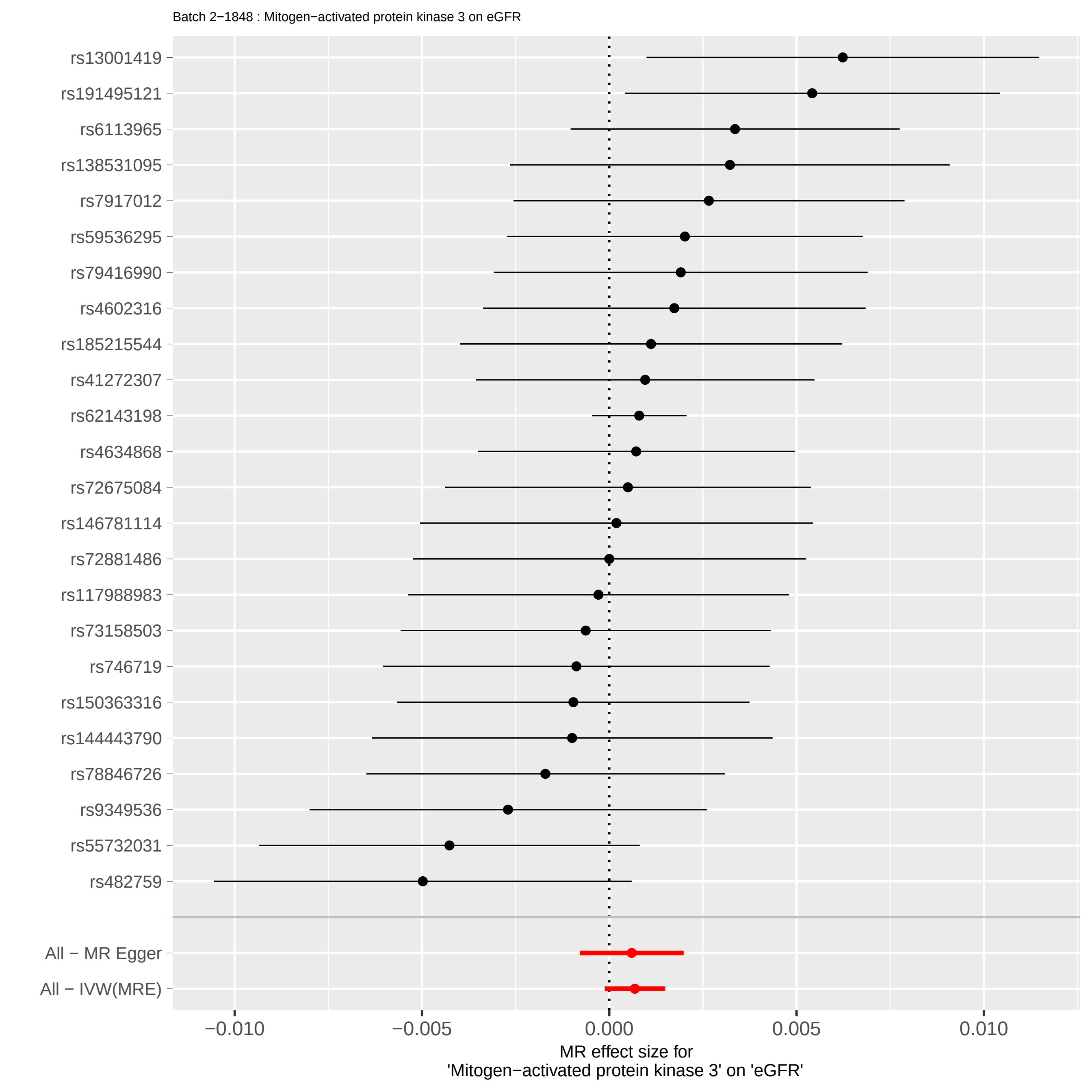

Batch 2−1848 : Mitogen−activated protein kinase 3 on eGFR
rs13001419
rs191495121
rs6113965
rs138531095
rs7917012
rs59536295
rs79416990
rs4602316
rs185215544
rs41272307
rs62143198
rs4634868
rs72675084
rs146781114
rs72881486
rs117988983
rs73158503
rs746719
rs150363316
rs144443790
rs78846726
rs9349536
rs55732031
rs482759
All − MR Egger
All − IVW(MRE)
−0.010
−0.005
0.000
0.005
0.010
MR effect size for
'Mitogen−activated protein kinase 3' on 'eGFR'

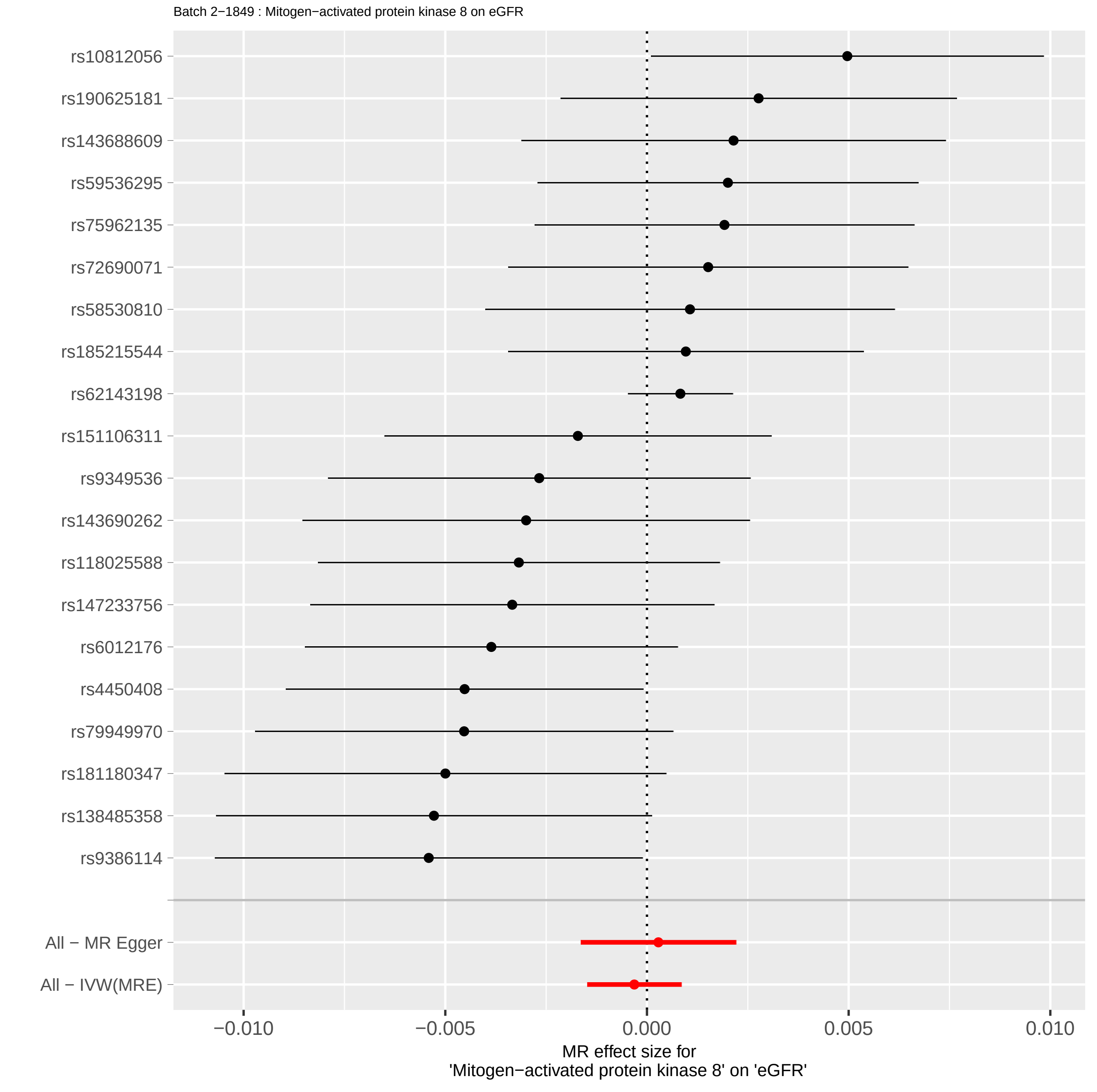

Batch 2−1849 : Mitogen−activated protein kinase 8 on eGFR
rs10812056
rs190625181
rs143688609
rs59536295
rs75962135
rs72690071
rs58530810
rs185215544
rs62143198
rs151106311
rs9349536
rs143690262
rs118025588
rs147233756
rs6012176
rs4450408
rs79949970
rs181180347
rs138485358
rs9386114
All − MR Egger
All − IVW(MRE)
−0.010
−0.005
0.000
0.005
0.010
MR effect size for
'Mitogen−activated protein kinase 8' on 'eGFR'

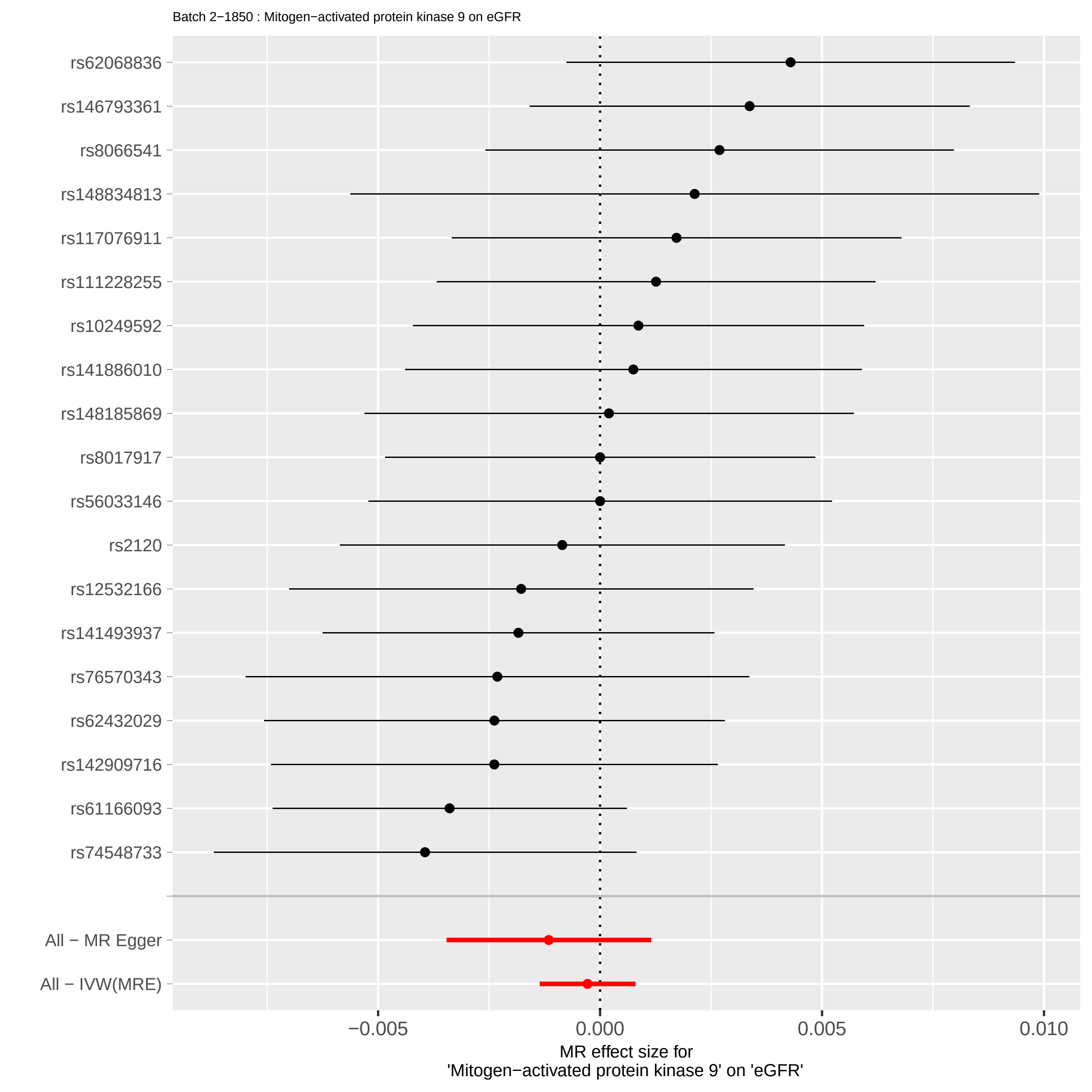

Batch 2−1850 : Mitogen−activated protein kinase 9 on eGFR
rs62068836
rs146793361
rs8066541
rs148834813
rs117076911
rs111228255
rs10249592
rs141886010
rs148185869
rs8017917
rs56033146
rs2120
rs12532166
rs141493937
rs76570343
rs62432029
rs142909716
rs61166093
rs74548733
All − MR Egger
All − IVW(MRE)
−0.005
0.000
0.005
0.010
MR effect size for
'Mitogen−activated protein kinase 9' on 'eGFR'

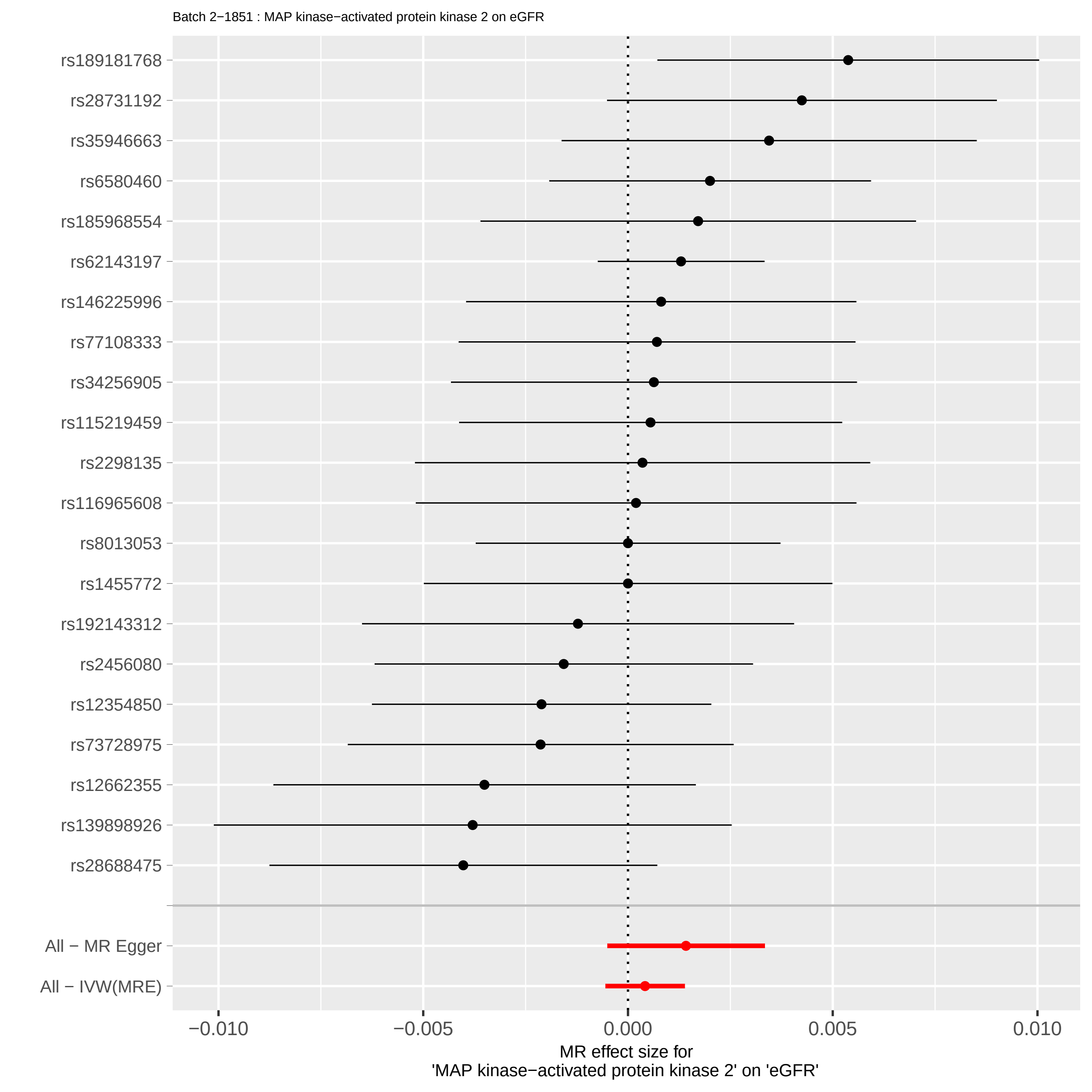

Batch 2−1851 : MAP kinase−activated protein kinase 2 on eGFR
rs189181768
rs28731192
rs35946663
rs6580460
rs185968554
rs62143197
rs146225996
rs77108333
rs34256905
rs115219459
rs2298135
rs116965608
rs8013053
rs1455772
rs192143312
rs2456080
rs12354850
rs73728975
rs12662355
rs139898926
rs28688475
All − MR Egger
All − IVW(MRE)
−0.010
−0.005
0.000
0.005
0.010
MR effect size for
'MAP kinase−activated protein kinase 2' on 'eGFR'

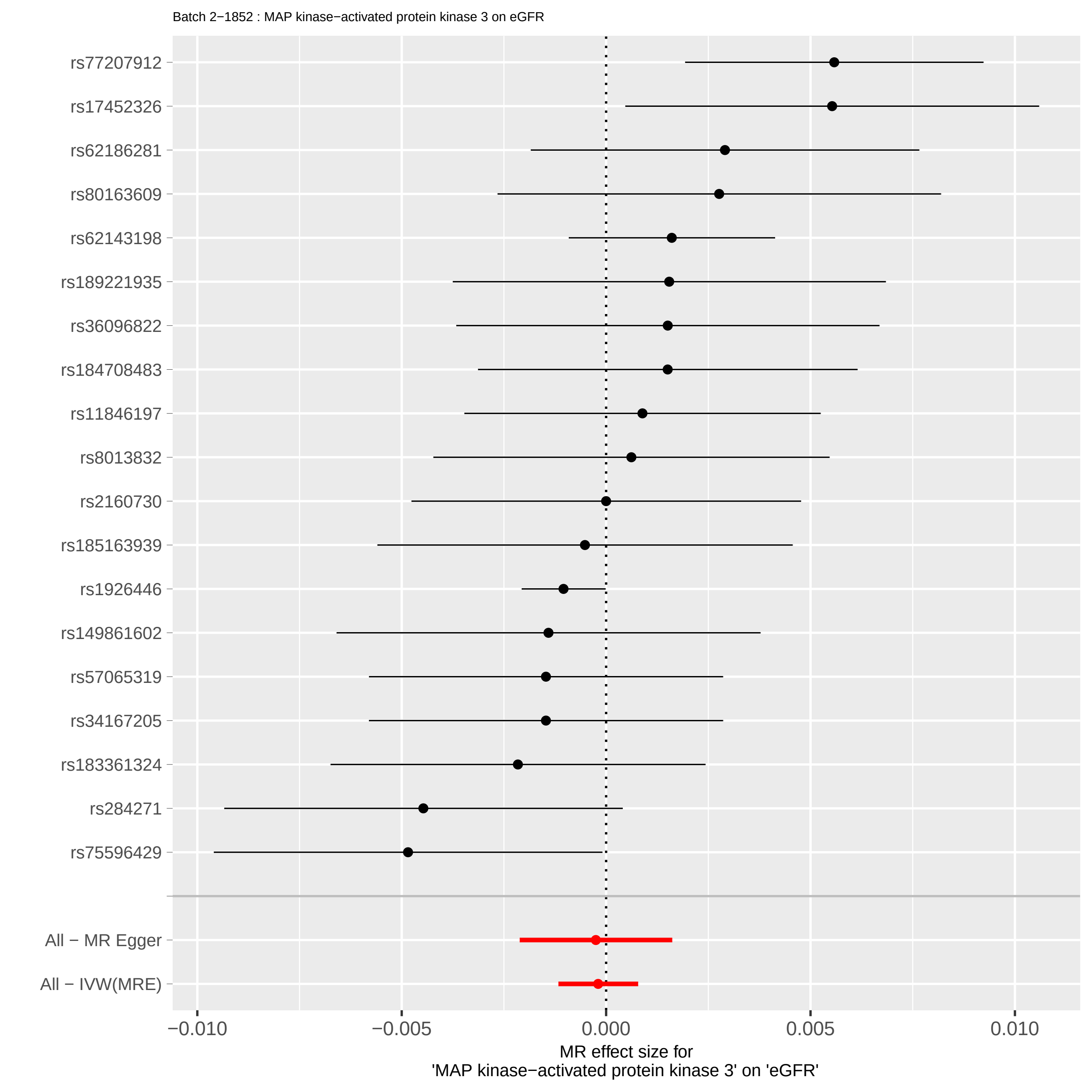

Batch 2−1852 : MAP kinase−activated protein kinase 3 on eGFR
rs77207912
rs17452326
rs62186281
rs80163609
rs62143198
rs189221935
rs36096822
rs184708483
rs11846197
rs8013832
rs2160730
rs185163939
rs1926446
rs149861602
rs57065319
rs34167205
rs183361324
rs284271
rs75596429
All − MR Egger
All − IVW(MRE)
−0.010
−0.005
0.000
0.005
0.010
MR effect size for
'MAP kinase−activated protein kinase 3' on 'eGFR'

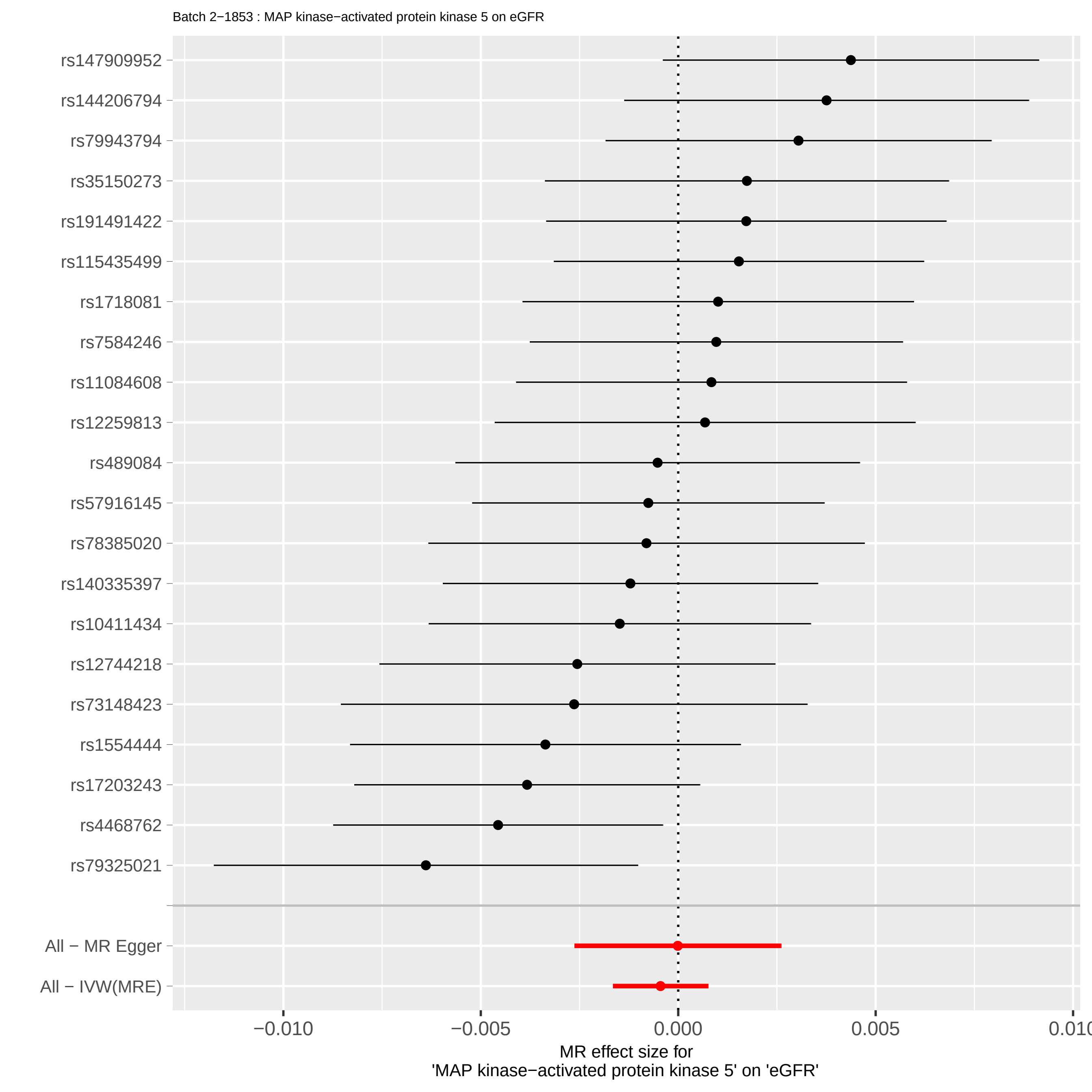

Batch 2−1853 : MAP kinase−activated protein kinase 5 on eGFR
rs147909952
rs144206794
rs79943794
rs35150273
rs191491422
rs115435499
rs1718081
rs7584246
rs11084608
rs12259813
rs489084
rs57916145
rs78385020
rs140335397
rs10411434
rs12744218
rs73148423
rs1554444
rs17203243
rs4468762
rs79325021
All − MR Egger
All − IVW(MRE)
−0.010
−0.005
0.000
0.005
0.010
MR effect size for
'MAP kinase−activated protein kinase 5' on 'eGFR'

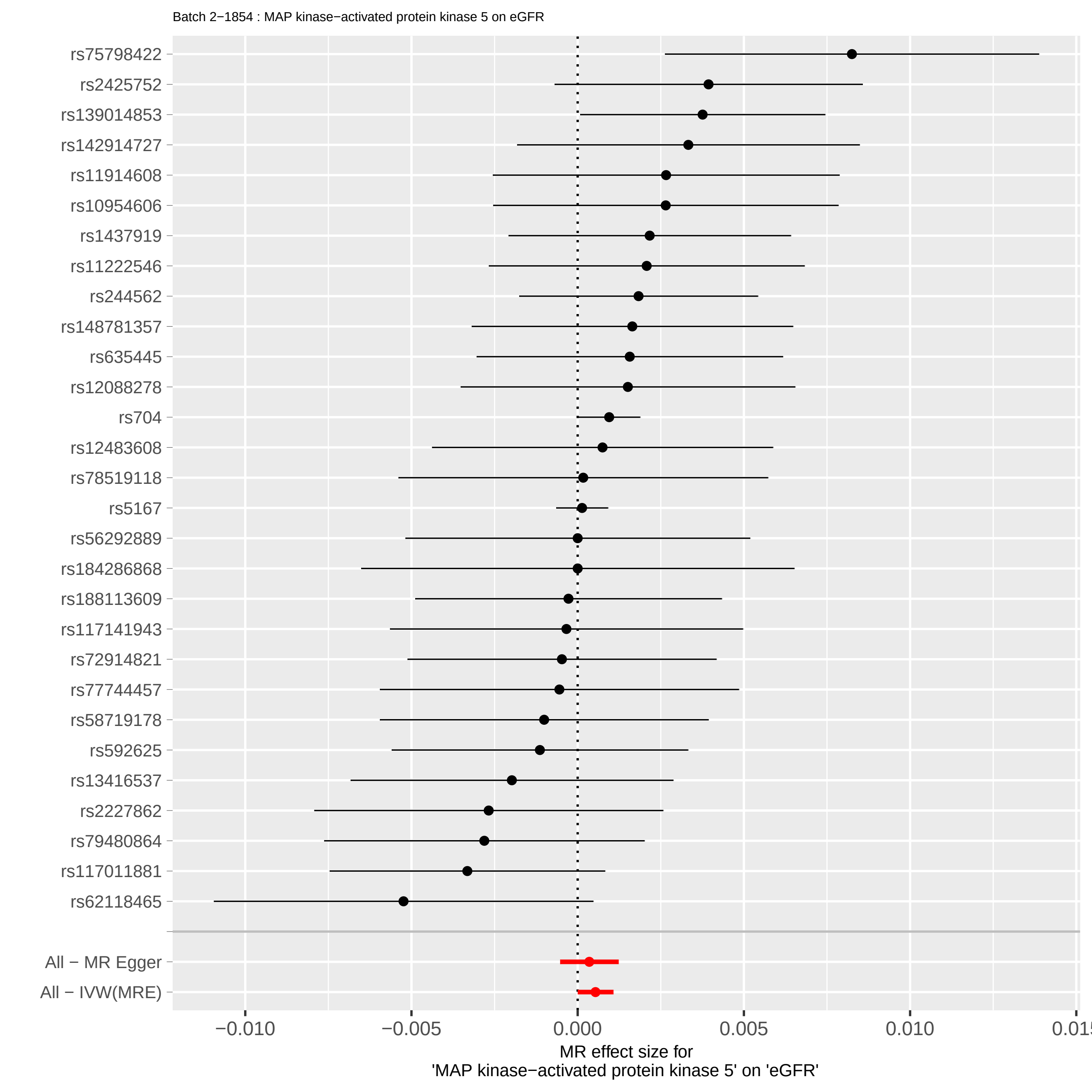
Batch 2−1854 : MAP kinase−activated protein kinase 5 on eGFR
rs75798422
rs2425752
rs139014853
rs142914727
rs11914608
rs10954606
rs1437919
rs11222546
rs244562
rs148781357
rs635445
rs12088278
rs704
rs12483608
rs78519118
rs5167
rs56292889
rs184286868
rs188113609
rs117141943
rs72914821
rs77744457
rs58719178
rs592625
rs13416537
rs2227862
rs79480864
rs117011881
rs62118465
All − MR Egger
All − IVW(MRE)
−0.010
−0.005
0.000
0.005
0.010
0.015
MR effect size for
'MAP kinase−activated protein kinase 5' on 'eGFR'

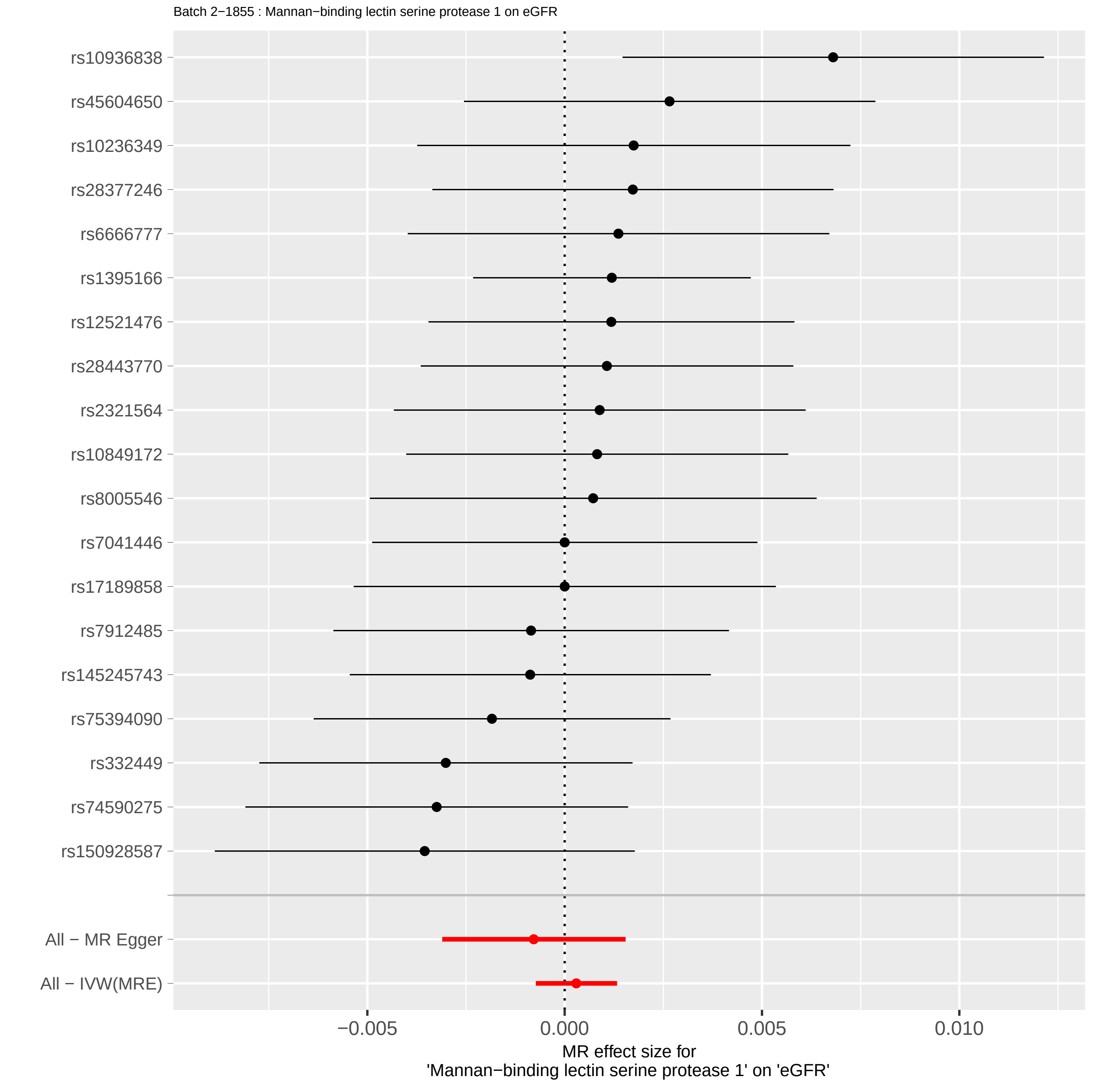
Batch 2−1855 : Mannan−binding lectin serine protease 1 on eGFR
rs10936838
rs45604650
rs10236349
rs28377246
rs6666777
rs1395166
rs12521476
rs28443770
rs2321564
rs10849172
rs8005546
rs7041446
rs17189858
rs7912485
rs145245743
rs75394090
rs332449
rs74590275
rs150928587
All − MR Egger
All − IVW(MRE)
−0.005
0.000
0.005
0.010
MR effect size for
'Mannan−binding lectin serine protease 1' on 'eGFR'

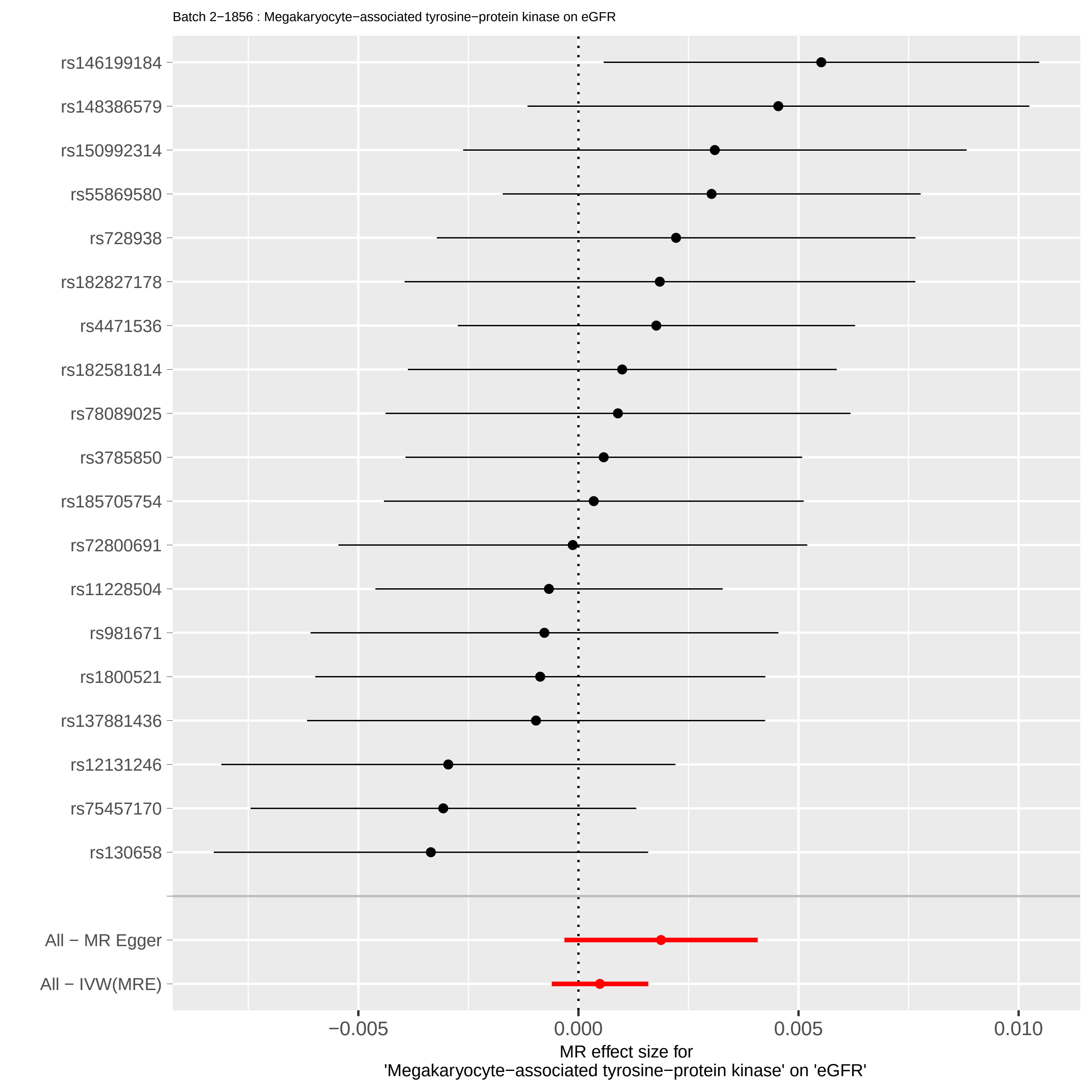

Batch 2−1856 : Megakaryocyte−associated tyrosine−protein kinase on eGFR
rs146199184
rs148386579
rs150992314
rs55869580
rs728938
rs182827178
rs4471536
rs182581814
rs78089025
rs3785850
rs185705754
rs72800691
rs11228504
rs981671
rs1800521
rs137881436
rs12131246
rs75457170
rs130658
All − MR Egger
All − IVW(MRE)
−0.005
0.000
0.005
0.010
MR effect size for
'Megakaryocyte−associated tyrosine−protein kinase' on 'eGFR'

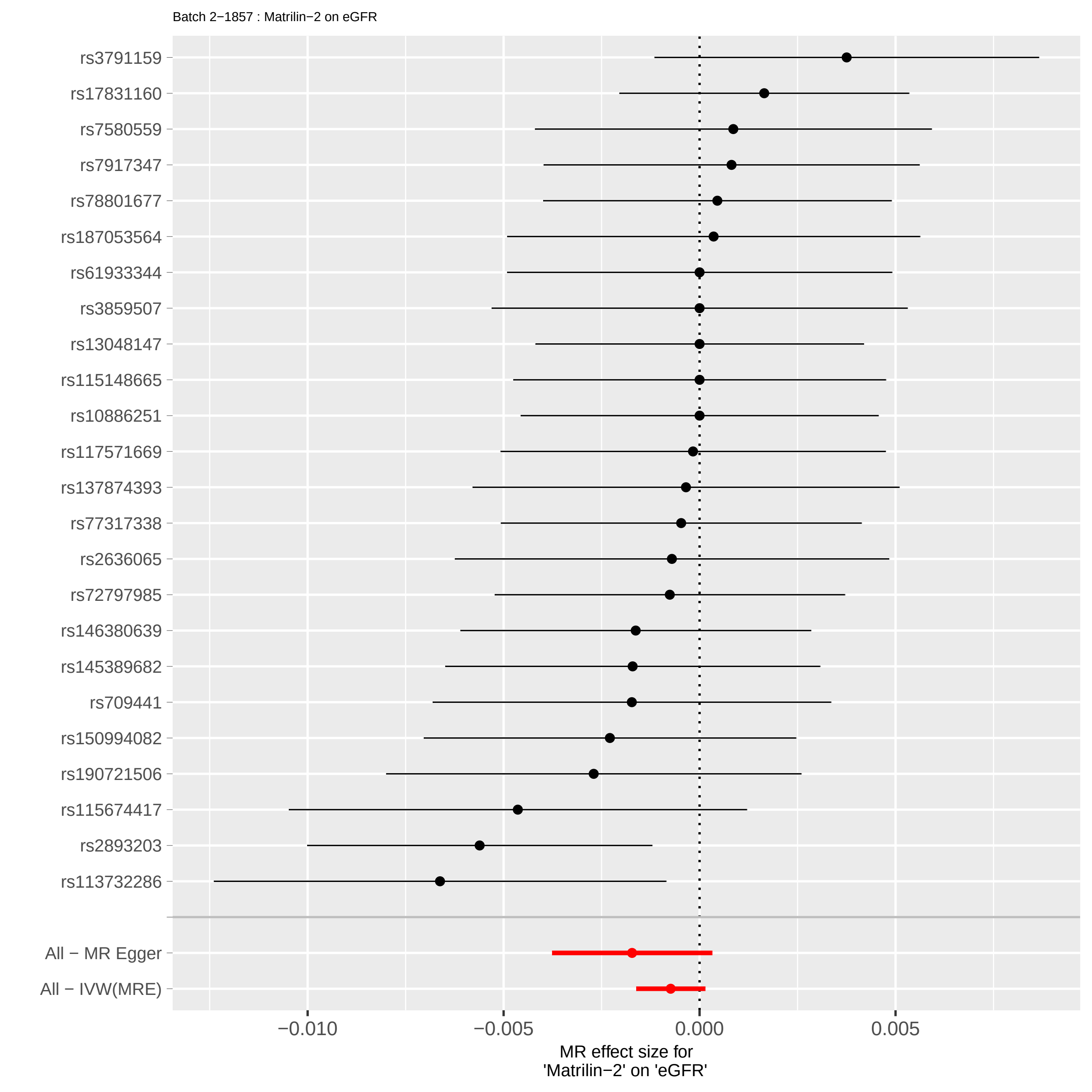
Batch 2−1857 : Matrilin−2 on eGFR
rs3791159
rs17831160
rs7580559
rs7917347
rs78801677
rs187053564
rs61933344
rs3859507
rs13048147
rs115148665
rs10886251
rs117571669
rs137874393
rs77317338
rs2636065
rs72797985
rs146380639
rs145389682
rs709441
rs150994082
rs190721506
rs115674417
rs2893203
rs113732286
All − MR Egger
All − IVW(MRE)
−0.010
−0.005
0.000
0.005
MR effect size for
'Matrilin−2' on 'eGFR'

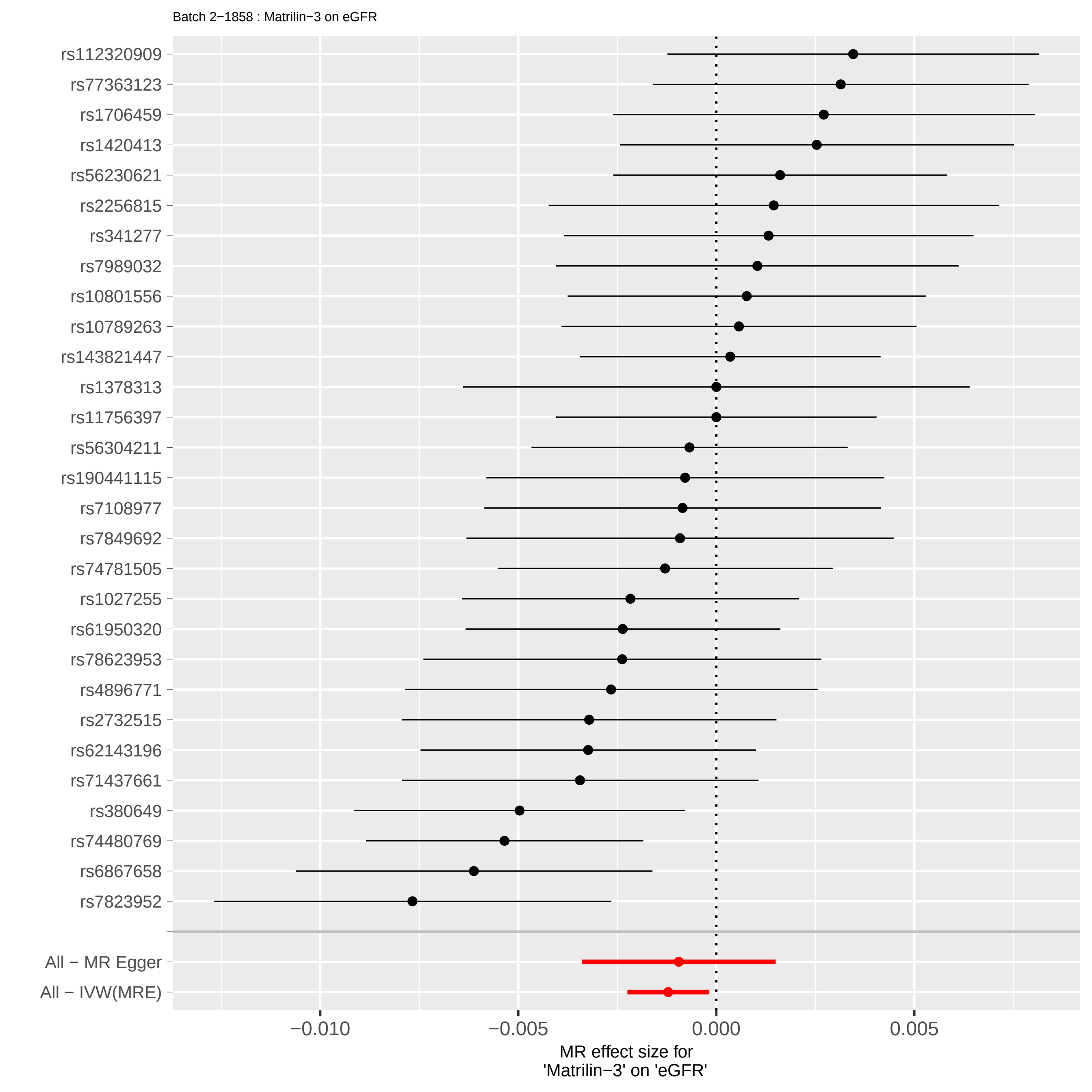

Batch 2−1858 : Matrilin−3 on eGFR
rs112320909
rs77363123
rs1706459
rs1420413
rs56230621
rs2256815
rs341277
rs7989032
rs10801556
rs10789263
rs143821447
rs1378313
rs11756397
rs56304211
rs190441115
rs7108977
rs7849692
rs74781505
rs1027255
rs61950320
rs78623953
rs4896771
rs2732515
rs62143196
rs71437661
rs380649
rs74480769
rs6867658
rs7823952
All − MR Egger
All − IVW(MRE)
−0.010
−0.005
0.000
0.005
MR effect size for
'Matrilin−3' on 'eGFR'

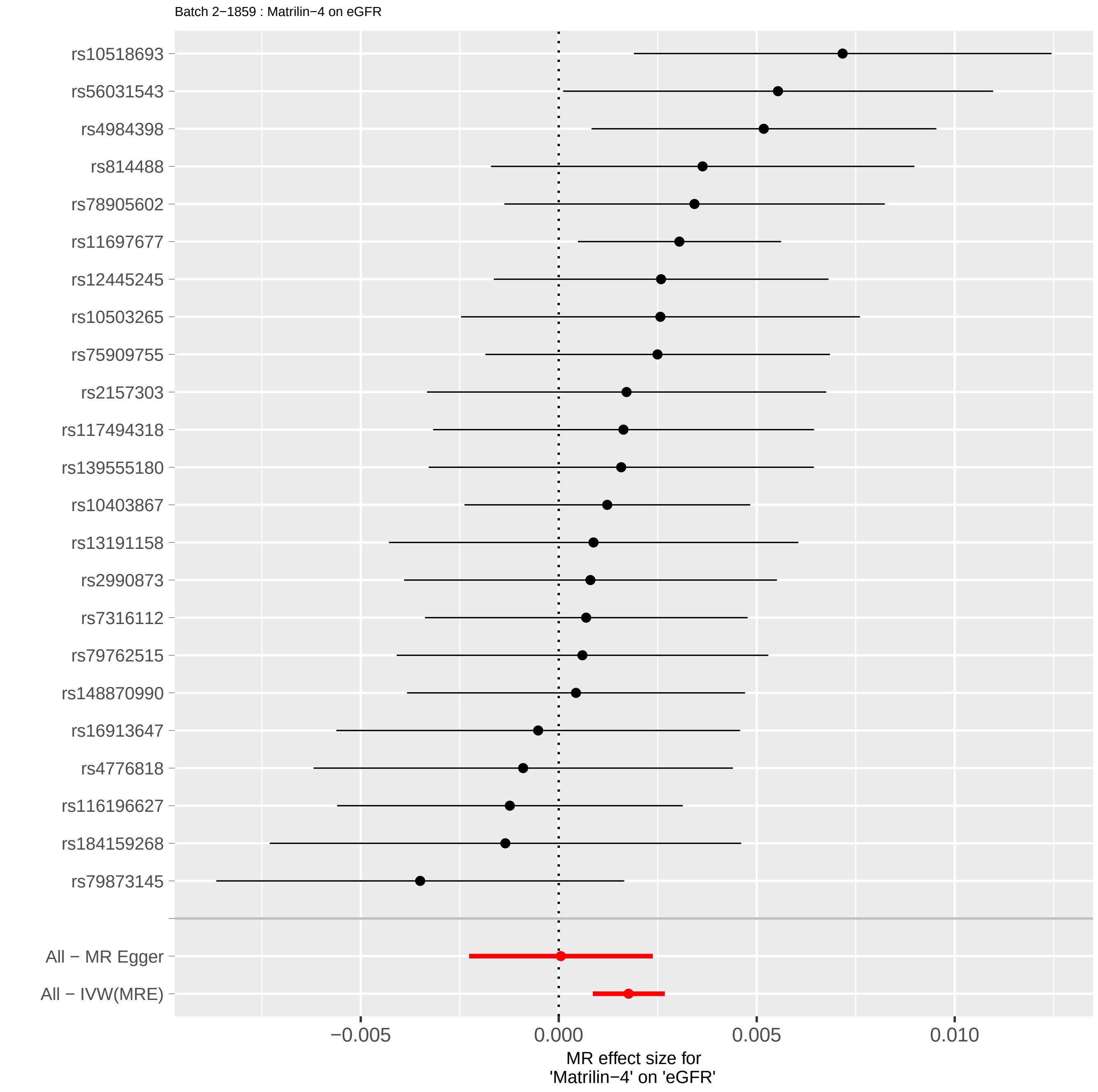

Batch 2−1859 : Matrilin−4 on eGFR
rs10518693
rs56031543
rs4984398
rs814488
rs78905602
rs11697677
rs12445245
rs10503265
rs75909755
rs2157303
rs117494318
rs139555180
rs10403867
rs13191158
rs2990873
rs7316112
rs79762515
rs148870990
rs16913647
rs4776818
rs116196627
rs184159268
rs79873145
All − MR Egger
All − IVW(MRE)
−0.005
0.000
0.005
0.010
MR effect size for
'Matrilin−4' on 'eGFR'

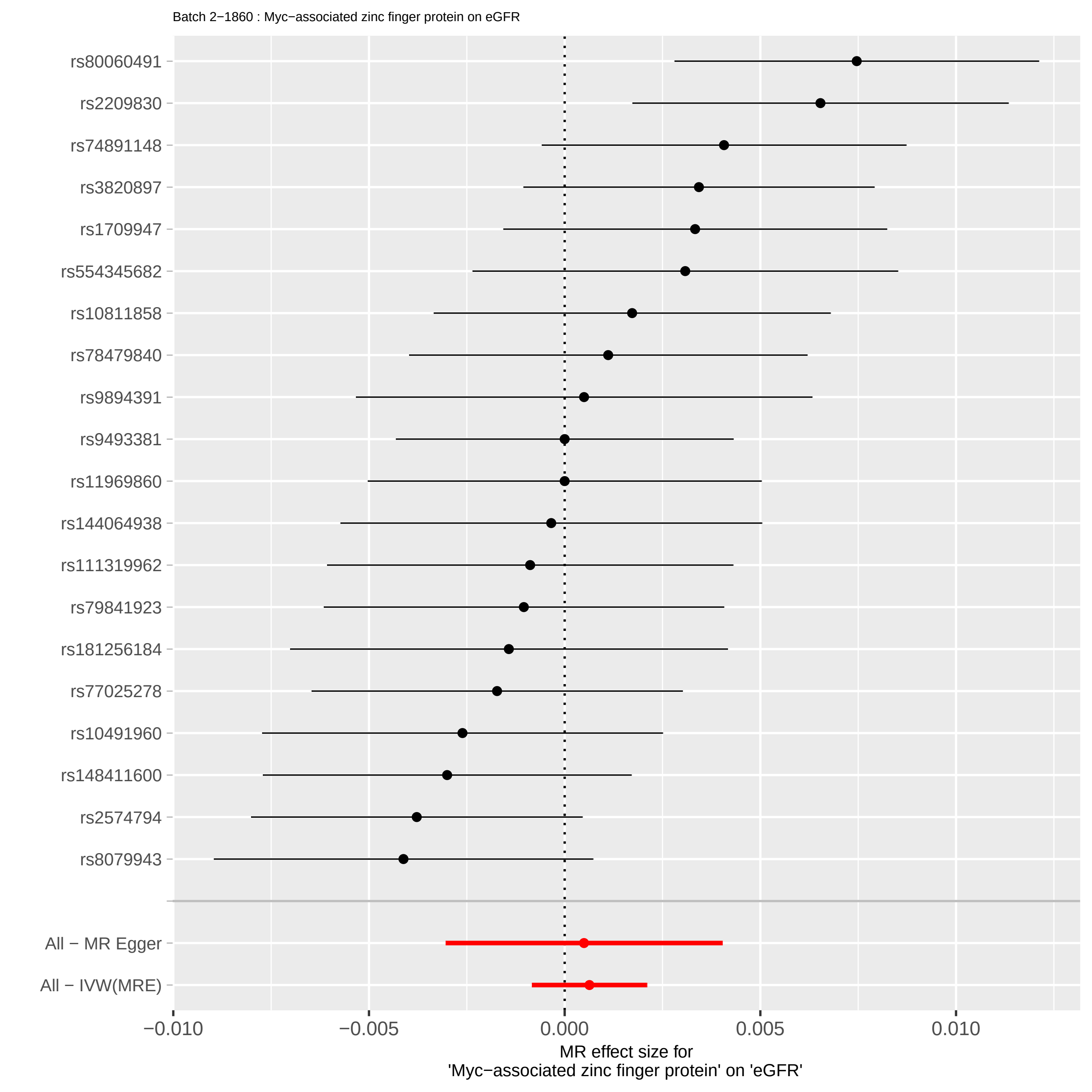

Batch 2−1860 : Myc−associated zinc finger protein on eGFR
rs80060491
rs2209830
rs74891148
rs3820897
rs1709947
rs554345682
rs10811858
rs78479840
rs9894391
rs9493381
rs11969860
rs144064938
rs111319962
rs79841923
rs181256184
rs77025278
rs10491960
rs148411600
rs2574794
rs8079943
All − MR Egger
All − IVW(MRE)
−0.010
−0.005
0.000
0.005
0.010
MR effect size for
'Myc−associated zinc finger protein' on 'eGFR'

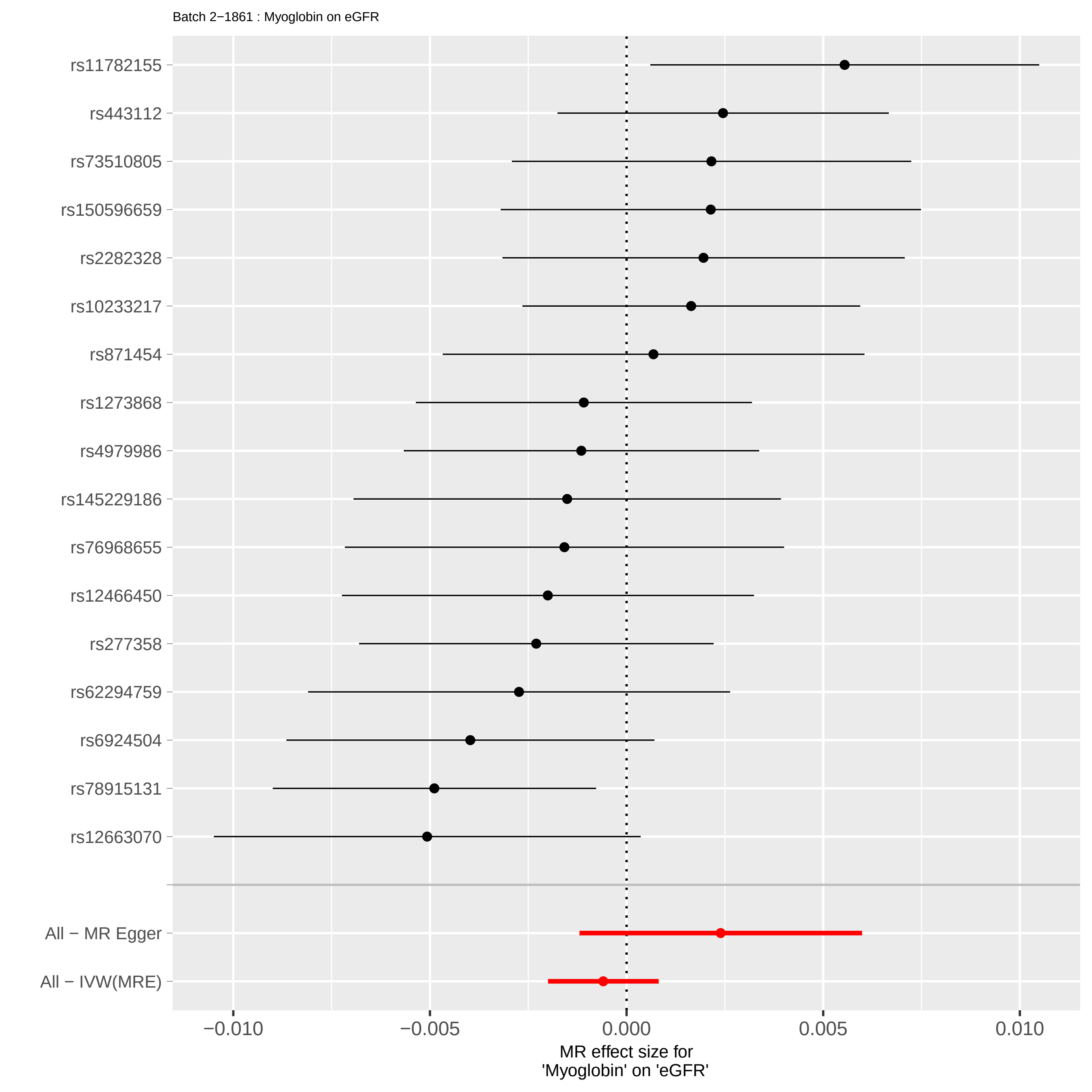
Batch 2−1861 : Myoglobin on eGFR
rs11782155
rs443112
rs73510805
rs150596659
rs2282328
rs10233217
rs871454
rs1273868
rs4979986
rs145229186
rs76968655
rs12466450
rs277358
rs62294759
rs6924504
rs78915131
rs12663070
All − MR Egger
All − IVW(MRE)
−0.010
−0.005
0.000
0.005
0.010
MR effect size for
'Myoglobin' on 'eGFR'

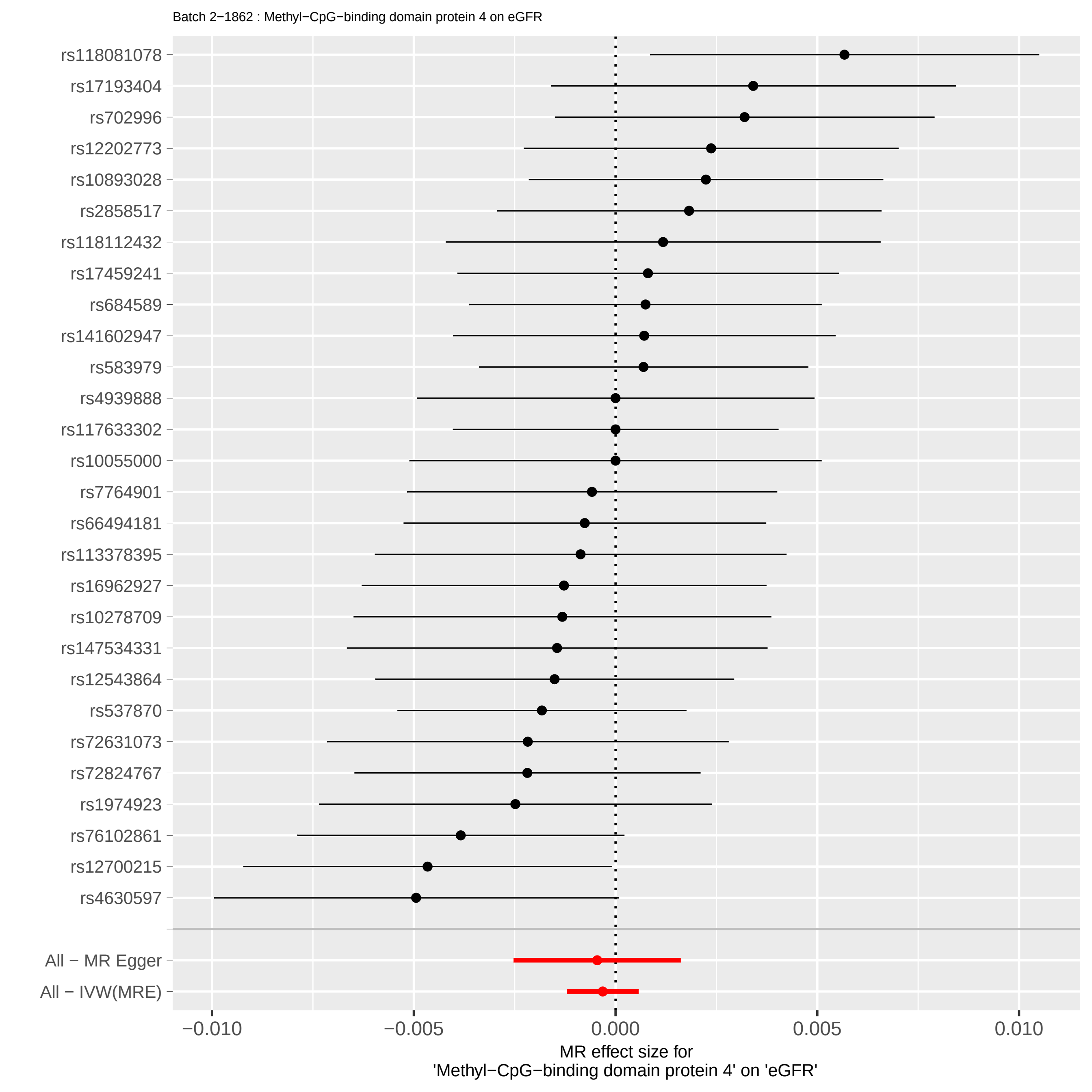

Batch 2−1862 : Methyl−CpG−binding domain protein 4 on eGFR
rs118081078
rs17193404
rs702996
rs12202773
rs10893028
rs2858517
rs118112432
rs17459241
rs684589
rs141602947
rs583979
rs4939888
rs117633302
rs10055000
rs7764901
rs66494181
rs113378395
rs16962927
rs10278709
rs147534331
rs12543864
rs537870
rs72631073
rs72824767
rs1974923
rs76102861
rs12700215
rs4630597
All − MR Egger
All − IVW(MRE)
−0.010
−0.005
0.000
0.005
0.010
MR effect size for
'Methyl−CpG−binding domain protein 4' on 'eGFR'

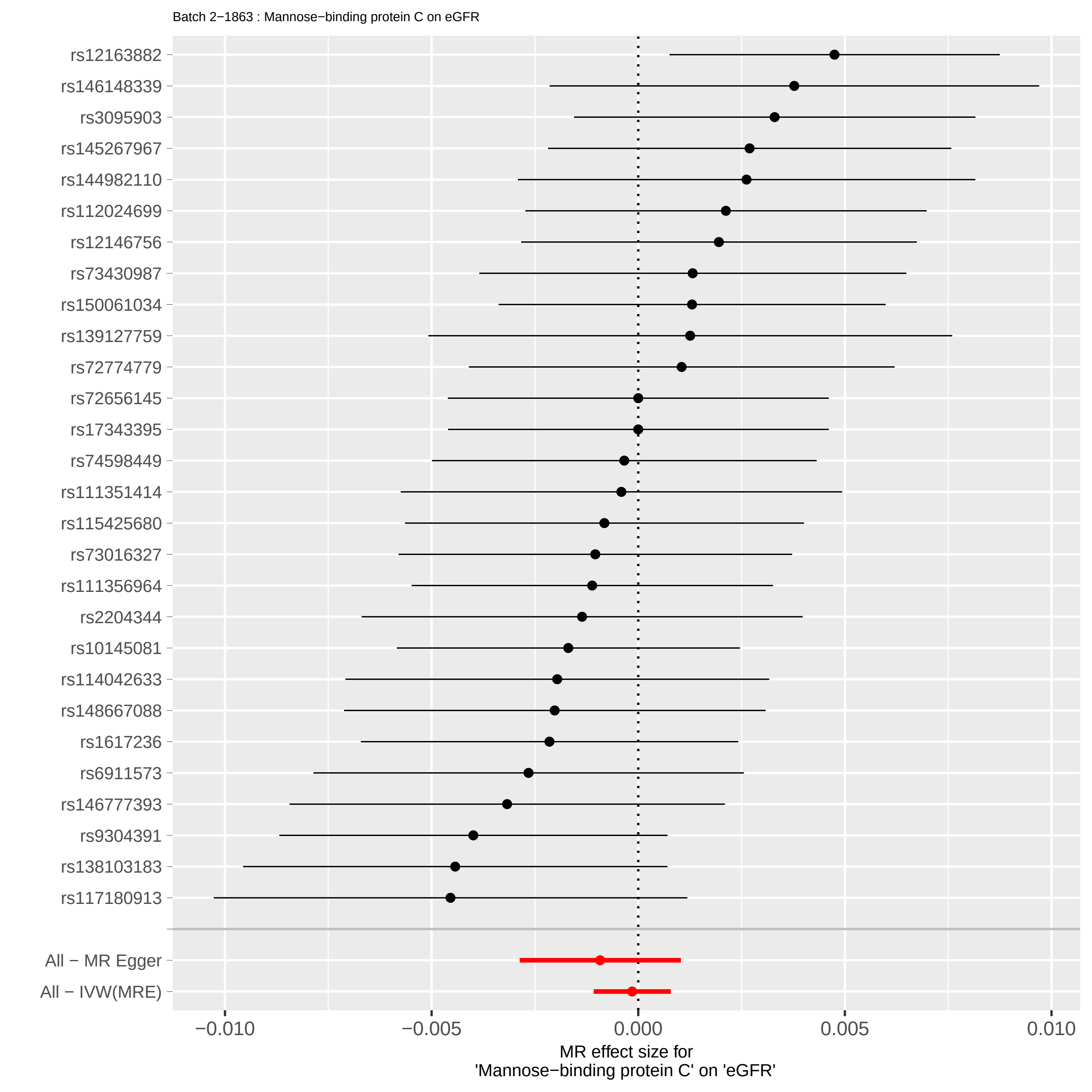
Batch 2−1863 : Mannose−binding protein C on eGFR
rs12163882
rs146148339
rs3095903
rs145267967
rs144982110
rs112024699
rs12146756
rs73430987
rs150061034
rs139127759
rs72774779
rs72656145
rs17343395
rs74598449
rs111351414
rs115425680
rs73016327
rs111356964
rs2204344
rs10145081
rs114042633
rs148667088
rs1617236
rs6911573
rs146777393
rs9304391
rs138103183
rs117180913
All − MR Egger
All − IVW(MRE)
−0.010
−0.005
0.000
0.005
0.010
MR effect size for
'Mannose−binding protein C' on 'eGFR'

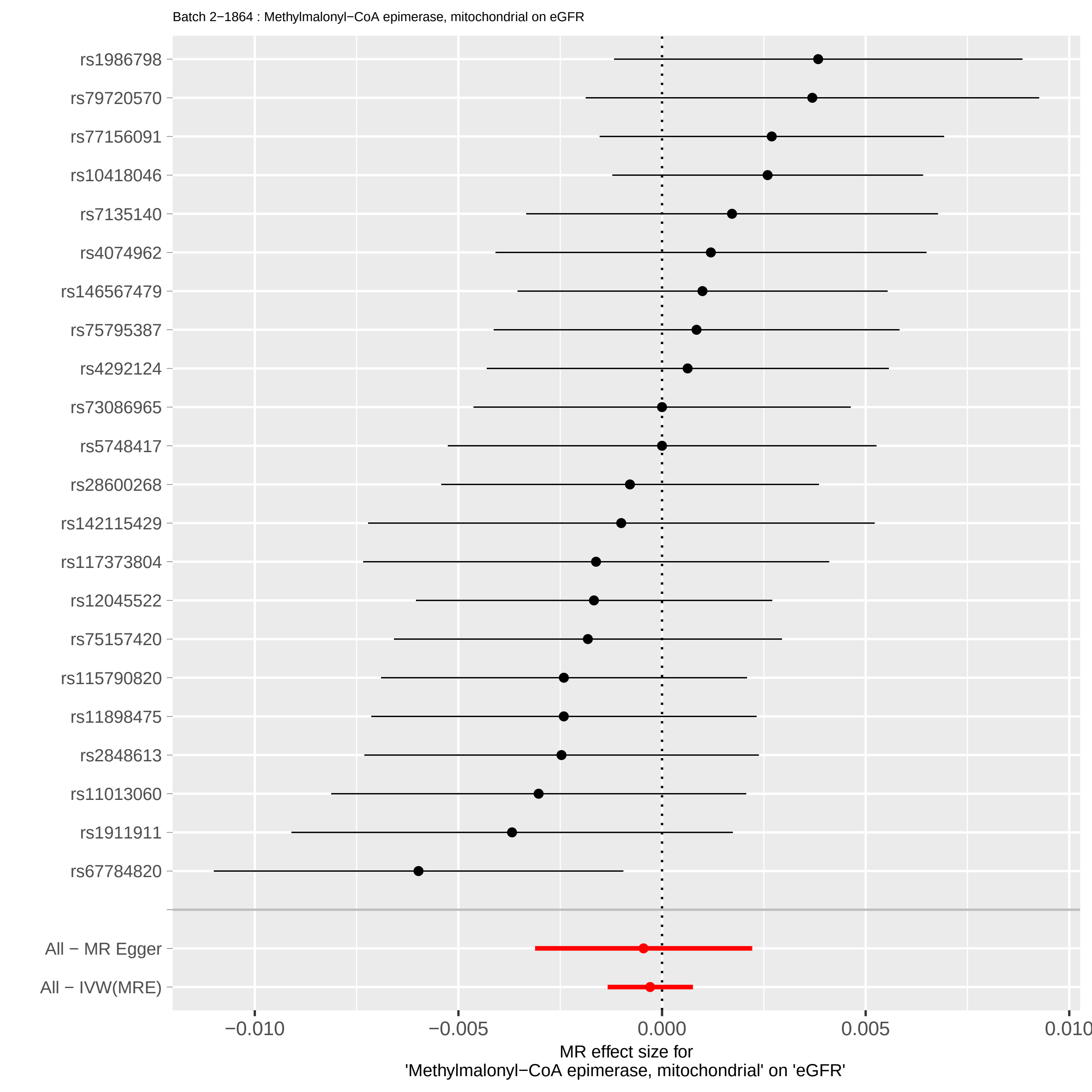
Batch 2−1864 : Methylmalonyl−CoA epimerase, mitochondrial on eGFR
rs1986798
rs79720570
rs77156091
rs10418046
rs7135140
rs4074962
rs146567479
rs75795387
rs4292124
rs73086965
rs5748417
rs28600268
rs142115429
rs117373804
rs12045522
rs75157420
rs115790820
rs11898475
rs2848613
rs11013060
rs1911911
rs67784820
All − MR Egger
All − IVW(MRE)
−0.010
−0.005
0.000
0.005
0.010
MR effect size for
'Methylmalonyl−CoA epimerase, mitochondrial' on 'eGFR'

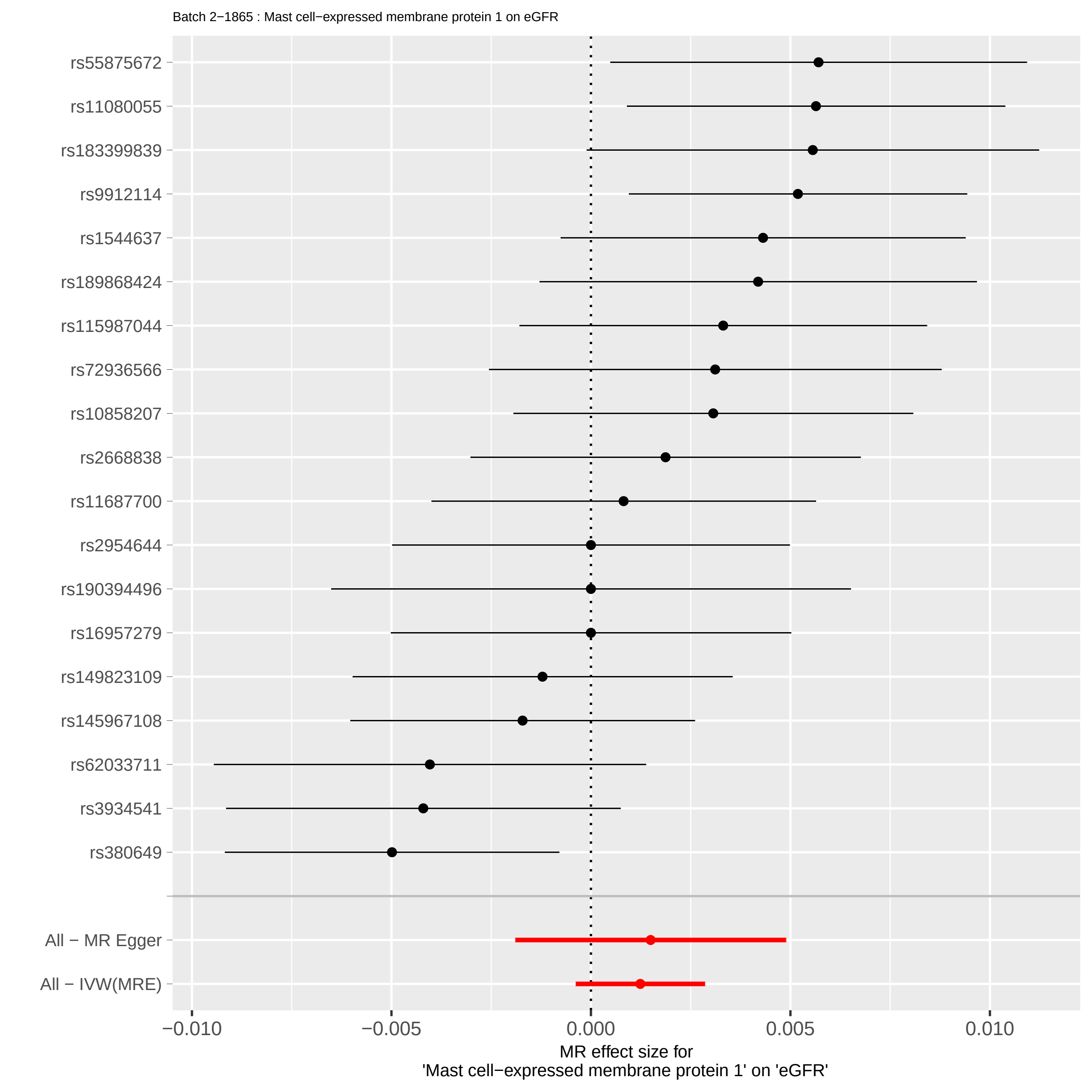

Batch 2−1865 : Mast cell−expressed membrane protein 1 on eGFR
rs55875672
rs11080055
rs183399839
rs9912114
rs1544637
rs189868424
rs115987044
rs72936566
rs10858207
rs2668838
rs11687700
rs2954644
rs190394496
rs16957279
rs149823109
rs145967108
rs62033711
rs3934541
rs380649
All − MR Egger
All − IVW(MRE)
−0.010
−0.005
0.000
0.005
0.010
MR effect size for
'Mast cell−expressed membrane protein 1' on 'eGFR'

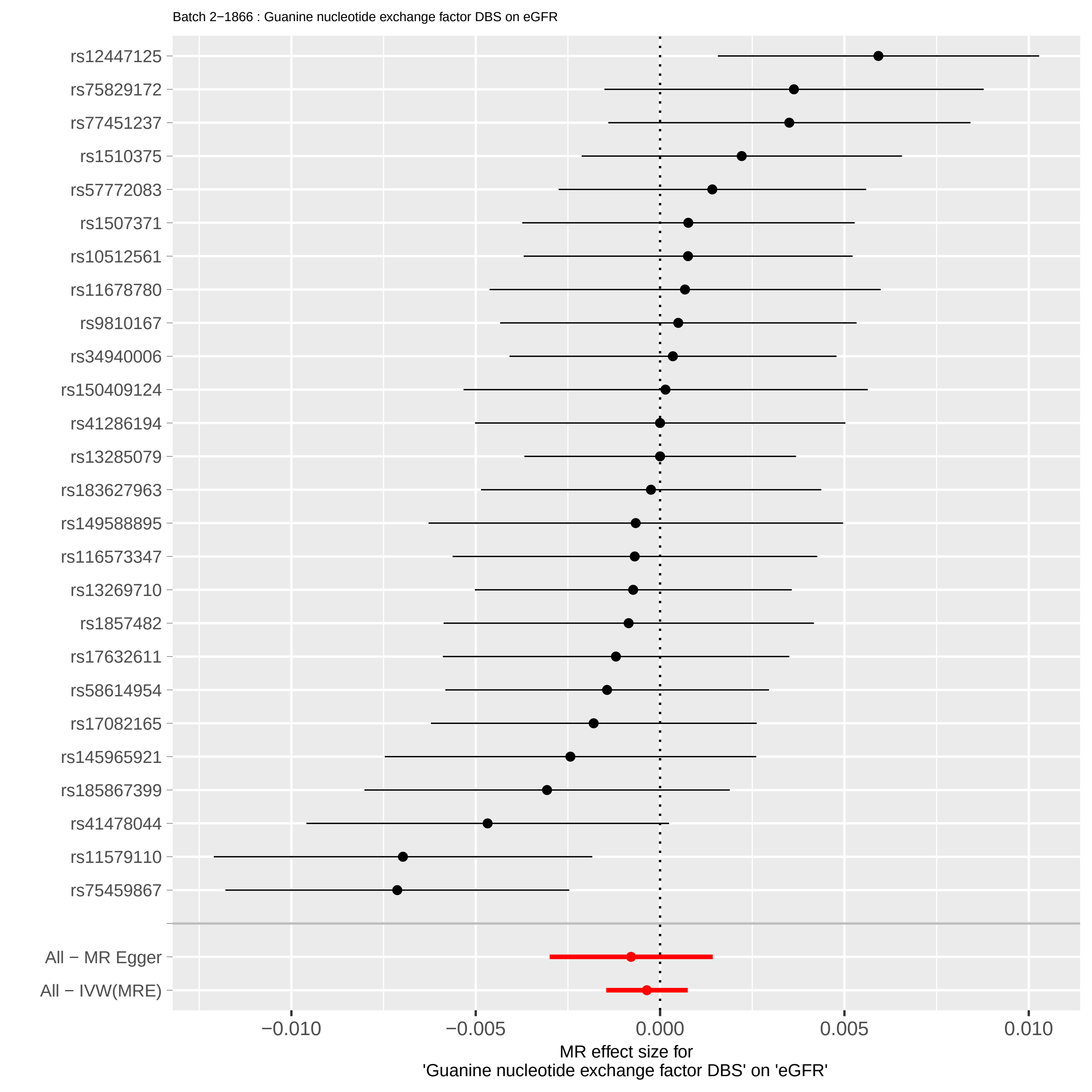

Batch 2−1866 : Guanine nucleotide exchange factor DBS on eGFR
rs12447125
rs75829172
rs77451237
rs1510375
rs57772083
rs1507371
rs10512561
rs11678780
rs9810167
rs34940006
rs150409124
rs41286194
rs13285079
rs183627963
rs149588895
rs116573347
rs13269710
rs1857482
rs17632611
rs58614954
rs17082165
rs145965921
rs185867399
rs41478044
rs11579110
rs75459867
All − MR Egger
All − IVW(MRE)
−0.010
−0.005
0.000
0.005
0.010
MR effect size for
'Guanine nucleotide exchange factor DBS' on 'eGFR'

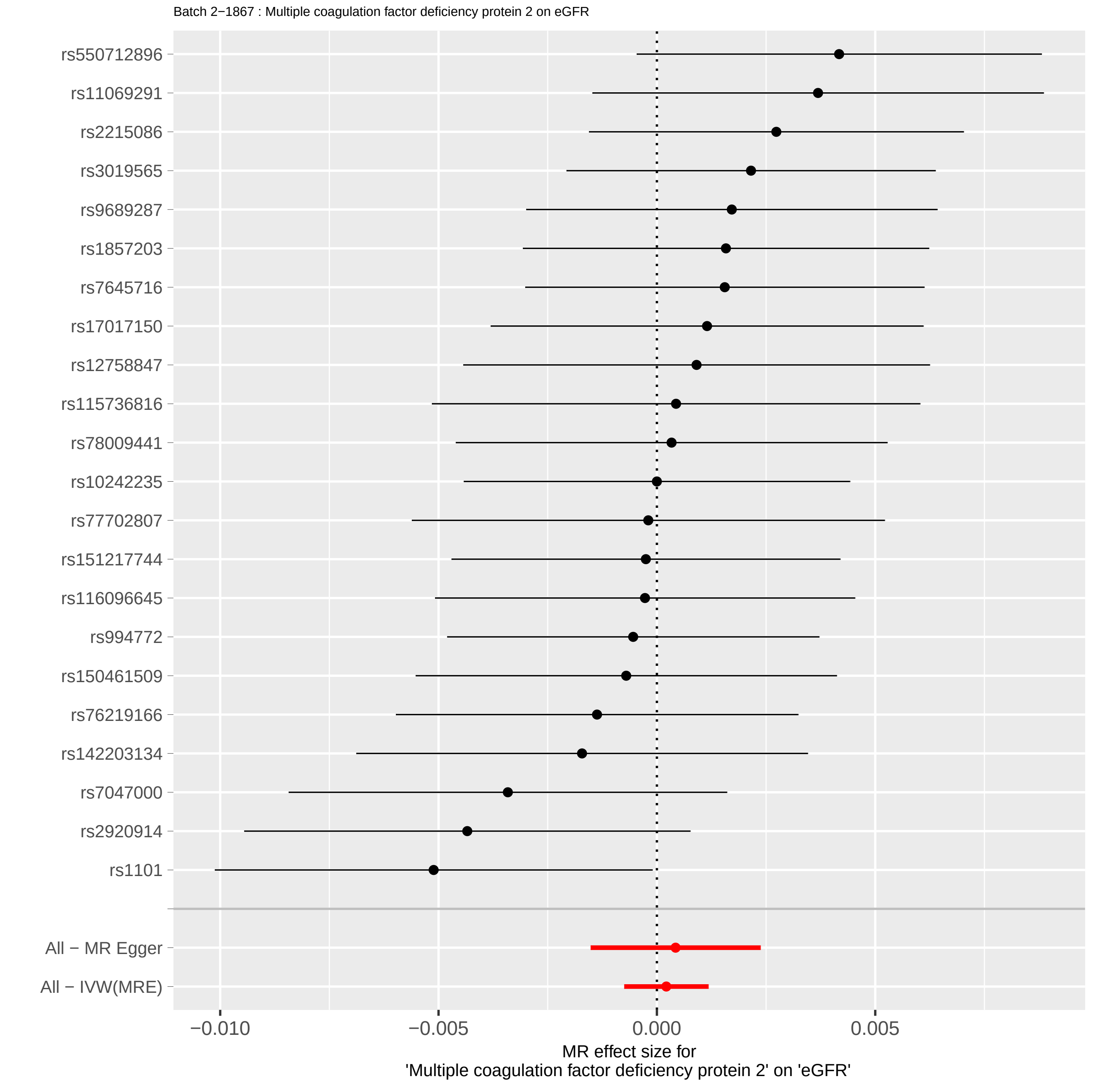

Batch 2−1867 : Multiple coagulation factor deficiency protein 2 on eGFR
rs550712896
rs11069291
rs2215086
rs3019565
rs9689287
rs1857203
rs7645716
rs17017150
rs12758847
rs115736816
rs78009441
rs10242235
rs77702807
rs151217744
rs116096645
rs994772
rs150461509
rs76219166
rs142203134
rs7047000
rs2920914
rs1101
All − MR Egger
All − IVW(MRE)
−0.010
−0.005
0.000
0.005
MR effect size for
'Multiple coagulation factor deficiency protein 2' on 'eGFR'

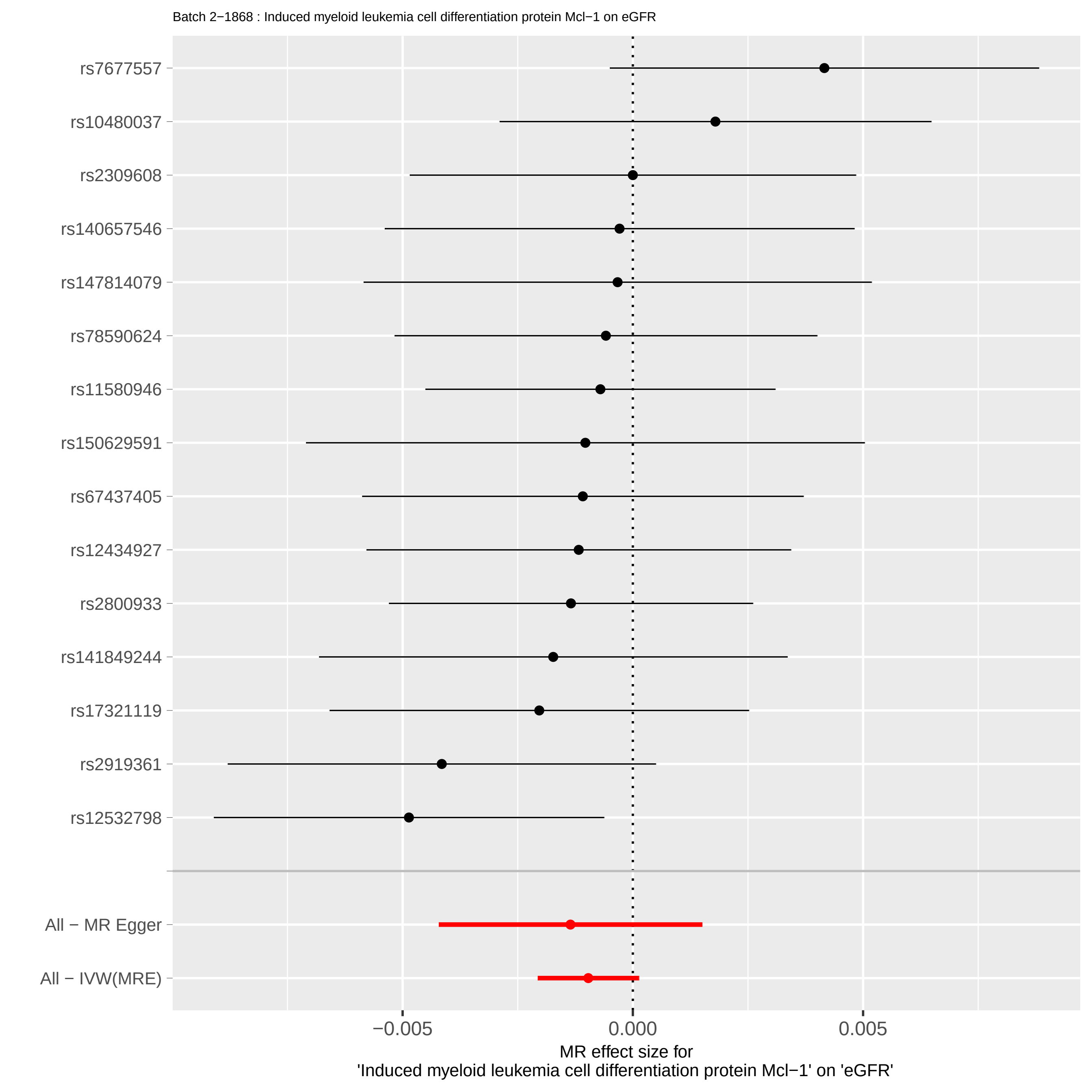

Batch 2−1868 : Induced myeloid leukemia cell differentiation protein Mcl−1 on eGFR
rs7677557
rs10480037
rs2309608
rs140657546
rs147814079
rs78590624
rs11580946
rs150629591
rs67437405
rs12434927
rs2800933
rs141849244
rs17321119
rs2919361
rs12532798
All − MR Egger
All − IVW(MRE)
−0.005
0.000
0.005
MR effect size for
'Induced myeloid leukemia cell differentiation protein Mcl−1' on 'eGFR'

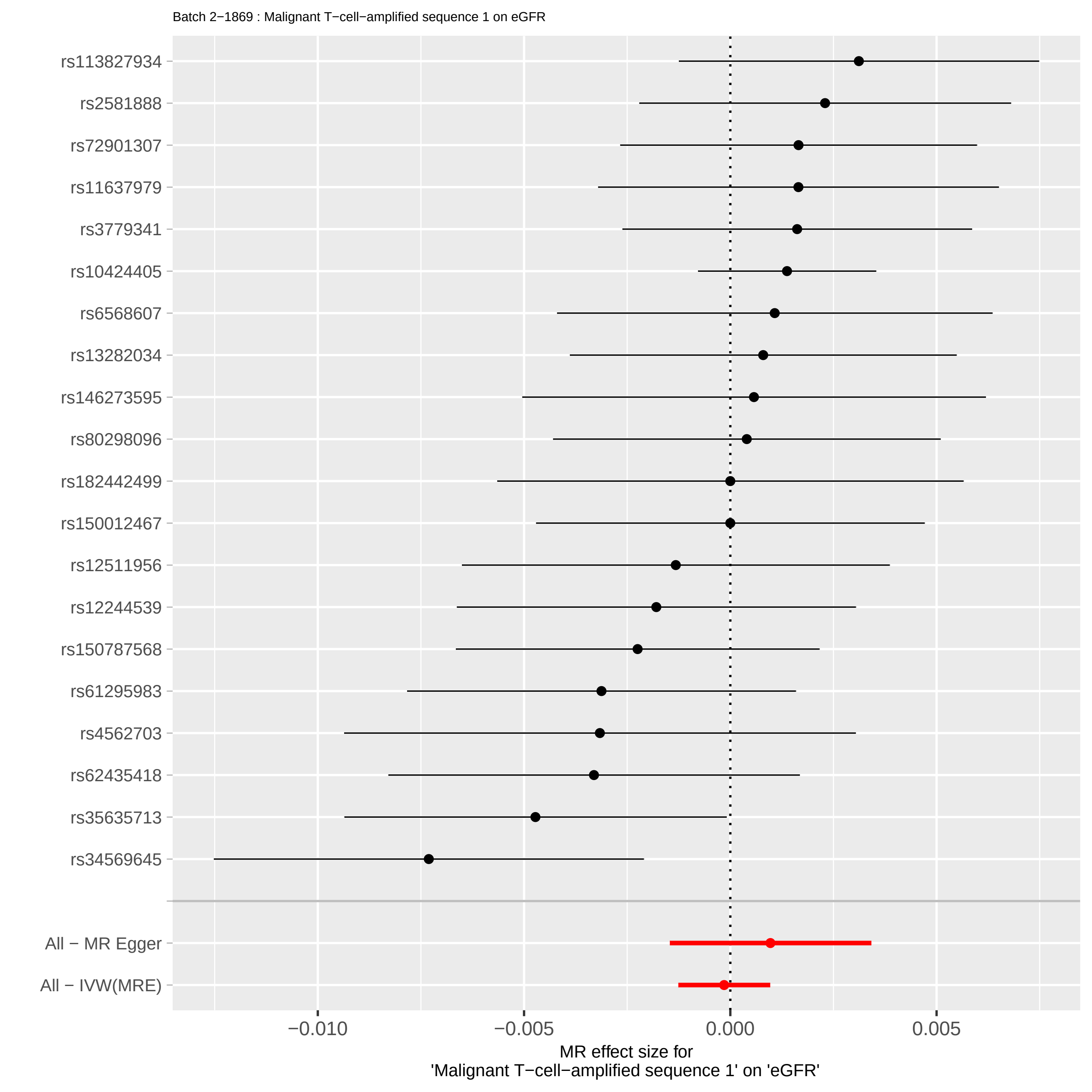

Batch 2−1869 : Malignant T−cell−amplified sequence 1 on eGFR
rs113827934
rs2581888
rs72901307
rs11637979
rs3779341
rs10424405
rs6568607
rs13282034
rs146273595
rs80298096
rs182442499
rs150012467
rs12511956
rs12244539
rs150787568
rs61295983
rs4562703
rs62435418
rs35635713
rs34569645
All − MR Egger
All − IVW(MRE)
−0.010
−0.005
0.000
0.005
MR effect size for
'Malignant T−cell−amplified sequence 1' on 'eGFR'

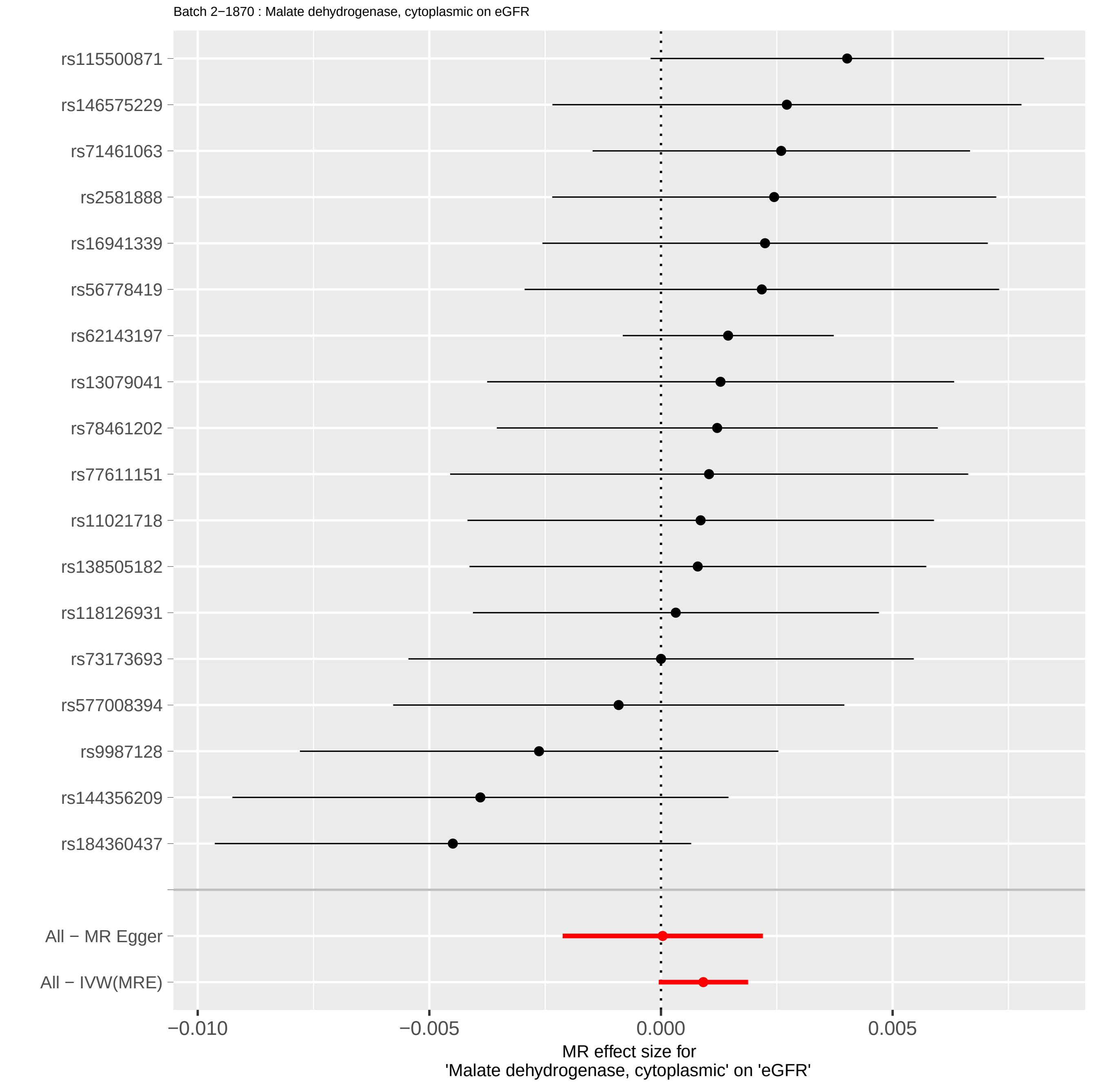

Batch 2−1870 : Malate dehydrogenase, cytoplasmic on eGFR
rs115500871
rs146575229
rs71461063
rs2581888
rs16941339
rs56778419
rs62143197
rs13079041
rs78461202
rs77611151
rs11021718
rs138505182
rs118126931
rs73173693
rs577008394
rs9987128
rs144356209
rs184360437
All − MR Egger
All − IVW(MRE)
−0.010
−0.005
0.000
0.005
MR effect size for
'Malate dehydrogenase, cytoplasmic' on 'eGFR'

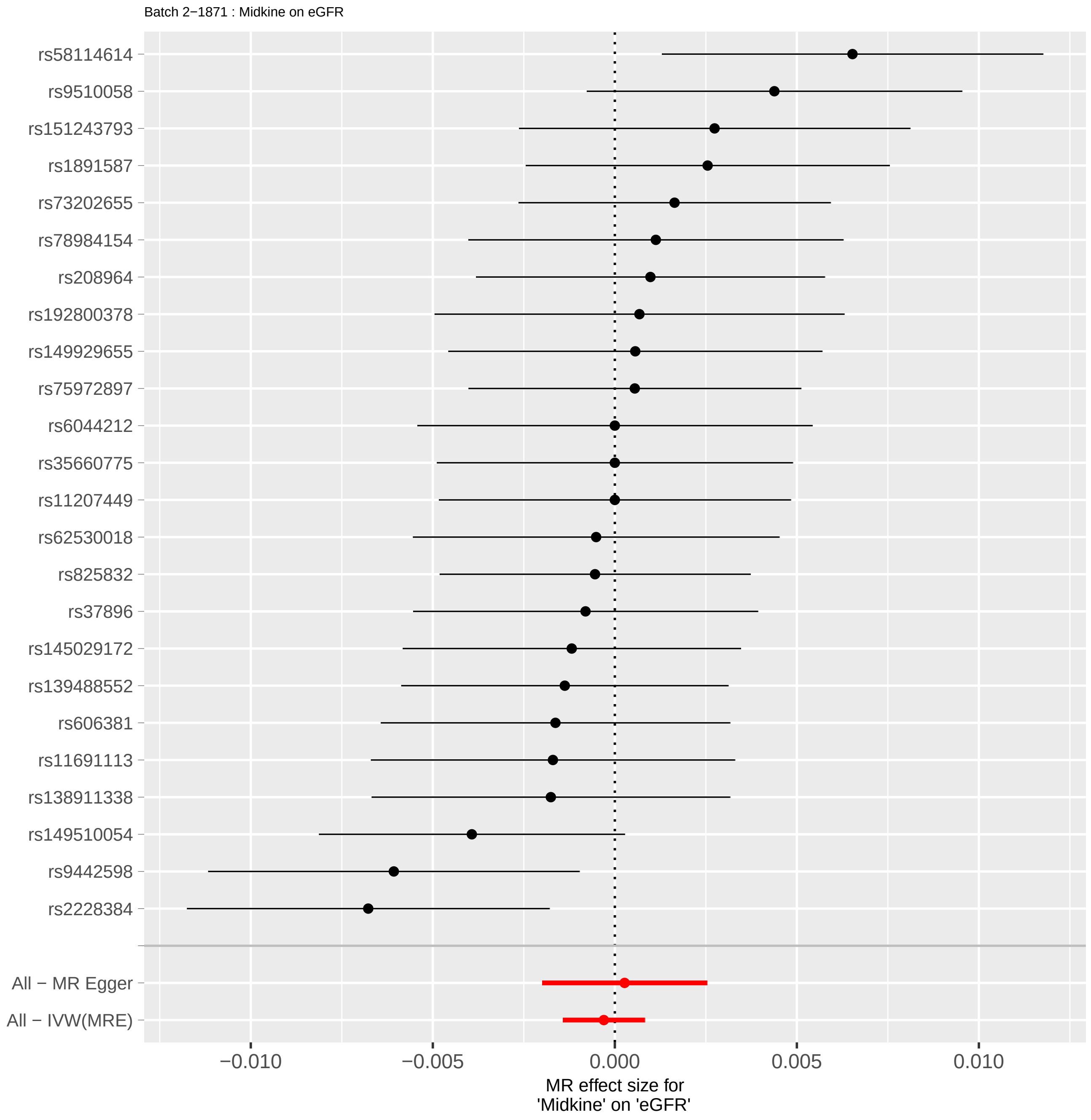

Batch 2−1871 : Midkine on eGFR
rs58114614
rs9510058
rs151243793
rs1891587
rs73202655
rs78984154
rs208964
rs192800378
rs149929655
rs75972897
rs6044212
rs35660775
rs11207449
rs62530018
rs825832
rs37896
rs145029172
rs139488552
rs606381
rs11691113
rs138911338
rs149510054
rs9442598
rs2228384
All − MR Egger
All − IVW(MRE)
−0.010
−0.005
0.000
0.005
0.010
MR effect size for
'Midkine' on 'eGFR'

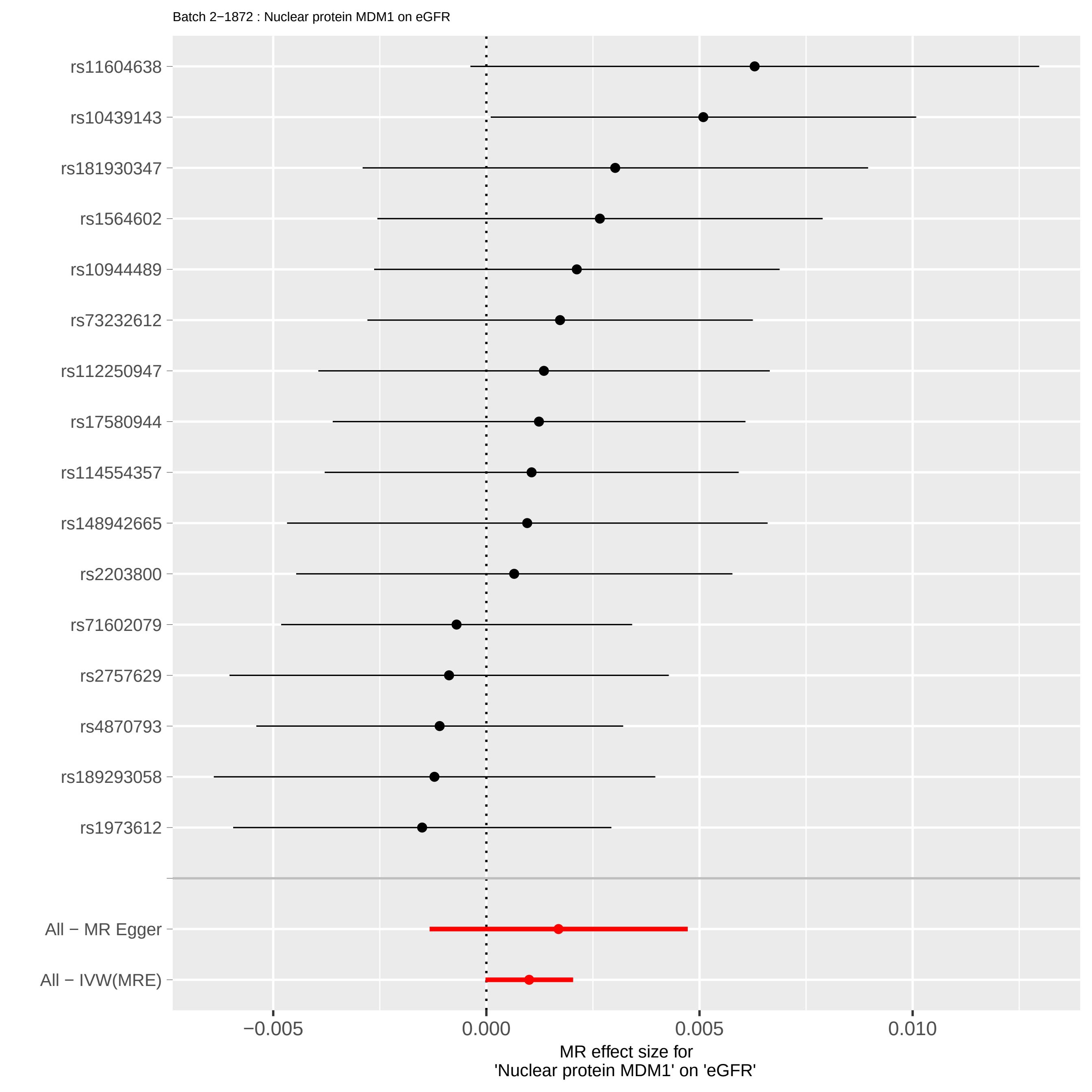

Batch 2−1872 : Nuclear protein MDM1 on eGFR
rs11604638
rs10439143
rs181930347
rs1564602
rs10944489
rs73232612
rs112250947
rs17580944
rs114554357
rs148942665
rs2203800
rs71602079
rs2757629
rs4870793
rs189293058
rs1973612
All − MR Egger
All − IVW(MRE)
−0.005
0.000
0.005
0.010
MR effect size for
'Nuclear protein MDM1' on 'eGFR'

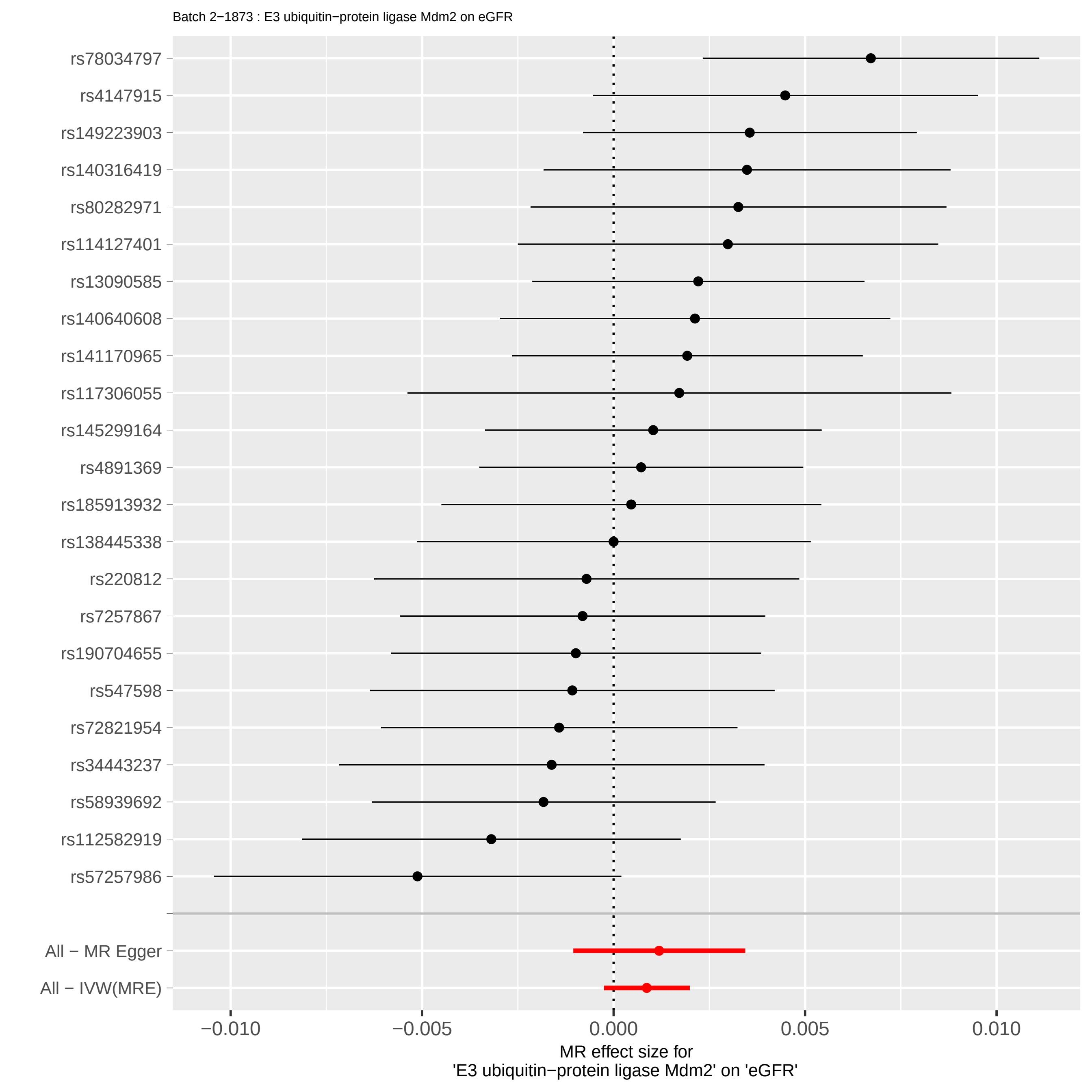

Batch 2−1873 : E3 ubiquitin−protein ligase Mdm2 on eGFR
rs78034797
rs4147915
rs149223903
rs140316419
rs80282971
rs114127401
rs13090585
rs140640608
rs141170965
rs117306055
rs145299164
rs4891369
rs185913932
rs138445338
rs220812
rs7257867
rs190704655
rs547598
rs72821954
rs34443237
rs58939692
rs112582919
rs57257986
All − MR Egger
All − IVW(MRE)
−0.010
−0.005
0.000
0.005
0.010
MR effect size for
'E3 ubiquitin−protein ligase Mdm2' on 'eGFR'

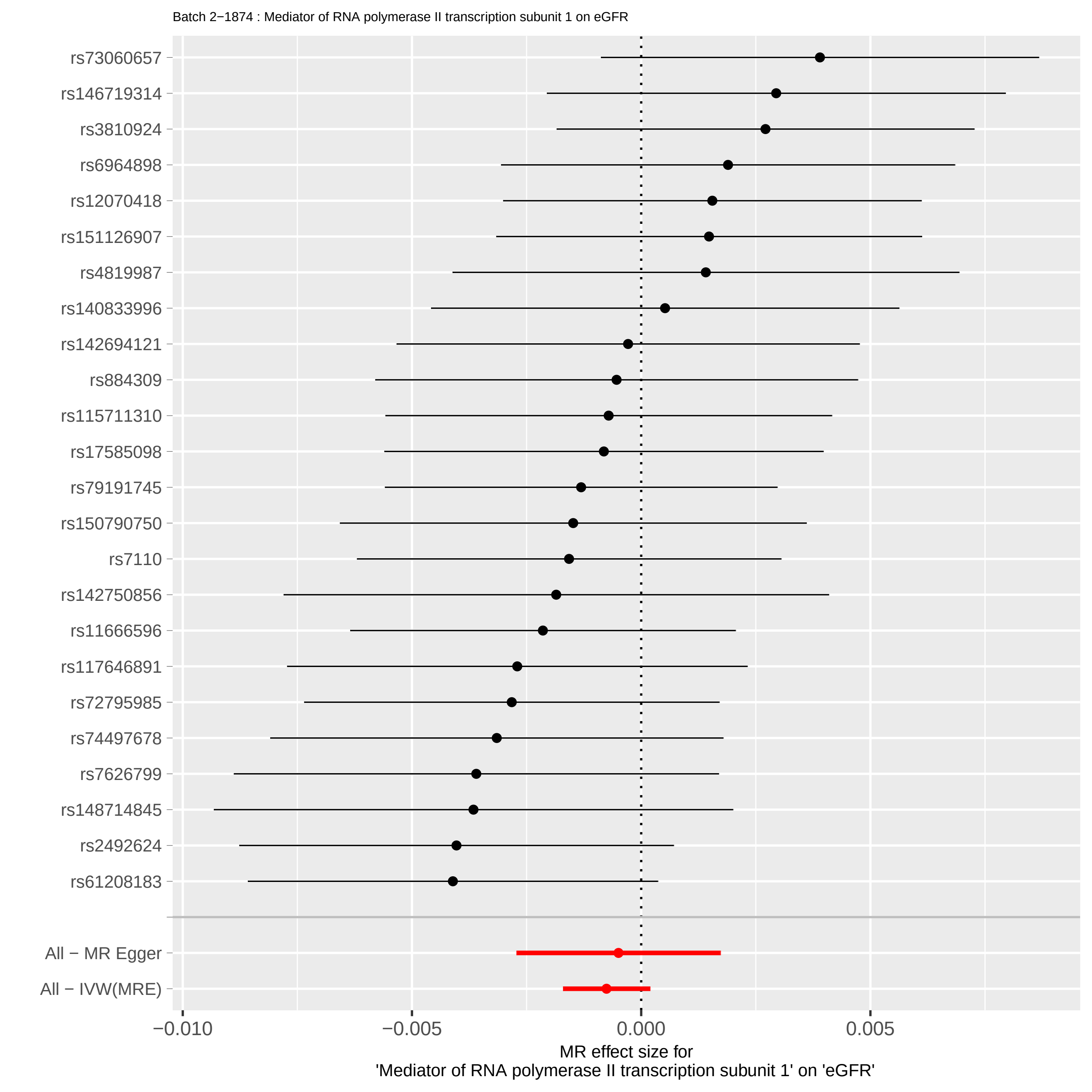

Batch 2−1874 : Mediator of RNA polymerase II transcription subunit 1 on eGFR
rs73060657
rs146719314
rs3810924
rs6964898
rs12070418
rs151126907
rs4819987
rs140833996
rs142694121
rs884309
rs115711310
rs17585098
rs79191745
rs150790750
rs7110
rs142750856
rs11666596
rs117646891
rs72795985
rs74497678
rs7626799
rs148714845
rs2492624
rs61208183
All − MR Egger
All − IVW(MRE)
−0.010
−0.005
0.000
0.005
MR effect size for
'Mediator of RNA polymerase II transcription subunit 1' on 'eGFR'

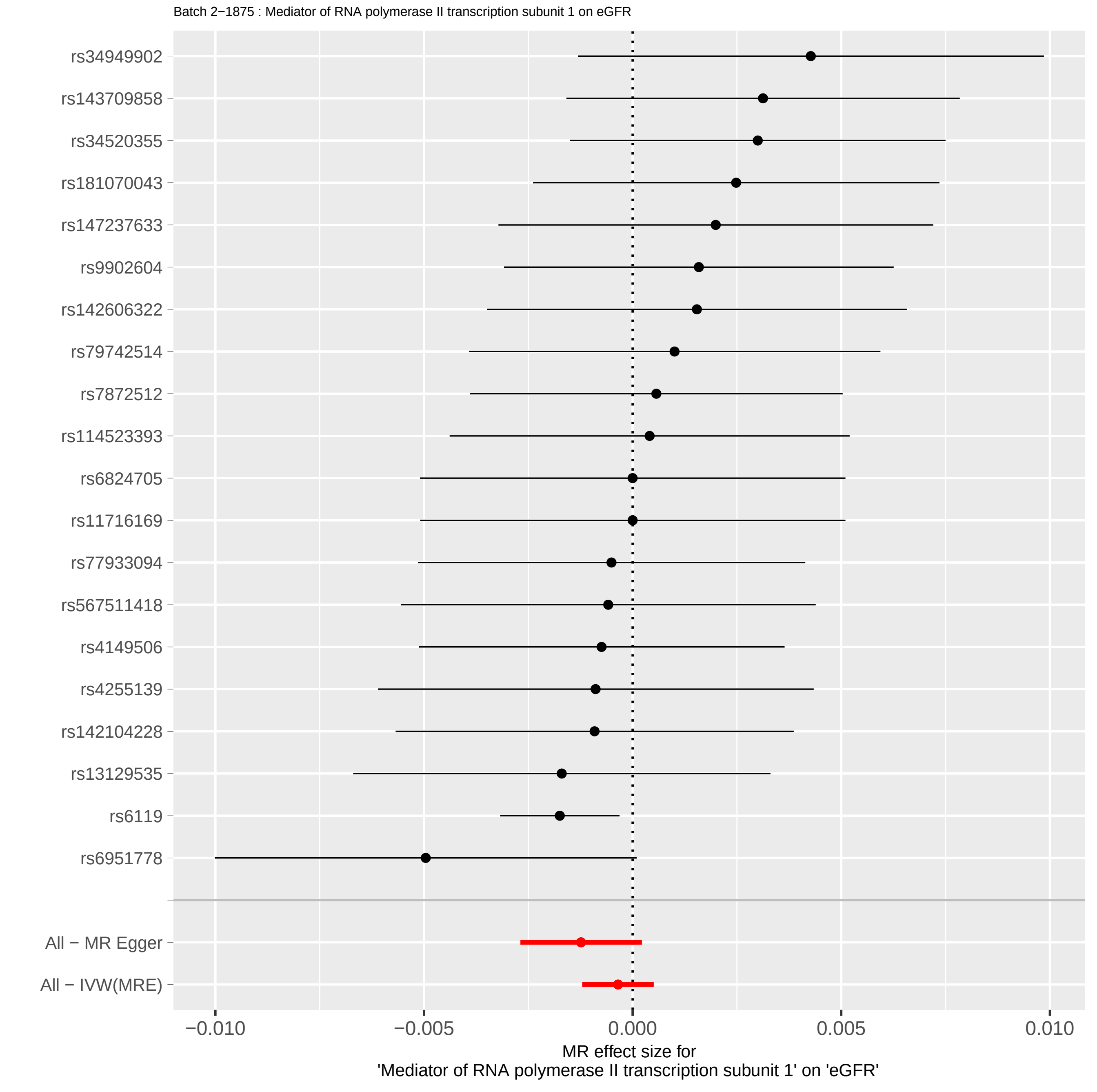

Batch 2−1875 : Mediator of RNA polymerase II transcription subunit 1 on eGFR
rs34949902
rs143709858
rs34520355
rs181070043
rs147237633
rs9902604
rs142606322
rs79742514
rs7872512
rs114523393
rs6824705
rs11716169
rs77933094
rs567511418
rs4149506
rs4255139
rs142104228
rs13129535
rs6119
rs6951778
All − MR Egger
All − IVW(MRE)
−0.010
−0.005
0.000
0.005
0.010
MR effect size for
'Mediator of RNA polymerase II transcription subunit 1' on 'eGFR'

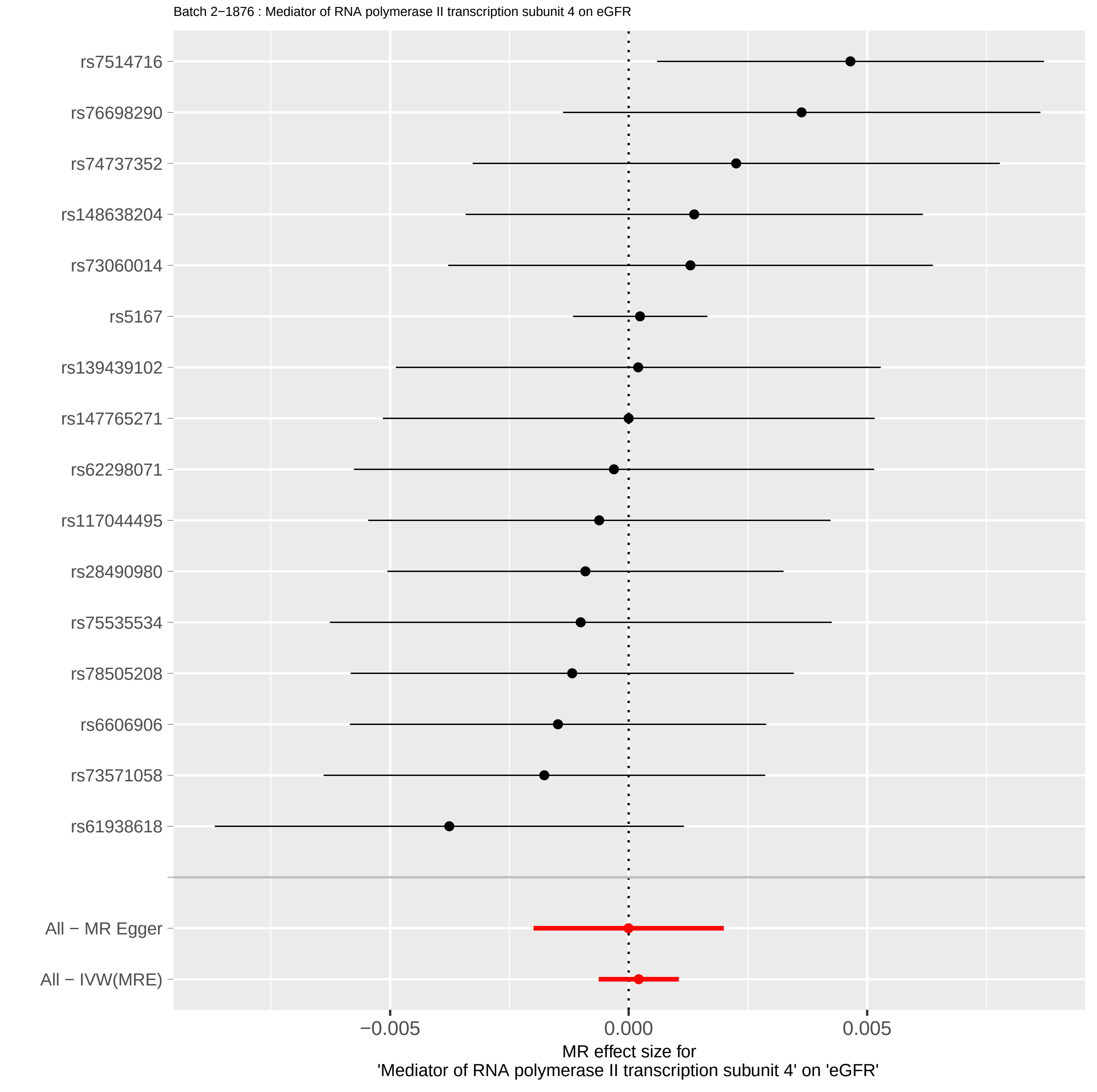

Batch 2−1876 : Mediator of RNA polymerase II transcription subunit 4 on eGFR
rs7514716
rs76698290
rs74737352
rs148638204
rs73060014
rs5167
rs139439102
rs147765271
rs62298071
rs117044495
rs28490980
rs75535534
rs78505208
rs6606906
rs73571058
rs61938618
All − MR Egger
All − IVW(MRE)
−0.005
0.000
0.005
MR effect size for
'Mediator of RNA polymerase II transcription subunit 4' on 'eGFR'

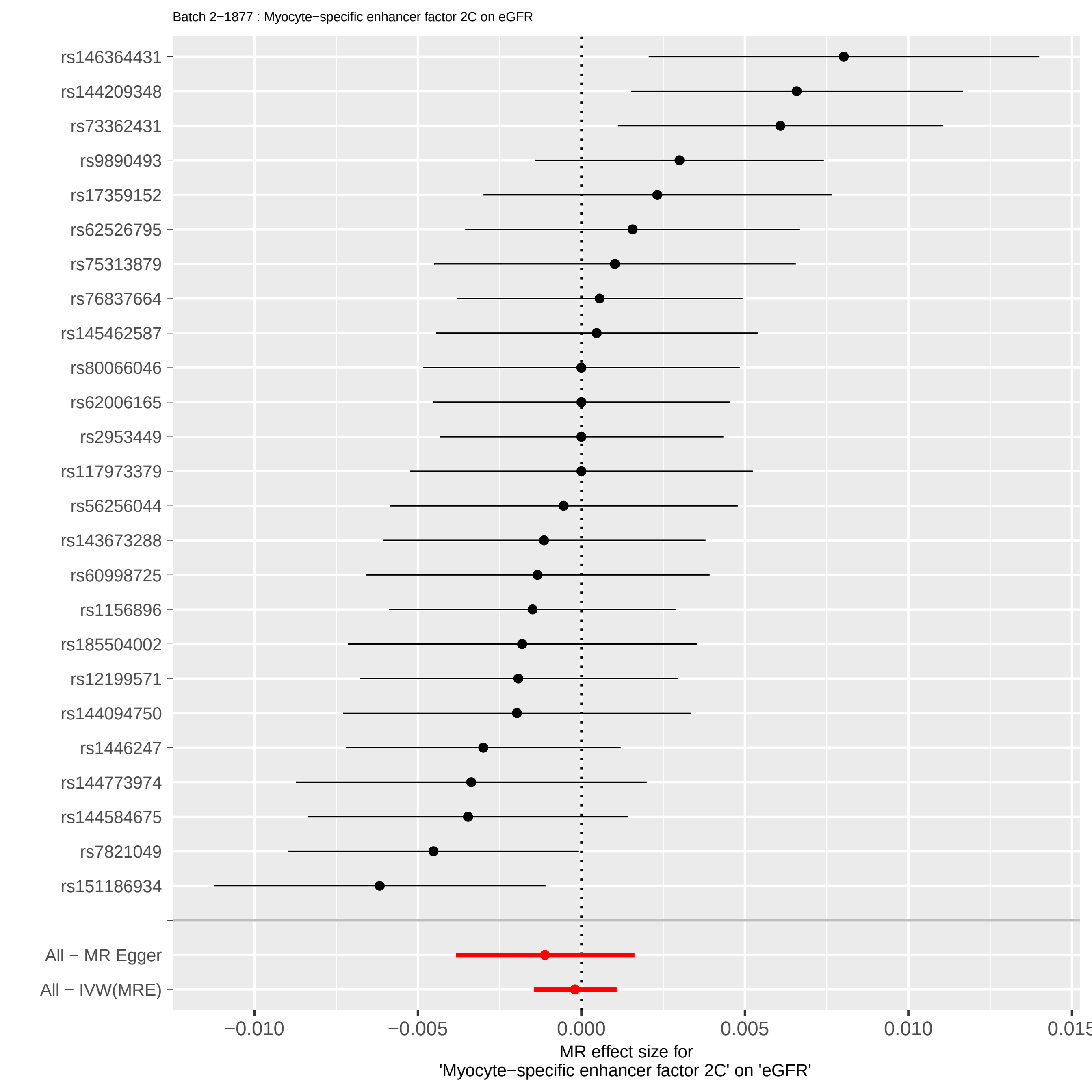

Batch 2−1877 : Myocyte−specific enhancer factor 2C on eGFR
rs146364431
rs144209348
rs73362431
rs9890493
rs17359152
rs62526795
rs75313879
rs76837664
rs145462587
rs80066046
rs62006165
rs2953449
rs117973379
rs56256044
rs143673288
rs60998725
rs1156896
rs185504002
rs12199571
rs144094750
rs1446247
rs144773974
rs144584675
rs7821049
rs151186934
All − MR Egger
All − IVW(MRE)
−0.010
−0.005
0.000
0.005
0.010
0.015
MR effect size for
'Myocyte−specific enhancer factor 2C' on 'eGFR'

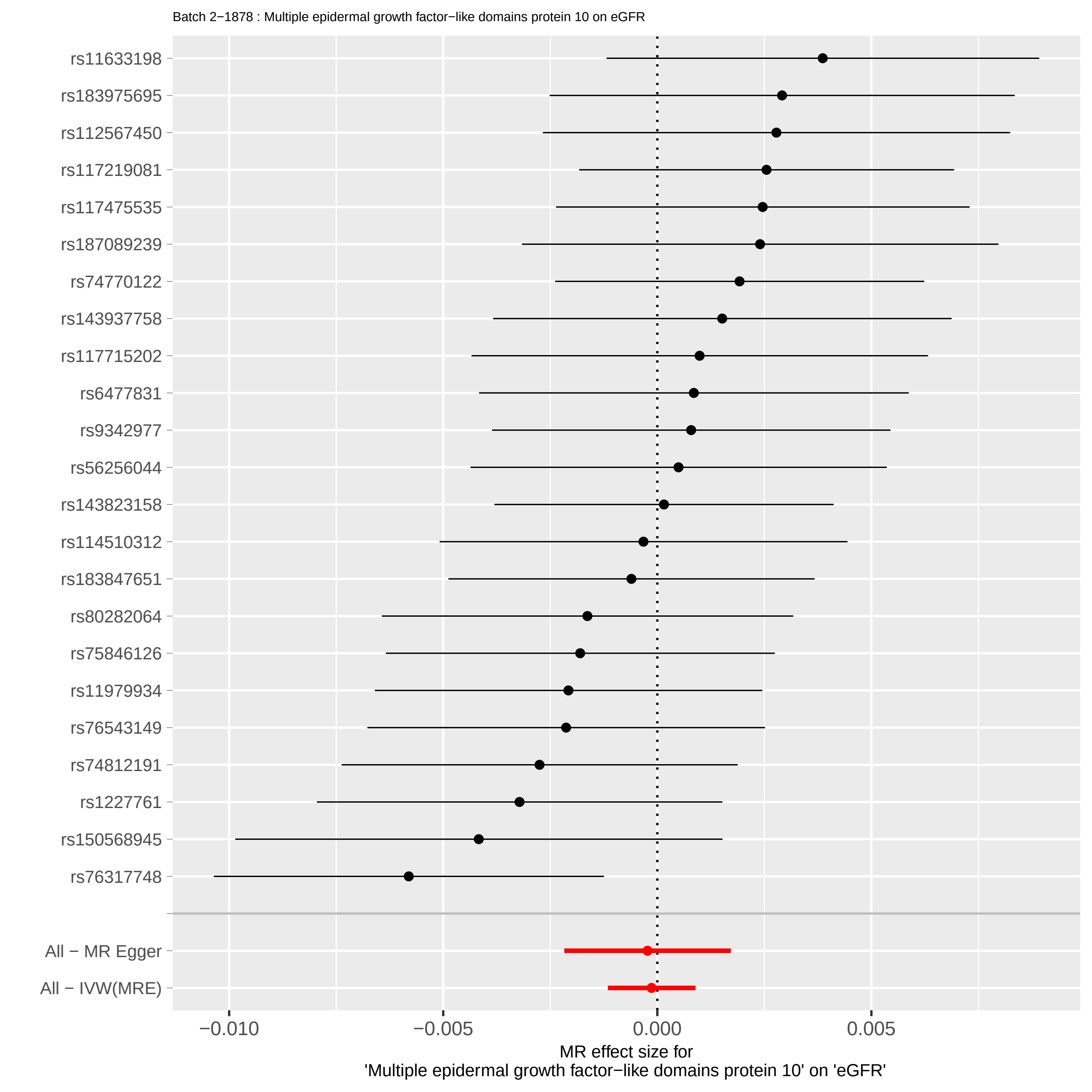
Batch 2−1878 : Multiple epidermal growth factor−like domains protein 10 on eGFR
rs11633198
rs183975695
rs112567450
rs117219081
rs117475535
rs187089239
rs74770122
rs143937758
rs117715202
rs6477831
rs9342977
rs56256044
rs143823158
rs114510312
rs183847651
rs80282064
rs75846126
rs11979934
rs76543149
rs74812191
rs1227761
rs150568945
rs76317748
All − MR Egger
All − IVW(MRE)
−0.010
−0.005
0.000
0.005
MR effect size for
'Multiple epidermal growth factor−like domains protein 10' on 'eGFR'

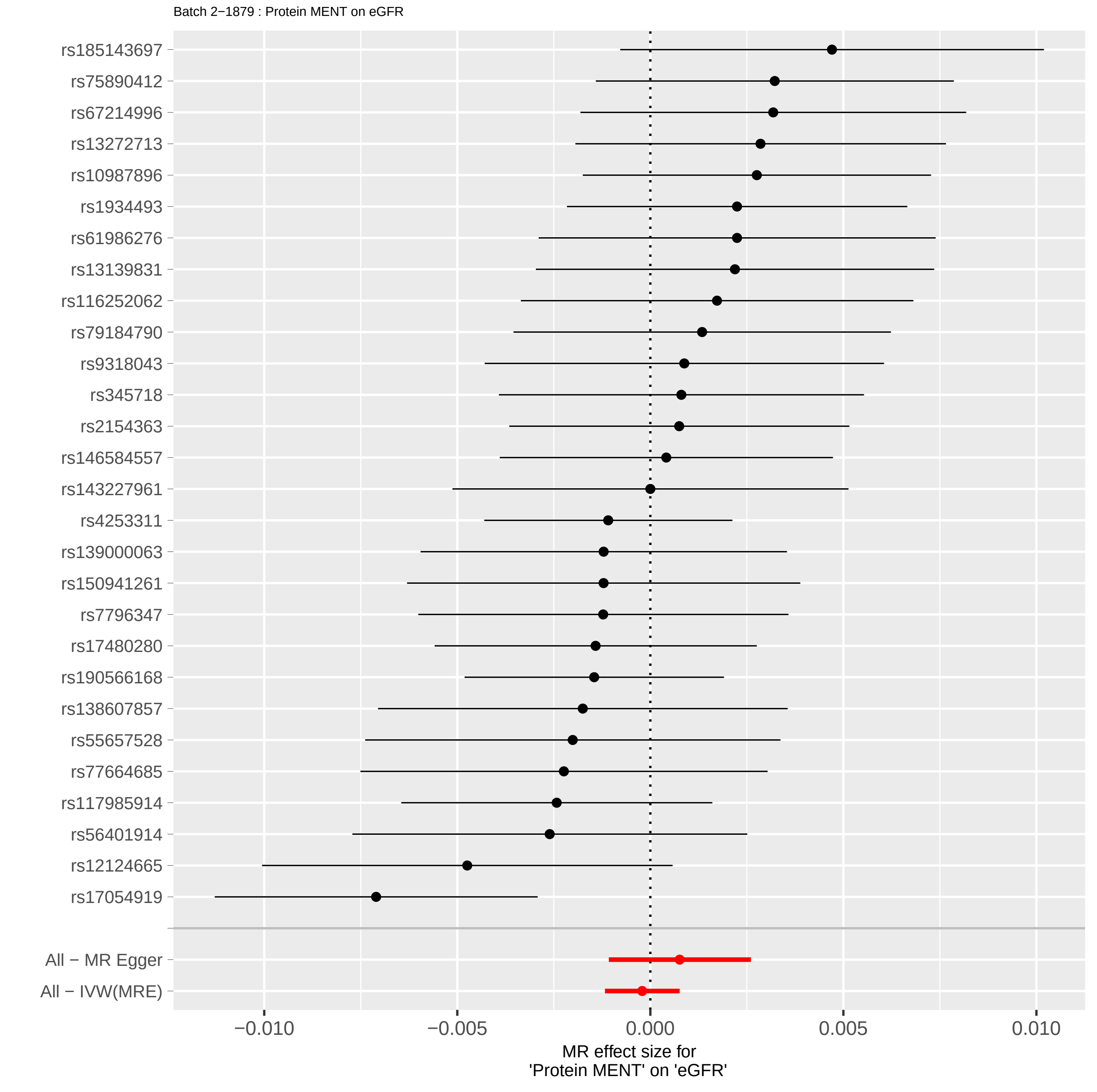

Batch 2−1879 : Protein MENT on eGFR
rs185143697
rs75890412
rs67214996
rs13272713
rs10987896
rs1934493
rs61986276
rs13139831
rs116252062
rs79184790
rs9318043
rs345718
rs2154363
rs146584557
rs143227961
rs4253311
rs139000063
rs150941261
rs7796347
rs17480280
rs190566168
rs138607857
rs55657528
rs77664685
rs117985914
rs56401914
rs12124665
rs17054919
All − MR Egger
All − IVW(MRE)
−0.010
−0.005
0.000
0.005
0.010
MR effect size for
'Protein MENT' on 'eGFR'

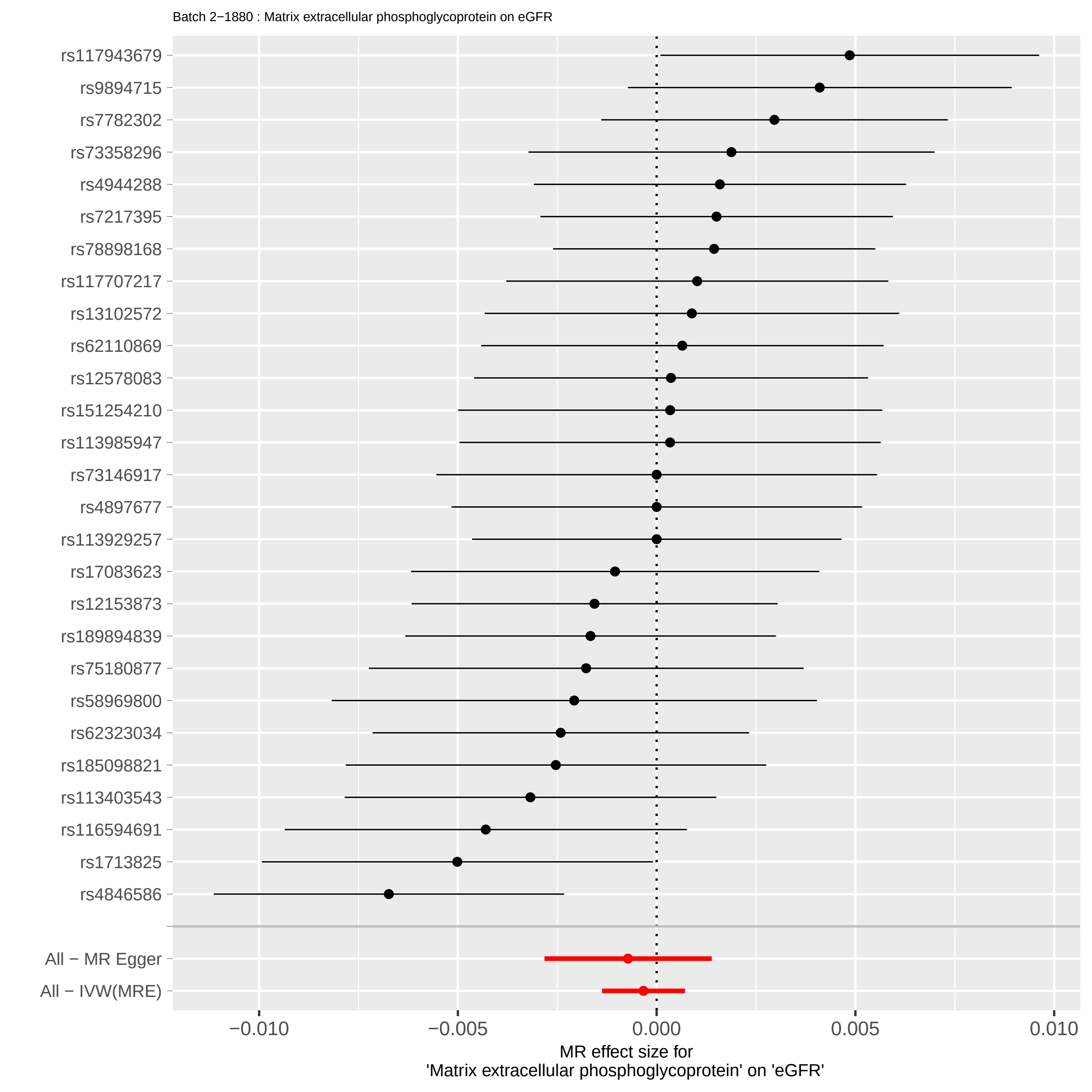

Batch 2−1880 : Matrix extracellular phosphoglycoprotein on eGFR
rs117943679
rs9894715
rs7782302
rs73358296
rs4944288
rs7217395
rs78898168
rs117707217
rs13102572
rs62110869
rs12578083
rs151254210
rs113985947
rs73146917
rs4897677
rs113929257
rs17083623
rs12153873
rs189894839
rs75180877
rs58969800
rs62323034
rs185098821
rs113403543
rs116594691
rs1713825
rs4846586
All − MR Egger
All − IVW(MRE)
−0.010
−0.005
0.000
0.005
0.010
MR effect size for
'Matrix extracellular phosphoglycoprotein' on 'eGFR'

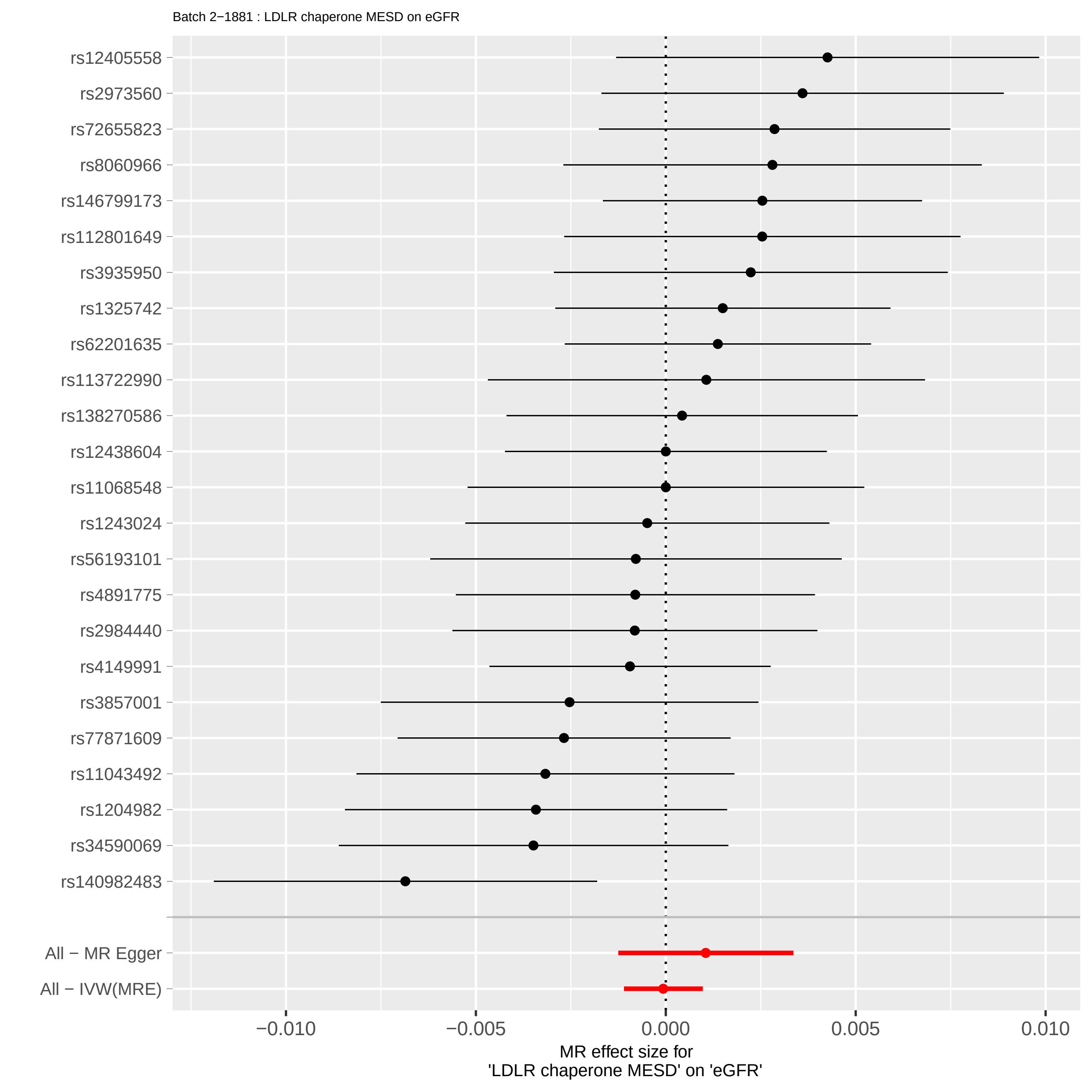
Batch 2−1881 : LDLR chaperone MESD on eGFR
rs12405558
rs2973560
rs72655823
rs8060966
rs146799173
rs112801649
rs3935950
rs1325742
rs62201635
rs113722990
rs138270586
rs12438604
rs11068548
rs1243024
rs56193101
rs4891775
rs2984440
rs4149991
rs3857001
rs77871609
rs11043492
rs1204982
rs34590069
rs140982483
All − MR Egger
All − IVW(MRE)
−0.010
−0.005
0.000
0.005
0.010
MR effect size for
'LDLR chaperone MESD' on 'eGFR'

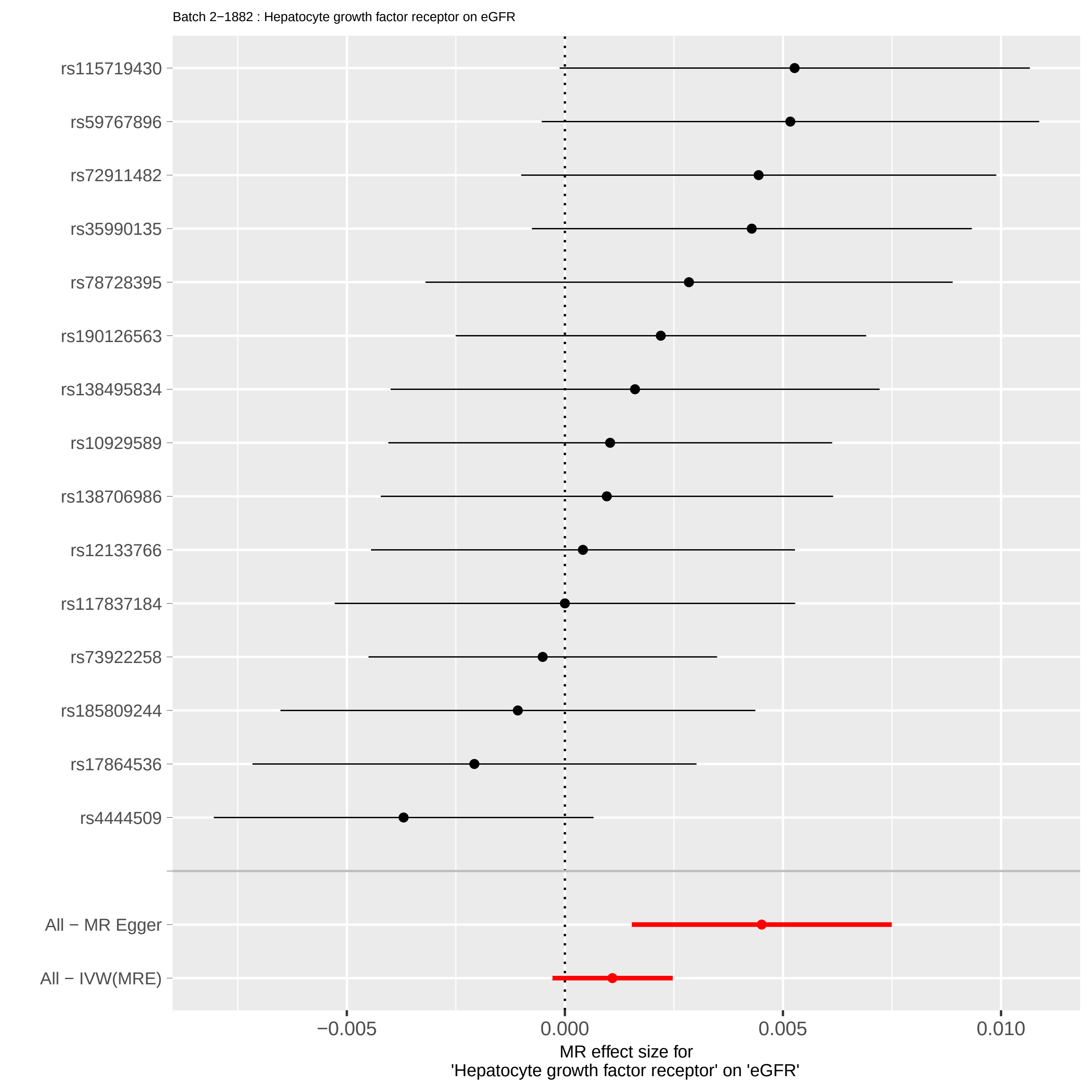

Batch 2−1882 : Hepatocyte growth factor receptor on eGFR
rs115719430
rs59767896
rs72911482
rs35990135
rs78728395
rs190126563
rs138495834
rs10929589
rs138706986
rs12133766
rs117837184
rs73922258
rs185809244
rs17864536
rs4444509
All − MR Egger
All − IVW(MRE)
−0.005
0.000
0.005
0.010
MR effect size for
'Hepatocyte growth factor receptor' on 'eGFR'

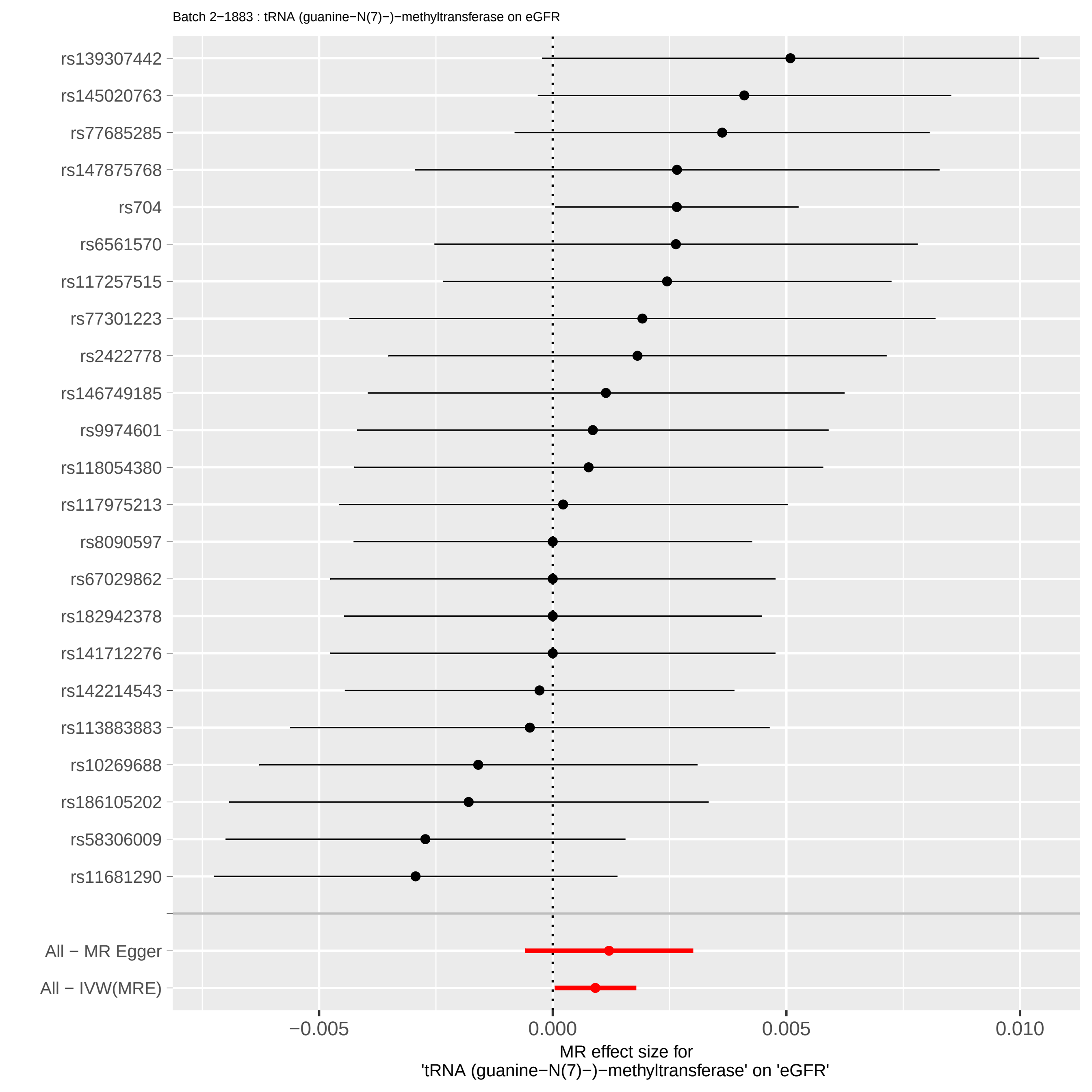
Batch 2−1883 : tRNA (guanine−N(7)−)−methyltransferase on eGFR
rs139307442
rs145020763
rs77685285
rs147875768
rs704
rs6561570
rs117257515
rs77301223
rs2422778
rs146749185
rs9974601
rs118054380
rs117975213
rs8090597
rs67029862
rs182942378
rs141712276
rs142214543
rs113883883
rs10269688
rs186105202
rs58306009
rs11681290
All − MR Egger
All − IVW(MRE)
−0.005
0.000
0.005
0.010
MR effect size for
'tRNA (guanine−N(7)−)−methyltransferase' on 'eGFR'

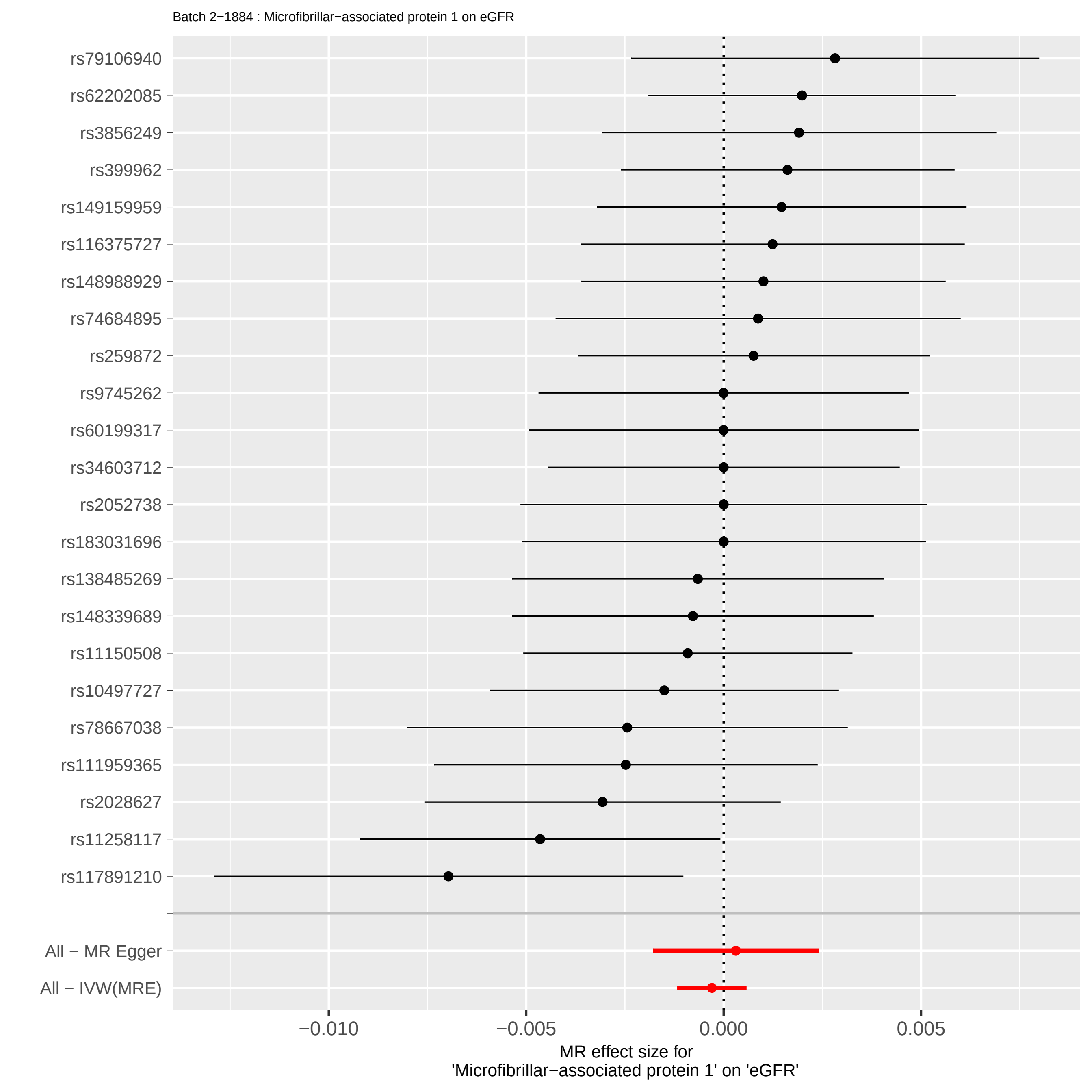

Batch 2−1884 : Microfibrillar−associated protein 1 on eGFR
rs79106940
rs62202085
rs3856249
rs399962
rs149159959
rs116375727
rs148988929
rs74684895
rs259872
rs9745262
rs60199317
rs34603712
rs2052738
rs183031696
rs138485269
rs148339689
rs11150508
rs10497727
rs78667038
rs111959365
rs2028627
rs11258117
rs117891210
All − MR Egger
All − IVW(MRE)
−0.010
−0.005
0.000
0.005
MR effect size for
'Microfibrillar−associated protein 1' on 'eGFR'

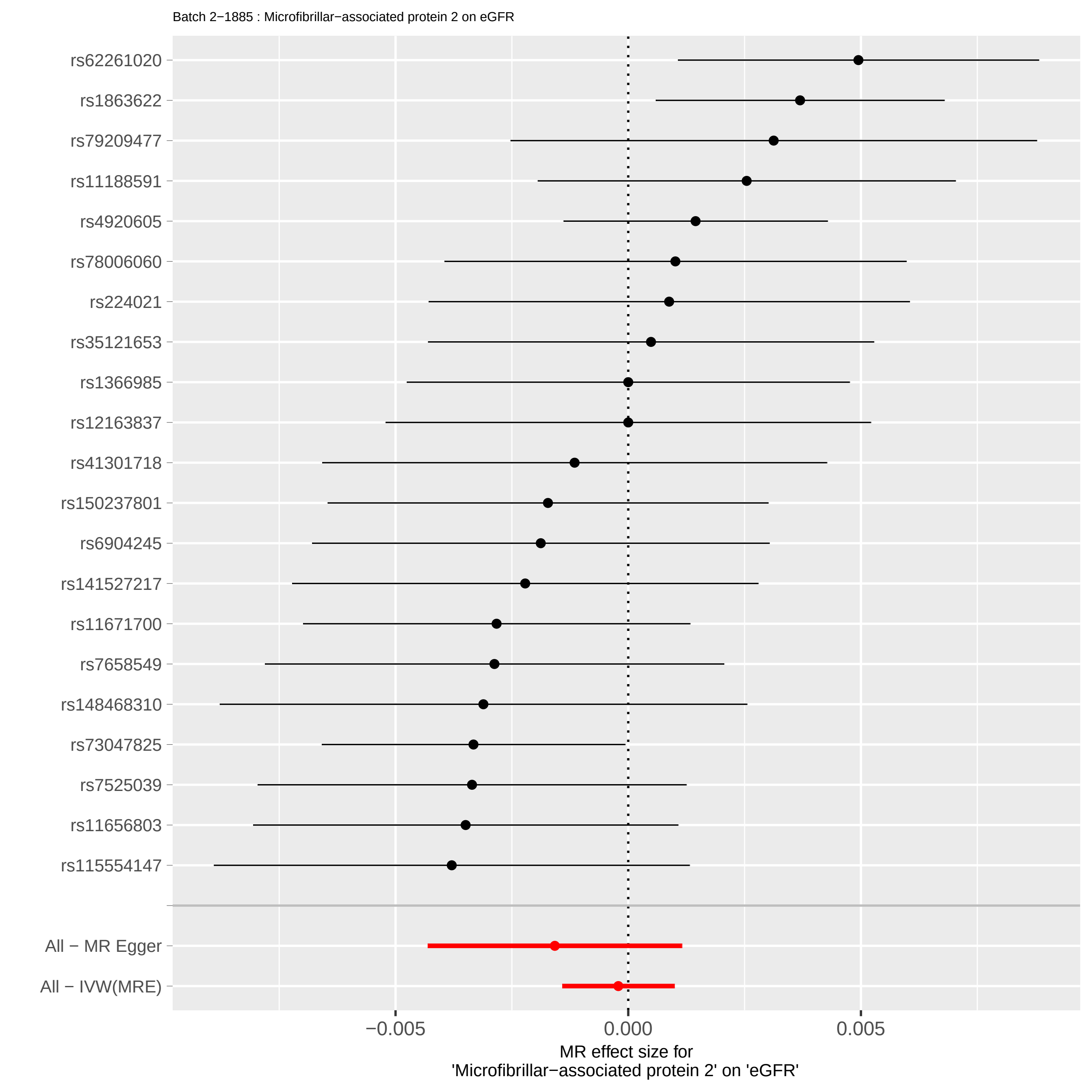

Batch 2−1885 : Microfibrillar−associated protein 2 on eGFR
rs62261020
rs1863622
rs79209477
rs11188591
rs4920605
rs78006060
rs224021
rs35121653
rs1366985
rs12163837
rs41301718
rs150237801
rs6904245
rs141527217
rs11671700
rs7658549
rs148468310
rs73047825
rs7525039
rs11656803
rs115554147
All − MR Egger
All − IVW(MRE)
−0.005
0.000
0.005
MR effect size for
'Microfibrillar−associated protein 2' on 'eGFR'

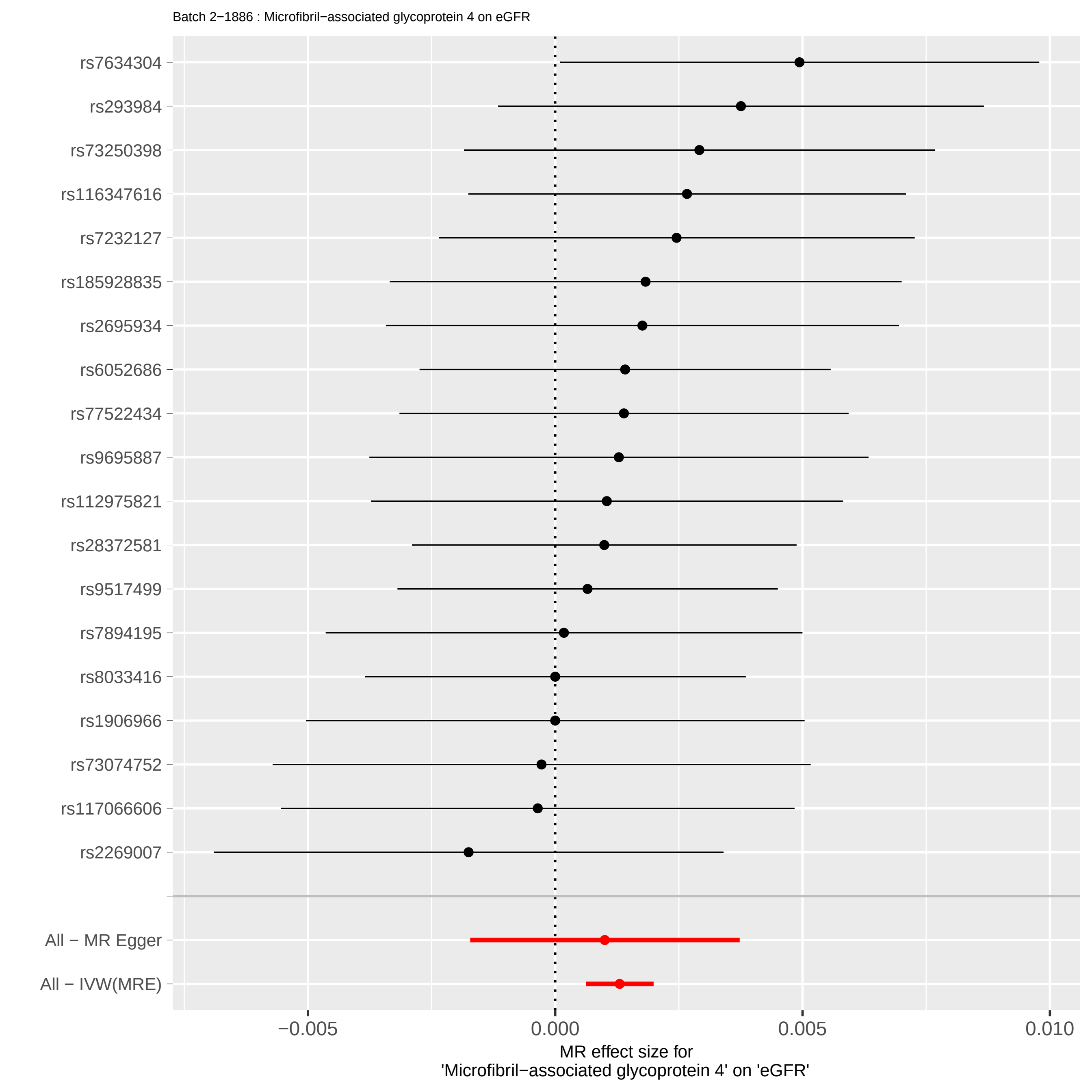

Batch 2−1886 : Microfibril−associated glycoprotein 4 on eGFR
rs7634304
rs293984
rs73250398
rs116347616
rs7232127
rs185928835
rs2695934
rs6052686
rs77522434
rs9695887
rs112975821
rs28372581
rs9517499
rs7894195
rs8033416
rs1906966
rs73074752
rs117066606
rs2269007
All − MR Egger
All − IVW(MRE)
−0.005
0.000
0.005
0.010
MR effect size for
'Microfibril−associated glycoprotein 4' on 'eGFR'

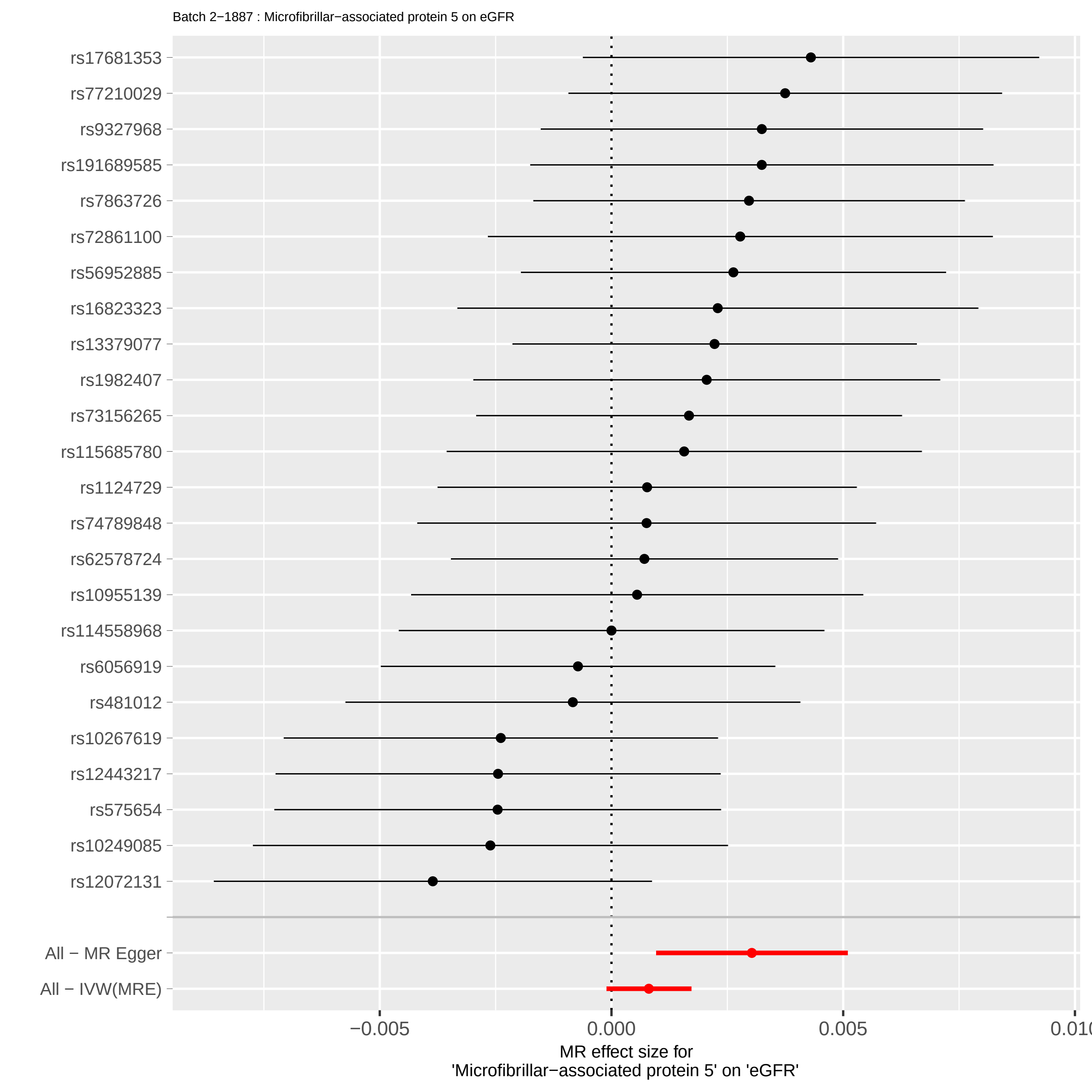

Batch 2−1887 : Microfibrillar−associated protein 5 on eGFR
rs17681353
rs77210029
rs9327968
rs191689585
rs7863726
rs72861100
rs56952885
rs16823323
rs13379077
rs1982407
rs73156265
rs115685780
rs1124729
rs74789848
rs62578724
rs10955139
rs114558968
rs6056919
rs481012
rs10267619
rs12443217
rs575654
rs10249085
rs12072131
All − MR Egger
All − IVW(MRE)
−0.005
0.000
0.005
0.010
MR effect size for
'Microfibrillar−associated protein 5' on 'eGFR'

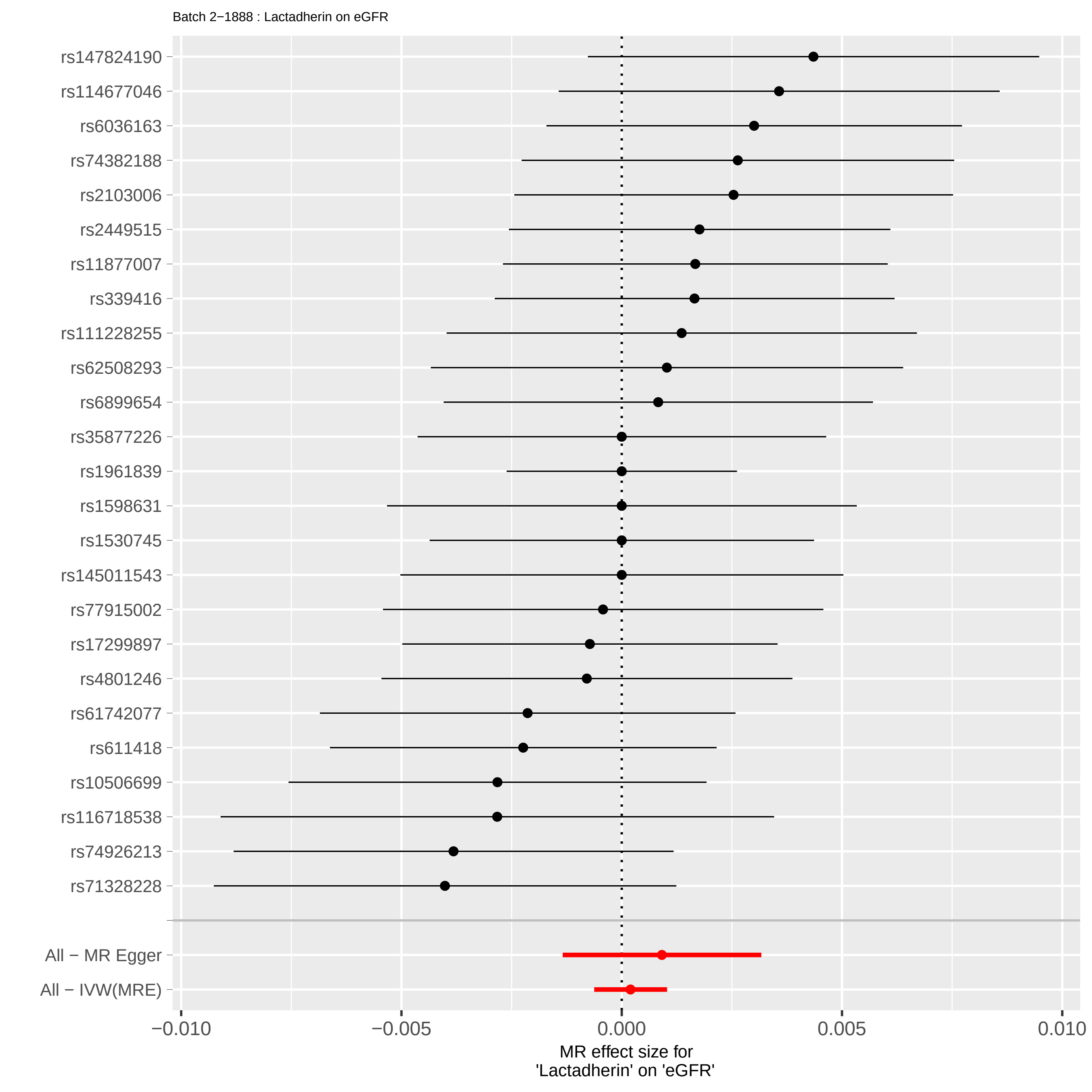
Batch 2−1888 : Lactadherin on eGFR
rs147824190
rs114677046
rs6036163
rs74382188
rs2103006
rs2449515
rs11877007
rs339416
rs111228255
rs62508293
rs6899654
rs35877226
rs1961839
rs1598631
rs1530745
rs145011543
rs77915002
rs17299897
rs4801246
rs61742077
rs611418
rs10506699
rs116718538
rs74926213
rs71328228
All − MR Egger
All − IVW(MRE)
−0.010
−0.005
0.000
0.005
0.010
MR effect size for
'Lactadherin' on 'eGFR'

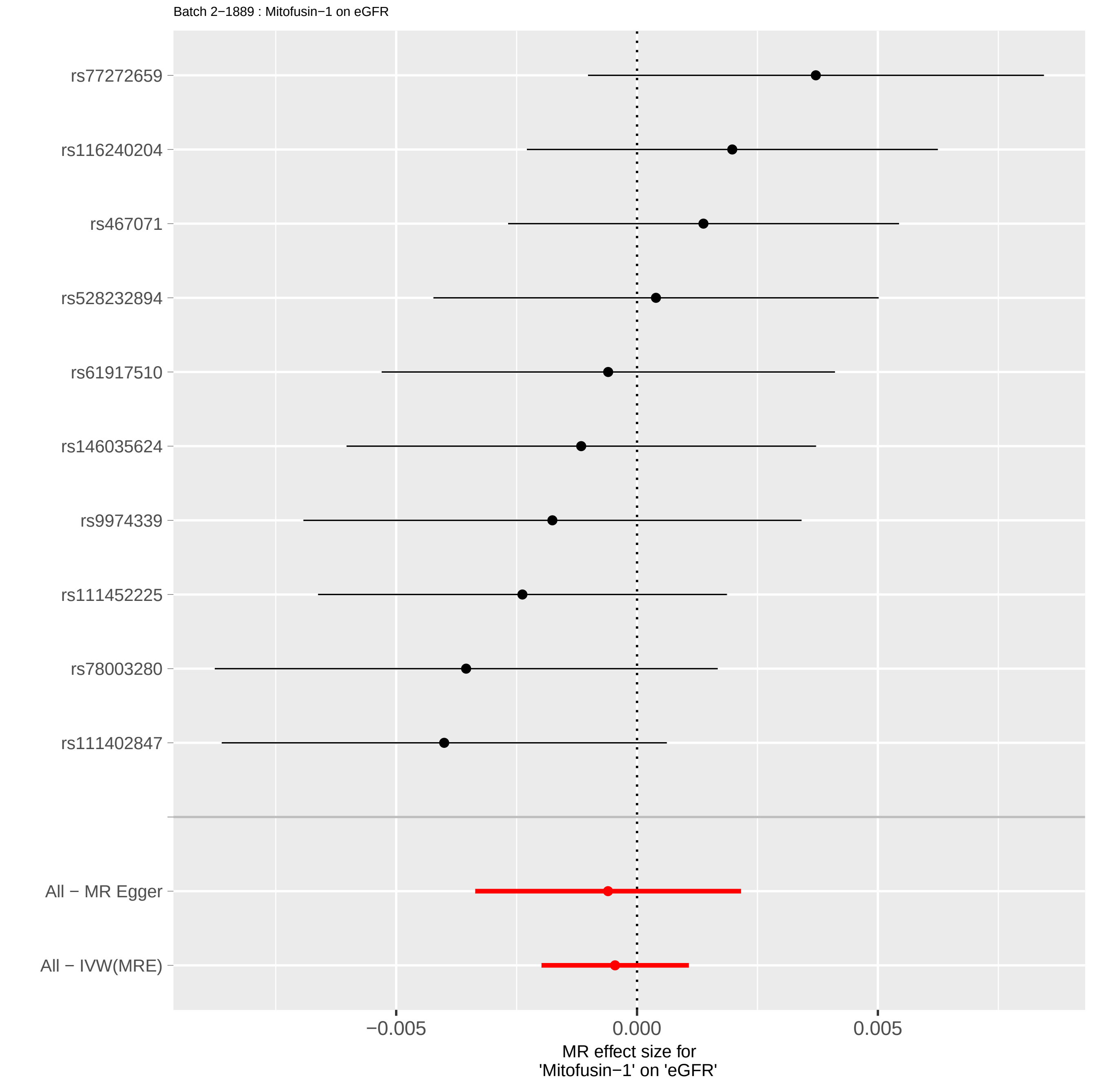
Batch 2−1889 : Mitofusin−1 on eGFR
rs77272659
rs116240204
rs467071
rs528232894
rs61917510
rs146035624
rs9974339
rs111452225
rs78003280
rs111402847
All − MR Egger
All − IVW(MRE)
−0.005
0.000
0.005
MR effect size for
'Mitofusin−1' on 'eGFR'

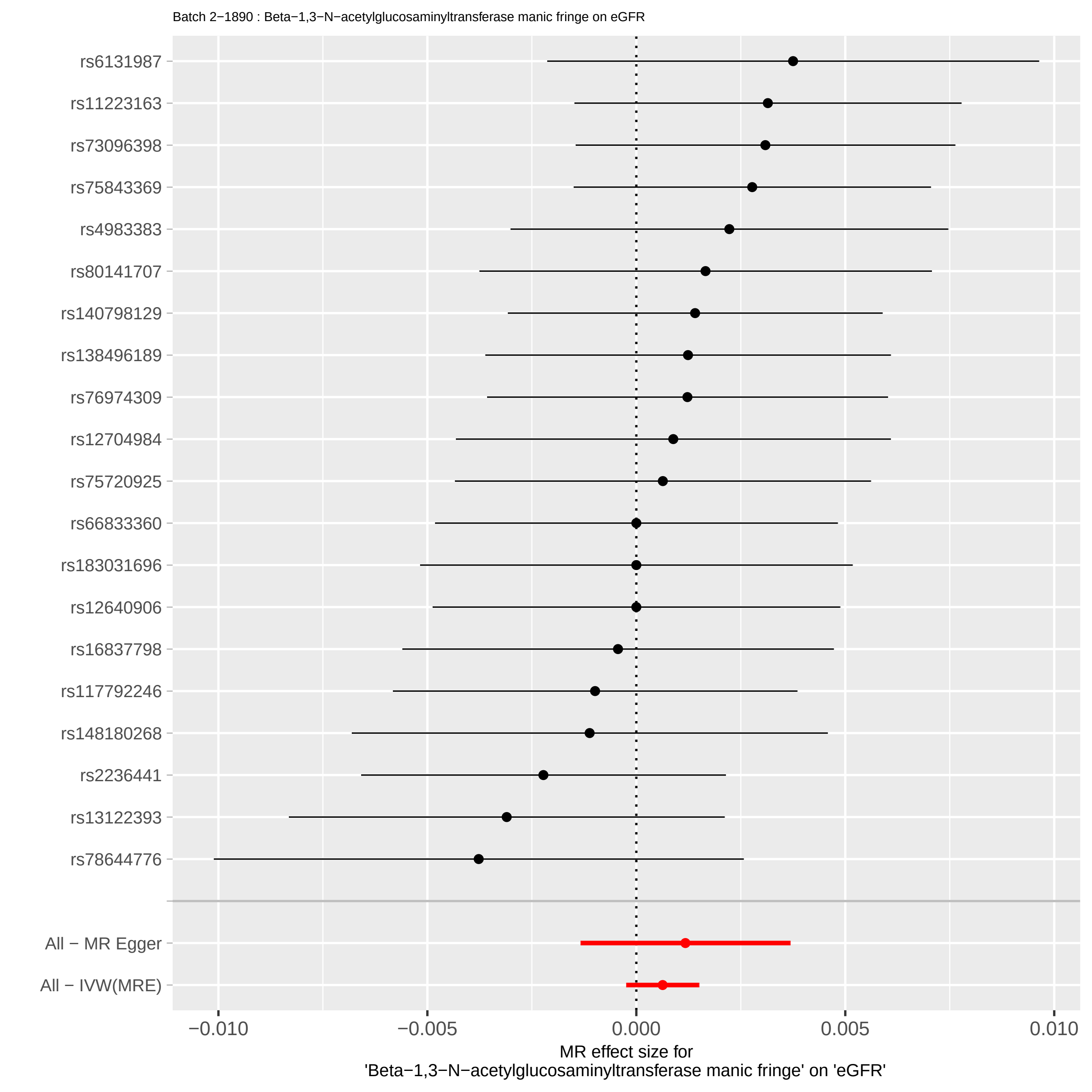
Batch 2−1890 : Beta−1,3−N−acetylglucosaminyltransferase manic fringe on eGFR
rs6131987
rs11223163
rs73096398
rs75843369
rs4983383
rs80141707
rs140798129
rs138496189
rs76974309
rs12704984
rs75720925
rs66833360
rs183031696
rs12640906
rs16837798
rs117792246
rs148180268
rs2236441
rs13122393
rs78644776
All − MR Egger
All − IVW(MRE)
−0.010
−0.005
0.000
0.005
0.010
MR effect size for
'Beta−1,3−N−acetylglucosaminyltransferase manic fringe' on 'eGFR'

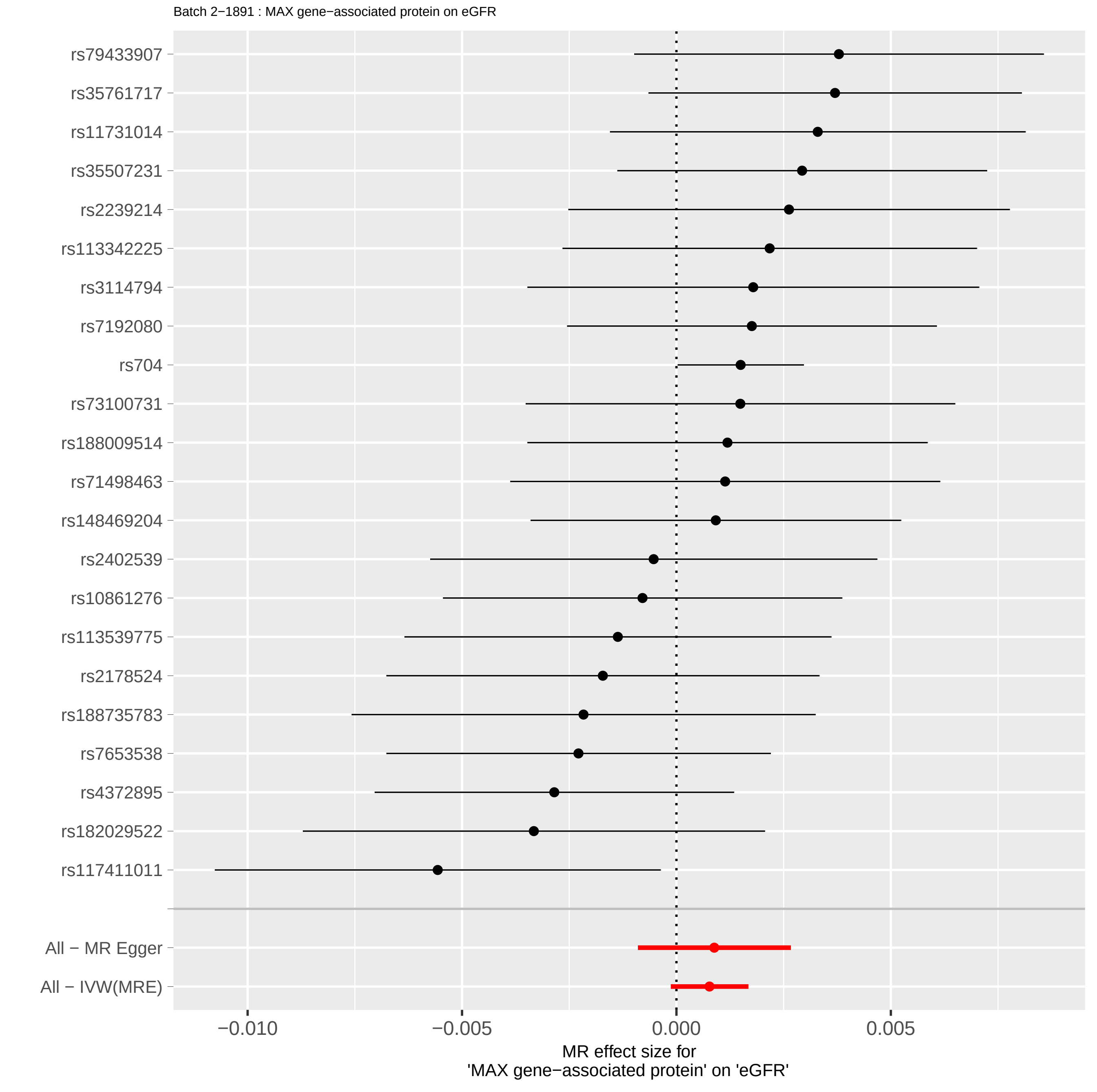
Batch 2−1891 : MAX gene−associated protein on eGFR
rs79433907
rs35761717
rs11731014
rs35507231
rs2239214
rs113342225
rs3114794
rs7192080
rs704
rs73100731
rs188009514
rs71498463
rs148469204
rs2402539
rs10861276
rs113539775
rs2178524
rs188735783
rs7653538
rs4372895
rs182029522
rs117411011
All − MR Egger
All − IVW(MRE)
−0.010
−0.005
0.000
0.005
MR effect size for
'MAX gene−associated protein' on 'eGFR'

Batch 2−1892 : Alpha−1,3−mannosyl−glycoprotein 2−beta−N−acetylglucosaminyltransferase on eGFR

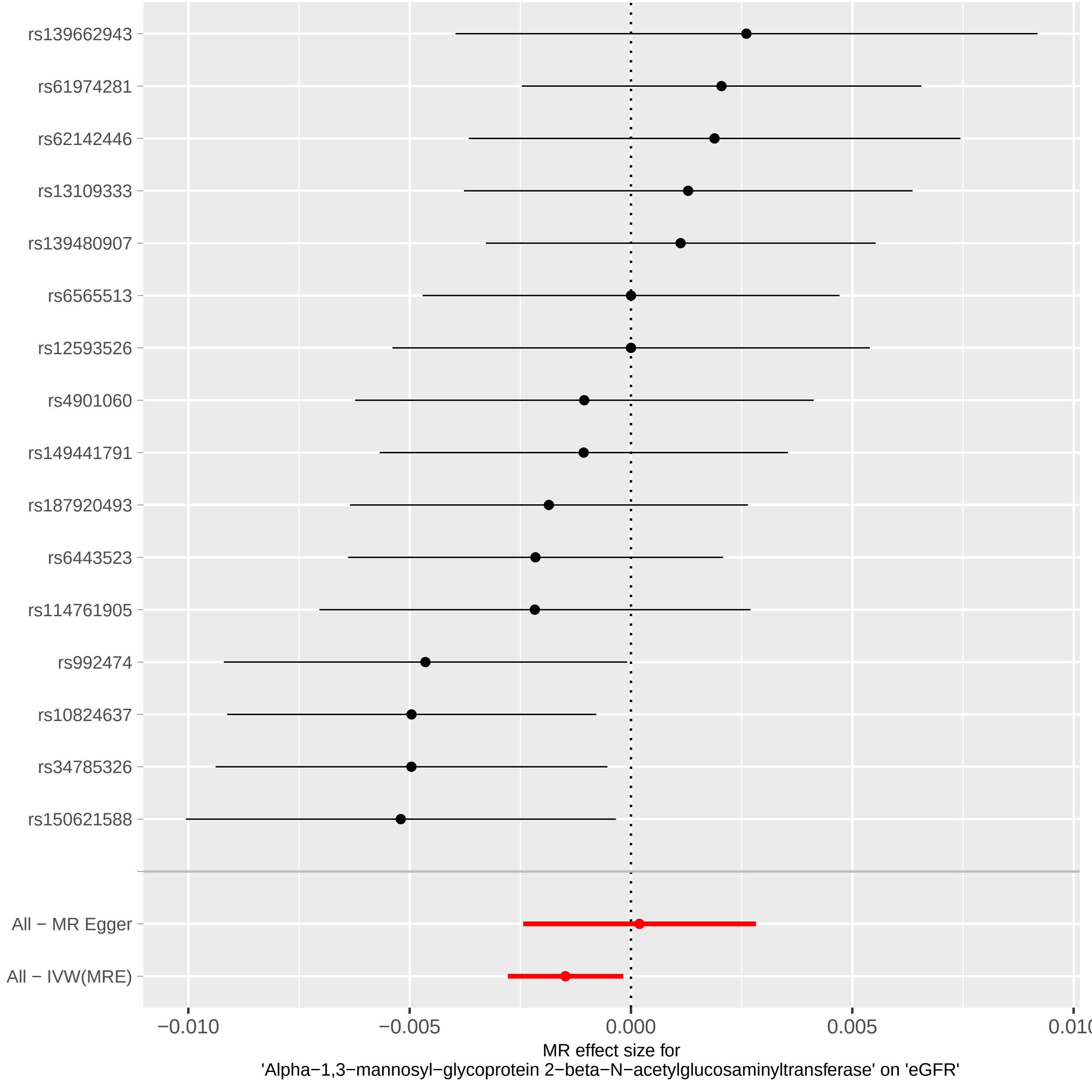

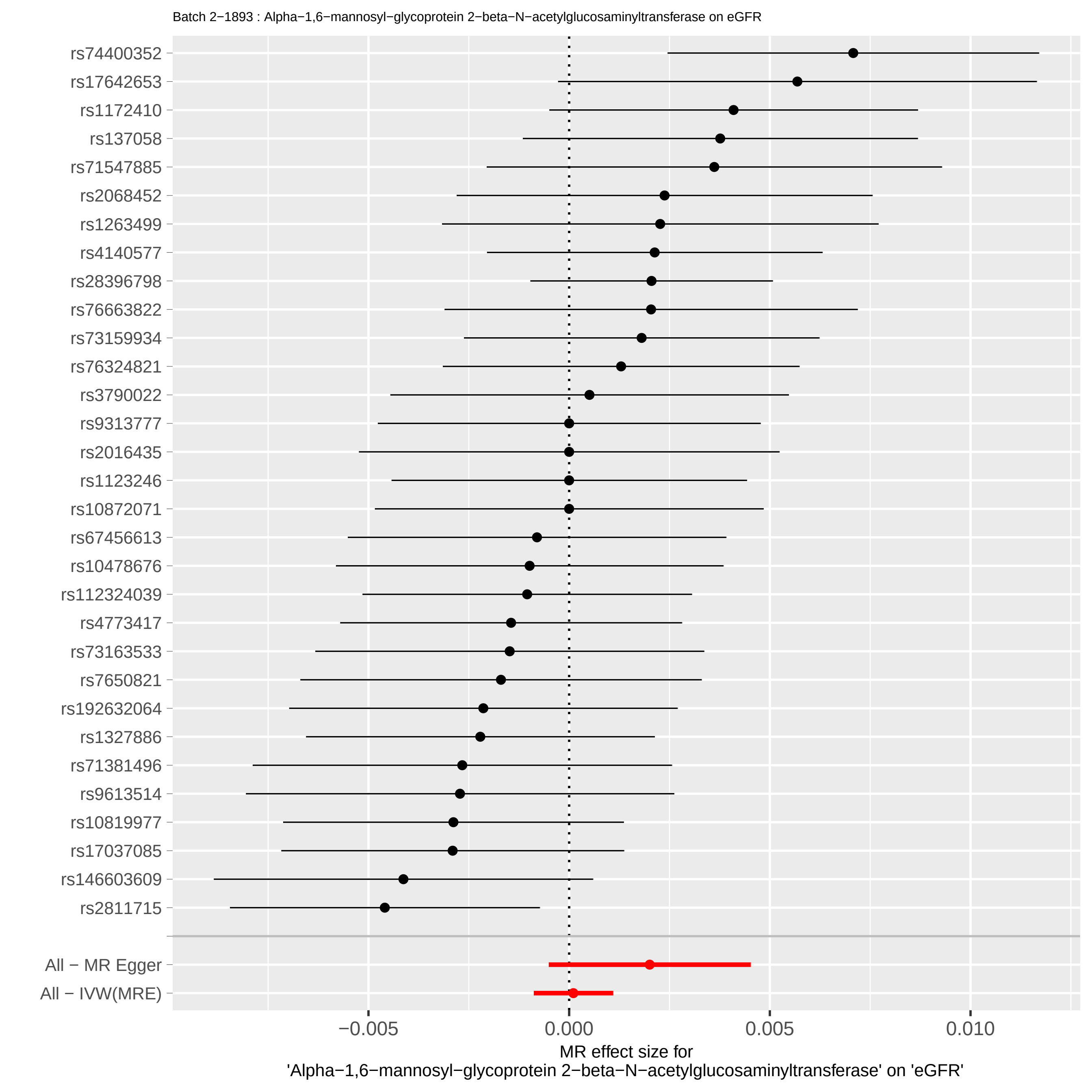

Batch 2−1893 : Alpha−1,6−mannosyl−glycoprotein 2−beta−N−acetylglucosaminyltransferase on eGFR
rs74400352
rs17642653
rs1172410
rs137058
rs71547885
rs2068452
rs1263499
rs4140577
rs28396798
rs76663822
rs73159934
rs76324821
rs3790022
rs9313777
rs2016435
rs1123246
rs10872071
rs67456613
rs10478676
rs112324039
rs4773417
rs73163533
rs7650821
rs192632064
rs1327886
rs71381496
rs9613514
rs10819977
rs17037085
rs146603609
rs2811715
All − MR Egger
All − IVW(MRE)
−0.005
0.000
0.005
0.010
MR effect size for
'Alpha−1,6−mannosyl−glycoprotein 2−beta−N−acetylglucosaminyltransferase' on 'eGFR'

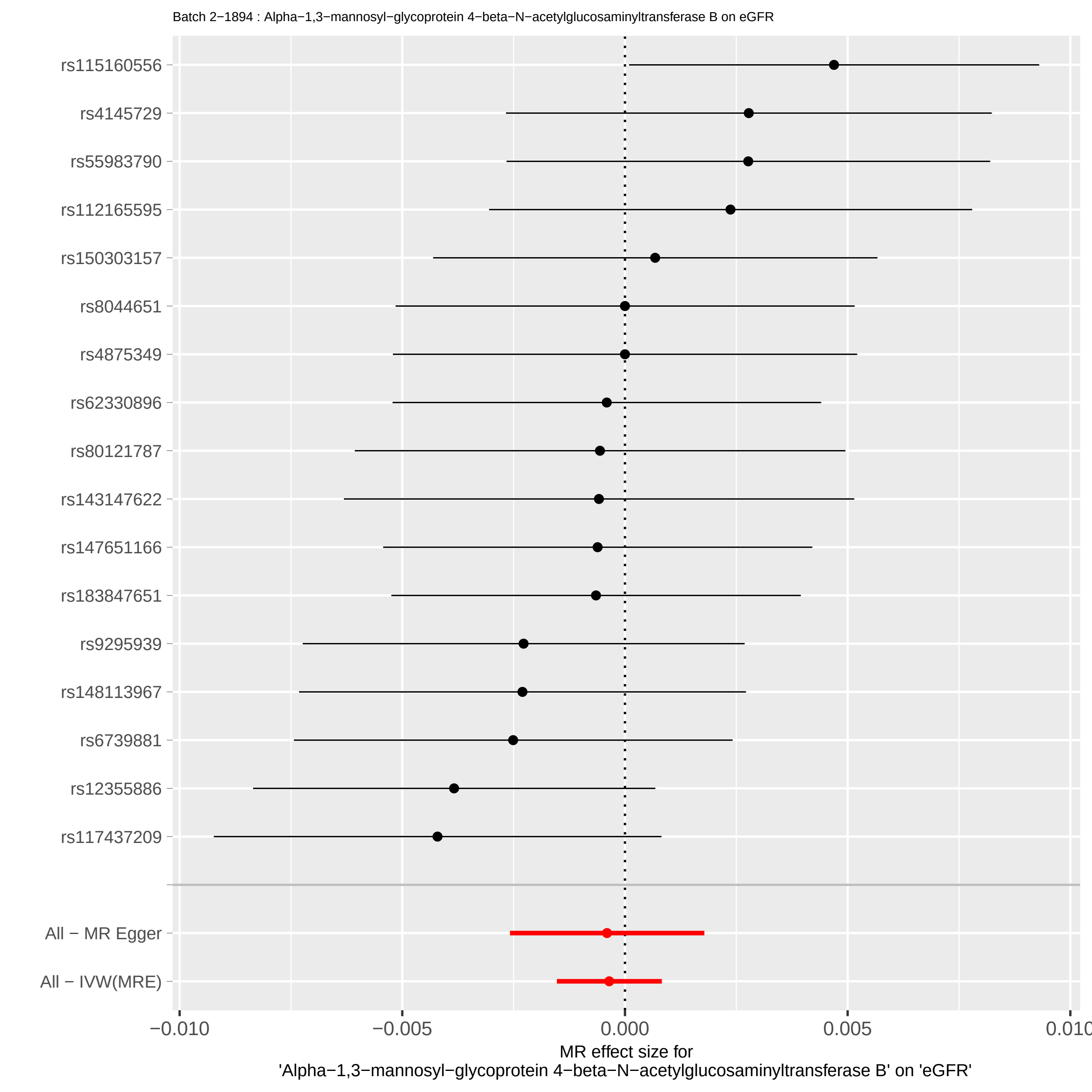

Batch 2−1894 : Alpha−1,3−mannosyl−glycoprotein 4−beta−N−acetylglucosaminyltransferase B on eGFR
rs115160556
rs4145729
rs55983790
rs112165595
rs150303157
rs8044651
rs4875349
rs62330896
rs80121787
rs143147622
rs147651166
rs183847651
rs9295939
rs148113967
rs6739881
rs12355886
rs117437209
All − MR Egger
All − IVW(MRE)
−0.010
−0.005
0.000
0.005
0.010
MR effect size for
'Alpha−1,3−mannosyl−glycoprotein 4−beta−N−acetylglucosaminyltransferase B' on 'eGFR'

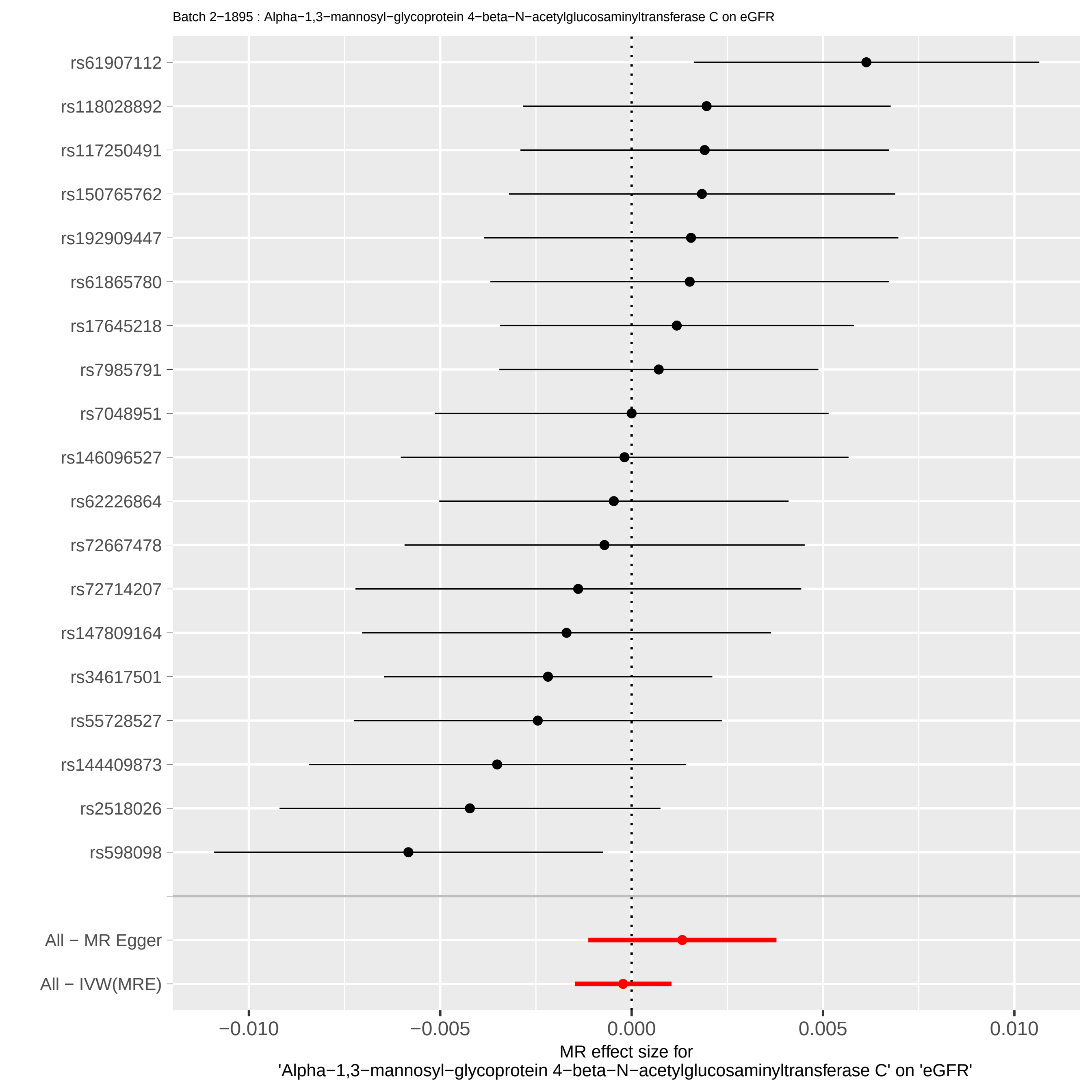
Batch 2−1895 : Alpha−1,3−mannosyl−glycoprotein 4−beta−N−acetylglucosaminyltransferase C on eGFR
rs61907112
rs118028892
rs117250491
rs150765762
rs192909447
rs61865780
rs17645218
rs7985791
rs7048951
rs146096527
rs62226864
rs72667478
rs72714207
rs147809164
rs34617501
rs55728527
rs144409873
rs2518026
rs598098
All − MR Egger
All − IVW(MRE)
−0.010
−0.005
0.000
0.005
0.010
MR effect size for
'Alpha−1,3−mannosyl−glycoprotein 4−beta−N−acetylglucosaminyltransferase C' on 'eGFR'

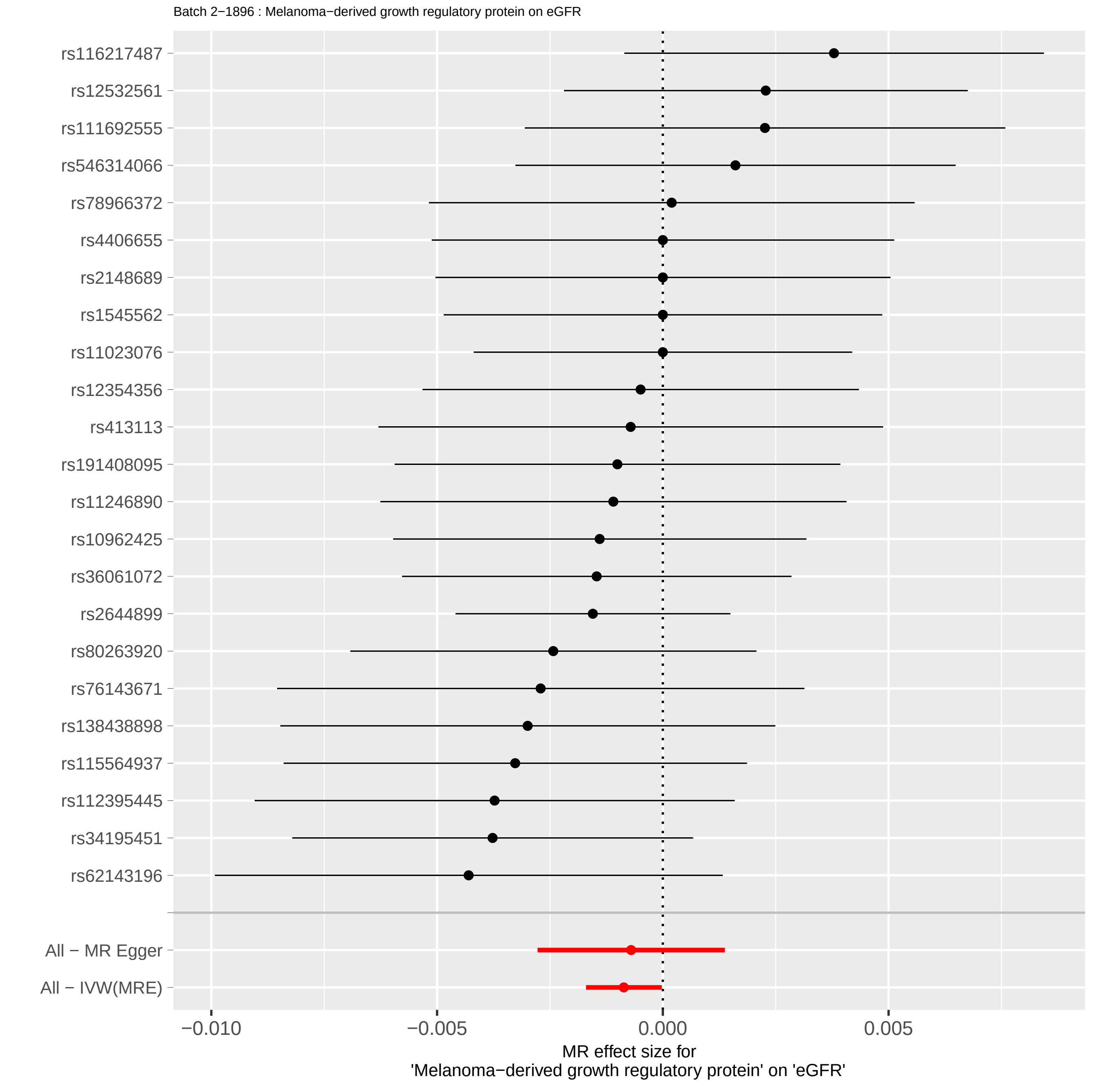
Batch 2−1896 : Melanoma−derived growth regulatory protein on eGFR
rs116217487
rs12532561
rs111692555
rs546314066
rs78966372
rs4406655
rs2148689
rs1545562
rs11023076
rs12354356
rs413113
rs191408095
rs11246890
rs10962425
rs36061072
rs2644899
rs80263920
rs76143671
rs138438898
rs115564937
rs112395445
rs34195451
rs62143196
All − MR Egger
All − IVW(MRE)
−0.010
−0.005
0.000
0.005
MR effect size for
'Melanoma−derived growth regulatory protein' on 'eGFR'

Batch 2−1897 : MICAL−like protein 2 on eGFR

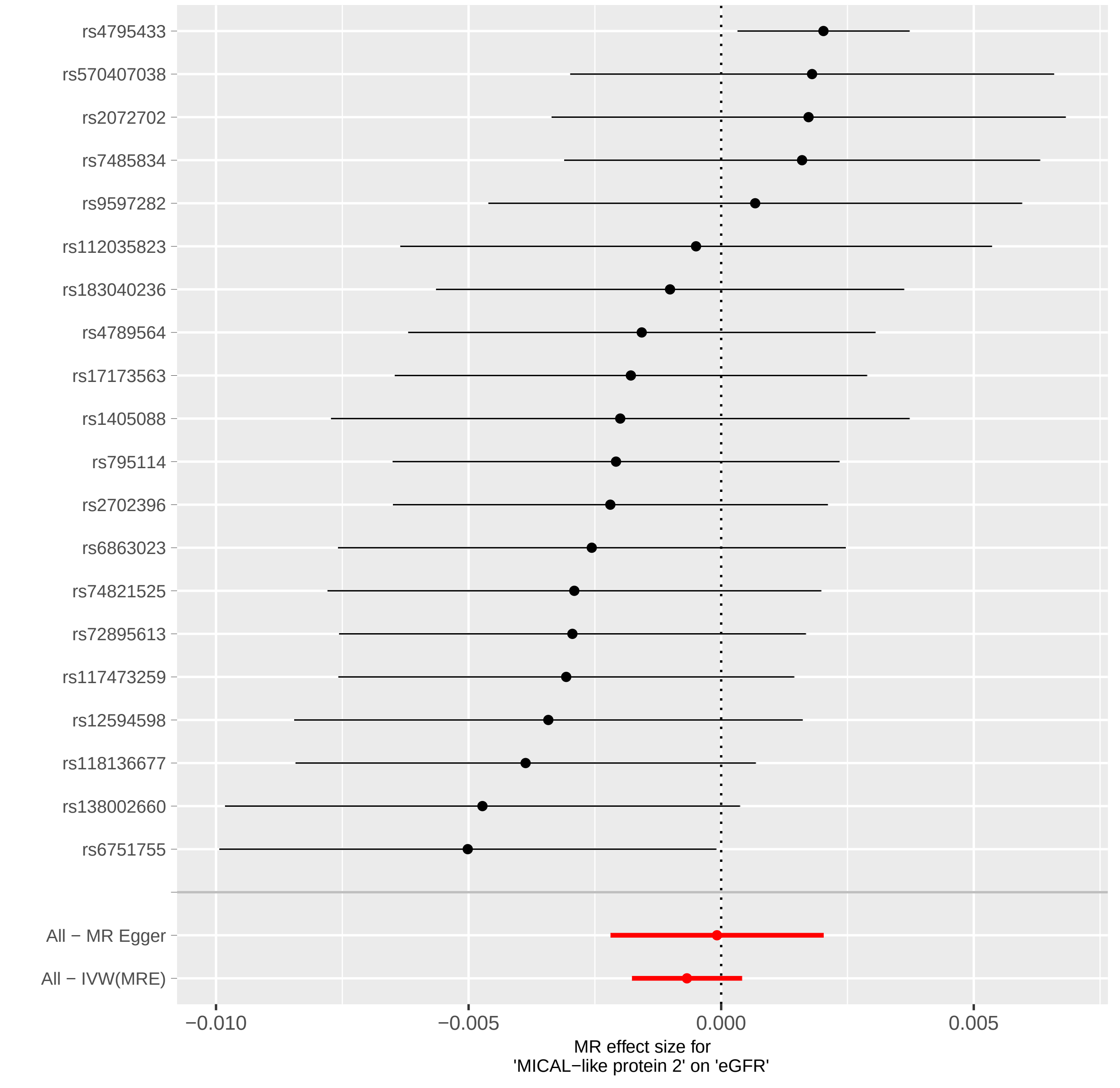

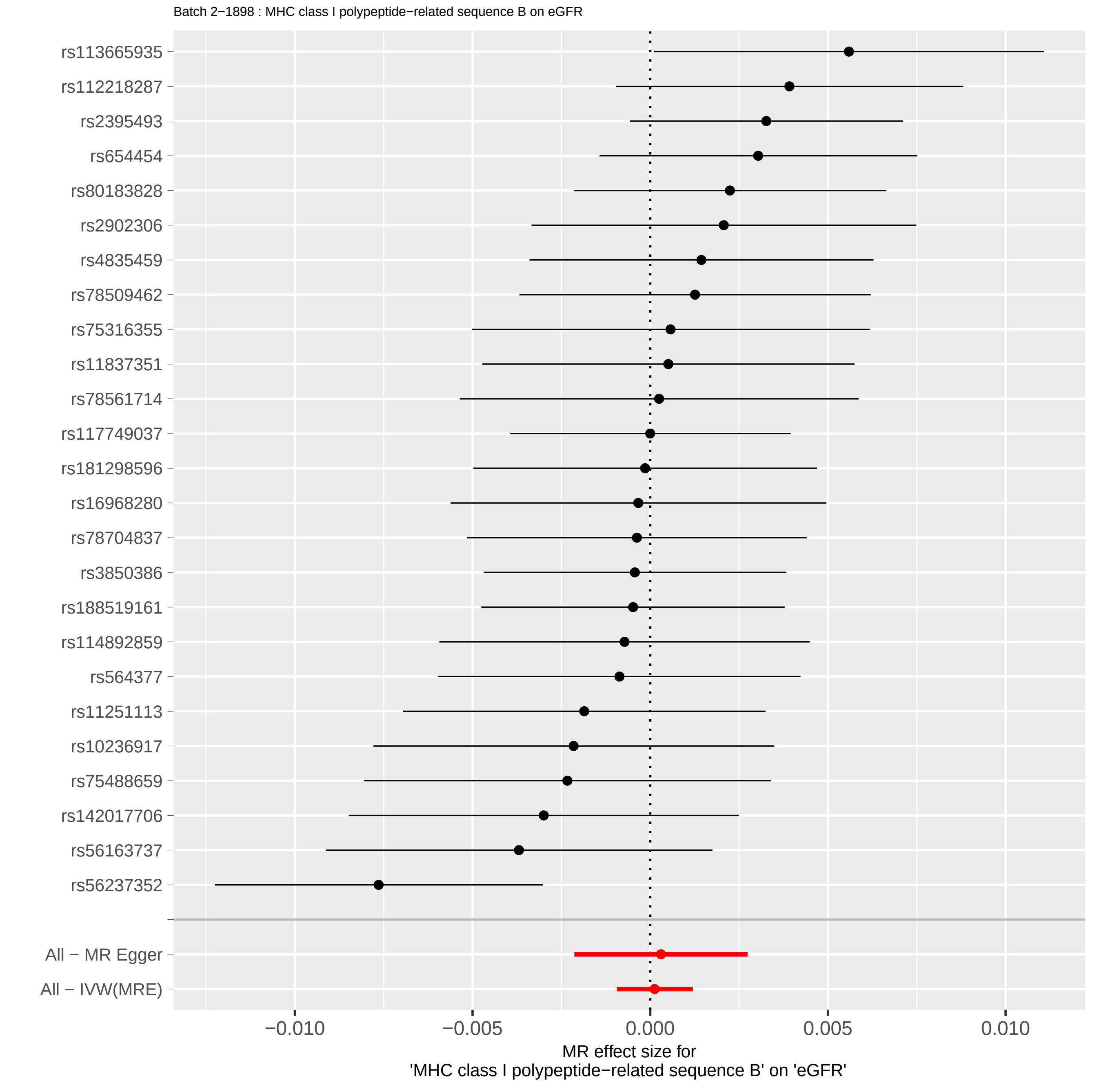

Batch 2−1898 : MHC class I polypeptide−related sequence B on eGFR
rs113665935
rs112218287
rs2395493
rs654454
rs80183828
rs2902306
rs4835459
rs78509462
rs75316355
rs11837351
rs78561714
rs117749037
rs181298596
rs16968280
rs78704837
rs3850386
rs188519161
rs114892859
rs564377
rs11251113
rs10236917
rs75488659
rs142017706
rs56163737
rs56237352
All − MR Egger
All − IVW(MRE)
−0.010
−0.005
0.000
0.005
0.010
MR effect size for
'MHC class I polypeptide−related sequence B' on 'eGFR'

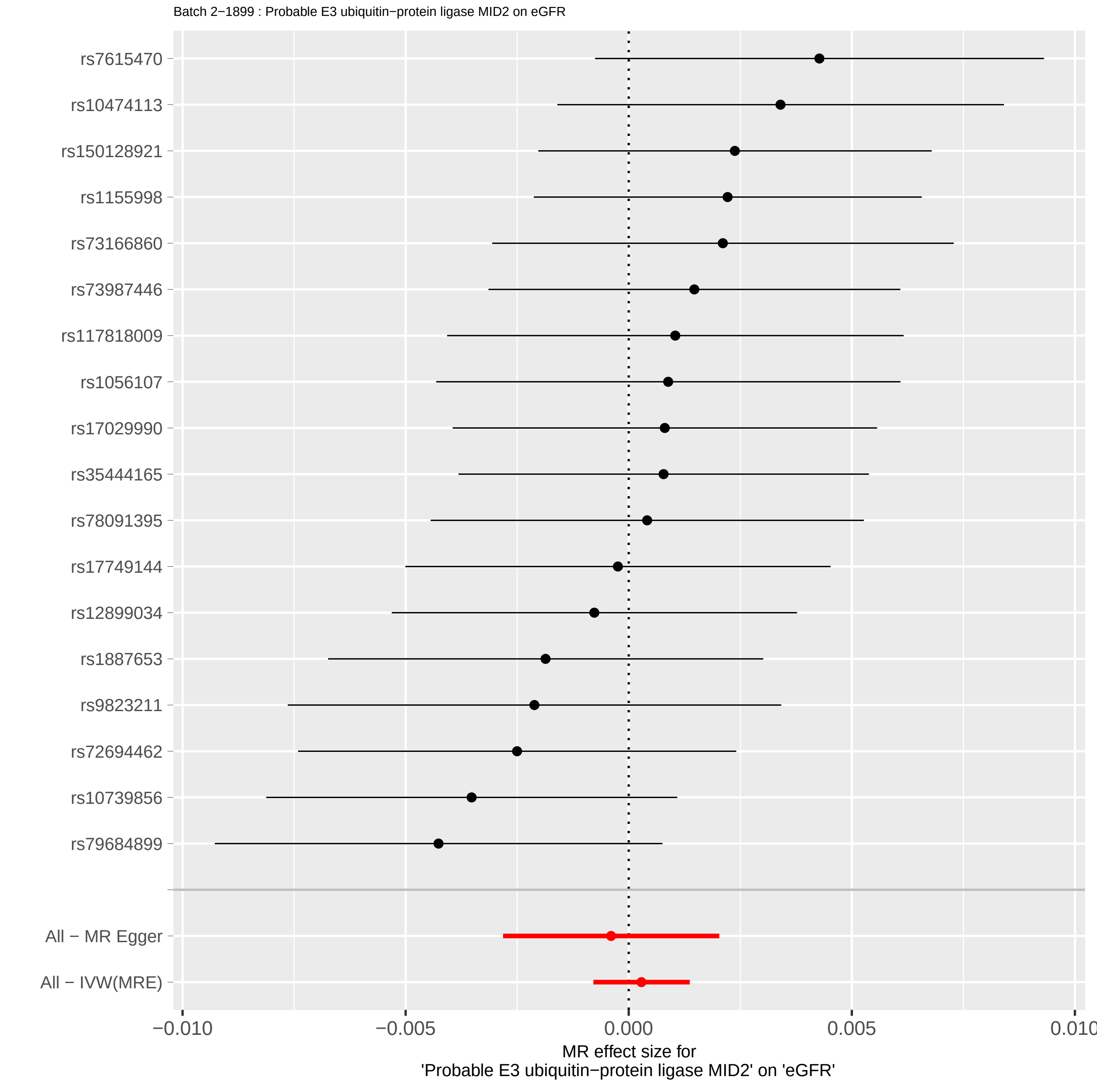

Batch 2−1899 : Probable E3 ubiquitin−protein ligase MID2 on eGFR
rs7615470
rs10474113
rs150128921
rs1155998
rs73166860
rs73987446
rs117818009
rs1056107
rs17029990
rs35444165
rs78091395
rs17749144
rs12899034
rs1887653
rs9823211
rs72694462
rs10739856
rs79684899
All − MR Egger
All − IVW(MRE)
−0.010
−0.005
0.000
0.005
0.010
MR effect size for
'Probable E3 ubiquitin−protein ligase MID2' on 'eGFR'

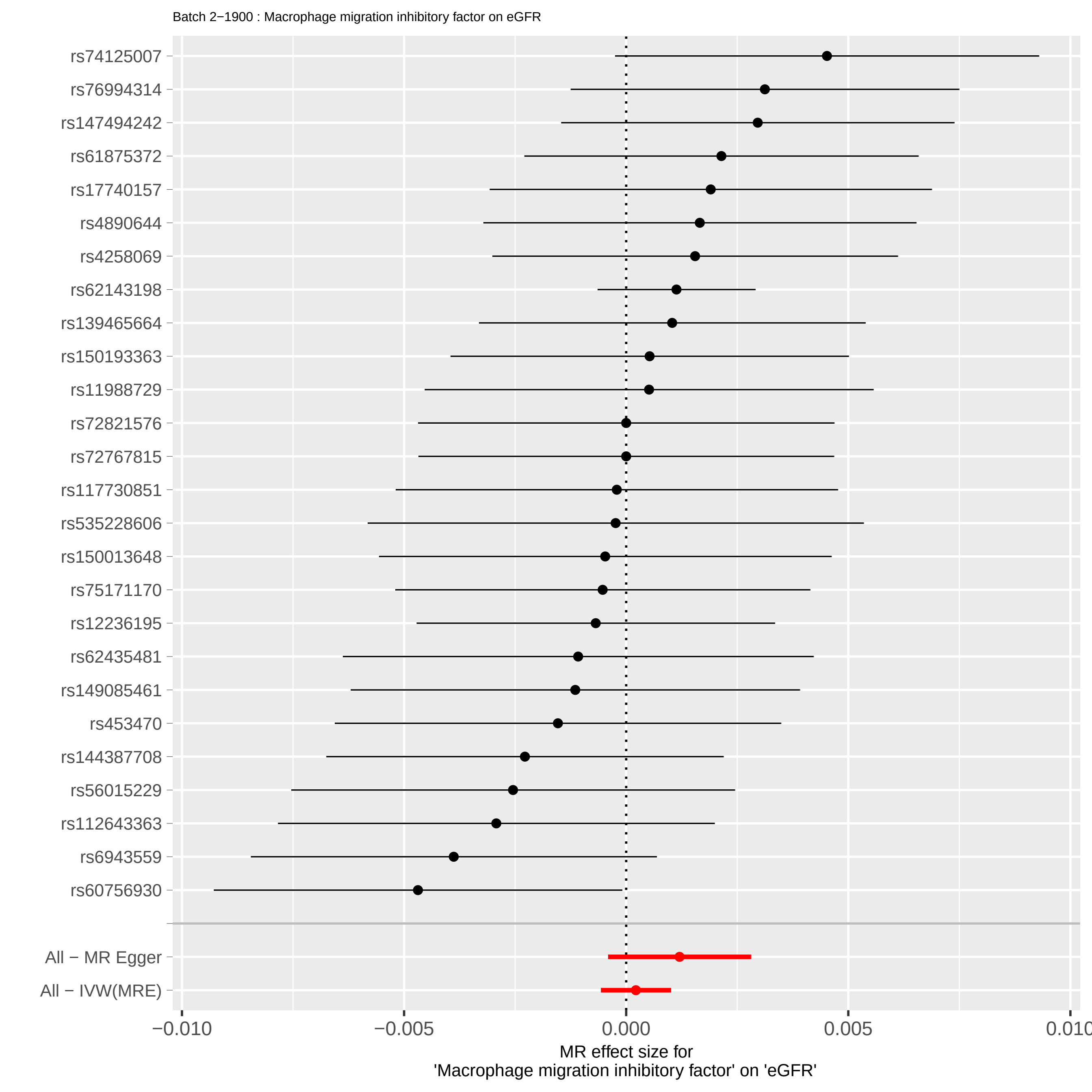
Batch 2−1900 : Macrophage migration inhibitory factor on eGFR
rs74125007
rs76994314
rs147494242
rs61875372
rs17740157
rs4890644
rs4258069
rs62143198
rs139465664
rs150193363
rs11988729
rs72821576
rs72767815
rs117730851
rs535228606
rs150013648
rs75171170
rs12236195
rs62435481
rs149085461
rs453470
rs144387708
rs56015229
rs112643363
rs6943559
rs60756930
All − MR Egger
All − IVW(MRE)
−0.010
−0.005
0.000
0.005
0.010
MR effect size for
'Macrophage migration inhibitory factor' on 'eGFR'

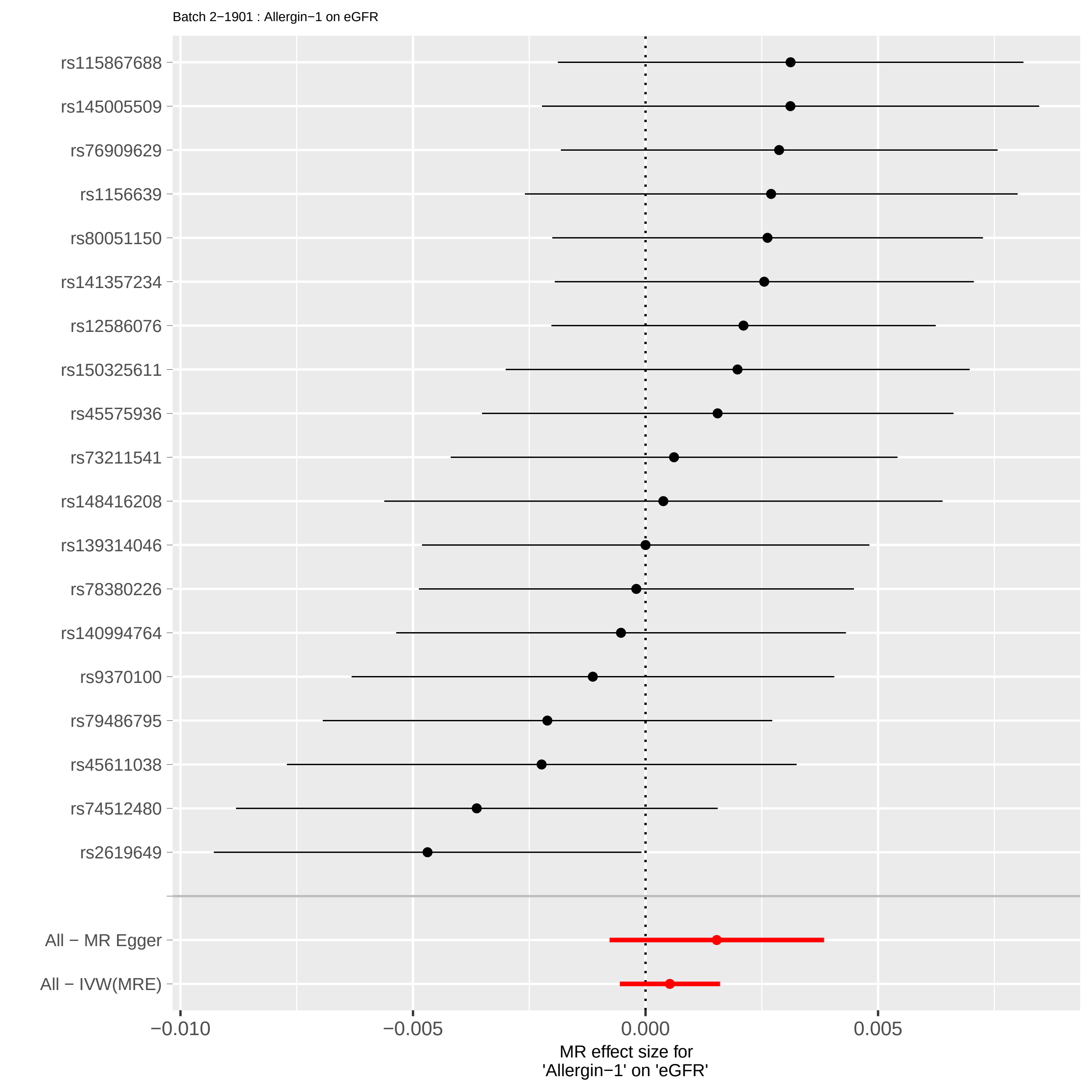

Batch 2−1901 : Allergin−1 on eGFR
rs115867688
rs145005509
rs76909629
rs1156639
rs80051150
rs141357234
rs12586076
rs150325611
rs45575936
rs73211541
rs148416208
rs139314046
rs78380226
rs140994764
rs9370100
rs79486795
rs45611038
rs74512480
rs2619649
All − MR Egger
All − IVW(MRE)
−0.010
−0.005
0.000
0.005
MR effect size for
'Allergin−1' on 'eGFR'

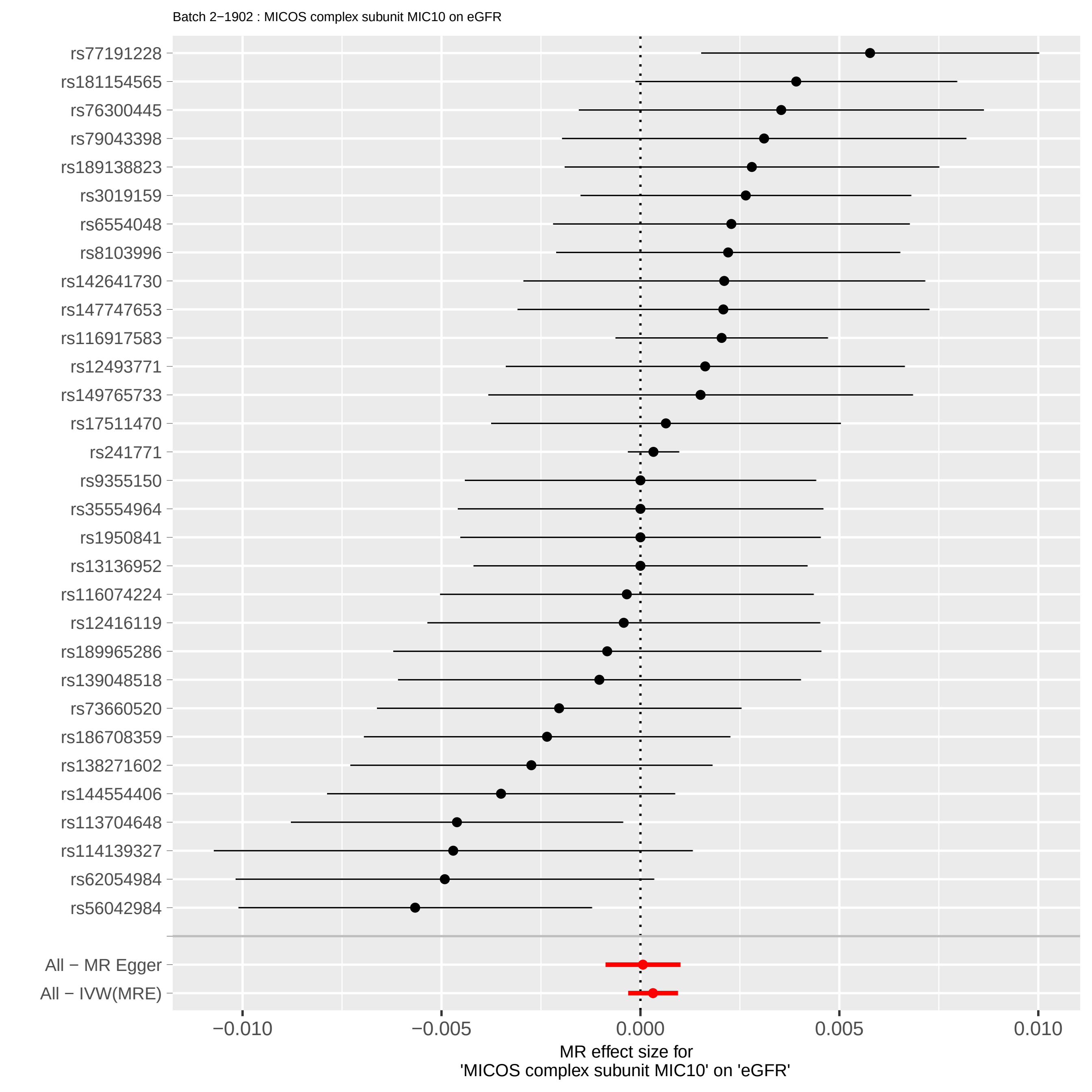

Batch 2−1902 : MICOS complex subunit MIC10 on eGFR
rs77191228
rs181154565
rs76300445
rs79043398
rs189138823
rs3019159
rs6554048
rs8103996
rs142641730
rs147747653
rs116917583
rs12493771
rs149765733
rs17511470
rs241771
rs9355150
rs35554964
rs1950841
rs13136952
rs116074224
rs12416119
rs189965286
rs139048518
rs73660520
rs186708359
rs138271602
rs144554406
rs113704648
rs114139327
rs62054984
rs56042984
All − MR Egger
All − IVW(MRE)
−0.010
−0.005
0.000
0.005
0.010
MR effect size for
'MICOS complex subunit MIC10' on 'eGFR'

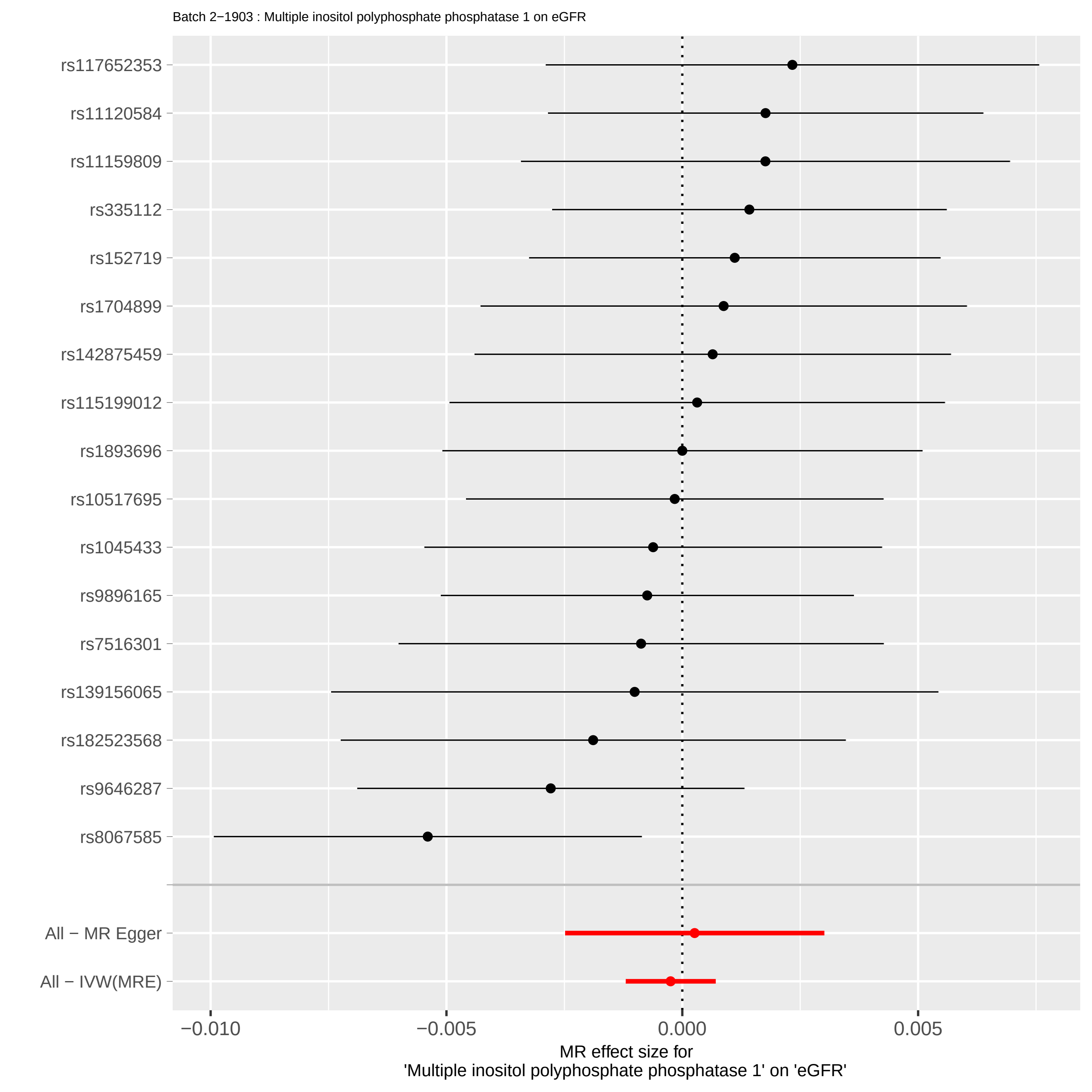

Batch 2−1903 : Multiple inositol polyphosphate phosphatase 1 on eGFR
rs117652353
rs11120584
rs11159809
rs335112
rs152719
rs1704899
rs142875459
rs115199012
rs1893696
rs10517695
rs1045433
rs9896165
rs7516301
rs139156065
rs182523568
rs9646287
rs8067585
All − MR Egger
All − IVW(MRE)
−0.010
−0.005
0.000
0.005
MR effect size for
'Multiple inositol polyphosphate phosphatase 1' on 'eGFR'

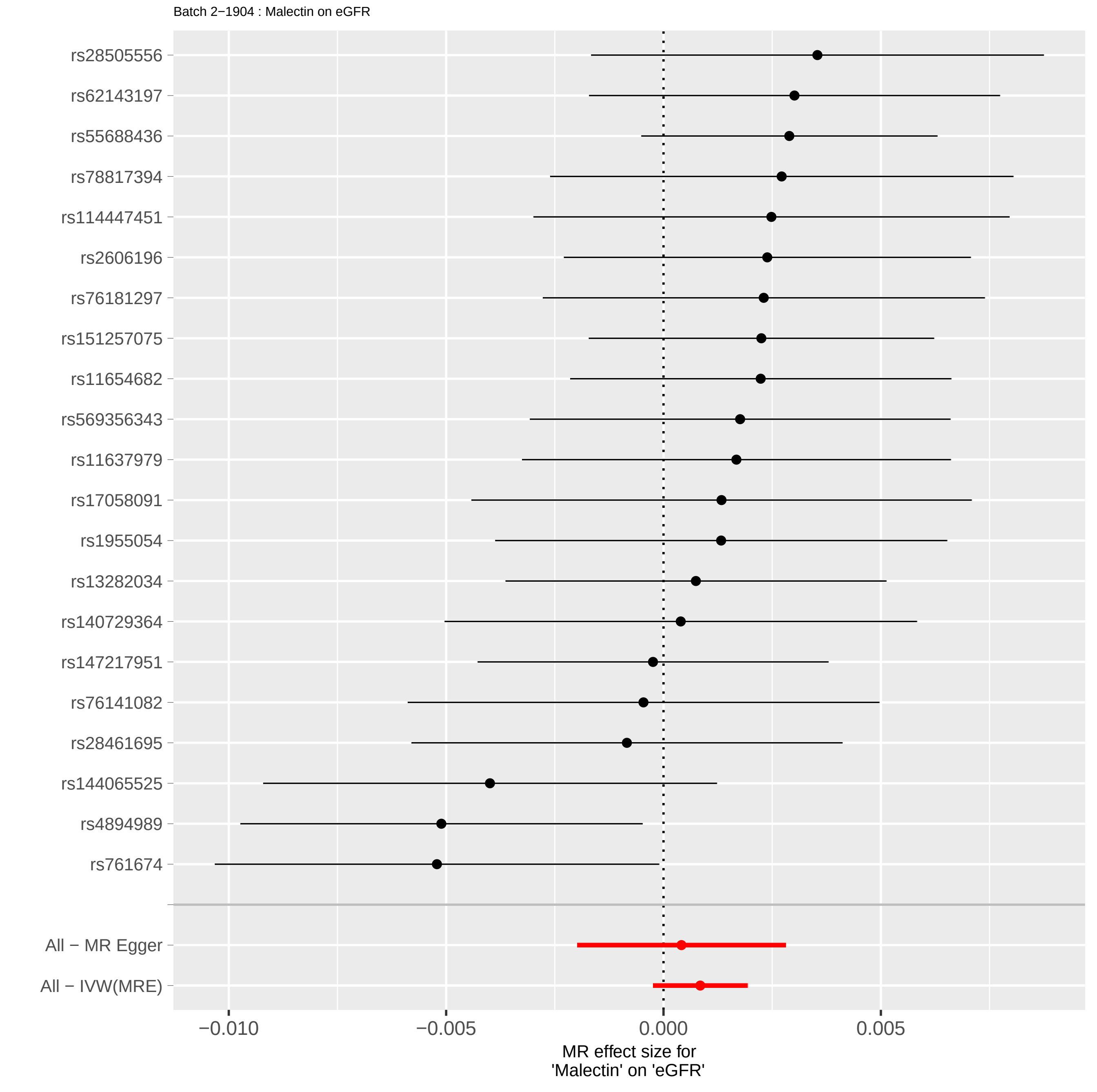

Batch 2−1904 : Malectin on eGFR
rs28505556
rs62143197
rs55688436
rs78817394
rs114447451
rs2606196
rs76181297
rs151257075
rs11654682
rs569356343
rs11637979
rs17058091
rs1955054
rs13282034
rs140729364
rs147217951
rs76141082
rs28461695
rs144065525
rs4894989
rs761674
All − MR Egger
All − IVW(MRE)
−0.010
−0.005
0.000
0.005
MR effect size for
'Malectin' on 'eGFR'

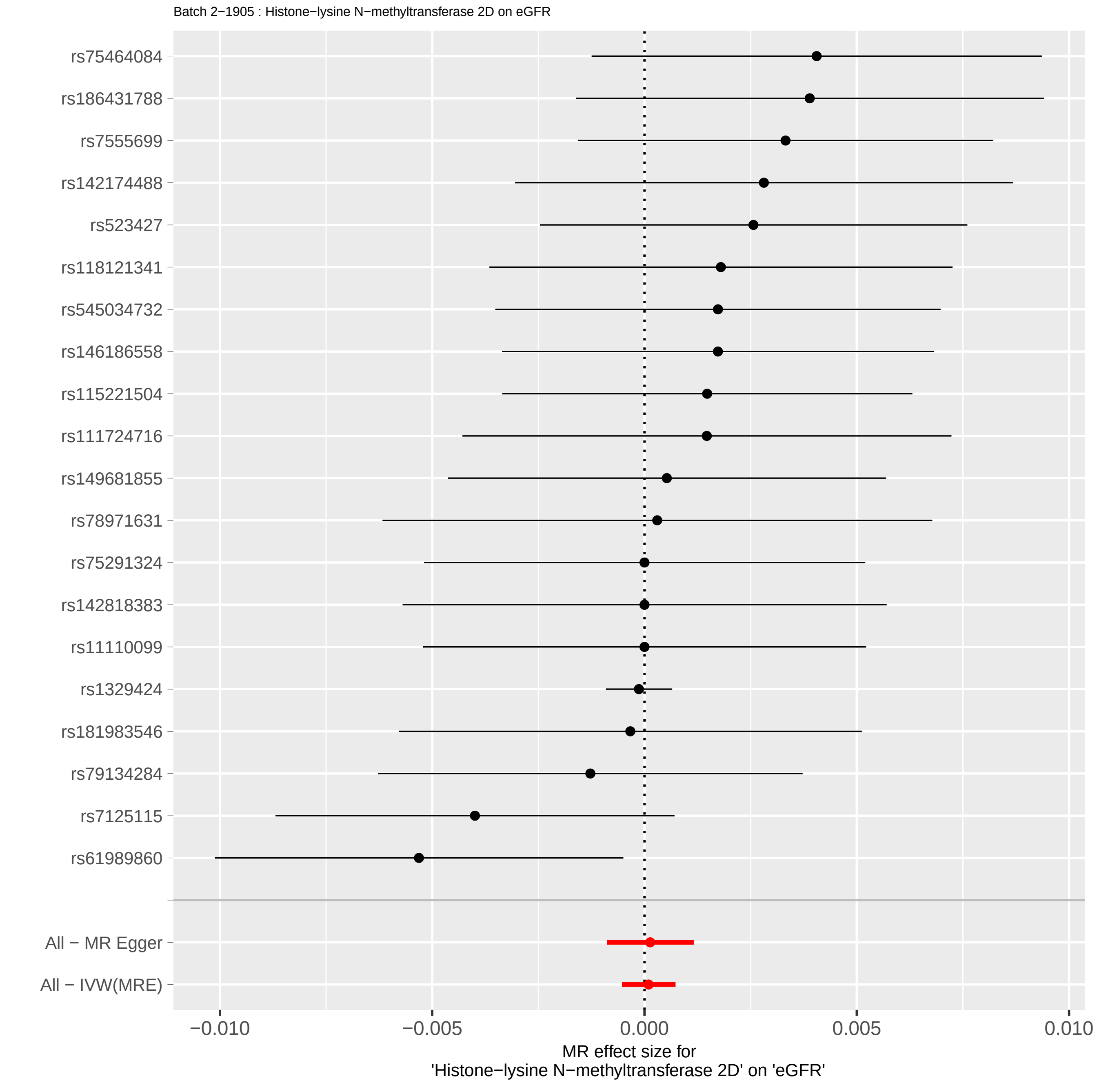
Batch 2−1905 : Histone−lysine N−methyltransferase 2D on eGFR
rs75464084
rs186431788
rs7555699
rs142174488
rs523427
rs118121341
rs545034732
rs146186558
rs115221504
rs111724716
rs149681855
rs78971631
rs75291324
rs142818383
rs11110099
rs1329424
rs181983546
rs79134284
rs7125115
rs61989860
All − MR Egger
All − IVW(MRE)
−0.010
−0.005
0.000
0.005
0.010
MR effect size for
'Histone−lysine N−methyltransferase 2D' on 'eGFR'

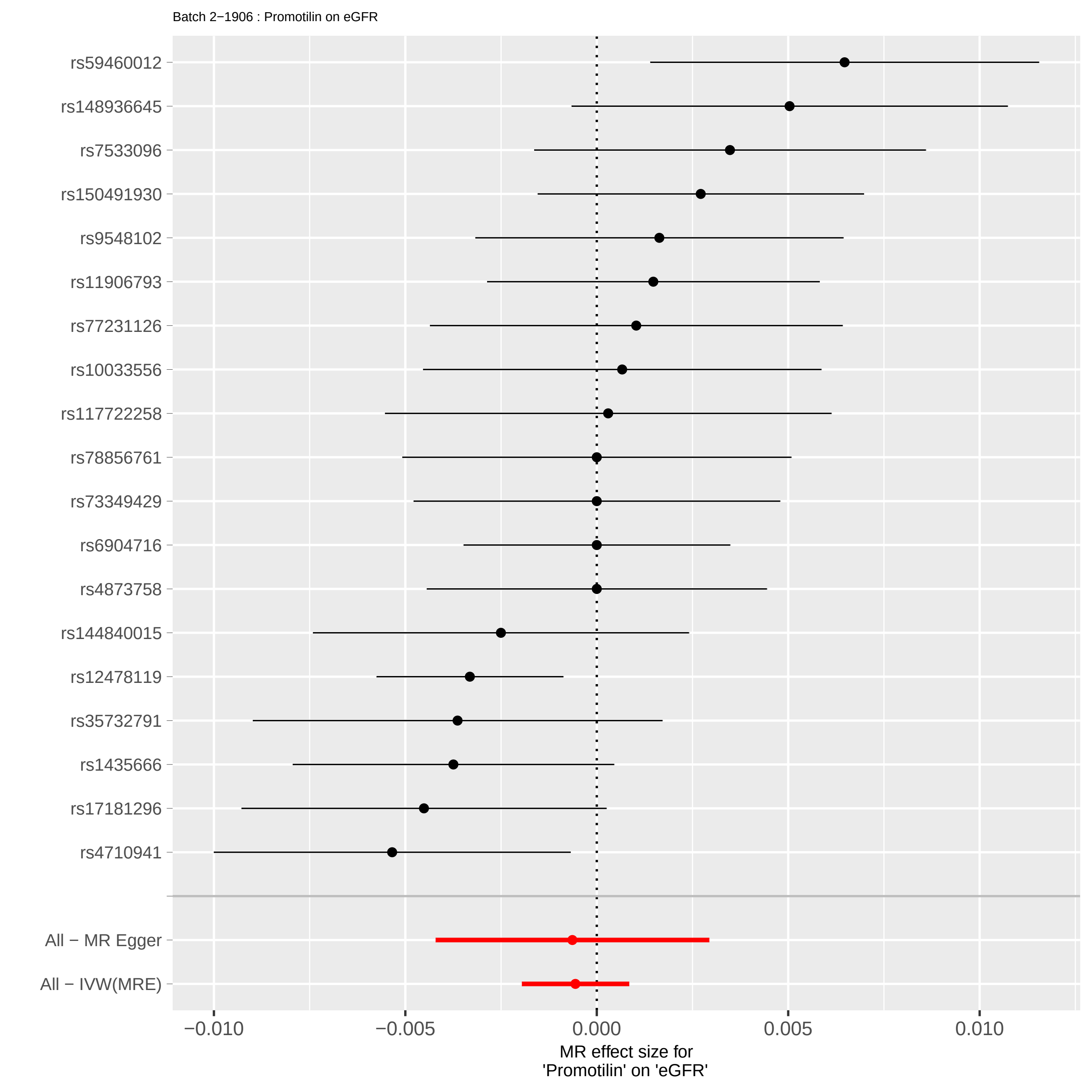

Batch 2−1906 : Promotilin on eGFR
rs59460012
rs148936645
rs7533096
rs150491930
rs9548102
rs11906793
rs77231126
rs10033556
rs117722258
rs78856761
rs73349429
rs6904716
rs4873758
rs144840015
rs12478119
rs35732791
rs1435666
rs17181296
rs4710941
All − MR Egger
All − IVW(MRE)
−0.010
−0.005
0.000
0.005
0.010
MR effect size for
'Promotilin' on 'eGFR'

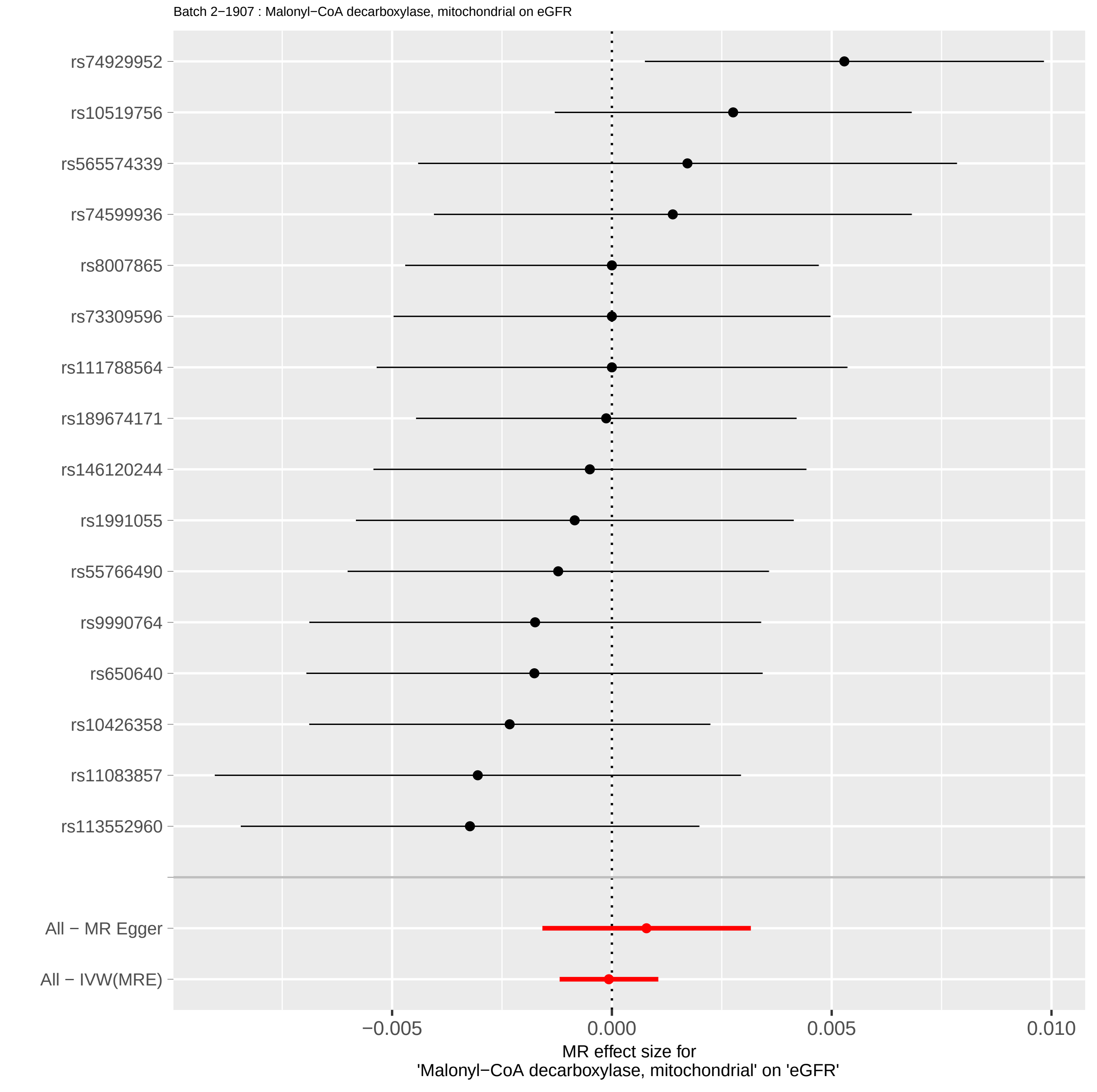

Batch 2−1907 : Malonyl−CoA decarboxylase, mitochondrial on eGFR
rs74929952
rs10519756
rs565574339
rs74599936
rs8007865
rs73309596
rs111788564
rs189674171
rs146120244
rs1991055
rs55766490
rs9990764
rs650640
rs10426358
rs11083857
rs113552960
All − MR Egger
All − IVW(MRE)
−0.005
0.000
0.005
0.010
MR effect size for
'Malonyl−CoA decarboxylase, mitochondrial' on 'eGFR'

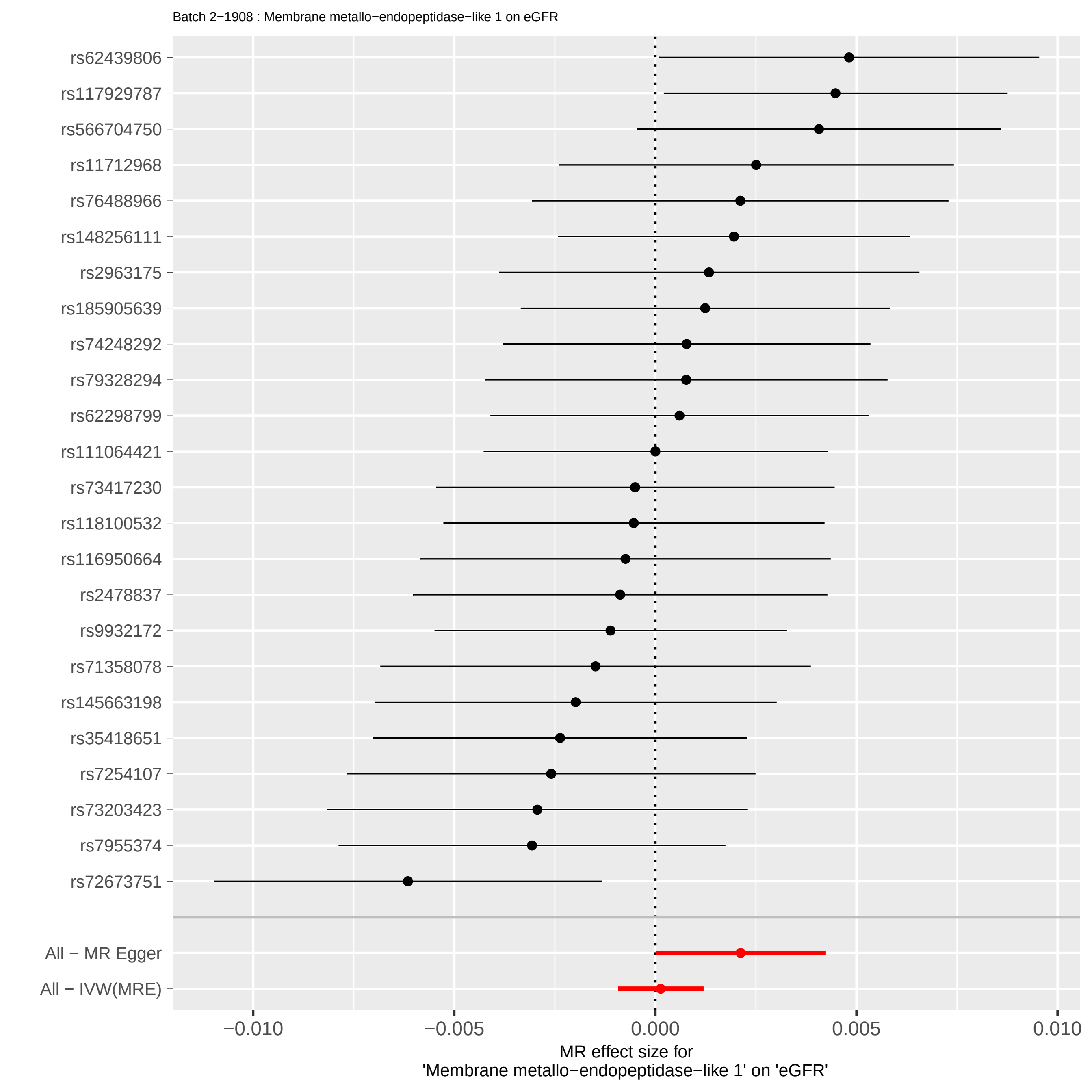
Batch 2−1908 : Membrane metallo−endopeptidase−like 1 on eGFR
rs62439806
rs117929787
rs566704750
rs11712968
rs76488966
rs148256111
rs2963175
rs185905639
rs74248292
rs79328294
rs62298799
rs111064421
rs73417230
rs118100532
rs116950664
rs2478837
rs9932172
rs71358078
rs145663198
rs35418651
rs7254107
rs73203423
rs7955374
rs72673751
All − MR Egger
All − IVW(MRE)
−0.010
−0.005
0.000
0.005
0.010
MR effect size for
'Membrane metallo−endopeptidase−like 1' on 'eGFR'

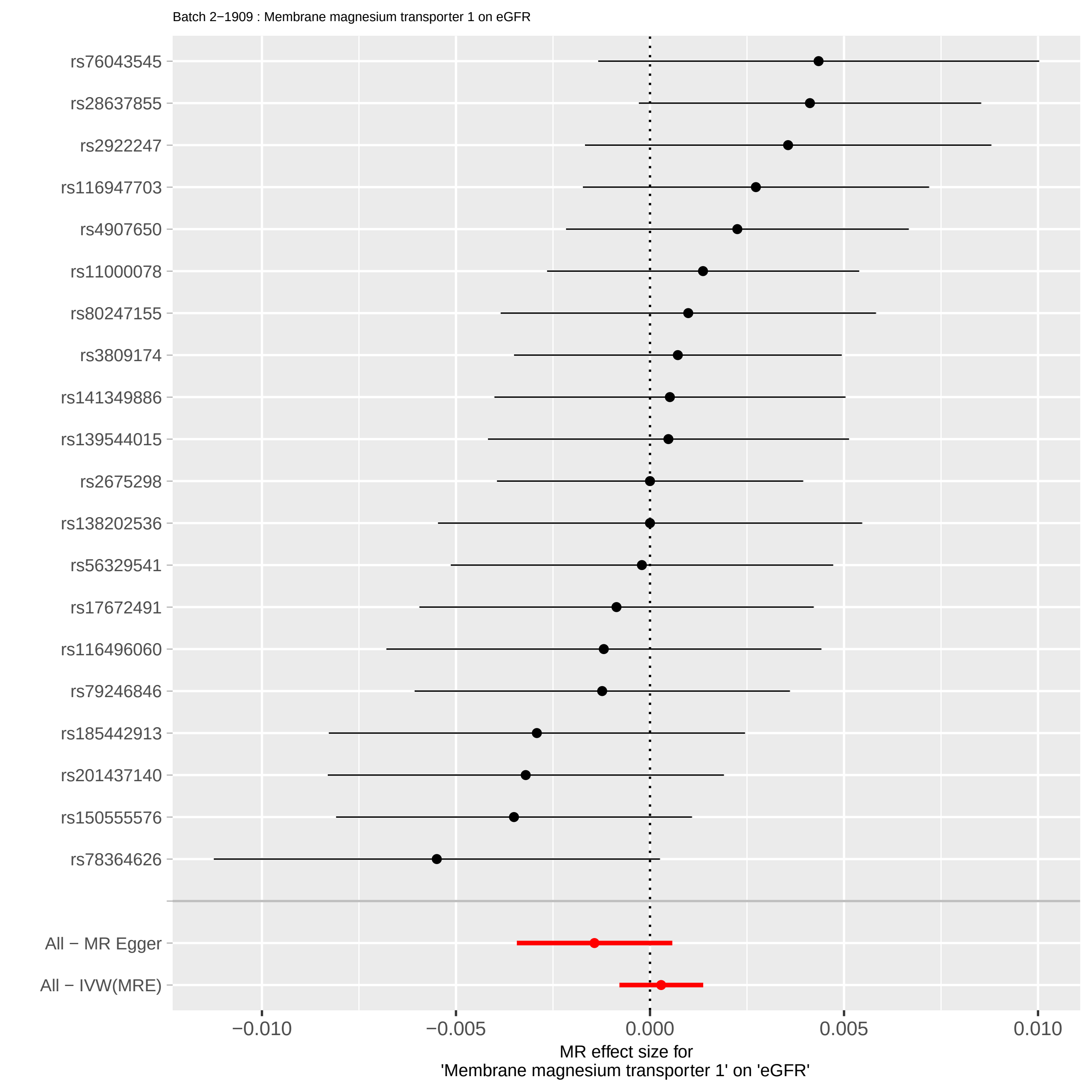

Batch 2−1909 : Membrane magnesium transporter 1 on eGFR
rs76043545
rs28637855
rs2922247
rs116947703
rs4907650
rs11000078
rs80247155
rs3809174
rs141349886
rs139544015
rs2675298
rs138202536
rs56329541
rs17672491
rs116496060
rs79246846
rs185442913
rs201437140
rs150555576
rs78364626
All − MR Egger
All − IVW(MRE)
−0.010
−0.005
0.000
0.005
0.010
MR effect size for
'Membrane magnesium transporter 1' on 'eGFR'

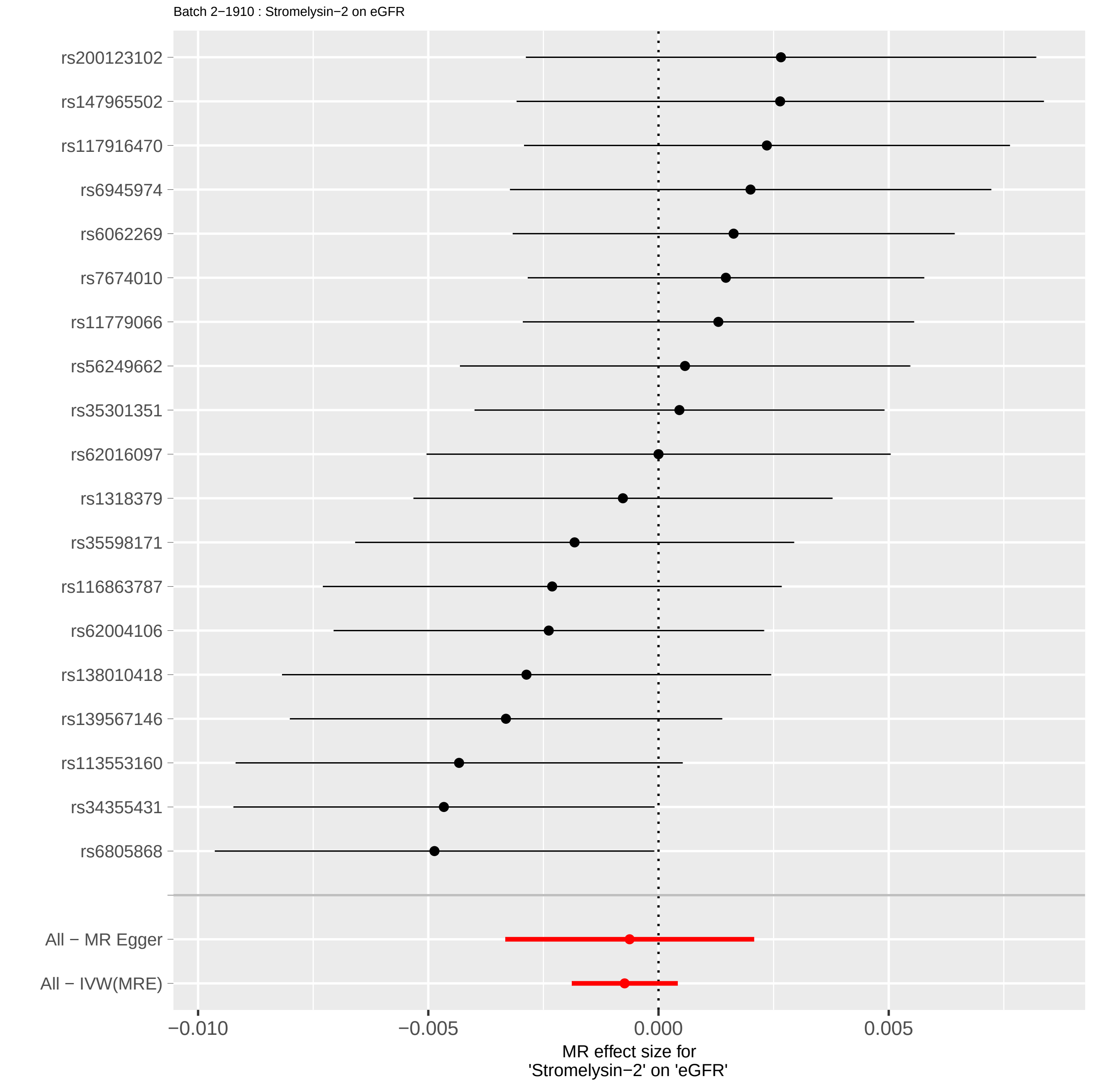

Batch 2−1910 : Stromelysin−2 on eGFR
rs200123102
rs147965502
rs117916470
rs6945974
rs6062269
rs7674010
rs11779066
rs56249662
rs35301351
rs62016097
rs1318379
rs35598171
rs116863787
rs62004106
rs138010418
rs139567146
rs113553160
rs34355431
rs6805868
All − MR Egger
All − IVW(MRE)
−0.010
−0.005
0.000
0.005
MR effect size for
'Stromelysin−2' on 'eGFR'

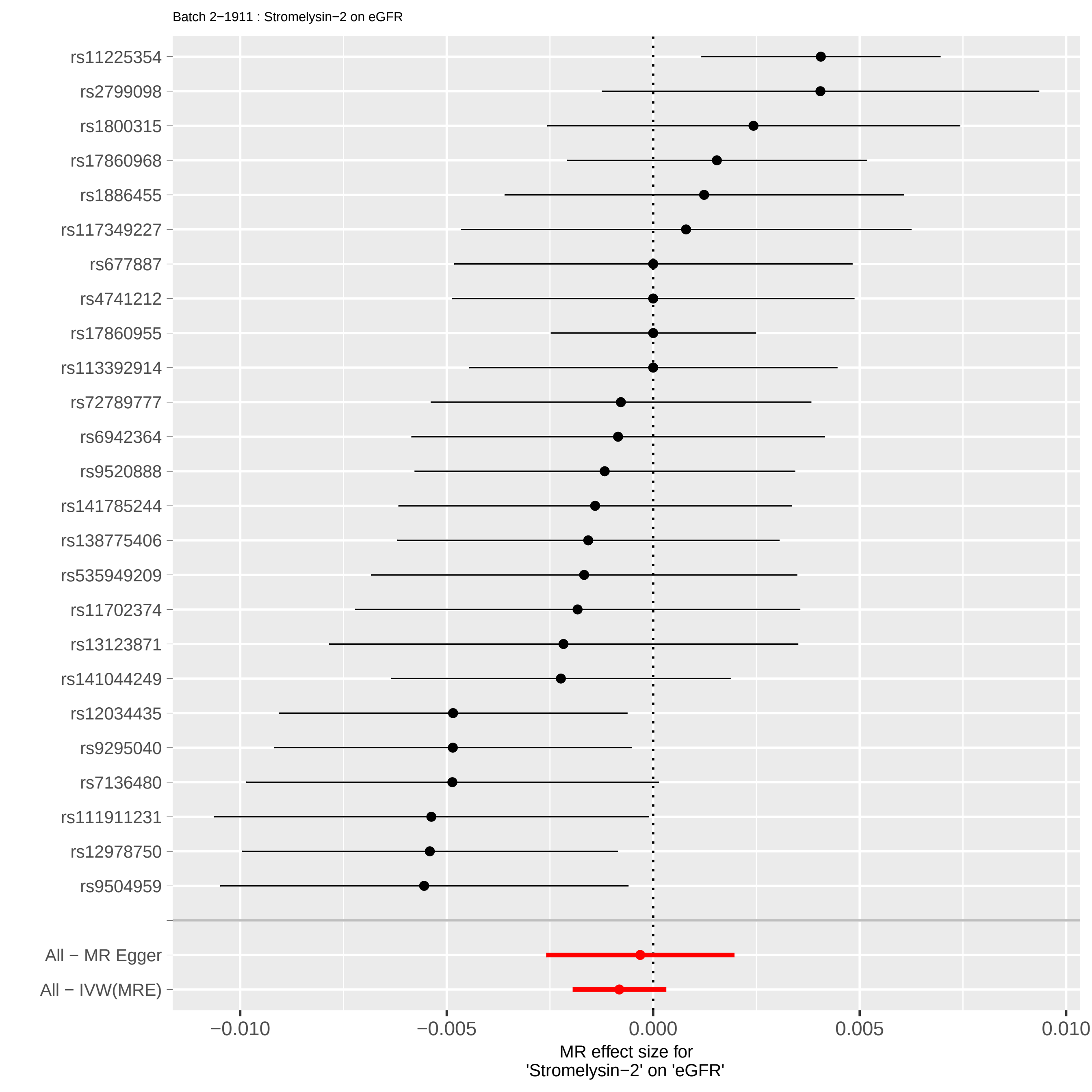

Batch 2−1911 : Stromelysin−2 on eGFR
rs11225354
rs2799098
rs1800315
rs17860968
rs1886455
rs117349227
rs677887
rs4741212
rs17860955
rs113392914
rs72789777
rs6942364
rs9520888
rs141785244
rs138775406
rs535949209
rs11702374
rs13123871
rs141044249
rs12034435
rs9295040
rs7136480
rs111911231
rs12978750
rs9504959
All − MR Egger
All − IVW(MRE)
−0.010
−0.005
0.000
0.005
0.010
MR effect size for
'Stromelysin−2' on 'eGFR'

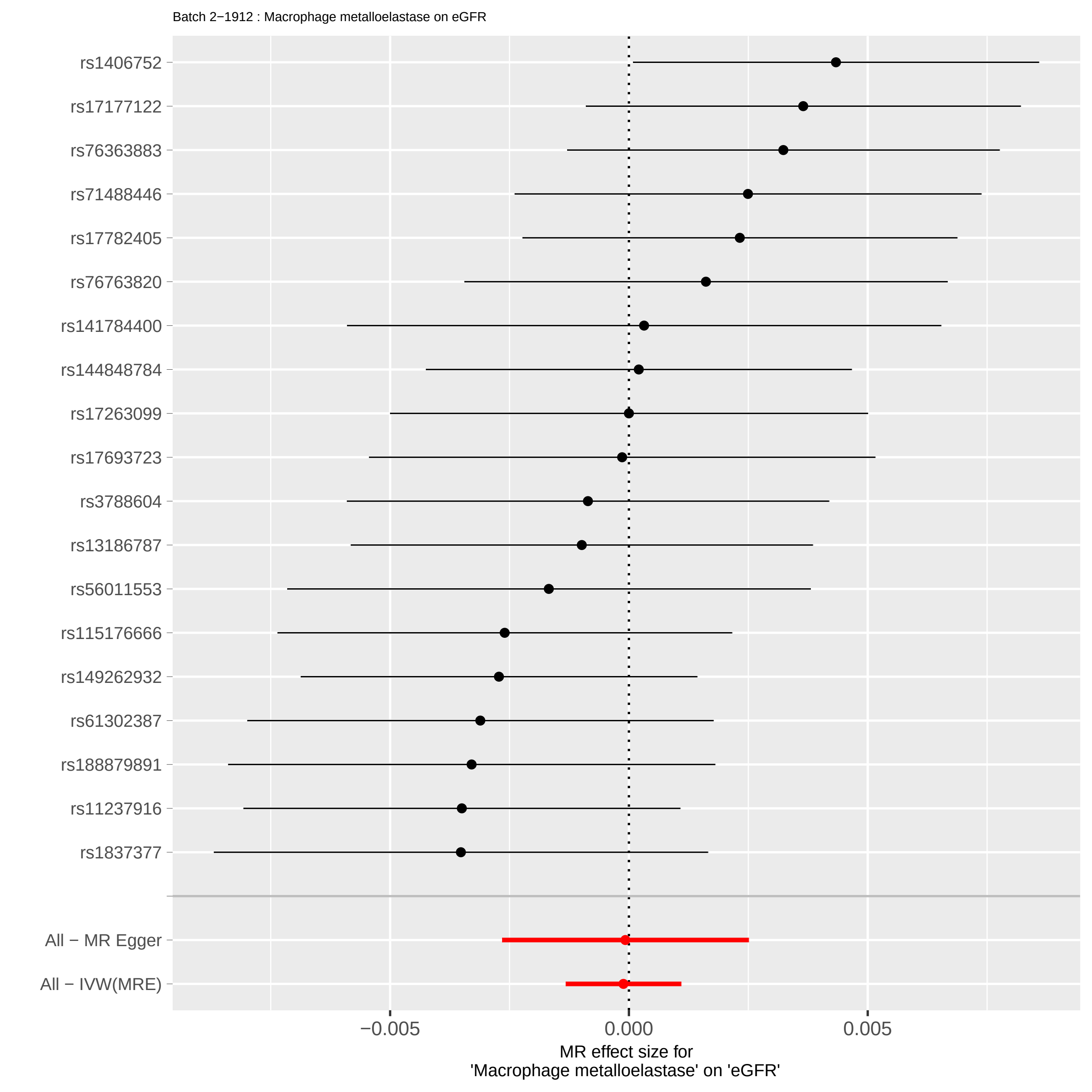
Batch 2−1912 : Macrophage metalloelastase on eGFR
rs1406752
rs17177122
rs76363883
rs71488446
rs17782405
rs76763820
rs141784400
rs144848784
rs17263099
rs17693723
rs3788604
rs13186787
rs56011553
rs115176666
rs149262932
rs61302387
rs188879891
rs11237916
rs1837377
All − MR Egger
All − IVW(MRE)
−0.005
0.000
0.005
MR effect size for
'Macrophage metalloelastase' on 'eGFR'

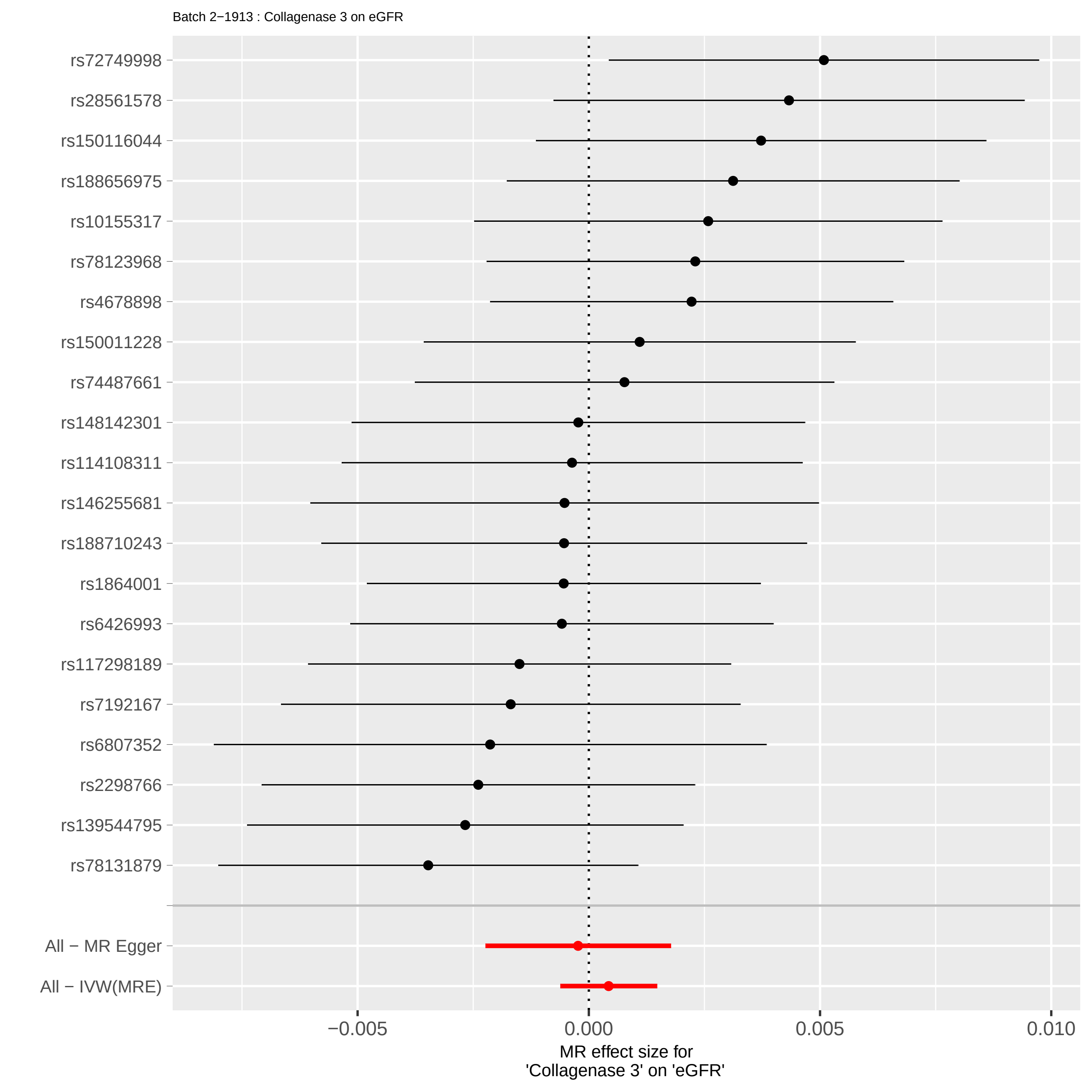
Batch 2−1913 : Collagenase 3 on eGFR
rs72749998
rs28561578
rs150116044
rs188656975
rs10155317
rs78123968
rs4678898
rs150011228
rs74487661
rs148142301
rs114108311
rs146255681
rs188710243
rs1864001
rs6426993
rs117298189
rs7192167
rs6807352
rs2298766
rs139544795
rs78131879
All − MR Egger
All − IVW(MRE)
−0.005
0.000
0.005
0.010
MR effect size for
'Collagenase 3' on 'eGFR'

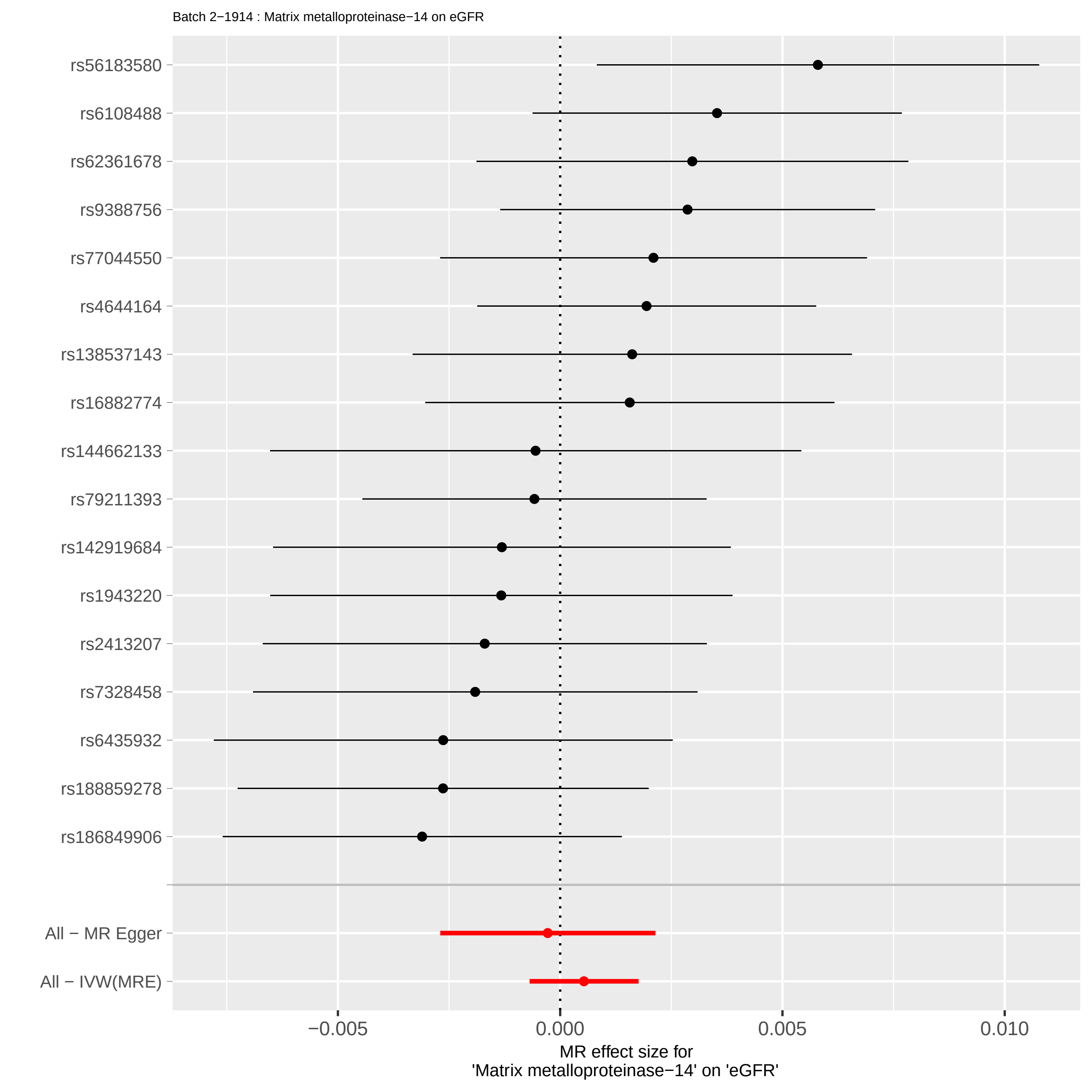

Batch 2−1914 : Matrix metalloproteinase−14 on eGFR
rs56183580
rs6108488
rs62361678
rs9388756
rs77044550
rs4644164
rs138537143
rs16882774
rs144662133
rs79211393
rs142919684
rs1943220
rs2413207
rs7328458
rs6435932
rs188859278
rs186849906
All − MR Egger
All − IVW(MRE)
−0.005
0.000
0.005
0.010
MR effect size for
'Matrix metalloproteinase−14' on 'eGFR'

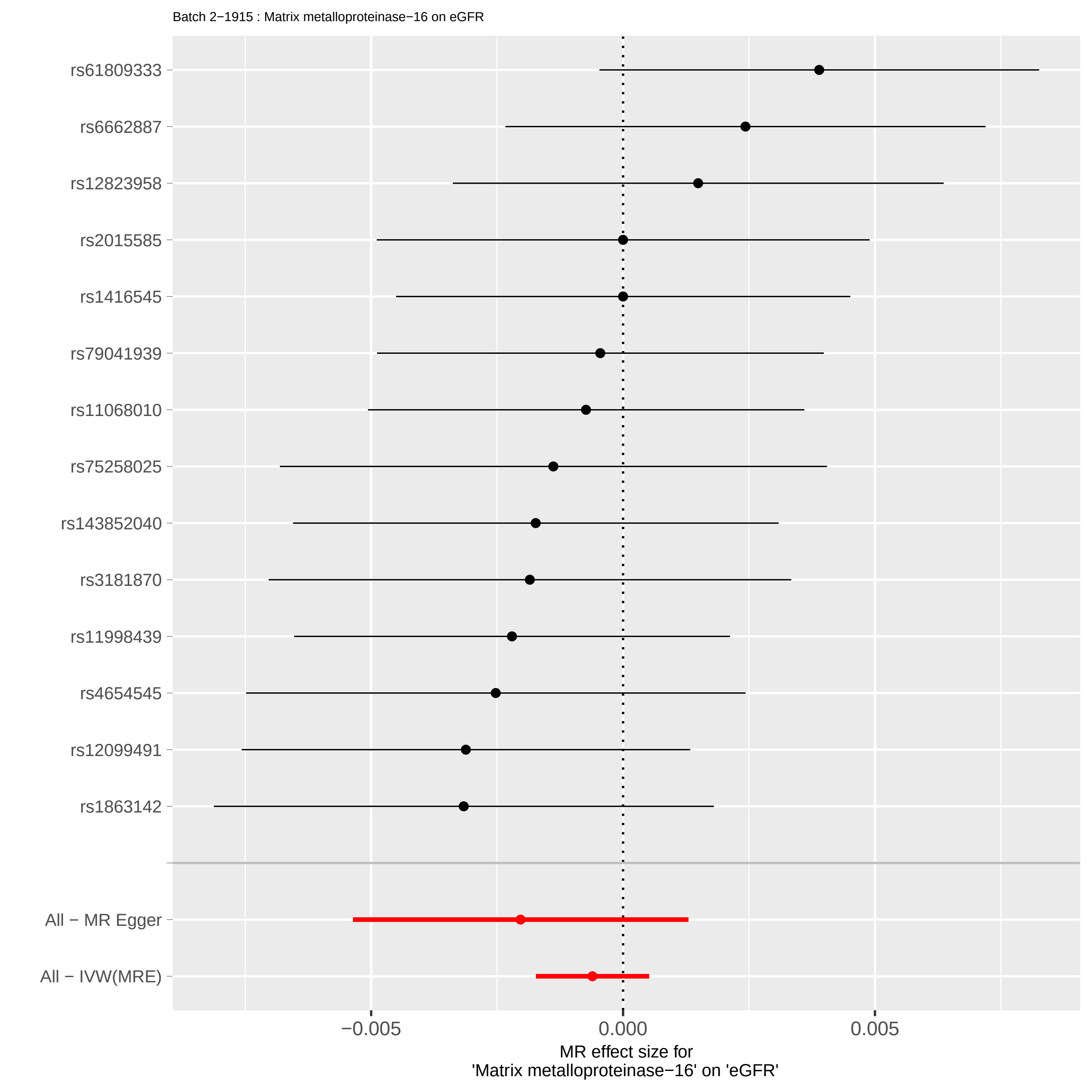

Batch 2−1915 : Matrix metalloproteinase−16 on eGFR
rs61809333
rs6662887
rs12823958
rs2015585
rs1416545
rs79041939
rs11068010
rs75258025
rs143852040
rs3181870
rs11998439
rs4654545
rs12099491
rs1863142
All − MR Egger
All − IVW(MRE)
−0.005
0.000
0.005
MR effect size for
'Matrix metalloproteinase−16' on 'eGFR'

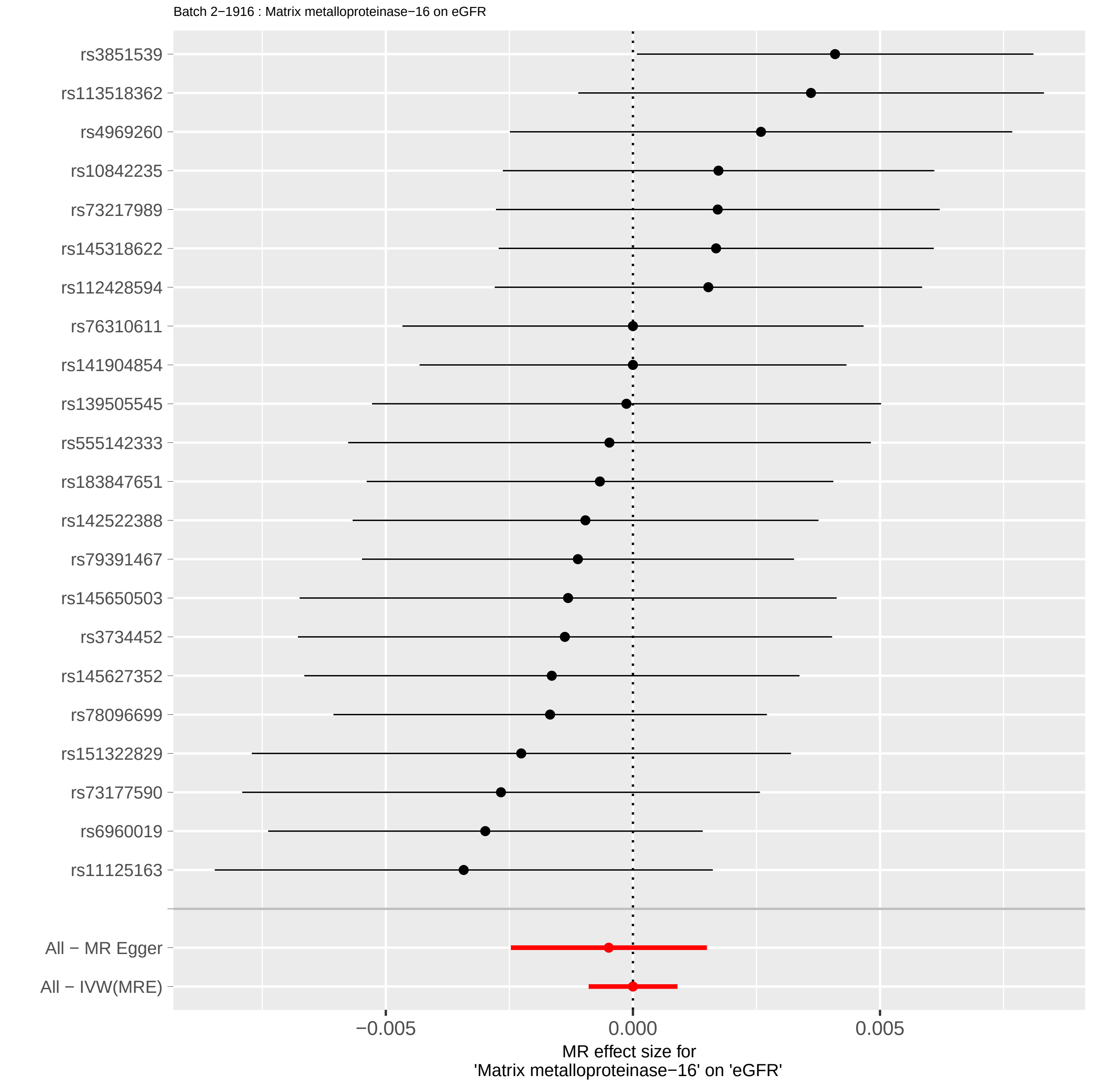

Batch 2−1916 : Matrix metalloproteinase−16 on eGFR
rs3851539
rs113518362
rs4969260
rs10842235
rs73217989
rs145318622
rs112428594
rs76310611
rs141904854
rs139505545
rs555142333
rs183847651
rs142522388
rs79391467
rs145650503
rs3734452
rs145627352
rs78096699
rs151322829
rs73177590
rs6960019
rs11125163
All − MR Egger
All − IVW(MRE)
−0.005
0.000
0.005
MR effect size for
'Matrix metalloproteinase−16' on 'eGFR'

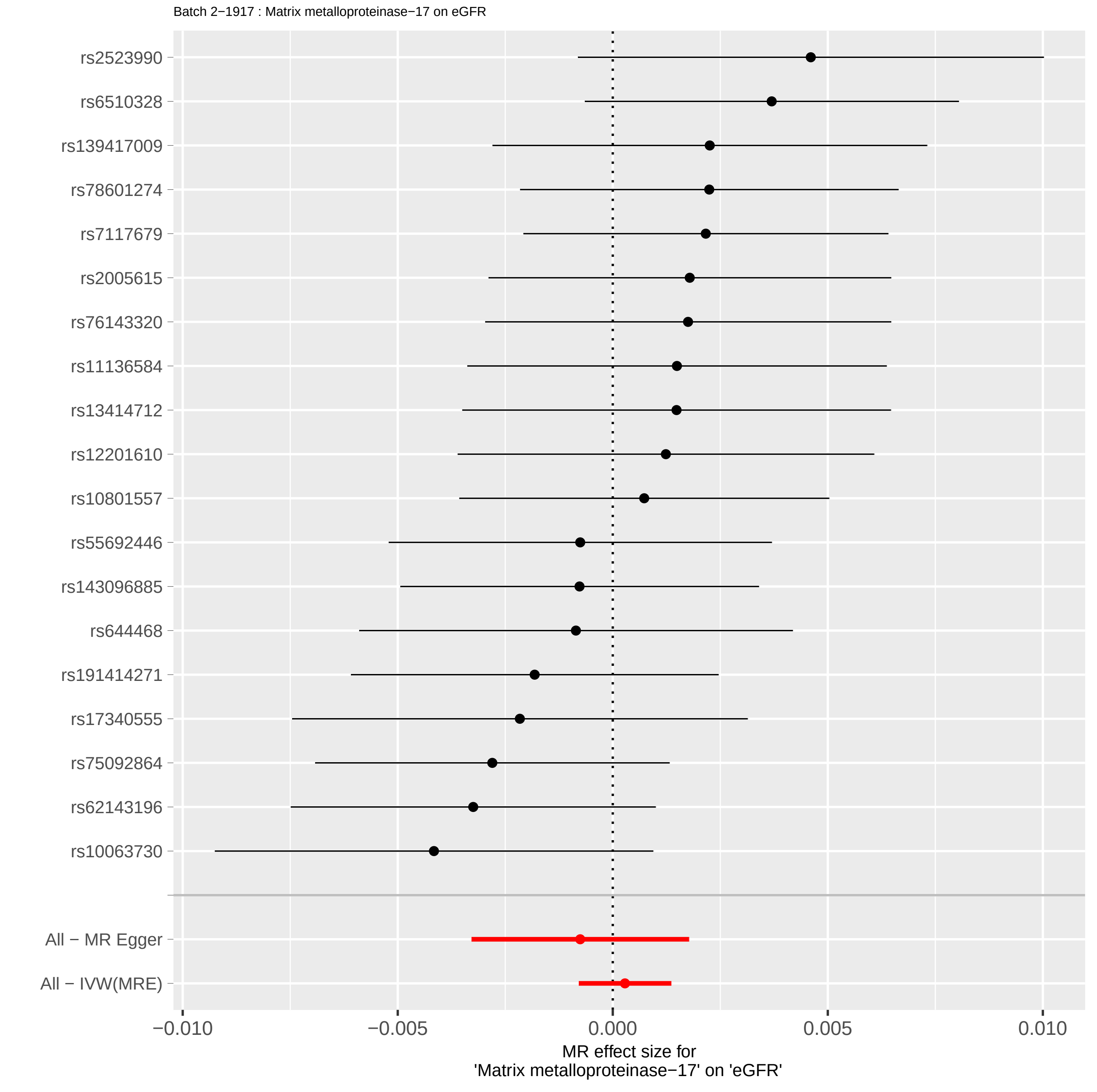

Batch 2−1917 : Matrix metalloproteinase−17 on eGFR
rs2523990
rs6510328
rs139417009
rs78601274
rs7117679
rs2005615
rs76143320
rs11136584
rs13414712
rs12201610
rs10801557
rs55692446
rs143096885
rs644468
rs191414271
rs17340555
rs75092864
rs62143196
rs10063730
All − MR Egger
All − IVW(MRE)
−0.010
−0.005
0.000
0.005
0.010
MR effect size for
'Matrix metalloproteinase−17' on 'eGFR'

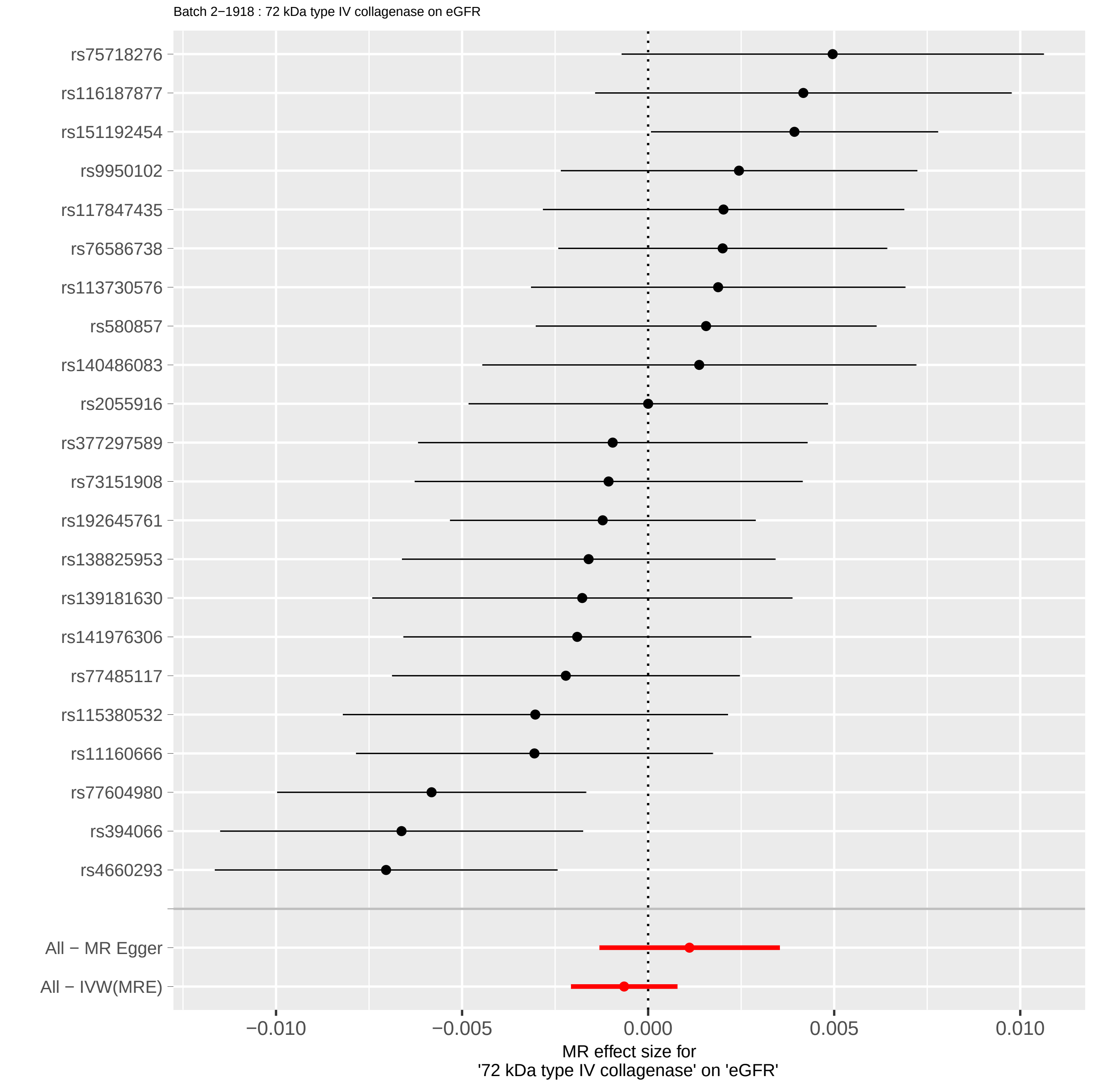

Batch 2−1918 : 72 kDa type IV collagenase on eGFR
rs75718276
rs116187877
rs151192454
rs9950102
rs117847435
rs76586738
rs113730576
rs580857
rs140486083
rs2055916
rs377297589
rs73151908
rs192645761
rs138825953
rs139181630
rs141976306
rs77485117
rs115380532
rs11160666
rs77604980
rs394066
rs4660293
All − MR Egger
All − IVW(MRE)
−0.010
−0.005
0.000
0.005
0.010
MR effect size for
'72 kDa type IV collagenase' on 'eGFR'

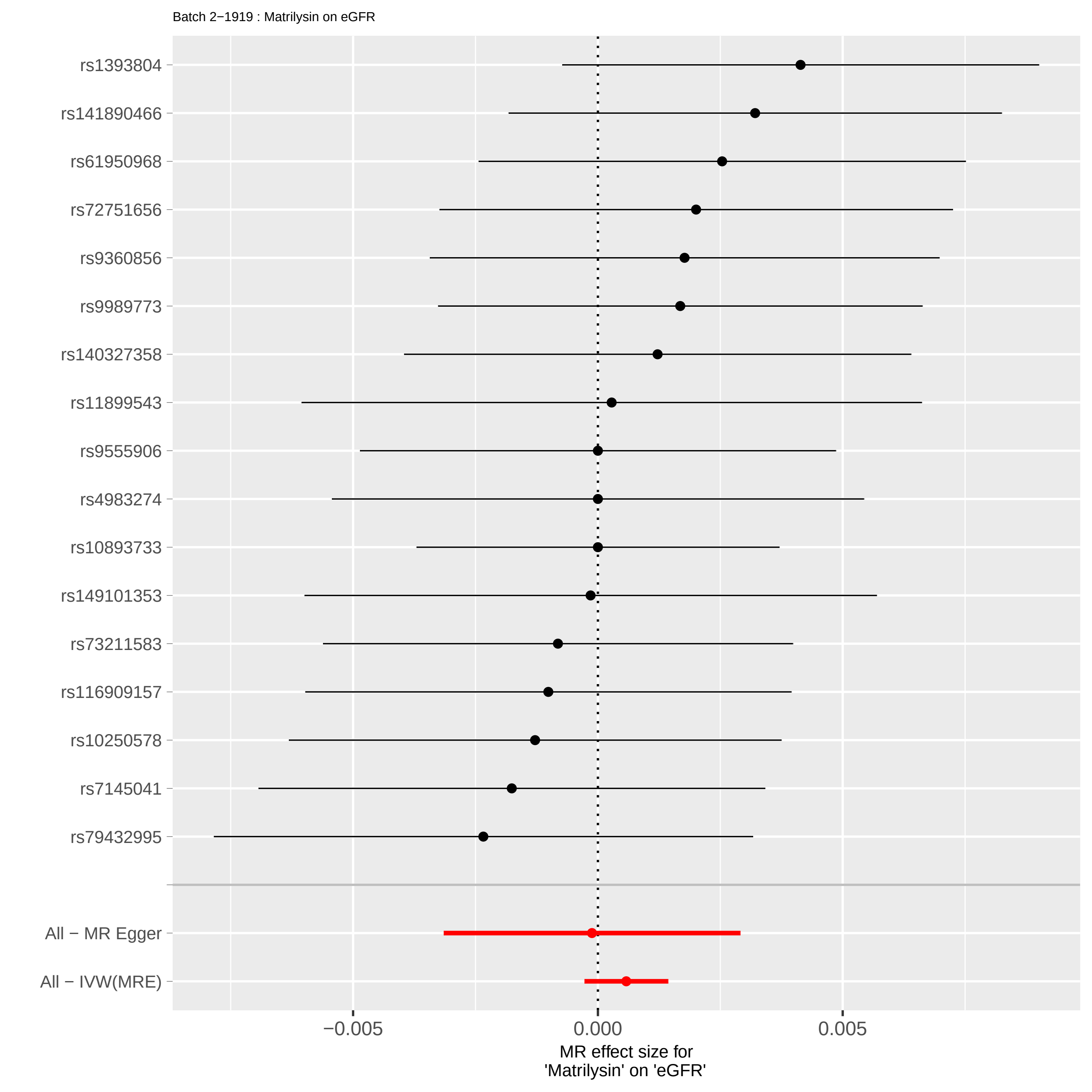

Batch 2−1919 : Matrilysin on eGFR
rs1393804
rs141890466
rs61950968
rs72751656
rs9360856
rs9989773
rs140327358
rs11899543
rs9555906
rs4983274
rs10893733
rs149101353
rs73211583
rs116909157
rs10250578
rs7145041
rs79432995
All − MR Egger
All − IVW(MRE)
−0.005
0.000
0.005
MR effect size for
'Matrilysin' on 'eGFR'

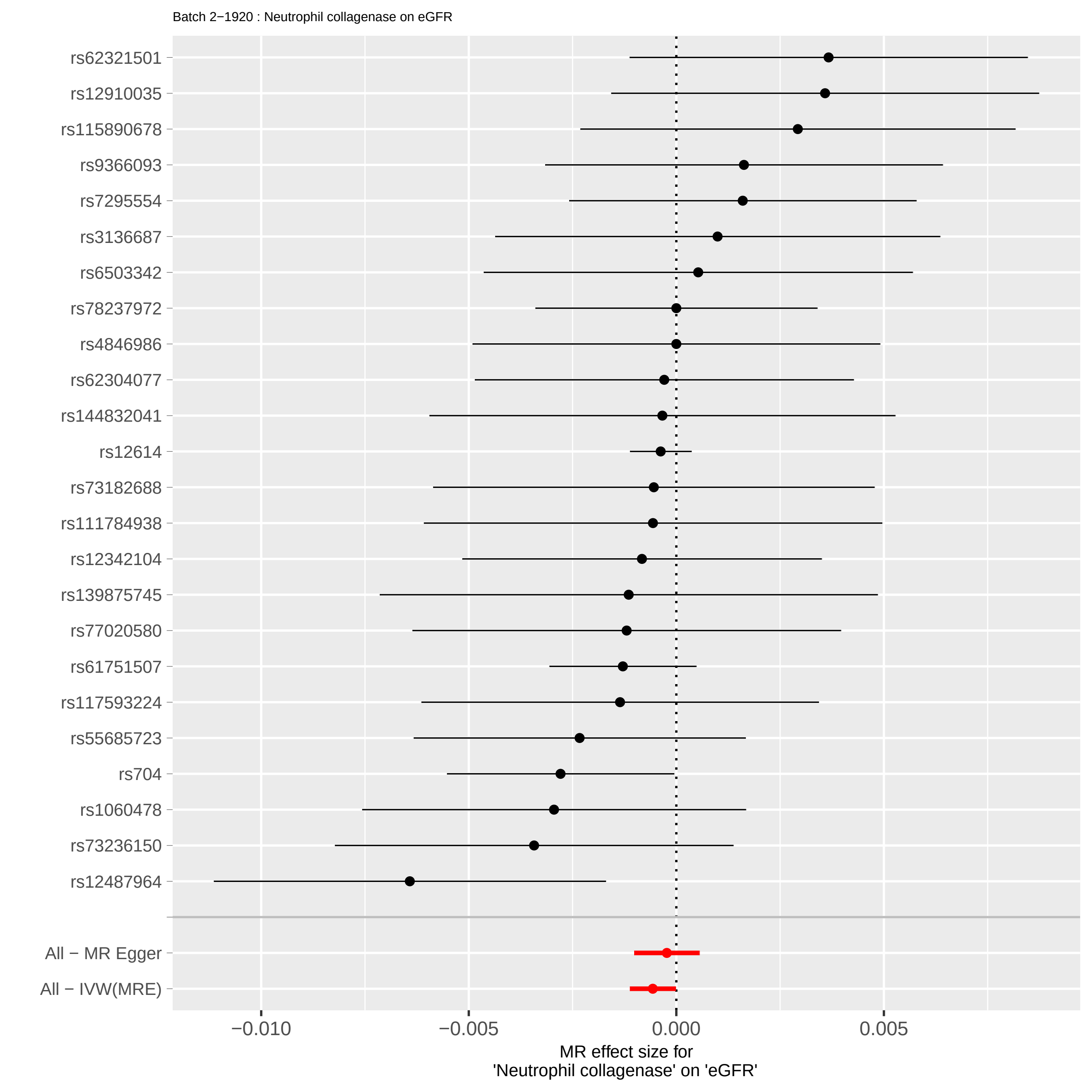
Batch 2−1920 : Neutrophil collagenase on eGFR
rs62321501
rs12910035
rs115890678
rs9366093
rs7295554
rs3136687
rs6503342
rs78237972
rs4846986
rs62304077
rs144832041
rs12614
rs73182688
rs111784938
rs12342104
rs139875745
rs77020580
rs61751507
rs117593224
rs55685723
rs704
rs1060478
rs73236150
rs12487964
All − MR Egger
All − IVW(MRE)
−0.010
−0.005
0.000
0.005
MR effect size for
'Neutrophil collagenase' on 'eGFR'

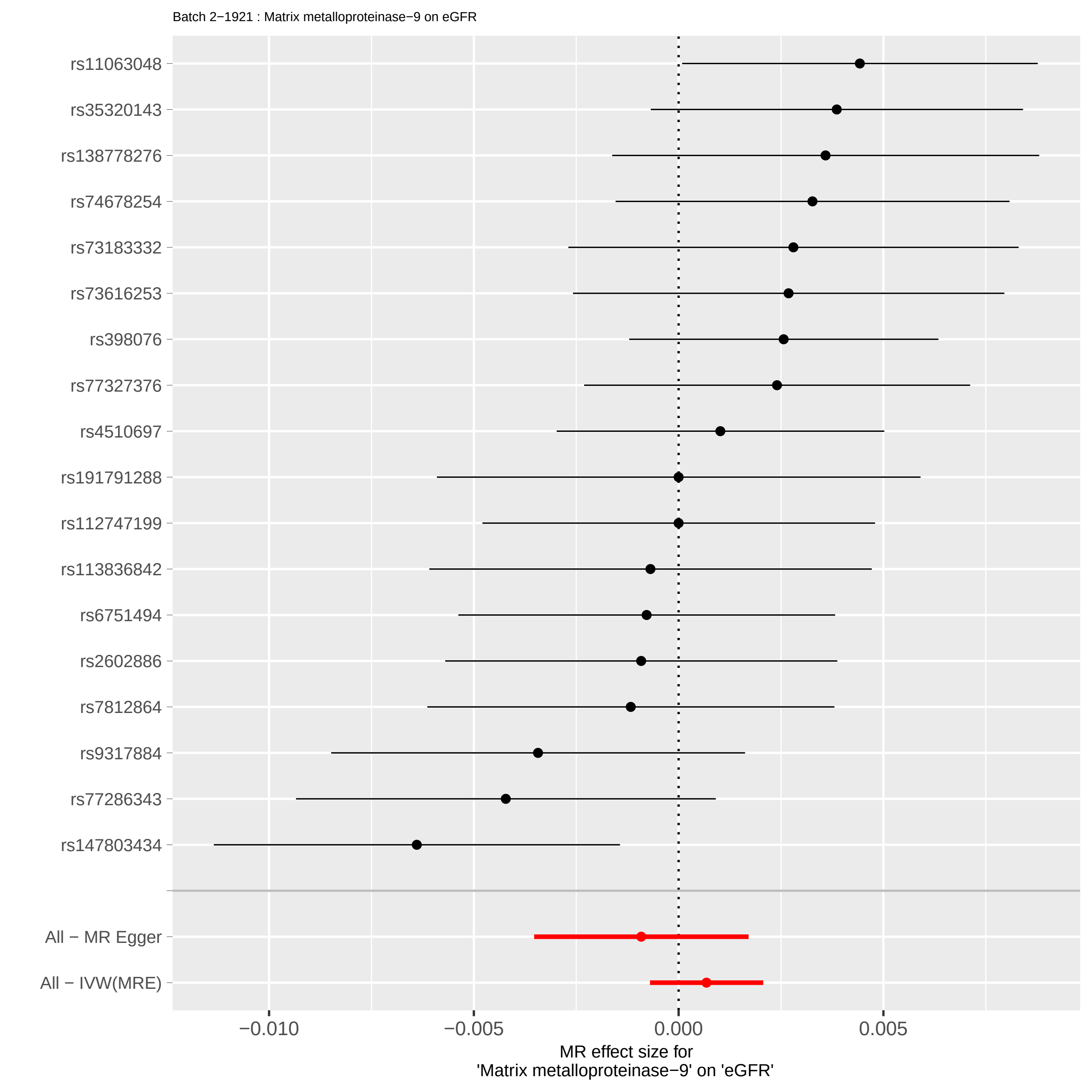
Batch 2−1921 : Matrix metalloproteinase−9 on eGFR
rs11063048
rs35320143
rs138778276
rs74678254
rs73183332
rs73616253
rs398076
rs77327376
rs4510697
rs191791288
rs112747199
rs113836842
rs6751494
rs2602886
rs7812864
rs9317884
rs77286343
rs147803434
All − MR Egger
All − IVW(MRE)
−0.010
−0.005
0.000
0.005
MR effect size for
'Matrix metalloproteinase−9' on 'eGFR'

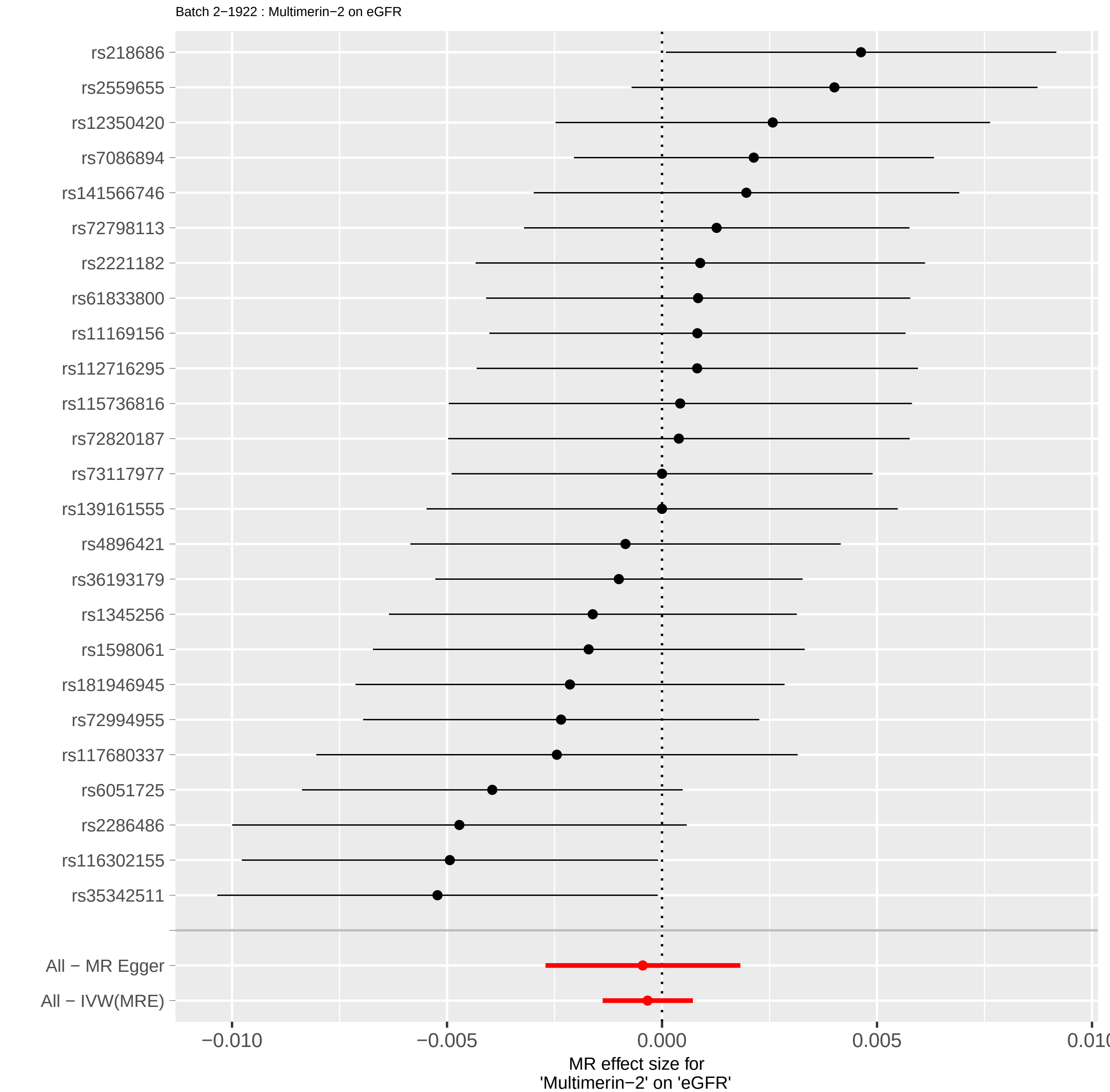
Batch 2−1922 : Multimerin−2 on eGFR
rs218686
rs2559655
rs12350420
rs7086894
rs141566746
rs72798113
rs2221182
rs61833800
rs11169156
rs112716295
rs115736816
rs72820187
rs73117977
rs139161555
rs4896421
rs36193179
rs1345256
rs1598061
rs181946945
rs72994955
rs117680337
rs6051725
rs2286486
rs116302155
rs35342511
All − MR Egger
All − IVW(MRE)
−0.010
−0.005
0.000
0.005
0.010
MR effect size for
'Multimerin−2' on 'eGFR'

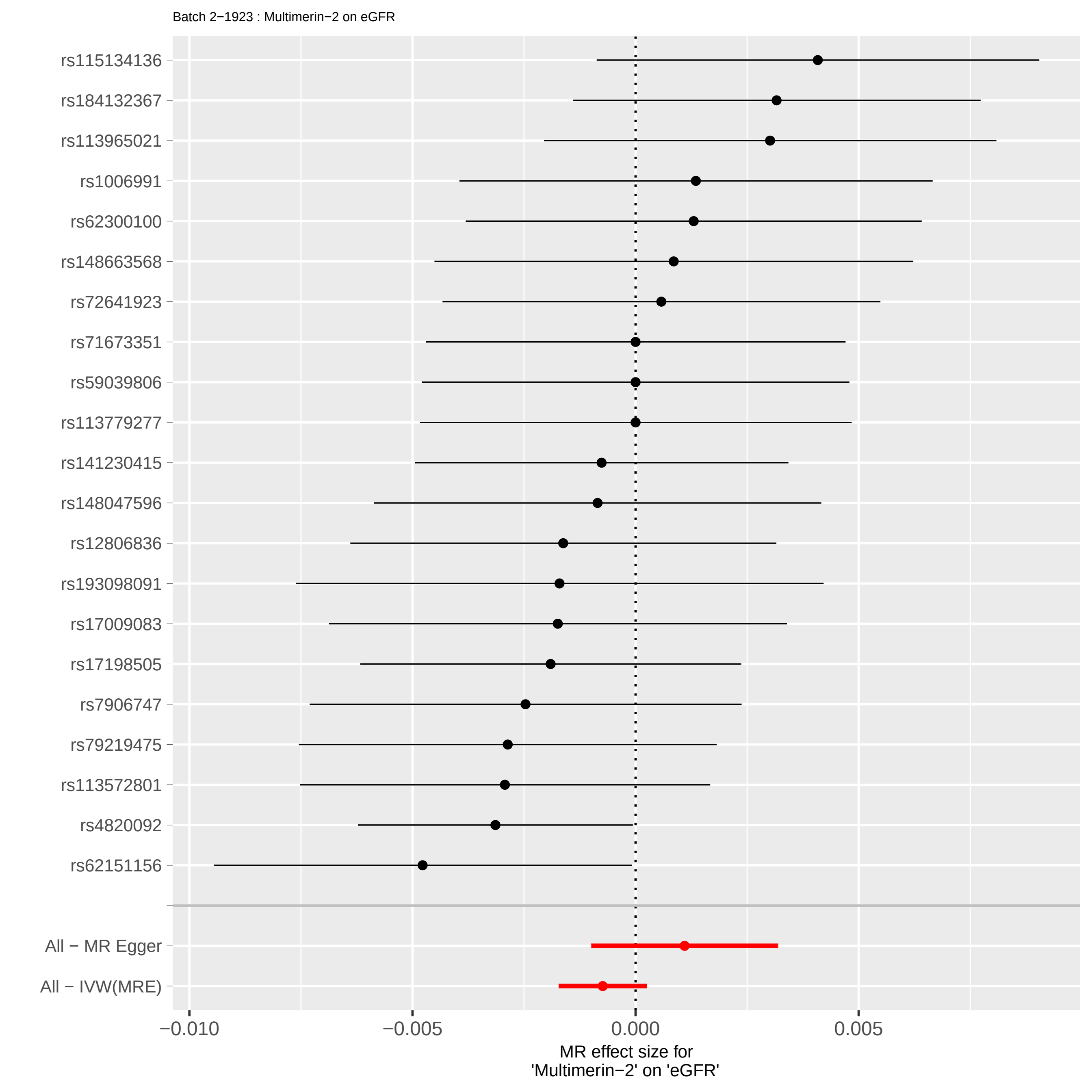

Batch 2−1923 : Multimerin−2 on eGFR
rs115134136
rs184132367
rs113965021
rs1006991
rs62300100
rs148663568
rs72641923
rs71673351
rs59039806
rs113779277
rs141230415
rs148047596
rs12806836
rs193098091
rs17009083
rs17198505
rs7906747
rs79219475
rs113572801
rs4820092
rs62151156
All − MR Egger
All − IVW(MRE)
−0.010
−0.005
0.000
0.005
MR effect size for
'Multimerin−2' on 'eGFR'

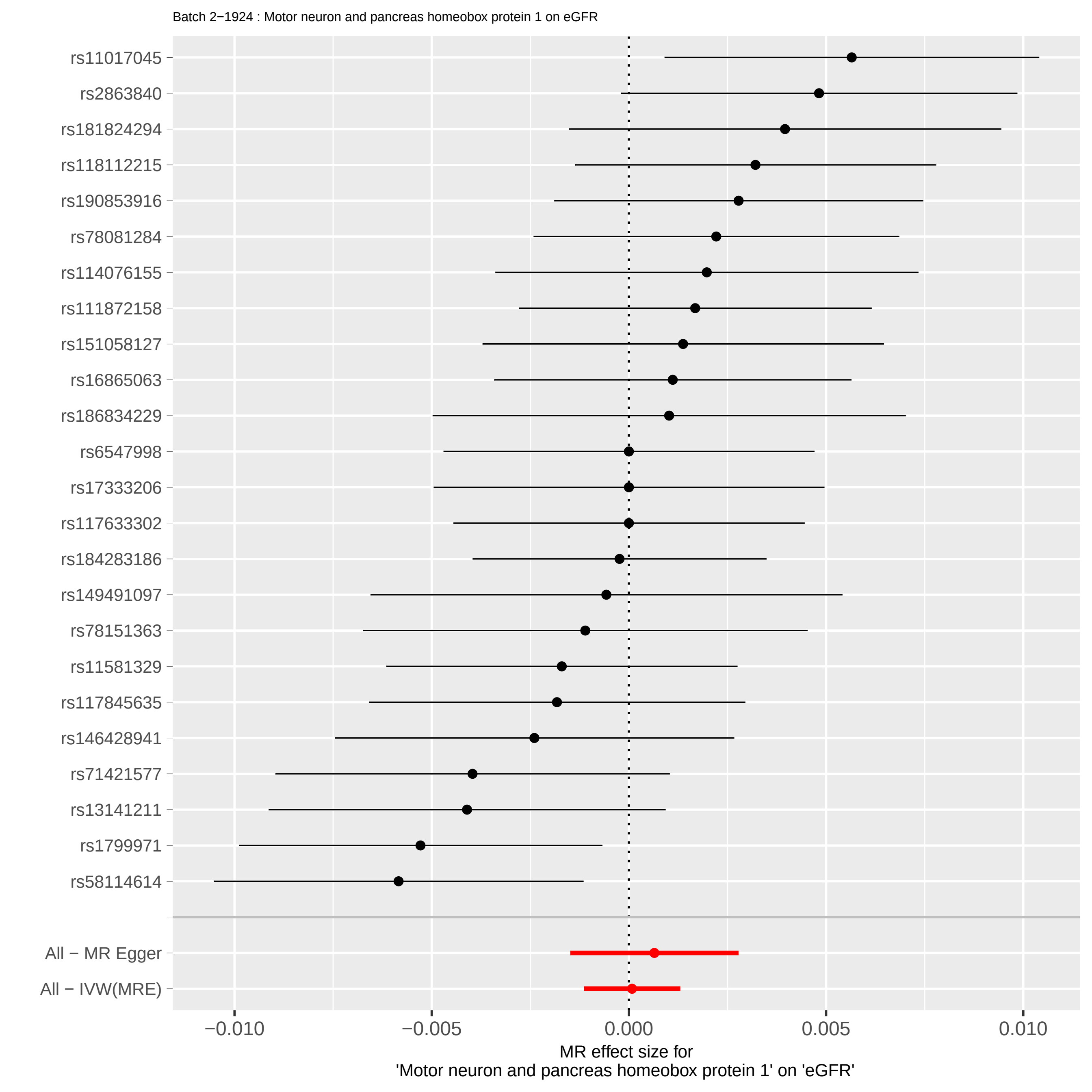

Batch 2−1924 : Motor neuron and pancreas homeobox protein 1 on eGFR
rs11017045
rs2863840
rs181824294
rs118112215
rs190853916
rs78081284
rs114076155
rs111872158
rs151058127
rs16865063
rs186834229
rs6547998
rs17333206
rs117633302
rs184283186
rs149491097
rs78151363
rs11581329
rs117845635
rs146428941
rs71421577
rs13141211
rs1799971
rs58114614
All − MR Egger
All − IVW(MRE)
−0.010
−0.005
0.000
0.005
0.010
MR effect size for
'Motor neuron and pancreas homeobox protein 1' on 'eGFR'

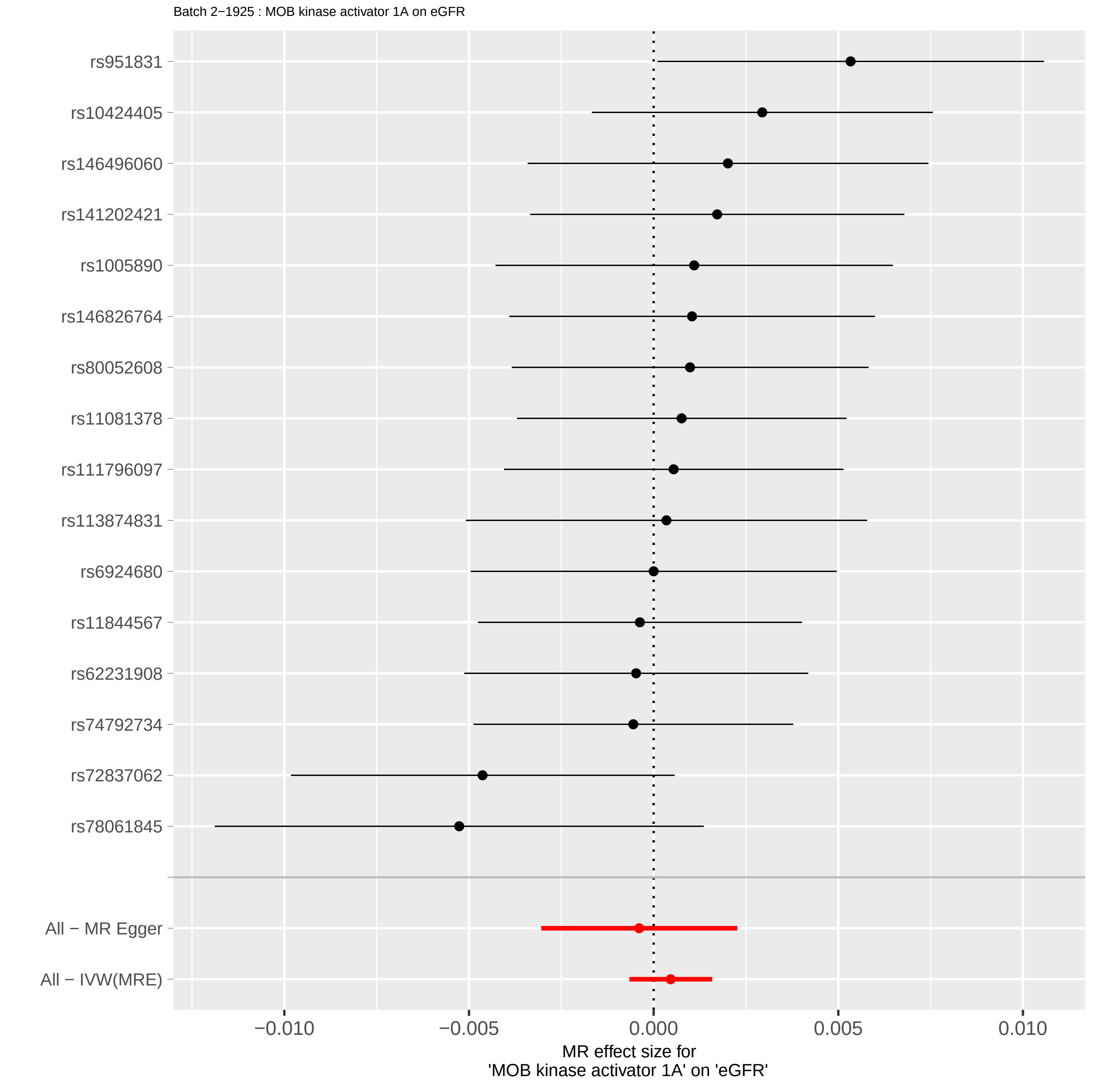

Batch 2−1925 : MOB kinase activator 1A on eGFR
rs951831
rs10424405
rs146496060
rs141202421
rs1005890
rs146826764
rs80052608
rs11081378
rs111796097
rs113874831
rs6924680
rs11844567
rs62231908
rs74792734
rs72837062
rs78061845
All − MR Egger
All − IVW(MRE)
−0.010
−0.005
0.000
0.005
0.010
MR effect size for
'MOB kinase activator 1A' on 'eGFR'

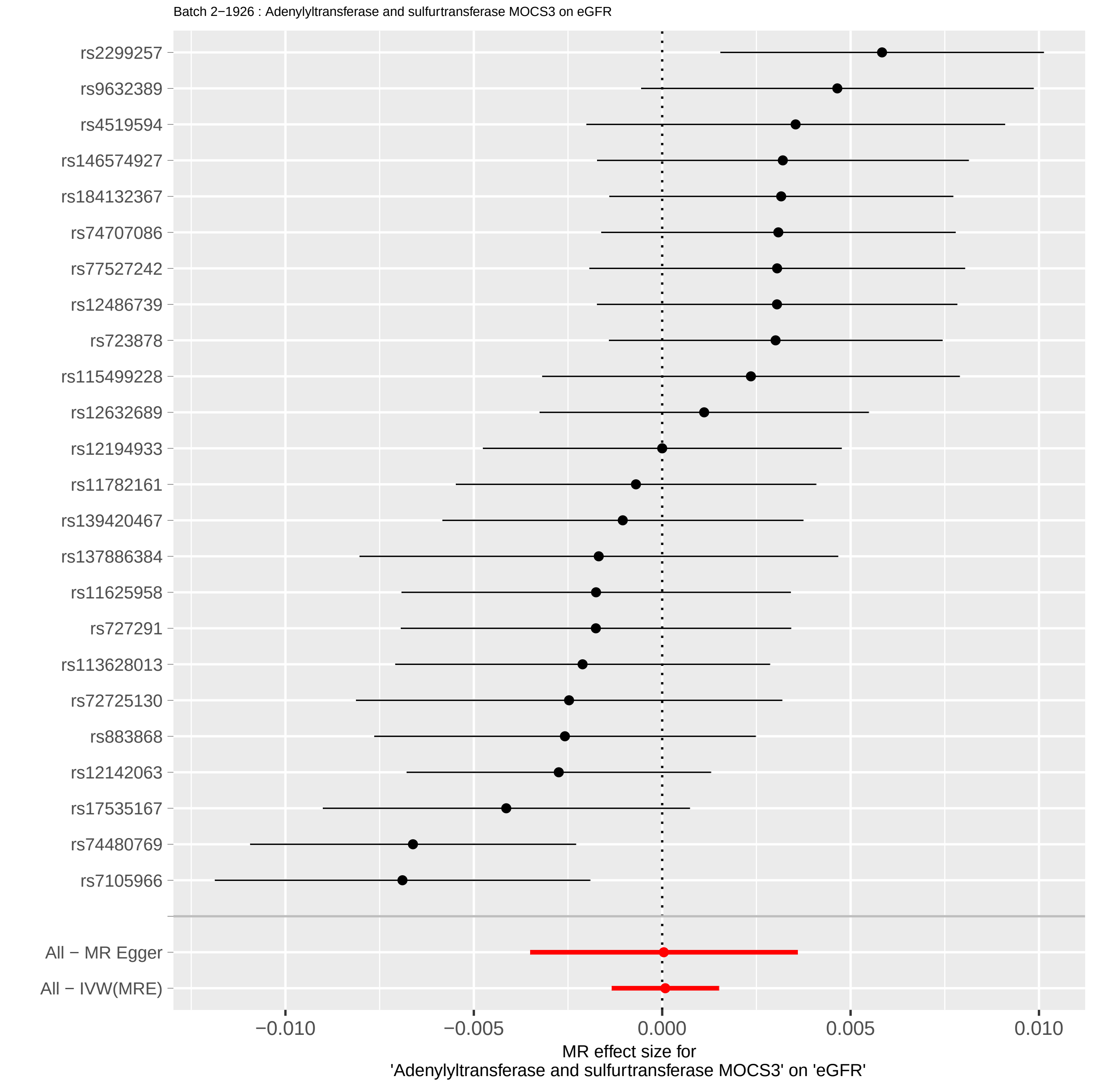

Batch 2−1926 : Adenylyltransferase and sulfurtransferase MOCS3 on eGFR
rs2299257
rs9632389
rs4519594
rs146574927
rs184132367
rs74707086
rs77527242
rs12486739
rs723878
rs115499228
rs12632689
rs12194933
rs11782161
rs139420467
rs137886384
rs11625958
rs727291
rs113628013
rs72725130
rs883868
rs12142063
rs17535167
rs74480769
rs7105966
All − MR Egger
All − IVW(MRE)
−0.010
−0.005
0.000
0.005
0.010
MR effect size for
'Adenylyltransferase and sulfurtransferase MOCS3' on 'eGFR'

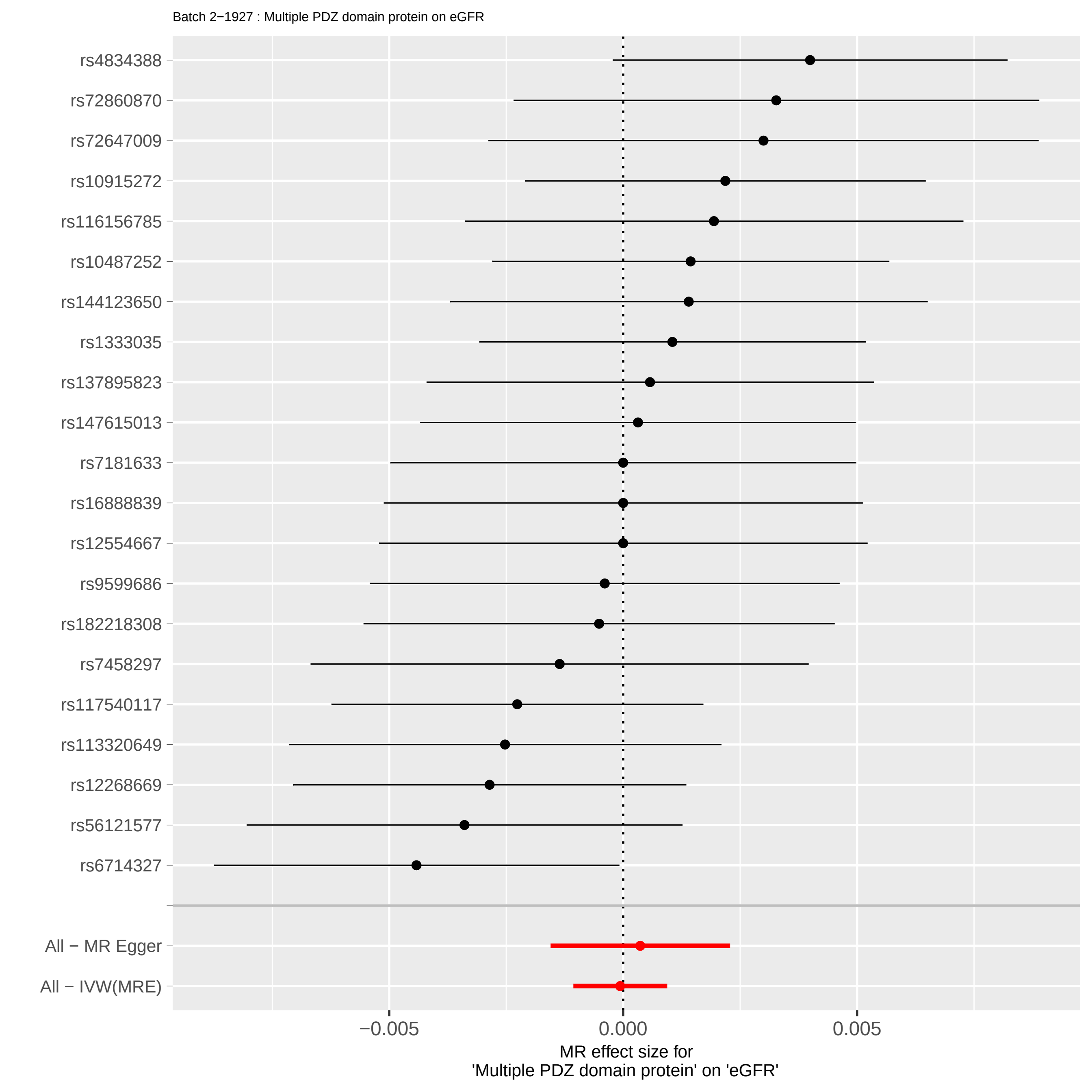

Batch 2−1927 : Multiple PDZ domain protein on eGFR
rs4834388
rs72860870
rs72647009
rs10915272
rs116156785
rs10487252
rs144123650
rs1333035
rs137895823
rs147615013
rs7181633
rs16888839
rs12554667
rs9599686
rs182218308
rs7458297
rs117540117
rs113320649
rs12268669
rs56121577
rs6714327
All − MR Egger
All − IVW(MRE)
−0.005
0.000
0.005
MR effect size for
'Multiple PDZ domain protein' on 'eGFR'

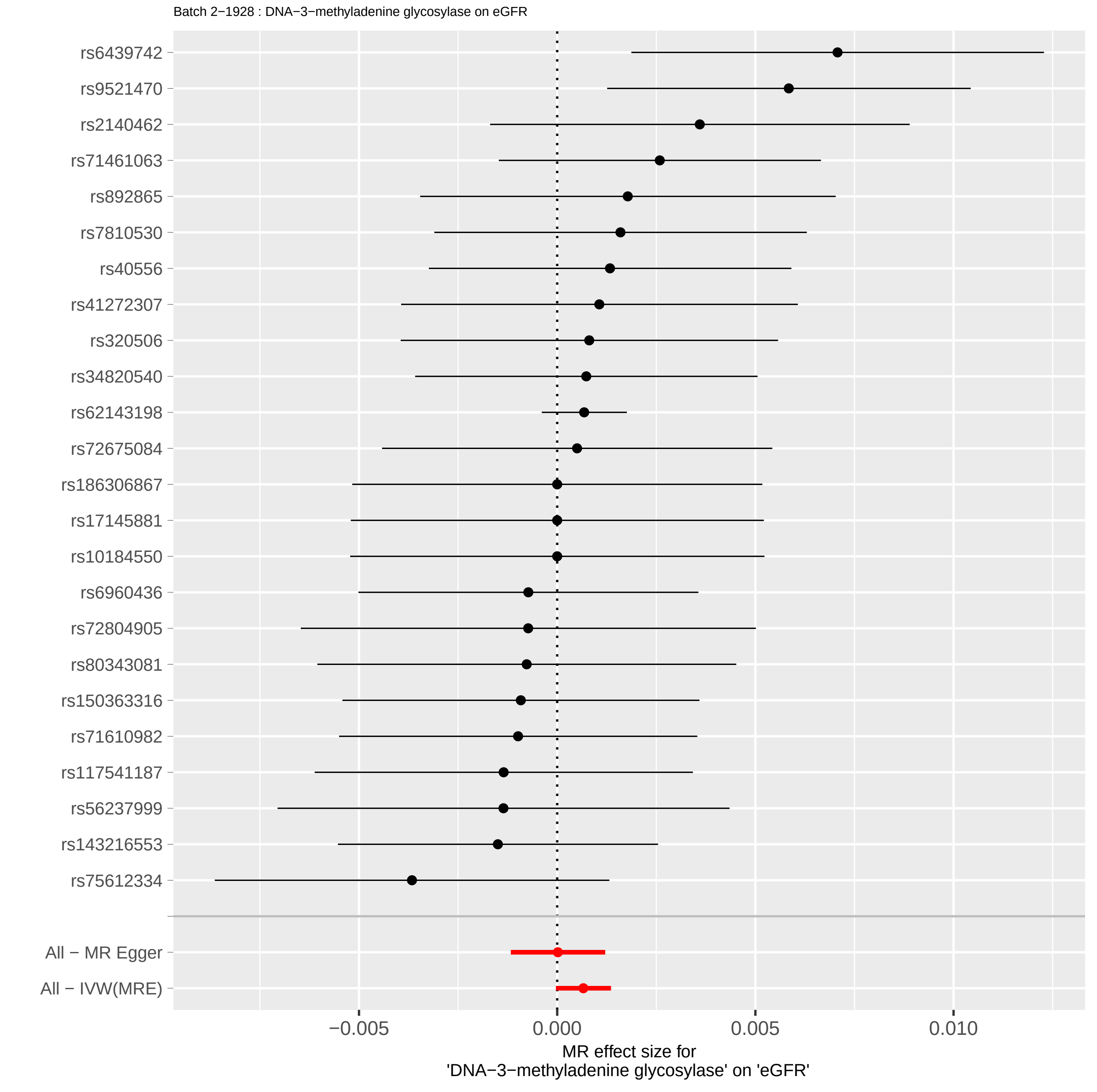

Batch 2−1928 : DNA−3−methyladenine glycosylase on eGFR
rs6439742
rs9521470
rs2140462
rs71461063
rs892865
rs7810530
rs40556
rs41272307
rs320506
rs34820540
rs62143198
rs72675084
rs186306867
rs17145881
rs10184550
rs6960436
rs72804905
rs80343081
rs150363316
rs71610982
rs117541187
rs56237999
rs143216553
rs75612334
All − MR Egger
All − IVW(MRE)
−0.005
0.000
0.005
0.010
MR effect size for
'DNA−3−methyladenine glycosylase' on 'eGFR'

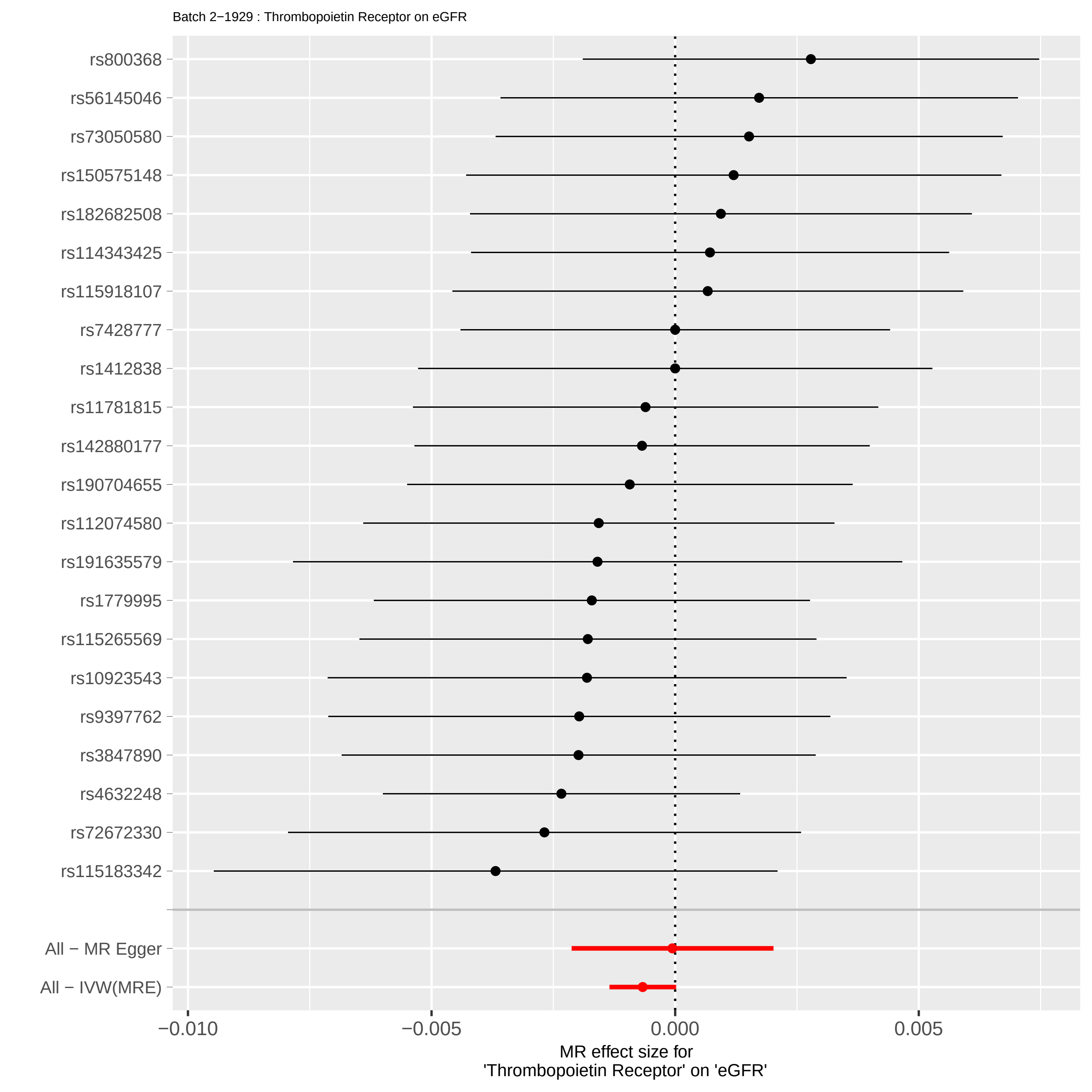
Batch 2−1929 : Thrombopoietin Receptor on eGFR
rs800368
rs56145046
rs73050580
rs150575148
rs182682508
rs114343425
rs115918107
rs7428777
rs1412838
rs11781815
rs142880177
rs190704655
rs112074580
rs191635579
rs1779995
rs115265569
rs10923543
rs9397762
rs3847890
rs4632248
rs72672330
rs115183342
All − MR Egger
All − IVW(MRE)
−0.010
−0.005
0.000
0.005
MR effect size for
'Thrombopoietin Receptor' on 'eGFR'

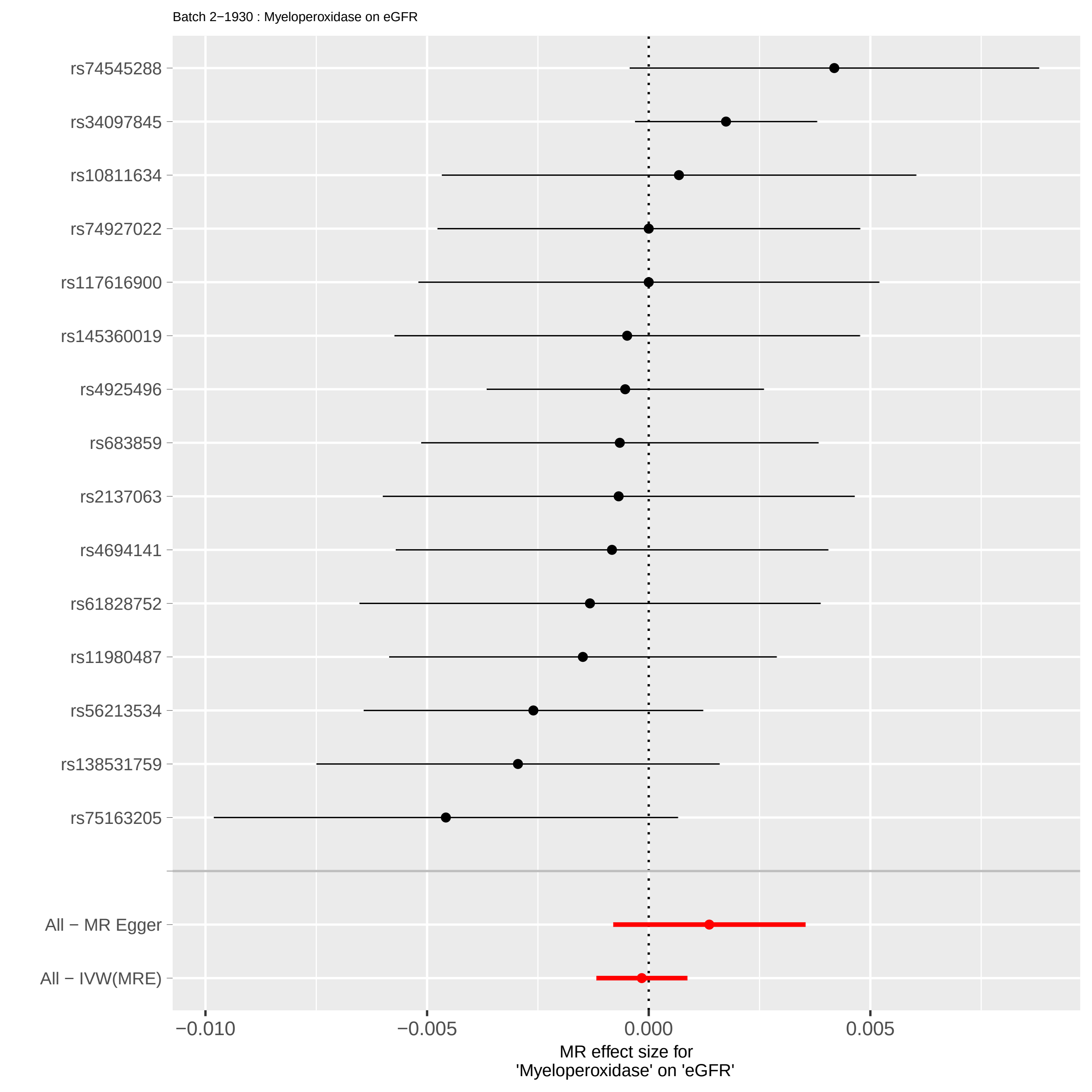

Batch 2−1930 : Myeloperoxidase on eGFR
rs74545288
rs34097845
rs10811634
rs74927022
rs117616900
rs145360019
rs4925496
rs683859
rs2137063
rs4694141
rs61828752
rs11980487
rs56213534
rs138531759
rs75163205
All − MR Egger
All − IVW(MRE)
−0.010
−0.005
0.000
0.005
MR effect size for
'Myeloperoxidase' on 'eGFR'

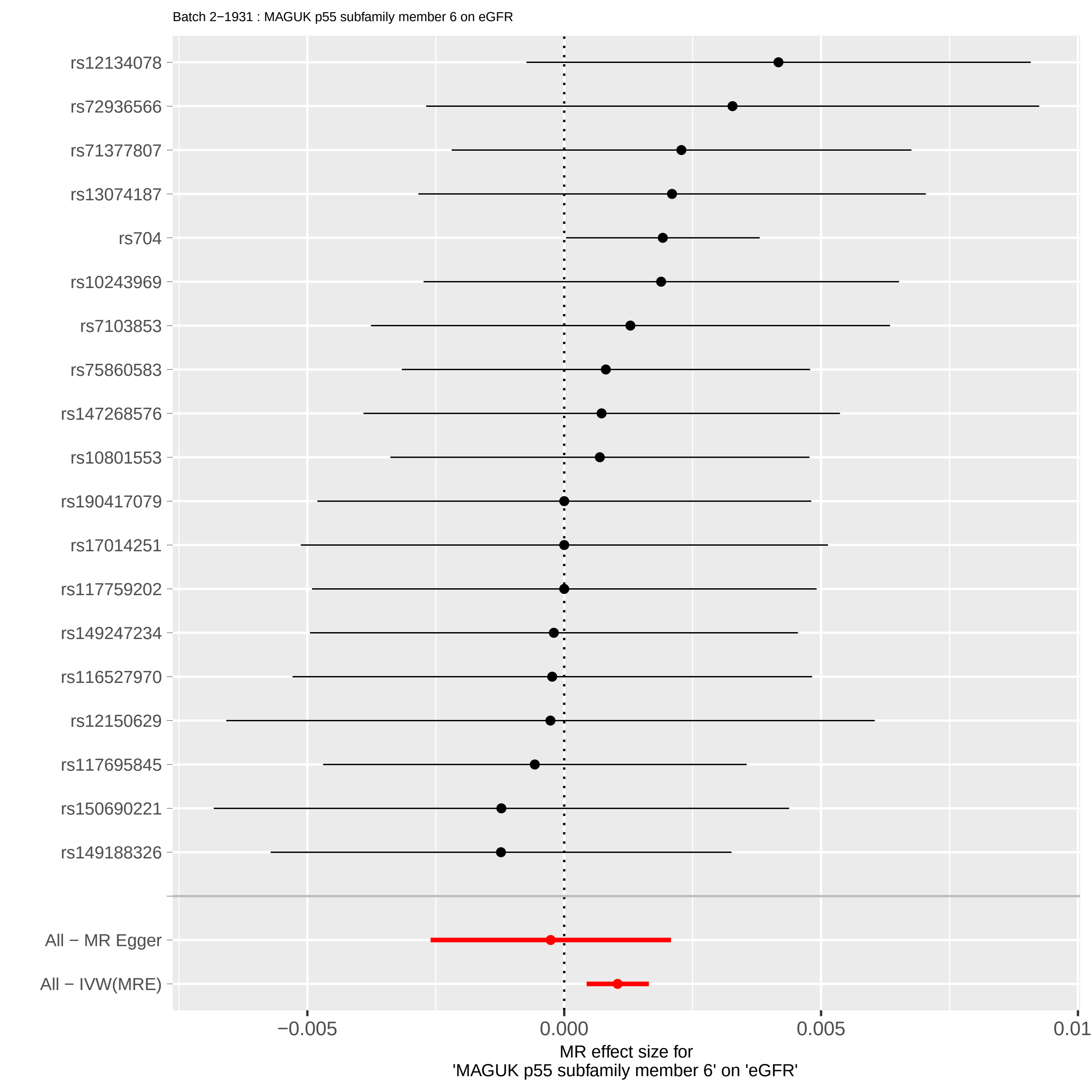

Batch 2−1931 : MAGUK p55 subfamily member 6 on eGFR
rs12134078
rs72936566
rs71377807
rs13074187
rs704
rs10243969
rs7103853
rs75860583
rs147268576
rs10801553
rs190417079
rs17014251
rs117759202
rs149247234
rs116527970
rs12150629
rs117695845
rs150690221
rs149188326
All − MR Egger
All − IVW(MRE)
−0.005
0.000
0.005
0.01
MR effect size for
'MAGUK p55 subfamily member 6' on 'eGFR'

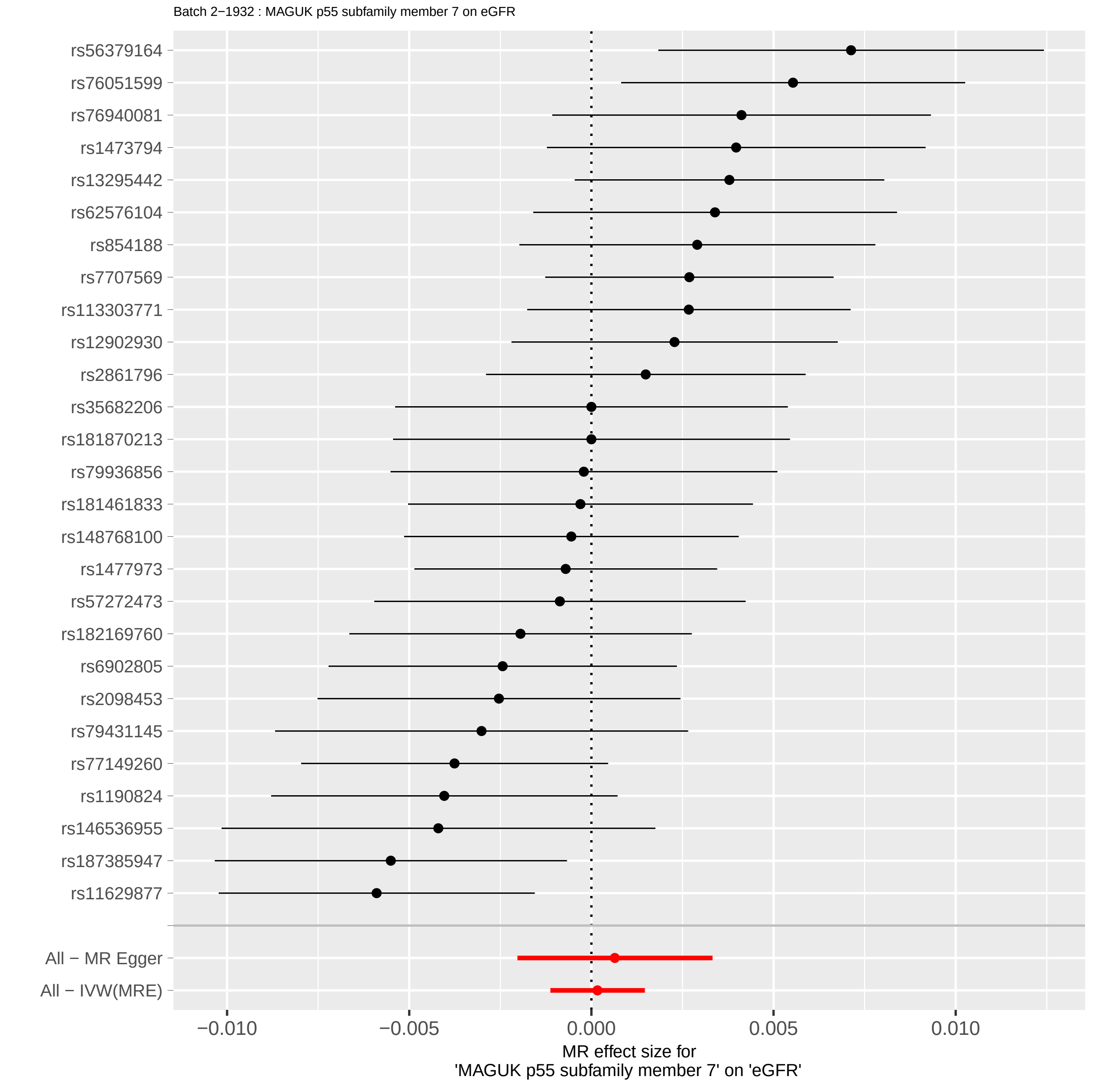

Batch 2−1932 : MAGUK p55 subfamily member 7 on eGFR
rs56379164
rs76051599
rs76940081
rs1473794
rs13295442
rs62576104
rs854188
rs7707569
rs113303771
rs12902930
rs2861796
rs35682206
rs181870213
rs79936856
rs181461833
rs148768100
rs1477973
rs57272473
rs182169760
rs6902805
rs2098453
rs79431145
rs77149260
rs1190824
rs146536955
rs187385947
rs11629877
All − MR Egger
All − IVW(MRE)
−0.010
−0.005
0.000
0.005
0.010
MR effect size for
'MAGUK p55 subfamily member 7' on 'eGFR'

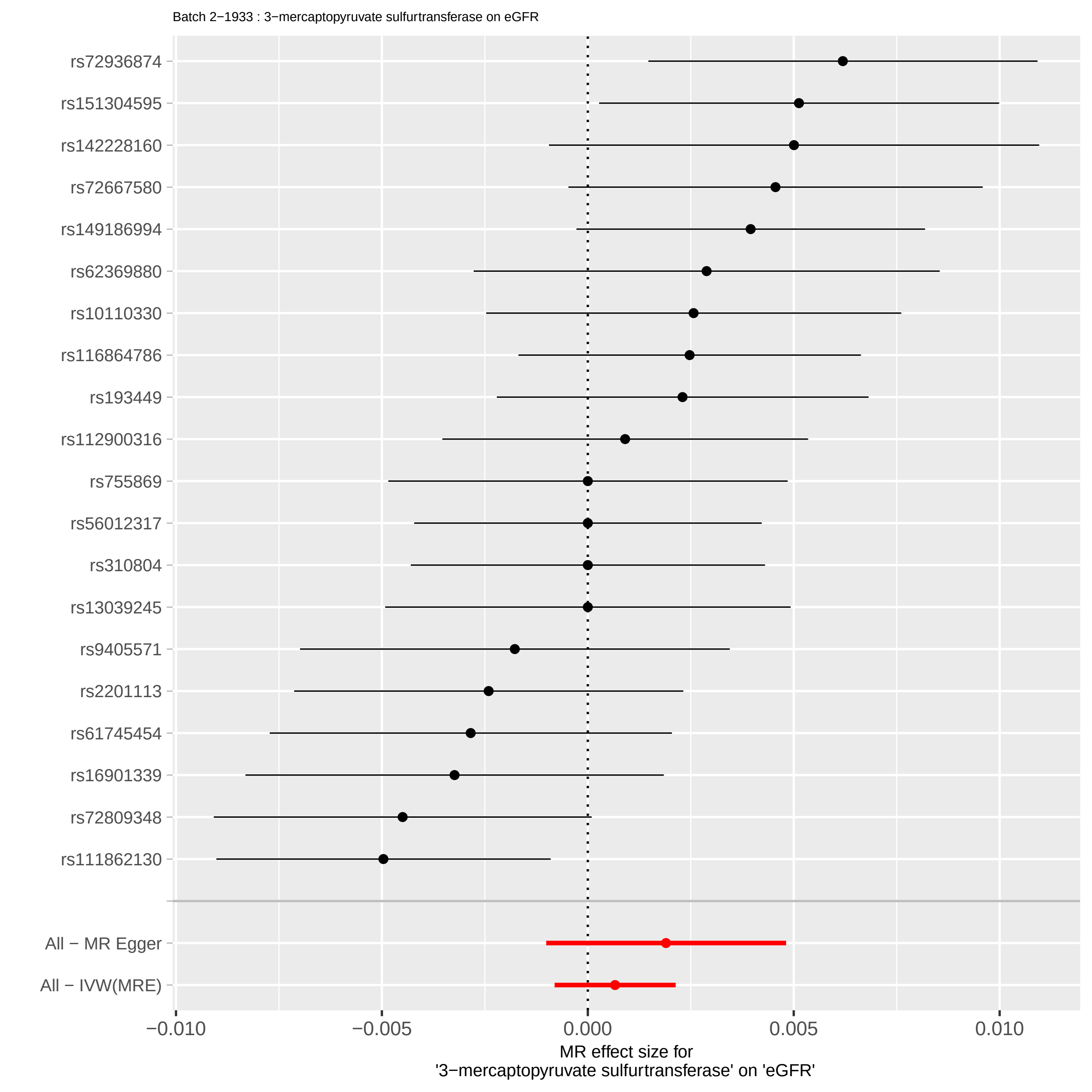
Batch 2−1933 : 3−mercaptopyruvate sulfurtransferase on eGFR
rs72936874
rs151304595
rs142228160
rs72667580
rs149186994
rs62369880
rs10110330
rs116864786
rs193449
rs112900316
rs755869
rs56012317
rs310804
rs13039245
rs9405571
rs2201113
rs61745454
rs16901339
rs72809348
rs111862130
All − MR Egger
All − IVW(MRE)
−0.010
−0.005
0.000
0.005
0.010
MR effect size for
'3−mercaptopyruvate sulfurtransferase' on 'eGFR'

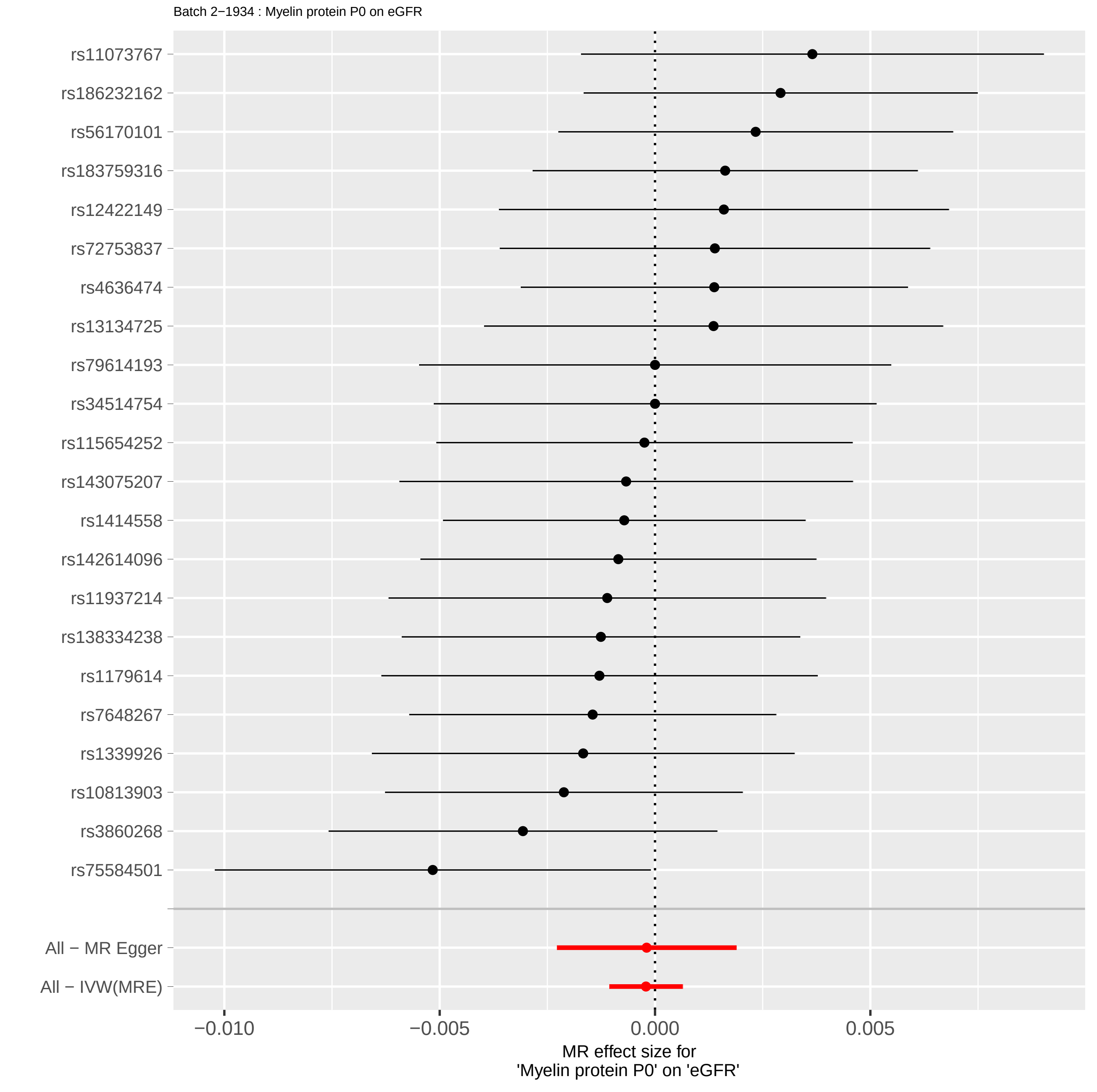
Batch 2−1934 : Myelin protein P0 on eGFR
rs11073767
rs186232162
rs56170101
rs183759316
rs12422149
rs72753837
rs4636474
rs13134725
rs79614193
rs34514754
rs115654252
rs143075207
rs1414558
rs142614096
rs11937214
rs138334238
rs1179614
rs7648267
rs1339926
rs10813903
rs3860268
rs75584501
All − MR Egger
All − IVW(MRE)
−0.010
−0.005
0.000
0.005
MR effect size for
'Myelin protein P0' on 'eGFR'

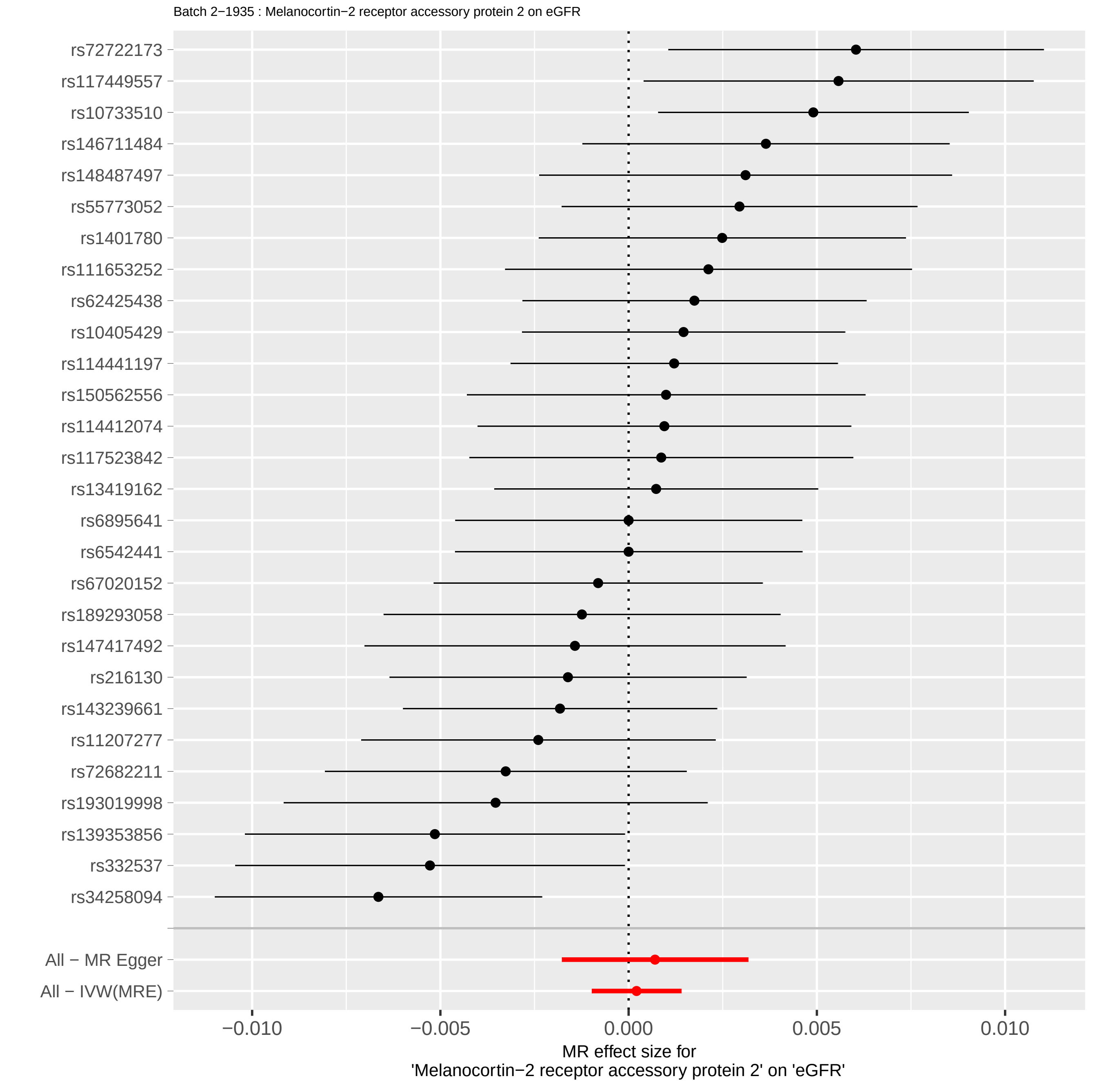

Batch 2−1935 : Melanocortin−2 receptor accessory protein 2 on eGFR
rs72722173
rs117449557
rs10733510
rs146711484
rs148487497
rs55773052
rs1401780
rs111653252
rs62425438
rs10405429
rs114441197
rs150562556
rs114412074
rs117523842
rs13419162
rs6895641
rs6542441
rs67020152
rs189293058
rs147417492
rs216130
rs143239661
rs11207277
rs72682211
rs193019998
rs139353856
rs332537
rs34258094
All − MR Egger
All − IVW(MRE)
−0.010
−0.005
0.000
0.005
0.010
MR effect size for
'Melanocortin−2 receptor accessory protein 2' on 'eGFR'

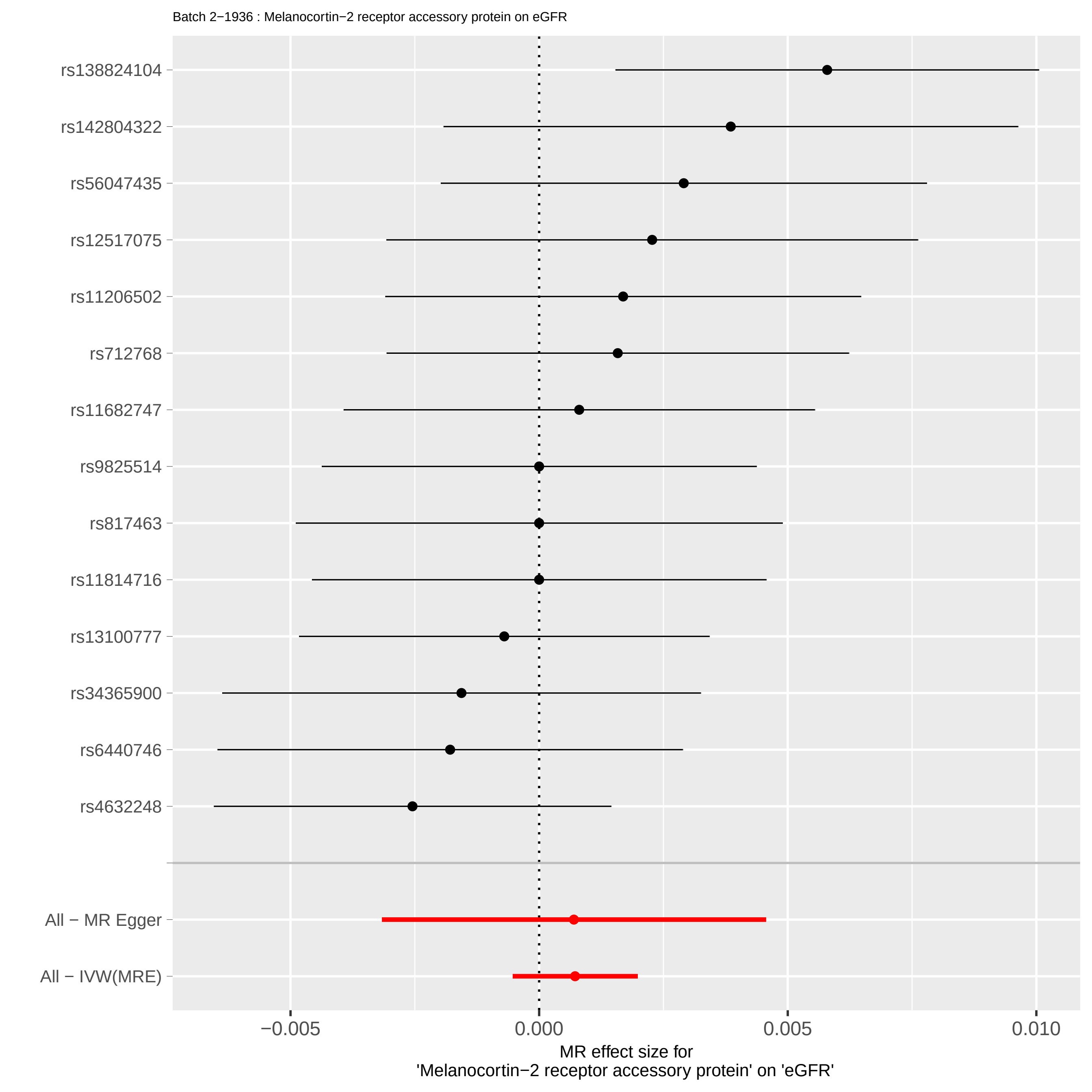
Batch 2−1936 : Melanocortin−2 receptor accessory protein on eGFR
rs138824104
rs142804322
rs56047435
rs12517075
rs11206502
rs712768
rs11682747
rs9825514
rs817463
rs11814716
rs13100777
rs34365900
rs6440746
rs4632248
All − MR Egger
All − IVW(MRE)
−0.005
0.000
0.005
0.010
MR effect size for
'Melanocortin−2 receptor accessory protein' on 'eGFR'

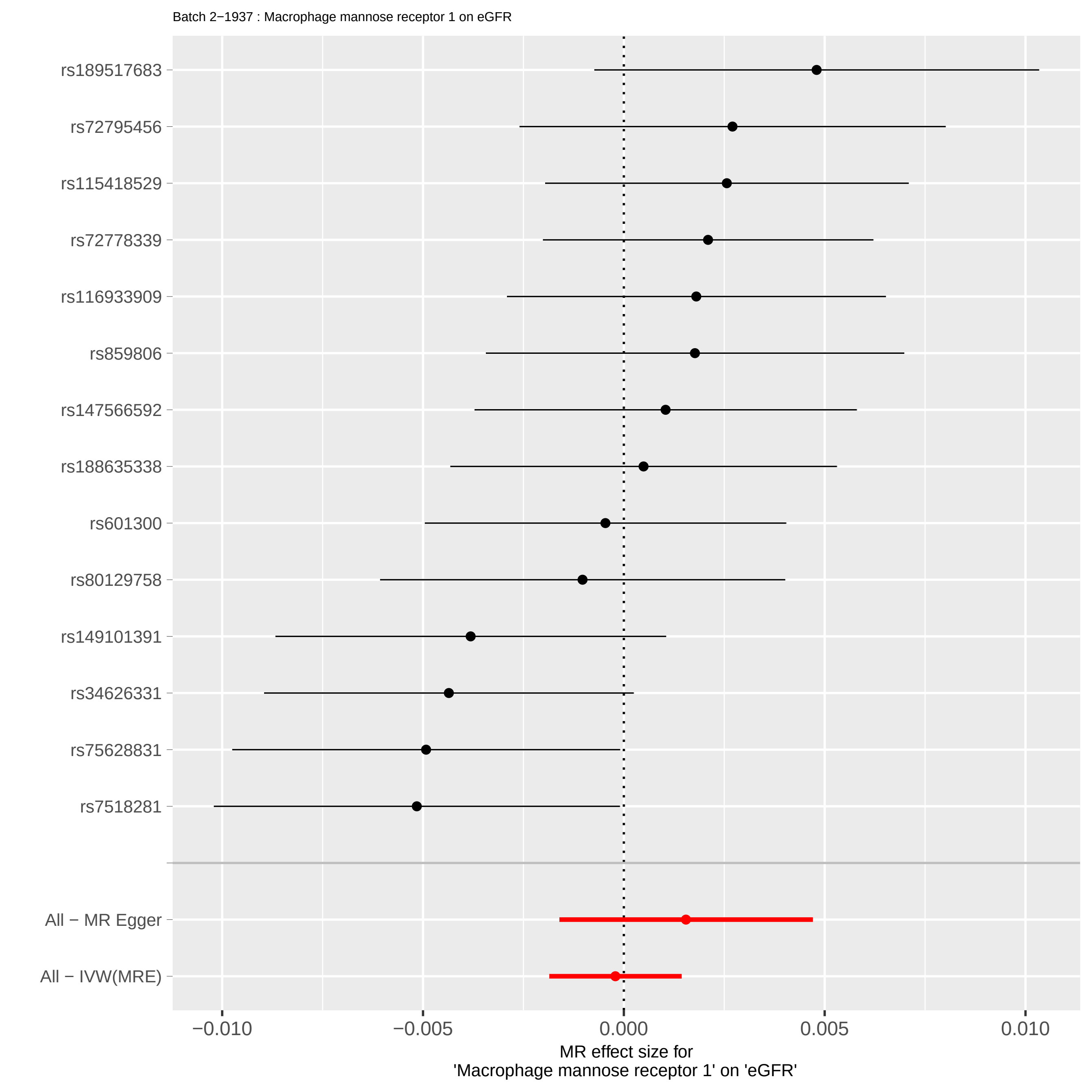

Batch 2−1937 : Macrophage mannose receptor 1 on eGFR
rs189517683
rs72795456
rs115418529
rs72778339
rs116933909
rs859806
rs147566592
rs188635338
rs601300
rs80129758
rs149101391
rs34626331
rs75628831
rs7518281
All − MR Egger
All − IVW(MRE)
−0.010
−0.005
0.000
0.005
0.010
MR effect size for
'Macrophage mannose receptor 1' on 'eGFR'

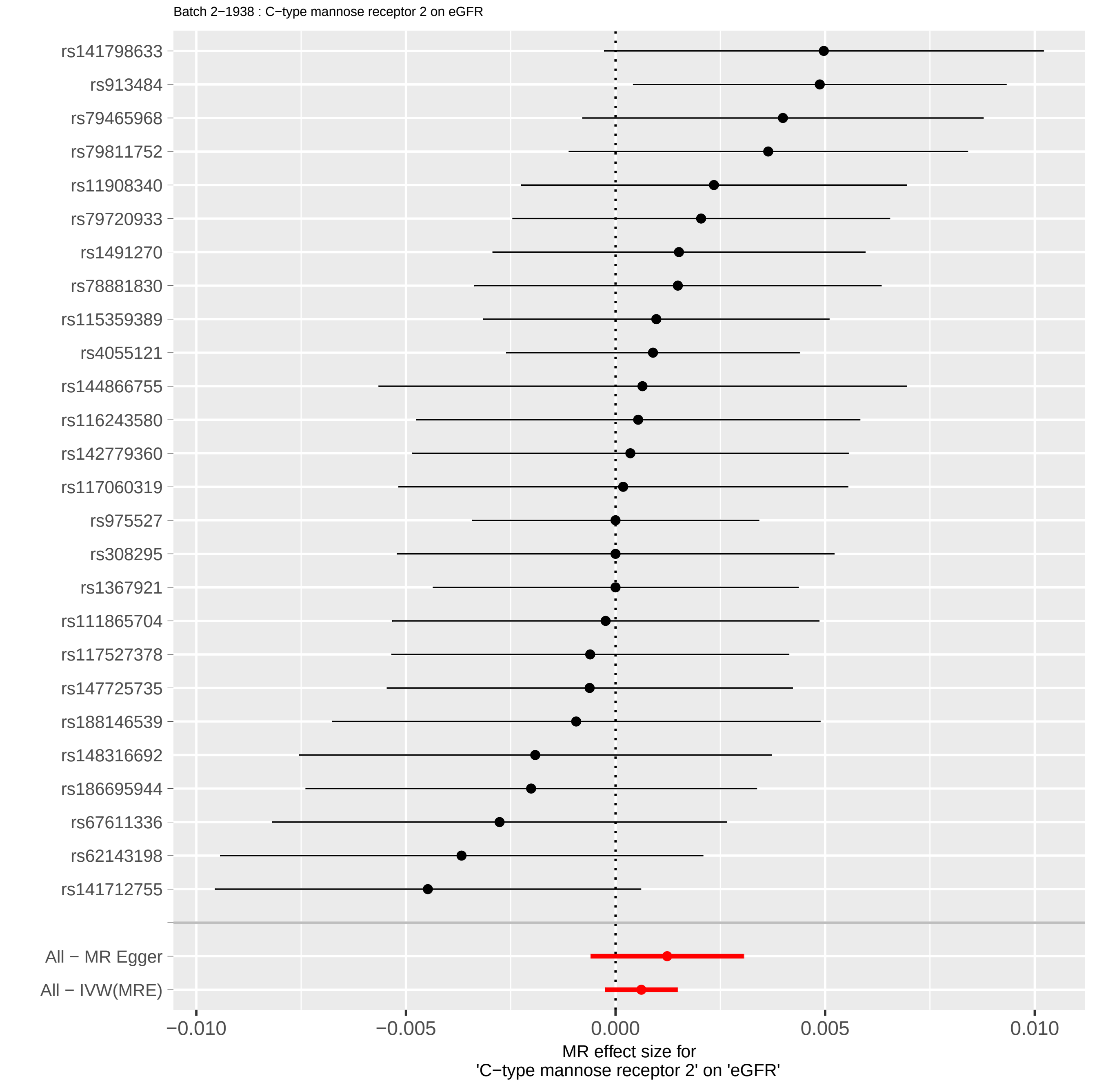

Batch 2−1938 : C−type mannose receptor 2 on eGFR
rs141798633
rs913484
rs79465968
rs79811752
rs11908340
rs79720933
rs1491270
rs78881830
rs115359389
rs4055121
rs144866755
rs116243580
rs142779360
rs117060319
rs975527
rs308295
rs1367921
rs111865704
rs117527378
rs147725735
rs188146539
rs148316692
rs186695944
rs67611336
rs62143198
rs141712755
All − MR Egger
All − IVW(MRE)
−0.010
−0.005
0.000
0.005
0.010
MR effect size for
'C−type mannose receptor 2' on 'eGFR'

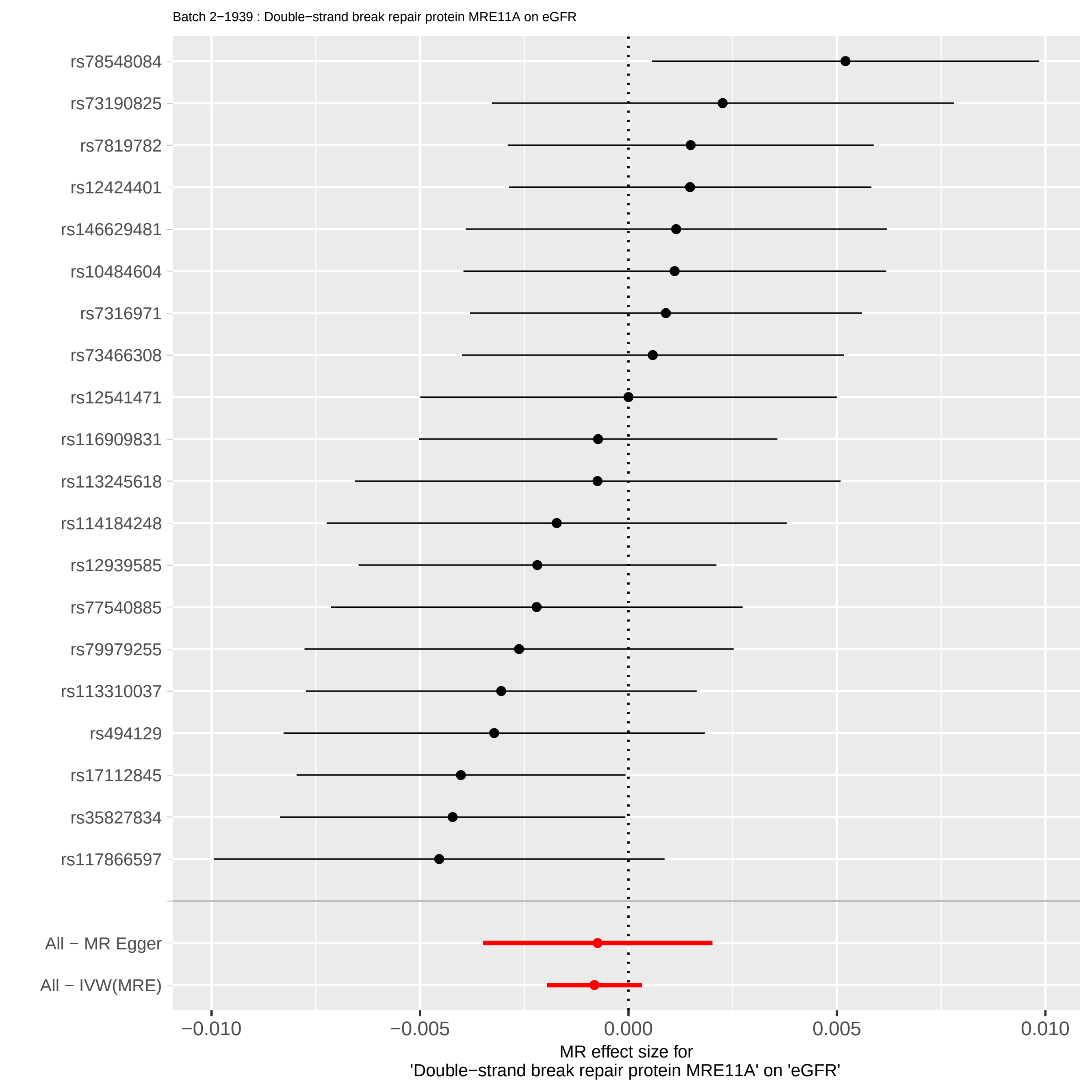

Batch 2−1939 : Double−strand break repair protein MRE11A on eGFR
rs78548084
rs73190825
rs7819782
rs12424401
rs146629481
rs10484604
rs7316971
rs73466308
rs12541471
rs116909831
rs113245618
rs114184248
rs12939585
rs77540885
rs79979255
rs113310037
rs494129
rs17112845
rs35827834
rs117866597
All − MR Egger
All − IVW(MRE)
−0.010
−0.005
0.000
0.005
0.010
MR effect size for
'Double−strand break repair protein MRE11A' on 'eGFR'

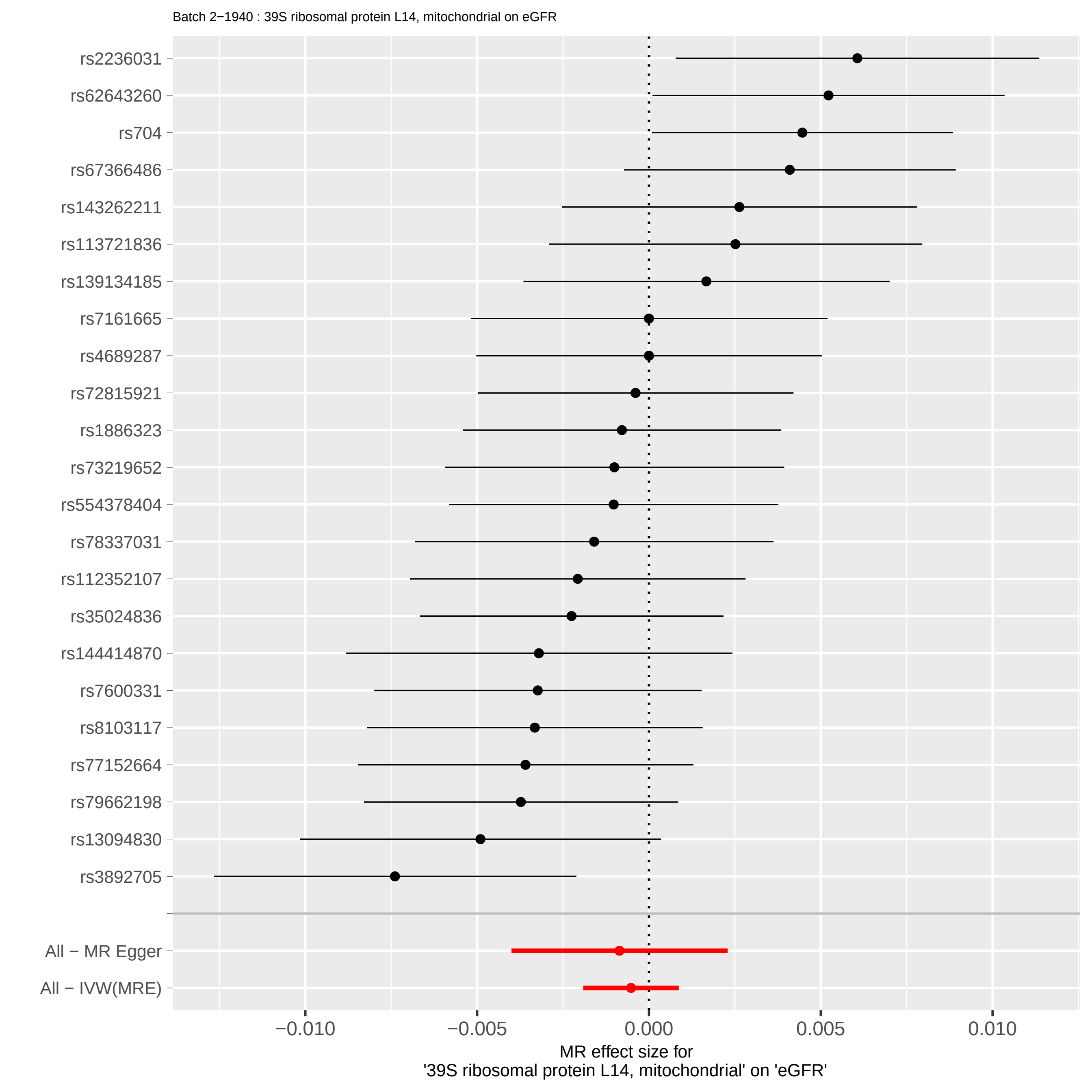

Batch 2−1940 : 39S ribosomal protein L14, mitochondrial on eGFR
rs2236031
rs62643260
rs704
rs67366486
rs143262211
rs113721836
rs139134185
rs7161665
rs4689287
rs72815921
rs1886323
rs73219652
rs554378404
rs78337031
rs112352107
rs35024836
rs144414870
rs7600331
rs8103117
rs77152664
rs79662198
rs13094830
rs3892705
All − MR Egger
All − IVW(MRE)
−0.010
−0.005
0.000
0.005
0.010
MR effect size for
'39S ribosomal protein L14, mitochondrial' on 'eGFR'

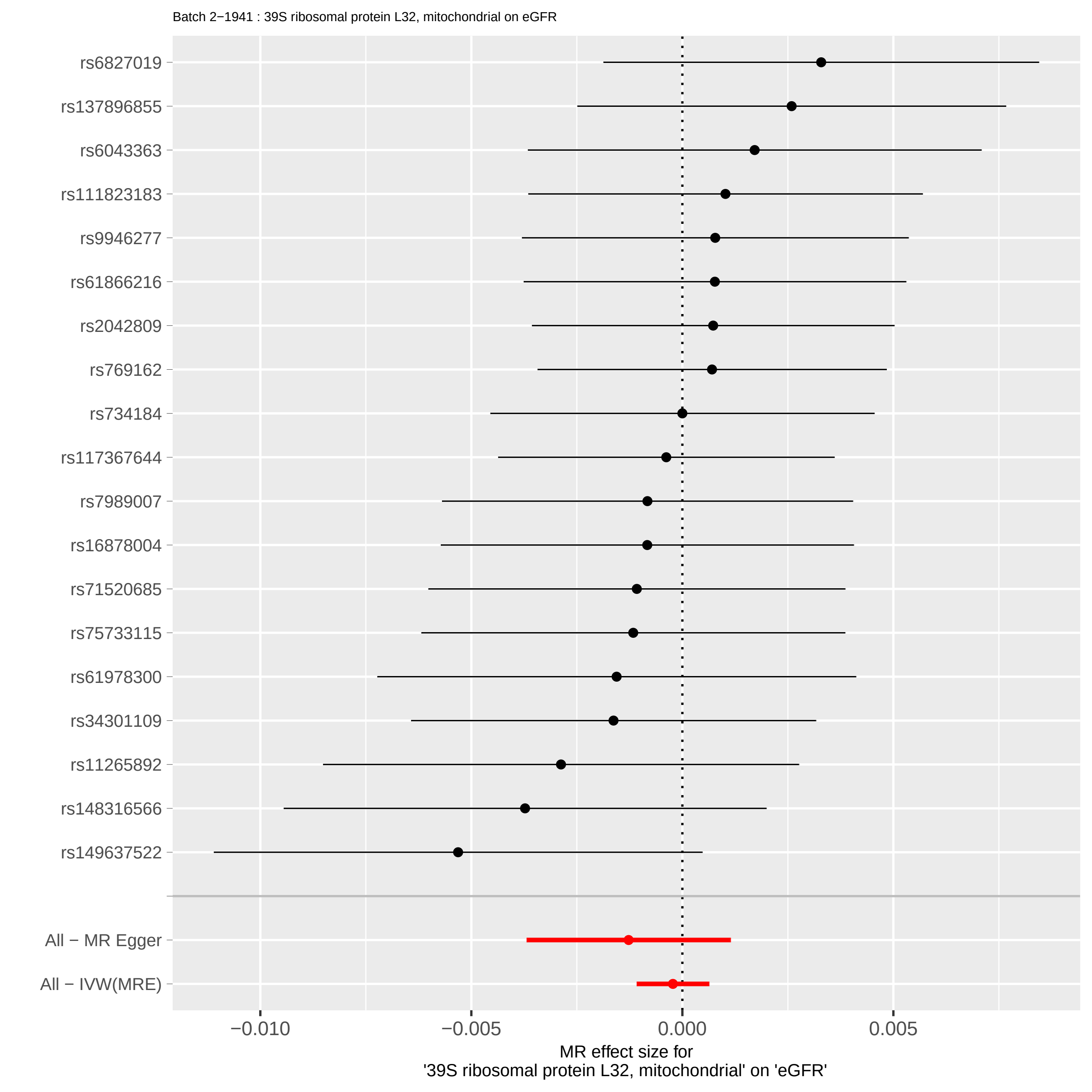

Batch 2−1941 : 39S ribosomal protein L32, mitochondrial on eGFR
rs6827019
rs137896855
rs6043363
rs111823183
rs9946277
rs61866216
rs2042809
rs769162
rs734184
rs117367644
rs7989007
rs16878004
rs71520685
rs75733115
rs61978300
rs34301109
rs11265892
rs148316566
rs149637522
All − MR Egger
All − IVW(MRE)
−0.010
−0.005
0.000
0.005
MR effect size for
'39S ribosomal protein L32, mitochondrial' on 'eGFR'

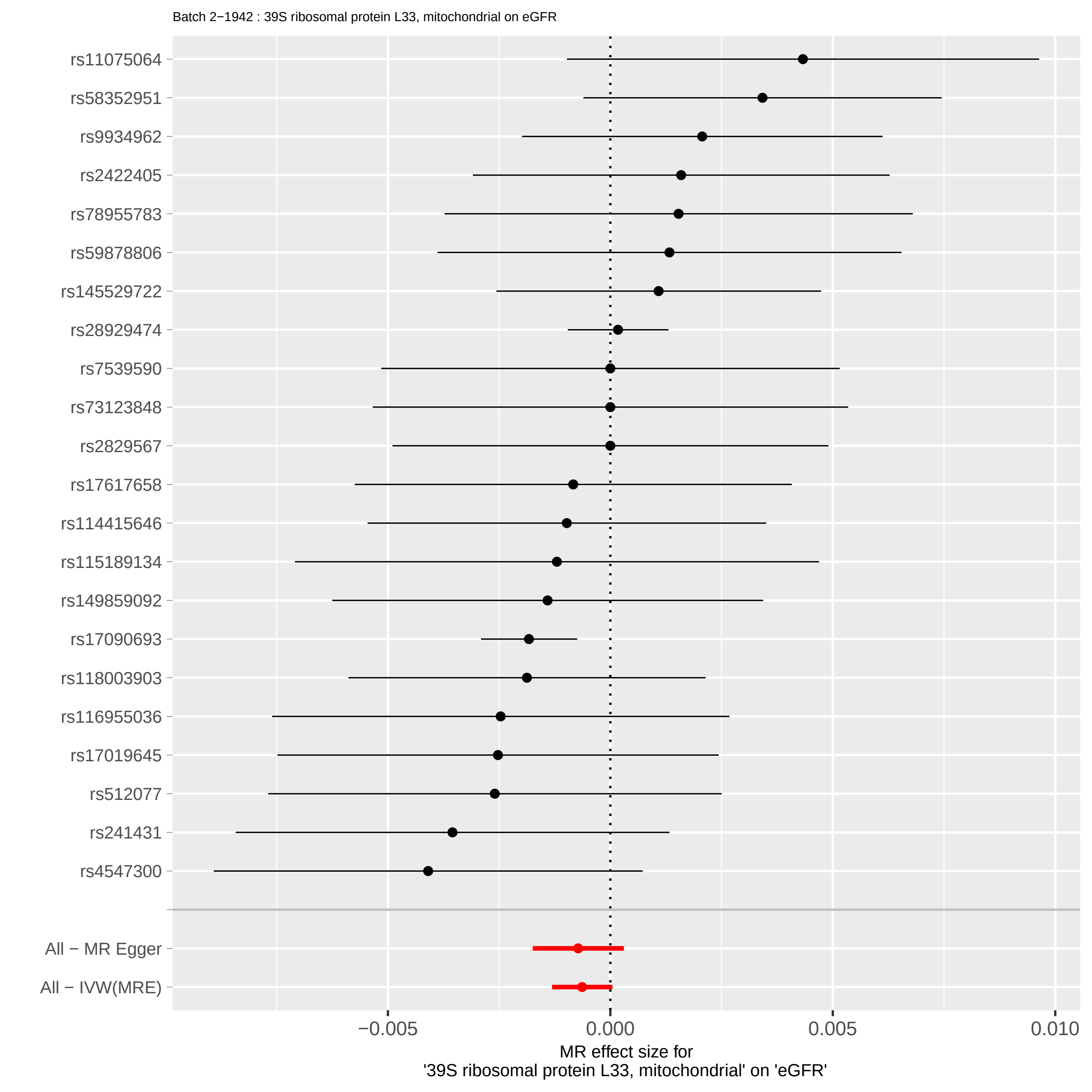
Batch 2−1942 : 39S ribosomal protein L33, mitochondrial on eGFR
rs11075064
rs58352951
rs9934962
rs2422405
rs78955783
rs59878806
rs145529722
rs28929474
rs7539590
rs73123848
rs2829567
rs17617658
rs114415646
rs115189134
rs149859092
rs17090693
rs118003903
rs116955036
rs17019645
rs512077
rs241431
rs4547300
All − MR Egger
All − IVW(MRE)
−0.005
0.000
0.005
0.010
MR effect size for
'39S ribosomal protein L33, mitochondrial' on 'eGFR'

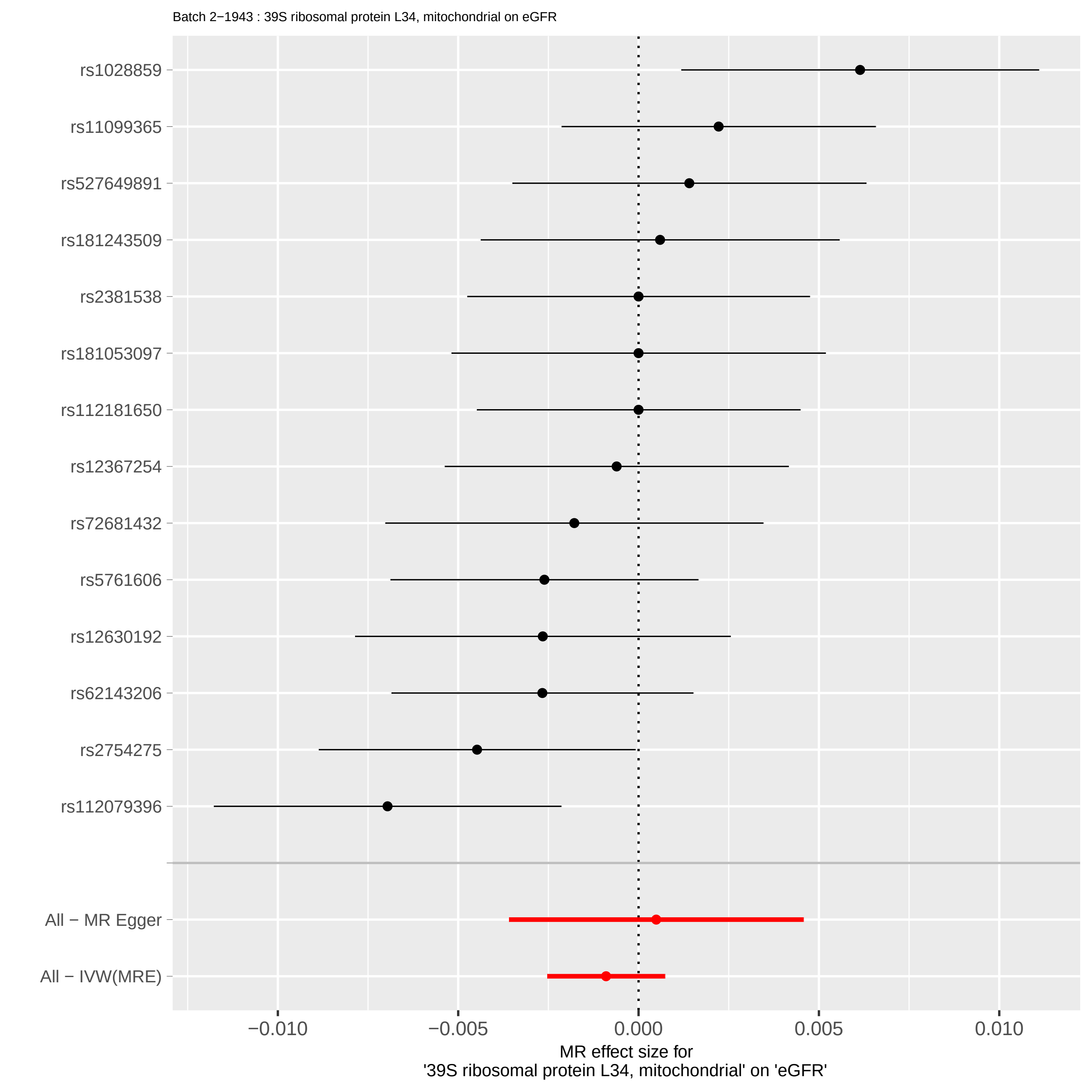
Batch 2−1943 : 39S ribosomal protein L34, mitochondrial on eGFR
rs1028859
rs11099365
rs527649891
rs181243509
rs2381538
rs181053097
rs112181650
rs12367254
rs72681432
rs5761606
rs12630192
rs62143206
rs2754275
rs112079396
All − MR Egger
All − IVW(MRE)
−0.010
−0.005
0.000
0.005
0.010
MR effect size for
'39S ribosomal protein L34, mitochondrial' on 'eGFR'

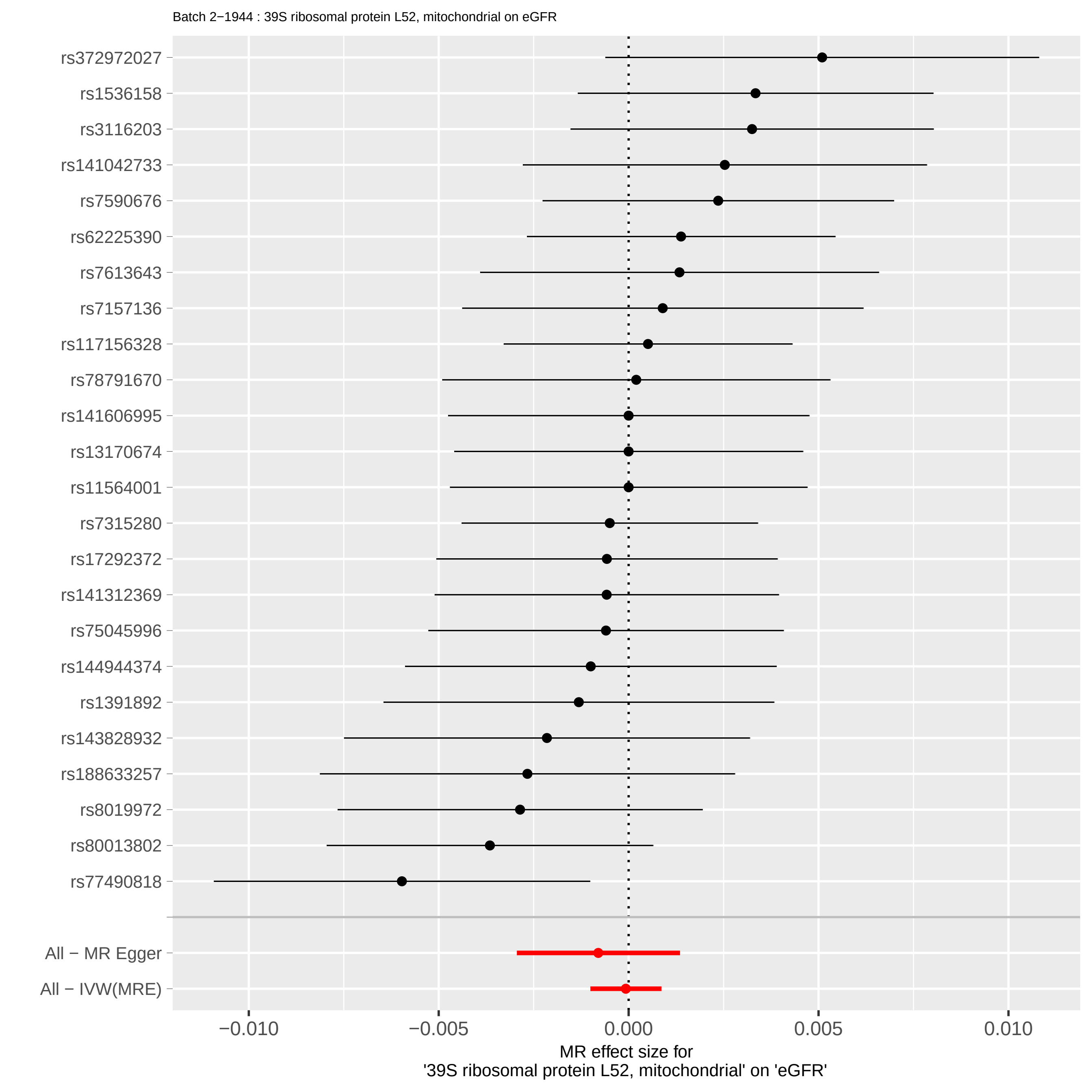
Batch 2−1944 : 39S ribosomal protein L52, mitochondrial on eGFR
rs372972027
rs1536158
rs3116203
rs141042733
rs7590676
rs62225390
rs7613643
rs7157136
rs117156328
rs78791670
rs141606995
rs13170674
rs11564001
rs7315280
rs17292372
rs141312369
rs75045996
rs144944374
rs1391892
rs143828932
rs188633257
rs8019972
rs80013802
rs77490818
All − MR Egger
All − IVW(MRE)
−0.010
−0.005
0.000
0.005
0.010
MR effect size for
'39S ribosomal protein L52, mitochondrial' on 'eGFR'

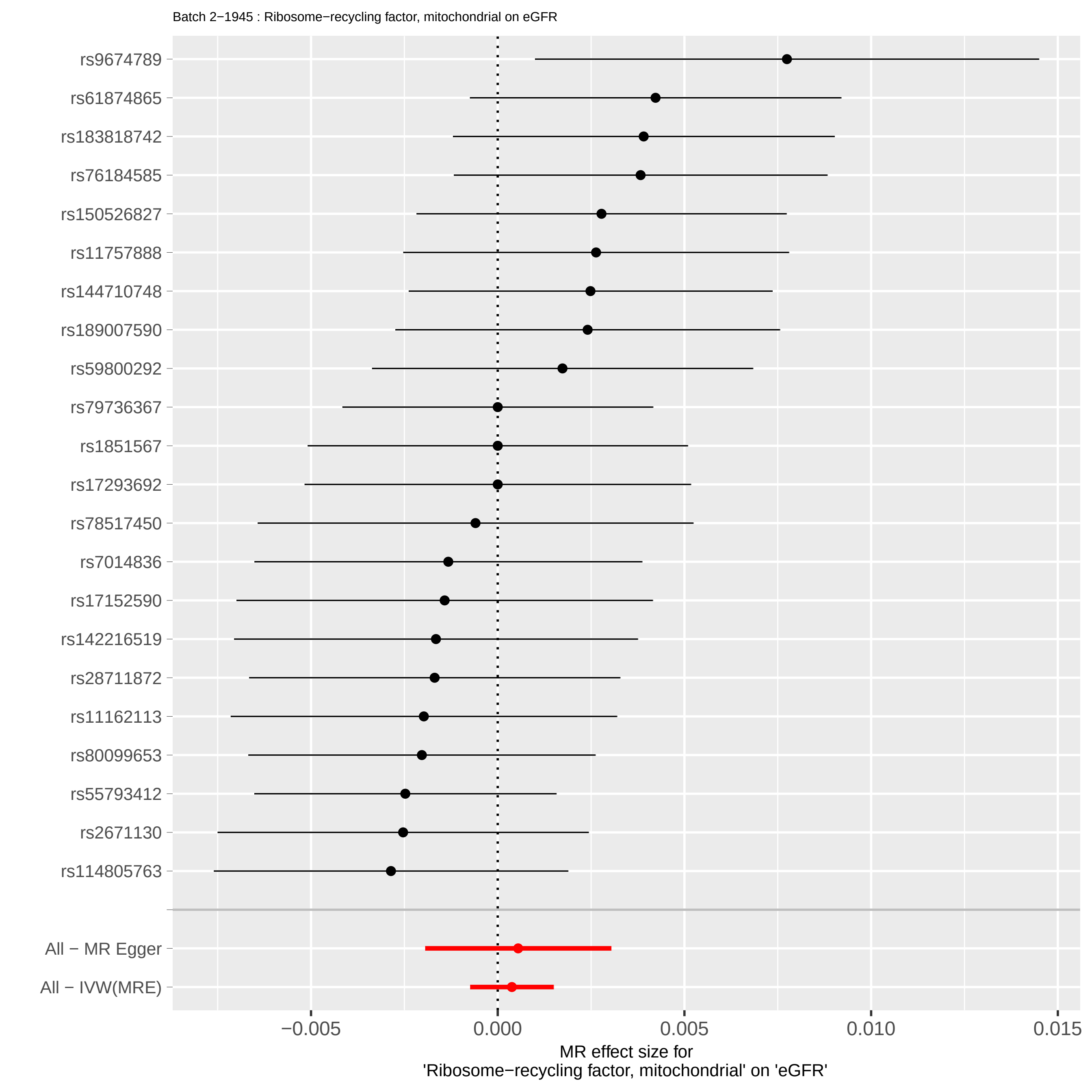

Batch 2−1945 : Ribosome−recycling factor, mitochondrial on eGFR
rs9674789
rs61874865
rs183818742
rs76184585
rs150526827
rs11757888
rs144710748
rs189007590
rs59800292
rs79736367
rs1851567
rs17293692
rs78517450
rs7014836
rs17152590
rs142216519
rs28711872
rs11162113
rs80099653
rs55793412
rs2671130
rs114805763
All − MR Egger
All − IVW(MRE)
−0.005
0.000
0.005
0.010
0.015
MR effect size for
'Ribosome−recycling factor, mitochondrial' on 'eGFR'

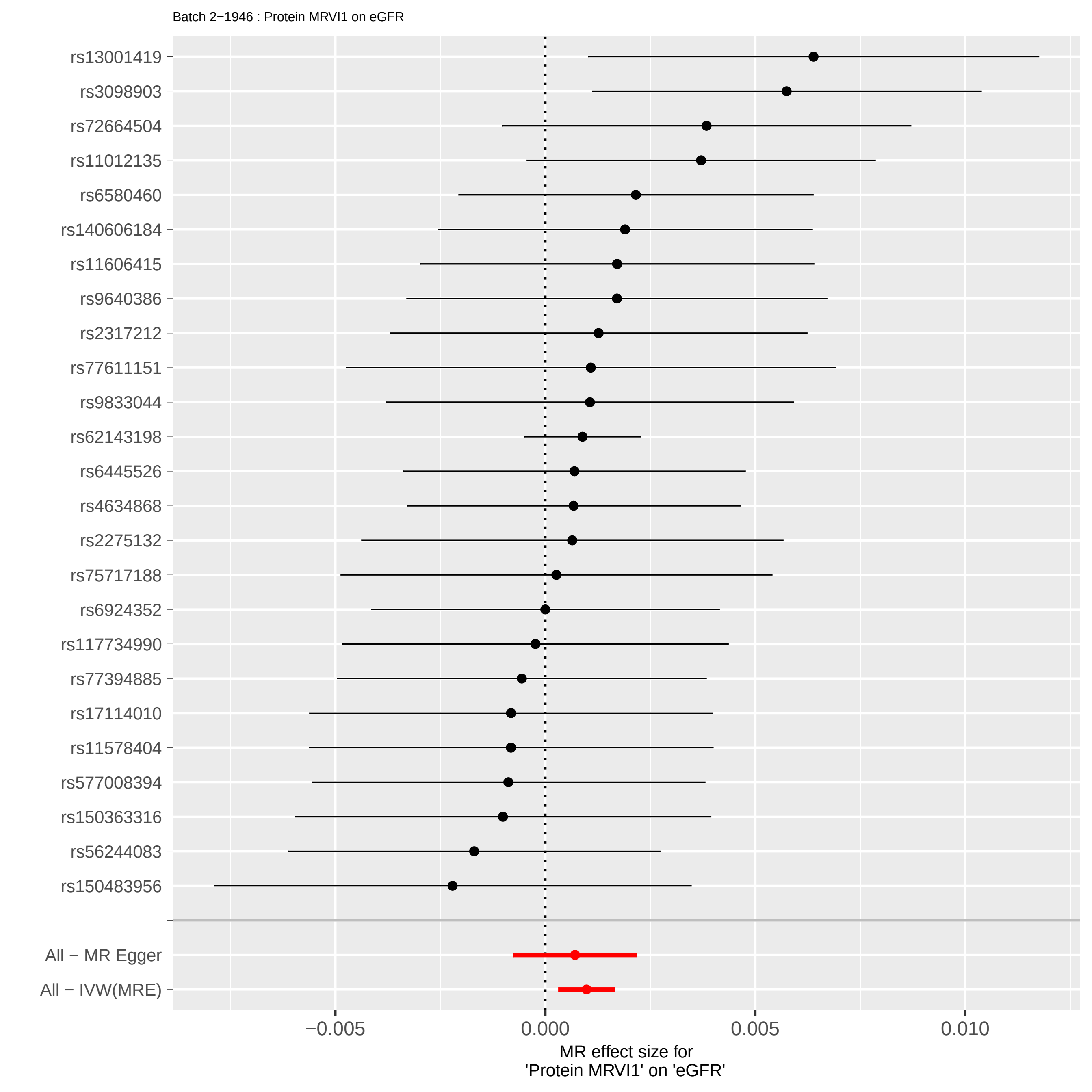

Batch 2−1946 : Protein MRVI1 on eGFR
rs13001419
rs3098903
rs72664504
rs11012135
rs6580460
rs140606184
rs11606415
rs9640386
rs2317212
rs77611151
rs9833044
rs62143198
rs6445526
rs4634868
rs2275132
rs75717188
rs6924352
rs117734990
rs77394885
rs17114010
rs11578404
rs577008394
rs150363316
rs56244083
rs150483956
All − MR Egger
All − IVW(MRE)
−0.005
0.000
0.005
0.010
MR effect size for
'Protein MRVI1' on 'eGFR'

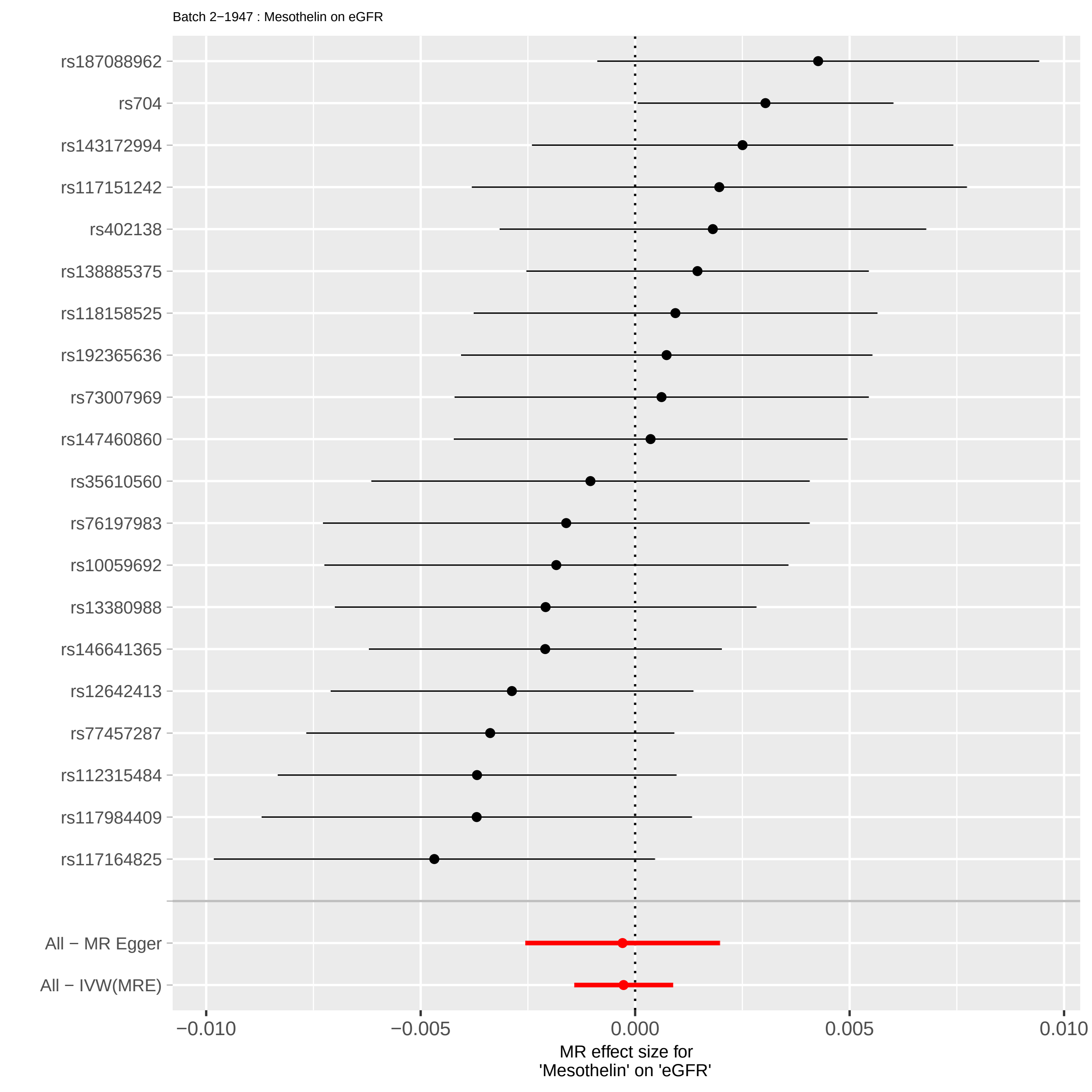

Batch 2−1947 : Mesothelin on eGFR
rs187088962
rs704
rs143172994
rs117151242
rs402138
rs138885375
rs118158525
rs192365636
rs73007969
rs147460860
rs35610560
rs76197983
rs10059692
rs13380988
rs146641365
rs12642413
rs77457287
rs112315484
rs117984409
rs117164825
All − MR Egger
All − IVW(MRE)
−0.010
−0.005
0.000
0.005
0.010
MR effect size for
'Mesothelin' on 'eGFR'

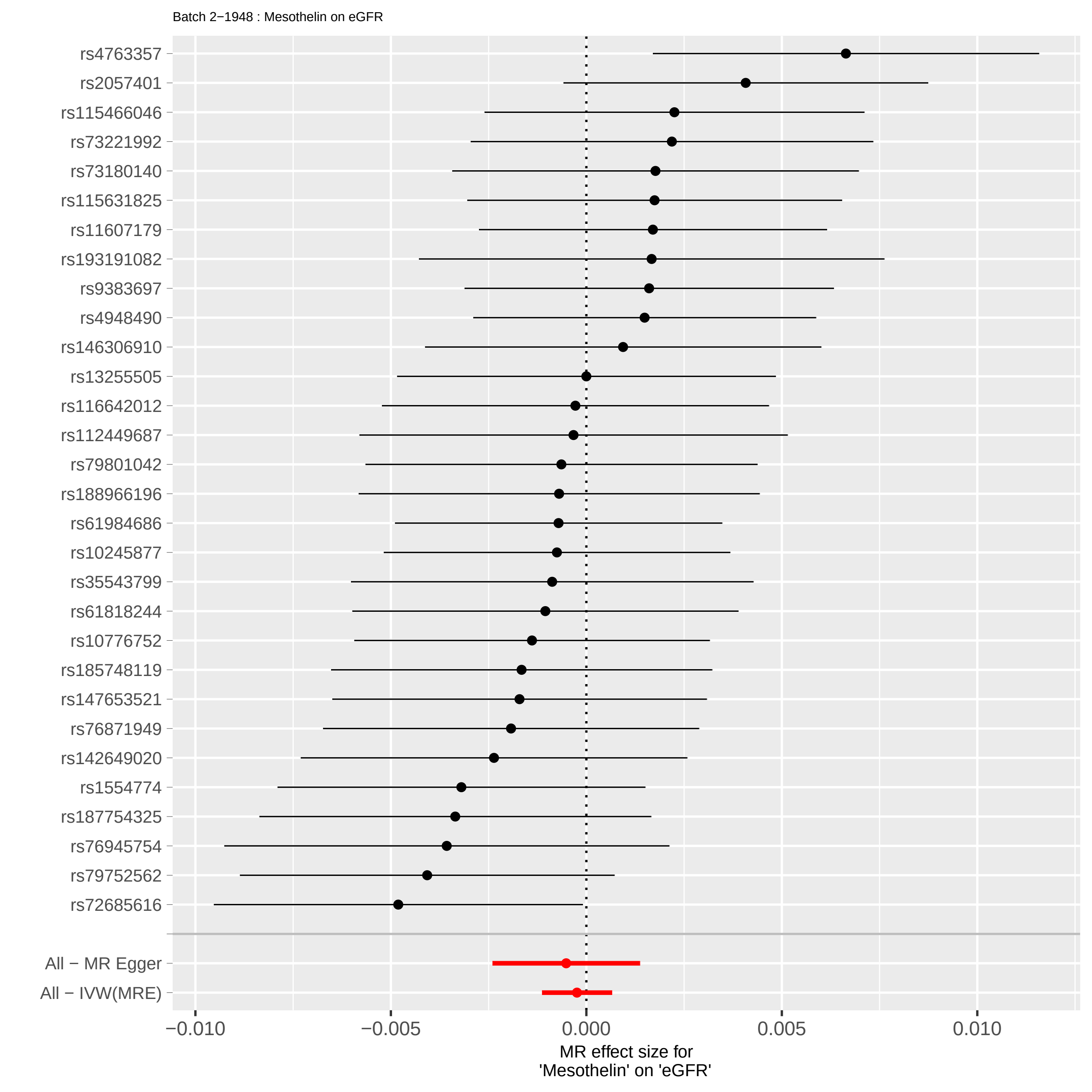

Batch 2−1948 : Mesothelin on eGFR
rs4763357
rs2057401
rs115466046
rs73221992
rs73180140
rs115631825
rs11607179
rs193191082
rs9383697
rs4948490
rs146306910
rs13255505
rs116642012
rs112449687
rs79801042
rs188966196
rs61984686
rs10245877
rs35543799
rs61818244
rs10776752
rs185748119
rs147653521
rs76871949
rs142649020
rs1554774
rs187754325
rs76945754
rs79752562
rs72685616
All − MR Egger
All − IVW(MRE)
−0.010
−0.005
0.000
0.005
0.010
MR effect size for
'Mesothelin' on 'eGFR'

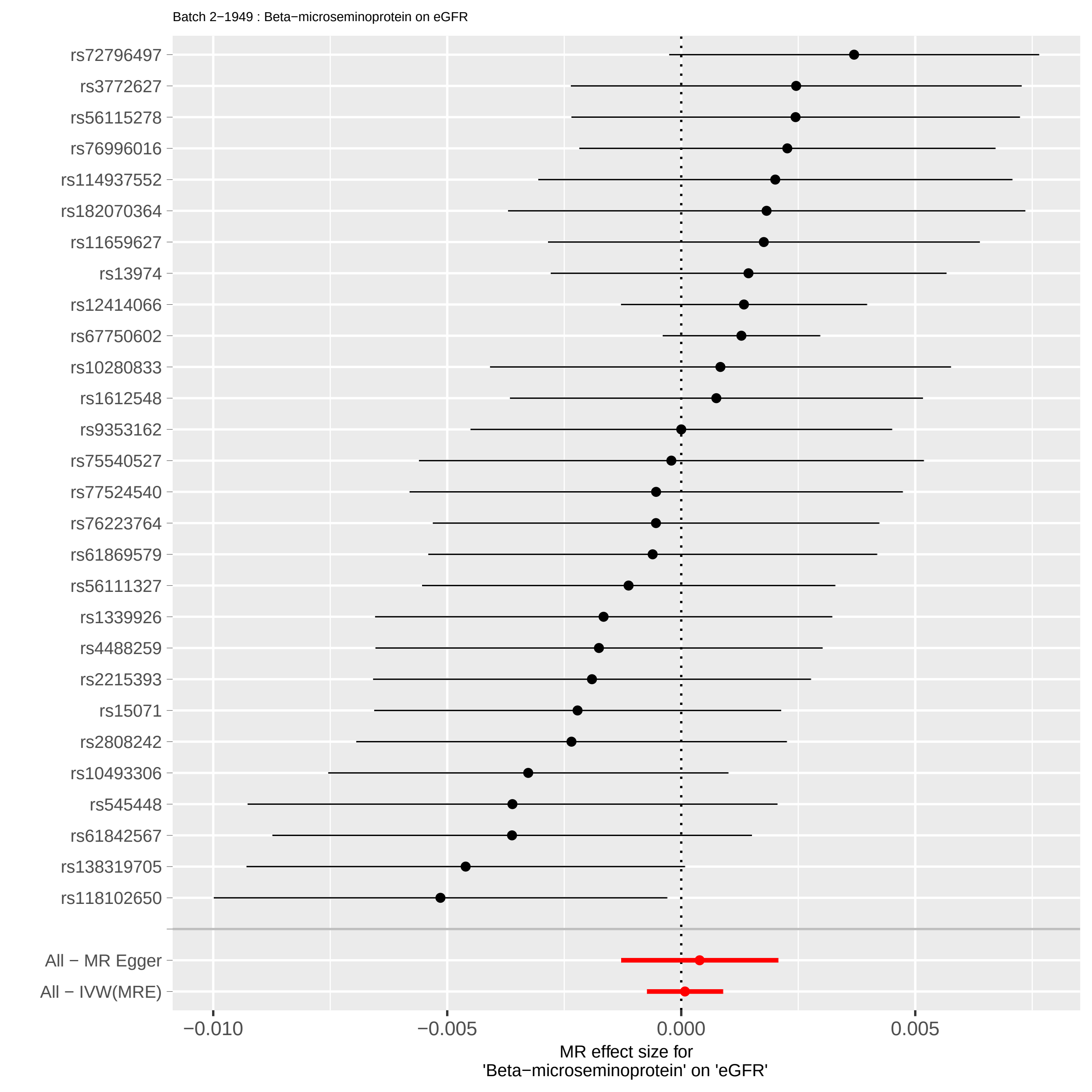
Batch 2−1949 : Beta−microseminoprotein on eGFR
rs72796497
rs3772627
rs56115278
rs76996016
rs114937552
rs182070364
rs11659627
rs13974
rs12414066
rs67750602
rs10280833
rs1612548
rs9353162
rs75540527
rs77524540
rs76223764
rs61869579
rs56111327
rs1339926
rs4488259
rs2215393
rs15071
rs2808242
rs10493306
rs545448
rs61842567
rs138319705
rs118102650
All − MR Egger
All − IVW(MRE)
−0.010
−0.005
0.000
0.005
MR effect size for
'Beta−microseminoprotein' on 'eGFR'

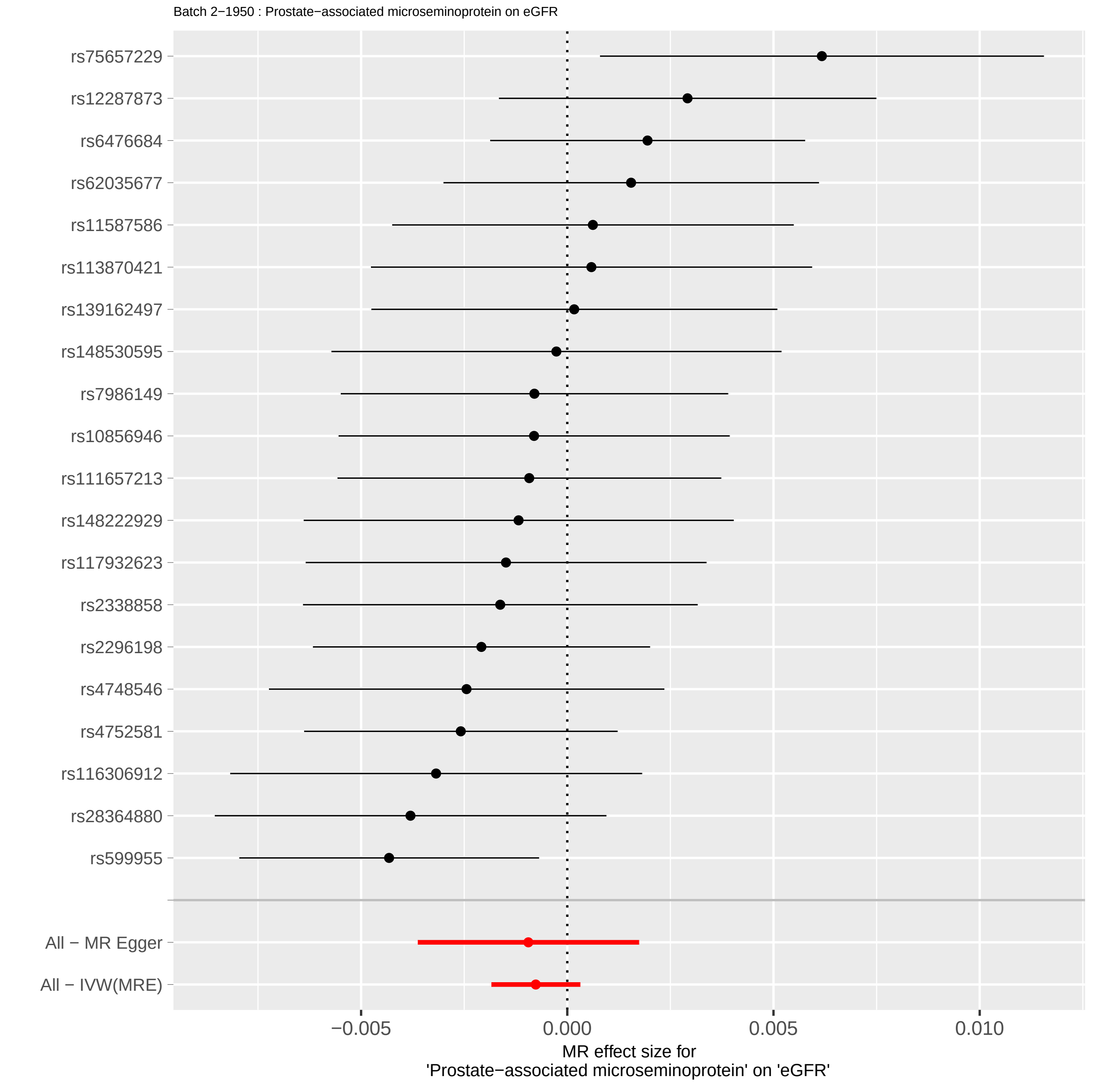

Batch 2−1950 : Prostate−associated microseminoprotein on eGFR
rs75657229
rs12287873
rs6476684
rs62035677
rs11587586
rs113870421
rs139162497
rs148530595
rs7986149
rs10856946
rs111657213
rs148222929
rs117932623
rs2338858
rs2296198
rs4748546
rs4752581
rs116306912
rs28364880
rs599955
All − MR Egger
All − IVW(MRE)
−0.005
0.000
0.005
0.010
MR effect size for
'Prostate−associated microseminoprotein' on 'eGFR'

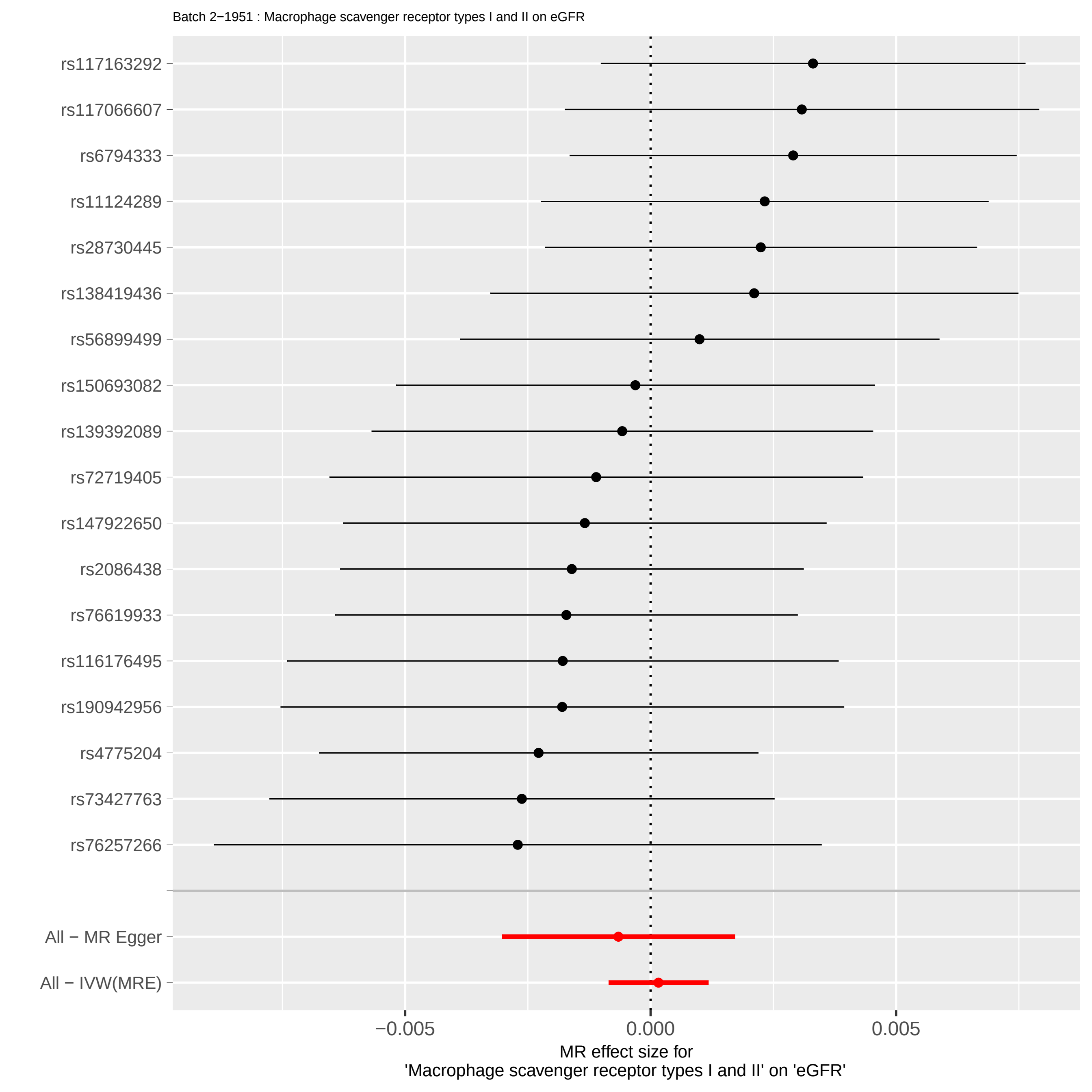

Batch 2−1951 : Macrophage scavenger receptor types I and II on eGFR
rs117163292
rs117066607
rs6794333
rs11124289
rs28730445
rs138419436
rs56899499
rs150693082
rs139392089
rs72719405
rs147922650
rs2086438
rs76619933
rs116176495
rs190942956
rs4775204
rs73427763
rs76257266
All − MR Egger
All − IVW(MRE)
−0.005
0.000
0.005
MR effect size for
'Macrophage scavenger receptor types I and II' on 'eGFR'

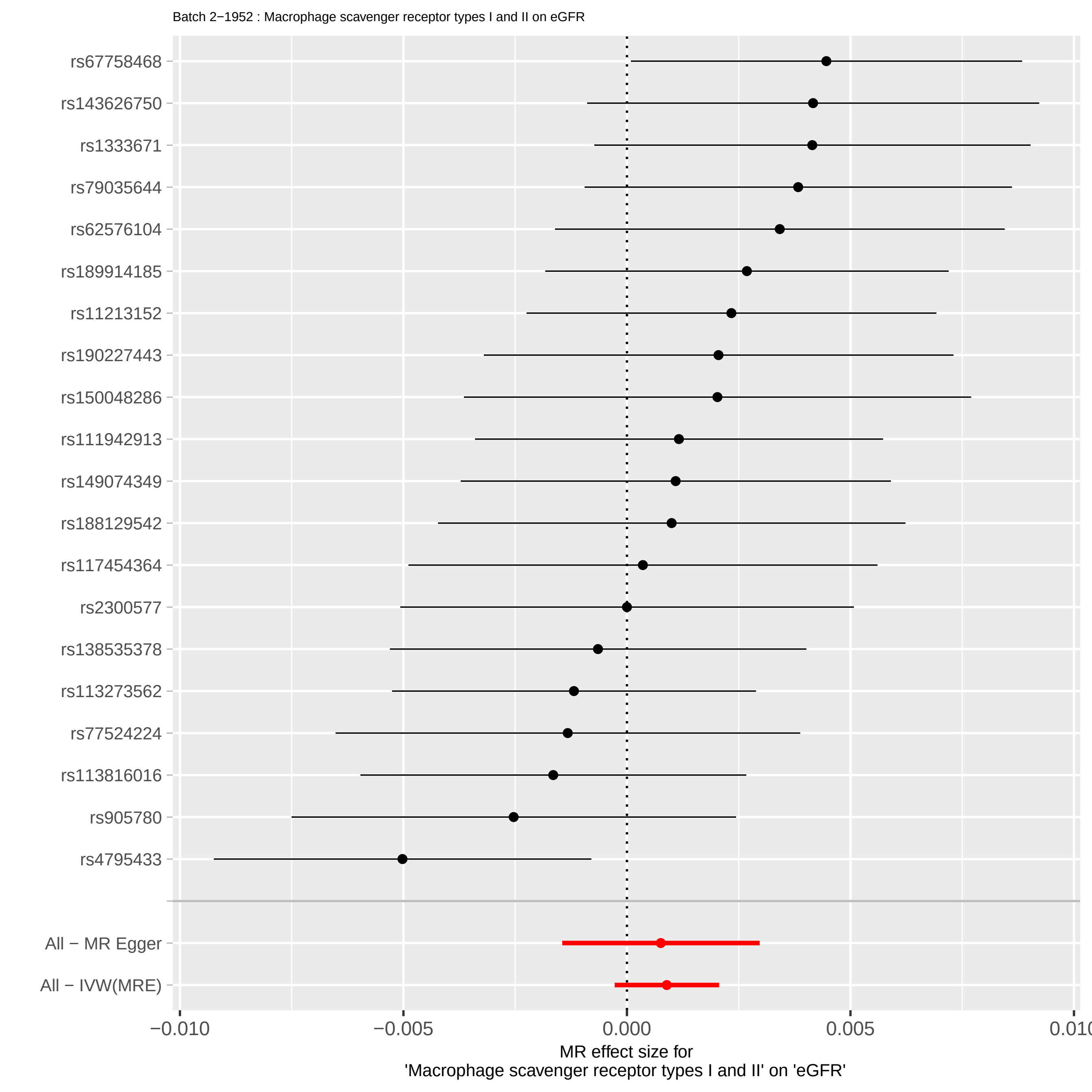

Batch 2−1952 : Macrophage scavenger receptor types I and II on eGFR
rs67758468
rs143626750
rs1333671
rs79035644
rs62576104
rs189914185
rs11213152
rs190227443
rs150048286
rs111942913
rs149074349
rs188129542
rs117454364
rs2300577
rs138535378
rs113273562
rs77524224
rs113816016
rs905780
rs4795433
All − MR Egger
All − IVW(MRE)
−0.010
−0.005
0.000
0.005
MR effect size for
'Macrophage scavenger receptor types I and II' on 'eGFR'

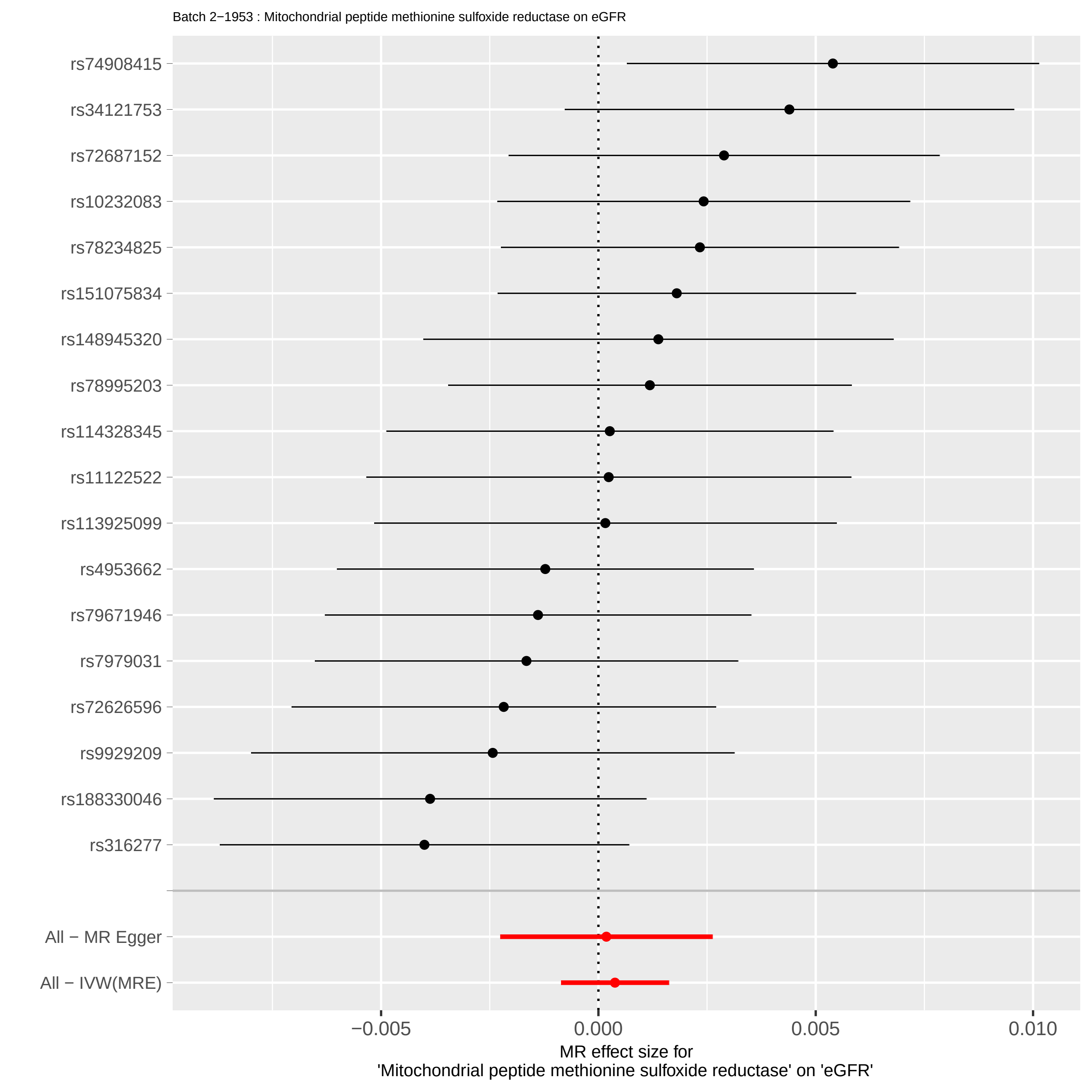

Batch 2−1953 : Mitochondrial peptide methionine sulfoxide reductase on eGFR
rs74908415
rs34121753
rs72687152
rs10232083
rs78234825
rs151075834
rs148945320
rs78995203
rs114328345
rs11122522
rs113925099
rs4953662
rs79671946
rs7979031
rs72626596
rs9929209
rs188330046
rs316277
All − MR Egger
All − IVW(MRE)
−0.005
0.000
0.005
0.010
MR effect size for
'Mitochondrial peptide methionine sulfoxide reductase' on 'eGFR'

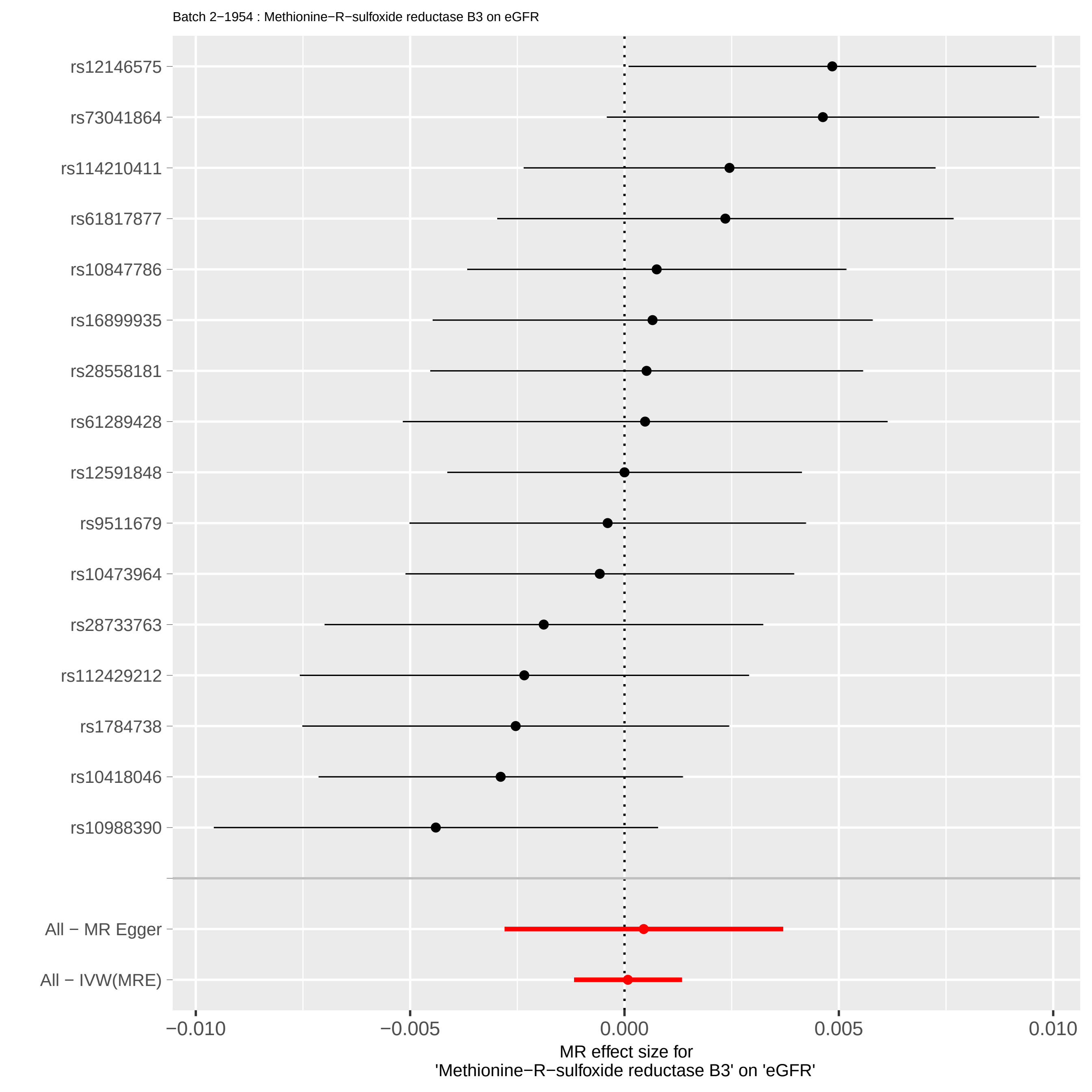
Batch 2−1954 : Methionine−R−sulfoxide reductase B3 on eGFR
rs12146575
rs73041864
rs114210411
rs61817877
rs10847786
rs16899935
rs28558181
rs61289428
rs12591848
rs9511679
rs10473964
rs28733763
rs112429212
rs1784738
rs10418046
rs10988390
All − MR Egger
All − IVW(MRE)
−0.010
−0.005
0.000
0.005
0.010
MR effect size for
'Methionine−R−sulfoxide reductase B3' on 'eGFR'

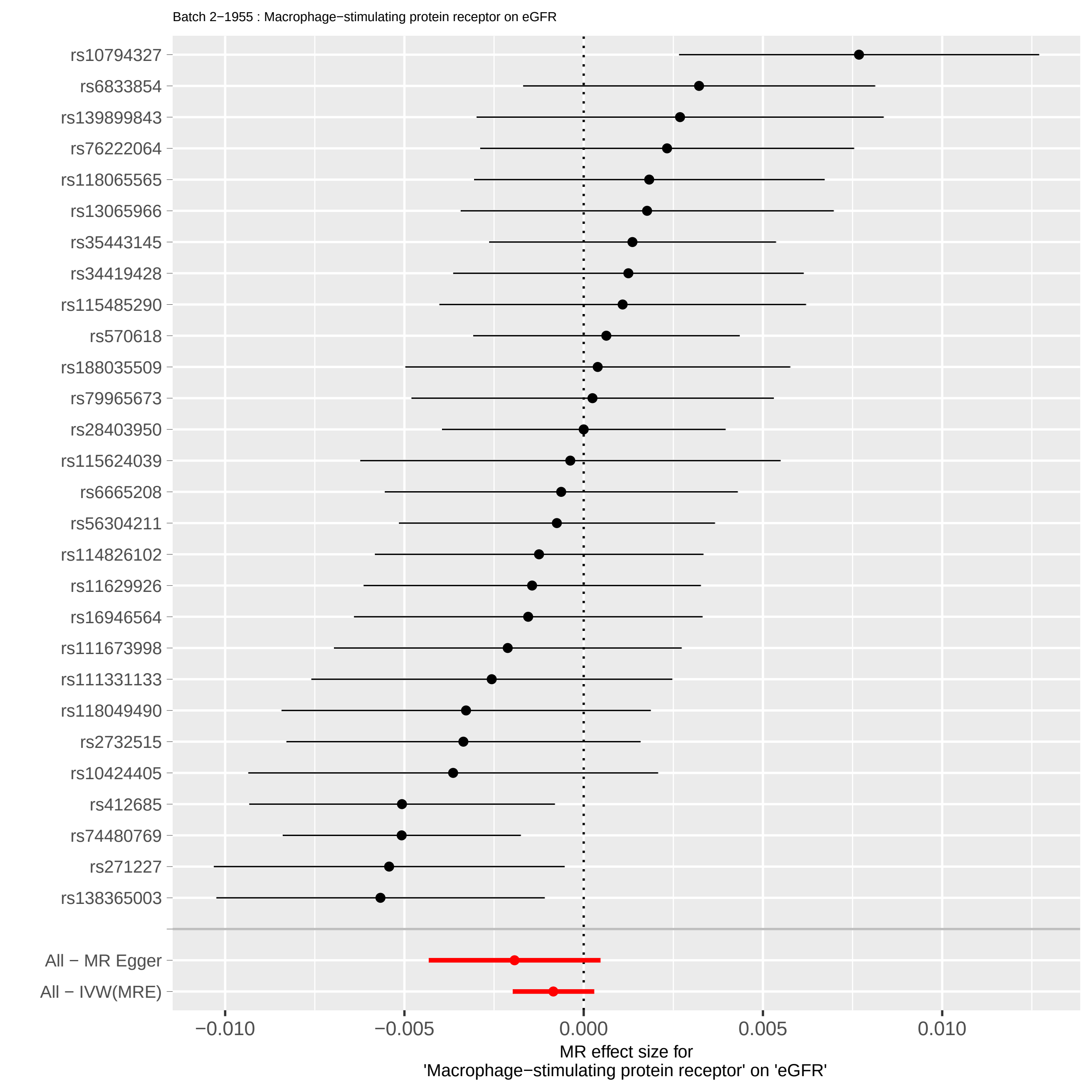

Batch 2−1955 : Macrophage−stimulating protein receptor on eGFR
rs10794327
rs6833854
rs139899843
rs76222064
rs118065565
rs13065966
rs35443145
rs34419428
rs115485290
rs570618
rs188035509
rs79965673
rs28403950
rs115624039
rs6665208
rs56304211
rs114826102
rs11629926
rs16946564
rs111673998
rs111331133
rs118049490
rs2732515
rs10424405
rs412685
rs74480769
rs271227
rs138365003
All − MR Egger
All − IVW(MRE)
−0.010
−0.005
0.000
0.005
0.010
MR effect size for
'Macrophage−stimulating protein receptor' on 'eGFR'

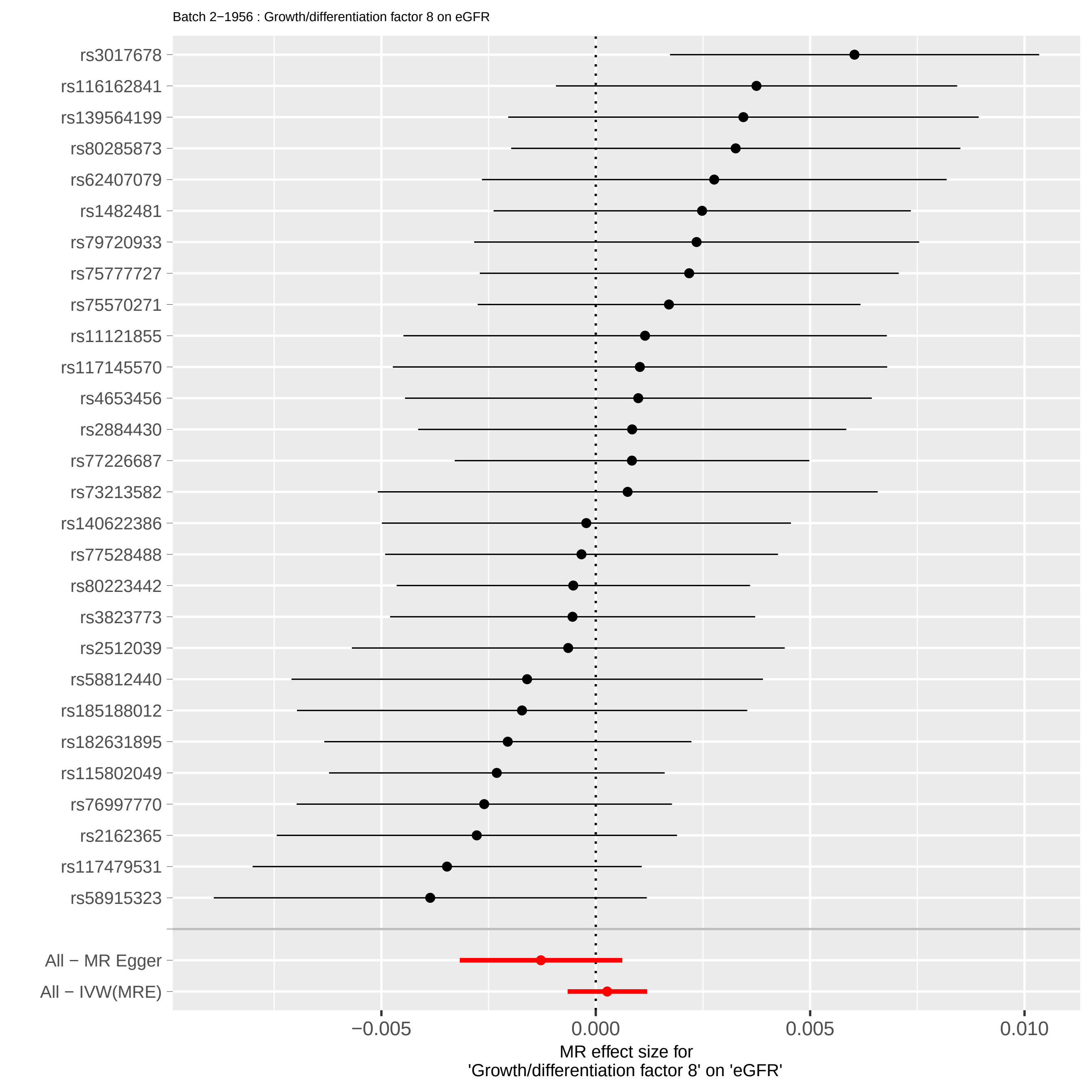
Batch 2−1956 : Growth/differentiation factor 8 on eGFR
rs3017678
rs116162841
rs139564199
rs80285873
rs62407079
rs1482481
rs79720933
rs75777727
rs75570271
rs11121855
rs117145570
rs4653456
rs2884430
rs77226687
rs73213582
rs140622386
rs77528488
rs80223442
rs3823773
rs2512039
rs58812440
rs185188012
rs182631895
rs115802049
rs76997770
rs2162365
rs117479531
rs58915323
All − MR Egger
All − IVW(MRE)
−0.005
0.000
0.005
0.010
MR effect size for
'Growth/differentiation factor 8' on 'eGFR'

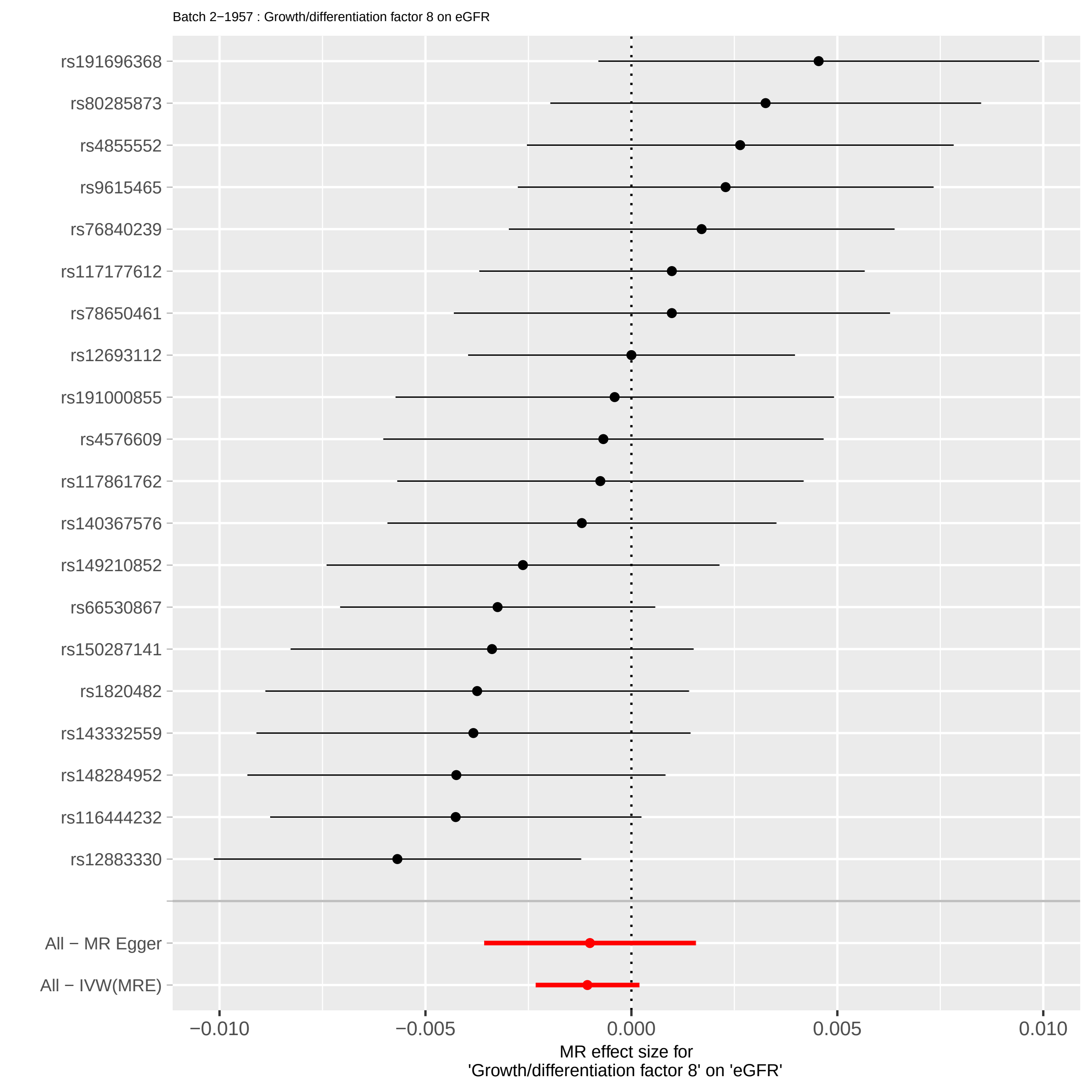
Batch 2−1957 : Growth/differentiation factor 8 on eGFR
rs191696368
rs80285873
rs4855552
rs9615465
rs76840239
rs117177612
rs78650461
rs12693112
rs191000855
rs4576609
rs117861762
rs140367576
rs149210852
rs66530867
rs150287141
rs1820482
rs143332559
rs148284952
rs116444232
rs12883330
All − MR Egger
All − IVW(MRE)
−0.010
−0.005
0.000
0.005
0.010
MR effect size for
'Growth/differentiation factor 8' on 'eGFR'

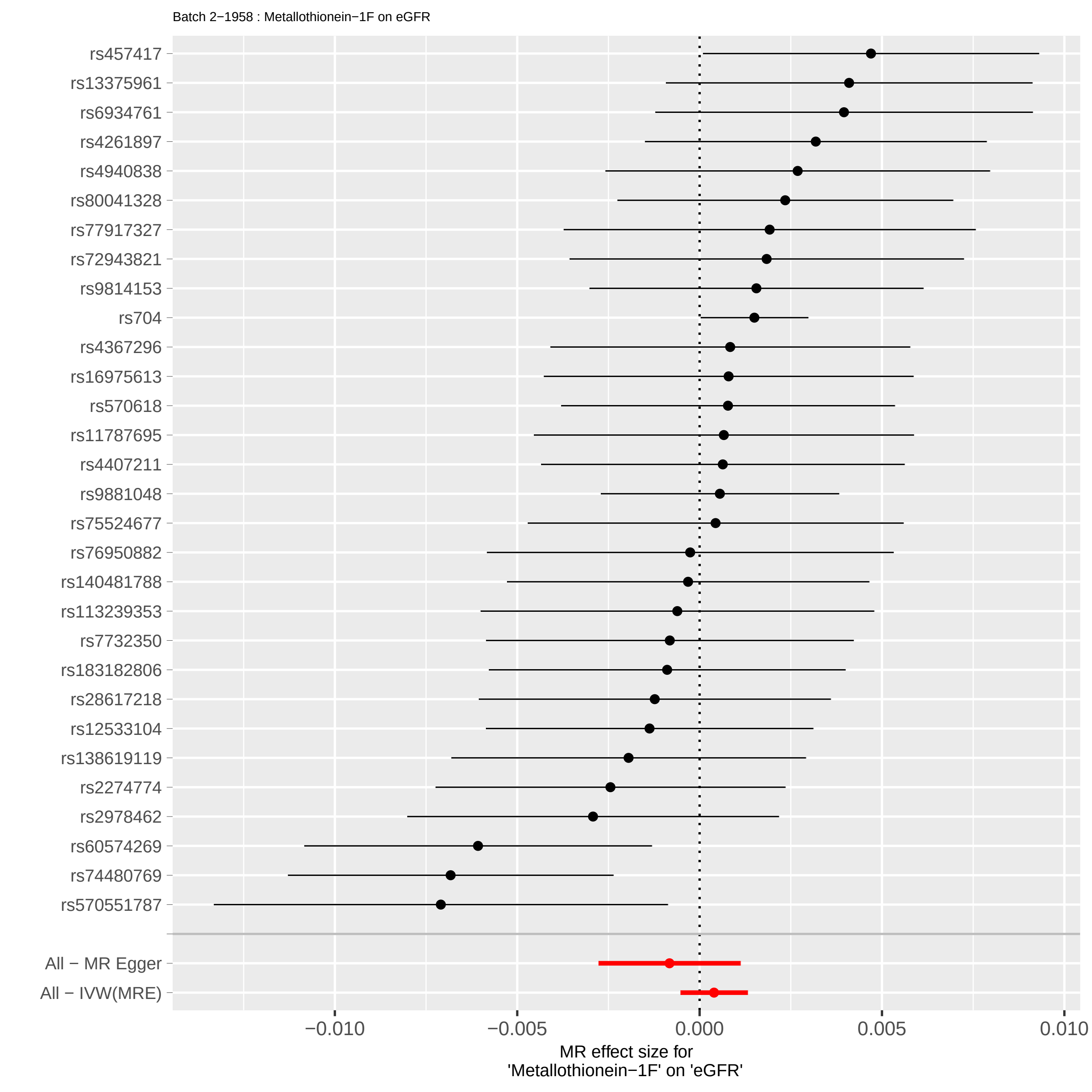

Batch 2−1958 : Metallothionein−1F on eGFR
rs457417
rs13375961
rs6934761
rs4261897
rs4940838
rs80041328
rs77917327
rs72943821
rs9814153
rs704
rs4367296
rs16975613
rs570618
rs11787695
rs4407211
rs9881048
rs75524677
rs76950882
rs140481788
rs113239353
rs7732350
rs183182806
rs28617218
rs12533104
rs138619119
rs2274774
rs2978462
rs60574269
rs74480769
rs570551787
All − MR Egger
All − IVW(MRE)
−0.010
−0.005
0.000
0.005
0.010
MR effect size for
'Metallothionein−1F' on 'eGFR'

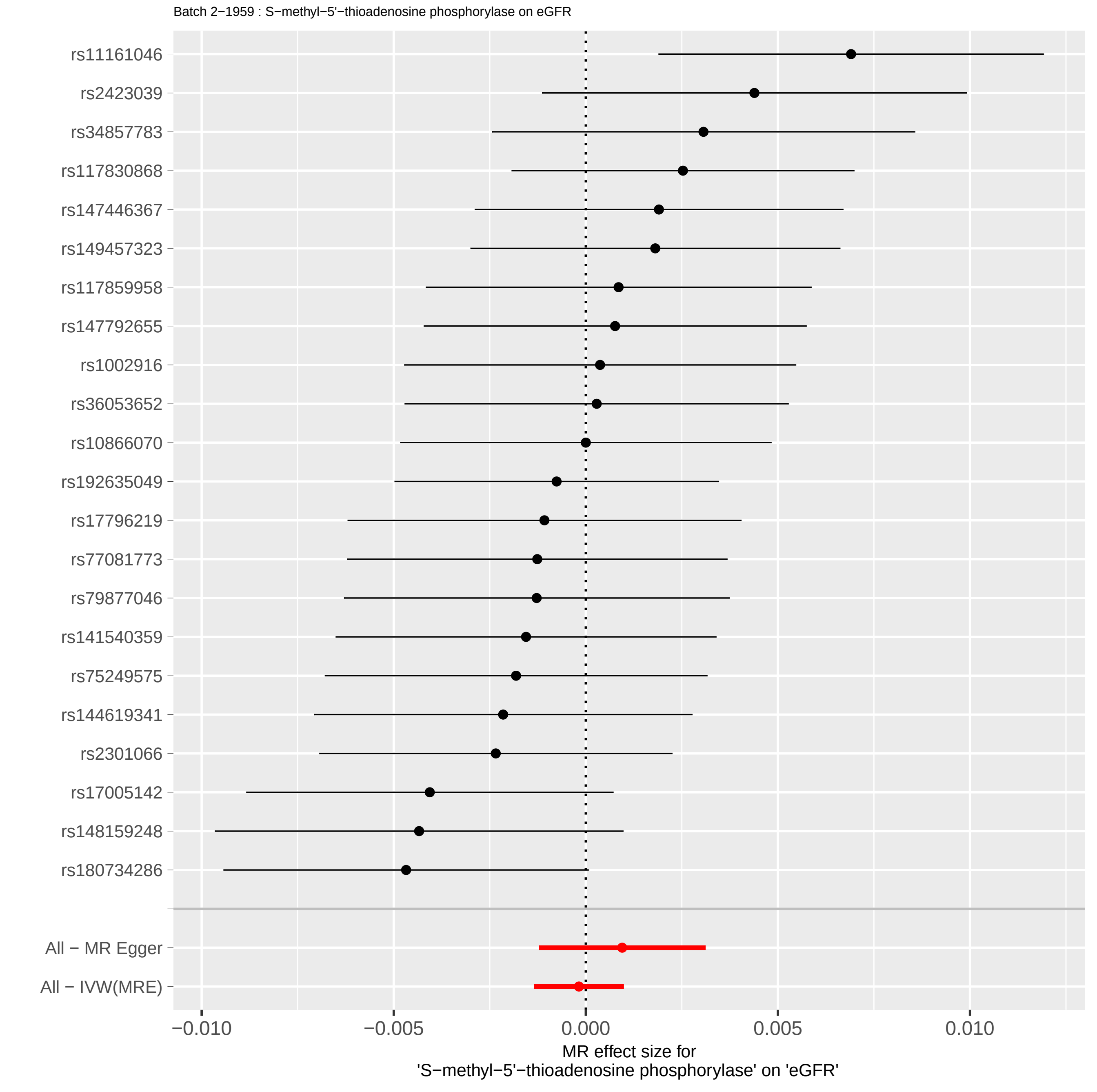

Batch 2−1959 : S−methyl−5'−thioadenosine phosphorylase on eGFR
rs11161046
rs2423039
rs34857783
rs117830868
rs147446367
rs149457323
rs117859958
rs147792655
rs1002916
rs36053652
rs10866070
rs192635049
rs17796219
rs77081773
rs79877046
rs141540359
rs75249575
rs144619341
rs2301066
rs17005142
rs148159248
rs180734286
All − MR Egger
All − IVW(MRE)
−0.010
−0.005
0.000
0.005
0.010
MR effect size for
'S−methyl−5'−thioadenosine phosphorylase' on 'eGFR'

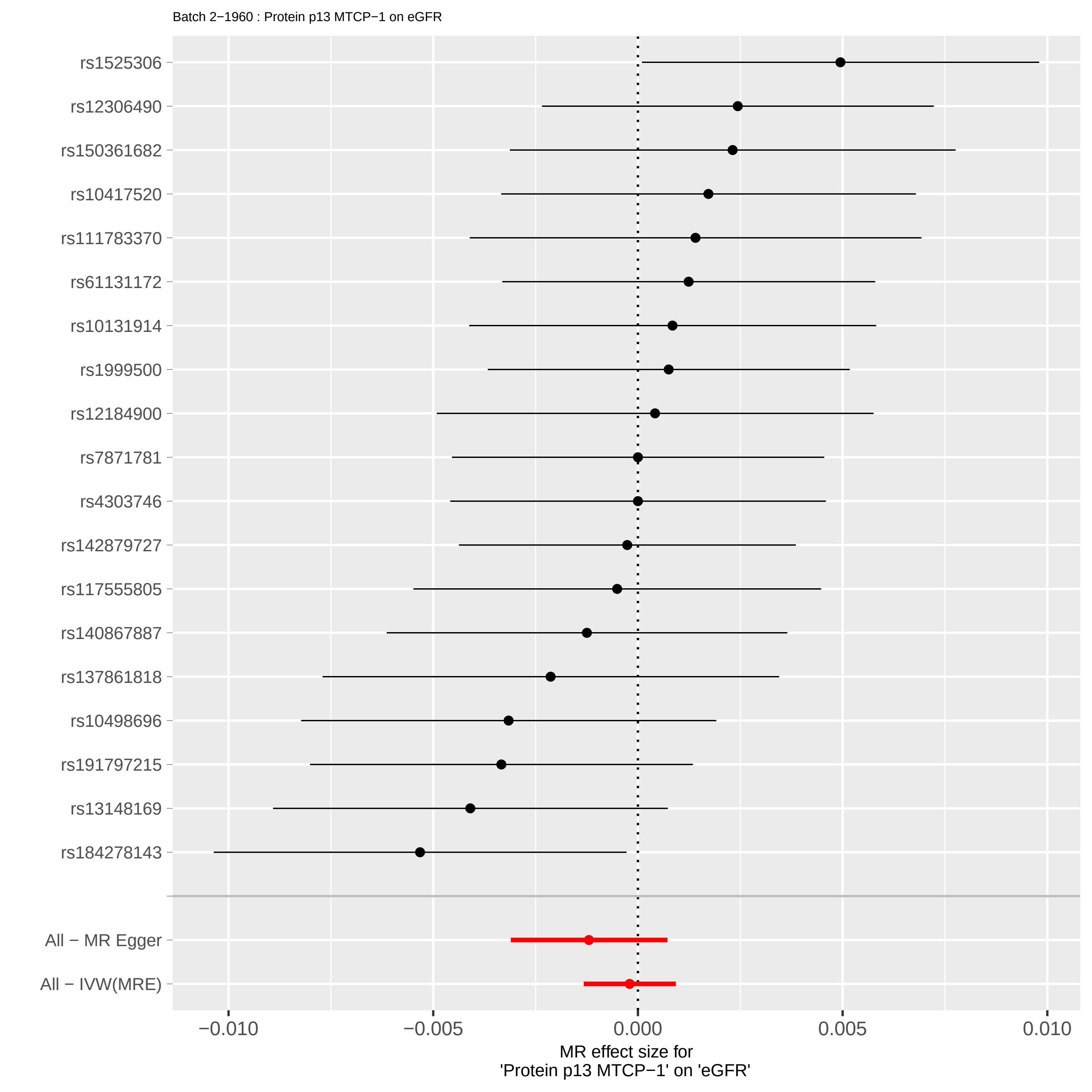
Batch 2−1960 : Protein p13 MTCP−1 on eGFR
rs1525306
rs12306490
rs150361682
rs10417520
rs111783370
rs61131172
rs10131914
rs1999500
rs12184900
rs7871781
rs4303746
rs142879727
rs117555805
rs140867887
rs137861818
rs10498696
rs191797215
rs13148169
rs184278143
All − MR Egger
All − IVW(MRE)
−0.010
−0.005
0.000
0.005
0.010
MR effect size for
'Protein p13 MTCP−1' on 'eGFR'

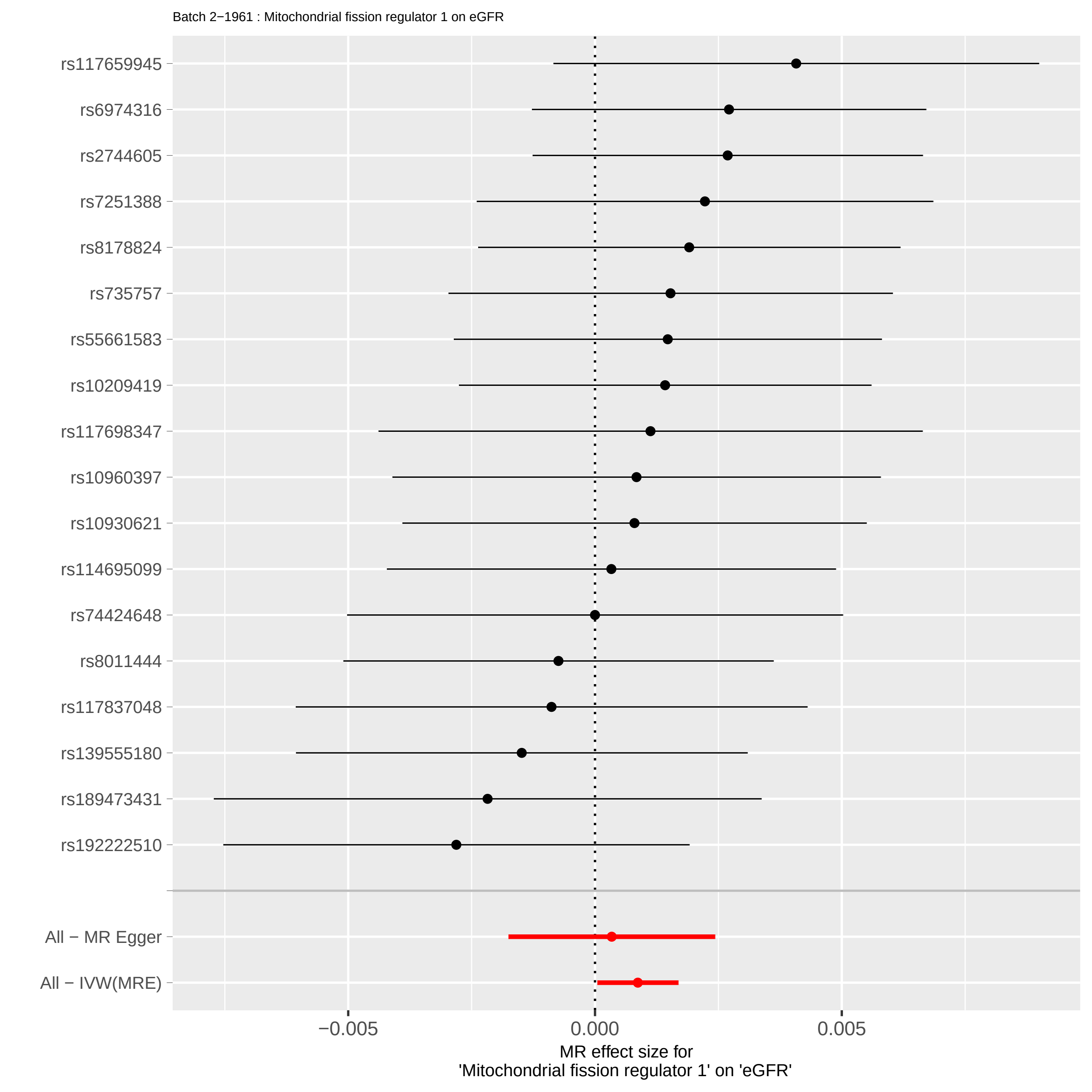

Batch 2−1961 : Mitochondrial fission regulator 1 on eGFR
rs117659945
rs6974316
rs2744605
rs7251388
rs8178824
rs735757
rs55661583
rs10209419
rs117698347
rs10960397
rs10930621
rs114695099
rs74424648
rs8011444
rs117837048
rs139555180
rs189473431
rs192222510
All − MR Egger
All − IVW(MRE)
−0.005
0.000
0.005
MR effect size for
'Mitochondrial fission regulator 1' on 'eGFR'

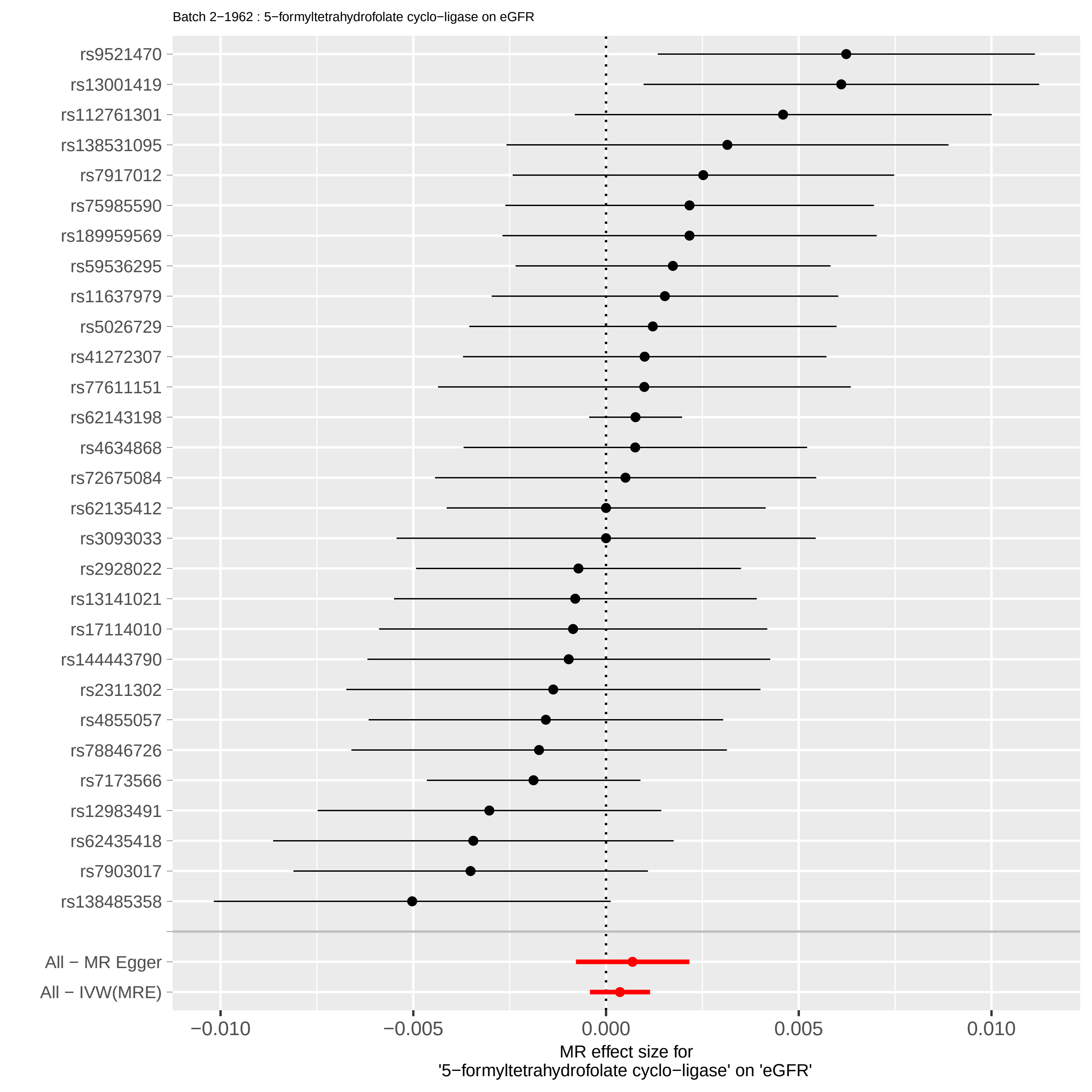

Batch 2−1962 : 5−formyltetrahydrofolate cyclo−ligase on eGFR
rs9521470
rs13001419
rs112761301
rs138531095
rs7917012
rs75985590
rs189959569
rs59536295
rs11637979
rs5026729
rs41272307
rs77611151
rs62143198
rs4634868
rs72675084
rs62135412
rs3093033
rs2928022
rs13141021
rs17114010
rs144443790
rs2311302
rs4855057
rs78846726
rs7173566
rs12983491
rs62435418
rs7903017
rs138485358
All − MR Egger
All − IVW(MRE)
−0.010
−0.005
0.000
0.005
0.010
MR effect size for
'5−formyltetrahydrofolate cyclo−ligase' on 'eGFR'

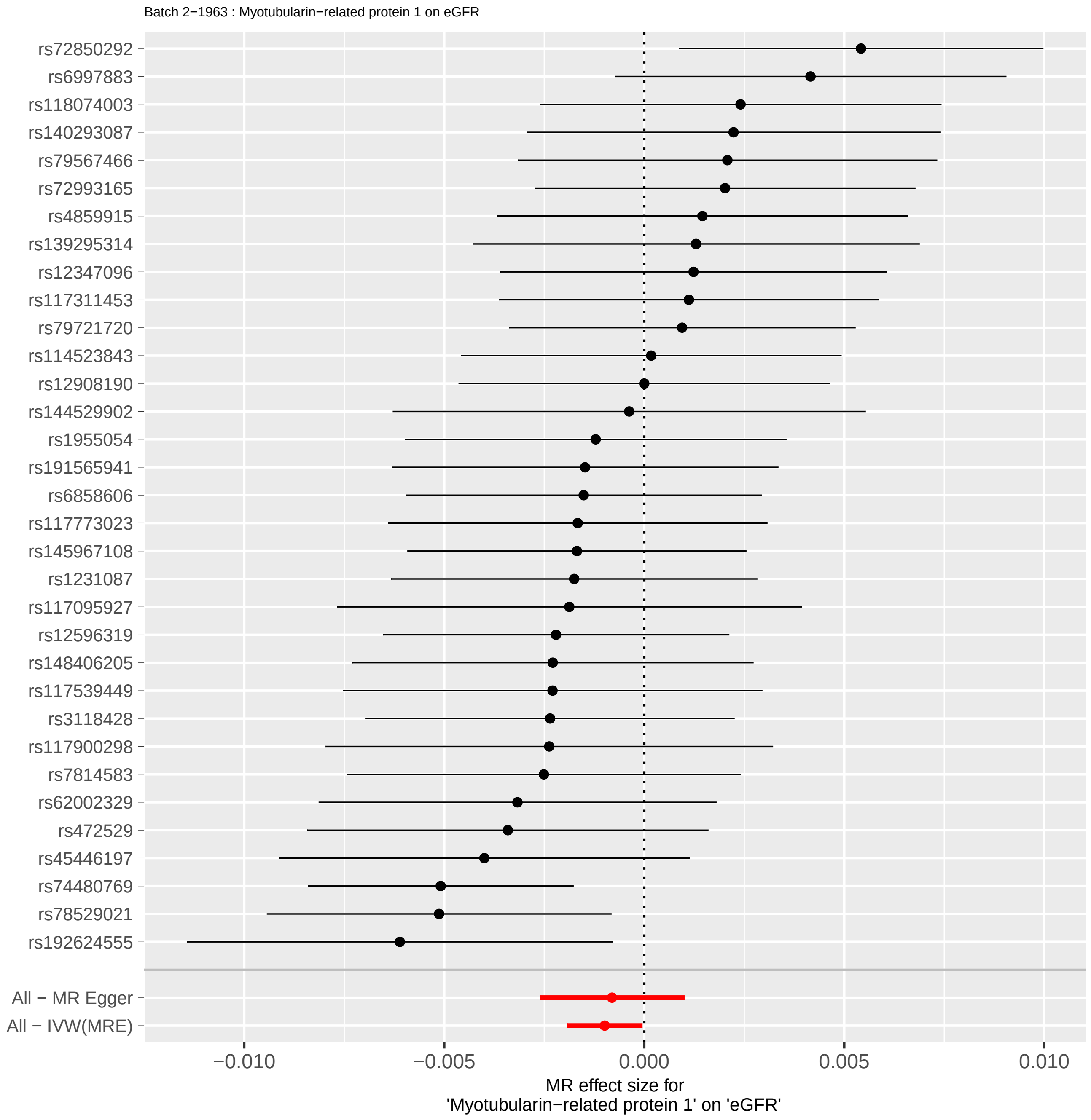

Batch 2−1963 : Myotubularin−related protein 1 on eGFR
rs72850292
rs6997883
rs118074003
rs140293087
rs79567466
rs72993165
rs4859915
rs139295314
rs12347096
rs117311453
rs79721720
rs114523843
rs12908190
rs144529902
rs1955054
rs191565941
rs6858606
rs117773023
rs145967108
rs1231087
rs117095927
rs12596319
rs148406205
rs117539449
rs3118428
rs117900298
rs7814583
rs62002329
rs472529
rs45446197
rs74480769
rs78529021
rs192624555
All − MR Egger
All − IVW(MRE)
−0.010
−0.005
0.000
0.005
0.010
MR effect size for
'Myotubularin−related protein 1' on 'eGFR'

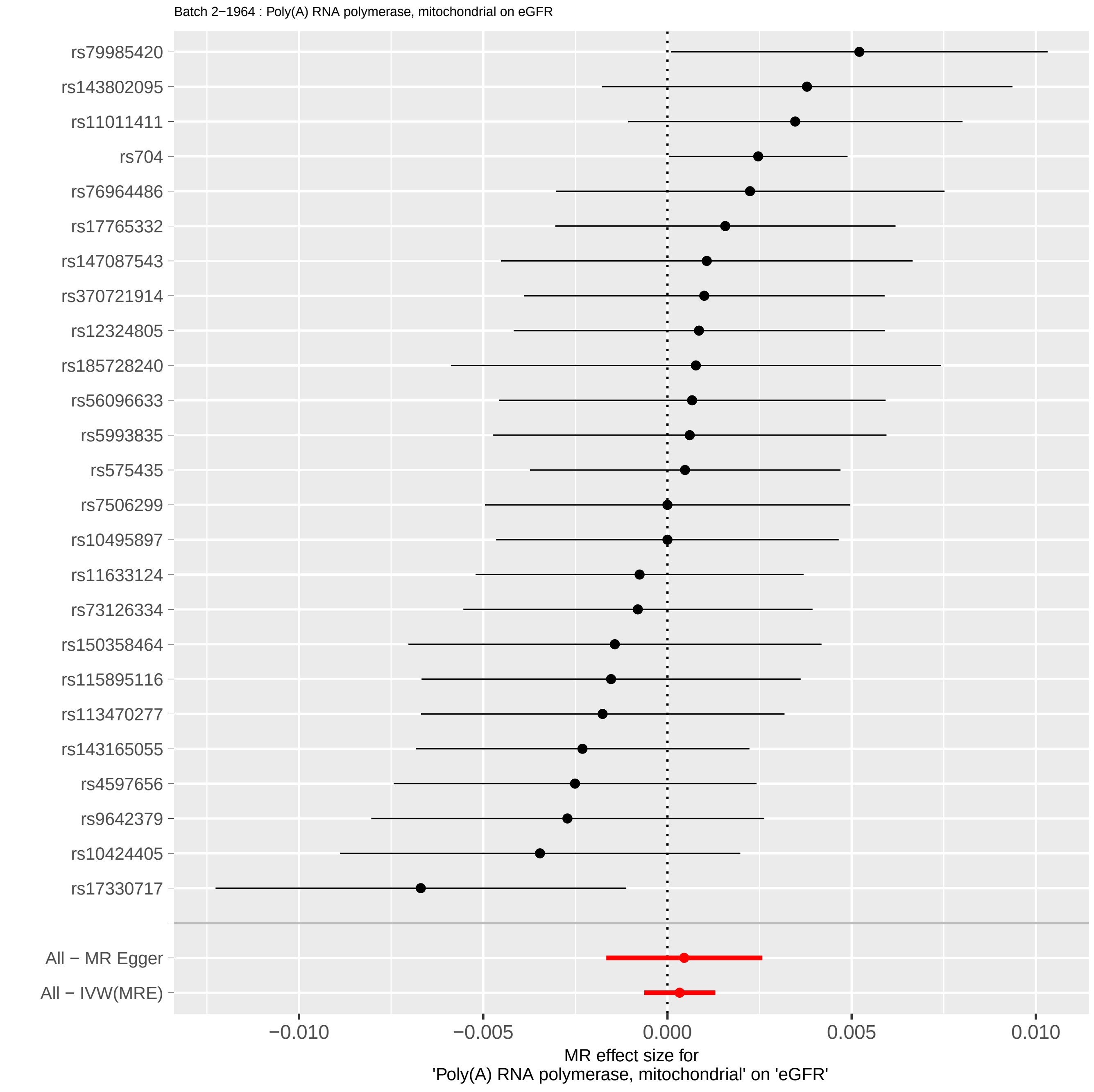
Batch 2−1964 : Poly(A) RNA polymerase, mitochondrial on eGFR
rs79985420
rs143802095
rs11011411
rs704
rs76964486
rs17765332
rs147087543
rs370721914
rs12324805
rs185728240
rs56096633
rs5993835
rs575435
rs7506299
rs10495897
rs11633124
rs73126334
rs150358464
rs115895116
rs113470277
rs143165055
rs4597656
rs9642379
rs10424405
rs17330717
All − MR Egger
All − IVW(MRE)
−0.010
−0.005
0.000
0.005
0.010
MR effect size for
'Poly(A) RNA polymerase, mitochondrial' on 'eGFR'

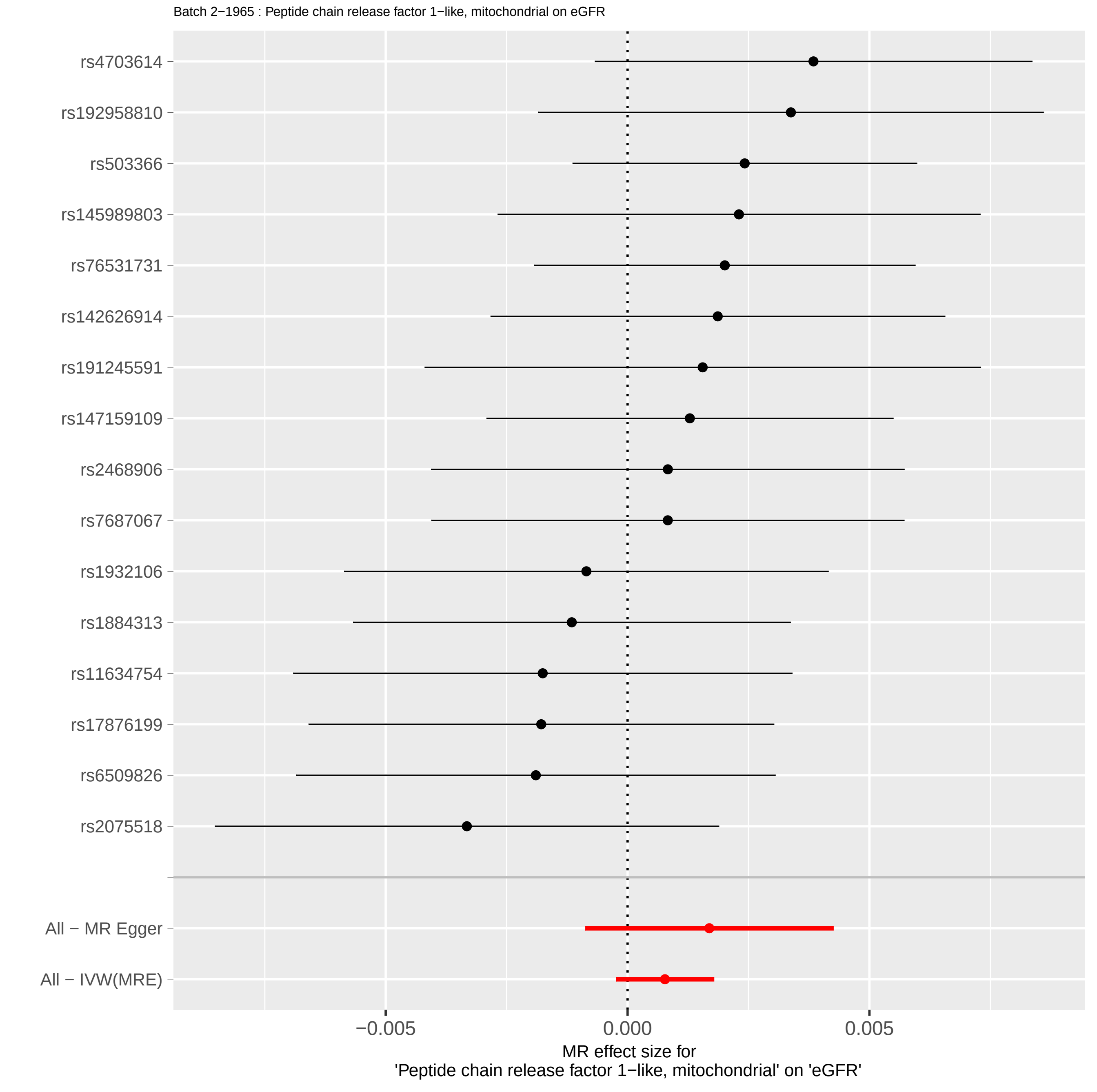

Batch 2−1965 : Peptide chain release factor 1−like, mitochondrial on eGFR
rs4703614
rs192958810
rs503366
rs145989803
rs76531731
rs142626914
rs191245591
rs147159109
rs2468906
rs7687067
rs1932106
rs1884313
rs11634754
rs17876199
rs6509826
rs2075518
All − MR Egger
All − IVW(MRE)
−0.005
0.000
0.005
MR effect size for
'Peptide chain release factor 1−like, mitochondrial' on 'eGFR'

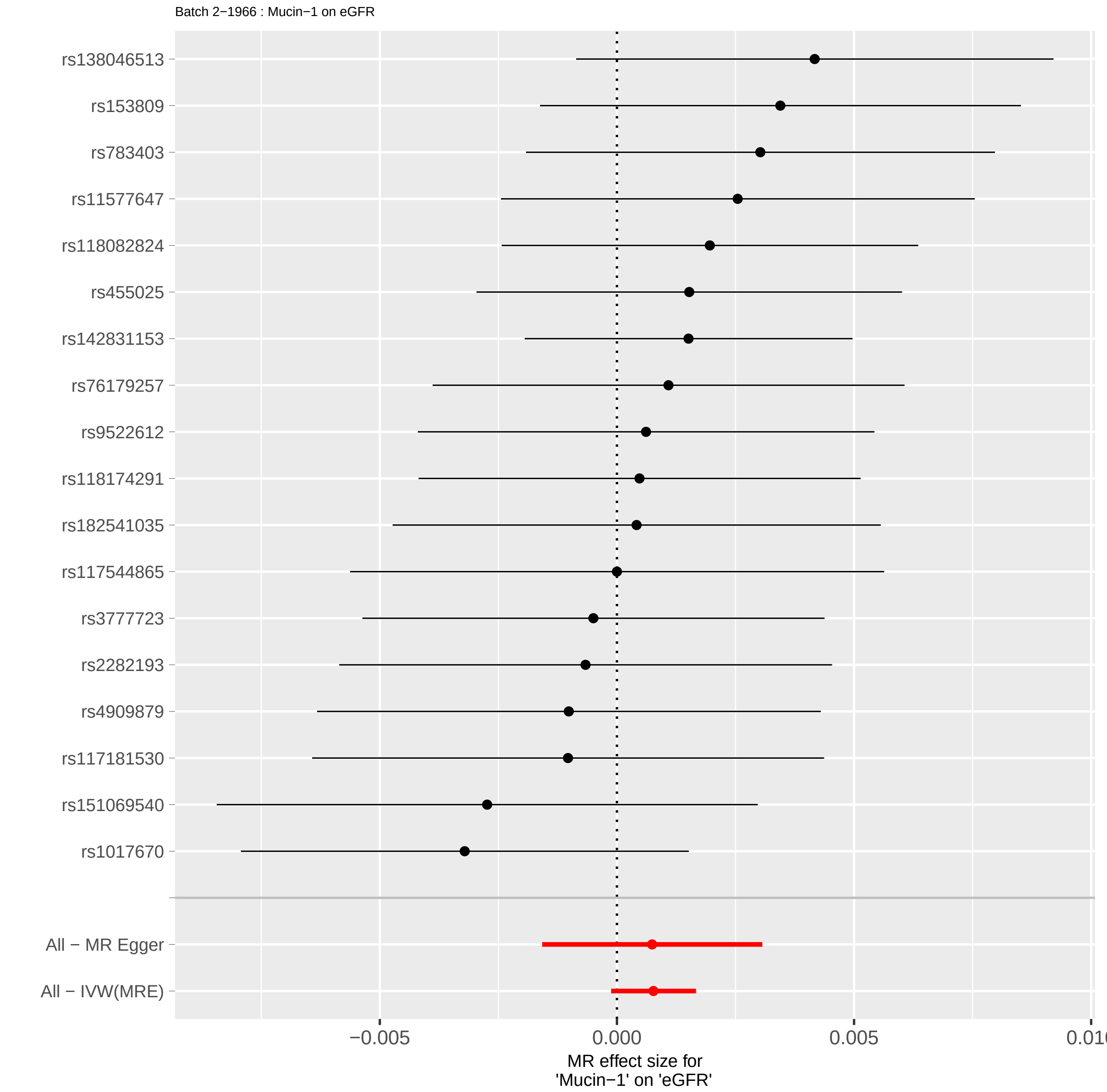
Batch 2−1966 : Mucin−1 on eGFR
rs138046513
rs153809
rs783403
rs11577647
rs118082824
rs455025
rs142831153
rs76179257
rs9522612
rs118174291
rs182541035
rs117544865
rs3777723
rs2282193
rs4909879
rs117181530
rs151069540
rs1017670
All − MR Egger
All − IVW(MRE)
−0.005
0.000
0.005
MR effect size for
'Mucin−1' on 'eGFR'

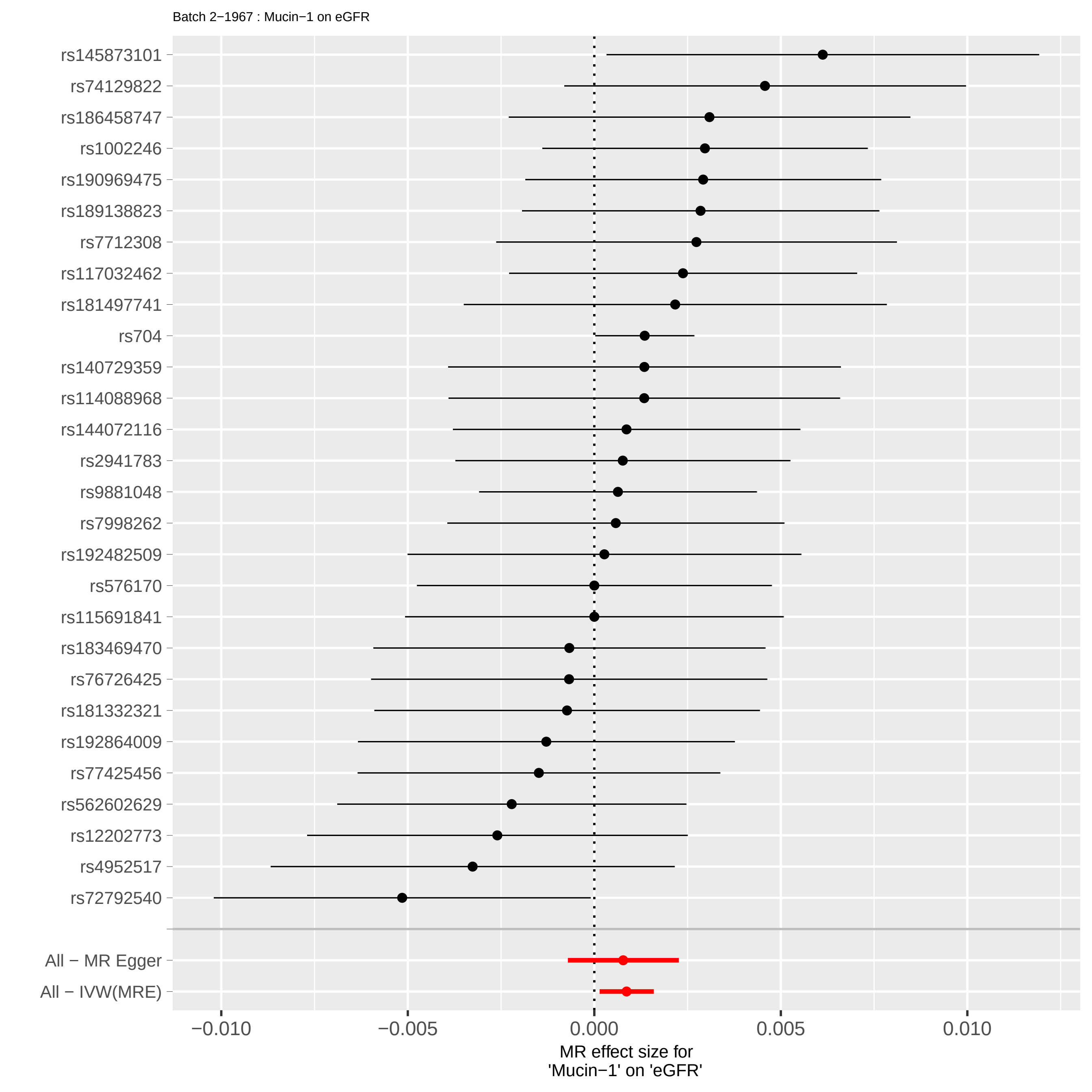

Batch 2−1967 : Mucin−1 on eGFR
rs145873101
rs74129822
rs186458747
rs1002246
rs190969475
rs189138823
rs7712308
rs117032462
rs181497741
rs704
rs140729359
rs114088968
rs144072116
rs2941783
rs9881048
rs7998262
rs192482509
rs576170
rs115691841
rs183469470
rs76726425
rs181332321
rs192864009
rs77425456
rs562602629
rs12202773
rs4952517
rs72792540
All − MR Egger
All − IVW(MRE)
−0.010
−0.005
0.000
0.005
0.010
MR effect size for
'Mucin−1' on 'eGFR'

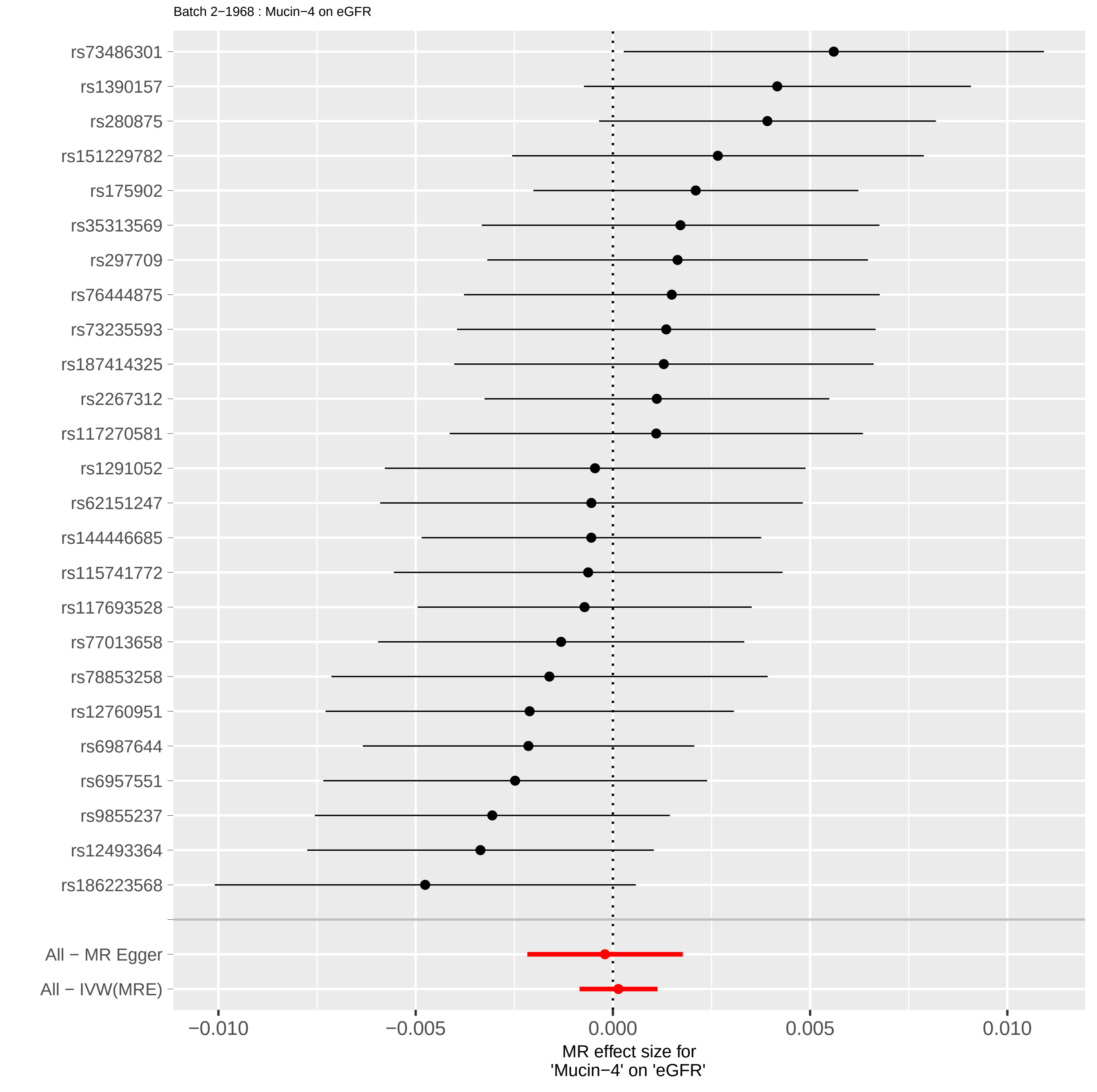
Batch 2−1968 : Mucin−4 on eGFR
rs73486301
rs1390157
rs280875
rs151229782
rs175902
rs35313569
rs297709
rs76444875
rs73235593
rs187414325
rs2267312
rs117270581
rs1291052
rs62151247
rs144446685
rs115741772
rs117693528
rs77013658
rs78853258
rs12760951
rs6987644
rs6957551
rs9855237
rs12493364
rs186223568
All − MR Egger
All − IVW(MRE)
−0.010
−0.005
0.000
0.005
0.010
MR effect size for
'Mucin−4' on 'eGFR'

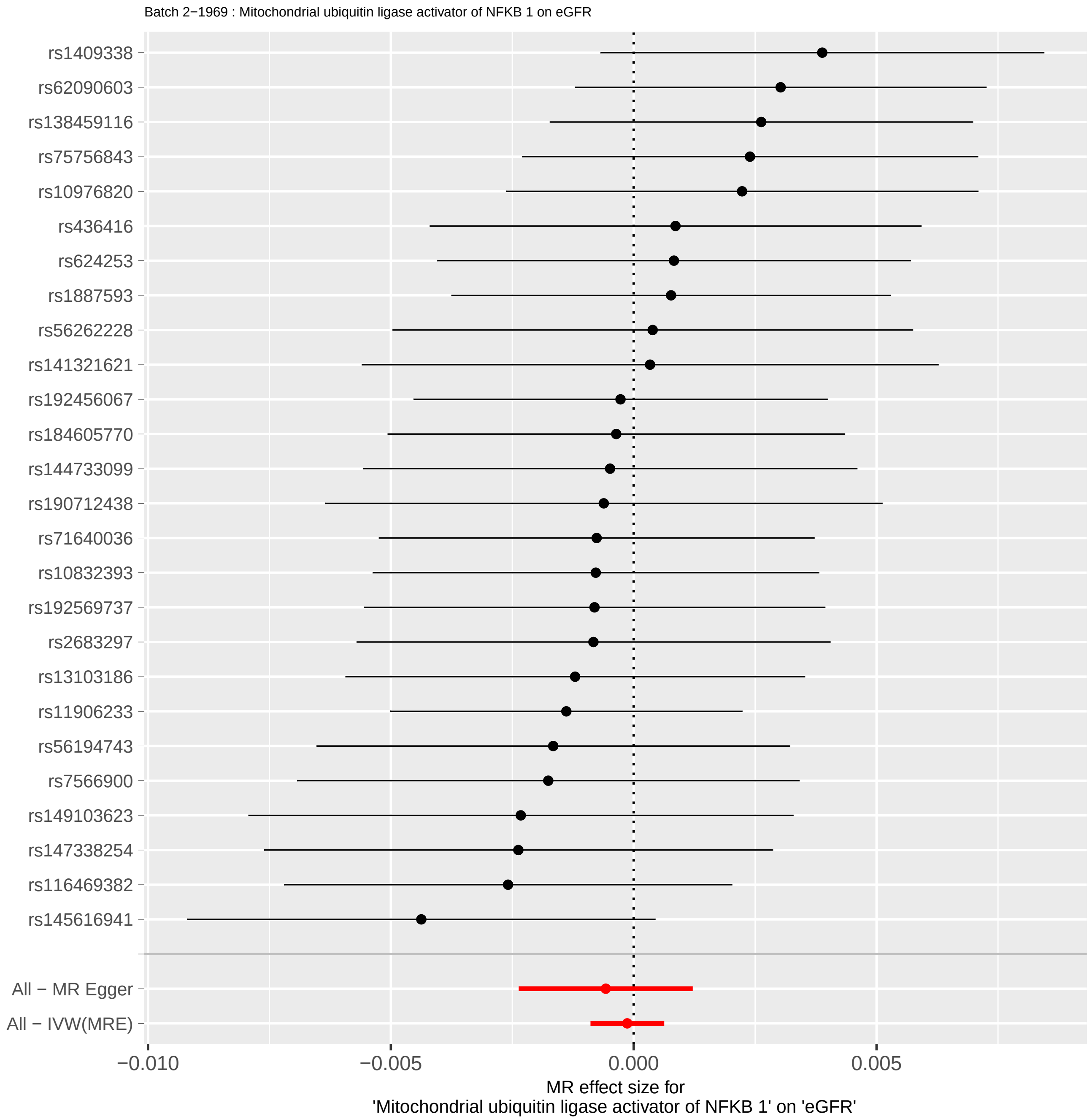

Batch 2−1969 : Mitochondrial ubiquitin ligase activator of NFKB 1 on eGFR
rs1409338
rs62090603
rs138459116
rs75756843
rs10976820
rs436416
rs624253
rs1887593
rs56262228
rs141321621
rs192456067
rs184605770
rs144733099
rs190712438
rs71640036
rs10832393
rs192569737
rs2683297
rs13103186
rs11906233
rs56194743
rs7566900
rs149103623
rs147338254
rs116469382
rs145616941
All − MR Egger
All − IVW(MRE)
−0.010
−0.005
0.000
0.005
MR effect size for
'Mitochondrial ubiquitin ligase activator of NFKB 1' on 'eGFR'

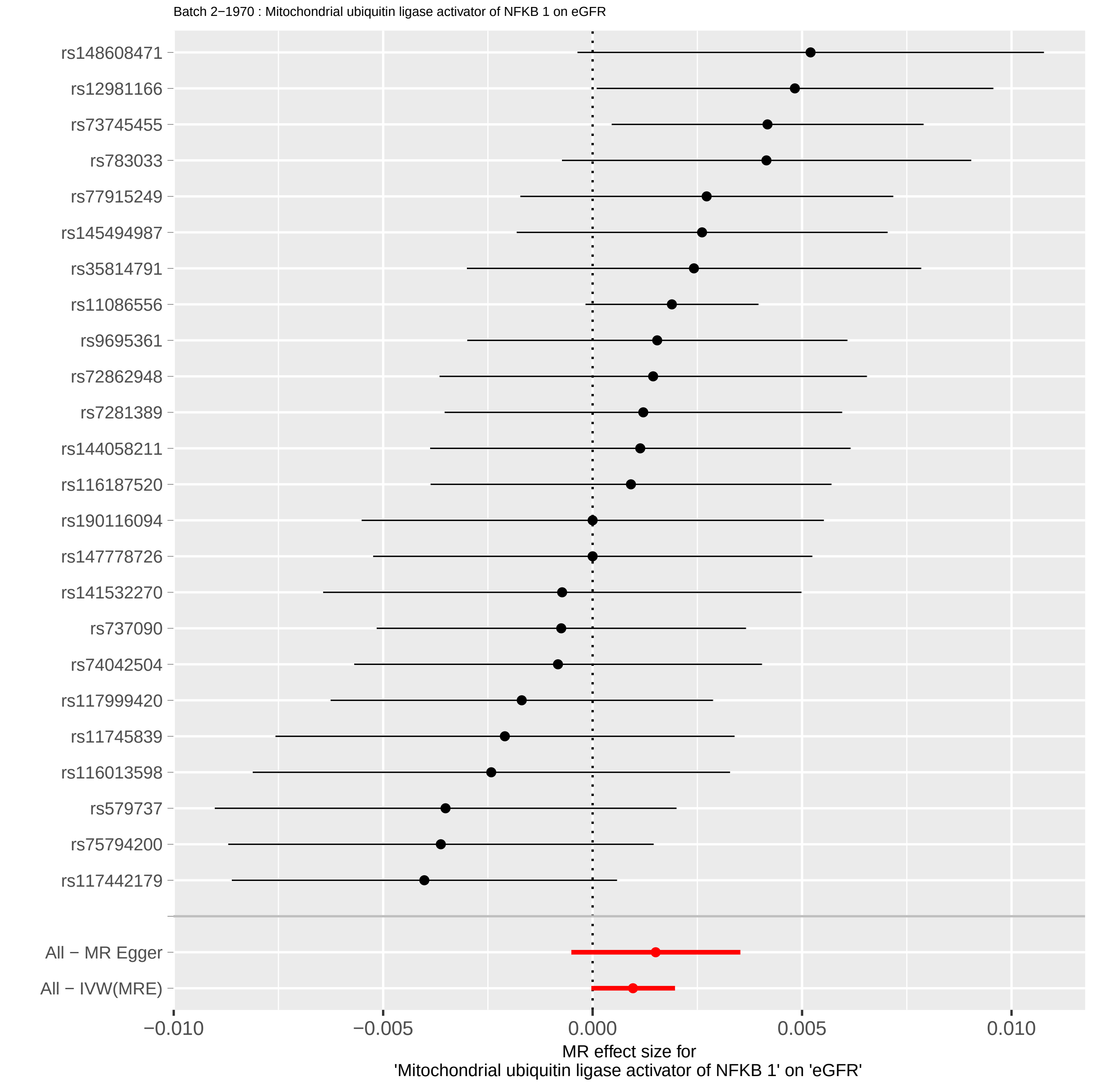
Batch 2−1970 : Mitochondrial ubiquitin ligase activator of NFKB 1 on eGFR
rs148608471
rs12981166
rs73745455
rs783033
rs77915249
rs145494987
rs35814791
rs11086556
rs9695361
rs72862948
rs7281389
rs144058211
rs116187520
rs190116094
rs147778726
rs141532270
rs737090
rs74042504
rs117999420
rs11745839
rs116013598
rs579737
rs75794200
rs117442179
All − MR Egger
All − IVW(MRE)
−0.010
−0.005
0.000
0.005
0.010
MR effect size for
'Mitochondrial ubiquitin ligase activator of NFKB 1' on 'eGFR'

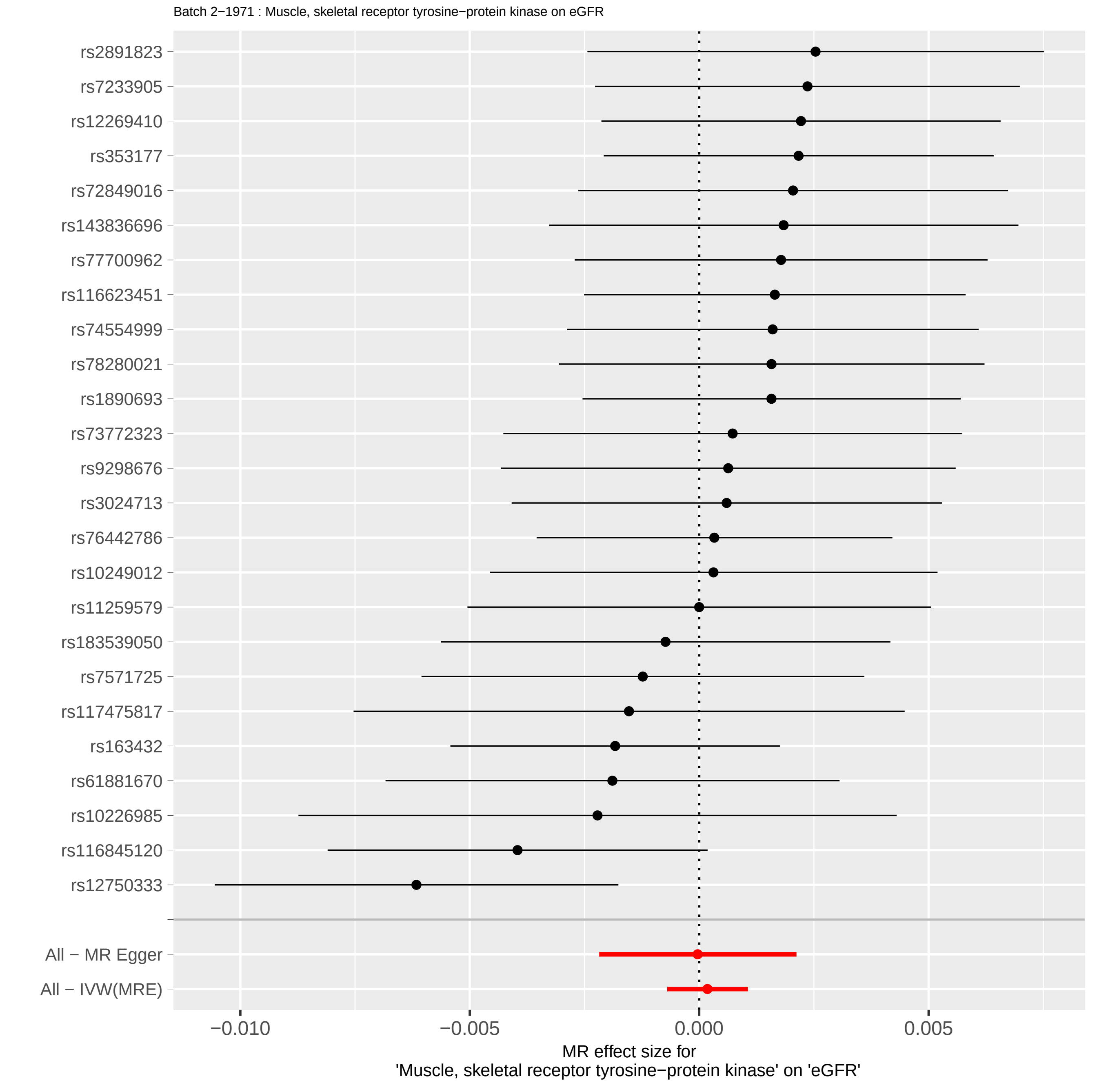

Batch 2−1971 : Muscle, skeletal receptor tyrosine−protein kinase on eGFR
rs2891823
rs7233905
rs12269410
rs353177
rs72849016
rs143836696
rs77700962
rs116623451
rs74554999
rs78280021
rs1890693
rs73772323
rs9298676
rs3024713
rs76442786
rs10249012
rs11259579
rs183539050
rs7571725
rs117475817
rs163432
rs61881670
rs10226985
rs116845120
rs12750333
All − MR Egger
All − IVW(MRE)
−0.010
−0.005
0.000
0.005
MR effect size for
'Muscle, skeletal receptor tyrosine−protein kinase' on 'eGFR'

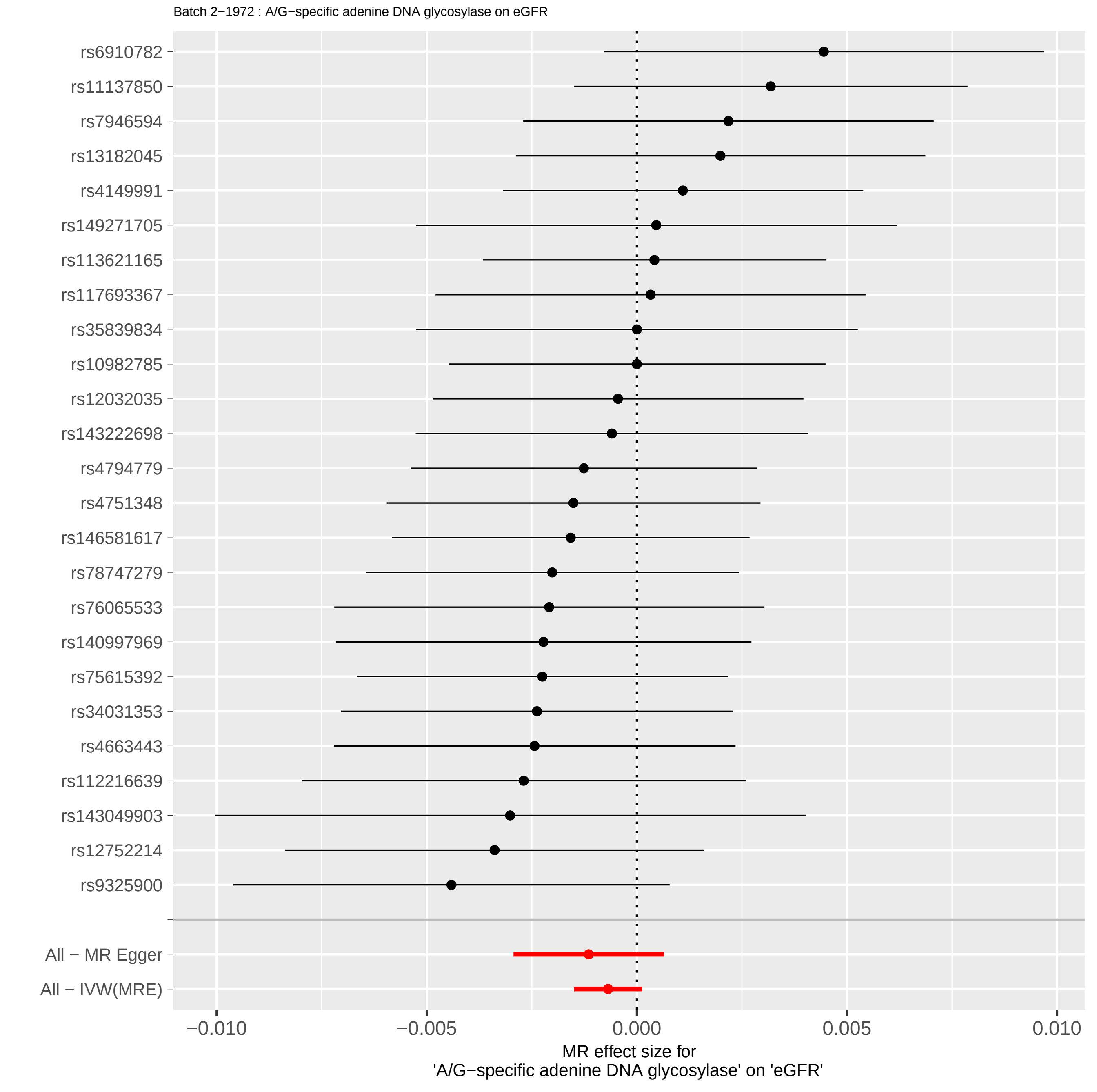
Batch 2−1972 : A/G−specific adenine DNA glycosylase on eGFR
rs6910782
rs11137850
rs7946594
rs13182045
rs4149991
rs149271705
rs113621165
rs117693367
rs35839834
rs10982785
rs12032035
rs143222698
rs4794779
rs4751348
rs146581617
rs78747279
rs76065533
rs140997969
rs75615392
rs34031353
rs4663443
rs112216639
rs143049903
rs12752214
rs9325900
All − MR Egger
All − IVW(MRE)
−0.010
−0.005
0.000
0.005
0.010
MR effect size for
'A/G−specific adenine DNA glycosylase' on 'eGFR'

Batch 2−1973 : MAX−interacting protein 1 on eGFR

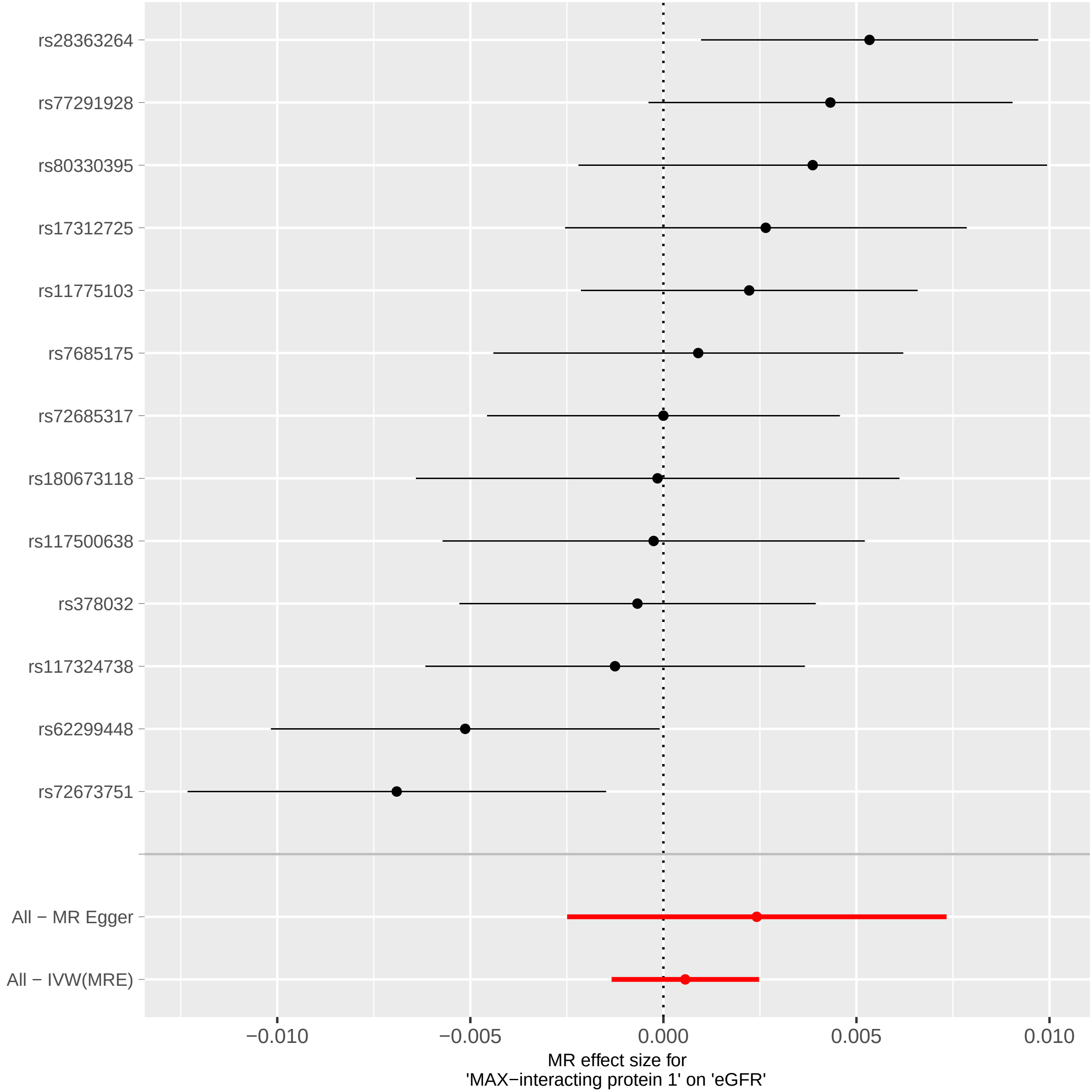

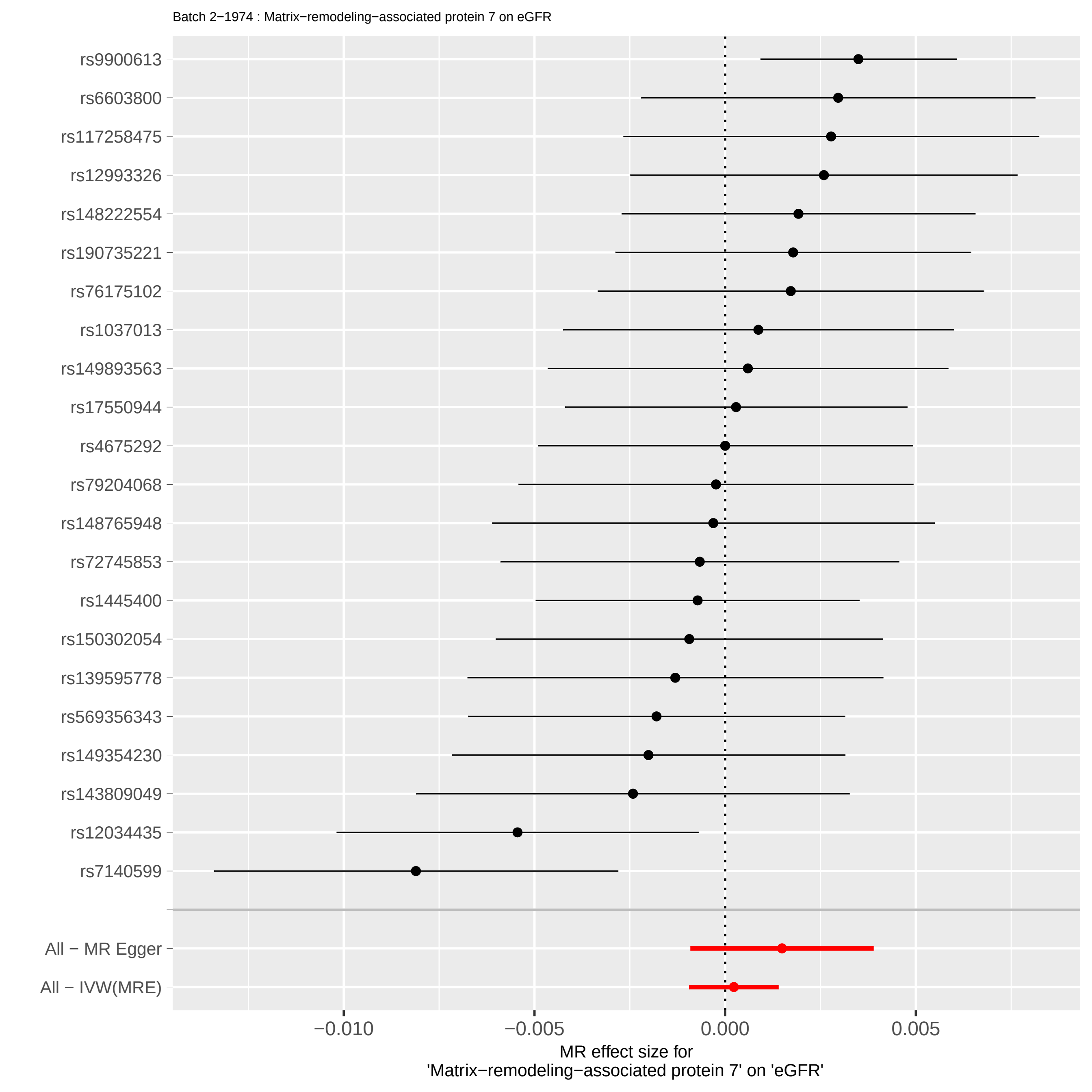
Batch 2−1974 : Matrix−remodeling−associated protein 7 on eGFR
rs9900613
rs6603800
rs117258475
rs12993326
rs148222554
rs190735221
rs76175102
rs1037013
rs149893563
rs17550944
rs4675292
rs79204068
rs148765948
rs72745853
rs1445400
rs150302054
rs139595778
rs569356343
rs149354230
rs143809049
rs12034435
rs7140599
All − MR Egger
All − IVW(MRE)
−0.010
−0.005
0.000
0.005
MR effect size for
'Matrix−remodeling−associated protein 7' on 'eGFR'

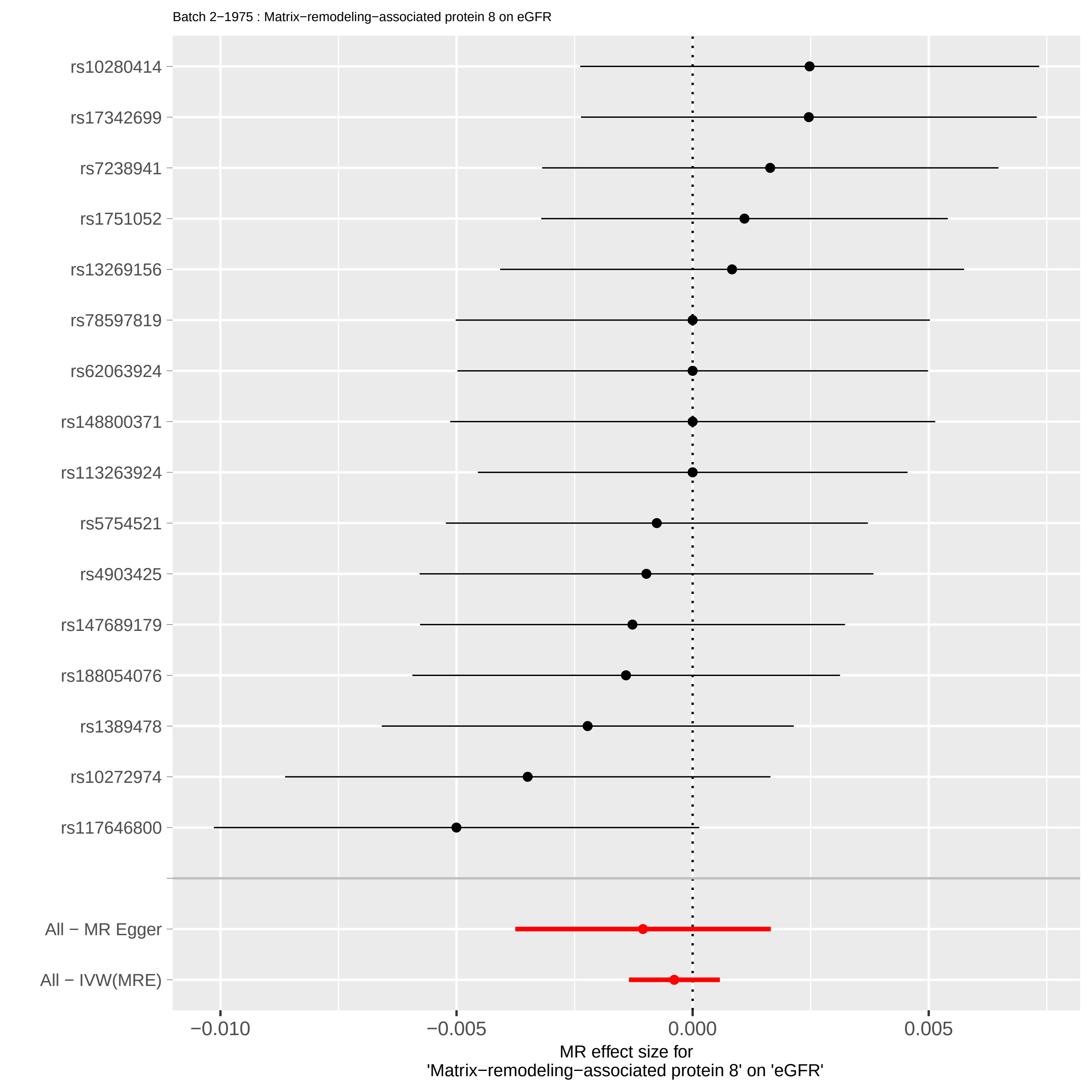

Batch 2−1975 : Matrix−remodeling−associated protein 8 on eGFR
rs10280414
rs17342699
rs7238941
rs1751052
rs13269156
rs78597819
rs62063924
rs148800371
rs113263924
rs5754521
rs4903425
rs147689179
rs188054076
rs1389478
rs10272974
rs117646800
All − MR Egger
All − IVW(MRE)
−0.010
−0.005
0.000
0.005
MR effect size for
'Matrix−remodeling−associated protein 8' on 'eGFR'

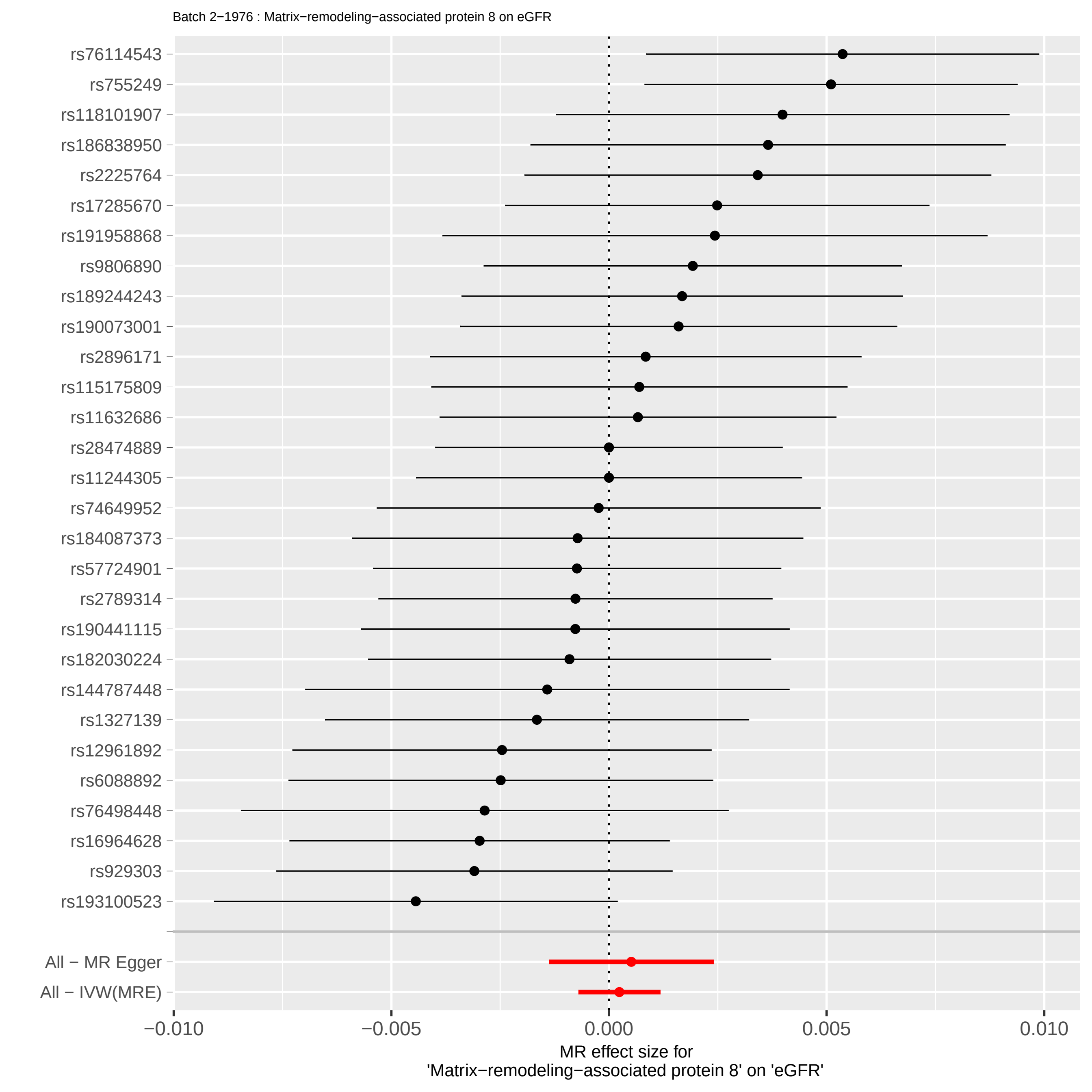
Batch 2−1976 : Matrix−remodeling−associated protein 8 on eGFR
rs76114543
rs755249
rs118101907
rs186838950
rs2225764
rs17285670
rs191958868
rs9806890
rs189244243
rs190073001
rs2896171
rs115175809
rs11632686
rs28474889
rs11244305
rs74649952
rs184087373
rs57724901
rs2789314
rs190441115
rs182030224
rs144787448
rs1327139
rs12961892
rs6088892
rs76498448
rs16964628
rs929303
rs193100523
All − MR Egger
All − IVW(MRE)
−0.010
−0.005
0.000
0.005
0.010
MR effect size for
'Matrix−remodeling−associated protein 8' on 'eGFR'

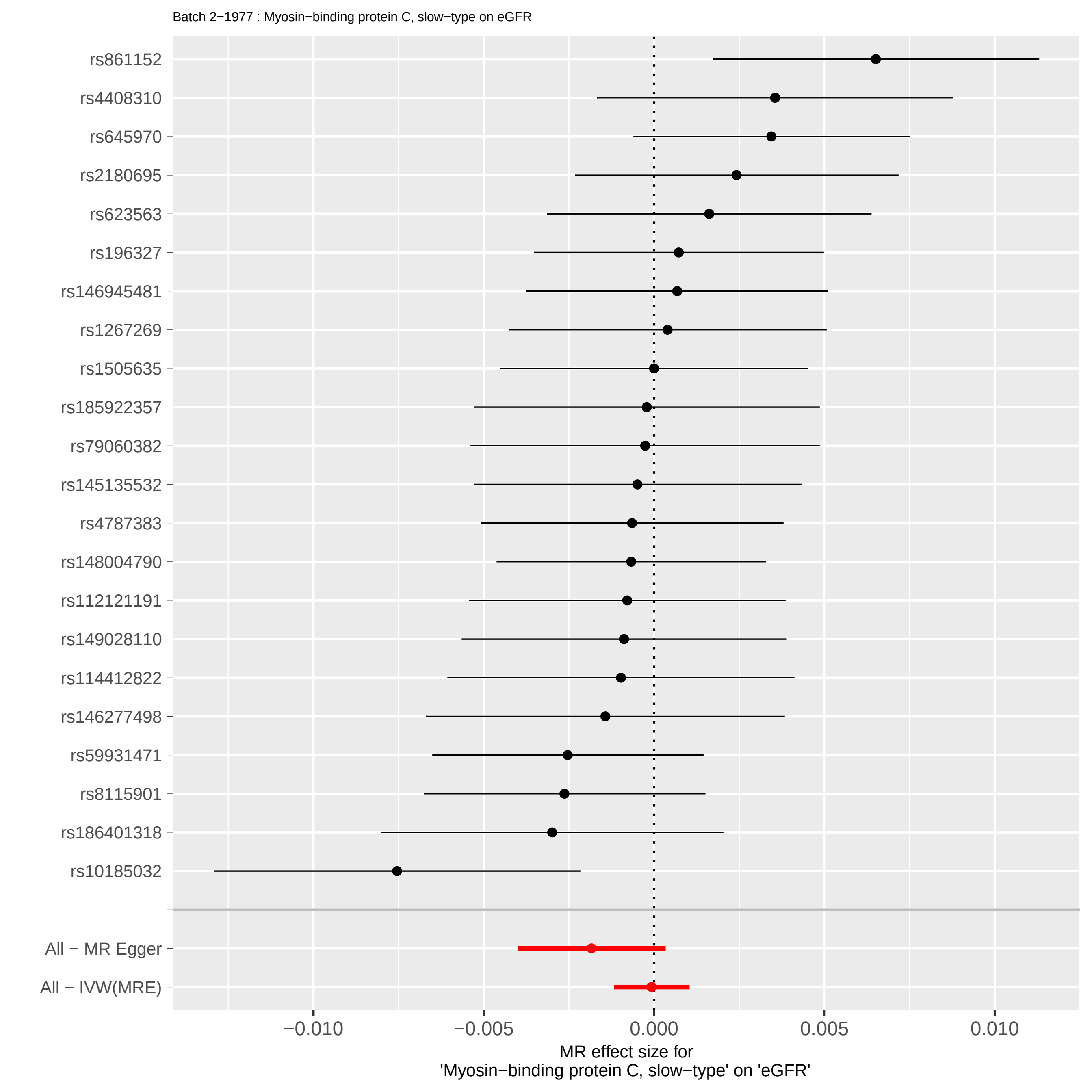

Batch 2−1977 : Myosin−binding protein C, slow−type on eGFR
rs861152
rs4408310
rs645970
rs2180695
rs623563
rs196327
rs146945481
rs1267269
rs1505635
rs185922357
rs79060382
rs145135532
rs4787383
rs148004790
rs112121191
rs149028110
rs114412822
rs146277498
rs59931471
rs8115901
rs186401318
rs10185032
All − MR Egger
All − IVW(MRE)
−0.010
−0.005
0.000
0.005
0.010
MR effect size for
'Myosin−binding protein C, slow−type' on 'eGFR'

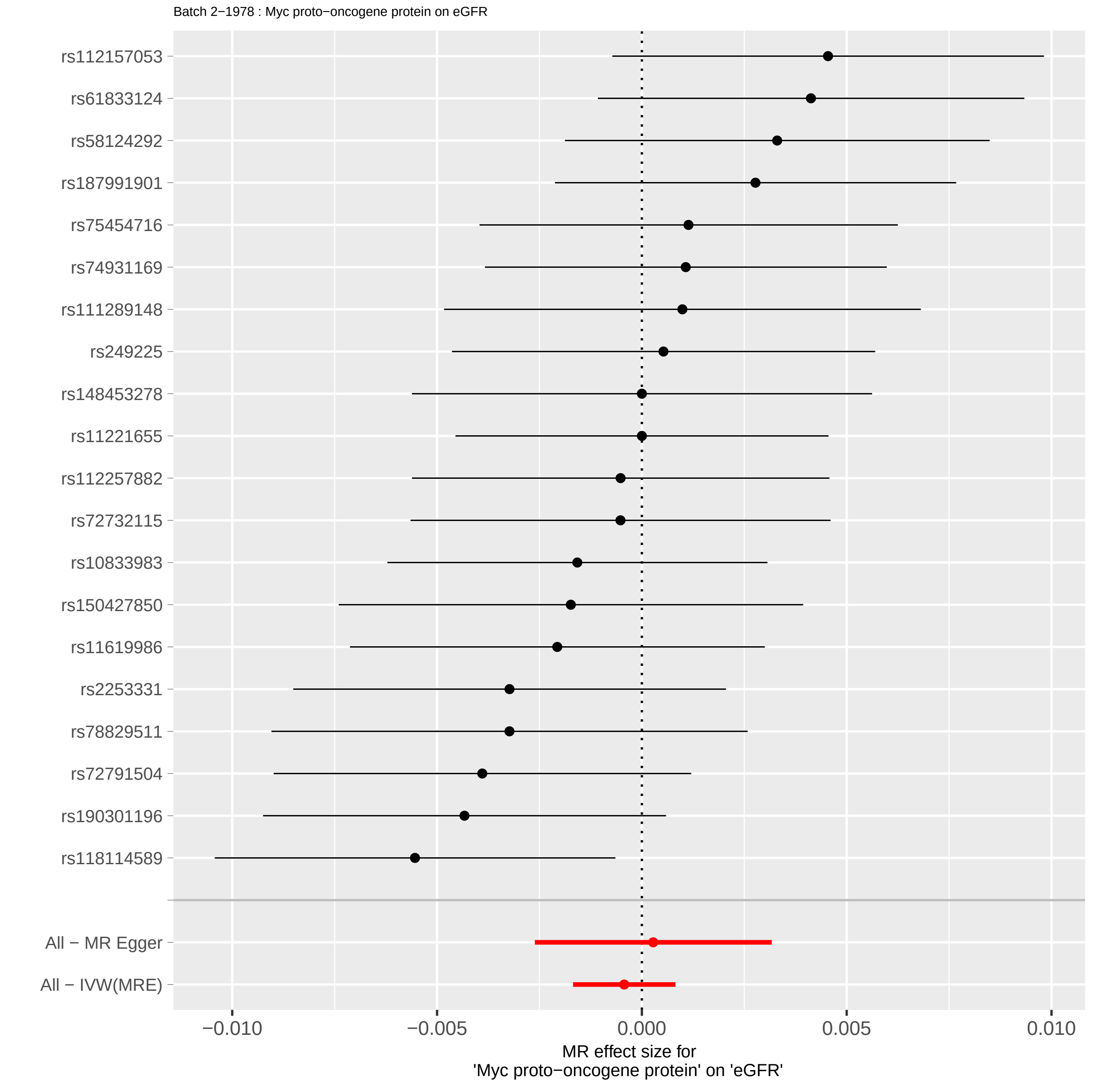

Batch 2−1978 : Myc proto−oncogene protein on eGFR
rs112157053
rs61833124
rs58124292
rs187991901
rs75454716
rs74931169
rs111289148
rs249225
rs148453278
rs11221655
rs112257882
rs72732115
rs10833983
rs150427850
rs11619986
rs2253331
rs78829511
rs72791504
rs190301196
rs118114589
All − MR Egger
All − IVW(MRE)
−0.010
−0.005
0.000
0.005
0.010
MR effect size for
'Myc proto−oncogene protein' on 'eGFR'

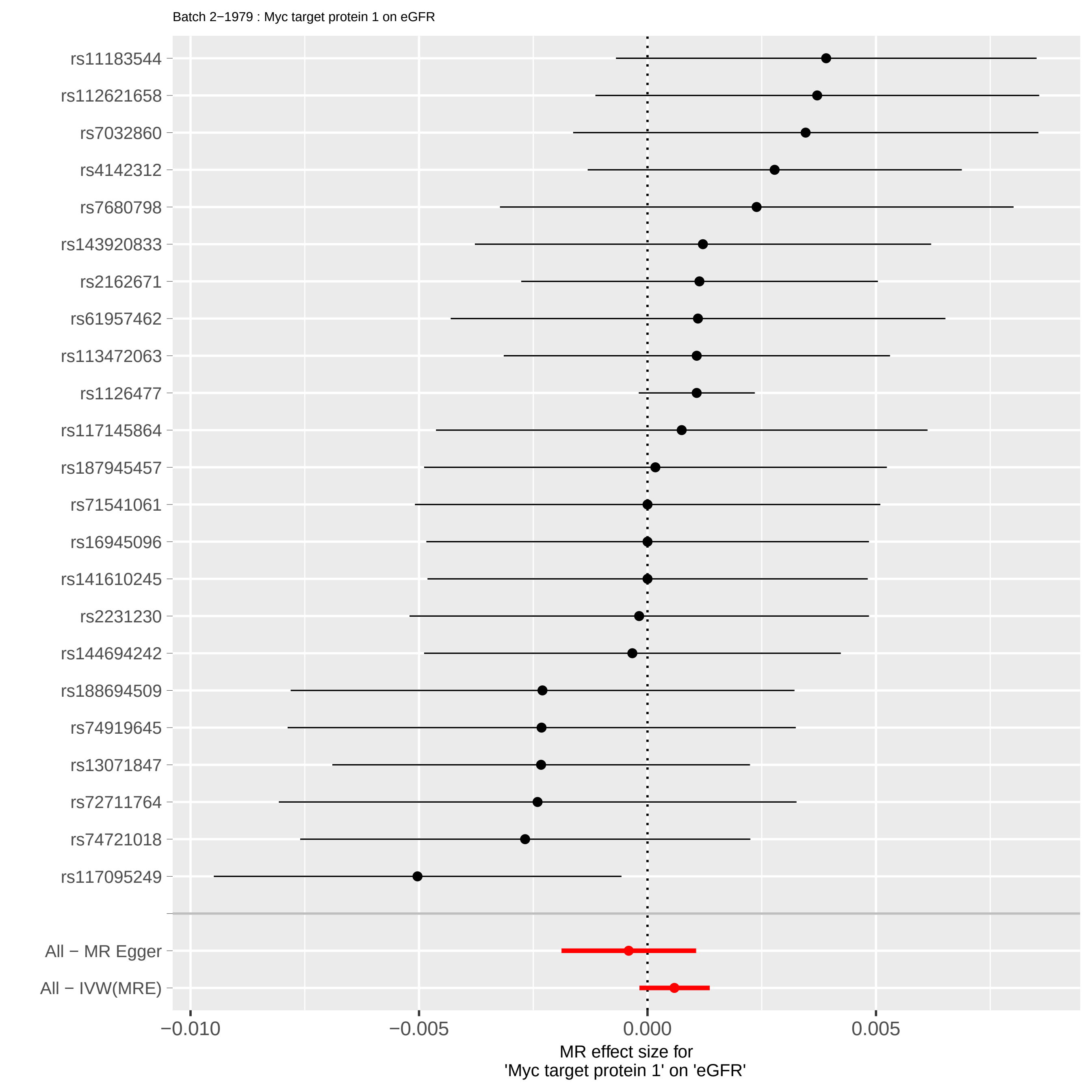

Batch 2−1979 : Myc target protein 1 on eGFR
rs11183544
rs112621658
rs7032860
rs4142312
rs7680798
rs143920833
rs2162671
rs61957462
rs113472063
rs1126477
rs117145864
rs187945457
rs71541061
rs16945096
rs141610245
rs2231230
rs144694242
rs188694509
rs74919645
rs13071847
rs72711764
rs74721018
rs117095249
All − MR Egger
All − IVW(MRE)
−0.010
−0.005
0.000
0.005
MR effect size for
'Myc target protein 1' on 'eGFR'

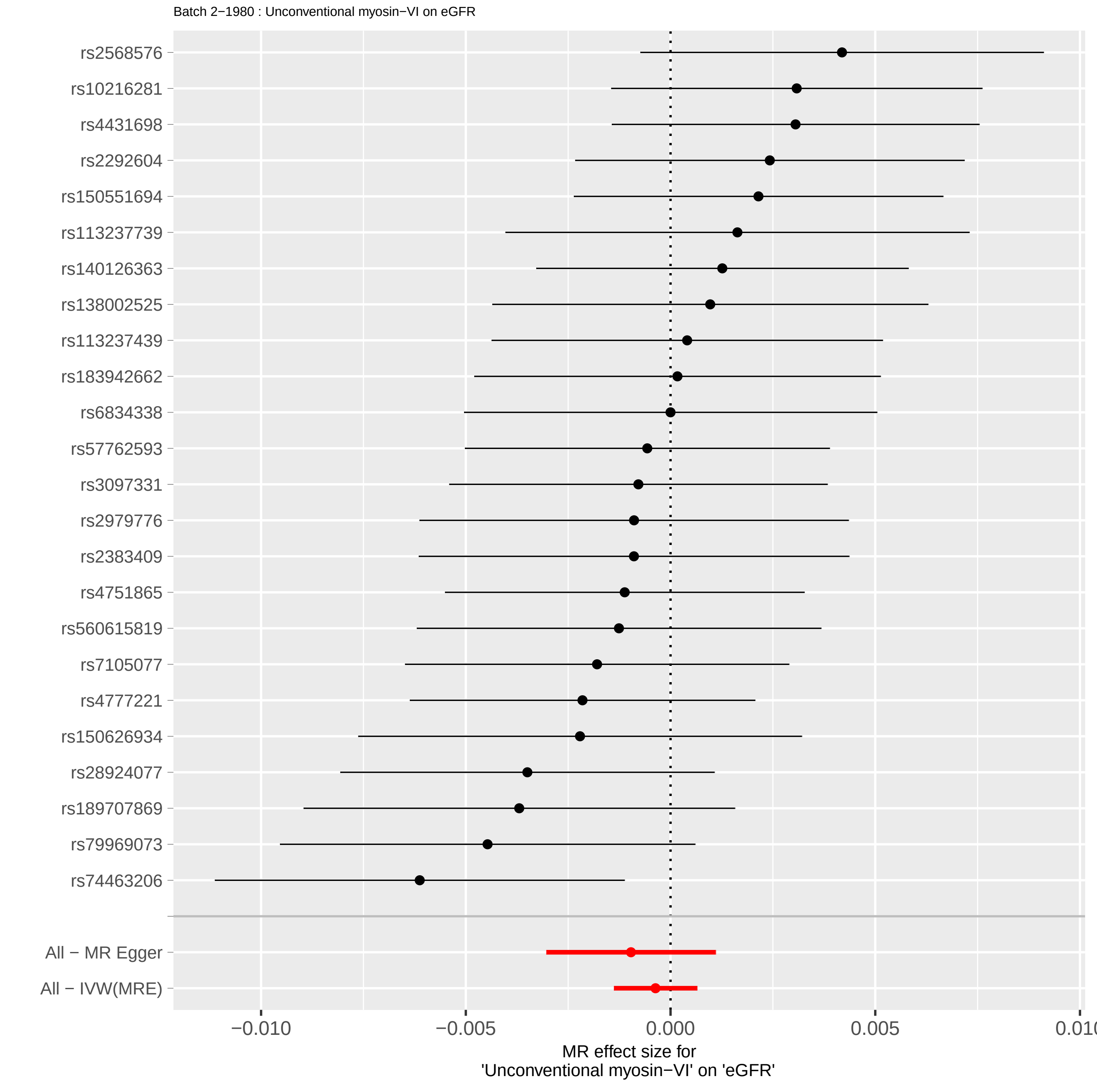

Batch 2−1980 : Unconventional myosin−VI on eGFR
rs2568576
rs10216281
rs4431698
rs2292604
rs150551694
rs113237739
rs140126363
rs138002525
rs113237439
rs183942662
rs6834338
rs57762593
rs3097331
rs2979776
rs2383409
rs4751865
rs560615819
rs7105077
rs4777221
rs150626934
rs28924077
rs189707869
rs79969073
rs74463206
All − MR Egger
All − IVW(MRE)
−0.010
−0.005
0.000
0.005
MR effect size for
'Unconventional myosin−VI' on 'eGFR'

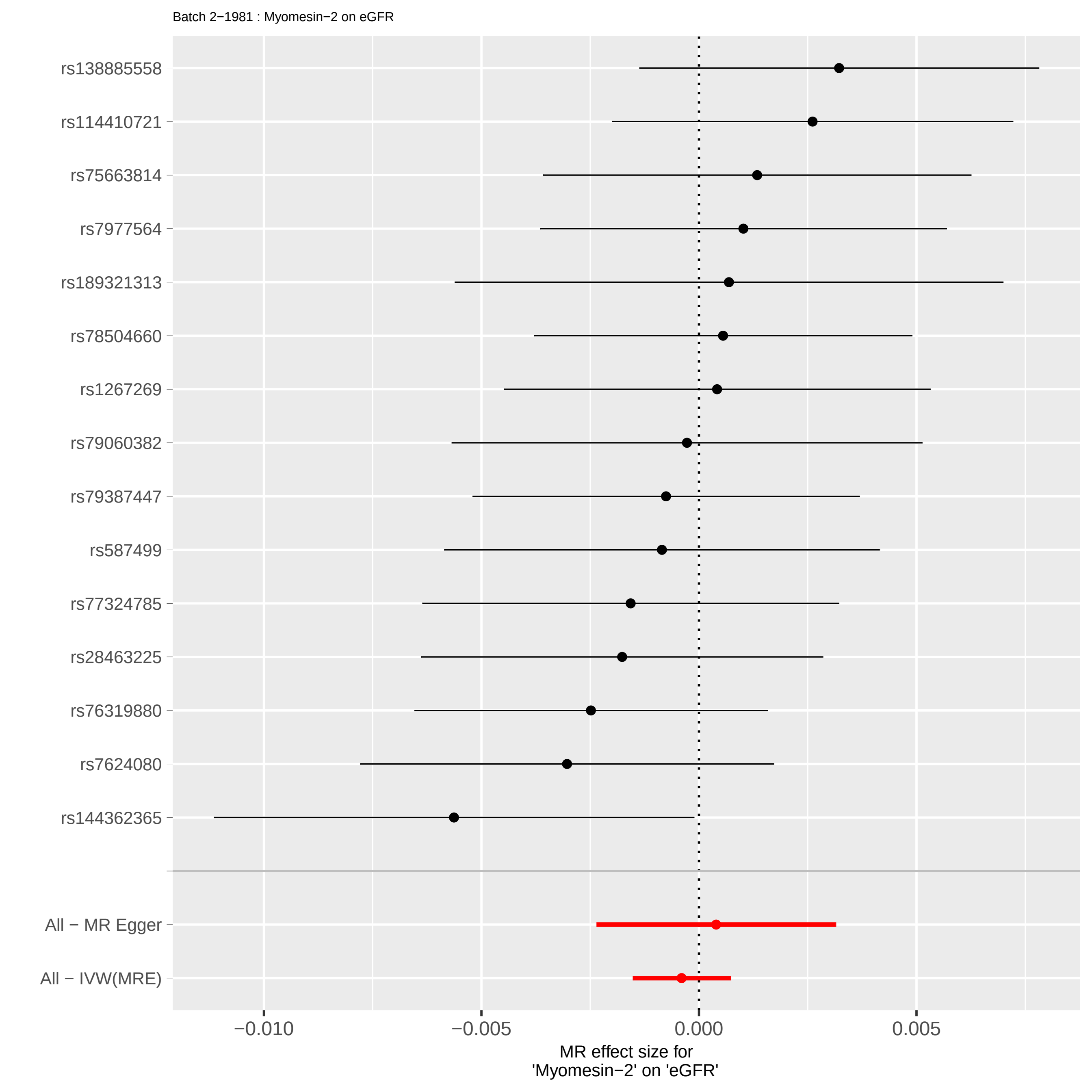
Batch 2−1981 : Myomesin−2 on eGFR
rs138885558
rs114410721
rs75663814
rs7977564
rs189321313
rs78504660
rs1267269
rs79060382
rs79387447
rs587499
rs77324785
rs28463225
rs76319880
rs7624080
rs144362365
All − MR Egger
All − IVW(MRE)
−0.010
−0.005
0.000
0.005
MR effect size for
'Myomesin−2' on 'eGFR'

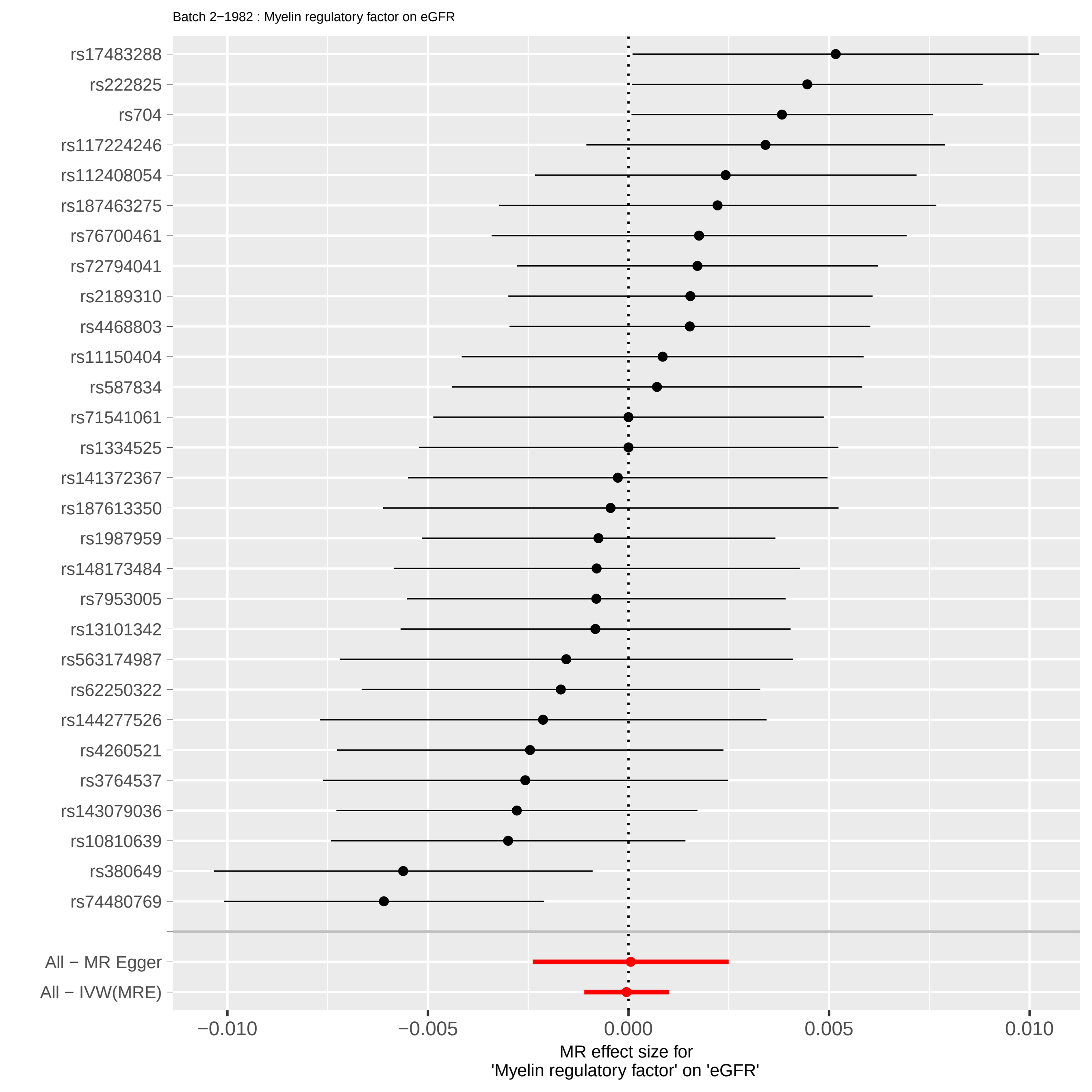
Batch 2−1982 : Myelin regulatory factor on eGFR
rs17483288
rs222825
rs704
rs117224246
rs112408054
rs187463275
rs76700461
rs72794041
rs2189310
rs4468803
rs11150404
rs587834
rs71541061
rs1334525
rs141372367
rs187613350
rs1987959
rs148173484
rs7953005
rs13101342
rs563174987
rs62250322
rs144277526
rs4260521
rs3764537
rs143079036
rs10810639
rs380649
rs74480769
All − MR Egger
All − IVW(MRE)
−0.010
−0.005
0.000
0.005
0.010
MR effect size for
'Myelin regulatory factor' on 'eGFR'

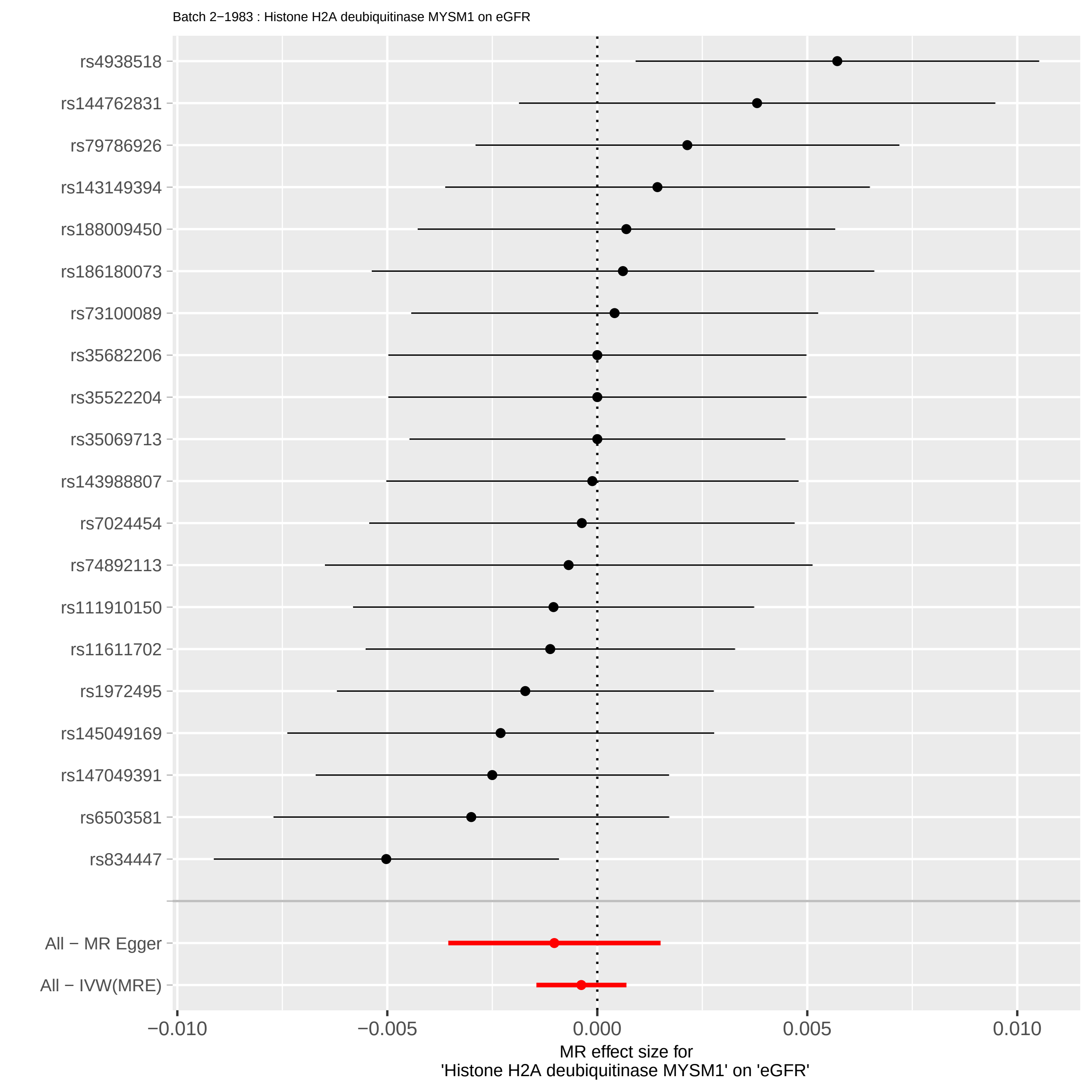

Batch 2−1983 : Histone H2A deubiquitinase MYSM1 on eGFR
rs4938518
rs144762831
rs79786926
rs143149394
rs188009450
rs186180073
rs73100089
rs35682206
rs35522204
rs35069713
rs143988807
rs7024454
rs74892113
rs111910150
rs11611702
rs1972495
rs145049169
rs147049391
rs6503581
rs834447
All − MR Egger
All − IVW(MRE)
−0.010
−0.005
0.000
0.005
0.010
MR effect size for
'Histone H2A deubiquitinase MYSM1' on 'eGFR'

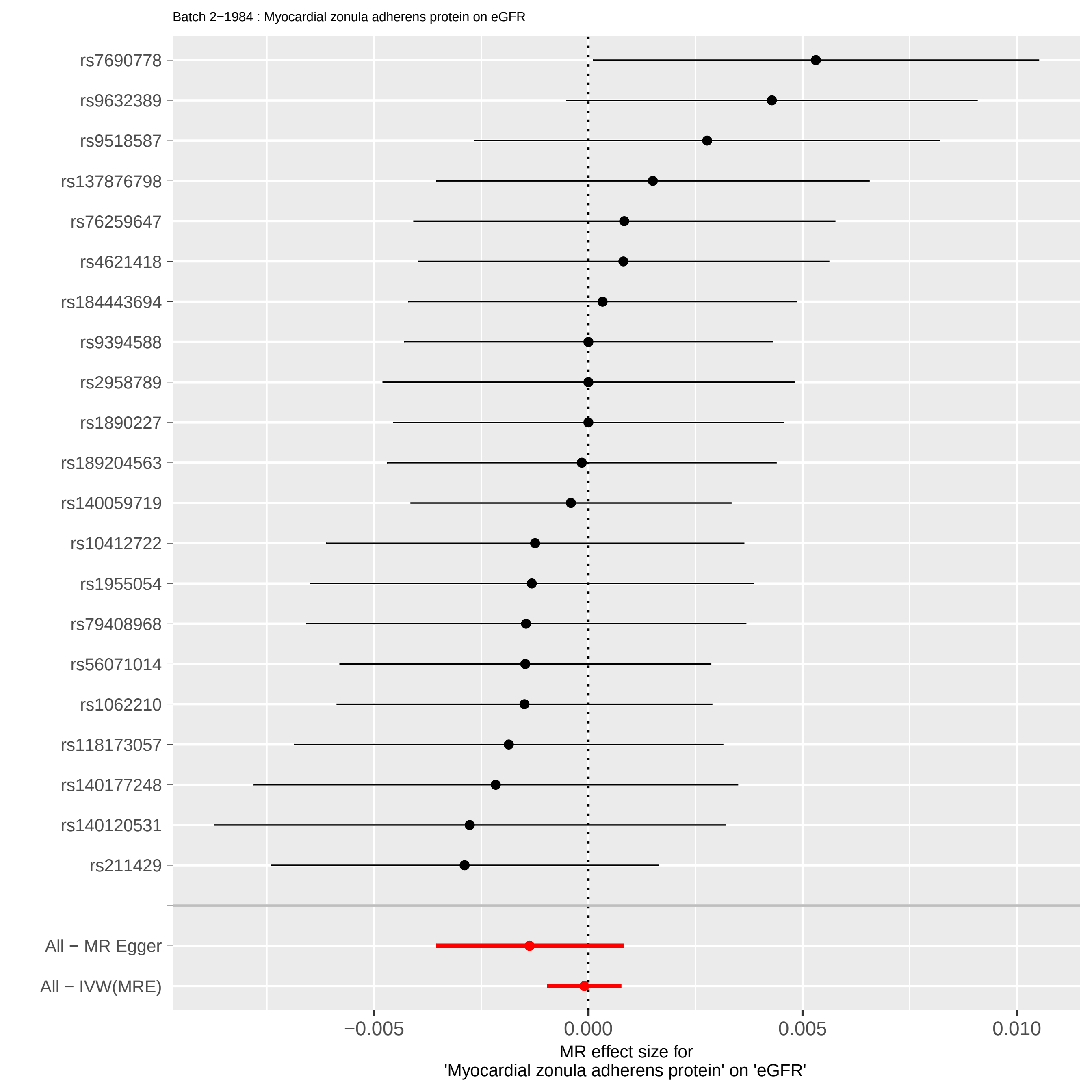

Batch 2−1984 : Myocardial zonula adherens protein on eGFR
rs7690778
rs9632389
rs9518587
rs137876798
rs76259647
rs4621418
rs184443694
rs9394588
rs2958789
rs1890227
rs189204563
rs140059719
rs10412722
rs1955054
rs79408968
rs56071014
rs1062210
rs118173057
rs140177248
rs140120531
rs211429
All − MR Egger
All − IVW(MRE)
−0.005
0.000
0.005
0.010
MR effect size for
'Myocardial zonula adherens protein' on 'eGFR'

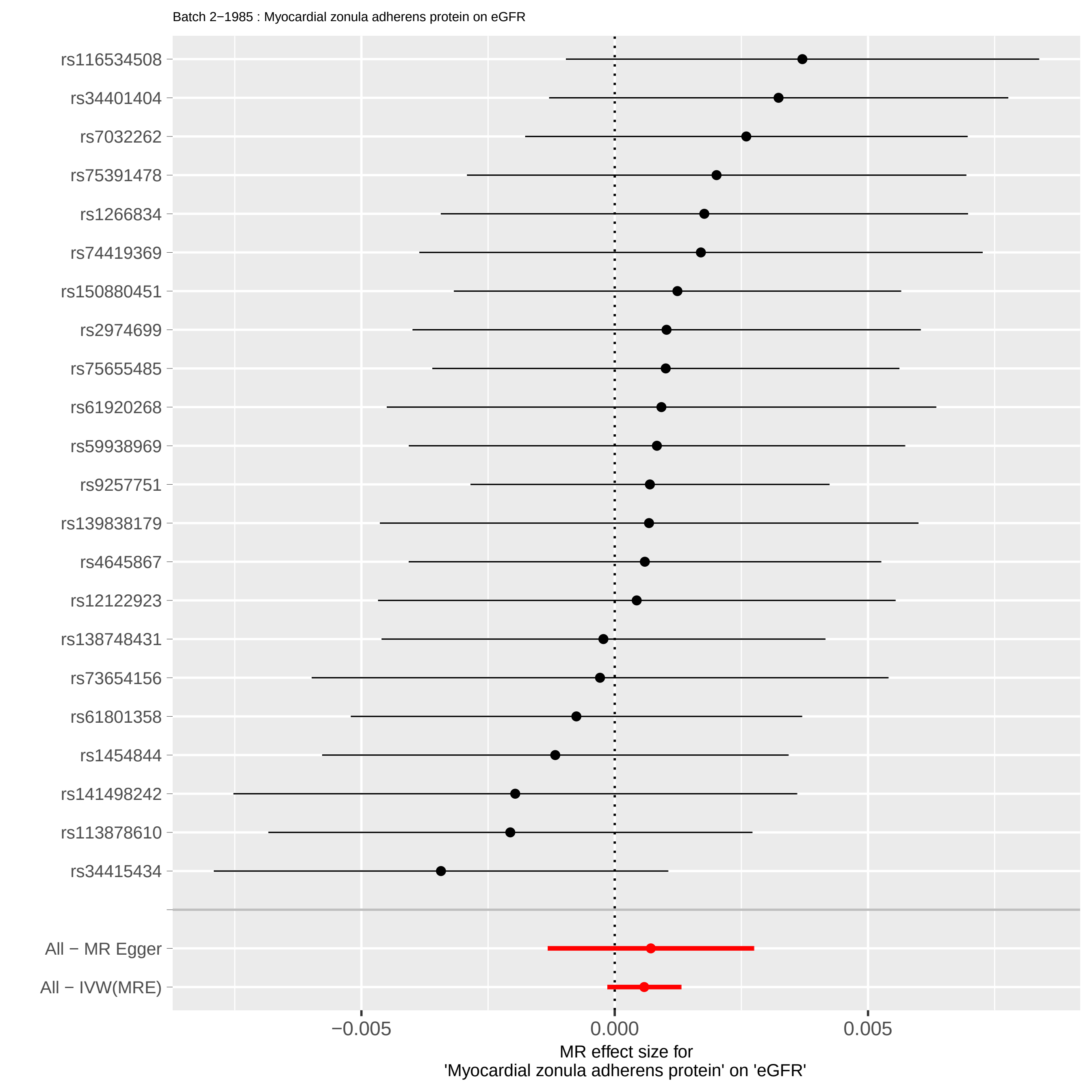

Batch 2−1985 : Myocardial zonula adherens protein on eGFR
rs116534508
rs34401404
rs7032262
rs75391478
rs1266834
rs74419369
rs150880451
rs2974699
rs75655485
rs61920268
rs59938969
rs9257751
rs139838179
rs4645867
rs12122923
rs138748431
rs73654156
rs61801358
rs1454844
rs141498242
rs113878610
rs34415434
All − MR Egger
All − IVW(MRE)
−0.005
0.000
0.005
MR effect size for
'Myocardial zonula adherens protein' on 'eGFR'

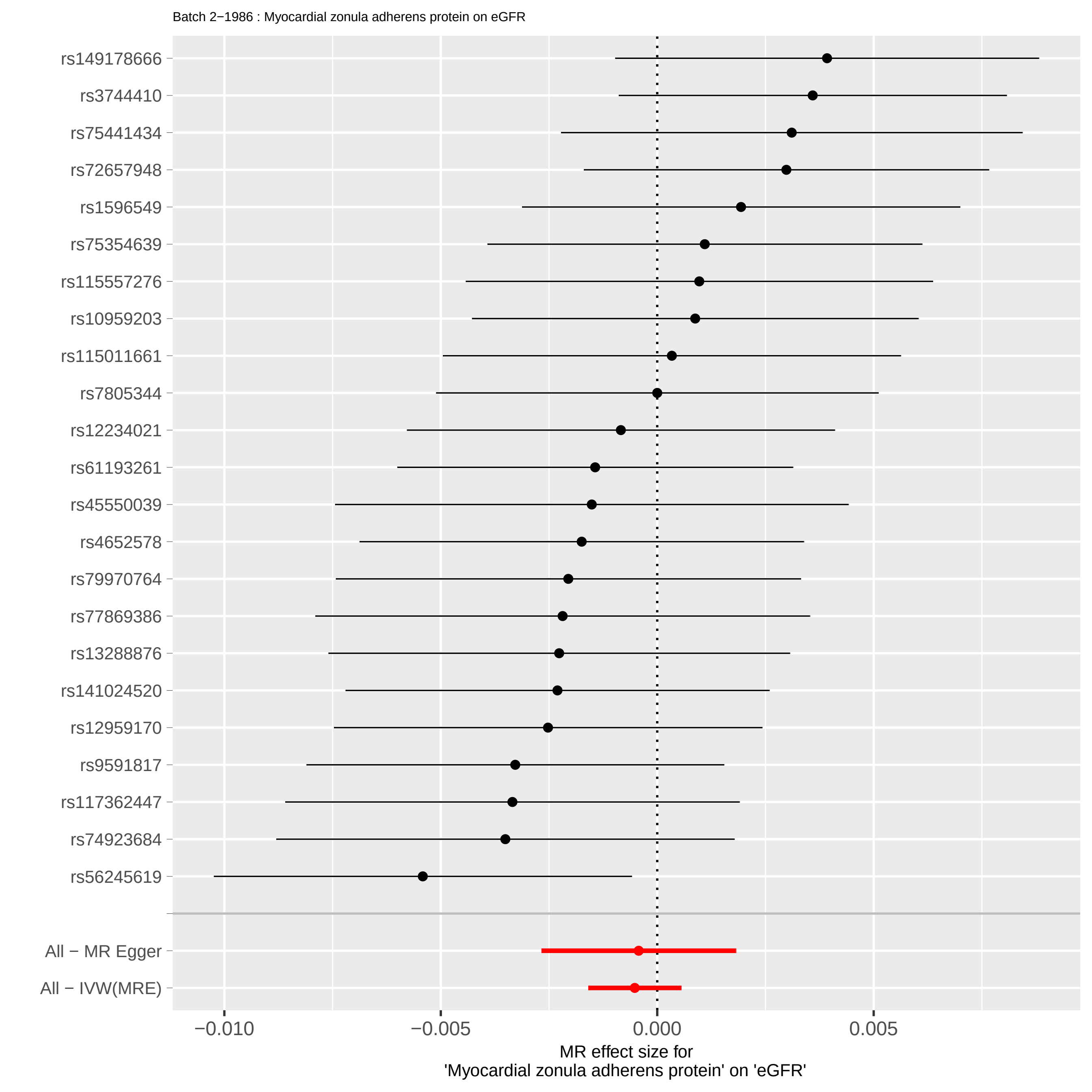

Batch 2−1986 : Myocardial zonula adherens protein on eGFR
rs149178666
rs3744410
rs75441434
rs72657948
rs1596549
rs75354639
rs115557276
rs10959203
rs115011661
rs7805344
rs12234021
rs61193261
rs45550039
rs4652578
rs79970764
rs77869386
rs13288876
rs141024520
rs12959170
rs9591817
rs117362447
rs74923684
rs56245619
All − MR Egger
All − IVW(MRE)
−0.010
−0.005
0.000
0.005
MR effect size for
'Myocardial zonula adherens protein' on 'eGFR'

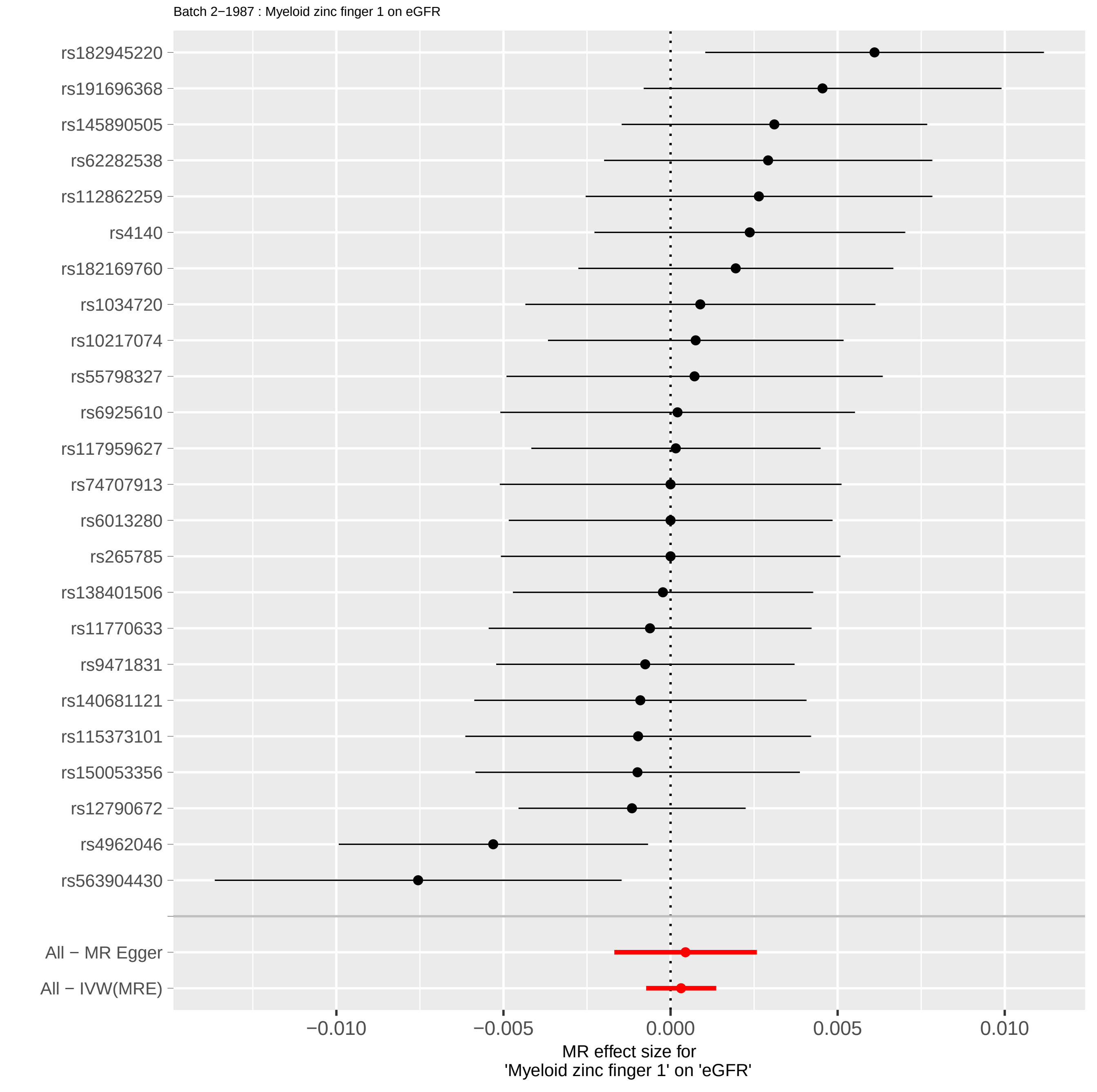
Batch 2−1987 : Myeloid zinc finger 1 on eGFR
rs182945220
rs191696368
rs145890505
rs62282538
rs112862259
rs4140
rs182169760
rs1034720
rs10217074
rs55798327
rs6925610
rs117959627
rs74707913
rs6013280
rs265785
rs138401506
rs11770633
rs9471831
rs140681121
rs115373101
rs150053356
rs12790672
rs4962046
rs563904430
All − MR Egger
All − IVW(MRE)
−0.010
−0.005
0.000
0.005
0.010
MR effect size for
'Myeloid zinc finger 1' on 'eGFR'

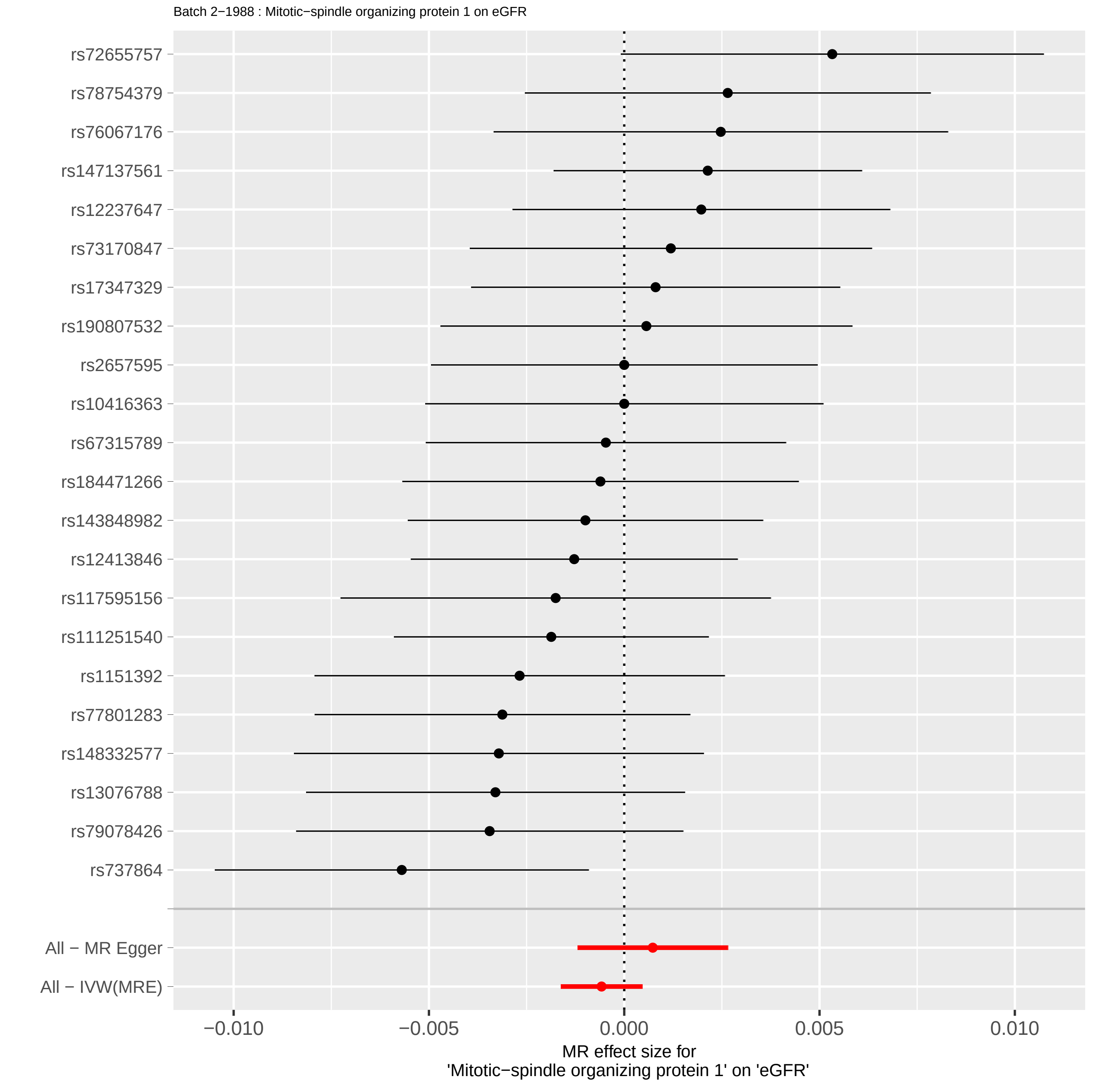
Batch 2−1988 : Mitotic−spindle organizing protein 1 on eGFR
rs72655757
rs78754379
rs76067176
rs147137561
rs12237647
rs73170847
rs17347329
rs190807532
rs2657595
rs10416363
rs67315789
rs184471266
rs143848982
rs12413846
rs117595156
rs111251540
rs1151392
rs77801283
rs148332577
rs13076788
rs79078426
rs737864
All − MR Egger
All − IVW(MRE)
−0.010
−0.005
0.000
0.005
0.010
MR effect size for
'Mitotic−spindle organizing protein 1' on 'eGFR'

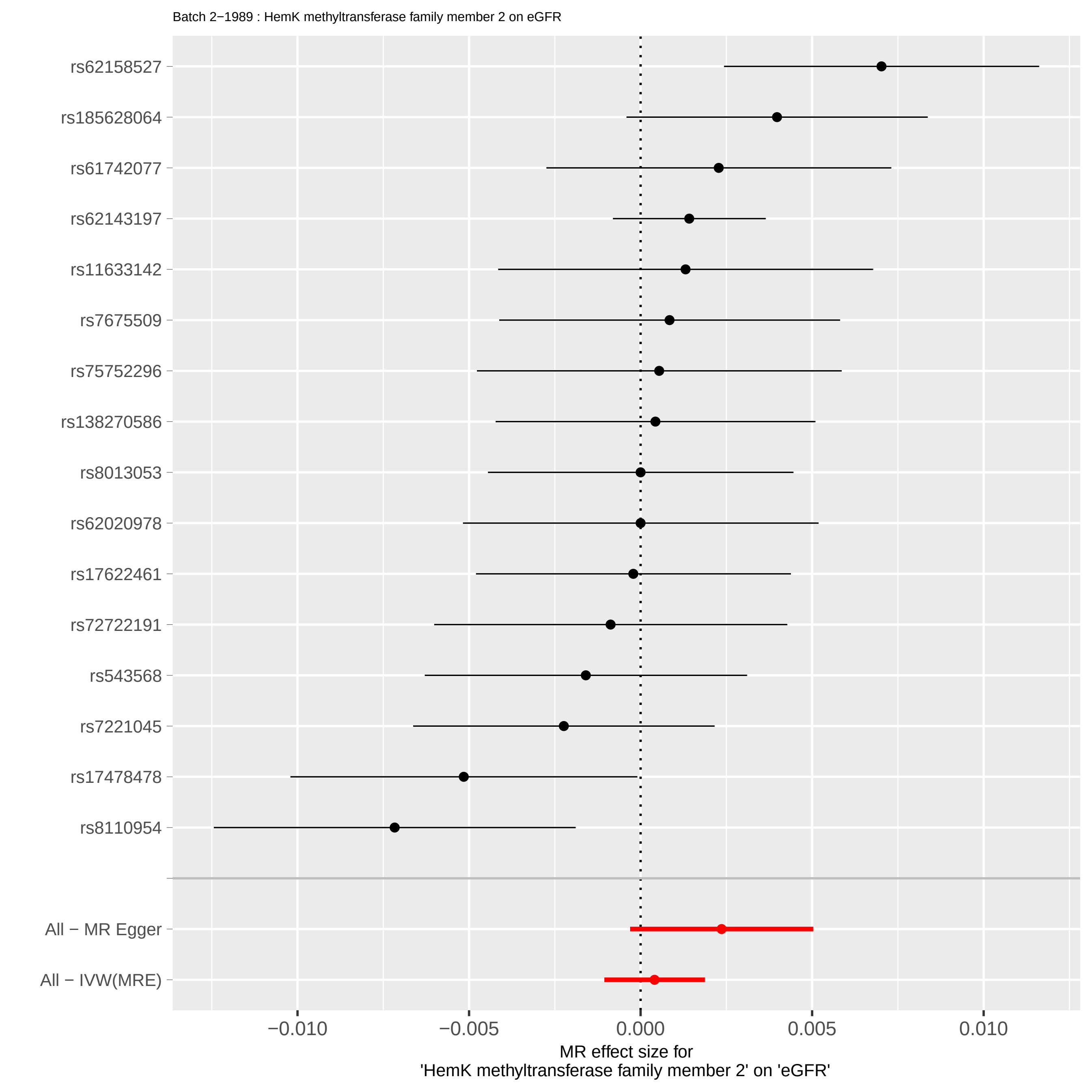

Batch 2−1989 : HemK methyltransferase family member 2 on eGFR
rs62158527
rs185628064
rs61742077
rs62143197
rs11633142
rs7675509
rs75752296
rs138270586
rs8013053
rs62020978
rs17622461
rs72722191
rs543568
rs7221045
rs17478478
rs8110954
All − MR Egger
All − IVW(MRE)
−0.010
−0.005
0.000
0.005
0.010
MR effect size for
'HemK methyltransferase family member 2' on 'eGFR'

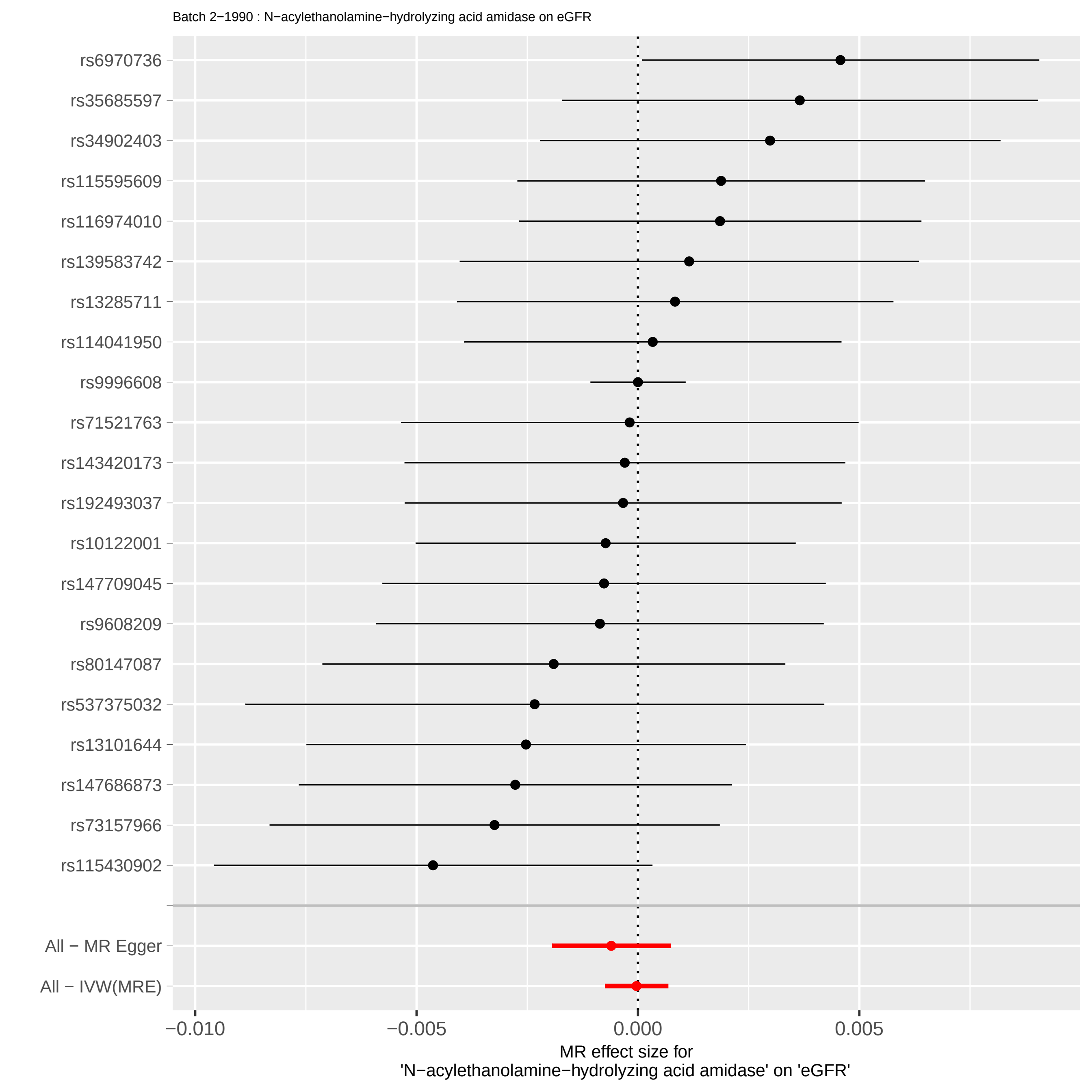

Batch 2−1990 : N−acylethanolamine−hydrolyzing acid amidase on eGFR
rs6970736
rs35685597
rs34902403
rs115595609
rs116974010
rs139583742
rs13285711
rs114041950
rs9996608
rs71521763
rs143420173
rs192493037
rs10122001
rs147709045
rs9608209
rs80147087
rs537375032
rs13101644
rs147686873
rs73157966
rs115430902
All − MR Egger
All − IVW(MRE)
−0.010
−0.005
0.000
0.005
MR effect size for
'N−acylethanolamine−hydrolyzing acid amidase' on 'eGFR'

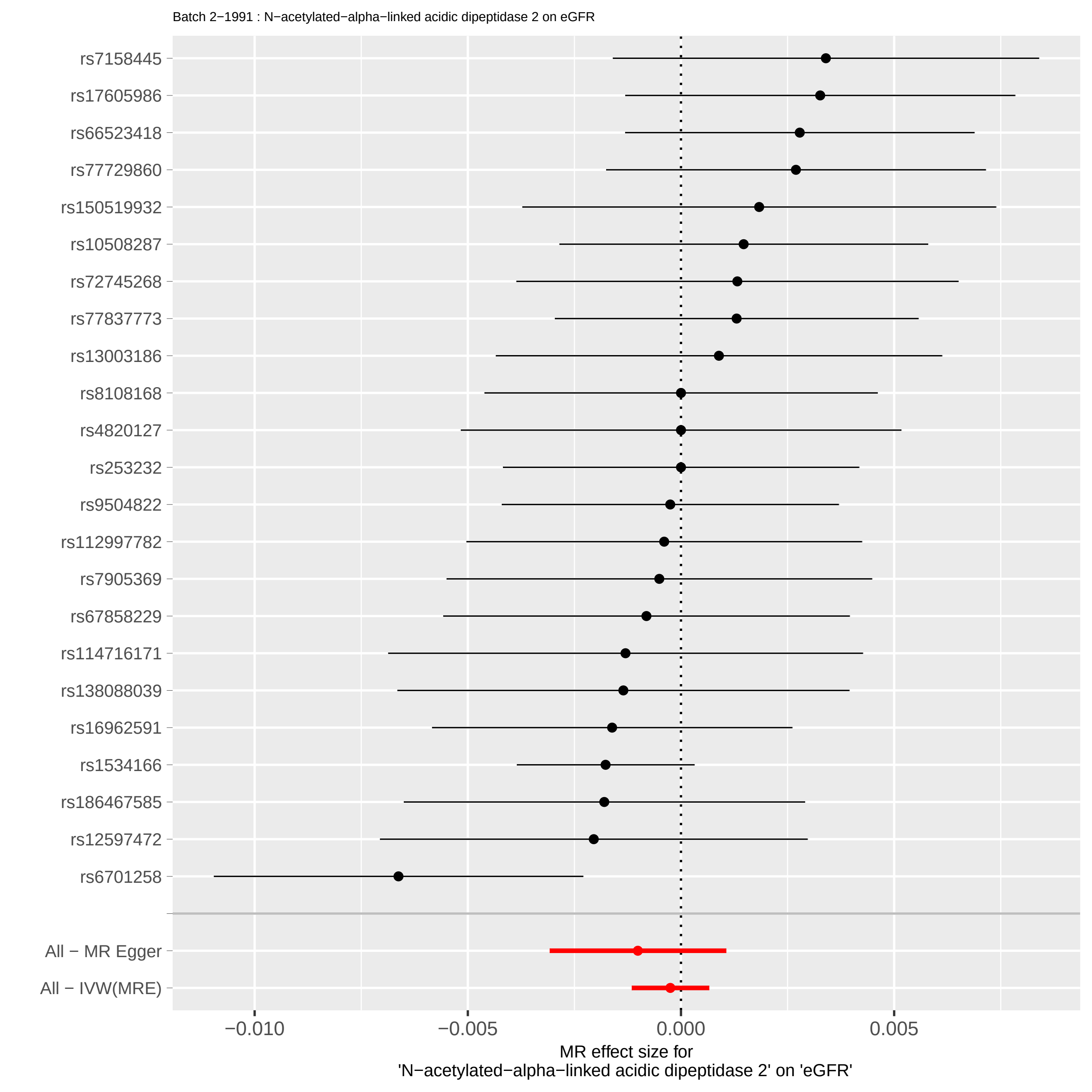

Batch 2−1991 : N−acetylated−alpha−linked acidic dipeptidase 2 on eGFR
rs7158445
rs17605986
rs66523418
rs77729860
rs150519932
rs10508287
rs72745268
rs77837773
rs13003186
rs8108168
rs4820127
rs253232
rs9504822
rs112997782
rs7905369
rs67858229
rs114716171
rs138088039
rs16962591
rs1534166
rs186467585
rs12597472
rs6701258
All − MR Egger
All − IVW(MRE)
−0.010
−0.005
0.000
0.005
MR effect size for
'N−acetylated−alpha−linked acidic dipeptidase 2' on 'eGFR'

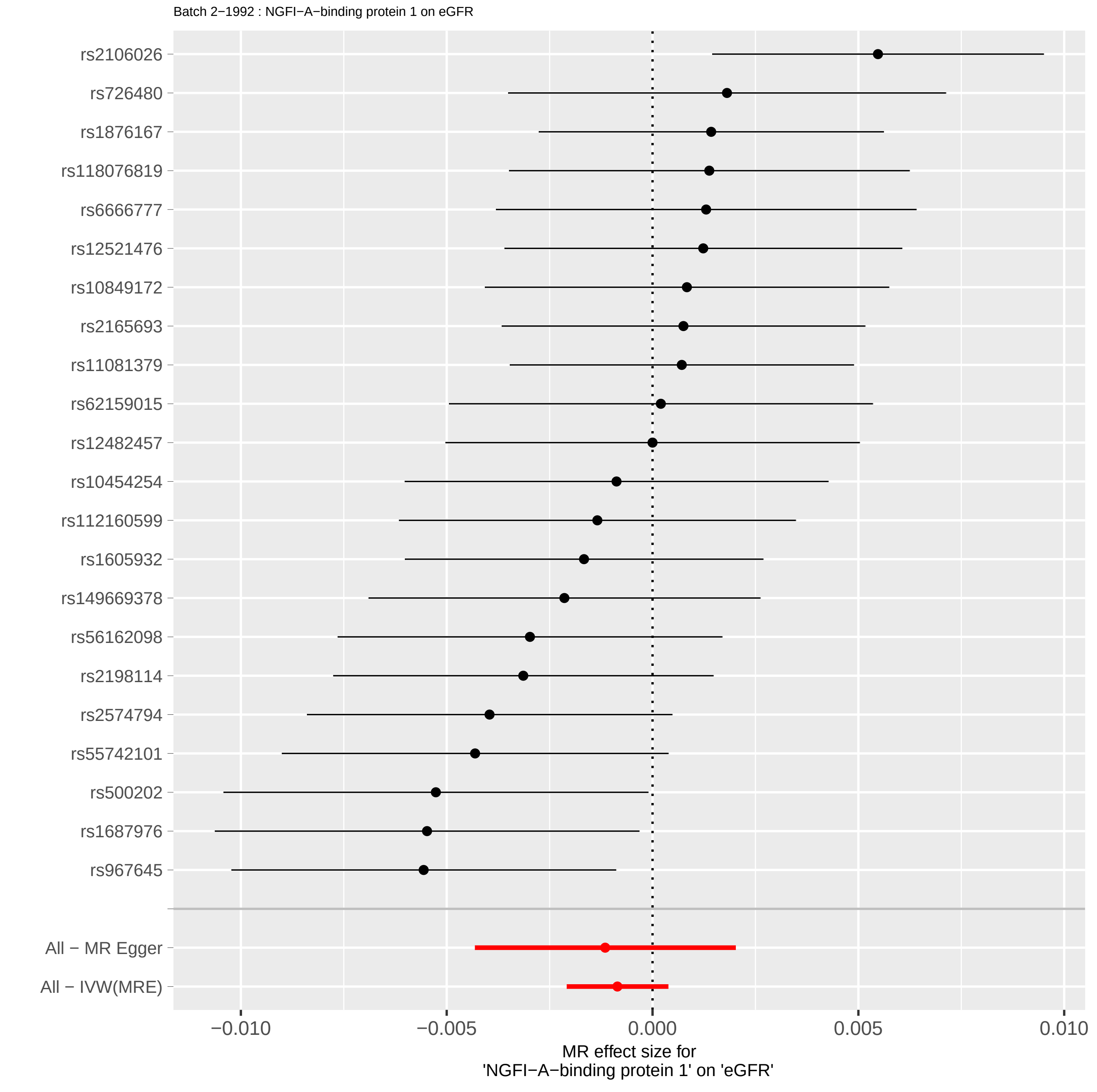

Batch 2−1992 : NGFI−A−binding protein 1 on eGFR
rs2106026
rs726480
rs1876167
rs118076819
rs6666777
rs12521476
rs10849172
rs2165693
rs11081379
rs62159015
rs12482457
rs10454254
rs112160599
rs1605932
rs149669378
rs56162098
rs2198114
rs2574794
rs55742101
rs500202
rs1687976
rs967645
All − MR Egger
All − IVW(MRE)
−0.010
−0.005
0.000
0.005
0.010
MR effect size for
'NGFI−A−binding protein 1' on 'eGFR'

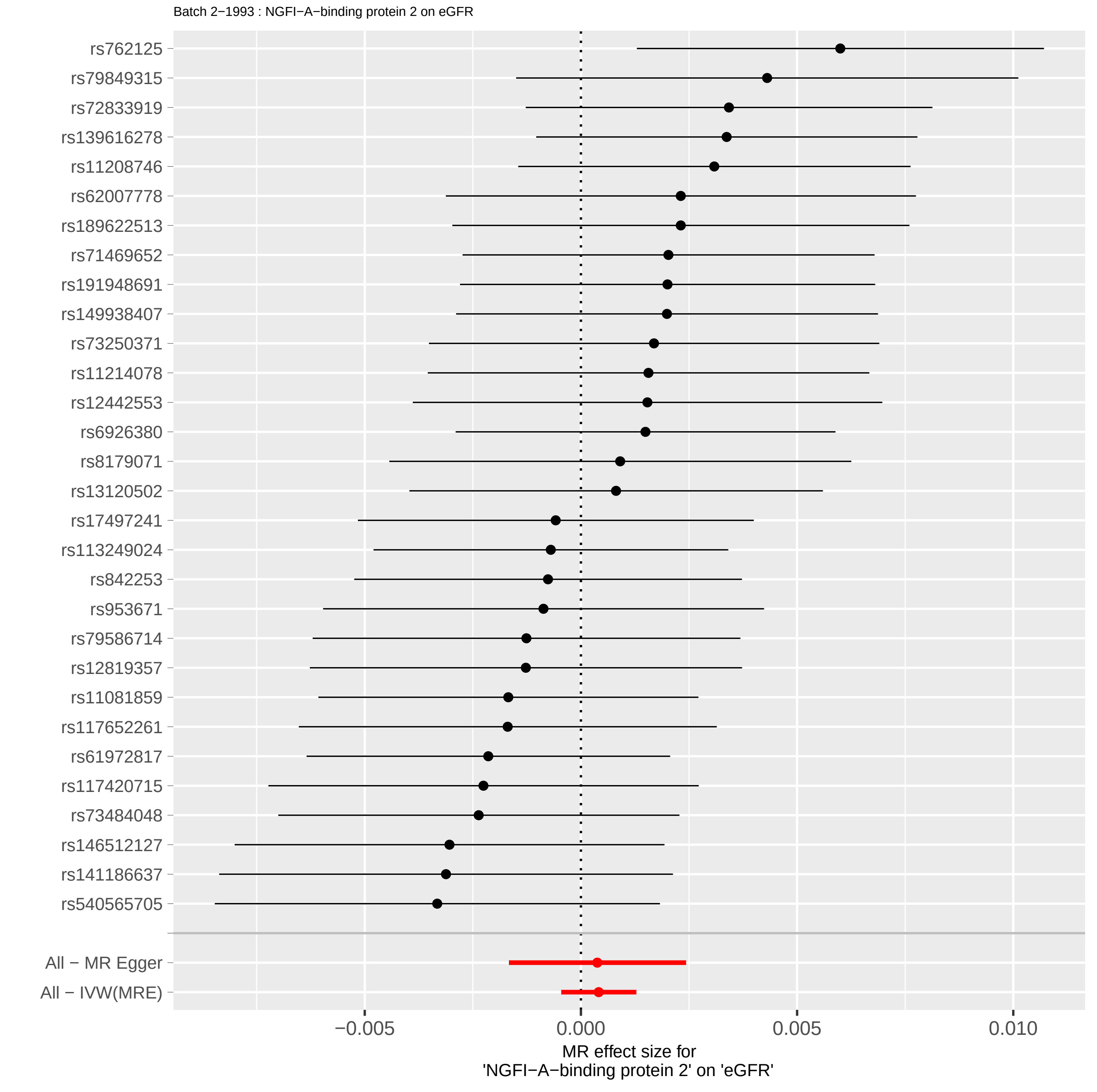
Batch 2−1993 : NGFI−A−binding protein 2 on eGFR
rs762125
rs79849315
rs72833919
rs139616278
rs11208746
rs62007778
rs189622513
rs71469652
rs191948691
rs149938407
rs73250371
rs11214078
rs12442553
rs6926380
rs8179071
rs13120502
rs17497241
rs113249024
rs842253
rs953671
rs79586714
rs12819357
rs11081859
rs117652261
rs61972817
rs117420715
rs73484048
rs146512127
rs141186637
rs540565705
All − MR Egger
All − IVW(MRE)
−0.005
0.000
0.005
0.010
MR effect size for
'NGFI−A−binding protein 2' on 'eGFR'

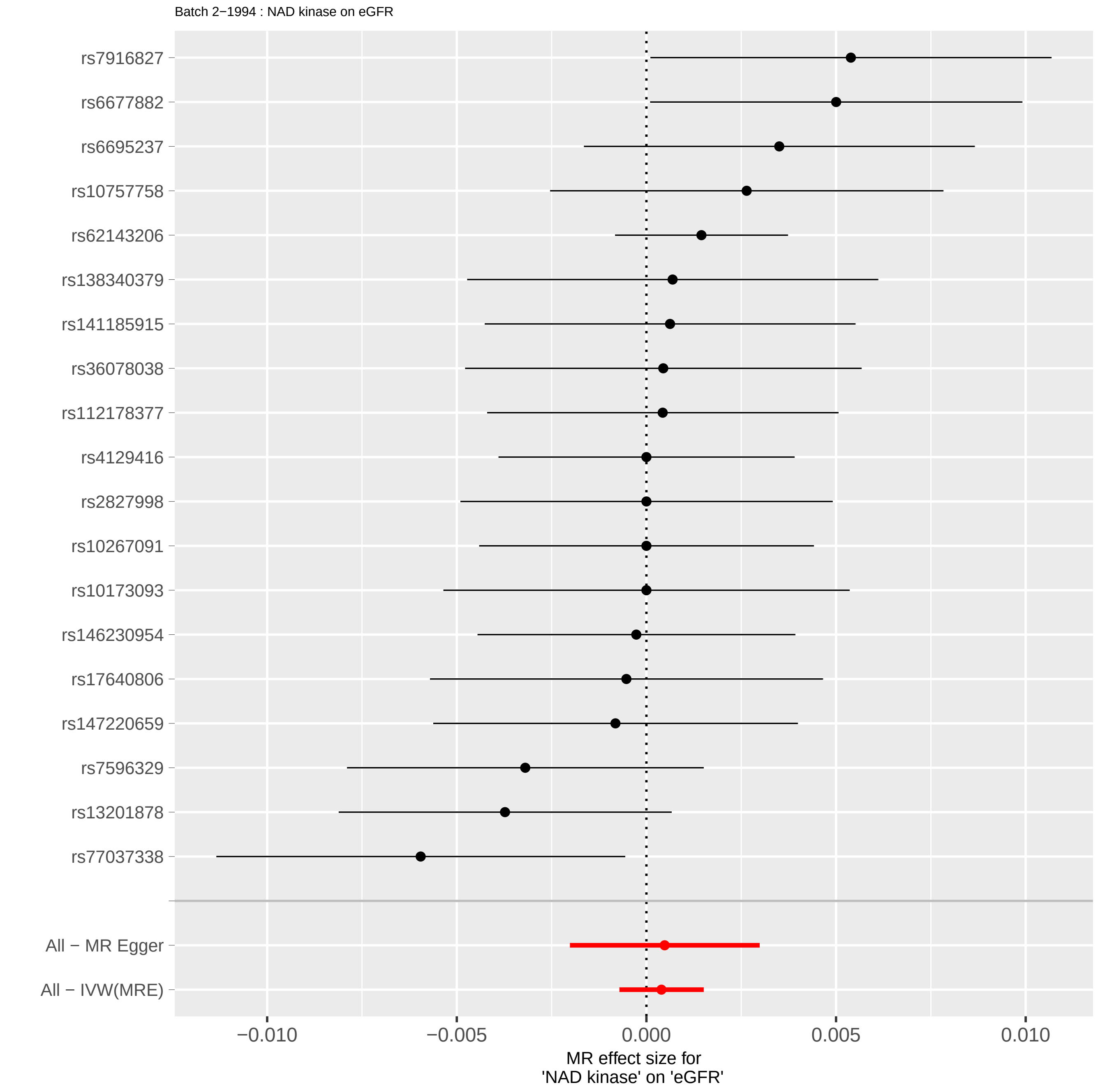

Batch 2−1994 : NAD kinase on eGFR
rs7916827
rs6677882
rs6695237
rs10757758
rs62143206
rs138340379
rs141185915
rs36078038
rs112178377
rs4129416
rs2827998
rs10267091
rs10173093
rs146230954
rs17640806
rs147220659
rs7596329
rs13201878
rs77037338
All − MR Egger
All − IVW(MRE)
−0.010
−0.005
0.000
0.005
0.010
MR effect size for
'NAD kinase' on 'eGFR'

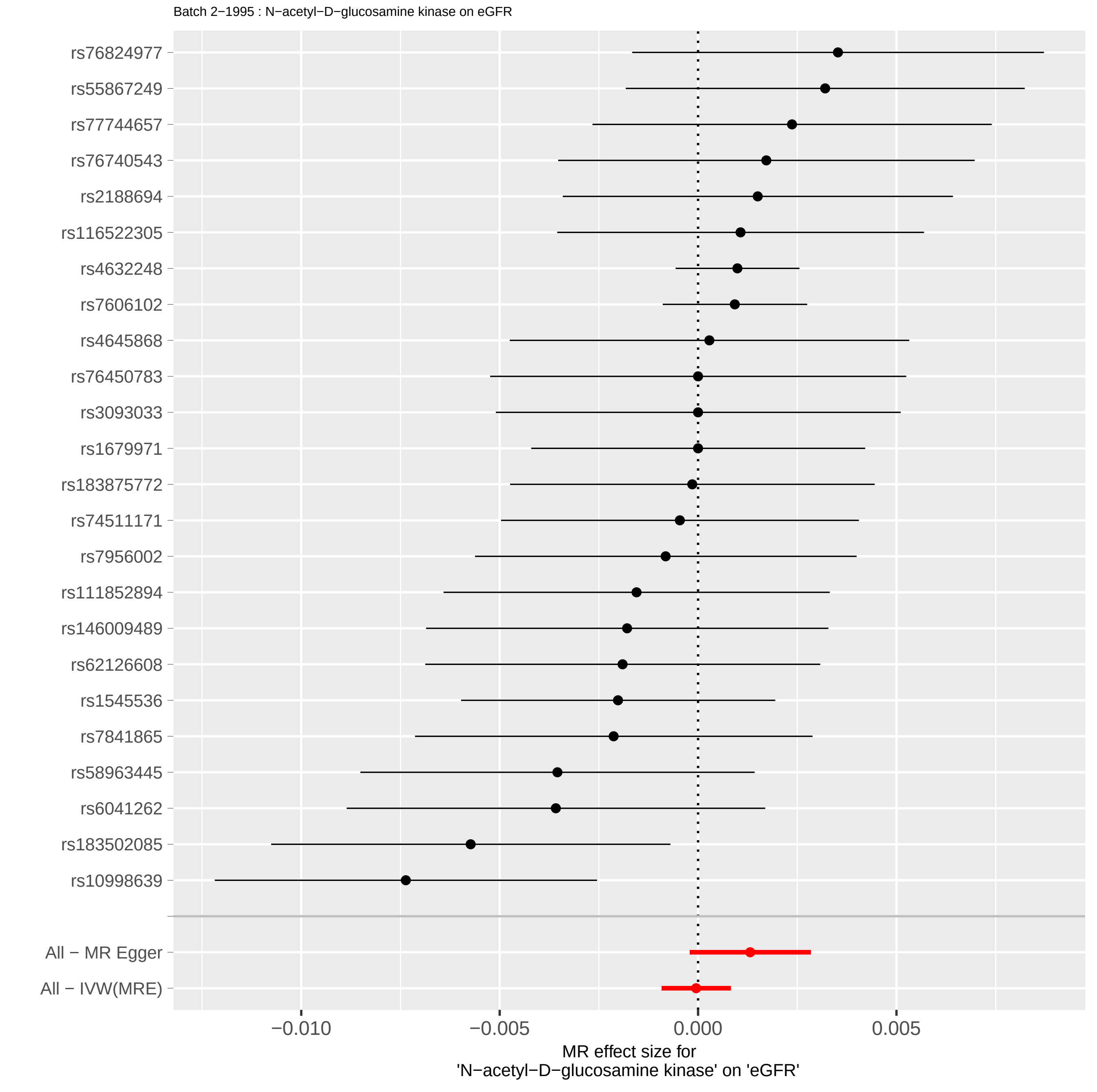

Batch 2−1995 : N−acetyl−D−glucosamine kinase on eGFR
rs76824977
rs55867249
rs77744657
rs76740543
rs2188694
rs116522305
rs4632248
rs7606102
rs4645868
rs76450783
rs3093033
rs1679971
rs183875772
rs74511171
rs7956002
rs111852894
rs146009489
rs62126608
rs1545536
rs7841865
rs58963445
rs6041262
rs183502085
rs10998639
All − MR Egger
All − IVW(MRE)
−0.010
−0.005
0.000
0.005
MR effect size for
'N−acetyl−D−glucosamine kinase' on 'eGFR'

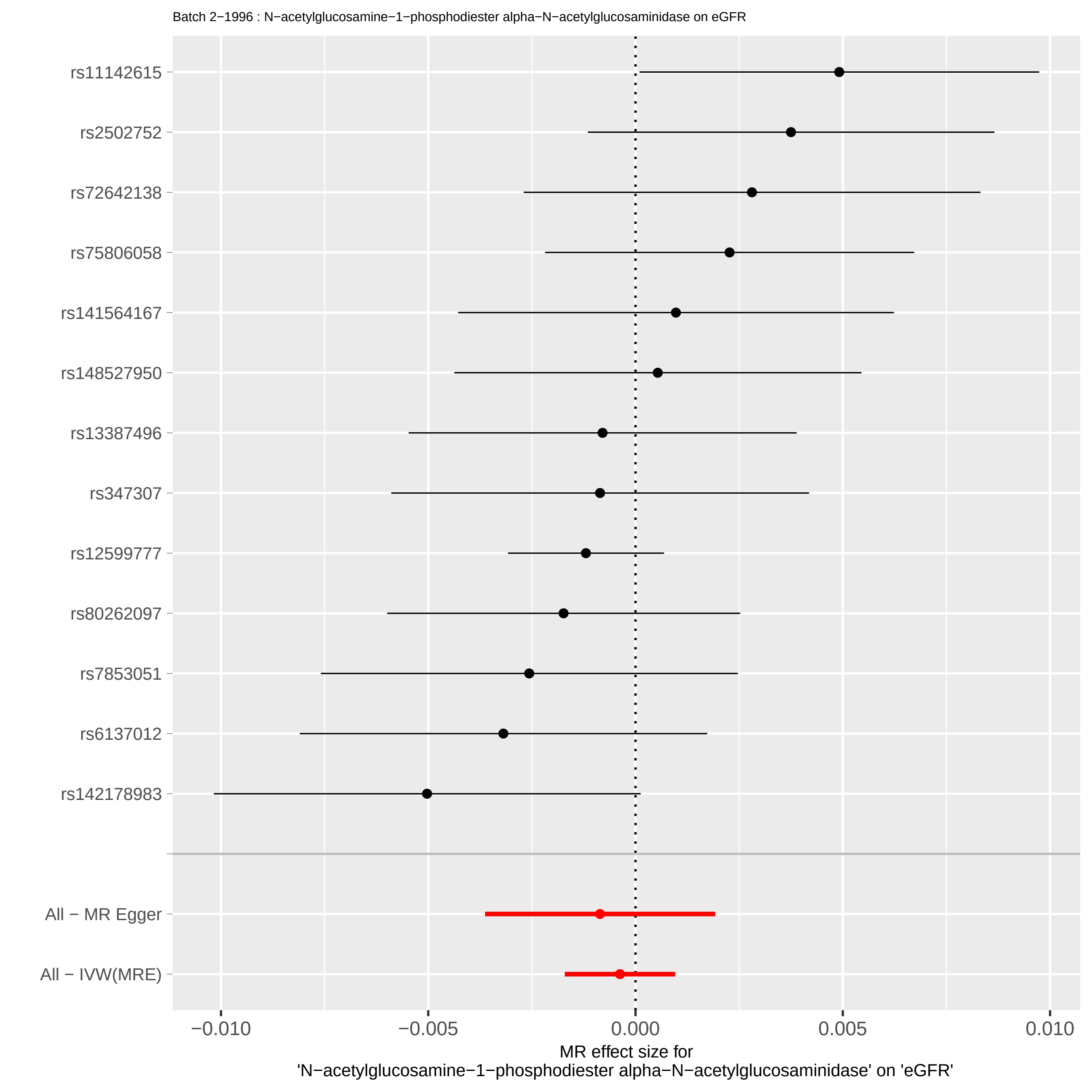
Batch 2−1996 : N−acetylglucosamine−1−phosphodiester alpha−N−acetylglucosaminidase on eGFR
rs11142615
rs2502752
rs72642138
rs75806058
rs141564167
rs148527950
rs13387496
rs347307
rs12599777
rs80262097
rs7853051
rs6137012
rs142178983
All − MR Egger
All − IVW(MRE)
−0.010
−0.005
0.000
0.005
0.010
MR effect size for
'N−acetylglucosamine−1−phosphodiester alpha−N−acetylglucosaminidase' on 'eGFR'

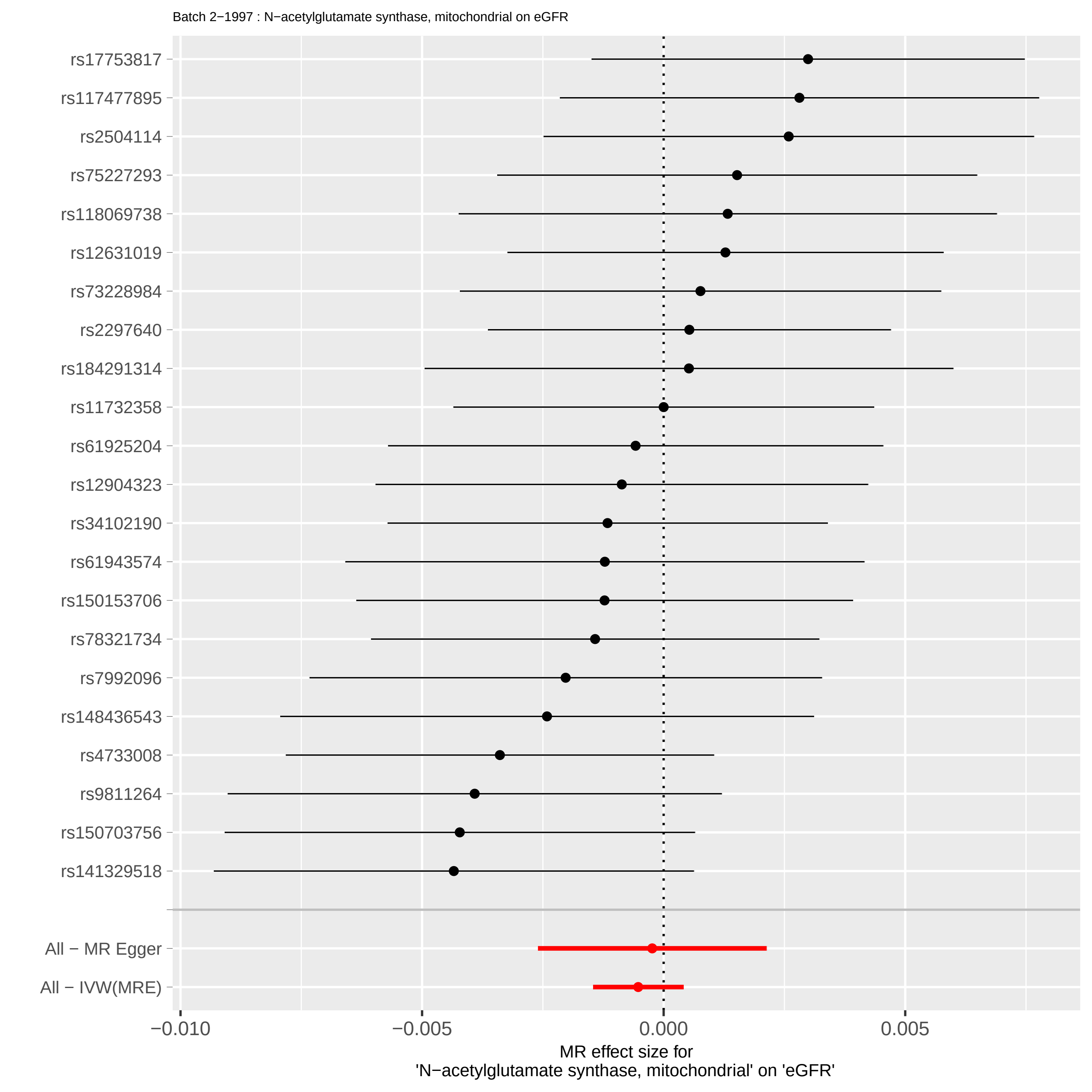

Batch 2−1997 : N−acetylglutamate synthase, mitochondrial on eGFR
rs17753817
rs117477895
rs2504114
rs75227293
rs118069738
rs12631019
rs73228984
rs2297640
rs184291314
rs11732358
rs61925204
rs12904323
rs34102190
rs61943574
rs150153706
rs78321734
rs7992096
rs148436543
rs4733008
rs9811264
rs150703756
rs141329518
All − MR Egger
All − IVW(MRE)
−0.010
−0.005
0.000
0.005
MR effect size for
'N−acetylglutamate synthase, mitochondrial' on 'eGFR'

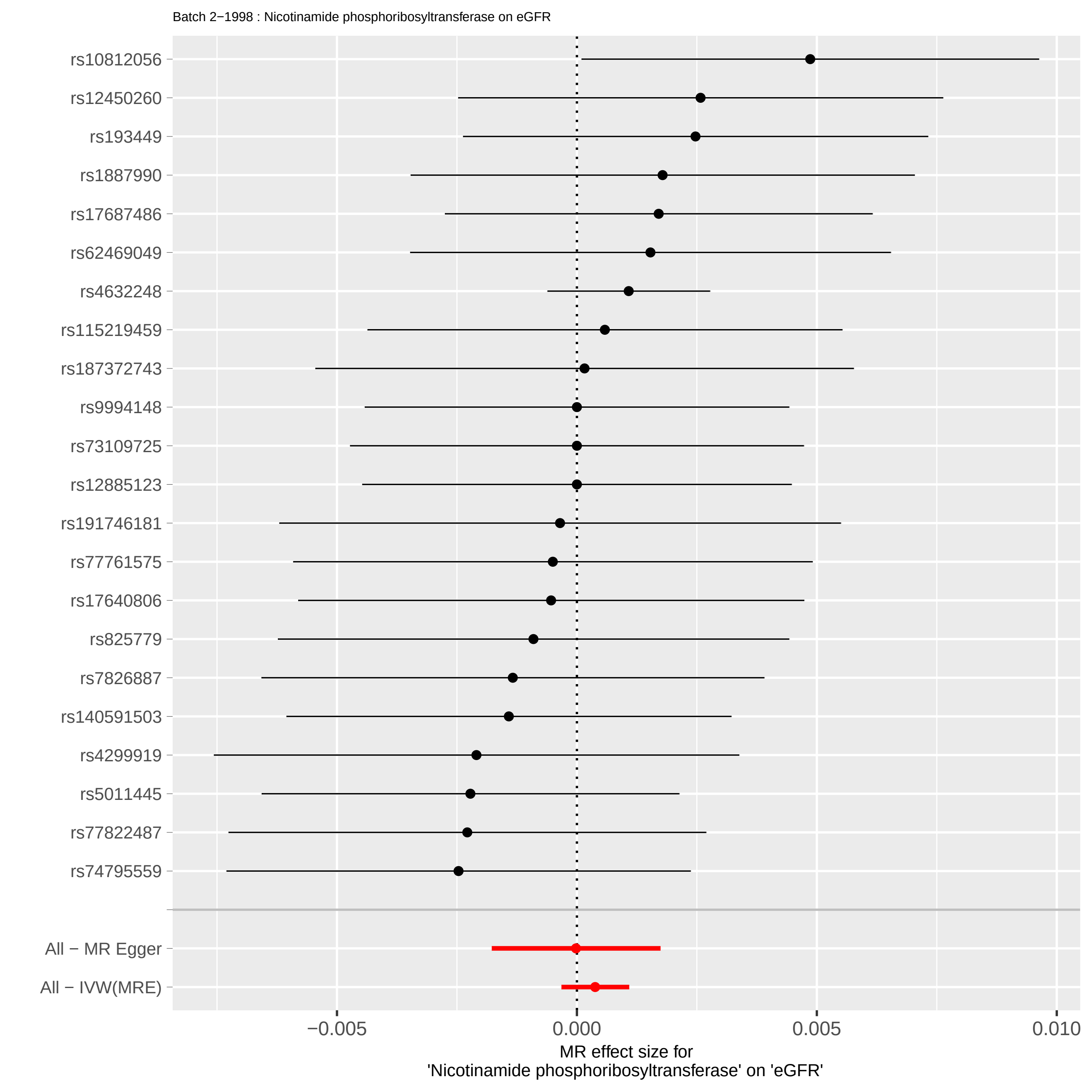

Batch 2−1998 : Nicotinamide phosphoribosyltransferase on eGFR
rs10812056
rs12450260
rs193449
rs1887990
rs17687486
rs62469049
rs4632248
rs115219459
rs187372743
rs9994148
rs73109725
rs12885123
rs191746181
rs77761575
rs17640806
rs825779
rs7826887
rs140591503
rs4299919
rs5011445
rs77822487
rs74795559
All − MR Egger
All − IVW(MRE)
−0.005
0.000
0.005
0.010
MR effect size for
'Nicotinamide phosphoribosyltransferase' on 'eGFR'

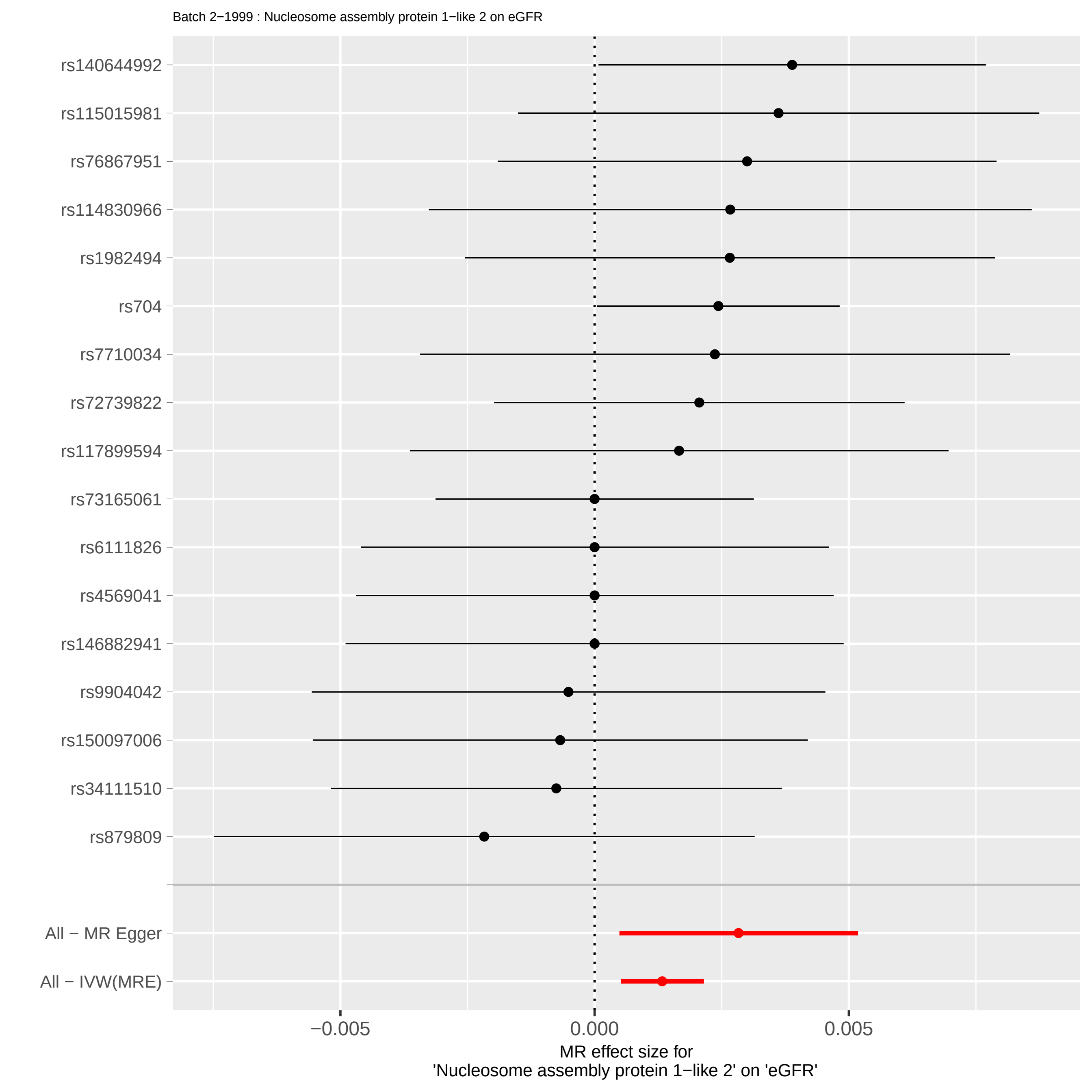
Batch 2−1999 : Nucleosome assembly protein 1−like 2 on eGFR
rs140644992
rs115015981
rs76867951
rs114830966
rs1982494
rs704
rs7710034
rs72739822
rs117899594
rs73165061
rs6111826
rs4569041
rs146882941
rs9904042
rs150097006
rs34111510
rs879809
All − MR Egger
All − IVW(MRE)
−0.005
0.000
0.005
MR effect size for
'Nucleosome assembly protein 1−like 2' on 'eGFR'

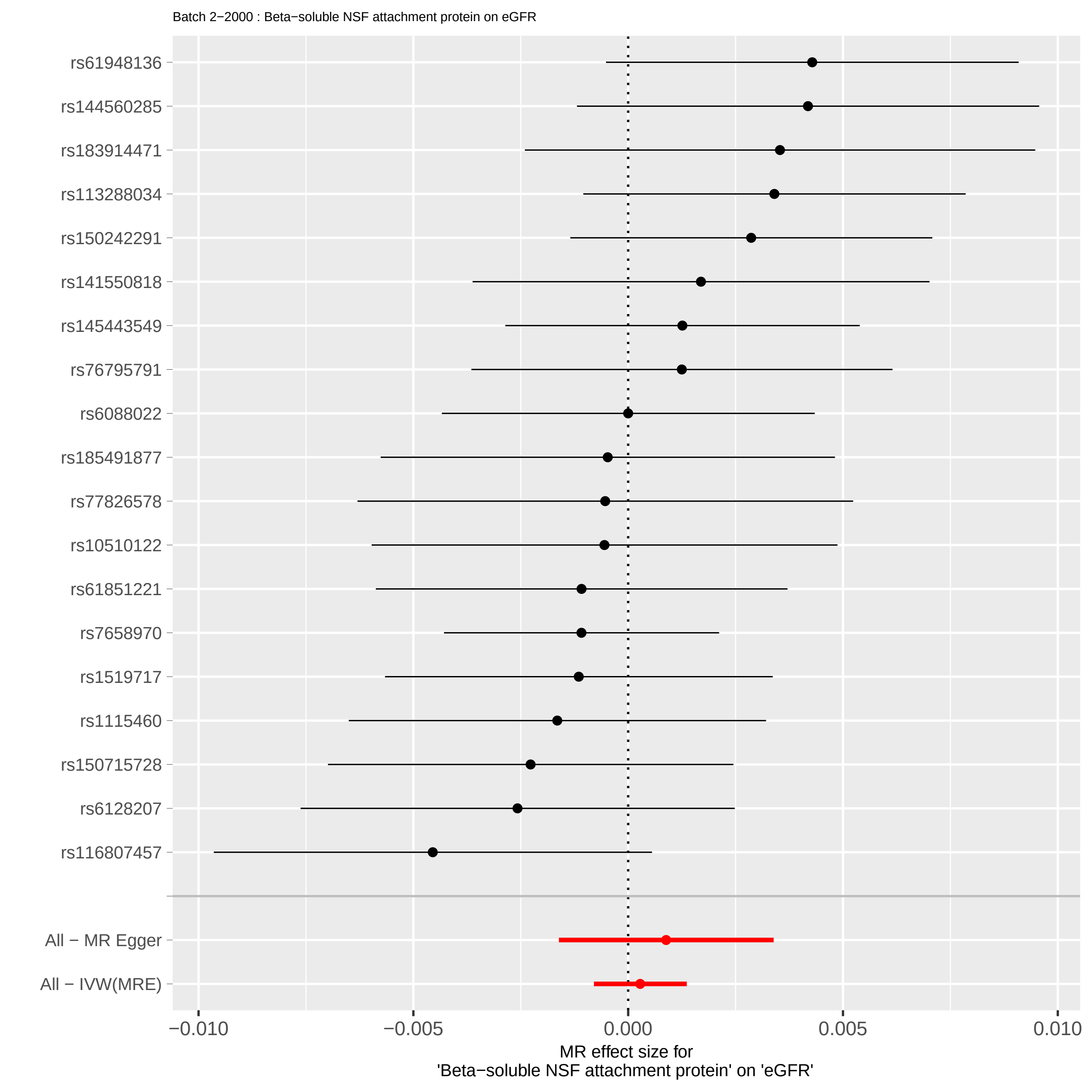

Batch 2−2000 : Beta−soluble NSF attachment protein on eGFR
rs61948136
rs144560285
rs183914471
rs113288034
rs150242291
rs141550818
rs145443549
rs76795791
rs6088022
rs185491877
rs77826578
rs10510122
rs61851221
rs7658970
rs1519717
rs1115460
rs150715728
rs6128207
rs116807457
All − MR Egger
All − IVW(MRE)
−0.010
−0.005
0.000
0.005
0.010
MR effect size for
'Beta−soluble NSF attachment protein' on 'eGFR'

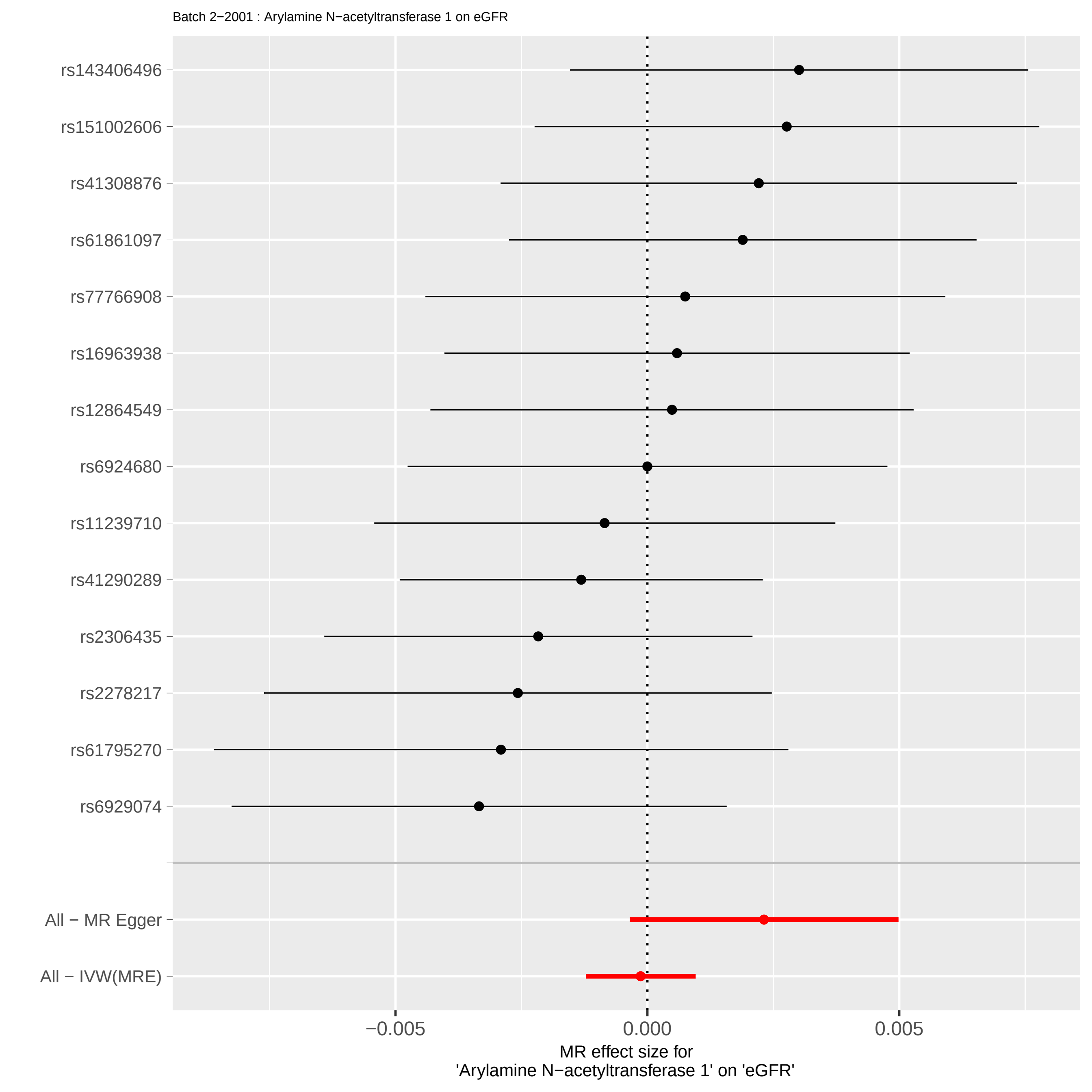

Batch 2−2001 : Arylamine N−acetyltransferase 1 on eGFR
rs143406496
rs151002606
rs41308876
rs61861097
rs77766908
rs16963938
rs12864549
rs6924680
rs11239710
rs41290289
rs2306435
rs2278217
rs61795270
rs6929074
All − MR Egger
All − IVW(MRE)
−0.005
0.000
0.005
MR effect size for
'Arylamine N−acetyltransferase 1' on 'eGFR'

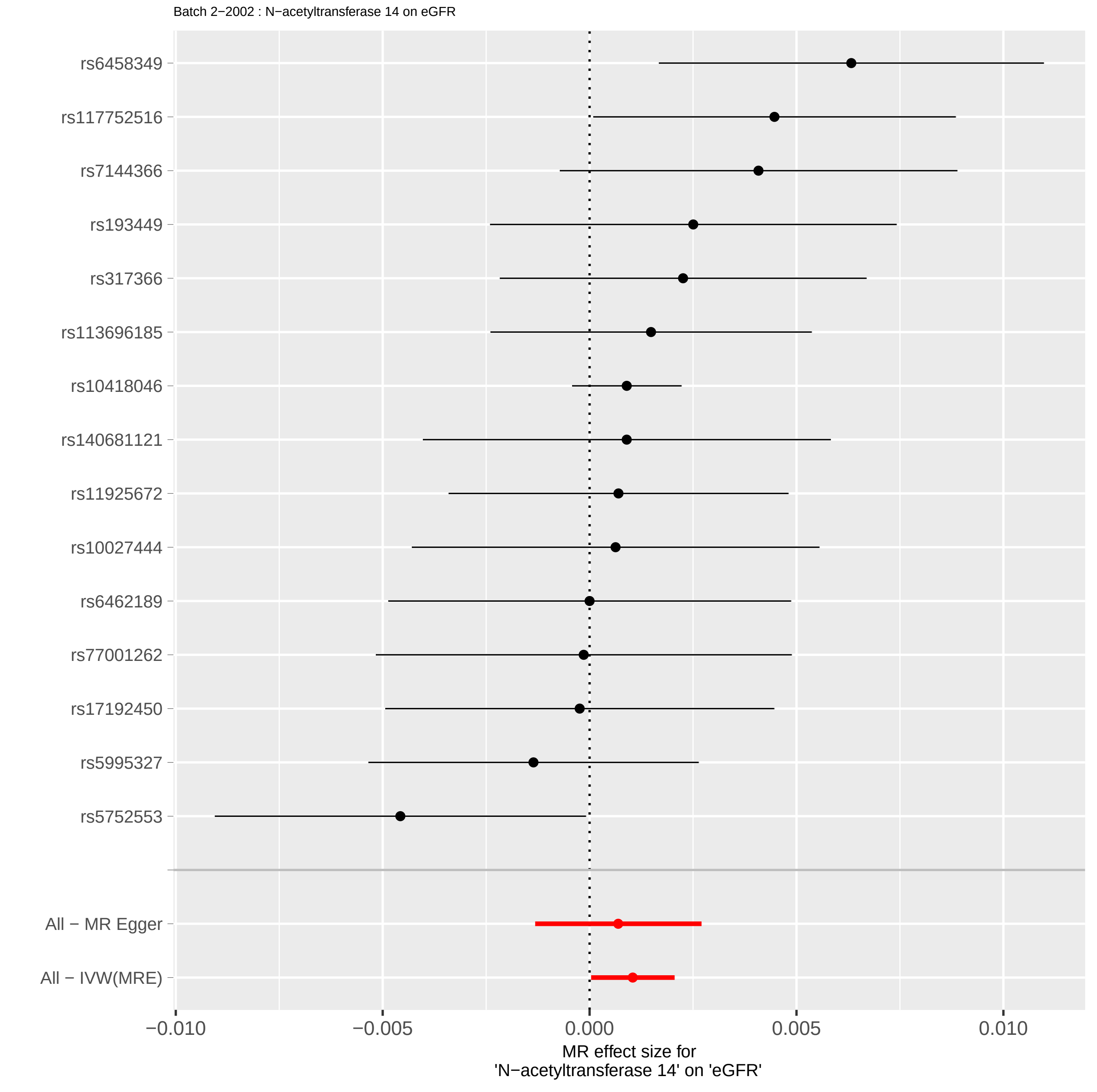

Batch 2−2002 : N−acetyltransferase 14 on eGFR
rs6458349
rs117752516
rs7144366
rs193449
rs317366
rs113696185
rs10418046
rs140681121
rs11925672
rs10027444
rs6462189
rs77001262
rs17192450
rs5995327
rs5752553
All − MR Egger
All − IVW(MRE)
−0.010
−0.005
0.000
0.005
0.010
MR effect size for
'N−acetyltransferase 14' on 'eGFR'

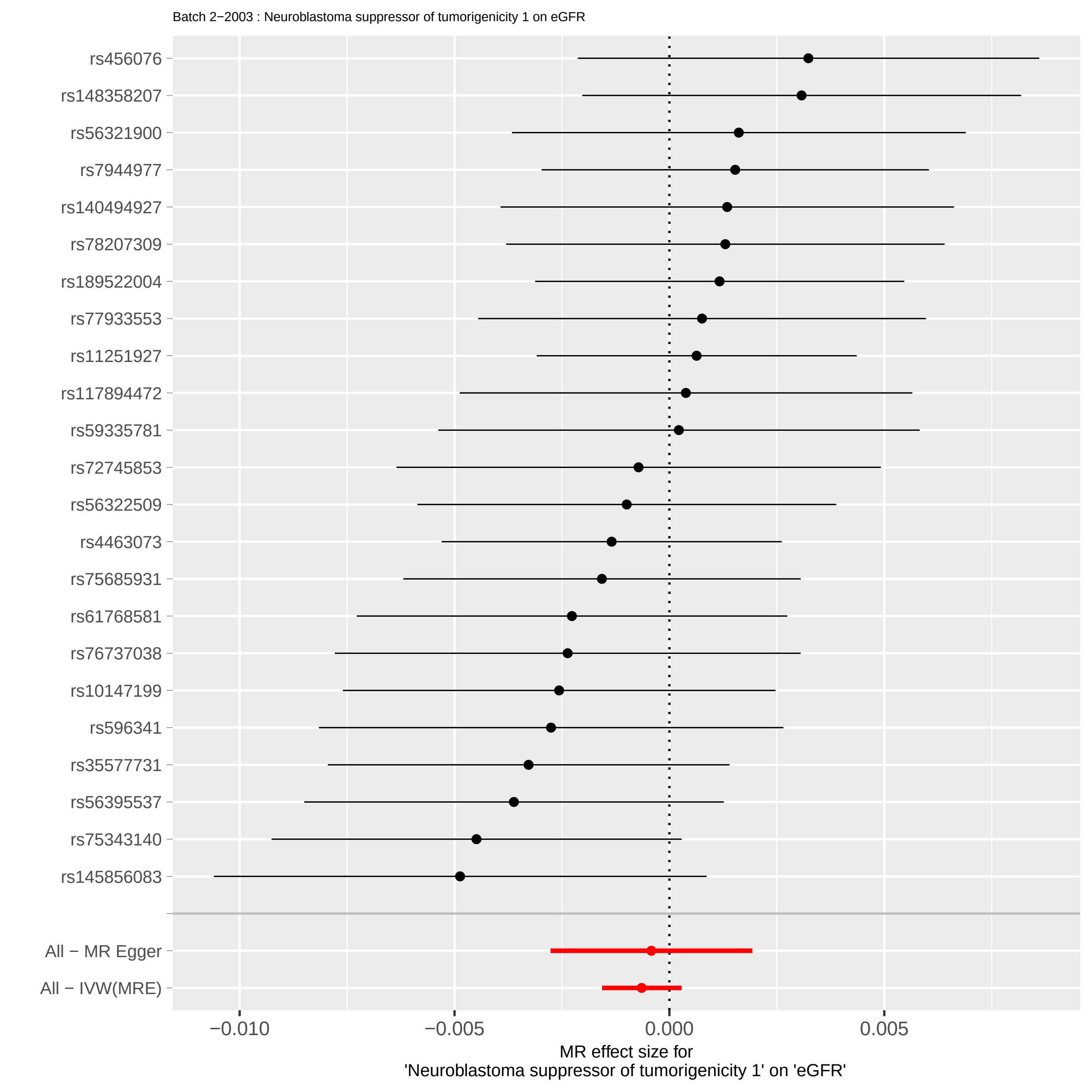

Batch 2−2003 : Neuroblastoma suppressor of tumorigenicity 1 on eGFR
rs456076
rs148358207
rs56321900
rs7944977
rs140494927
rs78207309
rs189522004
rs77933553
rs11251927
rs117894472
rs59335781
rs72745853
rs56322509
rs4463073
rs75685931
rs61768581
rs76737038
rs10147199
rs596341
rs35577731
rs56395537
rs75343140
rs145856083
All − MR Egger
All − IVW(MRE)
−0.010
−0.005
0.000
0.005
MR effect size for
'Neuroblastoma suppressor of tumorigenicity 1' on 'eGFR'

Batch 2−2004 : Next to BRCA1 gene 1 protein on eGFR

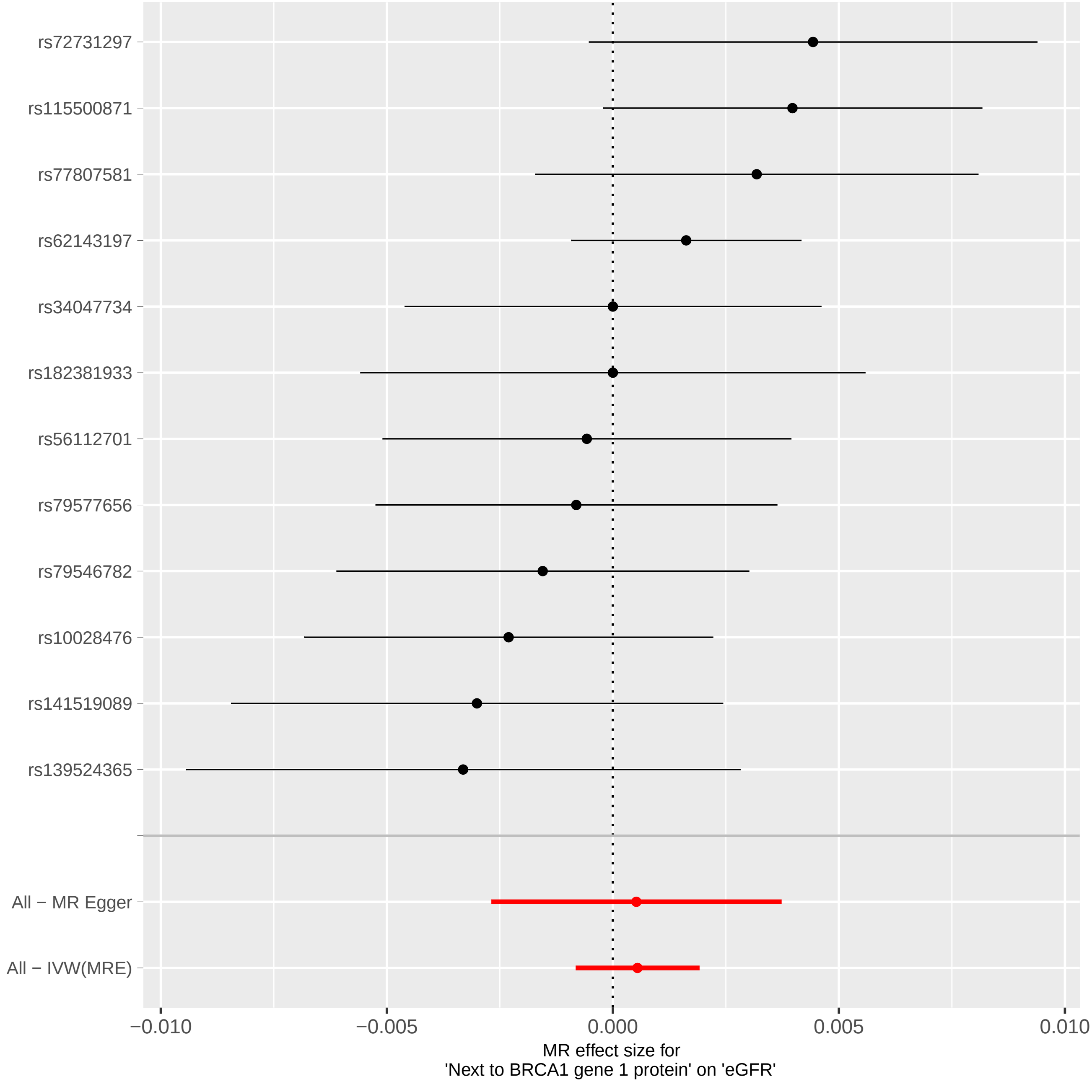

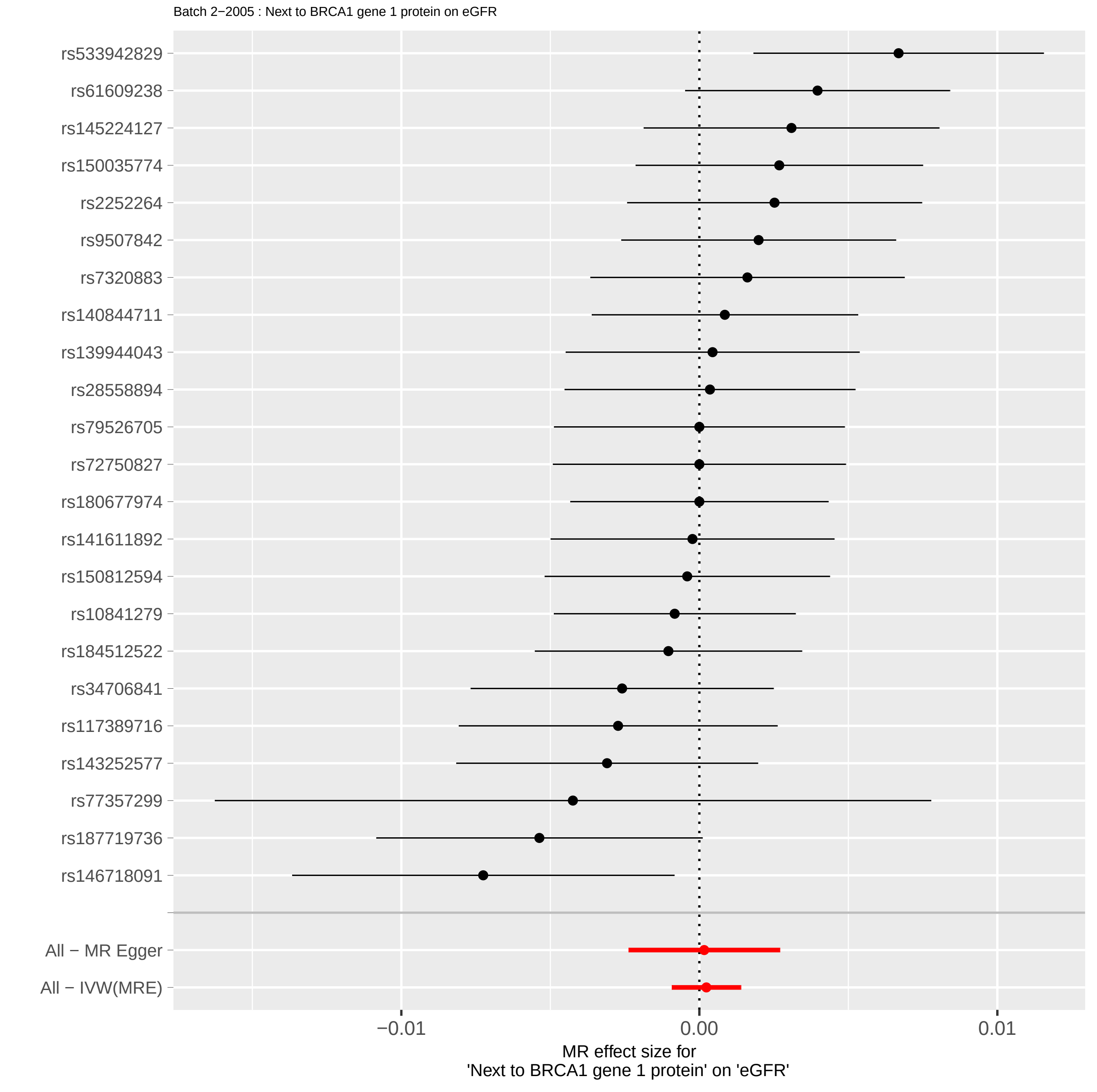

Batch 2−2005 : Next to BRCA1 gene 1 protein on eGFR
rs533942829
rs61609238
rs145224127
rs150035774
rs2252264
rs9507842
rs7320883
rs140844711
rs139944043
rs28558894
rs79526705
rs72750827
rs180677974
rs141611892
rs150812594
rs10841279
rs184512522
rs34706841
rs117389716
rs143252577
rs77357299
rs187719736
rs146718091
All − MR Egger
All − IVW(MRE)
−0.01
0.00
0.01
MR effect size for
'Next to BRCA1 gene 1 protein' on 'eGFR'

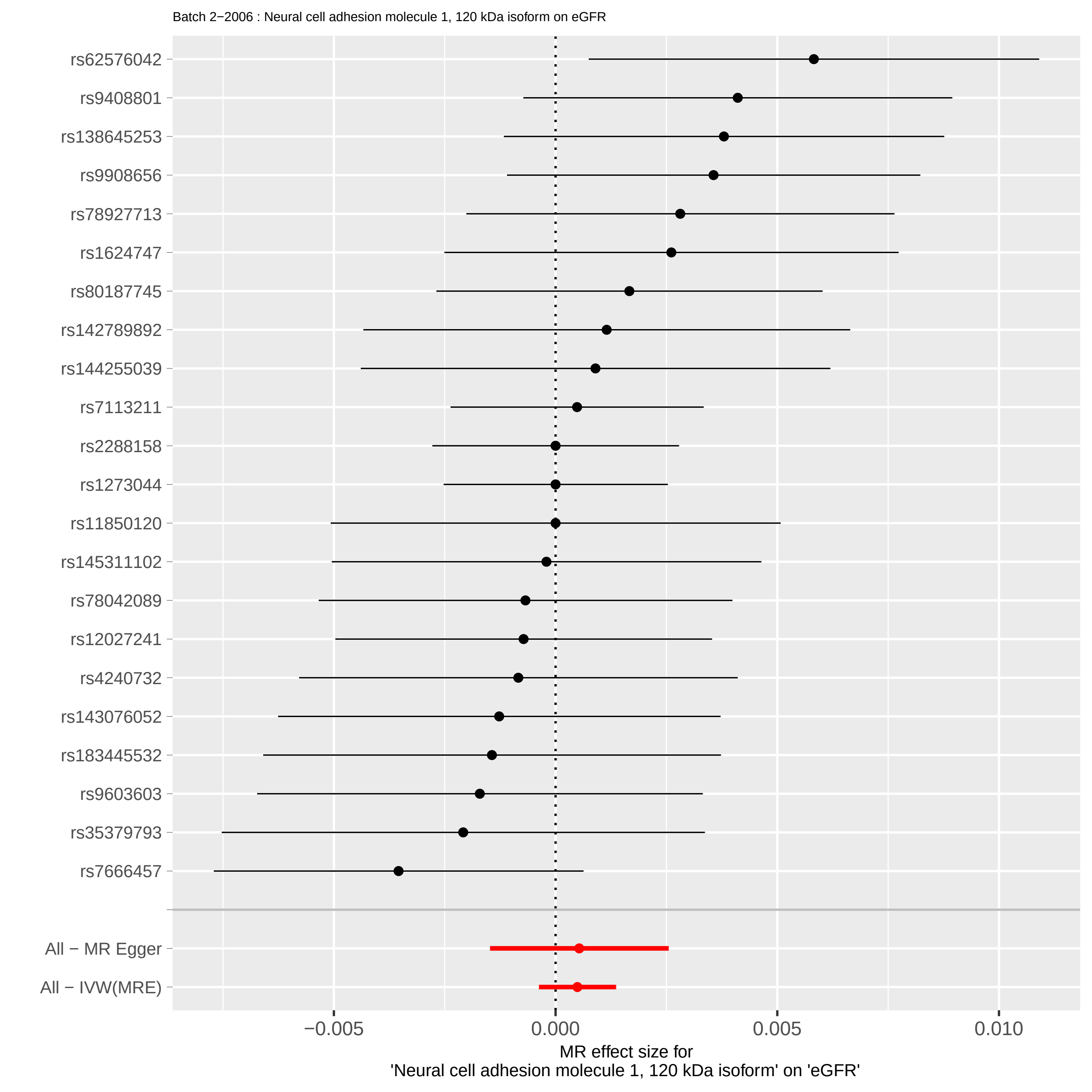

Batch 2−2006 : Neural cell adhesion molecule 1, 120 kDa isoform on eGFR
rs62576042
rs9408801
rs138645253
rs9908656
rs78927713
rs1624747
rs80187745
rs142789892
rs144255039
rs7113211
rs2288158
rs1273044
rs11850120
rs145311102
rs78042089
rs12027241
rs4240732
rs143076052
rs183445532
rs9603603
rs35379793
rs7666457
All − MR Egger
All − IVW(MRE)
−0.005
0.000
0.005
0.010
MR effect size for
'Neural cell adhesion molecule 1, 120 kDa isoform' on 'eGFR'

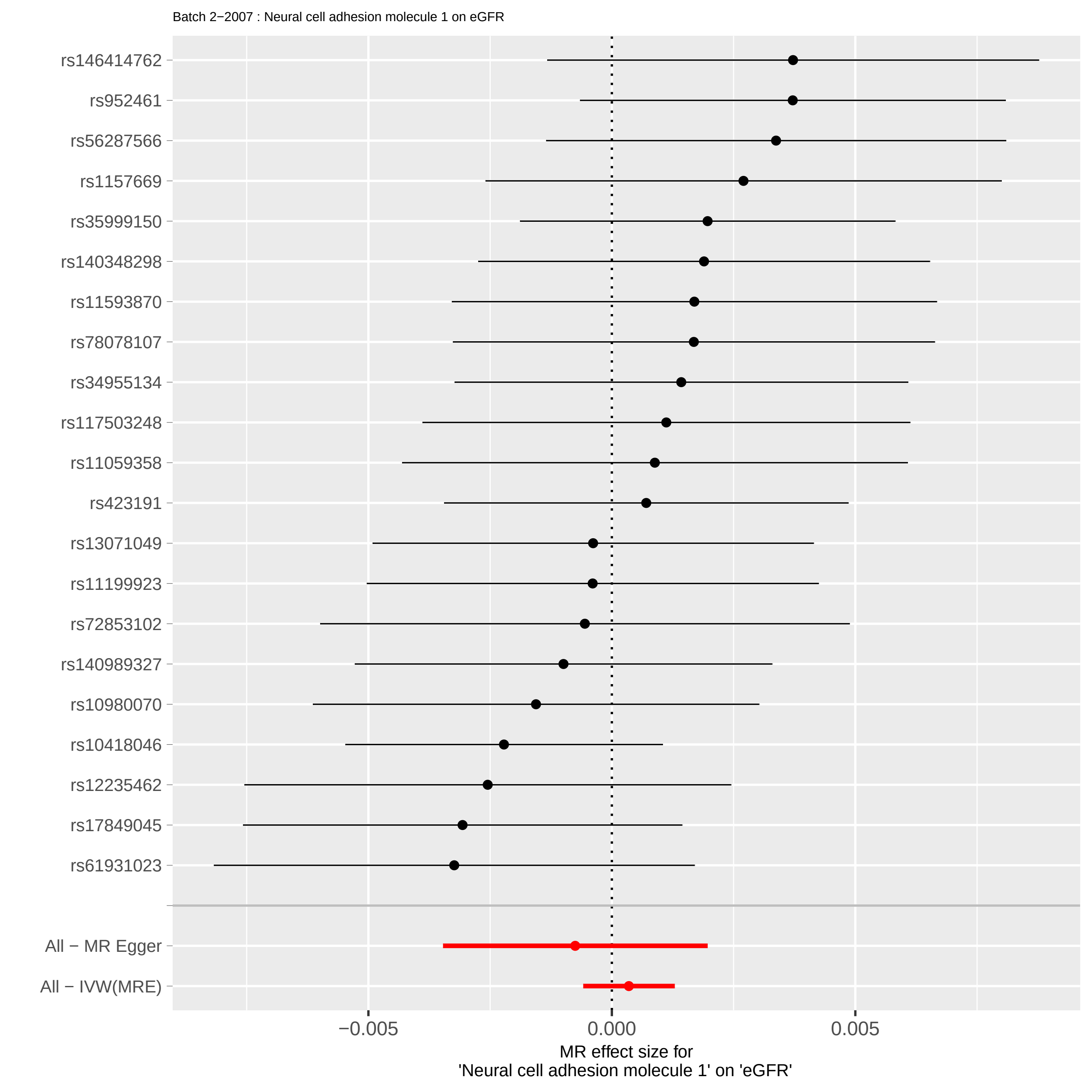
Batch 2−2007 : Neural cell adhesion molecule 1 on eGFR
rs146414762
rs952461
rs56287566
rs1157669
rs35999150
rs140348298
rs11593870
rs78078107
rs34955134
rs117503248
rs11059358
rs423191
rs13071049
rs11199923
rs72853102
rs140989327
rs10980070
rs10418046
rs12235462
rs17849045
rs61931023
All − MR Egger
All − IVW(MRE)
−0.005
0.000
0.005
MR effect size for
'Neural cell adhesion molecule 1' on 'eGFR'

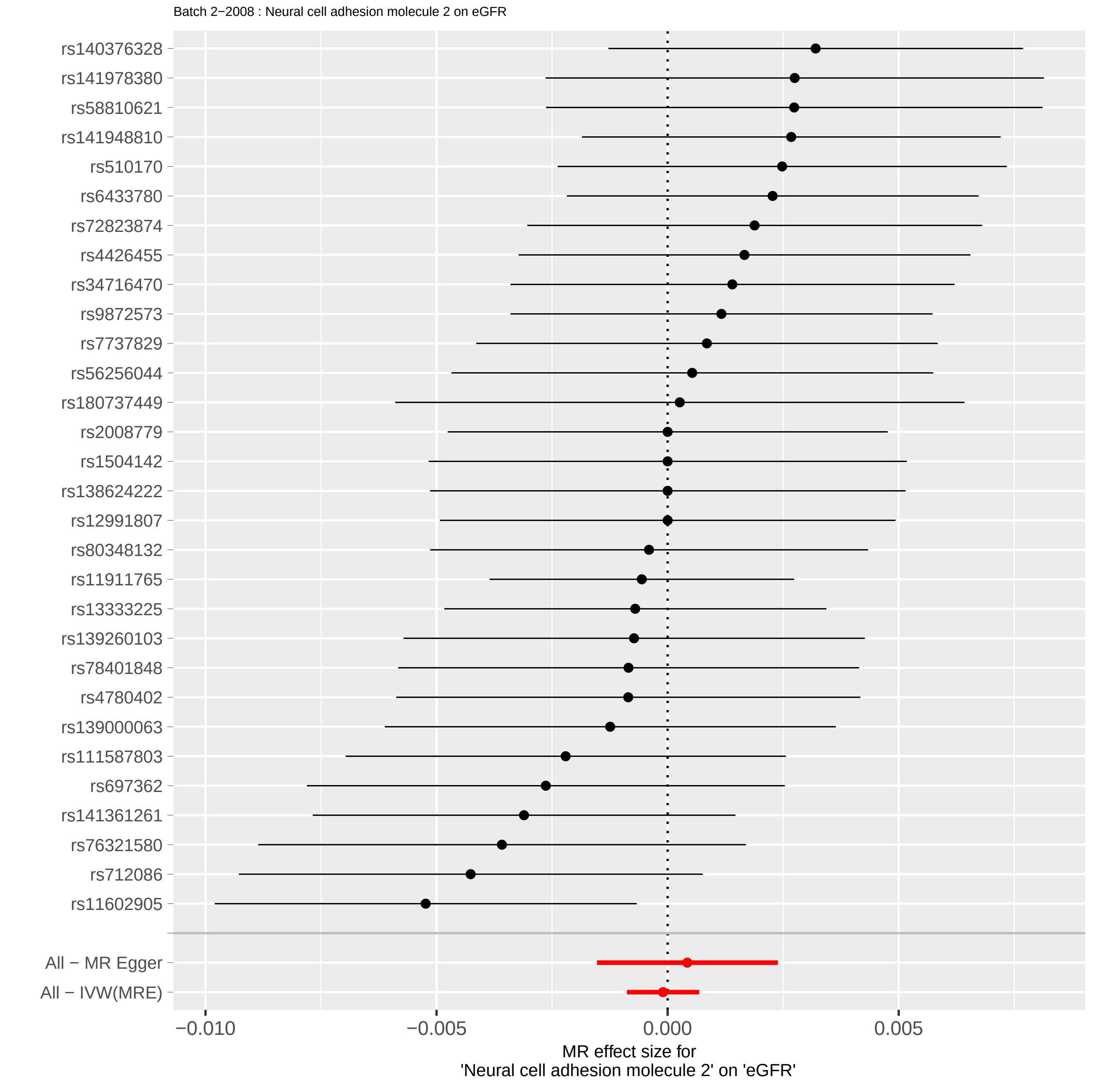

Batch 2−2008 : Neural cell adhesion molecule 2 on eGFR
rs140376328
rs141978380
rs58810621
rs141948810
rs510170
rs6433780
rs72823874
rs4426455
rs34716470
rs9872573
rs7737829
rs56256044
rs180737449
rs2008779
rs1504142
rs138624222
rs12991807
rs80348132
rs11911765
rs13333225
rs139260103
rs78401848
rs4780402
rs139000063
rs111587803
rs697362
rs141361261
rs76321580
rs712086
rs11602905
All − MR Egger
All − IVW(MRE)
−0.010
−0.005
0.000
0.005
MR effect size for
'Neural cell adhesion molecule 2' on 'eGFR'

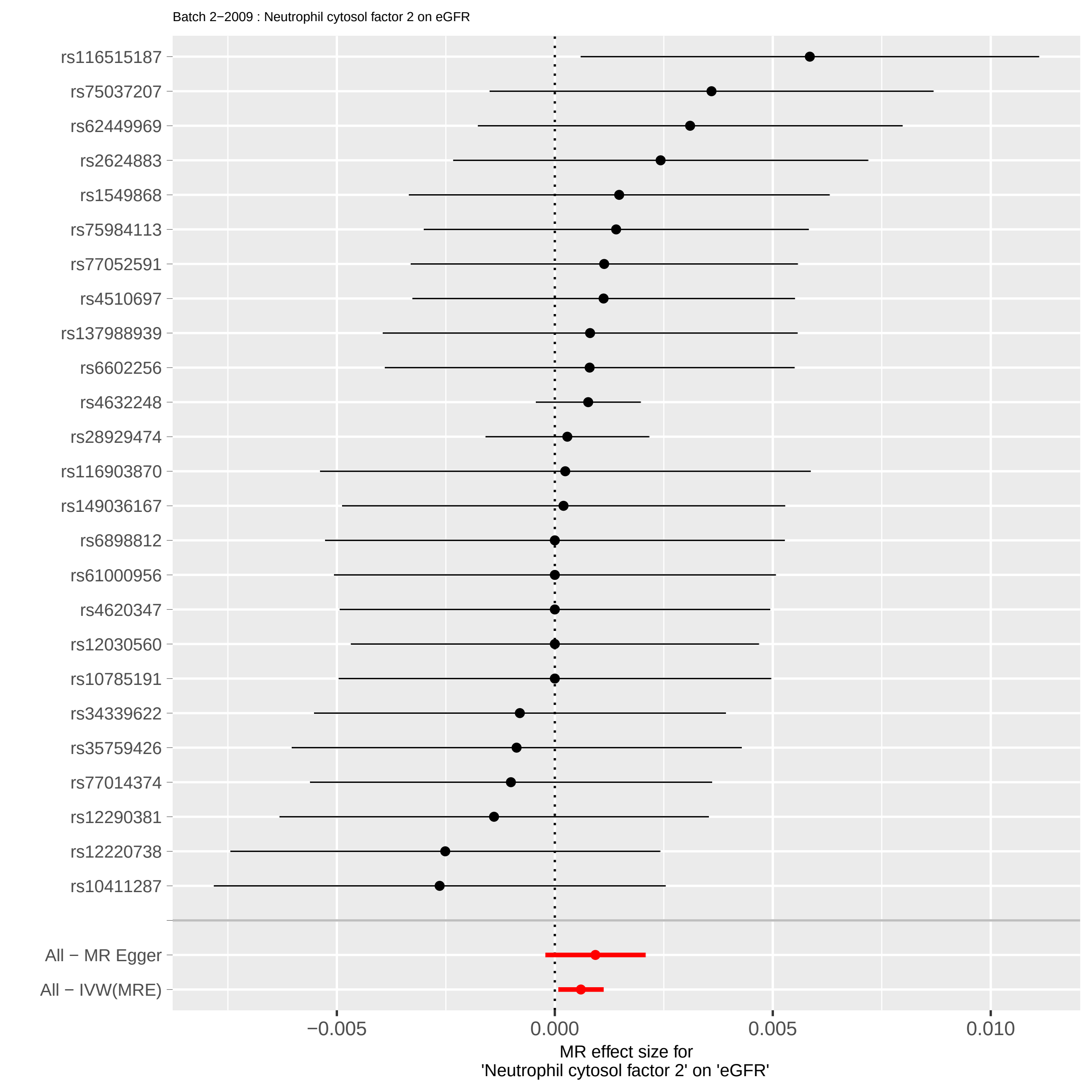

Batch 2−2009 : Neutrophil cytosol factor 2 on eGFR
rs116515187
rs75037207
rs62449969
rs2624883
rs1549868
rs75984113
rs77052591
rs4510697
rs137988939
rs6602256
rs4632248
rs28929474
rs116903870
rs149036167
rs6898812
rs61000956
rs4620347
rs12030560
rs10785191
rs34339622
rs35759426
rs77014374
rs12290381
rs12220738
rs10411287
All − MR Egger
All − IVW(MRE)
−0.005
0.000
0.005
0.010
MR effect size for
'Neutrophil cytosol factor 2' on 'eGFR'

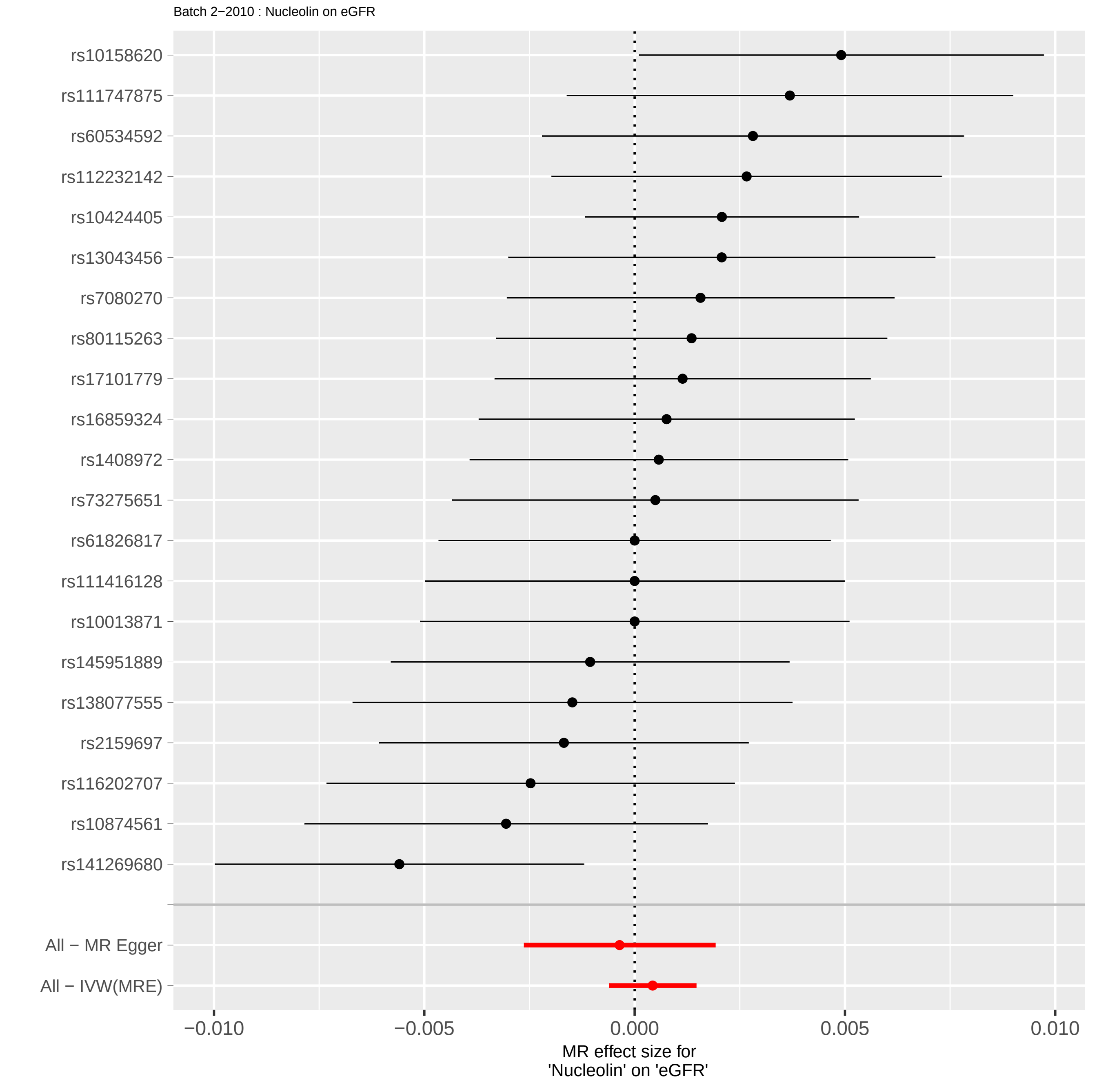

Batch 2−2010 : Nucleolin on eGFR
rs10158620
rs111747875
rs60534592
rs112232142
rs10424405
rs13043456
rs7080270
rs80115263
rs17101779
rs16859324
rs1408972
rs73275651
rs61826817
rs111416128
rs10013871
rs145951889
rs138077555
rs2159697
rs116202707
rs10874561
rs141269680
All − MR Egger
All − IVW(MRE)
−0.010
−0.005
0.000
0.005
0.010
MR effect size for
'Nucleolin' on 'eGFR'

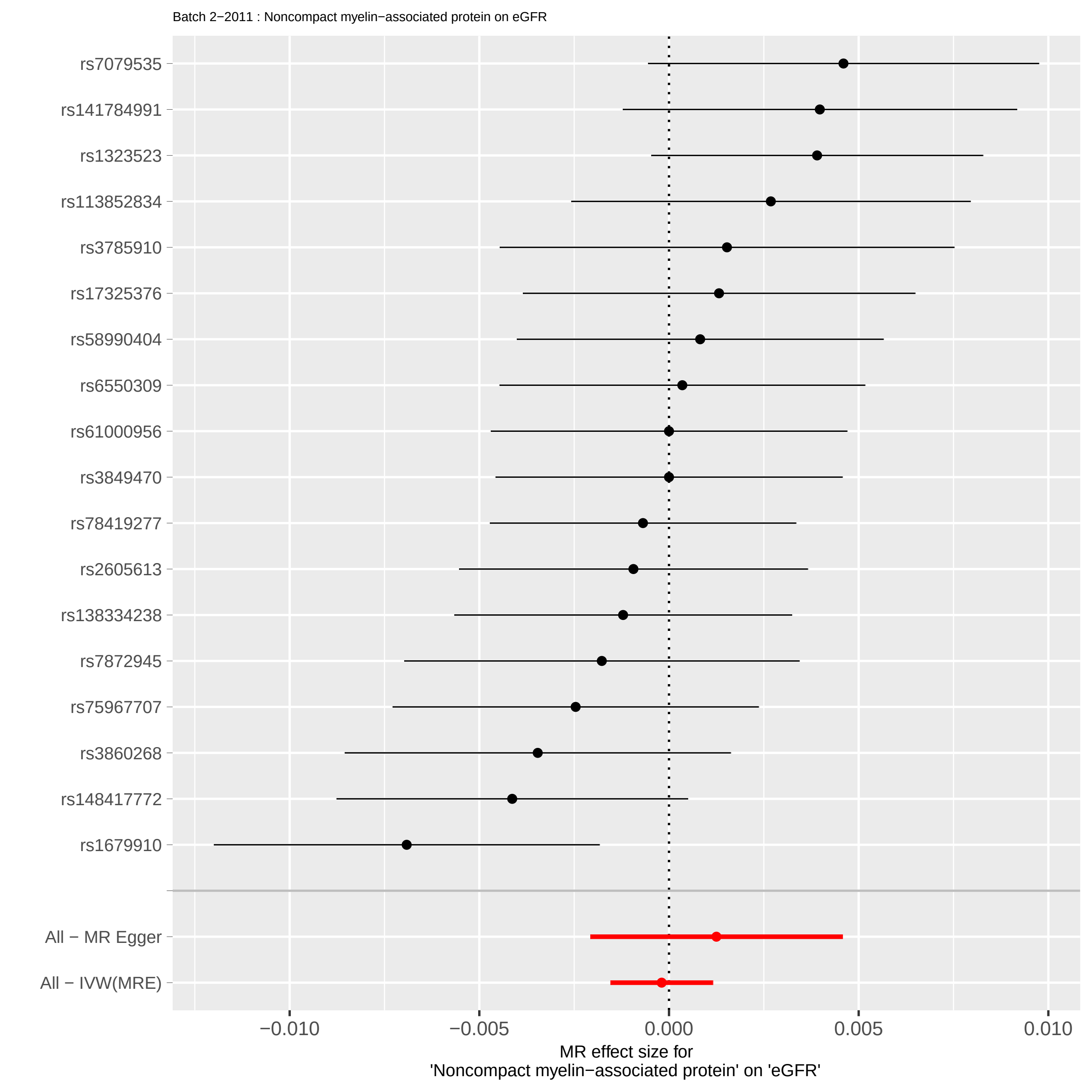

Batch 2−2011 : Noncompact myelin−associated protein on eGFR
rs7079535
rs141784991
rs1323523
rs113852834
rs3785910
rs17325376
rs58990404
rs6550309
rs61000956
rs3849470
rs78419277
rs2605613
rs138334238
rs7872945
rs75967707
rs3860268
rs148417772
rs1679910
All − MR Egger
All − IVW(MRE)
−0.010
−0.005
0.000
0.005
0.010
MR effect size for
'Noncompact myelin−associated protein' on 'eGFR'

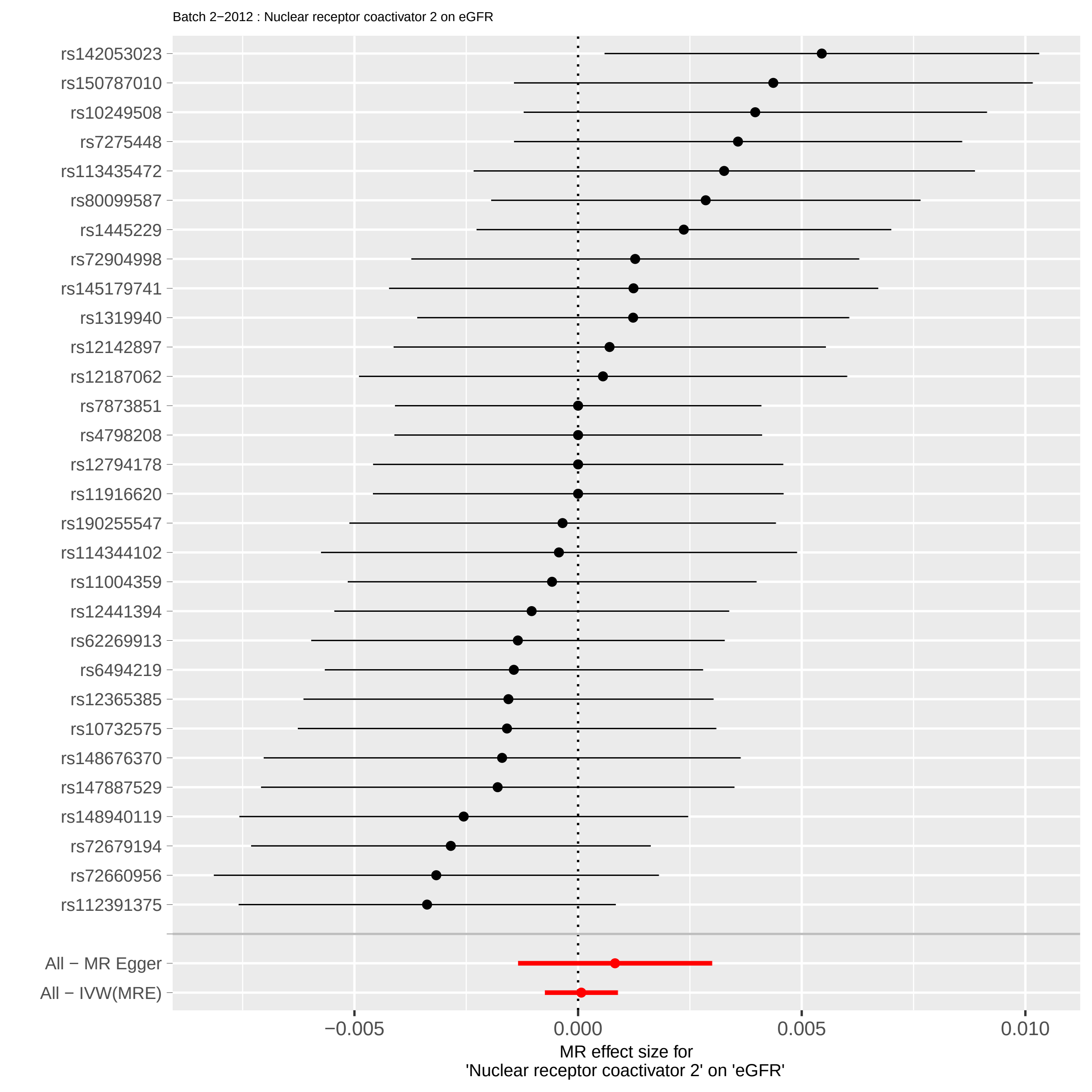
Batch 2−2012 : Nuclear receptor coactivator 2 on eGFR
rs142053023
rs150787010
rs10249508
rs7275448
rs113435472
rs80099587
rs1445229
rs72904998
rs145179741
rs1319940
rs12142897
rs12187062
rs7873851
rs4798208
rs12794178
rs11916620
rs190255547
rs114344102
rs11004359
rs12441394
rs62269913
rs6494219
rs12365385
rs10732575
rs148676370
rs147887529
rs148940119
rs72679194
rs72660956
rs112391375
All − MR Egger
All − IVW(MRE)
−0.005
0.000
0.005
0.010
MR effect size for
'Nuclear receptor coactivator 2' on 'eGFR'

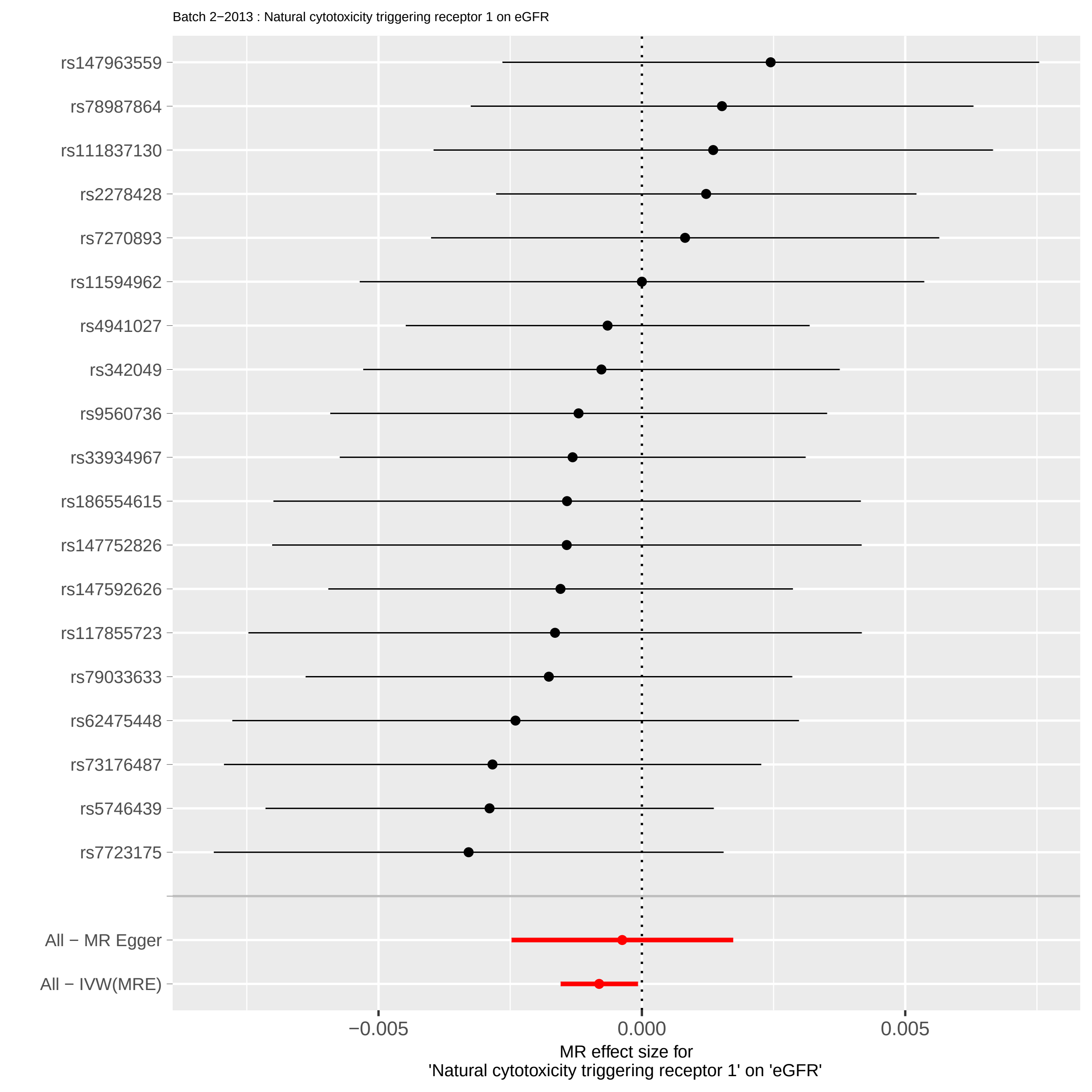
Batch 2−2013 : Natural cytotoxicity triggering receptor 1 on eGFR
rs147963559
rs78987864
rs111837130
rs2278428
rs7270893
rs11594962
rs4941027
rs342049
rs9560736
rs33934967
rs186554615
rs147752826
rs147592626
rs117855723
rs79033633
rs62475448
rs73176487
rs5746439
rs7723175
All − MR Egger
All − IVW(MRE)
−0.005
0.000
0.005
MR effect size for
'Natural cytotoxicity triggering receptor 1' on 'eGFR'

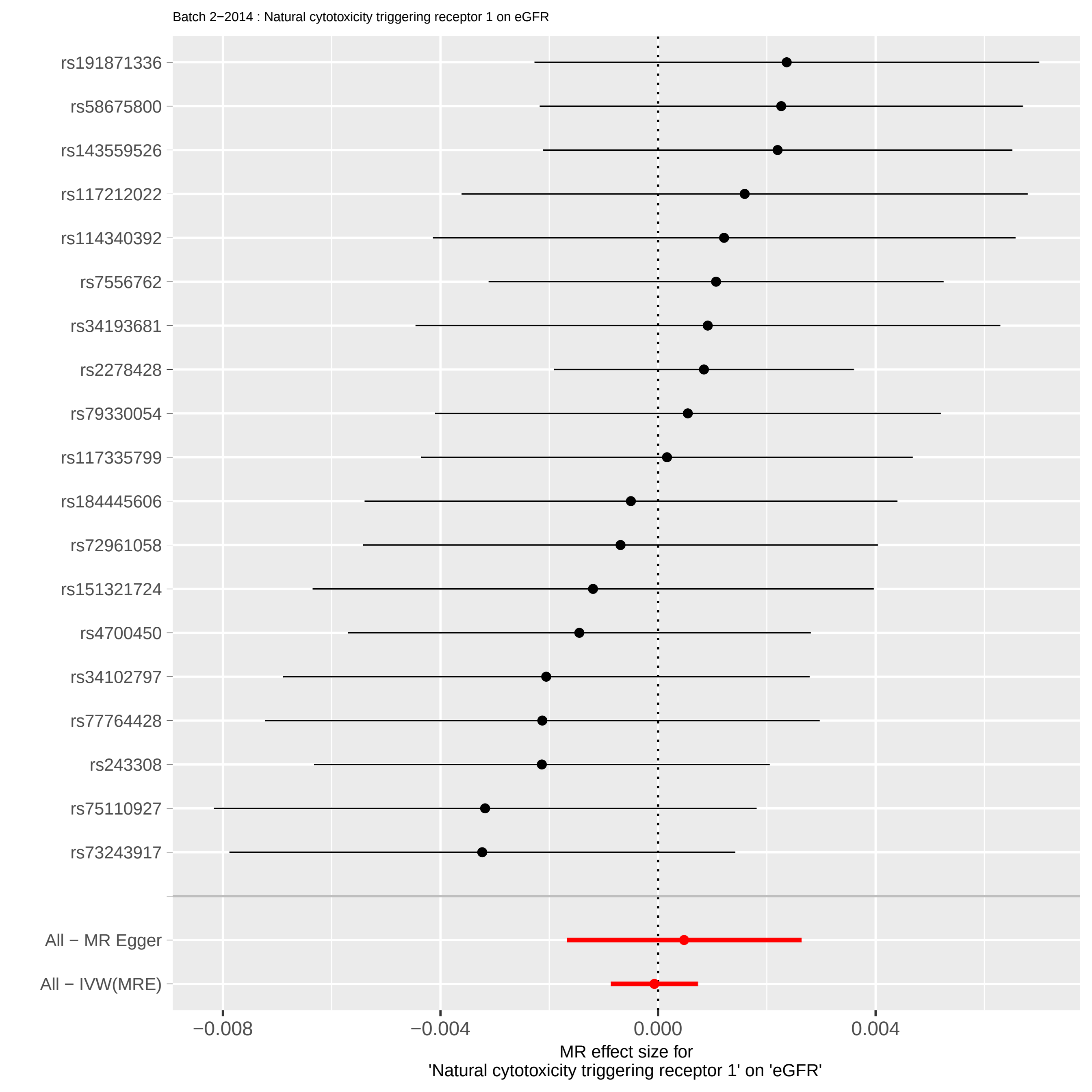

Batch 2−2014 : Natural cytotoxicity triggering receptor 1 on eGFR
rs191871336
rs58675800
rs143559526
rs117212022
rs114340392
rs7556762
rs34193681
rs2278428
rs79330054
rs117335799
rs184445606
rs72961058
rs151321724
rs4700450
rs34102797
rs77764428
rs243308
rs75110927
rs73243917
All − MR Egger
All − IVW(MRE)
−0.008
−0.004
0.000
0.004
MR effect size for
'Natural cytotoxicity triggering receptor 1' on 'eGFR'

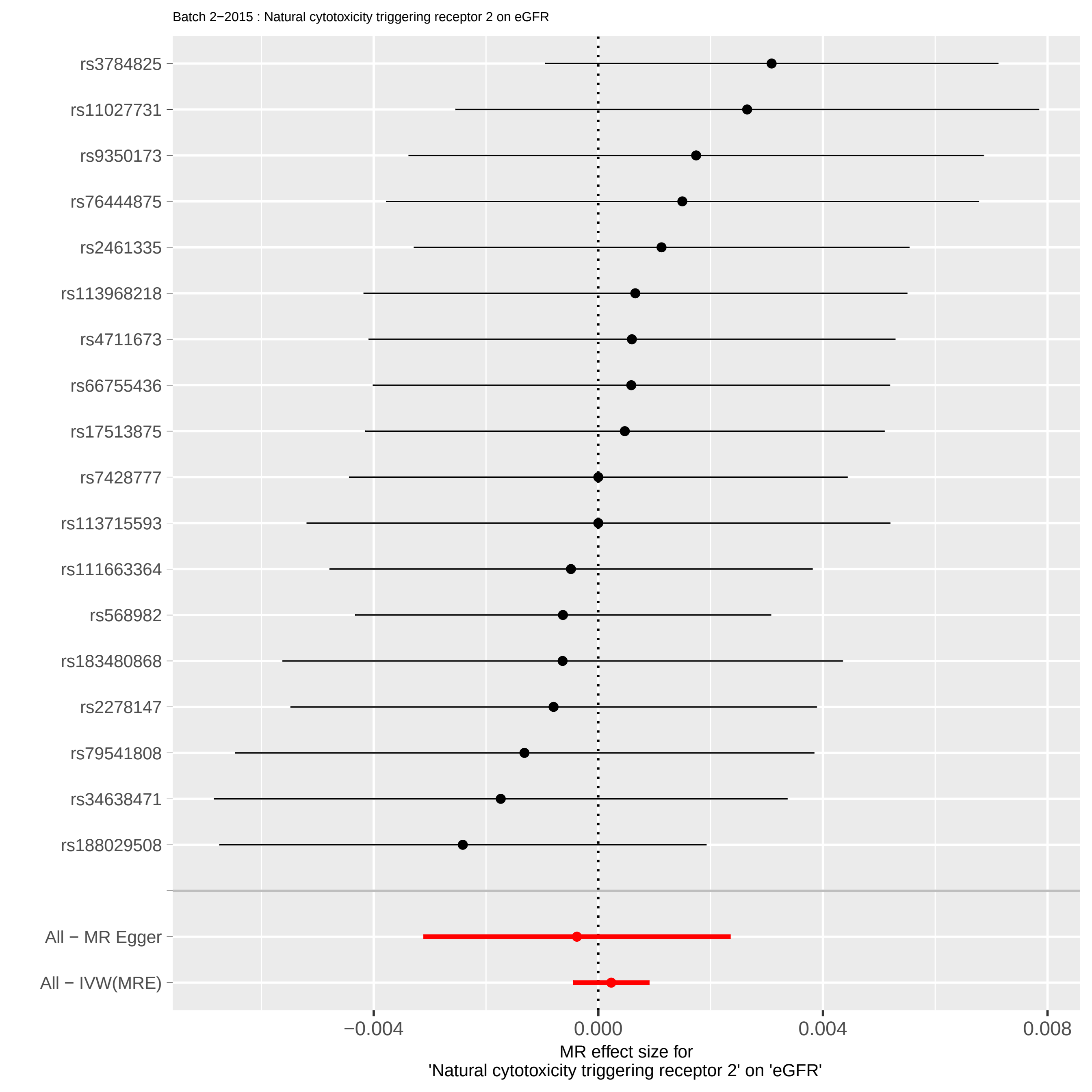
Batch 2−2015 : Natural cytotoxicity triggering receptor 2 on eGFR
rs3784825
rs11027731
rs9350173
rs76444875
rs2461335
rs113968218
rs4711673
rs66755436
rs17513875
rs7428777
rs113715593
rs111663364
rs568982
rs183480868
rs2278147
rs79541808
rs34638471
rs188029508
All − MR Egger
All − IVW(MRE)
−0.004
0.000
0.004
0.008
MR effect size for
'Natural cytotoxicity triggering receptor 2' on 'eGFR'

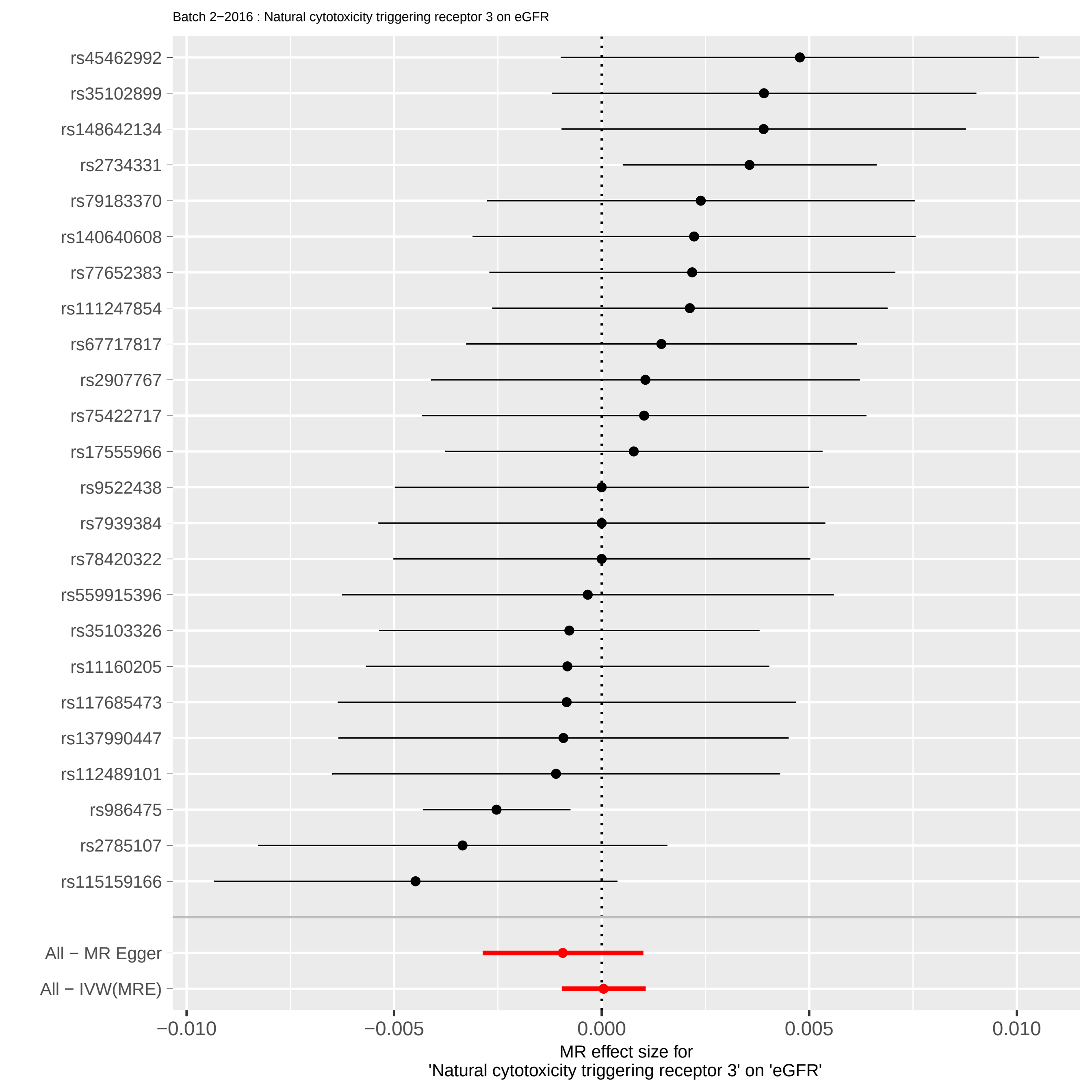

Batch 2−2016 : Natural cytotoxicity triggering receptor 3 on eGFR
rs45462992
rs35102899
rs148642134
rs2734331
rs79183370
rs140640608
rs77652383
rs111247854
rs67717817
rs2907767
rs75422717
rs17555966
rs9522438
rs7939384
rs78420322
rs559915396
rs35103326
rs11160205
rs117685473
rs137990447
rs112489101
rs986475
rs2785107
rs115159166
All − MR Egger
All − IVW(MRE)
−0.010
−0.005
0.000
0.005
0.010
MR effect size for
'Natural cytotoxicity triggering receptor 3' on 'eGFR'

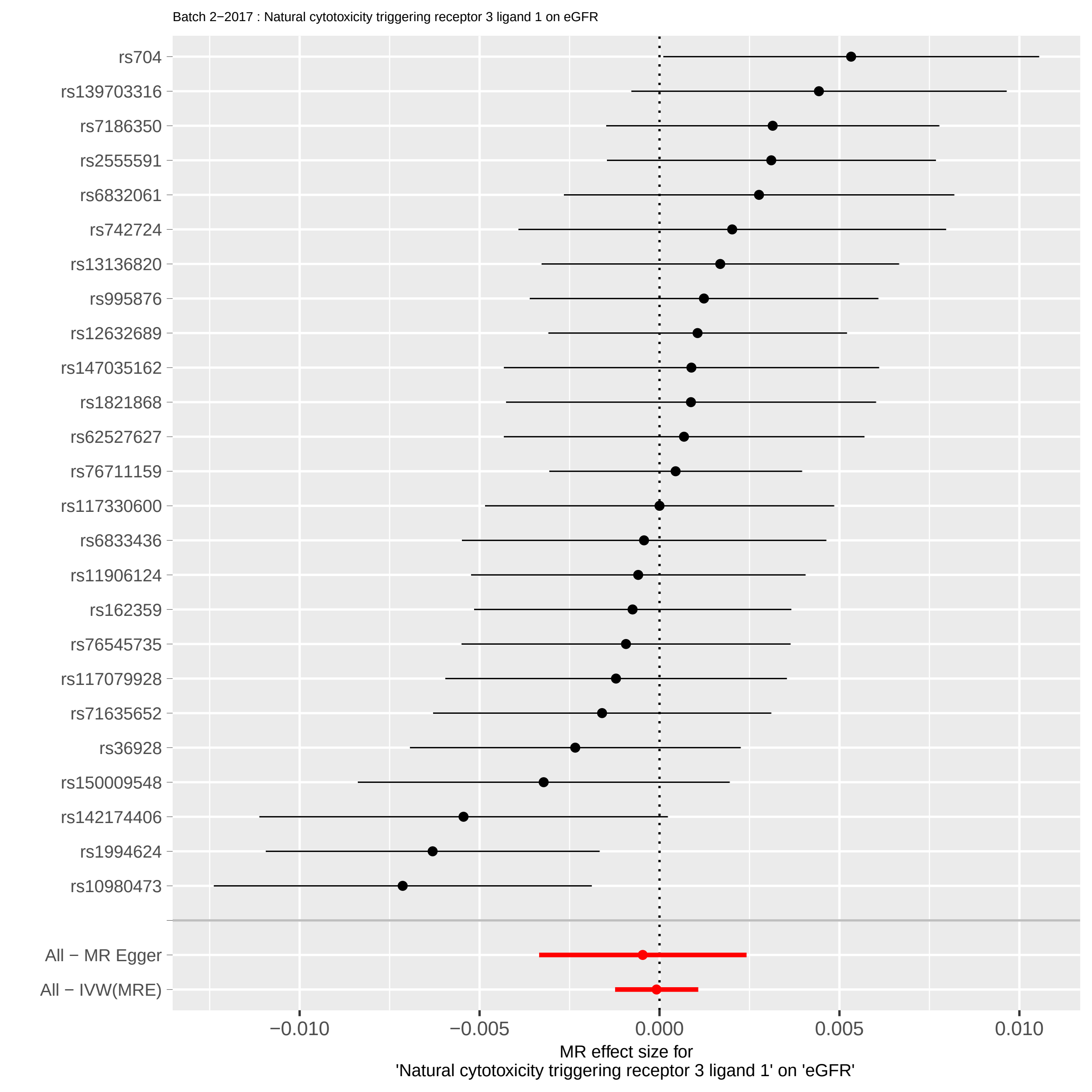
Batch 2−2017 : Natural cytotoxicity triggering receptor 3 ligand 1 on eGFR
rs704
rs139703316
rs7186350
rs2555591
rs6832061
rs742724
rs13136820
rs995876
rs12632689
rs147035162
rs1821868
rs62527627
rs76711159
rs117330600
rs6833436
rs11906124
rs162359
rs76545735
rs117079928
rs71635652
rs36928
rs150009548
rs142174406
rs1994624
rs10980473
All − MR Egger
All − IVW(MRE)
−0.010
−0.005
0.000
0.005
0.010
MR effect size for
'Natural cytotoxicity triggering receptor 3 ligand 1' on 'eGFR'

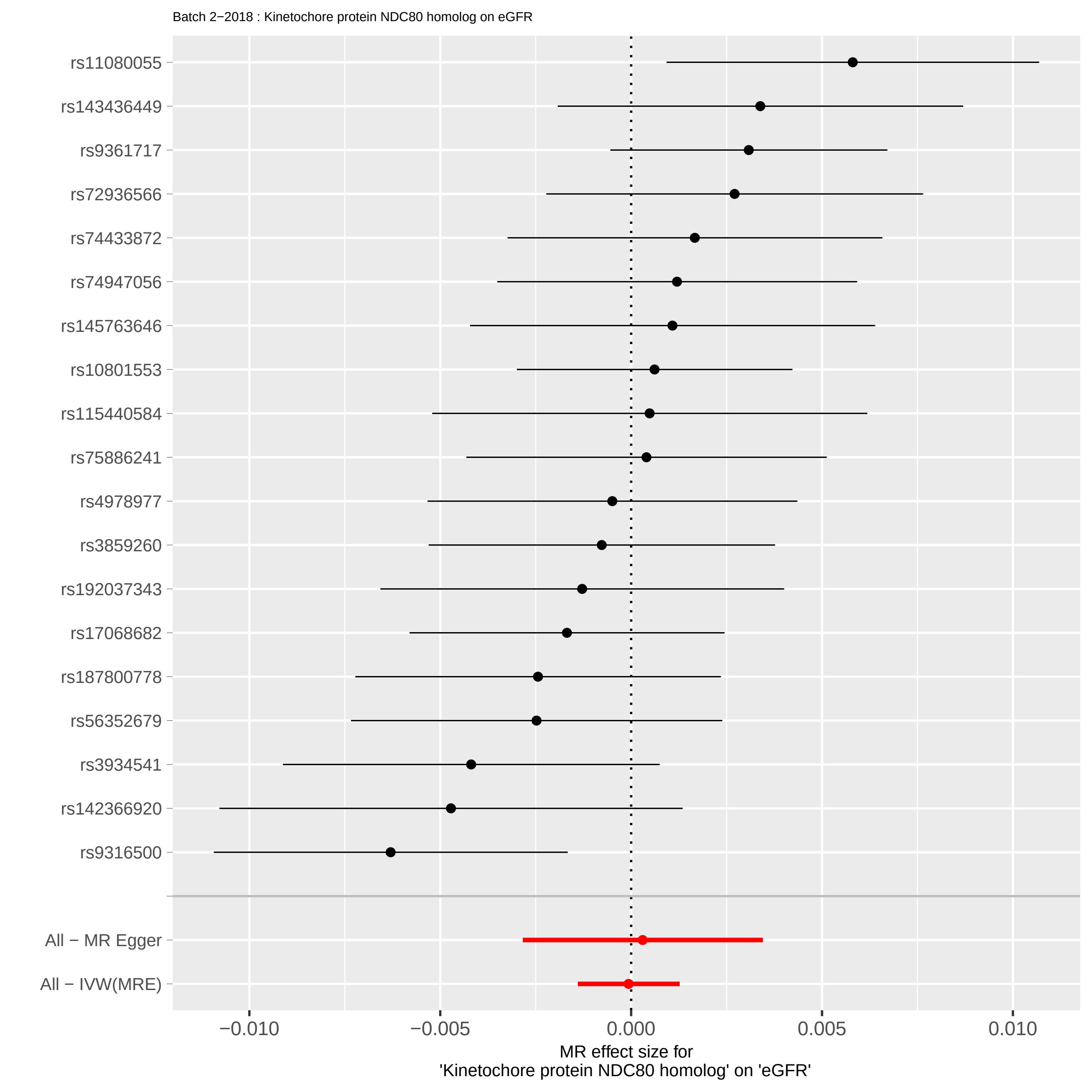

Batch 2−2018 : Kinetochore protein NDC80 homolog on eGFR
rs11080055
rs143436449
rs9361717
rs72936566
rs74433872
rs74947056
rs145763646
rs10801553
rs115440584
rs75886241
rs4978977
rs3859260
rs192037343
rs17068682
rs187800778
rs56352679
rs3934541
rs142366920
rs9316500
All − MR Egger
All − IVW(MRE)
−0.010
−0.005
0.000
0.005
0.010
MR effect size for
'Kinetochore protein NDC80 homolog' on 'eGFR'

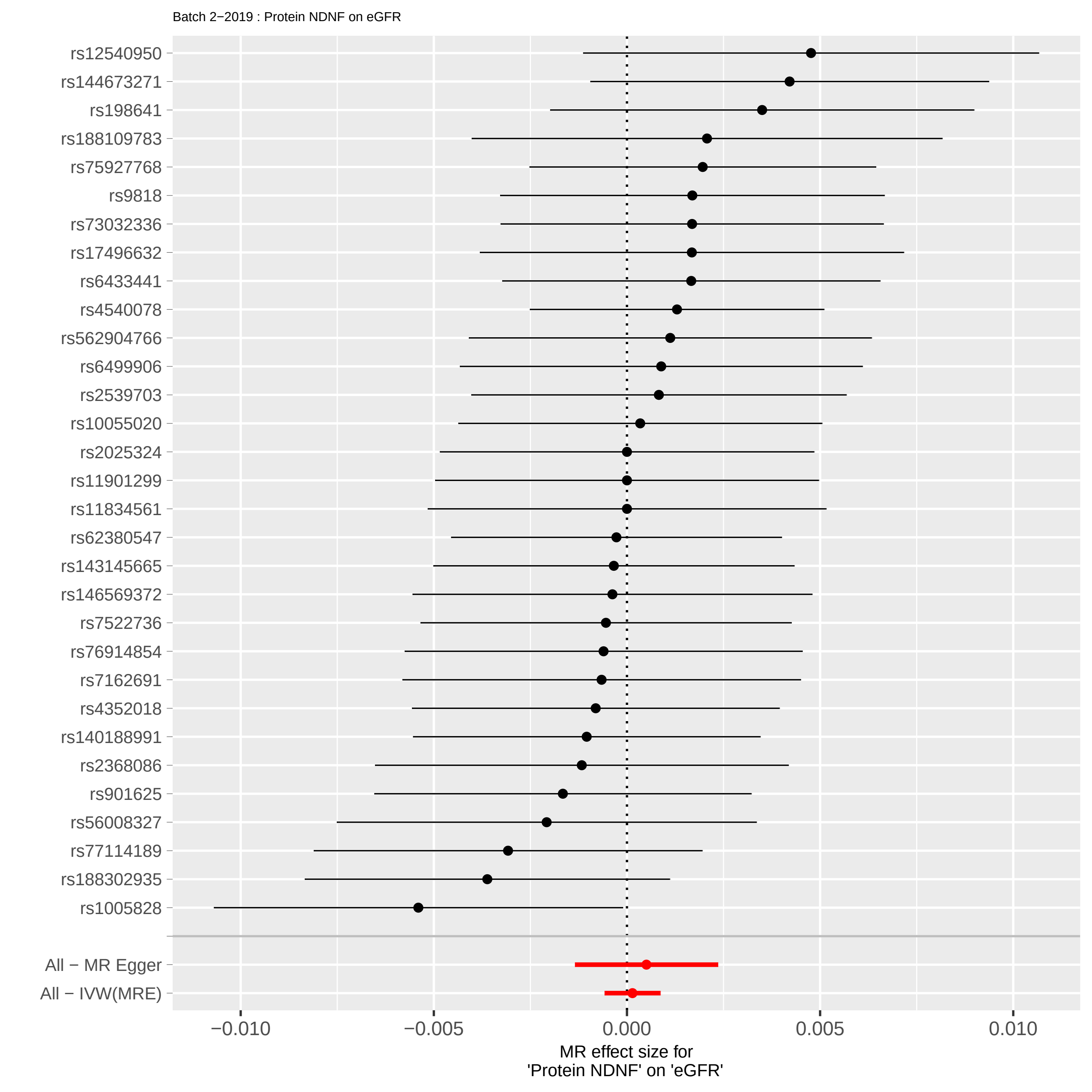

Batch 2−2019 : Protein NDNF on eGFR
rs12540950
rs144673271
rs198641
rs188109783
rs75927768
rs9818
rs73032336
rs17496632
rs6433441
rs4540078
rs562904766
rs6499906
rs2539703
rs10055020
rs2025324
rs11901299
rs11834561
rs62380547
rs143145665
rs146569372
rs7522736
rs76914854
rs7162691
rs4352018
rs140188991
rs2368086
rs901625
rs56008327
rs77114189
rs188302935
rs1005828
All − MR Egger
All − IVW(MRE)
−0.010
−0.005
0.000
0.005
0.010
MR effect size for
'Protein NDNF' on 'eGFR'

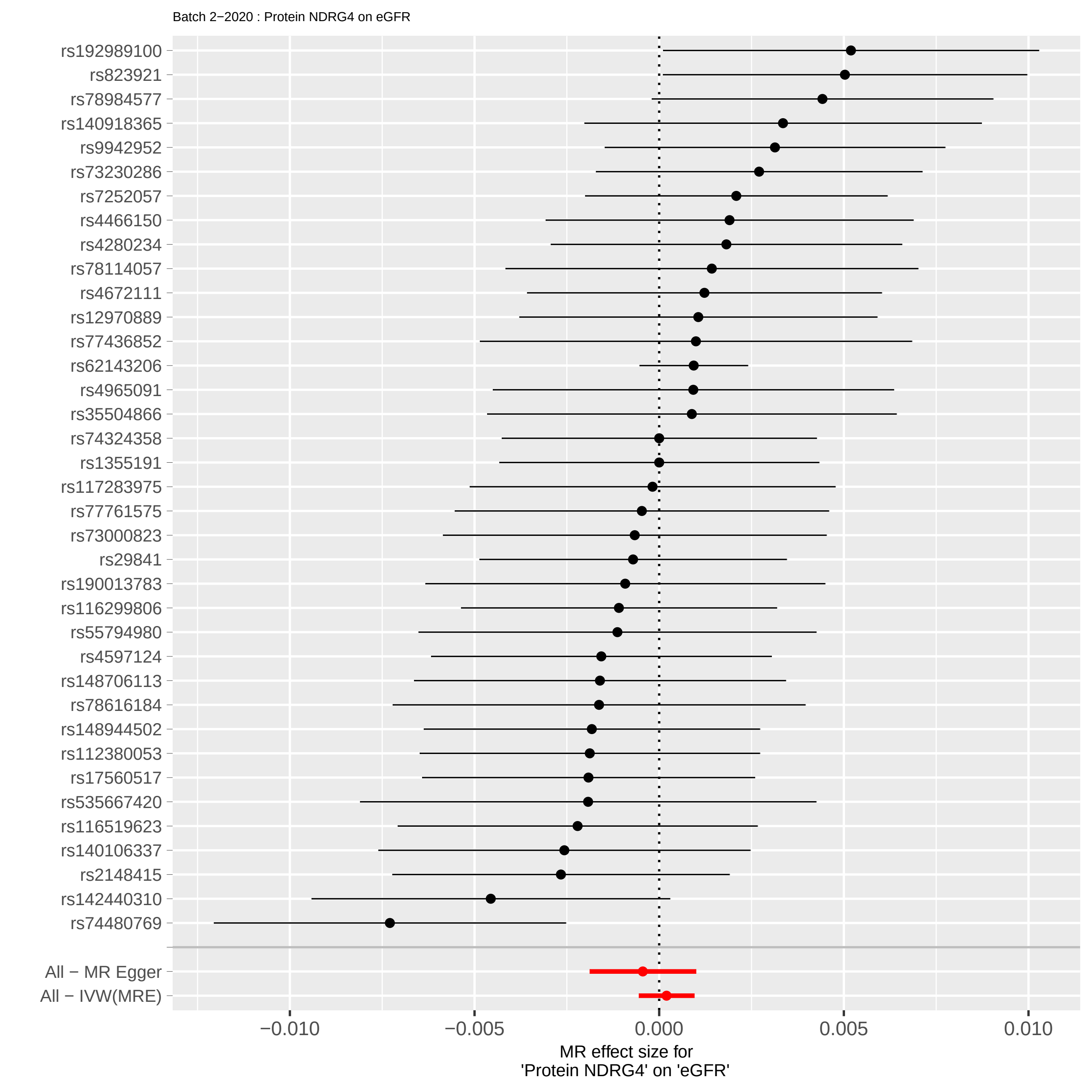
Batch 2−2020 : Protein NDRG4 on eGFR
rs192989100
rs823921
rs78984577
rs140918365
rs9942952
rs73230286
rs7252057
rs4466150
rs4280234
rs78114057
rs4672111
rs12970889
rs77436852
rs62143206
rs4965091
rs35504866
rs74324358
rs1355191
rs117283975
rs77761575
rs73000823
rs29841
rs190013783
rs116299806
rs55794980
rs4597124
rs148706113
rs78616184
rs148944502
rs112380053
rs17560517
rs535667420
rs116519623
rs140106337
rs2148415
rs142440310
rs74480769
All − MR Egger
All − IVW(MRE)
−0.010
−0.005
0.000
0.005
0.010
MR effect size for
'Protein NDRG4' on 'eGFR'

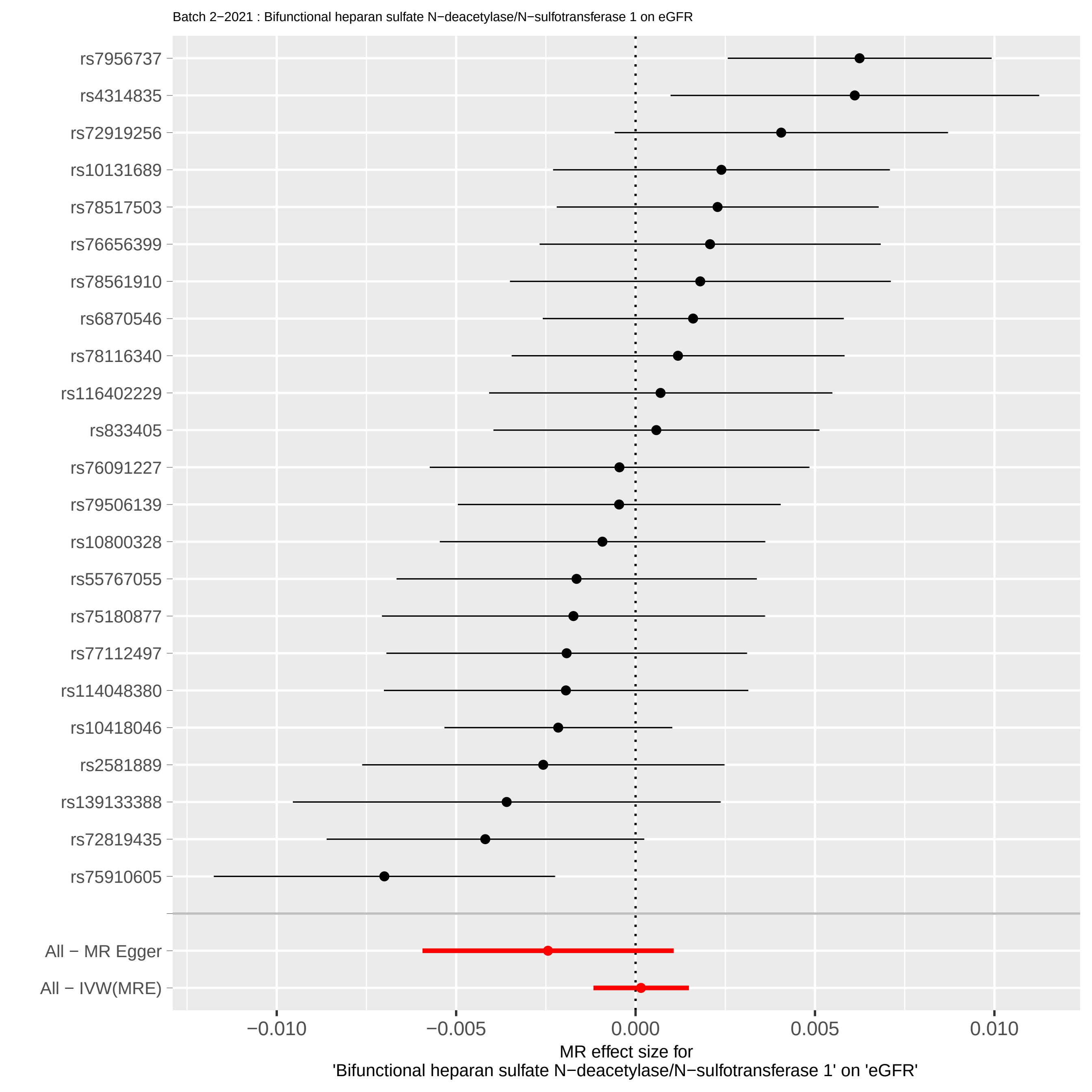

Batch 2−2021 : Bifunctional heparan sulfate N−deacetylase/N−sulfotransferase 1 on eGFR
rs7956737
rs4314835
rs72919256
rs10131689
rs78517503
rs76656399
rs78561910
rs6870546
rs78116340
rs116402229
rs833405
rs76091227
rs79506139
rs10800328
rs55767055
rs75180877
rs77112497
rs114048380
rs10418046
rs2581889
rs139133388
rs72819435
rs75910605
All − MR Egger
All − IVW(MRE)
−0.010
−0.005
0.000
0.005
0.010
MR effect size for
'Bifunctional heparan sulfate N−deacetylase/N−sulfotransferase 1' on 'eGFR'

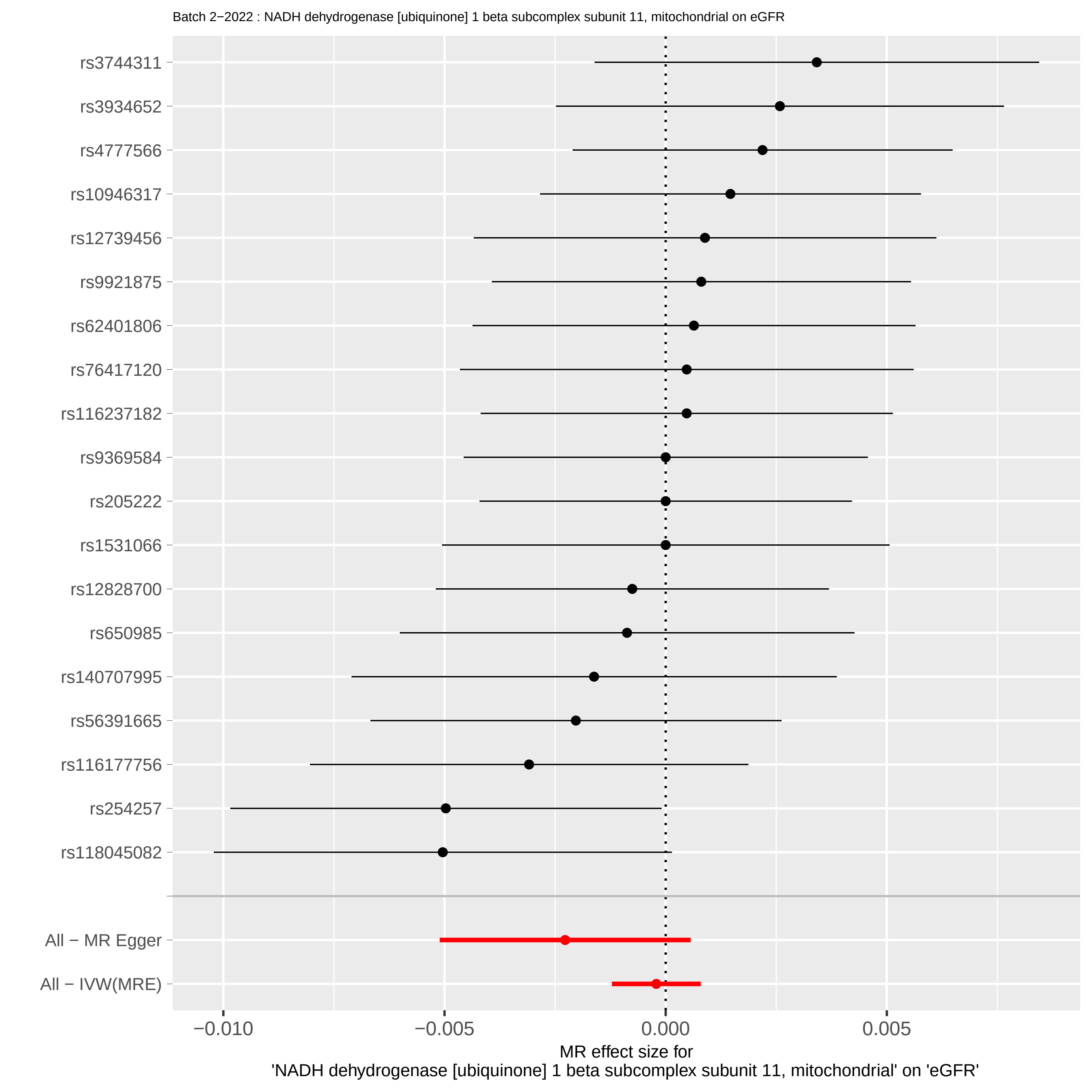

Batch 2−2022 : NADH dehydrogenase [ubiquinone] 1 beta subcomplex subunit 11, mitochondrial on eGFR
rs3744311
rs3934652
rs4777566
rs10946317
rs12739456
rs9921875
rs62401806
rs76417120
rs116237182
rs9369584
rs205222
rs1531066
rs12828700
rs650985
rs140707995
rs56391665
rs116177756
rs254257
rs118045082
All − MR Egger
All − IVW(MRE)
−0.010
−0.005
0.000
0.005
MR effect size for
'NADH dehydrogenase [ubiquinone] 1 beta subcomplex subunit 11, mitochondrial' on 'eGFR'

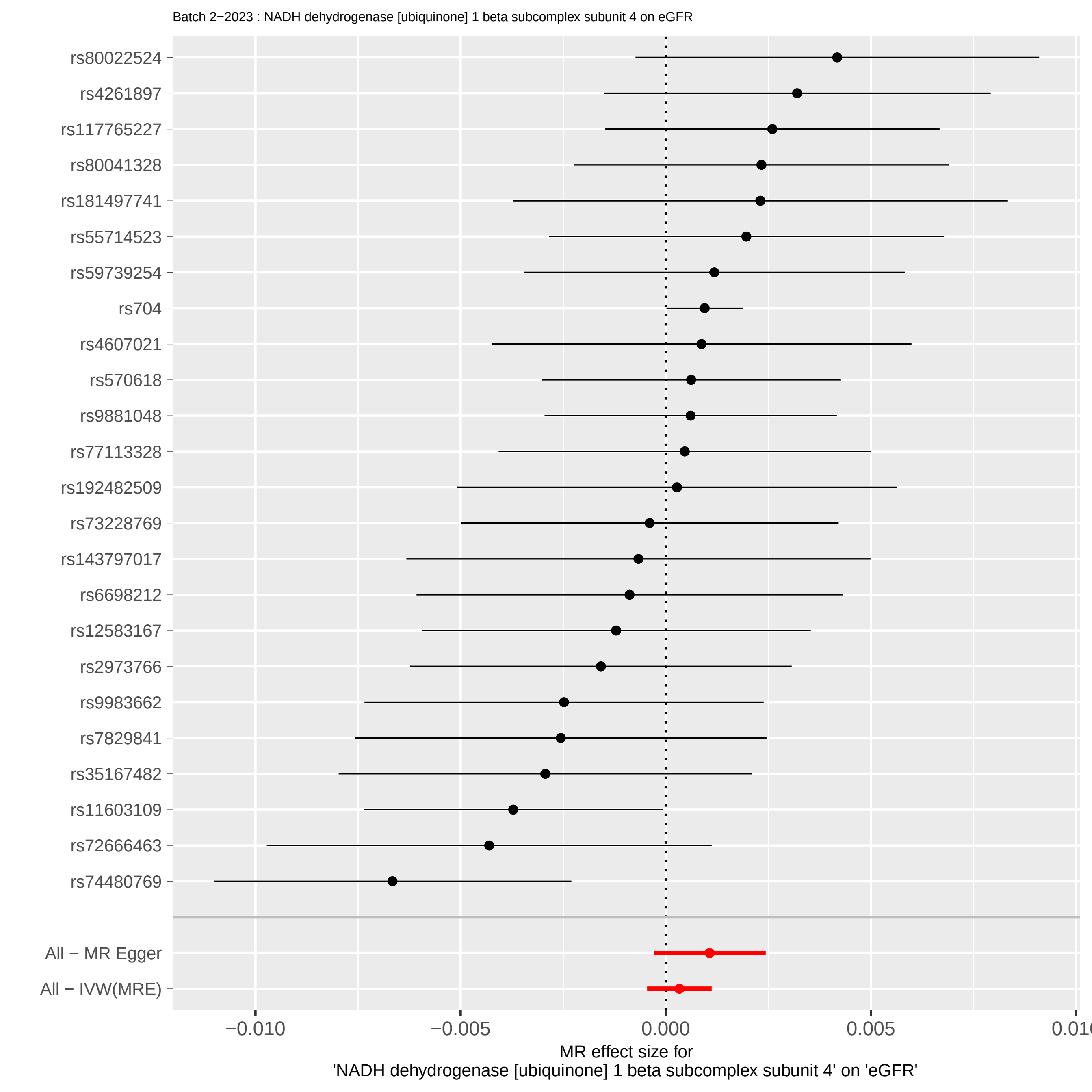
Batch 2−2023 : NADH dehydrogenase [ubiquinone] 1 beta subcomplex subunit 4 on eGFR
rs80022524
rs4261897
rs117765227
rs80041328
rs181497741
rs55714523
rs59739254
rs704
rs4607021
rs570618
rs9881048
rs77113328
rs192482509
rs73228769
rs143797017
rs6698212
rs12583167
rs2973766
rs9983662
rs7829841
rs35167482
rs11603109
rs72666463
rs74480769
All − MR Egger
All − IVW(MRE)
−0.010
−0.005
0.000
0.005
MR effect size for
'NADH dehydrogenase [ubiquinone] 1 beta subcomplex subunit 4' on 'eGFR'

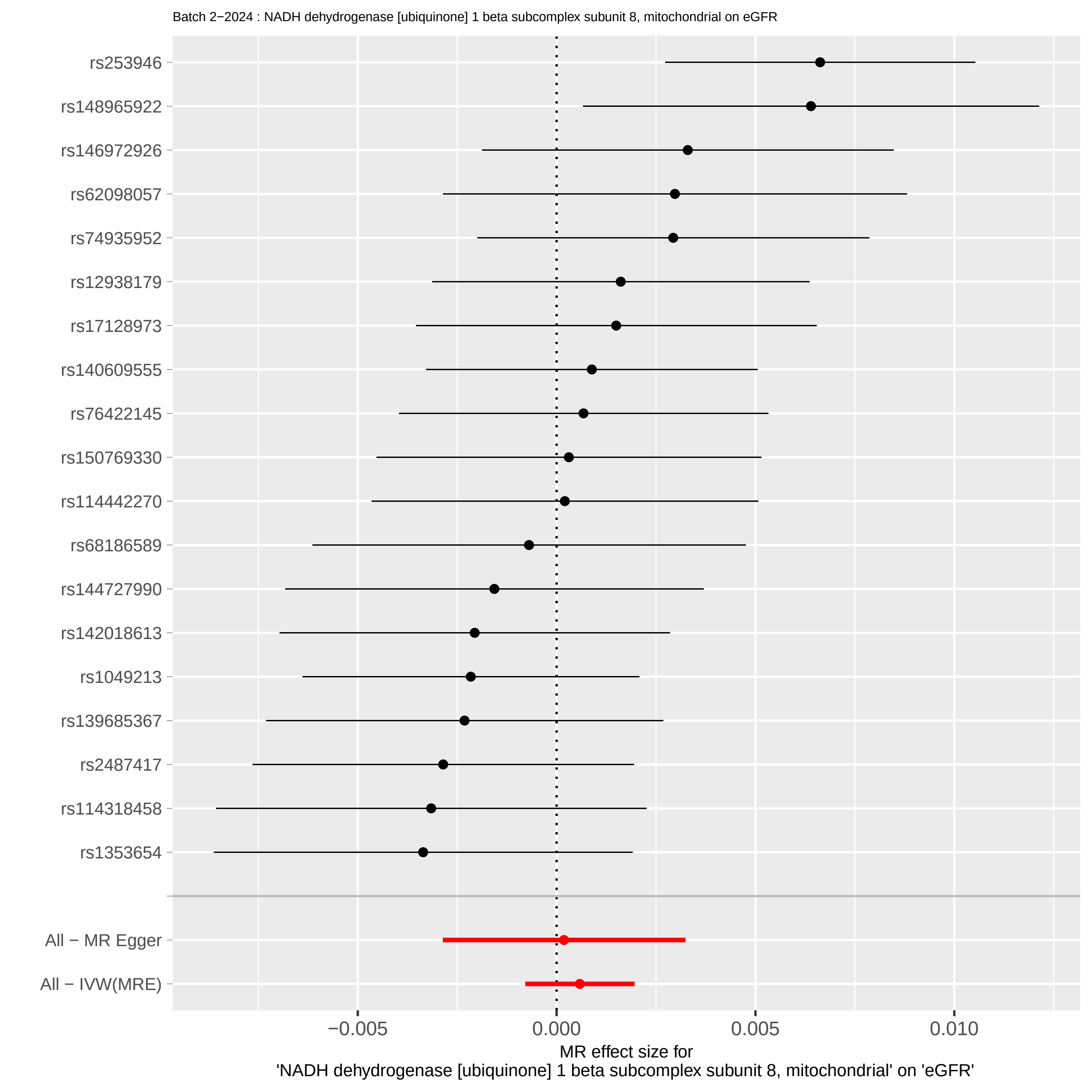

Batch 2−2024 : NADH dehydrogenase [ubiquinone] 1 beta subcomplex subunit 8, mitochondrial on eGFR
rs253946
rs148965922
rs146972926
rs62098057
rs74935952
rs12938179
rs17128973
rs140609555
rs76422145
rs150769330
rs114442270
rs68186589
rs144727990
rs142018613
rs1049213
rs139685367
rs2487417
rs114318458
rs1353654
All − MR Egger
All − IVW(MRE)
−0.005
0.000
0.005
0.010
MR effect size for
'NADH dehydrogenase [ubiquinone] 1 beta subcomplex subunit 8, mitochondrial' on 'eGFR'

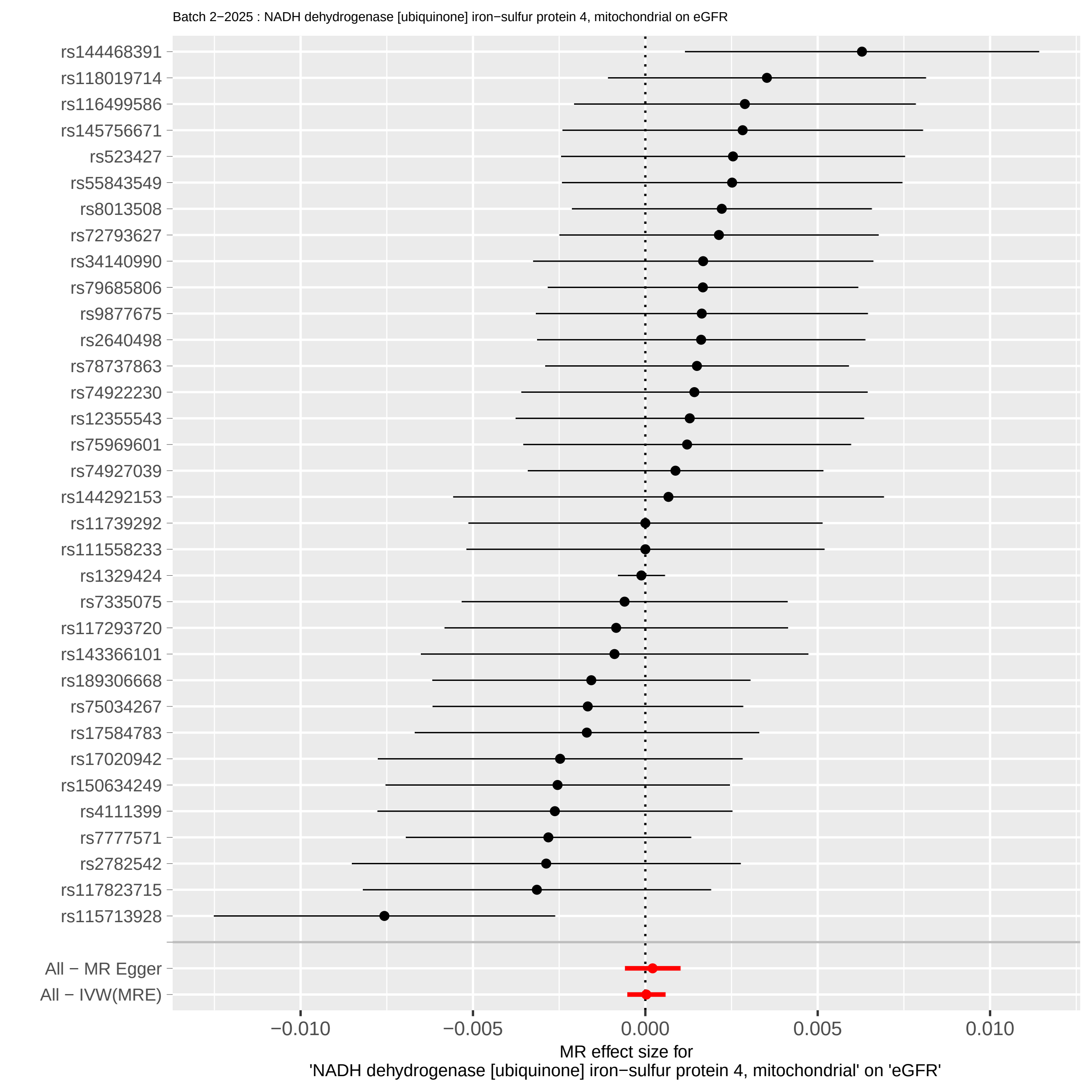

Batch 2−2025 : NADH dehydrogenase [ubiquinone] iron−sulfur protein 4, mitochondrial on eGFR
rs144468391
rs118019714
rs116499586
rs145756671
rs523427
rs55843549
rs8013508
rs72793627
rs34140990
rs79685806
rs9877675
rs2640498
rs78737863
rs74922230
rs12355543
rs75969601
rs74927039
rs144292153
rs11739292
rs111558233
rs1329424
rs7335075
rs117293720
rs143366101
rs189306668
rs75034267
rs17584783
rs17020942
rs150634249
rs4111399
rs7777571
rs2782542
rs117823715
rs115713928
All − MR Egger
All − IVW(MRE)
−0.010
−0.005
0.000
0.005
0.010
MR effect size for
'NADH dehydrogenase [ubiquinone] iron−sulfur protein 4, mitochondrial' on 'eGFR'

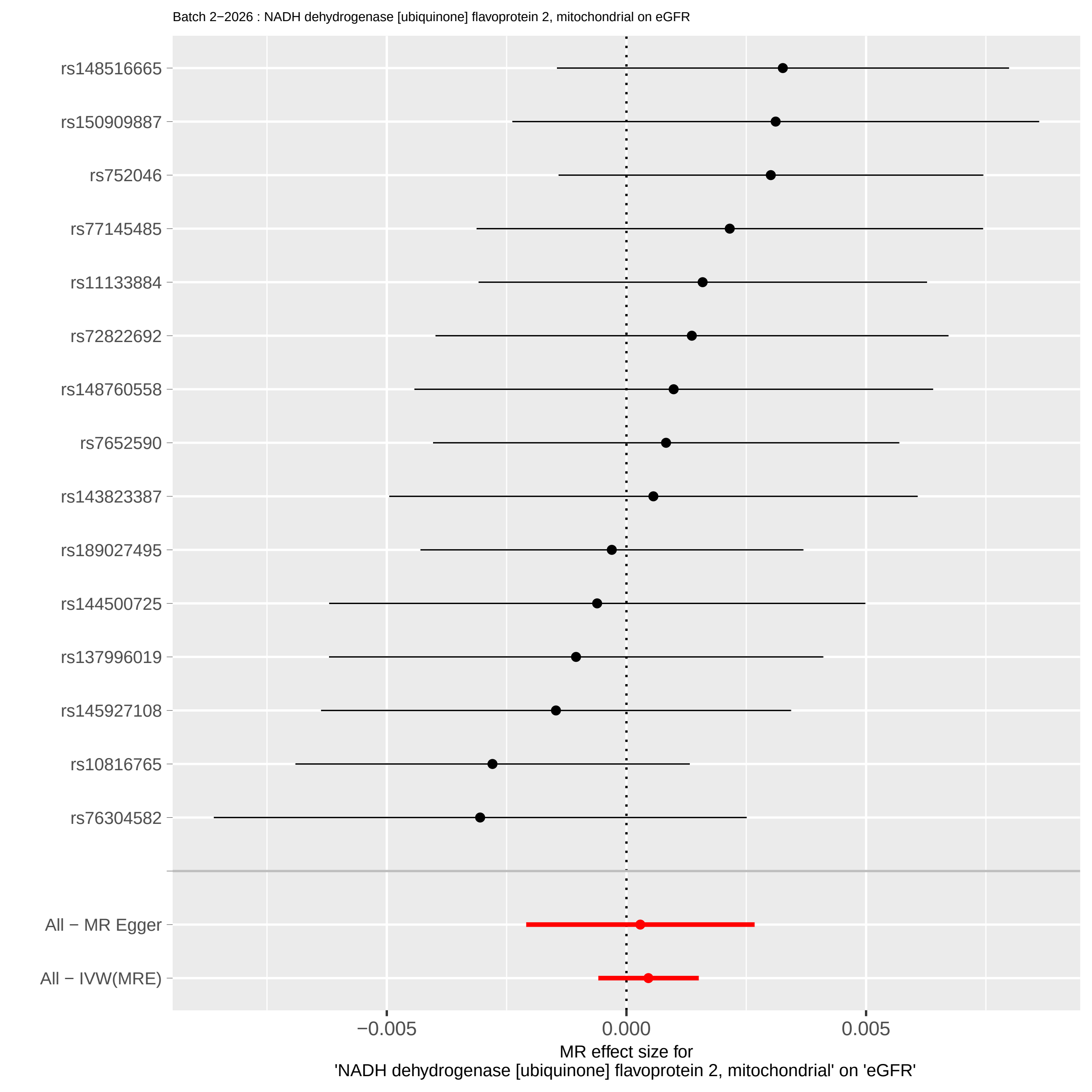
Batch 2−2026 : NADH dehydrogenase [ubiquinone] flavoprotein 2, mitochondrial on eGFR
rs148516665
rs150909887
rs752046
rs77145485
rs11133884
rs72822692
rs148760558
rs7652590
rs143823387
rs189027495
rs144500725
rs137996019
rs145927108
rs10816765
rs76304582
All − MR Egger
All − IVW(MRE)
−0.005
0.000
0.005
MR effect size for
'NADH dehydrogenase [ubiquinone] flavoprotein 2, mitochondrial' on 'eGFR'

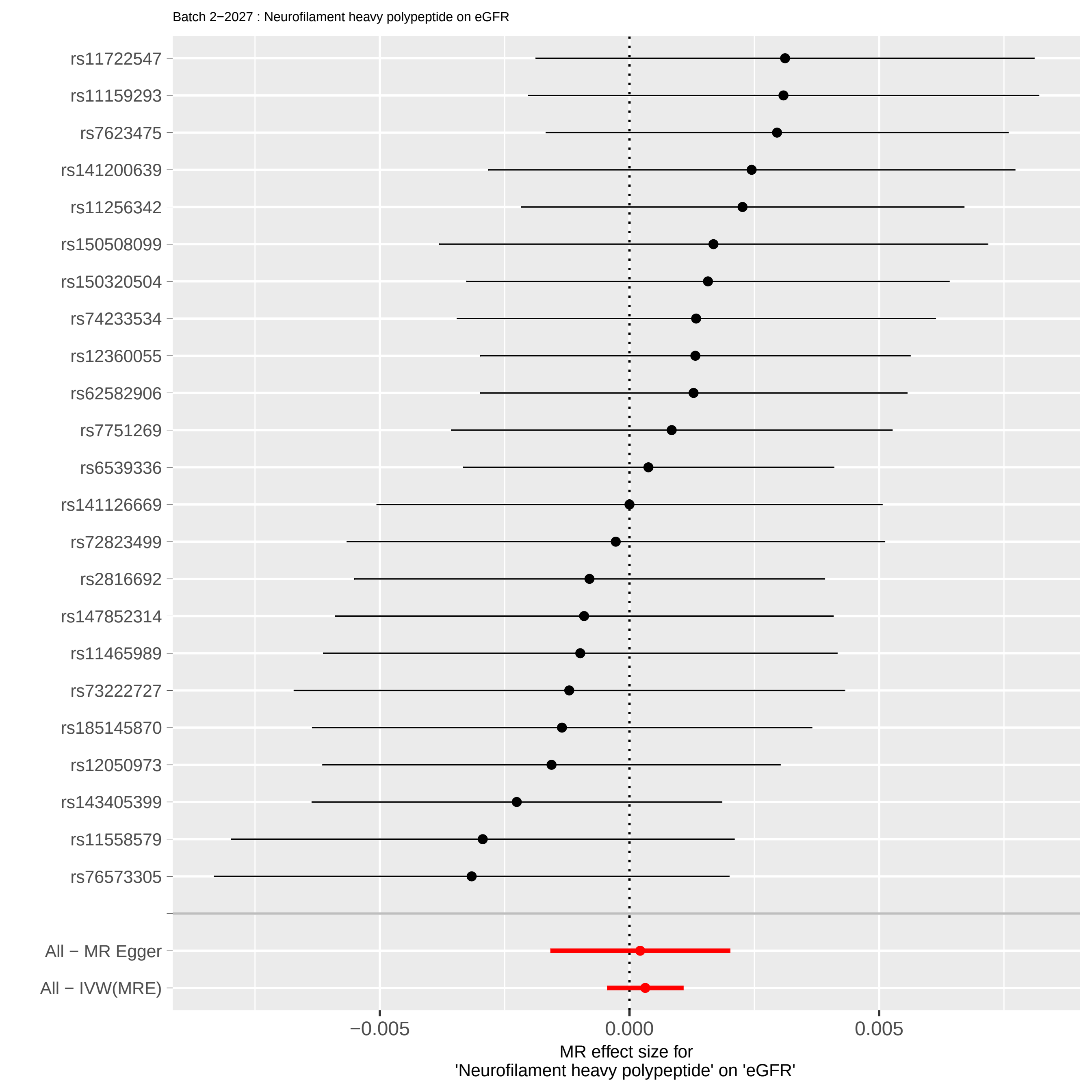

Batch 2−2027 : Neurofilament heavy polypeptide on eGFR
rs11722547
rs11159293
rs7623475
rs141200639
rs11256342
rs150508099
rs150320504
rs74233534
rs12360055
rs62582906
rs7751269
rs6539336
rs141126669
rs72823499
rs2816692
rs147852314
rs11465989
rs73222727
rs185145870
rs12050973
rs143405399
rs11558579
rs76573305
All − MR Egger
All − IVW(MRE)
−0.005
0.000
0.005
MR effect size for
'Neurofilament heavy polypeptide' on 'eGFR'

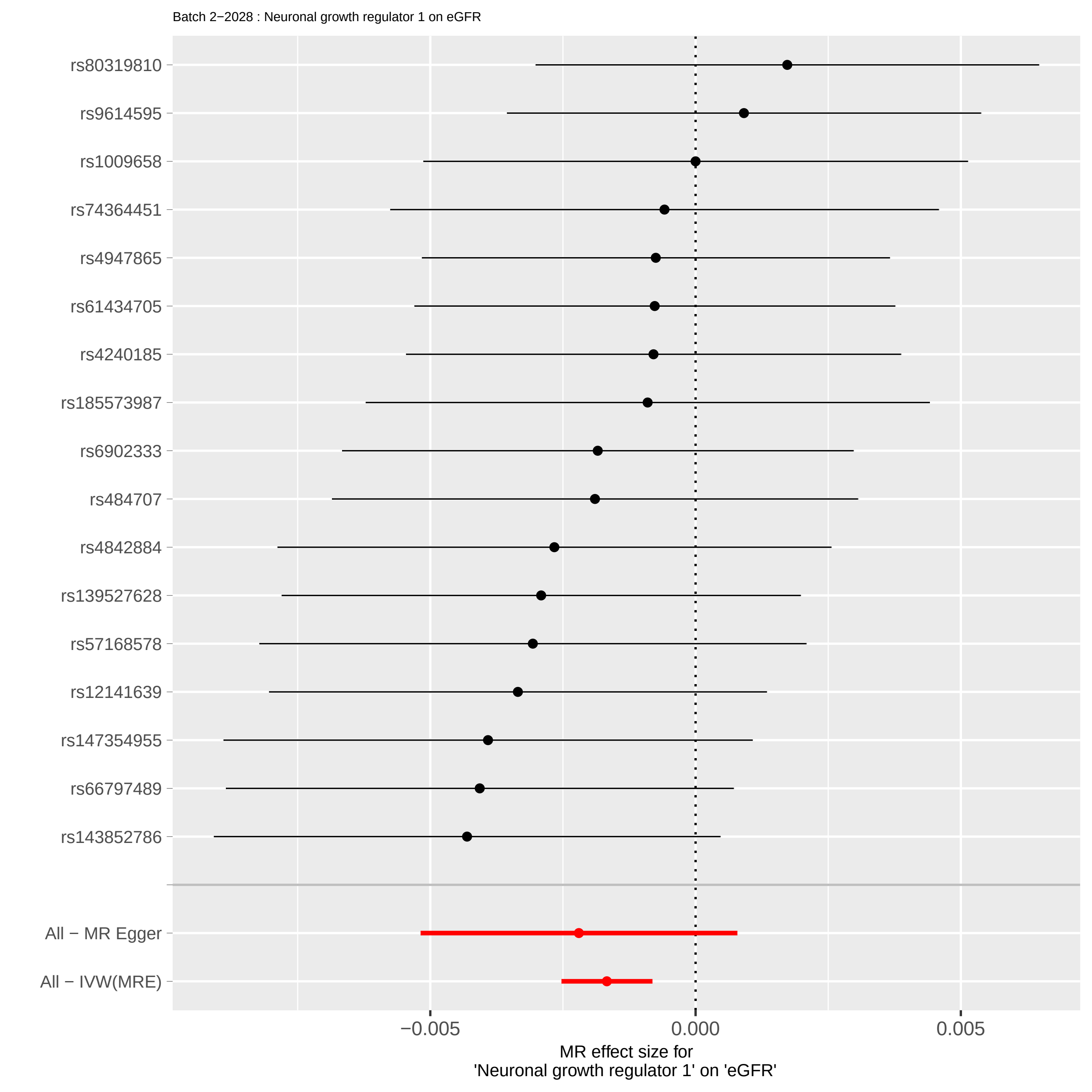
Batch 2−2028 : Neuronal growth regulator 1 on eGFR
rs80319810
rs9614595
rs1009658
rs74364451
rs4947865
rs61434705
rs4240185
rs185573987
rs6902333
rs484707
rs4842884
rs139527628
rs57168578
rs12141639
rs147354955
rs66797489
rs143852786
All − MR Egger
All − IVW(MRE)
−0.005
0.000
0.005
MR effect size for
'Neuronal growth regulator 1' on 'eGFR'

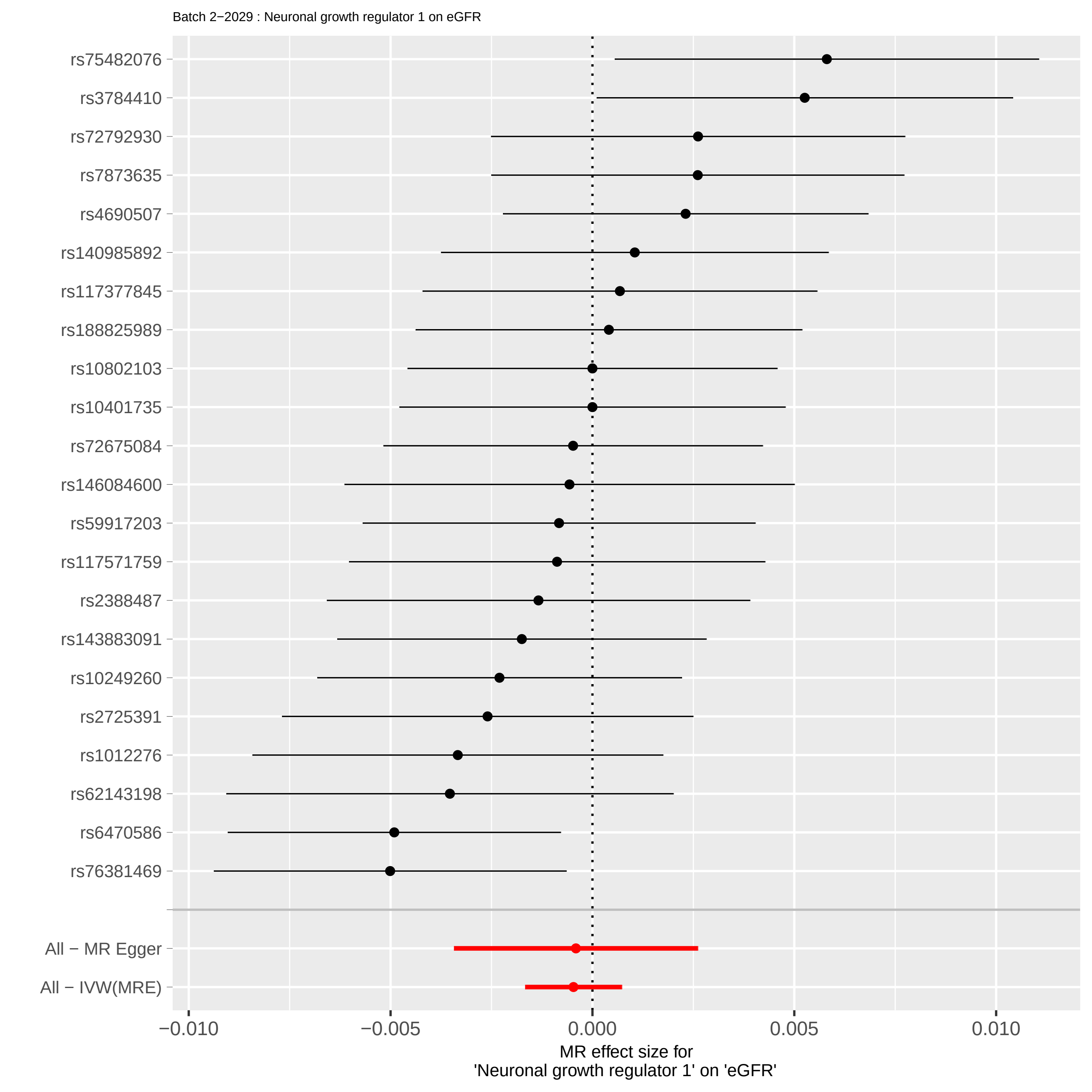
Batch 2−2029 : Neuronal growth regulator 1 on eGFR
rs75482076
rs3784410
rs72792930
rs7873635
rs4690507
rs140985892
rs117377845
rs188825989
rs10802103
rs10401735
rs72675084
rs146084600
rs59917203
rs117571759
rs2388487
rs143883091
rs10249260
rs2725391
rs1012276
rs62143198
rs6470586
rs76381469
All − MR Egger
All − IVW(MRE)
−0.010
−0.005
0.000
0.005
0.010
MR effect size for
'Neuronal growth regulator 1' on 'eGFR'

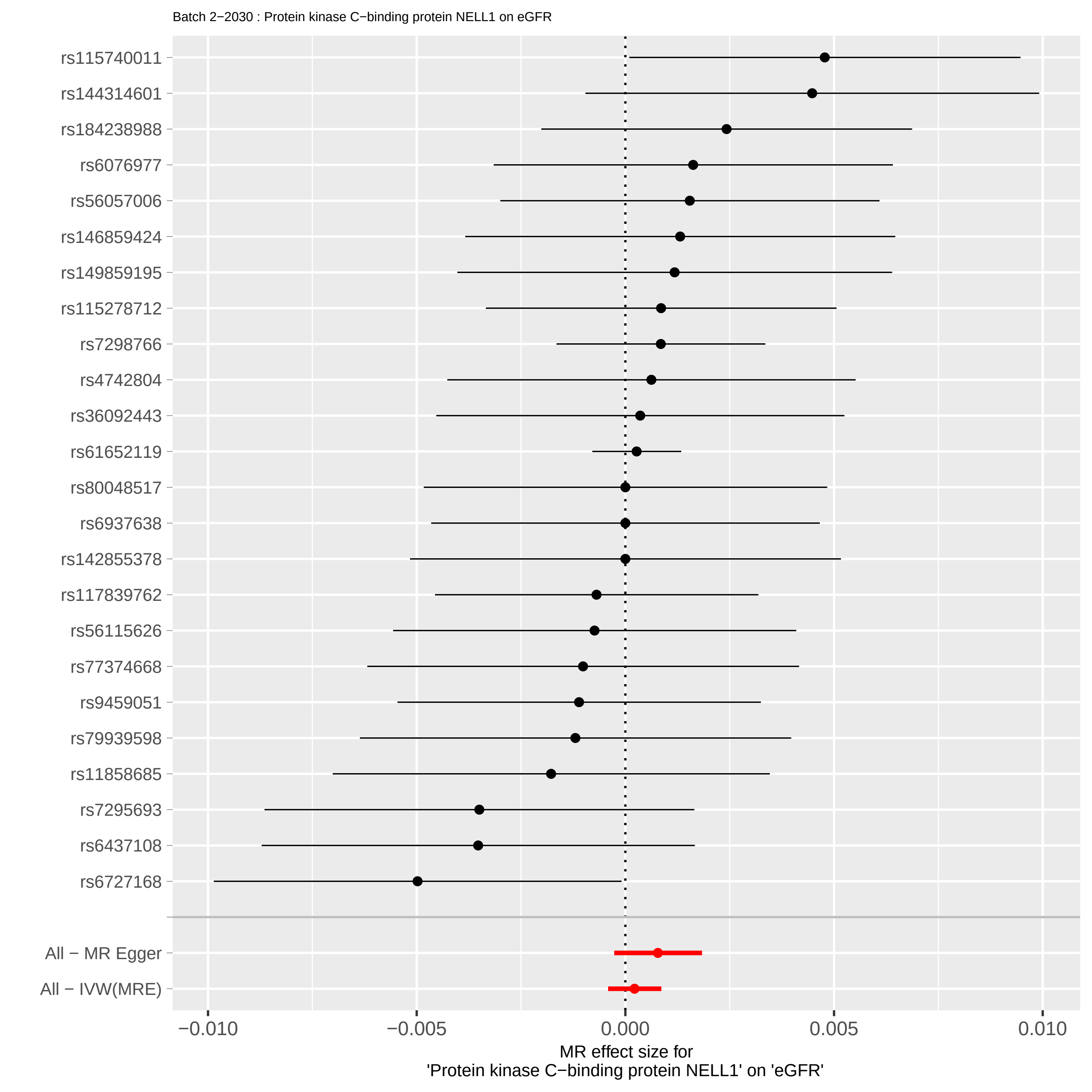

Batch 2−2030 : Protein kinase C−binding protein NELL1 on eGFR
rs115740011
rs144314601
rs184238988
rs6076977
rs56057006
rs146859424
rs149859195
rs115278712
rs7298766
rs4742804
rs36092443
rs61652119
rs80048517
rs6937638
rs142855378
rs117839762
rs56115626
rs77374668
rs9459051
rs79939598
rs11858685
rs7295693
rs6437108
rs6727168
All − MR Egger
All − IVW(MRE)
−0.010
−0.005
0.000
0.005
0.010
MR effect size for
'Protein kinase C−binding protein NELL1' on 'eGFR'

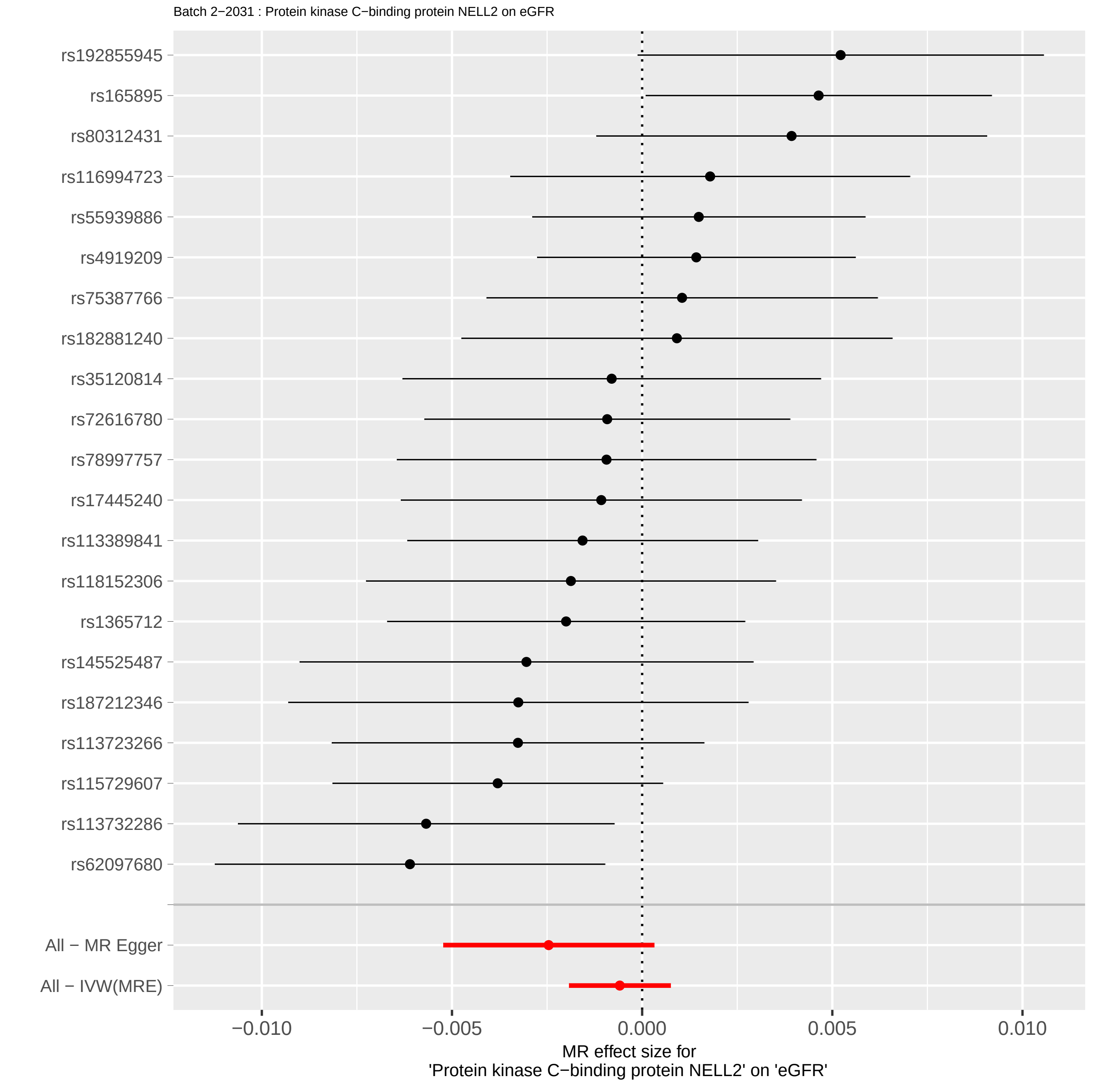

Batch 2−2031 : Protein kinase C−binding protein NELL2 on eGFR
rs192855945
rs165895
rs80312431
rs116994723
rs55939886
rs4919209
rs75387766
rs182881240
rs35120814
rs72616780
rs78997757
rs17445240
rs113389841
rs118152306
rs1365712
rs145525487
rs187212346
rs113723266
rs115729607
rs113732286
rs62097680
All − MR Egger
All − IVW(MRE)
−0.010
−0.005
0.000
0.005
0.010
MR effect size for
'Protein kinase C−binding protein NELL2' on 'eGFR'

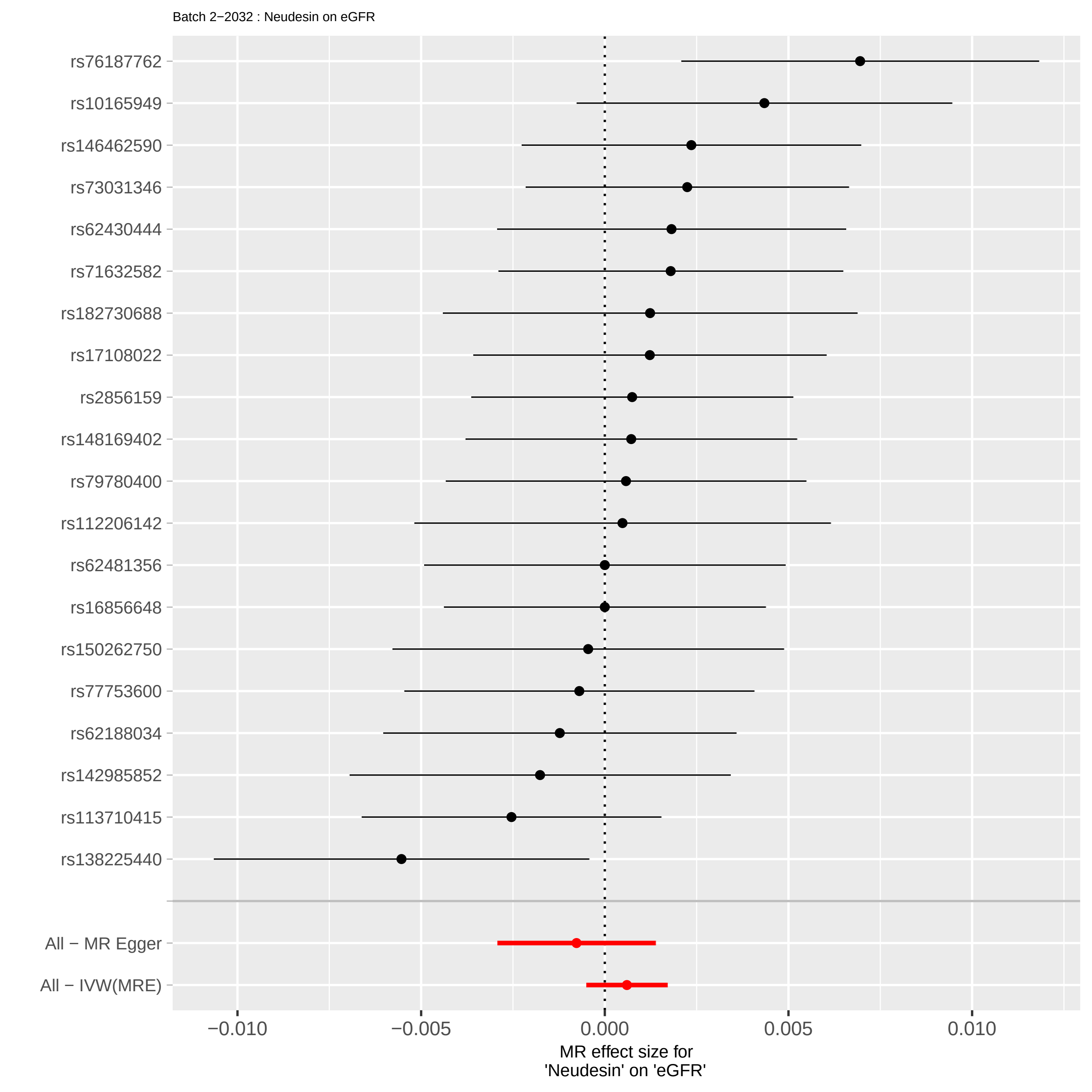

Batch 2−2032 : Neudesin on eGFR
rs76187762
rs10165949
rs146462590
rs73031346
rs62430444
rs71632582
rs182730688
rs17108022
rs2856159
rs148169402
rs79780400
rs112206142
rs62481356
rs16856648
rs150262750
rs77753600
rs62188034
rs142985852
rs113710415
rs138225440
All − MR Egger
All − IVW(MRE)
−0.010
−0.005
0.000
0.005
0.010
MR effect size for
'Neudesin' on 'eGFR'

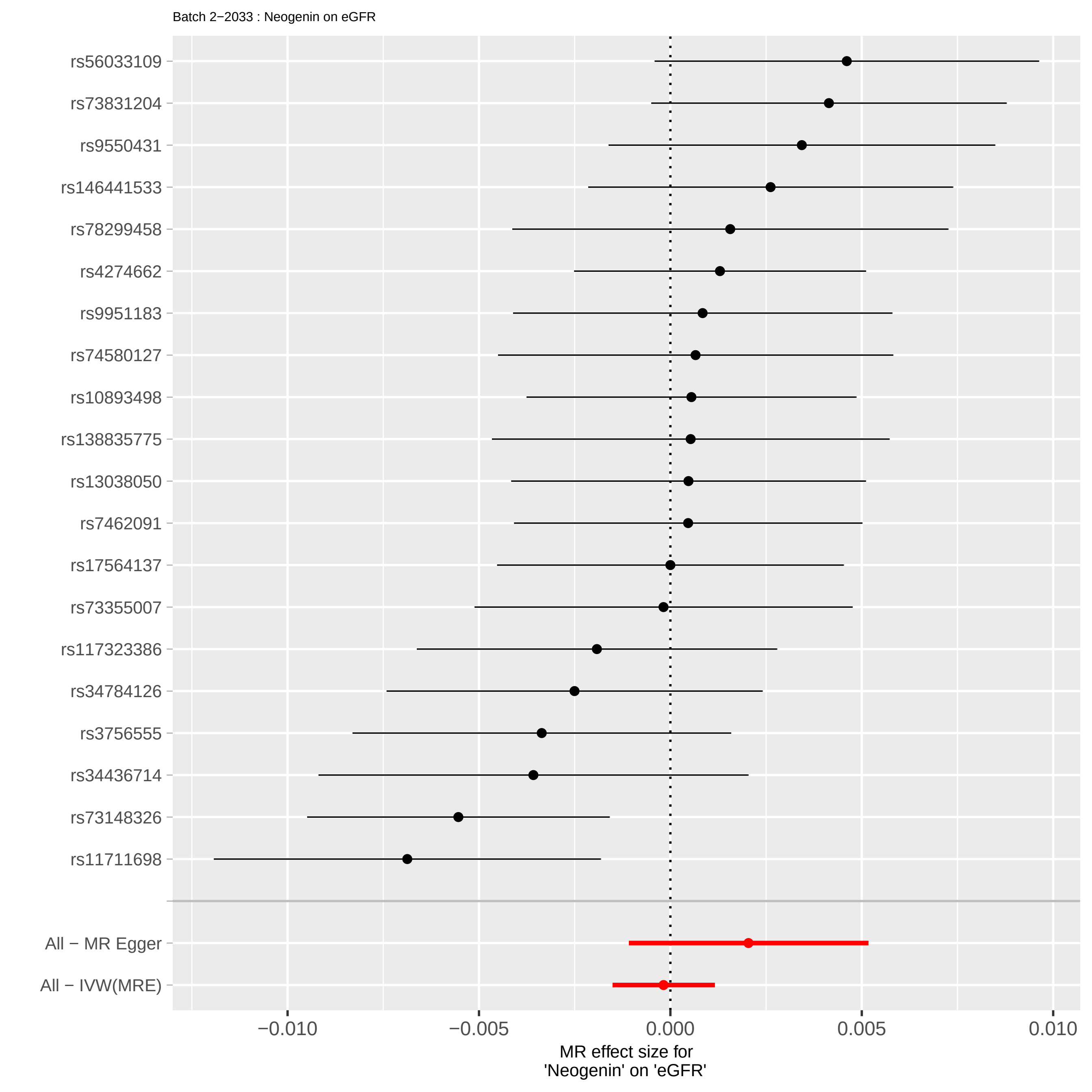

Batch 2−2033 : Neogenin on eGFR
rs56033109
rs73831204
rs9550431
rs146441533
rs78299458
rs4274662
rs9951183
rs74580127
rs10893498
rs138835775
rs13038050
rs7462091
rs17564137
rs73355007
rs117323386
rs34784126
rs3756555
rs34436714
rs73148326
rs11711698
All − MR Egger
All − IVW(MRE)
−0.010
−0.005
0.000
0.005
0.010
MR effect size for
'Neogenin' on 'eGFR'

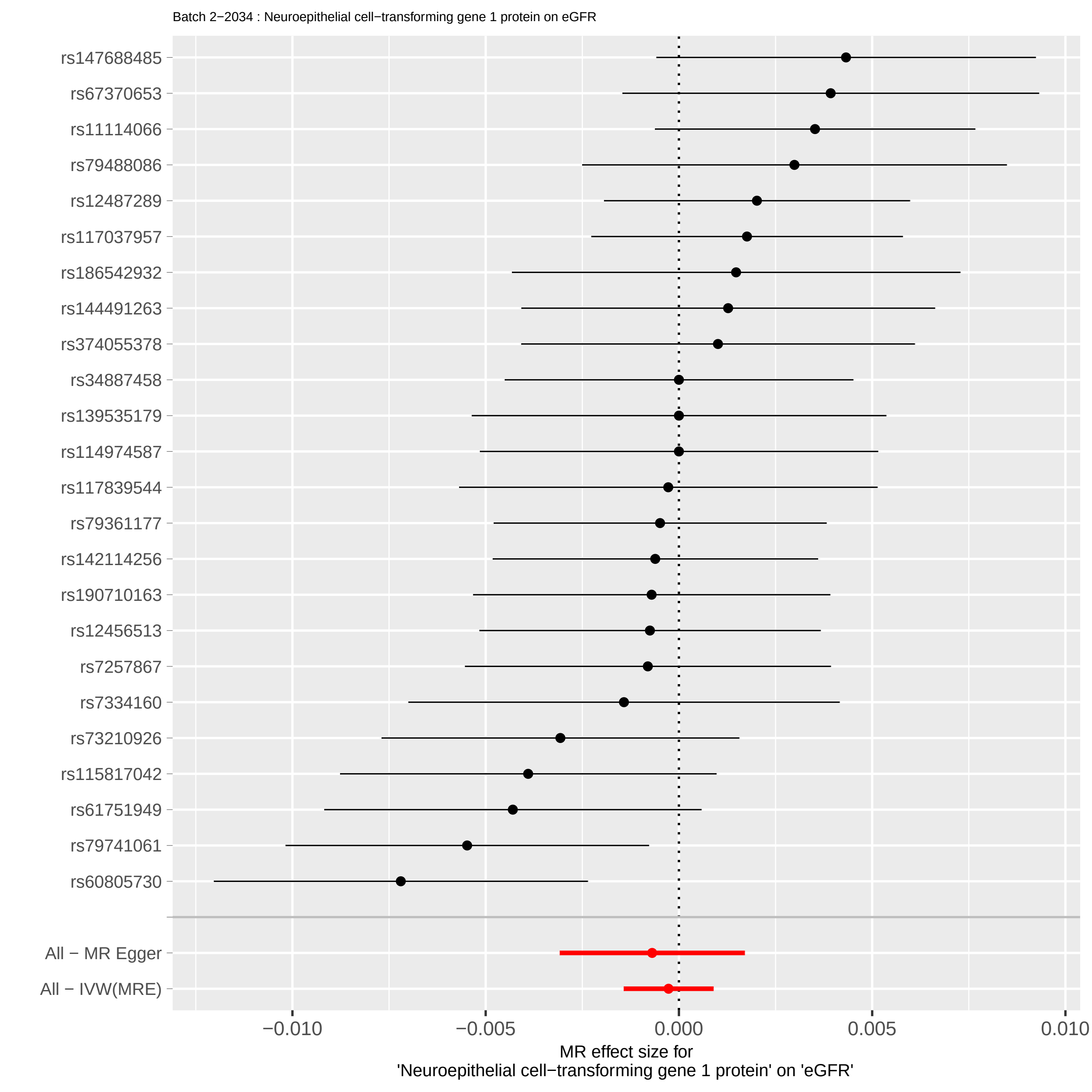

Batch 2−2034 : Neuroepithelial cell−transforming gene 1 protein on eGFR
rs147688485
rs67370653
rs11114066
rs79488086
rs12487289
rs117037957
rs186542932
rs144491263
rs374055378
rs34887458
rs139535179
rs114974587
rs117839544
rs79361177
rs142114256
rs190710163
rs12456513
rs7257867
rs7334160
rs73210926
rs115817042
rs61751949
rs79741061
rs60805730
All − MR Egger
All − IVW(MRE)
−0.010
−0.005
0.000
0.005
0.010
MR effect size for
'Neuroepithelial cell−transforming gene 1 protein' on 'eGFR'

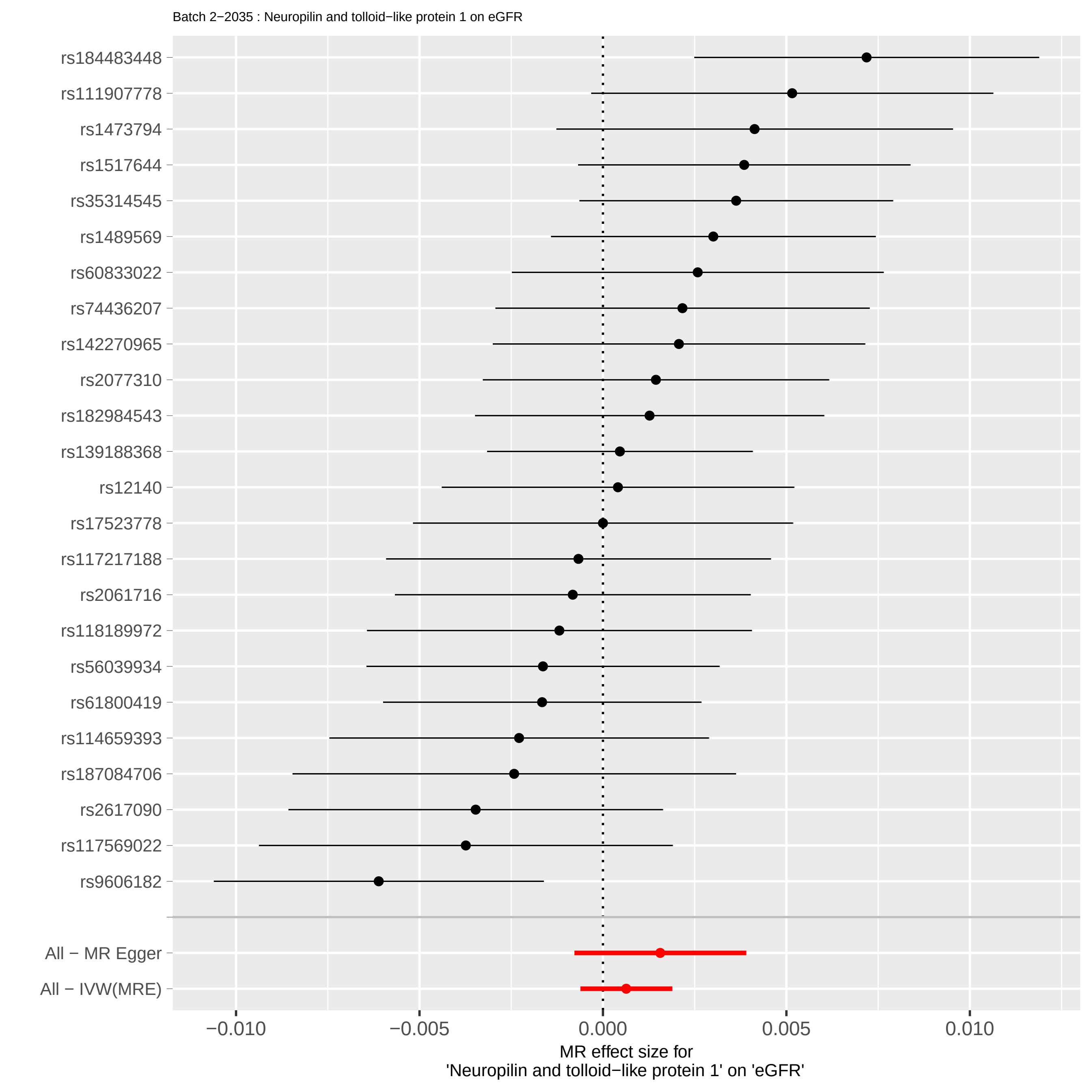

Batch 2−2035 : Neuropilin and tolloid−like protein 1 on eGFR
rs184483448
rs111907778
rs1473794
rs1517644
rs35314545
rs1489569
rs60833022
rs74436207
rs142270965
rs2077310
rs182984543
rs139188368
rs12140
rs17523778
rs117217188
rs2061716
rs118189972
rs56039934
rs61800419
rs114659393
rs187084706
rs2617090
rs117569022
rs9606182
All − MR Egger
All − IVW(MRE)
−0.010
−0.005
0.000
0.005
0.010
MR effect size for
'Neuropilin and tolloid−like protein 1' on 'eGFR'

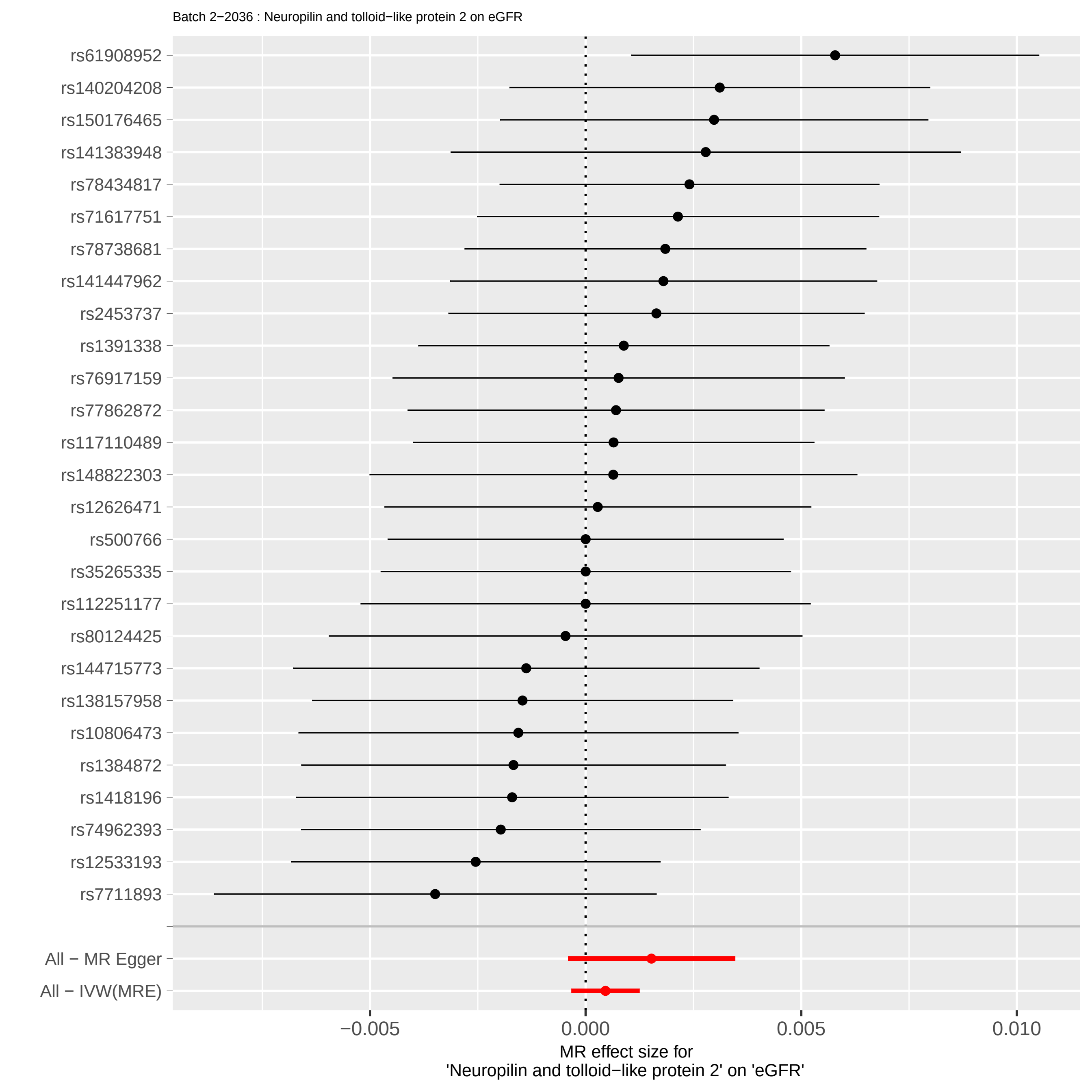
Batch 2−2036 : Neuropilin and tolloid−like protein 2 on eGFR
rs61908952
rs140204208
rs150176465
rs141383948
rs78434817
rs71617751
rs78738681
rs141447962
rs2453737
rs1391338
rs76917159
rs77862872
rs117110489
rs148822303
rs12626471
rs500766
rs35265335
rs112251177
rs80124425
rs144715773
rs138157958
rs10806473
rs1384872
rs1418196
rs74962393
rs12533193
rs7711893
All − MR Egger
All − IVW(MRE)
−0.005
0.000
0.005
0.010
MR effect size for
'Neuropilin and tolloid−like protein 2' on 'eGFR'

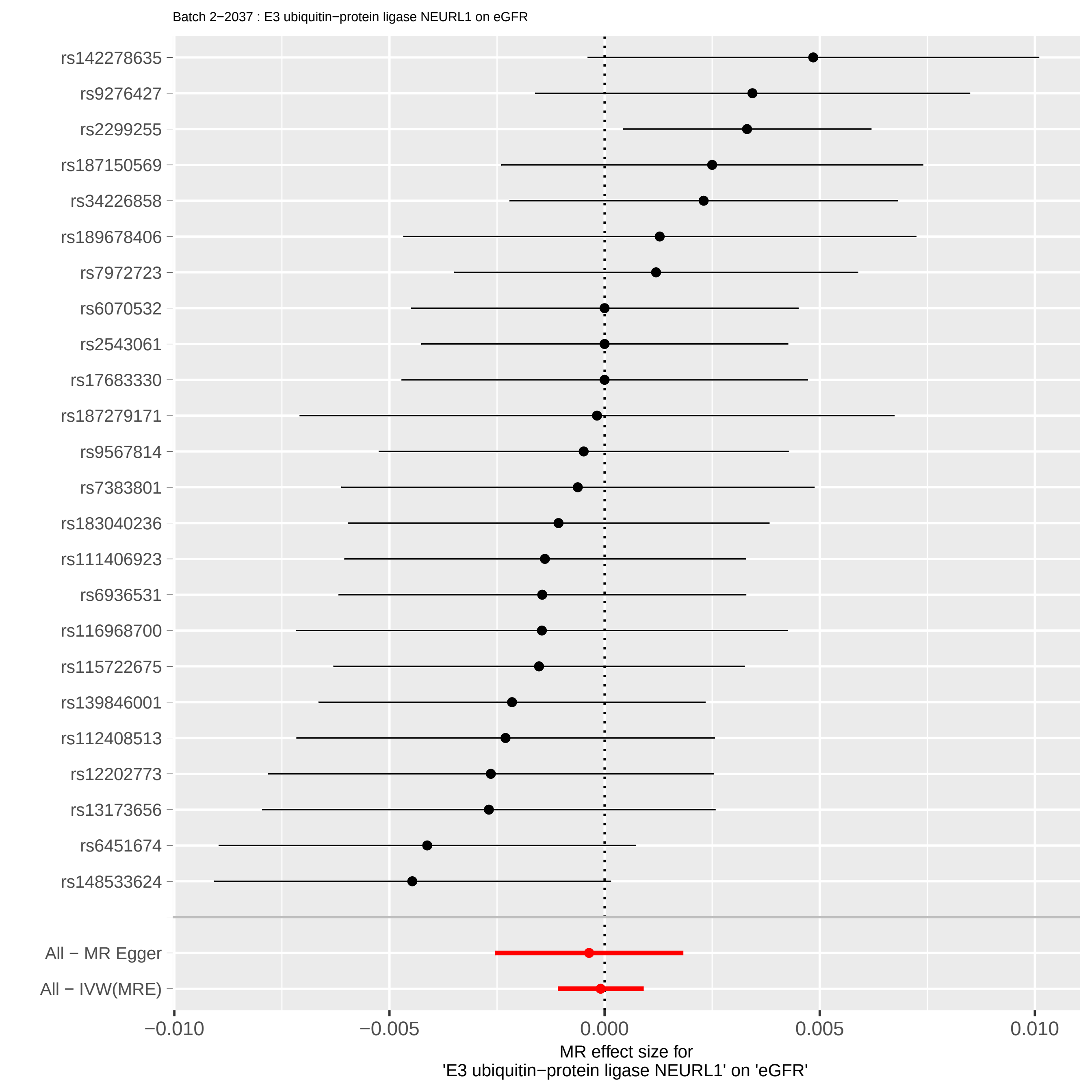

Batch 2−2037 : E3 ubiquitin−protein ligase NEURL1 on eGFR
rs142278635
rs9276427
rs2299255
rs187150569
rs34226858
rs189678406
rs7972723
rs6070532
rs2543061
rs17683330
rs187279171
rs9567814
rs7383801
rs183040236
rs111406923
rs6936531
rs116968700
rs115722675
rs139846001
rs112408513
rs12202773
rs13173656
rs6451674
rs148533624
All − MR Egger
All − IVW(MRE)
−0.010
−0.005
0.000
0.005
0.010
MR effect size for
'E3 ubiquitin−protein ligase NEURL1' on 'eGFR'

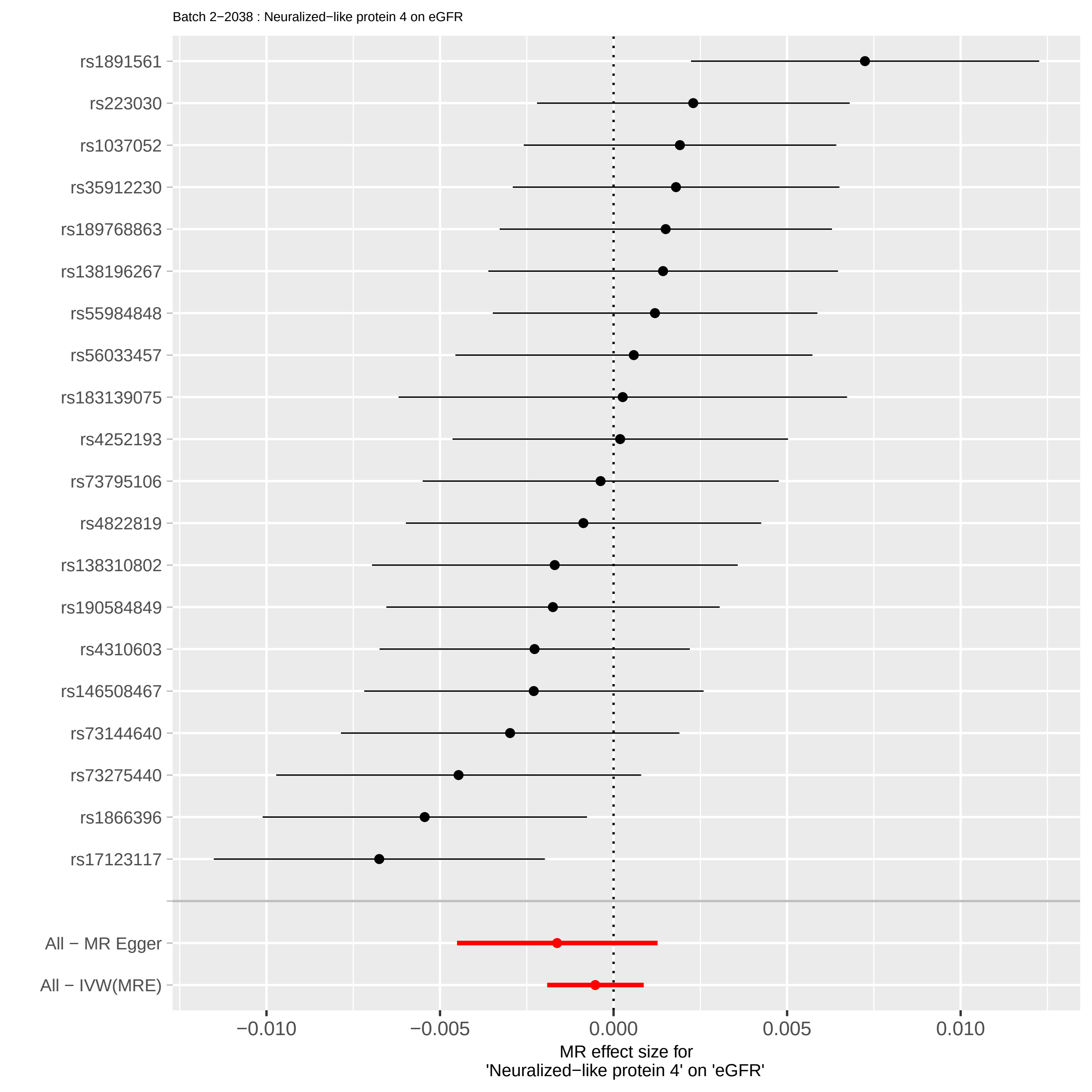

Batch 2−2038 : Neuralized−like protein 4 on eGFR
rs1891561
rs223030
rs1037052
rs35912230
rs189768863
rs138196267
rs55984848
rs56033457
rs183139075
rs4252193
rs73795106
rs4822819
rs138310802
rs190584849
rs4310603
rs146508467
rs73144640
rs73275440
rs1866396
rs17123117
All − MR Egger
All − IVW(MRE)
−0.010
−0.005
0.000
0.005
0.010
MR effect size for
'Neuralized−like protein 4' on 'eGFR'

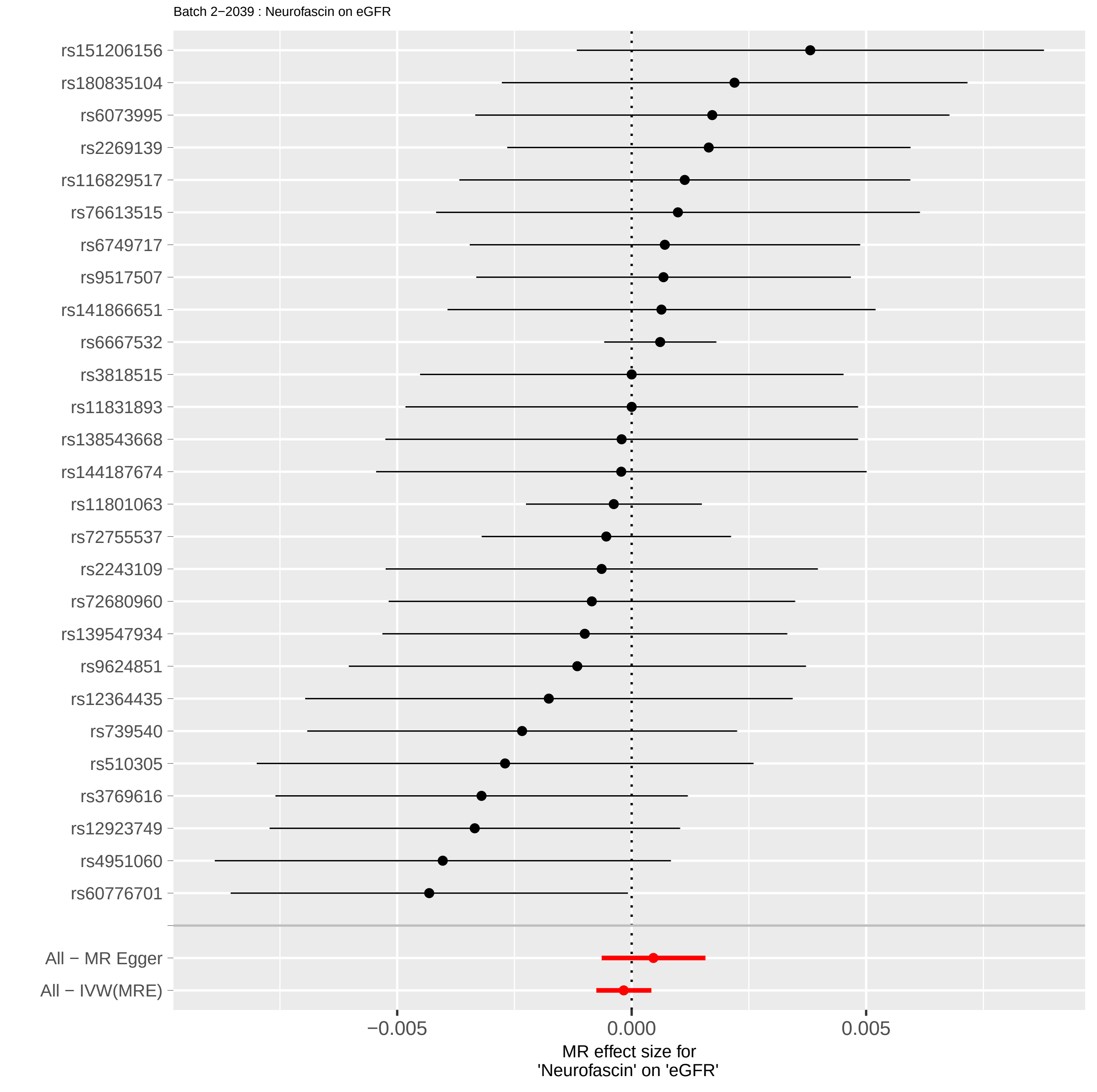

Batch 2−2039 : Neurofascin on eGFR
rs151206156
rs180835104
rs6073995
rs2269139
rs116829517
rs76613515
rs6749717
rs9517507
rs141866651
rs6667532
rs3818515
rs11831893
rs138543668
rs144187674
rs11801063
rs72755537
rs2243109
rs72680960
rs139547934
rs9624851
rs12364435
rs739540
rs510305
rs3769616
rs12923749
rs4951060
rs60776701
All − MR Egger
All − IVW(MRE)
−0.005
0.000
0.005
MR effect size for
'Neurofascin' on 'eGFR'

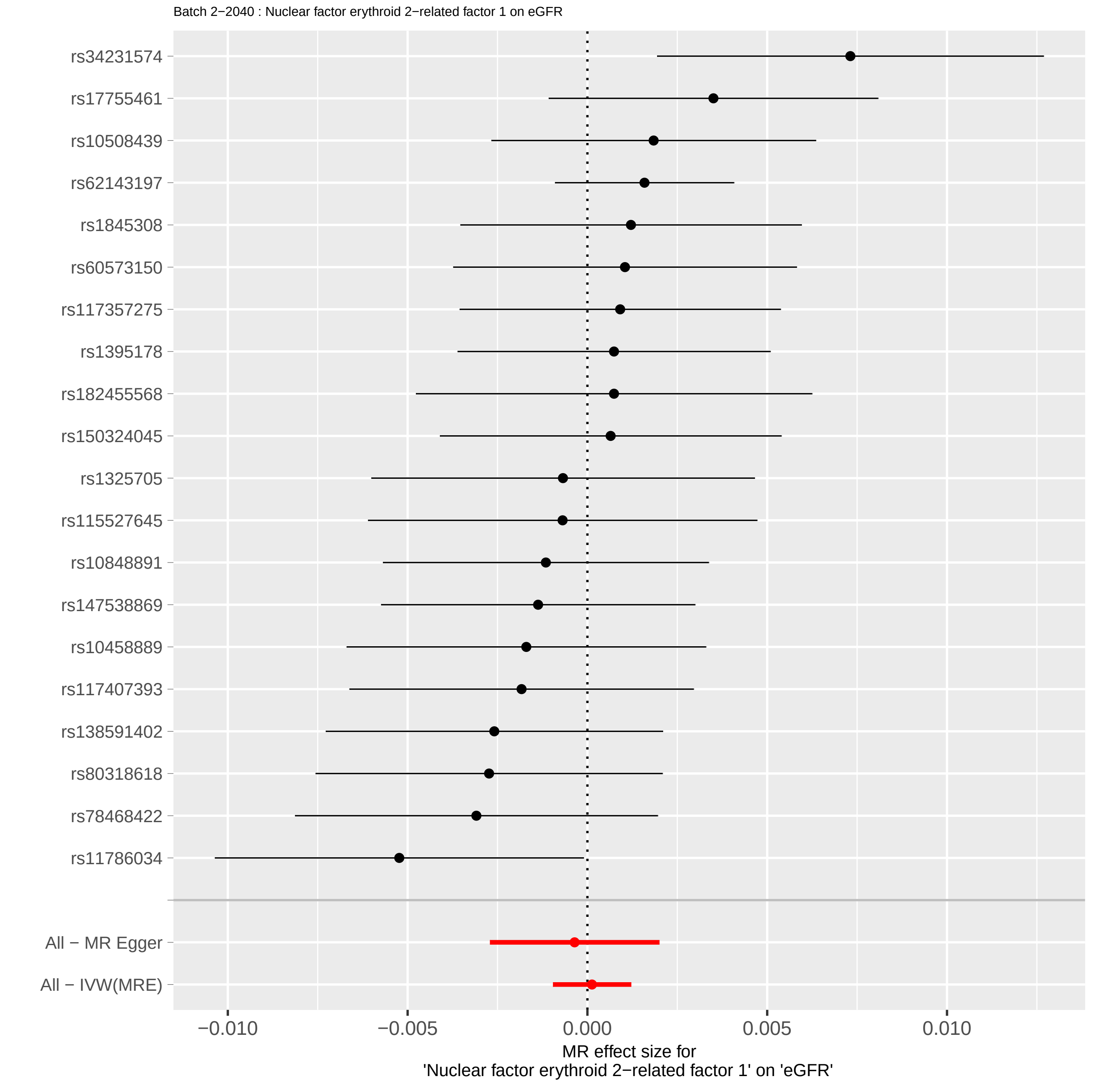

Batch 2−2040 : Nuclear factor erythroid 2−related factor 1 on eGFR
rs34231574
rs17755461
rs10508439
rs62143197
rs1845308
rs60573150
rs117357275
rs1395178
rs182455568
rs150324045
rs1325705
rs115527645
rs10848891
rs147538869
rs10458889
rs117407393
rs138591402
rs80318618
rs78468422
rs11786034
All − MR Egger
All − IVW(MRE)
−0.010
−0.005
0.000
0.005
0.010
MR effect size for
'Nuclear factor erythroid 2−related factor 1' on 'eGFR'

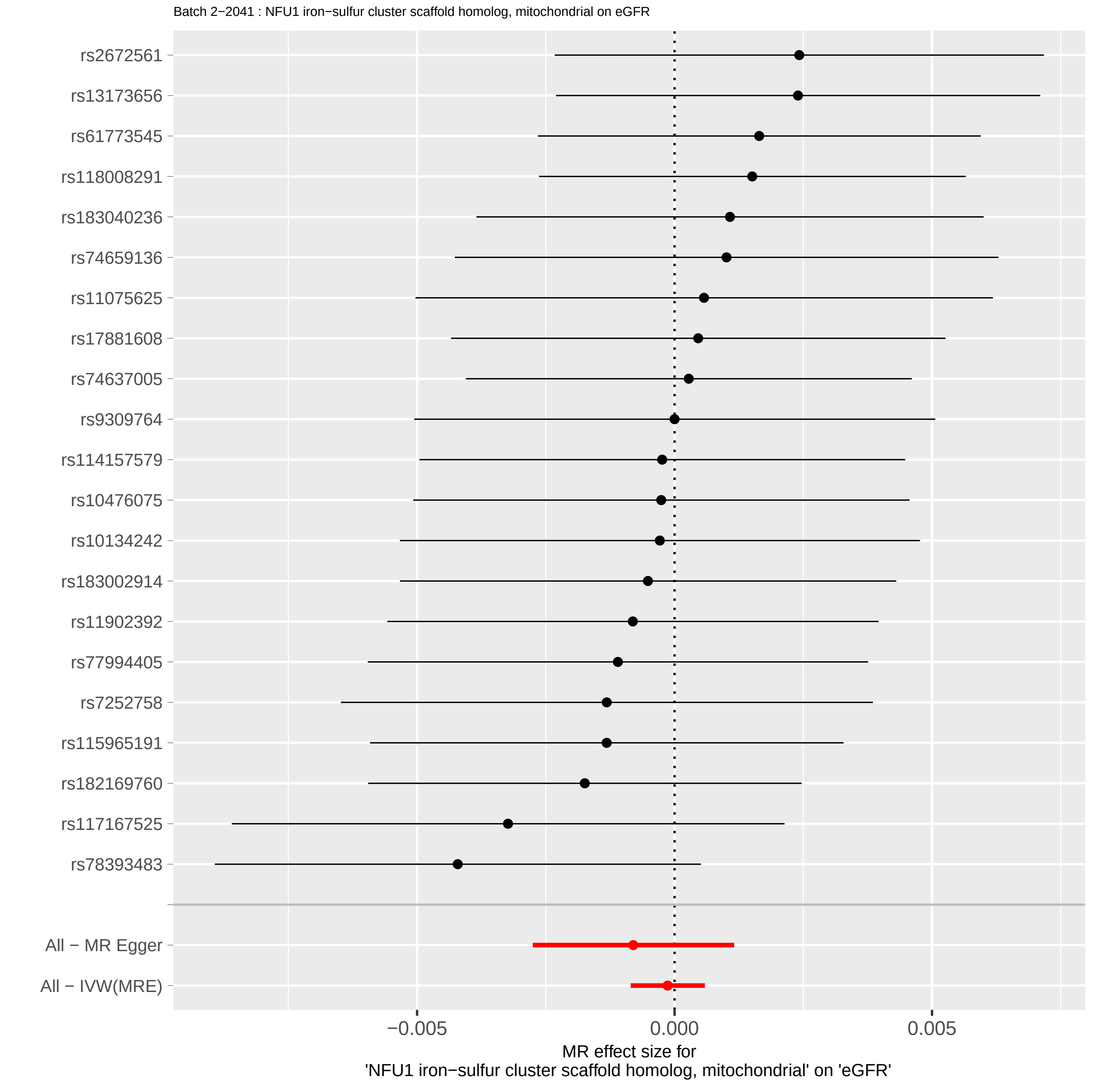

Batch 2−2041 : NFU1 iron−sulfur cluster scaffold homolog, mitochondrial on eGFR
rs2672561
rs13173656
rs61773545
rs118008291
rs183040236
rs74659136
rs11075625
rs17881608
rs74637005
rs9309764
rs114157579
rs10476075
rs10134242
rs183002914
rs11902392
rs77994405
rs7252758
rs115965191
rs182169760
rs117167525
rs78393483
All − MR Egger
All − IVW(MRE)
−0.005
0.000
0.005
MR effect size for
'NFU1 iron−sulfur cluster scaffold homolog, mitochondrial' on 'eGFR'

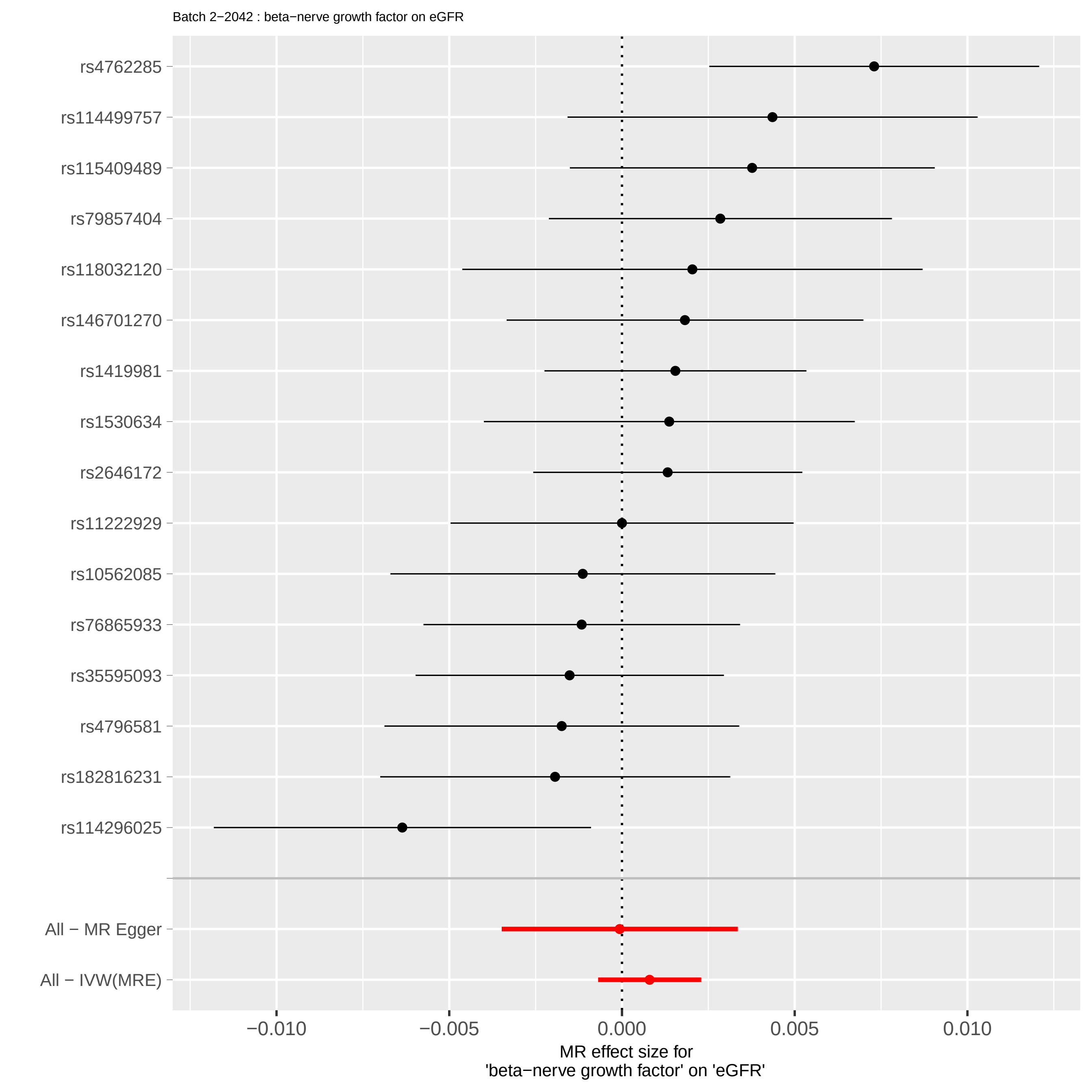

Batch 2−2042 : beta−nerve growth factor on eGFR
rs4762285
rs114499757
rs115409489
rs79857404
rs118032120
rs146701270
rs1419981
rs1530634
rs2646172
rs11222929
rs10562085
rs76865933
rs35595093
rs4796581
rs182816231
rs114296025
All − MR Egger
All − IVW(MRE)
−0.010
−0.005
0.000
0.005
0.010
MR effect size for
'beta−nerve growth factor' on 'eGFR'

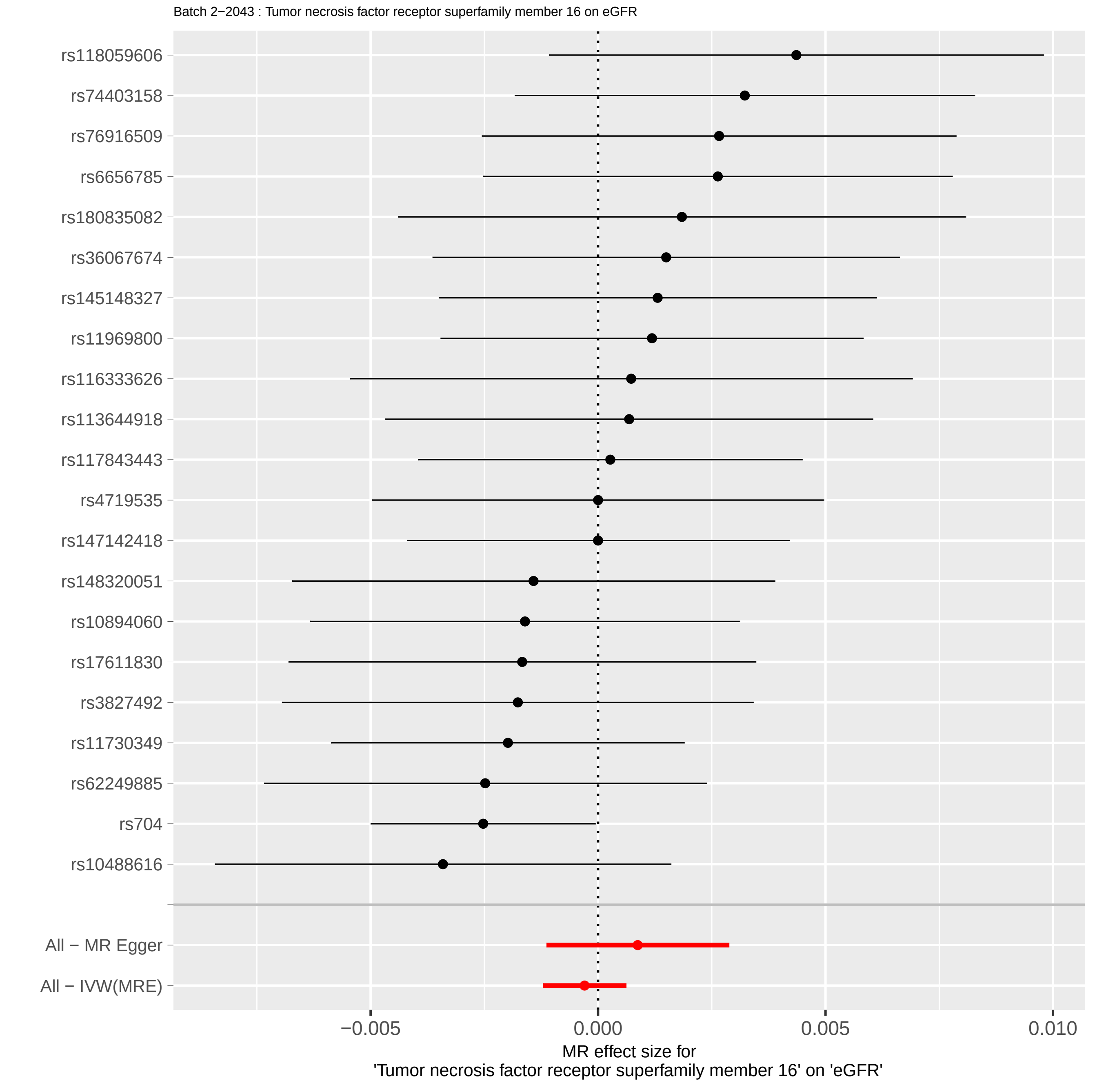

Batch 2−2043 : Tumor necrosis factor receptor superfamily member 16 on eGFR
rs118059606
rs74403158
rs76916509
rs6656785
rs180835082
rs36067674
rs145148327
rs11969800
rs116333626
rs113644918
rs117843443
rs4719535
rs147142418
rs148320051
rs10894060
rs17611830
rs3827492
rs11730349
rs62249885
rs704
rs10488616
All − MR Egger
All − IVW(MRE)
−0.005
0.000
0.005
0.010
MR effect size for
'Tumor necrosis factor receptor superfamily member 16' on 'eGFR'

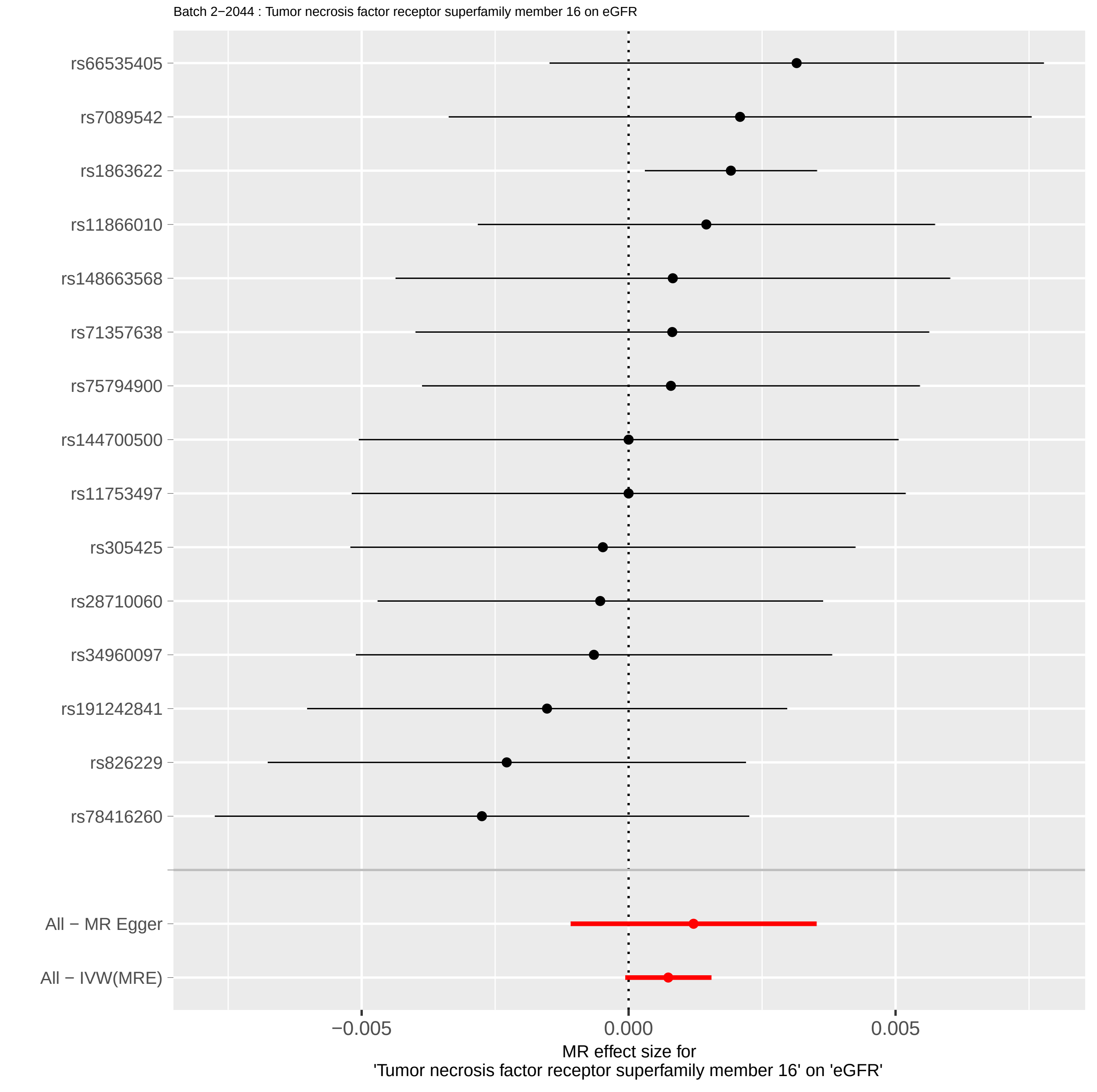

Batch 2−2044 : Tumor necrosis factor receptor superfamily member 16 on eGFR
rs66535405
rs7089542
rs1863622
rs11866010
rs148663568
rs71357638
rs75794900
rs144700500
rs11753497
rs305425
rs28710060
rs34960097
rs191242841
rs826229
rs78416260
All − MR Egger
All − IVW(MRE)
−0.005
0.000
0.005
MR effect size for
'Tumor necrosis factor receptor superfamily member 16' on 'eGFR'

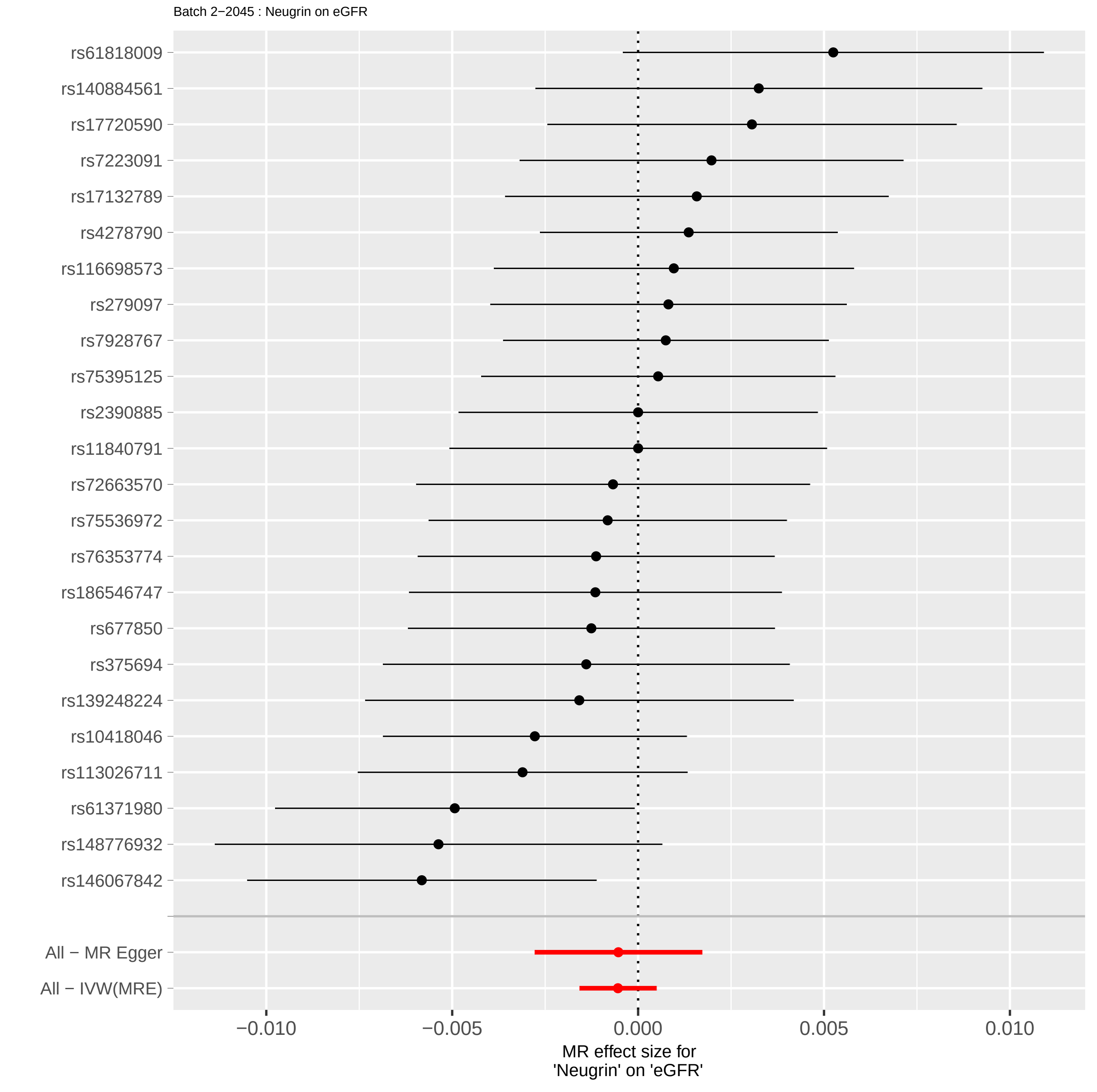
Batch 2−2045 : Neugrin on eGFR
rs61818009
rs140884561
rs17720590
rs7223091
rs17132789
rs4278790
rs116698573
rs279097
rs7928767
rs75395125
rs2390885
rs11840791
rs72663570
rs75536972
rs76353774
rs186546747
rs677850
rs375694
rs139248224
rs10418046
rs113026711
rs61371980
rs148776932
rs146067842
All − MR Egger
All − IVW(MRE)
−0.010
−0.005
0.000
0.005
0.010
MR effect size for
'Neugrin' on 'eGFR'

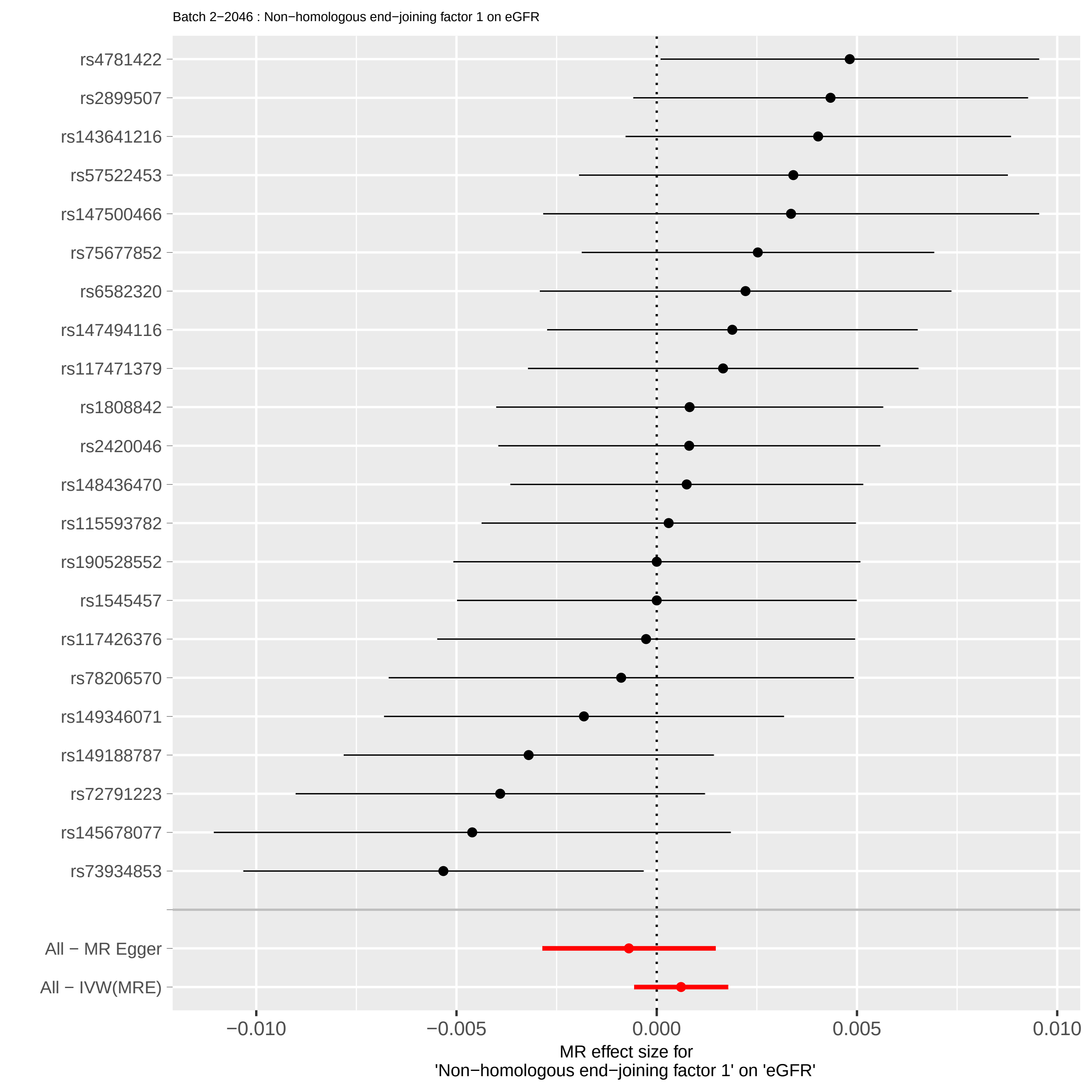

Batch 2−2046 : Non−homologous end−joining factor 1 on eGFR
rs4781422
rs2899507
rs143641216
rs57522453
rs147500466
rs75677852
rs6582320
rs147494116
rs117471379
rs1808842
rs2420046
rs148436470
rs115593782
rs190528552
rs1545457
rs117426376
rs78206570
rs149346071
rs149188787
rs72791223
rs145678077
rs73934853
All − MR Egger
All − IVW(MRE)
−0.010
−0.005
0.000
0.005
0.010
MR effect size for
'Non−homologous end−joining factor 1' on 'eGFR'

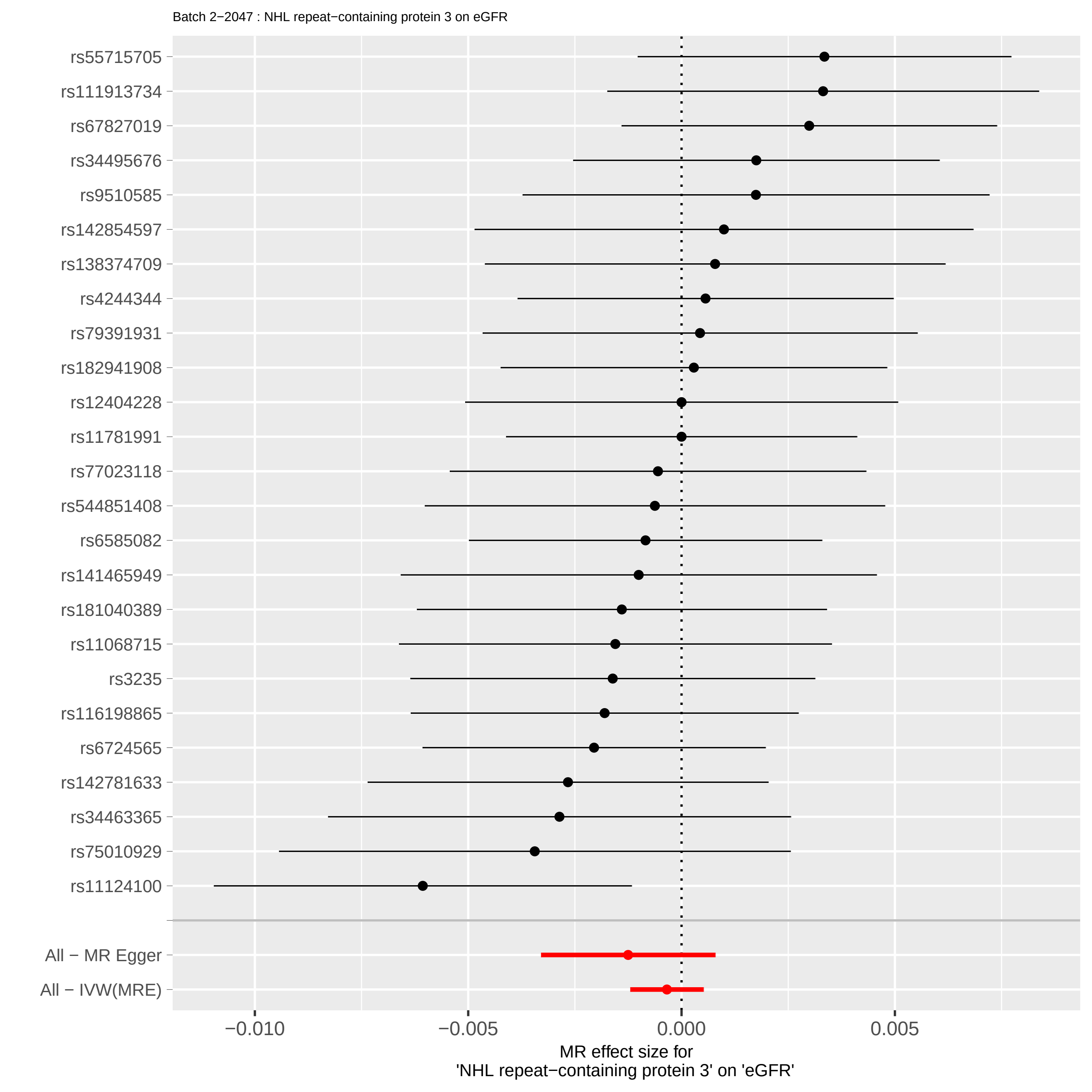

Batch 2−2047 : NHL repeat−containing protein 3 on eGFR
rs55715705
rs111913734
rs67827019
rs34495676
rs9510585
rs142854597
rs138374709
rs4244344
rs79391931
rs182941908
rs12404228
rs11781991
rs77023118
rs544851408
rs6585082
rs141465949
rs181040389
rs11068715
rs3235
rs116198865
rs6724565
rs142781633
rs34463365
rs75010929
rs11124100
All − MR Egger
All − IVW(MRE)
−0.010
−0.005
0.000
0.005
MR effect size for
'NHL repeat−containing protein 3' on 'eGFR'

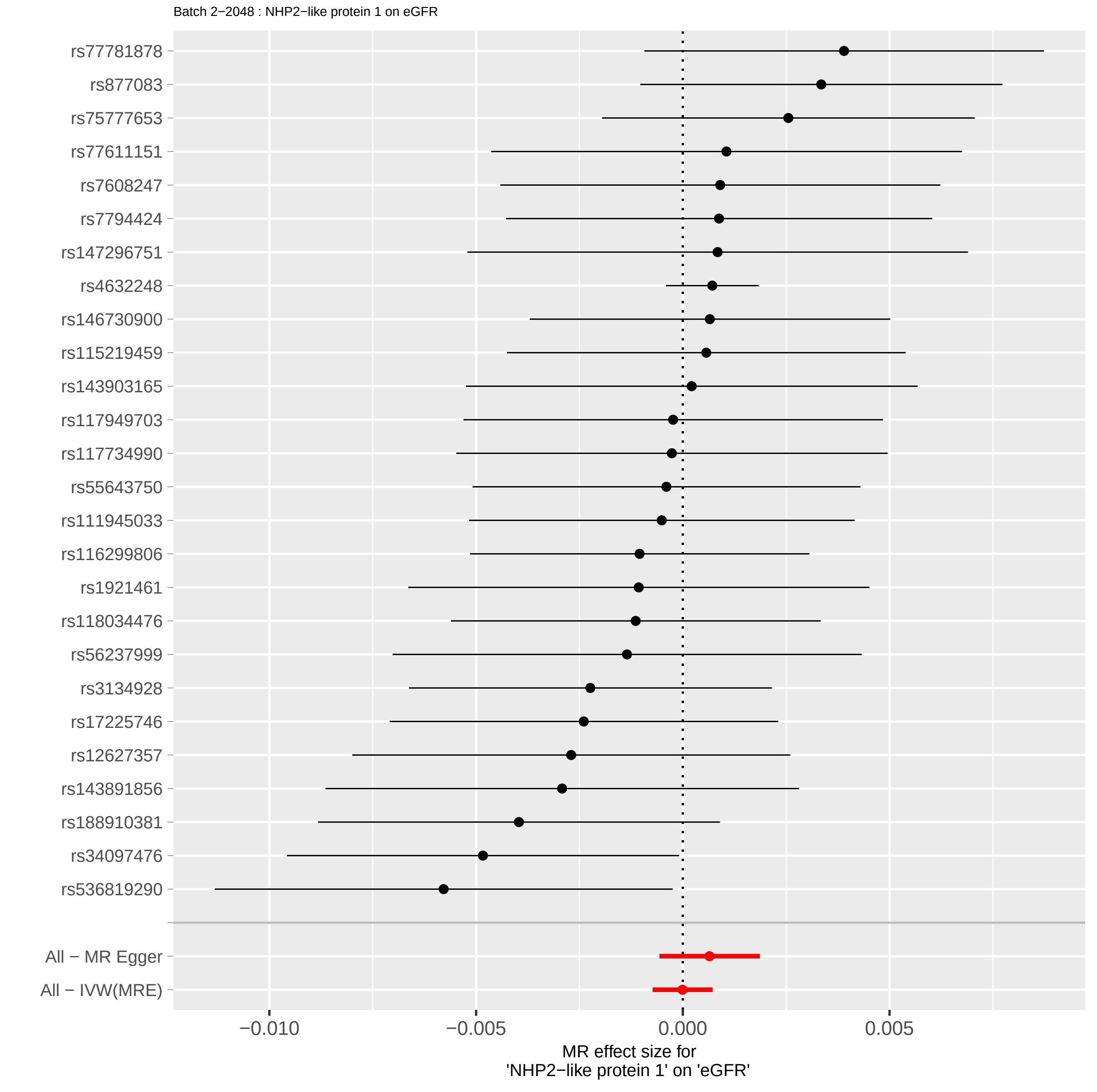

Batch 2−2048 : NHP2−like protein 1 on eGFR
rs77781878
rs877083
rs75777653
rs77611151
rs7608247
rs7794424
rs147296751
rs4632248
rs146730900
rs115219459
rs143903165
rs117949703
rs117734990
rs55643750
rs111945033
rs116299806
rs1921461
rs118034476
rs56237999
rs3134928
rs17225746
rs12627357
rs143891856
rs188910381
rs34097476
rs536819290
All − MR Egger
All − IVW(MRE)
−0.010
−0.005
0.000
0.005
MR effect size for
'NHP2−like protein 1' on 'eGFR'

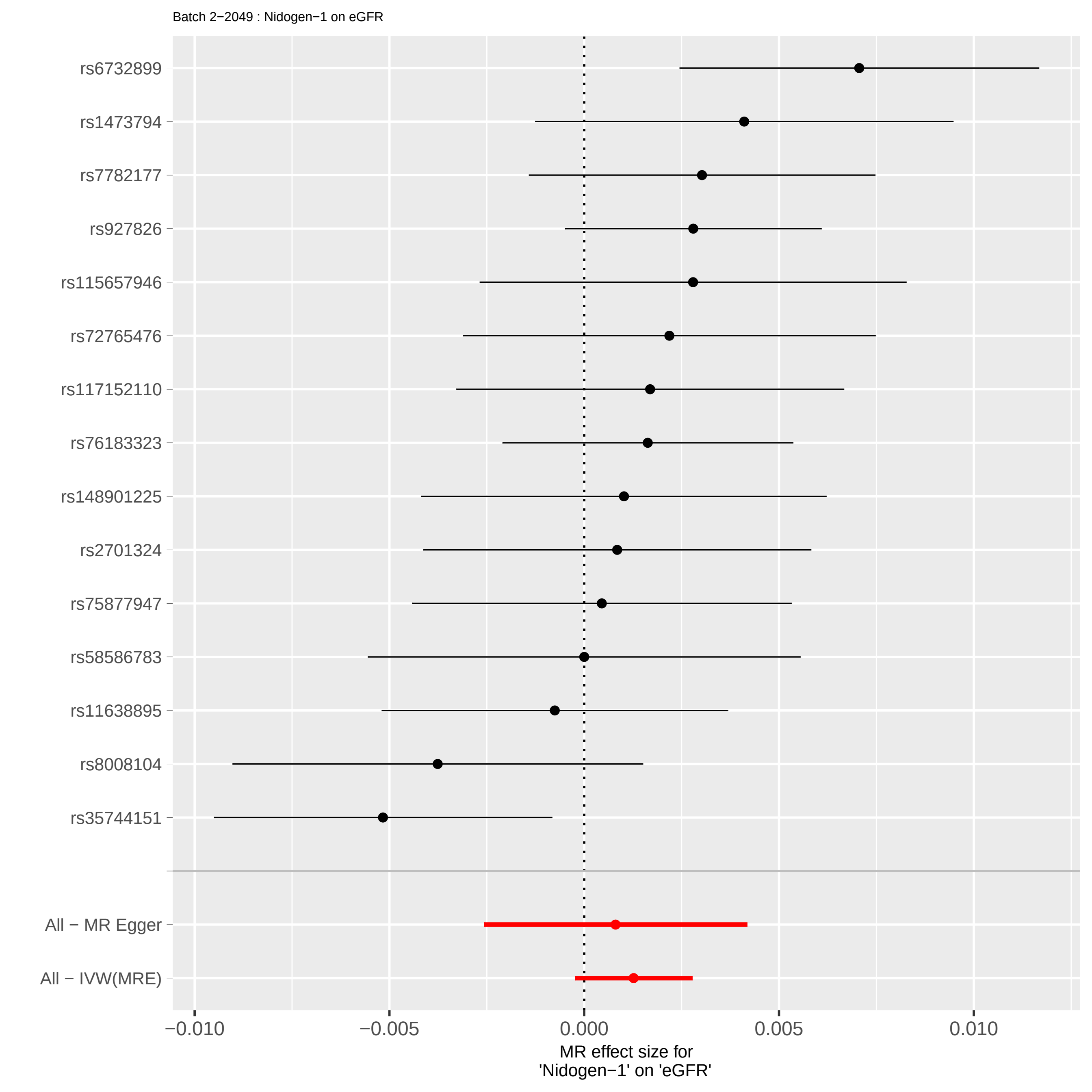

Batch 2−2049 : Nidogen−1 on eGFR
rs6732899
rs1473794
rs7782177
rs927826
rs115657946
rs72765476
rs117152110
rs76183323
rs148901225
rs2701324
rs75877947
rs58586783
rs11638895
rs8008104
rs35744151
All − MR Egger
All − IVW(MRE)
−0.010
−0.005
0.000
0.005
0.010
MR effect size for
'Nidogen−1' on 'eGFR'

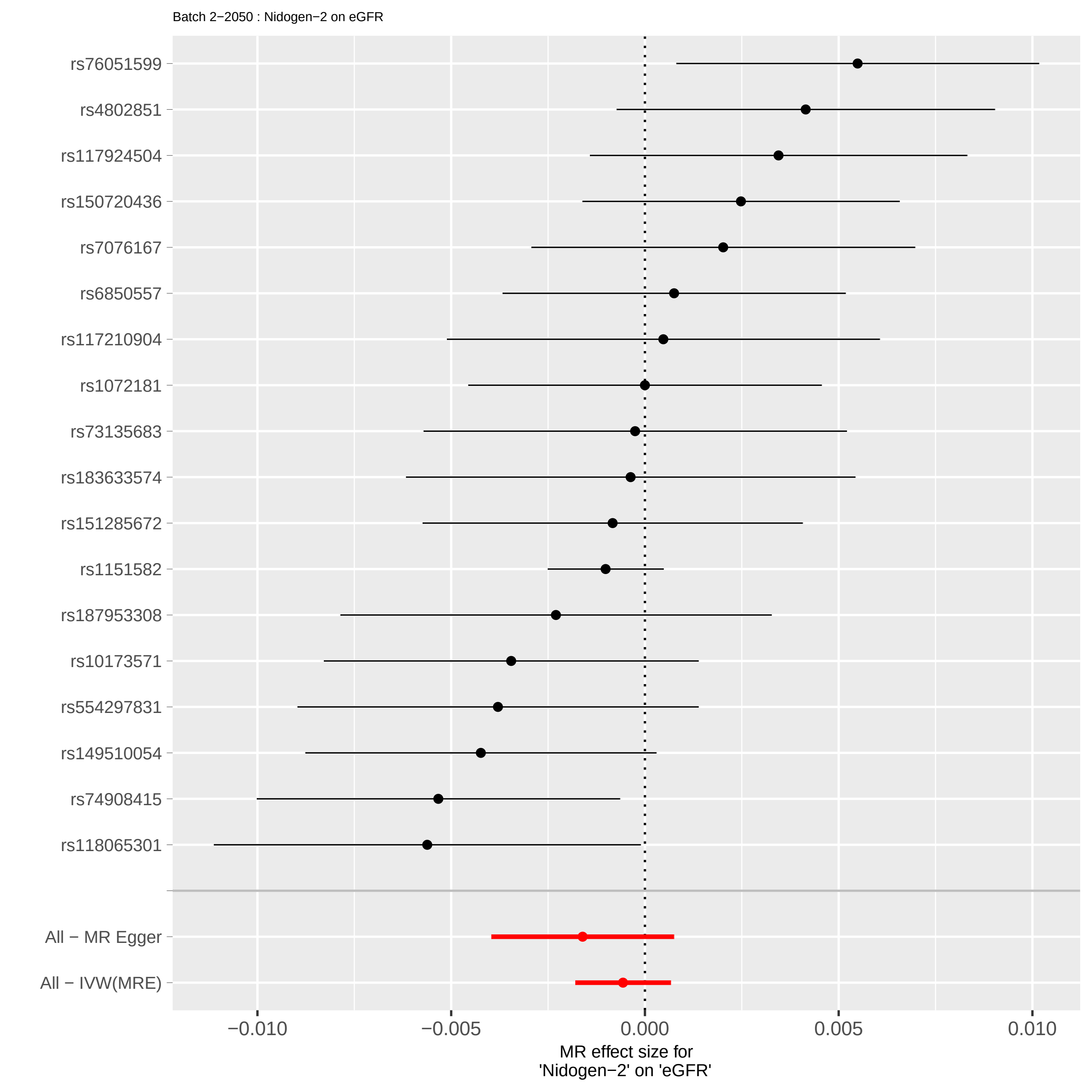

Batch 2−2050 : Nidogen−2 on eGFR
rs76051599
rs4802851
rs117924504
rs150720436
rs7076167
rs6850557
rs117210904
rs1072181
rs73135683
rs183633574
rs151285672
rs1151582
rs187953308
rs10173571
rs554297831
rs149510054
rs74908415
rs118065301
All − MR Egger
All − IVW(MRE)
−0.010
−0.005
0.000
0.005
0.010
MR effect size for
'Nidogen−2' on 'eGFR'

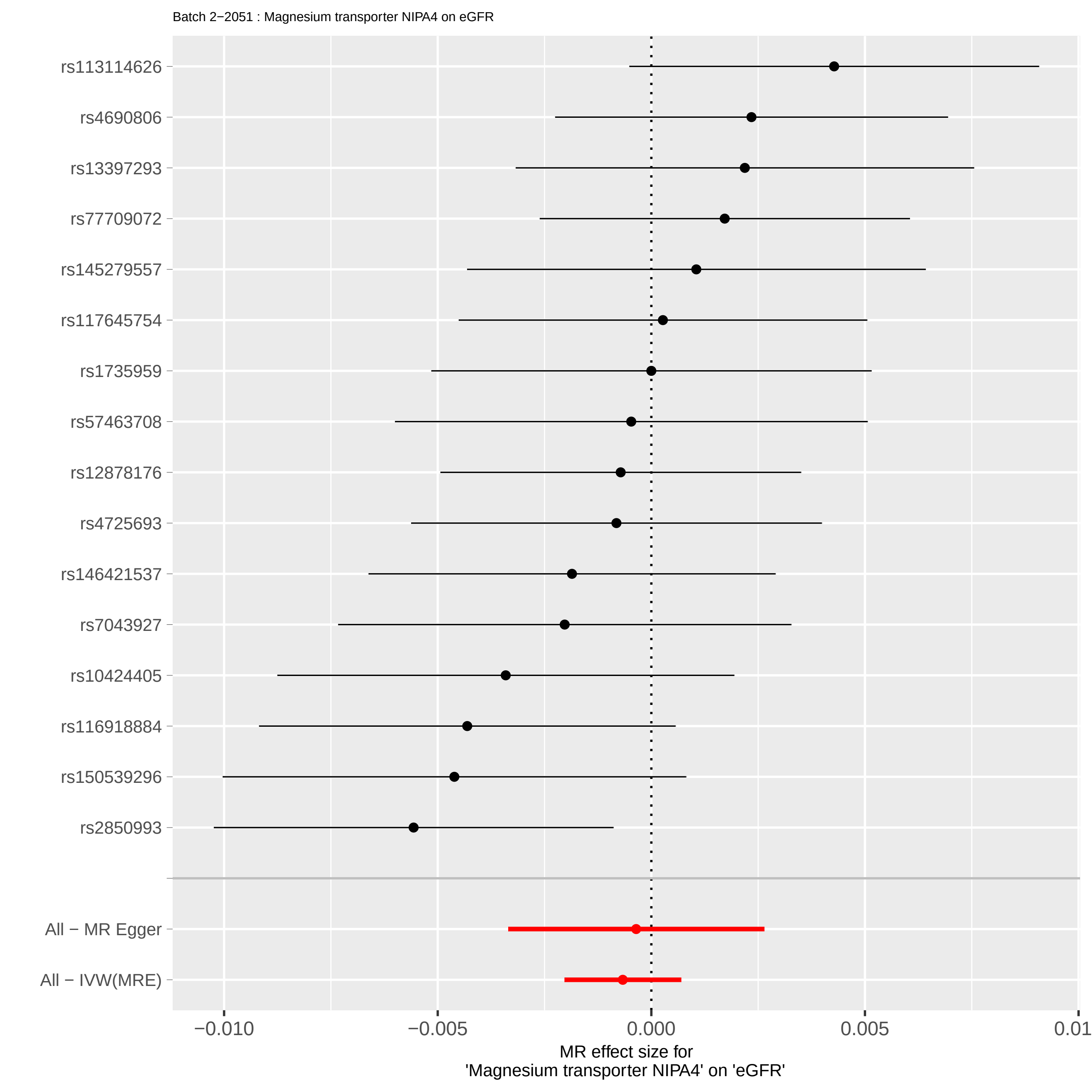

Batch 2−2051 : Magnesium transporter NIPA4 on eGFR
rs113114626
rs4690806
rs13397293
rs77709072
rs145279557
rs117645754
rs1735959
rs57463708
rs12878176
rs4725693
rs146421537
rs7043927
rs10424405
rs116918884
rs150539296
rs2850993
All − MR Egger
All − IVW(MRE)
−0.010
−0.005
0.000
0.005
0.01
MR effect size for
'Magnesium transporter NIPA4' on 'eGFR'

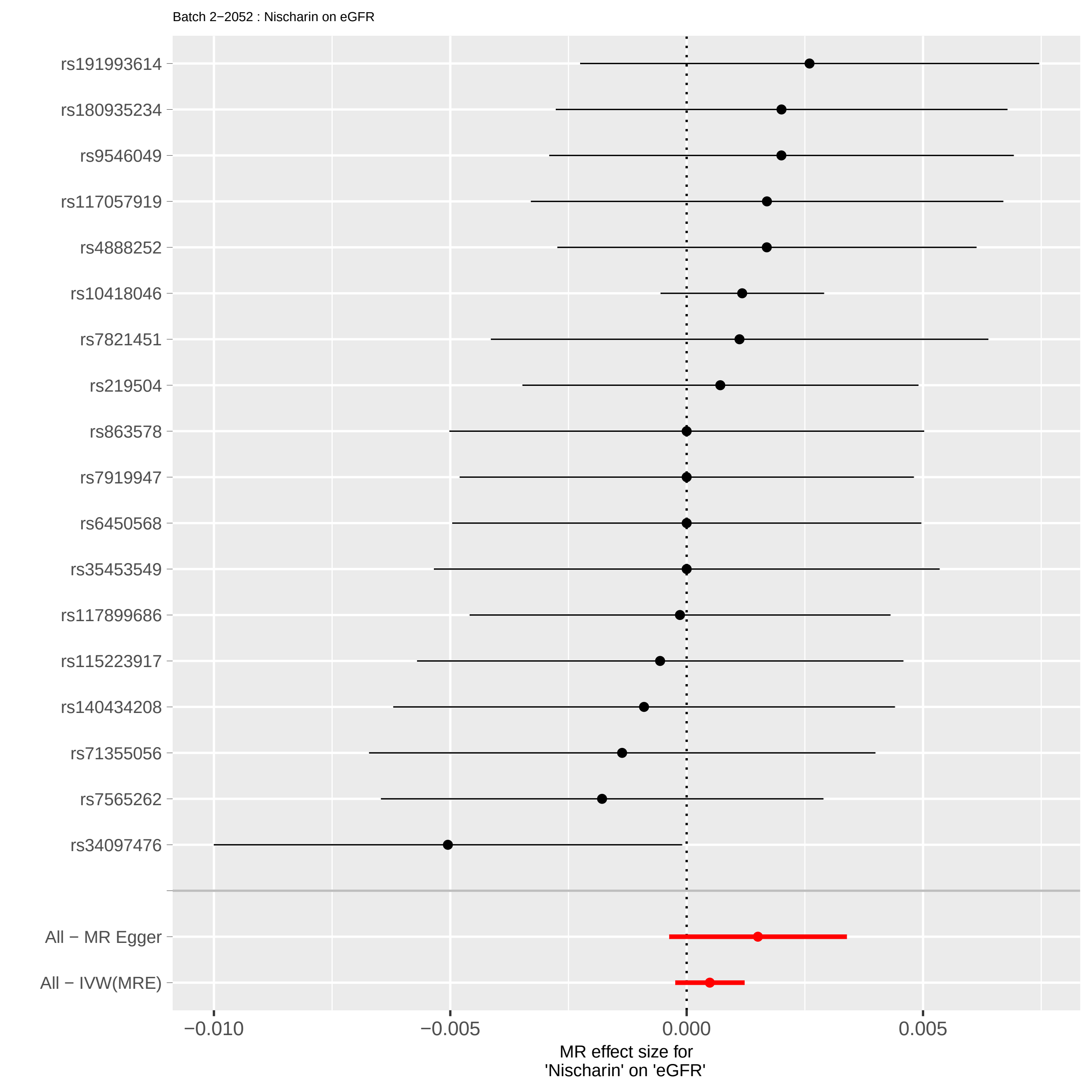
Batch 2−2052 : Nischarin on eGFR
rs191993614
rs180935234
rs9546049
rs117057919
rs4888252
rs10418046
rs7821451
rs219504
rs863578
rs7919947
rs6450568
rs35453549
rs117899686
rs115223917
rs140434208
rs71355056
rs7565262
rs34097476
All − MR Egger
All − IVW(MRE)
−0.010
−0.005
0.000
0.005
MR effect size for
'Nischarin' on 'eGFR'

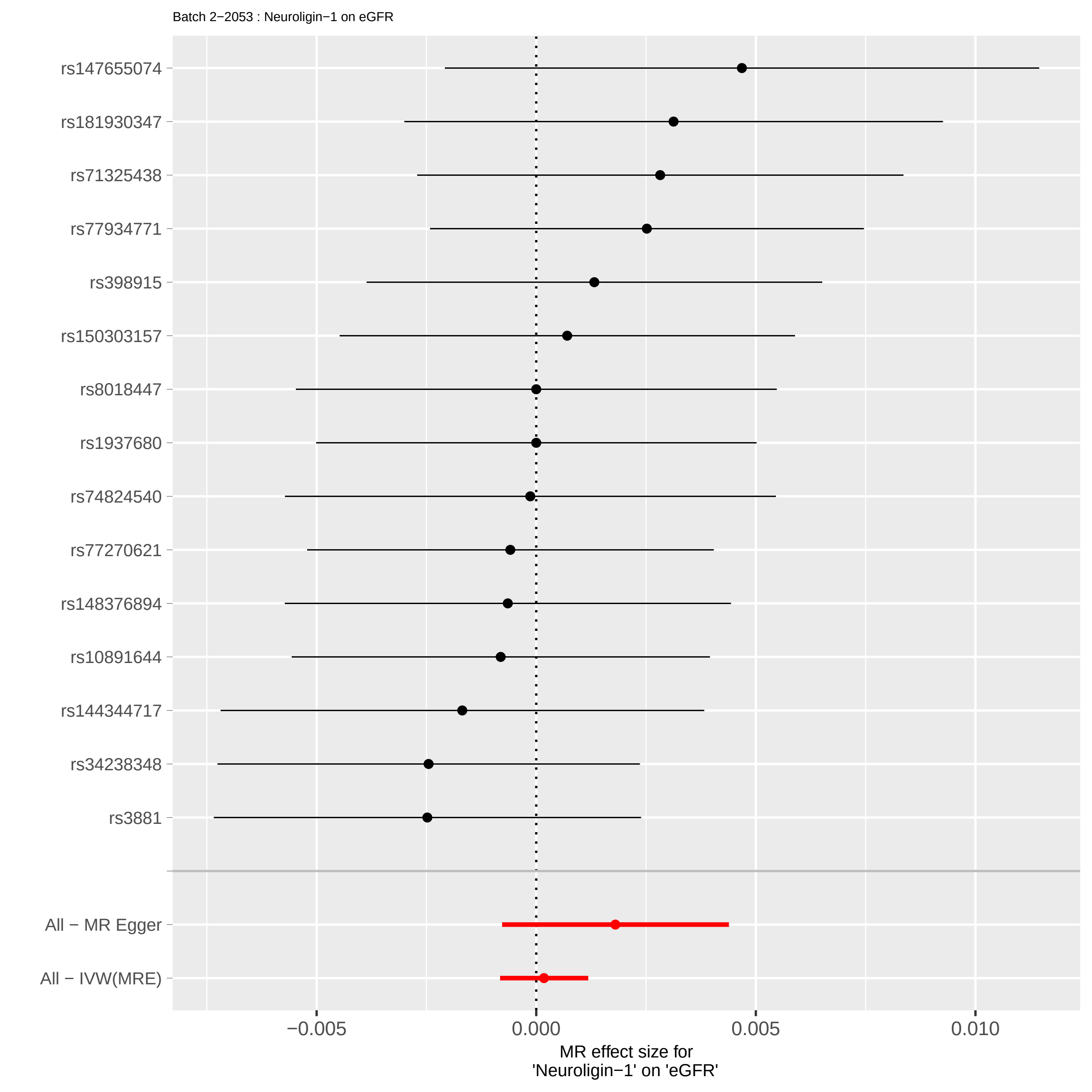

Batch 2−2053 : Neuroligin−1 on eGFR
rs147655074
rs181930347
rs71325438
rs77934771
rs398915
rs150303157
rs8018447
rs1937680
rs74824540
rs77270621
rs148376894
rs10891644
rs144344717
rs34238348
rs3881
All − MR Egger
All − IVW(MRE)
−0.005
0.000
0.005
0.010
MR effect size for
'Neuroligin−1' on 'eGFR'

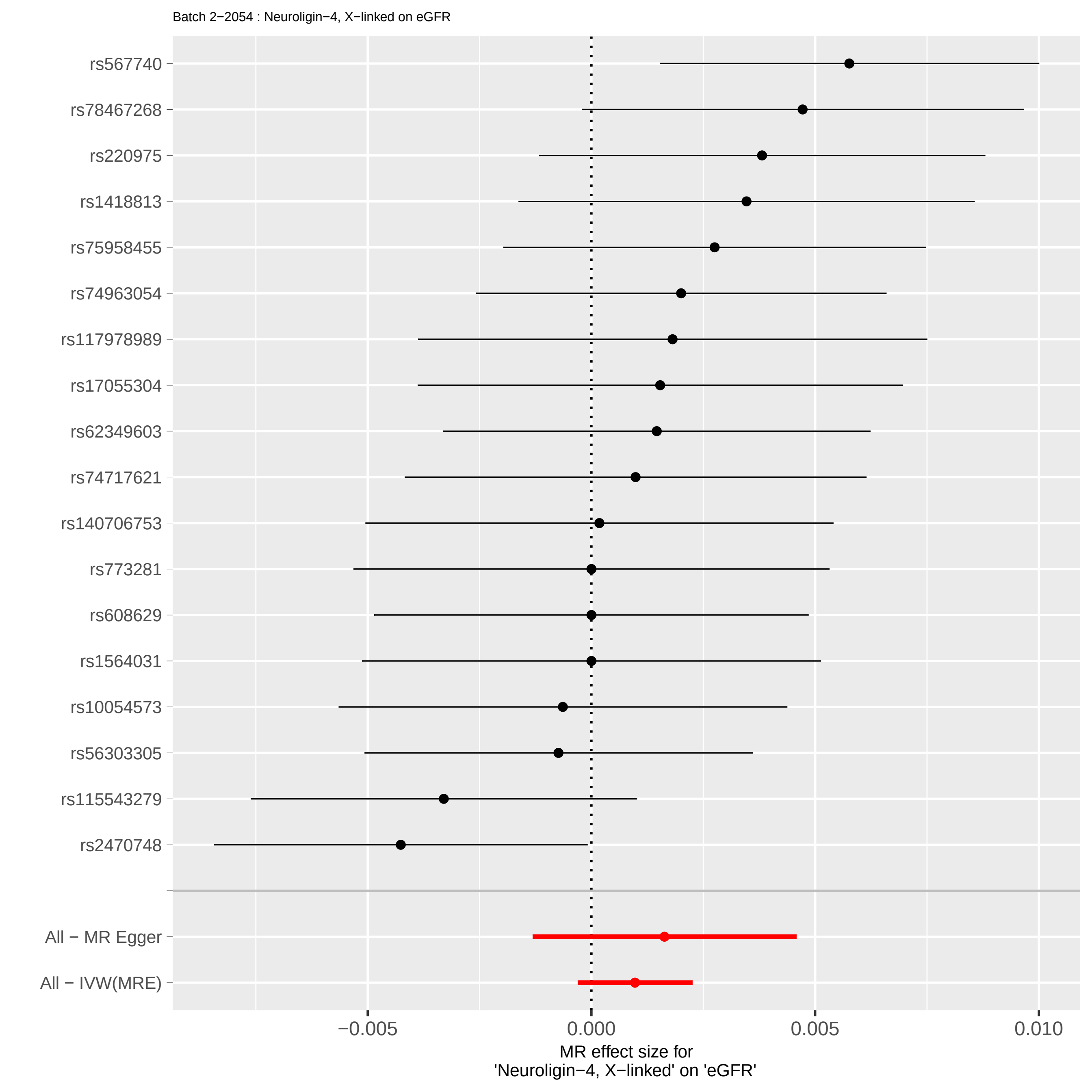
Batch 2−2054 : Neuroligin−4, X−linked on eGFR
rs567740
rs78467268
rs220975
rs1418813
rs75958455
rs74963054
rs117978989
rs17055304
rs62349603
rs74717621
rs140706753
rs773281
rs608629
rs1564031
rs10054573
rs56303305
rs115543279
rs2470748
All − MR Egger
All − IVW(MRE)
−0.005
0.000
0.005
0.010
MR effect size for
'Neuroligin−4, X−linked' on 'eGFR'

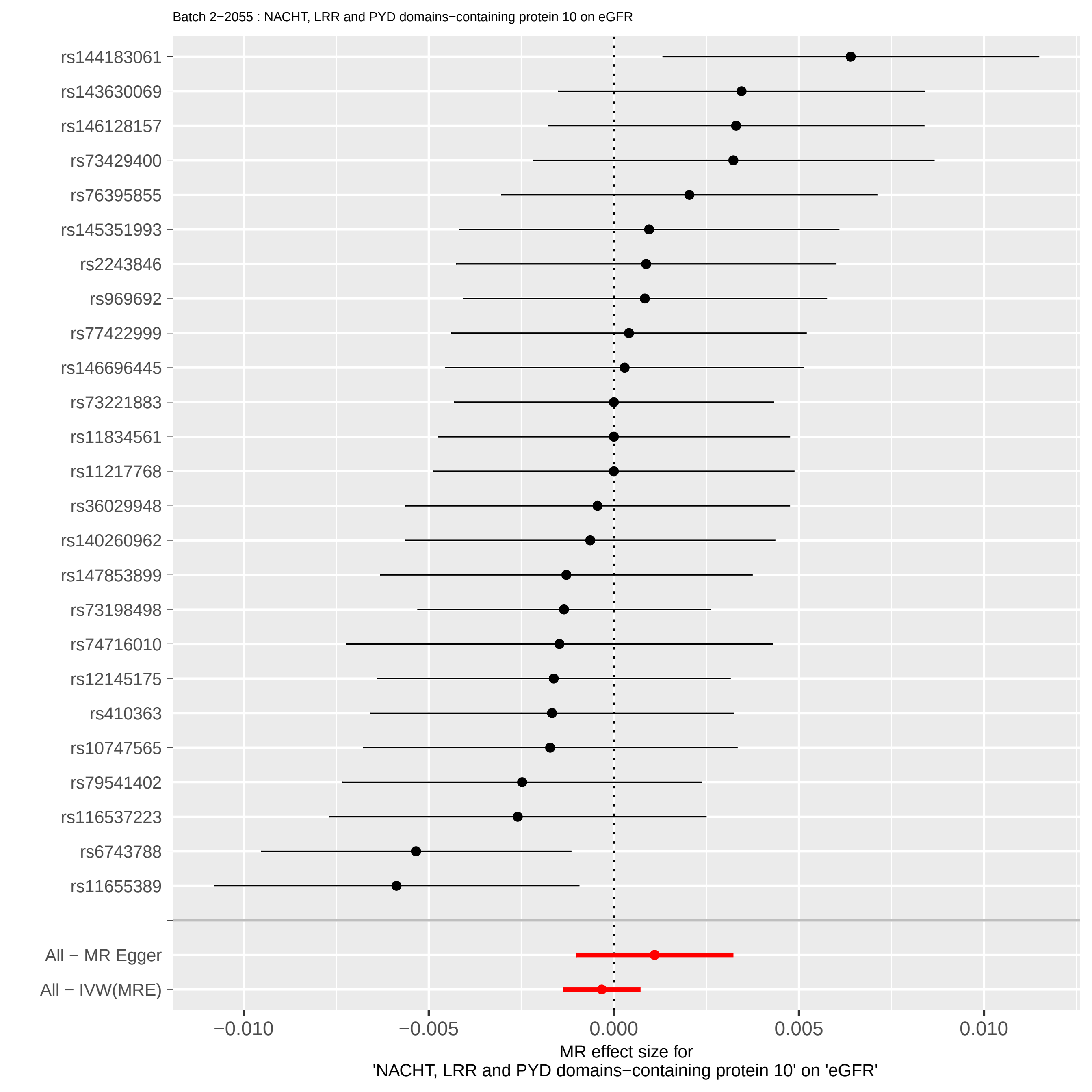

Batch 2−2055 : NACHT, LRR and PYD domains−containing protein 10 on eGFR
rs144183061
rs143630069
rs146128157
rs73429400
rs76395855
rs145351993
rs2243846
rs969692
rs77422999
rs146696445
rs73221883
rs11834561
rs11217768
rs36029948
rs140260962
rs147853899
rs73198498
rs74716010
rs12145175
rs410363
rs10747565
rs79541402
rs116537223
rs6743788
rs11655389
All − MR Egger
All − IVW(MRE)
−0.010
−0.005
0.000
0.005
0.010
MR effect size for
'NACHT, LRR and PYD domains−containing protein 10' on 'eGFR'

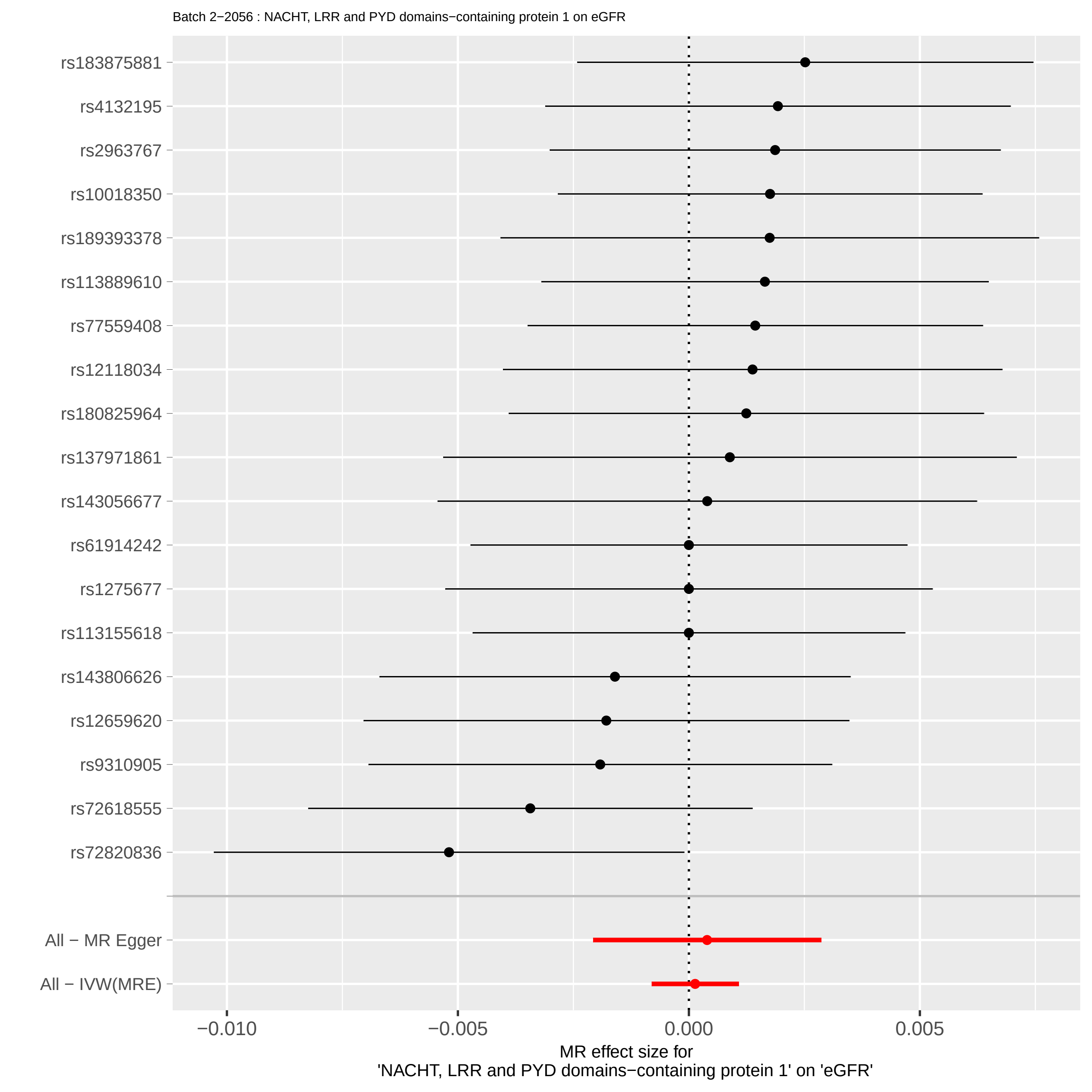
Batch 2−2056 : NACHT, LRR and PYD domains−containing protein 1 on eGFR
rs183875881
rs4132195
rs2963767
rs10018350
rs189393378
rs113889610
rs77559408
rs12118034
rs180825964
rs137971861
rs143056677
rs61914242
rs1275677
rs113155618
rs143806626
rs12659620
rs9310905
rs72618555
rs72820836
All − MR Egger
All − IVW(MRE)
−0.010
−0.005
0.000
0.005
MR effect size for
'NACHT, LRR and PYD domains−containing protein 1' on 'eGFR'

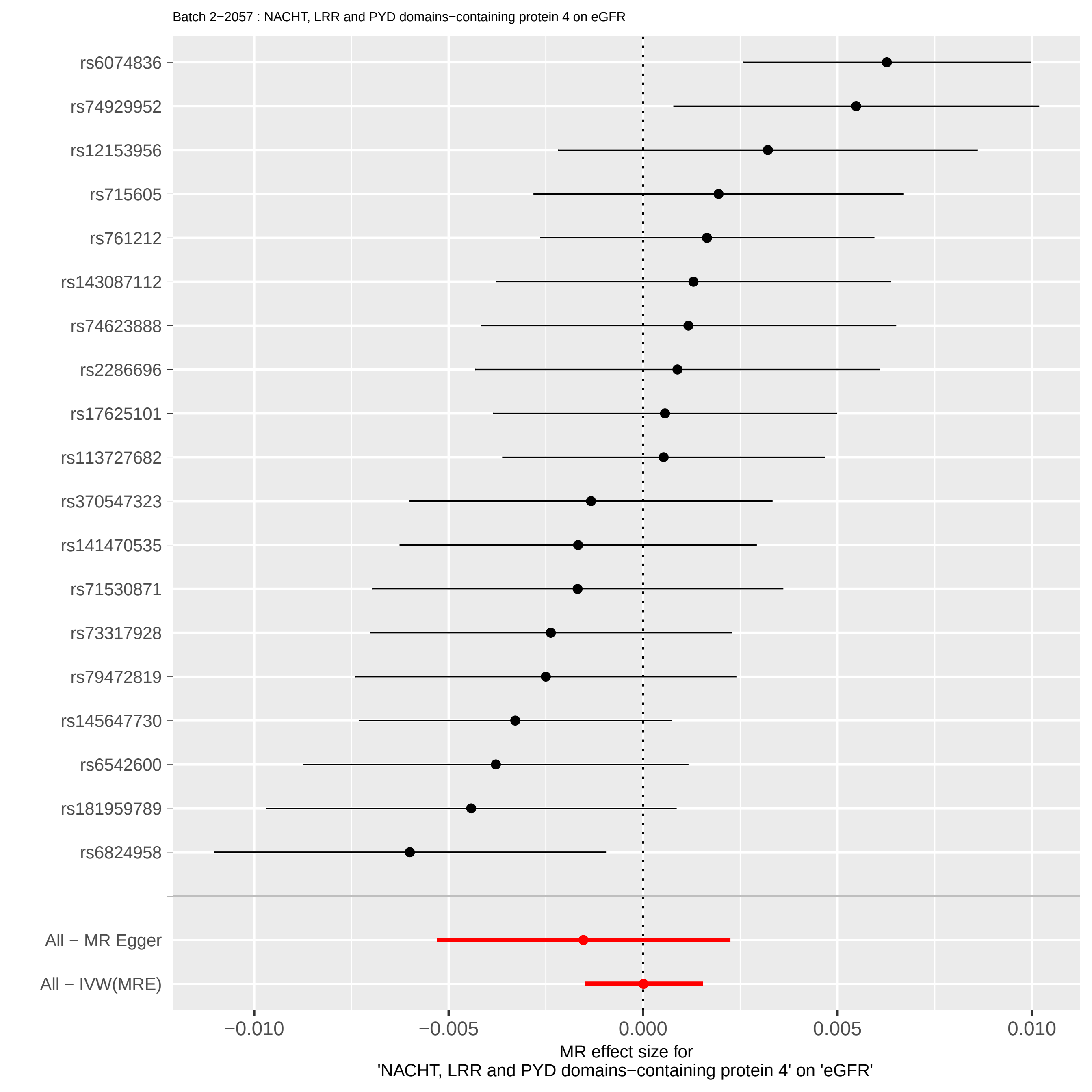

Batch 2−2057 : NACHT, LRR and PYD domains−containing protein 4 on eGFR
rs6074836
rs74929952
rs12153956
rs715605
rs761212
rs143087112
rs74623888
rs2286696
rs17625101
rs113727682
rs370547323
rs141470535
rs71530871
rs73317928
rs79472819
rs145647730
rs6542600
rs181959789
rs6824958
All − MR Egger
All − IVW(MRE)
−0.010
−0.005
0.000
0.005
0.010
MR effect size for
'NACHT, LRR and PYD domains−containing protein 4' on 'eGFR'

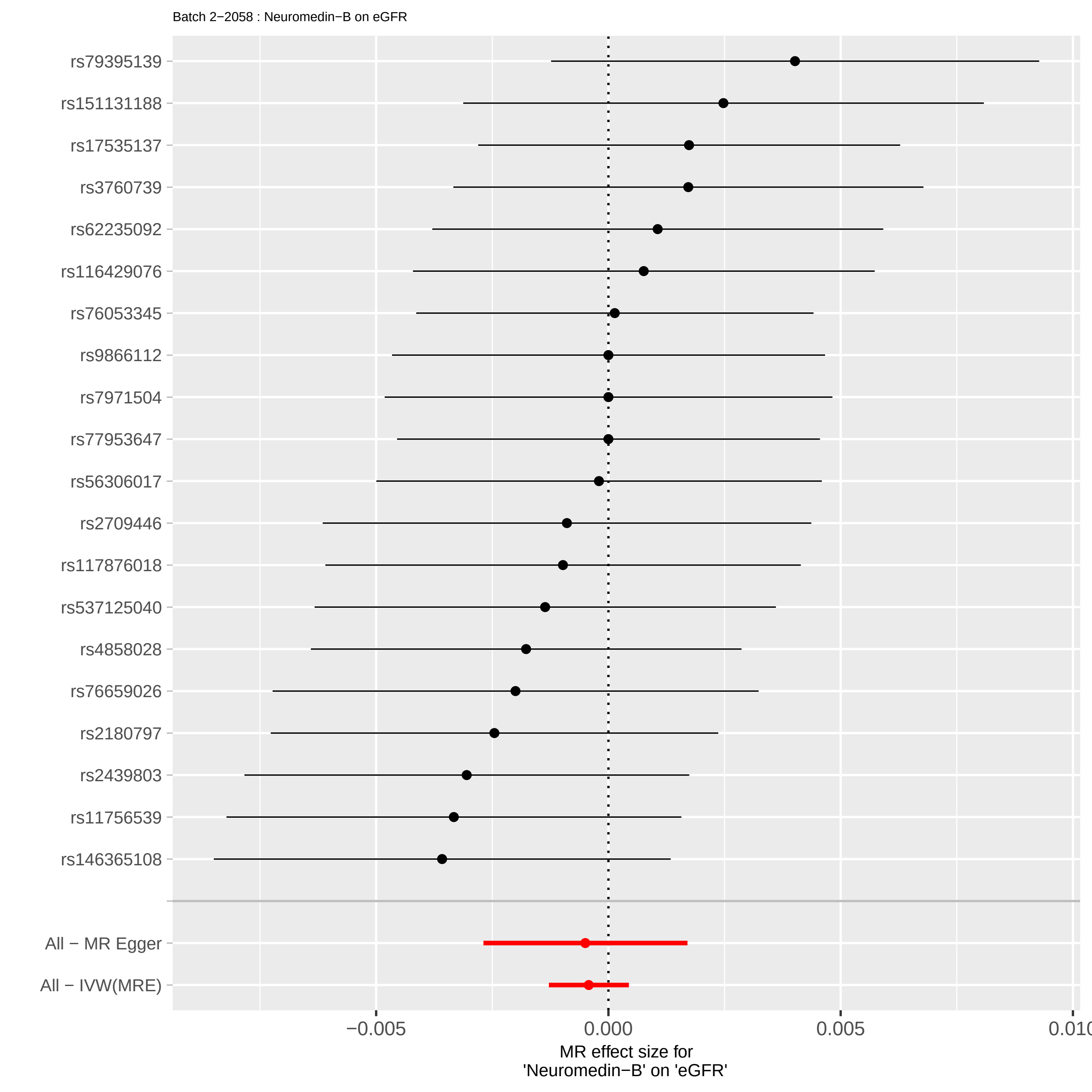

Batch 2−2058 : Neuromedin−B on eGFR
rs79395139
rs151131188
rs17535137
rs3760739
rs62235092
rs116429076
rs76053345
rs9866112
rs7971504
rs77953647
rs56306017
rs2709446
rs117876018
rs537125040
rs4858028
rs76659026
rs2180797
rs2439803
rs11756539
rs146365108
All − MR Egger
All − IVW(MRE)
−0.005
0.000
0.005
0.010
MR effect size for
'Neuromedin−B' on 'eGFR'

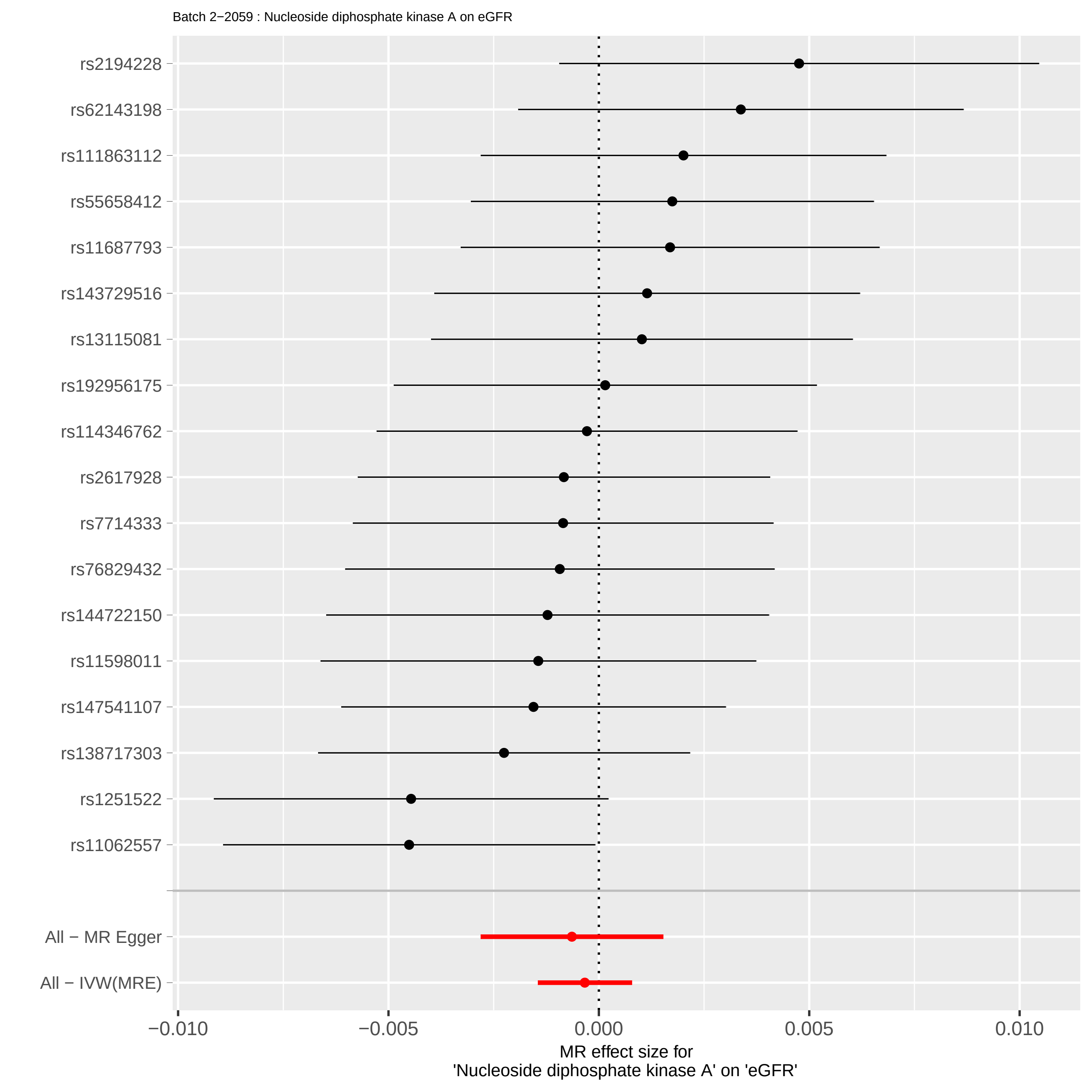

Batch 2−2059 : Nucleoside diphosphate kinase A on eGFR
rs2194228
rs62143198
rs111863112
rs55658412
rs11687793
rs143729516
rs13115081
rs192956175
rs114346762
rs2617928
rs7714333
rs76829432
rs144722150
rs11598011
rs147541107
rs138717303
rs1251522
rs11062557
All − MR Egger
All − IVW(MRE)
−0.010
−0.005
0.000
0.005
0.010
MR effect size for
'Nucleoside diphosphate kinase A' on 'eGFR'

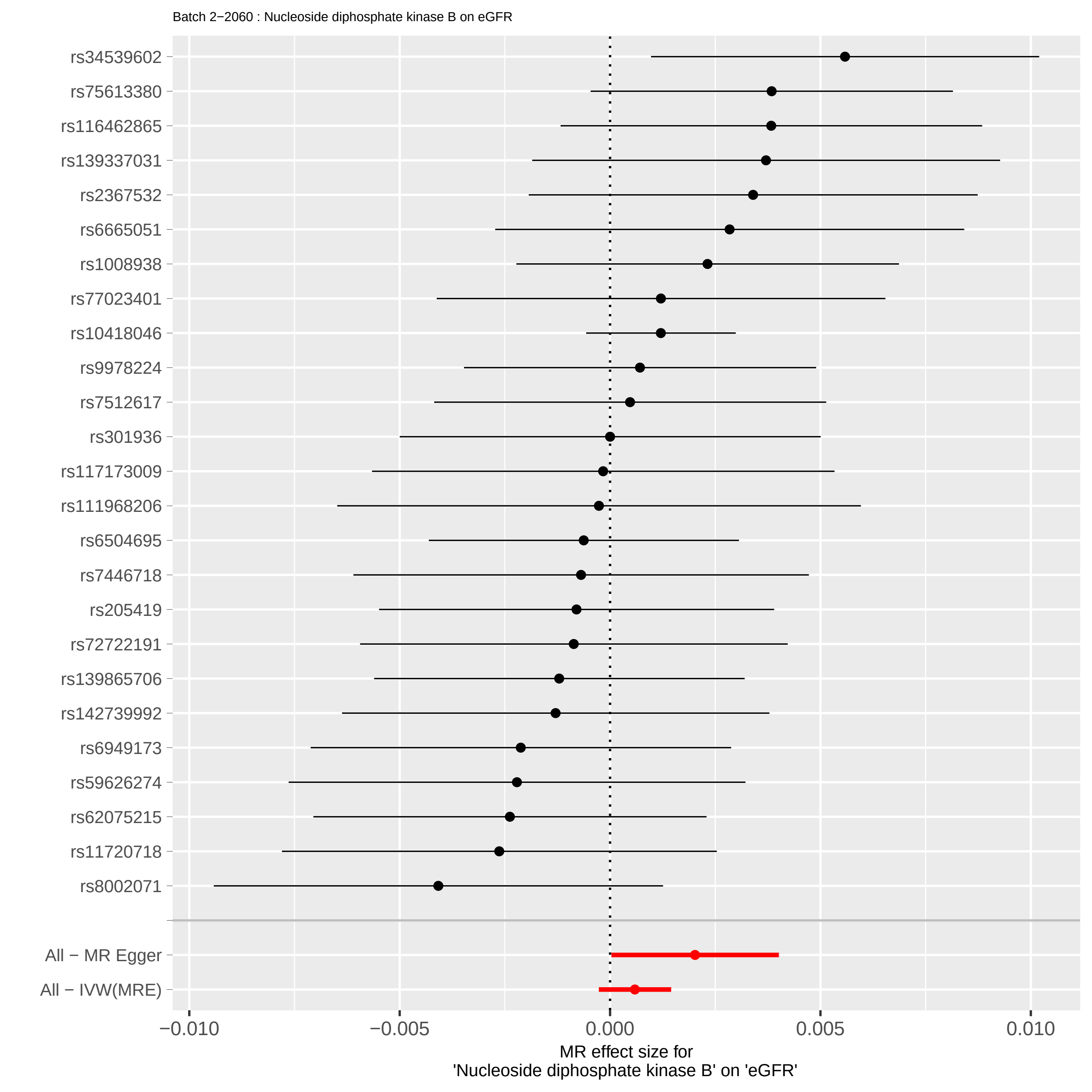

Batch 2−2060 : Nucleoside diphosphate kinase B on eGFR
rs34539602
rs75613380
rs116462865
rs139337031
rs2367532
rs6665051
rs1008938
rs77023401
rs10418046
rs9978224
rs7512617
rs301936
rs117173009
rs111968206
rs6504695
rs7446718
rs205419
rs72722191
rs139865706
rs142739992
rs6949173
rs59626274
rs62075215
rs11720718
rs8002071
All − MR Egger
All − IVW(MRE)
−0.010
−0.005
0.000
0.005
0.010
MR effect size for
'Nucleoside diphosphate kinase B' on 'eGFR'

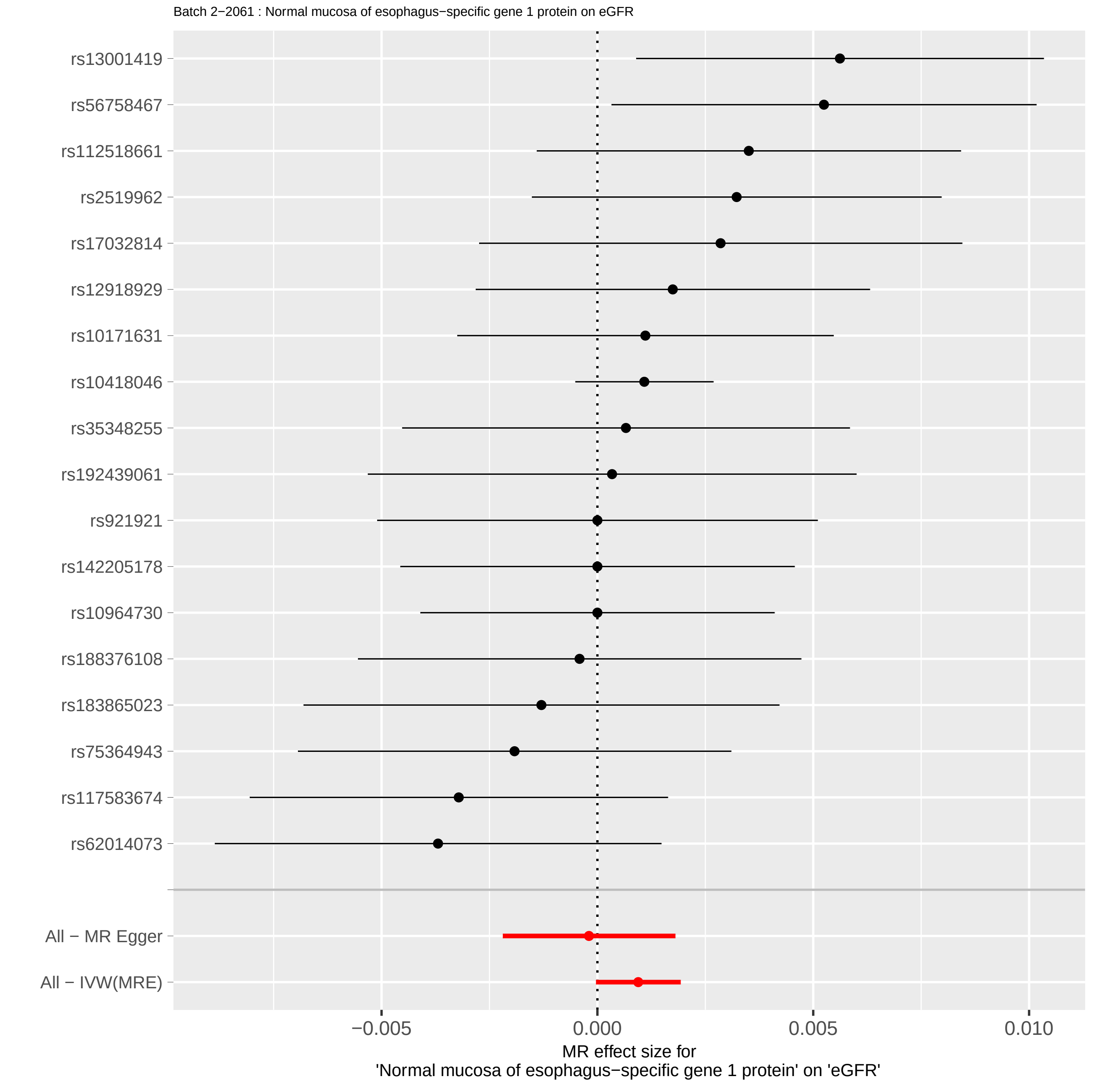

Batch 2−2061 : Normal mucosa of esophagus−specific gene 1 protein on eGFR
rs13001419
rs56758467
rs112518661
rs2519962
rs17032814
rs12918929
rs10171631
rs10418046
rs35348255
rs192439061
rs921921
rs142205178
rs10964730
rs188376108
rs183865023
rs75364943
rs117583674
rs62014073
All − MR Egger
All − IVW(MRE)
−0.005
0.000
0.005
0.010
MR effect size for
'Normal mucosa of esophagus−specific gene 1 protein' on 'eGFR'

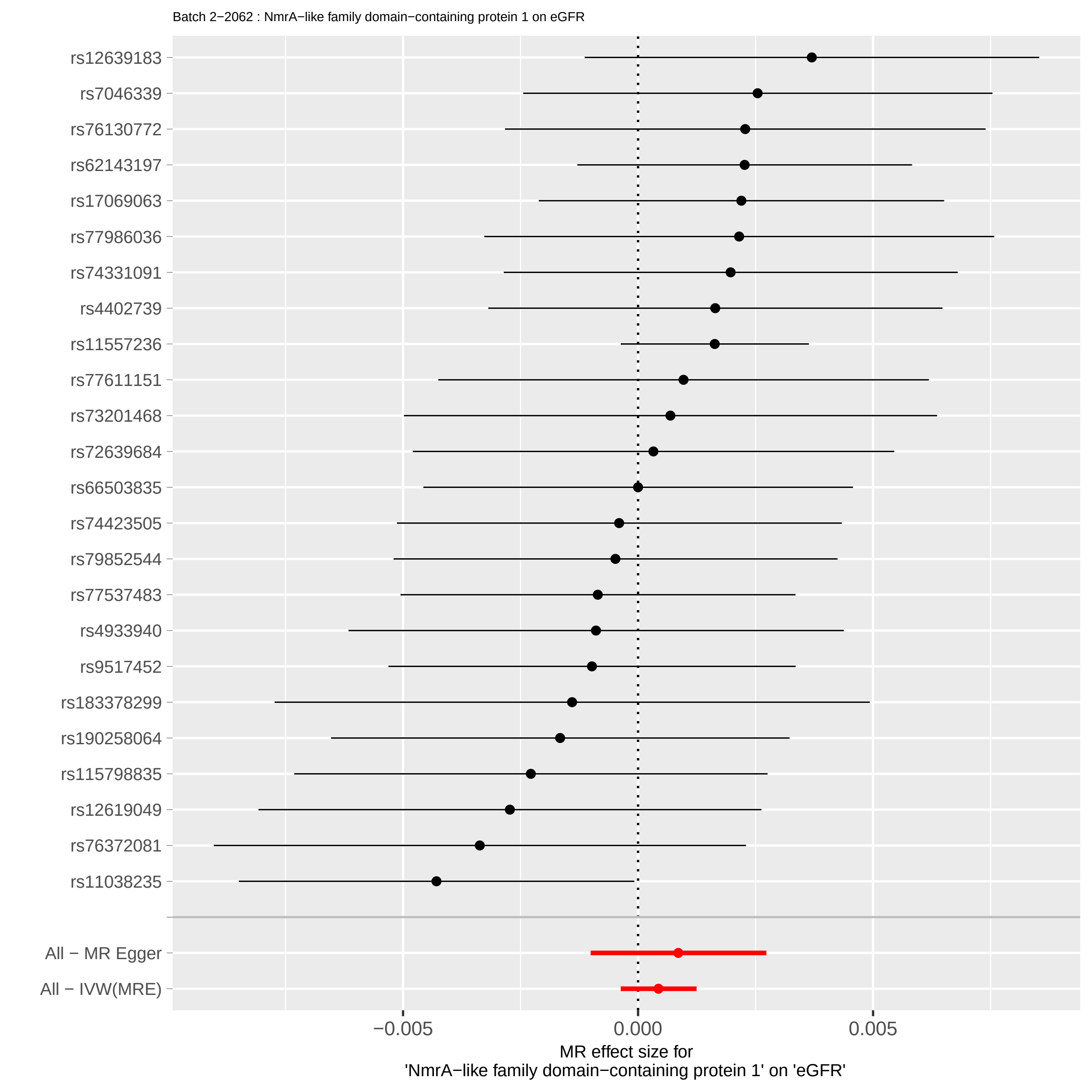
Batch 2−2062 : NmrA−like family domain−containing protein 1 on eGFR
rs12639183
rs7046339
rs76130772
rs62143197
rs17069063
rs77986036
rs74331091
rs4402739
rs11557236
rs77611151
rs73201468
rs72639684
rs66503835
rs74423505
rs79852544
rs77537483
rs4933940
rs9517452
rs183378299
rs190258064
rs115798835
rs12619049
rs76372081
rs11038235
All − MR Egger
All − IVW(MRE)
−0.005
0.000
0.005
MR effect size for
'NmrA−like family domain−containing protein 1' on 'eGFR'

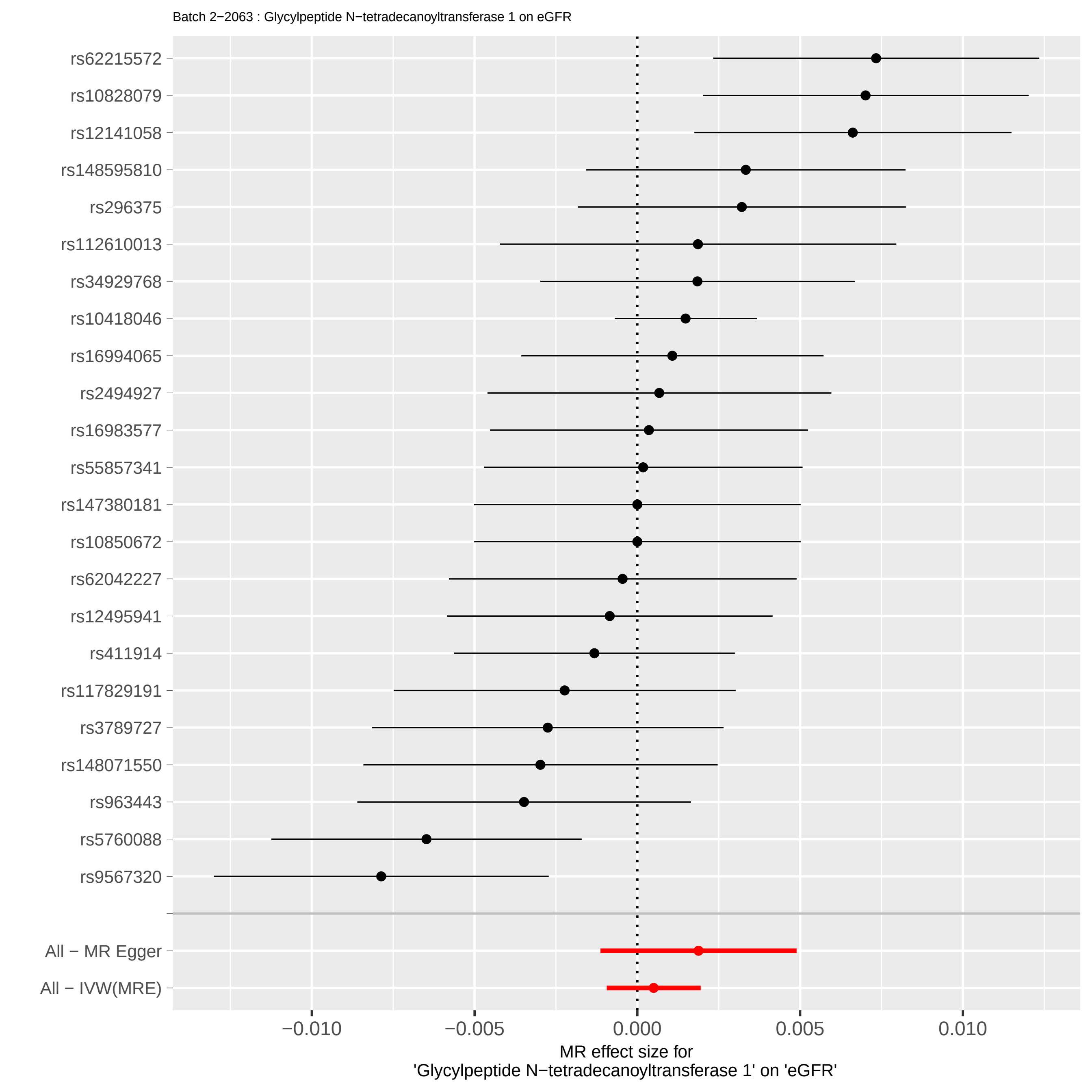

Batch 2−2063 : Glycylpeptide N−tetradecanoyltransferase 1 on eGFR
rs62215572
rs10828079
rs12141058
rs148595810
rs296375
rs112610013
rs34929768
rs10418046
rs16994065
rs2494927
rs16983577
rs55857341
rs147380181
rs10850672
rs62042227
rs12495941
rs411914
rs117829191
rs3789727
rs148071550
rs963443
rs5760088
rs9567320
All − MR Egger
All − IVW(MRE)
−0.010
−0.005
0.000
0.005
0.010
MR effect size for
'Glycylpeptide N−tetradecanoyltransferase 1' on 'eGFR'

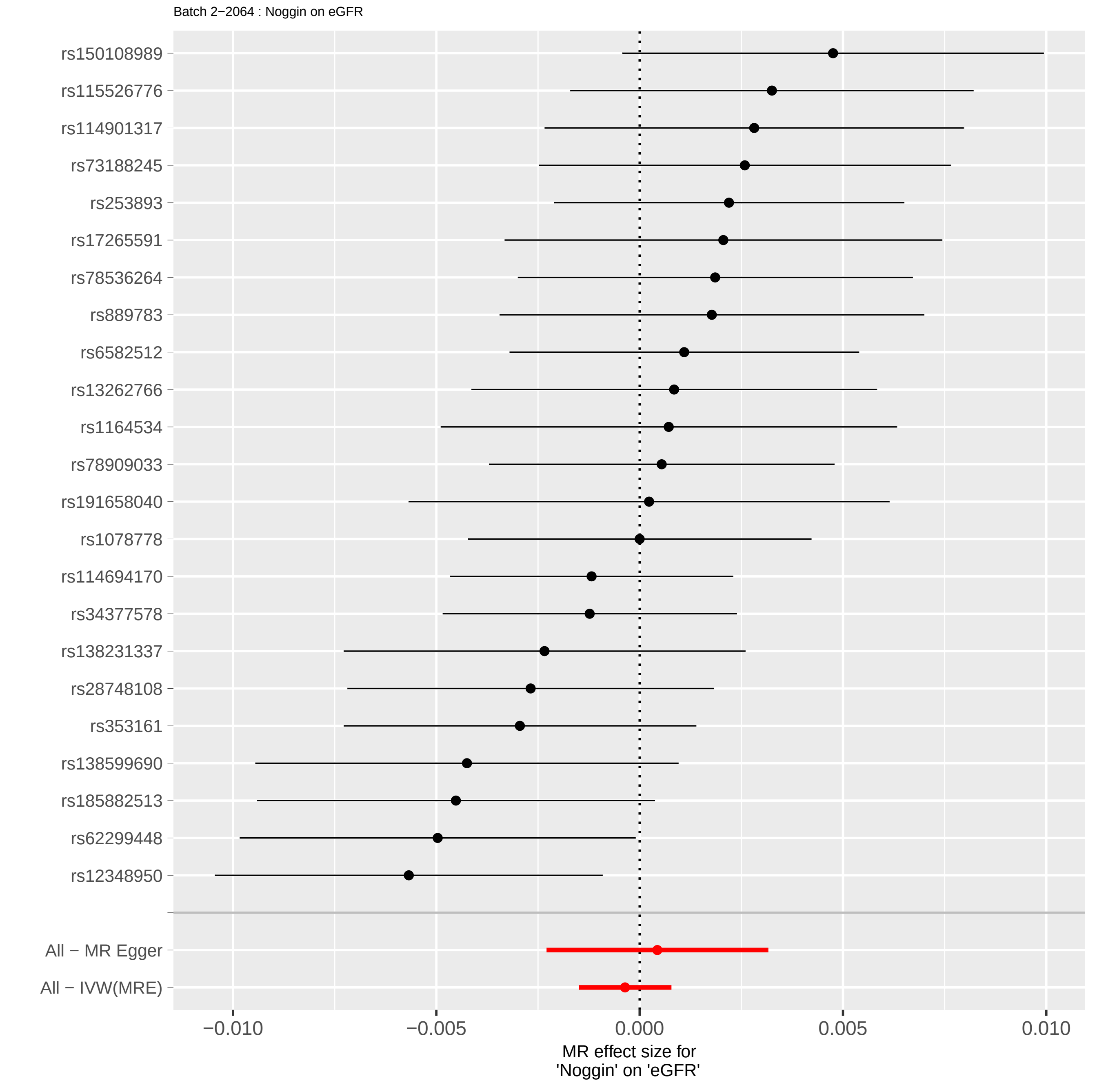
Batch 2−2064 : Noggin on eGFR
rs150108989
rs115526776
rs114901317
rs73188245
rs253893
rs17265591
rs78536264
rs889783
rs6582512
rs13262766
rs1164534
rs78909033
rs191658040
rs1078778
rs114694170
rs34377578
rs138231337
rs28748108
rs353161
rs138599690
rs185882513
rs62299448
rs12348950
All − MR Egger
All − IVW(MRE)
−0.010
−0.005
0.000
0.005
0.010
MR effect size for
'Noggin' on 'eGFR'

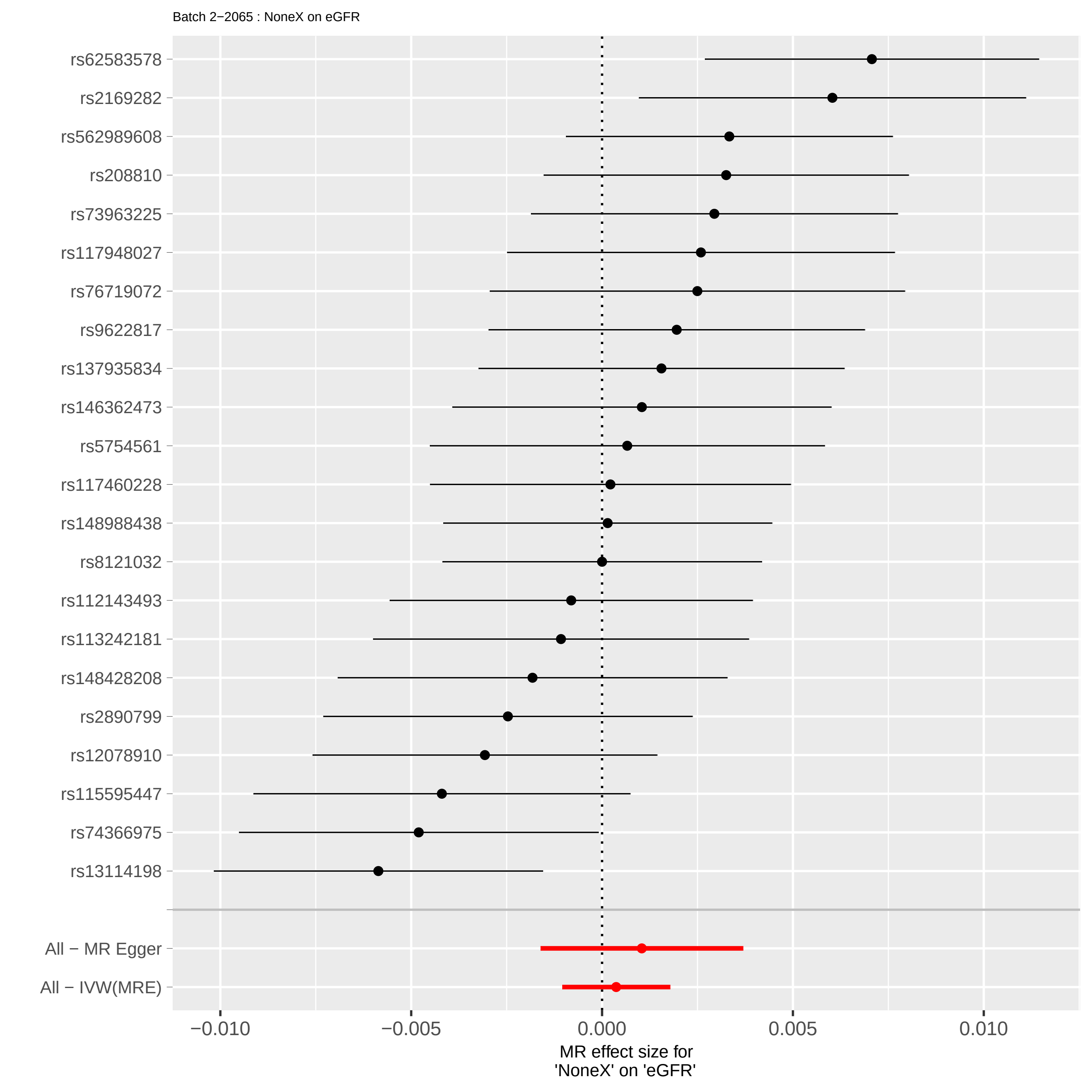

Batch 2−2065 : NoneX on eGFR
rs62583578
rs2169282
rs562989608
rs208810
rs73963225
rs117948027
rs76719072
rs9622817
rs137935834
rs146362473
rs5754561
rs117460228
rs148988438
rs8121032
rs112143493
rs113242181
rs148428208
rs2890799
rs12078910
rs115595447
rs74366975
rs13114198
All − MR Egger
All − IVW(MRE)
−0.010
−0.005
0.000
0.005
0.010
MR effect size for
'NoneX' on 'eGFR'

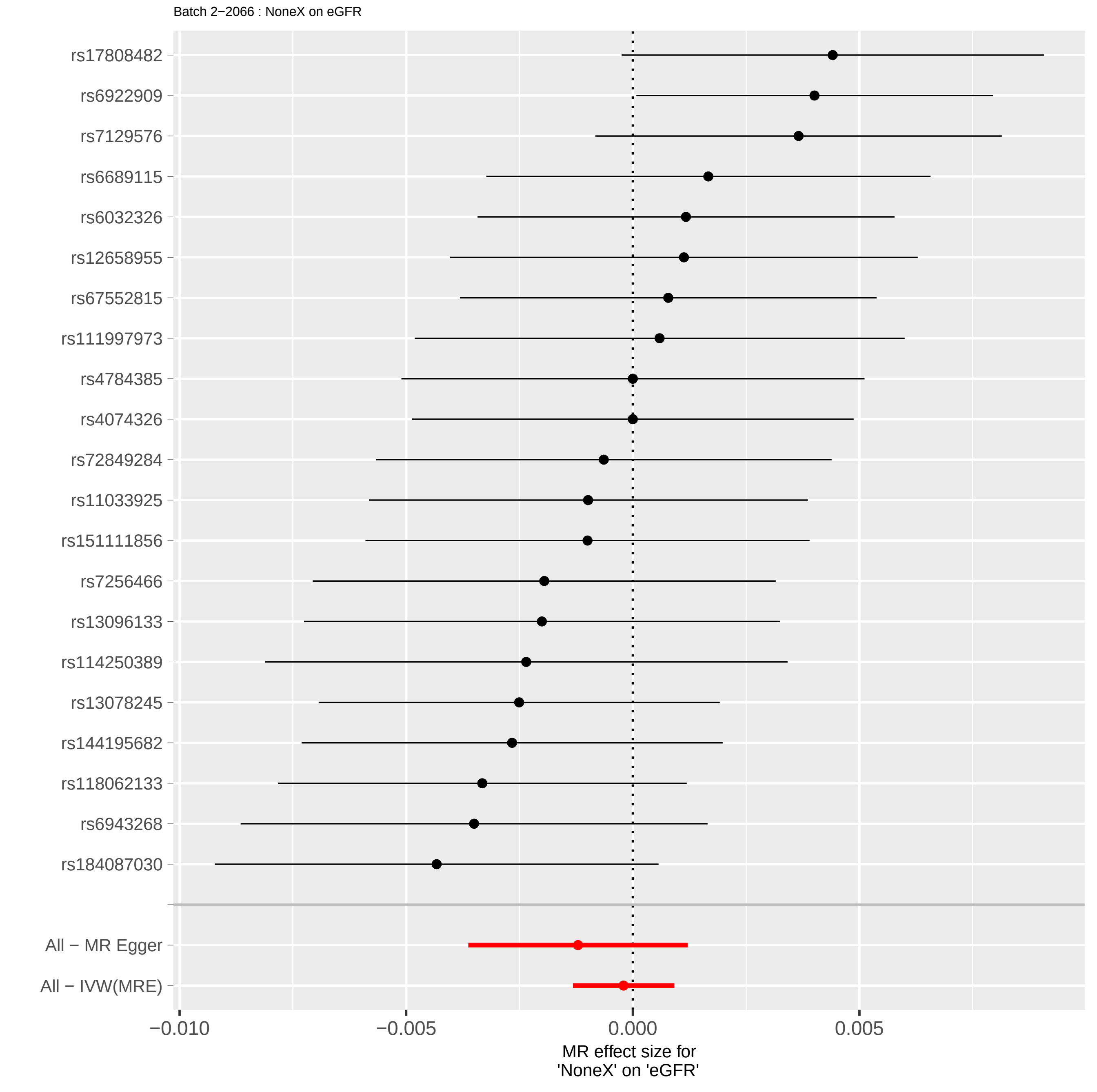

Batch 2−2066 : NoneX on eGFR
rs17808482
rs6922909
rs7129576
rs6689115
rs6032326
rs12658955
rs67552815
rs111997973
rs4784385
rs4074326
rs72849284
rs11033925
rs151111856
rs7256466
rs13096133
rs114250389
rs13078245
rs144195682
rs118062133
rs6943268
rs184087030
All − MR Egger
All − IVW(MRE)
−0.010
−0.005
0.000
0.005
MR effect size for
'NoneX' on 'eGFR'

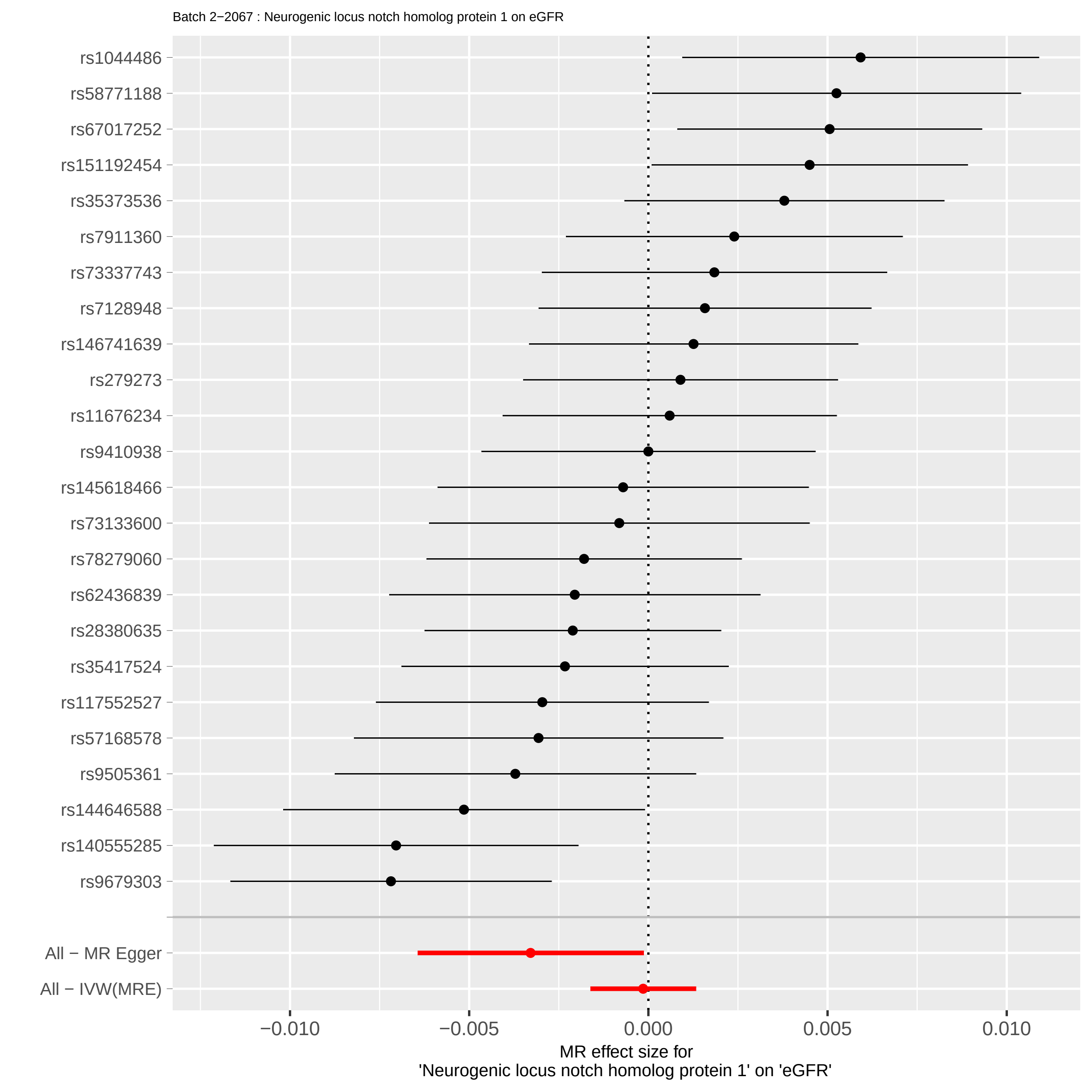

Batch 2−2067 : Neurogenic locus notch homolog protein 1 on eGFR
rs1044486
rs58771188
rs67017252
rs151192454
rs35373536
rs7911360
rs73337743
rs7128948
rs146741639
rs279273
rs11676234
rs9410938
rs145618466
rs73133600
rs78279060
rs62436839
rs28380635
rs35417524
rs117552527
rs57168578
rs9505361
rs144646588
rs140555285
rs9679303
All − MR Egger
All − IVW(MRE)
−0.010
−0.005
0.000
0.005
0.010
MR effect size for
'Neurogenic locus notch homolog protein 1' on 'eGFR'

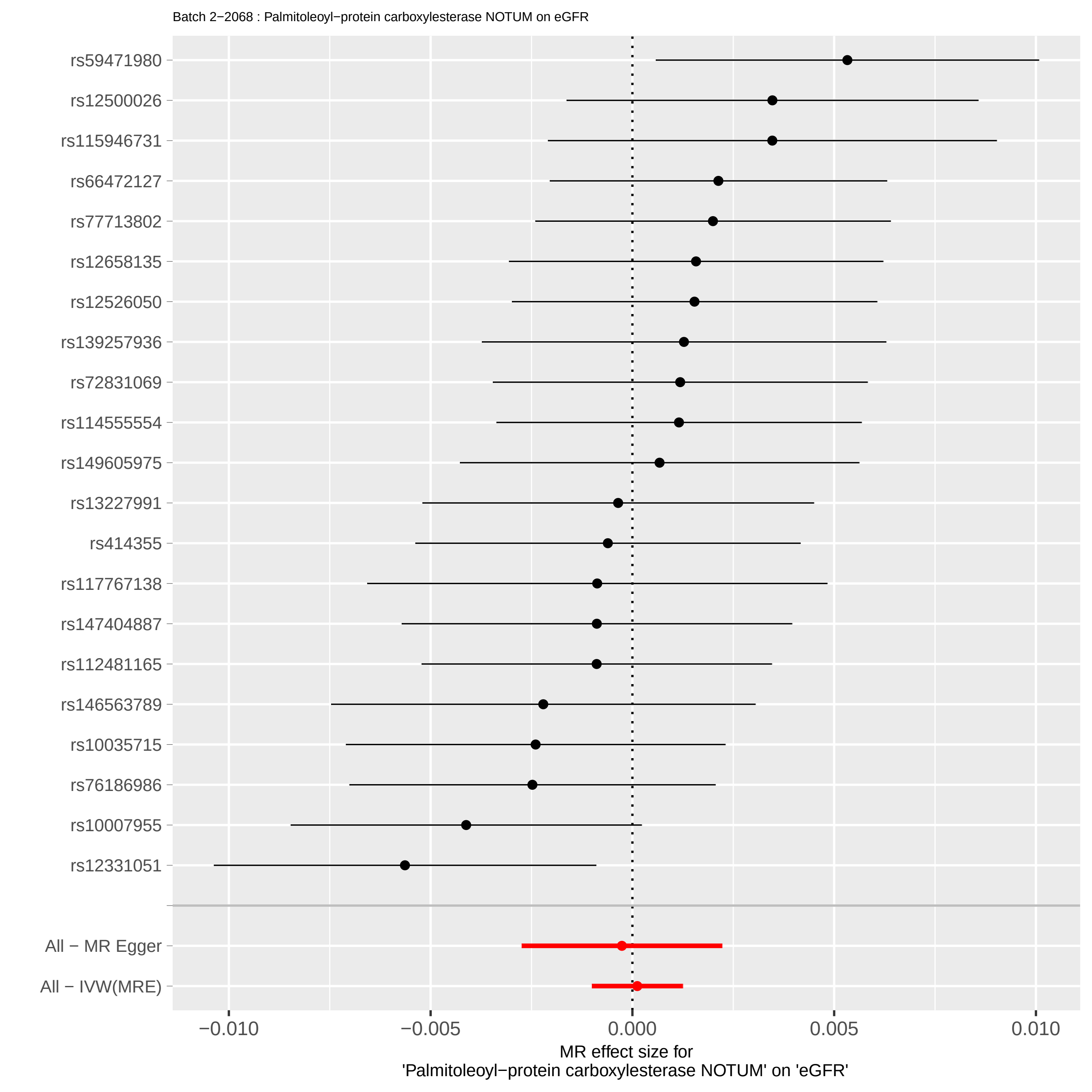

Batch 2−2068 : Palmitoleoyl−protein carboxylesterase NOTUM on eGFR
rs59471980
rs12500026
rs115946731
rs66472127
rs77713802
rs12658135
rs12526050
rs139257936
rs72831069
rs114555554
rs149605975
rs13227991
rs414355
rs117767138
rs147404887
rs112481165
rs146563789
rs10035715
rs76186986
rs10007955
rs12331051
All − MR Egger
All − IVW(MRE)
−0.010
−0.005
0.000
0.005
0.010
MR effect size for
'Palmitoleoyl−protein carboxylesterase NOTUM' on 'eGFR'

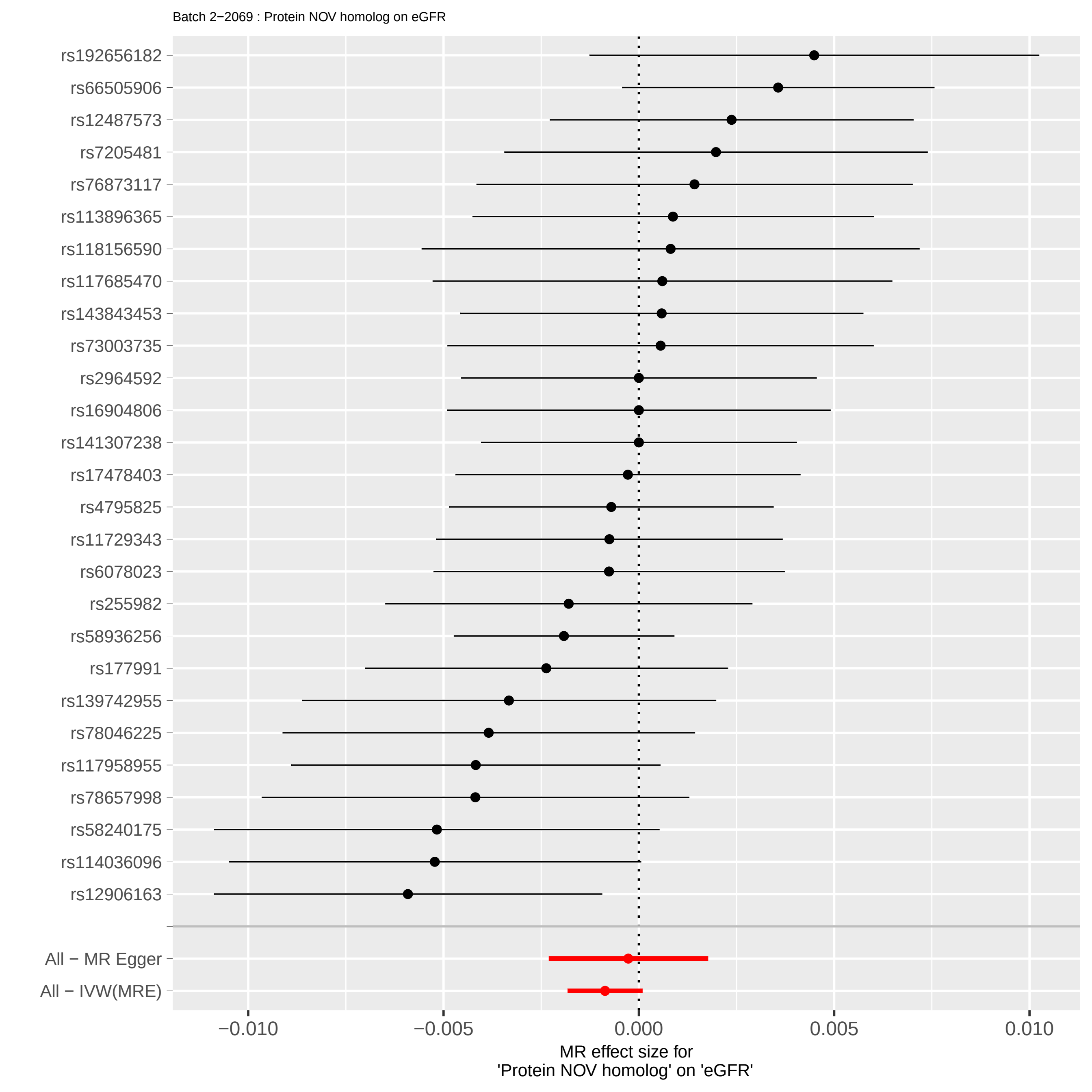

Batch 2−2069 : Protein NOV homolog on eGFR
rs192656182
rs66505906
rs12487573
rs7205481
rs76873117
rs113896365
rs118156590
rs117685470
rs143843453
rs73003735
rs2964592
rs16904806
rs141307238
rs17478403
rs4795825
rs11729343
rs6078023
rs255982
rs58936256
rs177991
rs139742955
rs78046225
rs117958955
rs78657998
rs58240175
rs114036096
rs12906163
All − MR Egger
All − IVW(MRE)
−0.010
−0.005
0.000
0.005
0.010
MR effect size for
'Protein NOV homolog' on 'eGFR'

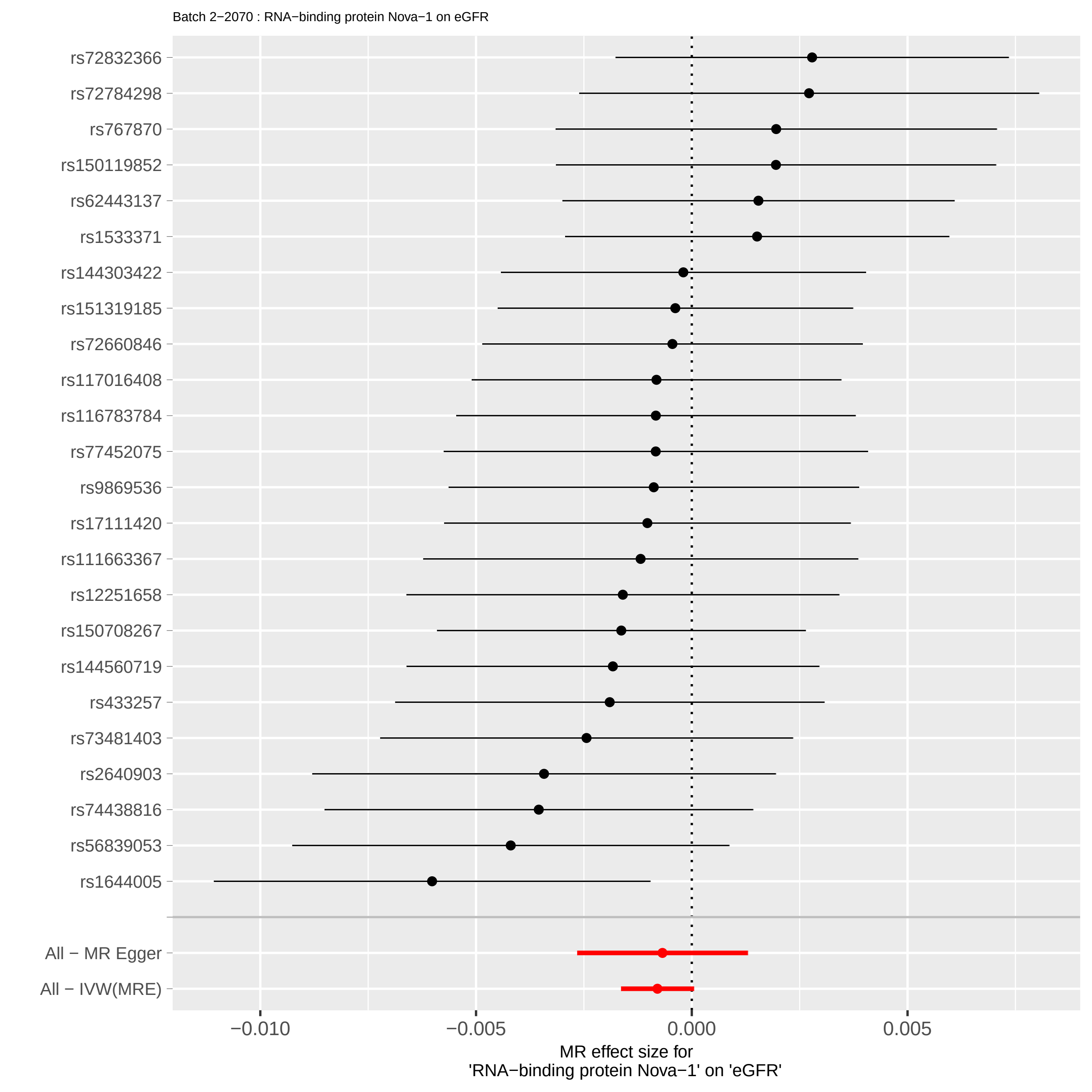

Batch 2−2070 : RNA−binding protein Nova−1 on eGFR
rs72832366
rs72784298
rs767870
rs150119852
rs62443137
rs1533371
rs144303422
rs151319185
rs72660846
rs117016408
rs116783784
rs77452075
rs9869536
rs17111420
rs111663367
rs12251658
rs150708267
rs144560719
rs433257
rs73481403
rs2640903
rs74438816
rs56839053
rs1644005
All − MR Egger
All − IVW(MRE)
−0.010
−0.005
0.000
0.005
MR effect size for
'RNA−binding protein Nova−1' on 'eGFR'

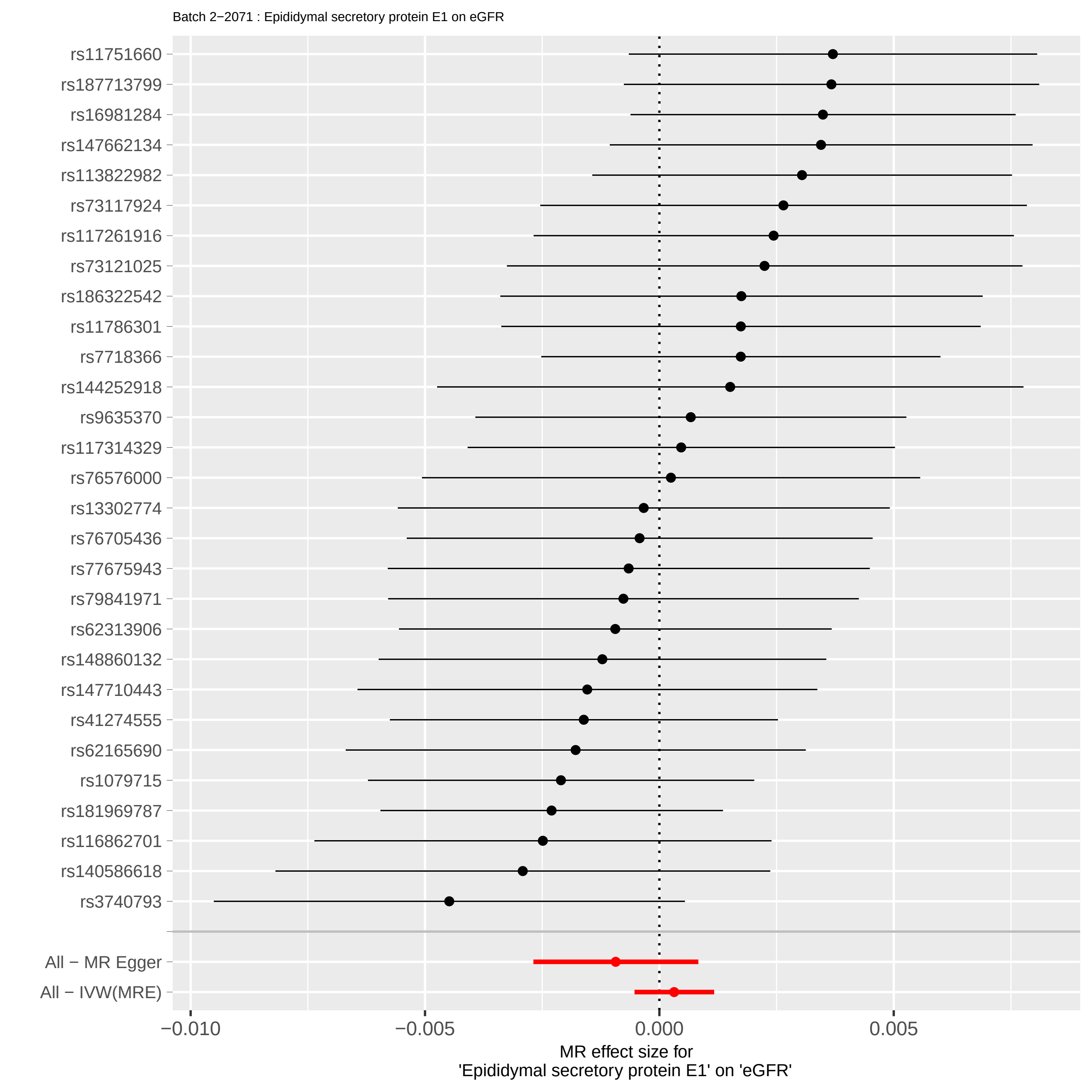
Batch 2−2071 : Epididymal secretory protein E1 on eGFR
rs11751660
rs187713799
rs16981284
rs147662134
rs113822982
rs73117924
rs117261916
rs73121025
rs186322542
rs11786301
rs7718366
rs144252918
rs9635370
rs117314329
rs76576000
rs13302774
rs76705436
rs77675943
rs79841971
rs62313906
rs148860132
rs147710443
rs41274555
rs62165690
rs1079715
rs181969787
rs116862701
rs140586618
rs3740793
All − MR Egger
All − IVW(MRE)
−0.010
−0.005
0.000
0.005
MR effect size for
'Epididymal secretory protein E1' on 'eGFR'

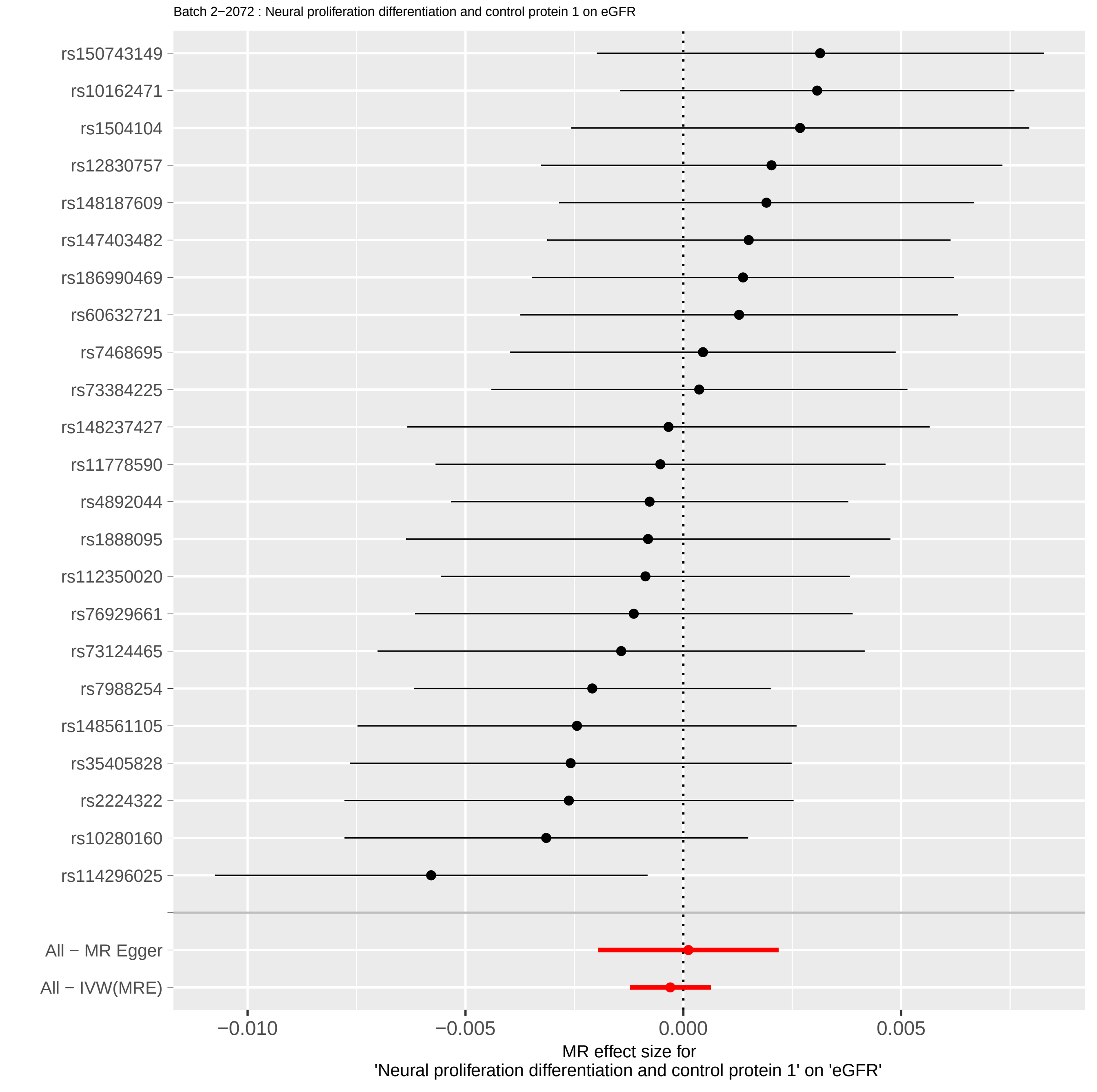
Batch 2−2072 : Neural proliferation differentiation and control protein 1 on eGFR
rs150743149
rs10162471
rs1504104
rs12830757
rs148187609
rs147403482
rs186990469
rs60632721
rs7468695
rs73384225
rs148237427
rs11778590
rs4892044
rs1888095
rs112350020
rs76929661
rs73124465
rs7988254
rs148561105
rs35405828
rs2224322
rs10280160
rs114296025
All − MR Egger
All − IVW(MRE)
−0.010
−0.005
0.000
0.005
MR effect size for
'Neural proliferation differentiation and control protein 1' on 'eGFR'

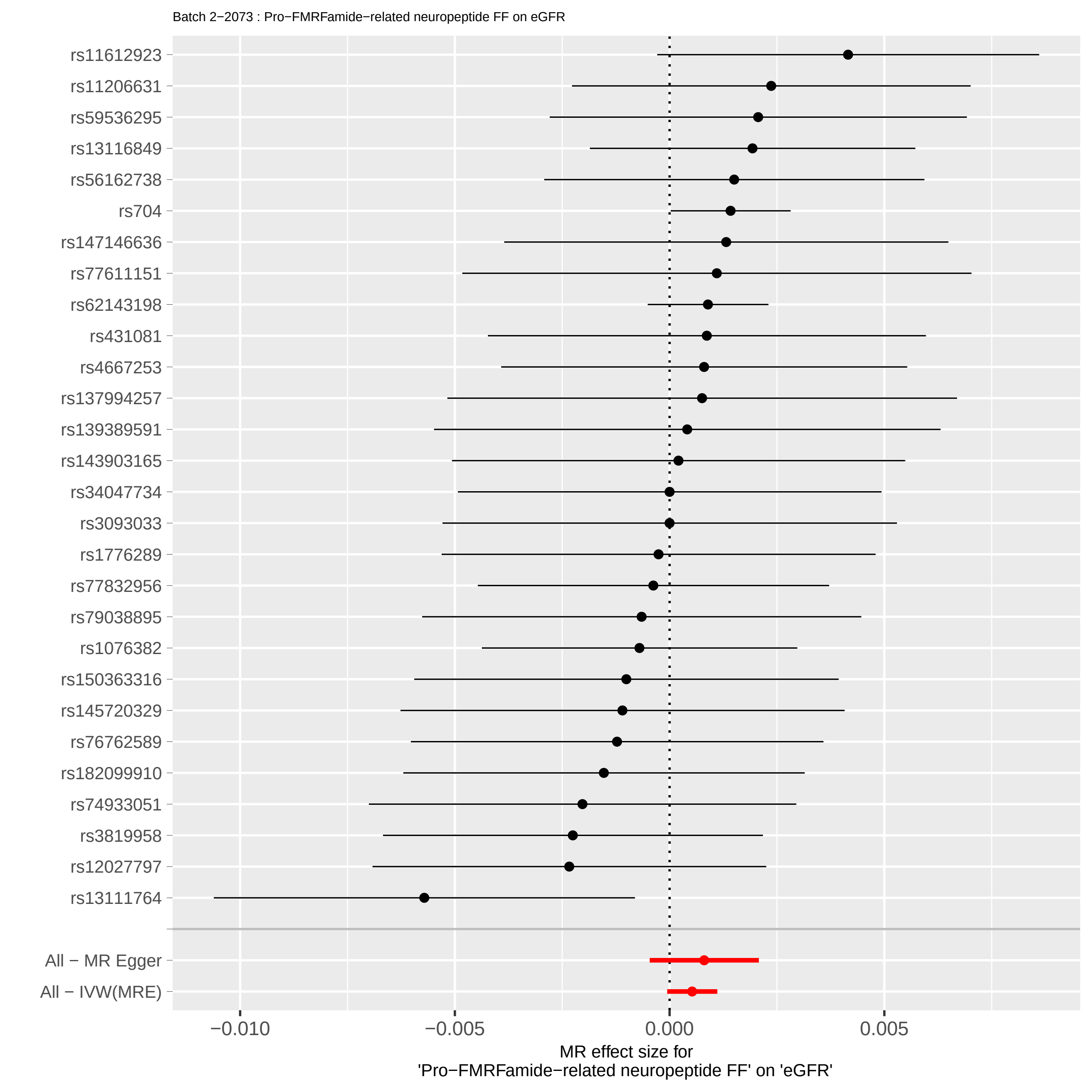

Batch 2−2073 : Pro−FMRFamide−related neuropeptide FF on eGFR
rs11612923
rs11206631
rs59536295
rs13116849
rs56162738
rs704
rs147146636
rs77611151
rs62143198
rs431081
rs4667253
rs137994257
rs139389591
rs143903165
rs34047734
rs3093033
rs1776289
rs77832956
rs79038895
rs1076382
rs150363316
rs145720329
rs76762589
rs182099910
rs74933051
rs3819958
rs12027797
rs13111764
All − MR Egger
All − IVW(MRE)
−0.010
−0.005
0.000
0.005
MR effect size for
'Pro−FMRFamide−related neuropeptide FF' on 'eGFR'

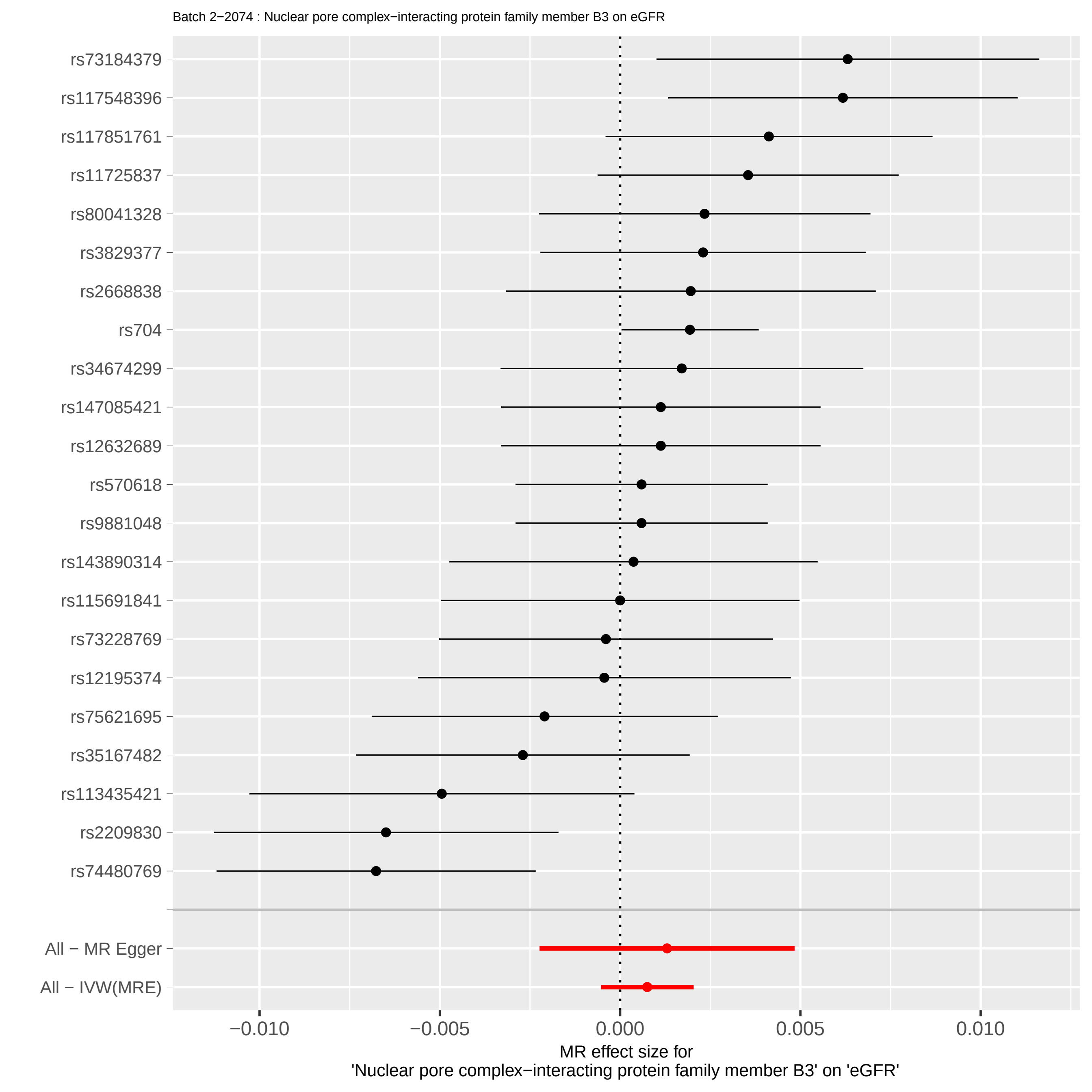

Batch 2−2074 : Nuclear pore complex−interacting protein family member B3 on eGFR
rs73184379
rs117548396
rs117851761
rs11725837
rs80041328
rs3829377
rs2668838
rs704
rs34674299
rs147085421
rs12632689
rs570618
rs9881048
rs143890314
rs115691841
rs73228769
rs12195374
rs75621695
rs35167482
rs113435421
rs2209830
rs74480769
All − MR Egger
All − IVW(MRE)
−0.010
−0.005
0.000
0.005
0.010
MR effect size for
'Nuclear pore complex−interacting protein family member B3' on 'eGFR'

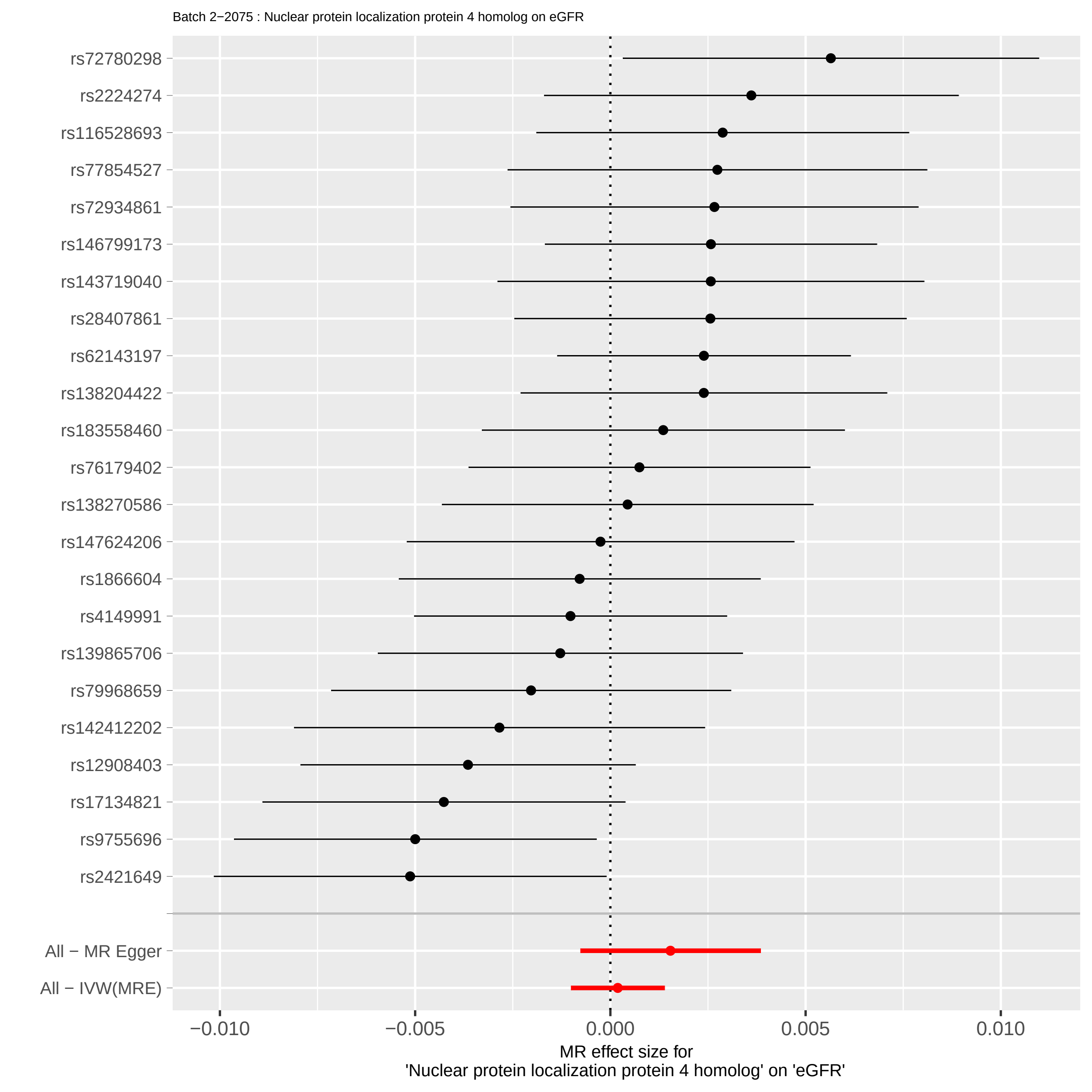

Batch 2−2075 : Nuclear protein localization protein 4 homolog on eGFR
rs72780298
rs2224274
rs116528693
rs77854527
rs72934861
rs146799173
rs143719040
rs28407861
rs62143197
rs138204422
rs183558460
rs76179402
rs138270586
rs147624206
rs1866604
rs4149991
rs139865706
rs79968659
rs142412202
rs12908403
rs17134821
rs9755696
rs2421649
All − MR Egger
All − IVW(MRE)
−0.010
−0.005
0.000
0.005
0.010
MR effect size for
'Nuclear protein localization protein 4 homolog' on 'eGFR'

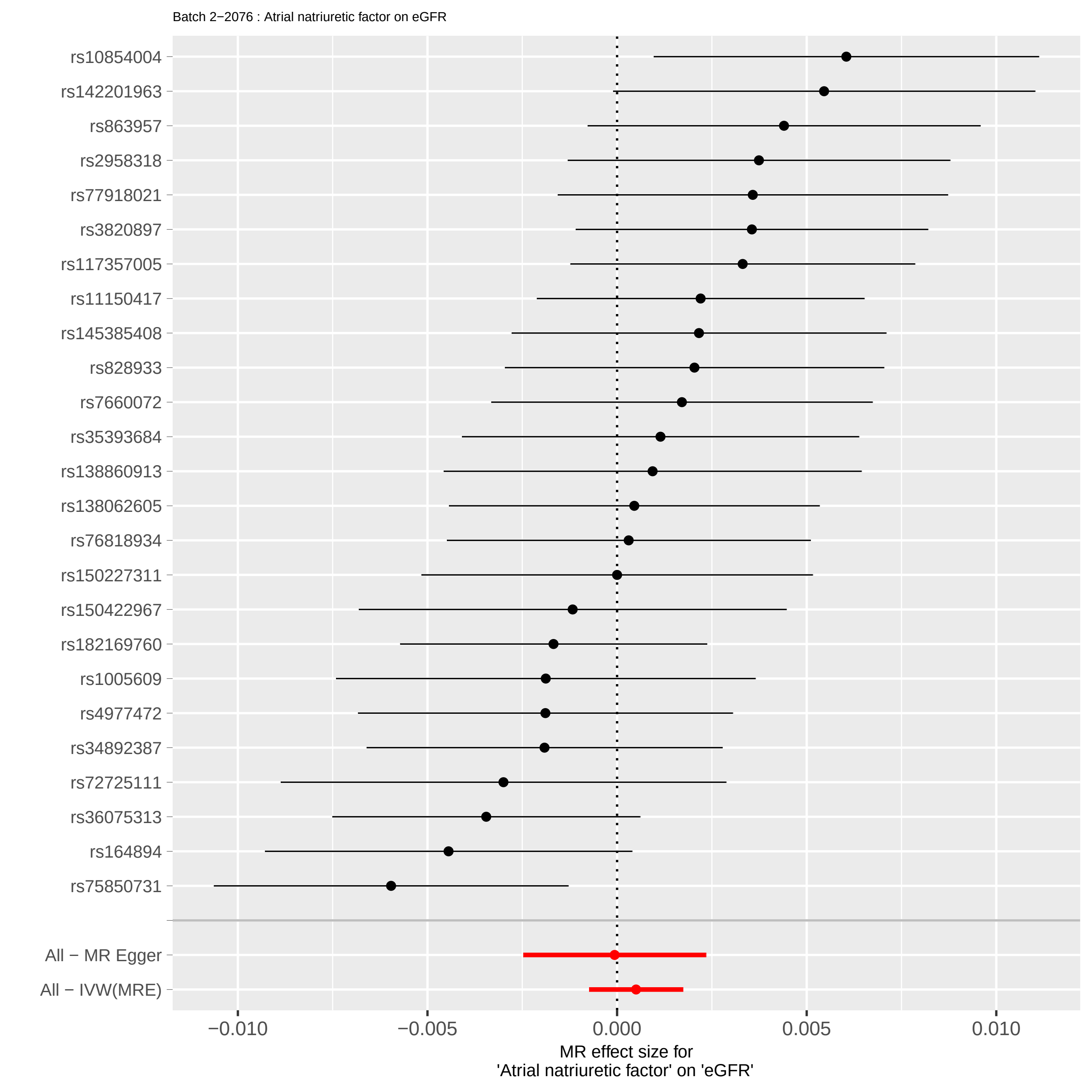

Batch 2−2076 : Atrial natriuretic factor on eGFR
rs10854004
rs142201963
rs863957
rs2958318
rs77918021
rs3820897
rs117357005
rs11150417
rs145385408
rs828933
rs7660072
rs35393684
rs138860913
rs138062605
rs76818934
rs150227311
rs150422967
rs182169760
rs1005609
rs4977472
rs34892387
rs72725111
rs36075313
rs164894
rs75850731
All − MR Egger
All − IVW(MRE)
−0.010
−0.005
0.000
0.005
0.010
MR effect size for
'Atrial natriuretic factor' on 'eGFR'

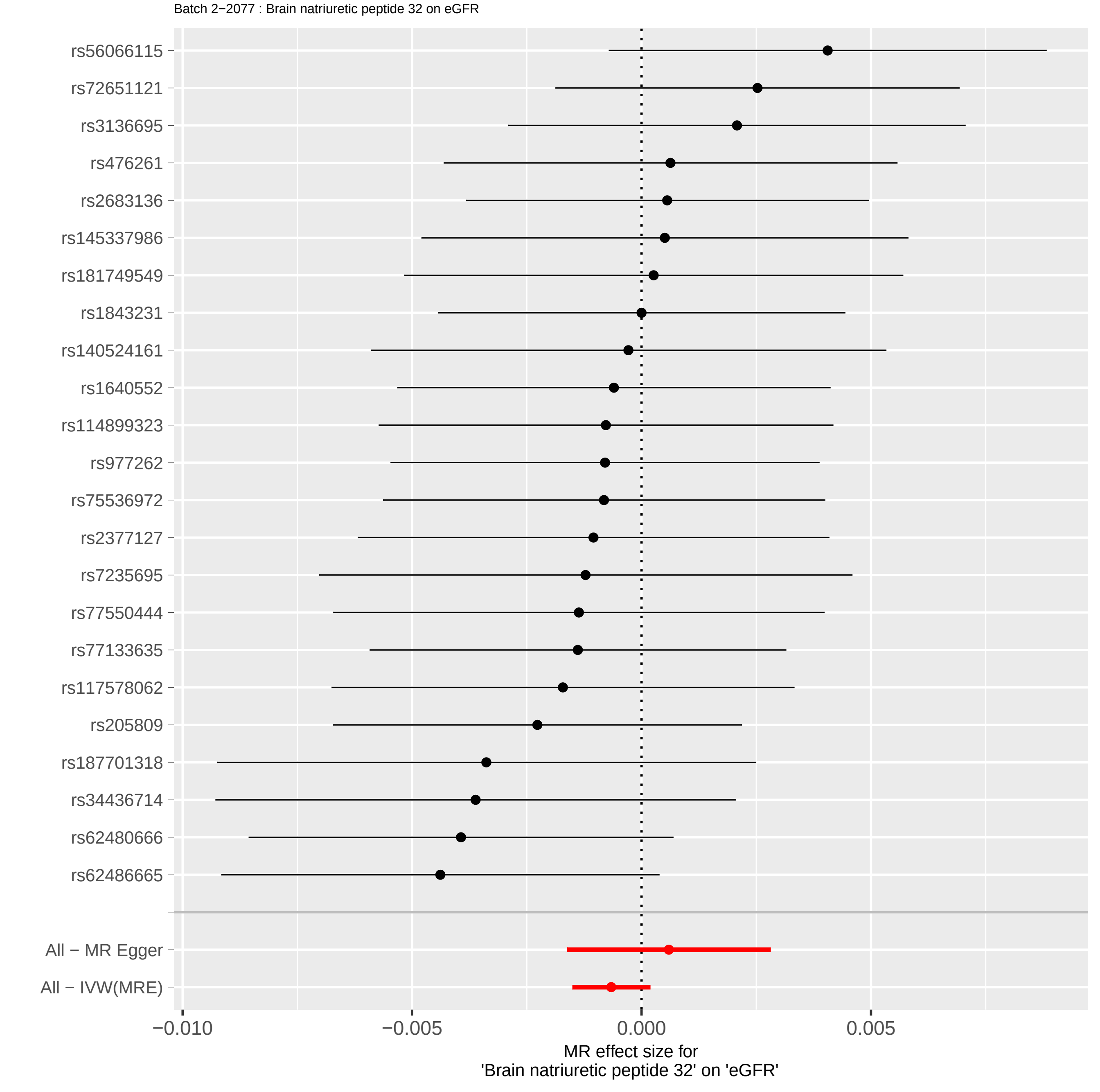

Batch 2−2077 : Brain natriuretic peptide 32 on eGFR
rs56066115
rs72651121
rs3136695
rs476261
rs2683136
rs145337986
rs181749549
rs1843231
rs140524161
rs1640552
rs114899323
rs977262
rs75536972
rs2377127
rs7235695
rs77550444
rs77133635
rs117578062
rs205809
rs187701318
rs34436714
rs62480666
rs62486665
All − MR Egger
All − IVW(MRE)
−0.010
−0.005
0.000
0.005
MR effect size for
'Brain natriuretic peptide 32' on 'eGFR'

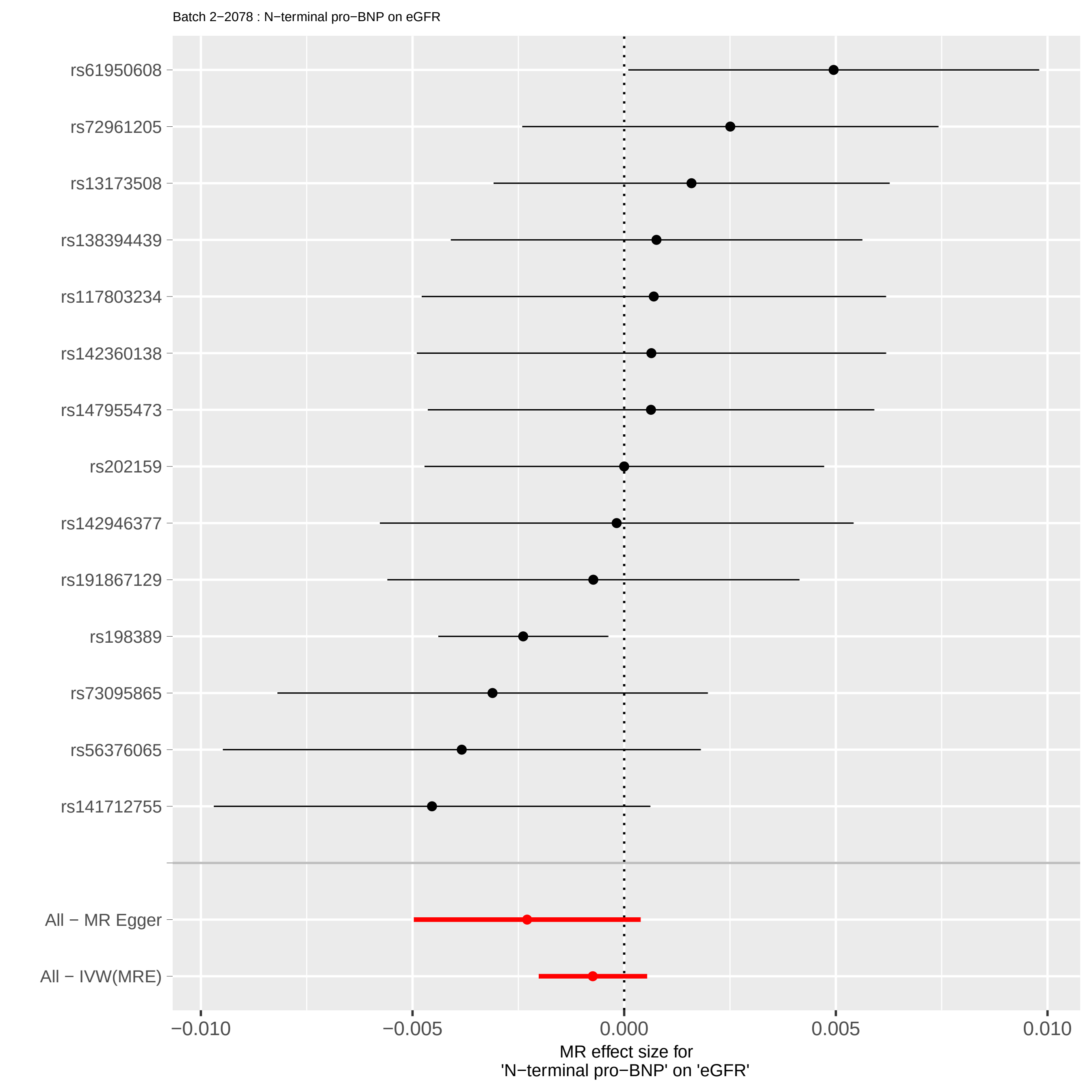

Batch 2−2078 : N−terminal pro−BNP on eGFR
rs61950608
rs72961205
rs13173508
rs138394439
rs117803234
rs142360138
rs147955473
rs202159
rs142946377
rs191867129
rs198389
rs73095865
rs56376065
rs141712755
All − MR Egger
All − IVW(MRE)
−0.010
−0.005
0.000
0.005
0.010
MR effect size for
'N−terminal pro−BNP' on 'eGFR'

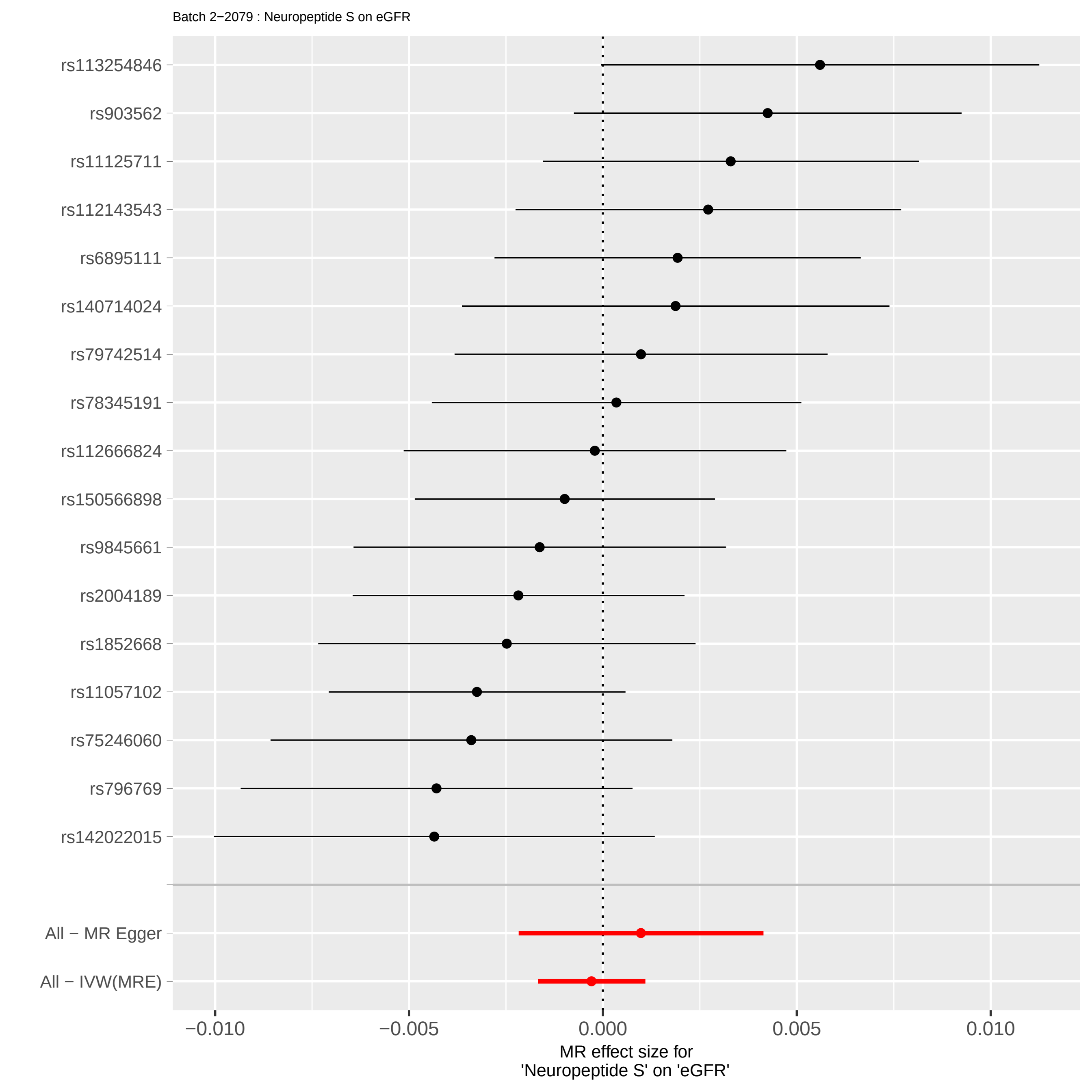

Batch 2−2079 : Neuropeptide S on eGFR
rs113254846
rs903562
rs11125711
rs112143543
rs6895111
rs140714024
rs79742514
rs78345191
rs112666824
rs150566898
rs9845661
rs2004189
rs1852668
rs11057102
rs75246060
rs796769
rs142022015
All − MR Egger
All − IVW(MRE)
−0.010
−0.005
0.000
0.005
0.010
MR effect size for
'Neuropeptide S' on 'eGFR'

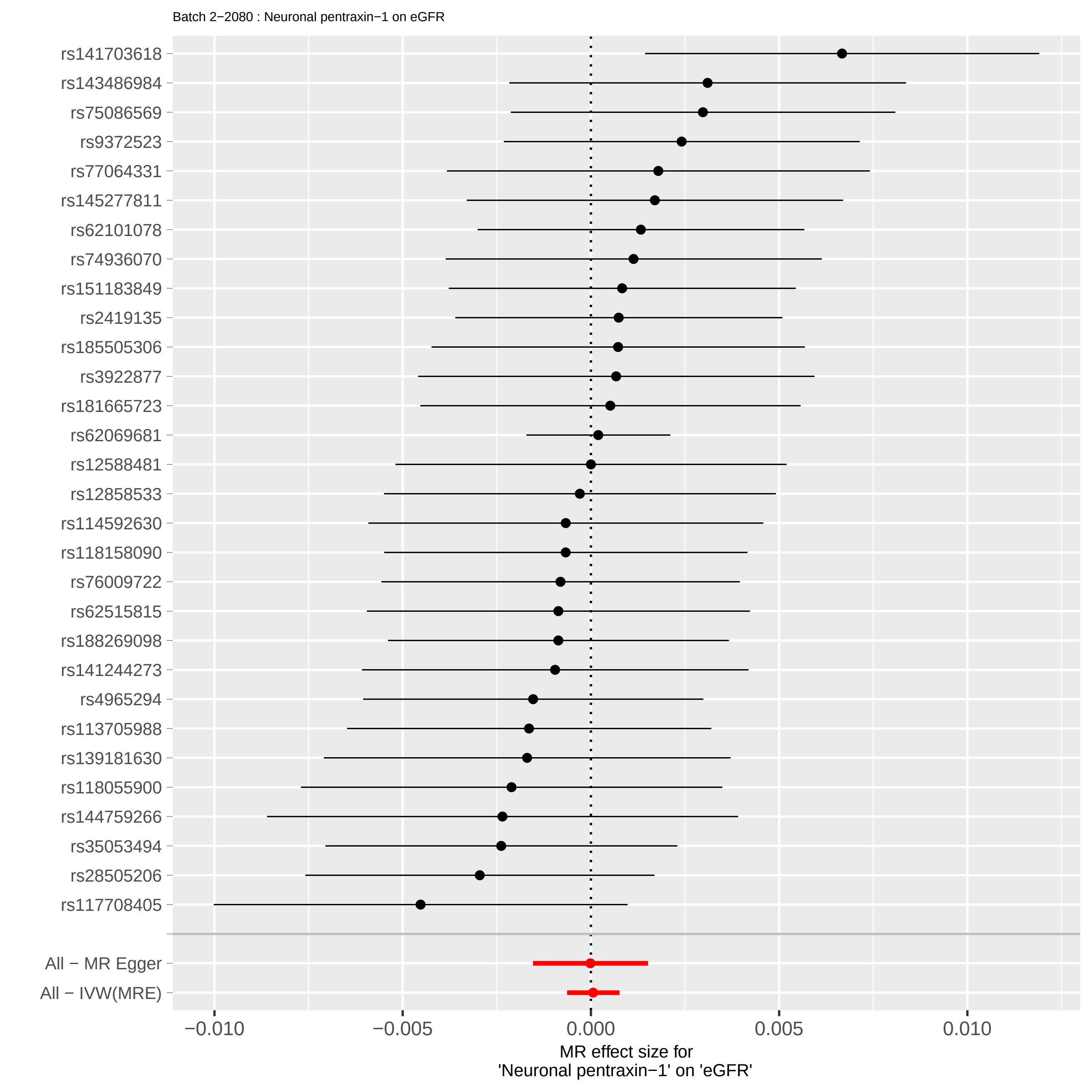
Batch 2−2080 : Neuronal pentraxin−1 on eGFR
rs141703618
rs143486984
rs75086569
rs9372523
rs77064331
rs145277811
rs62101078
rs74936070
rs151183849
rs2419135
rs185505306
rs3922877
rs181665723
rs62069681
rs12588481
rs12858533
rs114592630
rs118158090
rs76009722
rs62515815
rs188269098
rs141244273
rs4965294
rs113705988
rs139181630
rs118055900
rs144759266
rs35053494
rs28505206
rs117708405
All − MR Egger
All − IVW(MRE)
−0.010
−0.005
0.000
0.005
0.010
MR effect size for
'Neuronal pentraxin−1' on 'eGFR'

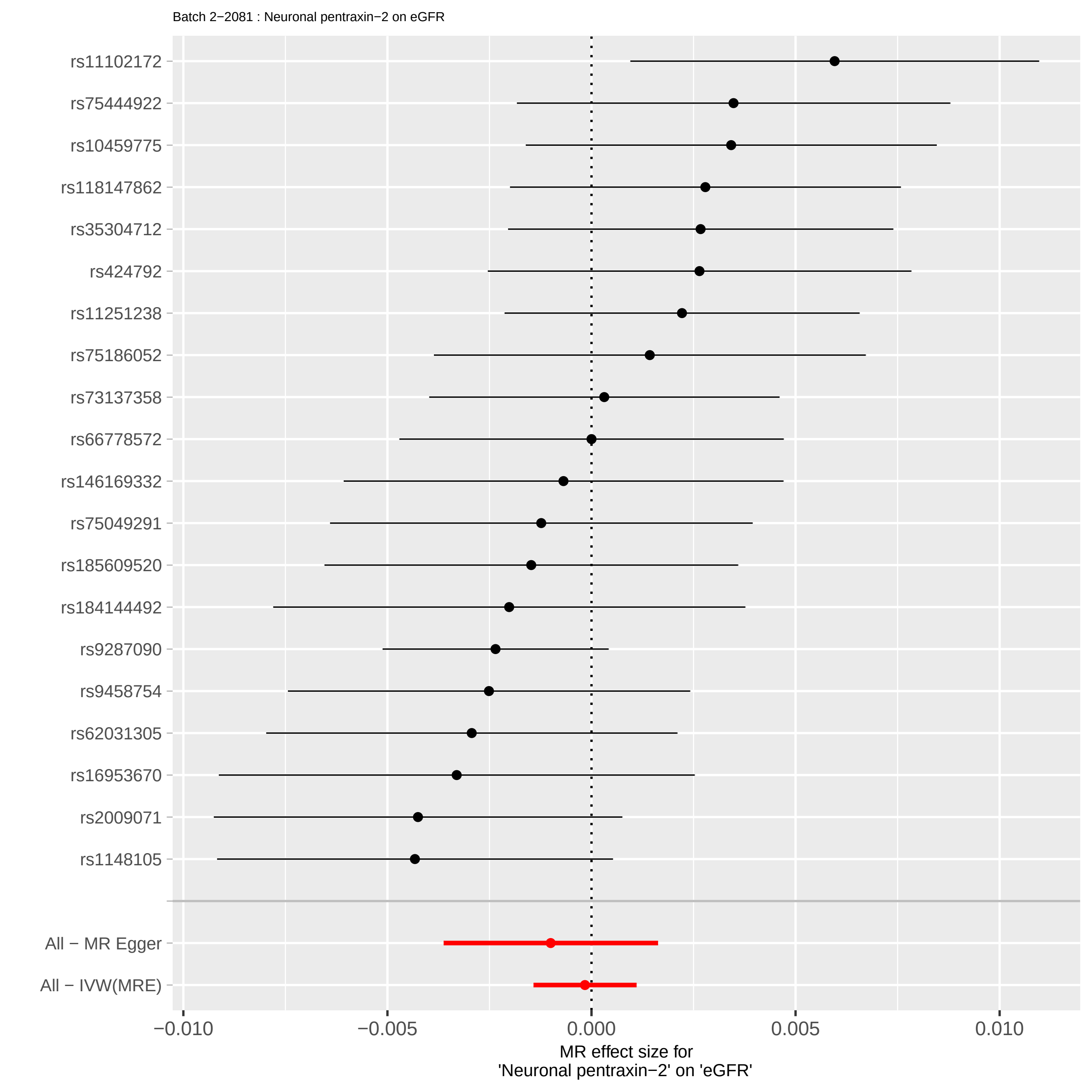
Batch 2−2081 : Neuronal pentraxin−2 on eGFR
rs11102172
rs75444922
rs10459775
rs118147862
rs35304712
rs424792
rs11251238
rs75186052
rs73137358
rs66778572
rs146169332
rs75049291
rs185609520
rs184144492
rs9287090
rs9458754
rs62031305
rs16953670
rs2009071
rs1148105
All − MR Egger
All − IVW(MRE)
−0.010
−0.005
0.000
0.005
0.010
MR effect size for
'Neuronal pentraxin−2' on 'eGFR'

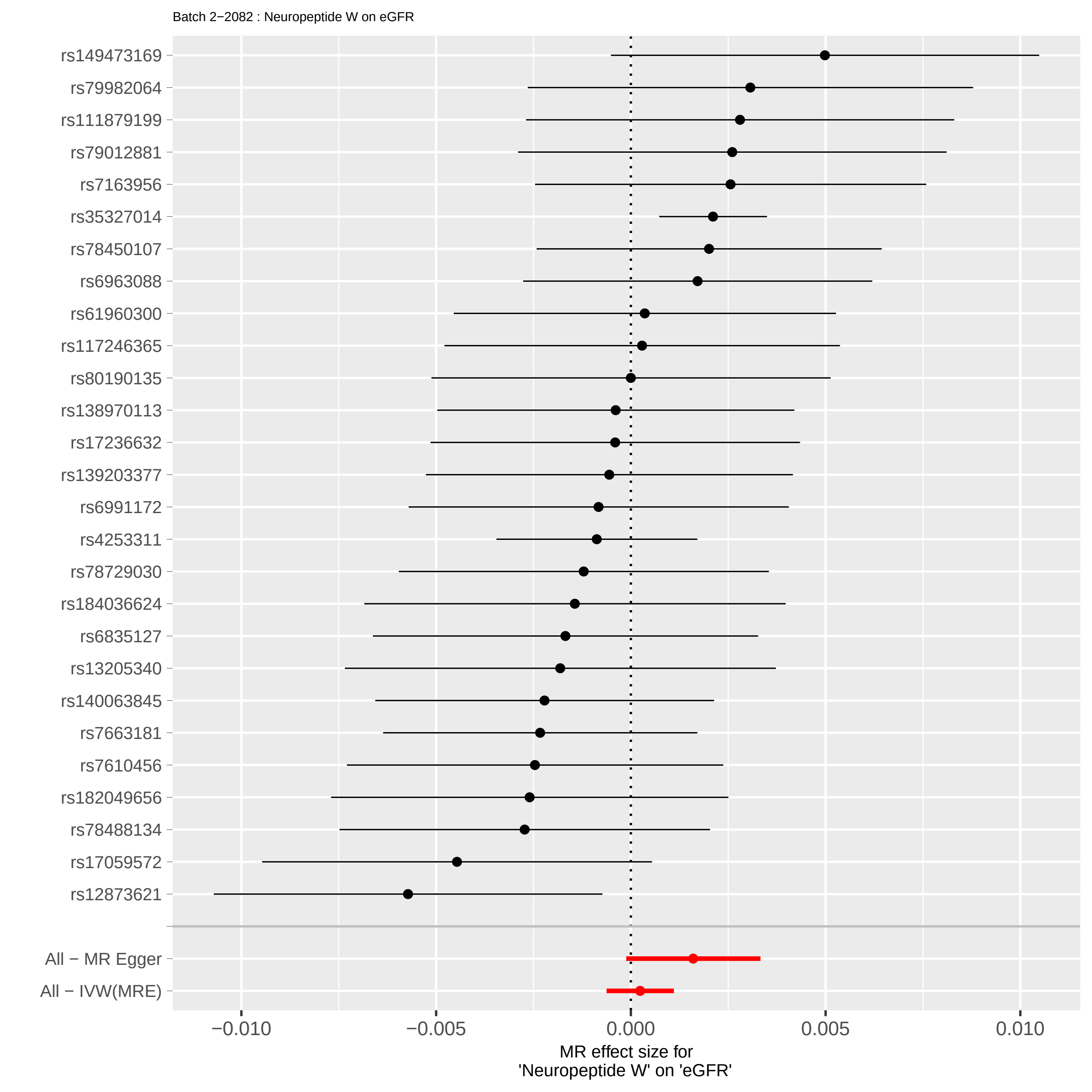

Batch 2−2082 : Neuropeptide W on eGFR
rs149473169
rs79982064
rs111879199
rs79012881
rs7163956
rs35327014
rs78450107
rs6963088
rs61960300
rs117246365
rs80190135
rs138970113
rs17236632
rs139203377
rs6991172
rs4253311
rs78729030
rs184036624
rs6835127
rs13205340
rs140063845
rs7663181
rs7610456
rs182049656
rs78488134
rs17059572
rs12873621
All − MR Egger
All − IVW(MRE)
−0.010
−0.005
0.000
0.005
0.010
MR effect size for
'Neuropeptide W' on 'eGFR'

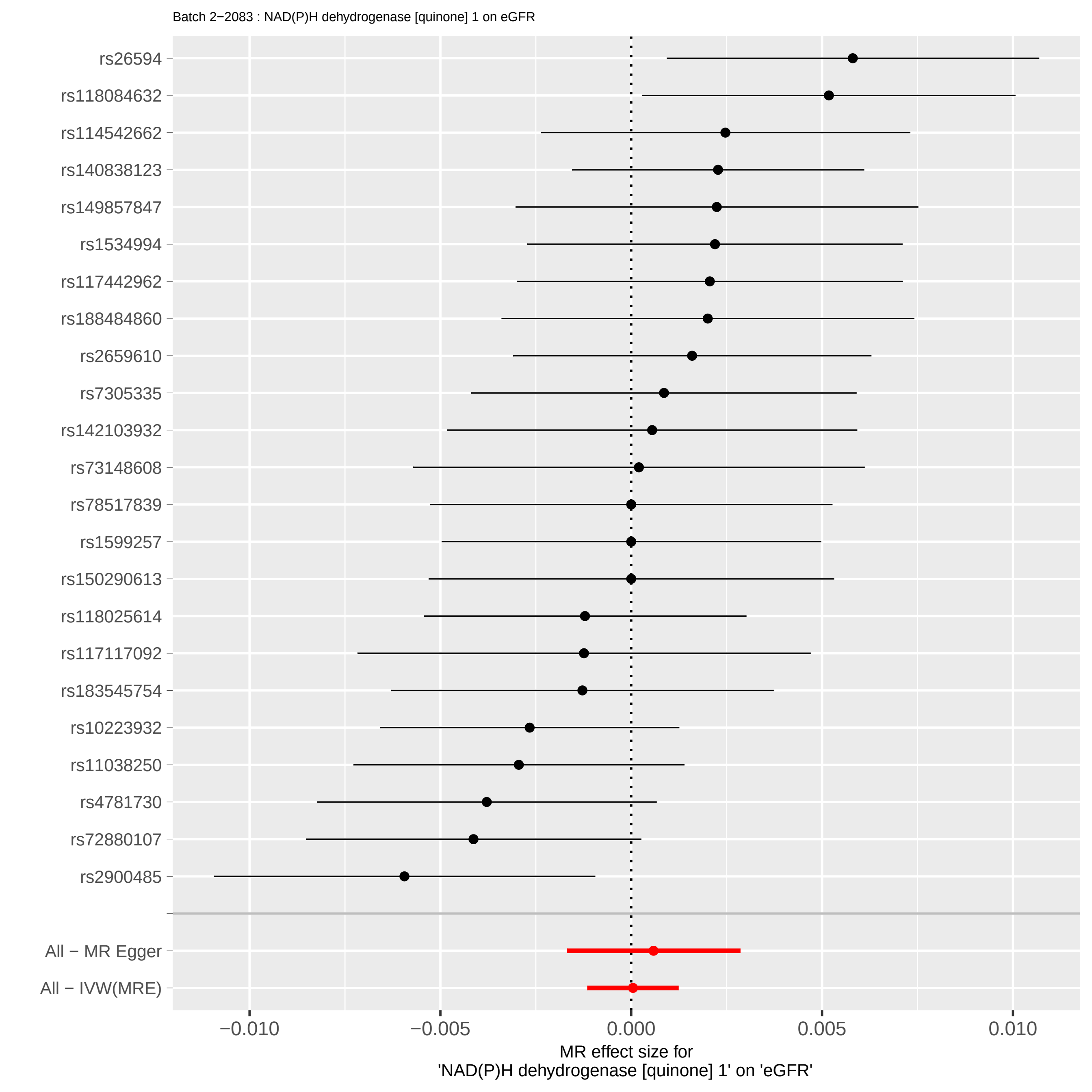
Batch 2−2083 : NAD(P)H dehydrogenase [quinone] 1 on eGFR
rs26594
rs118084632
rs114542662
rs140838123
rs149857847
rs1534994
rs117442962
rs188484860
rs2659610
rs7305335
rs142103932
rs73148608
rs78517839
rs1599257
rs150290613
rs118025614
rs117117092
rs183545754
rs10223932
rs11038250
rs4781730
rs72880107
rs2900485
All − MR Egger
All − IVW(MRE)
−0.010
−0.005
0.000
0.005
0.010
MR effect size for
'NAD(P)H dehydrogenase [quinone] 1' on 'eGFR'

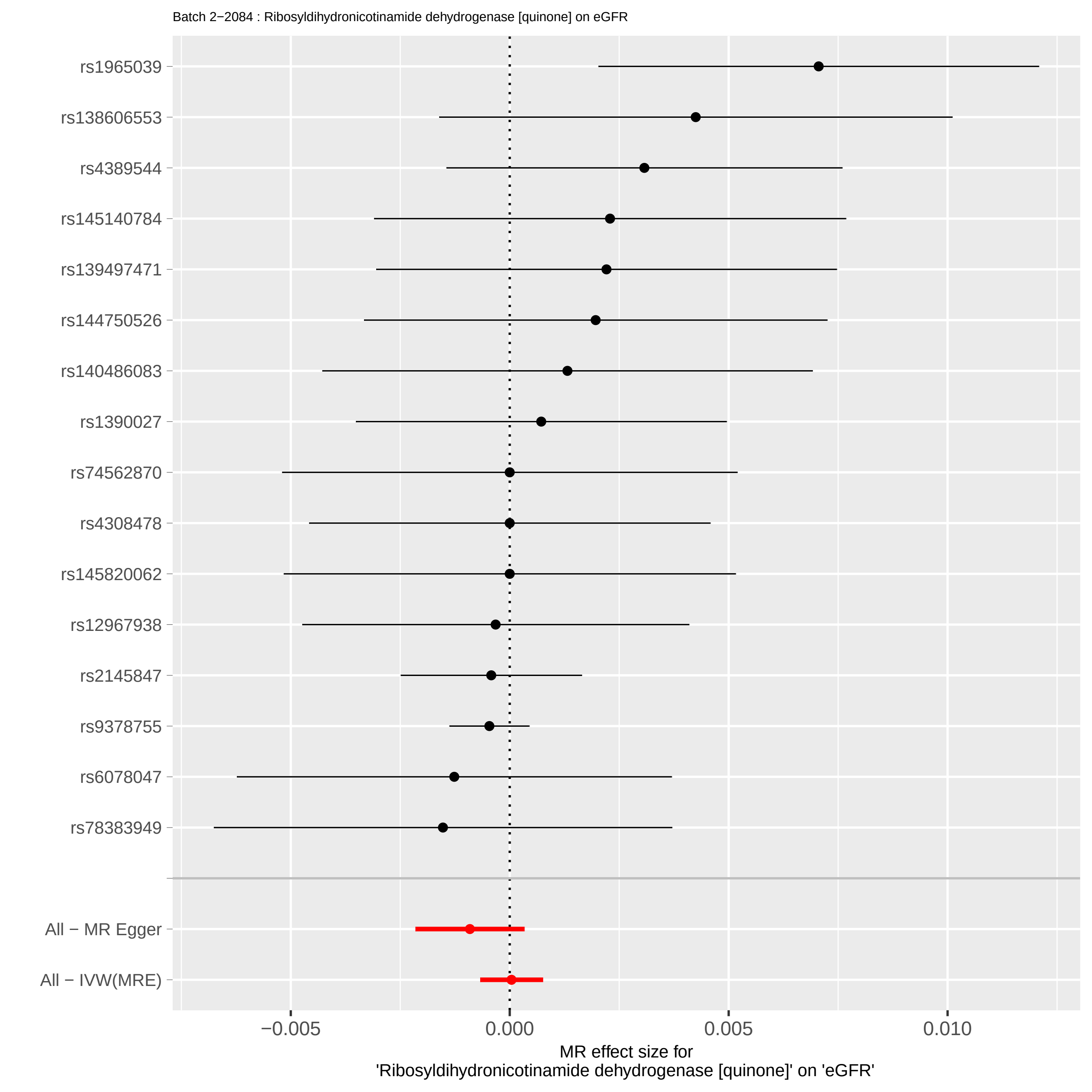

Batch 2−2084 : Ribosyldihydronicotinamide dehydrogenase [quinone] on eGFR
rs1965039
rs138606553
rs4389544
rs145140784
rs139497471
rs144750526
rs140486083
rs1390027
rs74562870
rs4308478
rs145820062
rs12967938
rs2145847
rs9378755
rs6078047
rs78383949
All − MR Egger
All − IVW(MRE)
−0.005
0.000
0.005
0.010
MR effect size for
'Ribosyldihydronicotinamide dehydrogenase [quinone]' on 'eGFR'

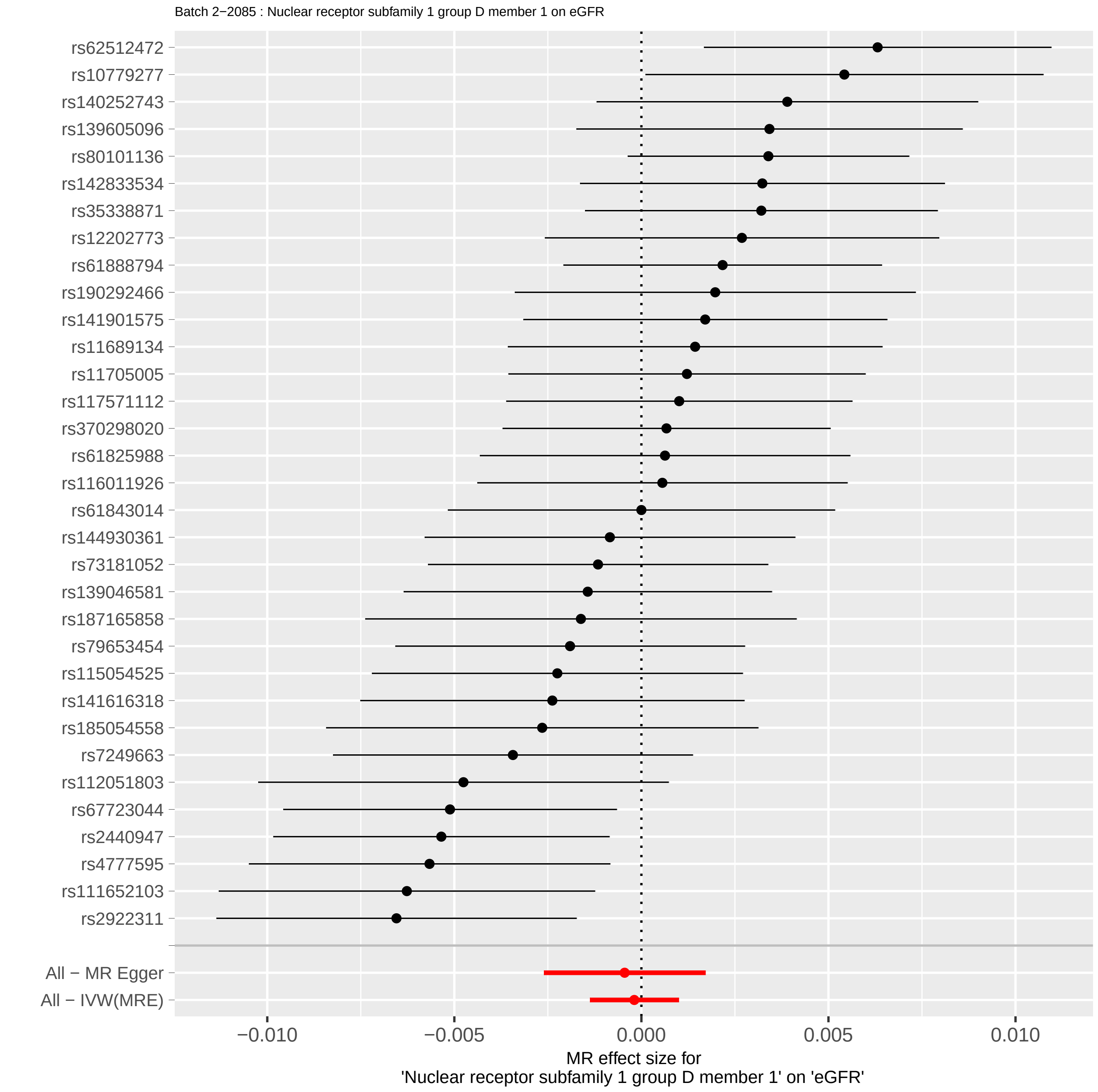

Batch 2−2085 : Nuclear receptor subfamily 1 group D member 1 on eGFR
rs62512472
rs10779277
rs140252743
rs139605096
rs80101136
rs142833534
rs35338871
rs12202773
rs61888794
rs190292466
rs141901575
rs11689134
rs11705005
rs117571112
rs370298020
rs61825988
rs116011926
rs61843014
rs144930361
rs73181052
rs139046581
rs187165858
rs79653454
rs115054525
rs141616318
rs185054558
rs7249663
rs112051803
rs67723044
rs2440947
rs4777595
rs111652103
rs2922311
All − MR Egger
All − IVW(MRE)
−0.010
−0.005
0.000
0.005
0.010
MR effect size for
'Nuclear receptor subfamily 1 group D member 1' on 'eGFR'

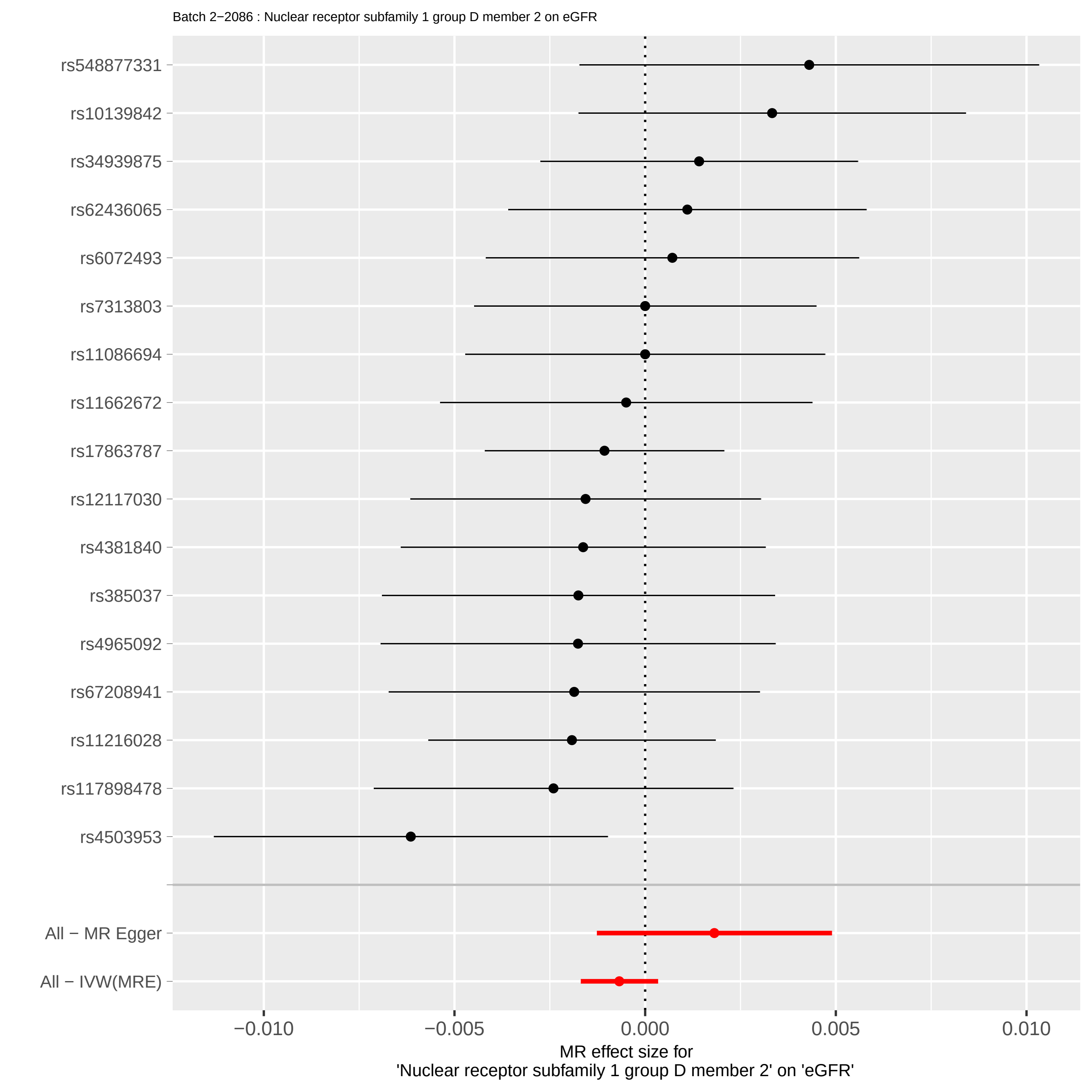
Batch 2−2086 : Nuclear receptor subfamily 1 group D member 2 on eGFR
rs548877331
rs10139842
rs34939875
rs62436065
rs6072493
rs7313803
rs11086694
rs11662672
rs17863787
rs12117030
rs4381840
rs385037
rs4965092
rs67208941
rs11216028
rs117898478
rs4503953
All − MR Egger
All − IVW(MRE)
−0.010
−0.005
0.000
0.005
0.010
MR effect size for
'Nuclear receptor subfamily 1 group D member 2' on 'eGFR'

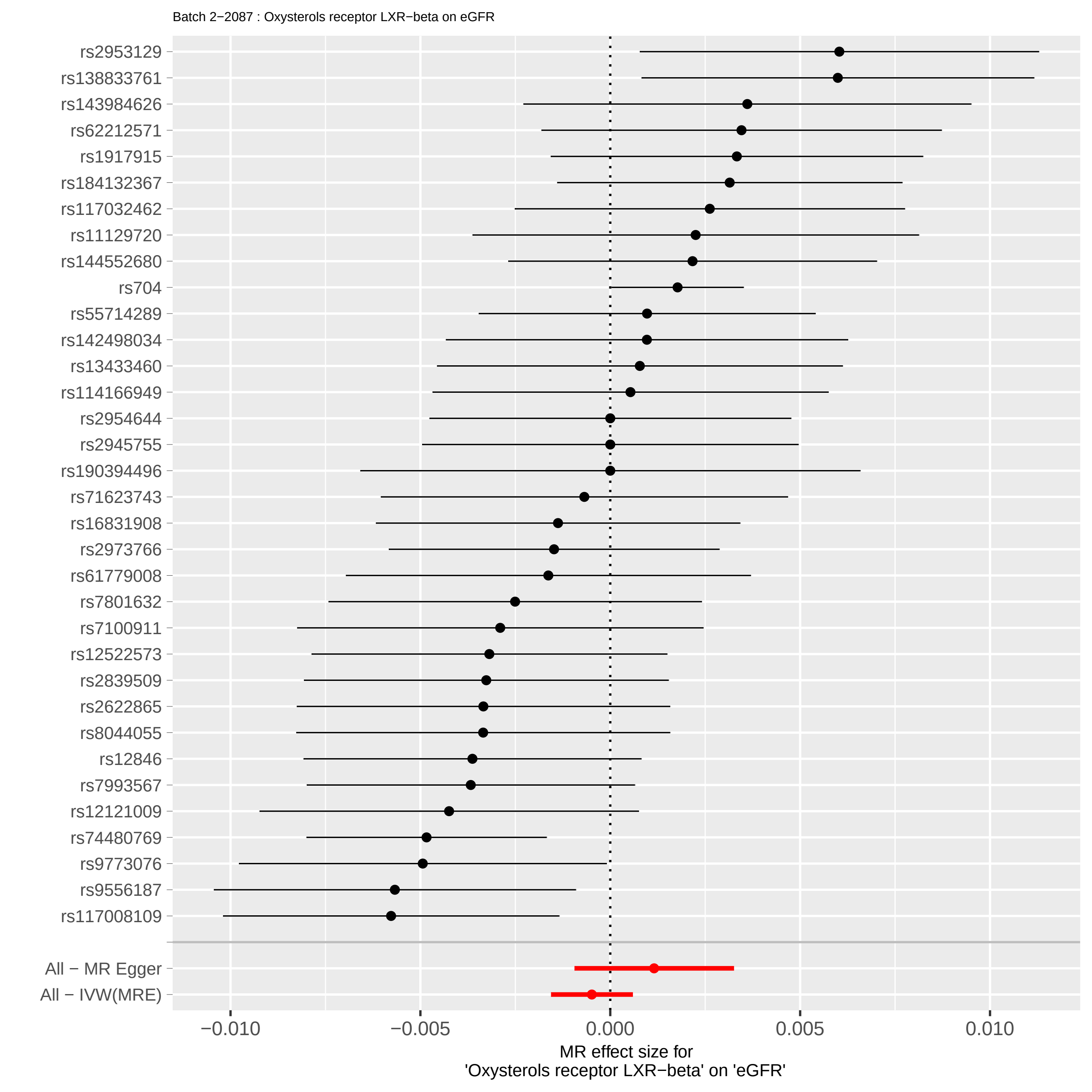

Batch 2−2087 : Oxysterols receptor LXR−beta on eGFR
rs2953129
rs138833761
rs143984626
rs62212571
rs1917915
rs184132367
rs117032462
rs11129720
rs144552680
rs704
rs55714289
rs142498034
rs13433460
rs114166949
rs2954644
rs2945755
rs190394496
rs71623743
rs16831908
rs2973766
rs61779008
rs7801632
rs7100911
rs12522573
rs2839509
rs2622865
rs8044055
rs12846
rs7993567
rs12121009
rs74480769
rs9773076
rs9556187
rs117008109
All − MR Egger
All − IVW(MRE)
−0.010
−0.005
0.000
0.005
0.010
MR effect size for
'Oxysterols receptor LXR−beta' on 'eGFR'

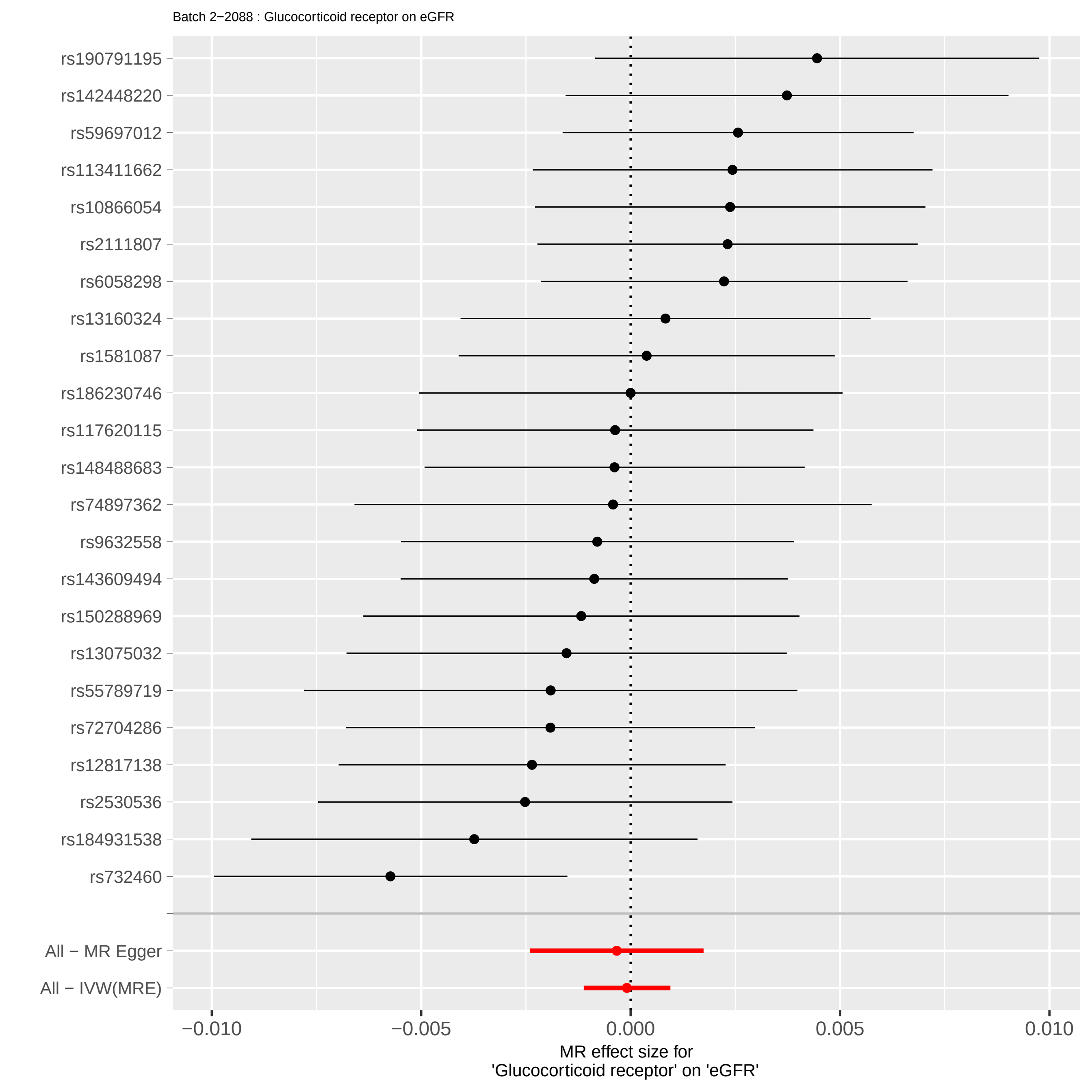

Batch 2−2088 : Glucocorticoid receptor on eGFR
rs190791195
rs142448220
rs59697012
rs113411662
rs10866054
rs2111807
rs6058298
rs13160324
rs1581087
rs186230746
rs117620115
rs148488683
rs74897362
rs9632558
rs143609494
rs150288969
rs13075032
rs55789719
rs72704286
rs12817138
rs2530536
rs184931538
rs732460
All − MR Egger
All − IVW(MRE)
−0.010
−0.005
0.000
0.005
0.010
MR effect size for
'Glucocorticoid receptor' on 'eGFR'

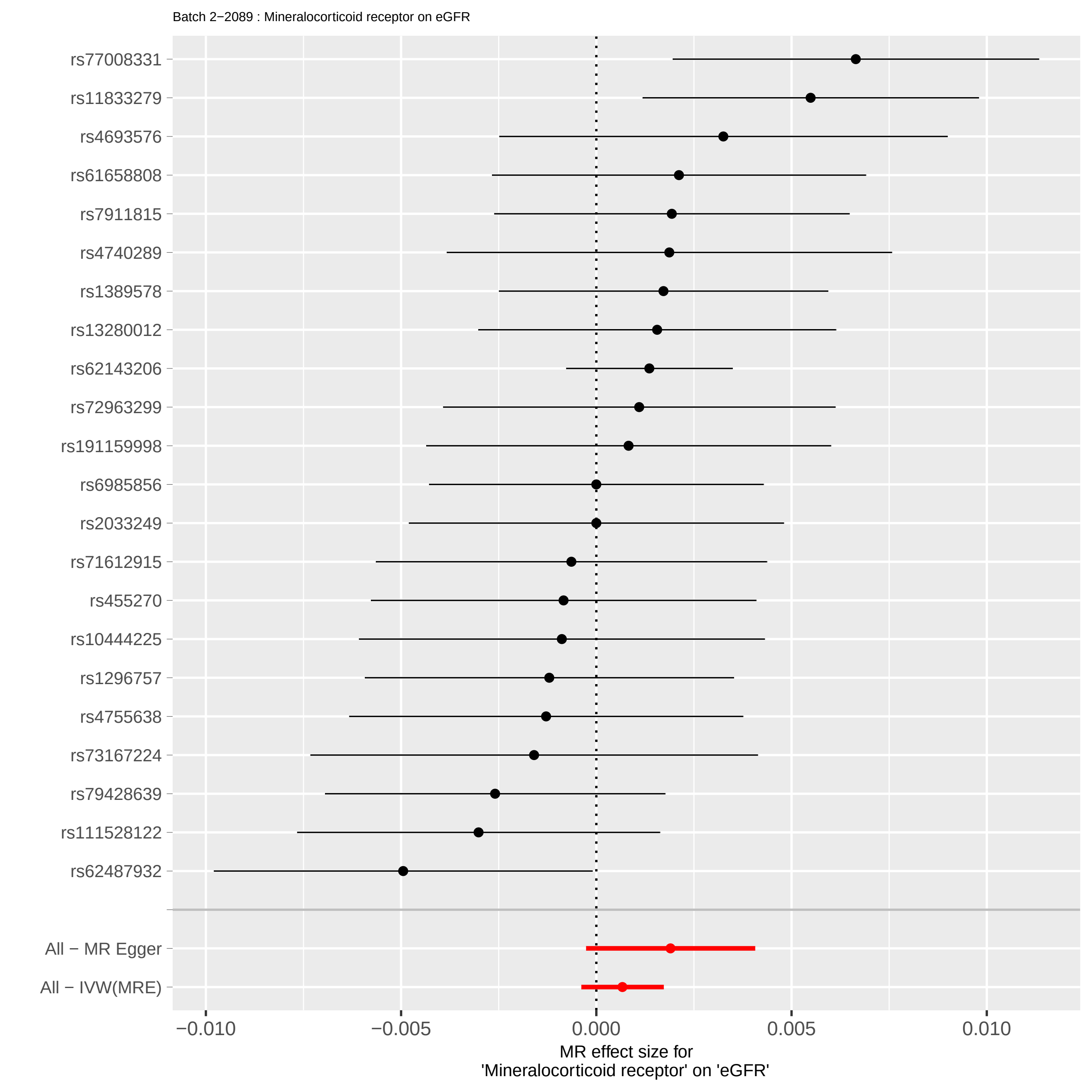

Batch 2−2089 : Mineralocorticoid receptor on eGFR
rs77008331
rs11833279
rs4693576
rs61658808
rs7911815
rs4740289
rs1389578
rs13280012
rs62143206
rs72963299
rs191159998
rs6985856
rs2033249
rs71612915
rs455270
rs10444225
rs1296757
rs4755638
rs73167224
rs79428639
rs111528122
rs62487932
All − MR Egger
All − IVW(MRE)
−0.010
−0.005
0.000
0.005
0.010
MR effect size for
'Mineralocorticoid receptor' on 'eGFR'

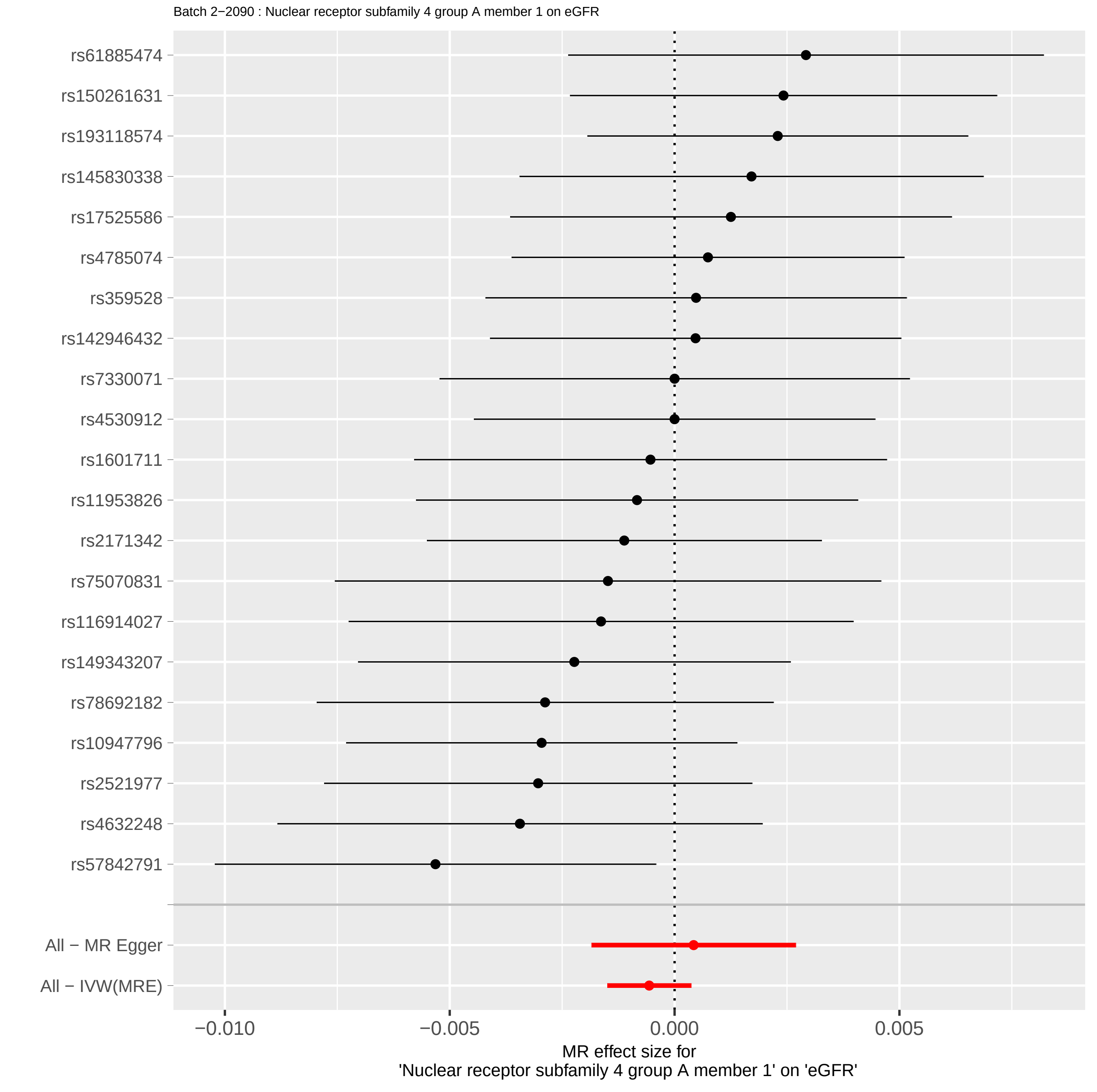

Batch 2−2090 : Nuclear receptor subfamily 4 group A member 1 on eGFR
rs61885474
rs150261631
rs193118574
rs145830338
rs17525586
rs4785074
rs359528
rs142946432
rs7330071
rs4530912
rs1601711
rs11953826
rs2171342
rs75070831
rs116914027
rs149343207
rs78692182
rs10947796
rs2521977
rs4632248
rs57842791
All − MR Egger
All − IVW(MRE)
−0.010
−0.005
0.000
0.005
MR effect size for
'Nuclear receptor subfamily 4 group A member 1' on 'eGFR'

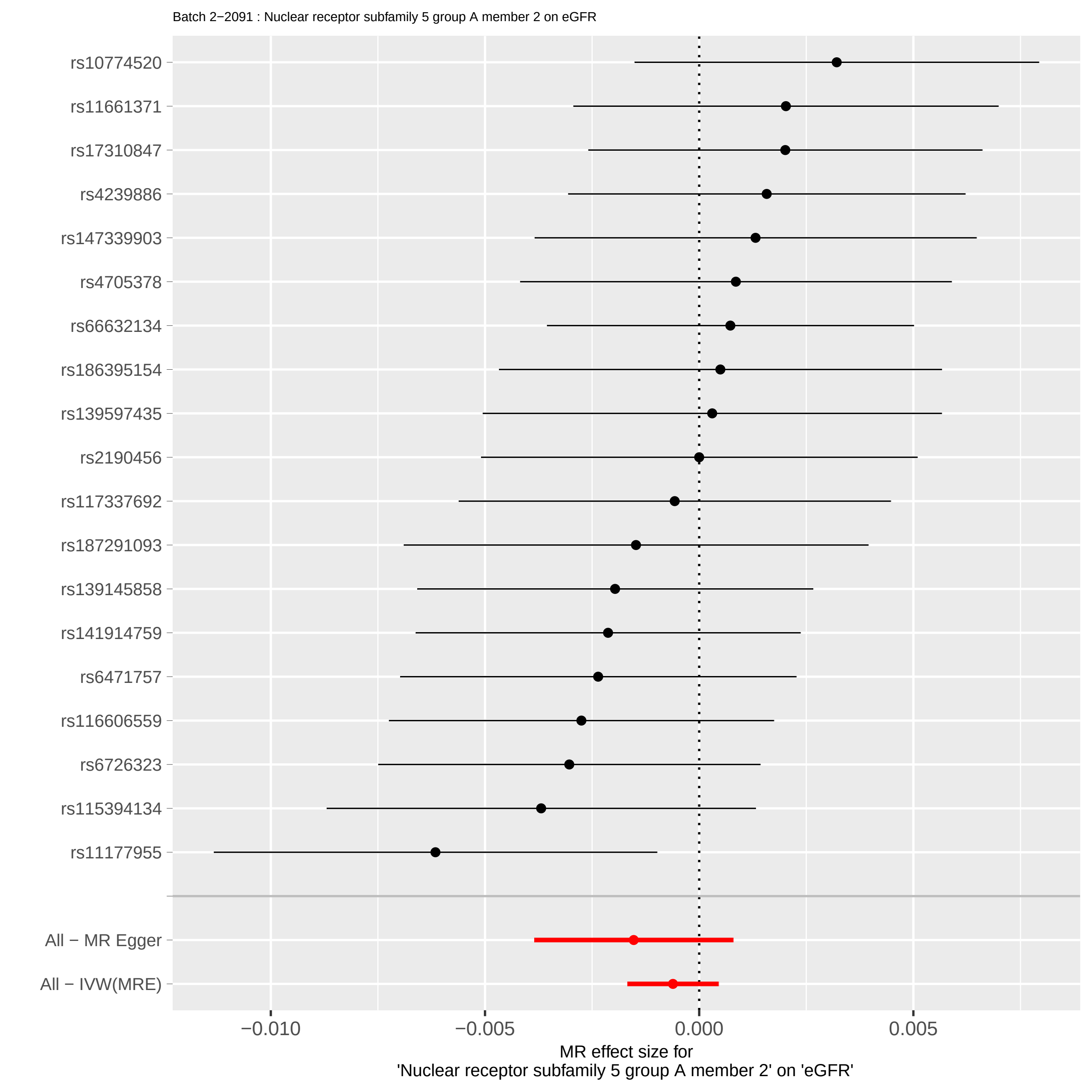

Batch 2−2091 : Nuclear receptor subfamily 5 group A member 2 on eGFR
rs10774520
rs11661371
rs17310847
rs4239886
rs147339903
rs4705378
rs66632134
rs186395154
rs139597435
rs2190456
rs117337692
rs187291093
rs139145858
rs141914759
rs6471757
rs116606559
rs6726323
rs115394134
rs11177955
All − MR Egger
All − IVW(MRE)
−0.010
−0.005
0.000
0.005
MR effect size for
'Nuclear receptor subfamily 5 group A member 2' on 'eGFR'

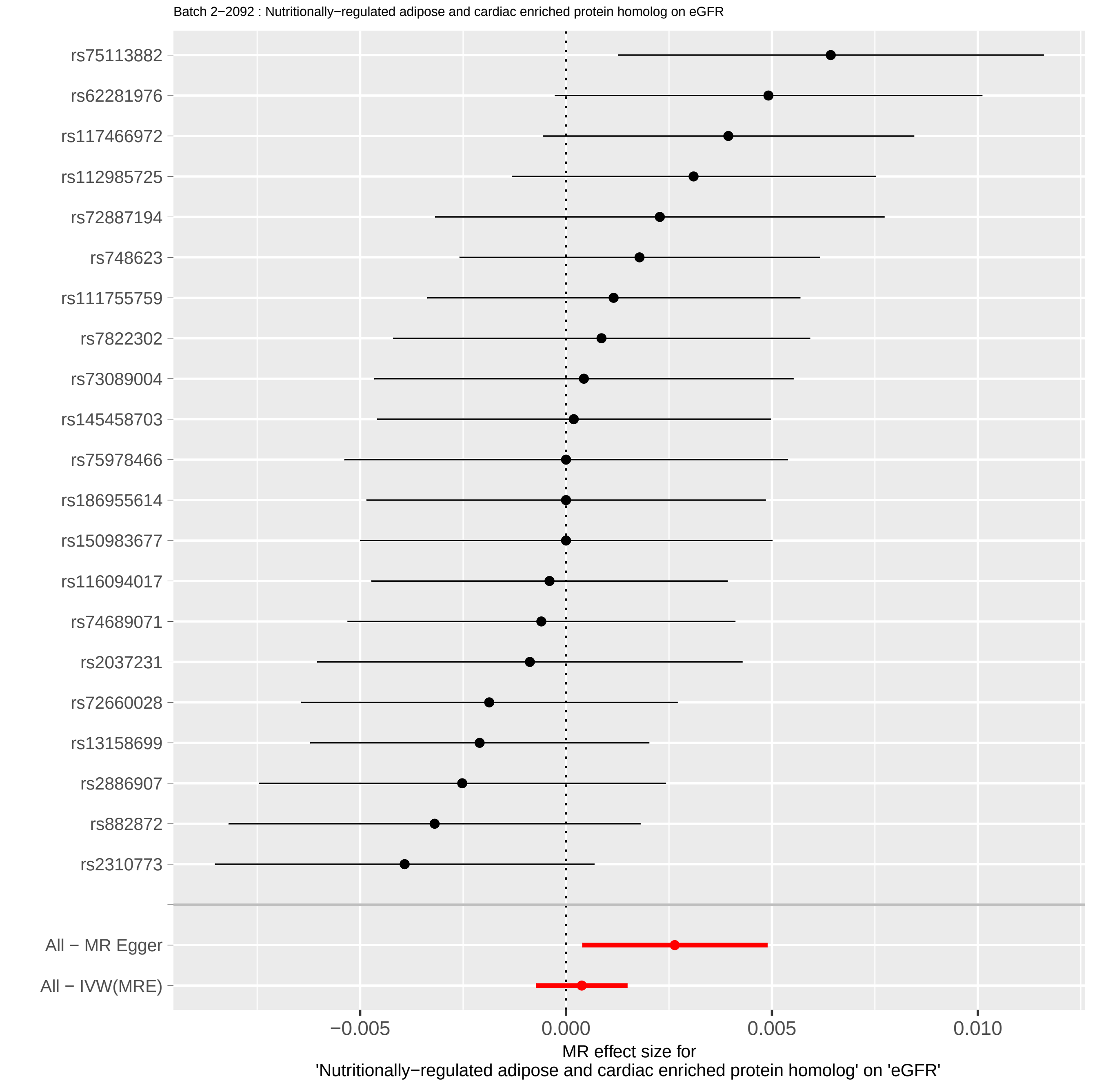
Batch 2−2092 : Nutritionally−regulated adipose and cardiac enriched protein homolog on eGFR
rs75113882
rs62281976
rs117466972
rs112985725
rs72887194
rs748623
rs111755759
rs7822302
rs73089004
rs145458703
rs75978466
rs186955614
rs150983677
rs116094017
rs74689071
rs2037231
rs72660028
rs13158699
rs2886907
rs882872
rs2310773
All − MR Egger
All − IVW(MRE)
−0.005
0.000
0.005
0.010
MR effect size for
'Nutritionally−regulated adipose and cardiac enriched protein homolog' on 'eGFR'

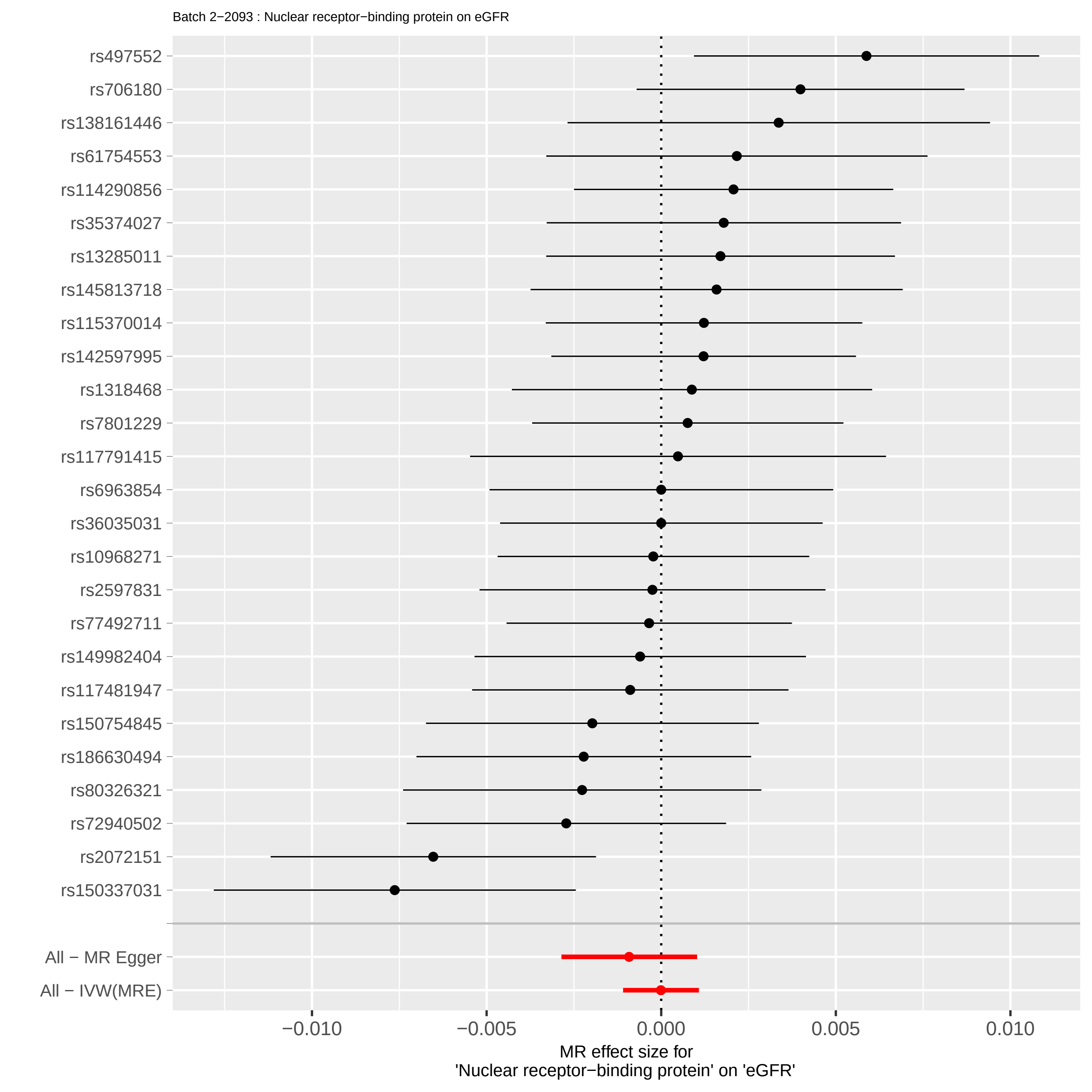

Batch 2−2093 : Nuclear receptor−binding protein on eGFR
rs497552
rs706180
rs138161446
rs61754553
rs114290856
rs35374027
rs13285011
rs145813718
rs115370014
rs142597995
rs1318468
rs7801229
rs117791415
rs6963854
rs36035031
rs10968271
rs2597831
rs77492711
rs149982404
rs117481947
rs150754845
rs186630494
rs80326321
rs72940502
rs2072151
rs150337031
All − MR Egger
All − IVW(MRE)
−0.010
−0.005
0.000
0.005
0.010
MR effect size for
'Nuclear receptor−binding protein' on 'eGFR'

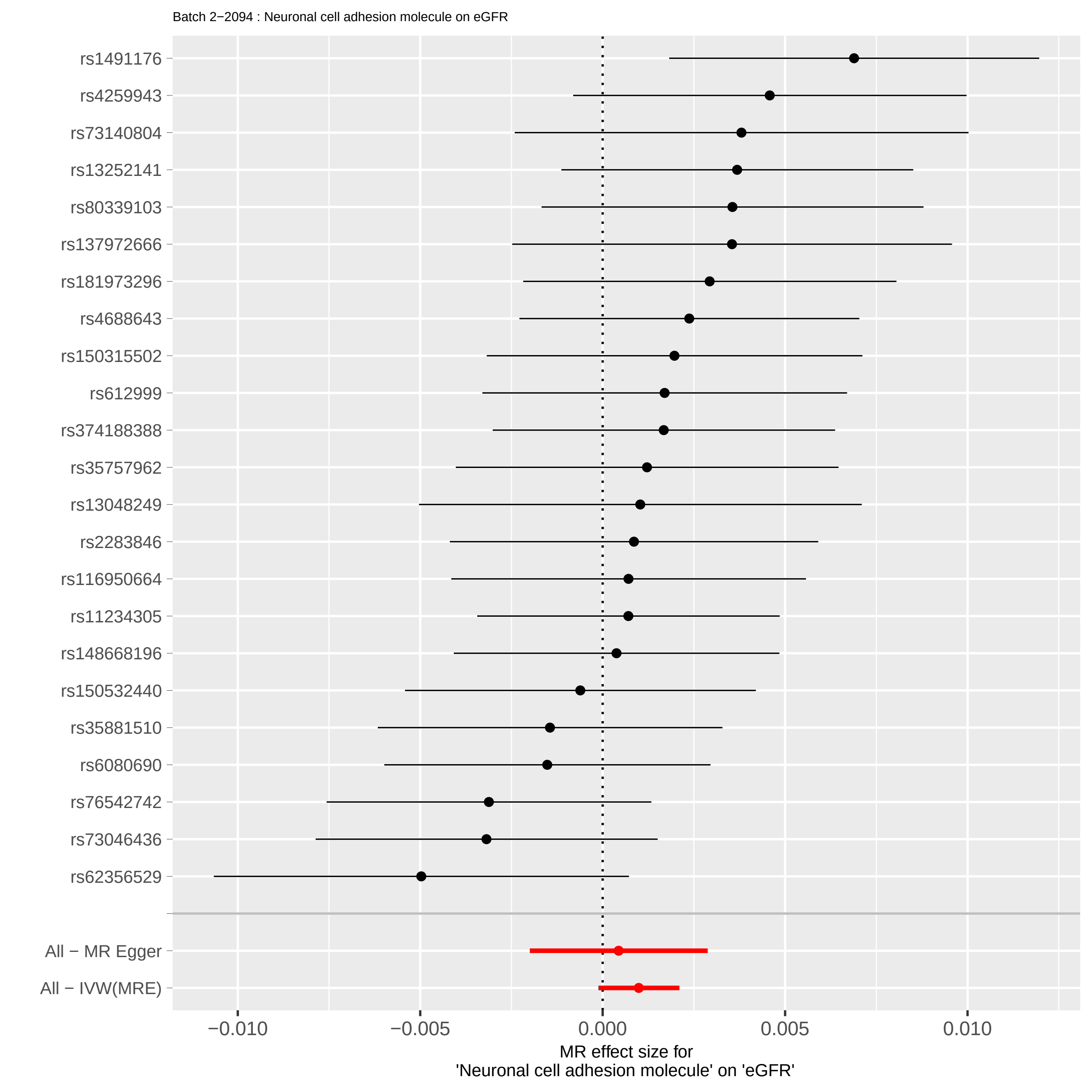

Batch 2−2094 : Neuronal cell adhesion molecule on eGFR
rs1491176
rs4259943
rs73140804
rs13252141
rs80339103
rs137972666
rs181973296
rs4688643
rs150315502
rs612999
rs374188388
rs35757962
rs13048249
rs2283846
rs116950664
rs11234305
rs148668196
rs150532440
rs35881510
rs6080690
rs76542742
rs73046436
rs62356529
All − MR Egger
All − IVW(MRE)
−0.010
−0.005
0.000
0.005
0.010
MR effect size for
'Neuronal cell adhesion molecule' on 'eGFR'

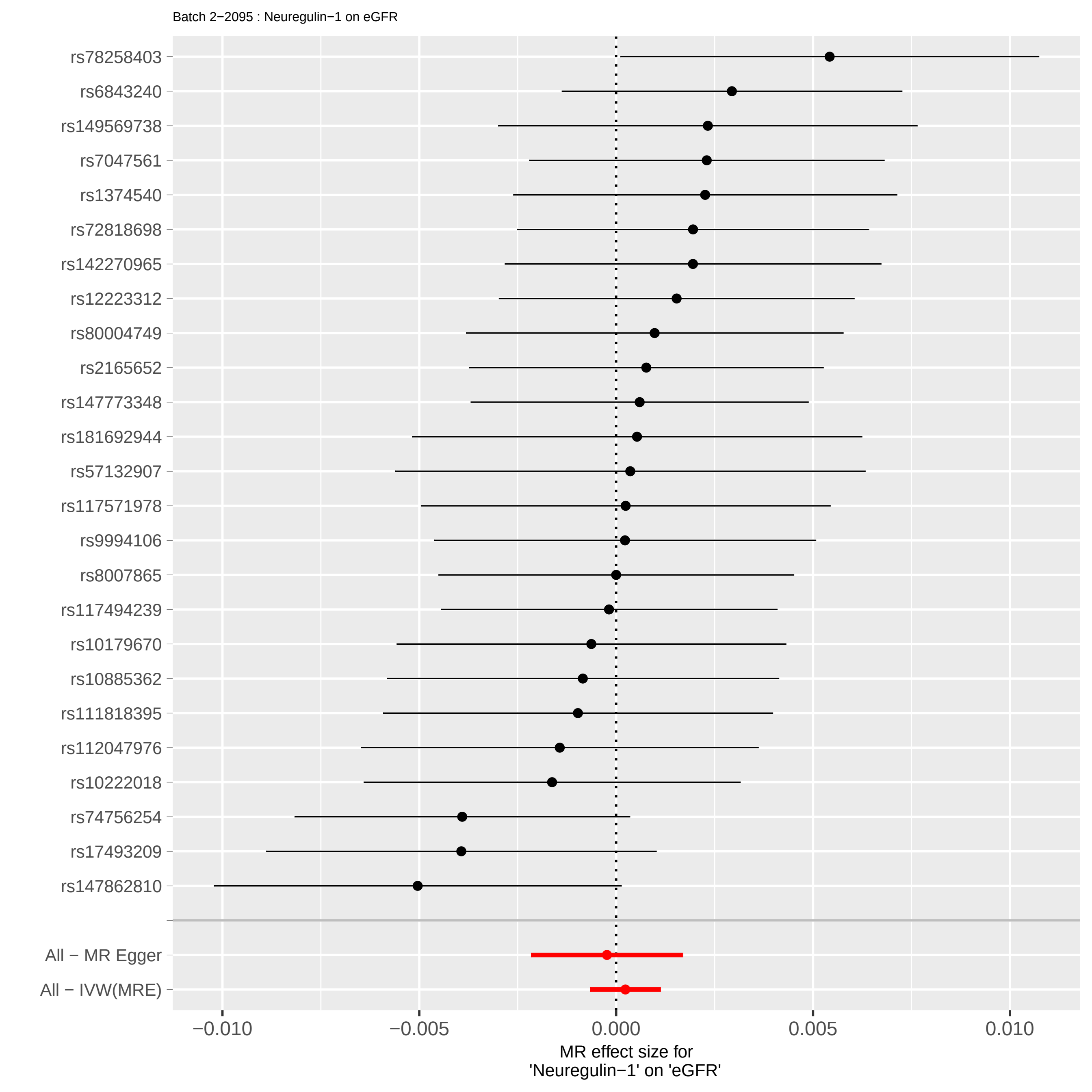

Batch 2−2095 : Neuregulin−1 on eGFR
rs78258403
rs6843240
rs149569738
rs7047561
rs1374540
rs72818698
rs142270965
rs12223312
rs80004749
rs2165652
rs147773348
rs181692944
rs57132907
rs117571978
rs9994106
rs8007865
rs117494239
rs10179670
rs10885362
rs111818395
rs112047976
rs10222018
rs74756254
rs17493209
rs147862810
All − MR Egger
All − IVW(MRE)
−0.010
−0.005
0.000
0.005
0.010
MR effect size for
'Neuregulin−1' on 'eGFR'

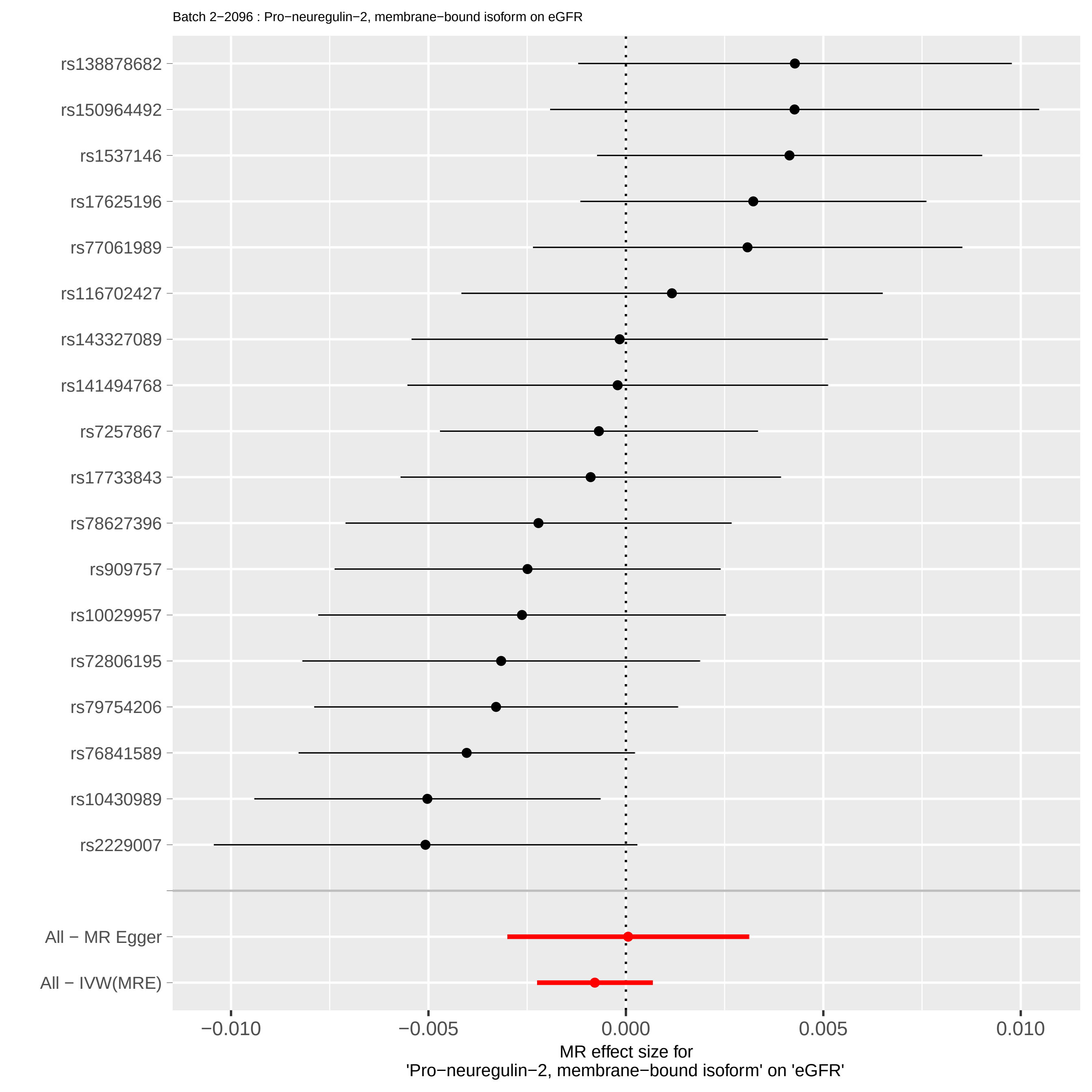

Batch 2−2096 : Pro−neuregulin−2, membrane−bound isoform on eGFR
rs138878682
rs150964492
rs1537146
rs17625196
rs77061989
rs116702427
rs143327089
rs141494768
rs7257867
rs17733843
rs78627396
rs909757
rs10029957
rs72806195
rs79754206
rs76841589
rs10430989
rs2229007
All − MR Egger
All − IVW(MRE)
−0.010
−0.005
0.000
0.005
0.010
MR effect size for
'Pro−neuregulin−2, membrane−bound isoform' on 'eGFR'

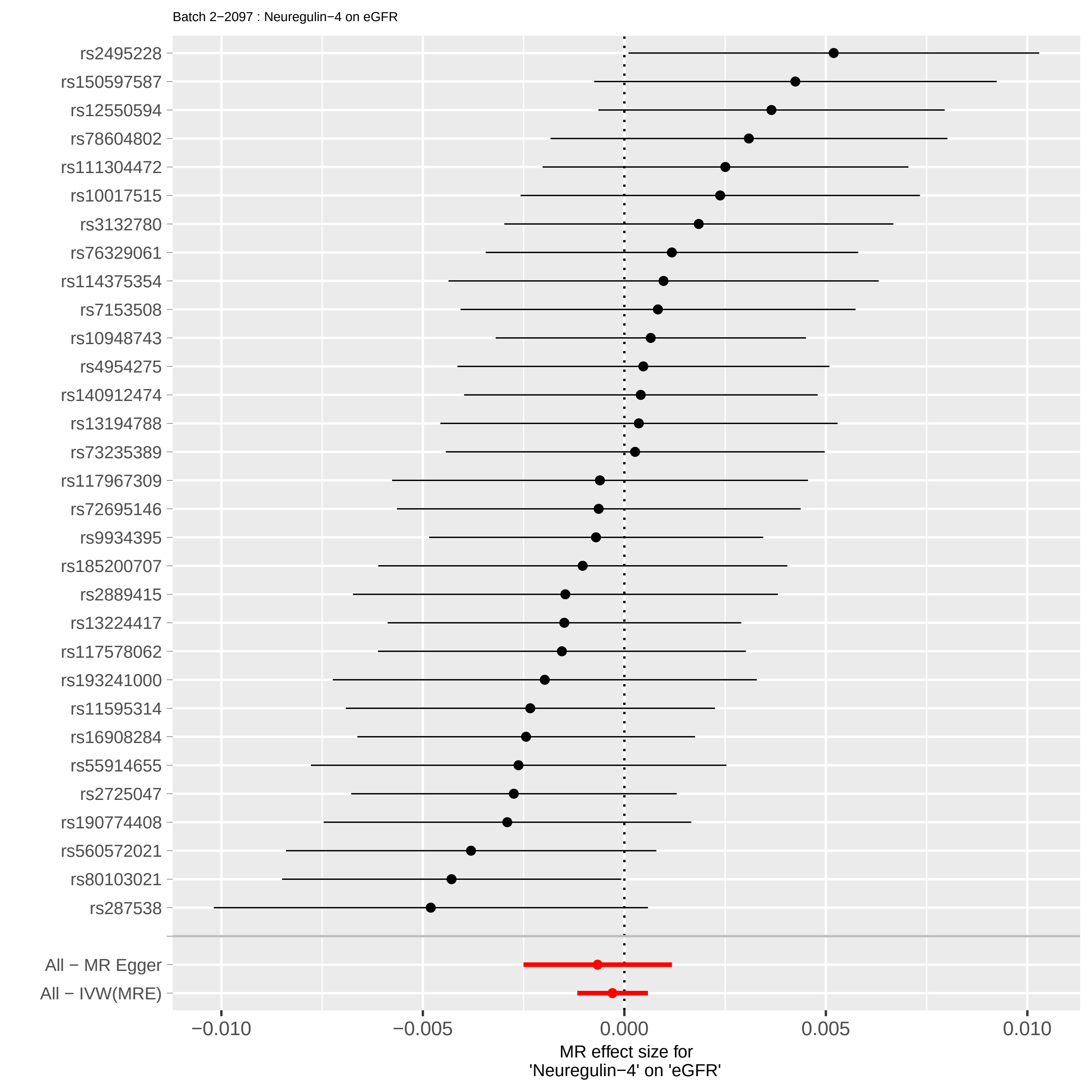

Batch 2−2097 : Neuregulin−4 on eGFR
rs2495228
rs150597587
rs12550594
rs78604802
rs111304472
rs10017515
rs3132780
rs76329061
rs114375354
rs7153508
rs10948743
rs4954275
rs140912474
rs13194788
rs73235389
rs117967309
rs72695146
rs9934395
rs185200707
rs2889415
rs13224417
rs117578062
rs193241000
rs11595314
rs16908284
rs55914655
rs2725047
rs190774408
rs560572021
rs80103021
rs287538
All − MR Egger
All − IVW(MRE)
−0.010
−0.005
0.000
0.005
0.010
MR effect size for
'Neuregulin−4' on 'eGFR'

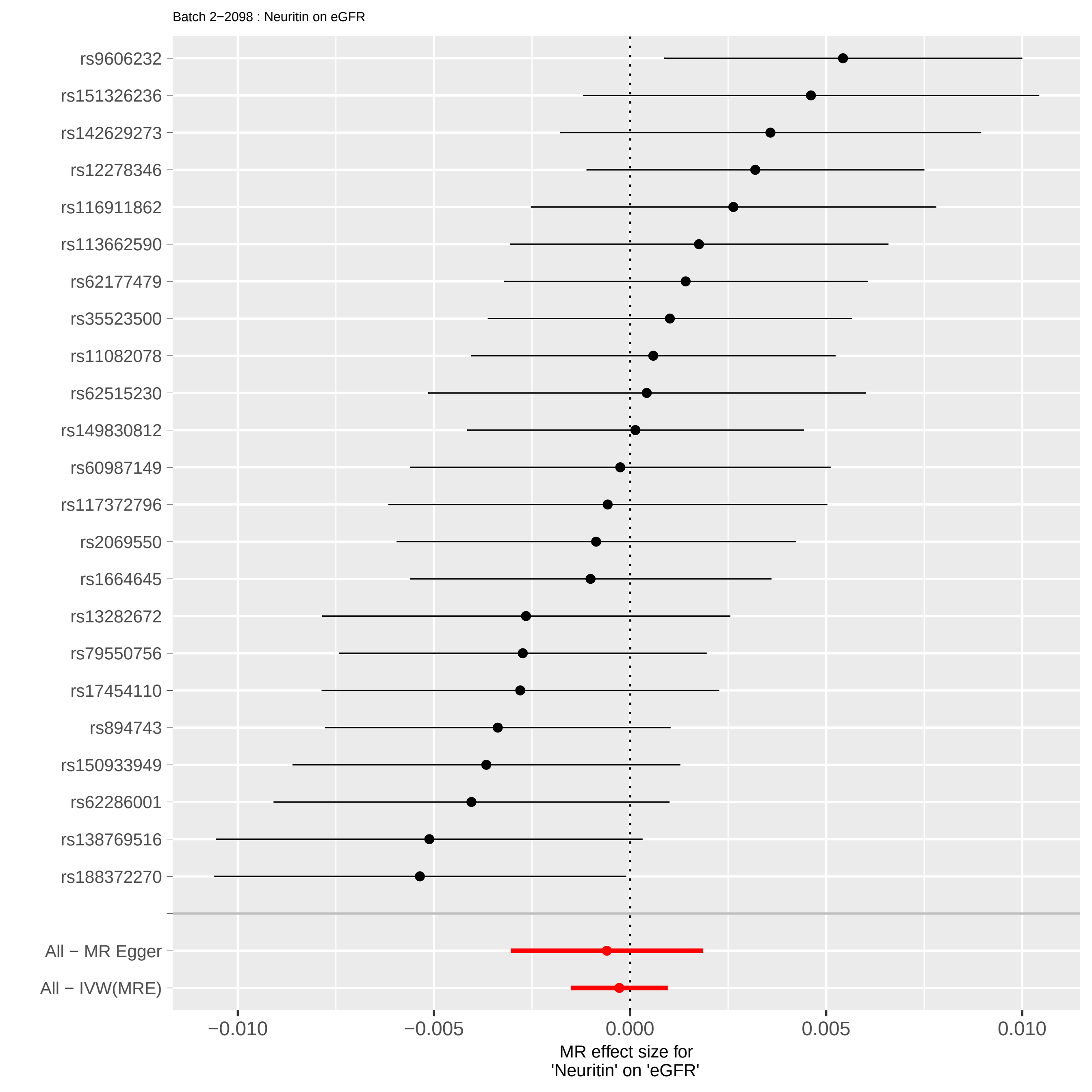
Batch 2−2098 : Neuritin on eGFR
rs9606232
rs151326236
rs142629273
rs12278346
rs116911862
rs113662590
rs62177479
rs35523500
rs11082078
rs62515230
rs149830812
rs60987149
rs117372796
rs2069550
rs1664645
rs13282672
rs79550756
rs17454110
rs894743
rs150933949
rs62286001
rs138769516
rs188372270
All − MR Egger
All − IVW(MRE)
−0.010
−0.005
0.000
0.005
0.010
MR effect size for
'Neuritin' on 'eGFR'

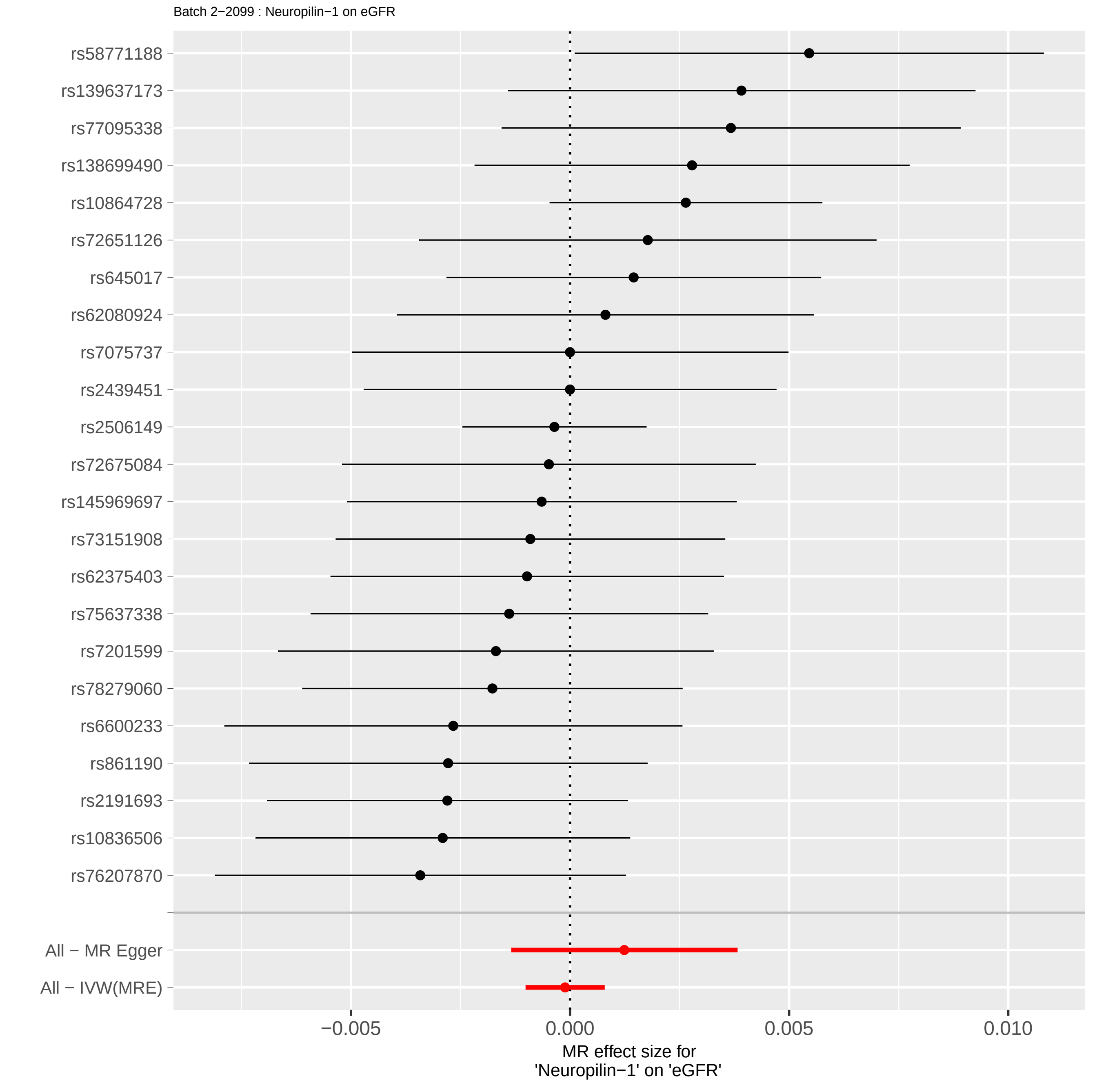
Batch 2−2099 : Neuropilin−1 on eGFR
rs58771188
rs139637173
rs77095338
rs138699490
rs10864728
rs72651126
rs645017
rs62080924
rs7075737
rs2439451
rs2506149
rs72675084
rs145969697
rs73151908
rs62375403
rs75637338
rs7201599
rs78279060
rs6600233
rs861190
rs2191693
rs10836506
rs76207870
All − MR Egger
All − IVW(MRE)
−0.005
0.000
0.005
0.010
MR effect size for
'Neuropilin−1' on 'eGFR'

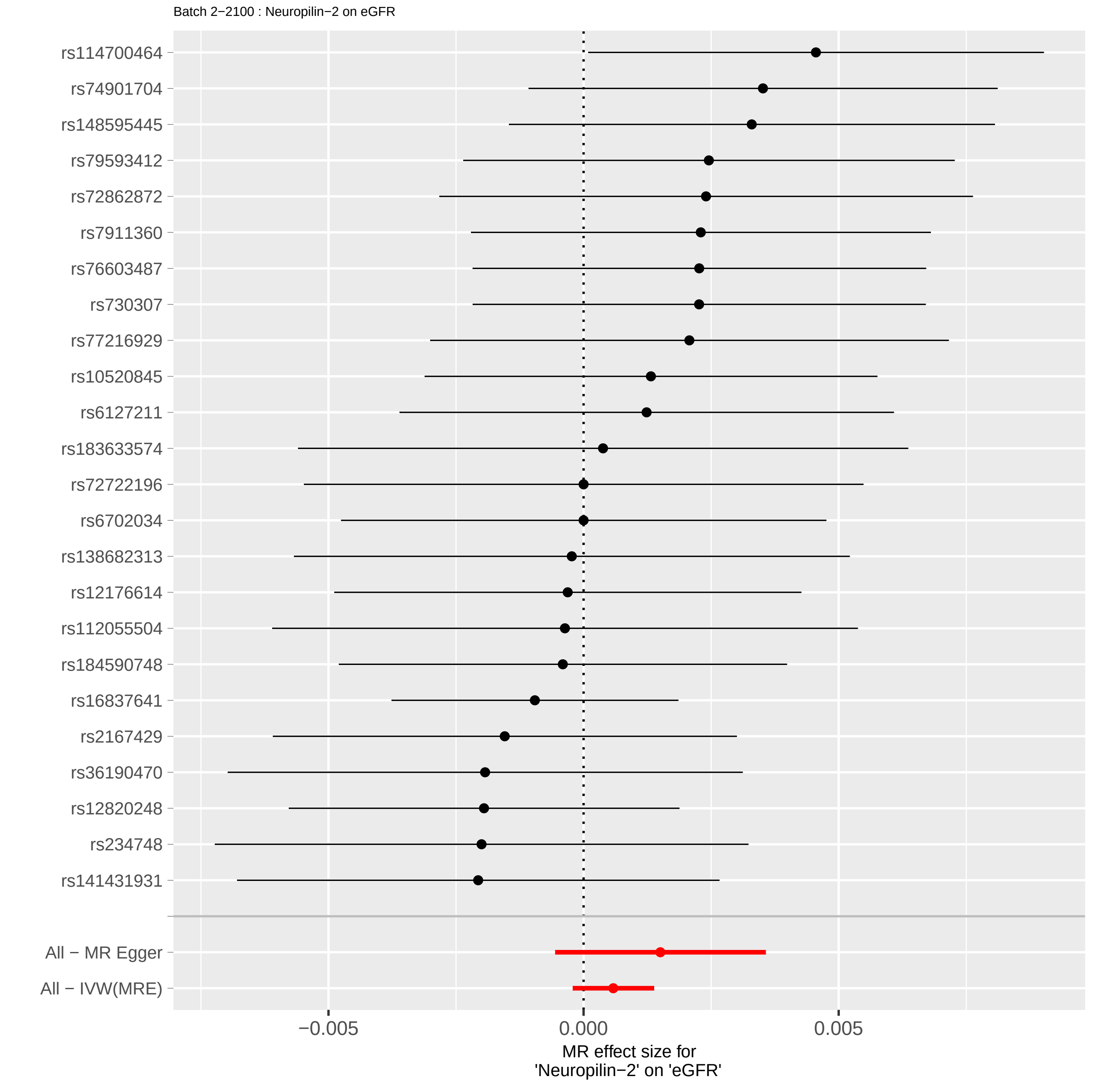

Batch 2−2100 : Neuropilin−2 on eGFR
rs114700464
rs74901704
rs148595445
rs79593412
rs72862872
rs7911360
rs76603487
rs730307
rs77216929
rs10520845
rs6127211
rs183633574
rs72722196
rs6702034
rs138682313
rs12176614
rs112055504
rs184590748
rs16837641
rs2167429
rs36190470
rs12820248
rs234748
rs141431931
All − MR Egger
All − IVW(MRE)
−0.005
0.000
0.005
MR effect size for
'Neuropilin−2' on 'eGFR'

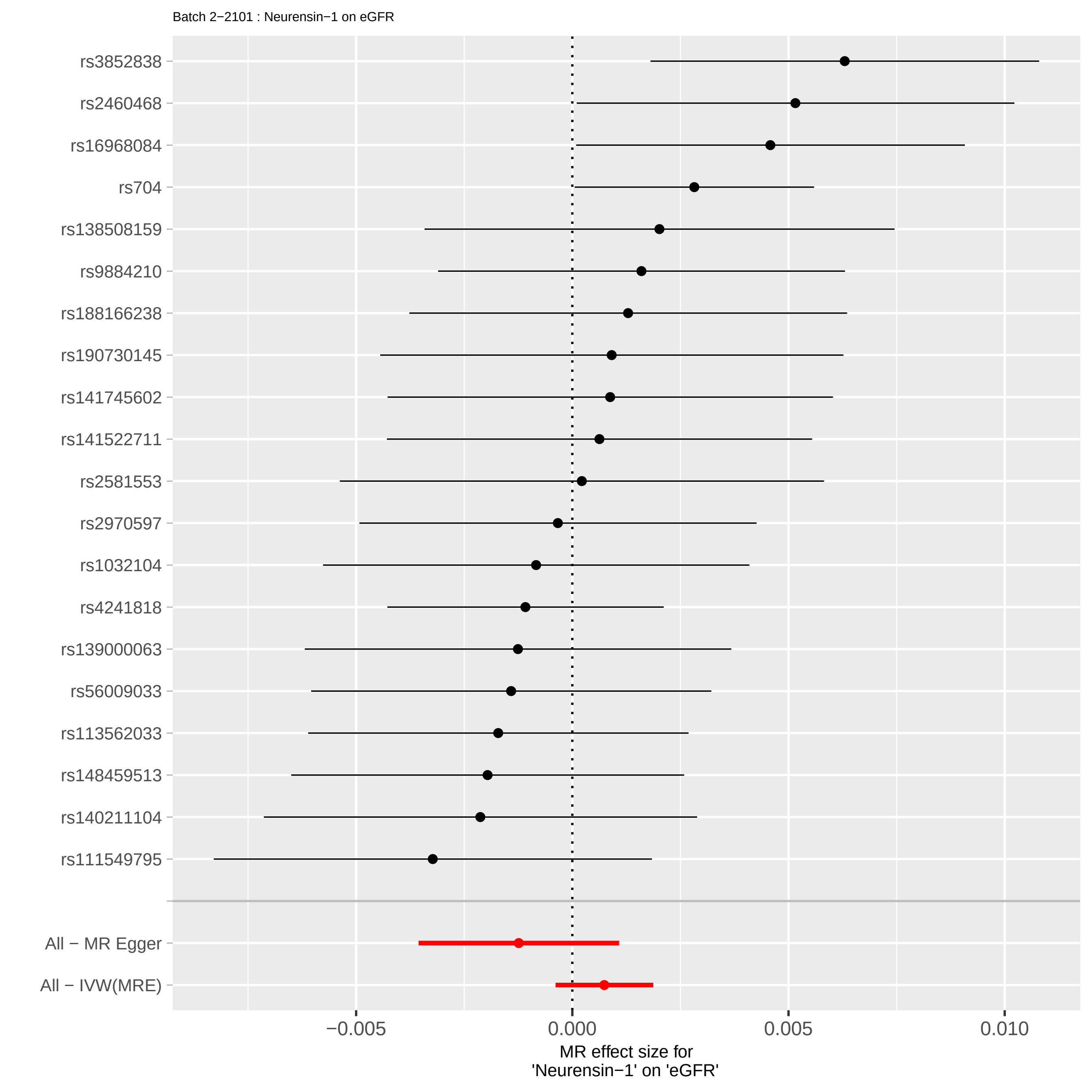

Batch 2−2101 : Neurensin−1 on eGFR
rs3852838
rs2460468
rs16968084
rs704
rs138508159
rs9884210
rs188166238
rs190730145
rs141745602
rs141522711
rs2581553
rs2970597
rs1032104
rs4241818
rs139000063
rs56009033
rs113562033
rs148459513
rs140211104
rs111549795
All − MR Egger
All − IVW(MRE)
−0.005
0.000
0.005
0.010
MR effect size for
'Neurensin−1' on 'eGFR'

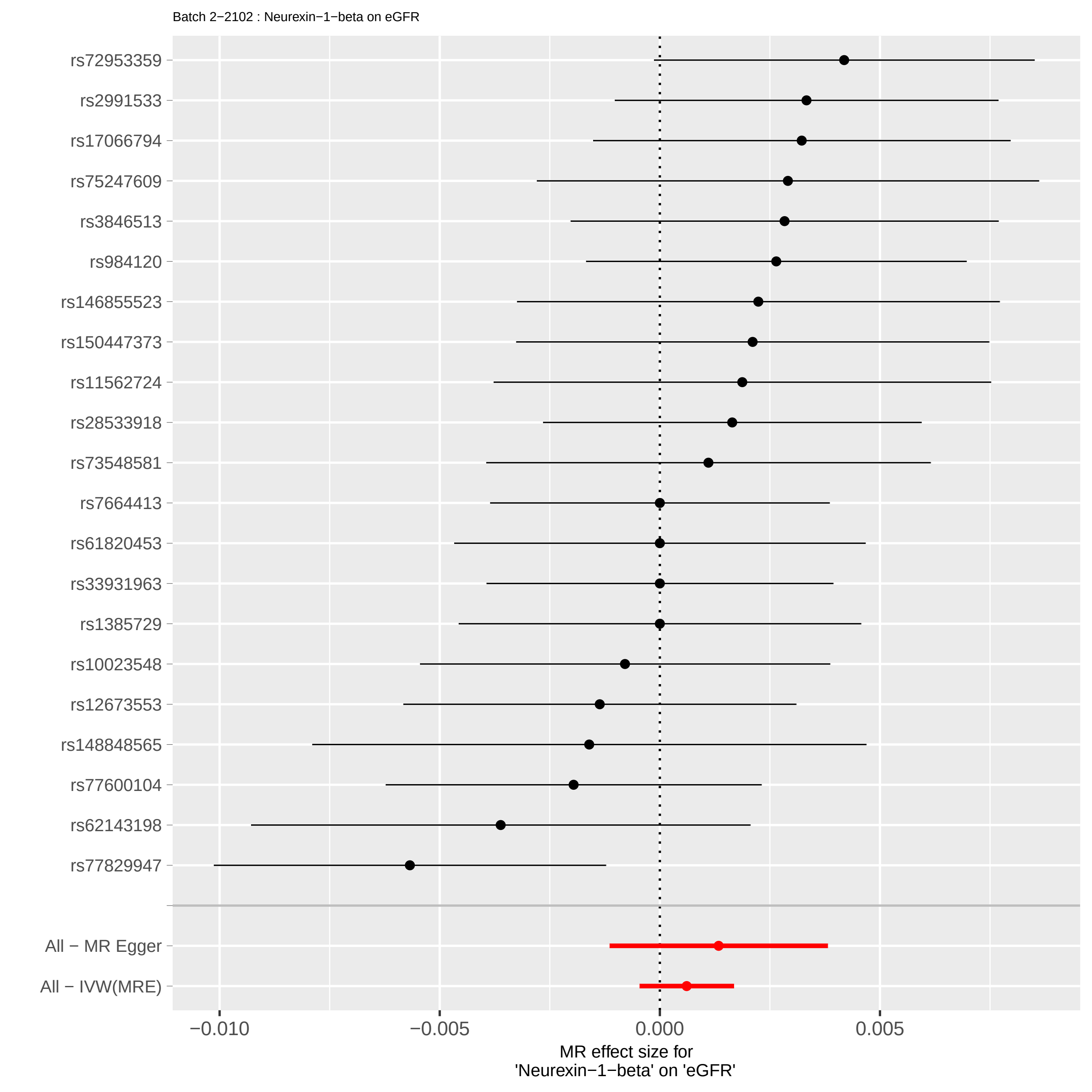

Batch 2−2102 : Neurexin−1−beta on eGFR
rs72953359
rs2991533
rs17066794
rs75247609
rs3846513
rs984120
rs146855523
rs150447373
rs11562724
rs28533918
rs73548581
rs7664413
rs61820453
rs33931963
rs1385729
rs10023548
rs12673553
rs148848565
rs77600104
rs62143198
rs77829947
All − MR Egger
All − IVW(MRE)
−0.010
−0.005
0.000
0.005
MR effect size for
'Neurexin−1−beta' on 'eGFR'

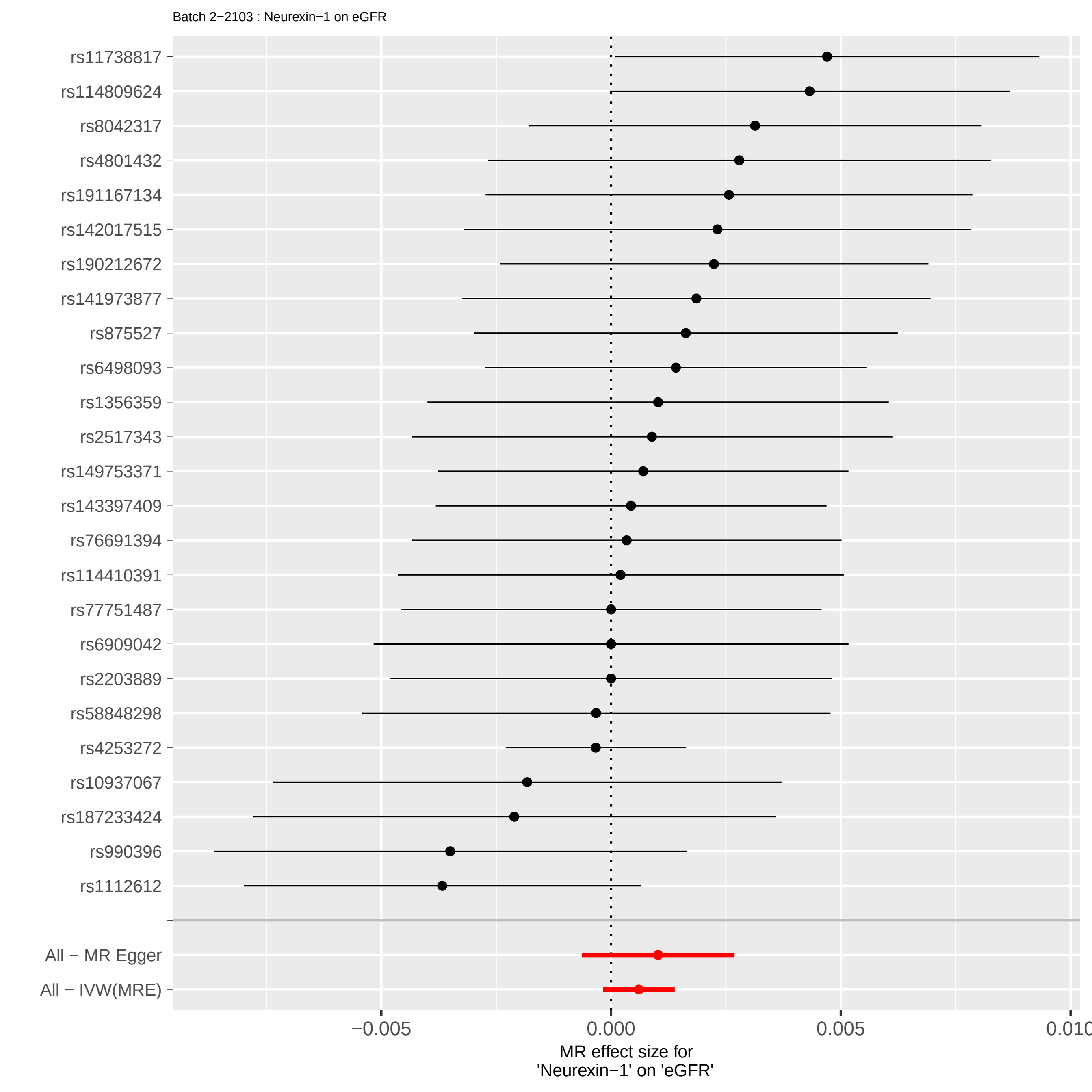

Batch 2−2103 : Neurexin−1 on eGFR
rs11738817
rs114809624
rs8042317
rs4801432
rs191167134
rs142017515
rs190212672
rs141973877
rs875527
rs6498093
rs1356359
rs2517343
rs149753371
rs143397409
rs76691394
rs114410391
rs77751487
rs6909042
rs2203889
rs58848298
rs4253272
rs10937067
rs187233424
rs990396
rs1112612
All − MR Egger
All − IVW(MRE)
−0.005
0.000
0.005
0.010
MR effect size for
'Neurexin−1' on 'eGFR'

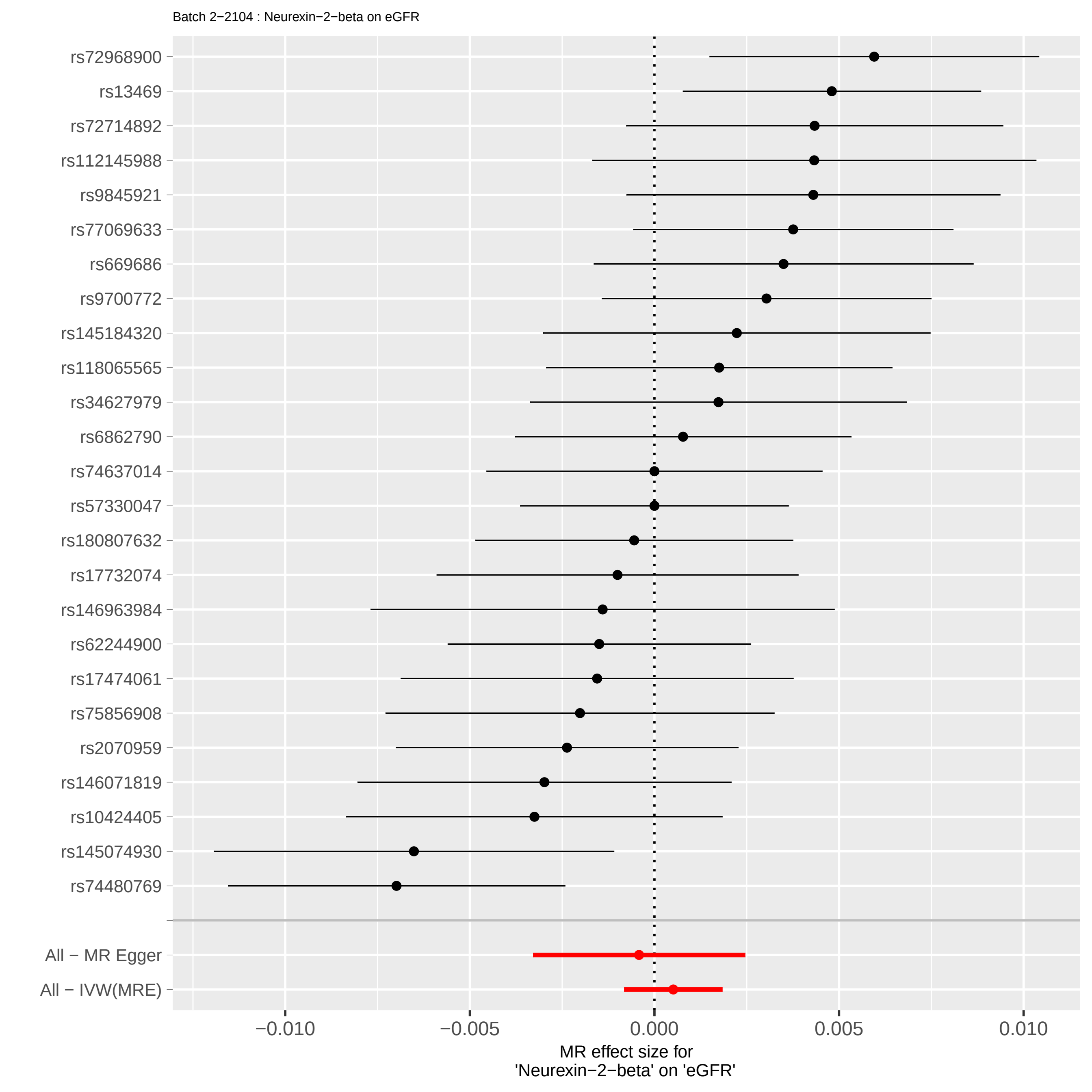
Batch 2−2104 : Neurexin−2−beta on eGFR
rs72968900
rs13469
rs72714892
rs112145988
rs9845921
rs77069633
rs669686
rs9700772
rs145184320
rs118065565
rs34627979
rs6862790
rs74637014
rs57330047
rs180807632
rs17732074
rs146963984
rs62244900
rs17474061
rs75856908
rs2070959
rs146071819
rs10424405
rs145074930
rs74480769
All − MR Egger
All − IVW(MRE)
−0.010
−0.005
0.000
0.005
0.010
MR effect size for
'Neurexin−2−beta' on 'eGFR'

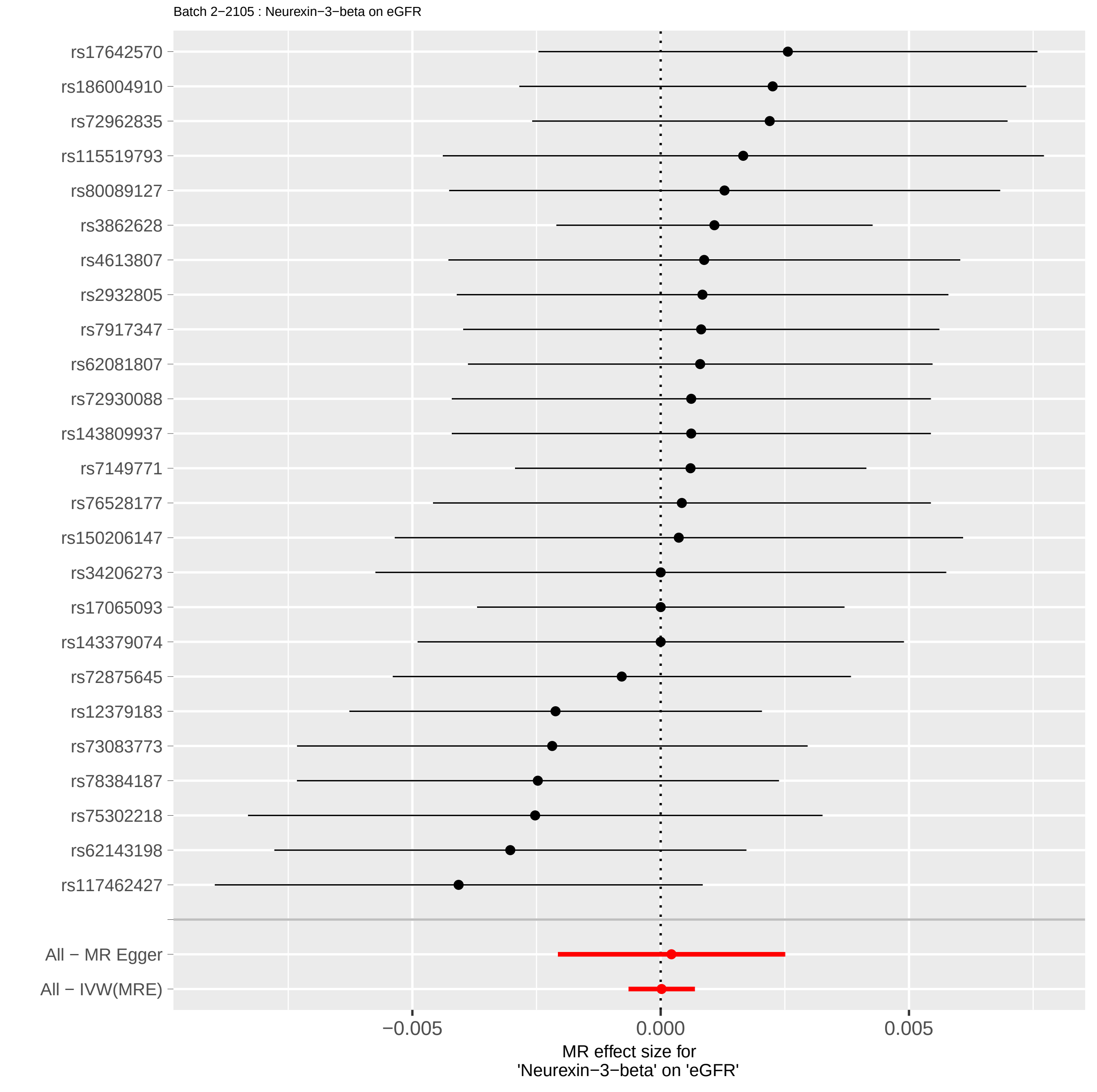
Batch 2−2105 : Neurexin−3−beta on eGFR
rs17642570
rs186004910
rs72962835
rs115519793
rs80089127
rs3862628
rs4613807
rs2932805
rs7917347
rs62081807
rs72930088
rs143809937
rs7149771
rs76528177
rs150206147
rs34206273
rs17065093
rs143379074
rs72875645
rs12379183
rs73083773
rs78384187
rs75302218
rs62143198
rs117462427
All − MR Egger
All − IVW(MRE)
−0.005
0.000
0.005
MR effect size for
'Neurexin−3−beta' on 'eGFR'

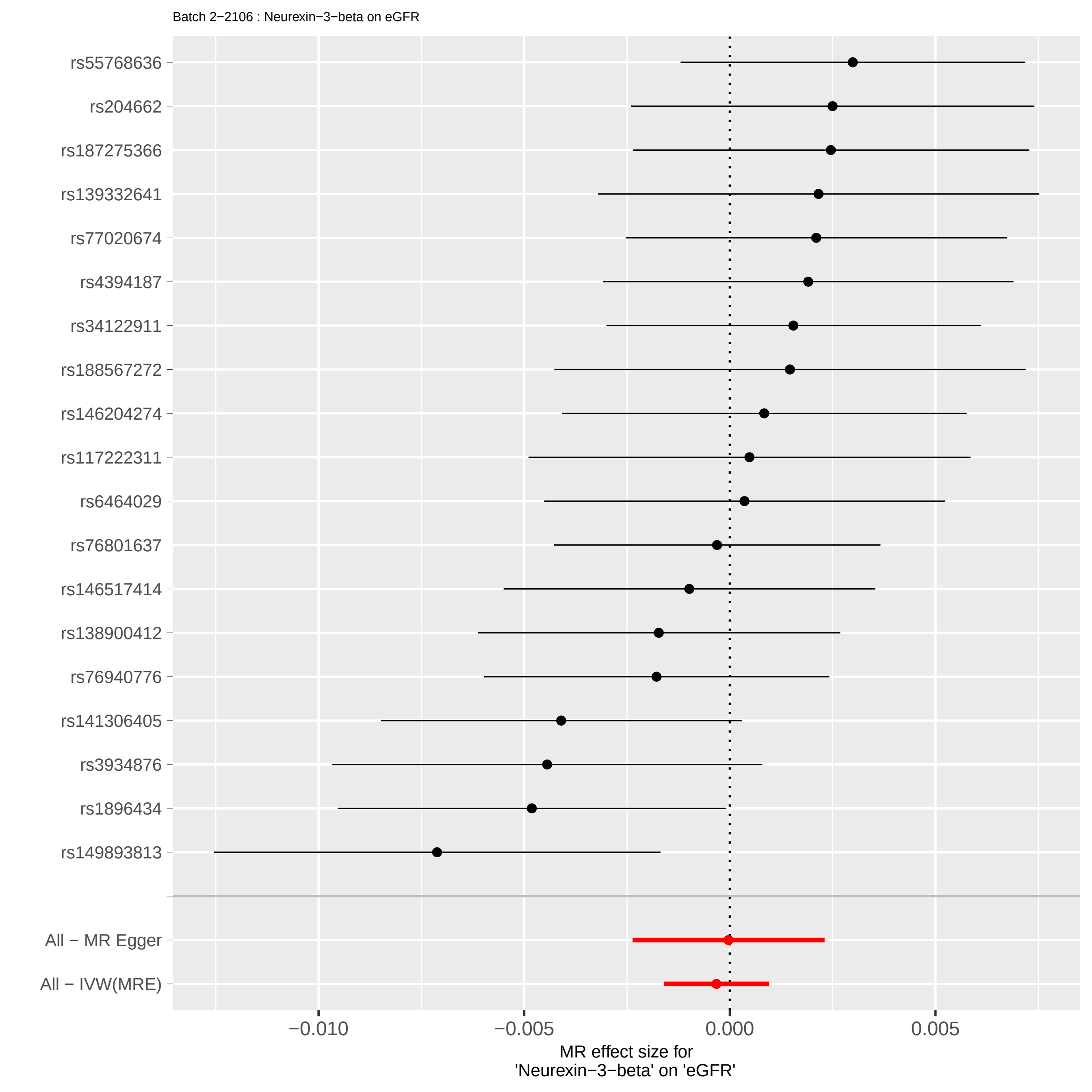

Batch 2−2106 : Neurexin−3−beta on eGFR
rs55768636
rs204662
rs187275366
rs139332641
rs77020674
rs4394187
rs34122911
rs188567272
rs146204274
rs117222311
rs6464029
rs76801637
rs146517414
rs138900412
rs76940776
rs141306405
rs3934876
rs1896434
rs149893813
All − MR Egger
All − IVW(MRE)
−0.010
−0.005
0.000
0.005
MR effect size for
'Neurexin−3−beta' on 'eGFR'

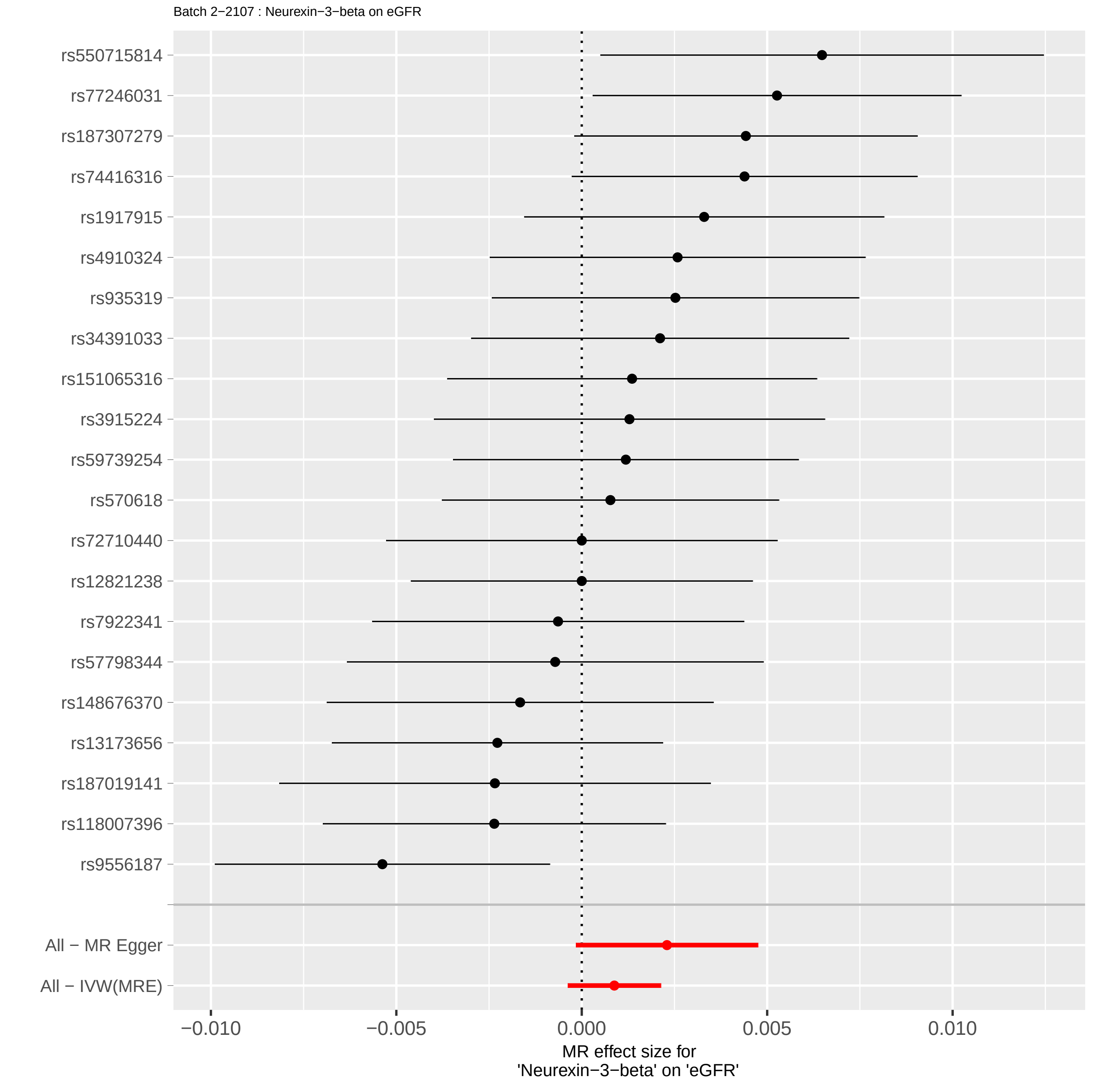
Batch 2−2107 : Neurexin−3−beta on eGFR
rs550715814
rs77246031
rs187307279
rs74416316
rs1917915
rs4910324
rs935319
rs34391033
rs151065316
rs3915224
rs59739254
rs570618
rs72710440
rs12821238
rs7922341
rs57798344
rs148676370
rs13173656
rs187019141
rs118007396
rs9556187
All − MR Egger
All − IVW(MRE)
−0.010
−0.005
0.000
0.005
0.010
MR effect size for
'Neurexin−3−beta' on 'eGFR'

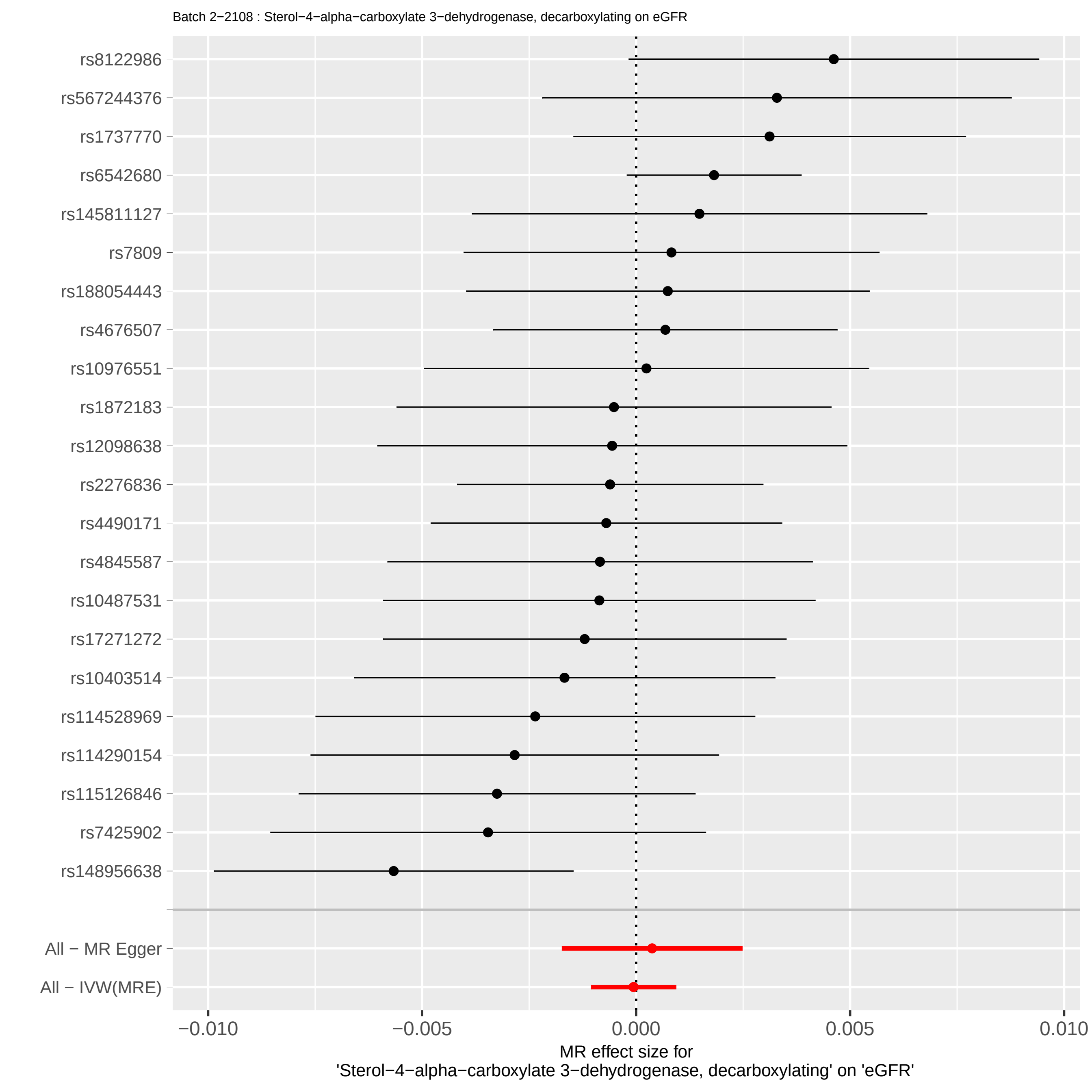

Batch 2−2108 : Sterol−4−alpha−carboxylate 3−dehydrogenase, decarboxylating on eGFR
rs8122986
rs567244376
rs1737770
rs6542680
rs145811127
rs7809
rs188054443
rs4676507
rs10976551
rs1872183
rs12098638
rs2276836
rs4490171
rs4845587
rs10487531
rs17271272
rs10403514
rs114528969
rs114290154
rs115126846
rs7425902
rs148956638
All − MR Egger
All − IVW(MRE)
−0.010
−0.005
0.000
0.005
0.010
MR effect size for
'Sterol−4−alpha−carboxylate 3−dehydrogenase, decarboxylating' on 'eGFR'

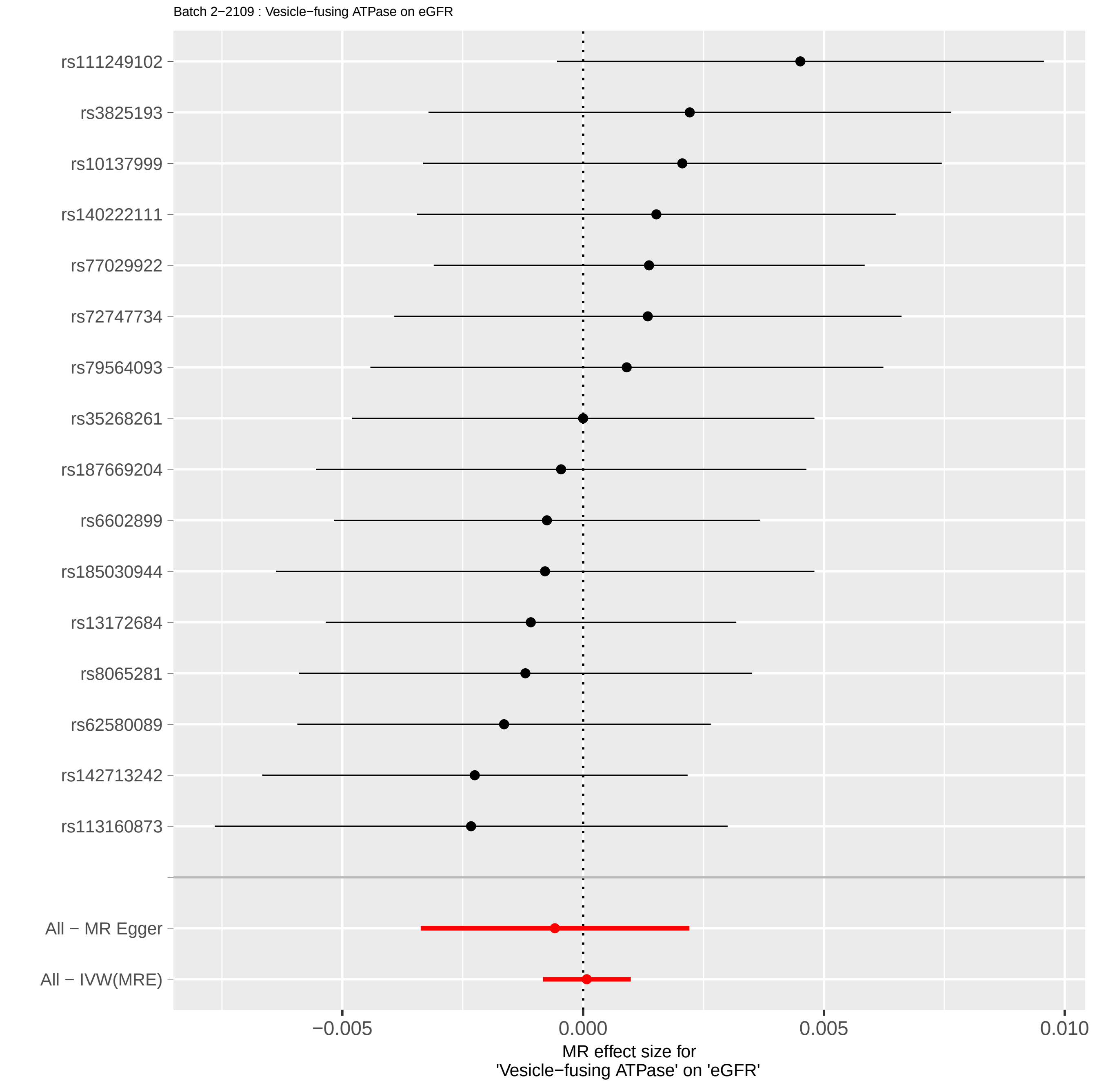

Batch 2−2109 : Vesicle−fusing ATPase on eGFR
rs111249102
rs3825193
rs10137999
rs140222111
rs77029922
rs72747734
rs79564093
rs35268261
rs187669204
rs6602899
rs185030944
rs13172684
rs8065281
rs62580089
rs142713242
rs113160873
All − MR Egger
All − IVW(MRE)
−0.005
0.000
0.005
0.010
MR effect size for
'Vesicle−fusing ATPase' on 'eGFR'

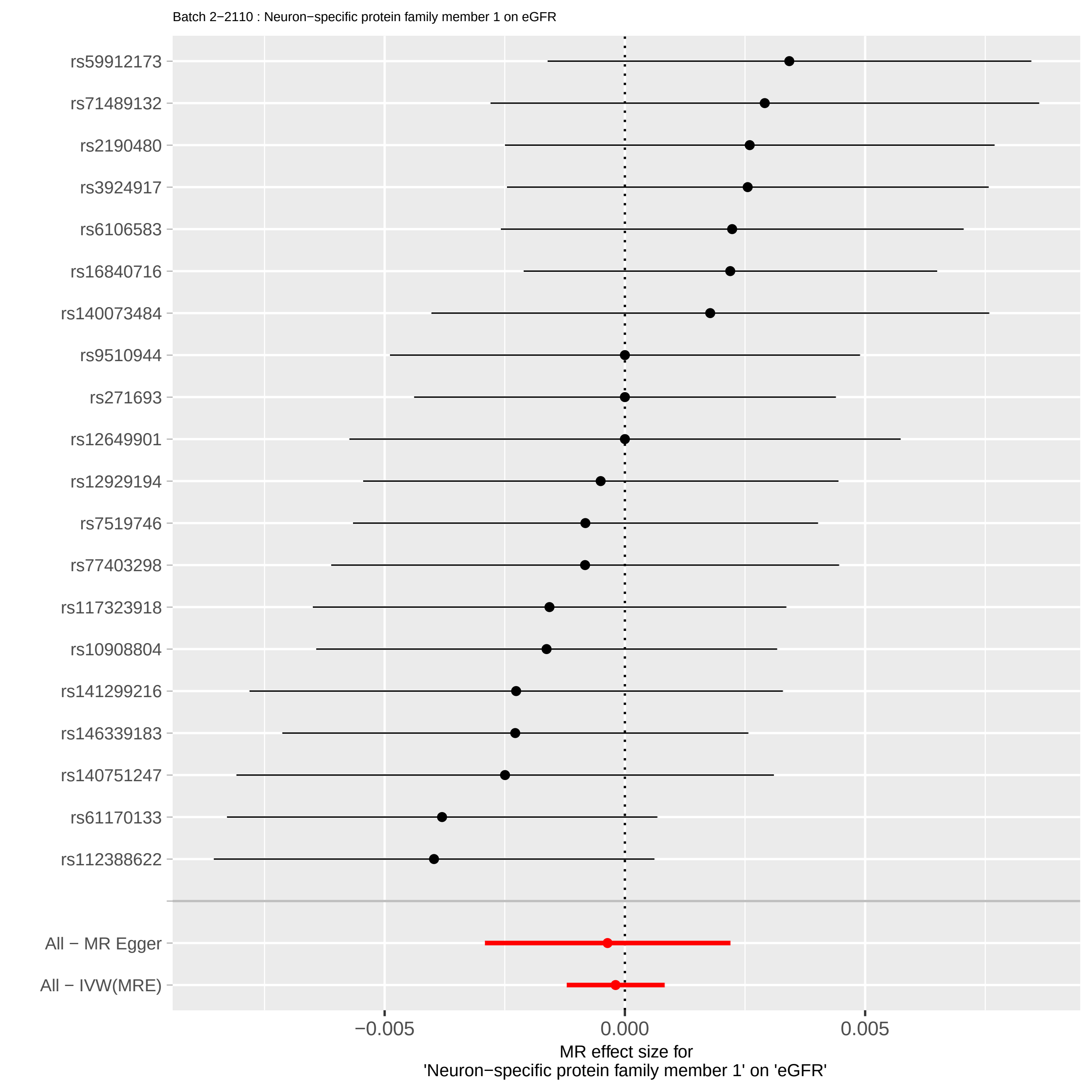

Batch 2−2110 : Neuron−specific protein family member 1 on eGFR
rs59912173
rs71489132
rs2190480
rs3924917
rs6106583
rs16840716
rs140073484
rs9510944
rs271693
rs12649901
rs12929194
rs7519746
rs77403298
rs117323918
rs10908804
rs141299216
rs146339183
rs140751247
rs61170133
rs112388622
All − MR Egger
All − IVW(MRE)
−0.005
0.000
0.005
MR effect size for
'Neuron−specific protein family member 1' on 'eGFR'

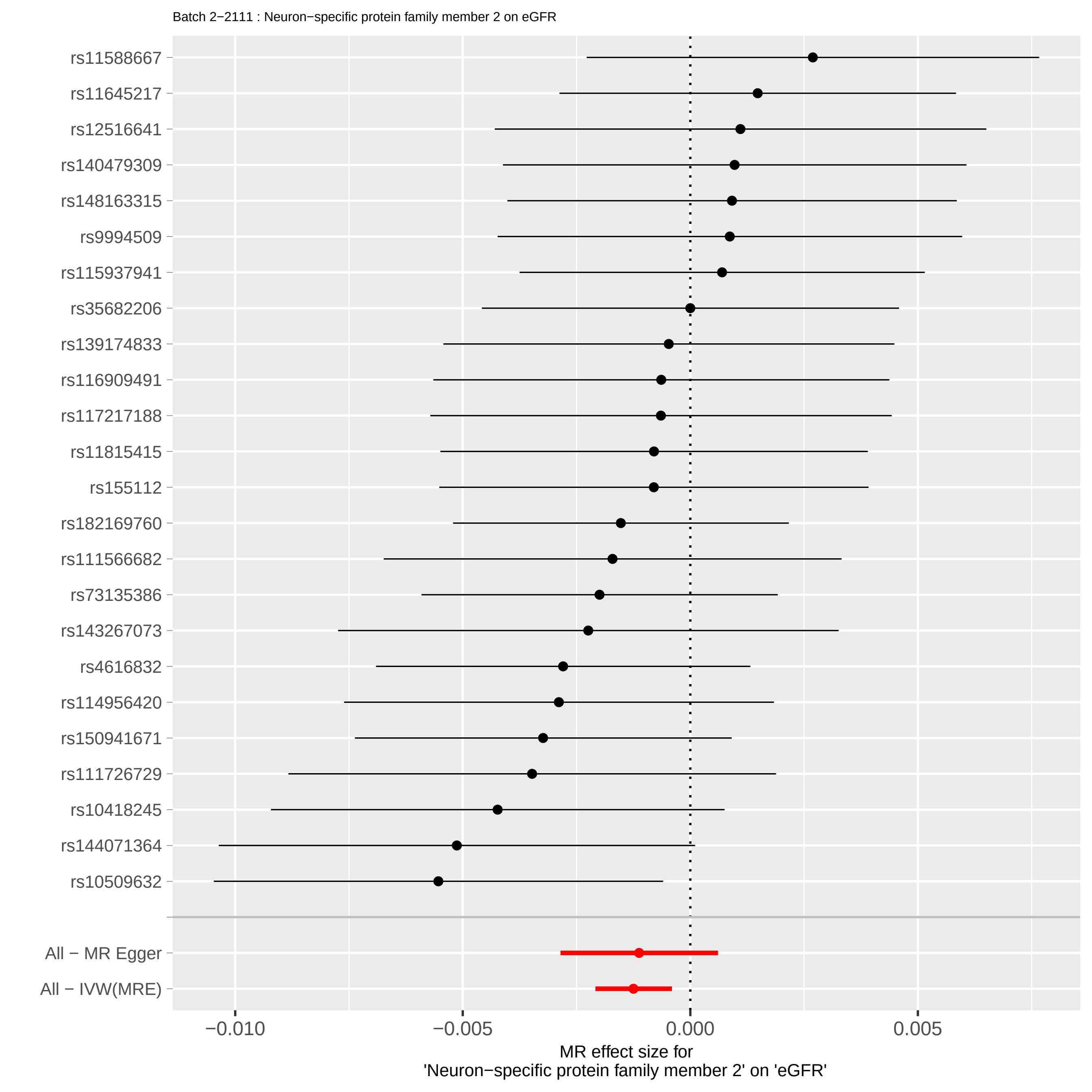
Batch 2−2111 : Neuron−specific protein family member 2 on eGFR
rs11588667
rs11645217
rs12516641
rs140479309
rs148163315
rs9994509
rs115937941
rs35682206
rs139174833
rs116909491
rs117217188
rs11815415
rs155112
rs182169760
rs111566682
rs73135386
rs143267073
rs4616832
rs114956420
rs150941671
rs111726729
rs10418245
rs144071364
rs10509632
All − MR Egger
All − IVW(MRE)
−0.010
−0.005
0.000
0.005
MR effect size for
'Neuron−specific protein family member 2' on 'eGFR'

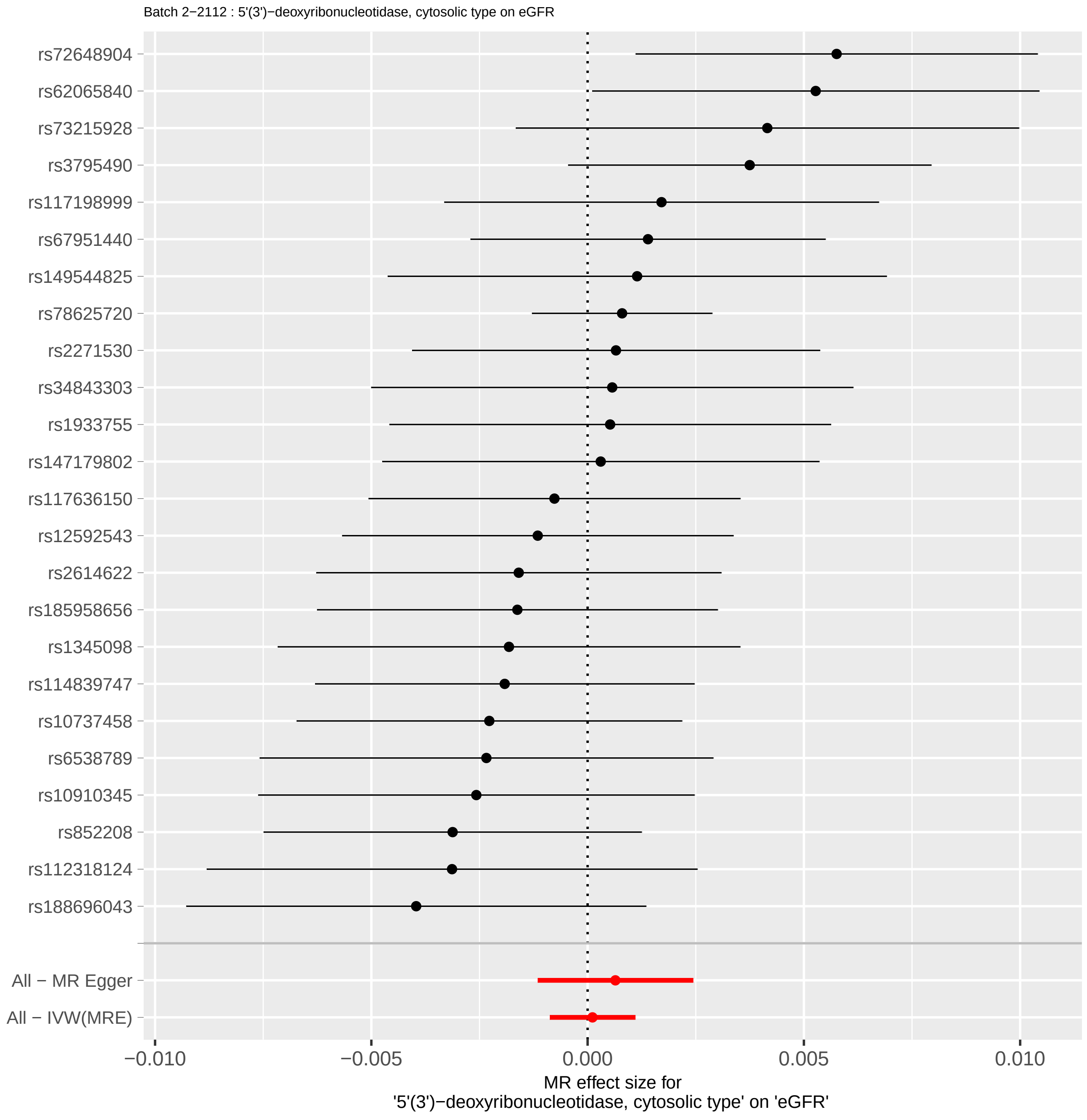

Batch 2−2112 : 5'(3')−deoxyribonucleotidase, cytosolic type on eGFR
rs72648904
rs62065840
rs73215928
rs3795490
rs117198999
rs67951440
rs149544825
rs78625720
rs2271530
rs34843303
rs1933755
rs147179802
rs117636150
rs12592543
rs2614622
rs185958656
rs1345098
rs114839747
rs10737458
rs6538789
rs10910345
rs852208
rs112318124
rs188696043
All − MR Egger
All − IVW(MRE)
−0.010
−0.005
0.000
0.005
0.010
MR effect size for
'5'(3')−deoxyribonucleotidase, cytosolic type' on 'eGFR'

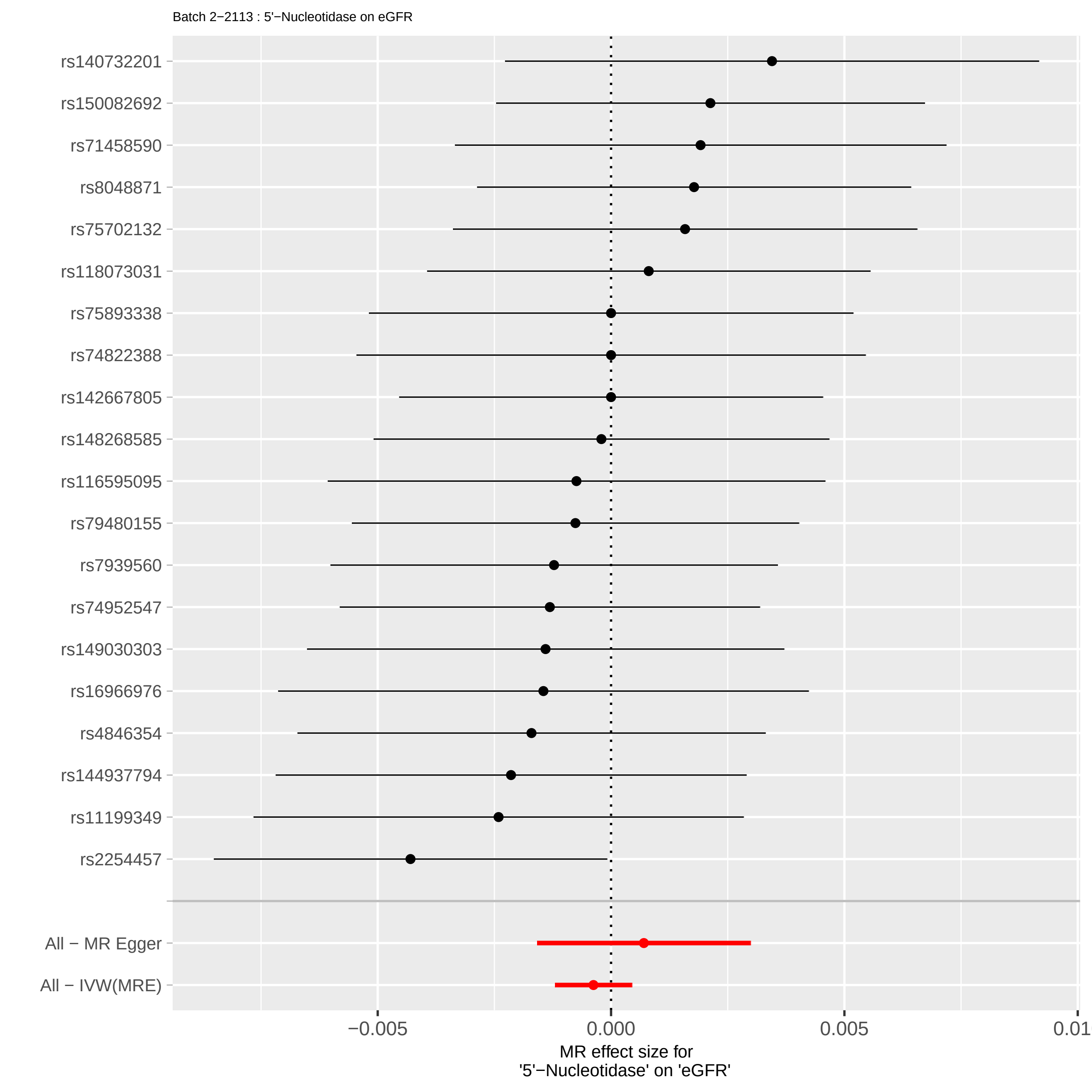

Batch 2−2113 : 5'−Nucleotidase on eGFR
rs140732201
rs150082692
rs71458590
rs8048871
rs75702132
rs118073031
rs75893338
rs74822388
rs142667805
rs148268585
rs116595095
rs79480155
rs7939560
rs74952547
rs149030303
rs16966976
rs4846354
rs144937794
rs11199349
rs2254457
All − MR Egger
All − IVW(MRE)
−0.005
0.000
0.005
0.01
MR effect size for
'5'−Nucleotidase' on 'eGFR'

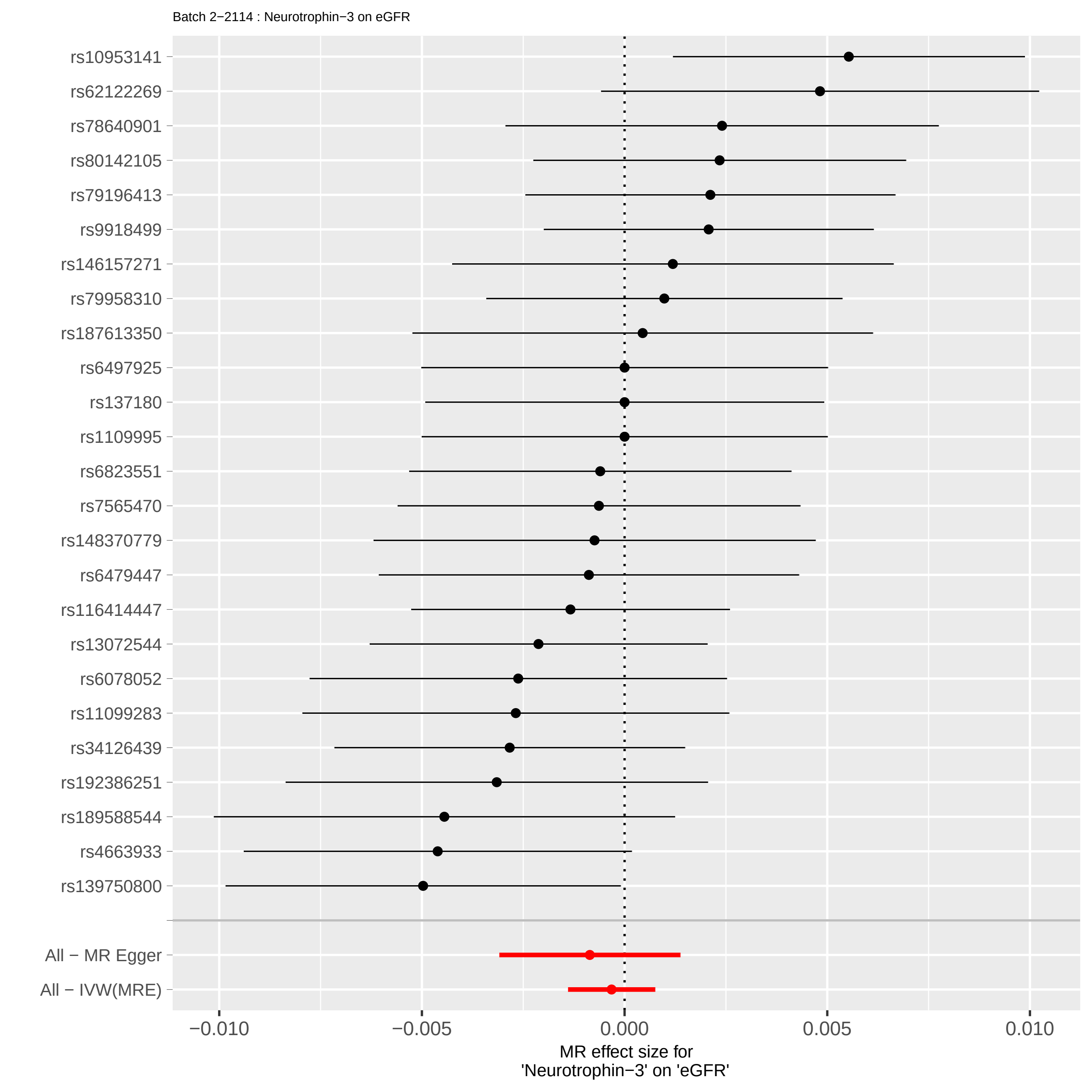

Batch 2−2114 : Neurotrophin−3 on eGFR
rs10953141
rs62122269
rs78640901
rs80142105
rs79196413
rs9918499
rs146157271
rs79958310
rs187613350
rs6497925
rs137180
rs1109995
rs6823551
rs7565470
rs148370779
rs6479447
rs116414447
rs13072544
rs6078052
rs11099283
rs34126439
rs192386251
rs189588544
rs4663933
rs139750800
All − MR Egger
All − IVW(MRE)
−0.010
−0.005
0.000
0.005
0.010
MR effect size for
'Neurotrophin−3' on 'eGFR'

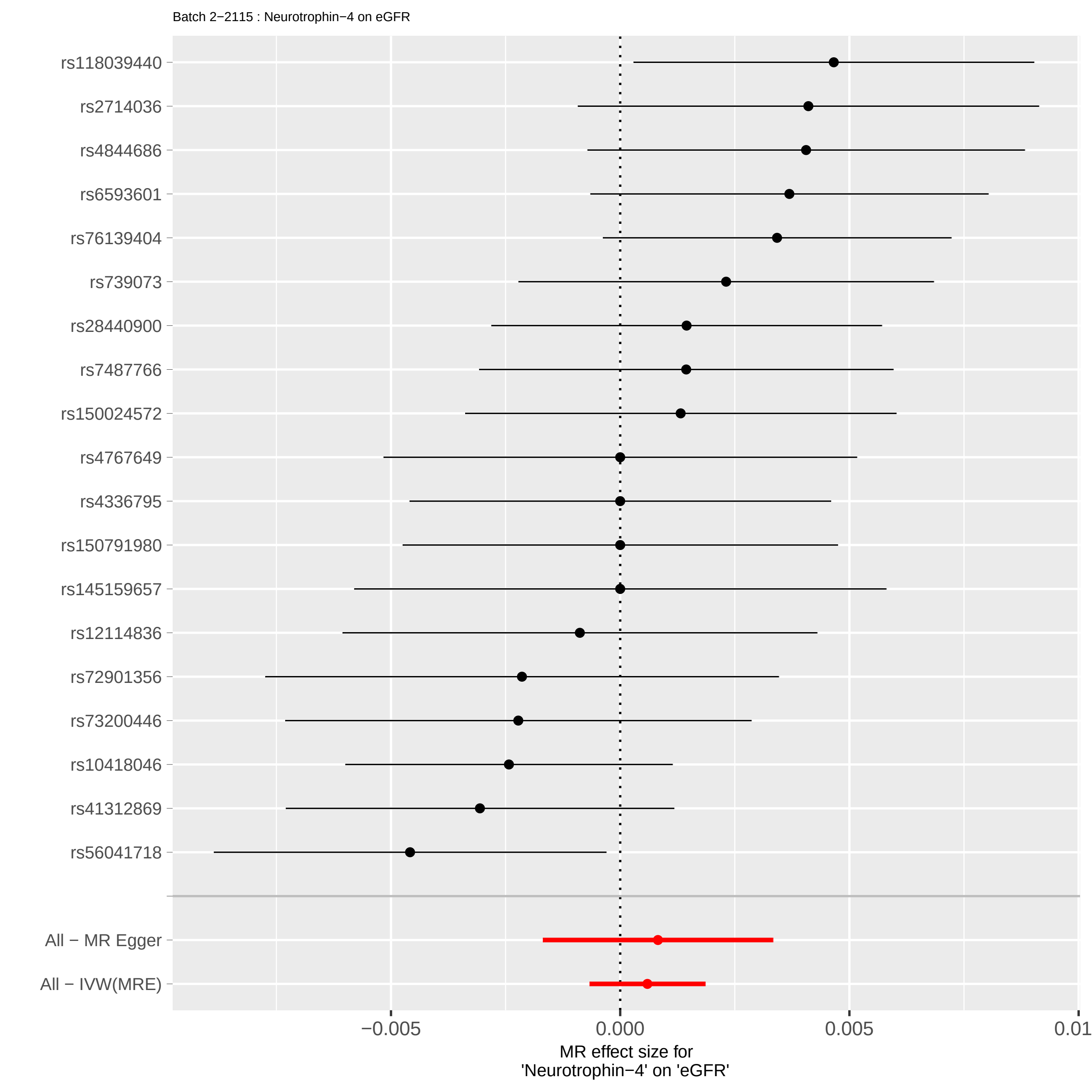

Batch 2−2115 : Neurotrophin−4 on eGFR
rs118039440
rs2714036
rs4844686
rs6593601
rs76139404
rs739073
rs28440900
rs7487766
rs150024572
rs4767649
rs4336795
rs150791980
rs145159657
rs12114836
rs72901356
rs73200446
rs10418046
rs41312869
rs56041718
All − MR Egger
All − IVW(MRE)
−0.005
0.000
0.005
0.01
MR effect size for
'Neurotrophin−4' on 'eGFR'

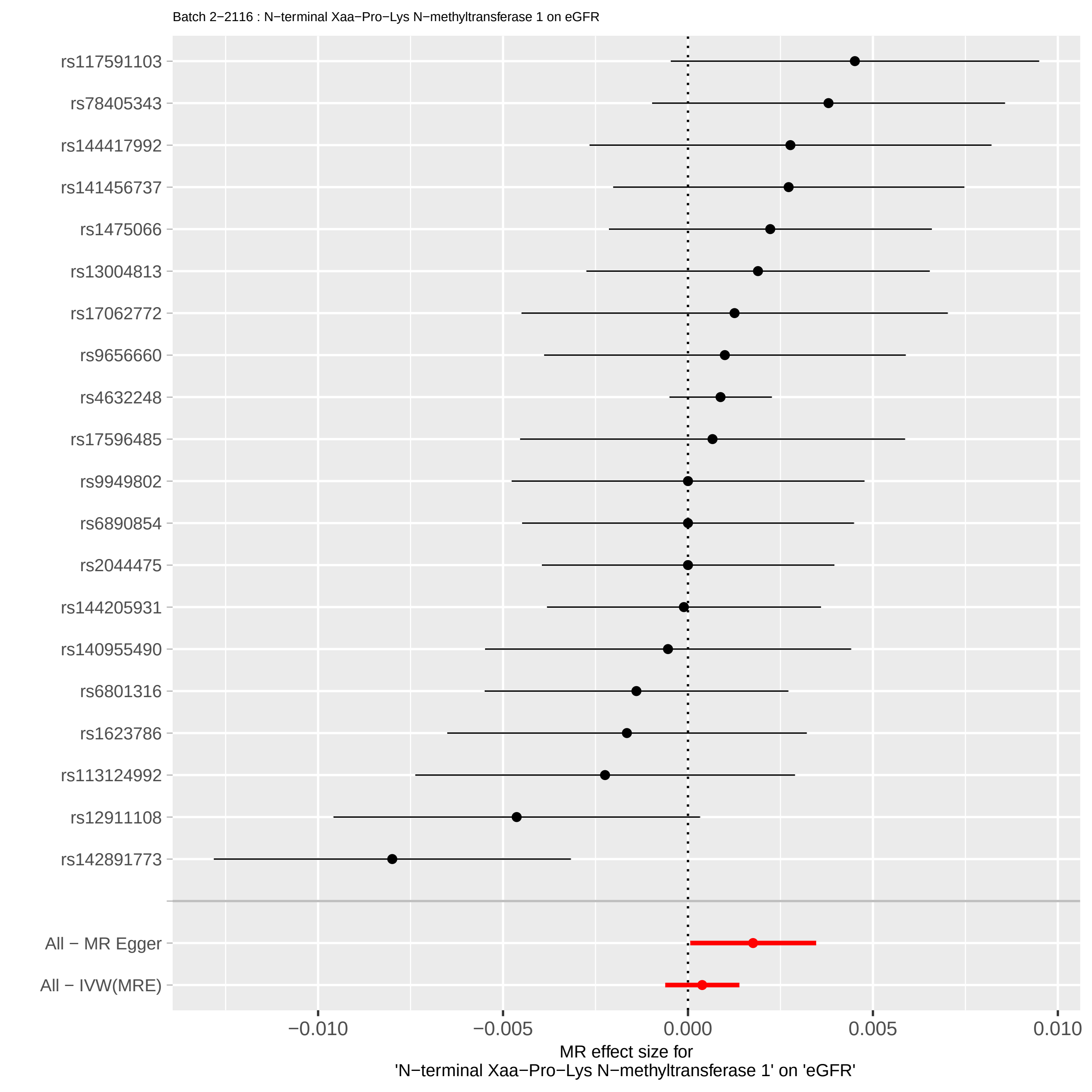

Batch 2−2116 : N−terminal Xaa−Pro−Lys N−methyltransferase 1 on eGFR
rs117591103
rs78405343
rs144417992
rs141456737
rs1475066
rs13004813
rs17062772
rs9656660
rs4632248
rs17596485
rs9949802
rs6890854
rs2044475
rs144205931
rs140955490
rs6801316
rs1623786
rs113124992
rs12911108
rs142891773
All − MR Egger
All − IVW(MRE)
−0.010
−0.005
0.000
0.005
0.010
MR effect size for
'N−terminal Xaa−Pro−Lys N−methyltransferase 1' on 'eGFR'

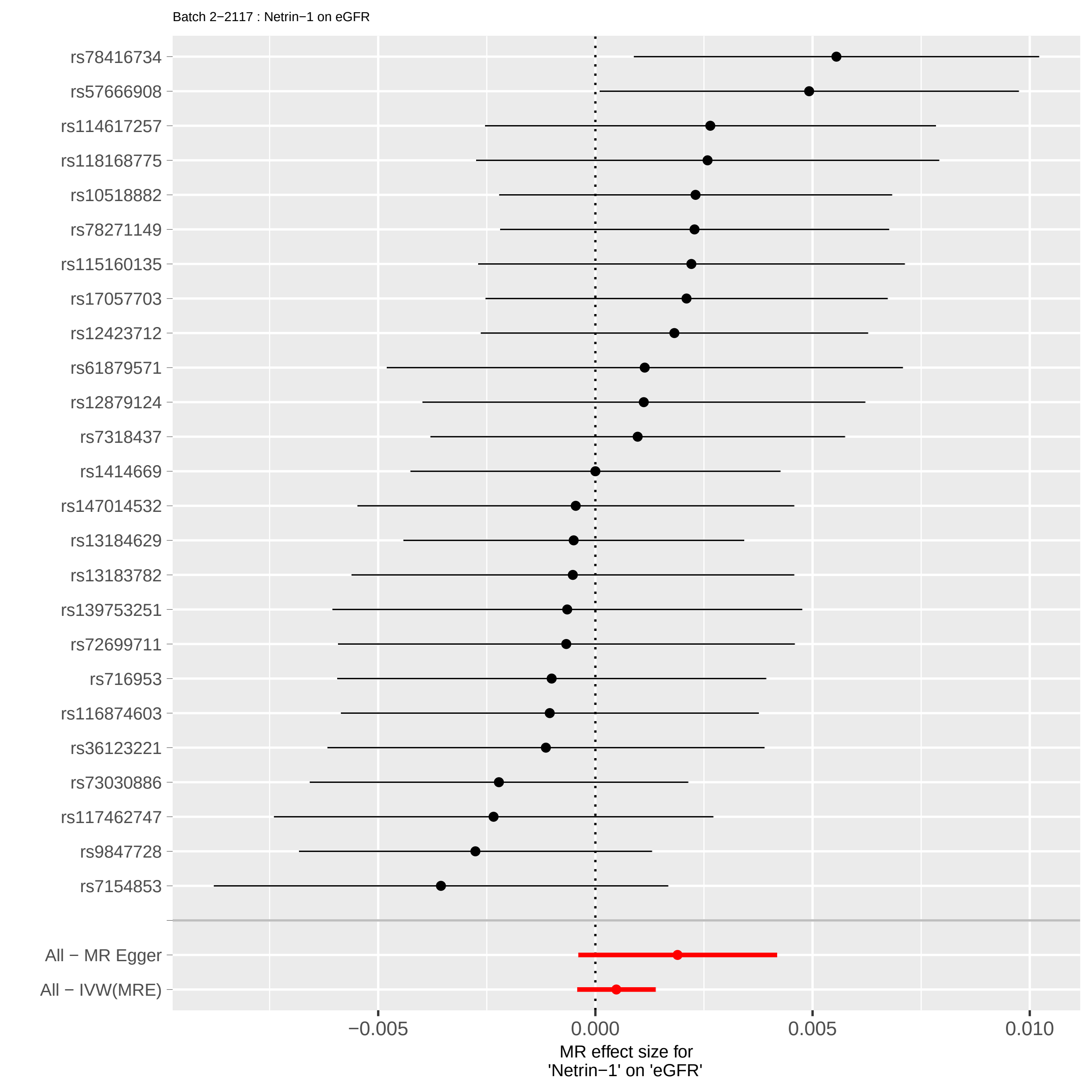

Batch 2−2117 : Netrin−1 on eGFR
rs78416734
rs57666908
rs114617257
rs118168775
rs10518882
rs78271149
rs115160135
rs17057703
rs12423712
rs61879571
rs12879124
rs7318437
rs1414669
rs147014532
rs13184629
rs13183782
rs139753251
rs72699711
rs716953
rs116874603
rs36123221
rs73030886
rs117462747
rs9847728
rs7154853
All − MR Egger
All − IVW(MRE)
−0.005
0.000
0.005
0.010
MR effect size for
'Netrin−1' on 'eGFR'

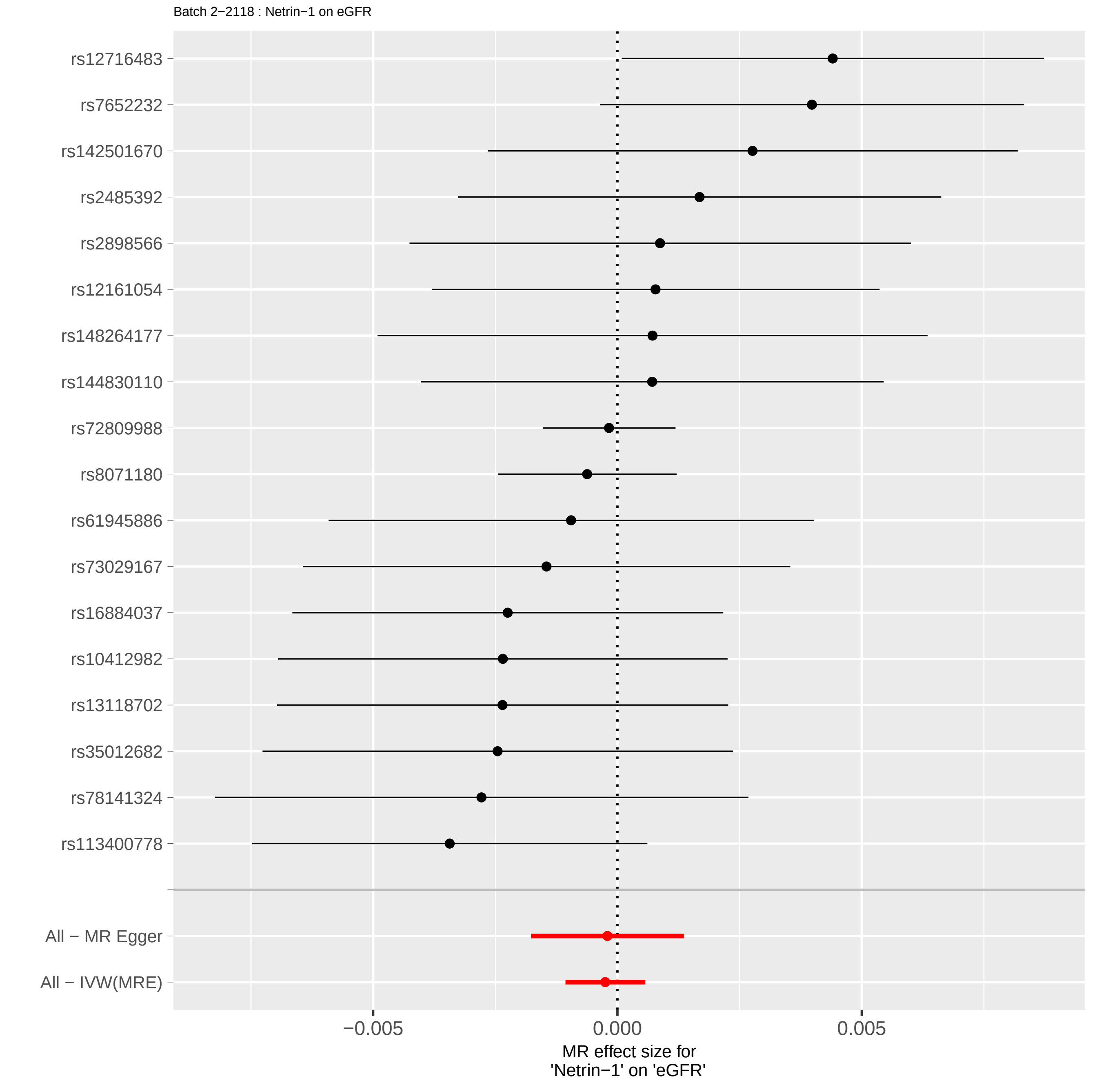

Batch 2−2118 : Netrin−1 on eGFR
rs12716483
rs7652232
rs142501670
rs2485392
rs2898566
rs12161054
rs148264177
rs144830110
rs72809988
rs8071180
rs61945886
rs73029167
rs16884037
rs10412982
rs13118702
rs35012682
rs78141324
rs113400778
All − MR Egger
All − IVW(MRE)
−0.005
0.000
0.005
MR effect size for
'Netrin−1' on 'eGFR'

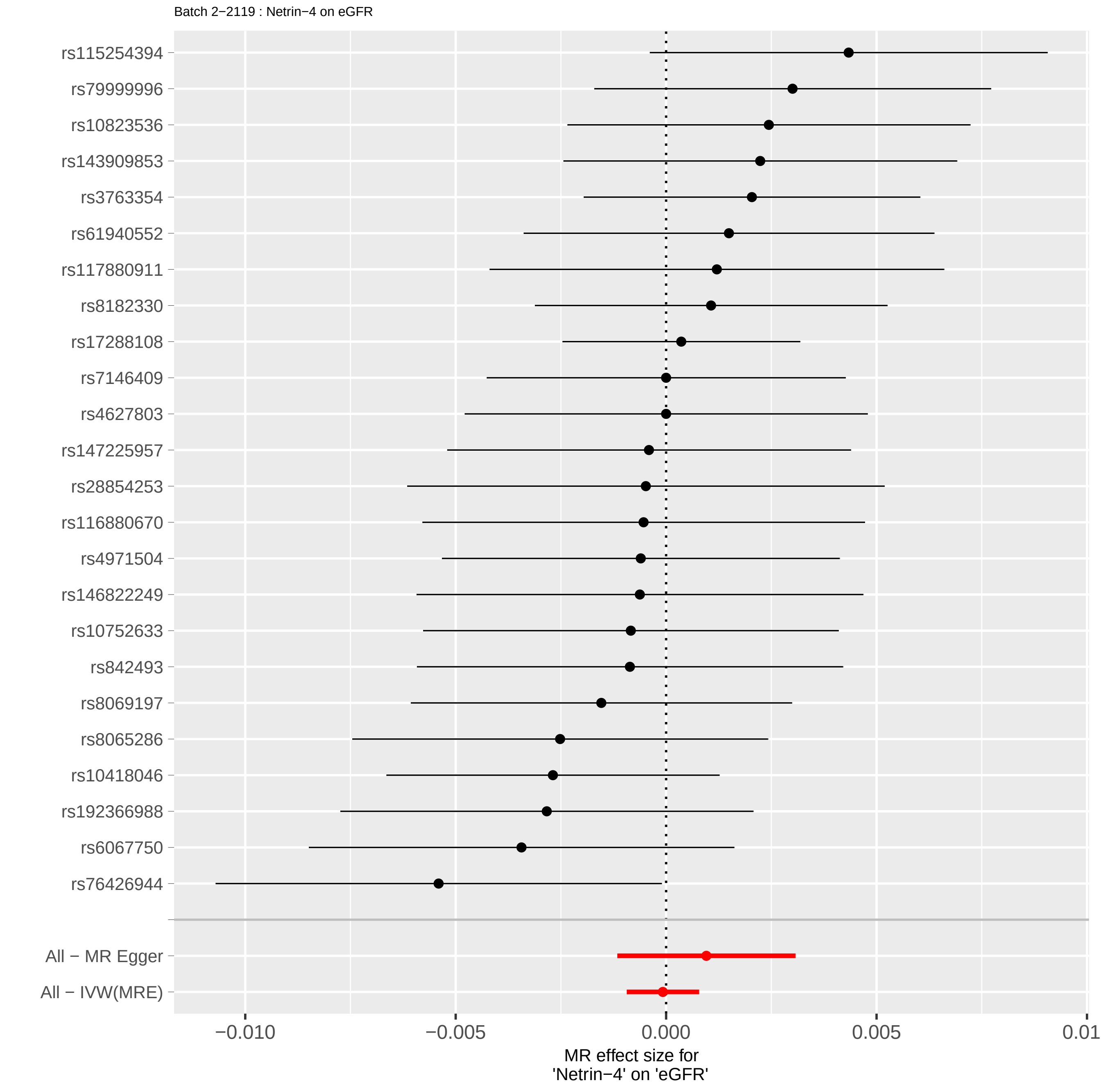
Batch 2−2119 : Netrin−4 on eGFR
rs115254394
rs79999996
rs10823536
rs143909853
rs3763354
rs61940552
rs117880911
rs8182330
rs17288108
rs7146409
rs4627803
rs147225957
rs28854253
rs116880670
rs4971504
rs146822249
rs10752633
rs842493
rs8069197
rs8065286
rs10418046
rs192366988
rs6067750
rs76426944
All − MR Egger
All − IVW(MRE)
−0.010
−0.005
0.000
0.005
0.01
MR effect size for
'Netrin−4' on 'eGFR'

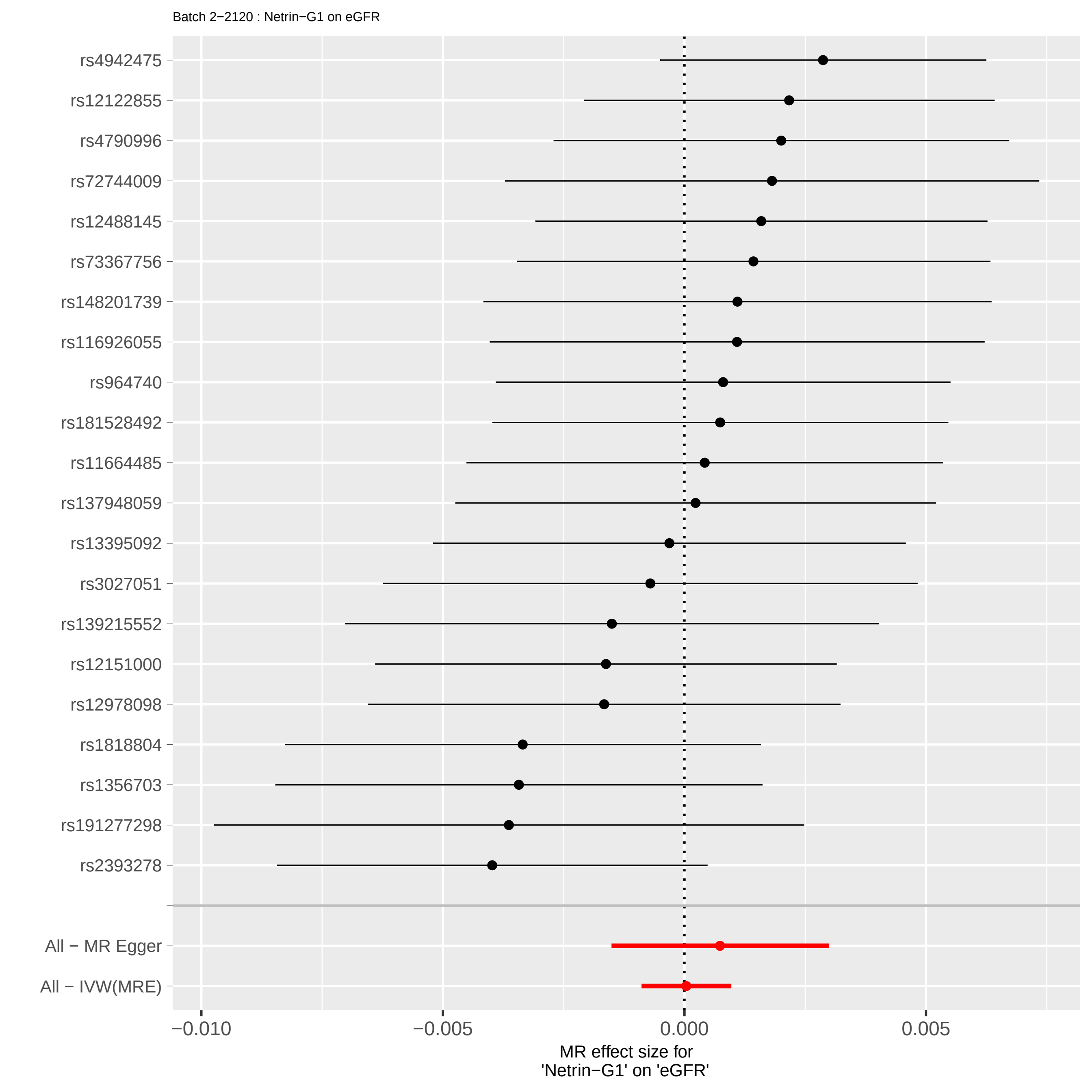

Batch 2−2120 : Netrin−G1 on eGFR
rs4942475
rs12122855
rs4790996
rs72744009
rs12488145
rs73367756
rs148201739
rs116926055
rs964740
rs181528492
rs11664485
rs137948059
rs13395092
rs3027051
rs139215552
rs12151000
rs12978098
rs1818804
rs1356703
rs191277298
rs2393278
All − MR Egger
All − IVW(MRE)
−0.010
−0.005
0.000
0.005
MR effect size for
'Netrin−G1' on 'eGFR'

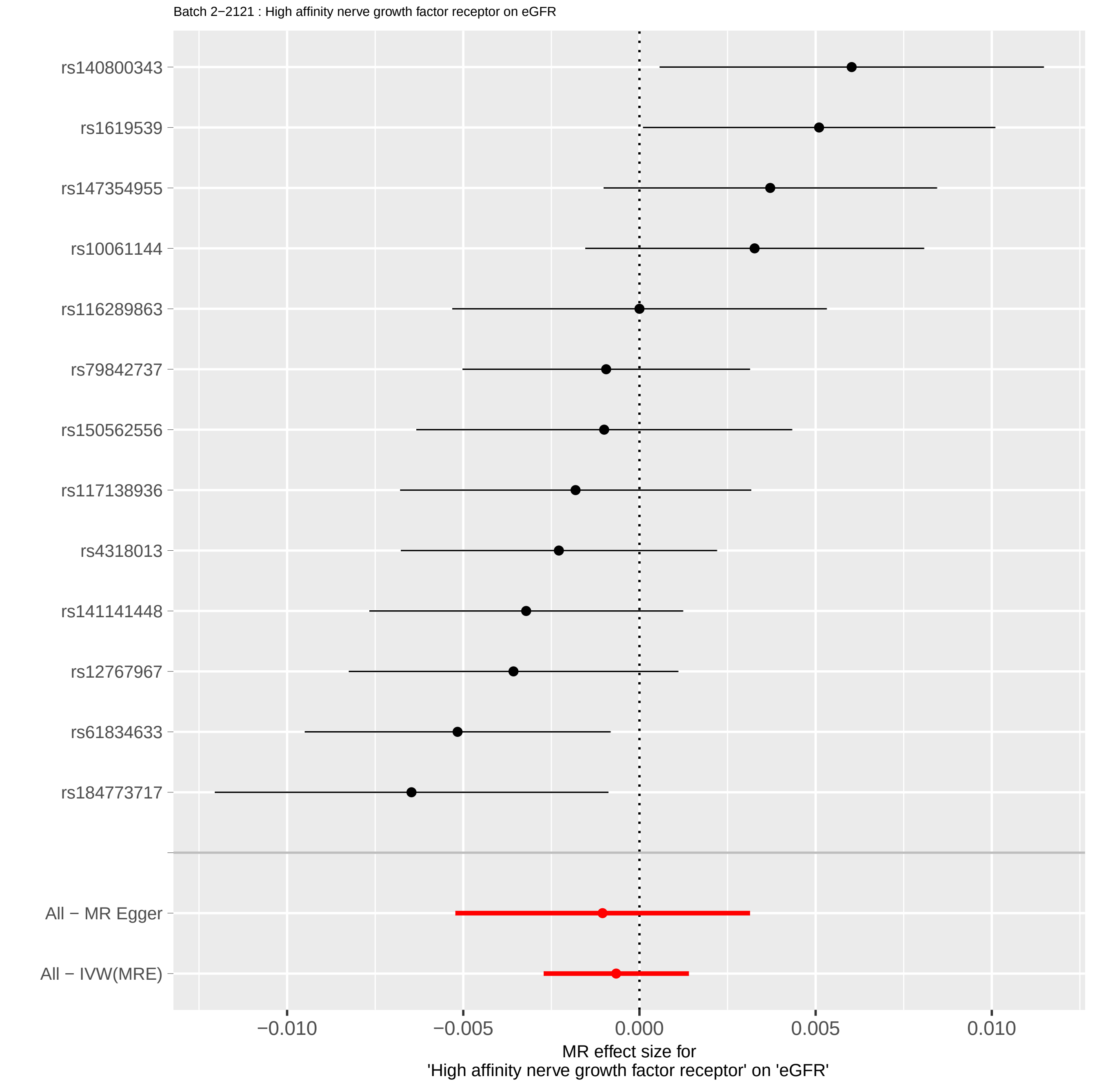

Batch 2−2121 : High affinity nerve growth factor receptor on eGFR
rs140800343
rs1619539
rs147354955
rs10061144
rs116289863
rs79842737
rs150562556
rs117138936
rs4318013
rs141141448
rs12767967
rs61834633
rs184773717
All − MR Egger
All − IVW(MRE)
−0.010
−0.005
0.000
0.005
0.010
MR effect size for
'High affinity nerve growth factor receptor' on 'eGFR'

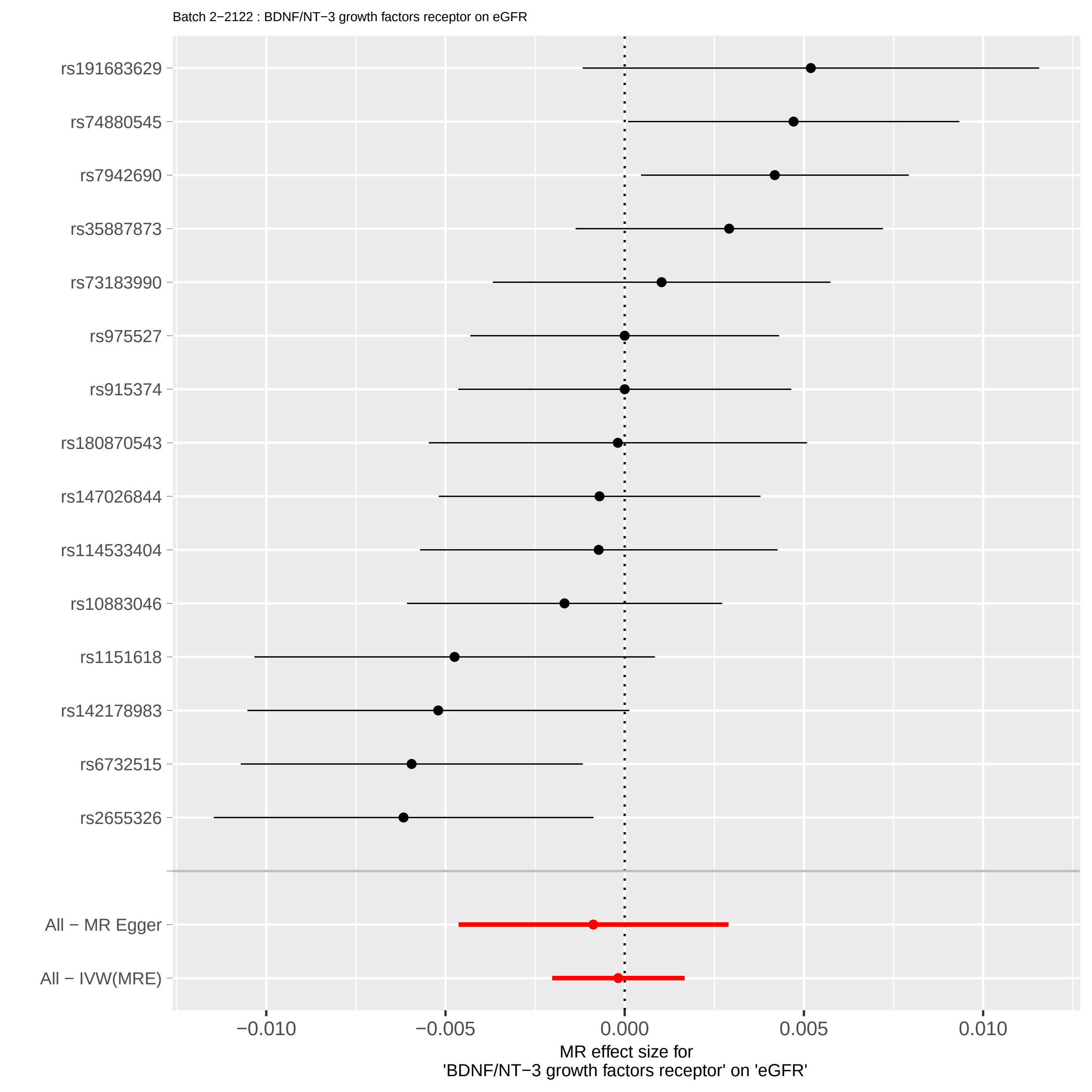

Batch 2−2122 : BDNF/NT−3 growth factors receptor on eGFR
rs191683629
rs74880545
rs7942690
rs35887873
rs73183990
rs975527
rs915374
rs180870543
rs147026844
rs114533404
rs10883046
rs1151618
rs142178983
rs6732515
rs2655326
All − MR Egger
All − IVW(MRE)
−0.010
−0.005
0.000
0.005
0.010
MR effect size for
'BDNF/NT−3 growth factors receptor' on 'eGFR'

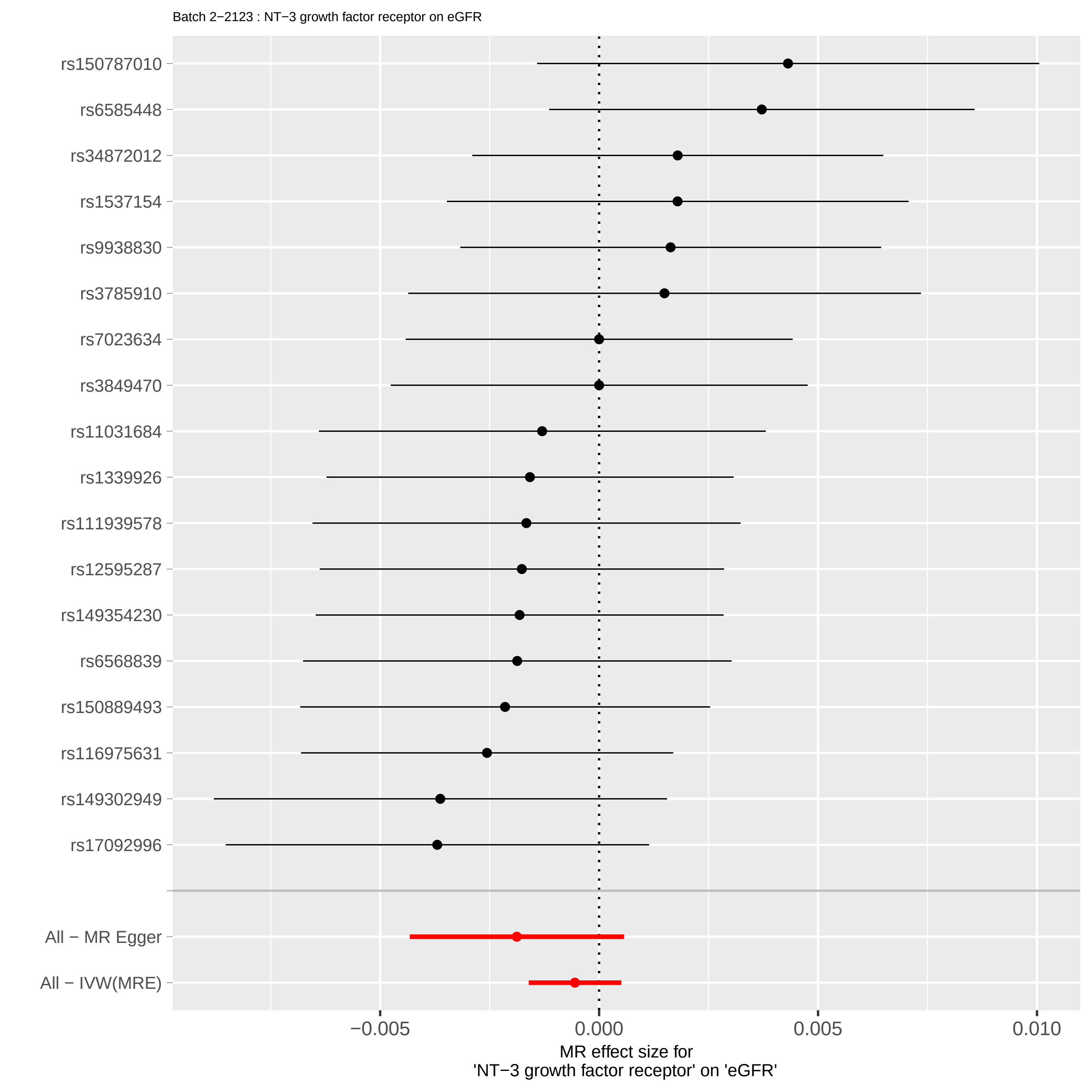

Batch 2−2123 : NT−3 growth factor receptor on eGFR
rs150787010
rs6585448
rs34872012
rs1537154
rs9938830
rs3785910
rs7023634
rs3849470
rs11031684
rs1339926
rs111939578
rs12595287
rs149354230
rs6568839
rs150889493
rs116975631
rs149302949
rs17092996
All − MR Egger
All − IVW(MRE)
−0.005
0.000
0.005
0.010
MR effect size for
'NT−3 growth factor receptor' on 'eGFR'

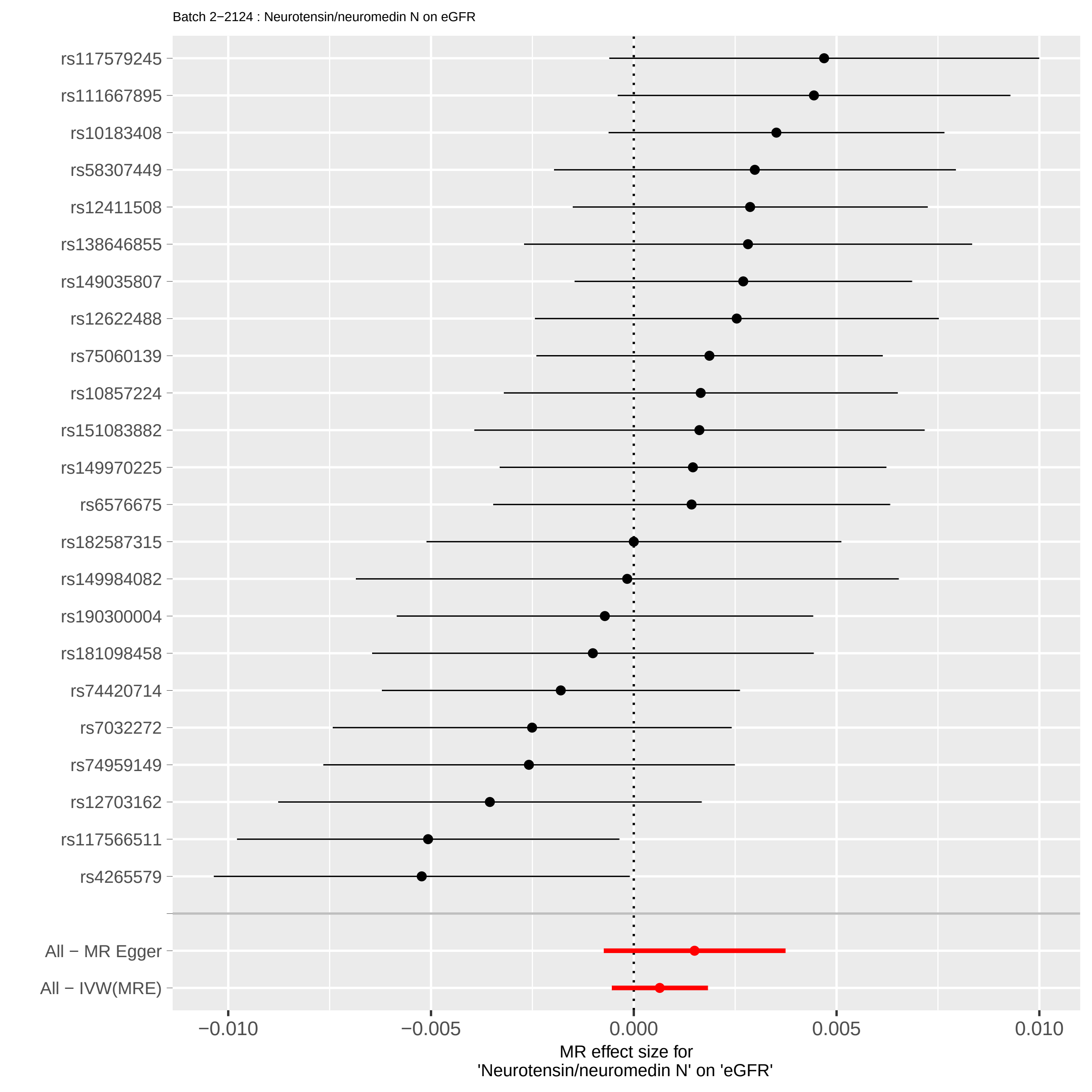
Batch 2−2124 : Neurotensin/neuromedin N on eGFR
rs117579245
rs111667895
rs10183408
rs58307449
rs12411508
rs138646855
rs149035807
rs12622488
rs75060139
rs10857224
rs151083882
rs149970225
rs6576675
rs182587315
rs149984082
rs190300004
rs181098458
rs74420714
rs7032272
rs74959149
rs12703162
rs117566511
rs4265579
All − MR Egger
All − IVW(MRE)
−0.010
−0.005
0.000
0.005
0.010
MR effect size for
'Neurotensin/neuromedin N' on 'eGFR'

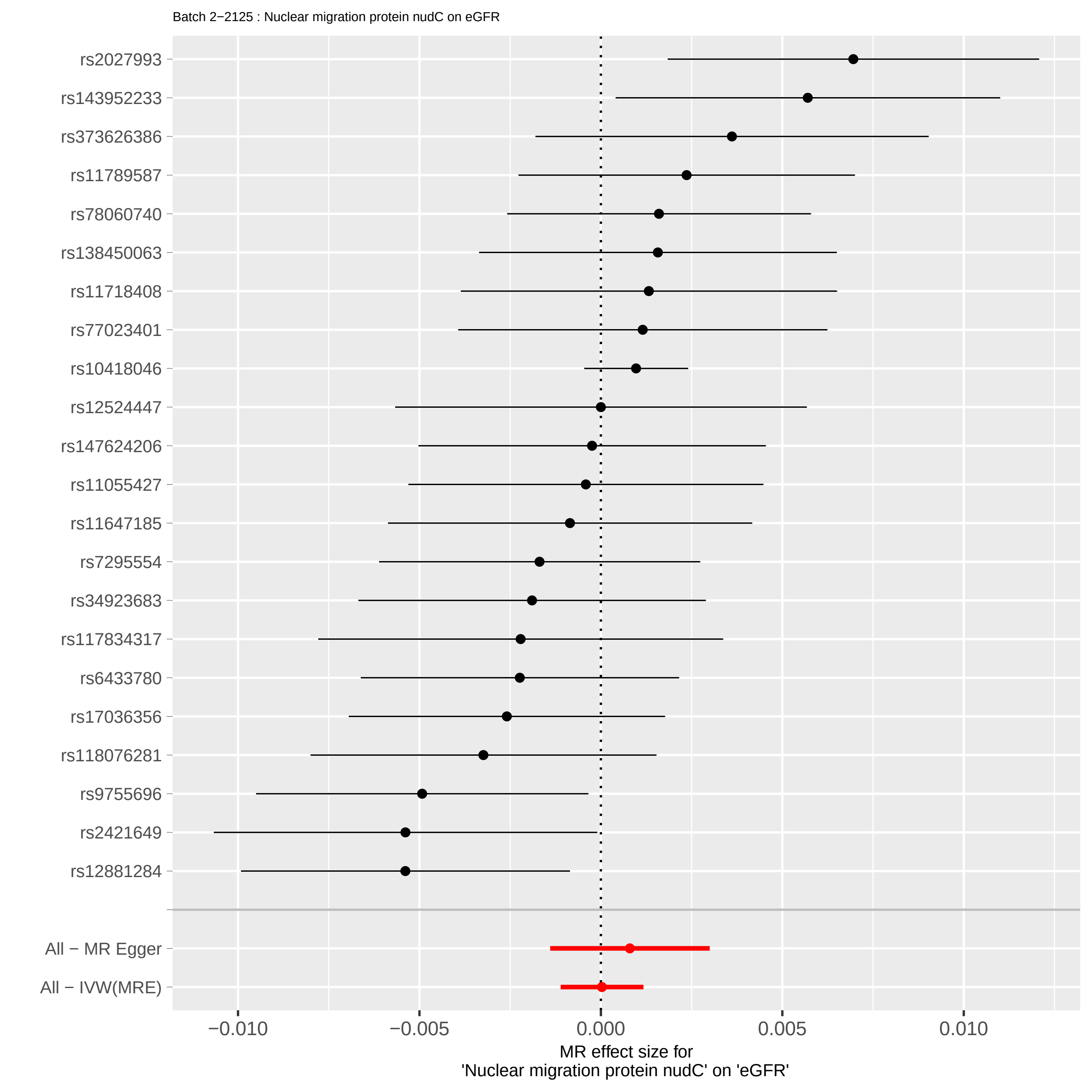

Batch 2−2125 : Nuclear migration protein nudC on eGFR
rs2027993
rs143952233
rs373626386
rs11789587
rs78060740
rs138450063
rs11718408
rs77023401
rs10418046
rs12524447
rs147624206
rs11055427
rs11647185
rs7295554
rs34923683
rs117834317
rs6433780
rs17036356
rs118076281
rs9755696
rs2421649
rs12881284
All − MR Egger
All − IVW(MRE)
−0.010
−0.005
0.000
0.005
0.010
MR effect size for
'Nuclear migration protein nudC' on 'eGFR'

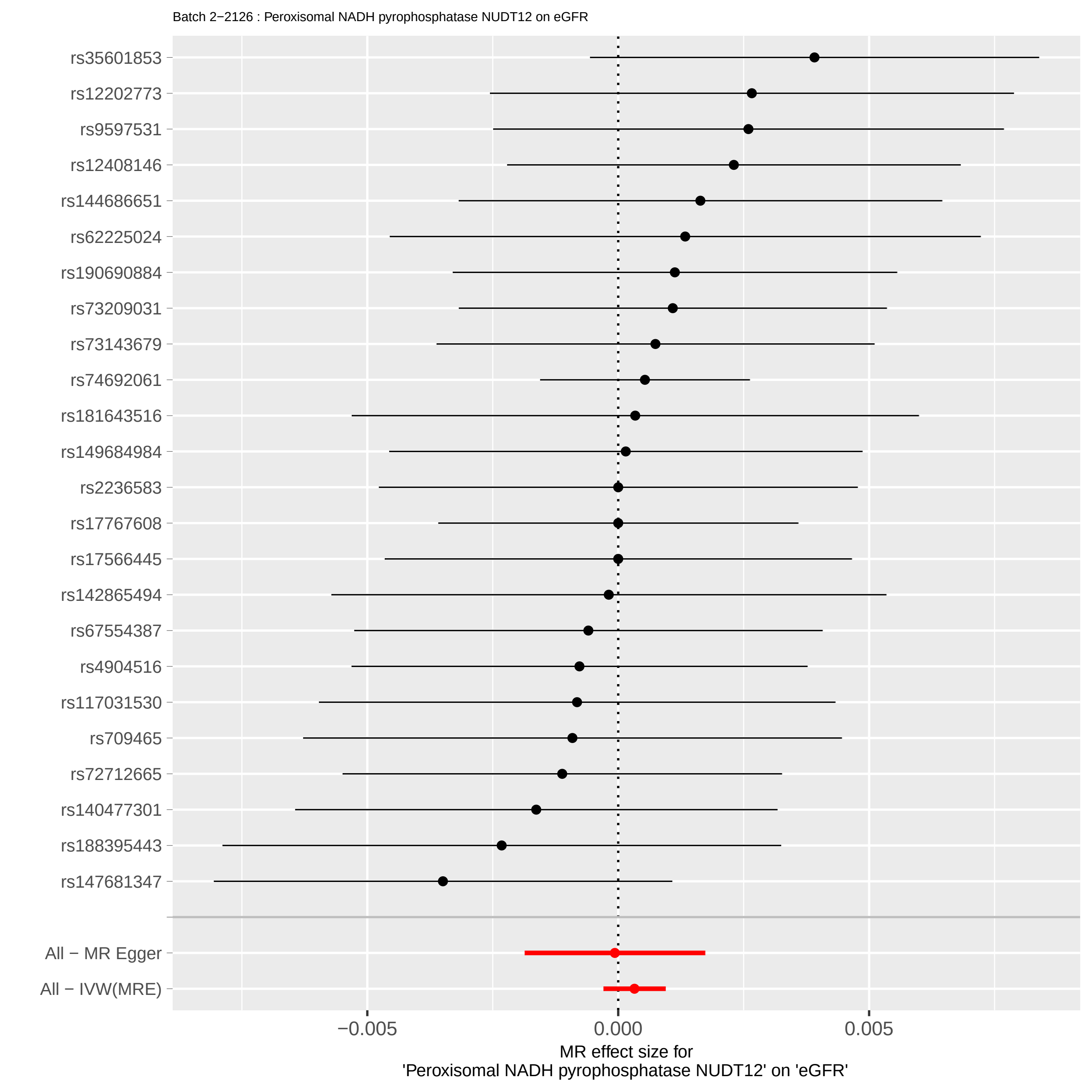

Batch 2−2126 : Peroxisomal NADH pyrophosphatase NUDT12 on eGFR
rs35601853
rs12202773
rs9597531
rs12408146
rs144686651
rs62225024
rs190690884
rs73209031
rs73143679
rs74692061
rs181643516
rs149684984
rs2236583
rs17767608
rs17566445
rs142865494
rs67554387
rs4904516
rs117031530
rs709465
rs72712665
rs140477301
rs188395443
rs147681347
All − MR Egger
All − IVW(MRE)
−0.005
0.000
0.005
MR effect size for
'Peroxisomal NADH pyrophosphatase NUDT12' on 'eGFR'

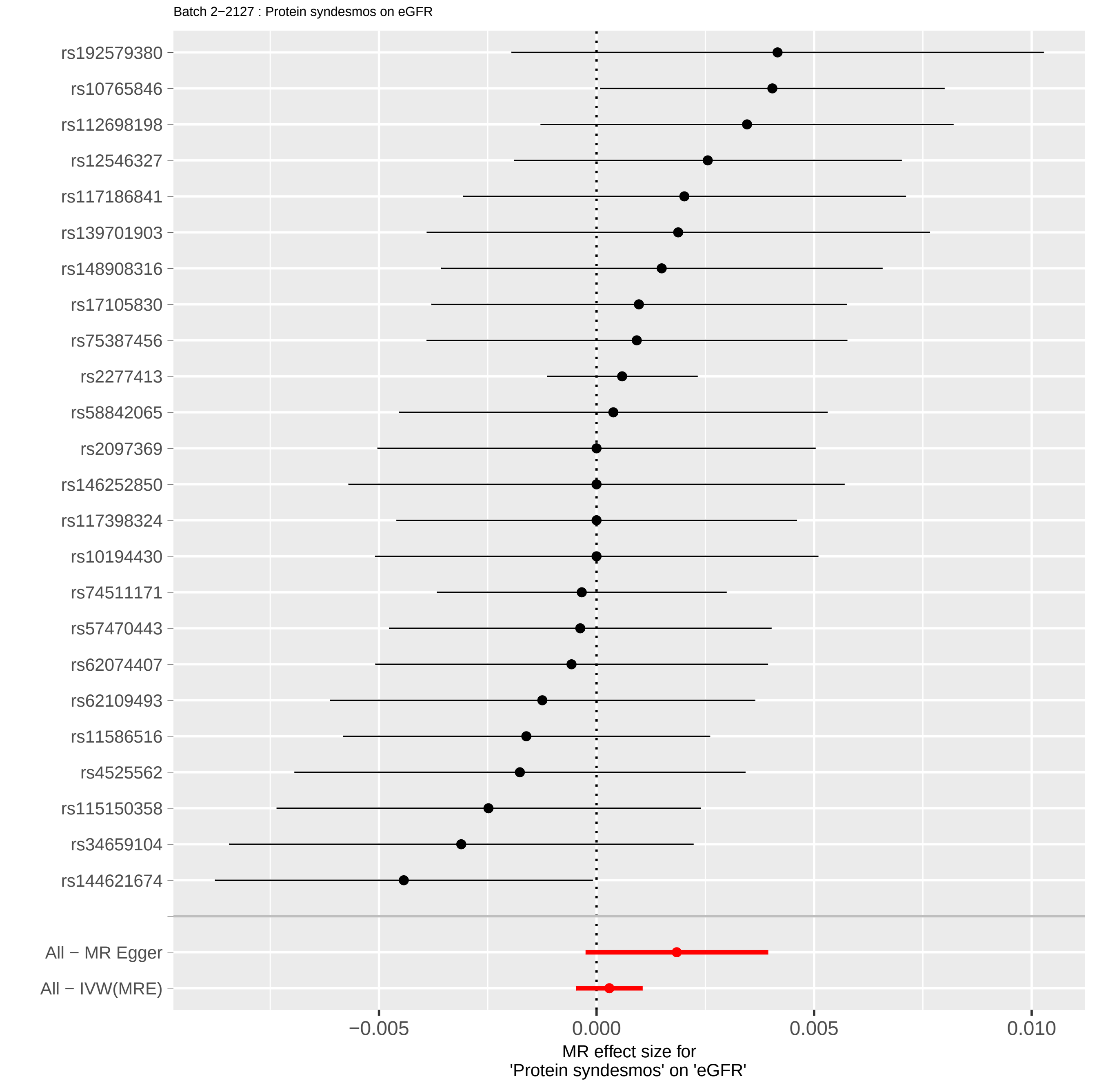

Batch 2−2127 : Protein syndesmos on eGFR
rs192579380
rs10765846
rs112698198
rs12546327
rs117186841
rs139701903
rs148908316
rs17105830
rs75387456
rs2277413
rs58842065
rs2097369
rs146252850
rs117398324
rs10194430
rs74511171
rs57470443
rs62074407
rs62109493
rs11586516
rs4525562
rs115150358
rs34659104
rs144621674
All − MR Egger
All − IVW(MRE)
−0.005
0.000
0.005
0.010
MR effect size for
'Protein syndesmos' on 'eGFR'

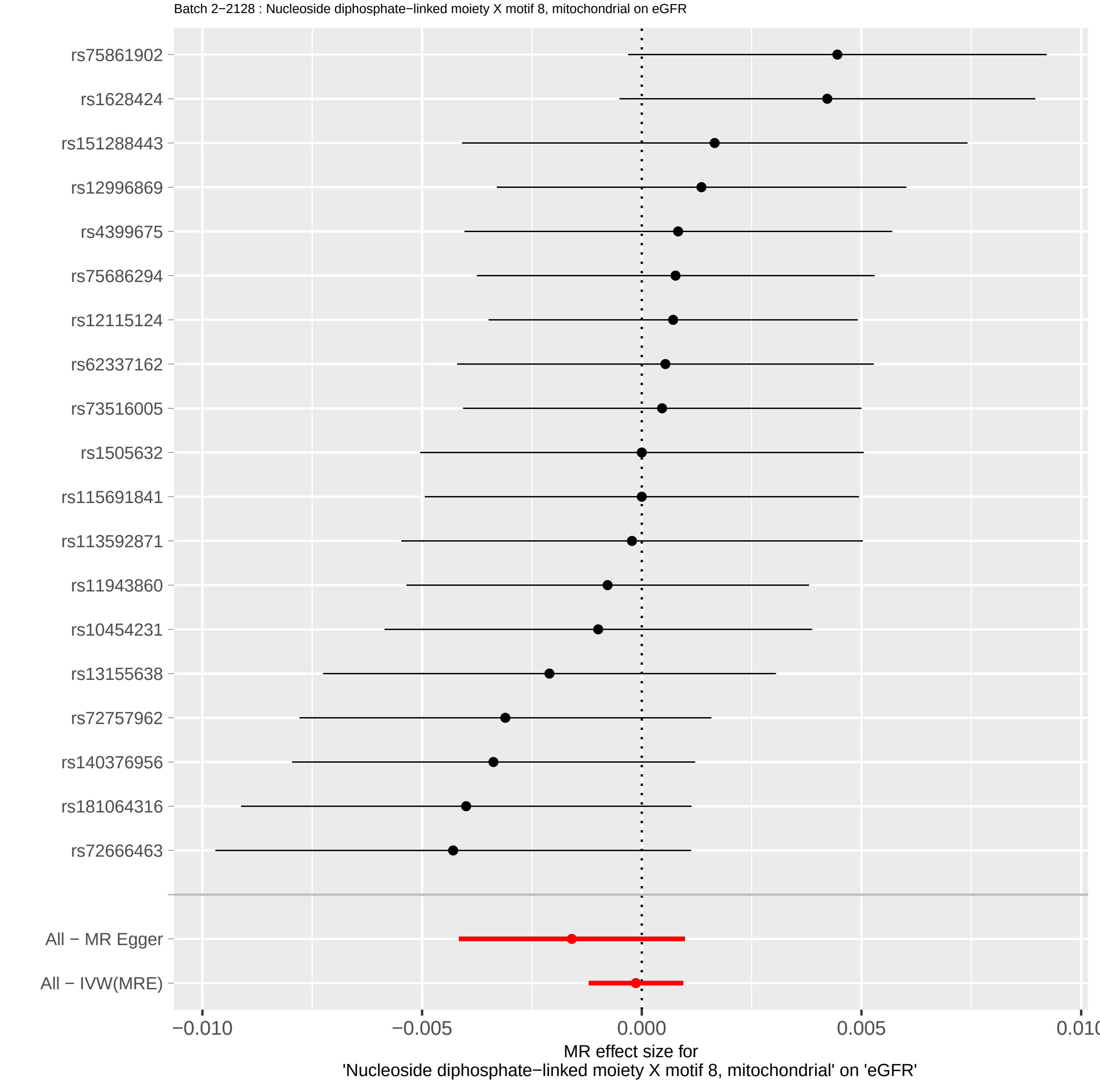
Batch 2−2128 : Nucleoside diphosphate−linked moiety X motif 8, mitochondrial on eGFR
rs75861902
rs1628424
rs151288443
rs12996869
rs4399675
rs75686294
rs12115124
rs62337162
rs73516005
rs1505632
rs115691841
rs113592871
rs11943860
rs10454231
rs13155638
rs72757962
rs140376956
rs181064316
rs72666463
All − MR Egger
All − IVW(MRE)
−0.010
−0.005
0.000
0.005
0.010
MR effect size for
'Nucleoside diphosphate−linked moiety X motif 8, mitochondrial' on 'eGFR'

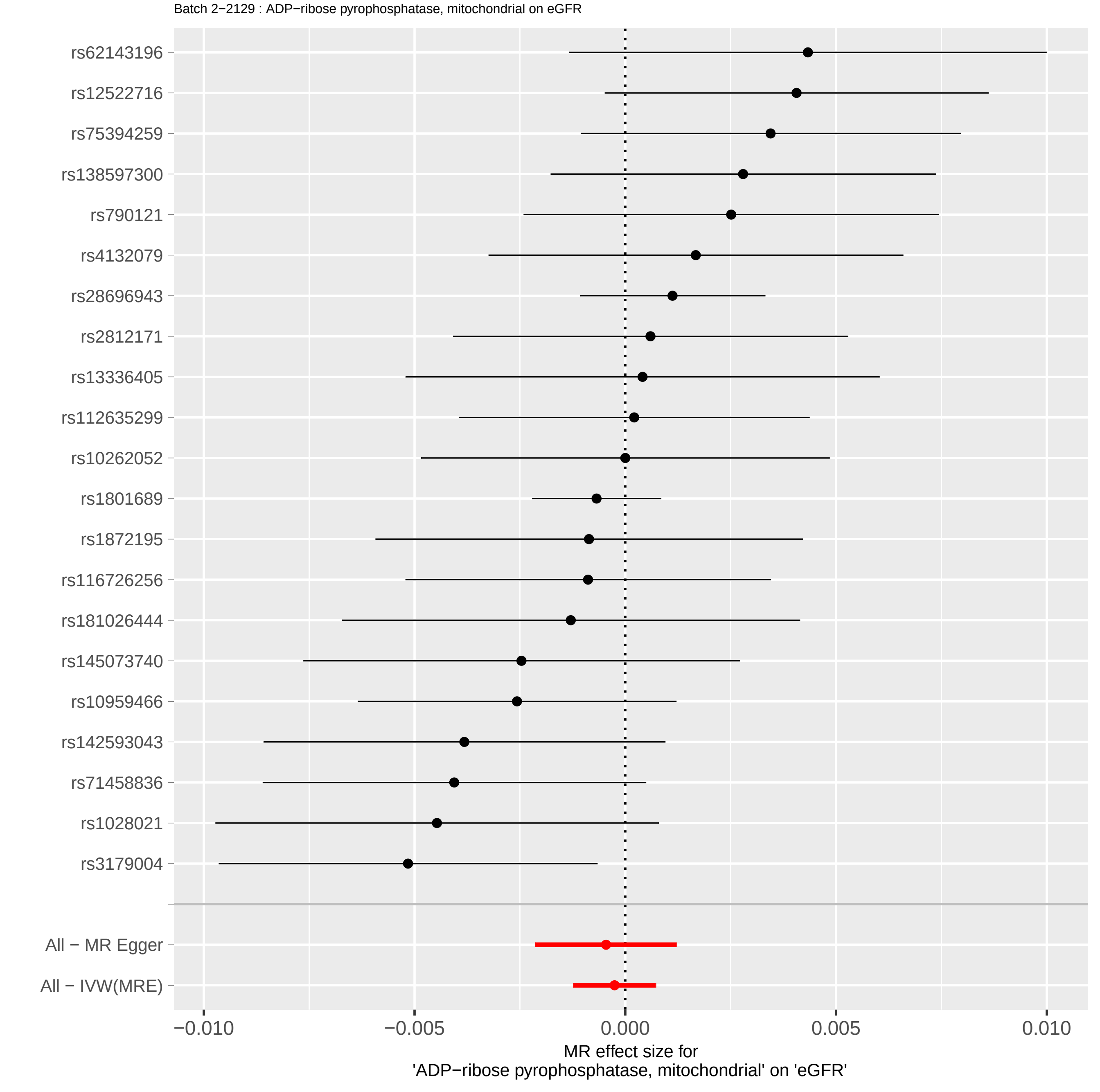

Batch 2−2129 : ADP−ribose pyrophosphatase, mitochondrial on eGFR
rs62143196
rs12522716
rs75394259
rs138597300
rs790121
rs4132079
rs28696943
rs2812171
rs13336405
rs112635299
rs10262052
rs1801689
rs1872195
rs116726256
rs181026444
rs145073740
rs10959466
rs142593043
rs71458836
rs1028021
rs3179004
All − MR Egger
All − IVW(MRE)
−0.010
−0.005
0.000
0.005
0.010
MR effect size for
'ADP−ribose pyrophosphatase, mitochondrial' on 'eGFR'

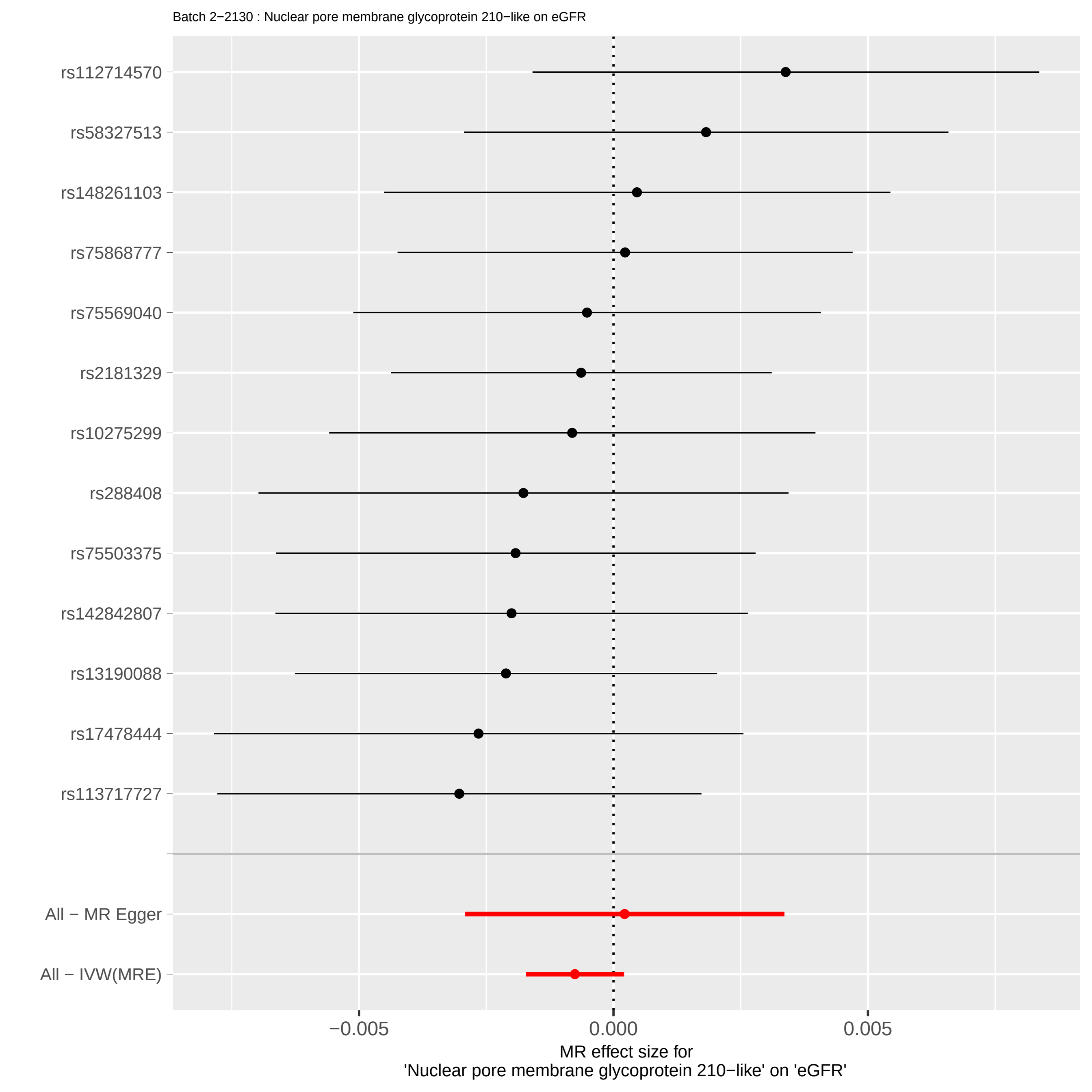

Batch 2−2130 : Nuclear pore membrane glycoprotein 210−like on eGFR
rs112714570
rs58327513
rs148261103
rs75868777
rs75569040
rs2181329
rs10275299
rs288408
rs75503375
rs142842807
rs13190088
rs17478444
rs113717727
All − MR Egger
All − IVW(MRE)
−0.005
0.000
0.005
MR effect size for
'Nuclear pore membrane glycoprotein 210−like' on 'eGFR'

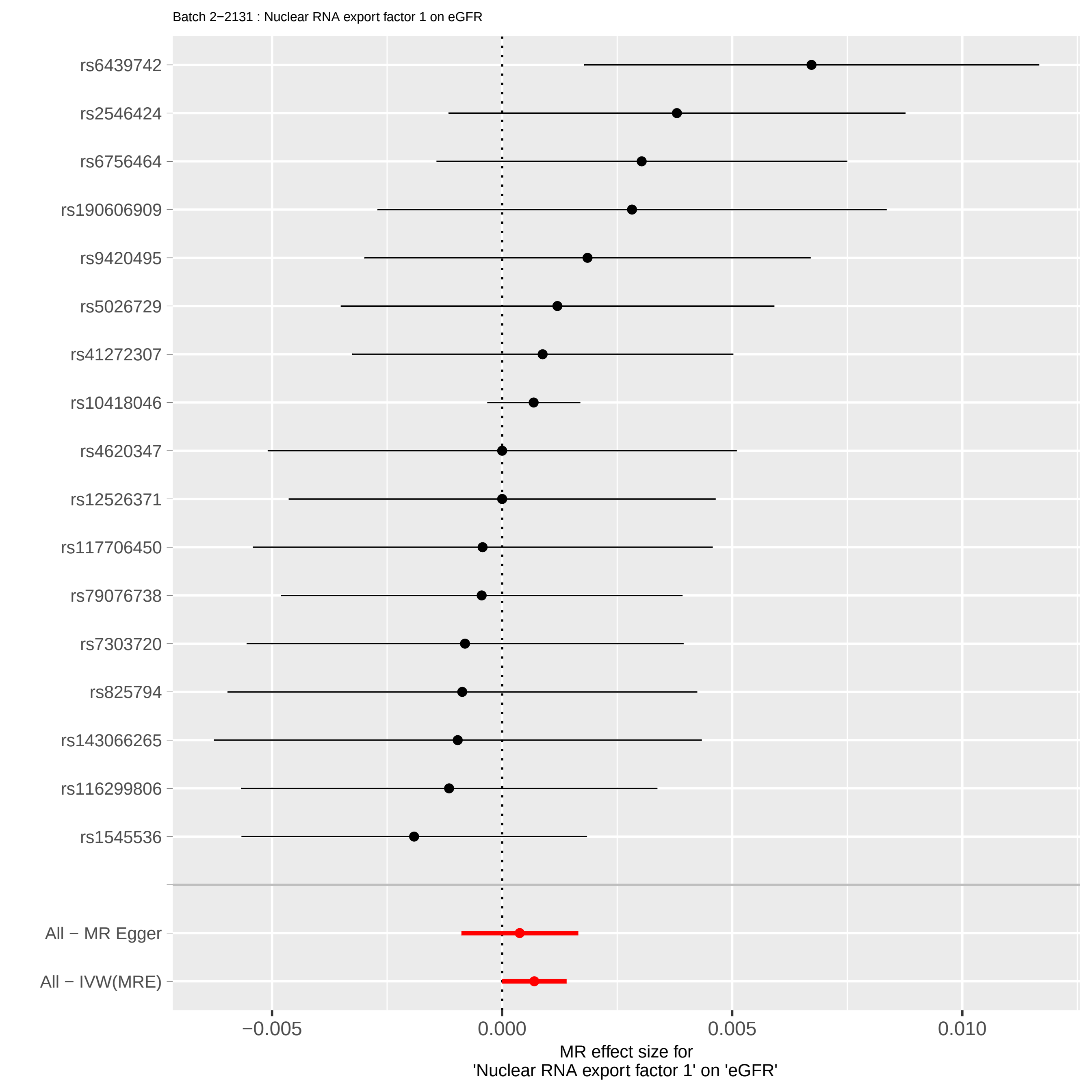

Batch 2−2131 : Nuclear RNA export factor 1 on eGFR
rs6439742
rs2546424
rs6756464
rs190606909
rs9420495
rs5026729
rs41272307
rs10418046
rs4620347
rs12526371
rs117706450
rs79076738
rs7303720
rs825794
rs143066265
rs116299806
rs1545536
All − MR Egger
All − IVW(MRE)
−0.005
0.000
0.005
0.010
MR effect size for
'Nuclear RNA export factor 1' on 'eGFR'

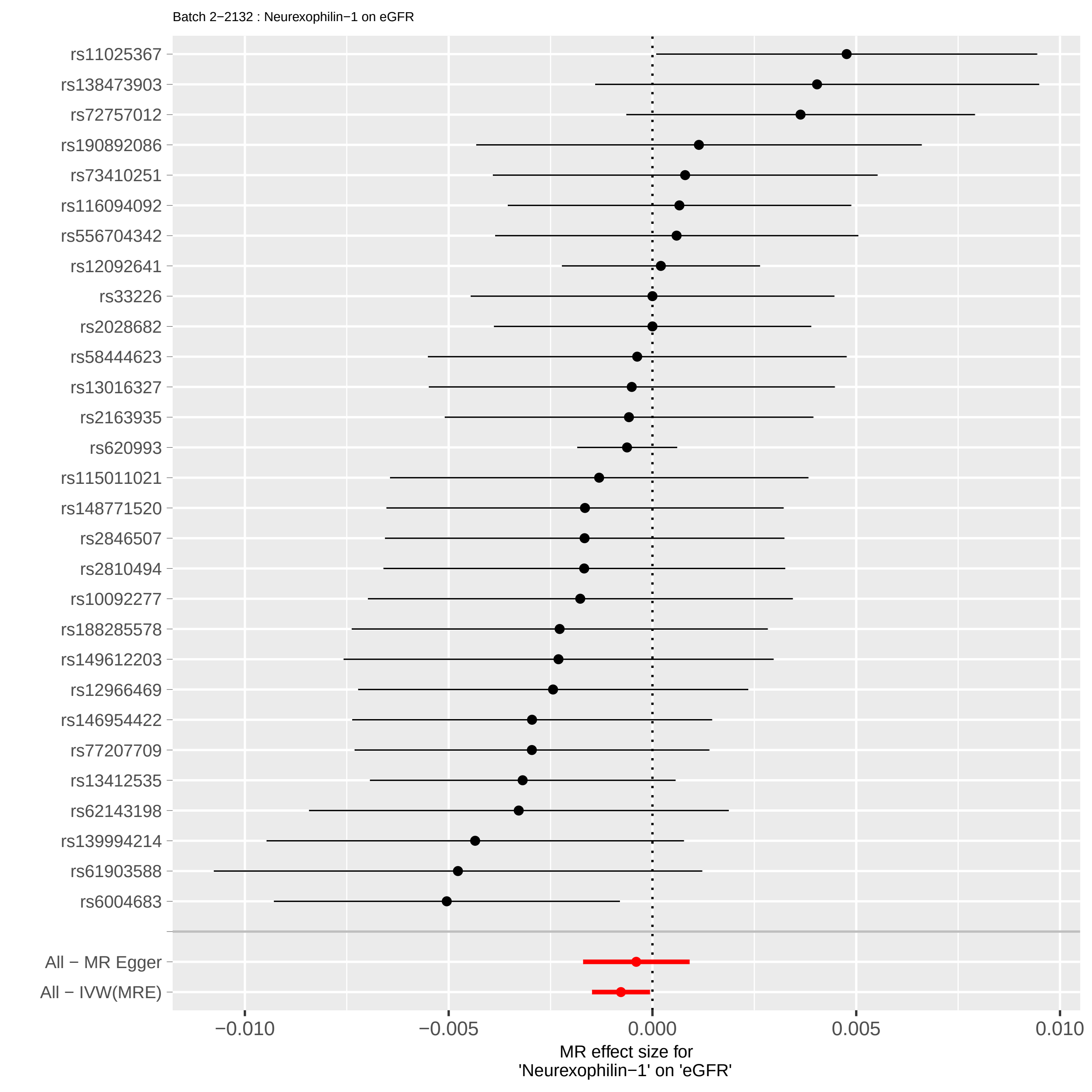

Batch 2−2132 : Neurexophilin−1 on eGFR
rs11025367
rs138473903
rs72757012
rs190892086
rs73410251
rs116094092
rs556704342
rs12092641
rs33226
rs2028682
rs58444623
rs13016327
rs2163935
rs620993
rs115011021
rs148771520
rs2846507
rs2810494
rs10092277
rs188285578
rs149612203
rs12966469
rs146954422
rs77207709
rs13412535
rs62143198
rs139994214
rs61903588
rs6004683
All − MR Egger
All − IVW(MRE)
−0.010
−0.005
0.000
0.005
0.010
MR effect size for
'Neurexophilin−1' on 'eGFR'

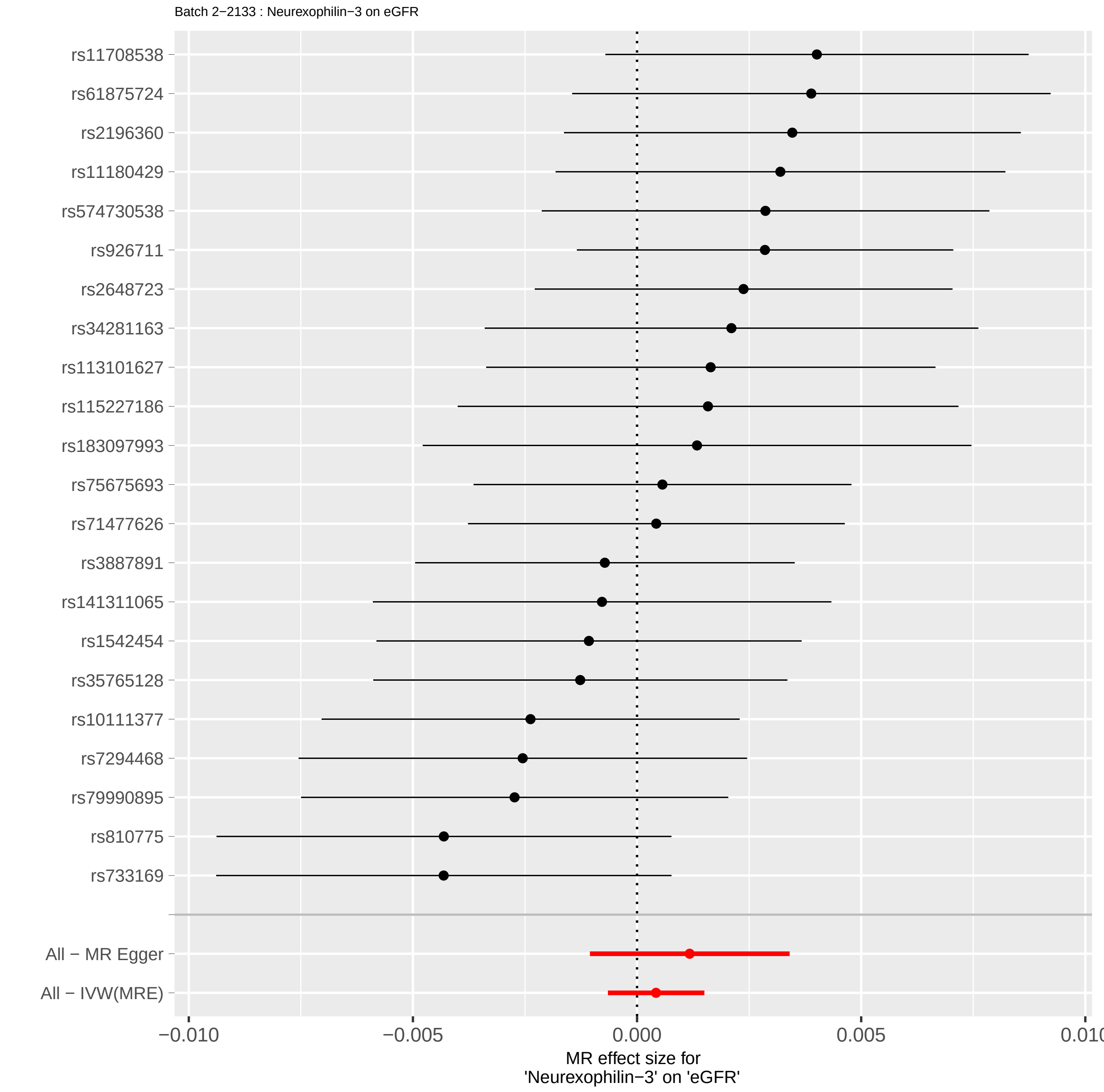

Batch 2−2133 : Neurexophilin−3 on eGFR
rs11708538
rs61875724
rs2196360
rs11180429
rs574730538
rs926711
rs2648723
rs34281163
rs113101627
rs115227186
rs183097993
rs75675693
rs71477626
rs3887891
rs141311065
rs1542454
rs35765128
rs10111377
rs7294468
rs79990895
rs810775
rs733169
All − MR Egger
All − IVW(MRE)
−0.010
−0.005
0.000
0.005
0.010
MR effect size for
'Neurexophilin−3' on 'eGFR'

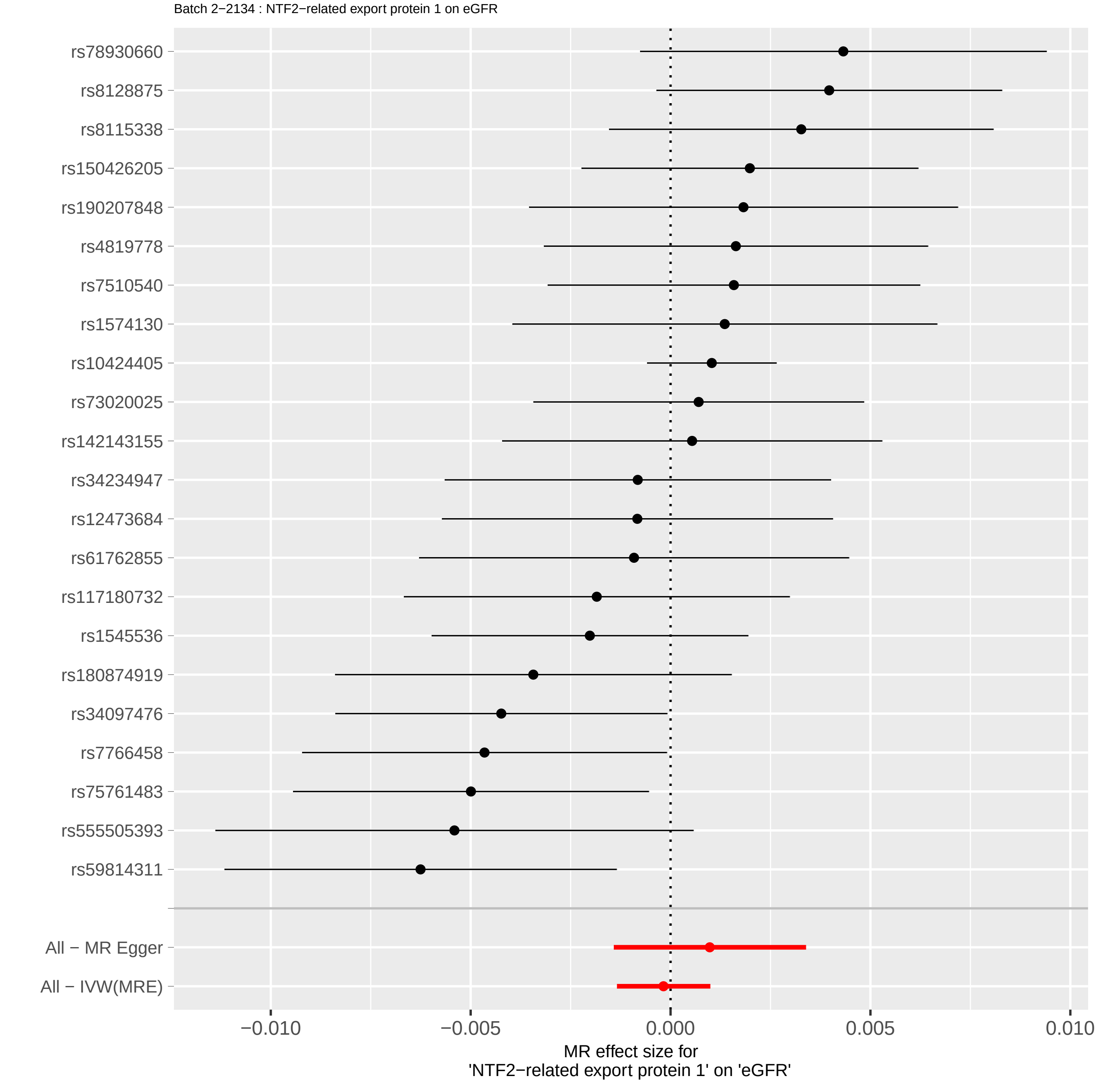

Batch 2−2134 : NTF2−related export protein 1 on eGFR
rs78930660
rs8128875
rs8115338
rs150426205
rs190207848
rs4819778
rs7510540
rs1574130
rs10424405
rs73020025
rs142143155
rs34234947
rs12473684
rs61762855
rs117180732
rs1545536
rs180874919
rs34097476
rs7766458
rs75761483
rs555505393
rs59814311
All − MR Egger
All − IVW(MRE)
−0.010
−0.005
0.000
0.005
0.010
MR effect size for
'NTF2−related export protein 1' on 'eGFR'

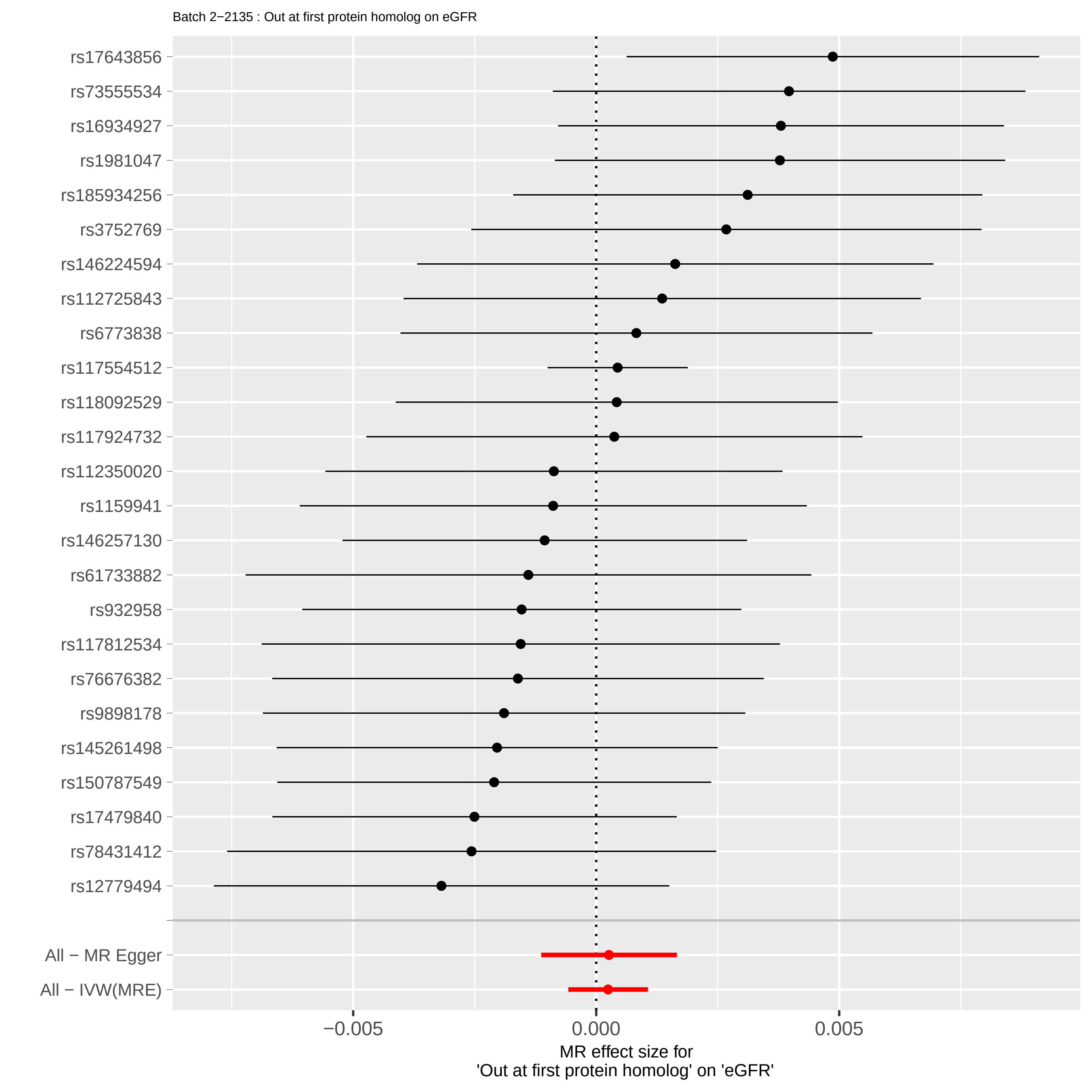

Batch 2−2135 : Out at first protein homolog on eGFR
rs17643856
rs73555534
rs16934927
rs1981047
rs185934256
rs3752769
rs146224594
rs112725843
rs6773838
rs117554512
rs118092529
rs117924732
rs112350020
rs1159941
rs146257130
rs61733882
rs932958
rs117812534
rs76676382
rs9898178
rs145261498
rs150787549
rs17479840
rs78431412
rs12779494
All − MR Egger
All − IVW(MRE)
−0.005
0.000
0.005
MR effect size for
'Out at first protein homolog' on 'eGFR'

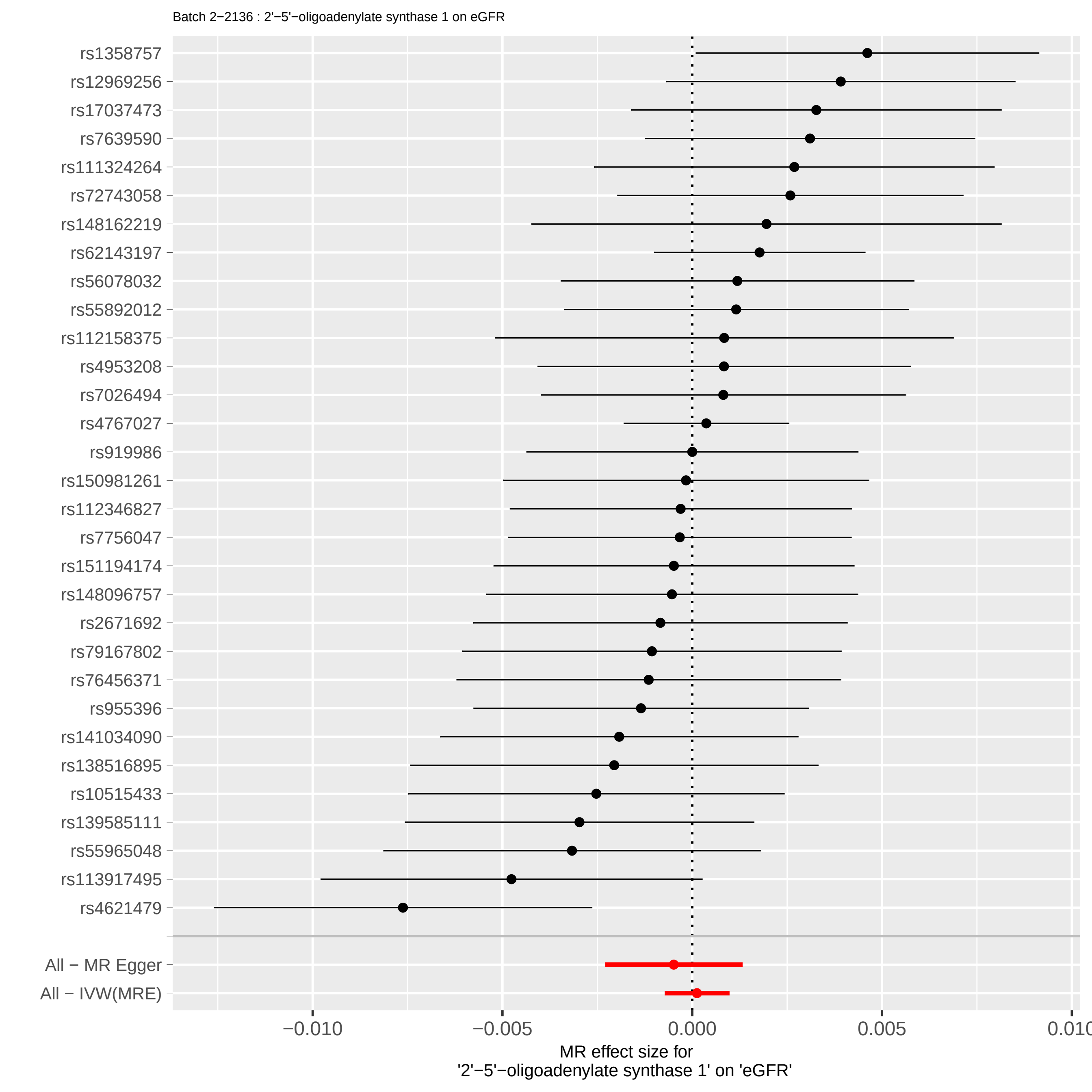

Batch 2−2136 : 2'−5'−oligoadenylate synthase 1 on eGFR
rs1358757
rs12969256
rs17037473
rs7639590
rs111324264
rs72743058
rs148162219
rs62143197
rs56078032
rs55892012
rs112158375
rs4953208
rs7026494
rs4767027
rs919986
rs150981261
rs112346827
rs7756047
rs151194174
rs148096757
rs2671692
rs79167802
rs76456371
rs955396
rs141034090
rs138516895
rs10515433
rs139585111
rs55965048
rs113917495
rs4621479
All − MR Egger
All − IVW(MRE)
−0.010
−0.005
0.000
0.005
0.010
MR effect size for
'2'−5'−oligoadenylate synthase 1' on 'eGFR'

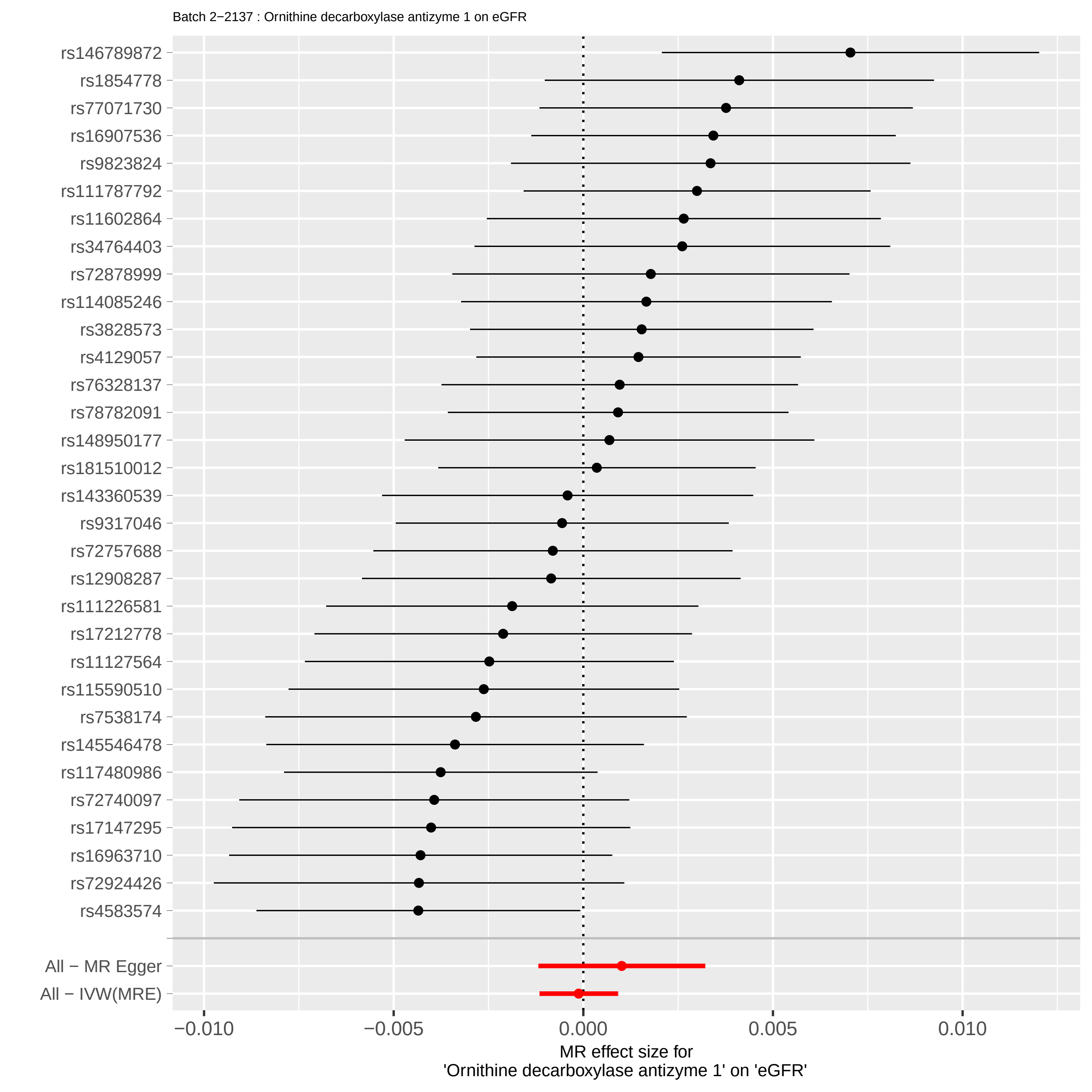

Batch 2−2137 : Ornithine decarboxylase antizyme 1 on eGFR
rs146789872
rs1854778
rs77071730
rs16907536
rs9823824
rs111787792
rs11602864
rs34764403
rs72878999
rs114085246
rs3828573
rs4129057
rs76328137
rs78782091
rs148950177
rs181510012
rs143360539
rs9317046
rs72757688
rs12908287
rs111226581
rs17212778
rs11127564
rs115590510
rs7538174
rs145546478
rs117480986
rs72740097
rs17147295
rs16963710
rs72924426
rs4583574
All − MR Egger
All − IVW(MRE)
−0.010
−0.005
0.000
0.005
0.010
MR effect size for
'Ornithine decarboxylase antizyme 1' on 'eGFR'

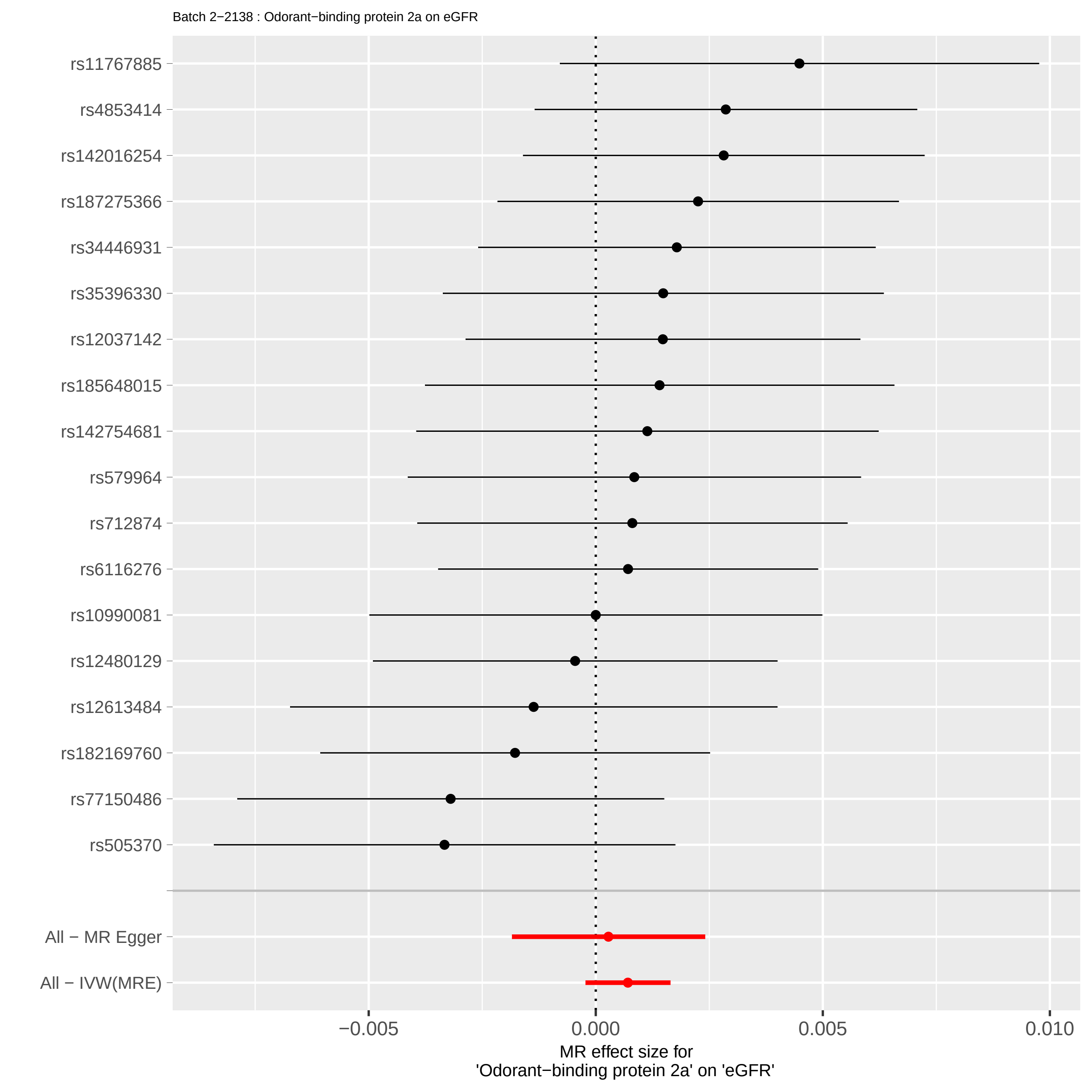
Batch 2−2138 : Odorant−binding protein 2a on eGFR
rs11767885
rs4853414
rs142016254
rs187275366
rs34446931
rs35396330
rs12037142
rs185648015
rs142754681
rs579964
rs712874
rs6116276
rs10990081
rs12480129
rs12613484
rs182169760
rs77150486
rs505370
All − MR Egger
All − IVW(MRE)
−0.005
0.000
0.005
0.010
MR effect size for
'Odorant−binding protein 2a' on 'eGFR'

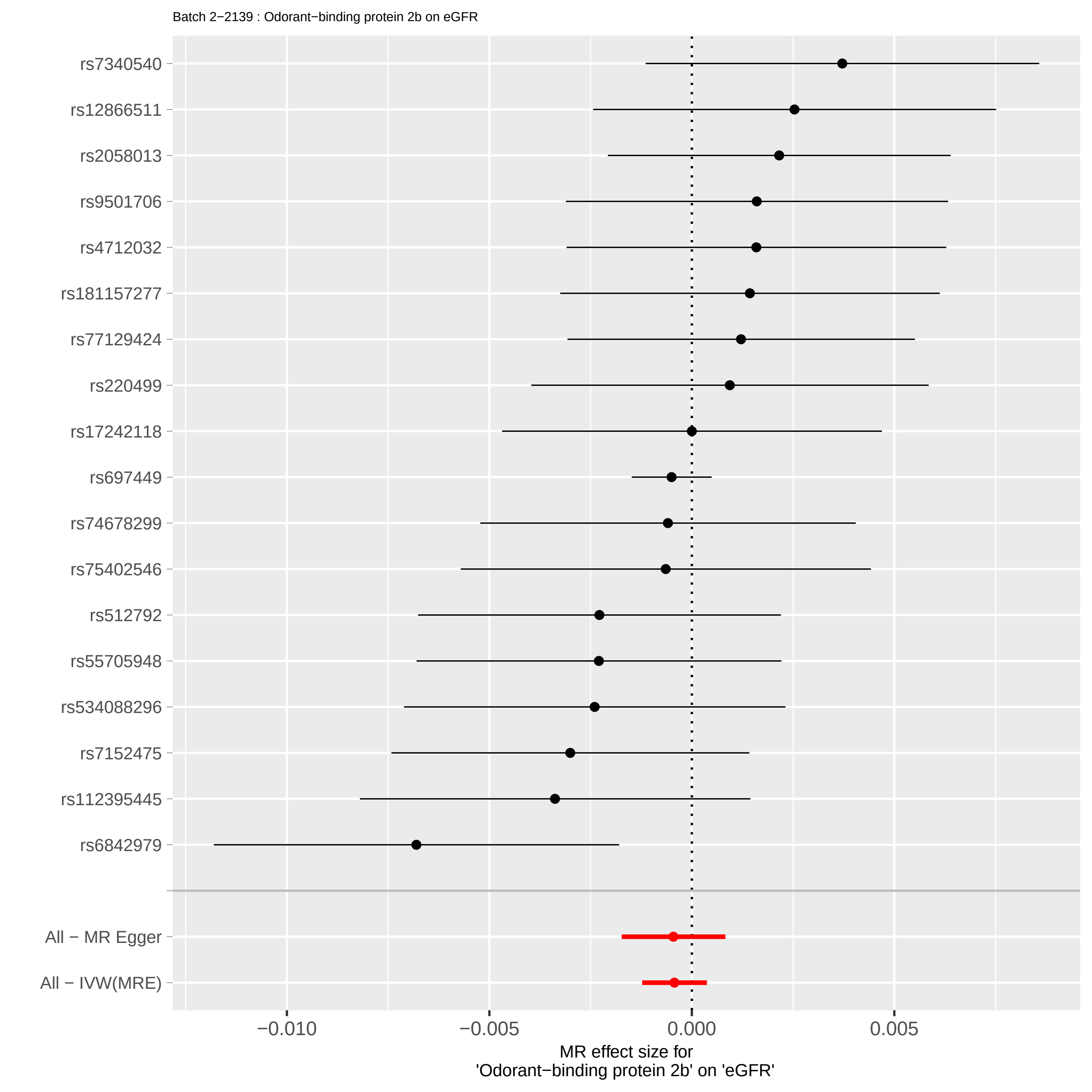

Batch 2−2139 : Odorant−binding protein 2b on eGFR
rs7340540
rs12866511
rs2058013
rs9501706
rs4712032
rs181157277
rs77129424
rs220499
rs17242118
rs697449
rs74678299
rs75402546
rs512792
rs55705948
rs534088296
rs7152475
rs112395445
rs6842979
All − MR Egger
All − IVW(MRE)
−0.010
−0.005
0.000
0.005
MR effect size for
'Odorant−binding protein 2b' on 'eGFR'

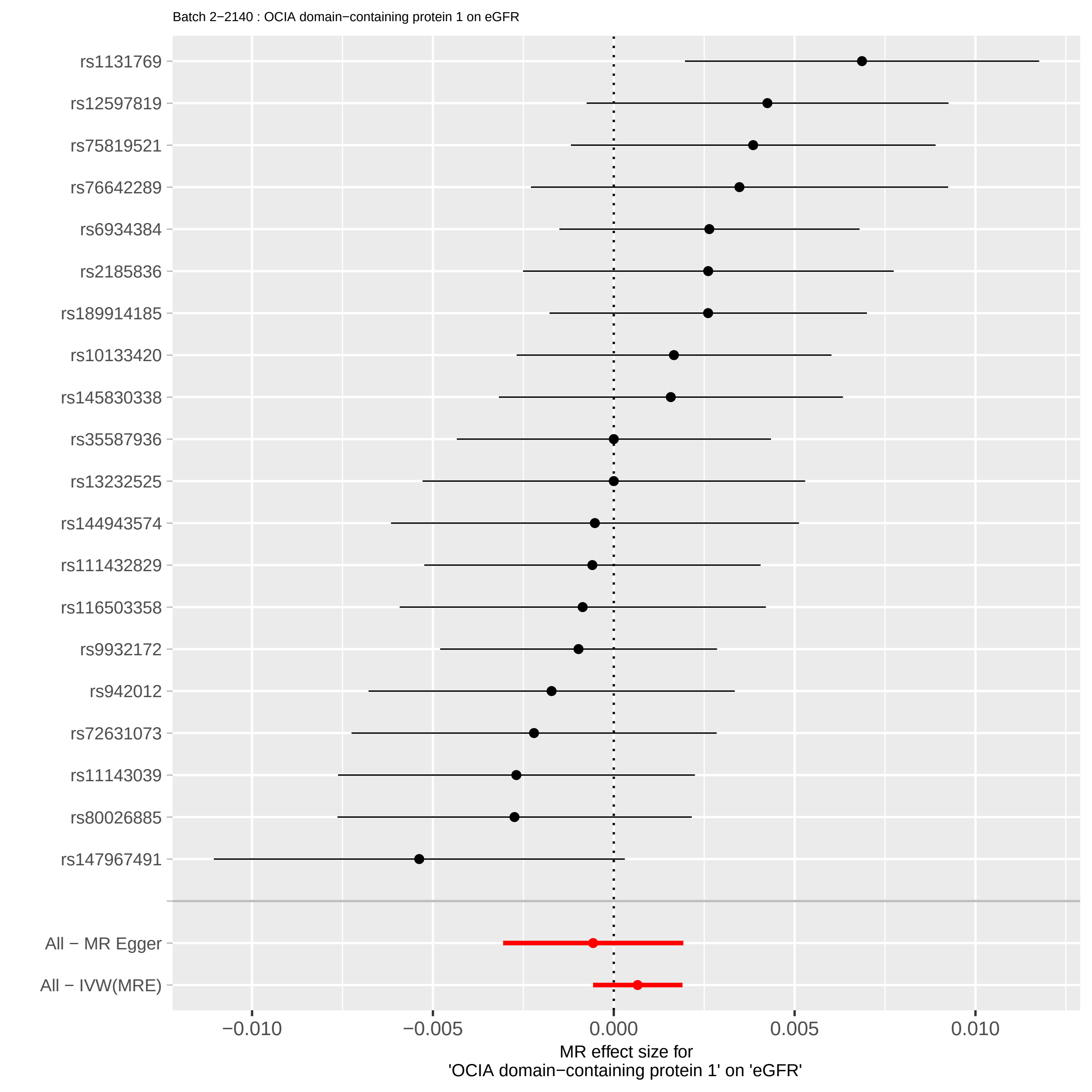
Batch 2−2140 : OCIA domain−containing protein 1 on eGFR
rs1131769
rs12597819
rs75819521
rs76642289
rs6934384
rs2185836
rs189914185
rs10133420
rs145830338
rs35587936
rs13232525
rs144943574
rs111432829
rs116503358
rs9932172
rs942012
rs72631073
rs11143039
rs80026885
rs147967491
All − MR Egger
All − IVW(MRE)
−0.010
−0.005
0.000
0.005
0.010
MR effect size for
'OCIA domain−containing protein 1' on 'eGFR'

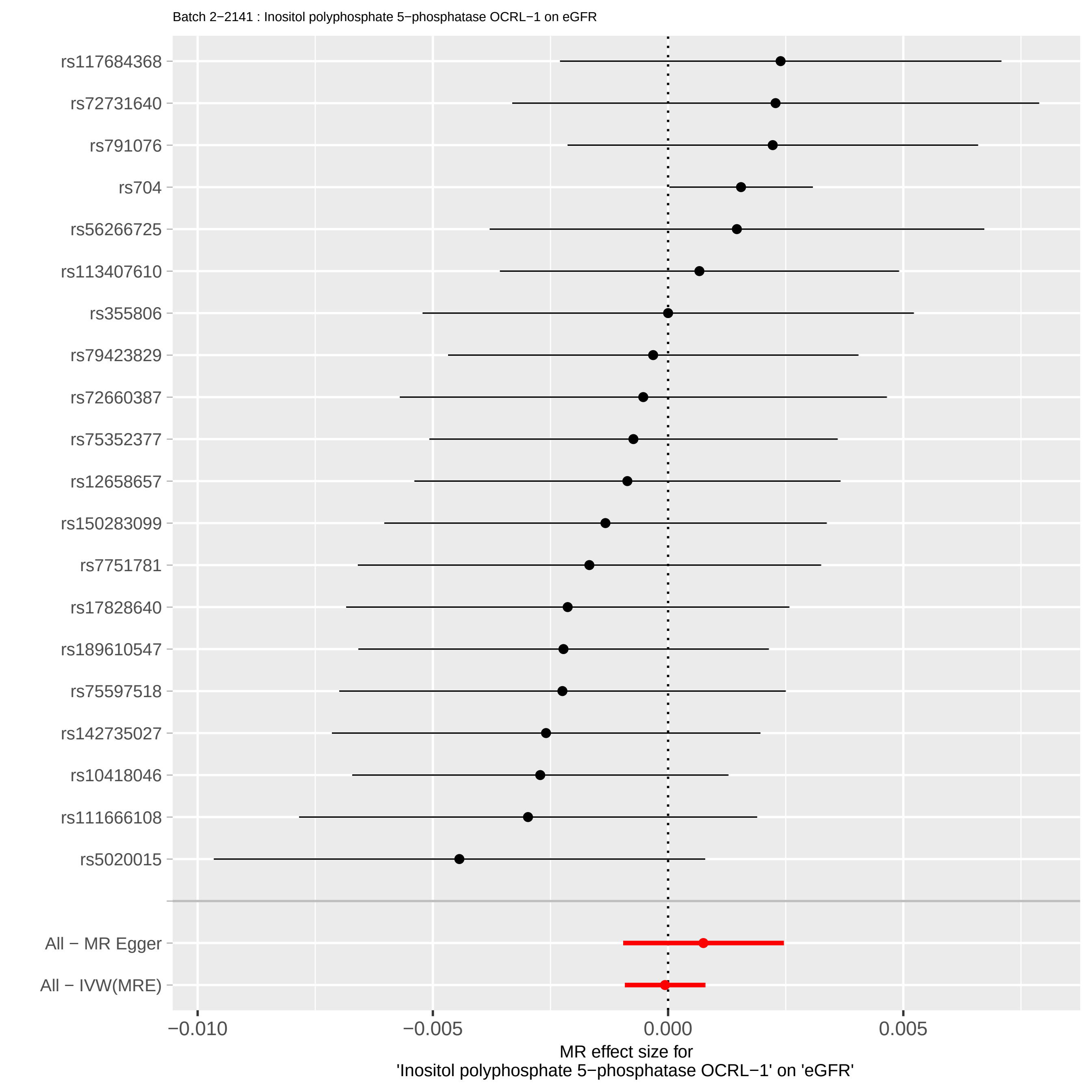

Batch 2−2141 : Inositol polyphosphate 5−phosphatase OCRL−1 on eGFR
rs117684368
rs72731640
rs791076
rs704
rs56266725
rs113407610
rs355806
rs79423829
rs72660387
rs75352377
rs12658657
rs150283099
rs7751781
rs17828640
rs189610547
rs75597518
rs142735027
rs10418046
rs111666108
rs5020015
All − MR Egger
All − IVW(MRE)
−0.010
−0.005
0.000
0.005
MR effect size for
'Inositol polyphosphate 5−phosphatase OCRL−1' on 'eGFR'

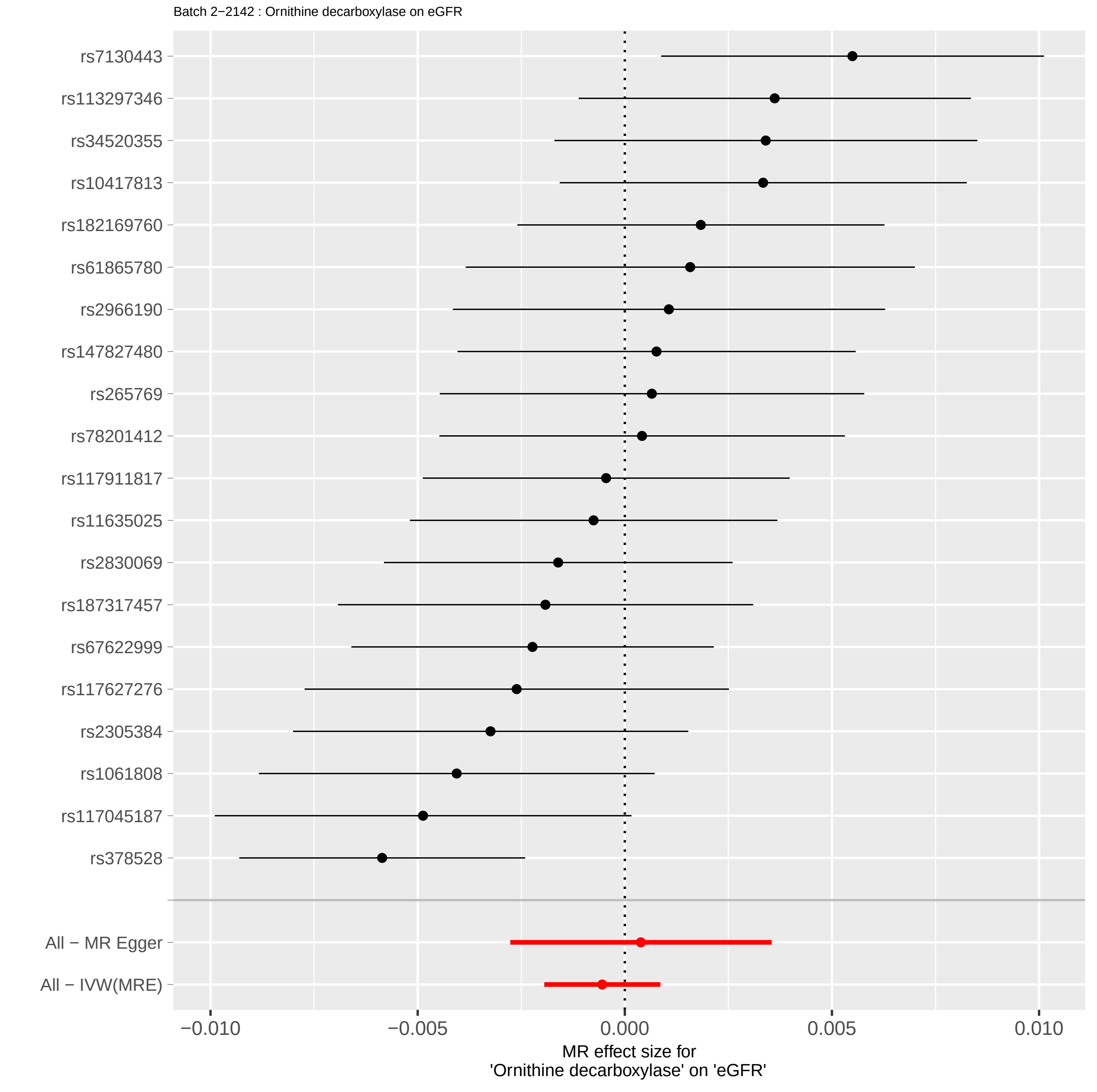

Batch 2−2142 : Ornithine decarboxylase on eGFR
rs7130443
rs113297346
rs34520355
rs10417813
rs182169760
rs61865780
rs2966190
rs147827480
rs265769
rs78201412
rs117911817
rs11635025
rs2830069
rs187317457
rs67622999
rs117627276
rs2305384
rs1061808
rs117045187
rs378528
All − MR Egger
All − IVW(MRE)
−0.010
−0.005
0.000
0.005
0.010
MR effect size for
'Ornithine decarboxylase' on 'eGFR'

Batch 2−2143 : Obg−like ATPase 1 on eGFR

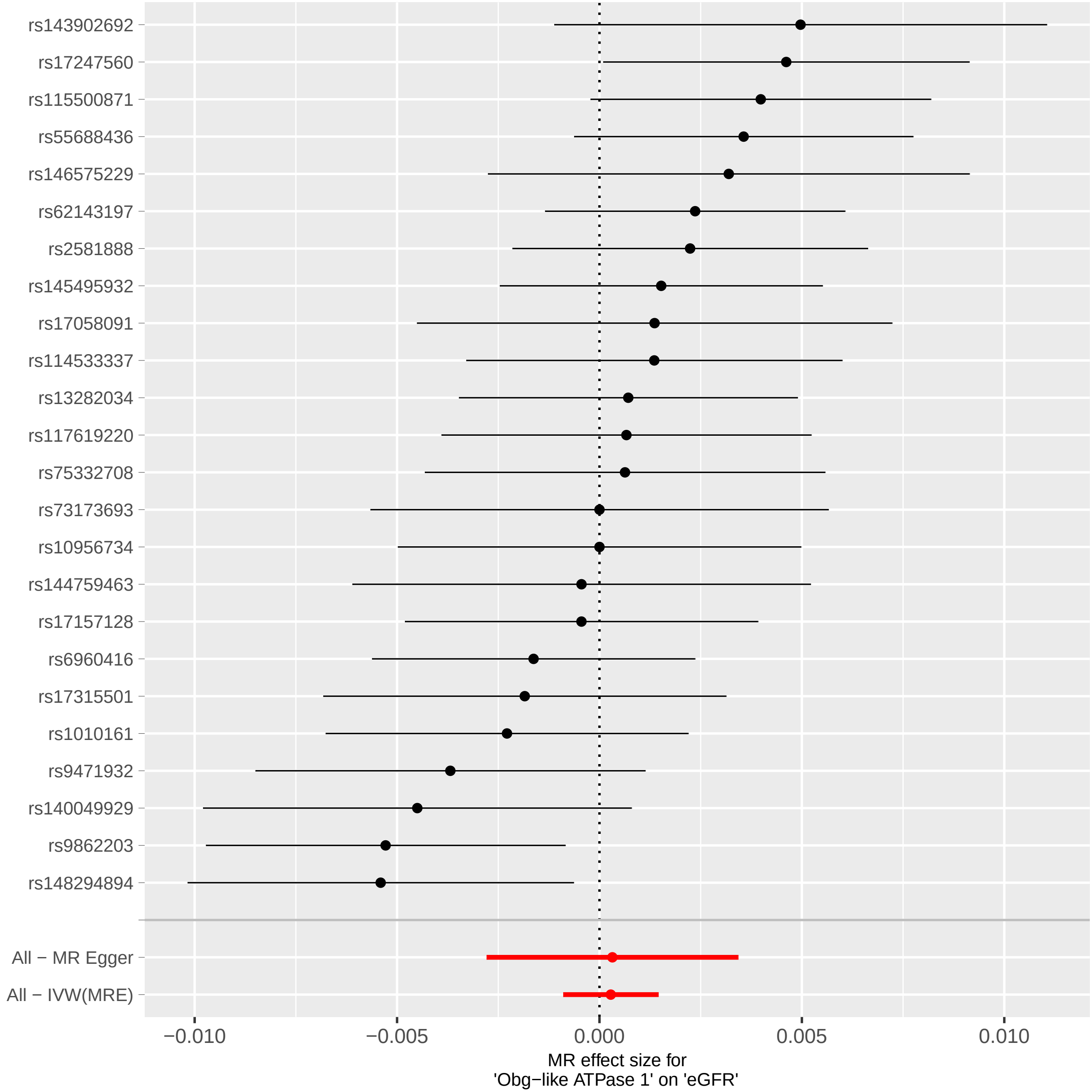

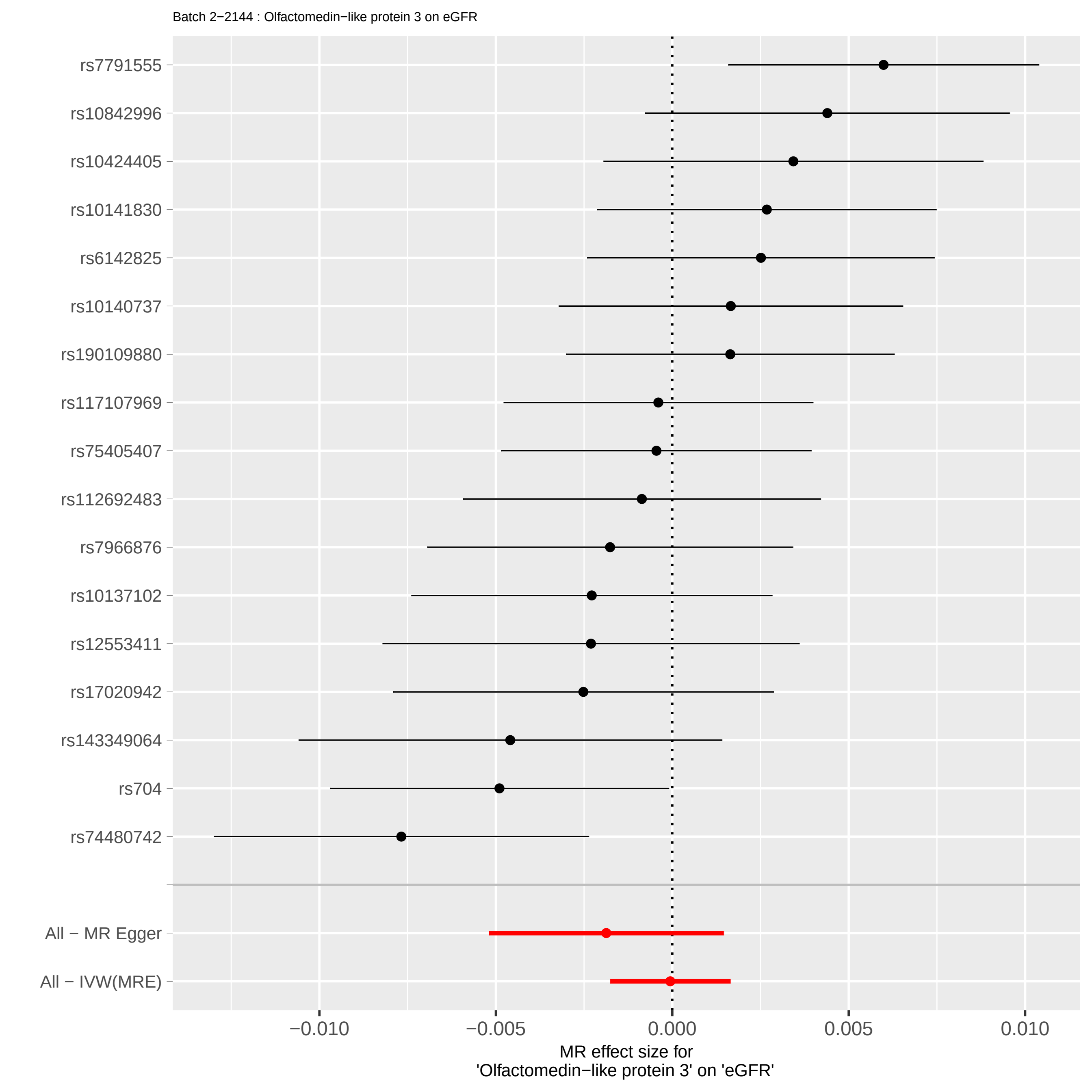

Batch 2−2144 : Olfactomedin−like protein 3 on eGFR
rs7791555
rs10842996
rs10424405
rs10141830
rs6142825
rs10140737
rs190109880
rs117107969
rs75405407
rs112692483
rs7966876
rs10137102
rs12553411
rs17020942
rs143349064
rs704
rs74480742
All − MR Egger
All − IVW(MRE)
−0.010
−0.005
0.000
0.005
0.010
MR effect size for
'Olfactomedin−like protein 3' on 'eGFR'

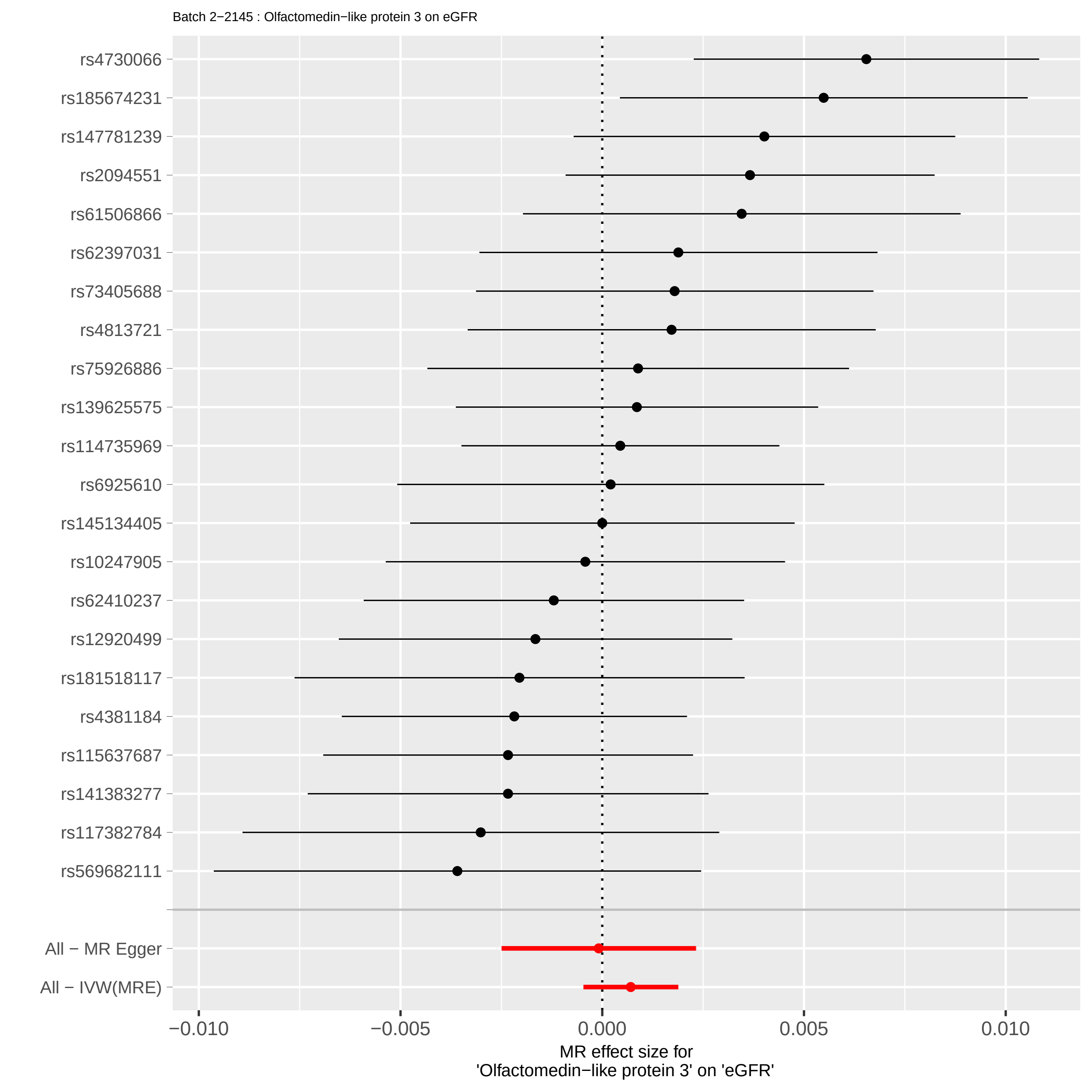

Batch 2−2145 : Olfactomedin−like protein 3 on eGFR
rs4730066
rs185674231
rs147781239
rs2094551
rs61506866
rs62397031
rs73405688
rs4813721
rs75926886
rs139625575
rs114735969
rs6925610
rs145134405
rs10247905
rs62410237
rs12920499
rs181518117
rs4381184
rs115637687
rs141383277
rs117382784
rs569682111
All − MR Egger
All − IVW(MRE)
−0.010
−0.005
0.000
0.005
0.010
MR effect size for
'Olfactomedin−like protein 3' on 'eGFR'

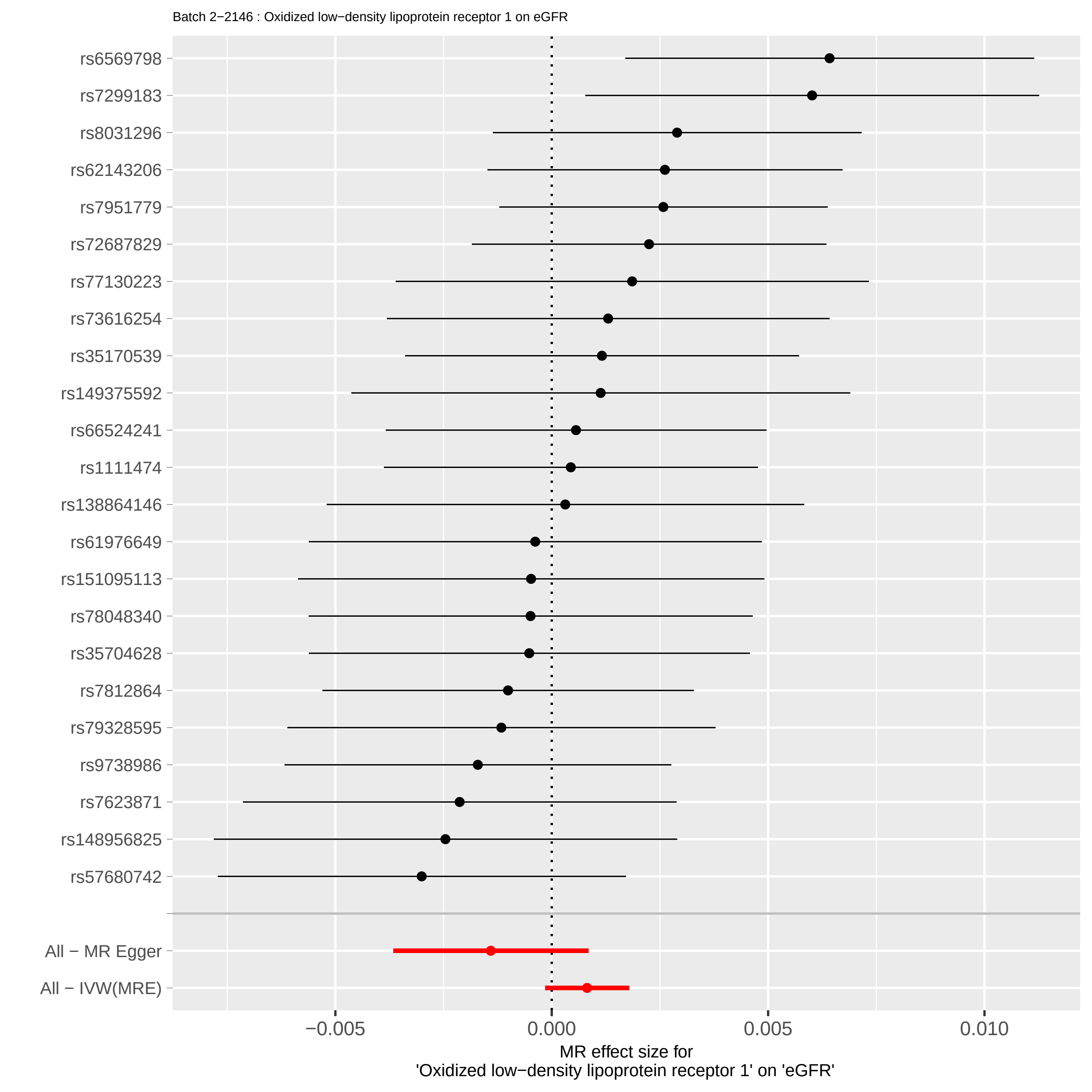

Batch 2−2146 : Oxidized low−density lipoprotein receptor 1 on eGFR
rs6569798
rs7299183
rs8031296
rs62143206
rs7951779
rs72687829
rs77130223
rs73616254
rs35170539
rs149375592
rs66524241
rs1111474
rs138864146
rs61976649
rs151095113
rs78048340
rs35704628
rs7812864
rs79328595
rs9738986
rs7623871
rs148956825
rs57680742
All − MR Egger
All − IVW(MRE)
−0.005
0.000
0.005
0.010
MR effect size for
'Oxidized low−density lipoprotein receptor 1' on 'eGFR'

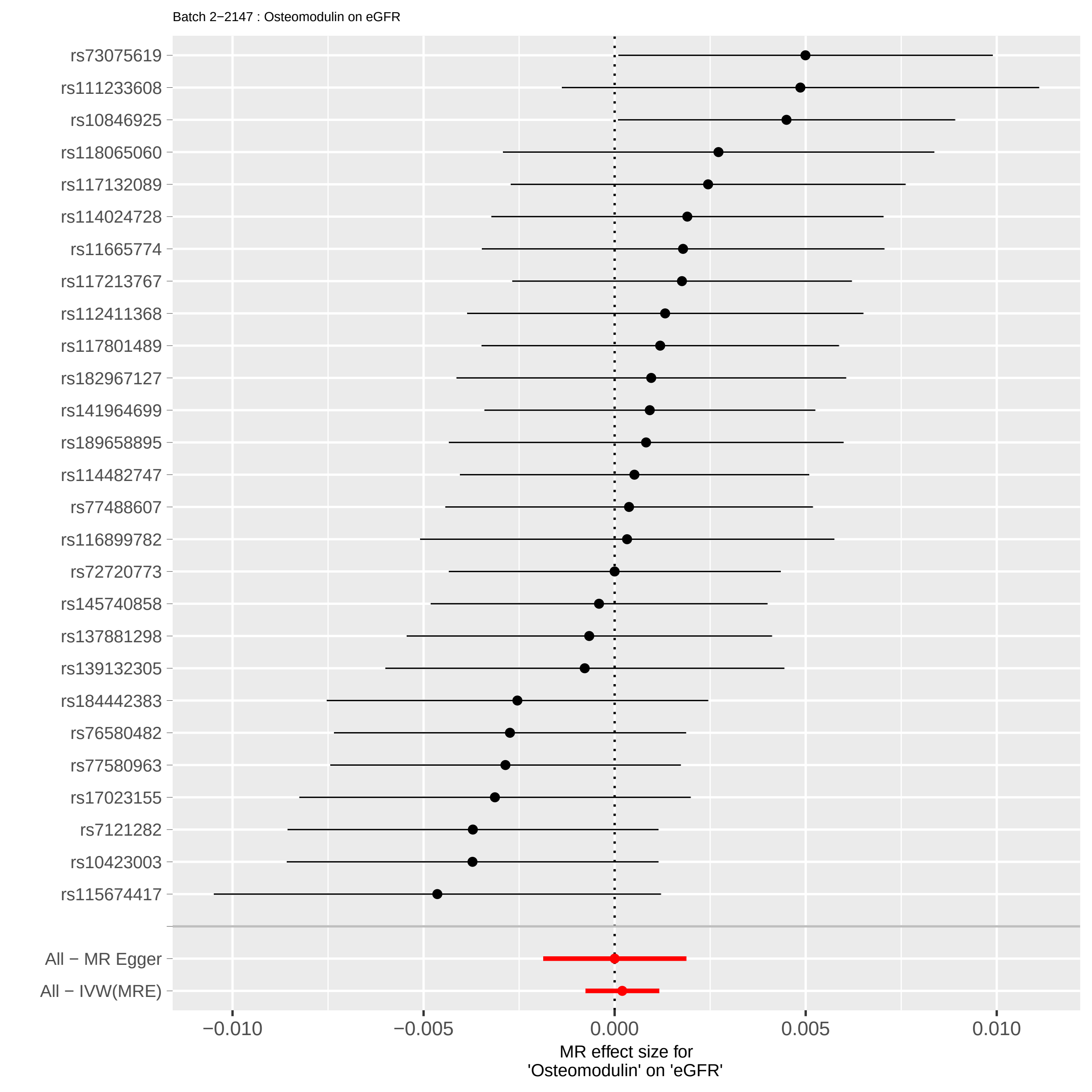
Batch 2−2147 : Osteomodulin on eGFR
rs73075619
rs111233608
rs10846925
rs118065060
rs117132089
rs114024728
rs11665774
rs117213767
rs112411368
rs117801489
rs182967127
rs141964699
rs189658895
rs114482747
rs77488607
rs116899782
rs72720773
rs145740858
rs137881298
rs139132305
rs184442383
rs76580482
rs77580963
rs17023155
rs7121282
rs10423003
rs115674417
All − MR Egger
All − IVW(MRE)
−0.010
−0.005
0.000
0.005
0.010
MR effect size for
'Osteomodulin' on 'eGFR'

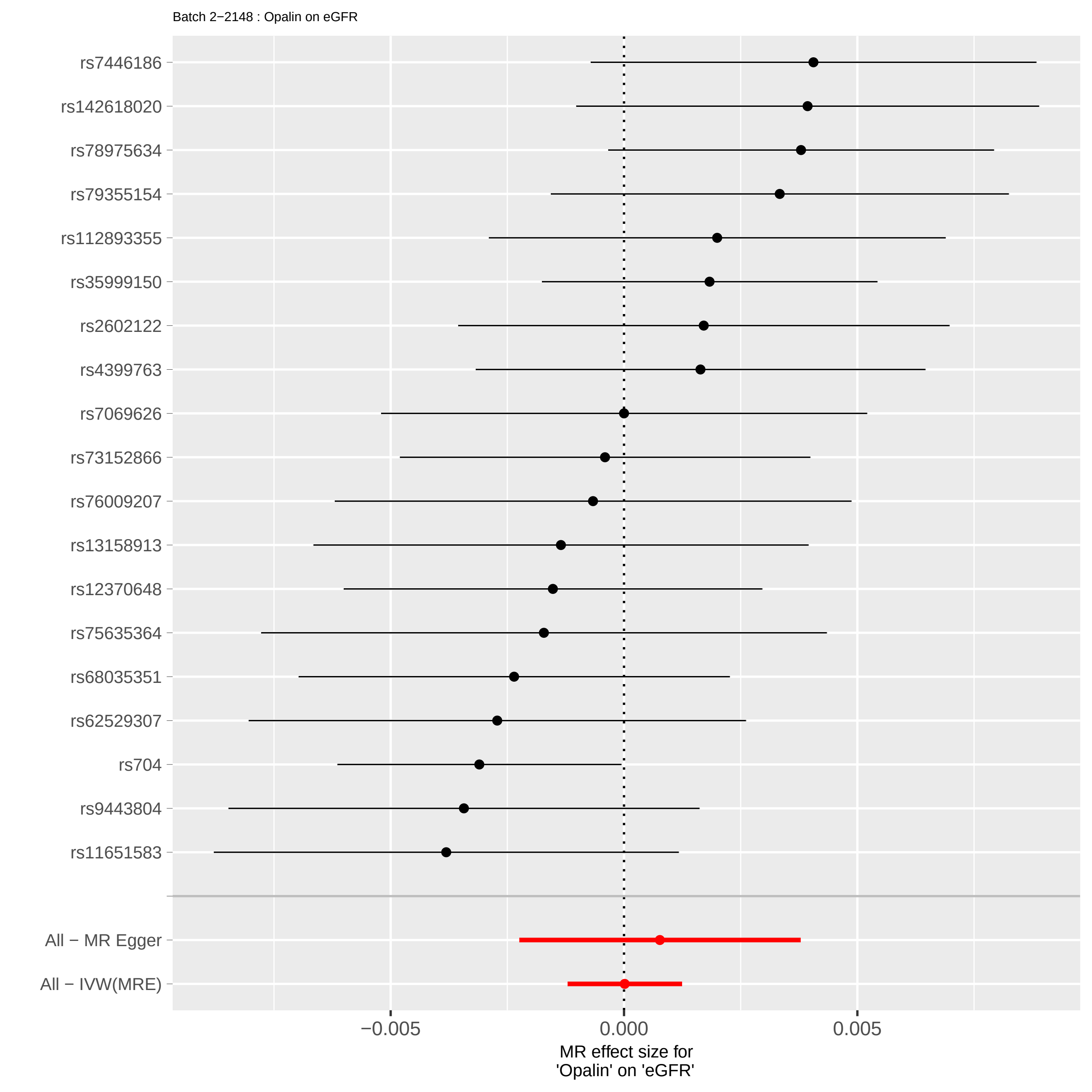

Batch 2−2148 : Opalin on eGFR
rs7446186
rs142618020
rs78975634
rs79355154
rs112893355
rs35999150
rs2602122
rs4399763
rs7069626
rs73152866
rs76009207
rs13158913
rs12370648
rs75635364
rs68035351
rs62529307
rs704
rs9443804
rs11651583
All − MR Egger
All − IVW(MRE)
−0.005
0.000
0.005
MR effect size for
'Opalin' on 'eGFR'

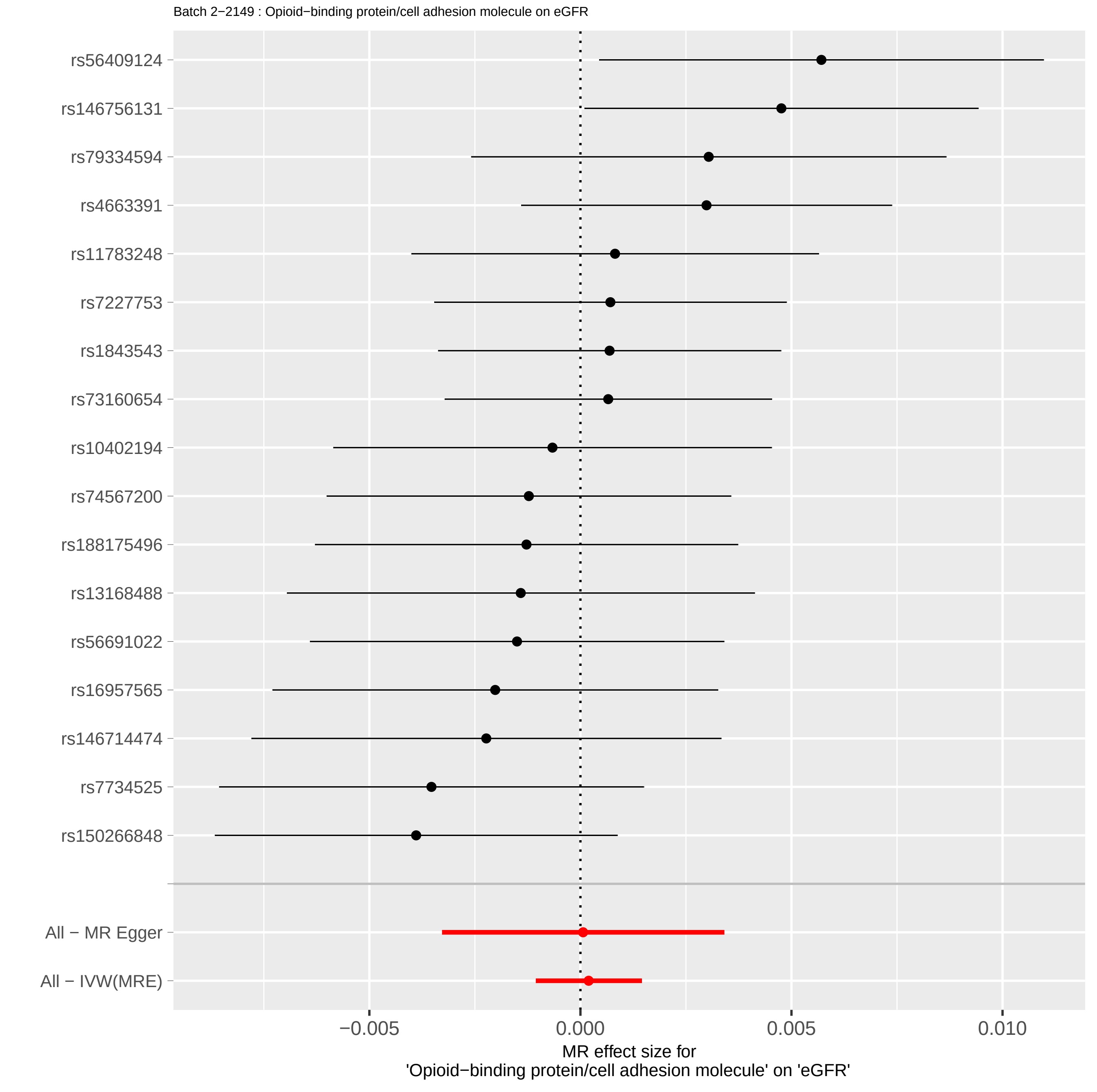
Batch 2−2149 : Opioid−binding protein/cell adhesion molecule on eGFR
rs56409124
rs146756131
rs79334594
rs4663391
rs11783248
rs7227753
rs1843543
rs73160654
rs10402194
rs74567200
rs188175496
rs13168488
rs56691022
rs16957565
rs146714474
rs7734525
rs150266848
All − MR Egger
All − IVW(MRE)
−0.005
0.000
0.005
0.010
MR effect size for
'Opioid−binding protein/cell adhesion molecule' on 'eGFR'

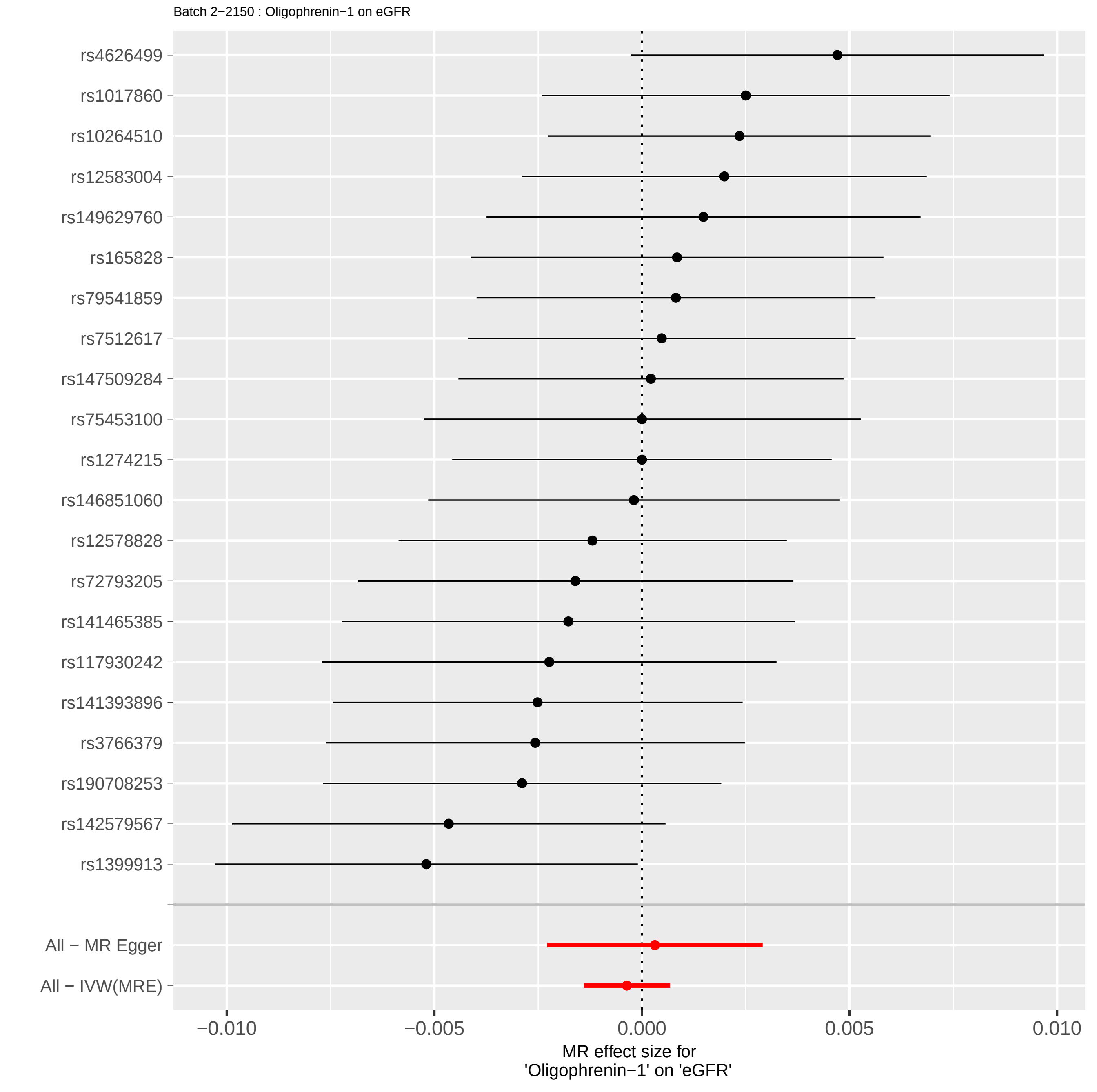

Batch 2−2150 : Oligophrenin−1 on eGFR
rs4626499
rs1017860
rs10264510
rs12583004
rs149629760
rs165828
rs79541859
rs7512617
rs147509284
rs75453100
rs1274215
rs146851060
rs12578828
rs72793205
rs141465385
rs117930242
rs141393896
rs3766379
rs190708253
rs142579567
rs1399913
All − MR Egger
All − IVW(MRE)
−0.010
−0.005
0.000
0.005
0.010
MR effect size for
'Oligophrenin−1' on 'eGFR'

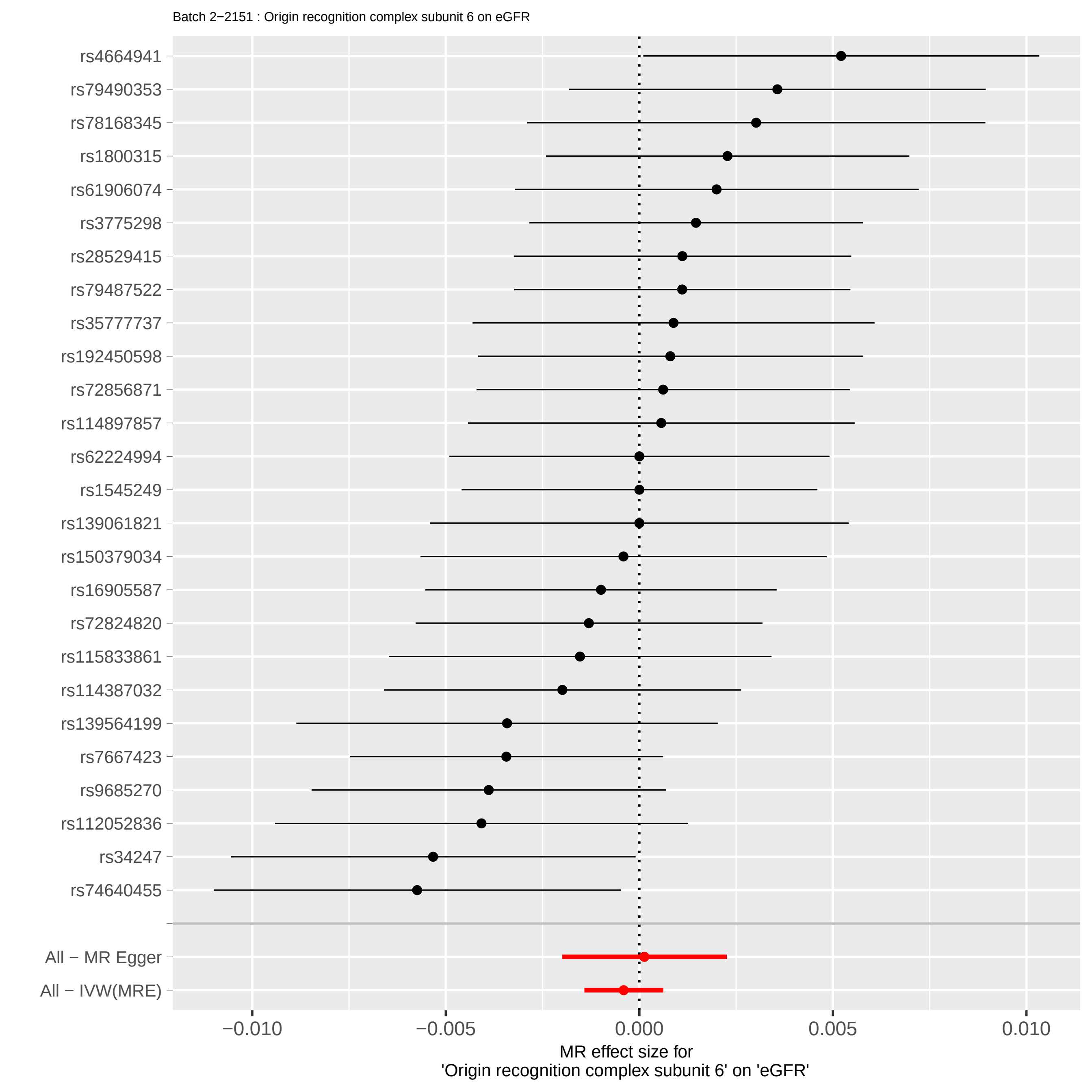

Batch 2−2151 : Origin recognition complex subunit 6 on eGFR
rs4664941
rs79490353
rs78168345
rs1800315
rs61906074
rs3775298
rs28529415
rs79487522
rs35777737
rs192450598
rs72856871
rs114897857
rs62224994
rs1545249
rs139061821
rs150379034
rs16905587
rs72824820
rs115833861
rs114387032
rs139564199
rs7667423
rs9685270
rs112052836
rs34247
rs74640455
All − MR Egger
All − IVW(MRE)
−0.010
−0.005
0.000
0.005
0.010
MR effect size for
'Origin recognition complex subunit 6' on 'eGFR'

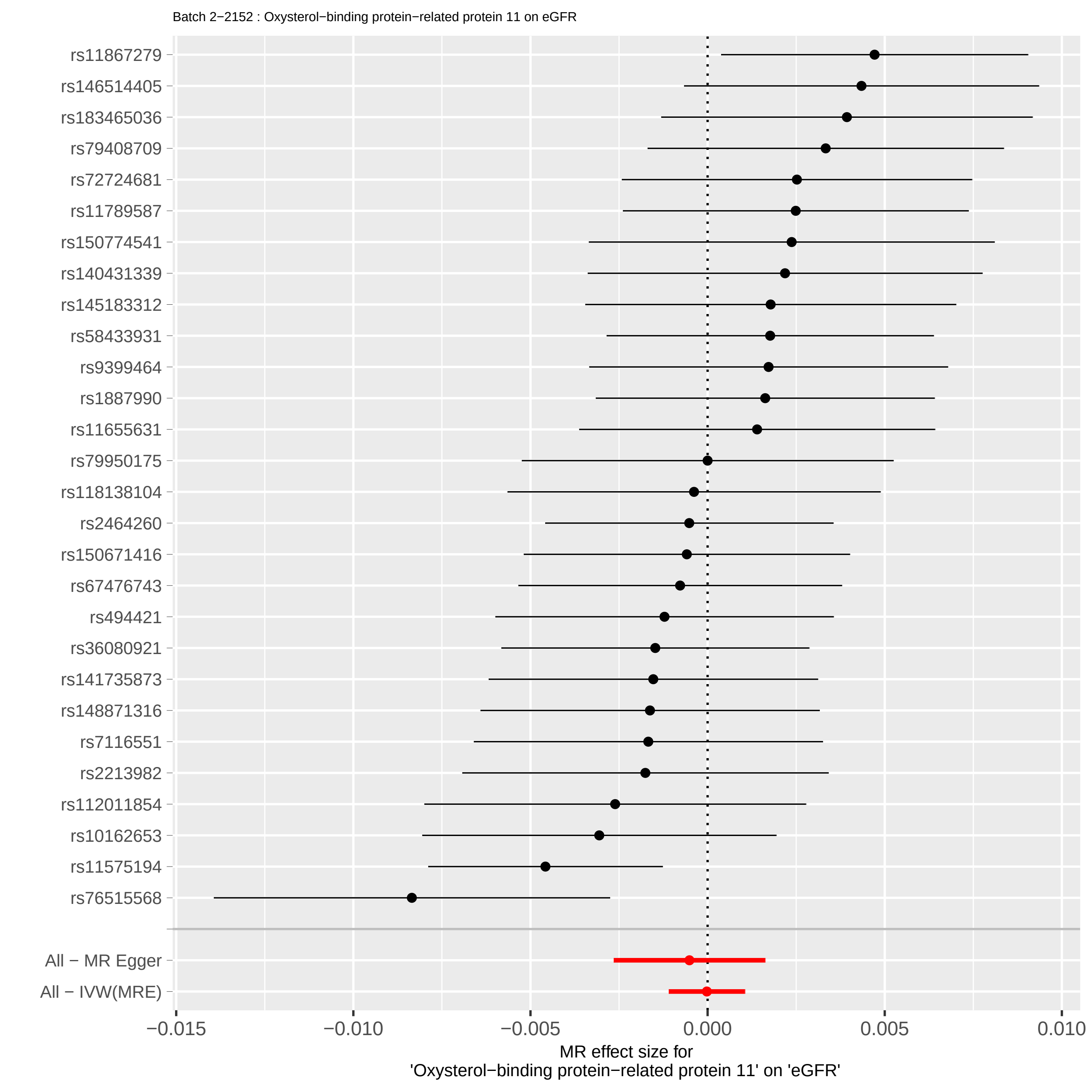
Batch 2−2152 : Oxysterol−binding protein−related protein 11 on eGFR
rs11867279
rs146514405
rs183465036
rs79408709
rs72724681
rs11789587
rs150774541
rs140431339
rs145183312
rs58433931
rs9399464
rs1887990
rs11655631
rs79950175
rs118138104
rs2464260
rs150671416
rs67476743
rs494421
rs36080921
rs141735873
rs148871316
rs7116551
rs2213982
rs112011854
rs10162653
rs11575194
rs76515568
All − MR Egger
All − IVW(MRE)
−0.015
−0.010
−0.005
0.000
0.005
0.010
MR effect size for
'Oxysterol−binding protein−related protein 11' on 'eGFR'

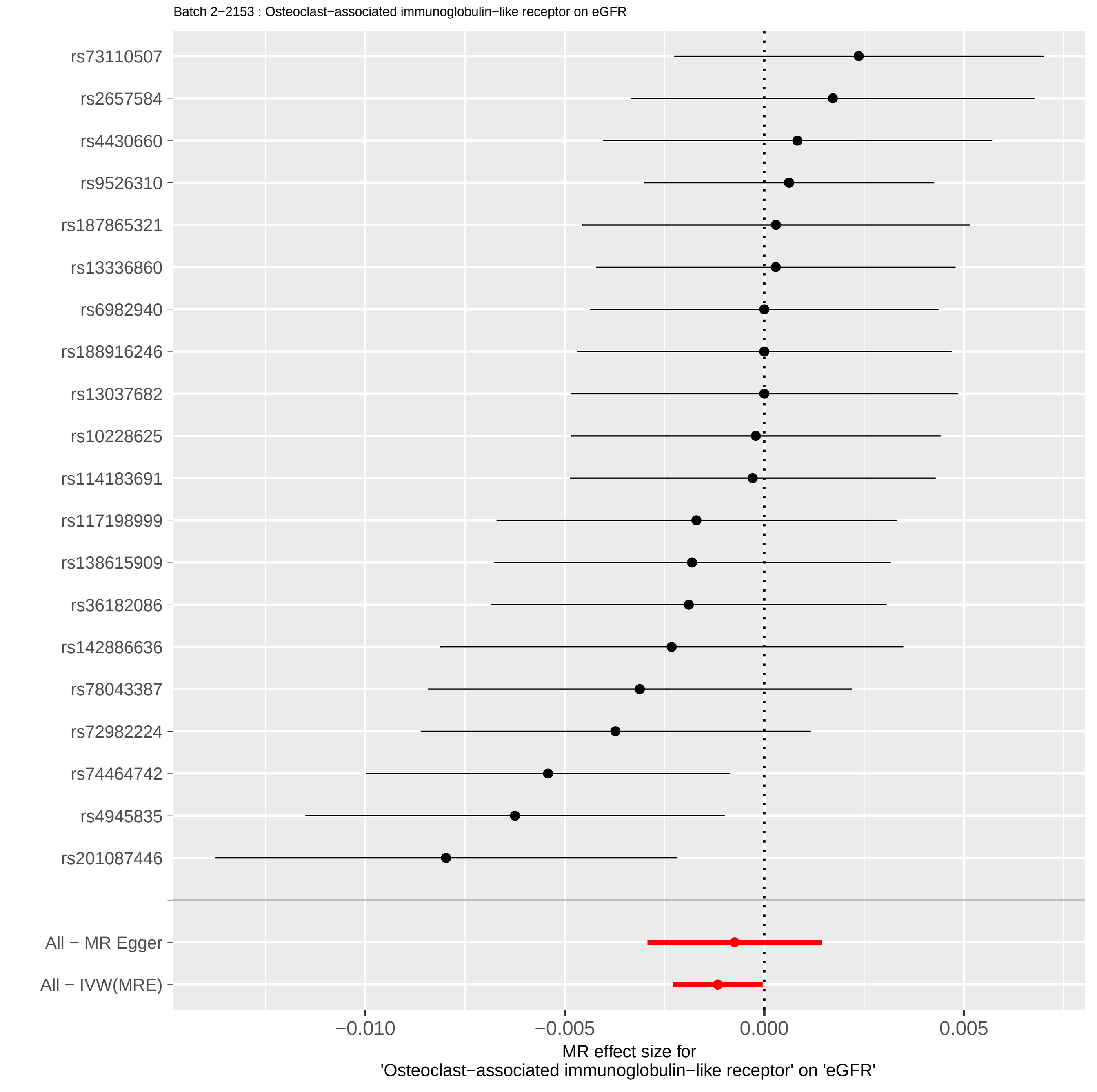
Batch 2−2153 : Osteoclast−associated immunoglobulin−like receptor on eGFR
rs73110507
rs2657584
rs4430660
rs9526310
rs187865321
rs13336860
rs6982940
rs188916246
rs13037682
rs10228625
rs114183691
rs117198999
rs138615909
rs36182086
rs142886636
rs78043387
rs72982224
rs74464742
rs4945835
rs201087446
All − MR Egger
All − IVW(MRE)
−0.010
−0.005
0.000
0.005
MR effect size for
'Osteoclast−associated immunoglobulin−like receptor' on 'eGFR'

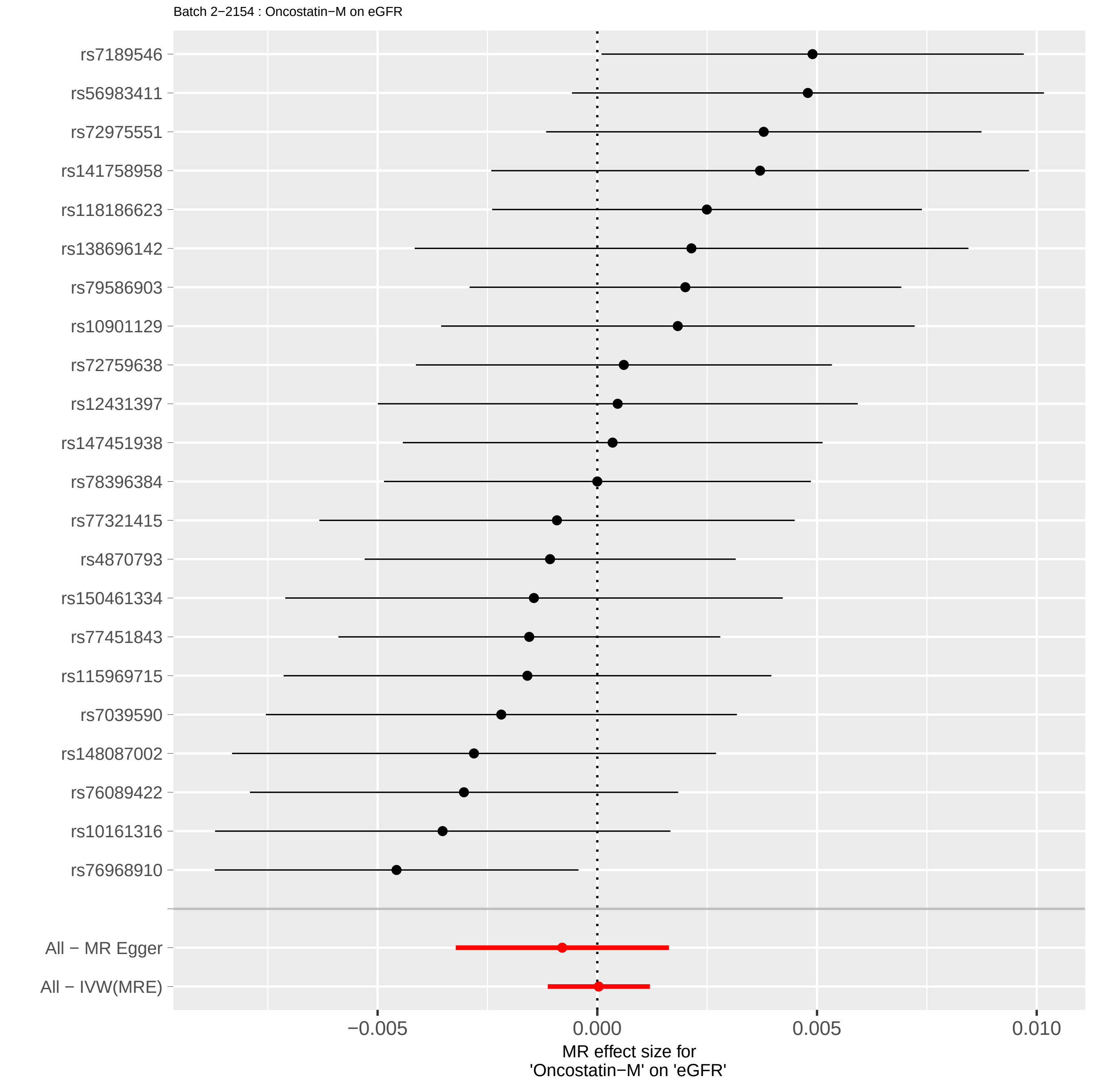
Batch 2−2154 : Oncostatin−M on eGFR
rs7189546
rs56983411
rs72975551
rs141758958
rs118186623
rs138696142
rs79586903
rs10901129
rs72759638
rs12431397
rs147451938
rs78396384
rs77321415
rs4870793
rs150461334
rs77451843
rs115969715
rs7039590
rs148087002
rs76089422
rs10161316
rs76968910
All − MR Egger
All − IVW(MRE)
−0.005
0.000
0.005
0.010
MR effect size for
'Oncostatin−M' on 'eGFR'

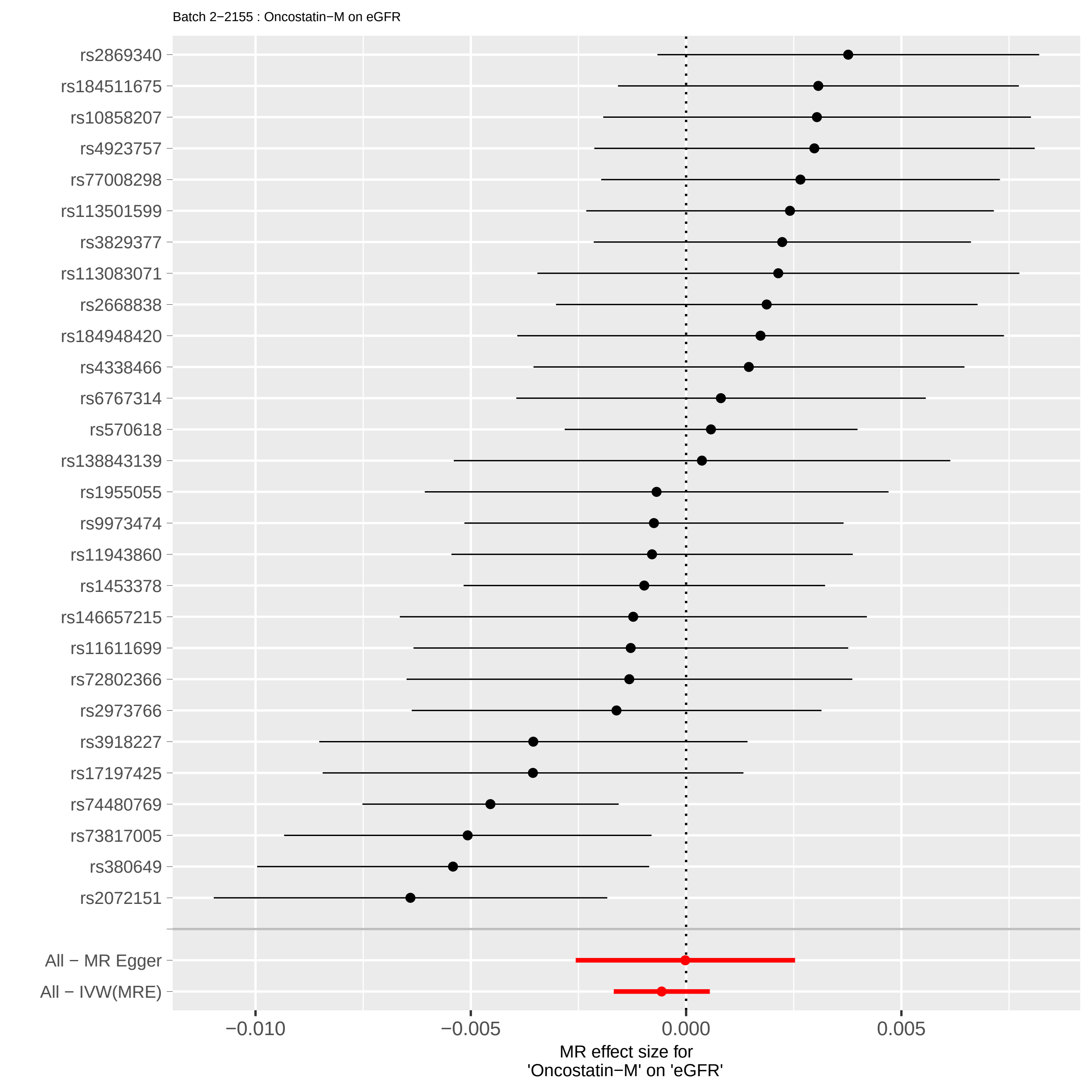

Batch 2−2155 : Oncostatin−M on eGFR
rs2869340
rs184511675
rs10858207
rs4923757
rs77008298
rs113501599
rs3829377
rs113083071
rs2668838
rs184948420
rs4338466
rs6767314
rs570618
rs138843139
rs1955055
rs9973474
rs11943860
rs1453378
rs146657215
rs11611699
rs72802366
rs2973766
rs3918227
rs17197425
rs74480769
rs73817005
rs380649
rs2072151
All − MR Egger
All − IVW(MRE)
−0.010
−0.005
0.000
0.005
MR effect size for
'Oncostatin−M' on 'eGFR'

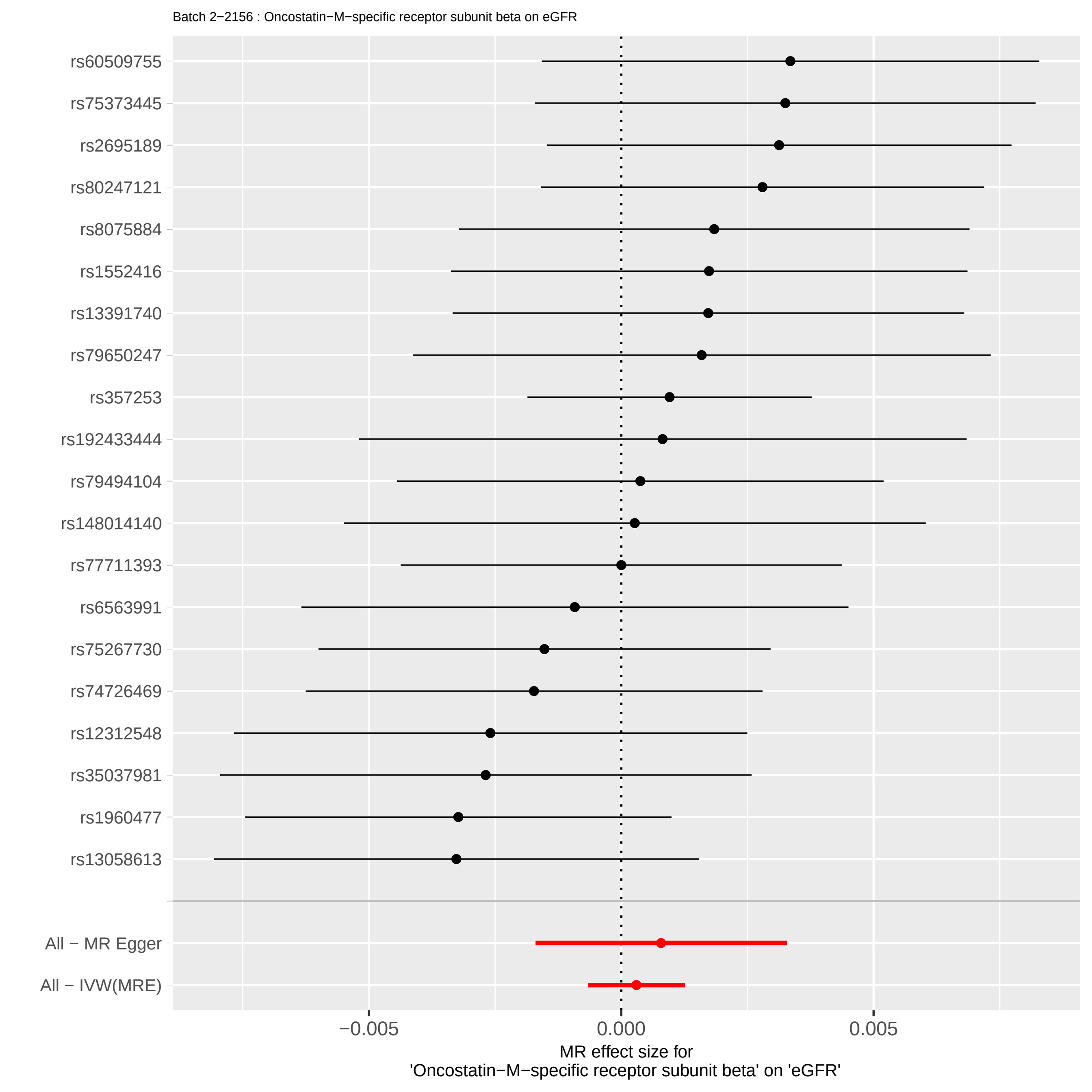
Batch 2−2156 : Oncostatin−M−specific receptor subunit beta on eGFR
rs60509755
rs75373445
rs2695189
rs80247121
rs8075884
rs1552416
rs13391740
rs79650247
rs357253
rs192433444
rs79494104
rs148014140
rs77711393
rs6563991
rs75267730
rs74726469
rs12312548
rs35037981
rs1960477
rs13058613
All − MR Egger
All − IVW(MRE)
−0.005
0.000
0.005
MR effect size for
'Oncostatin−M−specific receptor subunit beta' on 'eGFR'

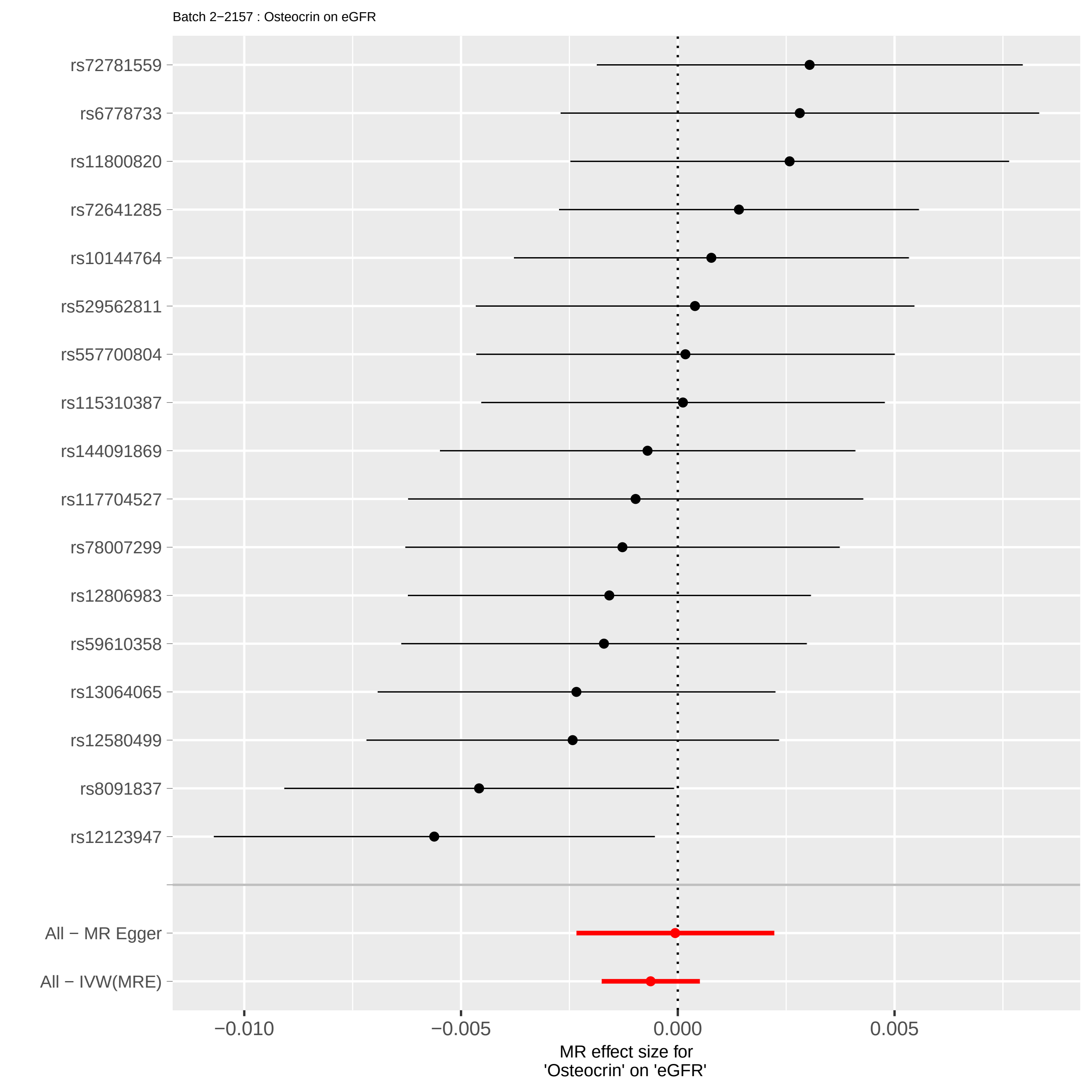

Batch 2−2157 : Osteocrin on eGFR
rs72781559
rs6778733
rs11800820
rs72641285
rs10144764
rs529562811
rs557700804
rs115310387
rs144091869
rs117704527
rs78007299
rs12806983
rs59610358
rs13064065
rs12580499
rs8091837
rs12123947
All − MR Egger
All − IVW(MRE)
−0.010
−0.005
0.000
0.005
MR effect size for
'Osteocrin' on 'eGFR'

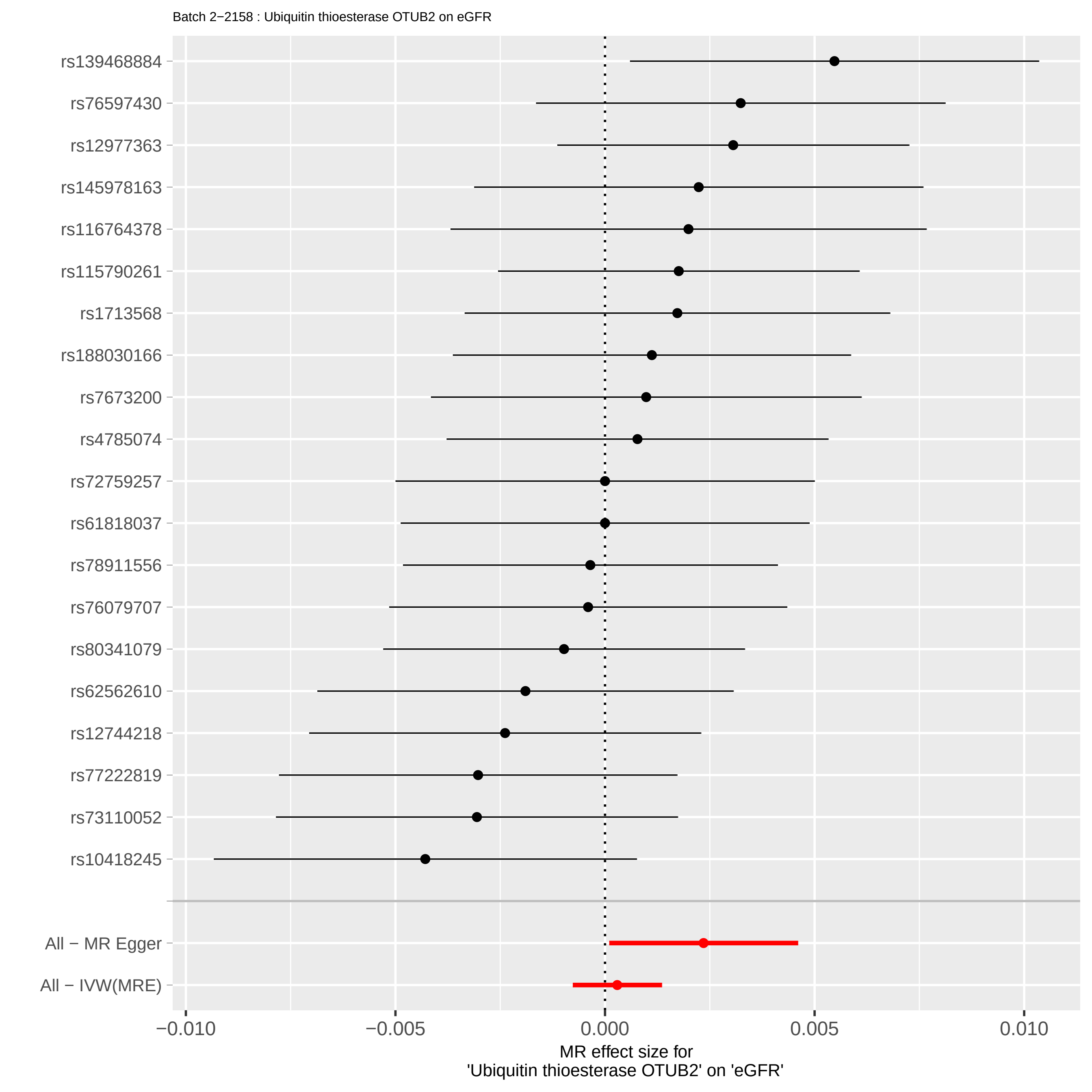
Batch 2−2158 : Ubiquitin thioesterase OTUB2 on eGFR
rs139468884
rs76597430
rs12977363
rs145978163
rs116764378
rs115790261
rs1713568
rs188030166
rs7673200
rs4785074
rs72759257
rs61818037
rs78911556
rs76079707
rs80341079
rs62562610
rs12744218
rs77222819
rs73110052
rs10418245
All − MR Egger
All − IVW(MRE)
−0.010
−0.005
0.000
0.005
0.010
MR effect size for
'Ubiquitin thioesterase OTUB2' on 'eGFR'

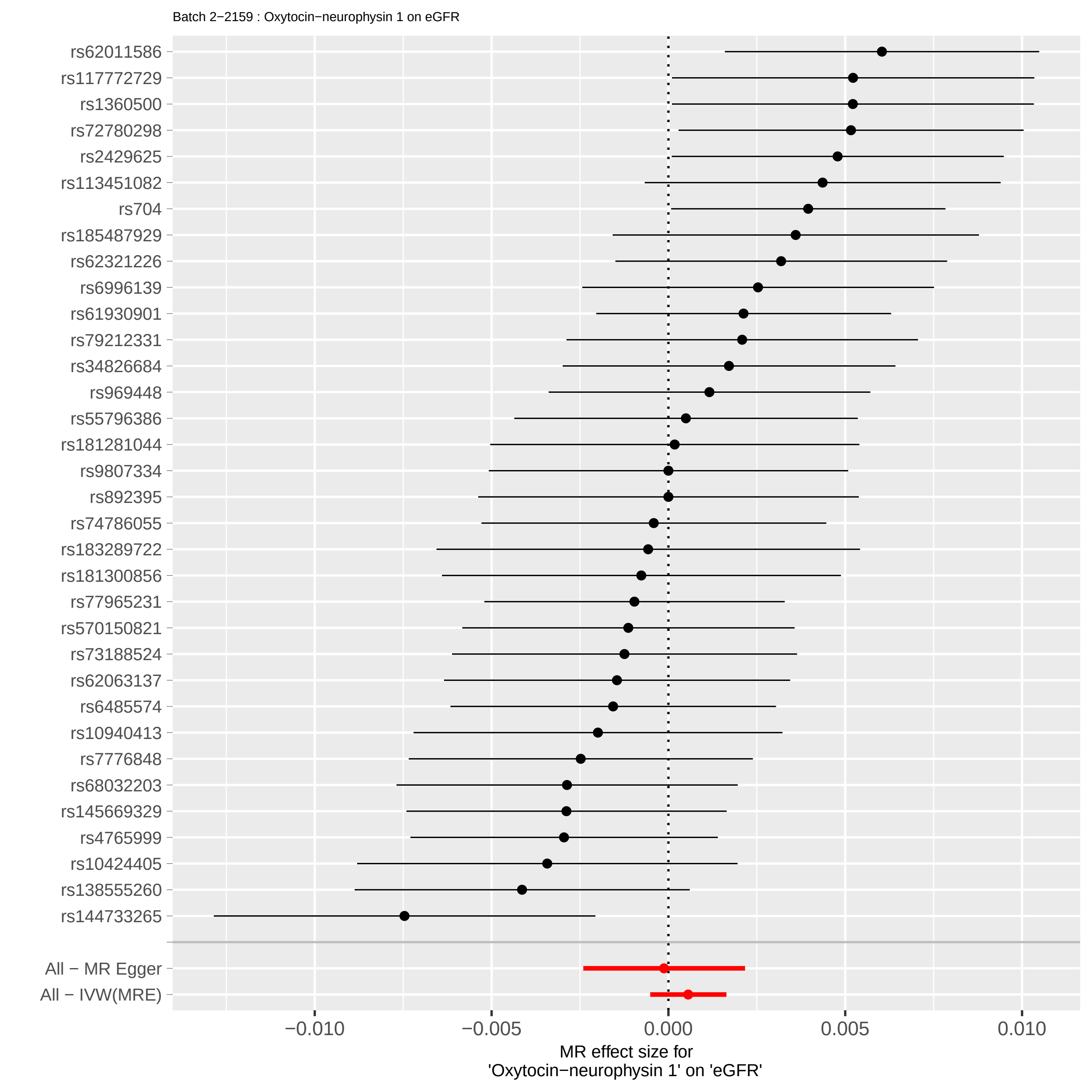

Batch 2−2159 : Oxytocin−neurophysin 1 on eGFR
rs62011586
rs117772729
rs1360500
rs72780298
rs2429625
rs113451082
rs704
rs185487929
rs62321226
rs6996139
rs61930901
rs79212331
rs34826684
rs969448
rs55796386
rs181281044
rs9807334
rs892395
rs74786055
rs183289722
rs181300856
rs77965231
rs570150821
rs73188524
rs62063137
rs6485574
rs10940413
rs7776848
rs68032203
rs145669329
rs4765999
rs10424405
rs138555260
rs144733265
All − MR Egger
All − IVW(MRE)
−0.010
−0.005
0.000
0.005
0.010
MR effect size for
'Oxytocin−neurophysin 1' on 'eGFR'

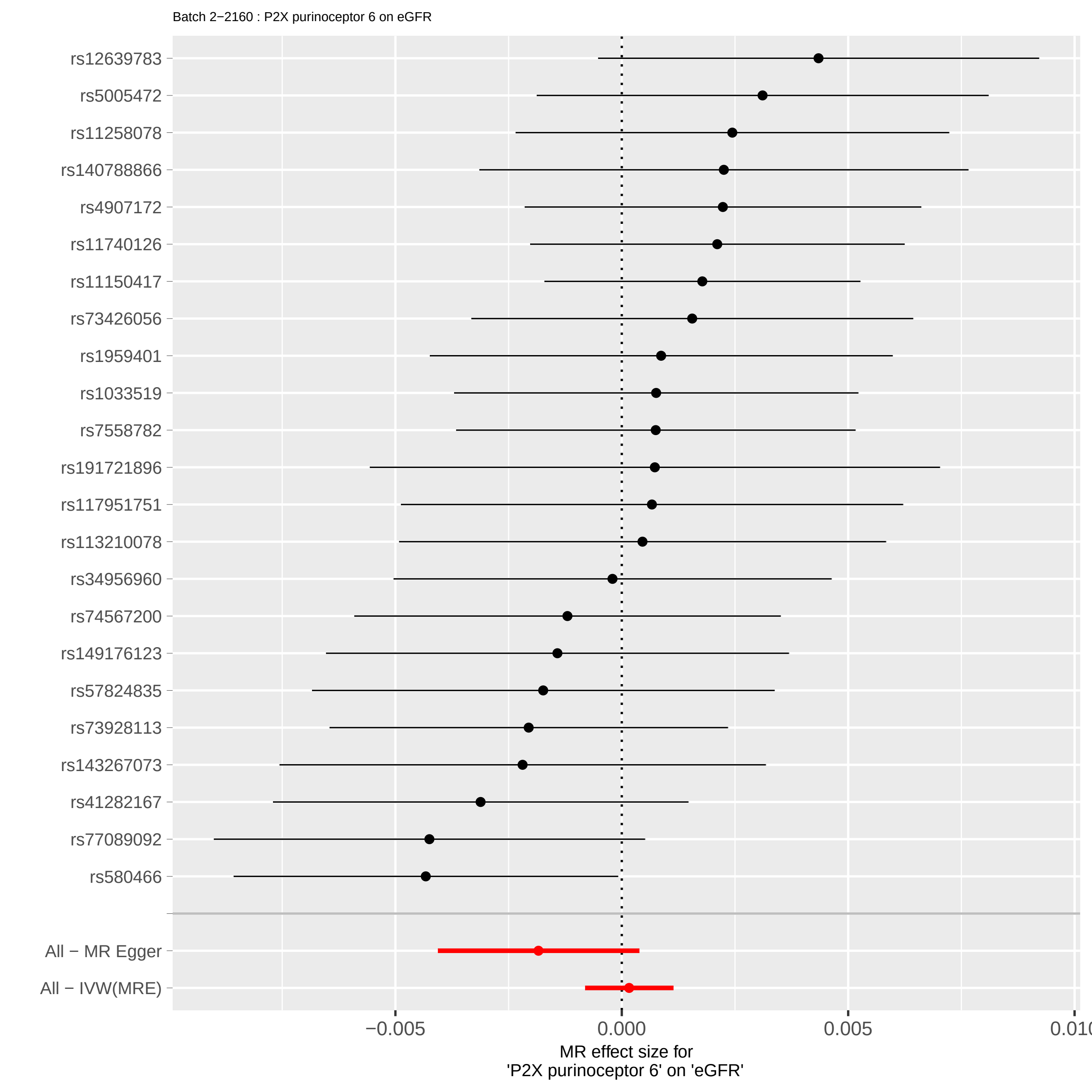

Batch 2−2160 : P2X purinoceptor 6 on eGFR
rs12639783
rs5005472
rs11258078
rs140788866
rs4907172
rs11740126
rs11150417
rs73426056
rs1959401
rs1033519
rs7558782
rs191721896
rs117951751
rs113210078
rs34956960
rs74567200
rs149176123
rs57824835
rs73928113
rs143267073
rs41282167
rs77089092
rs580466
All − MR Egger
All − IVW(MRE)
−0.005
0.000
0.005
0.010
MR effect size for
'P2X purinoceptor 6' on 'eGFR'

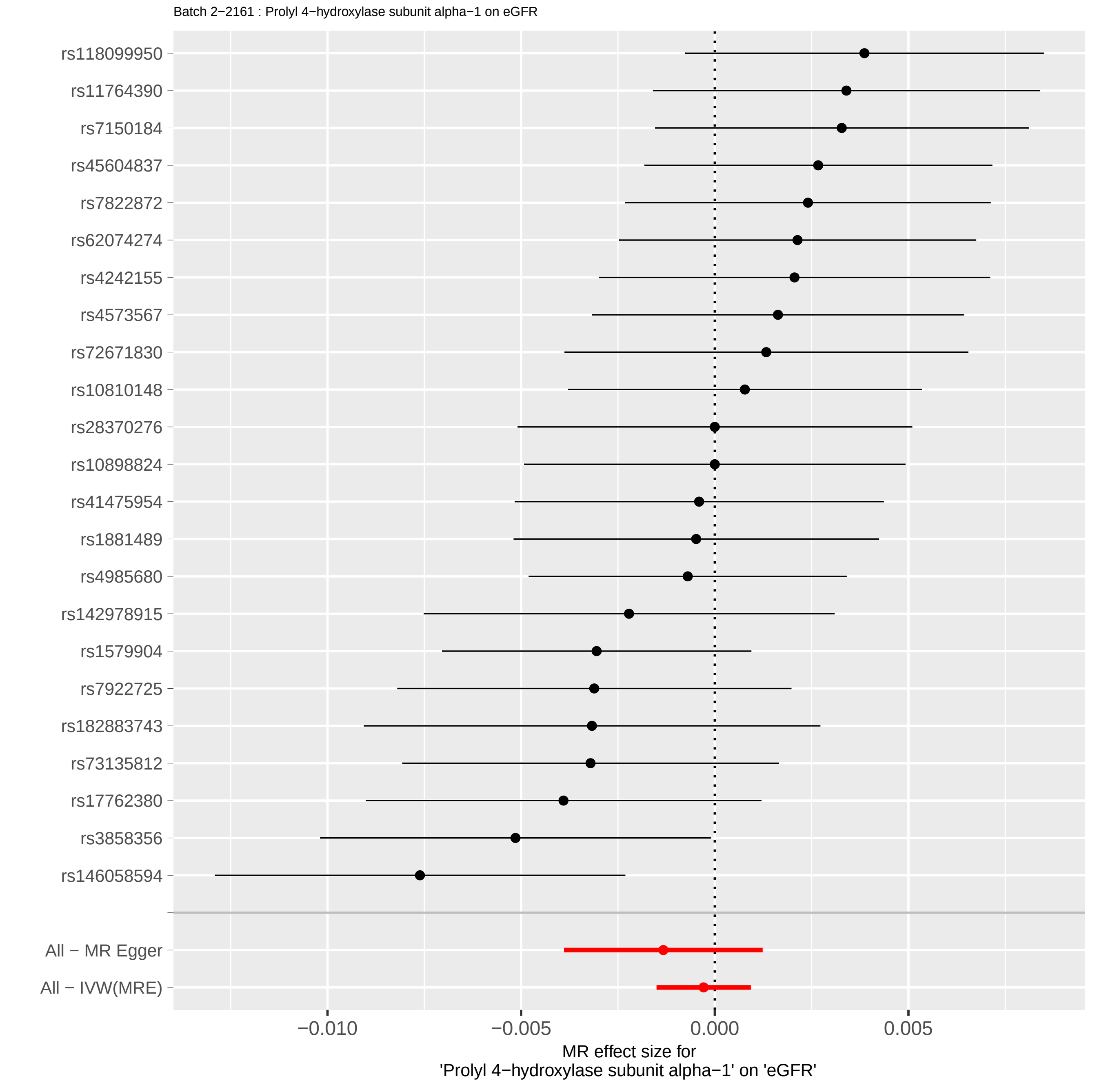

Batch 2−2161 : Prolyl 4−hydroxylase subunit alpha−1 on eGFR
rs118099950
rs11764390
rs7150184
rs45604837
rs7822872
rs62074274
rs4242155
rs4573567
rs72671830
rs10810148
rs28370276
rs10898824
rs41475954
rs1881489
rs4985680
rs142978915
rs1579904
rs7922725
rs182883743
rs73135812
rs17762380
rs3858356
rs146058594
All − MR Egger
All − IVW(MRE)
−0.010
−0.005
0.000
0.005
MR effect size for
'Prolyl 4−hydroxylase subunit alpha−1' on 'eGFR'

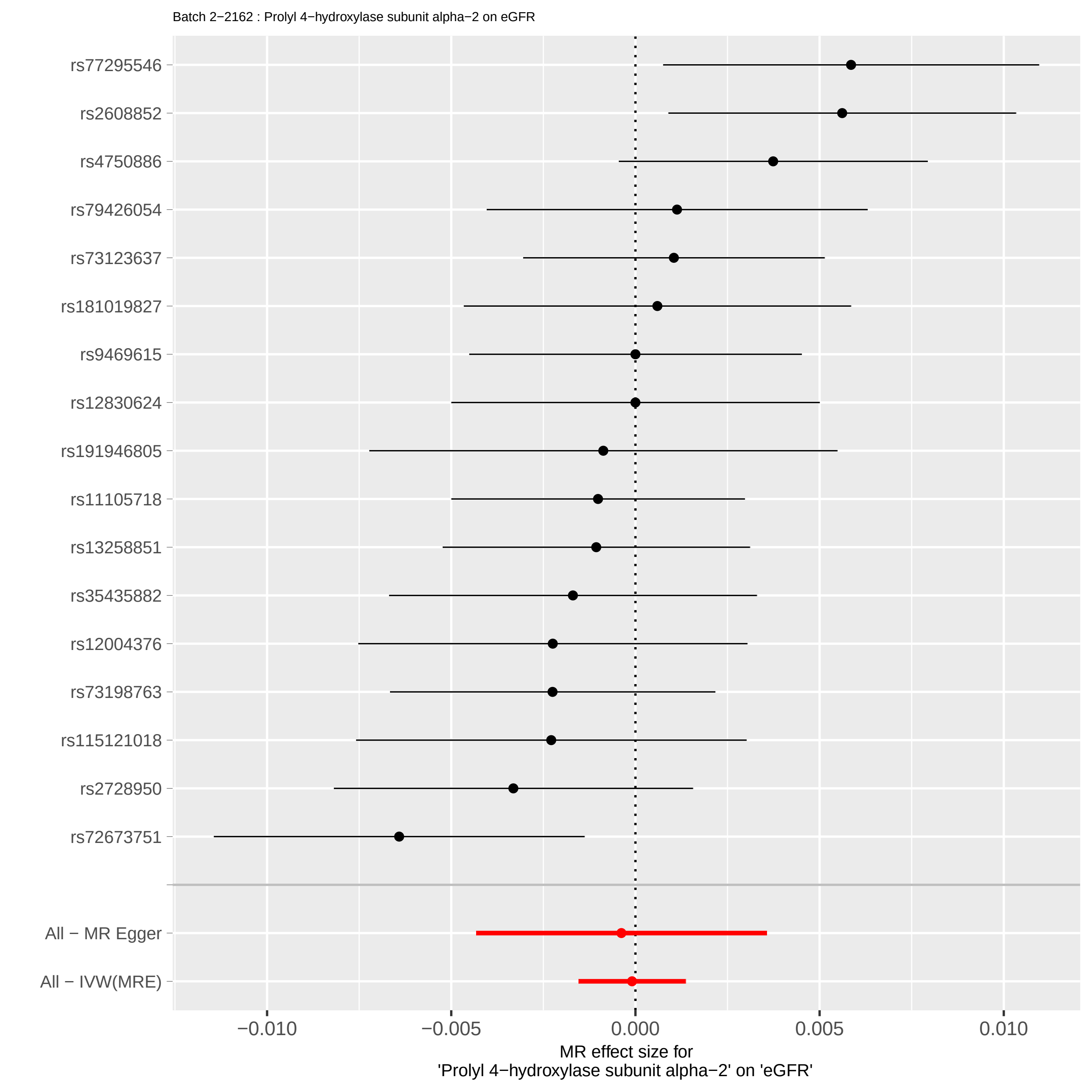
Batch 2−2162 : Prolyl 4−hydroxylase subunit alpha−2 on eGFR
rs77295546
rs2608852
rs4750886
rs79426054
rs73123637
rs181019827
rs9469615
rs12830624
rs191946805
rs11105718
rs13258851
rs35435882
rs12004376
rs73198763
rs115121018
rs2728950
rs72673751
All − MR Egger
All − IVW(MRE)
−0.010
−0.005
0.000
0.005
0.010
MR effect size for
'Prolyl 4−hydroxylase subunit alpha−2' on 'eGFR'

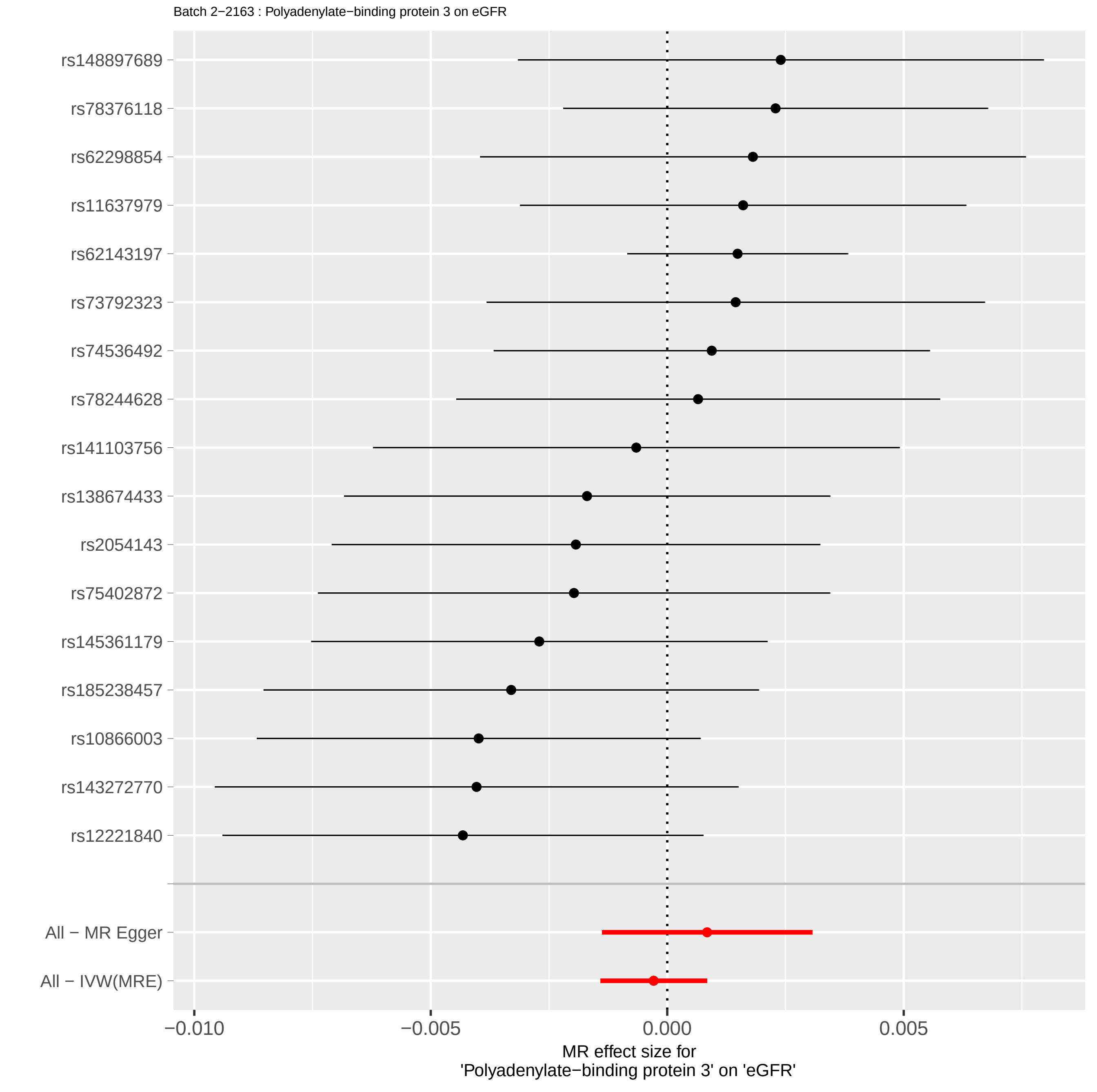

Batch 2−2163 : Polyadenylate−binding protein 3 on eGFR
rs148897689
rs78376118
rs62298854
rs11637979
rs62143197
rs73792323
rs74536492
rs78244628
rs141103756
rs138674433
rs2054143
rs75402872
rs145361179
rs185238457
rs10866003
rs143272770
rs12221840
All − MR Egger
All − IVW(MRE)
−0.010
−0.005
0.000
0.005
MR effect size for
'Polyadenylate−binding protein 3' on 'eGFR'

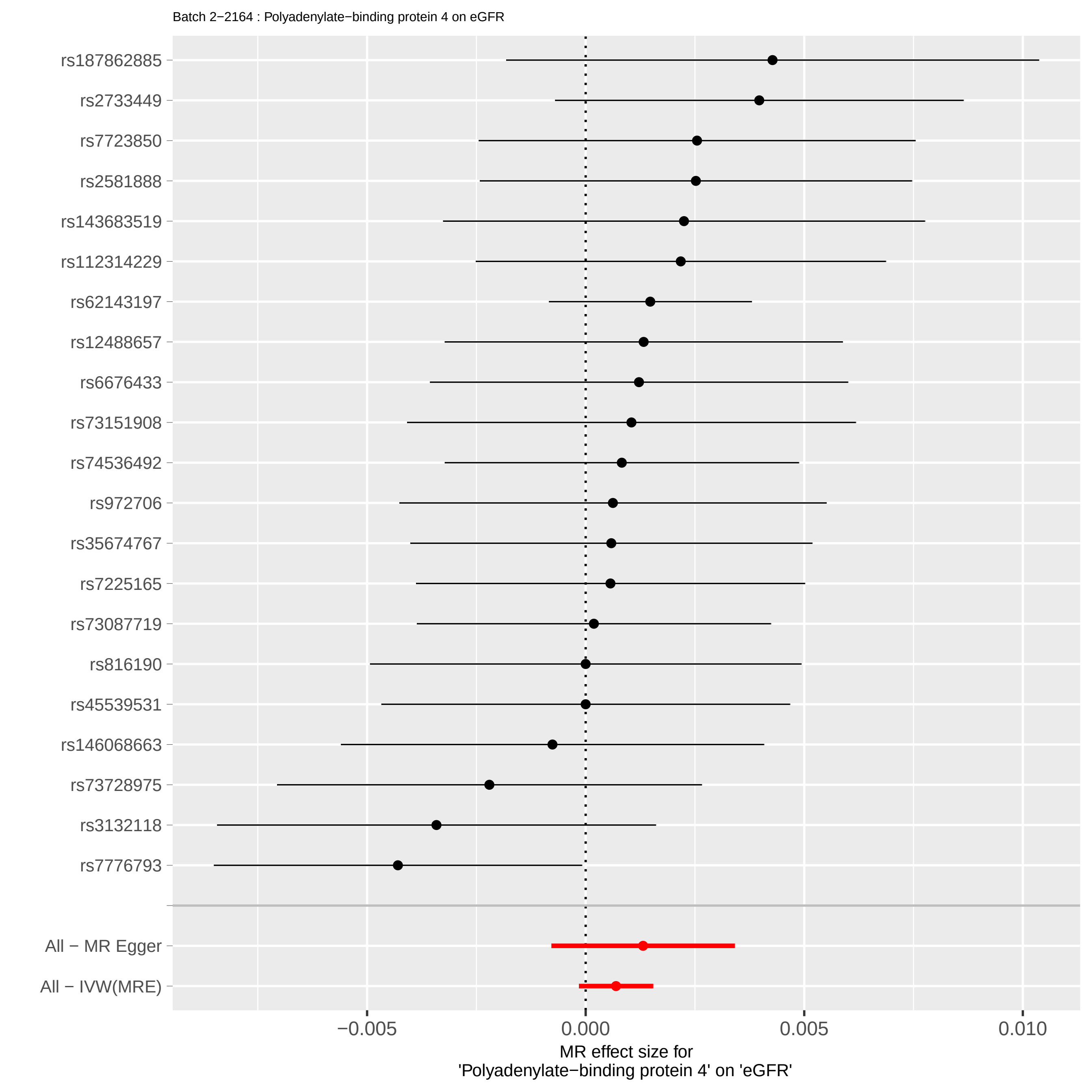
Batch 2−2164 : Polyadenylate−binding protein 4 on eGFR
rs187862885
rs2733449
rs7723850
rs2581888
rs143683519
rs112314229
rs62143197
rs12488657
rs6676433
rs73151908
rs74536492
rs972706
rs35674767
rs7225165
rs73087719
rs816190
rs45539531
rs146068663
rs73728975
rs3132118
rs7776793
All − MR Egger
All − IVW(MRE)
−0.005
0.000
0.005
0.010
MR effect size for
'Polyadenylate−binding protein 4' on 'eGFR'

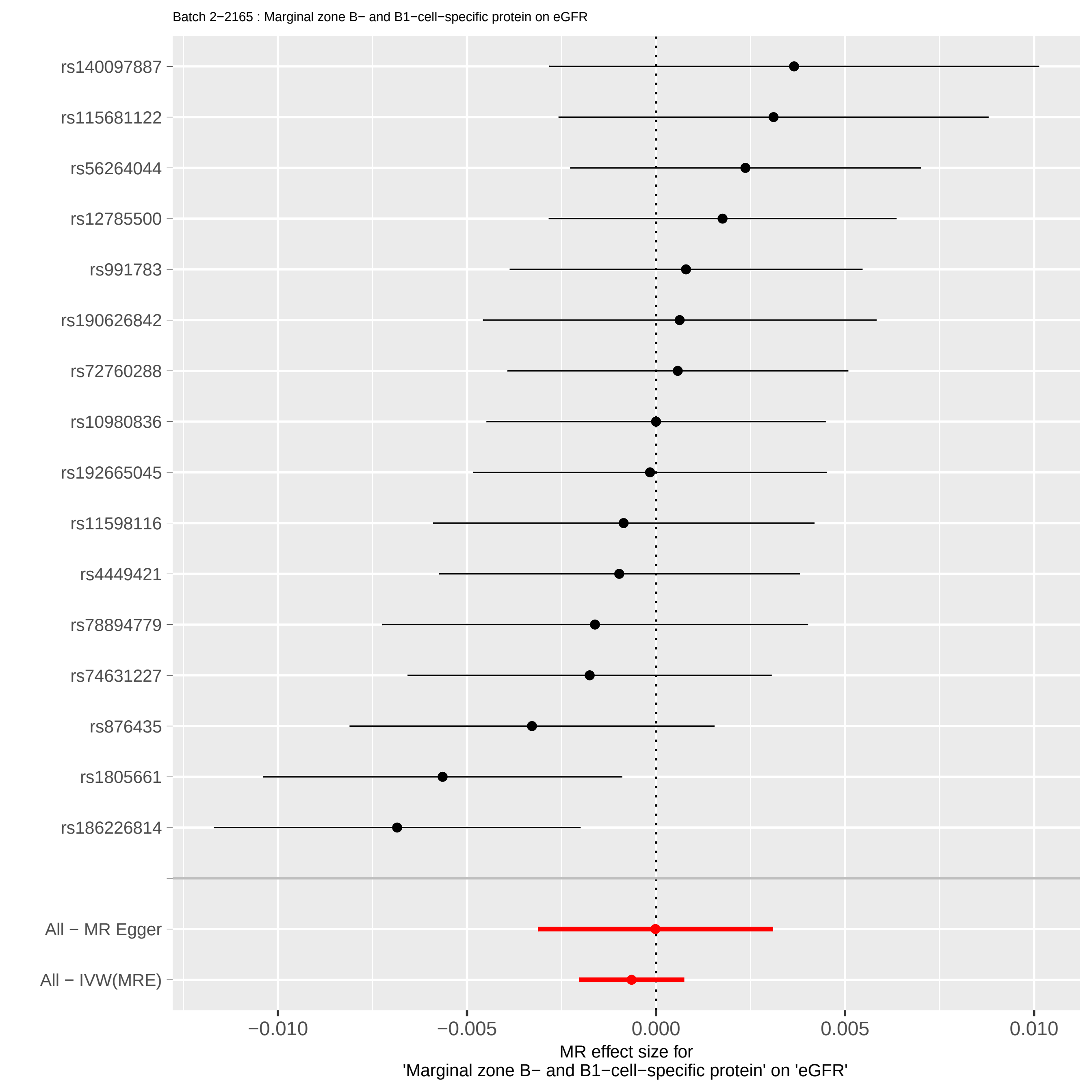

Batch 2−2165 : Marginal zone B− and B1−cell−specific protein on eGFR
rs140097887
rs115681122
rs56264044
rs12785500
rs991783
rs190626842
rs72760288
rs10980836
rs192665045
rs11598116
rs4449421
rs78894779
rs74631227
rs876435
rs1805661
rs186226814
All − MR Egger
All − IVW(MRE)
−0.010
−0.005
0.000
0.005
0.010
MR effect size for
'Marginal zone B− and B1−cell−specific protein' on 'eGFR'

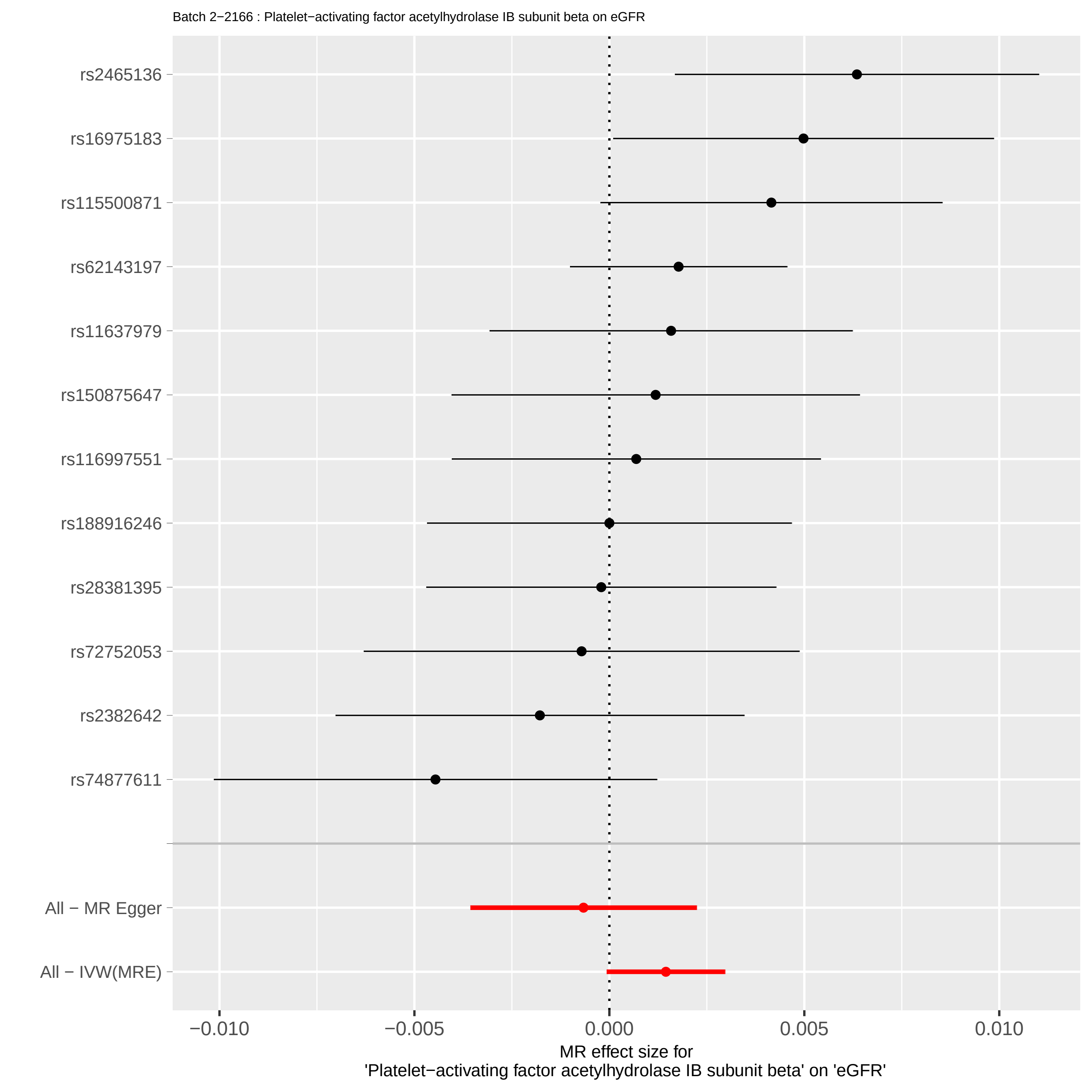

Batch 2−2166 : Platelet−activating factor acetylhydrolase IB subunit beta on eGFR
rs2465136
rs16975183
rs115500871
rs62143197
rs11637979
rs150875647
rs116997551
rs188916246
rs28381395
rs72752053
rs2382642
rs74877611
All − MR Egger
All − IVW(MRE)
−0.010
−0.005
0.000
0.005
0.010
MR effect size for
'Platelet−activating factor acetylhydrolase IB subunit beta' on 'eGFR'

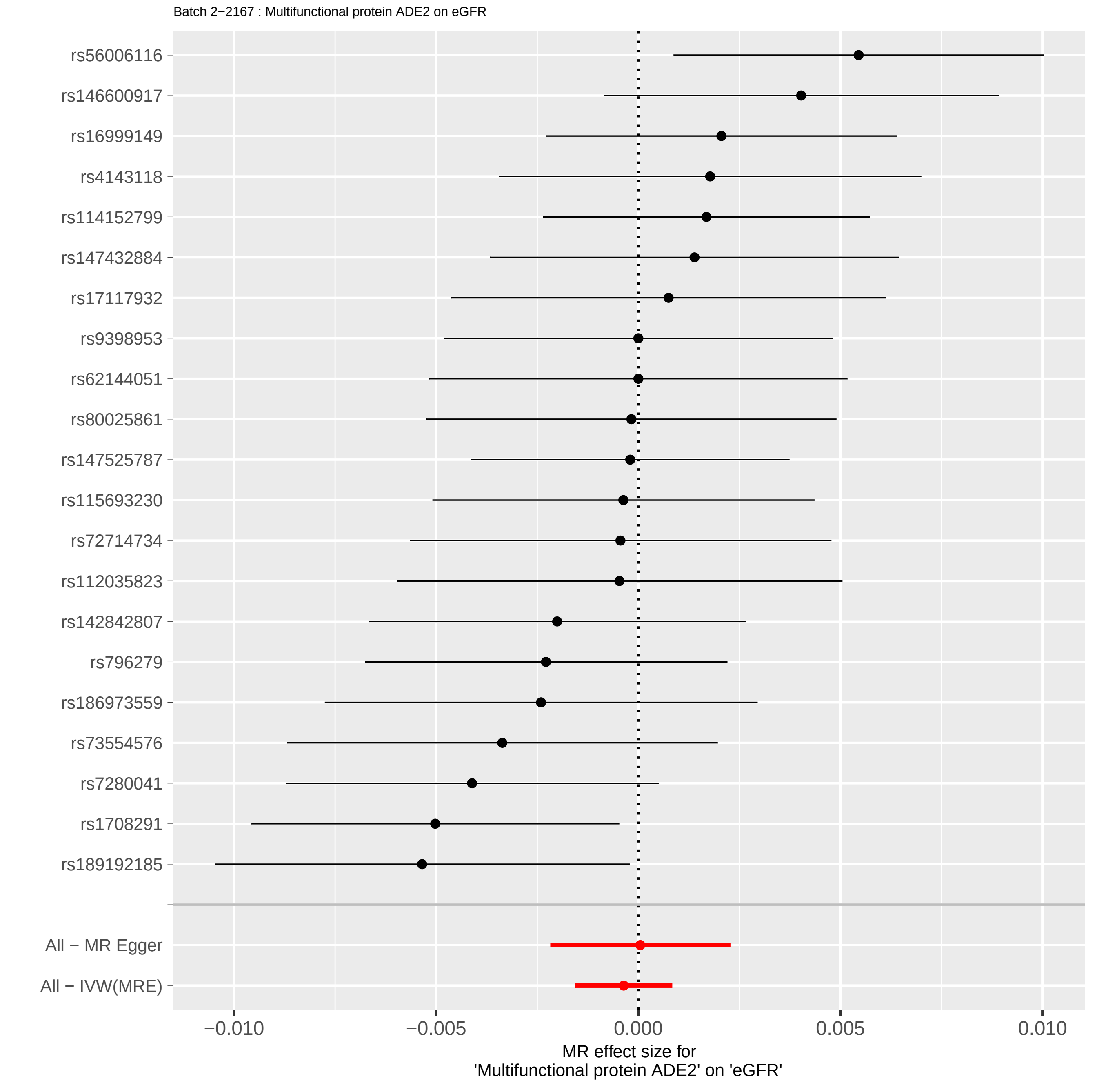

Batch 2−2167 : Multifunctional protein ADE2 on eGFR
rs56006116
rs146600917
rs16999149
rs4143118
rs114152799
rs147432884
rs17117932
rs9398953
rs62144051
rs80025861
rs147525787
rs115693230
rs72714734
rs112035823
rs142842807
rs796279
rs186973559
rs73554576
rs7280041
rs1708291
rs189192185
All − MR Egger
All − IVW(MRE)
−0.010
−0.005
0.000
0.005
0.010
MR effect size for
'Multifunctional protein ADE2' on 'eGFR'

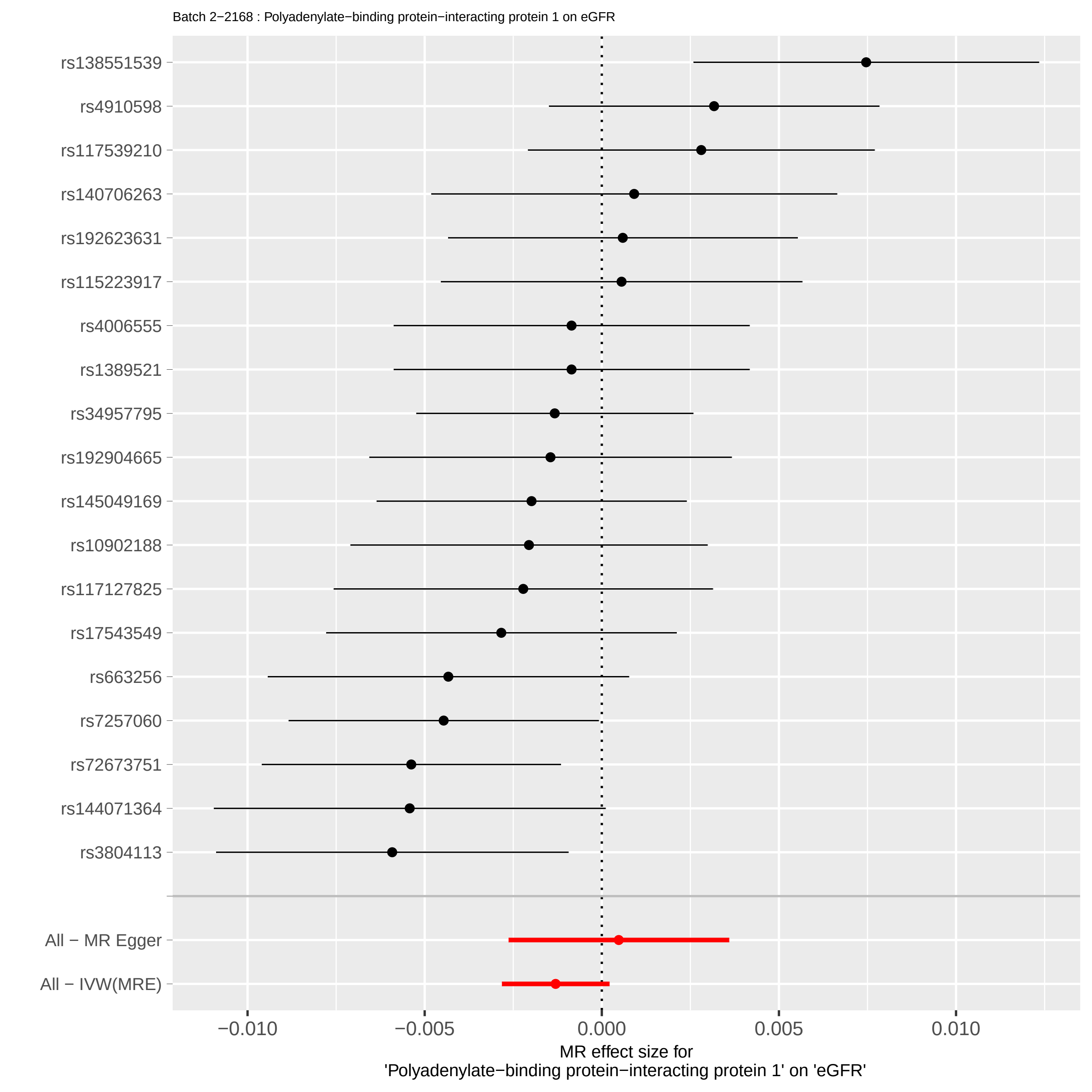

Batch 2−2168 : Polyadenylate−binding protein−interacting protein 1 on eGFR
rs138551539
rs4910598
rs117539210
rs140706263
rs192623631
rs115223917
rs4006555
rs1389521
rs34957795
rs192904665
rs145049169
rs10902188
rs117127825
rs17543549
rs663256
rs7257060
rs72673751
rs144071364
rs3804113
All − MR Egger
All − IVW(MRE)
−0.010
−0.005
0.000
0.005
0.010
MR effect size for
'Polyadenylate−binding protein−interacting protein 1' on 'eGFR'

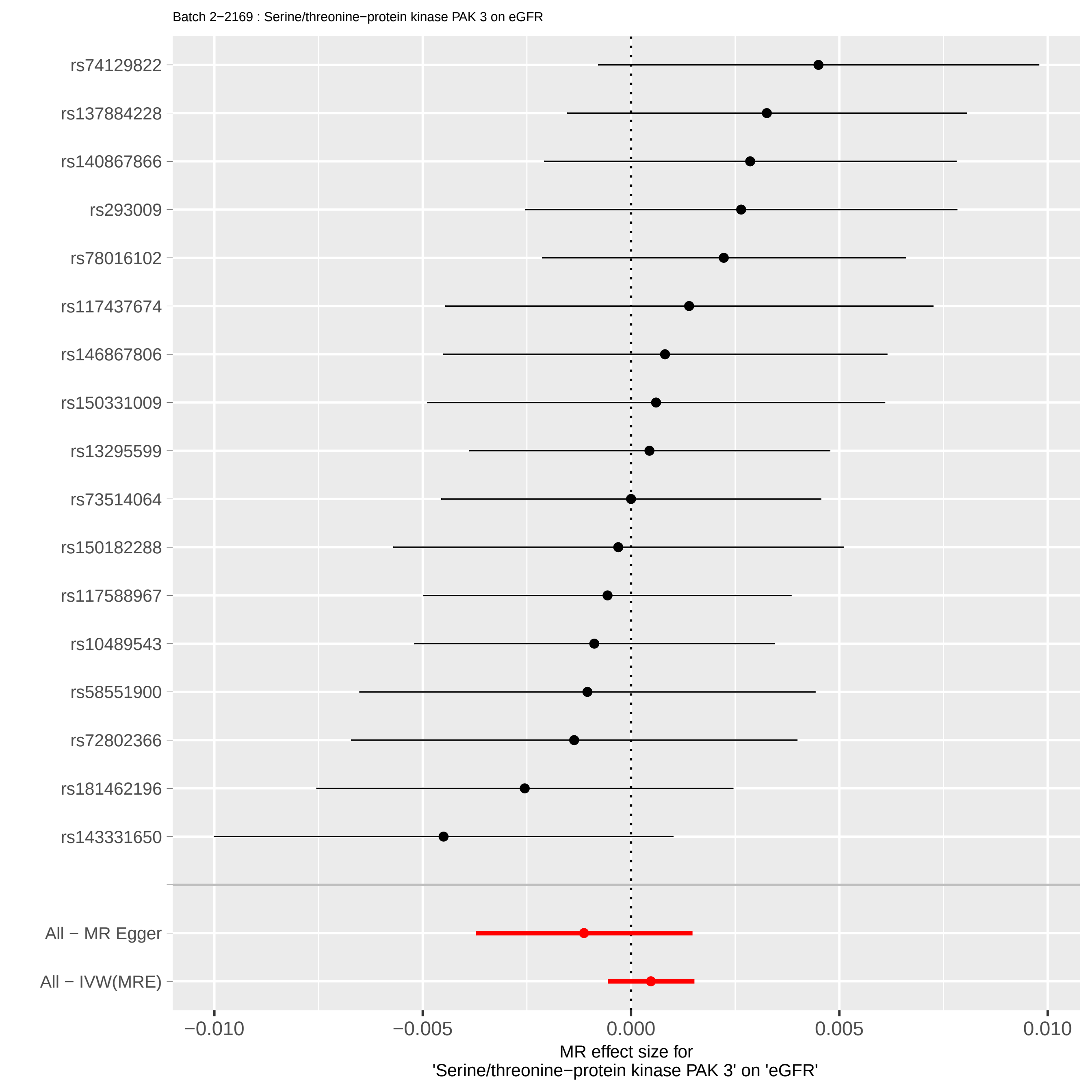

Batch 2−2169 : Serine/threonine−protein kinase PAK 3 on eGFR
rs74129822
rs137884228
rs140867866
rs293009
rs78016102
rs117437674
rs146867806
rs150331009
rs13295599
rs73514064
rs150182288
rs117588967
rs10489543
rs58551900
rs72802366
rs181462196
rs143331650
All − MR Egger
All − IVW(MRE)
−0.010
−0.005
0.000
0.005
0.010
MR effect size for
'Serine/threonine−protein kinase PAK 3' on 'eGFR'

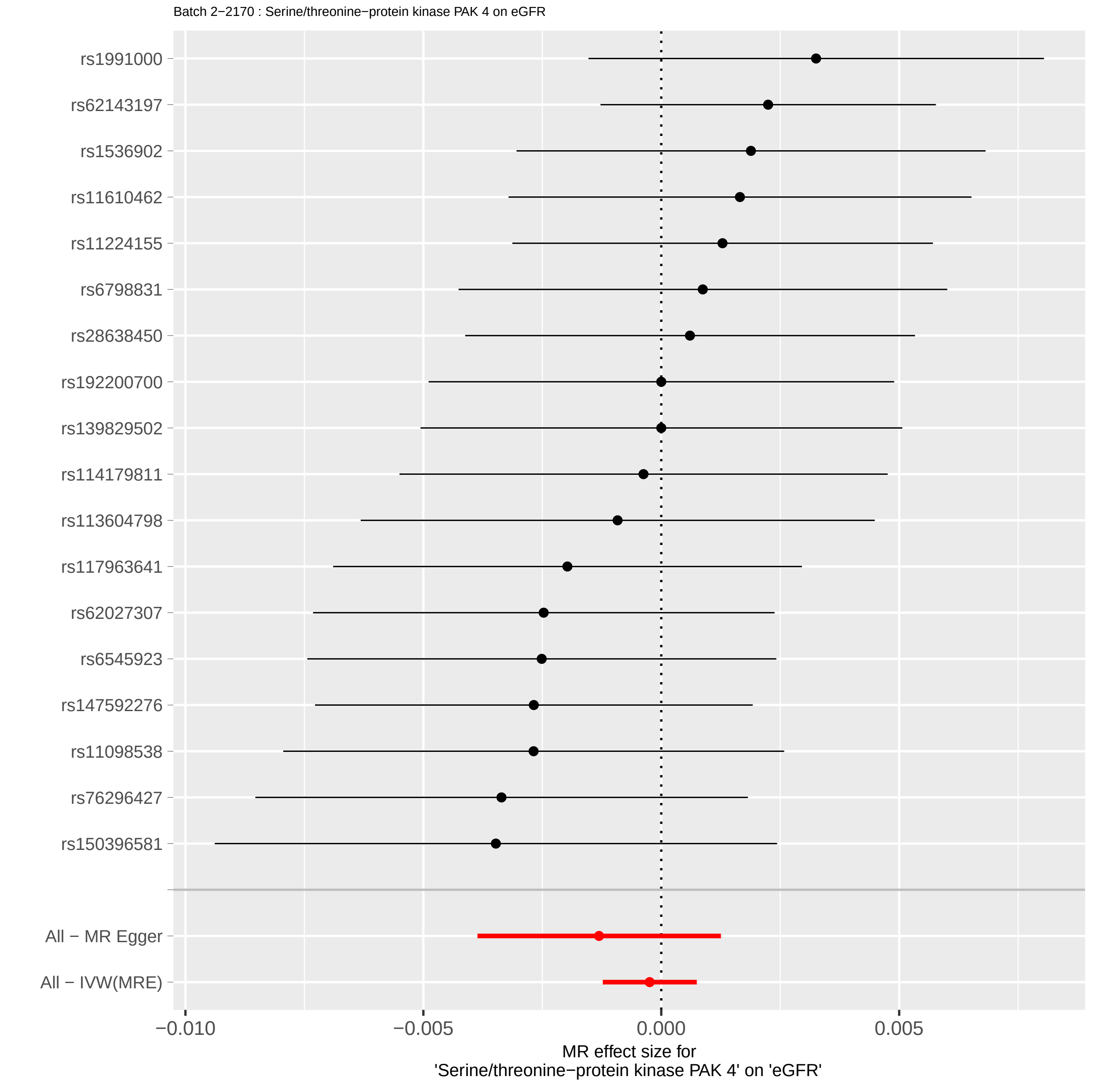

Batch 2−2170 : Serine/threonine−protein kinase PAK 4 on eGFR
rs1991000
rs62143197
rs1536902
rs11610462
rs11224155
rs6798831
rs28638450
rs192200700
rs139829502
rs114179811
rs113604798
rs117963641
rs62027307
rs6545923
rs147592276
rs11098538
rs76296427
rs150396581
All − MR Egger
All − IVW(MRE)
−0.010
−0.005
0.000
0.005
MR effect size for
'Serine/threonine−protein kinase PAK 4' on 'eGFR'

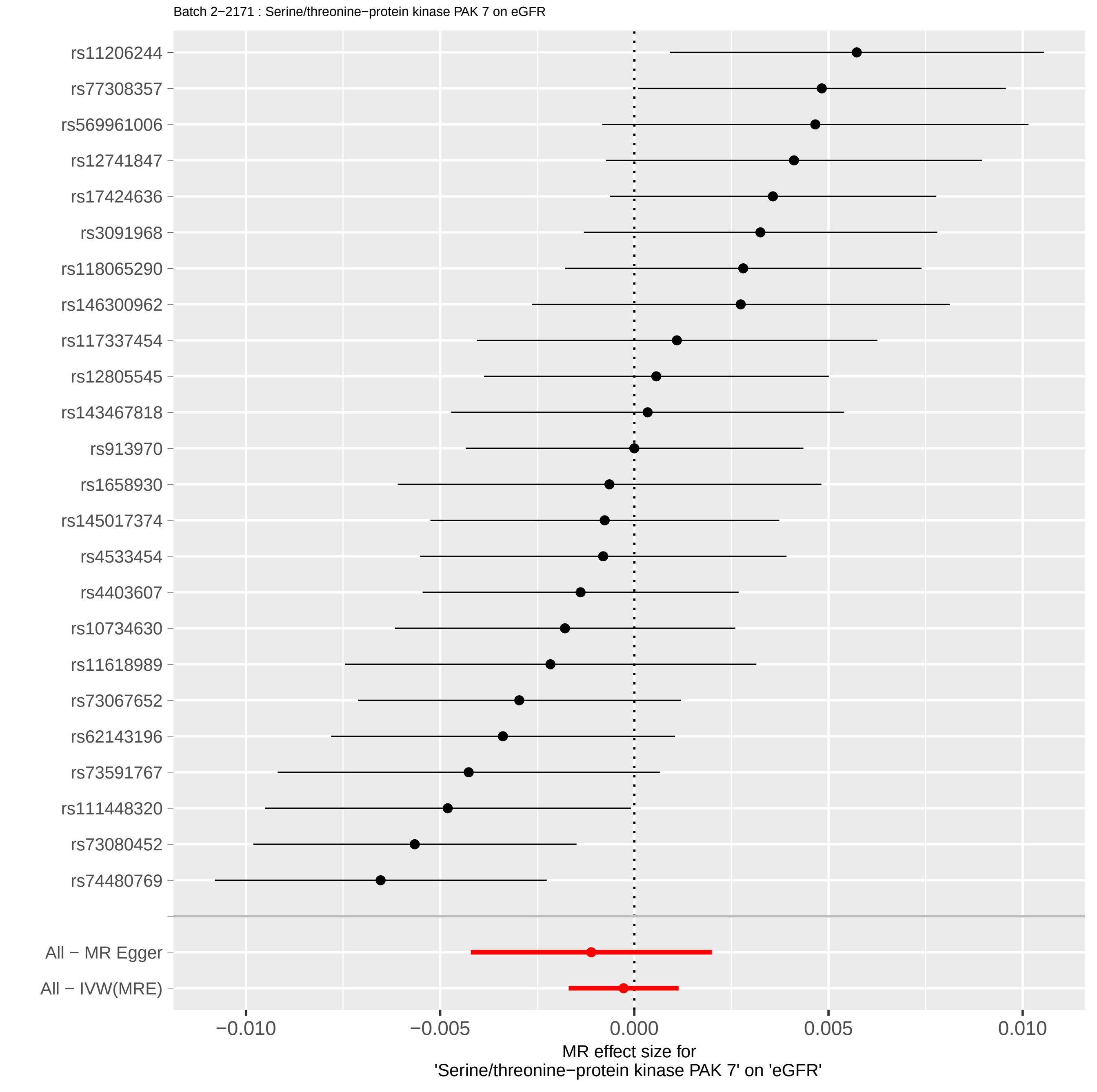
Batch 2−2171 : Serine/threonine−protein kinase PAK 7 on eGFR
rs11206244
rs77308357
rs569961006
rs12741847
rs17424636
rs3091968
rs118065290
rs146300962
rs117337454
rs12805545
rs143467818
rs913970
rs1658930
rs145017374
rs4533454
rs4403607
rs10734630
rs11618989
rs73067652
rs62143196
rs73591767
rs111448320
rs73080452
rs74480769
All − MR Egger
All − IVW(MRE)
−0.010
−0.005
0.000
0.005
0.010
MR effect size for
'Serine/threonine−protein kinase PAK 7' on 'eGFR'

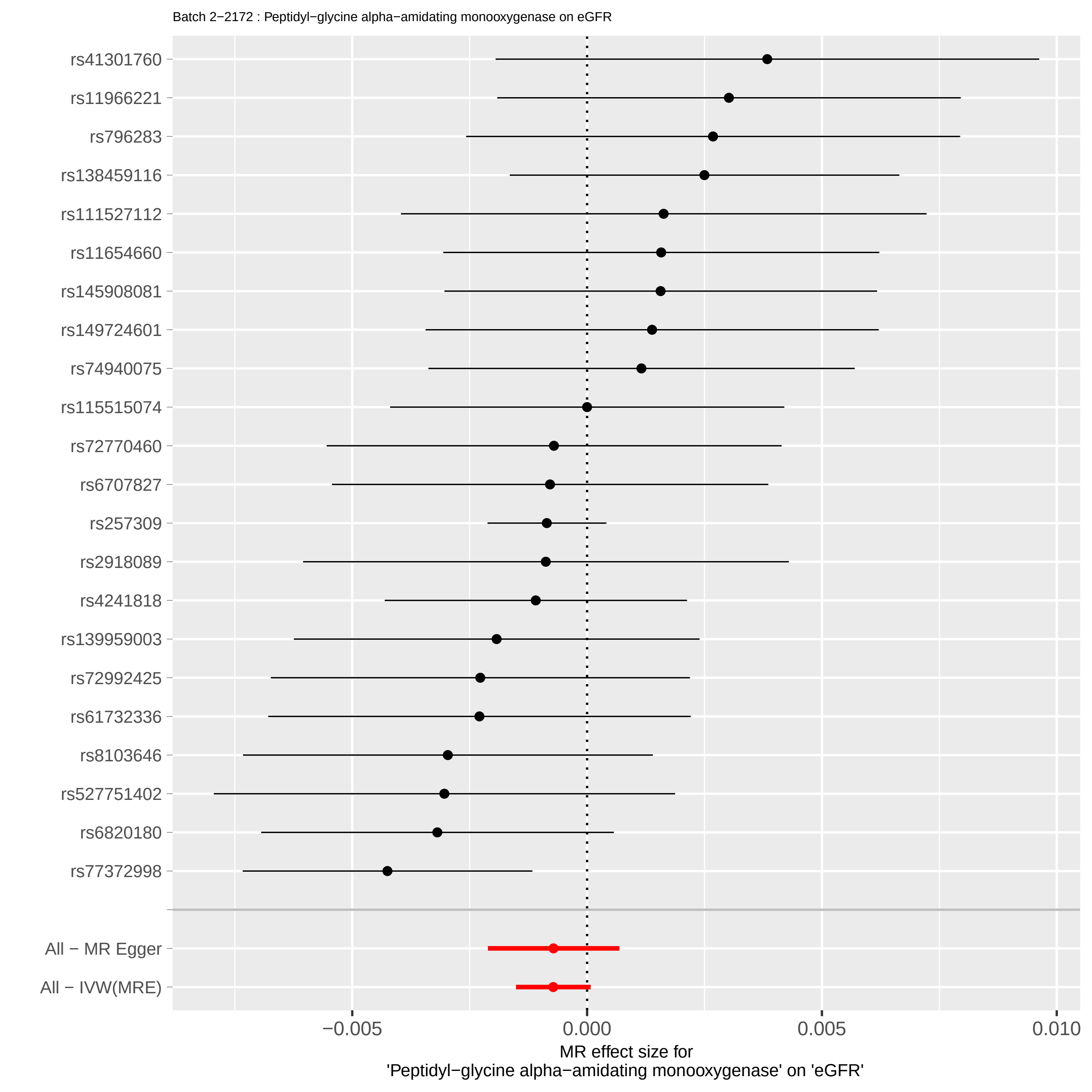

Batch 2−2172 : Peptidyl−glycine alpha−amidating monooxygenase on eGFR
rs41301760
rs11966221
rs796283
rs138459116
rs111527112
rs11654660
rs145908081
rs149724601
rs74940075
rs115515074
rs72770460
rs6707827
rs257309
rs2918089
rs4241818
rs139959003
rs72992425
rs61732336
rs8103646
rs527751402
rs6820180
rs77372998
All − MR Egger
All − IVW(MRE)
−0.005
0.000
0.005
0.010
MR effect size for
'Peptidyl−glycine alpha−amidating monooxygenase' on 'eGFR'

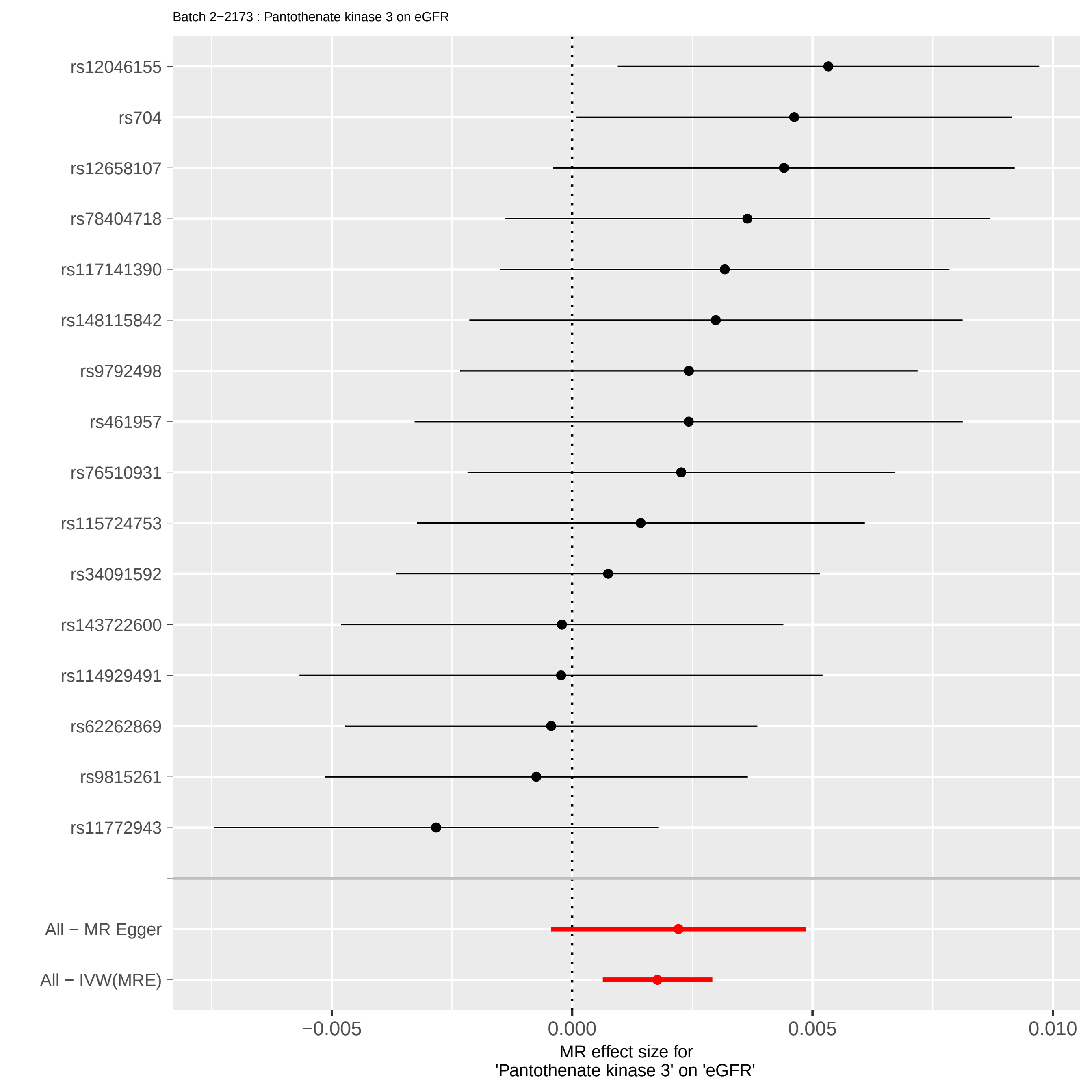

Batch 2−2173 : Pantothenate kinase 3 on eGFR
rs12046155
rs704
rs12658107
rs78404718
rs117141390
rs148115842
rs9792498
rs461957
rs76510931
rs115724753
rs34091592
rs143722600
rs114929491
rs62262869
rs9815261
rs11772943
All − MR Egger
All − IVW(MRE)
−0.005
0.000
0.005
0.010
MR effect size for
'Pantothenate kinase 3' on 'eGFR'

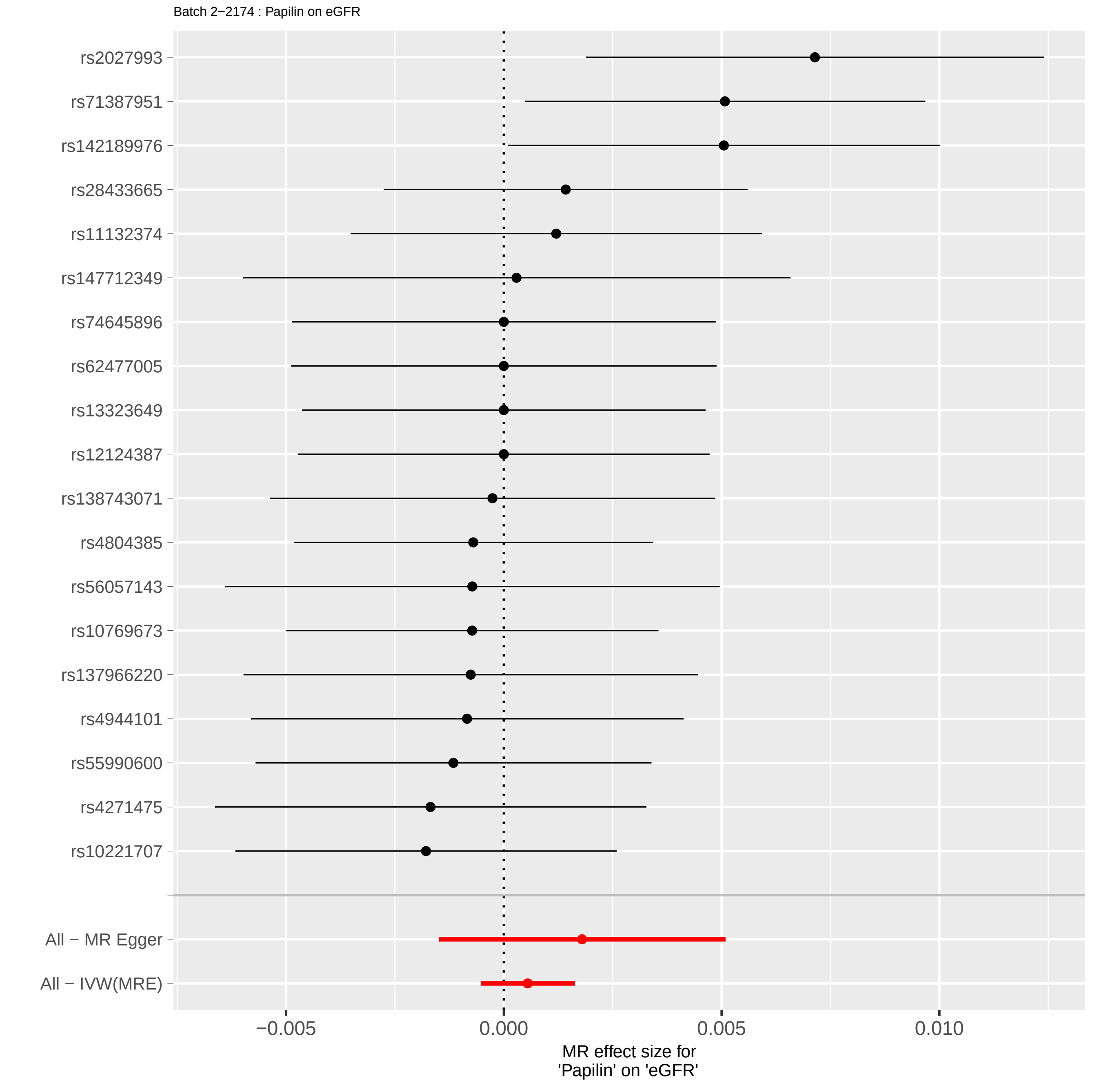

Batch 2−2174 : Papilin on eGFR
rs2027993
rs71387951
rs142189976
rs28433665
rs11132374
rs147712349
rs74645896
rs62477005
rs13323649
rs12124387
rs138743071
rs4804385
rs56057143
rs10769673
rs137966220
rs4944101
rs55990600
rs4271475
rs10221707
All − MR Egger
All − IVW(MRE)
−0.005
0.000
0.005
0.010
MR effect size for
'Papilin' on 'eGFR'

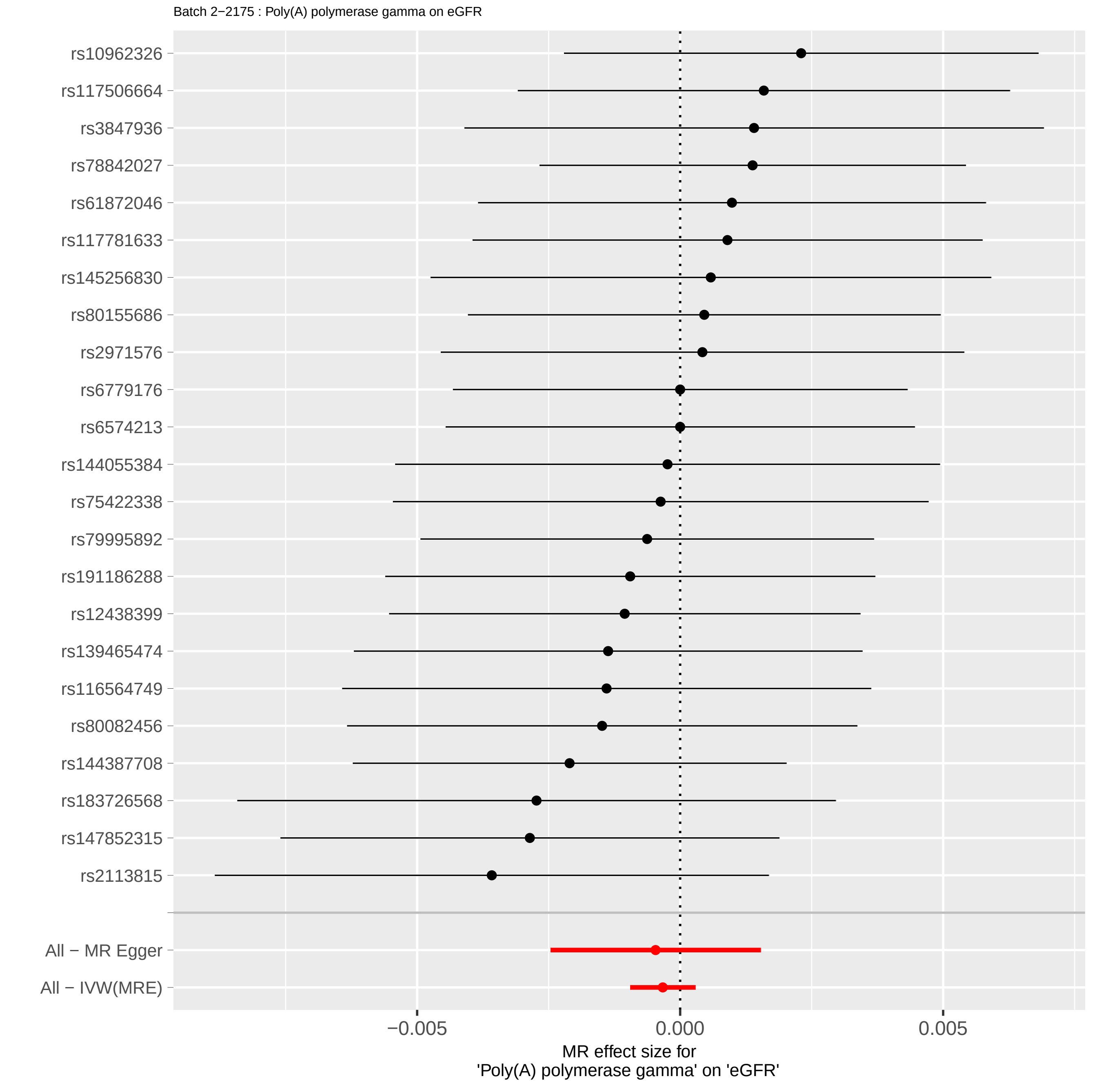

Batch 2−2175 : Poly(A) polymerase gamma on eGFR
rs10962326
rs117506664
rs3847936
rs78842027
rs61872046
rs117781633
rs145256830
rs80155686
rs2971576
rs6779176
rs6574213
rs144055384
rs75422338
rs79995892
rs191186288
rs12438399
rs139465474
rs116564749
rs80082456
rs144387708
rs183726568
rs147852315
rs2113815
All − MR Egger
All − IVW(MRE)
−0.005
0.000
0.005
MR effect size for
'Poly(A) polymerase gamma' on 'eGFR'

Batch 2−2176 : Pappalysin−2 on eGFR

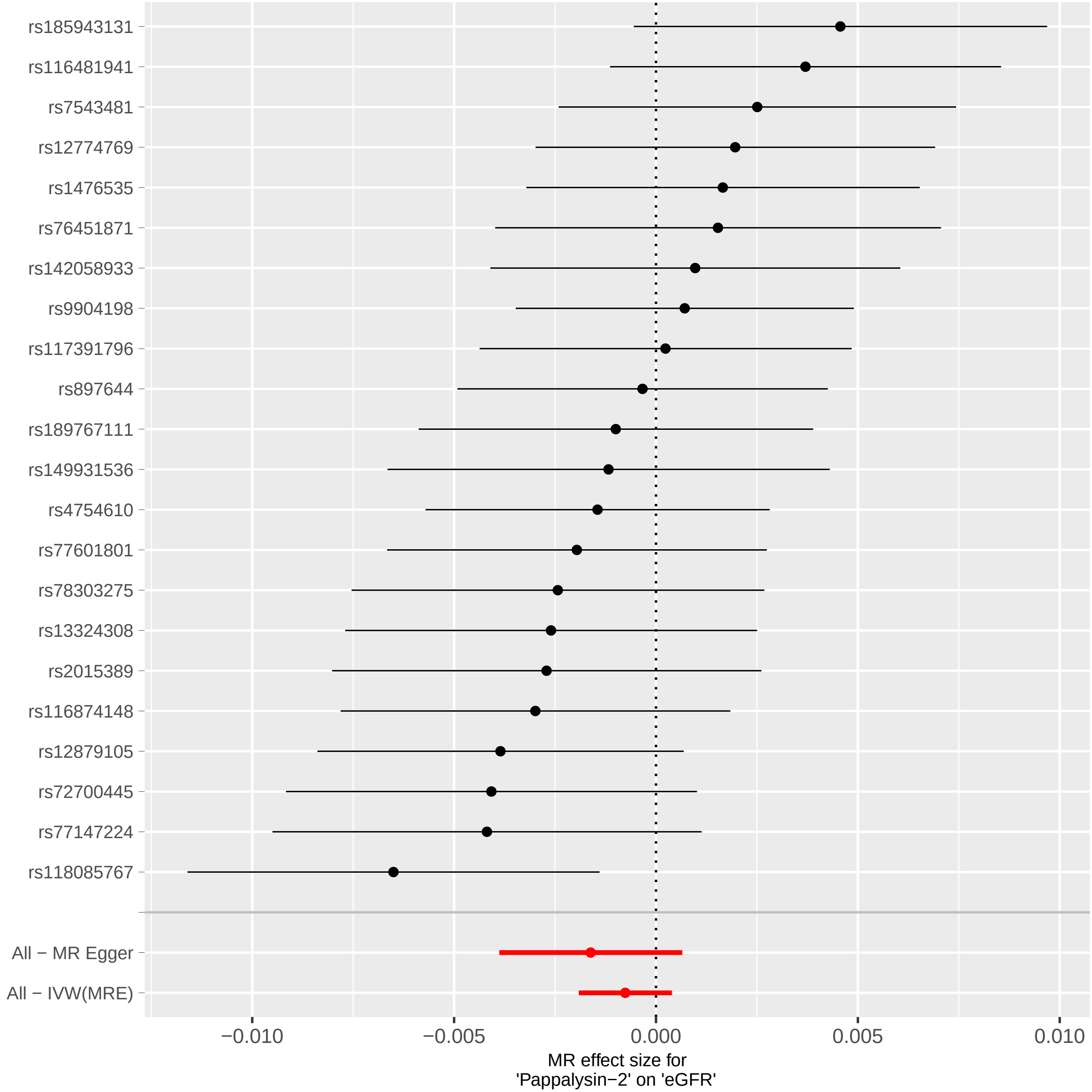

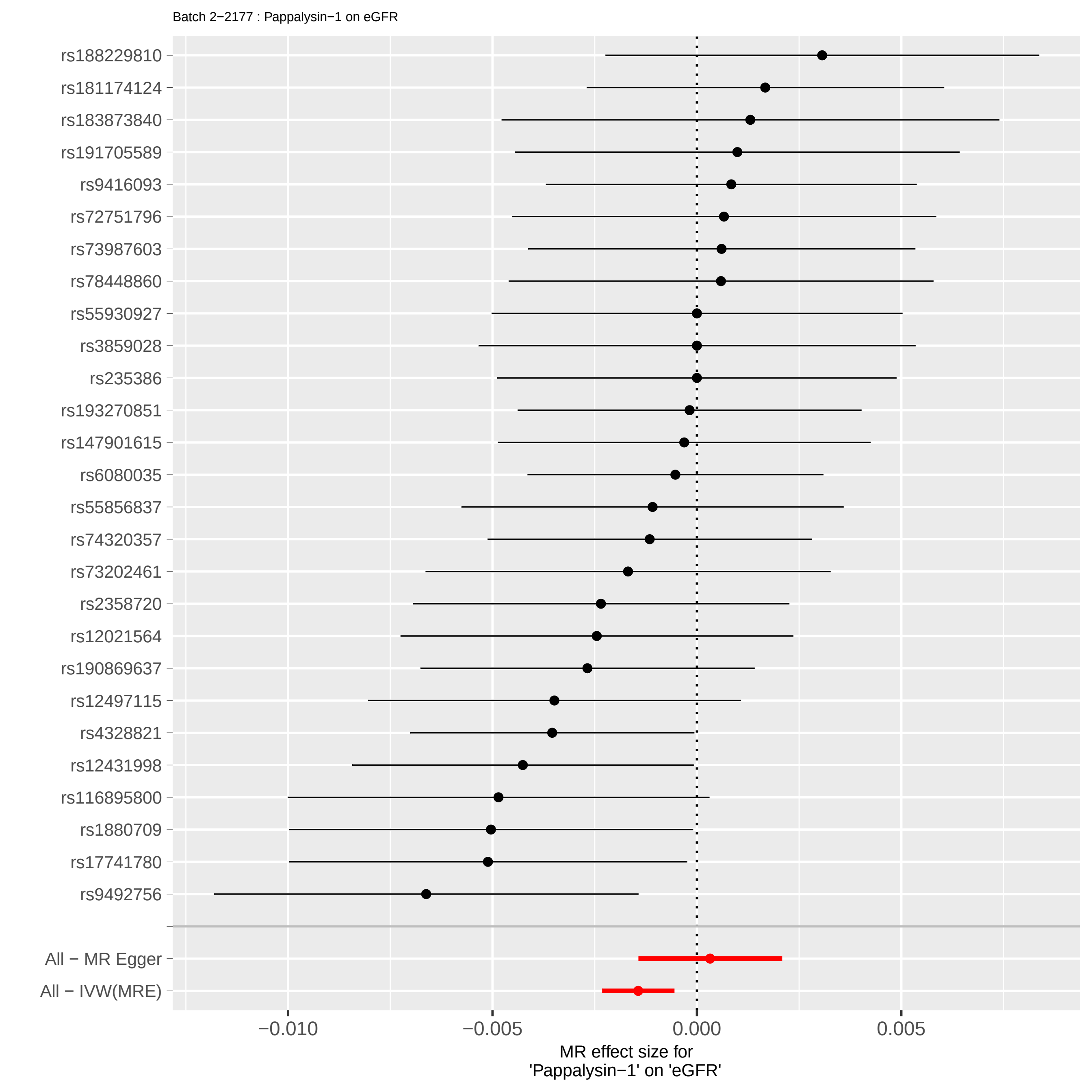

Batch 2−2177 : Pappalysin−1 on eGFR
rs188229810
rs181174124
rs183873840
rs191705589
rs9416093
rs72751796
rs73987603
rs78448860
rs55930927
rs3859028
rs235386
rs193270851
rs147901615
rs6080035
rs55856837
rs74320357
rs73202461
rs2358720
rs12021564
rs190869637
rs12497115
rs4328821
rs12431998
rs116895800
rs1880709
rs17741780
rs9492756
All − MR Egger
All − IVW(MRE)
−0.010
−0.005
0.000
0.005
MR effect size for
'Pappalysin−1' on 'eGFR'

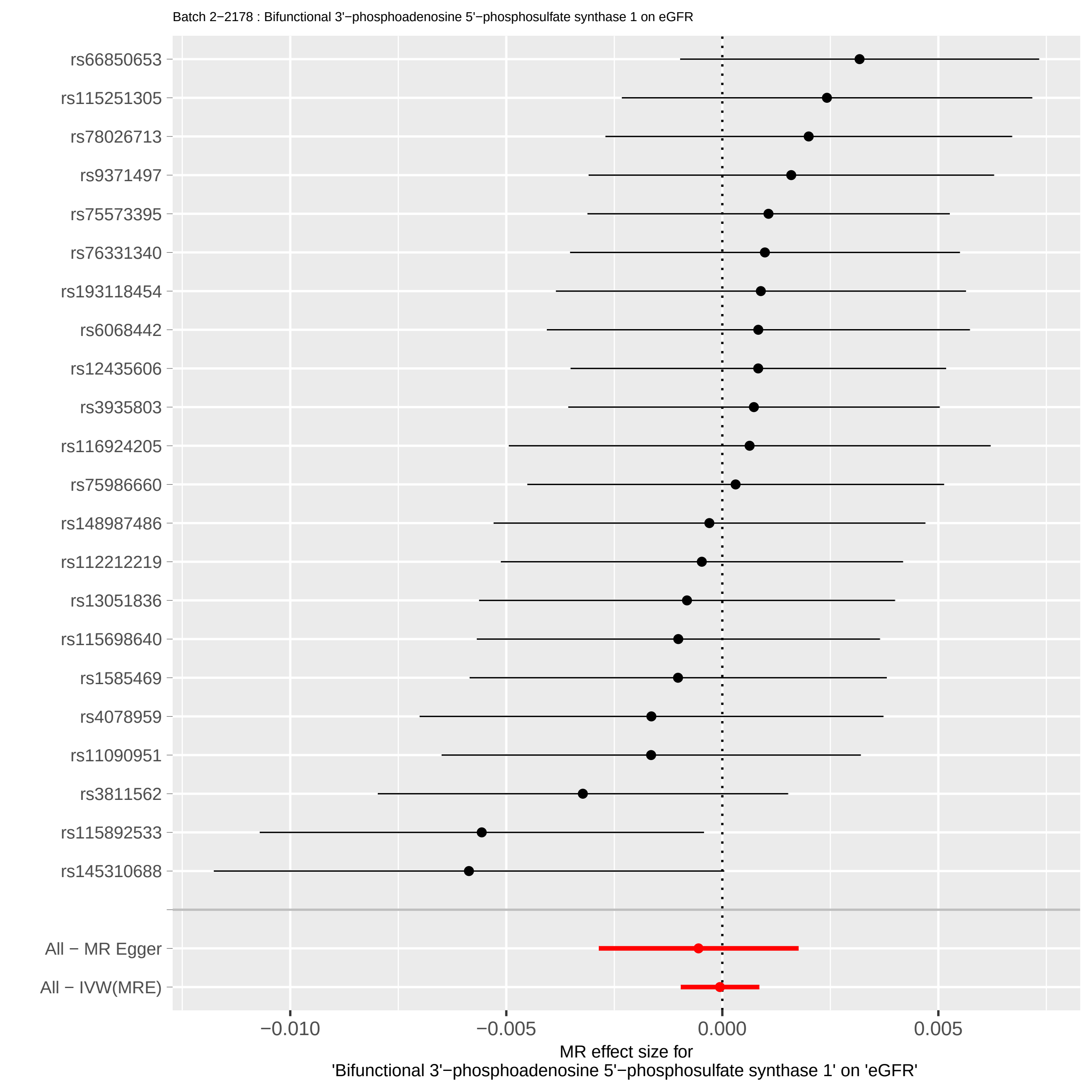

Batch 2−2178 : Bifunctional 3'−phosphoadenosine 5'−phosphosulfate synthase 1 on eGFR
rs66850653
rs115251305
rs78026713
rs9371497
rs75573395
rs76331340
rs193118454
rs6068442
rs12435606
rs3935803
rs116924205
rs75986660
rs148987486
rs112212219
rs13051836
rs115698640
rs1585469
rs4078959
rs11090951
rs3811562
rs115892533
rs145310688
All − MR Egger
All − IVW(MRE)
−0.010
−0.005
0.000
0.005
MR effect size for
'Bifunctional 3'−phosphoadenosine 5'−phosphosulfate synthase 1' on 'eGFR'

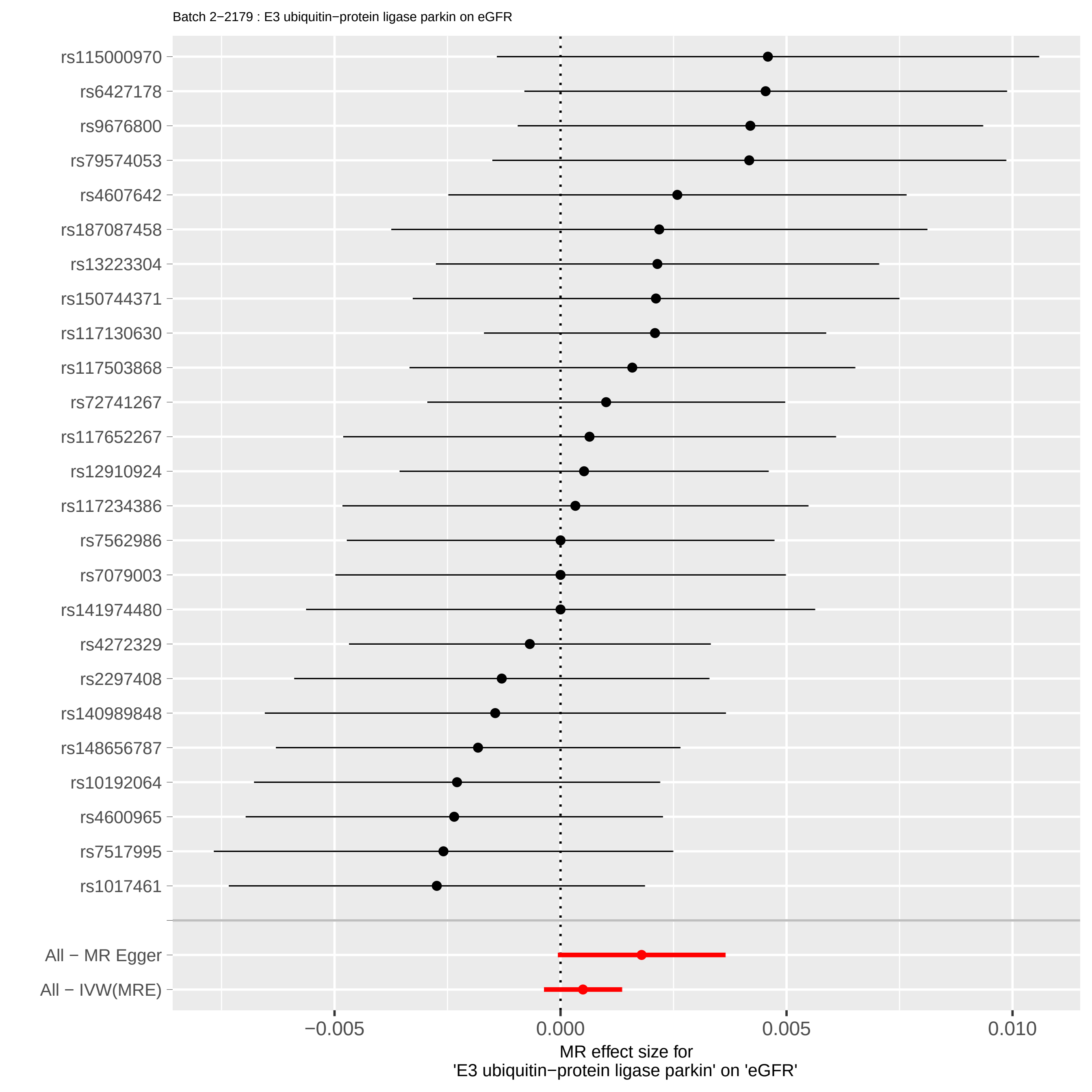

Batch 2−2179 : E3 ubiquitin−protein ligase parkin on eGFR
rs115000970
rs6427178
rs9676800
rs79574053
rs4607642
rs187087458
rs13223304
rs150744371
rs117130630
rs117503868
rs72741267
rs117652267
rs12910924
rs117234386
rs7562986
rs7079003
rs141974480
rs4272329
rs2297408
rs140989848
rs148656787
rs10192064
rs4600965
rs7517995
rs1017461
All − MR Egger
All − IVW(MRE)
−0.005
0.000
0.005
0.010
MR effect size for
'E3 ubiquitin−protein ligase parkin' on 'eGFR'

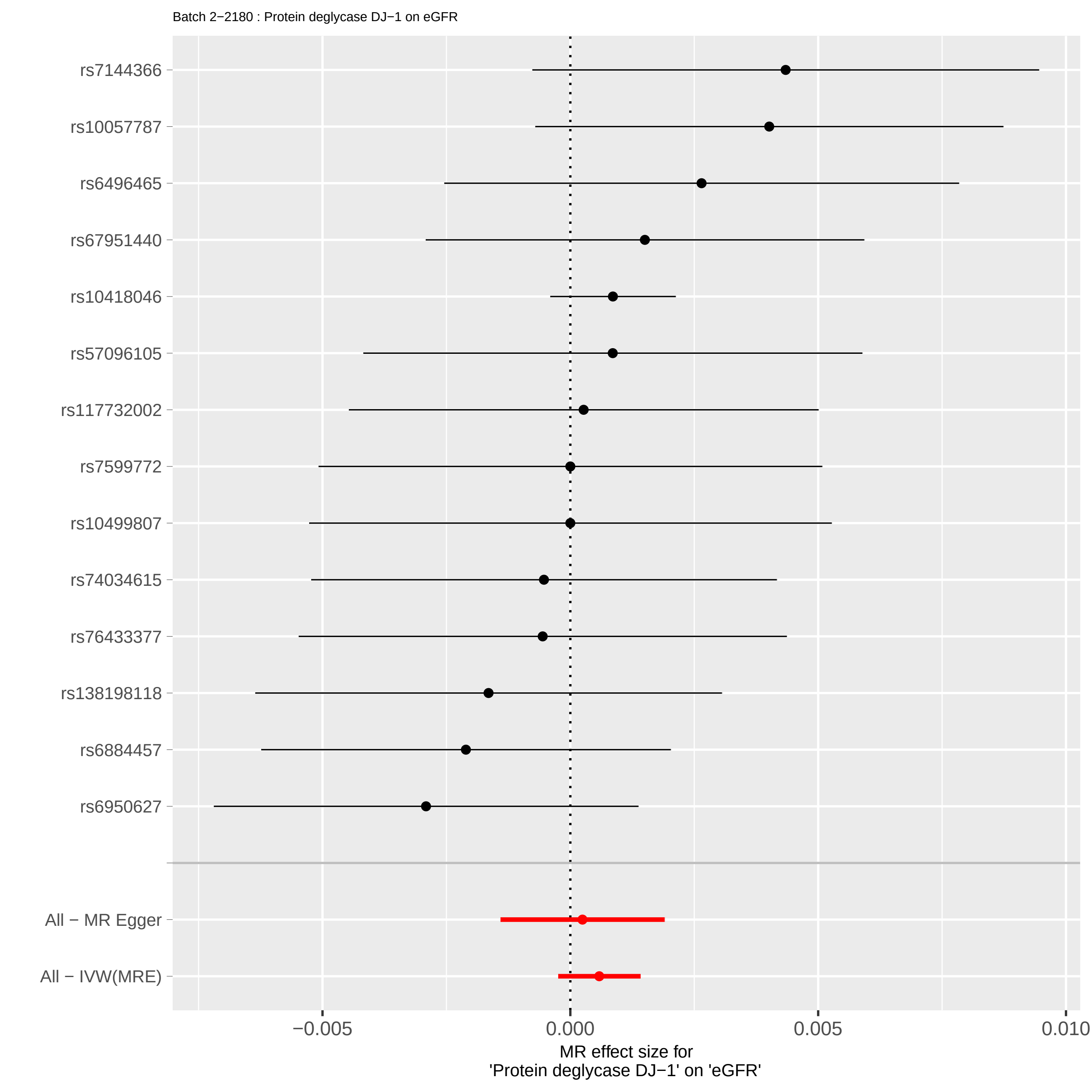

Batch 2−2180 : Protein deglycase DJ−1 on eGFR
rs7144366
rs10057787
rs6496465
rs67951440
rs10418046
rs57096105
rs117732002
rs7599772
rs10499807
rs74034615
rs76433377
rs138198118
rs6884457
rs6950627
All − MR Egger
All − IVW(MRE)
−0.005
0.000
0.005
0.010
MR effect size for
'Protein deglycase DJ−1' on 'eGFR'

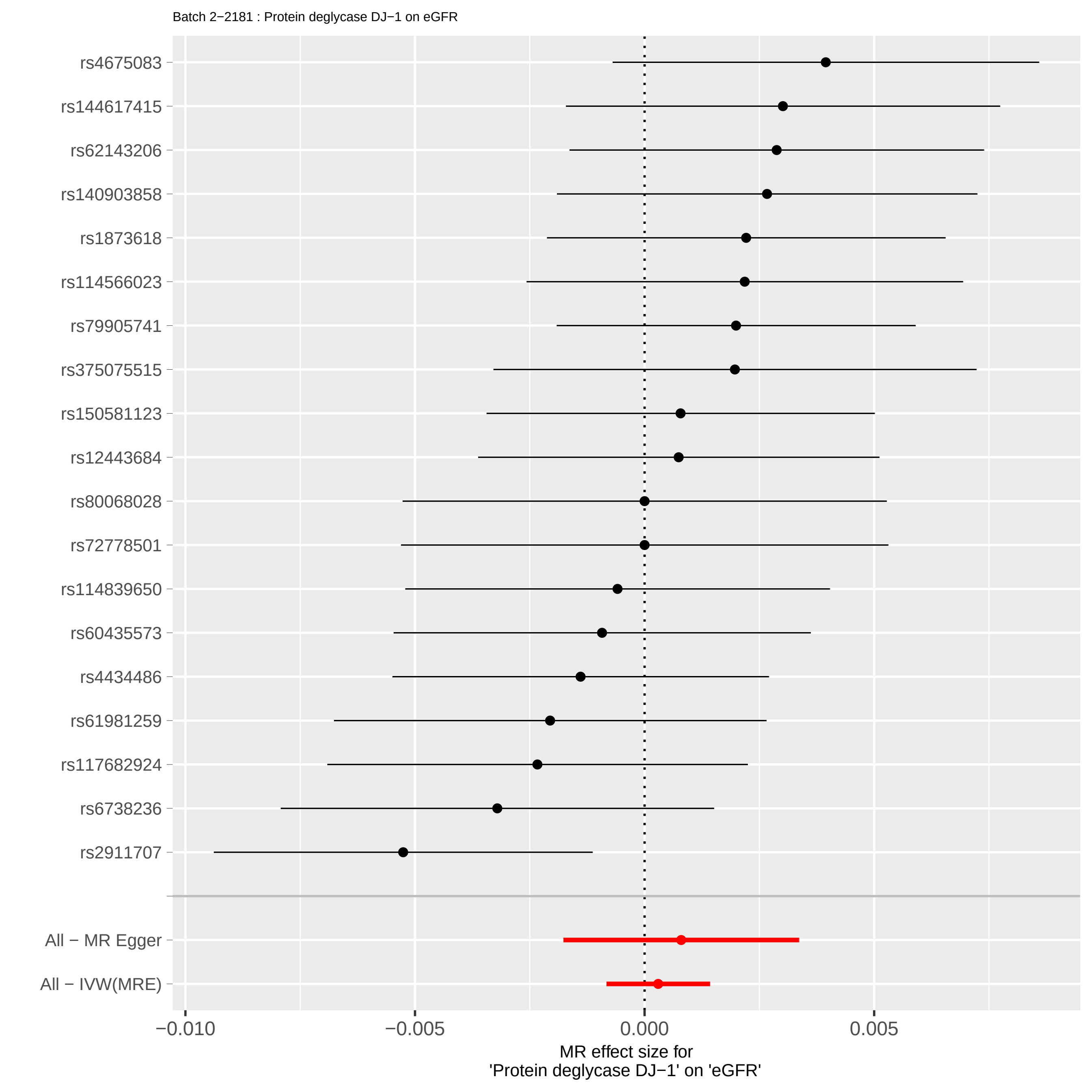

Batch 2−2181 : Protein deglycase DJ−1 on eGFR
rs4675083
rs144617415
rs62143206
rs140903858
rs1873618
rs114566023
rs79905741
rs375075515
rs150581123
rs12443684
rs80068028
rs72778501
rs114839650
rs60435573
rs4434486
rs61981259
rs117682924
rs6738236
rs2911707
All − MR Egger
All − IVW(MRE)
−0.010
−0.005
0.000
0.005
MR effect size for
'Protein deglycase DJ−1' on 'eGFR'

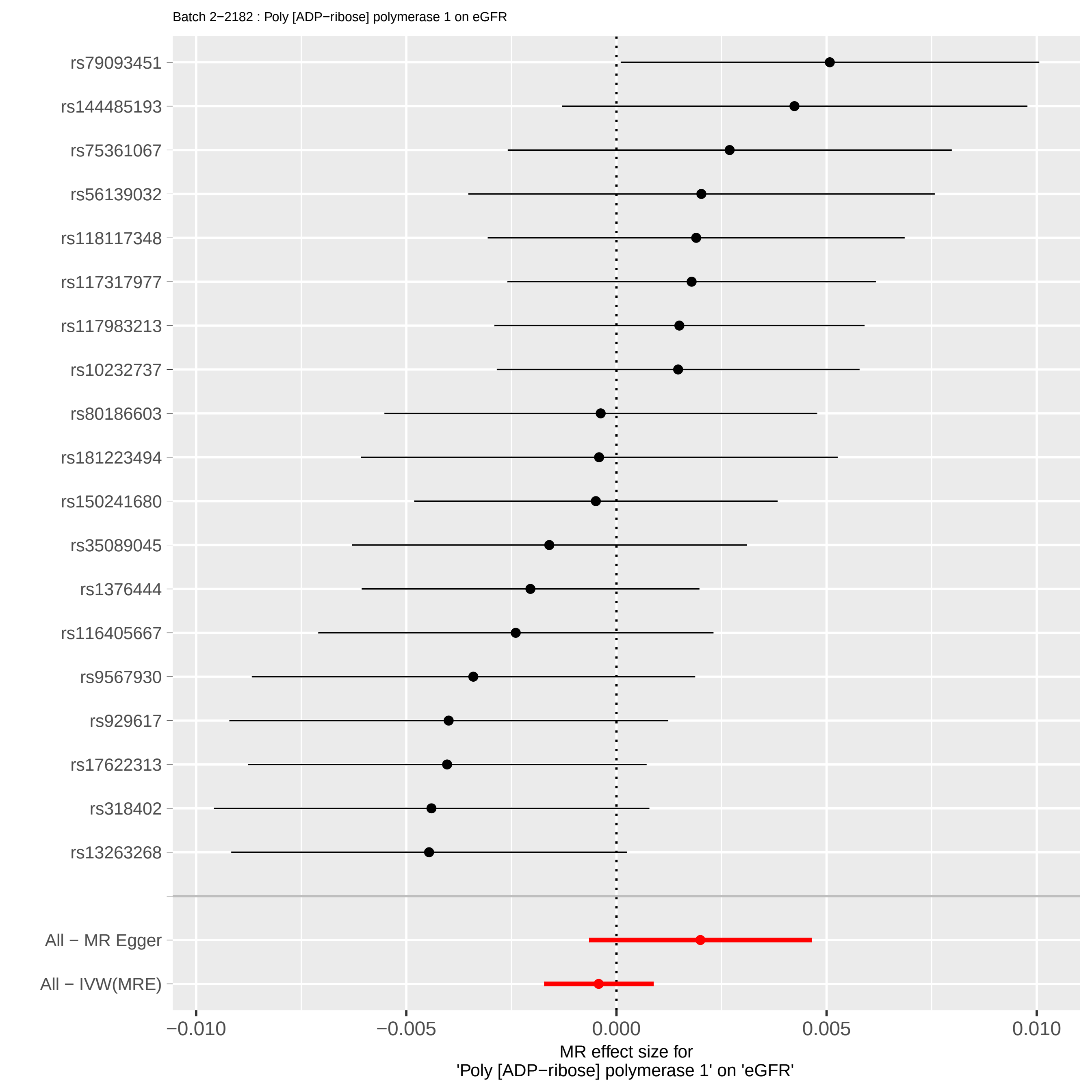

Batch 2−2182 : Poly [ADP−ribose] polymerase 1 on eGFR
rs79093451
rs144485193
rs75361067
rs56139032
rs118117348
rs117317977
rs117983213
rs10232737
rs80186603
rs181223494
rs150241680
rs35089045
rs1376444
rs116405667
rs9567930
rs929617
rs17622313
rs318402
rs13263268
All − MR Egger
All − IVW(MRE)
−0.010
−0.005
0.000
0.005
0.010
MR effect size for
'Poly [ADP−ribose] polymerase 1' on 'eGFR'

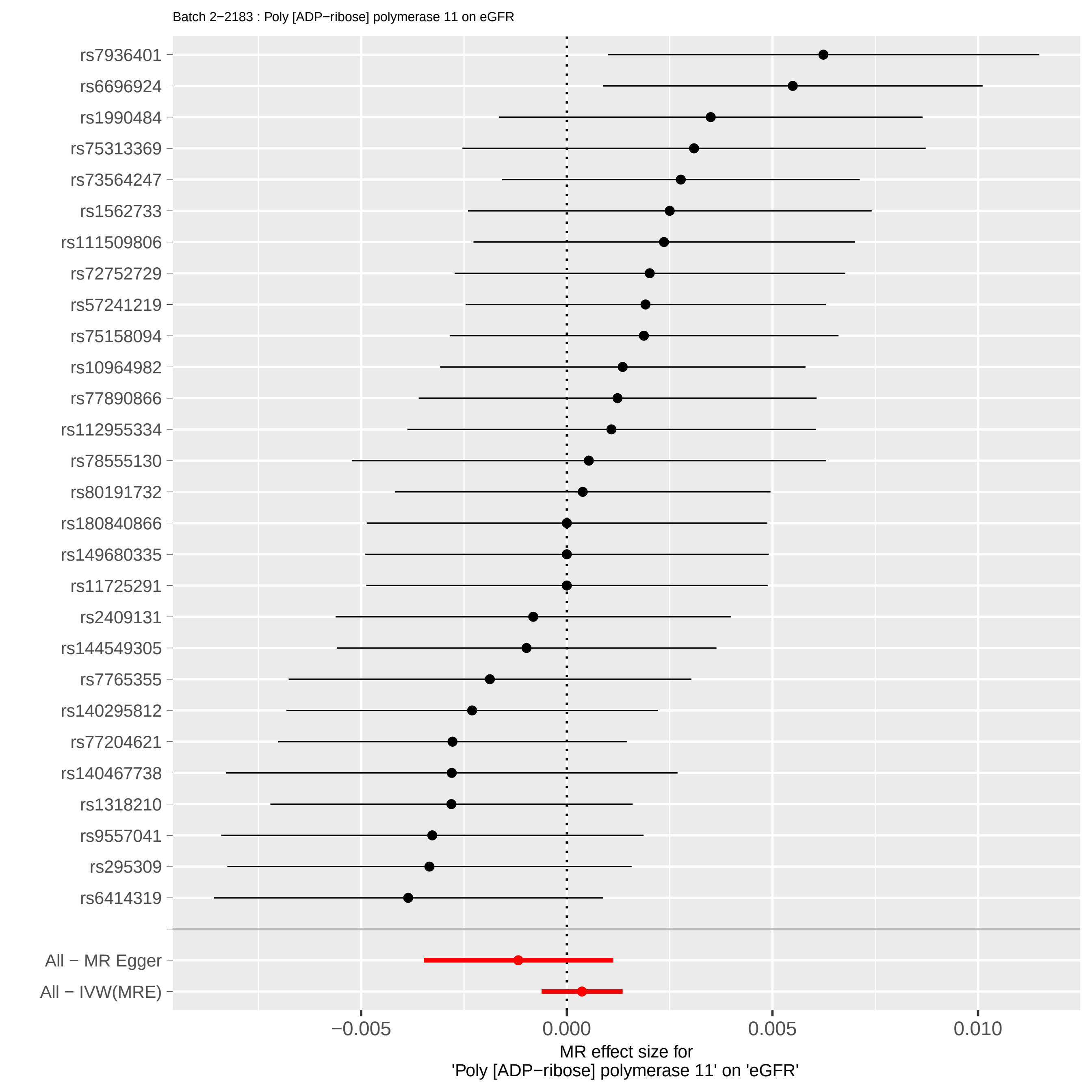
Batch 2−2183 : Poly [ADP−ribose] polymerase 11 on eGFR
rs7936401
rs6696924
rs1990484
rs75313369
rs73564247
rs1562733
rs111509806
rs72752729
rs57241219
rs75158094
rs10964982
rs77890866
rs112955334
rs78555130
rs80191732
rs180840866
rs149680335
rs11725291
rs2409131
rs144549305
rs7765355
rs140295812
rs77204621
rs140467738
rs1318210
rs9557041
rs295309
rs6414319
All − MR Egger
All − IVW(MRE)
−0.005
0.000
0.005
0.010
MR effect size for
'Poly [ADP−ribose] polymerase 11' on 'eGFR'

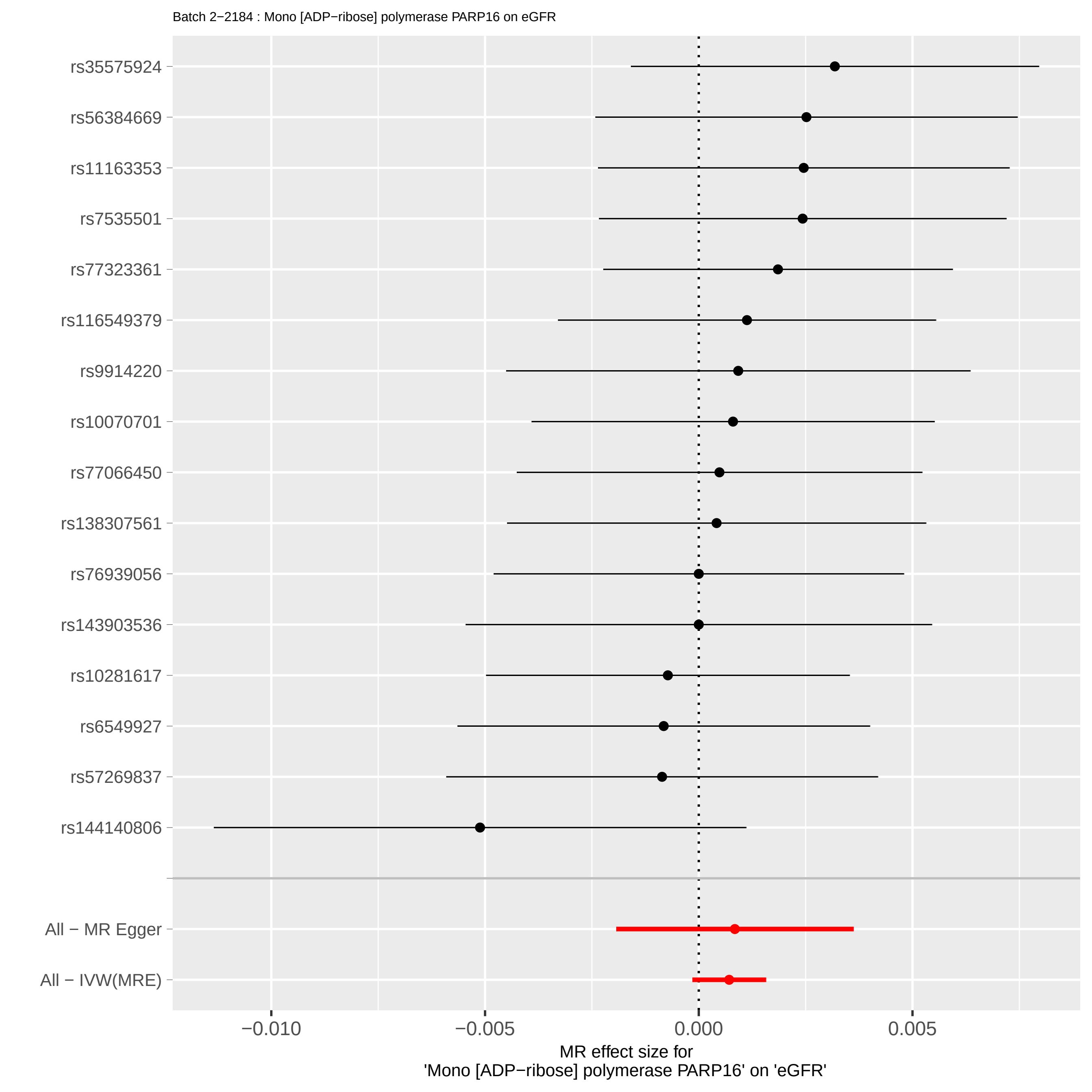

Batch 2−2184 : Mono [ADP−ribose] polymerase PARP16 on eGFR
rs35575924
rs56384669
rs11163353
rs7535501
rs77323361
rs116549379
rs9914220
rs10070701
rs77066450
rs138307561
rs76939056
rs143903536
rs10281617
rs6549927
rs57269837
rs144140806
All − MR Egger
All − IVW(MRE)
−0.010
−0.005
0.000
0.005
MR effect size for
'Mono [ADP−ribose] polymerase PARP16' on 'eGFR'

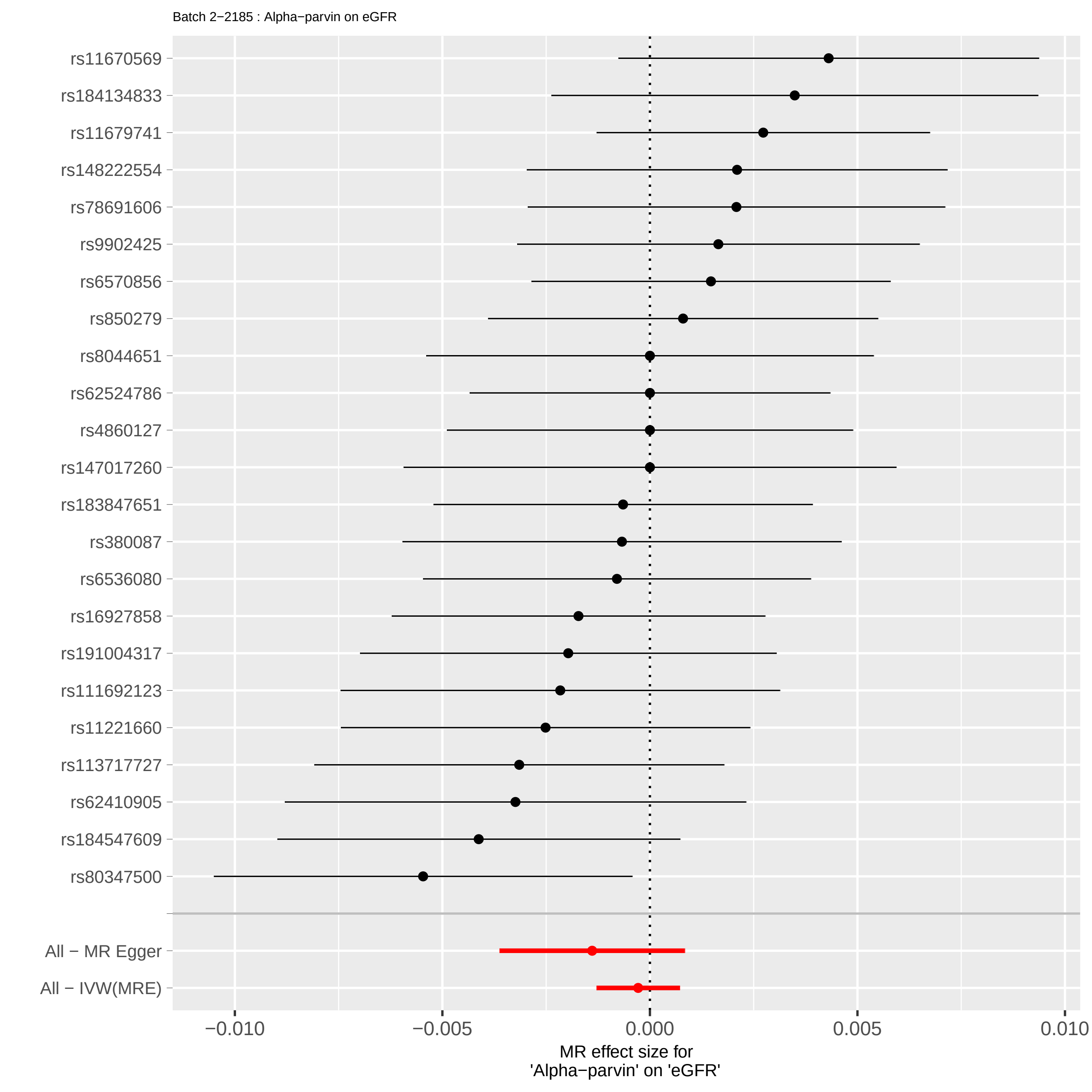

Batch 2−2185 : Alpha−parvin on eGFR
rs11670569
rs184134833
rs11679741
rs148222554
rs78691606
rs9902425
rs6570856
rs850279
rs8044651
rs62524786
rs4860127
rs147017260
rs183847651
rs380087
rs6536080
rs16927858
rs191004317
rs111692123
rs11221660
rs113717727
rs62410905
rs184547609
rs80347500
All − MR Egger
All − IVW(MRE)
−0.010
−0.005
0.000
0.005
0.010
MR effect size for
'Alpha−parvin' on 'eGFR'

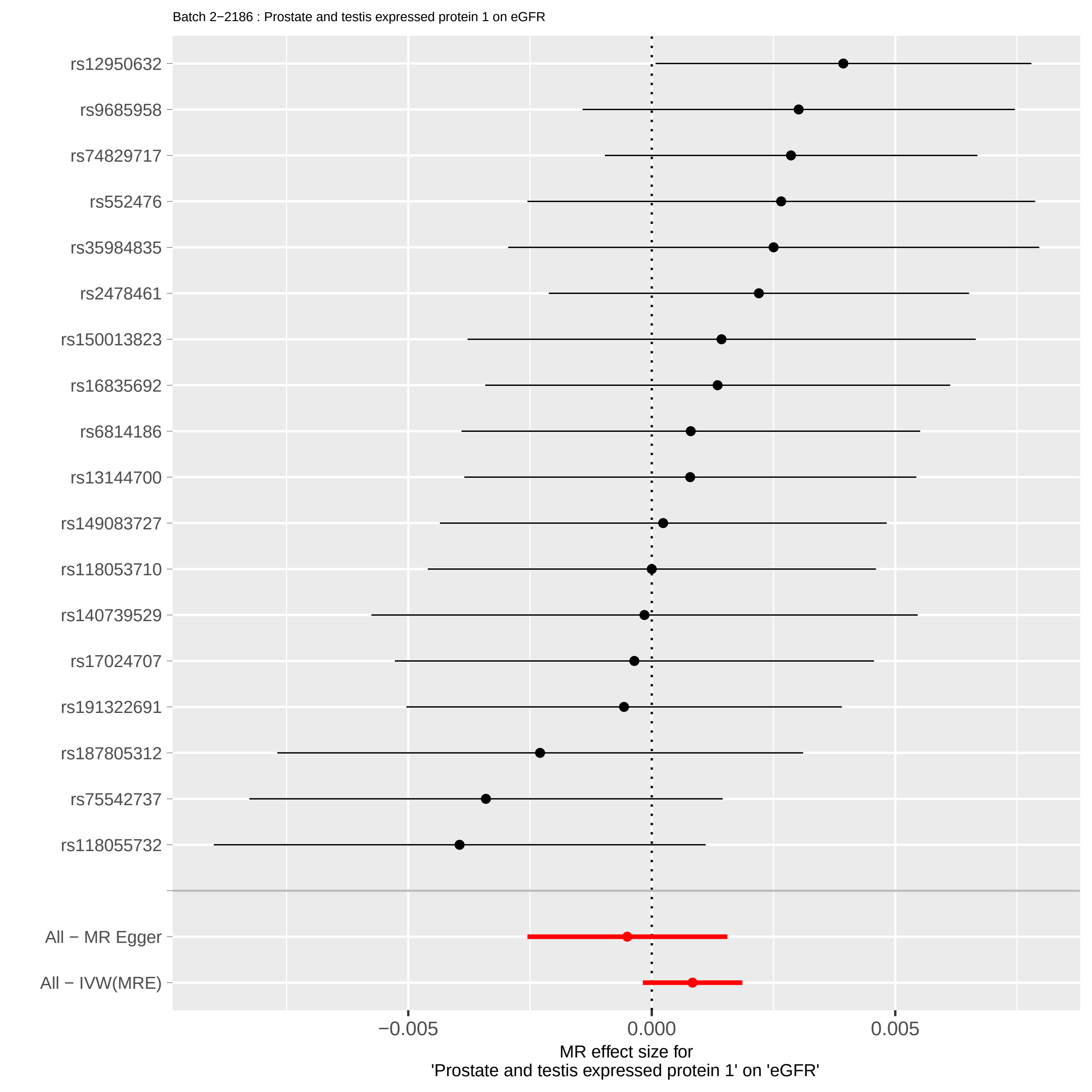

Batch 2−2186 : Prostate and testis expressed protein 1 on eGFR
rs12950632
rs9685958
rs74829717
rs552476
rs35984835
rs2478461
rs150013823
rs16835692
rs6814186
rs13144700
rs149083727
rs118053710
rs140739529
rs17024707
rs191322691
rs187805312
rs75542737
rs118055732
All − MR Egger
All − IVW(MRE)
−0.005
0.000
0.005
MR effect size for
'Prostate and testis expressed protein 1' on 'eGFR'

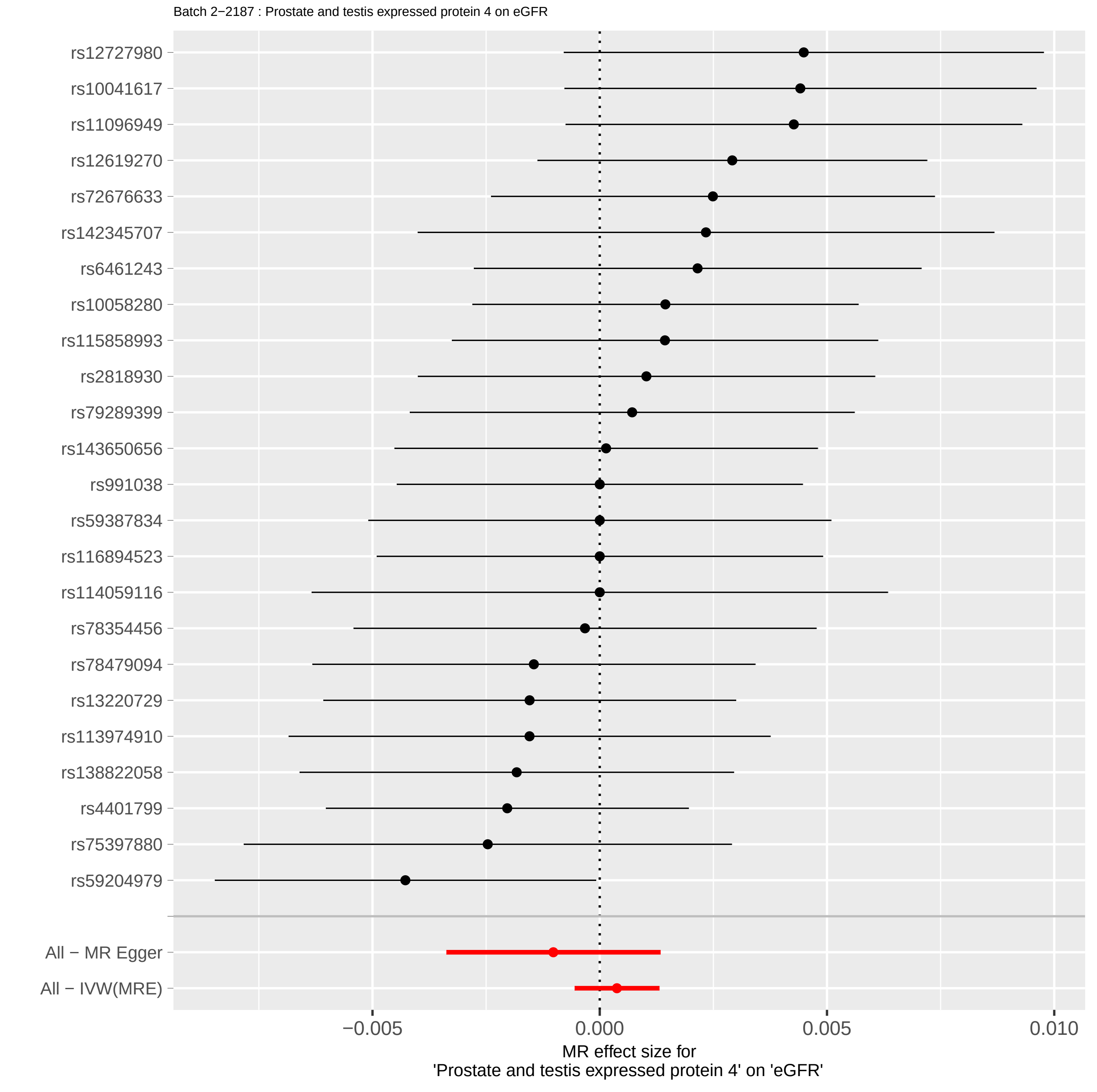

Batch 2−2187 : Prostate and testis expressed protein 4 on eGFR
rs12727980
rs10041617
rs11096949
rs12619270
rs72676633
rs142345707
rs6461243
rs10058280
rs115858993
rs2818930
rs79289399
rs143650656
rs991038
rs59387834
rs116894523
rs114059116
rs78354456
rs78479094
rs13220729
rs113974910
rs138822058
rs4401799
rs75397880
rs59204979
All − MR Egger
All − IVW(MRE)
−0.005
0.000
0.005
0.010
MR effect size for
'Prostate and testis expressed protein 4' on 'eGFR'

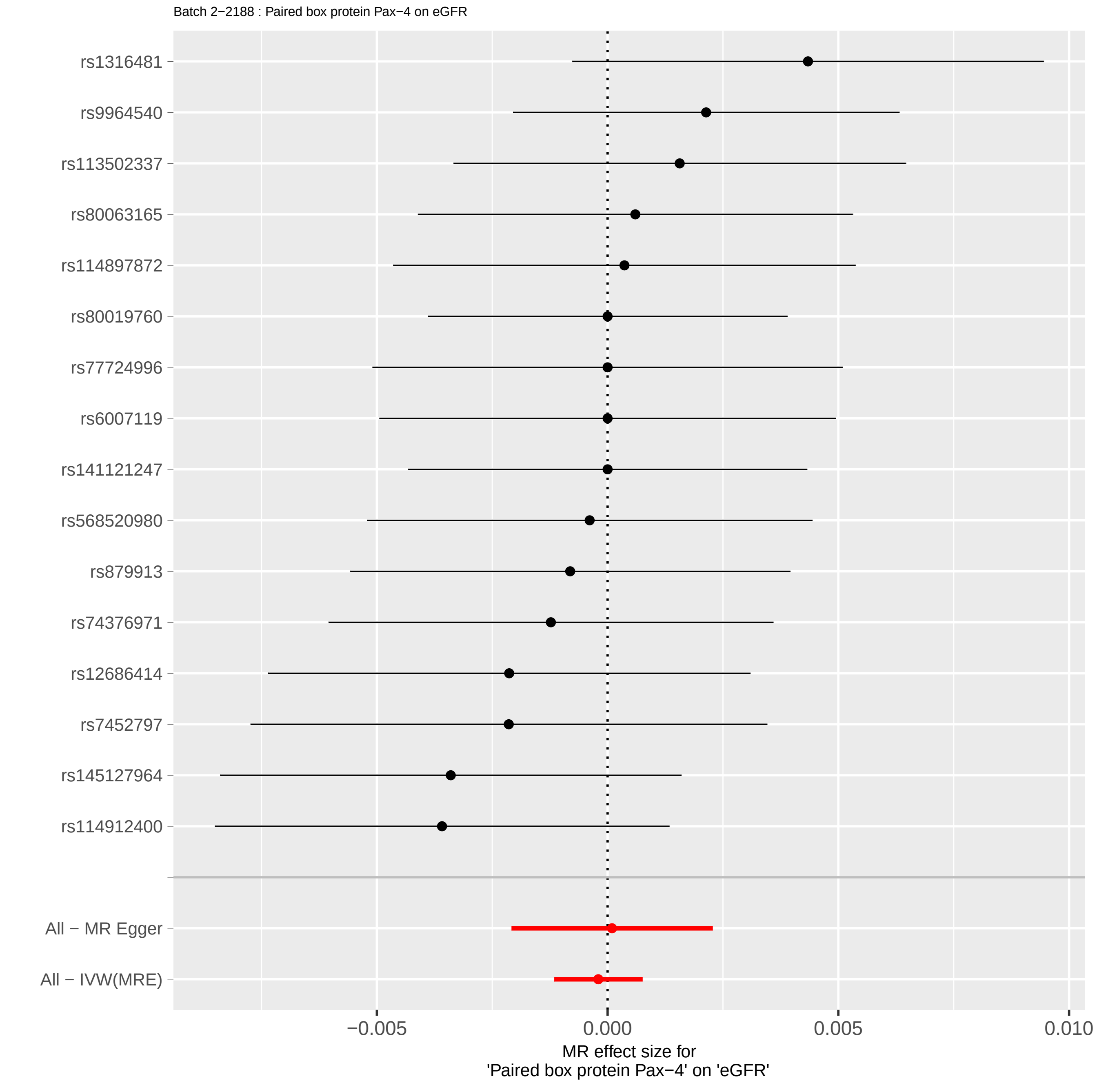

Batch 2−2188 : Paired box protein Pax−4 on eGFR
rs1316481
rs9964540
rs113502337
rs80063165
rs114897872
rs80019760
rs77724996
rs6007119
rs141121247
rs568520980
rs879913
rs74376971
rs12686414
rs7452797
rs145127964
rs114912400
All − MR Egger
All − IVW(MRE)
−0.005
0.000
0.005
0.010
MR effect size for
'Paired box protein Pax−4' on 'eGFR'

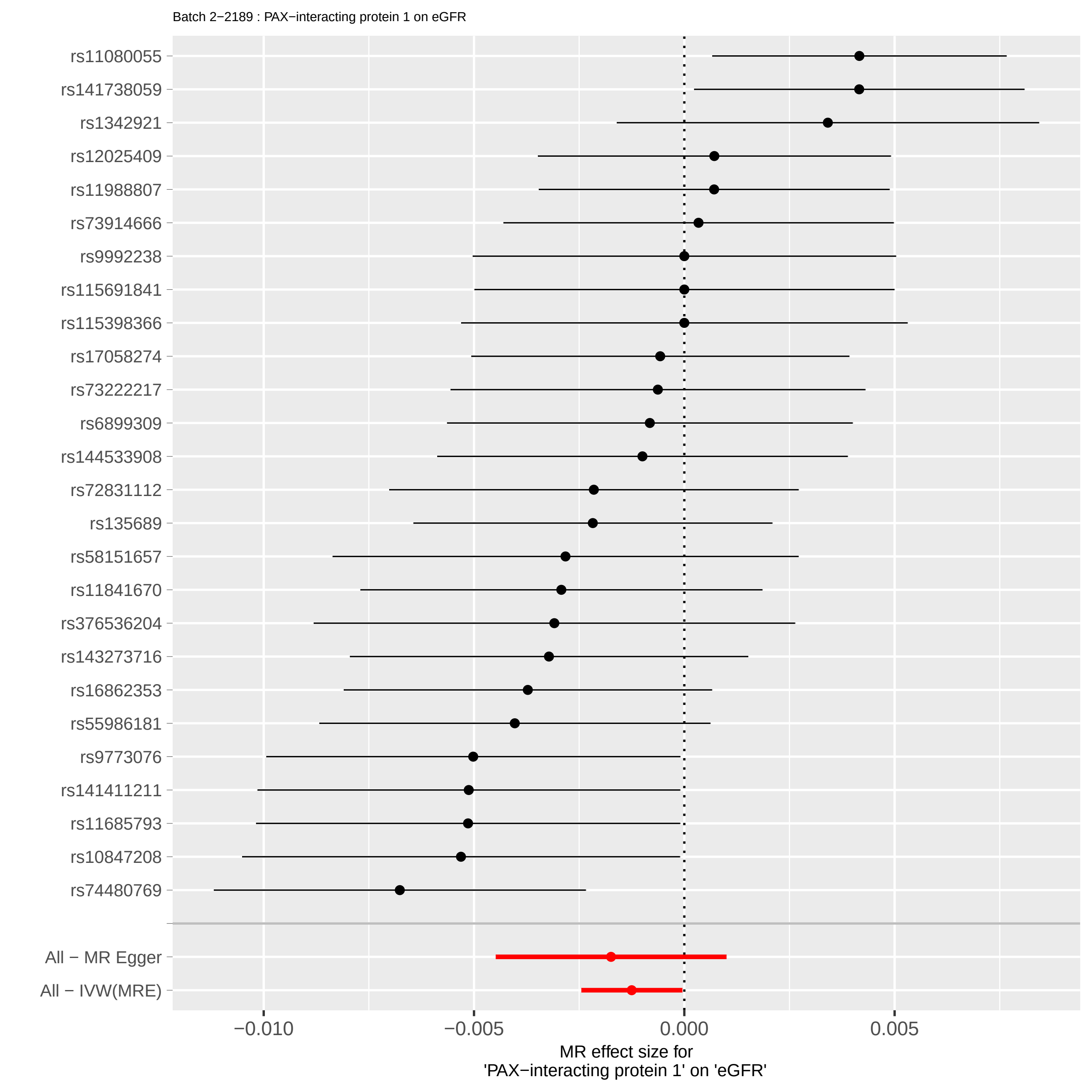

Batch 2−2189 : PAX−interacting protein 1 on eGFR
rs11080055
rs141738059
rs1342921
rs12025409
rs11988807
rs73914666
rs9992238
rs115691841
rs115398366
rs17058274
rs73222217
rs6899309
rs144533908
rs72831112
rs135689
rs58151657
rs11841670
rs376536204
rs143273716
rs16862353
rs55986181
rs9773076
rs141411211
rs11685793
rs10847208
rs74480769
All − MR Egger
All − IVW(MRE)
−0.010
−0.005
0.000
0.005
MR effect size for
'PAX−interacting protein 1' on 'eGFR'

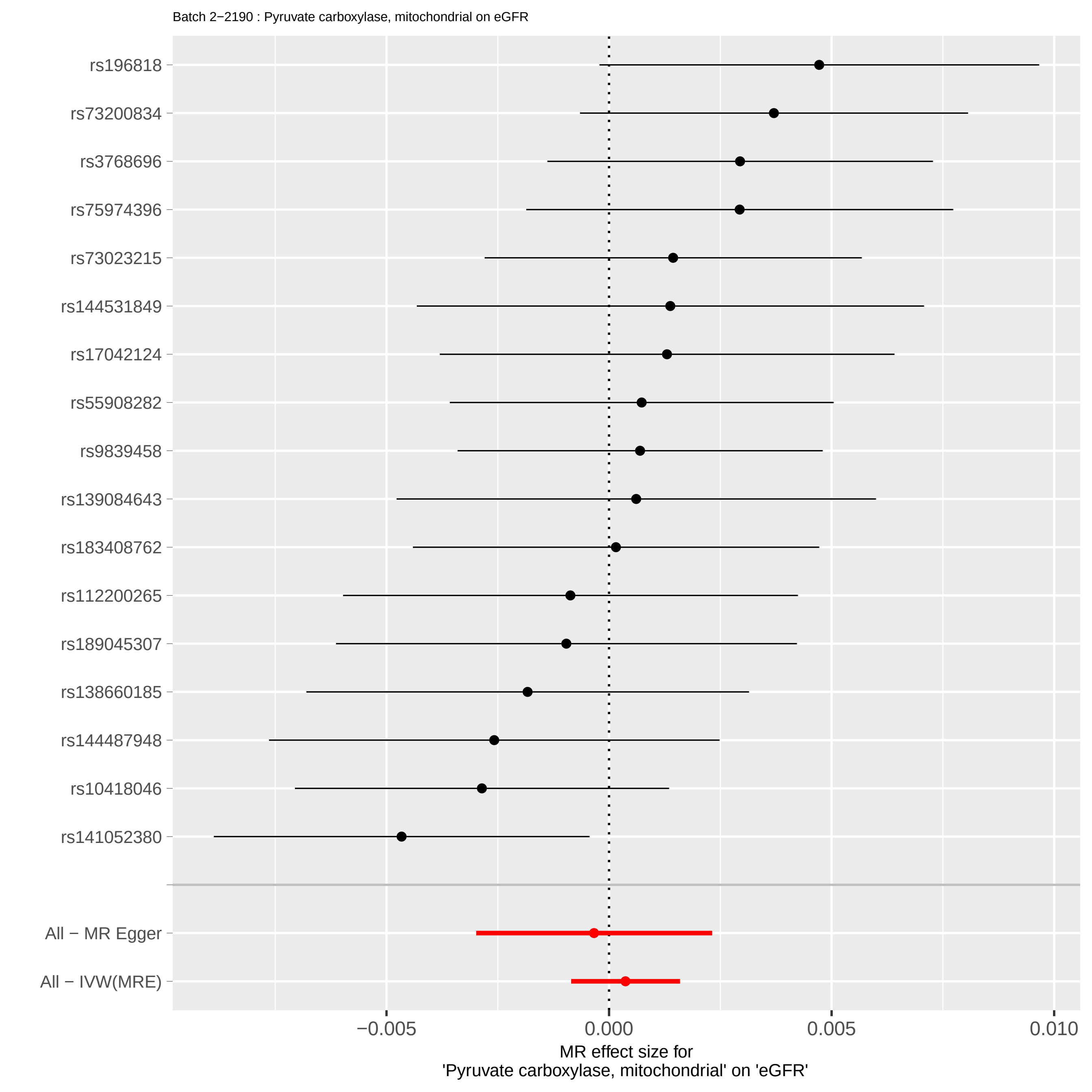
Batch 2−2190 : Pyruvate carboxylase, mitochondrial on eGFR
rs196818
rs73200834
rs3768696
rs75974396
rs73023215
rs144531849
rs17042124
rs55908282
rs9839458
rs139084643
rs183408762
rs112200265
rs189045307
rs138660185
rs144487948
rs10418046
rs141052380
All − MR Egger
All − IVW(MRE)
−0.005
0.000
0.005
0.010
MR effect size for
'Pyruvate carboxylase, mitochondrial' on 'eGFR'

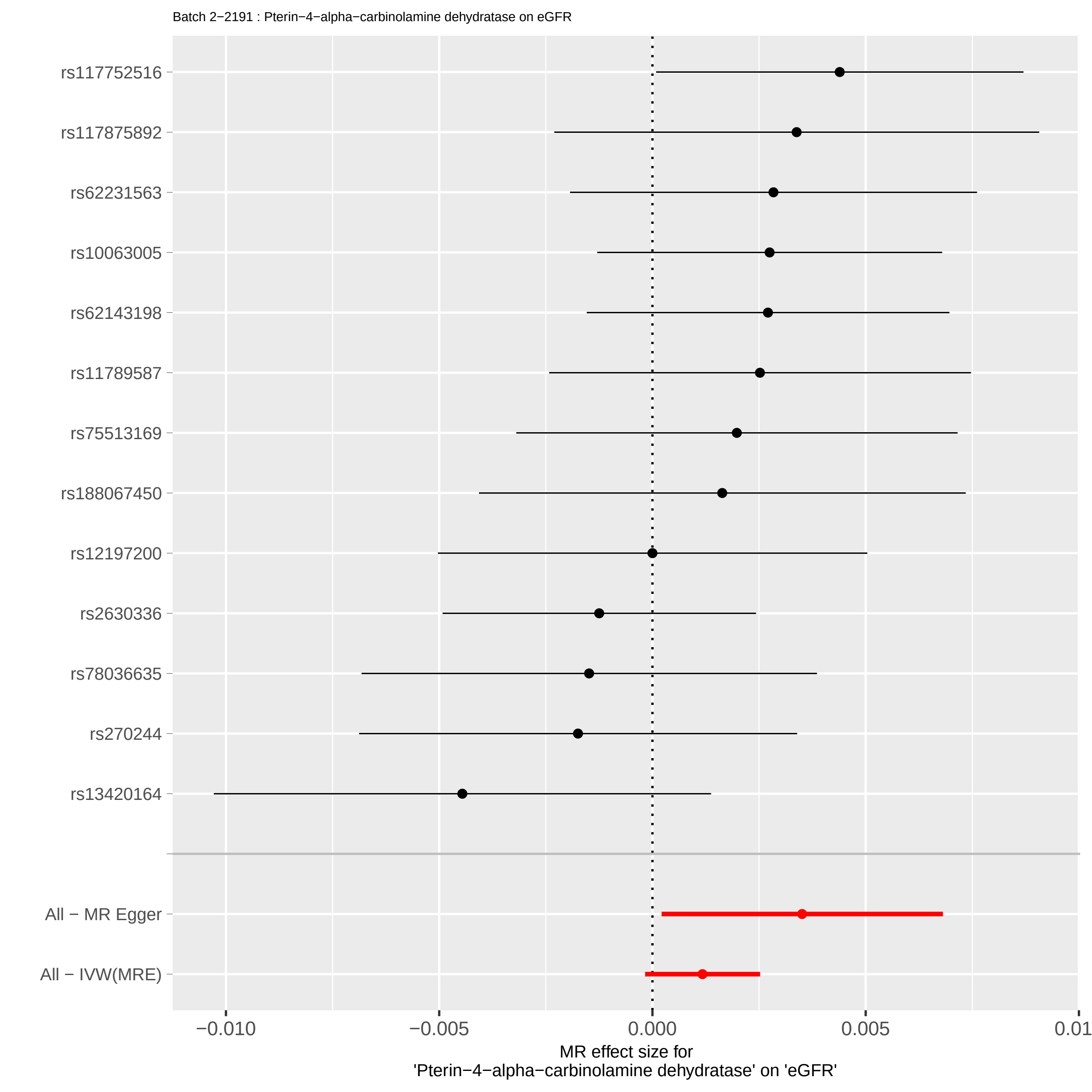

Batch 2−2191 : Pterin−4−alpha−carbinolamine dehydratase on eGFR
rs117752516
rs117875892
rs62231563
rs10063005
rs62143198
rs11789587
rs75513169
rs188067450
rs12197200
rs2630336
rs78036635
rs270244
rs13420164
All − MR Egger
All − IVW(MRE)
−0.010
−0.005
0.000
0.005
0.01
MR effect size for
'Pterin−4−alpha−carbinolamine dehydratase' on 'eGFR'

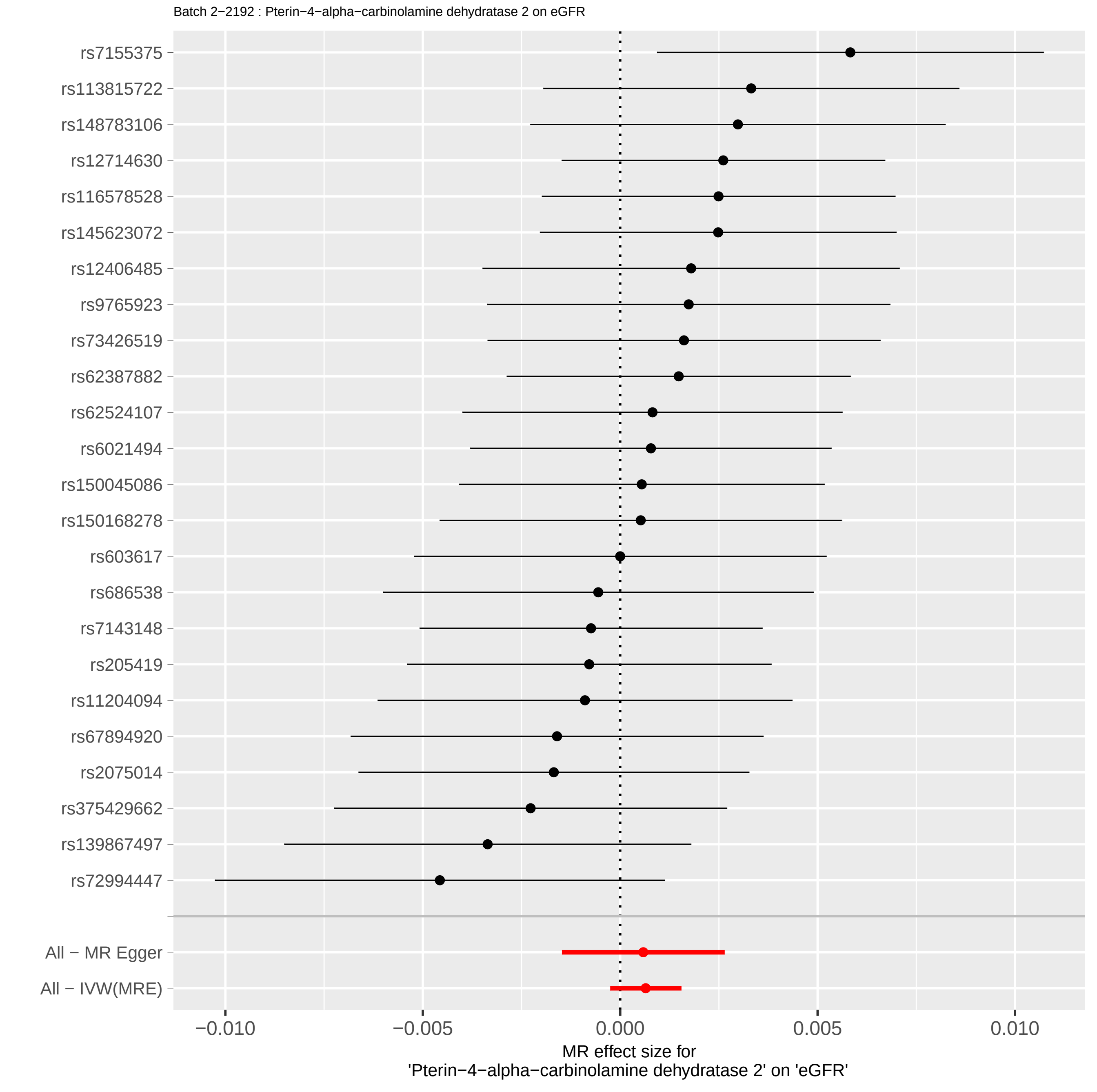

Batch 2−2192 : Pterin−4−alpha−carbinolamine dehydratase 2 on eGFR
rs7155375
rs113815722
rs148783106
rs12714630
rs116578528
rs145623072
rs12406485
rs9765923
rs73426519
rs62387882
rs62524107
rs6021494
rs150045086
rs150168278
rs603617
rs686538
rs7143148
rs205419
rs11204094
rs67894920
rs2075014
rs375429662
rs139867497
rs72994447
All − MR Egger
All − IVW(MRE)
−0.010
−0.005
0.000
0.005
0.010
MR effect size for
'Pterin−4−alpha−carbinolamine dehydratase 2' on 'eGFR'

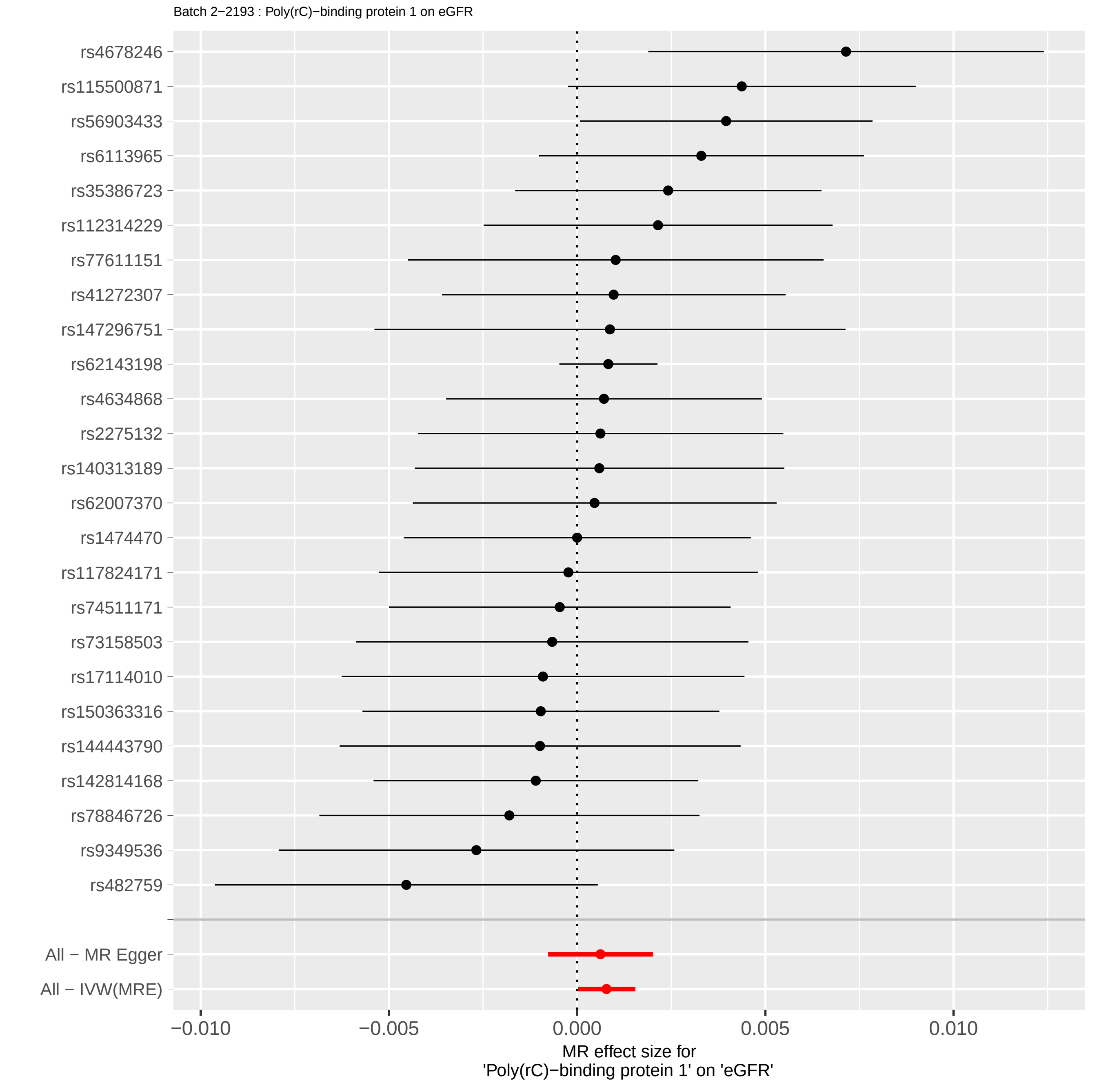
Batch 2−2193 : Poly(rC)−binding protein 1 on eGFR
rs4678246
rs115500871
rs56903433
rs6113965
rs35386723
rs112314229
rs77611151
rs41272307
rs147296751
rs62143198
rs4634868
rs2275132
rs140313189
rs62007370
rs1474470
rs117824171
rs74511171
rs73158503
rs17114010
rs150363316
rs144443790
rs142814168
rs78846726
rs9349536
rs482759
All − MR Egger
All − IVW(MRE)
−0.010
−0.005
0.000
0.005
0.010
MR effect size for
'Poly(rC)−binding protein 1' on 'eGFR'

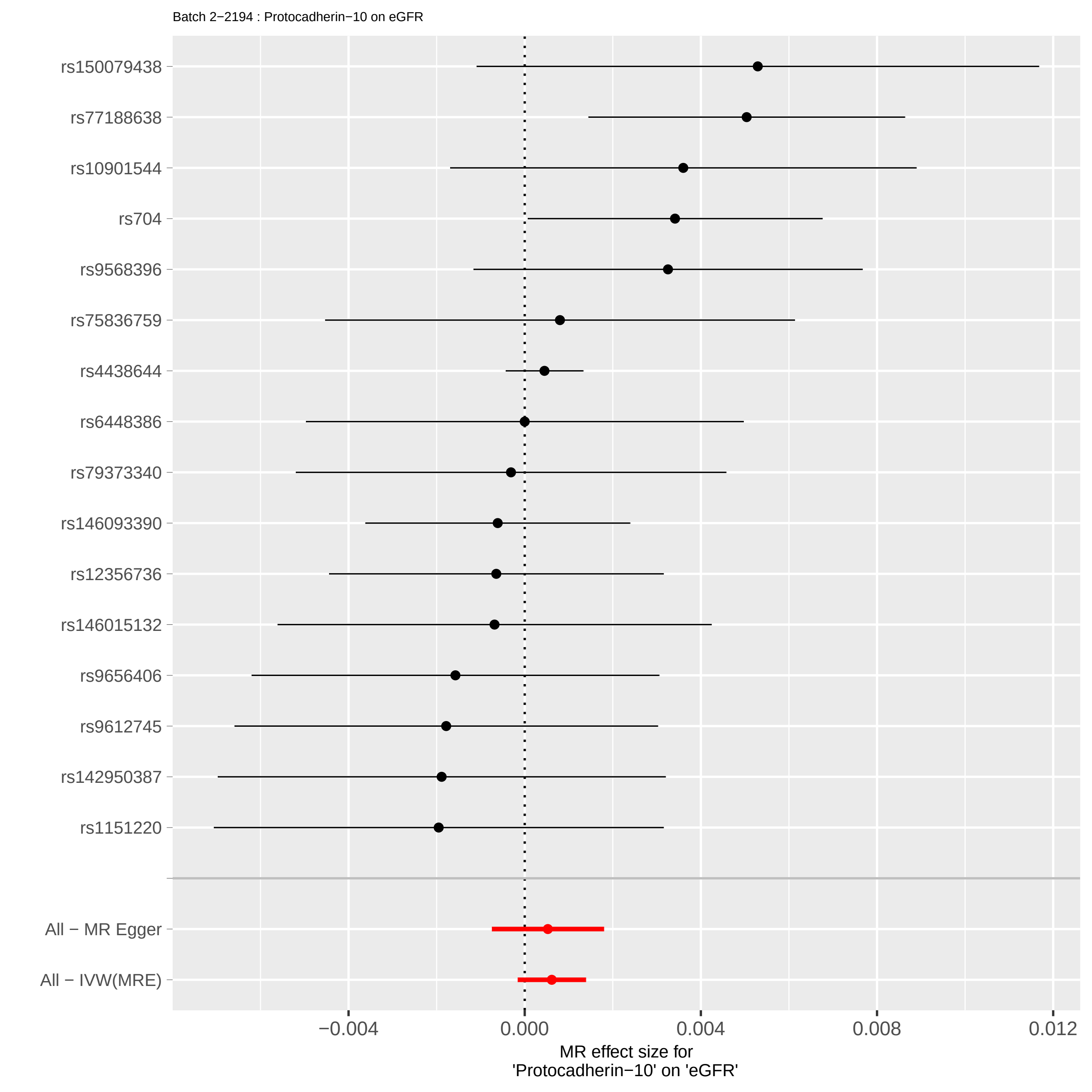
Batch 2−2194 : Protocadherin−10 on eGFR
rs150079438
rs77188638
rs10901544
rs704
rs9568396
rs75836759
rs4438644
rs6448386
rs79373340
rs146093390
rs12356736
rs146015132
rs9656406
rs9612745
rs142950387
rs1151220
All − MR Egger
All − IVW(MRE)
−0.004
0.000
0.004
0.008
0.012
MR effect size for
'Protocadherin−10' on 'eGFR'

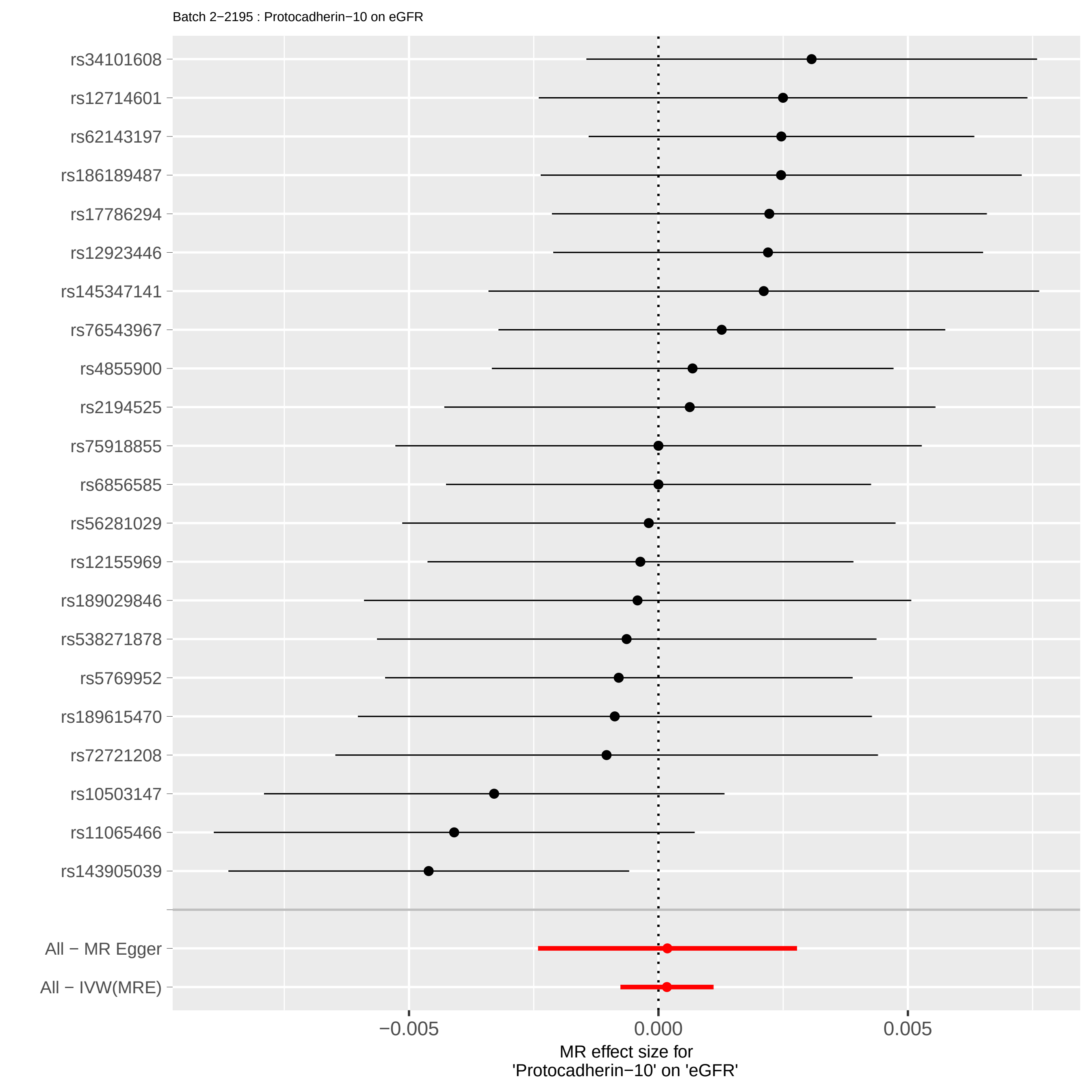

Batch 2−2195 : Protocadherin−10 on eGFR
rs34101608
rs12714601
rs62143197
rs186189487
rs17786294
rs12923446
rs145347141
rs76543967
rs4855900
rs2194525
rs75918855
rs6856585
rs56281029
rs12155969
rs189029846
rs538271878
rs5769952
rs189615470
rs72721208
rs10503147
rs11065466
rs143905039
All − MR Egger
All − IVW(MRE)
−0.005
0.000
0.005
MR effect size for
'Protocadherin−10' on 'eGFR'

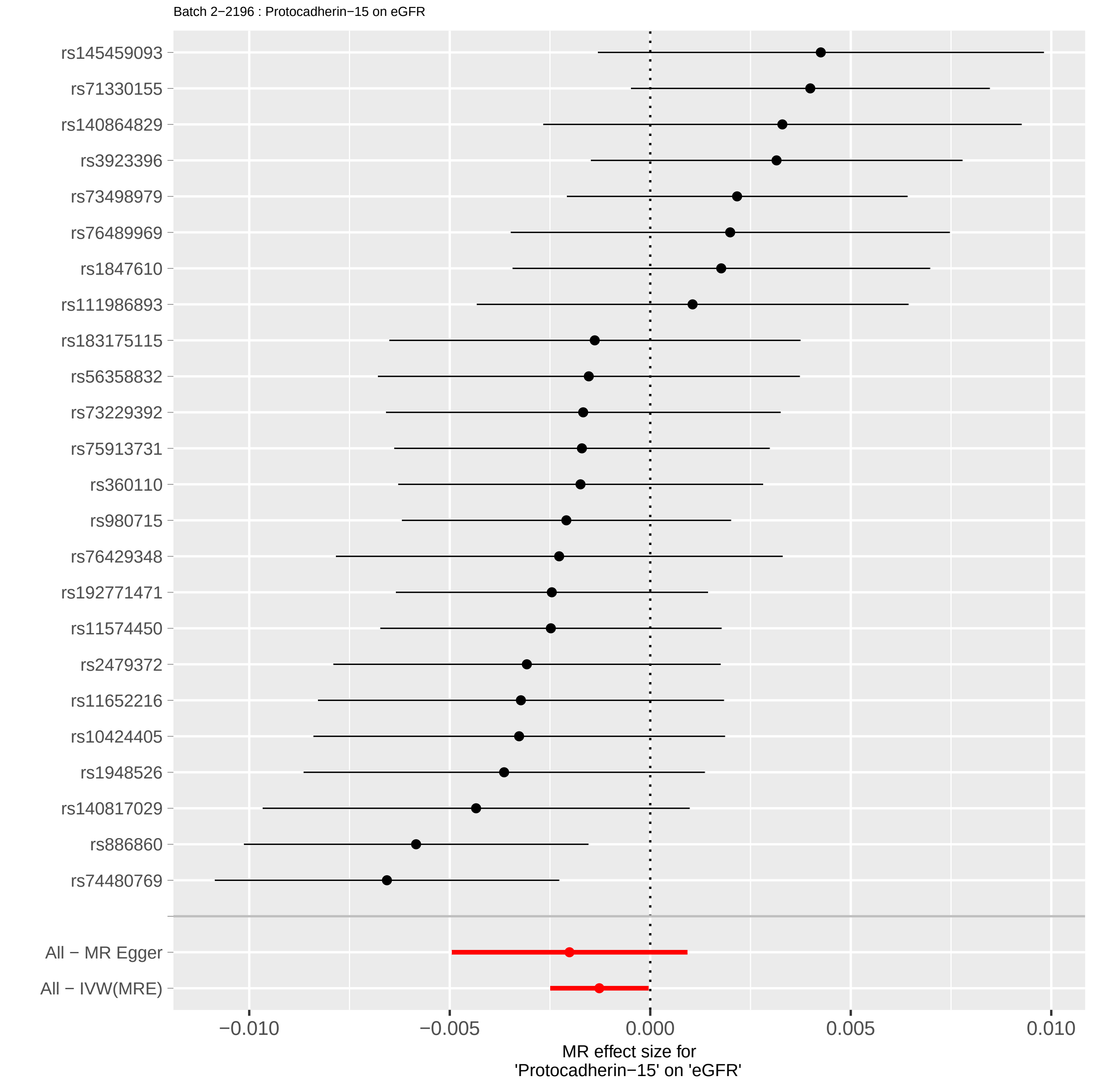

Batch 2−2196 : Protocadherin−15 on eGFR
rs145459093
rs71330155
rs140864829
rs3923396
rs73498979
rs76489969
rs1847610
rs111986893
rs183175115
rs56358832
rs73229392
rs75913731
rs360110
rs980715
rs76429348
rs192771471
rs11574450
rs2479372
rs11652216
rs10424405
rs1948526
rs140817029
rs886860
rs74480769
All − MR Egger
All − IVW(MRE)
−0.010
−0.005
0.000
0.005
0.010
MR effect size for
'Protocadherin−15' on 'eGFR'

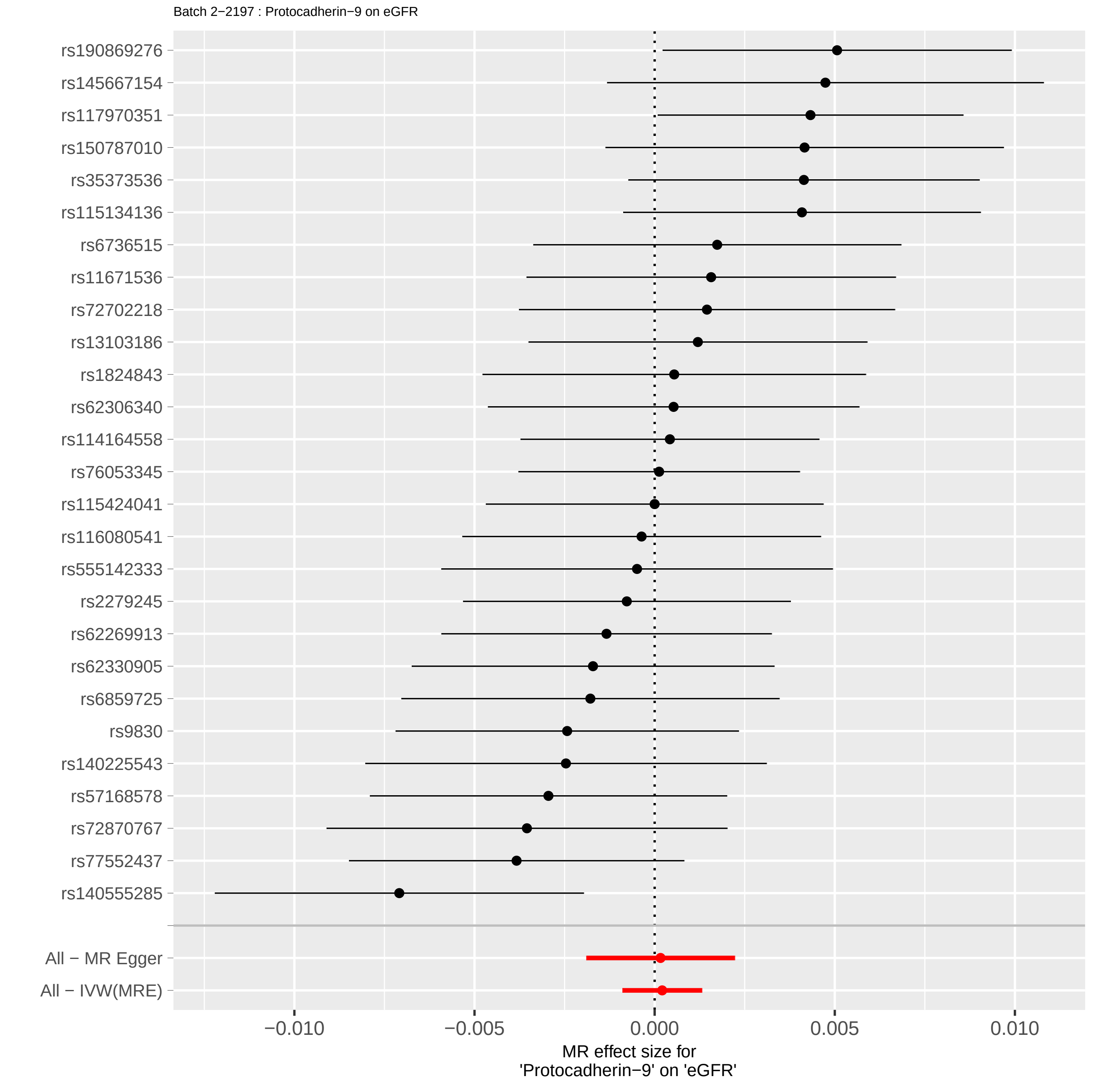

Batch 2−2197 : Protocadherin−9 on eGFR
rs190869276
rs145667154
rs117970351
rs150787010
rs35373536
rs115134136
rs6736515
rs11671536
rs72702218
rs13103186
rs1824843
rs62306340
rs114164558
rs76053345
rs115424041
rs116080541
rs555142333
rs2279245
rs62269913
rs62330905
rs6859725
rs9830
rs140225543
rs57168578
rs72870767
rs77552437
rs140555285
All − MR Egger
All − IVW(MRE)
−0.010
−0.005
0.000
0.005
0.010
MR effect size for
'Protocadherin−9' on 'eGFR'

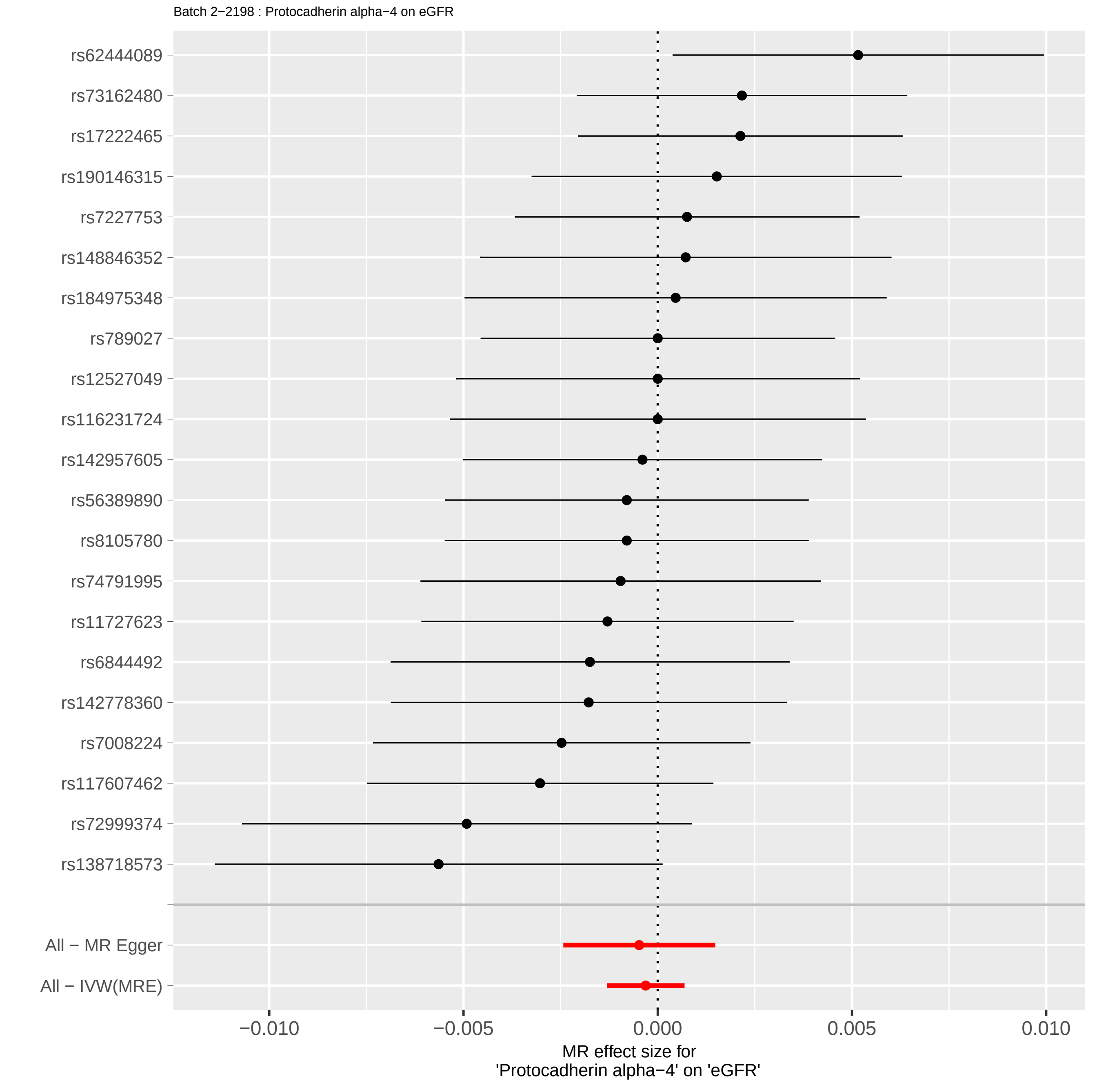
Batch 2−2198 : Protocadherin alpha−4 on eGFR
rs62444089
rs73162480
rs17222465
rs190146315
rs7227753
rs148846352
rs184975348
rs789027
rs12527049
rs116231724
rs142957605
rs56389890
rs8105780
rs74791995
rs11727623
rs6844492
rs142778360
rs7008224
rs117607462
rs72999374
rs138718573
All − MR Egger
All − IVW(MRE)
−0.010
−0.005
0.000
0.005
0.010
MR effect size for
'Protocadherin alpha−4' on 'eGFR'

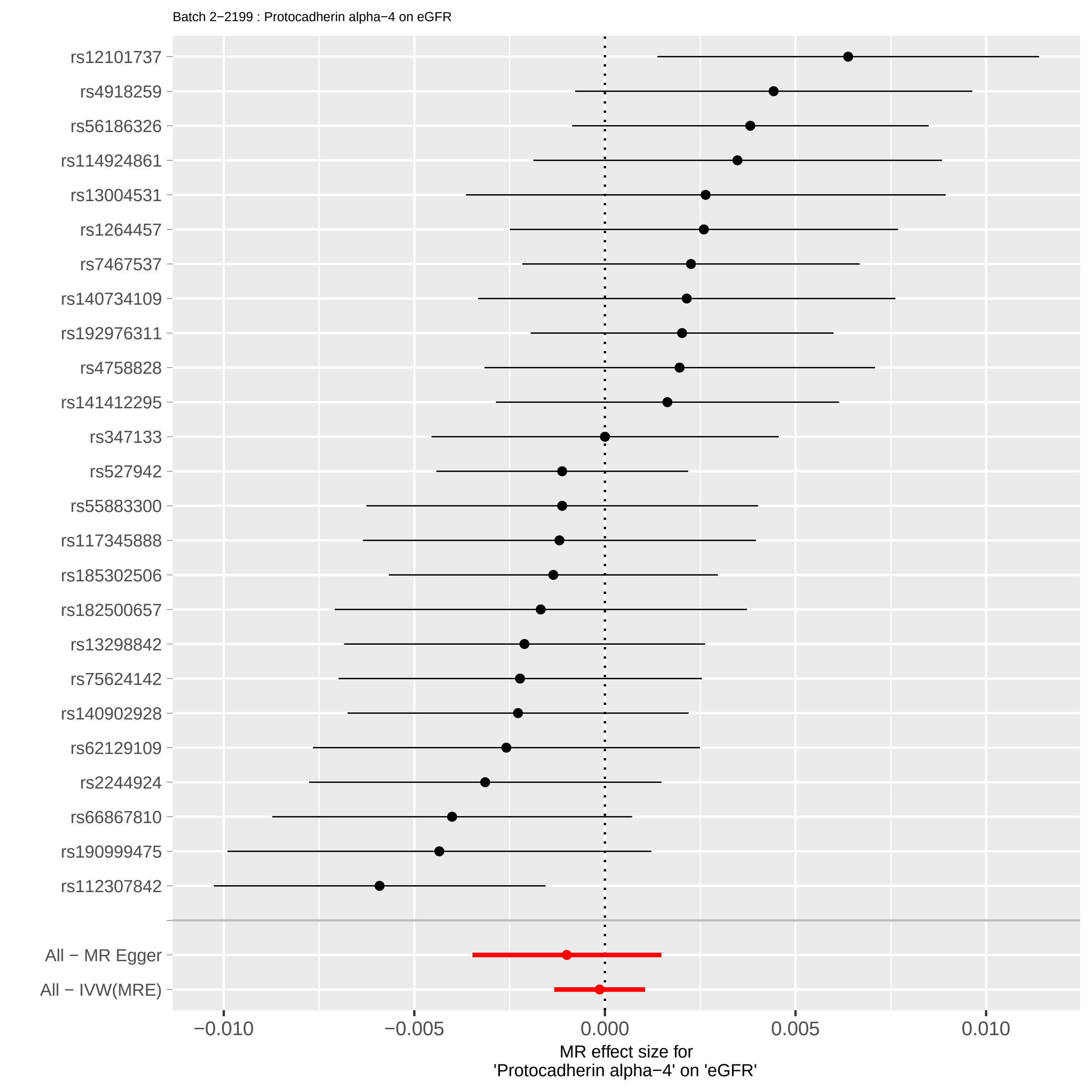
Batch 2−2199 : Protocadherin alpha−4 on eGFR
rs12101737
rs4918259
rs56186326
rs114924861
rs13004531
rs1264457
rs7467537
rs140734109
rs192976311
rs4758828
rs141412295
rs347133
rs527942
rs55883300
rs117345888
rs185302506
rs182500657
rs13298842
rs75624142
rs140902928
rs62129109
rs2244924
rs66867810
rs190999475
rs112307842
All − MR Egger
All − IVW(MRE)
−0.010
−0.005
0.000
0.005
0.010
MR effect size for
'Protocadherin alpha−4' on 'eGFR'

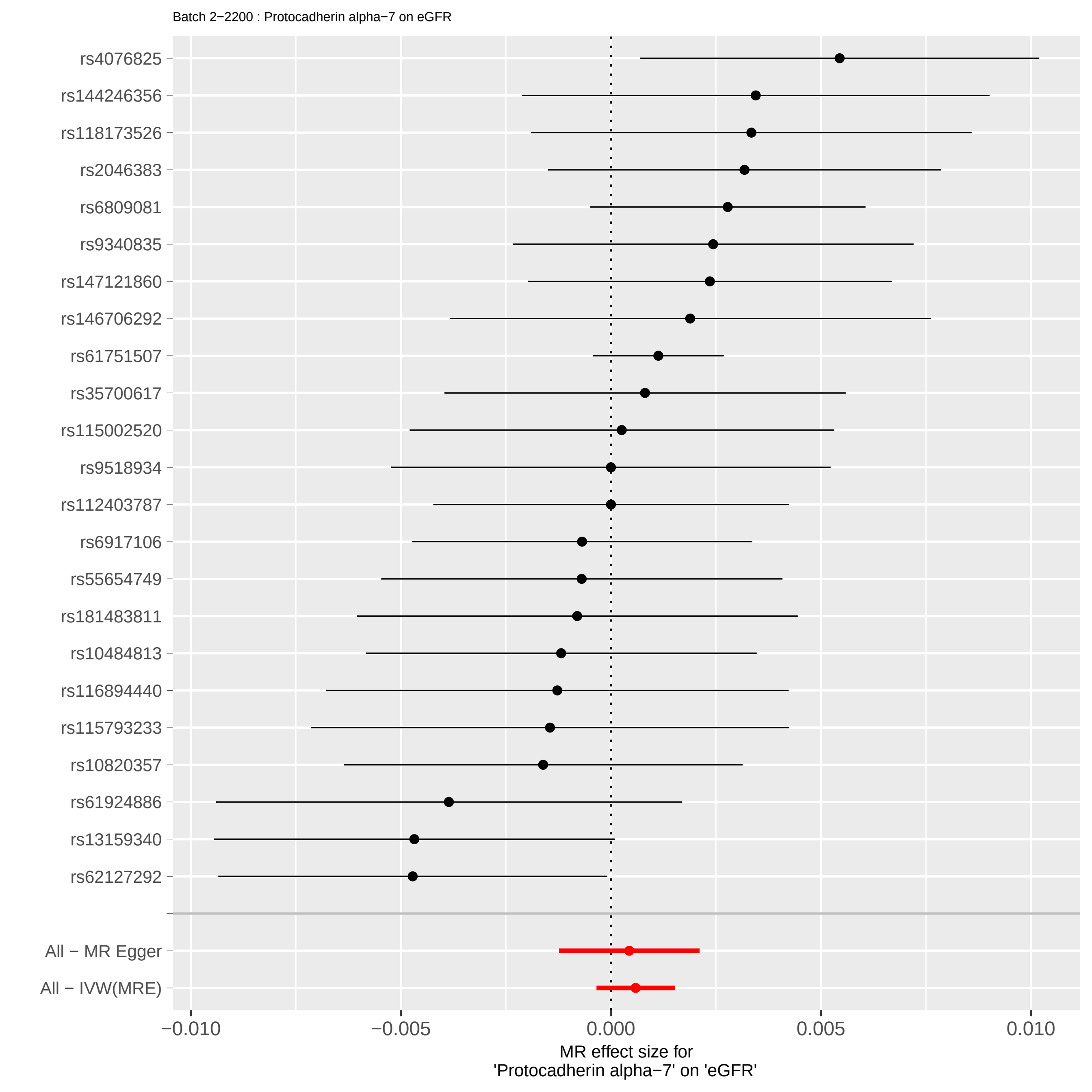

Batch 2−2200 : Protocadherin alpha−7 on eGFR
rs4076825
rs144246356
rs118173526
rs2046383
rs6809081
rs9340835
rs147121860
rs146706292
rs61751507
rs35700617
rs115002520
rs9518934
rs112403787
rs6917106
rs55654749
rs181483811
rs10484813
rs116894440
rs115793233
rs10820357
rs61924886
rs13159340
rs62127292
All − MR Egger
All − IVW(MRE)
−0.010
−0.005
0.000
0.005
0.010
MR effect size for
'Protocadherin alpha−7' on 'eGFR'

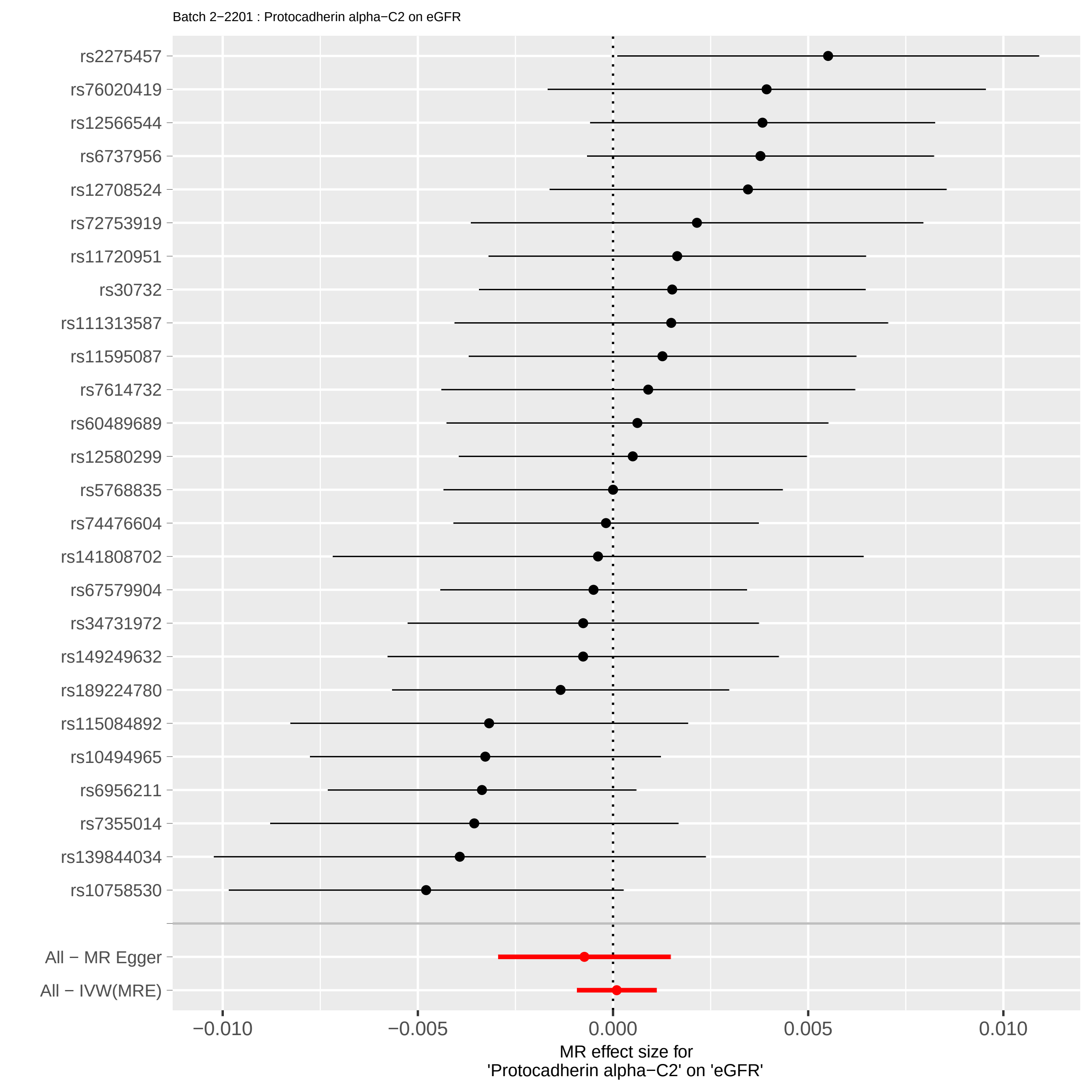

Batch 2−2201 : Protocadherin alpha−C2 on eGFR
rs2275457
rs76020419
rs12566544
rs6737956
rs12708524
rs72753919
rs11720951
rs30732
rs111313587
rs11595087
rs7614732
rs60489689
rs12580299
rs5768835
rs74476604
rs141808702
rs67579904
rs34731972
rs149249632
rs189224780
rs115084892
rs10494965
rs6956211
rs7355014
rs139844034
rs10758530
All − MR Egger
All − IVW(MRE)
−0.010
−0.005
0.000
0.005
0.010
MR effect size for
'Protocadherin alpha−C2' on 'eGFR'

Batch 2−2202 : Protocadherin beta−10 on eGFR

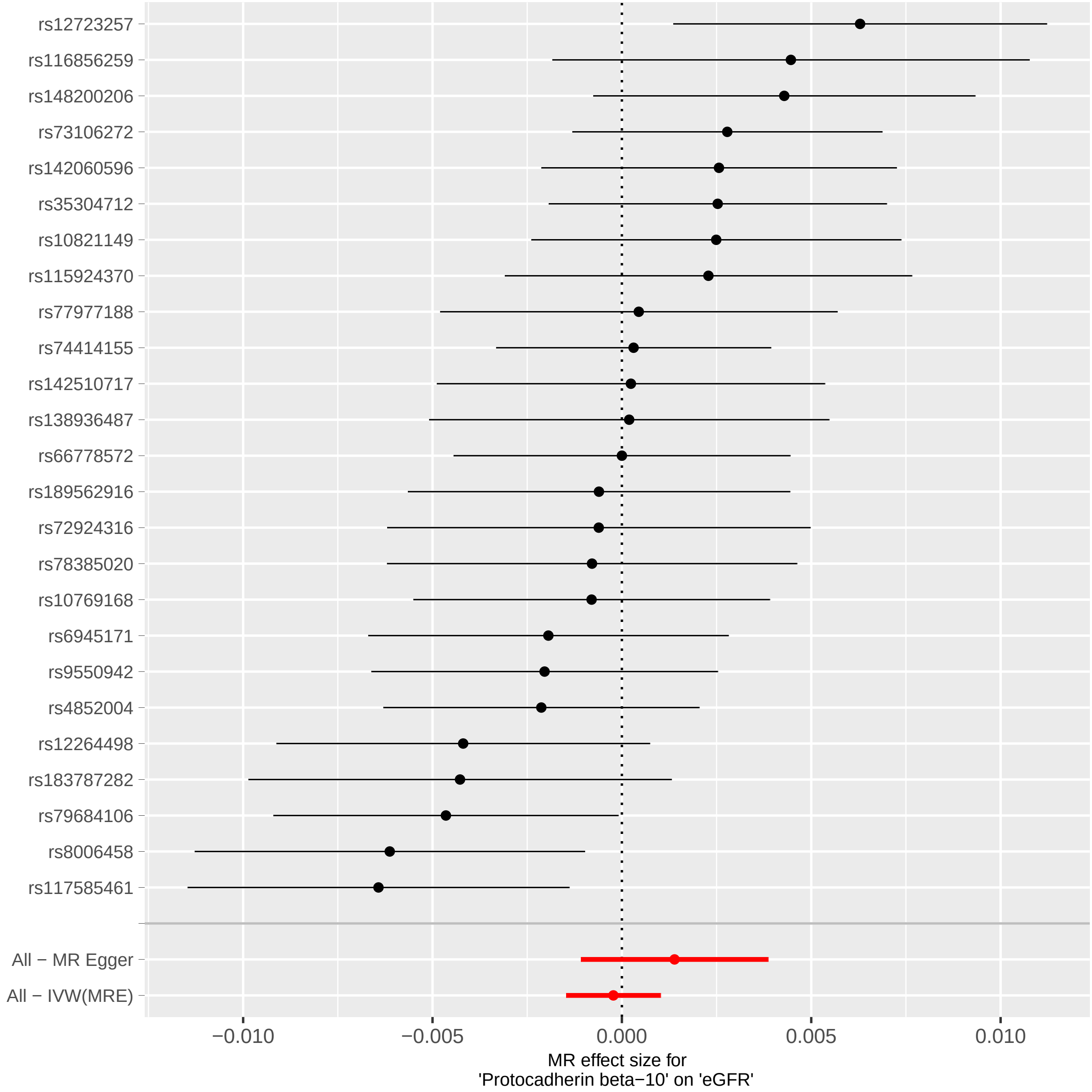

Batch 2−2203 : Protocadherin beta−1 on eGFR

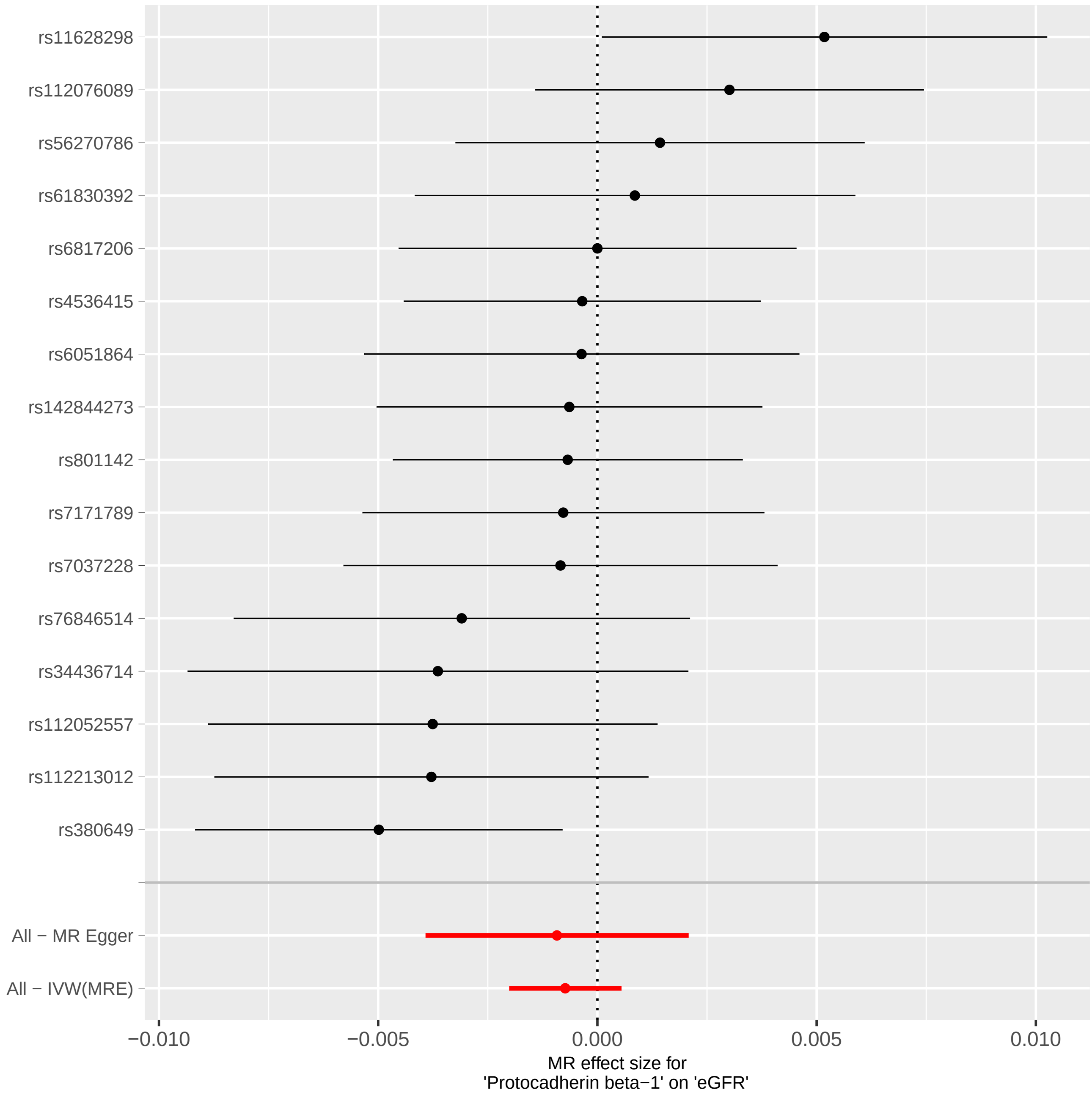

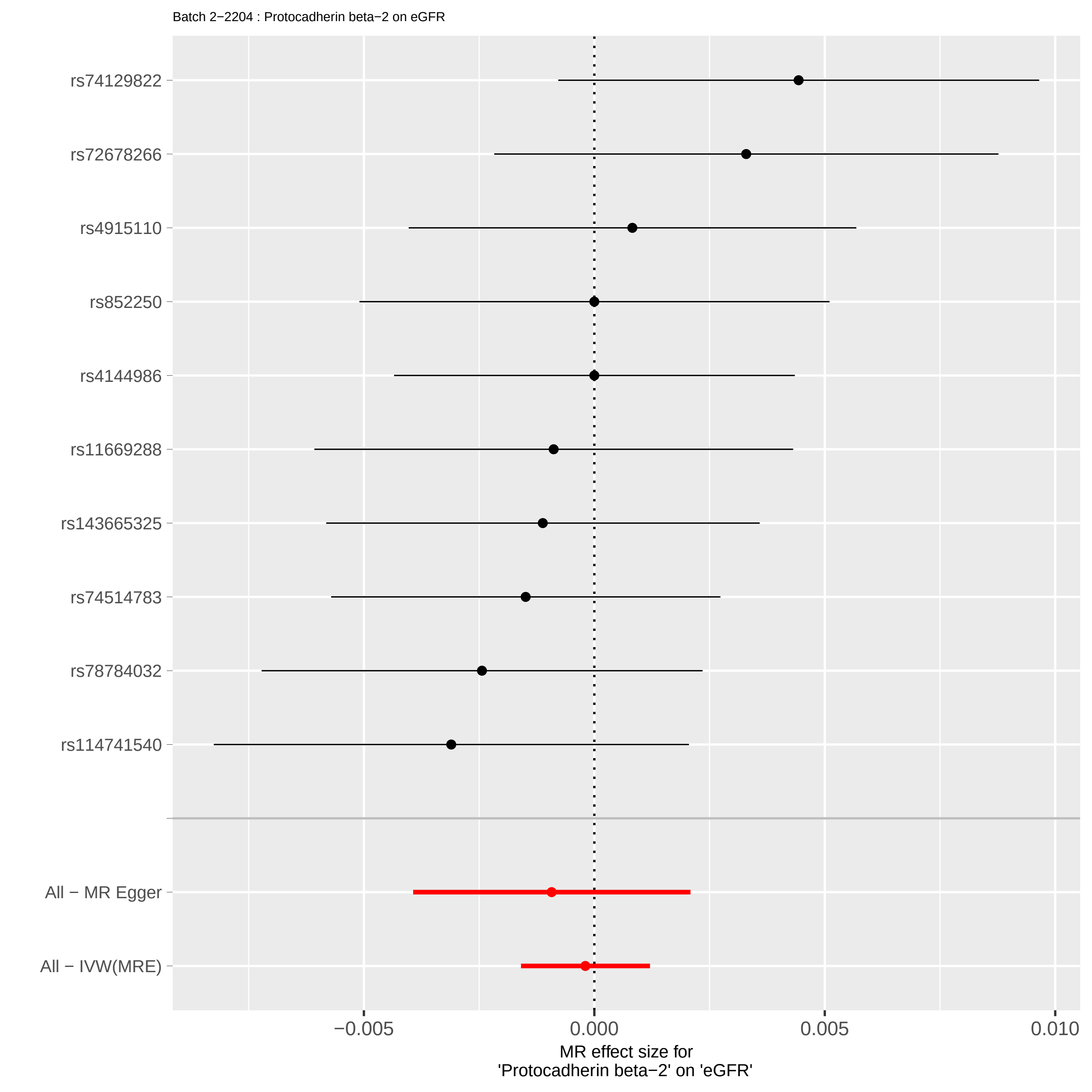

Batch 2−2204 : Protocadherin beta−2 on eGFR
rs74129822
rs72678266
rs4915110
rs852250
rs4144986
rs11669288
rs143665325
rs74514783
rs78784032
rs114741540
All − MR Egger
All − IVW(MRE)
−0.005
0.000
0.005
0.010
MR effect size for
'Protocadherin beta−2' on 'eGFR'

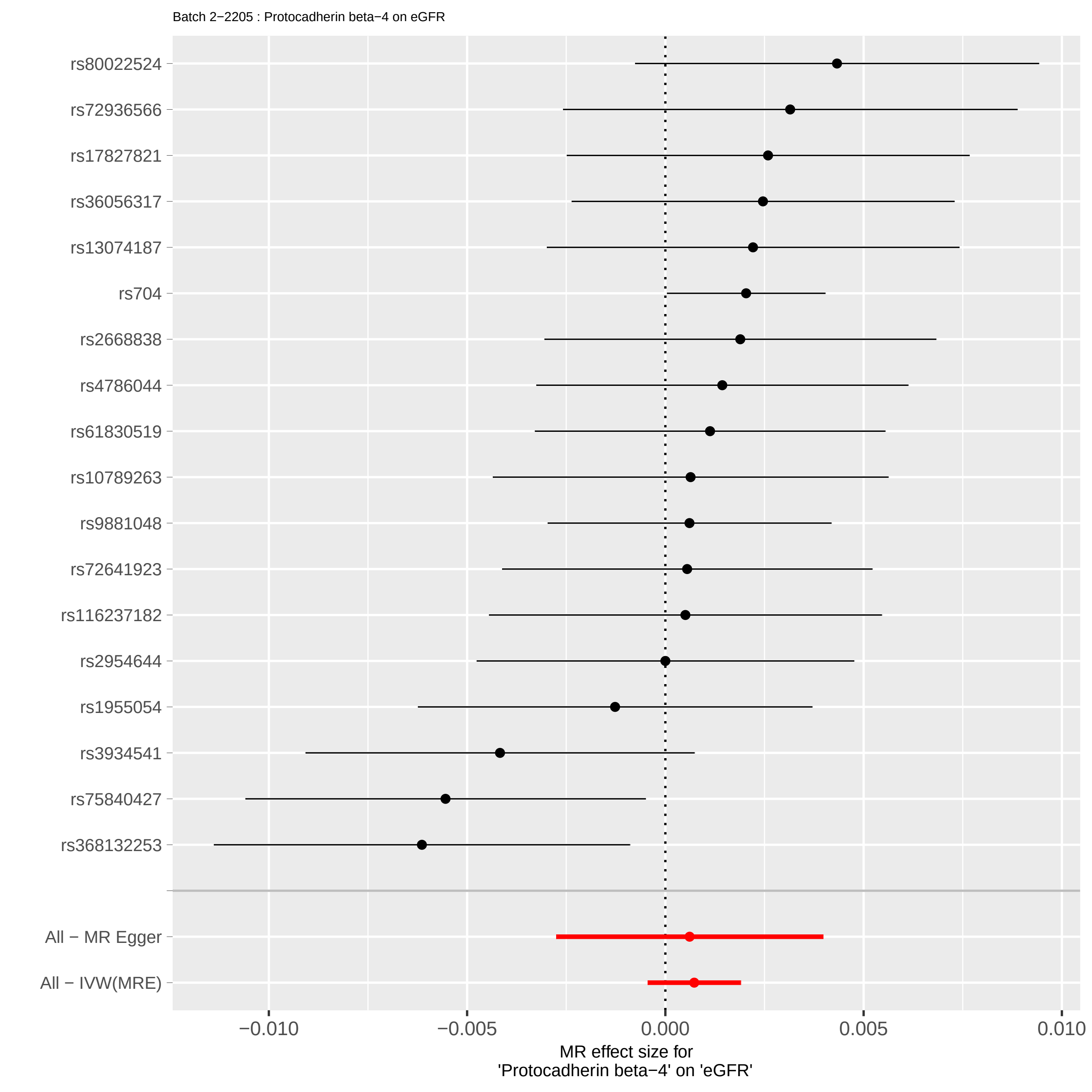
Batch 2−2205 : Protocadherin beta−4 on eGFR
rs80022524
rs72936566
rs17827821
rs36056317
rs13074187
rs704
rs2668838
rs4786044
rs61830519
rs10789263
rs9881048
rs72641923
rs116237182
rs2954644
rs1955054
rs3934541
rs75840427
rs368132253
All − MR Egger
All − IVW(MRE)
−0.010
−0.005
0.000
0.005
0.010
MR effect size for
'Protocadherin beta−4' on 'eGFR'

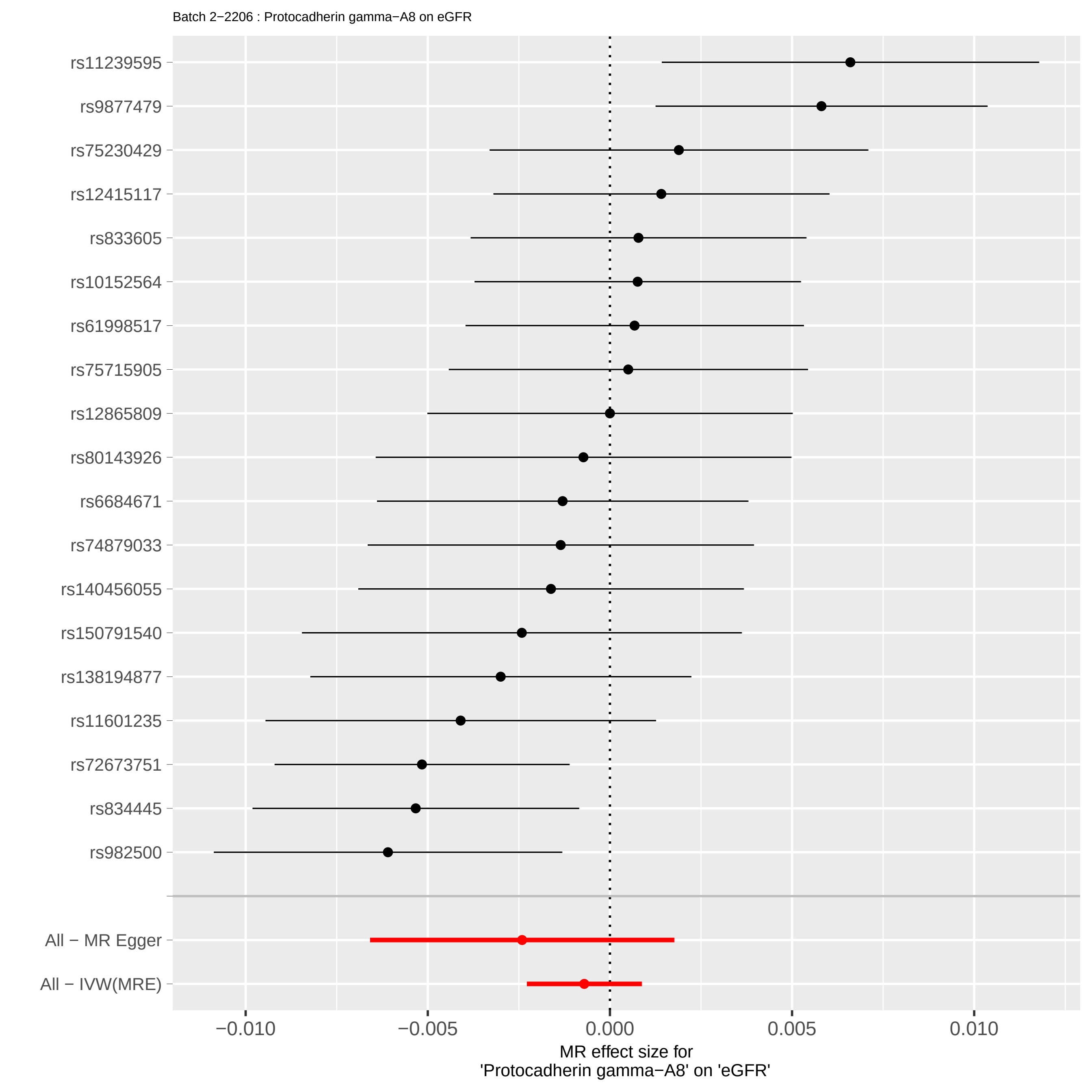

Batch 2−2206 : Protocadherin gamma−A8 on eGFR
rs11239595
rs9877479
rs75230429
rs12415117
rs833605
rs10152564
rs61998517
rs75715905
rs12865809
rs80143926
rs6684671
rs74879033
rs140456055
rs150791540
rs138194877
rs11601235
rs72673751
rs834445
rs982500
All − MR Egger
All − IVW(MRE)
−0.010
−0.005
0.000
0.005
0.010
MR effect size for
'Protocadherin gamma−A8' on 'eGFR'

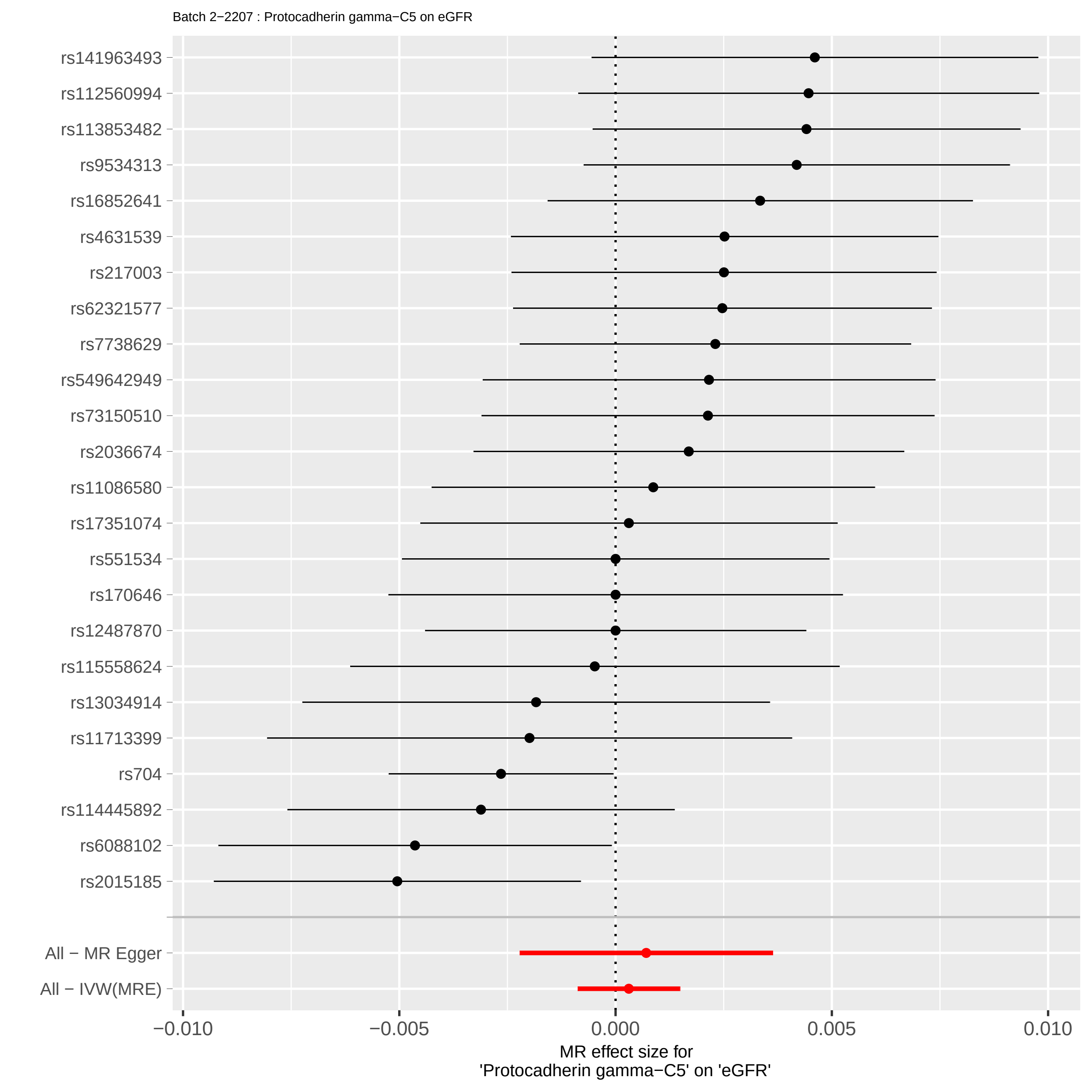
Batch 2−2207 : Protocadherin gamma−C5 on eGFR
rs141963493
rs112560994
rs113853482
rs9534313
rs16852641
rs4631539
rs217003
rs62321577
rs7738629
rs549642949
rs73150510
rs2036674
rs11086580
rs17351074
rs551534
rs170646
rs12487870
rs115558624
rs13034914
rs11713399
rs704
rs114445892
rs6088102
rs2015185
All − MR Egger
All − IVW(MRE)
−0.010
−0.005
0.000
0.005
0.010
MR effect size for
'Protocadherin gamma−C5' on 'eGFR'

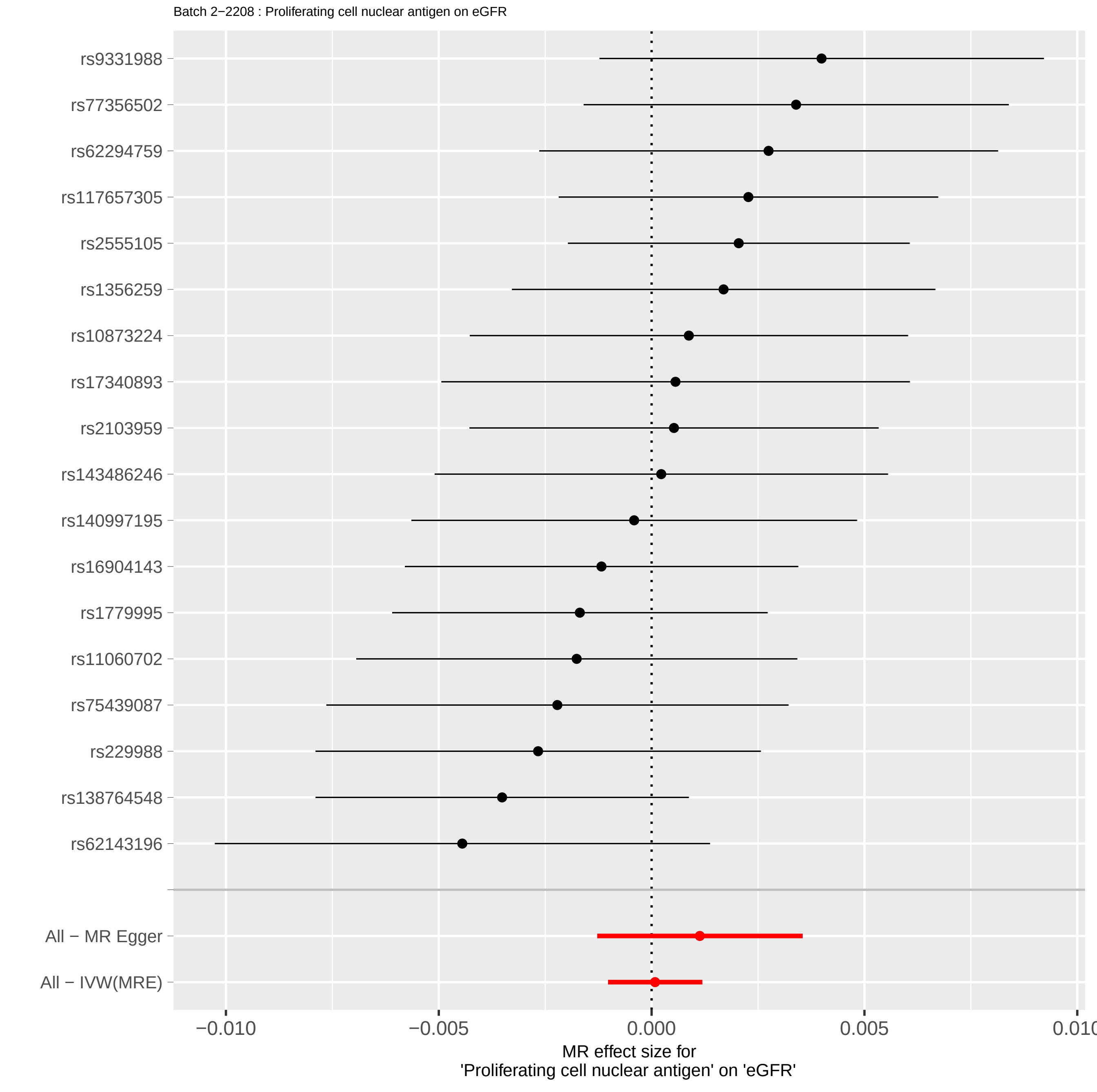
Batch 2−2208 : Proliferating cell nuclear antigen on eGFR
rs9331988
rs77356502
rs62294759
rs117657305
rs2555105
rs1356259
rs10873224
rs17340893
rs2103959
rs143486246
rs140997195
rs16904143
rs1779995
rs11060702
rs75439087
rs229988
rs138764548
rs62143196
All − MR Egger
All − IVW(MRE)
−0.010
−0.005
0.000
0.005
0.010
MR effect size for
'Proliferating cell nuclear antigen' on 'eGFR'

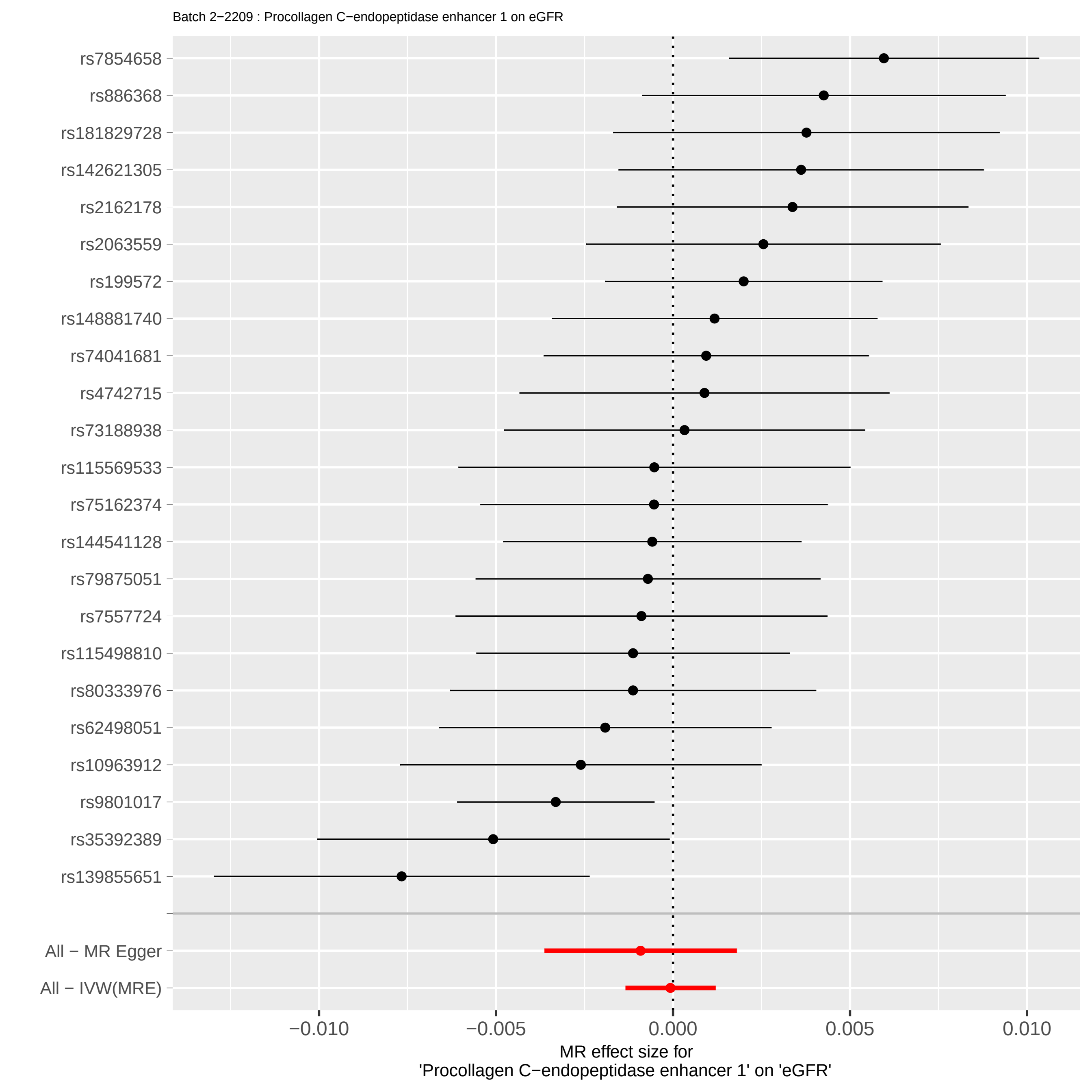
Batch 2−2209 : Procollagen C−endopeptidase enhancer 1 on eGFR
rs7854658
rs886368
rs181829728
rs142621305
rs2162178
rs2063559
rs199572
rs148881740
rs74041681
rs4742715
rs73188938
rs115569533
rs75162374
rs144541128
rs79875051
rs7557724
rs115498810
rs80333976
rs62498051
rs10963912
rs9801017
rs35392389
rs139855651
All − MR Egger
All − IVW(MRE)
−0.010
−0.005
0.000
0.005
0.010
MR effect size for
'Procollagen C−endopeptidase enhancer 1' on 'eGFR'

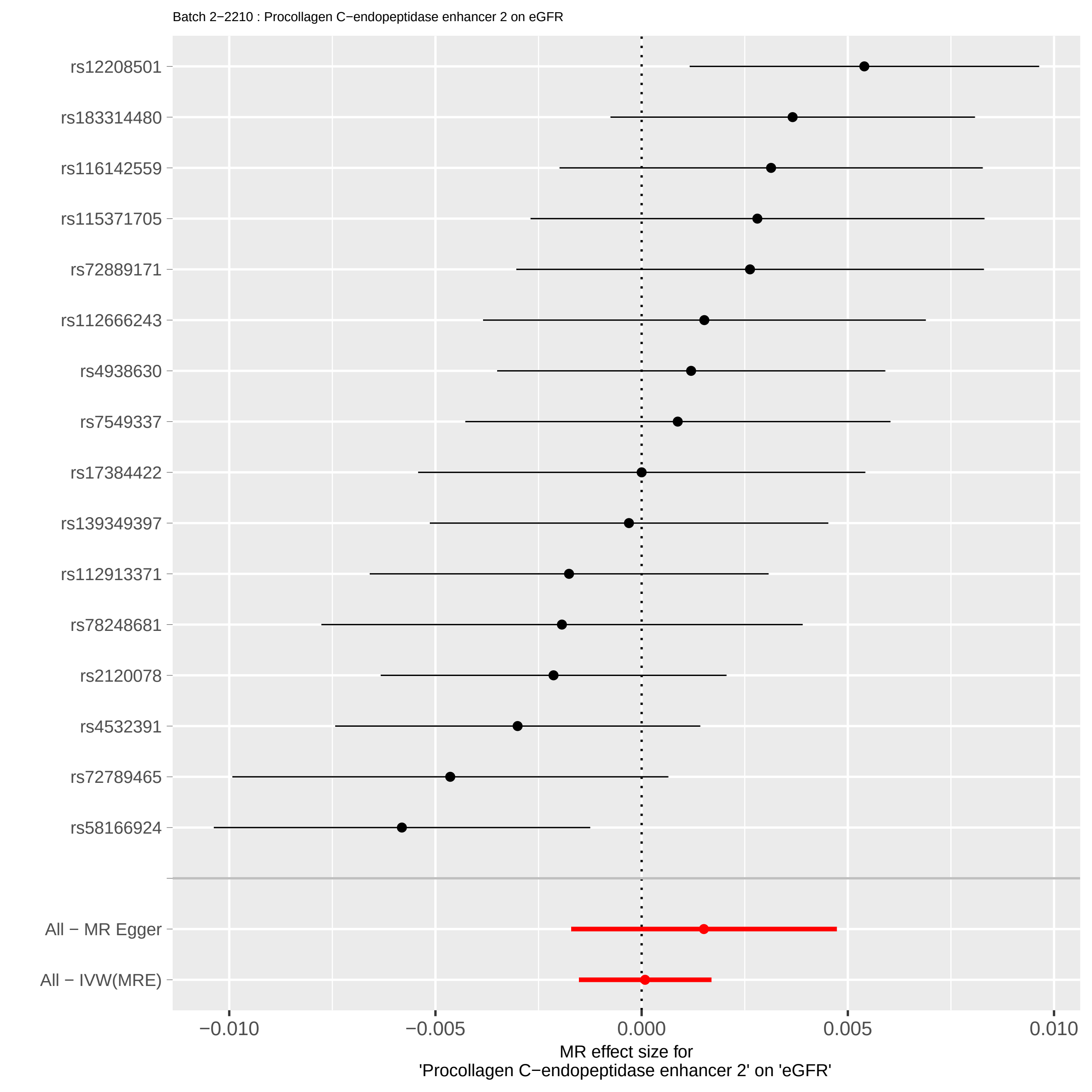

Batch 2−2210 : Procollagen C−endopeptidase enhancer 2 on eGFR
rs12208501
rs183314480
rs116142559
rs115371705
rs72889171
rs112666243
rs4938630
rs7549337
rs17384422
rs139349397
rs112913371
rs78248681
rs2120078
rs4532391
rs72789465
rs58166924
All − MR Egger
All − IVW(MRE)
−0.010
−0.005
0.000
0.005
0.010
MR effect size for
'Procollagen C−endopeptidase enhancer 2' on 'eGFR'

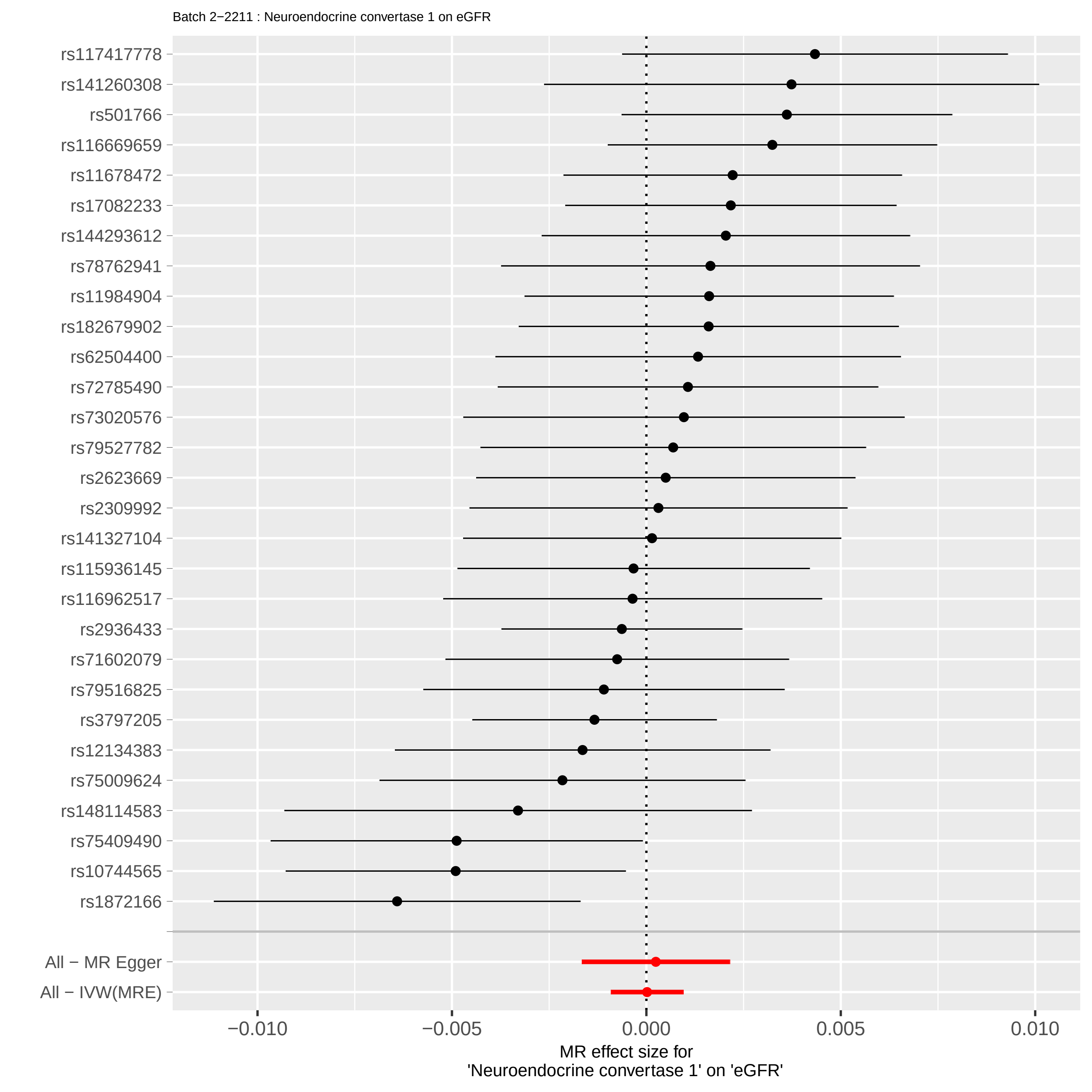

Batch 2−2211 : Neuroendocrine convertase 1 on eGFR
rs117417778
rs141260308
rs501766
rs116669659
rs11678472
rs17082233
rs144293612
rs78762941
rs11984904
rs182679902
rs62504400
rs72785490
rs73020576
rs79527782
rs2623669
rs2309992
rs141327104
rs115936145
rs116962517
rs2936433
rs71602079
rs79516825
rs3797205
rs12134383
rs75009624
rs148114583
rs75409490
rs10744565
rs1872166
All − MR Egger
All − IVW(MRE)
−0.010
−0.005
0.000
0.005
0.010
MR effect size for
'Neuroendocrine convertase 1' on 'eGFR'

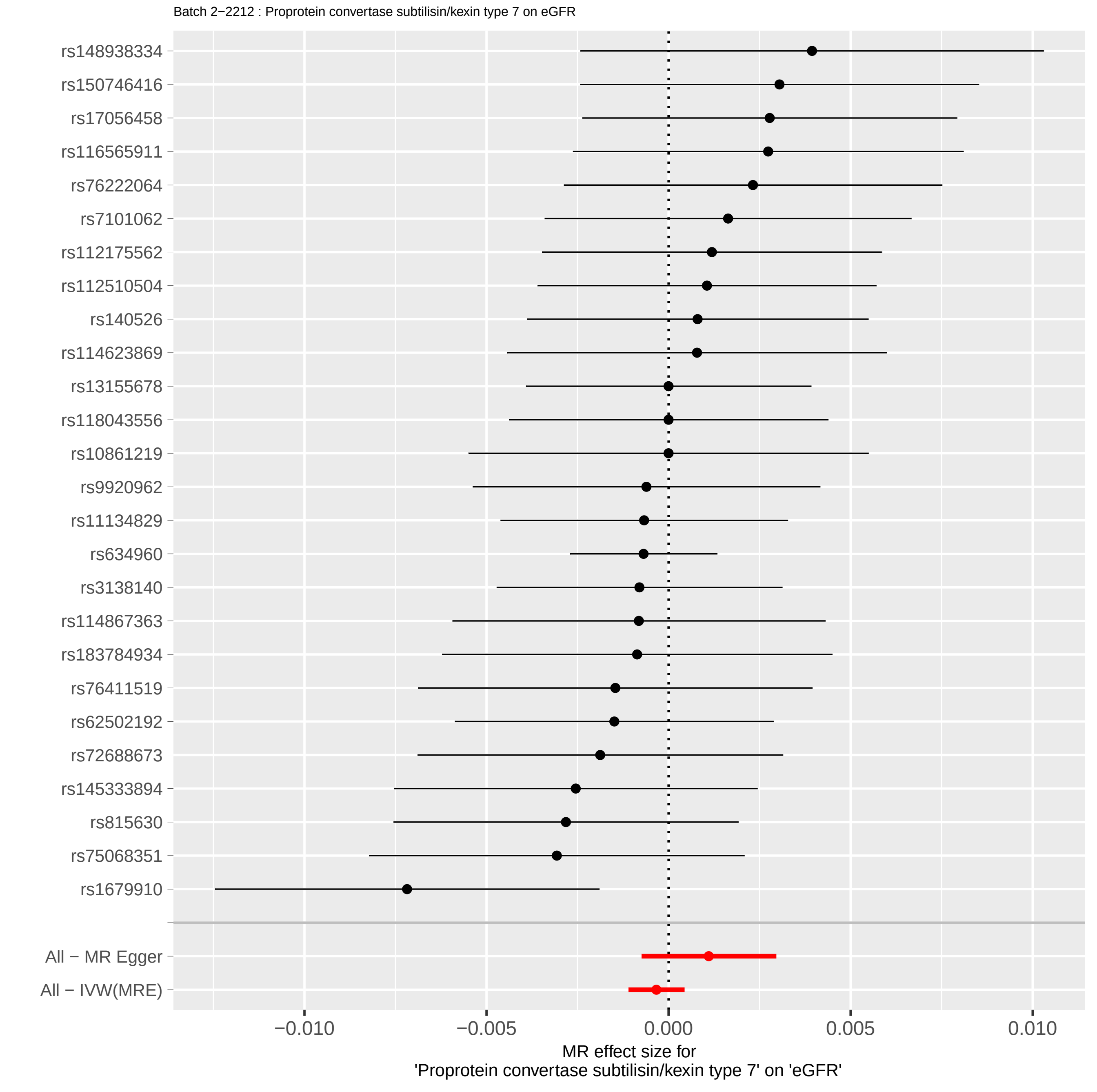
Batch 2−2212 : Proprotein convertase subtilisin/kexin type 7 on eGFR
rs148938334
rs150746416
rs17056458
rs116565911
rs76222064
rs7101062
rs112175562
rs112510504
rs140526
rs114623869
rs13155678
rs118043556
rs10861219
rs9920962
rs11134829
rs634960
rs3138140
rs114867363
rs183784934
rs76411519
rs62502192
rs72688673
rs145333894
rs815630
rs75068351
rs1679910
All − MR Egger
All − IVW(MRE)
−0.010
−0.005
0.000
0.005
0.010
MR effect size for
'Proprotein convertase subtilisin/kexin type 7' on 'eGFR'

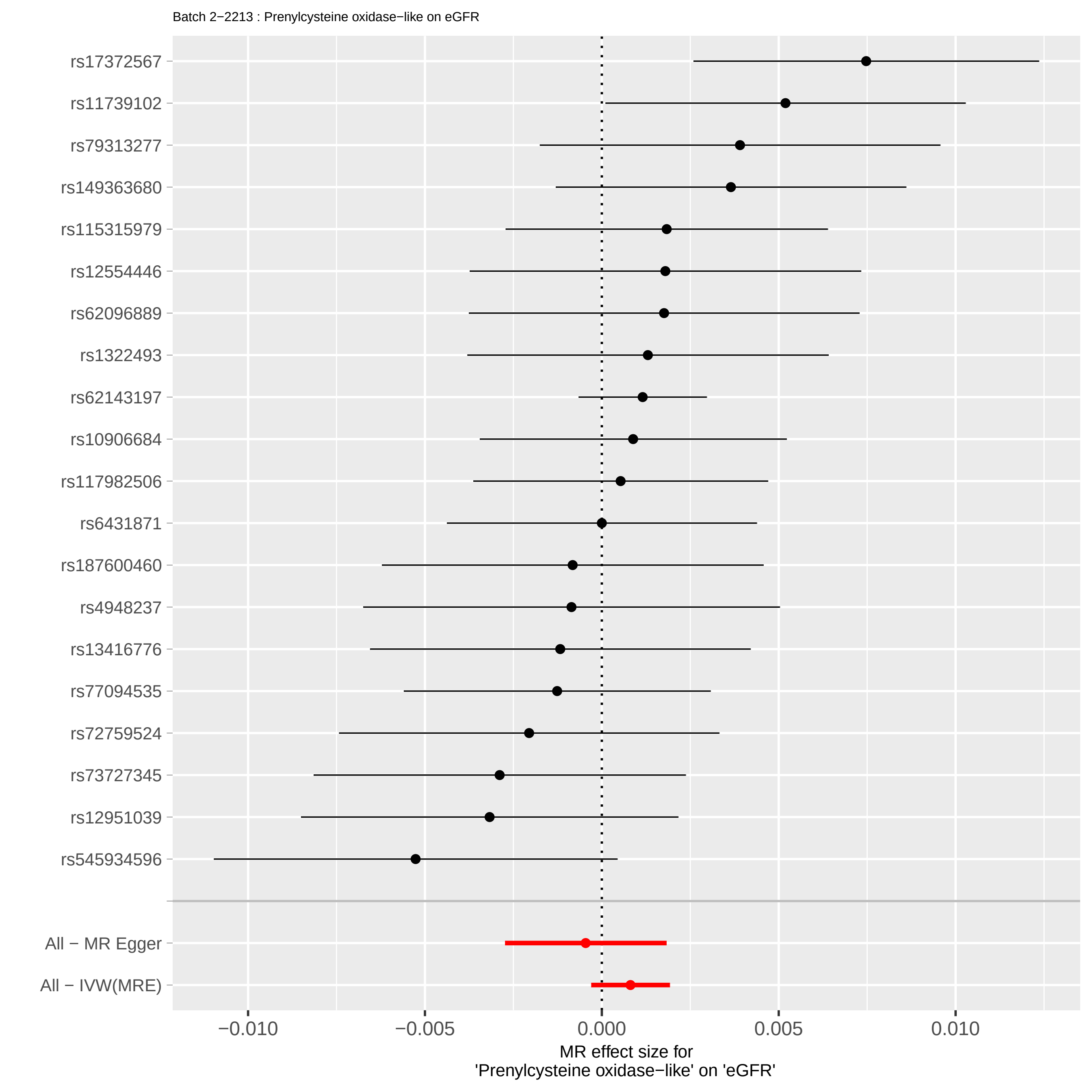

Batch 2−2213 : Prenylcysteine oxidase−like on eGFR
rs17372567
rs11739102
rs79313277
rs149363680
rs115315979
rs12554446
rs62096889
rs1322493
rs62143197
rs10906684
rs117982506
rs6431871
rs187600460
rs4948237
rs13416776
rs77094535
rs72759524
rs73727345
rs12951039
rs545934596
All − MR Egger
All − IVW(MRE)
−0.010
−0.005
0.000
0.005
0.010
MR effect size for
'Prenylcysteine oxidase−like' on 'eGFR'

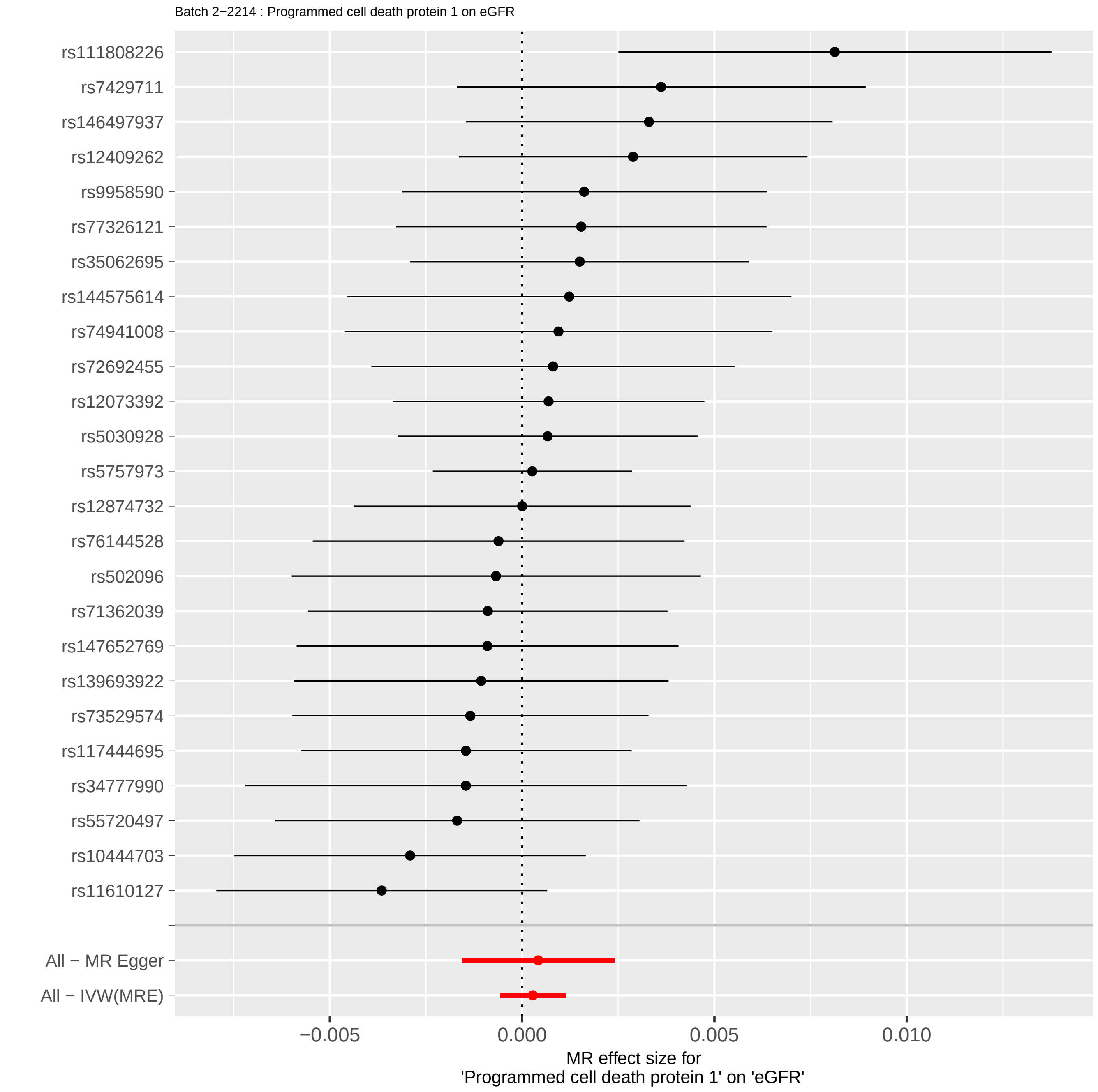

Batch 2−2214 : Programmed cell death protein 1 on eGFR
rs111808226
rs7429711
rs146497937
rs12409262
rs9958590
rs77326121
rs35062695
rs144575614
rs74941008
rs72692455
rs12073392
rs5030928
rs5757973
rs12874732
rs76144528
rs502096
rs71362039
rs147652769
rs139693922
rs73529574
rs117444695
rs34777990
rs55720497
rs10444703
rs11610127
All − MR Egger
All − IVW(MRE)
−0.005
0.000
0.005
0.010
MR effect size for
'Programmed cell death protein 1' on 'eGFR'

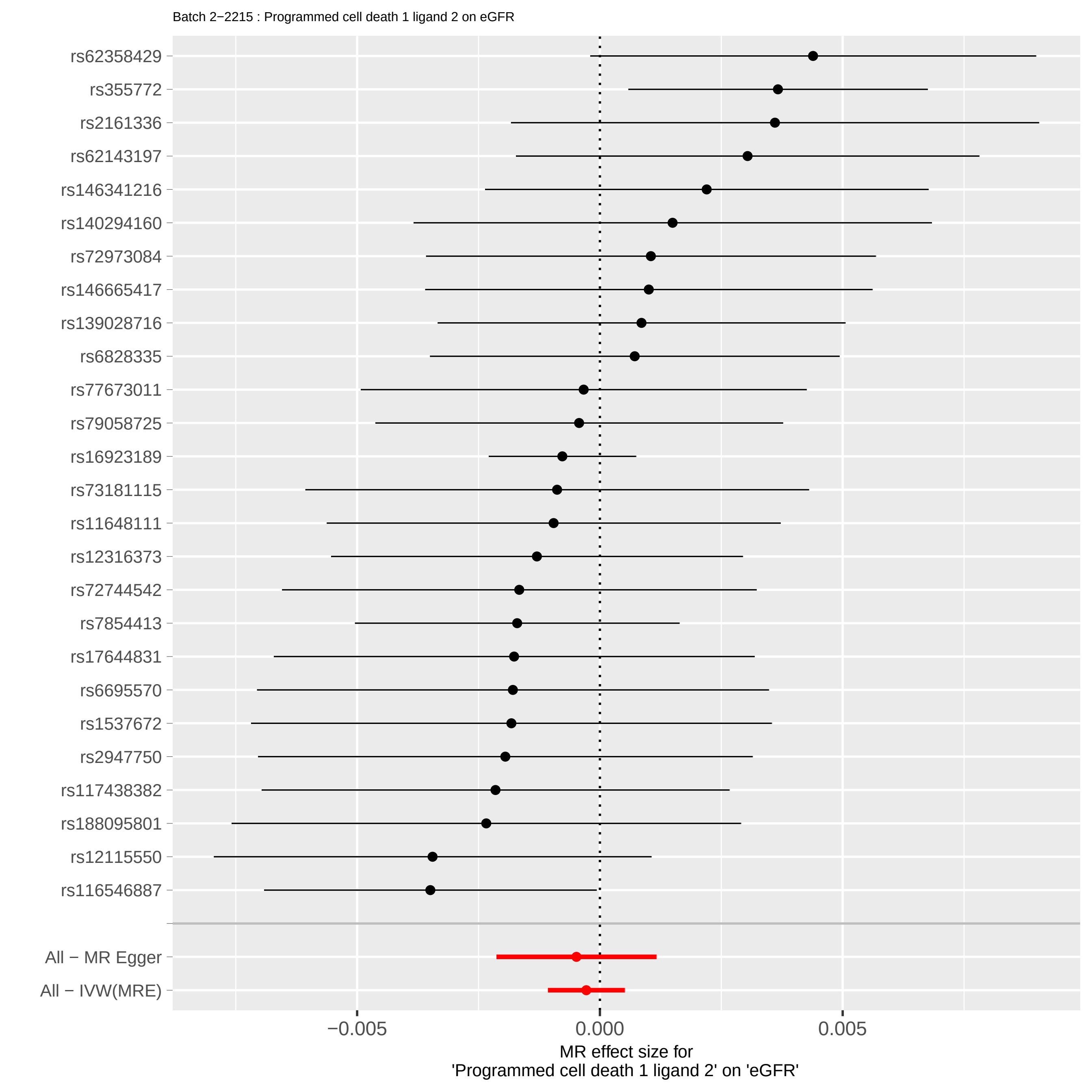
Batch 2−2215 : Programmed cell death 1 ligand 2 on eGFR
rs62358429
rs355772
rs2161336
rs62143197
rs146341216
rs140294160
rs72973084
rs146665417
rs139028716
rs6828335
rs77673011
rs79058725
rs16923189
rs73181115
rs11648111
rs12316373
rs72744542
rs7854413
rs17644831
rs6695570
rs1537672
rs2947750
rs117438382
rs188095801
rs12115550
rs116546887
All − MR Egger
All − IVW(MRE)
−0.005
0.000
0.005
MR effect size for
'Programmed cell death 1 ligand 2' on 'eGFR'

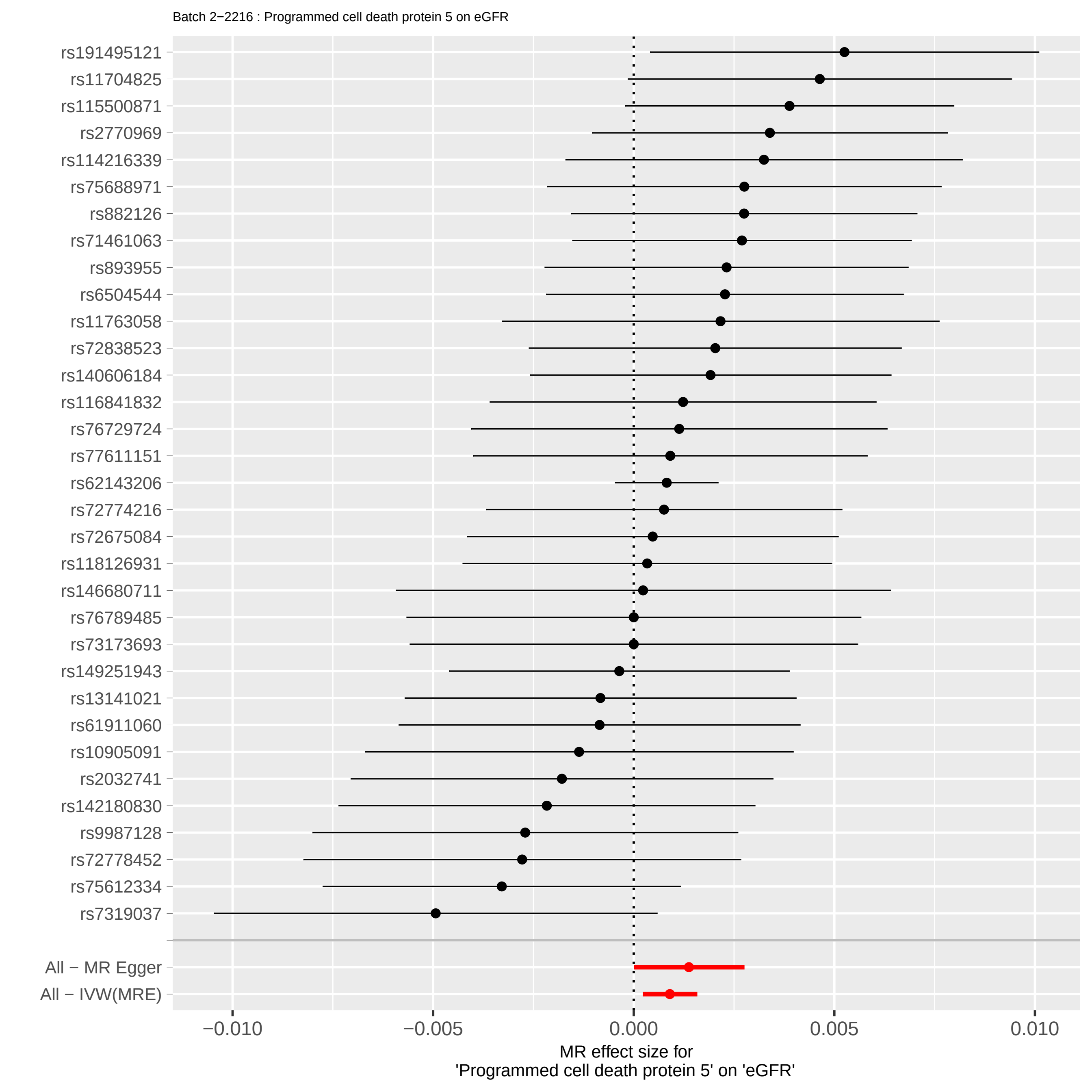

Batch 2−2216 : Programmed cell death protein 5 on eGFR
rs191495121
rs11704825
rs115500871
rs2770969
rs114216339
rs75688971
rs882126
rs71461063
rs893955
rs6504544
rs11763058
rs72838523
rs140606184
rs116841832
rs76729724
rs77611151
rs62143206
rs72774216
rs72675084
rs118126931
rs146680711
rs76789485
rs73173693
rs149251943
rs13141021
rs61911060
rs10905091
rs2032741
rs142180830
rs9987128
rs72778452
rs75612334
rs7319037
All − MR Egger
All − IVW(MRE)
−0.010
−0.005
0.000
0.005
0.010
MR effect size for
'Programmed cell death protein 5' on 'eGFR'

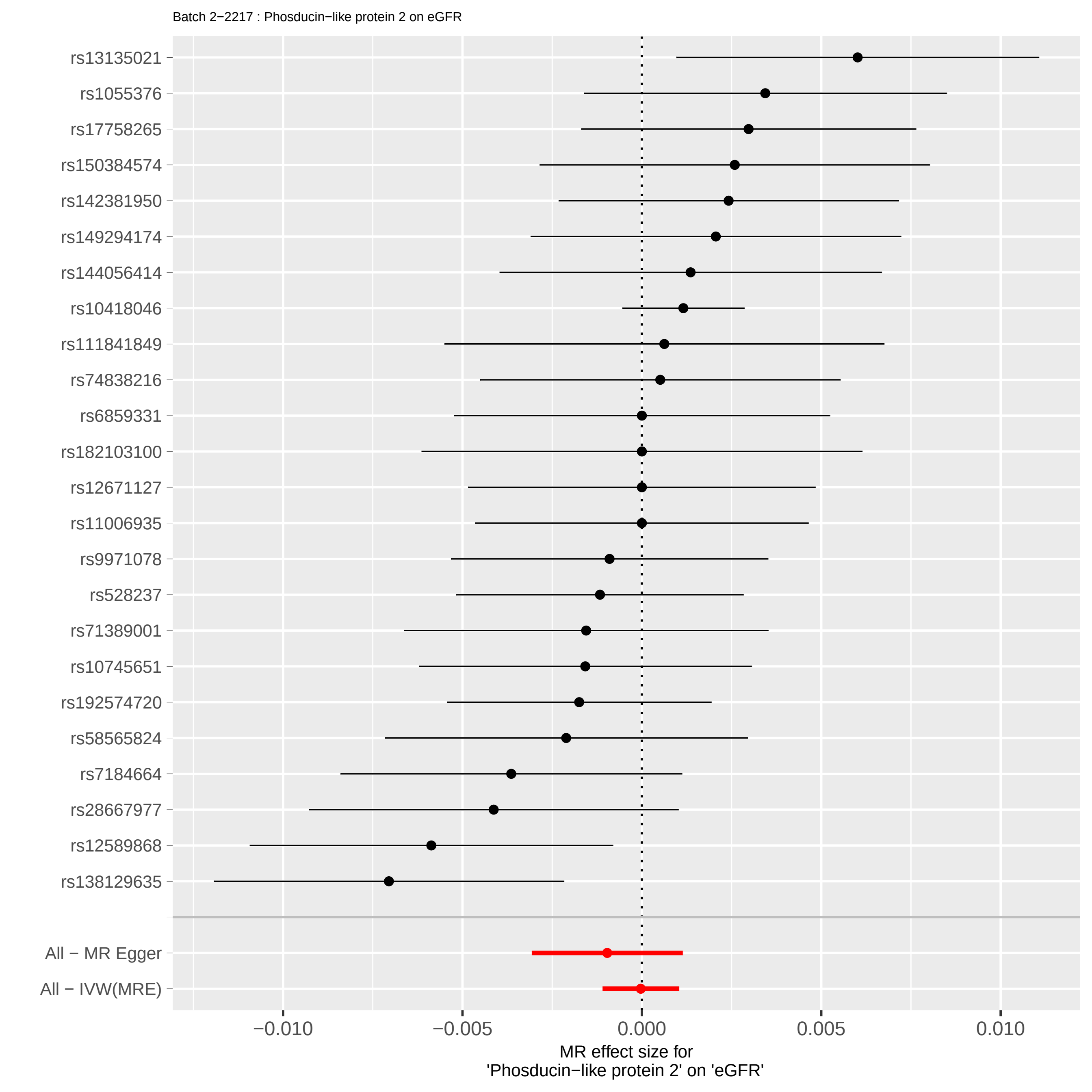
Batch 2−2217 : Phosducin−like protein 2 on eGFR
rs13135021
rs1055376
rs17758265
rs150384574
rs142381950
rs149294174
rs144056414
rs10418046
rs111841849
rs74838216
rs6859331
rs182103100
rs12671127
rs11006935
rs9971078
rs528237
rs71389001
rs10745651
rs192574720
rs58565824
rs7184664
rs28667977
rs12589868
rs138129635
All − MR Egger
All − IVW(MRE)
−0.010
−0.005
0.000
0.005
0.010
MR effect size for
'Phosducin−like protein 2' on 'eGFR'

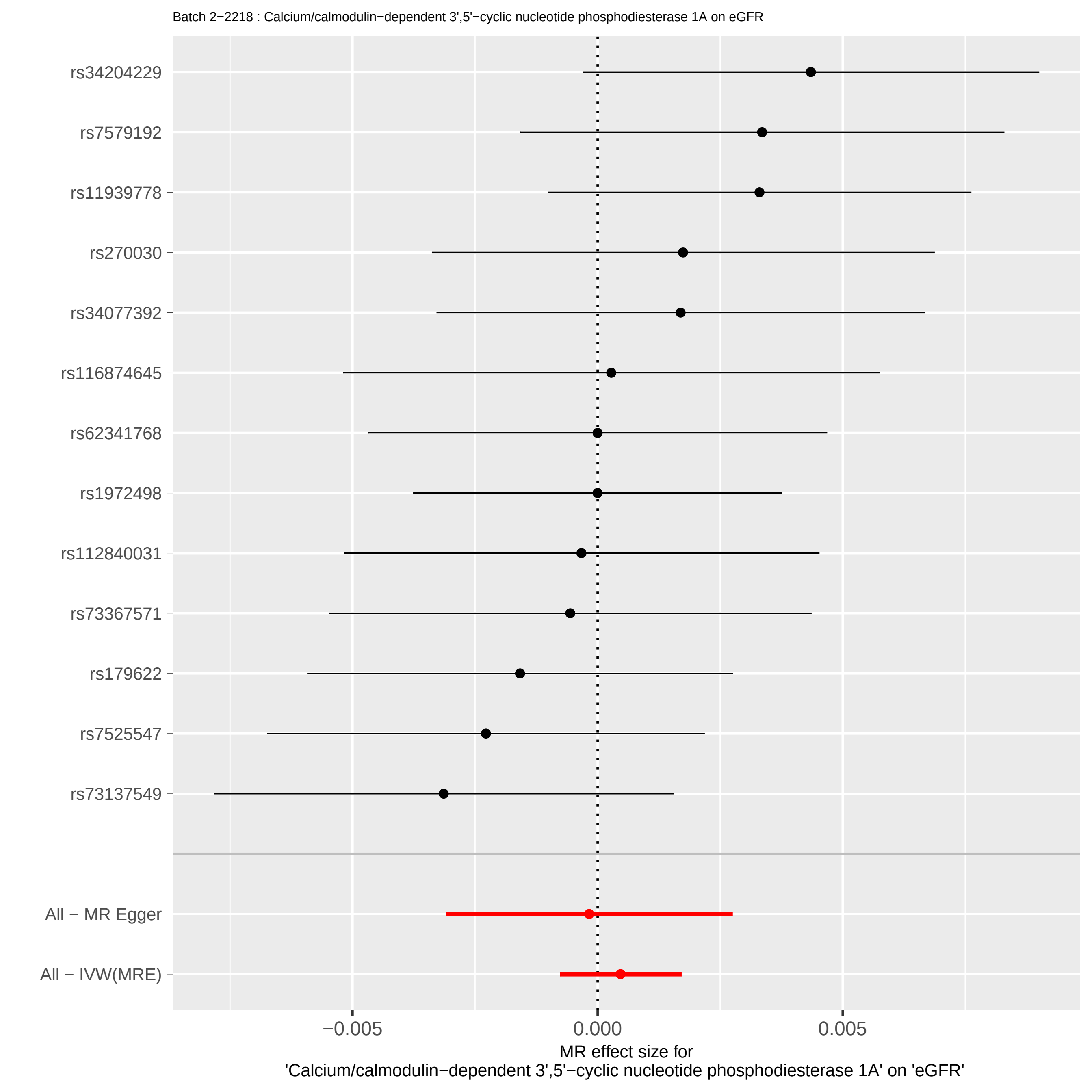

Batch 2−2218 : Calcium/calmodulin−dependent 3',5'−cyclic nucleotide phosphodiesterase 1A on eGFR
rs34204229
rs7579192
rs11939778
rs270030
rs34077392
rs116874645
rs62341768
rs1972498
rs112840031
rs73367571
rs179622
rs7525547
rs73137549
All − MR Egger
All − IVW(MRE)
−0.005
0.000
0.005
MR effect size for
'Calcium/calmodulin−dependent 3',5'−cyclic nucleotide phosphodiesterase 1A' on 'eGFR'

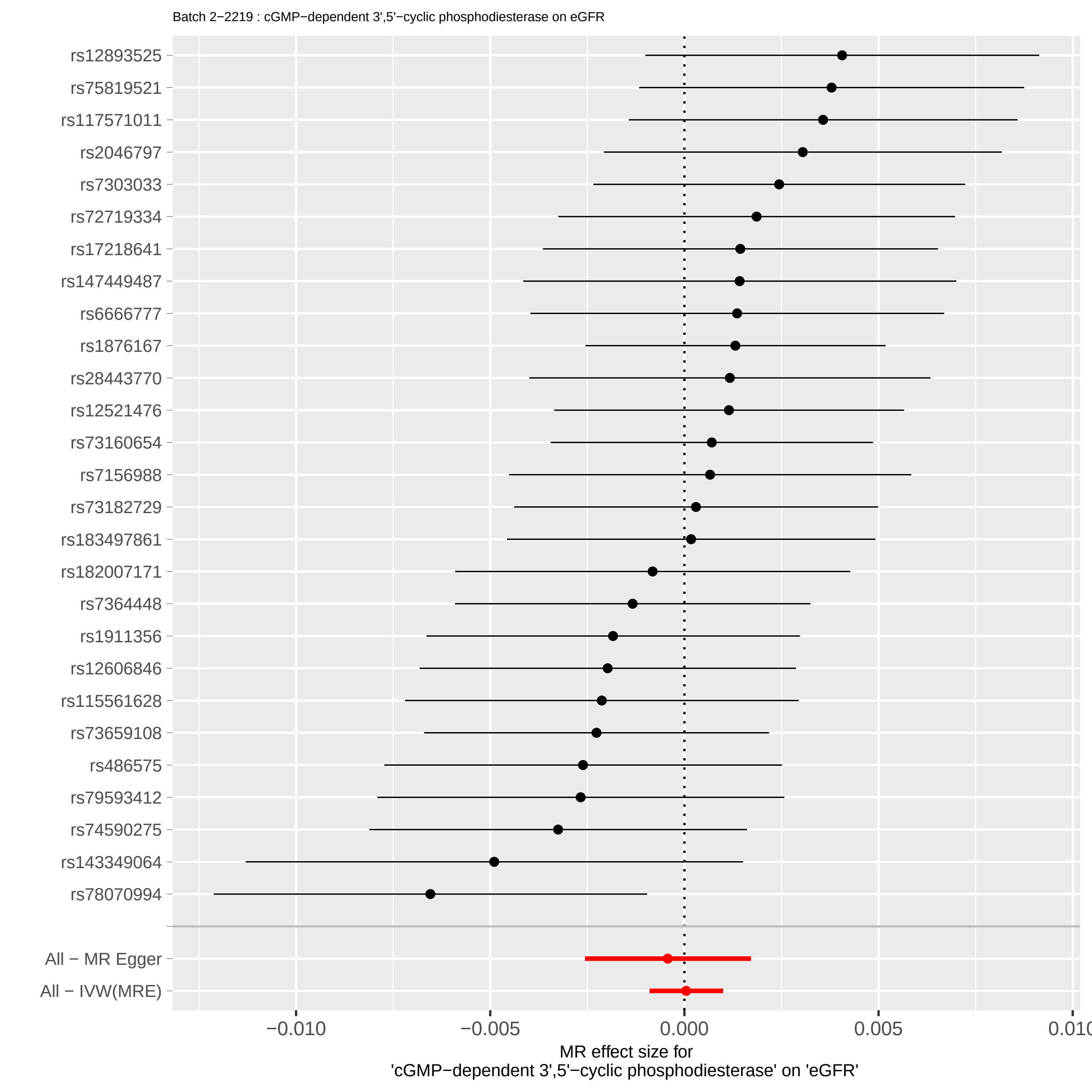
Batch 2−2219 : cGMP−dependent 3',5'−cyclic phosphodiesterase on eGFR
rs12893525
rs75819521
rs117571011
rs2046797
rs7303033
rs72719334
rs17218641
rs147449487
rs6666777
rs1876167
rs28443770
rs12521476
rs73160654
rs7156988
rs73182729
rs183497861
rs182007171
rs7364448
rs1911356
rs12606846
rs115561628
rs73659108
rs486575
rs79593412
rs74590275
rs143349064
rs78070994
All − MR Egger
All − IVW(MRE)
−0.010
−0.005
0.000
0.005
0.010
MR effect size for
'cGMP−dependent 3',5'−cyclic phosphodiesterase' on 'eGFR'

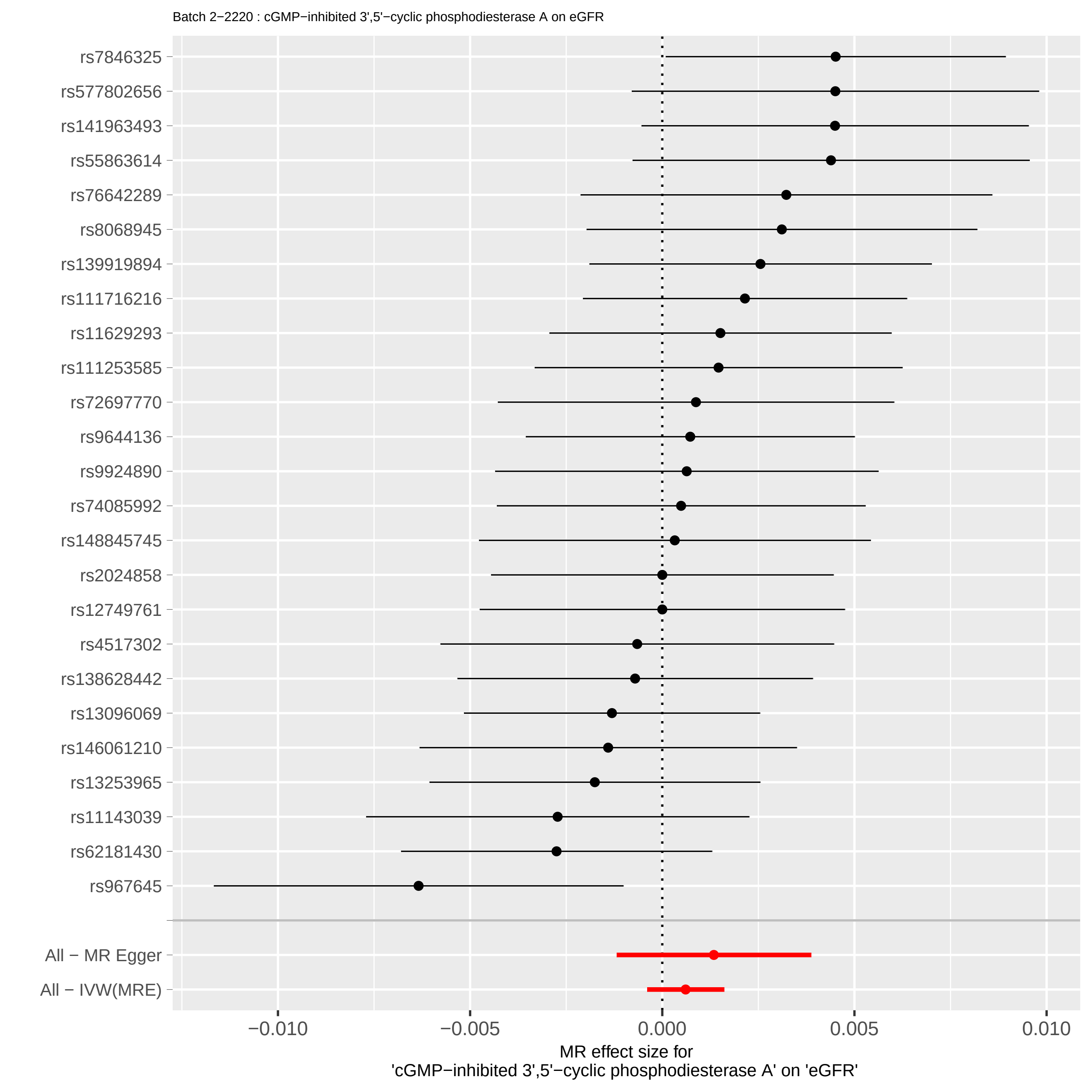

Batch 2−2220 : cGMP−inhibited 3',5'−cyclic phosphodiesterase A on eGFR
rs7846325
rs577802656
rs141963493
rs55863614
rs76642289
rs8068945
rs139919894
rs111716216
rs11629293
rs111253585
rs72697770
rs9644136
rs9924890
rs74085992
rs148845745
rs2024858
rs12749761
rs4517302
rs138628442
rs13096069
rs146061210
rs13253965
rs11143039
rs62181430
rs967645
All − MR Egger
All − IVW(MRE)
−0.010
−0.005
0.000
0.005
0.010
MR effect size for
'cGMP−inhibited 3',5'−cyclic phosphodiesterase A' on 'eGFR'

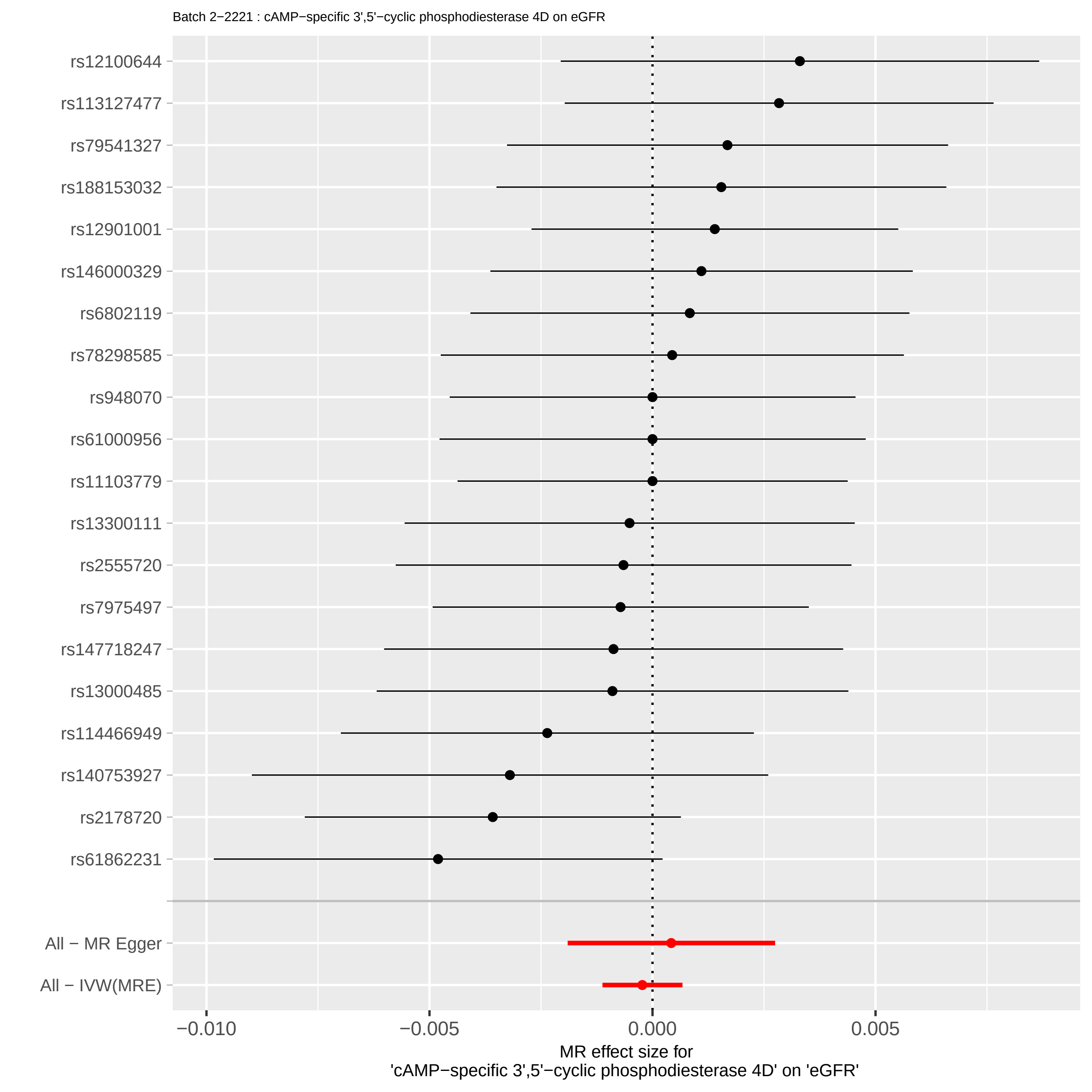
Batch 2−2221 : cAMP−specific 3',5'−cyclic phosphodiesterase 4D on eGFR
rs12100644
rs113127477
rs79541327
rs188153032
rs12901001
rs146000329
rs6802119
rs78298585
rs948070
rs61000956
rs11103779
rs13300111
rs2555720
rs7975497
rs147718247
rs13000485
rs114466949
rs140753927
rs2178720
rs61862231
All − MR Egger
All − IVW(MRE)
−0.010
−0.005
0.000
0.005
MR effect size for
'cAMP−specific 3',5'−cyclic phosphodiesterase 4D' on 'eGFR'

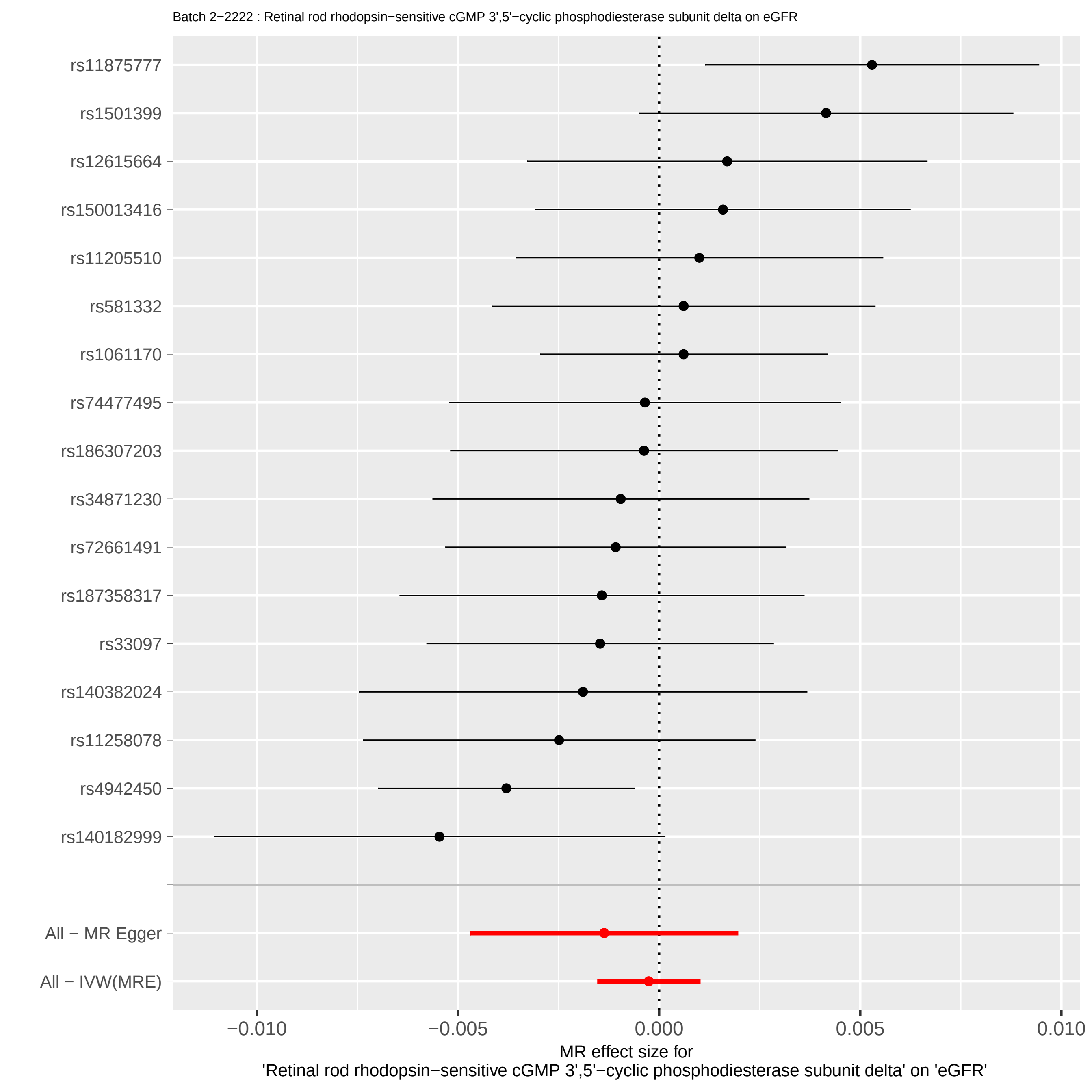
Batch 2−2222 : Retinal rod rhodopsin−sensitive cGMP 3',5'−cyclic phosphodiesterase subunit delta on eGFR
rs11875777
rs1501399
rs12615664
rs150013416
rs11205510
rs581332
rs1061170
rs74477495
rs186307203
rs34871230
rs72661491
rs187358317
rs33097
rs140382024
rs11258078
rs4942450
rs140182999
All − MR Egger
All − IVW(MRE)
−0.010
−0.005
0.000
0.005
0.010
MR effect size for
'Retinal rod rhodopsin−sensitive cGMP 3',5'−cyclic phosphodiesterase subunit delta' on 'eGFR'

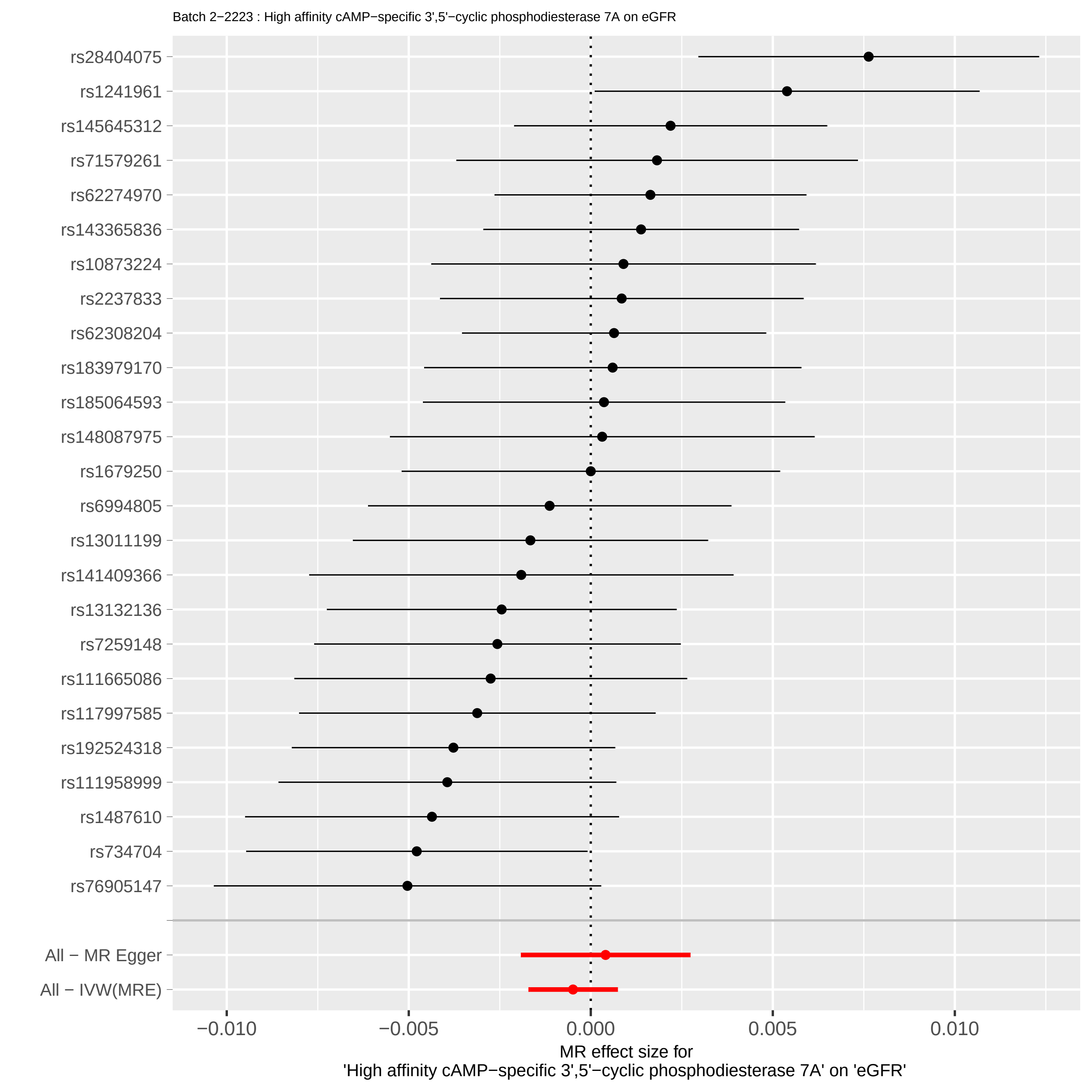

Batch 2−2223 : High affinity cAMP−specific 3',5'−cyclic phosphodiesterase 7A on eGFR
rs28404075
rs1241961
rs145645312
rs71579261
rs62274970
rs143365836
rs10873224
rs2237833
rs62308204
rs183979170
rs185064593
rs148087975
rs1679250
rs6994805
rs13011199
rs141409366
rs13132136
rs7259148
rs111665086
rs117997585
rs192524318
rs111958999
rs1487610
rs734704
rs76905147
All − MR Egger
All − IVW(MRE)
−0.010
−0.005
0.000
0.005
0.010
MR effect size for
'High affinity cAMP−specific 3',5'−cyclic phosphodiesterase 7A' on 'eGFR'

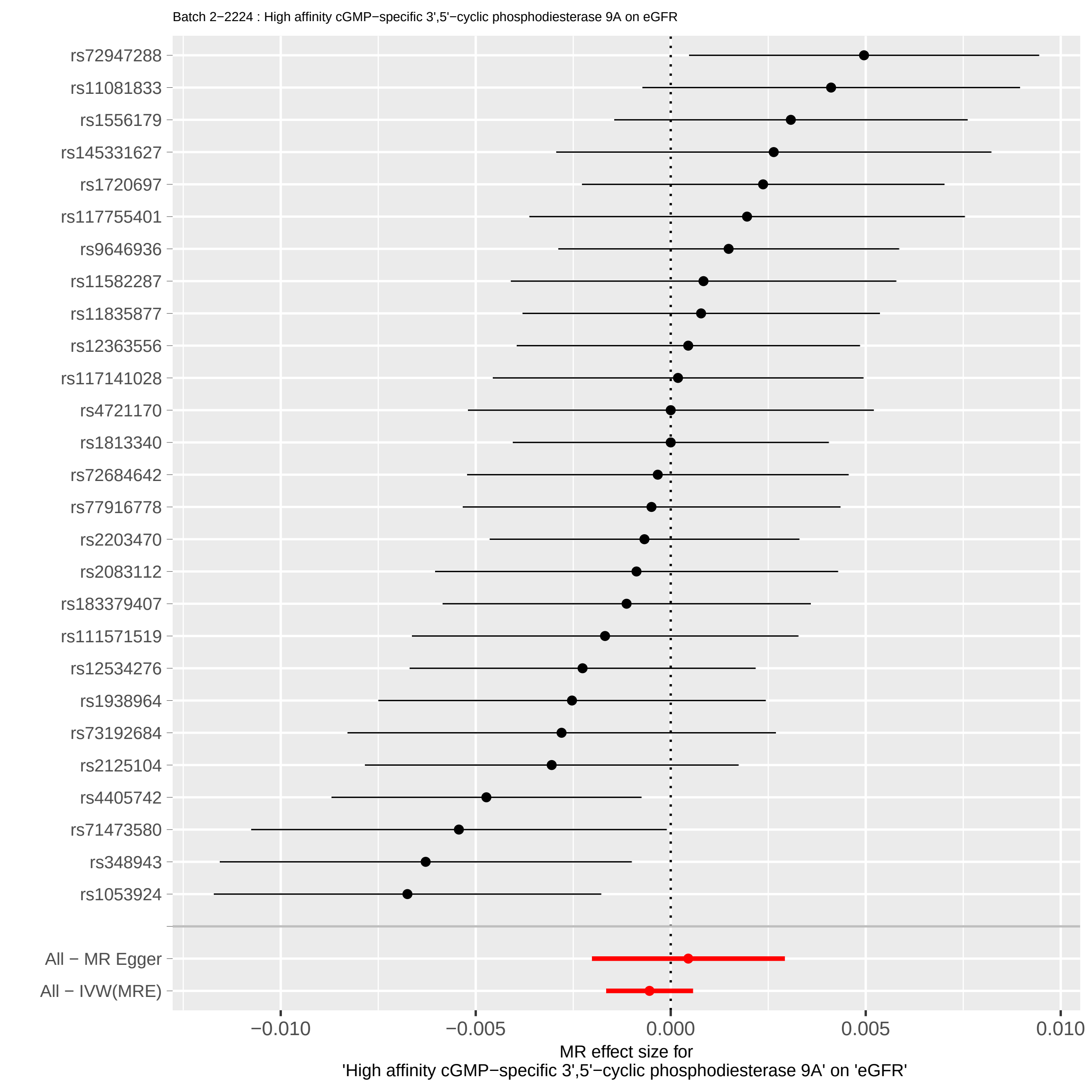
Batch 2−2224 : High affinity cGMP−specific 3',5'−cyclic phosphodiesterase 9A on eGFR
rs72947288
rs11081833
rs1556179
rs145331627
rs1720697
rs117755401
rs9646936
rs11582287
rs11835877
rs12363556
rs117141028
rs4721170
rs1813340
rs72684642
rs77916778
rs2203470
rs2083112
rs183379407
rs111571519
rs12534276
rs1938964
rs73192684
rs2125104
rs4405742
rs71473580
rs348943
rs1053924
All − MR Egger
All − IVW(MRE)
−0.010
−0.005
0.000
0.005
0.010
MR effect size for
'High affinity cGMP−specific 3',5'−cyclic phosphodiesterase 9A' on 'eGFR'

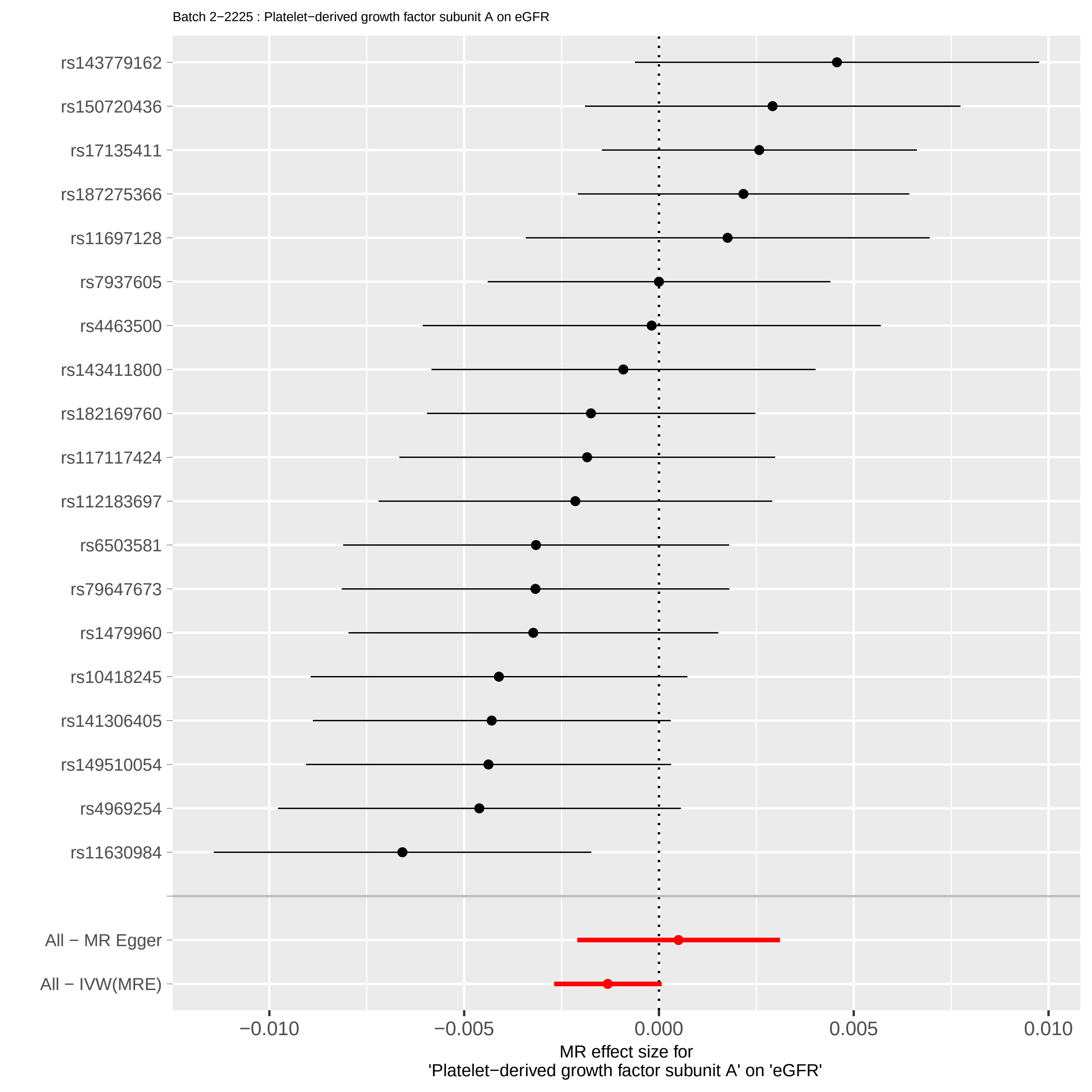

Batch 2−2225 : Platelet−derived growth factor subunit A on eGFR
rs143779162
rs150720436
rs17135411
rs187275366
rs11697128
rs7937605
rs4463500
rs143411800
rs182169760
rs117117424
rs112183697
rs6503581
rs79647673
rs1479960
rs10418245
rs141306405
rs149510054
rs4969254
rs11630984
All − MR Egger
All − IVW(MRE)
−0.010
−0.005
0.000
0.005
0.010
MR effect size for
'Platelet−derived growth factor subunit A' on 'eGFR'

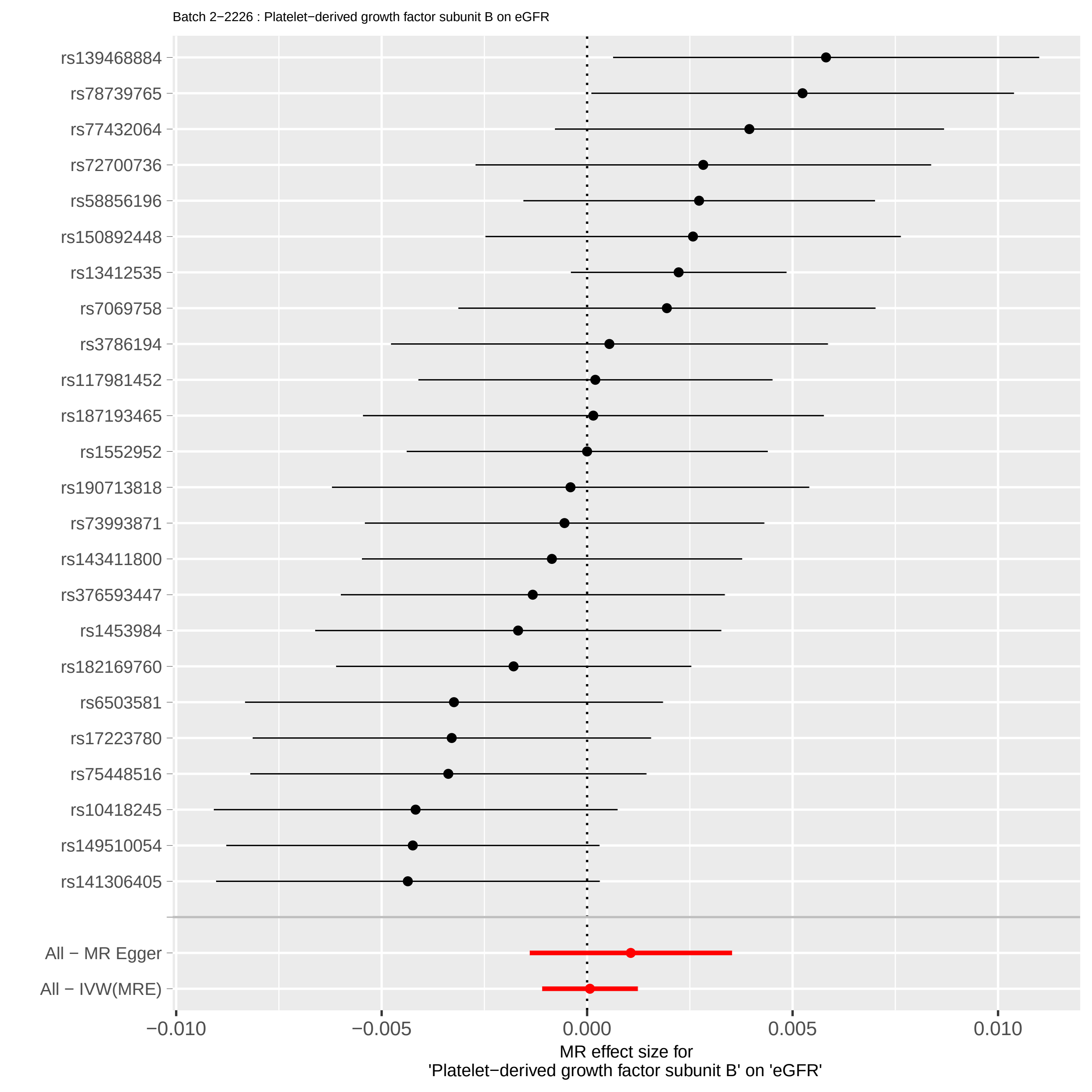
Batch 2−2226 : Platelet−derived growth factor subunit B on eGFR
rs139468884
rs78739765
rs77432064
rs72700736
rs58856196
rs150892448
rs13412535
rs7069758
rs3786194
rs117981452
rs187193465
rs1552952
rs190713818
rs73993871
rs143411800
rs376593447
rs1453984
rs182169760
rs6503581
rs17223780
rs75448516
rs10418245
rs149510054
rs141306405
All − MR Egger
All − IVW(MRE)
−0.010
−0.005
0.000
0.005
0.010
MR effect size for
'Platelet−derived growth factor subunit B' on 'eGFR'

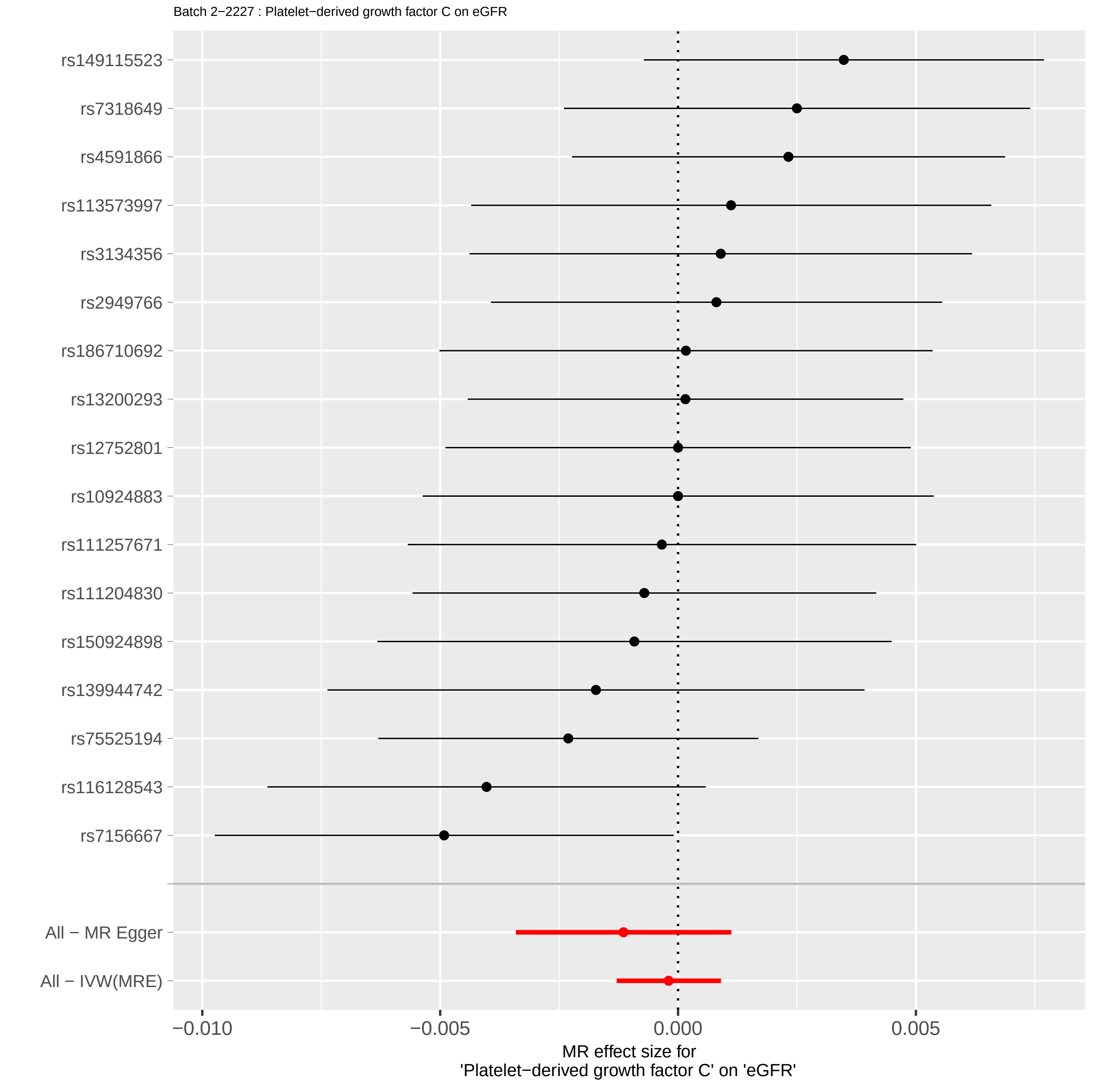
Batch 2−2227 : Platelet−derived growth factor C on eGFR
rs149115523
rs7318649
rs4591866
rs113573997
rs3134356
rs2949766
rs186710692
rs13200293
rs12752801
rs10924883
rs111257671
rs111204830
rs150924898
rs139944742
rs75525194
rs116128543
rs7156667
All − MR Egger
All − IVW(MRE)
−0.010
−0.005
0.000
0.005
MR effect size for
'Platelet−derived growth factor C' on 'eGFR'

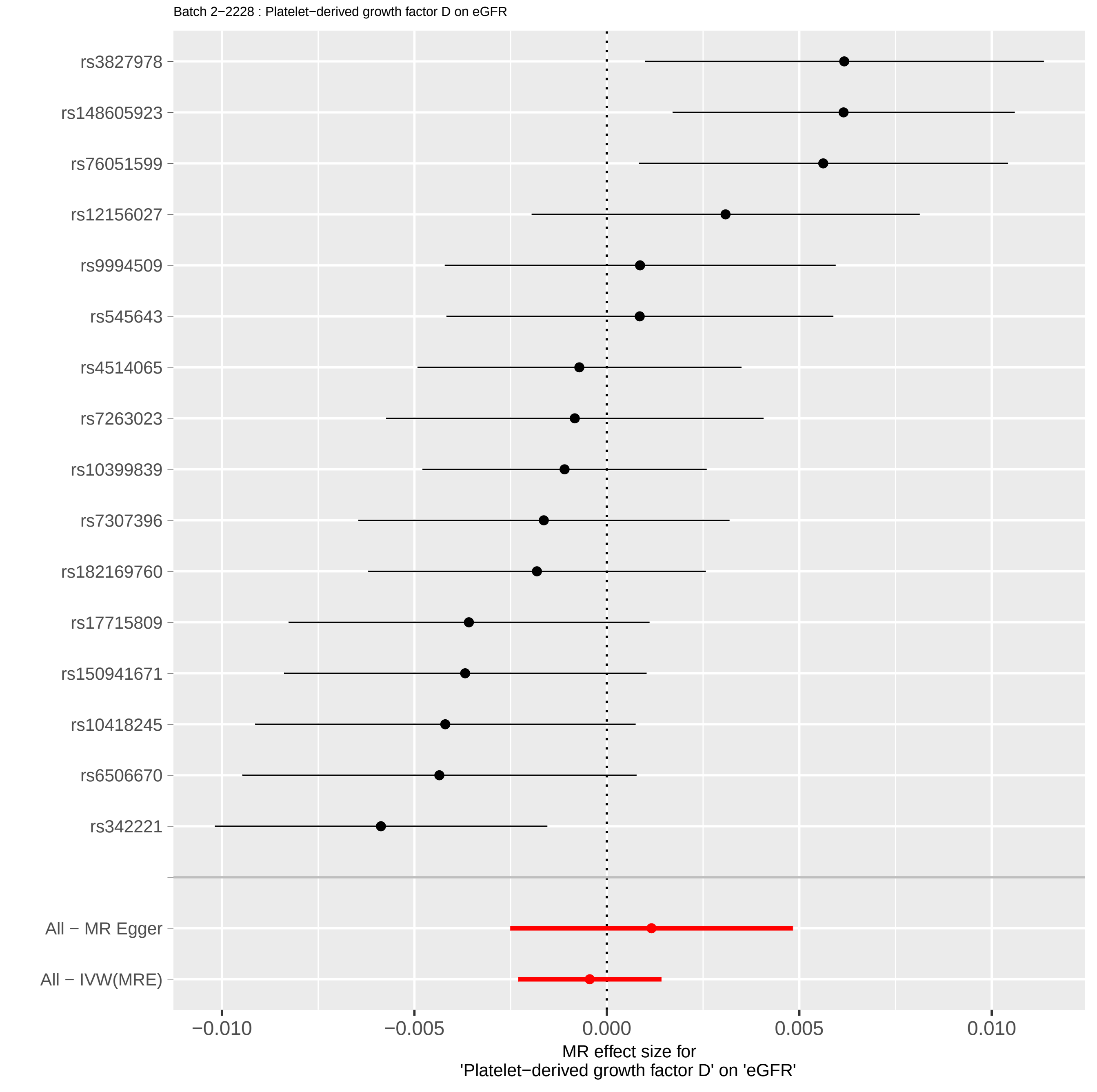
Batch 2−2228 : Platelet−derived growth factor D on eGFR
rs3827978
rs148605923
rs76051599
rs12156027
rs9994509
rs545643
rs4514065
rs7263023
rs10399839
rs7307396
rs182169760
rs17715809
rs150941671
rs10418245
rs6506670
rs342221
All − MR Egger
All − IVW(MRE)
−0.010
−0.005
0.000
0.005
0.010
MR effect size for
'Platelet−derived growth factor D' on 'eGFR'

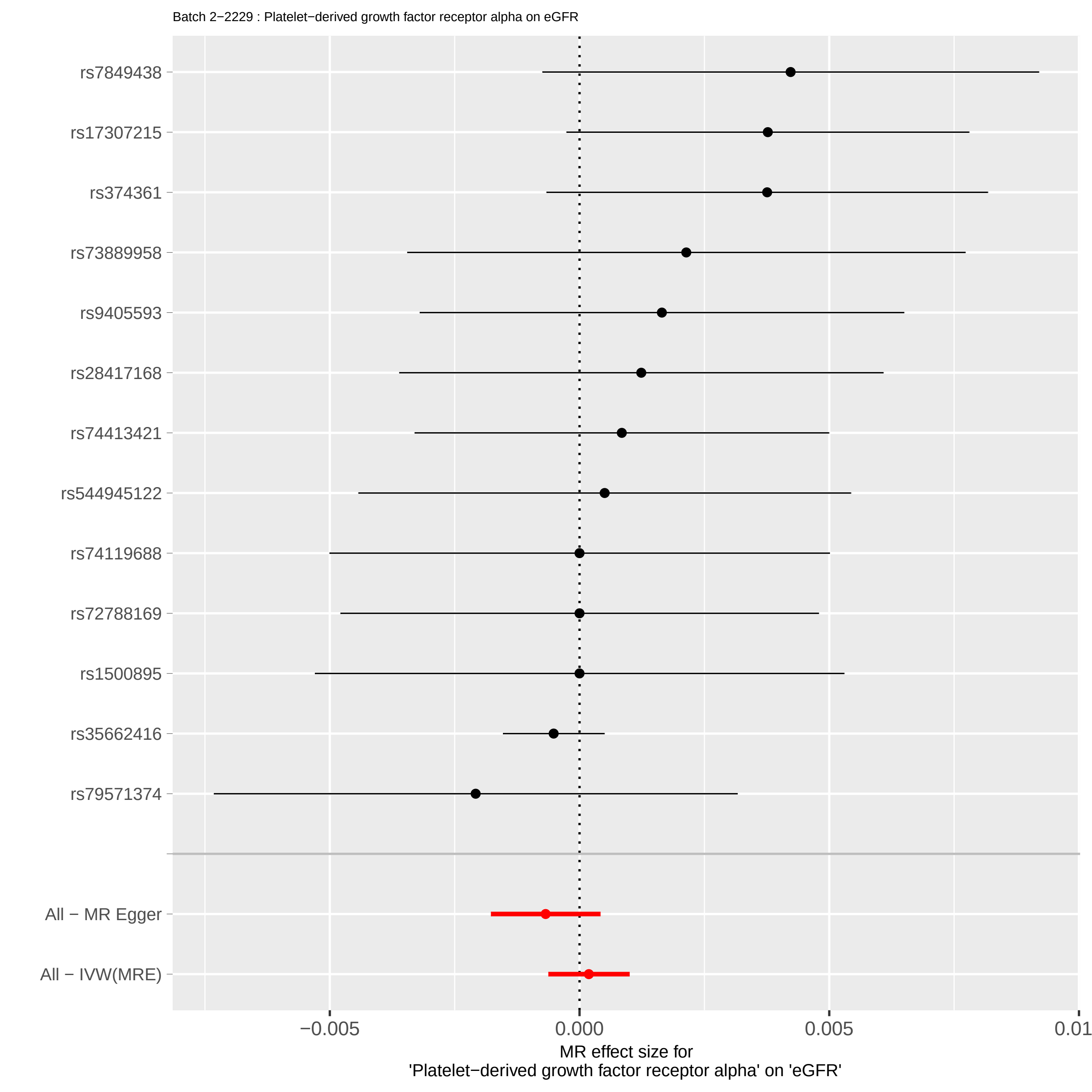

Batch 2−2229 : Platelet−derived growth factor receptor alpha on eGFR
rs7849438
rs17307215
rs374361
rs73889958
rs9405593
rs28417168
rs74413421
rs544945122
rs74119688
rs72788169
rs1500895
rs35662416
rs79571374
All − MR Egger
All − IVW(MRE)
−0.005
0.000
0.005
0.01
MR effect size for
'Platelet−derived growth factor receptor alpha' on 'eGFR'

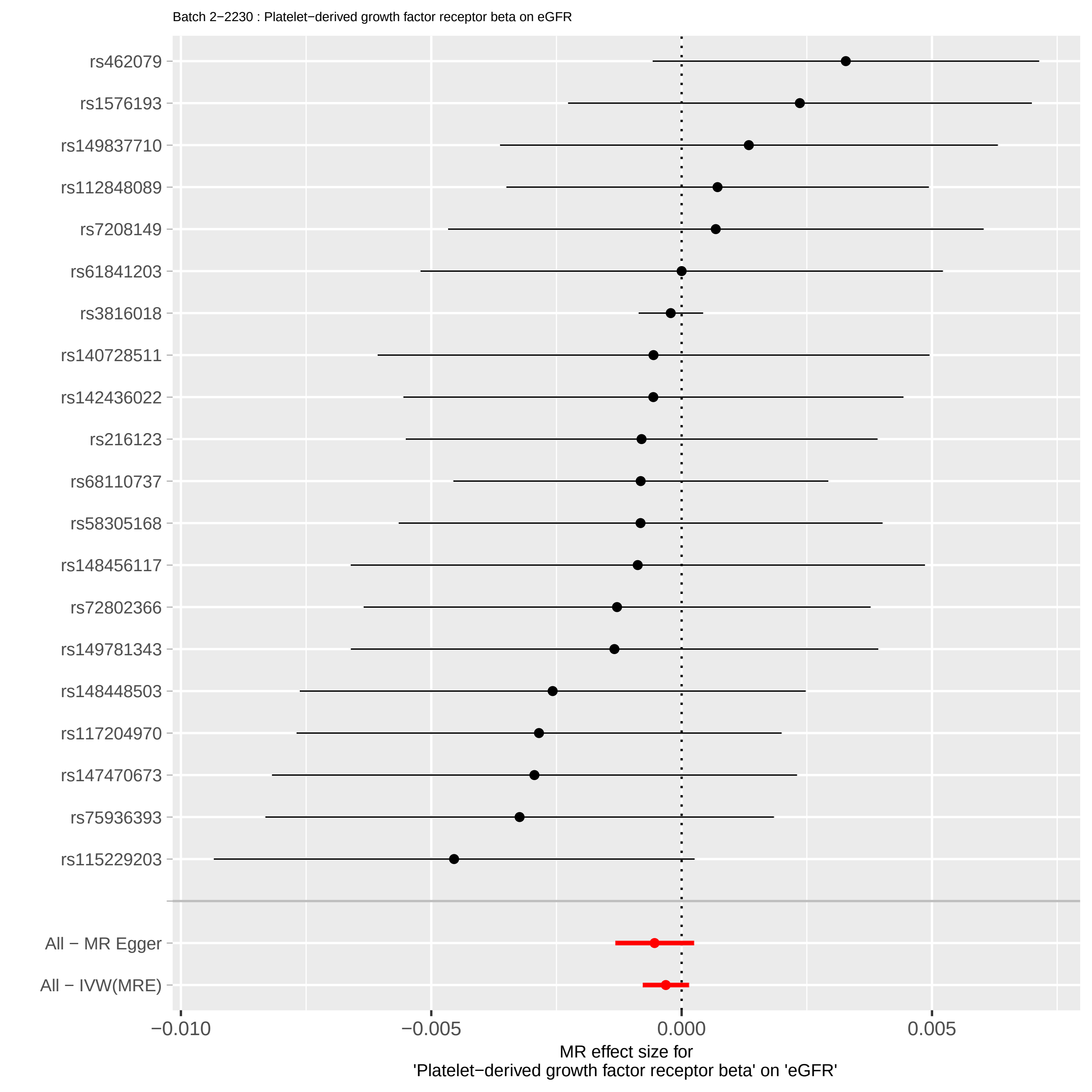

Batch 2−2230 : Platelet−derived growth factor receptor beta on eGFR
rs462079
rs1576193
rs149837710
rs112848089
rs7208149
rs61841203
rs3816018
rs140728511
rs142436022
rs216123
rs68110737
rs58305168
rs148456117
rs72802366
rs149781343
rs148448503
rs117204970
rs147470673
rs75936393
rs115229203
All − MR Egger
All − IVW(MRE)
−0.010
−0.005
0.000
0.005
MR effect size for
'Platelet−derived growth factor receptor beta' on 'eGFR'

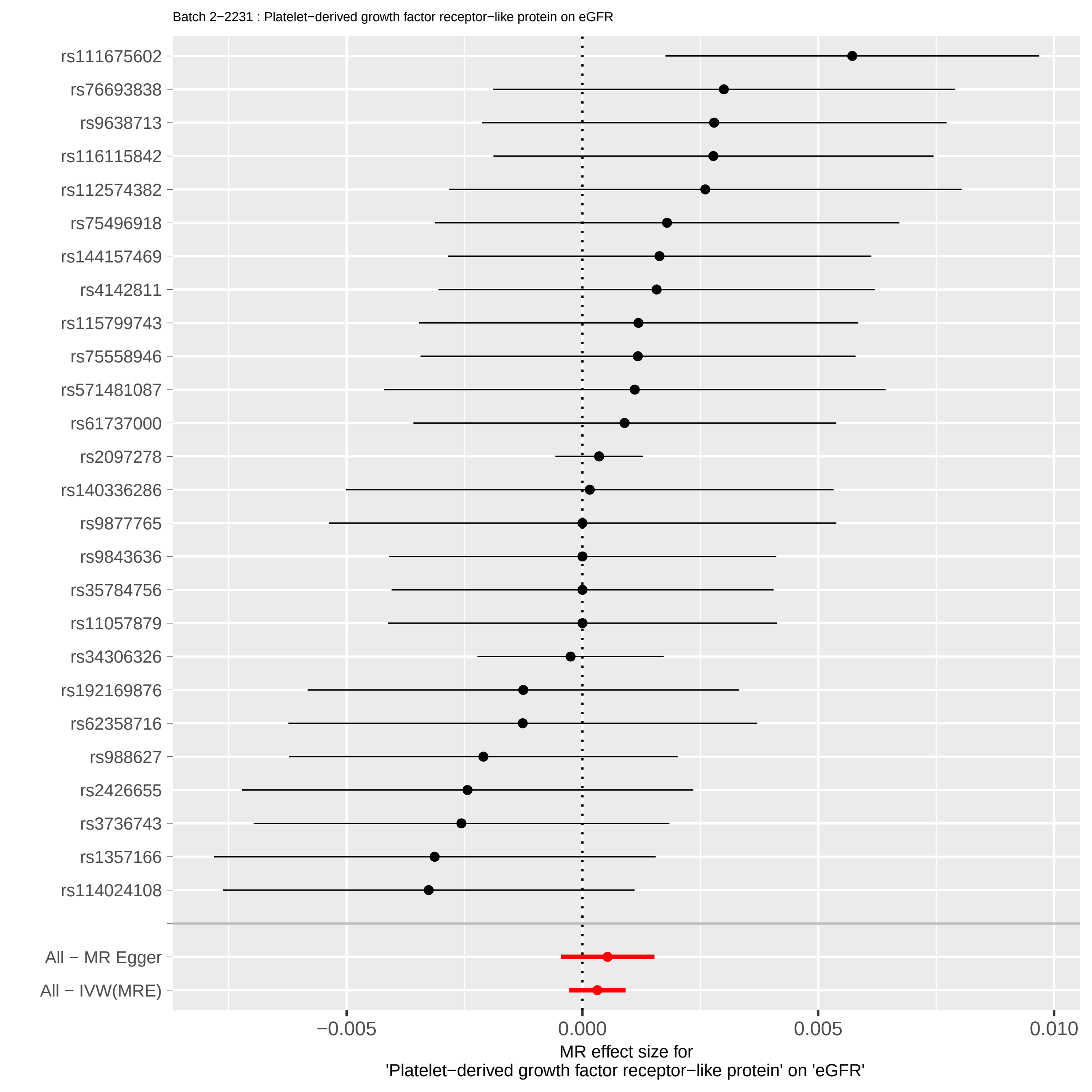

Batch 2−2231 : Platelet−derived growth factor receptor−like protein on eGFR
rs111675602
rs76693838
rs9638713
rs116115842
rs112574382
rs75496918
rs144157469
rs4142811
rs115799743
rs75558946
rs571481087
rs61737000
rs2097278
rs140336286
rs9877765
rs9843636
rs35784756
rs11057879
rs34306326
rs192169876
rs62358716
rs988627
rs2426655
rs3736743
rs1357166
rs114024108
All − MR Egger
All − IVW(MRE)
−0.005
0.000
0.005
0.010
MR effect size for
'Platelet−derived growth factor receptor−like protein' on 'eGFR'

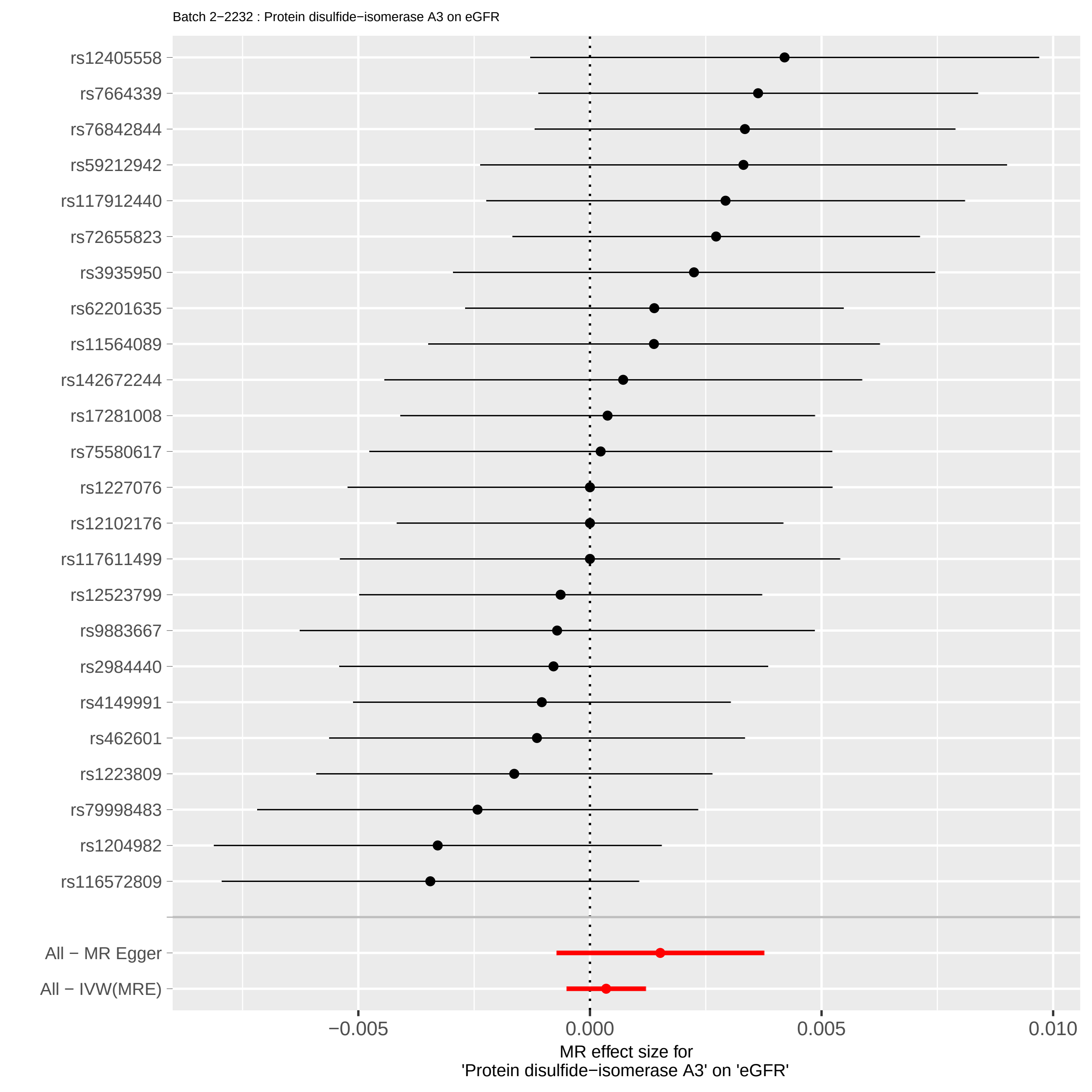

Batch 2−2232 : Protein disulfide−isomerase A3 on eGFR
rs12405558
rs7664339
rs76842844
rs59212942
rs117912440
rs72655823
rs3935950
rs62201635
rs11564089
rs142672244
rs17281008
rs75580617
rs1227076
rs12102176
rs117611499
rs12523799
rs9883667
rs2984440
rs4149991
rs462601
rs1223809
rs79998483
rs1204982
rs116572809
All − MR Egger
All − IVW(MRE)
−0.005
0.000
0.005
0.010
MR effect size for
'Protein disulfide−isomerase A3' on 'eGFR'

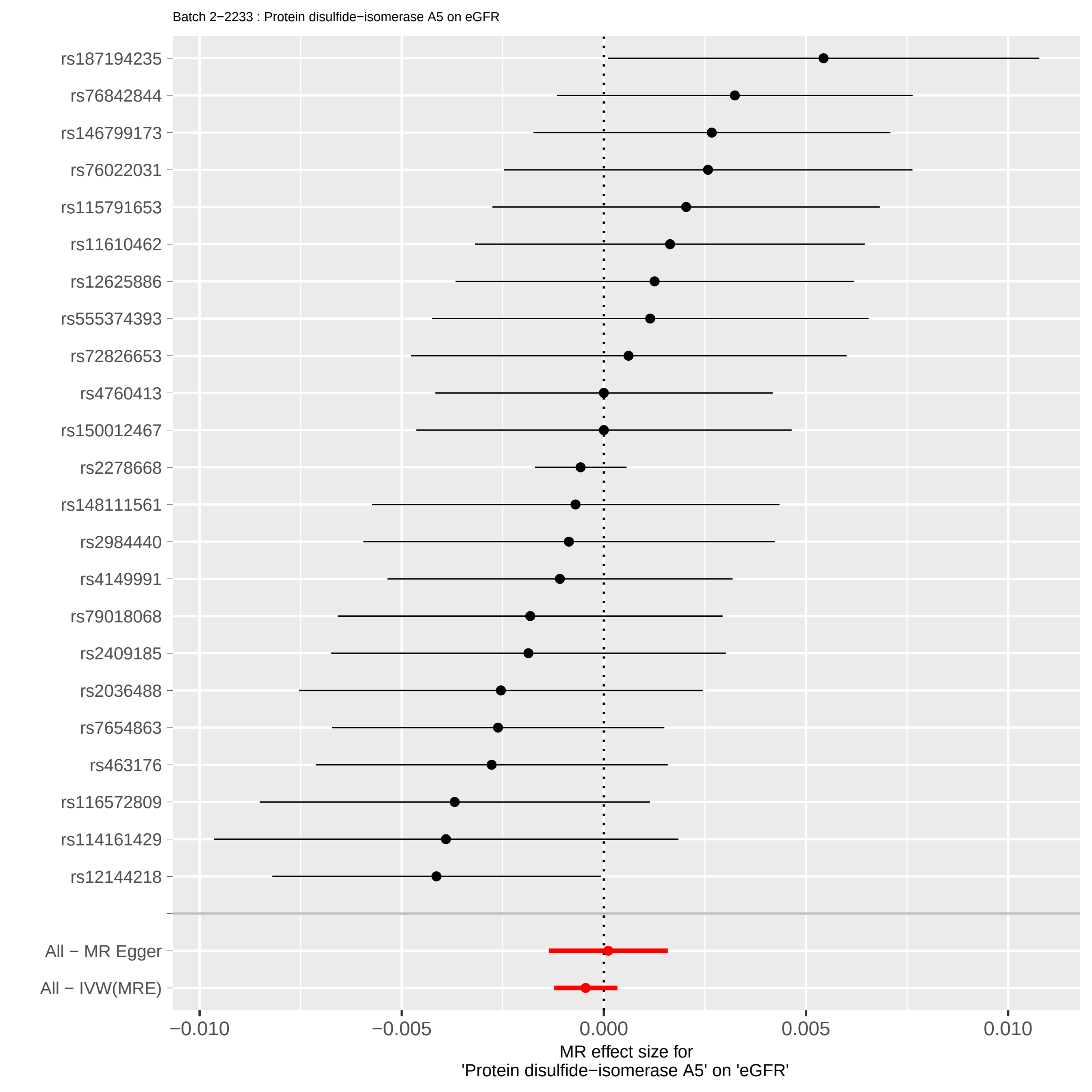

Batch 2−2233 : Protein disulfide−isomerase A5 on eGFR
rs187194235
rs76842844
rs146799173
rs76022031
rs115791653
rs11610462
rs12625886
rs555374393
rs72826653
rs4760413
rs150012467
rs2278668
rs148111561
rs2984440
rs4149991
rs79018068
rs2409185
rs2036488
rs7654863
rs463176
rs116572809
rs114161429
rs12144218
All − MR Egger
All − IVW(MRE)
−0.010
−0.005
0.000
0.005
0.010
MR effect size for
'Protein disulfide−isomerase A5' on 'eGFR'

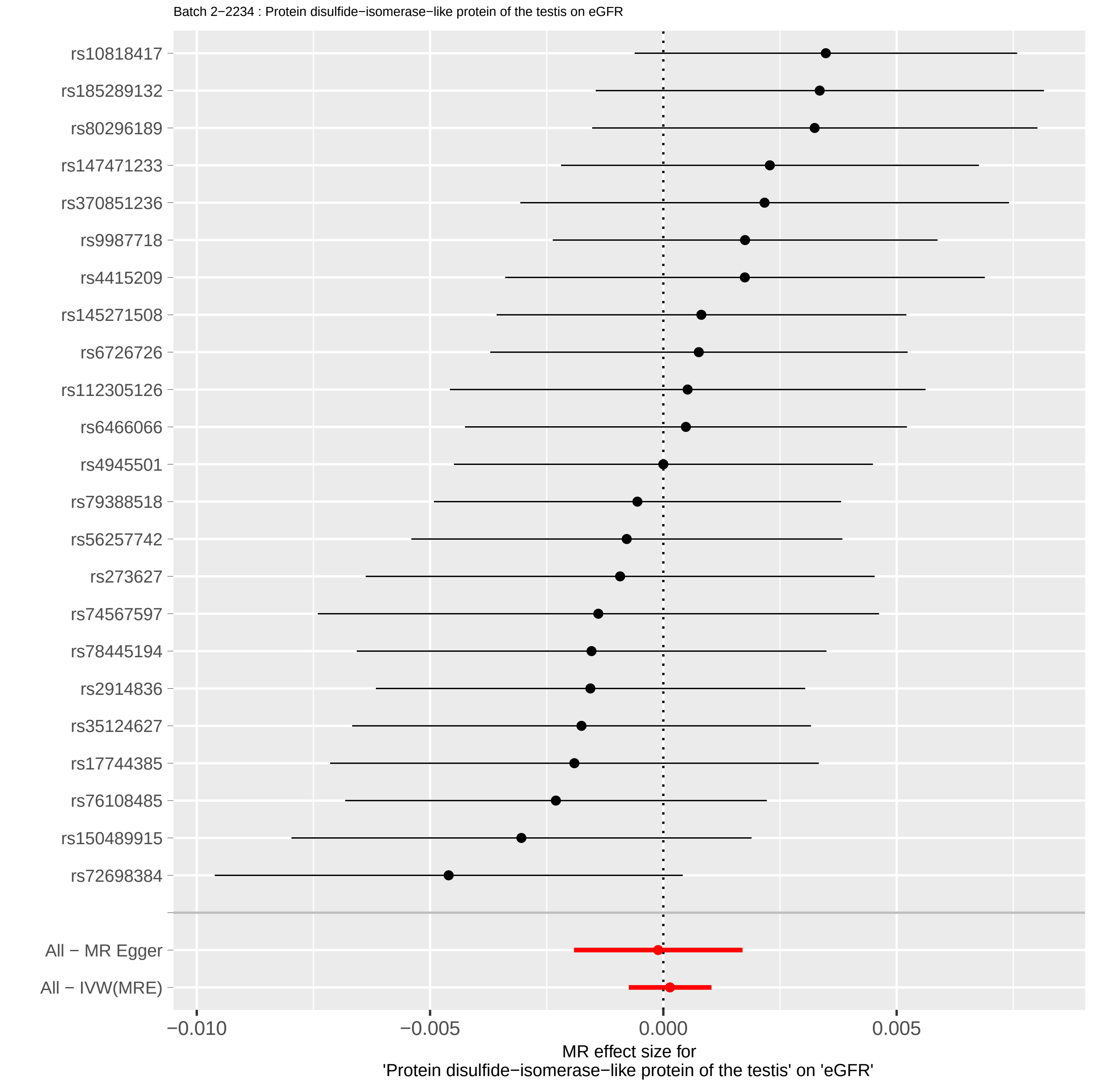

Batch 2−2234 : Protein disulfide−isomerase−like protein of the testis on eGFR
rs10818417
rs185289132
rs80296189
rs147471233
rs370851236
rs9987718
rs4415209
rs145271508
rs6726726
rs112305126
rs6466066
rs4945501
rs79388518
rs56257742
rs273627
rs74567597
rs78445194
rs2914836
rs35124627
rs17744385
rs76108485
rs150489915
rs72698384
All − MR Egger
All − IVW(MRE)
−0.010
−0.005
0.000
0.005
MR effect size for
'Protein disulfide−isomerase−like protein of the testis' on 'eGFR'

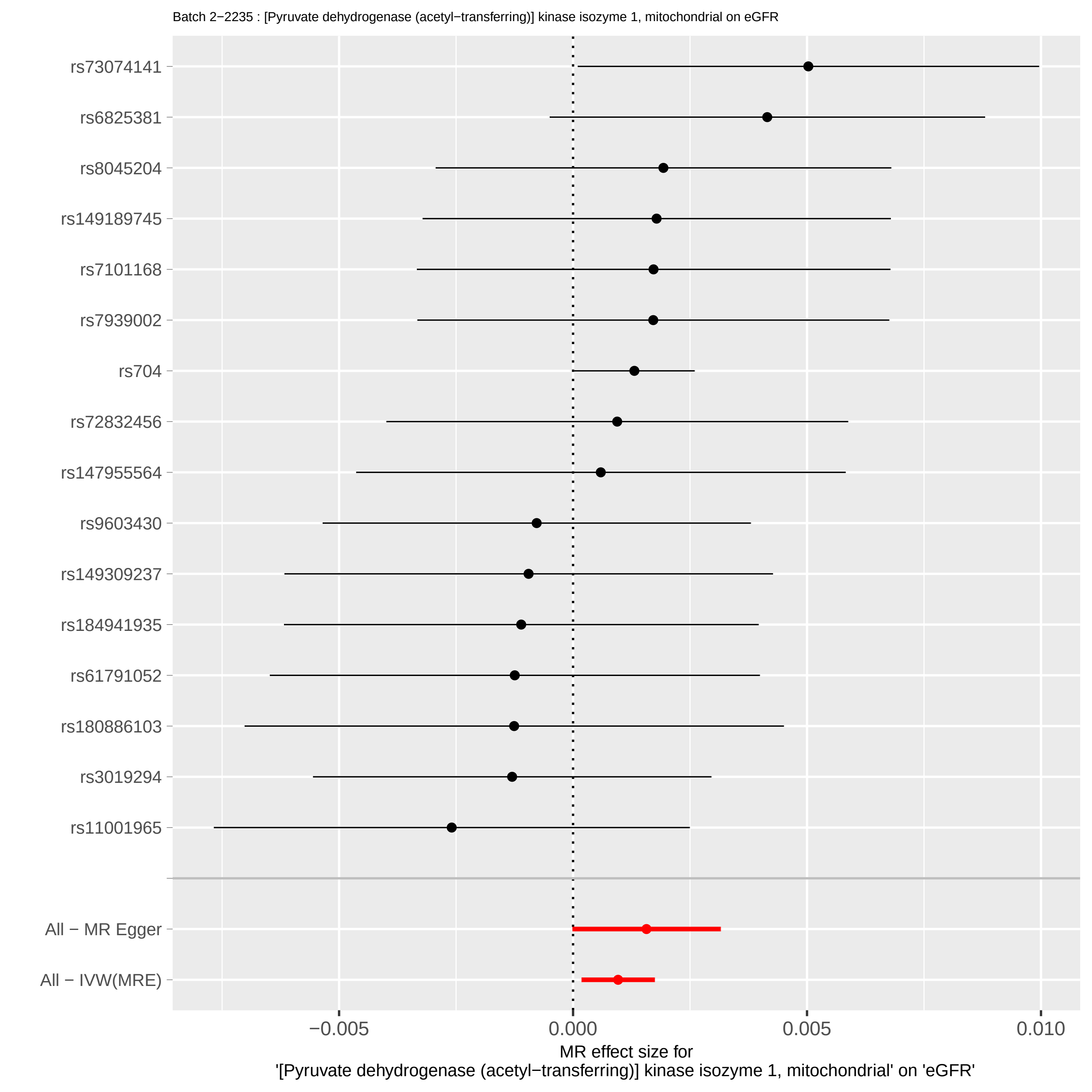

Batch 2−2235 : [Pyruvate dehydrogenase (acetyl−transferring)] kinase isozyme 1, mitochondrial on eGFR
rs73074141
rs6825381
rs8045204
rs149189745
rs7101168
rs7939002
rs704
rs72832456
rs147955564
rs9603430
rs149309237
rs184941935
rs61791052
rs180886103
rs3019294
rs11001965
All − MR Egger
All − IVW(MRE)
−0.005
0.000
0.005
0.010
MR effect size for
'[Pyruvate dehydrogenase (acetyl−transferring)] kinase isozyme 1, mitochondrial' on 'eGFR'

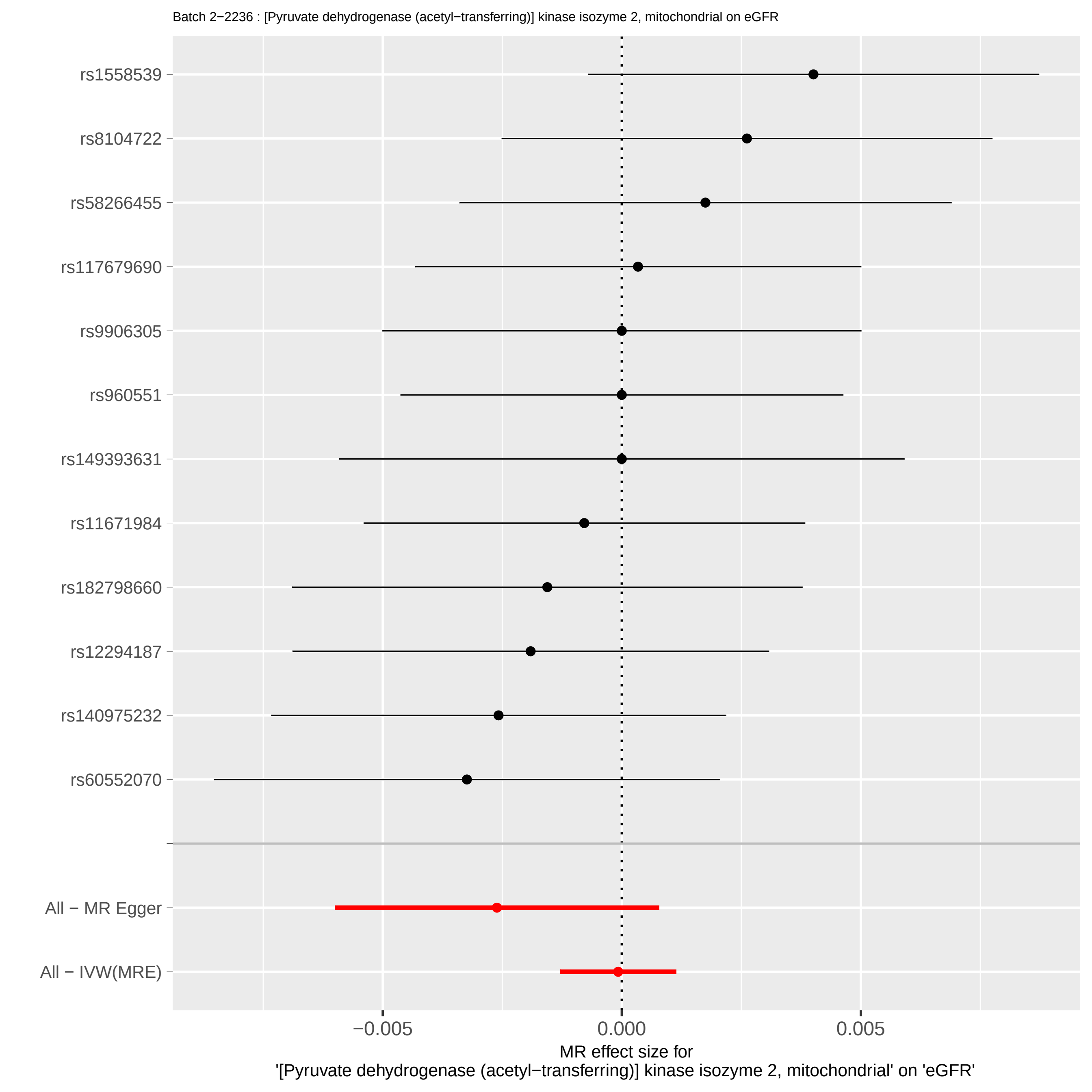

Batch 2−2236 : [Pyruvate dehydrogenase (acetyl−transferring)] kinase isozyme 2, mitochondrial on eGFR
rs1558539
rs8104722
rs58266455
rs117679690
rs9906305
rs960551
rs149393631
rs11671984
rs182798660
rs12294187
rs140975232
rs60552070
All − MR Egger
All − IVW(MRE)
−0.005
0.000
0.005
MR effect size for
'[Pyruvate dehydrogenase (acetyl−transferring)] kinase isozyme 2, mitochondrial' on 'eGFR'

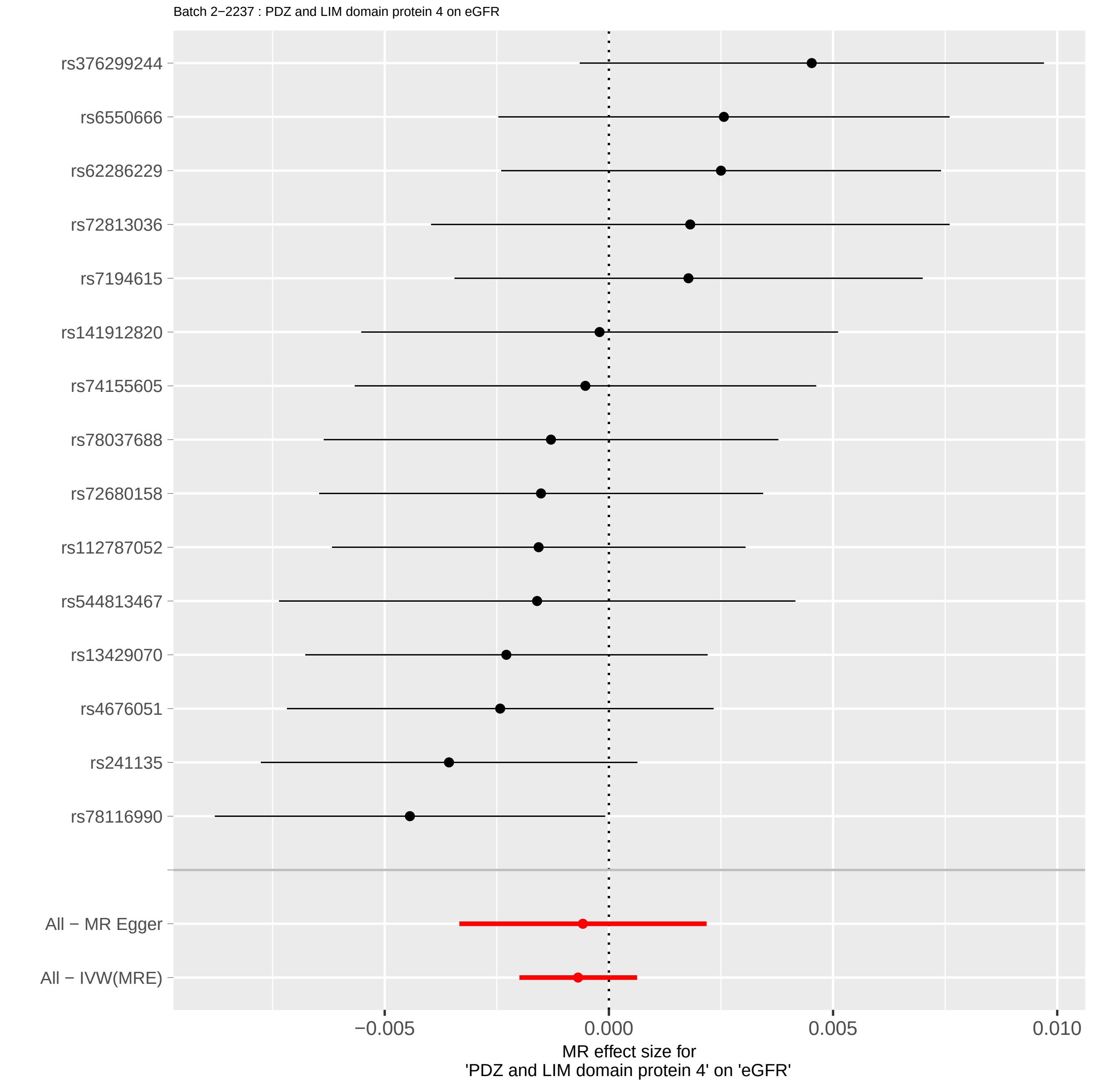
Batch 2−2237 : PDZ and LIM domain protein 4 on eGFR
rs376299244
rs6550666
rs62286229
rs72813036
rs7194615
rs141912820
rs74155605
rs78037688
rs72680158
rs112787052
rs544813467
rs13429070
rs4676051
rs241135
rs78116990
All − MR Egger
All − IVW(MRE)
−0.005
0.000
0.005
0.010
MR effect size for
'PDZ and LIM domain protein 4' on 'eGFR'

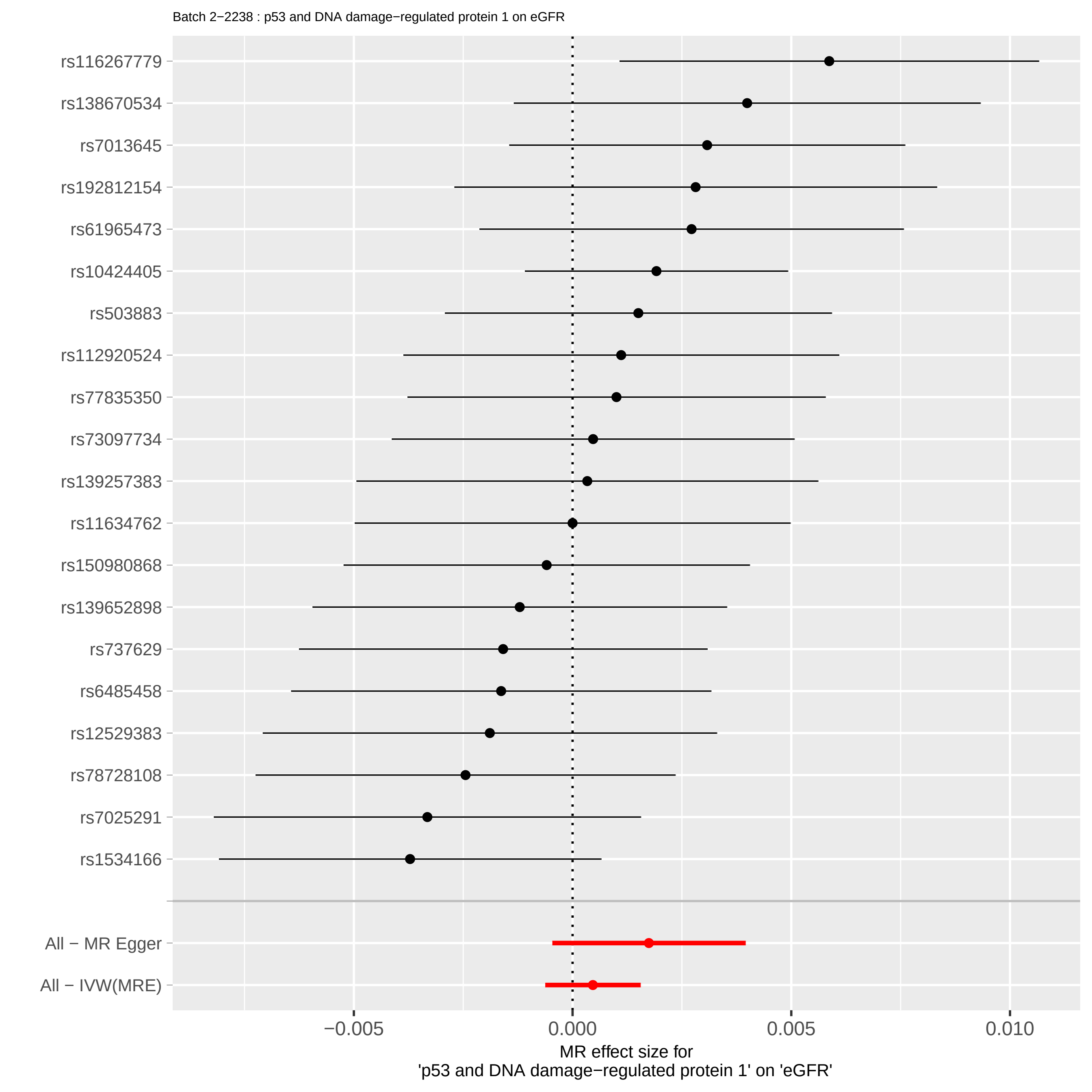

Batch 2−2238 : p53 and DNA damage−regulated protein 1 on eGFR
rs116267779
rs138670534
rs7013645
rs192812154
rs61965473
rs10424405
rs503883
rs112920524
rs77835350
rs73097734
rs139257383
rs11634762
rs150980868
rs139652898
rs737629
rs6485458
rs12529383
rs78728108
rs7025291
rs1534166
All − MR Egger
All − IVW(MRE)
−0.005
0.000
0.005
0.010
MR effect size for
'p53 and DNA damage−regulated protein 1' on 'eGFR'

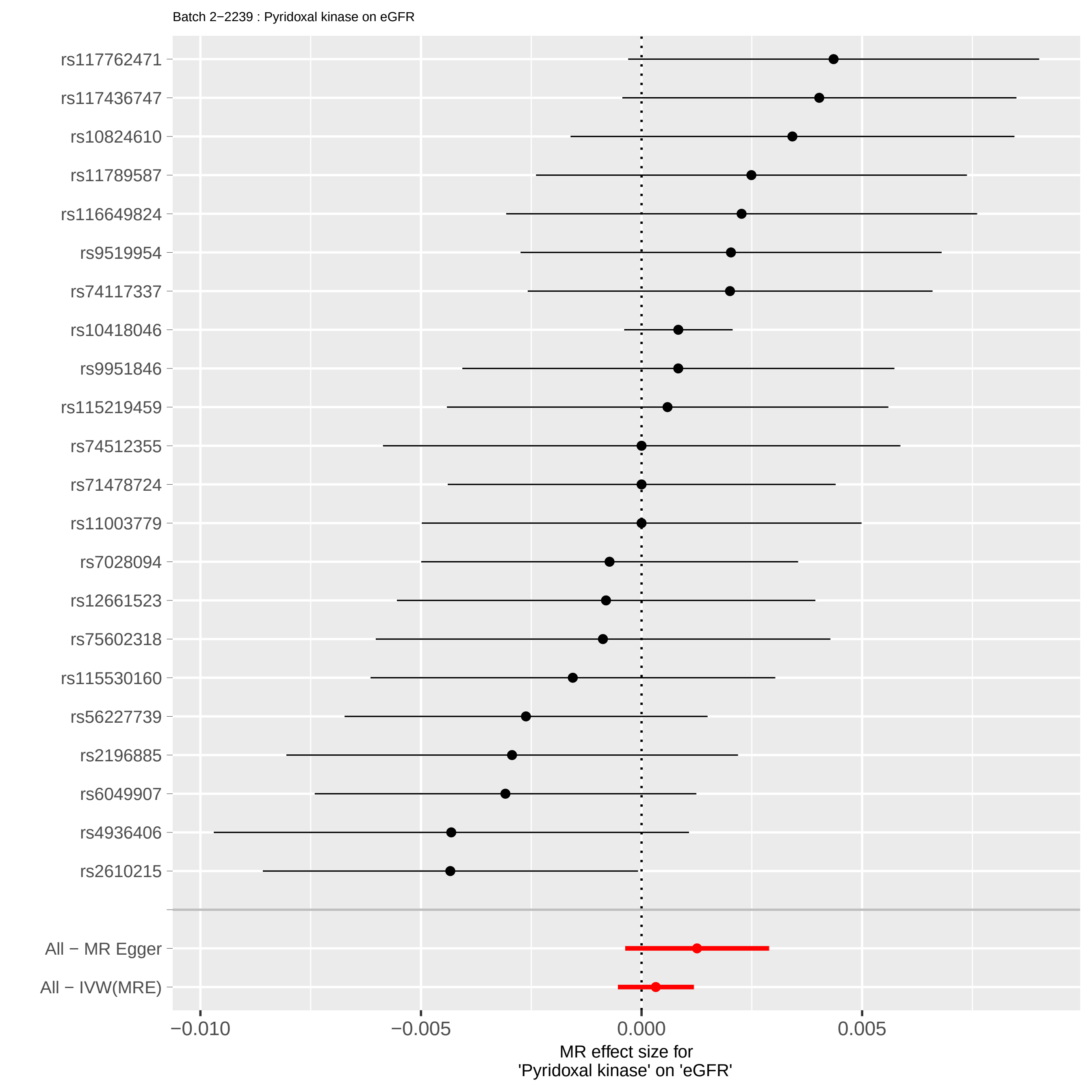

Batch 2−2239 : Pyridoxal kinase on eGFR
rs117762471
rs117436747
rs10824610
rs11789587
rs116649824
rs9519954
rs74117337
rs10418046
rs9951846
rs115219459
rs74512355
rs71478724
rs11003779
rs7028094
rs12661523
rs75602318
rs115530160
rs56227739
rs2196885
rs6049907
rs4936406
rs2610215
All − MR Egger
All − IVW(MRE)
−0.010
−0.005
0.000
0.005
MR effect size for
'Pyridoxal kinase' on 'eGFR'

Batch 2−2240 : Pyridoxal phosphate phosphatase on eGFR

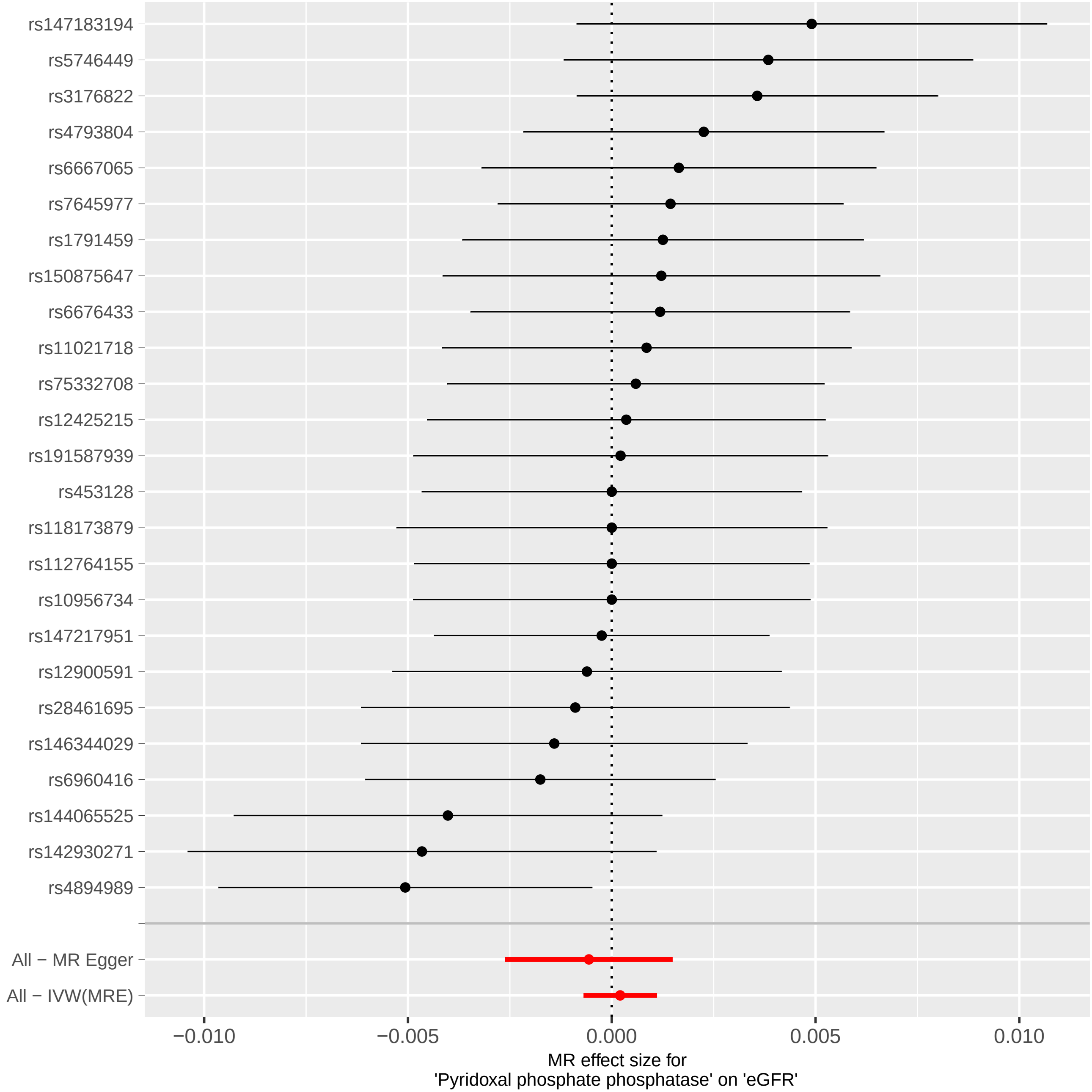

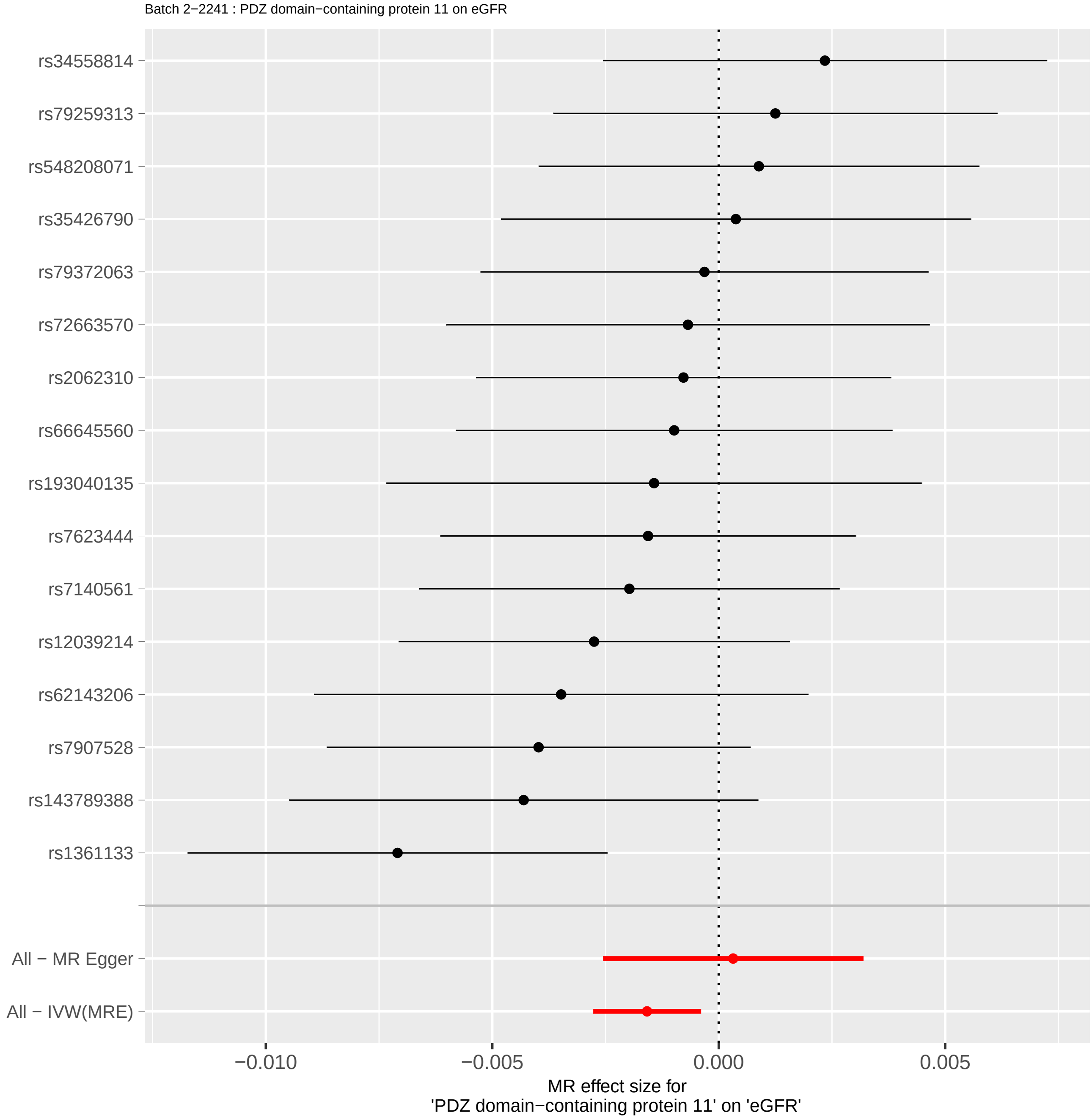

Batch 2−2241 : PDZ domain−containing protein 11 on eGFR
rs34558814
rs79259313
rs548208071
rs35426790
rs79372063
rs72663570
rs2062310
rs66645560
rs193040135
rs7623444
rs7140561
rs12039214
rs62143206
rs7907528
rs143789388
rs1361133
All − MR Egger
All − IVW(MRE)
−0.010
−0.005
0.000
0.005
MR effect size for
'PDZ domain−containing protein 11' on 'eGFR'

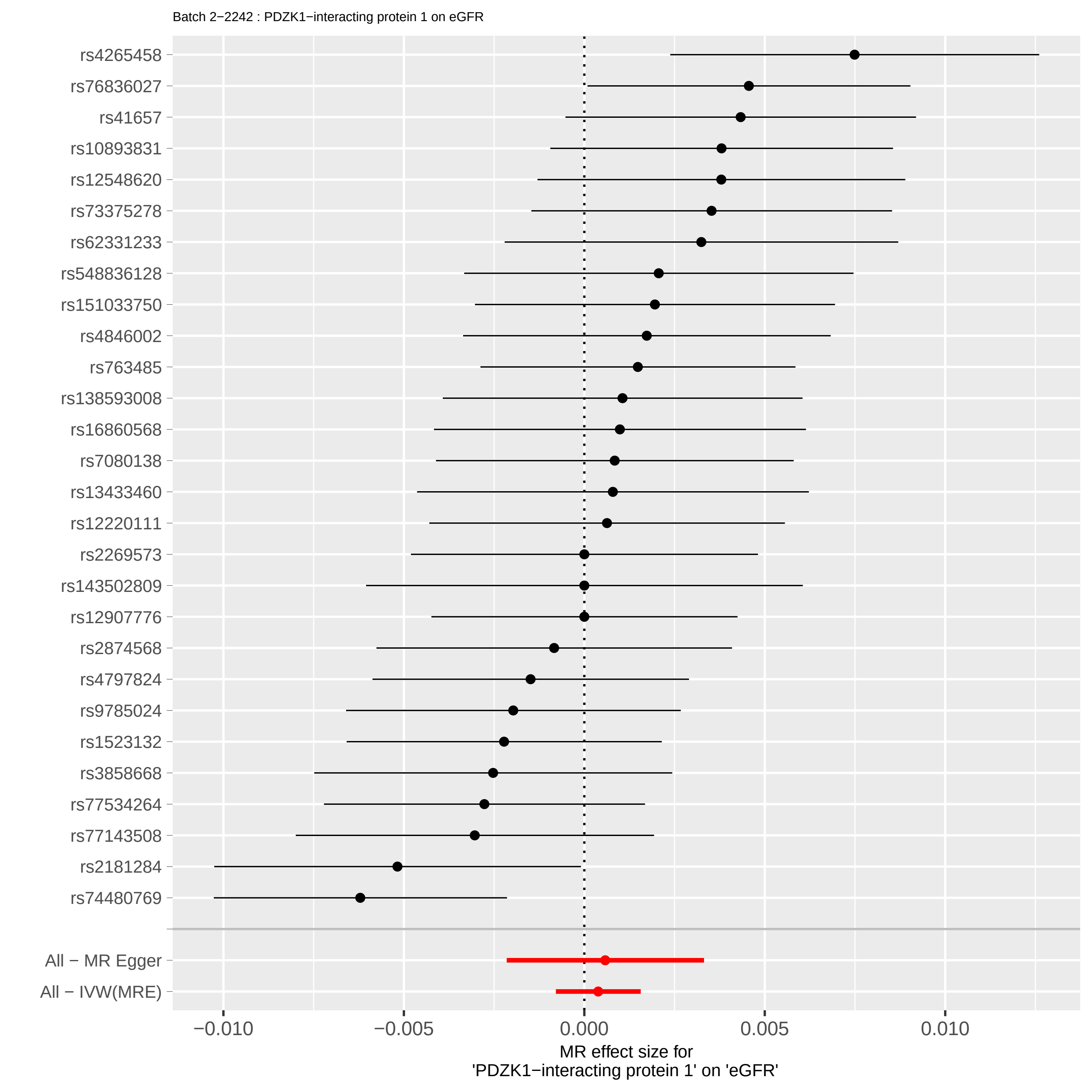
Batch 2−2242 : PDZK1−interacting protein 1 on eGFR
rs4265458
rs76836027
rs41657
rs10893831
rs12548620
rs73375278
rs62331233
rs548836128
rs151033750
rs4846002
rs763485
rs138593008
rs16860568
rs7080138
rs13433460
rs12220111
rs2269573
rs143502809
rs12907776
rs2874568
rs4797824
rs9785024
rs1523132
rs3858668
rs77534264
rs77143508
rs2181284
rs74480769
All − MR Egger
All − IVW(MRE)
−0.010
−0.005
0.000
0.005
0.010
MR effect size for
'PDZK1−interacting protein 1' on 'eGFR'

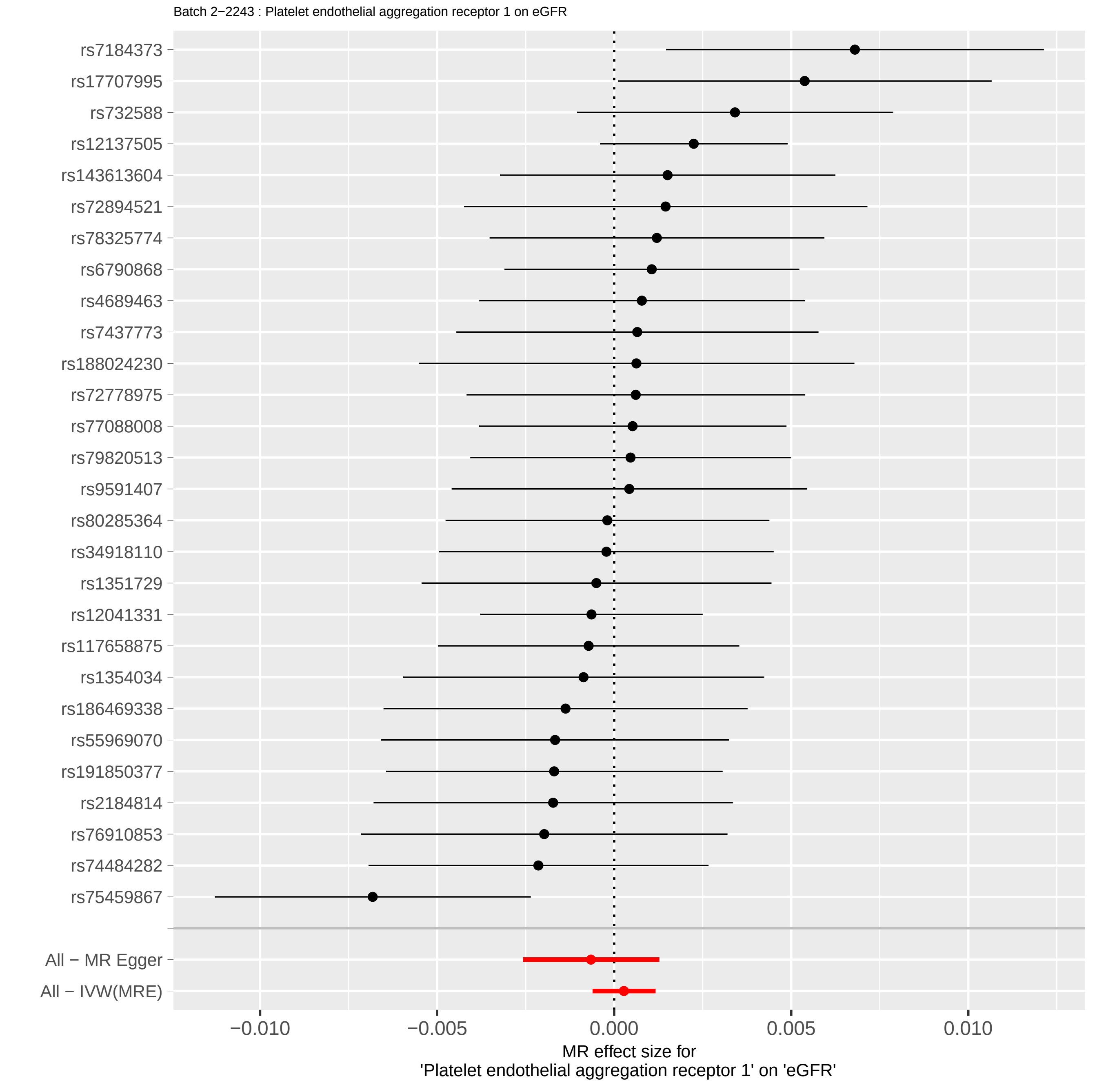
Batch 2−2243 : Platelet endothelial aggregation receptor 1 on eGFR
rs7184373
rs17707995
rs732588
rs12137505
rs143613604
rs72894521
rs78325774
rs6790868
rs4689463
rs7437773
rs188024230
rs72778975
rs77088008
rs79820513
rs9591407
rs80285364
rs34918110
rs1351729
rs12041331
rs117658875
rs1354034
rs186469338
rs55969070
rs191850377
rs2184814
rs76910853
rs74484282
rs75459867
All − MR Egger
All − IVW(MRE)
−0.010
−0.005
0.000
0.005
0.010
MR effect size for
'Platelet endothelial aggregation receptor 1' on 'eGFR'

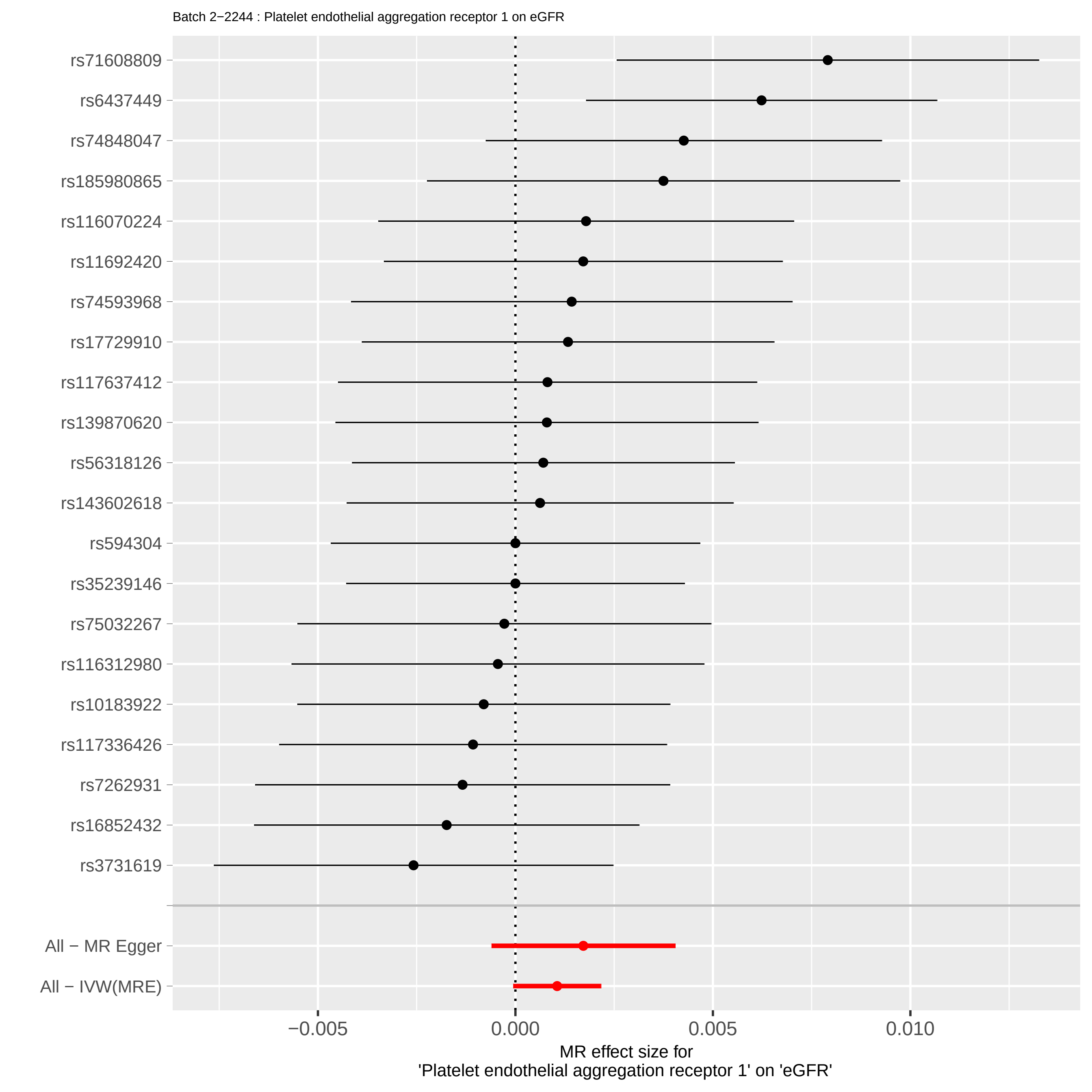

Batch 2−2244 : Platelet endothelial aggregation receptor 1 on eGFR
rs71608809
rs6437449
rs74848047
rs185980865
rs116070224
rs11692420
rs74593968
rs17729910
rs117637412
rs139870620
rs56318126
rs143602618
rs594304
rs35239146
rs75032267
rs116312980
rs10183922
rs117336426
rs7262931
rs16852432
rs3731619
All − MR Egger
All − IVW(MRE)
−0.005
0.000
0.005
0.010
MR effect size for
'Platelet endothelial aggregation receptor 1' on 'eGFR'

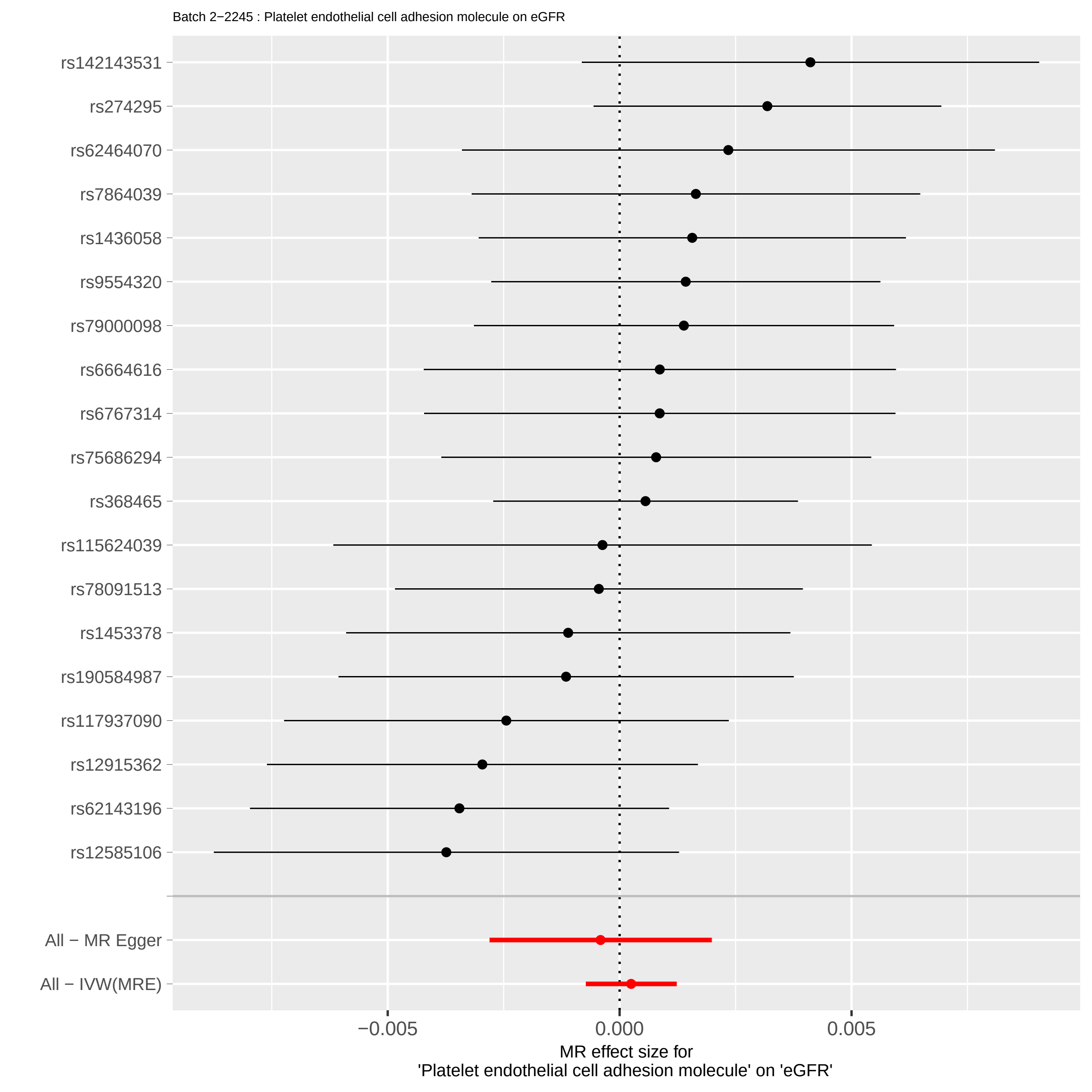
Batch 2−2245 : Platelet endothelial cell adhesion molecule on eGFR
rs142143531
rs274295
rs62464070
rs7864039
rs1436058
rs9554320
rs79000098
rs6664616
rs6767314
rs75686294
rs368465
rs115624039
rs78091513
rs1453378
rs190584987
rs117937090
rs12915362
rs62143196
rs12585106
All − MR Egger
All − IVW(MRE)
−0.005
0.000
0.005
MR effect size for
'Platelet endothelial cell adhesion molecule' on 'eGFR'

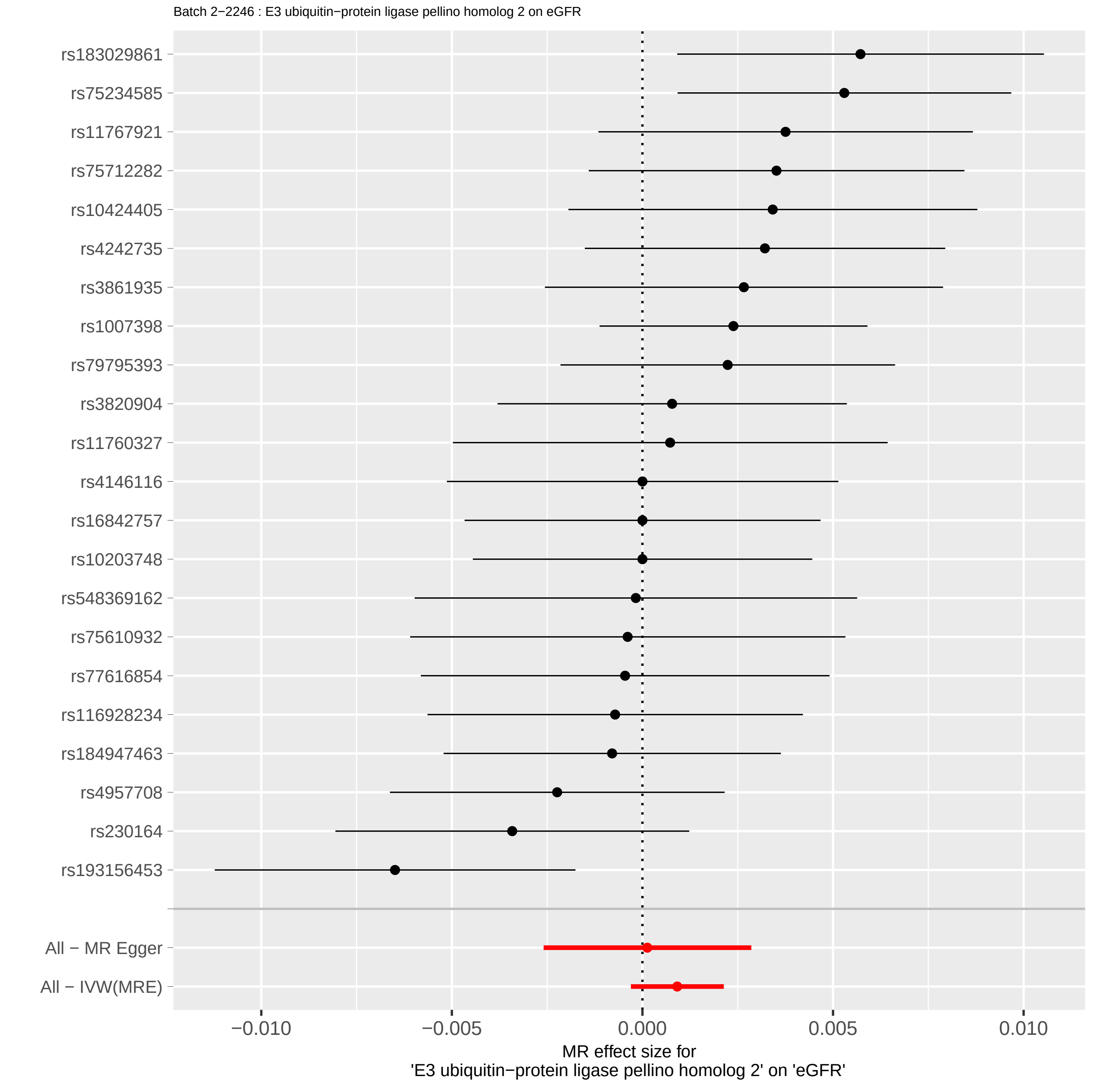
Batch 2−2246 : E3 ubiquitin−protein ligase pellino homolog 2 on eGFR
rs183029861
rs75234585
rs11767921
rs75712282
rs10424405
rs4242735
rs3861935
rs1007398
rs79795393
rs3820904
rs11760327
rs4146116
rs16842757
rs10203748
rs548369162
rs75610932
rs77616854
rs116928234
rs184947463
rs4957708
rs230164
rs193156453
All − MR Egger
All − IVW(MRE)
−0.010
−0.005
0.000
0.005
0.010
MR effect size for
'E3 ubiquitin−protein ligase pellino homolog 2' on 'eGFR'

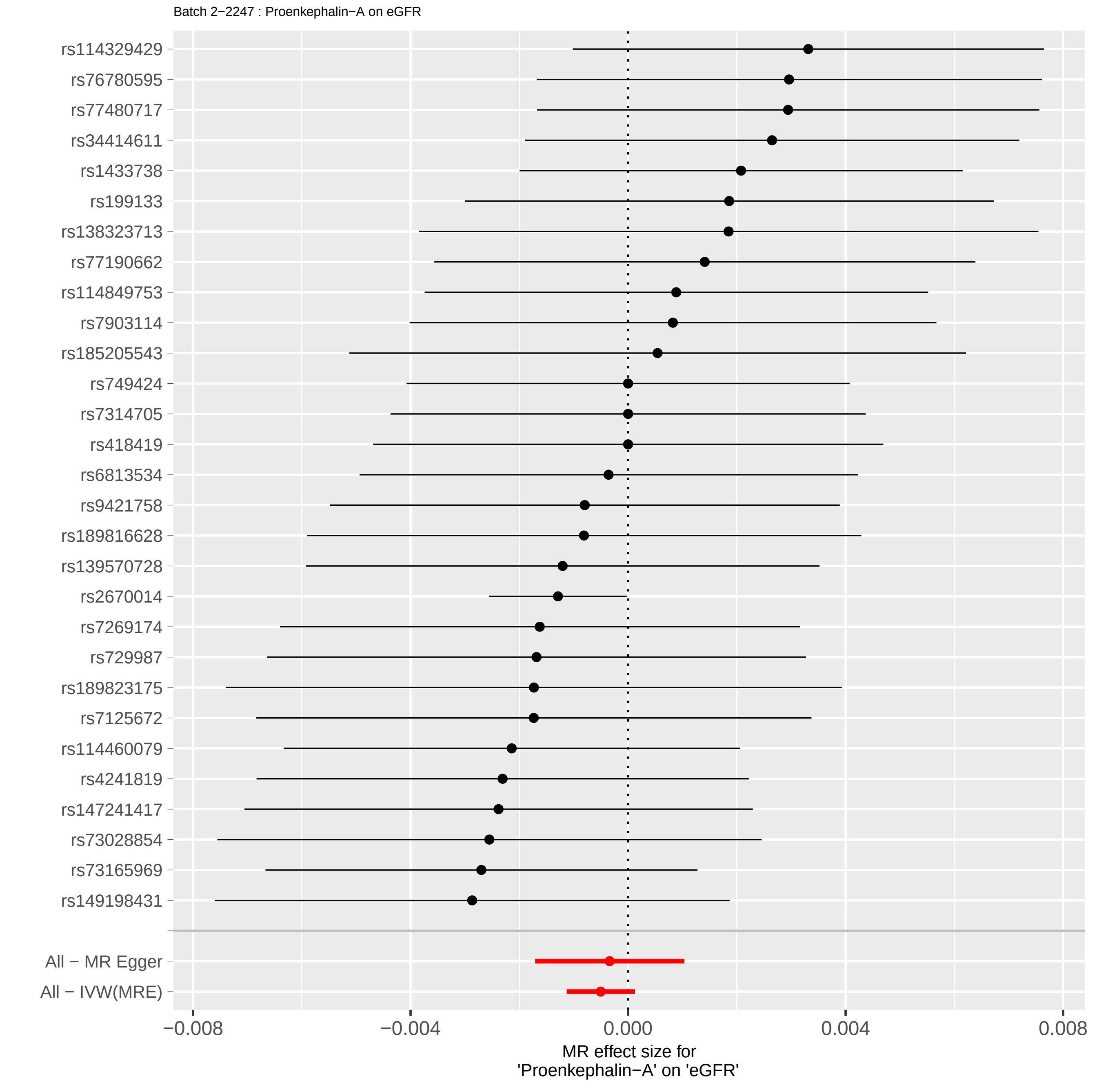
Batch 2−2247 : Proenkephalin−A on eGFR
rs114329429
rs76780595
rs77480717
rs34414611
rs1433738
rs199133
rs138323713
rs77190662
rs114849753
rs7903114
rs185205543
rs749424
rs7314705
rs418419
rs6813534
rs9421758
rs189816628
rs139570728
rs2670014
rs7269174
rs729987
rs189823175
rs7125672
rs114460079
rs4241819
rs147241417
rs73028854
rs73165969
rs149198431
All − MR Egger
All − IVW(MRE)
−0.008
−0.004
0.000
0.004
0.008
MR effect size for
'Proenkephalin−A' on 'eGFR'

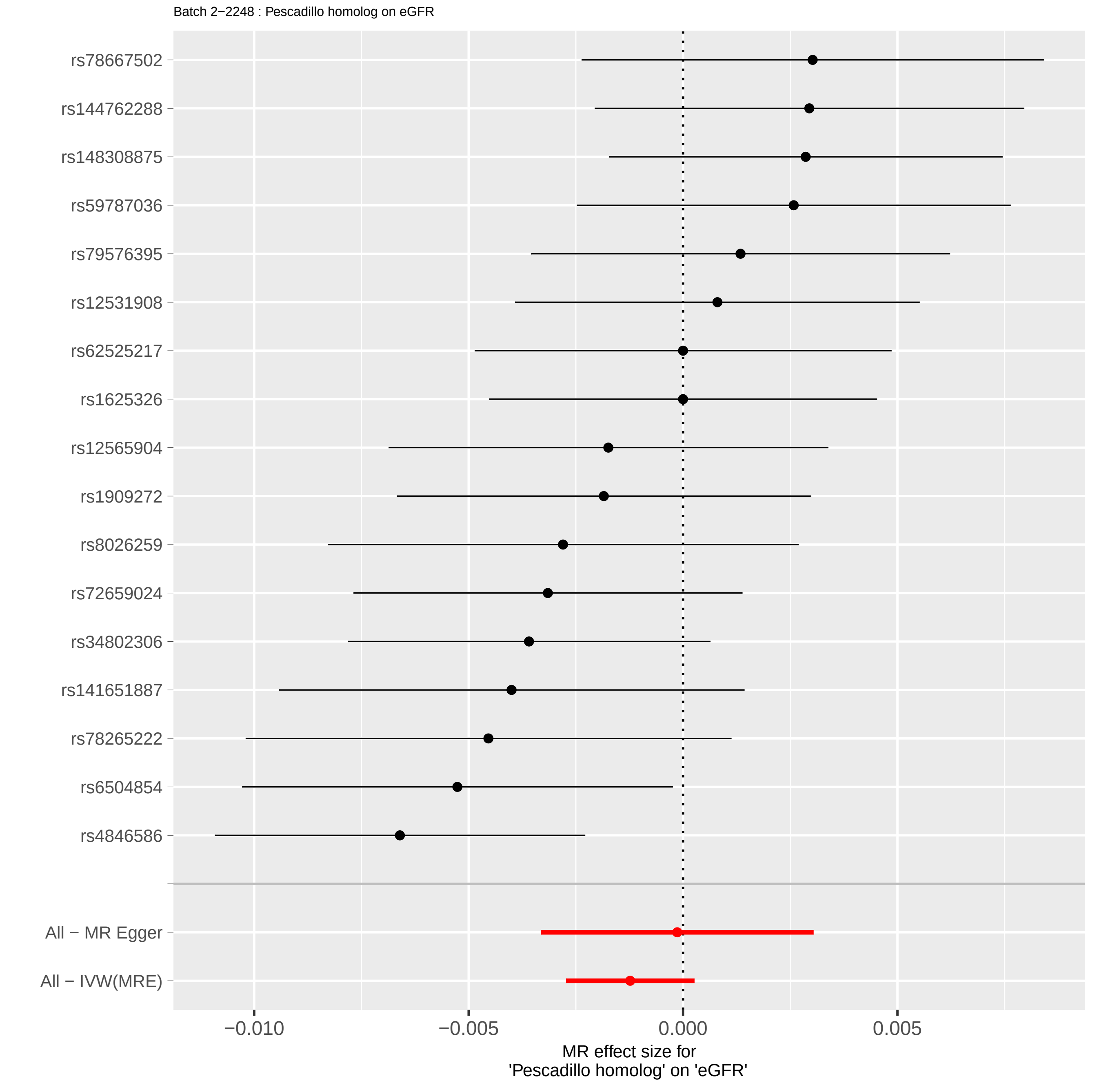

Batch 2−2248 : Pescadillo homolog on eGFR
rs78667502
rs144762288
rs148308875
rs59787036
rs79576395
rs12531908
rs62525217
rs1625326
rs12565904
rs1909272
rs8026259
rs72659024
rs34802306
rs141651887
rs78265222
rs6504854
rs4846586
All − MR Egger
All − IVW(MRE)
−0.010
−0.005
0.000
0.005
MR effect size for
'Pescadillo homolog' on 'eGFR'

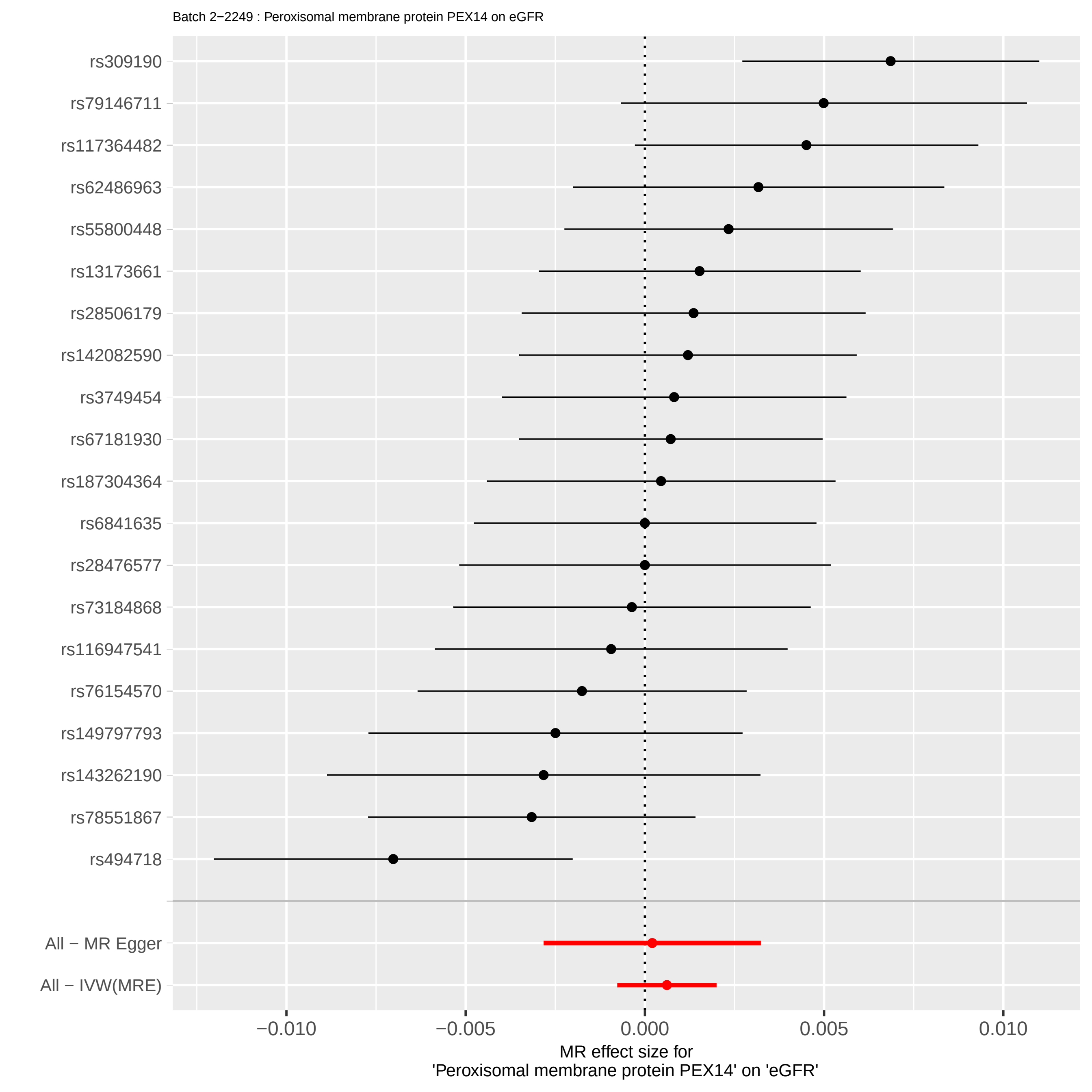

Batch 2−2249 : Peroxisomal membrane protein PEX14 on eGFR
rs309190
rs79146711
rs117364482
rs62486963
rs55800448
rs13173661
rs28506179
rs142082590
rs3749454
rs67181930
rs187304364
rs6841635
rs28476577
rs73184868
rs116947541
rs76154570
rs149797793
rs143262190
rs78551867
rs494718
All − MR Egger
All − IVW(MRE)
−0.010
−0.005
0.000
0.005
0.010
MR effect size for
'Peroxisomal membrane protein PEX14' on 'eGFR'

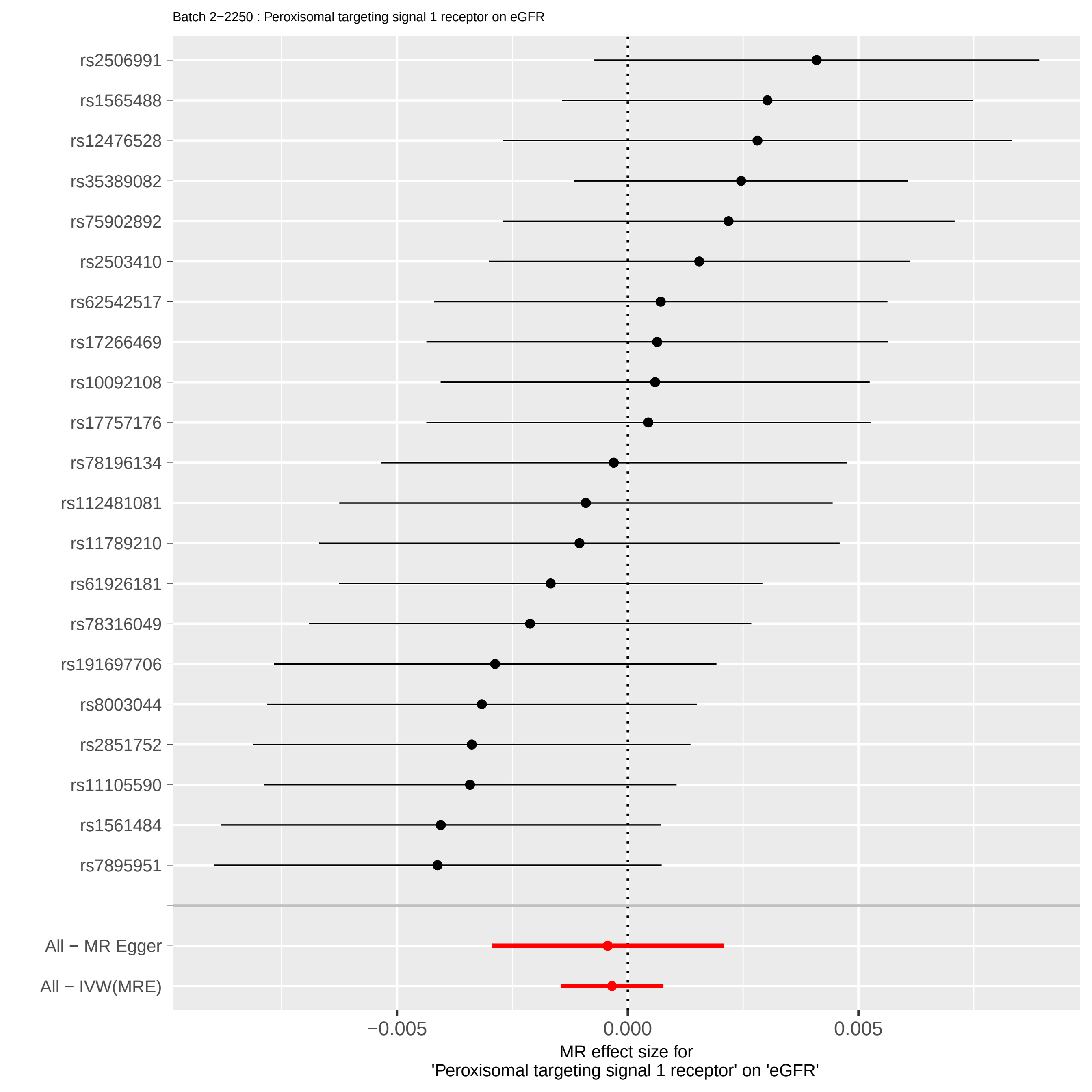
Batch 2−2250 : Peroxisomal targeting signal 1 receptor on eGFR
rs2506991
rs1565488
rs12476528
rs35389082
rs75902892
rs2503410
rs62542517
rs17266469
rs10092108
rs17757176
rs78196134
rs112481081
rs11789210
rs61926181
rs78316049
rs191697706
rs8003044
rs2851752
rs11105590
rs1561484
rs7895951
All − MR Egger
All − IVW(MRE)
−0.005
0.000
0.005
MR effect size for
'Peroxisomal targeting signal 1 receptor' on 'eGFR'

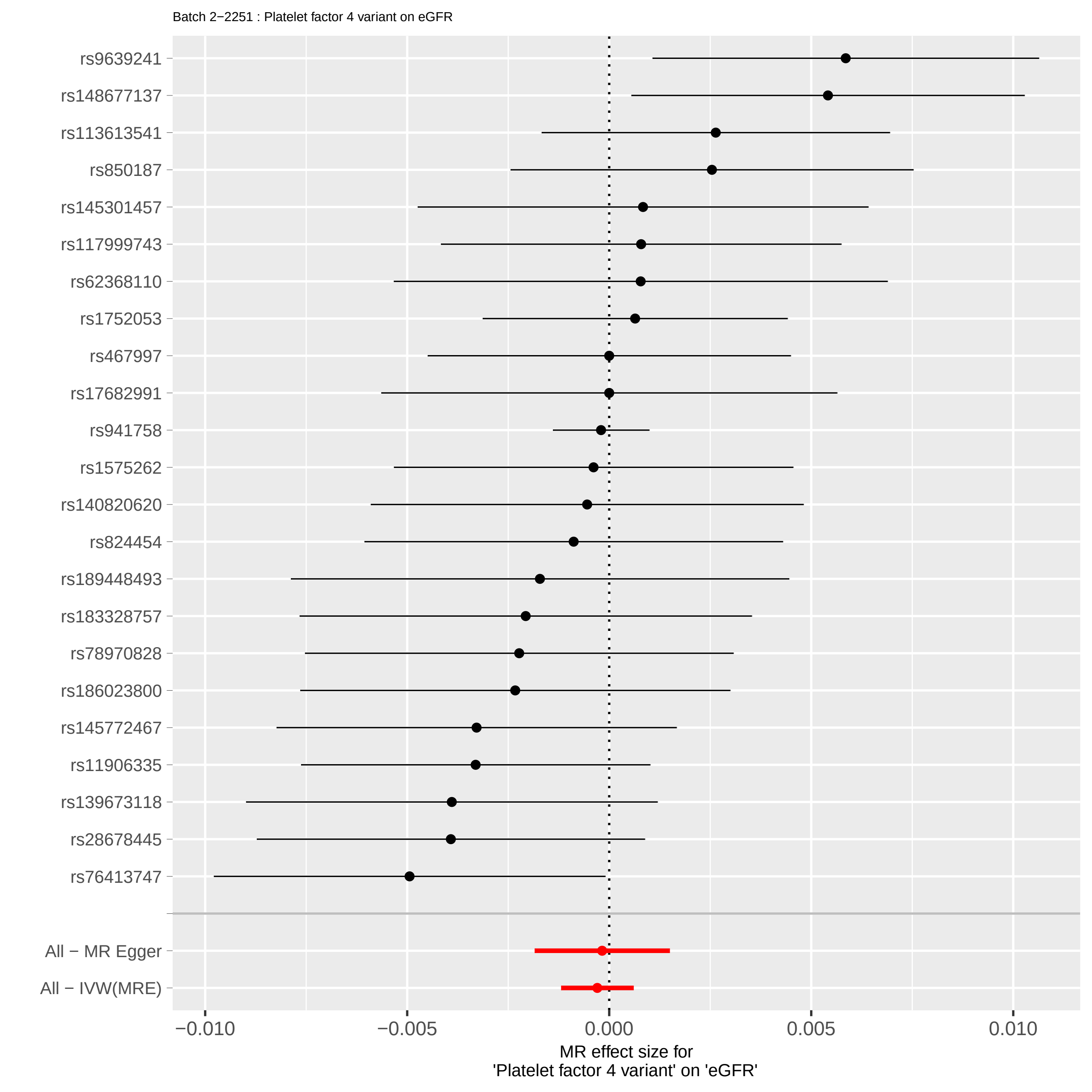
Batch 2−2251 : Platelet factor 4 variant on eGFR
rs9639241
rs148677137
rs113613541
rs850187
rs145301457
rs117999743
rs62368110
rs1752053
rs467997
rs17682991
rs941758
rs1575262
rs140820620
rs824454
rs189448493
rs183328757
rs78970828
rs186023800
rs145772467
rs11906335
rs139673118
rs28678445
rs76413747
All − MR Egger
All − IVW(MRE)
−0.010
−0.005
0.000
0.005
0.010
MR effect size for
'Platelet factor 4 variant' on 'eGFR'

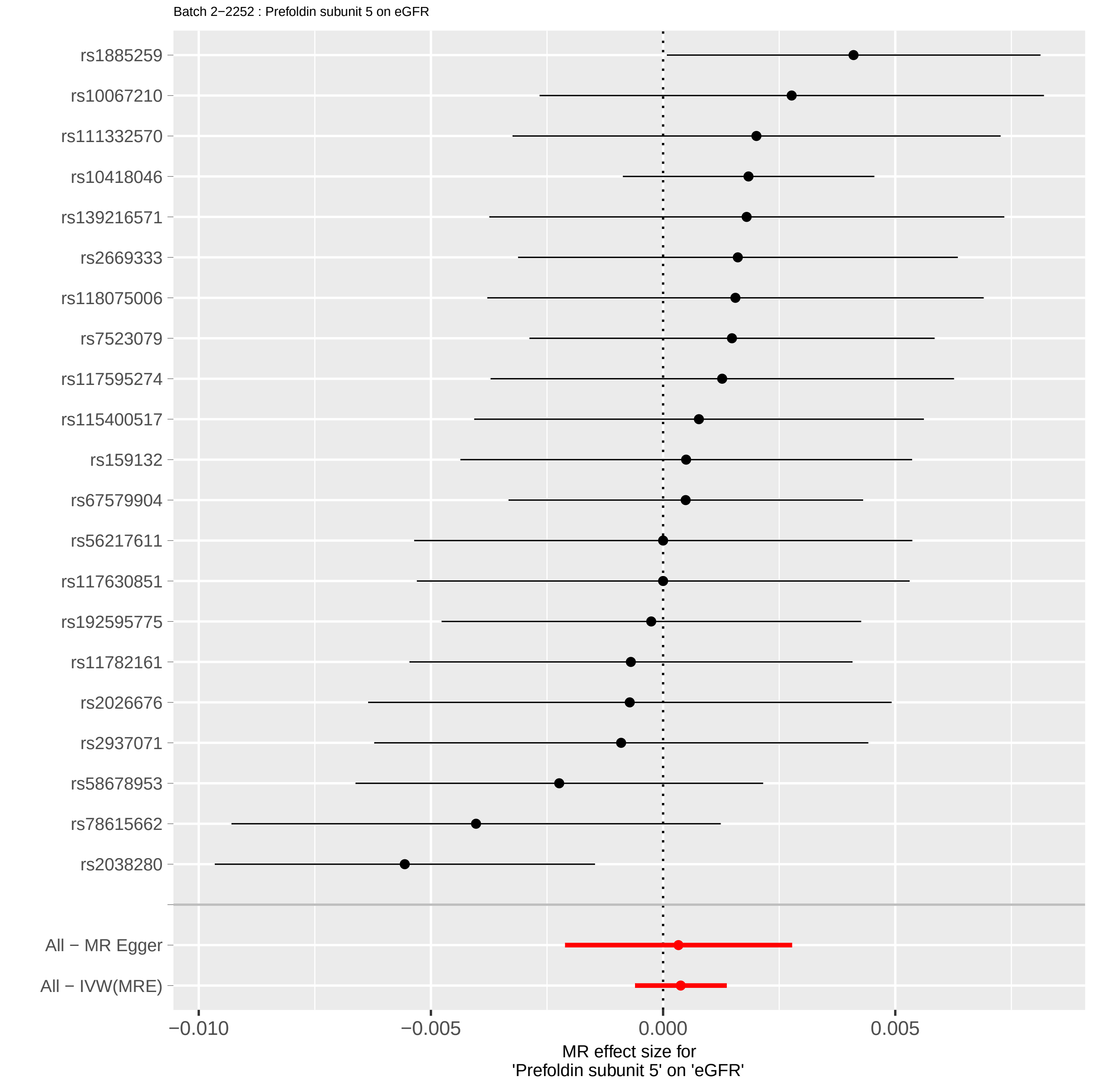

Batch 2−2252 : Prefoldin subunit 5 on eGFR
rs1885259
rs10067210
rs111332570
rs10418046
rs139216571
rs2669333
rs118075006
rs7523079
rs117595274
rs115400517
rs159132
rs67579904
rs56217611
rs117630851
rs192595775
rs11782161
rs2026676
rs2937071
rs58678953
rs78615662
rs2038280
All − MR Egger
All − IVW(MRE)
−0.010
−0.005
0.000
0.005
MR effect size for
'Prefoldin subunit 5' on 'eGFR'

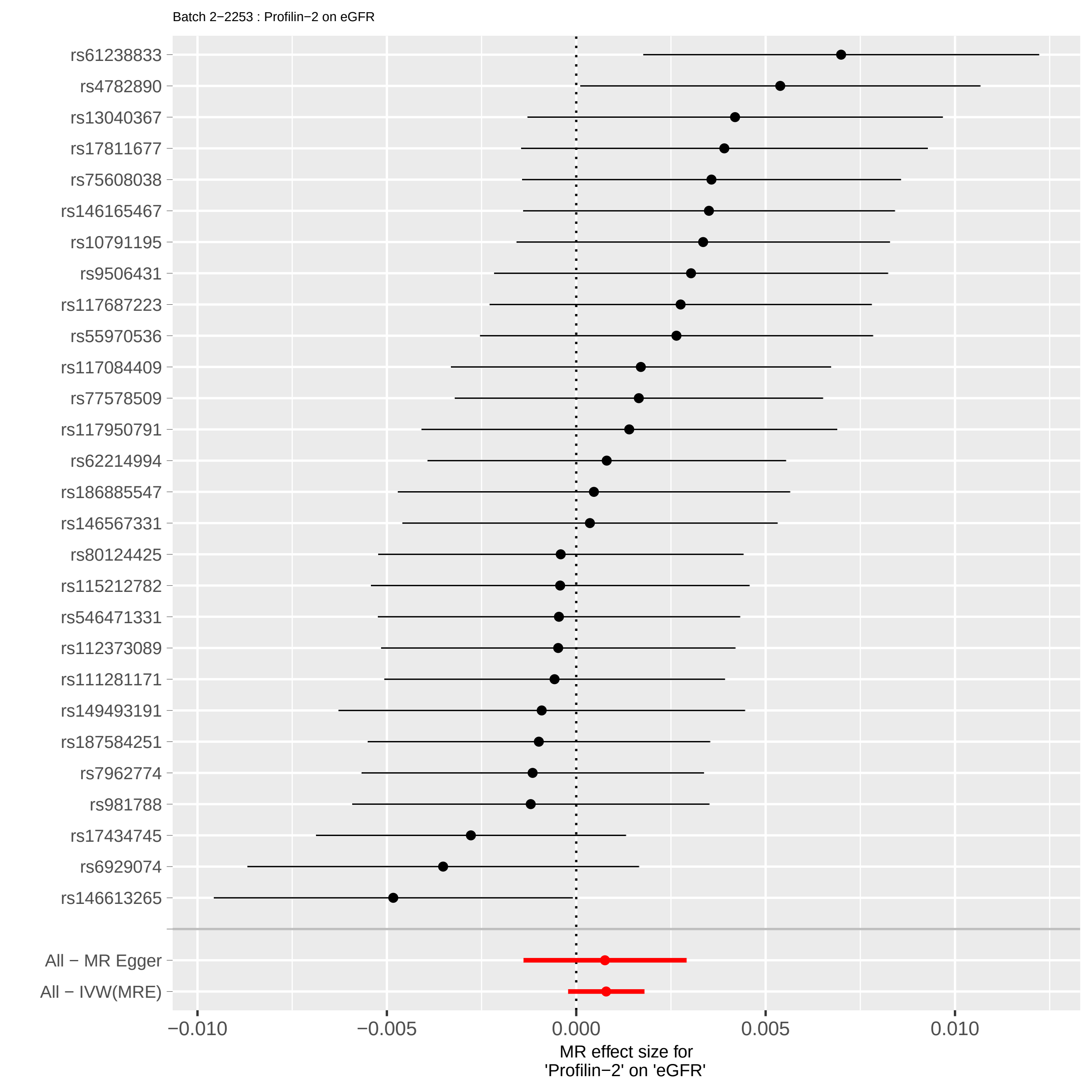
Batch 2−2253 : Profilin−2 on eGFR
rs61238833
rs4782890
rs13040367
rs17811677
rs75608038
rs146165467
rs10791195
rs9506431
rs117687223
rs55970536
rs117084409
rs77578509
rs117950791
rs62214994
rs186885547
rs146567331
rs80124425
rs115212782
rs546471331
rs112373089
rs111281171
rs149493191
rs187584251
rs7962774
rs981788
rs17434745
rs6929074
rs146613265
All − MR Egger
All − IVW(MRE)
−0.010
−0.005
0.000
0.005
0.010
MR effect size for
'Profilin−2' on 'eGFR'

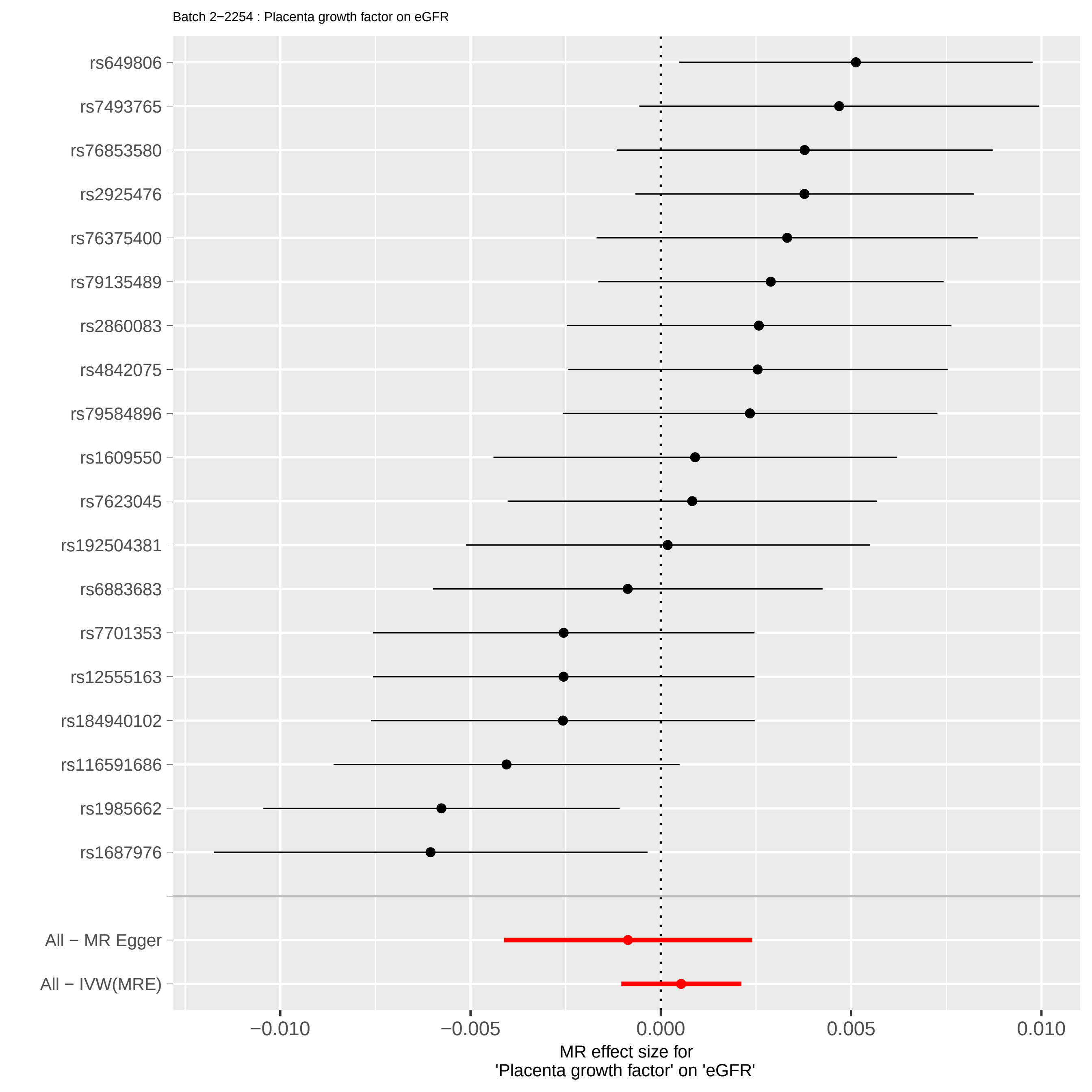

Batch 2−2254 : Placenta growth factor on eGFR
rs649806
rs7493765
rs76853580
rs2925476
rs76375400
rs79135489
rs2860083
rs4842075
rs79584896
rs1609550
rs7623045
rs192504381
rs6883683
rs7701353
rs12555163
rs184940102
rs116591686
rs1985662
rs1687976
All − MR Egger
All − IVW(MRE)
−0.010
−0.005
0.000
0.005
0.010
MR effect size for
'Placenta growth factor' on 'eGFR'

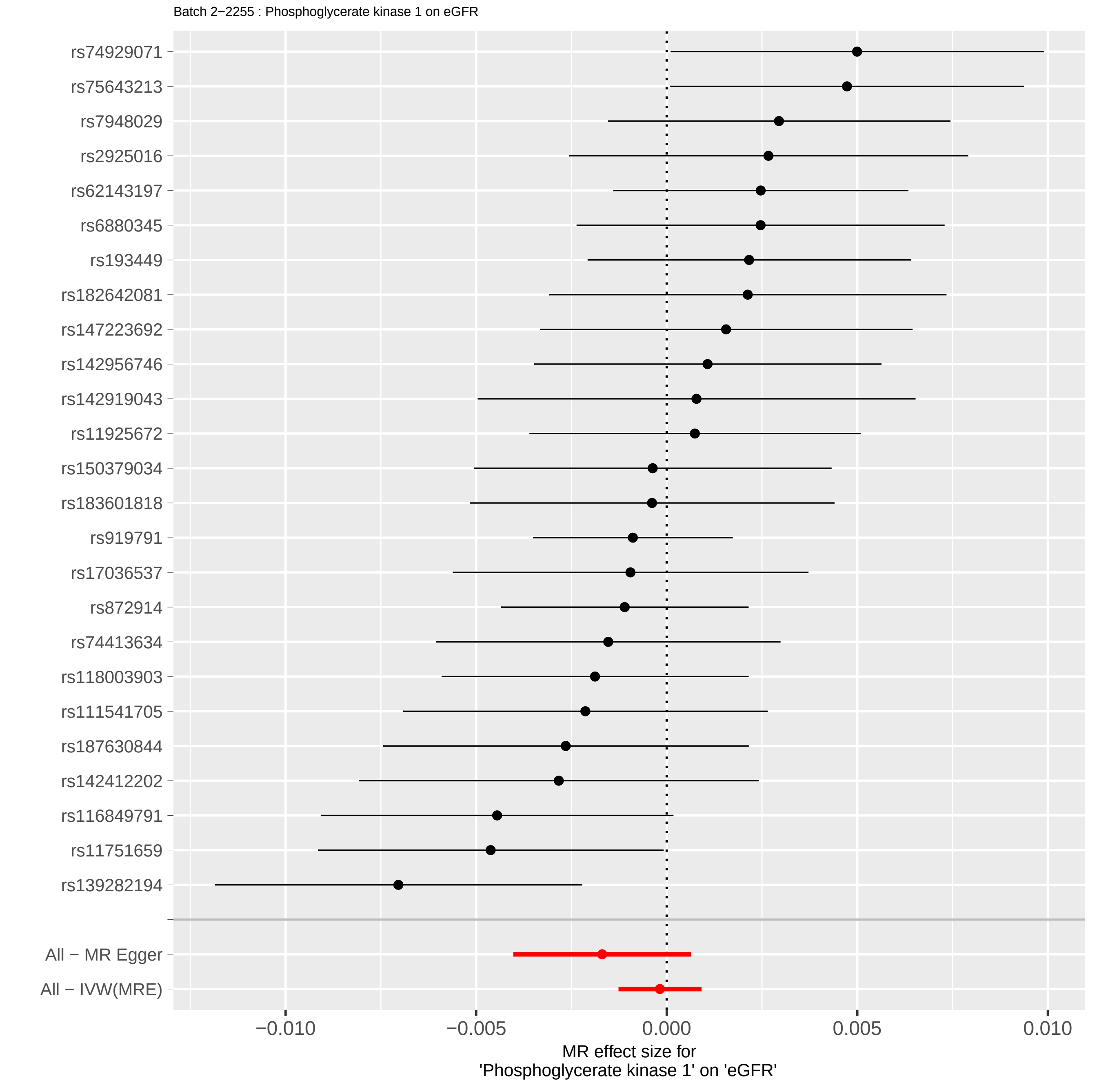

Batch 2−2255 : Phosphoglycerate kinase 1 on eGFR
rs74929071
rs75643213
rs7948029
rs2925016
rs62143197
rs6880345
rs193449
rs182642081
rs147223692
rs142956746
rs142919043
rs11925672
rs150379034
rs183601818
rs919791
rs17036537
rs872914
rs74413634
rs118003903
rs111541705
rs187630844
rs142412202
rs116849791
rs11751659
rs139282194
All − MR Egger
All − IVW(MRE)
−0.010
−0.005
0.000
0.005
0.010
MR effect size for
'Phosphoglycerate kinase 1' on 'eGFR'

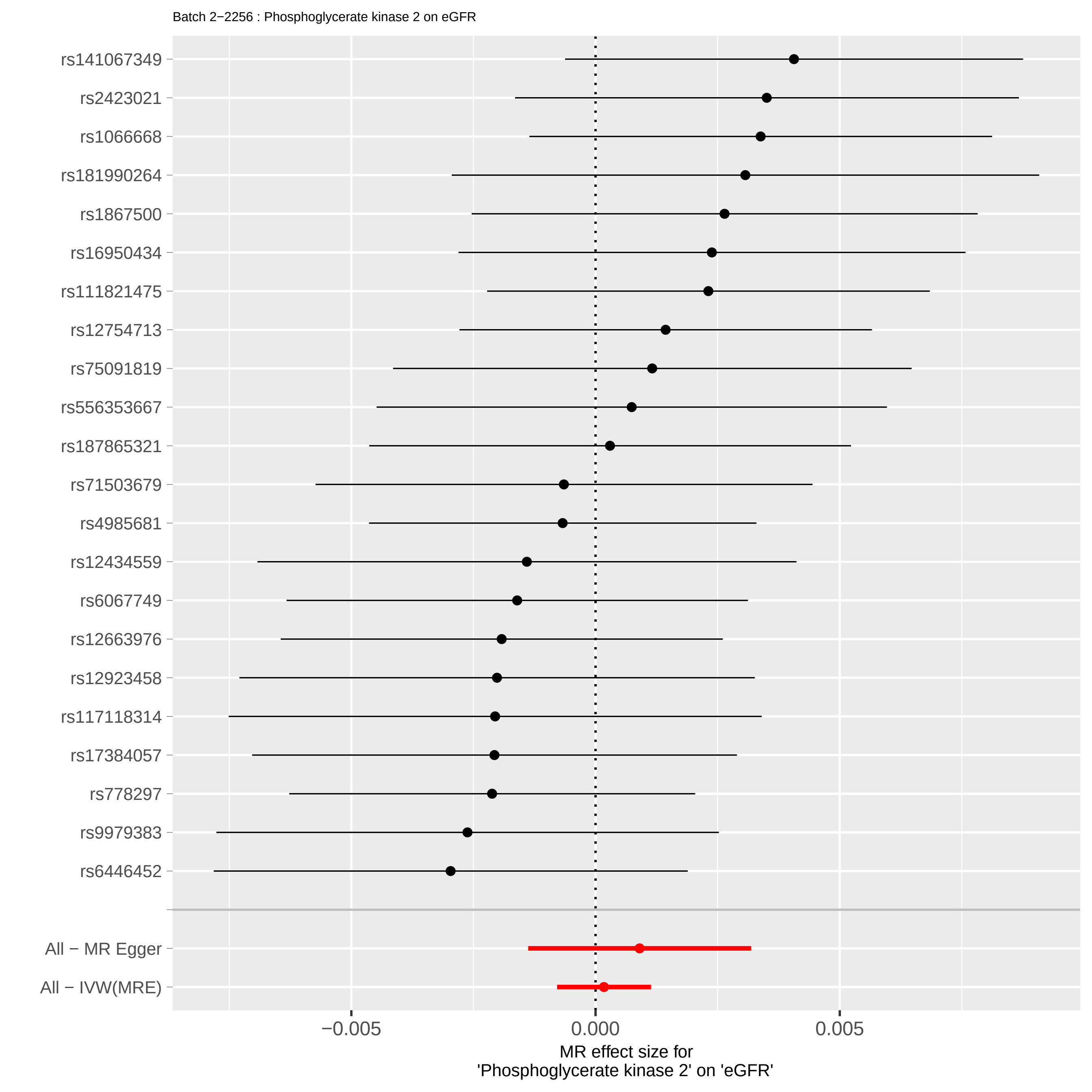

Batch 2−2256 : Phosphoglycerate kinase 2 on eGFR
rs141067349
rs2423021
rs1066668
rs181990264
rs1867500
rs16950434
rs111821475
rs12754713
rs75091819
rs556353667
rs187865321
rs71503679
rs4985681
rs12434559
rs6067749
rs12663976
rs12923458
rs117118314
rs17384057
rs778297
rs9979383
rs6446452
All − MR Egger
All − IVW(MRE)
−0.005
0.000
0.005
MR effect size for
'Phosphoglycerate kinase 2' on 'eGFR'

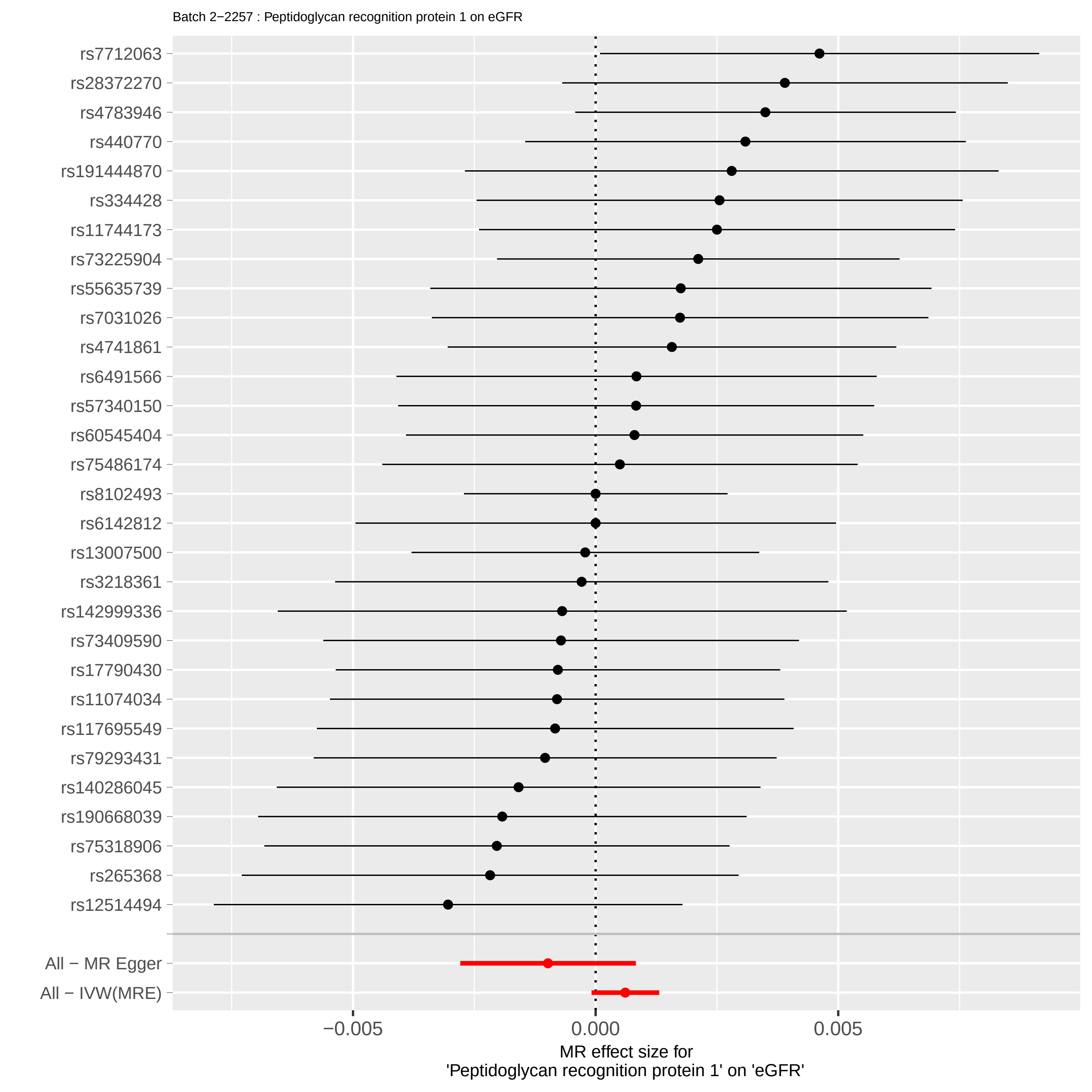

Batch 2−2257 : Peptidoglycan recognition protein 1 on eGFR
rs7712063
rs28372270
rs4783946
rs440770
rs191444870
rs334428
rs11744173
rs73225904
rs55635739
rs7031026
rs4741861
rs6491566
rs57340150
rs60545404
rs75486174
rs8102493
rs6142812
rs13007500
rs3218361
rs142999336
rs73409590
rs17790430
rs11074034
rs117695549
rs79293431
rs140286045
rs190668039
rs75318906
rs265368
rs12514494
All − MR Egger
All − IVW(MRE)
−0.005
0.000
0.005
MR effect size for
'Peptidoglycan recognition protein 1' on 'eGFR'

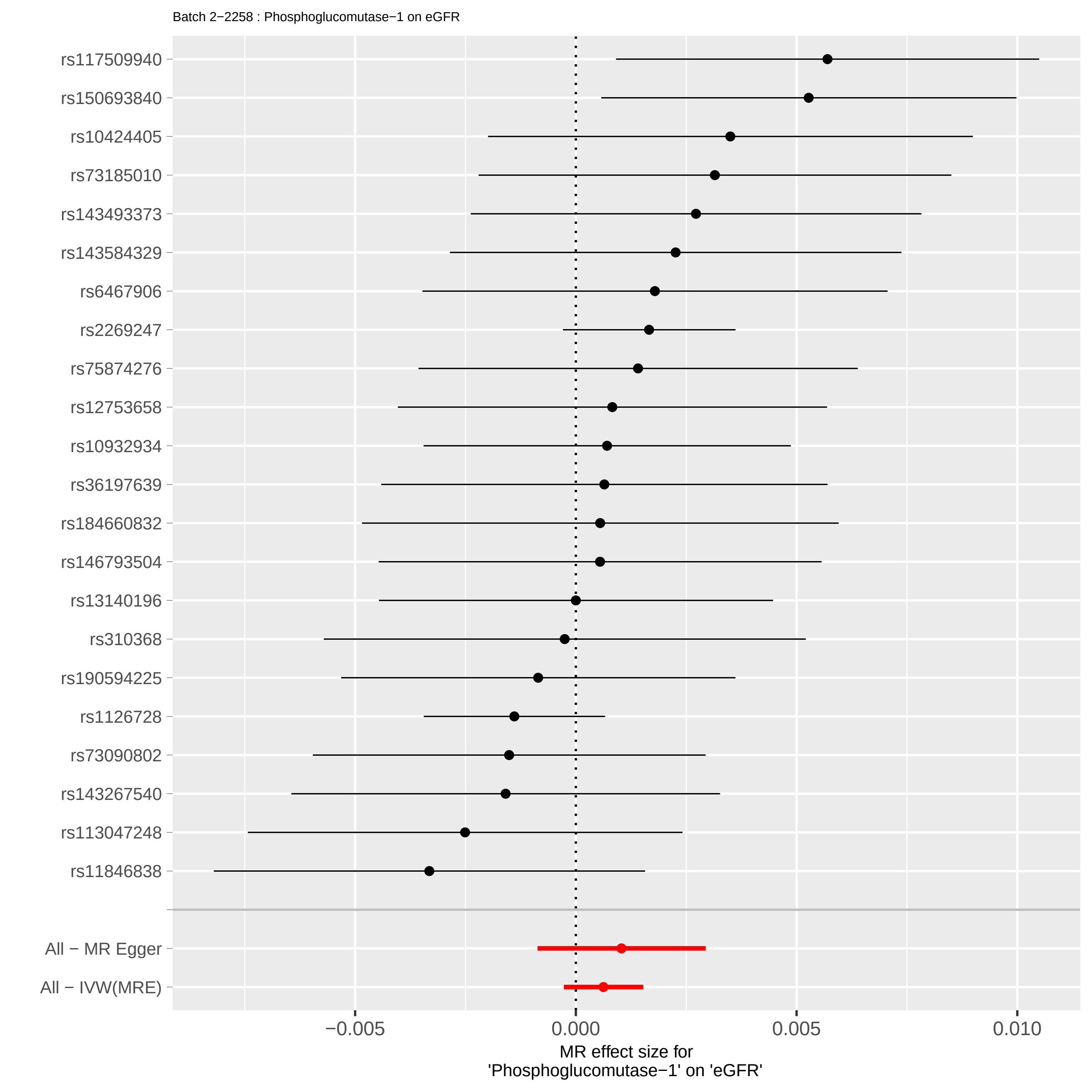

Batch 2−2258 : Phosphoglucomutase−1 on eGFR
rs117509940
rs150693840
rs10424405
rs73185010
rs143493373
rs143584329
rs6467906
rs2269247
rs75874276
rs12753658
rs10932934
rs36197639
rs184660832
rs146793504
rs13140196
rs310368
rs190594225
rs1126728
rs73090802
rs143267540
rs113047248
rs11846838
All − MR Egger
All − IVW(MRE)
−0.005
0.000
0.005
0.010
MR effect size for
'Phosphoglucomutase−1' on 'eGFR'

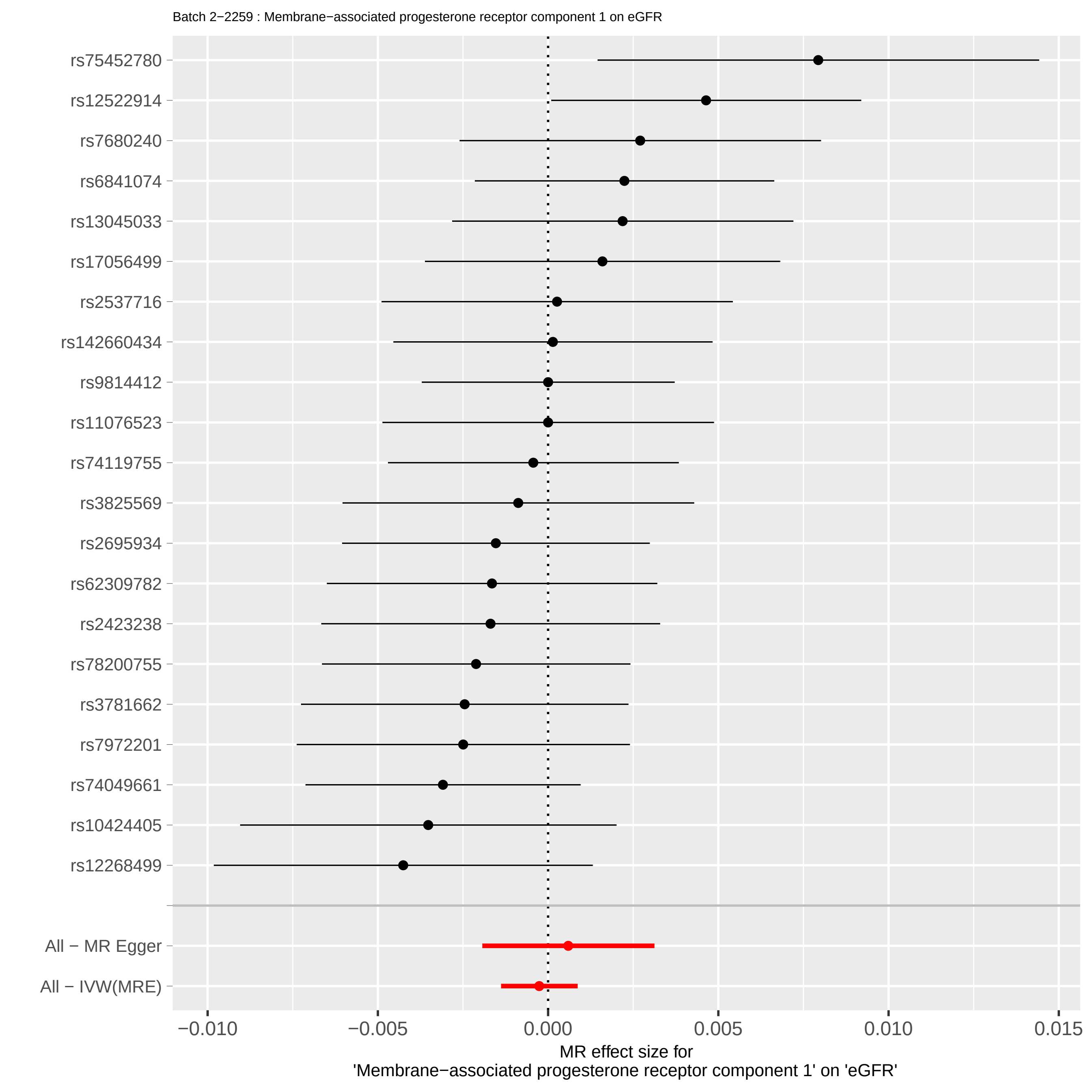

Batch 2−2259 : Membrane−associated progesterone receptor component 1 on eGFR
rs75452780
rs12522914
rs7680240
rs6841074
rs13045033
rs17056499
rs2537716
rs142660434
rs9814412
rs11076523
rs74119755
rs3825569
rs2695934
rs62309782
rs2423238
rs78200755
rs3781662
rs7972201
rs74049661
rs10424405
rs12268499
All − MR Egger
All − IVW(MRE)
−0.010
−0.005
0.000
0.005
0.010
0.015
MR effect size for
'Membrane−associated progesterone receptor component 1' on 'eGFR'

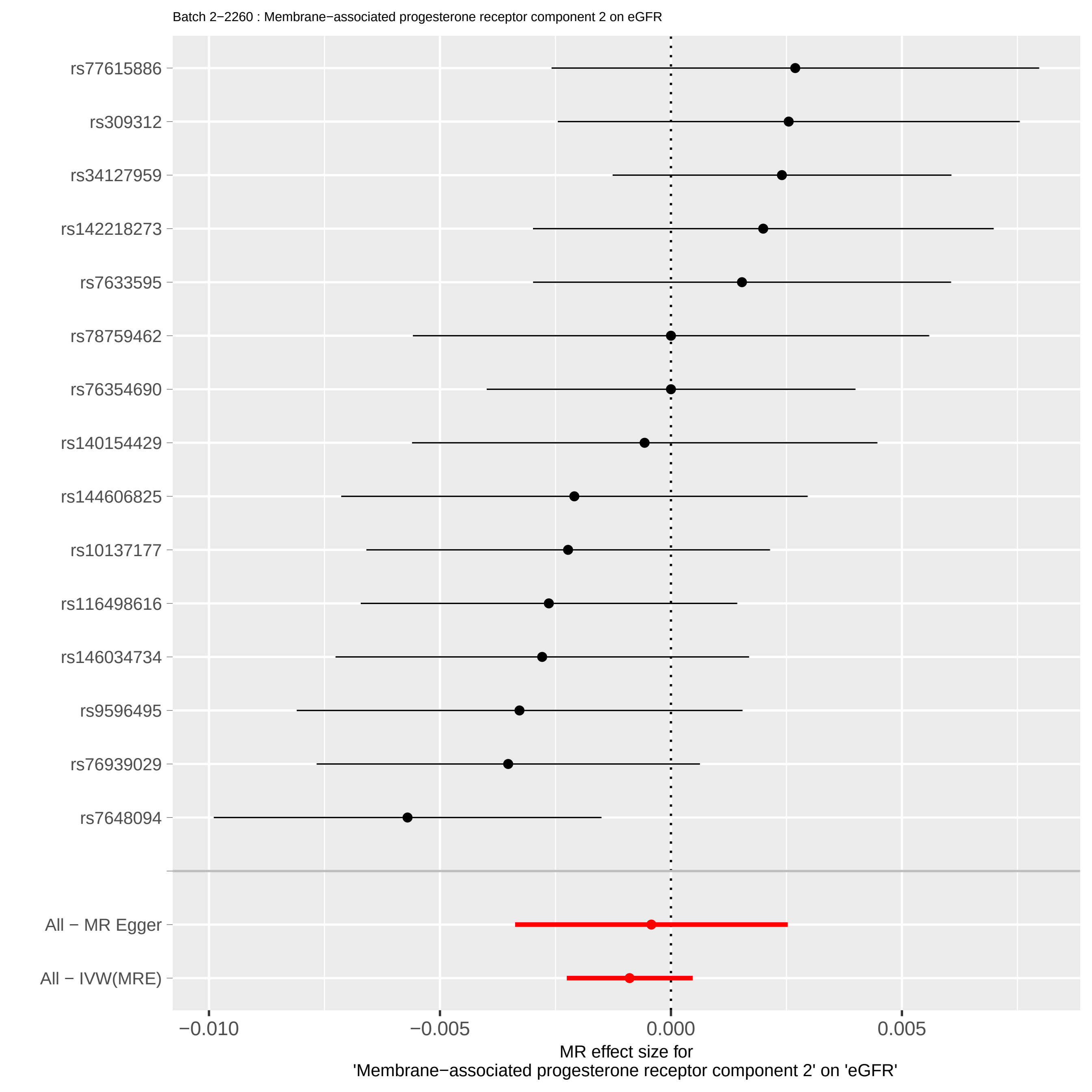
Batch 2−2260 : Membrane−associated progesterone receptor component 2 on eGFR
rs77615886
rs309312
rs34127959
rs142218273
rs7633595
rs78759462
rs76354690
rs140154429
rs144606825
rs10137177
rs116498616
rs146034734
rs9596495
rs76939029
rs7648094
All − MR Egger
All − IVW(MRE)
−0.010
−0.005
0.000
0.005
MR effect size for
'Membrane−associated progesterone receptor component 2' on 'eGFR'

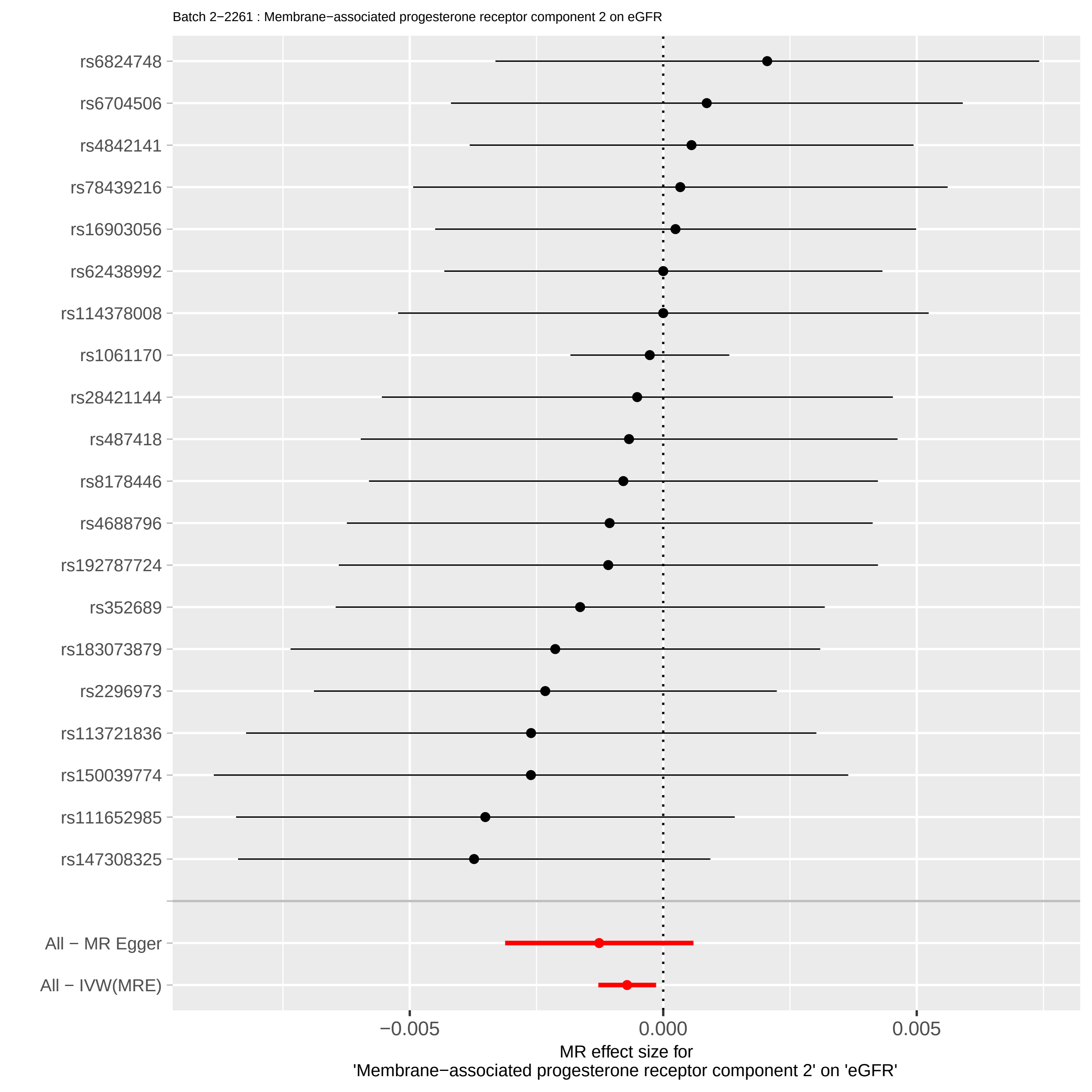

Batch 2−2261 : Membrane−associated progesterone receptor component 2 on eGFR
rs6824748
rs6704506
rs4842141
rs78439216
rs16903056
rs62438992
rs114378008
rs1061170
rs28421144
rs487418
rs8178446
rs4688796
rs192787724
rs352689
rs183073879
rs2296973
rs113721836
rs150039774
rs111652985
rs147308325
All − MR Egger
All − IVW(MRE)
−0.005
0.000
0.005
MR effect size for
'Membrane−associated progesterone receptor component 2' on 'eGFR'

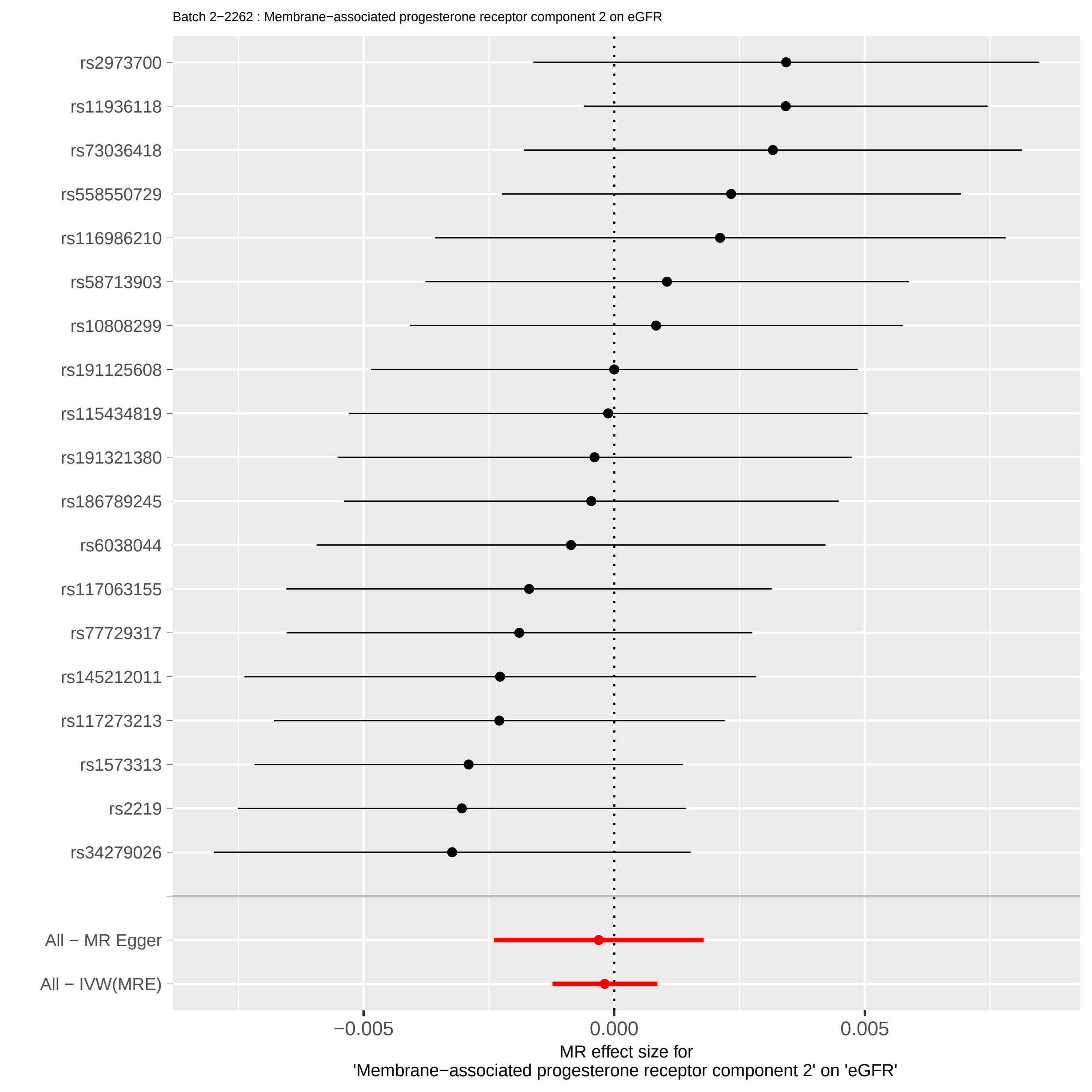
Batch 2−2262 : Membrane−associated progesterone receptor component 2 on eGFR
rs2973700
rs11936118
rs73036418
rs558550729
rs116986210
rs58713903
rs10808299
rs191125608
rs115434819
rs191321380
rs186789245
rs6038044
rs117063155
rs77729317
rs145212011
rs117273213
rs1573313
rs2219
rs34279026
All − MR Egger
All − IVW(MRE)
−0.005
0.000
0.005
MR effect size for
'Membrane−associated progesterone receptor component 2' on 'eGFR'

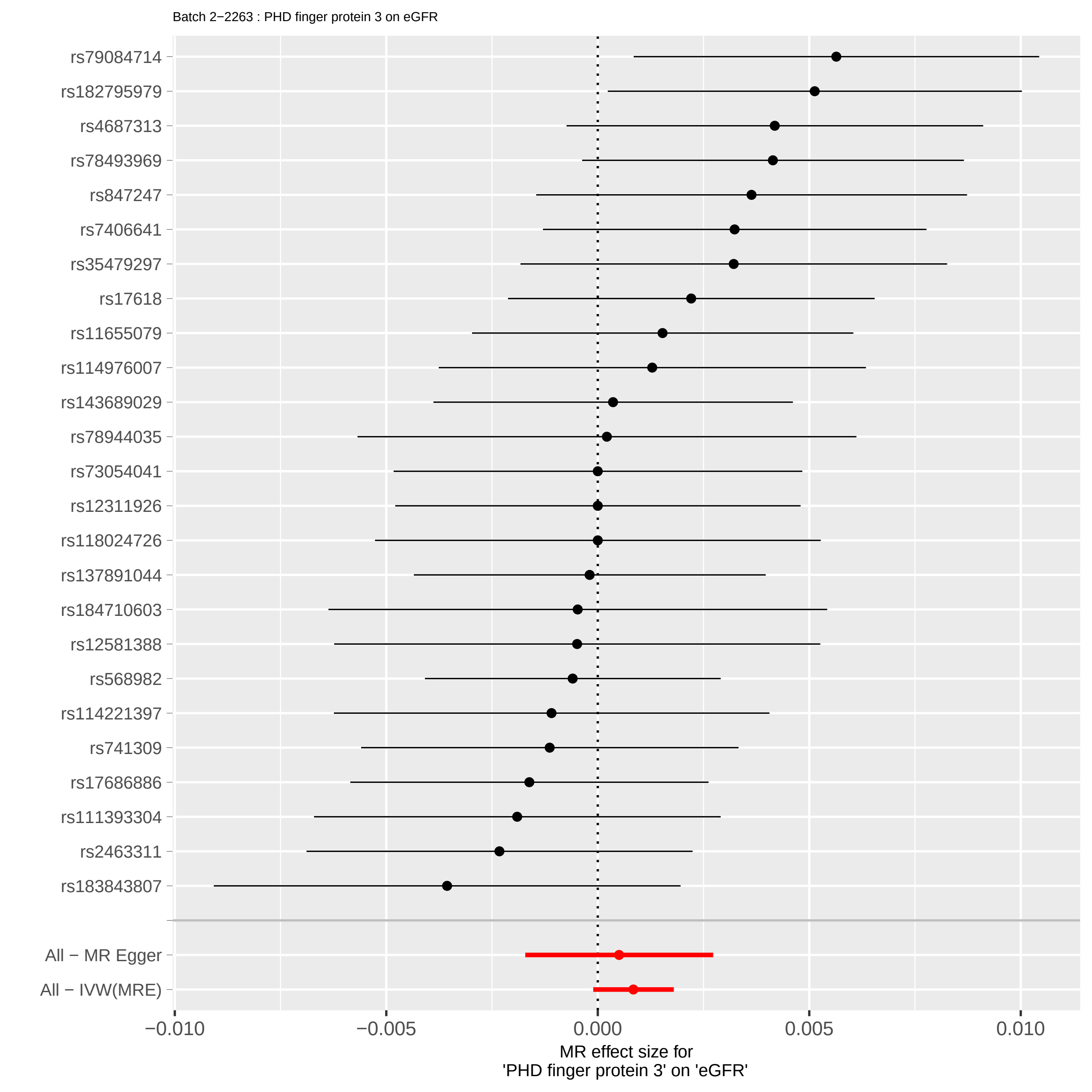

Batch 2−2263 : PHD finger protein 3 on eGFR
rs79084714
rs182795979
rs4687313
rs78493969
rs847247
rs7406641
rs35479297
rs17618
rs11655079
rs114976007
rs143689029
rs78944035
rs73054041
rs12311926
rs118024726
rs137891044
rs184710603
rs12581388
rs568982
rs114221397
rs741309
rs17686886
rs111393304
rs2463311
rs183843807
All − MR Egger
All − IVW(MRE)
−0.010
−0.005
0.000
0.005
0.010
MR effect size for
'PHD finger protein 3' on 'eGFR'

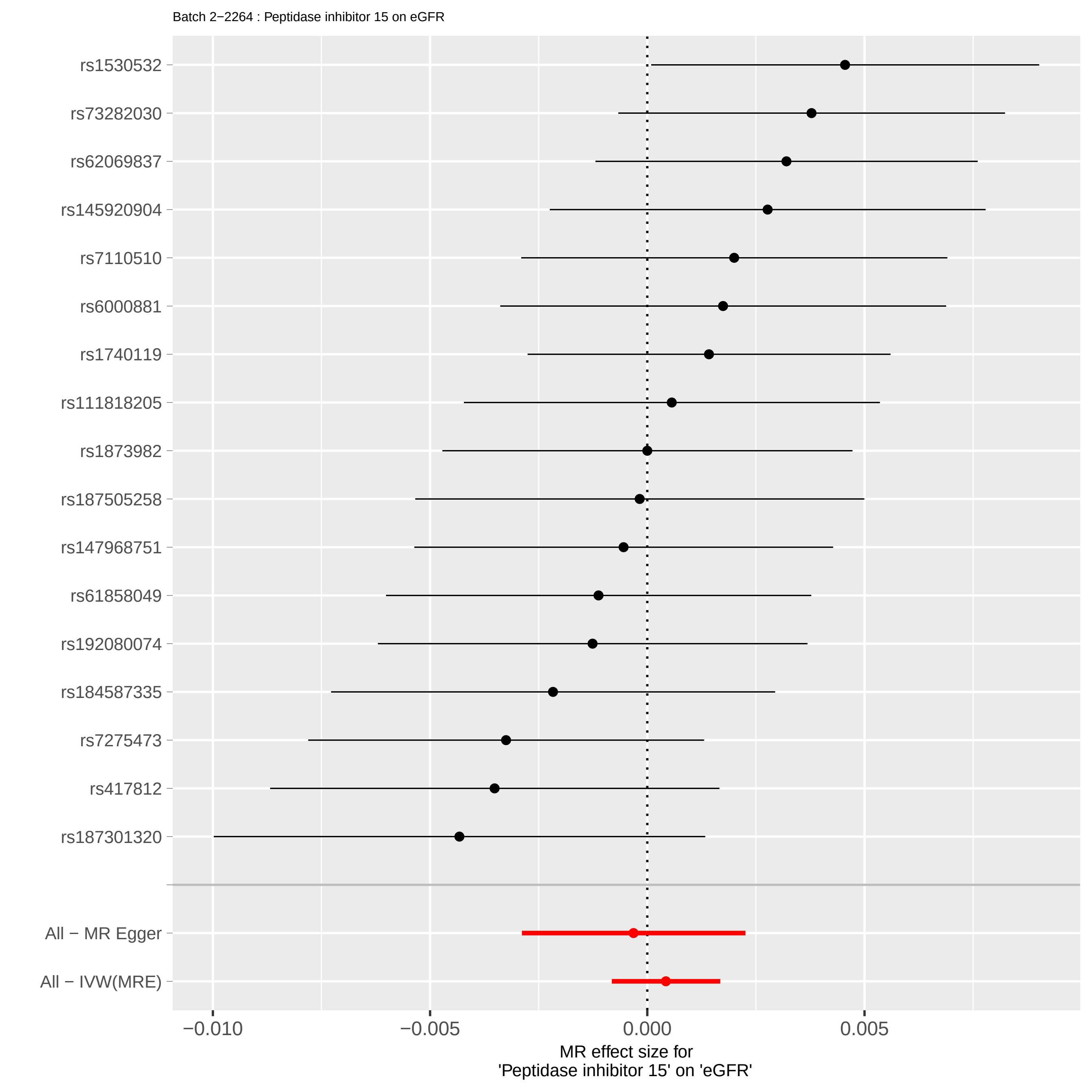
Batch 2−2264 : Peptidase inhibitor 15 on eGFR
rs1530532
rs73282030
rs62069837
rs145920904
rs7110510
rs6000881
rs1740119
rs111818205
rs1873982
rs187505258
rs147968751
rs61858049
rs192080074
rs184587335
rs7275473
rs417812
rs187301320
All − MR Egger
All − IVW(MRE)
−0.010
−0.005
0.000
0.005
MR effect size for
'Peptidase inhibitor 15' on 'eGFR'

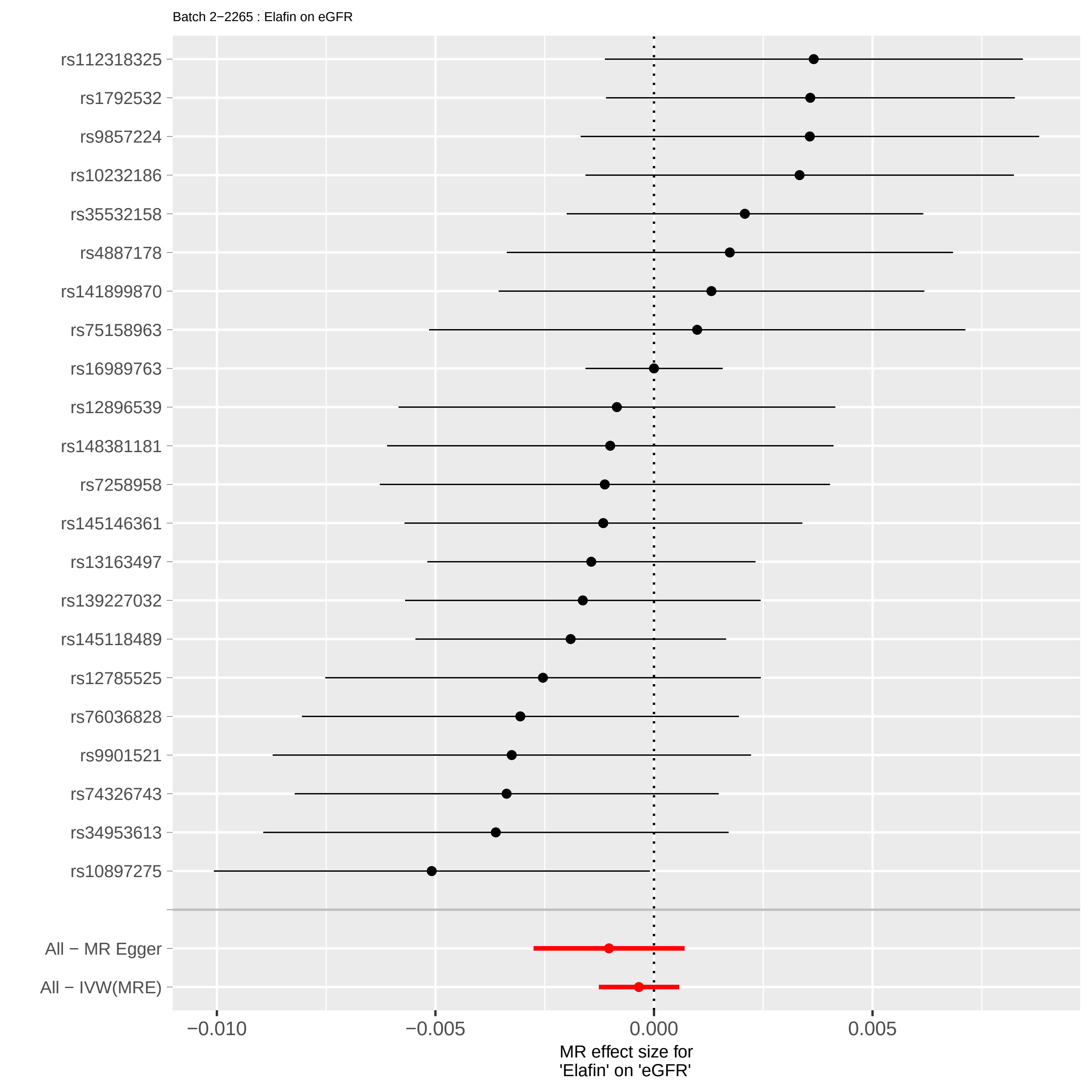

Batch 2−2265 : Elafin on eGFR
rs112318325
rs1792532
rs9857224
rs10232186
rs35532158
rs4887178
rs141899870
rs75158963
rs16989763
rs12896539
rs148381181
rs7258958
rs145146361
rs13163497
rs139227032
rs145118489
rs12785525
rs76036828
rs9901521
rs74326743
rs34953613
rs10897275
All − MR Egger
All − IVW(MRE)
−0.010
−0.005
0.000
0.005
MR effect size for
'Elafin' on 'eGFR'

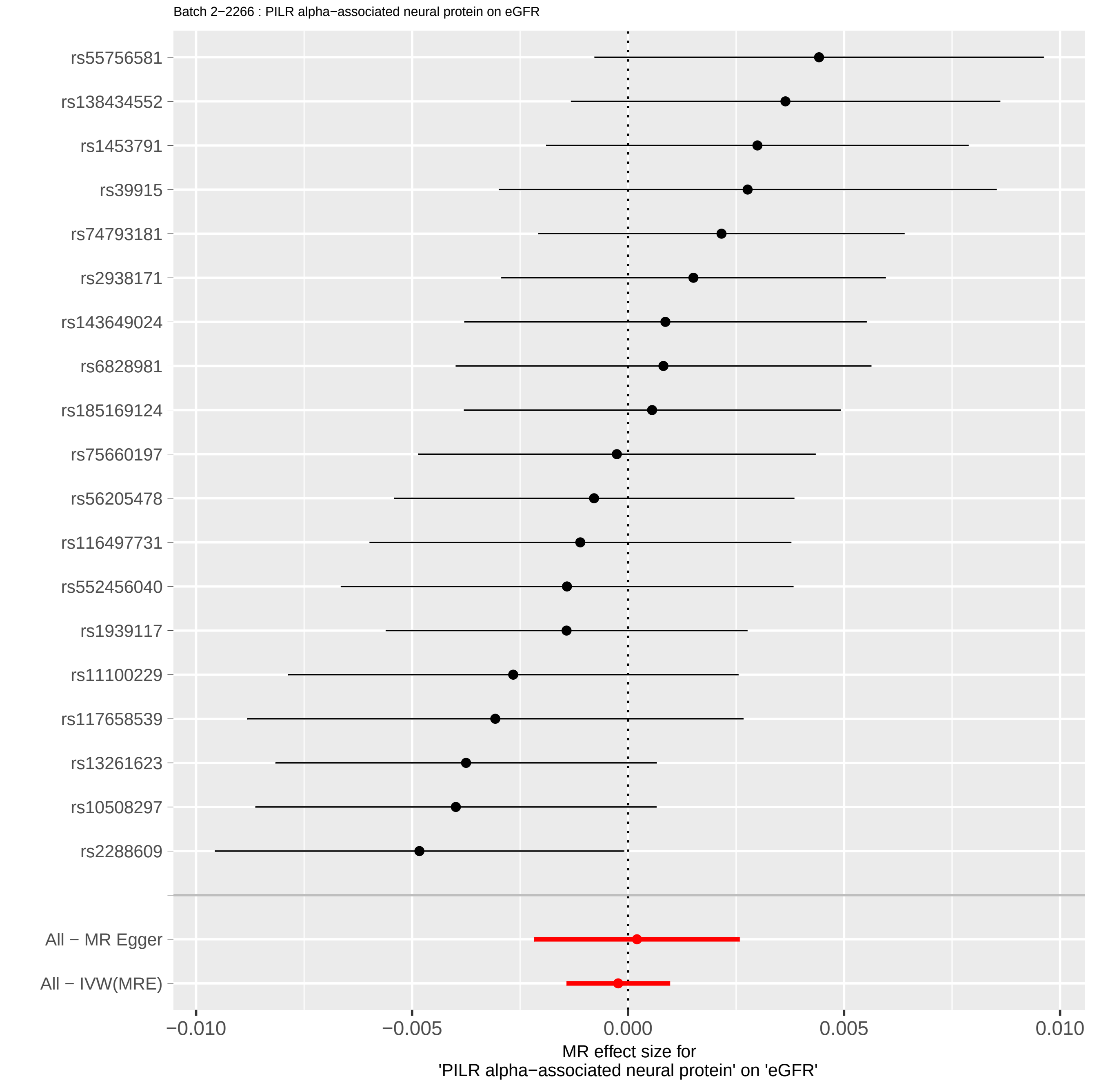
Batch 2−2266 : PILR alpha−associated neural protein on eGFR
rs55756581
rs138434552
rs1453791
rs39915
rs74793181
rs2938171
rs143649024
rs6828981
rs185169124
rs75660197
rs56205478
rs116497731
rs552456040
rs1939117
rs11100229
rs117658539
rs13261623
rs10508297
rs2288609
All − MR Egger
All − IVW(MRE)
−0.010
−0.005
0.000
0.005
0.010
MR effect size for
'PILR alpha−associated neural protein' on 'eGFR'

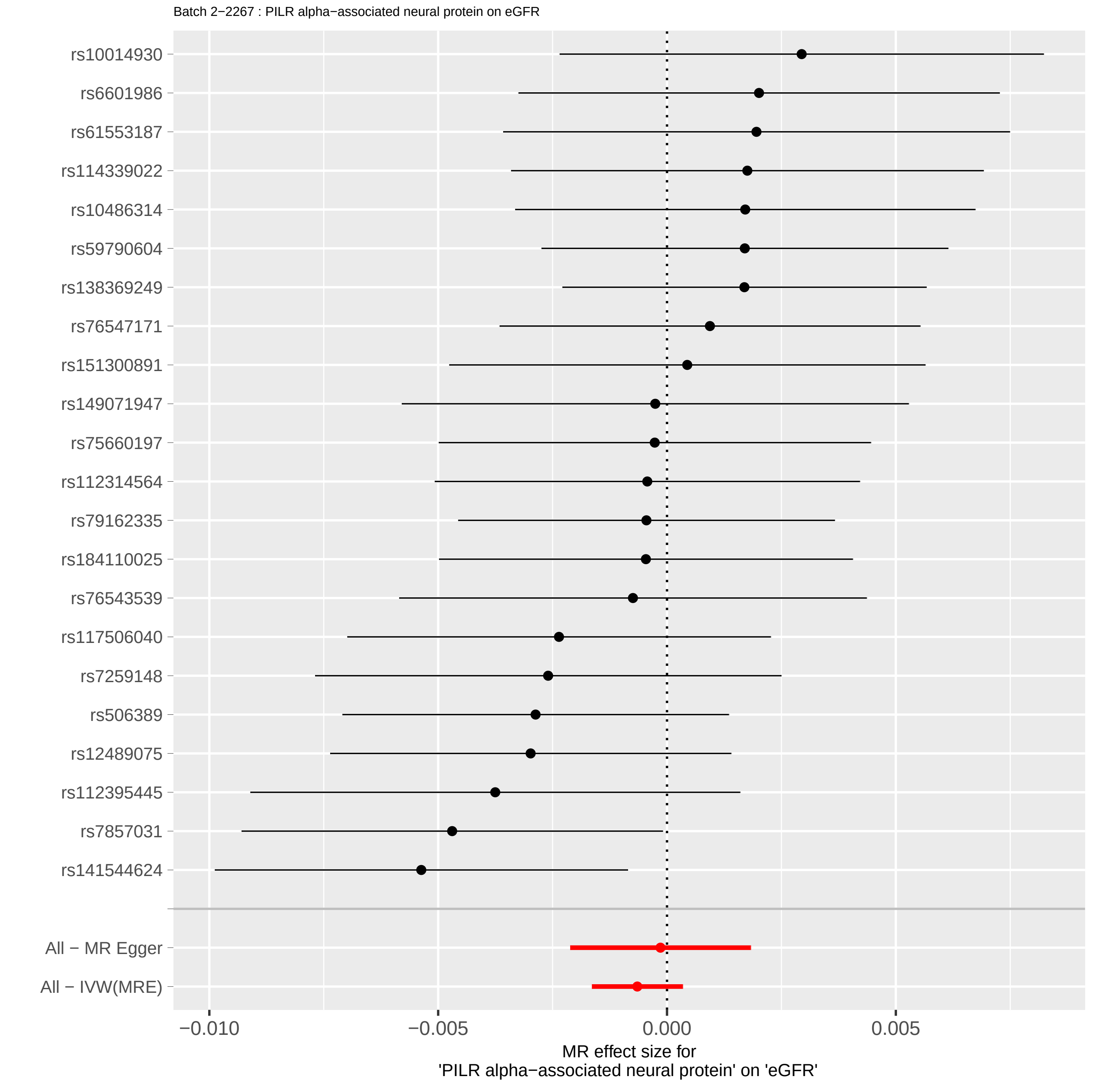

Batch 2−2267 : PILR alpha−associated neural protein on eGFR
rs10014930
rs6601986
rs61553187
rs114339022
rs10486314
rs59790604
rs138369249
rs76547171
rs151300891
rs149071947
rs75660197
rs112314564
rs79162335
rs184110025
rs76543539
rs117506040
rs7259148
rs506389
rs12489075
rs112395445
rs7857031
rs141544624
All − MR Egger
All − IVW(MRE)
−0.010
−0.005
0.000
0.005
MR effect size for
'PILR alpha−associated neural protein' on 'eGFR'

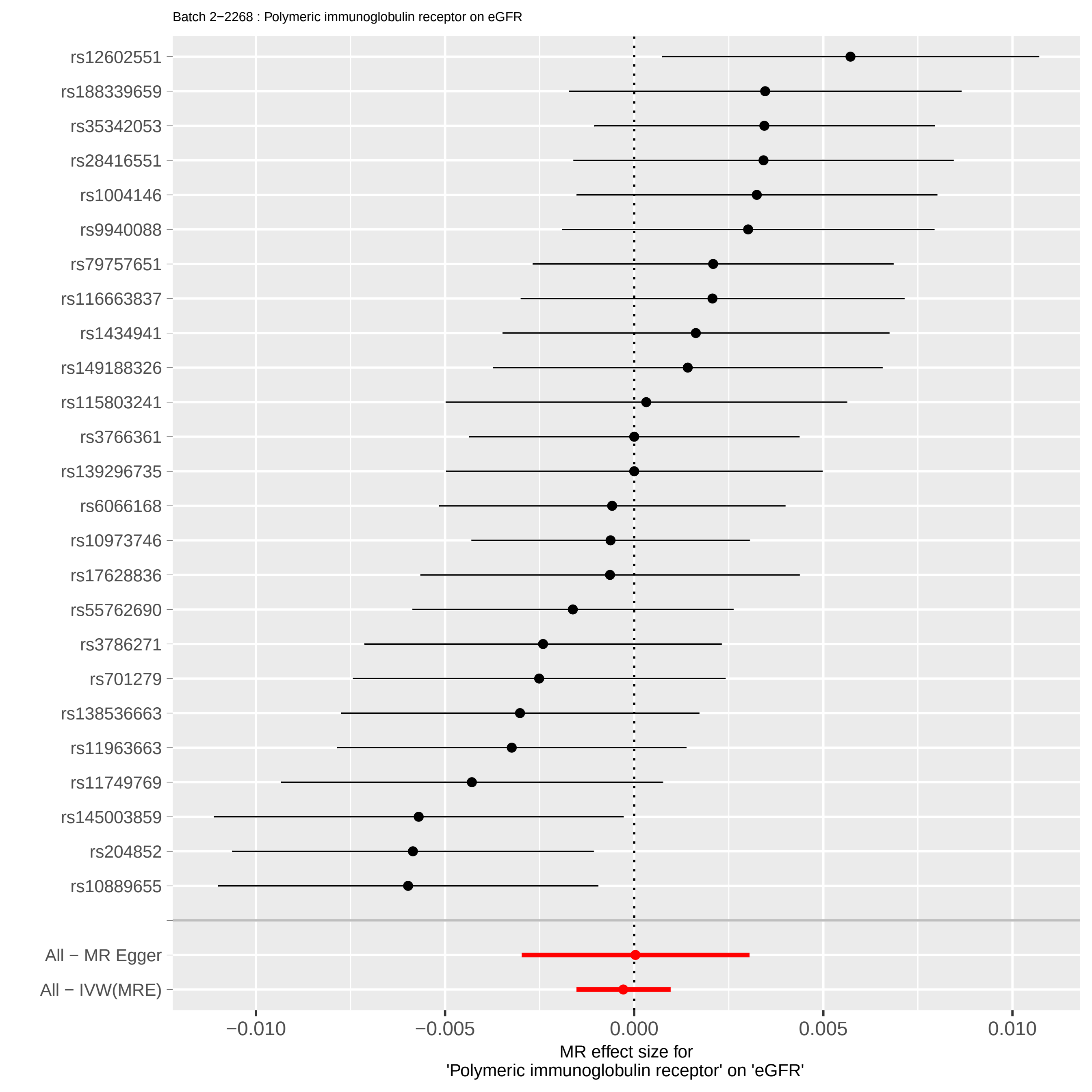
Batch 2−2268 : Polymeric immunoglobulin receptor on eGFR
rs12602551
rs188339659
rs35342053
rs28416551
rs1004146
rs9940088
rs79757651
rs116663837
rs1434941
rs149188326
rs115803241
rs3766361
rs139296735
rs6066168
rs10973746
rs17628836
rs55762690
rs3786271
rs701279
rs138536663
rs11963663
rs11749769
rs145003859
rs204852
rs10889655
All − MR Egger
All − IVW(MRE)
−0.010
−0.005
0.000
0.005
0.010
MR effect size for
'Polymeric immunoglobulin receptor' on 'eGFR'

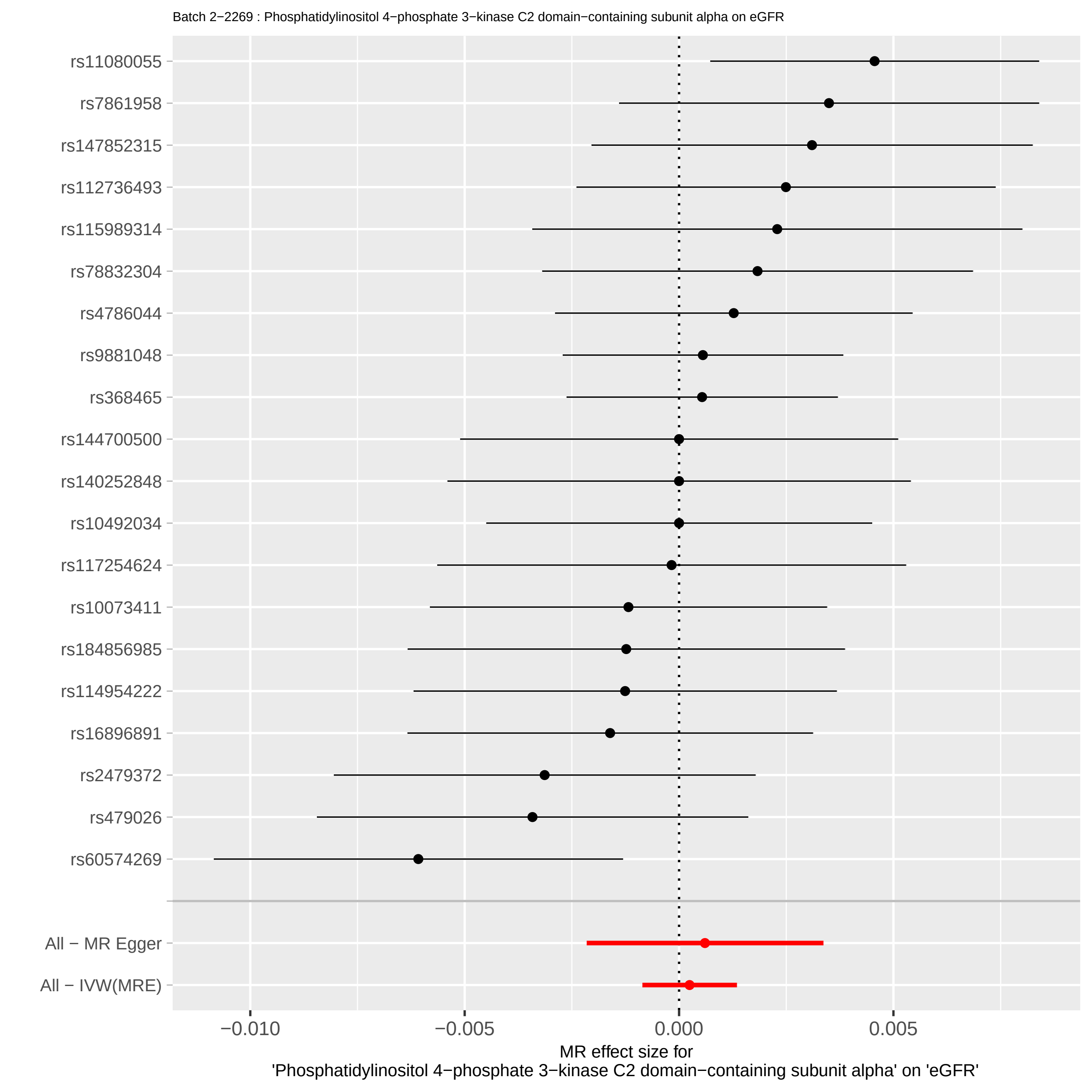

Batch 2−2269 : Phosphatidylinositol 4−phosphate 3−kinase C2 domain−containing subunit alpha on eGFR
rs11080055
rs7861958
rs147852315
rs112736493
rs115989314
rs78832304
rs4786044
rs9881048
rs368465
rs144700500
rs140252848
rs10492034
rs117254624
rs10073411
rs184856985
rs114954222
rs16896891
rs2479372
rs479026
rs60574269
All − MR Egger
All − IVW(MRE)
−0.010
−0.005
0.000
0.005
MR effect size for
'Phosphatidylinositol 4−phosphate 3−kinase C2 domain−containing subunit alpha' on 'eGFR'

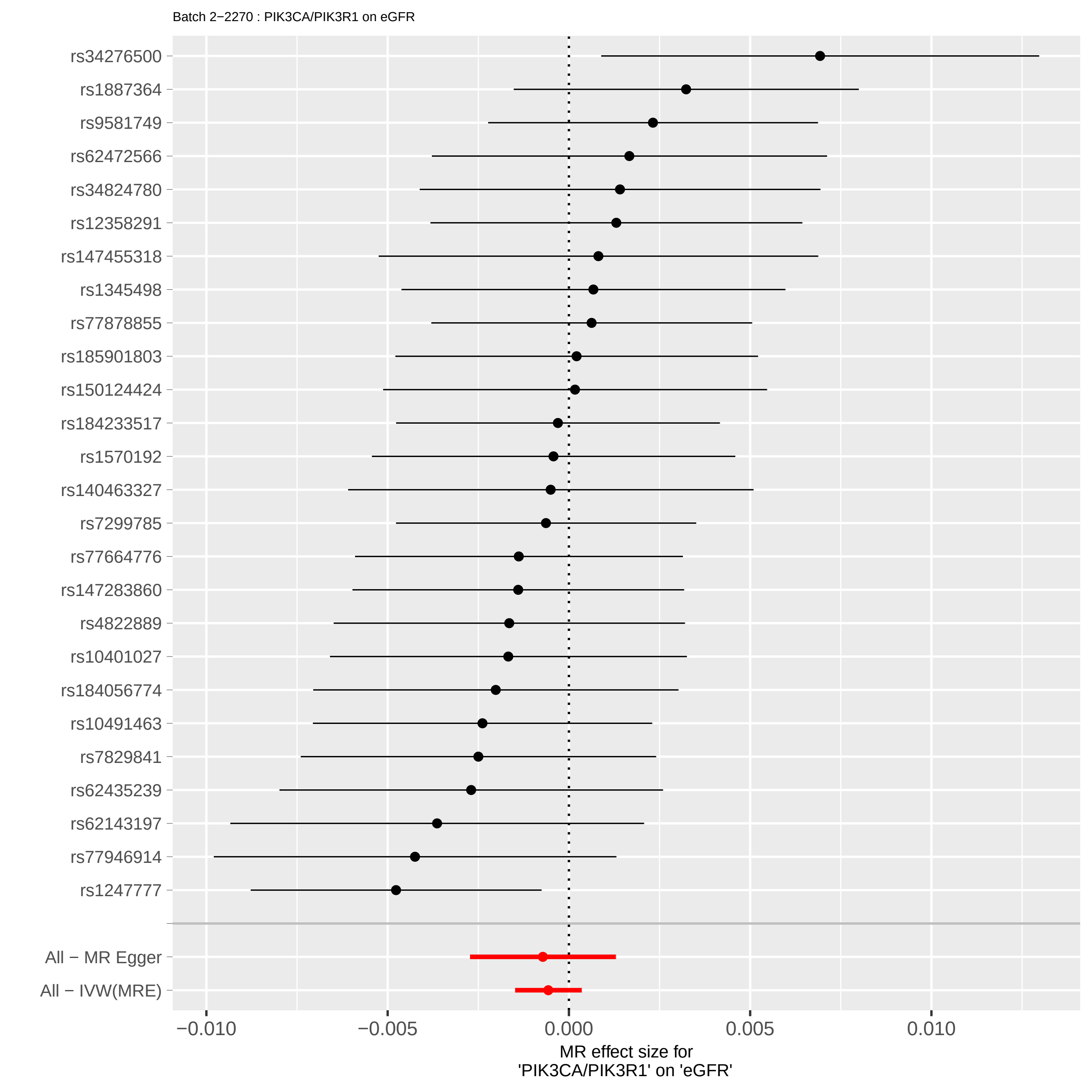

Batch 2−2270 : PIK3CA/PIK3R1 on eGFR
rs34276500
rs1887364
rs9581749
rs62472566
rs34824780
rs12358291
rs147455318
rs1345498
rs77878855
rs185901803
rs150124424
rs184233517
rs1570192
rs140463327
rs7299785
rs77664776
rs147283860
rs4822889
rs10401027
rs184056774
rs10491463
rs7829841
rs62435239
rs62143197
rs77946914
rs1247777
All − MR Egger
All − IVW(MRE)
−0.010
−0.005
0.000
0.005
0.010
MR effect size for
'PIK3CA/PIK3R1' on 'eGFR'

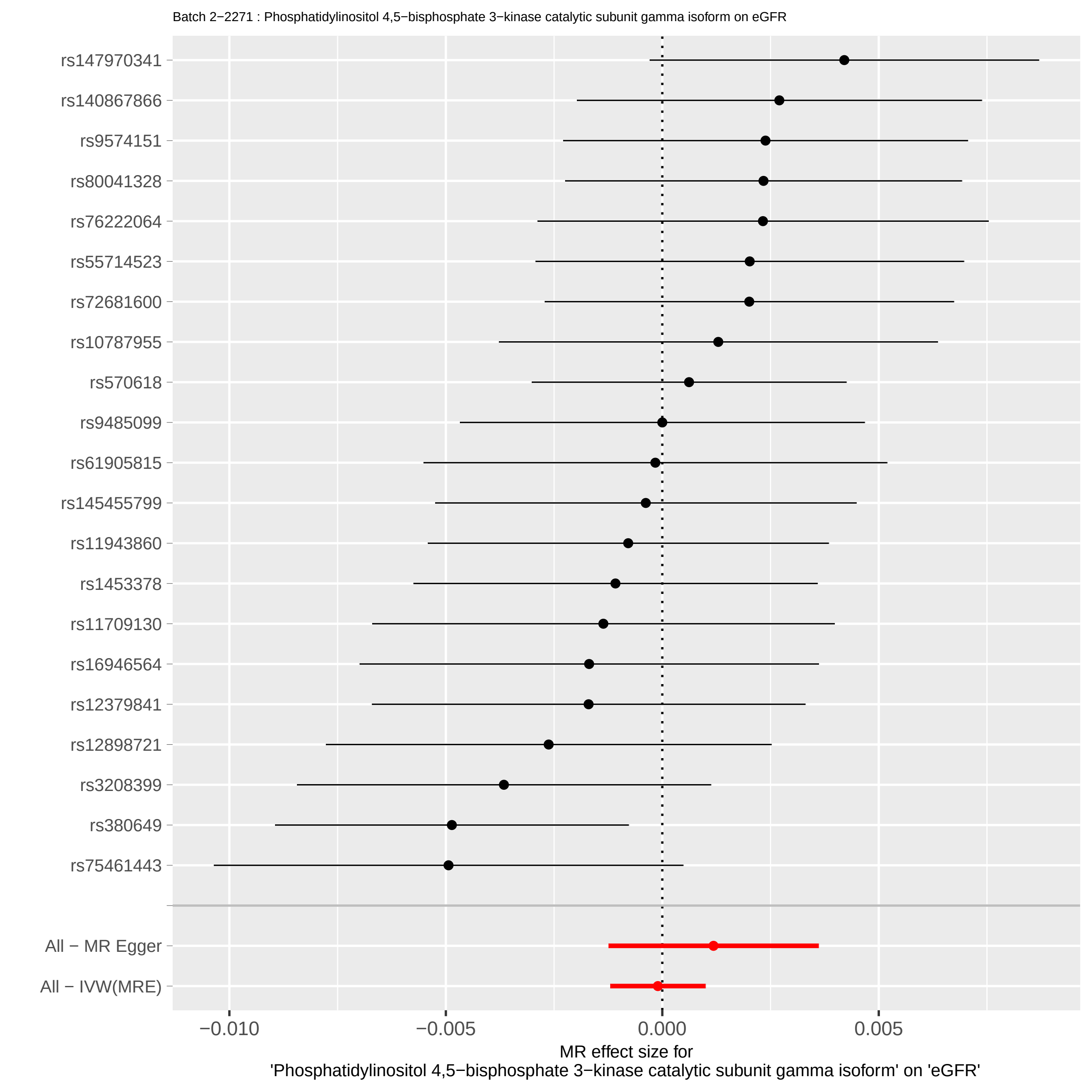

Batch 2−2271 : Phosphatidylinositol 4,5−bisphosphate 3−kinase catalytic subunit gamma isoform on eGFR
rs147970341
rs140867866
rs9574151
rs80041328
rs76222064
rs55714523
rs72681600
rs10787955
rs570618
rs9485099
rs61905815
rs145455799
rs11943860
rs1453378
rs11709130
rs16946564
rs12379841
rs12898721
rs3208399
rs380649
rs75461443
All − MR Egger
All − IVW(MRE)
−0.010
−0.005
0.000
0.005
MR effect size for
'Phosphatidylinositol 4,5−bisphosphate 3−kinase catalytic subunit gamma isoform' on 'eGFR'

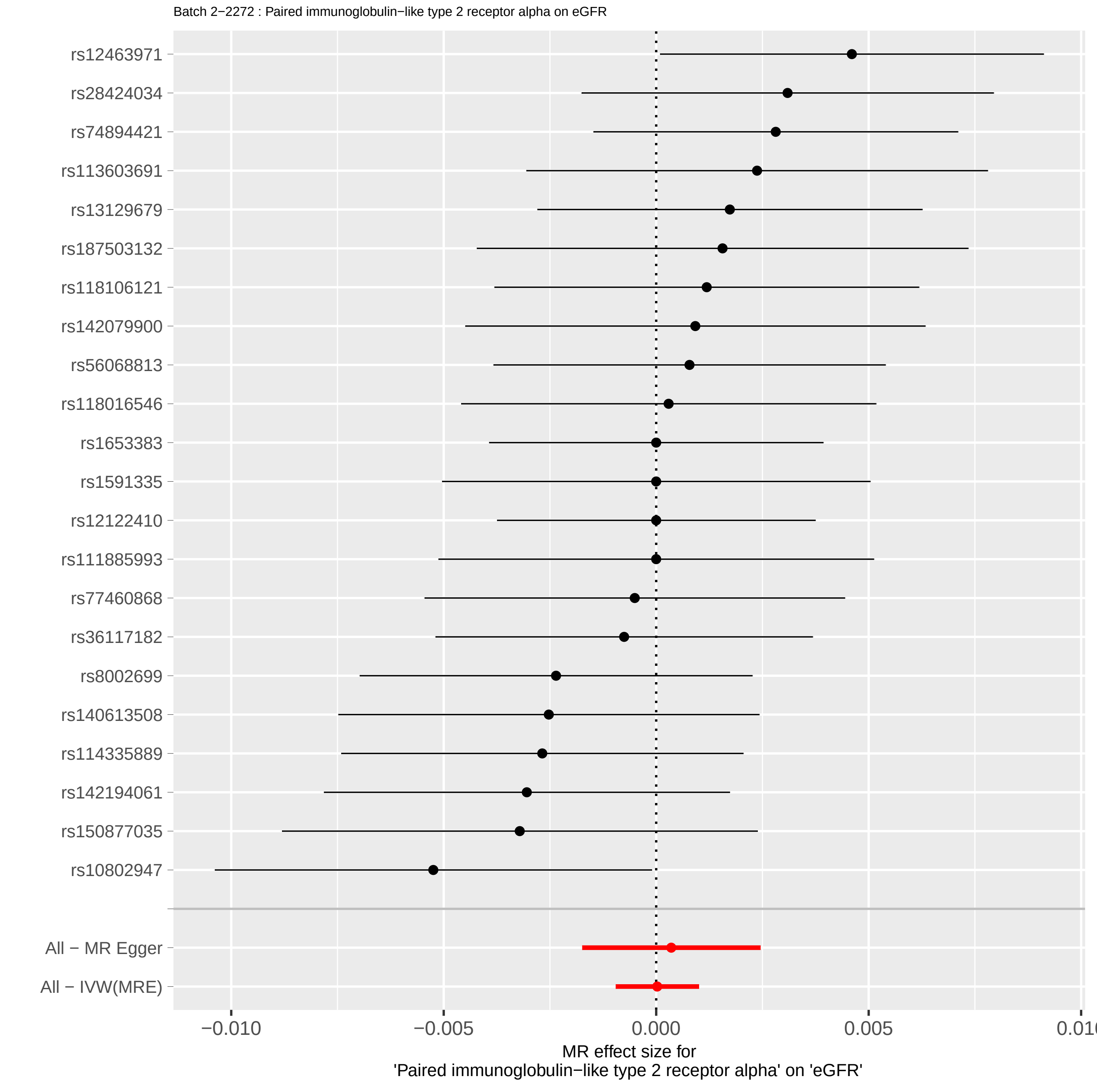

Batch 2−2272 : Paired immunoglobulin−like type 2 receptor alpha on eGFR
rs12463971
rs28424034
rs74894421
rs113603691
rs13129679
rs187503132
rs118106121
rs142079900
rs56068813
rs118016546
rs1653383
rs1591335
rs12122410
rs111885993
rs77460868
rs36117182
rs8002699
rs140613508
rs114335889
rs142194061
rs150877035
rs10802947
All − MR Egger
All − IVW(MRE)
−0.010
−0.005
0.000
0.005
0.010
MR effect size for
'Paired immunoglobulin−like type 2 receptor alpha' on 'eGFR'

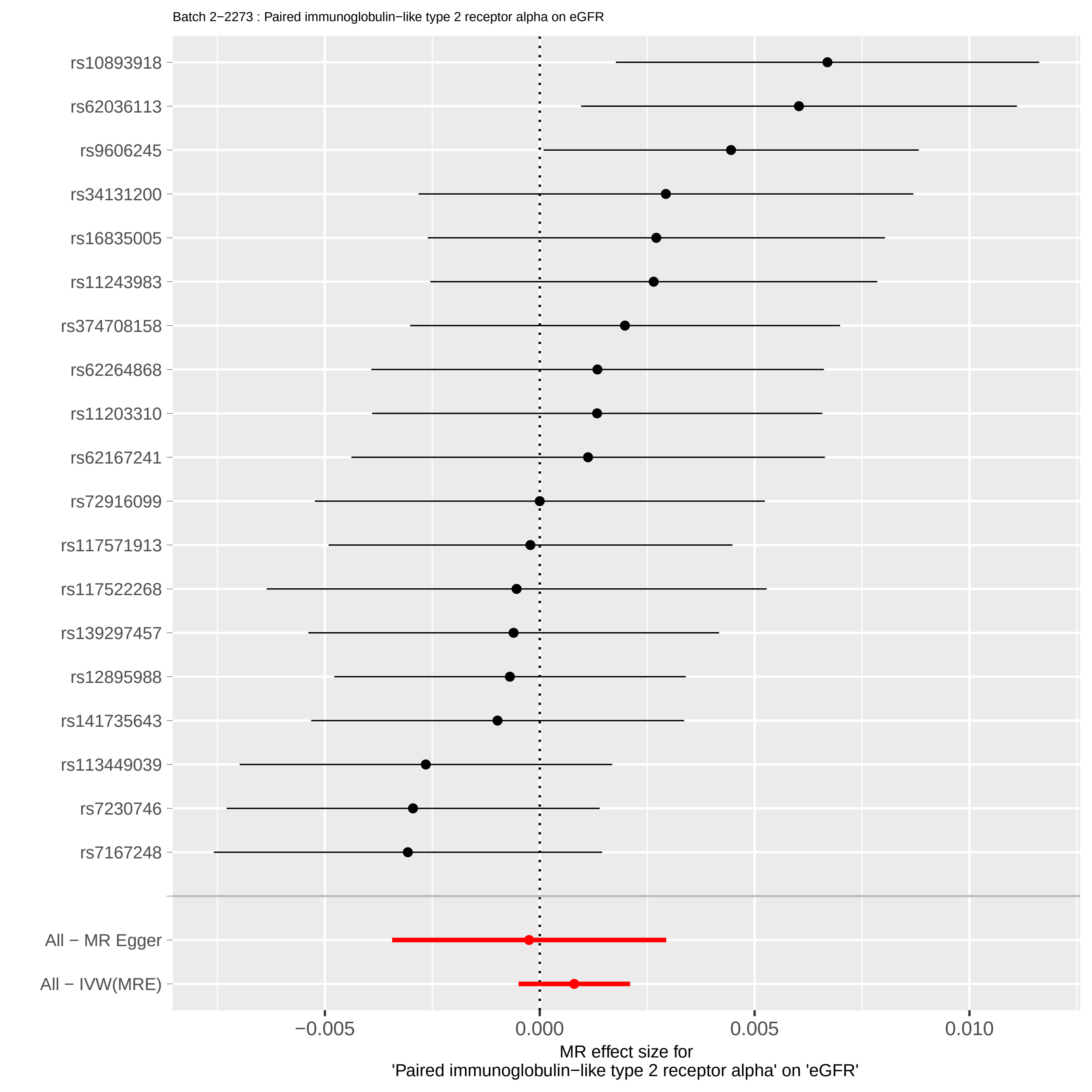
Batch 2−2273 : Paired immunoglobulin−like type 2 receptor alpha on eGFR
rs10893918
rs62036113
rs9606245
rs34131200
rs16835005
rs11243983
rs374708158
rs62264868
rs11203310
rs62167241
rs72916099
rs117571913
rs117522268
rs139297457
rs12895988
rs141735643
rs113449039
rs7230746
rs7167248
All − MR Egger
All − IVW(MRE)
−0.005
0.000
0.005
0.010
MR effect size for
'Paired immunoglobulin−like type 2 receptor alpha' on 'eGFR'

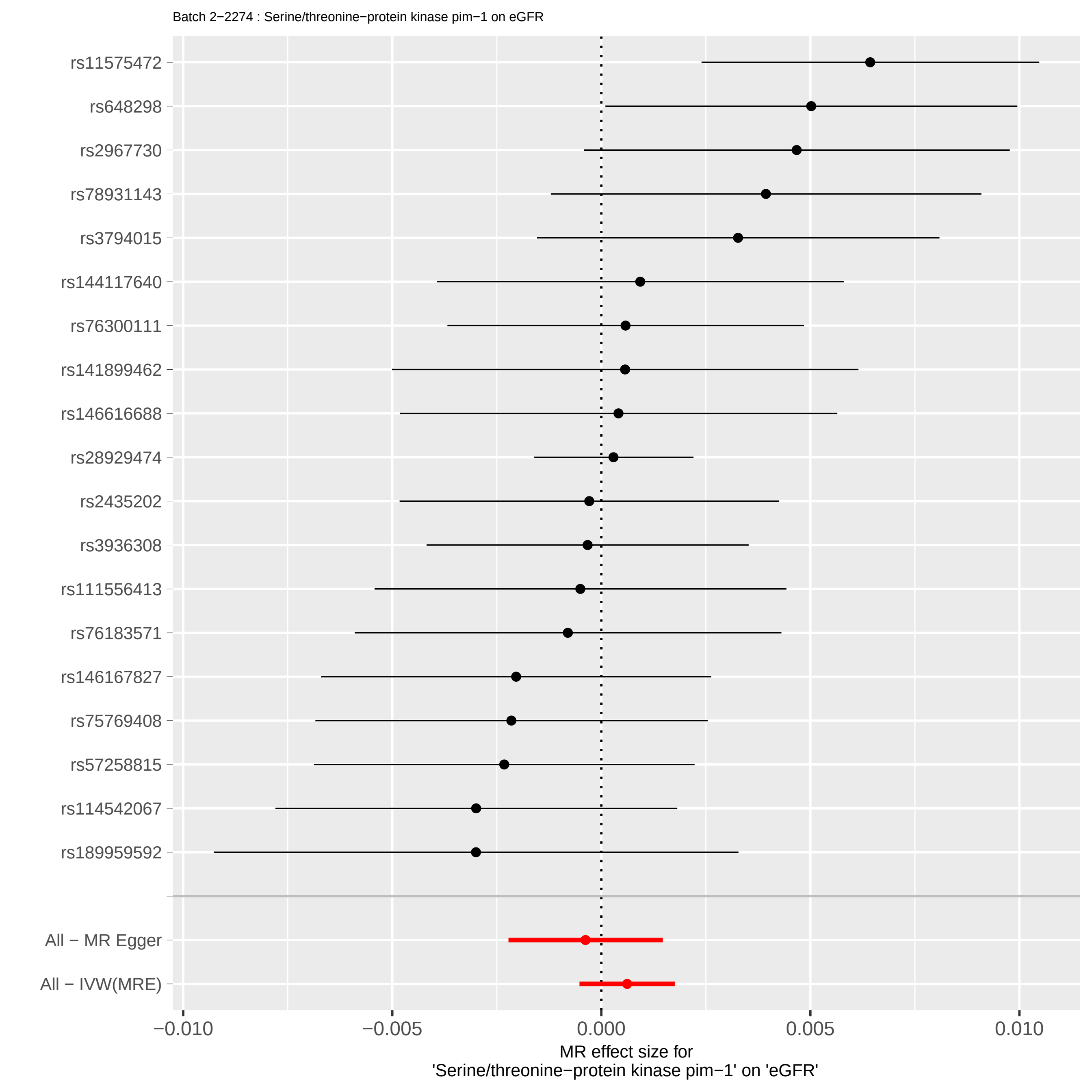

Batch 2−2274 : Serine/threonine−protein kinase pim−1 on eGFR
rs11575472
rs648298
rs2967730
rs78931143
rs3794015
rs144117640
rs76300111
rs141899462
rs146616688
rs28929474
rs2435202
rs3936308
rs111556413
rs76183571
rs146167827
rs75769408
rs57258815
rs114542067
rs189959592
All − MR Egger
All − IVW(MRE)
−0.010
−0.005
0.000
0.005
0.010
MR effect size for
'Serine/threonine−protein kinase pim−1' on 'eGFR'

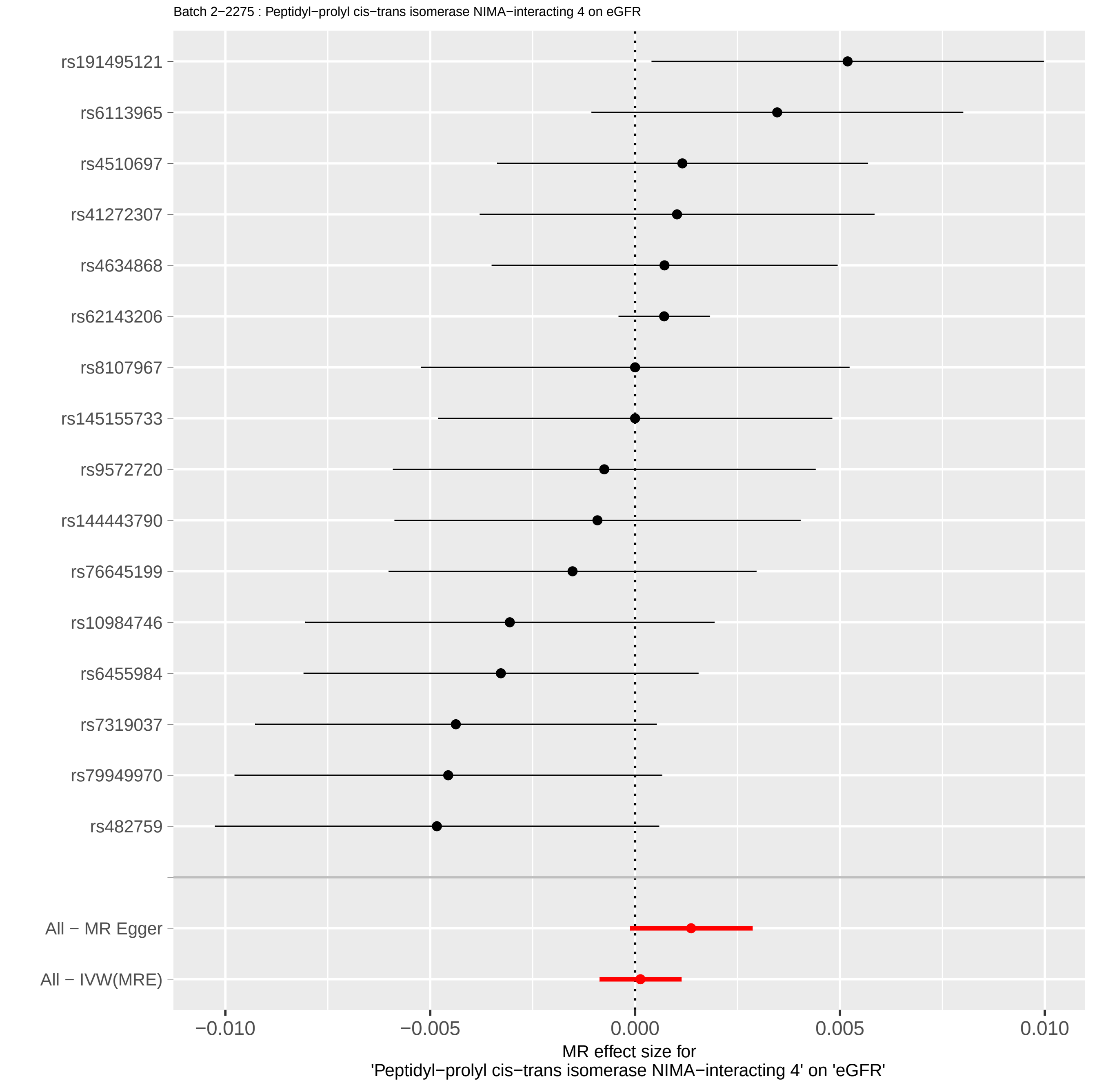
Batch 2−2275 : Peptidyl−prolyl cis−trans isomerase NIMA−interacting 4 on eGFR
rs191495121
rs6113965
rs4510697
rs41272307
rs4634868
rs62143206
rs8107967
rs145155733
rs9572720
rs144443790
rs76645199
rs10984746
rs6455984
rs7319037
rs79949970
rs482759
All − MR Egger
All − IVW(MRE)
−0.010
−0.005
0.000
0.005
0.010
MR effect size for
'Peptidyl−prolyl cis−trans isomerase NIMA−interacting 4' on 'eGFR'

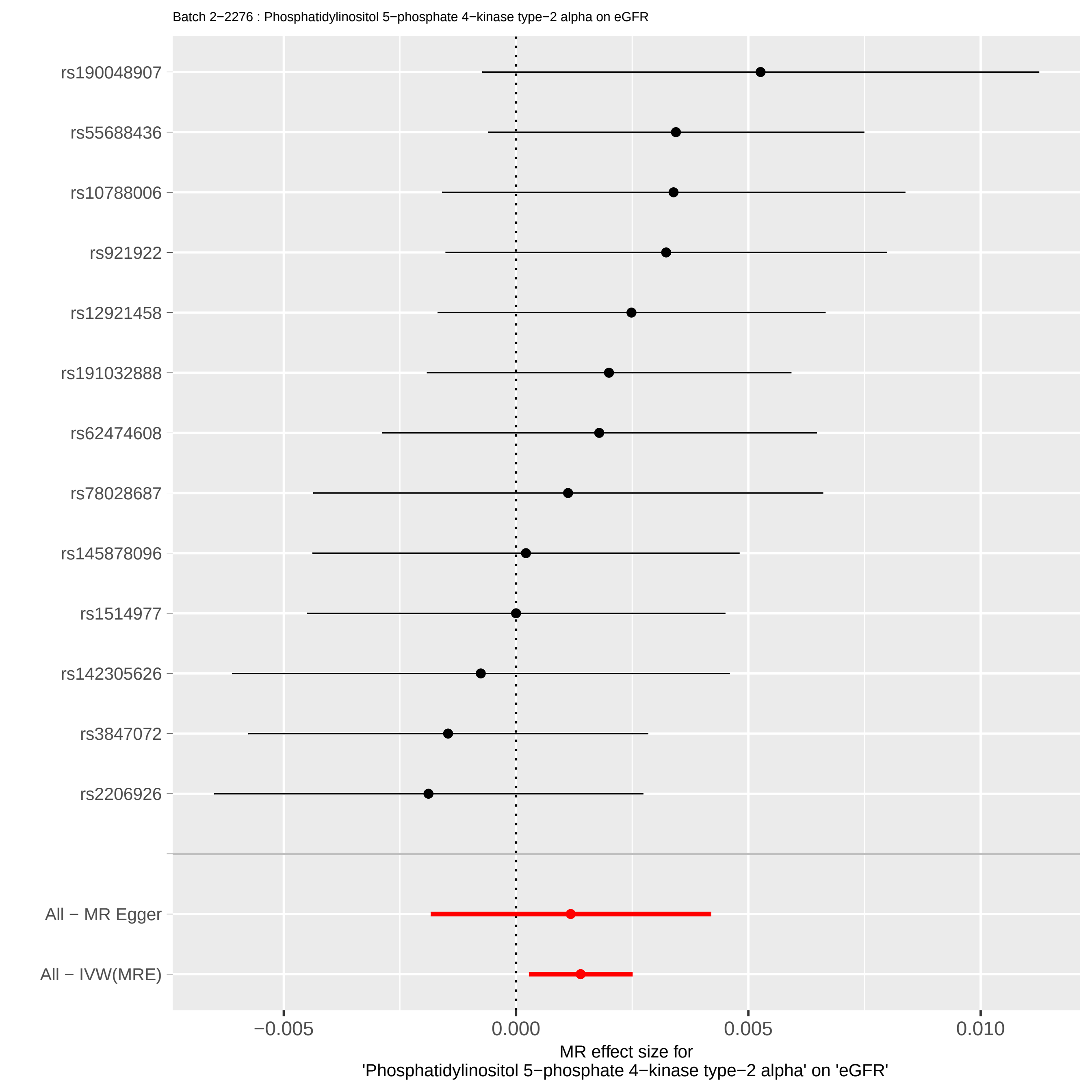

Batch 2−2276 : Phosphatidylinositol 5−phosphate 4−kinase type−2 alpha on eGFR
rs190048907
rs55688436
rs10788006
rs921922
rs12921458
rs191032888
rs62474608
rs78028687
rs145878096
rs1514977
rs142305626
rs3847072
rs2206926
All − MR Egger
All − IVW(MRE)
−0.005
0.000
0.005
0.010
MR effect size for
'Phosphatidylinositol 5−phosphate 4−kinase type−2 alpha' on 'eGFR'

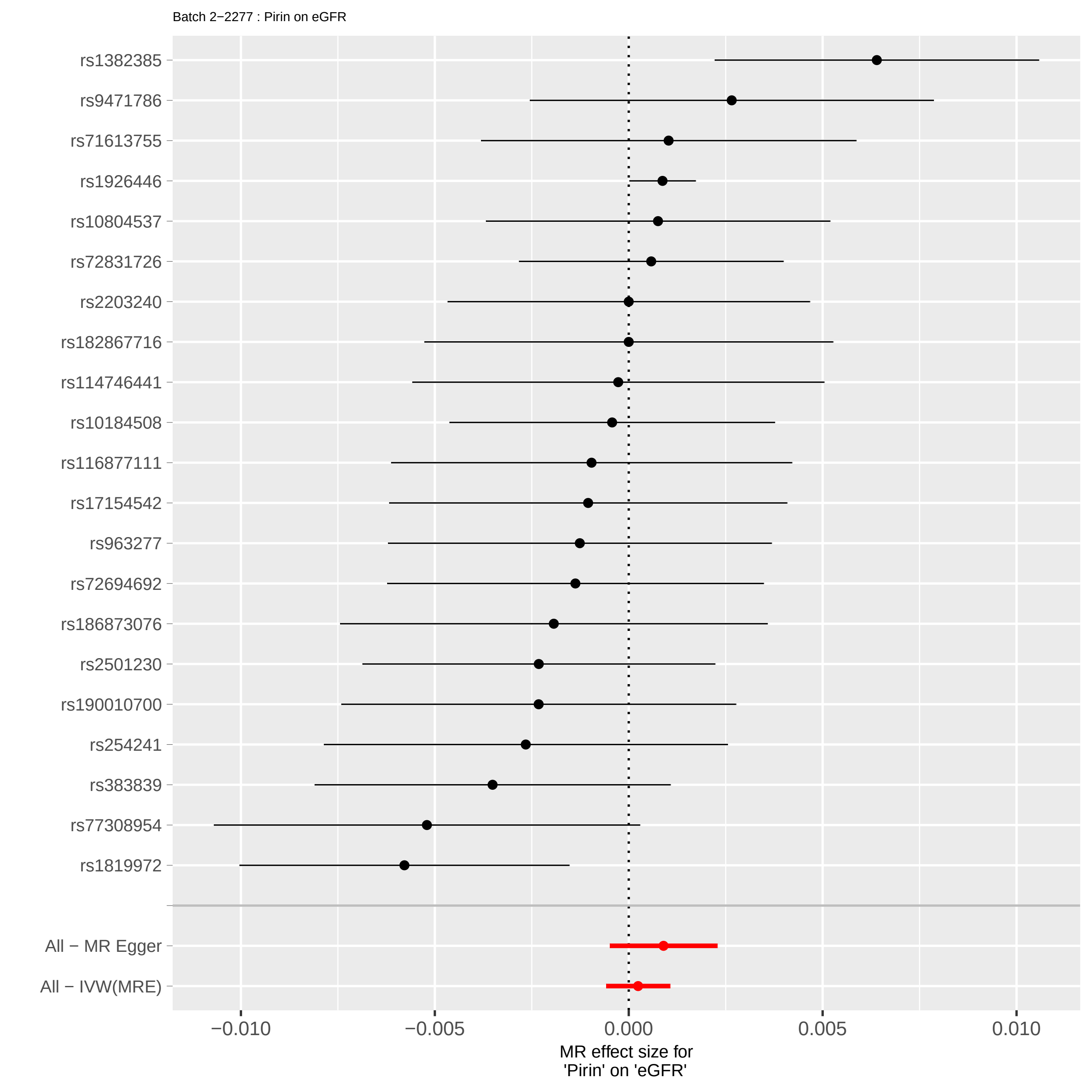

Batch 2−2277 : Pirin on eGFR
rs1382385
rs9471786
rs71613755
rs1926446
rs10804537
rs72831726
rs2203240
rs182867716
rs114746441
rs10184508
rs116877111
rs17154542
rs963277
rs72694692
rs186873076
rs2501230
rs190010700
rs254241
rs383839
rs77308954
rs1819972
All − MR Egger
All − IVW(MRE)
−0.010
−0.005
0.000
0.005
0.010
MR effect size for
'Pirin' on 'eGFR'

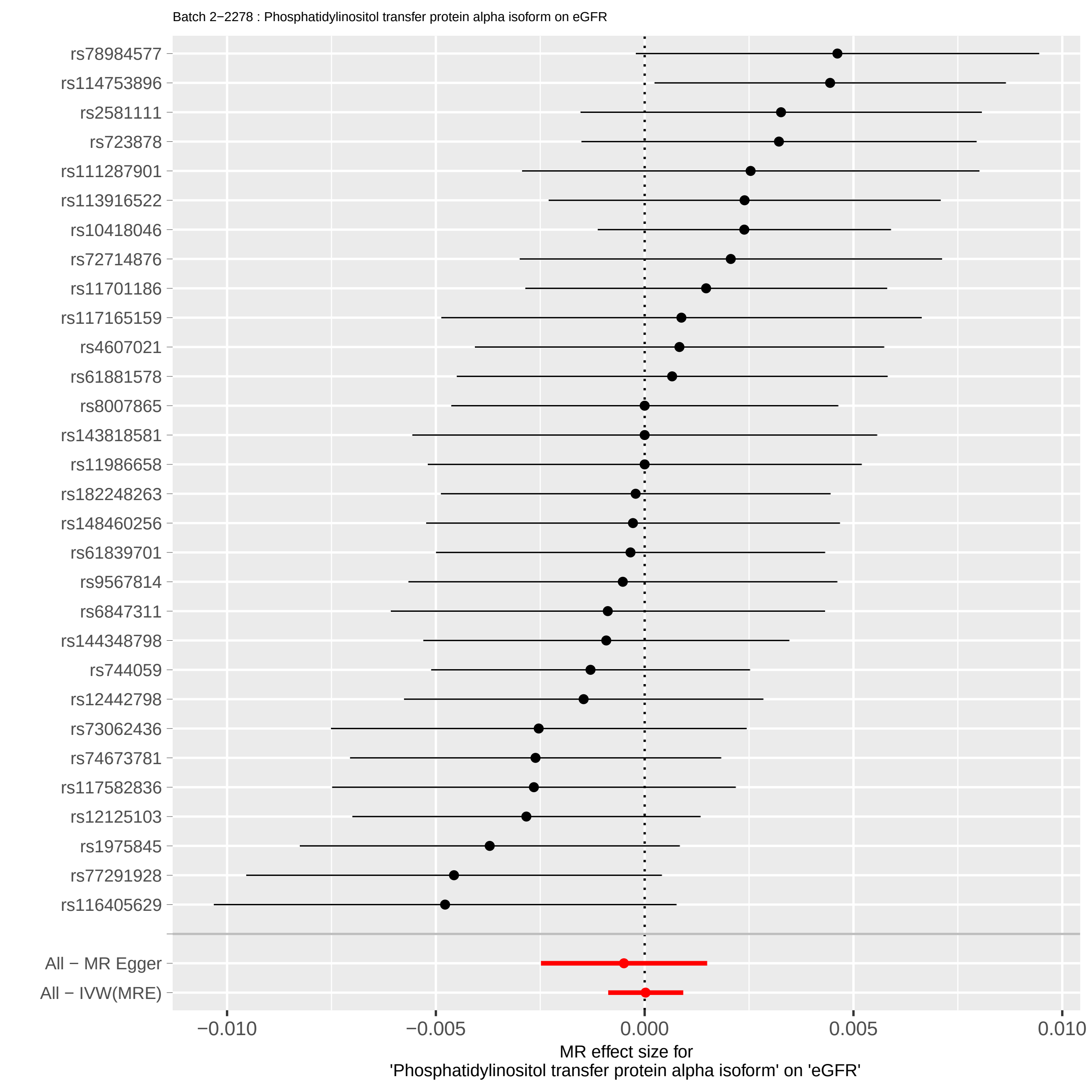

Batch 2−2278 : Phosphatidylinositol transfer protein alpha isoform on eGFR
rs78984577
rs114753896
rs2581111
rs723878
rs111287901
rs113916522
rs10418046
rs72714876
rs11701186
rs117165159
rs4607021
rs61881578
rs8007865
rs143818581
rs11986658
rs182248263
rs148460256
rs61839701
rs9567814
rs6847311
rs144348798
rs744059
rs12442798
rs73062436
rs74673781
rs117582836
rs12125103
rs1975845
rs77291928
rs116405629
All − MR Egger
All − IVW(MRE)
−0.010
−0.005
0.000
0.005
0.010
MR effect size for
'Phosphatidylinositol transfer protein alpha isoform' on 'eGFR'

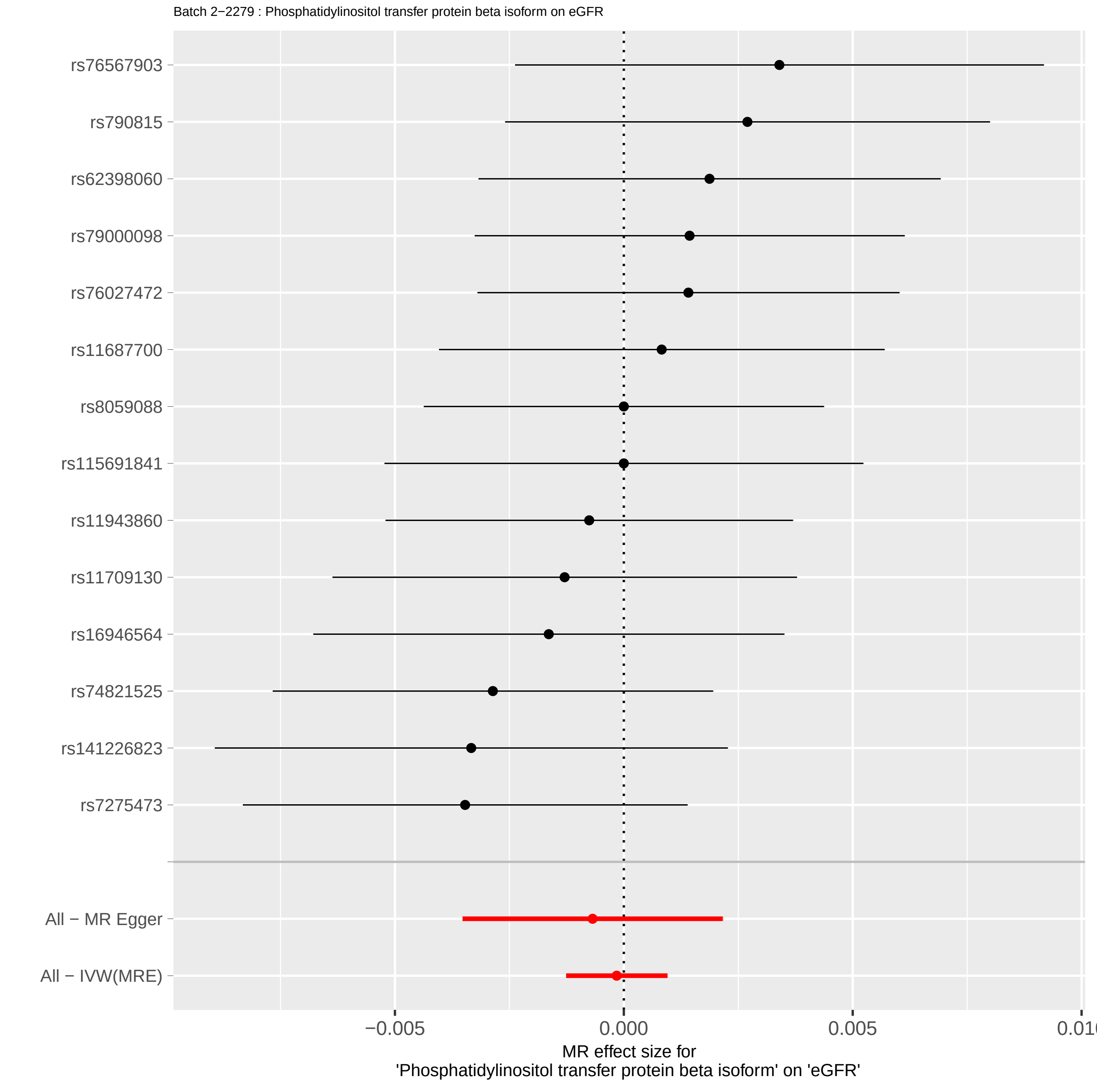

Batch 2−2279 : Phosphatidylinositol transfer protein beta isoform on eGFR
rs76567903
rs790815
rs62398060
rs79000098
rs76027472
rs11687700
rs8059088
rs115691841
rs11943860
rs11709130
rs16946564
rs74821525
rs141226823
rs7275473
All − MR Egger
All − IVW(MRE)
−0.005
0.000
0.005
MR effect size for
'Phosphatidylinositol transfer protein beta isoform' on 'eGFR'

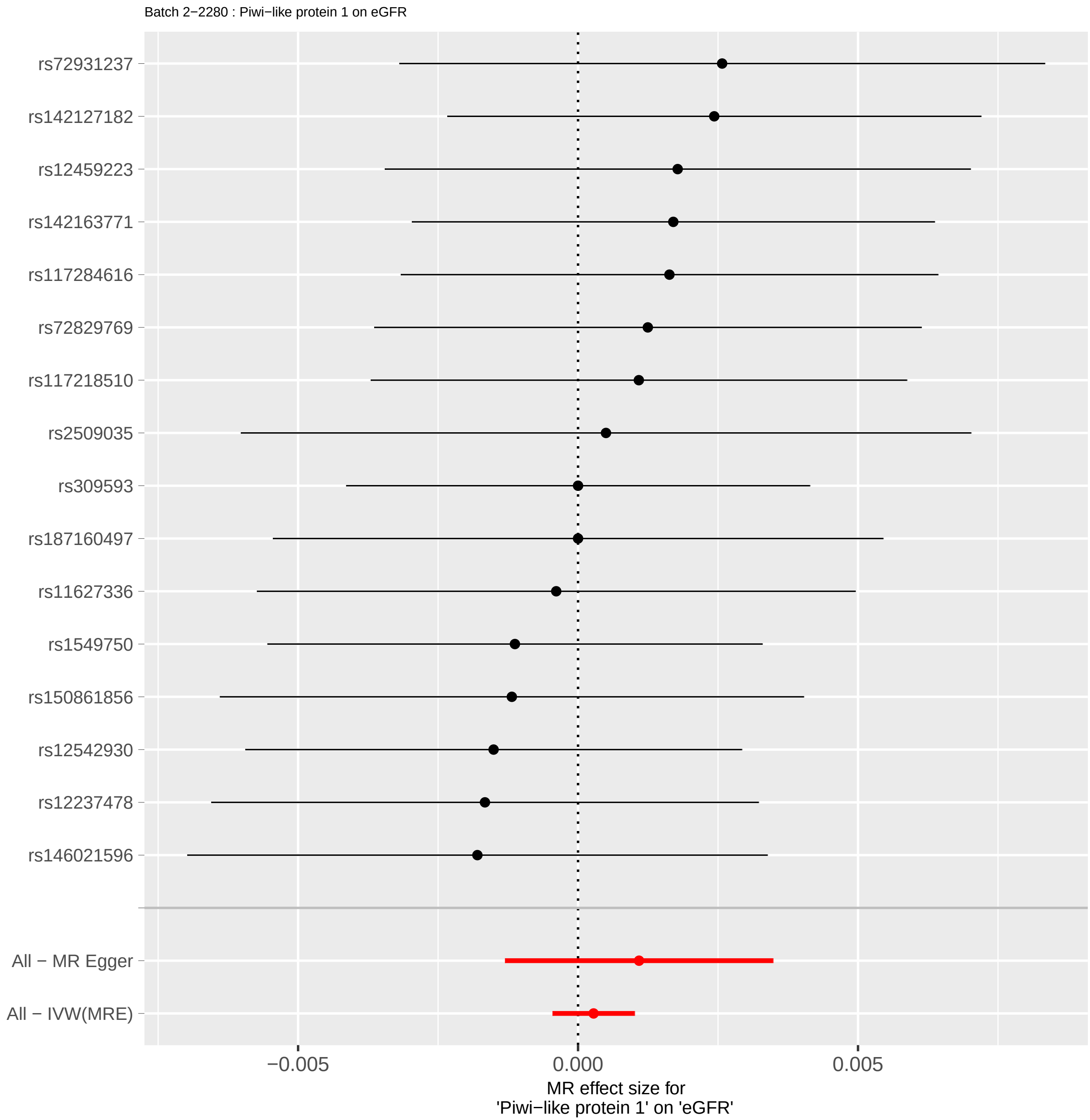
Batch 2−2280 : Piwi−like protein 1 on eGFR
rs72931237
rs142127182
rs12459223
rs142163771
rs117284616
rs72829769
rs117218510
rs2509035
rs309593
rs187160497
rs11627336
rs1549750
rs150861856
rs12542930
rs12237478
rs146021596
All − MR Egger
All − IVW(MRE)
−0.005
0.000
0.005
MR effect size for
'Piwi−like protein 1' on 'eGFR'

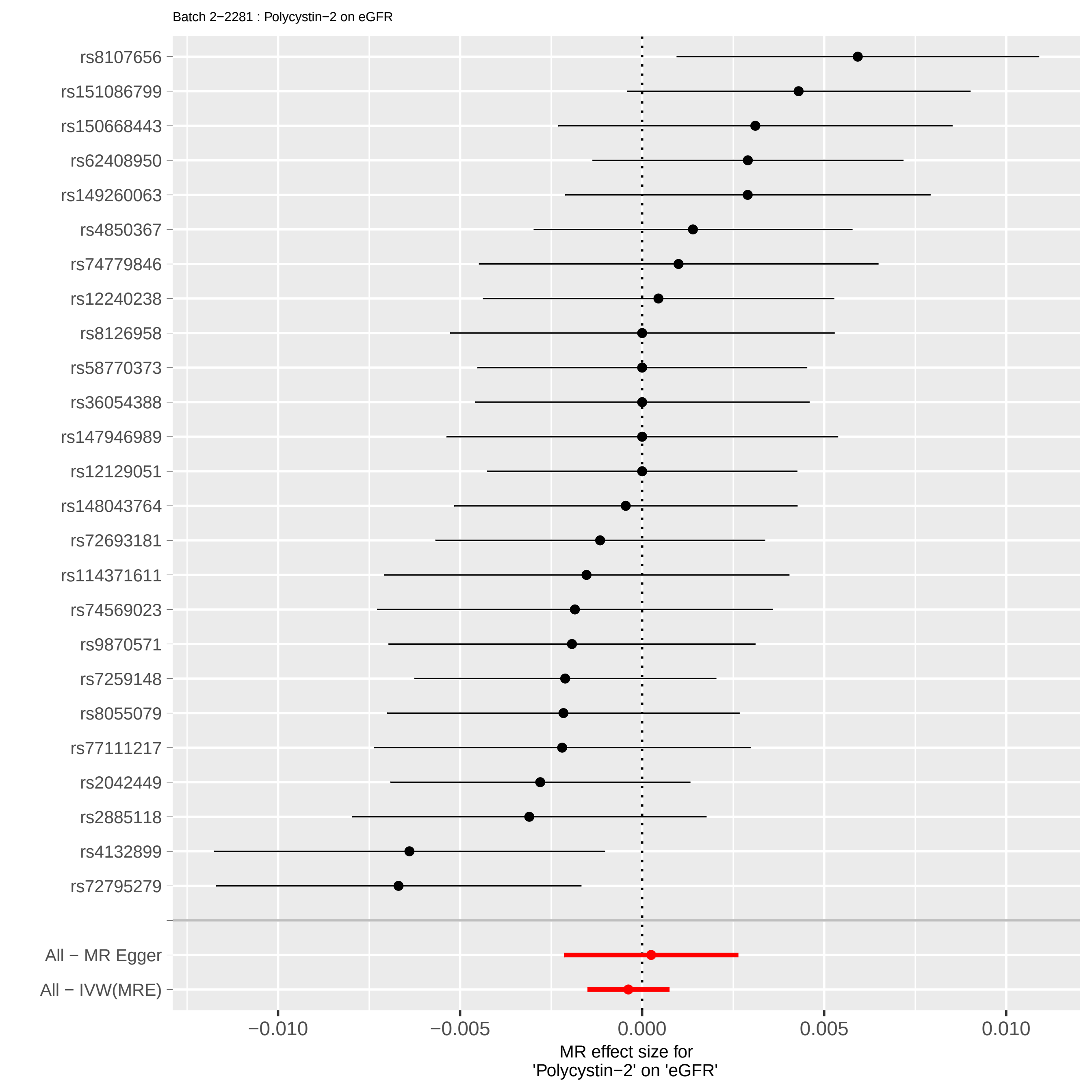
Batch 2−2281 : Polycystin−2 on eGFR
rs8107656
rs151086799
rs150668443
rs62408950
rs149260063
rs4850367
rs74779846
rs12240238
rs8126958
rs58770373
rs36054388
rs147946989
rs12129051
rs148043764
rs72693181
rs114371611
rs74569023
rs9870571
rs7259148
rs8055079
rs77111217
rs2042449
rs2885118
rs4132899
rs72795279
All − MR Egger
All − IVW(MRE)
−0.010
−0.005
0.000
0.005
0.010
MR effect size for
'Polycystin−2' on 'eGFR'

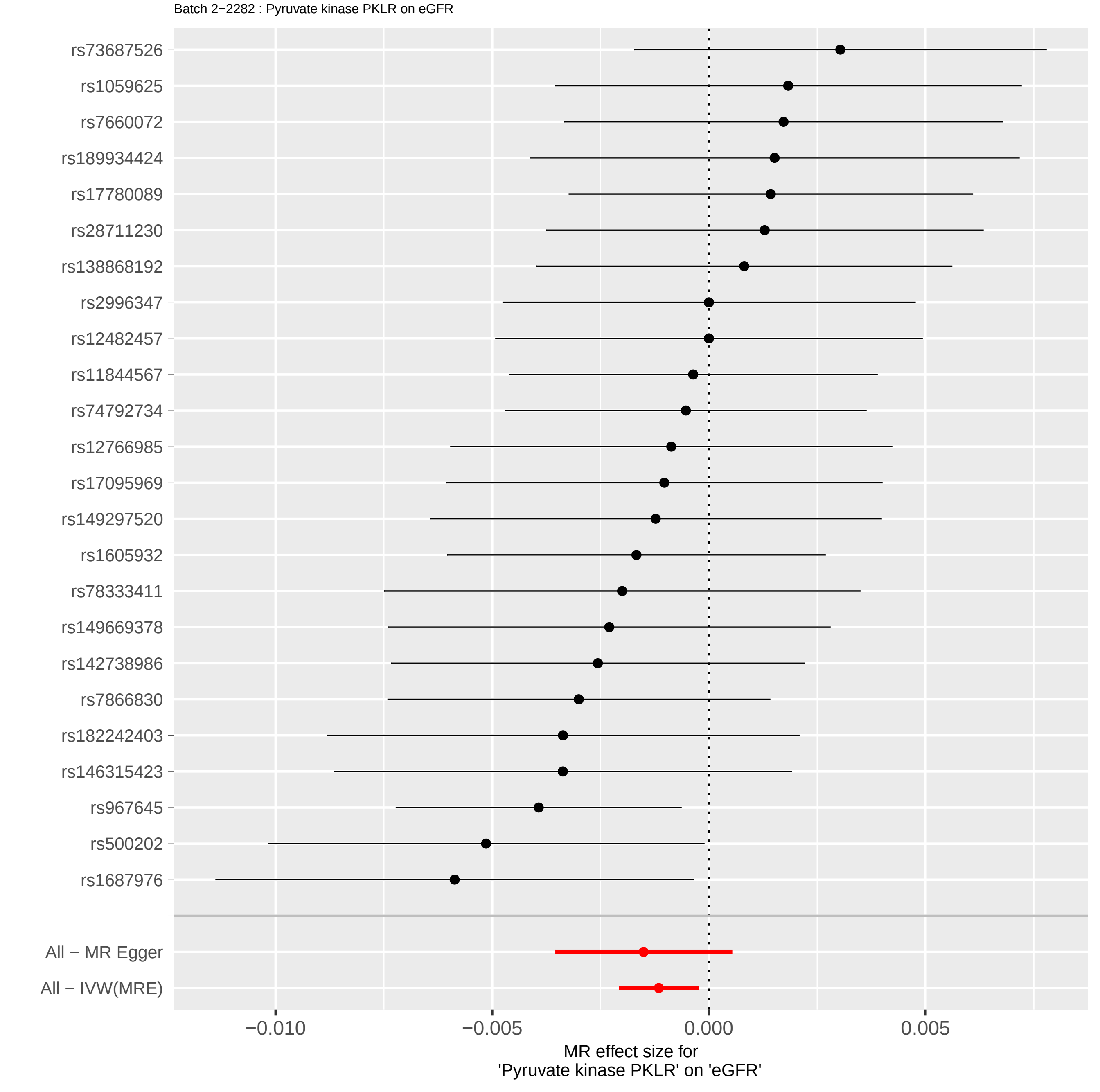

Batch 2−2282 : Pyruvate kinase PKLR on eGFR
rs73687526
rs1059625
rs7660072
rs189934424
rs17780089
rs28711230
rs138868192
rs2996347
rs12482457
rs11844567
rs74792734
rs12766985
rs17095969
rs149297520
rs1605932
rs78333411
rs149669378
rs142738986
rs7866830
rs182242403
rs146315423
rs967645
rs500202
rs1687976
All − MR Egger
All − IVW(MRE)
−0.010
−0.005
0.000
0.005
MR effect size for
'Pyruvate kinase PKLR' on 'eGFR'

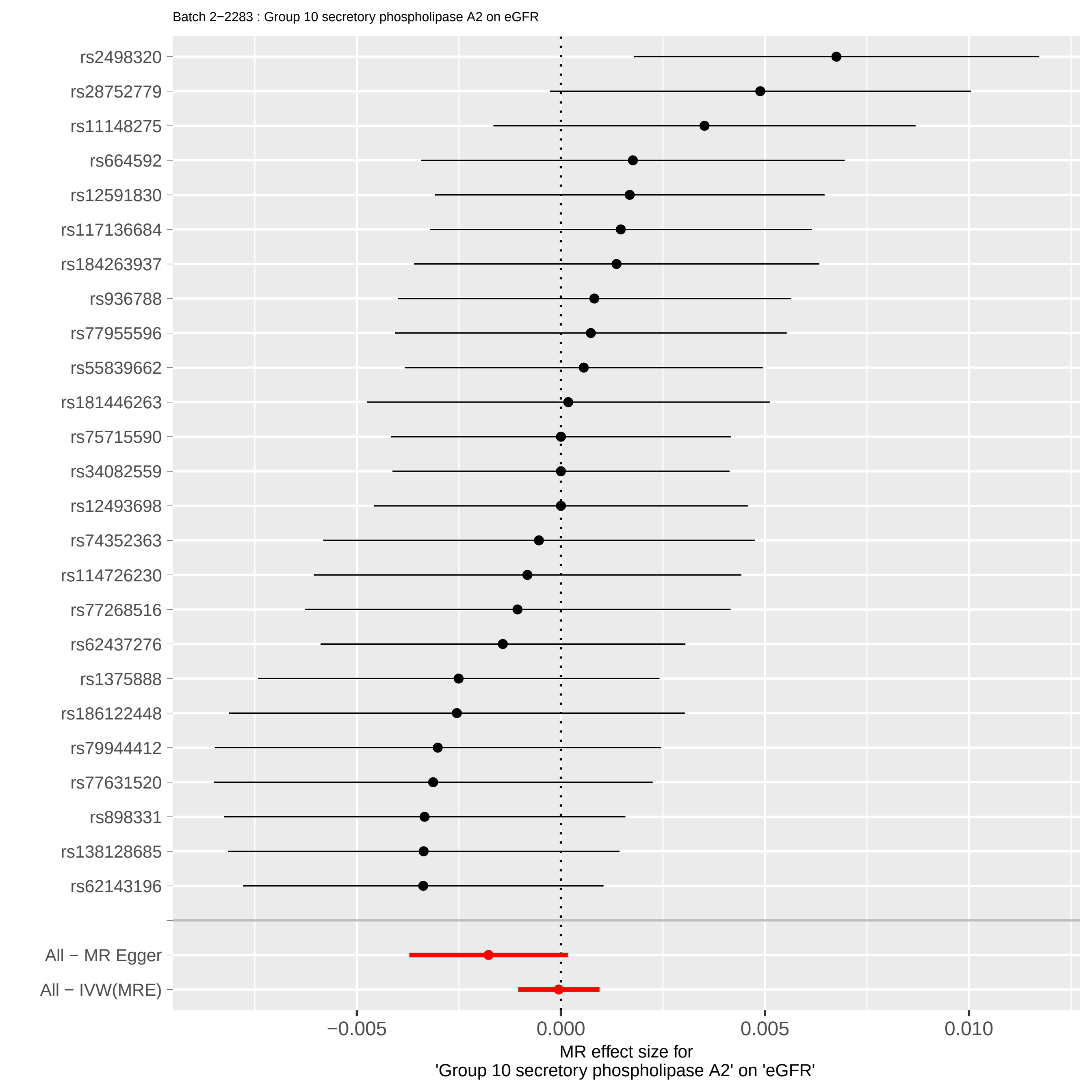

Batch 2−2283 : Group 10 secretory phospholipase A2 on eGFR
rs2498320
rs28752779
rs11148275
rs664592
rs12591830
rs117136684
rs184263937
rs936788
rs77955596
rs55839662
rs181446263
rs75715590
rs34082559
rs12493698
rs74352363
rs114726230
rs77268516
rs62437276
rs1375888
rs186122448
rs79944412
rs77631520
rs898331
rs138128685
rs62143196
All − MR Egger
All − IVW(MRE)
−0.005
0.000
0.005
0.010
MR effect size for
'Group 10 secretory phospholipase A2' on 'eGFR'

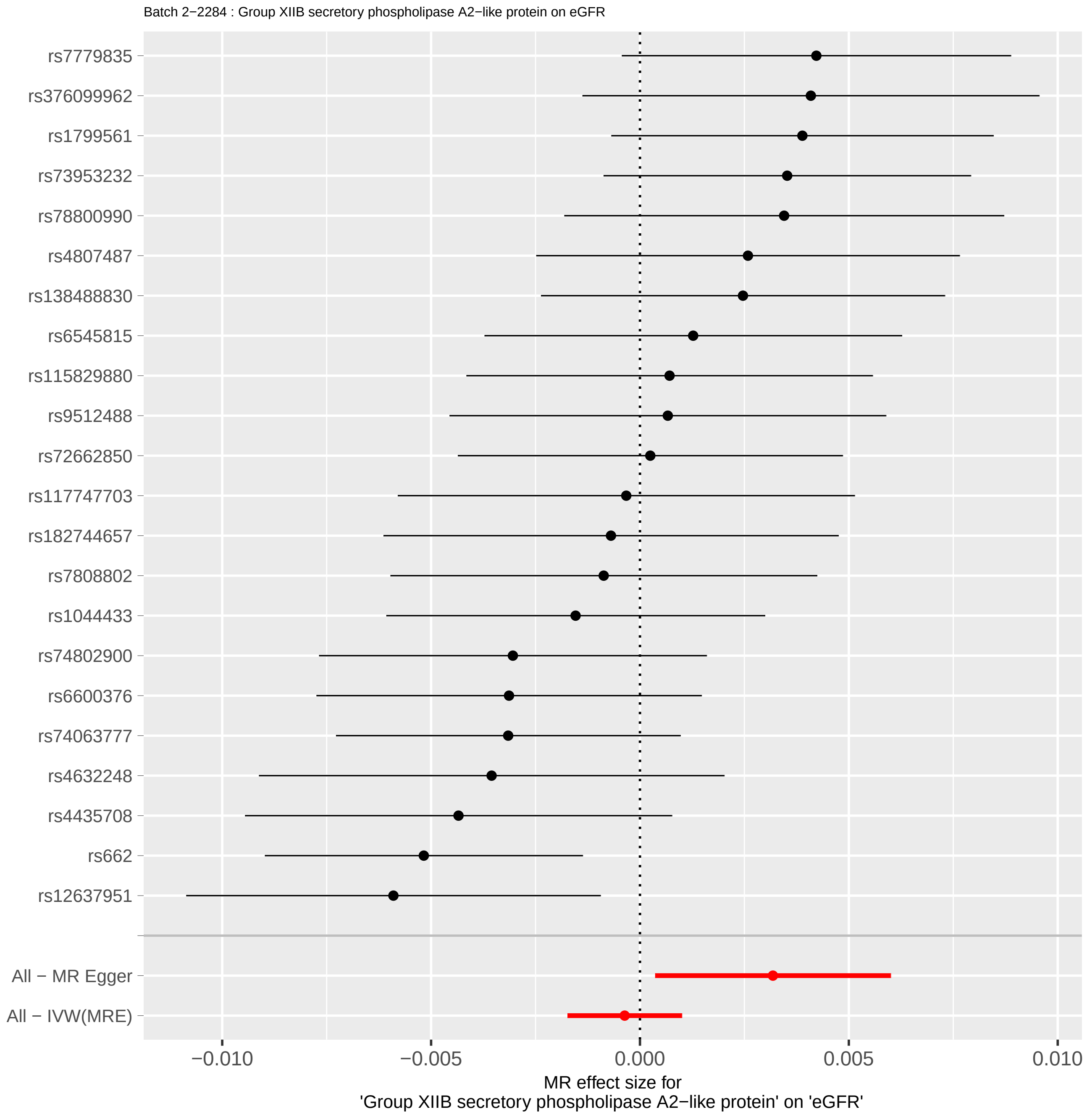

Batch 2−2284 : Group XIIB secretory phospholipase A2−like protein on eGFR
rs7779835
rs376099962
rs1799561
rs73953232
rs78800990
rs4807487
rs138488830
rs6545815
rs115829880
rs9512488
rs72662850
rs117747703
rs182744657
rs7808802
rs1044433
rs74802900
rs6600376
rs74063777
rs4632248
rs4435708
rs662
rs12637951
All − MR Egger
All − IVW(MRE)
−0.010
−0.005
0.000
0.005
0.010
MR effect size for
'Group XIIB secretory phospholipase A2−like protein' on 'eGFR'

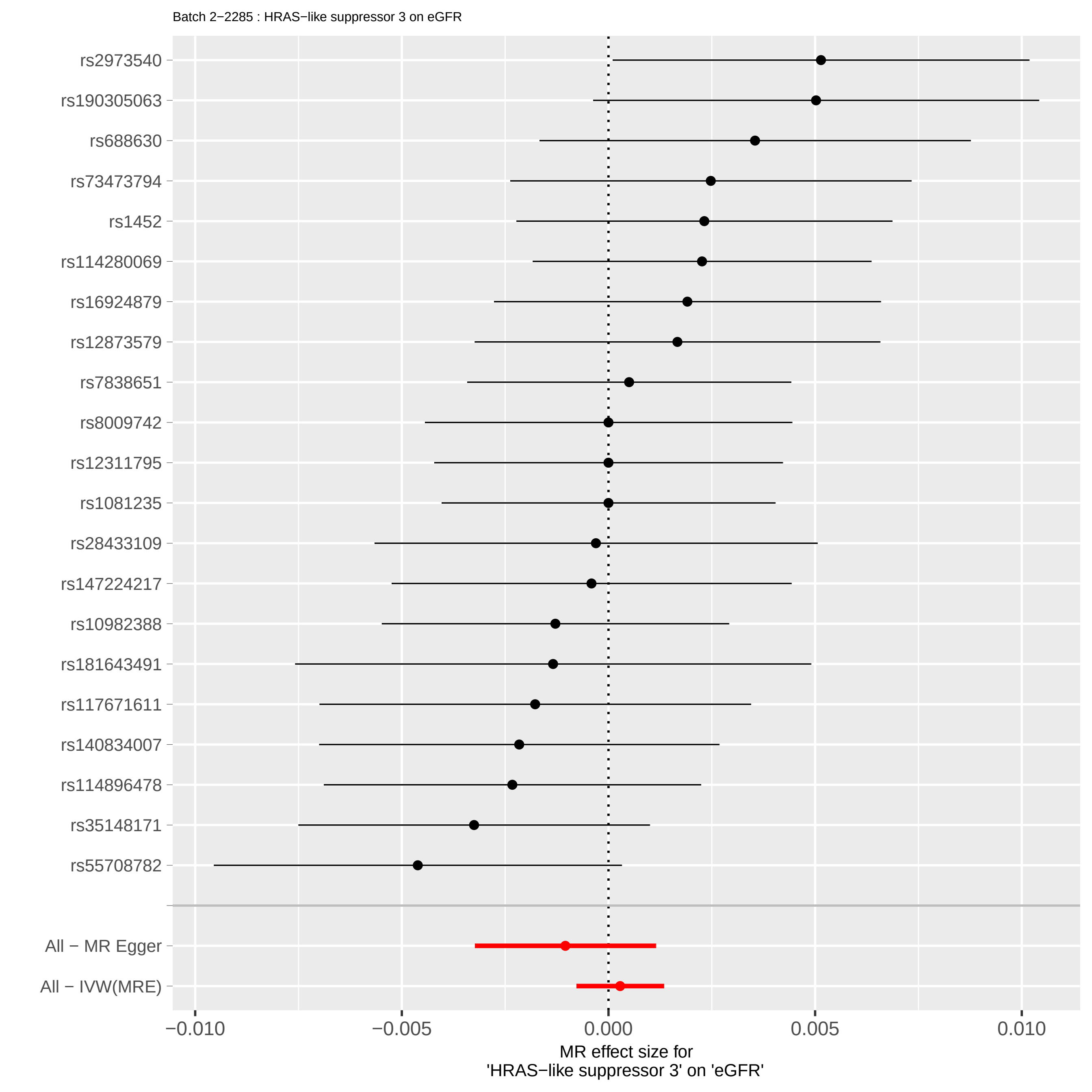
Batch 2−2285 : HRAS−like suppressor 3 on eGFR
rs2973540
rs190305063
rs688630
rs73473794
rs1452
rs114280069
rs16924879
rs12873579
rs7838651
rs8009742
rs12311795
rs1081235
rs28433109
rs147224217
rs10982388
rs181643491
rs117671611
rs140834007
rs114896478
rs35148171
rs55708782
All − MR Egger
All − IVW(MRE)
−0.010
−0.005
0.000
0.005
0.010
MR effect size for
'HRAS−like suppressor 3' on 'eGFR'

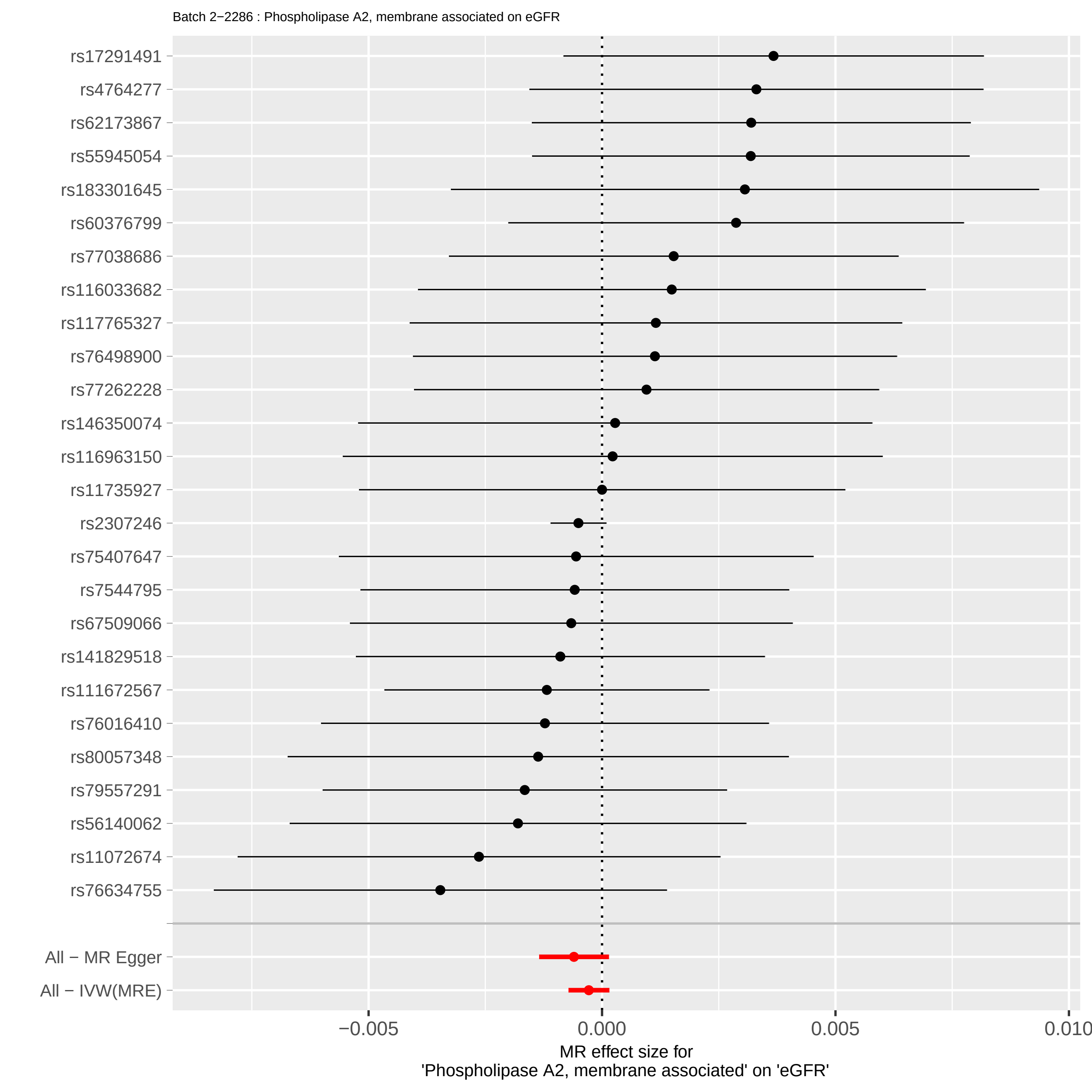
Batch 2−2286 : Phospholipase A2, membrane associated on eGFR
rs17291491
rs4764277
rs62173867
rs55945054
rs183301645
rs60376799
rs77038686
rs116033682
rs117765327
rs76498900
rs77262228
rs146350074
rs116963150
rs11735927
rs2307246
rs75407647
rs7544795
rs67509066
rs141829518
rs111672567
rs76016410
rs80057348
rs79557291
rs56140062
rs11072674
rs76634755
All − MR Egger
All − IVW(MRE)
−0.005
0.000
0.005
0.010
MR effect size for
'Phospholipase A2, membrane associated' on 'eGFR'

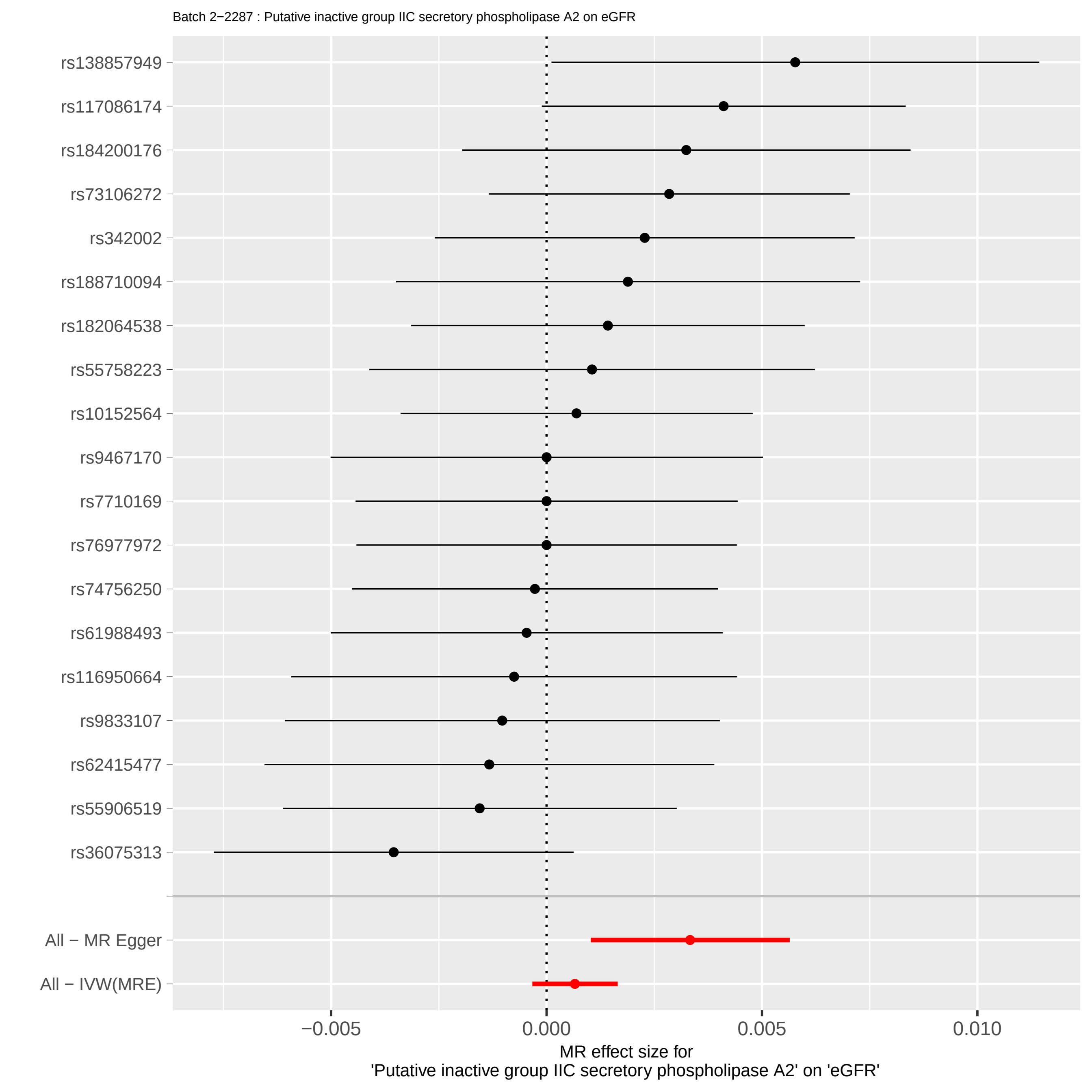
Batch 2−2287 : Putative inactive group IIC secretory phospholipase A2 on eGFR
rs138857949
rs117086174
rs184200176
rs73106272
rs342002
rs188710094
rs182064538
rs55758223
rs10152564
rs9467170
rs7710169
rs76977972
rs74756250
rs61988493
rs116950664
rs9833107
rs62415477
rs55906519
rs36075313
All − MR Egger
All − IVW(MRE)
−0.005
0.000
0.005
0.010
MR effect size for
'Putative inactive group IIC secretory phospholipase A2' on 'eGFR'

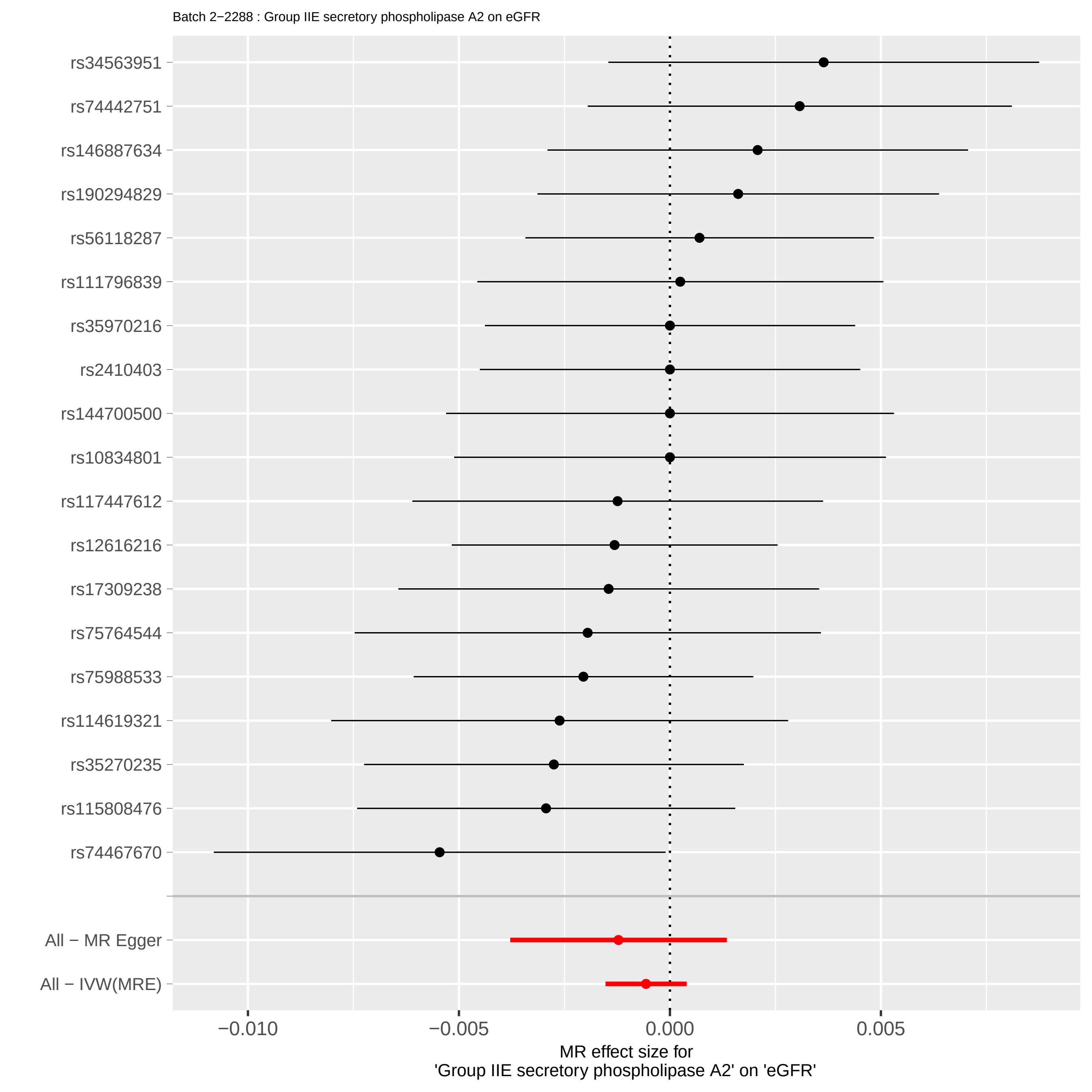
Batch 2−2288 : Group IIE secretory phospholipase A2 on eGFR
rs34563951
rs74442751
rs146887634
rs190294829
rs56118287
rs111796839
rs35970216
rs2410403
rs144700500
rs10834801
rs117447612
rs12616216
rs17309238
rs75764544
rs75988533
rs114619321
rs35270235
rs115808476
rs74467670
All − MR Egger
All − IVW(MRE)
−0.010
−0.005
0.000
0.005
MR effect size for
'Group IIE secretory phospholipase A2' on 'eGFR'

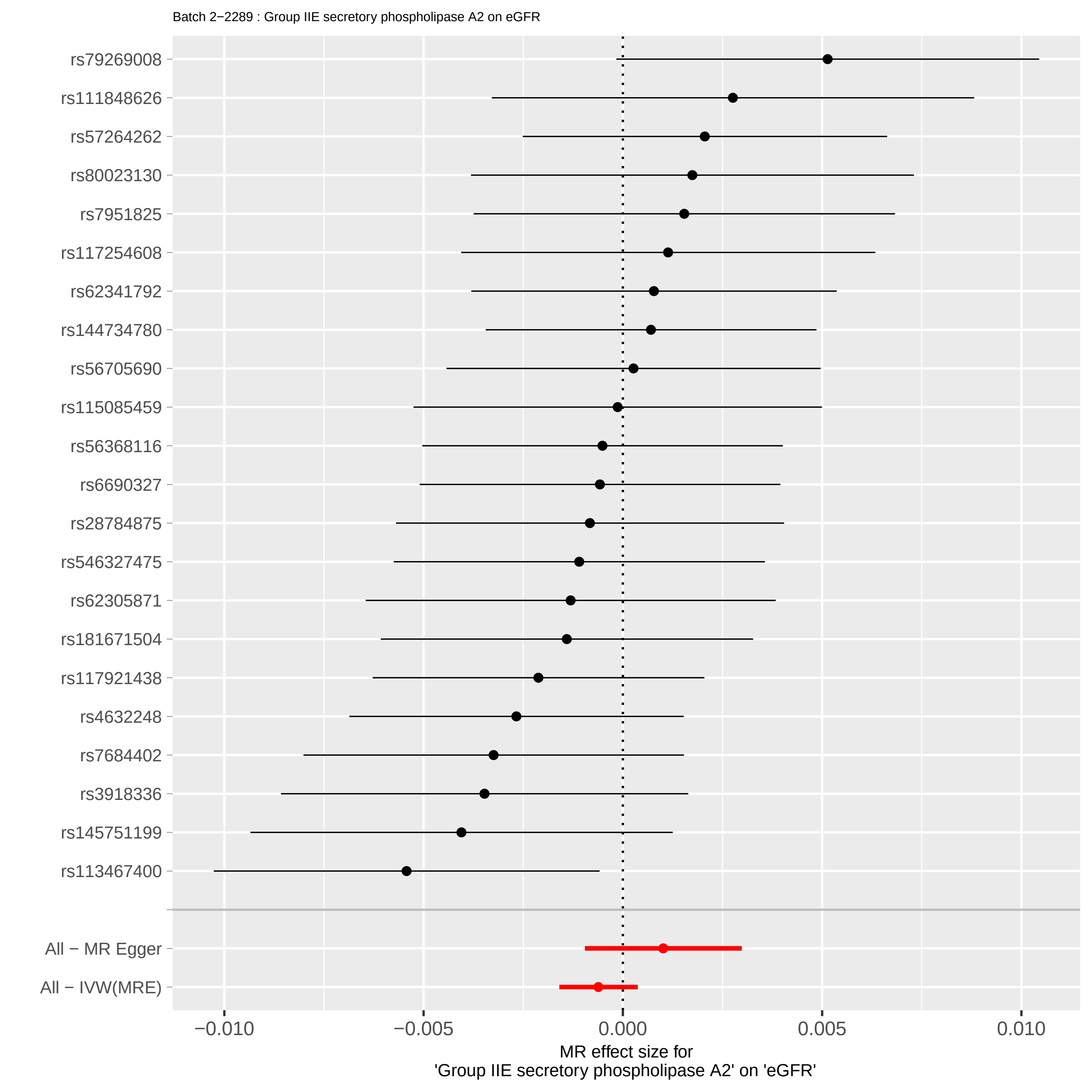
Batch 2−2289 : Group IIE secretory phospholipase A2 on eGFR
rs79269008
rs111848626
rs57264262
rs80023130
rs7951825
rs117254608
rs62341792
rs144734780
rs56705690
rs115085459
rs56368116
rs6690327
rs28784875
rs546327475
rs62305871
rs181671504
rs117921438
rs4632248
rs7684402
rs3918336
rs145751199
rs113467400
All − MR Egger
All − IVW(MRE)
−0.010
−0.005
0.000
0.005
0.010
MR effect size for
'Group IIE secretory phospholipase A2' on 'eGFR'

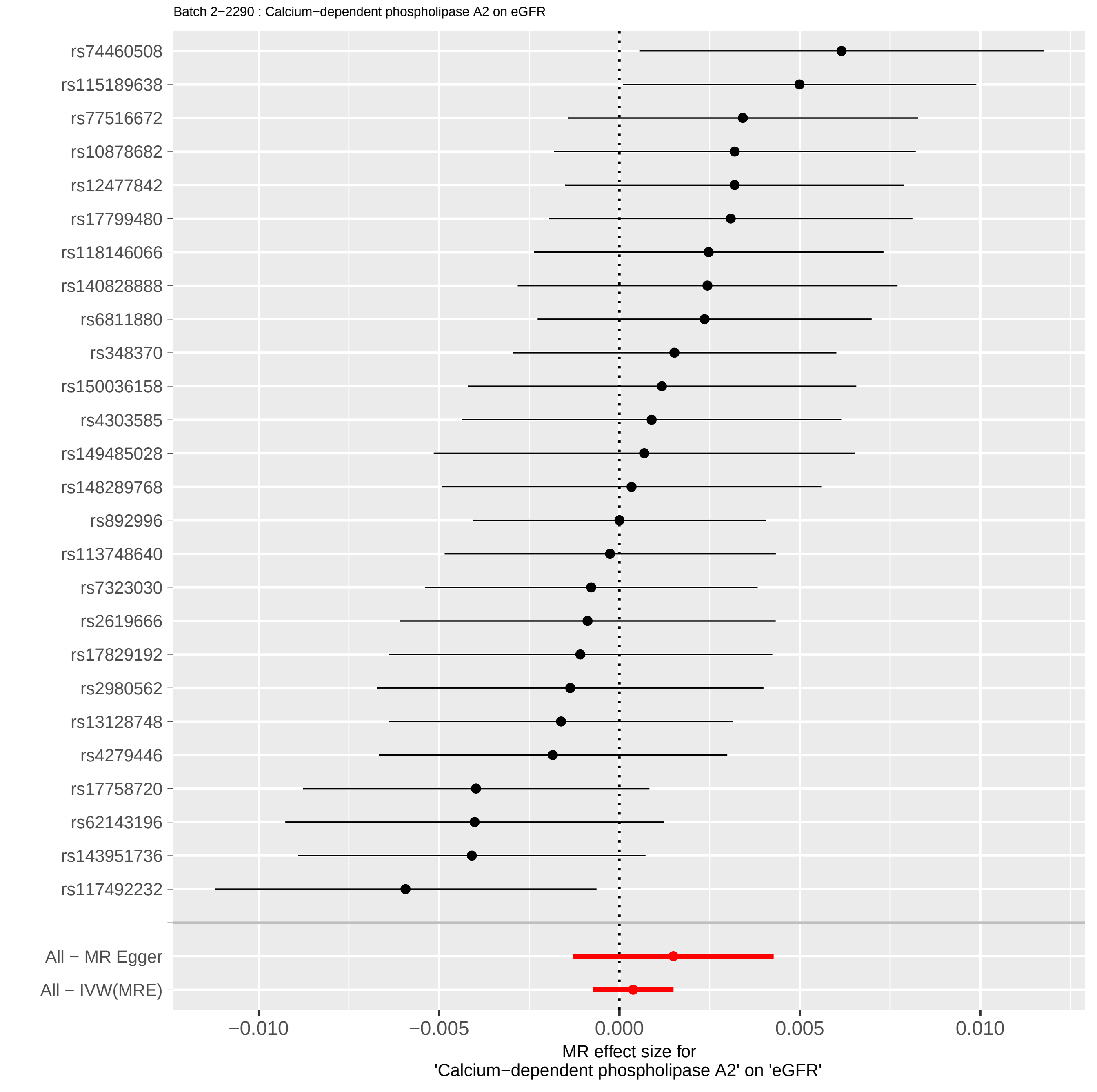

Batch 2−2290 : Calcium−dependent phospholipase A2 on eGFR
rs74460508
rs115189638
rs77516672
rs10878682
rs12477842
rs17799480
rs118146066
rs140828888
rs6811880
rs348370
rs150036158
rs4303585
rs149485028
rs148289768
rs892996
rs113748640
rs7323030
rs2619666
rs17829192
rs2980562
rs13128748
rs4279446
rs17758720
rs62143196
rs143951736
rs117492232
All − MR Egger
All − IVW(MRE)
−0.010
−0.005
0.000
0.005
0.010
MR effect size for
'Calcium−dependent phospholipase A2' on 'eGFR'

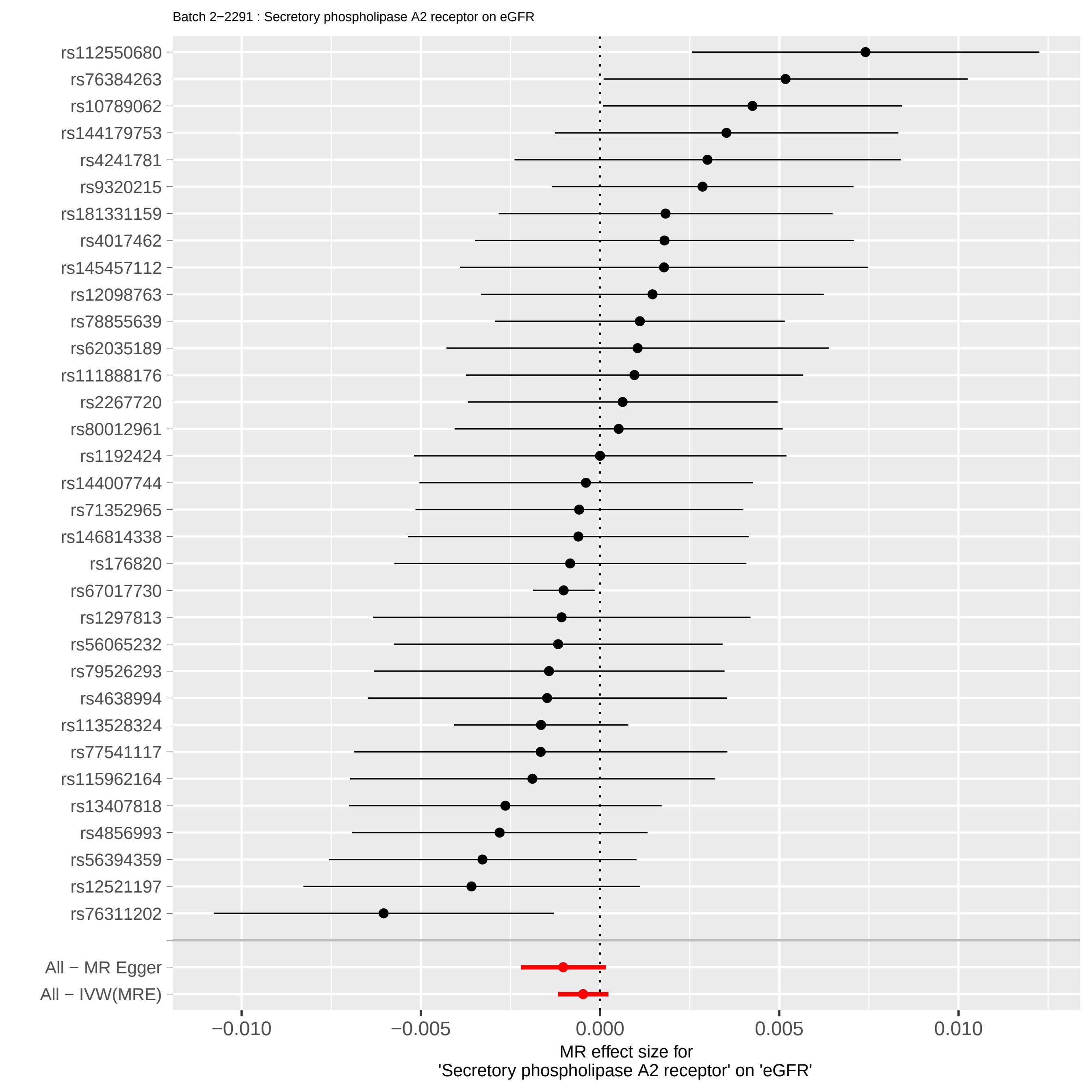

Batch 2−2291 : Secretory phospholipase A2 receptor on eGFR
rs112550680
rs76384263
rs10789062
rs144179753
rs4241781
rs9320215
rs181331159
rs4017462
rs145457112
rs12098763
rs78855639
rs62035189
rs111888176
rs2267720
rs80012961
rs1192424
rs144007744
rs71352965
rs146814338
rs176820
rs67017730
rs1297813
rs56065232
rs79526293
rs4638994
rs113528324
rs77541117
rs115962164
rs13407818
rs4856993
rs56394359
rs12521197
rs76311202
All − MR Egger
All − IVW(MRE)
−0.010
−0.005
0.000
0.005
0.010
MR effect size for
'Secretory phospholipase A2 receptor' on 'eGFR'

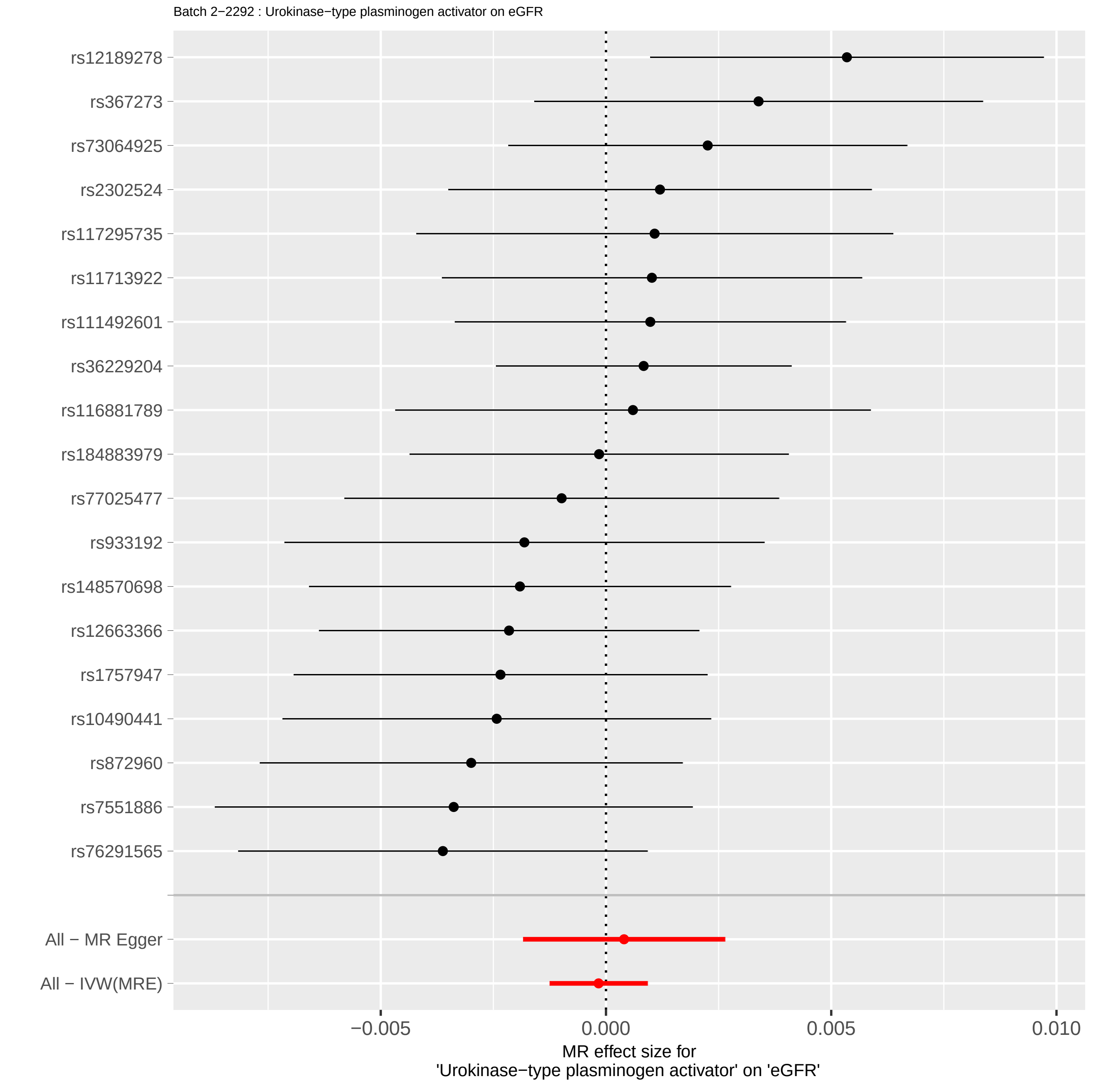

Batch 2−2292 : Urokinase−type plasminogen activator on eGFR
rs12189278
rs367273
rs73064925
rs2302524
rs117295735
rs11713922
rs111492601
rs36229204
rs116881789
rs184883979
rs77025477
rs933192
rs148570698
rs12663366
rs1757947
rs10490441
rs872960
rs7551886
rs76291565
All − MR Egger
All − IVW(MRE)
−0.005
0.000
0.005
0.010
MR effect size for
'Urokinase−type plasminogen activator' on 'eGFR'

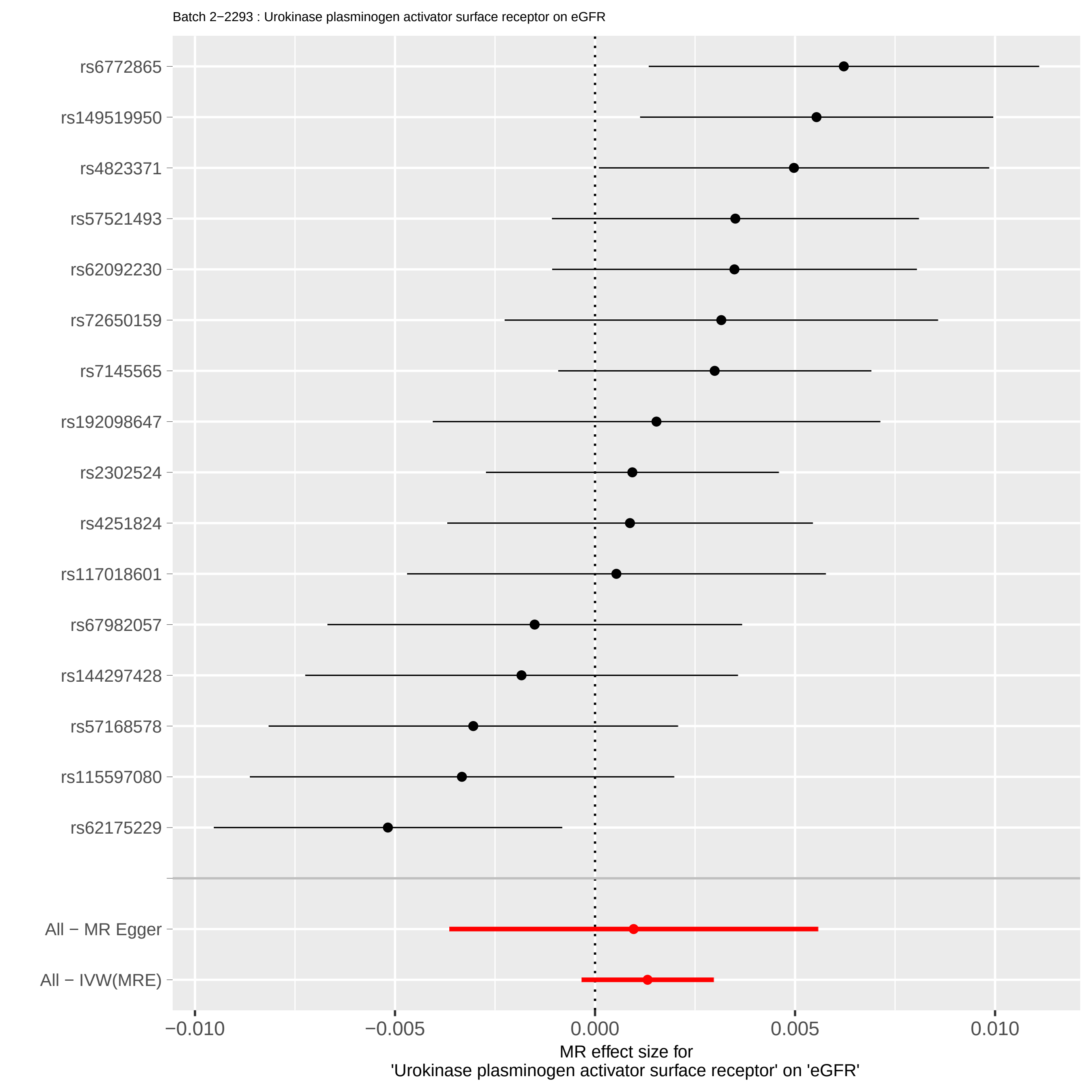
Batch 2−2293 : Urokinase plasminogen activator surface receptor on eGFR
rs6772865
rs149519950
rs4823371
rs57521493
rs62092230
rs72650159
rs7145565
rs192098647
rs2302524
rs4251824
rs117018601
rs67982057
rs144297428
rs57168578
rs115597080
rs62175229
All − MR Egger
All − IVW(MRE)
−0.010
−0.005
0.000
0.005
0.010
MR effect size for
'Urokinase plasminogen activator surface receptor' on 'eGFR'

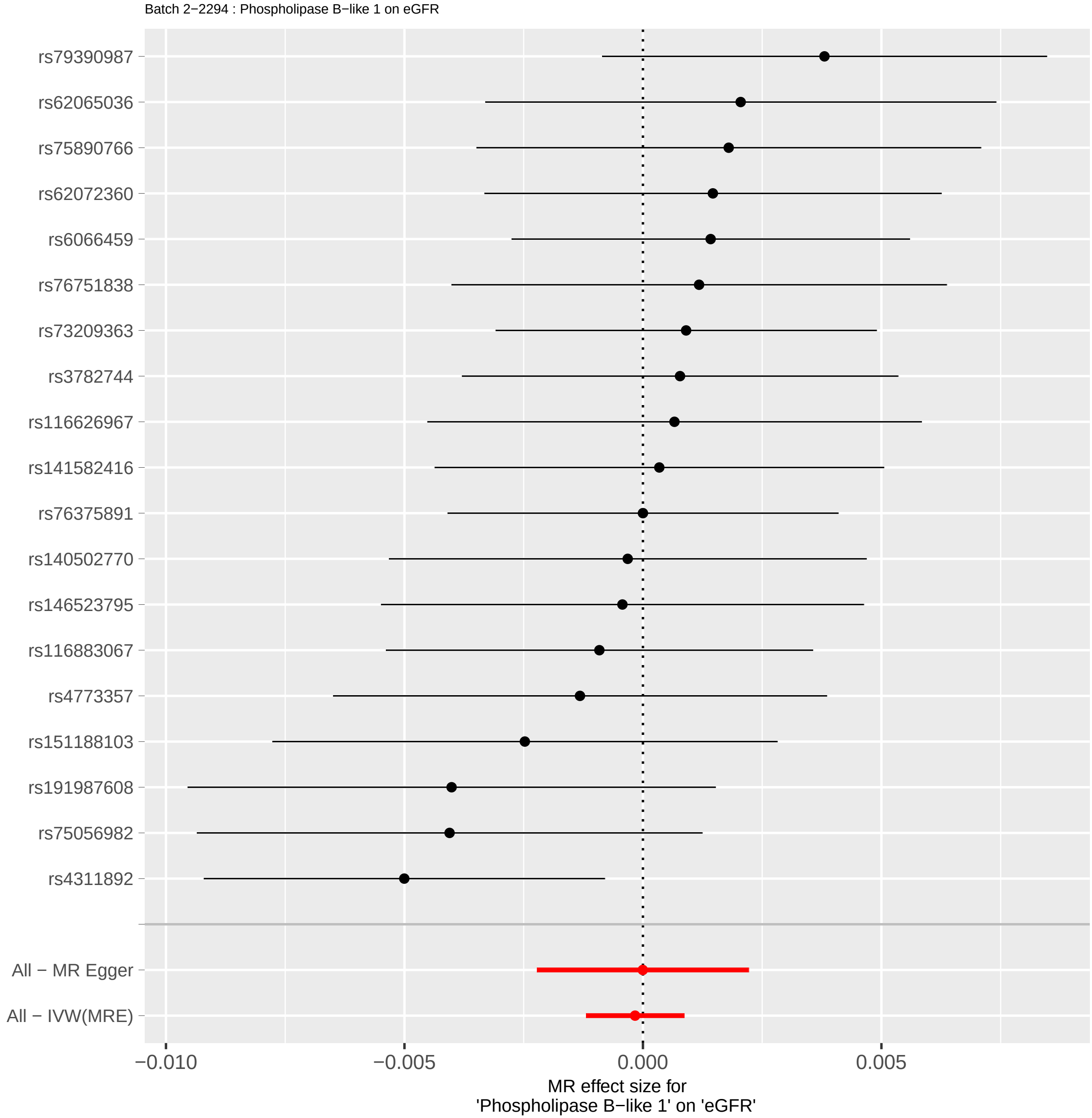
Batch 2−2294 : Phospholipase B−like 1 on eGFR
rs79390987
rs62065036
rs75890766
rs62072360
rs6066459
rs76751838
rs73209363
rs3782744
rs116626967
rs141582416
rs76375891
rs140502770
rs146523795
rs116883067
rs4773357
rs151188103
rs191987608
rs75056982
rs4311892
All − MR Egger
All − IVW(MRE)
−0.010
−0.005
0.000
0.005
MR effect size for
'Phospholipase B−like 1' on 'eGFR'

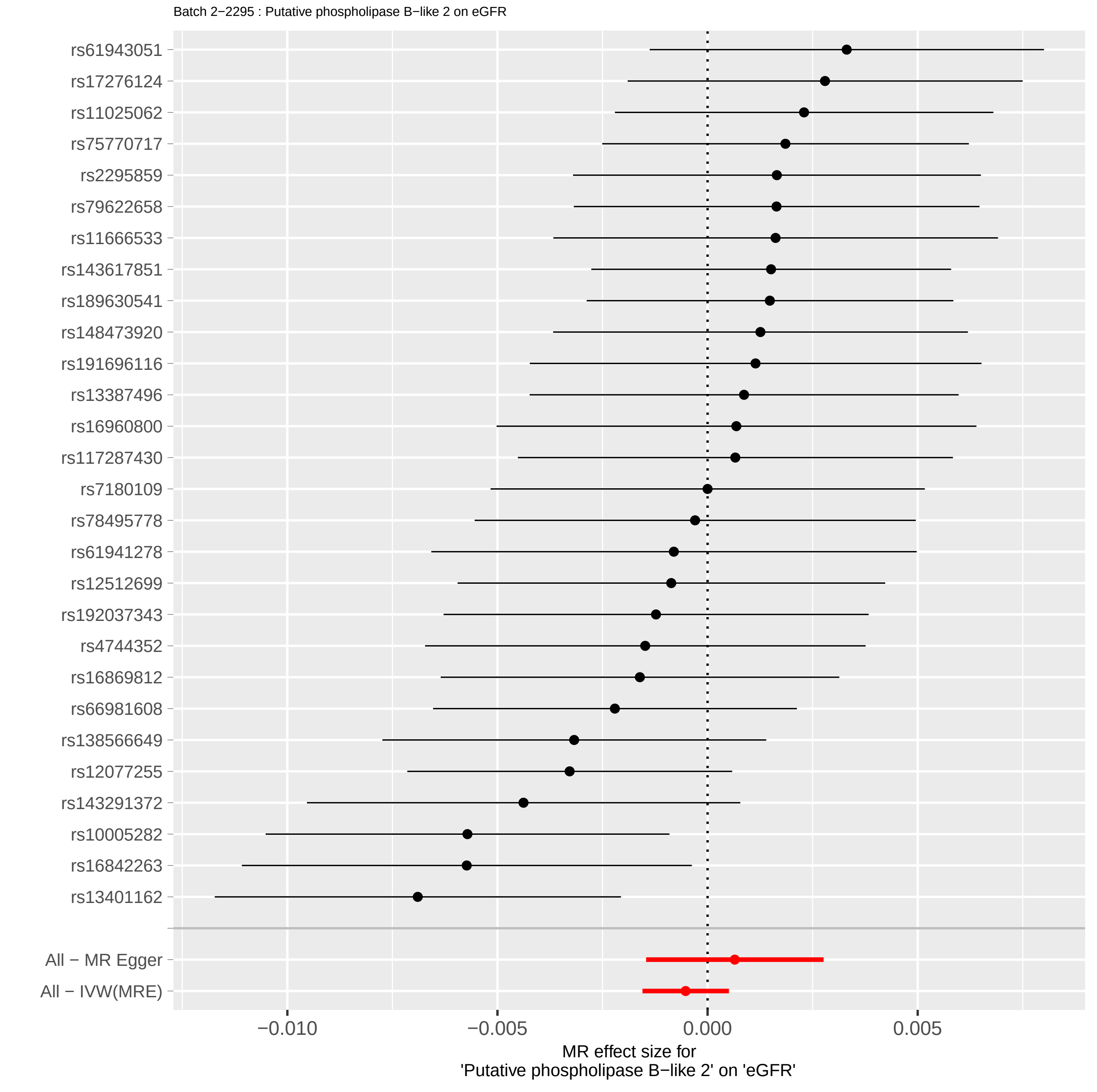

Batch 2−2295 : Putative phospholipase B−like 2 on eGFR
rs61943051
rs17276124
rs11025062
rs75770717
rs2295859
rs79622658
rs11666533
rs143617851
rs189630541
rs148473920
rs191696116
rs13387496
rs16960800
rs117287430
rs7180109
rs78495778
rs61941278
rs12512699
rs192037343
rs4744352
rs16869812
rs66981608
rs138566649
rs12077255
rs143291372
rs10005282
rs16842263
rs13401162
All − MR Egger
All − IVW(MRE)
−0.010
−0.005
0.000
0.005
MR effect size for
'Putative phospholipase B−like 2' on 'eGFR'

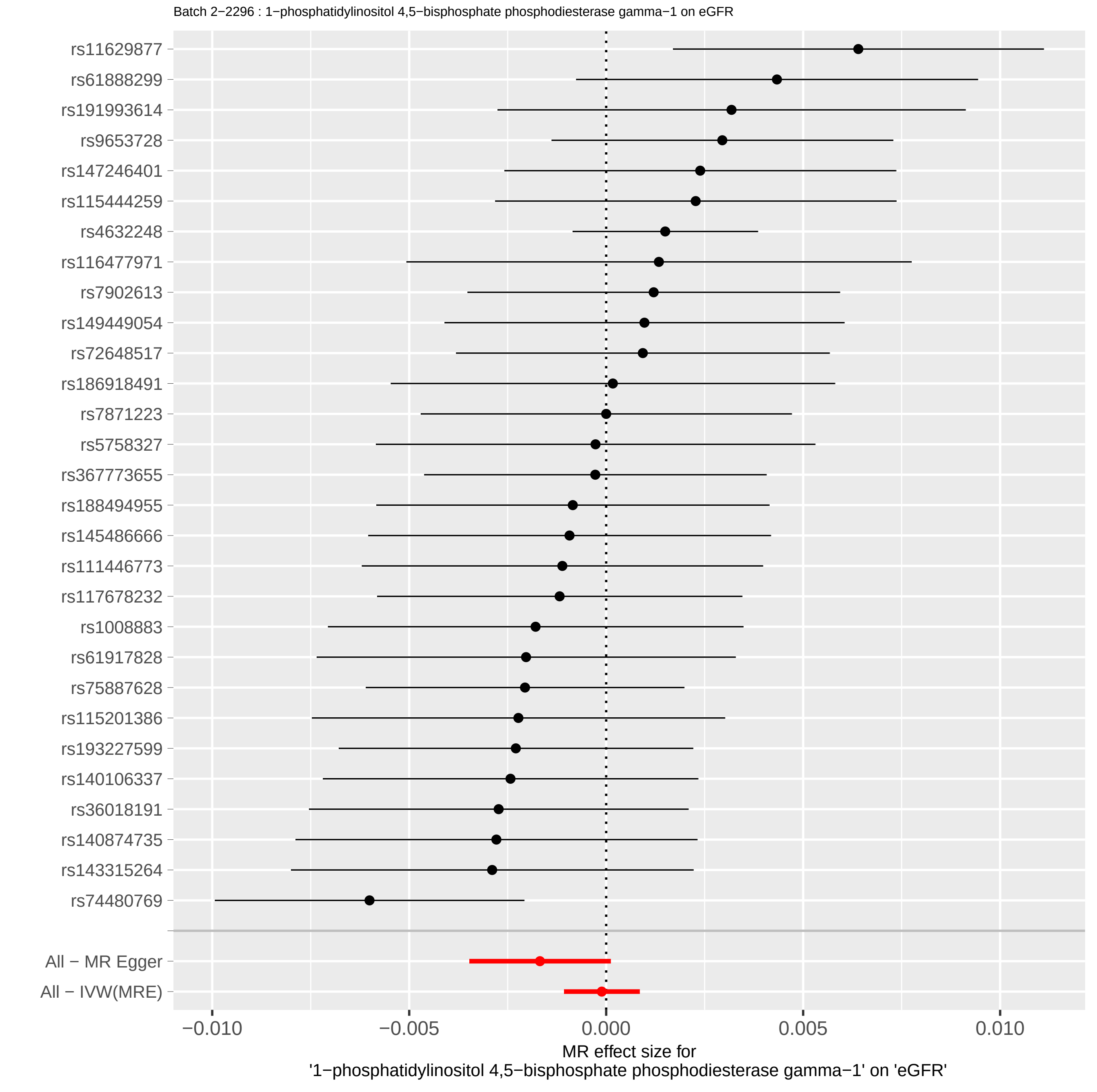
Batch 2−2296 : 1−phosphatidylinositol 4,5−bisphosphate phosphodiesterase gamma−1 on eGFR
rs11629877
rs61888299
rs191993614
rs9653728
rs147246401
rs115444259
rs4632248
rs116477971
rs7902613
rs149449054
rs72648517
rs186918491
rs7871223
rs5758327
rs367773655
rs188494955
rs145486666
rs111446773
rs117678232
rs1008883
rs61917828
rs75887628
rs115201386
rs193227599
rs140106337
rs36018191
rs140874735
rs143315264
rs74480769
All − MR Egger
All − IVW(MRE)
−0.010
−0.005
0.000
0.005
0.010
MR effect size for
'1−phosphatidylinositol 4,5−bisphosphate phosphodiesterase gamma−1' on 'eGFR'

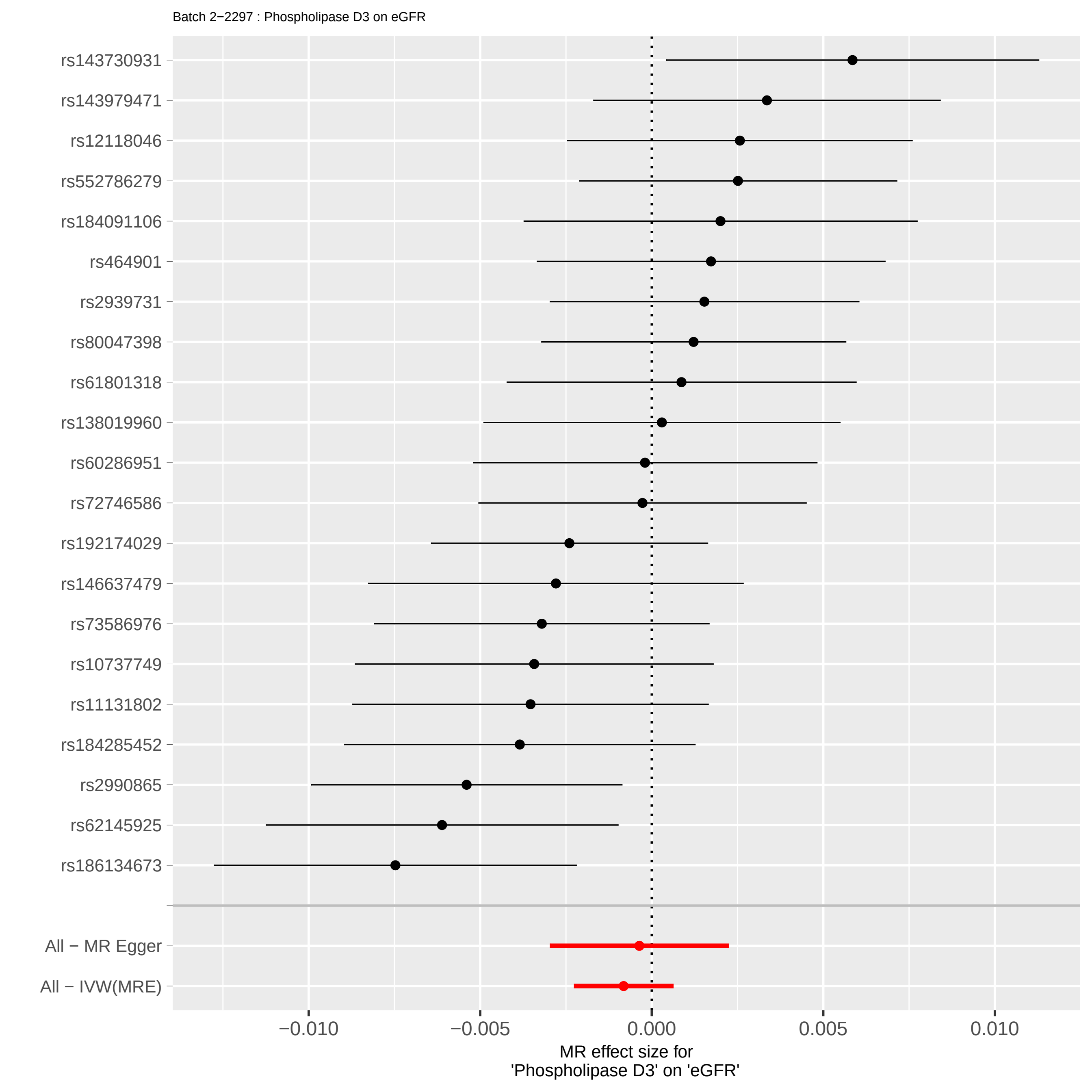

Batch 2−2297 : Phospholipase D3 on eGFR
rs143730931
rs143979471
rs12118046
rs552786279
rs184091106
rs464901
rs2939731
rs80047398
rs61801318
rs138019960
rs60286951
rs72746586
rs192174029
rs146637479
rs73586976
rs10737749
rs11131802
rs184285452
rs2990865
rs62145925
rs186134673
All − MR Egger
All − IVW(MRE)
−0.010
−0.005
0.000
0.005
0.010
MR effect size for
'Phospholipase D3' on 'eGFR'

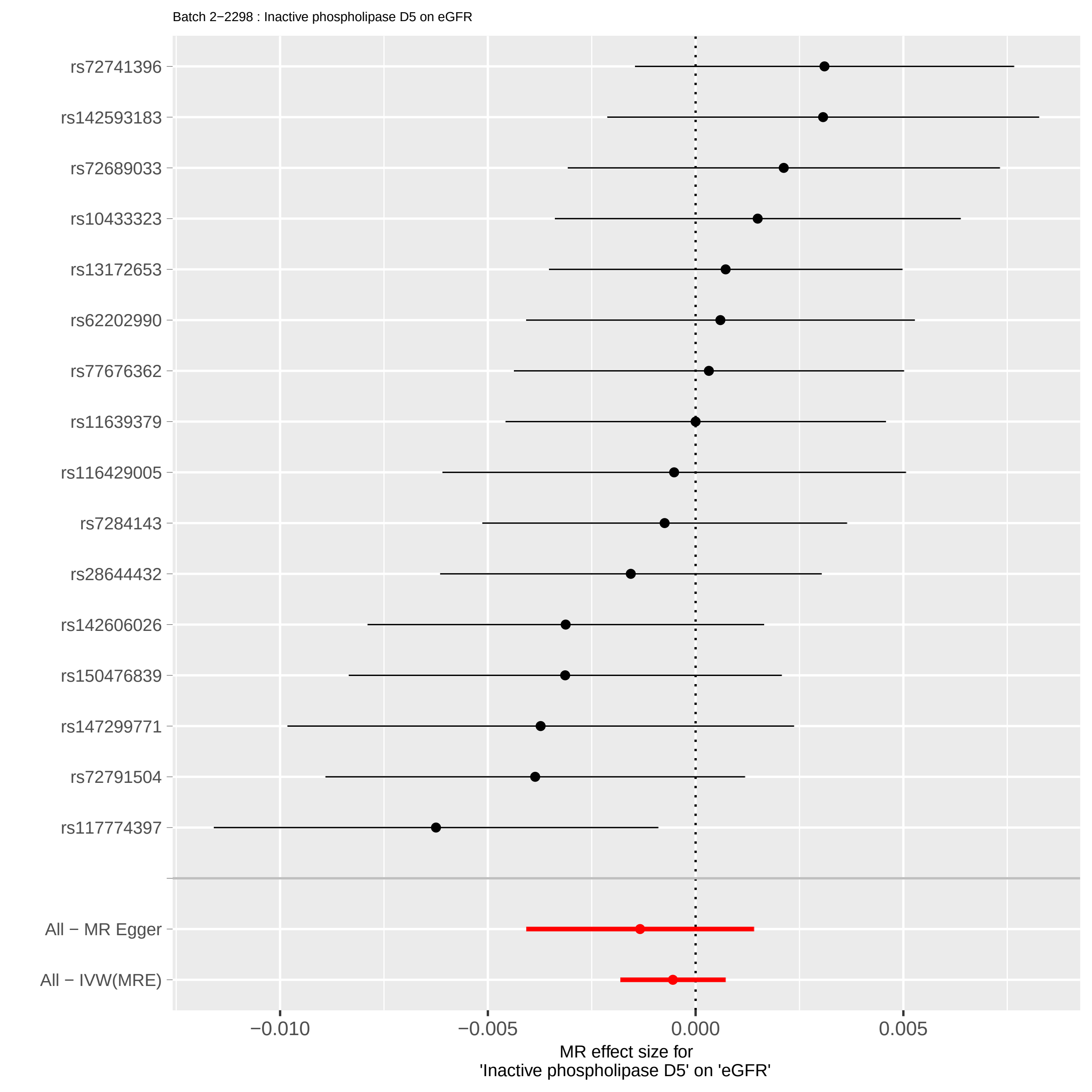

Batch 2−2298 : Inactive phospholipase D5 on eGFR
rs72741396
rs142593183
rs72689033
rs10433323
rs13172653
rs62202990
rs77676362
rs11639379
rs116429005
rs7284143
rs28644432
rs142606026
rs150476839
rs147299771
rs72791504
rs117774397
All − MR Egger
All − IVW(MRE)
−0.010
−0.005
0.000
0.005
MR effect size for
'Inactive phospholipase D5' on 'eGFR'

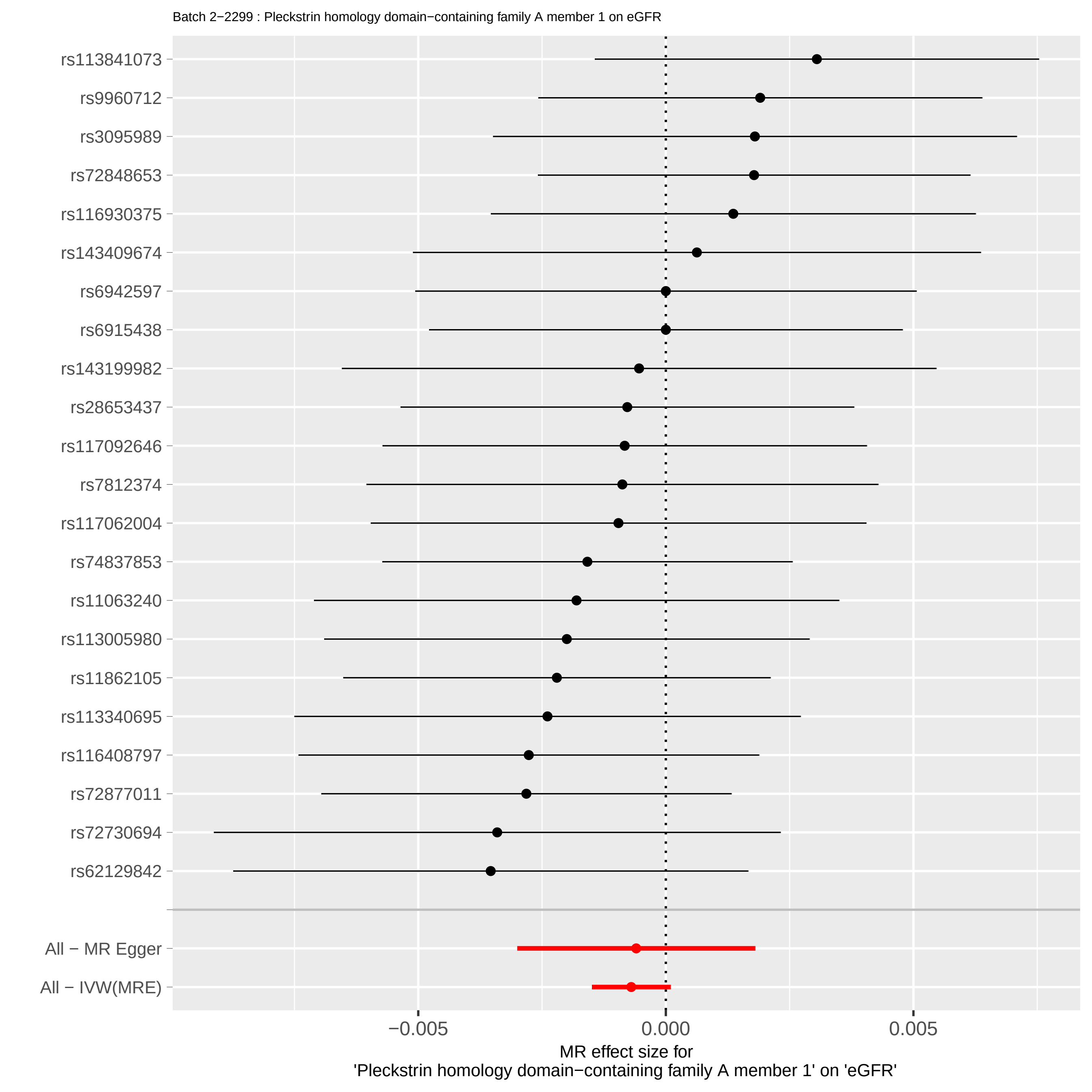

Batch 2−2299 : Pleckstrin homology domain−containing family A member 1 on eGFR
rs113841073
rs9960712
rs3095989
rs72848653
rs116930375
rs143409674
rs6942597
rs6915438
rs143199982
rs28653437
rs117092646
rs7812374
rs117062004
rs74837853
rs11063240
rs113005980
rs11862105
rs113340695
rs116408797
rs72877011
rs72730694
rs62129842
All − MR Egger
All − IVW(MRE)
−0.005
0.000
0.005
MR effect size for
'Pleckstrin homology domain−containing family A member 1' on 'eGFR'

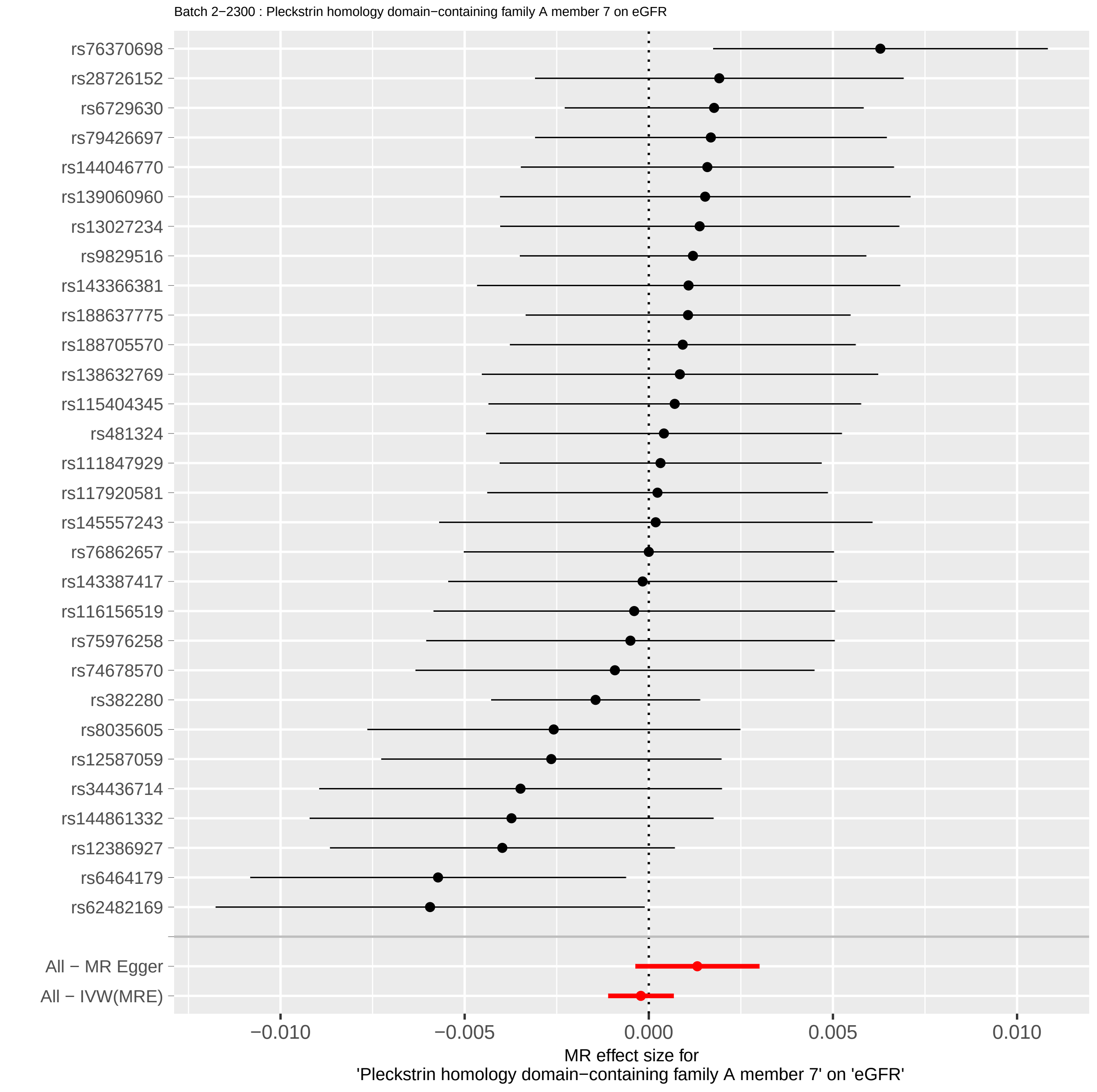

Batch 2−2300 : Pleckstrin homology domain−containing family A member 7 on eGFR
rs76370698
rs28726152
rs6729630
rs79426697
rs144046770
rs139060960
rs13027234
rs9829516
rs143366381
rs188637775
rs188705570
rs138632769
rs115404345
rs481324
rs111847929
rs117920581
rs145557243
rs76862657
rs143387417
rs116156519
rs75976258
rs74678570
rs382280
rs8035605
rs12587059
rs34436714
rs144861332
rs12386927
rs6464179
rs62482169
All − MR Egger
All − IVW(MRE)
−0.010
−0.005
0.000
0.005
0.010
MR effect size for
'Pleckstrin homology domain−containing family A member 7' on 'eGFR'

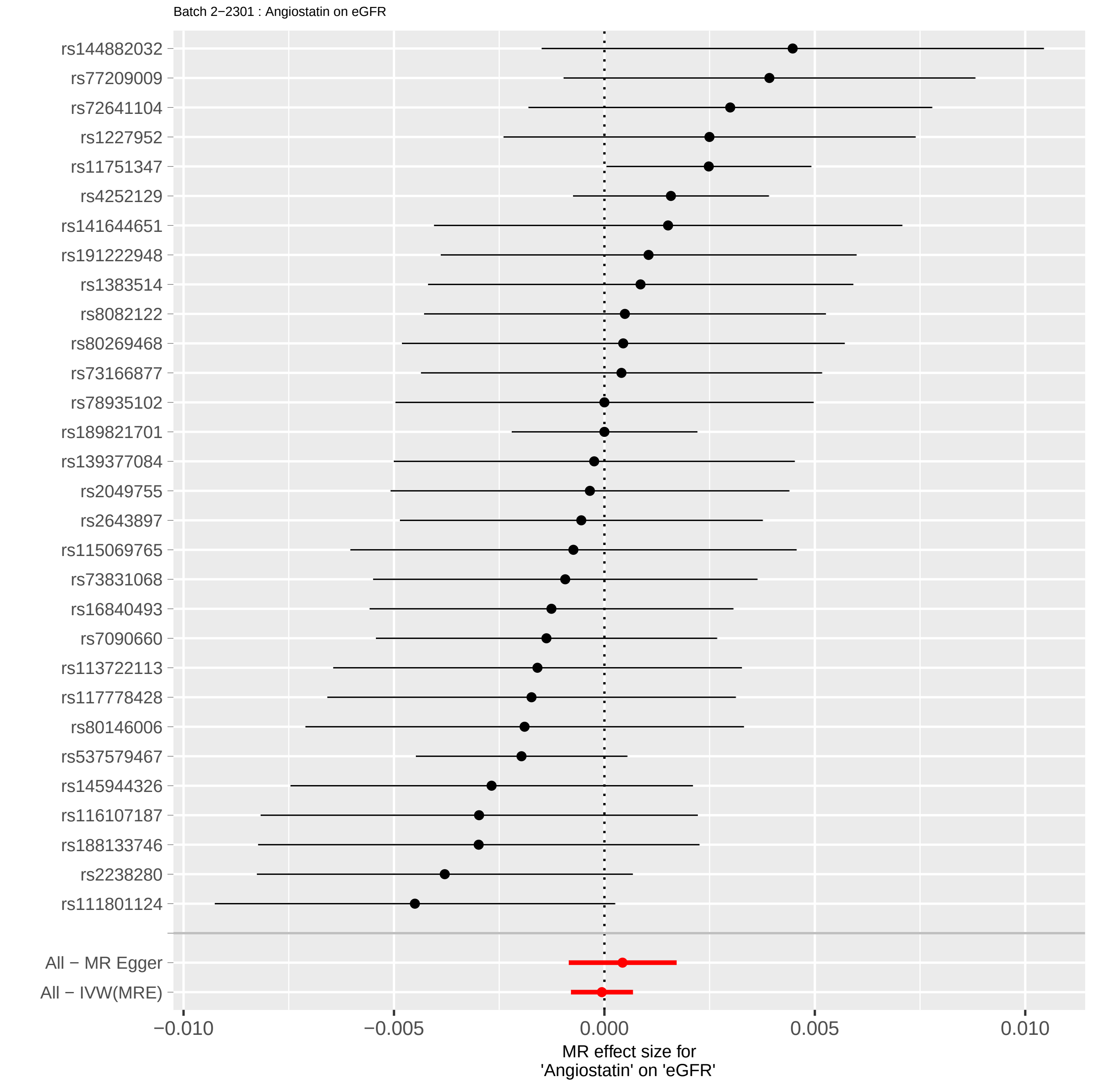

Batch 2−2301 : Angiostatin on eGFR
rs144882032
rs77209009
rs72641104
rs1227952
rs11751347
rs4252129
rs141644651
rs191222948
rs1383514
rs8082122
rs80269468
rs73166877
rs78935102
rs189821701
rs139377084
rs2049755
rs2643897
rs115069765
rs73831068
rs16840493
rs7090660
rs113722113
rs117778428
rs80146006
rs537579467
rs145944326
rs116107187
rs188133746
rs2238280
rs111801124
All − MR Egger
All − IVW(MRE)
−0.010
−0.005
0.000
0.005
0.010
MR effect size for
'Angiostatin' on 'eGFR'

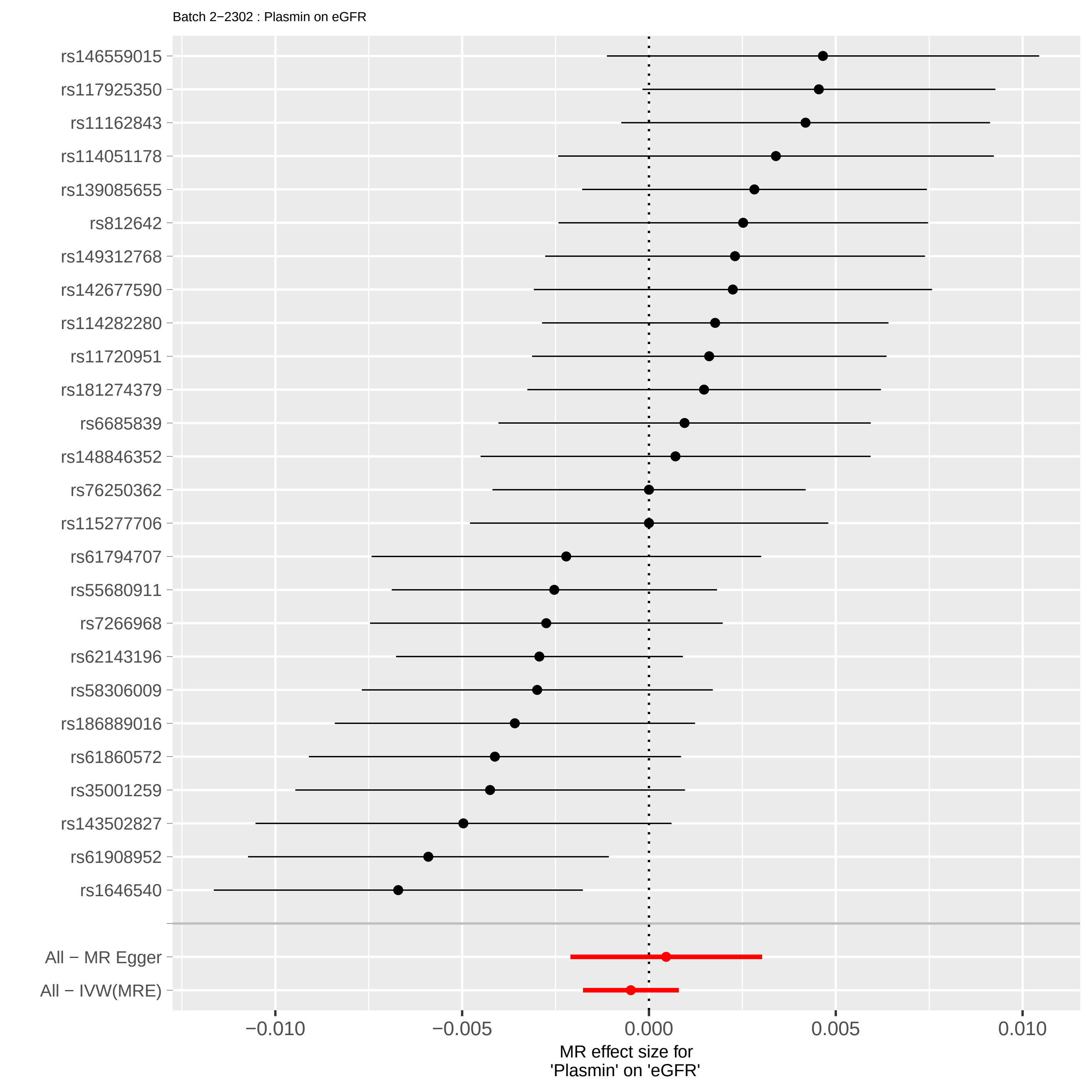

Batch 2−2302 : Plasmin on eGFR
rs146559015
rs117925350
rs11162843
rs114051178
rs139085655
rs812642
rs149312768
rs142677590
rs114282280
rs11720951
rs181274379
rs6685839
rs148846352
rs76250362
rs115277706
rs61794707
rs55680911
rs7266968
rs62143196
rs58306009
rs186889016
rs61860572
rs35001259
rs143502827
rs61908952
rs1646540
All − MR Egger
All − IVW(MRE)
−0.010
−0.005
0.000
0.005
0.010
MR effect size for
'Plasmin' on 'eGFR'

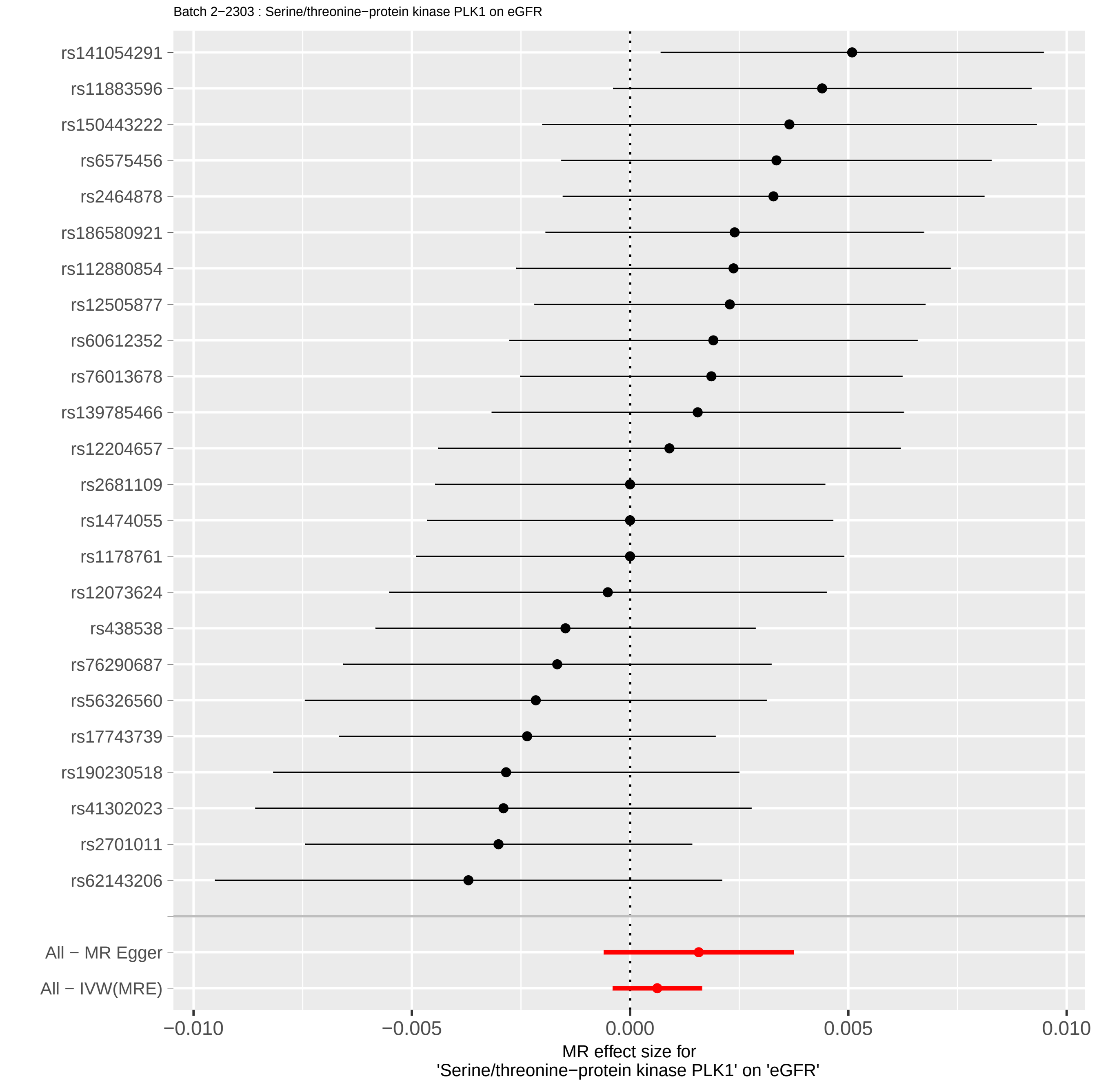

Batch 2−2303 : Serine/threonine−protein kinase PLK1 on eGFR
rs141054291
rs11883596
rs150443222
rs6575456
rs2464878
rs186580921
rs112880854
rs12505877
rs60612352
rs76013678
rs139785466
rs12204657
rs2681109
rs1474055
rs1178761
rs12073624
rs438538
rs76290687
rs56326560
rs17743739
rs190230518
rs41302023
rs2701011
rs62143206
All − MR Egger
All − IVW(MRE)
−0.010
−0.005
0.000
0.005
0.010
MR effect size for
'Serine/threonine−protein kinase PLK1' on 'eGFR'

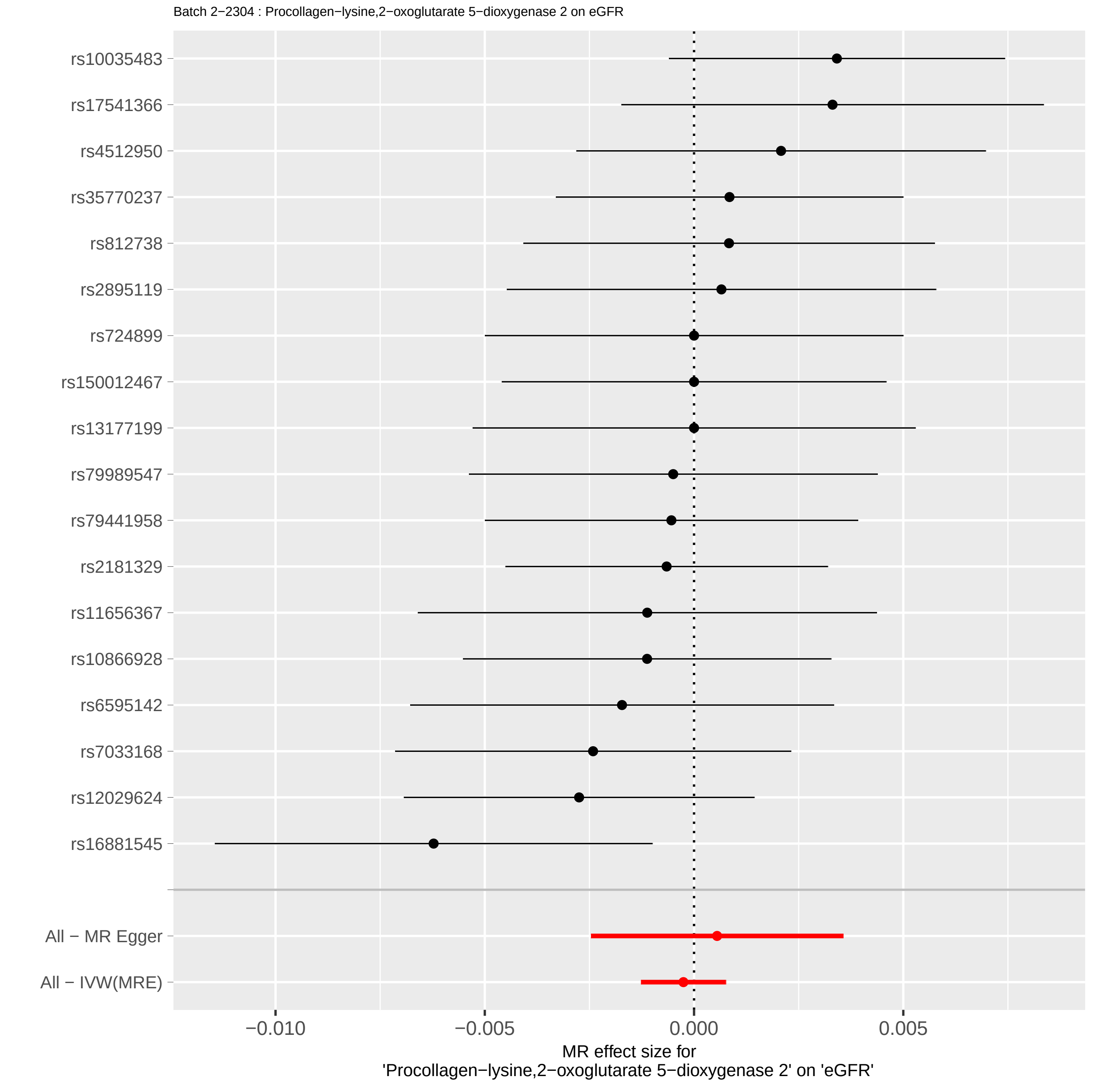

Batch 2−2304 : Procollagen−lysine,2−oxoglutarate 5−dioxygenase 2 on eGFR
rs10035483
rs17541366
rs4512950
rs35770237
rs812738
rs2895119
rs724899
rs150012467
rs13177199
rs79989547
rs79441958
rs2181329
rs11656367
rs10866928
rs6595142
rs7033168
rs12029624
rs16881545
All − MR Egger
All − IVW(MRE)
−0.010
−0.005
0.000
0.005
MR effect size for
'Procollagen−lysine,2−oxoglutarate 5−dioxygenase 2' on 'eGFR'

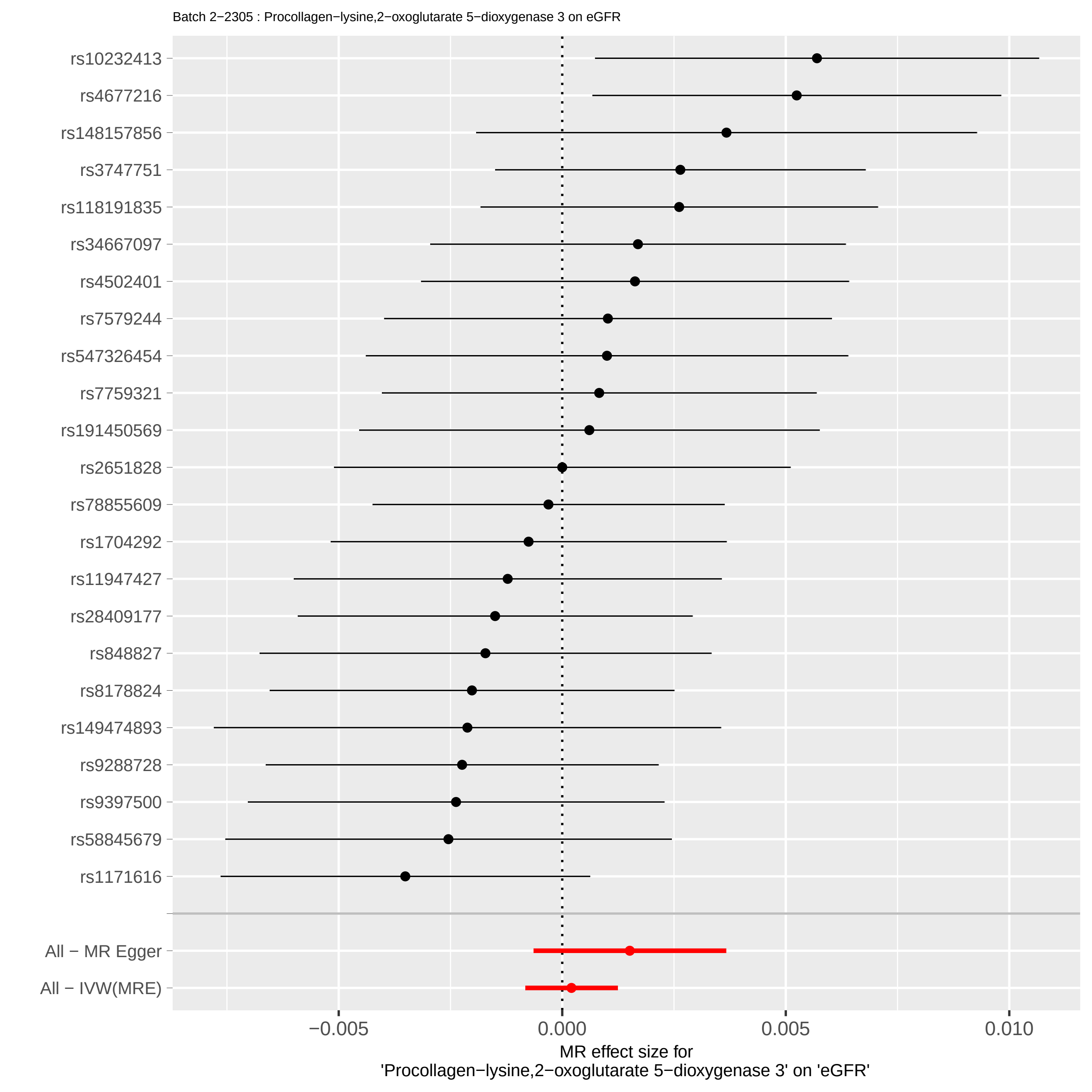

Batch 2−2305 : Procollagen−lysine,2−oxoglutarate 5−dioxygenase 3 on eGFR
rs10232413
rs4677216
rs148157856
rs3747751
rs118191835
rs34667097
rs4502401
rs7579244
rs547326454
rs7759321
rs191450569
rs2651828
rs78855609
rs1704292
rs11947427
rs28409177
rs848827
rs8178824
rs149474893
rs9288728
rs9397500
rs58845679
rs1171616
All − MR Egger
All − IVW(MRE)
−0.005
0.000
0.005
0.010
MR effect size for
'Procollagen−lysine,2−oxoglutarate 5−dioxygenase 3' on 'eGFR'

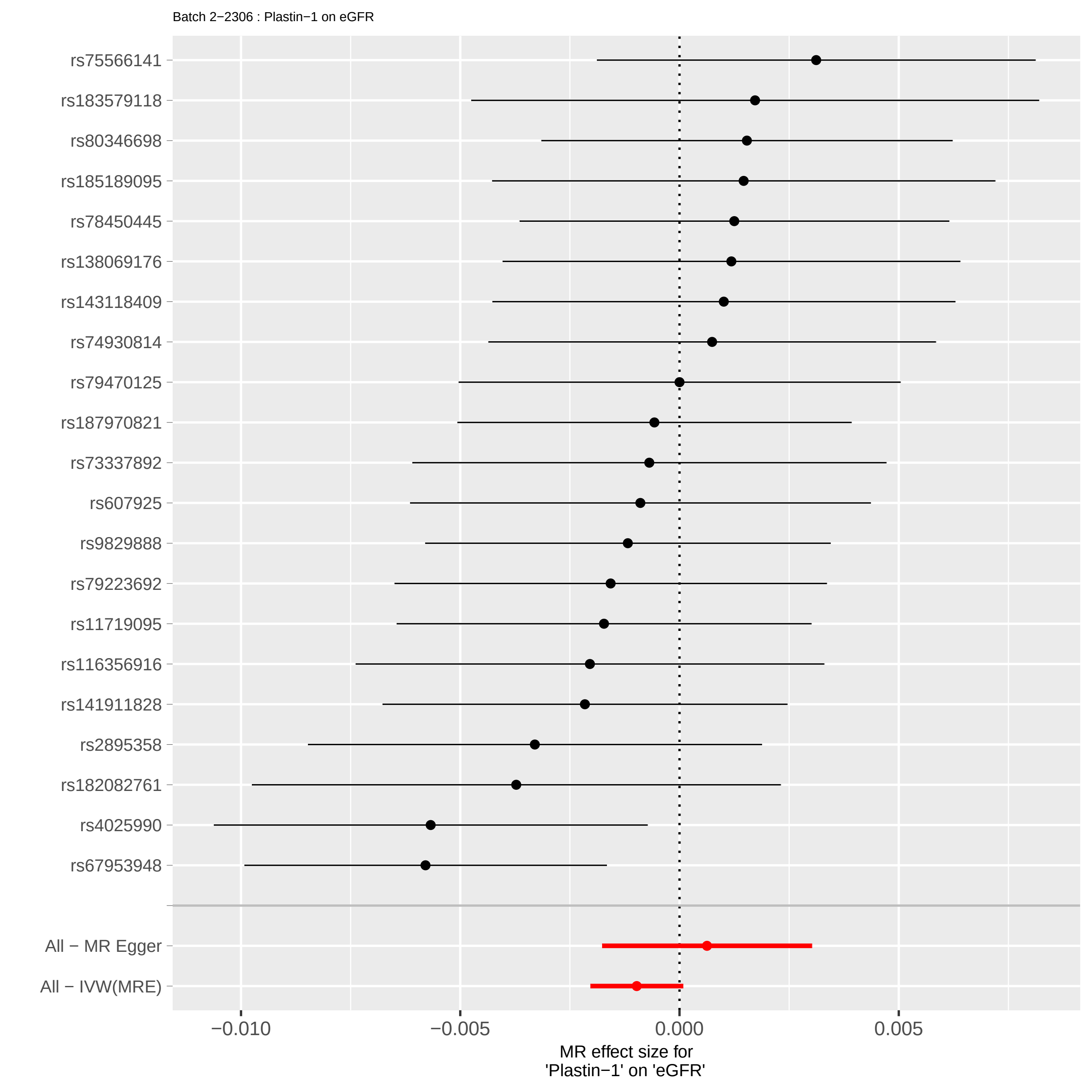
Batch 2−2306 : Plastin−1 on eGFR
rs75566141
rs183579118
rs80346698
rs185189095
rs78450445
rs138069176
rs143118409
rs74930814
rs79470125
rs187970821
rs73337892
rs607925
rs9829888
rs79223692
rs11719095
rs116356916
rs141911828
rs2895358
rs182082761
rs4025990
rs67953948
All − MR Egger
All − IVW(MRE)
−0.010
−0.005
0.000
0.005
MR effect size for
'Plastin−1' on 'eGFR'

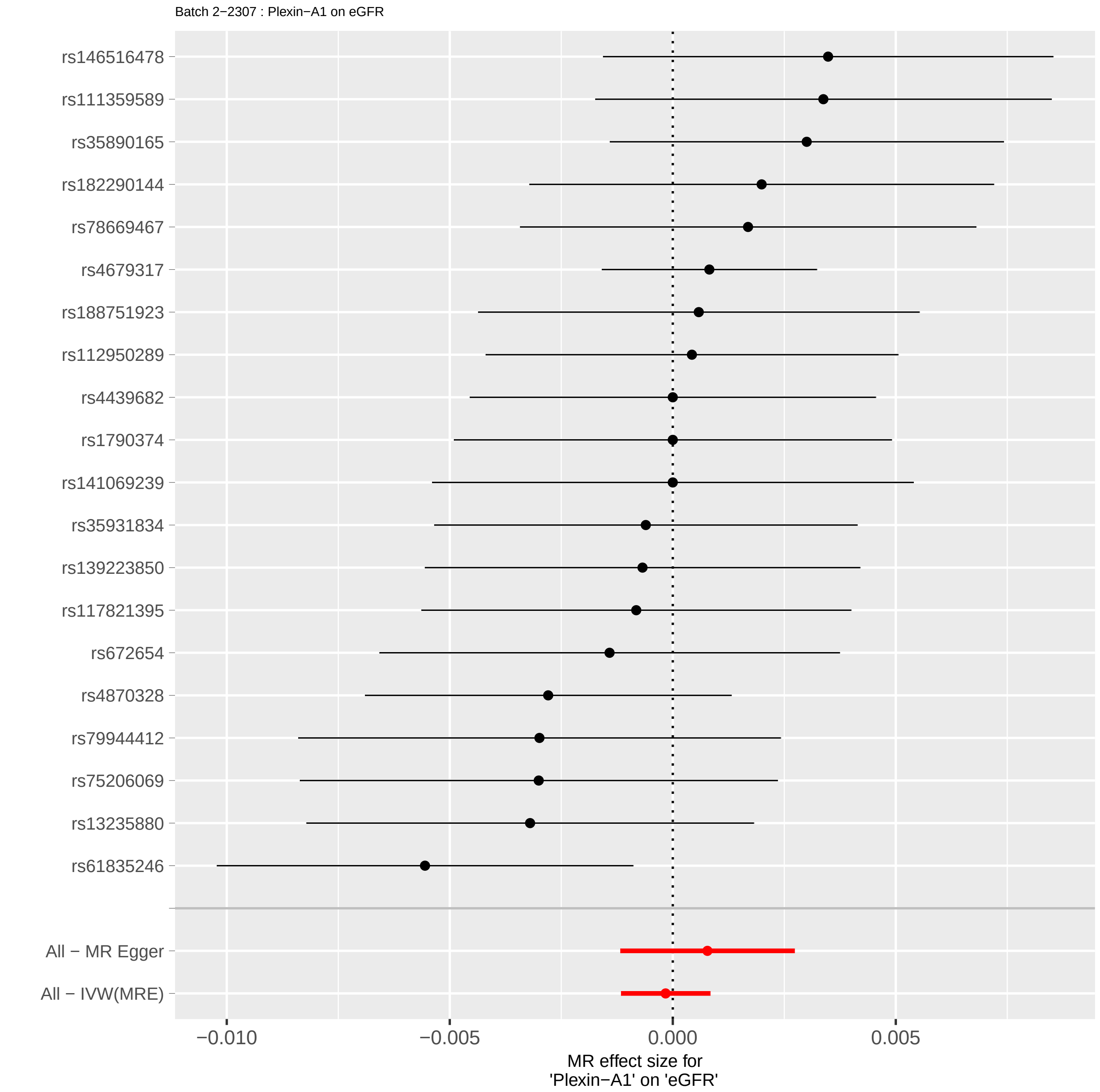

Batch 2−2307 : Plexin−A1 on eGFR
rs146516478
rs111359589
rs35890165
rs182290144
rs78669467
rs4679317
rs188751923
rs112950289
rs4439682
rs1790374
rs141069239
rs35931834
rs139223850
rs117821395
rs672654
rs4870328
rs79944412
rs75206069
rs13235880
rs61835246
All − MR Egger
All − IVW(MRE)
−0.010
−0.005
0.000
0.005
MR effect size for
'Plexin−A1' on 'eGFR'

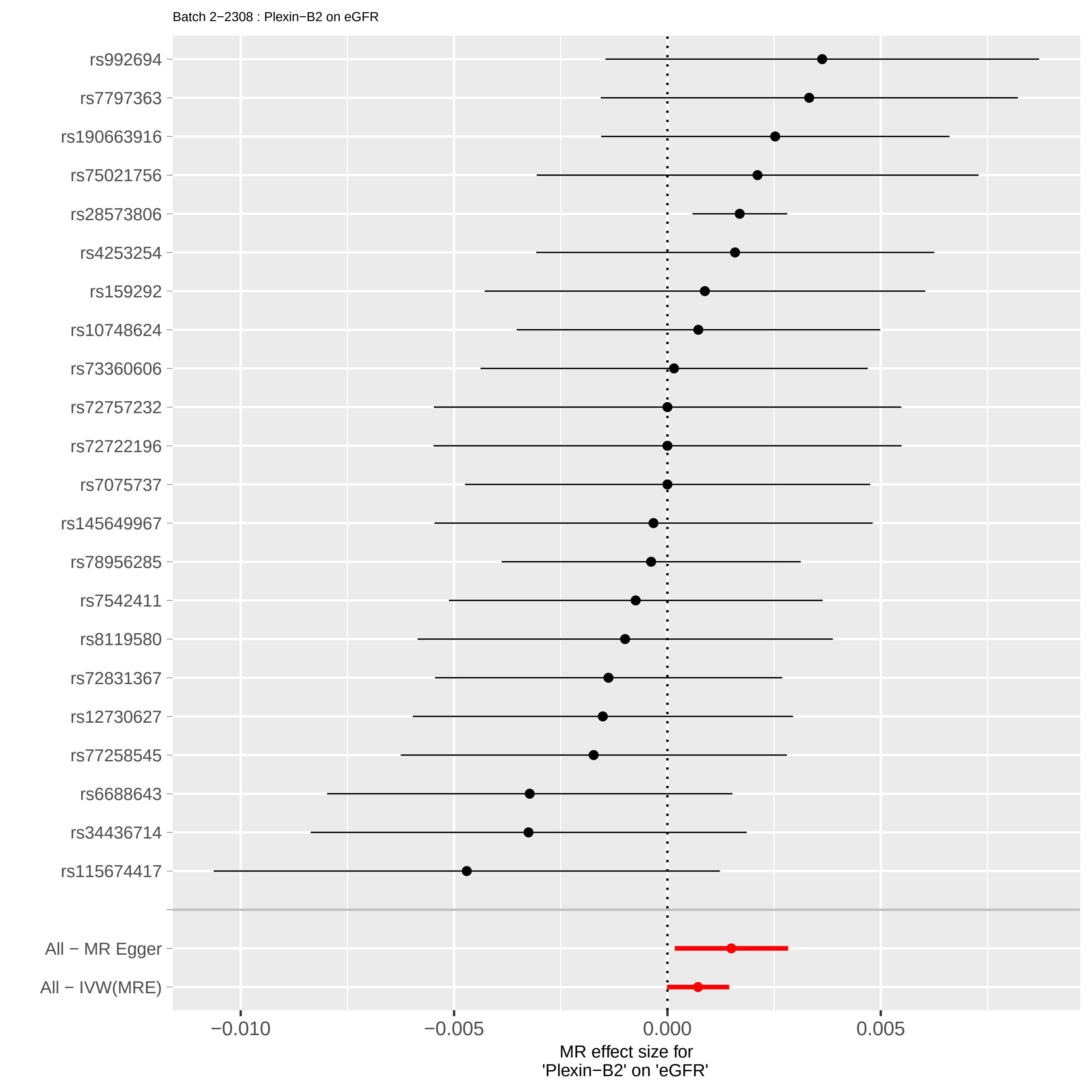

Batch 2−2308 : Plexin−B2 on eGFR
rs992694
rs7797363
rs190663916
rs75021756
rs28573806
rs4253254
rs159292
rs10748624
rs73360606
rs72757232
rs72722196
rs7075737
rs145649967
rs78956285
rs7542411
rs8119580
rs72831367
rs12730627
rs77258545
rs6688643
rs34436714
rs115674417
All − MR Egger
All − IVW(MRE)
−0.010
−0.005
0.000
0.005
MR effect size for
'Plexin−B2' on 'eGFR'

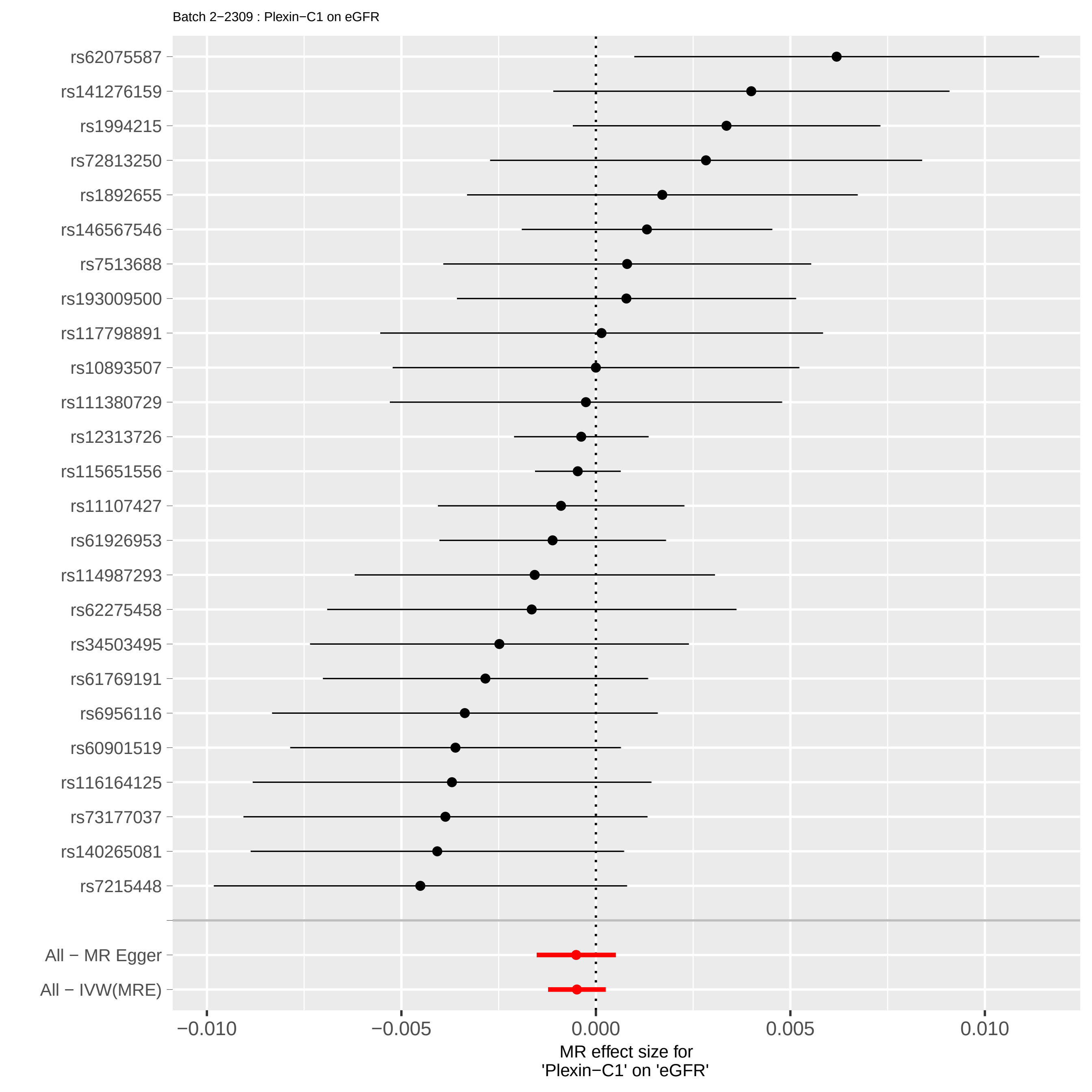
Batch 2−2309 : Plexin−C1 on eGFR
rs62075587
rs141276159
rs1994215
rs72813250
rs1892655
rs146567546
rs7513688
rs193009500
rs117798891
rs10893507
rs111380729
rs12313726
rs115651556
rs11107427
rs61926953
rs114987293
rs62275458
rs34503495
rs61769191
rs6956116
rs60901519
rs116164125
rs73177037
rs140265081
rs7215448
All − MR Egger
All − IVW(MRE)
−0.010
−0.005
0.000
0.005
0.010
MR effect size for
'Plexin−C1' on 'eGFR'

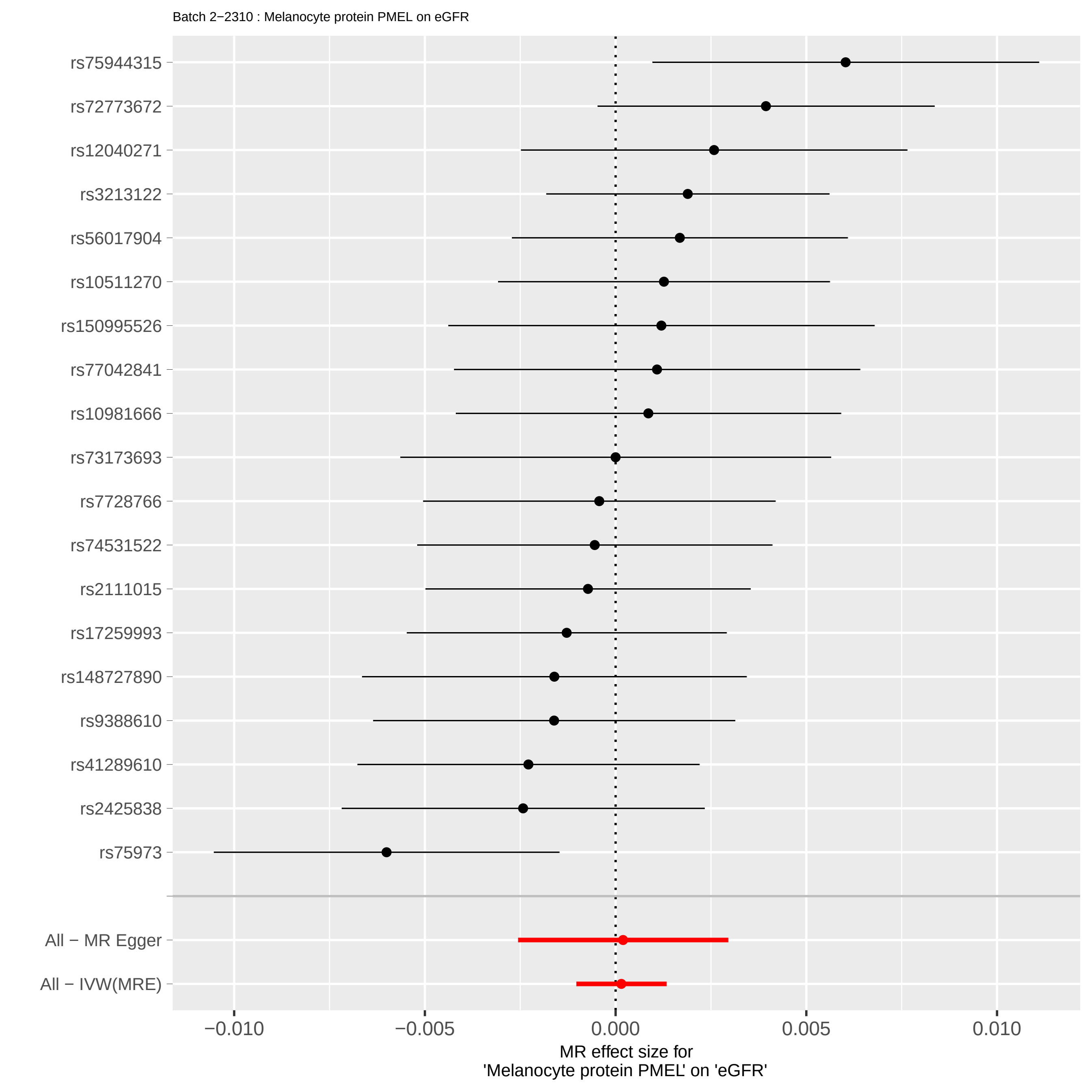

Batch 2−2310 : Melanocyte protein PMEL on eGFR
rs75944315
rs72773672
rs12040271
rs3213122
rs56017904
rs10511270
rs150995526
rs77042841
rs10981666
rs73173693
rs7728766
rs74531522
rs2111015
rs17259993
rs148727890
rs9388610
rs41289610
rs2425838
rs75973
All − MR Egger
All − IVW(MRE)
−0.010
−0.005
0.000
0.005
0.010
MR effect size for
'Melanocyte protein PMEL' on 'eGFR'

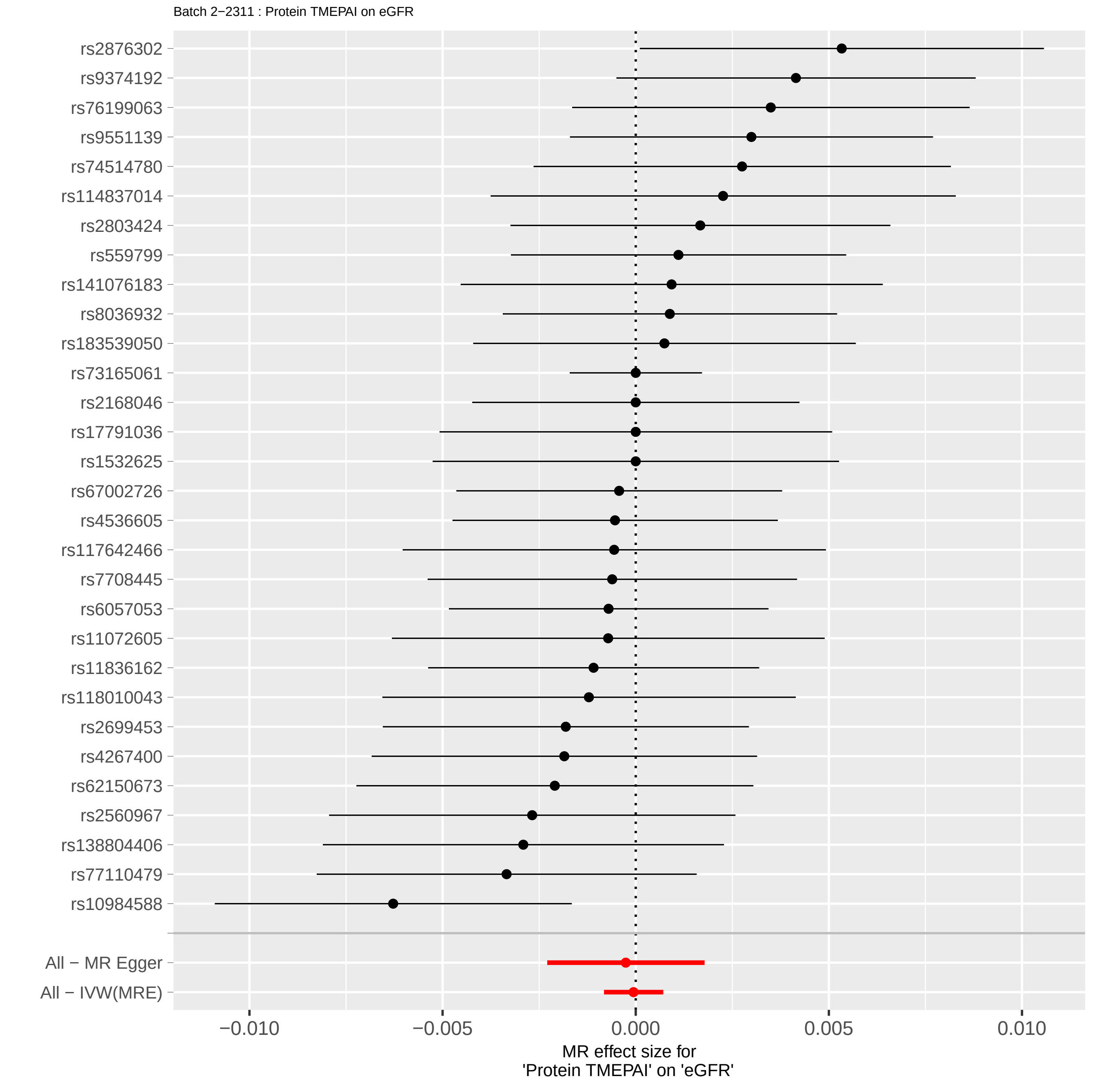

Batch 2−2311 : Protein TMEPAI on eGFR
rs2876302
rs9374192
rs76199063
rs9551139
rs74514780
rs114837014
rs2803424
rs559799
rs141076183
rs8036932
rs183539050
rs73165061
rs2168046
rs17791036
rs1532625
rs67002726
rs4536605
rs117642466
rs7708445
rs6057053
rs11072605
rs11836162
rs118010043
rs2699453
rs4267400
rs62150673
rs2560967
rs138804406
rs77110479
rs10984588
All − MR Egger
All − IVW(MRE)
−0.010
−0.005
0.000
0.005
0.010
MR effect size for
'Protein TMEPAI' on 'eGFR'

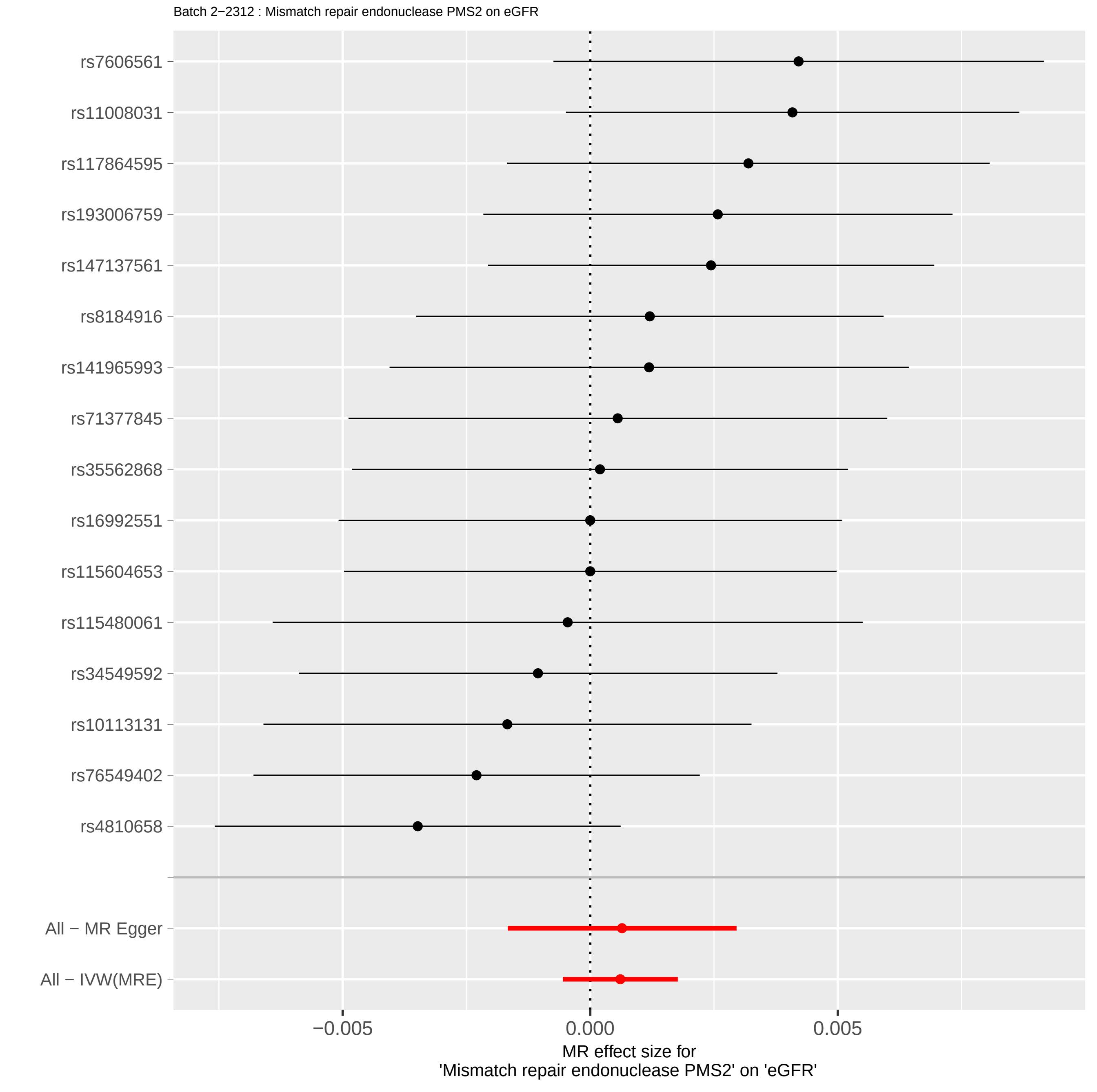

Batch 2−2312 : Mismatch repair endonuclease PMS2 on eGFR
rs7606561
rs11008031
rs117864595
rs193006759
rs147137561
rs8184916
rs141965993
rs71377845
rs35562868
rs16992551
rs115604653
rs115480061
rs34549592
rs10113131
rs76549402
rs4810658
All − MR Egger
All − IVW(MRE)
−0.005
0.000
0.005
MR effect size for
'Mismatch repair endonuclease PMS2' on 'eGFR'

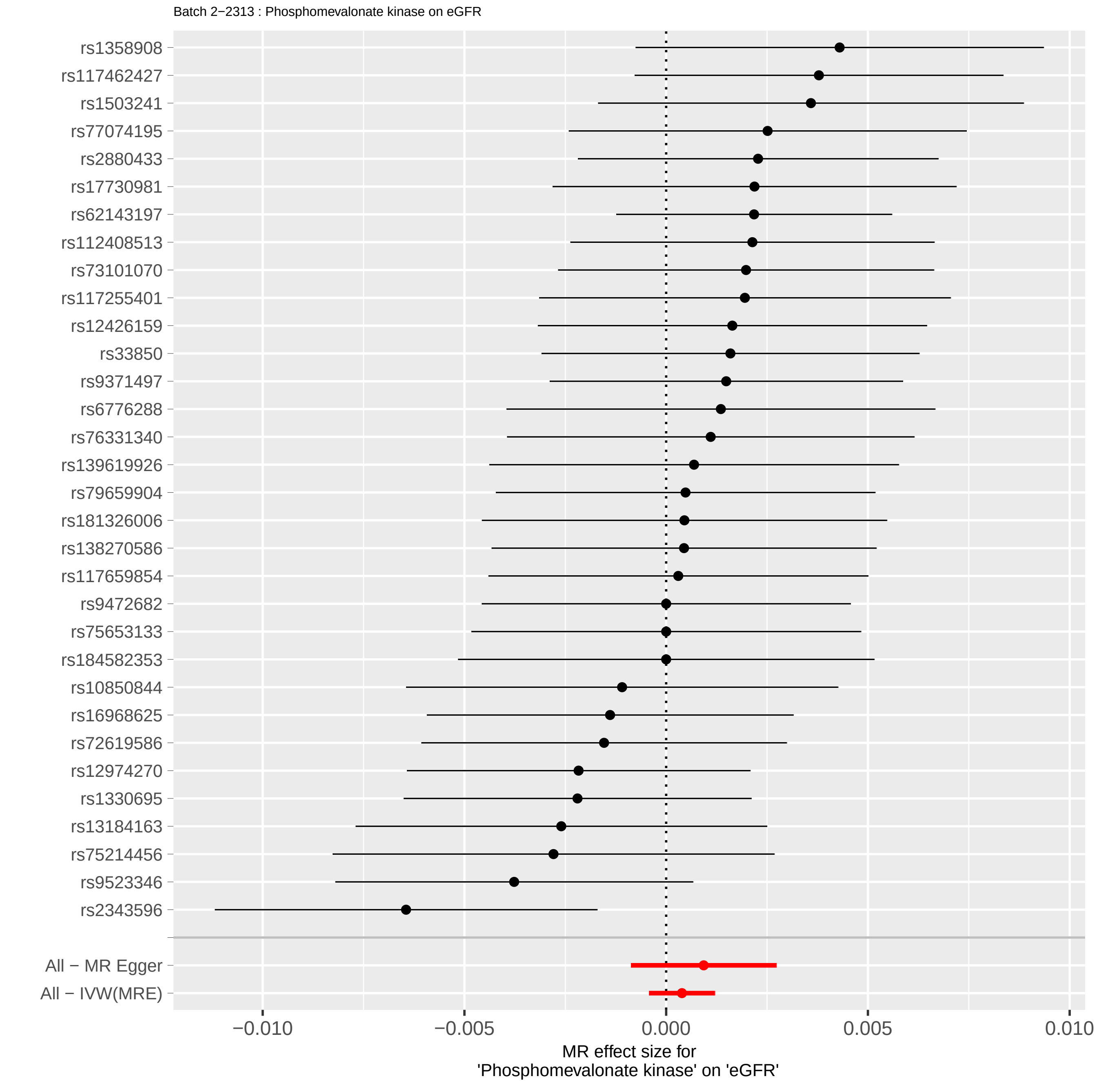

Batch 2−2313 : Phosphomevalonate kinase on eGFR
rs1358908
rs117462427
rs1503241
rs77074195
rs2880433
rs17730981
rs62143197
rs112408513
rs73101070
rs117255401
rs12426159
rs33850
rs9371497
rs6776288
rs76331340
rs139619926
rs79659904
rs181326006
rs138270586
rs117659854
rs9472682
rs75653133
rs184582353
rs10850844
rs16968625
rs72619586
rs12974270
rs1330695
rs13184163
rs75214456
rs9523346
rs2343596
All − MR Egger
All − IVW(MRE)
−0.010
−0.005
0.000
0.005
0.010
MR effect size for
'Phosphomevalonate kinase' on 'eGFR'

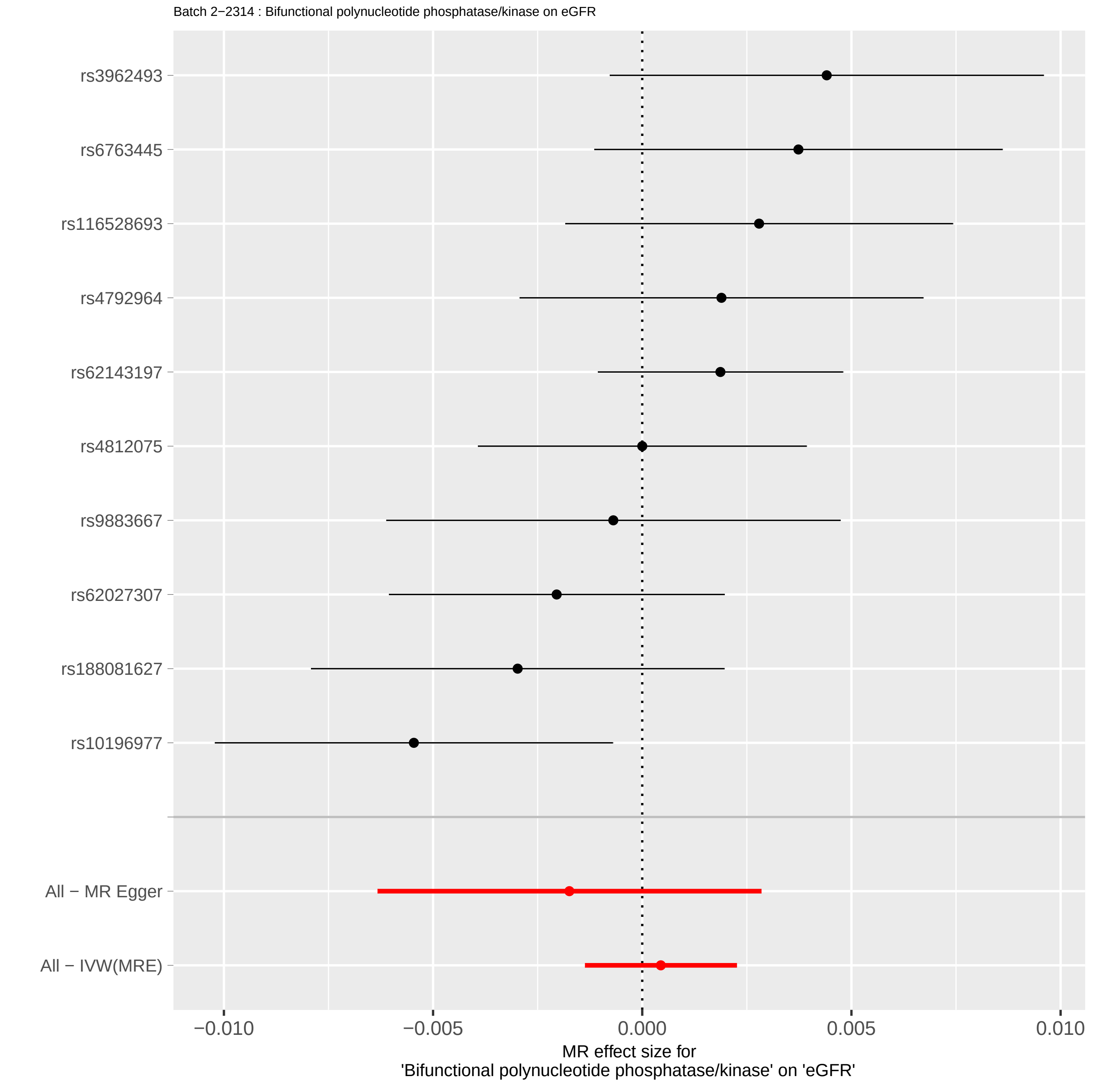
Batch 2−2314 : Bifunctional polynucleotide phosphatase/kinase on eGFR
rs3962493
rs6763445
rs116528693
rs4792964
rs62143197
rs4812075
rs9883667
rs62027307
rs188081627
rs10196977
All − MR Egger
All − IVW(MRE)
−0.010
−0.005
0.000
0.005
0.010
MR effect size for
'Bifunctional polynucleotide phosphatase/kinase' on 'eGFR'

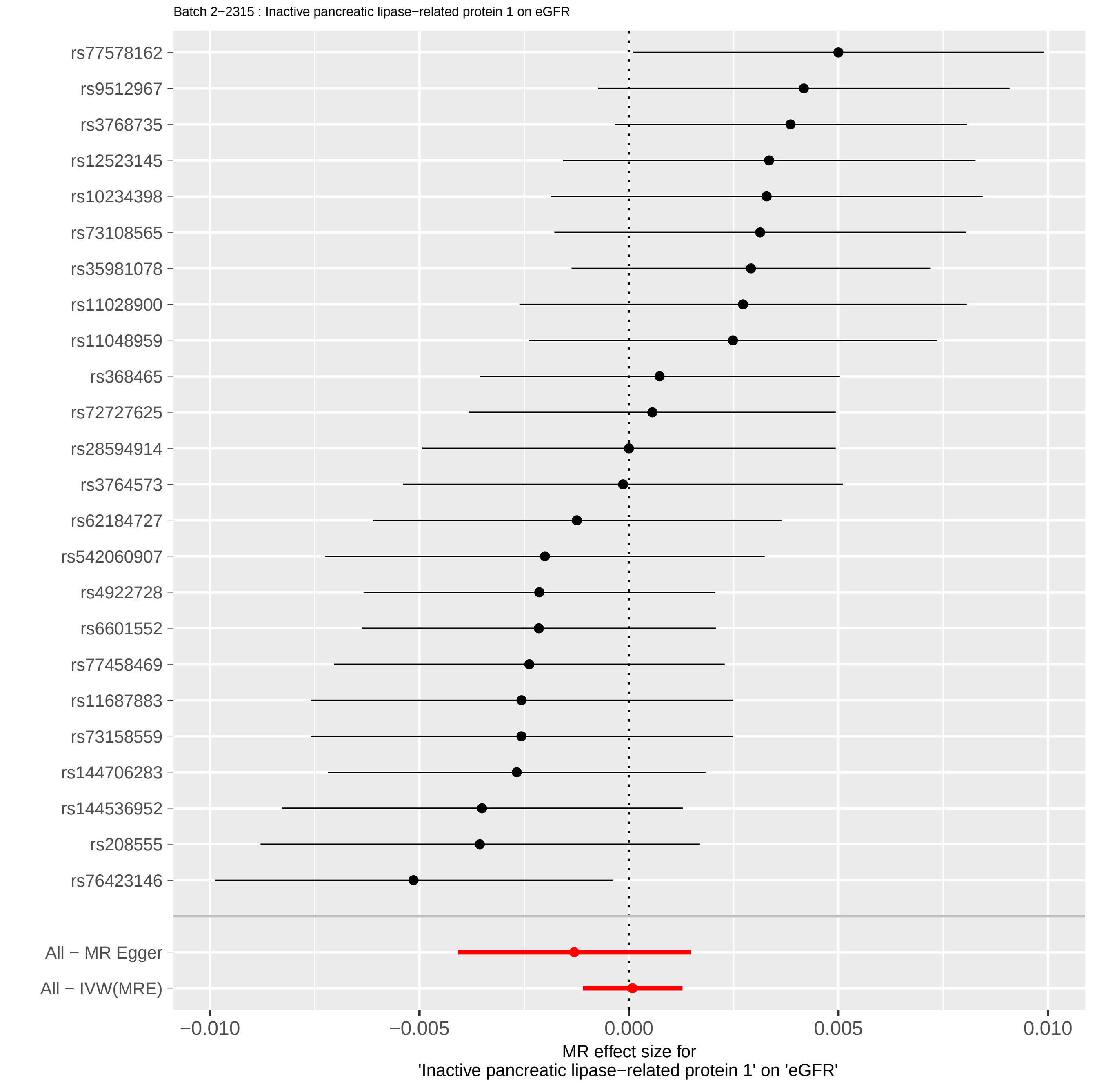
Batch 2−2315 : Inactive pancreatic lipase−related protein 1 on eGFR
rs77578162
rs9512967
rs3768735
rs12523145
rs10234398
rs73108565
rs35981078
rs11028900
rs11048959
rs368465
rs72727625
rs28594914
rs3764573
rs62184727
rs542060907
rs4922728
rs6601552
rs77458469
rs11687883
rs73158559
rs144706283
rs144536952
rs208555
rs76423146
All − MR Egger
All − IVW(MRE)
−0.010
−0.005
0.000
0.005
0.010
MR effect size for
'Inactive pancreatic lipase−related protein 1' on 'eGFR'

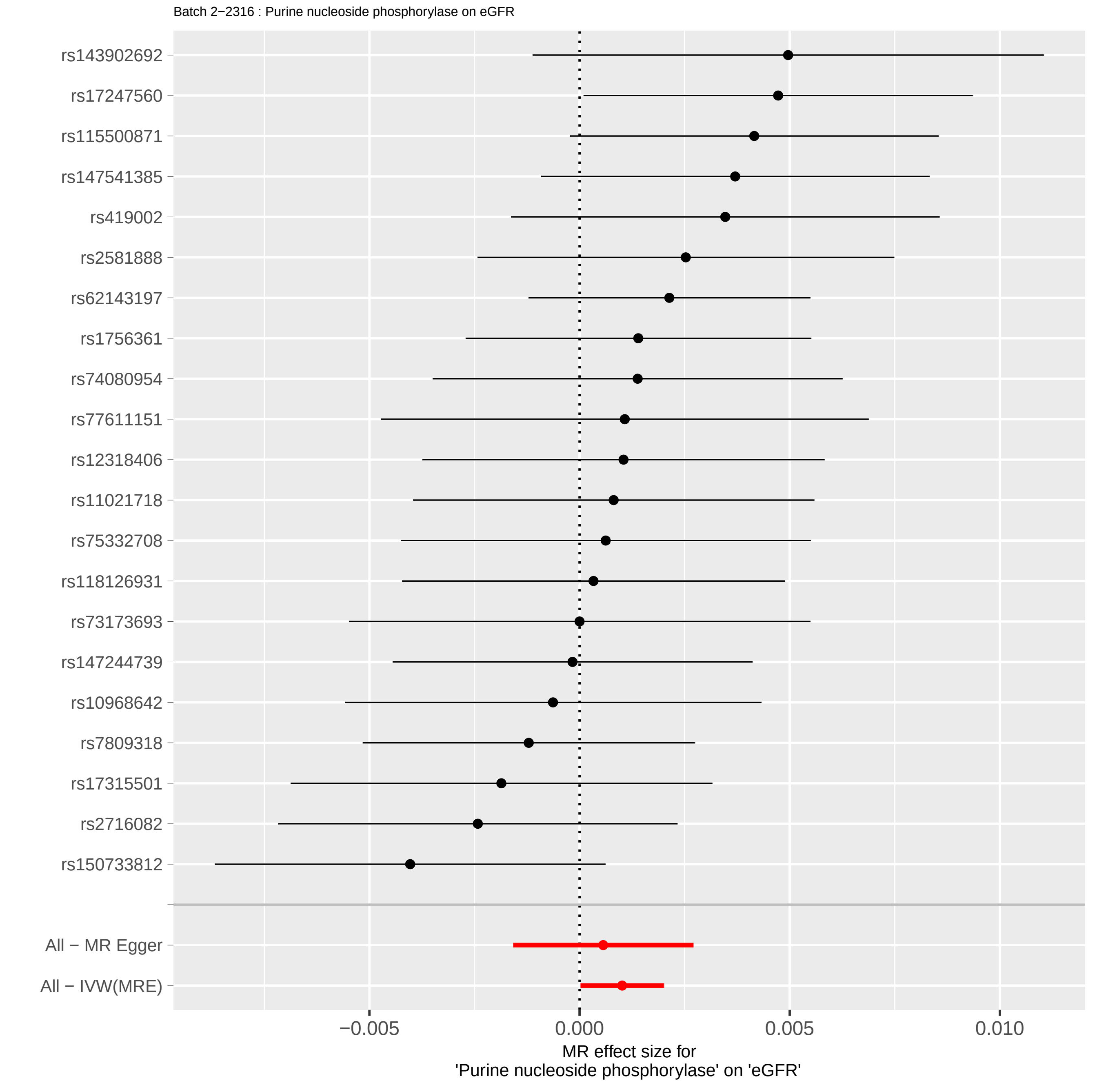
Batch 2−2316 : Purine nucleoside phosphorylase on eGFR
rs143902692
rs17247560
rs115500871
rs147541385
rs419002
rs2581888
rs62143197
rs1756361
rs74080954
rs77611151
rs12318406
rs11021718
rs75332708
rs118126931
rs73173693
rs147244739
rs10968642
rs7809318
rs17315501
rs2716082
rs150733812
All − MR Egger
All − IVW(MRE)
−0.005
0.000
0.005
0.010
MR effect size for
'Purine nucleoside phosphorylase' on 'eGFR'

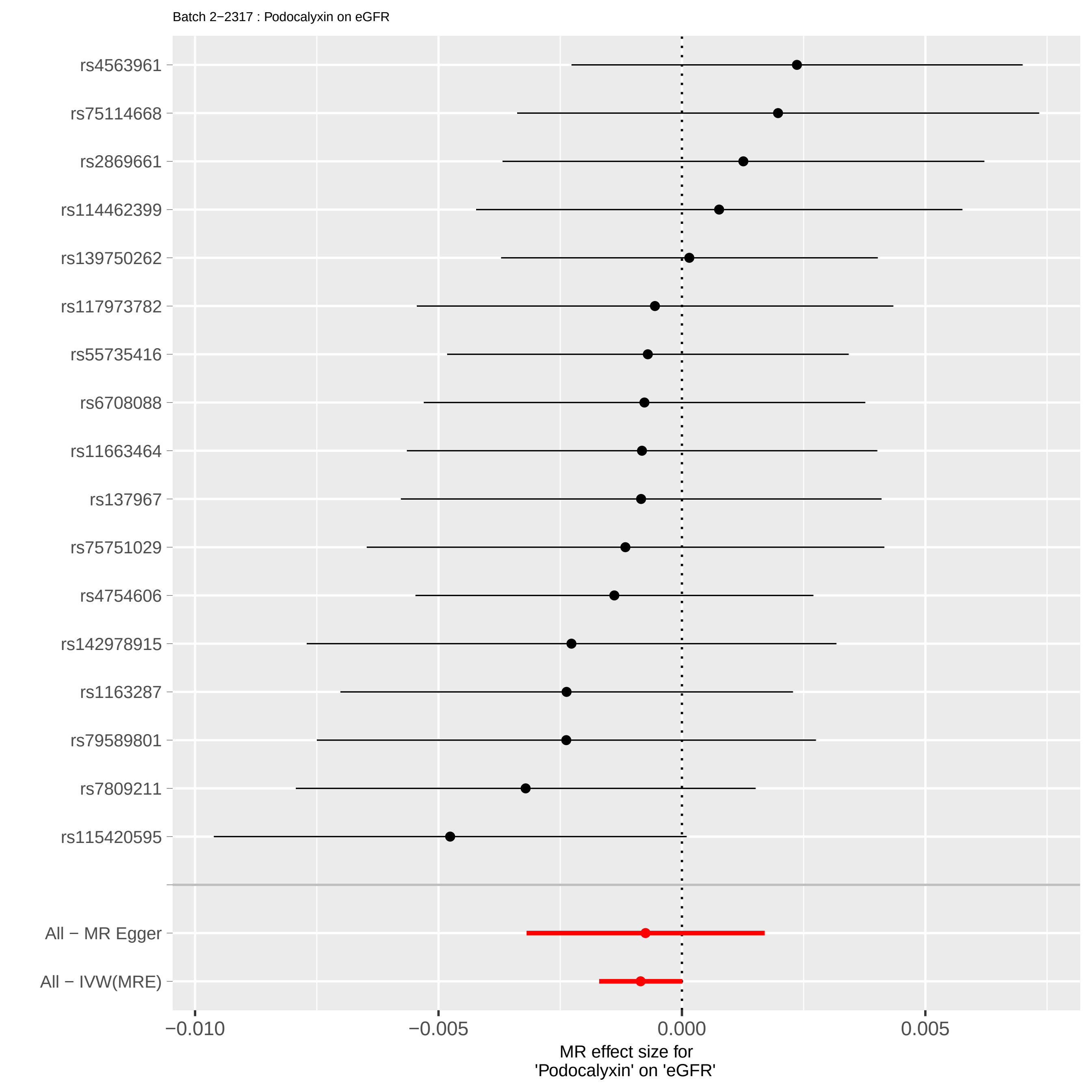
Batch 2−2317 : Podocalyxin on eGFR
rs4563961
rs75114668
rs2869661
rs114462399
rs139750262
rs117973782
rs55735416
rs6708088
rs11663464
rs137967
rs75751029
rs4754606
rs142978915
rs1163287
rs79589801
rs7809211
rs115420595
All − MR Egger
All − IVW(MRE)
−0.010
−0.005
0.000
0.005
MR effect size for
'Podocalyxin' on 'eGFR'

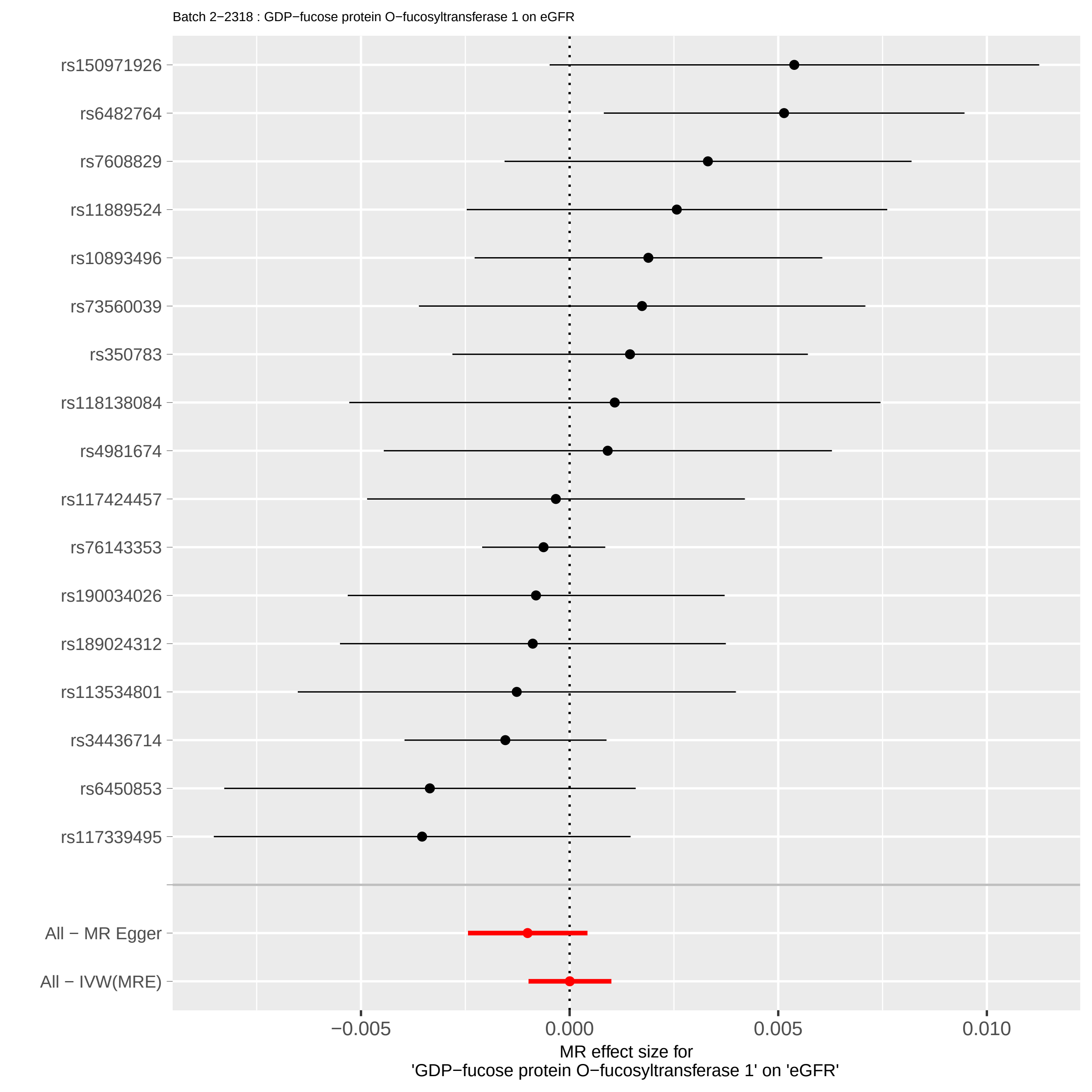

Batch 2−2318 : GDP−fucose protein O−fucosyltransferase 1 on eGFR
rs150971926
rs6482764
rs7608829
rs11889524
rs10893496
rs73560039
rs350783
rs118138084
rs4981674
rs117424457
rs76143353
rs190034026
rs189024312
rs113534801
rs34436714
rs6450853
rs117339495
All − MR Egger
All − IVW(MRE)
−0.005
0.000
0.005
0.010
MR effect size for
'GDP−fucose protein O−fucosyltransferase 1' on 'eGFR'

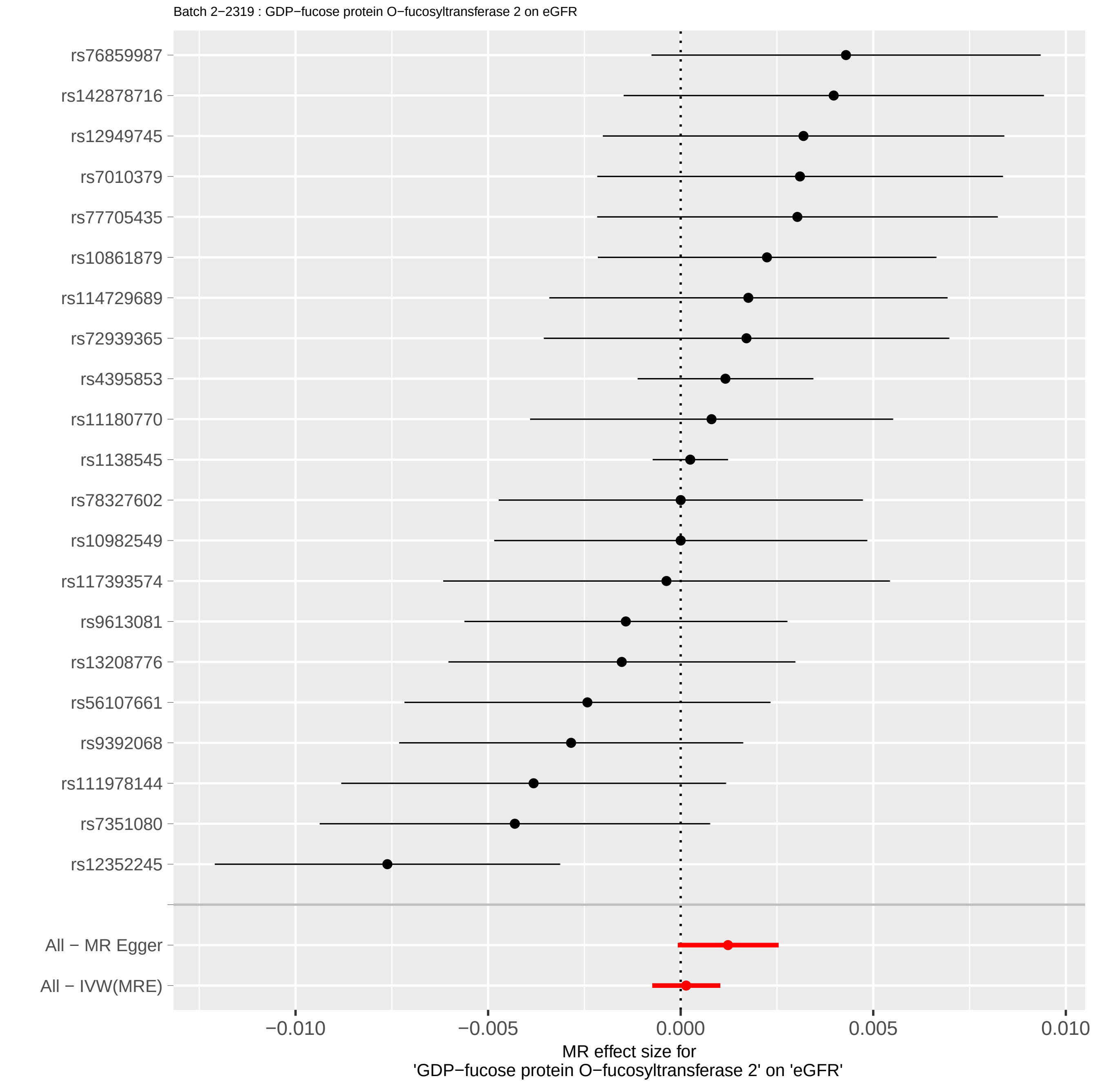

Batch 2−2319 : GDP−fucose protein O−fucosyltransferase 2 on eGFR
rs76859987
rs142878716
rs12949745
rs7010379
rs77705435
rs10861879
rs114729689
rs72939365
rs4395853
rs11180770
rs1138545
rs78327602
rs10982549
rs117393574
rs9613081
rs13208776
rs56107661
rs9392068
rs111978144
rs7351080
rs12352245
All − MR Egger
All − IVW(MRE)
−0.010
−0.005
0.000
0.005
0.010
MR effect size for
'GDP−fucose protein O−fucosyltransferase 2' on 'eGFR'

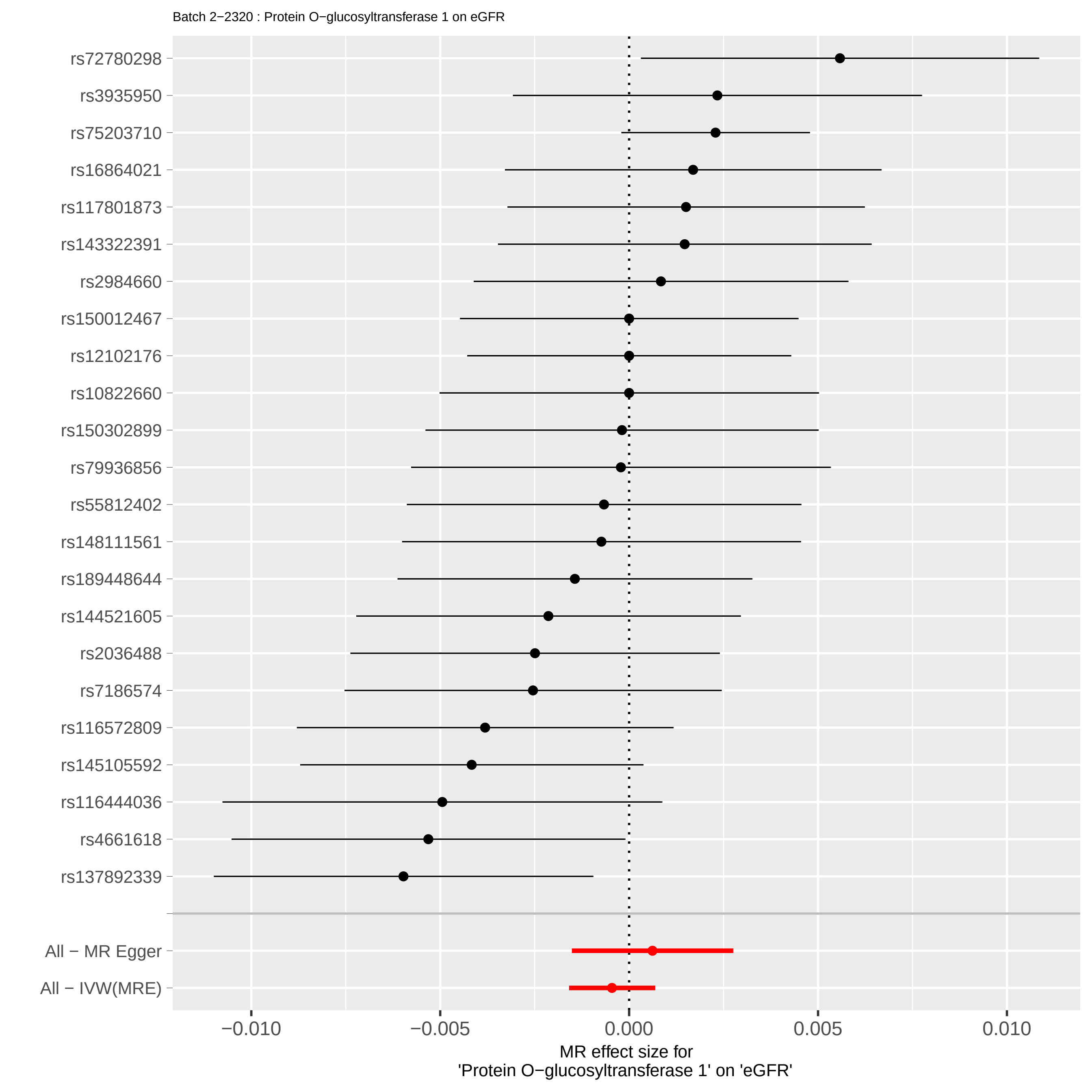
Batch 2−2320 : Protein O−glucosyltransferase 1 on eGFR
rs72780298
rs3935950
rs75203710
rs16864021
rs117801873
rs143322391
rs2984660
rs150012467
rs12102176
rs10822660
rs150302899
rs79936856
rs55812402
rs148111561
rs189448644
rs144521605
rs2036488
rs7186574
rs116572809
rs145105592
rs116444036
rs4661618
rs137892339
All − MR Egger
All − IVW(MRE)
−0.010
−0.005
0.000
0.005
0.010
MR effect size for
'Protein O−glucosyltransferase 1' on 'eGFR'

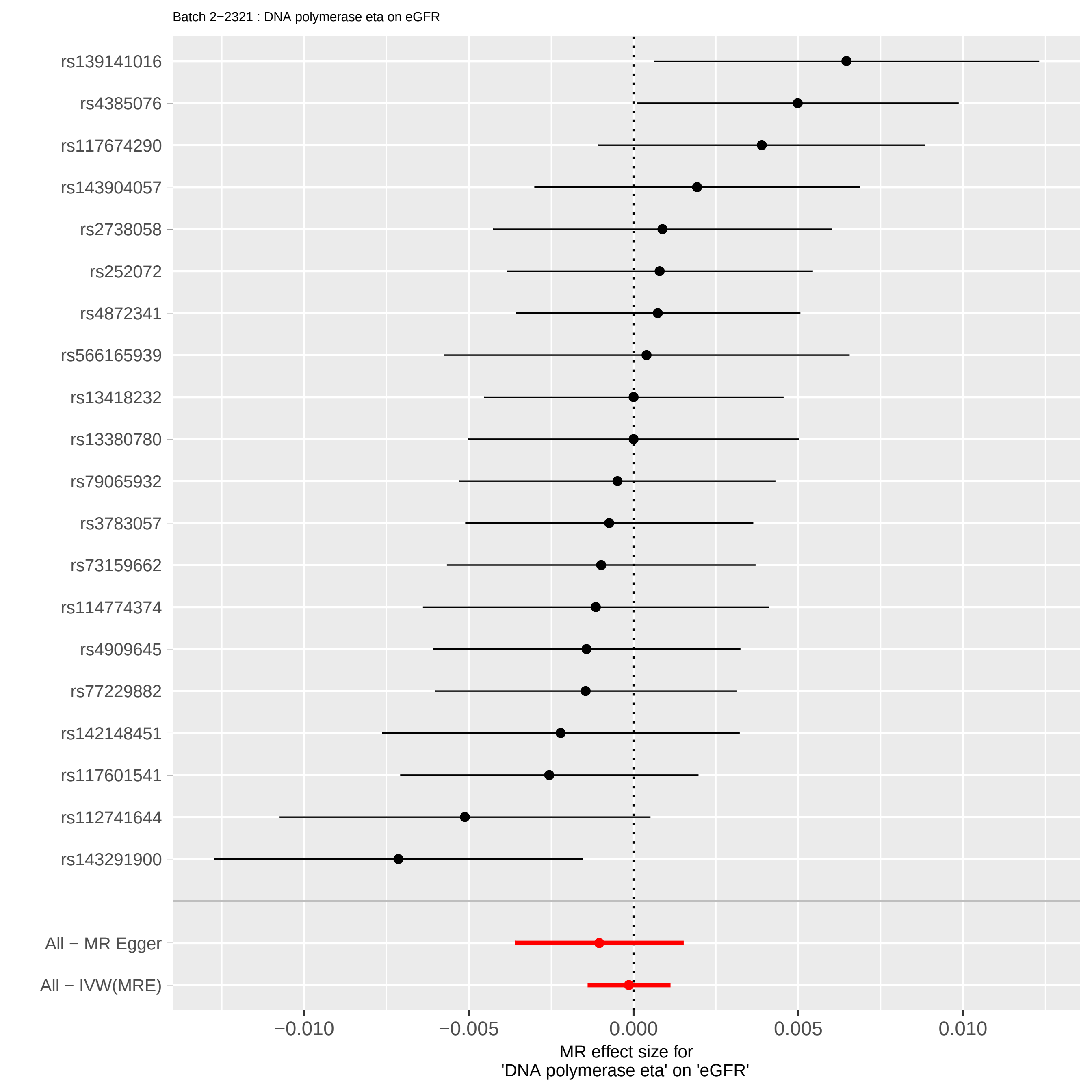

Batch 2−2321 : DNA polymerase eta on eGFR
rs139141016
rs4385076
rs117674290
rs143904057
rs2738058
rs252072
rs4872341
rs566165939
rs13418232
rs13380780
rs79065932
rs3783057
rs73159662
rs114774374
rs4909645
rs77229882
rs142148451
rs117601541
rs112741644
rs143291900
All − MR Egger
All − IVW(MRE)
−0.010
−0.005
0.000
0.005
0.010
MR effect size for
'DNA polymerase eta' on 'eGFR'

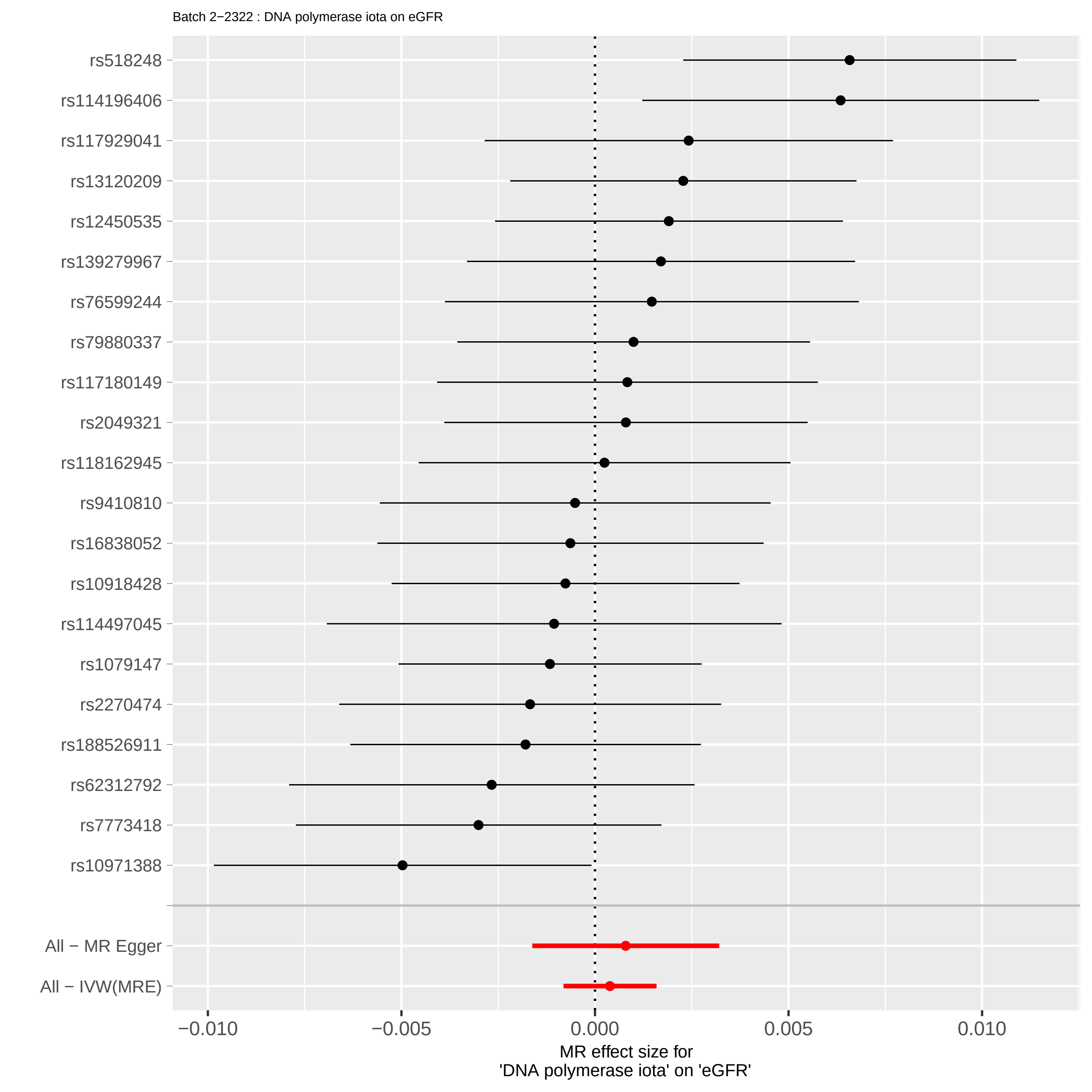

Batch 2−2322 : DNA polymerase iota on eGFR
rs518248
rs114196406
rs117929041
rs13120209
rs12450535
rs139279967
rs76599244
rs79880337
rs117180149
rs2049321
rs118162945
rs9410810
rs16838052
rs10918428
rs114497045
rs1079147
rs2270474
rs188526911
rs62312792
rs7773418
rs10971388
All − MR Egger
All − IVW(MRE)
−0.010
−0.005
0.000
0.005
0.010
MR effect size for
'DNA polymerase iota' on 'eGFR'

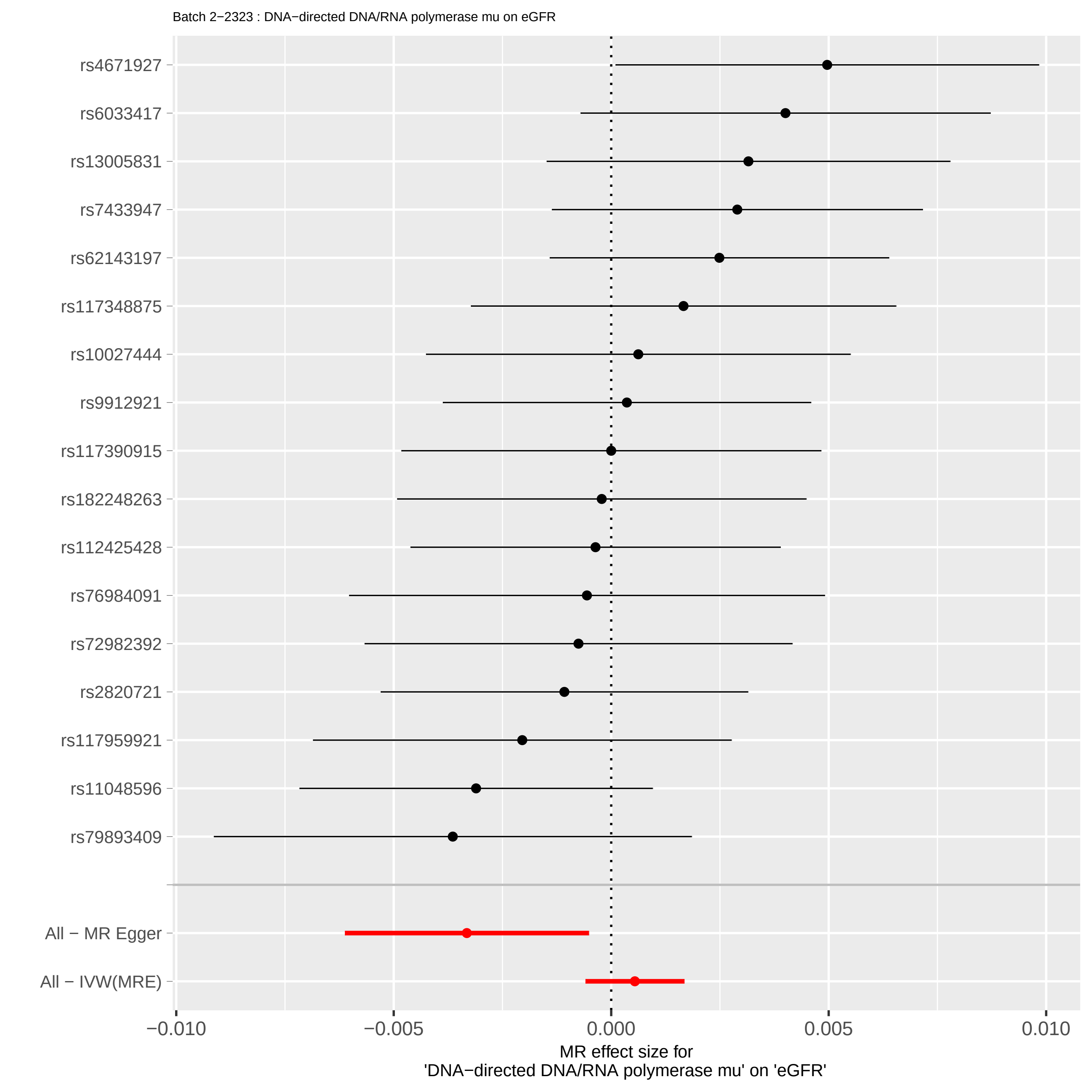

Batch 2−2323 : DNA−directed DNA/RNA polymerase mu on eGFR
rs4671927
rs6033417
rs13005831
rs7433947
rs62143197
rs117348875
rs10027444
rs9912921
rs117390915
rs182248263
rs112425428
rs76984091
rs72982392
rs2820721
rs117959921
rs11048596
rs79893409
All − MR Egger
All − IVW(MRE)
−0.010
−0.005
0.000
0.005
0.010
MR effect size for
'DNA−directed DNA/RNA polymerase mu' on 'eGFR'

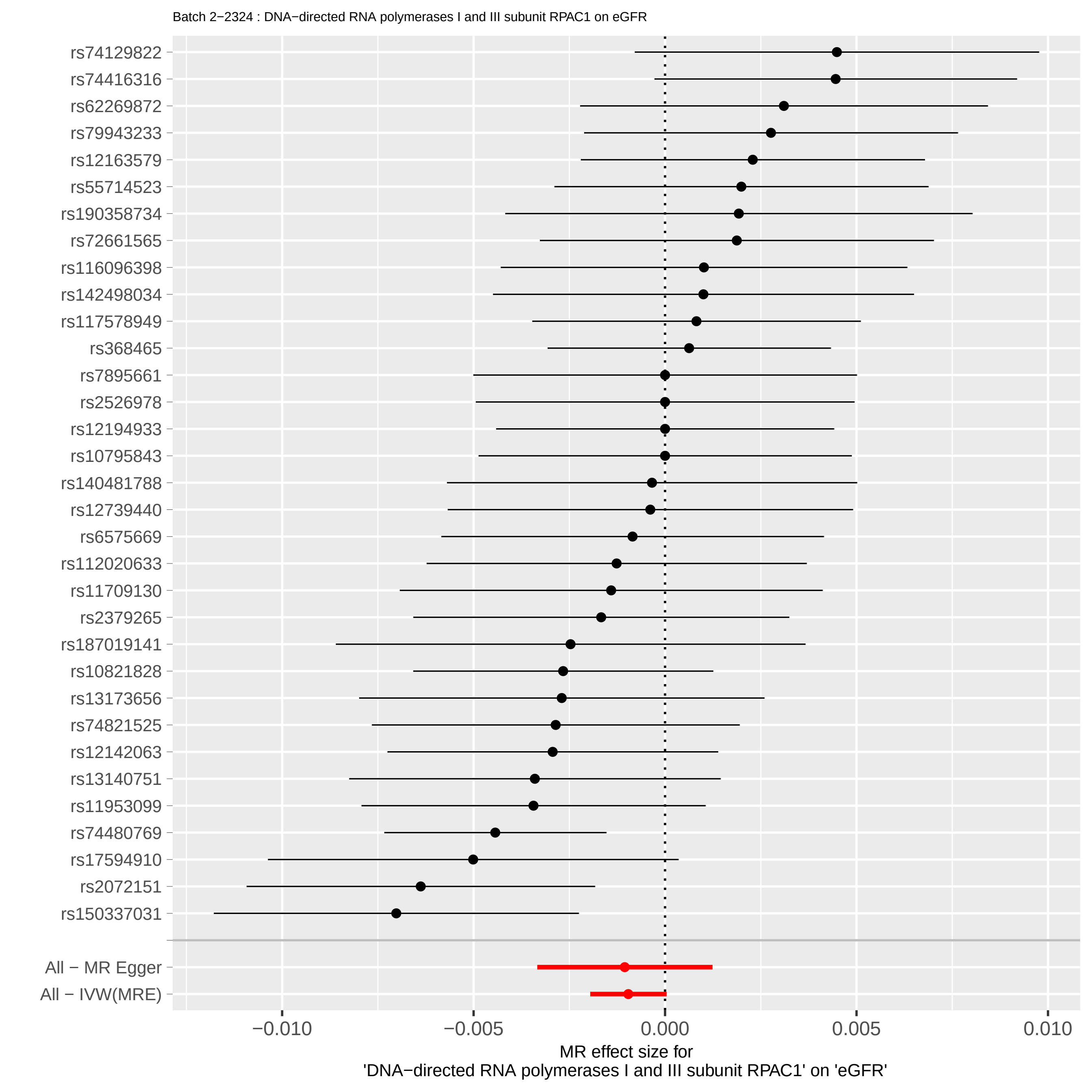
Batch 2−2324 : DNA−directed RNA polymerases I and III subunit RPAC1 on eGFR
rs74129822
rs74416316
rs62269872
rs79943233
rs12163579
rs55714523
rs190358734
rs72661565
rs116096398
rs142498034
rs117578949
rs368465
rs7895661
rs2526978
rs12194933
rs10795843
rs140481788
rs12739440
rs6575669
rs112020633
rs11709130
rs2379265
rs187019141
rs10821828
rs13173656
rs74821525
rs12142063
rs13140751
rs11953099
rs74480769
rs17594910
rs2072151
rs150337031
All − MR Egger
All − IVW(MRE)
−0.010
−0.005
0.000
0.005
0.010
MR effect size for
'DNA−directed RNA polymerases I and III subunit RPAC1' on 'eGFR'

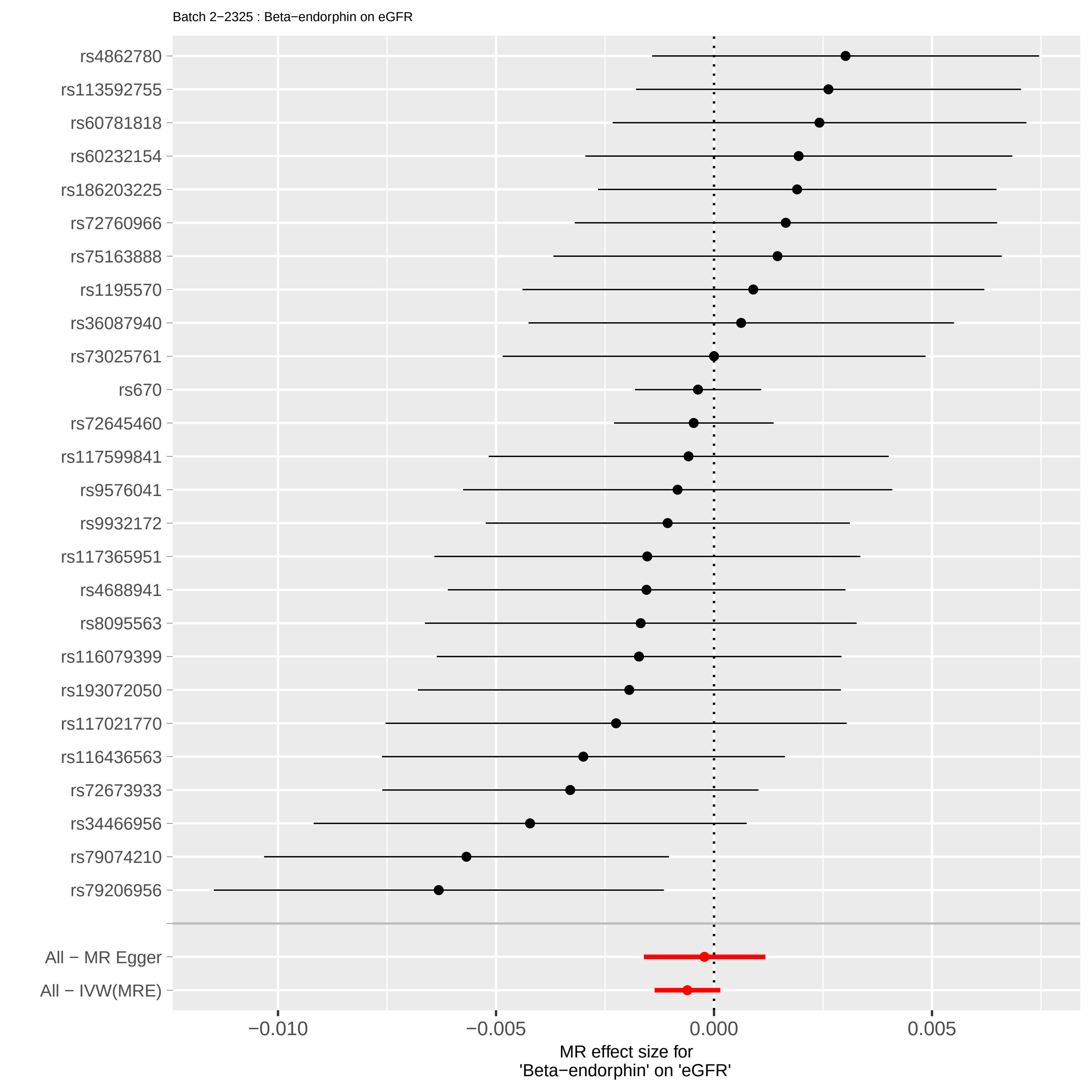

Batch 2−2325 : Beta−endorphin on eGFR
rs4862780
rs113592755
rs60781818
rs60232154
rs186203225
rs72760966
rs75163888
rs1195570
rs36087940
rs73025761
rs670
rs72645460
rs117599841
rs9576041
rs9932172
rs117365951
rs4688941
rs8095563
rs116079399
rs193072050
rs117021770
rs116436563
rs72673933
rs34466956
rs79074210
rs79206956
All − MR Egger
All − IVW(MRE)
−0.010
−0.005
0.000
0.005
MR effect size for
'Beta−endorphin' on 'eGFR'

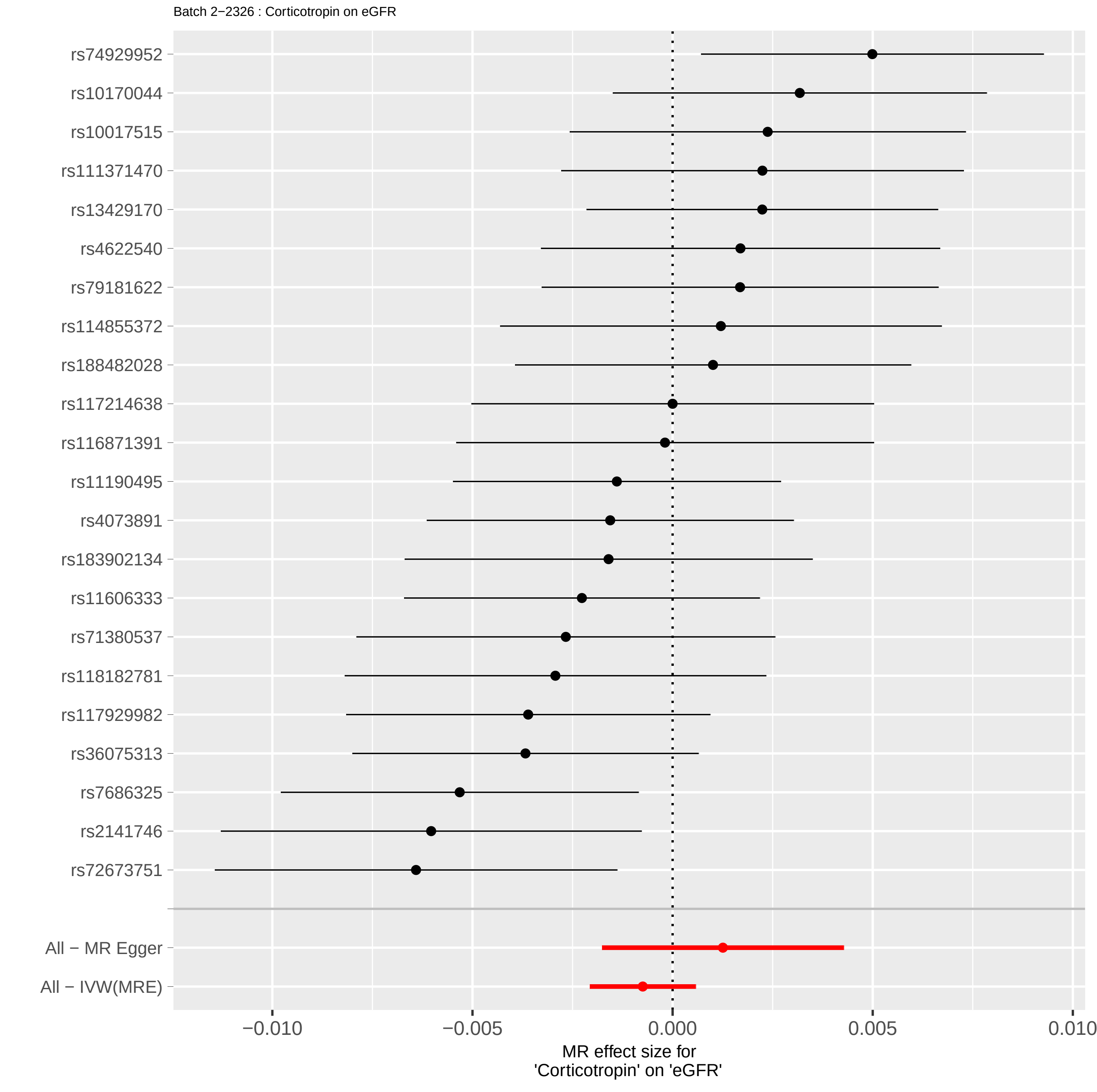
Batch 2−2326 : Corticotropin on eGFR
rs74929952
rs10170044
rs10017515
rs111371470
rs13429170
rs4622540
rs79181622
rs114855372
rs188482028
rs117214638
rs116871391
rs11190495
rs4073891
rs183902134
rs11606333
rs71380537
rs118182781
rs117929982
rs36075313
rs7686325
rs2141746
rs72673751
All − MR Egger
All − IVW(MRE)
−0.010
−0.005
0.000
0.005
0.010
MR effect size for
'Corticotropin' on 'eGFR'

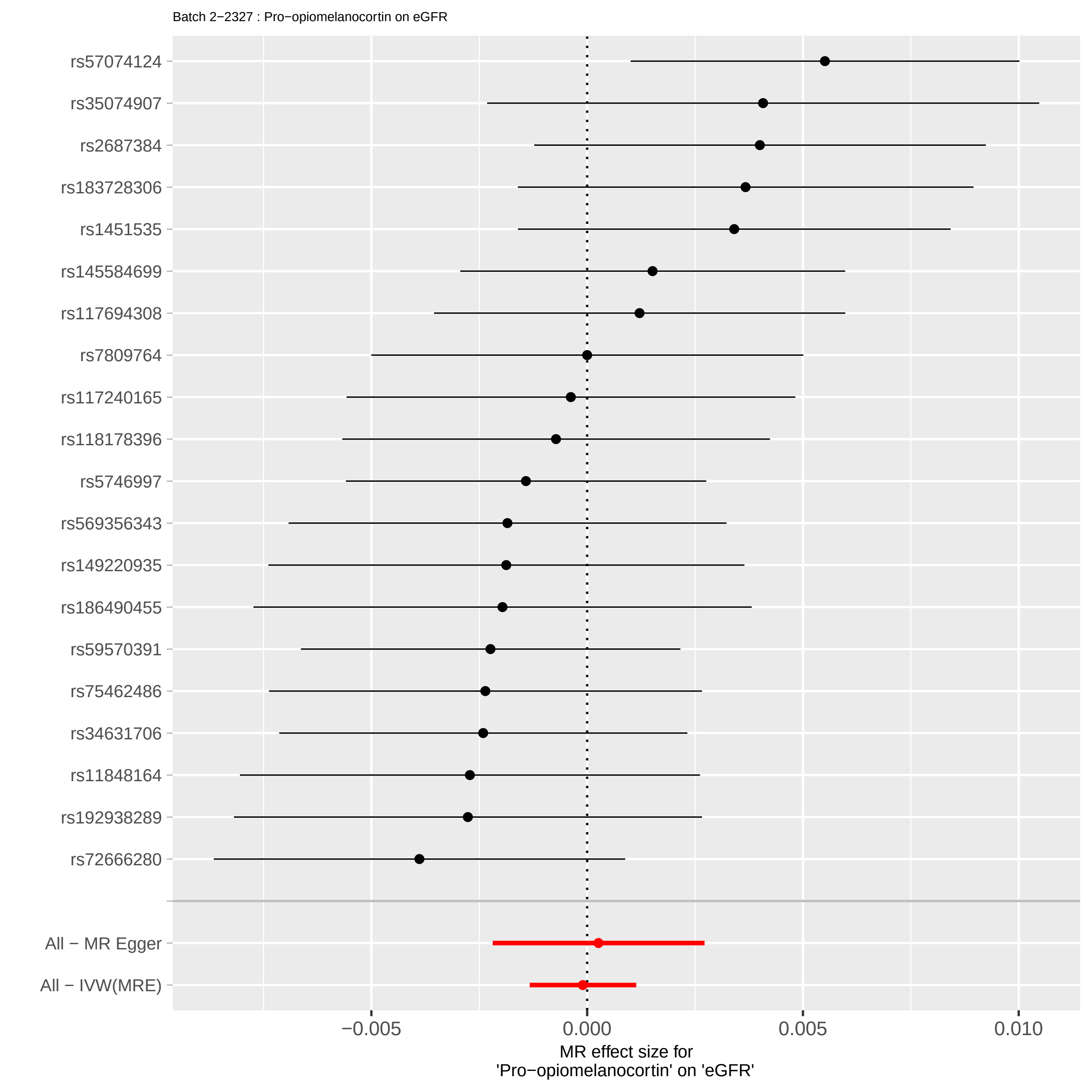

Batch 2−2327 : Pro−opiomelanocortin on eGFR
rs57074124
rs35074907
rs2687384
rs183728306
rs1451535
rs145584699
rs117694308
rs7809764
rs117240165
rs118178396
rs5746997
rs569356343
rs149220935
rs186490455
rs59570391
rs75462486
rs34631706
rs11848164
rs192938289
rs72666280
All − MR Egger
All − IVW(MRE)
−0.005
0.000
0.005
0.010
MR effect size for
'Pro−opiomelanocortin' on 'eGFR'

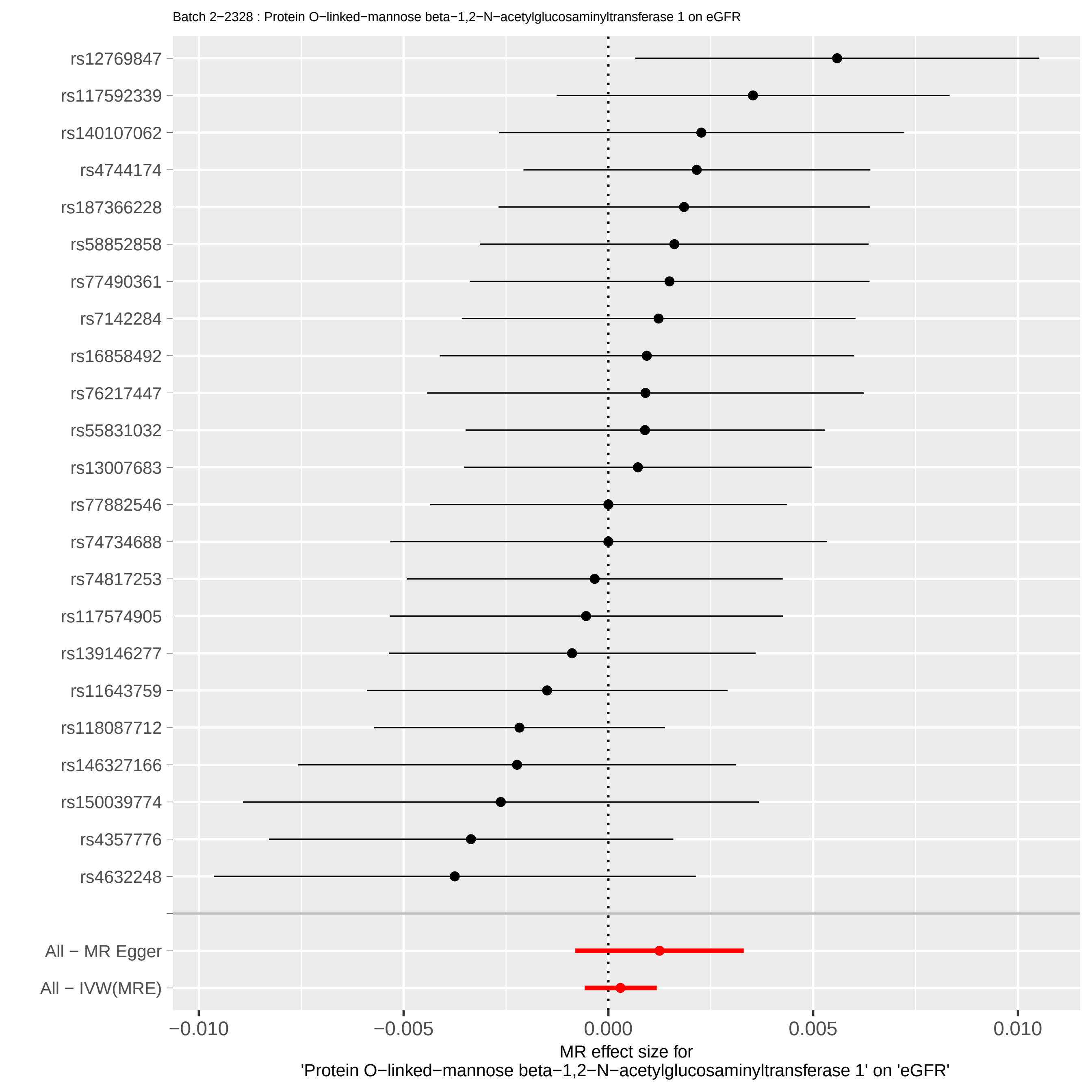

Batch 2−2328 : Protein O−linked−mannose beta−1,2−N−acetylglucosaminyltransferase 1 on eGFR
rs12769847
rs117592339
rs140107062
rs4744174
rs187366228
rs58852858
rs77490361
rs7142284
rs16858492
rs76217447
rs55831032
rs13007683
rs77882546
rs74734688
rs74817253
rs117574905
rs139146277
rs11643759
rs118087712
rs146327166
rs150039774
rs4357776
rs4632248
All − MR Egger
All − IVW(MRE)
−0.010
−0.005
0.000
0.005
0.010
MR effect size for
'Protein O−linked−mannose beta−1,2−N−acetylglucosaminyltransferase 1' on 'eGFR'

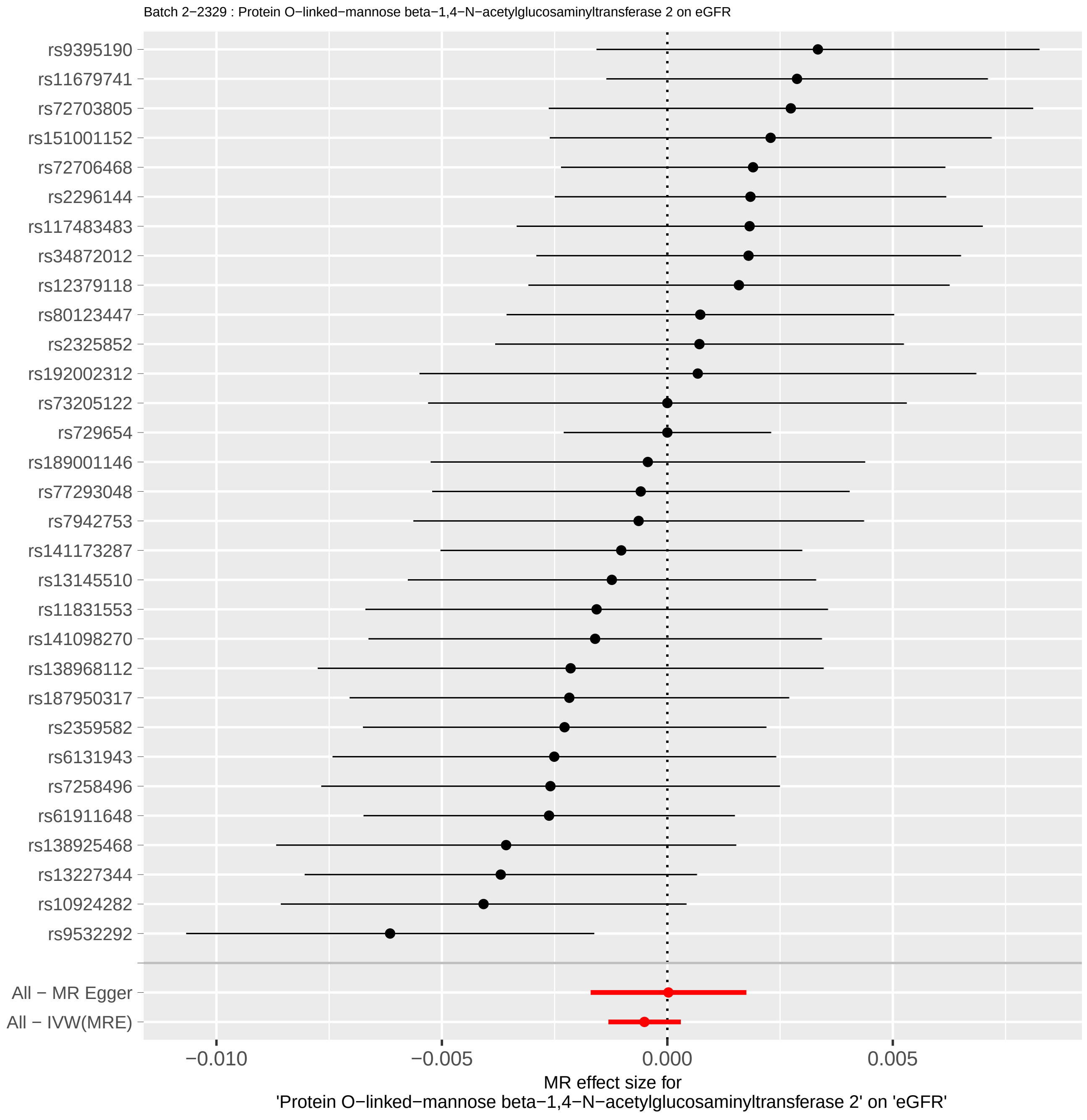
Batch 2−2329 : Protein O−linked−mannose beta−1,4−N−acetylglucosaminyltransferase 2 on eGFR
rs9395190
rs11679741
rs72703805
rs151001152
rs72706468
rs2296144
rs117483483
rs34872012
rs12379118
rs80123447
rs2325852
rs192002312
rs73205122
rs729654
rs189001146
rs77293048
rs7942753
rs141173287
rs13145510
rs11831553
rs141098270
rs138968112
rs187950317
rs2359582
rs6131943
rs7258496
rs61911648
rs138925468
rs13227344
rs10924282
rs9532292
All − MR Egger
All − IVW(MRE)
−0.010
−0.005
0.000
0.005
MR effect size for
'Protein O−linked−mannose beta−1,4−N−acetylglucosaminyltransferase 2' on 'eGFR'

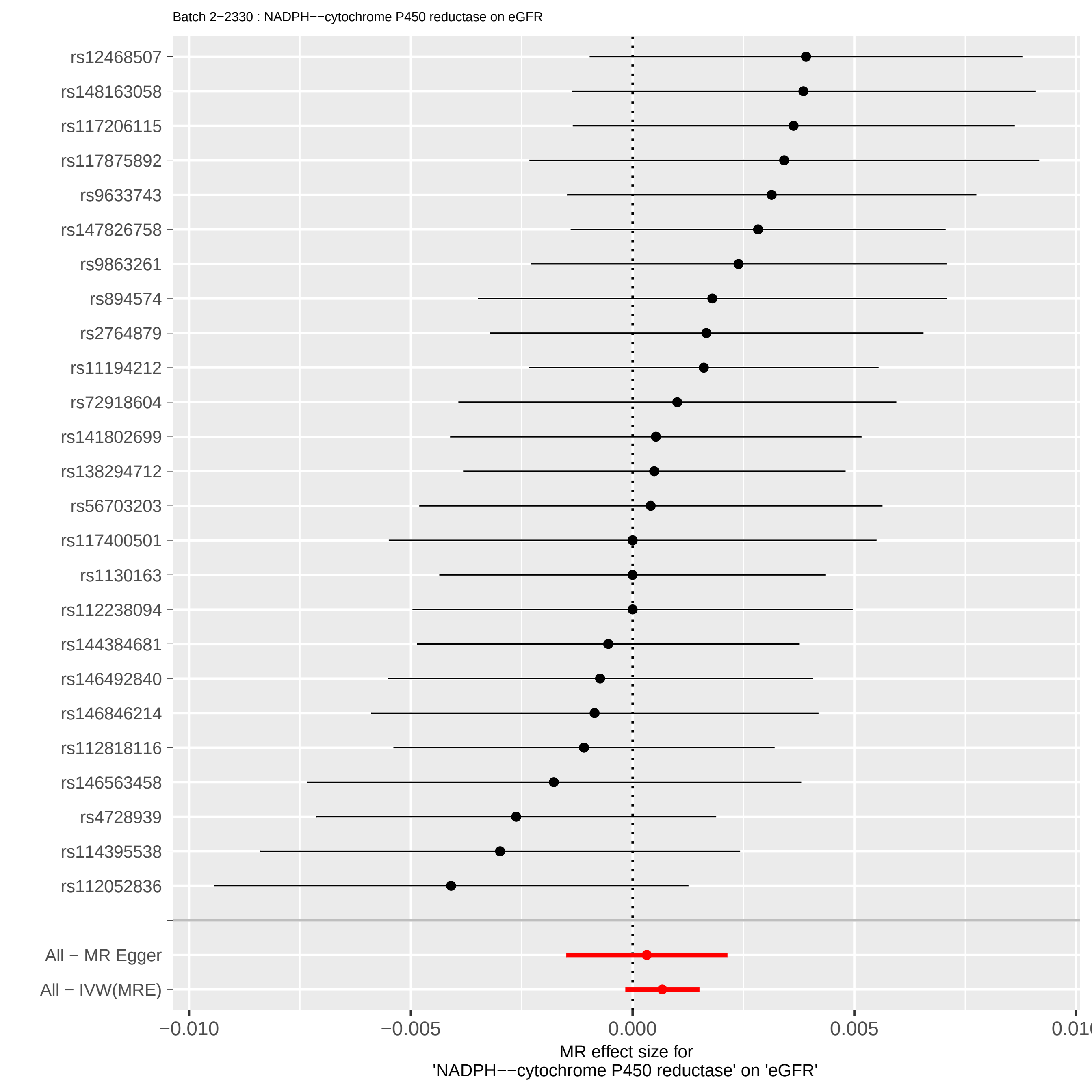

Batch 2−2330 : NADPH−−cytochrome P450 reductase on eGFR
rs12468507
rs148163058
rs117206115
rs117875892
rs9633743
rs147826758
rs9863261
rs894574
rs2764879
rs11194212
rs72918604
rs141802699
rs138294712
rs56703203
rs117400501
rs1130163
rs112238094
rs144384681
rs146492840
rs146846214
rs112818116
rs146563458
rs4728939
rs114395538
rs112052836
All − MR Egger
All − IVW(MRE)
−0.010
−0.005
0.000
0.005
MR effect size for
'NADPH−−cytochrome P450 reductase' on 'eGFR'

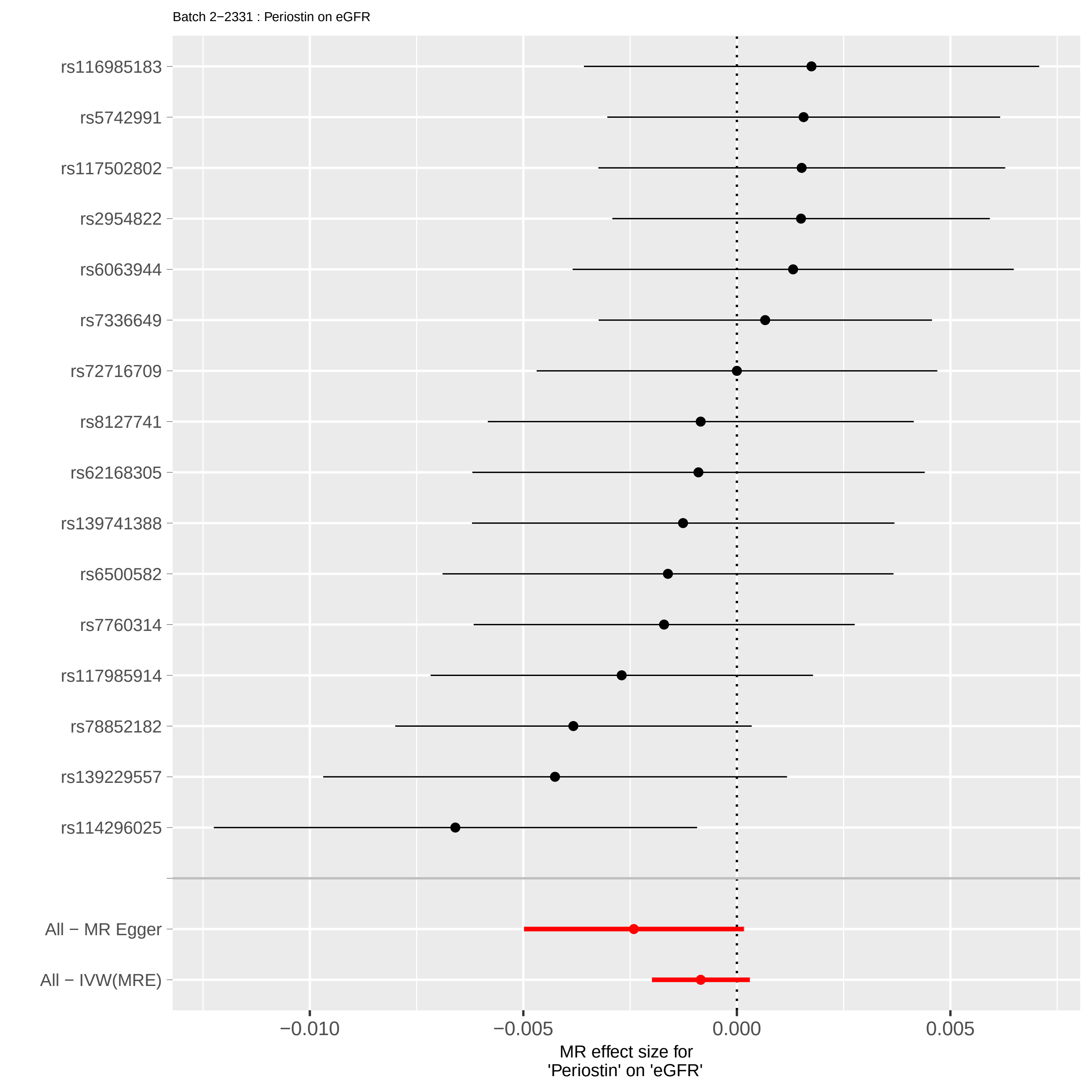
Batch 2−2331 : Periostin on eGFR
rs116985183
rs5742991
rs117502802
rs2954822
rs6063944
rs7336649
rs72716709
rs8127741
rs62168305
rs139741388
rs6500582
rs7760314
rs117985914
rs78852182
rs139229557
rs114296025
All − MR Egger
All − IVW(MRE)
−0.010
−0.005
0.000
0.005
MR effect size for
'Periostin' on 'eGFR'

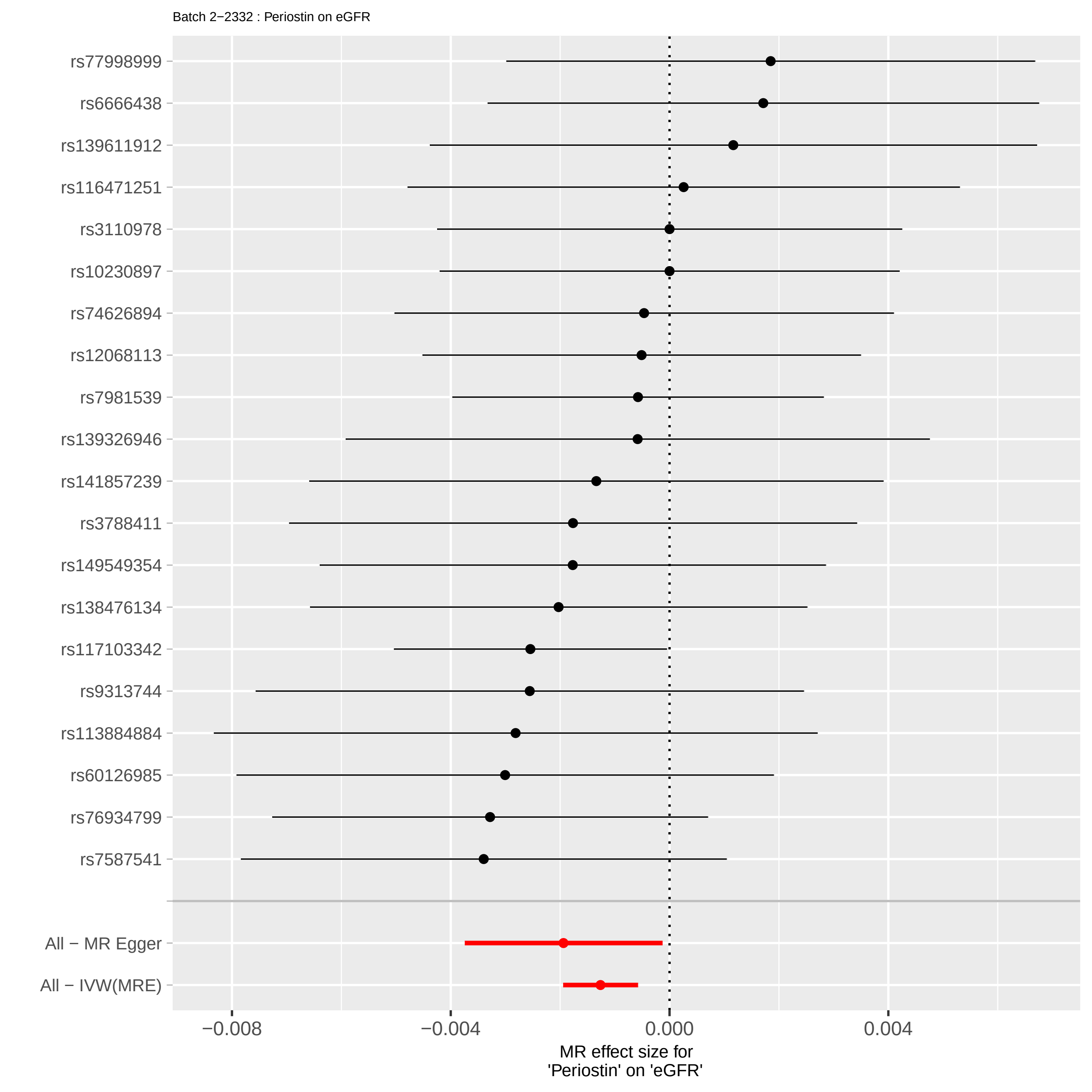

Batch 2−2332 : Periostin on eGFR
rs77998999
rs6666438
rs139611912
rs116471251
rs3110978
rs10230897
rs74626894
rs12068113
rs7981539
rs139326946
rs141857239
rs3788411
rs149549354
rs138476134
rs117103342
rs9313744
rs113884884
rs60126985
rs76934799
rs7587541
All − MR Egger
All − IVW(MRE)
−0.008
−0.004
0.000
0.004
MR effect size for
'Periostin' on 'eGFR'

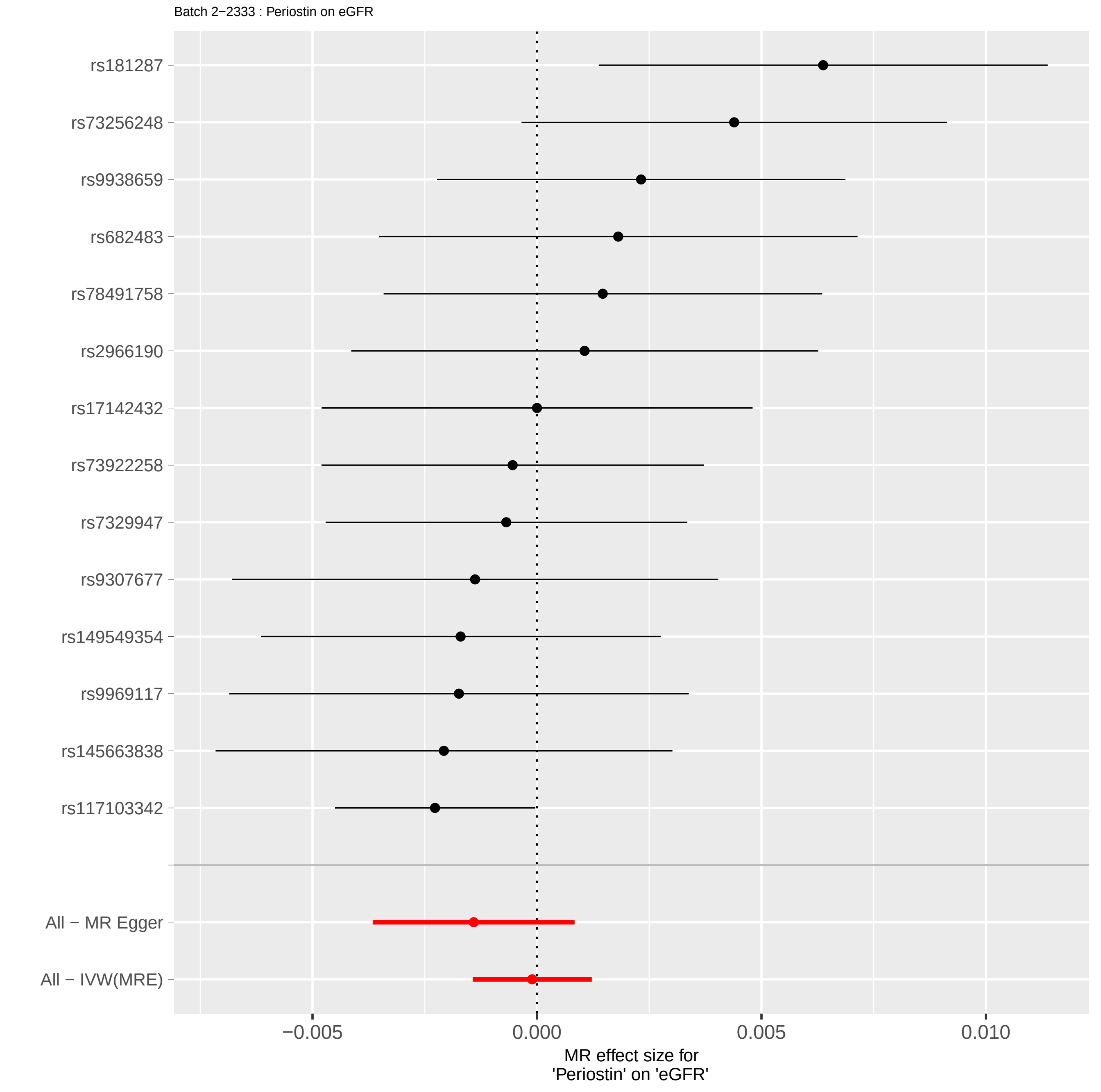
Batch 2−2333 : Periostin on eGFR
rs181287
rs73256248
rs9938659
rs682483
rs78491758
rs2966190
rs17142432
rs73922258
rs7329947
rs9307677
rs149549354
rs9969117
rs145663838
rs117103342
All − MR Egger
All − IVW(MRE)
−0.005
0.000
0.005
0.010
MR effect size for
'Periostin' on 'eGFR'

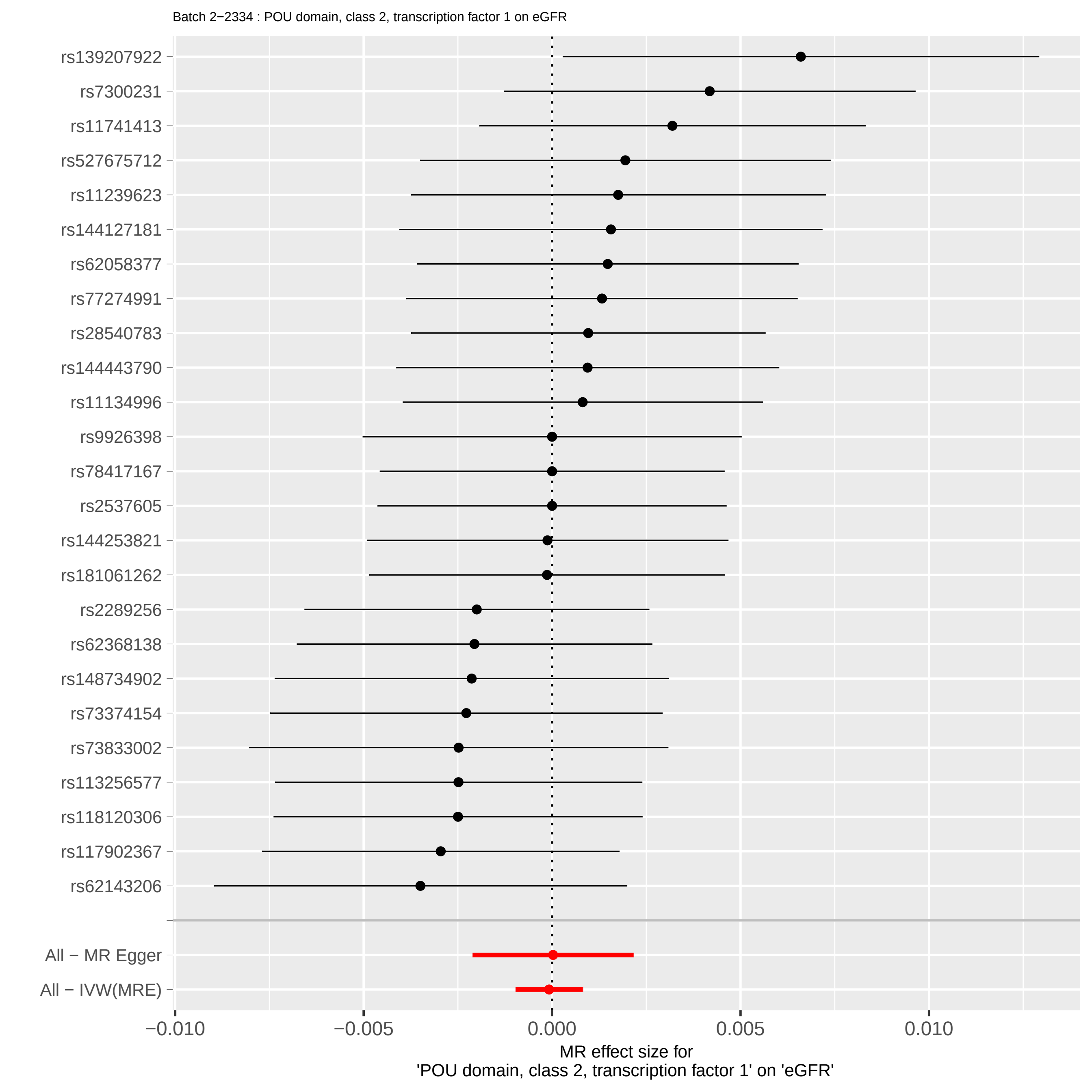
Batch 2−2334 : POU domain, class 2, transcription factor 1 on eGFR
rs139207922
rs7300231
rs11741413
rs527675712
rs11239623
rs144127181
rs62058377
rs77274991
rs28540783
rs144443790
rs11134996
rs9926398
rs78417167
rs2537605
rs144253821
rs181061262
rs2289256
rs62368138
rs148734902
rs73374154
rs73833002
rs113256577
rs118120306
rs117902367
rs62143206
All − MR Egger
All − IVW(MRE)
−0.010
−0.005
0.000
0.005
0.010
MR effect size for
'POU domain, class 2, transcription factor 1' on 'eGFR'

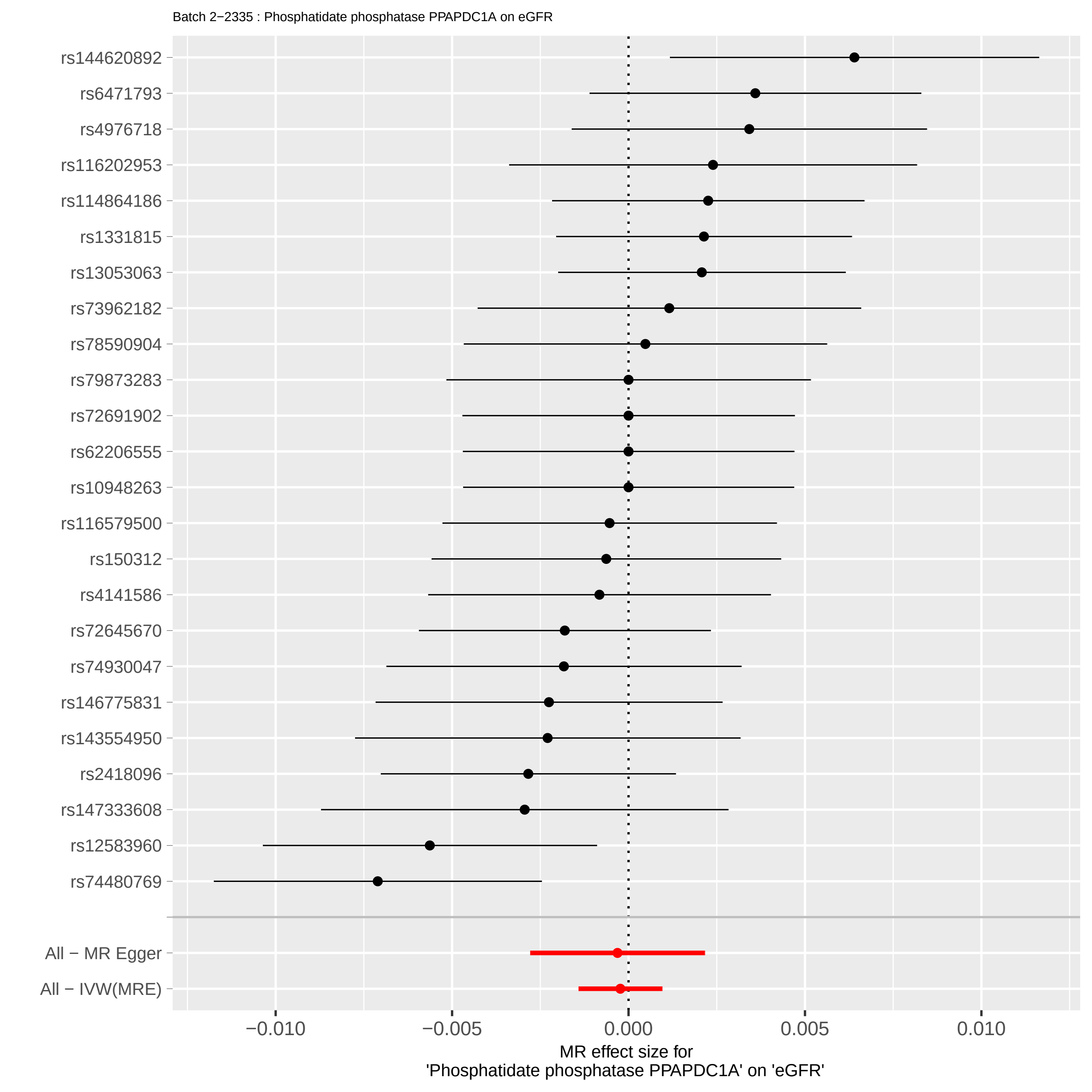

Batch 2−2335 : Phosphatidate phosphatase PPAPDC1A on eGFR
rs144620892
rs6471793
rs4976718
rs116202953
rs114864186
rs1331815
rs13053063
rs73962182
rs78590904
rs79873283
rs72691902
rs62206555
rs10948263
rs116579500
rs150312
rs4141586
rs72645670
rs74930047
rs146775831
rs143554950
rs2418096
rs147333608
rs12583960
rs74480769
All − MR Egger
All − IVW(MRE)
−0.010
−0.005
0.000
0.005
0.010
MR effect size for
'Phosphatidate phosphatase PPAPDC1A' on 'eGFR'

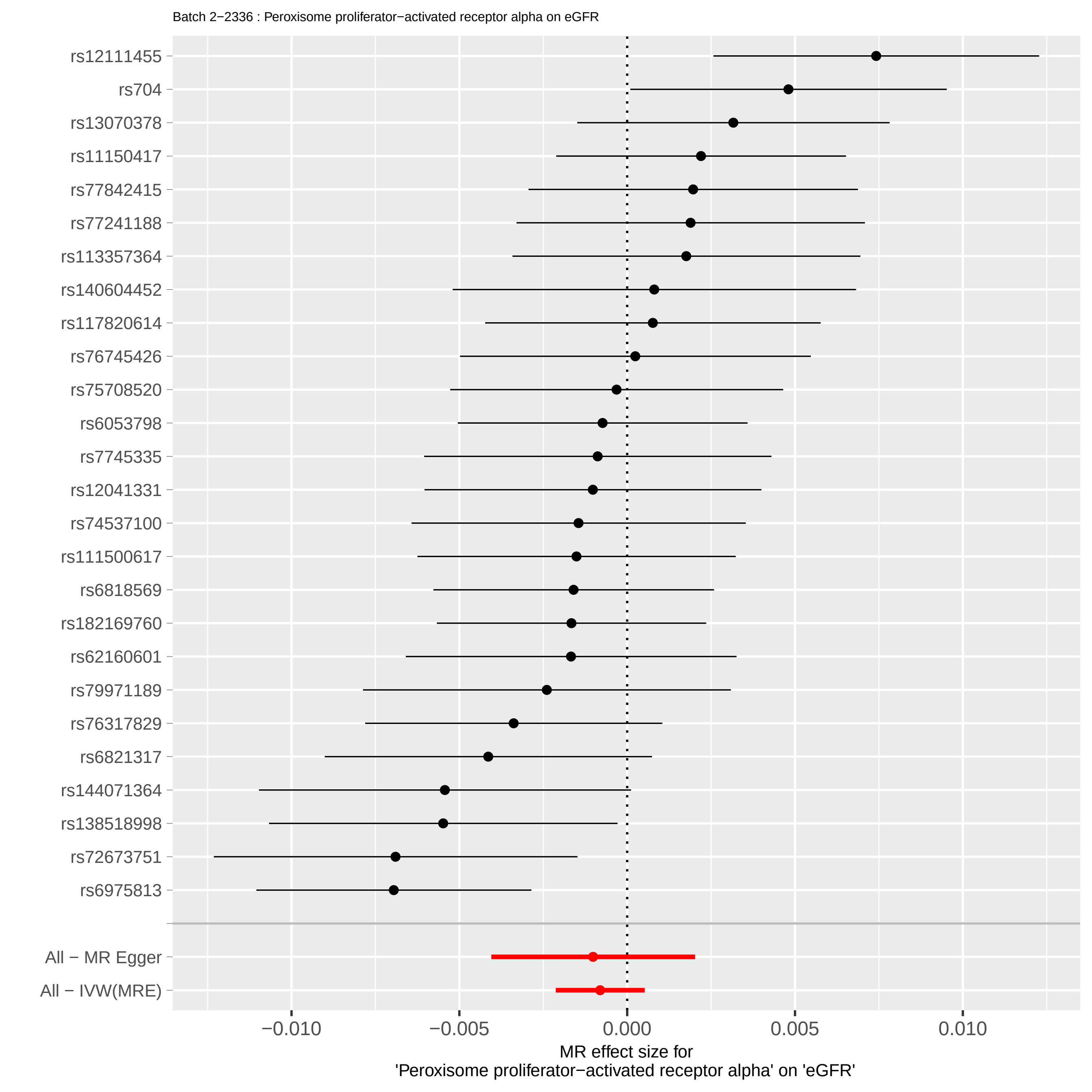

Batch 2−2336 : Peroxisome proliferator−activated receptor alpha on eGFR
rs12111455
rs704
rs13070378
rs11150417
rs77842415
rs77241188
rs113357364
rs140604452
rs117820614
rs76745426
rs75708520
rs6053798
rs7745335
rs12041331
rs74537100
rs111500617
rs6818569
rs182169760
rs62160601
rs79971189
rs76317829
rs6821317
rs144071364
rs138518998
rs72673751
rs6975813
All − MR Egger
All − IVW(MRE)
−0.010
−0.005
0.000
0.005
0.010
MR effect size for
'Peroxisome proliferator−activated receptor alpha' on 'eGFR'

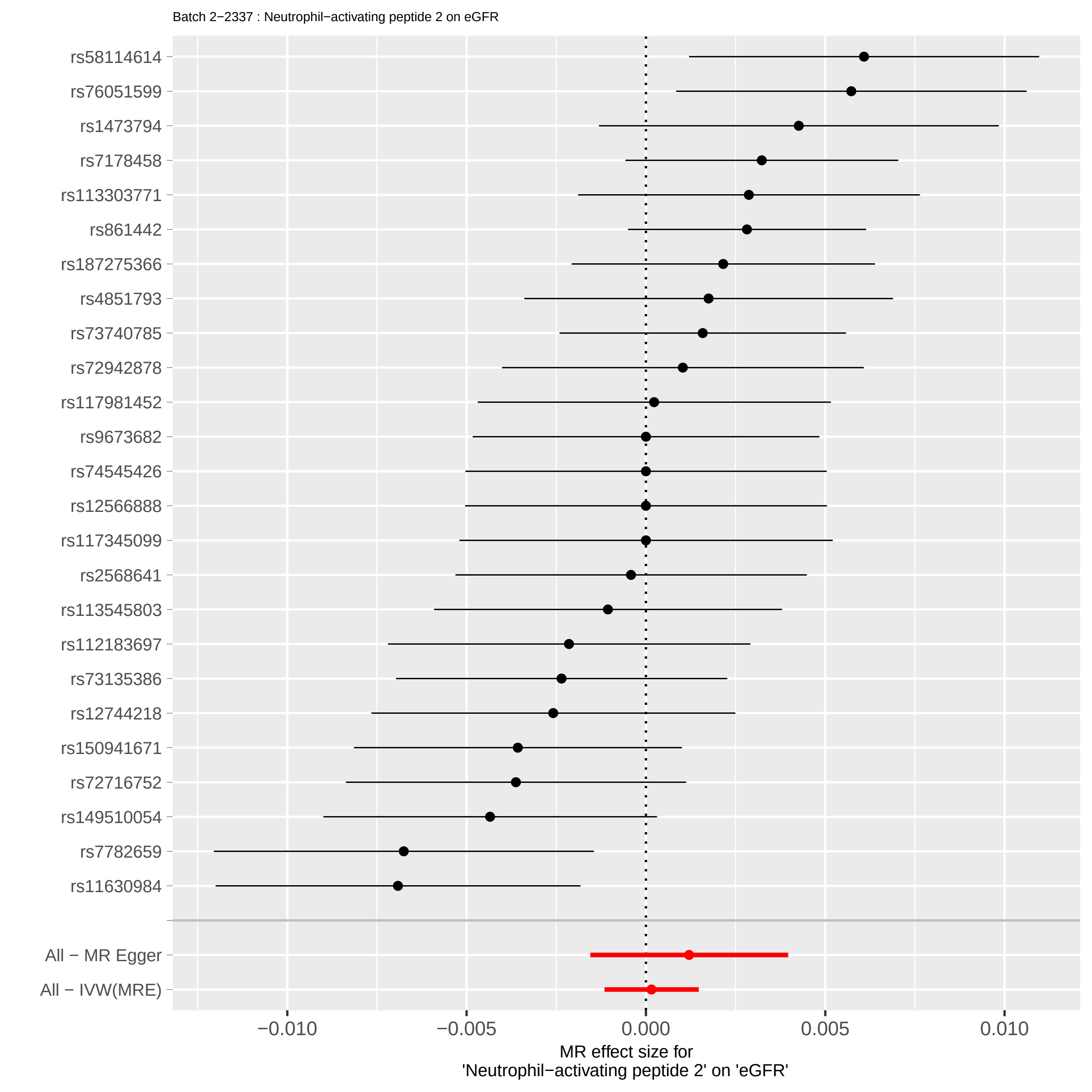

Batch 2−2337 : Neutrophil−activating peptide 2 on eGFR
rs58114614
rs76051599
rs1473794
rs7178458
rs113303771
rs861442
rs187275366
rs4851793
rs73740785
rs72942878
rs117981452
rs9673682
rs74545426
rs12566888
rs117345099
rs2568641
rs113545803
rs112183697
rs73135386
rs12744218
rs150941671
rs72716752
rs149510054
rs7782659
rs11630984
All − MR Egger
All − IVW(MRE)
−0.010
−0.005
0.000
0.005
0.010
MR effect size for
'Neutrophil−activating peptide 2' on 'eGFR'

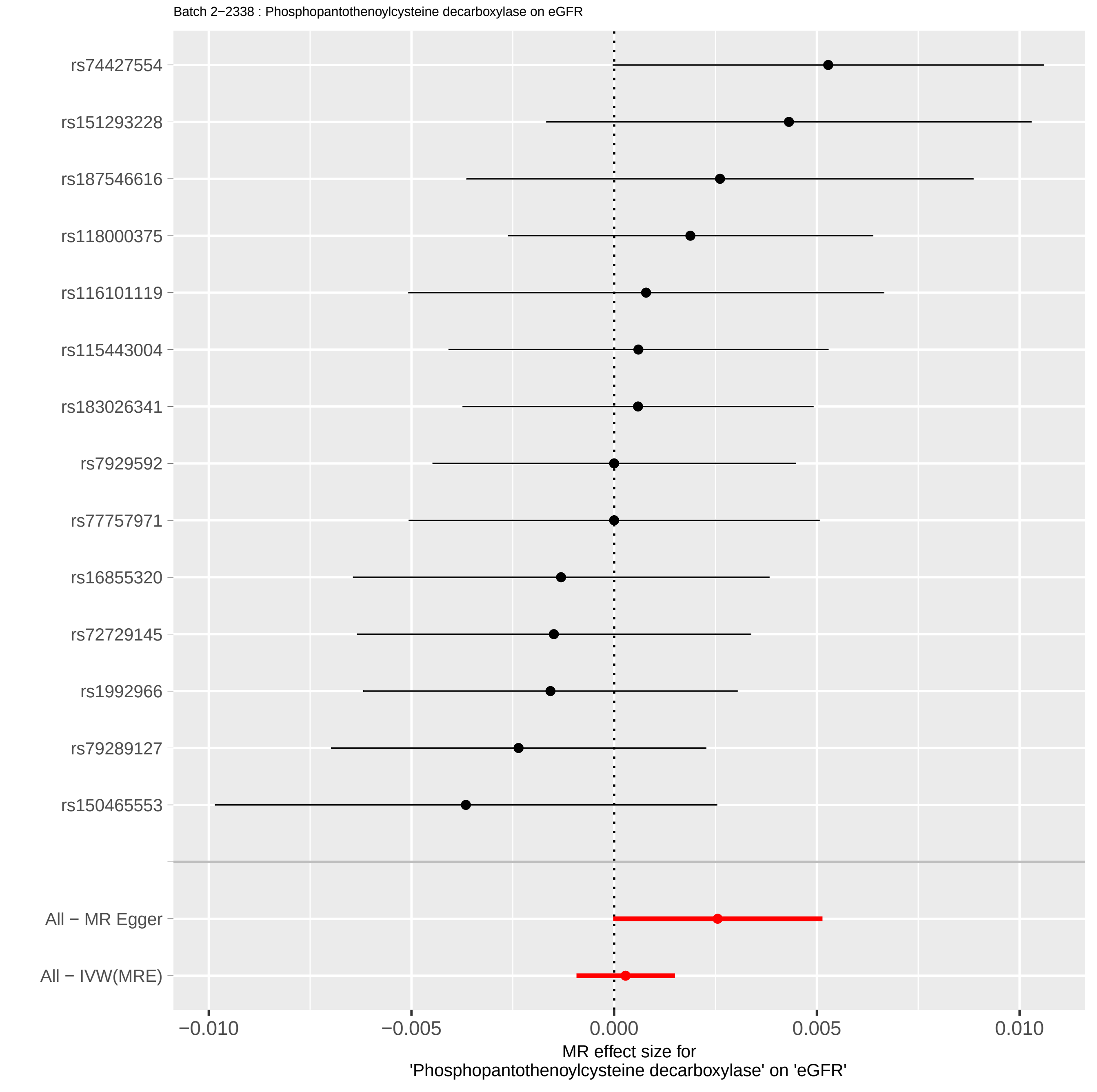

Batch 2−2338 : Phosphopantothenoylcysteine decarboxylase on eGFR
rs74427554
rs151293228
rs187546616
rs118000375
rs116101119
rs115443004
rs183026341
rs7929592
rs77757971
rs16855320
rs72729145
rs1992966
rs79289127
rs150465553
All − MR Egger
All − IVW(MRE)
−0.010
−0.005
0.000
0.005
0.010
MR effect size for
'Phosphopantothenoylcysteine decarboxylase' on 'eGFR'

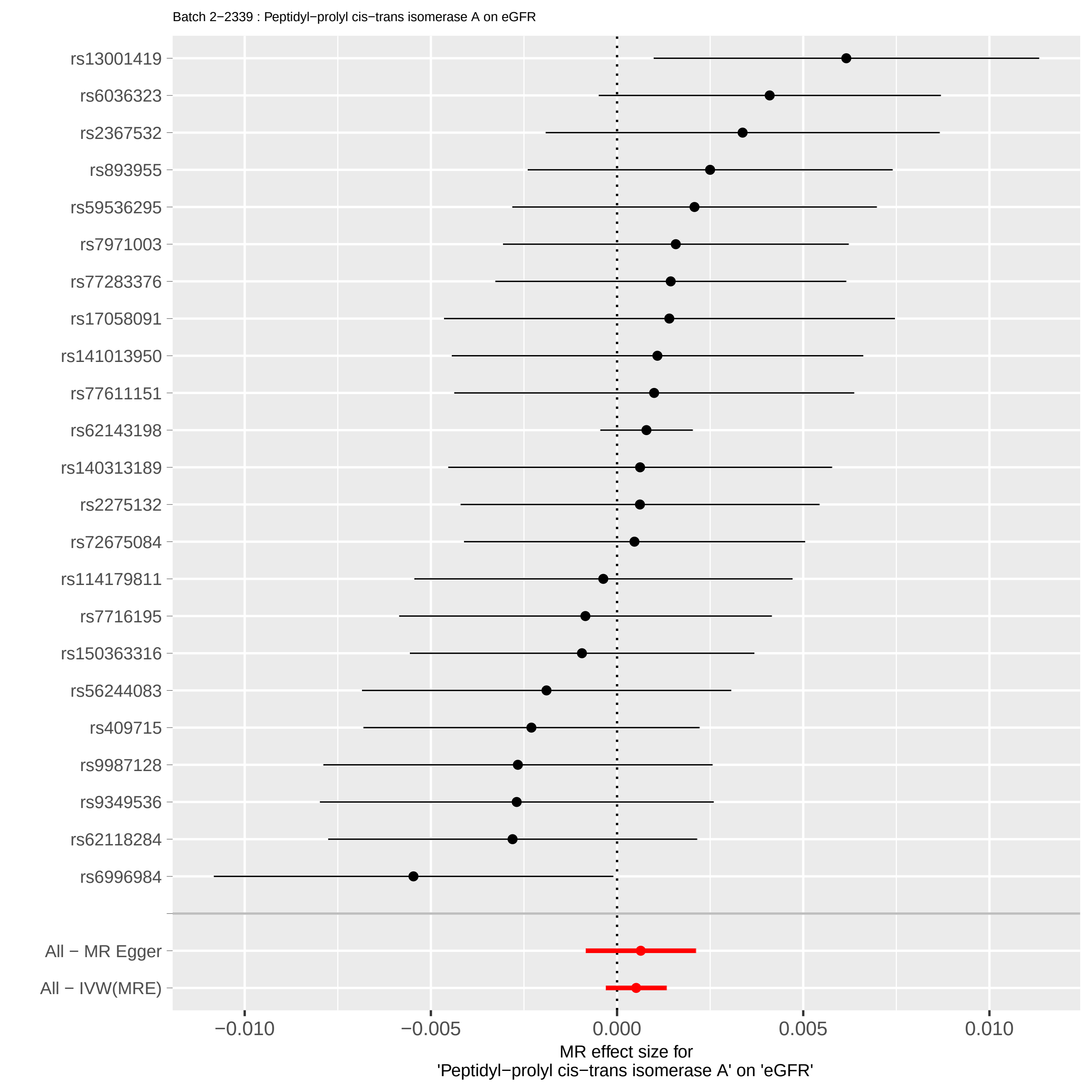
Batch 2−2339 : Peptidyl−prolyl cis−trans isomerase A on eGFR
rs13001419
rs6036323
rs2367532
rs893955
rs59536295
rs7971003
rs77283376
rs17058091
rs141013950
rs77611151
rs62143198
rs140313189
rs2275132
rs72675084
rs114179811
rs7716195
rs150363316
rs56244083
rs409715
rs9987128
rs9349536
rs62118284
rs6996984
All − MR Egger
All − IVW(MRE)
−0.010
−0.005
0.000
0.005
0.010
MR effect size for
'Peptidyl−prolyl cis−trans isomerase A' on 'eGFR'

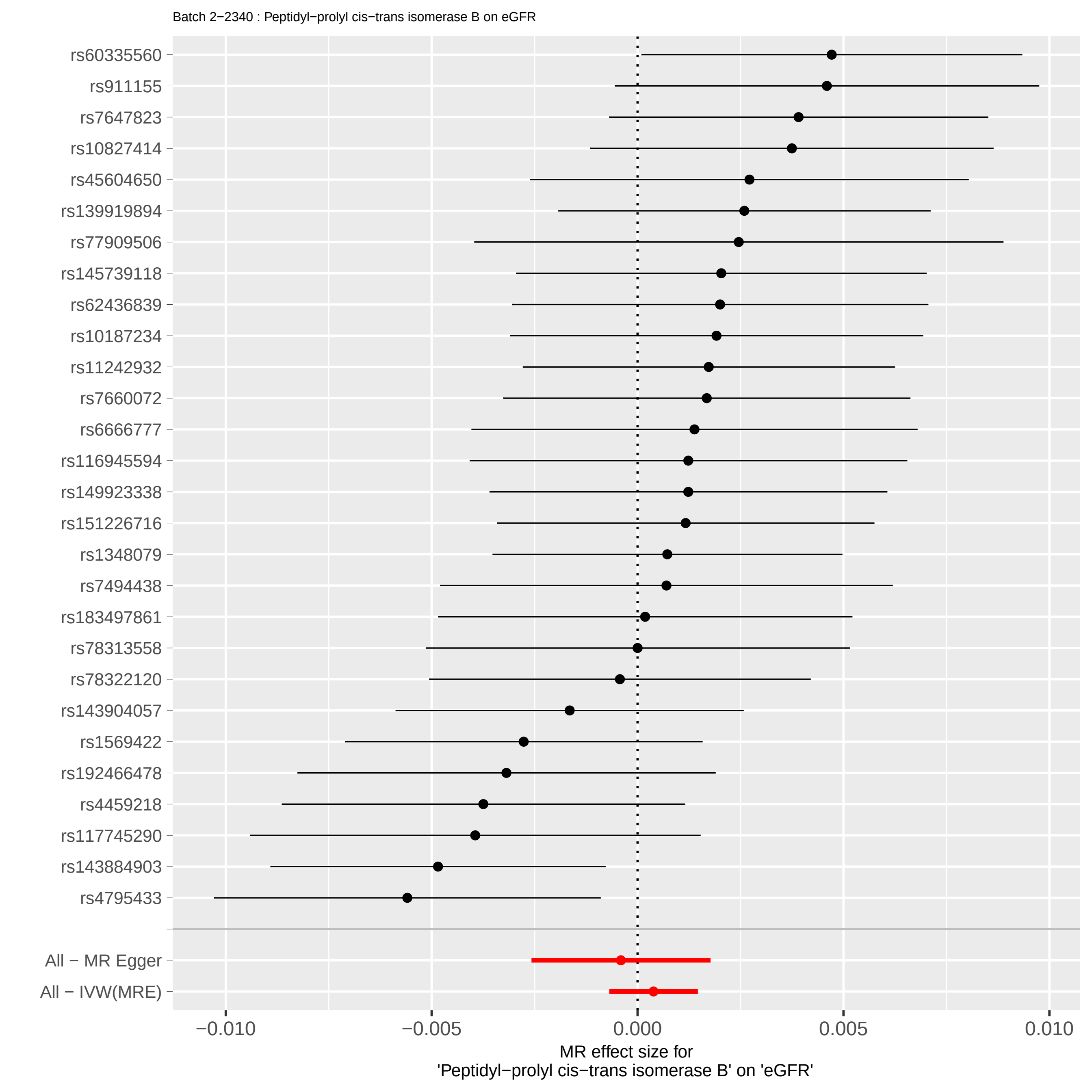
Batch 2−2340 : Peptidyl−prolyl cis−trans isomerase B on eGFR
rs60335560
rs911155
rs7647823
rs10827414
rs45604650
rs139919894
rs77909506
rs145739118
rs62436839
rs10187234
rs11242932
rs7660072
rs6666777
rs116945594
rs149923338
rs151226716
rs1348079
rs7494438
rs183497861
rs78313558
rs78322120
rs143904057
rs1569422
rs192466478
rs4459218
rs117745290
rs143884903
rs4795433
All − MR Egger
All − IVW(MRE)
−0.010
−0.005
0.000
0.005
0.010
MR effect size for
'Peptidyl−prolyl cis−trans isomerase B' on 'eGFR'

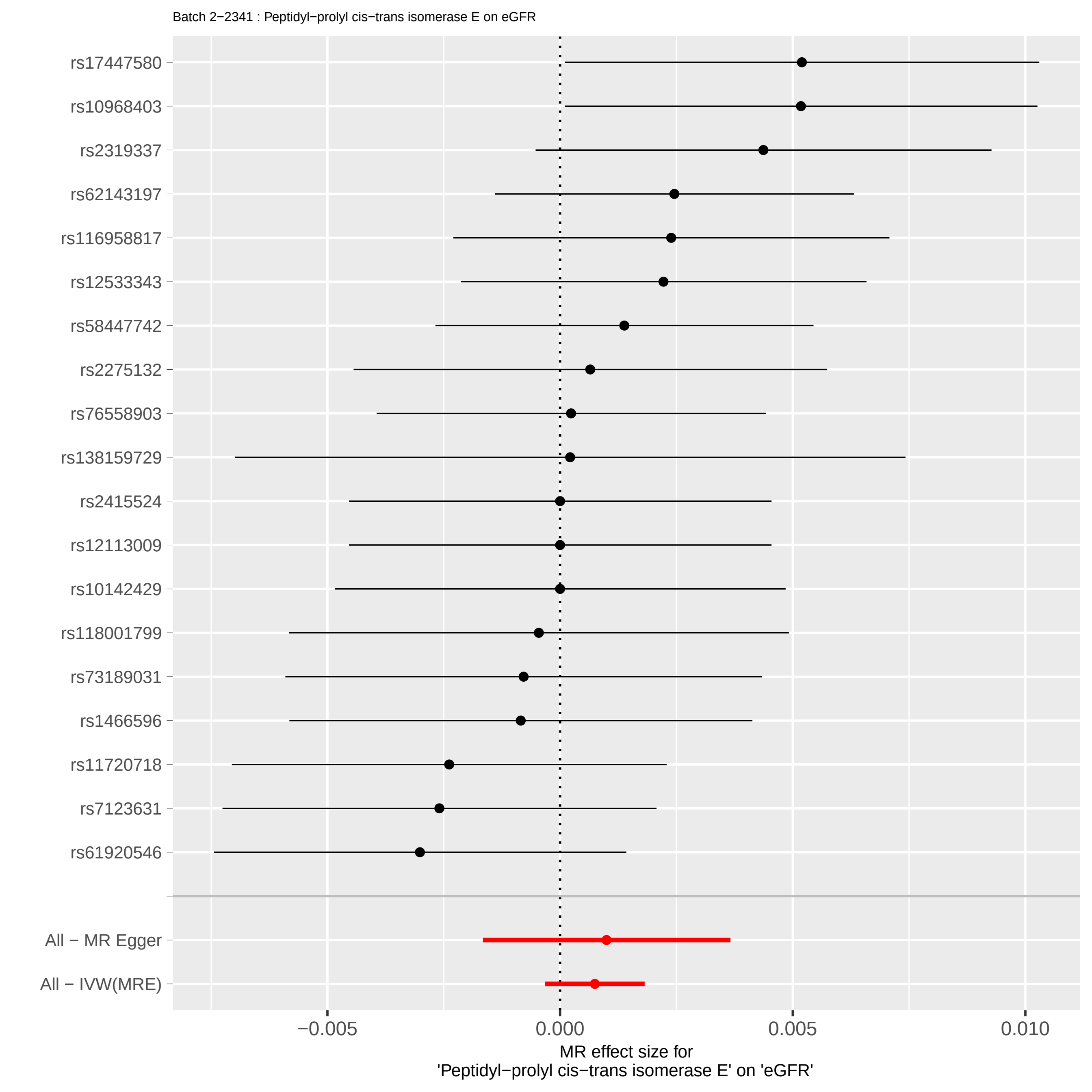

Batch 2−2341 : Peptidyl−prolyl cis−trans isomerase E on eGFR
rs17447580
rs10968403
rs2319337
rs62143197
rs116958817
rs12533343
rs58447742
rs2275132
rs76558903
rs138159729
rs2415524
rs12113009
rs10142429
rs118001799
rs73189031
rs1466596
rs11720718
rs7123631
rs61920546
All − MR Egger
All − IVW(MRE)
−0.005
0.000
0.005
0.010
MR effect size for
'Peptidyl−prolyl cis−trans isomerase E' on 'eGFR'

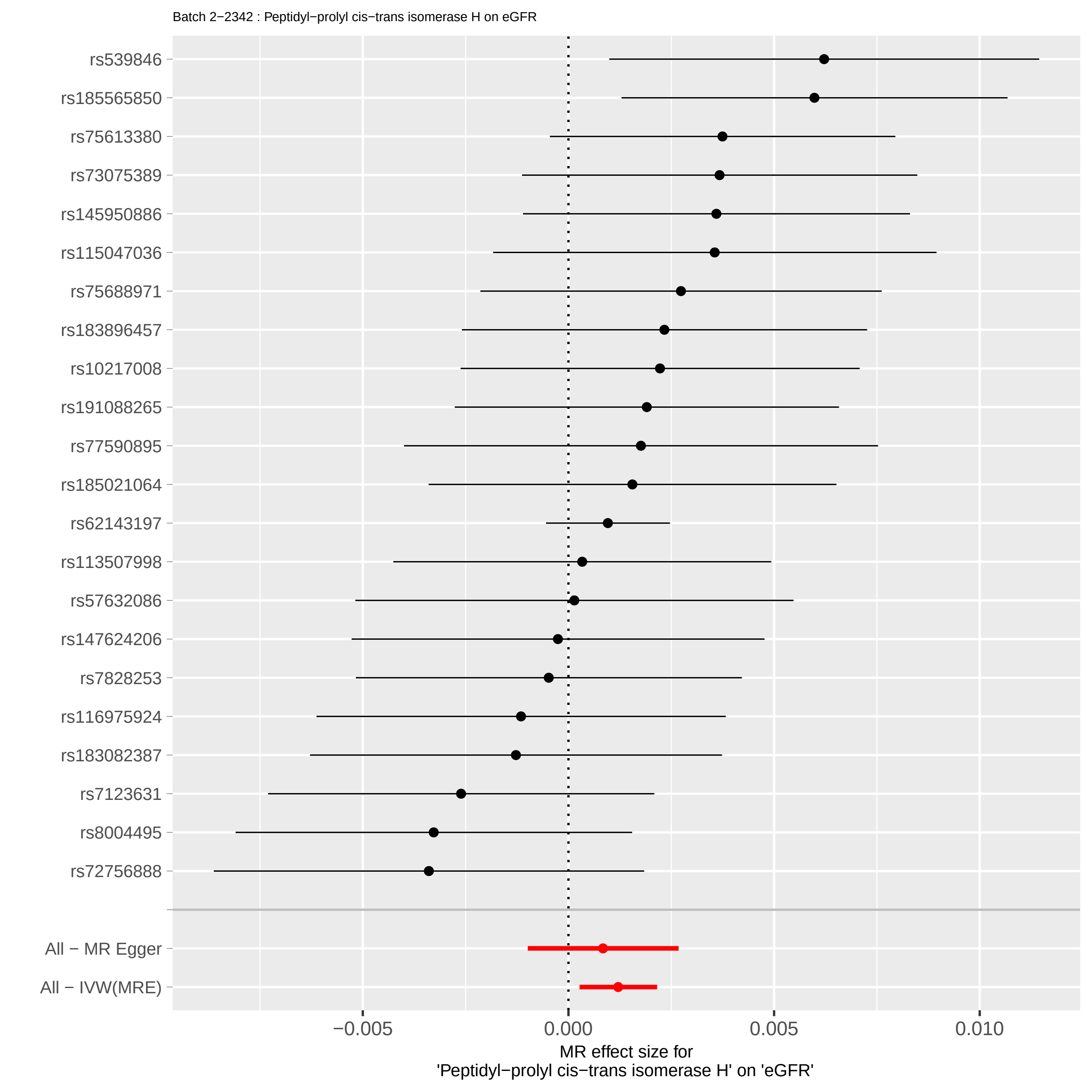

Batch 2−2342 : Peptidyl−prolyl cis−trans isomerase H on eGFR
rs539846
rs185565850
rs75613380
rs73075389
rs145950886
rs115047036
rs75688971
rs183896457
rs10217008
rs191088265
rs77590895
rs185021064
rs62143197
rs113507998
rs57632086
rs147624206
rs7828253
rs116975924
rs183082387
rs7123631
rs8004495
rs72756888
All − MR Egger
All − IVW(MRE)
−0.005
0.000
0.005
0.010
MR effect size for
'Peptidyl−prolyl cis−trans isomerase H' on 'eGFR'

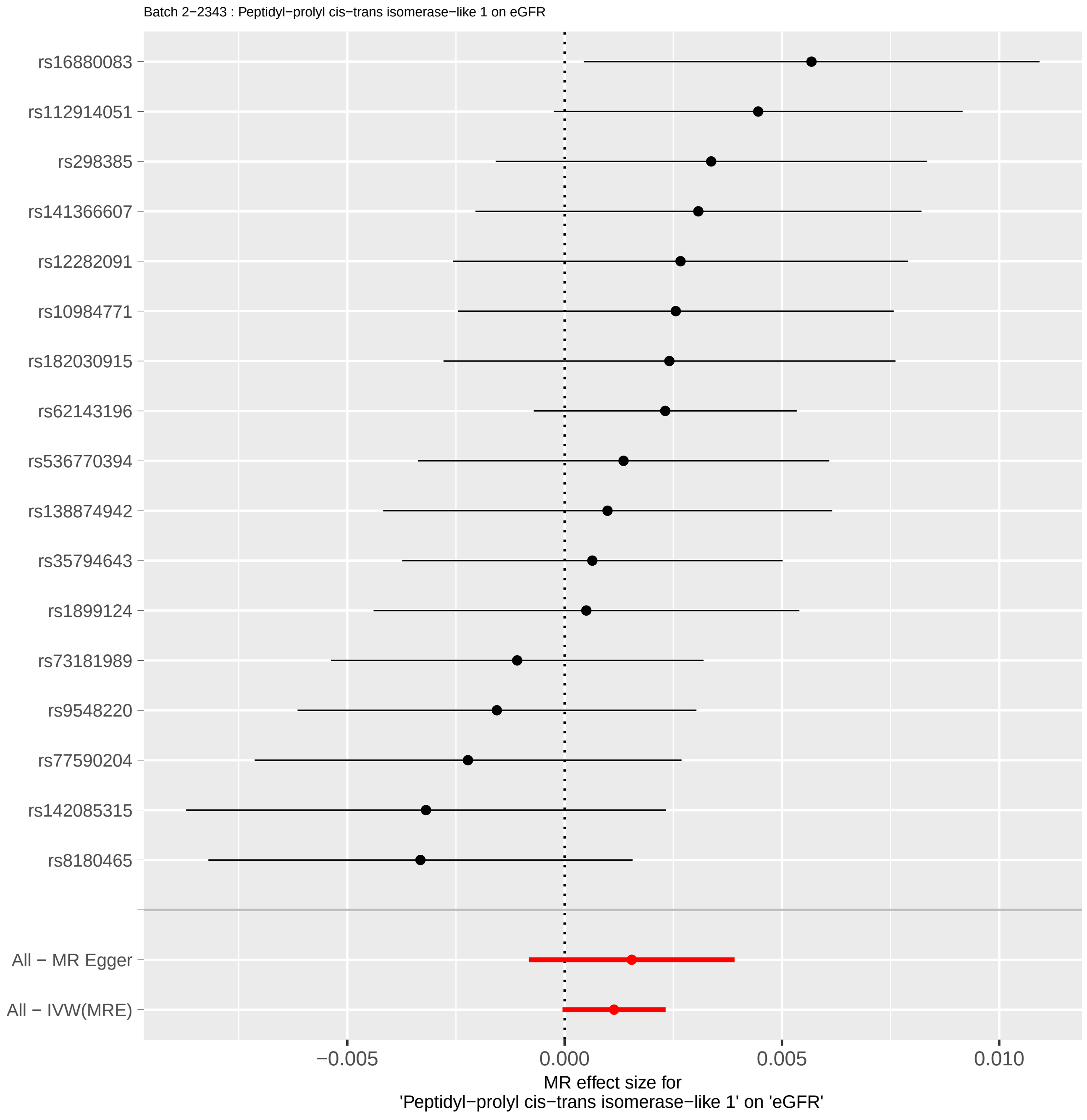

Batch 2−2343 : Peptidyl−prolyl cis−trans isomerase−like 1 on eGFR
rs16880083
rs112914051
rs298385
rs141366607
rs12282091
rs10984771
rs182030915
rs62143196
rs536770394
rs138874942
rs35794643
rs1899124
rs73181989
rs9548220
rs77590204
rs142085315
rs8180465
All − MR Egger
All − IVW(MRE)
−0.005
0.000
0.005
0.010
MR effect size for
'Peptidyl−prolyl cis−trans isomerase−like 1' on 'eGFR'

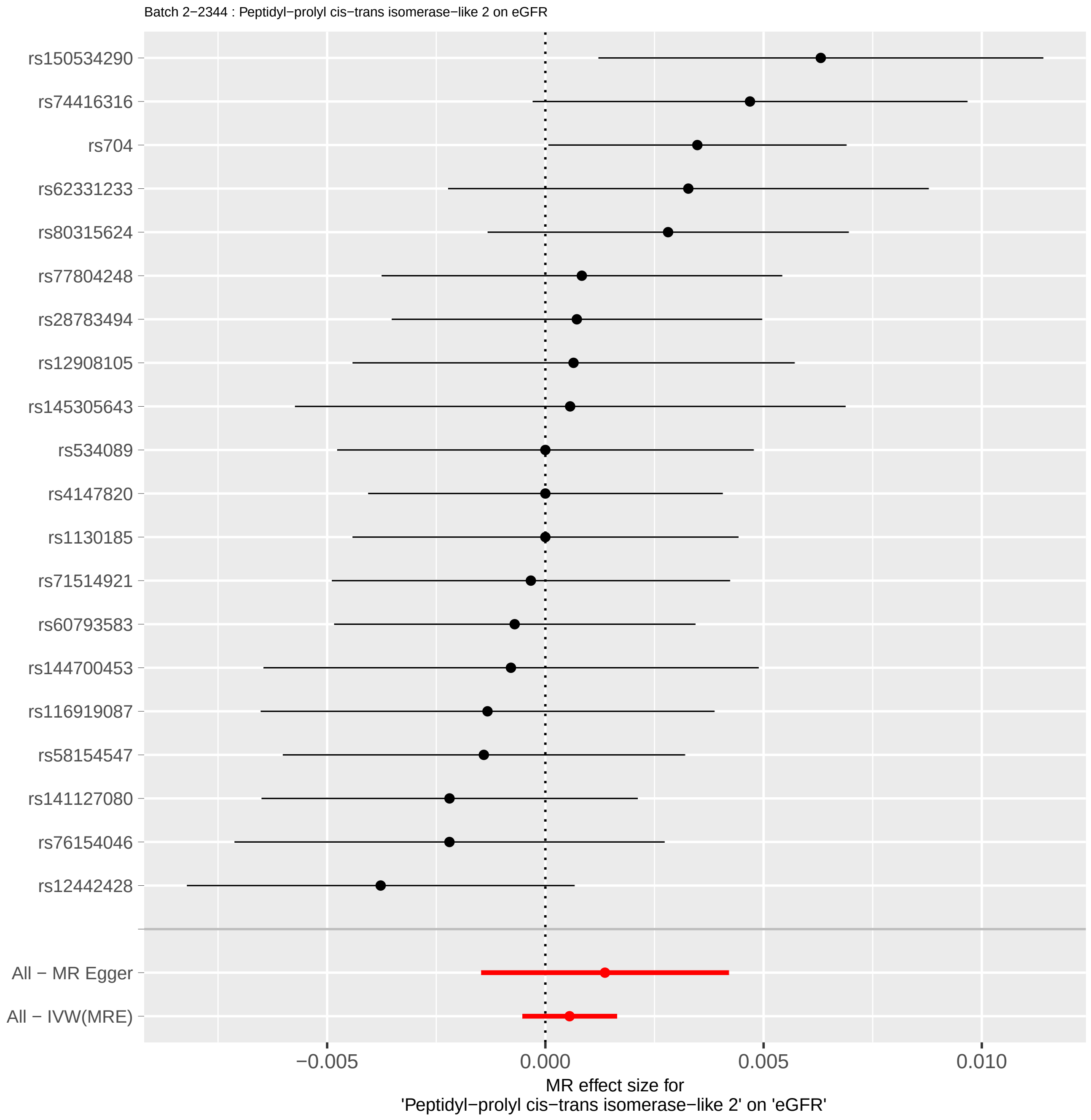

Batch 2−2344 : Peptidyl−prolyl cis−trans isomerase−like 2 on eGFR
rs150534290
rs74416316
rs704
rs62331233
rs80315624
rs77804248
rs28783494
rs12908105
rs145305643
rs534089
rs4147820
rs1130185
rs71514921
rs60793583
rs144700453
rs116919087
rs58154547
rs141127080
rs76154046
rs12442428
All − MR Egger
All − IVW(MRE)
−0.005
0.000
0.005
0.010
MR effect size for
'Peptidyl−prolyl cis−trans isomerase−like 2' on 'eGFR'

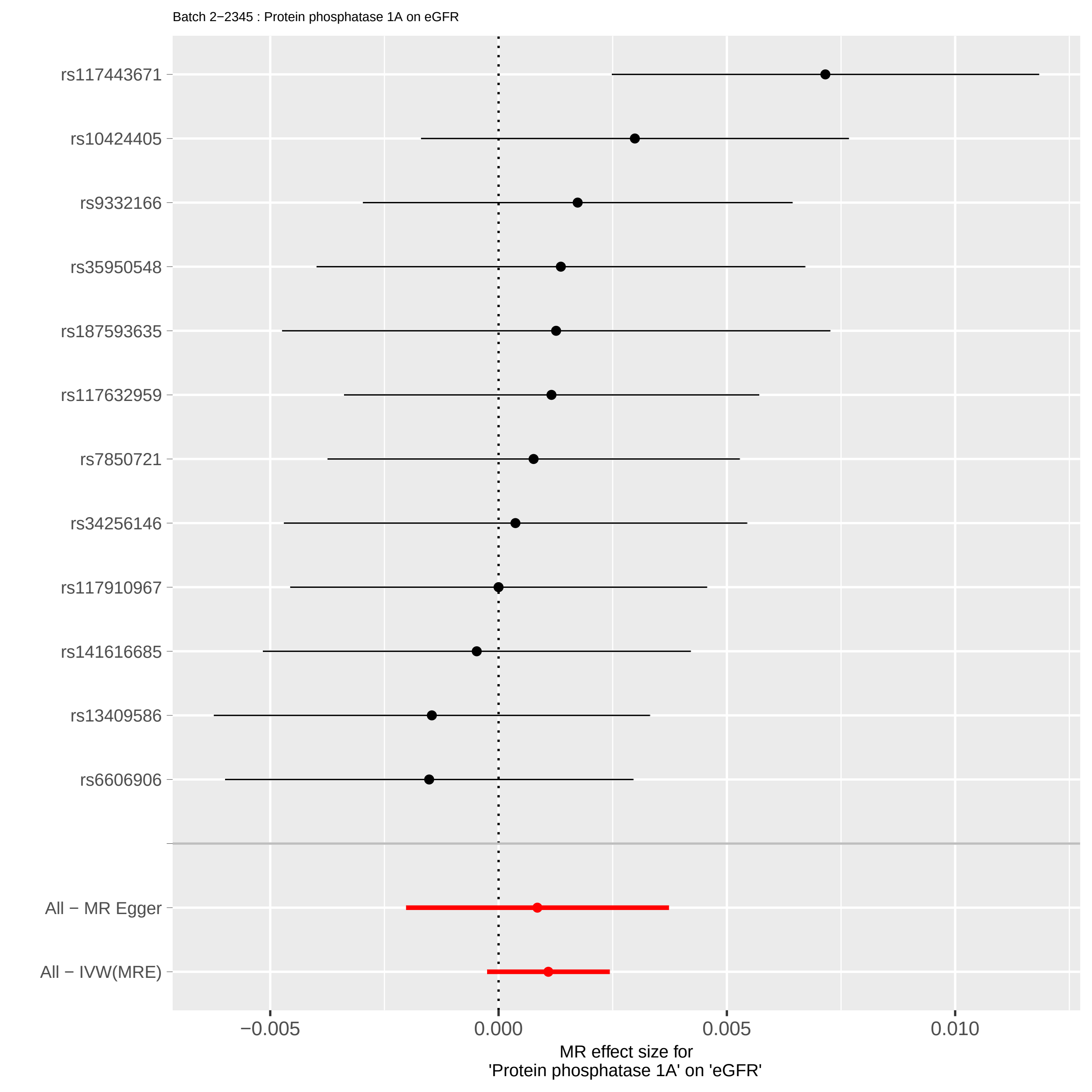

Batch 2−2345 : Protein phosphatase 1A on eGFR
rs117443671
rs10424405
rs9332166
rs35950548
rs187593635
rs117632959
rs7850721
rs34256146
rs117910967
rs141616685
rs13409586
rs6606906
All − MR Egger
All − IVW(MRE)
−0.005
0.000
0.005
0.010
MR effect size for
'Protein phosphatase 1A' on 'eGFR'

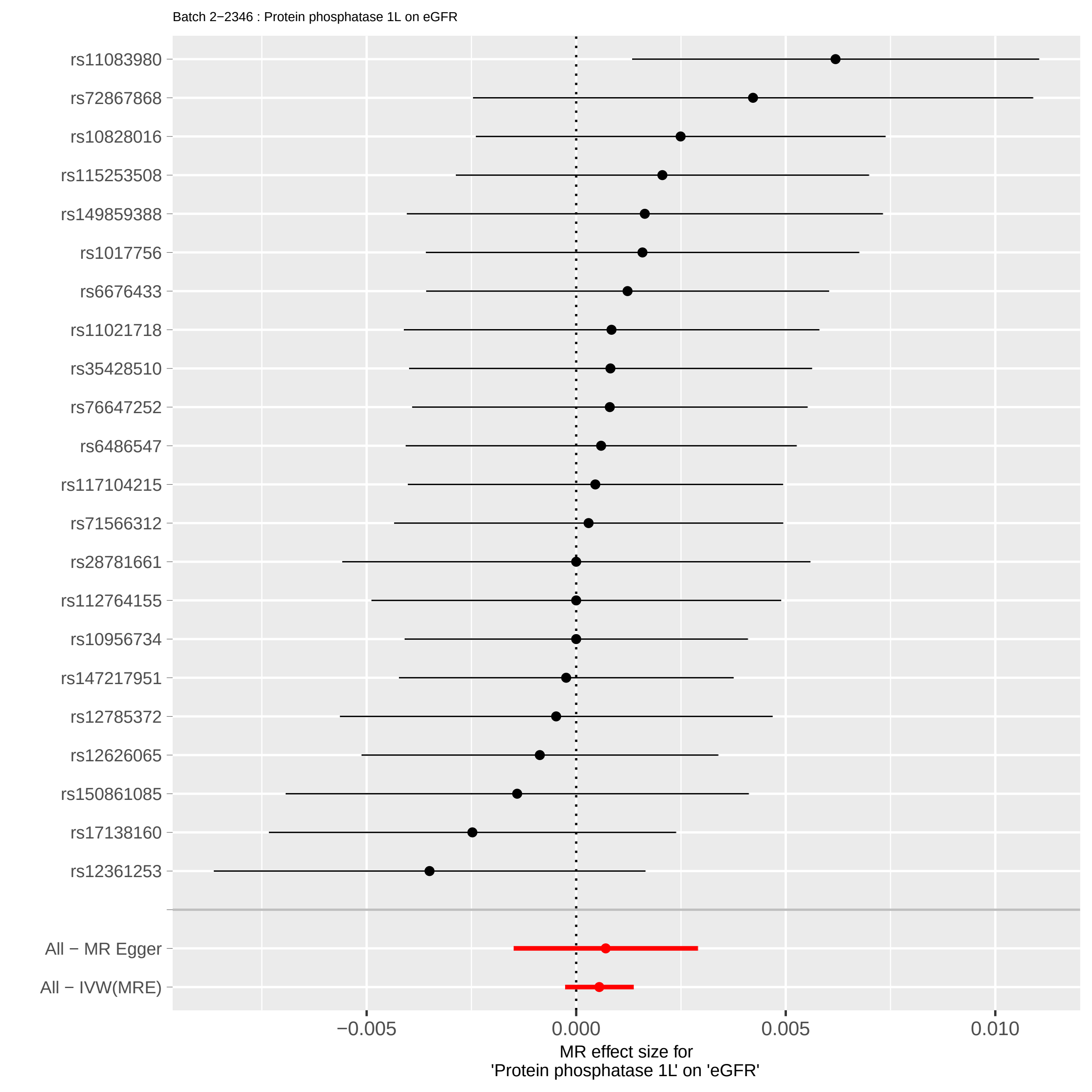

Batch 2−2346 : Protein phosphatase 1L on eGFR
rs11083980
rs72867868
rs10828016
rs115253508
rs149859388
rs1017756
rs6676433
rs11021718
rs35428510
rs76647252
rs6486547
rs117104215
rs71566312
rs28781661
rs112764155
rs10956734
rs147217951
rs12785372
rs12626065
rs150861085
rs17138160
rs12361253
All − MR Egger
All − IVW(MRE)
−0.005
0.000
0.005
0.010
MR effect size for
'Protein phosphatase 1L' on 'eGFR'

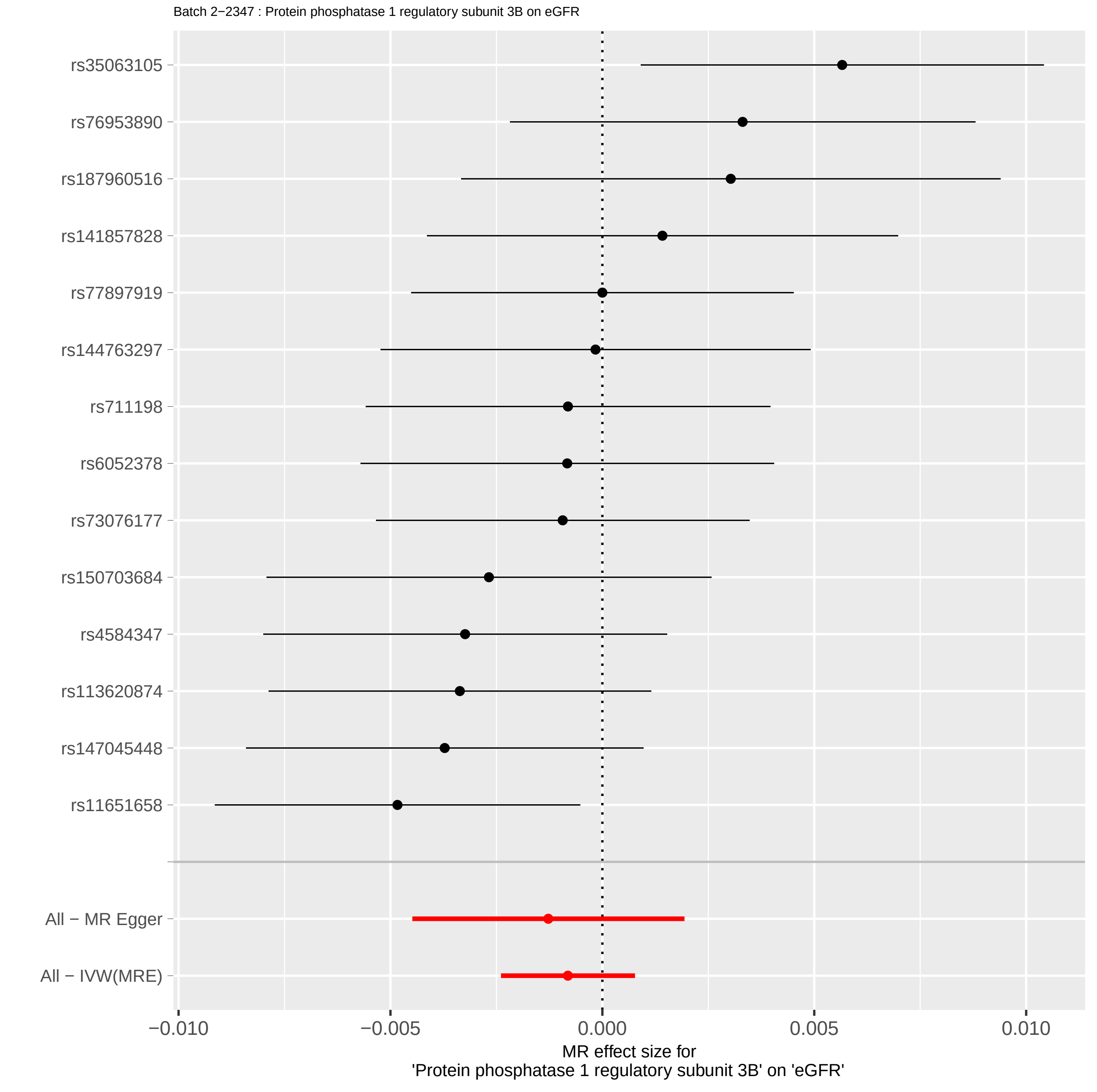
Batch 2−2347 : Protein phosphatase 1 regulatory subunit 3B on eGFR
rs35063105
rs76953890
rs187960516
rs141857828
rs77897919
rs144763297
rs711198
rs6052378
rs73076177
rs150703684
rs4584347
rs113620874
rs147045448
rs11651658
All − MR Egger
All − IVW(MRE)
−0.010
−0.005
0.000
0.005
0.010
MR effect size for
'Protein phosphatase 1 regulatory subunit 3B' on 'eGFR'

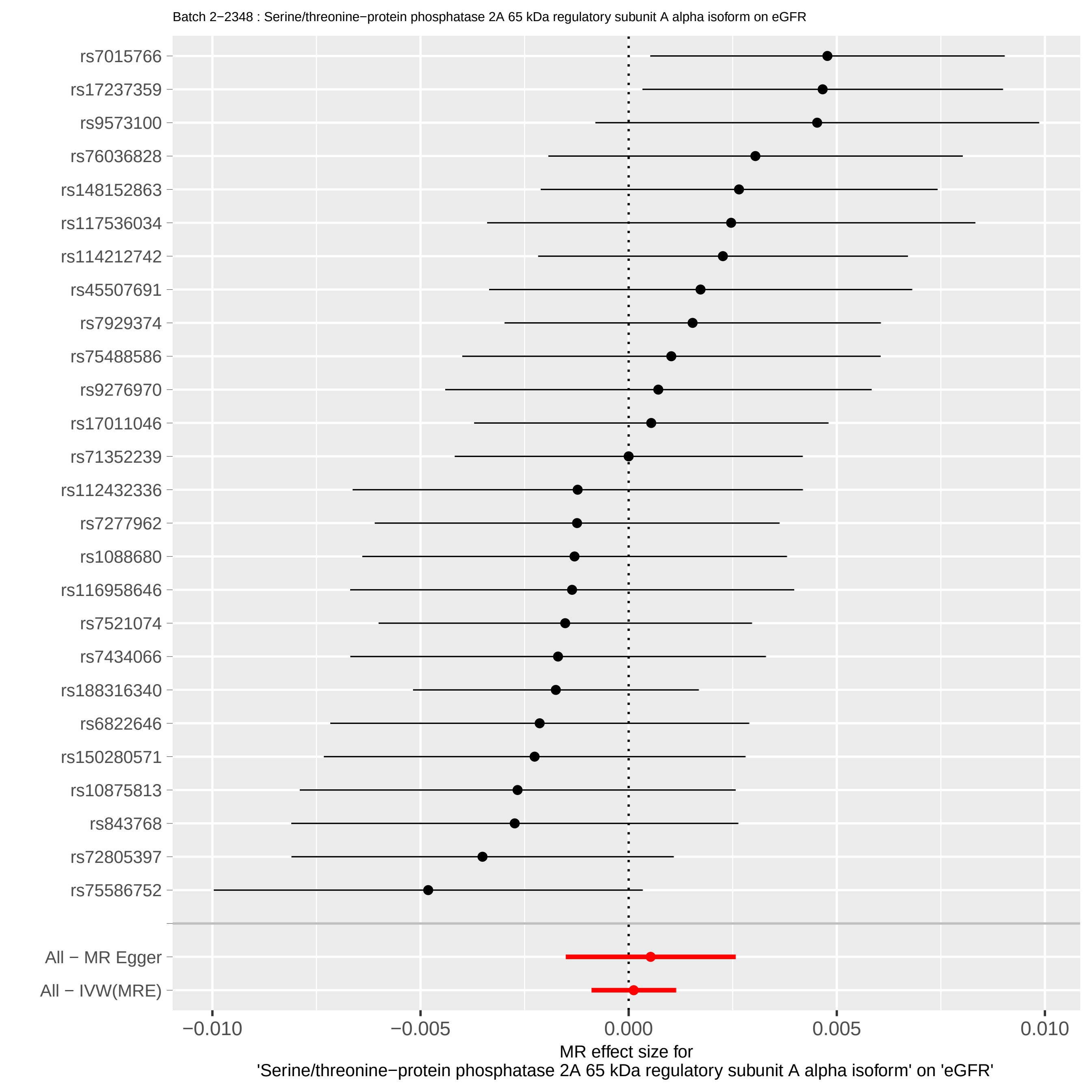

Batch 2−2348 : Serine/threonine−protein phosphatase 2A 65 kDa regulatory subunit A alpha isoform on eGFR
rs7015766
rs17237359
rs9573100
rs76036828
rs148152863
rs117536034
rs114212742
rs45507691
rs7929374
rs75488586
rs9276970
rs17011046
rs71352239
rs112432336
rs7277962
rs1088680
rs116958646
rs7521074
rs7434066
rs188316340
rs6822646
rs150280571
rs10875813
rs843768
rs72805397
rs75586752
All − MR Egger
All − IVW(MRE)
−0.010
−0.005
0.000
0.005
0.010
MR effect size for
'Serine/threonine−protein phosphatase 2A 65 kDa regulatory subunit A alpha isoform' on 'eGFR'

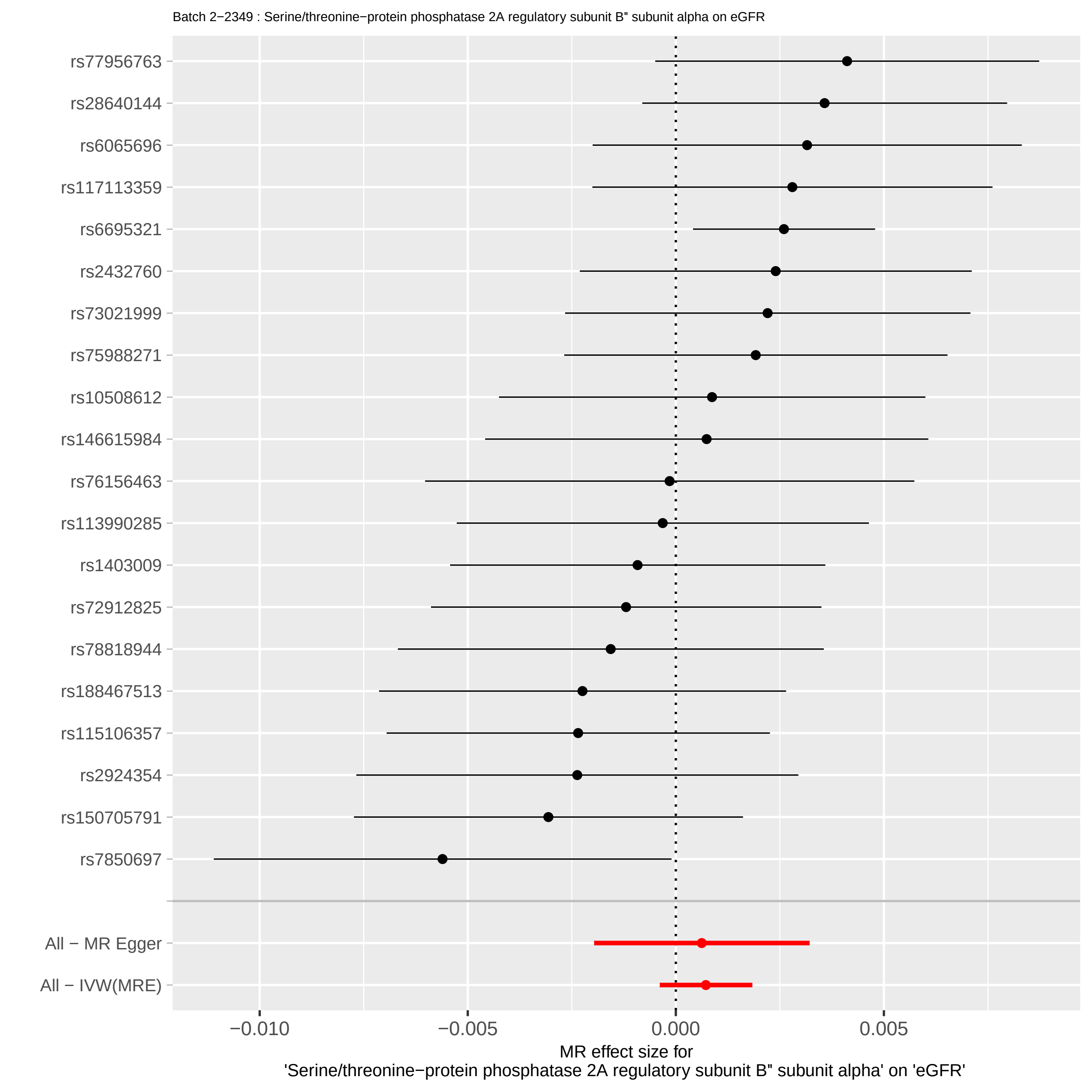

Batch 2−2349 : Serine/threonine−protein phosphatase 2A regulatory subunit B" subunit alpha on eGFR
rs77956763
rs28640144
rs6065696
rs117113359
rs6695321
rs2432760
rs73021999
rs75988271
rs10508612
rs146615984
rs76156463
rs113990285
rs1403009
rs72912825
rs78818944
rs188467513
rs115106357
rs2924354
rs150705791
rs7850697
All − MR Egger
All − IVW(MRE)
−0.010
−0.005
0.000
0.005
MR effect size for
'Serine/threonine−protein phosphatase 2A regulatory subunit B" subunit alpha' on 'eGFR'

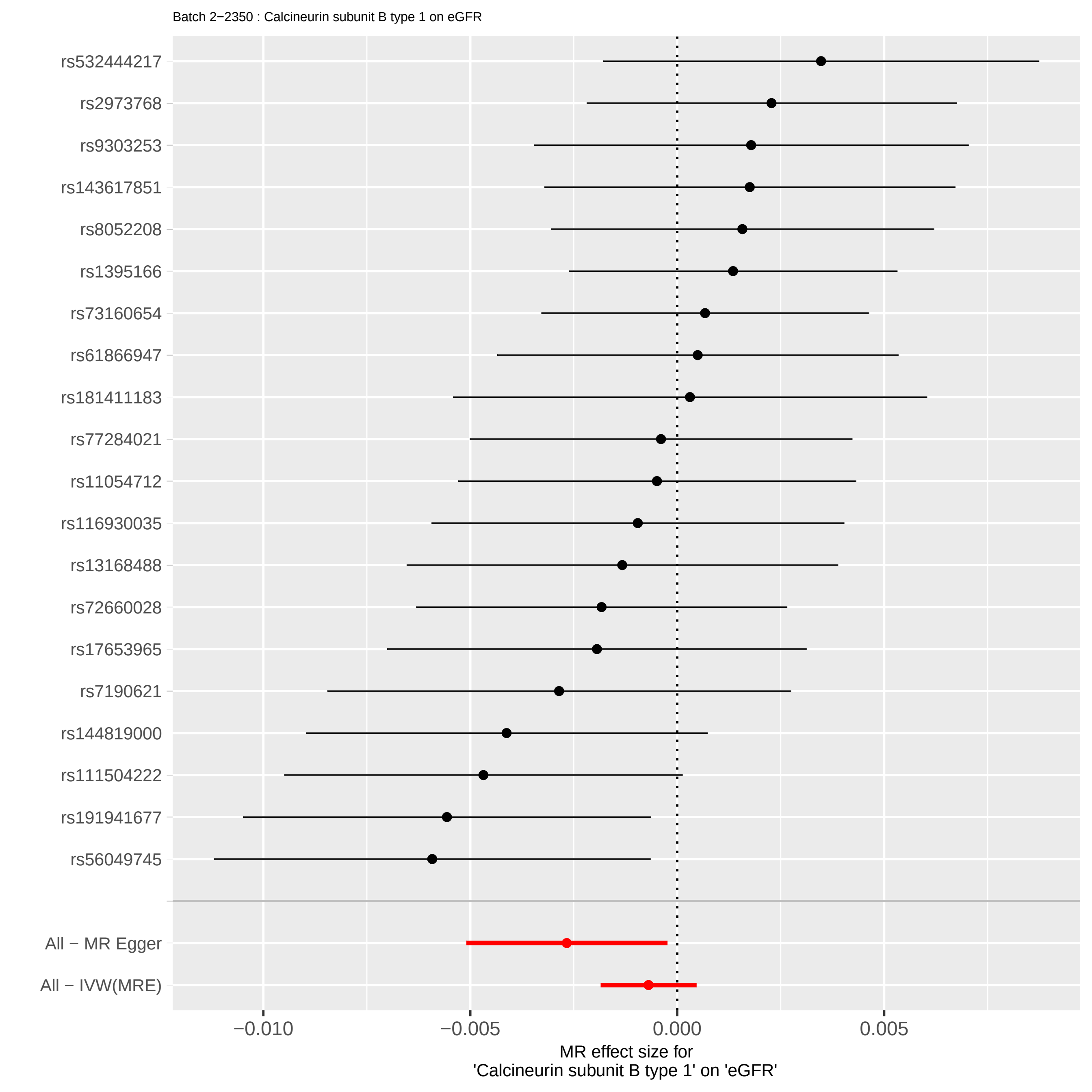

Batch 2−2350 : Calcineurin subunit B type 1 on eGFR
rs532444217
rs2973768
rs9303253
rs143617851
rs8052208
rs1395166
rs73160654
rs61866947
rs181411183
rs77284021
rs11054712
rs116930035
rs13168488
rs72660028
rs17653965
rs7190621
rs144819000
rs111504222
rs191941677
rs56049745
All − MR Egger
All − IVW(MRE)
−0.010
−0.005
0.000
0.005
MR effect size for
'Calcineurin subunit B type 1' on 'eGFR'

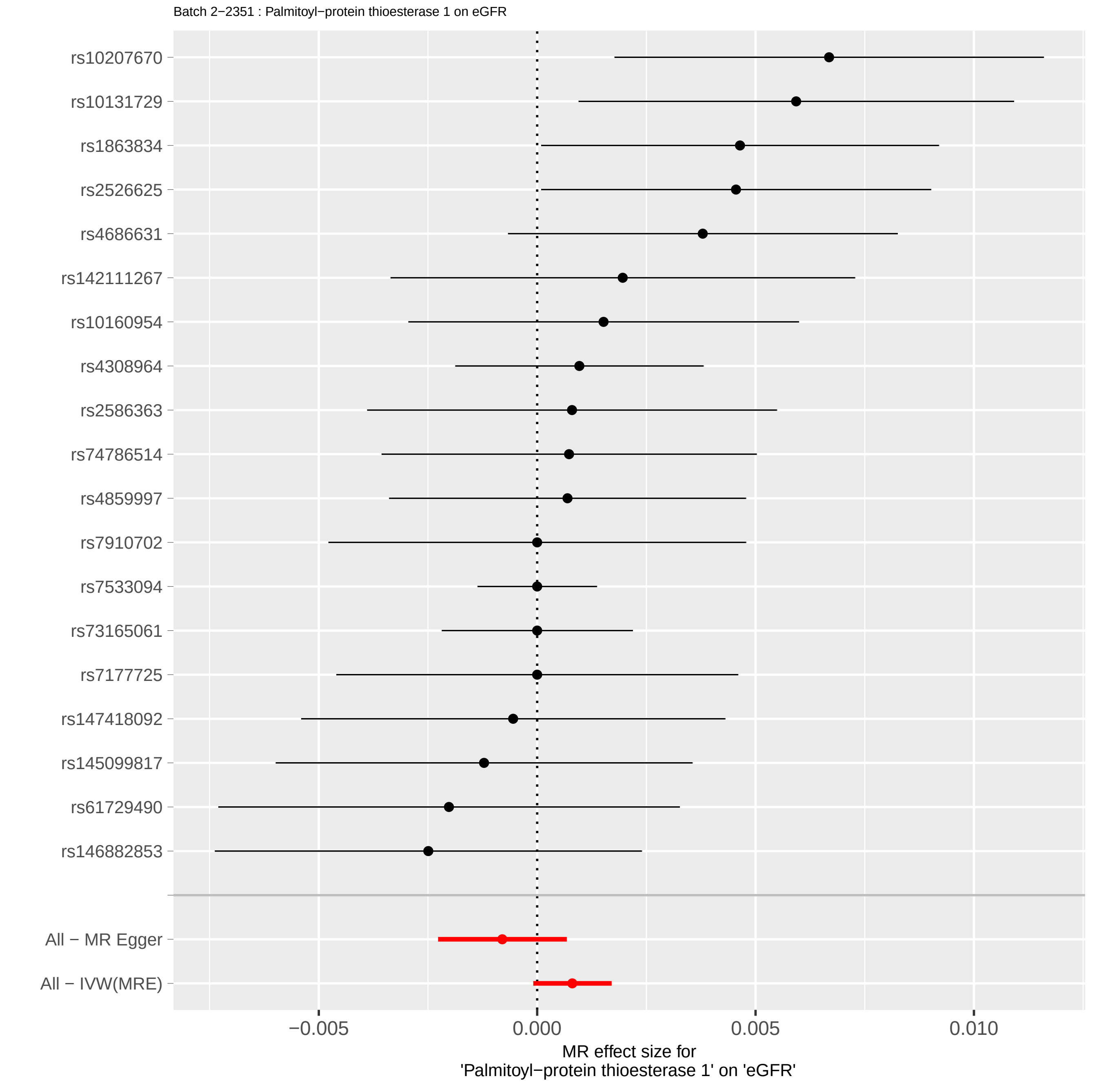
Batch 2−2351 : Palmitoyl−protein thioesterase 1 on eGFR
rs10207670
rs10131729
rs1863834
rs2526625
rs4686631
rs142111267
rs10160954
rs4308964
rs2586363
rs74786514
rs4859997
rs7910702
rs7533094
rs73165061
rs7177725
rs147418092
rs145099817
rs61729490
rs146882853
All − MR Egger
All − IVW(MRE)
−0.005
0.000
0.005
0.010
MR effect size for
'Palmitoyl−protein thioesterase 1' on 'eGFR'

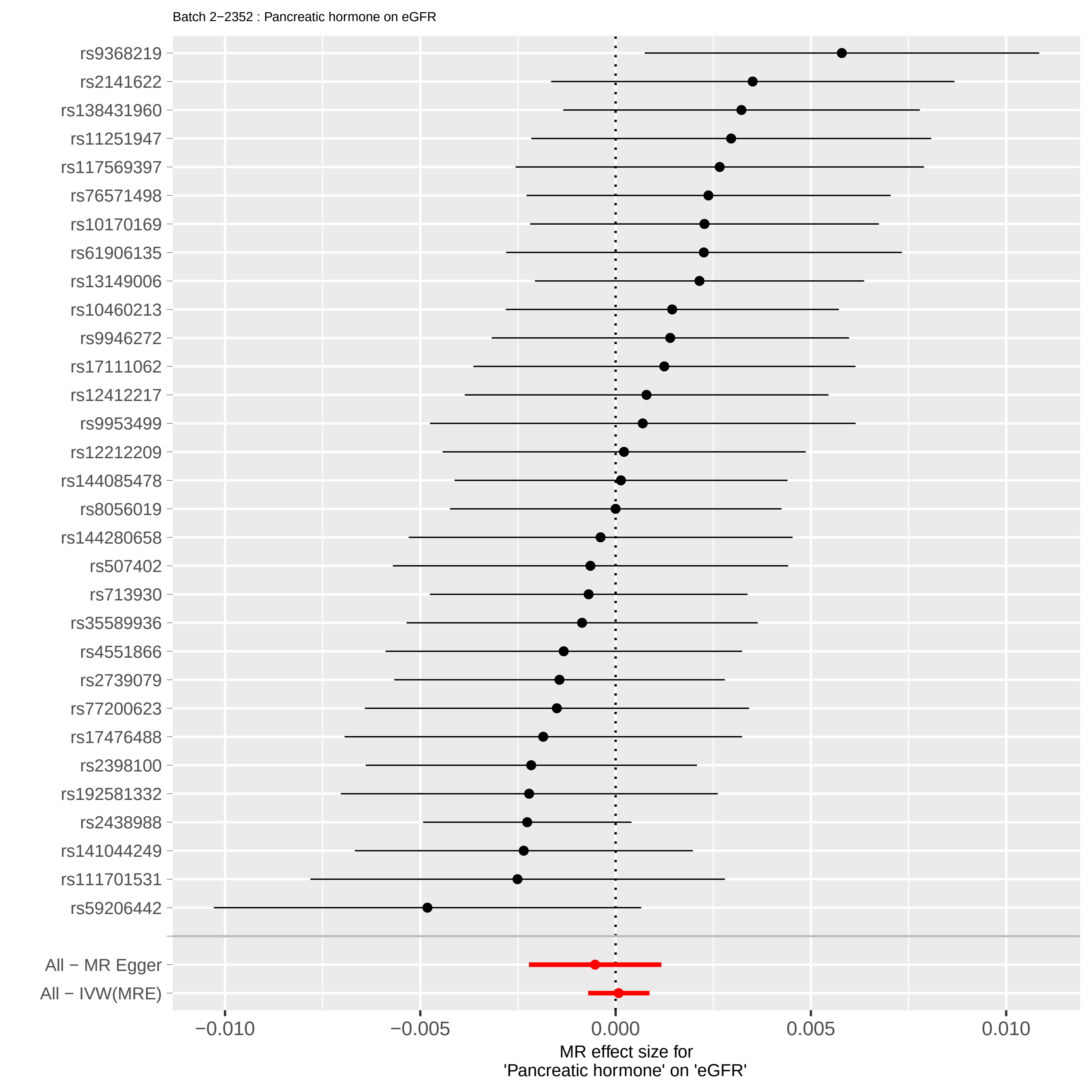
Batch 2−2352 : Pancreatic hormone on eGFR
rs9368219
rs2141622
rs138431960
rs11251947
rs117569397
rs76571498
rs10170169
rs61906135
rs13149006
rs10460213
rs9946272
rs17111062
rs12412217
rs9953499
rs12212209
rs144085478
rs8056019
rs144280658
rs507402
rs713930
rs35589936
rs4551866
rs2739079
rs77200623
rs17476488
rs2398100
rs192581332
rs2438988
rs141044249
rs111701531
rs59206442
All − MR Egger
All − IVW(MRE)
−0.010
−0.005
0.000
0.005
0.010
MR effect size for
'Pancreatic hormone' on 'eGFR'

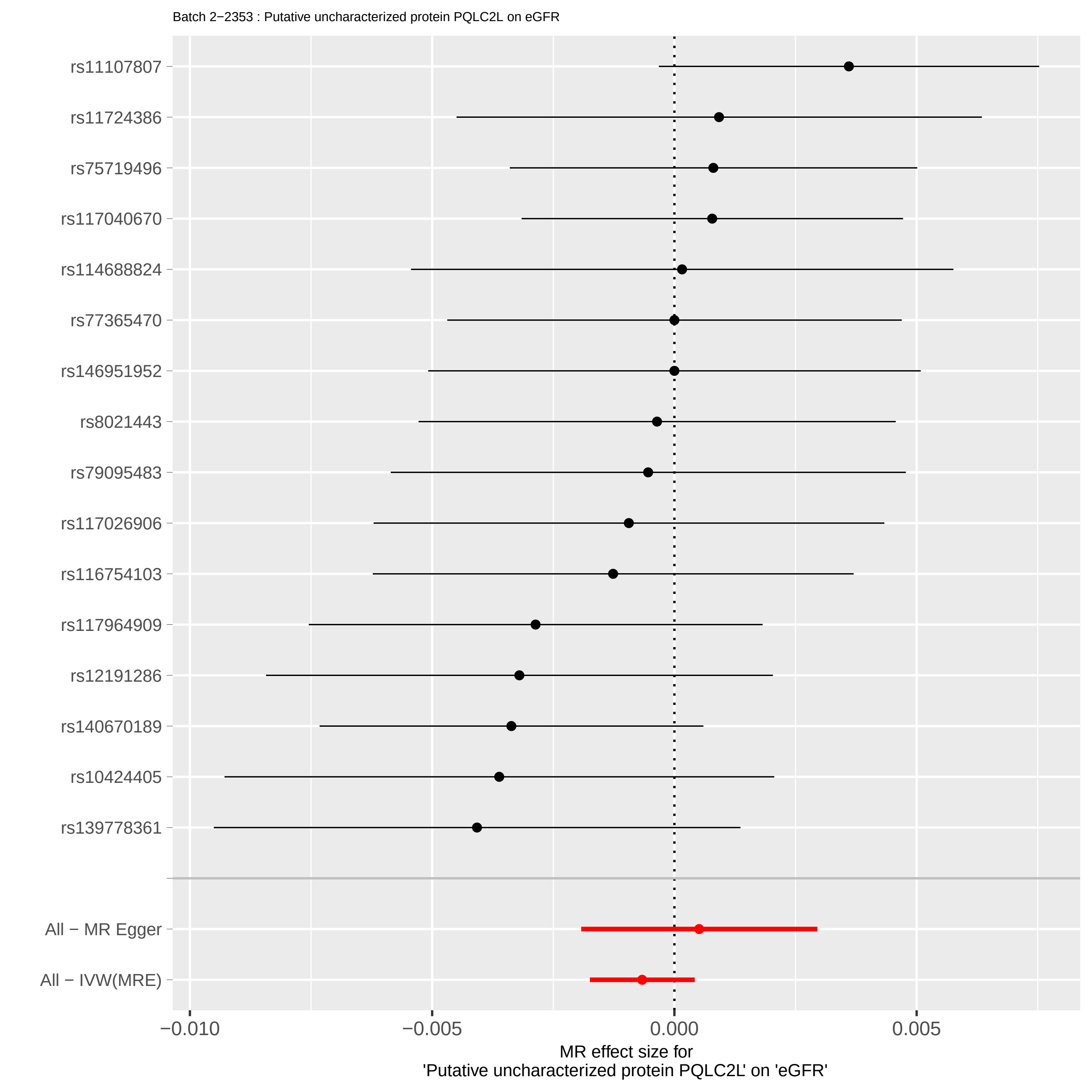

Batch 2−2353 : Putative uncharacterized protein PQLC2L on eGFR
rs11107807
rs11724386
rs75719496
rs117040670
rs114688824
rs77365470
rs146951952
rs8021443
rs79095483
rs117026906
rs116754103
rs117964909
rs12191286
rs140670189
rs10424405
rs139778361
All − MR Egger
All − IVW(MRE)
−0.010
−0.005
0.000
0.005
MR effect size for
'Putative uncharacterized protein PQLC2L' on 'eGFR'

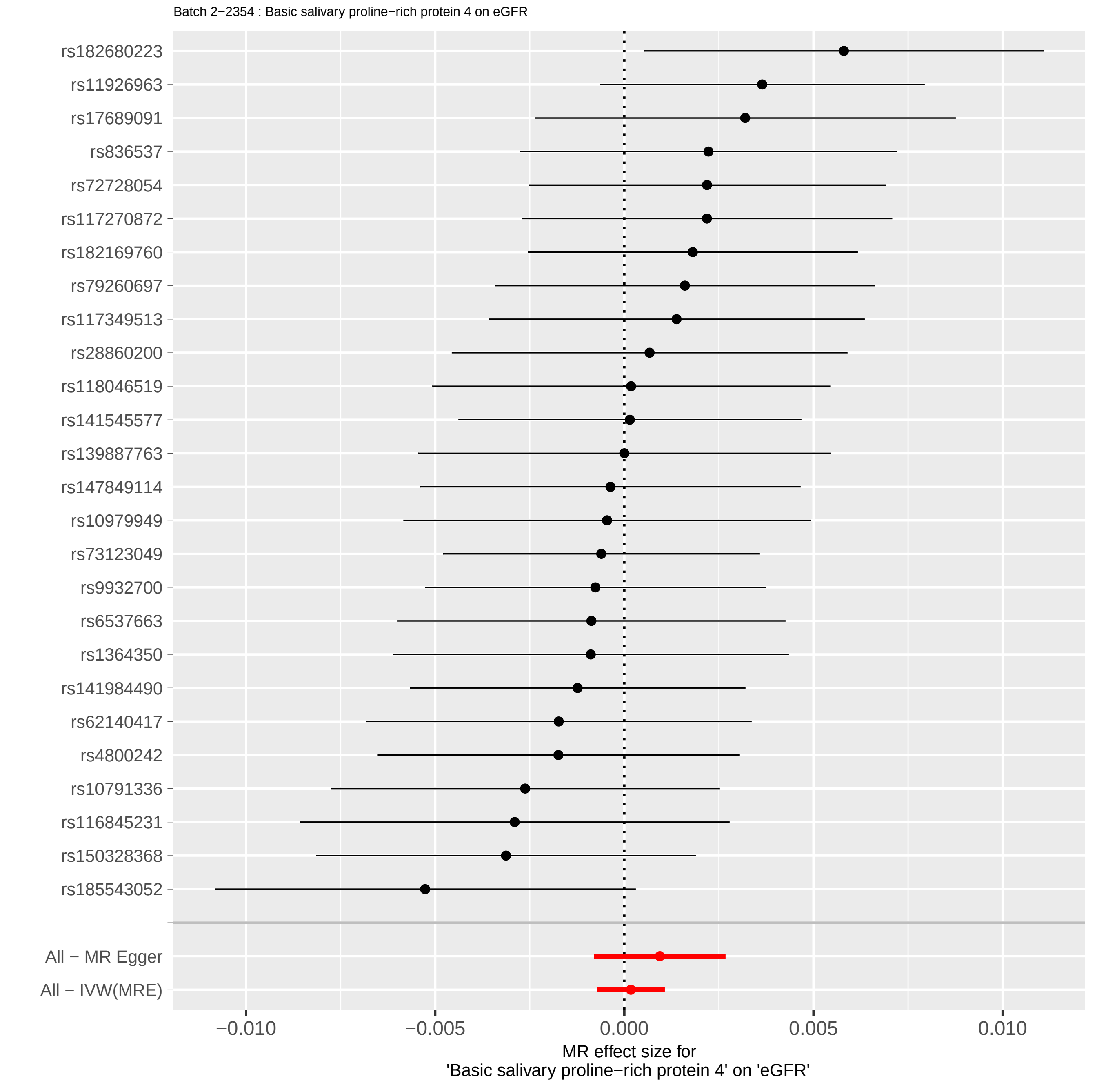
Batch 2−2354 : Basic salivary proline−rich protein 4 on eGFR
rs182680223
rs11926963
rs17689091
rs836537
rs72728054
rs117270872
rs182169760
rs79260697
rs117349513
rs28860200
rs118046519
rs141545577
rs139887763
rs147849114
rs10979949
rs73123049
rs9932700
rs6537663
rs1364350
rs141984490
rs62140417
rs4800242
rs10791336
rs116845231
rs150328368
rs185543052
All − MR Egger
All − IVW(MRE)
−0.010
−0.005
0.000
0.005
0.010
MR effect size for
'Basic salivary proline−rich protein 4' on 'eGFR'

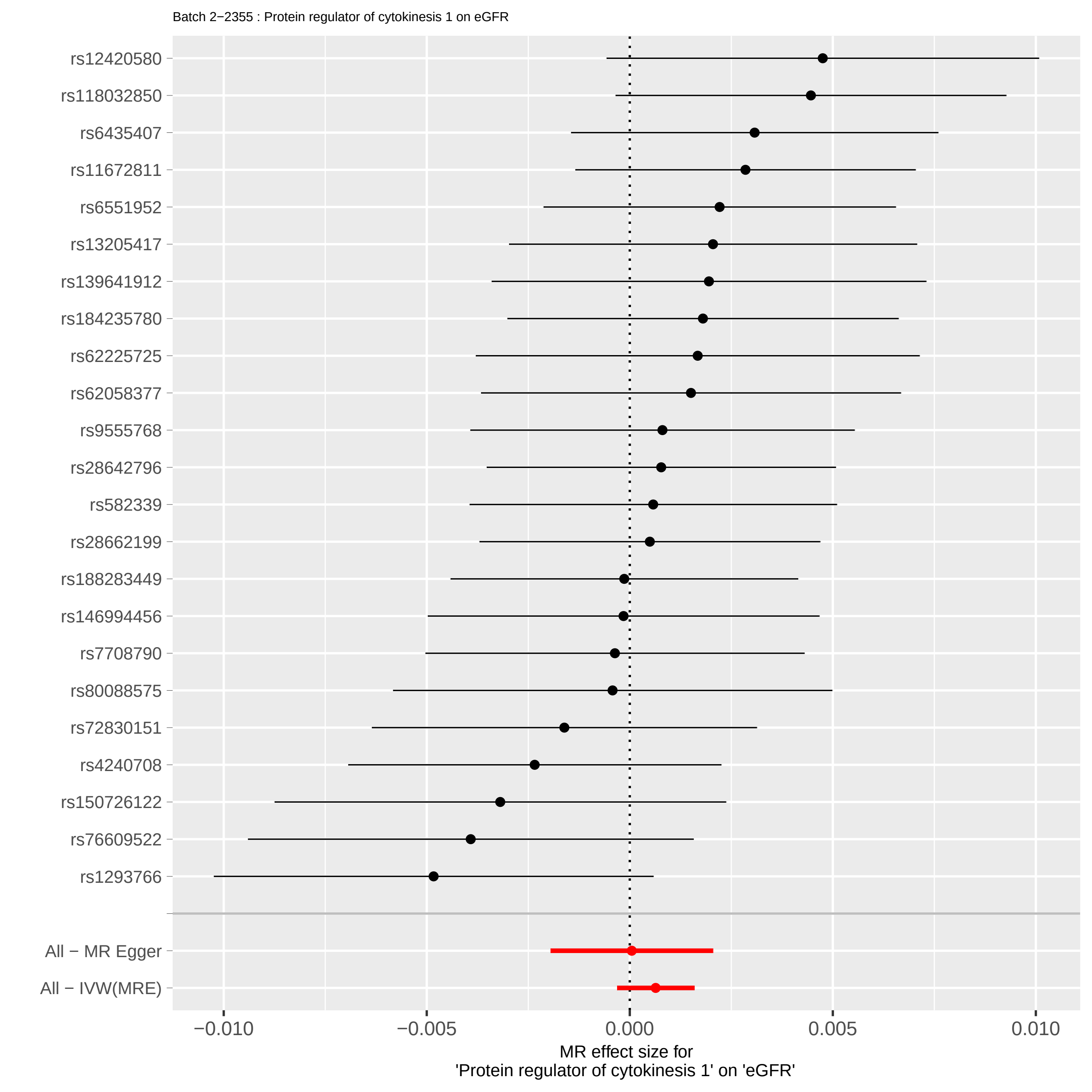

Batch 2−2355 : Protein regulator of cytokinesis 1 on eGFR
rs12420580
rs118032850
rs6435407
rs11672811
rs6551952
rs13205417
rs139641912
rs184235780
rs62225725
rs62058377
rs9555768
rs28642796
rs582339
rs28662199
rs188283449
rs146994456
rs7708790
rs80088575
rs72830151
rs4240708
rs150726122
rs76609522
rs1293766
All − MR Egger
All − IVW(MRE)
−0.010
−0.005
0.000
0.005
0.010
MR effect size for
'Protein regulator of cytokinesis 1' on 'eGFR'

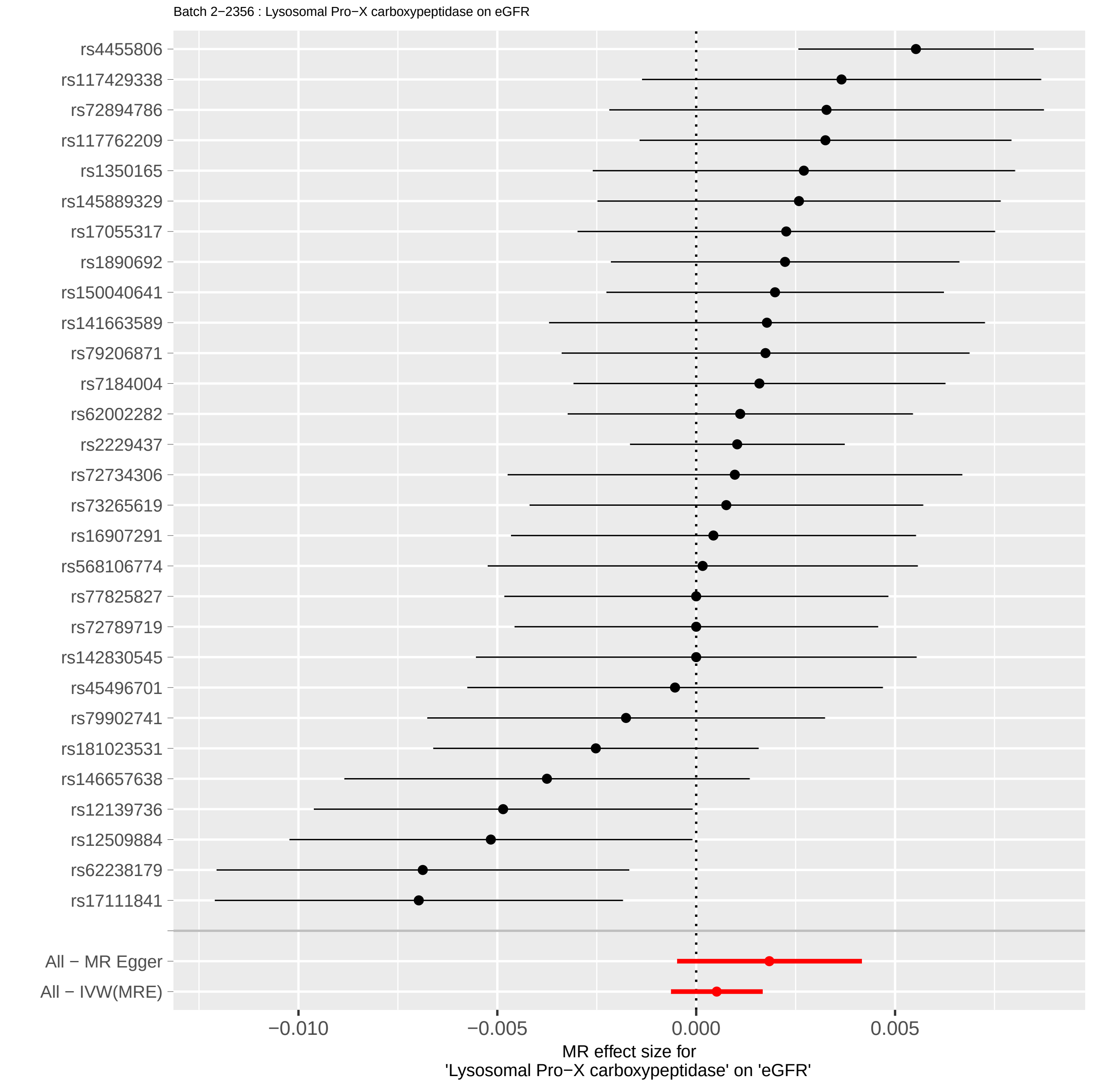
Batch 2−2356 : Lysosomal Pro−X carboxypeptidase on eGFR
rs4455806
rs117429338
rs72894786
rs117762209
rs1350165
rs145889329
rs17055317
rs1890692
rs150040641
rs141663589
rs79206871
rs7184004
rs62002282
rs2229437
rs72734306
rs73265619
rs16907291
rs568106774
rs77825827
rs72789719
rs142830545
rs45496701
rs79902741
rs181023531
rs146657638
rs12139736
rs12509884
rs62238179
rs17111841
All − MR Egger
All − IVW(MRE)
−0.010
−0.005
0.000
0.005
MR effect size for
'Lysosomal Pro−X carboxypeptidase' on 'eGFR'

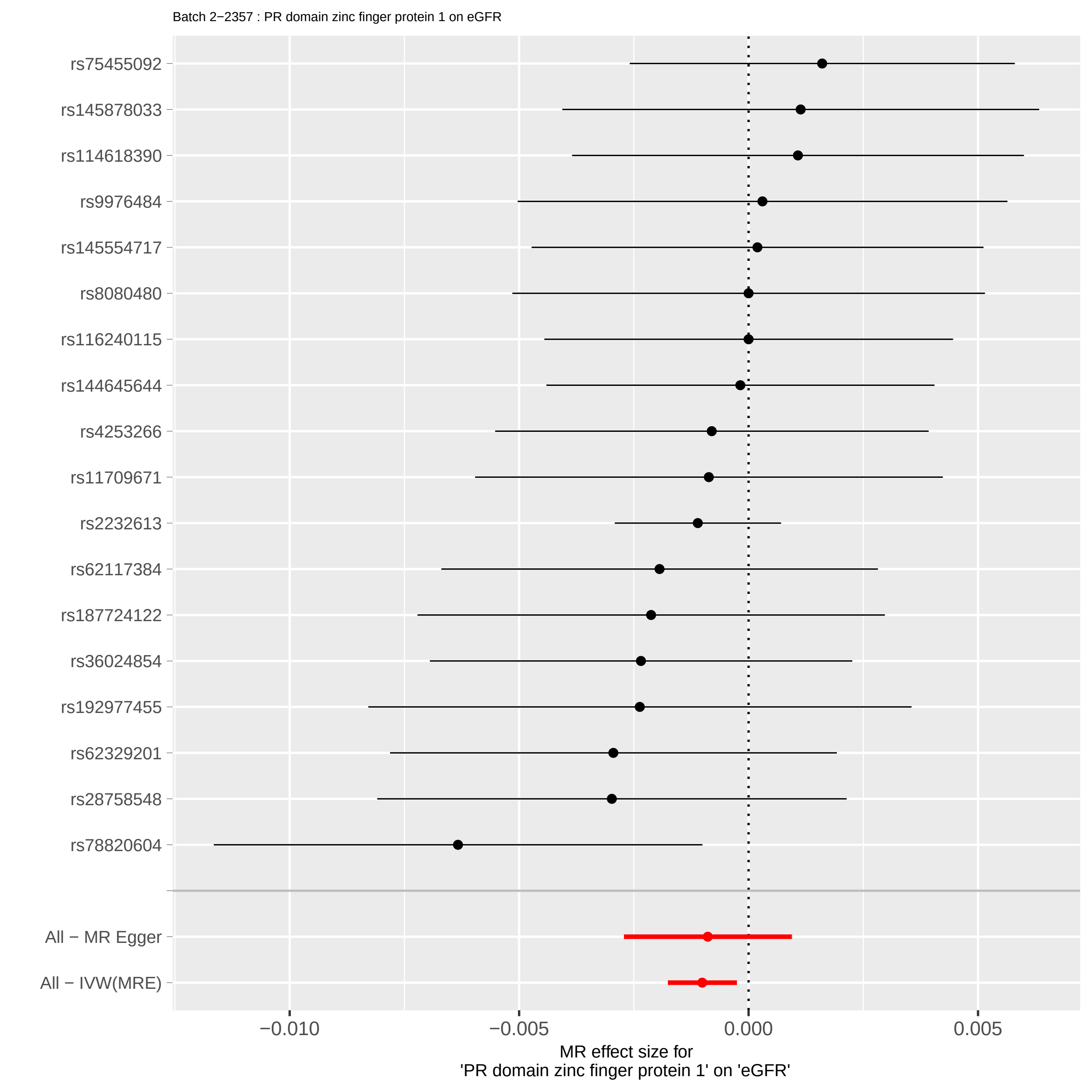

Batch 2−2357 : PR domain zinc finger protein 1 on eGFR
rs75455092
rs145878033
rs114618390
rs9976484
rs145554717
rs8080480
rs116240115
rs144645644
rs4253266
rs11709671
rs2232613
rs62117384
rs187724122
rs36024854
rs192977455
rs62329201
rs28758548
rs78820604
All − MR Egger
All − IVW(MRE)
−0.010
−0.005
0.000
0.005
MR effect size for
'PR domain zinc finger protein 1' on 'eGFR'

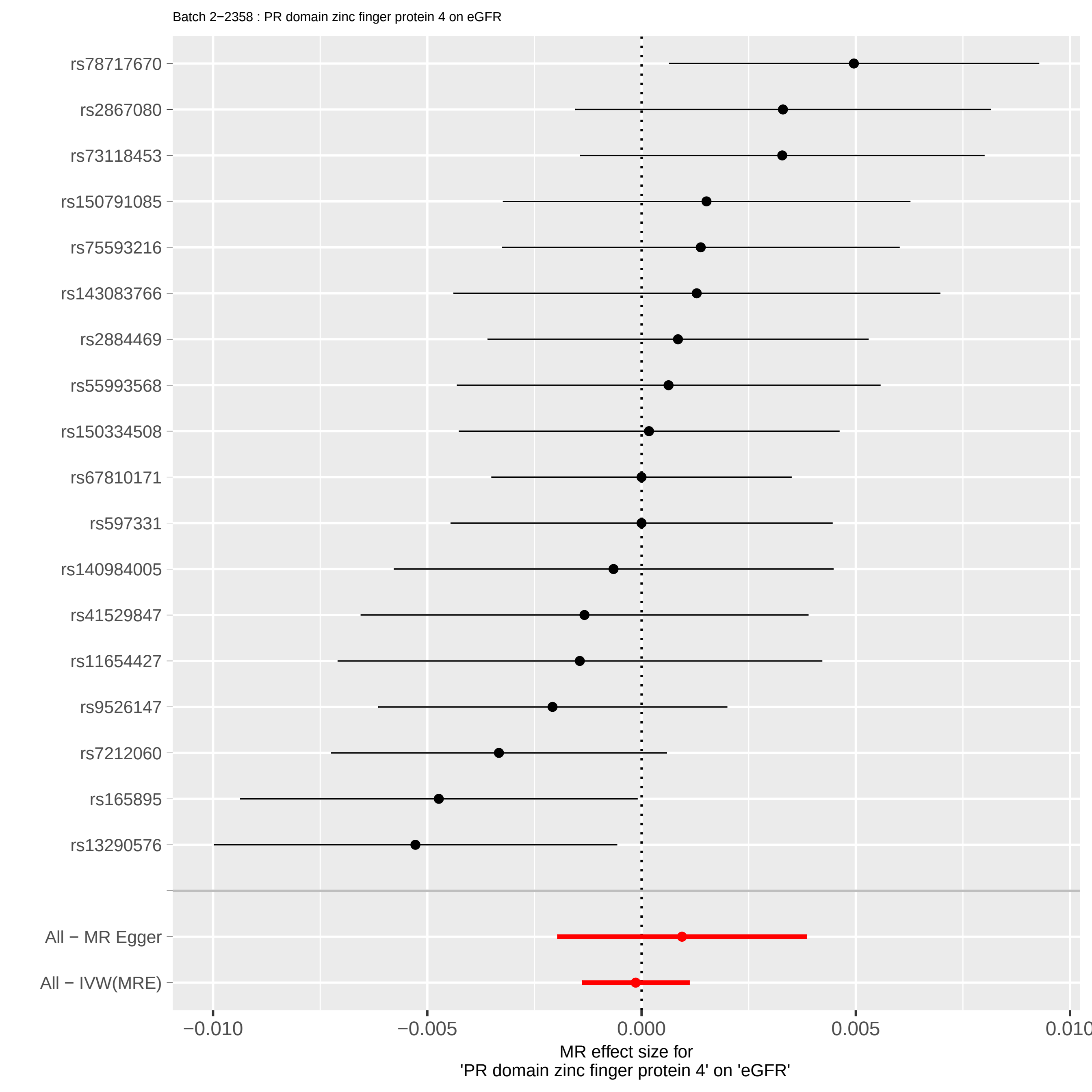
Batch 2−2358 : PR domain zinc finger protein 4 on eGFR
rs78717670
rs2867080
rs73118453
rs150791085
rs75593216
rs143083766
rs2884469
rs55993568
rs150334508
rs67810171
rs597331
rs140984005
rs41529847
rs11654427
rs9526147
rs7212060
rs165895
rs13290576
All − MR Egger
All − IVW(MRE)
−0.010
−0.005
0.000
0.005
0.010
MR effect size for
'PR domain zinc finger protein 4' on 'eGFR'

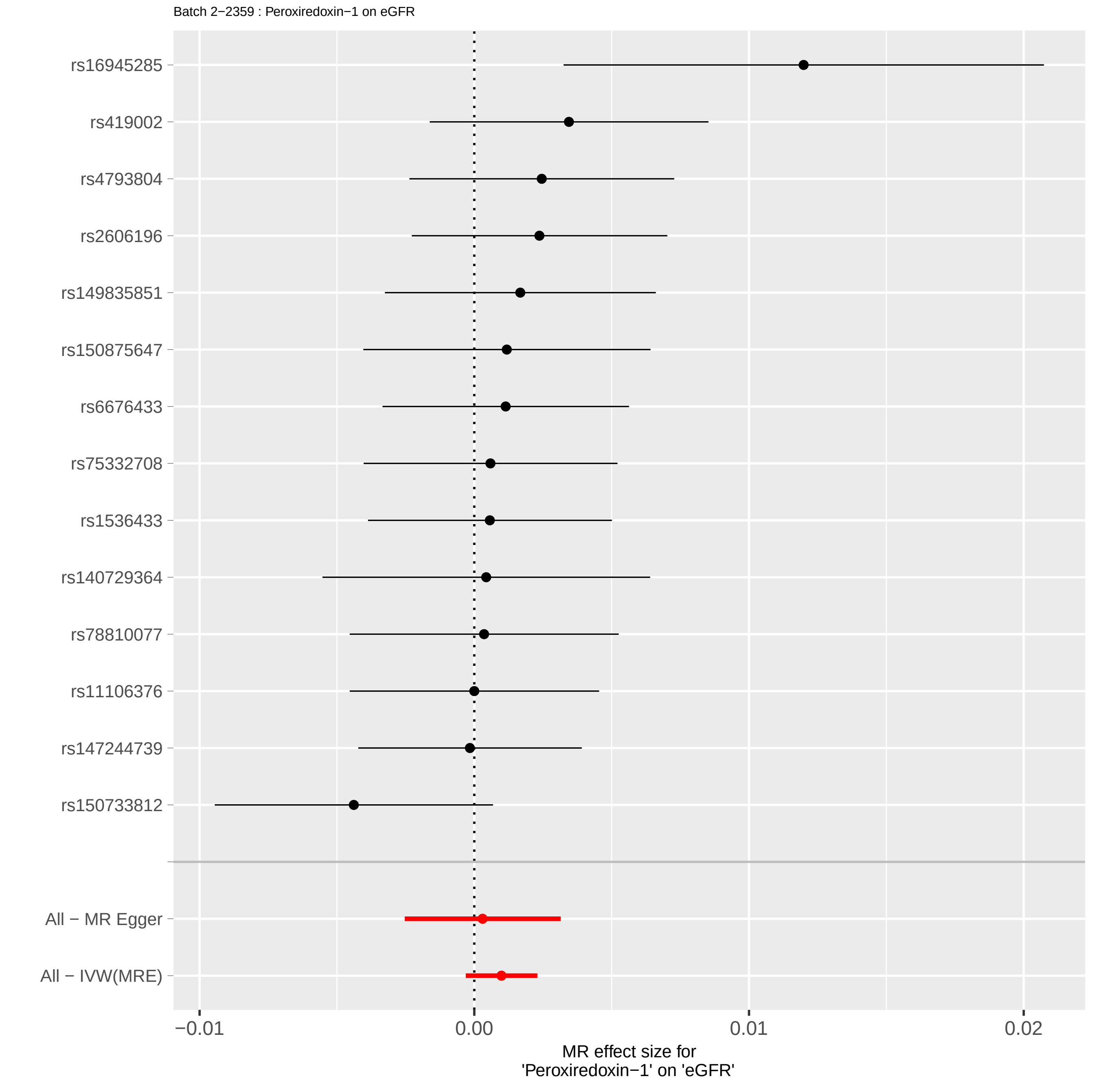

Batch 2−2359 : Peroxiredoxin−1 on eGFR
rs16945285
rs419002
rs4793804
rs2606196
rs149835851
rs150875647
rs6676433
rs75332708
rs1536433
rs140729364
rs78810077
rs11106376
rs147244739
rs150733812
All − MR Egger
All − IVW(MRE)
−0.01
0.00
0.01
0.02
MR effect size for
'Peroxiredoxin−1' on 'eGFR'

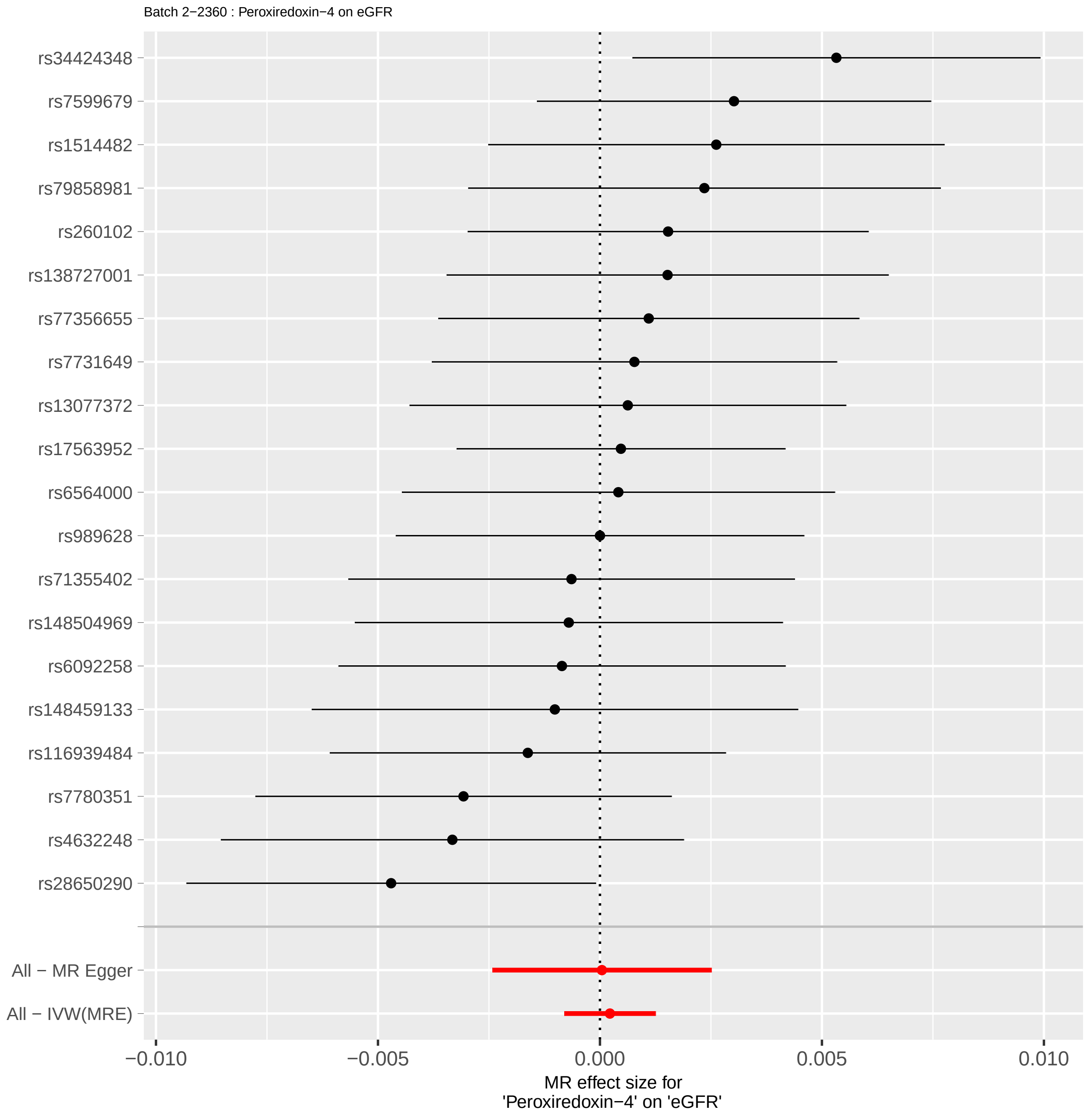

Batch 2−2360 : Peroxiredoxin−4 on eGFR
rs34424348
rs7599679
rs1514482
rs79858981
rs260102
rs138727001
rs77356655
rs7731649
rs13077372
rs17563952
rs6564000
rs989628
rs71355402
rs148504969
rs6092258
rs148459133
rs116939484
rs7780351
rs4632248
rs28650290
All − MR Egger
All − IVW(MRE)
−0.010
−0.005
0.000
0.005
0.010
MR effect size for
'Peroxiredoxin−4' on 'eGFR'

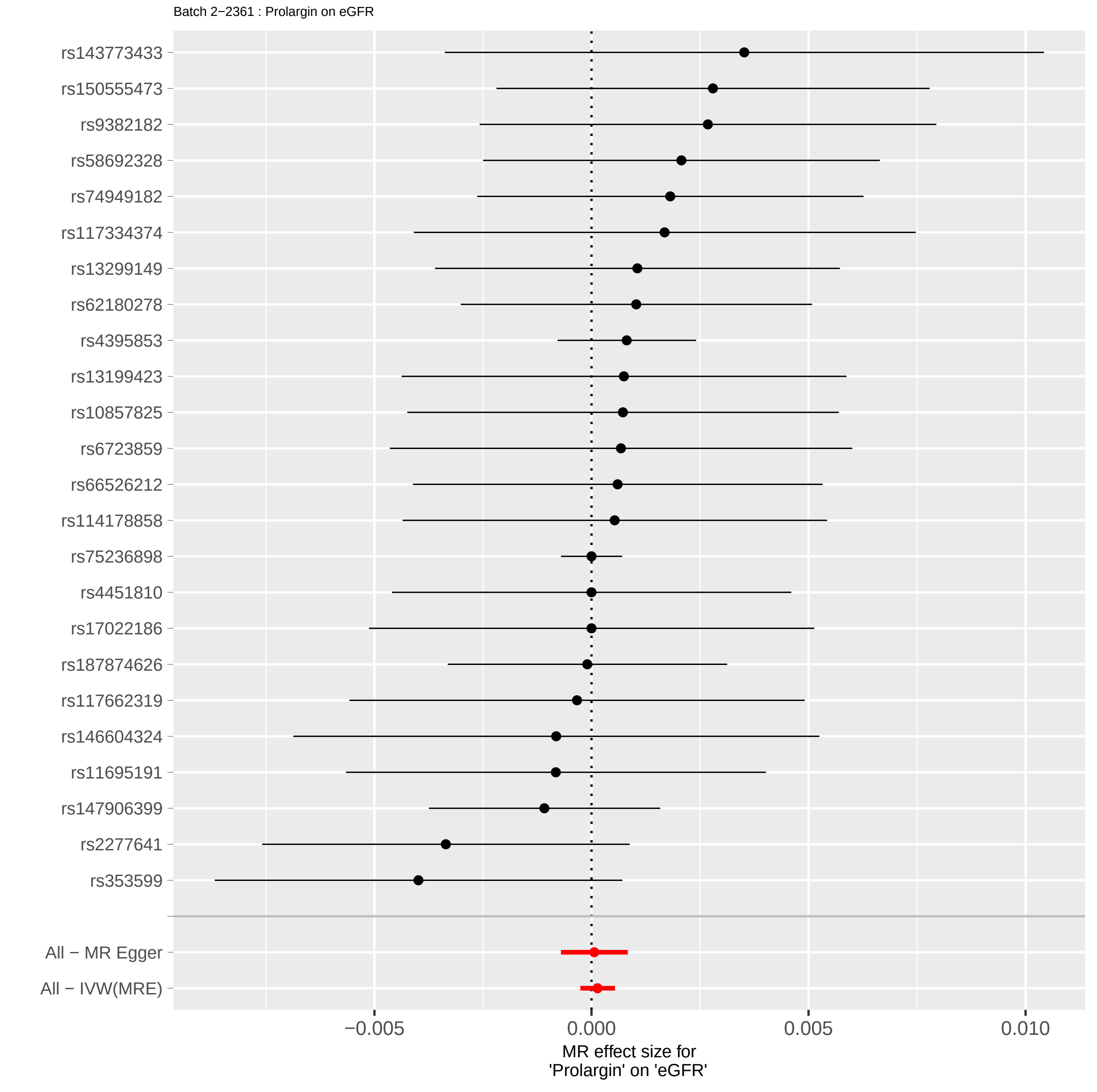

Batch 2−2361 : Prolargin on eGFR
rs143773433
rs150555473
rs9382182
rs58692328
rs74949182
rs117334374
rs13299149
rs62180278
rs4395853
rs13199423
rs10857825
rs6723859
rs66526212
rs114178858
rs75236898
rs4451810
rs17022186
rs187874626
rs117662319
rs146604324
rs11695191
rs147906399
rs2277641
rs353599
All − MR Egger
All − IVW(MRE)
−0.005
0.000
0.005
0.010
MR effect size for
'Prolargin' on 'eGFR'

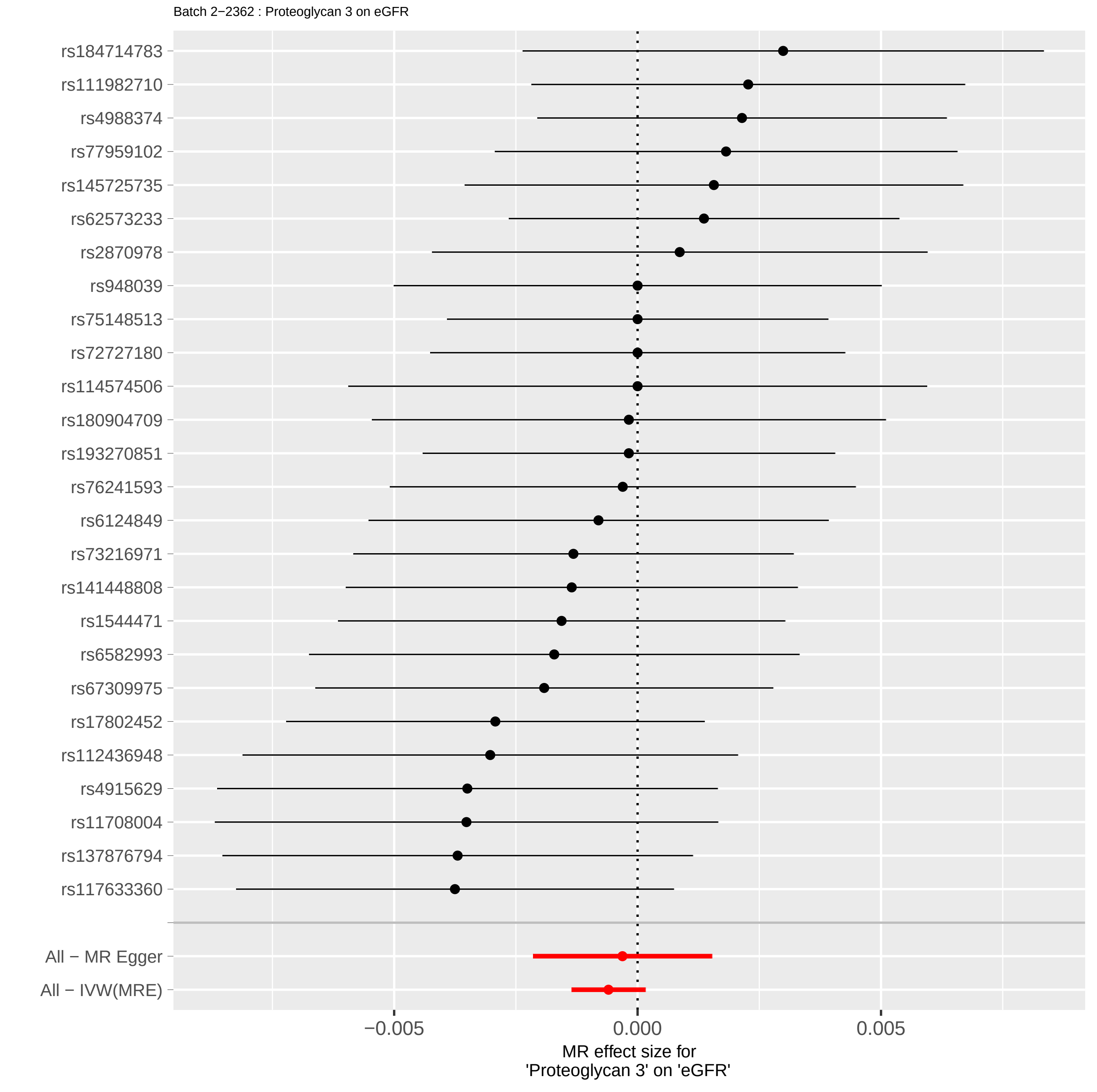
Batch 2−2362 : Proteoglycan 3 on eGFR
rs184714783
rs111982710
rs4988374
rs77959102
rs145725735
rs62573233
rs2870978
rs948039
rs75148513
rs72727180
rs114574506
rs180904709
rs193270851
rs76241593
rs6124849
rs73216971
rs141448808
rs1544471
rs6582993
rs67309975
rs17802452
rs112436948
rs4915629
rs11708004
rs137876794
rs117633360
All − MR Egger
All − IVW(MRE)
−0.005
0.000
0.005
MR effect size for
'Proteoglycan 3' on 'eGFR'

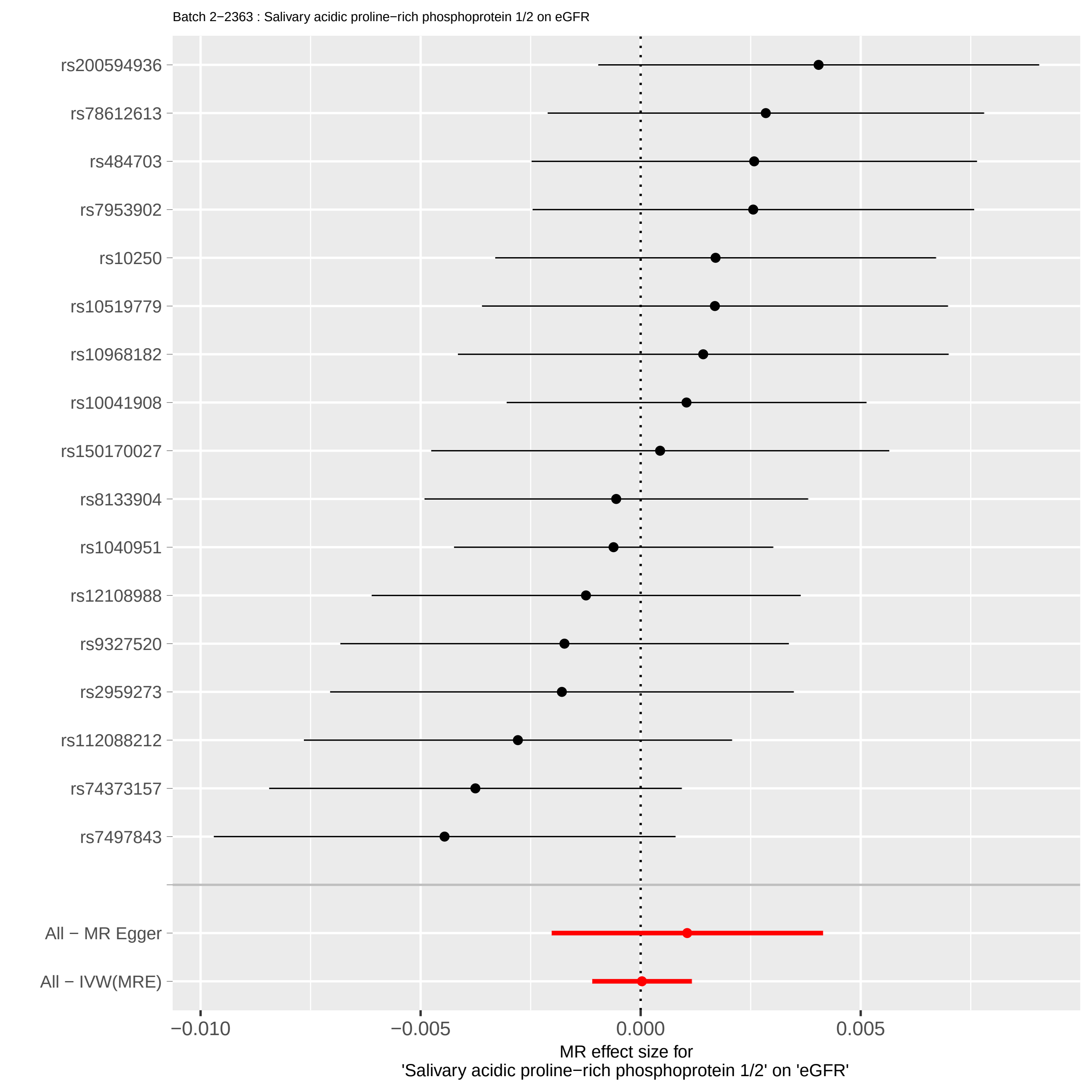

Batch 2−2363 : Salivary acidic proline−rich phosphoprotein 1/2 on eGFR
rs200594936
rs78612613
rs484703
rs7953902
rs10250
rs10519779
rs10968182
rs10041908
rs150170027
rs8133904
rs1040951
rs12108988
rs9327520
rs2959273
rs112088212
rs74373157
rs7497843
All − MR Egger
All − IVW(MRE)
−0.010
−0.005
0.000
0.005
MR effect size for
'Salivary acidic proline−rich phosphoprotein 1/2' on 'eGFR'

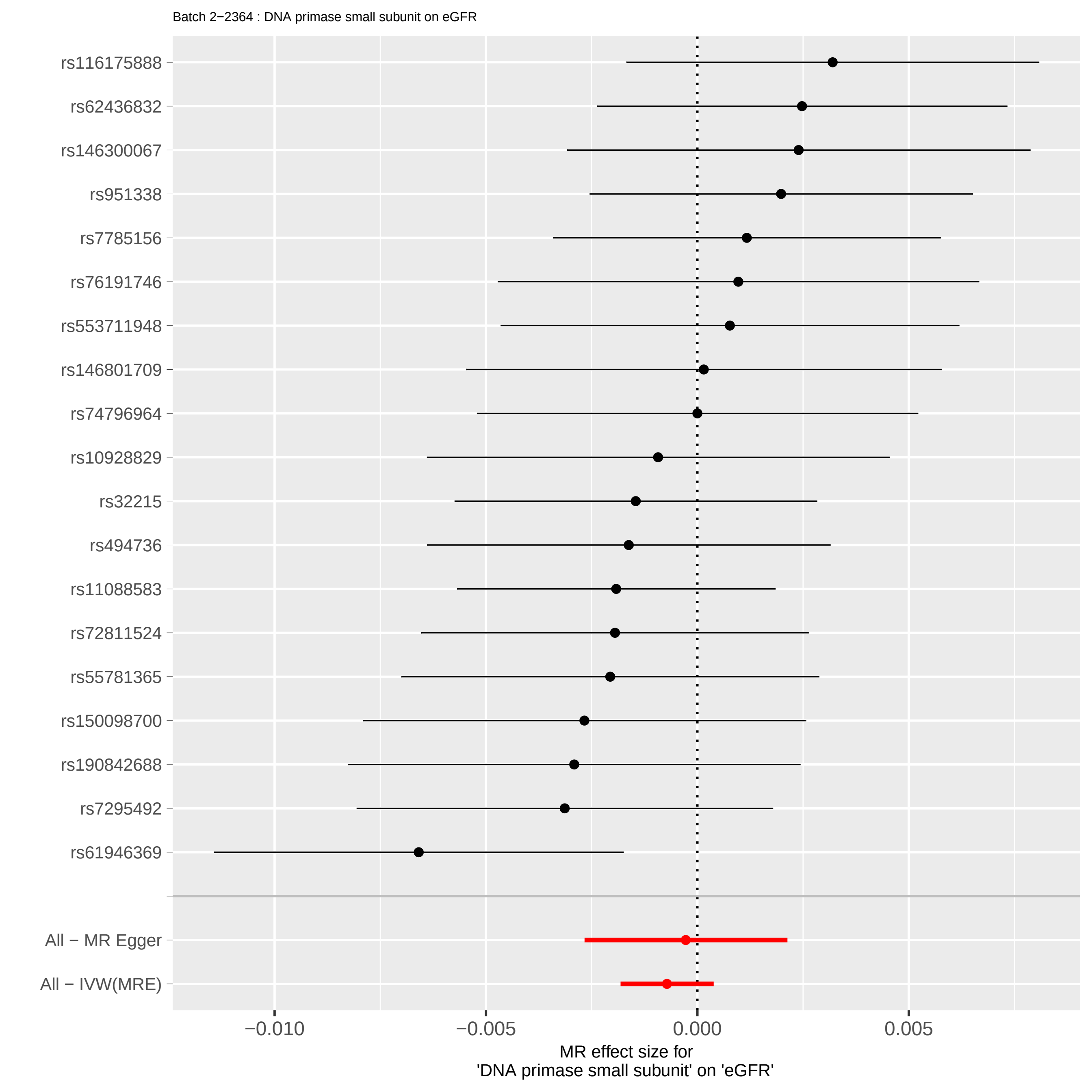

Batch 2−2364 : DNA primase small subunit on eGFR
rs116175888
rs62436832
rs146300067
rs951338
rs7785156
rs76191746
rs553711948
rs146801709
rs74796964
rs10928829
rs32215
rs494736
rs11088583
rs72811524
rs55781365
rs150098700
rs190842688
rs7295492
rs61946369
All − MR Egger
All − IVW(MRE)
−0.010
−0.005
0.000
0.005
MR effect size for
'DNA primase small subunit' on 'eGFR'

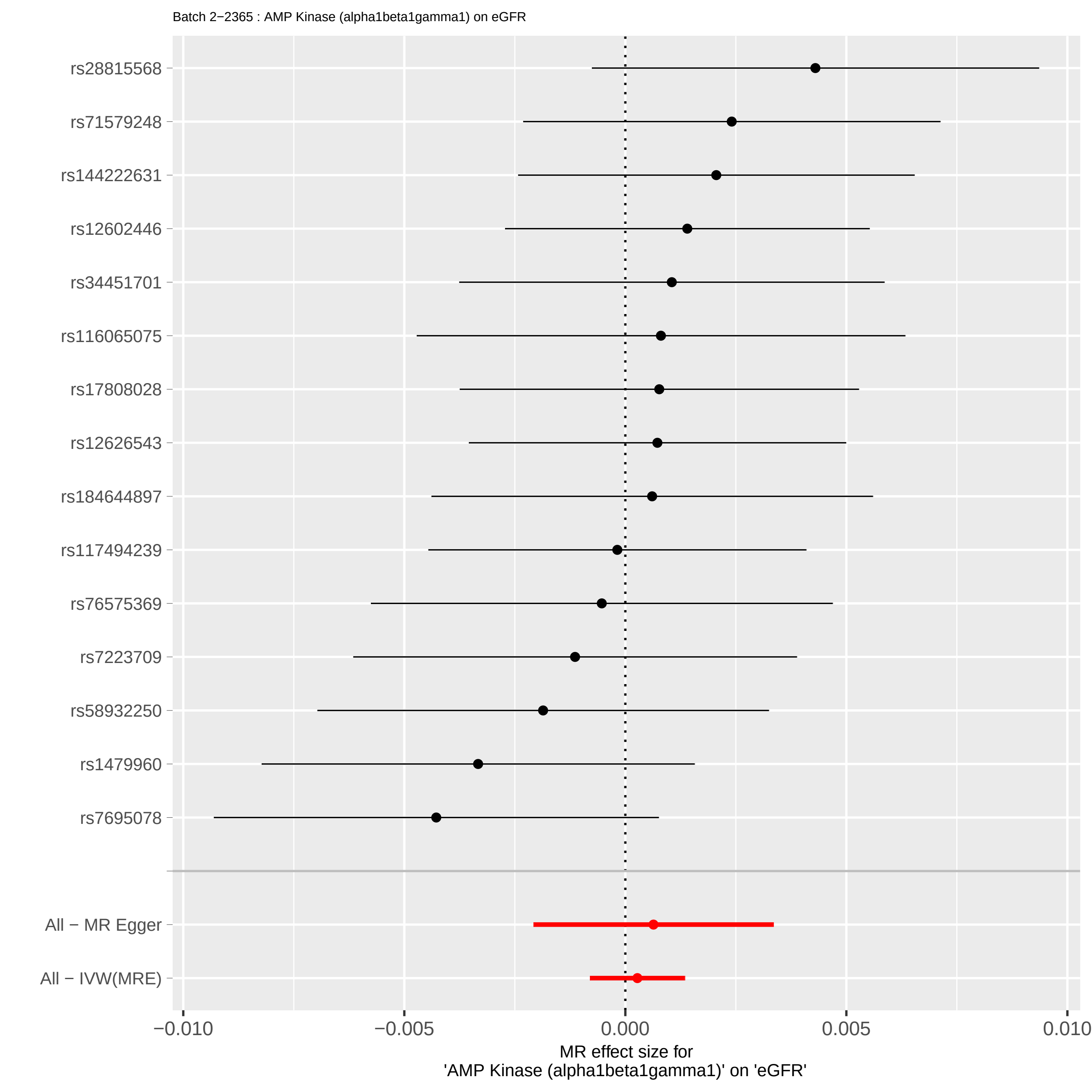
Batch 2−2365 : AMP Kinase (alpha1beta1gamma1) on eGFR
rs28815568
rs71579248
rs144222631
rs12602446
rs34451701
rs116065075
rs17808028
rs12626543
rs184644897
rs117494239
rs76575369
rs7223709
rs58932250
rs1479960
rs7695078
All − MR Egger
All − IVW(MRE)
−0.010
−0.005
0.000
0.005
0.010
MR effect size for
'AMP Kinase (alpha1beta1gamma1)' on 'eGFR'

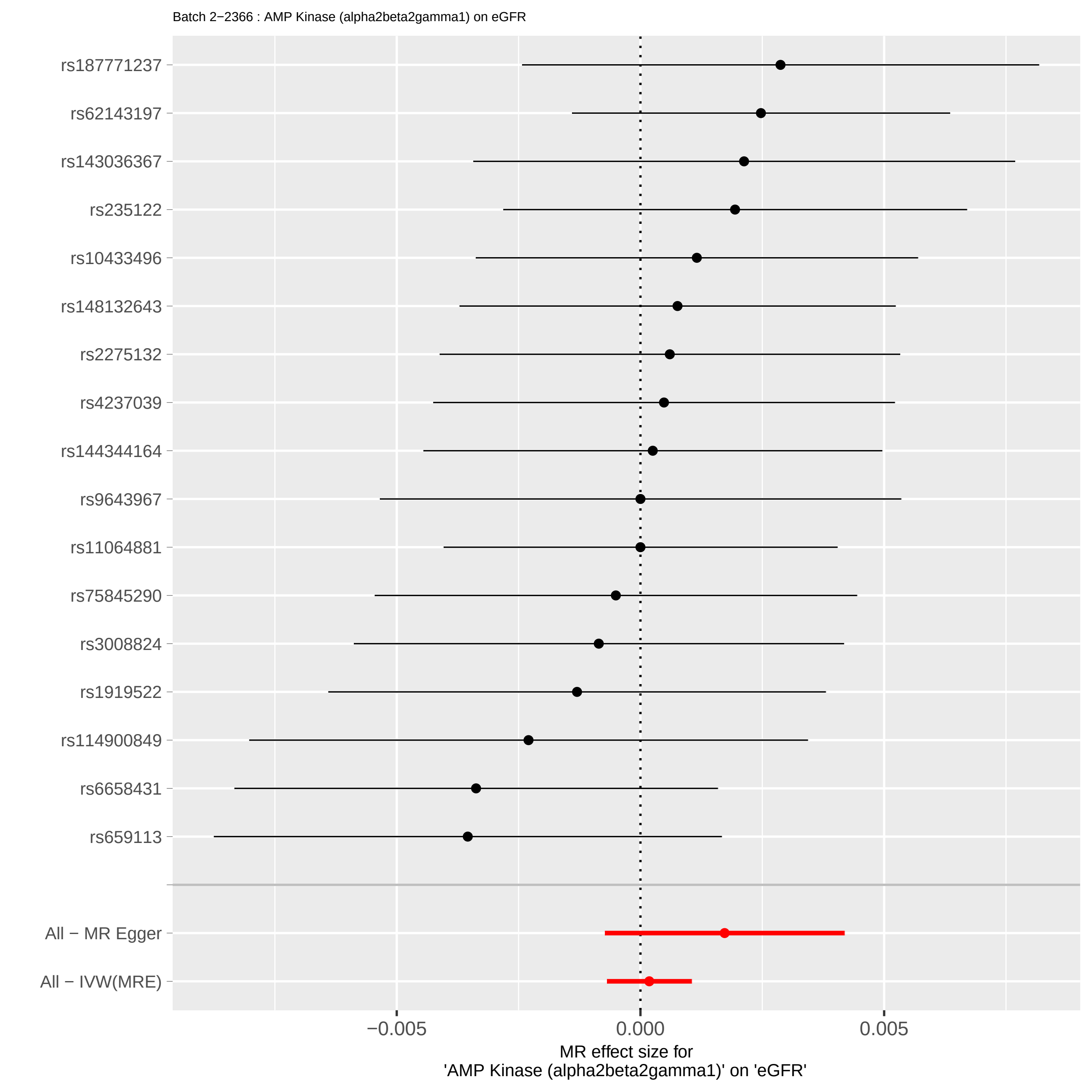

Batch 2−2366 : AMP Kinase (alpha2beta2gamma1) on eGFR
rs187771237
rs62143197
rs143036367
rs235122
rs10433496
rs148132643
rs2275132
rs4237039
rs144344164
rs9643967
rs11064881
rs75845290
rs3008824
rs1919522
rs114900849
rs6658431
rs659113
All − MR Egger
All − IVW(MRE)
−0.005
0.000
0.005
MR effect size for
'AMP Kinase (alpha2beta2gamma1)' on 'eGFR'

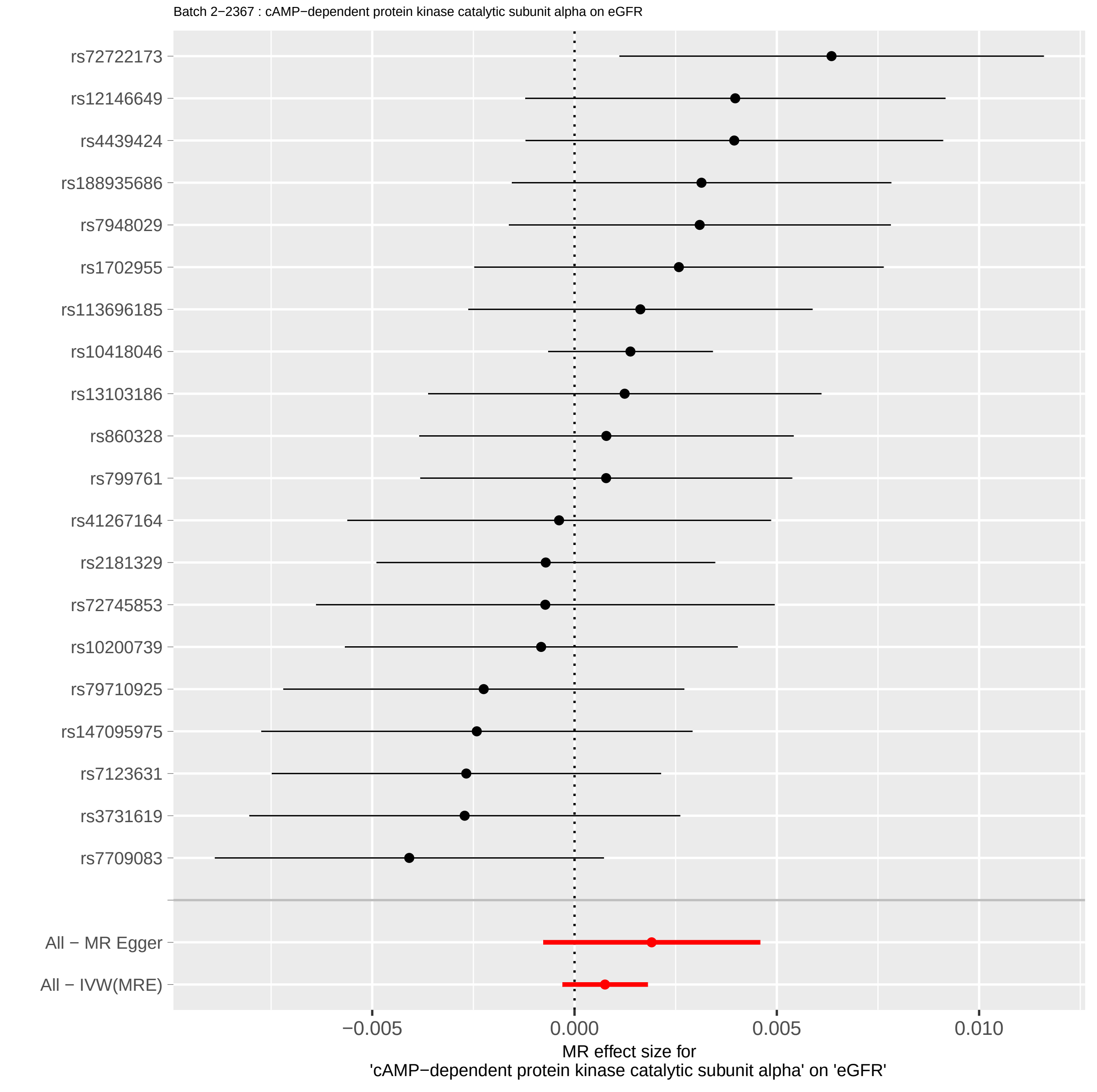

Batch 2−2367 : cAMP−dependent protein kinase catalytic subunit alpha on eGFR
rs72722173
rs12146649
rs4439424
rs188935686
rs7948029
rs1702955
rs113696185
rs10418046
rs13103186
rs860328
rs799761
rs41267164
rs2181329
rs72745853
rs10200739
rs79710925
rs147095975
rs7123631
rs3731619
rs7709083
All − MR Egger
All − IVW(MRE)
−0.005
0.000
0.005
0.010
MR effect size for
'cAMP−dependent protein kinase catalytic subunit alpha' on 'eGFR'

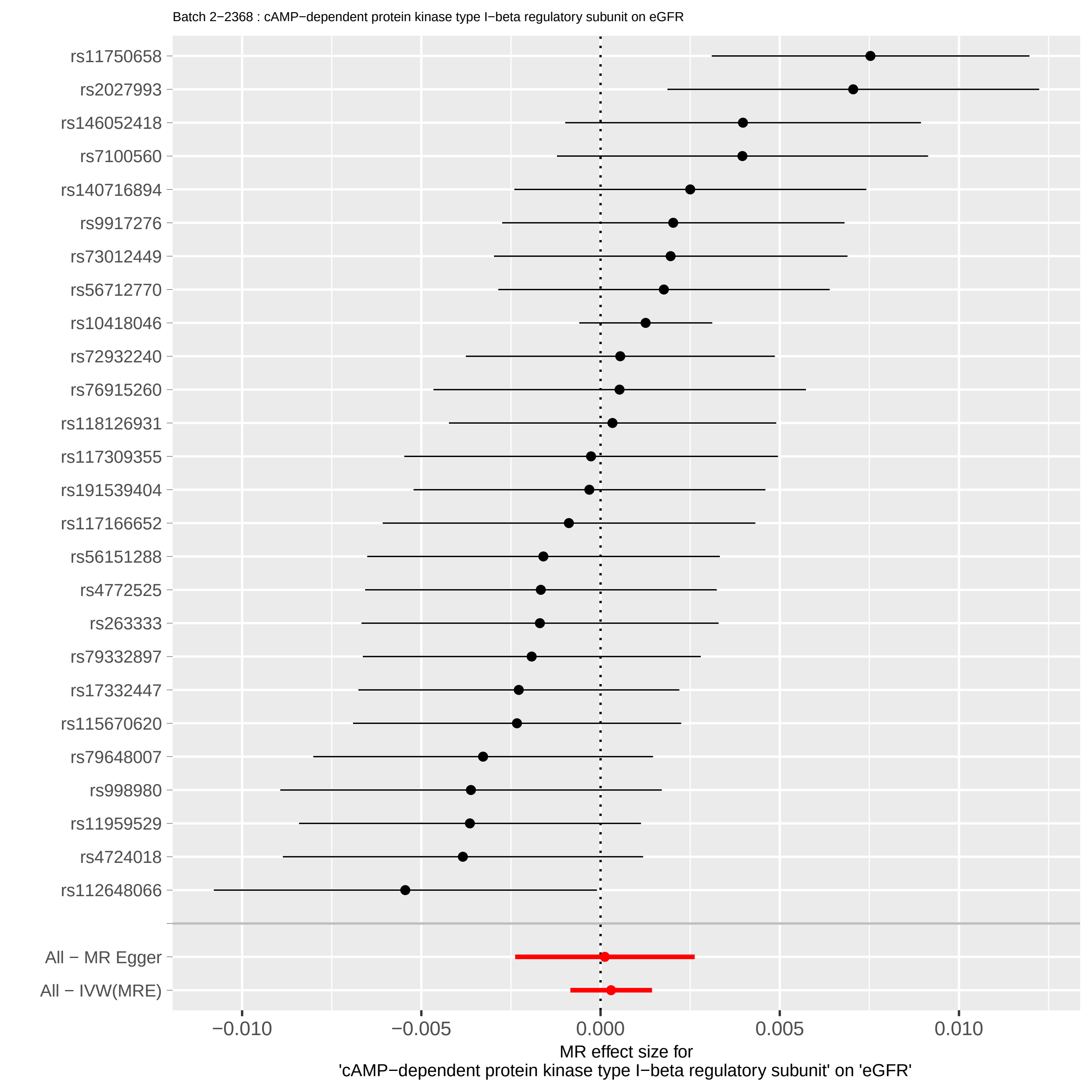

Batch 2−2368 : cAMP−dependent protein kinase type I−beta regulatory subunit on eGFR
rs11750658
rs2027993
rs146052418
rs7100560
rs140716894
rs9917276
rs73012449
rs56712770
rs10418046
rs72932240
rs76915260
rs118126931
rs117309355
rs191539404
rs117166652
rs56151288
rs4772525
rs263333
rs79332897
rs17332447
rs115670620
rs79648007
rs998980
rs11959529
rs4724018
rs112648066
All − MR Egger
All − IVW(MRE)
−0.010
−0.005
0.000
0.005
0.010
MR effect size for
'cAMP−dependent protein kinase type I−beta regulatory subunit' on 'eGFR'

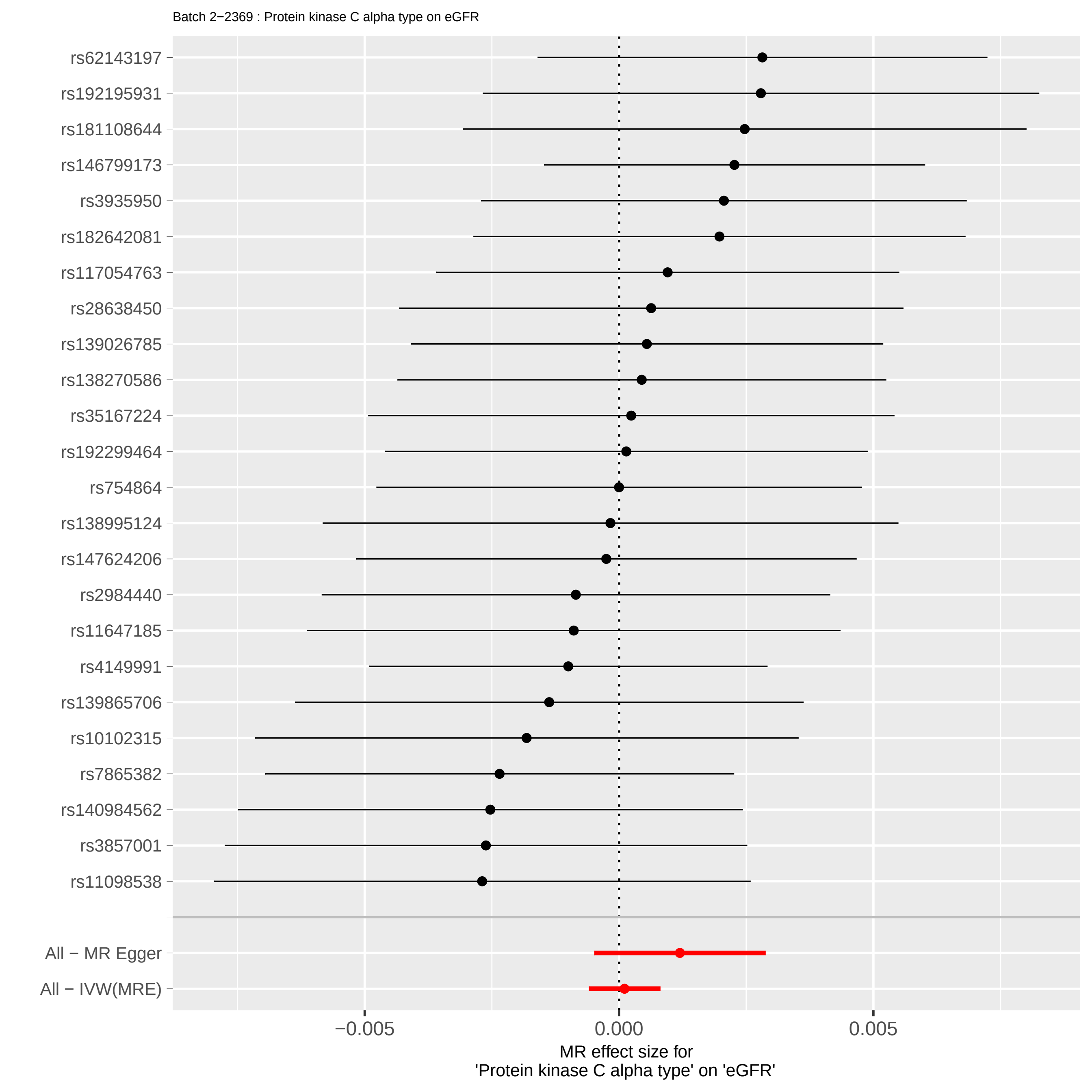

Batch 2−2369 : Protein kinase C alpha type on eGFR
rs62143197
rs192195931
rs181108644
rs146799173
rs3935950
rs182642081
rs117054763
rs28638450
rs139026785
rs138270586
rs35167224
rs192299464
rs754864
rs138995124
rs147624206
rs2984440
rs11647185
rs4149991
rs139865706
rs10102315
rs7865382
rs140984562
rs3857001
rs11098538
All − MR Egger
All − IVW(MRE)
−0.005
0.000
0.005
MR effect size for
'Protein kinase C alpha type' on 'eGFR'

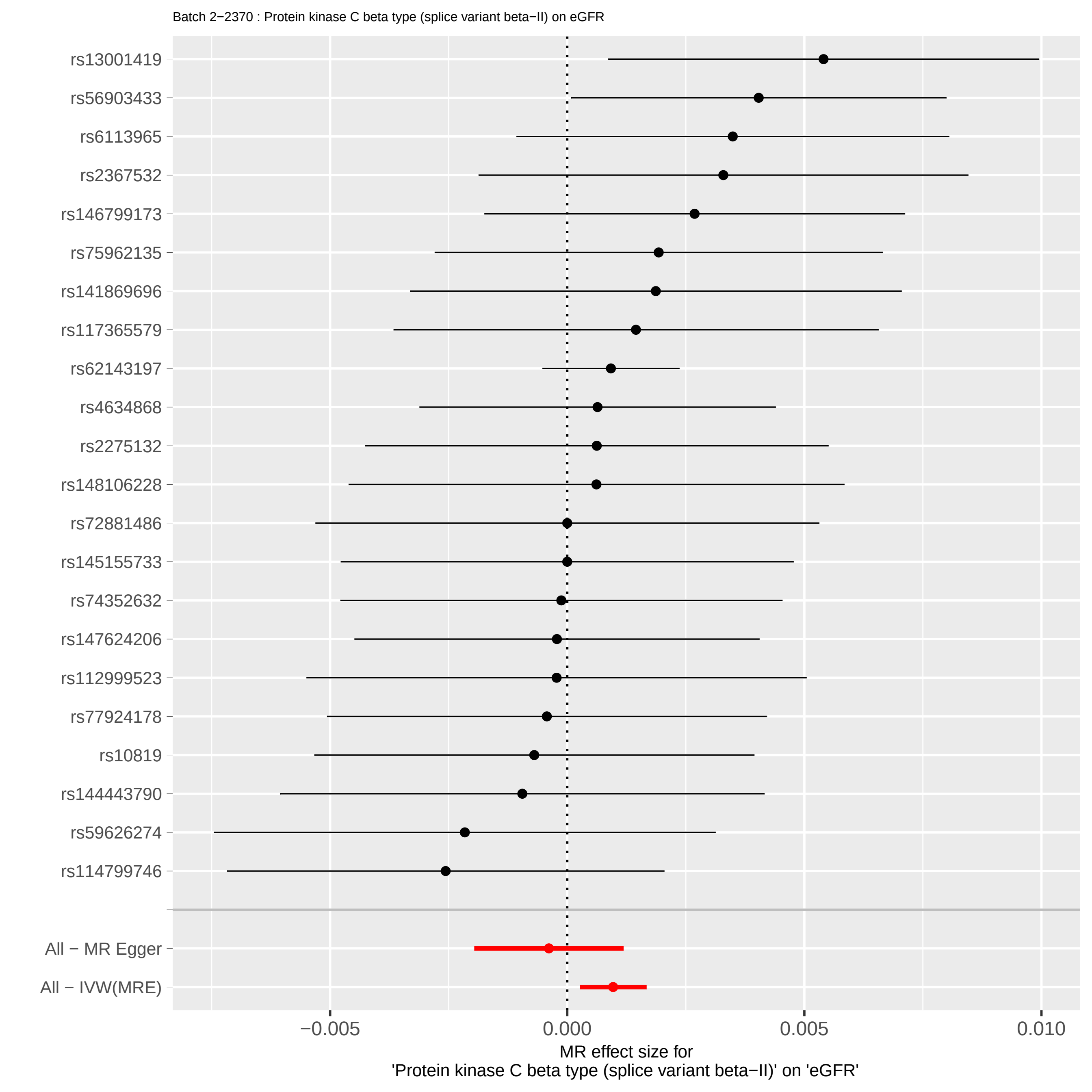

Batch 2−2370 : Protein kinase C beta type (splice variant beta−II) on eGFR
rs13001419
rs56903433
rs6113965
rs2367532
rs146799173
rs75962135
rs141869696
rs117365579
rs62143197
rs4634868
rs2275132
rs148106228
rs72881486
rs145155733
rs74352632
rs147624206
rs112999523
rs77924178
rs10819
rs144443790
rs59626274
rs114799746
All − MR Egger
All − IVW(MRE)
−0.005
0.000
0.005
0.010
MR effect size for
'Protein kinase C beta type (splice variant beta−II)' on 'eGFR'

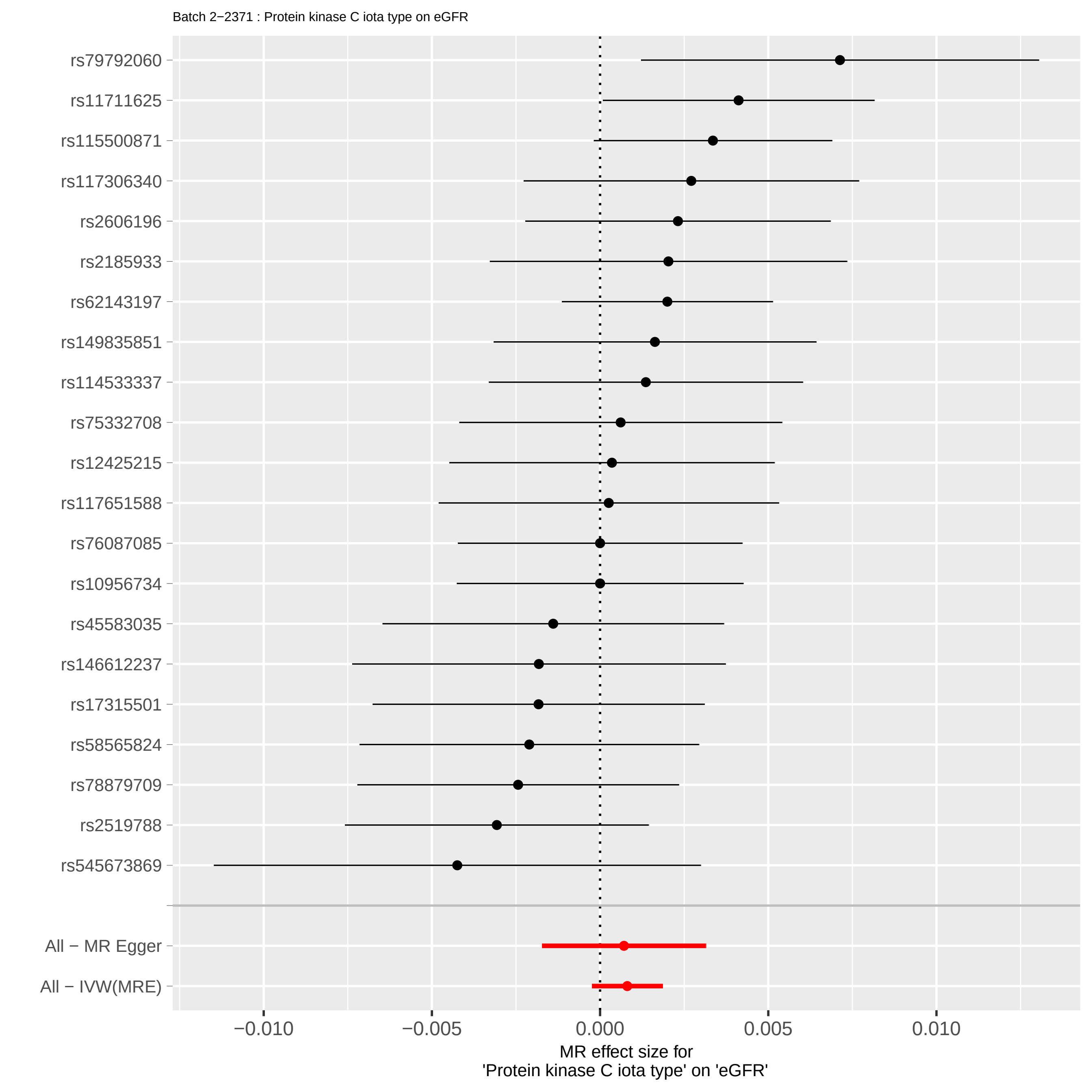

Batch 2−2371 : Protein kinase C iota type on eGFR
rs79792060
rs11711625
rs115500871
rs117306340
rs2606196
rs2185933
rs62143197
rs149835851
rs114533337
rs75332708
rs12425215
rs117651588
rs76087085
rs10956734
rs45583035
rs146612237
rs17315501
rs58565824
rs78879709
rs2519788
rs545673869
All − MR Egger
All − IVW(MRE)
−0.010
−0.005
0.000
0.005
0.010
MR effect size for
'Protein kinase C iota type' on 'eGFR'

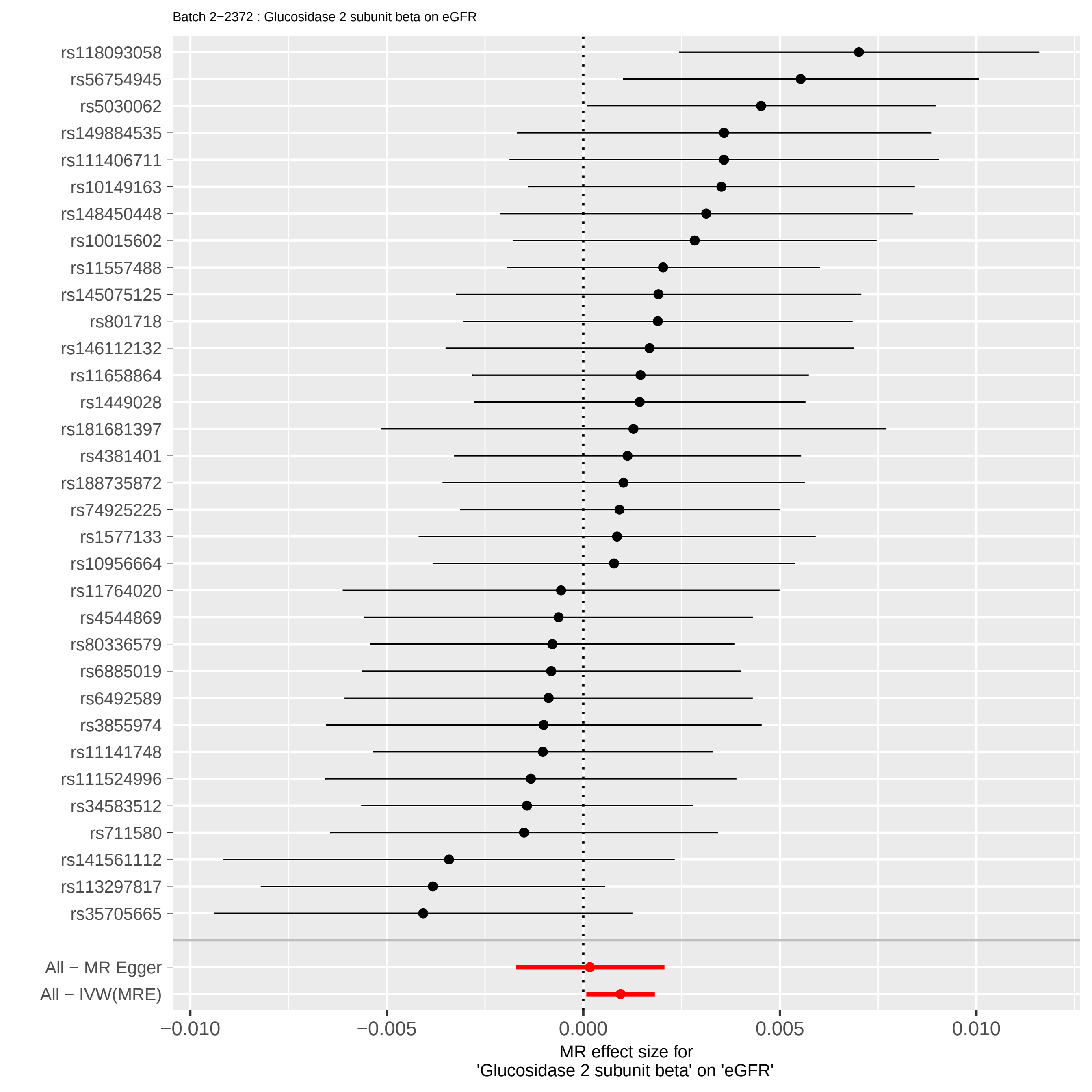

Batch 2−2372 : Glucosidase 2 subunit beta on eGFR
rs118093058
rs56754945
rs5030062
rs149884535
rs111406711
rs10149163
rs148450448
rs10015602
rs11557488
rs145075125
rs801718
rs146112132
rs11658864
rs1449028
rs181681397
rs4381401
rs188735872
rs74925225
rs1577133
rs10956664
rs11764020
rs4544869
rs80336579
rs6885019
rs6492589
rs3855974
rs11141748
rs111524996
rs34583512
rs711580
rs141561112
rs113297817
rs35705665
All − MR Egger
All − IVW(MRE)
−0.010
−0.005
0.000
0.005
0.010
MR effect size for
'Glucosidase 2 subunit beta' on 'eGFR'

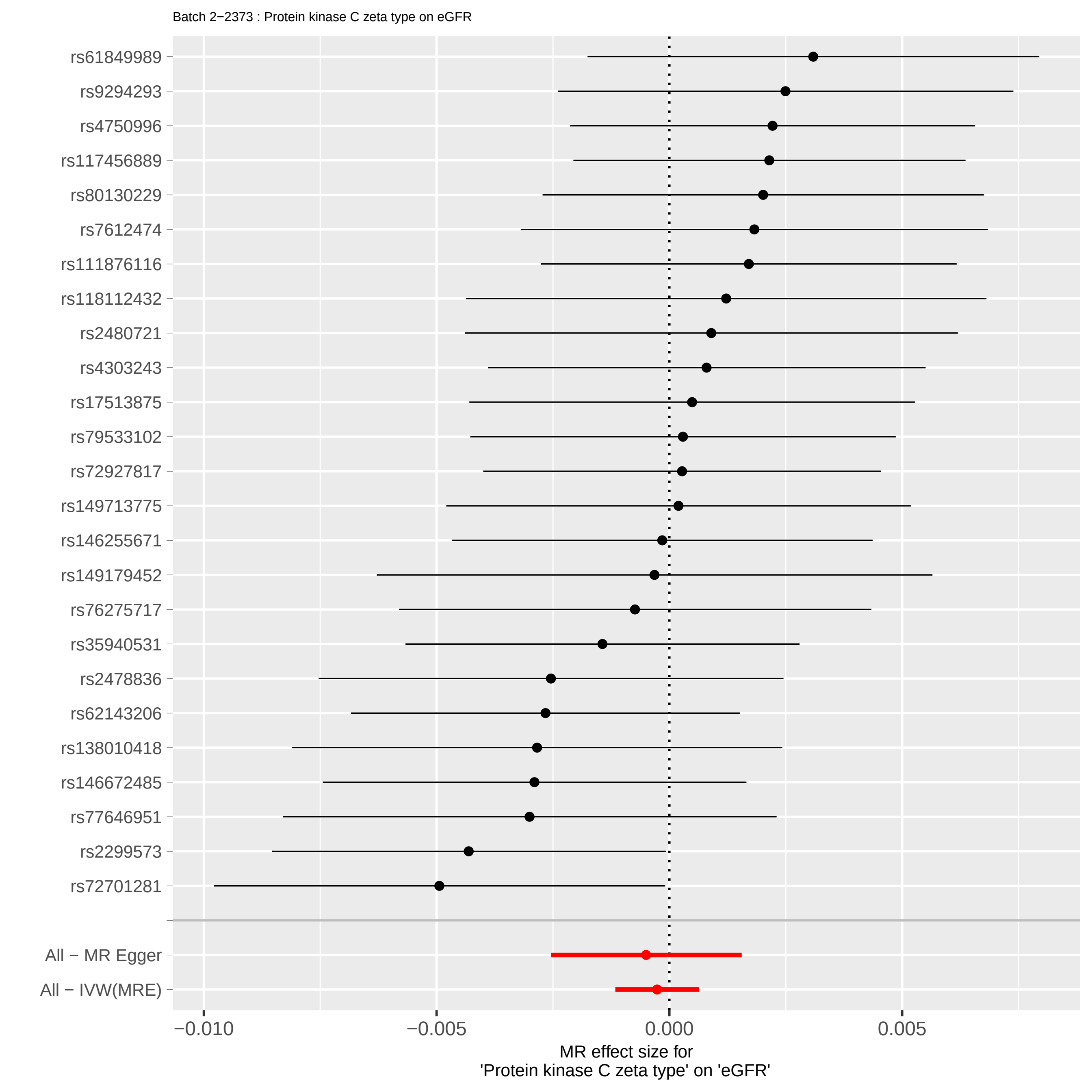

Batch 2−2373 : Protein kinase C zeta type on eGFR
rs61849989
rs9294293
rs4750996
rs117456889
rs80130229
rs7612474
rs111876116
rs118112432
rs2480721
rs4303243
rs17513875
rs79533102
rs72927817
rs149713775
rs146255671
rs149179452
rs76275717
rs35940531
rs2478836
rs62143206
rs138010418
rs146672485
rs77646951
rs2299573
rs72701281
All − MR Egger
All − IVW(MRE)
−0.010
−0.005
0.000
0.005
MR effect size for
'Protein kinase C zeta type' on 'eGFR'

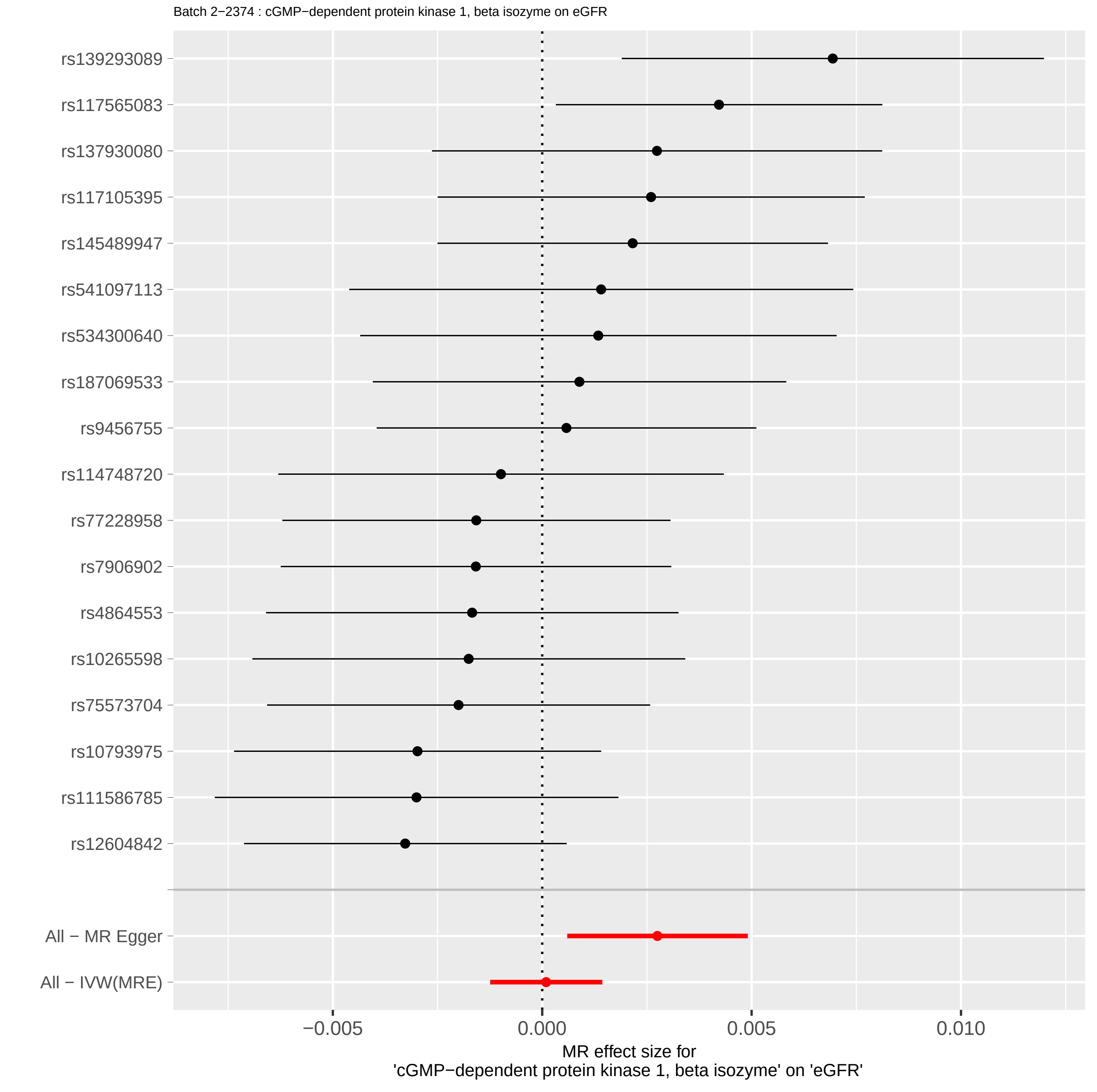

Batch 2−2374 : cGMP−dependent protein kinase 1, beta isozyme on eGFR
rs139293089
rs117565083
rs137930080
rs117105395
rs145489947
rs541097113
rs534300640
rs187069533
rs9456755
rs114748720
rs77228958
rs7906902
rs4864553
rs10265598
rs75573704
rs10793975
rs111586785
rs12604842
All − MR Egger
All − IVW(MRE)
−0.005
0.000
0.005
0.010
MR effect size for
'cGMP−dependent protein kinase 1, beta isozyme' on 'eGFR'

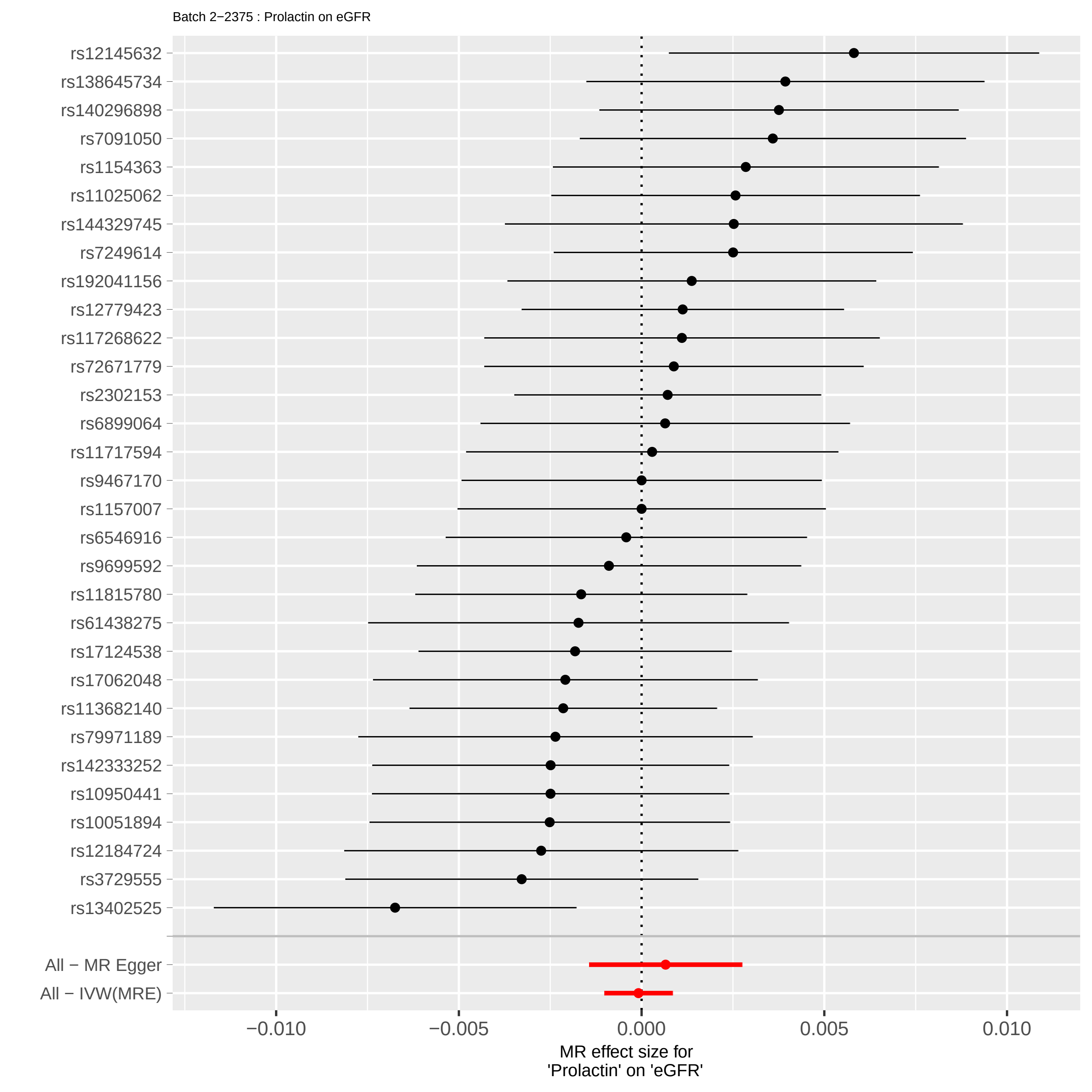

Batch 2−2375 : Prolactin on eGFR
rs12145632
rs138645734
rs140296898
rs7091050
rs1154363
rs11025062
rs144329745
rs7249614
rs192041156
rs12779423
rs117268622
rs72671779
rs2302153
rs6899064
rs11717594
rs9467170
rs1157007
rs6546916
rs9699592
rs11815780
rs61438275
rs17124538
rs17062048
rs113682140
rs79971189
rs142333252
rs10950441
rs10051894
rs12184724
rs3729555
rs13402525
All − MR Egger
All − IVW(MRE)
−0.010
−0.005
0.000
0.005
0.010
MR effect size for
'Prolactin' on 'eGFR'

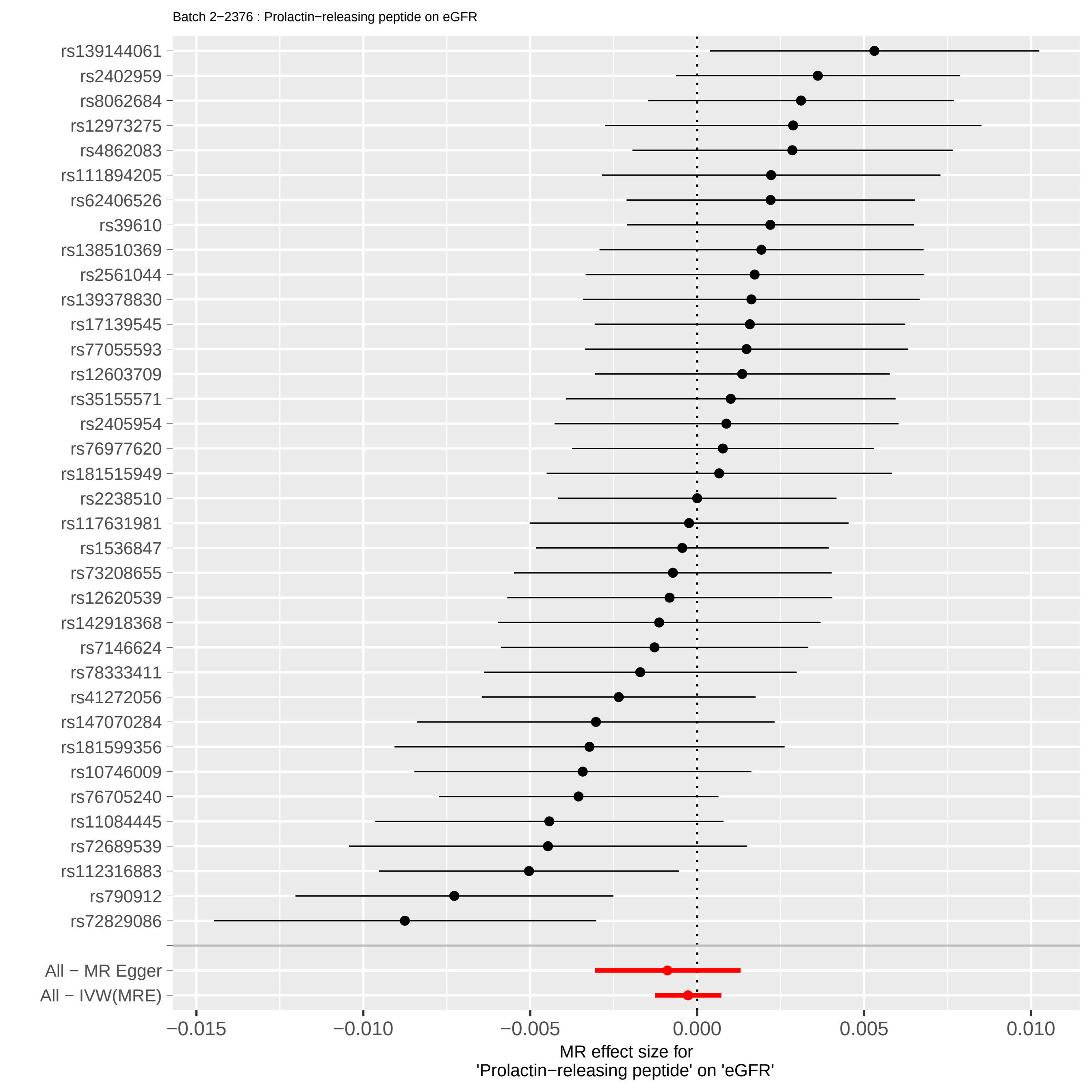

Batch 2−2376 : Prolactin−releasing peptide on eGFR
rs139144061
rs2402959
rs8062684
rs12973275
rs4862083
rs111894205
rs62406526
rs39610
rs138510369
rs2561044
rs139378830
rs17139545
rs77055593
rs12603709
rs35155571
rs2405954
rs76977620
rs181515949
rs2238510
rs117631981
rs1536847
rs73208655
rs12620539
rs142918368
rs7146624
rs78333411
rs41272056
rs147070284
rs181599356
rs10746009
rs76705240
rs11084445
rs72689539
rs112316883
rs790912
rs72829086
All − MR Egger
All − IVW(MRE)
−0.015
−0.010
−0.005
0.000
0.005
0.010
MR effect size for
'Prolactin−releasing peptide' on 'eGFR'

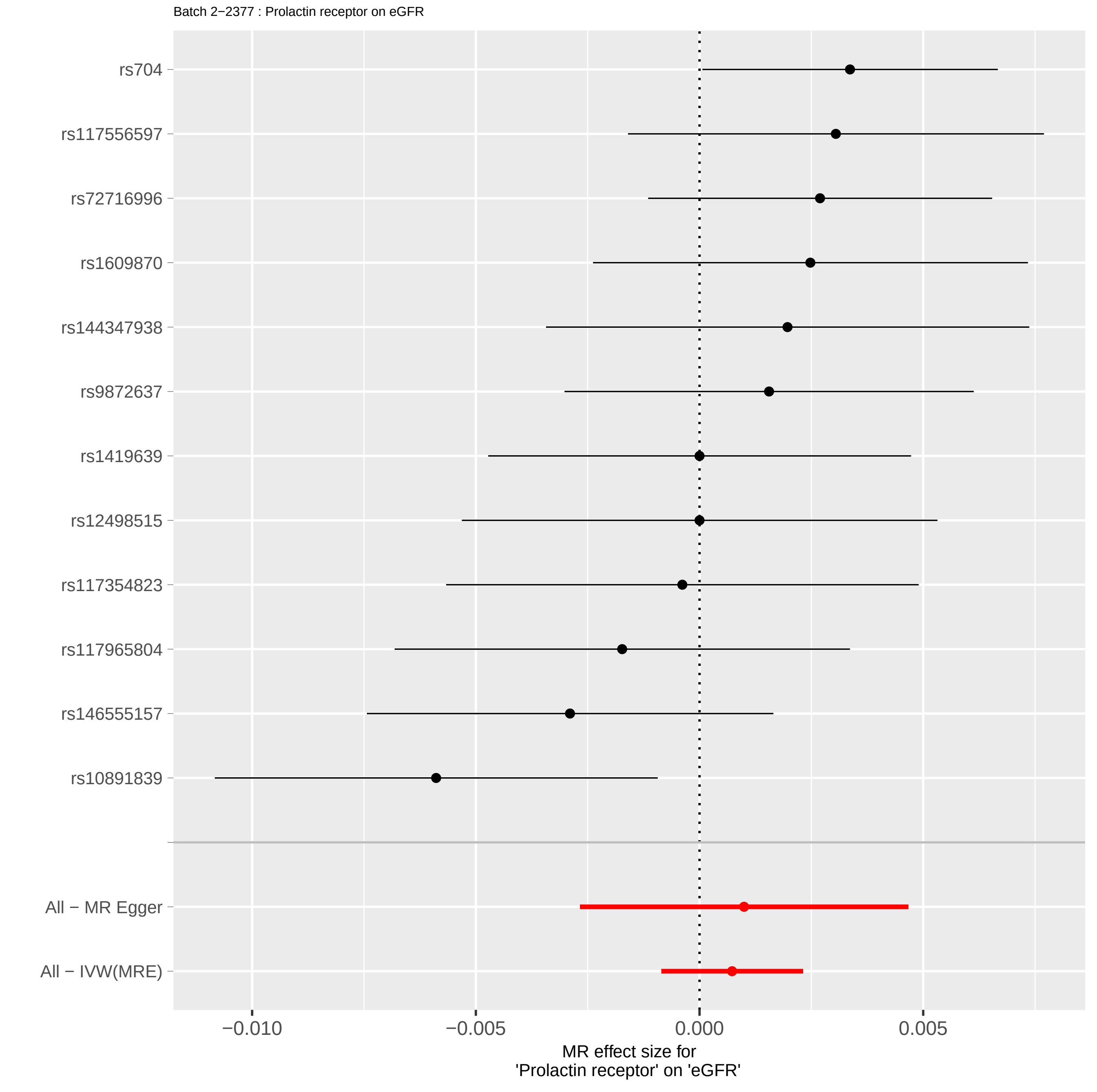

Batch 2−2377 : Prolactin receptor on eGFR
rs704
rs117556597
rs72716996
rs1609870
rs144347938
rs9872637
rs1419639
rs12498515
rs117354823
rs117965804
rs146555157
rs10891839
All − MR Egger
All − IVW(MRE)
−0.010
−0.005
0.000
0.005
MR effect size for
'Prolactin receptor' on 'eGFR'

Batch 2−2378 : Protein arginine N−methyltransferase 3 on eGFR

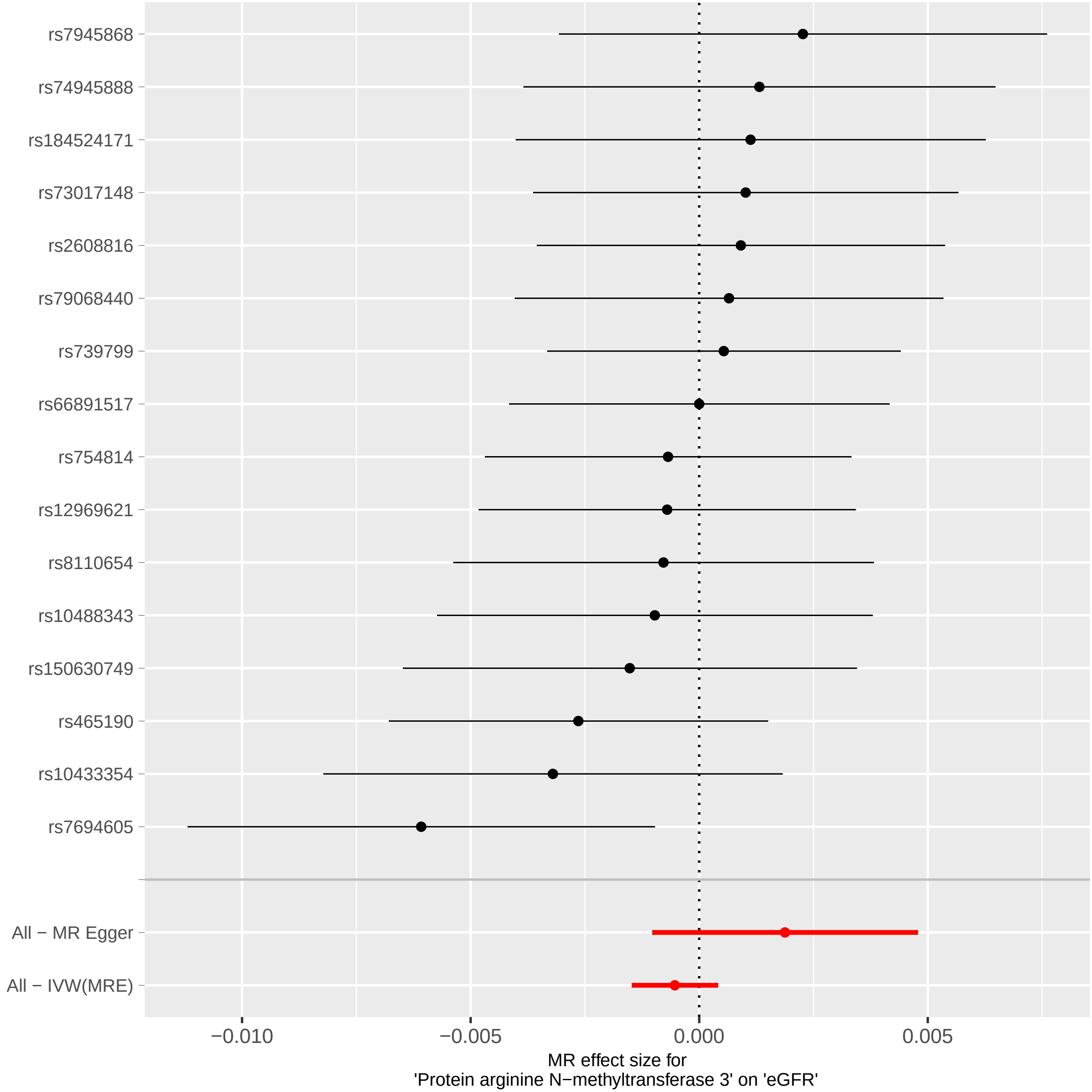

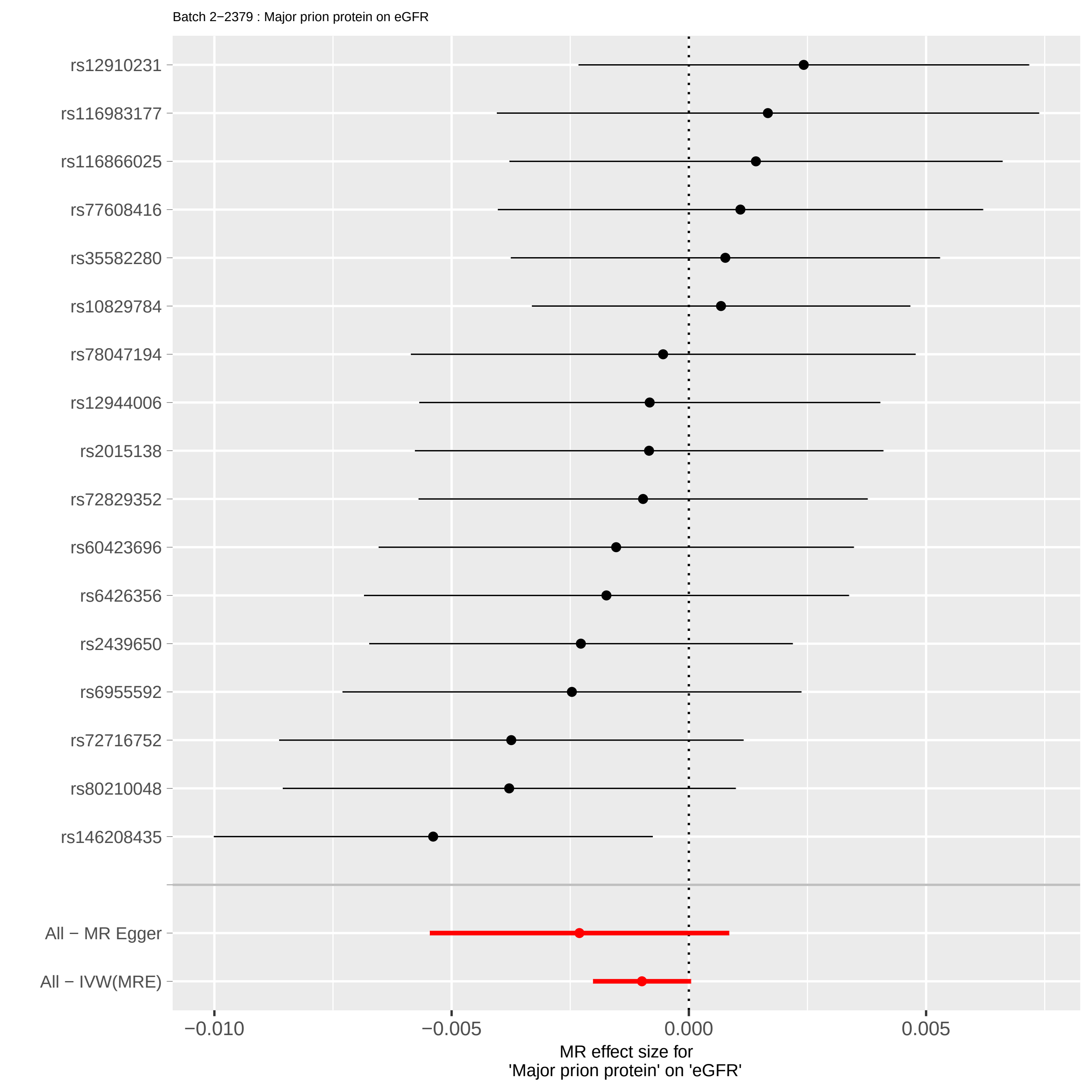
Batch 2−2379 : Major prion protein on eGFR
rs12910231
rs116983177
rs116866025
rs77608416
rs35582280
rs10829784
rs78047194
rs12944006
rs2015138
rs72829352
rs60423696
rs6426356
rs2439650
rs6955592
rs72716752
rs80210048
rs146208435
All − MR Egger
All − IVW(MRE)
−0.010
−0.005
0.000
0.005
MR effect size for
'Major prion protein' on 'eGFR'

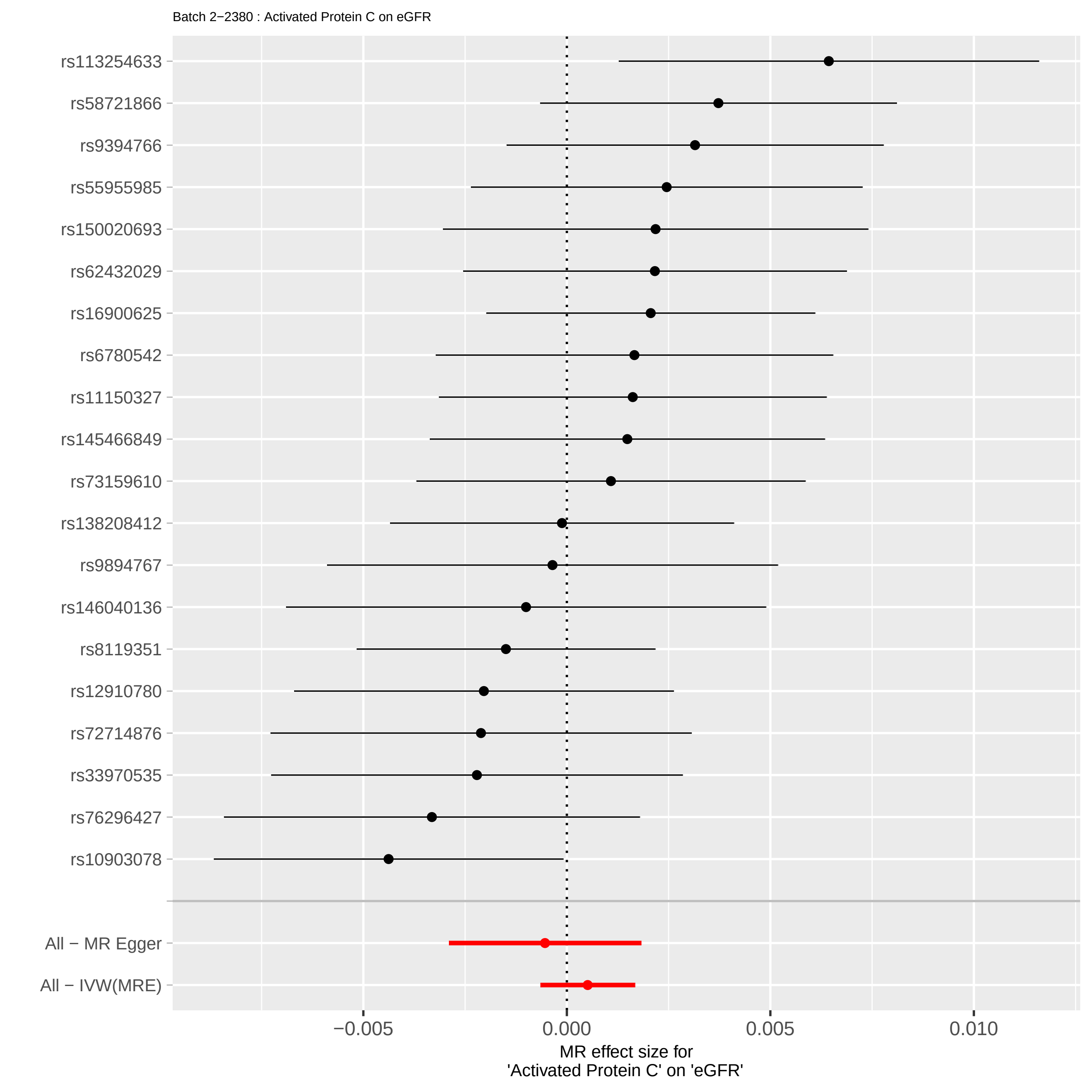

Batch 2−2380 : Activated Protein C on eGFR
rs113254633
rs58721866
rs9394766
rs55955985
rs150020693
rs62432029
rs16900625
rs6780542
rs11150327
rs145466849
rs73159610
rs138208412
rs9894767
rs146040136
rs8119351
rs12910780
rs72714876
rs33970535
rs76296427
rs10903078
All − MR Egger
All − IVW(MRE)
−0.005
0.000
0.005
0.010
MR effect size for
'Activated Protein C' on 'eGFR'

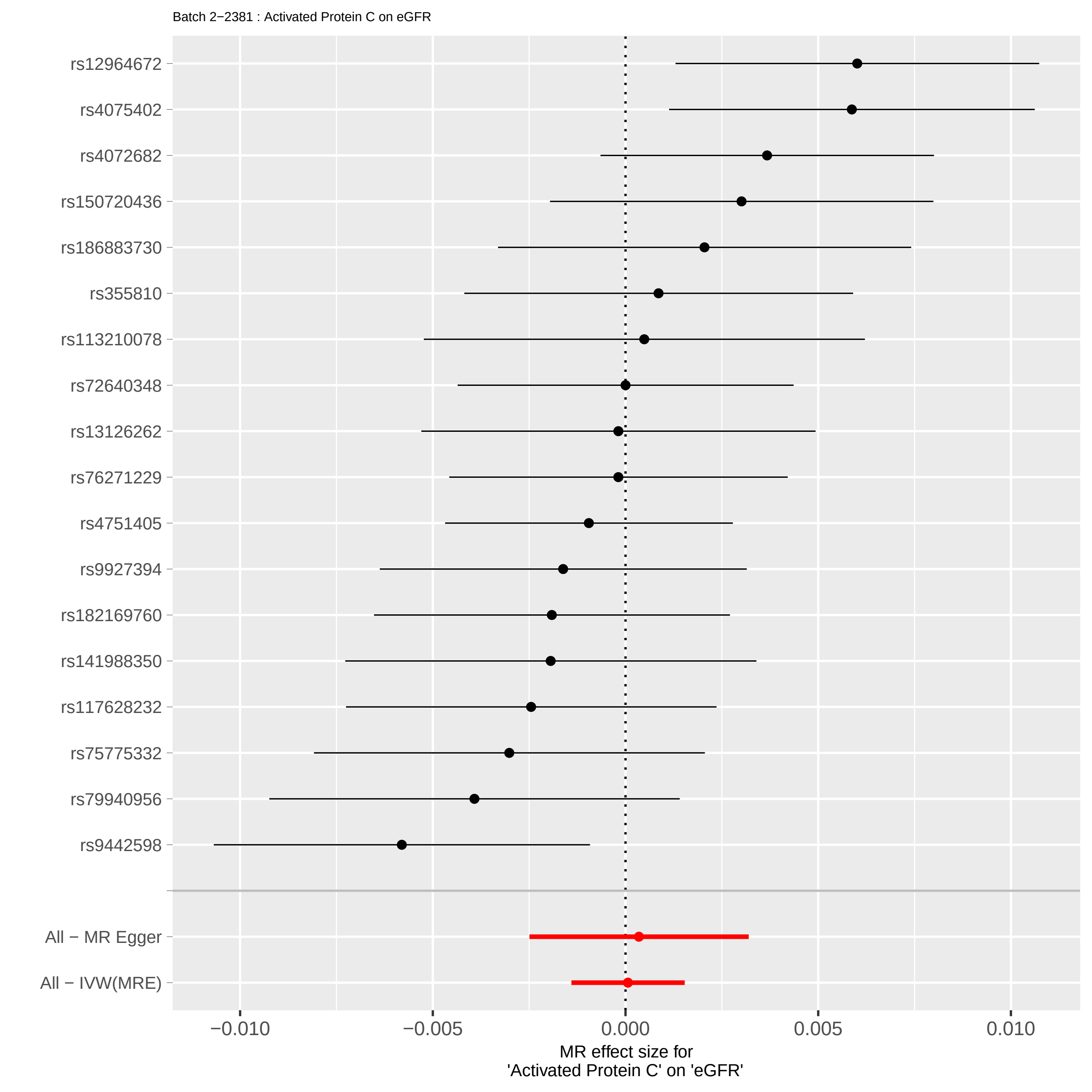

Batch 2−2381 : Activated Protein C on eGFR
rs12964672
rs4075402
rs4072682
rs150720436
rs186883730
rs355810
rs113210078
rs72640348
rs13126262
rs76271229
rs4751405
rs9927394
rs182169760
rs141988350
rs117628232
rs75775332
rs79940956
rs9442598
All − MR Egger
All − IVW(MRE)
−0.010
−0.005
0.000
0.005
0.010
MR effect size for
'Activated Protein C' on 'eGFR'

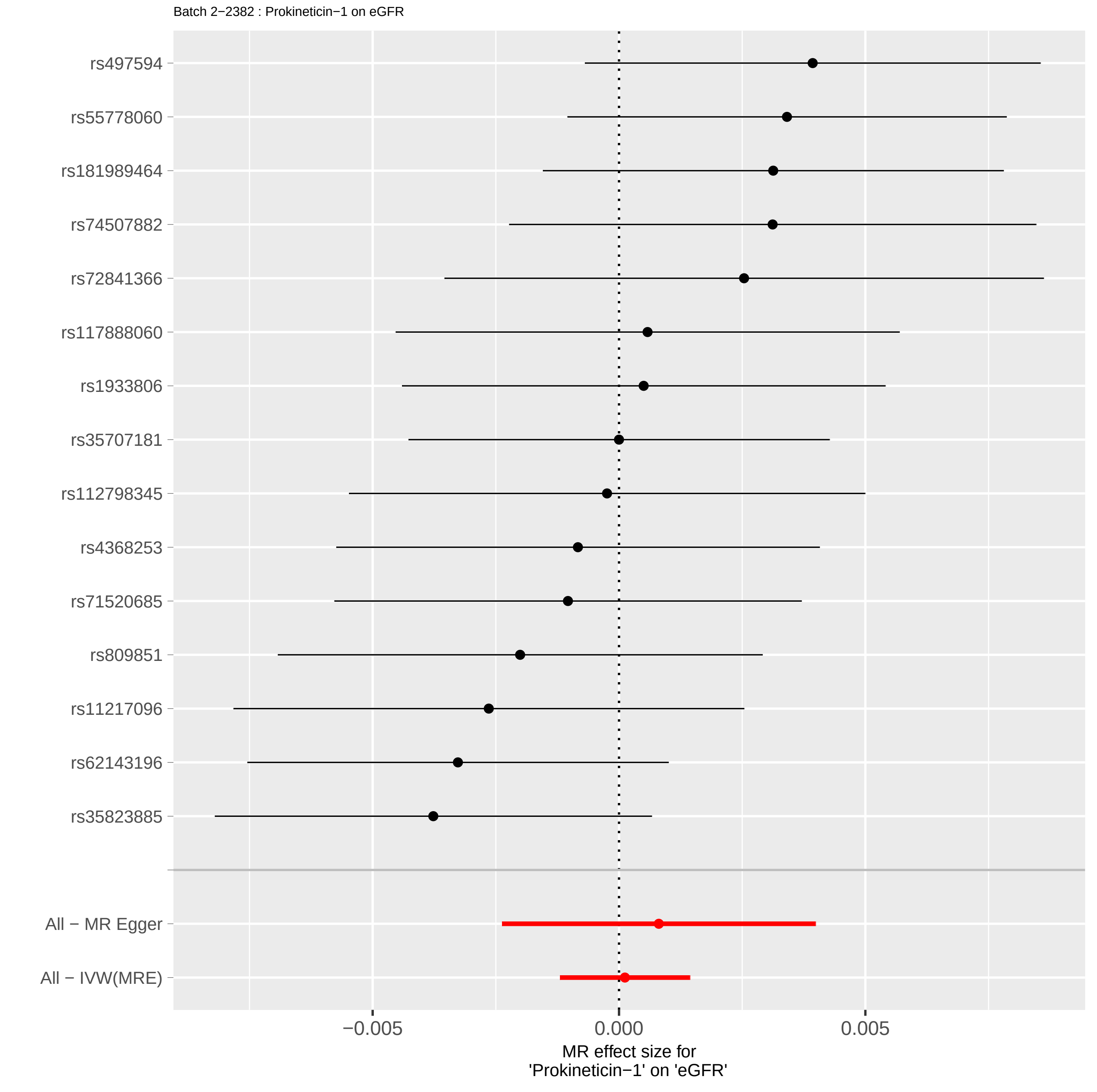

Batch 2−2382 : Prokineticin−1 on eGFR
rs497594
rs55778060
rs181989464
rs74507882
rs72841366
rs117888060
rs1933806
rs35707181
rs112798345
rs4368253
rs71520685
rs809851
rs11217096
rs62143196
rs35823885
All − MR Egger
All − IVW(MRE)
−0.005
0.000
0.005
MR effect size for
'Prokineticin−1' on 'eGFR'

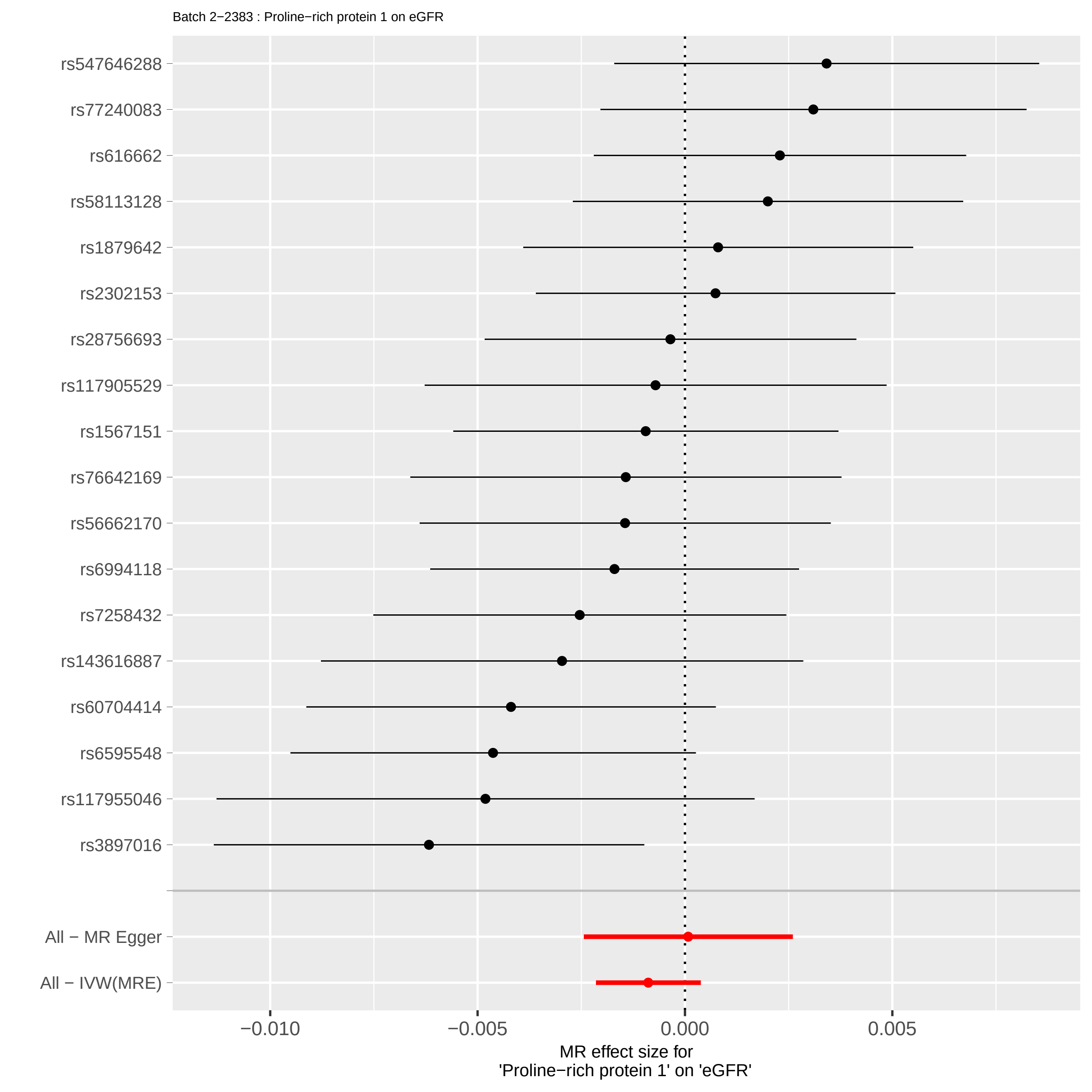

Batch 2−2383 : Proline−rich protein 1 on eGFR
rs547646288
rs77240083
rs616662
rs58113128
rs1879642
rs2302153
rs28756693
rs117905529
rs1567151
rs76642169
rs56662170
rs6994118
rs7258432
rs143616887
rs60704414
rs6595548
rs117955046
rs3897016
All − MR Egger
All − IVW(MRE)
−0.010
−0.005
0.000
0.005
MR effect size for
'Proline−rich protein 1' on 'eGFR'

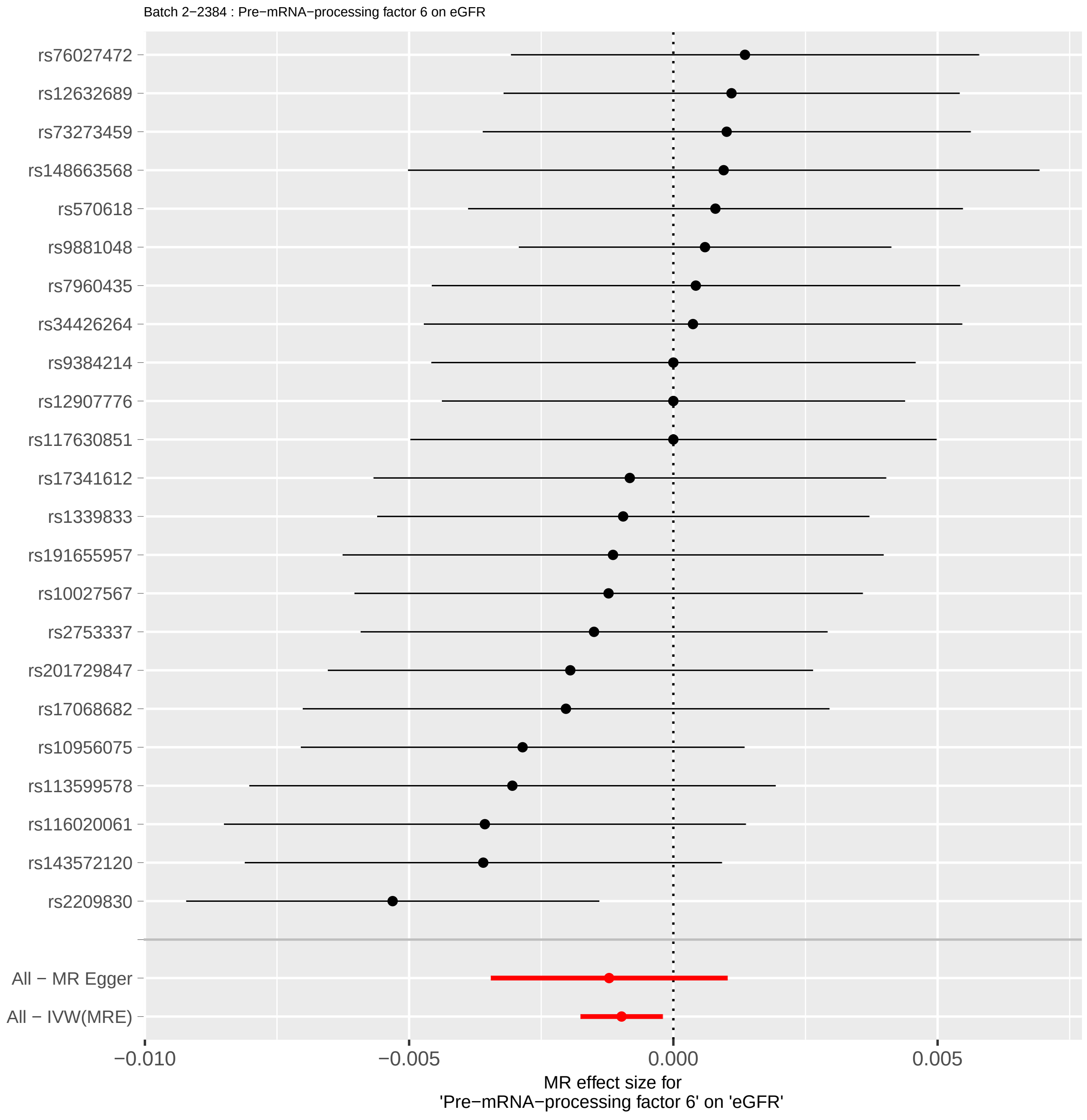

Batch 2−2384 : Pre−mRNA−processing factor 6 on eGFR
rs76027472
rs12632689
rs73273459
rs148663568
rs570618
rs9881048
rs7960435
rs34426264
rs9384214
rs12907776
rs117630851
rs17341612
rs1339833
rs191655957
rs10027567
rs2753337
rs201729847
rs17068682
rs10956075
rs113599578
rs116020061
rs143572120
rs2209830
All − MR Egger
All − IVW(MRE)
−0.010
−0.005
0.000
0.005
MR effect size for
'Pre−mRNA−processing factor 6' on 'eGFR'

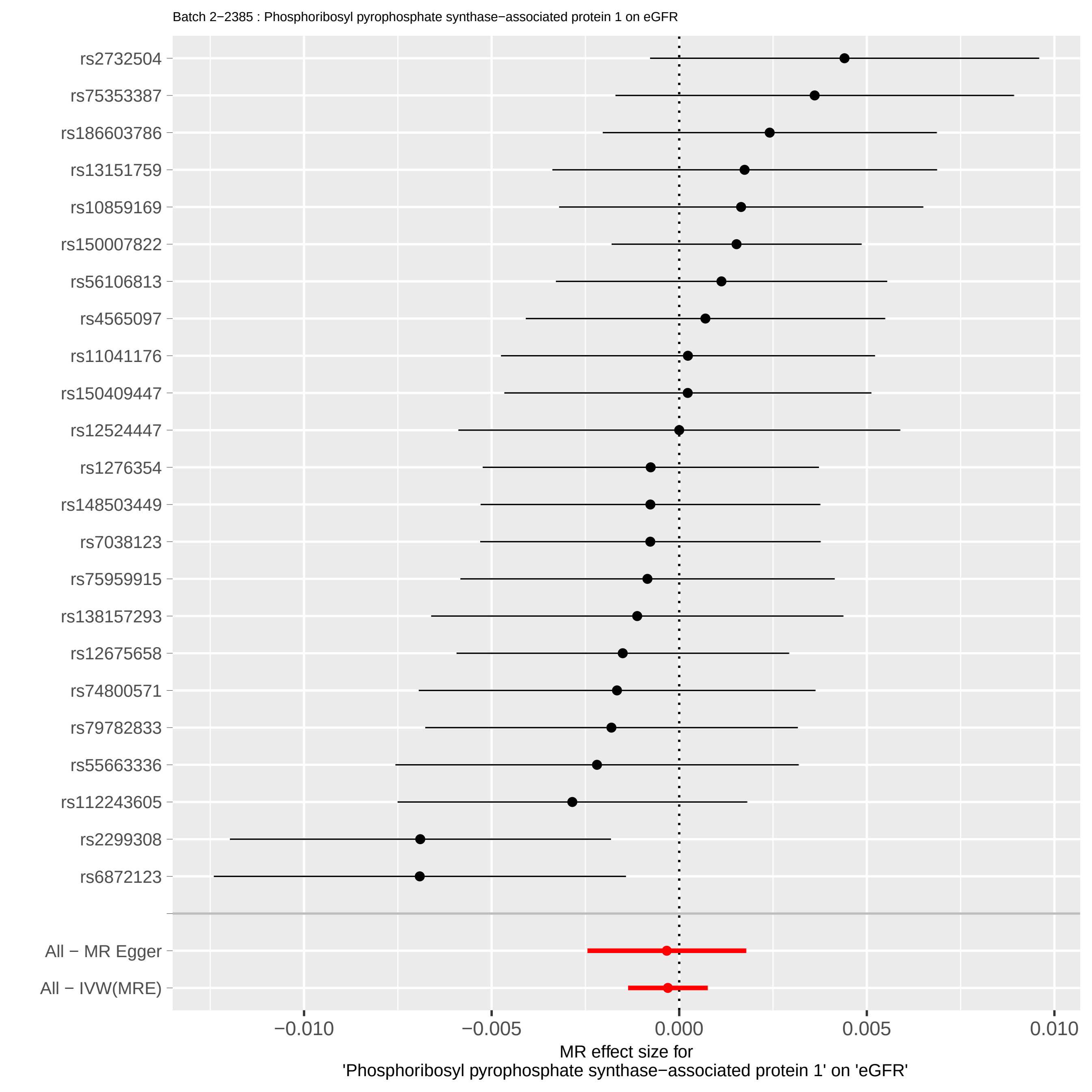

Batch 2−2385 : Phosphoribosyl pyrophosphate synthase−associated protein 1 on eGFR
rs2732504
rs75353387
rs186603786
rs13151759
rs10859169
rs150007822
rs56106813
rs4565097
rs11041176
rs150409447
rs12524447
rs1276354
rs148503449
rs7038123
rs75959915
rs138157293
rs12675658
rs74800571
rs79782833
rs55663336
rs112243605
rs2299308
rs6872123
All − MR Egger
All − IVW(MRE)
−0.010
−0.005
0.000
0.005
0.010
MR effect size for
'Phosphoribosyl pyrophosphate synthase−associated protein 1' on 'eGFR'

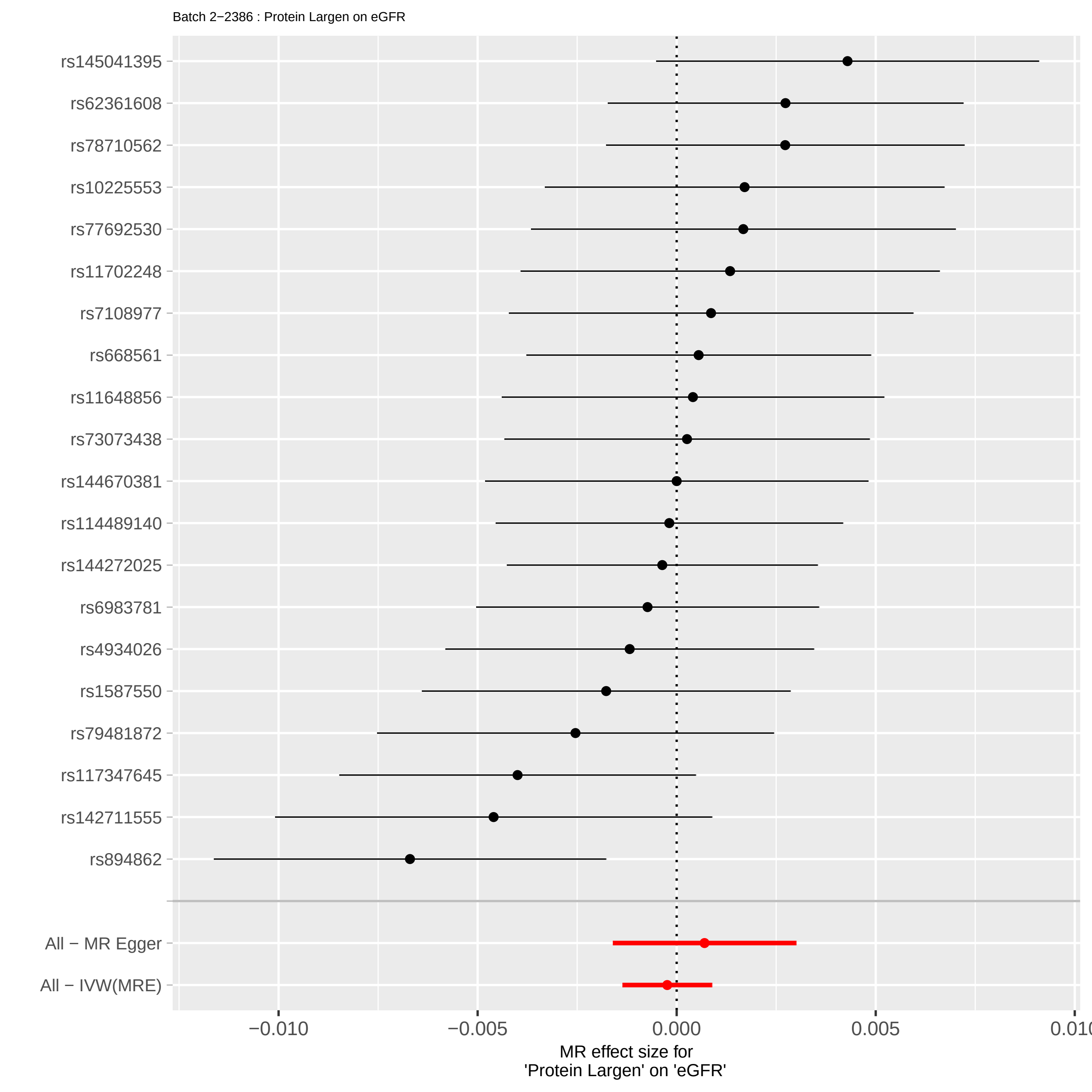

Batch 2−2386 : Protein Largen on eGFR
rs145041395
rs62361608
rs78710562
rs10225553
rs77692530
rs11702248
rs7108977
rs668561
rs11648856
rs73073438
rs144670381
rs114489140
rs144272025
rs6983781
rs4934026
rs1587550
rs79481872
rs117347645
rs142711555
rs894862
All − MR Egger
All − IVW(MRE)
−0.010
−0.005
0.000
0.005
0.010
MR effect size for
'Protein Largen' on 'eGFR'

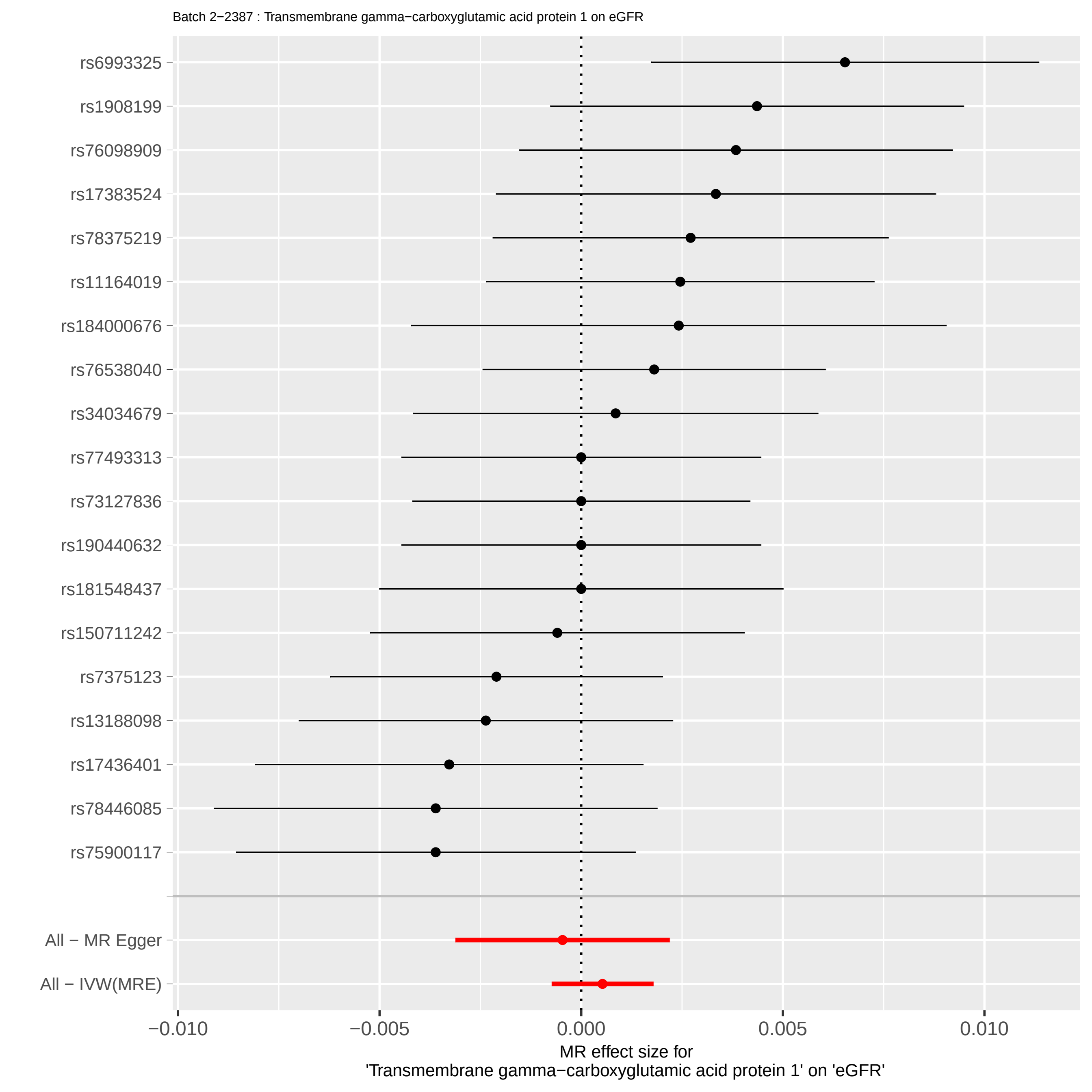
Batch 2−2387 : Transmembrane gamma−carboxyglutamic acid protein 1 on eGFR
rs6993325
rs1908199
rs76098909
rs17383524
rs78375219
rs11164019
rs184000676
rs76538040
rs34034679
rs77493313
rs73127836
rs190440632
rs181548437
rs150711242
rs7375123
rs13188098
rs17436401
rs78446085
rs75900117
All − MR Egger
All − IVW(MRE)
−0.010
−0.005
0.000
0.005
0.010
MR effect size for
'Transmembrane gamma−carboxyglutamic acid protein 1' on 'eGFR'

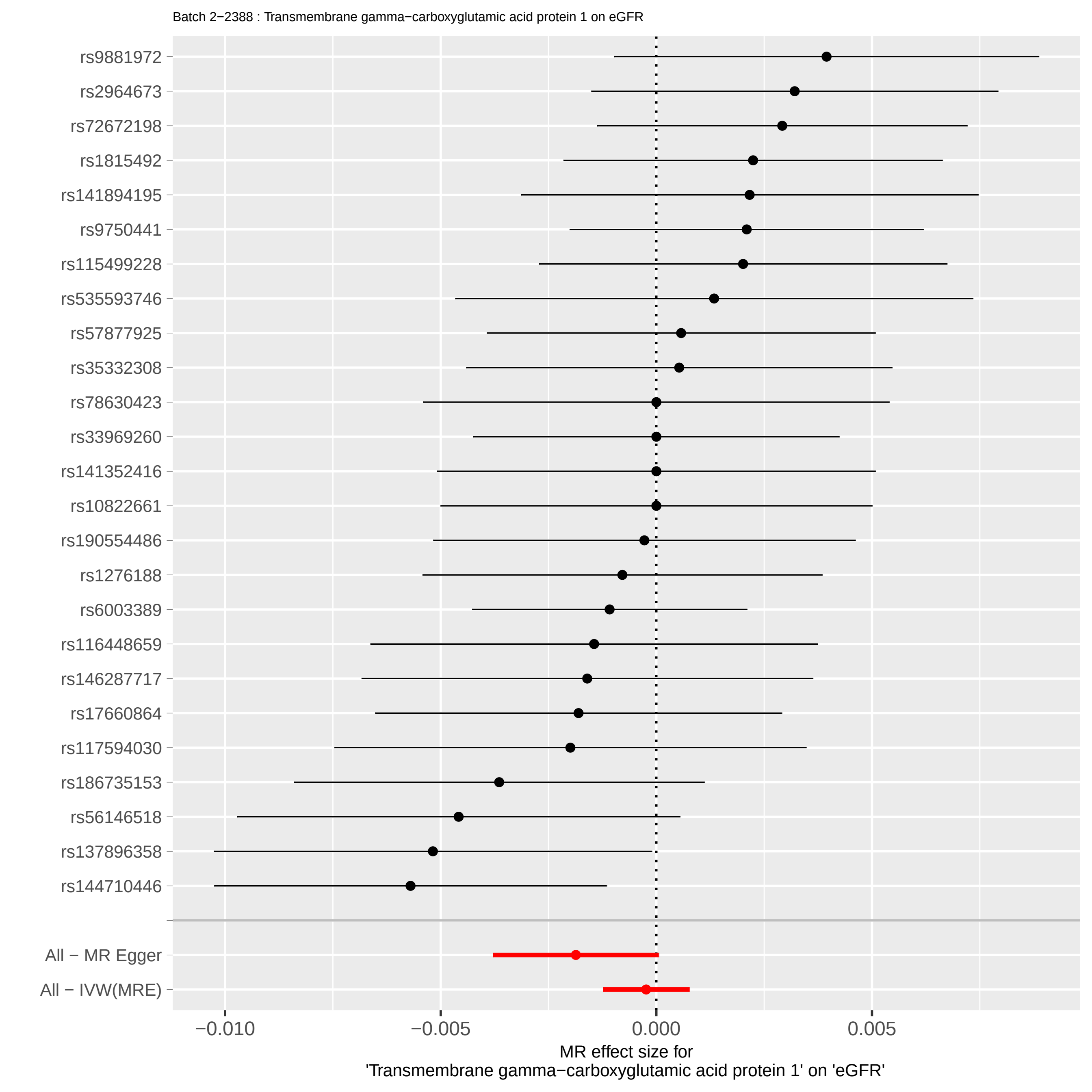

Batch 2−2388 : Transmembrane gamma−carboxyglutamic acid protein 1 on eGFR
rs9881972
rs2964673
rs72672198
rs1815492
rs141894195
rs9750441
rs115499228
rs535593746
rs57877925
rs35332308
rs78630423
rs33969260
rs141352416
rs10822661
rs190554486
rs1276188
rs6003389
rs116448659
rs146287717
rs17660864
rs117594030
rs186735153
rs56146518
rs137896358
rs144710446
All − MR Egger
All − IVW(MRE)
−0.010
−0.005
0.000
0.005
MR effect size for
'Transmembrane gamma−carboxyglutamic acid protein 1' on 'eGFR'

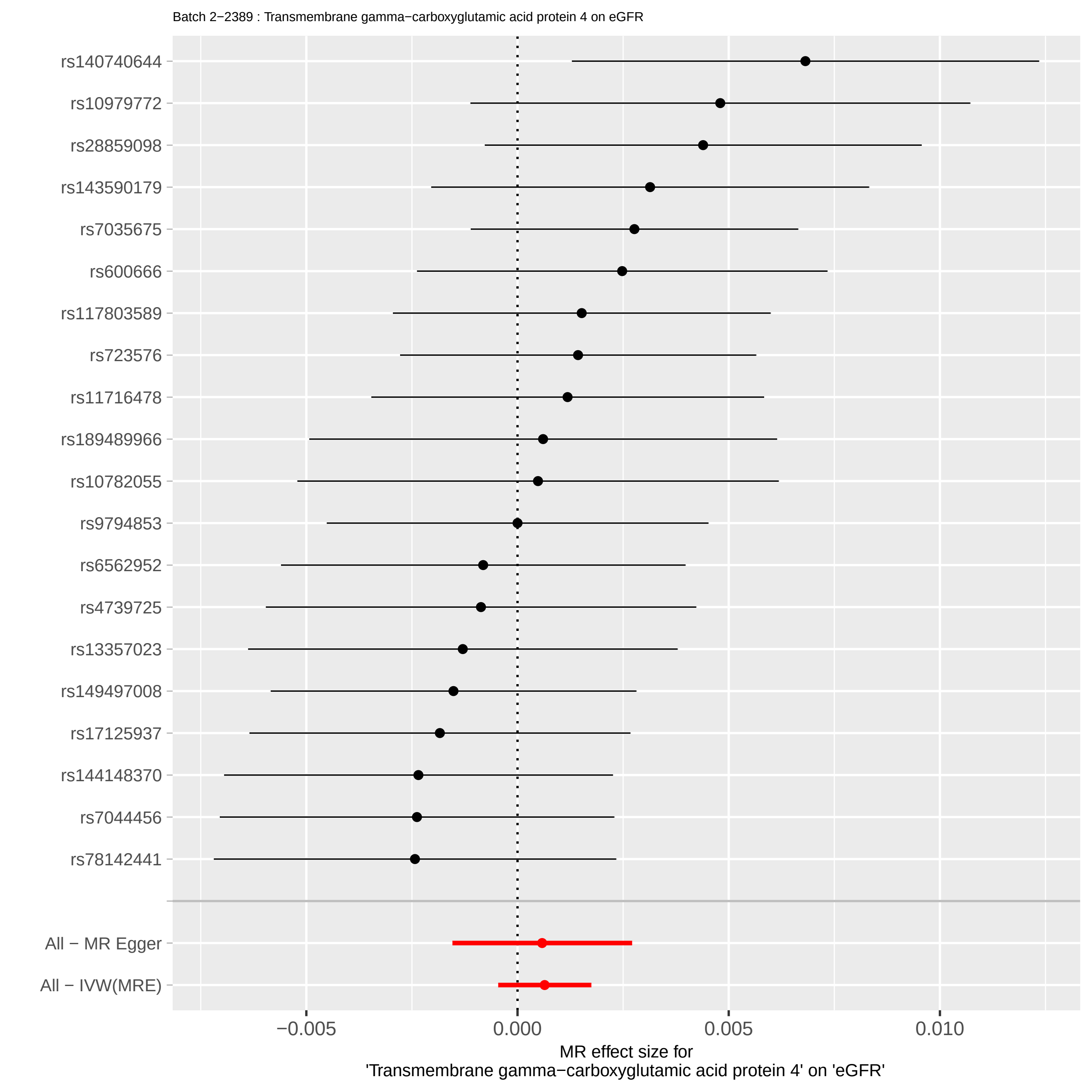

Batch 2−2389 : Transmembrane gamma−carboxyglutamic acid protein 4 on eGFR
rs140740644
rs10979772
rs28859098
rs143590179
rs7035675
rs600666
rs117803589
rs723576
rs11716478
rs189489966
rs10782055
rs9794853
rs6562952
rs4739725
rs13357023
rs149497008
rs17125937
rs144148370
rs7044456
rs78142441
All − MR Egger
All − IVW(MRE)
−0.005
0.000
0.005
0.010
MR effect size for
'Transmembrane gamma−carboxyglutamic acid protein 4' on 'eGFR'

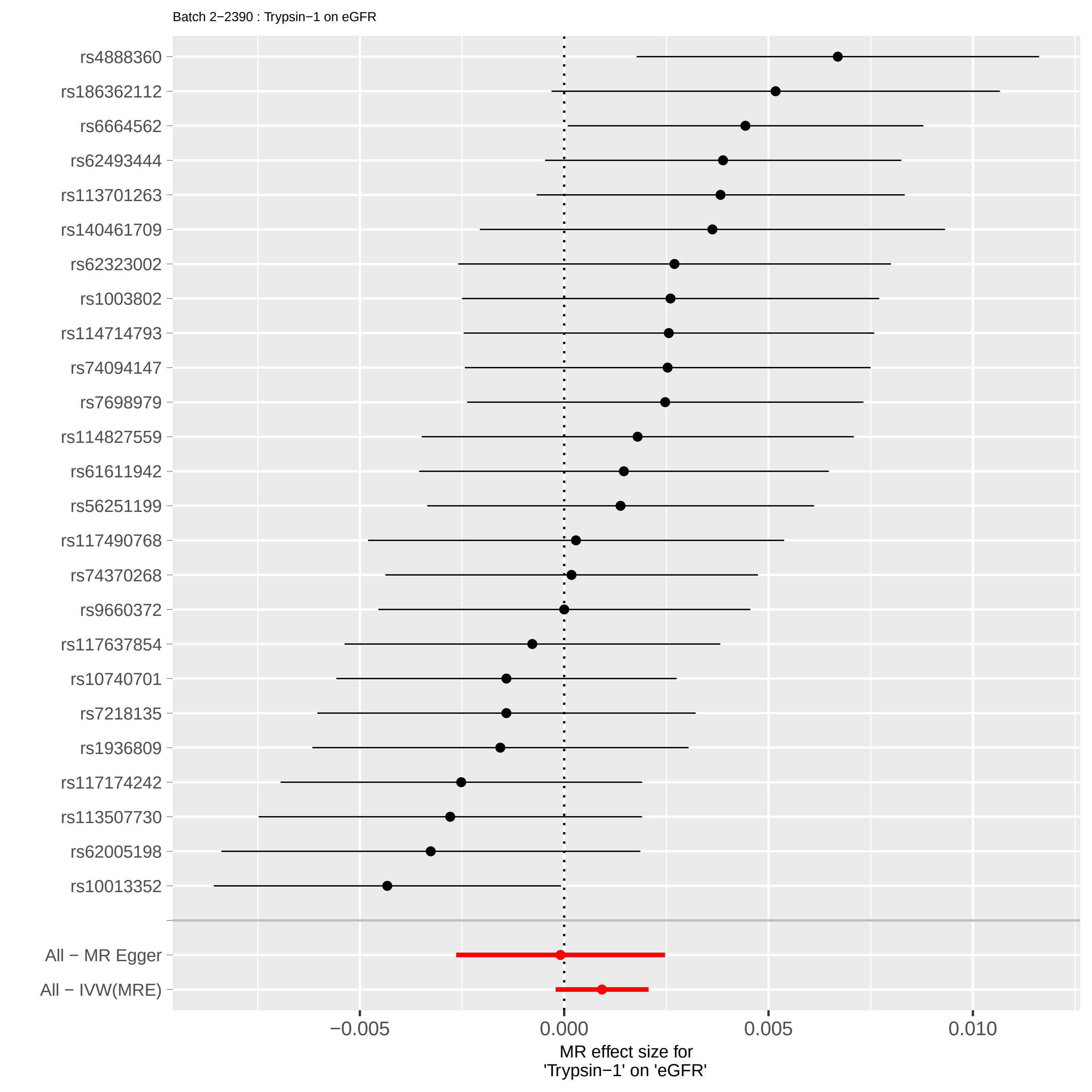

Batch 2−2390 : Trypsin−1 on eGFR
rs4888360
rs186362112
rs6664562
rs62493444
rs113701263
rs140461709
rs62323002
rs1003802
rs114714793
rs74094147
rs7698979
rs114827559
rs61611942
rs56251199
rs117490768
rs74370268
rs9660372
rs117637854
rs10740701
rs7218135
rs1936809
rs117174242
rs113507730
rs62005198
rs10013352
All − MR Egger
All − IVW(MRE)
−0.005
0.000
0.005
0.010
MR effect size for
'Trypsin−1' on 'eGFR'

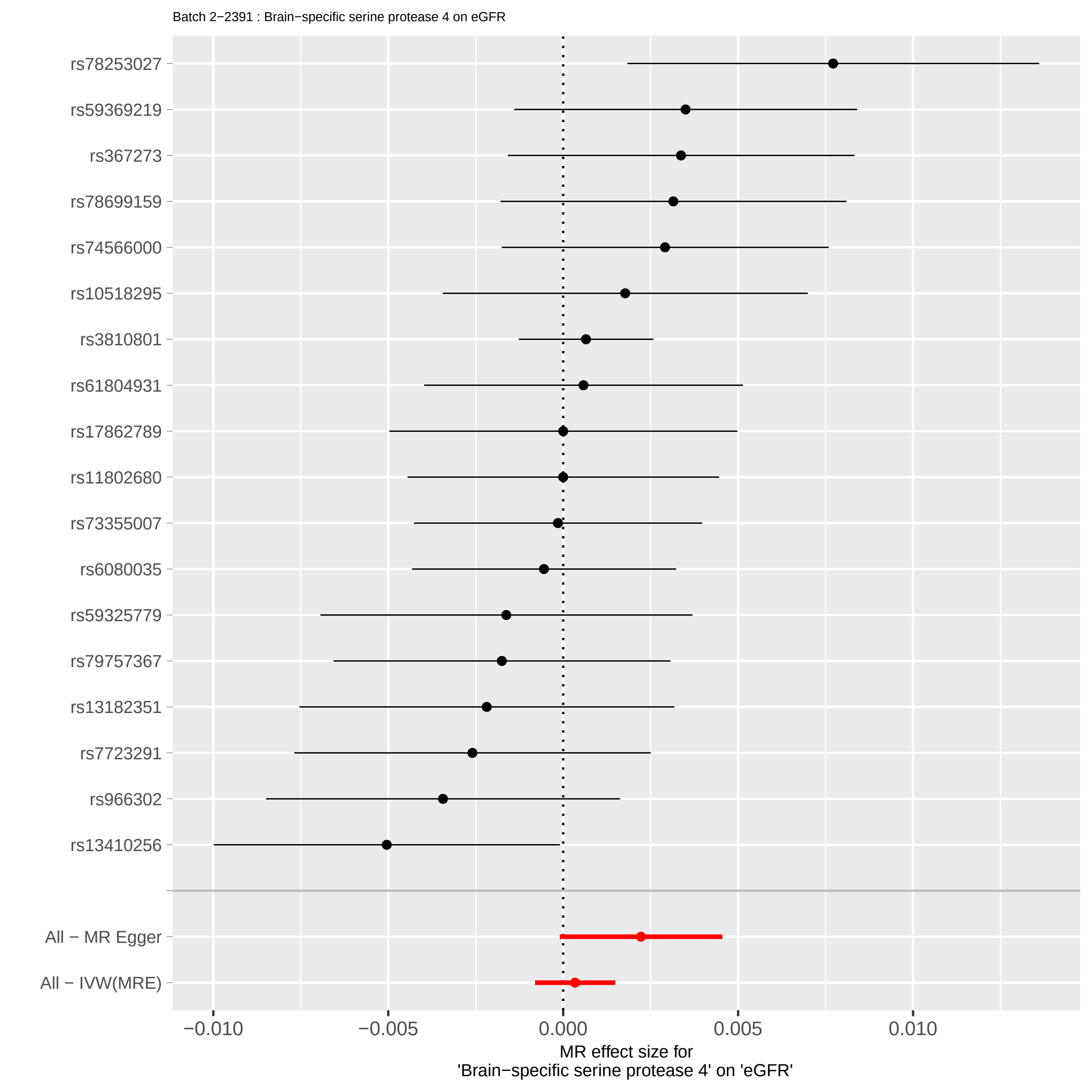

Batch 2−2391 : Brain−specific serine protease 4 on eGFR
rs78253027
rs59369219
rs367273
rs78699159
rs74566000
rs10518295
rs3810801
rs61804931
rs17862789
rs11802680
rs73355007
rs6080035
rs59325779
rs79757367
rs13182351
rs7723291
rs966302
rs13410256
All − MR Egger
All − IVW(MRE)
−0.010
−0.005
0.000
0.005
0.010
MR effect size for
'Brain−specific serine protease 4' on 'eGFR'

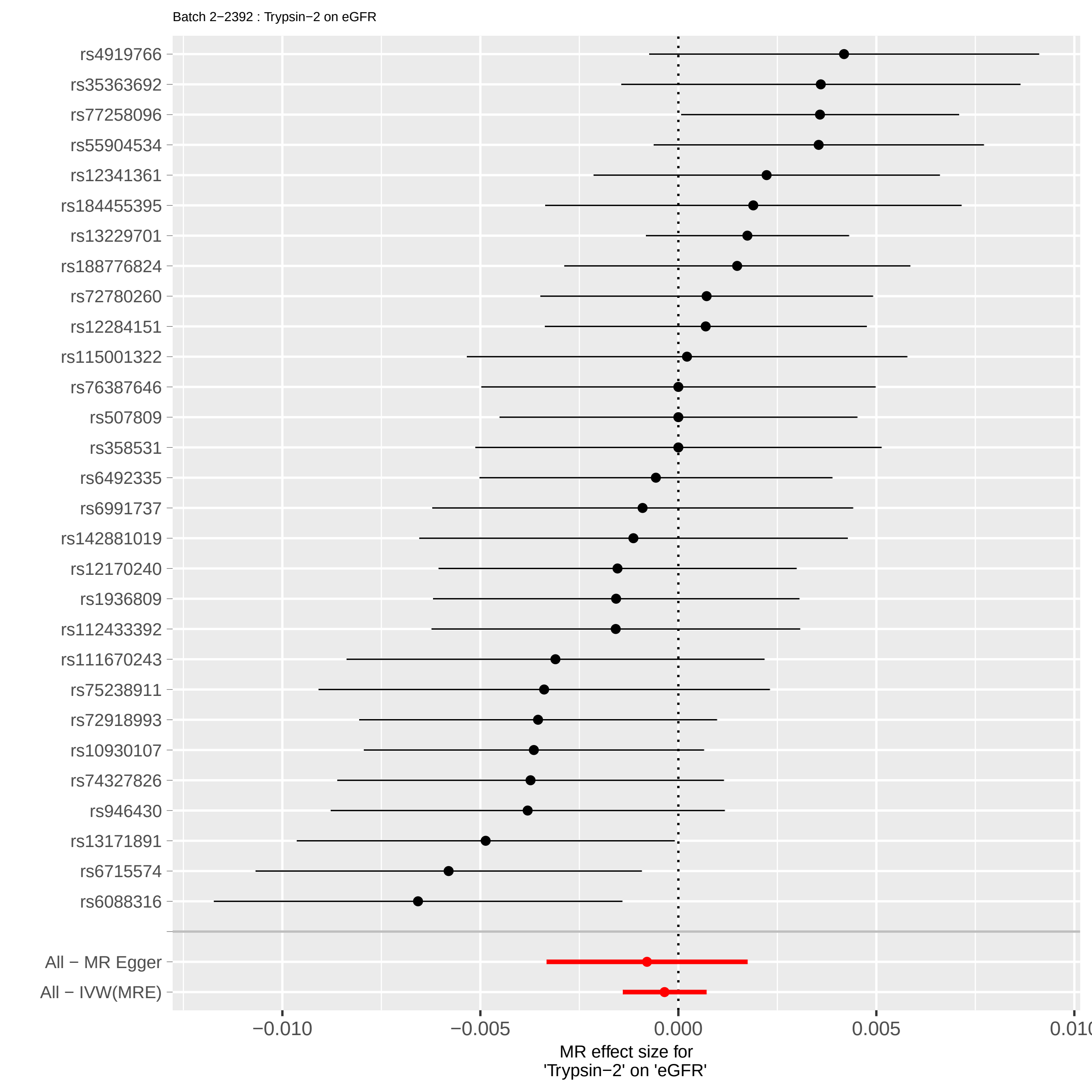

Batch 2−2392 : Trypsin−2 on eGFR
rs4919766
rs35363692
rs77258096
rs55904534
rs12341361
rs184455395
rs13229701
rs188776824
rs72780260
rs12284151
rs115001322
rs76387646
rs507809
rs358531
rs6492335
rs6991737
rs142881019
rs12170240
rs1936809
rs112433392
rs111670243
rs75238911
rs72918993
rs10930107
rs74327826
rs946430
rs13171891
rs6715574
rs6088316
All − MR Egger
All − IVW(MRE)
−0.010
−0.005
0.000
0.005
0.010
MR effect size for
'Trypsin−2' on 'eGFR'

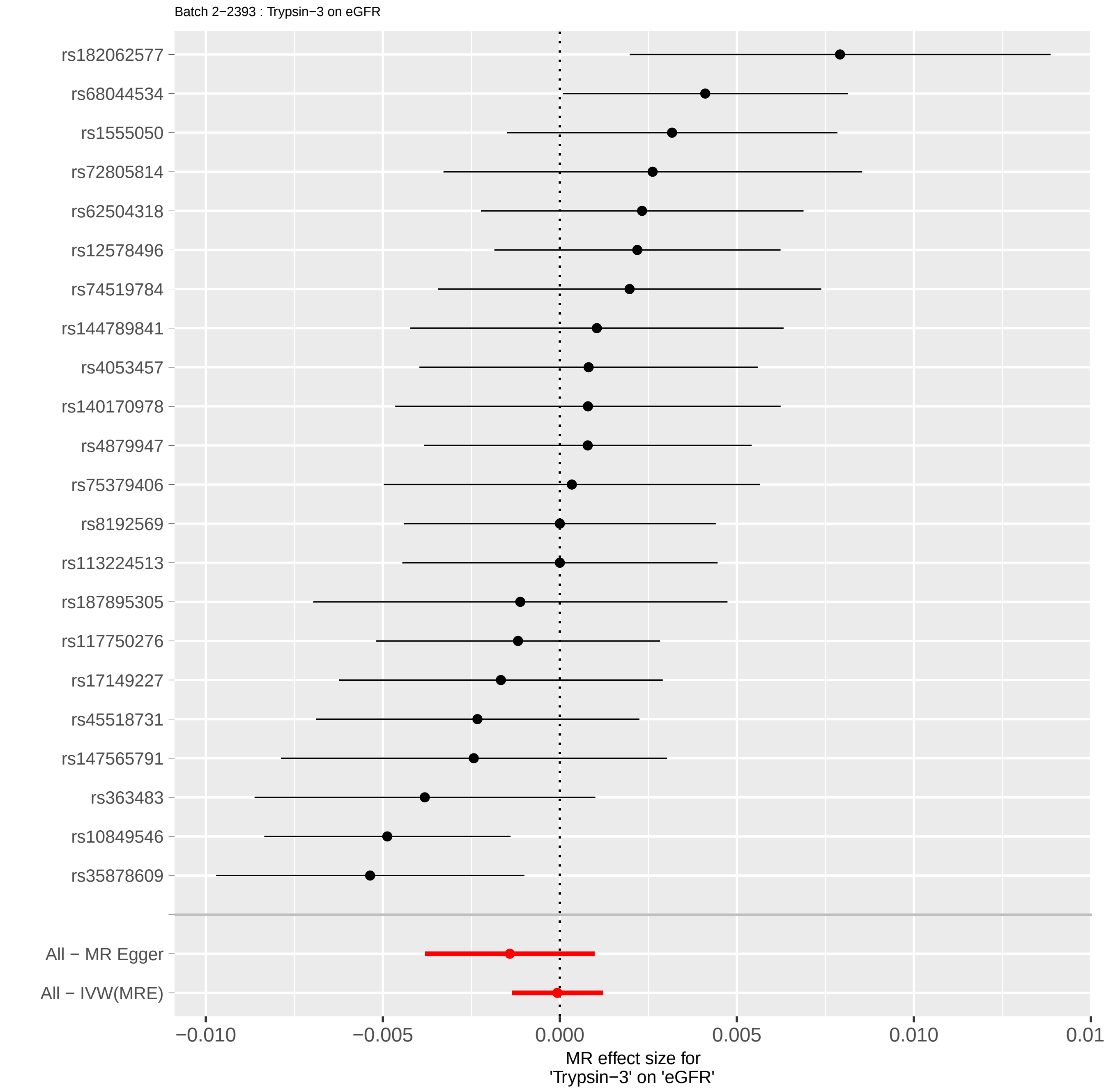

Batch 2−2393 : Trypsin−3 on eGFR
rs182062577
rs68044534
rs1555050
rs72805814
rs62504318
rs12578496
rs74519784
rs144789841
rs4053457
rs140170978
rs4879947
rs75379406
rs8192569
rs113224513
rs187895305
rs117750276
rs17149227
rs45518731
rs147565791
rs363483
rs10849546
rs35878609
All − MR Egger
All − IVW(MRE)
−0.010
−0.005
0.000
0.005
0.010
MR effect size for
'Trypsin−3' on 'eGFR'

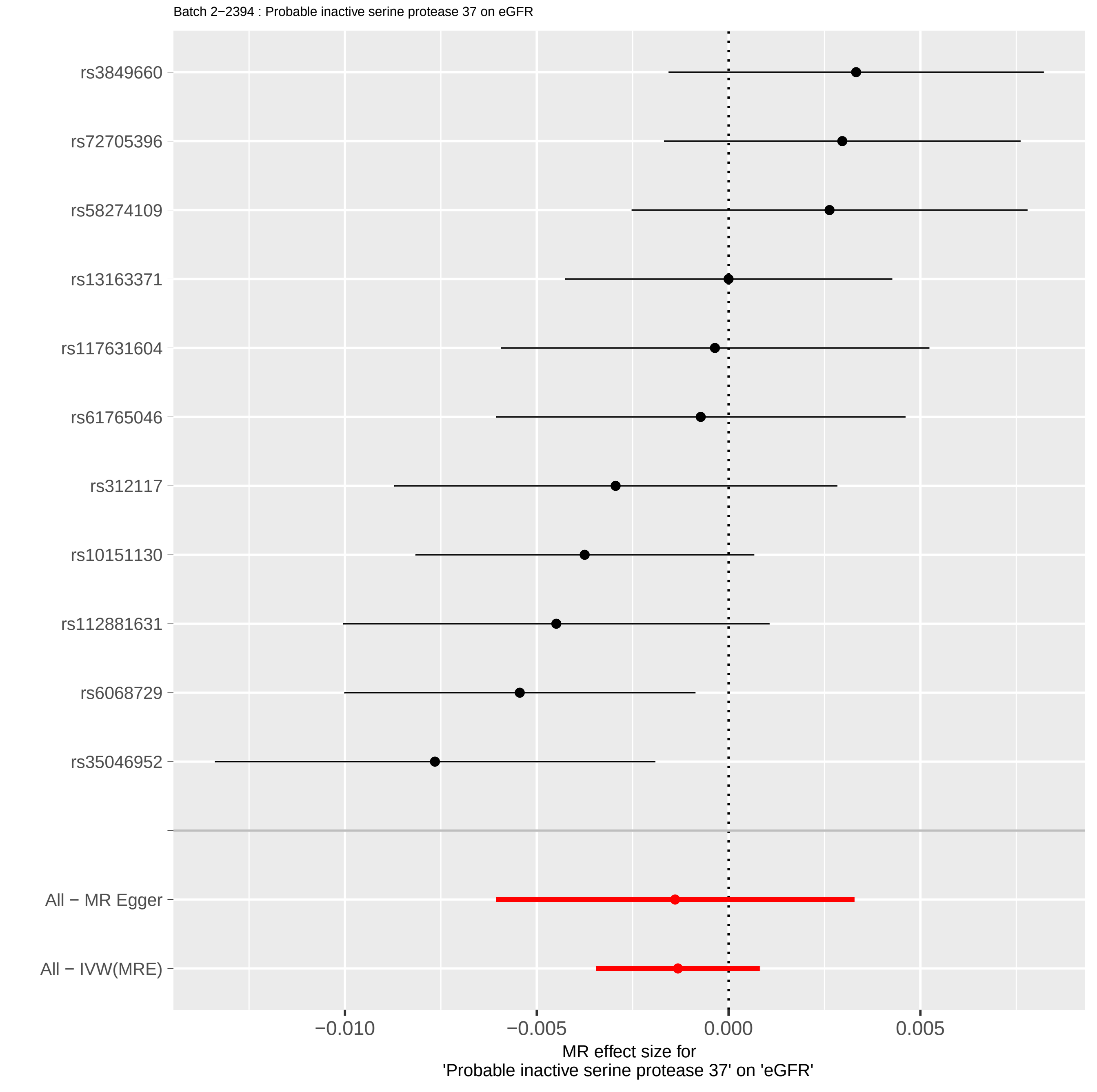
Batch 2−2394 : Probable inactive serine protease 37 on eGFR
rs3849660
rs72705396
rs58274109
rs13163371
rs117631604
rs61765046
rs312117
rs10151130
rs112881631
rs6068729
rs35046952
All − MR Egger
All − IVW(MRE)
−0.010
−0.005
0.000
0.005
MR effect size for
'Probable inactive serine protease 37' on 'eGFR'

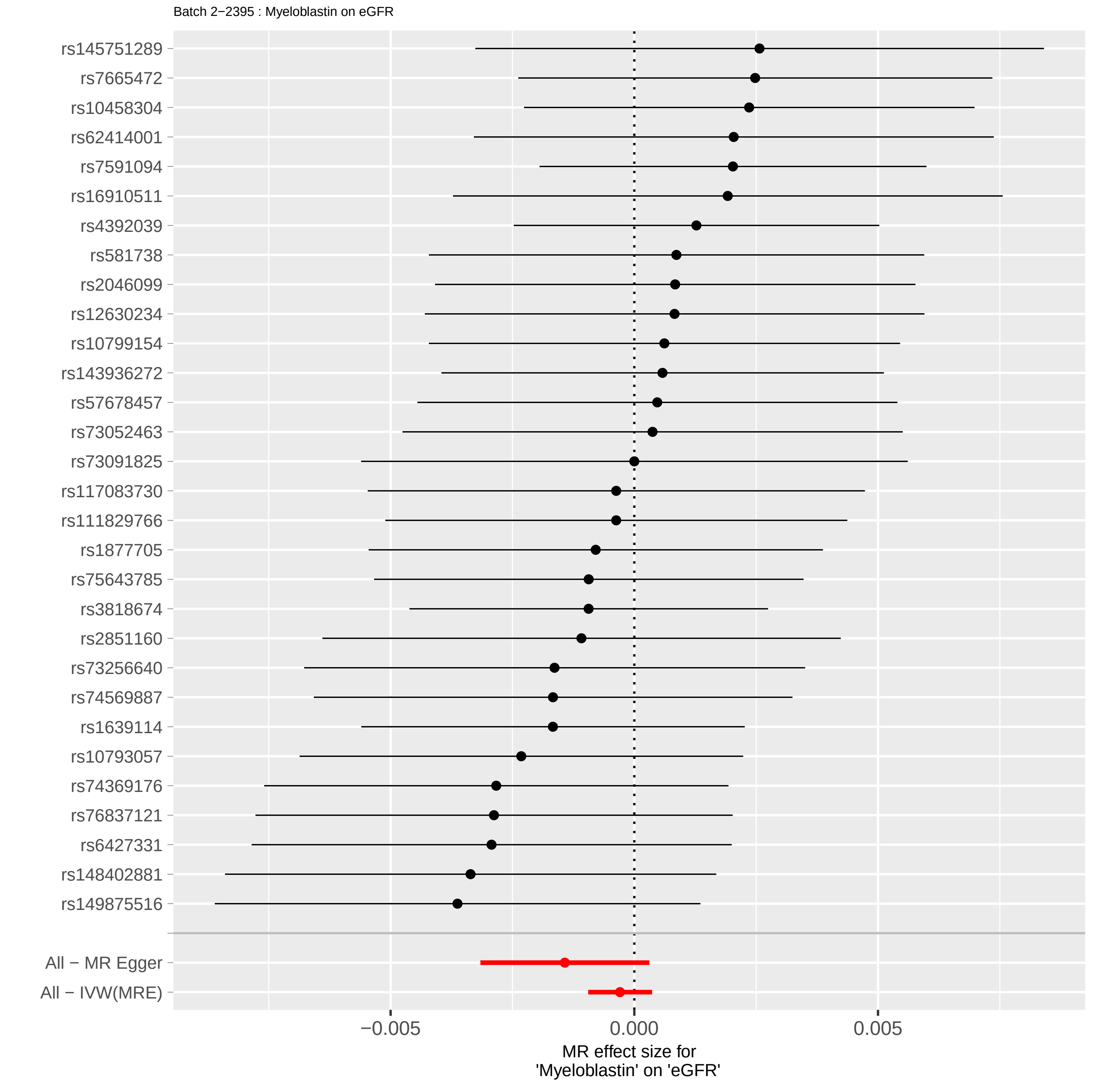
Batch 2−2395 : Myeloblastin on eGFR
rs145751289
rs7665472
rs10458304
rs62414001
rs7591094
rs16910511
rs4392039
rs581738
rs2046099
rs12630234
rs10799154
rs143936272
rs57678457
rs73052463
rs73091825
rs117083730
rs111829766
rs1877705
rs75643785
rs3818674
rs2851160
rs73256640
rs74569887
rs1639114
rs10793057
rs74369176
rs76837121
rs6427331
rs148402881
rs149875516
All − MR Egger
All − IVW(MRE)
−0.005
0.000
0.005
MR effect size for
'Myeloblastin' on 'eGFR'

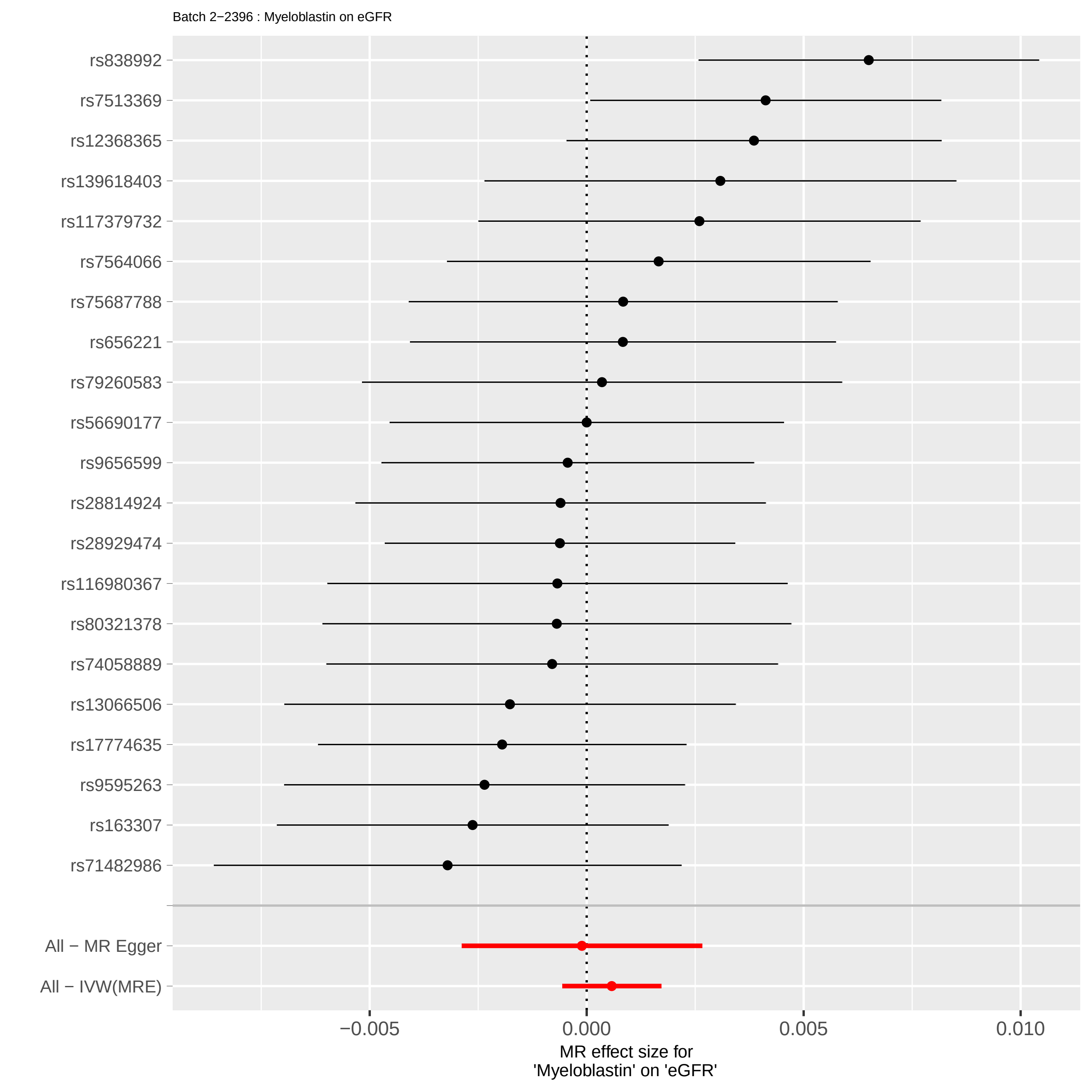
Batch 2−2396 : Myeloblastin on eGFR
rs838992
rs7513369
rs12368365
rs139618403
rs117379732
rs7564066
rs75687788
rs656221
rs79260583
rs56690177
rs9656599
rs28814924
rs28929474
rs116980367
rs80321378
rs74058889
rs13066506
rs17774635
rs9595263
rs163307
rs71482986
All − MR Egger
All − IVW(MRE)
−0.005
0.000
0.005
0.010
MR effect size for
'Myeloblastin' on 'eGFR'

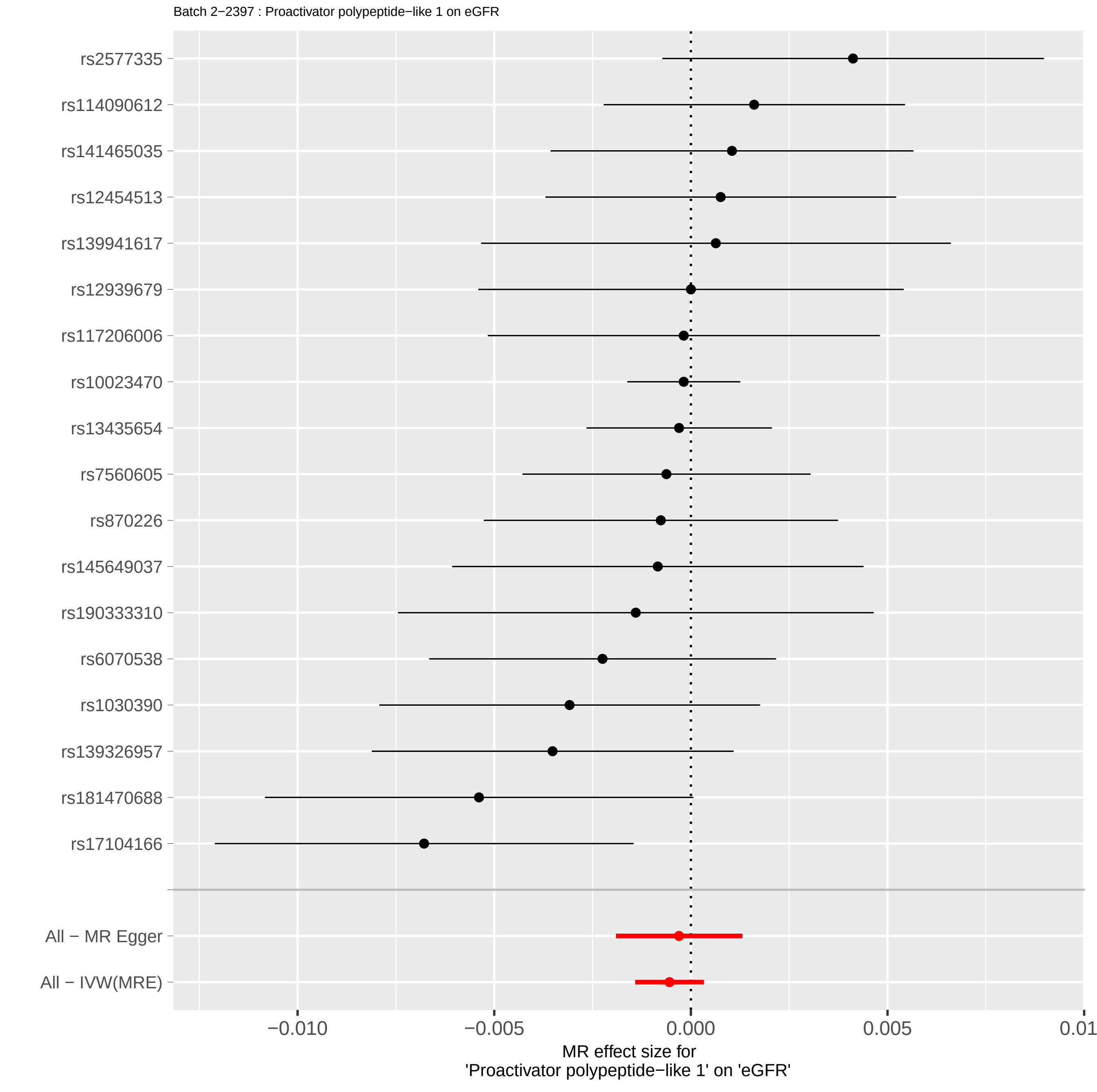

Batch 2−2397 : Proactivator polypeptide−like 1 on eGFR
rs2577335
rs114090612
rs141465035
rs12454513
rs139941617
rs12939679
rs117206006
rs10023470
rs13435654
rs7560605
rs870226
rs145649037
rs190333310
rs6070538
rs1030390
rs139326957
rs181470688
rs17104166
All − MR Egger
All − IVW(MRE)
−0.010
−0.005
0.000
0.005
0.01
MR effect size for
'Proactivator polypeptide−like 1' on 'eGFR'

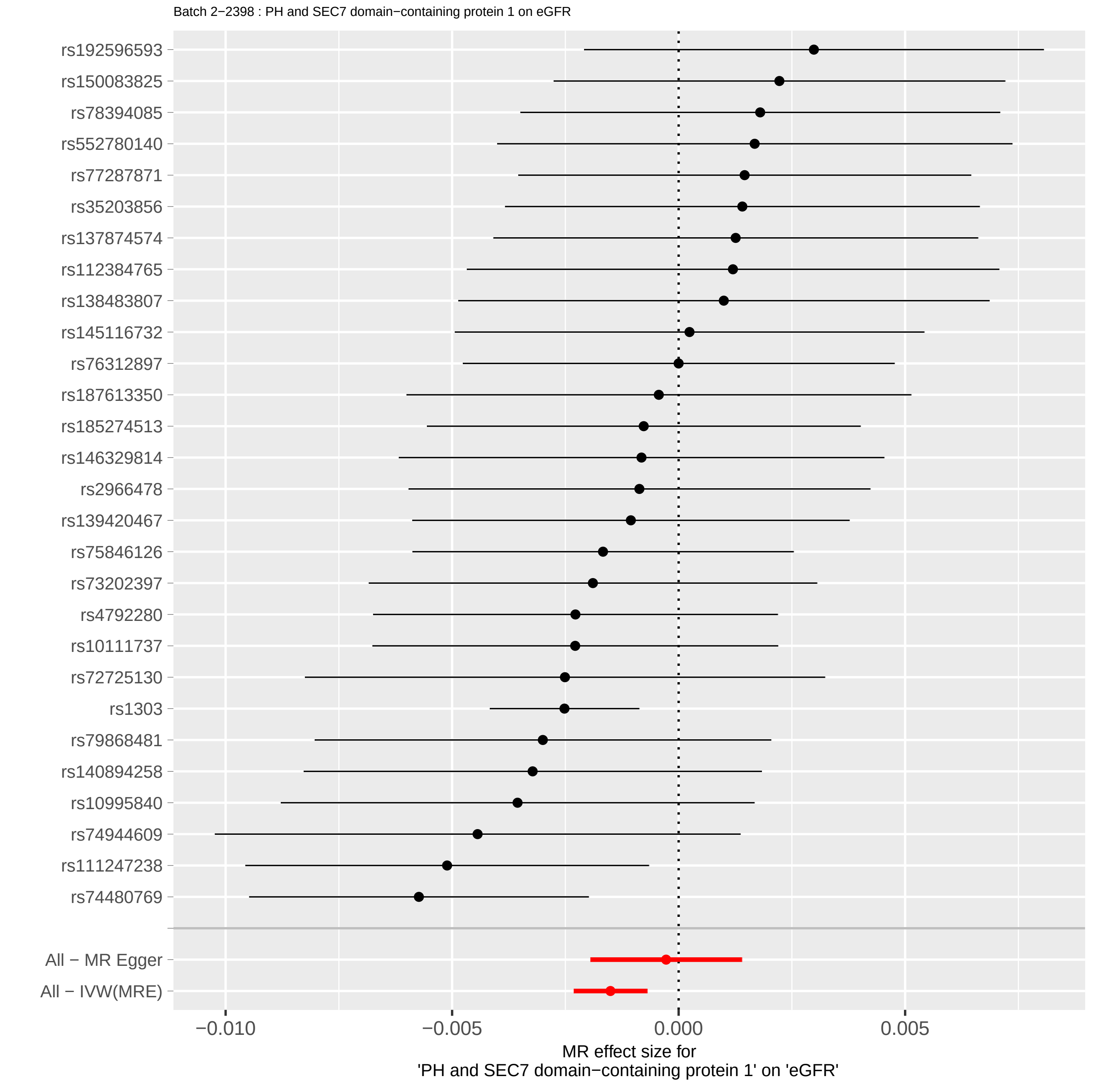
Batch 2−2398 : PH and SEC7 domain−containing protein 1 on eGFR
rs192596593
rs150083825
rs78394085
rs552780140
rs77287871
rs35203856
rs137874574
rs112384765
rs138483807
rs145116732
rs76312897
rs187613350
rs185274513
rs146329814
rs2966478
rs139420467
rs75846126
rs73202397
rs4792280
rs10111737
rs72725130
rs1303
rs79868481
rs140894258
rs10995840
rs74944609
rs111247238
rs74480769
All − MR Egger
All − IVW(MRE)
−0.010
−0.005
0.000
0.005
MR effect size for
'PH and SEC7 domain−containing protein 1' on 'eGFR'

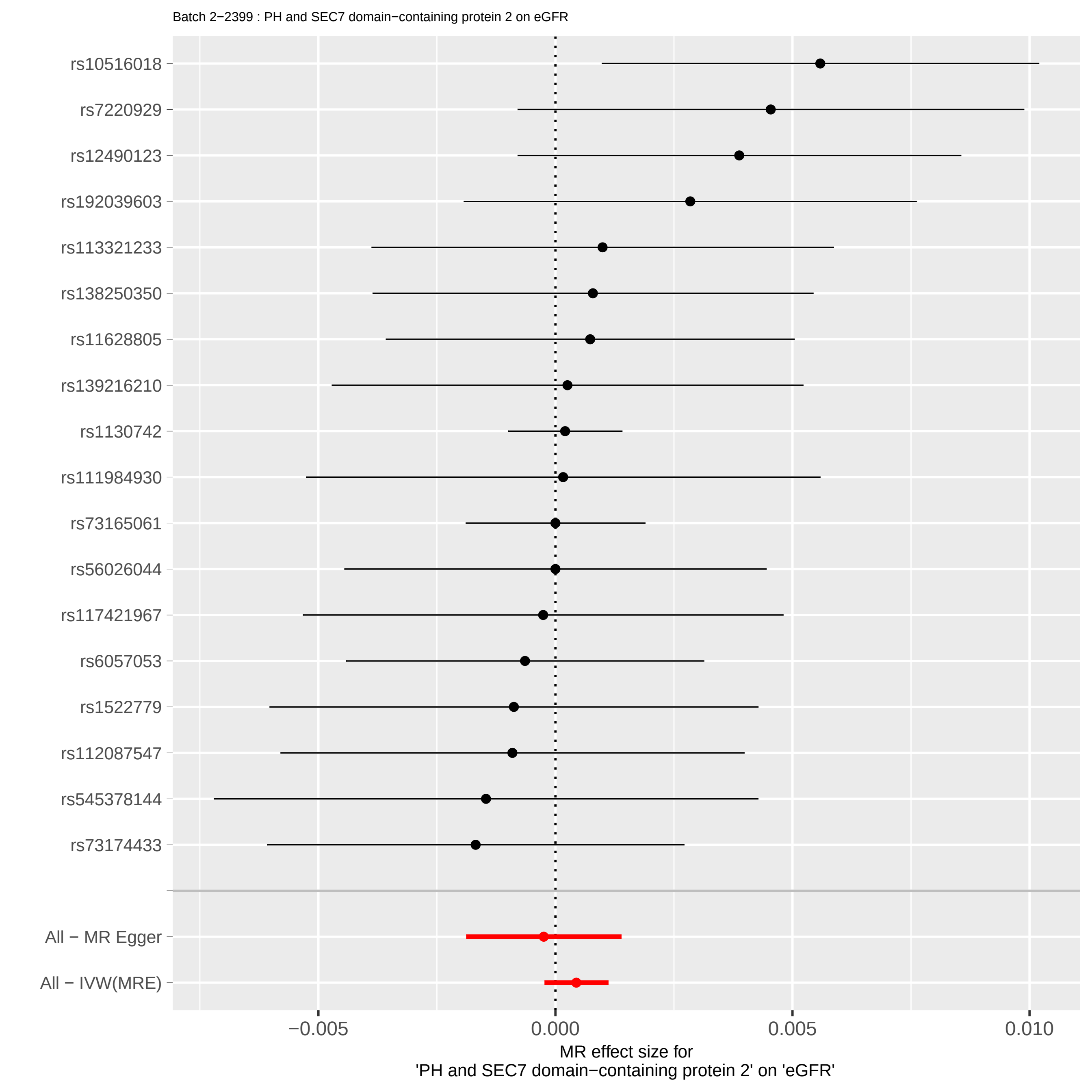
Batch 2−2399 : PH and SEC7 domain−containing protein 2 on eGFR
rs10516018
rs7220929
rs12490123
rs192039603
rs113321233
rs138250350
rs11628805
rs139216210
rs1130742
rs111984930
rs73165061
rs56026044
rs117421967
rs6057053
rs1522779
rs112087547
rs545378144
rs73174433
All − MR Egger
All − IVW(MRE)
−0.005
0.000
0.005
0.010
MR effect size for
'PH and SEC7 domain−containing protein 2' on 'eGFR'

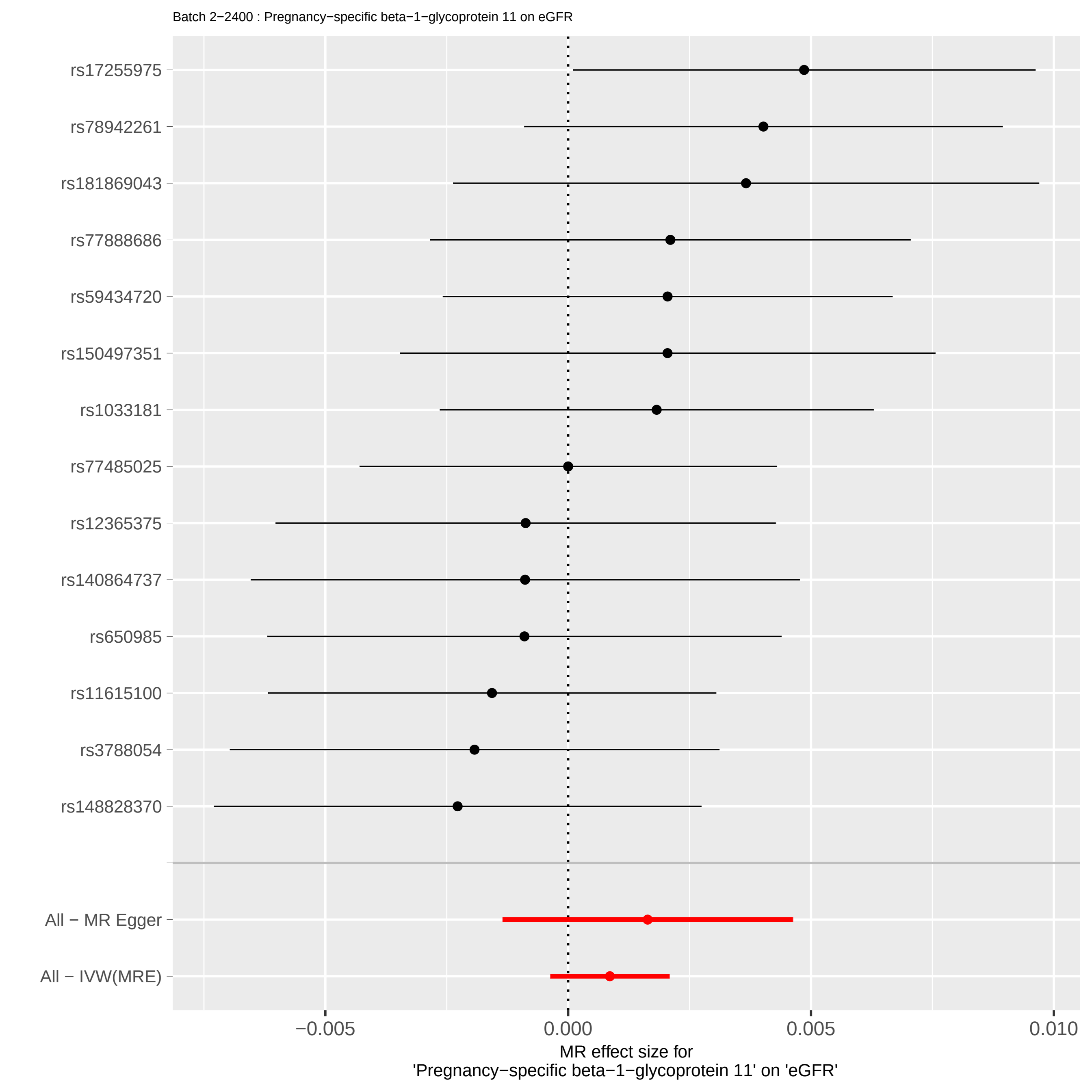

Batch 2−2400 : Pregnancy−specific beta−1−glycoprotein 11 on eGFR
rs17255975
rs78942261
rs181869043
rs77888686
rs59434720
rs150497351
rs1033181
rs77485025
rs12365375
rs140864737
rs650985
rs11615100
rs3788054
rs148828370
All − MR Egger
All − IVW(MRE)
−0.005
0.000
0.005
0.010
MR effect size for
'Pregnancy−specific beta−1−glycoprotein 11' on 'eGFR'

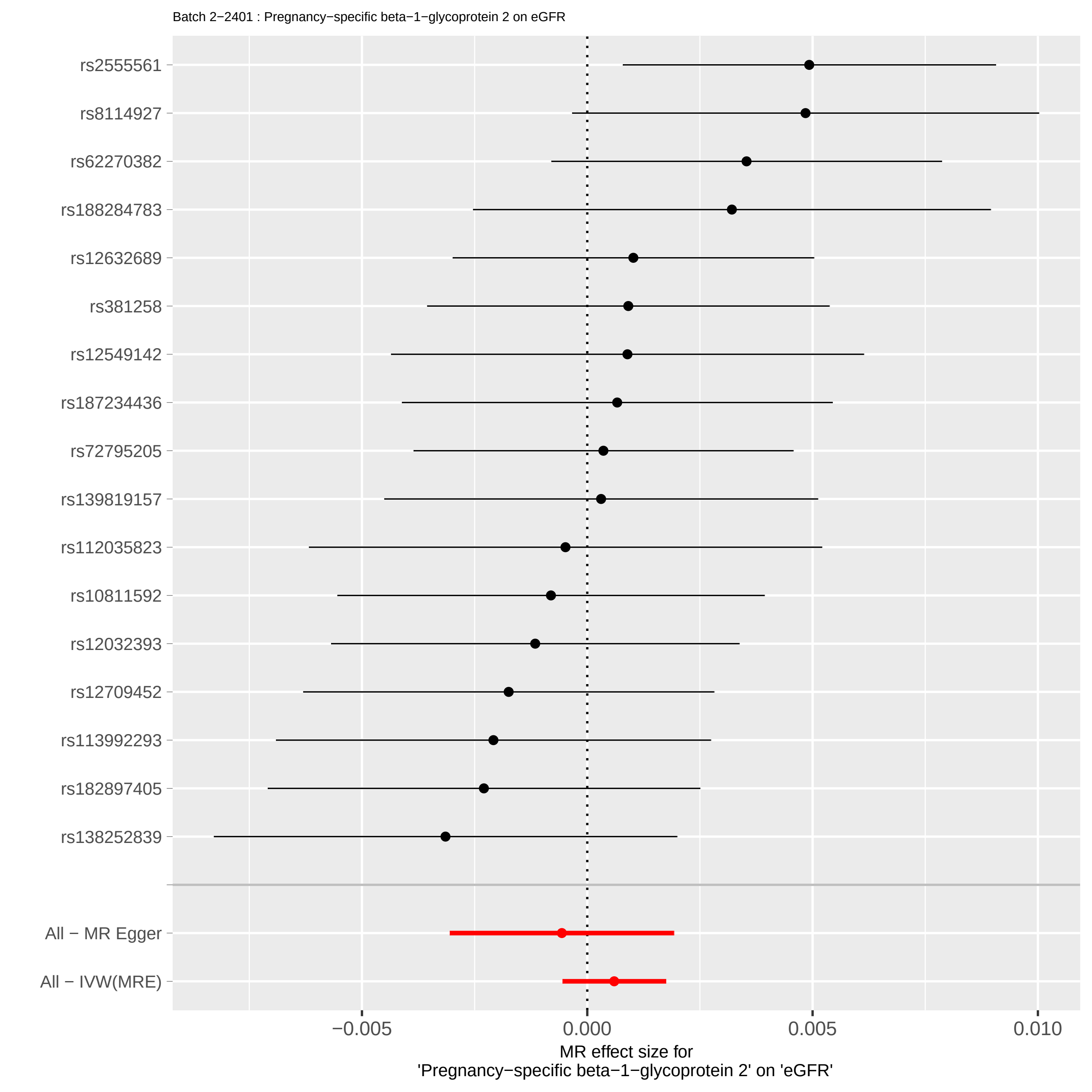

Batch 2−2401 : Pregnancy−specific beta−1−glycoprotein 2 on eGFR
rs2555561
rs8114927
rs62270382
rs188284783
rs12632689
rs381258
rs12549142
rs187234436
rs72795205
rs139819157
rs112035823
rs10811592
rs12032393
rs12709452
rs113992293
rs182897405
rs138252839
All − MR Egger
All − IVW(MRE)
−0.005
0.000
0.005
0.010
MR effect size for
'Pregnancy−specific beta−1−glycoprotein 2' on 'eGFR'

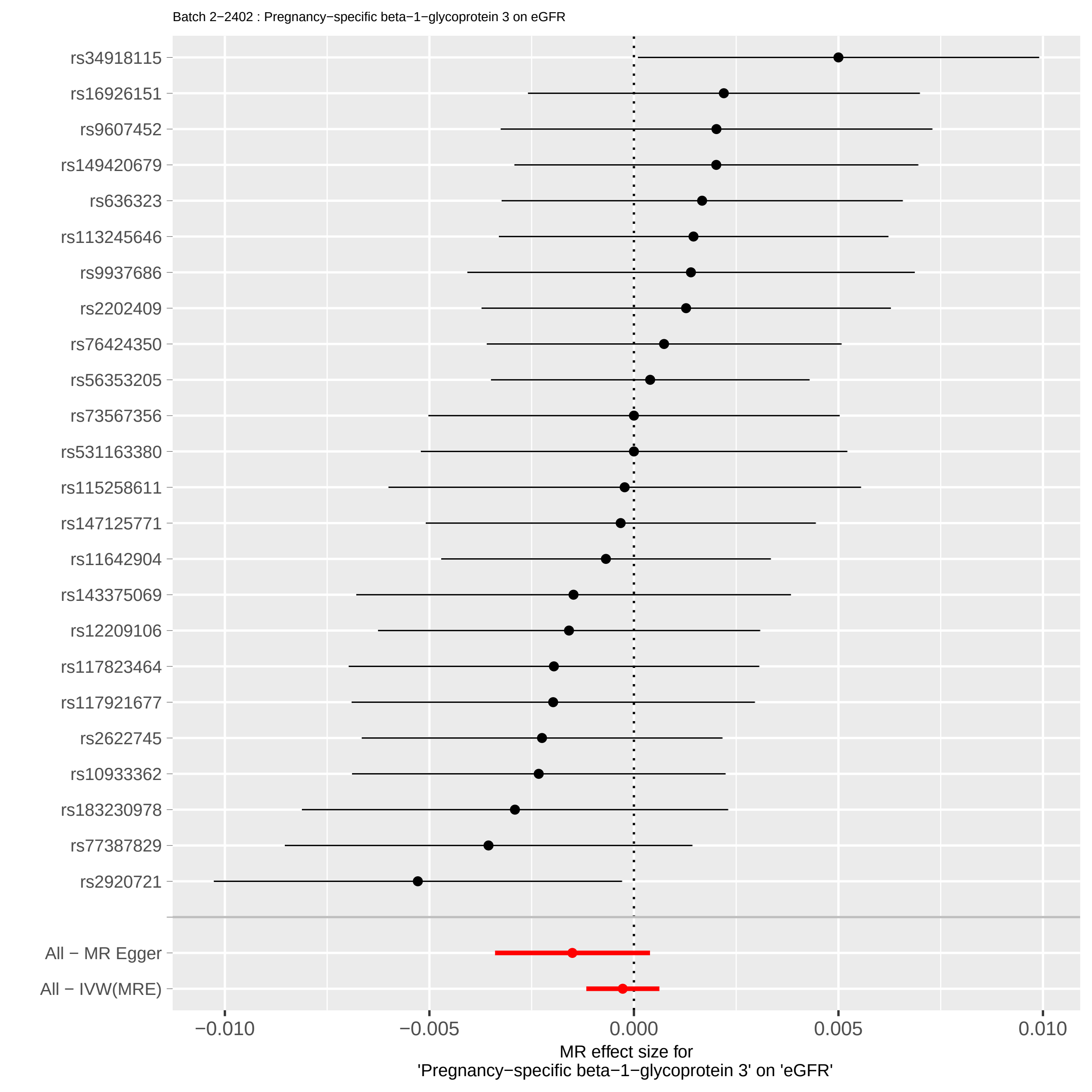

Batch 2−2402 : Pregnancy−specific beta−1−glycoprotein 3 on eGFR
rs34918115
rs16926151
rs9607452
rs149420679
rs636323
rs113245646
rs9937686
rs2202409
rs76424350
rs56353205
rs73567356
rs531163380
rs115258611
rs147125771
rs11642904
rs143375069
rs12209106
rs117823464
rs117921677
rs2622745
rs10933362
rs183230978
rs77387829
rs2920721
All − MR Egger
All − IVW(MRE)
−0.010
−0.005
0.000
0.005
0.010
MR effect size for
'Pregnancy−specific beta−1−glycoprotein 3' on 'eGFR'

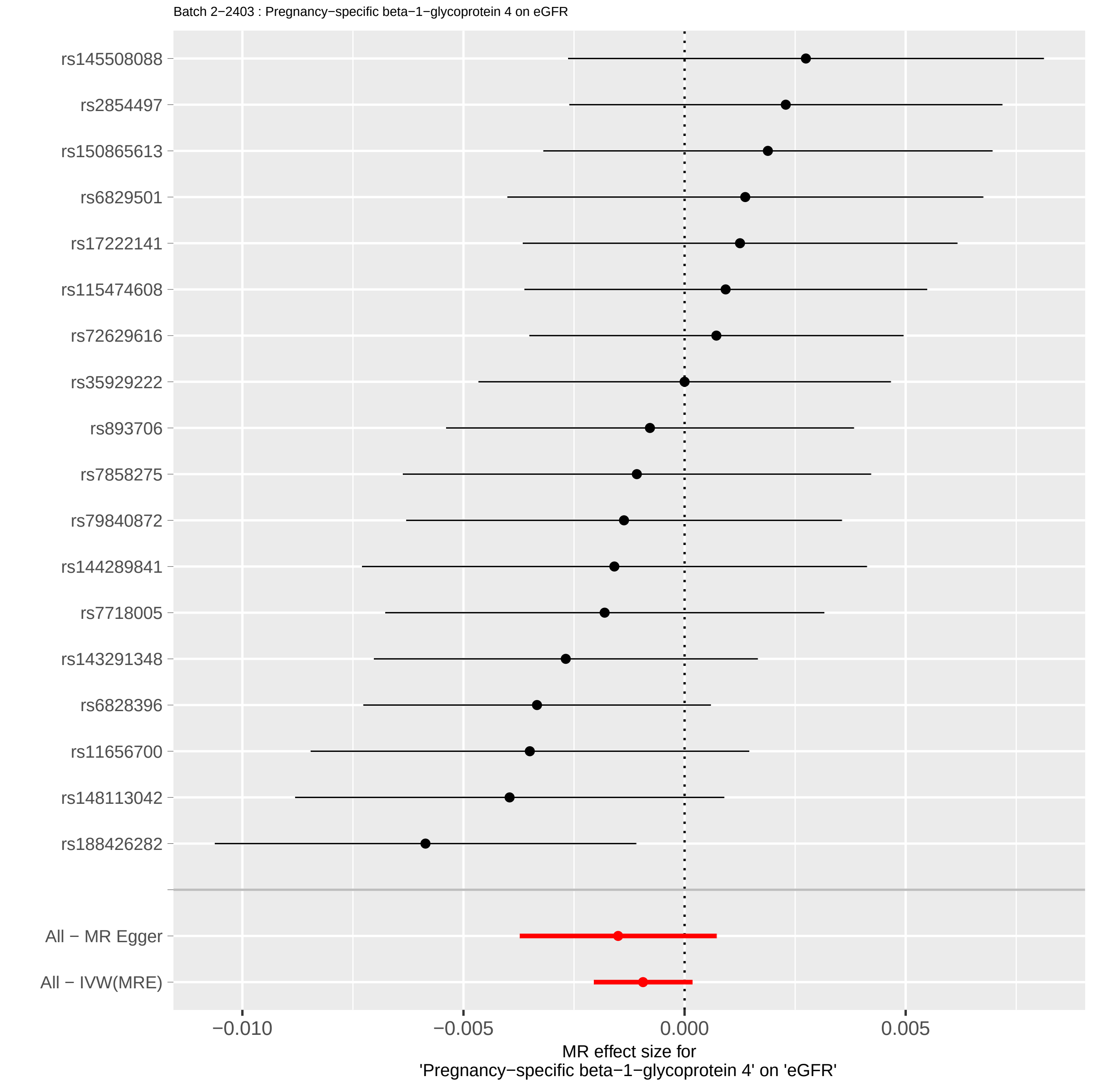

Batch 2−2403 : Pregnancy−specific beta−1−glycoprotein 4 on eGFR
rs145508088
rs2854497
rs150865613
rs6829501
rs17222141
rs115474608
rs72629616
rs35929222
rs893706
rs7858275
rs79840872
rs144289841
rs7718005
rs143291348
rs6828396
rs11656700
rs148113042
rs188426282
All − MR Egger
All − IVW(MRE)
−0.010
−0.005
0.000
0.005
MR effect size for
'Pregnancy−specific beta−1−glycoprotein 4' on 'eGFR'

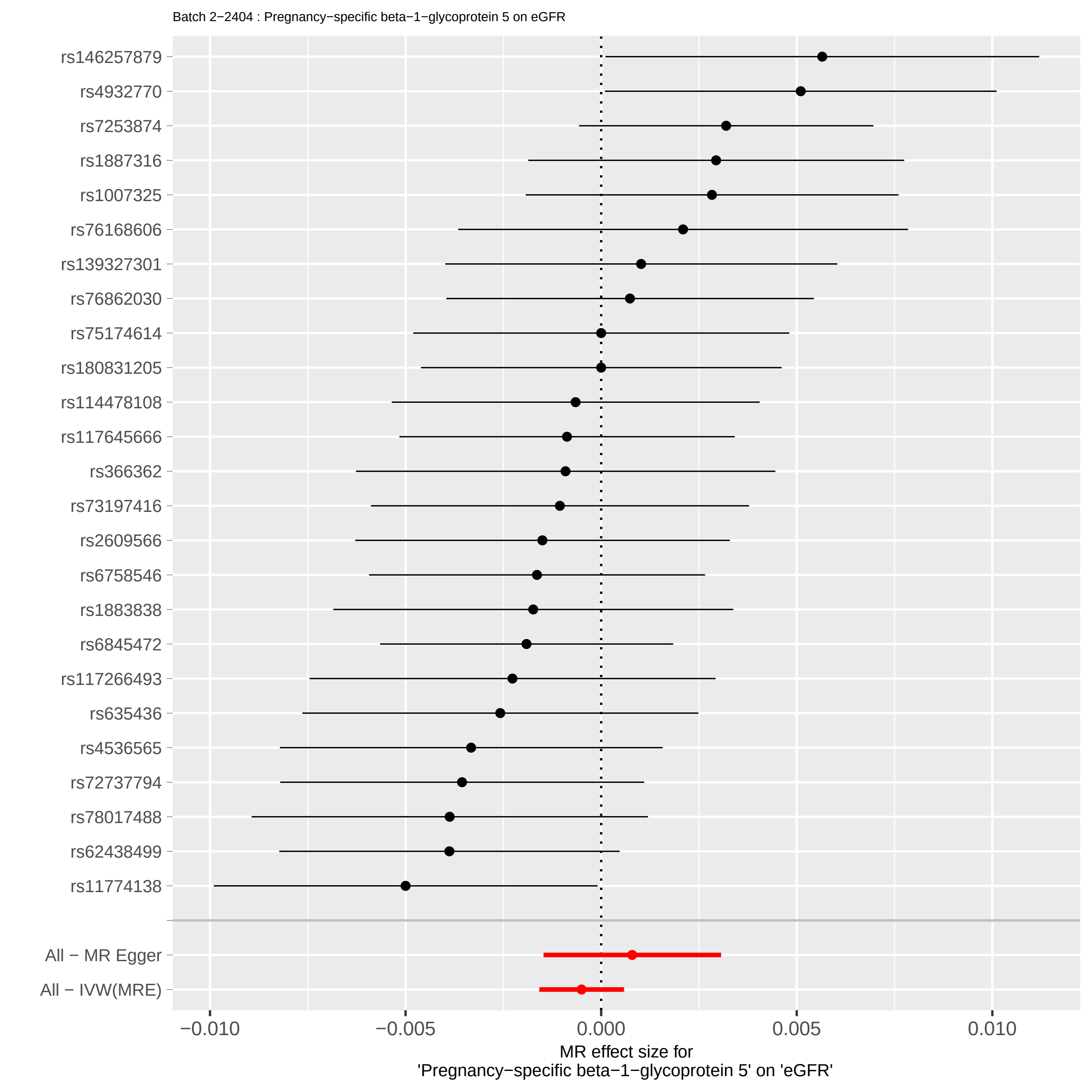

Batch 2−2404 : Pregnancy−specific beta−1−glycoprotein 5 on eGFR
rs146257879
rs4932770
rs7253874
rs1887316
rs1007325
rs76168606
rs139327301
rs76862030
rs75174614
rs180831205
rs114478108
rs117645666
rs366362
rs73197416
rs2609566
rs6758546
rs1883838
rs6845472
rs117266493
rs635436
rs4536565
rs72737794
rs78017488
rs62438499
rs11774138
All − MR Egger
All − IVW(MRE)
−0.010
−0.005
0.000
0.005
0.010
MR effect size for
'Pregnancy−specific beta−1−glycoprotein 5' on 'eGFR'

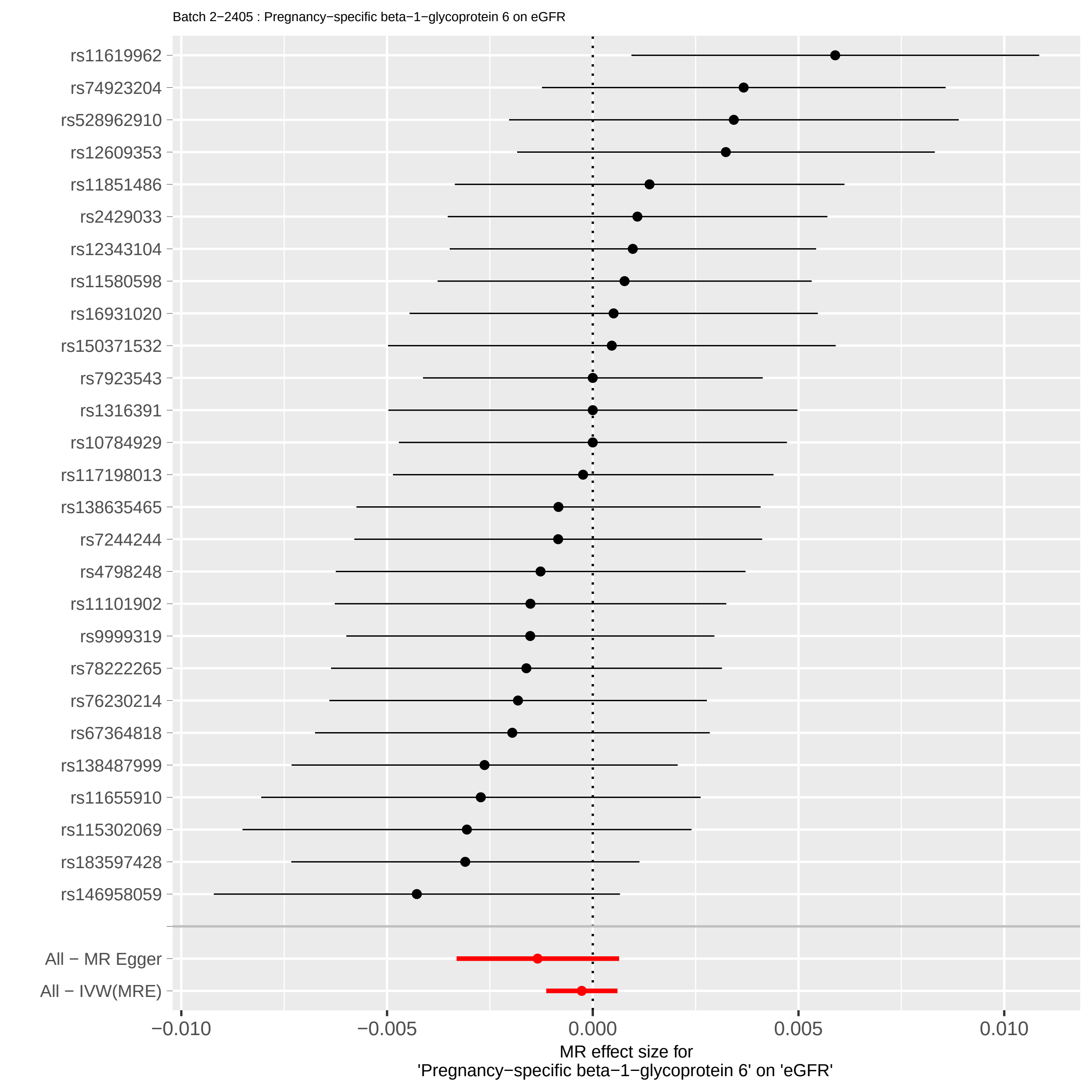

Batch 2−2405 : Pregnancy−specific beta−1−glycoprotein 6 on eGFR
rs11619962
rs74923204
rs528962910
rs12609353
rs11851486
rs2429033
rs12343104
rs11580598
rs16931020
rs150371532
rs7923543
rs1316391
rs10784929
rs117198013
rs138635465
rs7244244
rs4798248
rs11101902
rs9999319
rs78222265
rs76230214
rs67364818
rs138487999
rs11655910
rs115302069
rs183597428
rs146958059
All − MR Egger
All − IVW(MRE)
−0.010
−0.005
0.000
0.005
0.010
MR effect size for
'Pregnancy−specific beta−1−glycoprotein 6' on 'eGFR'

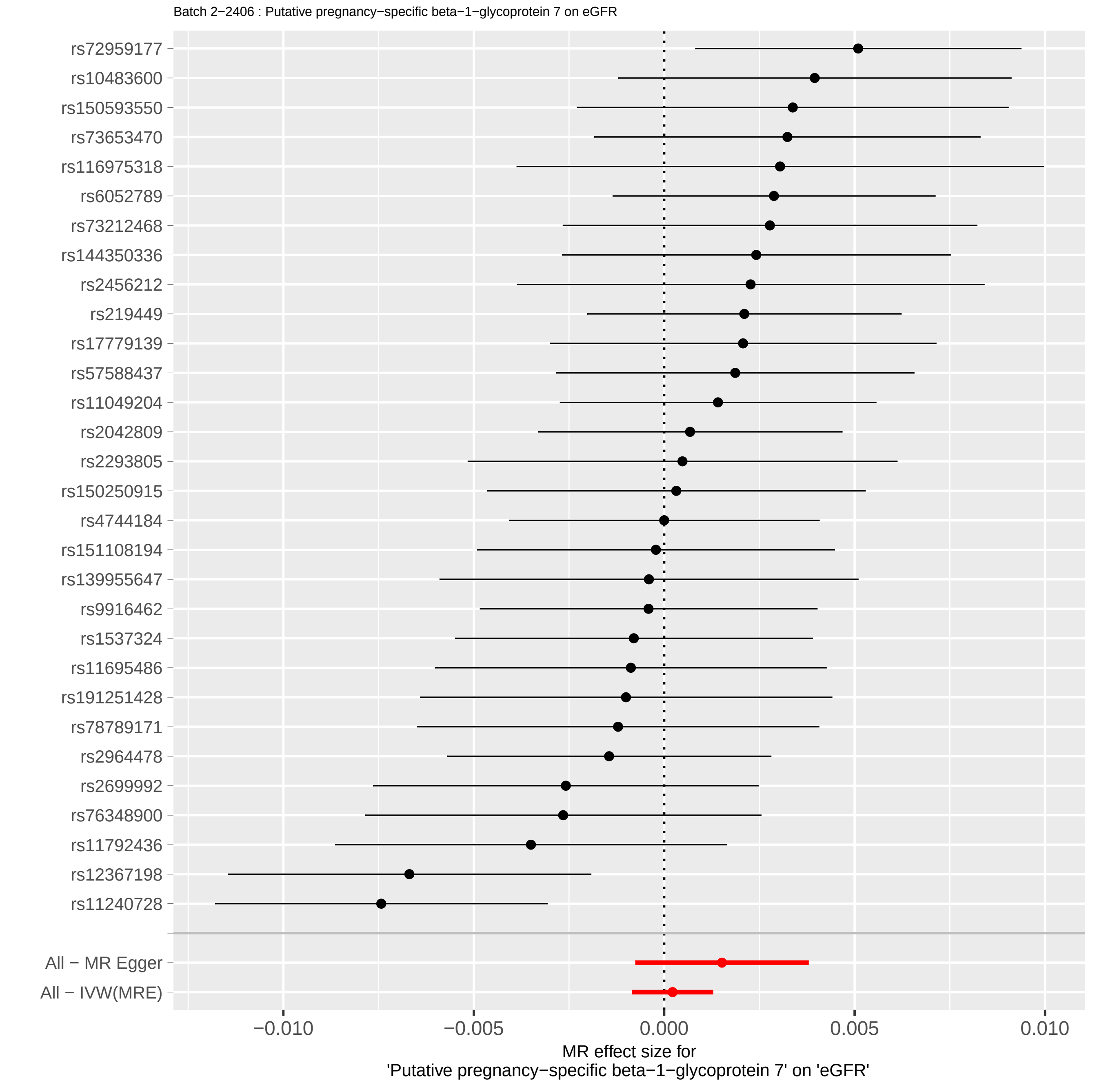

Batch 2−2406 : Putative pregnancy−specific beta−1−glycoprotein 7 on eGFR
rs72959177
rs10483600
rs150593550
rs73653470
rs116975318
rs6052789
rs73212468
rs144350336
rs2456212
rs219449
rs17779139
rs57588437
rs11049204
rs2042809
rs2293805
rs150250915
rs4744184
rs151108194
rs139955647
rs9916462
rs1537324
rs11695486
rs191251428
rs78789171
rs2964478
rs2699992
rs76348900
rs11792436
rs12367198
rs11240728
All − MR Egger
All − IVW(MRE)
−0.010
−0.005
0.000
0.005
0.010
MR effect size for
'Putative pregnancy−specific beta−1−glycoprotein 7' on 'eGFR'

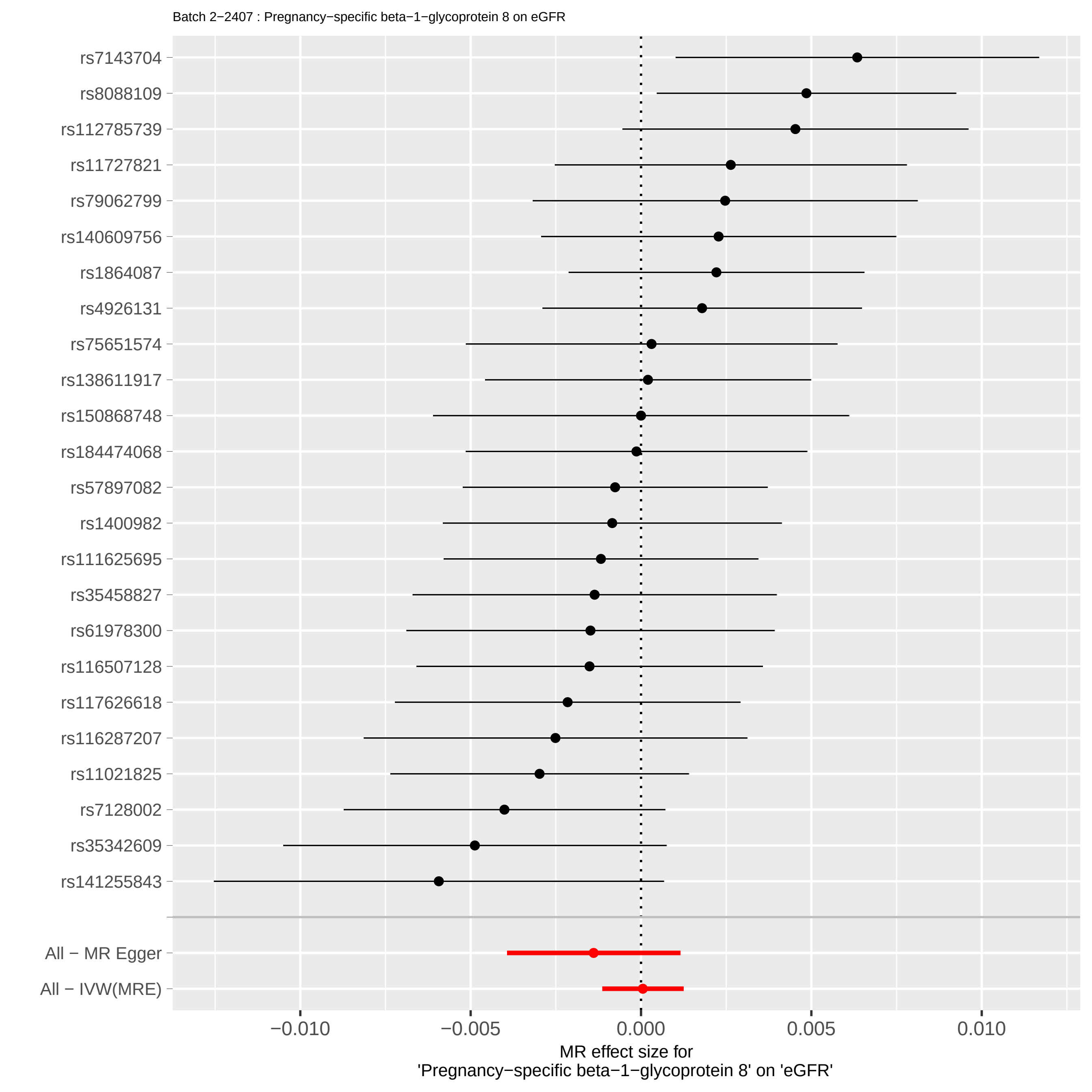

Batch 2−2407 : Pregnancy−specific beta−1−glycoprotein 8 on eGFR
rs7143704
rs8088109
rs112785739
rs11727821
rs79062799
rs140609756
rs1864087
rs4926131
rs75651574
rs138611917
rs150868748
rs184474068
rs57897082
rs1400982
rs111625695
rs35458827
rs61978300
rs116507128
rs117626618
rs116287207
rs11021825
rs7128002
rs35342609
rs141255843
All − MR Egger
All − IVW(MRE)
−0.010
−0.005
0.000
0.005
0.010
MR effect size for
'Pregnancy−specific beta−1−glycoprotein 8' on 'eGFR'

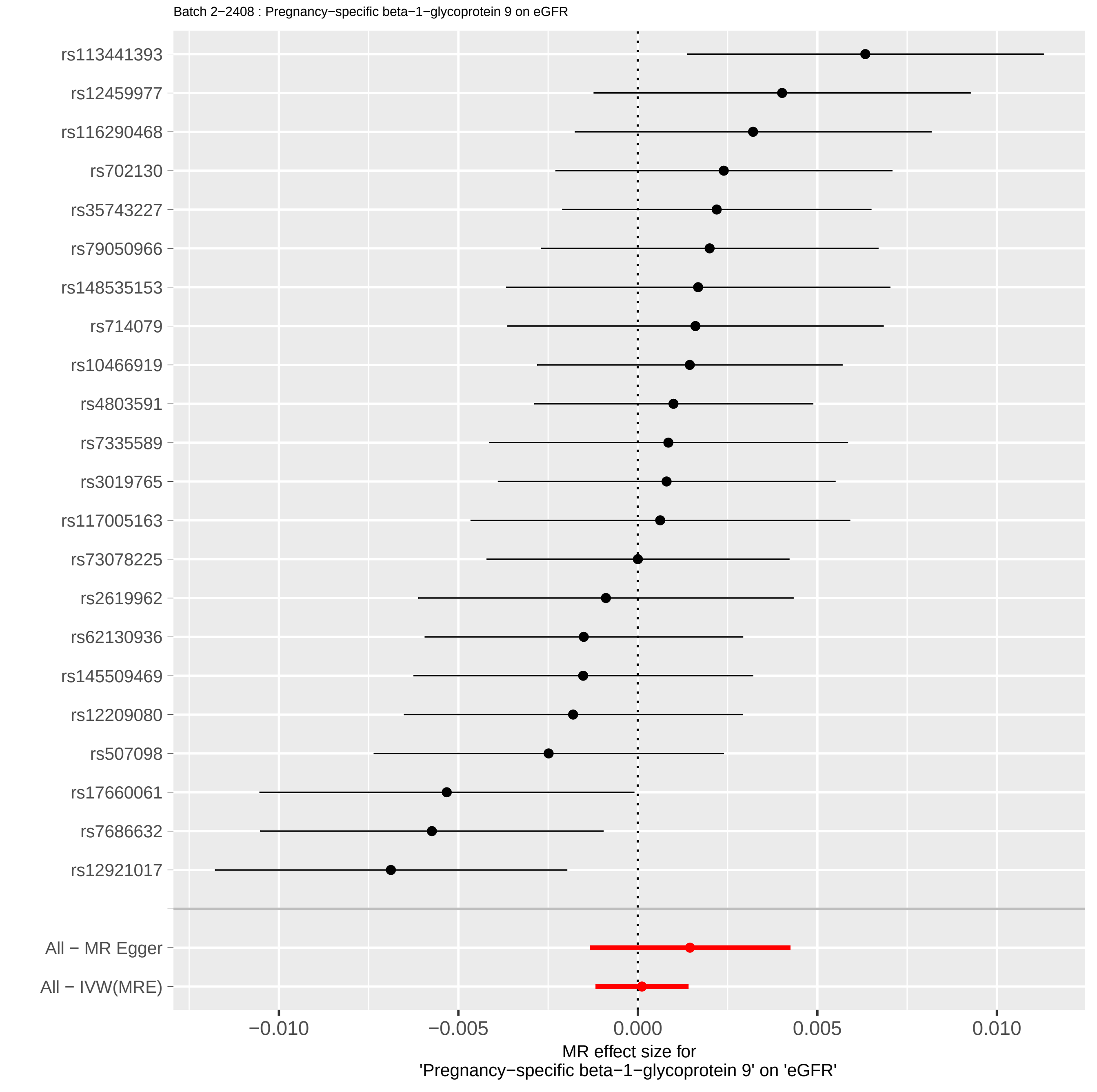
Batch 2−2408 : Pregnancy−specific beta−1−glycoprotein 9 on eGFR
rs113441393
rs12459977
rs116290468
rs702130
rs35743227
rs79050966
rs148535153
rs714079
rs10466919
rs4803591
rs7335589
rs3019765
rs117005163
rs73078225
rs2619962
rs62130936
rs145509469
rs12209080
rs507098
rs17660061
rs7686632
rs12921017
All − MR Egger
All − IVW(MRE)
−0.010
−0.005
0.000
0.005
0.010
MR effect size for
'Pregnancy−specific beta−1−glycoprotein 9' on 'eGFR'

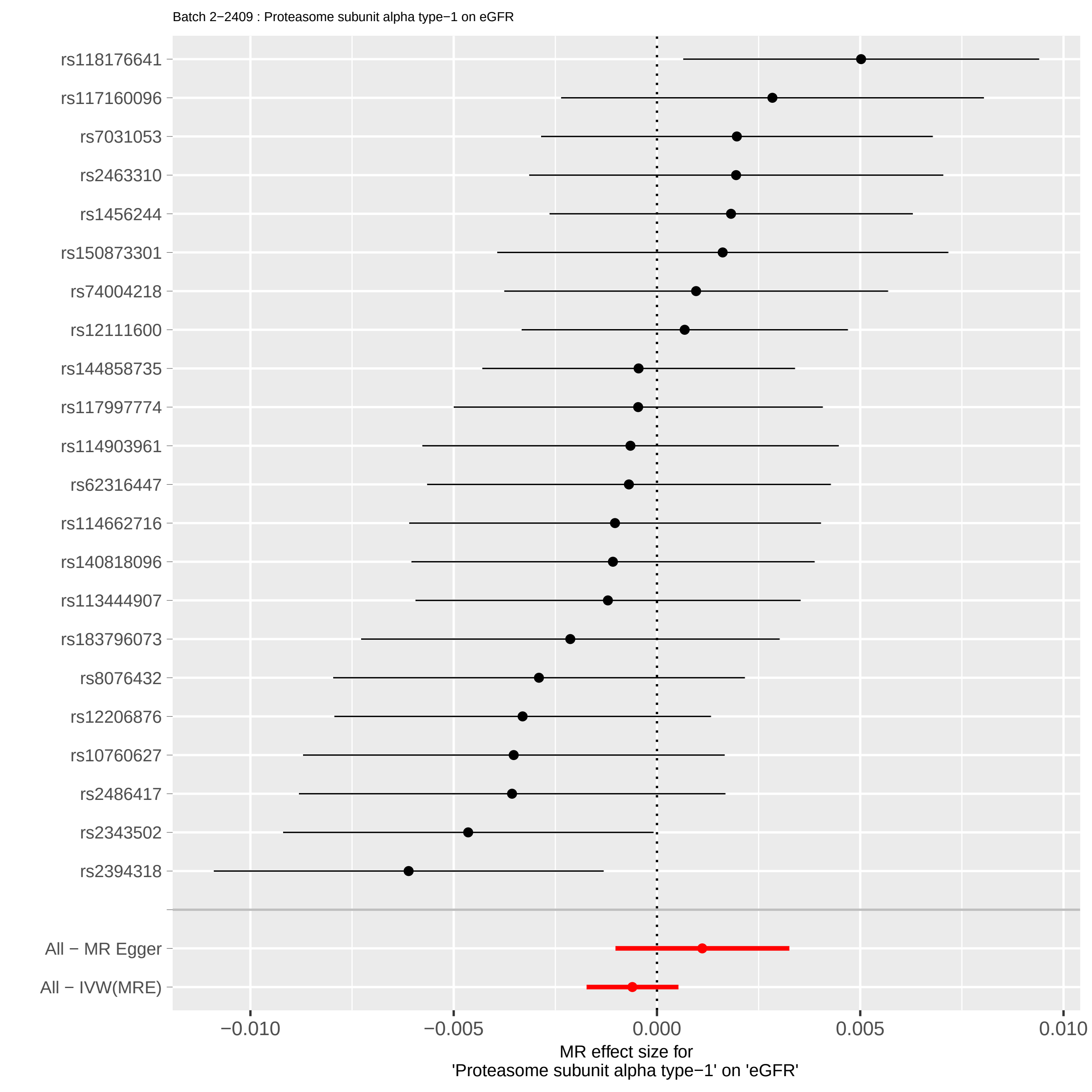
Batch 2−2409 : Proteasome subunit alpha type−1 on eGFR
rs118176641
rs117160096
rs7031053
rs2463310
rs1456244
rs150873301
rs74004218
rs12111600
rs144858735
rs117997774
rs114903961
rs62316447
rs114662716
rs140818096
rs113444907
rs183796073
rs8076432
rs12206876
rs10760627
rs2486417
rs2343502
rs2394318
All − MR Egger
All − IVW(MRE)
−0.010
−0.005
0.000
0.005
0.010
MR effect size for
'Proteasome subunit alpha type−1' on 'eGFR'

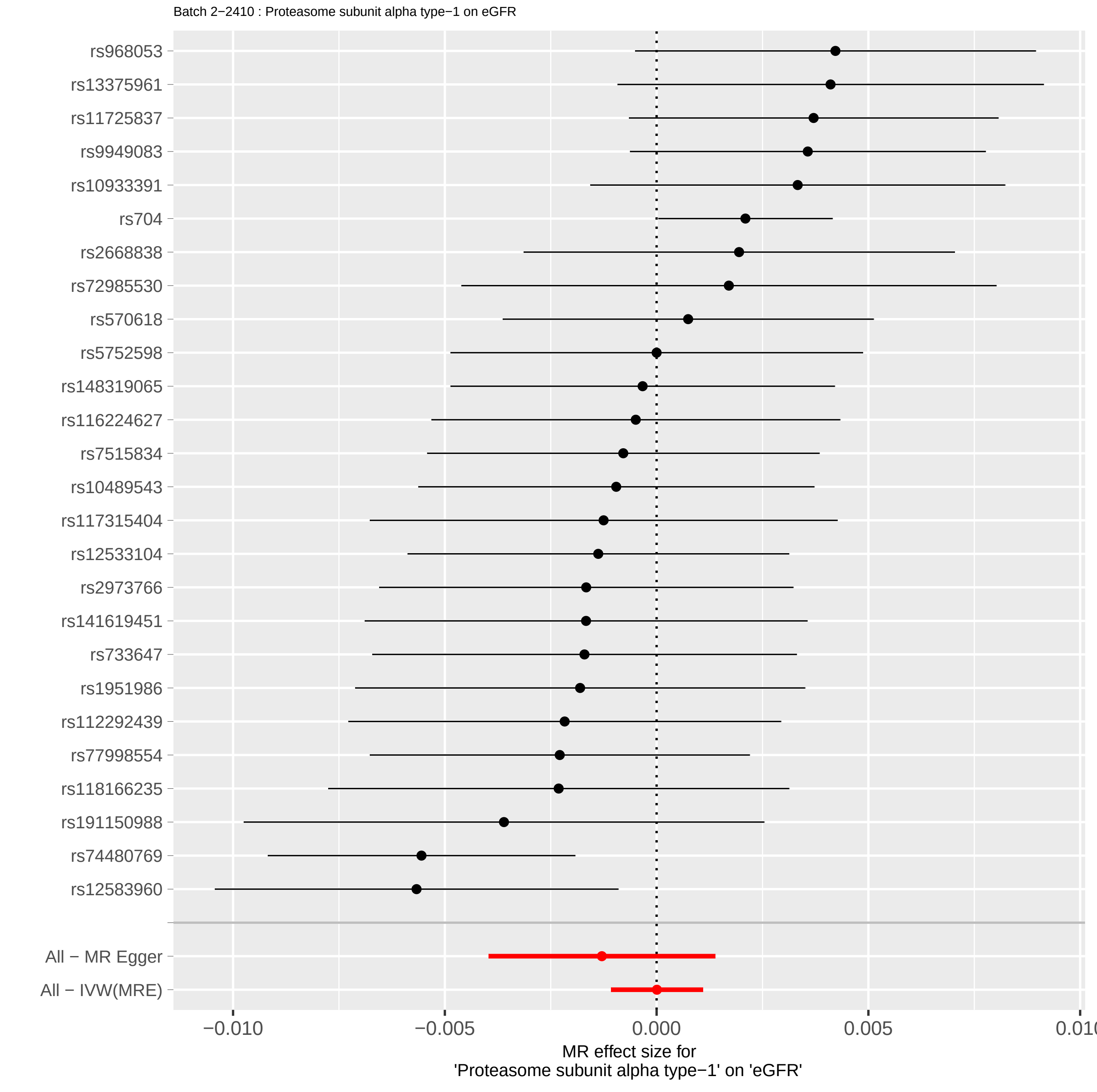

Batch 2−2410 : Proteasome subunit alpha type−1 on eGFR
rs968053
rs13375961
rs11725837
rs9949083
rs10933391
rs704
rs2668838
rs72985530
rs570618
rs5752598
rs148319065
rs116224627
rs7515834
rs10489543
rs117315404
rs12533104
rs2973766
rs141619451
rs733647
rs1951986
rs112292439
rs77998554
rs118166235
rs191150988
rs74480769
rs12583960
All − MR Egger
All − IVW(MRE)
−0.010
−0.005
0.000
0.005
0.010
MR effect size for
'Proteasome subunit alpha type−1' on 'eGFR'

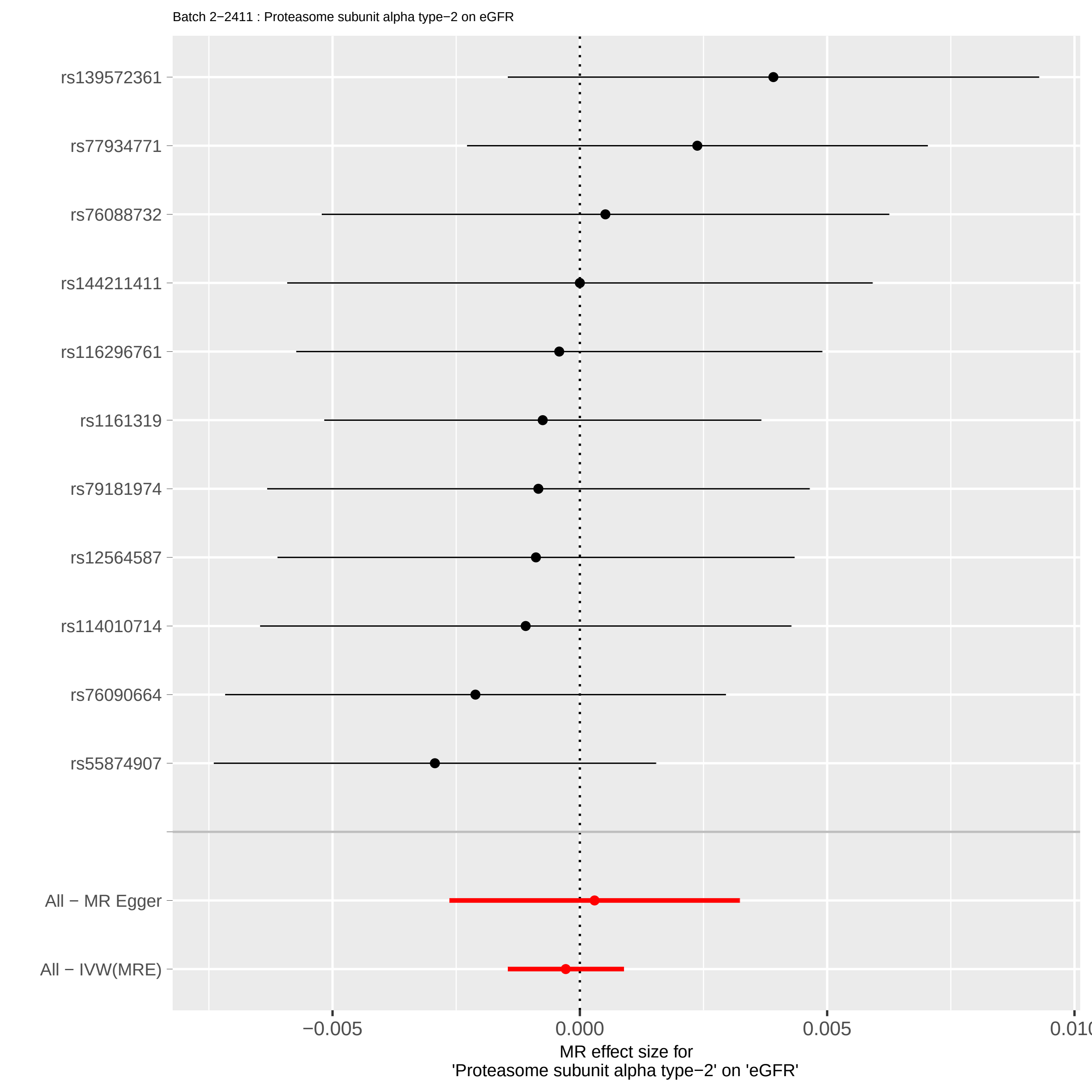

Batch 2−2411 : Proteasome subunit alpha type−2 on eGFR
rs139572361
rs77934771
rs76088732
rs144211411
rs116296761
rs1161319
rs79181974
rs12564587
rs114010714
rs76090664
rs55874907
All − MR Egger
All − IVW(MRE)
−0.005
0.000
0.005
0.010
MR effect size for
'Proteasome subunit alpha type−2' on 'eGFR'

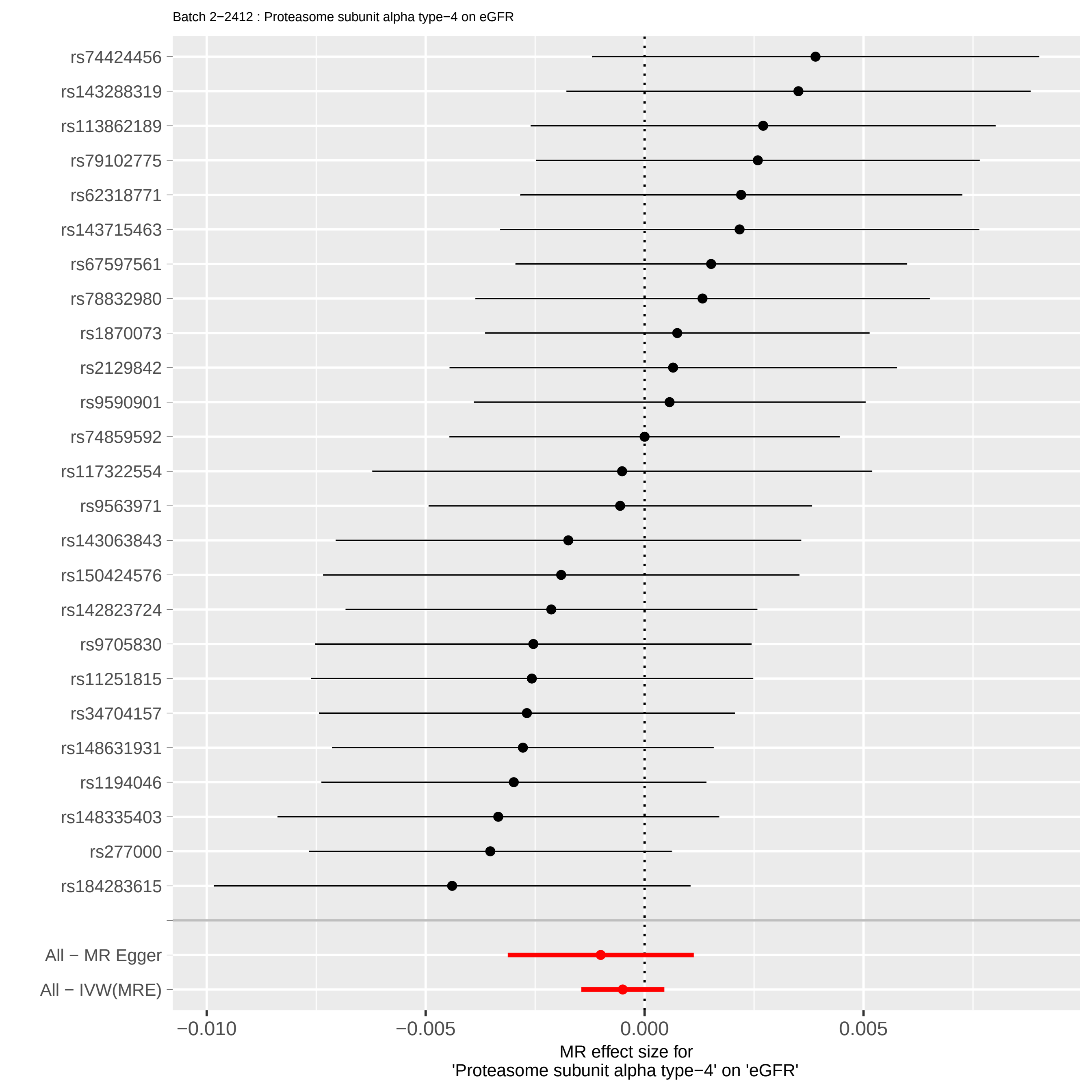
Batch 2−2412 : Proteasome subunit alpha type−4 on eGFR
rs74424456
rs143288319
rs113862189
rs79102775
rs62318771
rs143715463
rs67597561
rs78832980
rs1870073
rs2129842
rs9590901
rs74859592
rs117322554
rs9563971
rs143063843
rs150424576
rs142823724
rs9705830
rs11251815
rs34704157
rs148631931
rs1194046
rs148335403
rs277000
rs184283615
All − MR Egger
All − IVW(MRE)
−0.010
−0.005
0.000
0.005
MR effect size for
'Proteasome subunit alpha type−4' on 'eGFR'

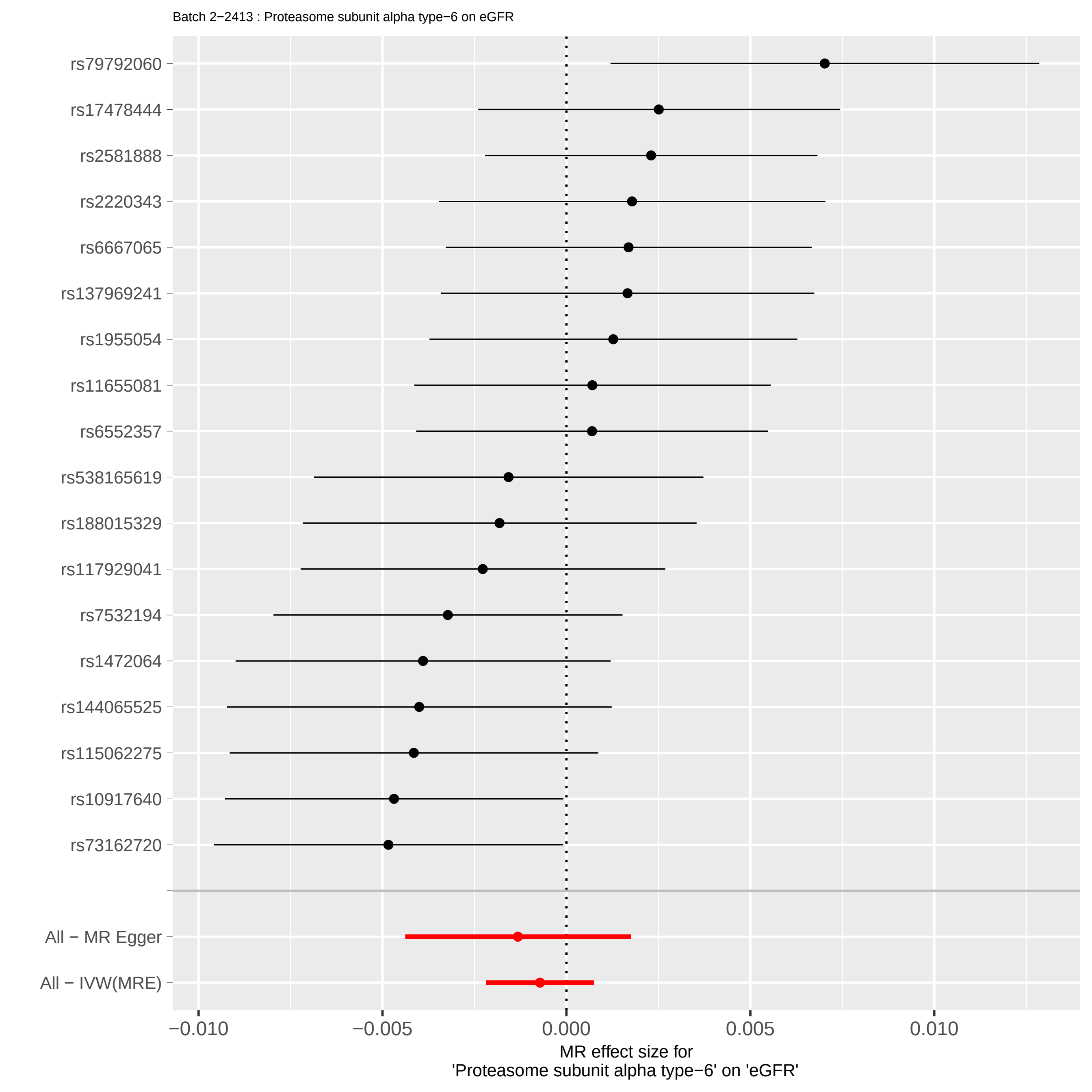

Batch 2−2413 : Proteasome subunit alpha type−6 on eGFR
rs79792060
rs17478444
rs2581888
rs2220343
rs6667065
rs137969241
rs1955054
rs11655081
rs6552357
rs538165619
rs188015329
rs117929041
rs7532194
rs1472064
rs144065525
rs115062275
rs10917640
rs73162720
All − MR Egger
All − IVW(MRE)
−0.010
−0.005
0.000
0.005
0.010
MR effect size for
'Proteasome subunit alpha type−6' on 'eGFR'

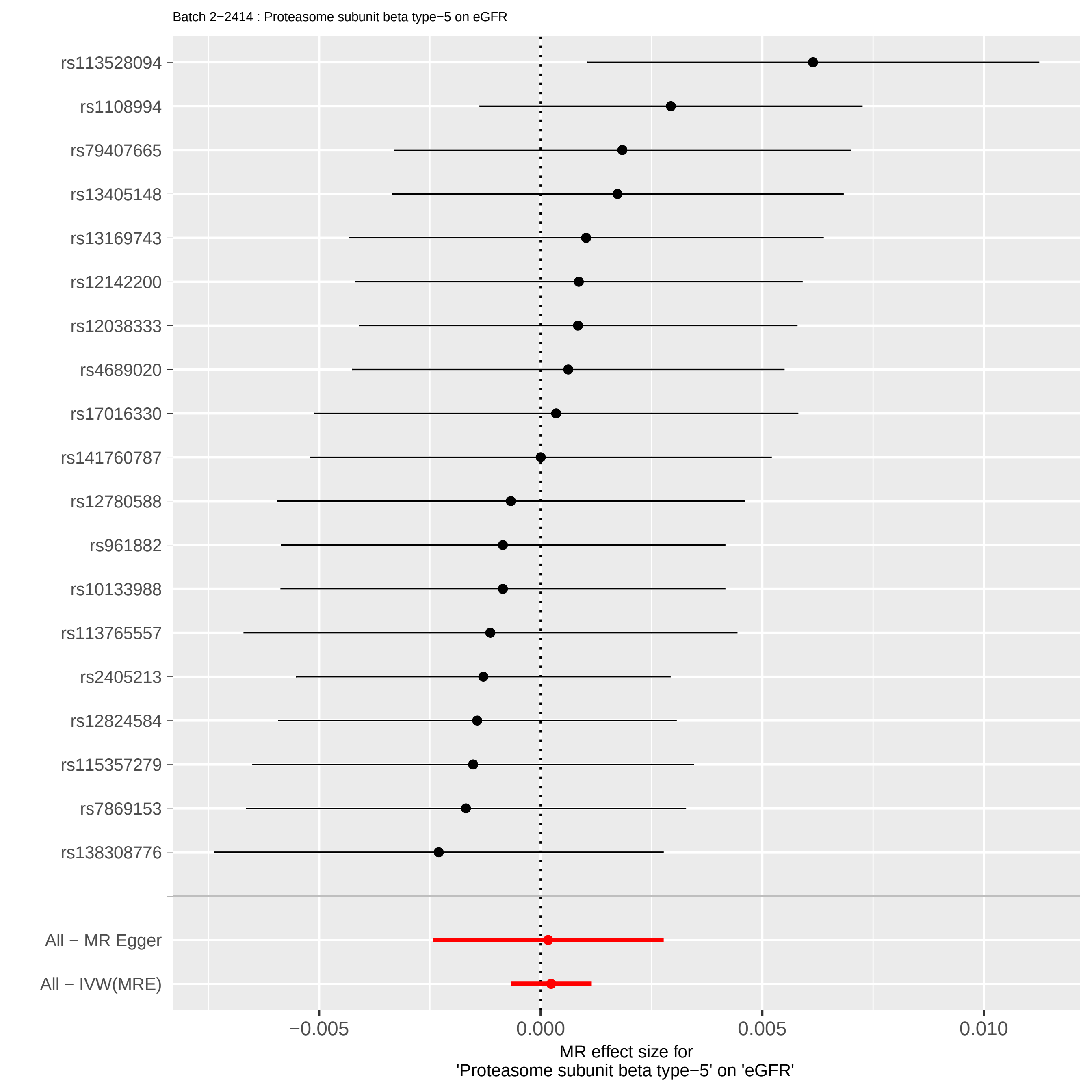

Batch 2−2414 : Proteasome subunit beta type−5 on eGFR
rs113528094
rs1108994
rs79407665
rs13405148
rs13169743
rs12142200
rs12038333
rs4689020
rs17016330
rs141760787
rs12780588
rs961882
rs10133988
rs113765557
rs2405213
rs12824584
rs115357279
rs7869153
rs138308776
All − MR Egger
All − IVW(MRE)
−0.005
0.000
0.005
0.010
MR effect size for
'Proteasome subunit beta type−5' on 'eGFR'

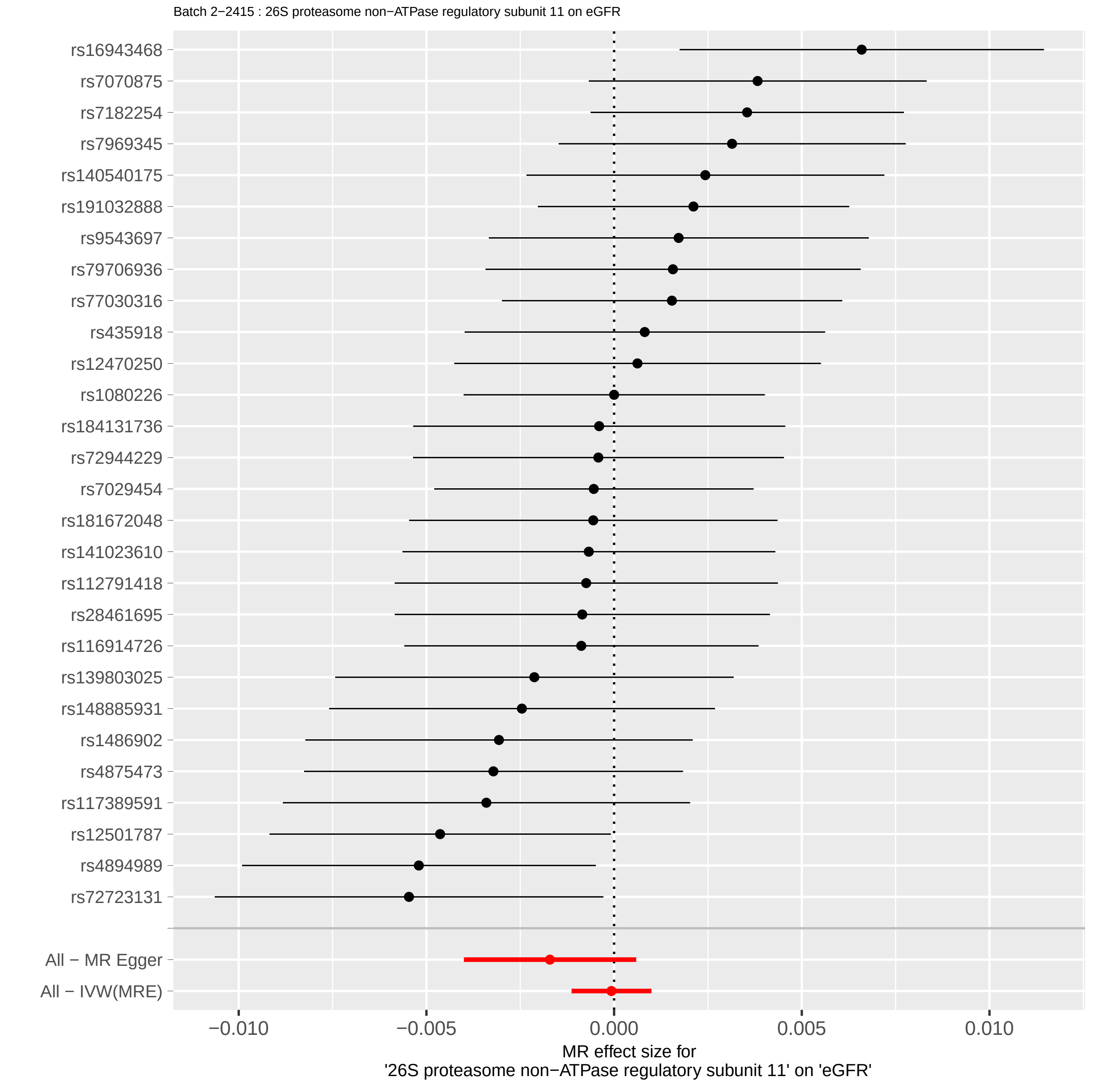

Batch 2−2415 : 26S proteasome non−ATPase regulatory subunit 11 on eGFR
rs16943468
rs7070875
rs7182254
rs7969345
rs140540175
rs191032888
rs9543697
rs79706936
rs77030316
rs435918
rs12470250
rs1080226
rs184131736
rs72944229
rs7029454
rs181672048
rs141023610
rs112791418
rs28461695
rs116914726
rs139803025
rs148885931
rs1486902
rs4875473
rs117389591
rs12501787
rs4894989
rs72723131
All − MR Egger
All − IVW(MRE)
−0.010
−0.005
0.000
0.005
0.010
MR effect size for
'26S proteasome non−ATPase regulatory subunit 11' on 'eGFR'

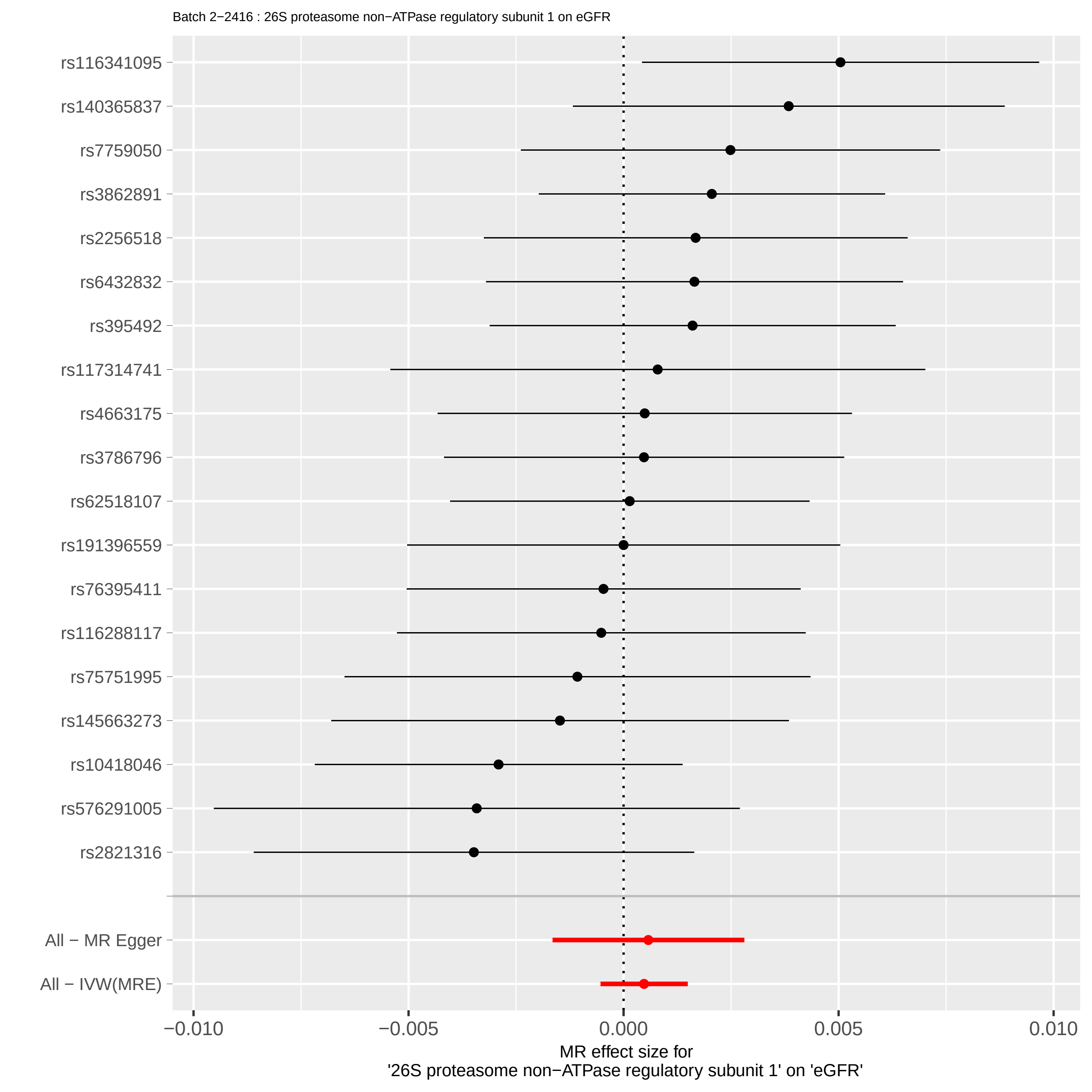

Batch 2−2416 : 26S proteasome non−ATPase regulatory subunit 1 on eGFR
rs116341095
rs140365837
rs7759050
rs3862891
rs2256518
rs6432832
rs395492
rs117314741
rs4663175
rs3786796
rs62518107
rs191396559
rs76395411
rs116288117
rs75751995
rs145663273
rs10418046
rs576291005
rs2821316
All − MR Egger
All − IVW(MRE)
−0.010
−0.005
0.000
0.005
0.010
MR effect size for
'26S proteasome non−ATPase regulatory subunit 1' on 'eGFR'

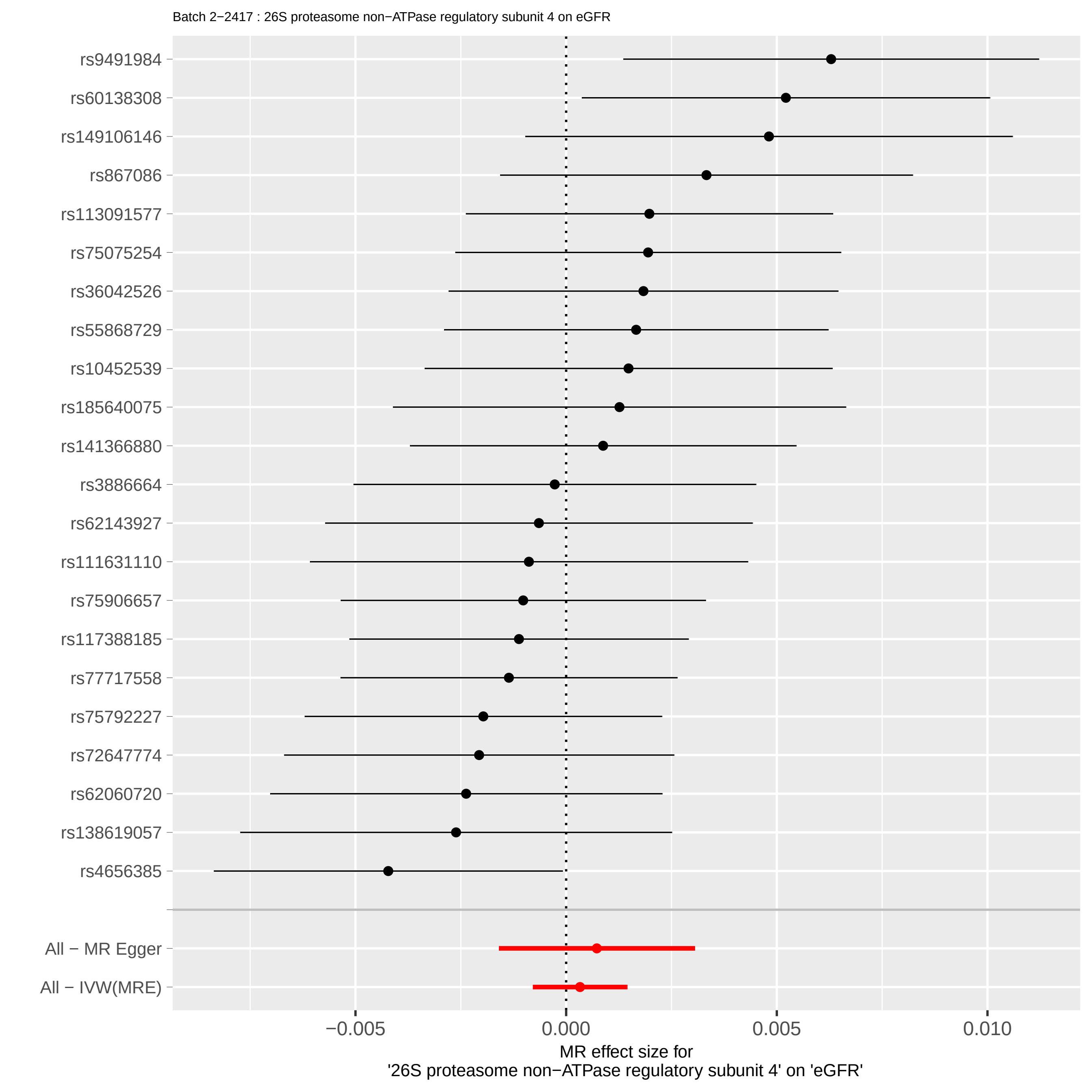

Batch 2−2417 : 26S proteasome non−ATPase regulatory subunit 4 on eGFR
rs9491984
rs60138308
rs149106146
rs867086
rs113091577
rs75075254
rs36042526
rs55868729
rs10452539
rs185640075
rs141366880
rs3886664
rs62143927
rs111631110
rs75906657
rs117388185
rs77717558
rs75792227
rs72647774
rs62060720
rs138619057
rs4656385
All − MR Egger
All − IVW(MRE)
−0.005
0.000
0.005
0.010
MR effect size for
'26S proteasome non−ATPase regulatory subunit 4' on 'eGFR'

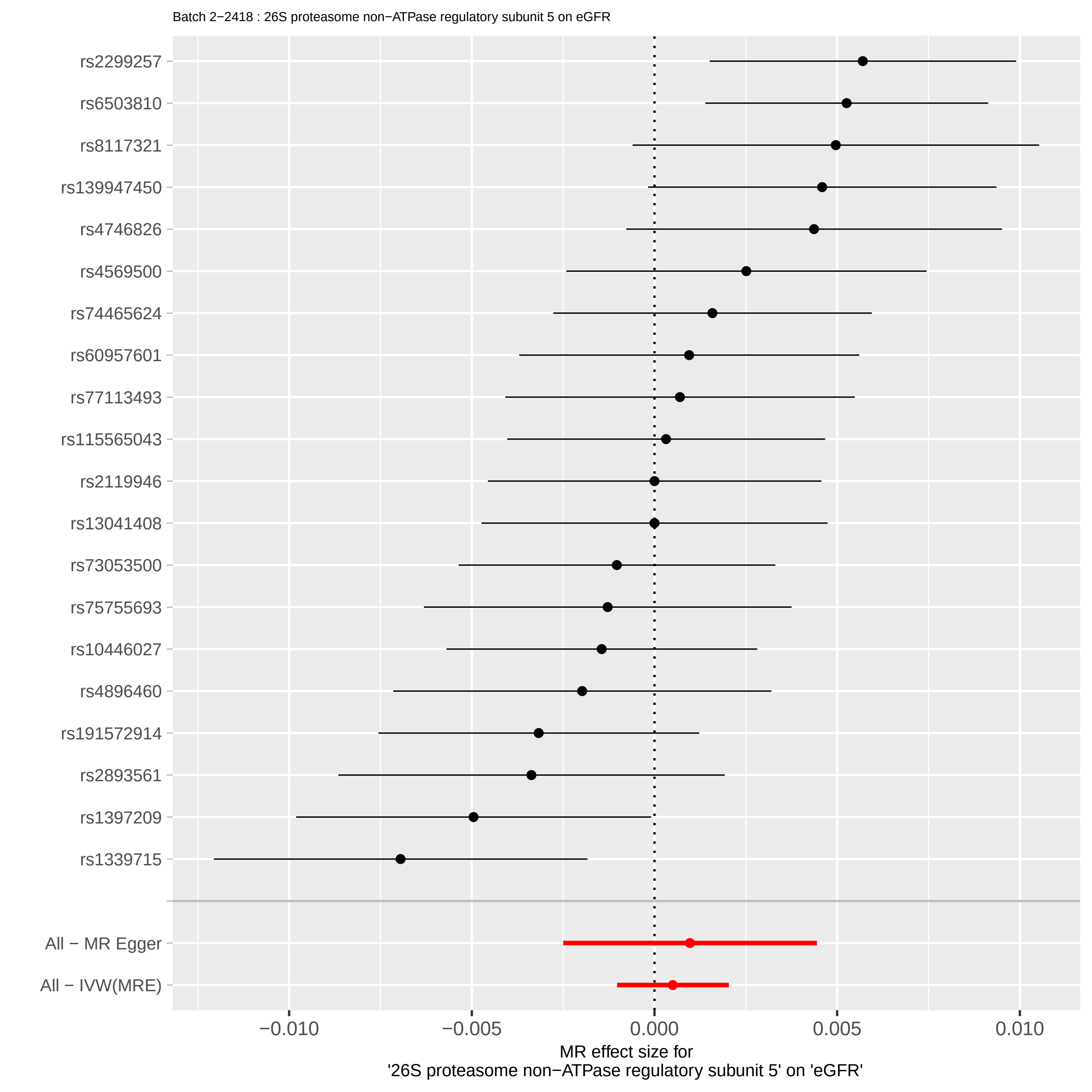

Batch 2−2418 : 26S proteasome non−ATPase regulatory subunit 5 on eGFR
rs2299257
rs6503810
rs8117321
rs139947450
rs4746826
rs4569500
rs74465624
rs60957601
rs77113493
rs115565043
rs2119946
rs13041408
rs73053500
rs75755693
rs10446027
rs4896460
rs191572914
rs2893561
rs1397209
rs1339715
All − MR Egger
All − IVW(MRE)
−0.010
−0.005
0.000
0.005
0.010
MR effect size for
'26S proteasome non−ATPase regulatory subunit 5' on 'eGFR'

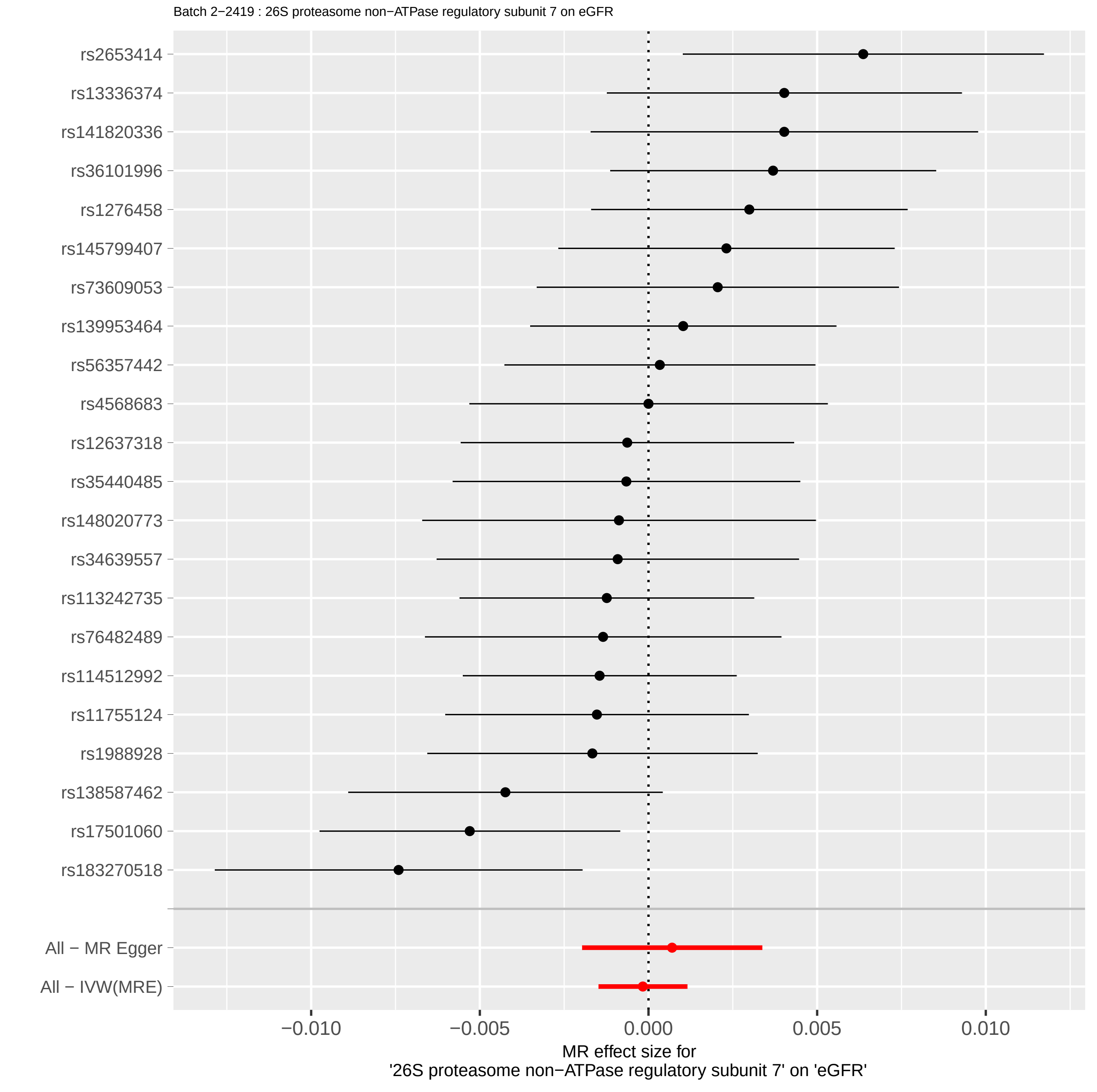

Batch 2−2419 : 26S proteasome non−ATPase regulatory subunit 7 on eGFR
rs2653414
rs13336374
rs141820336
rs36101996
rs1276458
rs145799407
rs73609053
rs139953464
rs56357442
rs4568683
rs12637318
rs35440485
rs148020773
rs34639557
rs113242735
rs76482489
rs114512992
rs11755124
rs1988928
rs138587462
rs17501060
rs183270518
All − MR Egger
All − IVW(MRE)
−0.010
−0.005
0.000
0.005
0.010
MR effect size for
'26S proteasome non−ATPase regulatory subunit 7' on 'eGFR'

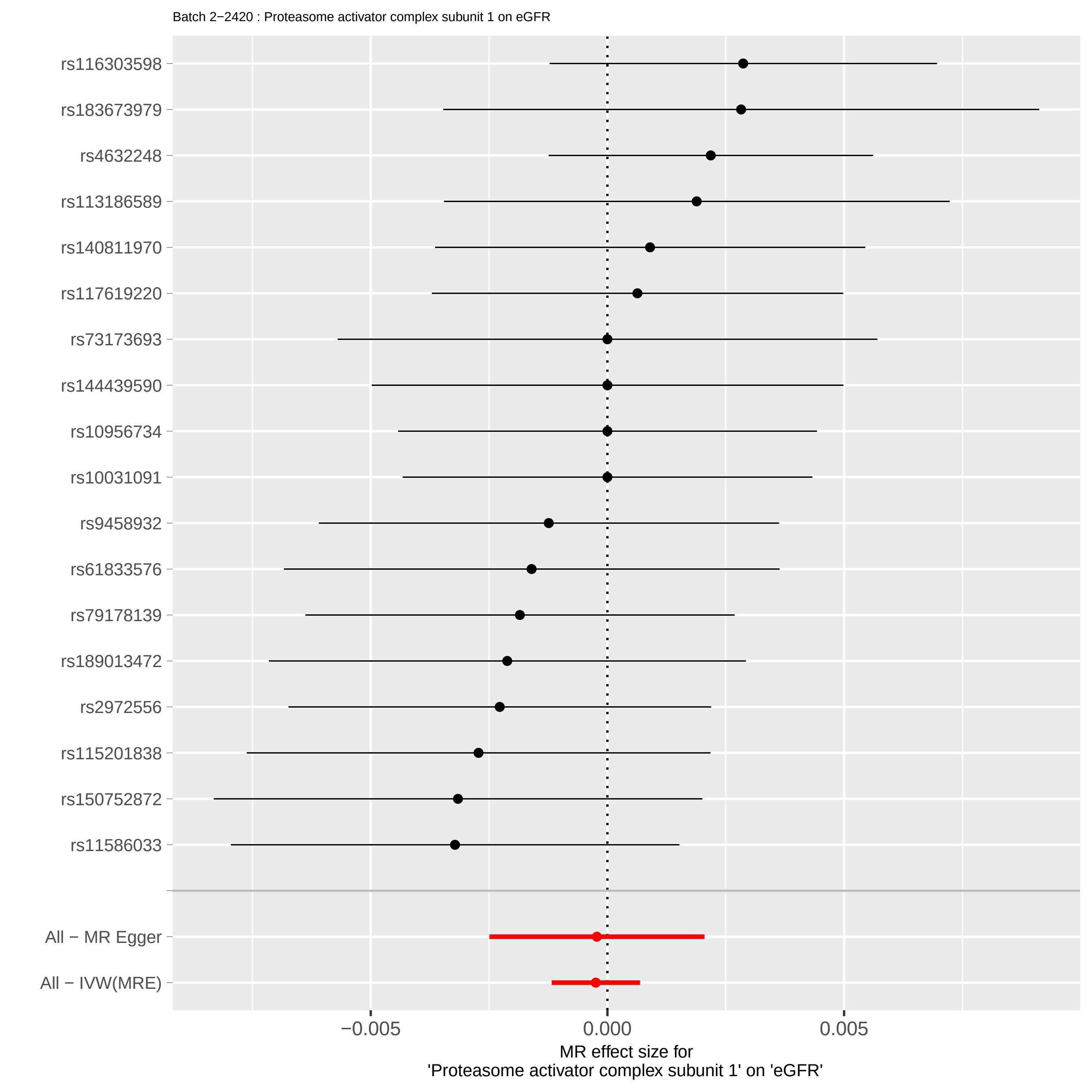

Batch 2−2420 : Proteasome activator complex subunit 1 on eGFR
rs116303598
rs183673979
rs4632248
rs113186589
rs140811970
rs117619220
rs73173693
rs144439590
rs10956734
rs10031091
rs9458932
rs61833576
rs79178139
rs189013472
rs2972556
rs115201838
rs150752872
rs11586033
All − MR Egger
All − IVW(MRE)
−0.005
0.000
0.005
MR effect size for
'Proteasome activator complex subunit 1' on 'eGFR'

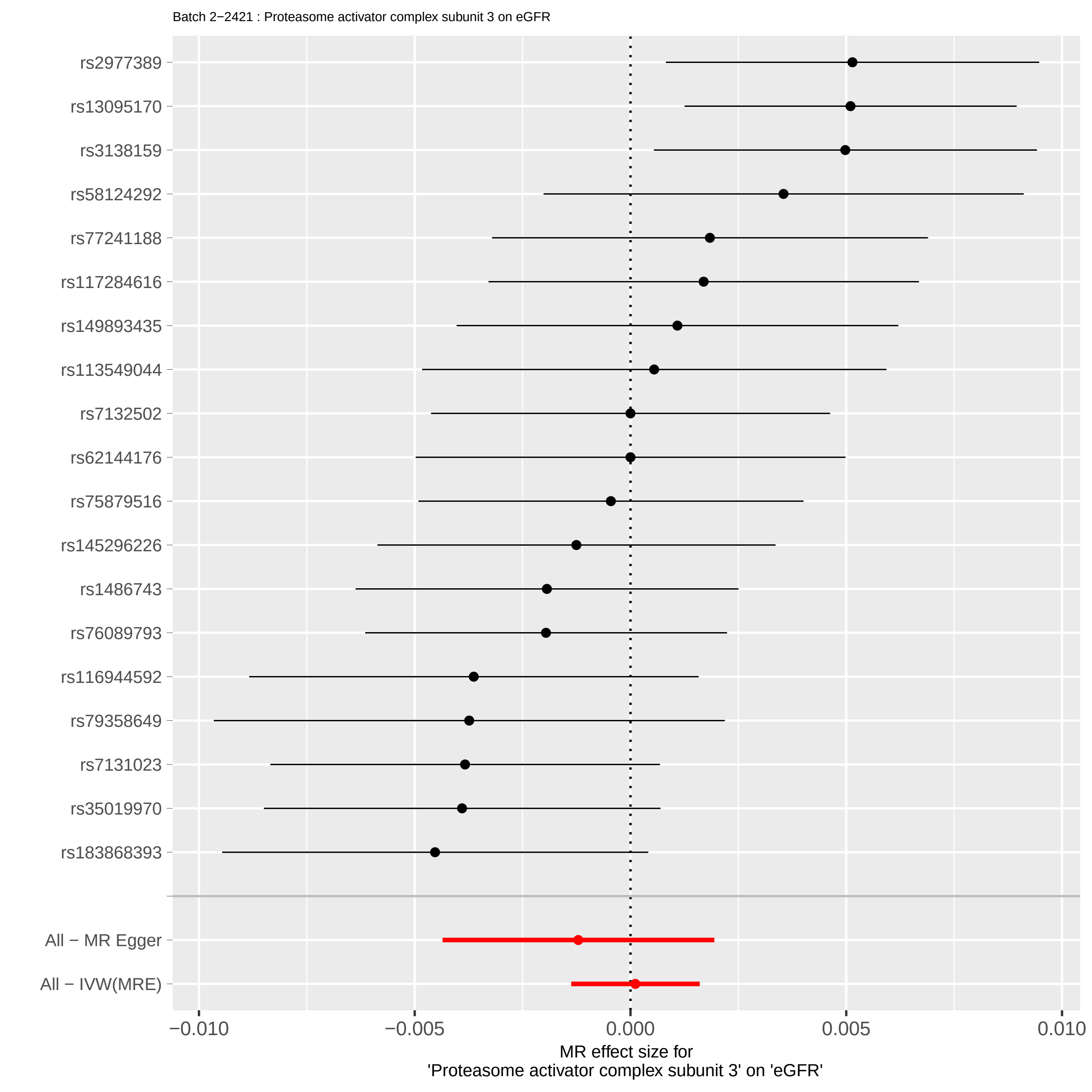

Batch 2−2421 : Proteasome activator complex subunit 3 on eGFR
rs2977389
rs13095170
rs3138159
rs58124292
rs77241188
rs117284616
rs149893435
rs113549044
rs7132502
rs62144176
rs75879516
rs145296226
rs1486743
rs76089793
rs116944592
rs79358649
rs7131023
rs35019970
rs183868393
All − MR Egger
All − IVW(MRE)
−0.010
−0.005
0.000
0.005
0.010
MR effect size for
'Proteasome activator complex subunit 3' on 'eGFR'

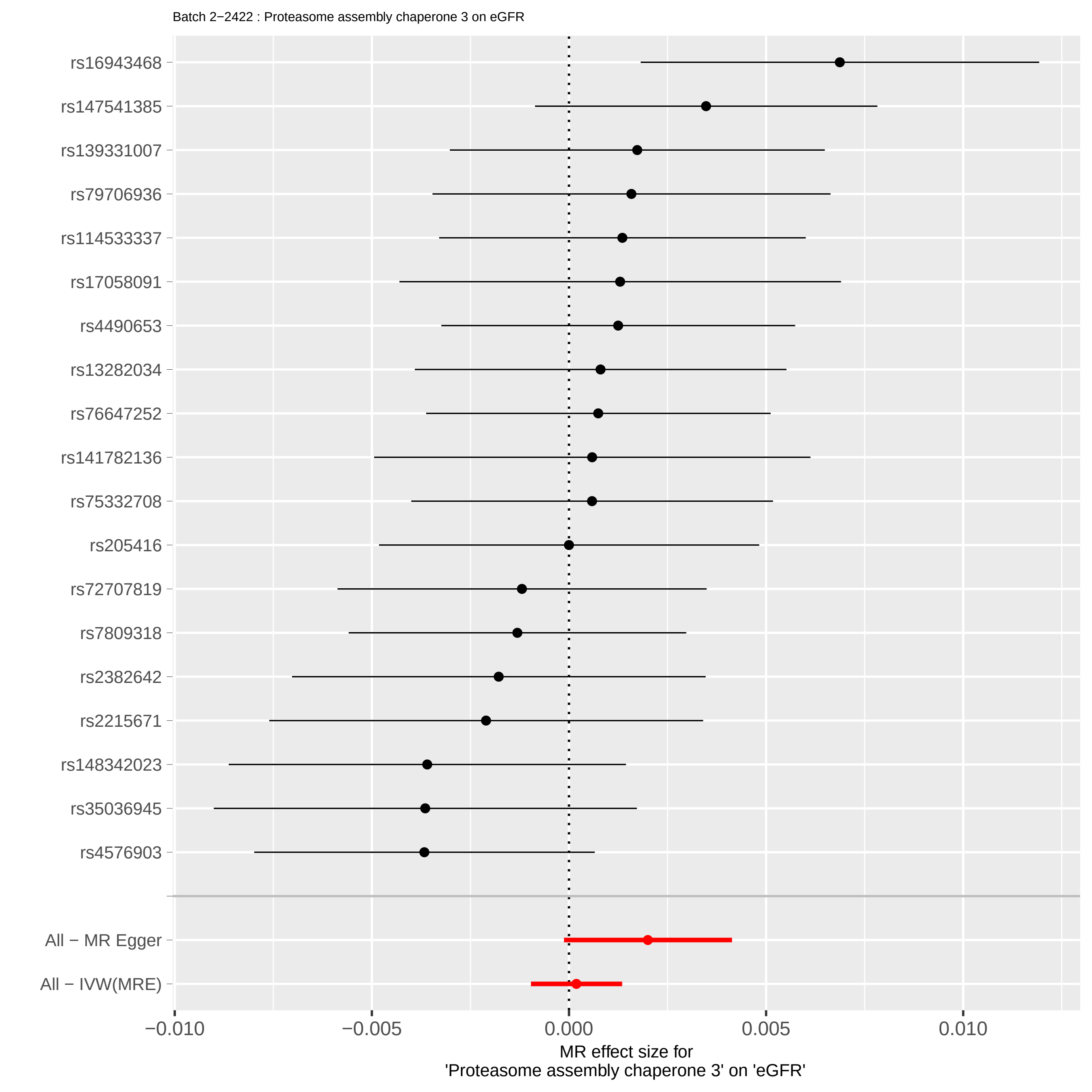

Batch 2−2422 : Proteasome assembly chaperone 3 on eGFR
rs16943468
rs147541385
rs139331007
rs79706936
rs114533337
rs17058091
rs4490653
rs13282034
rs76647252
rs141782136
rs75332708
rs205416
rs72707819
rs7809318
rs2382642
rs2215671
rs148342023
rs35036945
rs4576903
All − MR Egger
All − IVW(MRE)
−0.010
−0.005
0.000
0.005
0.010
MR effect size for
'Proteasome assembly chaperone 3' on 'eGFR'

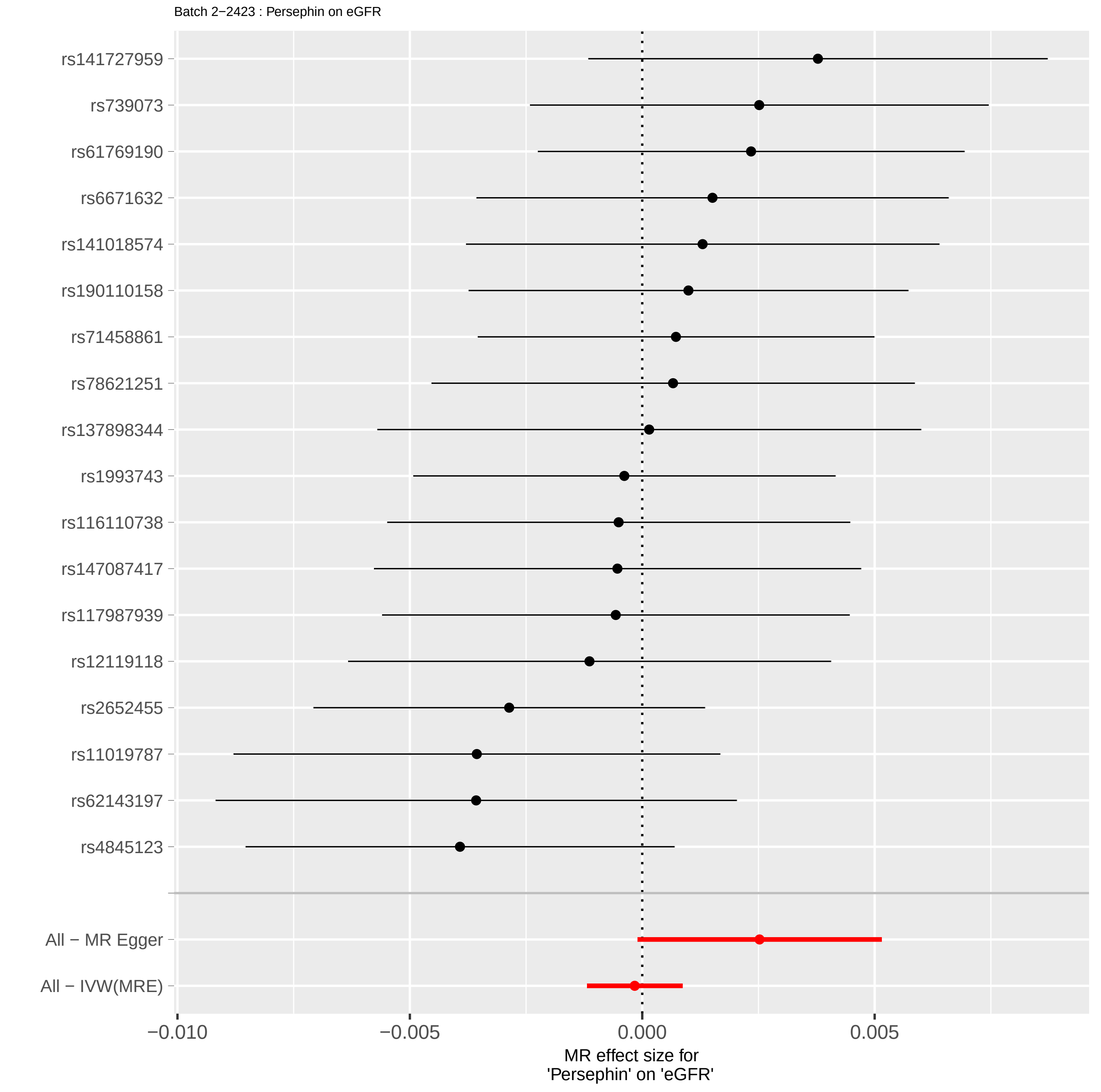

Batch 2−2423 : Persephin on eGFR
rs141727959
rs739073
rs61769190
rs6671632
rs141018574
rs190110158
rs71458861
rs78621251
rs137898344
rs1993743
rs116110738
rs147087417
rs117987939
rs12119118
rs2652455
rs11019787
rs62143197
rs4845123
All − MR Egger
All − IVW(MRE)
−0.010
−0.005
0.000
0.005
MR effect size for
'Persephin' on 'eGFR'

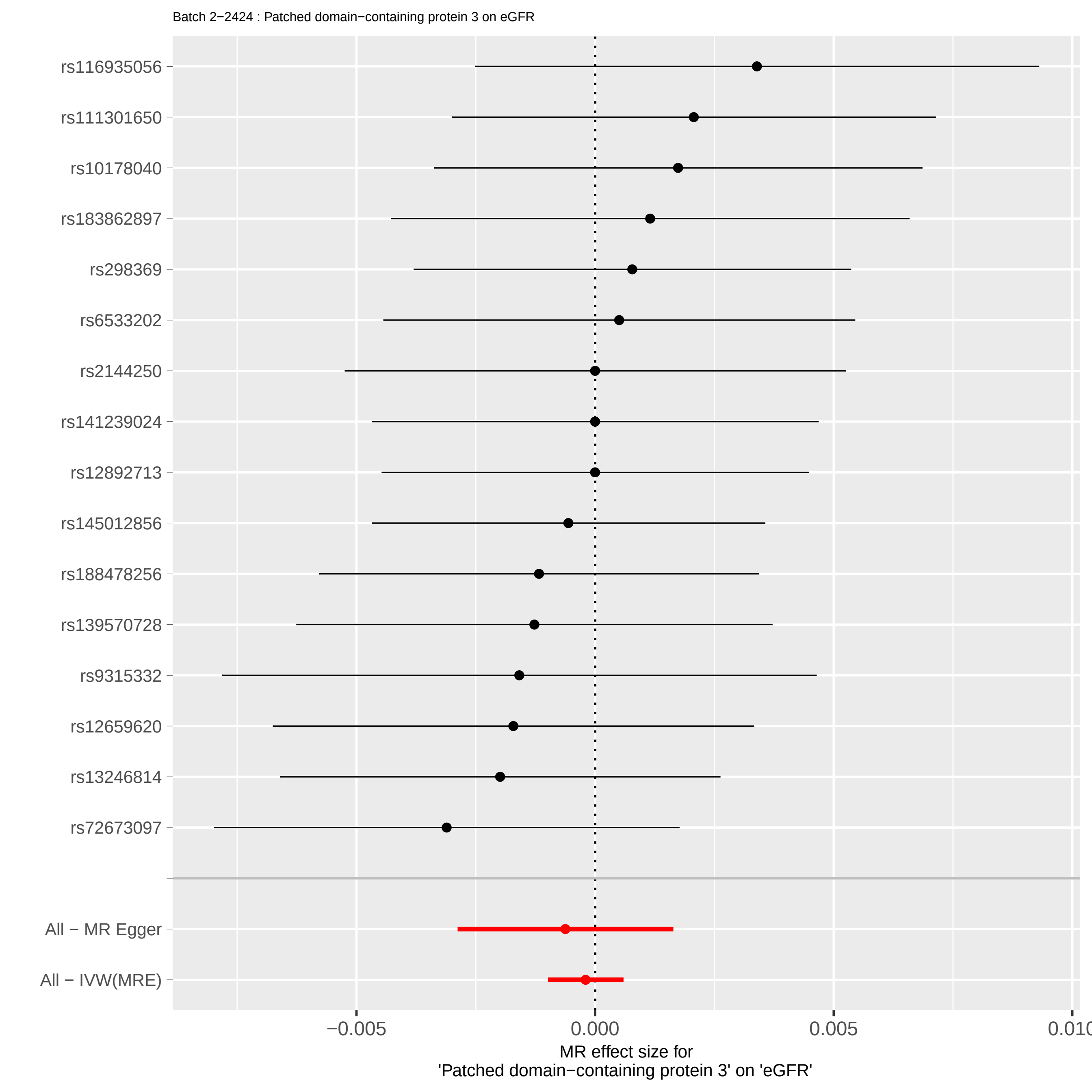

Batch 2−2424 : Patched domain−containing protein 3 on eGFR
rs116935056
rs111301650
rs10178040
rs183862897
rs298369
rs6533202
rs2144250
rs141239024
rs12892713
rs145012856
rs188478256
rs139570728
rs9315332
rs12659620
rs13246814
rs72673097
All − MR Egger
All − IVW(MRE)
−0.005
0.000
0.005
0.010
MR effect size for
'Patched domain−containing protein 3' on 'eGFR'

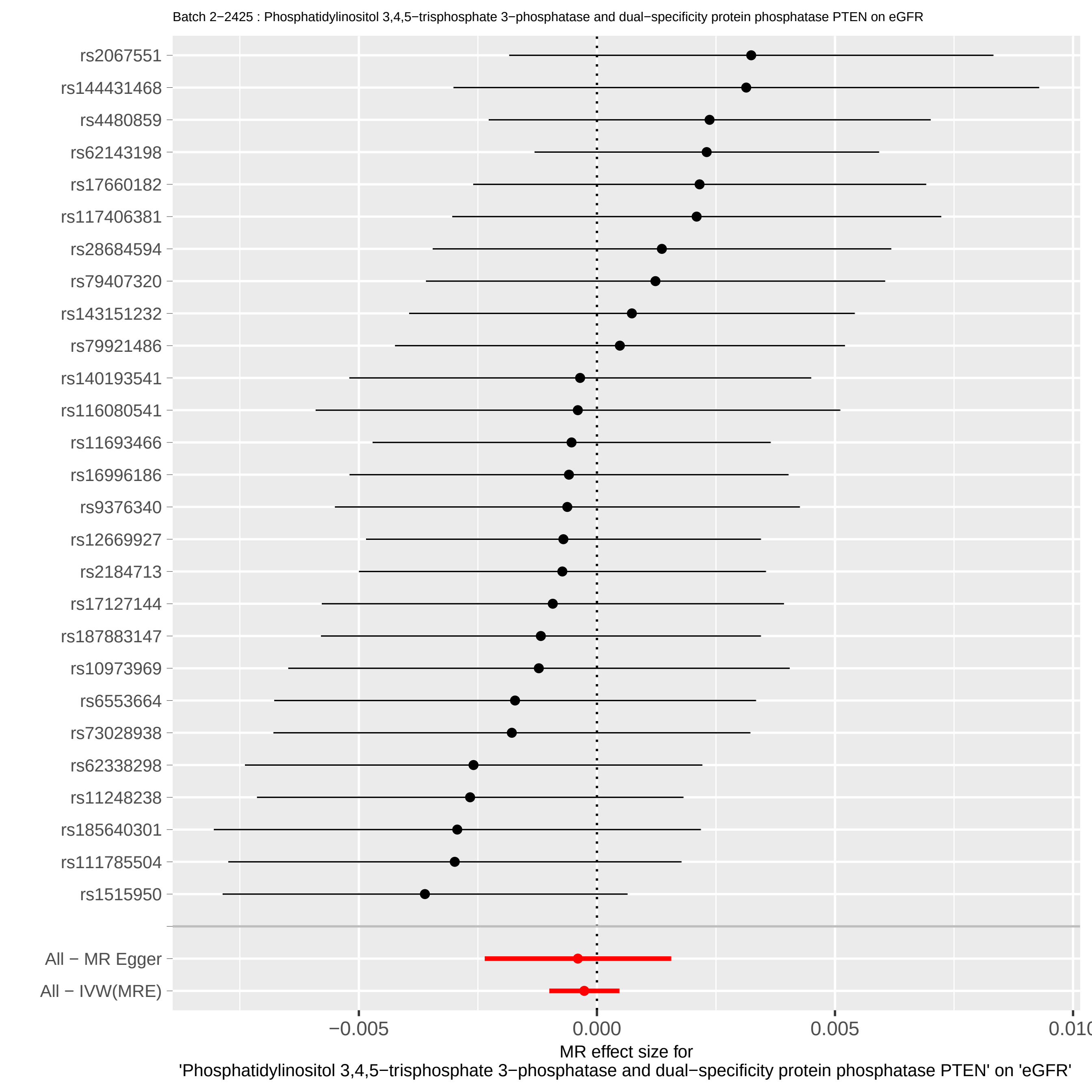

Batch 2−2425 : Phosphatidylinositol 3,4,5−trisphosphate 3−phosphatase and dual−specificity protein phosphatase PTEN on eGFR
rs2067551
rs144431468
rs4480859
rs62143198
rs17660182
rs117406381
rs28684594
rs79407320
rs143151232
rs79921486
rs140193541
rs116080541
rs11693466
rs16996186
rs9376340
rs12669927
rs2184713
rs17127144
rs187883147
rs10973969
rs6553664
rs73028938
rs62338298
rs11248238
rs185640301
rs111785504
rs1515950
All − MR Egger
All − IVW(MRE)
−0.005
0.000
0.005
0.010
MR effect size for
'Phosphatidylinositol 3,4,5−trisphosphate 3−phosphatase and dual−specificity protein phosphatase PTEN' on 'eGFR'

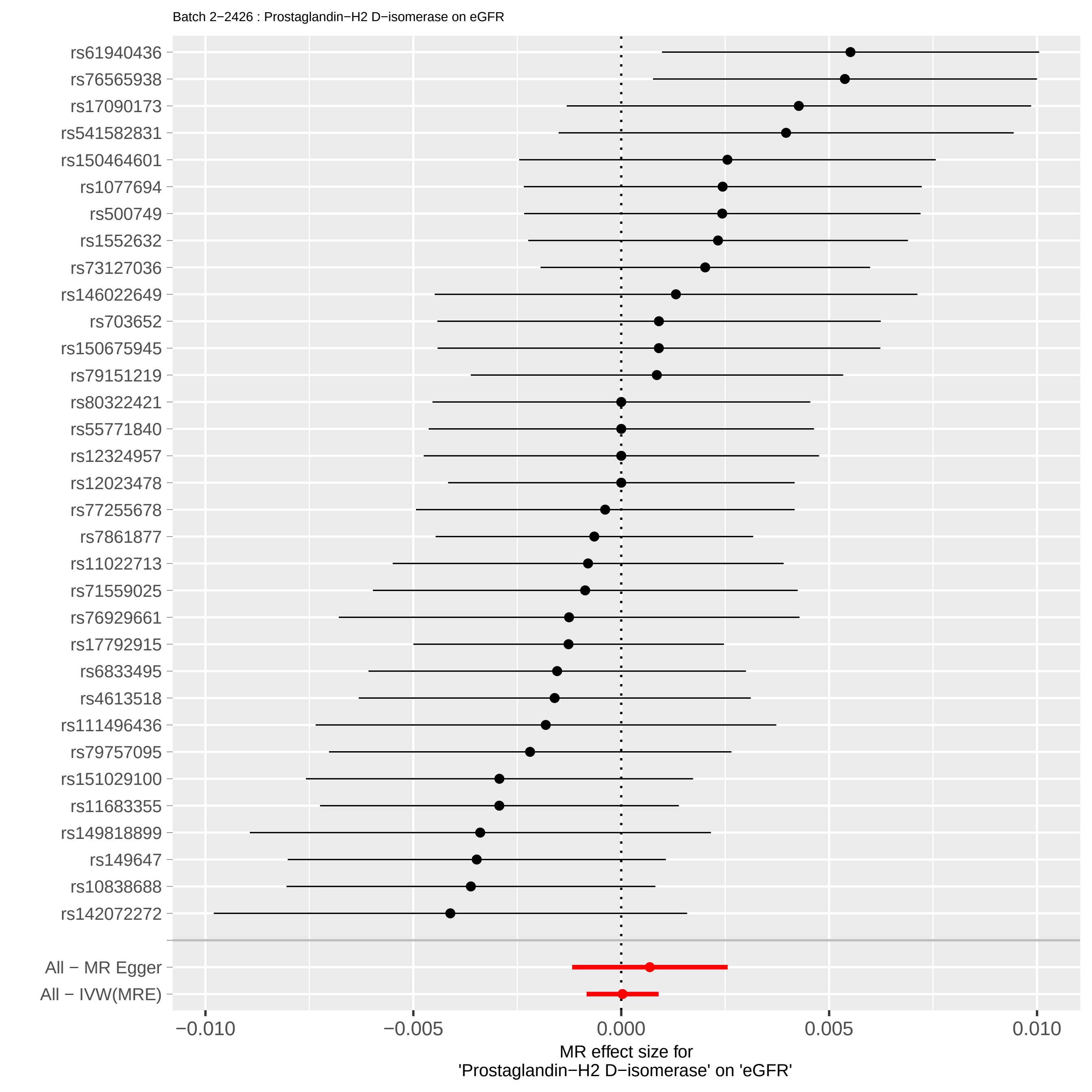

Batch 2−2426 : Prostaglandin−H2 D−isomerase on eGFR
rs61940436
rs76565938
rs17090173
rs541582831
rs150464601
rs1077694
rs500749
rs1552632
rs73127036
rs146022649
rs703652
rs150675945
rs79151219
rs80322421
rs55771840
rs12324957
rs12023478
rs77255678
rs7861877
rs11022713
rs71559025
rs76929661
rs17792915
rs6833495
rs4613518
rs111496436
rs79757095
rs151029100
rs11683355
rs149818899
rs149647
rs10838688
rs142072272
All − MR Egger
All − IVW(MRE)
−0.010
−0.005
0.000
0.005
0.010
MR effect size for
'Prostaglandin−H2 D−isomerase' on 'eGFR'

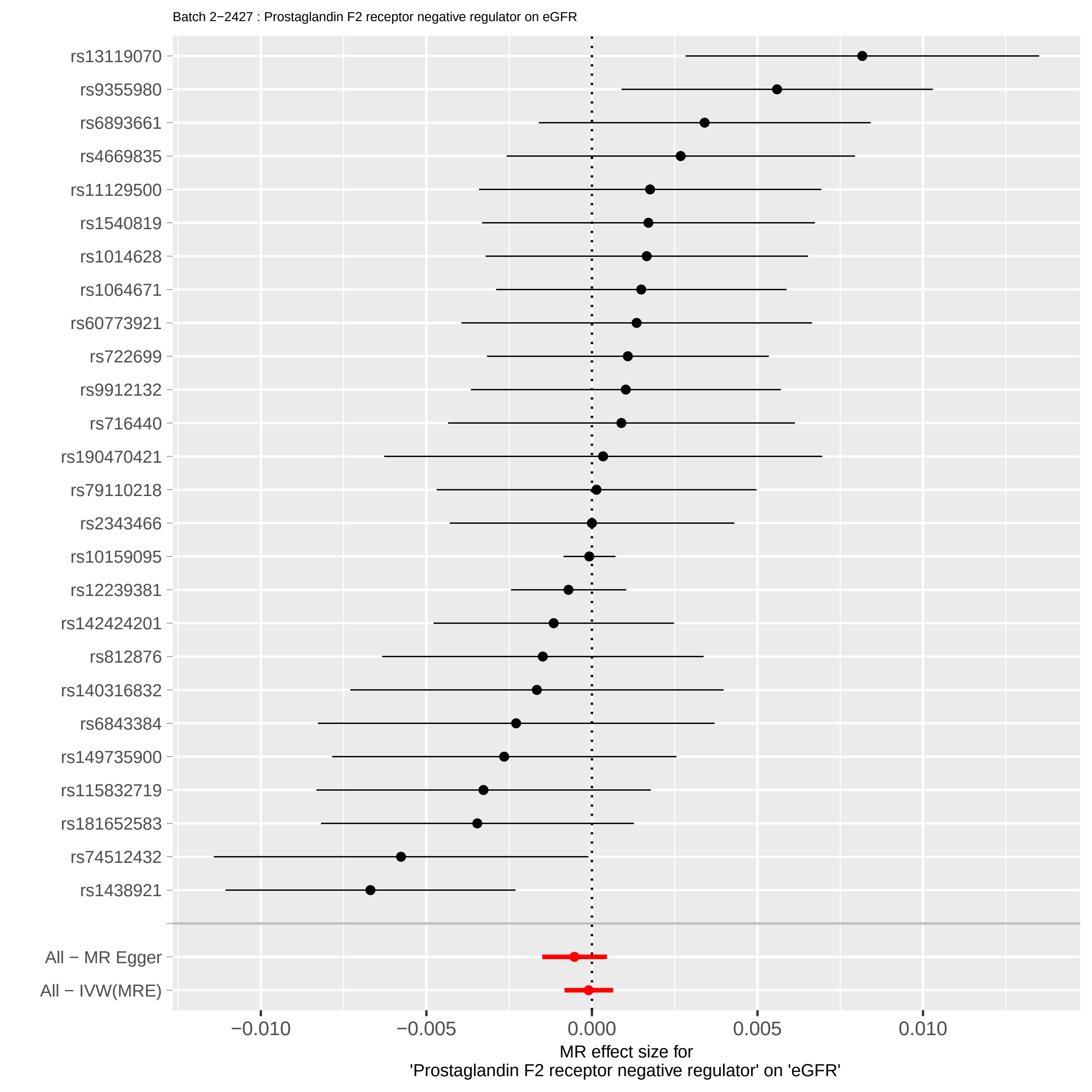

Batch 2−2427 : Prostaglandin F2 receptor negative regulator on eGFR
rs13119070
rs9355980
rs6893661
rs4669835
rs11129500
rs1540819
rs1014628
rs1064671
rs60773921
rs722699
rs9912132
rs716440
rs190470421
rs79110218
rs2343466
rs10159095
rs12239381
rs142424201
rs812876
rs140316832
rs6843384
rs149735900
rs115832719
rs181652583
rs74512432
rs1438921
All − MR Egger
All − IVW(MRE)
−0.010
−0.005
0.000
0.005
0.010
MR effect size for
'Prostaglandin F2 receptor negative regulator' on 'eGFR'

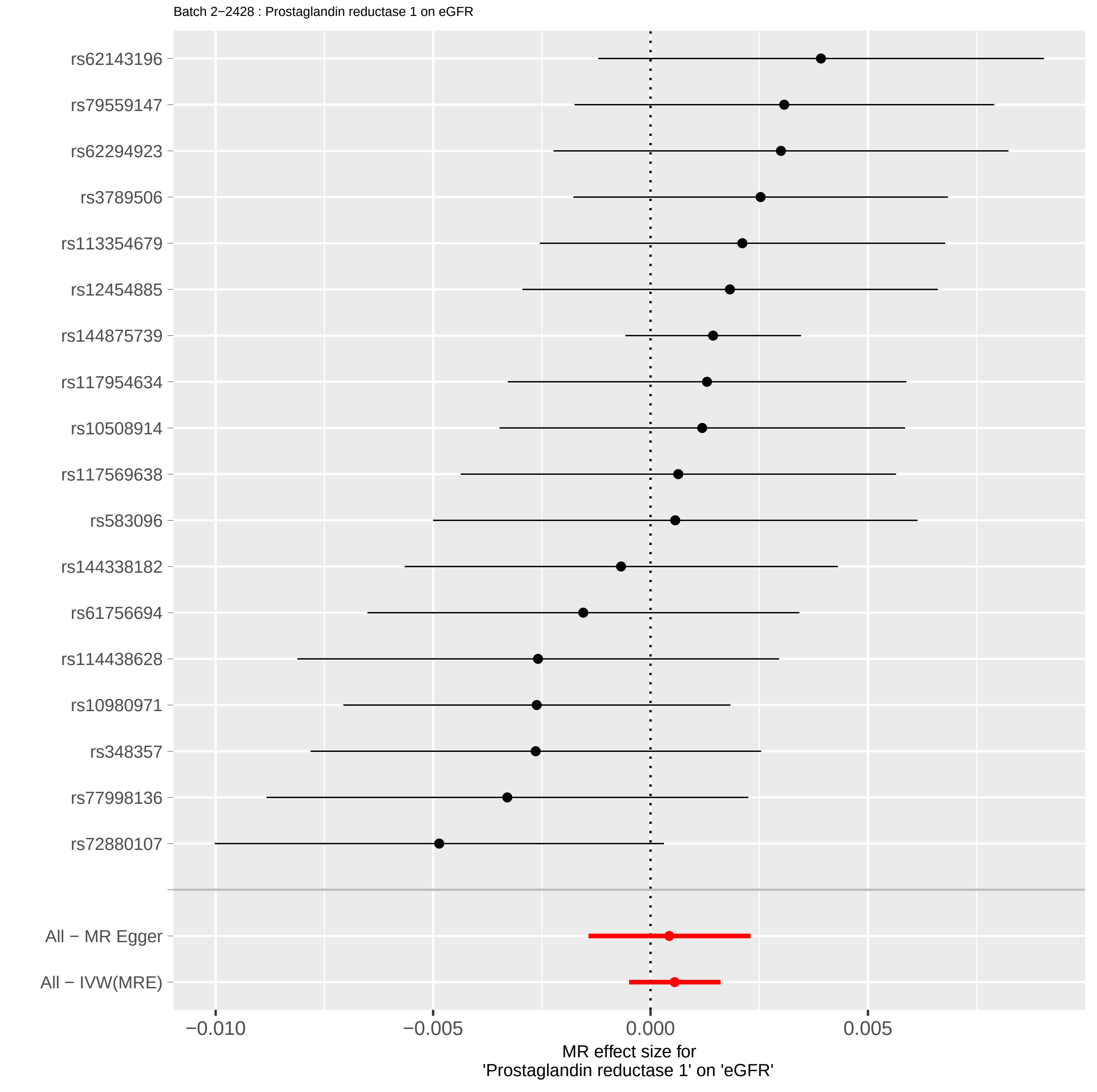

Batch 2−2428 : Prostaglandin reductase 1 on eGFR
rs62143196
rs79559147
rs62294923
rs3789506
rs113354679
rs12454885
rs144875739
rs117954634
rs10508914
rs117569638
rs583096
rs144338182
rs61756694
rs114438628
rs10980971
rs348357
rs77998136
rs72880107
All − MR Egger
All − IVW(MRE)
−0.010
−0.005
0.000
0.005
MR effect size for
'Prostaglandin reductase 1' on 'eGFR'

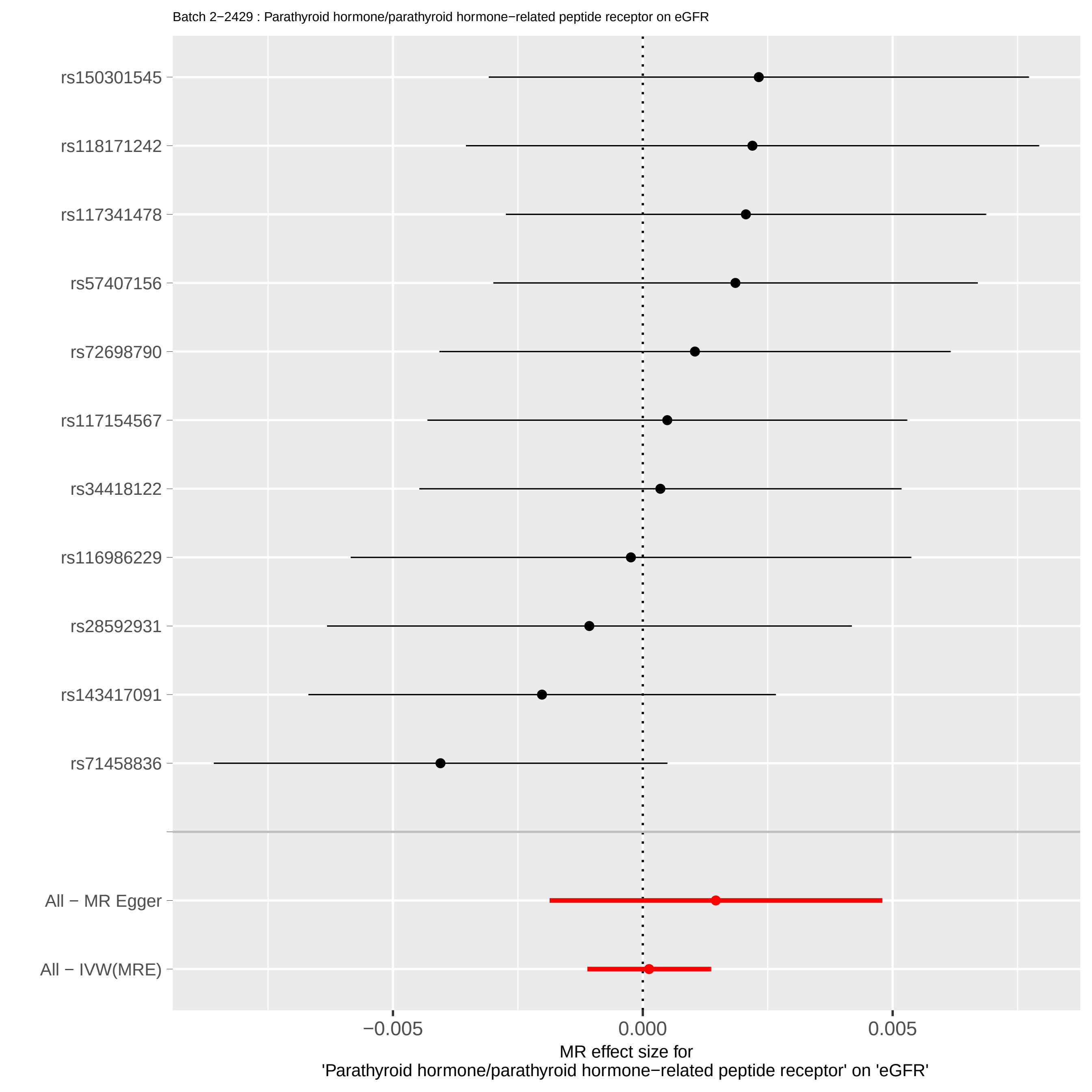

Batch 2−2429 : Parathyroid hormone/parathyroid hormone−related peptide receptor on eGFR
rs150301545
rs118171242
rs117341478
rs57407156
rs72698790
rs117154567
rs34418122
rs116986229
rs28592931
rs143417091
rs71458836
All − MR Egger
All − IVW(MRE)
−0.005
0.000
0.005
MR effect size for
'Parathyroid hormone/parathyroid hormone−related peptide receptor' on 'eGFR'

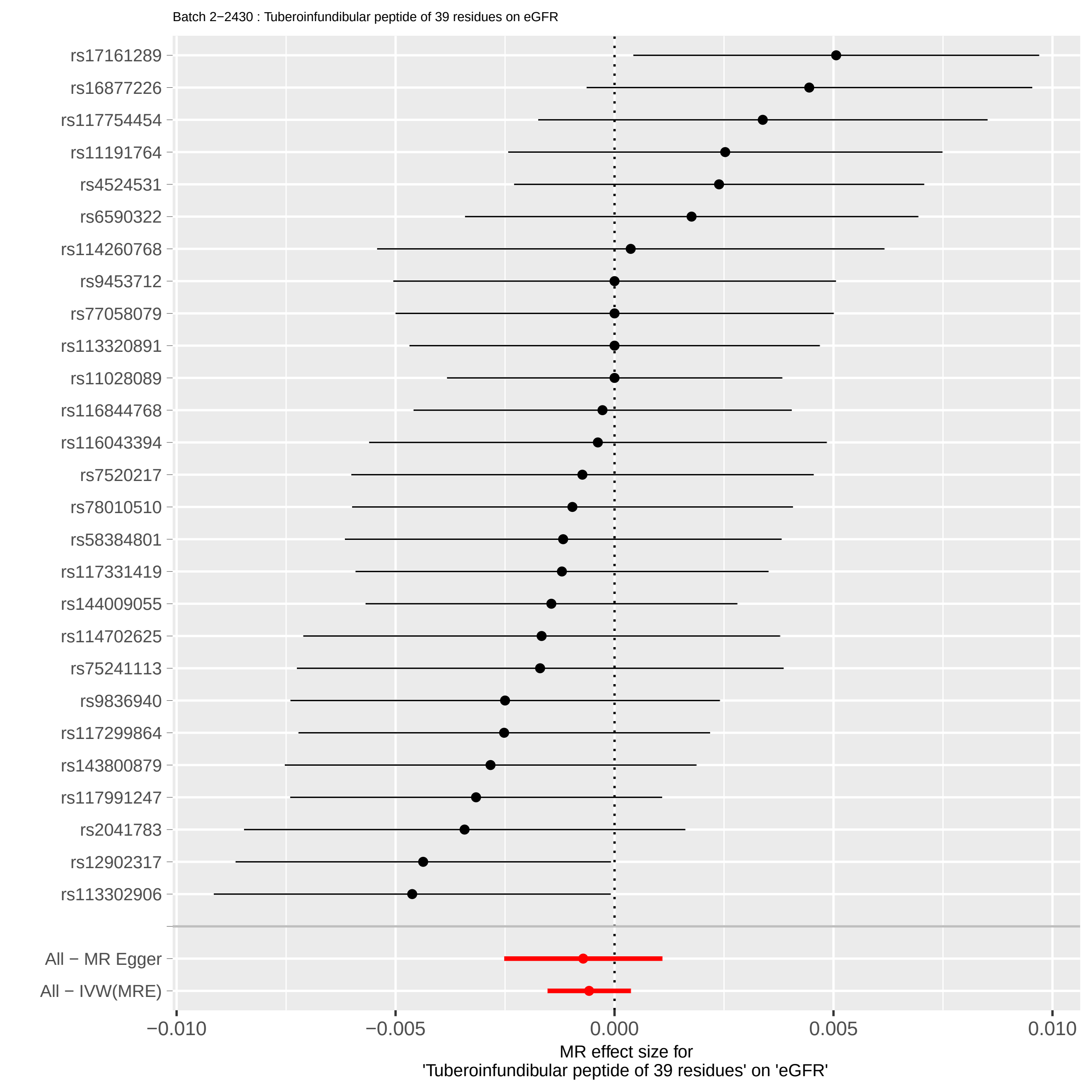

Batch 2−2430 : Tuberoinfundibular peptide of 39 residues on eGFR
rs17161289
rs16877226
rs117754454
rs11191764
rs4524531
rs6590322
rs114260768
rs9453712
rs77058079
rs113320891
rs11028089
rs116844768
rs116043394
rs7520217
rs78010510
rs58384801
rs117331419
rs144009055
rs114702625
rs75241113
rs9836940
rs117299864
rs143800879
rs117991247
rs2041783
rs12902317
rs113302906
All − MR Egger
All − IVW(MRE)
−0.010
−0.005
0.000
0.005
0.010
MR effect size for
'Tuberoinfundibular peptide of 39 residues' on 'eGFR'

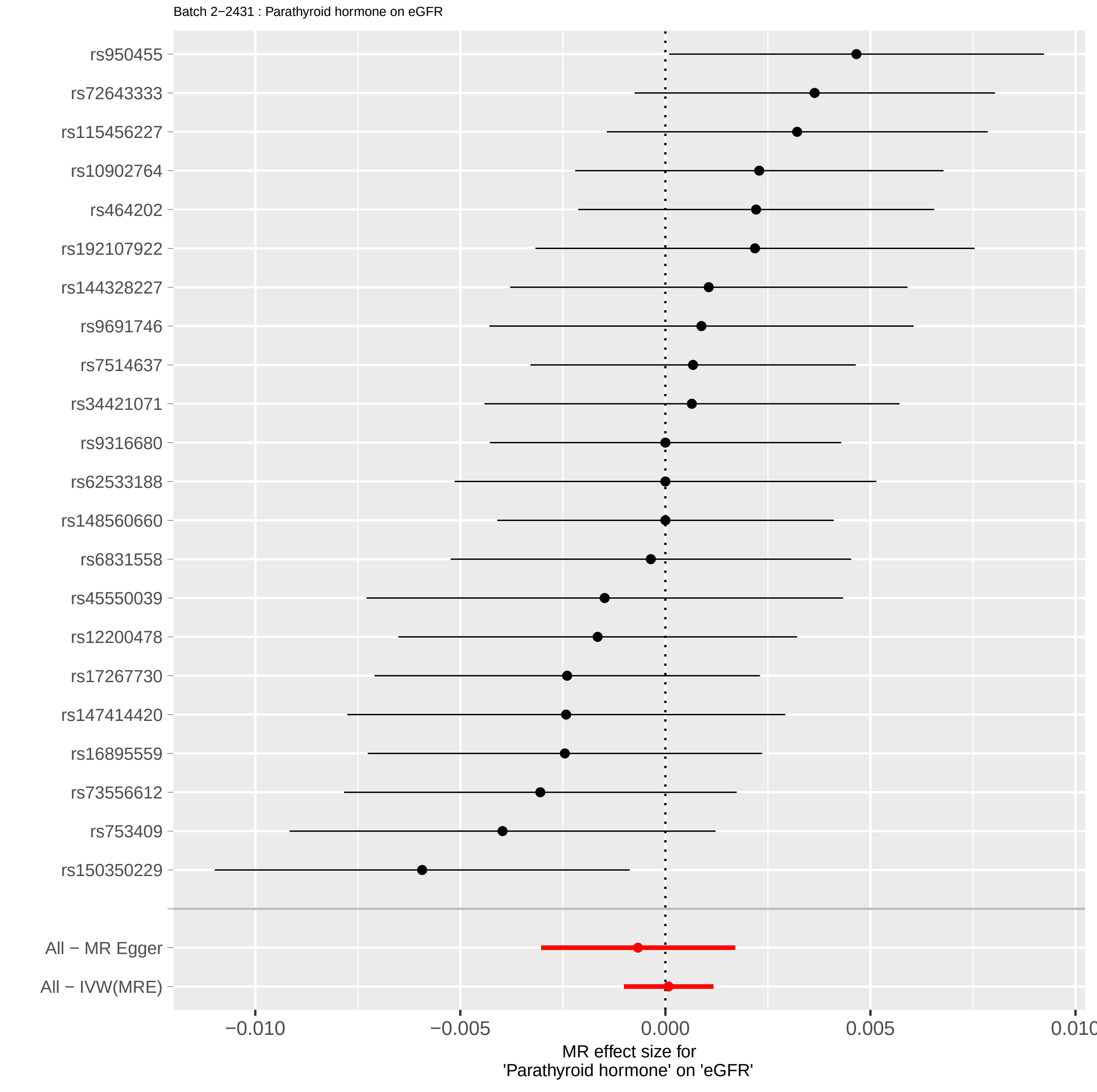

Batch 2−2431 : Parathyroid hormone on eGFR
rs950455
rs72643333
rs115456227
rs10902764
rs464202
rs192107922
rs144328227
rs9691746
rs7514637
rs34421071
rs9316680
rs62533188
rs148560660
rs6831558
rs45550039
rs12200478
rs17267730
rs147414420
rs16895559
rs73556612
rs753409
rs150350229
All − MR Egger
All − IVW(MRE)
−0.010
−0.005
0.000
0.005
0.010
MR effect size for
'Parathyroid hormone' on 'eGFR'

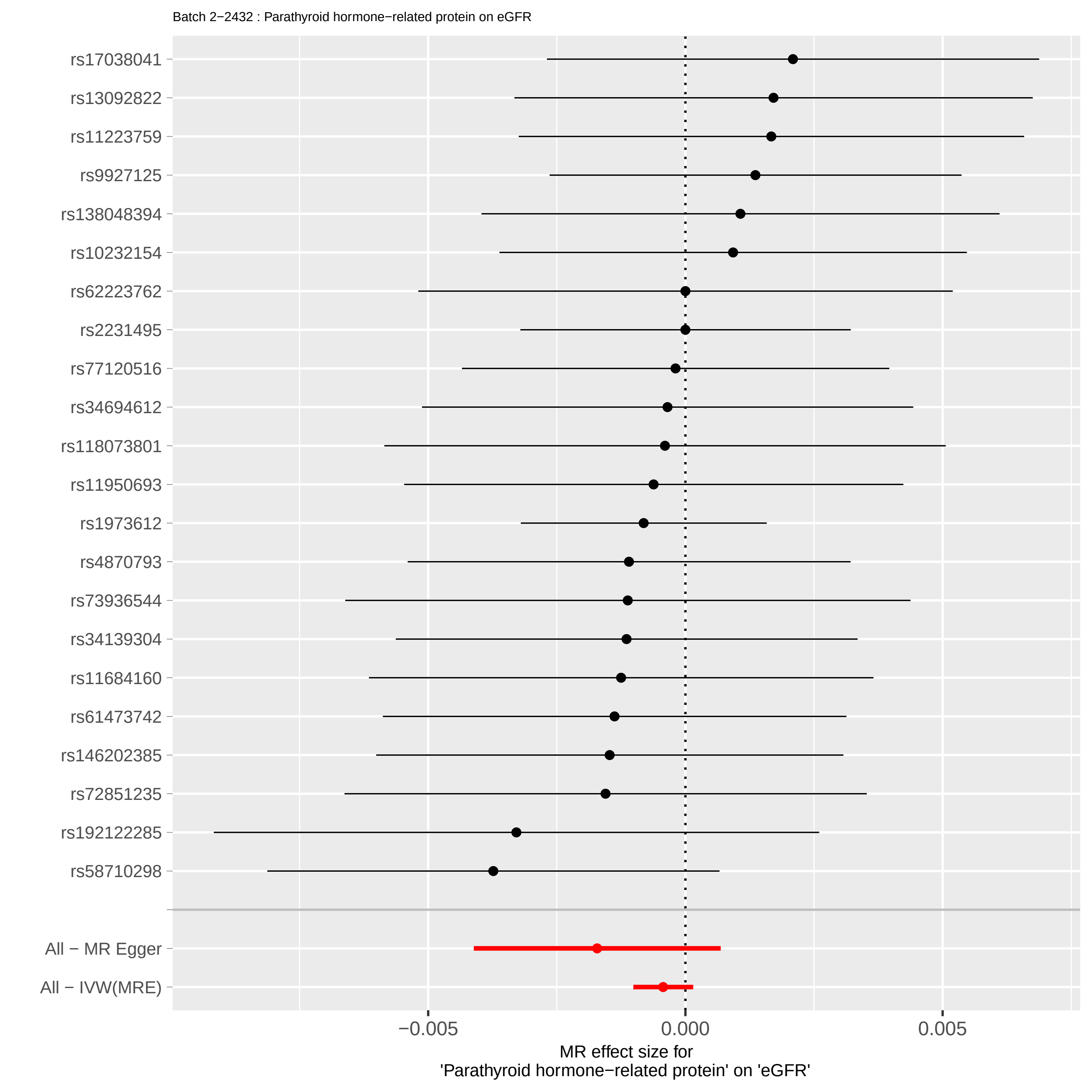

Batch 2−2432 : Parathyroid hormone−related protein on eGFR
rs17038041
rs13092822
rs11223759
rs9927125
rs138048394
rs10232154
rs62223762
rs2231495
rs77120516
rs34694612
rs118073801
rs11950693
rs1973612
rs4870793
rs73936544
rs34139304
rs11684160
rs61473742
rs146202385
rs72851235
rs192122285
rs58710298
All − MR Egger
All − IVW(MRE)
−0.005
0.000
0.005
MR effect size for
'Parathyroid hormone−related protein' on 'eGFR'

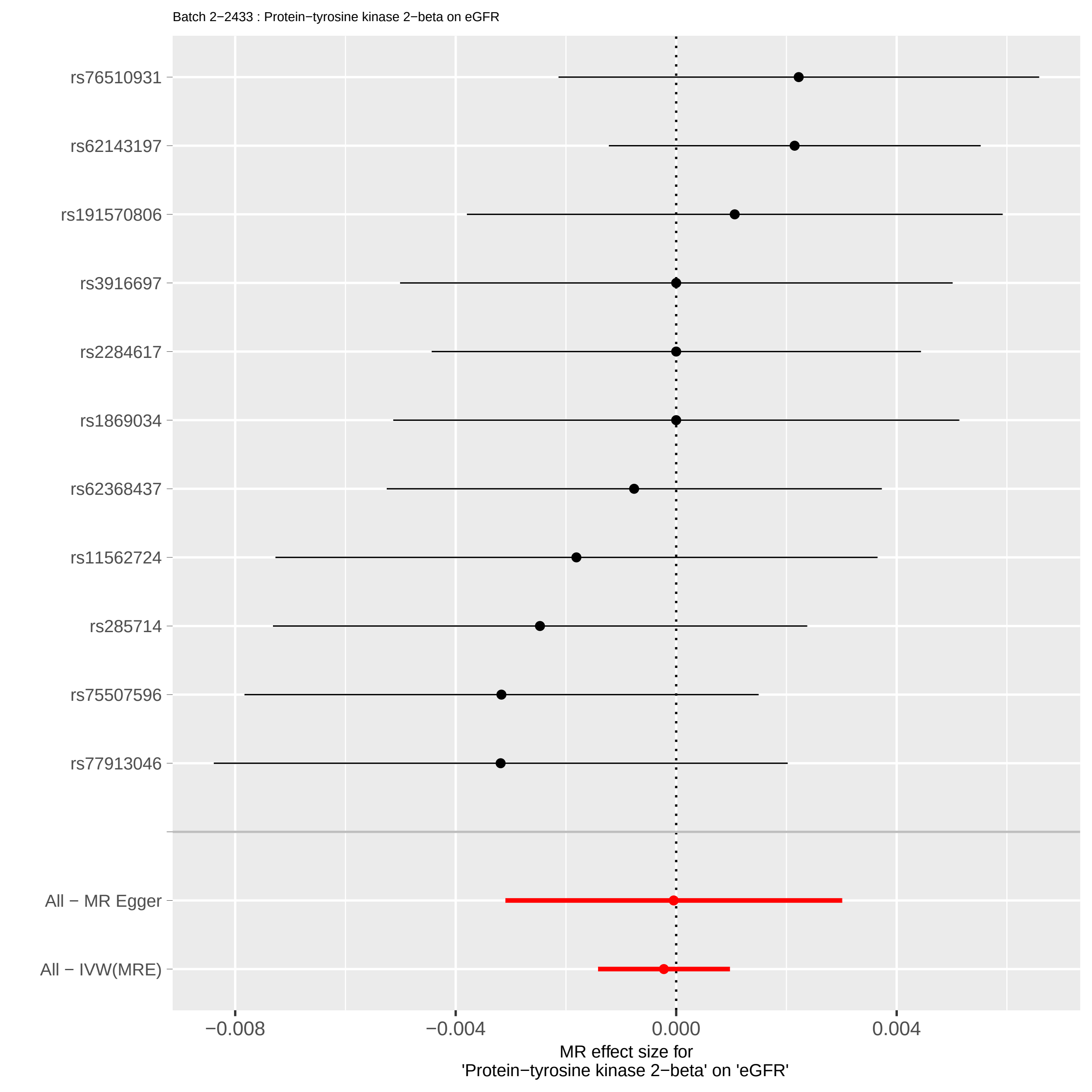

Batch 2−2433 : Protein−tyrosine kinase 2−beta on eGFR
rs76510931
rs62143197
rs191570806
rs3916697
rs2284617
rs1869034
rs62368437
rs11562724
rs285714
rs75507596
rs77913046
All − MR Egger
All − IVW(MRE)
−0.008
−0.004
0.000
0.004
MR effect size for
'Protein−tyrosine kinase 2−beta' on 'eGFR'

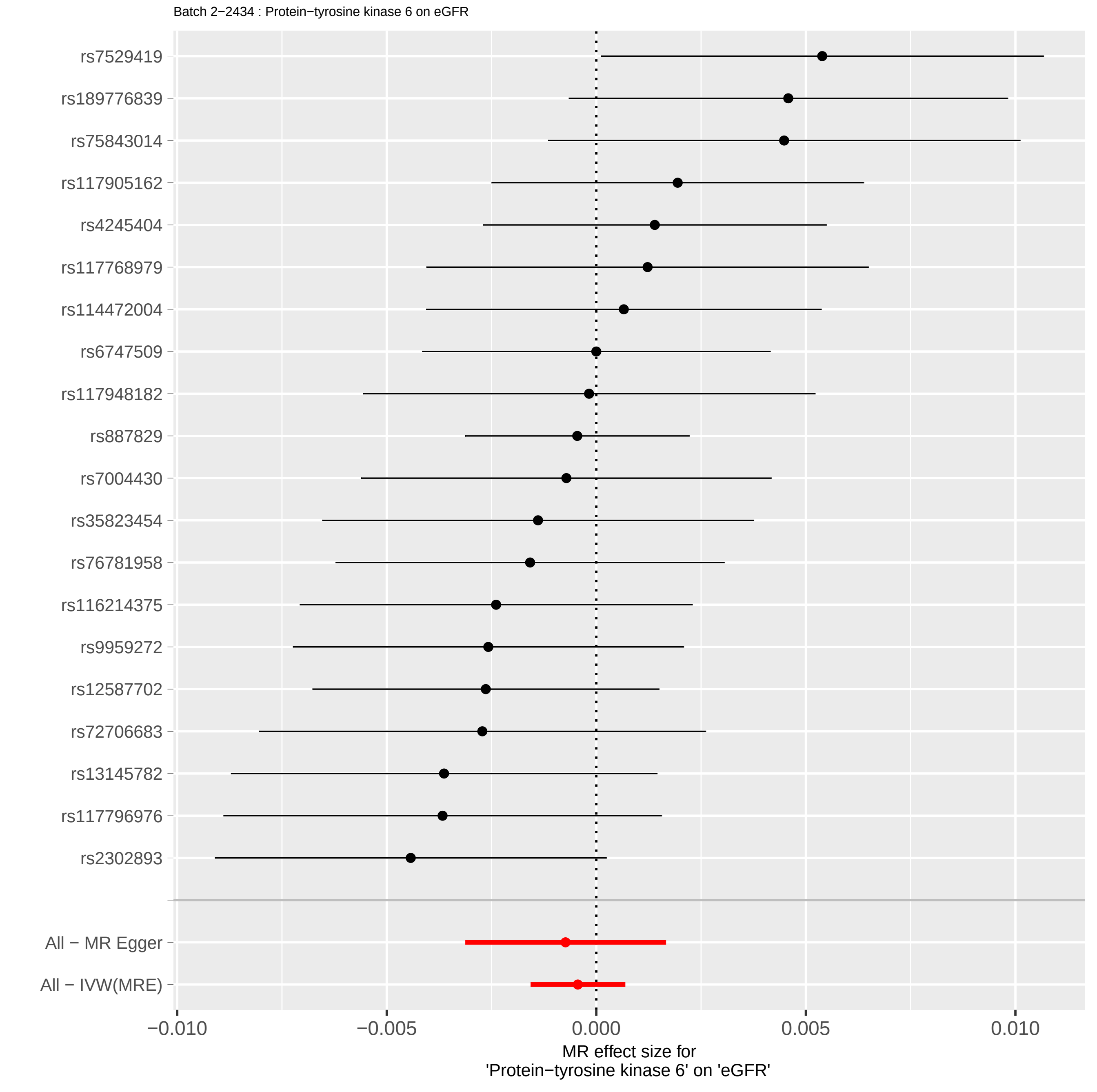

Batch 2−2434 : Protein−tyrosine kinase 6 on eGFR
rs7529419
rs189776839
rs75843014
rs117905162
rs4245404
rs117768979
rs114472004
rs6747509
rs117948182
rs887829
rs7004430
rs35823454
rs76781958
rs116214375
rs9959272
rs12587702
rs72706683
rs13145782
rs117796976
rs2302893
All − MR Egger
All − IVW(MRE)
−0.010
−0.005
0.000
0.005
0.010
MR effect size for
'Protein−tyrosine kinase 6' on 'eGFR'

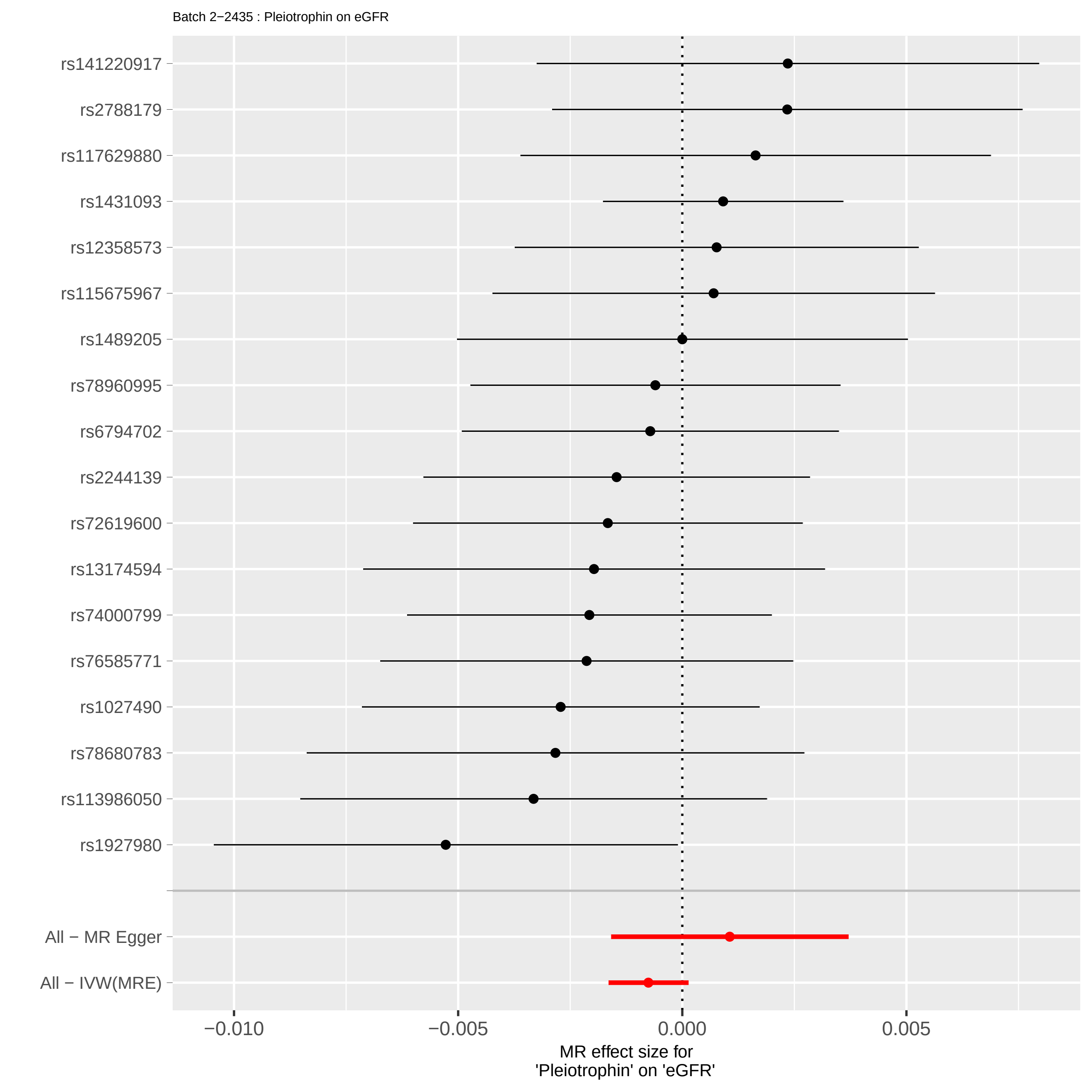
Batch 2−2435 : Pleiotrophin on eGFR
rs141220917
rs2788179
rs117629880
rs1431093
rs12358573
rs115675967
rs1489205
rs78960995
rs6794702
rs2244139
rs72619600
rs13174594
rs74000799
rs76585771
rs1027490
rs78680783
rs113986050
rs1927980
All − MR Egger
All − IVW(MRE)
−0.010
−0.005
0.000
0.005
MR effect size for
'Pleiotrophin' on 'eGFR'

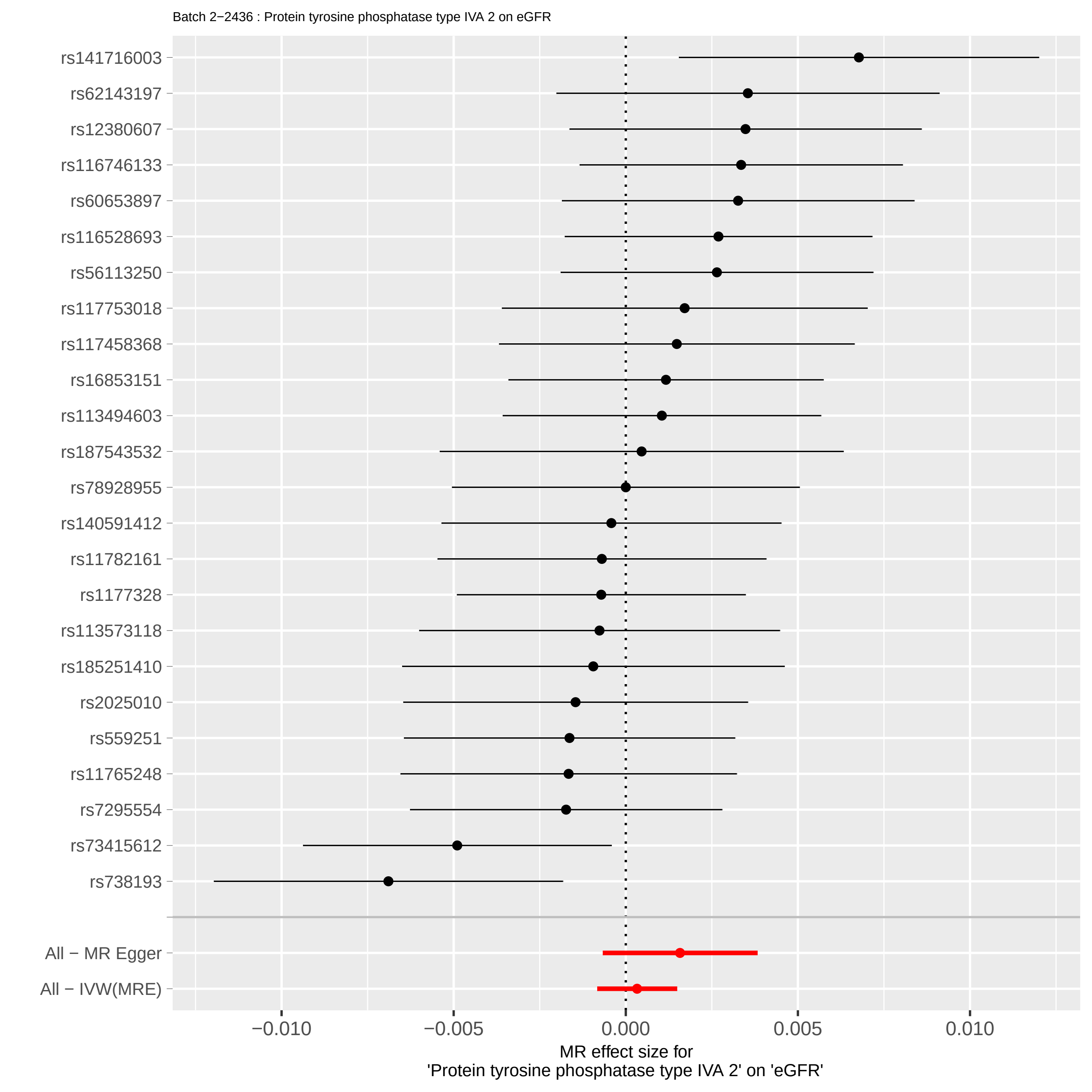

Batch 2−2436 : Protein tyrosine phosphatase type IVA 2 on eGFR
rs141716003
rs62143197
rs12380607
rs116746133
rs60653897
rs116528693
rs56113250
rs117753018
rs117458368
rs16853151
rs113494603
rs187543532
rs78928955
rs140591412
rs11782161
rs1177328
rs113573118
rs185251410
rs2025010
rs559251
rs11765248
rs7295554
rs73415612
rs738193
All − MR Egger
All − IVW(MRE)
−0.010
−0.005
0.000
0.005
0.010
MR effect size for
'Protein tyrosine phosphatase type IVA 2' on 'eGFR'

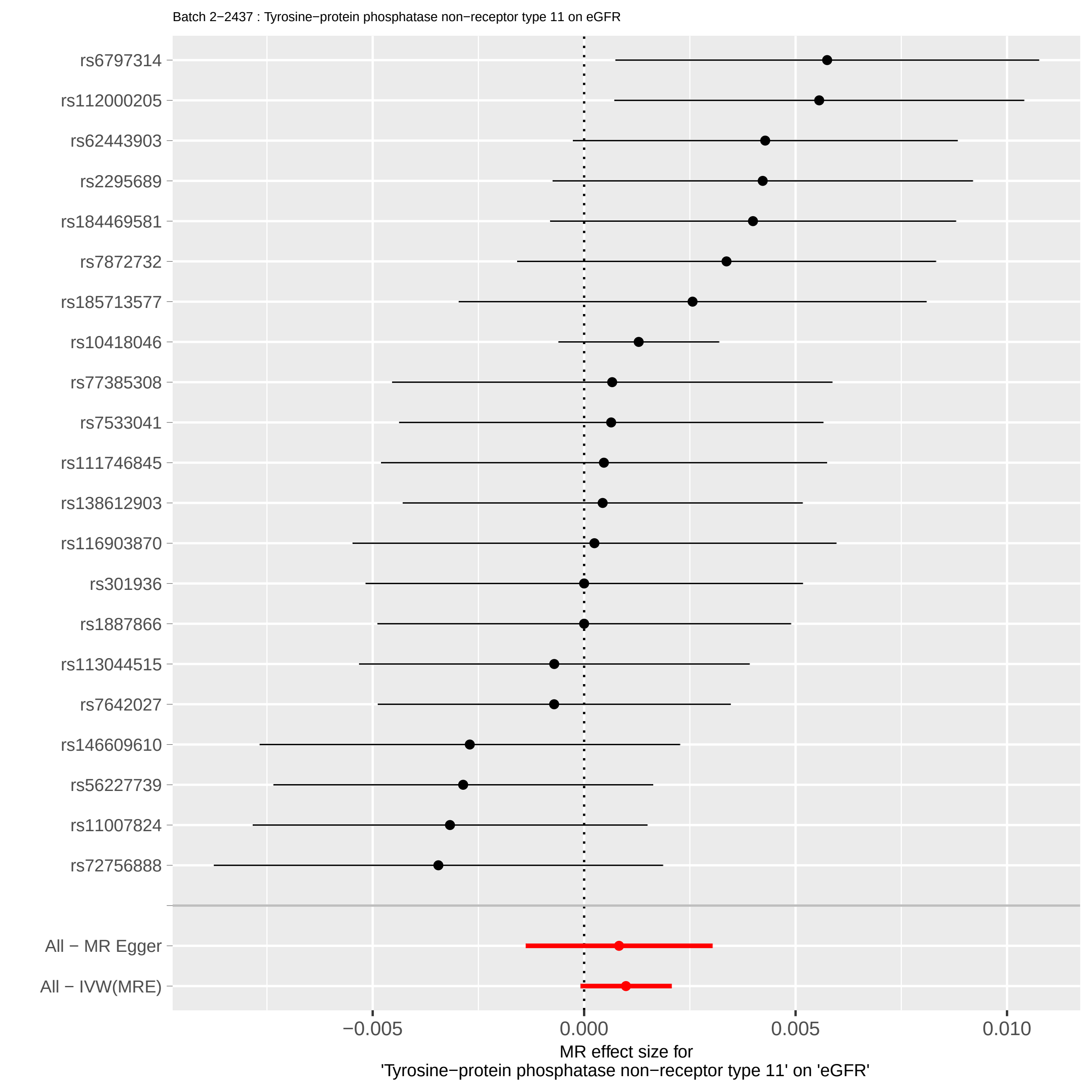

Batch 2−2437 : Tyrosine−protein phosphatase non−receptor type 11 on eGFR
rs6797314
rs112000205
rs62443903
rs2295689
rs184469581
rs7872732
rs185713577
rs10418046
rs77385308
rs7533041
rs111746845
rs138612903
rs116903870
rs301936
rs1887866
rs113044515
rs7642027
rs146609610
rs56227739
rs11007824
rs72756888
All − MR Egger
All − IVW(MRE)
−0.005
0.000
0.005
0.010
MR effect size for
'Tyrosine−protein phosphatase non−receptor type 11' on 'eGFR'

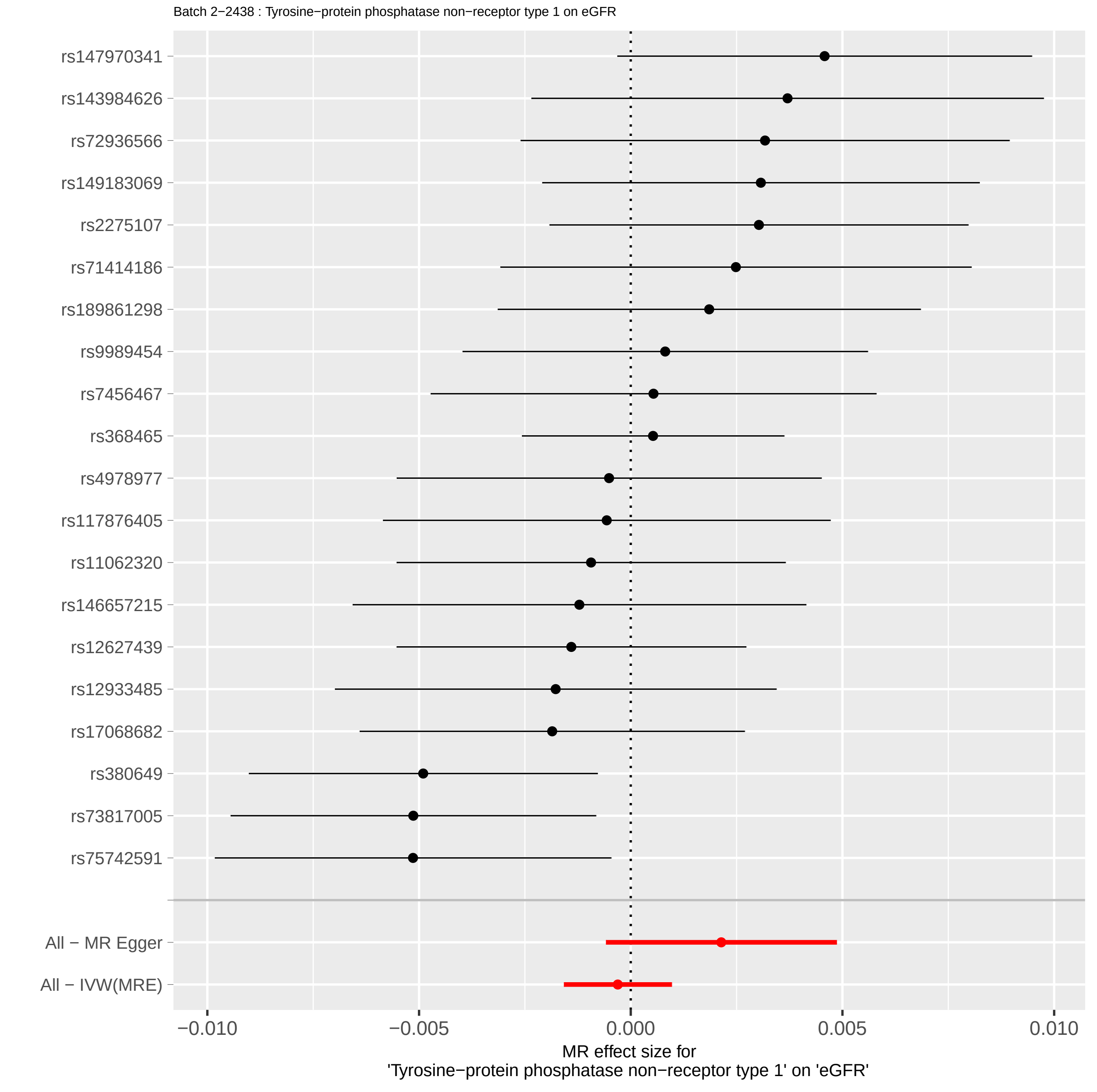

Batch 2−2438 : Tyrosine−protein phosphatase non−receptor type 1 on eGFR
rs147970341
rs143984626
rs72936566
rs149183069
rs2275107
rs71414186
rs189861298
rs9989454
rs7456467
rs368465
rs4978977
rs117876405
rs11062320
rs146657215
rs12627439
rs12933485
rs17068682
rs380649
rs73817005
rs75742591
All − MR Egger
All − IVW(MRE)
−0.010
−0.005
0.000
0.005
0.010
MR effect size for
'Tyrosine−protein phosphatase non−receptor type 1' on 'eGFR'

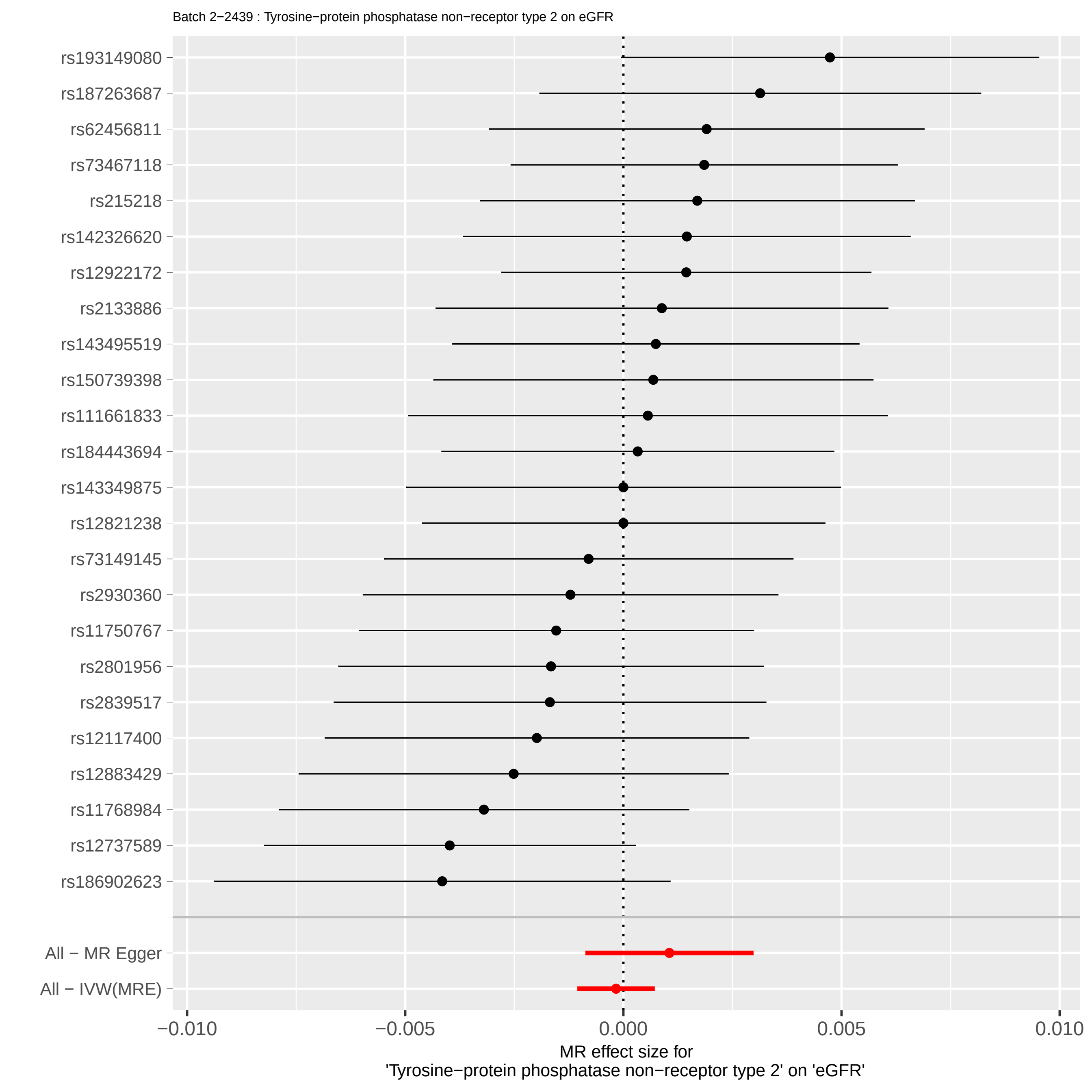

Batch 2−2439 : Tyrosine−protein phosphatase non−receptor type 2 on eGFR
rs193149080
rs187263687
rs62456811
rs73467118
rs215218
rs142326620
rs12922172
rs2133886
rs143495519
rs150739398
rs111661833
rs184443694
rs143349875
rs12821238
rs73149145
rs2930360
rs11750767
rs2801956
rs2839517
rs12117400
rs12883429
rs11768984
rs12737589
rs186902623
All − MR Egger
All − IVW(MRE)
−0.010
−0.005
0.000
0.005
0.010
MR effect size for
'Tyrosine−protein phosphatase non−receptor type 2' on 'eGFR'

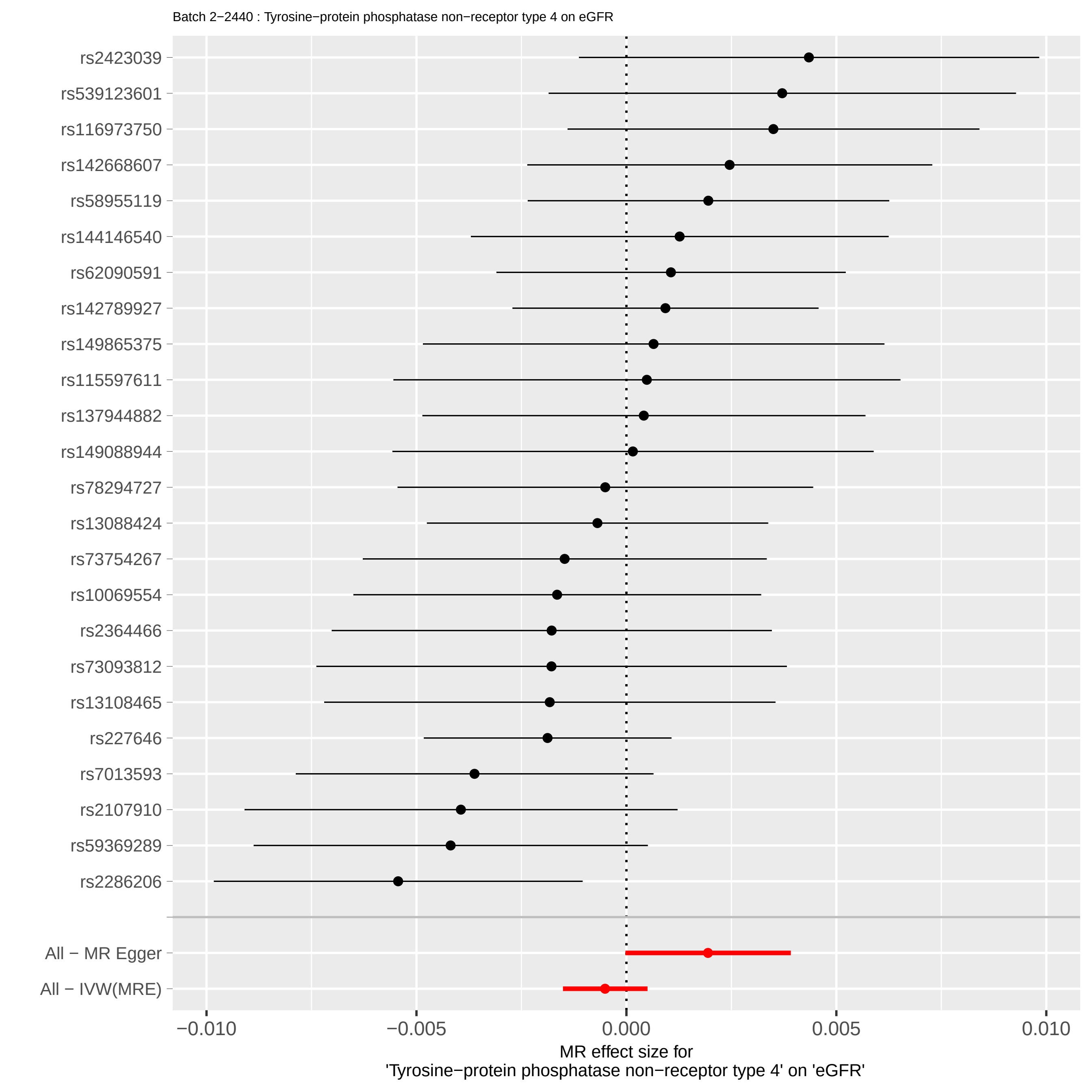
Batch 2−2440 : Tyrosine−protein phosphatase non−receptor type 4 on eGFR
rs2423039
rs539123601
rs116973750
rs142668607
rs58955119
rs144146540
rs62090591
rs142789927
rs149865375
rs115597611
rs137944882
rs149088944
rs78294727
rs13088424
rs73754267
rs10069554
rs2364466
rs73093812
rs13108465
rs227646
rs7013593
rs2107910
rs59369289
rs2286206
All − MR Egger
All − IVW(MRE)
−0.010
−0.005
0.000
0.005
0.010
MR effect size for
'Tyrosine−protein phosphatase non−receptor type 4' on 'eGFR'

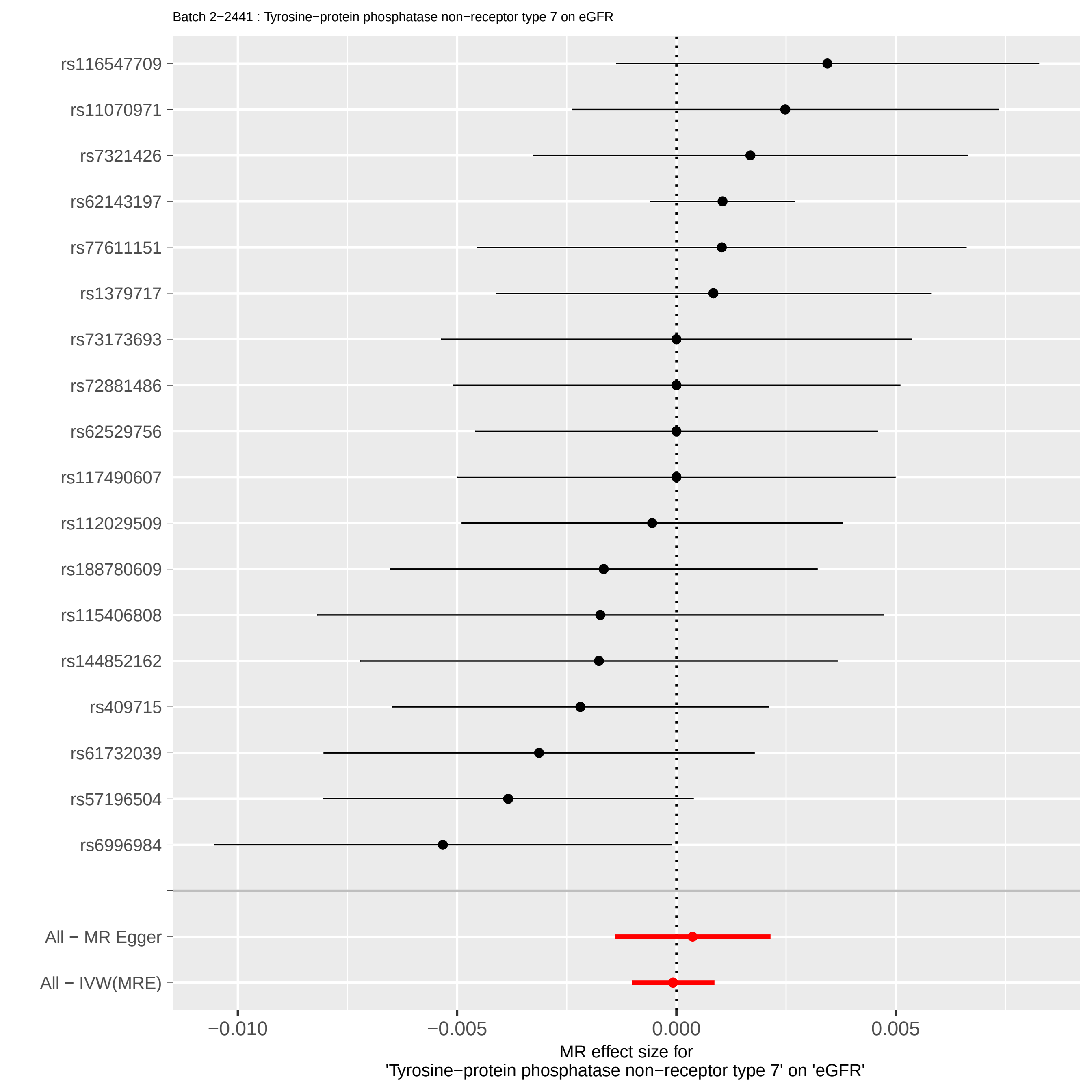
Batch 2−2441 : Tyrosine−protein phosphatase non−receptor type 7 on eGFR
rs116547709
rs11070971
rs7321426
rs62143197
rs77611151
rs1379717
rs73173693
rs72881486
rs62529756
rs117490607
rs112029509
rs188780609
rs115406808
rs144852162
rs409715
rs61732039
rs57196504
rs6996984
All − MR Egger
All − IVW(MRE)
−0.010
−0.005
0.000
0.005
MR effect size for
'Tyrosine−protein phosphatase non−receptor type 7' on 'eGFR'

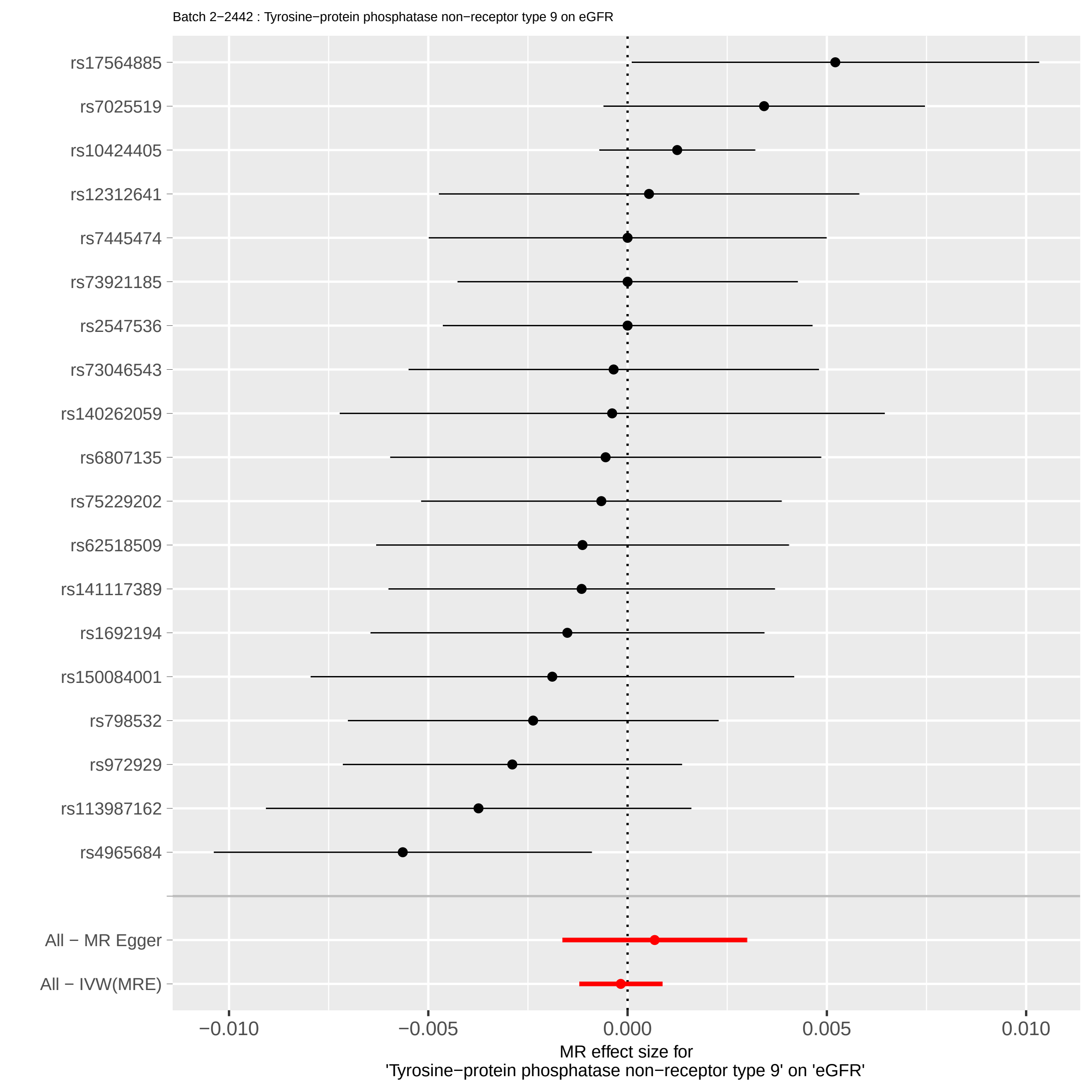

Batch 2−2442 : Tyrosine−protein phosphatase non−receptor type 9 on eGFR
rs17564885
rs7025519
rs10424405
rs12312641
rs7445474
rs73921185
rs2547536
rs73046543
rs140262059
rs6807135
rs75229202
rs62518509
rs141117389
rs1692194
rs150084001
rs798532
rs972929
rs113987162
rs4965684
All − MR Egger
All − IVW(MRE)
−0.010
−0.005
0.000
0.005
0.010
MR effect size for
'Tyrosine−protein phosphatase non−receptor type 9' on 'eGFR'

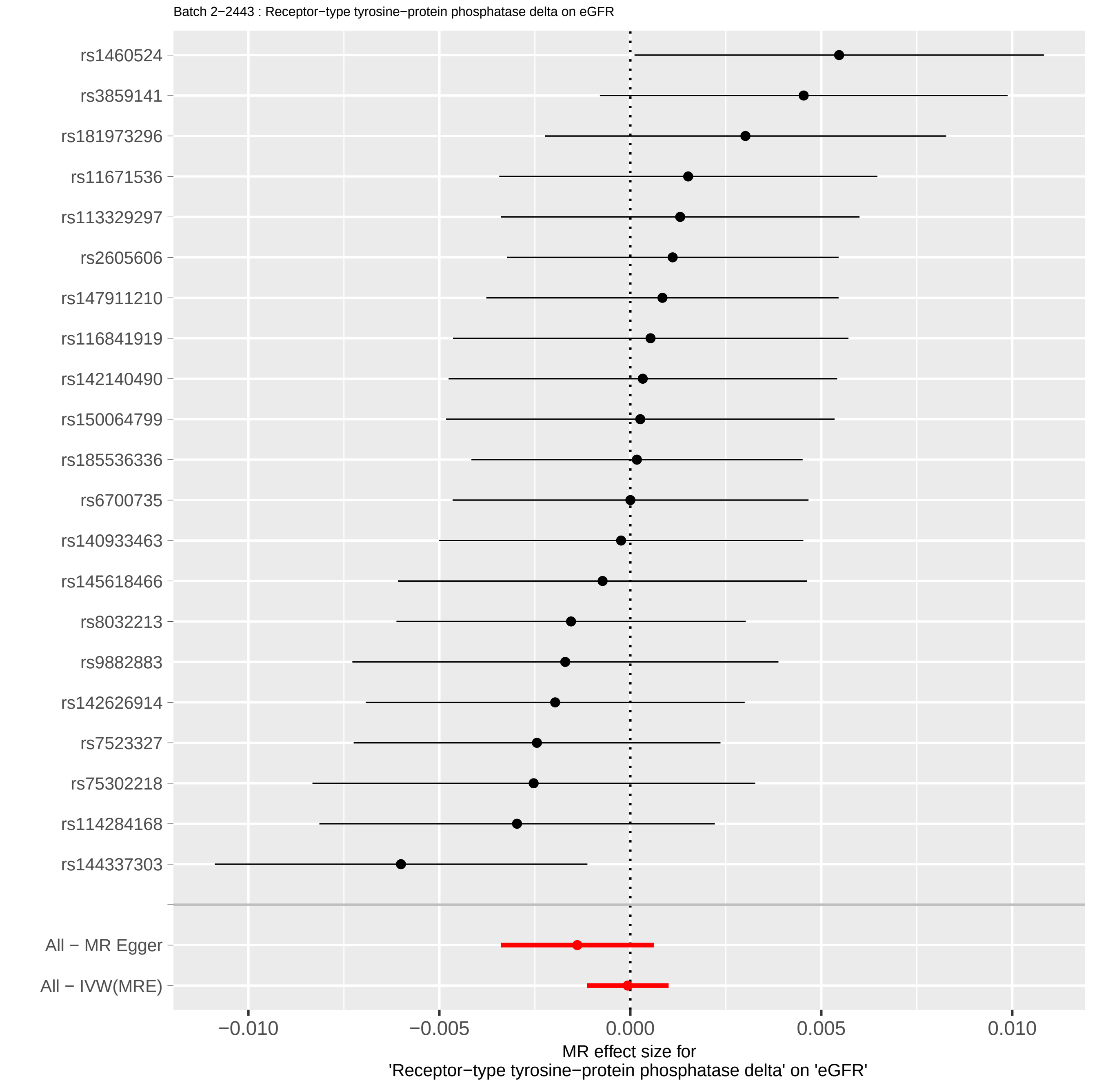

Batch 2−2443 : Receptor−type tyrosine−protein phosphatase delta on eGFR
rs1460524
rs3859141
rs181973296
rs11671536
rs113329297
rs2605606
rs147911210
rs116841919
rs142140490
rs150064799
rs185536336
rs6700735
rs140933463
rs145618466
rs8032213
rs9882883
rs142626914
rs7523327
rs75302218
rs114284168
rs144337303
All − MR Egger
All − IVW(MRE)
−0.010
−0.005
0.000
0.005
0.010
MR effect size for
'Receptor−type tyrosine−protein phosphatase delta' on 'eGFR'

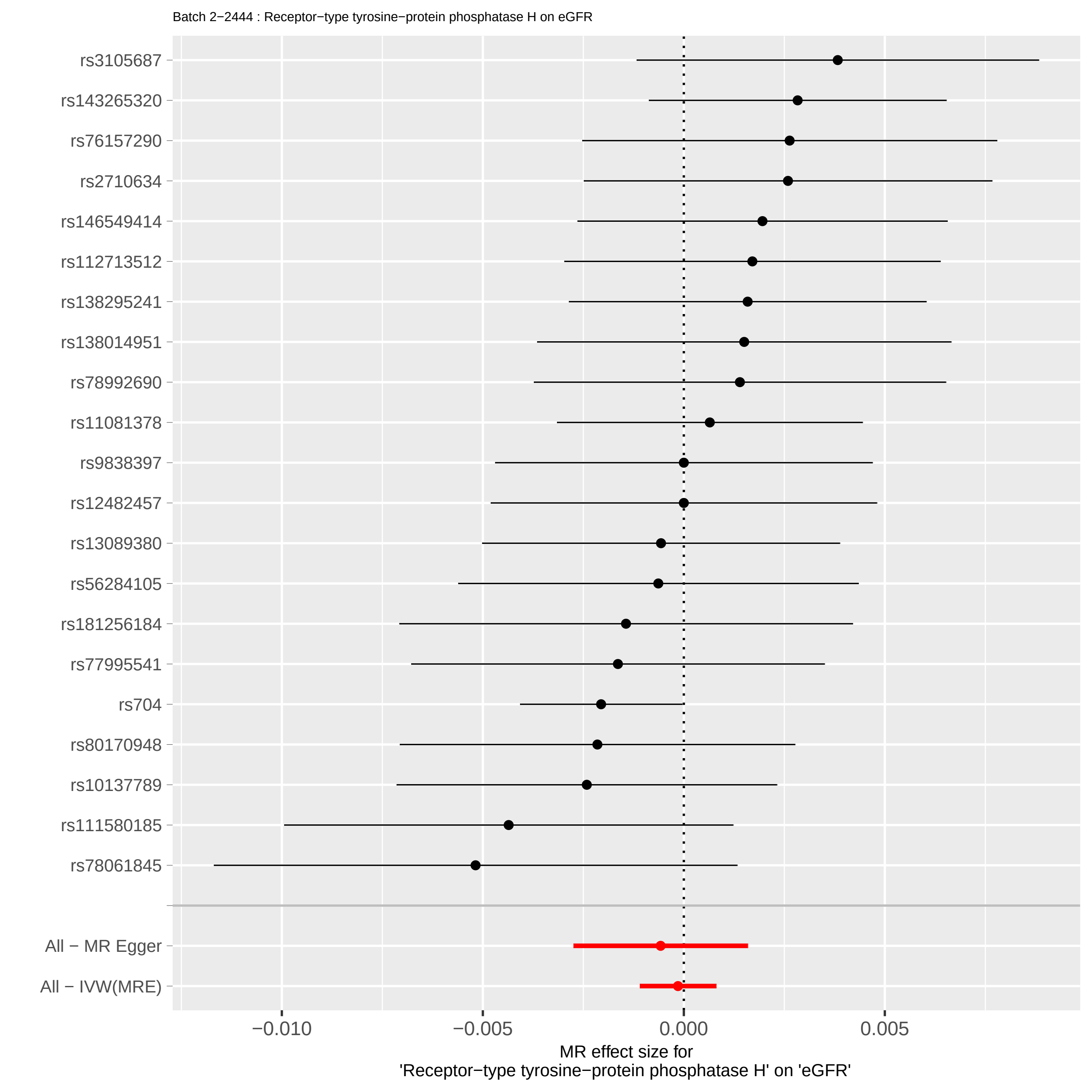

Batch 2−2444 : Receptor−type tyrosine−protein phosphatase H on eGFR
rs3105687
rs143265320
rs76157290
rs2710634
rs146549414
rs112713512
rs138295241
rs138014951
rs78992690
rs11081378
rs9838397
rs12482457
rs13089380
rs56284105
rs181256184
rs77995541
rs704
rs80170948
rs10137789
rs111580185
rs78061845
All − MR Egger
All − IVW(MRE)
−0.010
−0.005
0.000
0.005
MR effect size for
'Receptor−type tyrosine−protein phosphatase H' on 'eGFR'

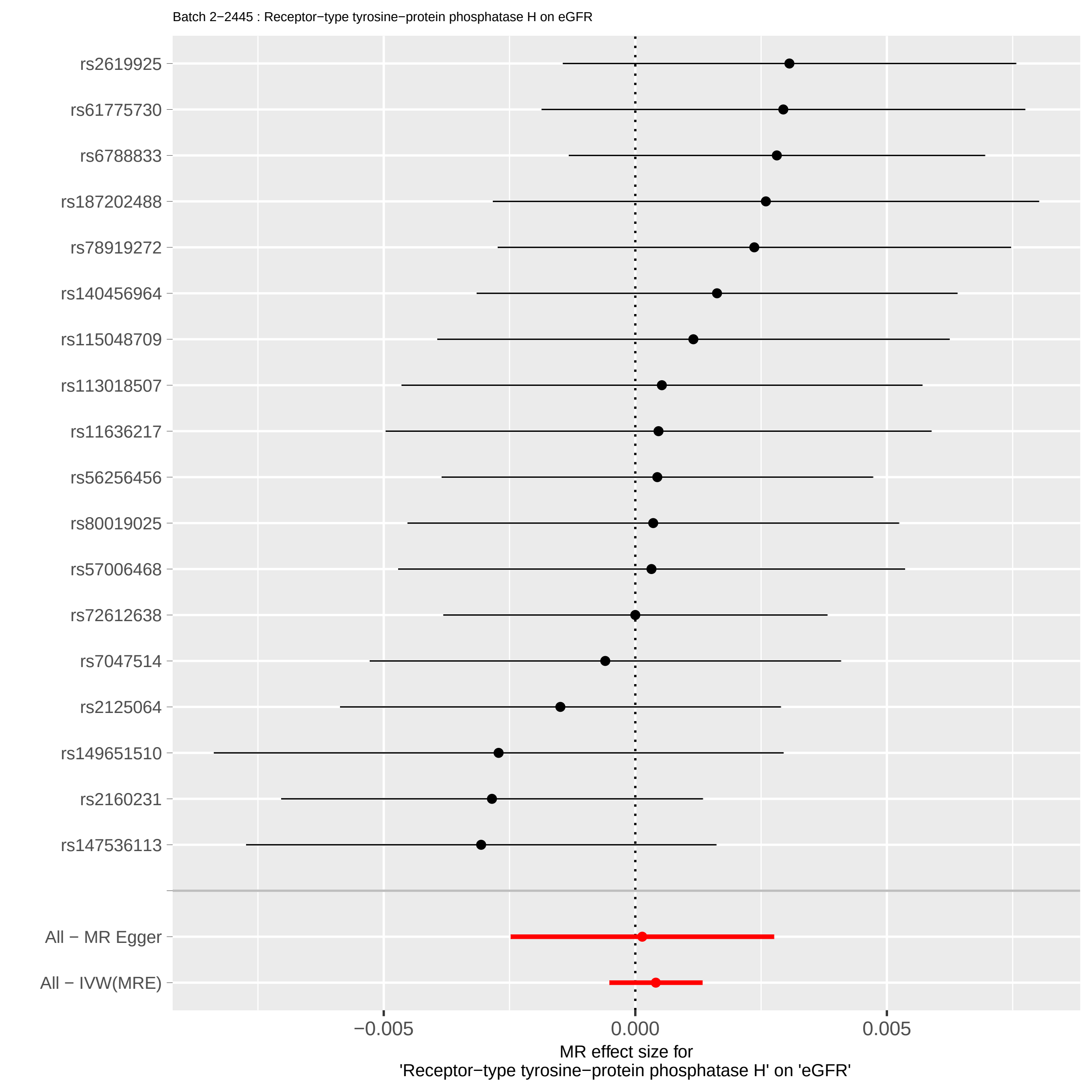
Batch 2−2445 : Receptor−type tyrosine−protein phosphatase H on eGFR
rs2619925
rs61775730
rs6788833
rs187202488
rs78919272
rs140456964
rs115048709
rs113018507
rs11636217
rs56256456
rs80019025
rs57006468
rs72612638
rs7047514
rs2125064
rs149651510
rs2160231
rs147536113
All − MR Egger
All − IVW(MRE)
−0.005
0.000
0.005
MR effect size for
'Receptor−type tyrosine−protein phosphatase H' on 'eGFR'

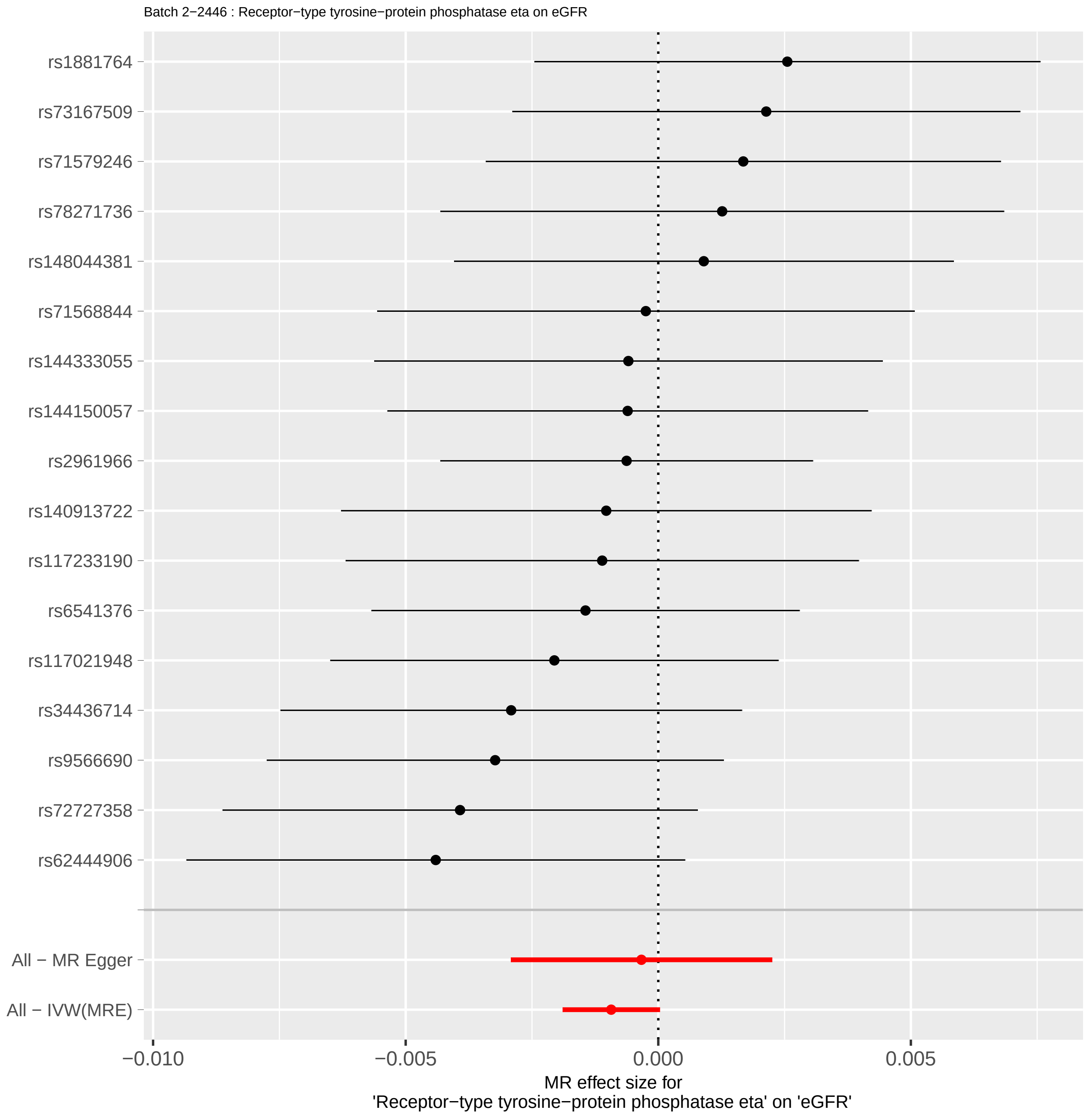
Batch 2−2446 : Receptor−type tyrosine−protein phosphatase eta on eGFR
rs1881764
rs73167509
rs71579246
rs78271736
rs148044381
rs71568844
rs144333055
rs144150057
rs2961966
rs140913722
rs117233190
rs6541376
rs117021948
rs34436714
rs9566690
rs72727358
rs62444906
All − MR Egger
All − IVW(MRE)
−0.010
−0.005
0.000
0.005
MR effect size for
'Receptor−type tyrosine−protein phosphatase eta' on 'eGFR'

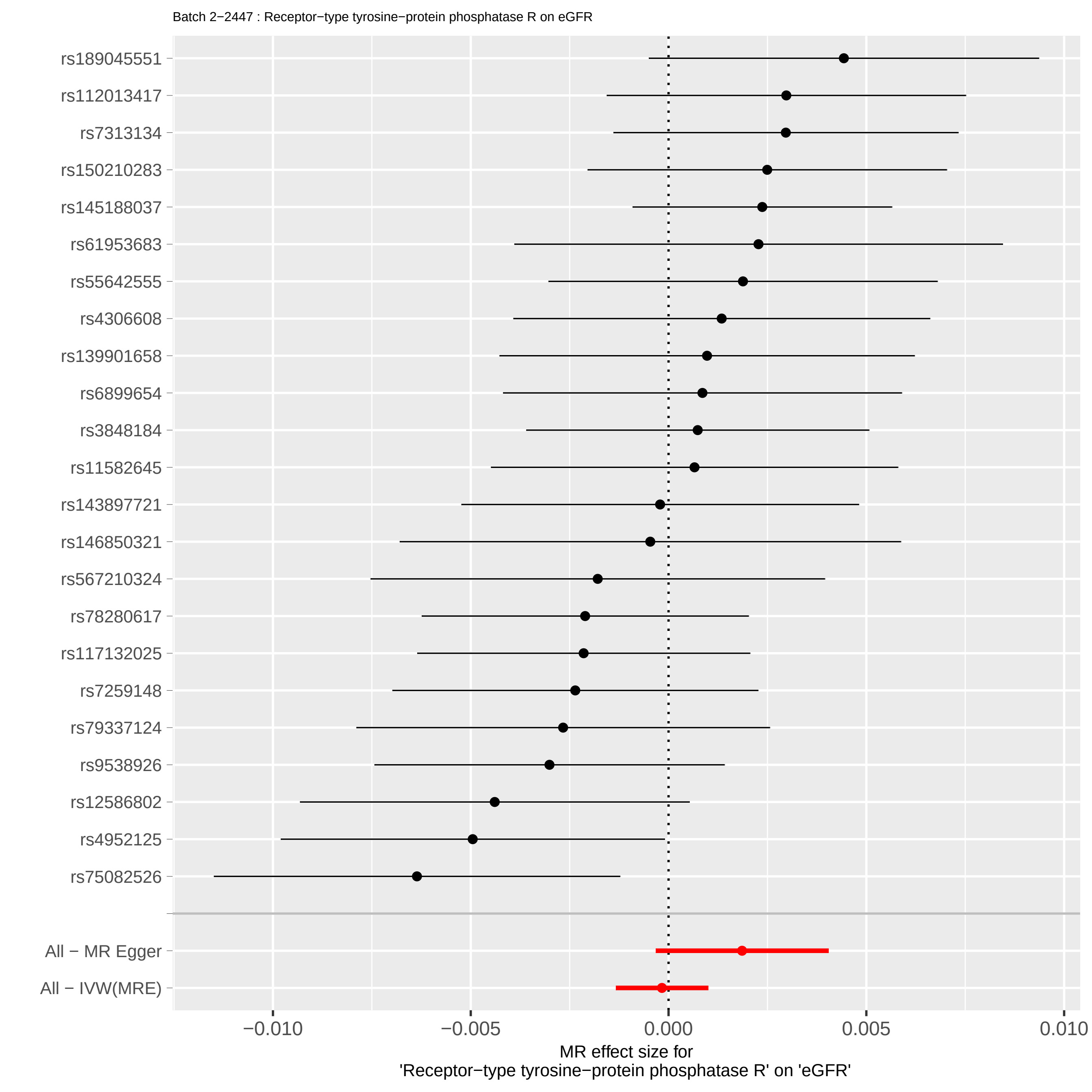

Batch 2−2447 : Receptor−type tyrosine−protein phosphatase R on eGFR
rs189045551
rs112013417
rs7313134
rs150210283
rs145188037
rs61953683
rs55642555
rs4306608
rs139901658
rs6899654
rs3848184
rs11582645
rs143897721
rs146850321
rs567210324
rs78280617
rs117132025
rs7259148
rs79337124
rs9538926
rs12586802
rs4952125
rs75082526
All − MR Egger
All − IVW(MRE)
−0.010
−0.005
0.000
0.005
0.010
MR effect size for
'Receptor−type tyrosine−protein phosphatase R' on 'eGFR'

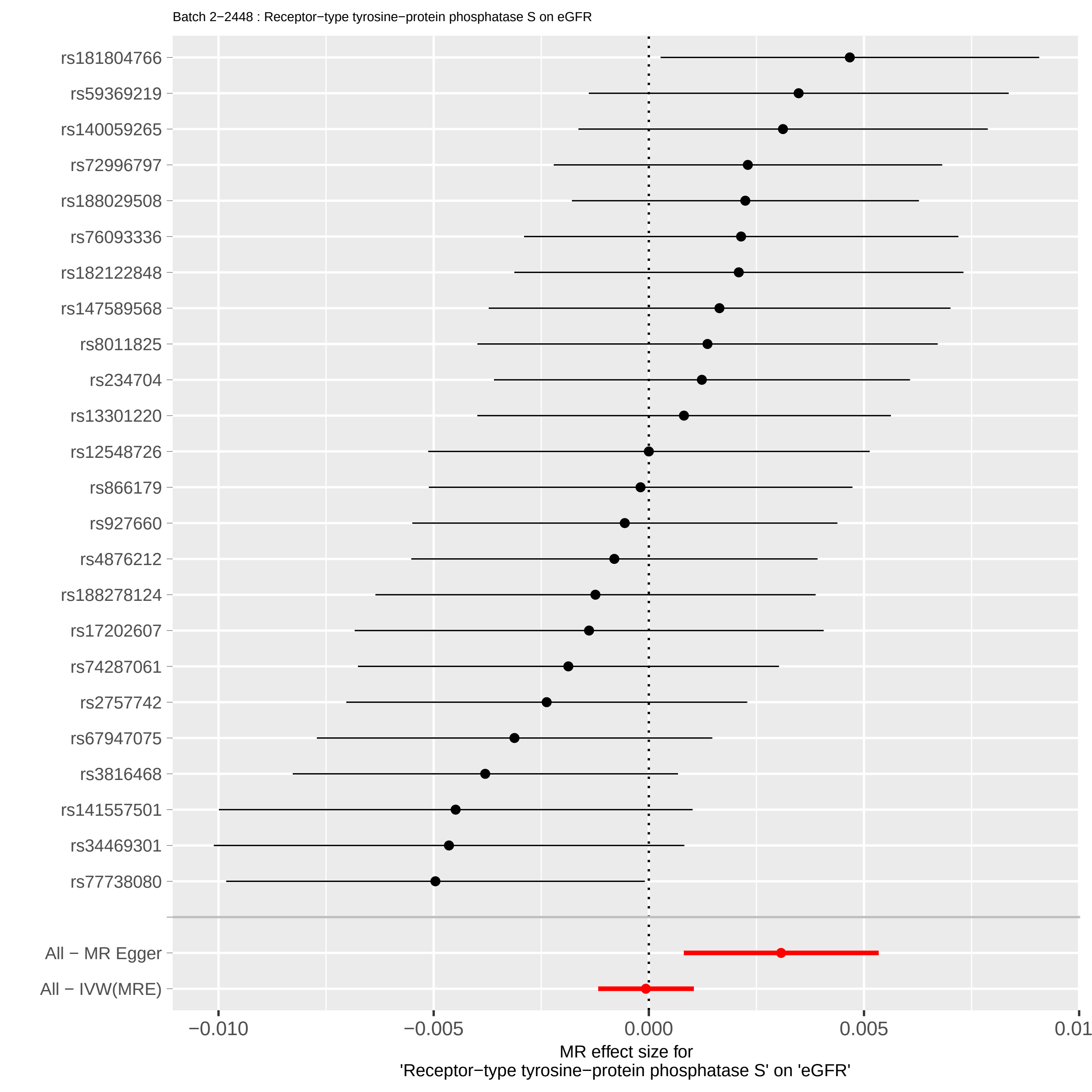

Batch 2−2448 : Receptor−type tyrosine−protein phosphatase S on eGFR
rs181804766
rs59369219
rs140059265
rs72996797
rs188029508
rs76093336
rs182122848
rs147589568
rs8011825
rs234704
rs13301220
rs12548726
rs866179
rs927660
rs4876212
rs188278124
rs17202607
rs74287061
rs2757742
rs67947075
rs3816468
rs141557501
rs34469301
rs77738080
All − MR Egger
All − IVW(MRE)
−0.010
−0.005
0.000
0.005
0.01
MR effect size for
'Receptor−type tyrosine−protein phosphatase S' on 'eGFR'

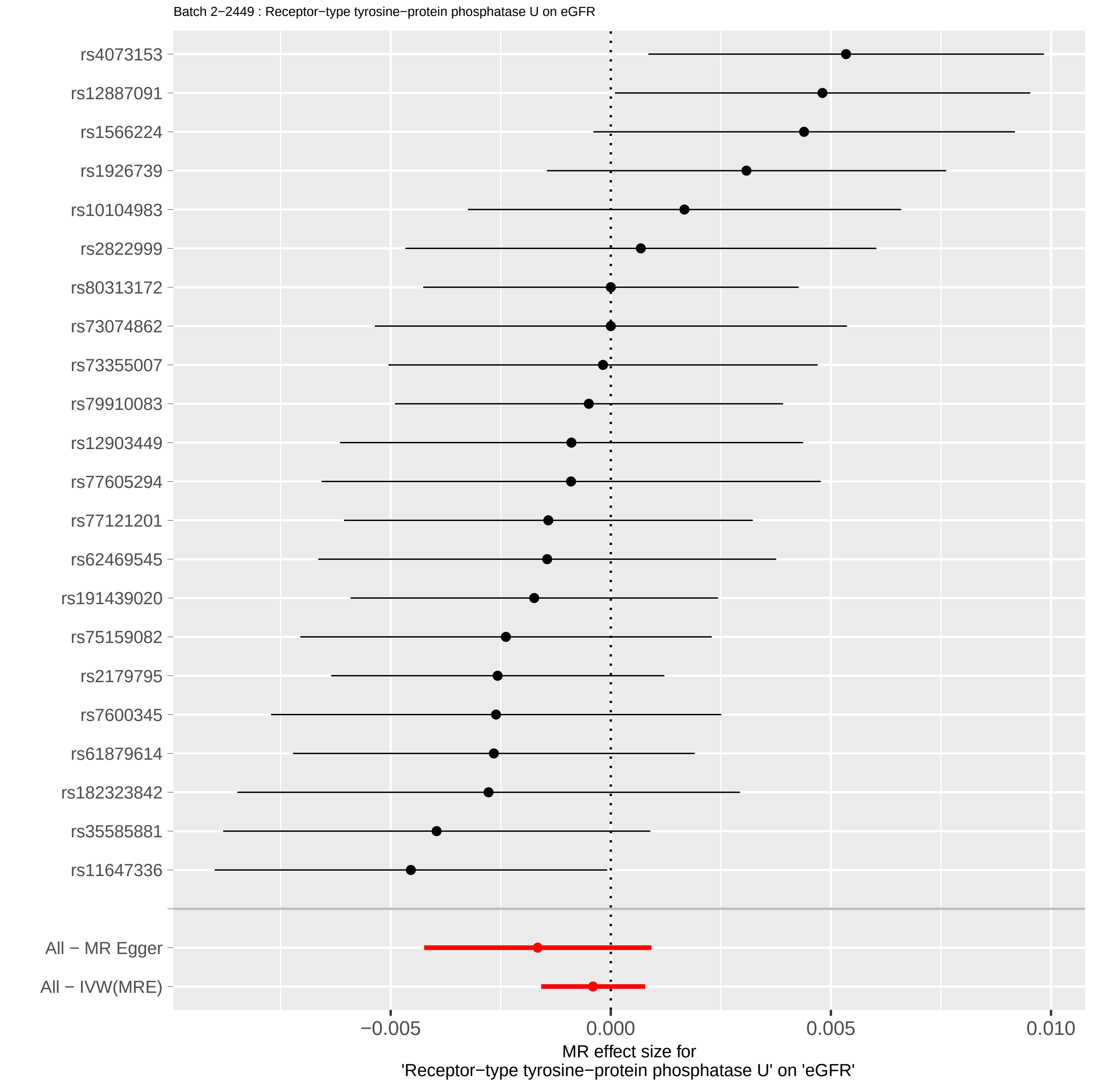

Batch 2−2449 : Receptor−type tyrosine−protein phosphatase U on eGFR
rs4073153
rs12887091
rs1566224
rs1926739
rs10104983
rs2822999
rs80313172
rs73074862
rs73355007
rs79910083
rs12903449
rs77605294
rs77121201
rs62469545
rs191439020
rs75159082
rs2179795
rs7600345
rs61879614
rs182323842
rs35585881
rs11647336
All − MR Egger
All − IVW(MRE)
−0.005
0.000
0.005
0.010
MR effect size for
'Receptor−type tyrosine−protein phosphatase U' on 'eGFR'

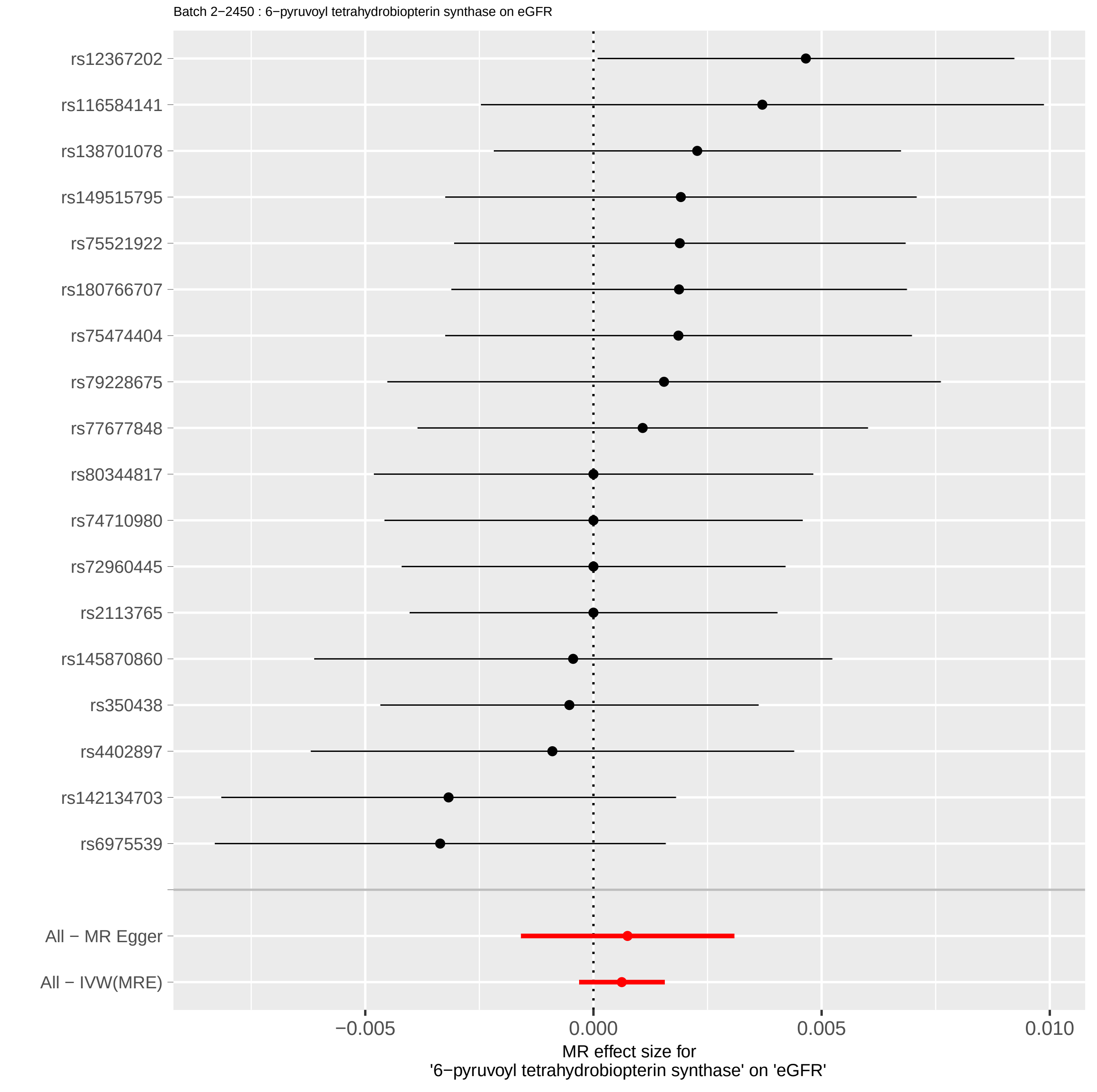

Batch 2−2450 : 6−pyruvoyl tetrahydrobiopterin synthase on eGFR
rs12367202
rs116584141
rs138701078
rs149515795
rs75521922
rs180766707
rs75474404
rs79228675
rs77677848
rs80344817
rs74710980
rs72960445
rs2113765
rs145870860
rs350438
rs4402897
rs142134703
rs6975539
All − MR Egger
All − IVW(MRE)
−0.005
0.000
0.005
0.010
MR effect size for
'6−pyruvoyl tetrahydrobiopterin synthase' on 'eGFR'

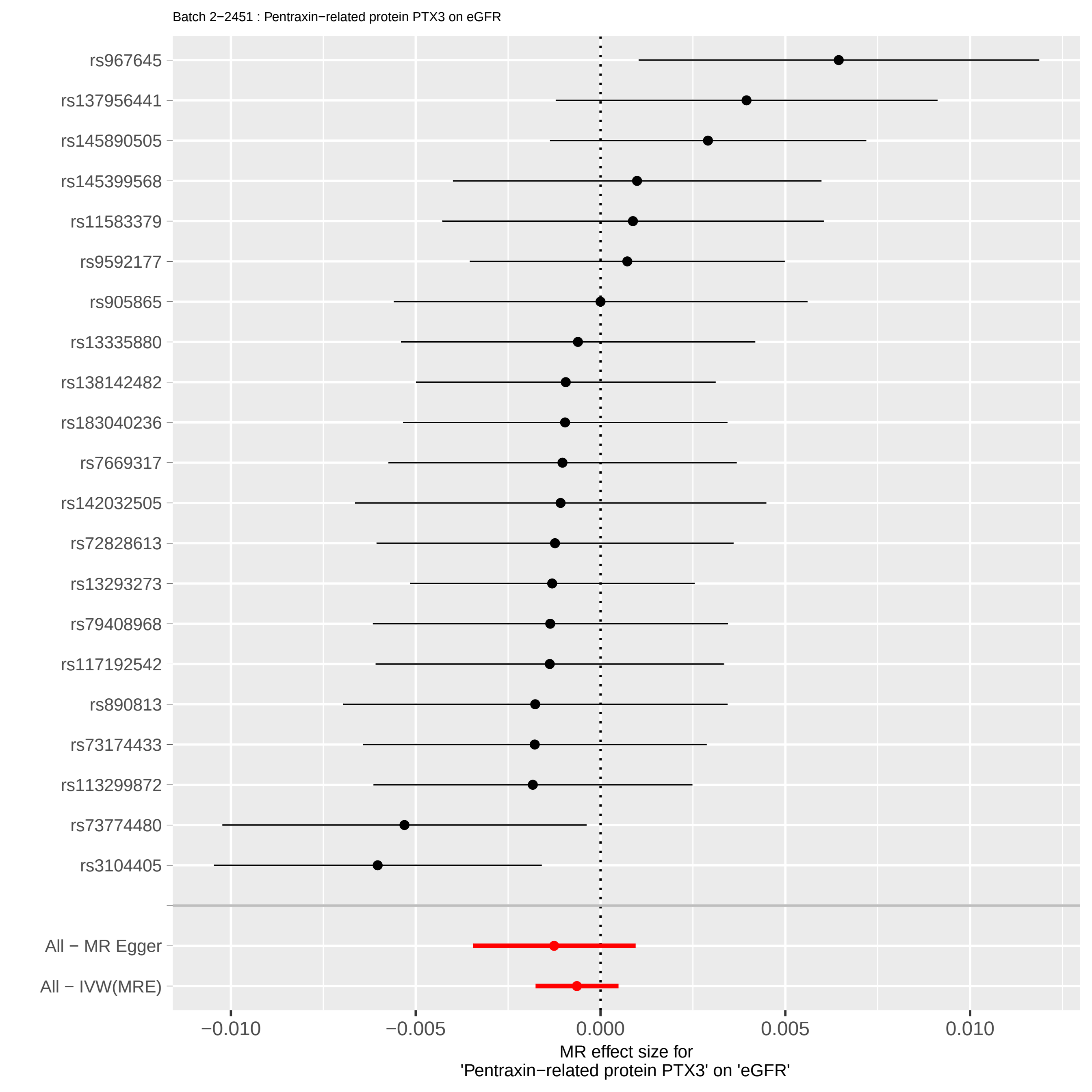

Batch 2−2451 : Pentraxin−related protein PTX3 on eGFR
rs967645
rs137956441
rs145890505
rs145399568
rs11583379
rs9592177
rs905865
rs13335880
rs138142482
rs183040236
rs7669317
rs142032505
rs72828613
rs13293273
rs79408968
rs117192542
rs890813
rs73174433
rs113299872
rs73774480
rs3104405
All − MR Egger
All − IVW(MRE)
−0.010
−0.005
0.000
0.005
0.010
MR effect size for
'Pentraxin−related protein PTX3' on 'eGFR'

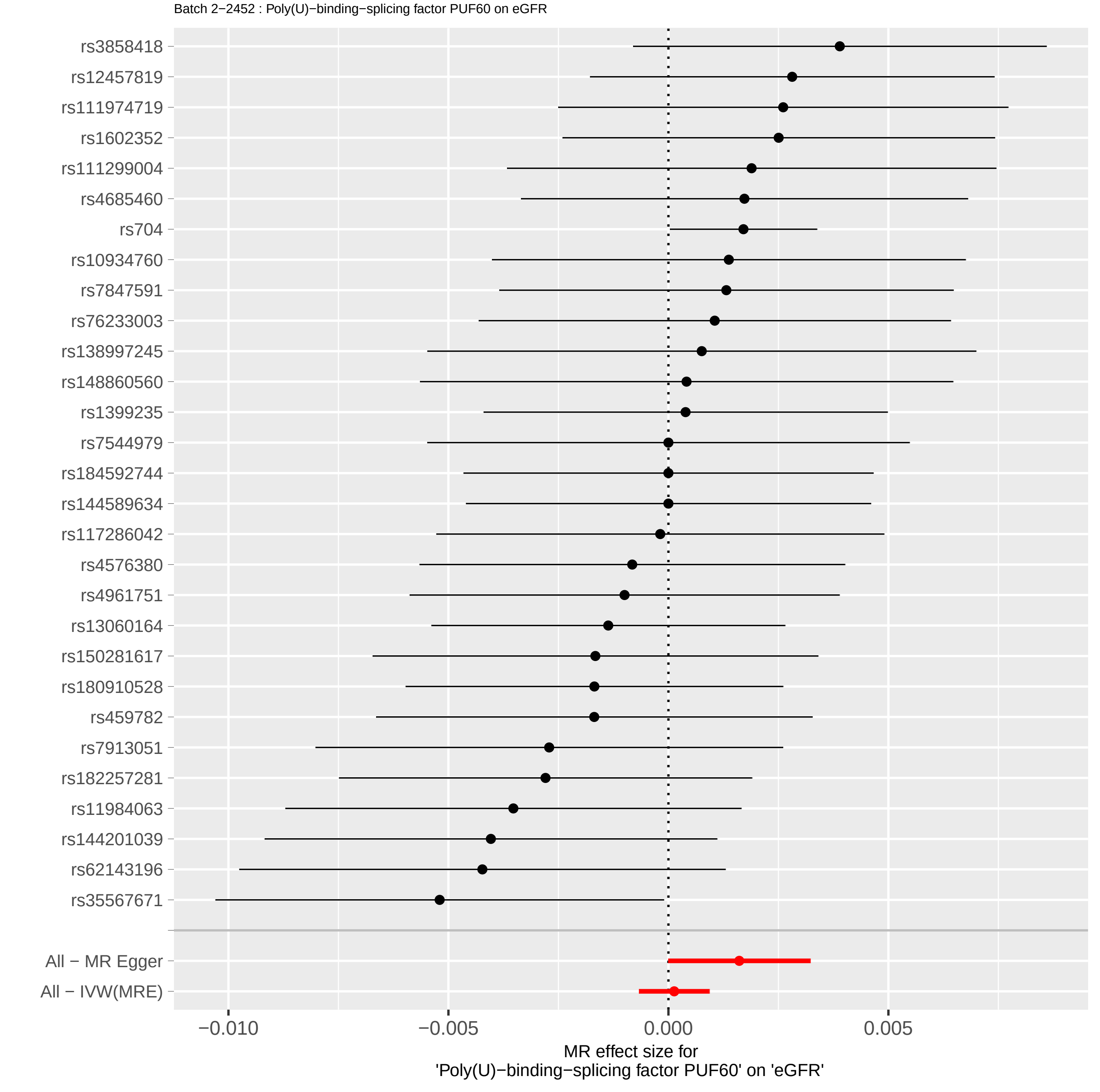

Batch 2−2452 : Poly(U)−binding−splicing factor PUF60 on eGFR
rs3858418
rs12457819
rs111974719
rs1602352
rs111299004
rs4685460
rs704
rs10934760
rs7847591
rs76233003
rs138997245
rs148860560
rs1399235
rs7544979
rs184592744
rs144589634
rs117286042
rs4576380
rs4961751
rs13060164
rs150281617
rs180910528
rs459782
rs7913051
rs182257281
rs11984063
rs144201039
rs62143196
rs35567671
All − MR Egger
All − IVW(MRE)
−0.010
−0.005
0.000
0.005
MR effect size for
'Poly(U)−binding−splicing factor PUF60' on 'eGFR'

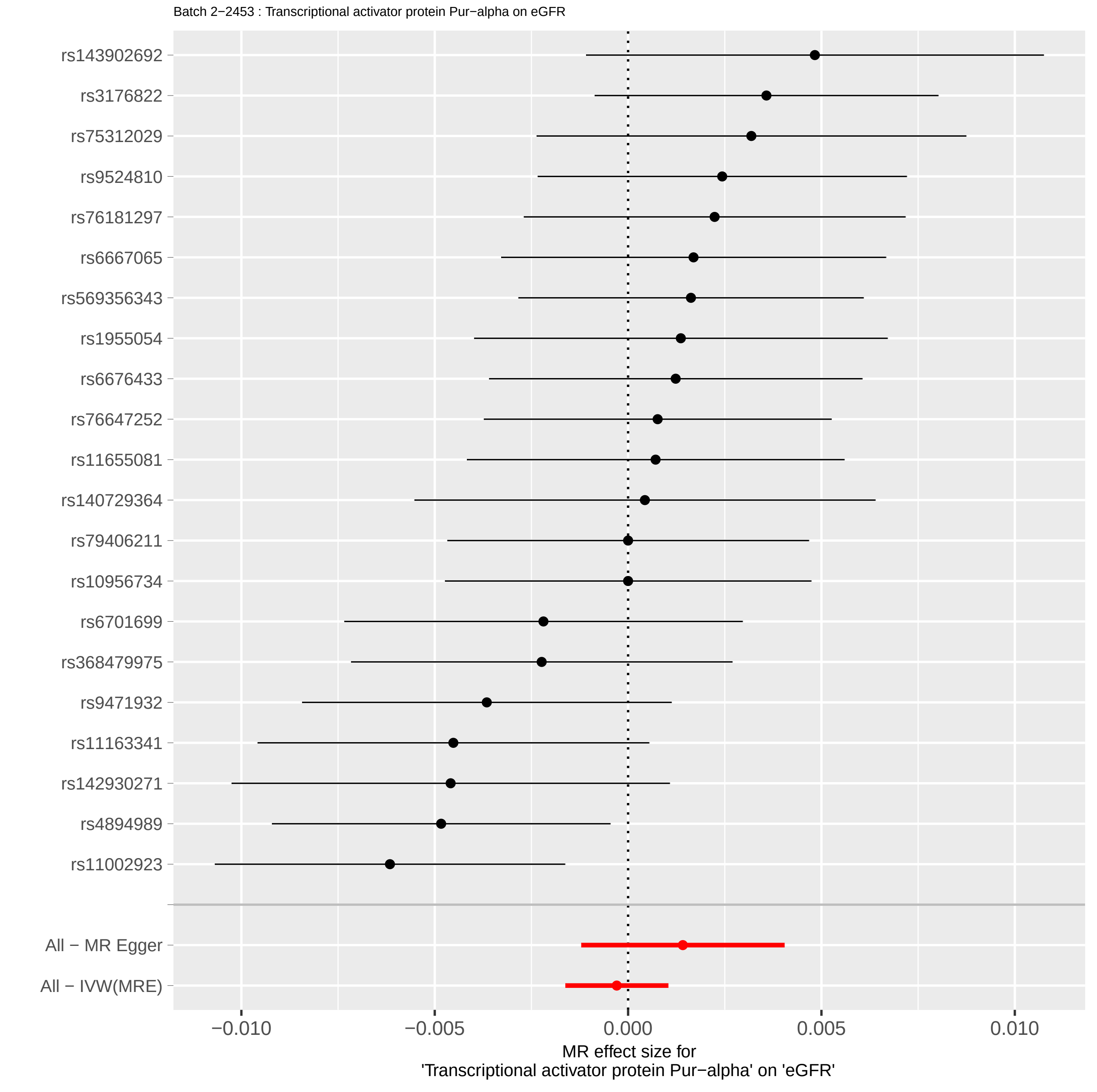

Batch 2−2453 : Transcriptional activator protein Pur−alpha on eGFR
rs143902692
rs3176822
rs75312029
rs9524810
rs76181297
rs6667065
rs569356343
rs1955054
rs6676433
rs76647252
rs11655081
rs140729364
rs79406211
rs10956734
rs6701699
rs368479975
rs9471932
rs11163341
rs142930271
rs4894989
rs11002923
All − MR Egger
All − IVW(MRE)
−0.010
−0.005
0.000
0.005
0.010
MR effect size for
'Transcriptional activator protein Pur−alpha' on 'eGFR'

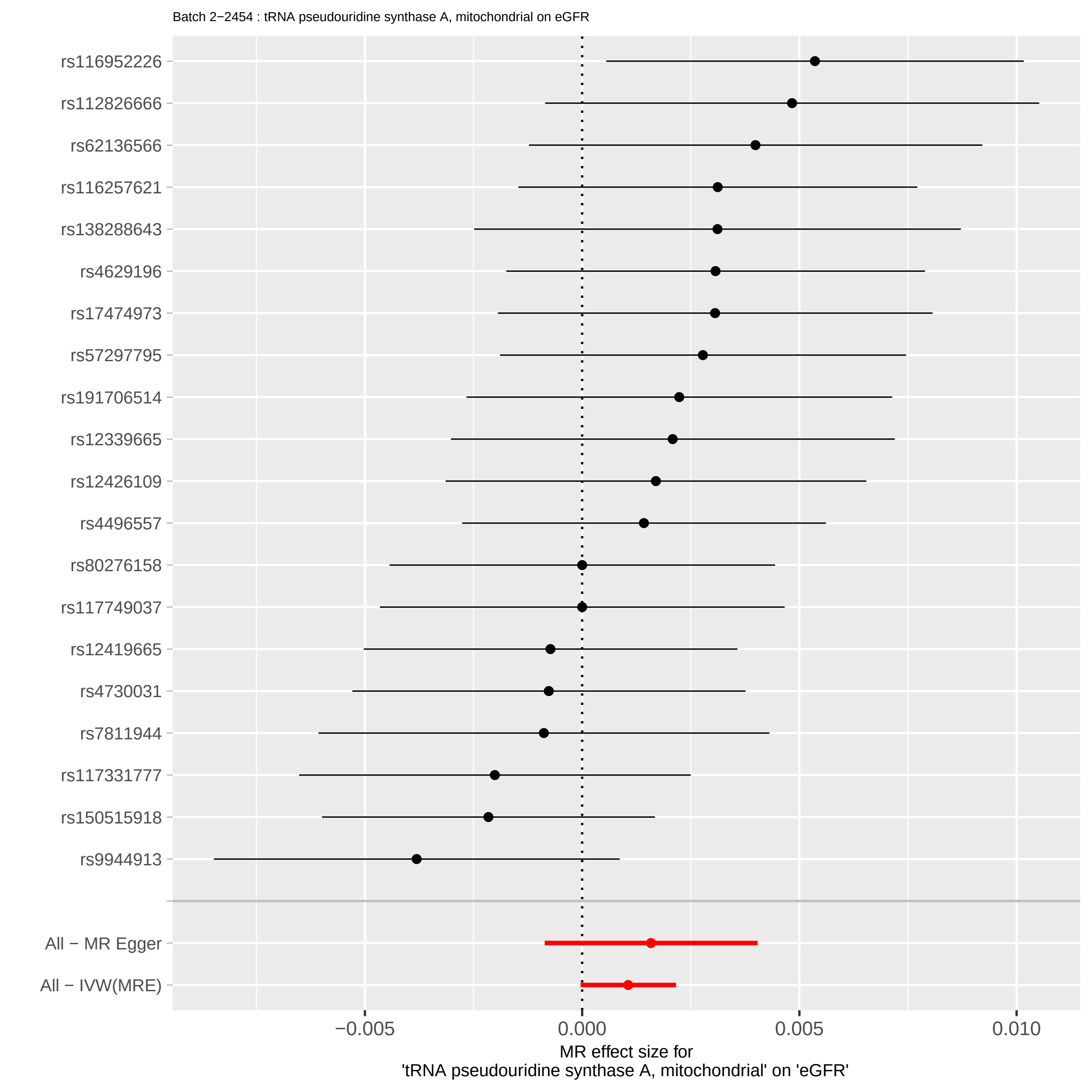

Batch 2−2454 : tRNA pseudouridine synthase A, mitochondrial on eGFR
rs116952226
rs112826666
rs62136566
rs116257621
rs138288643
rs4629196
rs17474973
rs57297795
rs191706514
rs12339665
rs12426109
rs4496557
rs80276158
rs117749037
rs12419665
rs4730031
rs7811944
rs117331777
rs150515918
rs9944913
All − MR Egger
All − IVW(MRE)
−0.005
0.000
0.005
0.010
MR effect size for
'tRNA pseudouridine synthase A, mitochondrial' on 'eGFR'

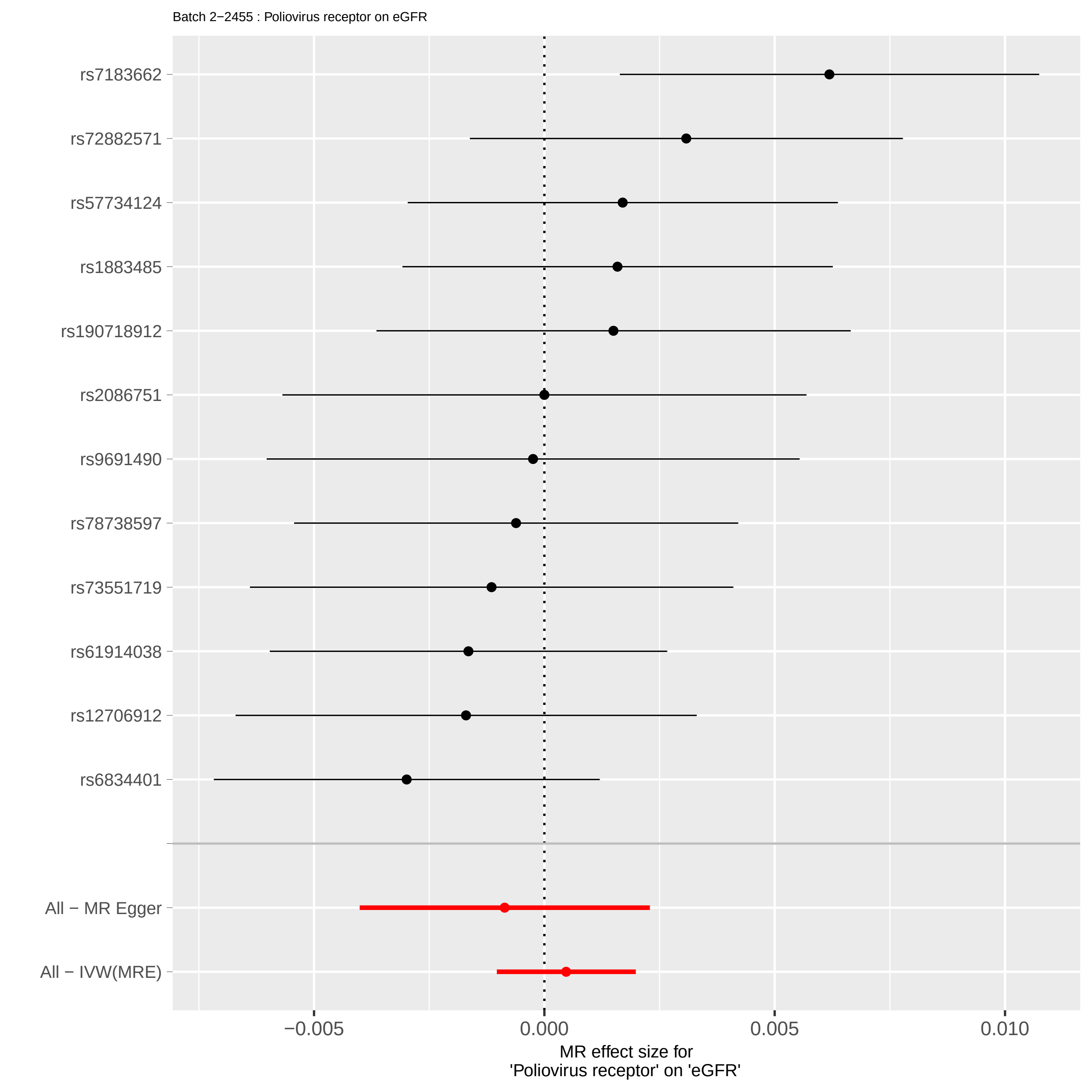

Batch 2−2455 : Poliovirus receptor on eGFR
rs7183662
rs72882571
rs57734124
rs1883485
rs190718912
rs2086751
rs9691490
rs78738597
rs73551719
rs61914038
rs12706912
rs6834401
All − MR Egger
All − IVW(MRE)
−0.005
0.000
0.005
0.010
MR effect size for
'Poliovirus receptor' on 'eGFR'

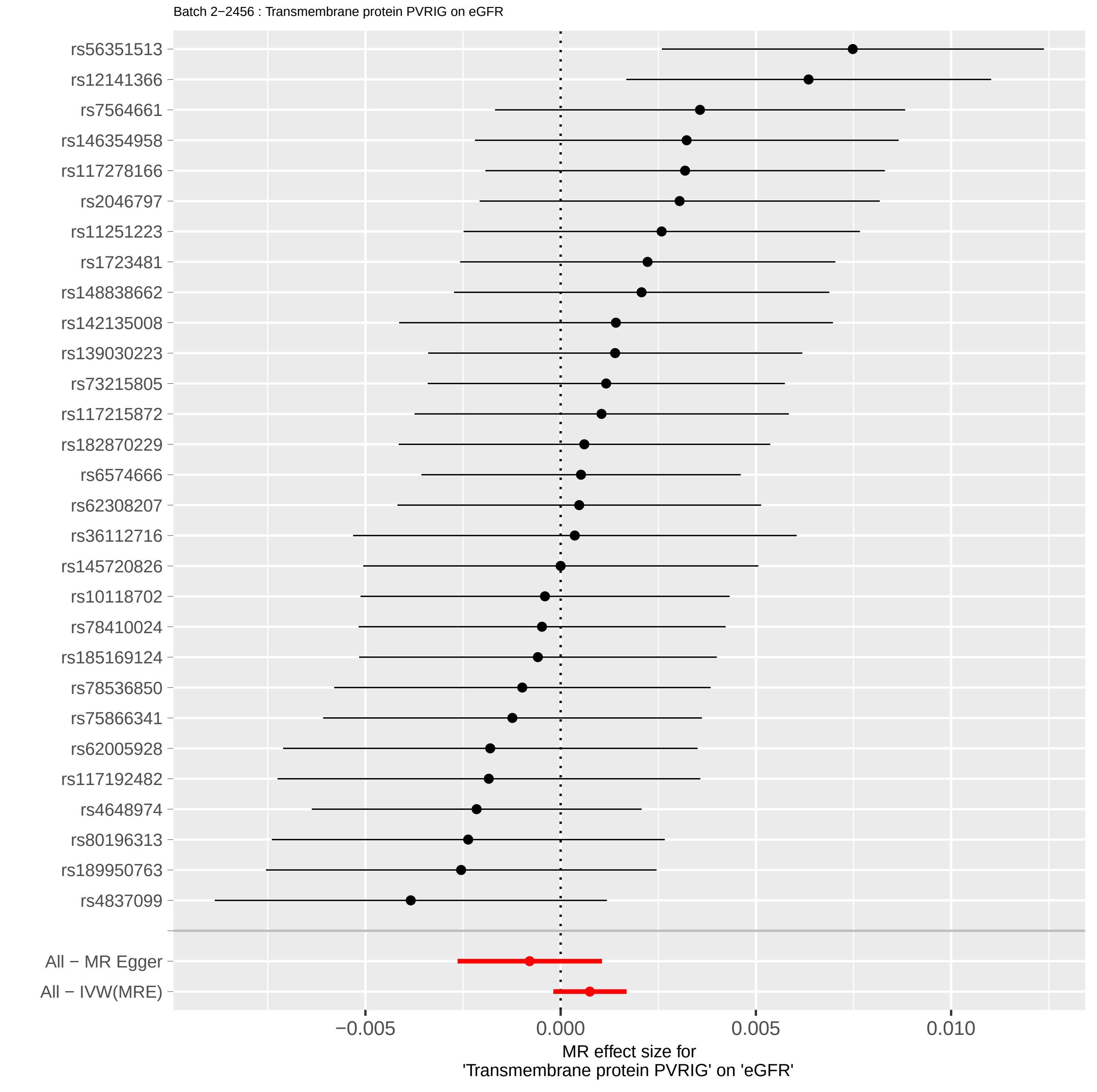
Batch 2−2456 : Transmembrane protein PVRIG on eGFR
rs56351513
rs12141366
rs7564661
rs146354958
rs117278166
rs2046797
rs11251223
rs1723481
rs148838662
rs142135008
rs139030223
rs73215805
rs117215872
rs182870229
rs6574666
rs62308207
rs36112716
rs145720826
rs10118702
rs78410024
rs185169124
rs78536850
rs75866341
rs62005928
rs117192482
rs4648974
rs80196313
rs189950763
rs4837099
All − MR Egger
All − IVW(MRE)
−0.005
0.000
0.005
0.010
MR effect size for
'Transmembrane protein PVRIG' on 'eGFR'

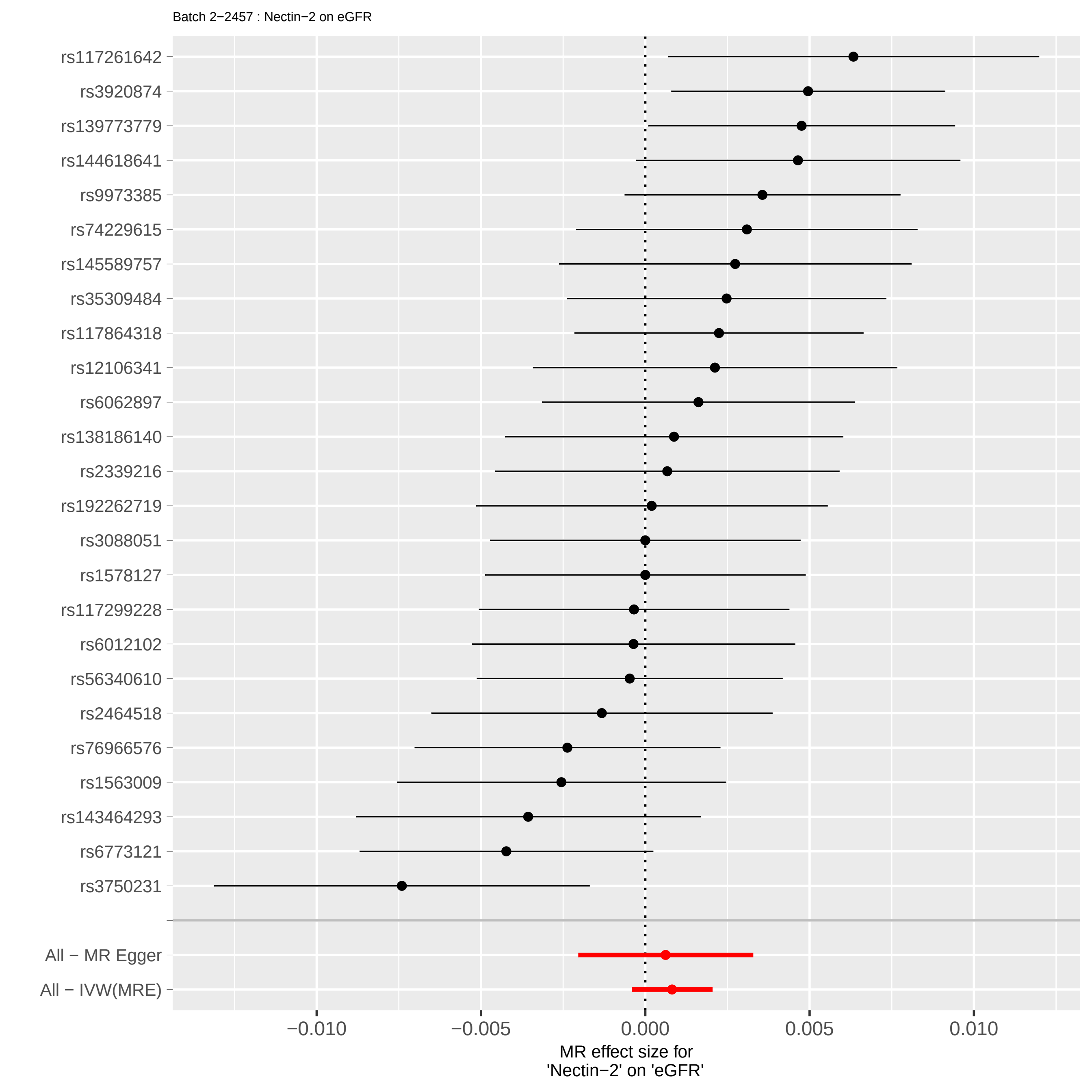
Batch 2−2457 : Nectin−2 on eGFR
rs117261642
rs3920874
rs139773779
rs144618641
rs9973385
rs74229615
rs145589757
rs35309484
rs117864318
rs12106341
rs6062897
rs138186140
rs2339216
rs192262719
rs3088051
rs1578127
rs117299228
rs6012102
rs56340610
rs2464518
rs76966576
rs1563009
rs143464293
rs6773121
rs3750231
All − MR Egger
All − IVW(MRE)
−0.010
−0.005
0.000
0.005
0.010
MR effect size for
'Nectin−2' on 'eGFR'

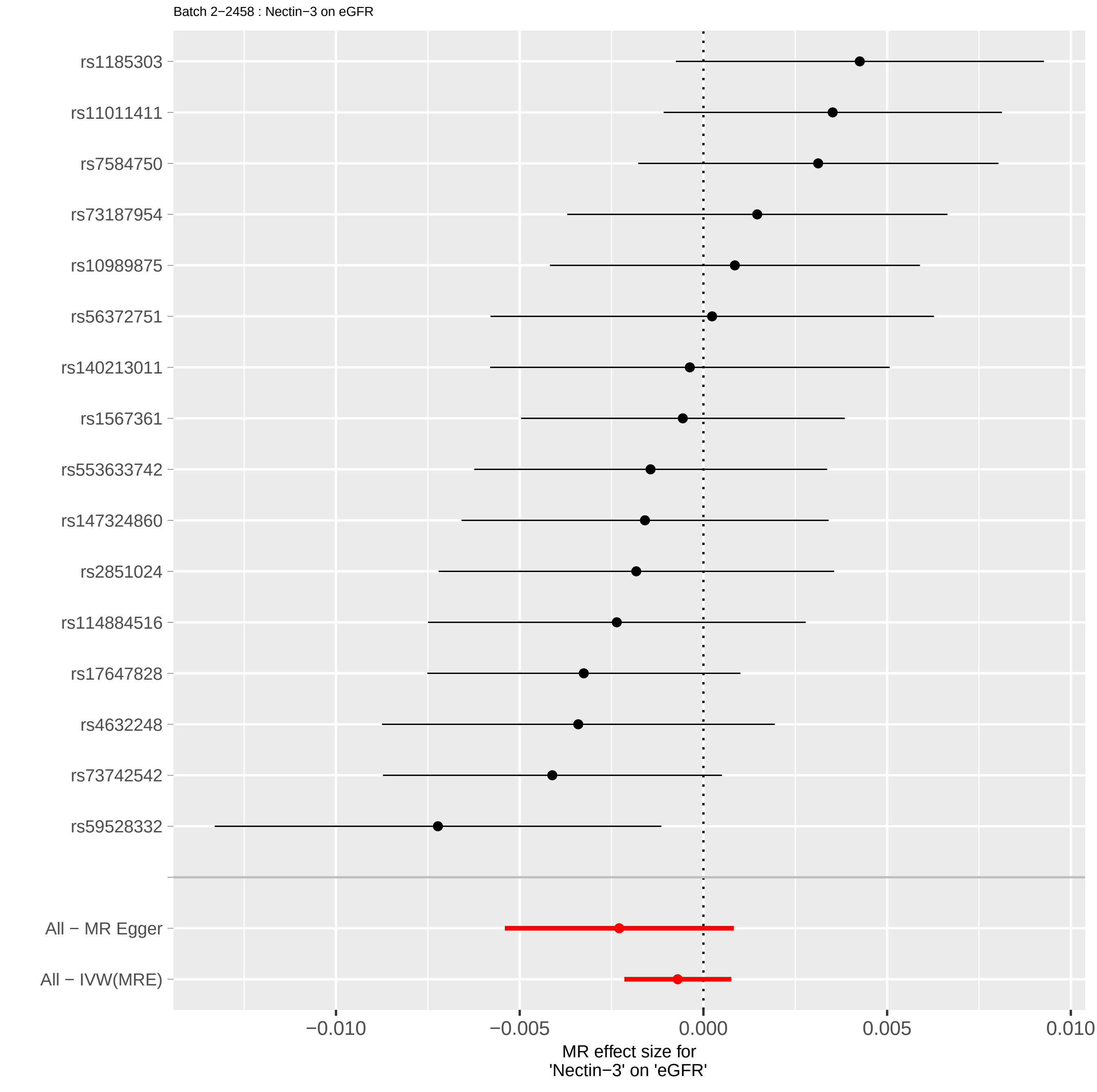

Batch 2−2458 : Nectin−3 on eGFR
rs1185303
rs11011411
rs7584750
rs73187954
rs10989875
rs56372751
rs140213011
rs1567361
rs553633742
rs147324860
rs2851024
rs114884516
rs17647828
rs4632248
rs73742542
rs59528332
All − MR Egger
All − IVW(MRE)
−0.010
−0.005
0.000
0.005
0.010
MR effect size for
'Nectin−3' on 'eGFR'

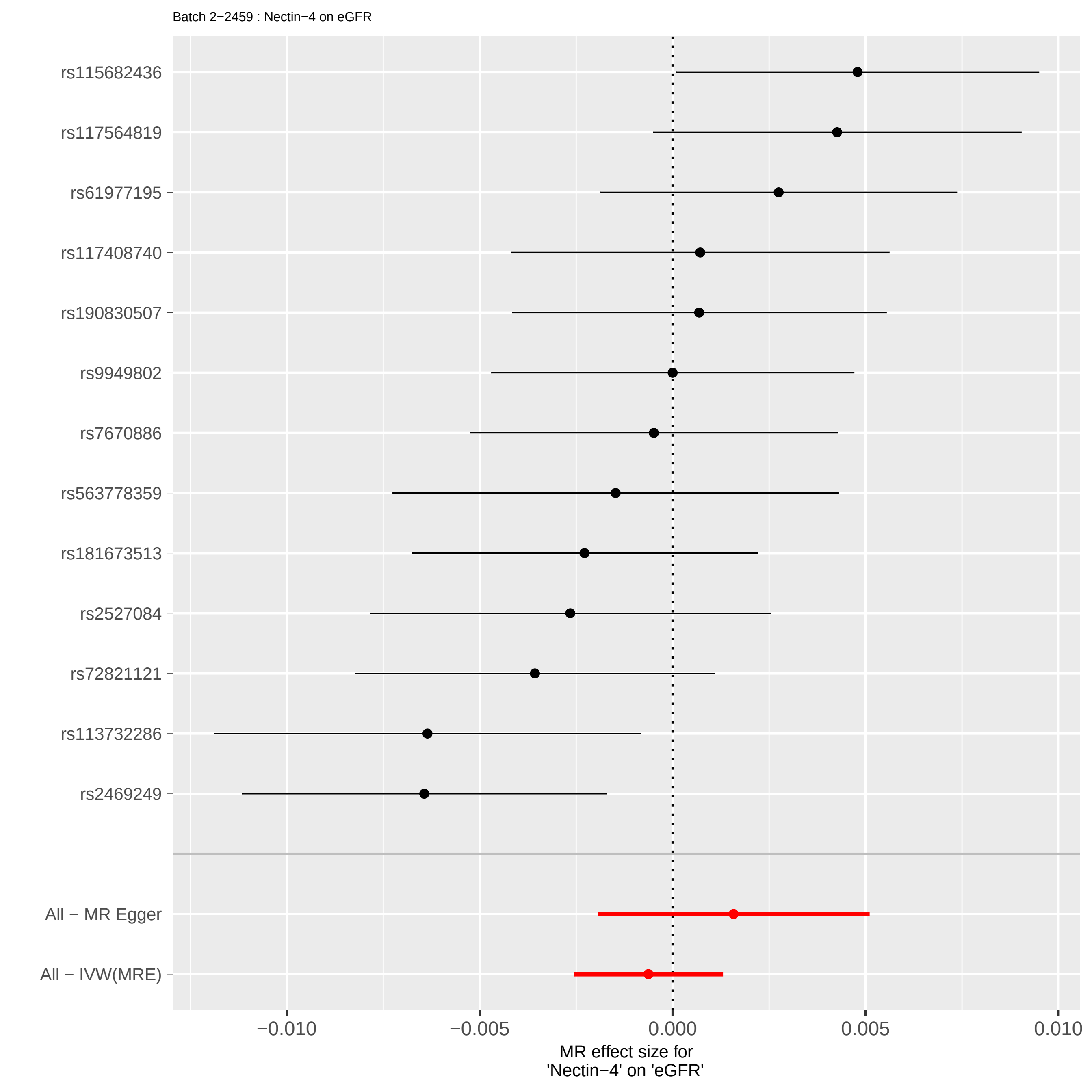

Batch 2−2459 : Nectin−4 on eGFR
rs115682436
rs117564819
rs61977195
rs117408740
rs190830507
rs9949802
rs7670886
rs563778359
rs181673513
rs2527084
rs72821121
rs113732286
rs2469249
All − MR Egger
All − IVW(MRE)
−0.010
−0.005
0.000
0.005
0.010
MR effect size for
'Nectin−4' on 'eGFR'

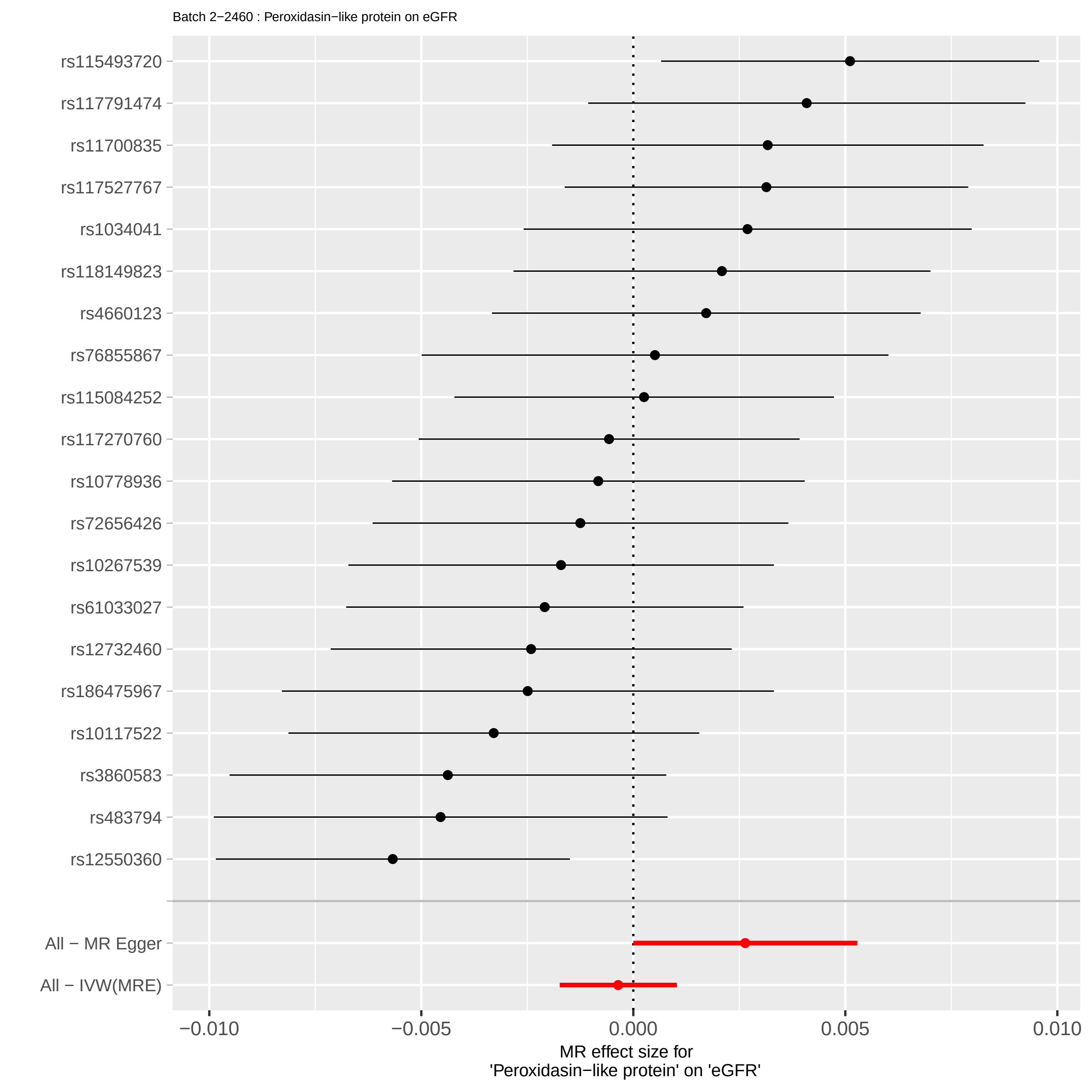

Batch 2−2460 : Peroxidasin−like protein on eGFR
rs115493720
rs117791474
rs11700835
rs117527767
rs1034041
rs118149823
rs4660123
rs76855867
rs115084252
rs117270760
rs10778936
rs72656426
rs10267539
rs61033027
rs12732460
rs186475967
rs10117522
rs3860583
rs483794
rs12550360
All − MR Egger
All − IVW(MRE)
−0.010
−0.005
0.000
0.005
0.010
MR effect size for
'Peroxidasin−like protein' on 'eGFR'

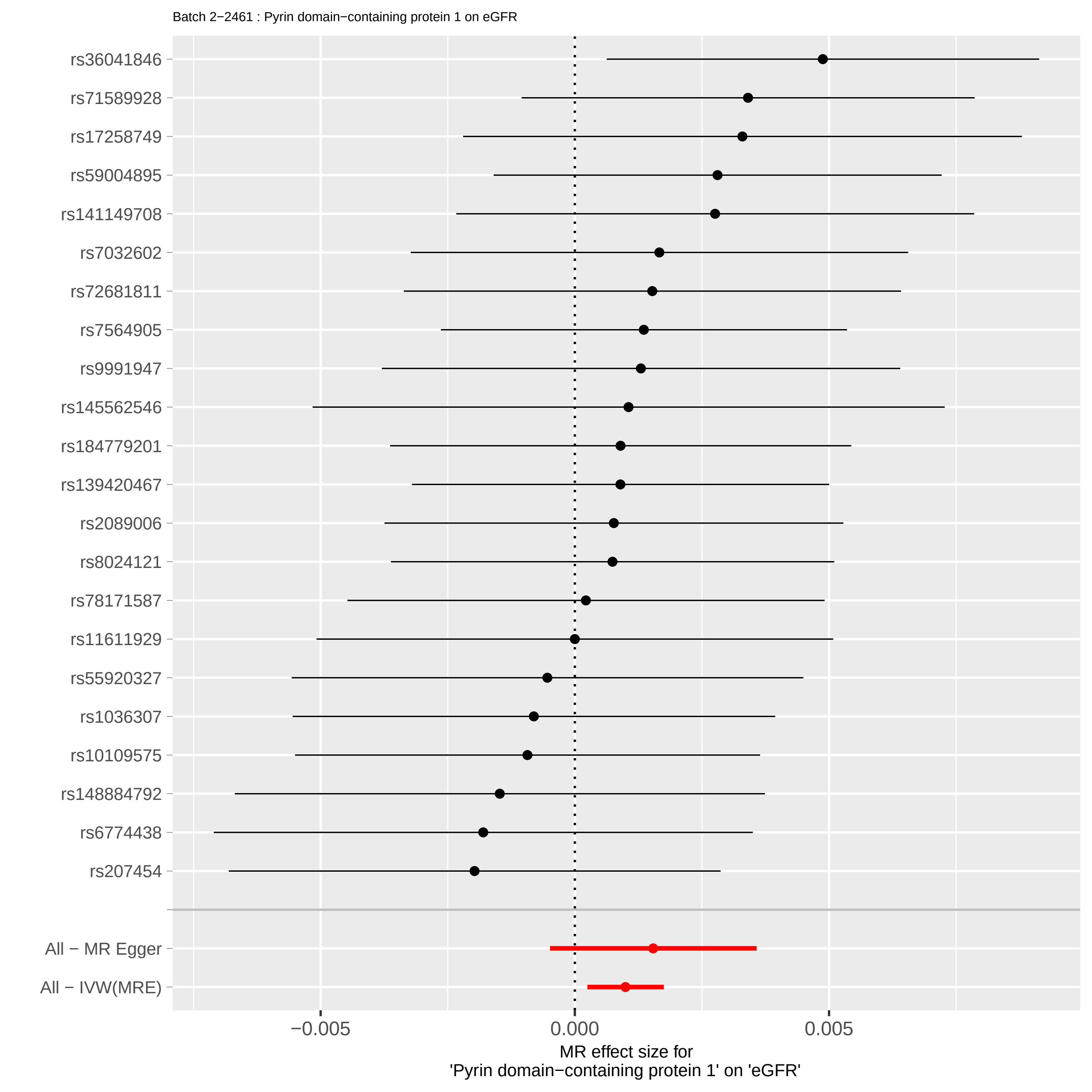

Batch 2−2461 : Pyrin domain−containing protein 1 on eGFR
rs36041846
rs71589928
rs17258749
rs59004895
rs141149708
rs7032602
rs72681811
rs7564905
rs9991947
rs145562546
rs184779201
rs139420467
rs2089006
rs8024121
rs78171587
rs11611929
rs55920327
rs1036307
rs10109575
rs148884792
rs6774438
rs207454
All − MR Egger
All − IVW(MRE)
−0.005
0.000
0.005
MR effect size for
'Pyrin domain−containing protein 1' on 'eGFR'

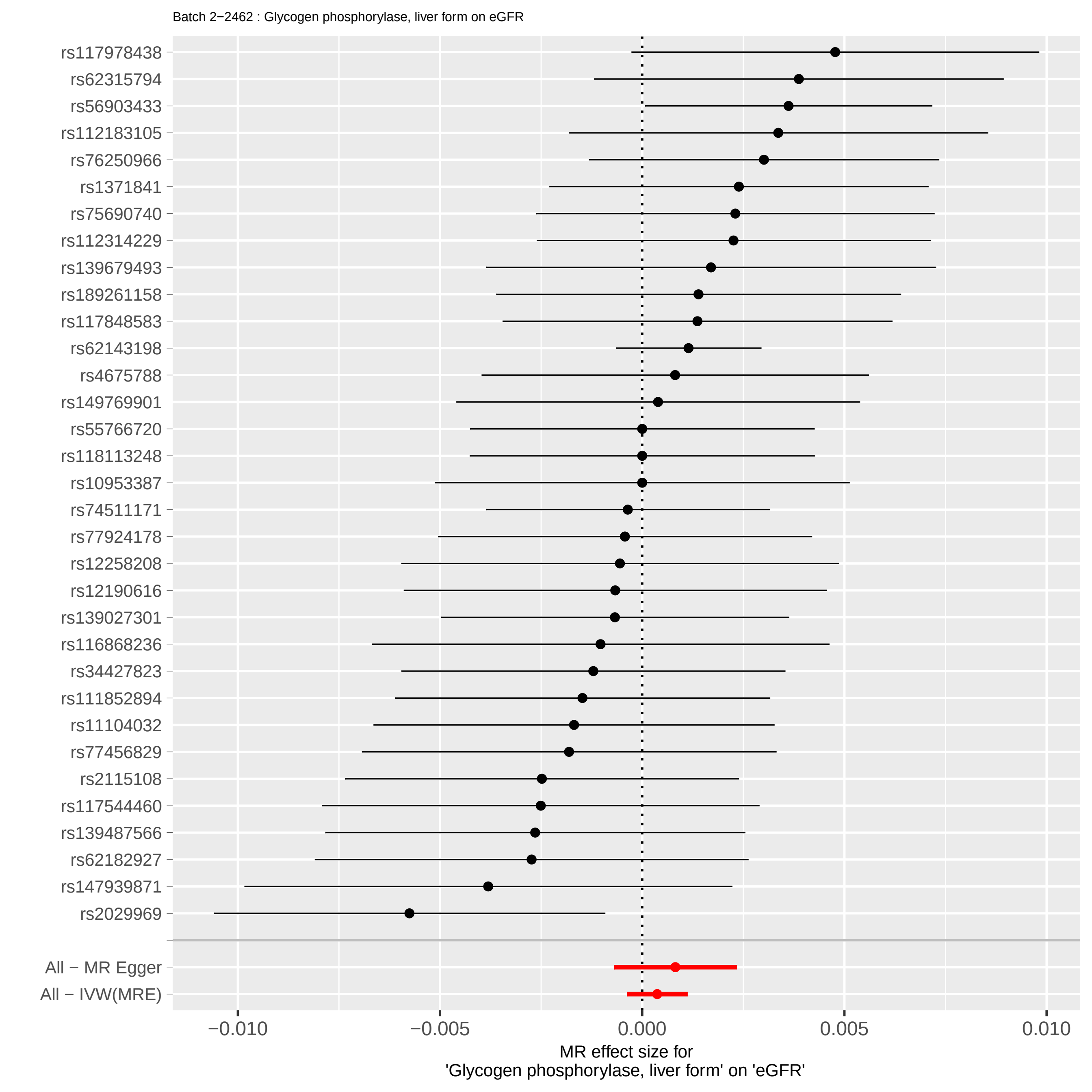
Batch 2−2462 : Glycogen phosphorylase, liver form on eGFR
rs117978438
rs62315794
rs56903433
rs112183105
rs76250966
rs1371841
rs75690740
rs112314229
rs139679493
rs189261158
rs117848583
rs62143198
rs4675788
rs149769901
rs55766720
rs118113248
rs10953387
rs74511171
rs77924178
rs12258208
rs12190616
rs139027301
rs116868236
rs34427823
rs111852894
rs11104032
rs77456829
rs2115108
rs117544460
rs139487566
rs62182927
rs147939871
rs2029969
All − MR Egger
All − IVW(MRE)
−0.010
−0.005
0.000
0.005
0.010
MR effect size for
'Glycogen phosphorylase, liver form' on 'eGFR'

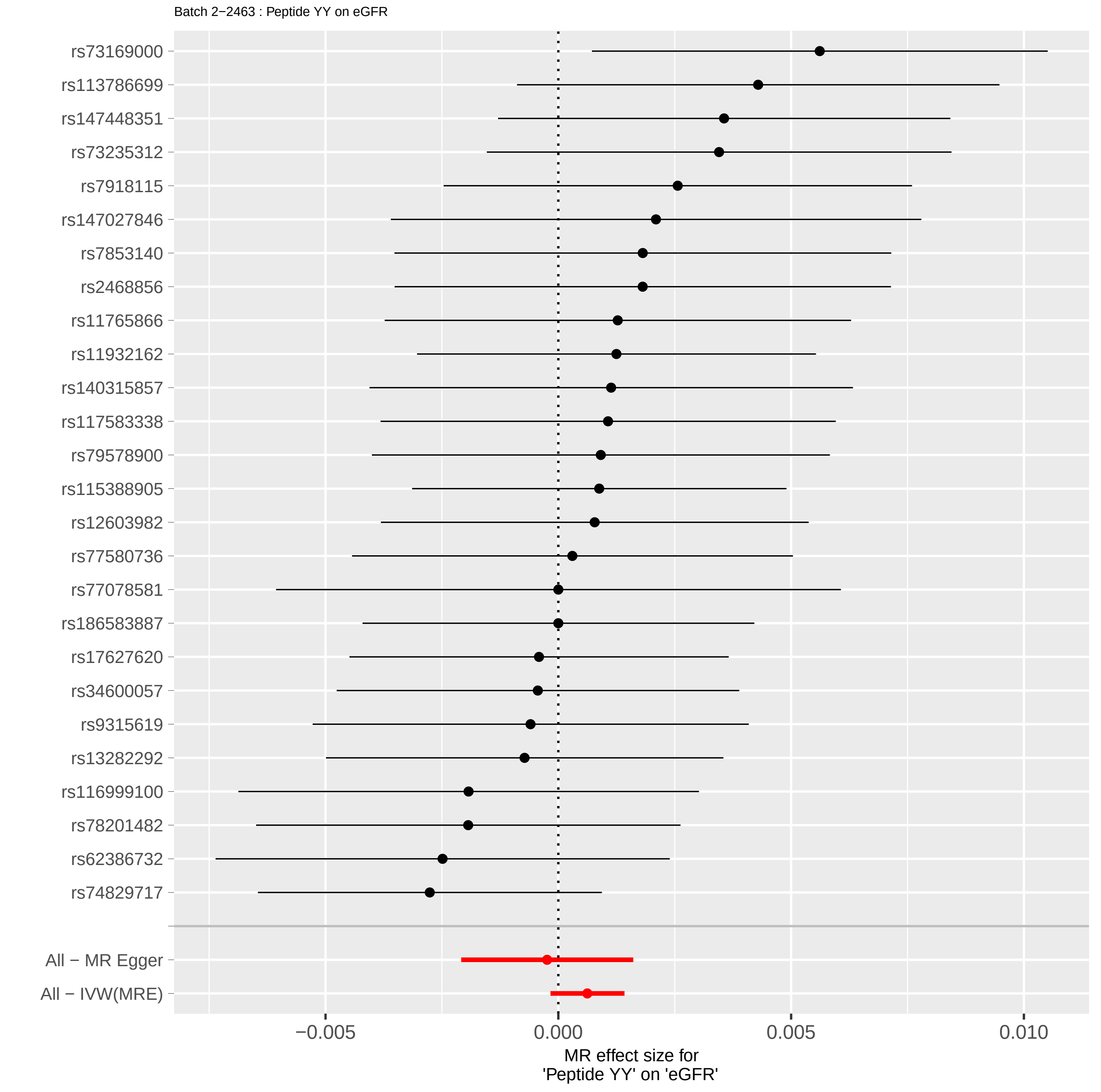

Batch 2−2463 : Peptide YY on eGFR
rs73169000
rs113786699
rs147448351
rs73235312
rs7918115
rs147027846
rs7853140
rs2468856
rs11765866
rs11932162
rs140315857
rs117583338
rs79578900
rs115388905
rs12603982
rs77580736
rs77078581
rs186583887
rs17627620
rs34600057
rs9315619
rs13282292
rs116999100
rs78201482
rs62386732
rs74829717
All − MR Egger
All − IVW(MRE)
−0.005
0.000
0.005
0.010
MR effect size for
'Peptide YY' on 'eGFR'

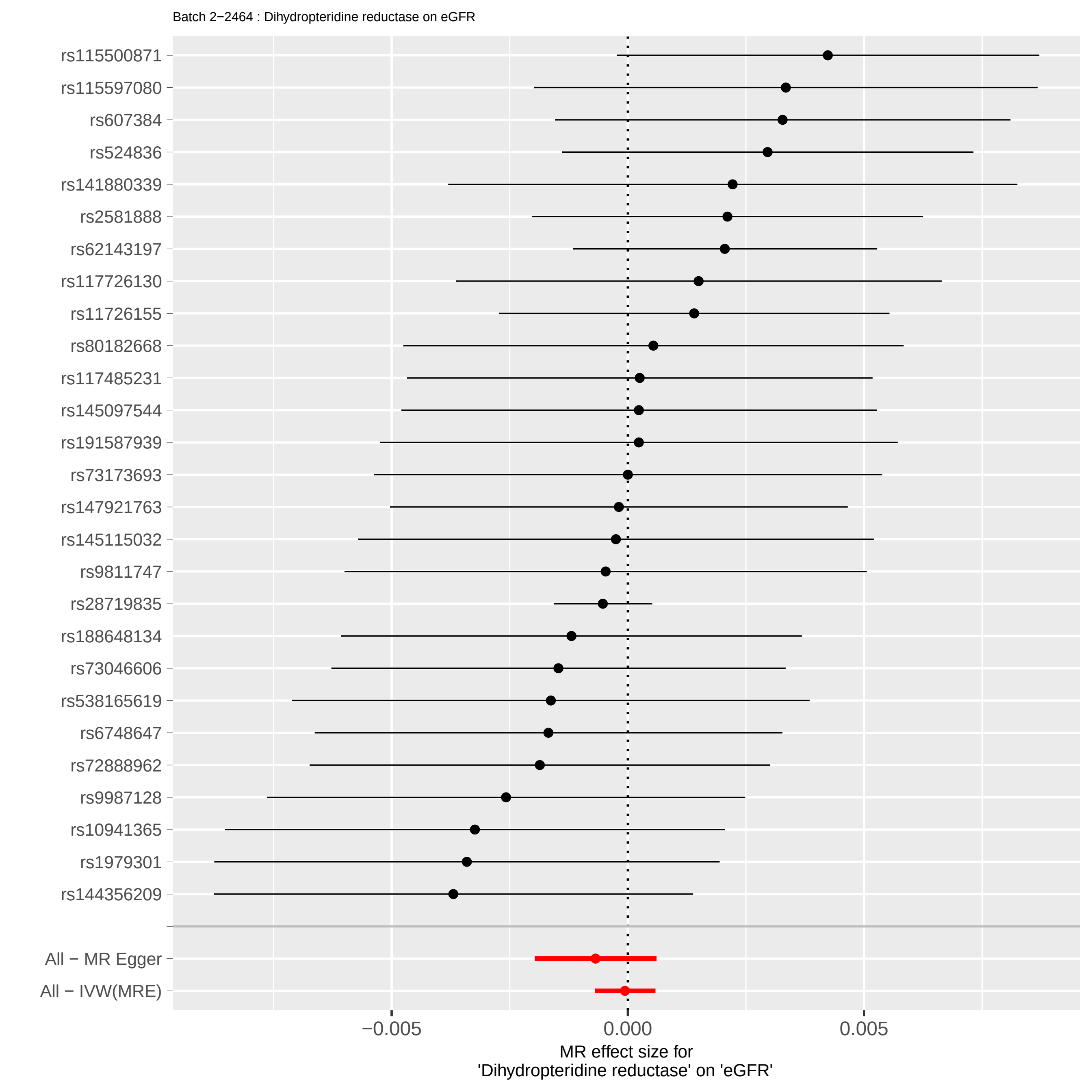

Batch 2−2464 : Dihydropteridine reductase on eGFR
rs115500871
rs115597080
rs607384
rs524836
rs141880339
rs2581888
rs62143197
rs117726130
rs11726155
rs80182668
rs117485231
rs145097544
rs191587939
rs73173693
rs147921763
rs145115032
rs9811747
rs28719835
rs188648134
rs73046606
rs538165619
rs6748647
rs72888962
rs9987128
rs10941365
rs1979301
rs144356209
All − MR Egger
All − IVW(MRE)
−0.005
0.000
0.005
MR effect size for
'Dihydropteridine reductase' on 'eGFR'

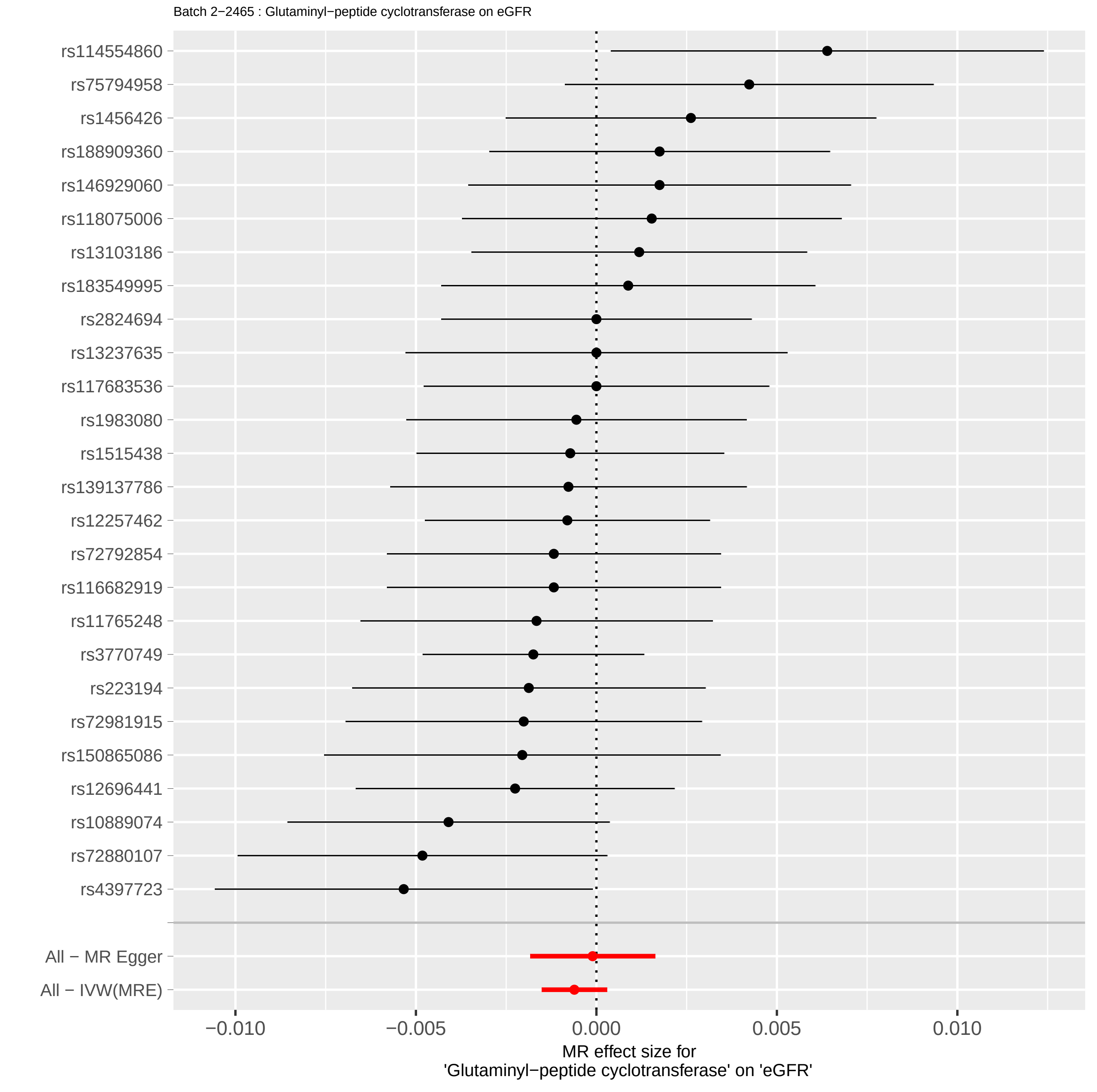
Batch 2−2465 : Glutaminyl−peptide cyclotransferase on eGFR
rs114554860
rs75794958
rs1456426
rs188909360
rs146929060
rs118075006
rs13103186
rs183549995
rs2824694
rs13237635
rs117683536
rs1983080
rs1515438
rs139137786
rs12257462
rs72792854
rs116682919
rs11765248
rs3770749
rs223194
rs72981915
rs150865086
rs12696441
rs10889074
rs72880107
rs4397723
All − MR Egger
All − IVW(MRE)
−0.010
−0.005
0.000
0.005
0.010
MR effect size for
'Glutaminyl−peptide cyclotransferase' on 'eGFR'

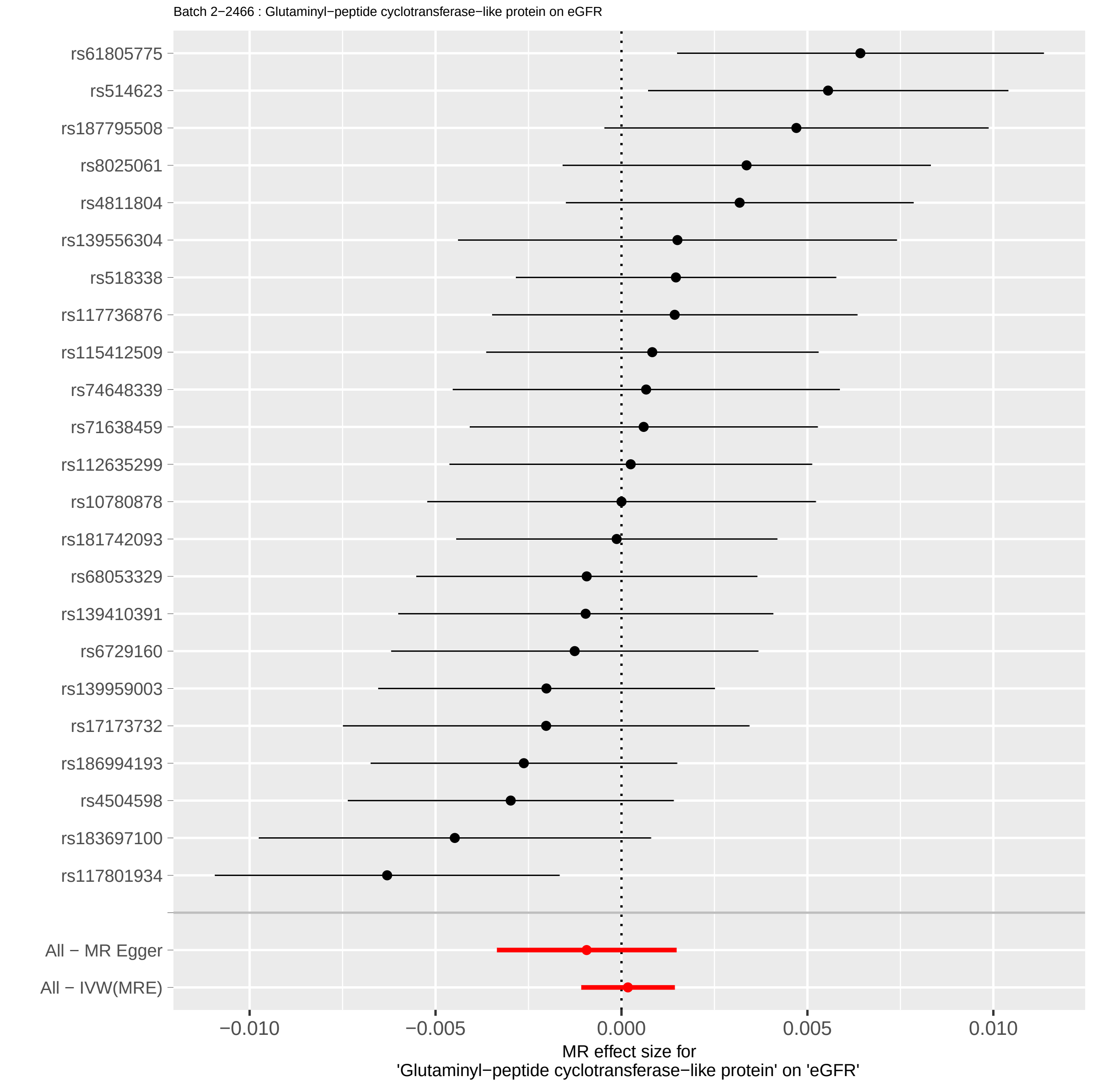

Batch 2−2466 : Glutaminyl−peptide cyclotransferase−like protein on eGFR
rs61805775
rs514623
rs187795508
rs8025061
rs4811804
rs139556304
rs518338
rs117736876
rs115412509
rs74648339
rs71638459
rs112635299
rs10780878
rs181742093
rs68053329
rs139410391
rs6729160
rs139959003
rs17173732
rs186994193
rs4504598
rs183697100
rs117801934
All − MR Egger
All − IVW(MRE)
−0.010
−0.005
0.000
0.005
0.010
MR effect size for
'Glutaminyl−peptide cyclotransferase−like protein' on 'eGFR'

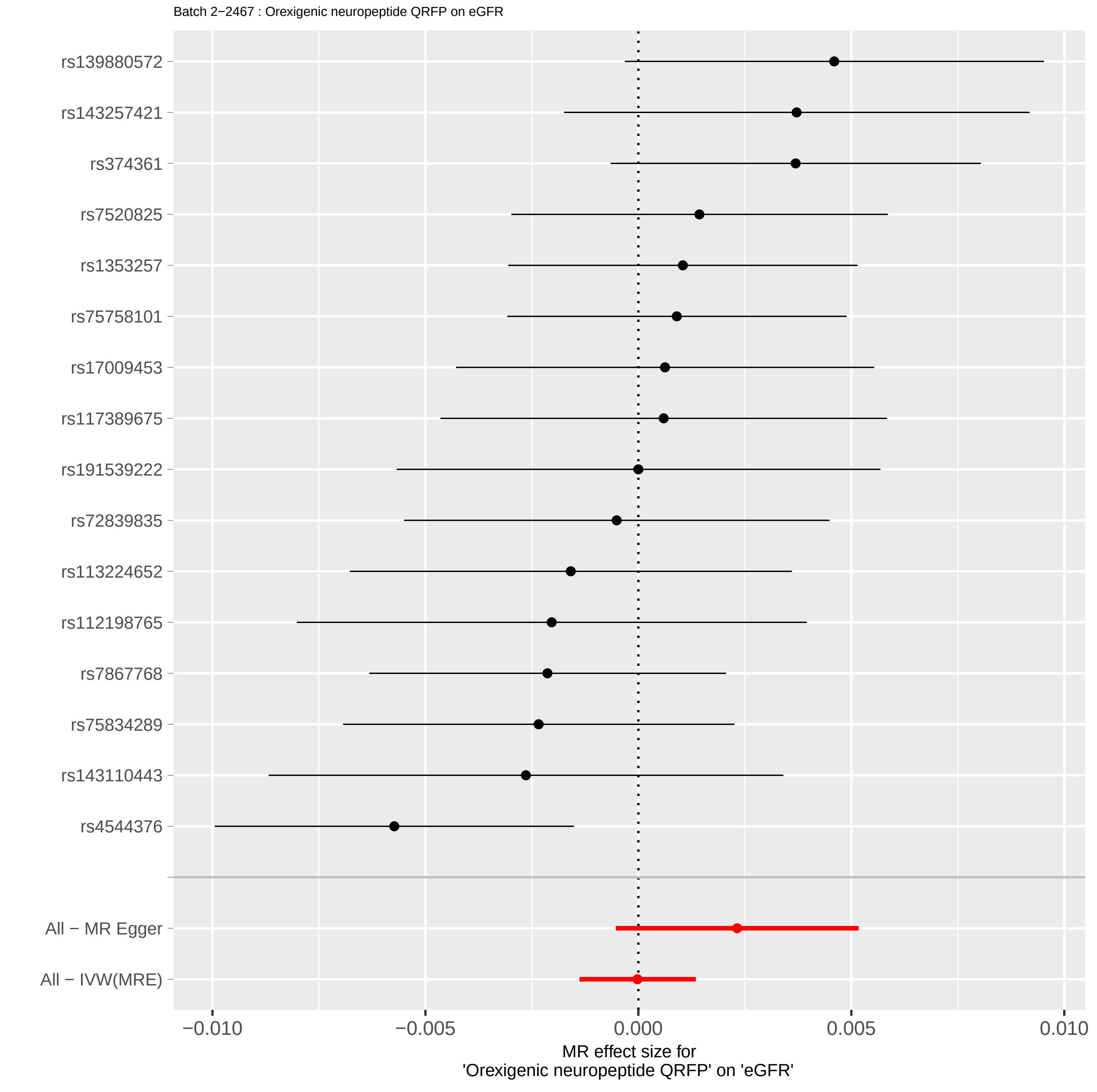
Batch 2−2467 : Orexigenic neuropeptide QRFP on eGFR
rs139880572
rs143257421
rs374361
rs7520825
rs1353257
rs75758101
rs17009453
rs117389675
rs191539222
rs72839835
rs113224652
rs112198765
rs7867768
rs75834289
rs143110443
rs4544376
All − MR Egger
All − IVW(MRE)
−0.010
−0.005
0.000
0.005
0.010
MR effect size for
'Orexigenic neuropeptide QRFP' on 'eGFR'

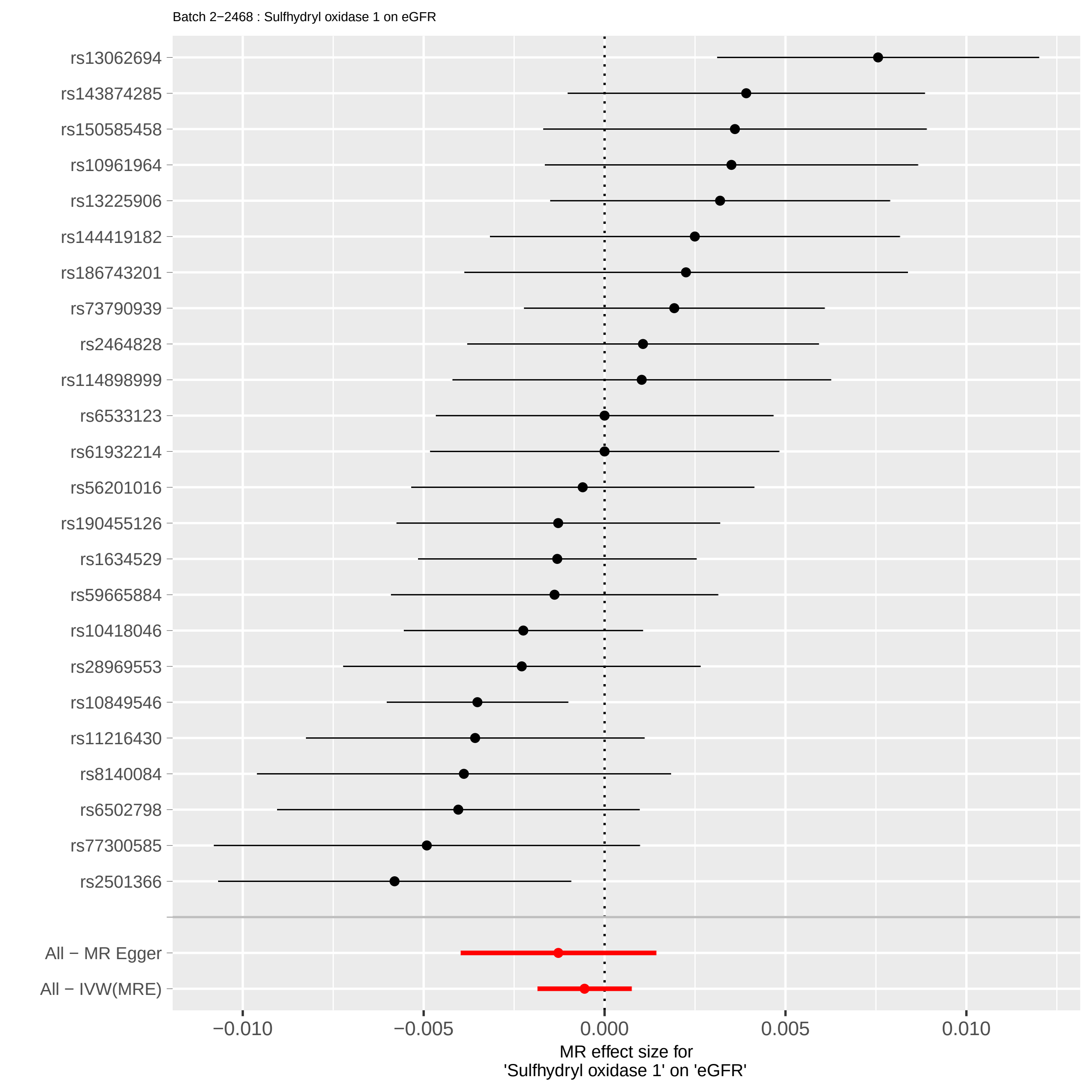

Batch 2−2468 : Sulfhydryl oxidase 1 on eGFR
rs13062694
rs143874285
rs150585458
rs10961964
rs13225906
rs144419182
rs186743201
rs73790939
rs2464828
rs114898999
rs6533123
rs61932214
rs56201016
rs190455126
rs1634529
rs59665884
rs10418046
rs28969553
rs10849546
rs11216430
rs8140084
rs6502798
rs77300585
rs2501366
All − MR Egger
All − IVW(MRE)
−0.010
−0.005
0.000
0.005
0.010
MR effect size for
'Sulfhydryl oxidase 1' on 'eGFR'

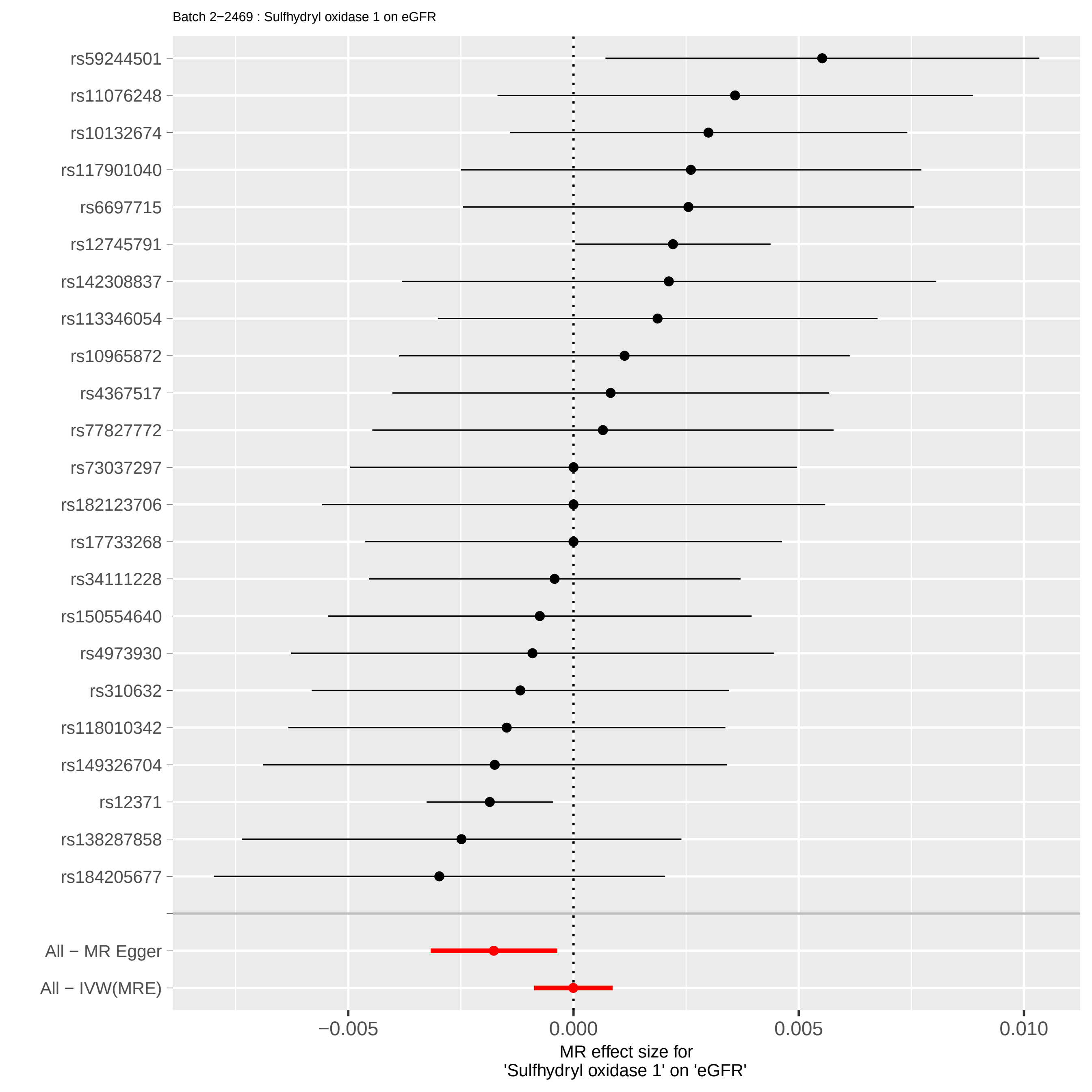
Batch 2−2469 : Sulfhydryl oxidase 1 on eGFR
rs59244501
rs11076248
rs10132674
rs117901040
rs6697715
rs12745791
rs142308837
rs113346054
rs10965872
rs4367517
rs77827772
rs73037297
rs182123706
rs17733268
rs34111228
rs150554640
rs4973930
rs310632
rs118010342
rs149326704
rs12371
rs138287858
rs184205677
All − MR Egger
All − IVW(MRE)
−0.005
0.000
0.005
0.010
MR effect size for
'Sulfhydryl oxidase 1' on 'eGFR'

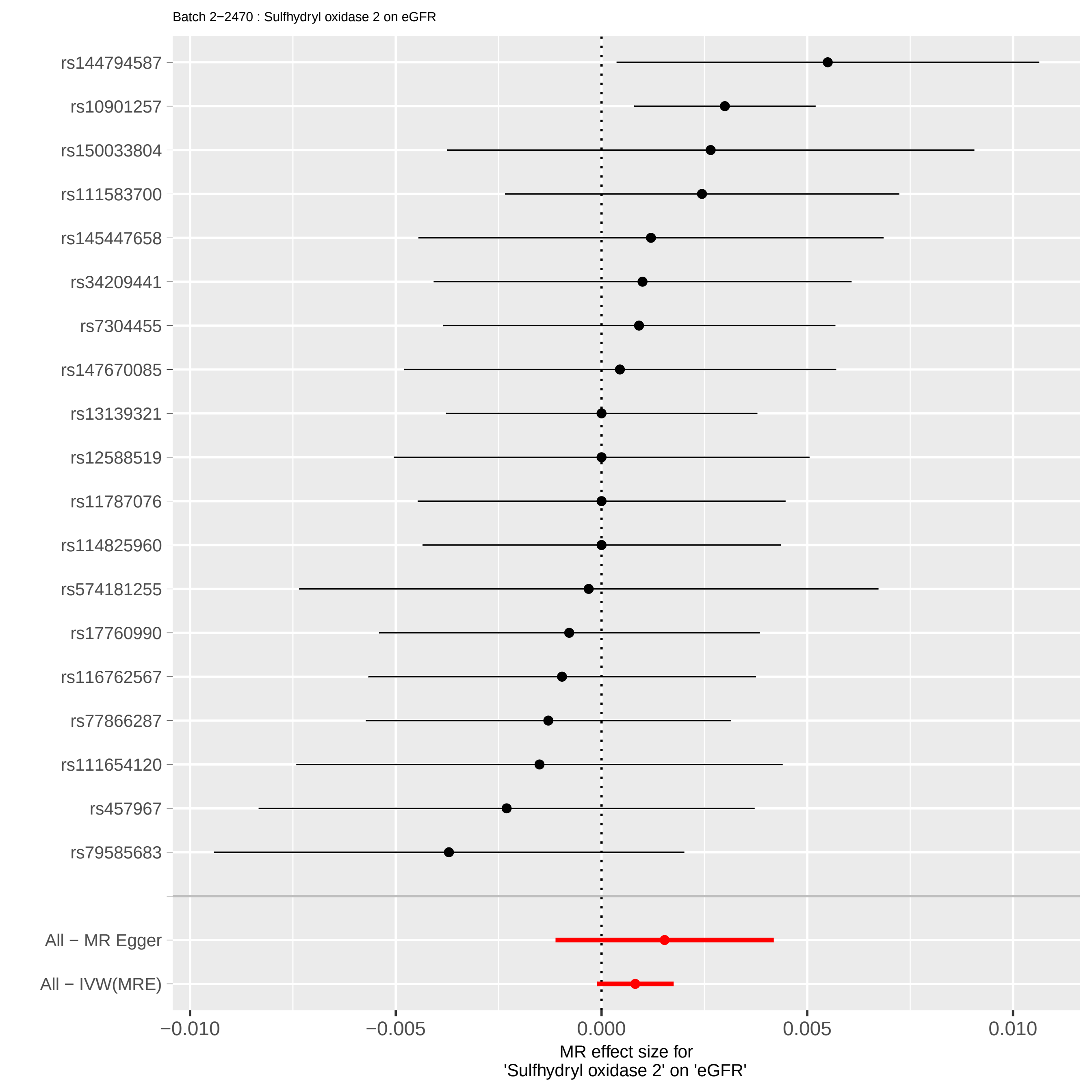

Batch 2−2470 : Sulfhydryl oxidase 2 on eGFR
rs144794587
rs10901257
rs150033804
rs111583700
rs145447658
rs34209441
rs7304455
rs147670085
rs13139321
rs12588519
rs11787076
rs114825960
rs574181255
rs17760990
rs116762567
rs77866287
rs111654120
rs457967
rs79585683
All − MR Egger
All − IVW(MRE)
−0.010
−0.005
0.000
0.005
0.010
MR effect size for
'Sulfhydryl oxidase 2' on 'eGFR'

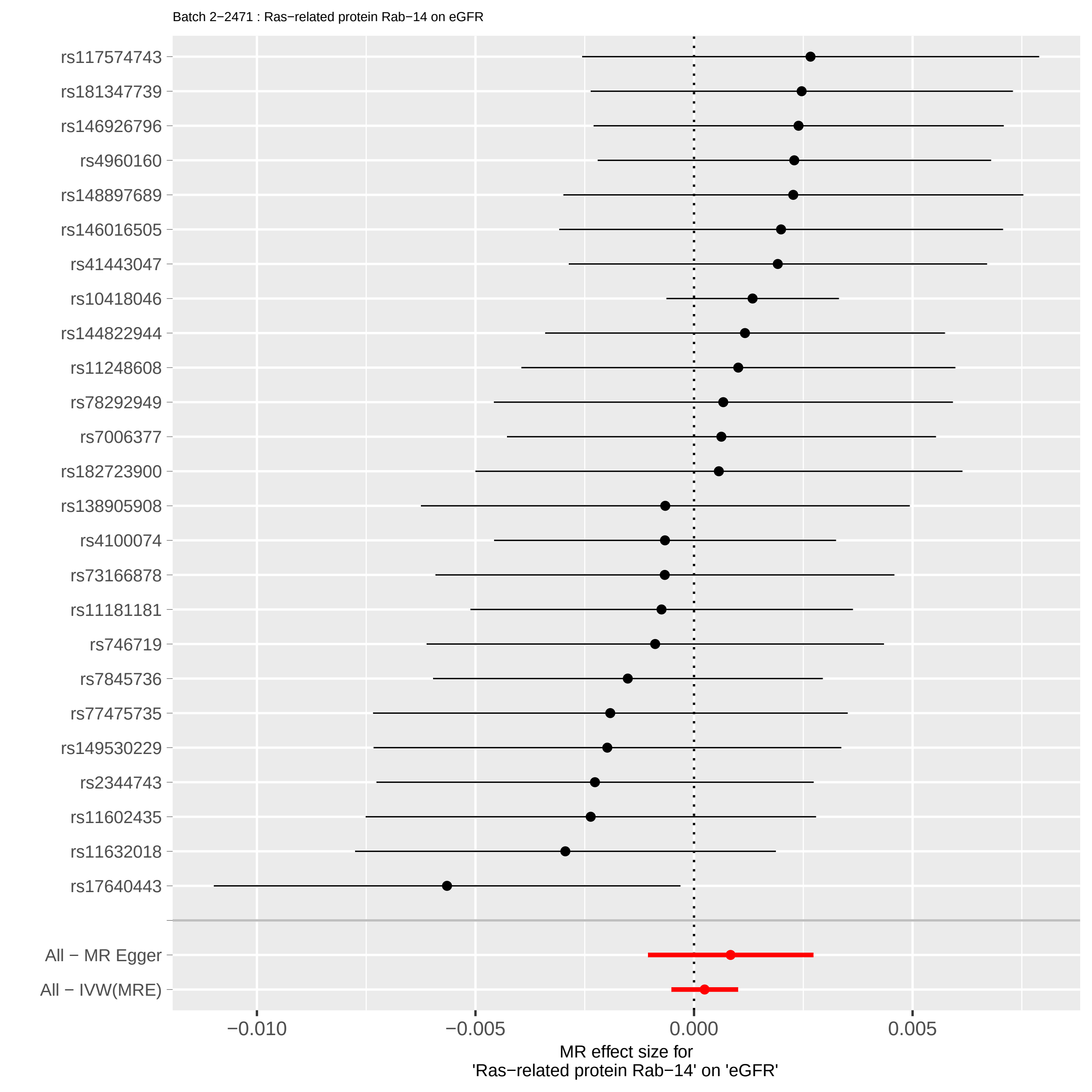
Batch 2−2471 : Ras−related protein Rab−14 on eGFR
rs117574743
rs181347739
rs146926796
rs4960160
rs148897689
rs146016505
rs41443047
rs10418046
rs144822944
rs11248608
rs78292949
rs7006377
rs182723900
rs138905908
rs4100074
rs73166878
rs11181181
rs746719
rs7845736
rs77475735
rs149530229
rs2344743
rs11602435
rs11632018
rs17640443
All − MR Egger
All − IVW(MRE)
−0.010
−0.005
0.000
0.005
MR effect size for
'Ras−related protein Rab−14' on 'eGFR'

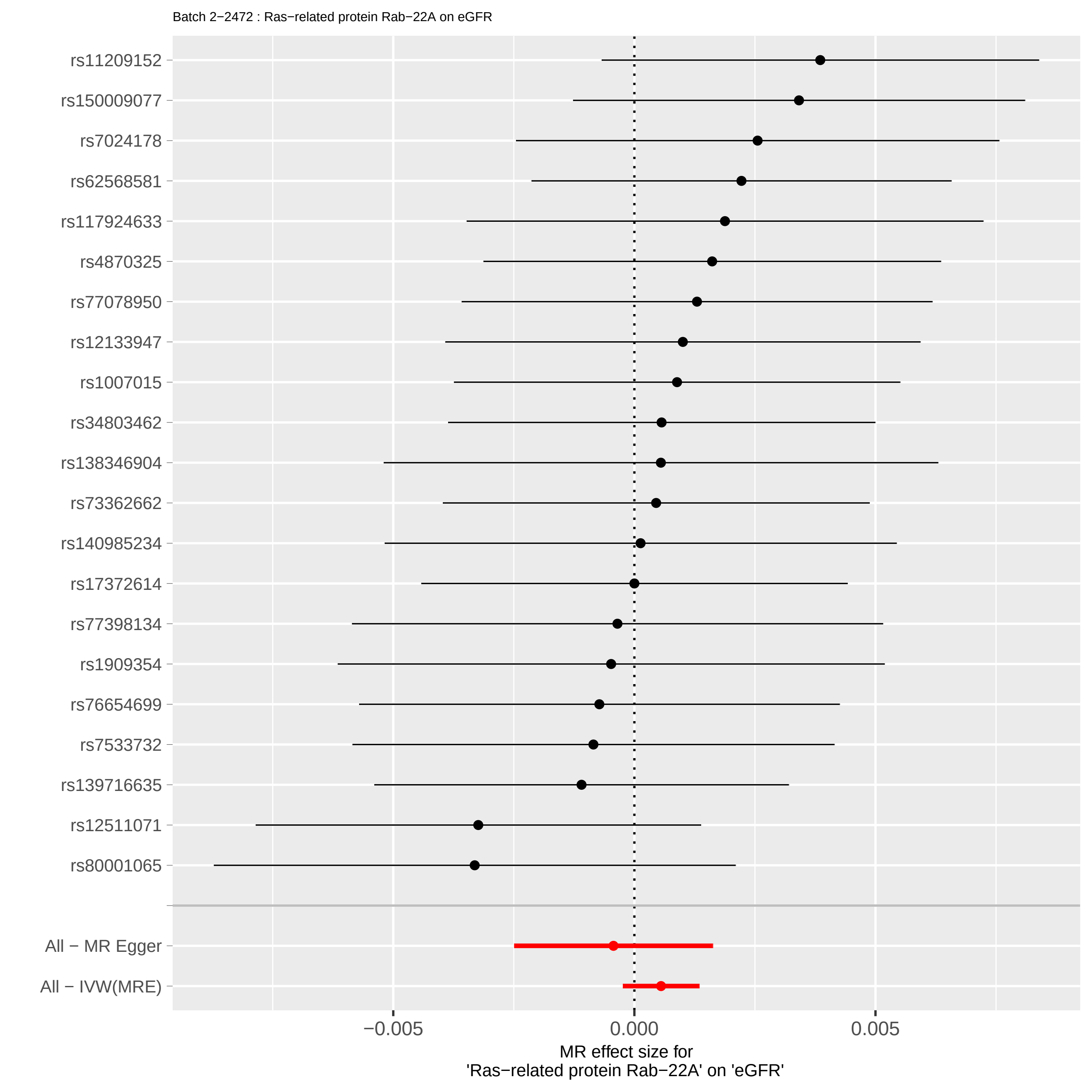
Batch 2−2472 : Ras−related protein Rab−22A on eGFR
rs11209152
rs150009077
rs7024178
rs62568581
rs117924633
rs4870325
rs77078950
rs12133947
rs1007015
rs34803462
rs138346904
rs73362662
rs140985234
rs17372614
rs77398134
rs1909354
rs76654699
rs7533732
rs139716635
rs12511071
rs80001065
All − MR Egger
All − IVW(MRE)
−0.005
0.000
0.005
MR effect size for
'Ras−related protein Rab−22A' on 'eGFR'

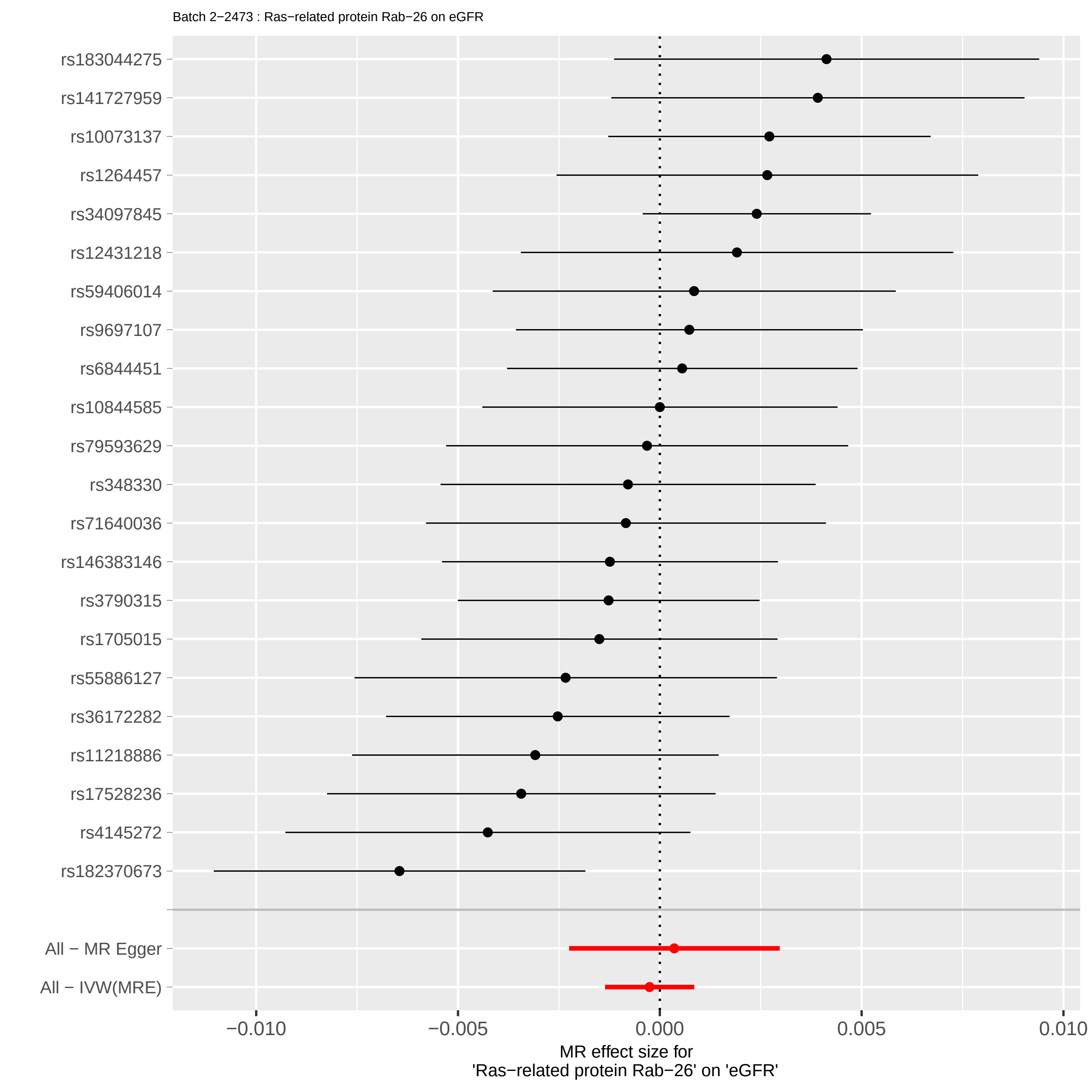

Batch 2−2473 : Ras−related protein Rab−26 on eGFR
rs183044275
rs141727959
rs10073137
rs1264457
rs34097845
rs12431218
rs59406014
rs9697107
rs6844451
rs10844585
rs79593629
rs348330
rs71640036
rs146383146
rs3790315
rs1705015
rs55886127
rs36172282
rs11218886
rs17528236
rs4145272
rs182370673
All − MR Egger
All − IVW(MRE)
−0.010
−0.005
0.000
0.005
0.010
MR effect size for
'Ras−related protein Rab−26' on 'eGFR'

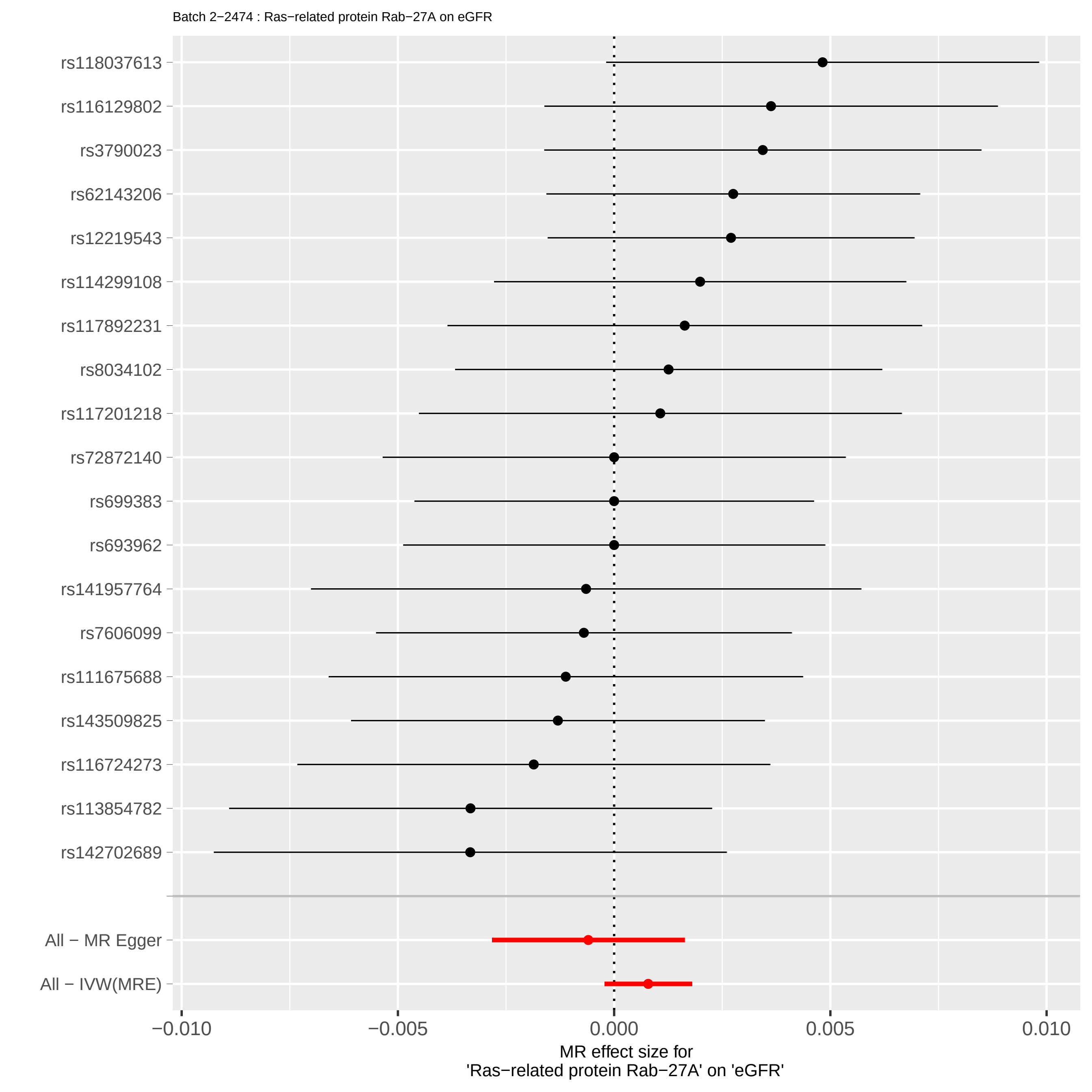
Batch 2−2474 : Ras−related protein Rab−27A on eGFR
rs118037613
rs116129802
rs3790023
rs62143206
rs12219543
rs114299108
rs117892231
rs8034102
rs117201218
rs72872140
rs699383
rs693962
rs141957764
rs7606099
rs111675688
rs143509825
rs116724273
rs113854782
rs142702689
All − MR Egger
All − IVW(MRE)
−0.010
−0.005
0.000
0.005
0.010
MR effect size for
'Ras−related protein Rab−27A' on 'eGFR'

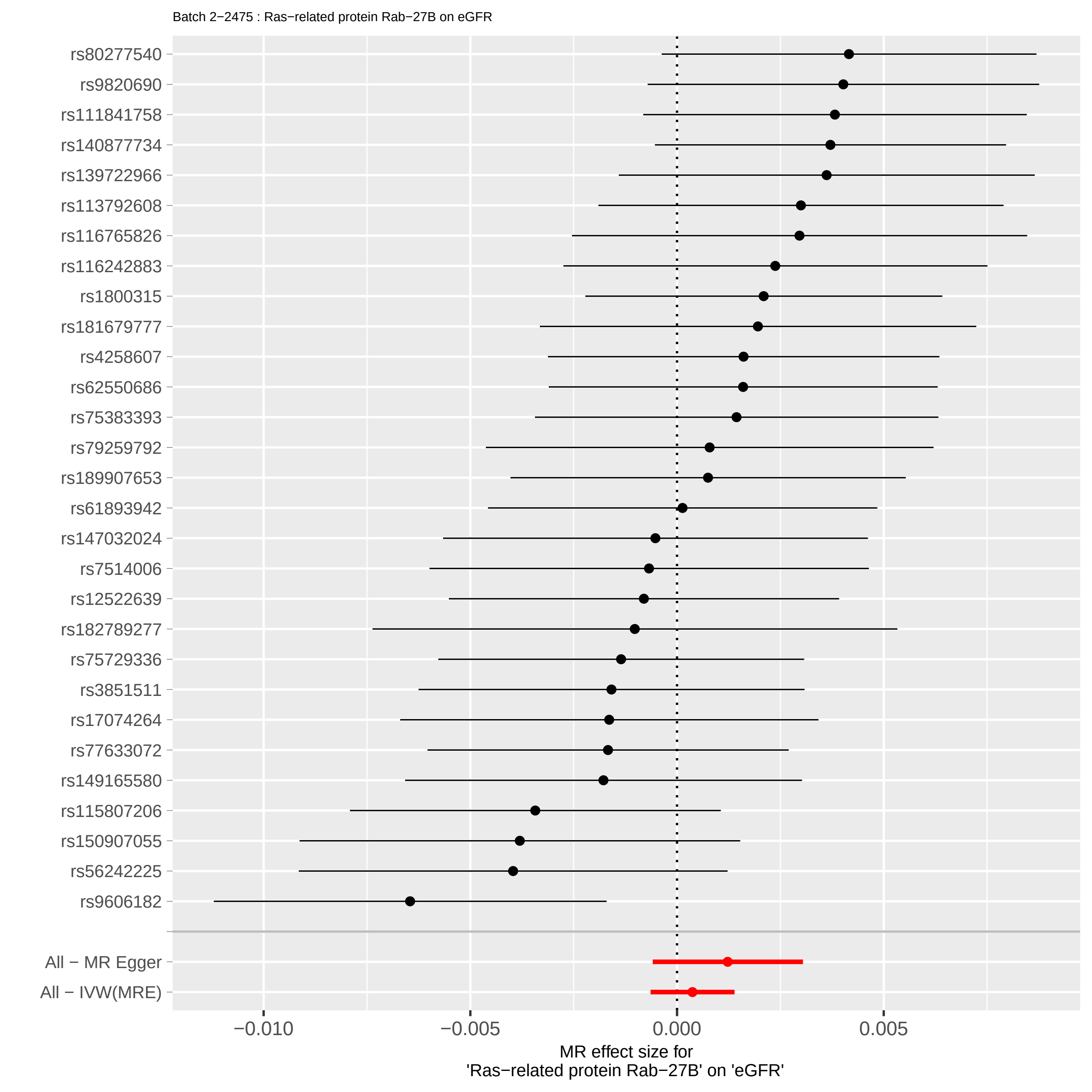
Batch 2−2475 : Ras−related protein Rab−27B on eGFR
rs80277540
rs9820690
rs111841758
rs140877734
rs139722966
rs113792608
rs116765826
rs116242883
rs1800315
rs181679777
rs4258607
rs62550686
rs75383393
rs79259792
rs189907653
rs61893942
rs147032024
rs7514006
rs12522639
rs182789277
rs75729336
rs3851511
rs17074264
rs77633072
rs149165580
rs115807206
rs150907055
rs56242225
rs9606182
All − MR Egger
All − IVW(MRE)
−0.010
−0.005
0.000
0.005
MR effect size for
'Ras−related protein Rab−27B' on 'eGFR'

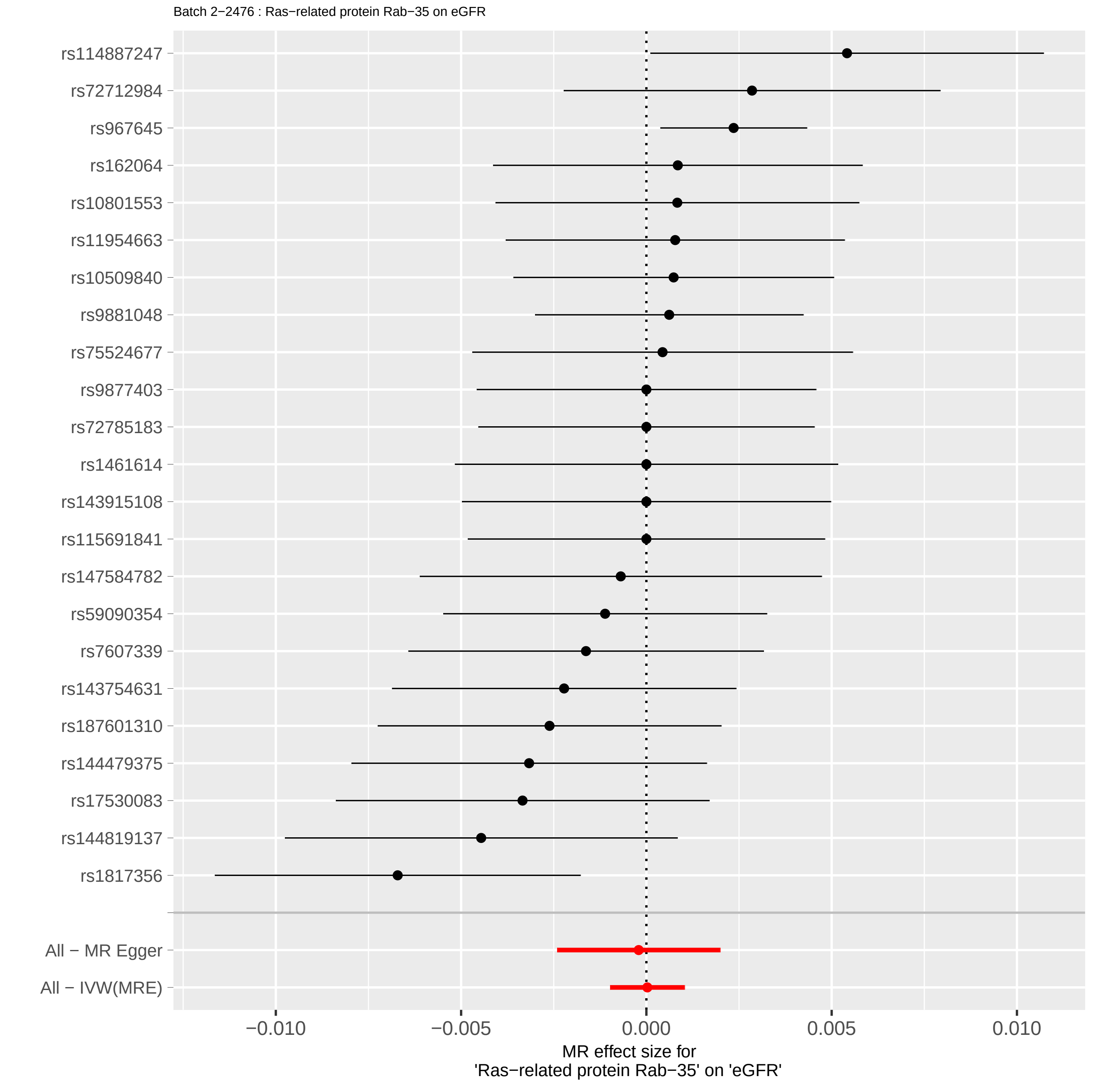

Batch 2−2476 : Ras−related protein Rab−35 on eGFR
rs114887247
rs72712984
rs967645
rs162064
rs10801553
rs11954663
rs10509840
rs9881048
rs75524677
rs9877403
rs72785183
rs1461614
rs143915108
rs115691841
rs147584782
rs59090354
rs7607339
rs143754631
rs187601310
rs144479375
rs17530083
rs144819137
rs1817356
All − MR Egger
All − IVW(MRE)
−0.010
−0.005
0.000
0.005
0.010
MR effect size for
'Ras−related protein Rab−35' on 'eGFR'

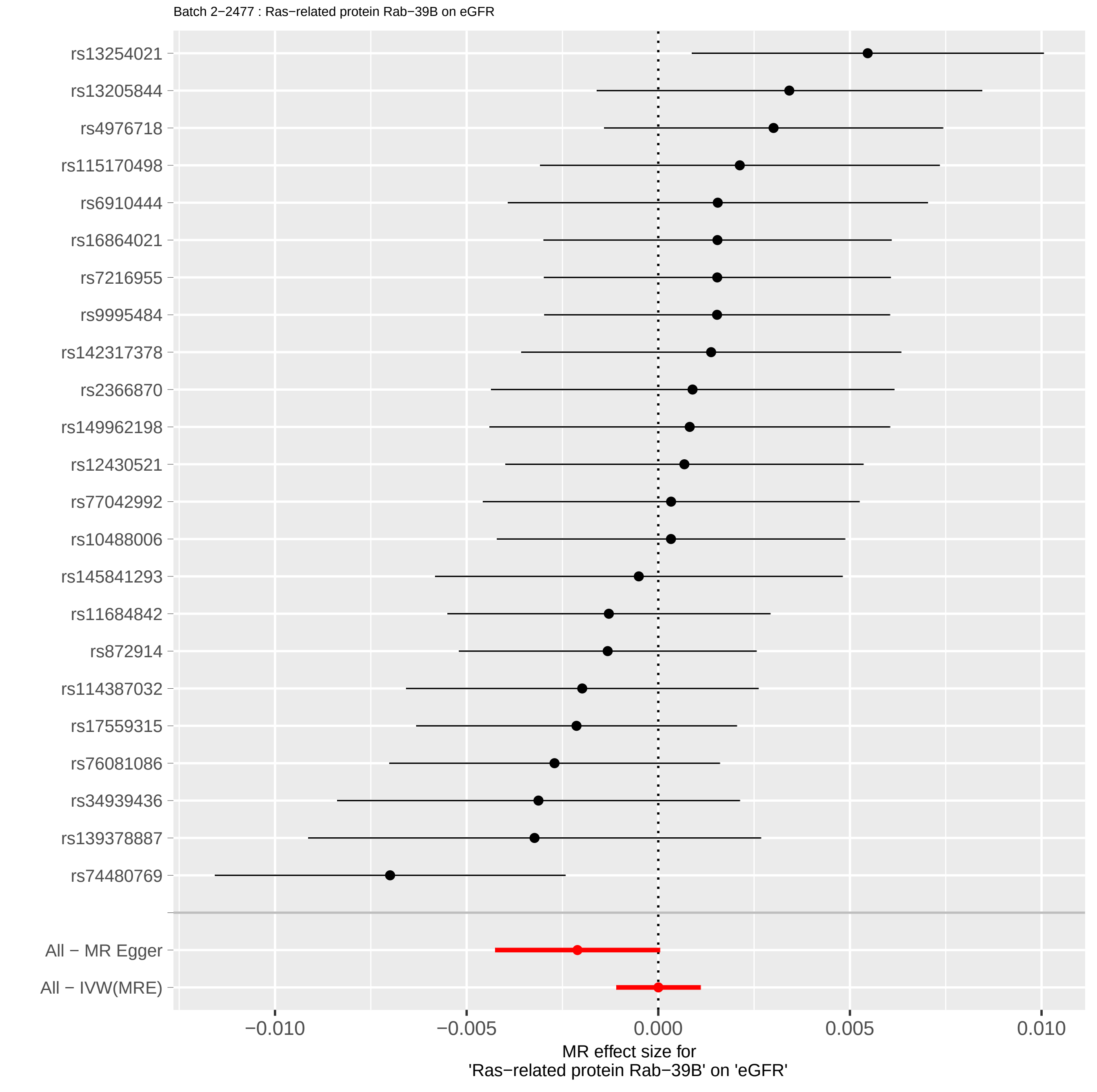

Batch 2−2477 : Ras−related protein Rab−39B on eGFR
rs13254021
rs13205844
rs4976718
rs115170498
rs6910444
rs16864021
rs7216955
rs9995484
rs142317378
rs2366870
rs149962198
rs12430521
rs77042992
rs10488006
rs145841293
rs11684842
rs872914
rs114387032
rs17559315
rs76081086
rs34939436
rs139378887
rs74480769
All − MR Egger
All − IVW(MRE)
−0.010
−0.005
0.000
0.005
0.010
MR effect size for
'Ras−related protein Rab−39B' on 'eGFR'

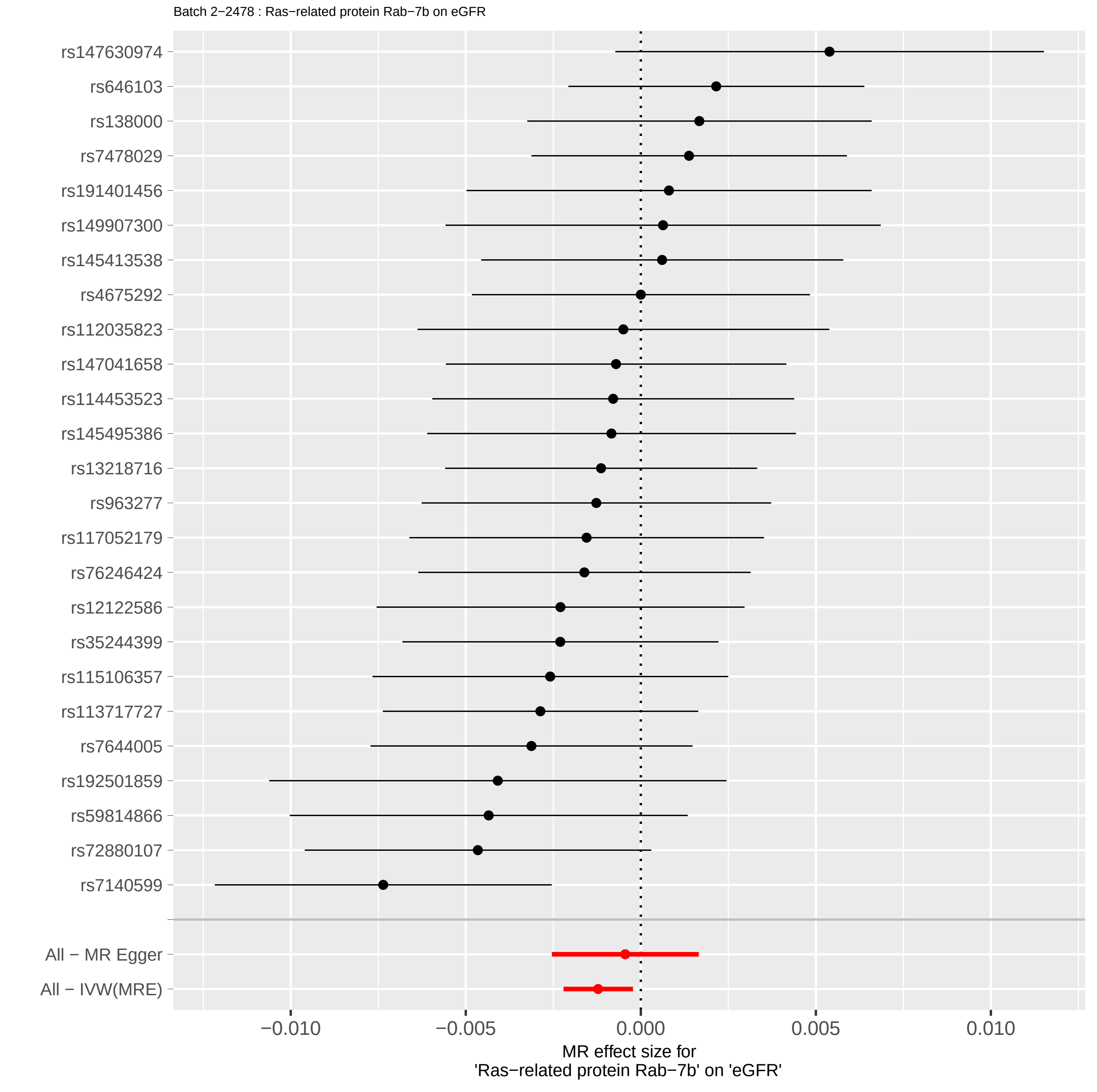

Batch 2−2478 : Ras−related protein Rab−7b on eGFR
rs147630974
rs646103
rs138000
rs7478029
rs191401456
rs149907300
rs145413538
rs4675292
rs112035823
rs147041658
rs114453523
rs145495386
rs13218716
rs963277
rs117052179
rs76246424
rs12122586
rs35244399
rs115106357
rs113717727
rs7644005
rs192501859
rs59814866
rs72880107
rs7140599
All − MR Egger
All − IVW(MRE)
−0.010
−0.005
0.000
0.005
0.010
MR effect size for
'Ras−related protein Rab−7b' on 'eGFR'

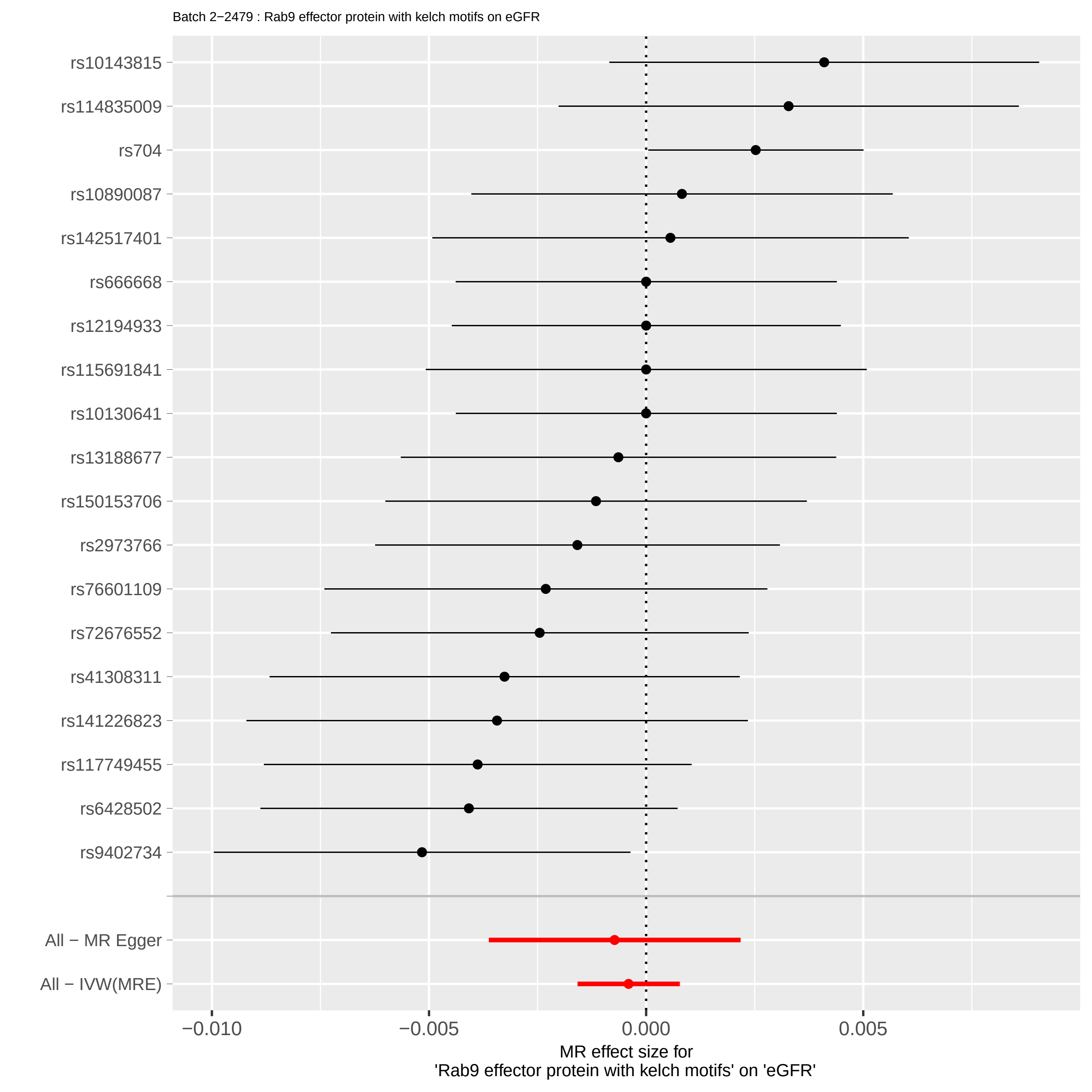
Batch 2−2479 : Rab9 effector protein with kelch motifs on eGFR
rs10143815
rs114835009
rs704
rs10890087
rs142517401
rs666668
rs12194933
rs115691841
rs10130641
rs13188677
rs150153706
rs2973766
rs76601109
rs72676552
rs41308311
rs141226823
rs117749455
rs6428502
rs9402734
All − MR Egger
All − IVW(MRE)
−0.010
−0.005
0.000
0.005
MR effect size for
'Rab9 effector protein with kelch motifs' on 'eGFR'

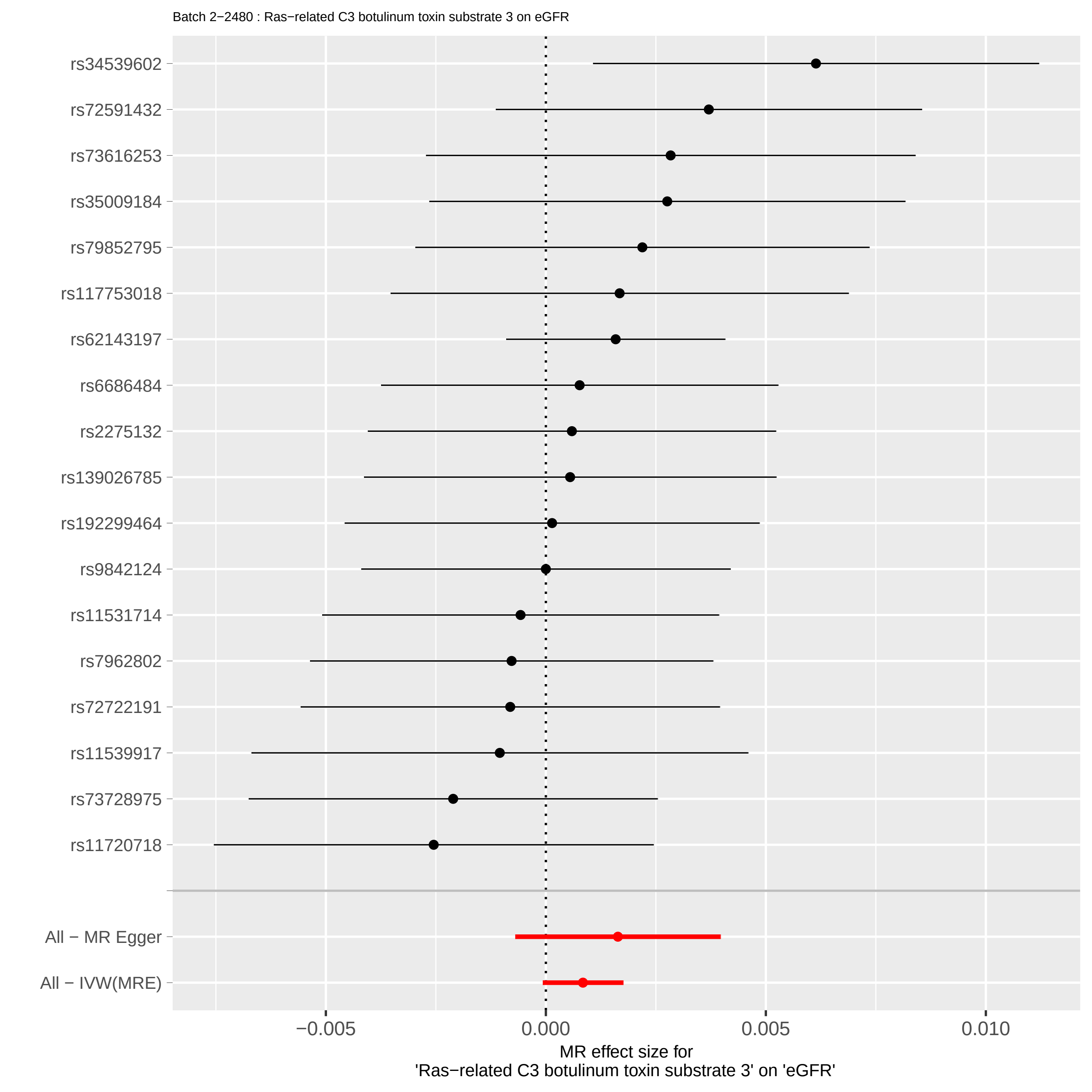

Batch 2−2480 : Ras−related C3 botulinum toxin substrate 3 on eGFR
rs34539602
rs72591432
rs73616253
rs35009184
rs79852795
rs117753018
rs62143197
rs6686484
rs2275132
rs139026785
rs192299464
rs9842124
rs11531714
rs7962802
rs72722191
rs11539917
rs73728975
rs11720718
All − MR Egger
All − IVW(MRE)
−0.005
0.000
0.005
0.010
MR effect size for
'Ras−related C3 botulinum toxin substrate 3' on 'eGFR'

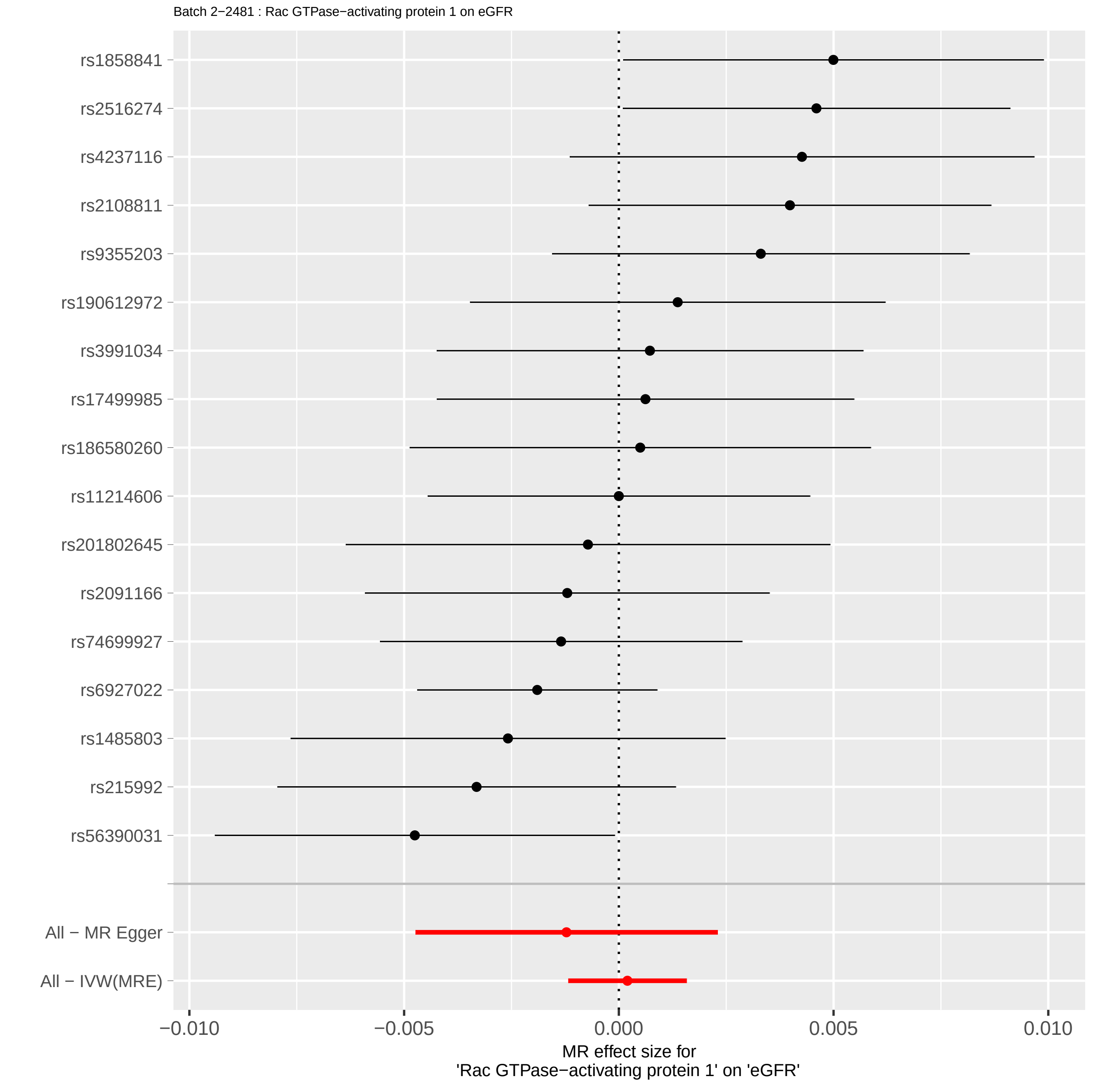

Batch 2−2481 : Rac GTPase−activating protein 1 on eGFR
rs1858841
rs2516274
rs4237116
rs2108811
rs9355203
rs190612972
rs3991034
rs17499985
rs186580260
rs11214606
rs201802645
rs2091166
rs74699927
rs6927022
rs1485803
rs215992
rs56390031
All − MR Egger
All − IVW(MRE)
−0.010
−0.005
0.000
0.005
0.010
MR effect size for
'Rac GTPase−activating protein 1' on 'eGFR'

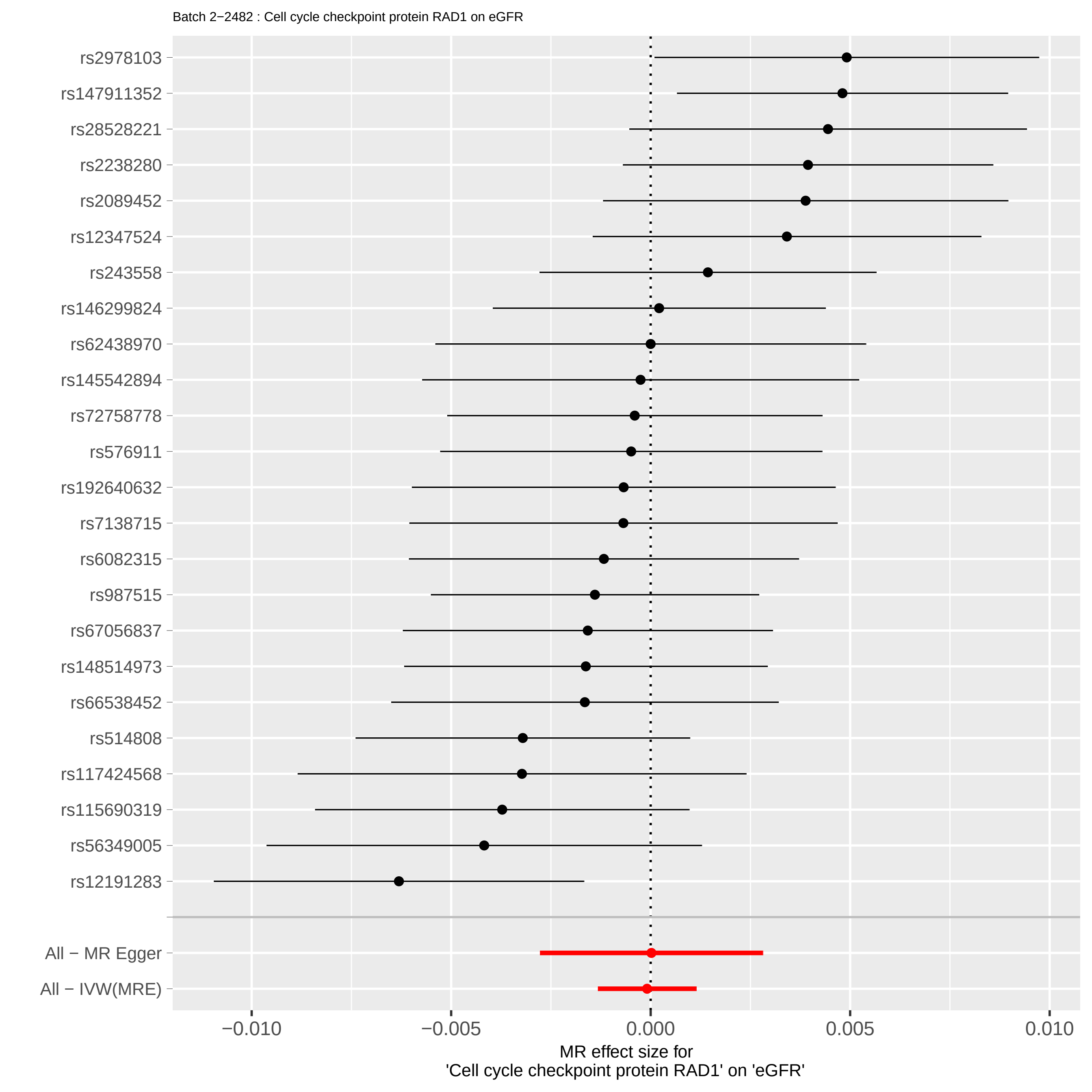

Batch 2−2482 : Cell cycle checkpoint protein RAD1 on eGFR
rs2978103
rs147911352
rs28528221
rs2238280
rs2089452
rs12347524
rs243558
rs146299824
rs62438970
rs145542894
rs72758778
rs576911
rs192640632
rs7138715
rs6082315
rs987515
rs67056837
rs148514973
rs66538452
rs514808
rs117424568
rs115690319
rs56349005
rs12191283
All − MR Egger
All − IVW(MRE)
−0.010
−0.005
0.000
0.005
0.010
MR effect size for
'Cell cycle checkpoint protein RAD1' on 'eGFR'

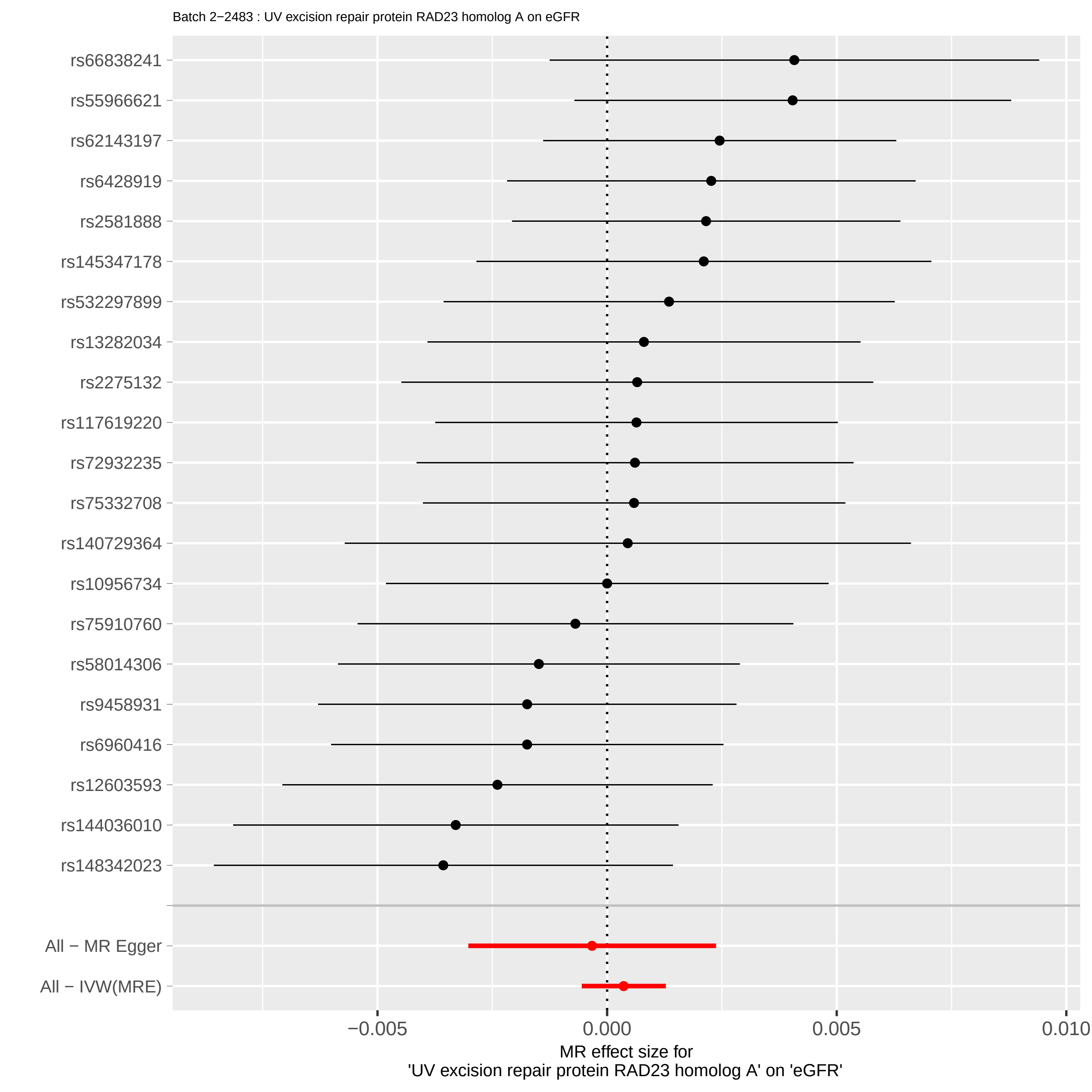

Batch 2−2483 : UV excision repair protein RAD23 homolog A on eGFR
rs66838241
rs55966621
rs62143197
rs6428919
rs2581888
rs145347178
rs532297899
rs13282034
rs2275132
rs117619220
rs72932235
rs75332708
rs140729364
rs10956734
rs75910760
rs58014306
rs9458931
rs6960416
rs12603593
rs144036010
rs148342023
All − MR Egger
All − IVW(MRE)
−0.005
0.000
0.005
0.010
MR effect size for
'UV excision repair protein RAD23 homolog A' on 'eGFR'

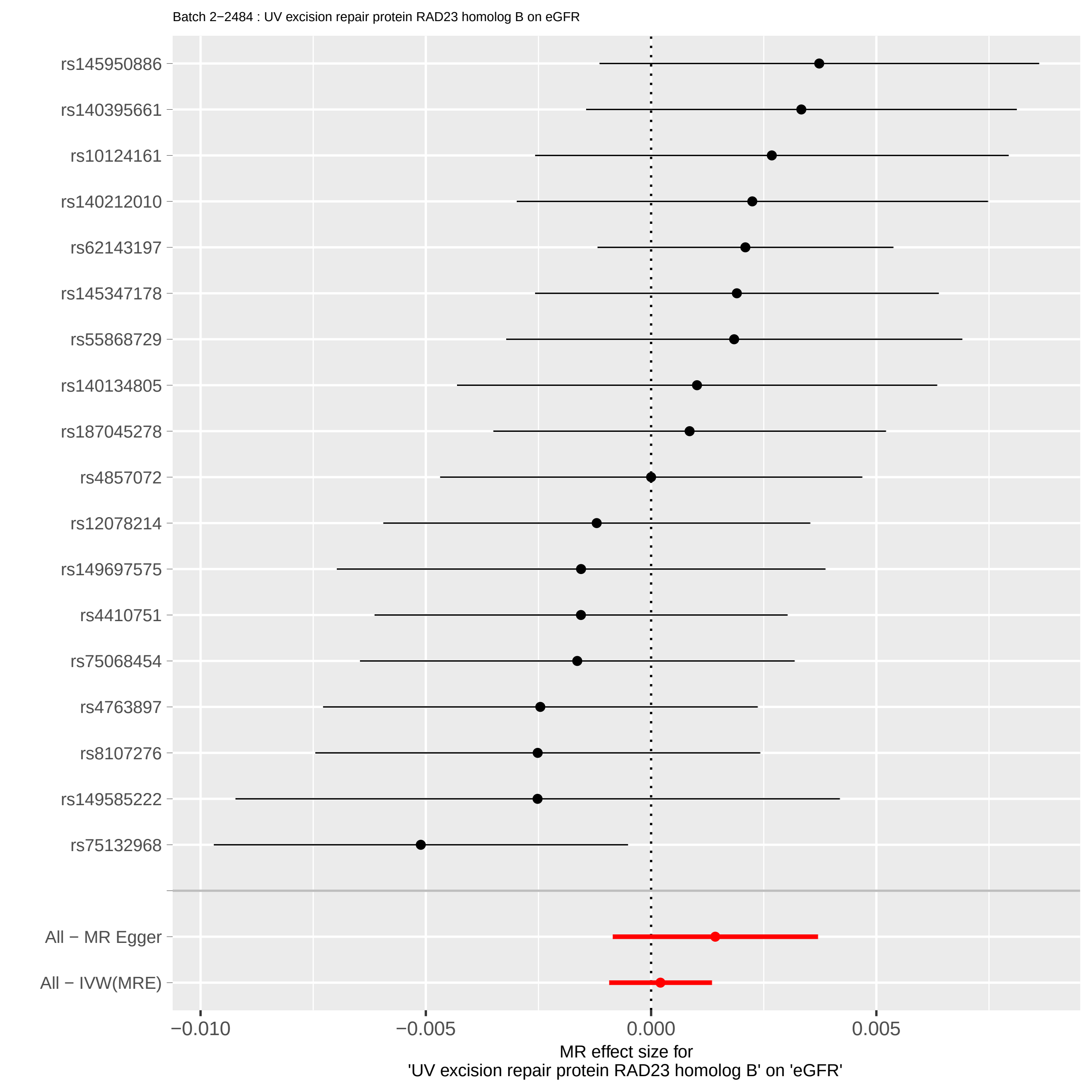

Batch 2−2484 : UV excision repair protein RAD23 homolog B on eGFR
rs145950886
rs140395661
rs10124161
rs140212010
rs62143197
rs145347178
rs55868729
rs140134805
rs187045278
rs4857072
rs12078214
rs149697575
rs4410751
rs75068454
rs4763897
rs8107276
rs149585222
rs75132968
All − MR Egger
All − IVW(MRE)
−0.010
−0.005
0.000
0.005
MR effect size for
'UV excision repair protein RAD23 homolog B' on 'eGFR'

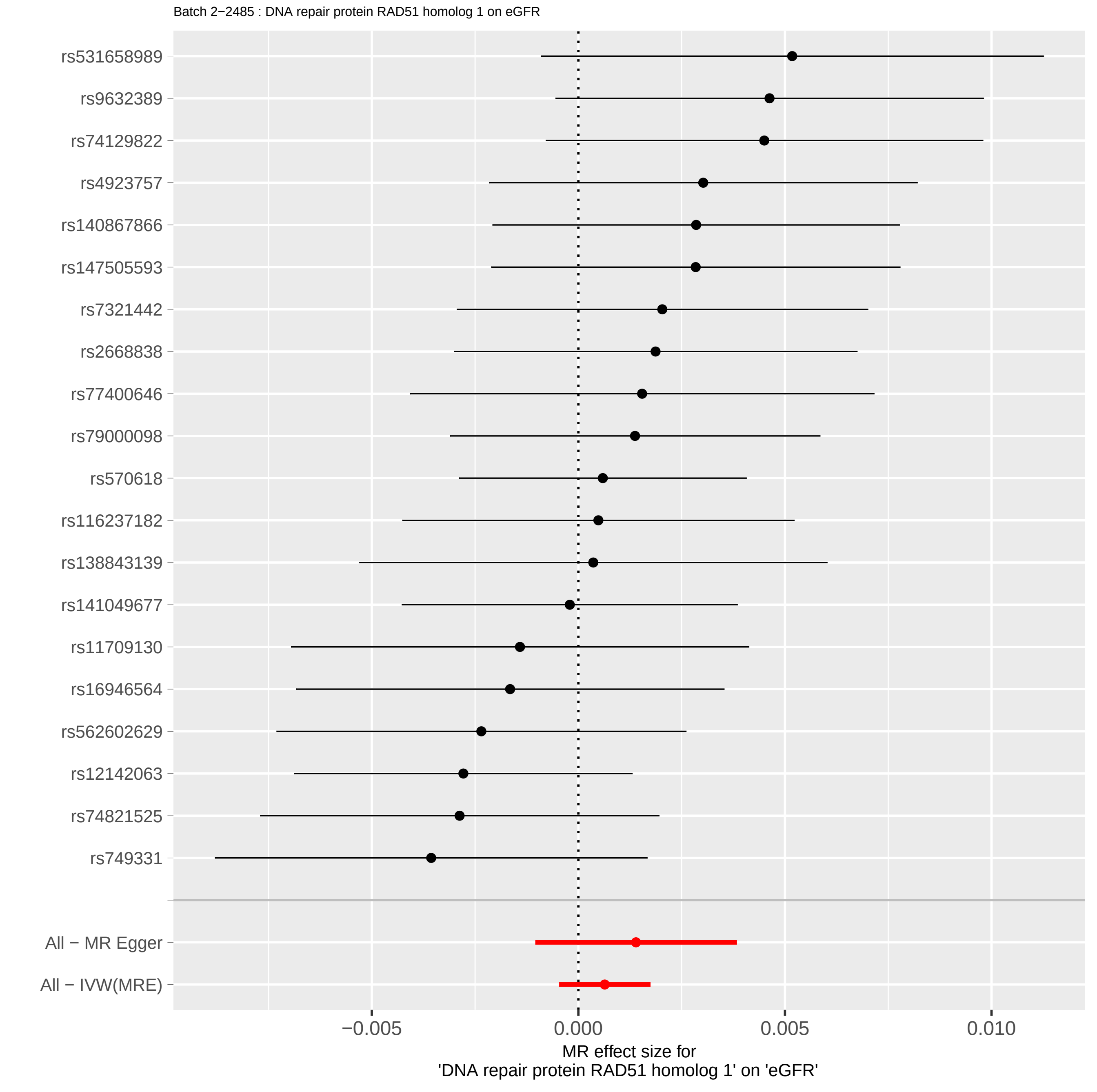

Batch 2−2485 : DNA repair protein RAD51 homolog 1 on eGFR
rs531658989
rs9632389
rs74129822
rs4923757
rs140867866
rs147505593
rs7321442
rs2668838
rs77400646
rs79000098
rs570618
rs116237182
rs138843139
rs141049677
rs11709130
rs16946564
rs562602629
rs12142063
rs74821525
rs749331
All − MR Egger
All − IVW(MRE)
−0.005
0.000
0.005
0.010
MR effect size for
'DNA repair protein RAD51 homolog 1' on 'eGFR'

Batch 2−2486 : DNA repair protein RAD51 homolog 4 on eGFR

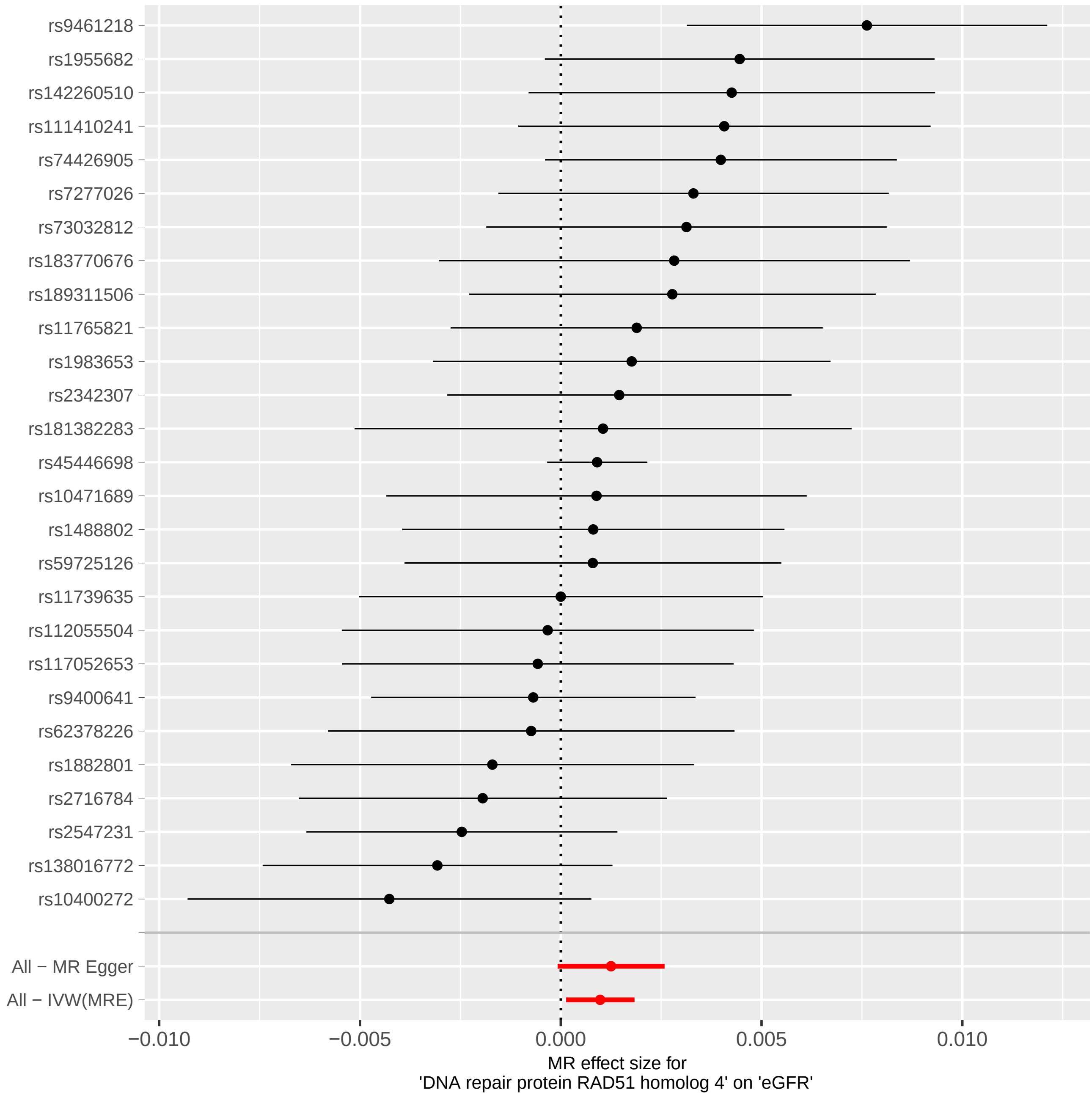

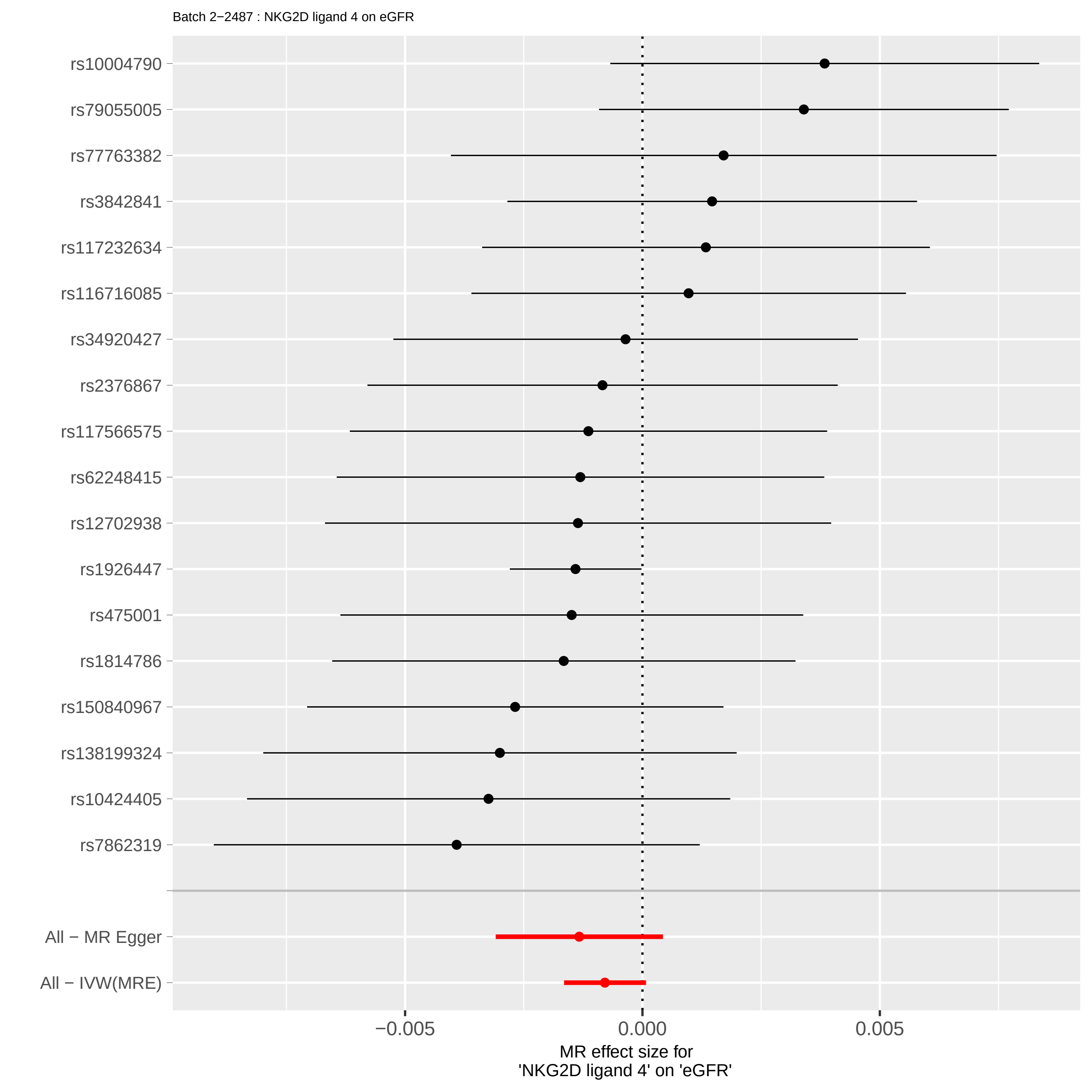

Batch 2−2487 : NKG2D ligand 4 on eGFR
rs10004790
rs79055005
rs77763382
rs3842841
rs117232634
rs116716085
rs34920427
rs2376867
rs117566575
rs62248415
rs12702938
rs1926447
rs475001
rs1814786
rs150840967
rs138199324
rs10424405
rs7862319
All − MR Egger
All − IVW(MRE)
−0.005
0.000
0.005
MR effect size for
'NKG2D ligand 4' on 'eGFR'

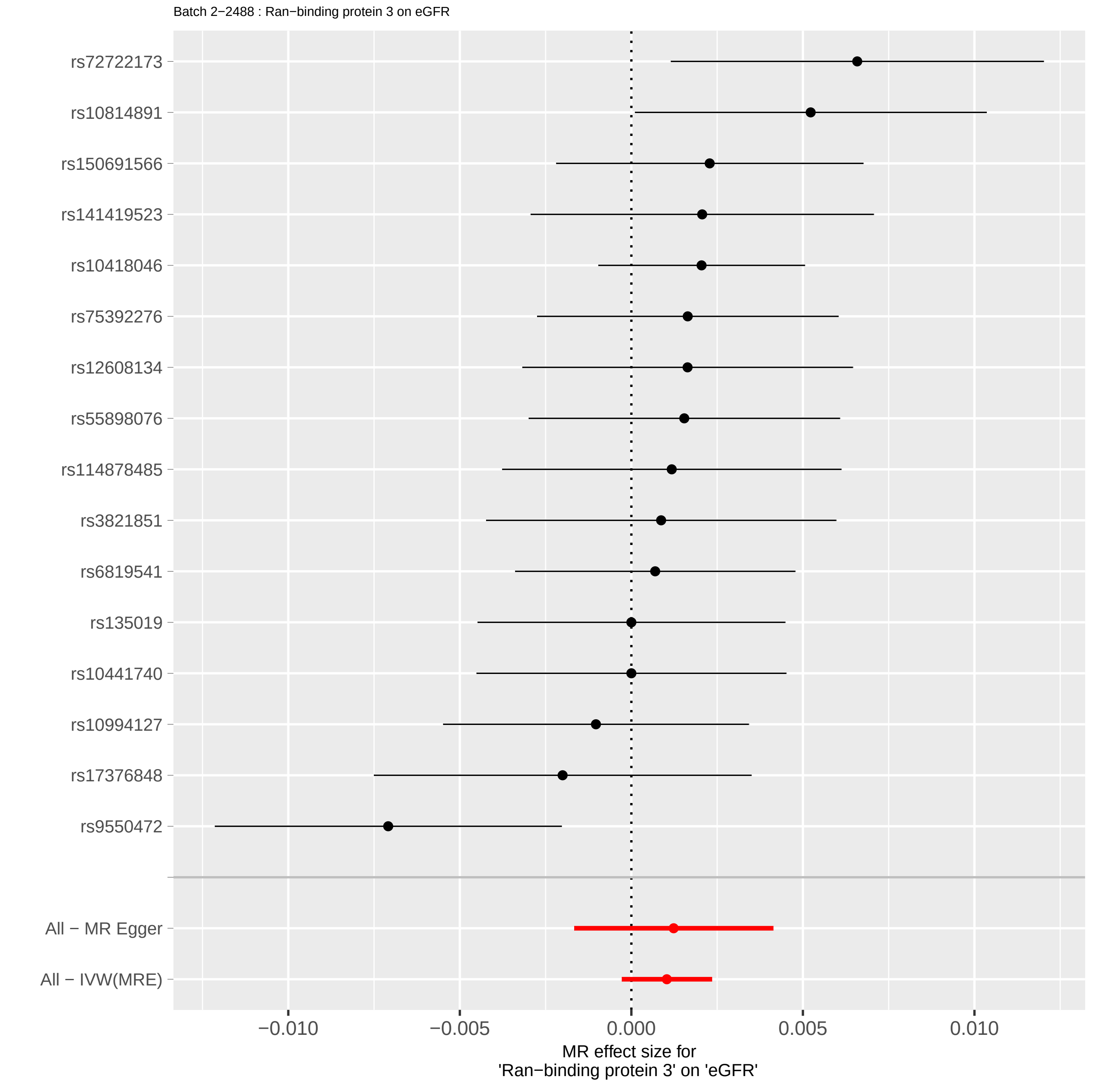

Batch 2−2488 : Ran−binding protein 3 on eGFR
rs72722173
rs10814891
rs150691566
rs141419523
rs10418046
rs75392276
rs12608134
rs55898076
rs114878485
rs3821851
rs6819541
rs135019
rs10441740
rs10994127
rs17376848
rs9550472
All − MR Egger
All − IVW(MRE)
−0.010
−0.005
0.000
0.005
0.010
MR effect size for
'Ran−binding protein 3' on 'eGFR'

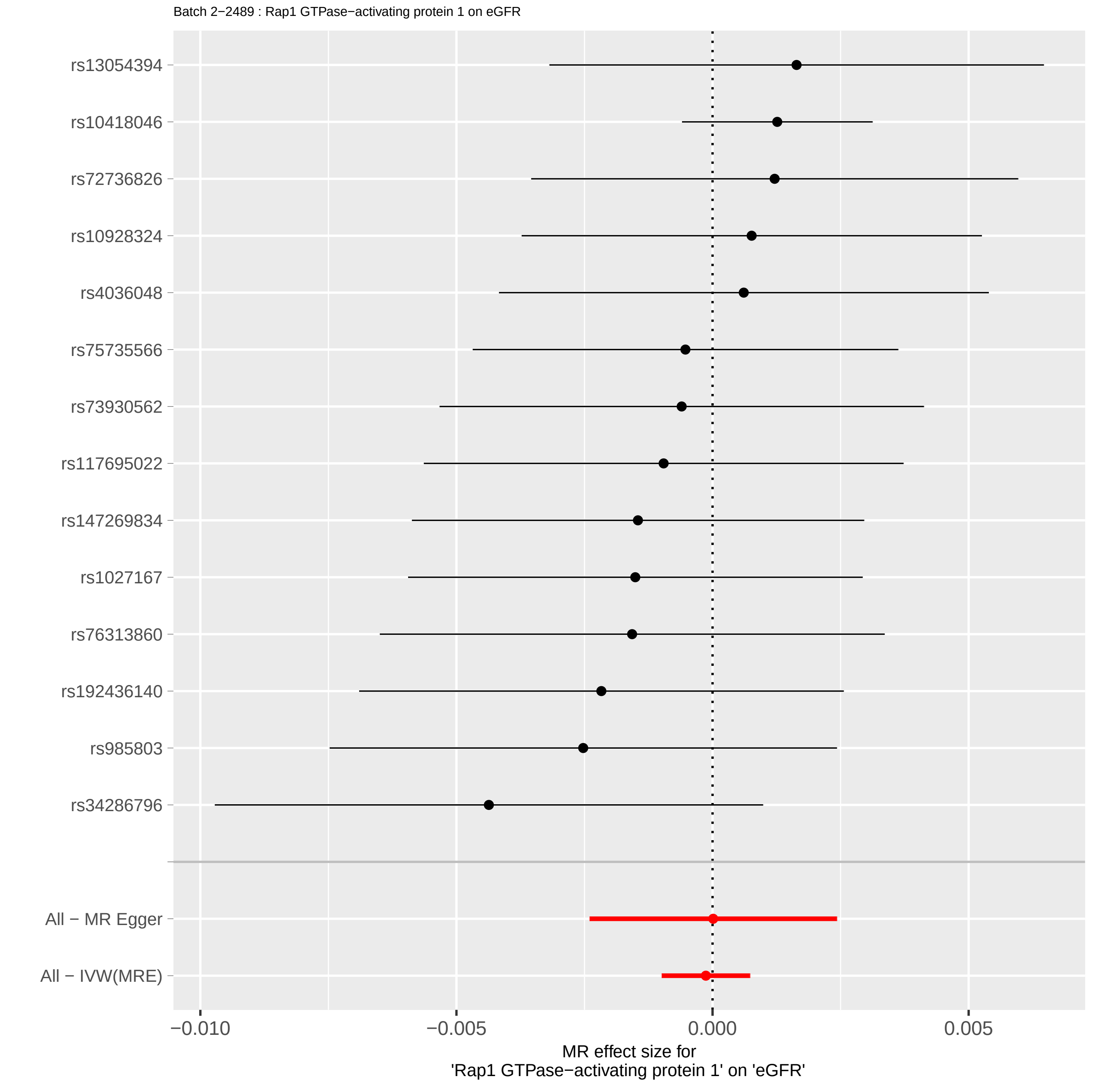

Batch 2−2489 : Rap1 GTPase−activating protein 1 on eGFR
rs13054394
rs10418046
rs72736826
rs10928324
rs4036048
rs75735566
rs73930562
rs117695022
rs147269834
rs1027167
rs76313860
rs192436140
rs985803
rs34286796
All − MR Egger
All − IVW(MRE)
−0.010
−0.005
0.000
0.005
MR effect size for
'Rap1 GTPase−activating protein 1' on 'eGFR'

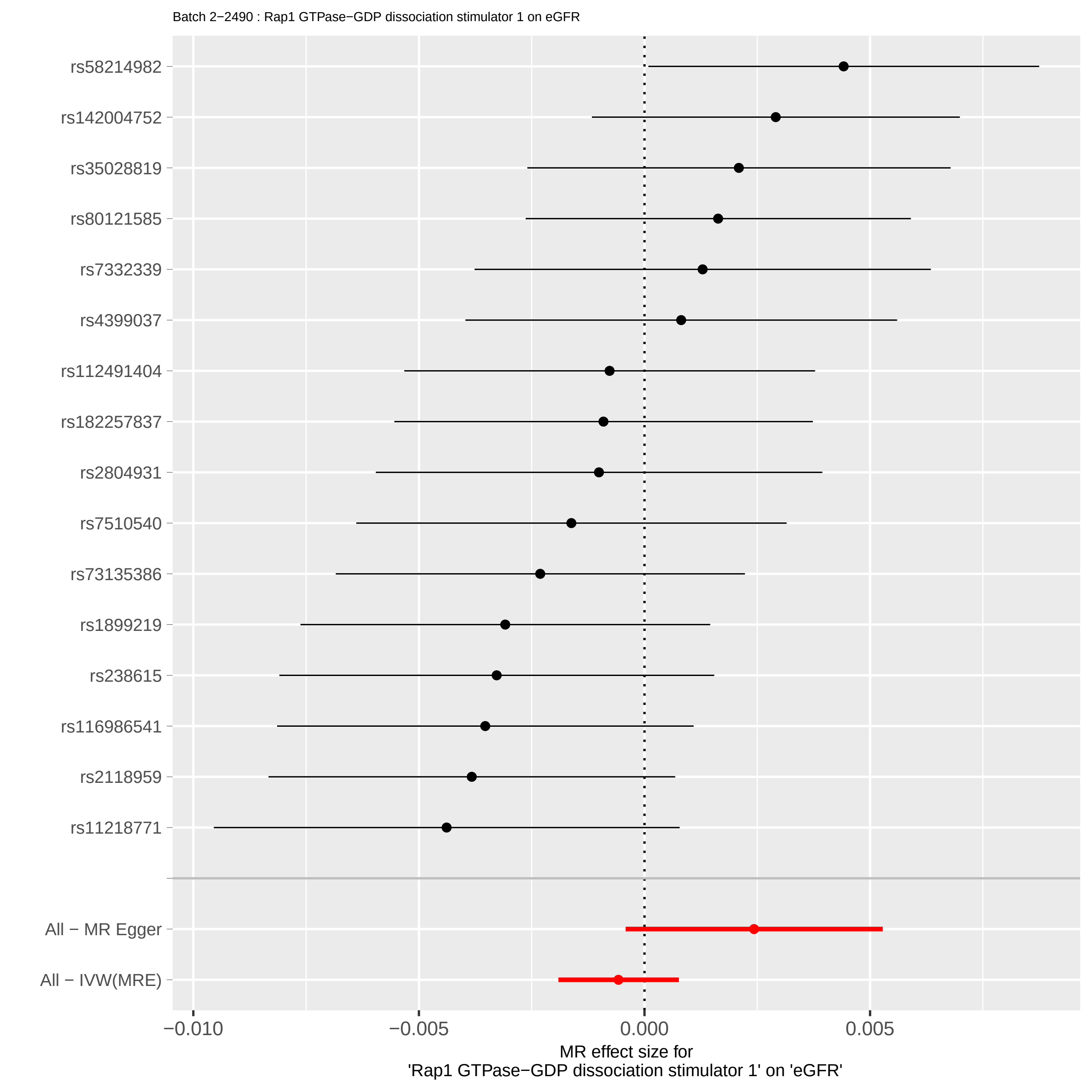

Batch 2−2490 : Rap1 GTPase−GDP dissociation stimulator 1 on eGFR
rs58214982
rs142004752
rs35028819
rs80121585
rs7332339
rs4399037
rs112491404
rs182257837
rs2804931
rs7510540
rs73135386
rs1899219
rs238615
rs116986541
rs2118959
rs11218771
All − MR Egger
All − IVW(MRE)
−0.010
−0.005
0.000
0.005
MR effect size for
'Rap1 GTPase−GDP dissociation stimulator 1' on 'eGFR'

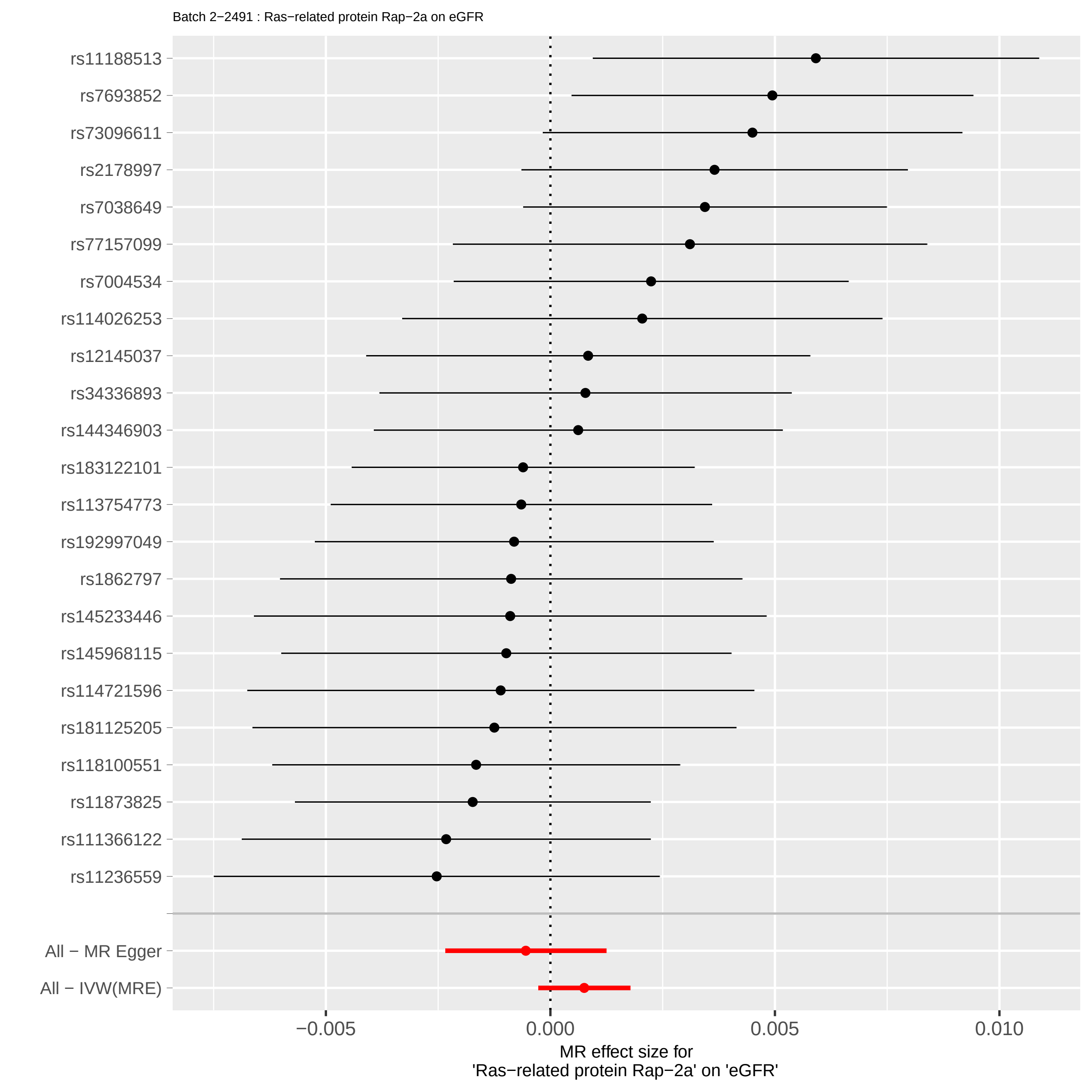

Batch 2−2491 : Ras−related protein Rap−2a on eGFR
rs11188513
rs7693852
rs73096611
rs2178997
rs7038649
rs77157099
rs7004534
rs114026253
rs12145037
rs34336893
rs144346903
rs183122101
rs113754773
rs192997049
rs1862797
rs145233446
rs145968115
rs114721596
rs181125205
rs118100551
rs11873825
rs111366122
rs11236559
All − MR Egger
All − IVW(MRE)
−0.005
0.000
0.005
0.010
MR effect size for
'Ras−related protein Rap−2a' on 'eGFR'

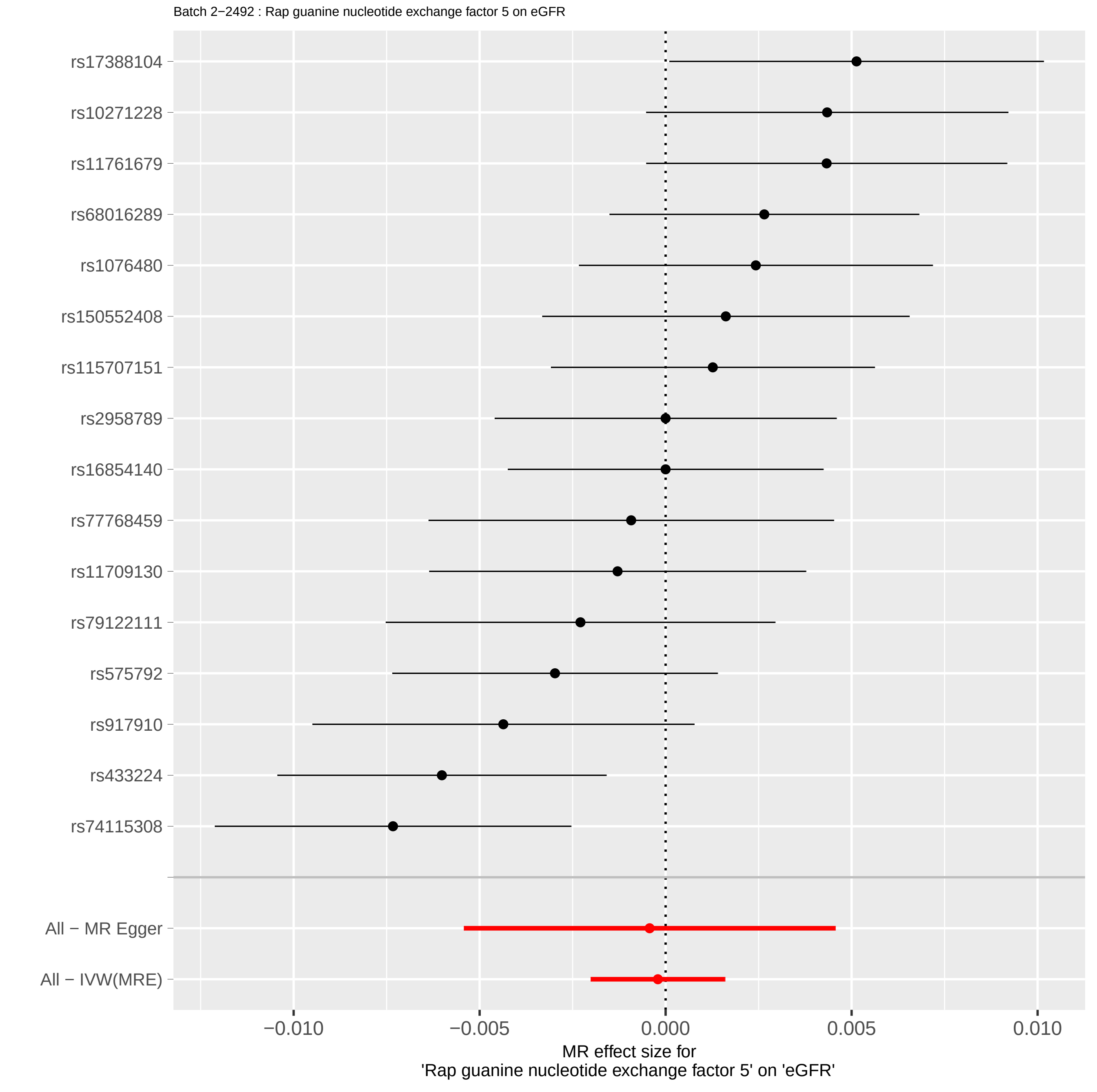
Batch 2−2492 : Rap guanine nucleotide exchange factor 5 on eGFR
rs17388104
rs10271228
rs11761679
rs68016289
rs1076480
rs150552408
rs115707151
rs2958789
rs16854140
rs77768459
rs11709130
rs79122111
rs575792
rs917910
rs433224
rs74115308
All − MR Egger
All − IVW(MRE)
−0.010
−0.005
0.000
0.005
0.010
MR effect size for
'Rap guanine nucleotide exchange factor 5' on 'eGFR'

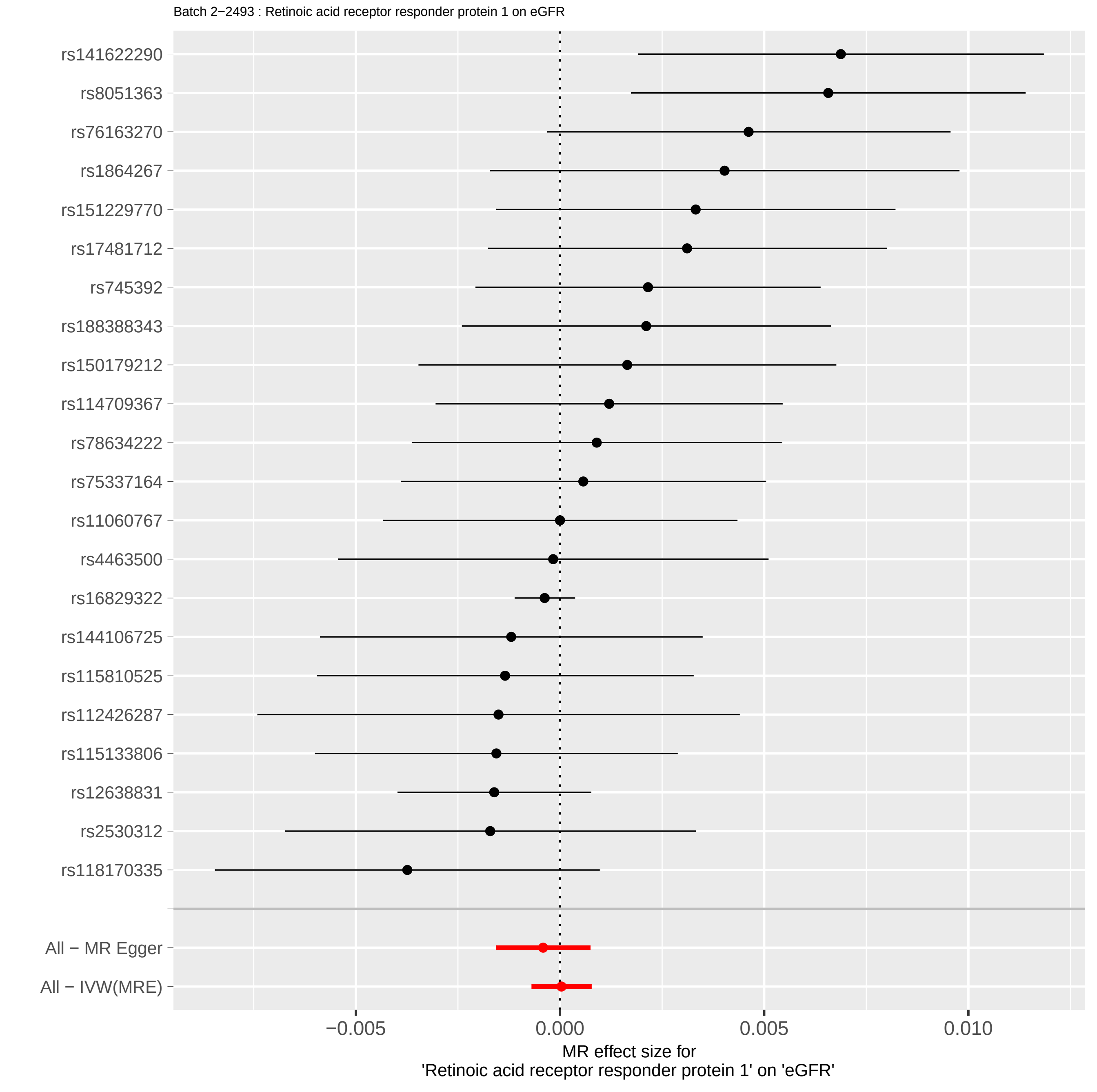

Batch 2−2493 : Retinoic acid receptor responder protein 1 on eGFR
rs141622290
rs8051363
rs76163270
rs1864267
rs151229770
rs17481712
rs745392
rs188388343
rs150179212
rs114709367
rs78634222
rs75337164
rs11060767
rs4463500
rs16829322
rs144106725
rs115810525
rs112426287
rs115133806
rs12638831
rs2530312
rs118170335
All − MR Egger
All − IVW(MRE)
−0.005
0.000
0.005
0.010
MR effect size for
'Retinoic acid receptor responder protein 1' on 'eGFR'

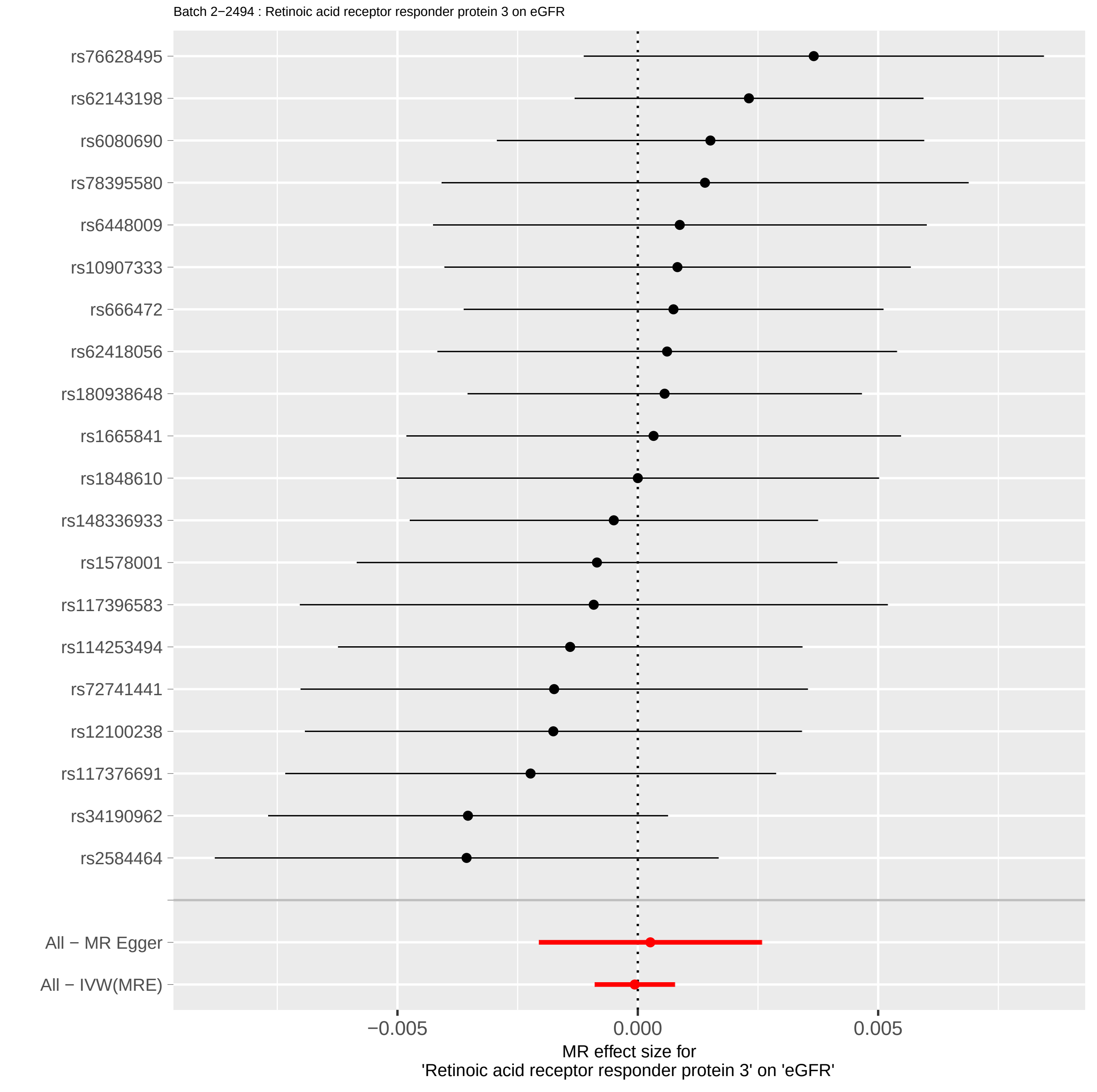

Batch 2−2494 : Retinoic acid receptor responder protein 3 on eGFR
rs76628495
rs62143198
rs6080690
rs78395580
rs6448009
rs10907333
rs666472
rs62418056
rs180938648
rs1665841
rs1848610
rs148336933
rs1578001
rs117396583
rs114253494
rs72741441
rs12100238
rs117376691
rs34190962
rs2584464
All − MR Egger
All − IVW(MRE)
−0.005
0.000
0.005
MR effect size for
'Retinoic acid receptor responder protein 3' on 'eGFR'

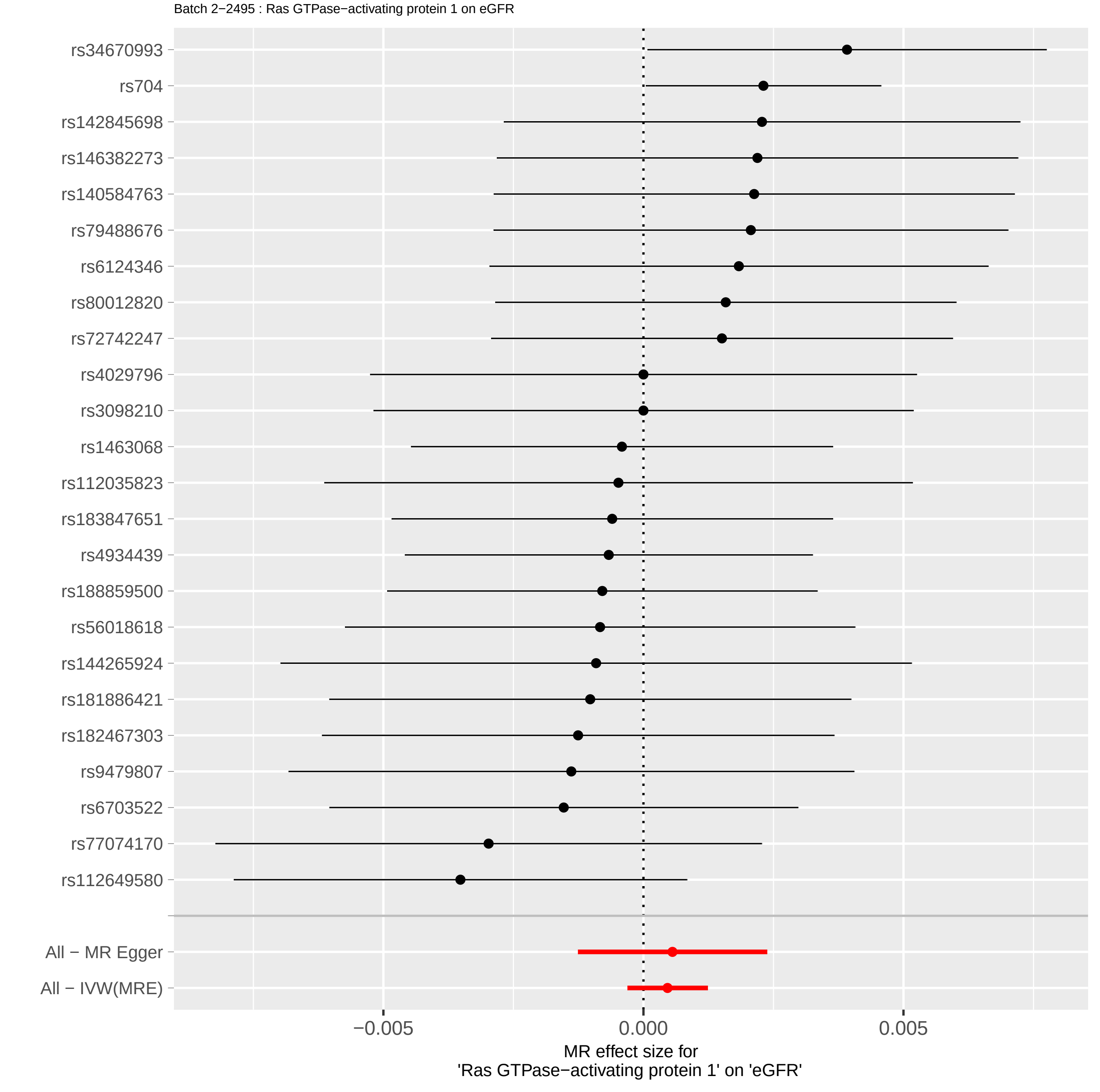

Batch 2−2495 : Ras GTPase−activating protein 1 on eGFR
rs34670993
rs704
rs142845698
rs146382273
rs140584763
rs79488676
rs6124346
rs80012820
rs72742247
rs4029796
rs3098210
rs1463068
rs112035823
rs183847651
rs4934439
rs188859500
rs56018618
rs144265924
rs181886421
rs182467303
rs9479807
rs6703522
rs77074170
rs112649580
All − MR Egger
All − IVW(MRE)
−0.005
0.000
0.005
MR effect size for
'Ras GTPase−activating protein 1' on 'eGFR'

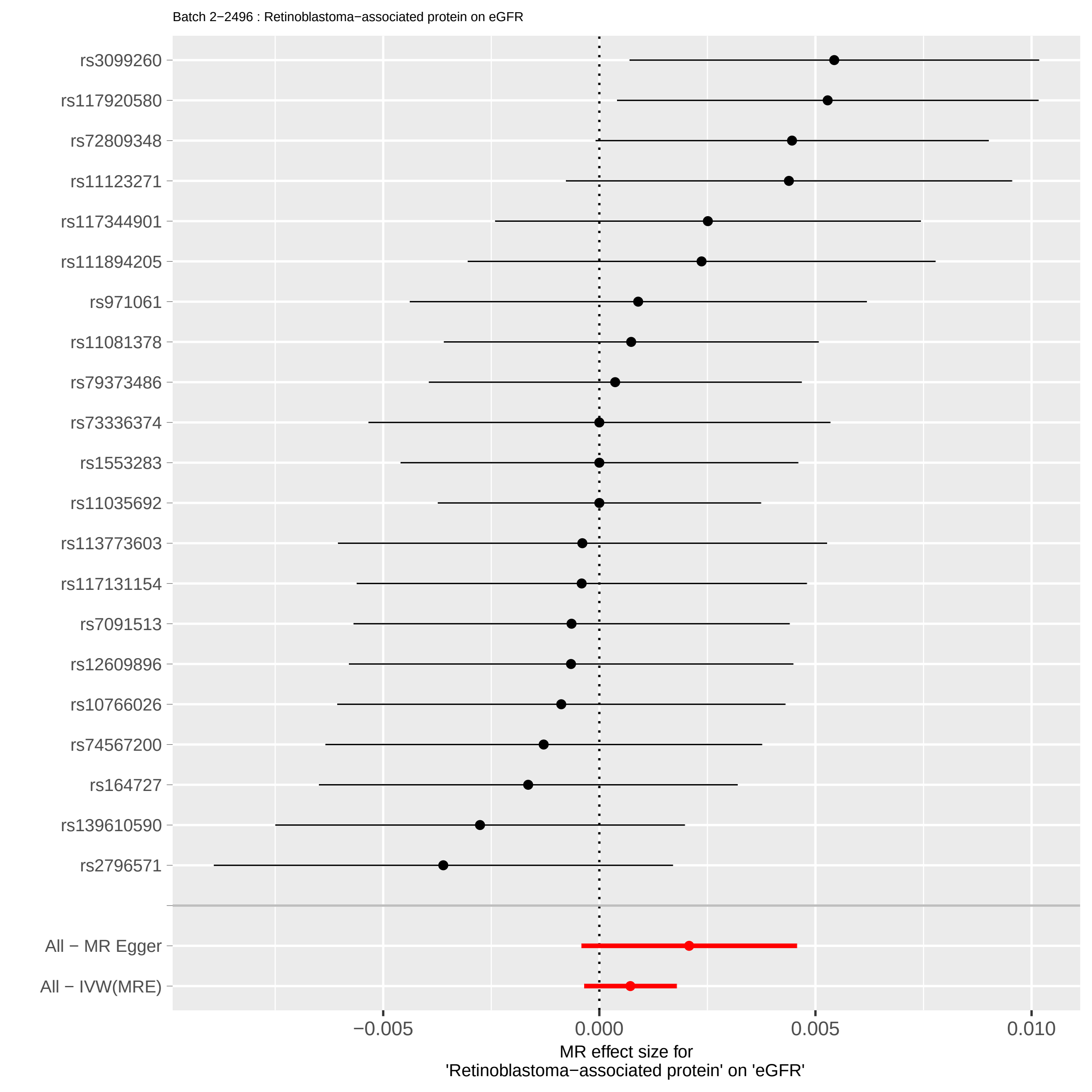

Batch 2−2496 : Retinoblastoma−associated protein on eGFR
rs3099260
rs117920580
rs72809348
rs11123271
rs117344901
rs111894205
rs971061
rs11081378
rs79373486
rs73336374
rs1553283
rs11035692
rs113773603
rs117131154
rs7091513
rs12609896
rs10766026
rs74567200
rs164727
rs139610590
rs2796571
All − MR Egger
All − IVW(MRE)
−0.005
0.000
0.005
0.010
MR effect size for
'Retinoblastoma−associated protein' on 'eGFR'

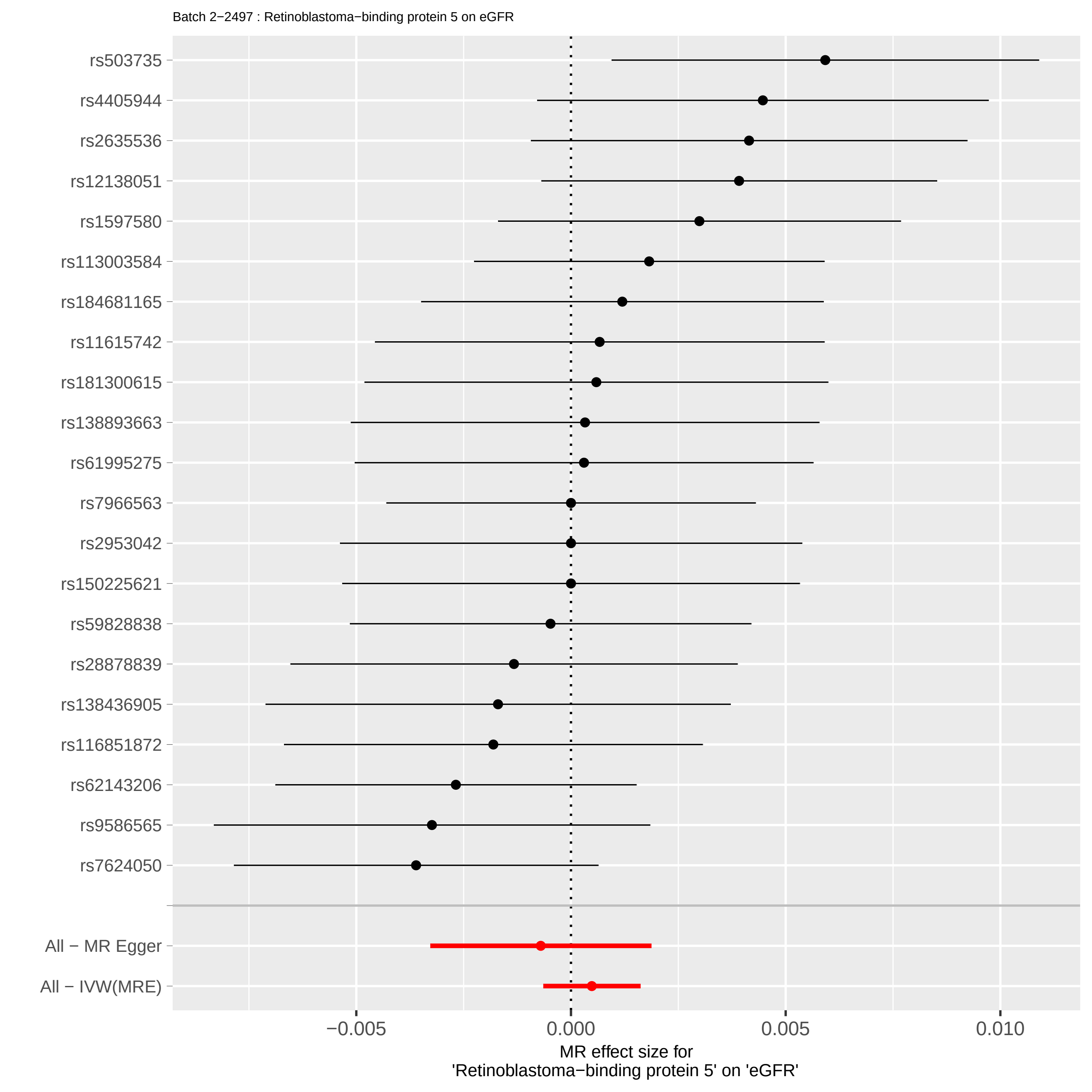

Batch 2−2497 : Retinoblastoma−binding protein 5 on eGFR
rs503735
rs4405944
rs2635536
rs12138051
rs1597580
rs113003584
rs184681165
rs11615742
rs181300615
rs138893663
rs61995275
rs7966563
rs2953042
rs150225621
rs59828838
rs28878839
rs138436905
rs116851872
rs62143206
rs9586565
rs7624050
All − MR Egger
All − IVW(MRE)
−0.005
0.000
0.005
0.010
MR effect size for
'Retinoblastoma−binding protein 5' on 'eGFR'

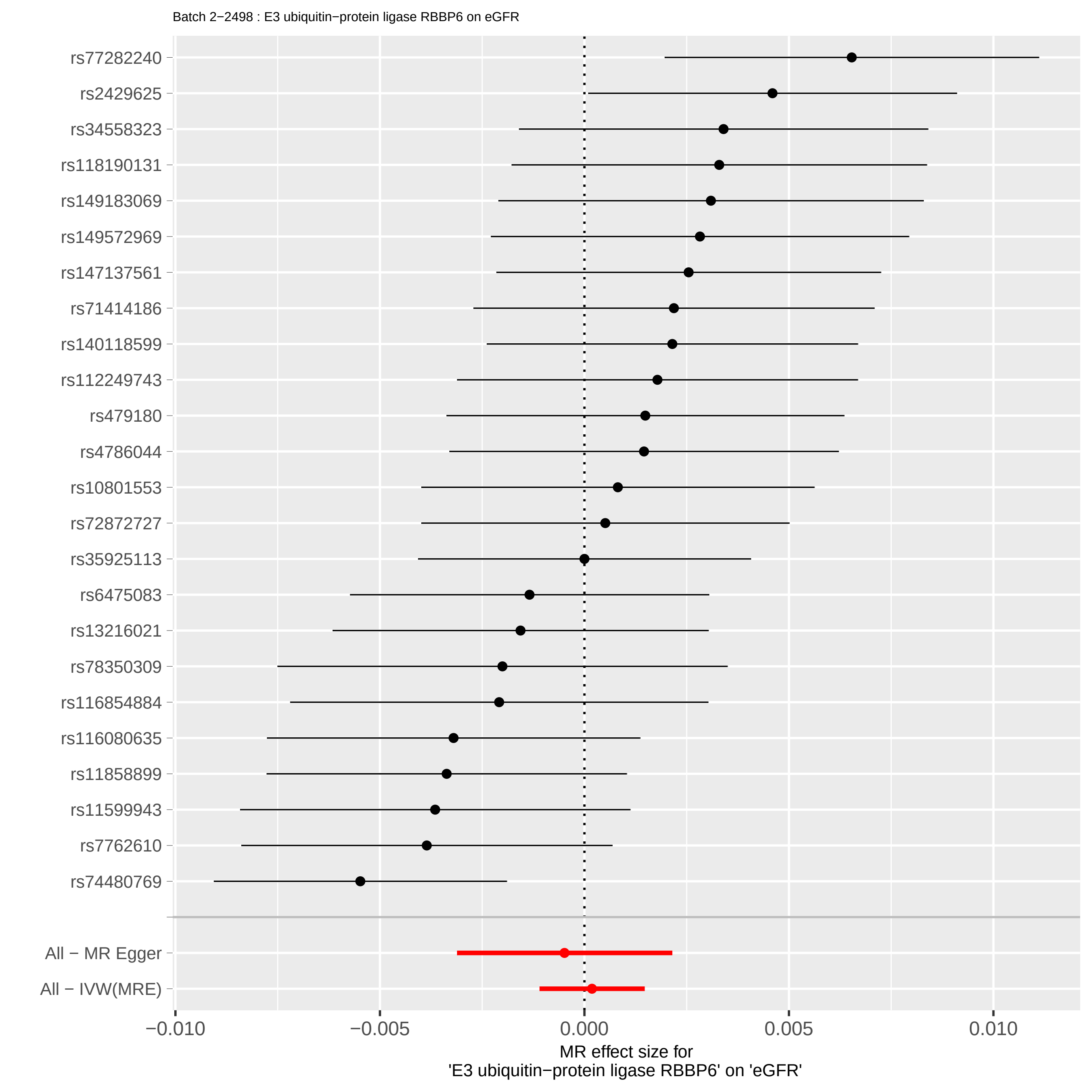

Batch 2−2498 : E3 ubiquitin−protein ligase RBBP6 on eGFR
rs77282240
rs2429625
rs34558323
rs118190131
rs149183069
rs149572969
rs147137561
rs71414186
rs140118599
rs112249743
rs479180
rs4786044
rs10801553
rs72872727
rs35925113
rs6475083
rs13216021
rs78350309
rs116854884
rs116080635
rs11858899
rs11599943
rs7762610
rs74480769
All − MR Egger
All − IVW(MRE)
−0.010
−0.005
0.000
0.005
0.010
MR effect size for
'E3 ubiquitin−protein ligase RBBP6' on 'eGFR'

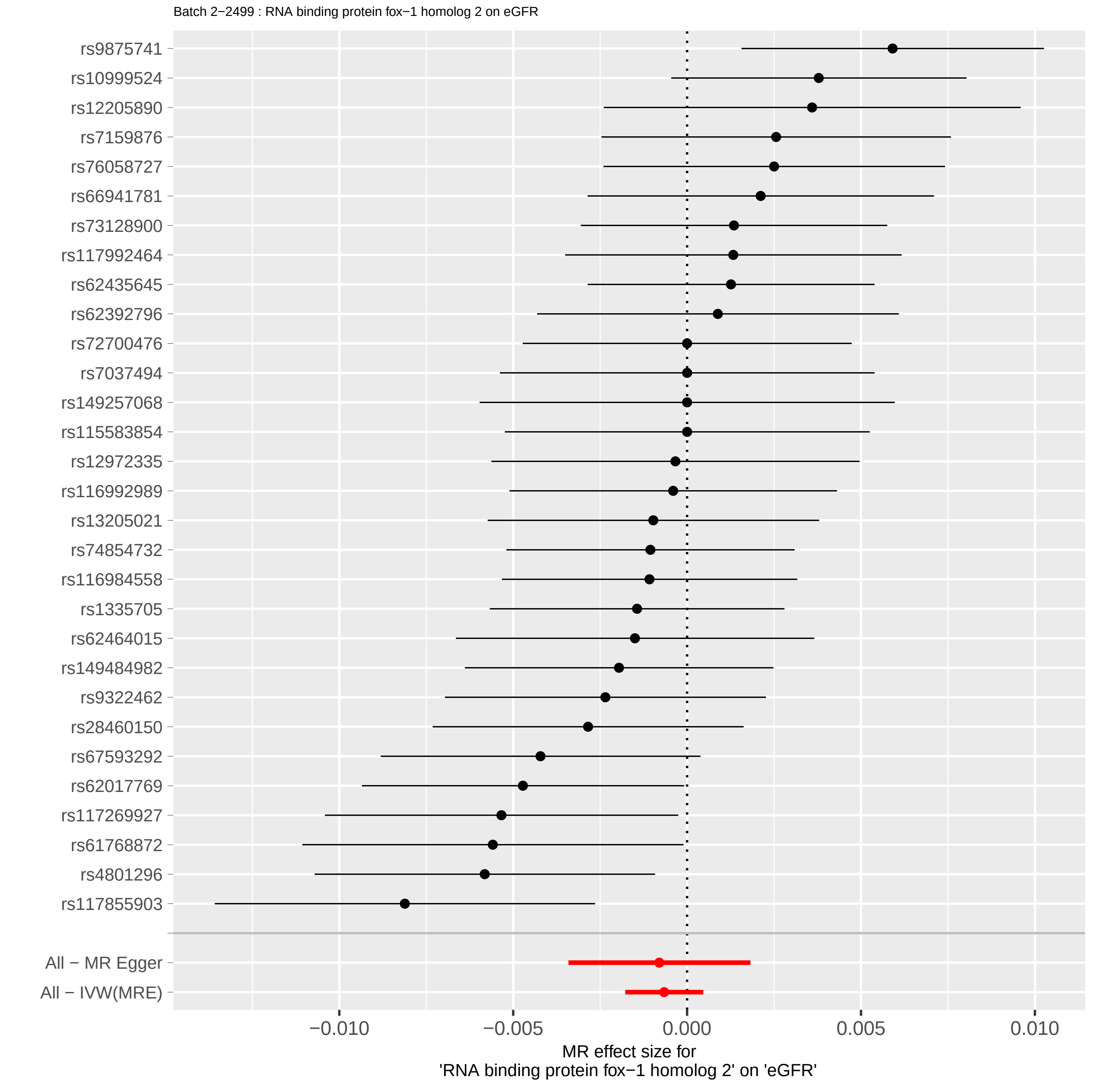
Batch 2−2499 : RNA binding protein fox−1 homolog 2 on eGFR
rs9875741
rs10999524
rs12205890
rs7159876
rs76058727
rs66941781
rs73128900
rs117992464
rs62435645
rs62392796
rs72700476
rs7037494
rs149257068
rs115583854
rs12972335
rs116992989
rs13205021
rs74854732
rs116984558
rs1335705
rs62464015
rs149484982
rs9322462
rs28460150
rs67593292
rs62017769
rs117269927
rs61768872
rs4801296
rs117855903
All − MR Egger
All − IVW(MRE)
−0.010
−0.005
0.000
0.005
0.010
MR effect size for
'RNA binding protein fox−1 homolog 2' on 'eGFR'

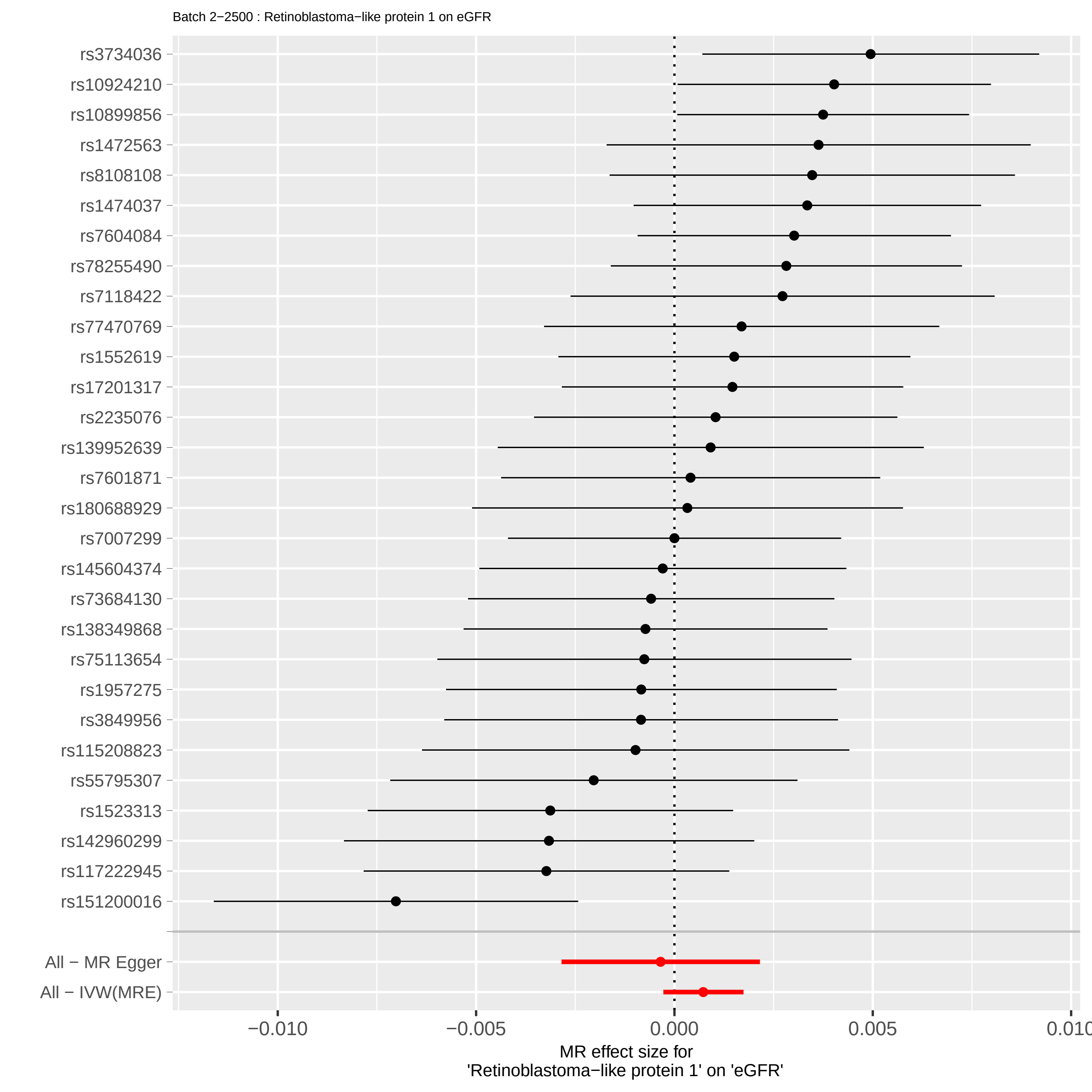
Batch 2−2500 : Retinoblastoma−like protein 1 on eGFR
rs3734036
rs10924210
rs10899856
rs1472563
rs8108108
rs1474037
rs7604084
rs78255490
rs7118422
rs77470769
rs1552619
rs17201317
rs2235076
rs139952639
rs7601871
rs180688929
rs7007299
rs145604374
rs73684130
rs138349868
rs75113654
rs1957275
rs3849956
rs115208823
rs55795307
rs1523313
rs142960299
rs117222945
rs151200016
All − MR Egger
All − IVW(MRE)
−0.010
−0.005
0.000
0.005
0.010
MR effect size for
'Retinoblastoma−like protein 1' on 'eGFR'

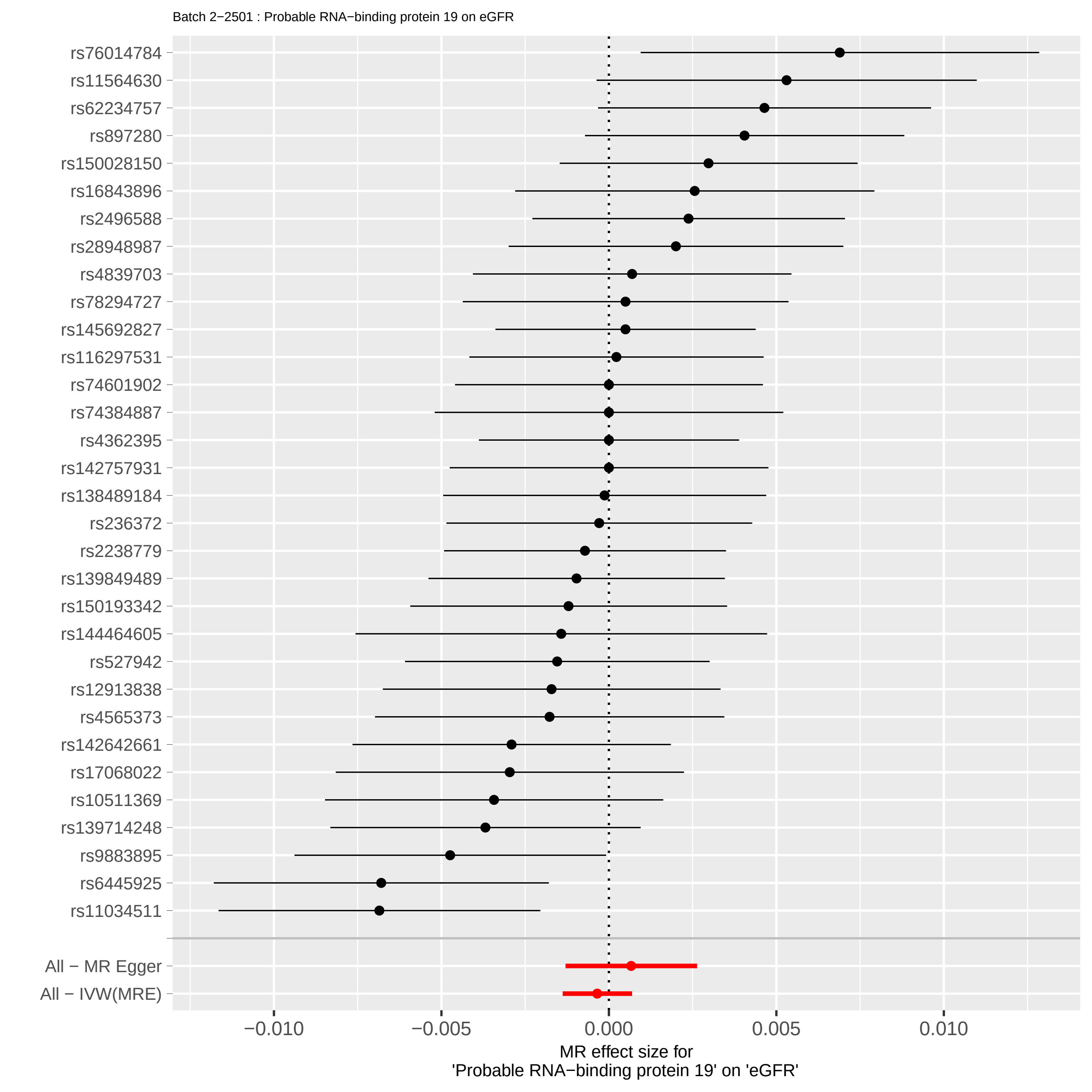

Batch 2−2501 : Probable RNA−binding protein 19 on eGFR
rs76014784
rs11564630
rs62234757
rs897280
rs150028150
rs16843896
rs2496588
rs28948987
rs4839703
rs78294727
rs145692827
rs116297531
rs74601902
rs74384887
rs4362395
rs142757931
rs138489184
rs236372
rs2238779
rs139849489
rs150193342
rs144464605
rs527942
rs12913838
rs4565373
rs142642661
rs17068022
rs10511369
rs139714248
rs9883895
rs6445925
rs11034511
All − MR Egger
All − IVW(MRE)
−0.010
−0.005
0.000
0.005
0.010
MR effect size for
'Probable RNA−binding protein 19' on 'eGFR'

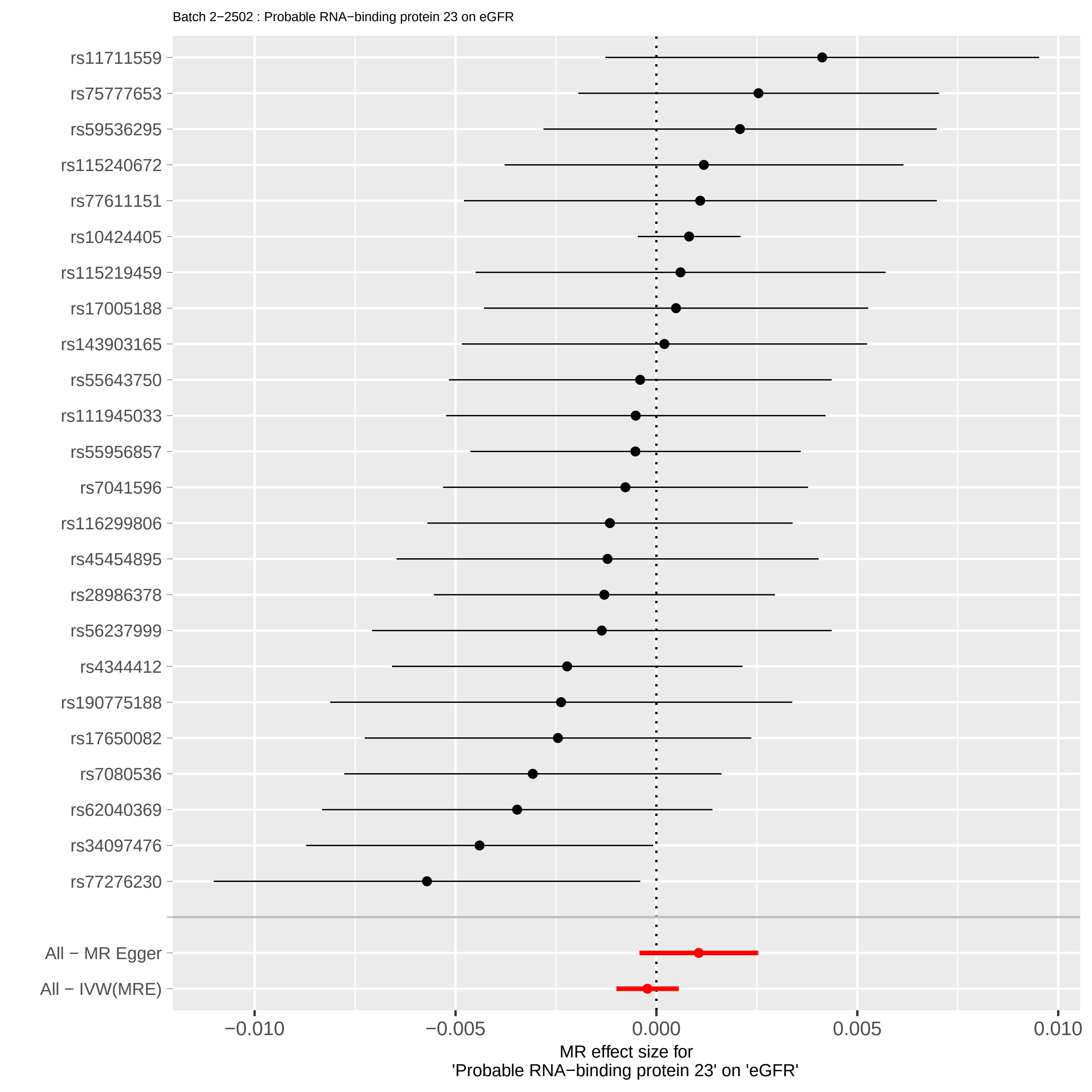

Batch 2−2502 : Probable RNA−binding protein 23 on eGFR
rs11711559
rs75777653
rs59536295
rs115240672
rs77611151
rs10424405
rs115219459
rs17005188
rs143903165
rs55643750
rs111945033
rs55956857
rs7041596
rs116299806
rs45454895
rs28986378
rs56237999
rs4344412
rs190775188
rs17650082
rs7080536
rs62040369
rs34097476
rs77276230
All − MR Egger
All − IVW(MRE)
−0.010
−0.005
0.000
0.005
0.010
MR effect size for
'Probable RNA−binding protein 23' on 'eGFR'

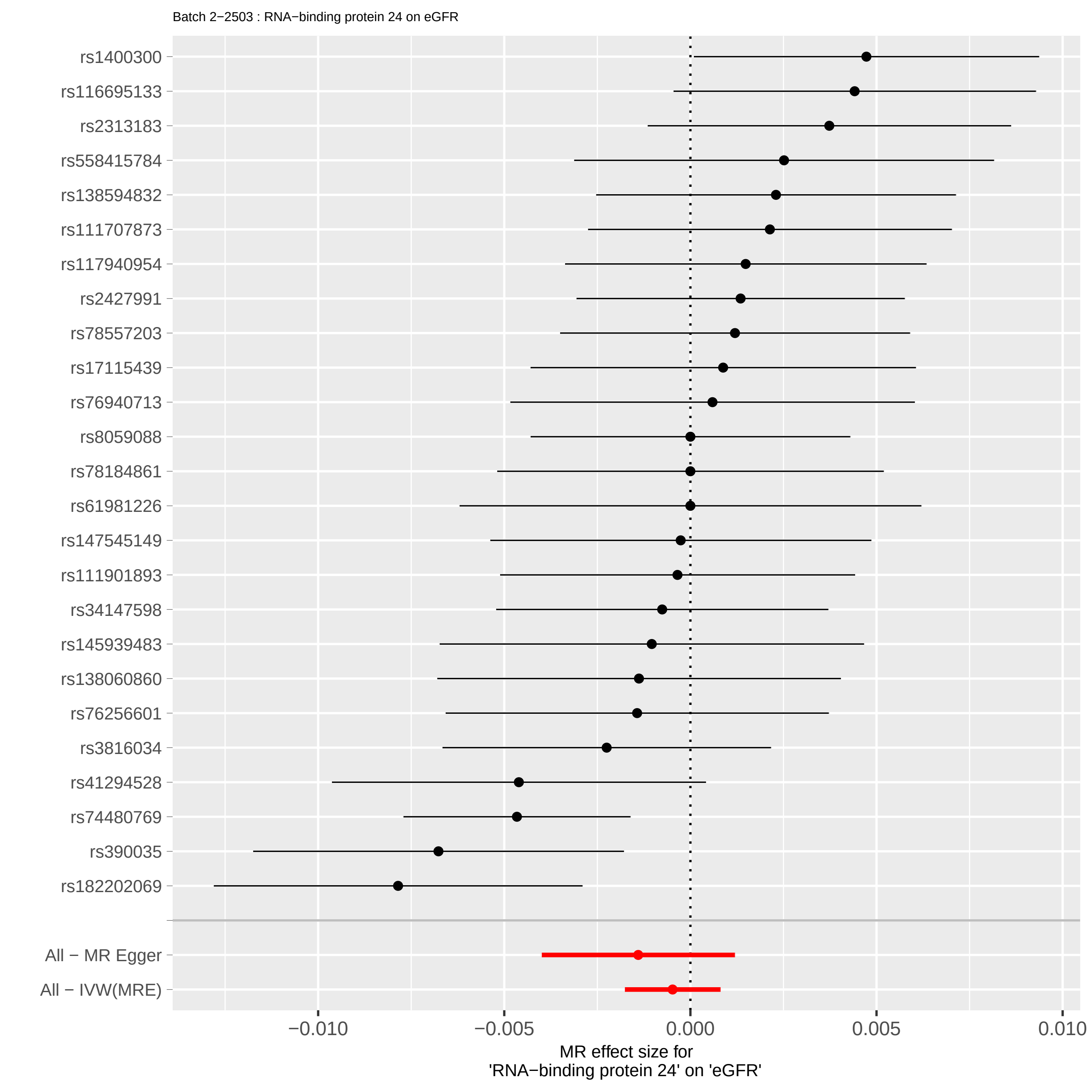
Batch 2−2503 : RNA−binding protein 24 on eGFR
rs1400300
rs116695133
rs2313183
rs558415784
rs138594832
rs111707873
rs117940954
rs2427991
rs78557203
rs17115439
rs76940713
rs8059088
rs78184861
rs61981226
rs147545149
rs111901893
rs34147598
rs145939483
rs138060860
rs76256601
rs3816034
rs41294528
rs74480769
rs390035
rs182202069
All − MR Egger
All − IVW(MRE)
−0.010
−0.005
0.000
0.005
0.010
MR effect size for
'RNA−binding protein 24' on 'eGFR'

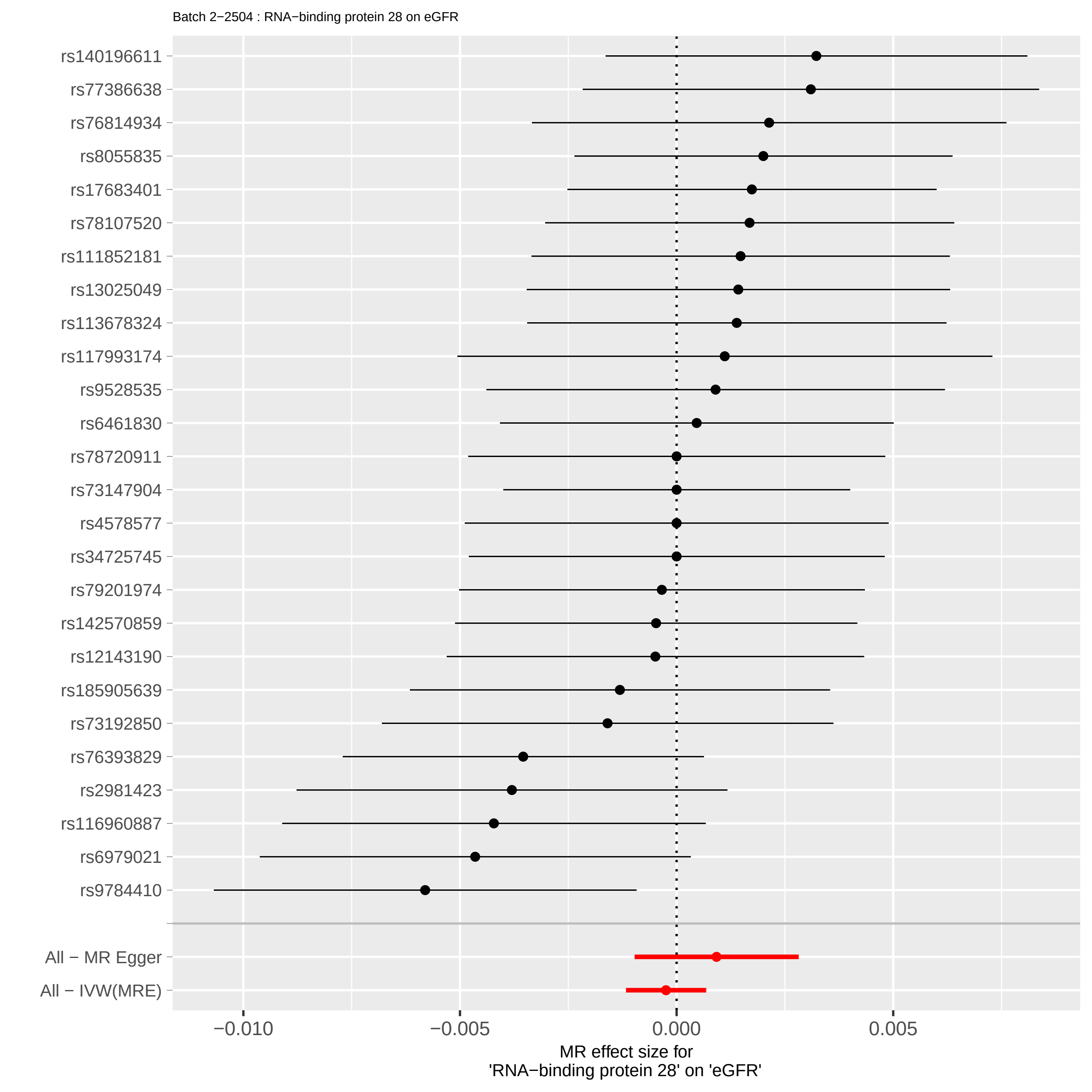

Batch 2−2504 : RNA−binding protein 28 on eGFR
rs140196611
rs77386638
rs76814934
rs8055835
rs17683401
rs78107520
rs111852181
rs13025049
rs113678324
rs117993174
rs9528535
rs6461830
rs78720911
rs73147904
rs4578577
rs34725745
rs79201974
rs142570859
rs12143190
rs185905639
rs73192850
rs76393829
rs2981423
rs116960887
rs6979021
rs9784410
All − MR Egger
All − IVW(MRE)
−0.010
−0.005
0.000
0.005
MR effect size for
'RNA−binding protein 28' on 'eGFR'

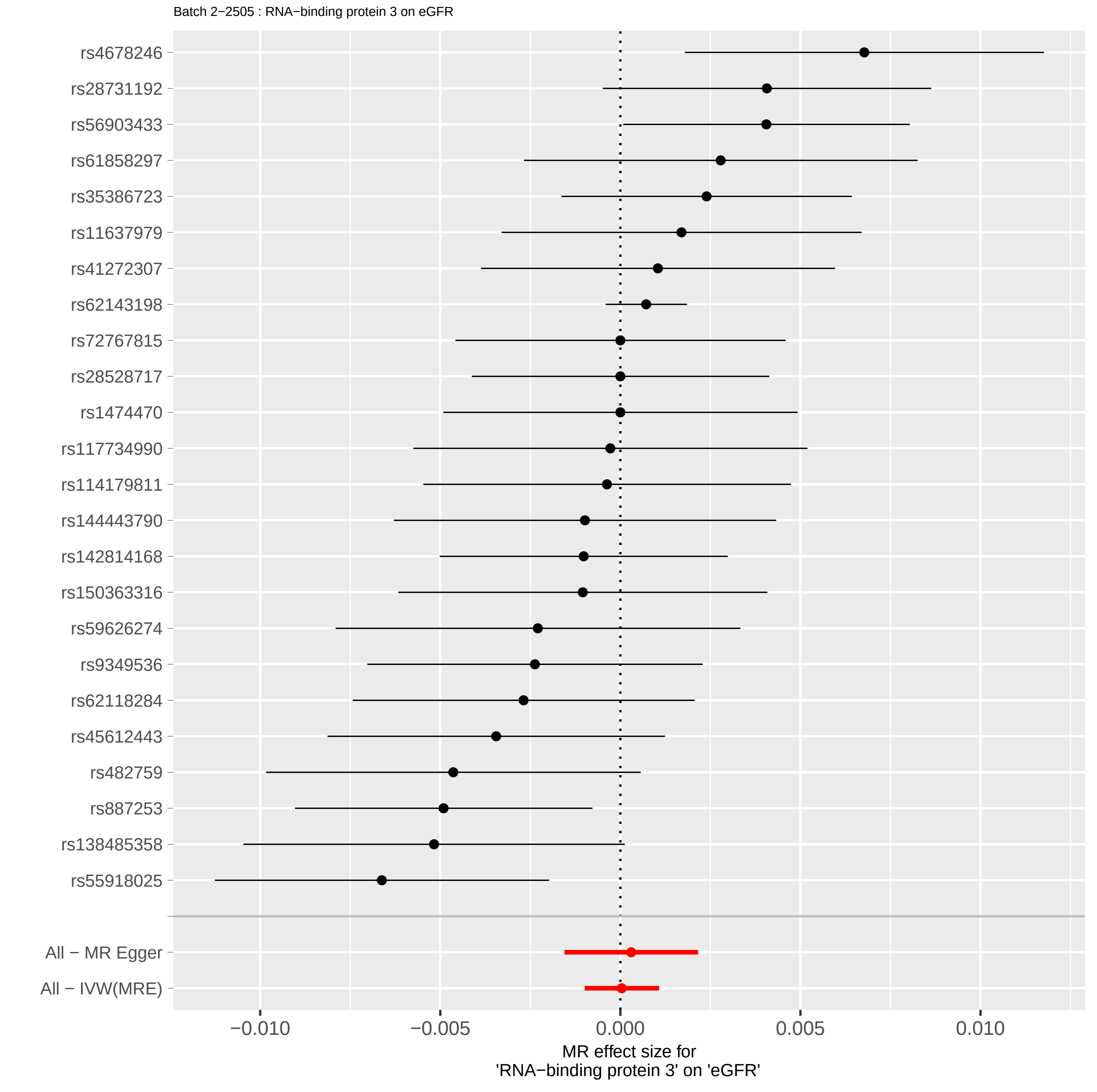
Batch 2−2505 : RNA−binding protein 3 on eGFR
rs4678246
rs28731192
rs56903433
rs61858297
rs35386723
rs11637979
rs41272307
rs62143198
rs72767815
rs28528717
rs1474470
rs117734990
rs114179811
rs144443790
rs142814168
rs150363316
rs59626274
rs9349536
rs62118284
rs45612443
rs482759
rs887253
rs138485358
rs55918025
All − MR Egger
All − IVW(MRE)
−0.010
−0.005
0.000
0.005
0.010
MR effect size for
'RNA−binding protein 3' on 'eGFR'

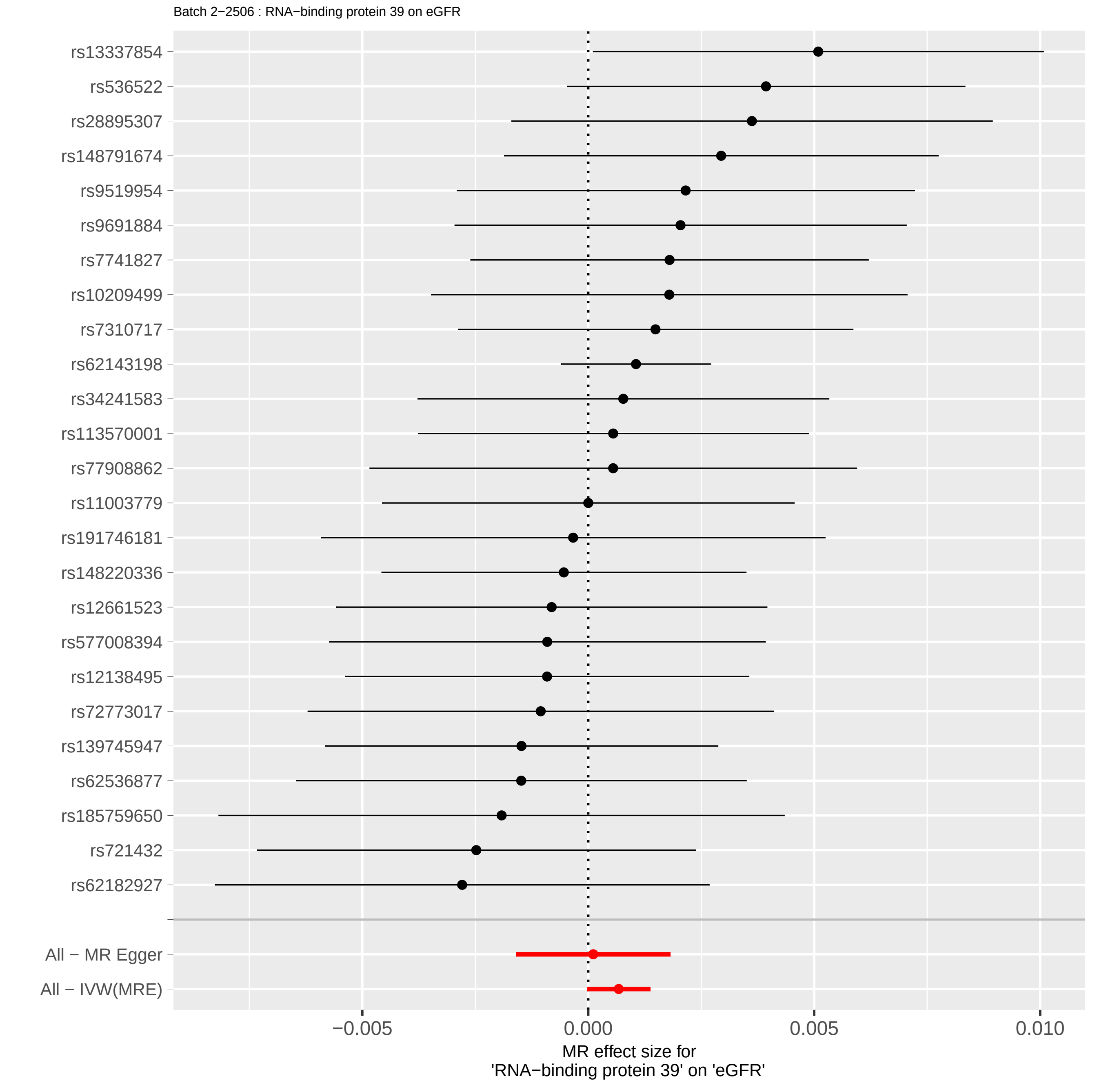
Batch 2−2506 : RNA−binding protein 39 on eGFR
rs13337854
rs536522
rs28895307
rs148791674
rs9519954
rs9691884
rs7741827
rs10209499
rs7310717
rs62143198
rs34241583
rs113570001
rs77908862
rs11003779
rs191746181
rs148220336
rs12661523
rs577008394
rs12138495
rs72773017
rs139745947
rs62536877
rs185759650
rs721432
rs62182927
All − MR Egger
All − IVW(MRE)
−0.005
0.000
0.005
0.010
MR effect size for
'RNA−binding protein 39' on 'eGFR'

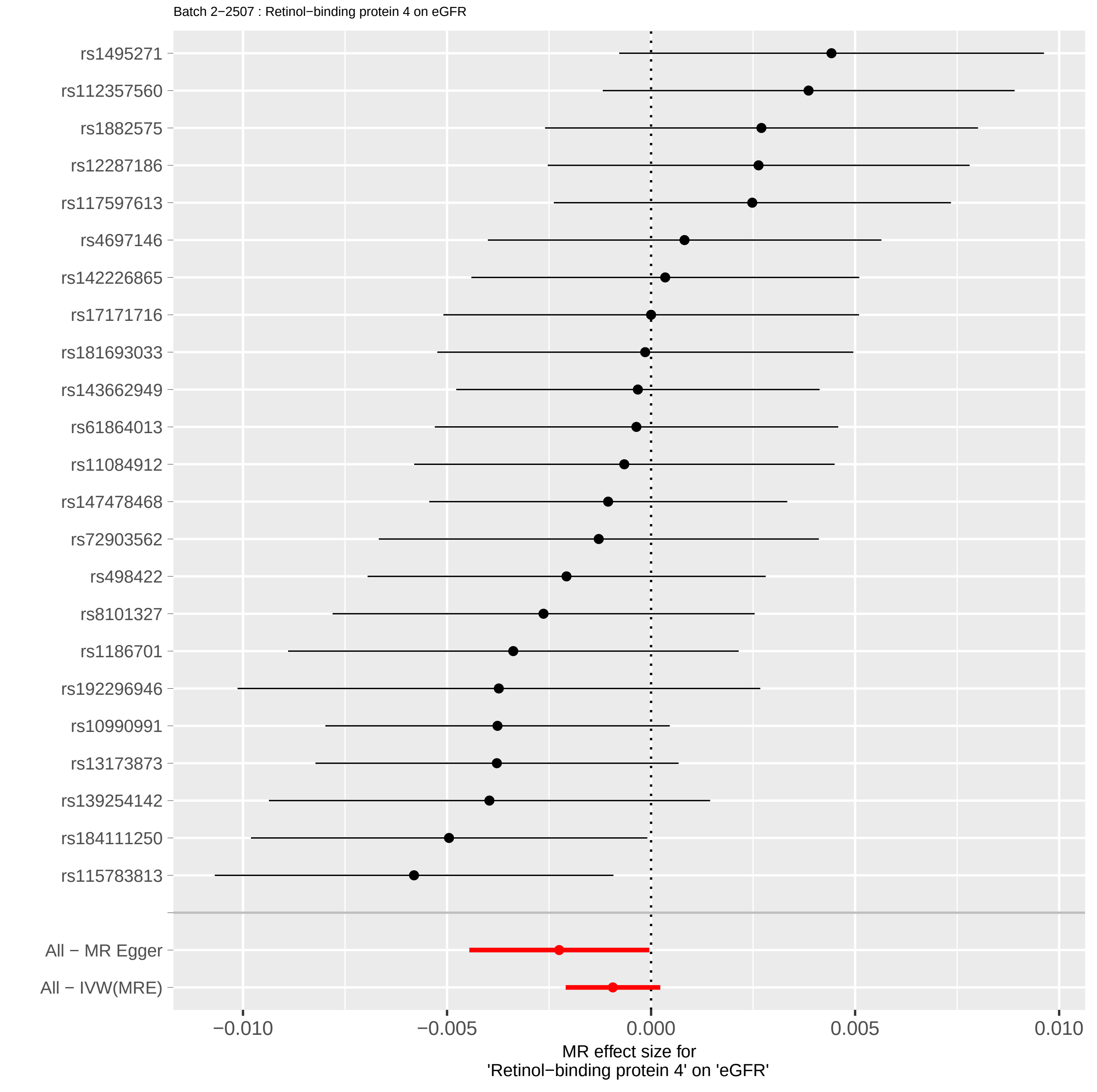

Batch 2−2507 : Retinol−binding protein 4 on eGFR
rs1495271
rs112357560
rs1882575
rs12287186
rs117597613
rs4697146
rs142226865
rs17171716
rs181693033
rs143662949
rs61864013
rs11084912
rs147478468
rs72903562
rs498422
rs8101327
rs1186701
rs192296946
rs10990991
rs13173873
rs139254142
rs184111250
rs115783813
All − MR Egger
All − IVW(MRE)
−0.010
−0.005
0.000
0.005
0.010
MR effect size for
'Retinol−binding protein 4' on 'eGFR'

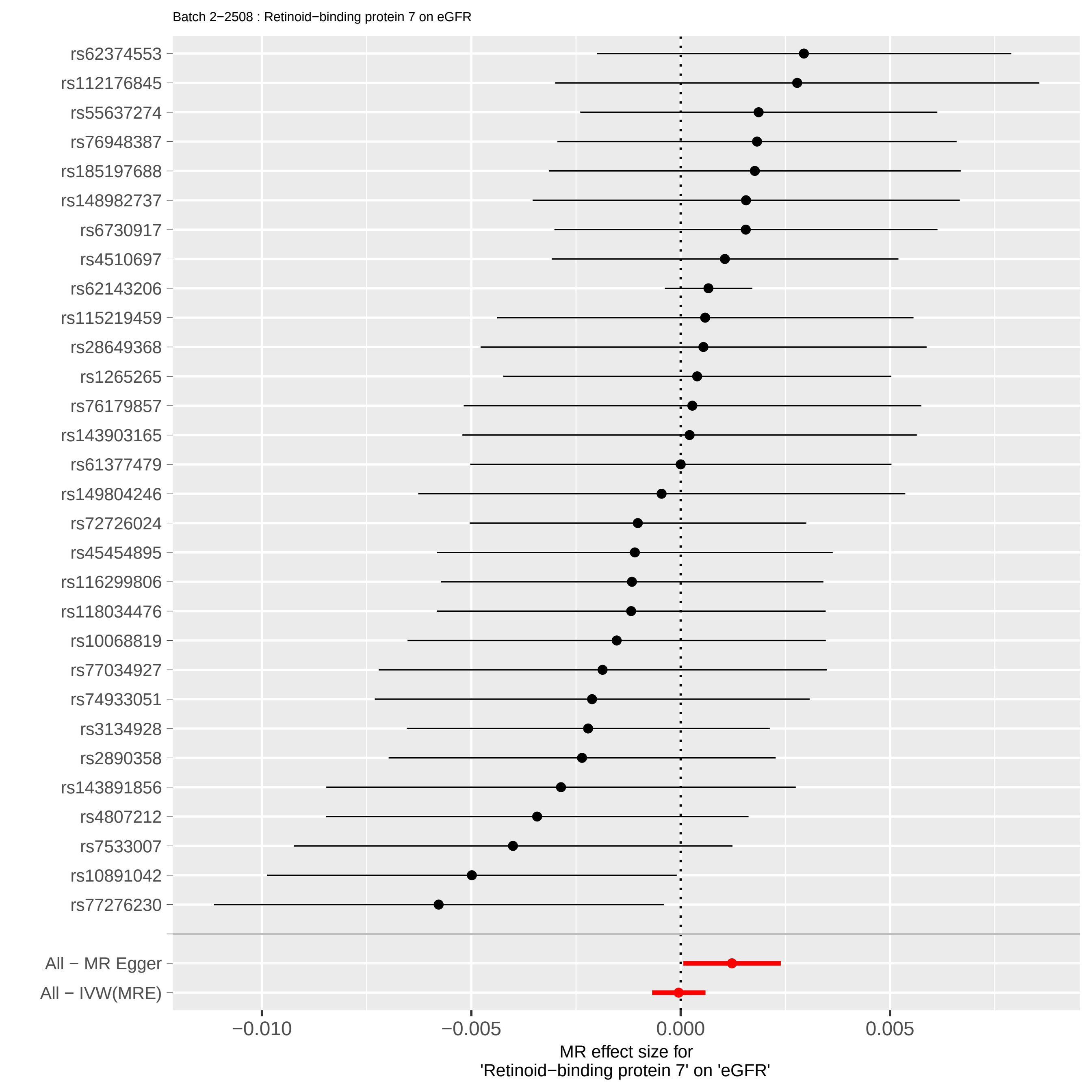

Batch 2−2508 : Retinoid−binding protein 7 on eGFR
rs62374553
rs112176845
rs55637274
rs76948387
rs185197688
rs148982737
rs6730917
rs4510697
rs62143206
rs115219459
rs28649368
rs1265265
rs76179857
rs143903165
rs61377479
rs149804246
rs72726024
rs45454895
rs116299806
rs118034476
rs10068819
rs77034927
rs74933051
rs3134928
rs2890358
rs143891856
rs4807212
rs7533007
rs10891042
rs77276230
All − MR Egger
All − IVW(MRE)
−0.010
−0.005
0.000
0.005
MR effect size for
'Retinoid−binding protein 7' on 'eGFR'

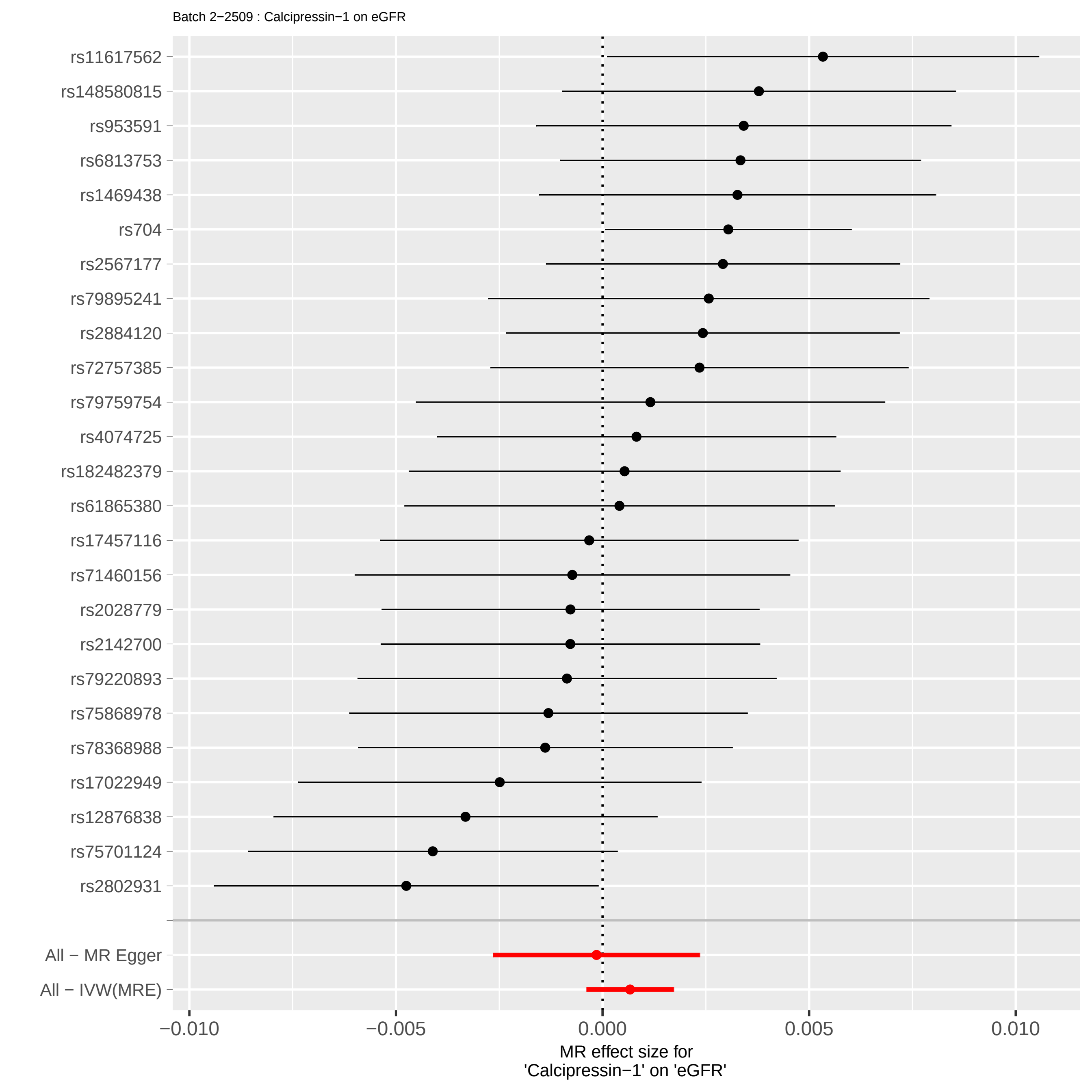

Batch 2−2509 : Calcipressin−1 on eGFR
rs11617562
rs148580815
rs953591
rs6813753
rs1469438
rs704
rs2567177
rs79895241
rs2884120
rs72757385
rs79759754
rs4074725
rs182482379
rs61865380
rs17457116
rs71460156
rs2028779
rs2142700
rs79220893
rs75868978
rs78368988
rs17022949
rs12876838
rs75701124
rs2802931
All − MR Egger
All − IVW(MRE)
−0.010
−0.005
0.000
0.005
0.010
MR effect size for
'Calcipressin−1' on 'eGFR'

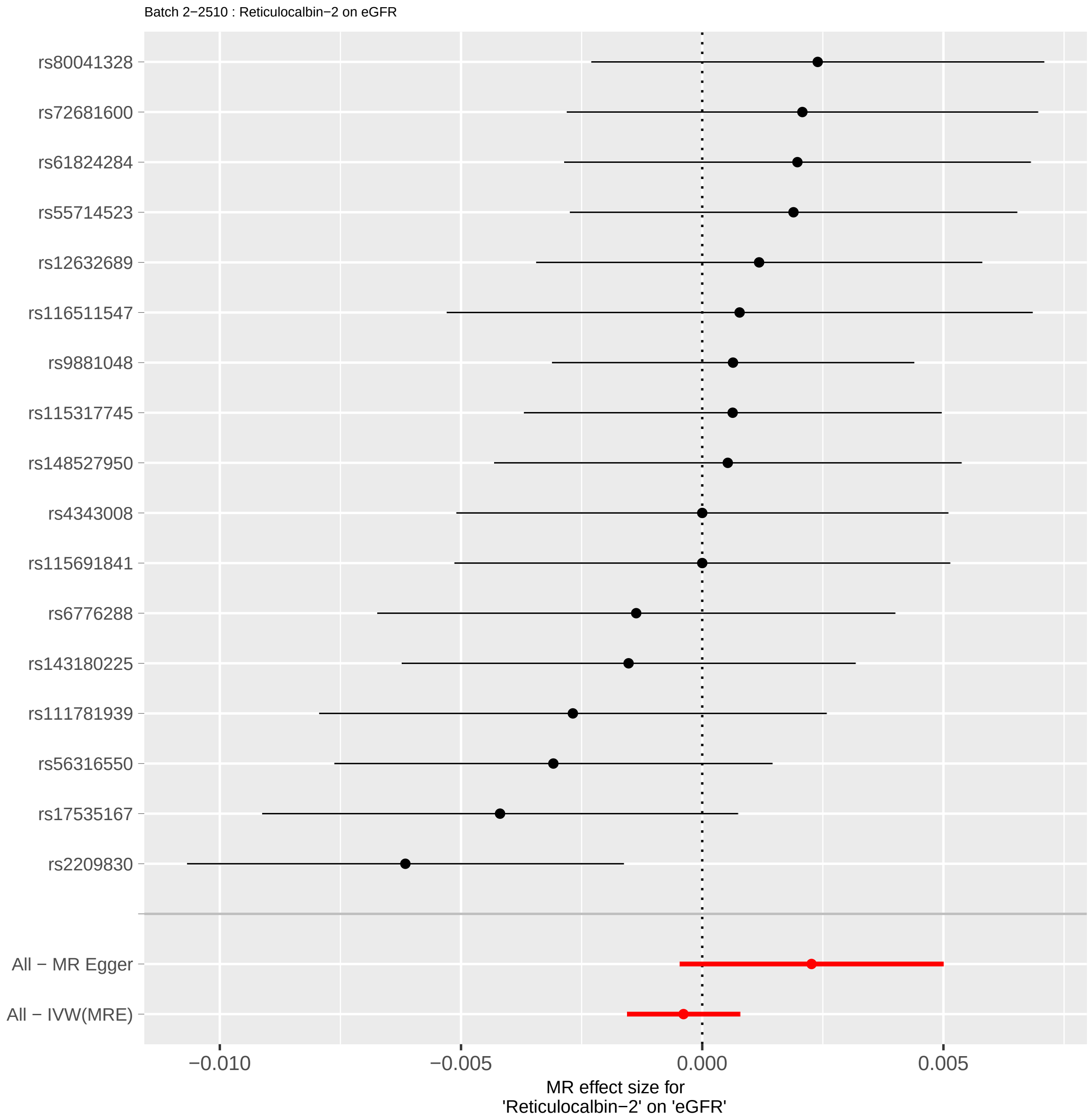

Batch 2−2510 : Reticulocalbin−2 on eGFR
rs80041328
rs72681600
rs61824284
rs55714523
rs12632689
rs116511547
rs9881048
rs115317745
rs148527950
rs4343008
rs115691841
rs6776288
rs143180225
rs111781939
rs56316550
rs17535167
rs2209830
All − MR Egger
All − IVW(MRE)
−0.010
−0.005
0.000
0.005
MR effect size for
'Reticulocalbin−2' on 'eGFR'

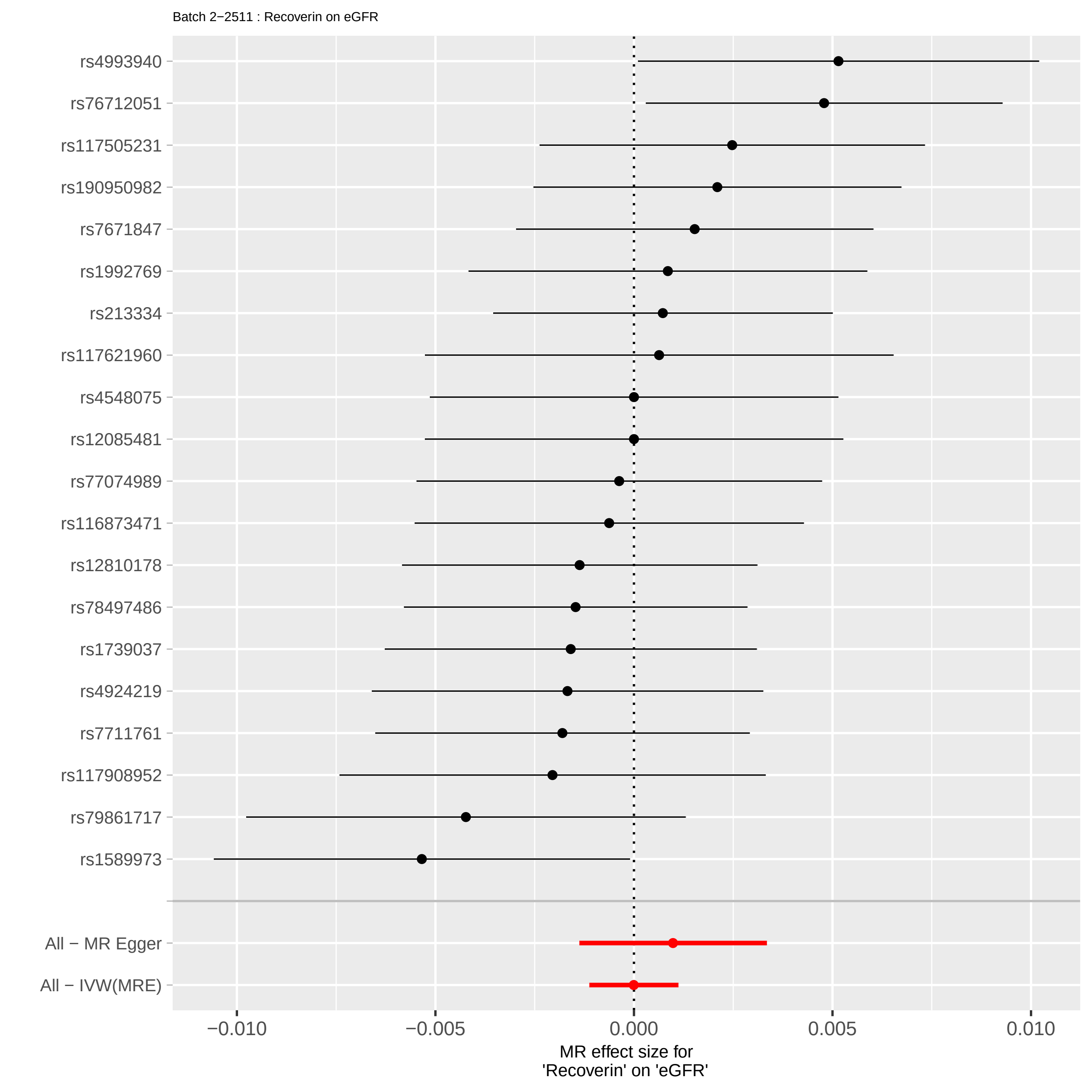
Batch 2−2511 : Recoverin on eGFR
rs4993940
rs76712051
rs117505231
rs190950982
rs7671847
rs1992769
rs213334
rs117621960
rs4548075
rs12085481
rs77074989
rs116873471
rs12810178
rs78497486
rs1739037
rs4924219
rs7711761
rs117908952
rs79861717
rs1589973
All − MR Egger
All − IVW(MRE)
−0.010
−0.005
0.000
0.005
0.010
MR effect size for
'Recoverin' on 'eGFR'

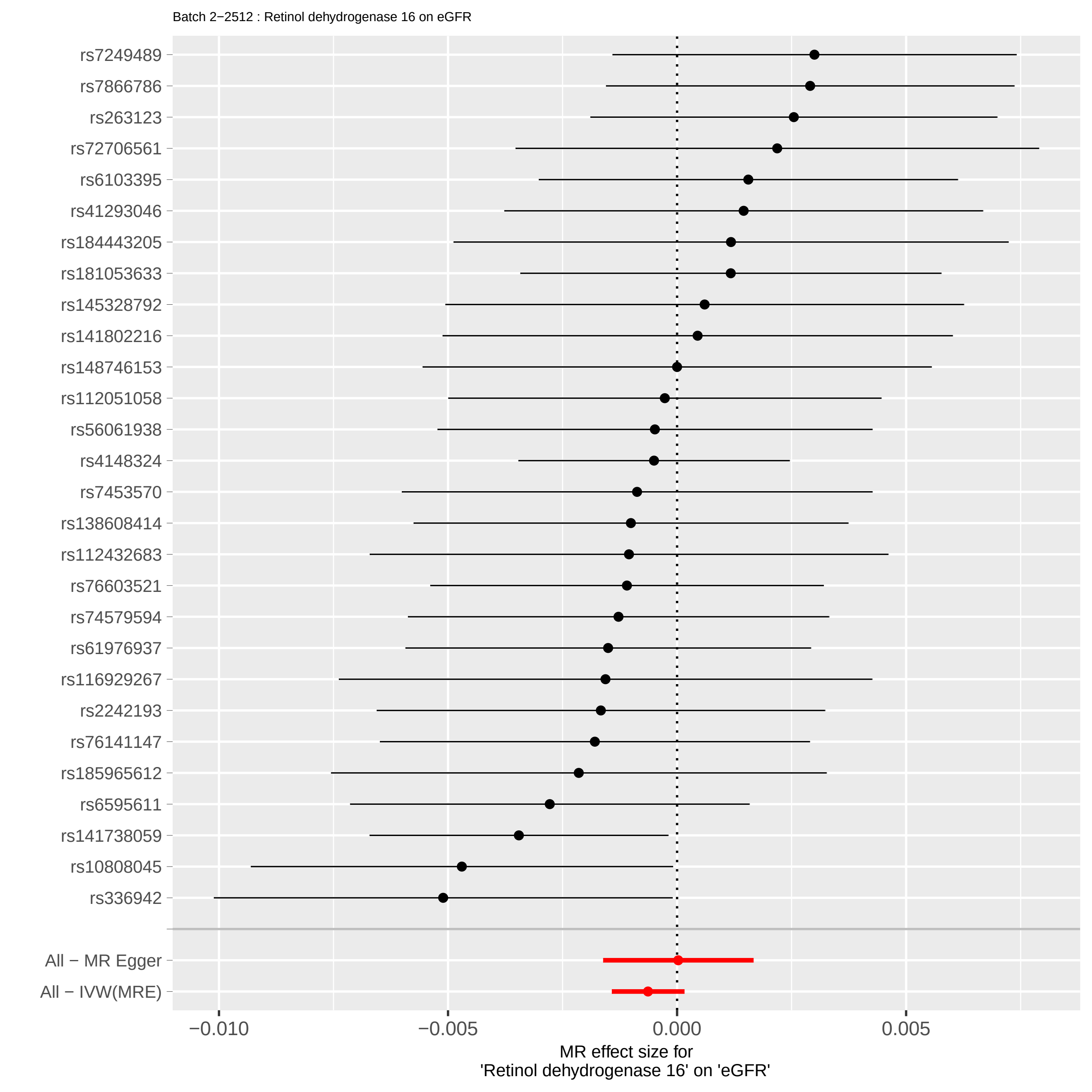
Batch 2−2512 : Retinol dehydrogenase 16 on eGFR
rs7249489
rs7866786
rs263123
rs72706561
rs6103395
rs41293046
rs184443205
rs181053633
rs145328792
rs141802216
rs148746153
rs112051058
rs56061938
rs4148324
rs7453570
rs138608414
rs112432683
rs76603521
rs74579594
rs61976937
rs116929267
rs2242193
rs76141147
rs185965612
rs6595611
rs141738059
rs10808045
rs336942
All − MR Egger
All − IVW(MRE)
−0.010
−0.005
0.000
0.005
MR effect size for
'Retinol dehydrogenase 16' on 'eGFR'

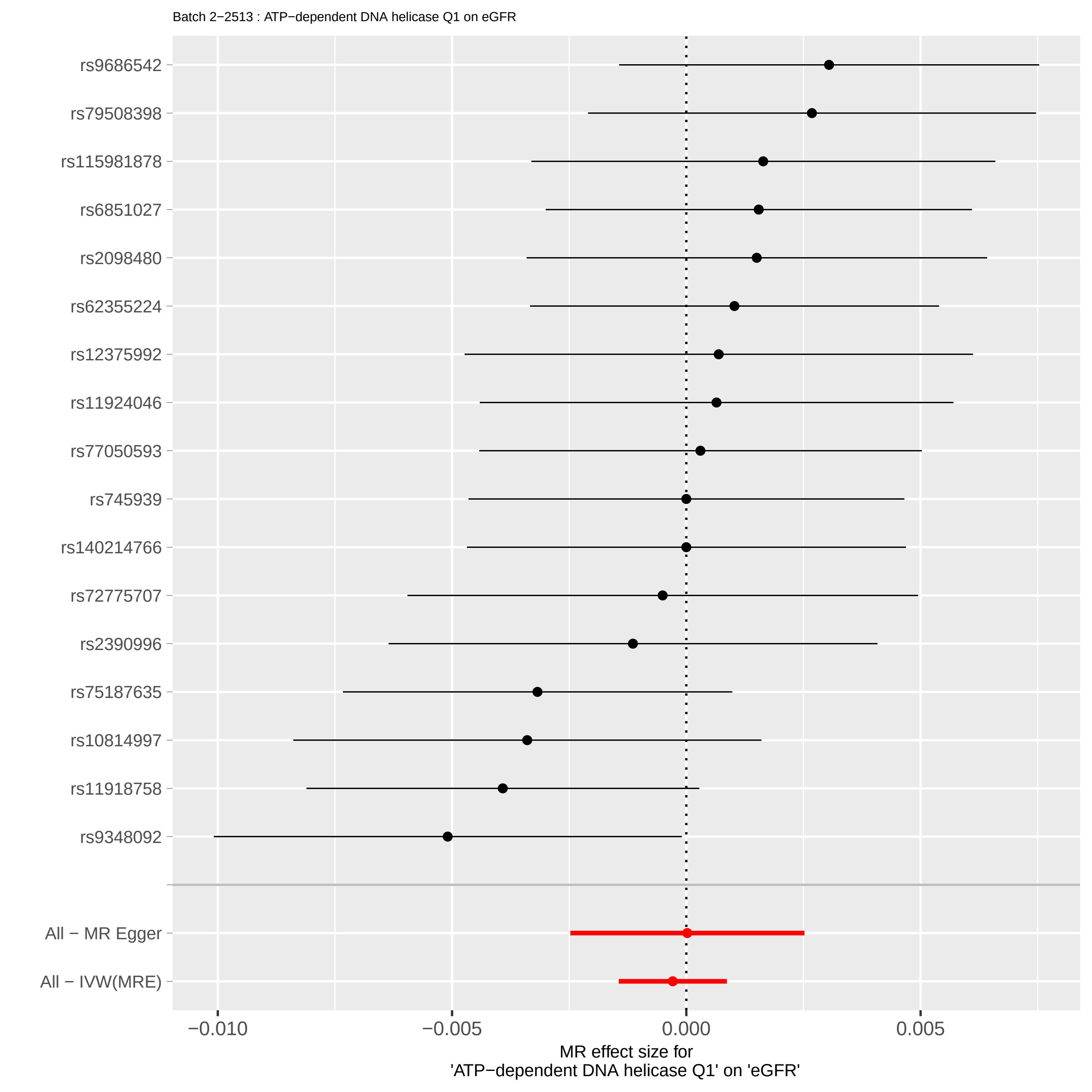
Batch 2−2513 : ATP−dependent DNA helicase Q1 on eGFR
rs9686542
rs79508398
rs115981878
rs6851027
rs2098480
rs62355224
rs12375992
rs11924046
rs77050593
rs745939
rs140214766
rs72775707
rs2390996
rs75187635
rs10814997
rs11918758
rs9348092
All − MR Egger
All − IVW(MRE)
−0.010
−0.005
0.000
0.005
MR effect size for
'ATP−dependent DNA helicase Q1' on 'eGFR'

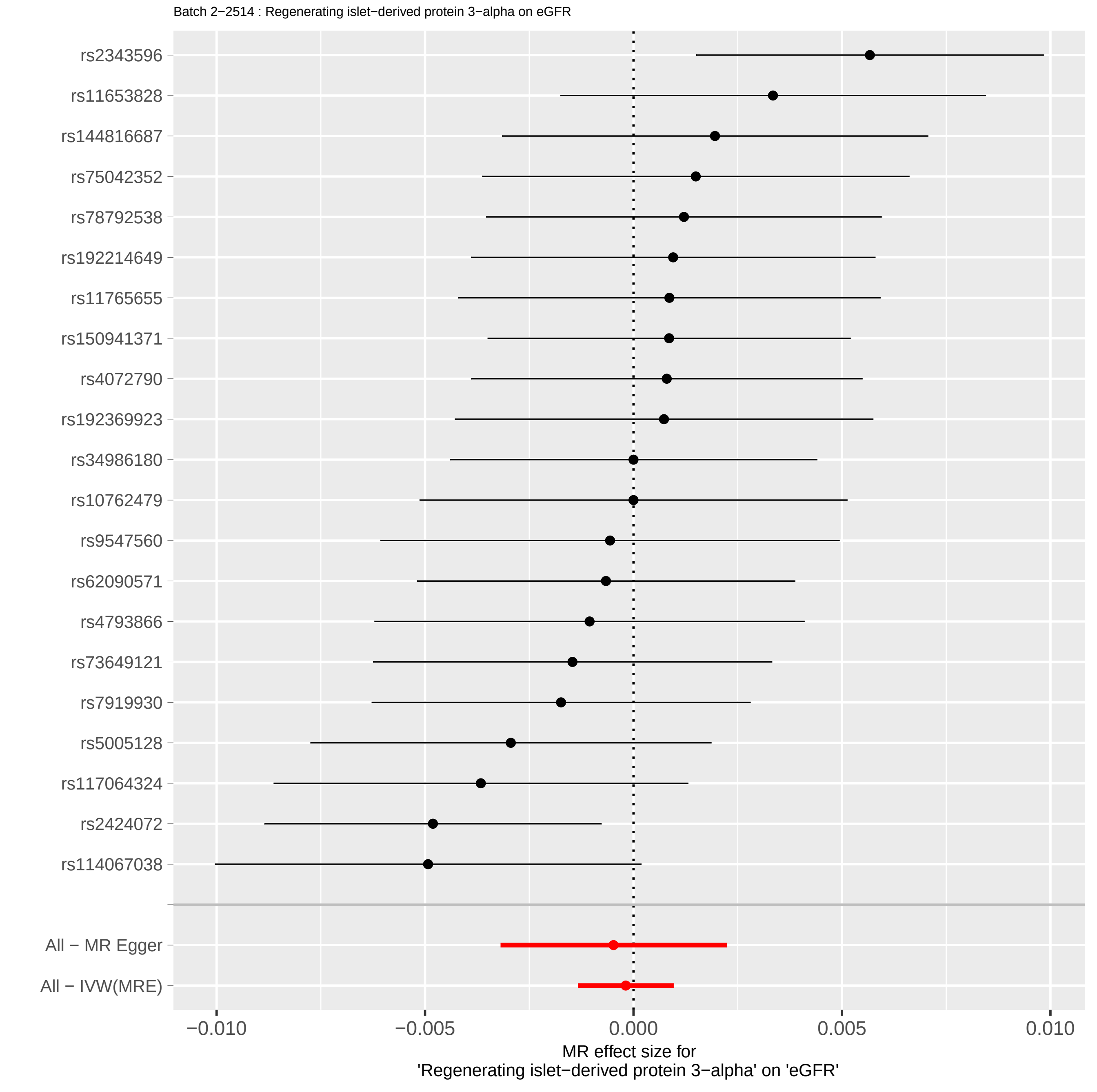
Batch 2−2514 : Regenerating islet−derived protein 3−alpha on eGFR
rs2343596
rs11653828
rs144816687
rs75042352
rs78792538
rs192214649
rs11765655
rs150941371
rs4072790
rs192369923
rs34986180
rs10762479
rs9547560
rs62090571
rs4793866
rs73649121
rs7919930
rs5005128
rs117064324
rs2424072
rs114067038
All − MR Egger
All − IVW(MRE)
−0.010
−0.005
0.000
0.005
0.010
MR effect size for
'Regenerating islet−derived protein 3−alpha' on 'eGFR'

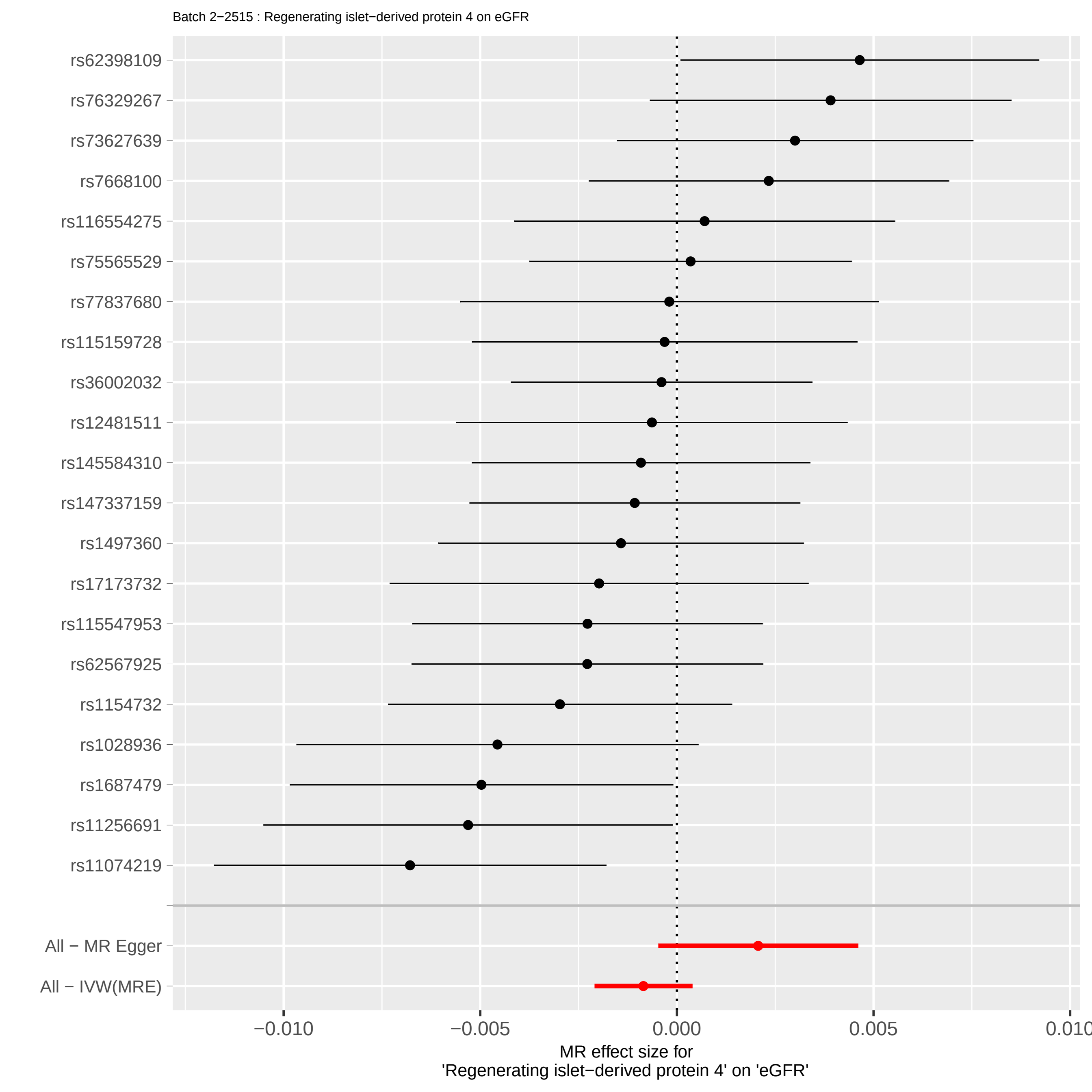

Batch 2−2515 : Regenerating islet−derived protein 4 on eGFR
rs62398109
rs76329267
rs73627639
rs7668100
rs116554275
rs75565529
rs77837680
rs115159728
rs36002032
rs12481511
rs145584310
rs147337159
rs1497360
rs17173732
rs115547953
rs62567925
rs1154732
rs1028936
rs1687479
rs11256691
rs11074219
All − MR Egger
All − IVW(MRE)
−0.010
−0.005
0.000
0.005
0.010
MR effect size for
'Regenerating islet−derived protein 4' on 'eGFR'

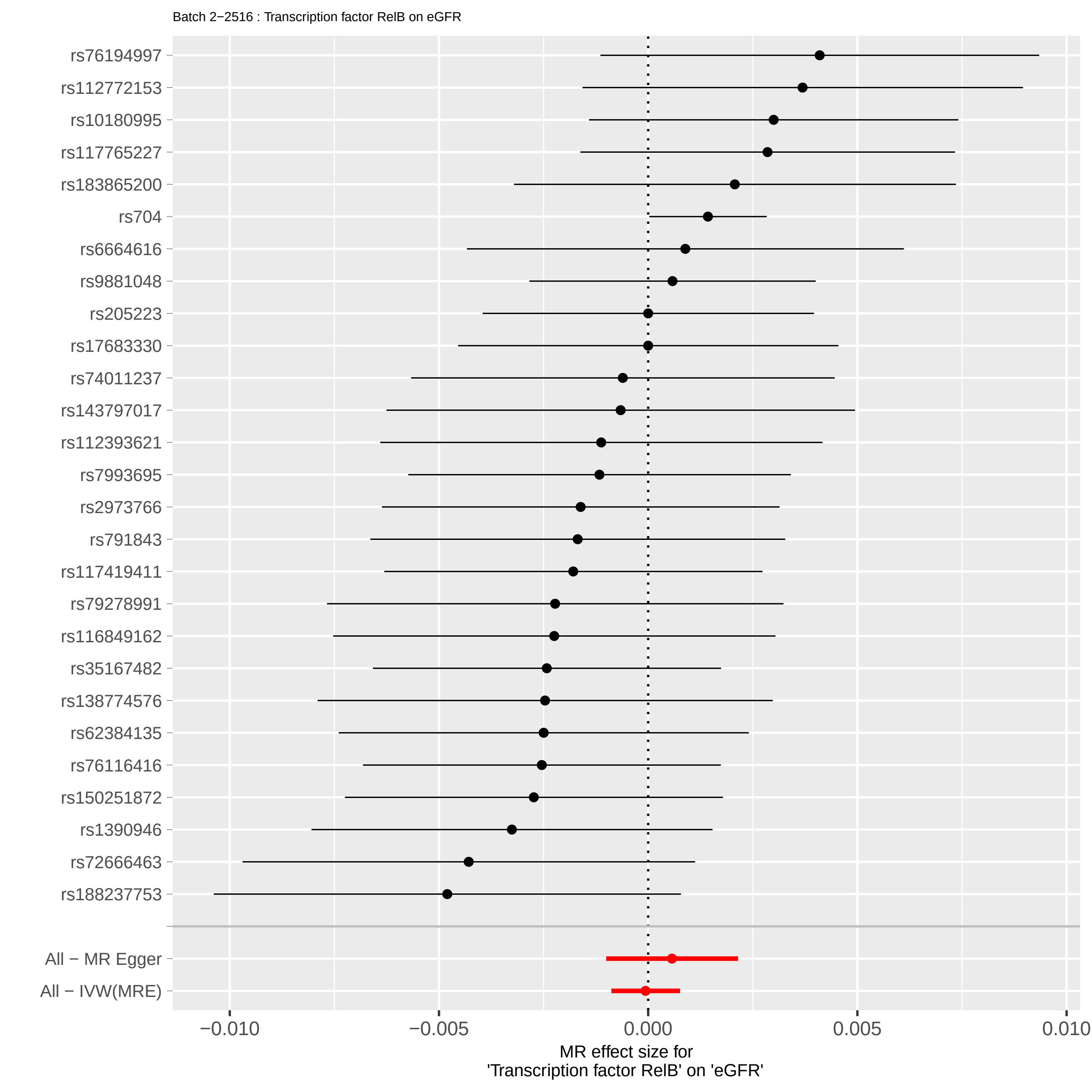

Batch 2−2516 : Transcription factor RelB on eGFR
rs76194997
rs112772153
rs10180995
rs117765227
rs183865200
rs704
rs6664616
rs9881048
rs205223
rs17683330
rs74011237
rs143797017
rs112393621
rs7993695
rs2973766
rs791843
rs117419411
rs79278991
rs116849162
rs35167482
rs138774576
rs62384135
rs76116416
rs150251872
rs1390946
rs72666463
rs188237753
All − MR Egger
All − IVW(MRE)
−0.010
−0.005
0.000
0.005
0.010
MR effect size for
'Transcription factor RelB' on 'eGFR'

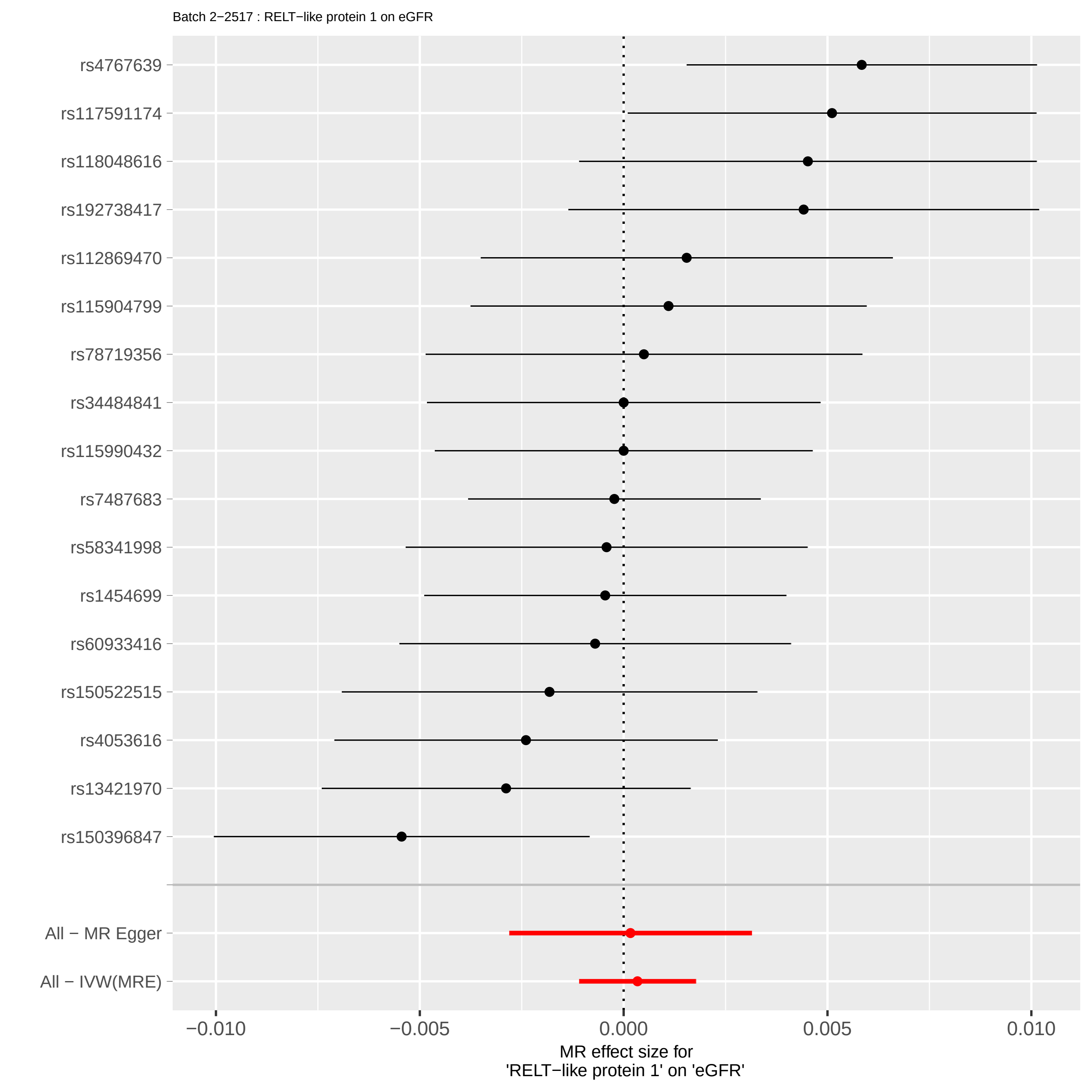

Batch 2−2517 : RELT−like protein 1 on eGFR
rs4767639
rs117591174
rs118048616
rs192738417
rs112869470
rs115904799
rs78719356
rs34484841
rs115990432
rs7487683
rs58341998
rs1454699
rs60933416
rs150522515
rs4053616
rs13421970
rs150396847
All − MR Egger
All − IVW(MRE)
−0.010
−0.005
0.000
0.005
0.010
MR effect size for
'RELT−like protein 1' on 'eGFR'

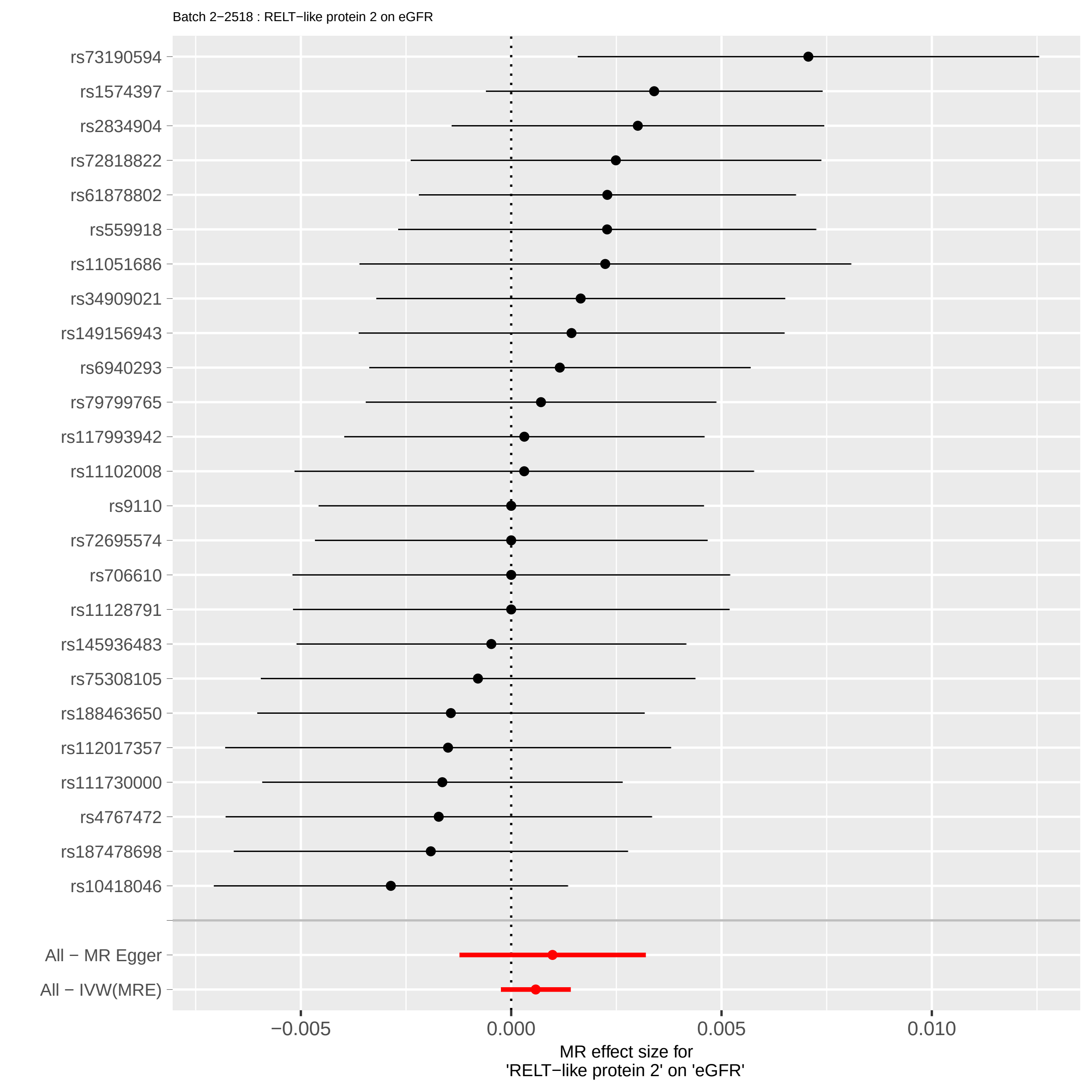

Batch 2−2518 : RELT−like protein 2 on eGFR
rs73190594
rs1574397
rs2834904
rs72818822
rs61878802
rs559918
rs11051686
rs34909021
rs149156943
rs6940293
rs79799765
rs117993942
rs11102008
rs9110
rs72695574
rs706610
rs11128791
rs145936483
rs75308105
rs188463650
rs112017357
rs111730000
rs4767472
rs187478698
rs10418046
All − MR Egger
All − IVW(MRE)
−0.005
0.000
0.005
0.010
MR effect size for
'RELT−like protein 2' on 'eGFR'

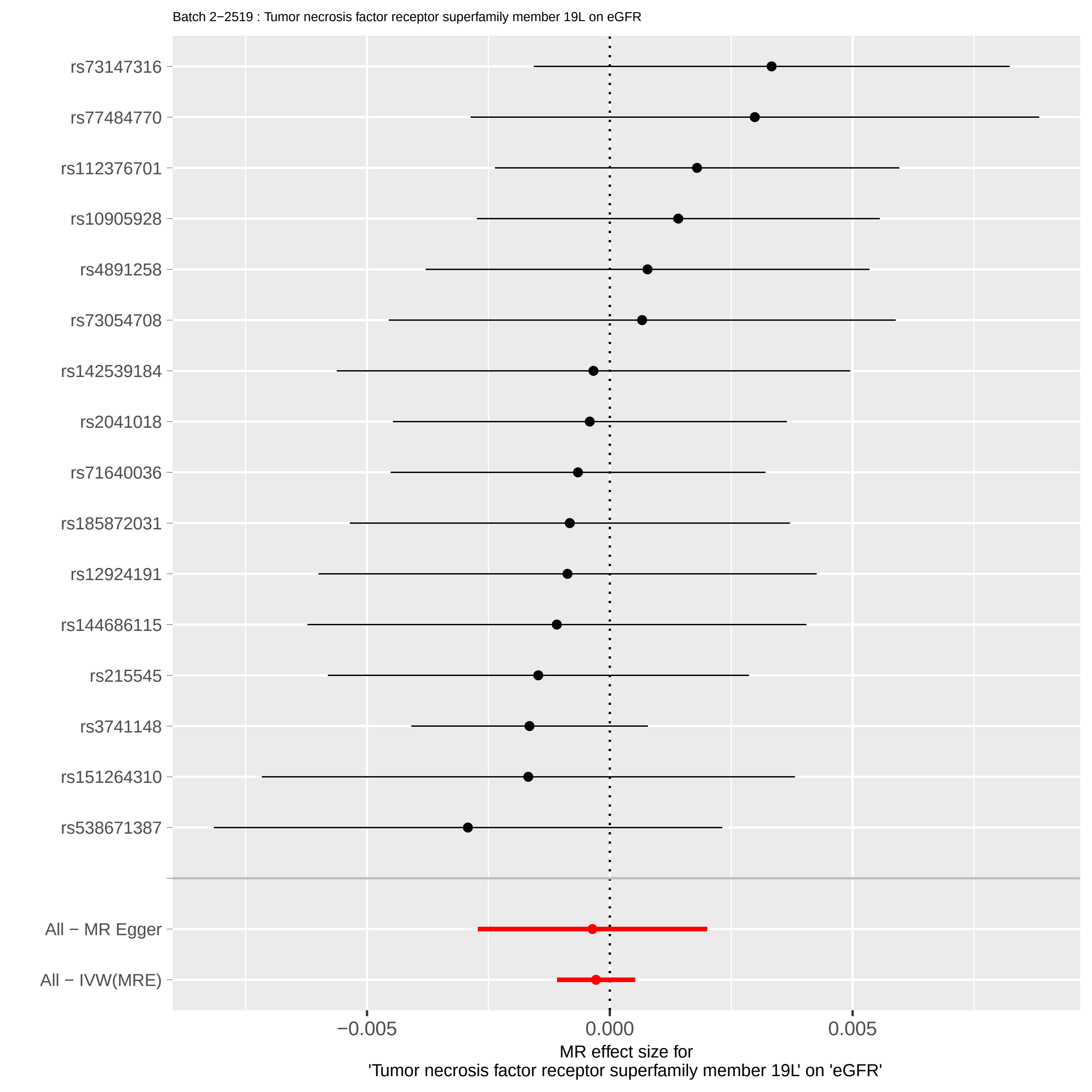

Batch 2−2519 : Tumor necrosis factor receptor superfamily member 19L on eGFR
rs73147316
rs77484770
rs112376701
rs10905928
rs4891258
rs73054708
rs142539184
rs2041018
rs71640036
rs185872031
rs12924191
rs144686115
rs215545
rs3741148
rs151264310
rs538671387
All − MR Egger
All − IVW(MRE)
−0.005
0.000
0.005
MR effect size for
'Tumor necrosis factor receptor superfamily member 19L' on 'eGFR'

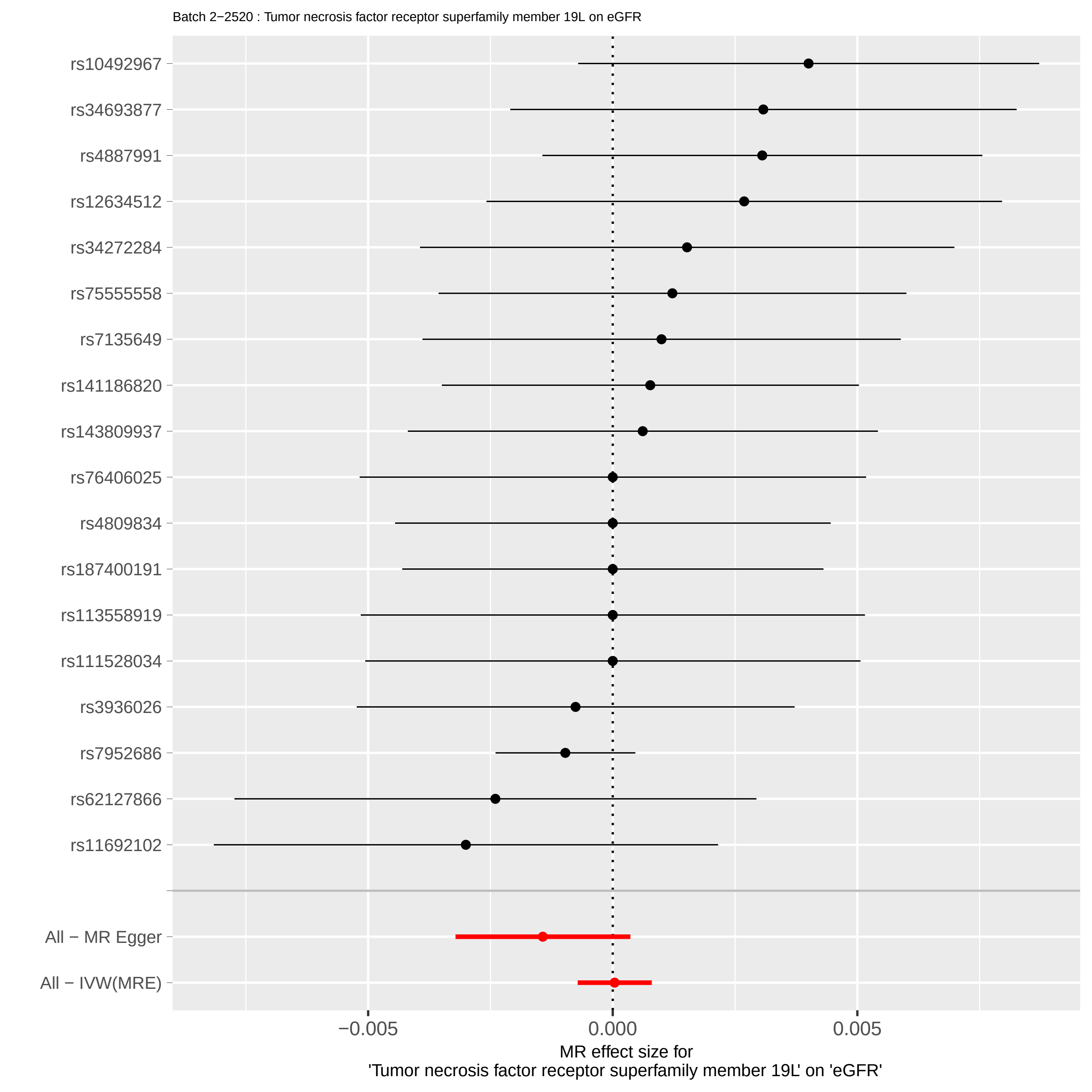

Batch 2−2520 : Tumor necrosis factor receptor superfamily member 19L on eGFR
rs10492967
rs34693877
rs4887991
rs12634512
rs34272284
rs75555558
rs7135649
rs141186820
rs143809937
rs76406025
rs4809834
rs187400191
rs113558919
rs111528034
rs3936026
rs7952686
rs62127866
rs11692102
All − MR Egger
All − IVW(MRE)
−0.005
0.000
0.005
MR effect size for
'Tumor necrosis factor receptor superfamily member 19L' on 'eGFR'

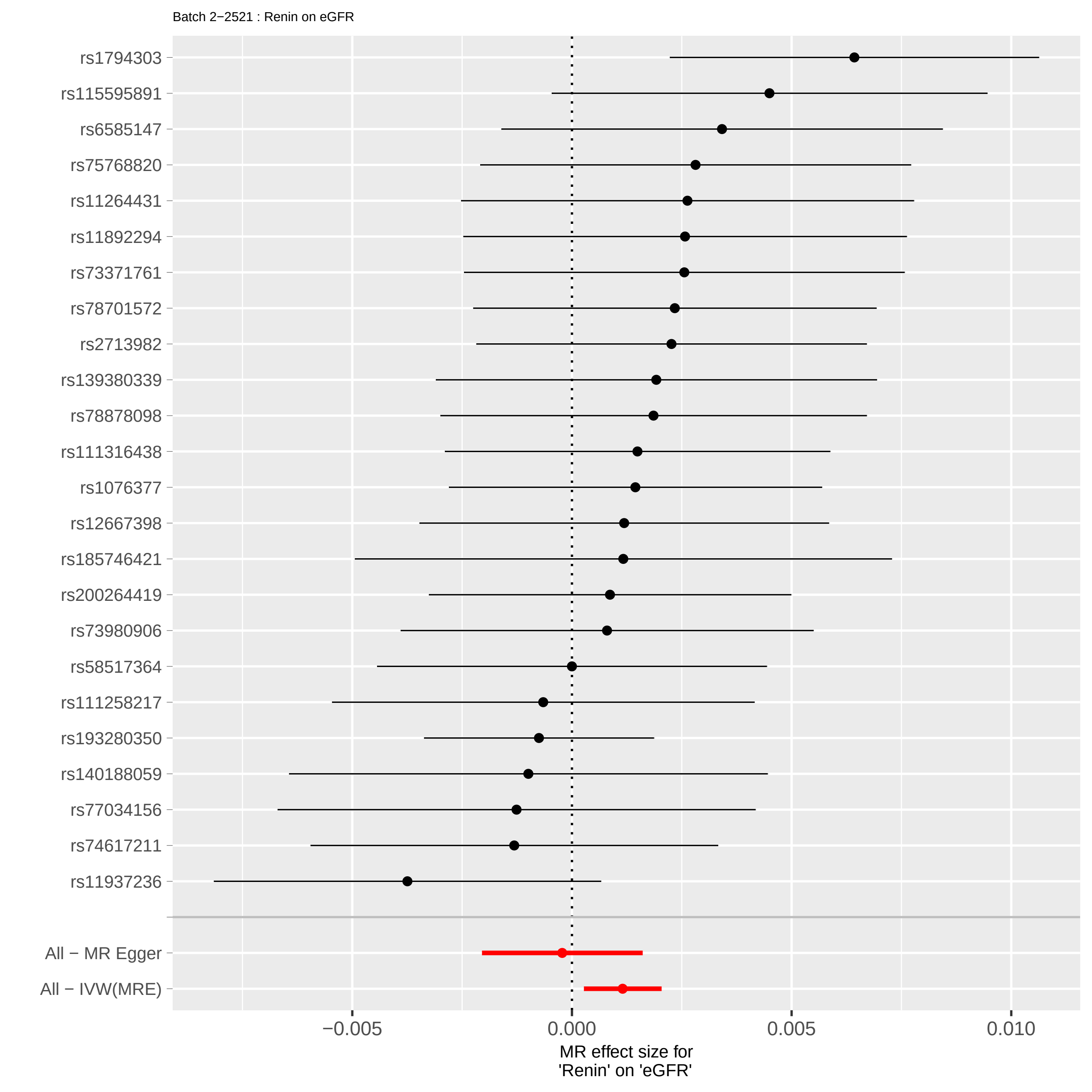
Batch 2−2521 : Renin on eGFR
rs1794303
rs115595891
rs6585147
rs75768820
rs11264431
rs11892294
rs73371761
rs78701572
rs2713982
rs139380339
rs78878098
rs111316438
rs1076377
rs12667398
rs185746421
rs200264419
rs73980906
rs58517364
rs111258217
rs193280350
rs140188059
rs77034156
rs74617211
rs11937236
All − MR Egger
All − IVW(MRE)
−0.005
0.000
0.005
0.010
MR effect size for
'Renin' on 'eGFR'

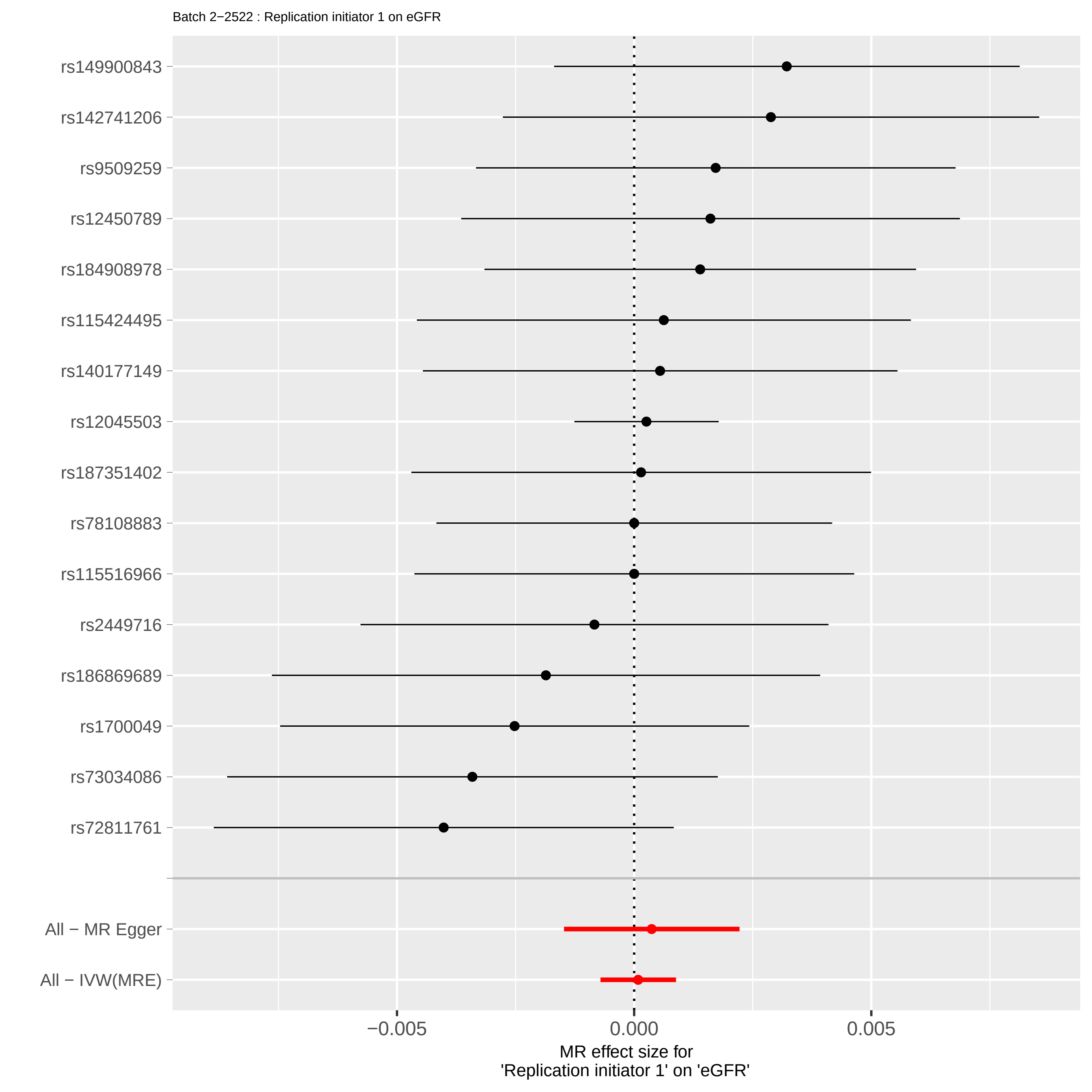

Batch 2−2522 : Replication initiator 1 on eGFR
rs149900843
rs142741206
rs9509259
rs12450789
rs184908978
rs115424495
rs140177149
rs12045503
rs187351402
rs78108883
rs115516966
rs2449716
rs186869689
rs1700049
rs73034086
rs72811761
All − MR Egger
All − IVW(MRE)
−0.005
0.000
0.005
MR effect size for
'Replication initiator 1' on 'eGFR'

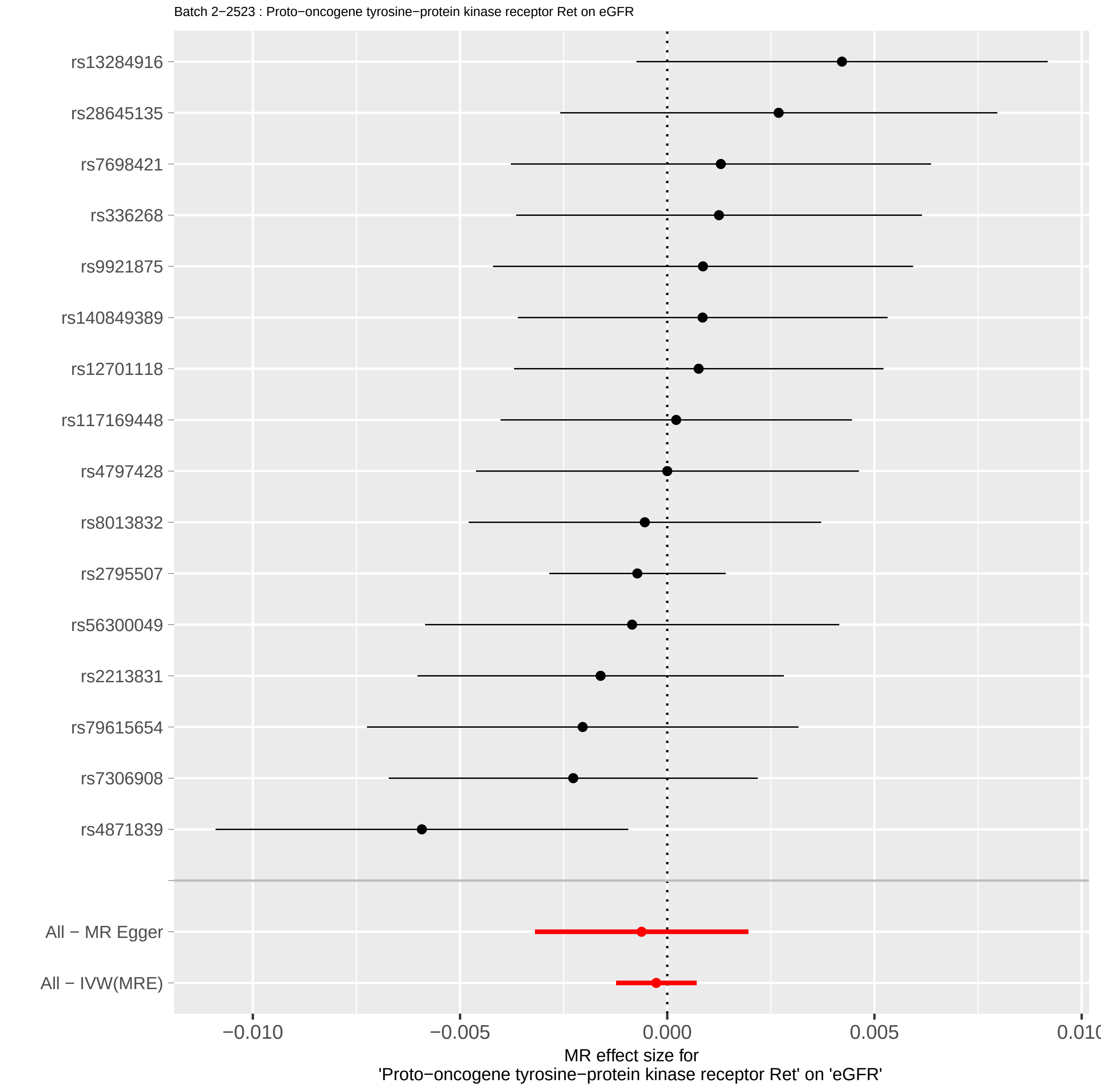
Batch 2−2523 : Proto−oncogene tyrosine−protein kinase receptor Ret on eGFR
rs13284916
rs28645135
rs7698421
rs336268
rs9921875
rs140849389
rs12701118
rs117169448
rs4797428
rs8013832
rs2795507
rs56300049
rs2213831
rs79615654
rs7306908
rs4871839
All − MR Egger
All − IVW(MRE)
−0.010
−0.005
0.000
0.005
0.010
MR effect size for
'Proto−oncogene tyrosine−protein kinase receptor Ret' on 'eGFR'

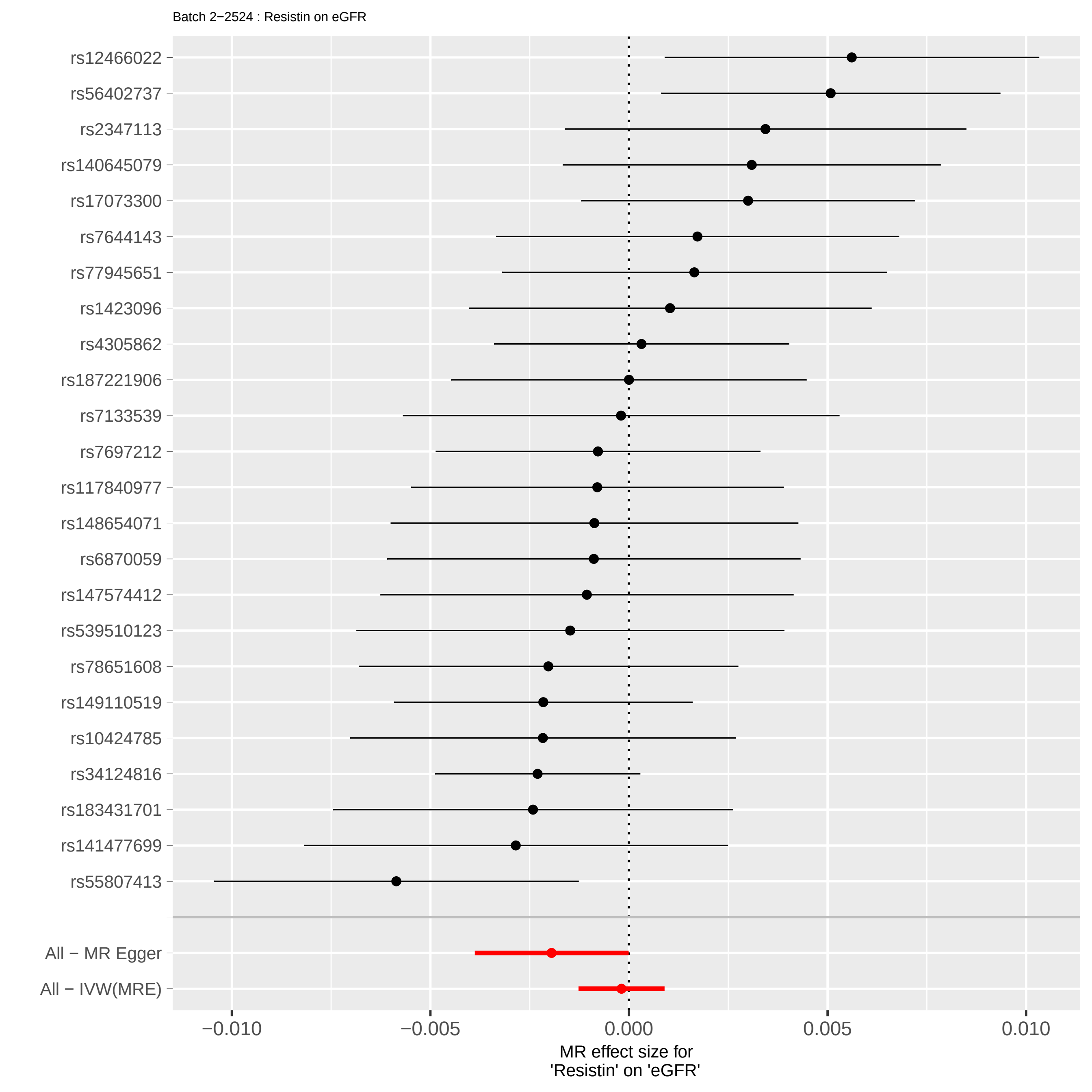

Batch 2−2524 : Resistin on eGFR
rs12466022
rs56402737
rs2347113
rs140645079
rs17073300
rs7644143
rs77945651
rs1423096
rs4305862
rs187221906
rs7133539
rs7697212
rs117840977
rs148654071
rs6870059
rs147574412
rs539510123
rs78651608
rs149110519
rs10424785
rs34124816
rs183431701
rs141477699
rs55807413
All − MR Egger
All − IVW(MRE)
−0.010
−0.005
0.000
0.005
0.010
MR effect size for
'Resistin' on 'eGFR'

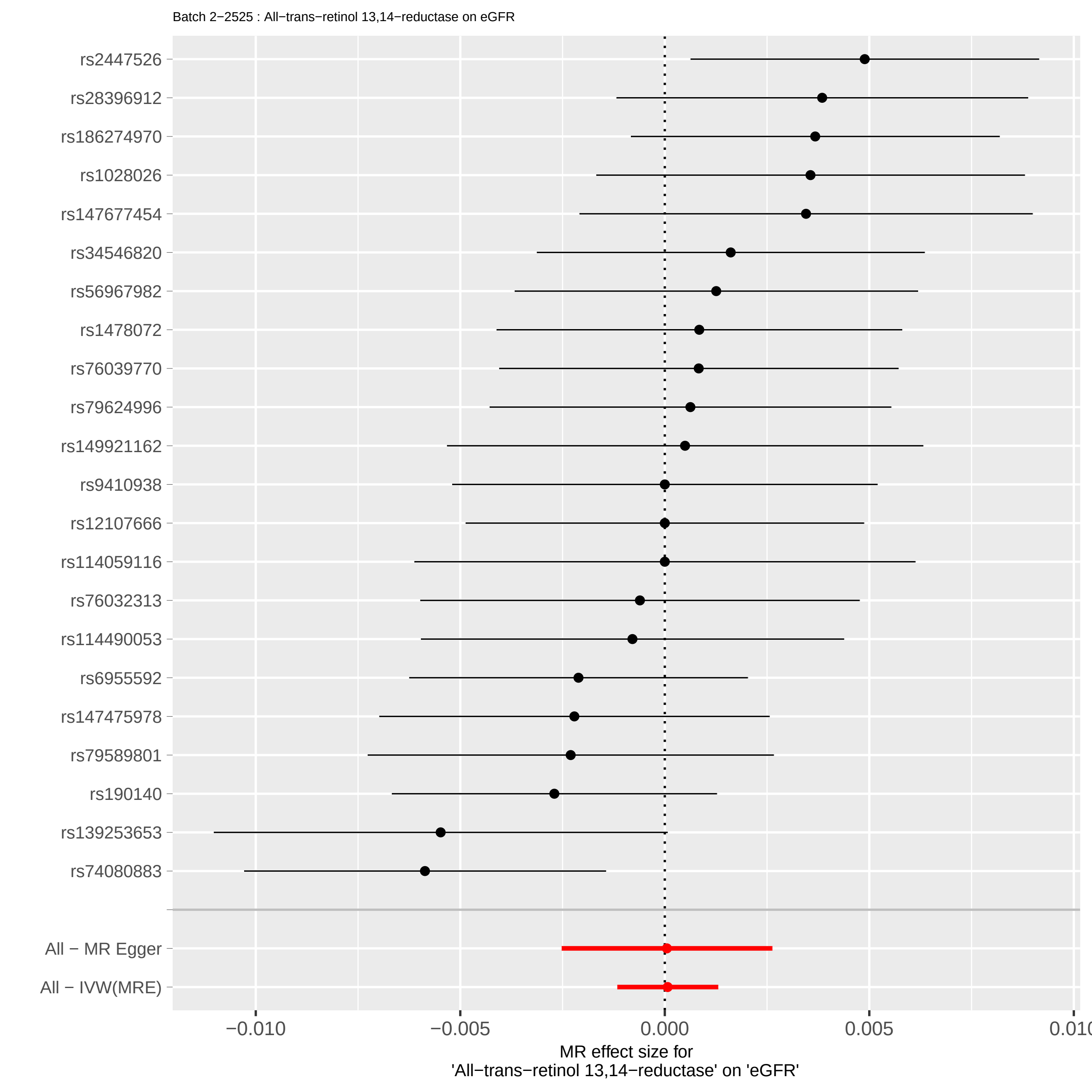

Batch 2−2525 : All−trans−retinol 13,14−reductase on eGFR
rs2447526
rs28396912
rs186274970
rs1028026
rs147677454
rs34546820
rs56967982
rs1478072
rs76039770
rs79624996
rs149921162
rs9410938
rs12107666
rs114059116
rs76032313
rs114490053
rs6955592
rs147475978
rs79589801
rs190140
rs139253653
rs74080883
All − MR Egger
All − IVW(MRE)
−0.010
−0.005
0.000
0.005
0.010
MR effect size for
'All−trans−retinol 13,14−reductase' on 'eGFR'

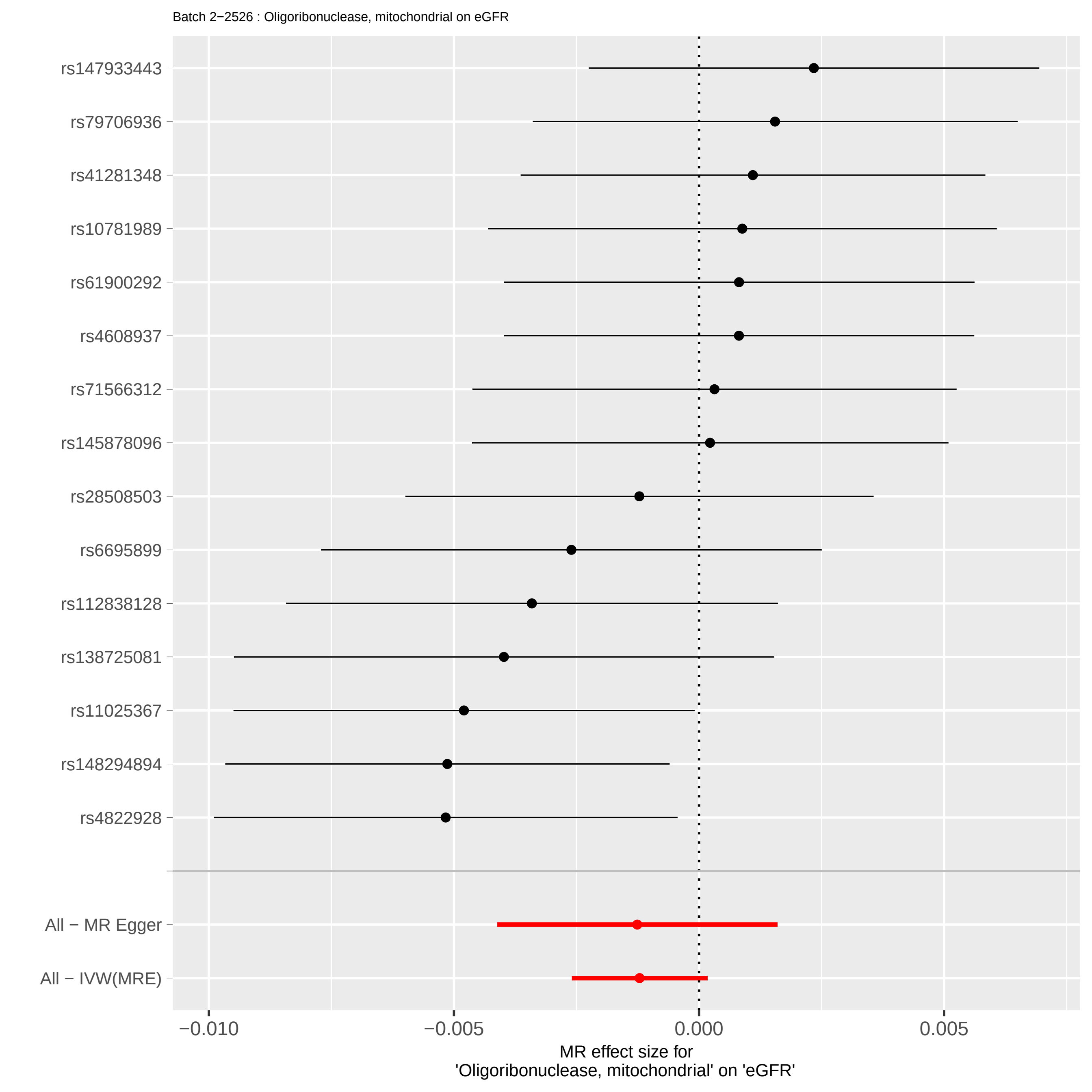

Batch 2−2526 : Oligoribonuclease, mitochondrial on eGFR
rs147933443
rs79706936
rs41281348
rs10781989
rs61900292
rs4608937
rs71566312
rs145878096
rs28508503
rs6695899
rs112838128
rs138725081
rs11025367
rs148294894
rs4822928
All − MR Egger
All − IVW(MRE)
−0.010
−0.005
0.000
0.005
MR effect size for
'Oligoribonuclease, mitochondrial' on 'eGFR'

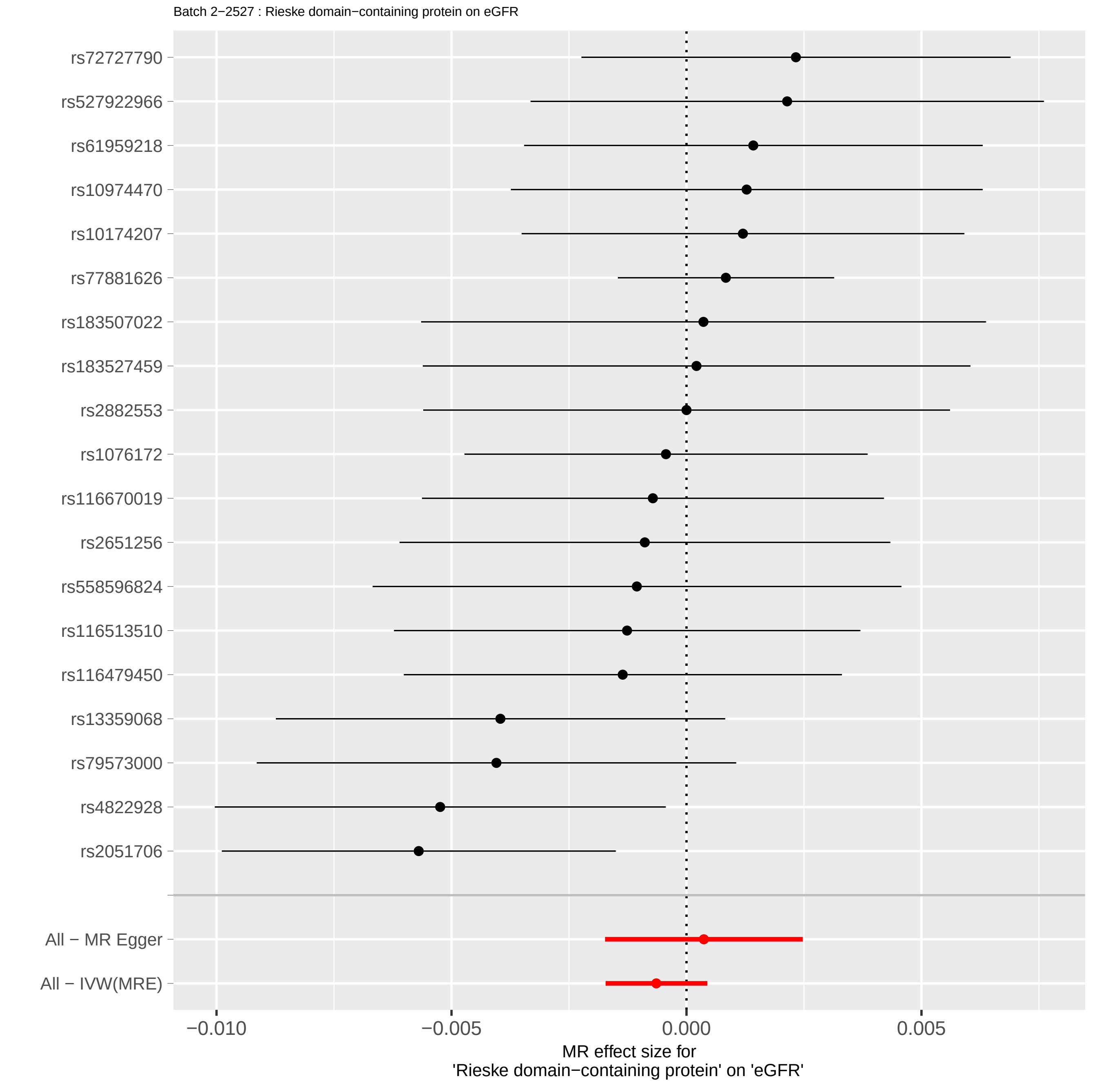

Batch 2−2527 : Rieske domain−containing protein on eGFR
rs72727790
rs527922966
rs61959218
rs10974470
rs10174207
rs77881626
rs183507022
rs183527459
rs2882553
rs1076172
rs116670019
rs2651256
rs558596824
rs116513510
rs116479450
rs13359068
rs79573000
rs4822928
rs2051706
All − MR Egger
All − IVW(MRE)
−0.010
−0.005
0.000
0.005
MR effect size for
'Rieske domain−containing protein' on 'eGFR'

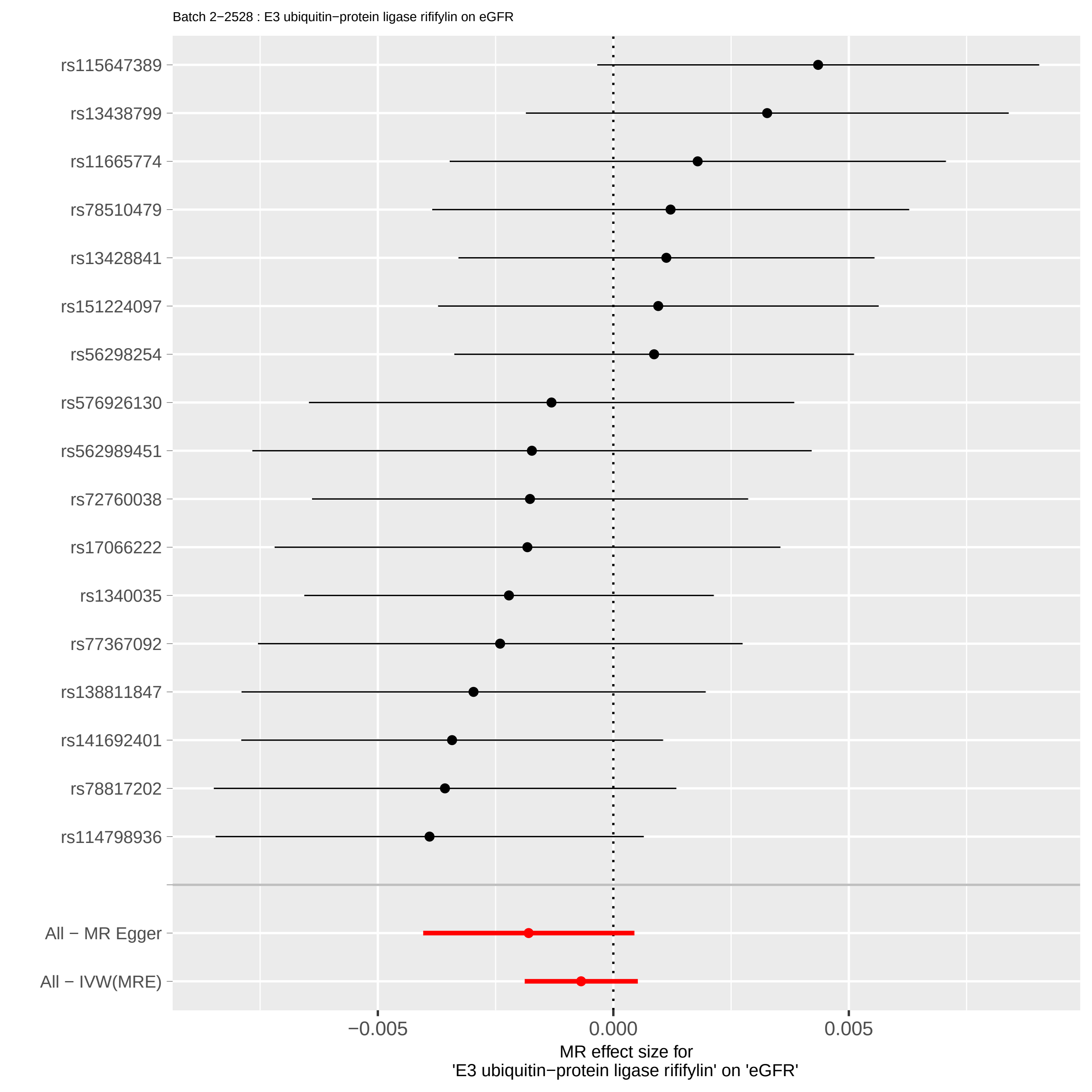

Batch 2−2528 : E3 ubiquitin−protein ligase rififylin on eGFR
rs115647389
rs13438799
rs11665774
rs78510479
rs13428841
rs151224097
rs56298254
rs576926130
rs562989451
rs72760038
rs17066222
rs1340035
rs77367092
rs138811847
rs141692401
rs78817202
rs114798936
All − MR Egger
All − IVW(MRE)
−0.005
0.000
0.005
MR effect size for
'E3 ubiquitin−protein ligase rififylin' on 'eGFR'

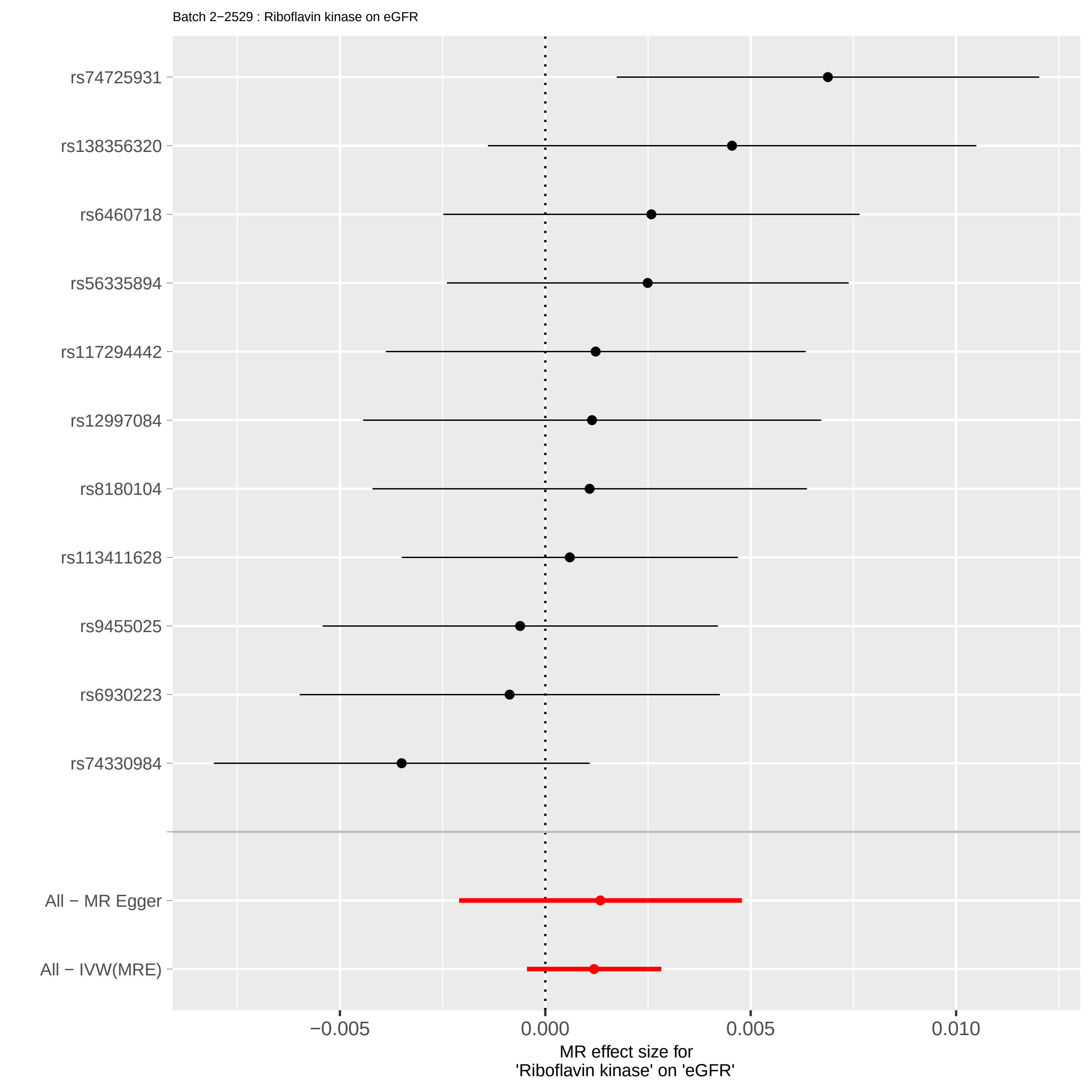
Batch 2−2529 : Riboflavin kinase on eGFR
rs74725931
rs138356320
rs6460718
rs56335894
rs117294442
rs12997084
rs8180104
rs113411628
rs9455025
rs6930223
rs74330984
All − MR Egger
All − IVW(MRE)
−0.005
0.000
0.005
0.010
MR effect size for
'Riboflavin kinase' on 'eGFR'

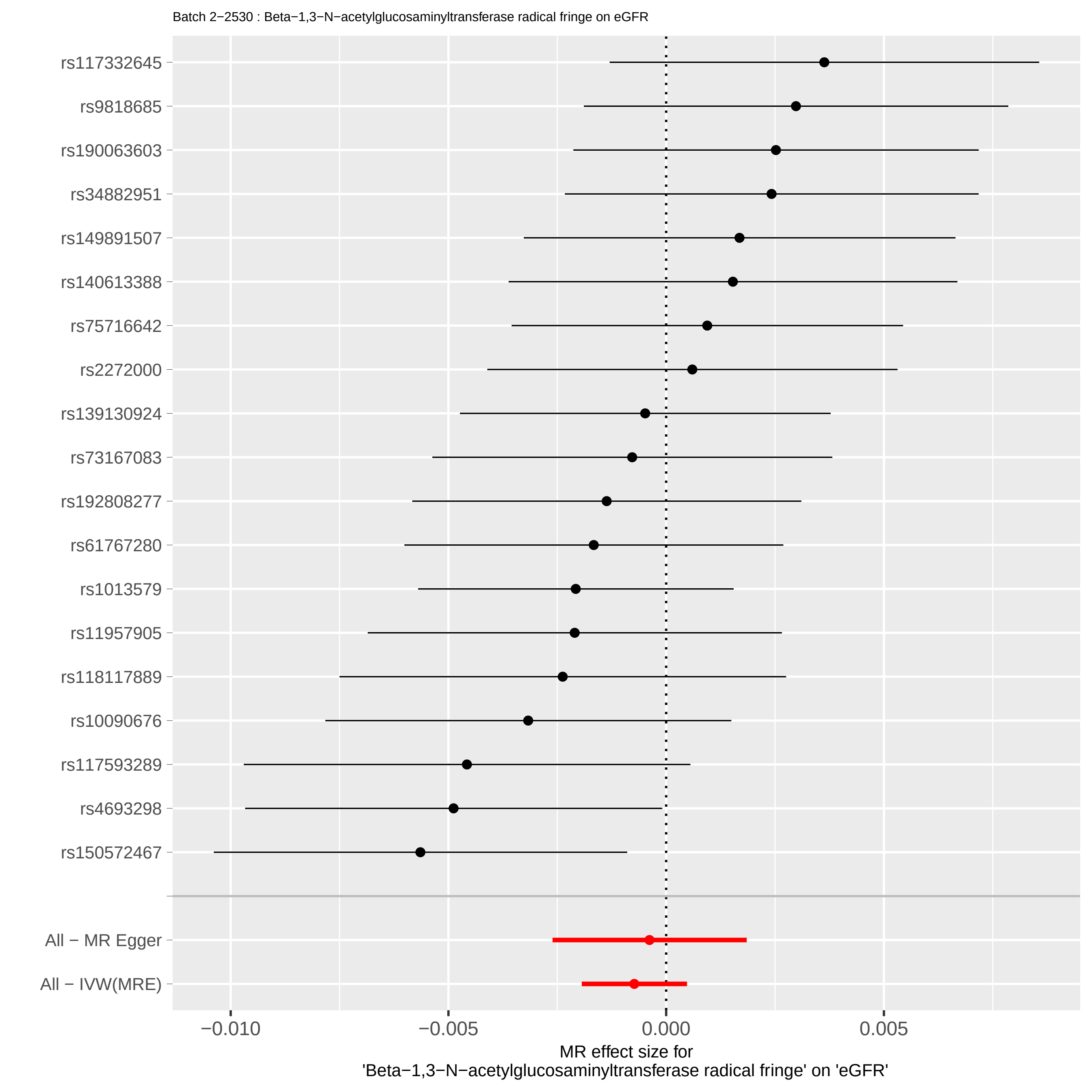

Batch 2−2530 : Beta−1,3−N−acetylglucosaminyltransferase radical fringe on eGFR
rs117332645
rs9818685
rs190063603
rs34882951
rs149891507
rs140613388
rs75716642
rs2272000
rs139130924
rs73167083
rs192808277
rs61767280
rs1013579
rs11957905
rs118117889
rs10090676
rs117593289
rs4693298
rs150572467
All − MR Egger
All − IVW(MRE)
−0.010
−0.005
0.000
0.005
MR effect size for
'Beta−1,3−N−acetylglucosaminyltransferase radical fringe' on 'eGFR'

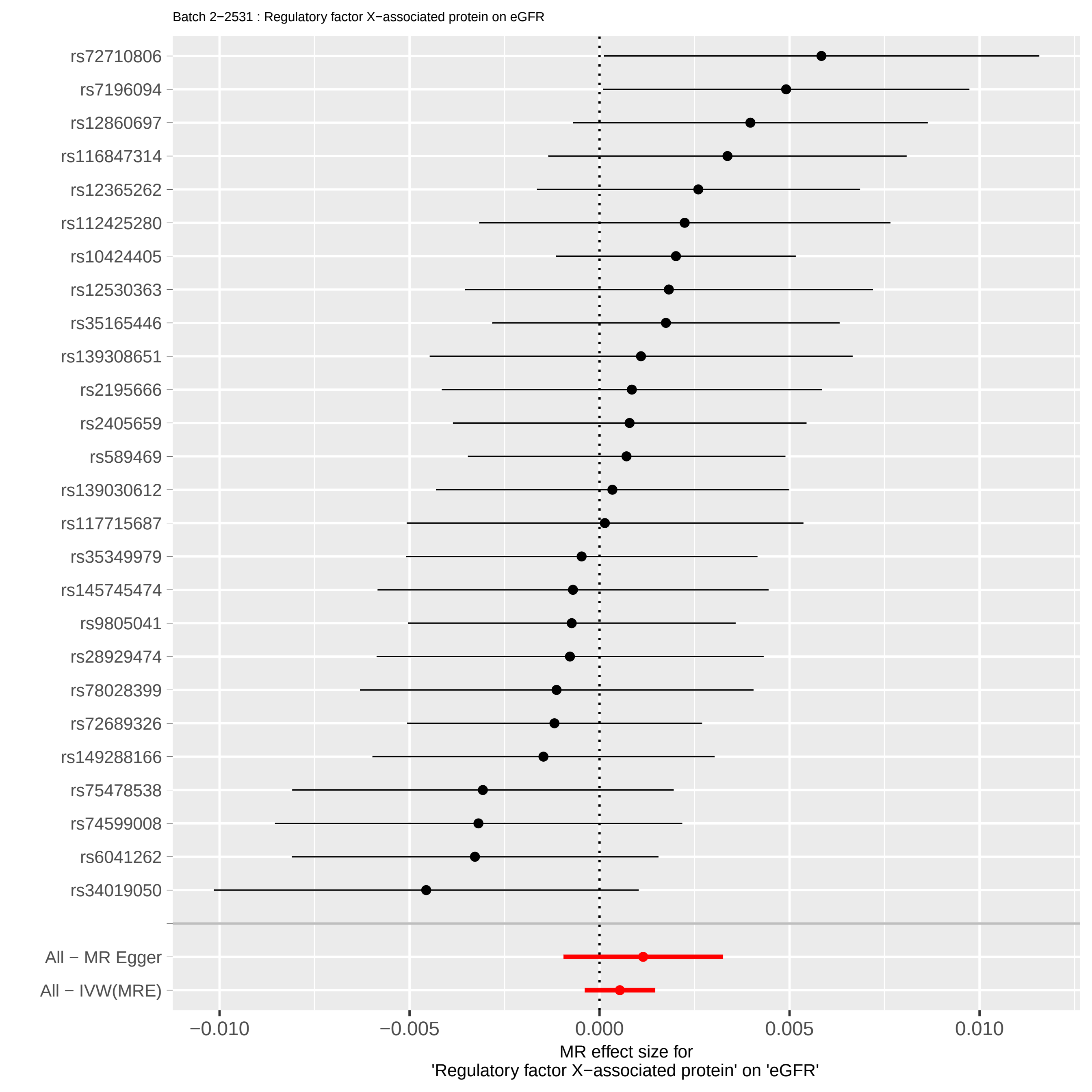

Batch 2−2531 : Regulatory factor X−associated protein on eGFR
rs72710806
rs7196094
rs12860697
rs116847314
rs12365262
rs112425280
rs10424405
rs12530363
rs35165446
rs139308651
rs2195666
rs2405659
rs589469
rs139030612
rs117715687
rs35349979
rs145745474
rs9805041
rs28929474
rs78028399
rs72689326
rs149288166
rs75478538
rs74599008
rs6041262
rs34019050
All − MR Egger
All − IVW(MRE)
−0.010
−0.005
0.000
0.005
0.010
MR effect size for
'Regulatory factor X−associated protein' on 'eGFR'

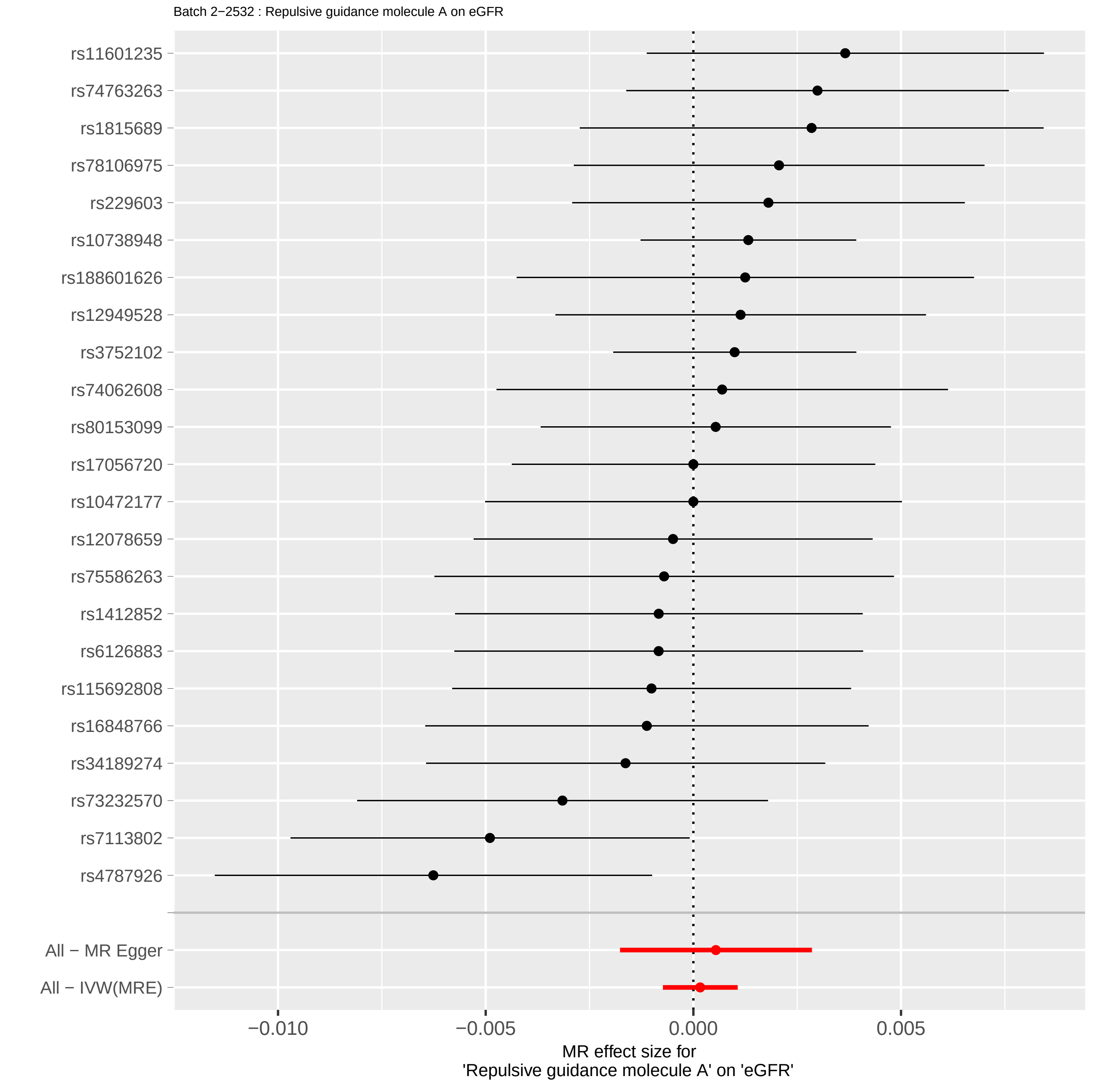

Batch 2−2532 : Repulsive guidance molecule A on eGFR
rs11601235
rs74763263
rs1815689
rs78106975
rs229603
rs10738948
rs188601626
rs12949528
rs3752102
rs74062608
rs80153099
rs17056720
rs10472177
rs12078659
rs75586263
rs1412852
rs6126883
rs115692808
rs16848766
rs34189274
rs73232570
rs7113802
rs4787926
All − MR Egger
All − IVW(MRE)
−0.010
−0.005
0.000
0.005
MR effect size for
'Repulsive guidance molecule A' on 'eGFR'

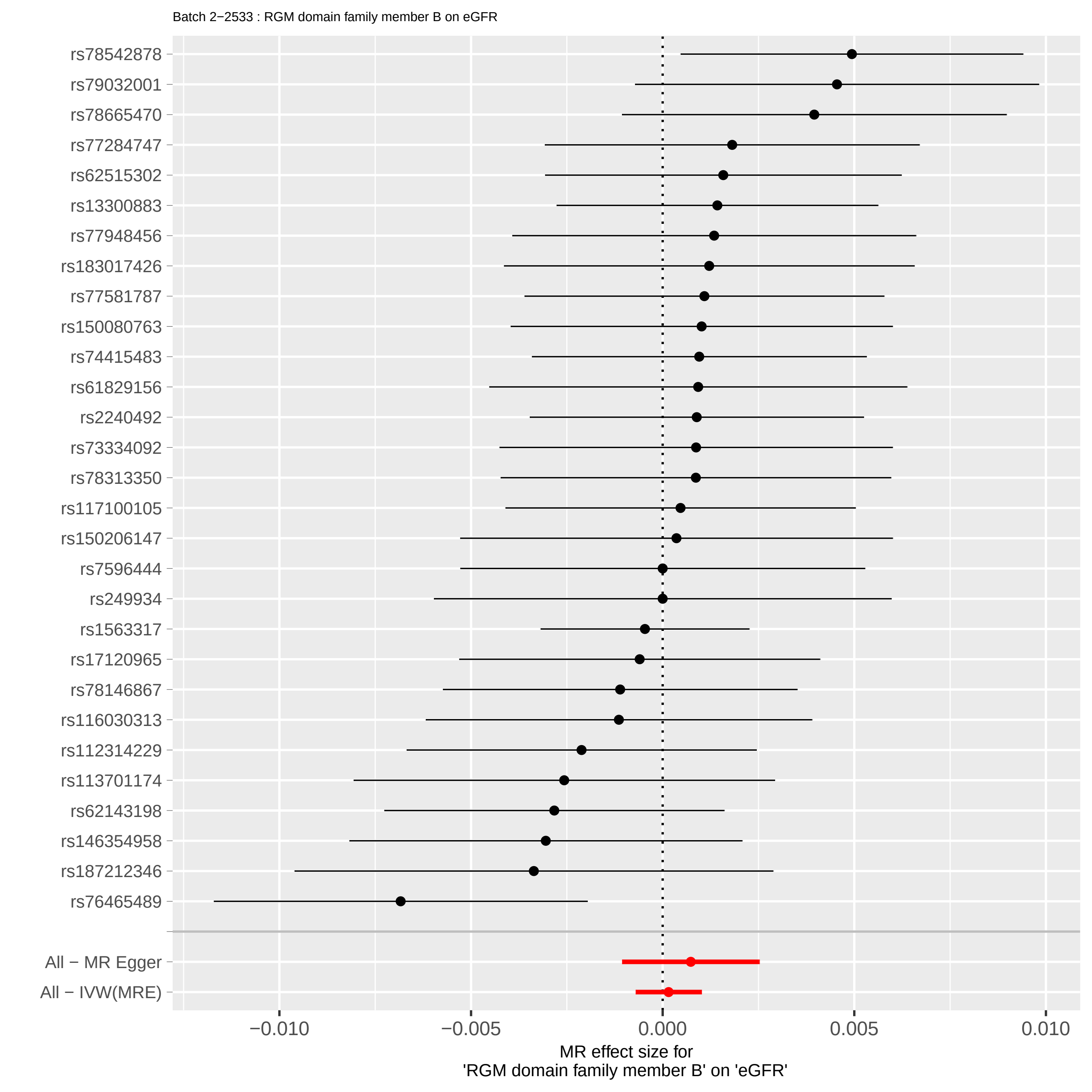

Batch 2−2533 : RGM domain family member B on eGFR
rs78542878
rs79032001
rs78665470
rs77284747
rs62515302
rs13300883
rs77948456
rs183017426
rs77581787
rs150080763
rs74415483
rs61829156
rs2240492
rs73334092
rs78313350
rs117100105
rs150206147
rs7596444
rs249934
rs1563317
rs17120965
rs78146867
rs116030313
rs112314229
rs113701174
rs62143198
rs146354958
rs187212346
rs76465489
All − MR Egger
All − IVW(MRE)
−0.010
−0.005
0.000
0.005
0.010
MR effect size for
'RGM domain family member B' on 'eGFR'

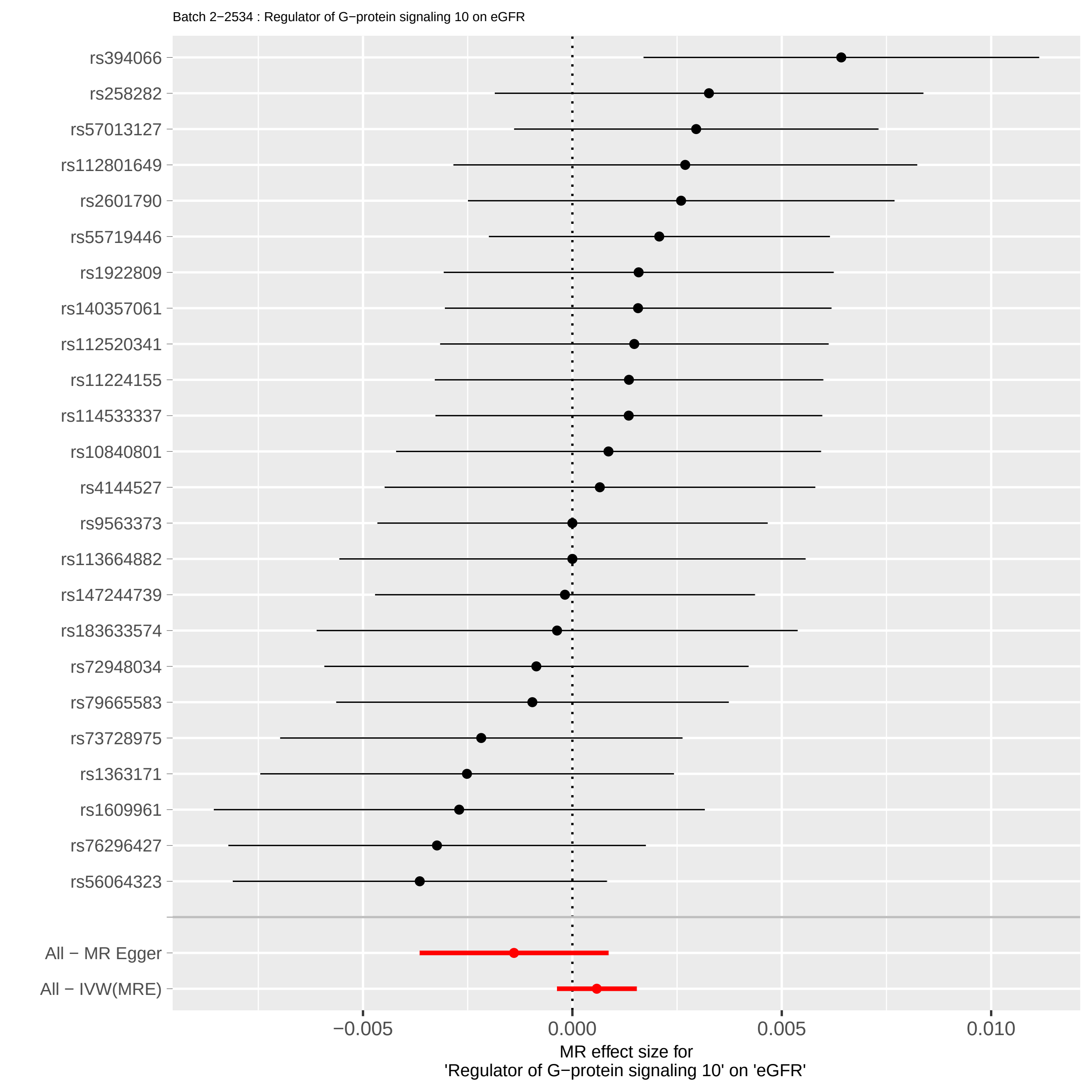

Batch 2−2534 : Regulator of G−protein signaling 10 on eGFR
rs394066
rs258282
rs57013127
rs112801649
rs2601790
rs55719446
rs1922809
rs140357061
rs112520341
rs11224155
rs114533337
rs10840801
rs4144527
rs9563373
rs113664882
rs147244739
rs183633574
rs72948034
rs79665583
rs73728975
rs1363171
rs1609961
rs76296427
rs56064323
All − MR Egger
All − IVW(MRE)
−0.005
0.000
0.005
0.010
MR effect size for
'Regulator of G−protein signaling 10' on 'eGFR'

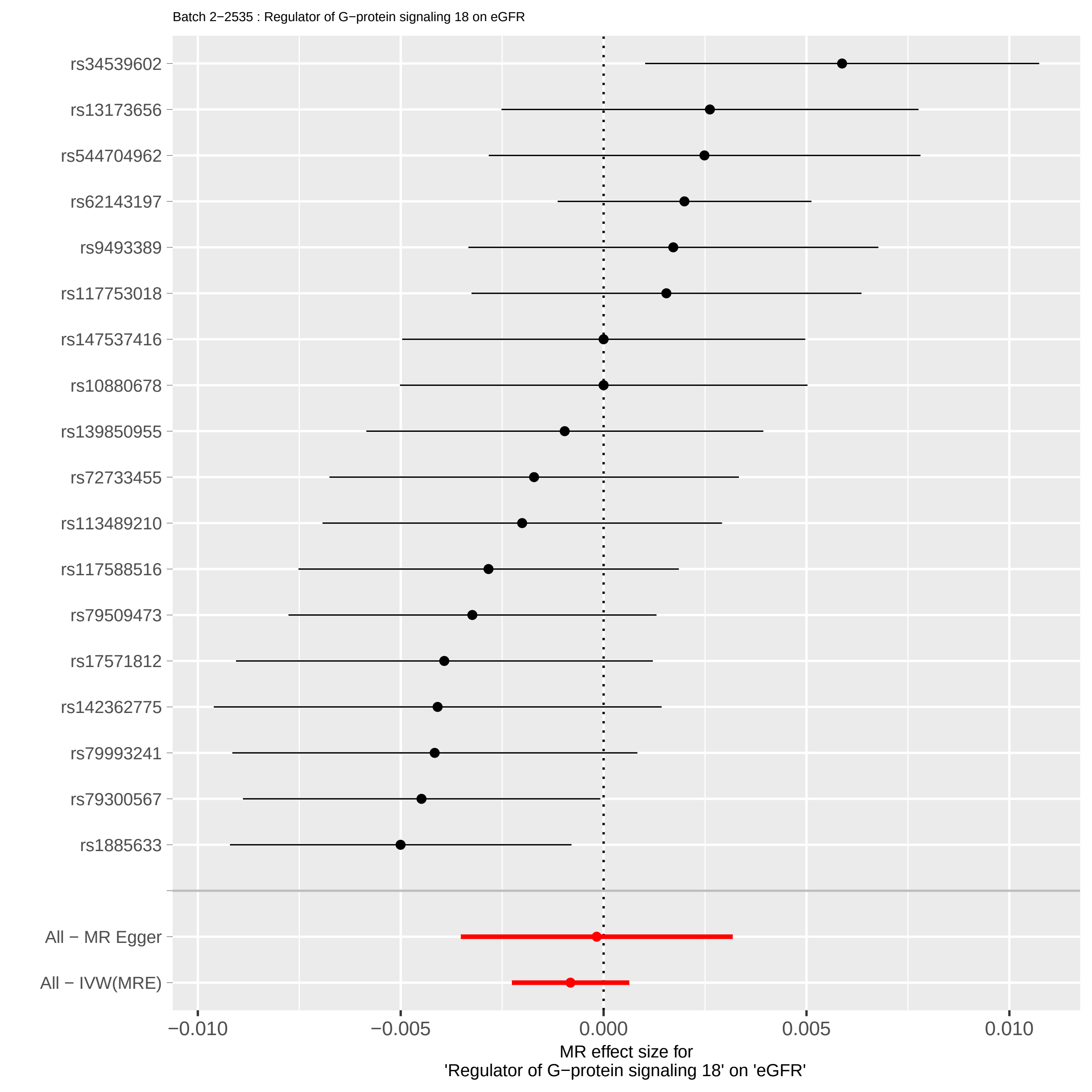

Batch 2−2535 : Regulator of G−protein signaling 18 on eGFR
rs34539602
rs13173656
rs544704962
rs62143197
rs9493389
rs117753018
rs147537416
rs10880678
rs139850955
rs72733455
rs113489210
rs117588516
rs79509473
rs17571812
rs142362775
rs79993241
rs79300567
rs1885633
All − MR Egger
All − IVW(MRE)
−0.010
−0.005
0.000
0.005
0.010
MR effect size for
'Regulator of G−protein signaling 18' on 'eGFR'

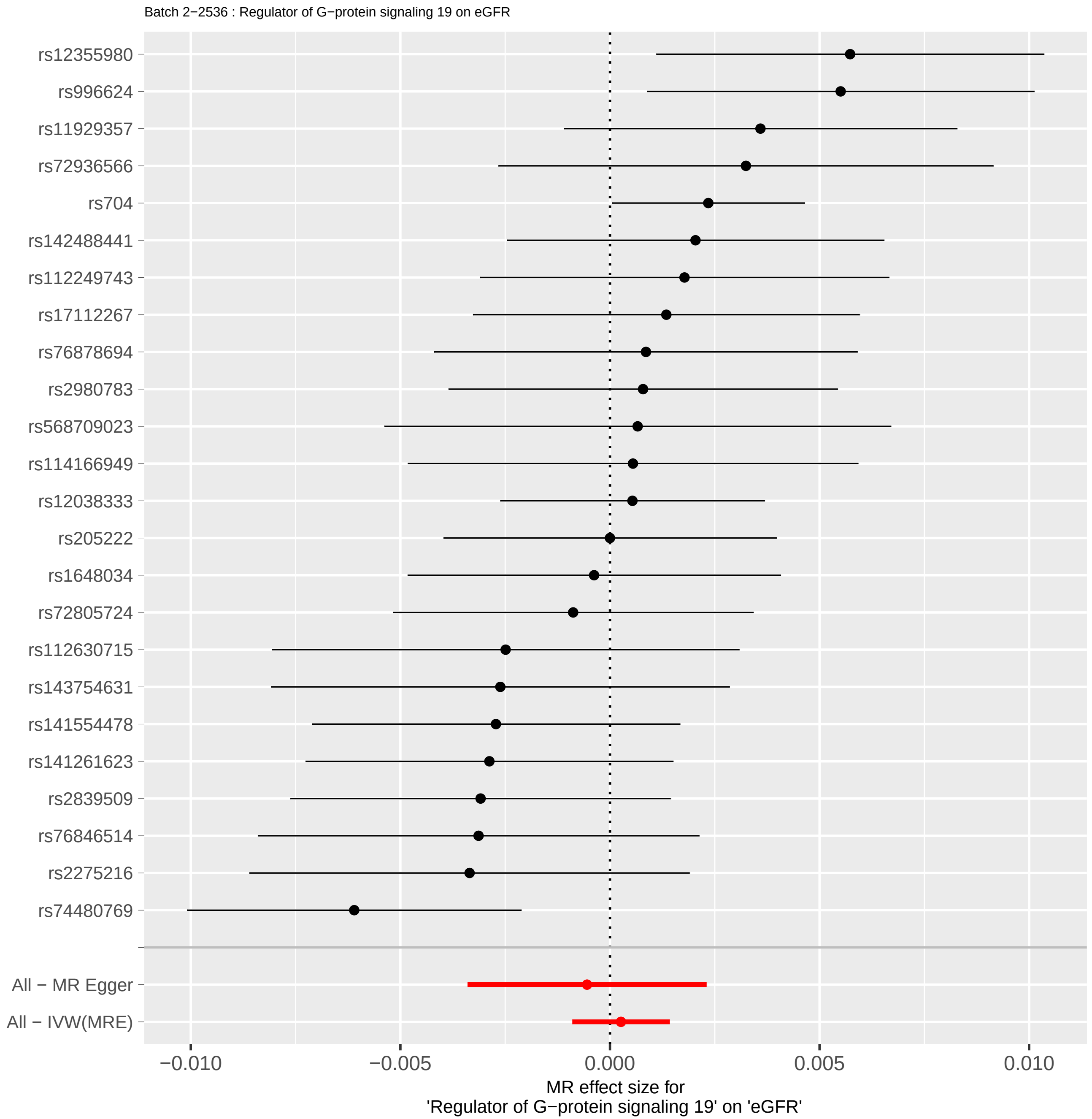

Batch 2−2536 : Regulator of G−protein signaling 19 on eGFR
rs12355980
rs996624
rs11929357
rs72936566
rs704
rs142488441
rs112249743
rs17112267
rs76878694
rs2980783
rs568709023
rs114166949
rs12038333
rs205222
rs1648034
rs72805724
rs112630715
rs143754631
rs141554478
rs141261623
rs2839509
rs76846514
rs2275216
rs74480769
All − MR Egger
All − IVW(MRE)
−0.010
−0.005
0.000
0.005
0.010
MR effect size for
'Regulator of G−protein signaling 19' on 'eGFR'

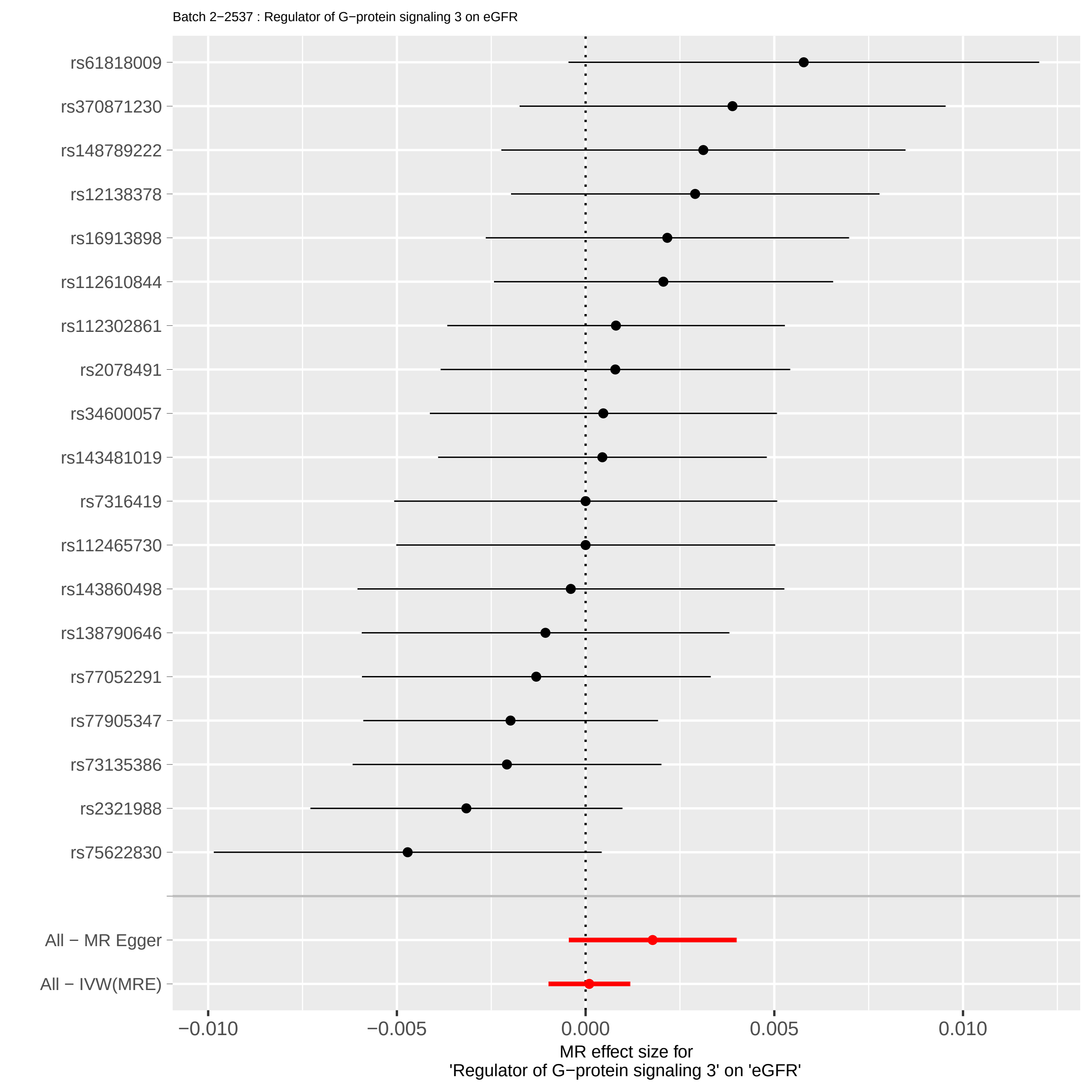

Batch 2−2537 : Regulator of G−protein signaling 3 on eGFR
rs61818009
rs370871230
rs148789222
rs12138378
rs16913898
rs112610844
rs112302861
rs2078491
rs34600057
rs143481019
rs7316419
rs112465730
rs143860498
rs138790646
rs77052291
rs77905347
rs73135386
rs2321988
rs75622830
All − MR Egger
All − IVW(MRE)
−0.010
−0.005
0.000
0.005
0.010
MR effect size for
'Regulator of G−protein signaling 3' on 'eGFR'

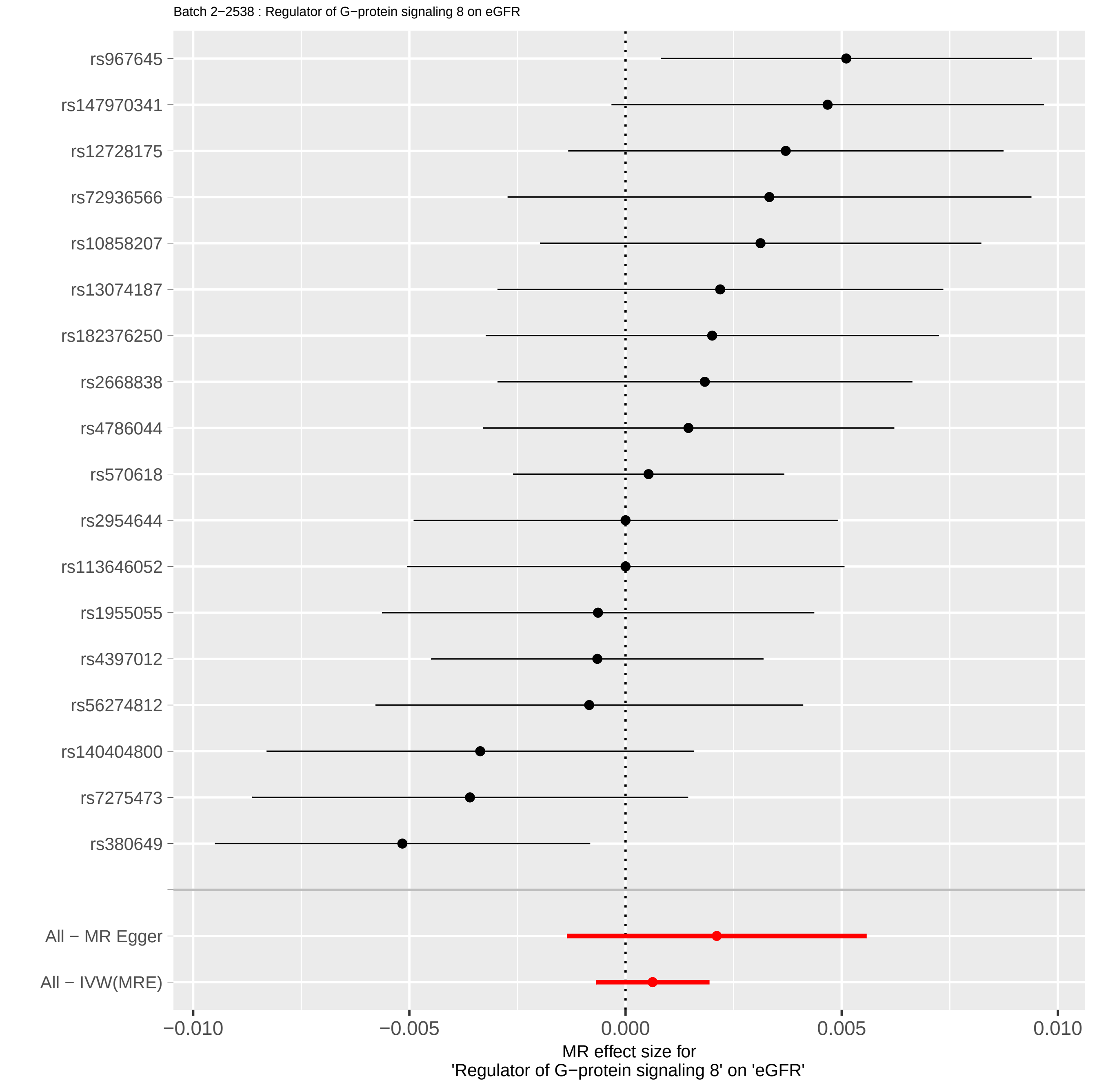

Batch 2−2538 : Regulator of G−protein signaling 8 on eGFR
rs967645
rs147970341
rs12728175
rs72936566
rs10858207
rs13074187
rs182376250
rs2668838
rs4786044
rs570618
rs2954644
rs113646052
rs1955055
rs4397012
rs56274812
rs140404800
rs7275473
rs380649
All − MR Egger
All − IVW(MRE)
−0.010
−0.005
0.000
0.005
0.010
MR effect size for
'Regulator of G−protein signaling 8' on 'eGFR'

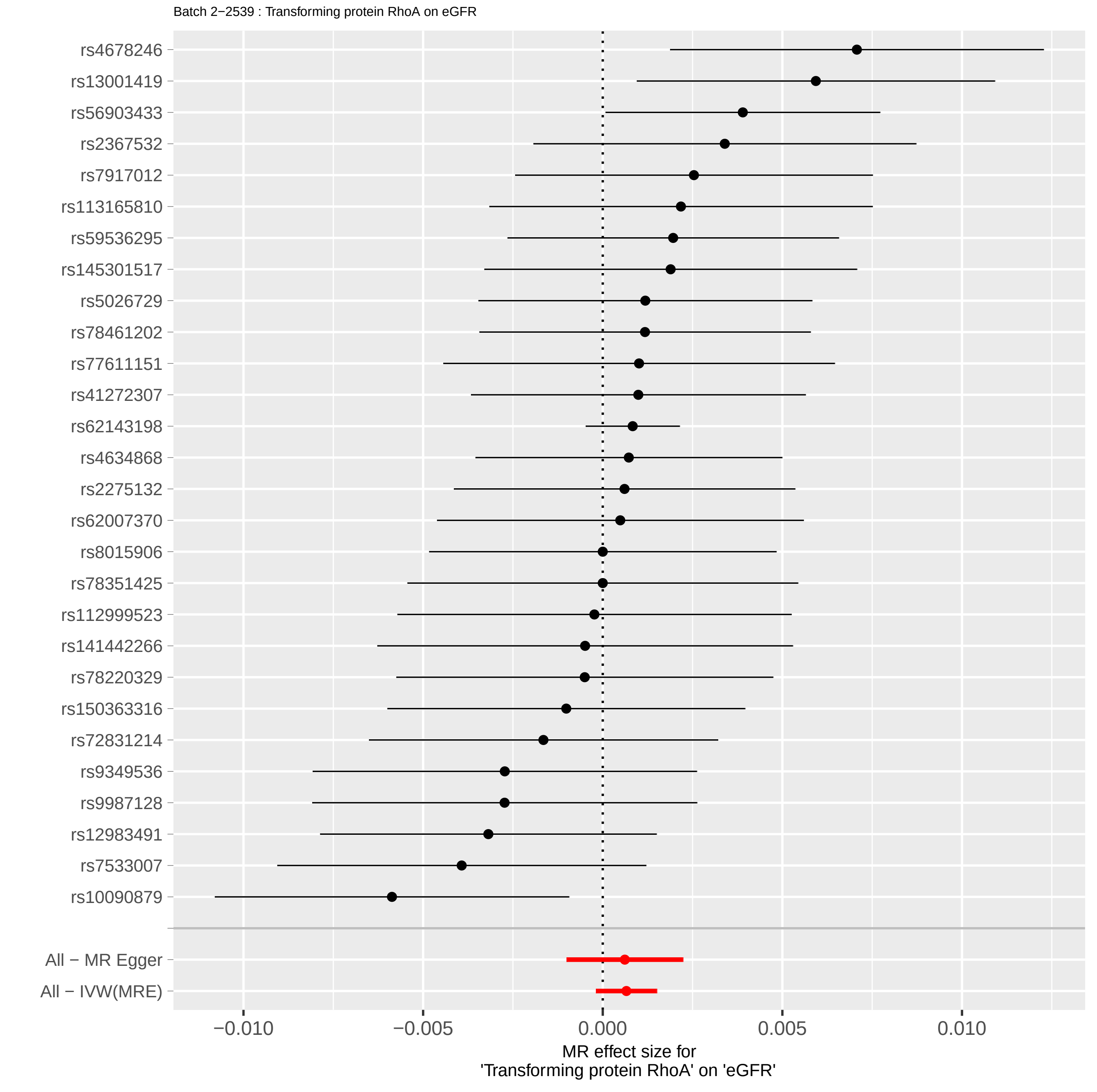

Batch 2−2539 : Transforming protein RhoA on eGFR
rs4678246
rs13001419
rs56903433
rs2367532
rs7917012
rs113165810
rs59536295
rs145301517
rs5026729
rs78461202
rs77611151
rs41272307
rs62143198
rs4634868
rs2275132
rs62007370
rs8015906
rs78351425
rs112999523
rs141442266
rs78220329
rs150363316
rs72831214
rs9349536
rs9987128
rs12983491
rs7533007
rs10090879
All − MR Egger
All − IVW(MRE)
−0.010
−0.005
0.000
0.005
0.010
MR effect size for
'Transforming protein RhoA' on 'eGFR'

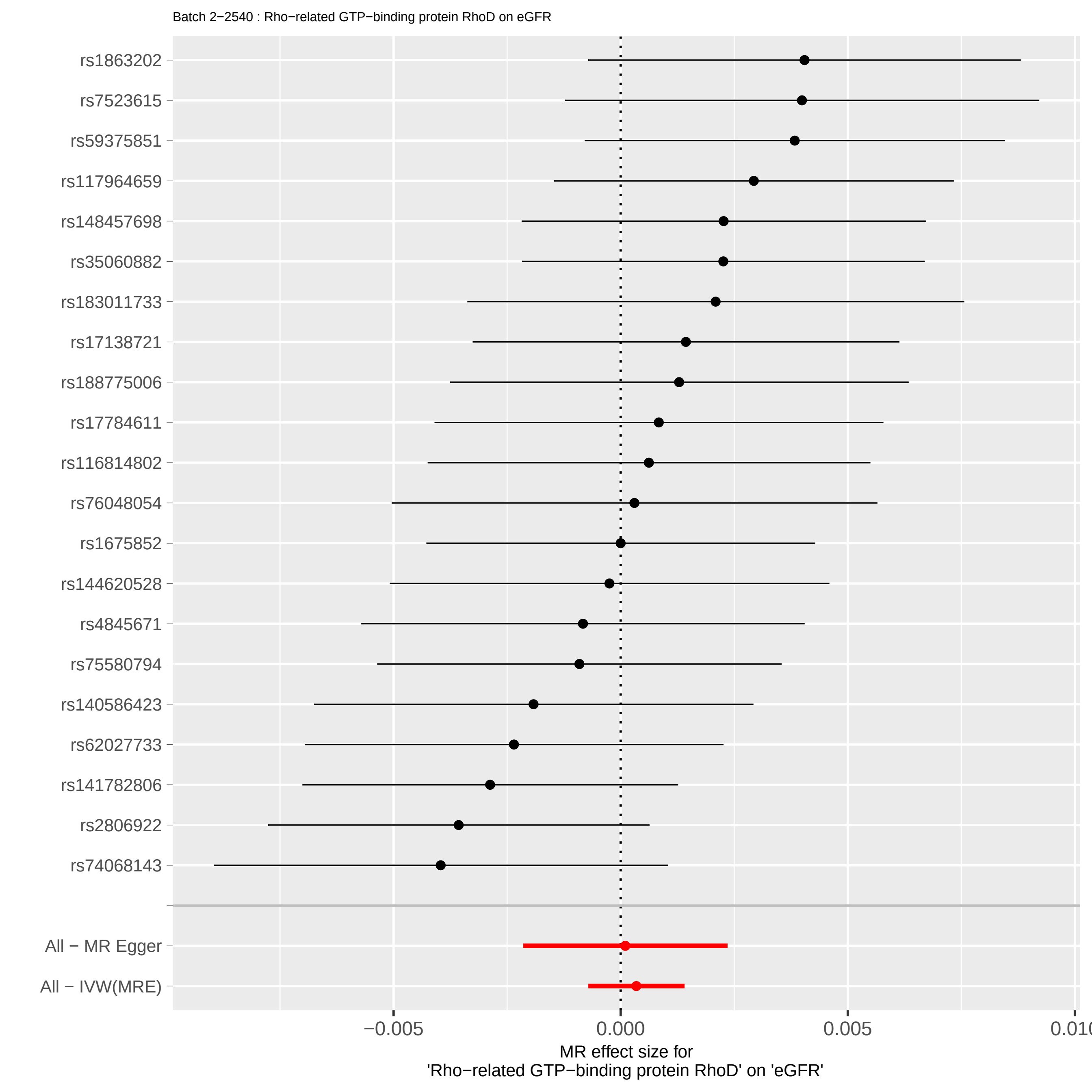

Batch 2−2540 : Rho−related GTP−binding protein RhoD on eGFR
rs1863202
rs7523615
rs59375851
rs117964659
rs148457698
rs35060882
rs183011733
rs17138721
rs188775006
rs17784611
rs116814802
rs76048054
rs1675852
rs144620528
rs4845671
rs75580794
rs140586423
rs62027733
rs141782806
rs2806922
rs74068143
All − MR Egger
All − IVW(MRE)
−0.005
0.000
0.005
0.010
MR effect size for
'Rho−related GTP−binding protein RhoD' on 'eGFR'

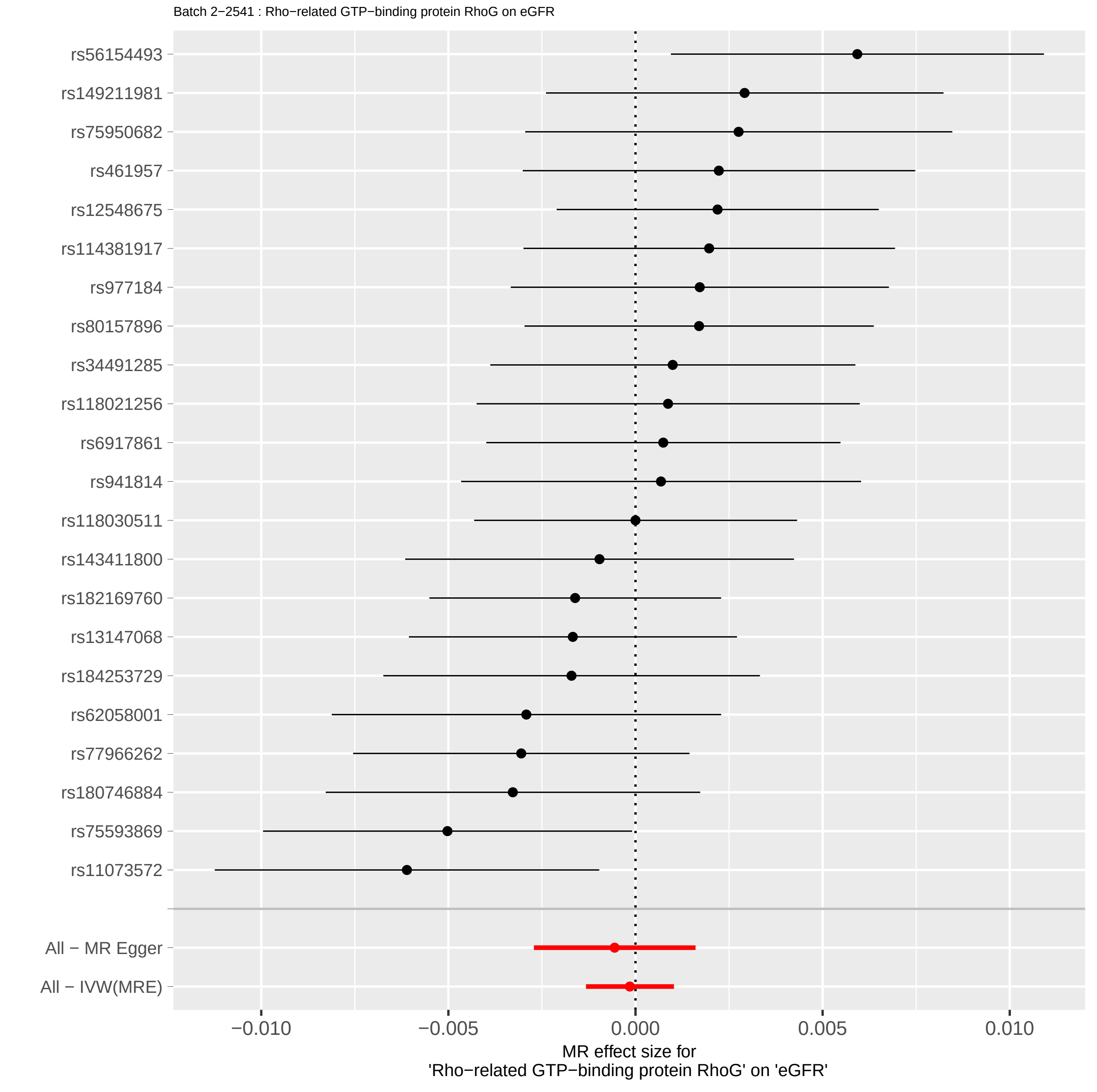
Batch 2−2541 : Rho−related GTP−binding protein RhoG on eGFR
rs56154493
rs149211981
rs75950682
rs461957
rs12548675
rs114381917
rs977184
rs80157896
rs34491285
rs118021256
rs6917861
rs941814
rs118030511
rs143411800
rs182169760
rs13147068
rs184253729
rs62058001
rs77966262
rs180746884
rs75593869
rs11073572
All − MR Egger
All − IVW(MRE)
−0.010
−0.005
0.000
0.005
0.010
MR effect size for
'Rho−related GTP−binding protein RhoG' on 'eGFR'

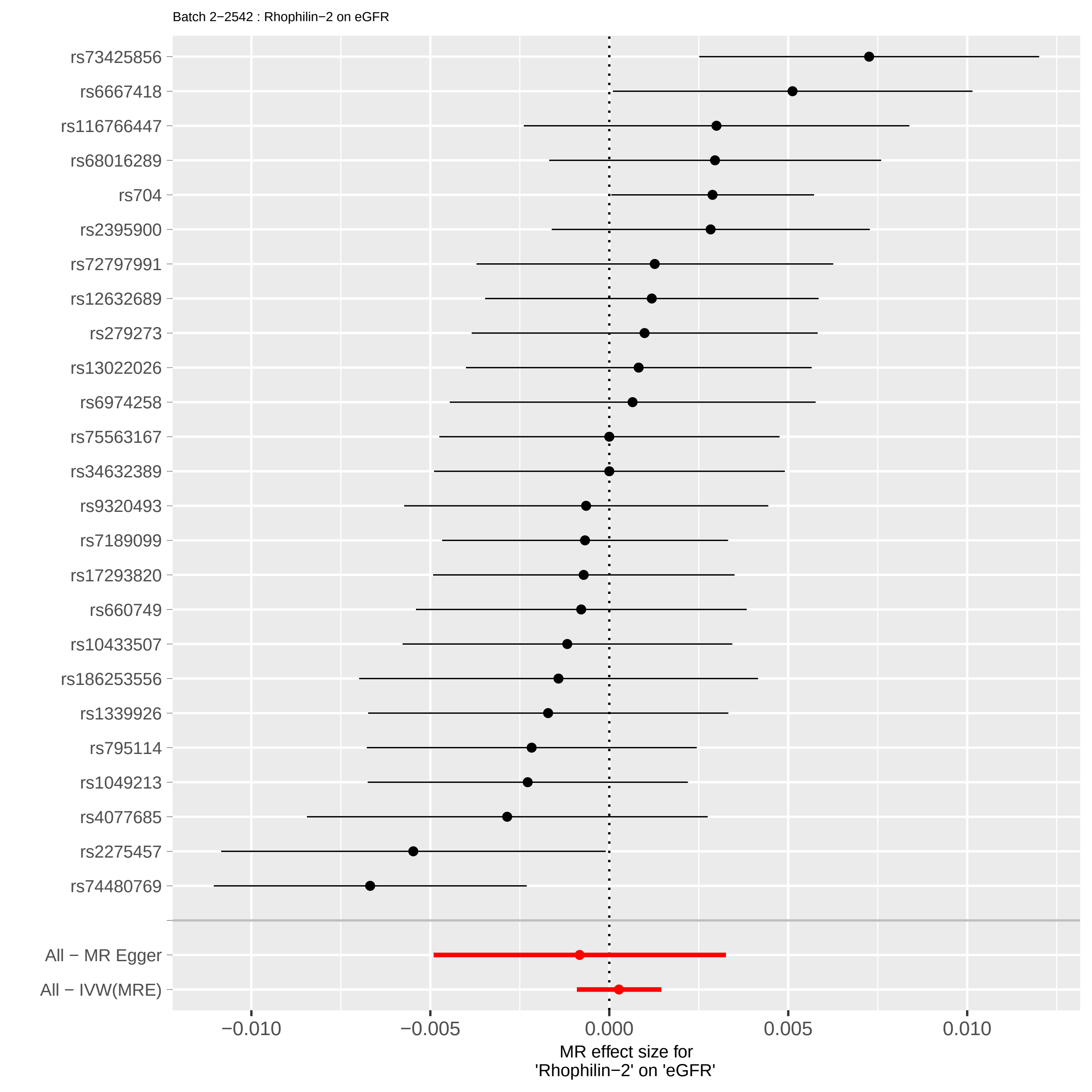
Batch 2−2542 : Rhophilin−2 on eGFR
rs73425856
rs6667418
rs116766447
rs68016289
rs704
rs2395900
rs72797991
rs12632689
rs279273
rs13022026
rs6974258
rs75563167
rs34632389
rs9320493
rs7189099
rs17293820
rs660749
rs10433507
rs186253556
rs1339926
rs795114
rs1049213
rs4077685
rs2275457
rs74480769
All − MR Egger
All − IVW(MRE)
−0.010
−0.005
0.000
0.005
0.010
MR effect size for
'Rhophilin−2' on 'eGFR'

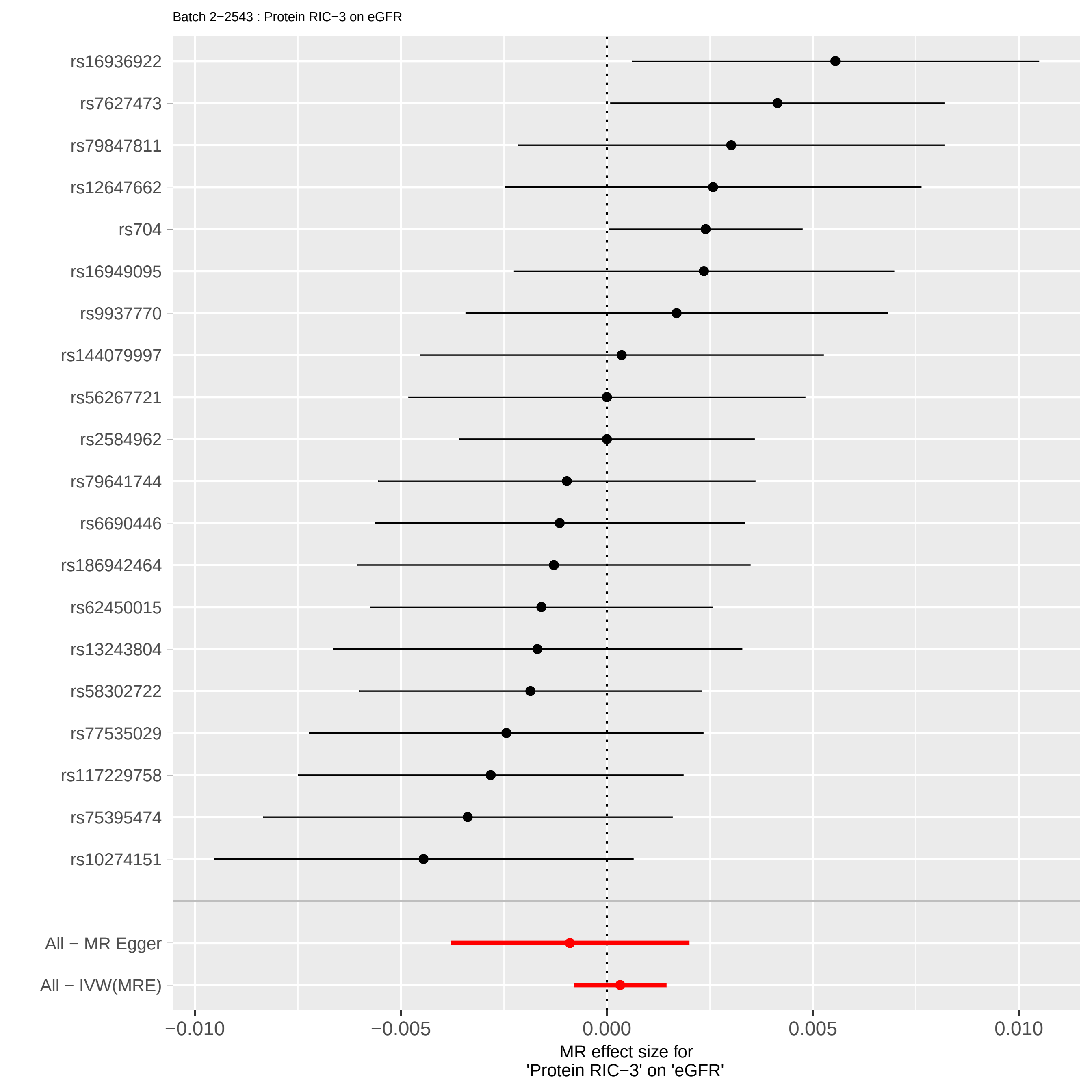

Batch 2−2543 : Protein RIC−3 on eGFR
rs16936922
rs7627473
rs79847811
rs12647662
rs704
rs16949095
rs9937770
rs144079997
rs56267721
rs2584962
rs79641744
rs6690446
rs186942464
rs62450015
rs13243804
rs58302722
rs77535029
rs117229758
rs75395474
rs10274151
All − MR Egger
All − IVW(MRE)
−0.010
−0.005
0.000
0.005
0.010
MR effect size for
'Protein RIC−3' on 'eGFR'

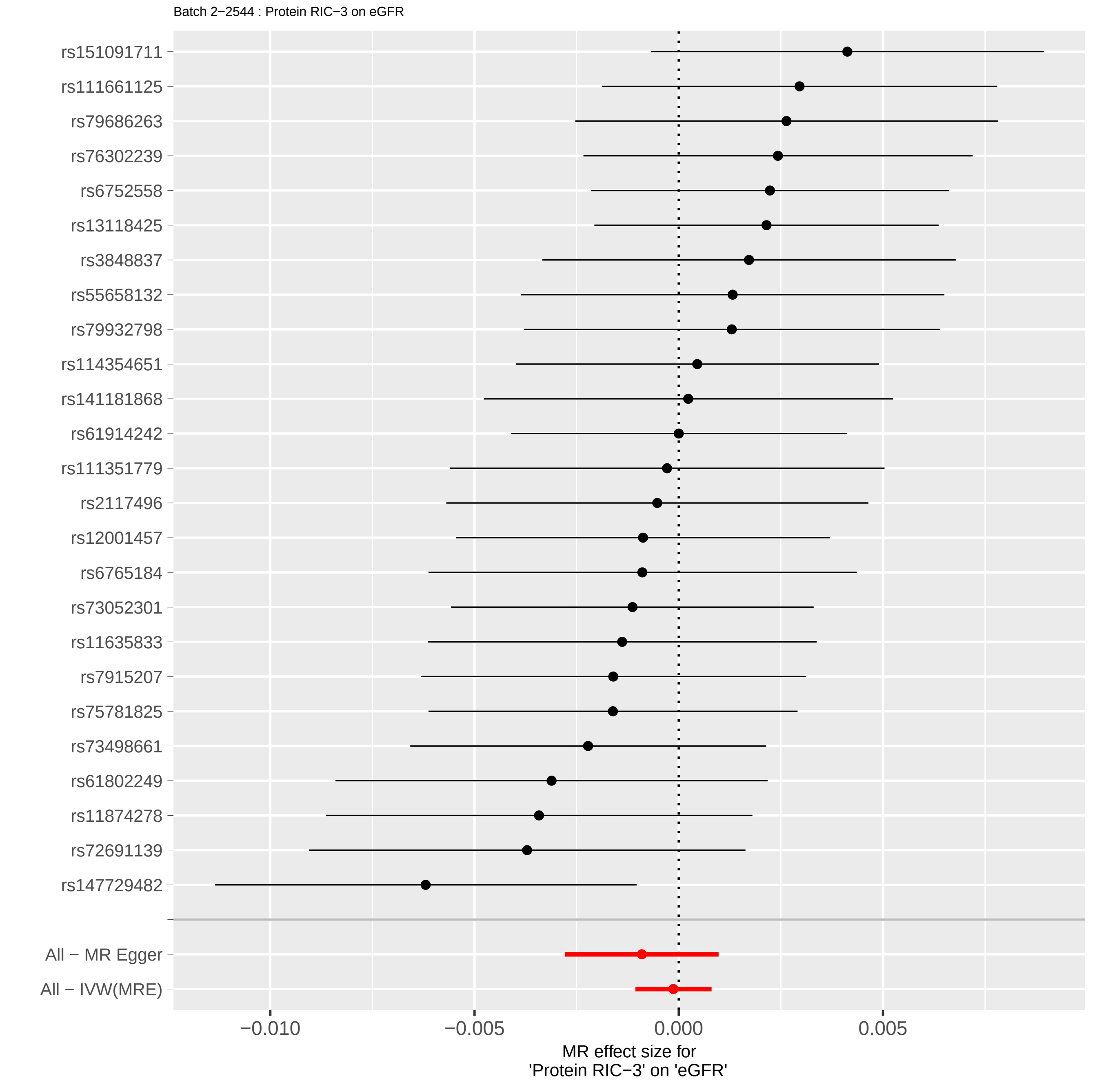

Batch 2−2544 : Protein RIC−3 on eGFR
rs151091711
rs111661125
rs79686263
rs76302239
rs6752558
rs13118425
rs3848837
rs55658132
rs79932798
rs114354651
rs141181868
rs61914242
rs111351779
rs2117496
rs12001457
rs6765184
rs73052301
rs11635833
rs7915207
rs75781825
rs73498661
rs61802249
rs11874278
rs72691139
rs147729482
All − MR Egger
All − IVW(MRE)
−0.010
−0.005
0.000
0.005
MR effect size for
'Protein RIC−3' on 'eGFR'

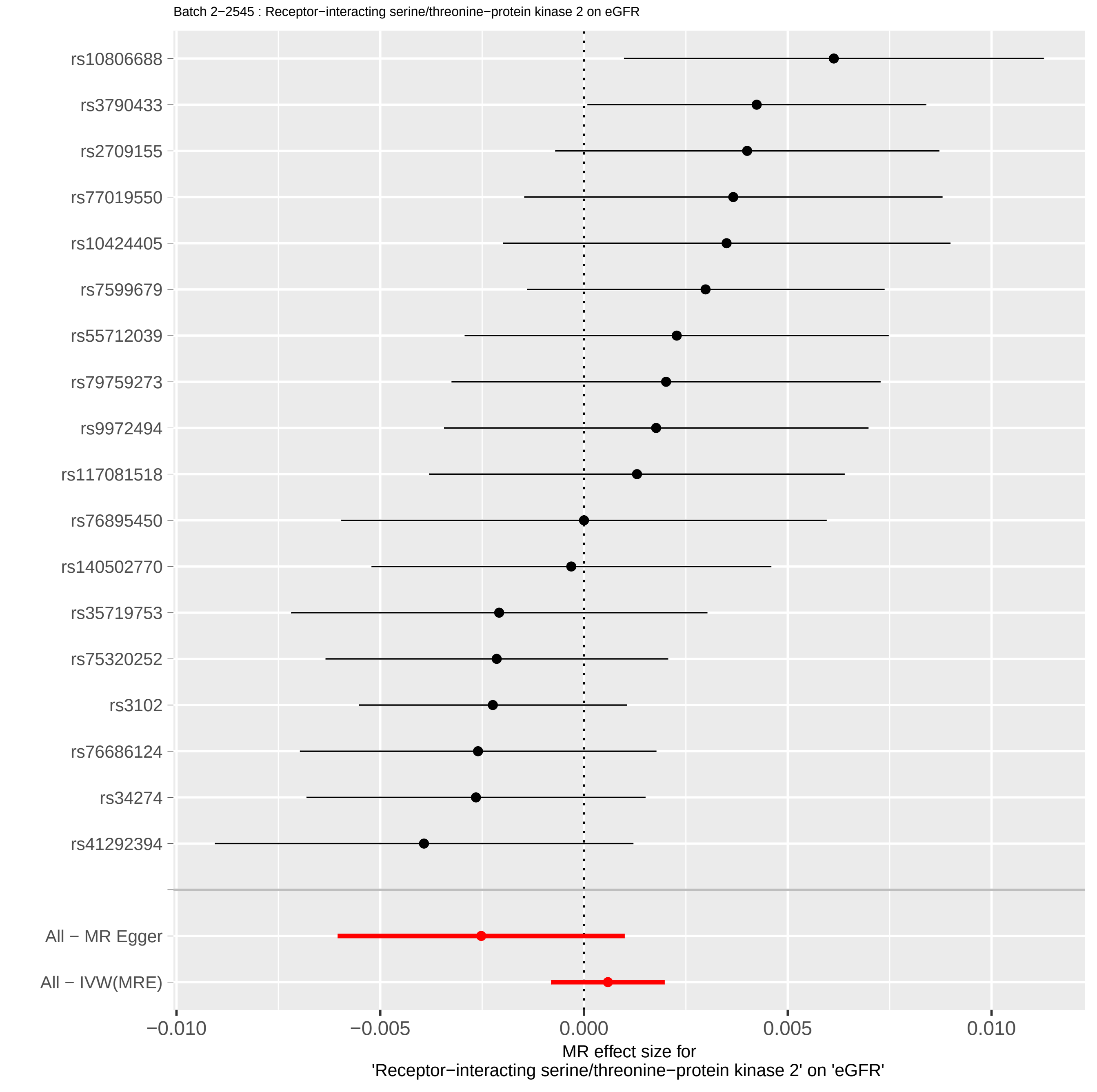
Batch 2−2545 : Receptor−interacting serine/threonine−protein kinase 2 on eGFR
rs10806688
rs3790433
rs2709155
rs77019550
rs10424405
rs7599679
rs55712039
rs79759273
rs9972494
rs117081518
rs76895450
rs140502770
rs35719753
rs75320252
rs3102
rs76686124
rs34274
rs41292394
All − MR Egger
All − IVW(MRE)
−0.010
−0.005
0.000
0.005
0.010
MR effect size for
'Receptor−interacting serine/threonine−protein kinase 2' on 'eGFR'

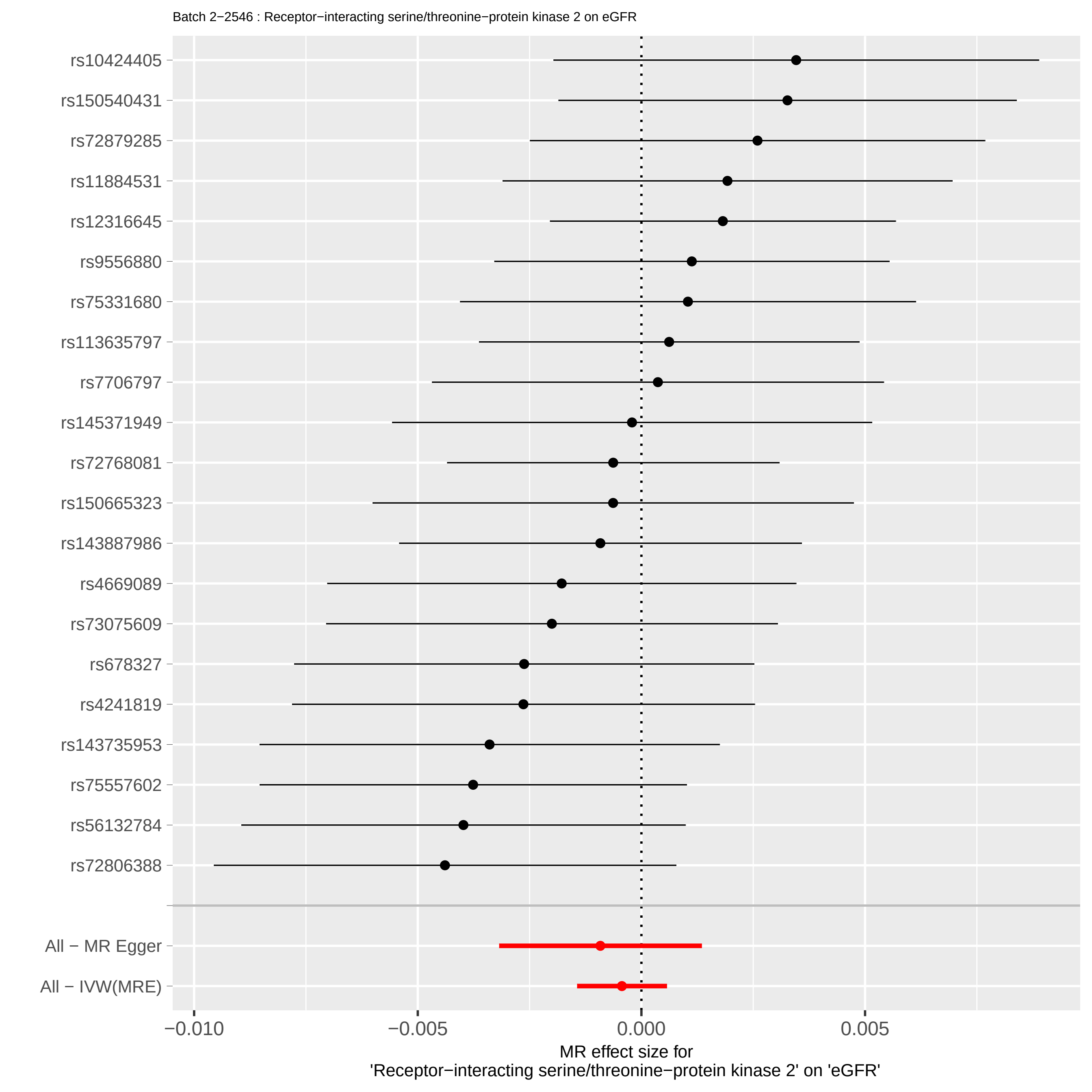

Batch 2−2546 : Receptor−interacting serine/threonine−protein kinase 2 on eGFR
rs10424405
rs150540431
rs72879285
rs11884531
rs12316645
rs9556880
rs75331680
rs113635797
rs7706797
rs145371949
rs72768081
rs150665323
rs143887986
rs4669089
rs73075609
rs678327
rs4241819
rs143735953
rs75557602
rs56132784
rs72806388
All − MR Egger
All − IVW(MRE)
−0.010
−0.005
0.000
0.005
MR effect size for
'Receptor−interacting serine/threonine−protein kinase 2' on 'eGFR'

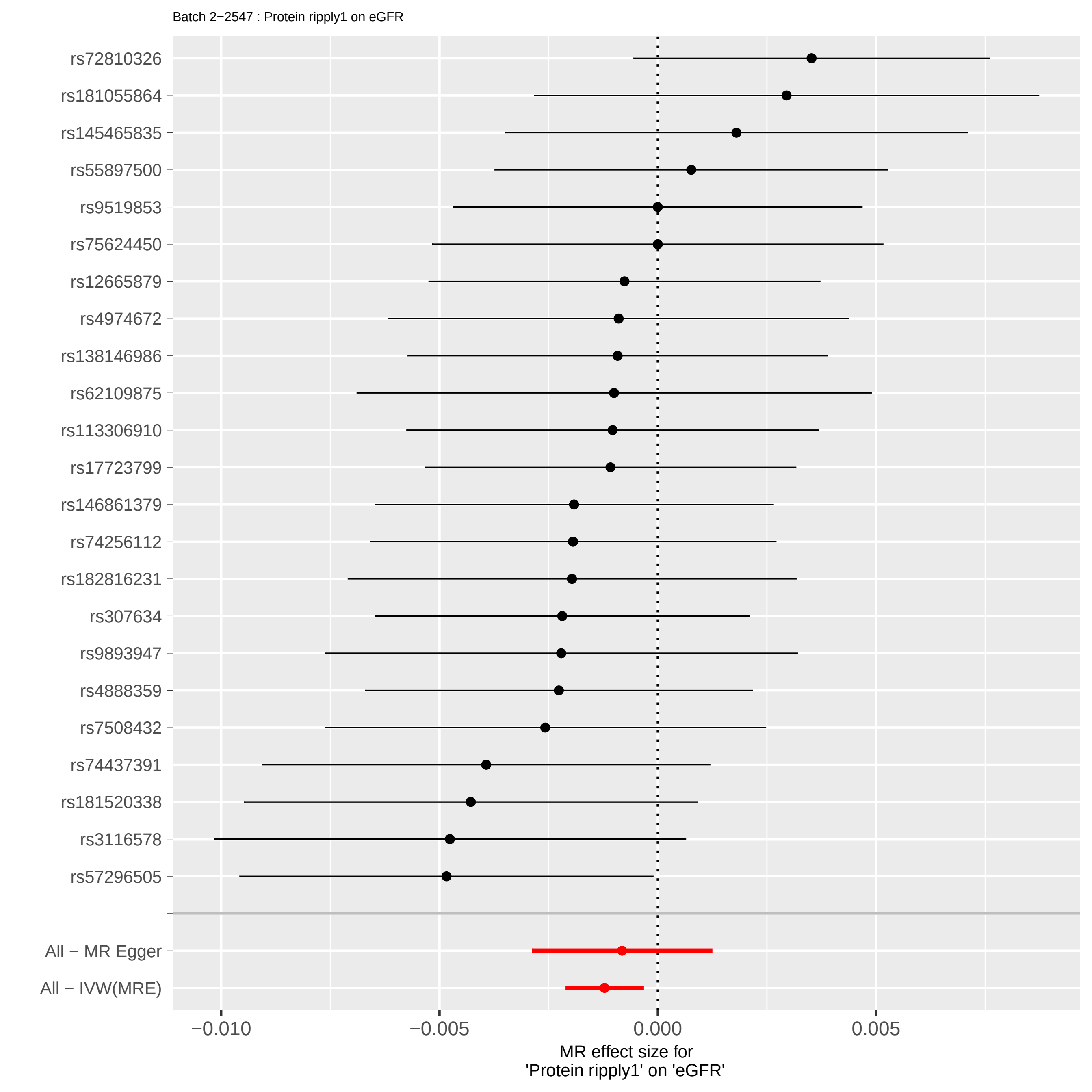

Batch 2−2547 : Protein ripply1 on eGFR
rs72810326
rs181055864
rs145465835
rs55897500
rs9519853
rs75624450
rs12665879
rs4974672
rs138146986
rs62109875
rs113306910
rs17723799
rs146861379
rs74256112
rs182816231
rs307634
rs9893947
rs4888359
rs7508432
rs74437391
rs181520338
rs3116578
rs57296505
All − MR Egger
All − IVW(MRE)
−0.010
−0.005
0.000
0.005
MR effect size for
'Protein ripply1' on 'eGFR'

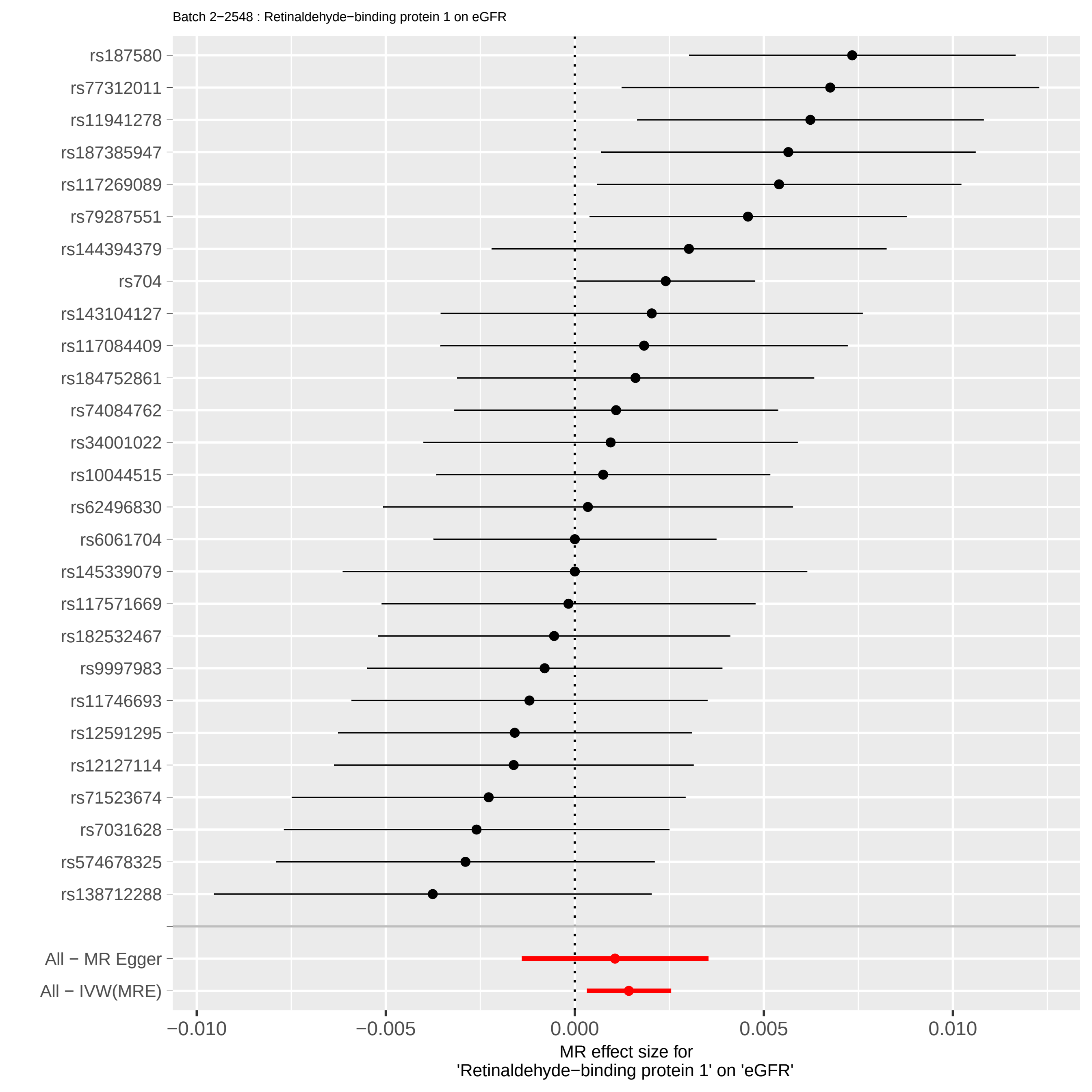

Batch 2−2548 : Retinaldehyde−binding protein 1 on eGFR
rs187580
rs77312011
rs11941278
rs187385947
rs117269089
rs79287551
rs144394379
rs704
rs143104127
rs117084409
rs184752861
rs74084762
rs34001022
rs10044515
rs62496830
rs6061704
rs145339079
rs117571669
rs182532467
rs9997983
rs11746693
rs12591295
rs12127114
rs71523674
rs7031628
rs574678325
rs138712288
All − MR Egger
All − IVW(MRE)
−0.010
−0.005
0.000
0.005
0.010
MR effect size for
'Retinaldehyde−binding protein 1' on 'eGFR'

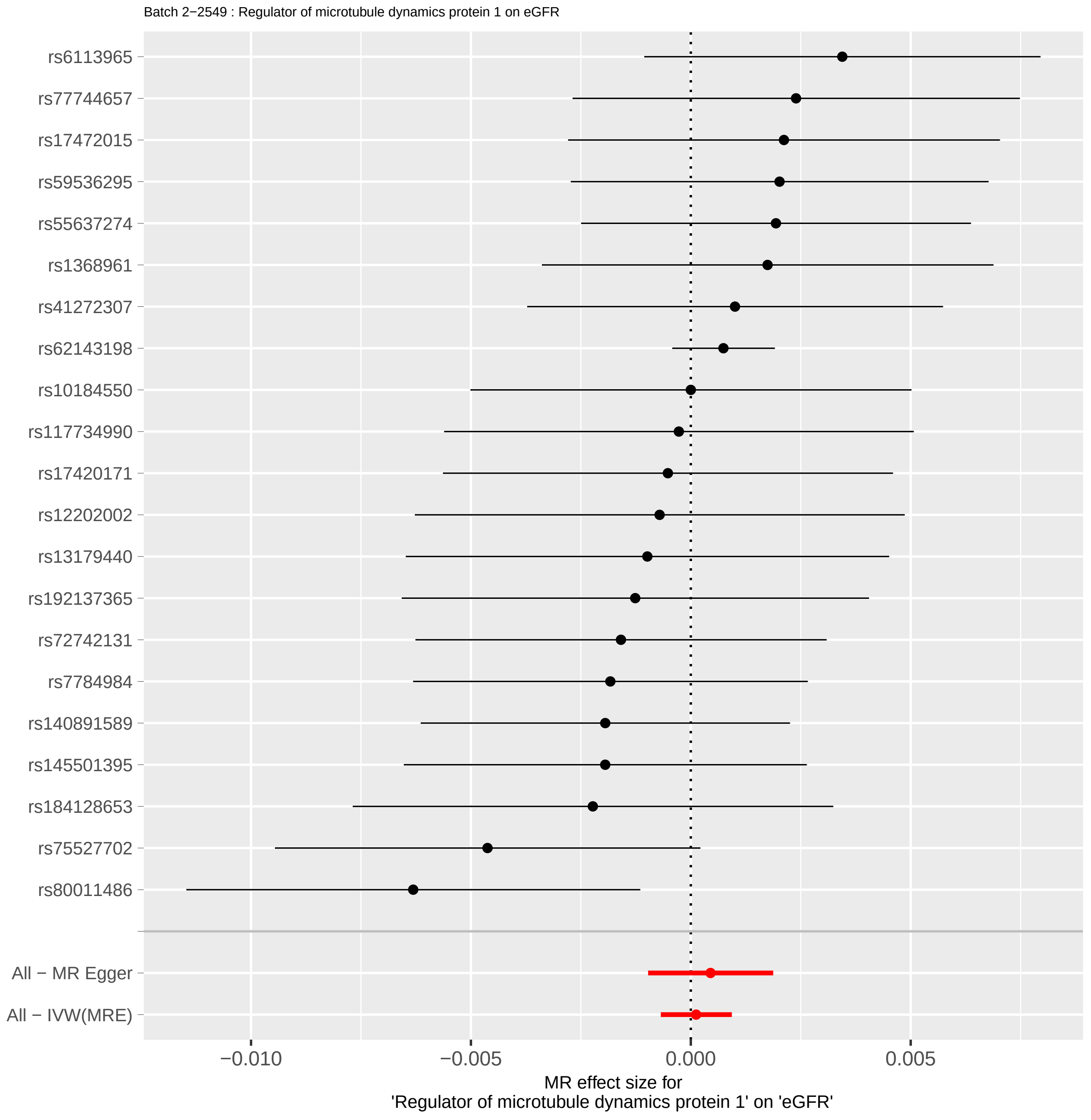
Batch 2−2549 : Regulator of microtubule dynamics protein 1 on eGFR
rs6113965
rs77744657
rs17472015
rs59536295
rs55637274
rs1368961
rs41272307
rs62143198
rs10184550
rs117734990
rs17420171
rs12202002
rs13179440
rs192137365
rs72742131
rs7784984
rs140891589
rs145501395
rs184128653
rs75527702
rs80011486
All − MR Egger
All − IVW(MRE)
−0.010
−0.005
0.000
0.005
MR effect size for
'Regulator of microtubule dynamics protein 1' on 'eGFR'

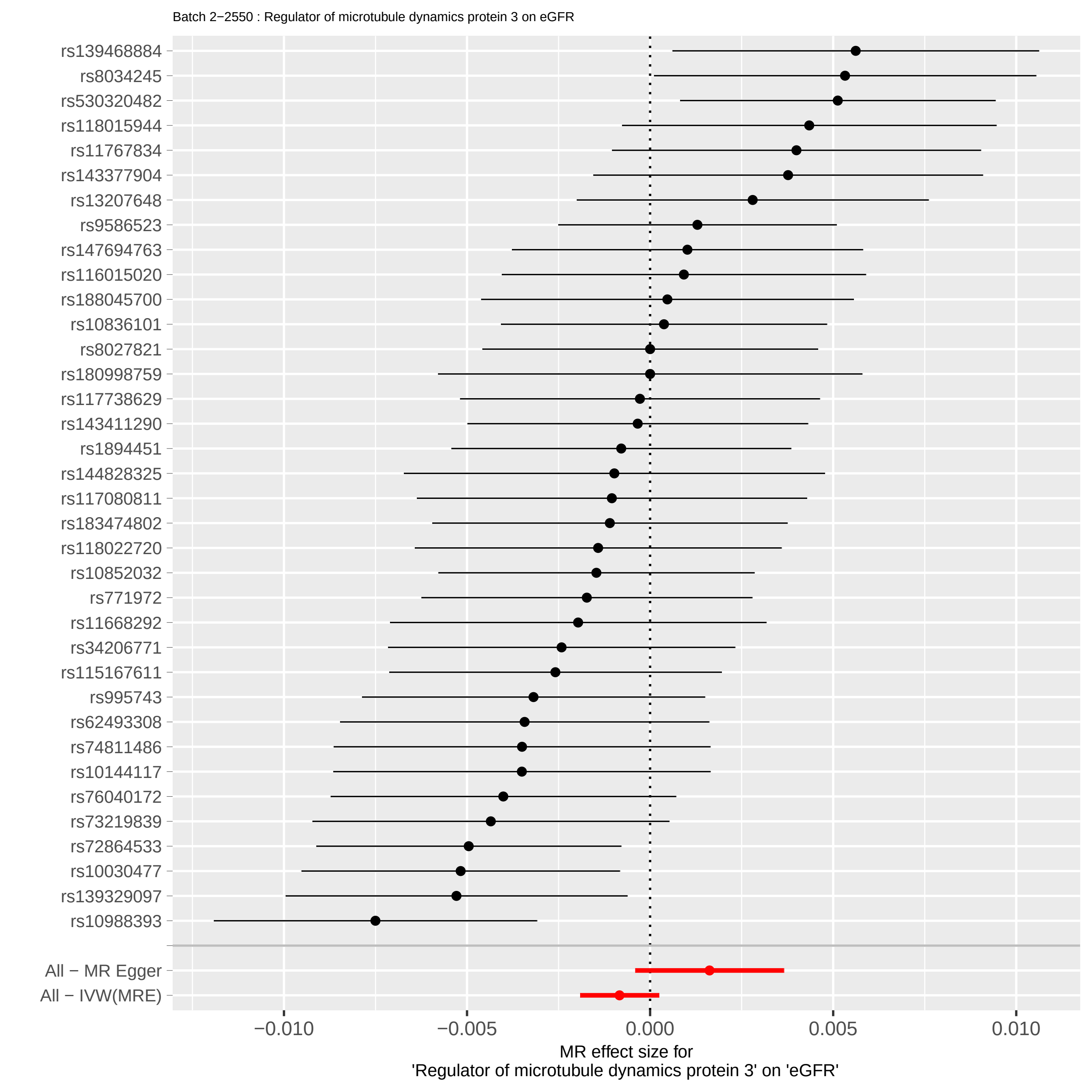

Batch 2−2550 : Regulator of microtubule dynamics protein 3 on eGFR
rs139468884
rs8034245
rs530320482
rs118015944
rs11767834
rs143377904
rs13207648
rs9586523
rs147694763
rs116015020
rs188045700
rs10836101
rs8027821
rs180998759
rs117738629
rs143411290
rs1894451
rs144828325
rs117080811
rs183474802
rs118022720
rs10852032
rs771972
rs11668292
rs34206771
rs115167611
rs995743
rs62493308
rs74811486
rs10144117
rs76040172
rs73219839
rs72864533
rs10030477
rs139329097
rs10988393
All − MR Egger
All − IVW(MRE)
−0.010
−0.005
0.000
0.005
0.010
MR effect size for
'Regulator of microtubule dynamics protein 3' on 'eGFR'

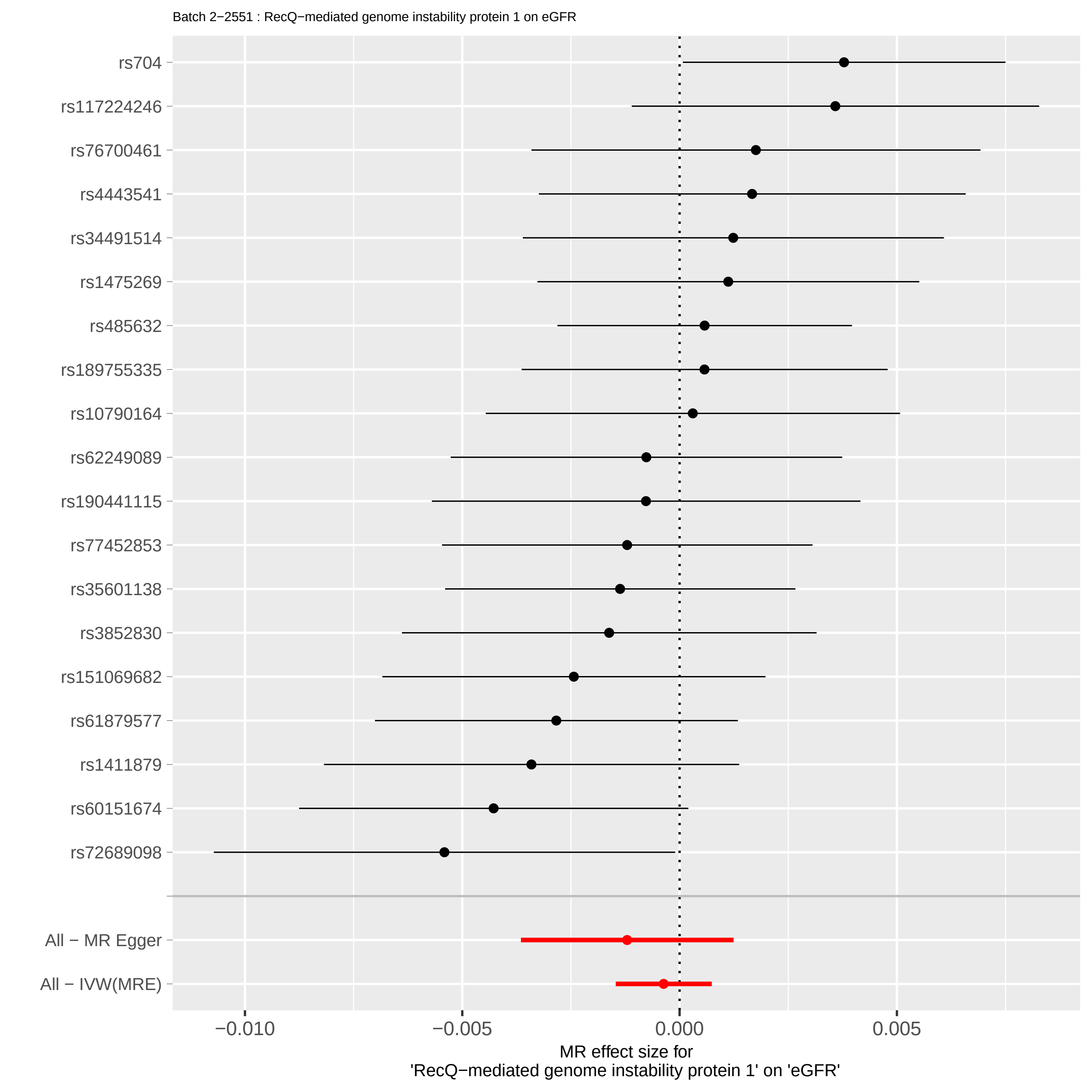

Batch 2−2551 : RecQ−mediated genome instability protein 1 on eGFR
rs704
rs117224246
rs76700461
rs4443541
rs34491514
rs1475269
rs485632
rs189755335
rs10790164
rs62249089
rs190441115
rs77452853
rs35601138
rs3852830
rs151069682
rs61879577
rs1411879
rs60151674
rs72689098
All − MR Egger
All − IVW(MRE)
−0.010
−0.005
0.000
0.005
MR effect size for
'RecQ−mediated genome instability protein 1' on 'eGFR'

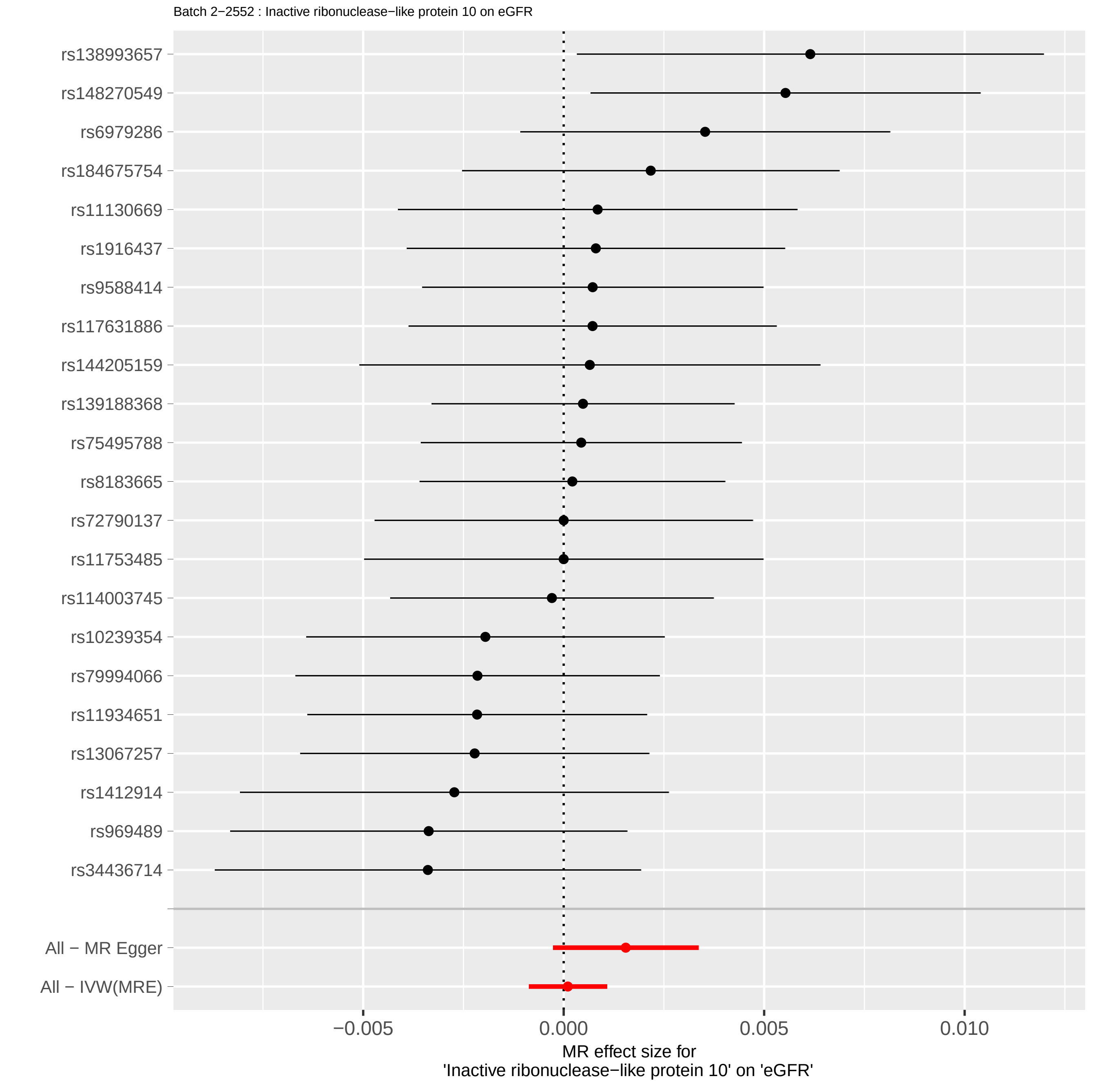
Batch 2−2552 : Inactive ribonuclease−like protein 10 on eGFR
rs138993657
rs148270549
rs6979286
rs184675754
rs11130669
rs1916437
rs9588414
rs117631886
rs144205159
rs139188368
rs75495788
rs8183665
rs72790137
rs11753485
rs114003745
rs10239354
rs79994066
rs11934651
rs13067257
rs1412914
rs969489
rs34436714
All − MR Egger
All − IVW(MRE)
−0.005
0.000
0.005
0.010
MR effect size for
'Inactive ribonuclease−like protein 10' on 'eGFR'

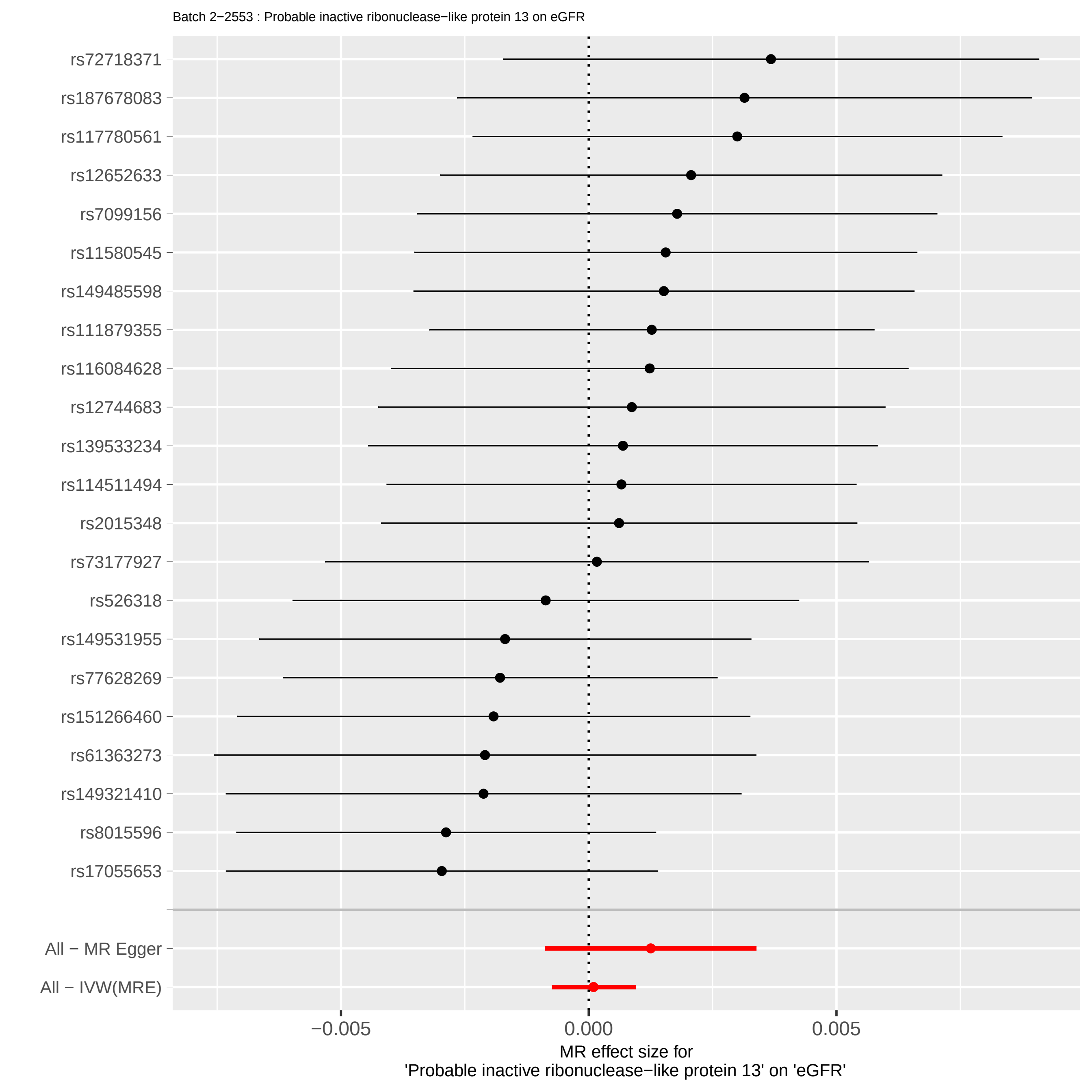

Batch 2−2553 : Probable inactive ribonuclease−like protein 13 on eGFR
rs72718371
rs187678083
rs117780561
rs12652633
rs7099156
rs11580545
rs149485598
rs111879355
rs116084628
rs12744683
rs139533234
rs114511494
rs2015348
rs73177927
rs526318
rs149531955
rs77628269
rs151266460
rs61363273
rs149321410
rs8015596
rs17055653
All − MR Egger
All − IVW(MRE)
−0.005
0.000
0.005
MR effect size for
'Probable inactive ribonuclease−like protein 13' on 'eGFR'

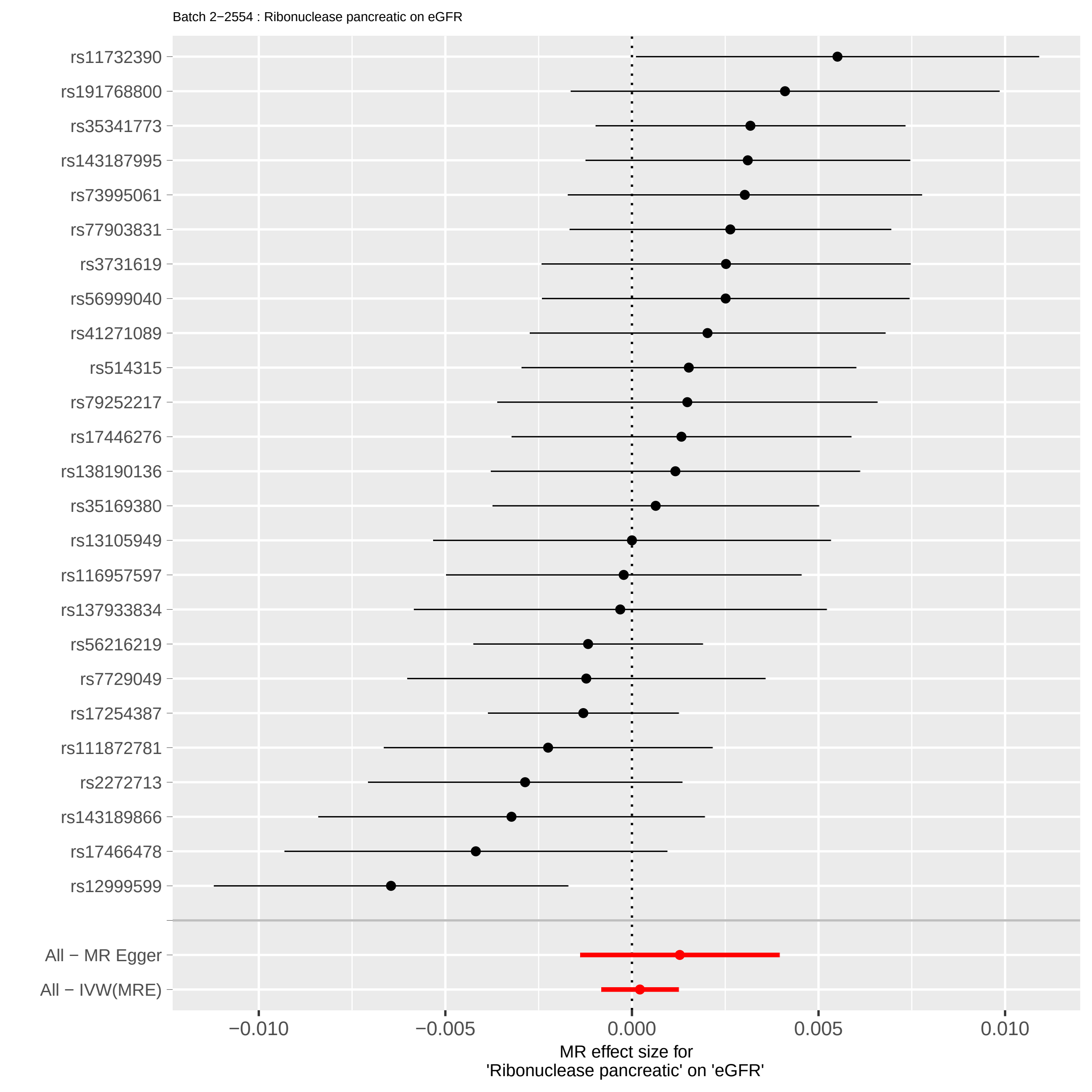

Batch 2−2554 : Ribonuclease pancreatic on eGFR
rs11732390
rs191768800
rs35341773
rs143187995
rs73995061
rs77903831
rs3731619
rs56999040
rs41271089
rs514315
rs79252217
rs17446276
rs138190136
rs35169380
rs13105949
rs116957597
rs137933834
rs56216219
rs7729049
rs17254387
rs111872781
rs2272713
rs143189866
rs17466478
rs12999599
All − MR Egger
All − IVW(MRE)
−0.010
−0.005
0.000
0.005
0.010
MR effect size for
'Ribonuclease pancreatic' on 'eGFR'

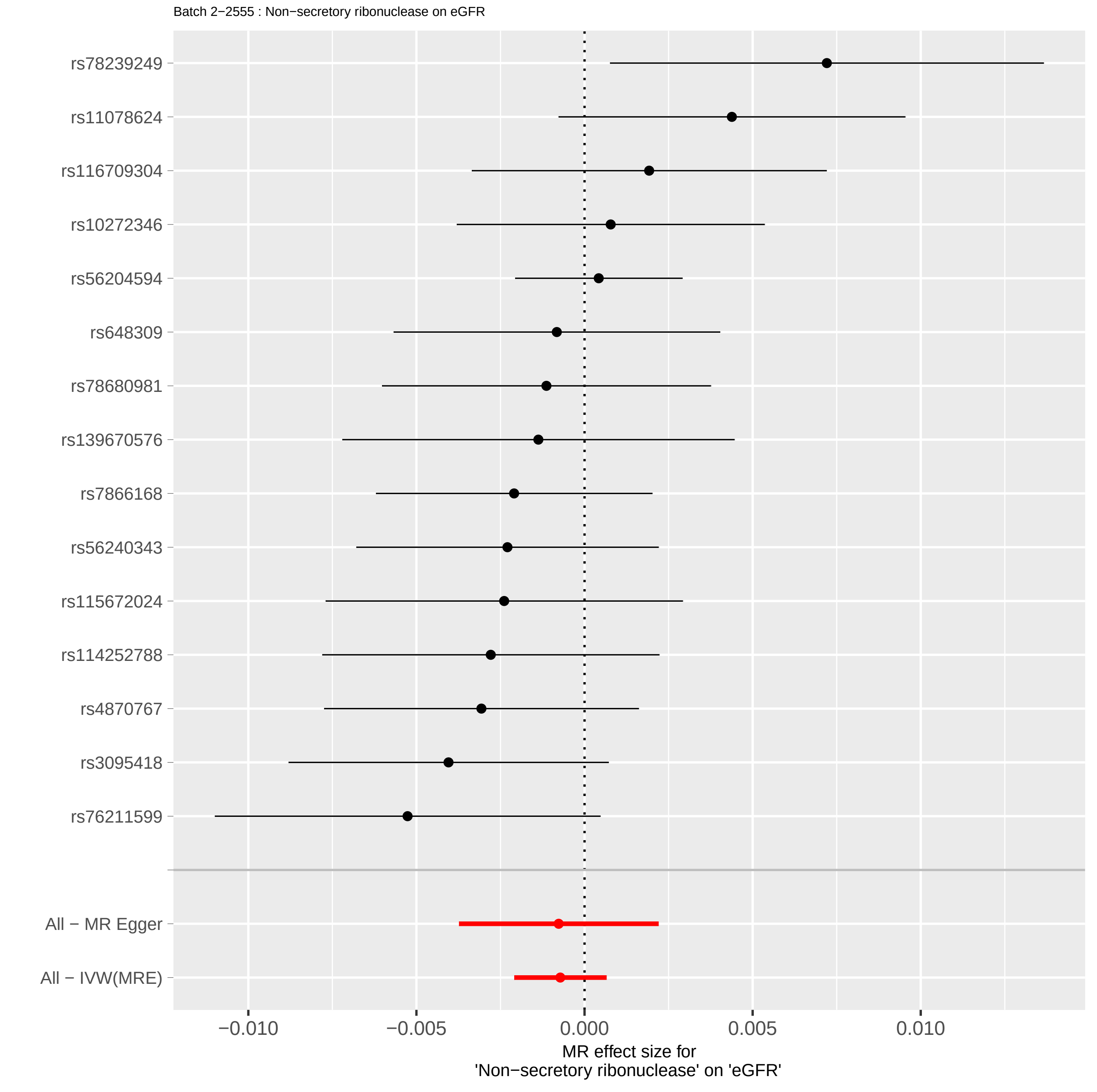

Batch 2−2555 : Non−secretory ribonuclease on eGFR
rs78239249
rs11078624
rs116709304
rs10272346
rs56204594
rs648309
rs78680981
rs139670576
rs7866168
rs56240343
rs115672024
rs114252788
rs4870767
rs3095418
rs76211599
All − MR Egger
All − IVW(MRE)
−0.010
−0.005
0.000
0.005
0.010
MR effect size for
'Non−secretory ribonuclease' on 'eGFR'

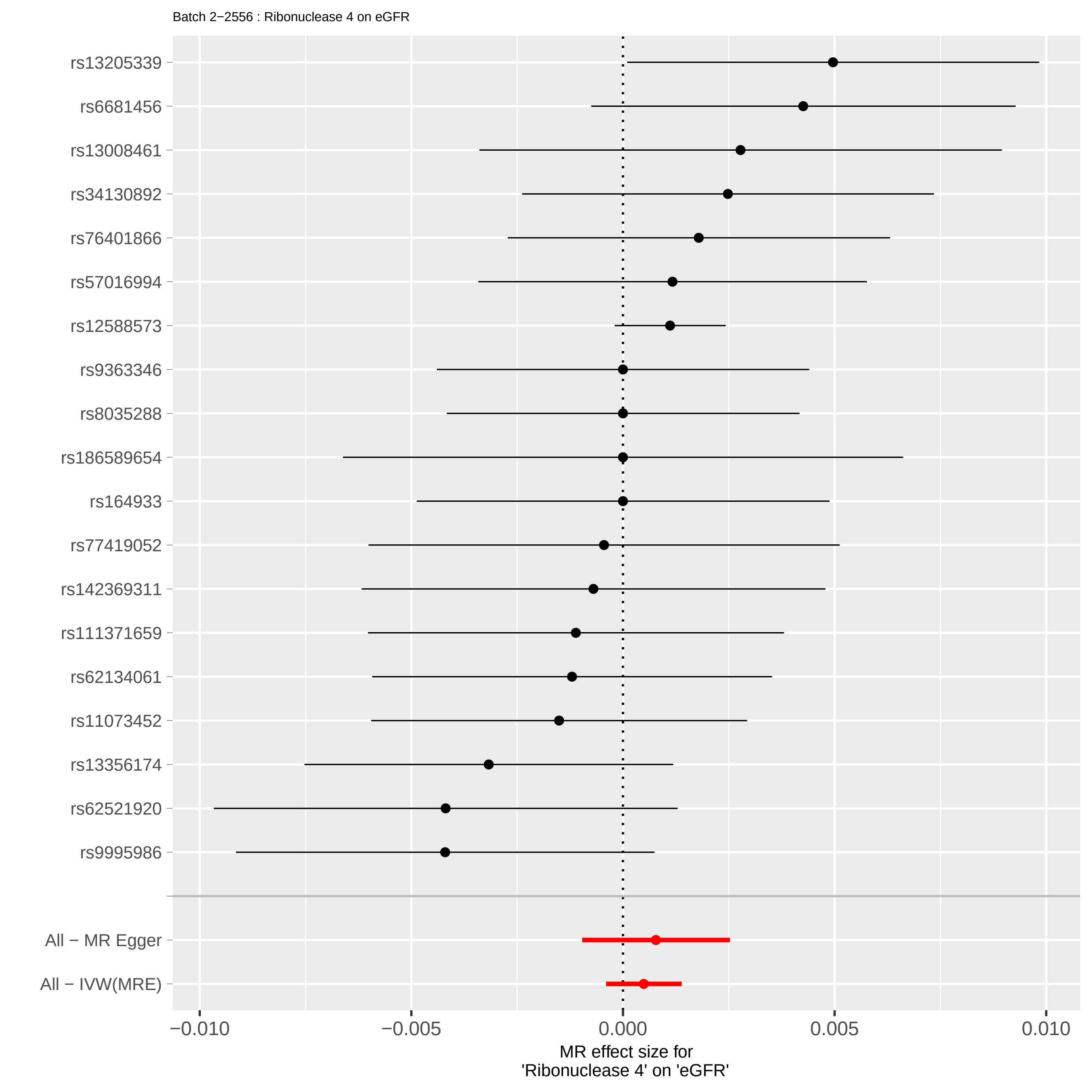

Batch 2−2556 : Ribonuclease 4 on eGFR
rs13205339
rs6681456
rs13008461
rs34130892
rs76401866
rs57016994
rs12588573
rs9363346
rs8035288
rs186589654
rs164933
rs77419052
rs142369311
rs111371659
rs62134061
rs11073452
rs13356174
rs62521920
rs9995986
All − MR Egger
All − IVW(MRE)
−0.010
−0.005
0.000
0.005
0.010
MR effect size for
'Ribonuclease 4' on 'eGFR'

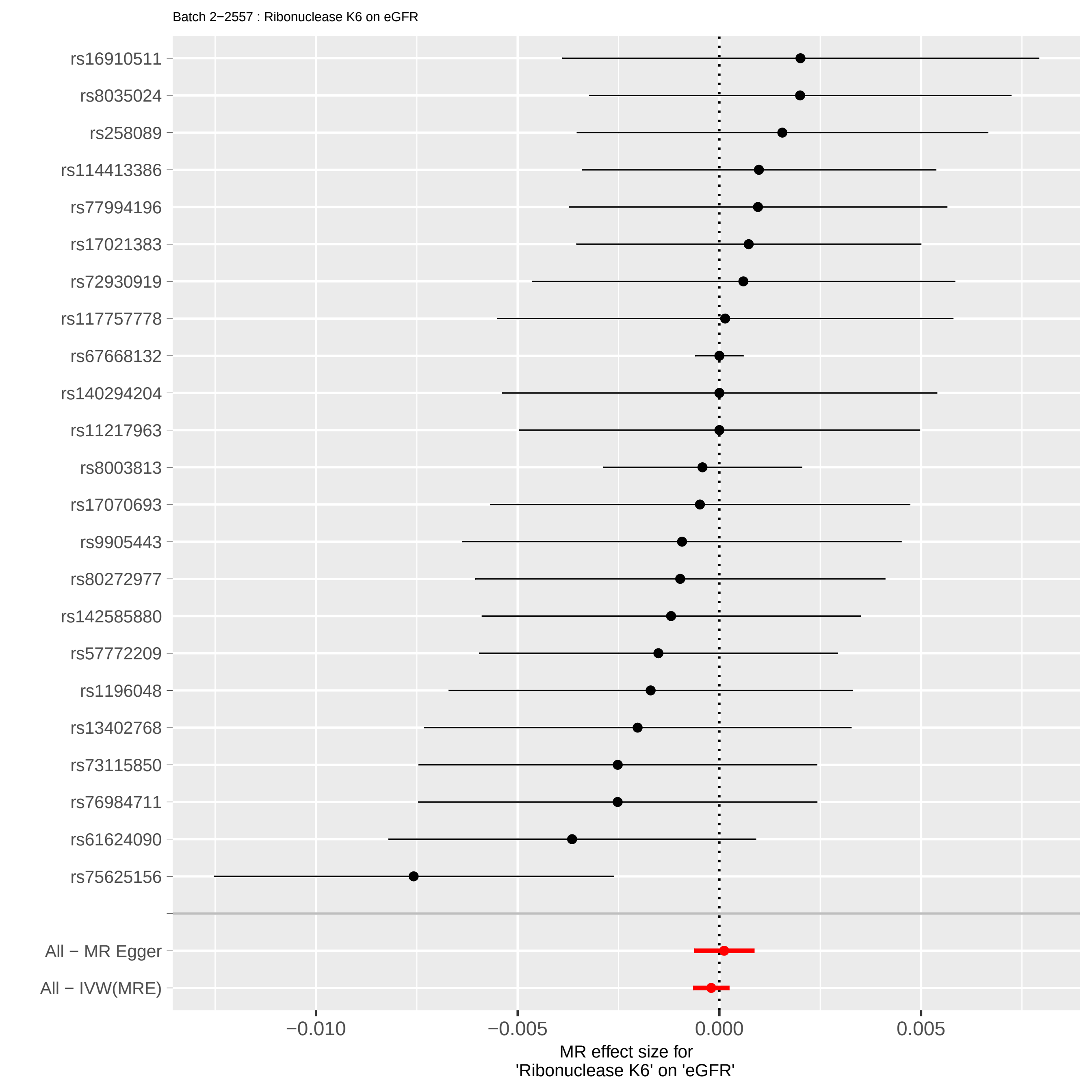

Batch 2−2557 : Ribonuclease K6 on eGFR
rs16910511
rs8035024
rs258089
rs114413386
rs77994196
rs17021383
rs72930919
rs117757778
rs67668132
rs140294204
rs11217963
rs8003813
rs17070693
rs9905443
rs80272977
rs142585880
rs57772209
rs1196048
rs13402768
rs73115850
rs76984711
rs61624090
rs75625156
All − MR Egger
All − IVW(MRE)
−0.010
−0.005
0.000
0.005
MR effect size for
'Ribonuclease K6' on 'eGFR'

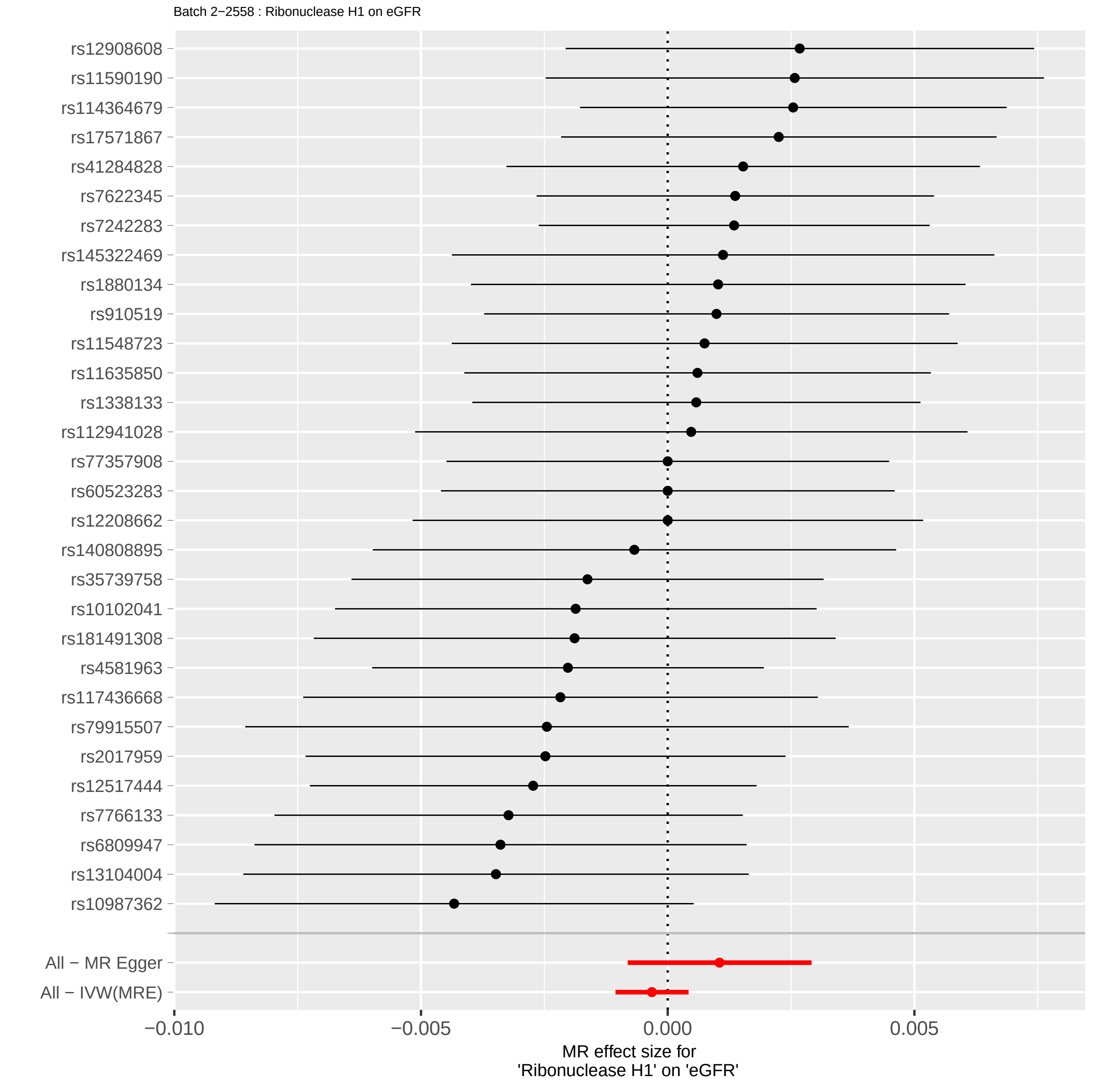

Batch 2−2558 : Ribonuclease H1 on eGFR
rs12908608
rs11590190
rs114364679
rs17571867
rs41284828
rs7622345
rs7242283
rs145322469
rs1880134
rs910519
rs11548723
rs11635850
rs1338133
rs112941028
rs77357908
rs60523283
rs12208662
rs140808895
rs35739758
rs10102041
rs181491308
rs4581963
rs117436668
rs79915507
rs2017959
rs12517444
rs7766133
rs6809947
rs13104004
rs10987362
All − MR Egger
All − IVW(MRE)
−0.010
−0.005
0.000
0.005
MR effect size for
'Ribonuclease H1' on 'eGFR'

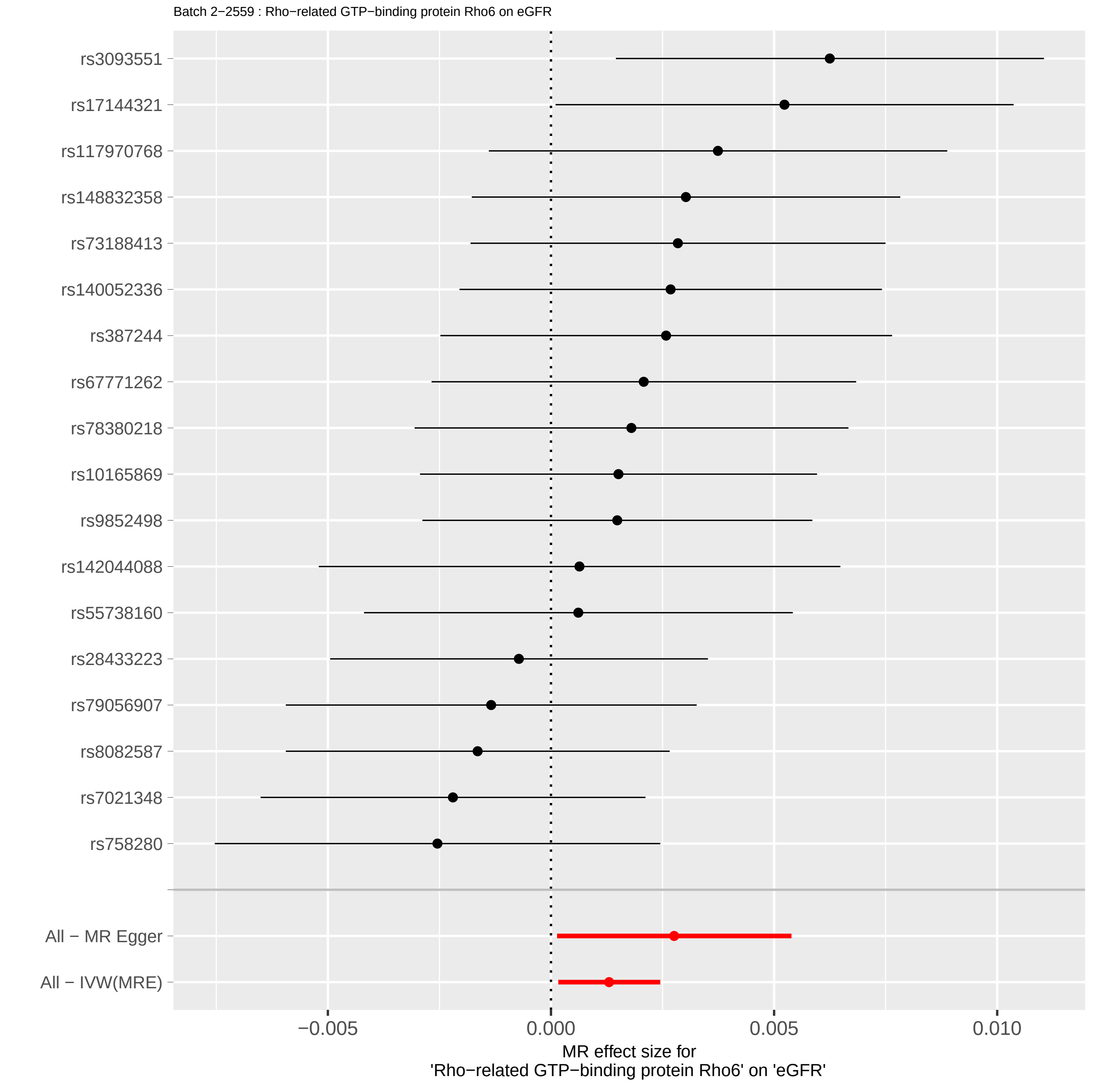

Batch 2−2559 : Rho−related GTP−binding protein Rho6 on eGFR
rs3093551
rs17144321
rs117970768
rs148832358
rs73188413
rs140052336
rs387244
rs67771262
rs78380218
rs10165869
rs9852498
rs142044088
rs55738160
rs28433223
rs79056907
rs8082587
rs7021348
rs758280
All − MR Egger
All − IVW(MRE)
−0.005
0.000
0.005
0.010
MR effect size for
'Rho−related GTP−binding protein Rho6' on 'eGFR'

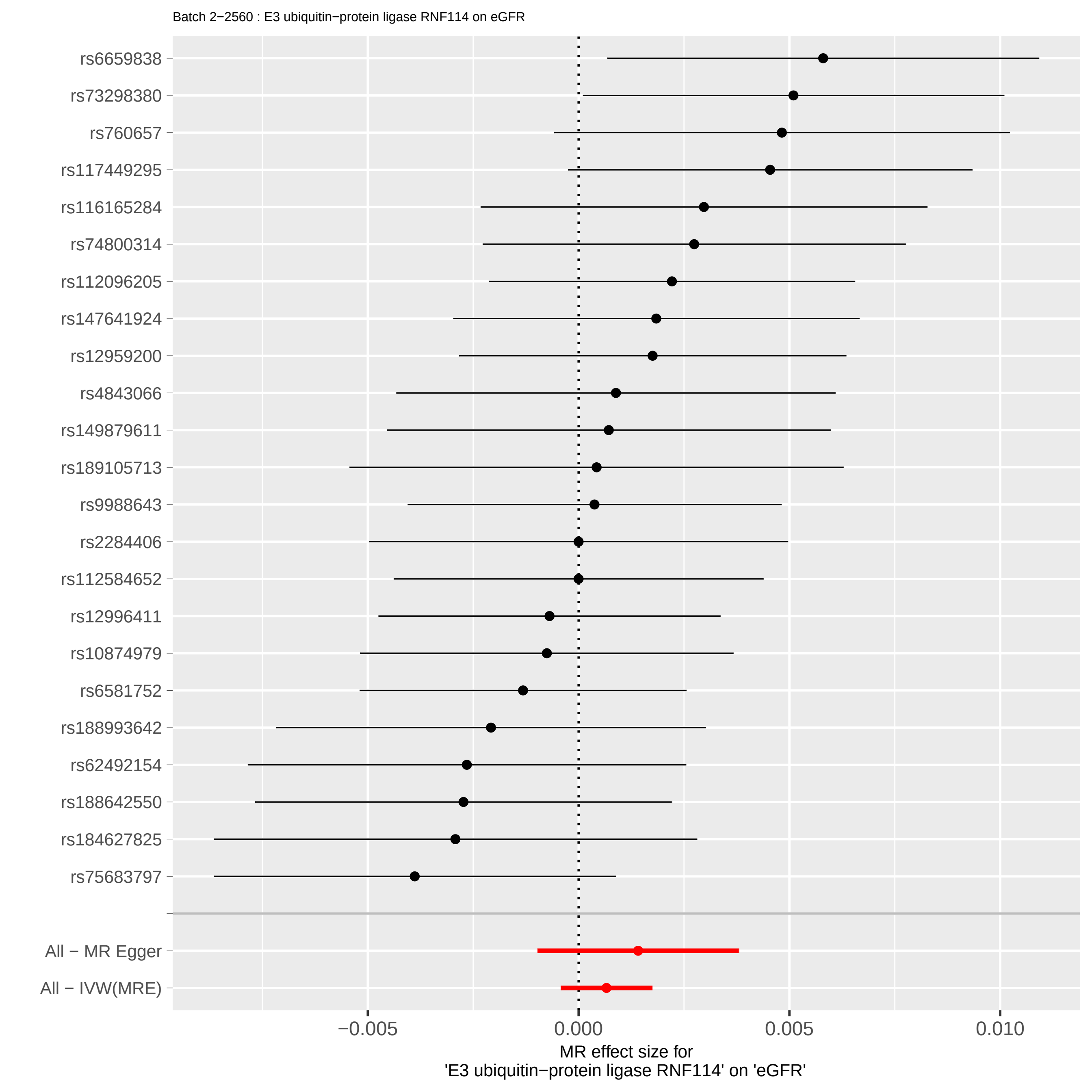
Batch 2−2560 : E3 ubiquitin−protein ligase RNF114 on eGFR
rs6659838
rs73298380
rs760657
rs117449295
rs116165284
rs74800314
rs112096205
rs147641924
rs12959200
rs4843066
rs149879611
rs189105713
rs9988643
rs2284406
rs112584652
rs12996411
rs10874979
rs6581752
rs188993642
rs62492154
rs188642550
rs184627825
rs75683797
All − MR Egger
All − IVW(MRE)
−0.005
0.000
0.005
0.010
MR effect size for
'E3 ubiquitin−protein ligase RNF114' on 'eGFR'

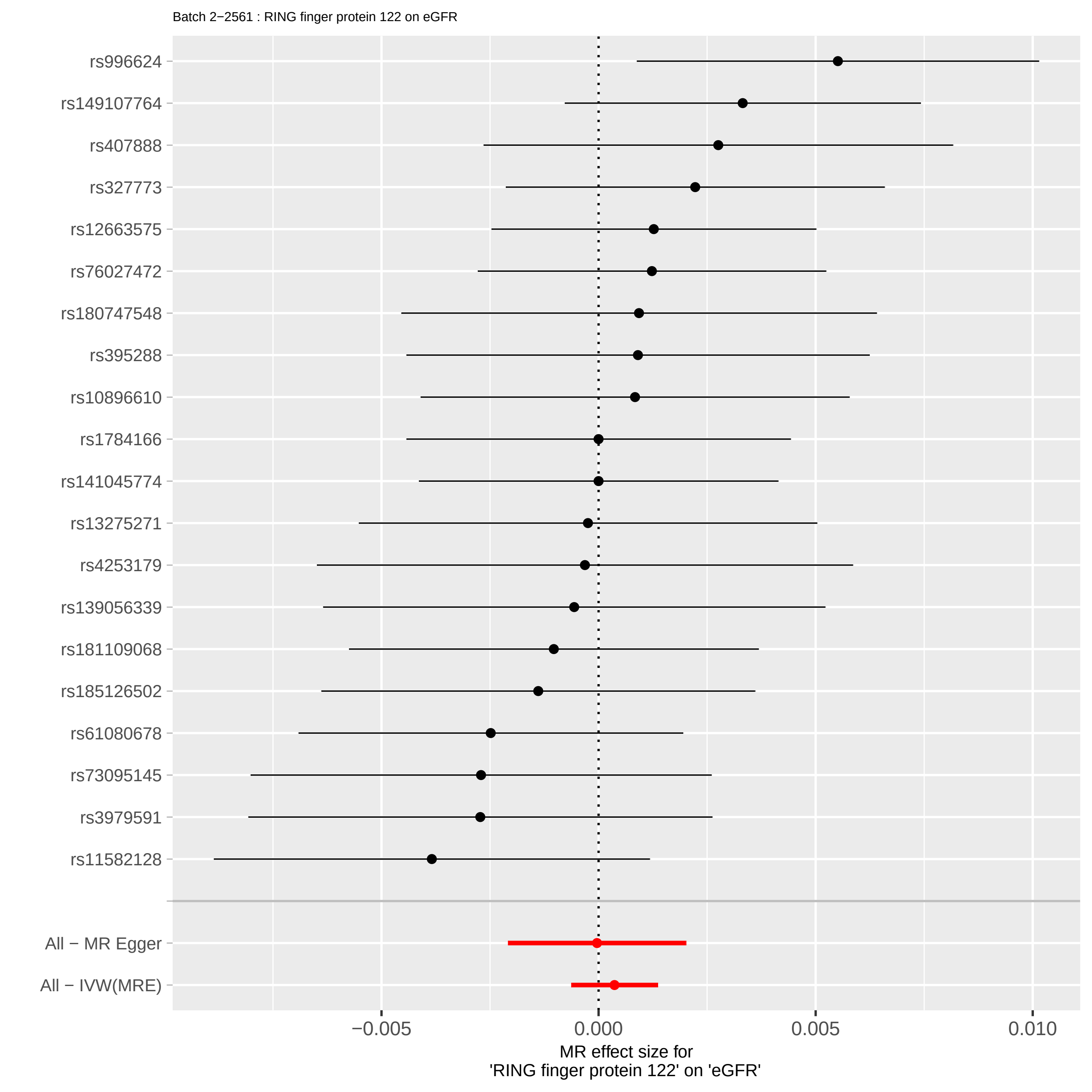
Batch 2−2561 : RING finger protein 122 on eGFR
rs996624
rs149107764
rs407888
rs327773
rs12663575
rs76027472
rs180747548
rs395288
rs10896610
rs1784166
rs141045774
rs13275271
rs4253179
rs139056339
rs181109068
rs185126502
rs61080678
rs73095145
rs3979591
rs11582128
All − MR Egger
All − IVW(MRE)
−0.005
0.000
0.005
0.010
MR effect size for
'RING finger protein 122' on 'eGFR'

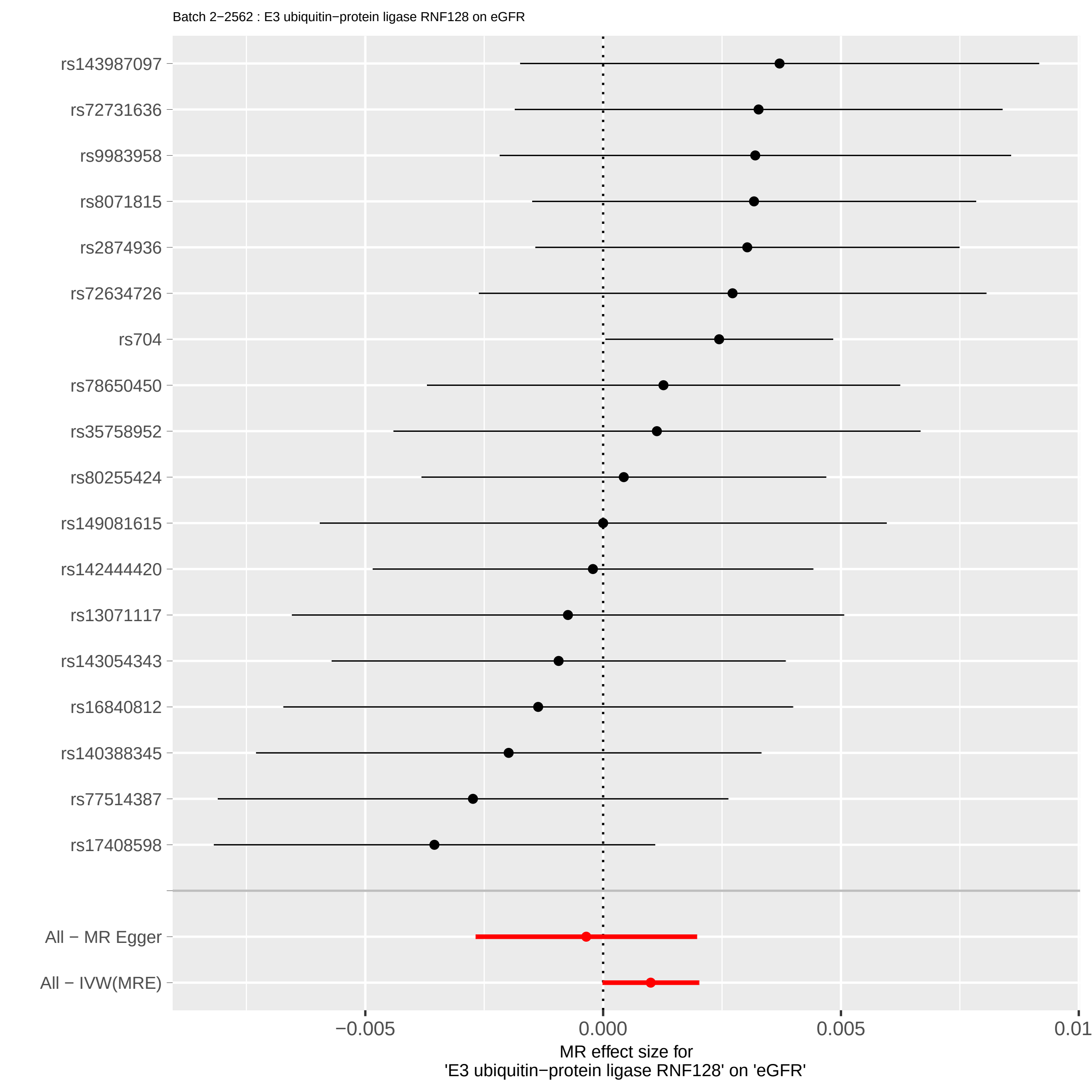

Batch 2−2562 : E3 ubiquitin−protein ligase RNF128 on eGFR
rs143987097
rs72731636
rs9983958
rs8071815
rs2874936
rs72634726
rs704
rs78650450
rs35758952
rs80255424
rs149081615
rs142444420
rs13071117
rs143054343
rs16840812
rs140388345
rs77514387
rs17408598
All − MR Egger
All − IVW(MRE)
−0.005
0.000
0.005
0.01
MR effect size for
'E3 ubiquitin−protein ligase RNF128' on 'eGFR'

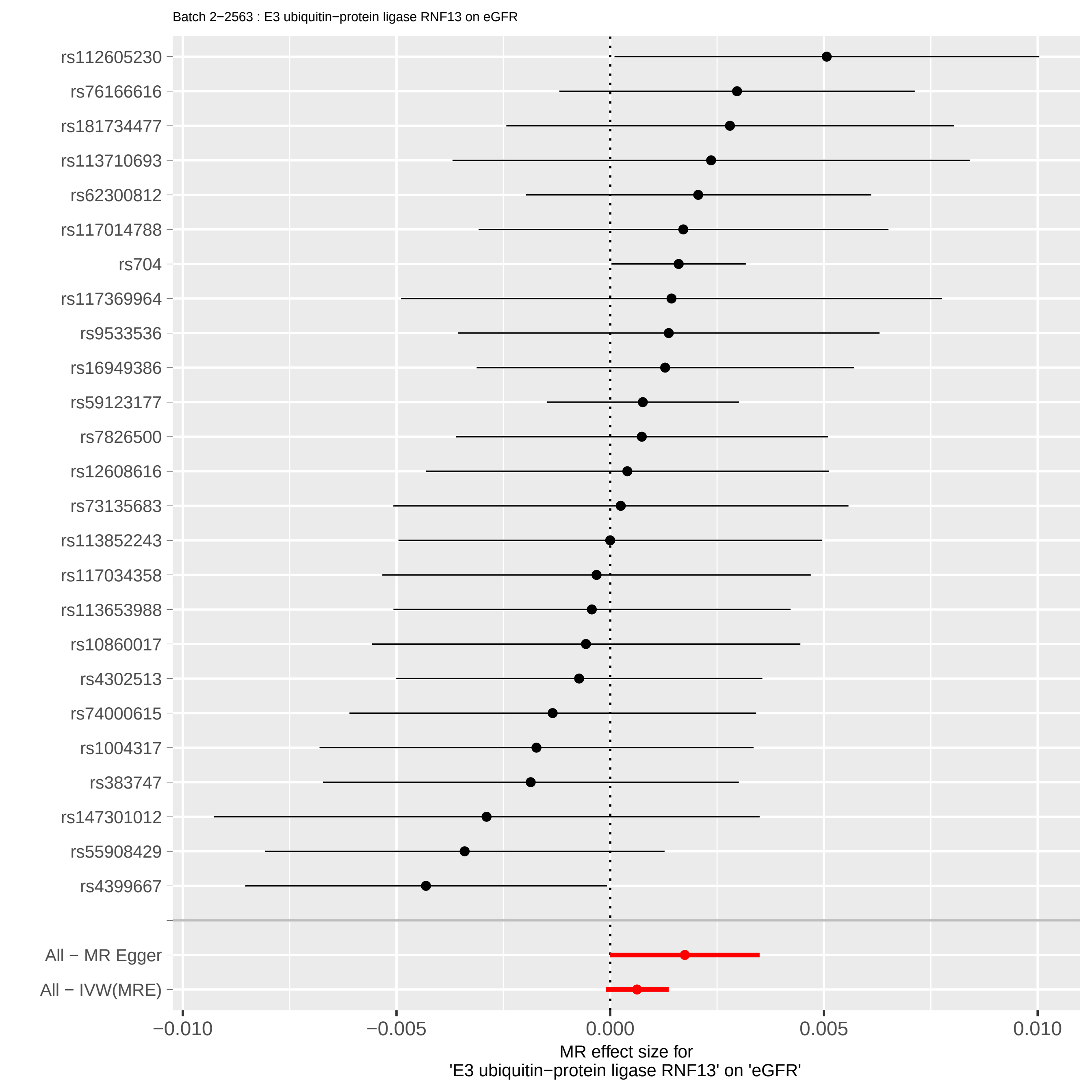

Batch 2−2563 : E3 ubiquitin−protein ligase RNF13 on eGFR
rs112605230
rs76166616
rs181734477
rs113710693
rs62300812
rs117014788
rs704
rs117369964
rs9533536
rs16949386
rs59123177
rs7826500
rs12608616
rs73135683
rs113852243
rs117034358
rs113653988
rs10860017
rs4302513
rs74000615
rs1004317
rs383747
rs147301012
rs55908429
rs4399667
All − MR Egger
All − IVW(MRE)
−0.010
−0.005
0.000
0.005
0.010
MR effect size for
'E3 ubiquitin−protein ligase RNF13' on 'eGFR'

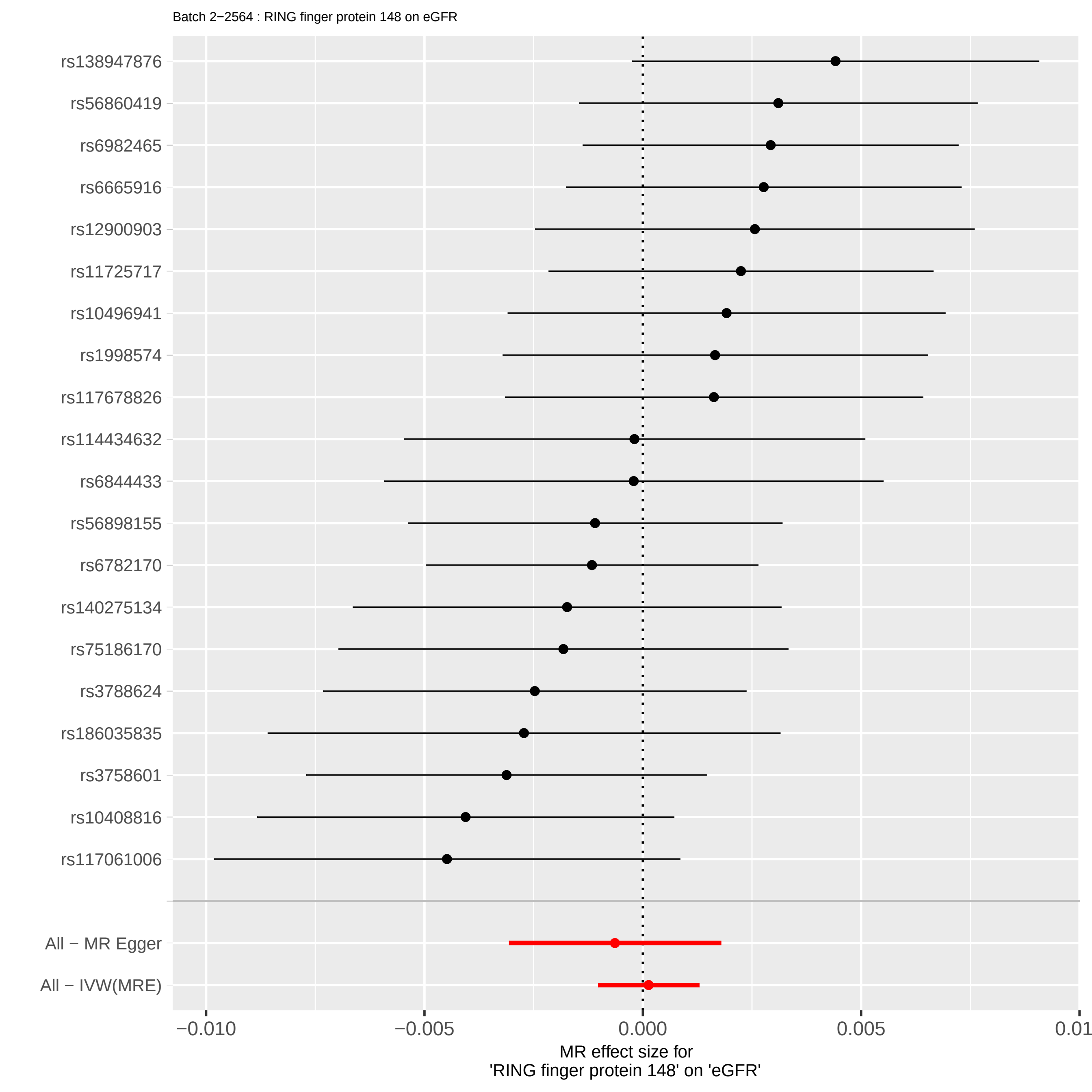

Batch 2−2564 : RING finger protein 148 on eGFR
rs138947876
rs56860419
rs6982465
rs6665916
rs12900903
rs11725717
rs10496941
rs1998574
rs117678826
rs114434632
rs6844433
rs56898155
rs6782170
rs140275134
rs75186170
rs3788624
rs186035835
rs3758601
rs10408816
rs117061006
All − MR Egger
All − IVW(MRE)
−0.010
−0.005
0.000
0.005
0.01
MR effect size for
'RING finger protein 148' on 'eGFR'

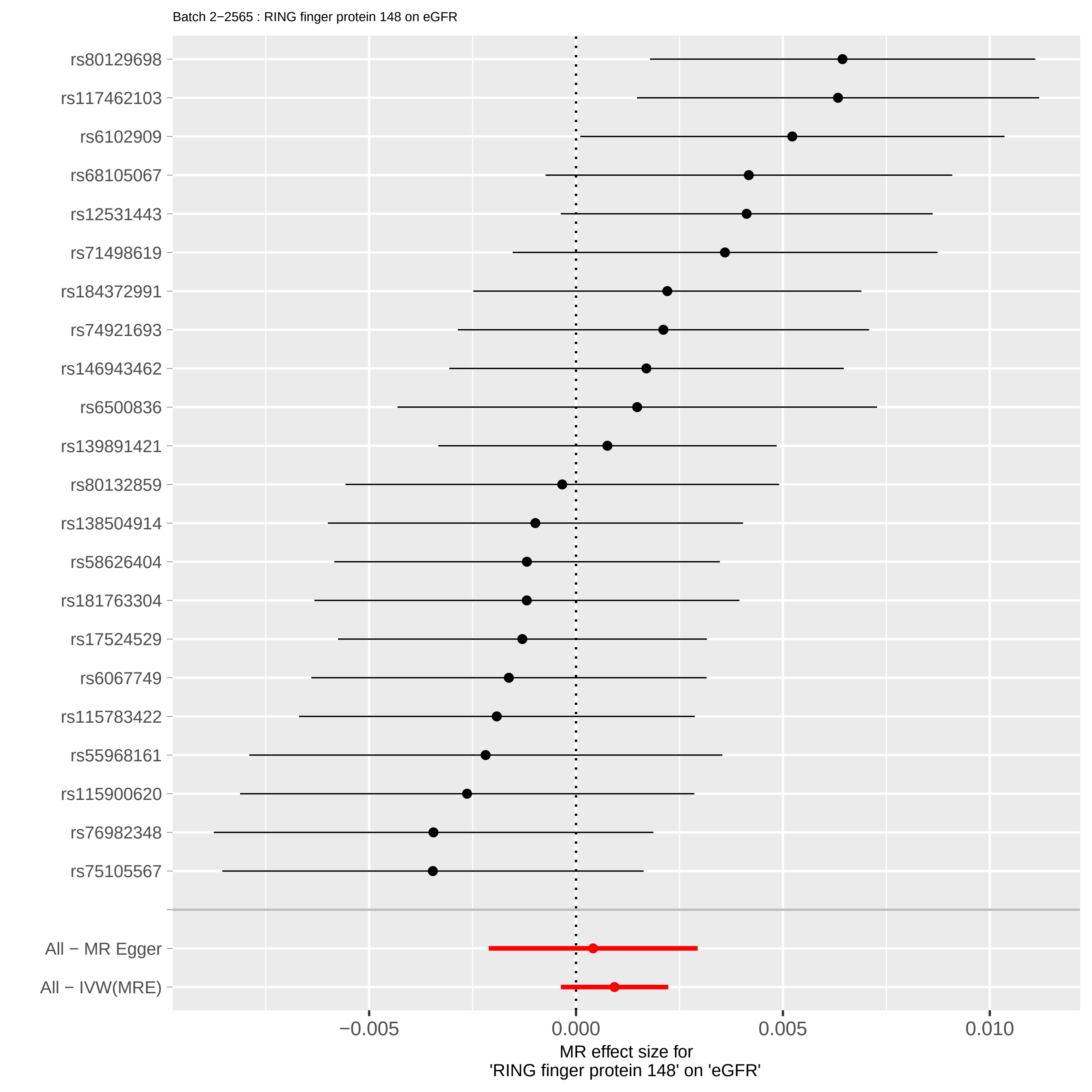

Batch 2−2565 : RING finger protein 148 on eGFR
rs80129698
rs117462103
rs6102909
rs68105067
rs12531443
rs71498619
rs184372991
rs74921693
rs146943462
rs6500836
rs139891421
rs80132859
rs138504914
rs58626404
rs181763304
rs17524529
rs6067749
rs115783422
rs55968161
rs115900620
rs76982348
rs75105567
All − MR Egger
All − IVW(MRE)
−0.005
0.000
0.005
0.010
MR effect size for
'RING finger protein 148' on 'eGFR'

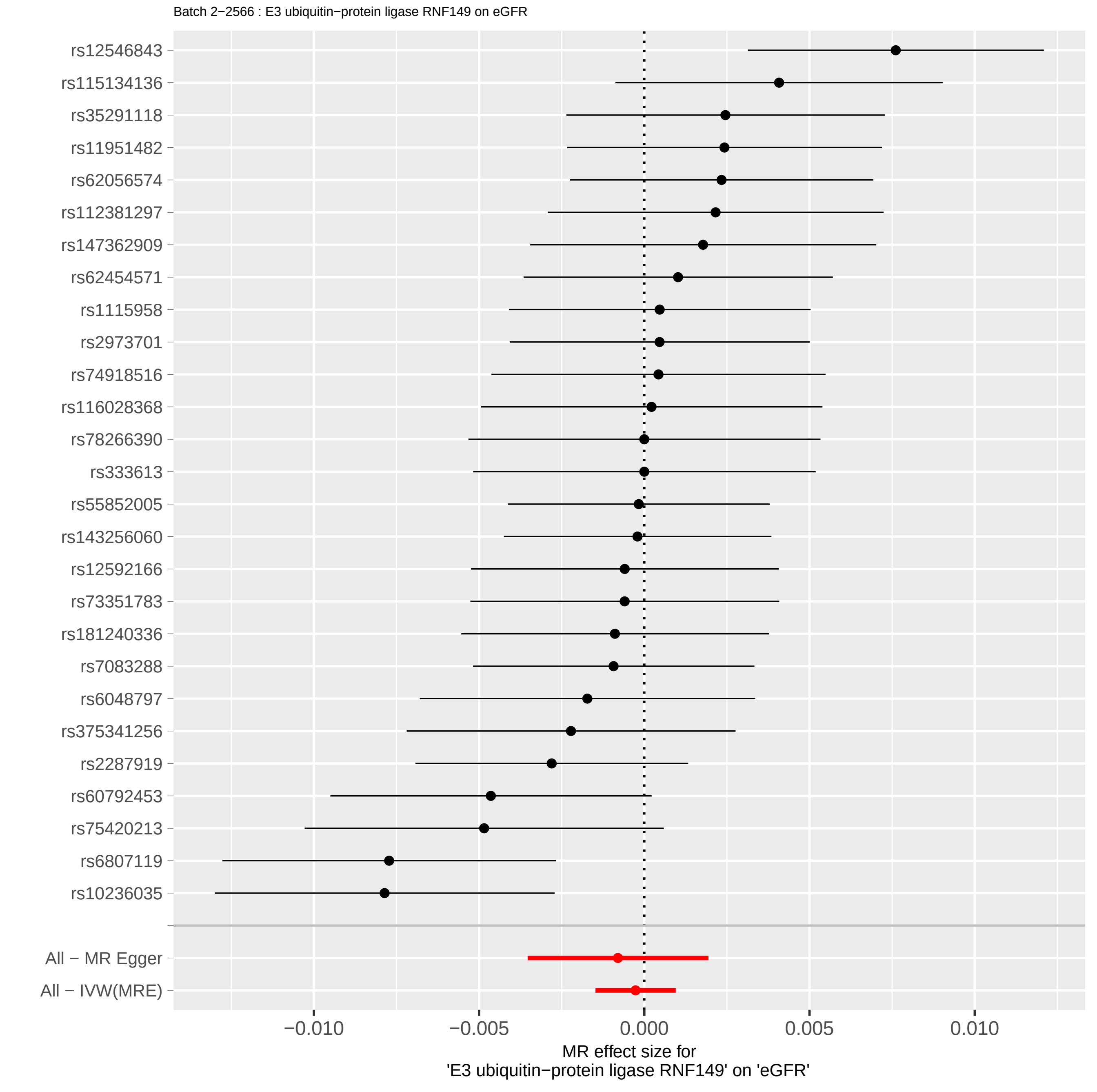

Batch 2−2566 : E3 ubiquitin−protein ligase RNF149 on eGFR
rs12546843
rs115134136
rs35291118
rs11951482
rs62056574
rs112381297
rs147362909
rs62454571
rs1115958
rs2973701
rs74918516
rs116028368
rs78266390
rs333613
rs55852005
rs143256060
rs12592166
rs73351783
rs181240336
rs7083288
rs6048797
rs375341256
rs2287919
rs60792453
rs75420213
rs6807119
rs10236035
All − MR Egger
All − IVW(MRE)
−0.010
−0.005
0.000
0.005
0.010
MR effect size for
'E3 ubiquitin−protein ligase RNF149' on 'eGFR'

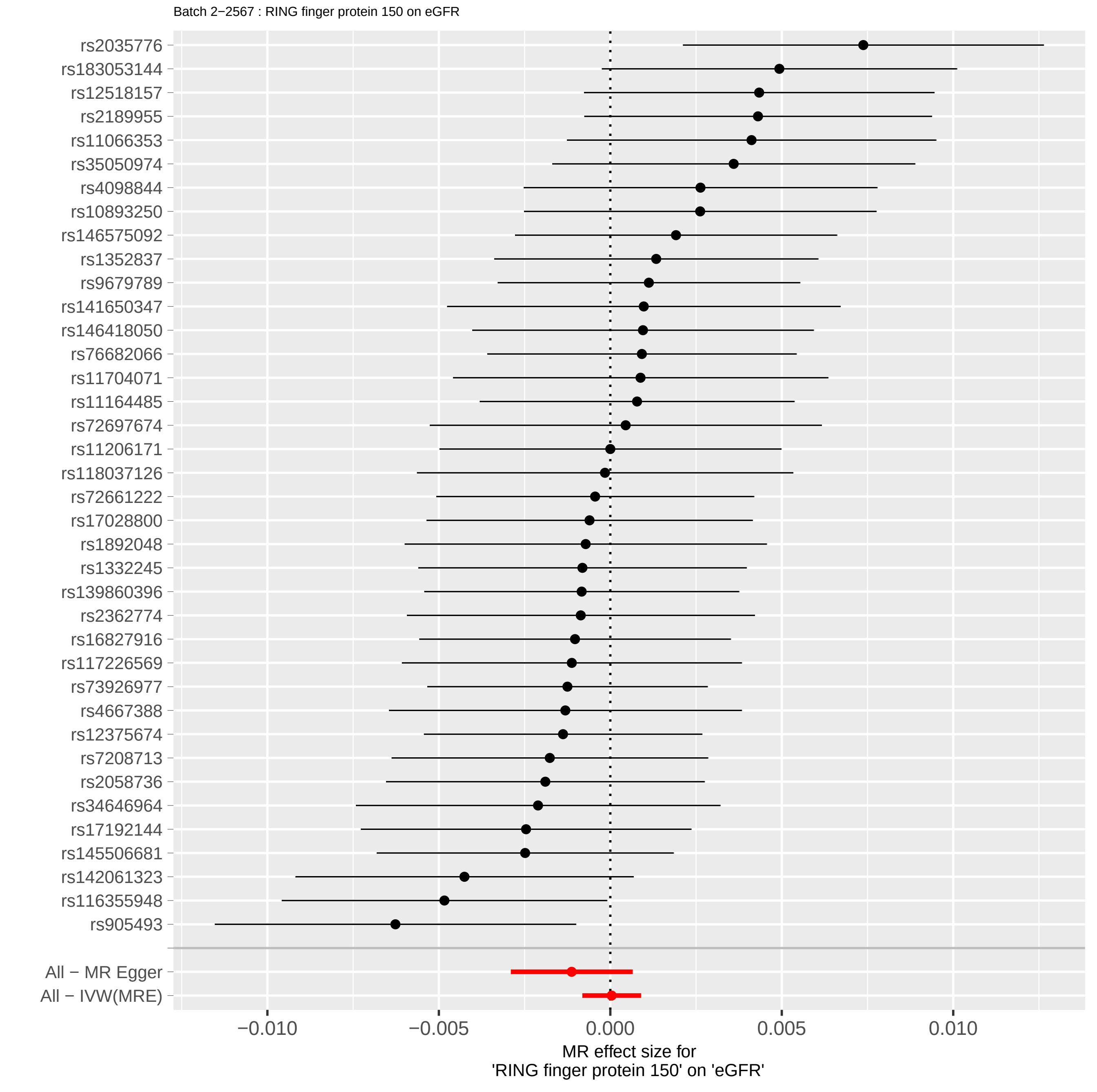

Batch 2−2567 : RING finger protein 150 on eGFR
rs2035776
rs183053144
rs12518157
rs2189955
rs11066353
rs35050974
rs4098844
rs10893250
rs146575092
rs1352837
rs9679789
rs141650347
rs146418050
rs76682066
rs11704071
rs11164485
rs72697674
rs11206171
rs118037126
rs72661222
rs17028800
rs1892048
rs1332245
rs139860396
rs2362774
rs16827916
rs117226569
rs73926977
rs4667388
rs12375674
rs7208713
rs2058736
rs34646964
rs17192144
rs145506681
rs142061323
rs116355948
rs905493
All − MR Egger
All − IVW(MRE)
−0.010
−0.005
0.000
0.005
0.010
MR effect size for
'RING finger protein 150' on 'eGFR'

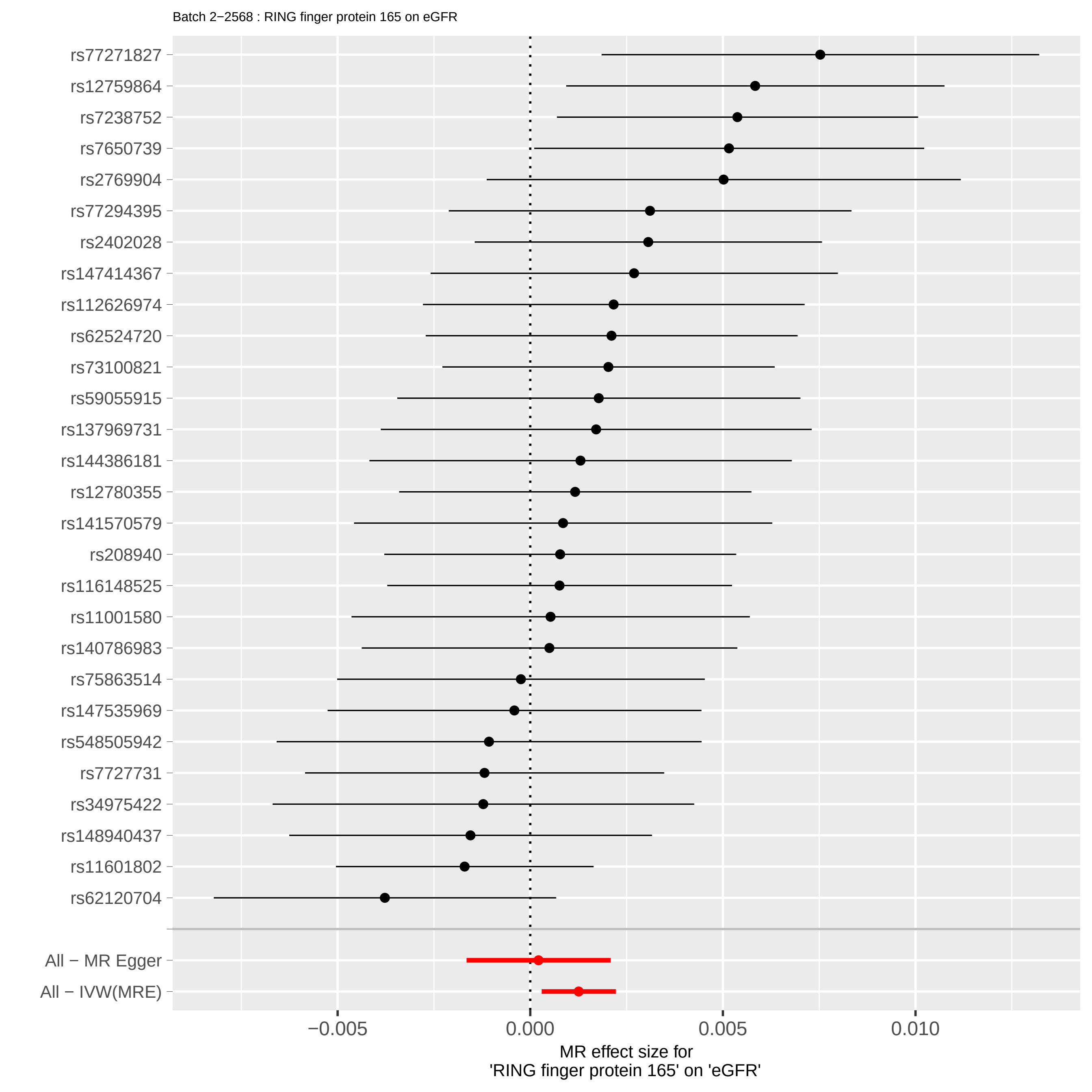
Batch 2−2568 : RING finger protein 165 on eGFR
rs77271827
rs12759864
rs7238752
rs7650739
rs2769904
rs77294395
rs2402028
rs147414367
rs112626974
rs62524720
rs73100821
rs59055915
rs137969731
rs144386181
rs12780355
rs141570579
rs208940
rs116148525
rs11001580
rs140786983
rs75863514
rs147535969
rs548505942
rs7727731
rs34975422
rs148940437
rs11601802
rs62120704
All − MR Egger
All − IVW(MRE)
−0.005
0.000
0.005
0.010
MR effect size for
'RING finger protein 165' on 'eGFR'

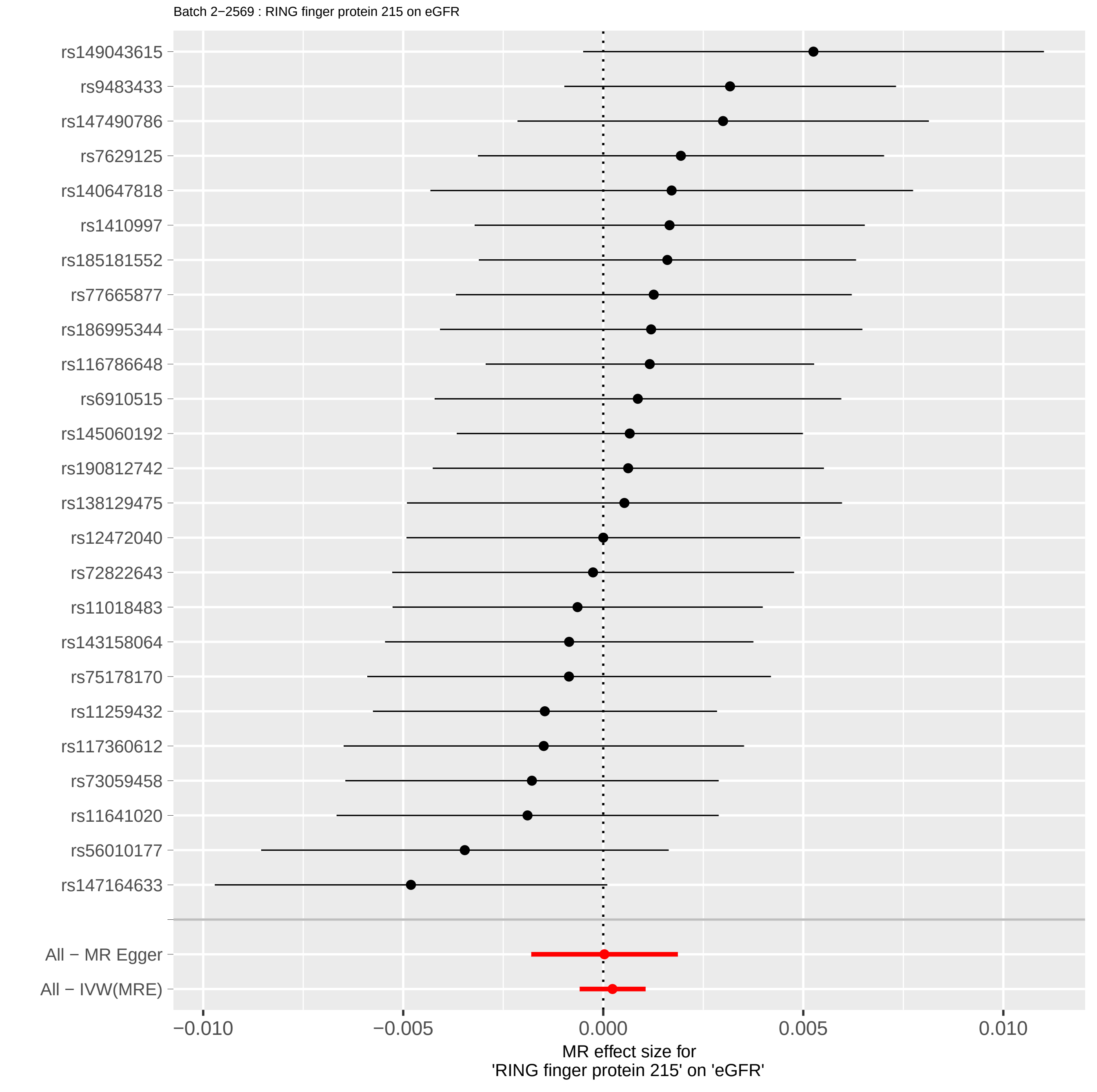

Batch 2−2569 : RING finger protein 215 on eGFR
rs149043615
rs9483433
rs147490786
rs7629125
rs140647818
rs1410997
rs185181552
rs77665877
rs186995344
rs116786648
rs6910515
rs145060192
rs190812742
rs138129475
rs12472040
rs72822643
rs11018483
rs143158064
rs75178170
rs11259432
rs117360612
rs73059458
rs11641020
rs56010177
rs147164633
All − MR Egger
All − IVW(MRE)
−0.010
−0.005
0.000
0.005
0.010
MR effect size for
'RING finger protein 215' on 'eGFR'

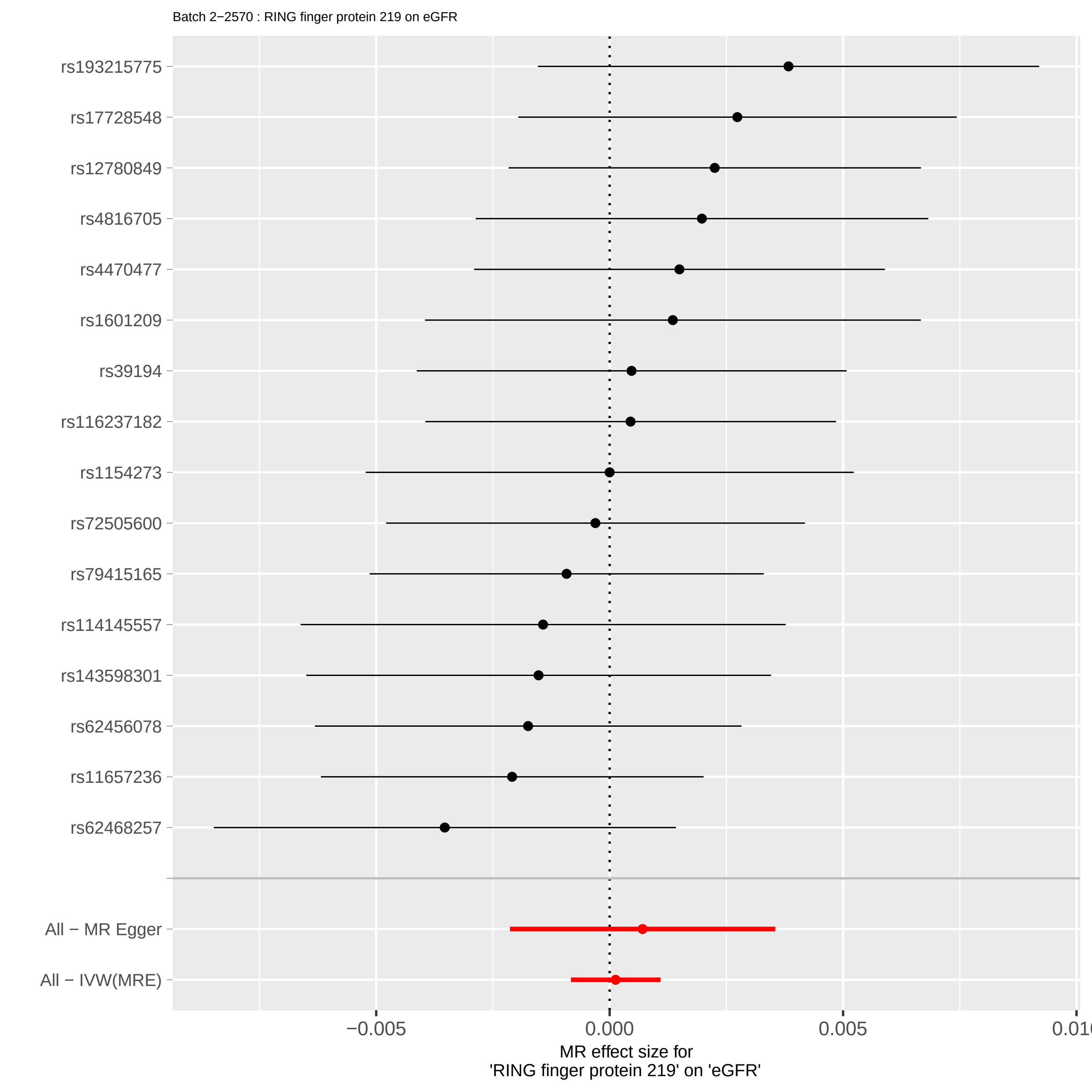

Batch 2−2570 : RING finger protein 219 on eGFR
rs193215775
rs17728548
rs12780849
rs4816705
rs4470477
rs1601209
rs39194
rs116237182
rs1154273
rs72505600
rs79415165
rs114145557
rs143598301
rs62456078
rs11657236
rs62468257
All − MR Egger
All − IVW(MRE)
−0.005
0.000
0.005
MR effect size for
'RING finger protein 219' on 'eGFR'

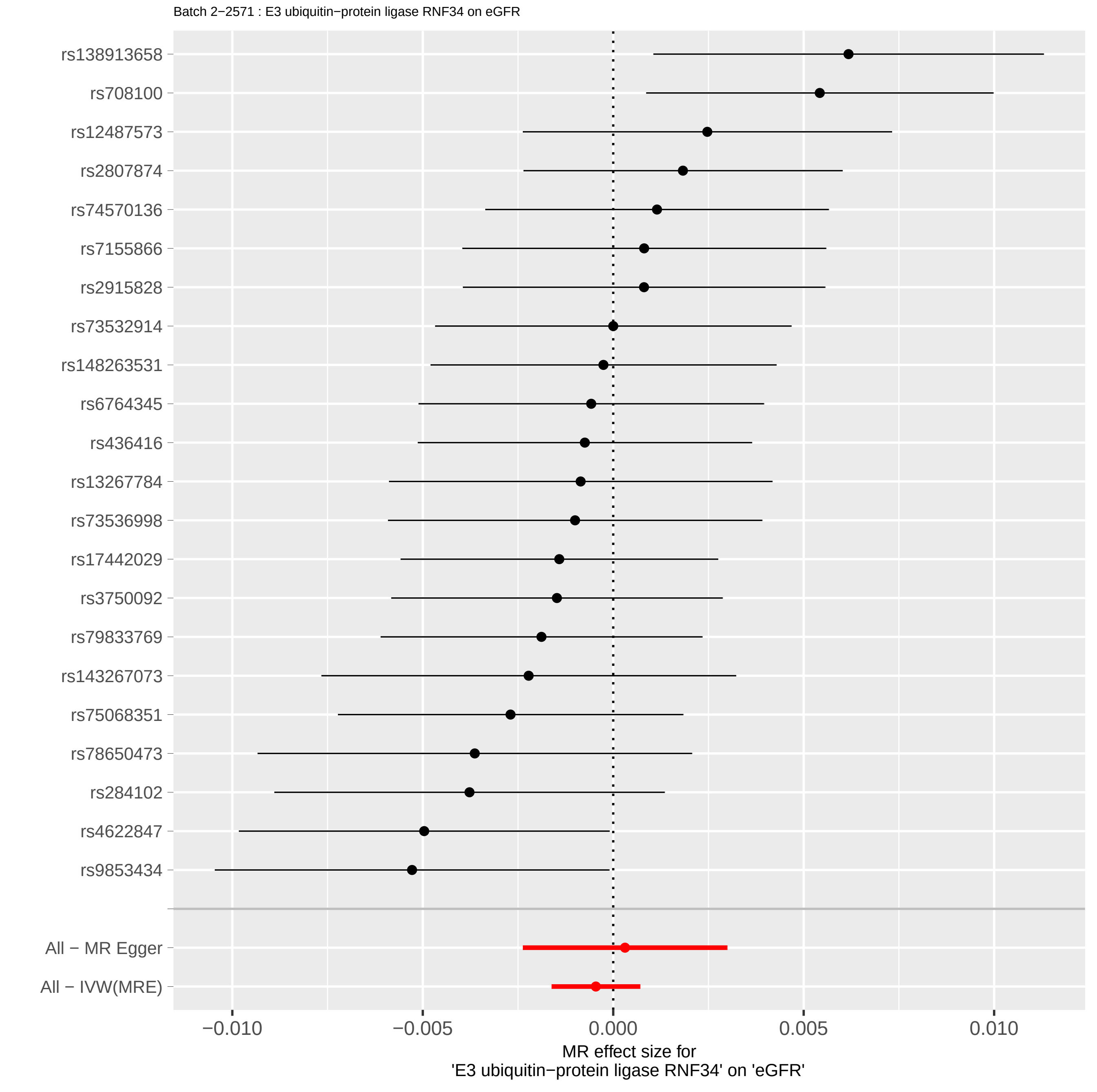

Batch 2−2571 : E3 ubiquitin−protein ligase RNF34 on eGFR
rs138913658
rs708100
rs12487573
rs2807874
rs74570136
rs7155866
rs2915828
rs73532914
rs148263531
rs6764345
rs436416
rs13267784
rs73536998
rs17442029
rs3750092
rs79833769
rs143267073
rs75068351
rs78650473
rs284102
rs4622847
rs9853434
All − MR Egger
All − IVW(MRE)
−0.010
−0.005
0.000
0.005
0.010
MR effect size for
'E3 ubiquitin−protein ligase RNF34' on 'eGFR'

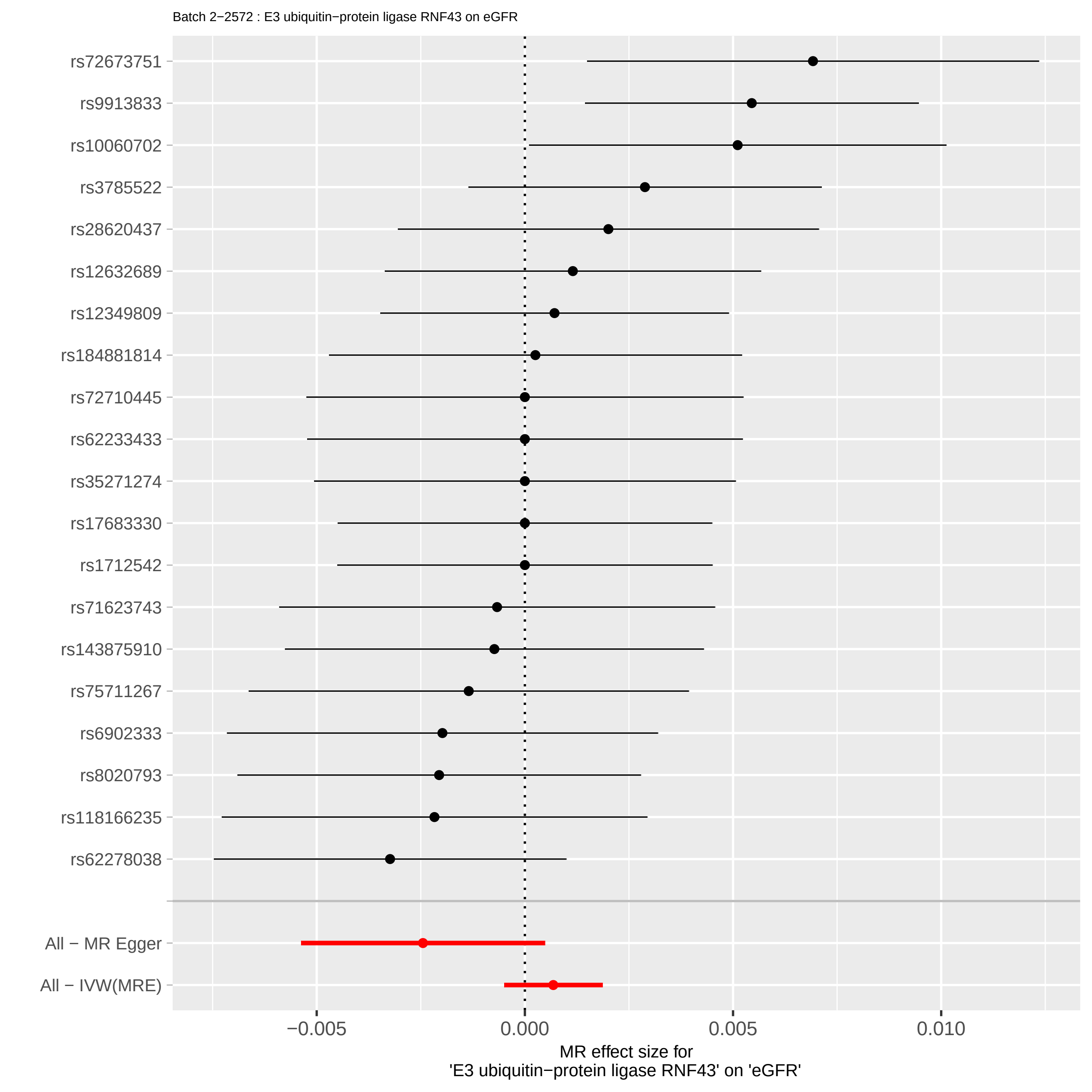
Batch 2−2572 : E3 ubiquitin−protein ligase RNF43 on eGFR
rs72673751
rs9913833
rs10060702
rs3785522
rs28620437
rs12632689
rs12349809
rs184881814
rs72710445
rs62233433
rs35271274
rs17683330
rs1712542
rs71623743
rs143875910
rs75711267
rs6902333
rs8020793
rs118166235
rs62278038
All − MR Egger
All − IVW(MRE)
−0.005
0.000
0.005
0.010
MR effect size for
'E3 ubiquitin−protein ligase RNF43' on 'eGFR'

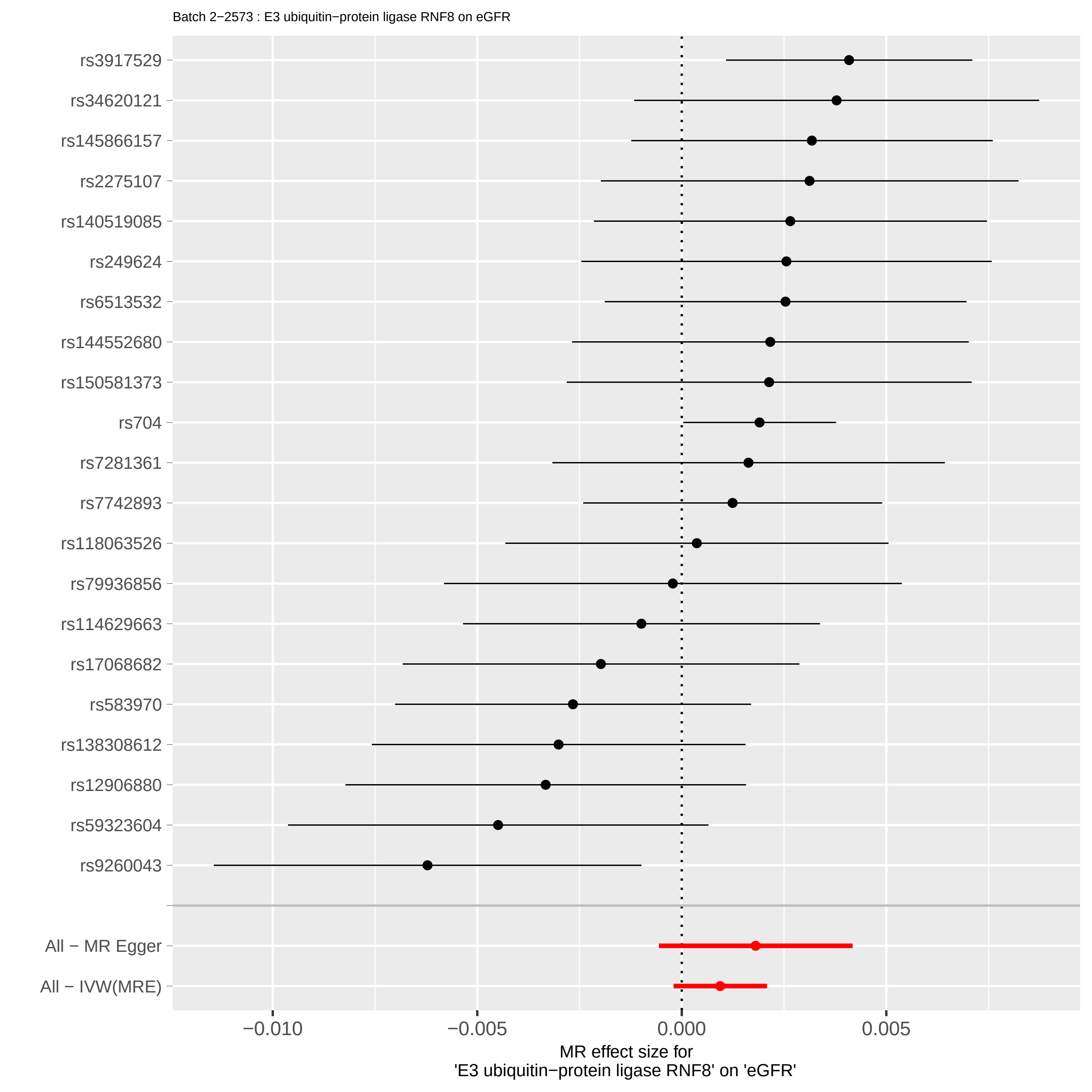

Batch 2−2573 : E3 ubiquitin−protein ligase RNF8 on eGFR
rs3917529
rs34620121
rs145866157
rs2275107
rs140519085
rs249624
rs6513532
rs144552680
rs150581373
rs704
rs7281361
rs7742893
rs118063526
rs79936856
rs114629663
rs17068682
rs583970
rs138308612
rs12906880
rs59323604
rs9260043
All − MR Egger
All − IVW(MRE)
−0.010
−0.005
0.000
0.005
MR effect size for
'E3 ubiquitin−protein ligase RNF8' on 'eGFR'

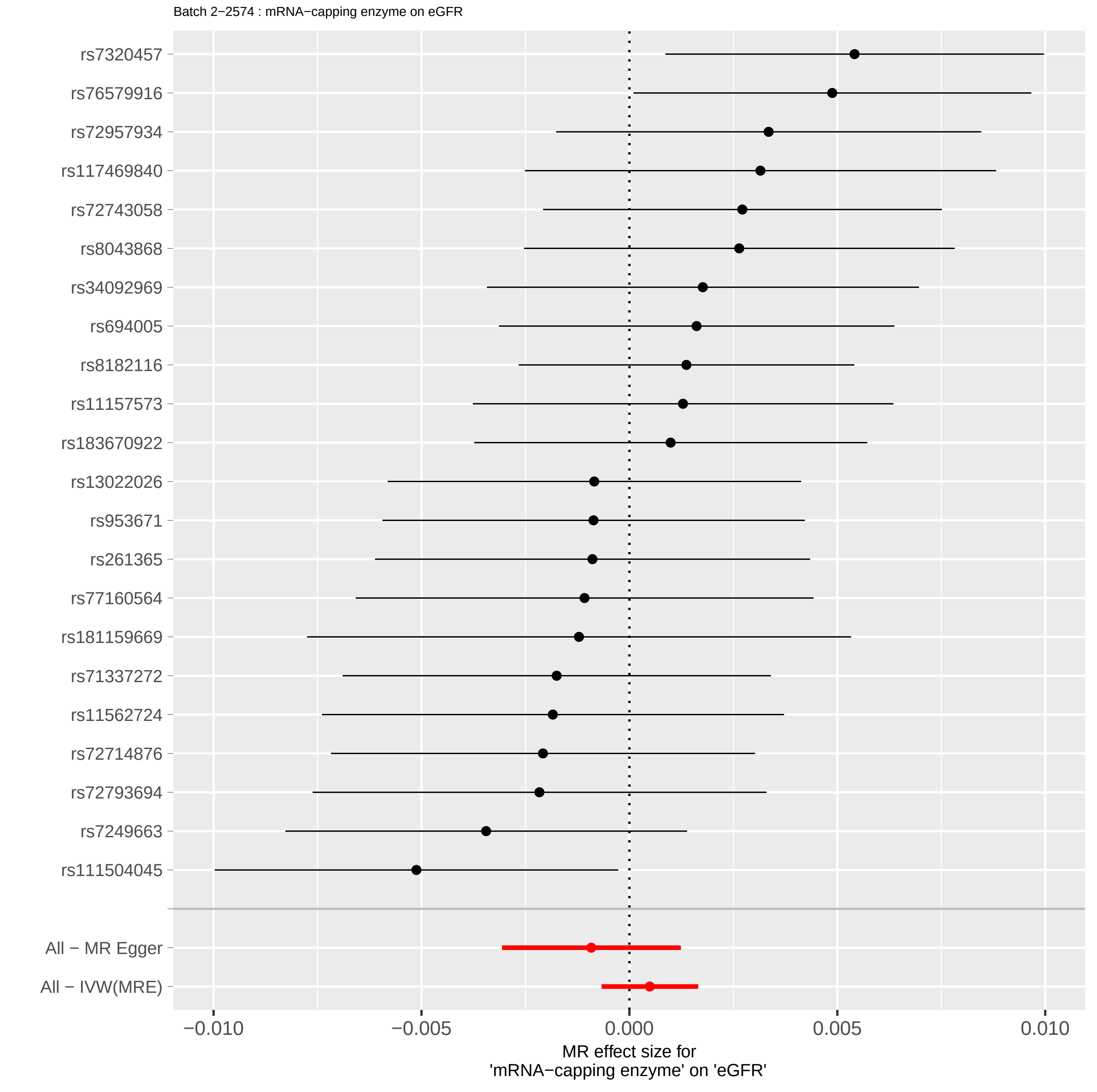

Batch 2−2574 : mRNA−capping enzyme on eGFR
rs7320457
rs76579916
rs72957934
rs117469840
rs72743058
rs8043868
rs34092969
rs694005
rs8182116
rs11157573
rs183670922
rs13022026
rs953671
rs261365
rs77160564
rs181159669
rs71337272
rs11562724
rs72714876
rs72793694
rs7249663
rs111504045
All − MR Egger
All − IVW(MRE)
−0.010
−0.005
0.000
0.005
0.010
MR effect size for
'mRNA−capping enzyme' on 'eGFR'

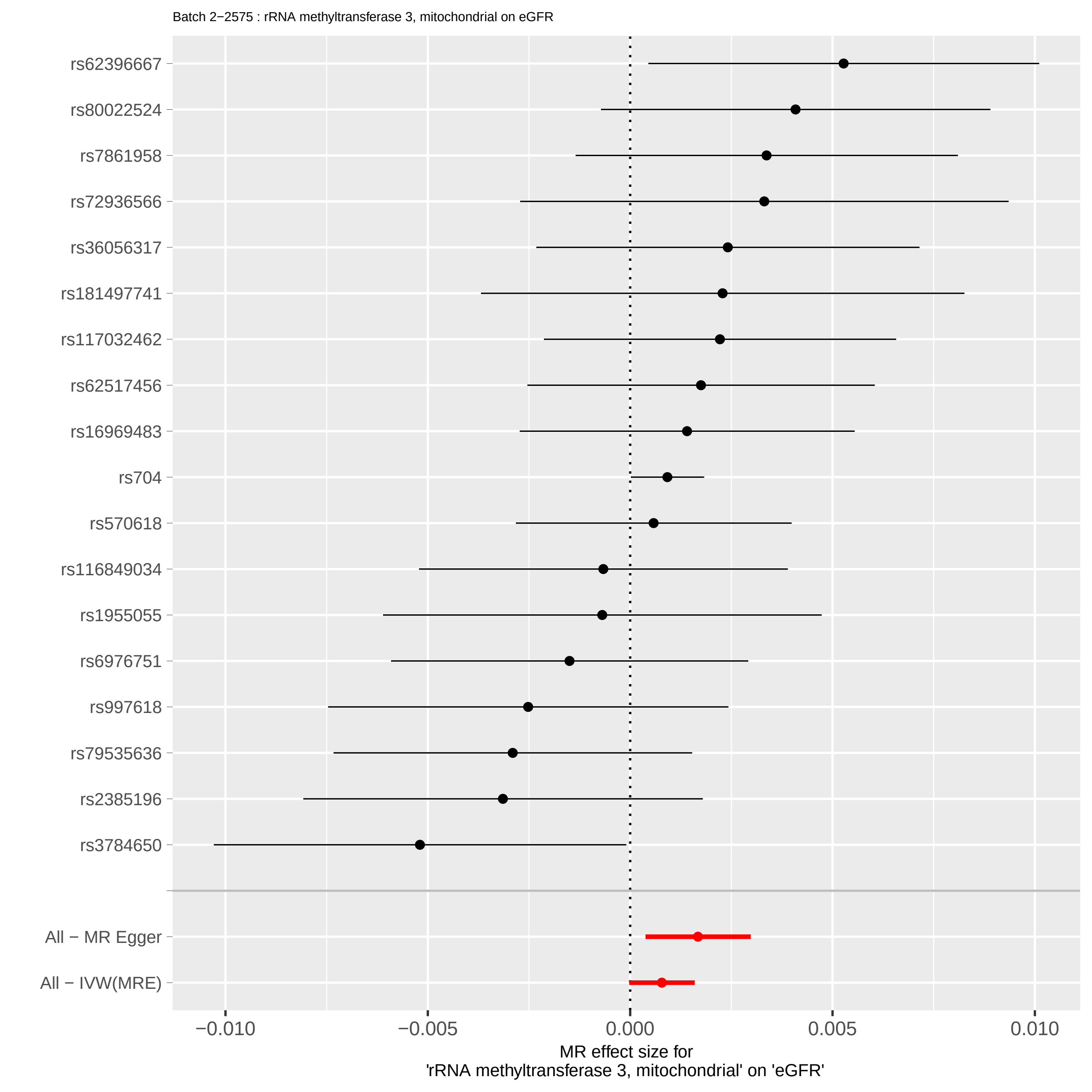

Batch 2−2575 : rRNA methyltransferase 3, mitochondrial on eGFR
rs62396667
rs80022524
rs7861958
rs72936566
rs36056317
rs181497741
rs117032462
rs62517456
rs16969483
rs704
rs570618
rs116849034
rs1955055
rs6976751
rs997618
rs79535636
rs2385196
rs3784650
All − MR Egger
All − IVW(MRE)
−0.010
−0.005
0.000
0.005
0.010
MR effect size for
'rRNA methyltransferase 3, mitochondrial' on 'eGFR'

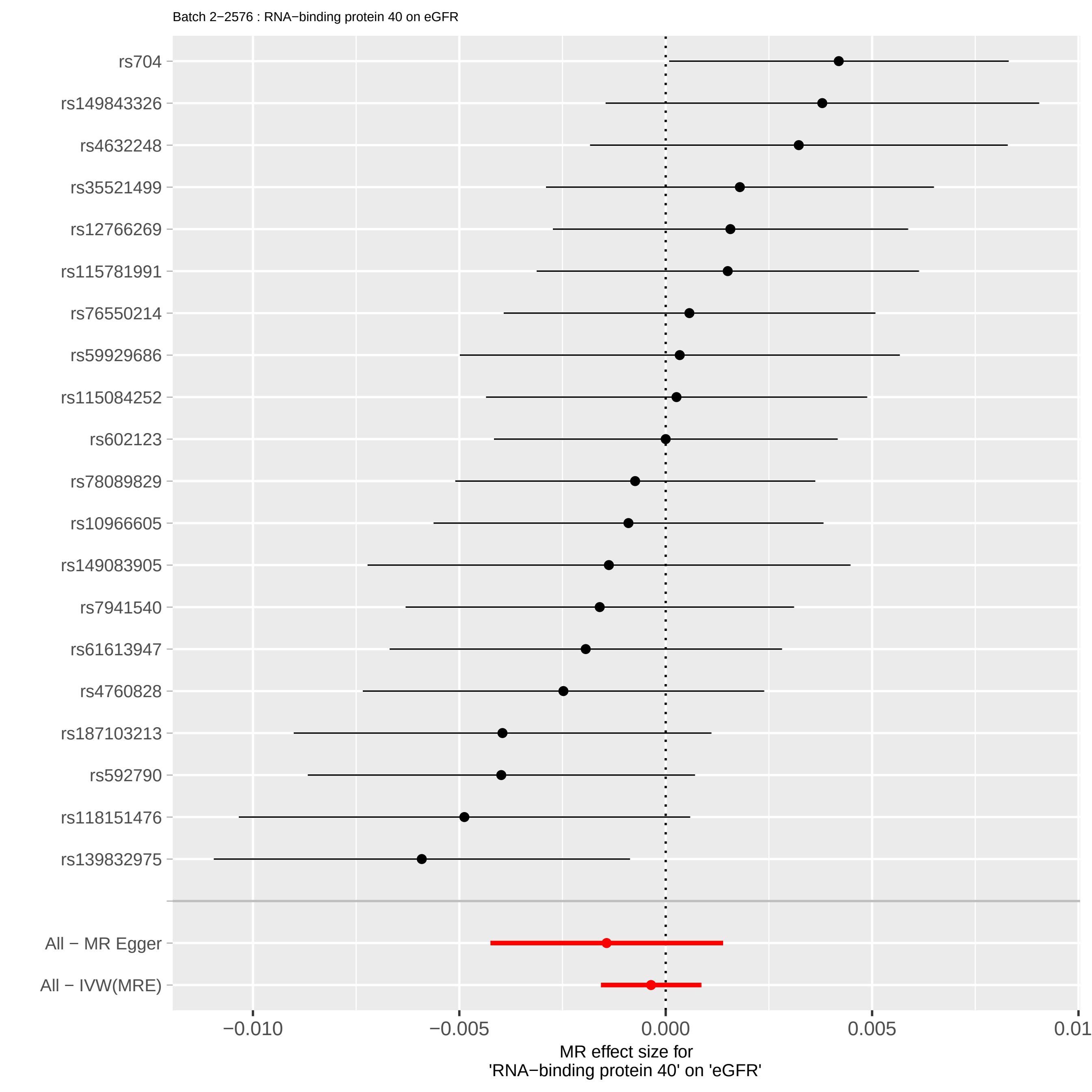

Batch 2−2576 : RNA−binding protein 40 on eGFR
rs704
rs149843326
rs4632248
rs35521499
rs12766269
rs115781991
rs76550214
rs59929686
rs115084252
rs602123
rs78089829
rs10966605
rs149083905
rs7941540
rs61613947
rs4760828
rs187103213
rs592790
rs118151476
rs139832975
All − MR Egger
All − IVW(MRE)
−0.010
−0.005
0.000
0.005
0.01
MR effect size for
'RNA−binding protein 40' on 'eGFR'

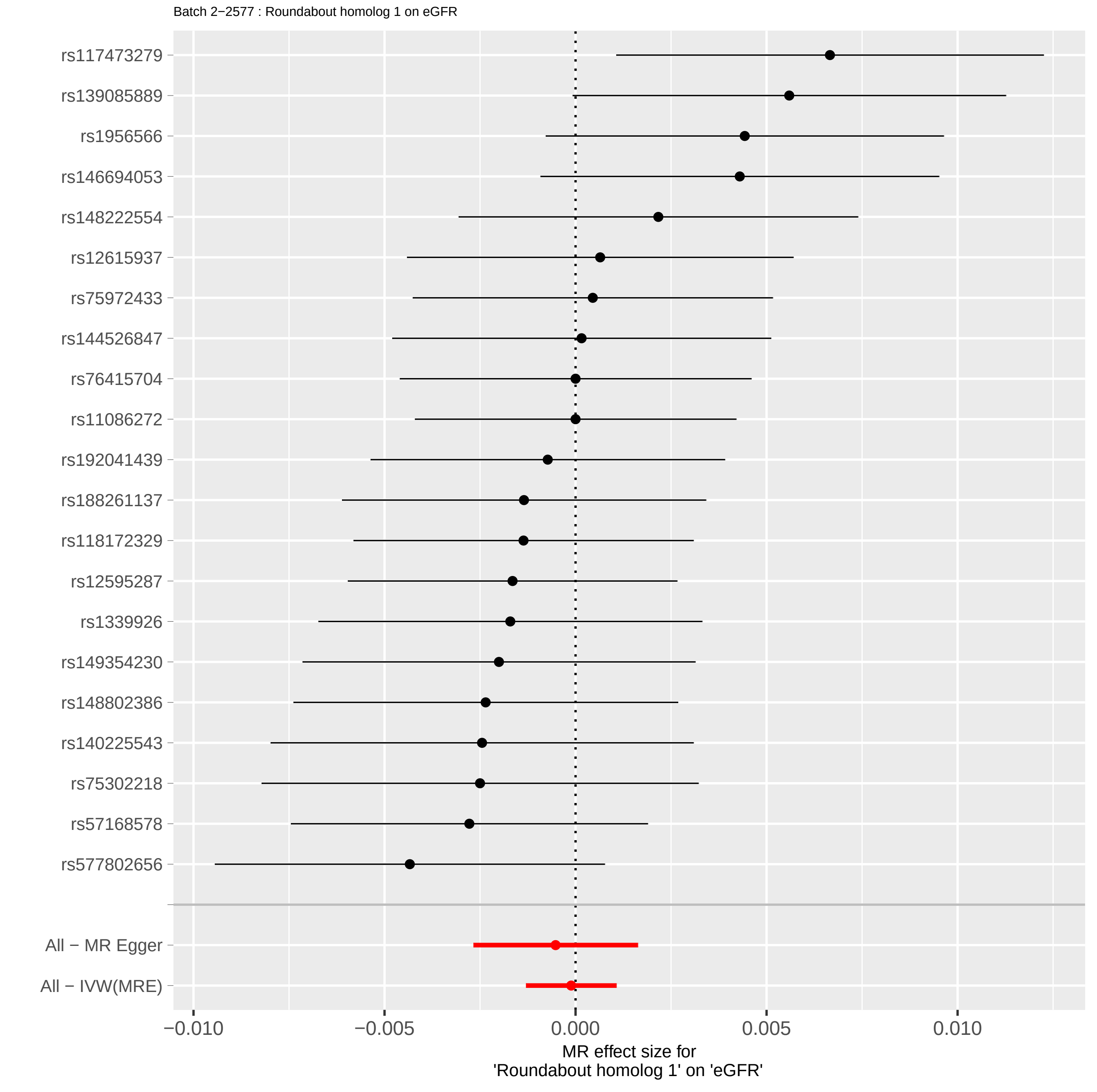

Batch 2−2577 : Roundabout homolog 1 on eGFR
rs117473279
rs139085889
rs1956566
rs146694053
rs148222554
rs12615937
rs75972433
rs144526847
rs76415704
rs11086272
rs192041439
rs188261137
rs118172329
rs12595287
rs1339926
rs149354230
rs148802386
rs140225543
rs75302218
rs57168578
rs577802656
All − MR Egger
All − IVW(MRE)
−0.010
−0.005
0.000
0.005
0.010
MR effect size for
'Roundabout homolog 1' on 'eGFR'

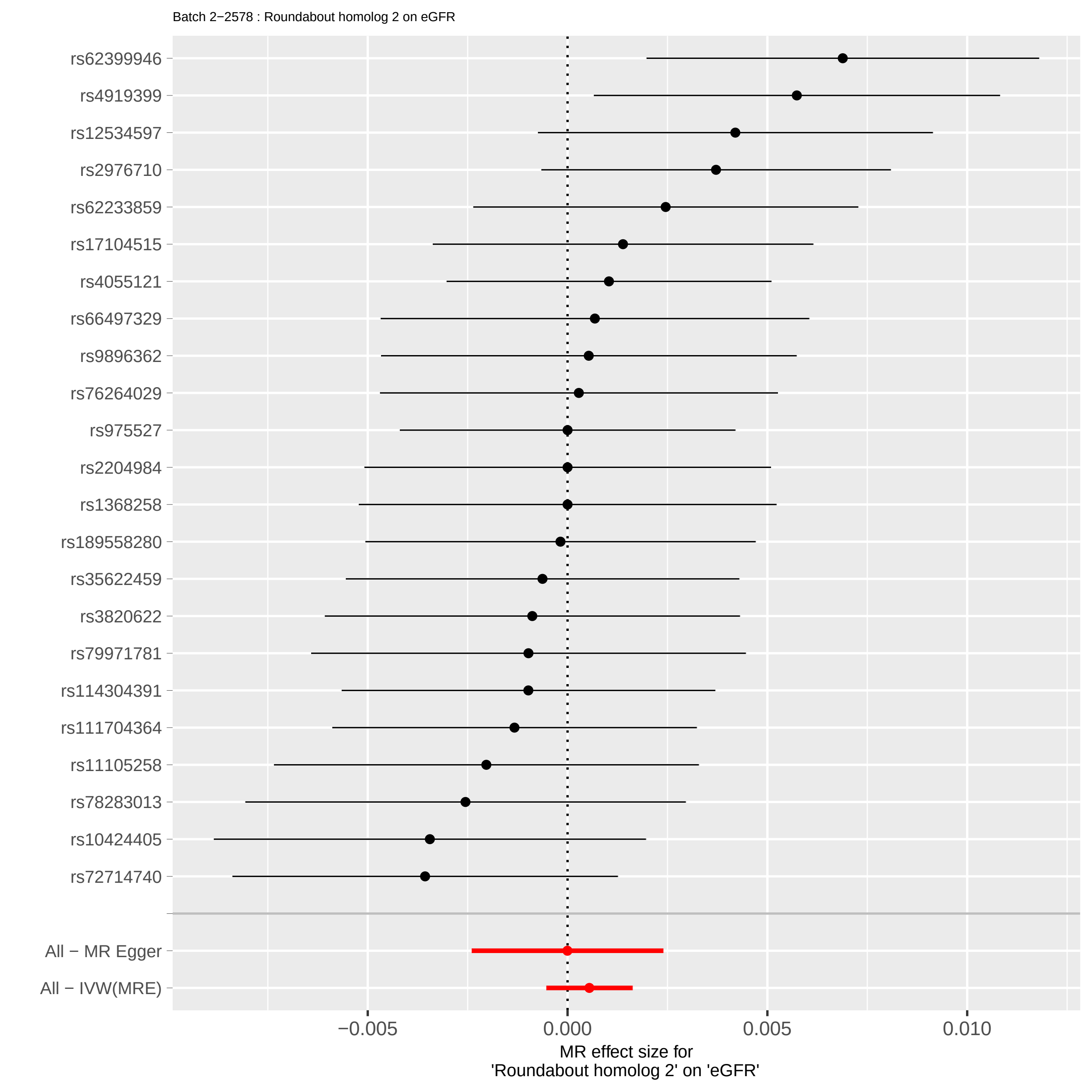

Batch 2−2578 : Roundabout homolog 2 on eGFR
rs62399946
rs4919399
rs12534597
rs2976710
rs62233859
rs17104515
rs4055121
rs66497329
rs9896362
rs76264029
rs975527
rs2204984
rs1368258
rs189558280
rs35622459
rs3820622
rs79971781
rs114304391
rs111704364
rs11105258
rs78283013
rs10424405
rs72714740
All − MR Egger
All − IVW(MRE)
−0.005
0.000
0.005
0.010
MR effect size for
'Roundabout homolog 2' on 'eGFR'

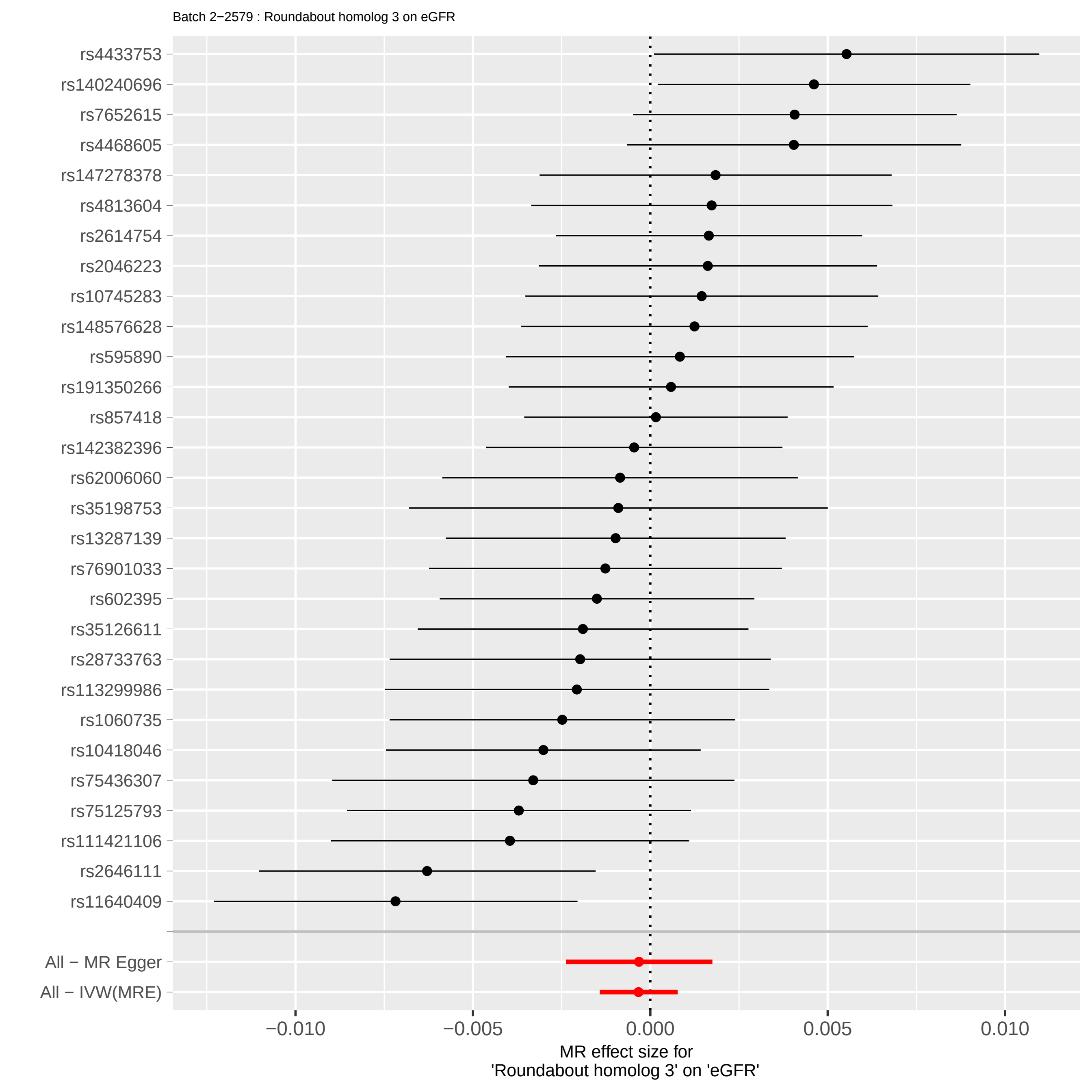

Batch 2−2579 : Roundabout homolog 3 on eGFR
rs4433753
rs140240696
rs7652615
rs4468605
rs147278378
rs4813604
rs2614754
rs2046223
rs10745283
rs148576628
rs595890
rs191350266
rs857418
rs142382396
rs62006060
rs35198753
rs13287139
rs76901033
rs602395
rs35126611
rs28733763
rs113299986
rs1060735
rs10418046
rs75436307
rs75125793
rs111421106
rs2646111
rs11640409
All − MR Egger
All − IVW(MRE)
−0.010
−0.005
0.000
0.005
0.010
MR effect size for
'Roundabout homolog 3' on 'eGFR'

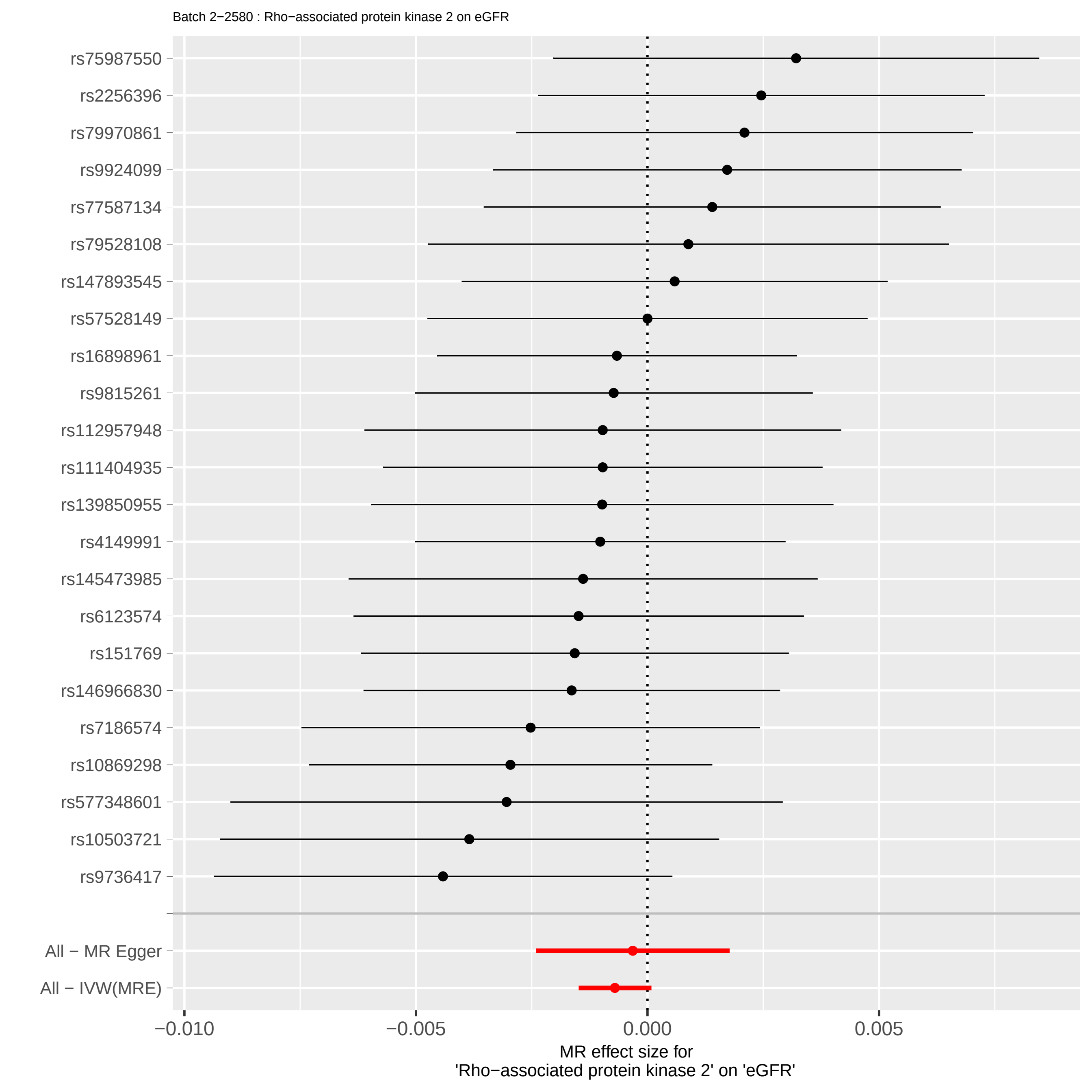

Batch 2−2580 : Rho−associated protein kinase 2 on eGFR
rs75987550
rs2256396
rs79970861
rs9924099
rs77587134
rs79528108
rs147893545
rs57528149
rs16898961
rs9815261
rs112957948
rs111404935
rs139850955
rs4149991
rs145473985
rs6123574
rs151769
rs146966830
rs7186574
rs10869298
rs577348601
rs10503721
rs9736417
All − MR Egger
All − IVW(MRE)
−0.010
−0.005
0.000
0.005
MR effect size for
'Rho−associated protein kinase 2' on 'eGFR'

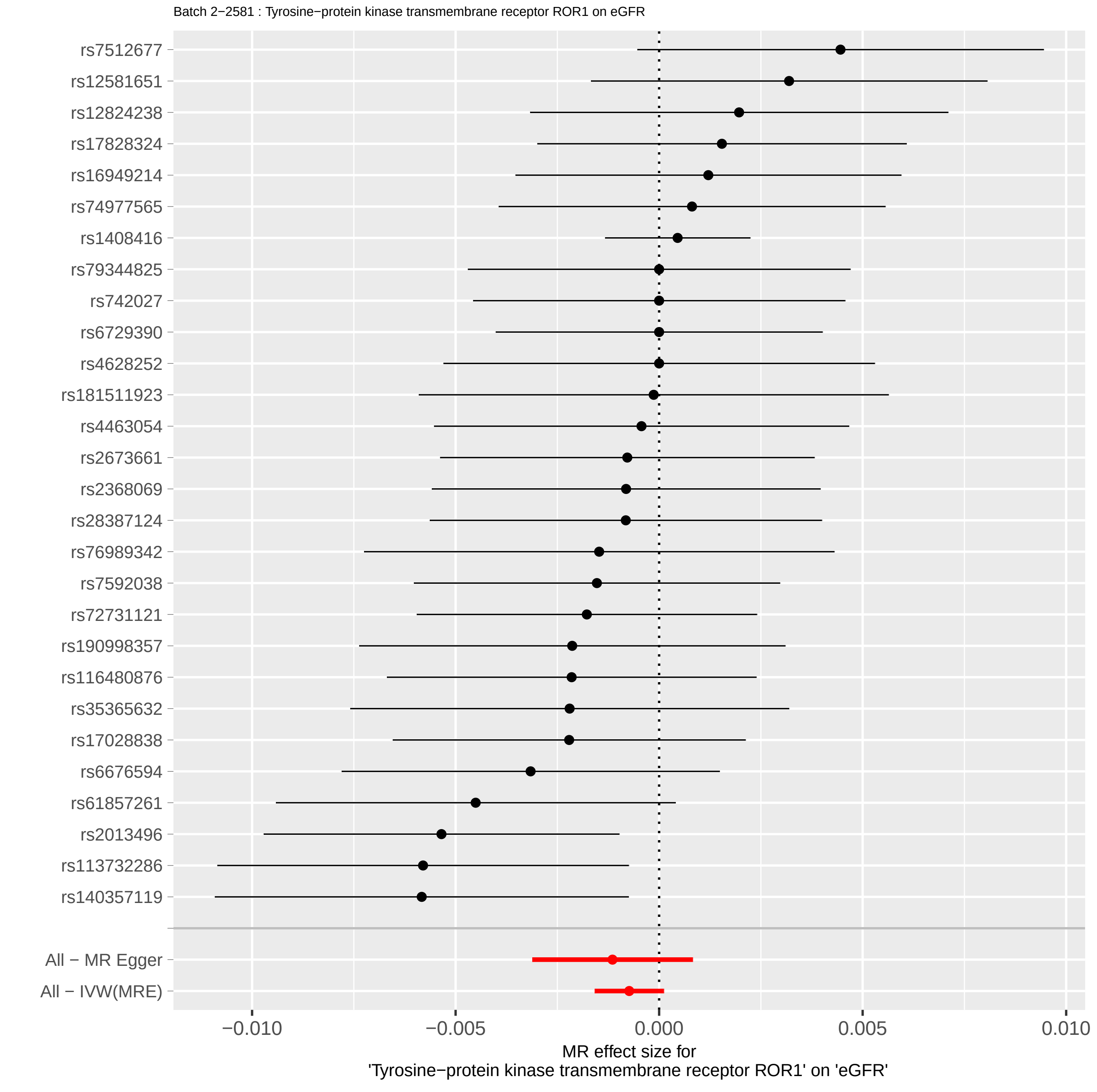
Batch 2−2581 : Tyrosine−protein kinase transmembrane receptor ROR1 on eGFR
rs7512677
rs12581651
rs12824238
rs17828324
rs16949214
rs74977565
rs1408416
rs79344825
rs742027
rs6729390
rs4628252
rs181511923
rs4463054
rs2673661
rs2368069
rs28387124
rs76989342
rs7592038
rs72731121
rs190998357
rs116480876
rs35365632
rs17028838
rs6676594
rs61857261
rs2013496
rs113732286
rs140357119
All − MR Egger
All − IVW(MRE)
−0.010
−0.005
0.000
0.005
0.010
MR effect size for
'Tyrosine−protein kinase transmembrane receptor ROR1' on 'eGFR'

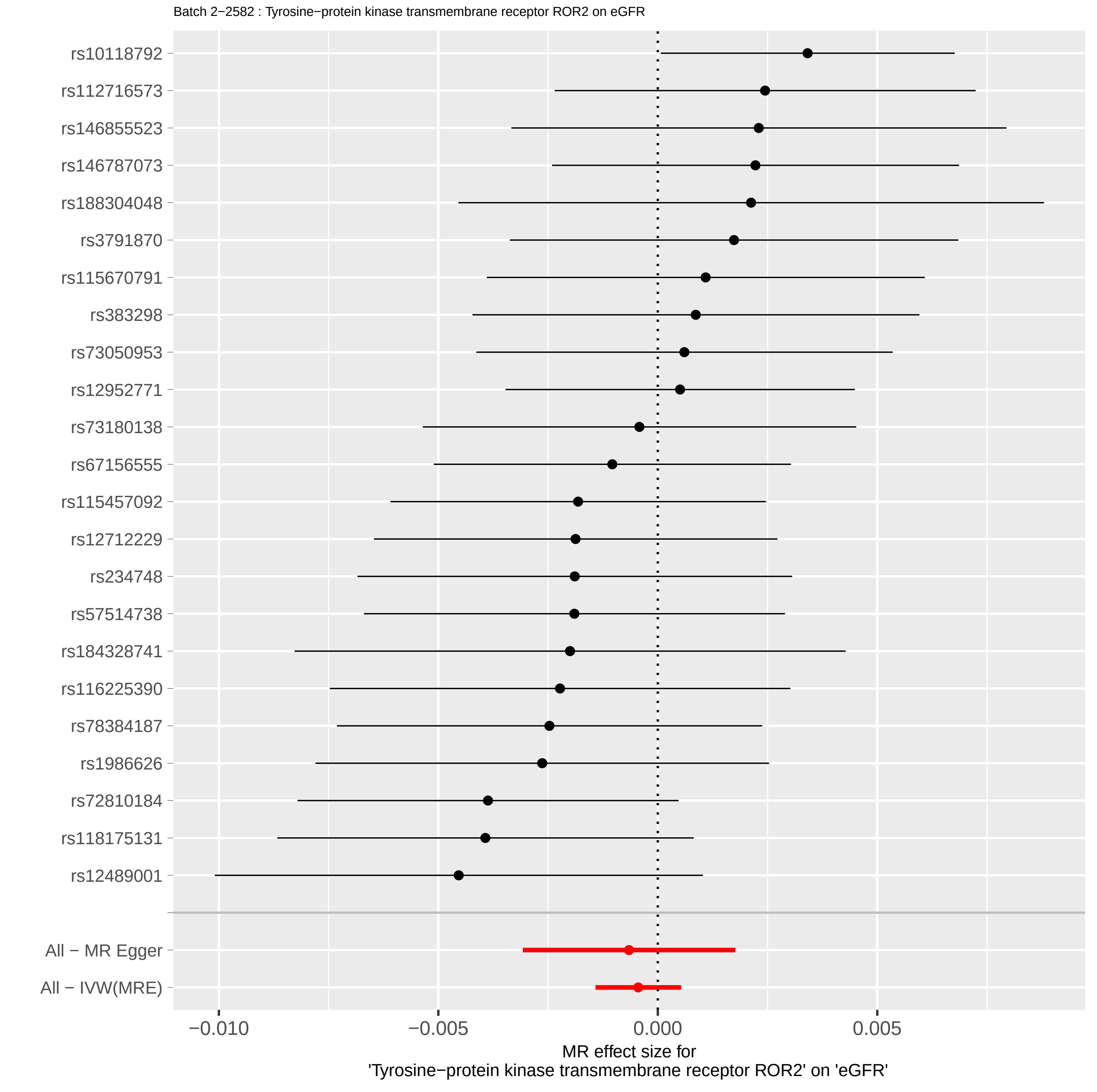

Batch 2−2582 : Tyrosine−protein kinase transmembrane receptor ROR2 on eGFR
rs10118792
rs112716573
rs146855523
rs146787073
rs188304048
rs3791870
rs115670791
rs383298
rs73050953
rs12952771
rs73180138
rs67156555
rs115457092
rs12712229
rs234748
rs57514738
rs184328741
rs116225390
rs78384187
rs1986626
rs72810184
rs118175131
rs12489001
All − MR Egger
All − IVW(MRE)
−0.010
−0.005
0.000
0.005
MR effect size for
'Tyrosine−protein kinase transmembrane receptor ROR2' on 'eGFR'

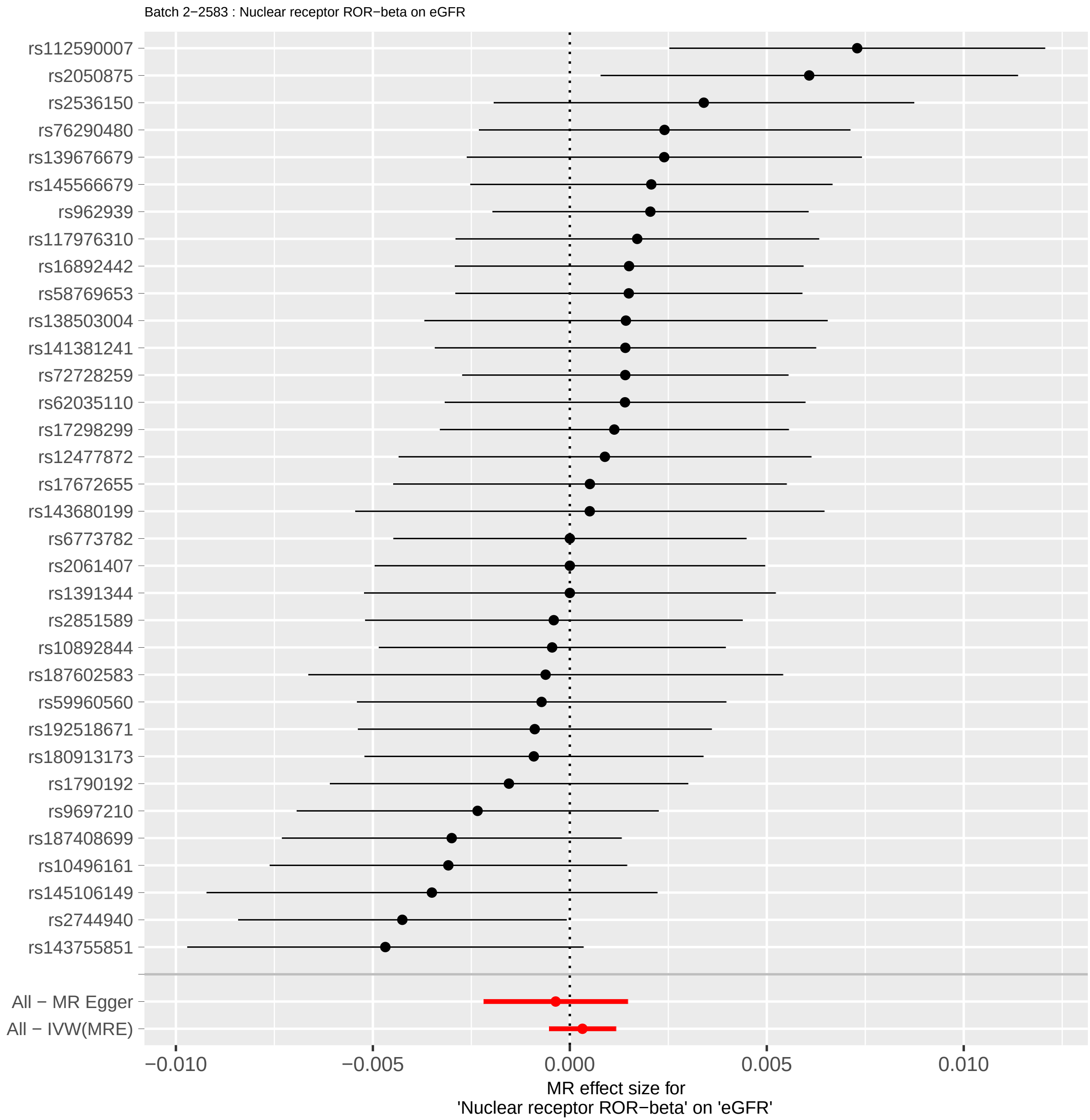

Batch 2−2583 : Nuclear receptor ROR−beta on eGFR
rs112590007
rs2050875
rs2536150
rs76290480
rs139676679
rs145566679
rs962939
rs117976310
rs16892442
rs58769653
rs138503004
rs141381241
rs72728259
rs62035110
rs17298299
rs12477872
rs17672655
rs143680199
rs6773782
rs2061407
rs1391344
rs2851589
rs10892844
rs187602583
rs59960560
rs192518671
rs180913173
rs1790192
rs9697210
rs187408699
rs10496161
rs145106149
rs2744940
rs143755851
All − MR Egger
All − IVW(MRE)
−0.010
−0.005
0.000
0.005
0.010
MR effect size for
'Nuclear receptor ROR−beta' on 'eGFR'

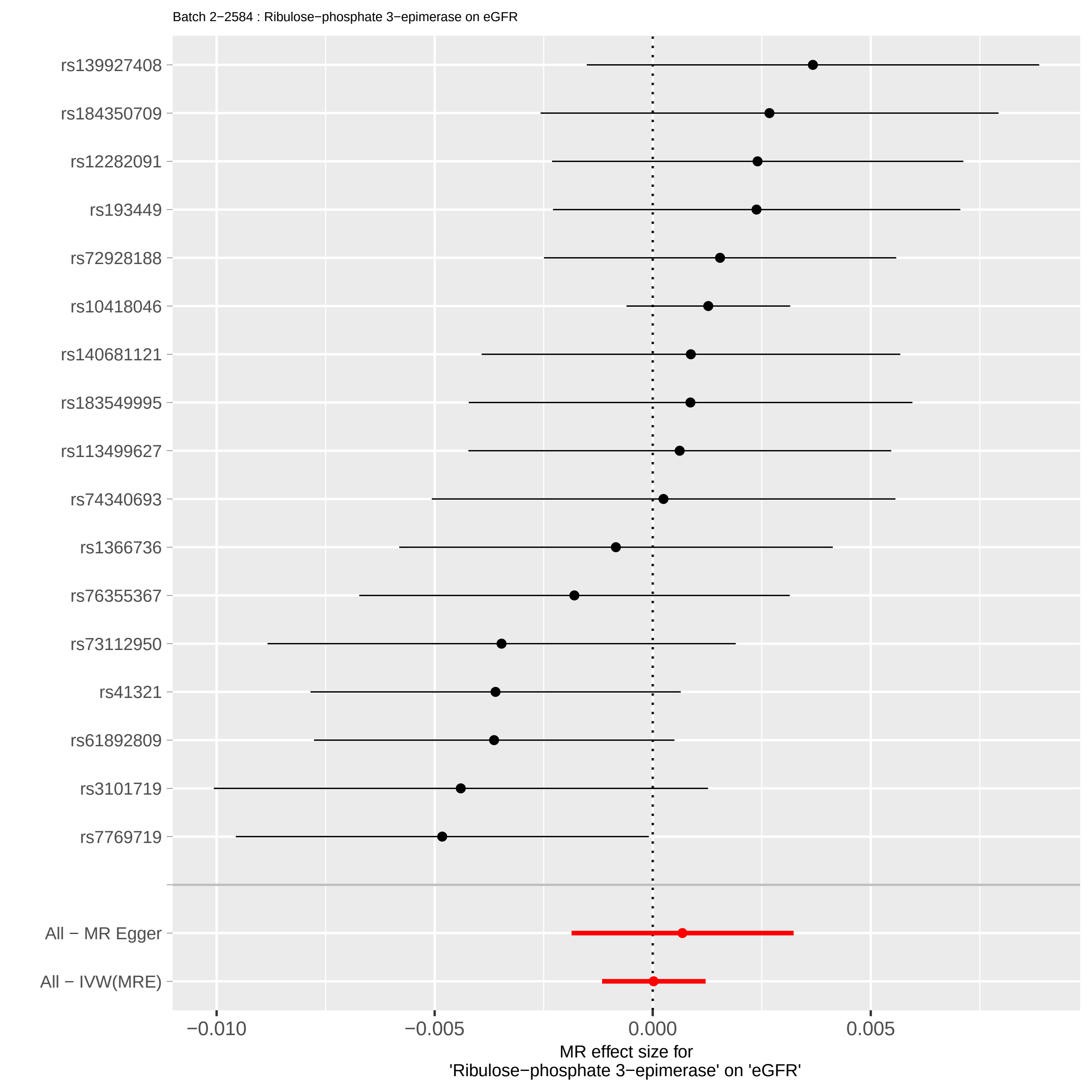

Batch 2−2584 : Ribulose−phosphate 3−epimerase on eGFR
rs139927408
rs184350709
rs12282091
rs193449
rs72928188
rs10418046
rs140681121
rs183549995
rs113499627
rs74340693
rs1366736
rs76355367
rs73112950
rs41321
rs61892809
rs3101719
rs7769719
All − MR Egger
All − IVW(MRE)
−0.010
−0.005
0.000
0.005
MR effect size for
'Ribulose−phosphate 3−epimerase' on 'eGFR'

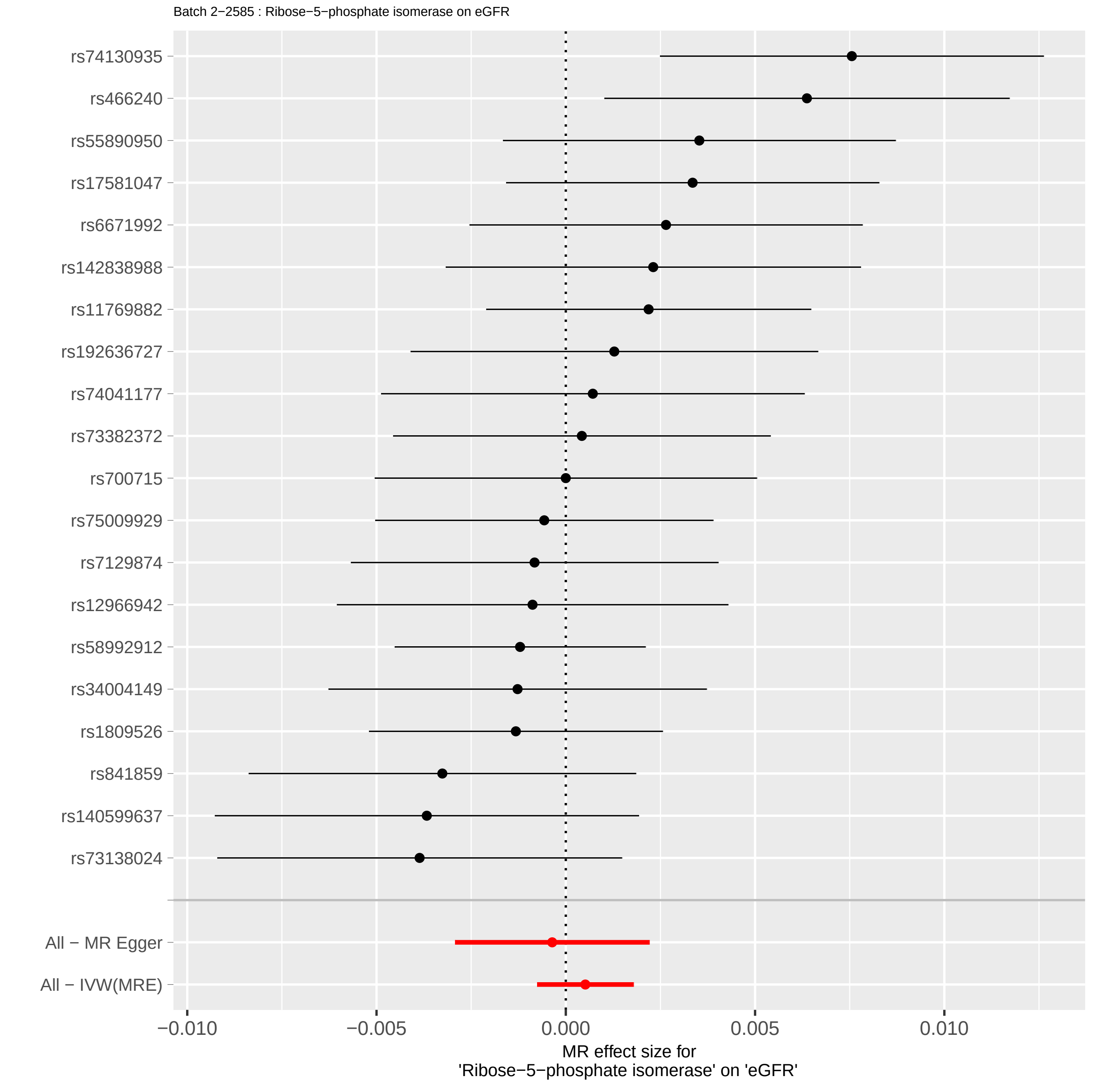

Batch 2−2585 : Ribose−5−phosphate isomerase on eGFR
rs74130935
rs466240
rs55890950
rs17581047
rs6671992
rs142838988
rs11769882
rs192636727
rs74041177
rs73382372
rs700715
rs75009929
rs7129874
rs12966942
rs58992912
rs34004149
rs1809526
rs841859
rs140599637
rs73138024
All − MR Egger
All − IVW(MRE)
−0.010
−0.005
0.000
0.005
0.010
MR effect size for
'Ribose−5−phosphate isomerase' on 'eGFR'

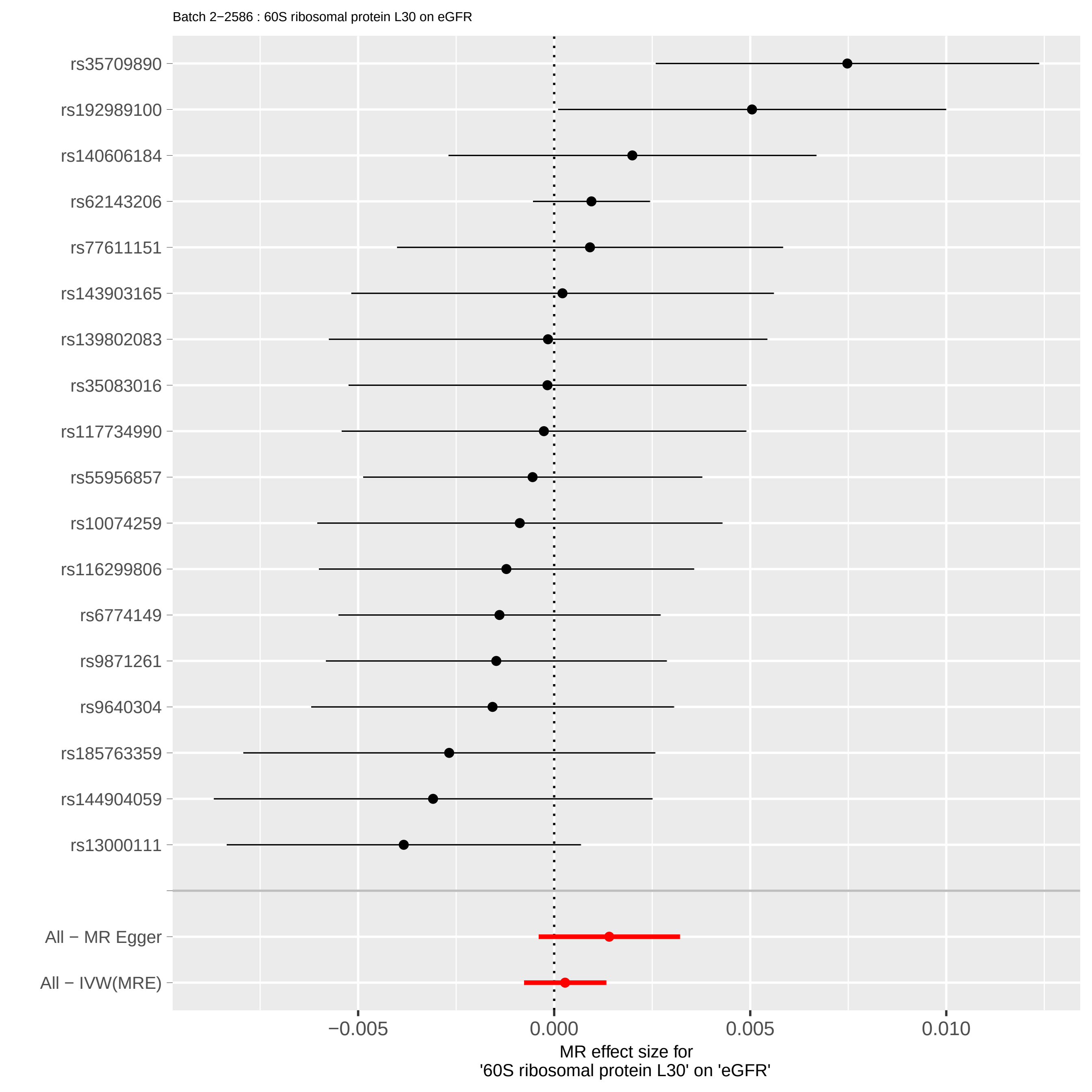

Batch 2−2586 : 60S ribosomal protein L30 on eGFR
rs35709890
rs192989100
rs140606184
rs62143206
rs77611151
rs143903165
rs139802083
rs35083016
rs117734990
rs55956857
rs10074259
rs116299806
rs6774149
rs9871261
rs9640304
rs185763359
rs144904059
rs13000111
All − MR Egger
All − IVW(MRE)
−0.005
0.000
0.005
0.010
MR effect size for
'60S ribosomal protein L30' on 'eGFR'

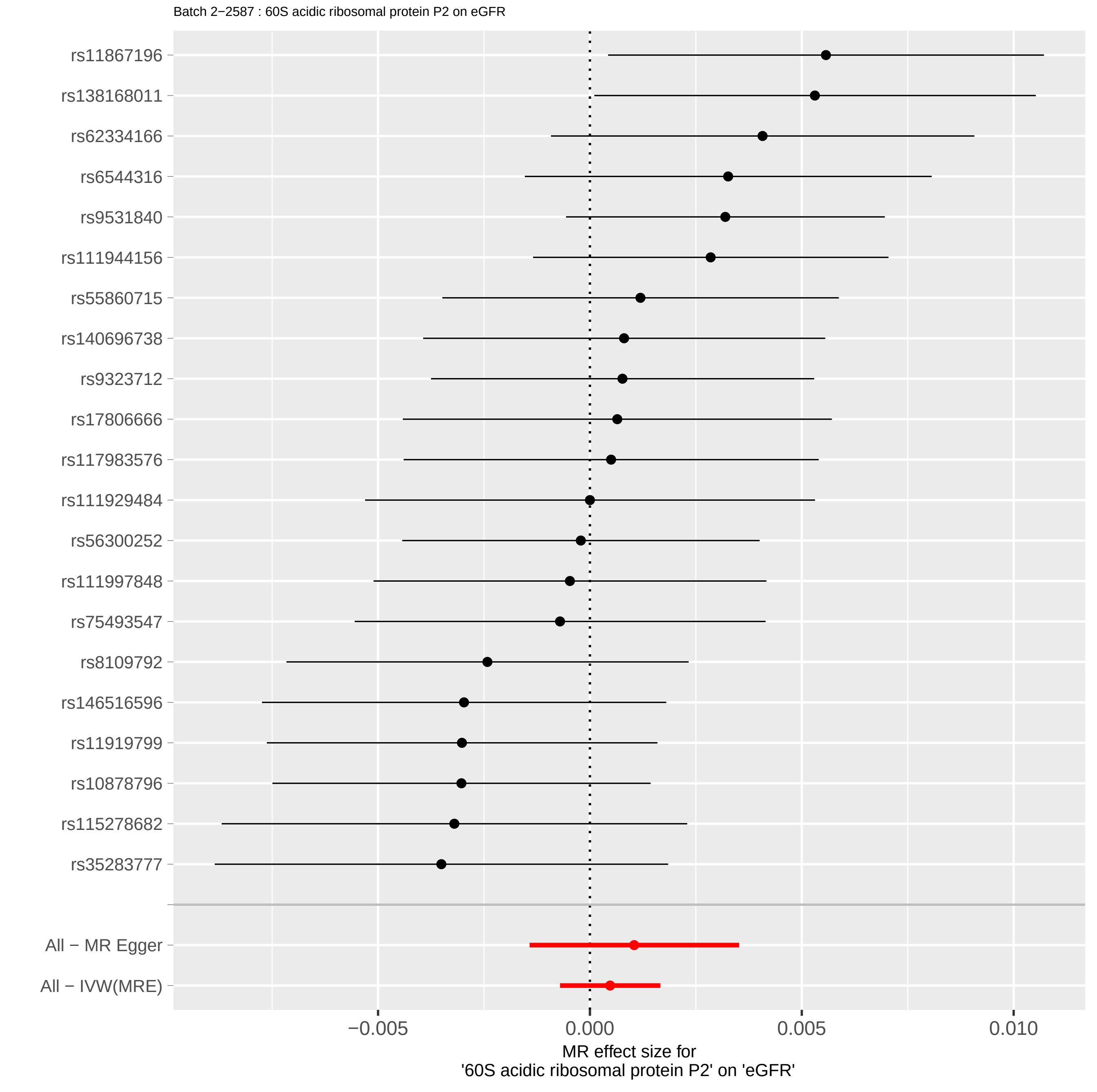
Batch 2−2587 : 60S acidic ribosomal protein P2 on eGFR
rs11867196
rs138168011
rs62334166
rs6544316
rs9531840
rs111944156
rs55860715
rs140696738
rs9323712
rs17806666
rs117983576
rs111929484
rs56300252
rs111997848
rs75493547
rs8109792
rs146516596
rs11919799
rs10878796
rs115278682
rs35283777
All − MR Egger
All − IVW(MRE)
−0.005
0.000
0.005
0.010
MR effect size for
'60S acidic ribosomal protein P2' on 'eGFR'

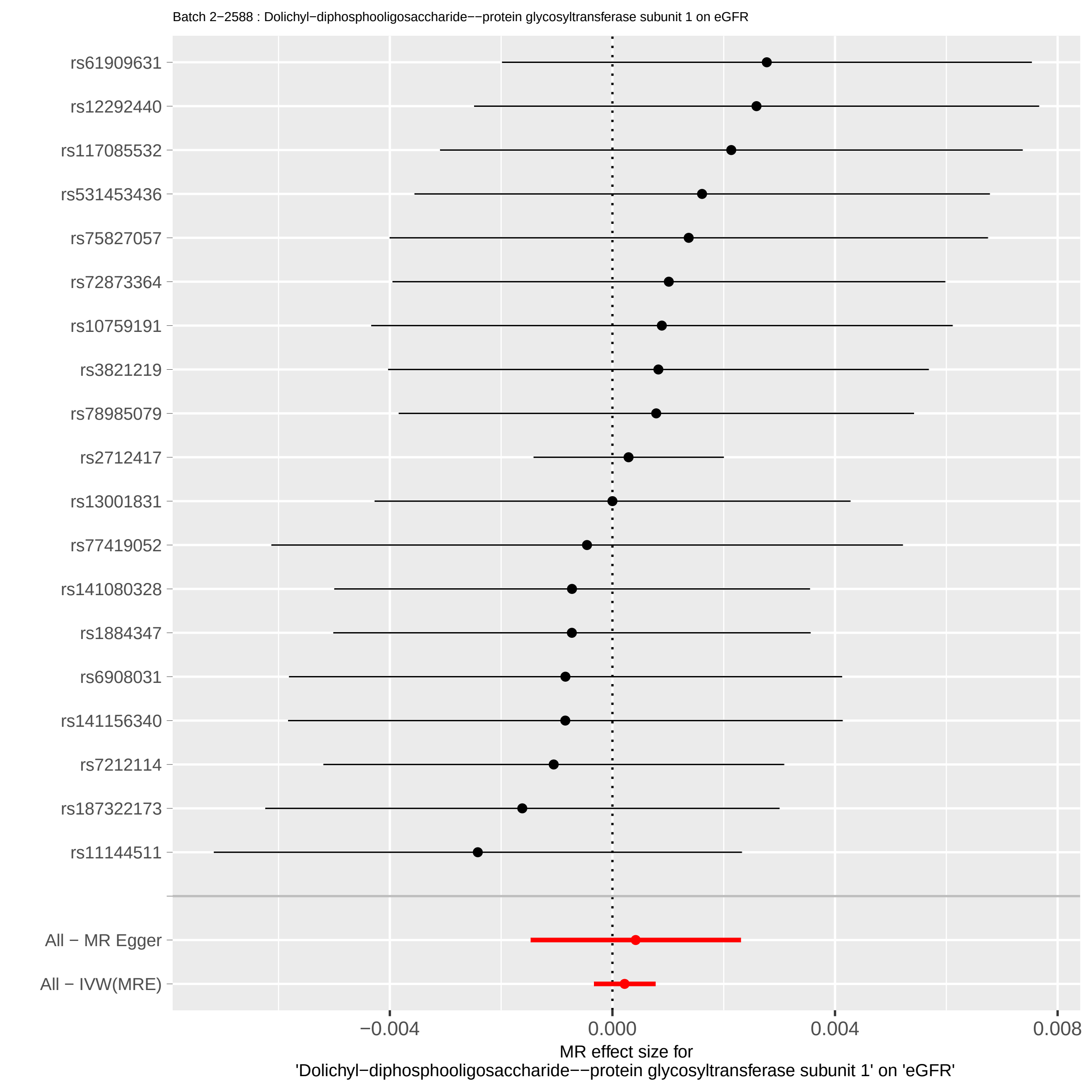

Batch 2−2588 : Dolichyl−diphosphooligosaccharide−−protein glycosyltransferase subunit 1 on eGFR
rs61909631
rs12292440
rs117085532
rs531453436
rs75827057
rs72873364
rs10759191
rs3821219
rs78985079
rs2712417
rs13001831
rs77419052
rs141080328
rs1884347
rs6908031
rs141156340
rs7212114
rs187322173
rs11144511
All − MR Egger
All − IVW(MRE)
−0.004
0.000
0.004
0.008
MR effect size for
'Dolichyl−diphosphooligosaccharide−−protein glycosyltransferase subunit 1' on 'eGFR'

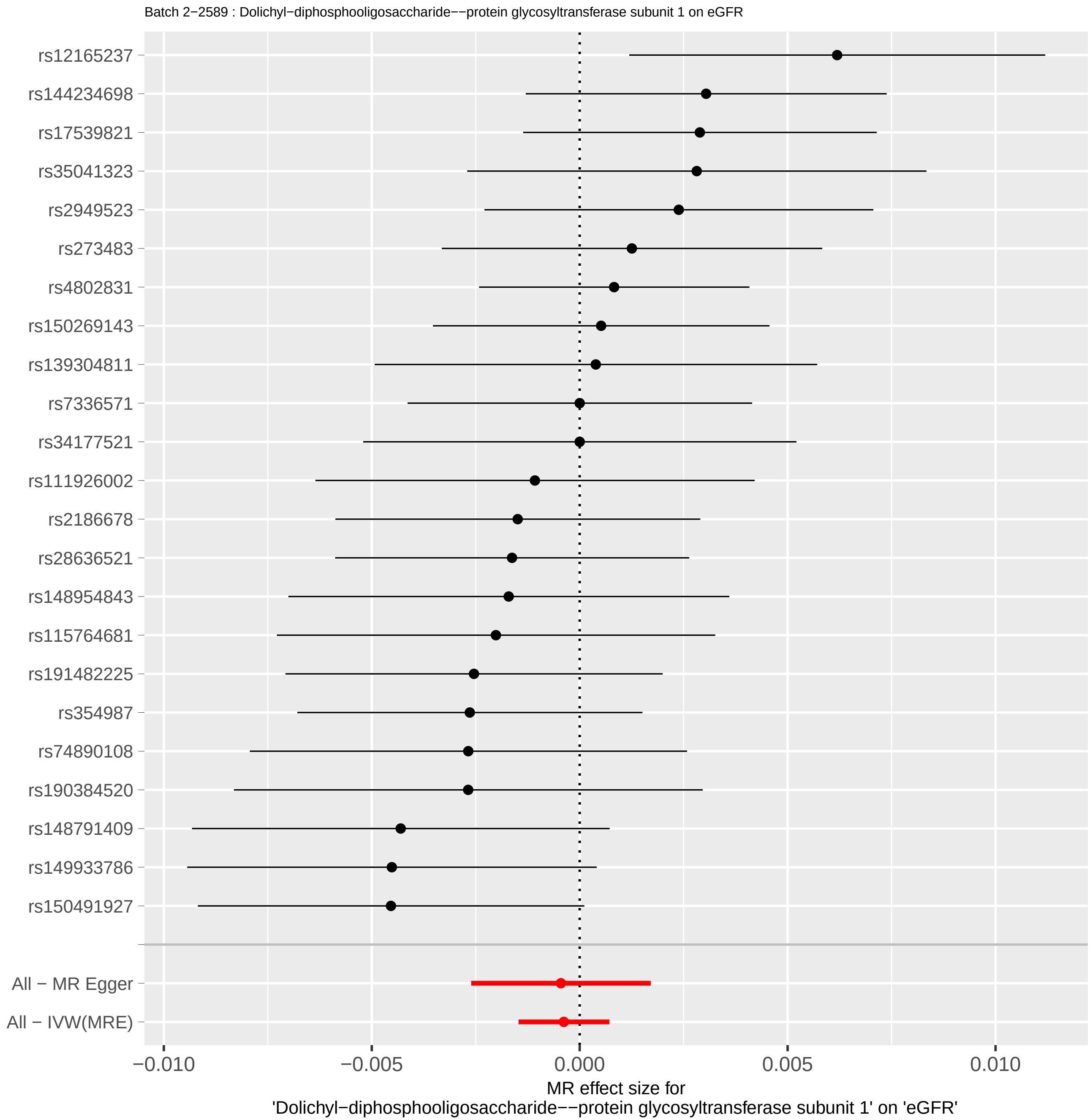

Batch 2−2589 : Dolichyl−diphosphooligosaccharide−−protein glycosyltransferase subunit 1 on eGFR
rs12165237
rs144234698
rs17539821
rs35041323
rs2949523
rs273483
rs4802831
rs150269143
rs139304811
rs7336571
rs34177521
rs111926002
rs2186678
rs28636521
rs148954843
rs115764681
rs191482225
rs354987
rs74890108
rs190384520
rs148791409
rs149933786
rs150491927
All − MR Egger
All − IVW(MRE)
−0.010
−0.005
0.000
0.005
0.010
MR effect size for
'Dolichyl−diphosphooligosaccharide−−protein glycosyltransferase subunit 1' on 'eGFR'

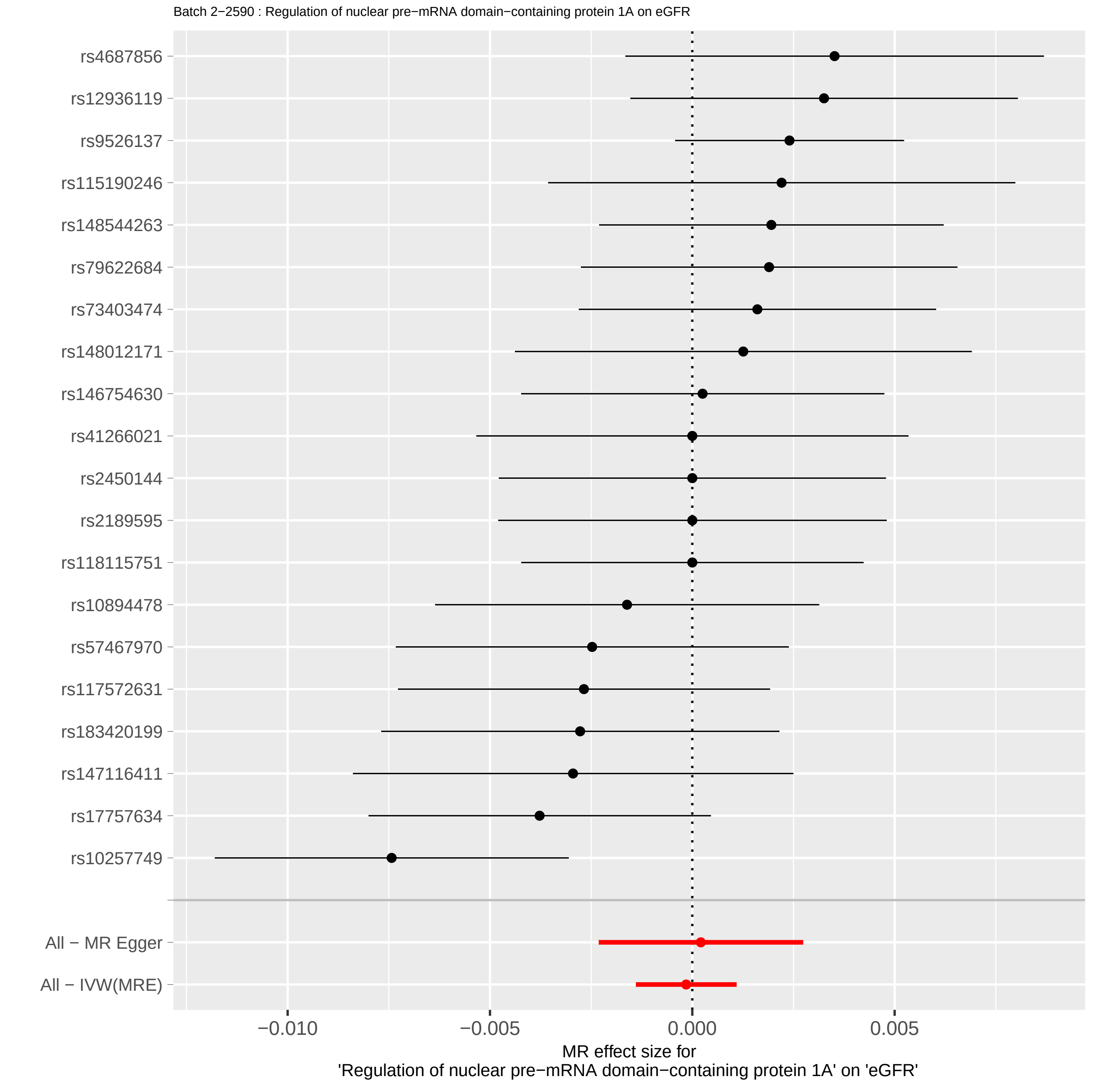

Batch 2−2590 : Regulation of nuclear pre−mRNA domain−containing protein 1A on eGFR
rs4687856
rs12936119
rs9526137
rs115190246
rs148544263
rs79622684
rs73403474
rs148012171
rs146754630
rs41266021
rs2450144
rs2189595
rs118115751
rs10894478
rs57467970
rs117572631
rs183420199
rs147116411
rs17757634
rs10257749
All − MR Egger
All − IVW(MRE)
−0.010
−0.005
0.000
0.005
MR effect size for
'Regulation of nuclear pre−mRNA domain−containing protein 1A' on 'eGFR'

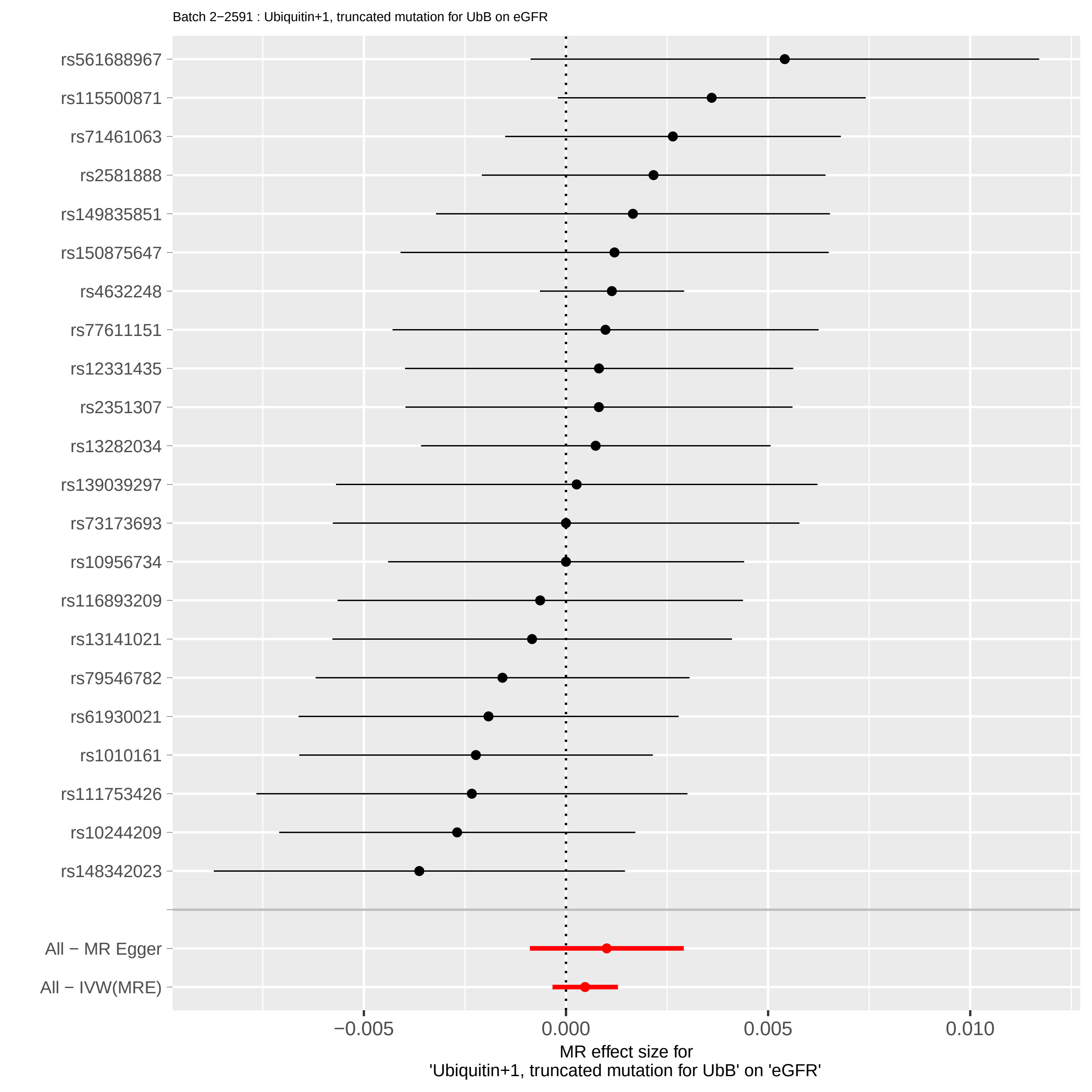
Batch 2−2591 : Ubiquitin+1, truncated mutation for UbB on eGFR
rs561688967
rs115500871
rs71461063
rs2581888
rs149835851
rs150875647
rs4632248
rs77611151
rs12331435
rs2351307
rs13282034
rs139039297
rs73173693
rs10956734
rs116893209
rs13141021
rs79546782
rs61930021
rs1010161
rs111753426
rs10244209
rs148342023
All − MR Egger
All − IVW(MRE)
−0.005
0.000
0.005
0.010
MR effect size for
'Ubiquitin+1, truncated mutation for UbB' on 'eGFR'

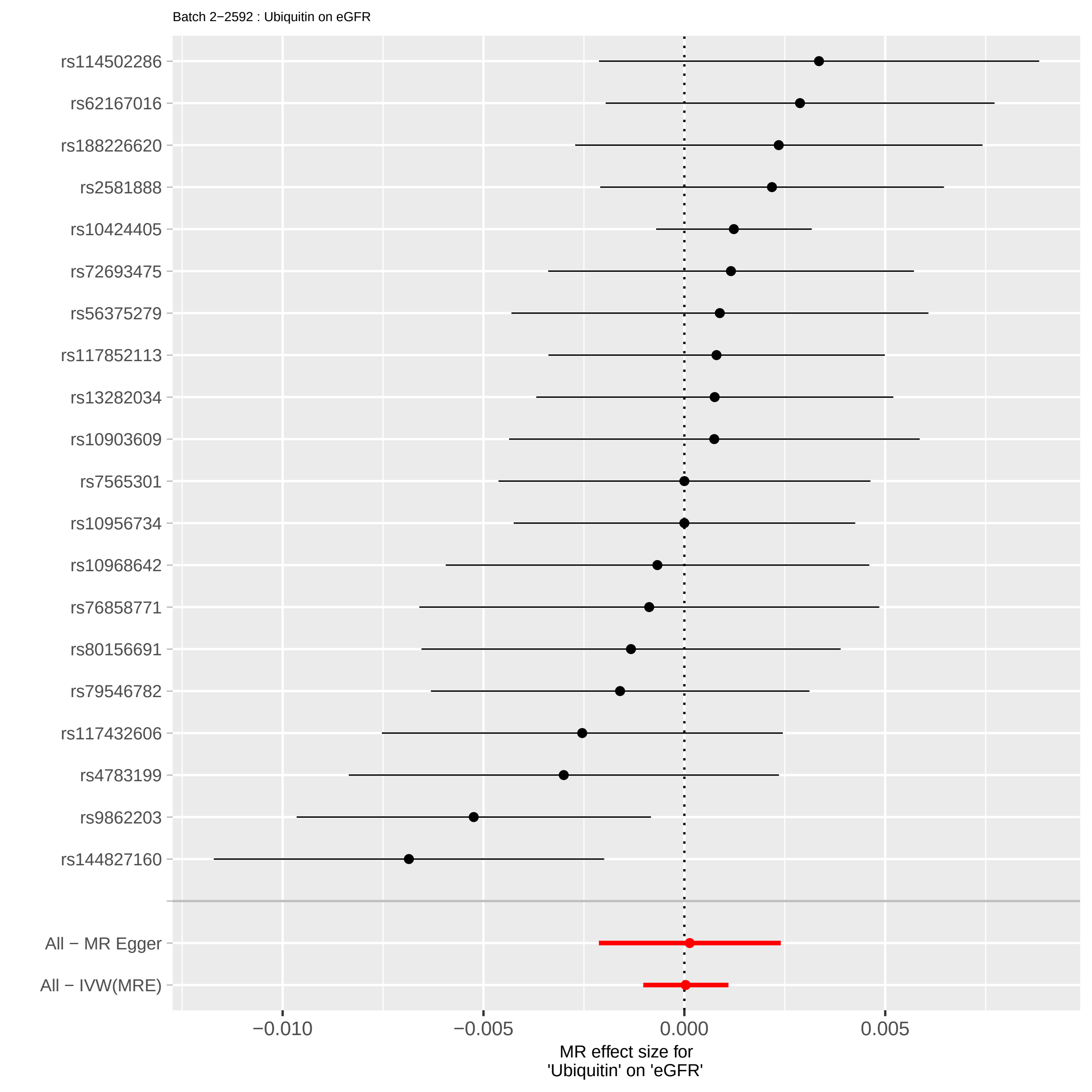
Batch 2−2592 : Ubiquitin on eGFR
rs114502286
rs62167016
rs188226620
rs2581888
rs10424405
rs72693475
rs56375279
rs117852113
rs13282034
rs10903609
rs7565301
rs10956734
rs10968642
rs76858771
rs80156691
rs79546782
rs117432606
rs4783199
rs9862203
rs144827160
All − MR Egger
All − IVW(MRE)
−0.010
−0.005
0.000
0.005
MR effect size for
'Ubiquitin' on 'eGFR'

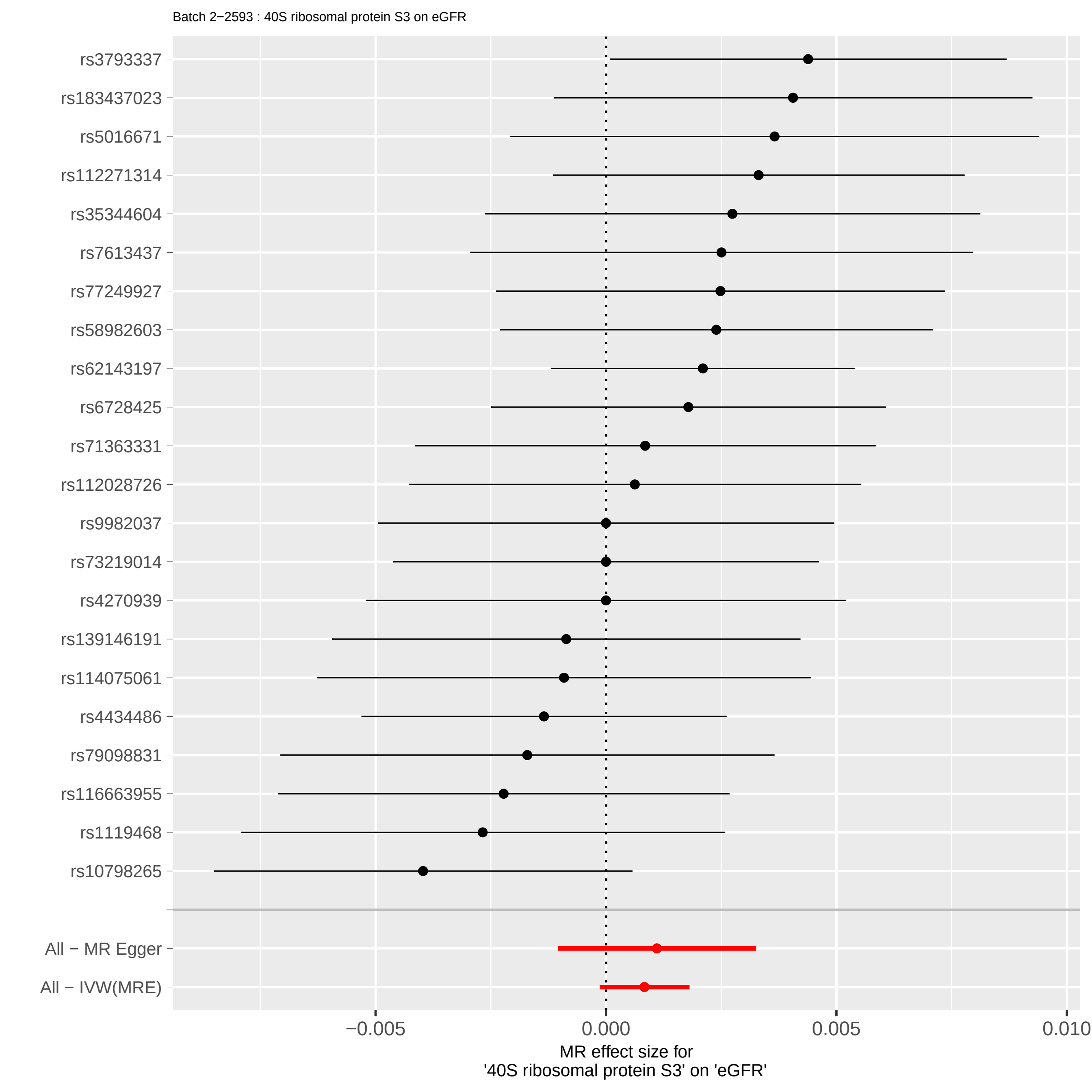

Batch 2−2593 : 40S ribosomal protein S3 on eGFR
rs3793337
rs183437023
rs5016671
rs112271314
rs35344604
rs7613437
rs77249927
rs58982603
rs62143197
rs6728425
rs71363331
rs112028726
rs9982037
rs73219014
rs4270939
rs139146191
rs114075061
rs4434486
rs79098831
rs116663955
rs1119468
rs10798265
All − MR Egger
All − IVW(MRE)
−0.005
0.000
0.005
0.010
MR effect size for
'40S ribosomal protein S3' on 'eGFR'

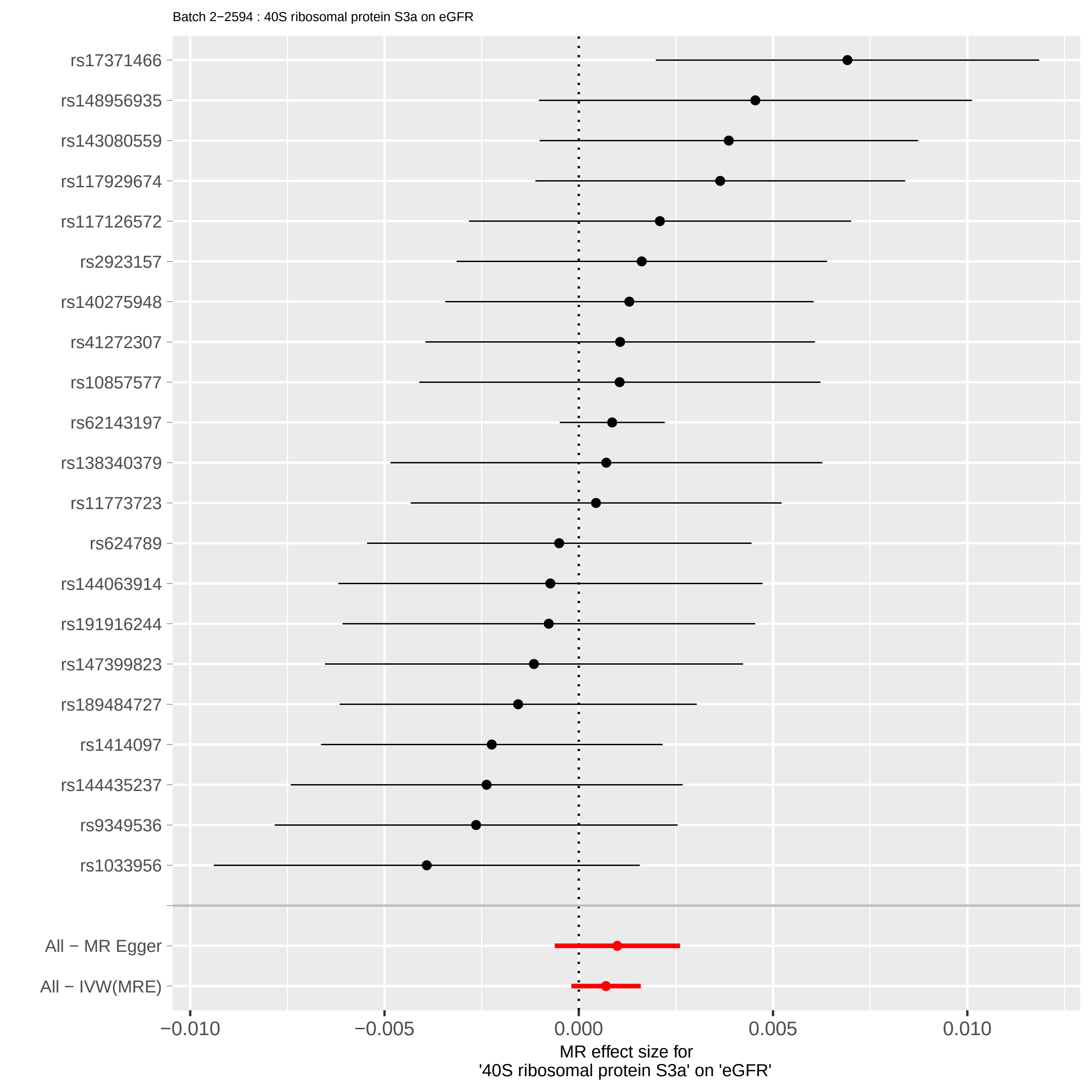

Batch 2−2594 : 40S ribosomal protein S3a on eGFR
rs17371466
rs148956935
rs143080559
rs117929674
rs117126572
rs2923157
rs140275948
rs41272307
rs10857577
rs62143197
rs138340379
rs11773723
rs624789
rs144063914
rs191916244
rs147399823
rs189484727
rs1414097
rs144435237
rs9349536
rs1033956
All − MR Egger
All − IVW(MRE)
−0.010
−0.005
0.000
0.005
0.010
MR effect size for
'40S ribosomal protein S3a' on 'eGFR'

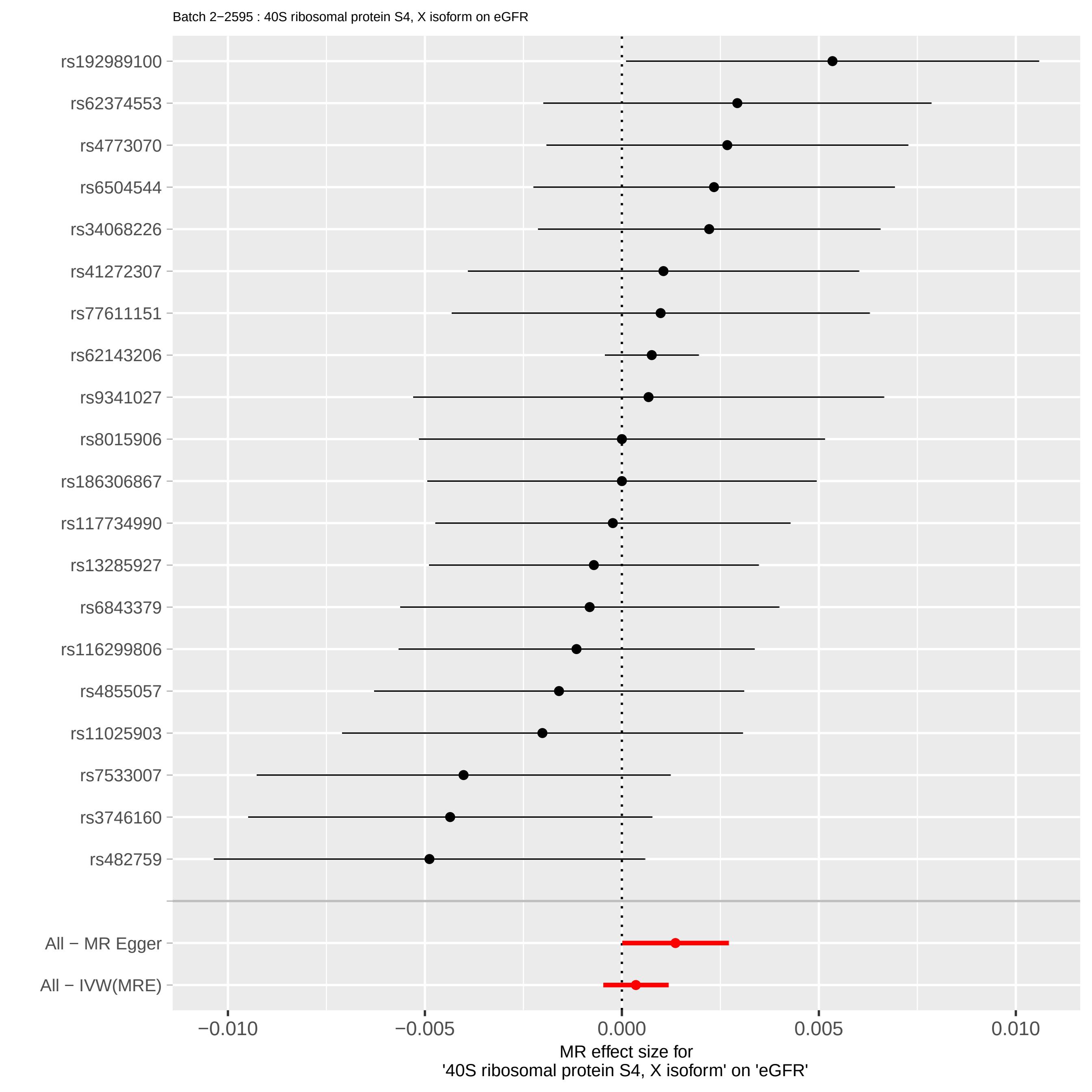
Batch 2−2595 : 40S ribosomal protein S4, X isoform on eGFR
rs192989100
rs62374553
rs4773070
rs6504544
rs34068226
rs41272307
rs77611151
rs62143206
rs9341027
rs8015906
rs186306867
rs117734990
rs13285927
rs6843379
rs116299806
rs4855057
rs11025903
rs7533007
rs3746160
rs482759
All − MR Egger
All − IVW(MRE)
−0.010
−0.005
0.000
0.005
0.010
MR effect size for
'40S ribosomal protein S4, X isoform' on 'eGFR'

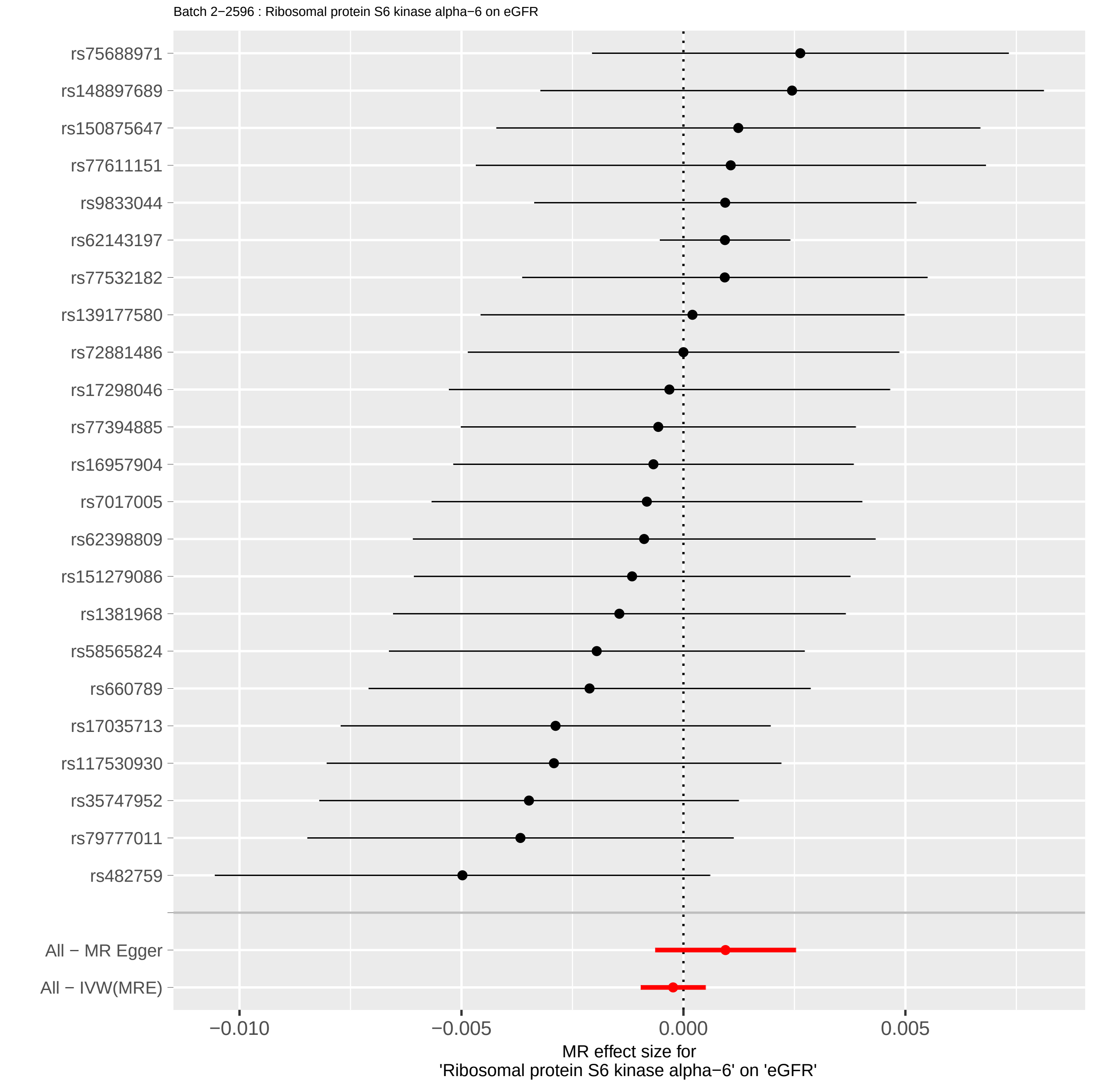

Batch 2−2596 : Ribosomal protein S6 kinase alpha−6 on eGFR
rs75688971
rs148897689
rs150875647
rs77611151
rs9833044
rs62143197
rs77532182
rs139177580
rs72881486
rs17298046
rs77394885
rs16957904
rs7017005
rs62398809
rs151279086
rs1381968
rs58565824
rs660789
rs17035713
rs117530930
rs35747952
rs79777011
rs482759
All − MR Egger
All − IVW(MRE)
−0.010
−0.005
0.000
0.005
MR effect size for
'Ribosomal protein S6 kinase alpha−6' on 'eGFR'

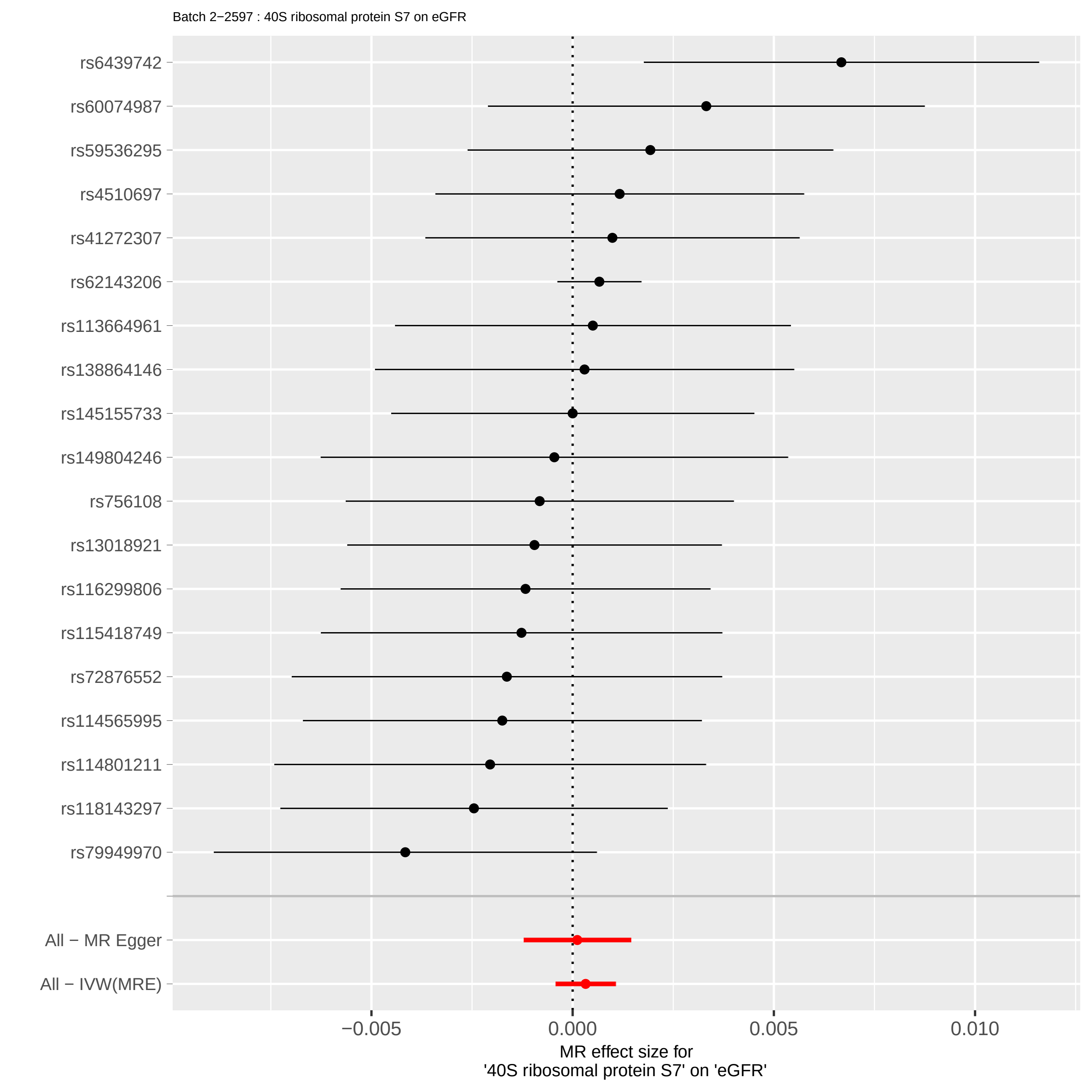

Batch 2−2597 : 40S ribosomal protein S7 on eGFR
rs6439742
rs60074987
rs59536295
rs4510697
rs41272307
rs62143206
rs113664961
rs138864146
rs145155733
rs149804246
rs756108
rs13018921
rs116299806
rs115418749
rs72876552
rs114565995
rs114801211
rs118143297
rs79949970
All − MR Egger
All − IVW(MRE)
−0.005
0.000
0.005
0.010
MR effect size for
'40S ribosomal protein S7' on 'eGFR'

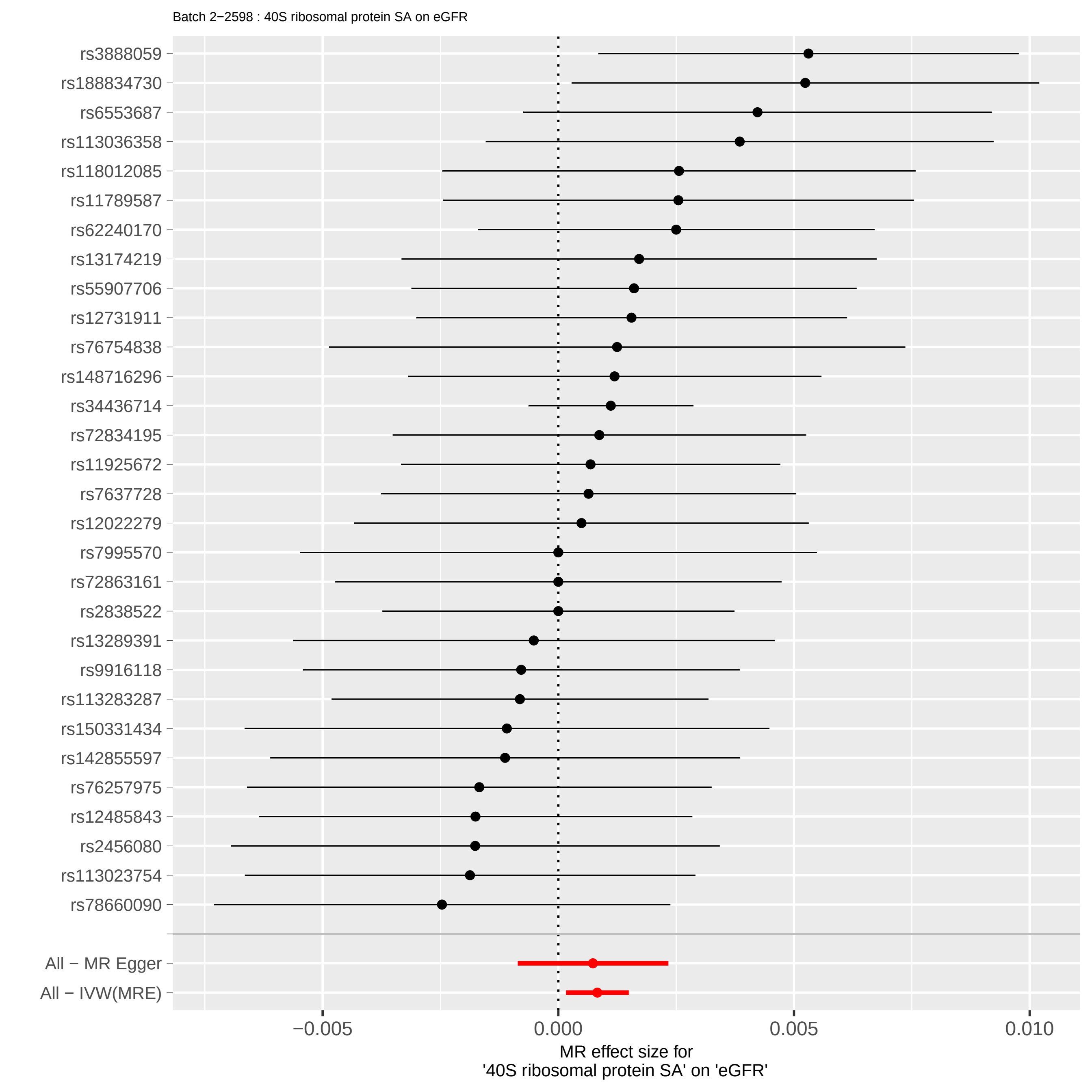

Batch 2−2598 : 40S ribosomal protein SA on eGFR
rs3888059
rs188834730
rs6553687
rs113036358
rs118012085
rs11789587
rs62240170
rs13174219
rs55907706
rs12731911
rs76754838
rs148716296
rs34436714
rs72834195
rs11925672
rs7637728
rs12022279
rs7995570
rs72863161
rs2838522
rs13289391
rs9916118
rs113283287
rs150331434
rs142855597
rs76257975
rs12485843
rs2456080
rs113023754
rs78660090
All − MR Egger
All − IVW(MRE)
−0.005
0.000
0.005
0.010
MR effect size for
'40S ribosomal protein SA' on 'eGFR'

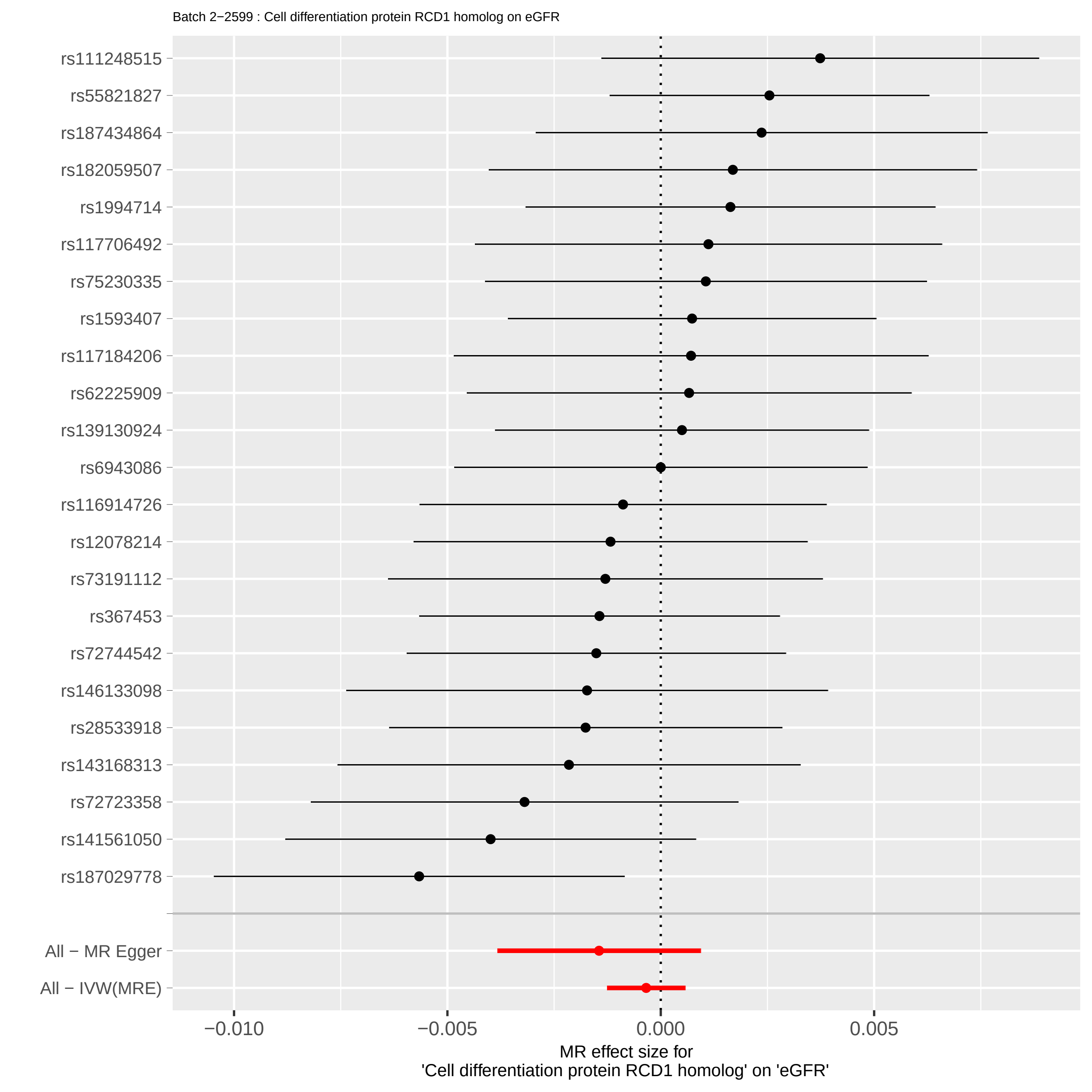

Batch 2−2599 : Cell differentiation protein RCD1 homolog on eGFR
rs111248515
rs55821827
rs187434864
rs182059507
rs1994714
rs117706492
rs75230335
rs1593407
rs117184206
rs62225909
rs139130924
rs6943086
rs116914726
rs12078214
rs73191112
rs367453
rs72744542
rs146133098
rs28533918
rs143168313
rs72723358
rs141561050
rs187029778
All − MR Egger
All − IVW(MRE)
−0.010
−0.005
0.000
0.005
MR effect size for
'Cell differentiation protein RCD1 homolog' on 'eGFR'

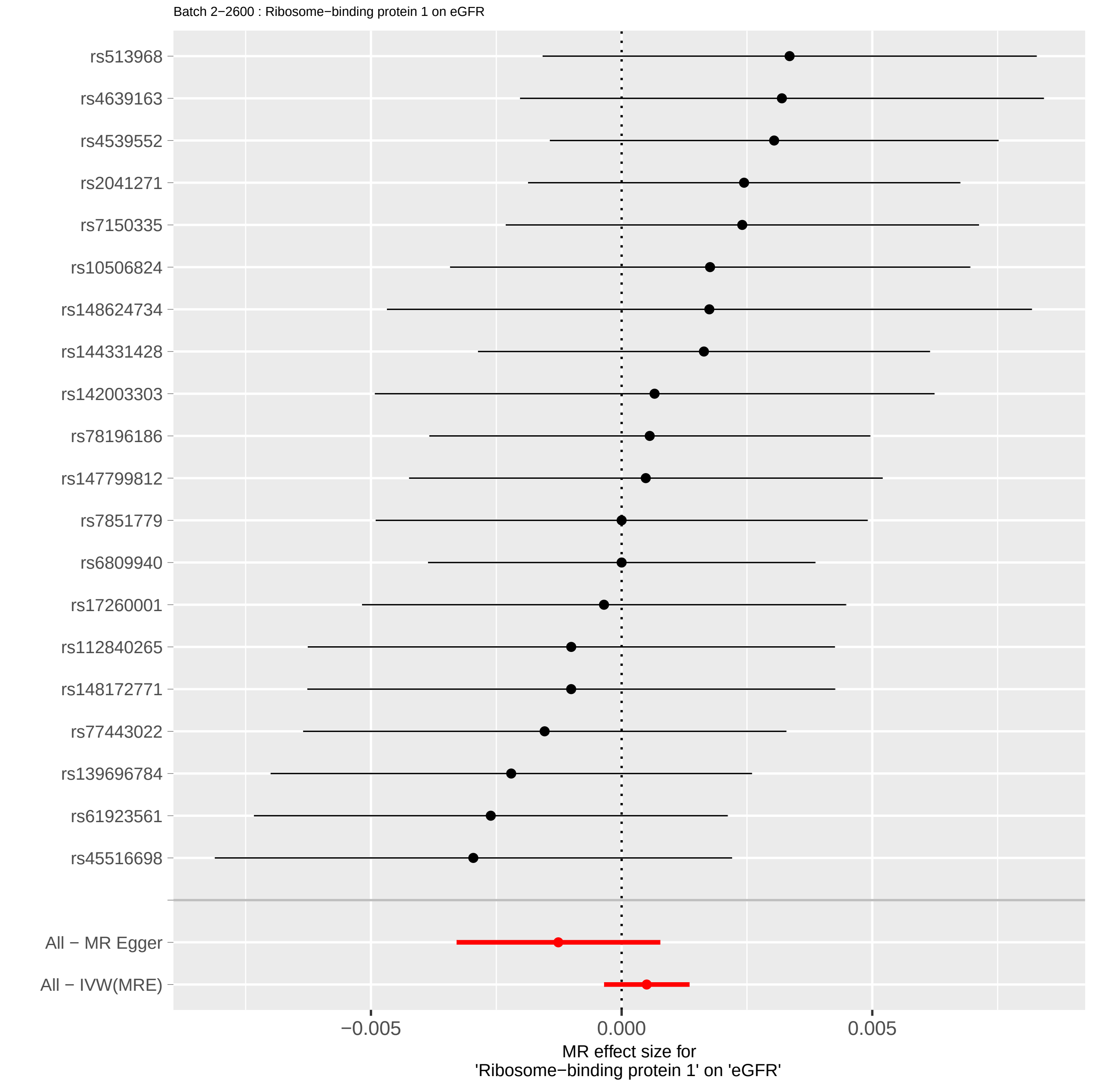
Batch 2−2600 : Ribosome−binding protein 1 on eGFR
rs513968
rs4639163
rs4539552
rs2041271
rs7150335
rs10506824
rs148624734
rs144331428
rs142003303
rs78196186
rs147799812
rs7851779
rs6809940
rs17260001
rs112840265
rs148172771
rs77443022
rs139696784
rs61923561
rs45516698
All − MR Egger
All − IVW(MRE)
−0.005
0.000
0.005
MR effect size for
'Ribosome−binding protein 1' on 'eGFR'

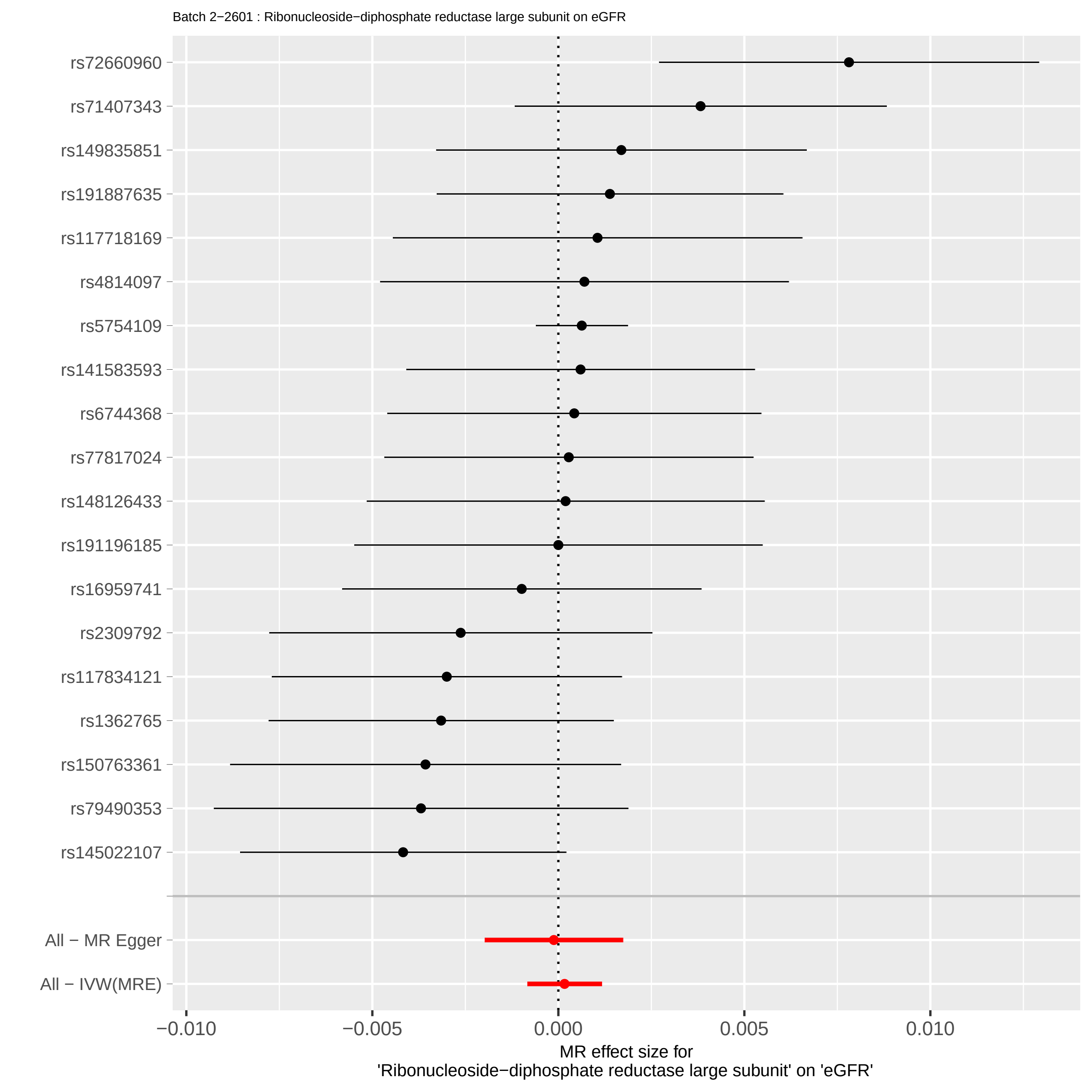

Batch 2−2601 : Ribonucleoside−diphosphate reductase large subunit on eGFR
rs72660960
rs71407343
rs149835851
rs191887635
rs117718169
rs4814097
rs5754109
rs141583593
rs6744368
rs77817024
rs148126433
rs191196185
rs16959741
rs2309792
rs117834121
rs1362765
rs150763361
rs79490353
rs145022107
All − MR Egger
All − IVW(MRE)
−0.010
−0.005
0.000
0.005
0.010
MR effect size for
'Ribonucleoside−diphosphate reductase large subunit' on 'eGFR'

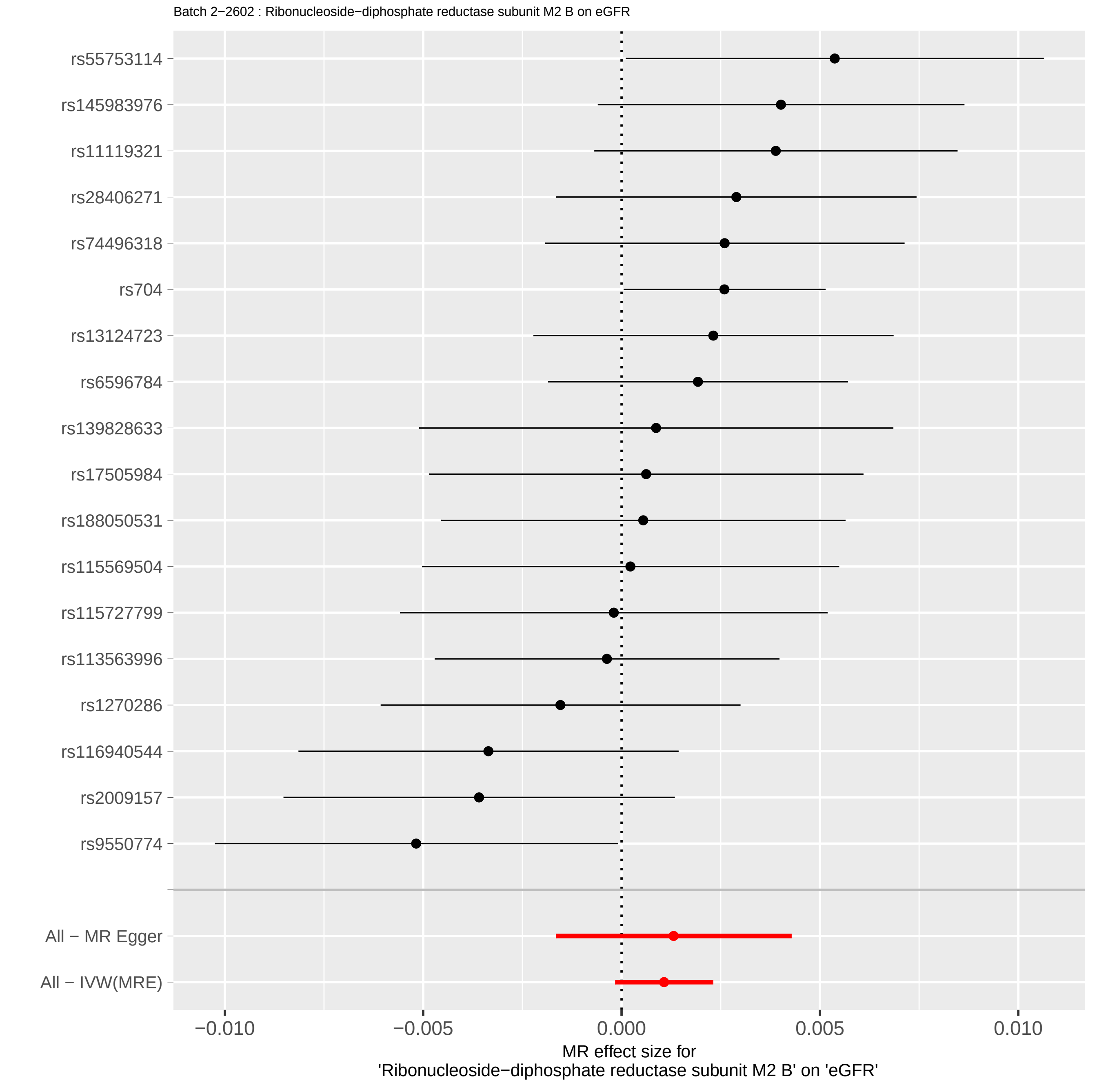

Batch 2−2602 : Ribonucleoside−diphosphate reductase subunit M2 B on eGFR
rs55753114
rs145983976
rs11119321
rs28406271
rs74496318
rs704
rs13124723
rs6596784
rs139828633
rs17505984
rs188050531
rs115569504
rs115727799
rs113563996
rs1270286
rs116940544
rs2009157
rs9550774
All − MR Egger
All − IVW(MRE)
−0.010
−0.005
0.000
0.005
0.010
MR effect size for
'Ribonucleoside−diphosphate reductase subunit M2 B' on 'eGFR'

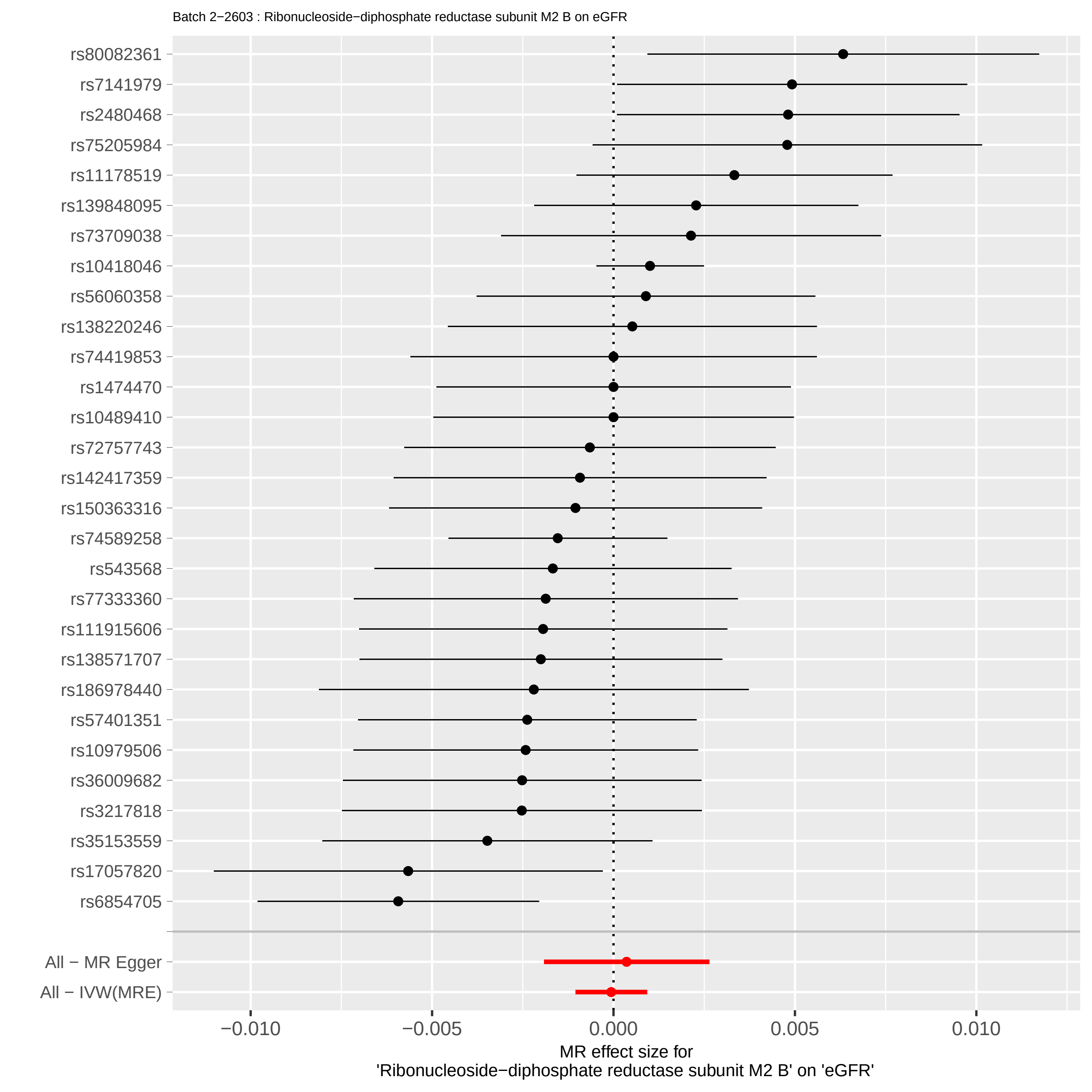

Batch 2−2603 : Ribonucleoside−diphosphate reductase subunit M2 B on eGFR
rs80082361
rs7141979
rs2480468
rs75205984
rs11178519
rs139848095
rs73709038
rs10418046
rs56060358
rs138220246
rs74419853
rs1474470
rs10489410
rs72757743
rs142417359
rs150363316
rs74589258
rs543568
rs77333360
rs111915606
rs138571707
rs186978440
rs57401351
rs10979506
rs36009682
rs3217818
rs35153559
rs17057820
rs6854705
All − MR Egger
All − IVW(MRE)
−0.010
−0.005
0.000
0.005
0.010
MR effect size for
'Ribonucleoside−diphosphate reductase subunit M2 B' on 'eGFR'

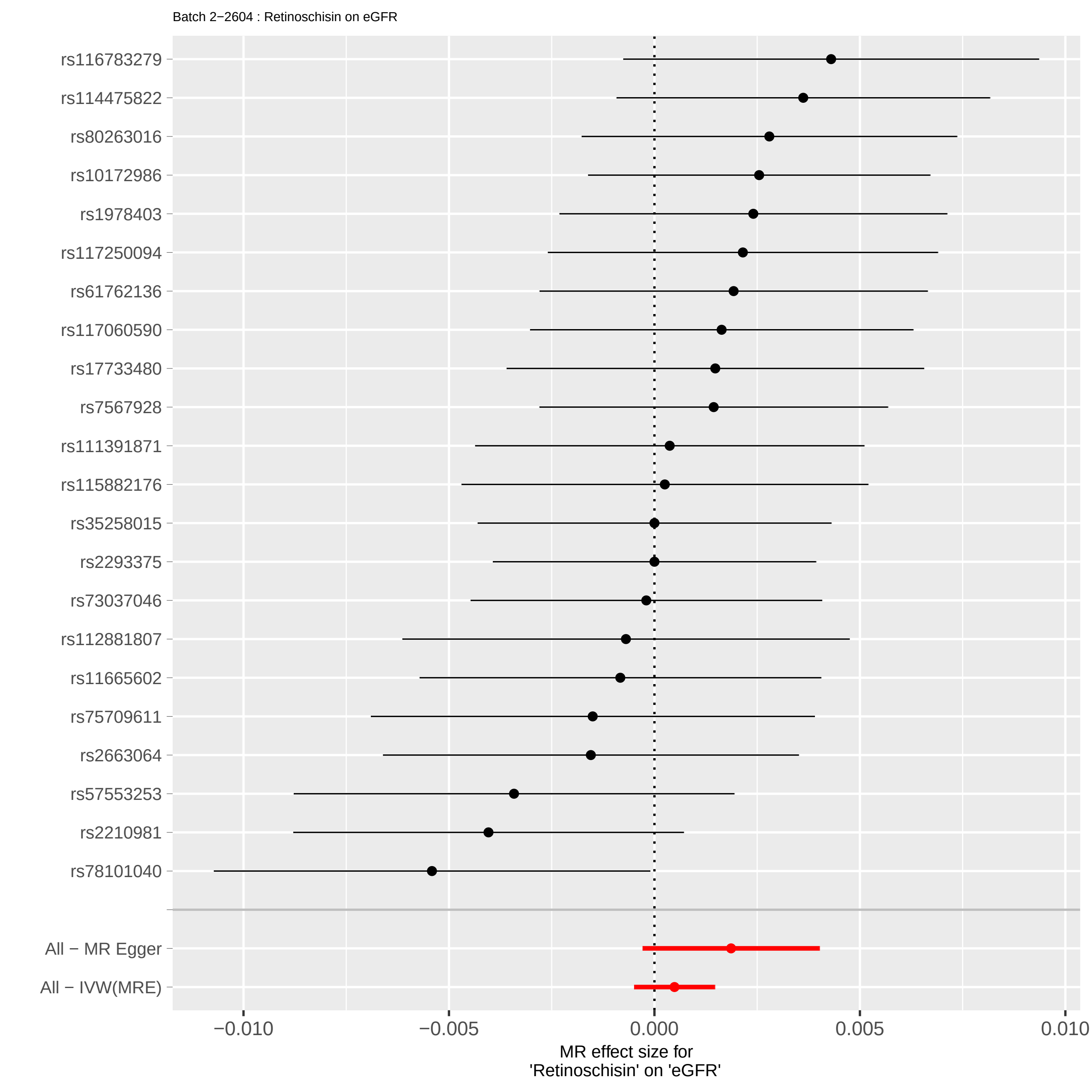
Batch 2−2604 : Retinoschisin on eGFR
rs116783279
rs114475822
rs80263016
rs10172986
rs1978403
rs117250094
rs61762136
rs117060590
rs17733480
rs7567928
rs111391871
rs115882176
rs35258015
rs2293375
rs73037046
rs112881807
rs11665602
rs75709611
rs2663064
rs57553253
rs2210981
rs78101040
All − MR Egger
All − IVW(MRE)
−0.010
−0.005
0.000
0.005
0.010
MR effect size for
'Retinoschisin' on 'eGFR'

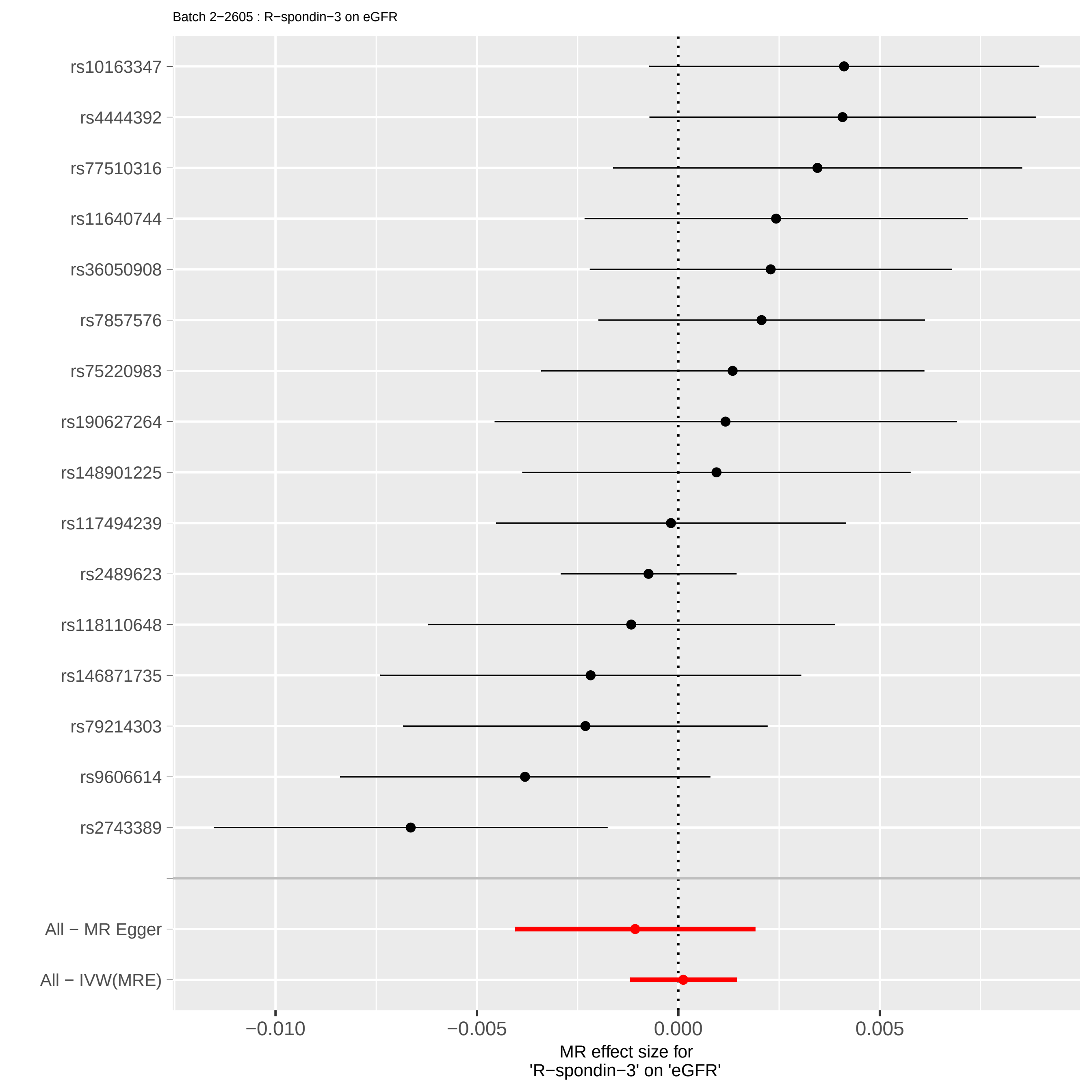
Batch 2−2605 : R−spondin−3 on eGFR
rs10163347
rs4444392
rs77510316
rs11640744
rs36050908
rs7857576
rs75220983
rs190627264
rs148901225
rs117494239
rs2489623
rs118110648
rs146871735
rs79214303
rs9606614
rs2743389
All − MR Egger
All − IVW(MRE)
−0.010
−0.005
0.000
0.005
MR effect size for
'R−spondin−3' on 'eGFR'

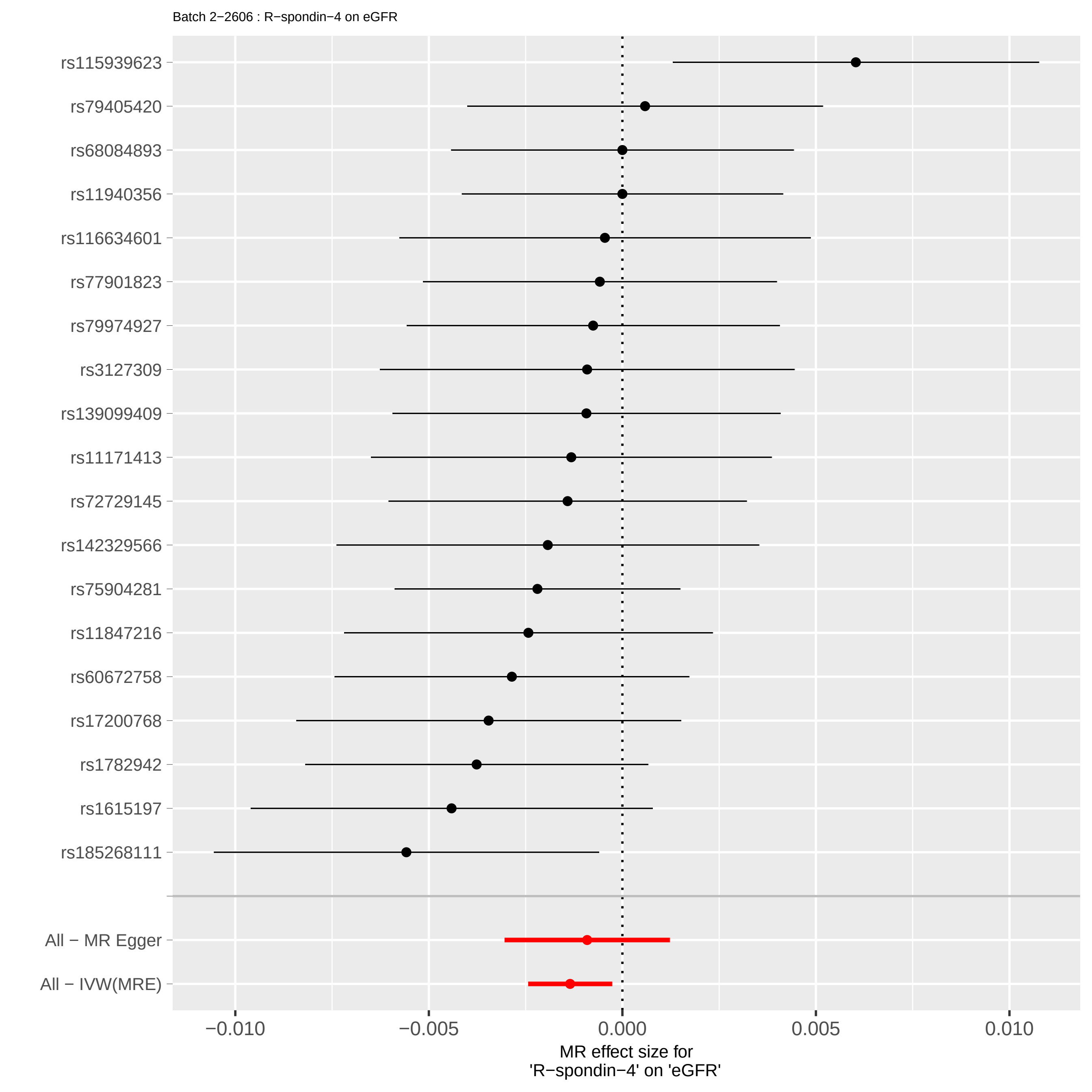

Batch 2−2606 : R−spondin−4 on eGFR
rs115939623
rs79405420
rs68084893
rs11940356
rs116634601
rs77901823
rs79974927
rs3127309
rs139099409
rs11171413
rs72729145
rs142329566
rs75904281
rs11847216
rs60672758
rs17200768
rs1782942
rs1615197
rs185268111
All − MR Egger
All − IVW(MRE)
−0.010
−0.005
0.000
0.005
0.010
MR effect size for
'R−spondin−4' on 'eGFR'

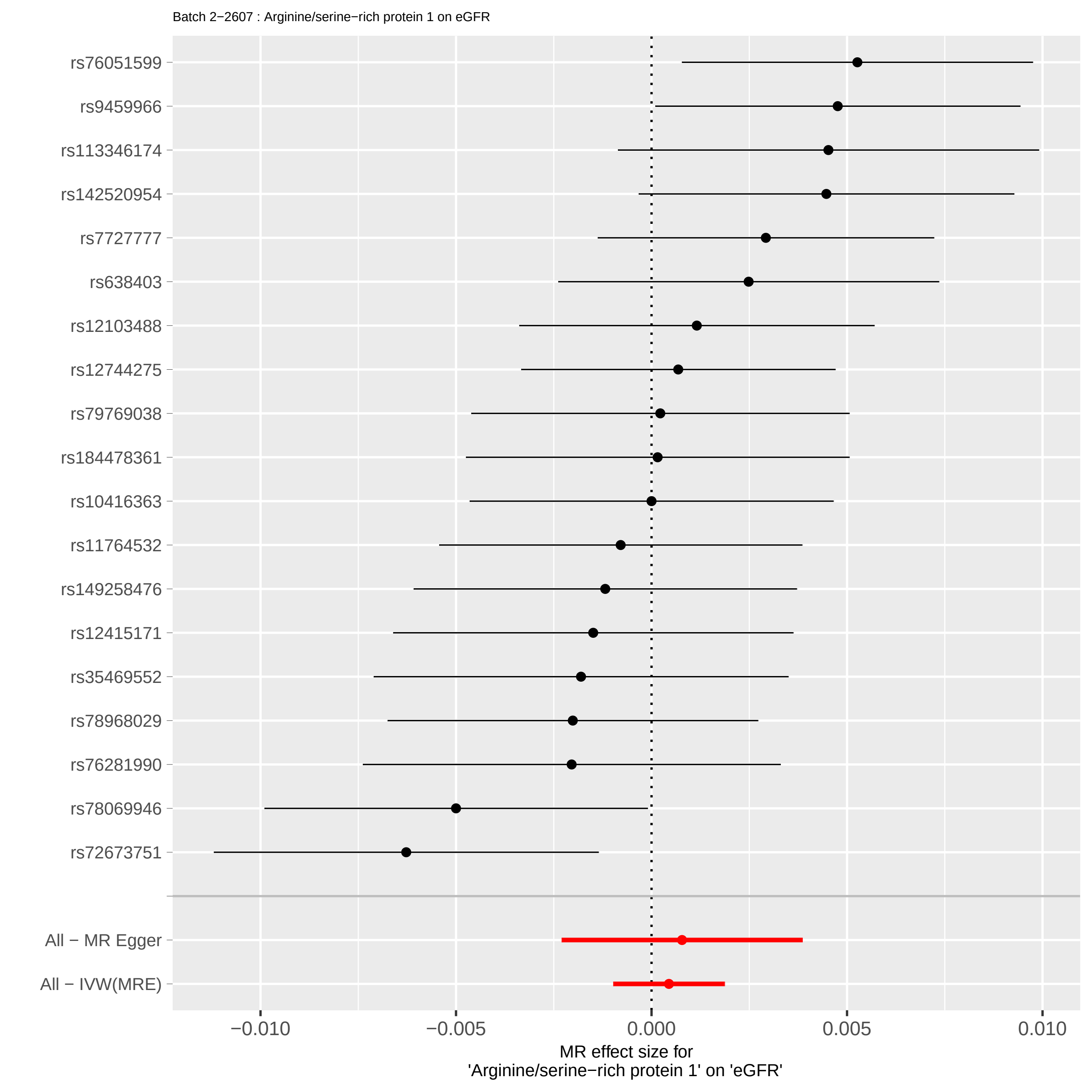

Batch 2−2607 : Arginine/serine−rich protein 1 on eGFR
rs76051599
rs9459966
rs113346174
rs142520954
rs7727777
rs638403
rs12103488
rs12744275
rs79769038
rs184478361
rs10416363
rs11764532
rs149258476
rs12415171
rs35469552
rs78968029
rs76281990
rs78069946
rs72673751
All − MR Egger
All − IVW(MRE)
−0.010
−0.005
0.000
0.005
0.010
MR effect size for
'Arginine/serine−rich protein 1' on 'eGFR'

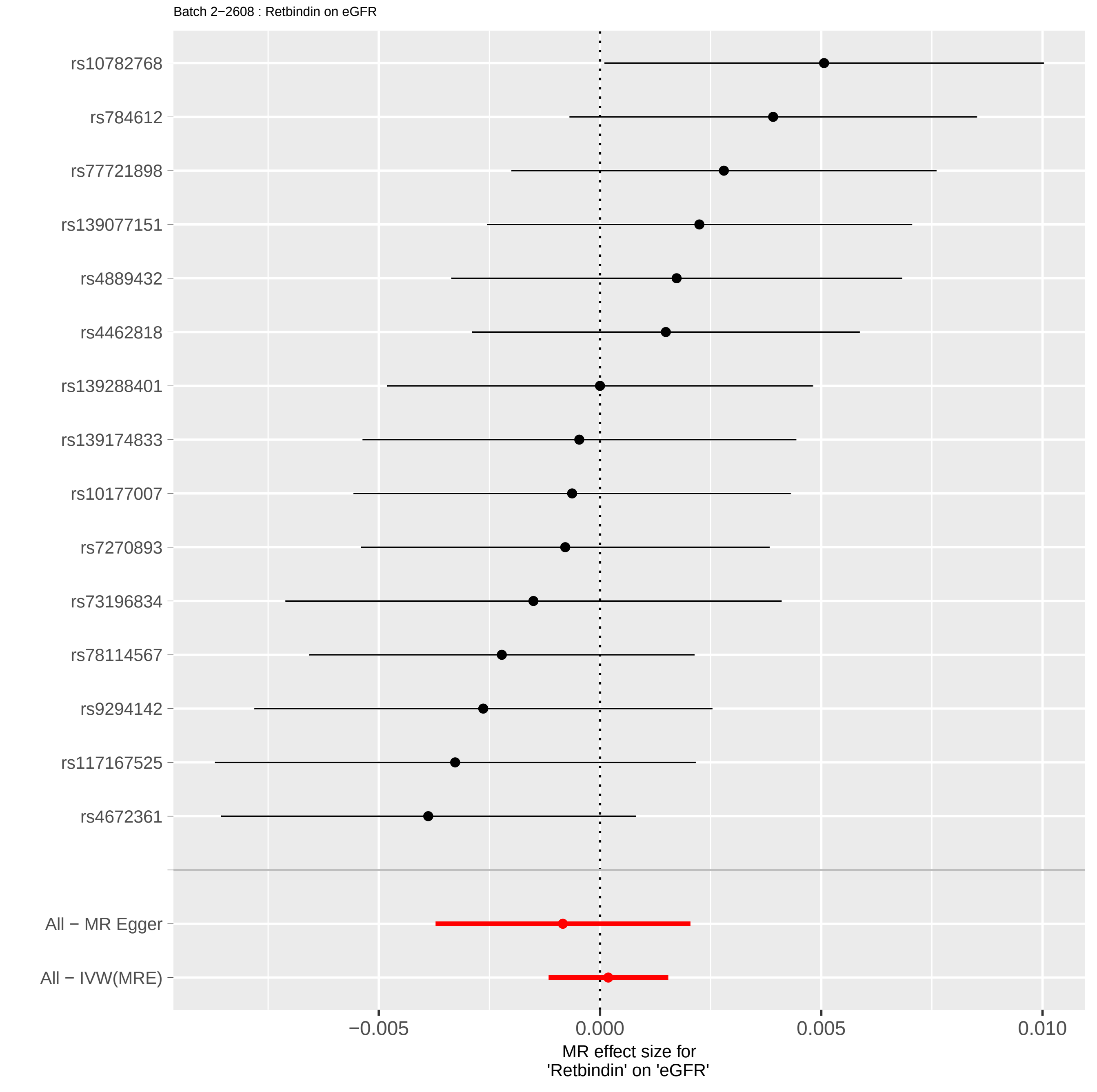

Batch 2−2608 : Retbindin on eGFR
rs10782768
rs784612
rs77721898
rs139077151
rs4889432
rs4462818
rs139288401
rs139174833
rs10177007
rs7270893
rs73196834
rs78114567
rs9294142
rs117167525
rs4672361
All − MR Egger
All − IVW(MRE)
−0.005
0.000
0.005
0.010
MR effect size for
'Retbindin' on 'eGFR'

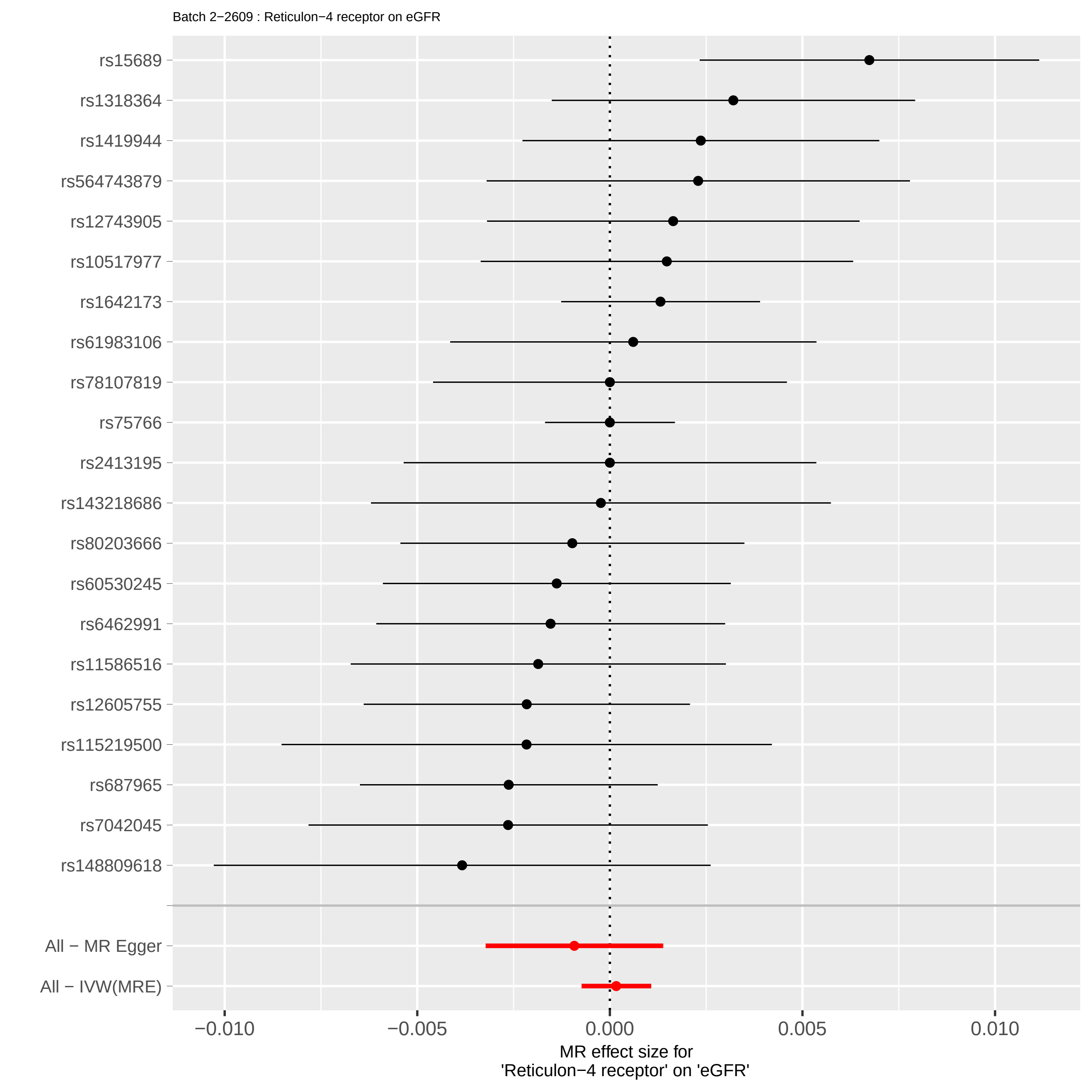
Batch 2−2609 : Reticulon−4 receptor on eGFR
rs15689
rs1318364
rs1419944
rs564743879
rs12743905
rs10517977
rs1642173
rs61983106
rs78107819
rs75766
rs2413195
rs143218686
rs80203666
rs60530245
rs6462991
rs11586516
rs12605755
rs115219500
rs687965
rs7042045
rs148809618
All − MR Egger
All − IVW(MRE)
−0.010
−0.005
0.000
0.005
0.010
MR effect size for
'Reticulon−4 receptor' on 'eGFR'

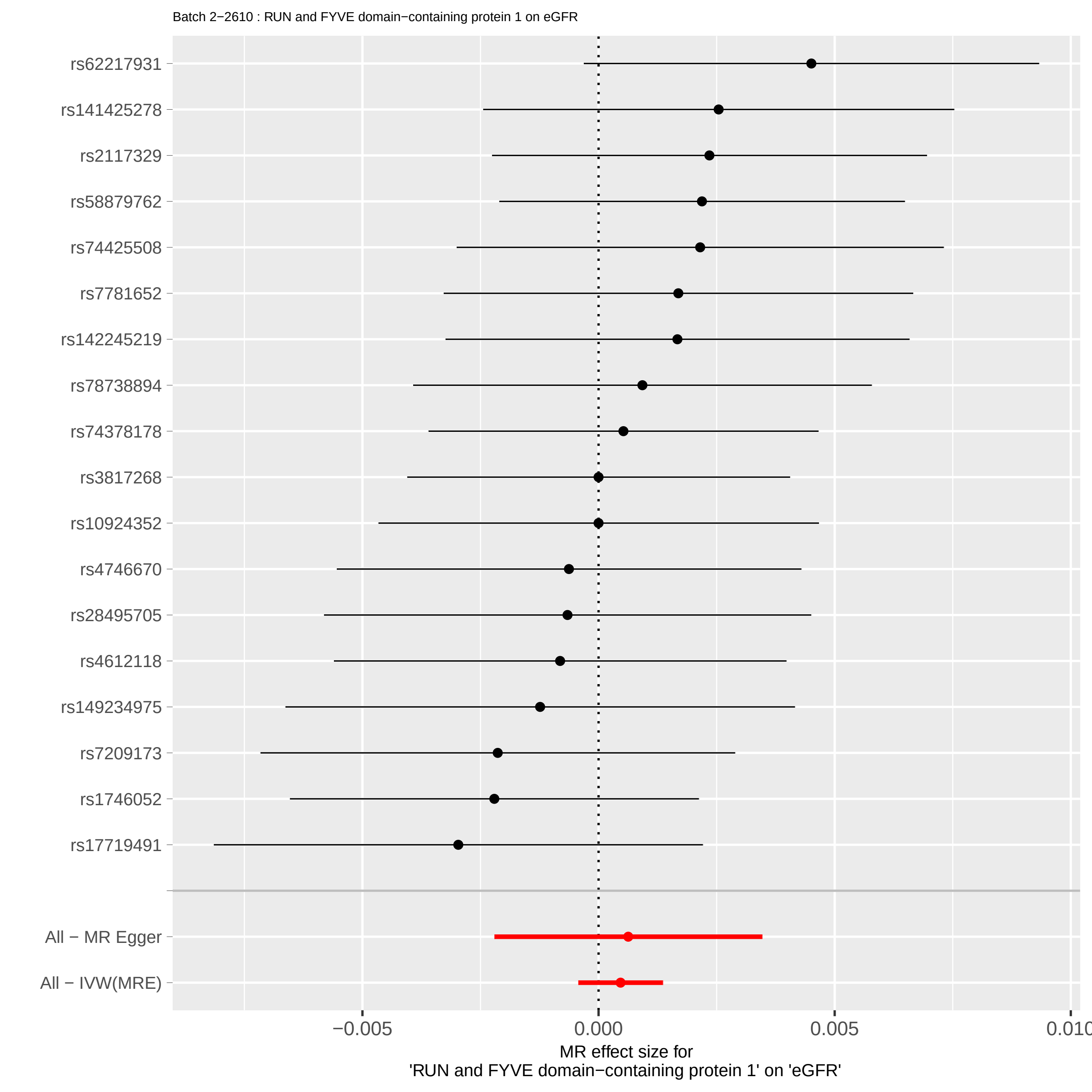
Batch 2−2610 : RUN and FYVE domain−containing protein 1 on eGFR
rs62217931
rs141425278
rs2117329
rs58879762
rs74425508
rs7781652
rs142245219
rs78738894
rs74378178
rs3817268
rs10924352
rs4746670
rs28495705
rs4612118
rs149234975
rs7209173
rs1746052
rs17719491
All − MR Egger
All − IVW(MRE)
−0.005
0.000
0.005
0.010
MR effect size for
'RUN and FYVE domain−containing protein 1' on 'eGFR'

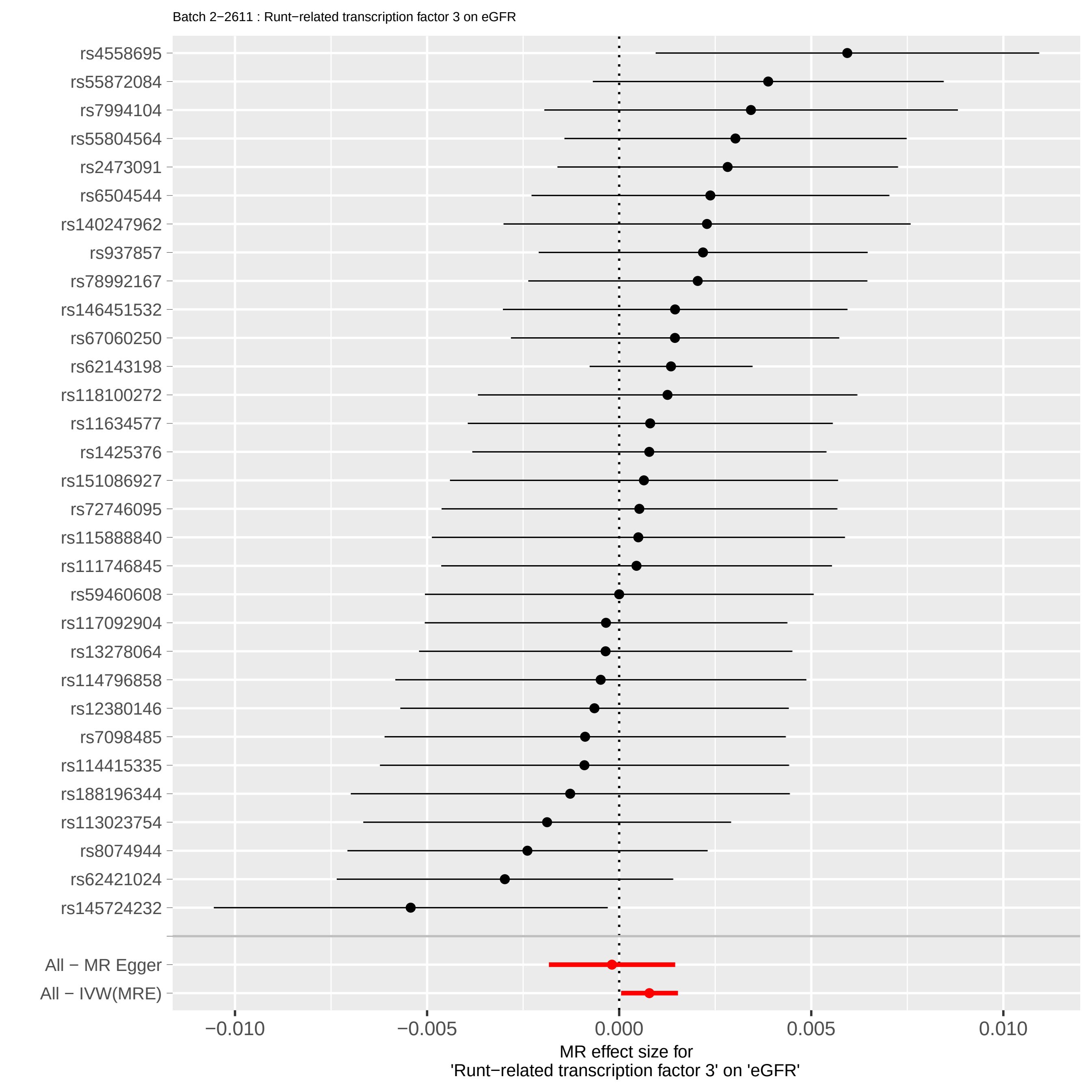
Batch 2−2611 : Runt−related transcription factor 3 on eGFR
rs4558695
rs55872084
rs7994104
rs55804564
rs2473091
rs6504544
rs140247962
rs937857
rs78992167
rs146451532
rs67060250
rs62143198
rs118100272
rs11634577
rs1425376
rs151086927
rs72746095
rs115888840
rs111746845
rs59460608
rs117092904
rs13278064
rs114796858
rs12380146
rs7098485
rs114415335
rs188196344
rs113023754
rs8074944
rs62421024
rs145724232
All − MR Egger
All − IVW(MRE)
−0.010
−0.005
0.000
0.005
0.010
MR effect size for
'Runt−related transcription factor 3' on 'eGFR'

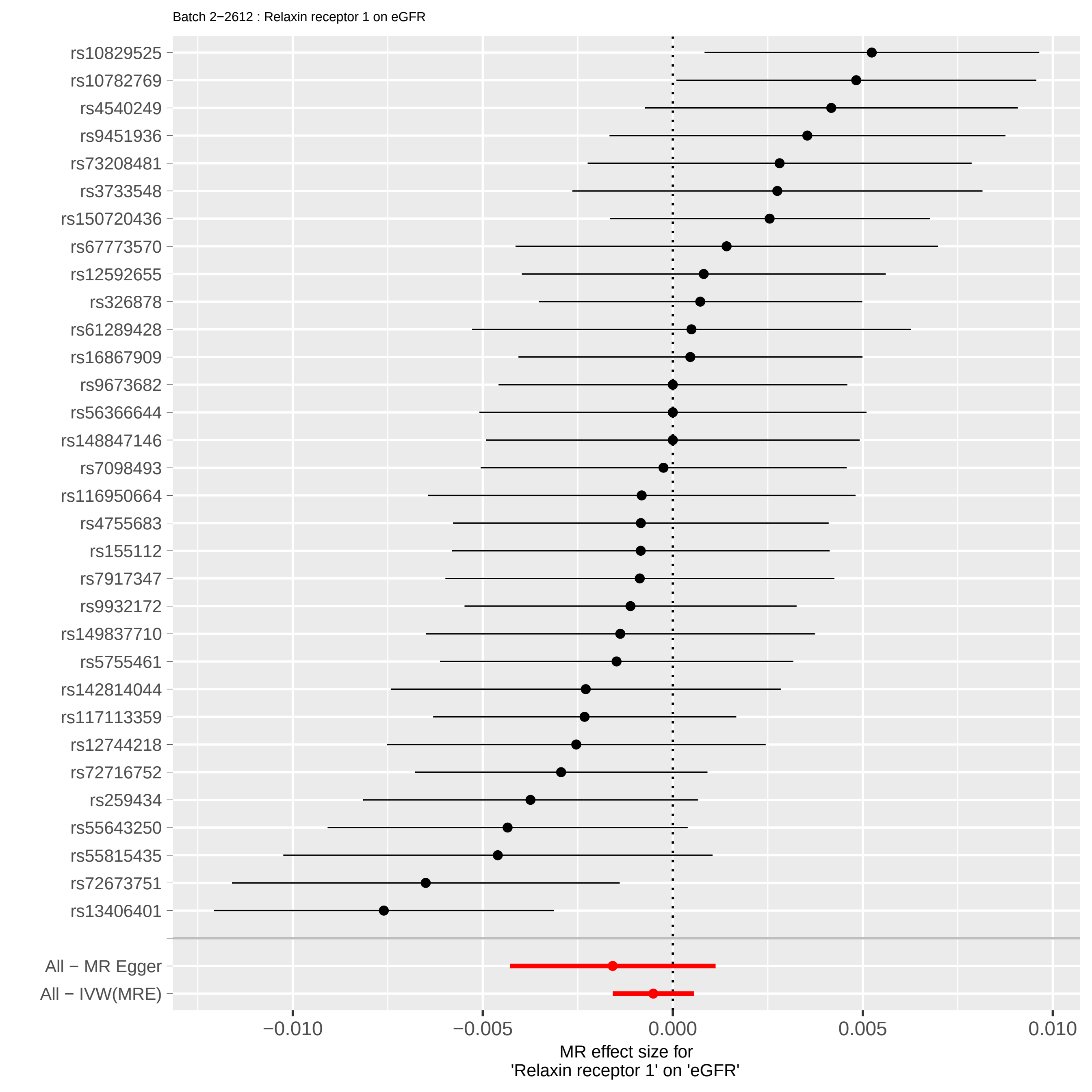

Batch 2−2612 : Relaxin receptor 1 on eGFR
rs10829525
rs10782769
rs4540249
rs9451936
rs73208481
rs3733548
rs150720436
rs67773570
rs12592655
rs326878
rs61289428
rs16867909
rs9673682
rs56366644
rs148847146
rs7098493
rs116950664
rs4755683
rs155112
rs7917347
rs9932172
rs149837710
rs5755461
rs142814044
rs117113359
rs12744218
rs72716752
rs259434
rs55643250
rs55815435
rs72673751
rs13406401
All − MR Egger
All − IVW(MRE)
−0.010
−0.005
0.000
0.005
0.010
MR effect size for
'Relaxin receptor 1' on 'eGFR'

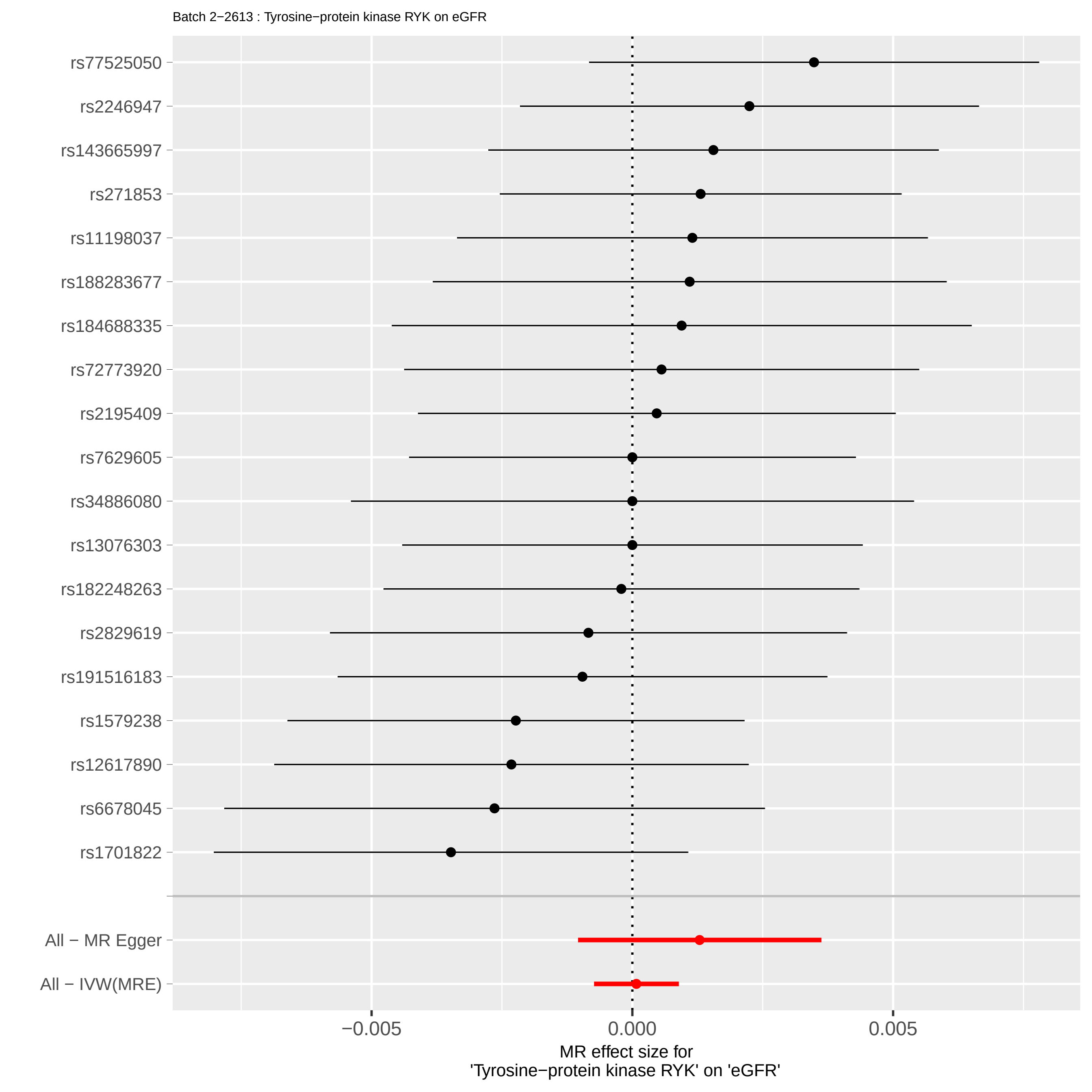

Batch 2−2613 : Tyrosine−protein kinase RYK on eGFR
rs77525050
rs2246947
rs143665997
rs271853
rs11198037
rs188283677
rs184688335
rs72773920
rs2195409
rs7629605
rs34886080
rs13076303
rs182248263
rs2829619
rs191516183
rs1579238
rs12617890
rs6678045
rs1701822
All − MR Egger
All − IVW(MRE)
−0.005
0.000
0.005
MR effect size for
'Tyrosine−protein kinase RYK' on 'eGFR'

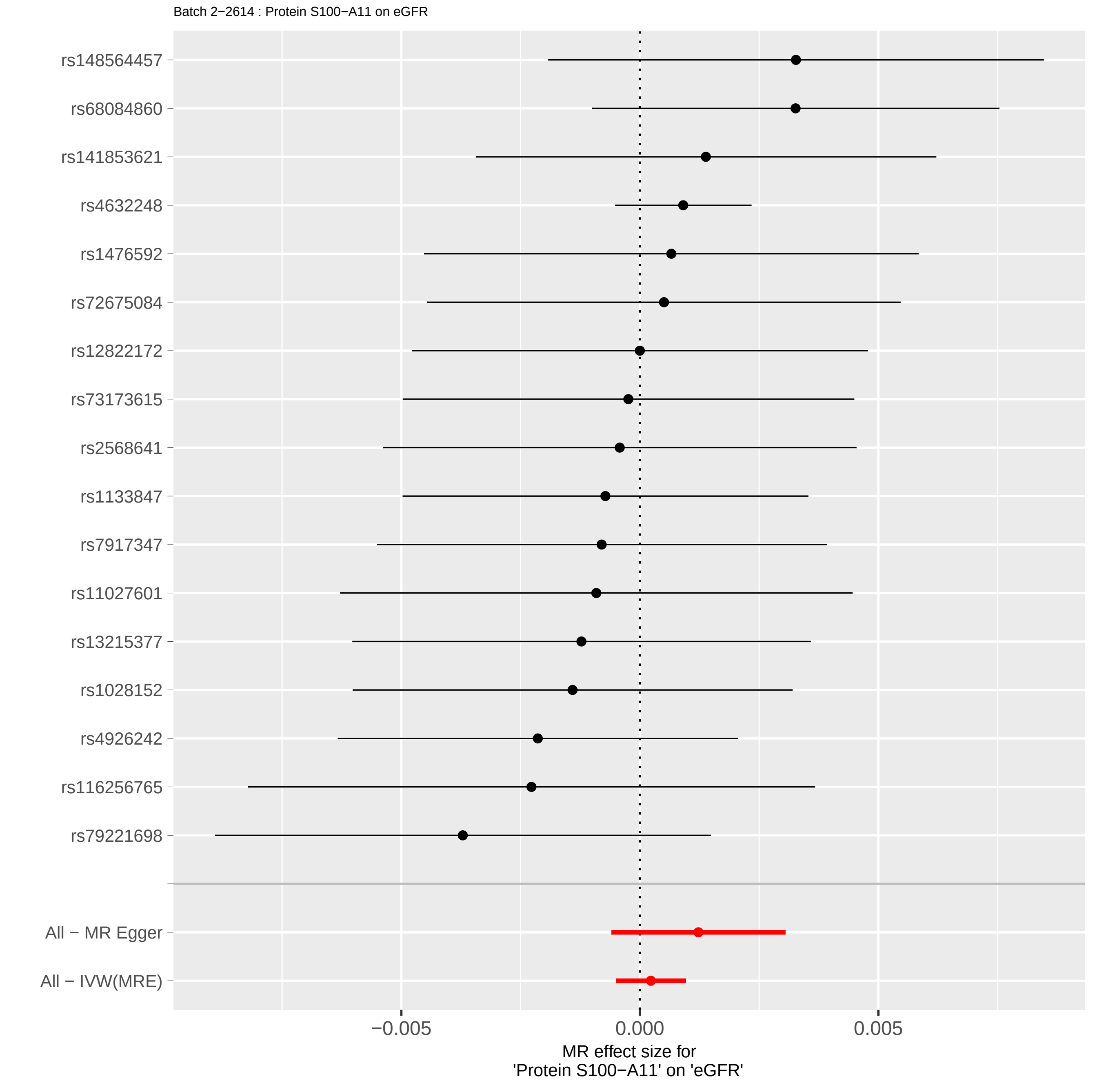
Batch 2−2614 : Protein S100−A11 on eGFR
rs148564457
rs68084860
rs141853621
rs4632248
rs1476592
rs72675084
rs12822172
rs73173615
rs2568641
rs1133847
rs7917347
rs11027601
rs13215377
rs1028152
rs4926242
rs116256765
rs79221698
All − MR Egger
All − IVW(MRE)
−0.005
0.000
0.005
MR effect size for
'Protein S100−A11' on 'eGFR'

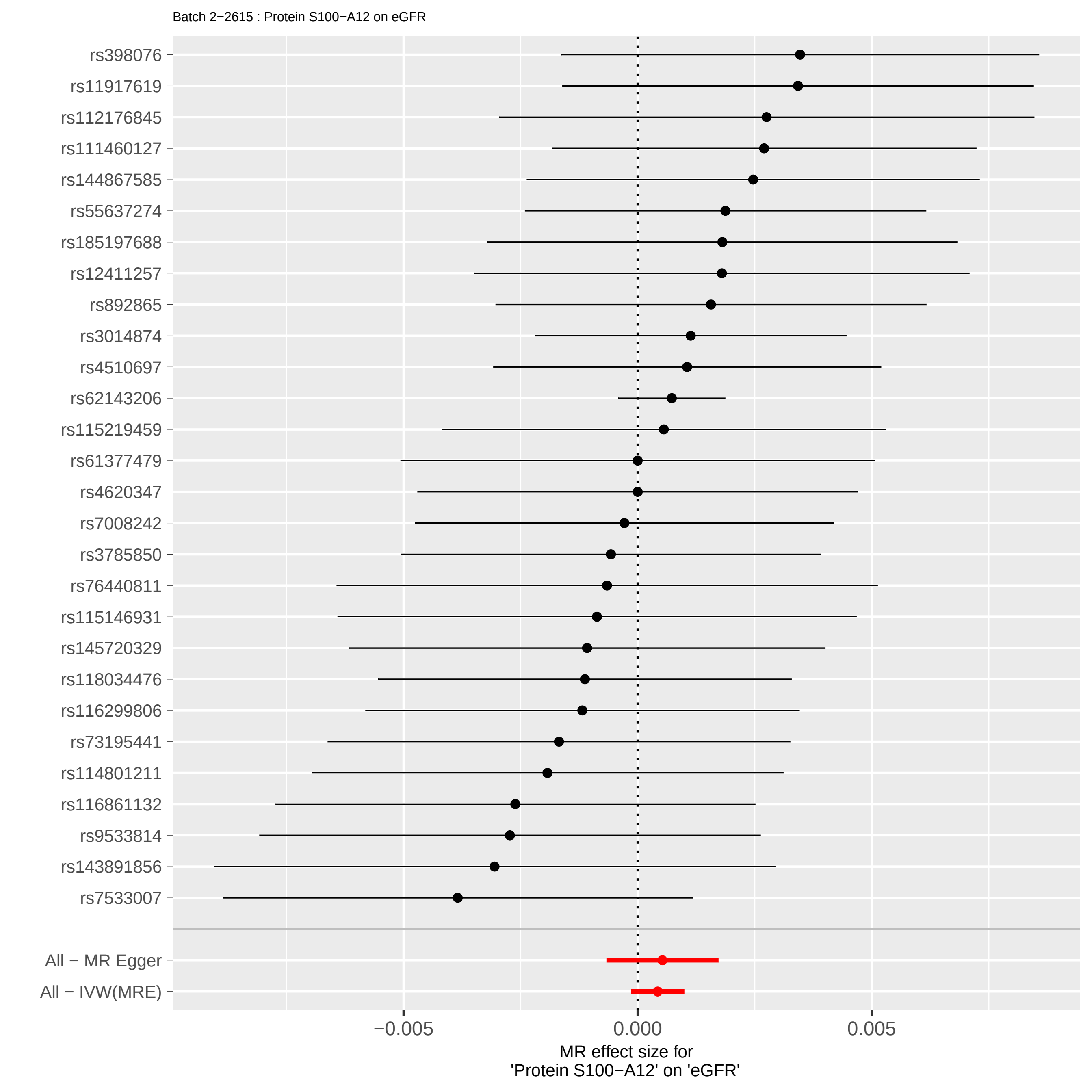

Batch 2−2615 : Protein S100−A12 on eGFR
rs398076
rs11917619
rs112176845
rs111460127
rs144867585
rs55637274
rs185197688
rs12411257
rs892865
rs3014874
rs4510697
rs62143206
rs115219459
rs61377479
rs4620347
rs7008242
rs3785850
rs76440811
rs115146931
rs145720329
rs118034476
rs116299806
rs73195441
rs114801211
rs116861132
rs9533814
rs143891856
rs7533007
All − MR Egger
All − IVW(MRE)
−0.005
0.000
0.005
MR effect size for
'Protein S100−A12' on 'eGFR'

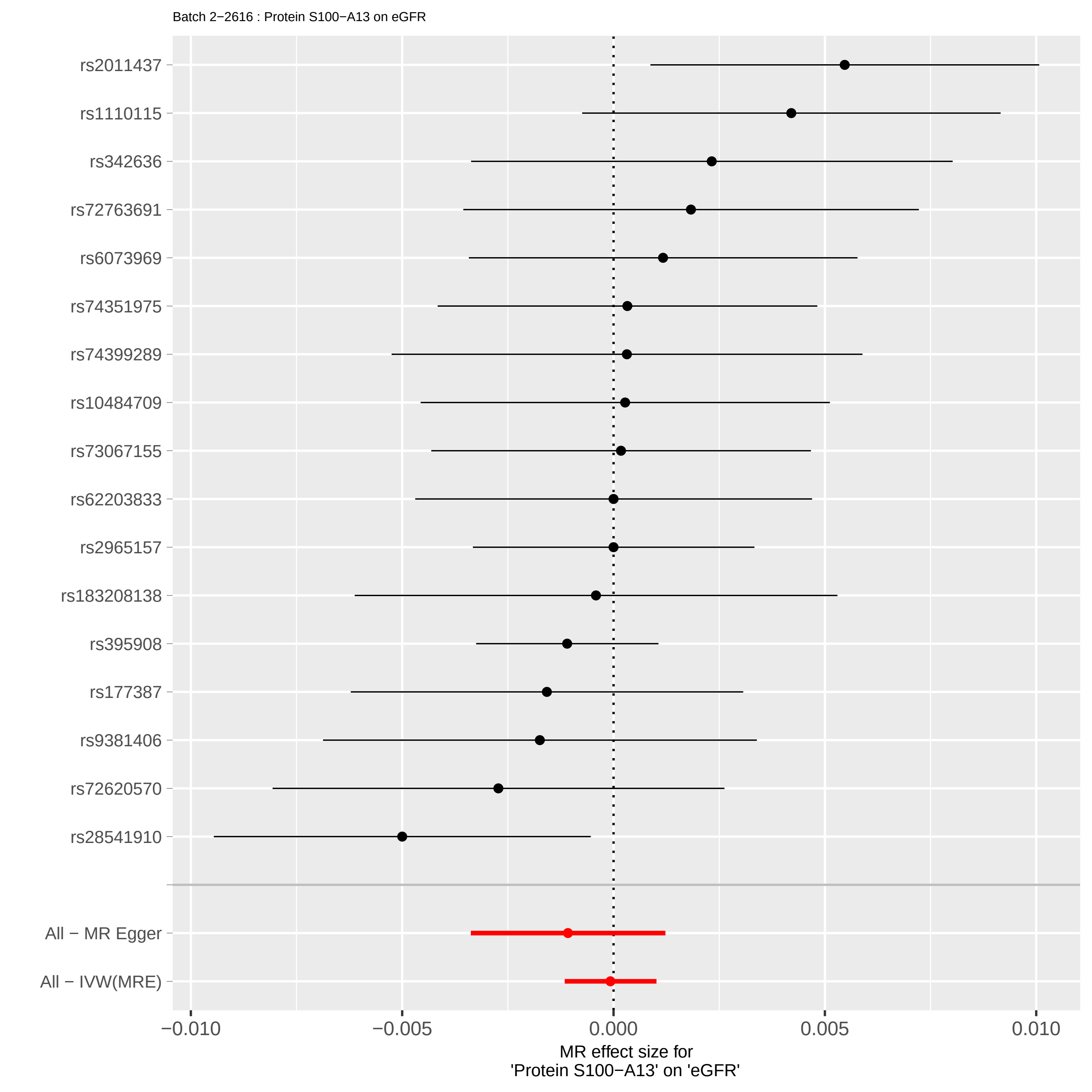

Batch 2−2616 : Protein S100−A13 on eGFR
rs2011437
rs1110115
rs342636
rs72763691
rs6073969
rs74351975
rs74399289
rs10484709
rs73067155
rs62203833
rs2965157
rs183208138
rs395908
rs177387
rs9381406
rs72620570
rs28541910
All − MR Egger
All − IVW(MRE)
−0.010
−0.005
0.000
0.005
0.010
MR effect size for
'Protein S100−A13' on 'eGFR'

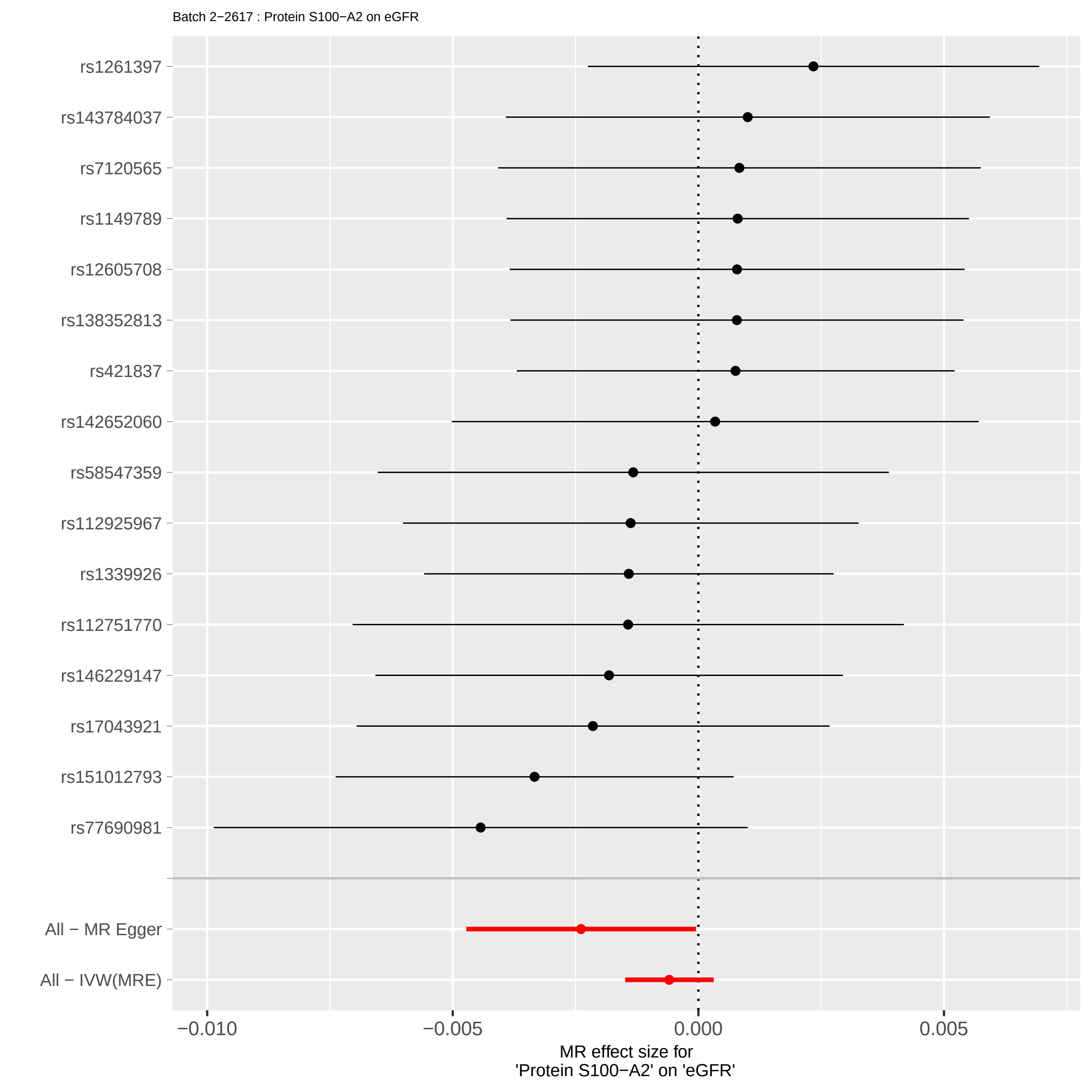

Batch 2−2617 : Protein S100−A2 on eGFR
rs1261397
rs143784037
rs7120565
rs1149789
rs12605708
rs138352813
rs421837
rs142652060
rs58547359
rs112925967
rs1339926
rs112751770
rs146229147
rs17043921
rs151012793
rs77690981
All − MR Egger
All − IVW(MRE)
−0.010
−0.005
0.000
0.005
MR effect size for
'Protein S100−A2' on 'eGFR'

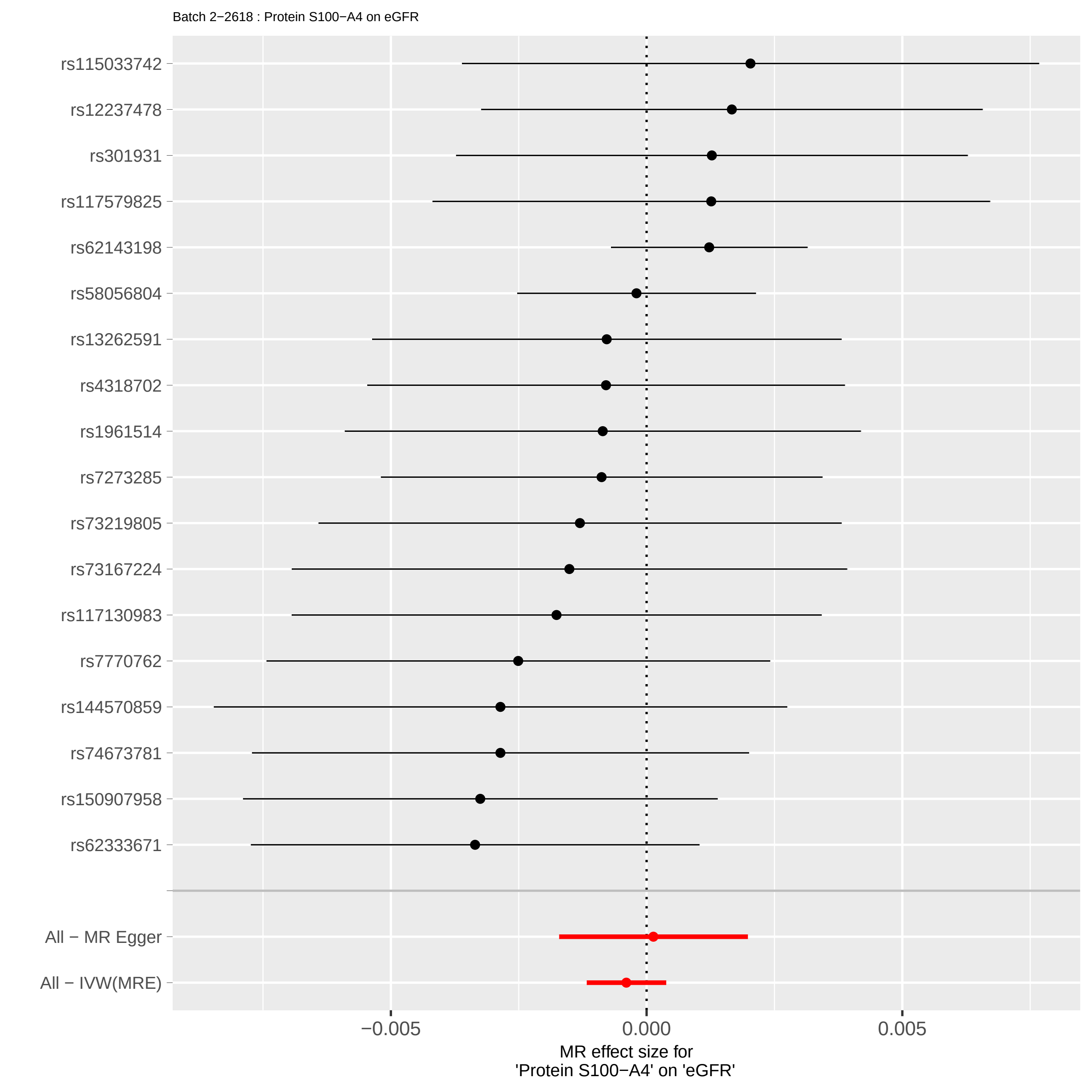
Batch 2−2618 : Protein S100−A4 on eGFR
rs115033742
rs12237478
rs301931
rs117579825
rs62143198
rs58056804
rs13262591
rs4318702
rs1961514
rs7273285
rs73219805
rs73167224
rs117130983
rs7770762
rs144570859
rs74673781
rs150907958
rs62333671
All − MR Egger
All − IVW(MRE)
−0.005
0.000
0.005
MR effect size for
'Protein S100−A4' on 'eGFR'

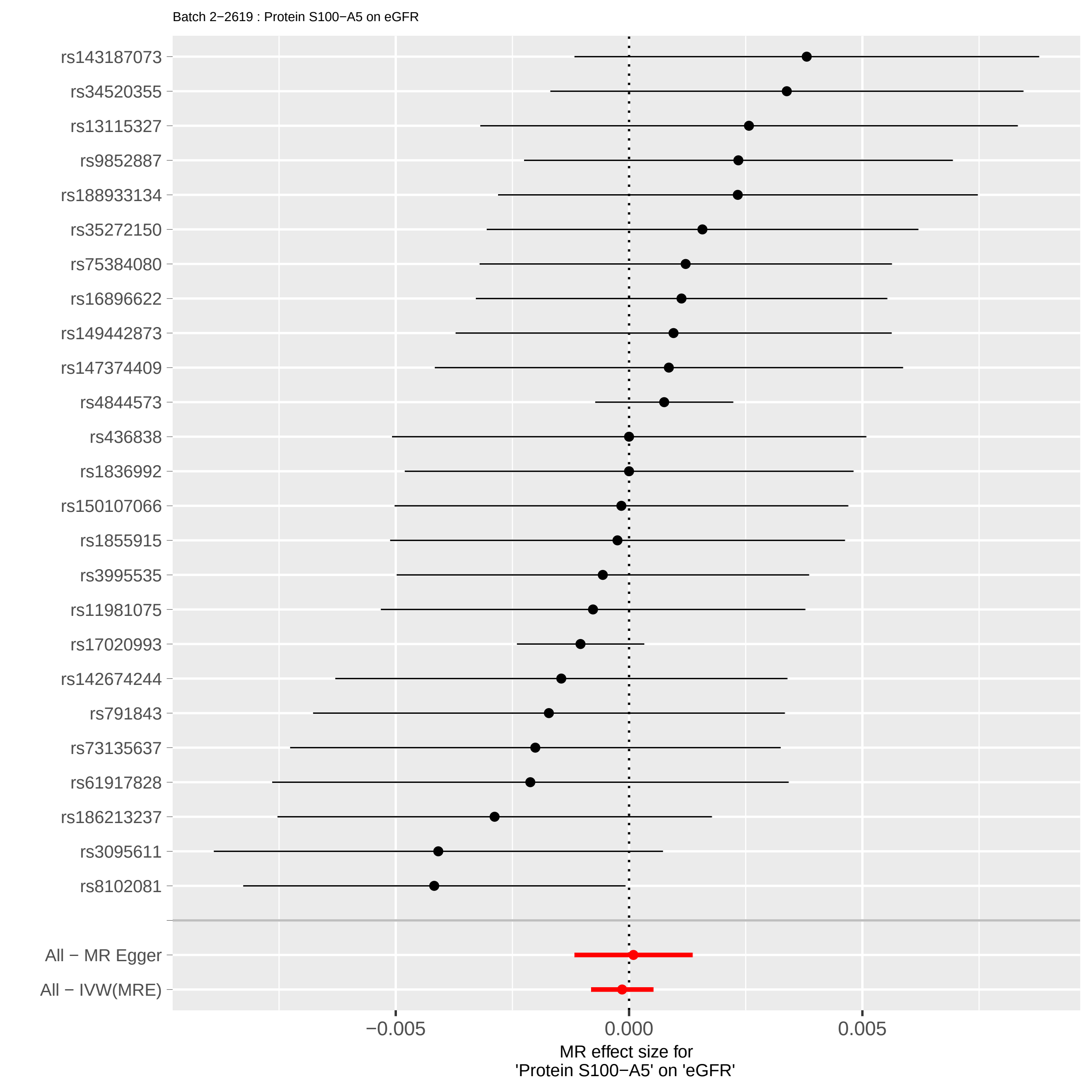
Batch 2−2619 : Protein S100−A5 on eGFR
rs143187073
rs34520355
rs13115327
rs9852887
rs188933134
rs35272150
rs75384080
rs16896622
rs149442873
rs147374409
rs4844573
rs436838
rs1836992
rs150107066
rs1855915
rs3995535
rs11981075
rs17020993
rs142674244
rs791843
rs73135637
rs61917828
rs186213237
rs3095611
rs8102081
All − MR Egger
All − IVW(MRE)
−0.005
0.000
0.005
MR effect size for
'Protein S100−A5' on 'eGFR'

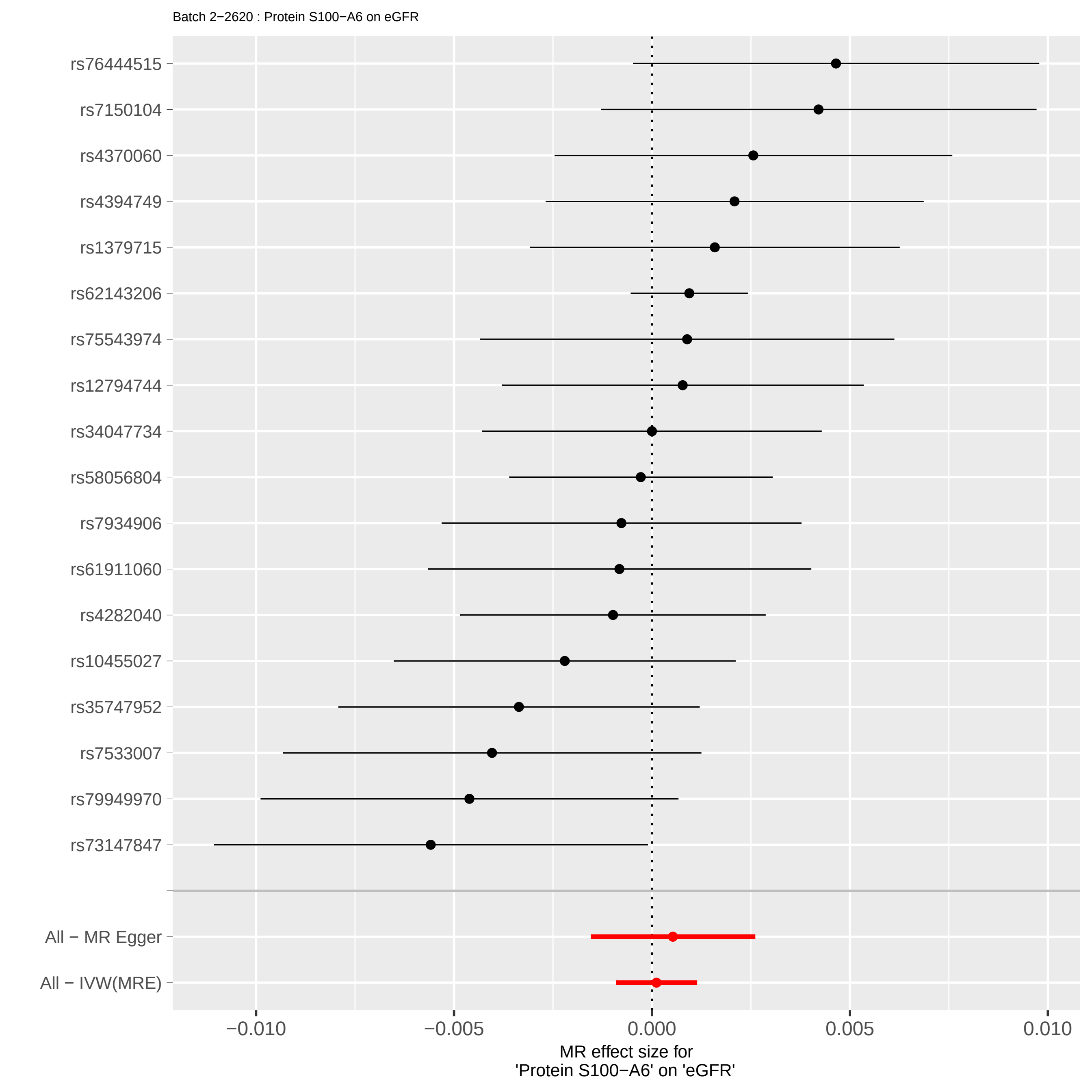

Batch 2−2620 : Protein S100−A6 on eGFR
rs76444515
rs7150104
rs4370060
rs4394749
rs1379715
rs62143206
rs75543974
rs12794744
rs34047734
rs58056804
rs7934906
rs61911060
rs4282040
rs10455027
rs35747952
rs7533007
rs79949970
rs73147847
All − MR Egger
All − IVW(MRE)
−0.010
−0.005
0.000
0.005
0.010
MR effect size for
'Protein S100−A6' on 'eGFR'

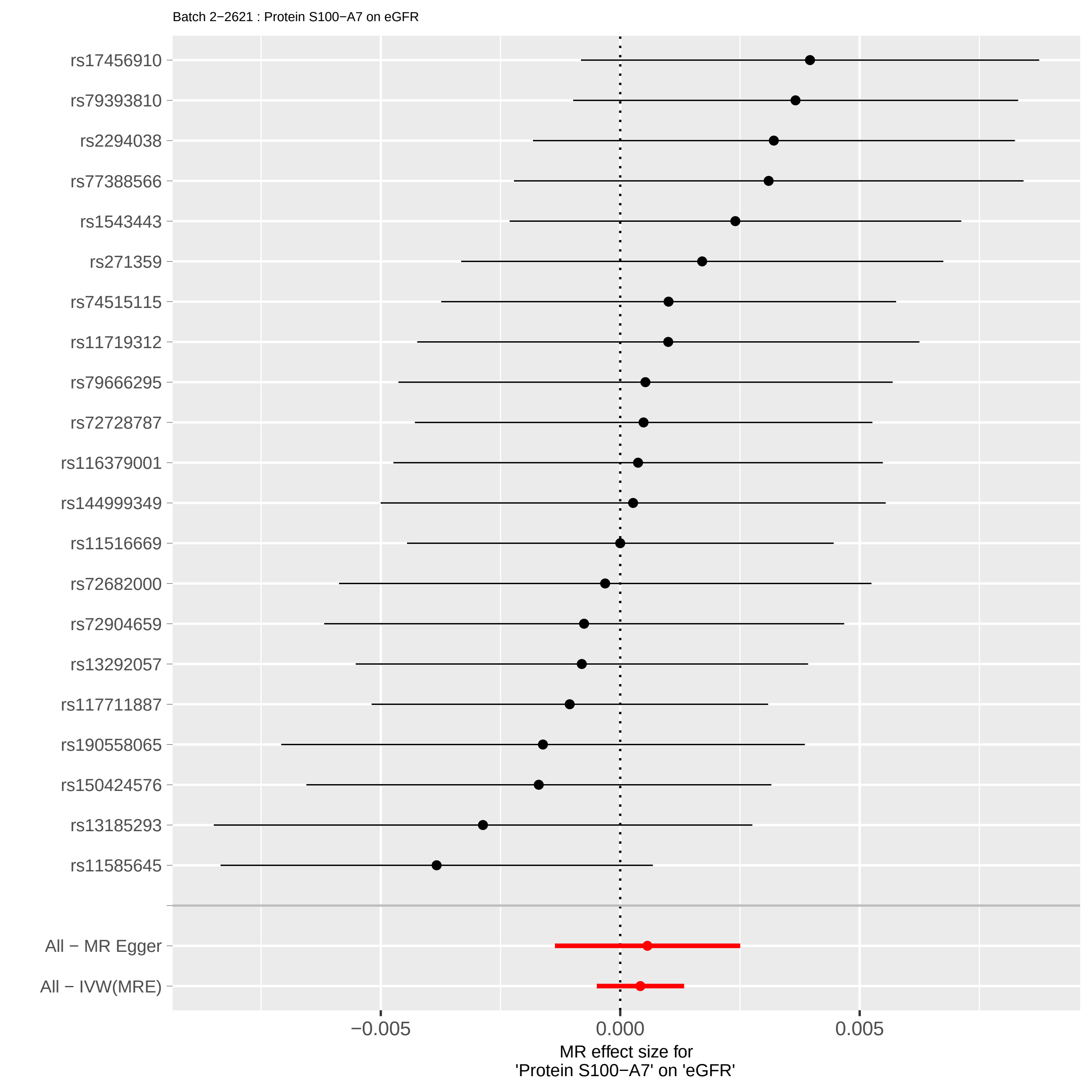
Batch 2−2621 : Protein S100−A7 on eGFR
rs17456910
rs79393810
rs2294038
rs77388566
rs1543443
rs271359
rs74515115
rs11719312
rs79666295
rs72728787
rs116379001
rs144999349
rs11516669
rs72682000
rs72904659
rs13292057
rs117711887
rs190558065
rs150424576
rs13185293
rs11585645
All − MR Egger
All − IVW(MRE)
−0.005
[truncated: 371,316 more chars]
