# Supplementary material for: A new perspective on selenium’s impact on renal function: European population-based analysis of plasma proteome-mediated Mendelian randomization study
Source: Front Endocrinol (Lausanne). 2024 Sep 12;15:1410463. doi: 10.3389/fendo.2024.1410463 (PMC11424436; doi:10.3389/fendo.2024.1410463)
Supplement: Supplementary Figure 2 — Scatter plots of all the mendelian randomization analyses in this study. [file DataSheet2.pdf]

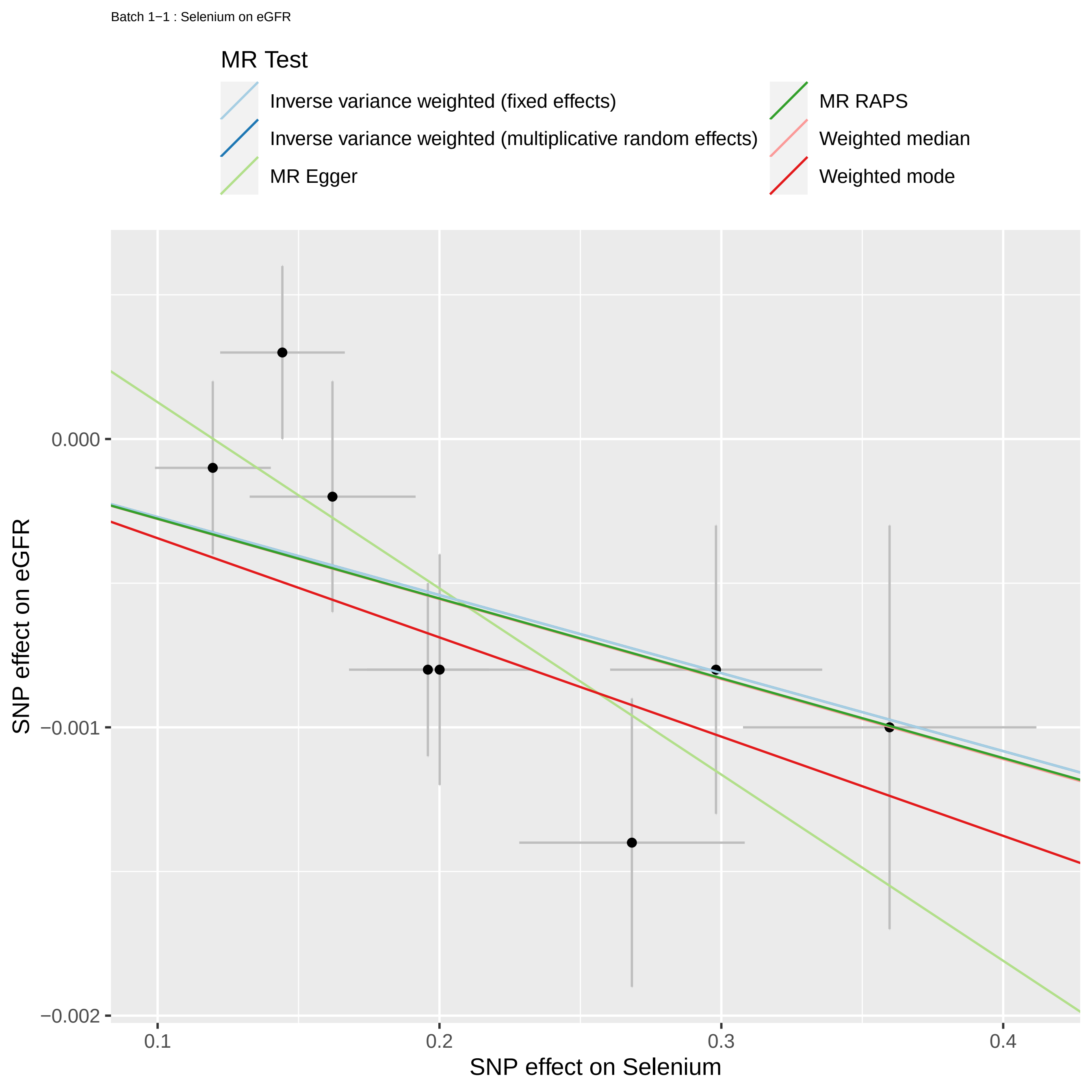

Batch 1−1 : Selenium on eGFR
MR Test
Inverse variance weighted (fixed effects)
Inverse variance weighted (multiplicative random effects)
MR Egger
MR RAPS
Weighted median
Weighted mode
0.000
−0.001
−0.002
0.1
0.2
0.3
0.4
SNP effect on eGFR
SNP effect on Selenium

Batch 3−1 : Selenium on APOBEC1 complementation factor

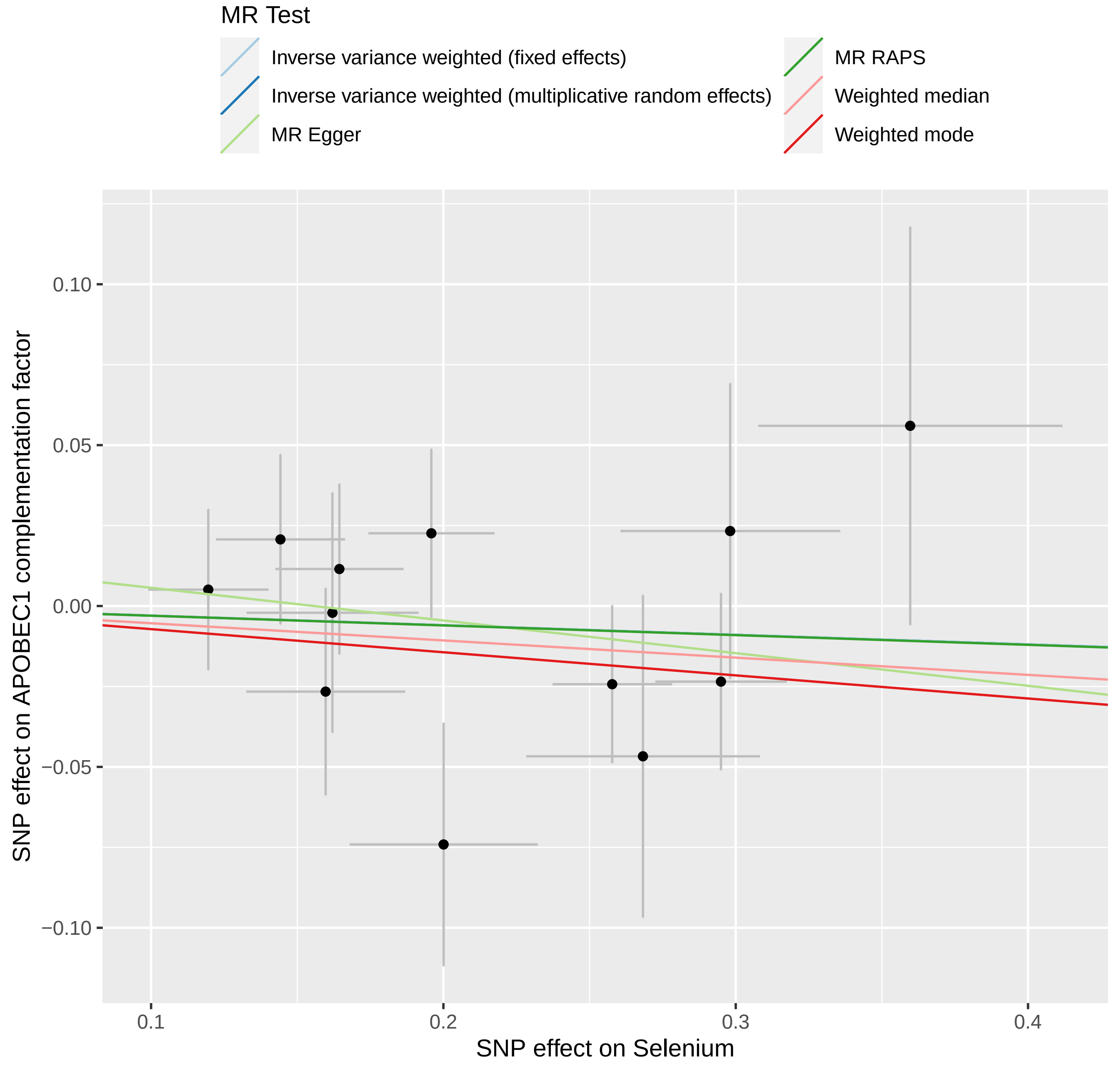

Batch 3−2 : Selenium on Lactosylceramide 4−alpha−galactosyltransferase

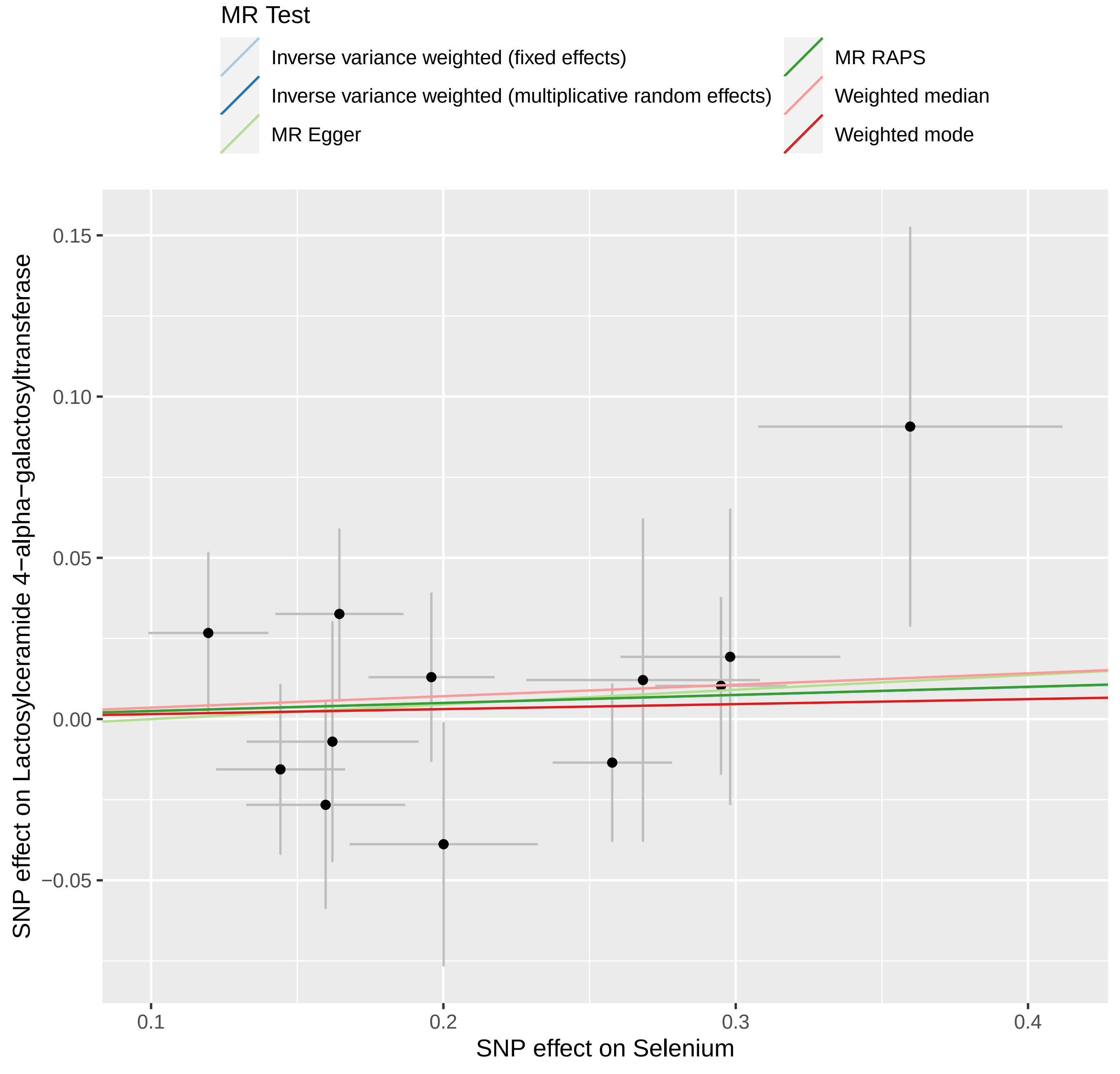

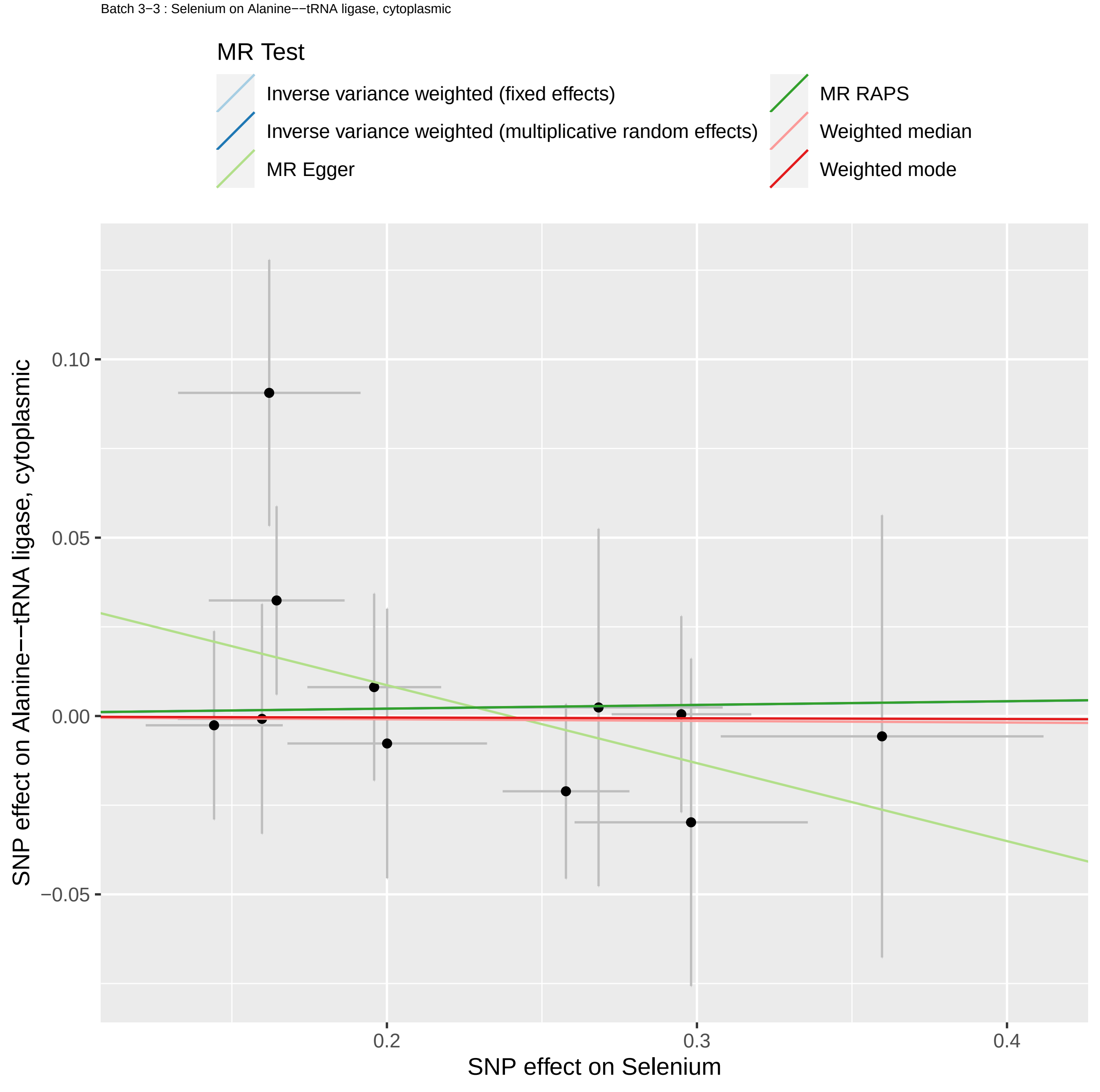

Batch 3−3 : Selenium on Alanine−−tRNA ligase, cytoplasmic
MR Test
Inverse variance weighted (fixed effects)
Inverse variance weighted (multiplicative random effects)
MR Egger
MR RAPS
Weighted median
Weighted mode
SNP effect on Alanine−−tRNA ligase, cytoplasmic
0.10
0.05
0.00
−0.05
0.2
0.3
0.4
SNP effect on Selenium

Batch 3−4 : Selenium on Multidrug resistance−associated protein 6

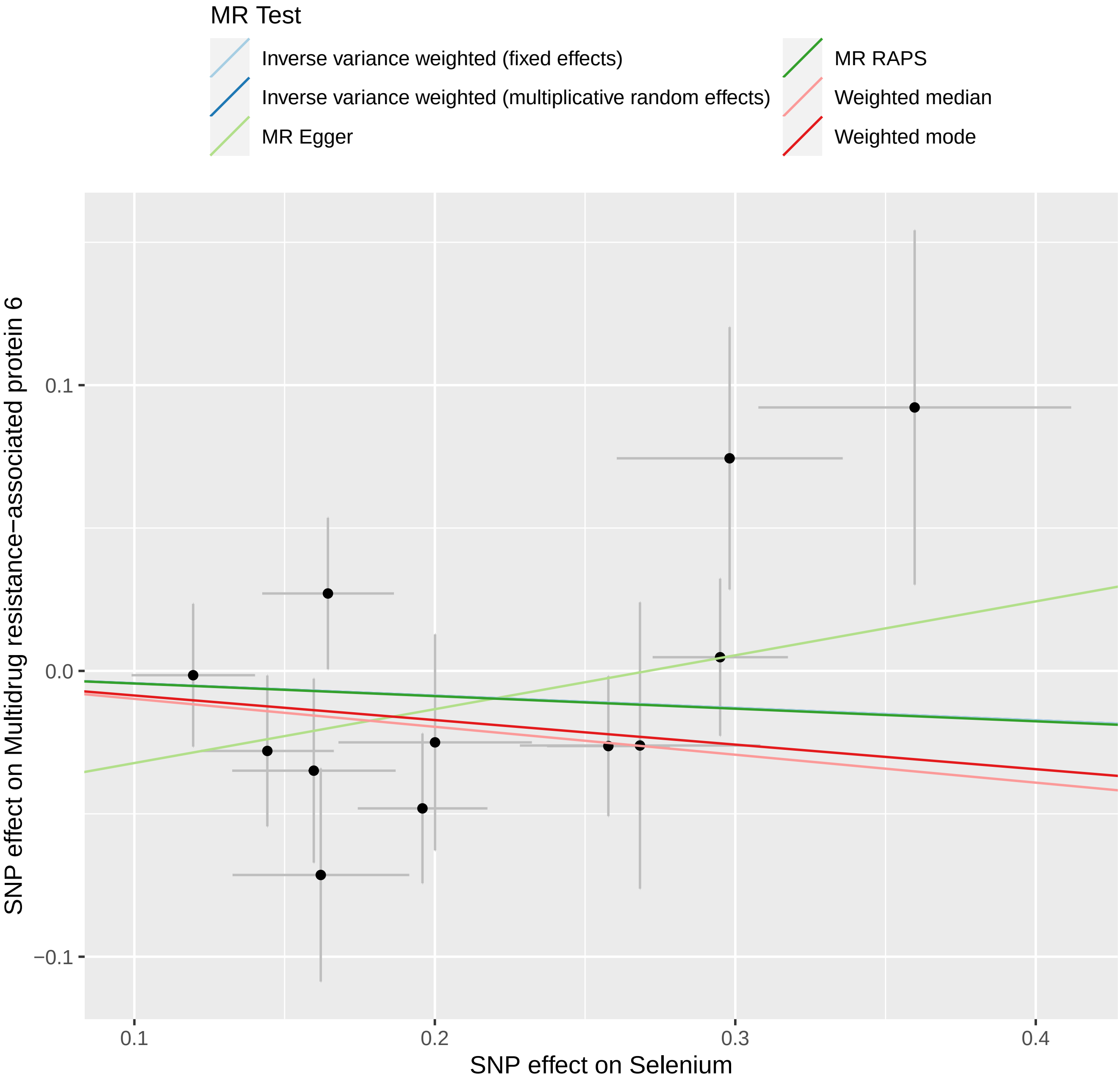

Batch 3−5 : Selenium on Monoacylglycerol lipase ABHD12

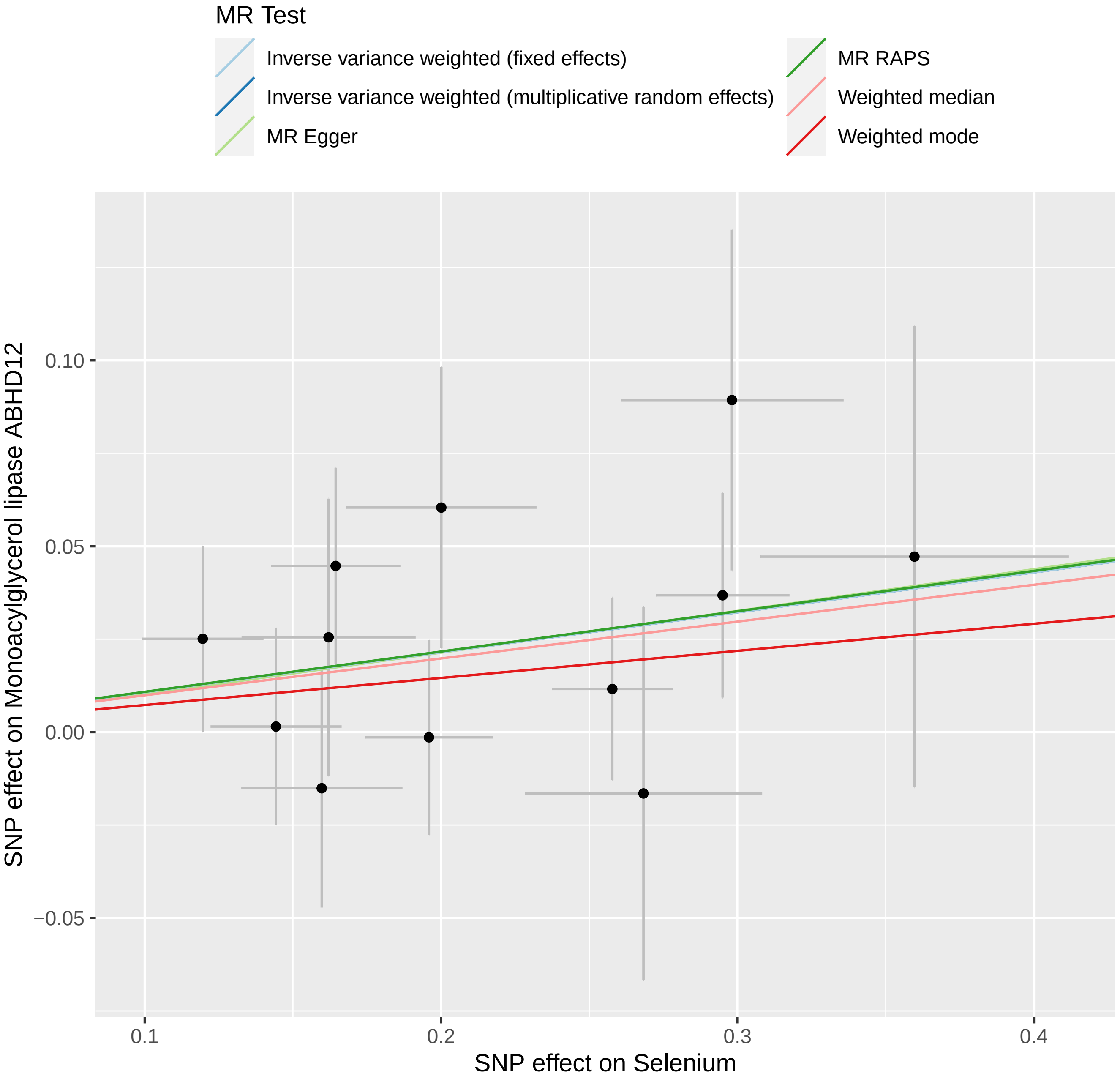

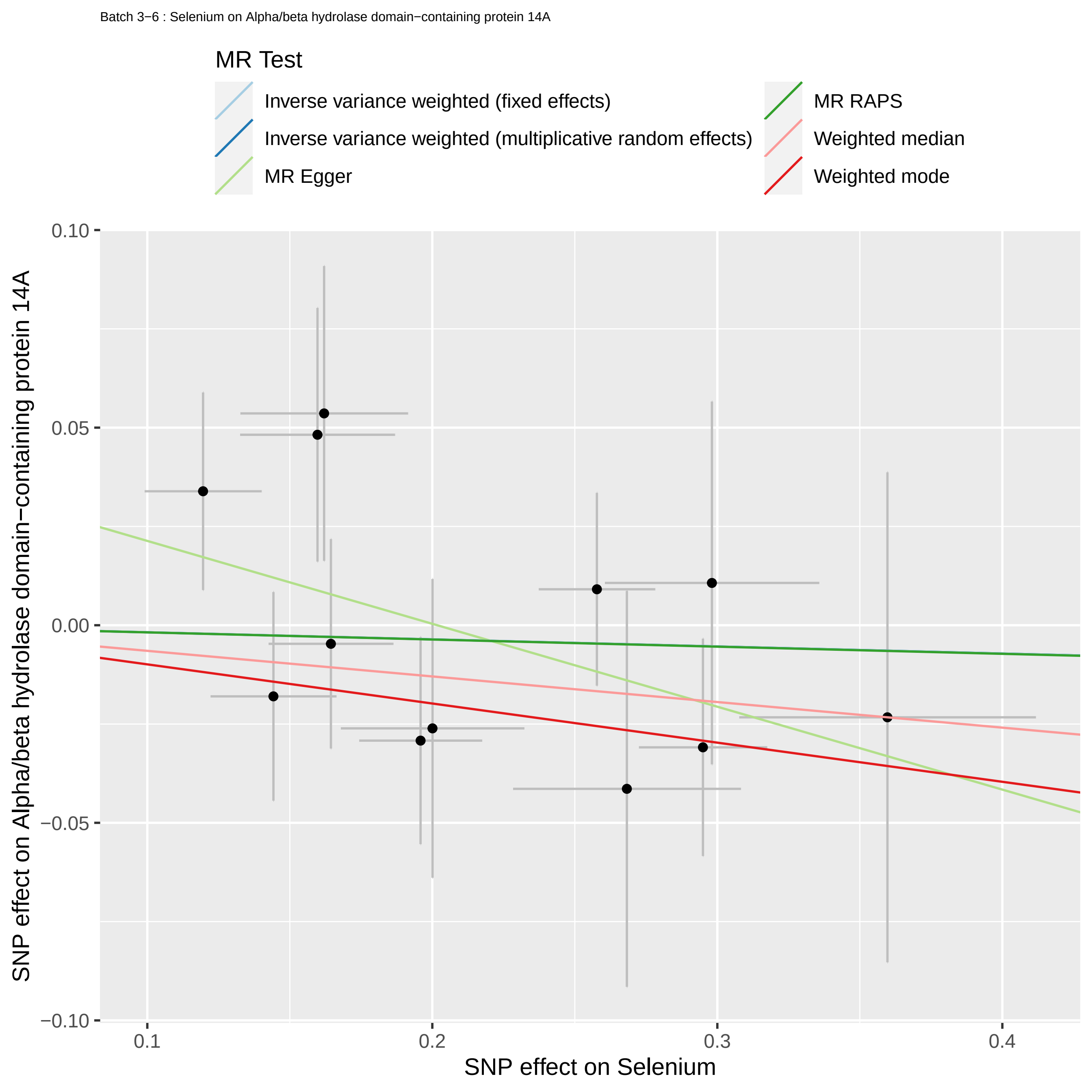
Batch 3−6 : Selenium on Alpha/beta hydrolase domain−containing protein 14A
MR Test
Inverse variance weighted (fixed effects)
Inverse variance weighted (multiplicative random effects)
MR Egger
MR RAPS
Weighted median
Weighted mode
0.10
0.05
0.00
−0.05
−0.10
0.1
0.2
0.3
0.4
SNP effect on Alpha/beta hydrolase domain−containing protein 14A
SNP effect on Selenium

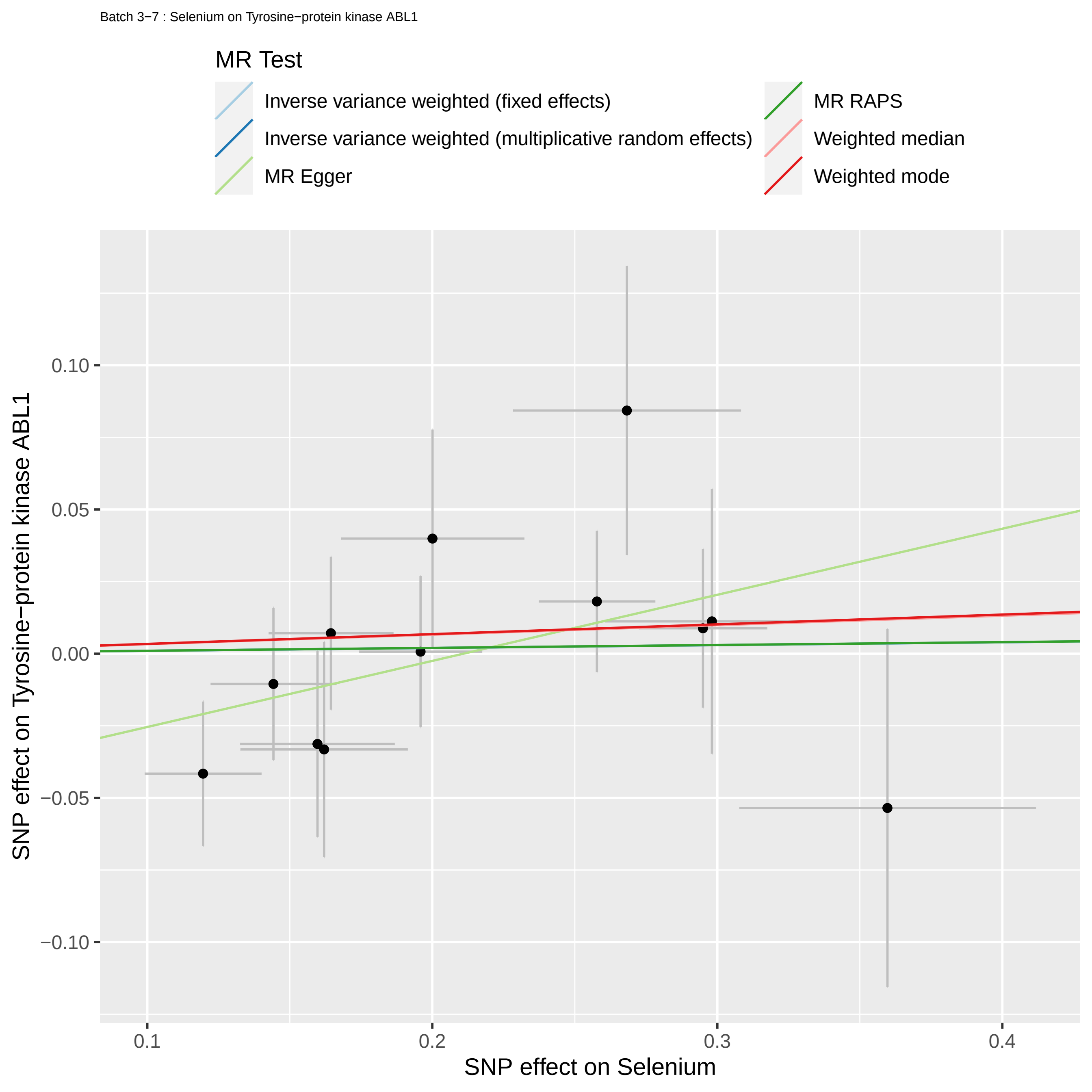
Batch 3−7 : Selenium on Tyrosine−protein kinase ABL1
MR Test
Inverse variance weighted (fixed effects)
Inverse variance weighted (multiplicative random effects)
MR Egger
MR RAPS
Weighted median
Weighted mode
0.10
0.05
0.00
−0.05
−0.10
SNP effect on Tyrosine−protein kinase ABL1
0.1
0.2
0.3
0.4
SNP effect on Selenium

Batch 3−8 : Selenium on Abelson tyrosine−protein kinase 2

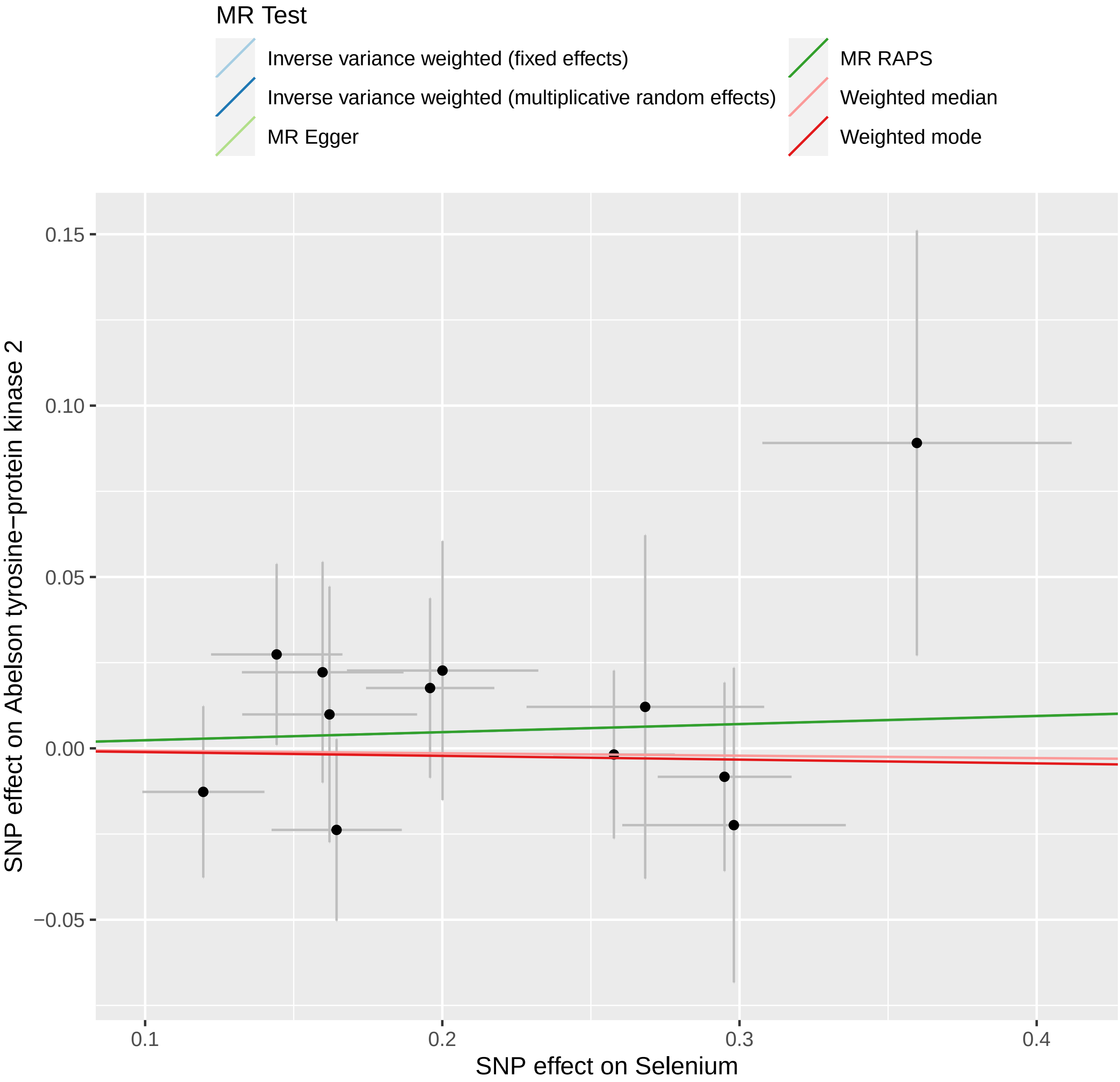

Batch 3−9 : Selenium on Actin−binding LIM protein 3

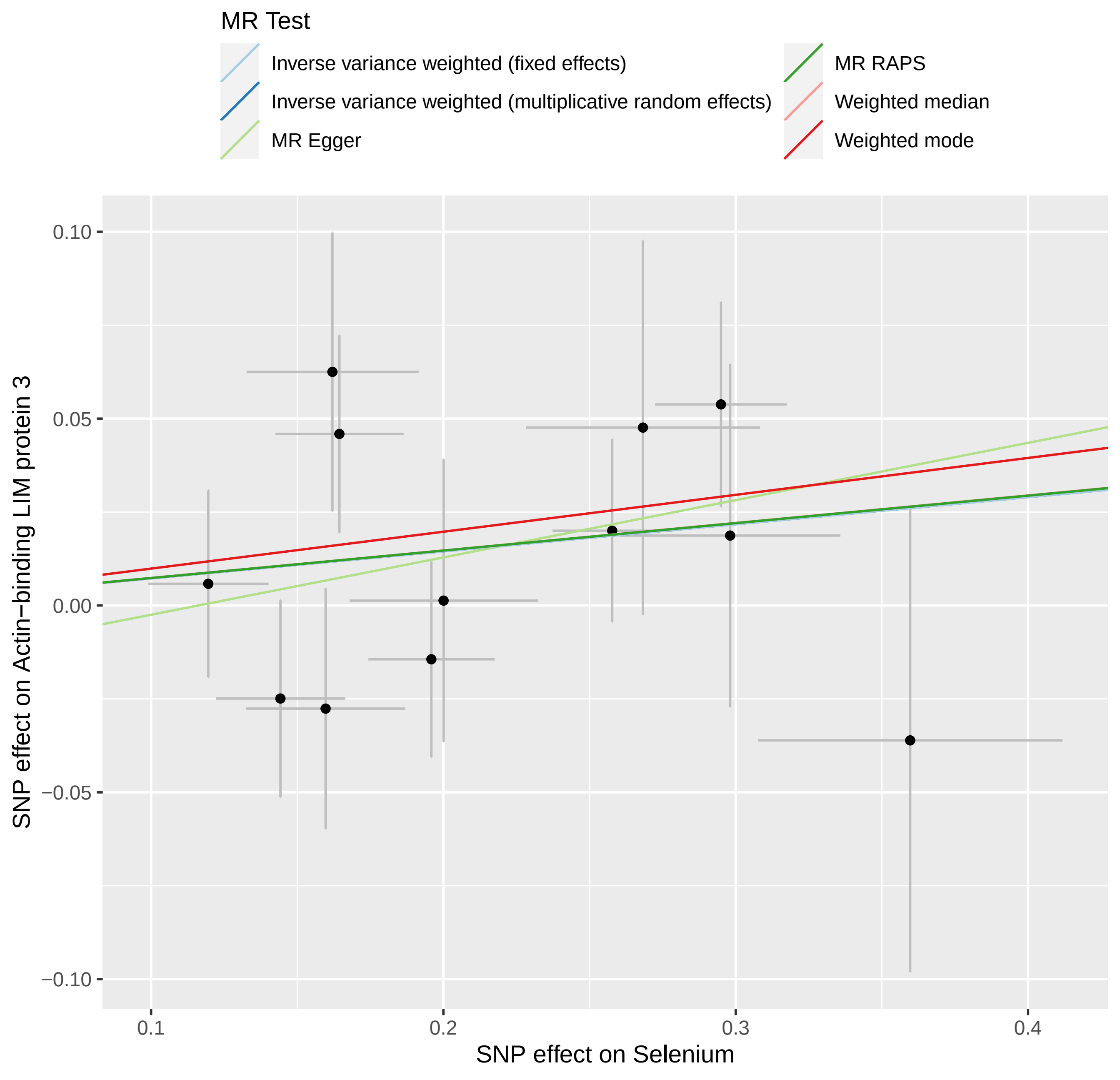

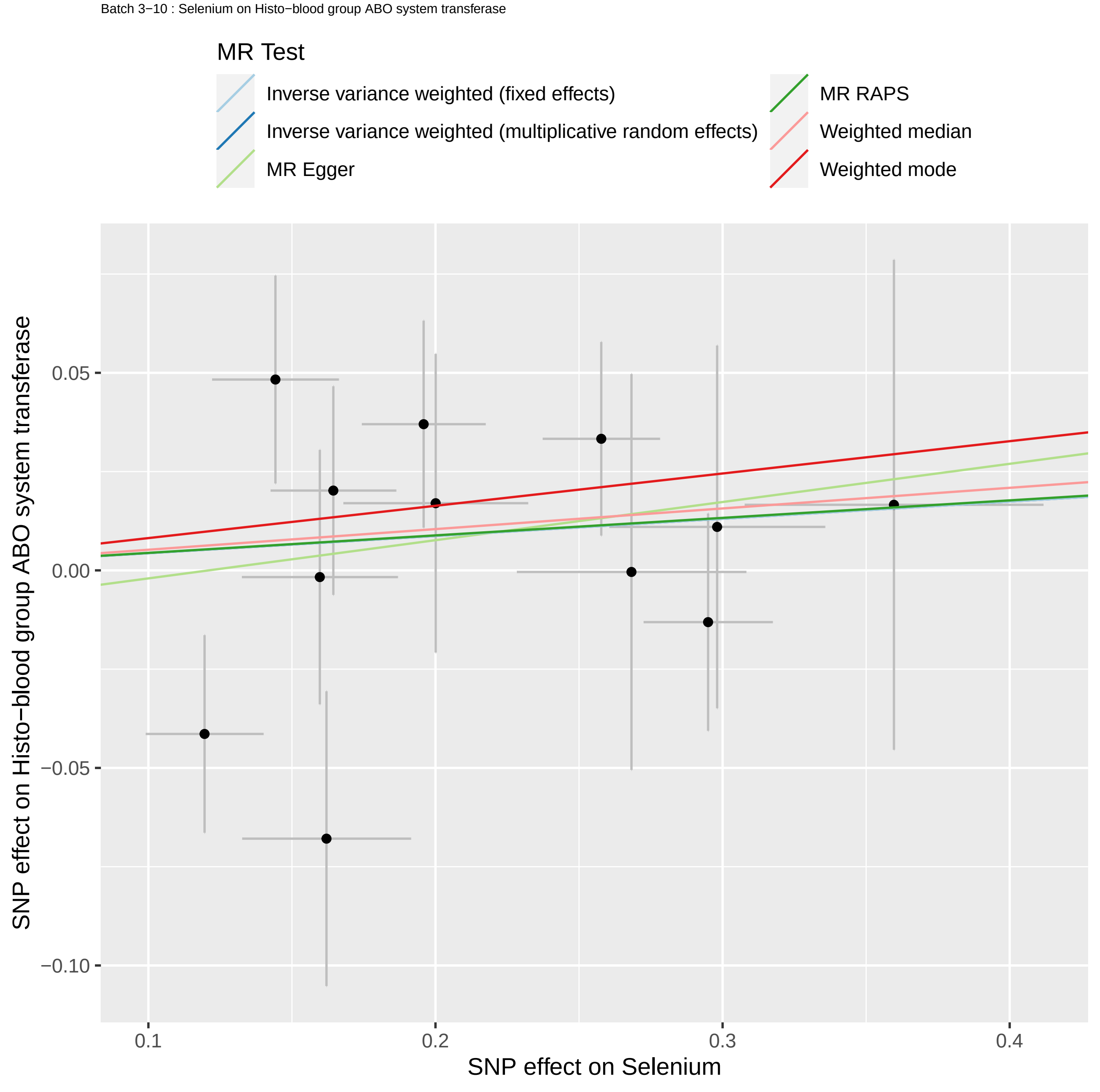
Batch 3−10 : Selenium on Histo−blood group ABO system transferase
MR Test
Inverse variance weighted (fixed effects)
Inverse variance weighted (multiplicative random effects)
MR Egger
MR RAPS
Weighted median
Weighted mode
0.05
0.00
−0.05
−0.10
0.1
0.2
0.3
0.4
SNP effect on Selenium
SNP effect on Histo−blood group ABO system transferase

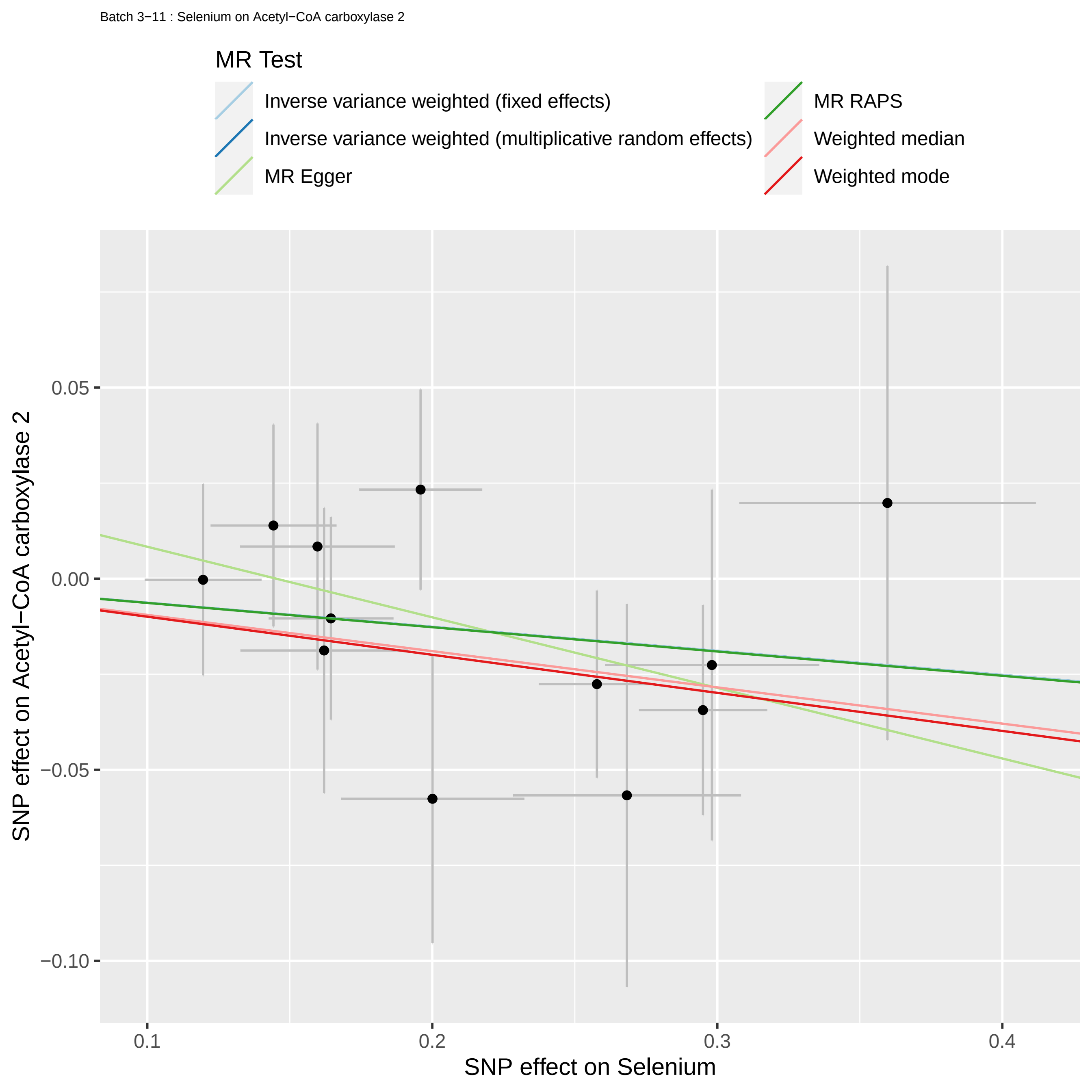

Batch 3−11 : Selenium on Acetyl−CoA carboxylase 2
MR Test
Inverse variance weighted (fixed effects)
Inverse variance weighted (multiplicative random effects)
MR Egger
MR RAPS
Weighted median
Weighted mode
0.05
0.00
−0.05
−0.10
0.1
0.2
0.3
0.4
SNP effect on Acetyl−CoA carboxylase 2
SNP effect on Selenium

Batch 3−12 : Selenium on Aggrecan core protein

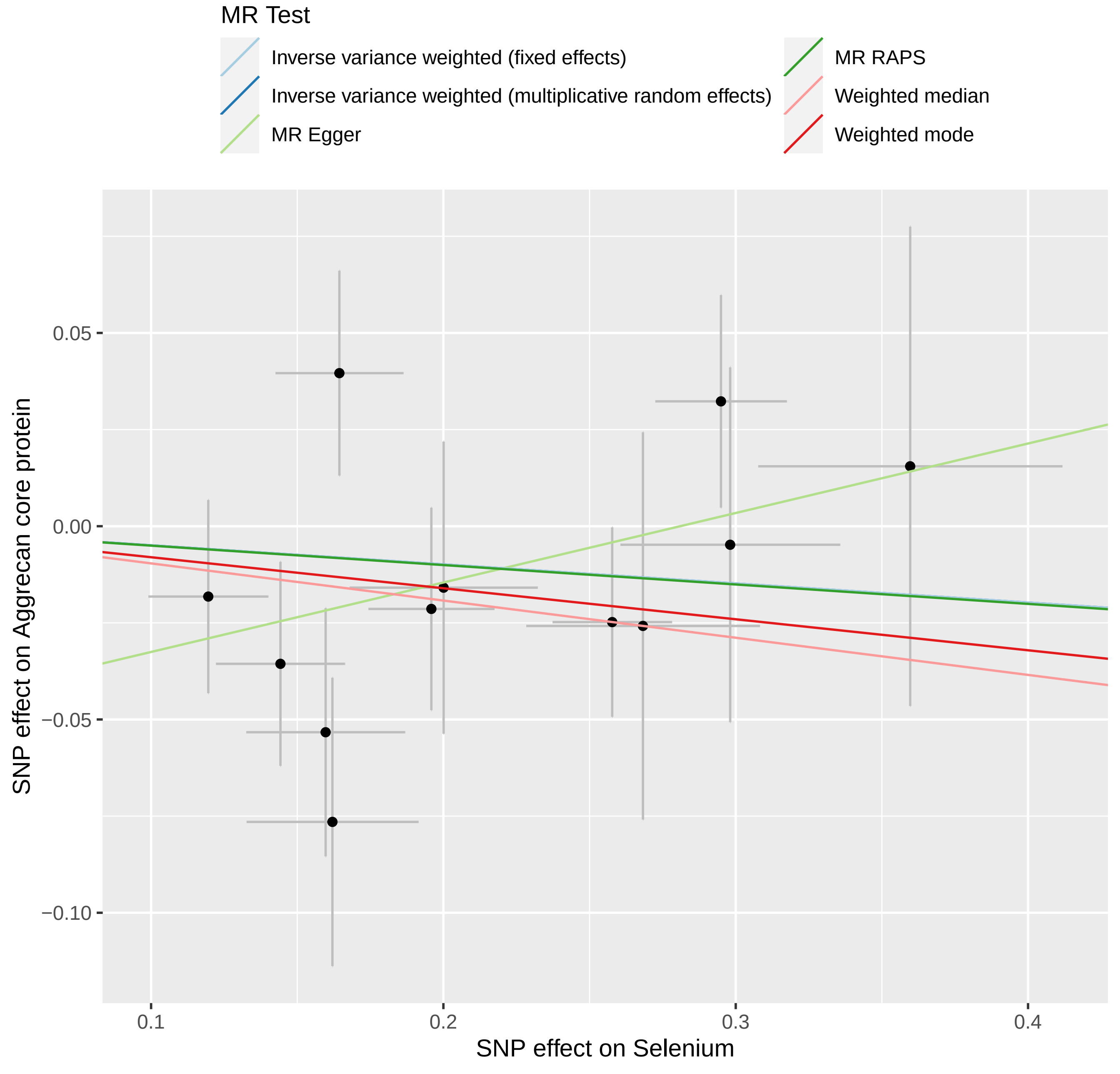

Batch 3−13 : Selenium on Arf−GAP with coiled−coil, ANK repeat and PH domain−containing protein 2

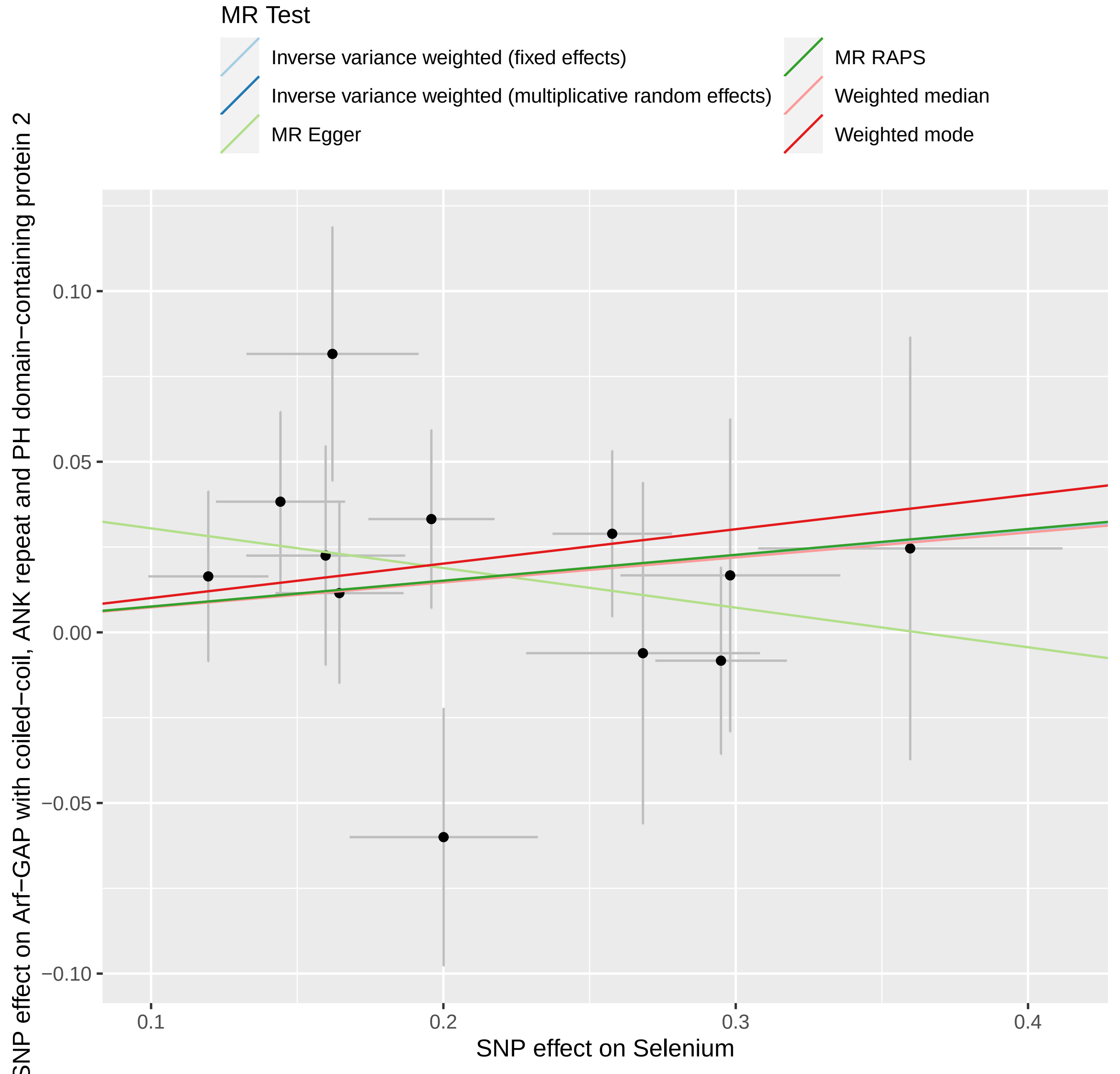

Batch 3−14 : Selenium on Acyl−CoA−binding domain−containing protein 6

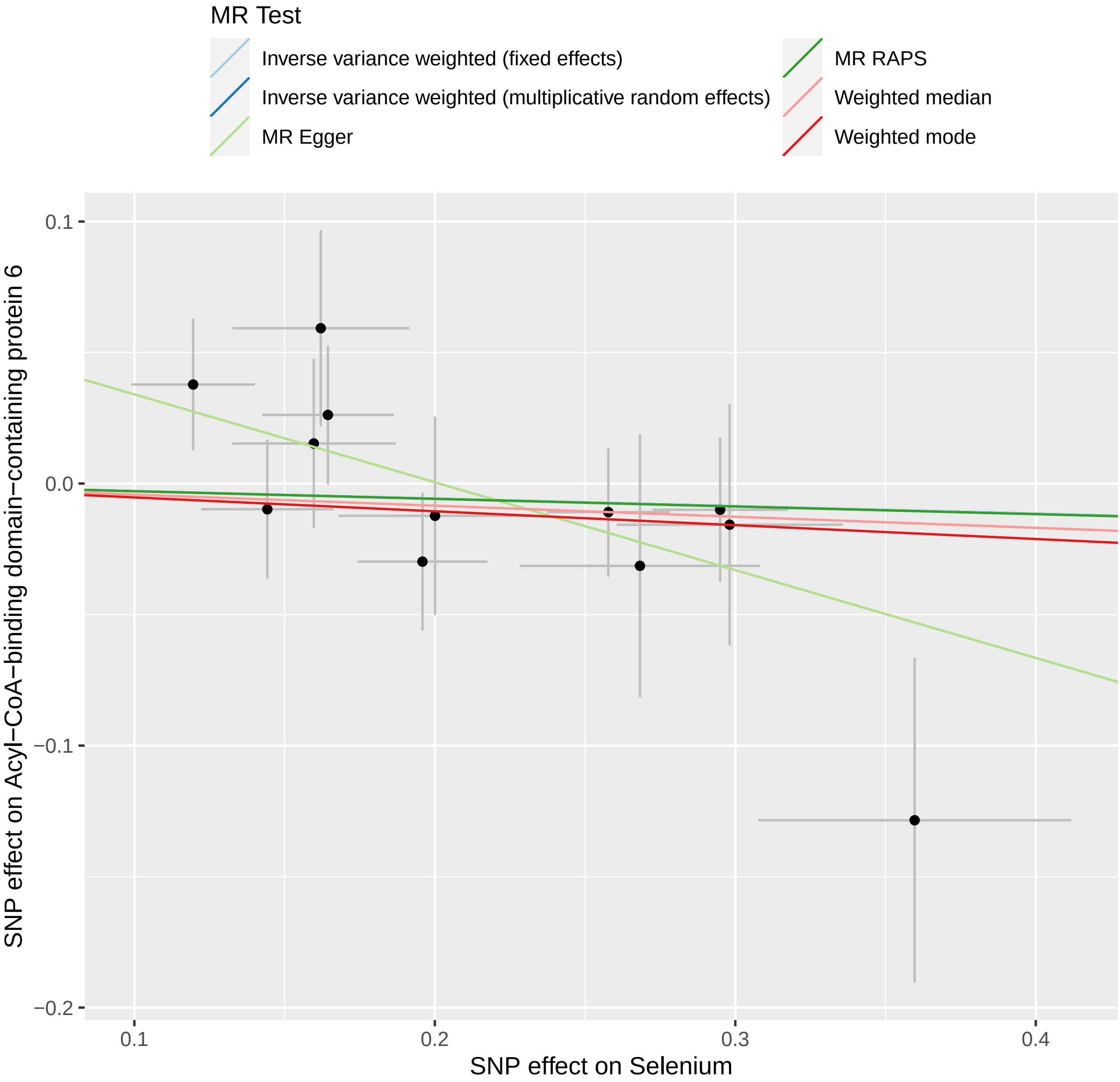

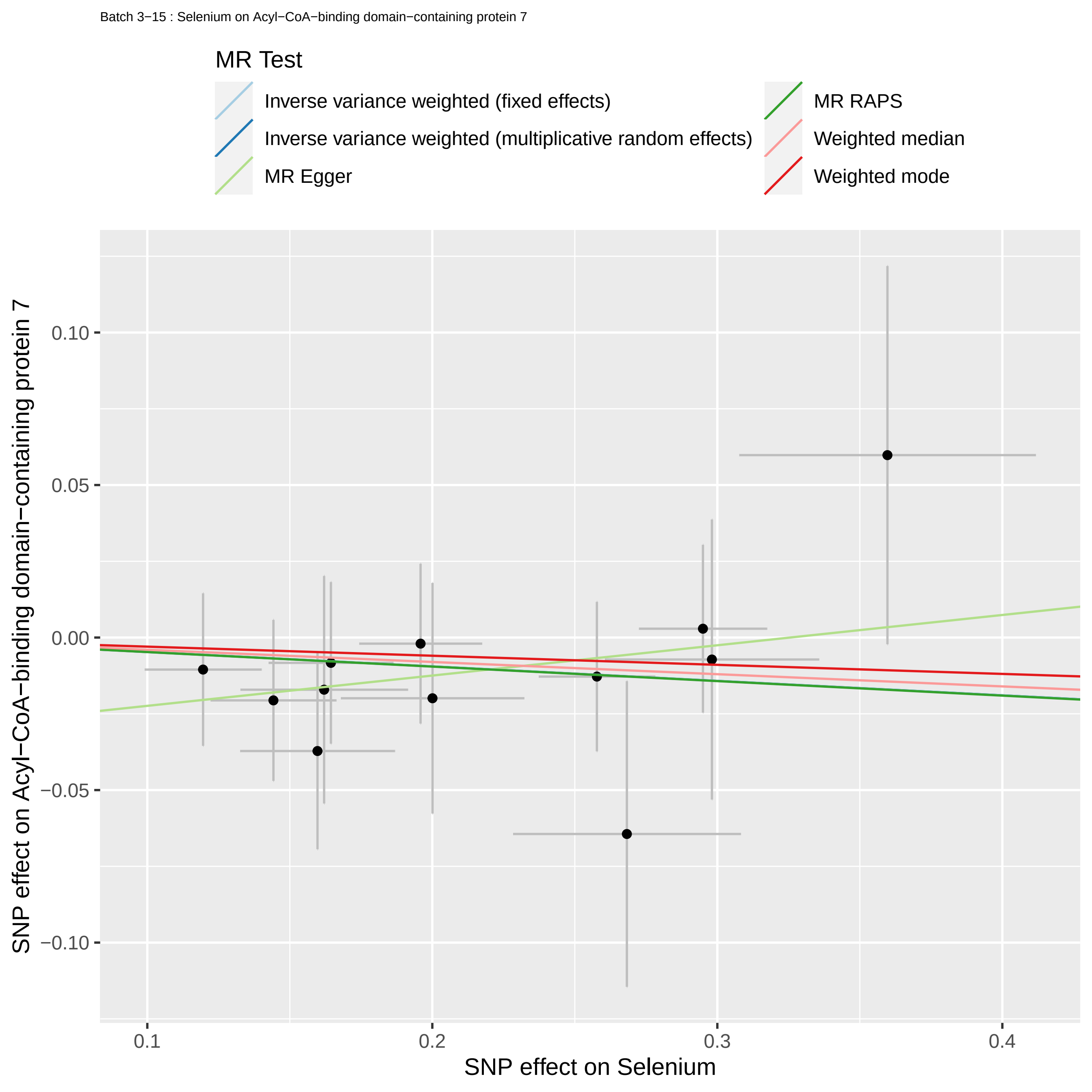

Batch 3−15 : Selenium on Acyl−CoA−binding domain−containing protein 7
MR Test
Inverse variance weighted (fixed effects)
Inverse variance weighted (multiplicative random effects)
MR Egger
MR RAPS
Weighted median
Weighted mode
SNP effect on Acyl−CoA−binding domain−containing protein 7
0.10
0.05
0.00
−0.05
−0.10
0.1
0.2
0.3
0.4
SNP effect on Selenium

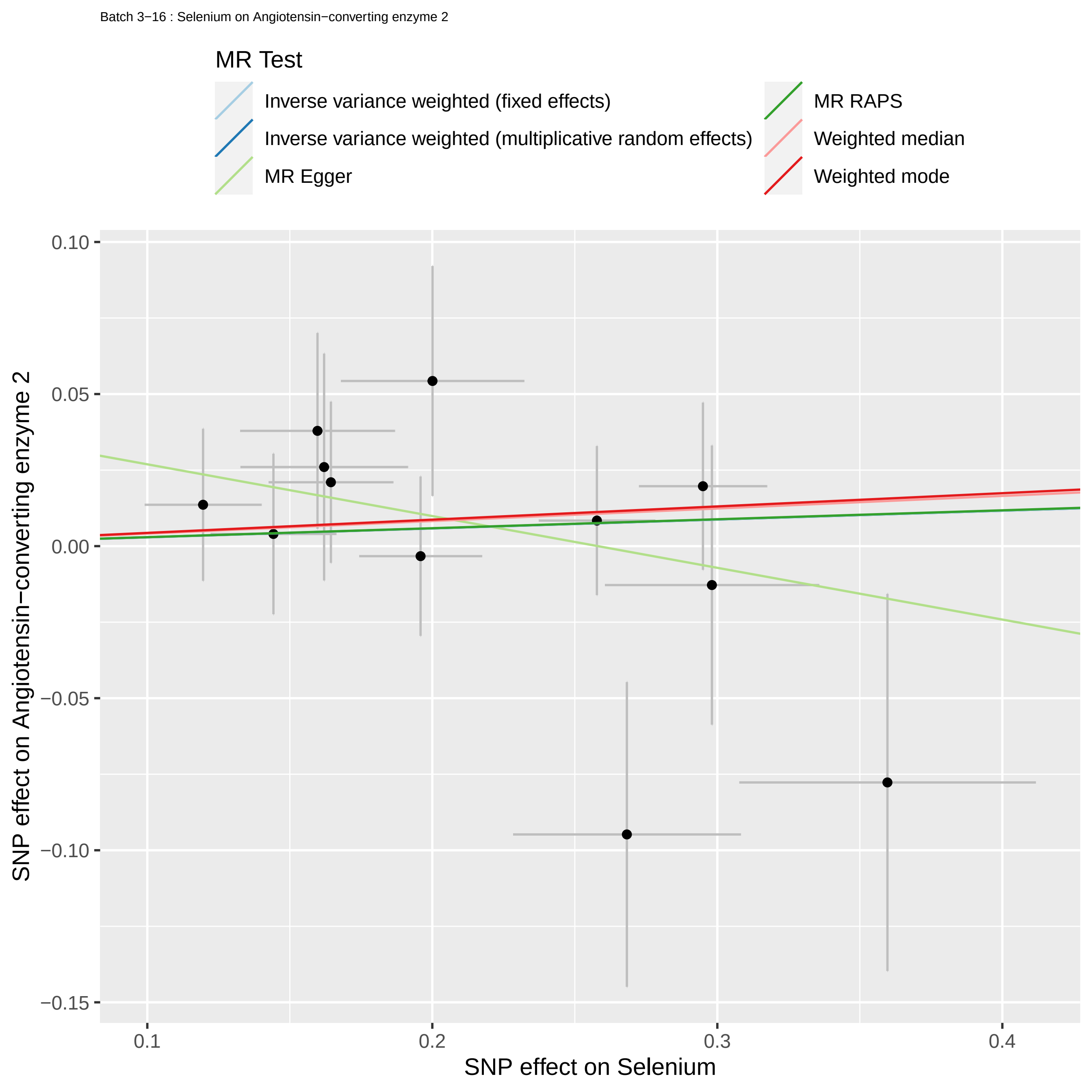
Batch 3−16 : Selenium on Angiotensin−converting enzyme 2
MR Test
Inverse variance weighted (fixed effects)
Inverse variance weighted (multiplicative random effects)
MR Egger
MR RAPS
Weighted median
Weighted mode
0.10
0.05
0.00
−0.05
−0.10
−0.15
0.1
0.2
0.3
0.4
SNP effect on Angiotensin−converting enzyme 2
SNP effect on Selenium

Batch 3−17 : Selenium on ATP−citrate synthase

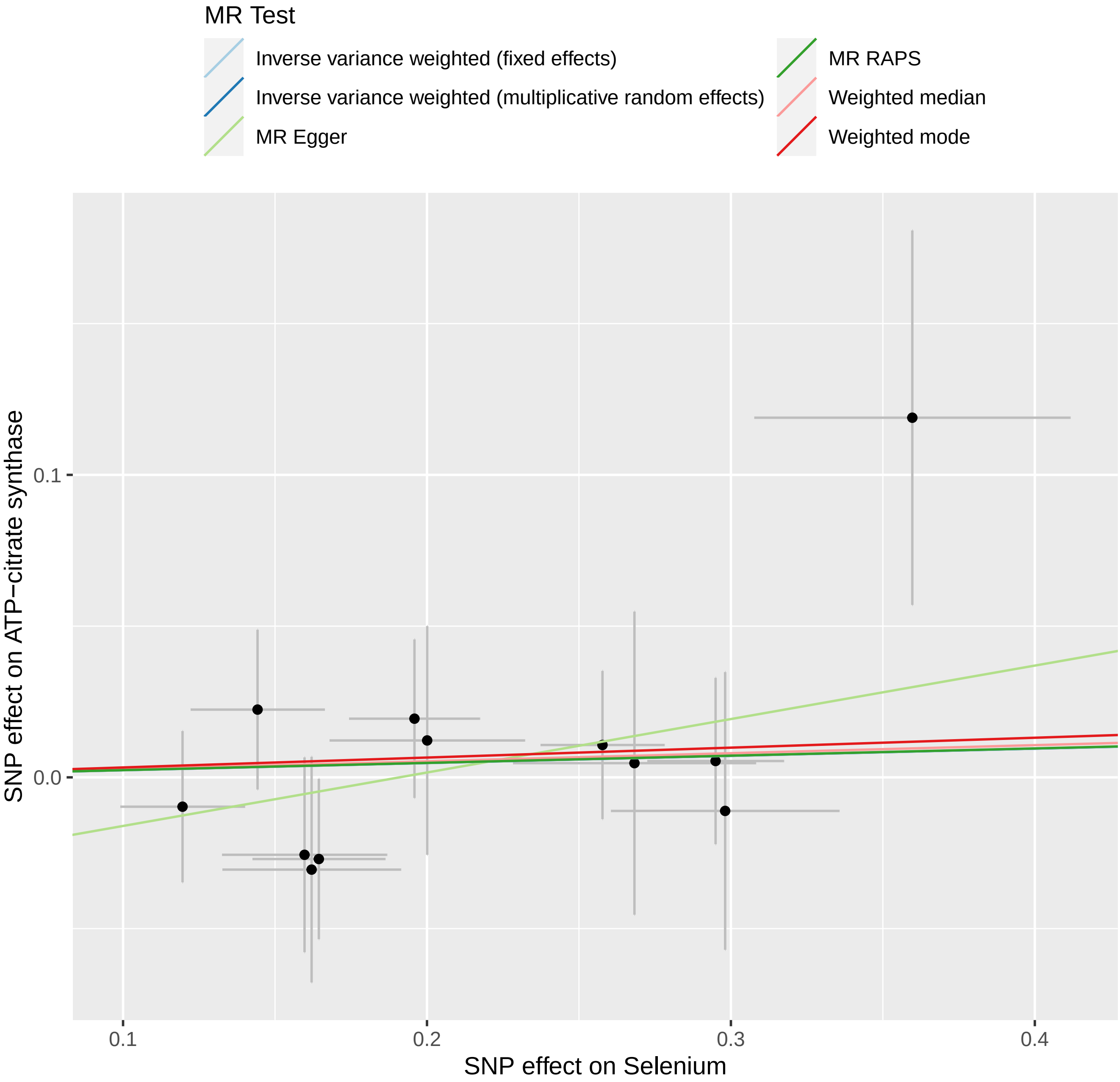

Batch 3−18 : Selenium on Low molecular weight phosphotyrosine protein phosphatase

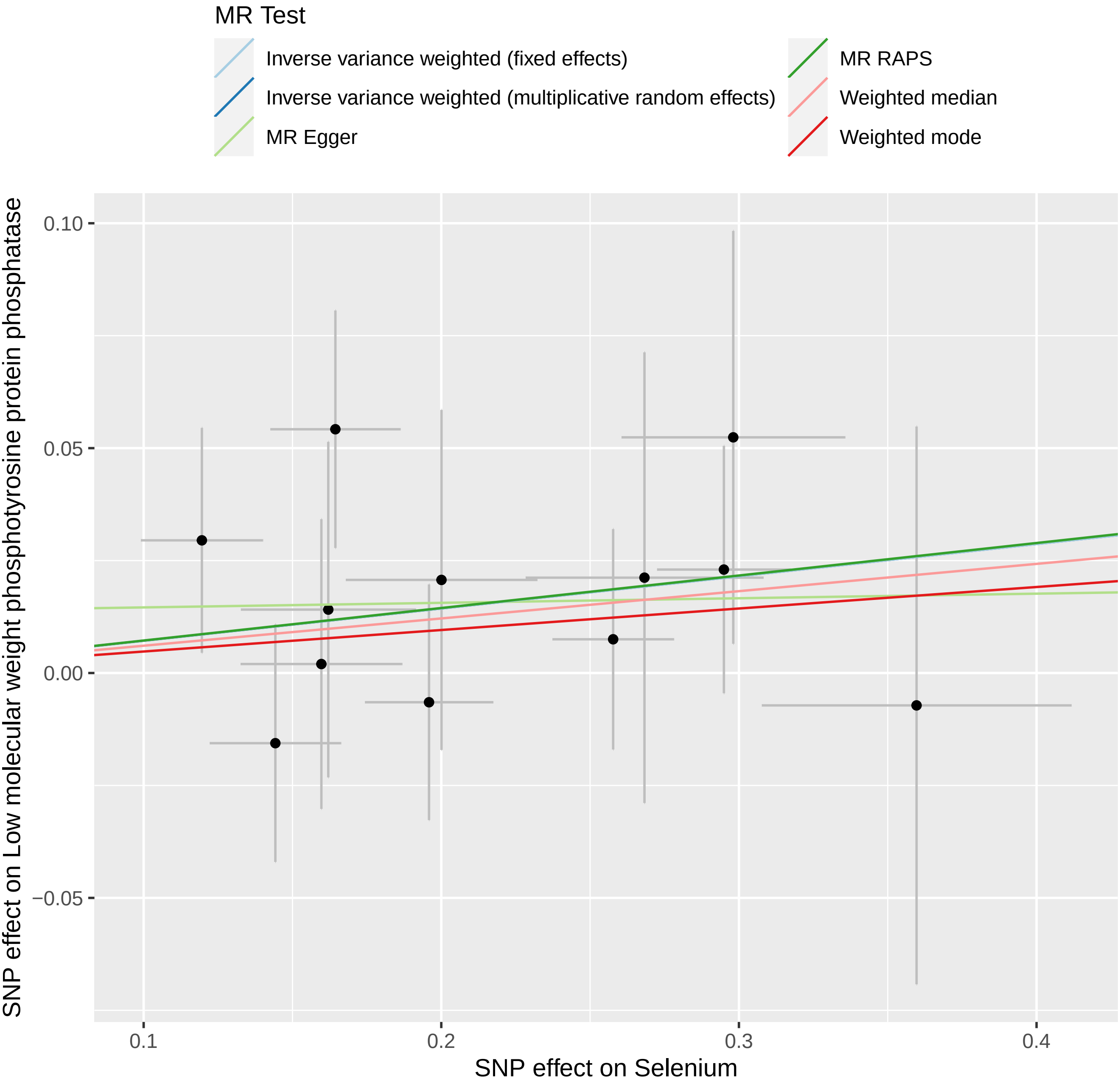

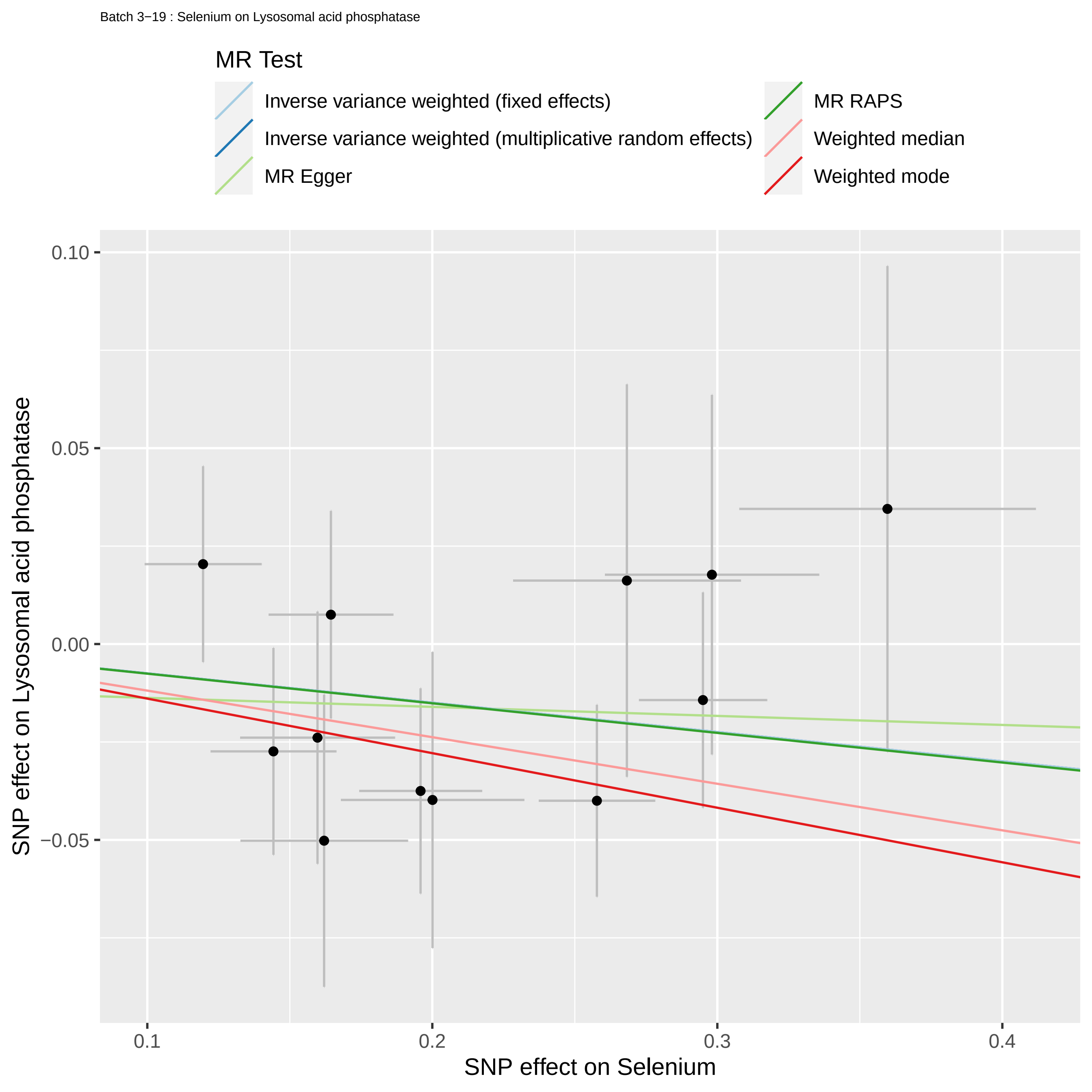
Batch 3−19 : Selenium on Lysosomal acid phosphatase
MR Test
Inverse variance weighted (fixed effects)
Inverse variance weighted (multiplicative random effects)
MR Egger
MR RAPS
Weighted median
Weighted mode
0.10
0.05
0.00
−0.05
0.1
0.2
0.3
0.4
SNP effect on Lysosomal acid phosphatase
SNP effect on Selenium

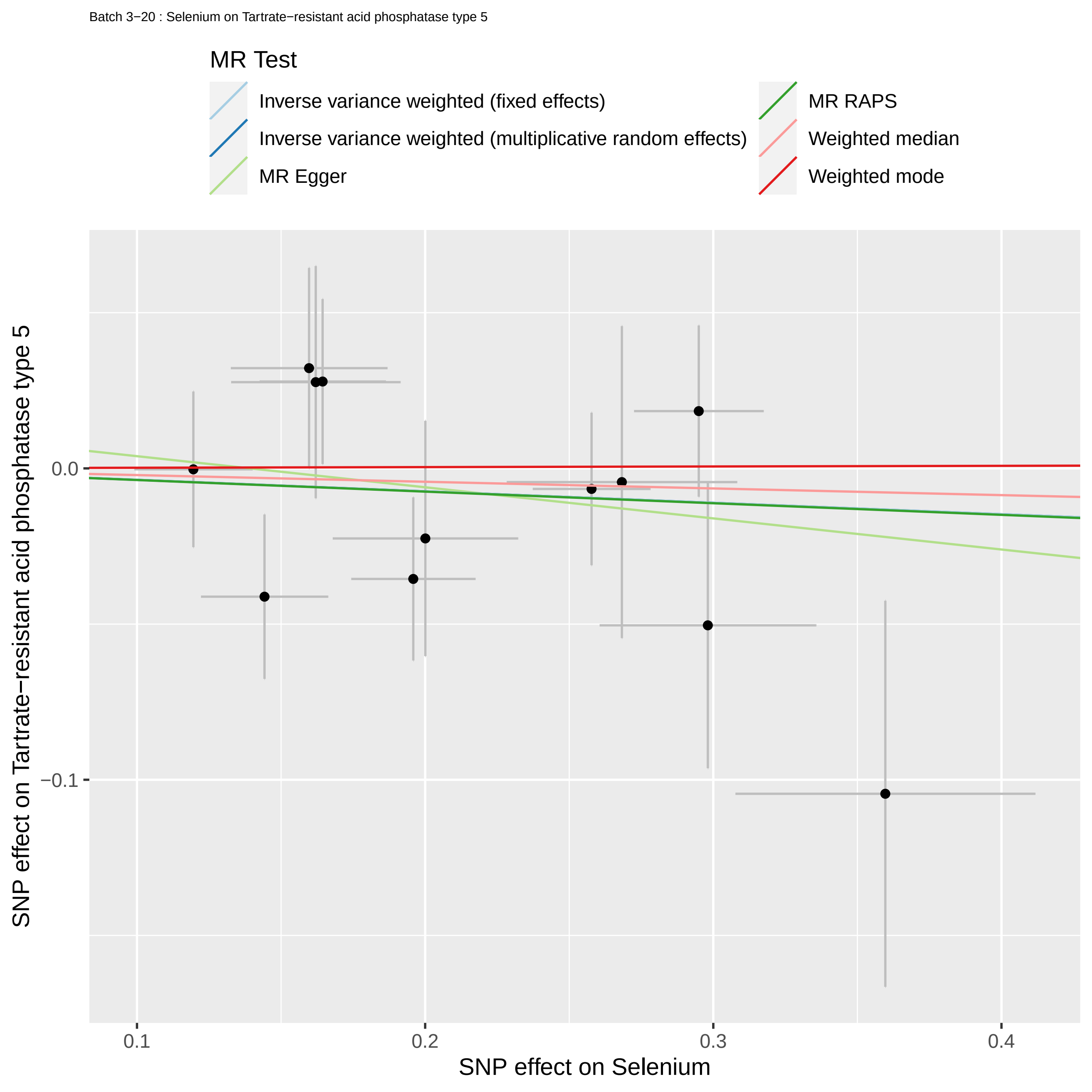

Batch 3−20 : Selenium on Tartrate−resistant acid phosphatase type 5
MR Test
Inverse variance weighted (fixed effects)
Inverse variance weighted (multiplicative random effects)
MR Egger
MR RAPS
Weighted median
Weighted mode
SNP effect on Tartrate−resistant acid phosphatase type 5
0.0
−0.1
0.1
0.2
0.3
0.4
SNP effect on Selenium

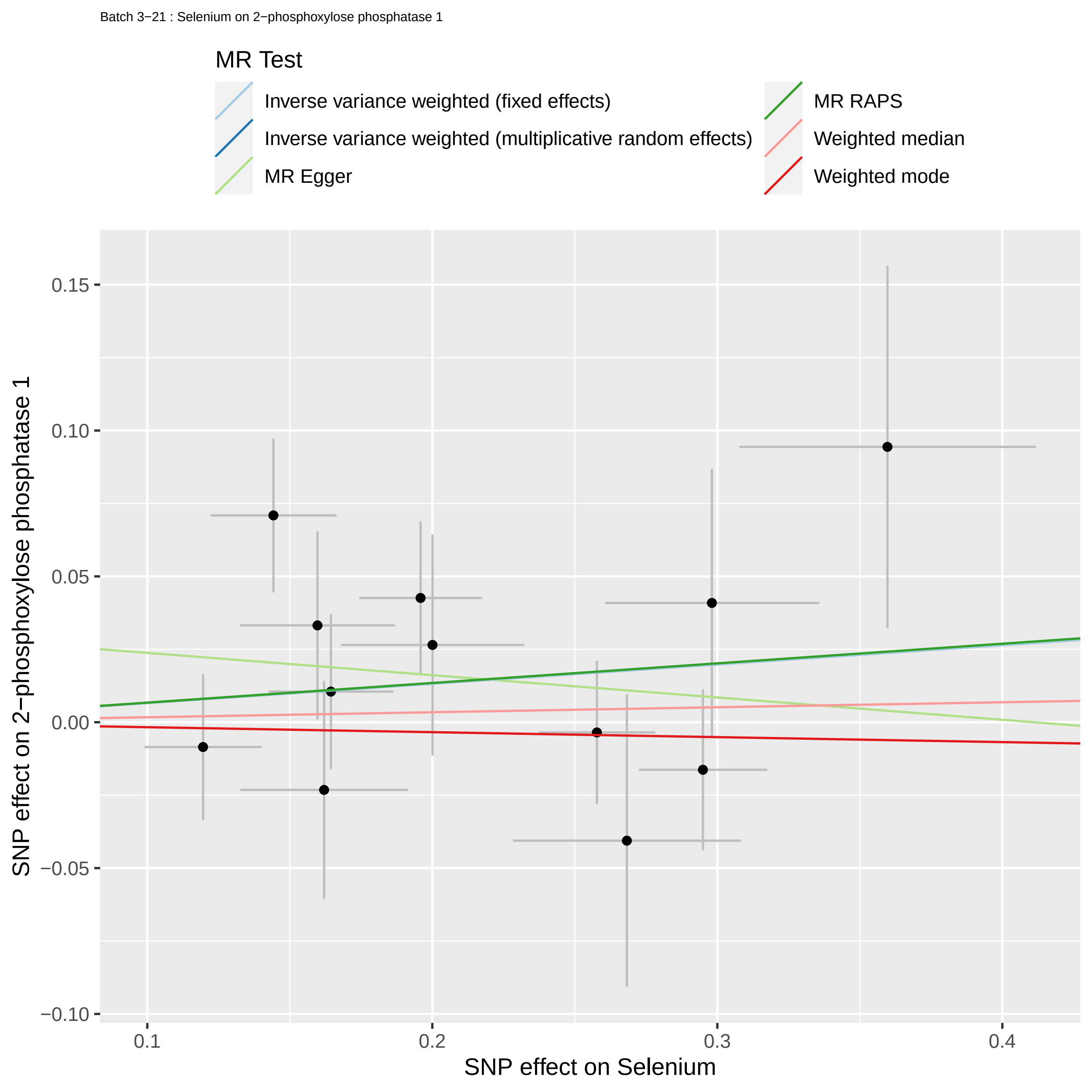

Batch 3−21 : Selenium on 2−phosphoxylose phosphatase 1
MR Test
Inverse variance weighted (fixed effects)
Inverse variance weighted (multiplicative random effects)
MR Egger
MR RAPS
Weighted median
Weighted mode
SNP effect on 2−phosphoxylose phosphatase 1
0.15
0.10
0.05
0.00
−0.05
−0.10
0.1
0.2
0.3
0.4
SNP effect on Selenium

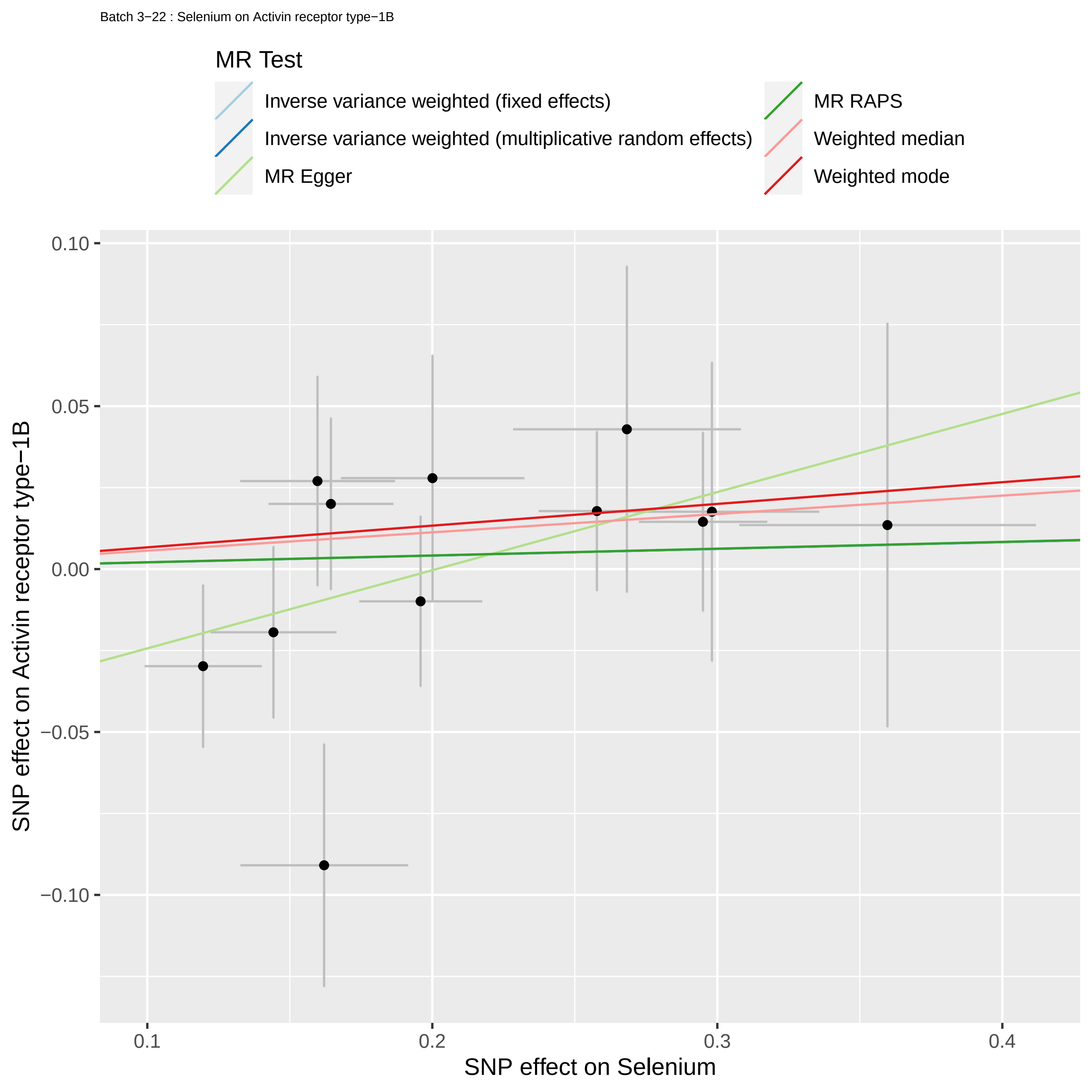
Batch 3−22 : Selenium on Activin receptor type−1B
MR Test
Inverse variance weighted (fixed effects)
Inverse variance weighted (multiplicative random effects)
MR Egger
MR RAPS
Weighted median
Weighted mode
0.10
0.05
0.00
−0.05
−0.10
0.1
0.2
0.3
0.4
SNP effect on Activin receptor type−1B
SNP effect on Selenium

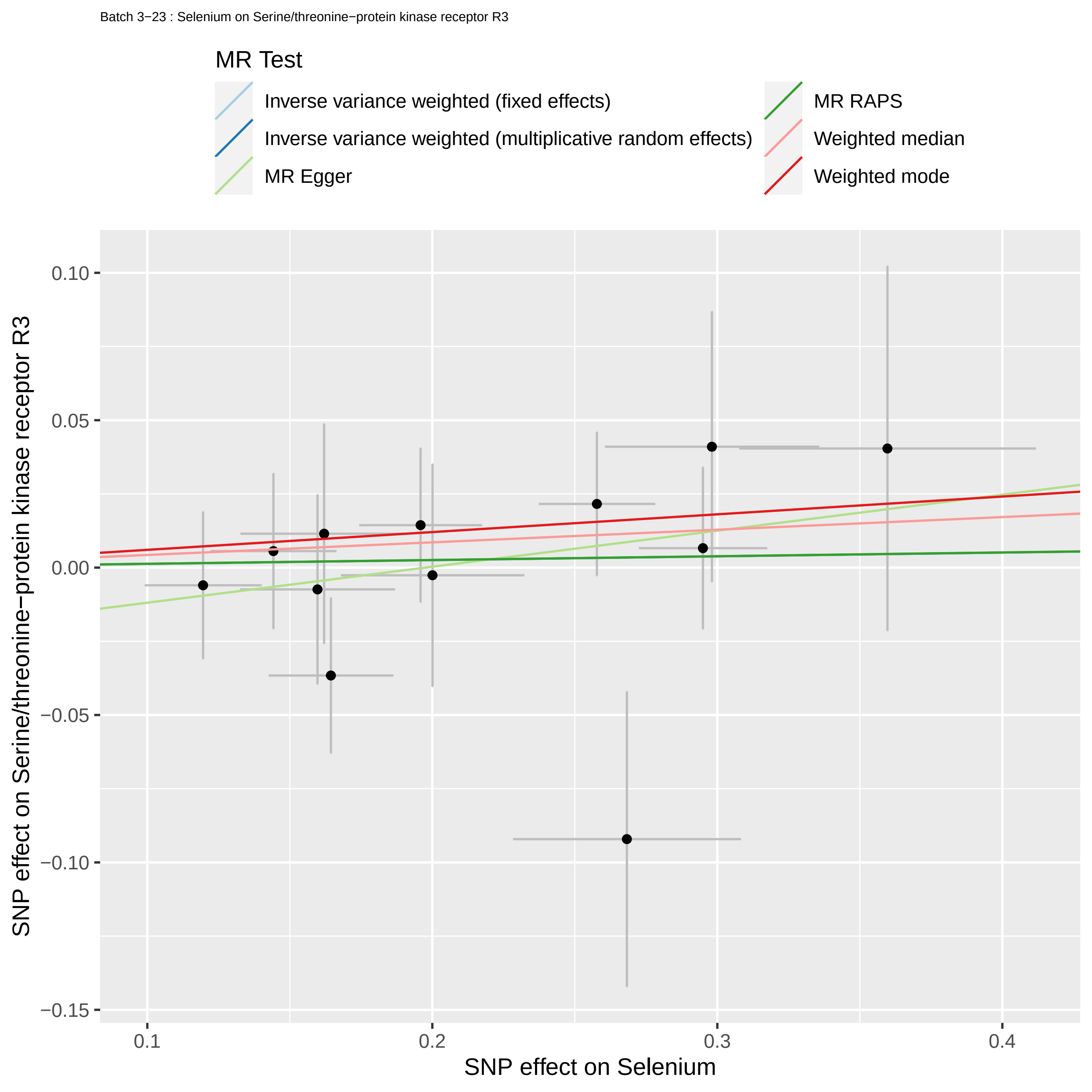
Batch 3−23 : Selenium on Serine/threonine−protein kinase receptor R3
MR Test
Inverse variance weighted (fixed effects)
Inverse variance weighted (multiplicative random effects)
MR Egger
MR RAPS
Weighted median
Weighted mode
0.10
0.05
0.00
−0.05
−0.10
−0.15
0.1
0.2
0.3
0.4
SNP effect on Serine/threonine−protein kinase receptor R3
SNP effect on Selenium

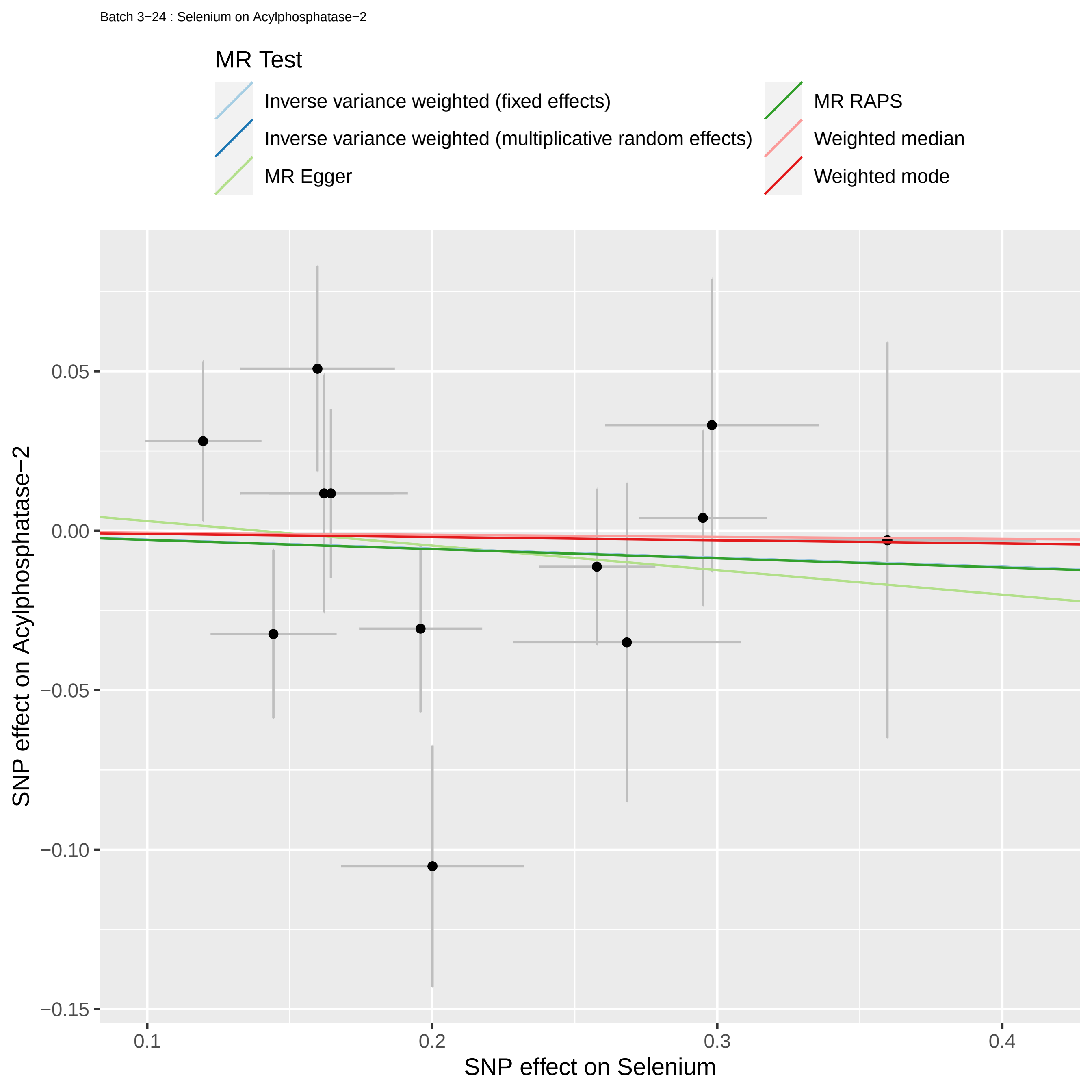

Batch 3−24 : Selenium on Acylphosphatase−2
MR Test
Inverse variance weighted (fixed effects)
Inverse variance weighted (multiplicative random effects)
MR Egger
MR RAPS
Weighted median
Weighted mode
0.05
0.00
−0.05
−0.10
−0.15
0.1
0.2
0.3
0.4
SNP effect on Acylphosphatase−2
SNP effect on Selenium

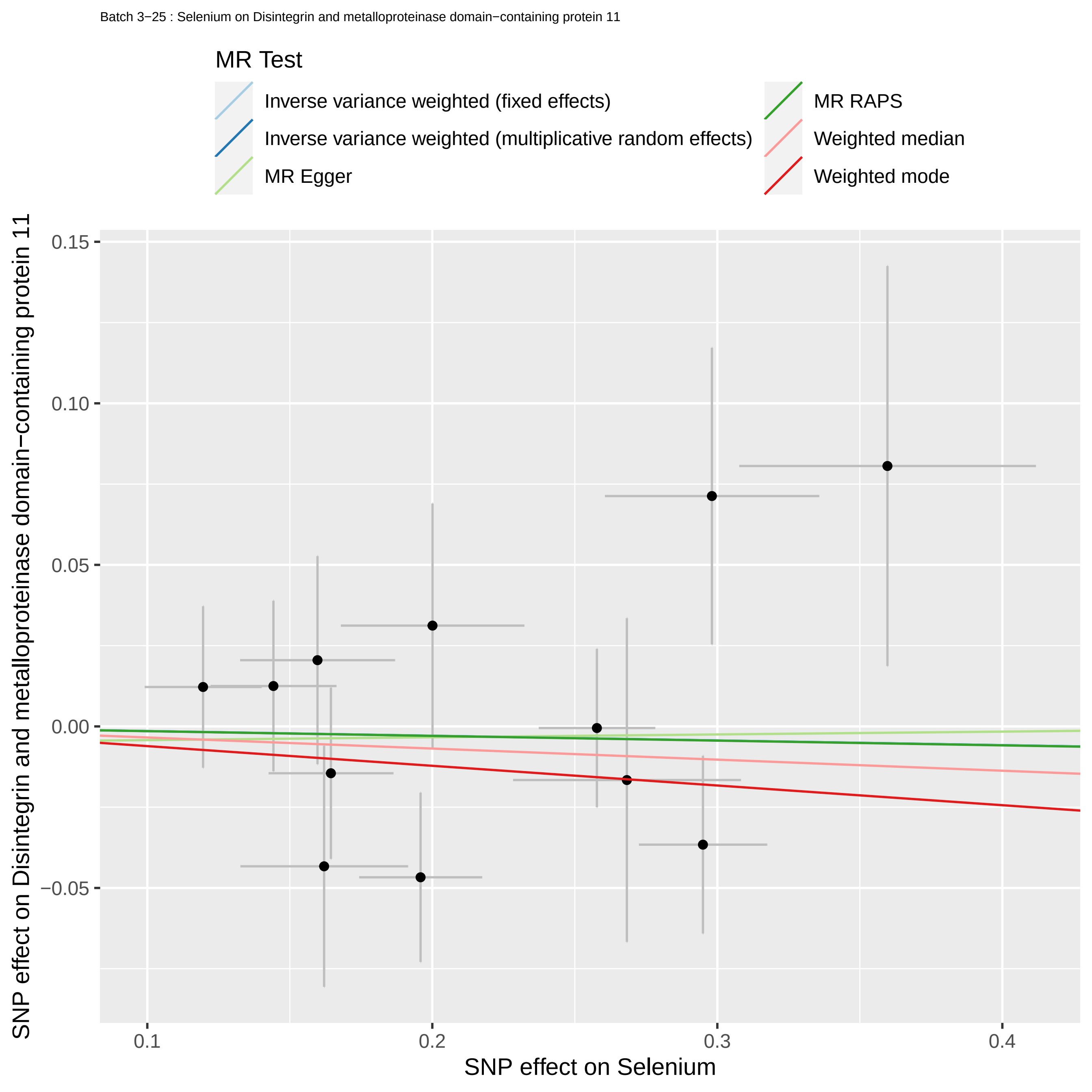
Batch 3−25 : Selenium on Disintegrin and metalloproteinase domain−containing protein 11
MR Test
Inverse variance weighted (fixed effects)
Inverse variance weighted (multiplicative random effects)
MR Egger
MR RAPS
Weighted median
Weighted mode
SNP effect on Disintegrin and metalloproteinase domain−containing protein 11
0.15
0.10
0.05
0.00
−0.05
0.1
0.2
0.3
0.4
SNP effect on Selenium

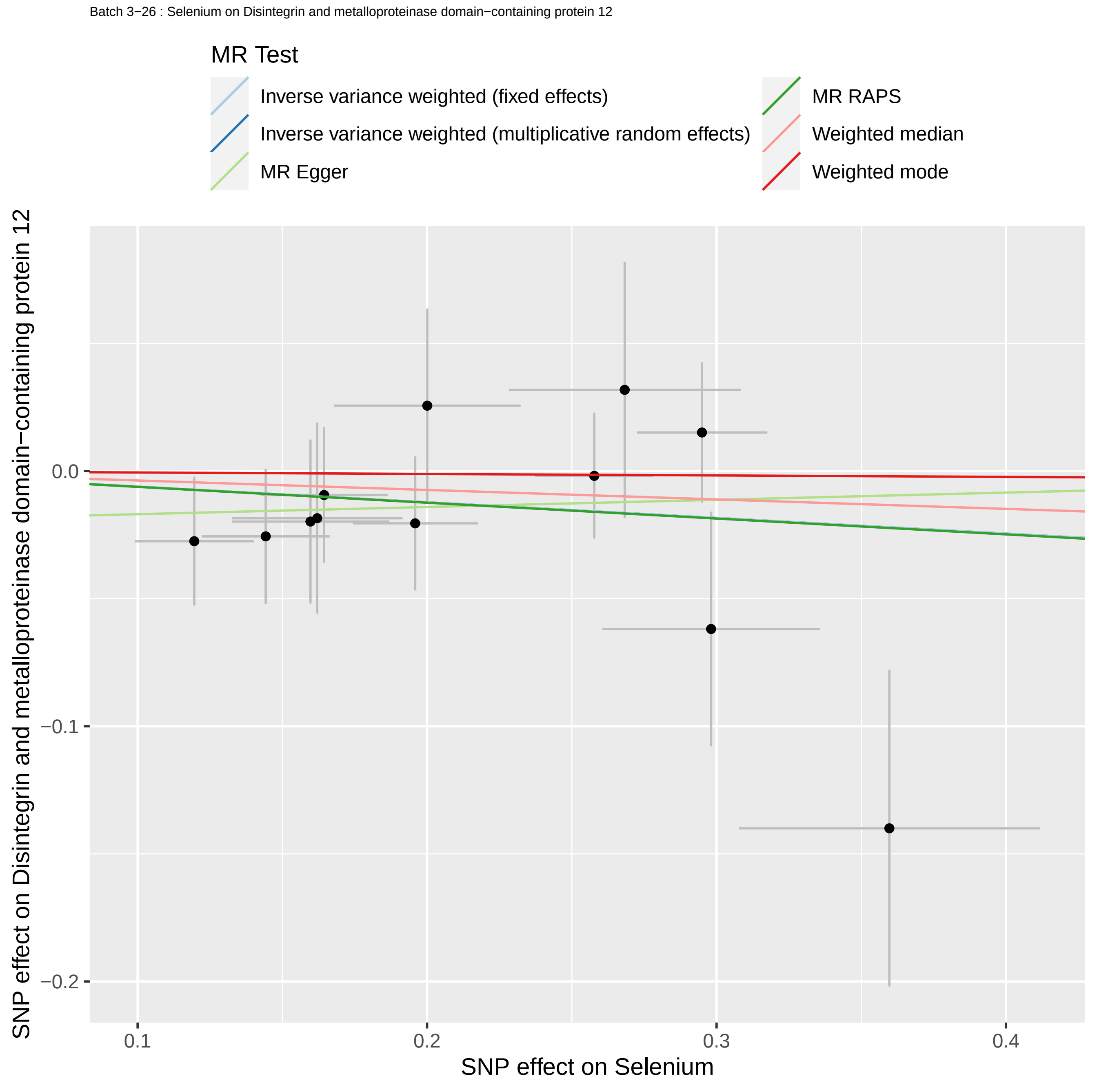
Batch 3−26 : Selenium on Disintegrin and metalloproteinase domain−containing protein 12
MR Test
Inverse variance weighted (fixed effects)
Inverse variance weighted (multiplicative random effects)
MR Egger
MR RAPS
Weighted median
Weighted mode
SNP effect on Disintegrin and metalloproteinase domain−containing protein 12
0.0
−0.1
−0.2
0.1
0.2
0.3
0.4
SNP effect on Selenium

Batch 3−27 : Selenium on Disintegrin and metalloproteinase domain−containing protein 19

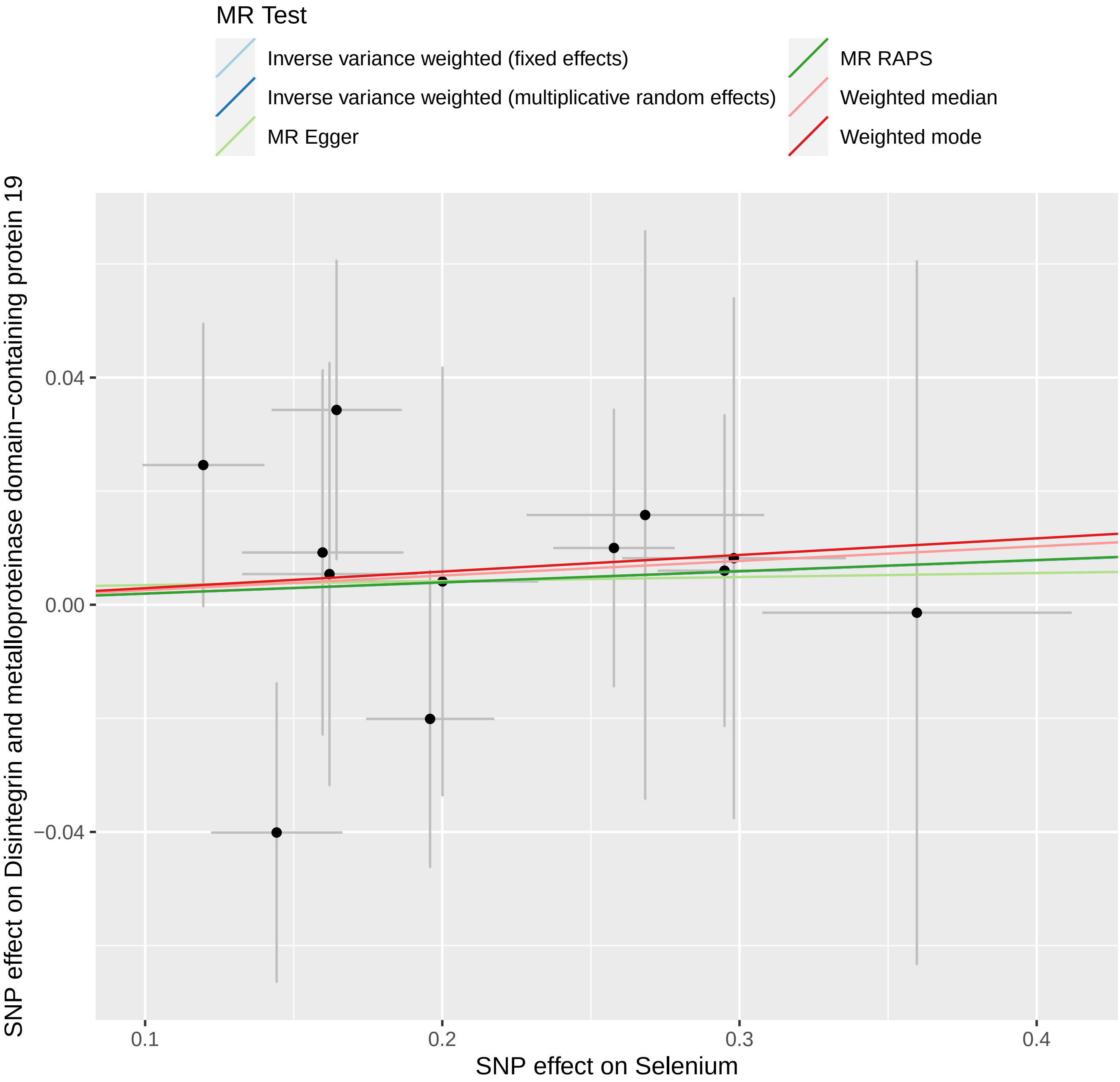

Batch 3−28 : Selenium on Disintegrin and metalloproteinase domain−containing protein 23

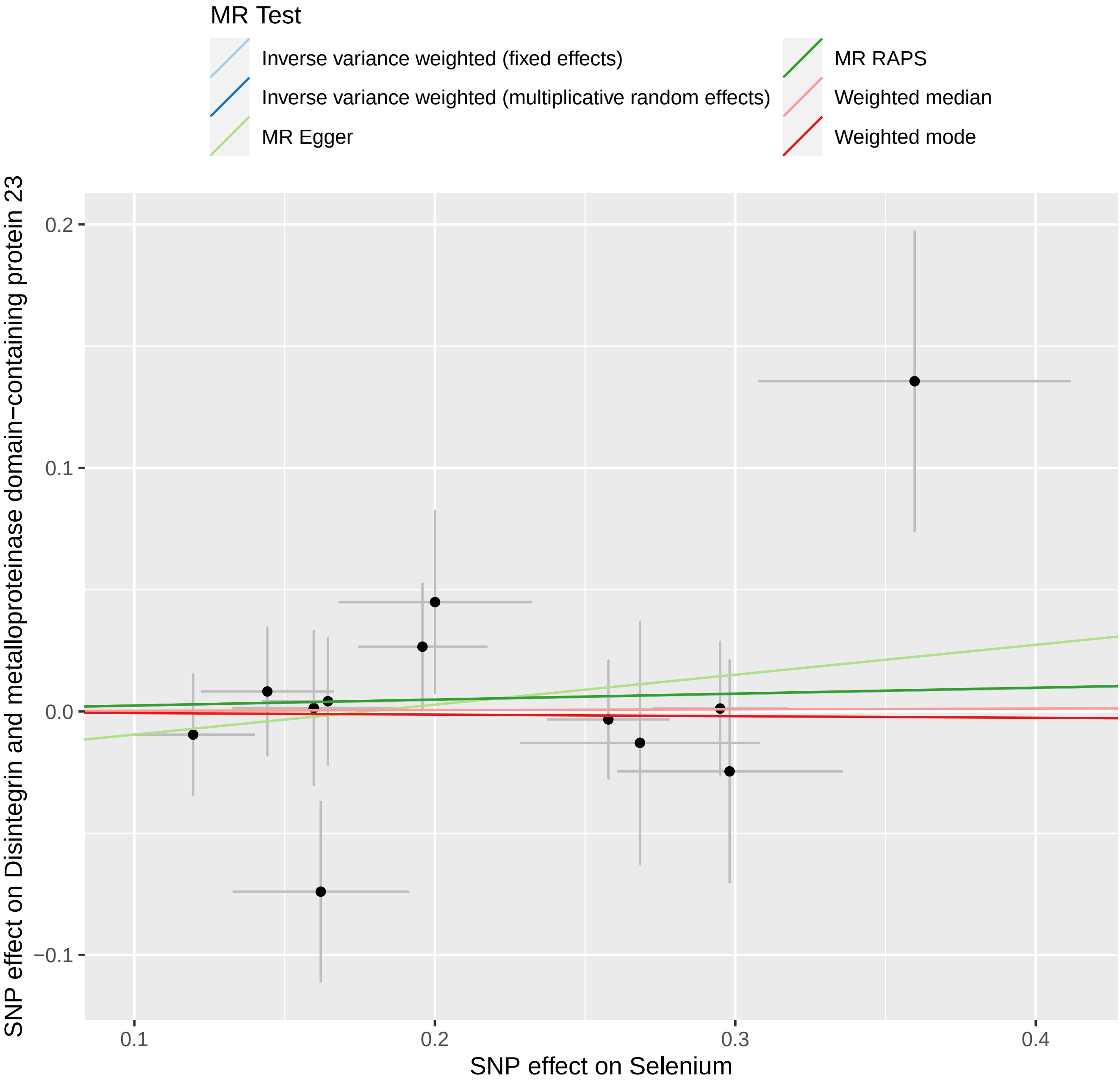

Batch 3−29 : Selenium on Disintegrin and metalloproteinase domain−containing protein 29

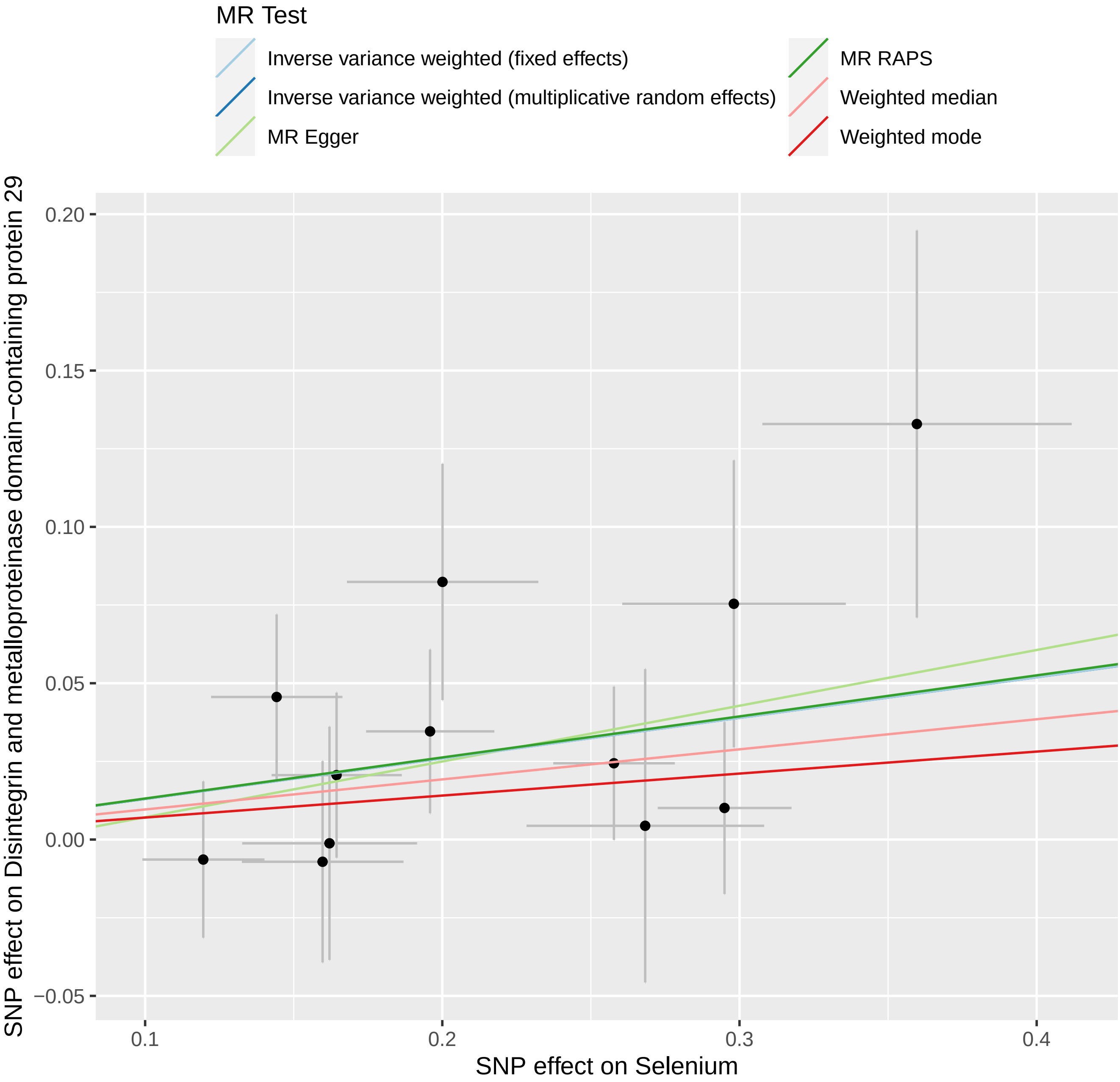

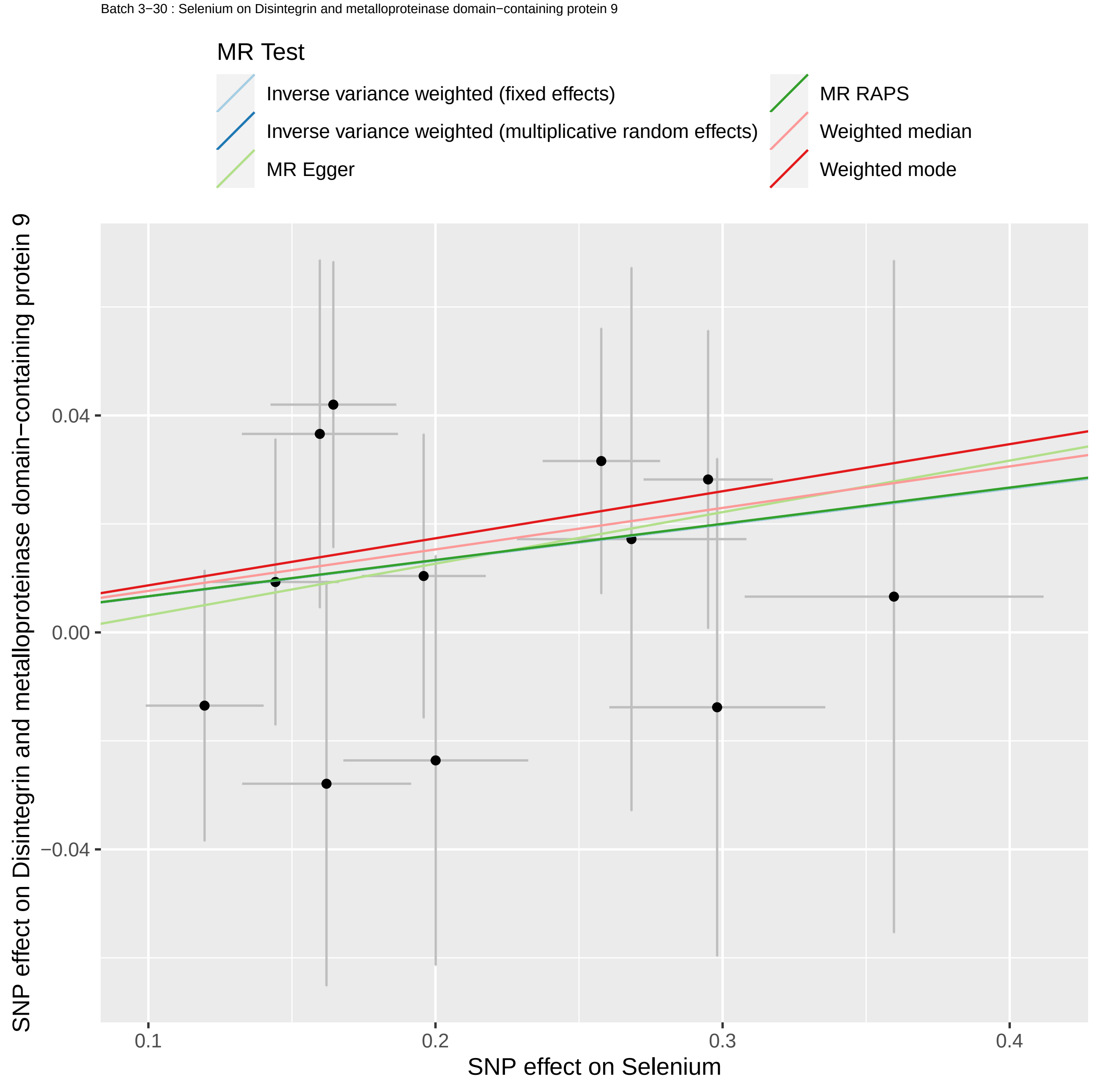
Batch 3−30 : Selenium on Disintegrin and metalloproteinase domain−containing protein 9
MR Test
Inverse variance weighted (fixed effects)
Inverse variance weighted (multiplicative random effects)
MR Egger
MR RAPS
Weighted median
Weighted mode
SNP effect on Disintegrin and metalloproteinase domain−containing protein 9
0.04
0.00
−0.04
0.1
0.2
0.3
0.4
SNP effect on Selenium

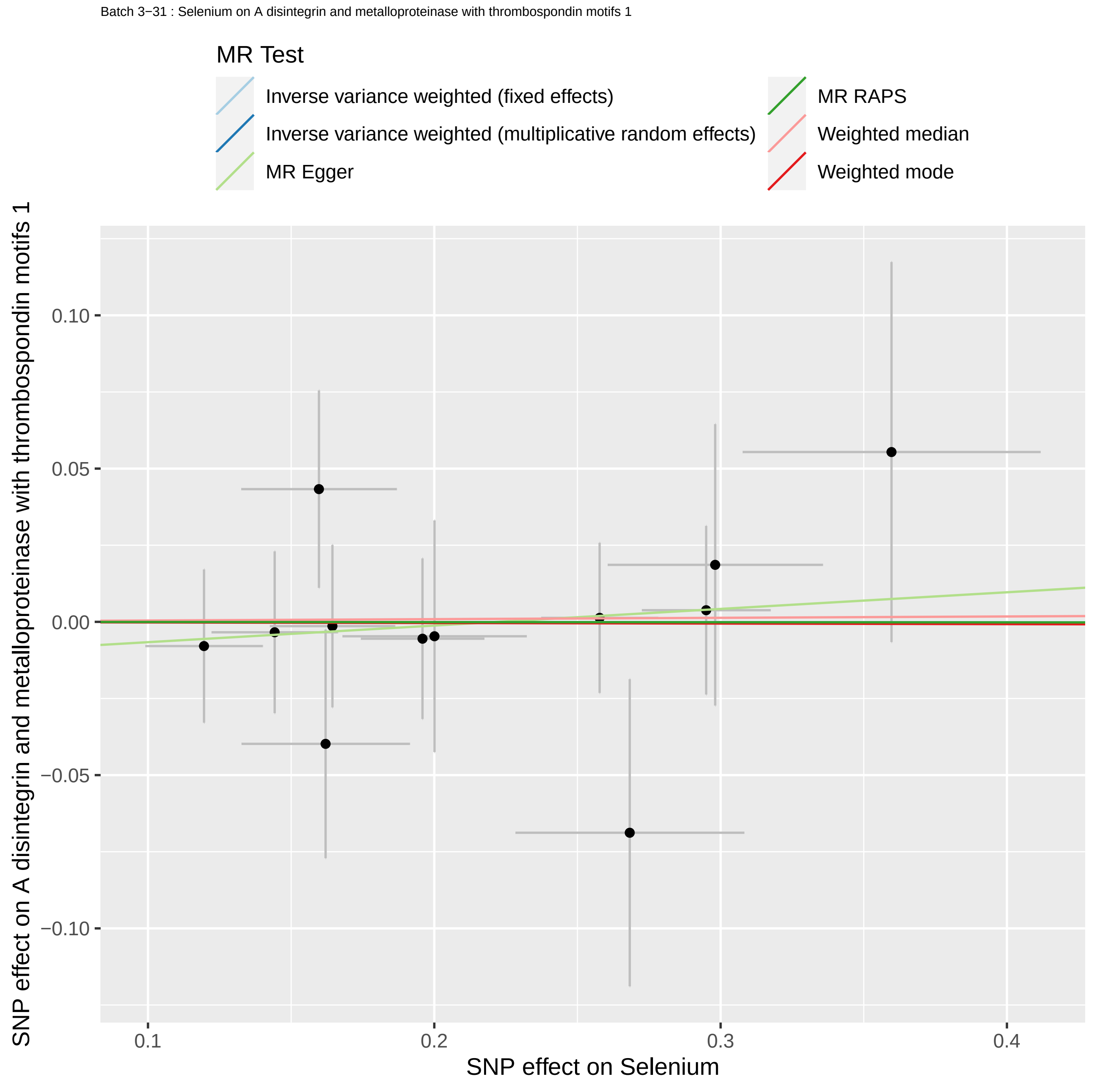

Batch 3−31 : Selenium on A disintegrin and metalloproteinase with thrombospondin motifs 1
MR Test
Inverse variance weighted (fixed effects)
Inverse variance weighted (multiplicative random effects)
MR Egger
MR RAPS
Weighted median
Weighted mode
SNP effect on A disintegrin and metalloproteinase with thrombospondin motifs 1
0.10
0.05
0.00
−0.05
−0.10
0.1
0.2
0.3
0.4
SNP effect on Selenium

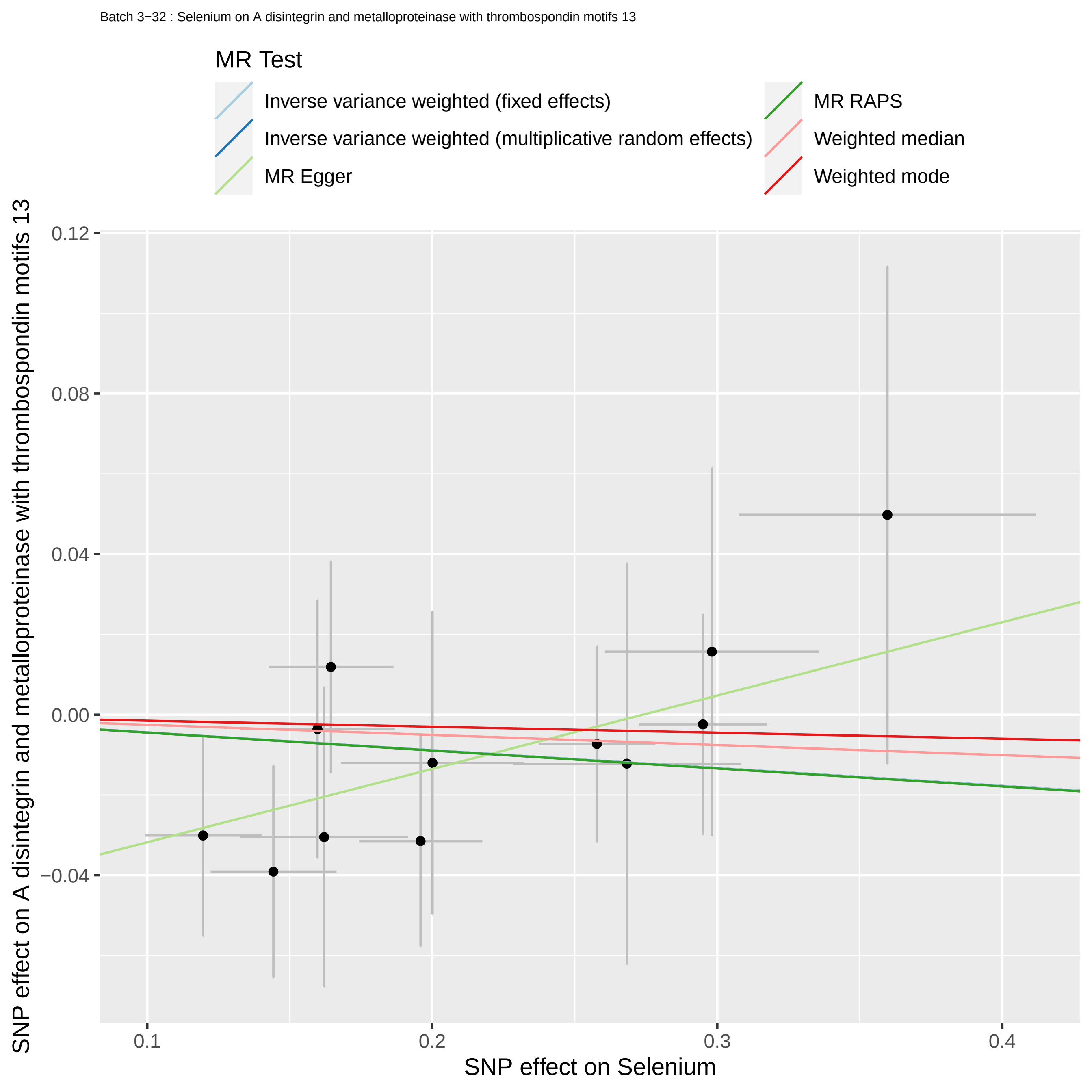

Batch 3−32 : Selenium on A disintegrin and metalloproteinase with thrombospondin motifs 13
MR Test
Inverse variance weighted (fixed effects)
Inverse variance weighted (multiplicative random effects)
MR Egger
MR RAPS
Weighted median
Weighted mode
SNP effect on A disintegrin and metalloproteinase with thrombospondin motifs 13
0.12
0.08
0.04
0.00
−0.04
0.1
0.2
0.3
0.4
SNP effect on Selenium

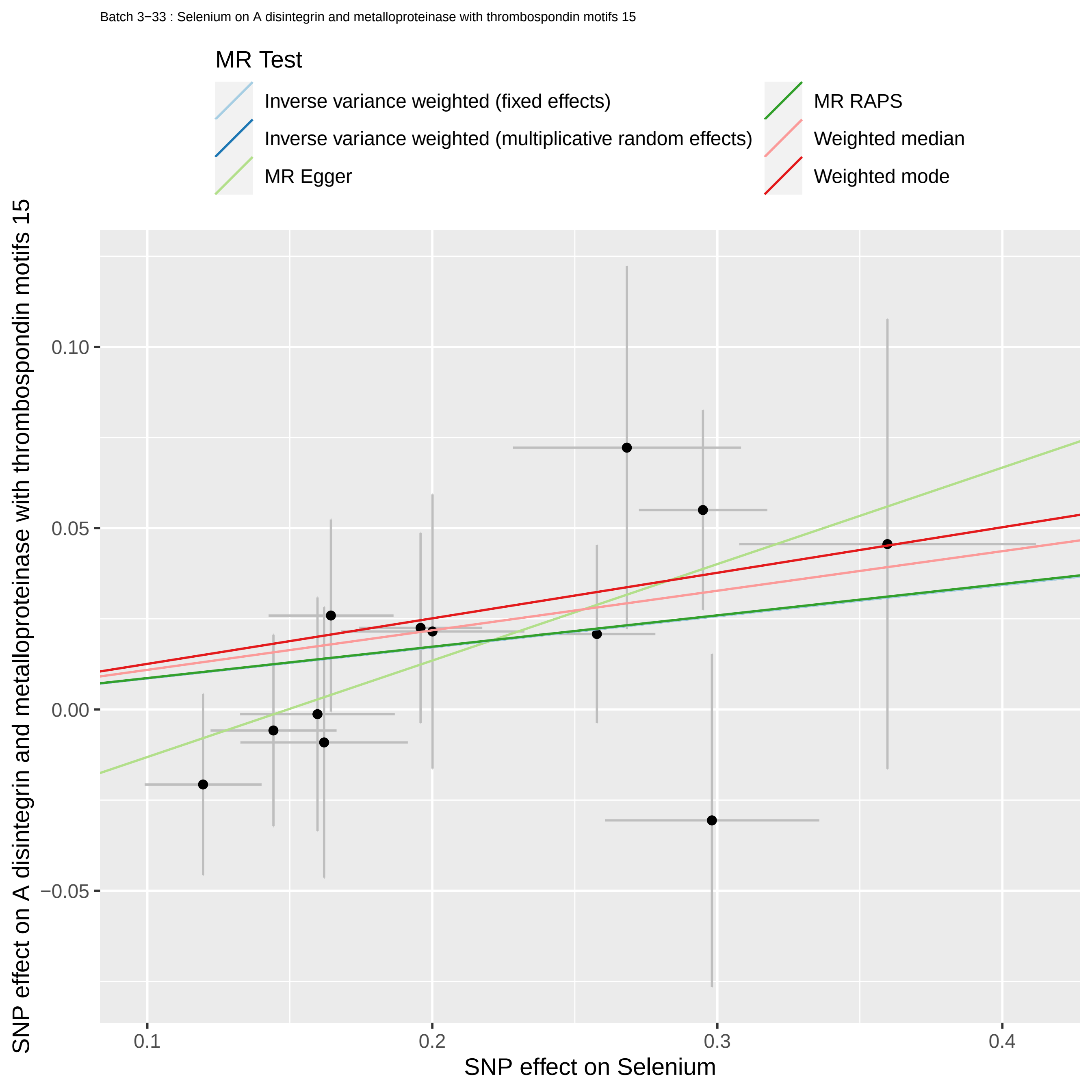

Batch 3−33 : Selenium on A disintegrin and metalloproteinase with thrombospondin motifs 15
MR Test
Inverse variance weighted (fixed effects)
Inverse variance weighted (multiplicative random effects)
MR Egger
MR RAPS
Weighted median
Weighted mode
SNP effect on A disintegrin and metalloproteinase with thrombospondin motifs 15
0.10
0.05
0.00
−0.05
0.1
0.2
0.3
0.4
SNP effect on Selenium

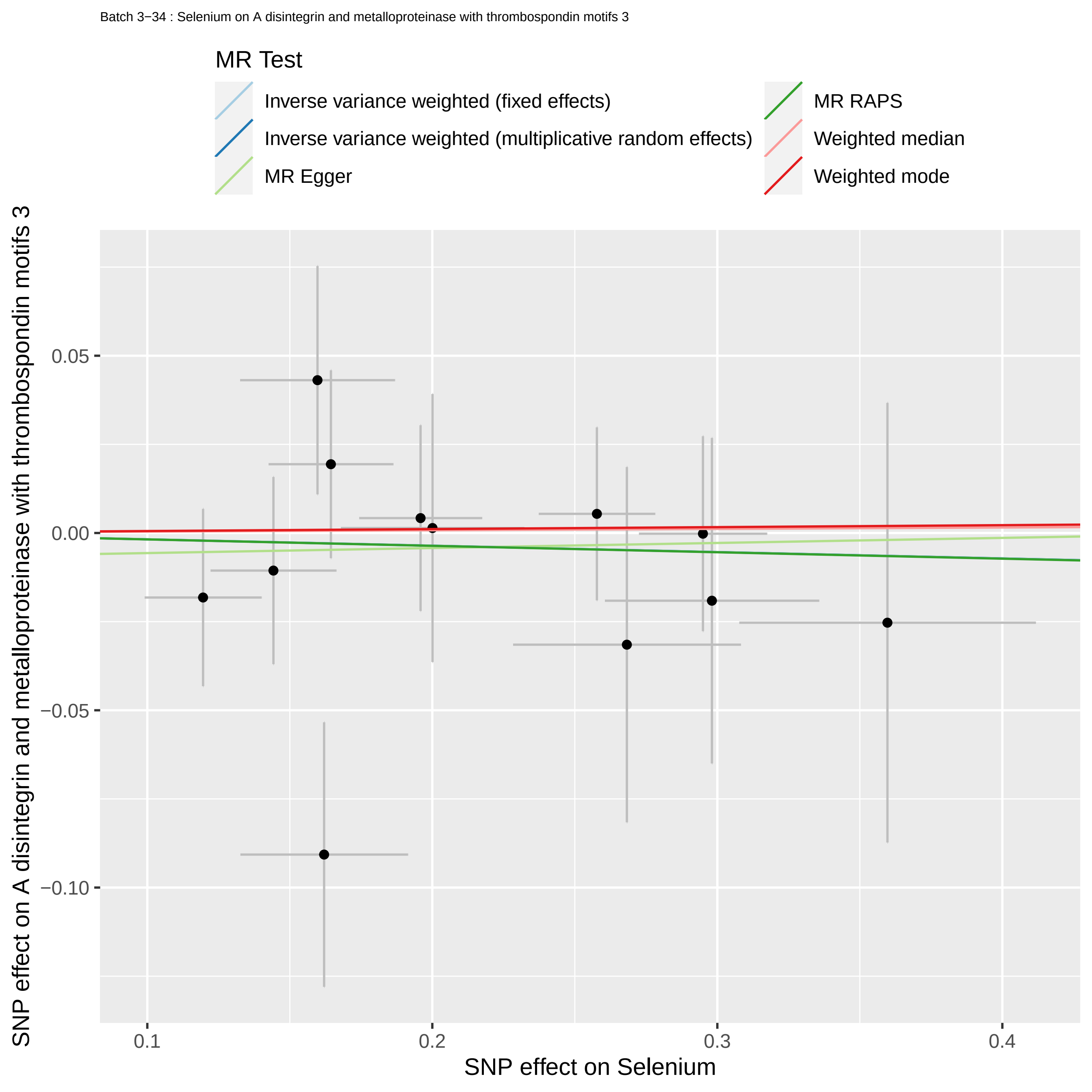
Batch 3−34 : Selenium on A disintegrin and metalloproteinase with thrombospondin motifs 3
MR Test
Inverse variance weighted (fixed effects)
Inverse variance weighted (multiplicative random effects)
MR Egger
MR RAPS
Weighted median
Weighted mode
SNP effect on A disintegrin and metalloproteinase with thrombospondin motifs 3
0.05
0.00
−0.05
−0.10
0.1
0.2
0.3
0.4
SNP effect on Selenium

Batch 3−35 : Selenium on A disintegrin and metalloproteinase with thrombospondin motifs 4

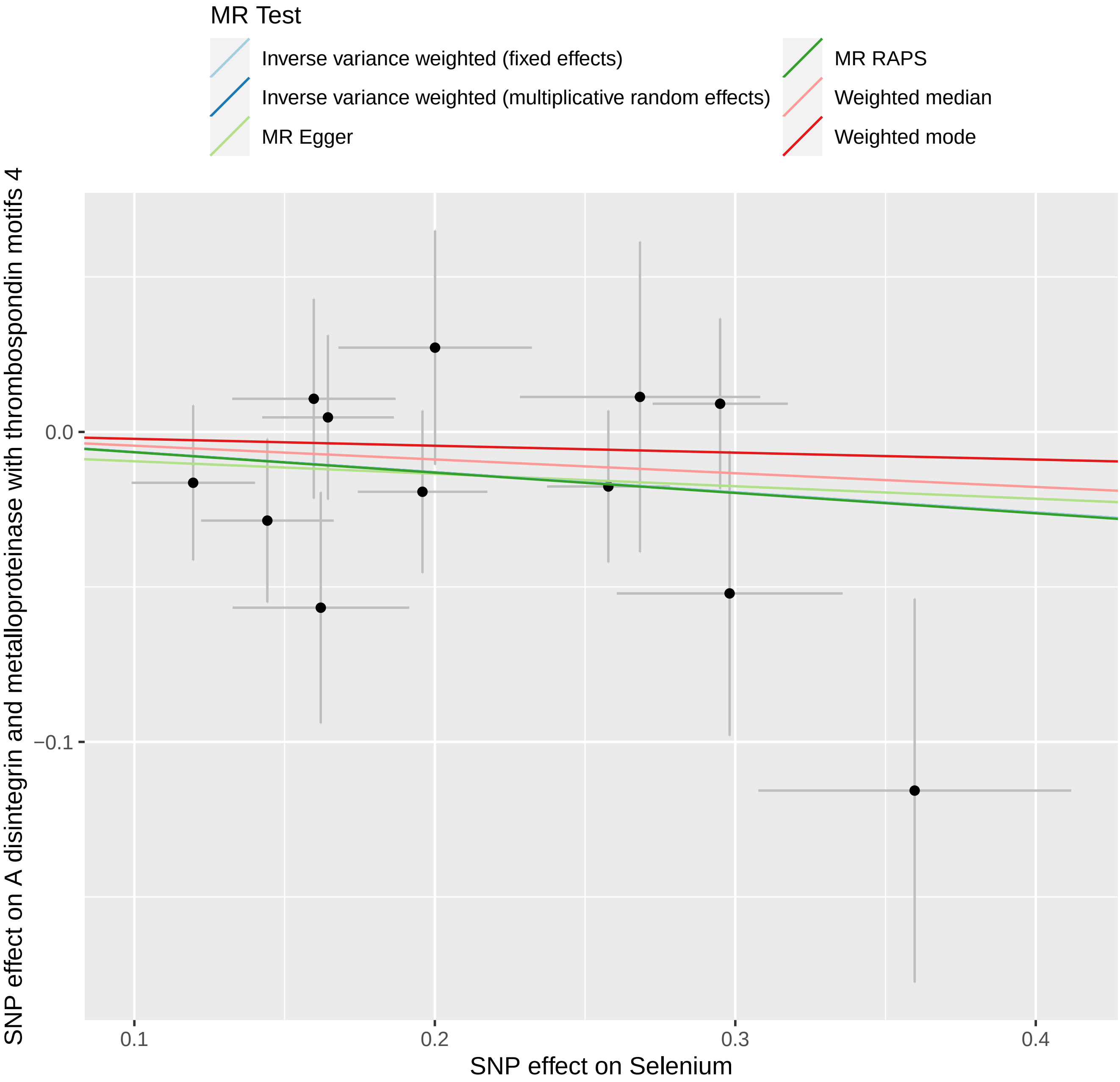

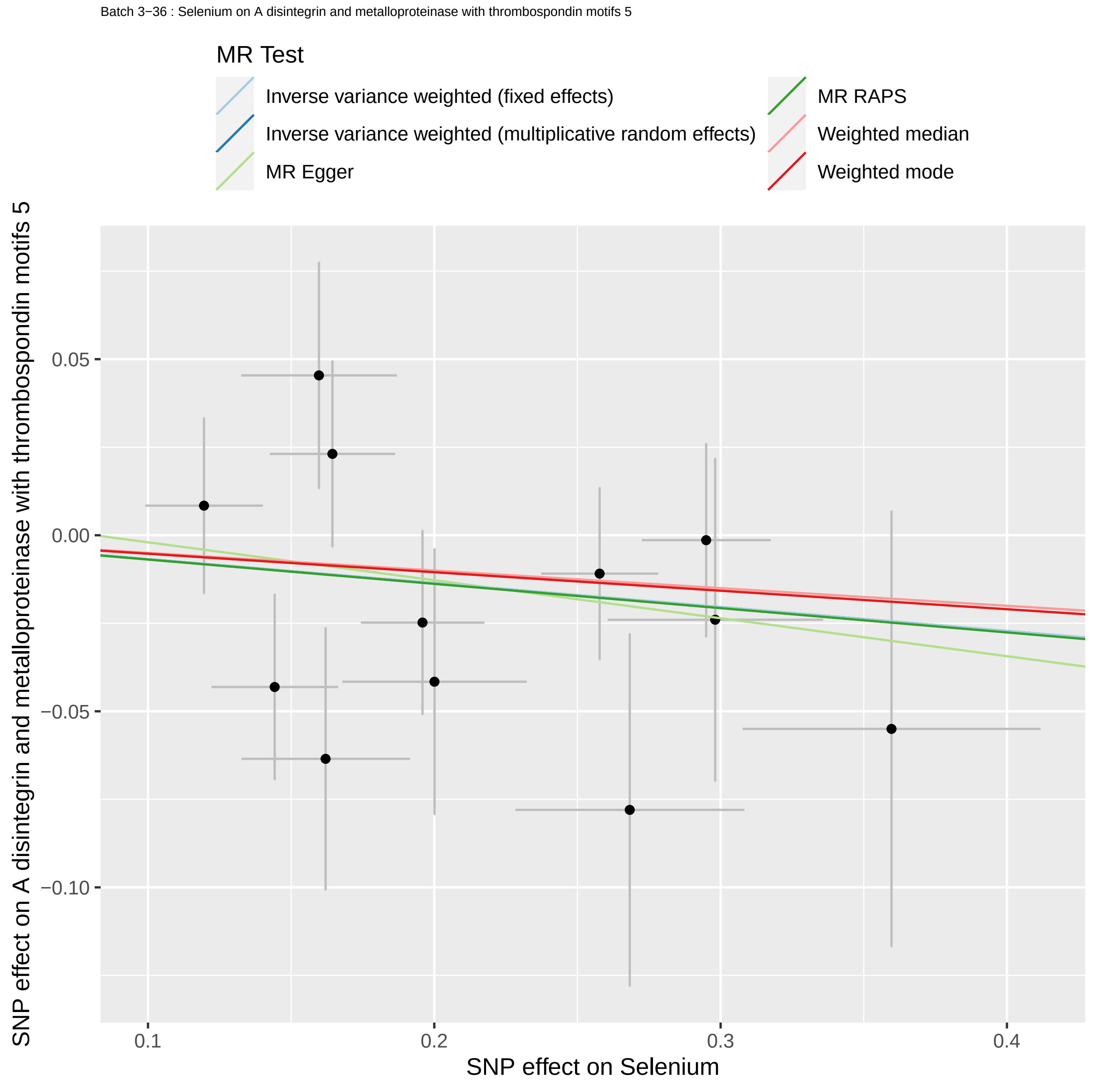
Batch 3−36 : Selenium on A disintegrin and metalloproteinase with thrombospondin motifs 5
MR Test
Inverse variance weighted (fixed effects)
Inverse variance weighted (multiplicative random effects)
MR Egger
MR RAPS
Weighted median
Weighted mode
SNP effect on A disintegrin and metalloproteinase with thrombospondin motifs 5
0.05
0.00
−0.05
−0.10
0.1
0.2
0.3
0.4
SNP effect on Selenium

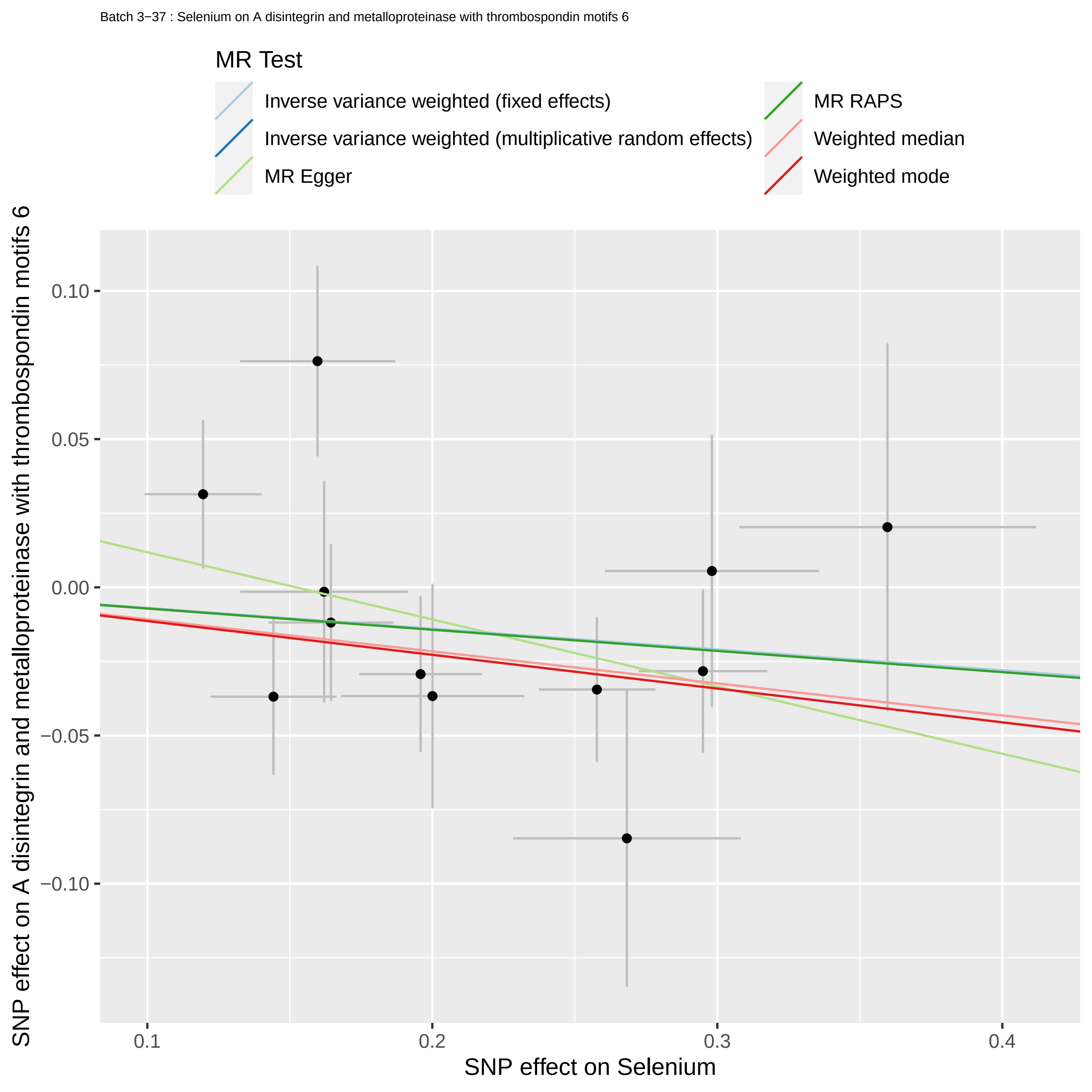

Batch 3−37 : Selenium on A disintegrin and metalloproteinase with thrombospondin motifs 6
MR Test
Inverse variance weighted (fixed effects)
Inverse variance weighted (multiplicative random effects)
MR Egger
MR RAPS
Weighted median
Weighted mode
SNP effect on A disintegrin and metalloproteinase with thrombospondin motifs 6
0.10
0.05
0.00
−0.05
−0.10
0.1
0.2
0.3
0.4
SNP effect on Selenium

Batch 3−38 : Selenium on ADAMTS−like protein 1

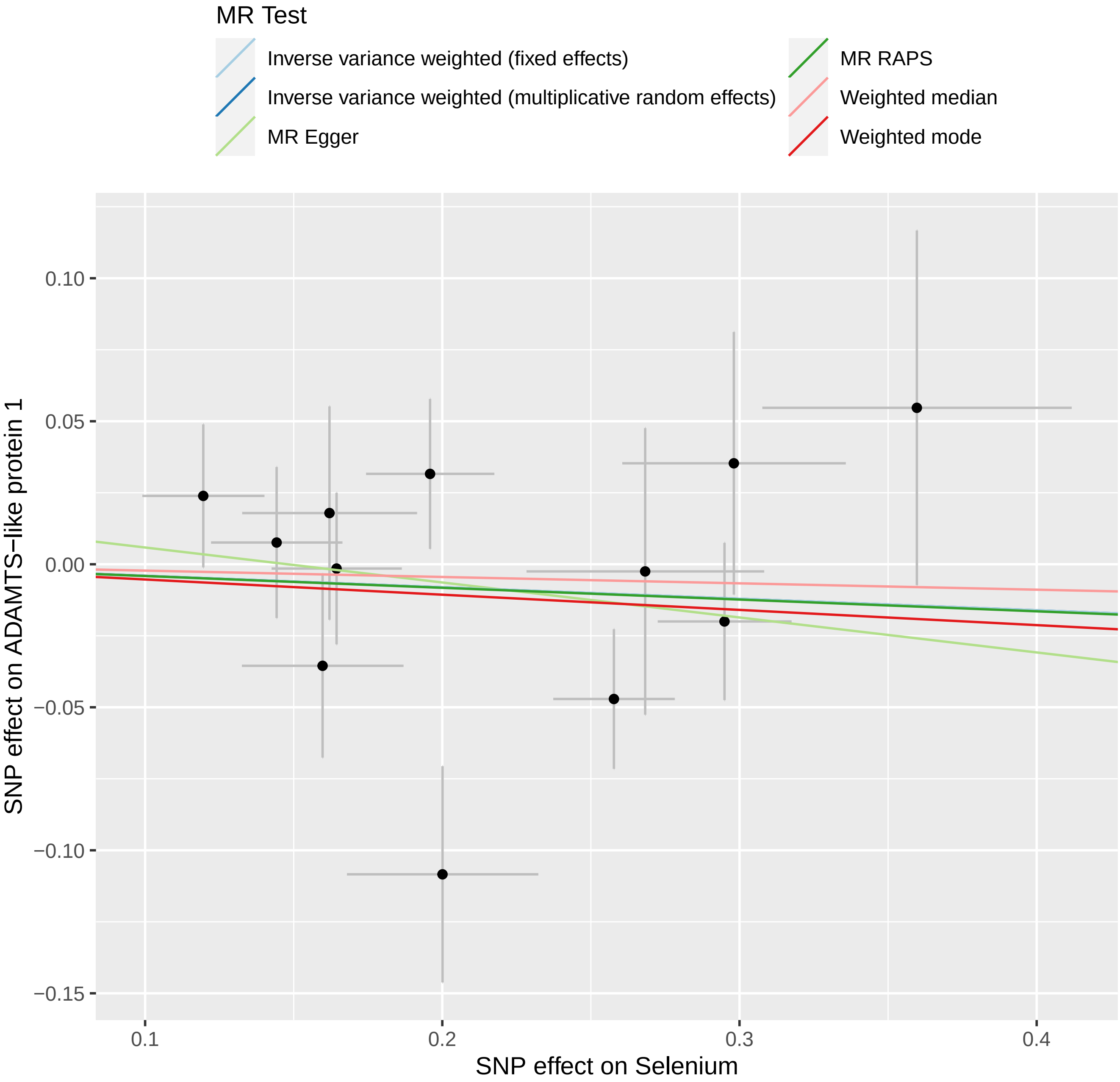

Batch 3−39 : Selenium on AarF domain−containing protein kinase 4

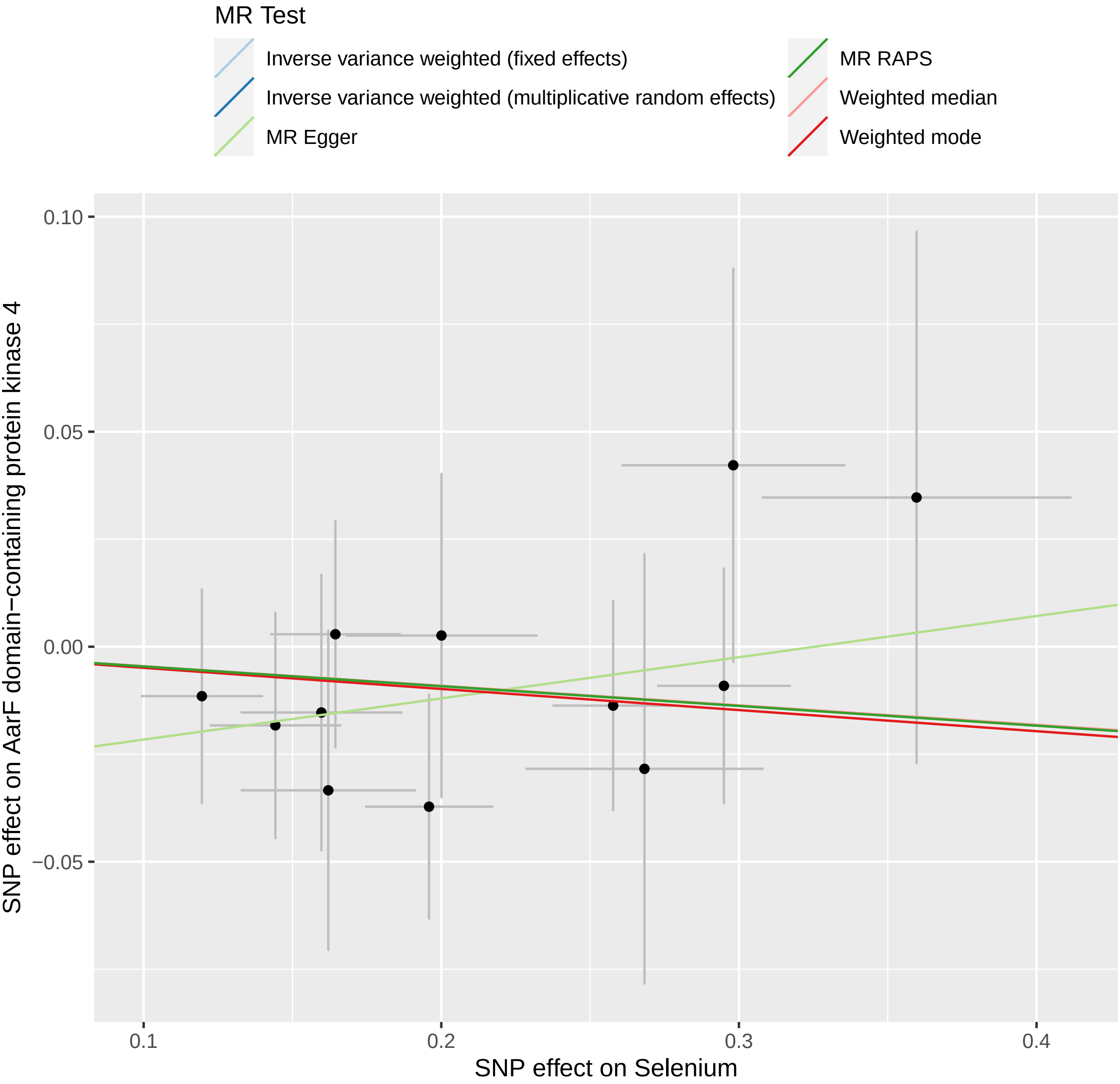

Batch 3−40 : Selenium on Pituitary adenylate cyclase−activating polypeptide 27

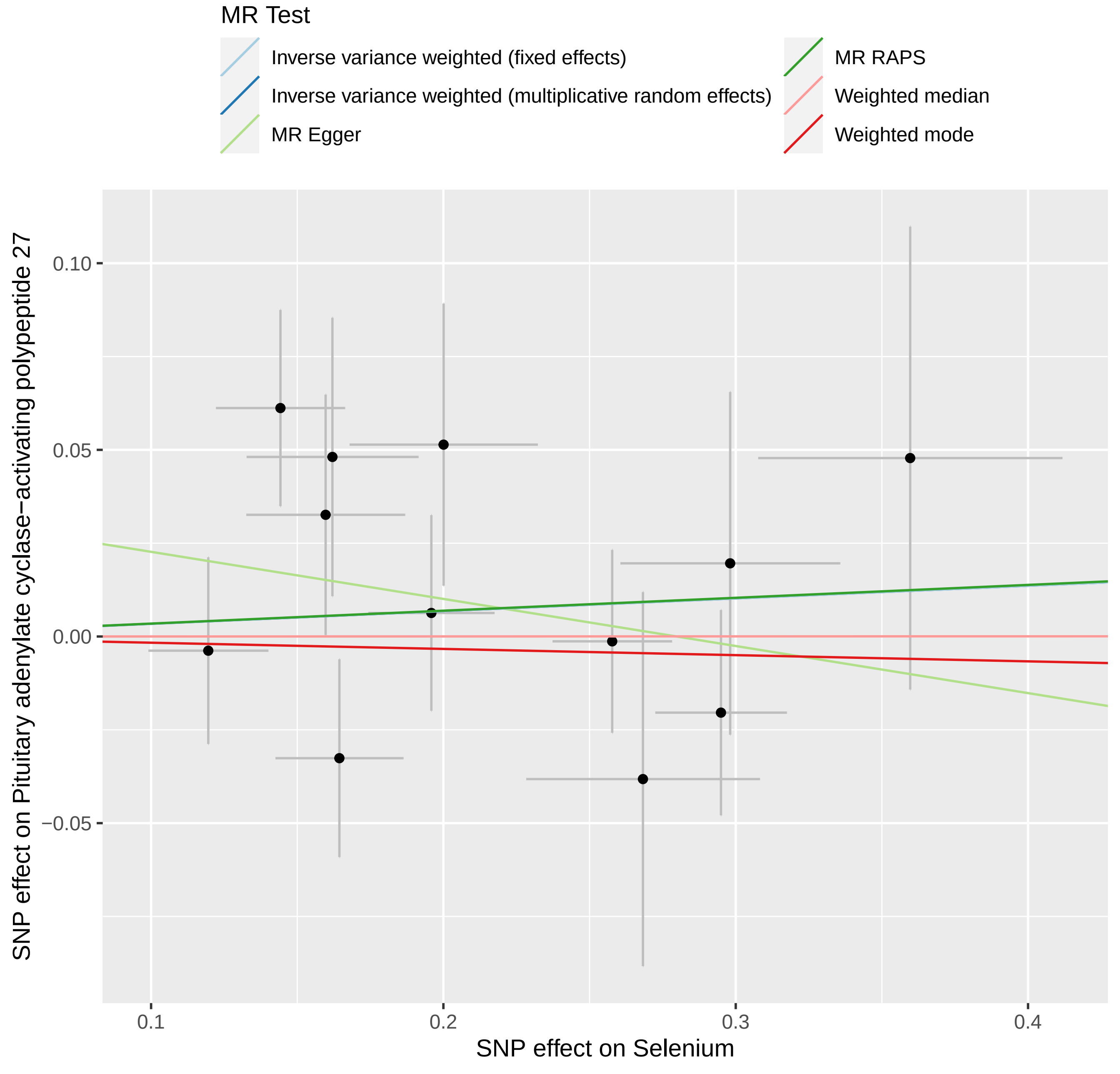

Batch 3−41 : Selenium on Pituitary adenylate cyclase−activating polypeptide 38

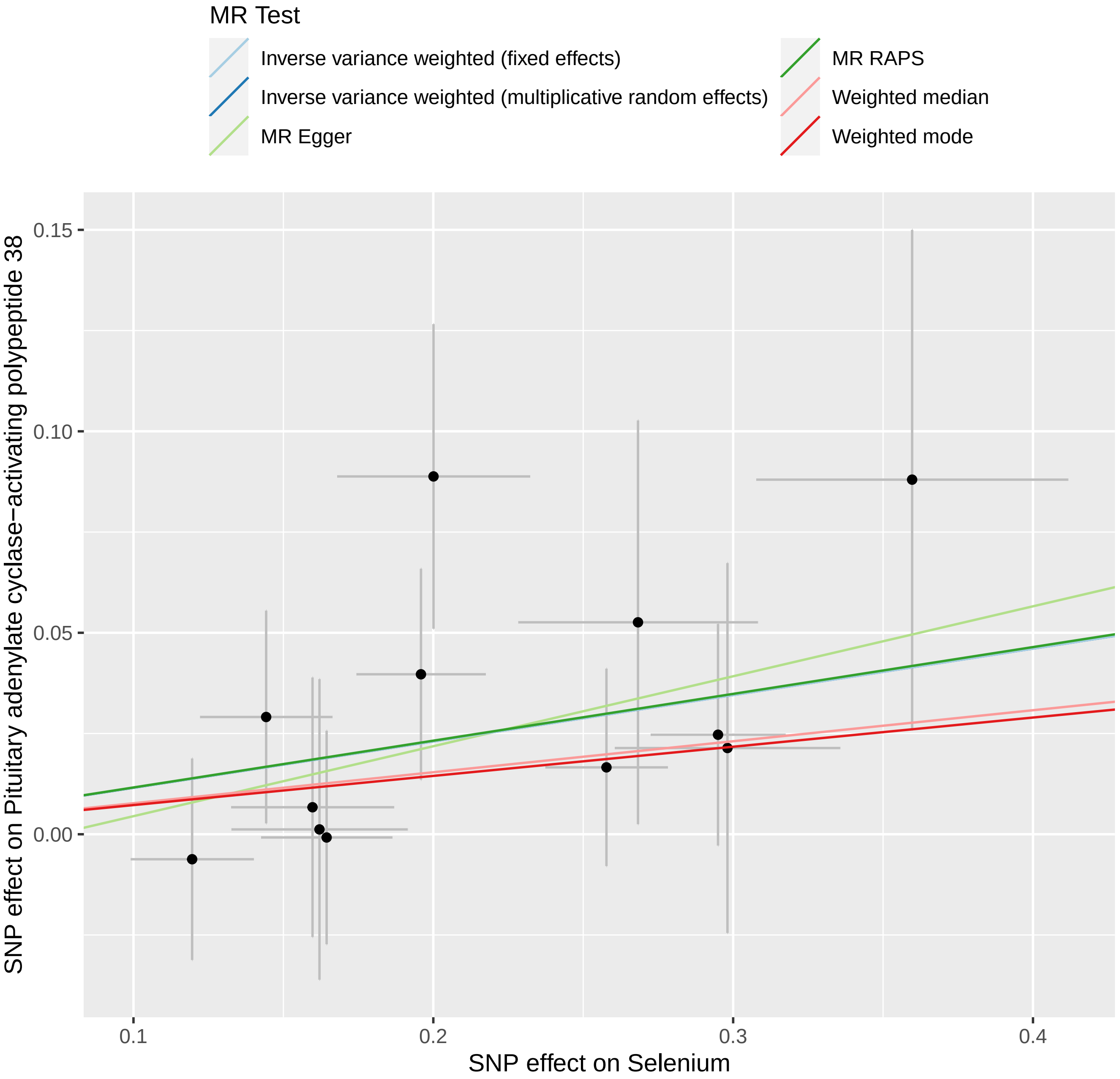

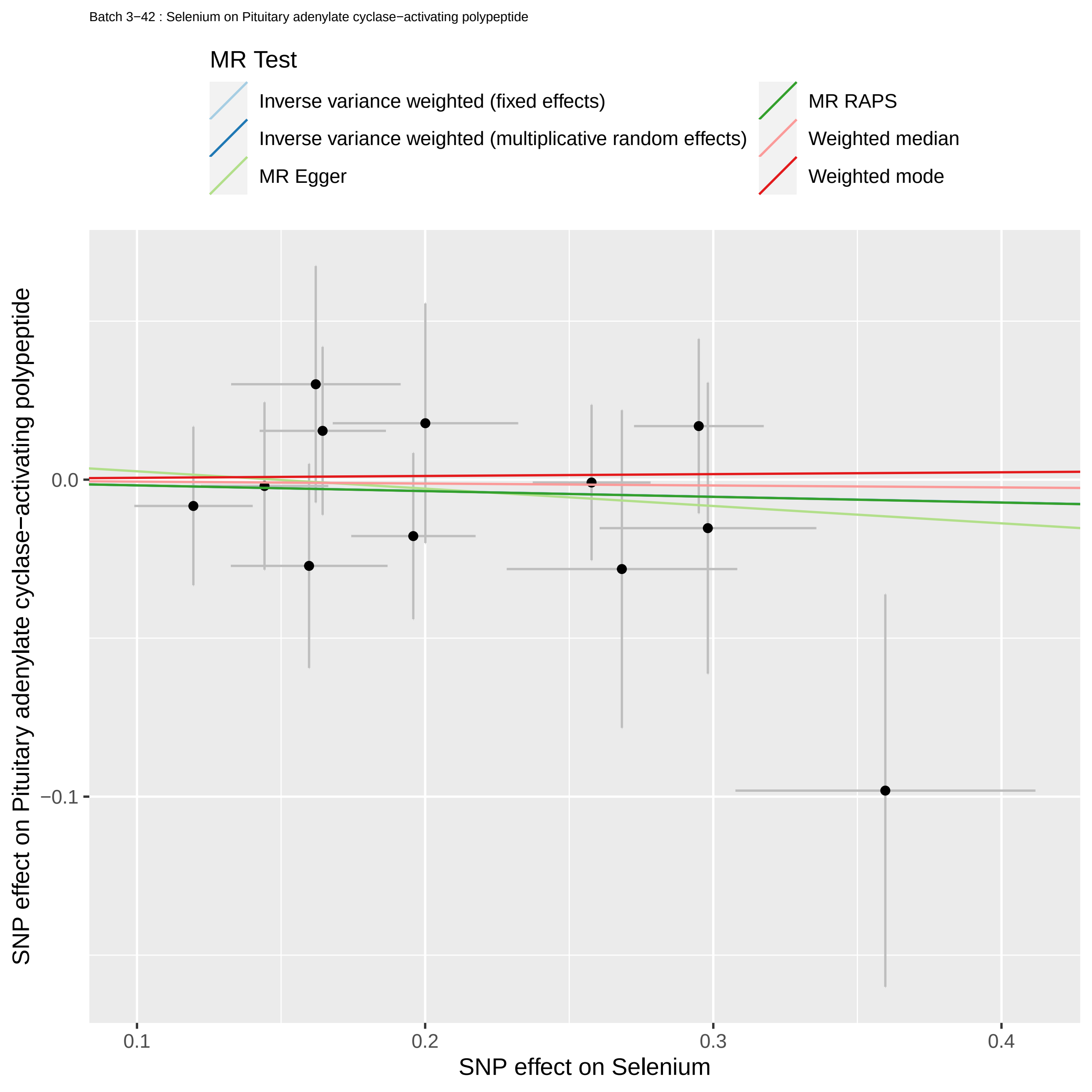
Batch 3−42 : Selenium on Pituitary adenylate cyclase−activating polypeptide
MR Test
Inverse variance weighted (fixed effects)
Inverse variance weighted (multiplicative random effects)
MR Egger
MR RAPS
Weighted median
Weighted mode
SNP effect on Pituitary adenylate cyclase−activating polypeptide
0.0
−0.1
0.1
0.2
0.3
0.4
SNP effect on Selenium

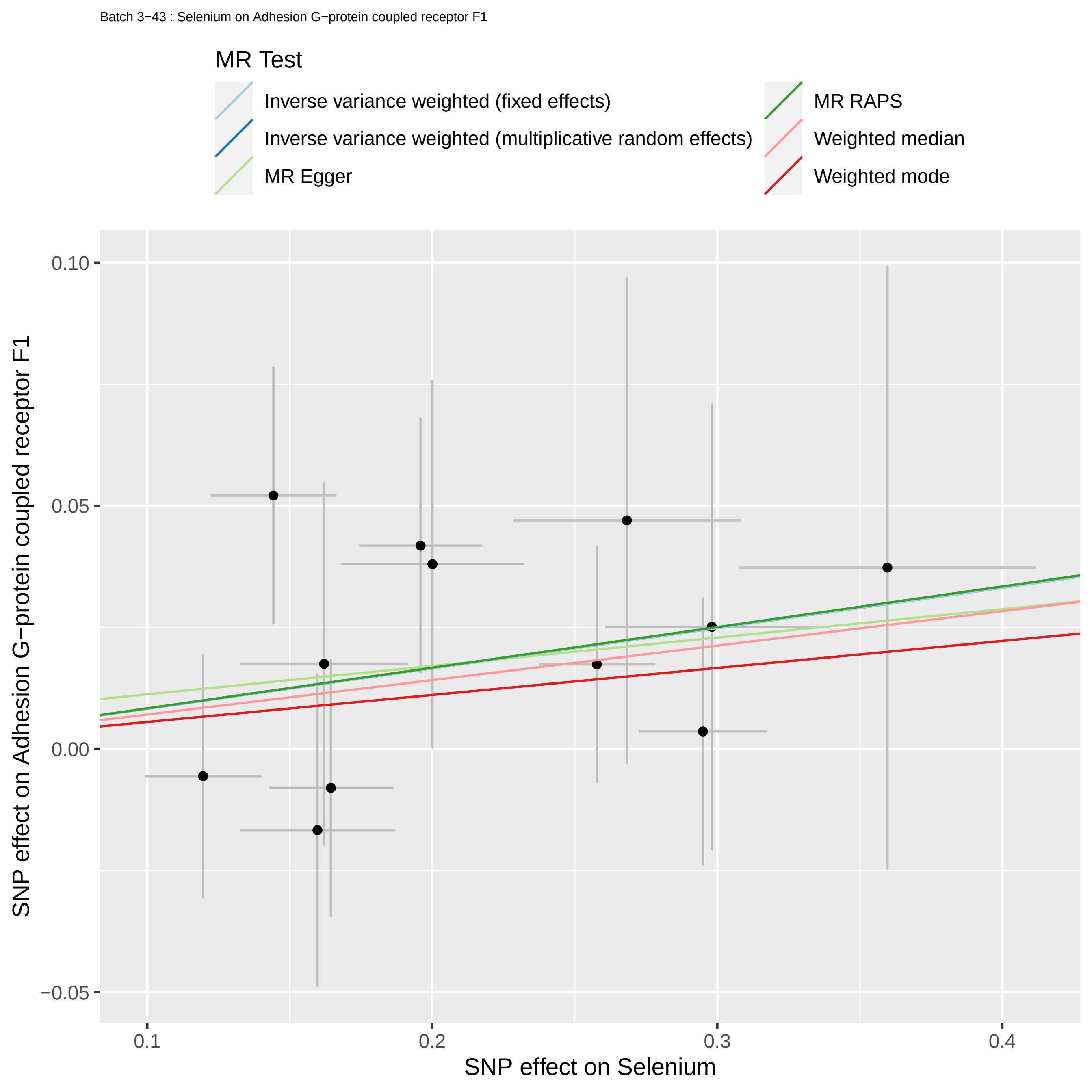
Batch 3−43 : Selenium on Adhesion G−protein coupled receptor F1
MR Test
Inverse variance weighted (fixed effects)
Inverse variance weighted (multiplicative random effects)
MR Egger
MR RAPS
Weighted median
Weighted mode
0.10
0.05
0.00
−0.05
0.1
0.2
0.3
0.4
SNP effect on Adhesion G−protein coupled receptor F1
SNP effect on Selenium

Batch 3−44 : Selenium on Adhesion G protein−coupled receptor F5

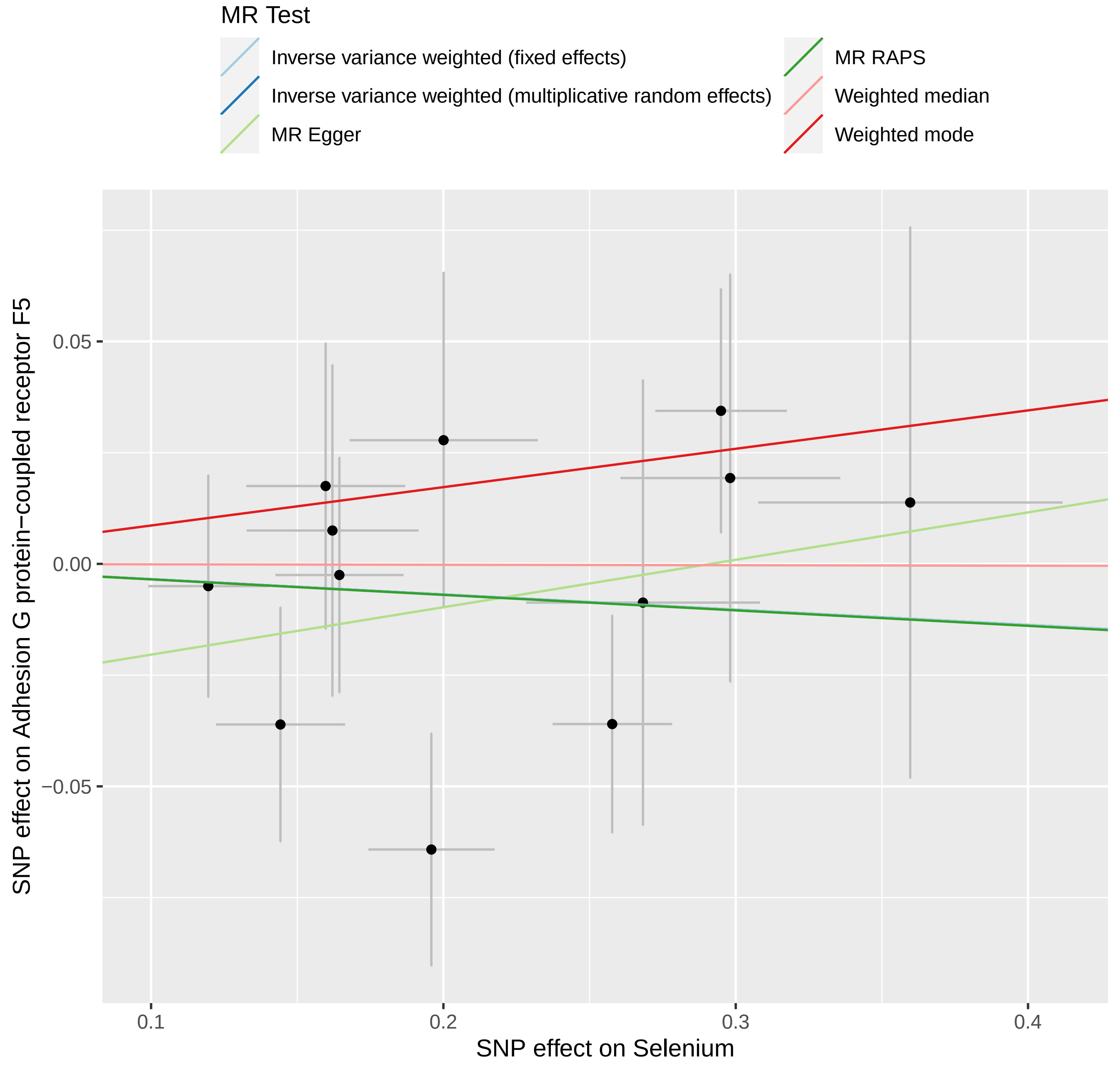

Batch 3−45 : Selenium on Adhesion G−protein coupled receptor G5

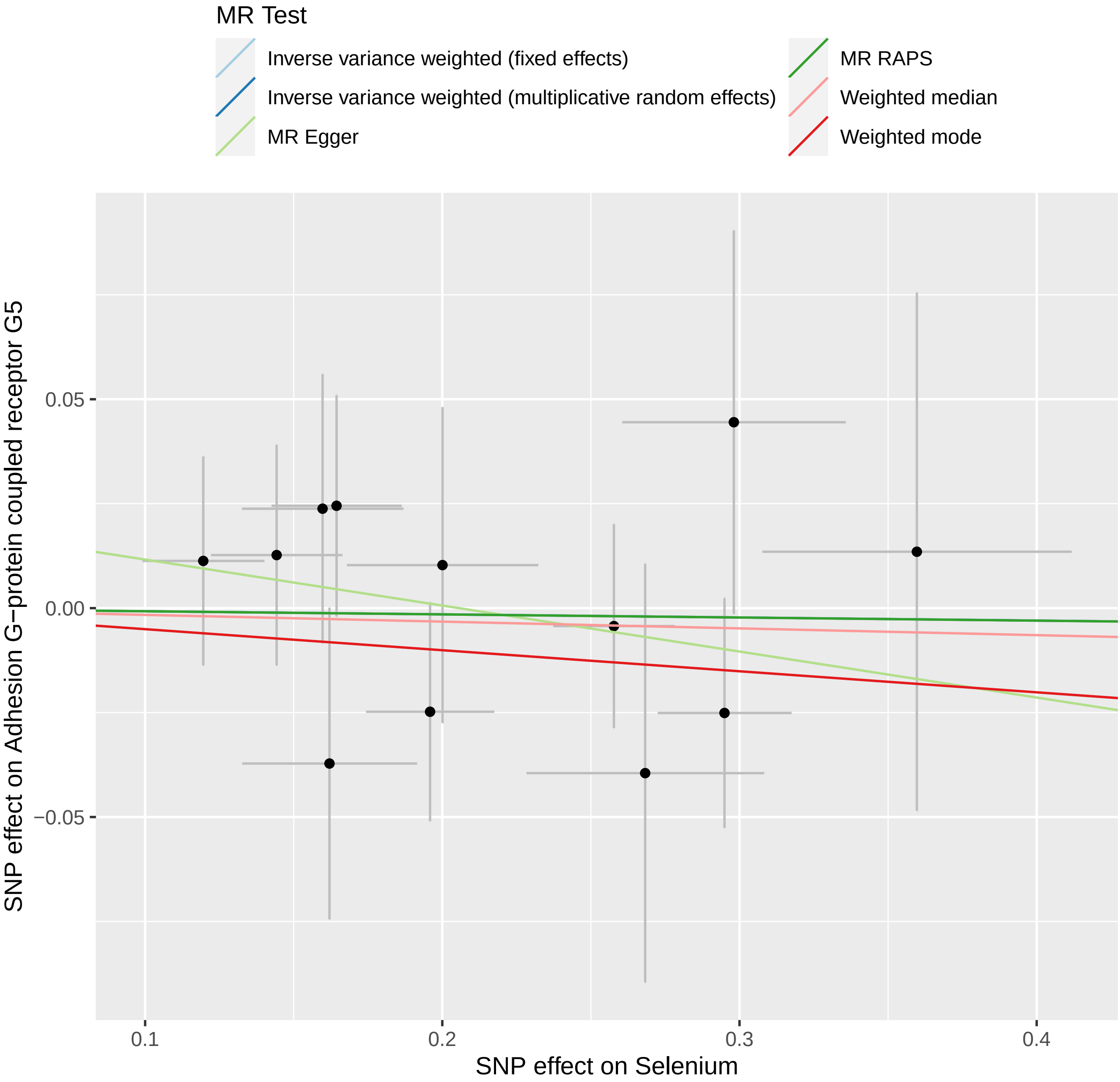

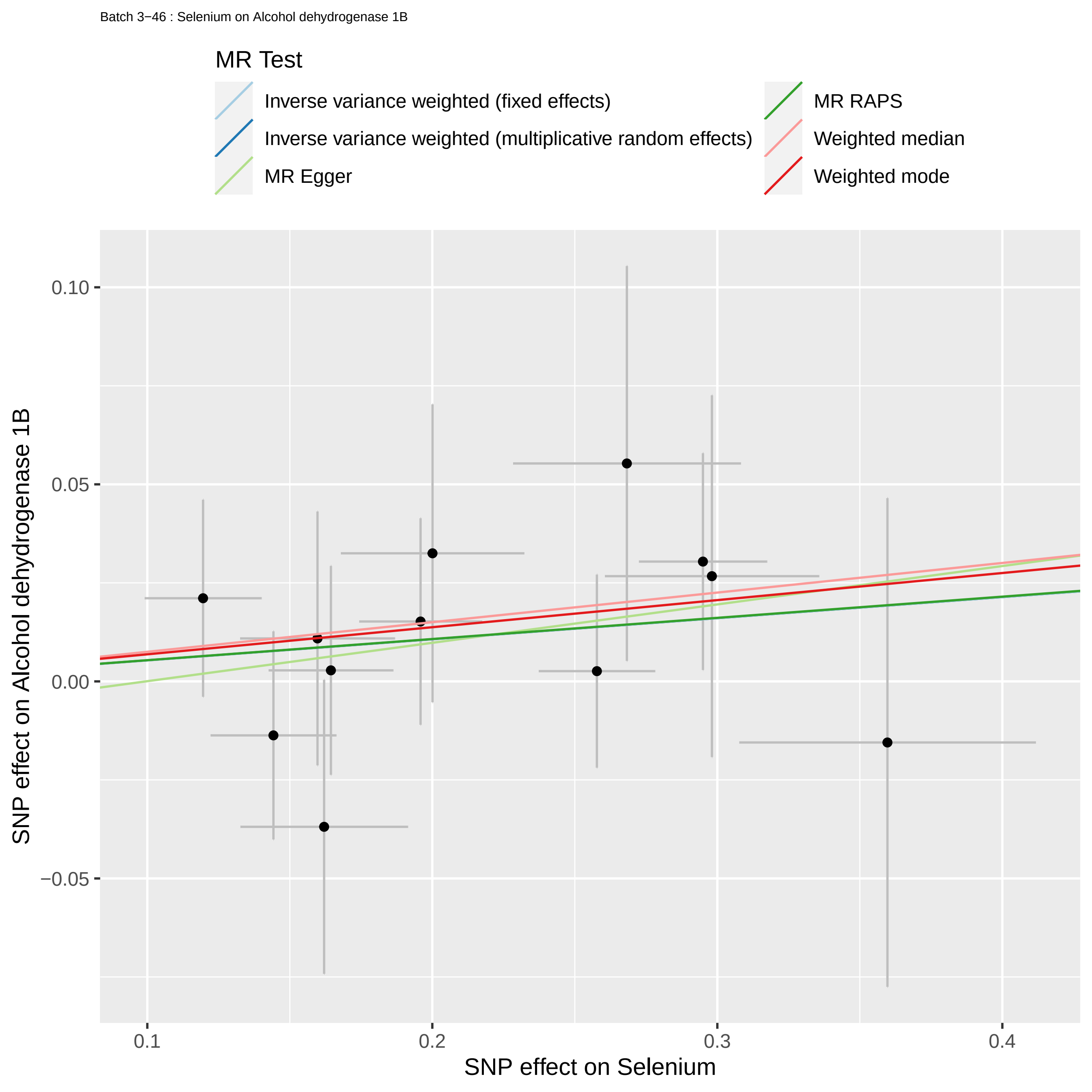
Batch 3−46 : Selenium on Alcohol dehydrogenase 1B
MR Test
Inverse variance weighted (fixed effects)
Inverse variance weighted (multiplicative random effects)
MR Egger
MR RAPS
Weighted median
Weighted mode
0.10
0.05
0.00
−0.05
0.1
0.2
0.3
0.4
SNP effect on Alcohol dehydrogenase 1B
SNP effect on Selenium

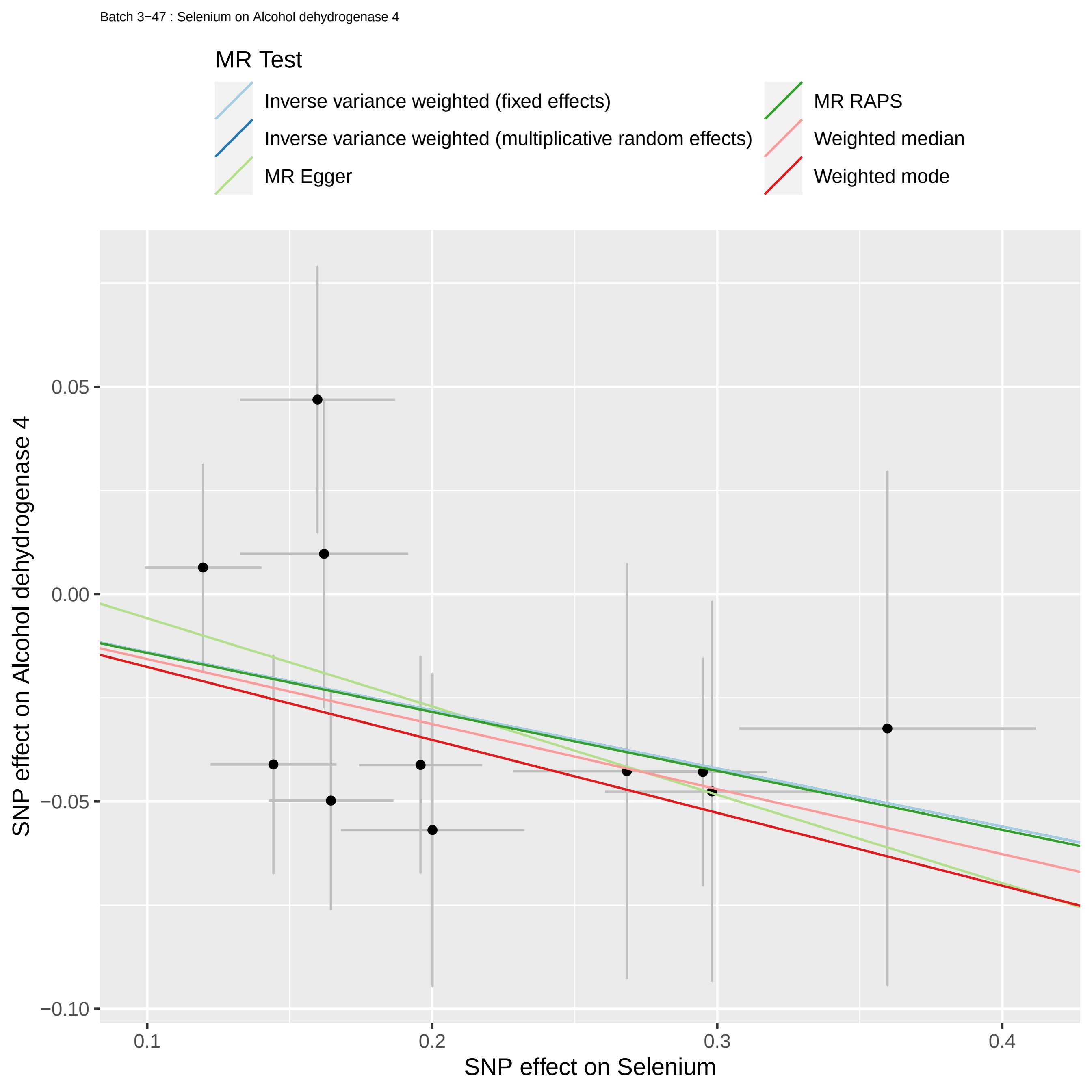
Batch 3−47 : Selenium on Alcohol dehydrogenase 4
MR Test
Inverse variance weighted (fixed effects)
Inverse variance weighted (multiplicative random effects)
MR Egger
MR RAPS
Weighted median
Weighted mode
0.05
0.00
−0.05
−0.10
0.1
0.2
0.3
0.4
SNP effect on Alcohol dehydrogenase 4
SNP effect on Selenium

Batch 3−48 : Selenium on Adrenomedullin

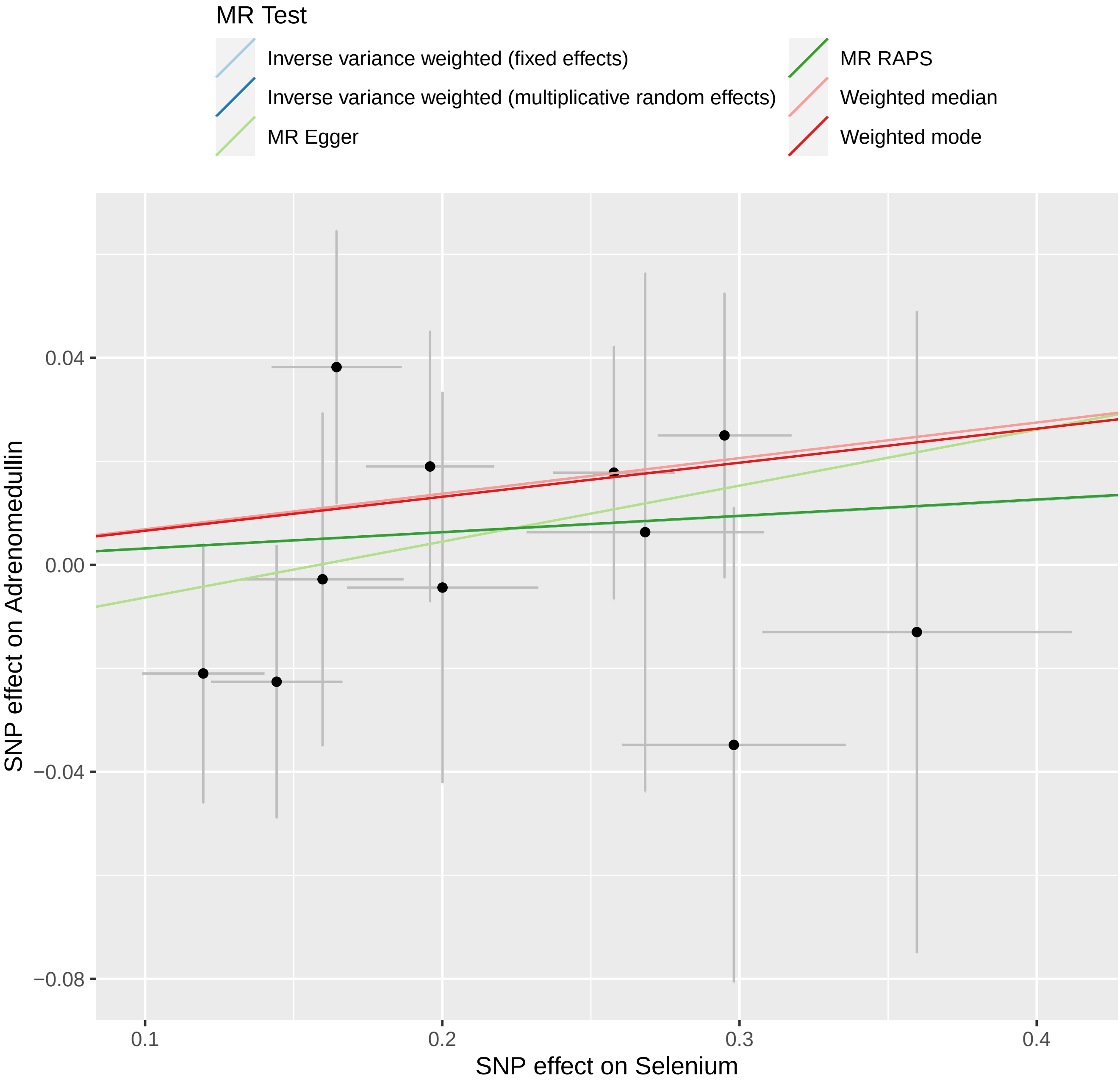

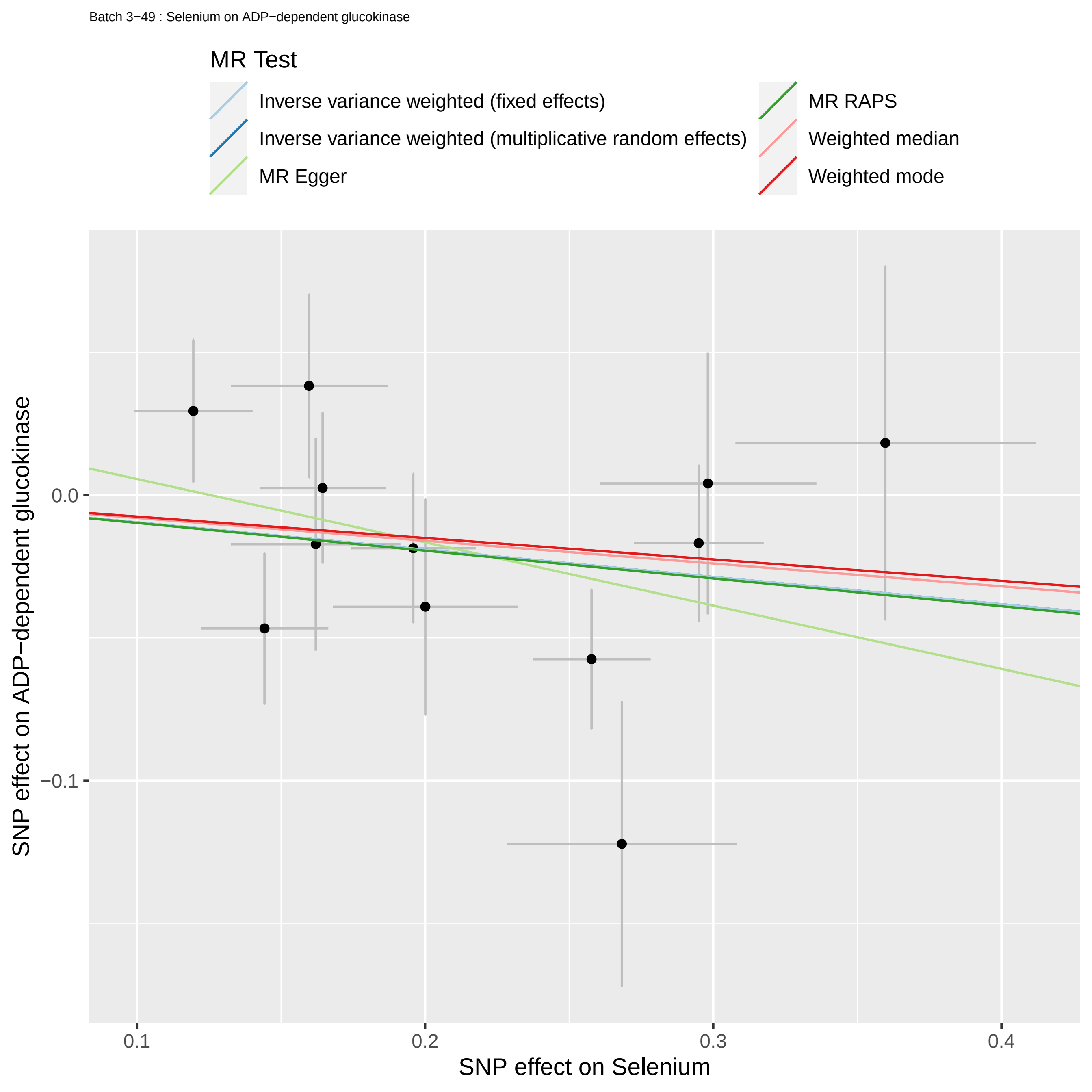
Batch 3−49 : Selenium on ADP−dependent glucokinase
MR Test
Inverse variance weighted (fixed effects)
Inverse variance weighted (multiplicative random effects)
MR Egger
MR RAPS
Weighted median
Weighted mode
SNP effect on ADP−dependent glucokinase
0.0
−0.1
0.1
0.2
0.3
0.4
SNP effect on Selenium

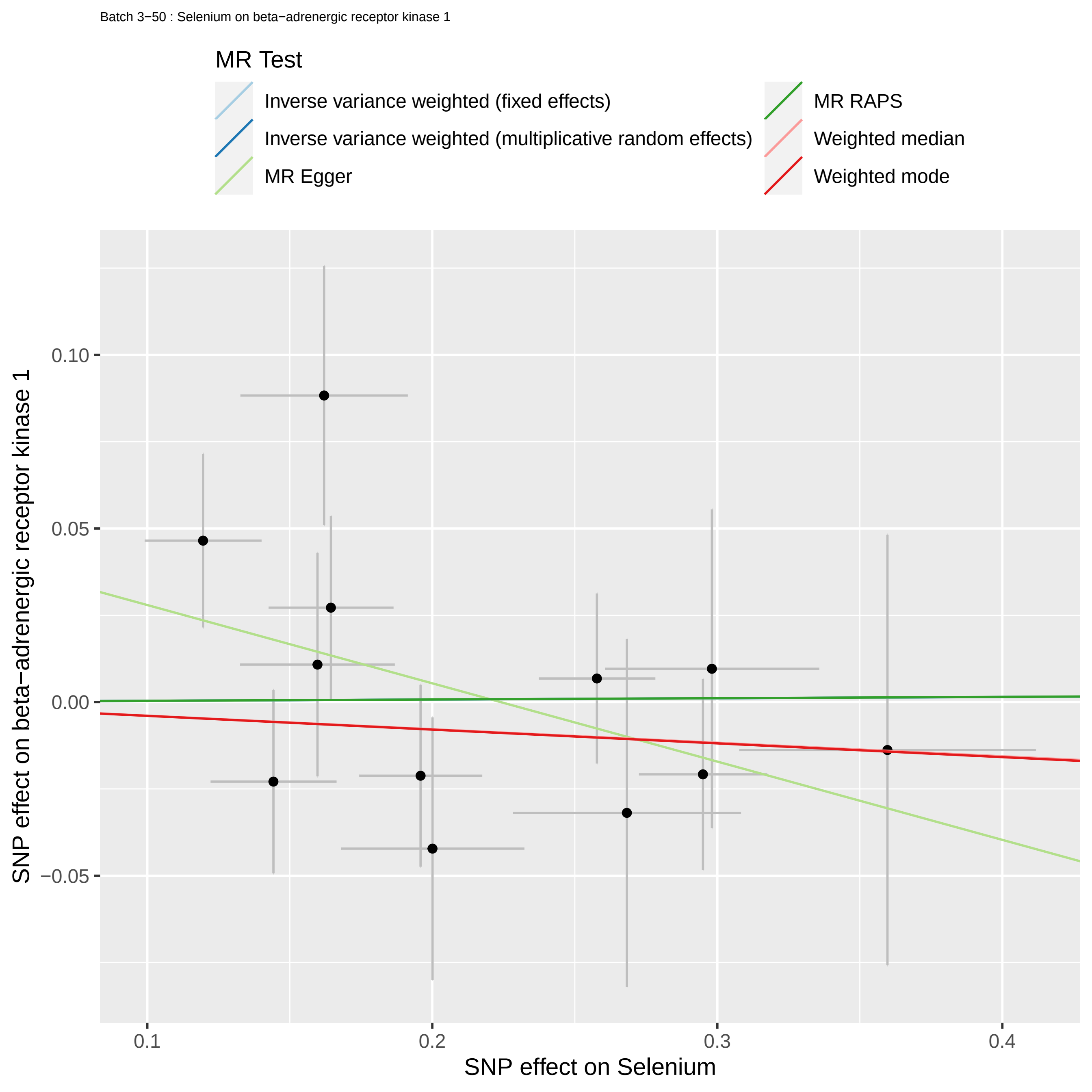
Batch 3−50 : Selenium on beta−adrenergic receptor kinase 1
MR Test
Inverse variance weighted (fixed effects)
Inverse variance weighted (multiplicative random effects)
MR Egger
MR RAPS
Weighted median
Weighted mode
SNP effect on beta−adrenergic receptor kinase 1
0.10
0.05
0.00
−0.05
SNP effect on Selenium
0.1
0.2
0.3
0.4

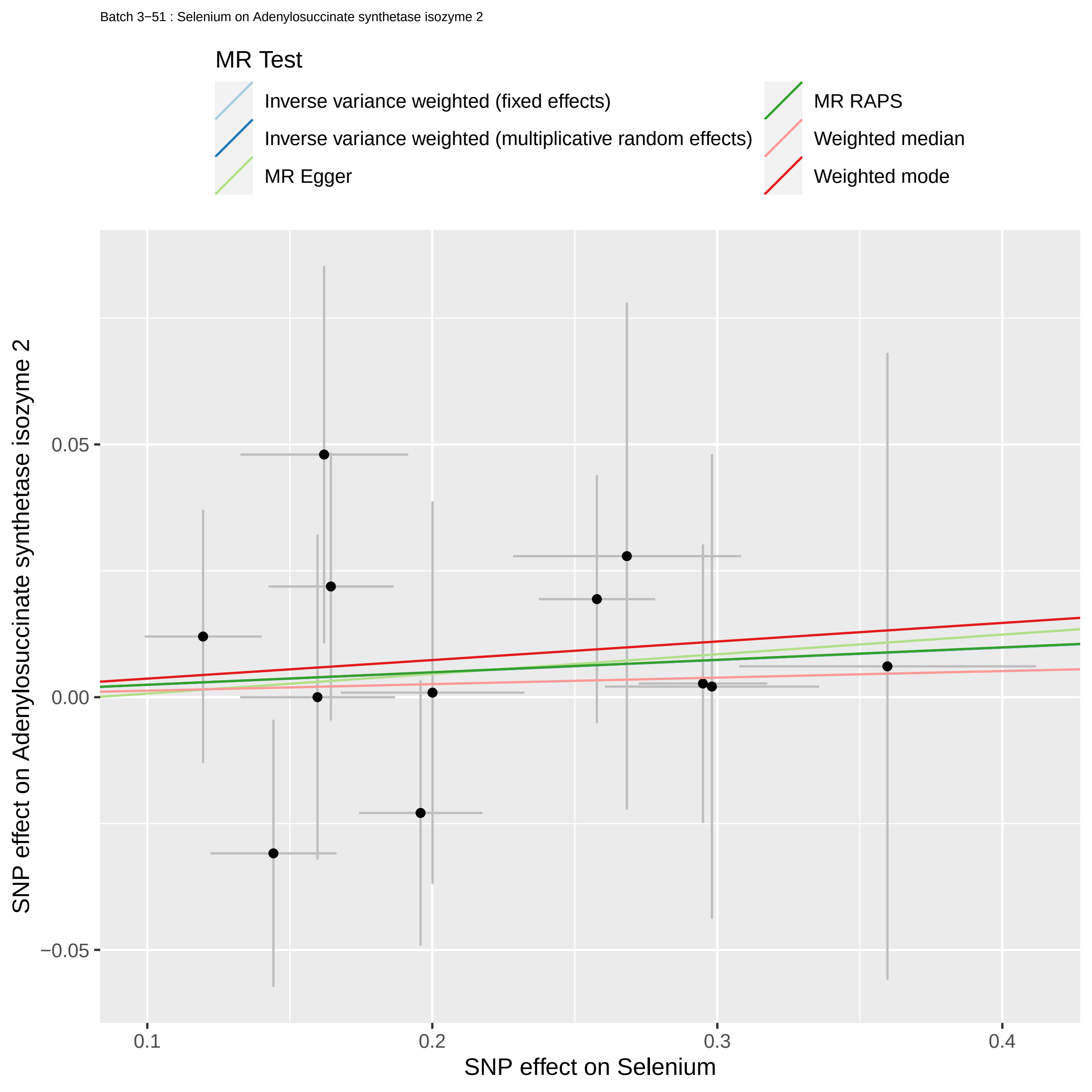
Batch 3−51 : Selenium on Adenylosuccinate synthetase isozyme 2
MR Test
Inverse variance weighted (fixed effects)
Inverse variance weighted (multiplicative random effects)
MR Egger
MR RAPS
Weighted median
Weighted mode
0.05
0.00
−0.05
0.1
0.2
0.3
0.4
SNP effect on Selenium
SNP effect on Adenylosuccinate synthetase isozyme 2

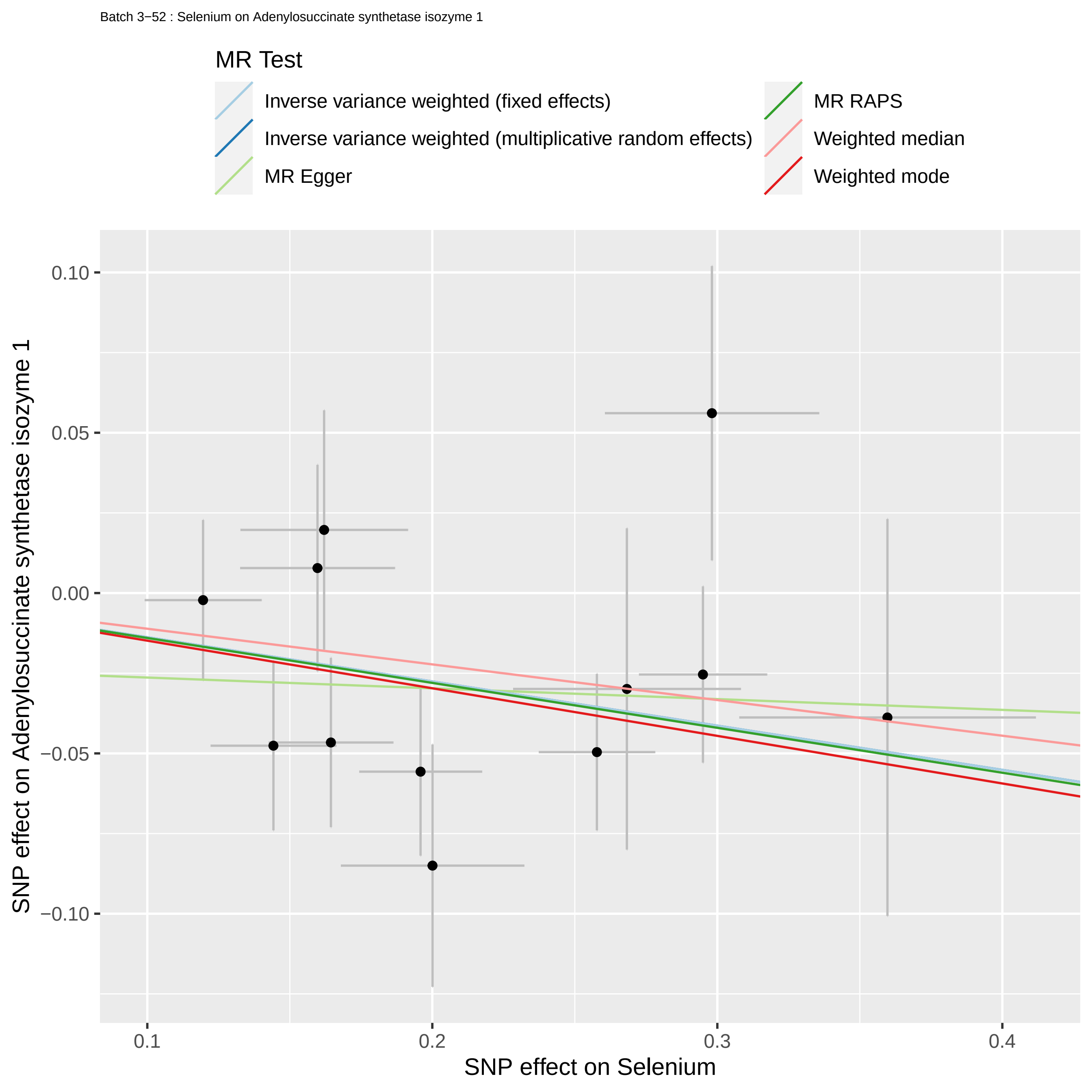
Batch 3−52 : Selenium on Adenylosuccinate synthetase isozyme 1
MR Test
Inverse variance weighted (fixed effects)
Inverse variance weighted (multiplicative random effects)
MR Egger
MR RAPS
Weighted median
Weighted mode
0.10
0.05
0.00
−0.05
−0.10
0.1
0.2
0.3
0.4
SNP effect on Adenylosuccinate synthetase isozyme 1
SNP effect on Selenium

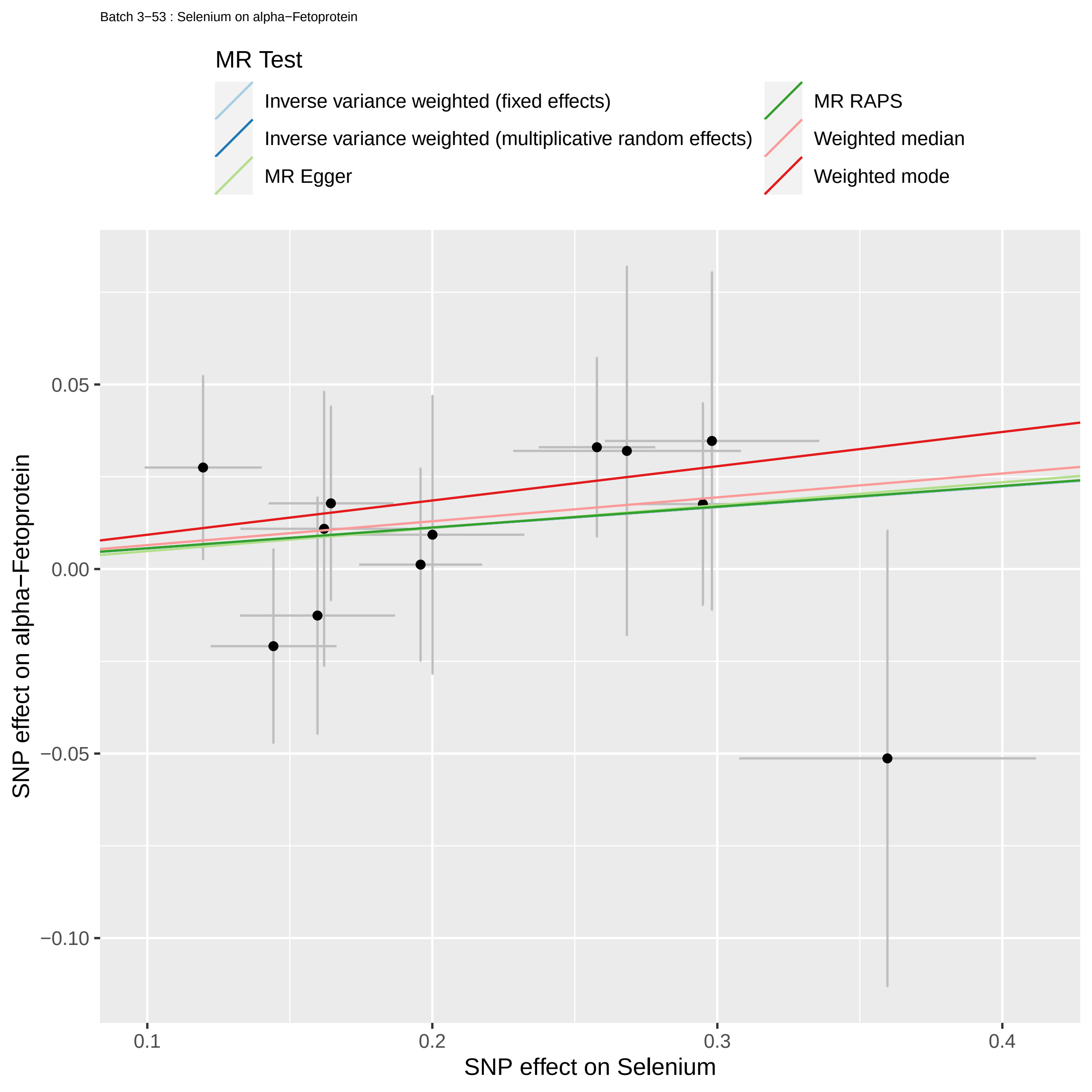
Batch 3−53 : Selenium on alpha−Fetoprotein
MR Test
Inverse variance weighted (fixed effects)
Inverse variance weighted (multiplicative random effects)
MR Egger
MR RAPS
Weighted median
Weighted mode
0.05
0.00
−0.05
−0.10
0.1
0.2
0.3
0.4
SNP effect on alpha−Fetoprotein
SNP effect on Selenium

Batch 3−54 : Selenium on Advanced glycosylation end product−specific receptor, soluble

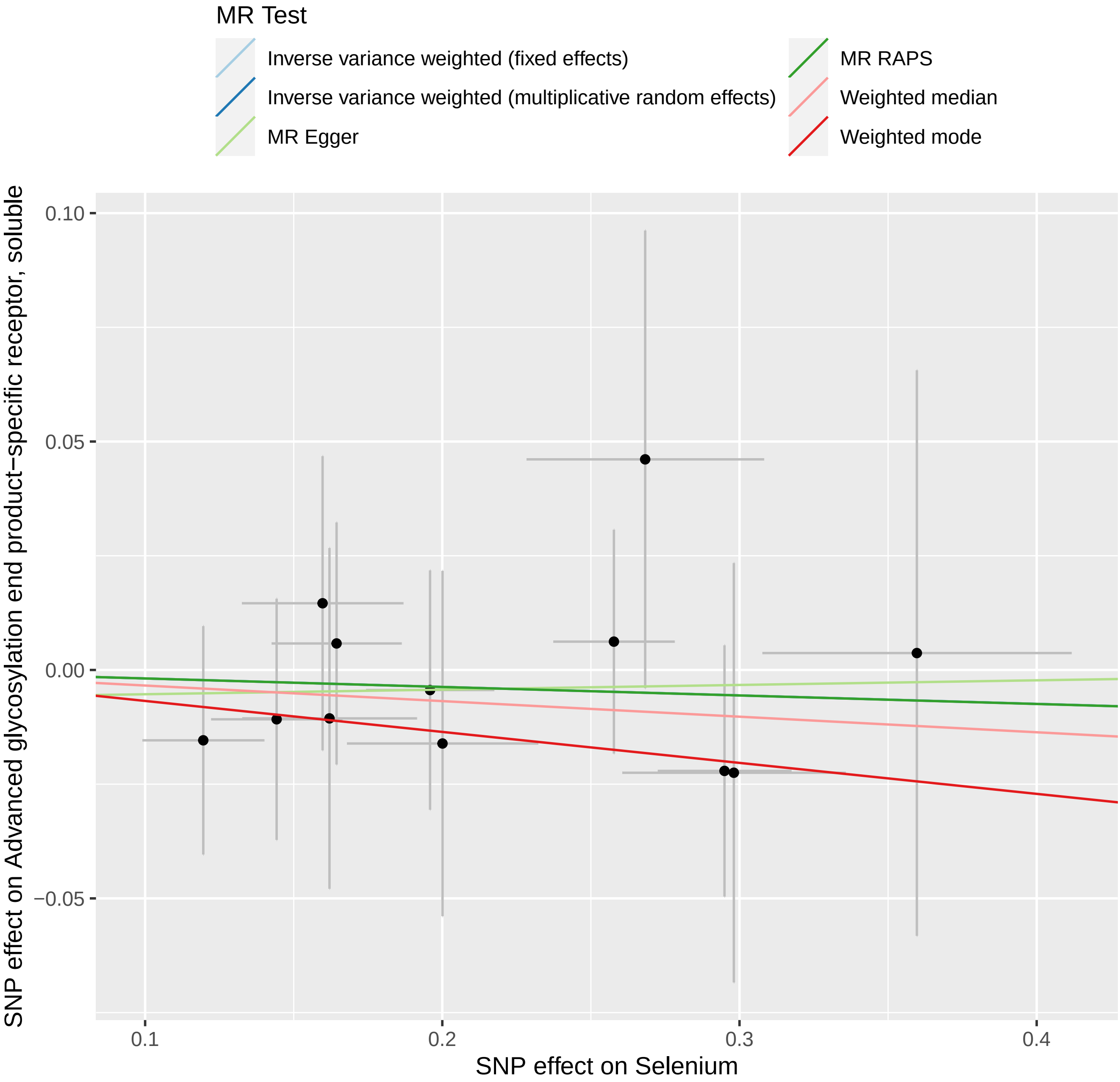

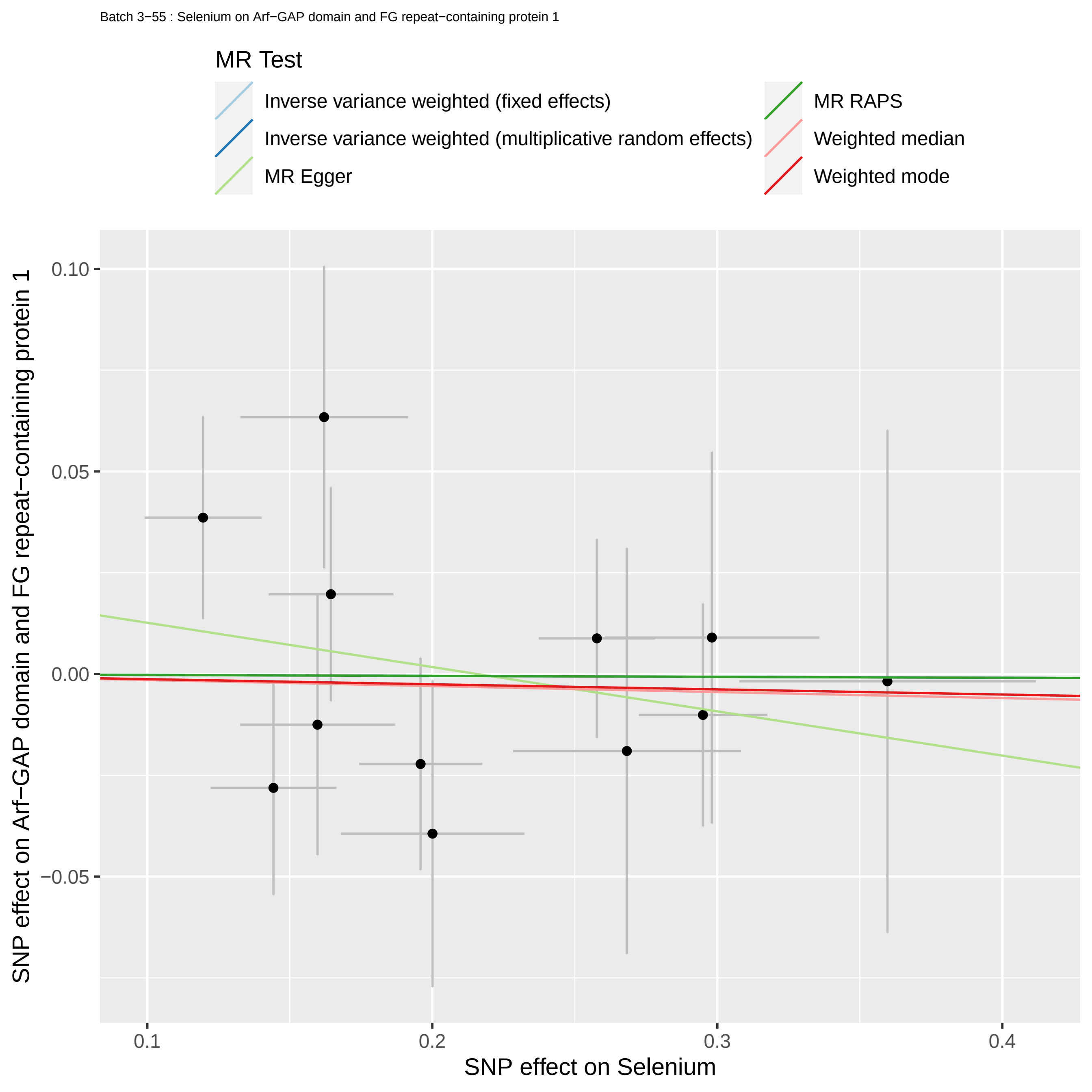

Batch 3−55 : Selenium on Arf−GAP domain and FG repeat−containing protein 1
MR Test
Inverse variance weighted (fixed effects)
Inverse variance weighted (multiplicative random effects)
MR Egger
MR RAPS
Weighted median
Weighted mode
0.10
0.05
0.00
−0.05
0.1
0.2
0.3
0.4
SNP effect on Arf−GAP domain and FG repeat−containing protein 1
SNP effect on Selenium

Batch 3−56 : Selenium on Angiogenic factor with G patch and FHA domains 1

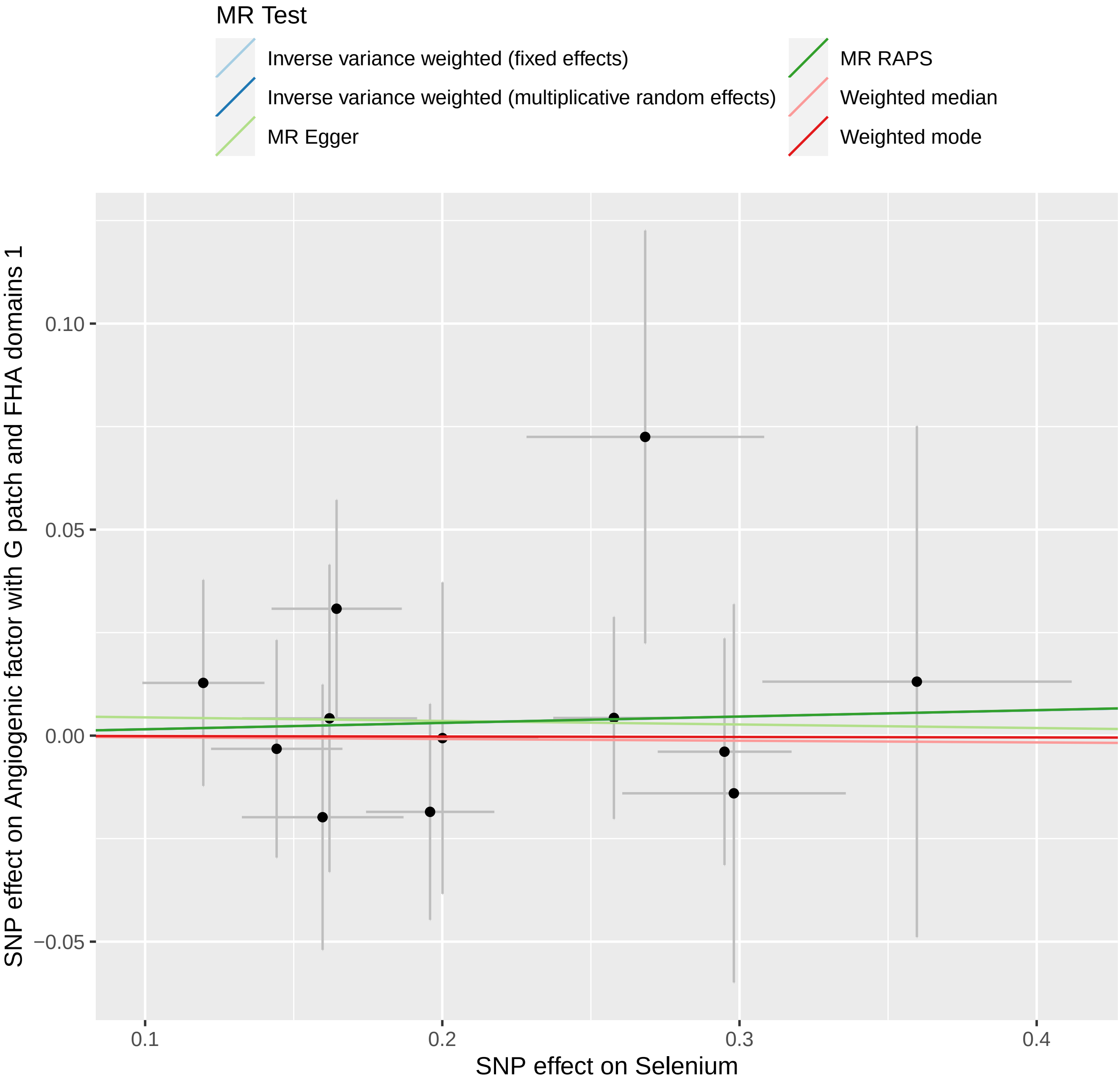

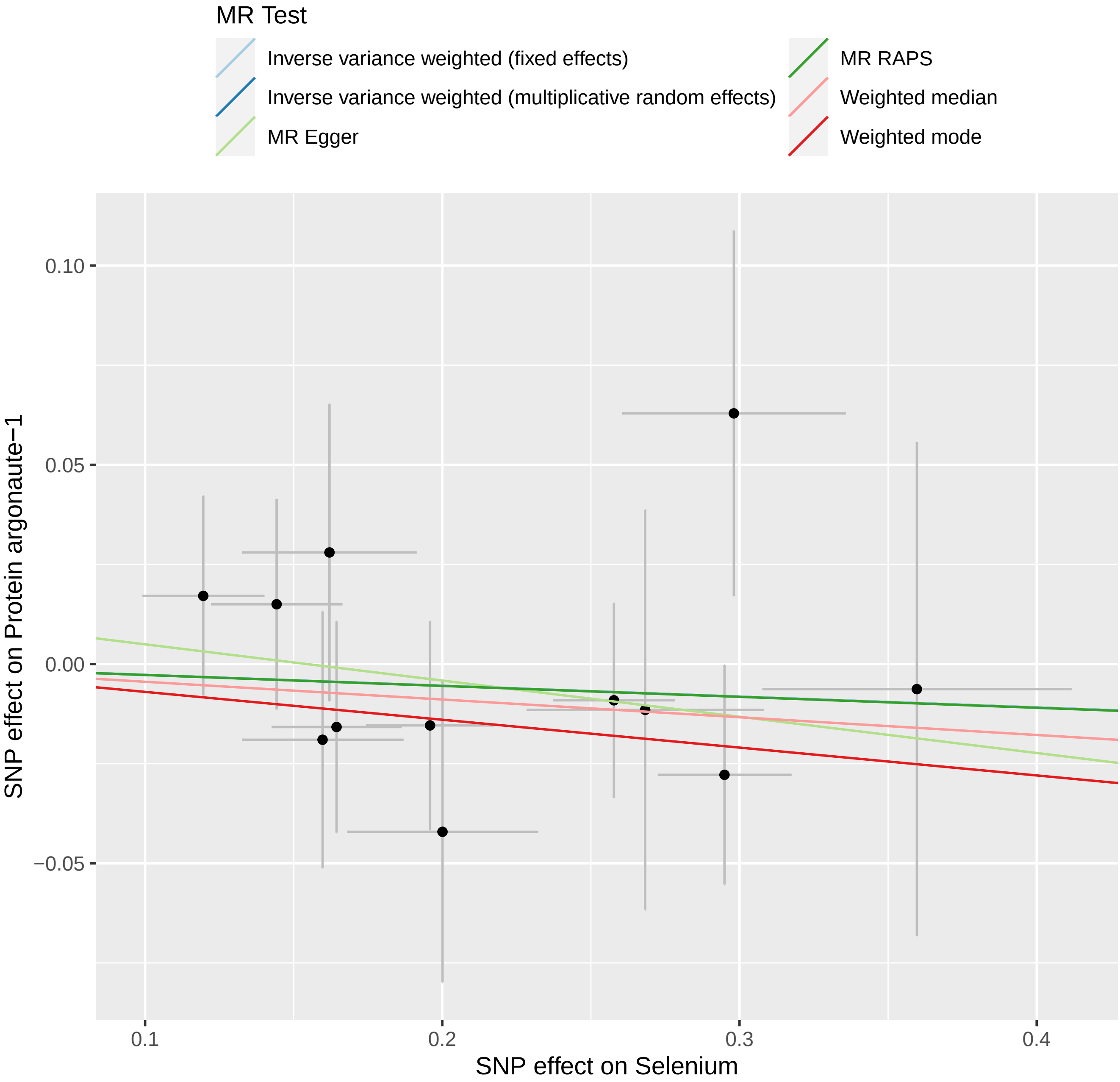
MR Test
Inverse variance weighted (fixed effects)
Inverse variance weighted (multiplicative random effects)
MR Egger
MR RAPS
Weighted median
Weighted mode
0.10
0.05
0.00
−0.05
0.1
0.2
0.3
0.4
SNP effect on Protein argonaute−1
SNP effect on Selenium

Batch 3−58 : Selenium on Anterior gradient protein 2 homolog

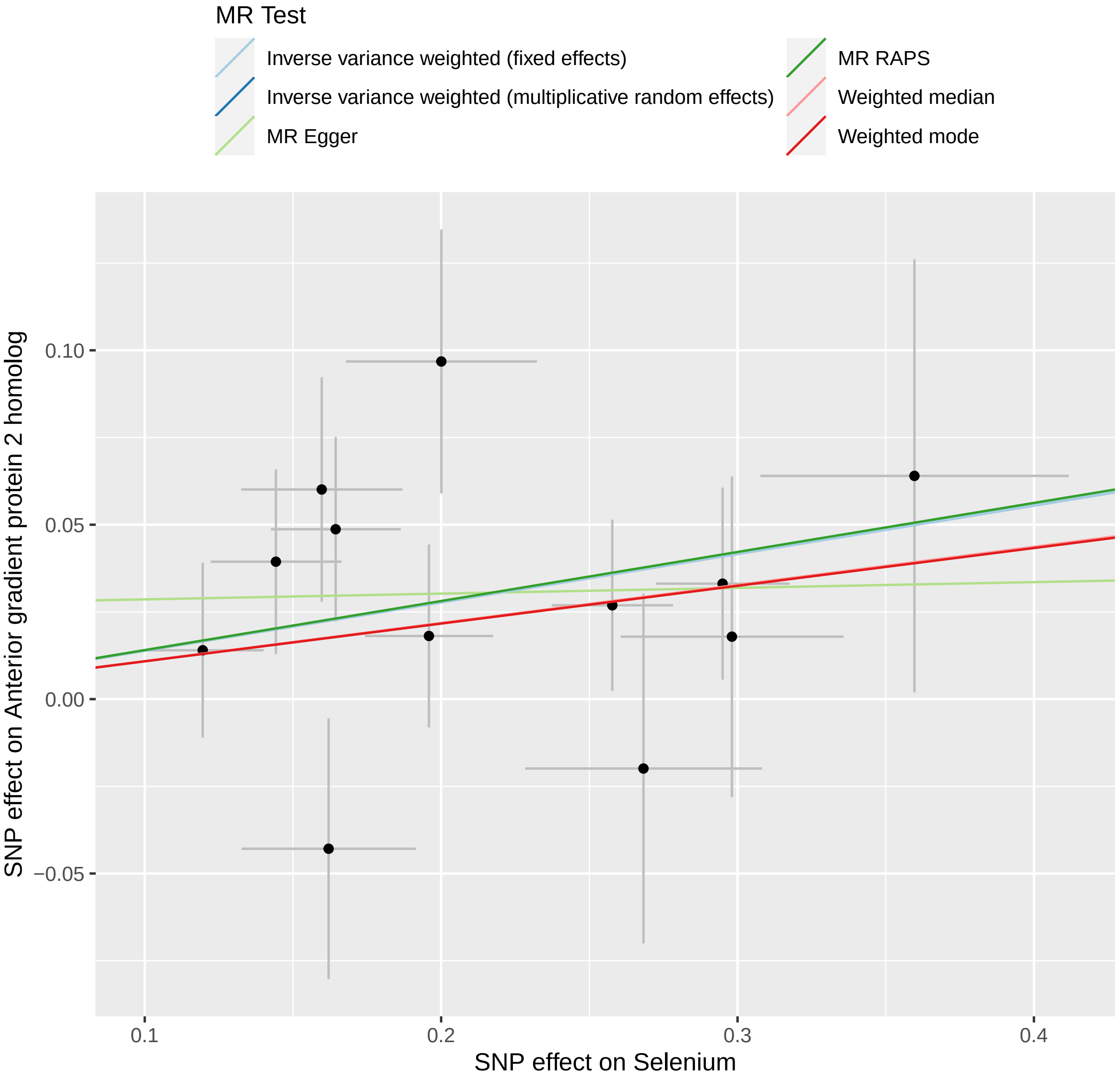

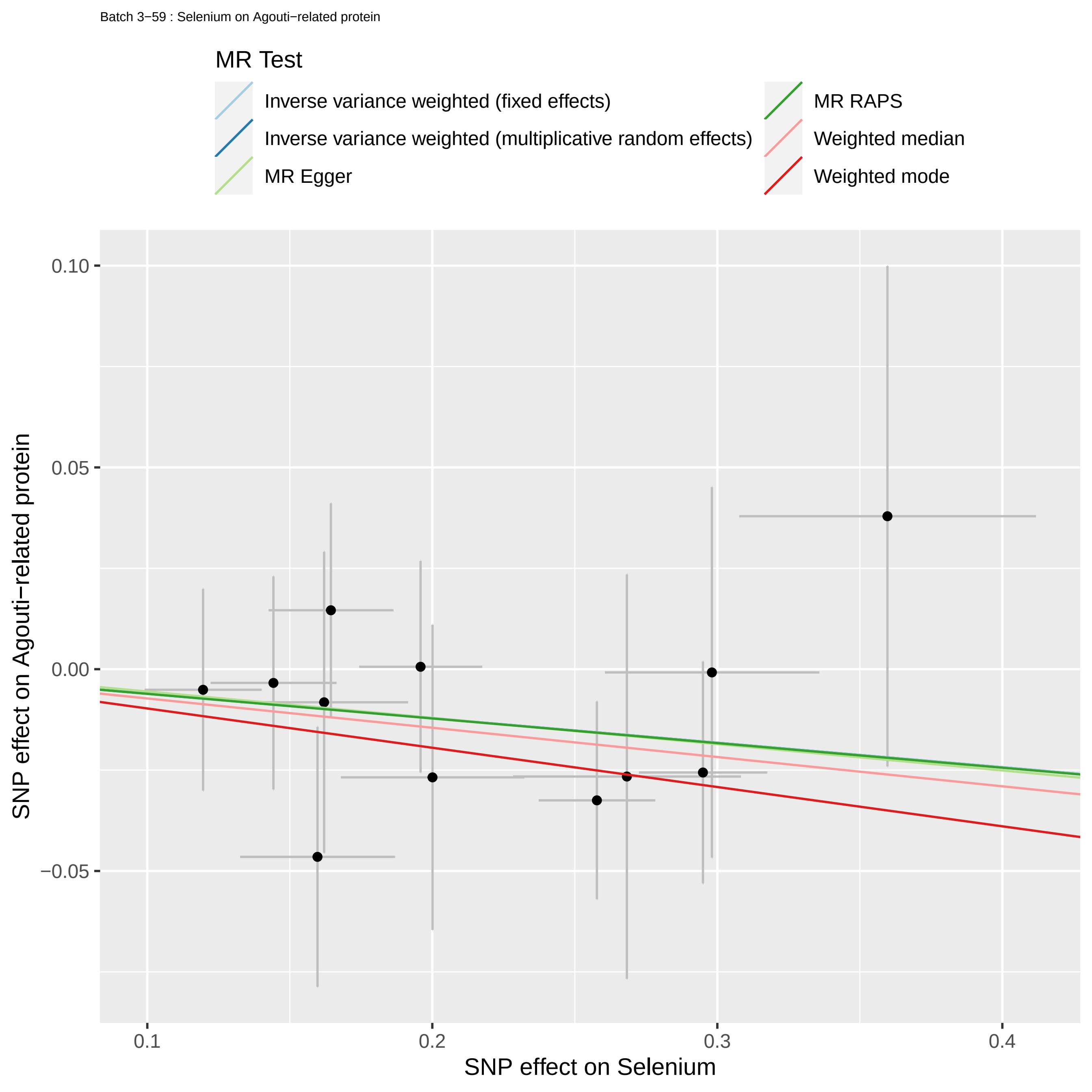

Batch 3−59 : Selenium on Agouti−related protein
MR Test
Inverse variance weighted (fixed effects)
Inverse variance weighted (multiplicative random effects)
MR Egger
MR RAPS
Weighted median
Weighted mode
0.10
0.05
0.00
−0.05
0.1
0.2
0.3
0.4
SNP effect on Agouti−related protein
SNP effect on Selenium

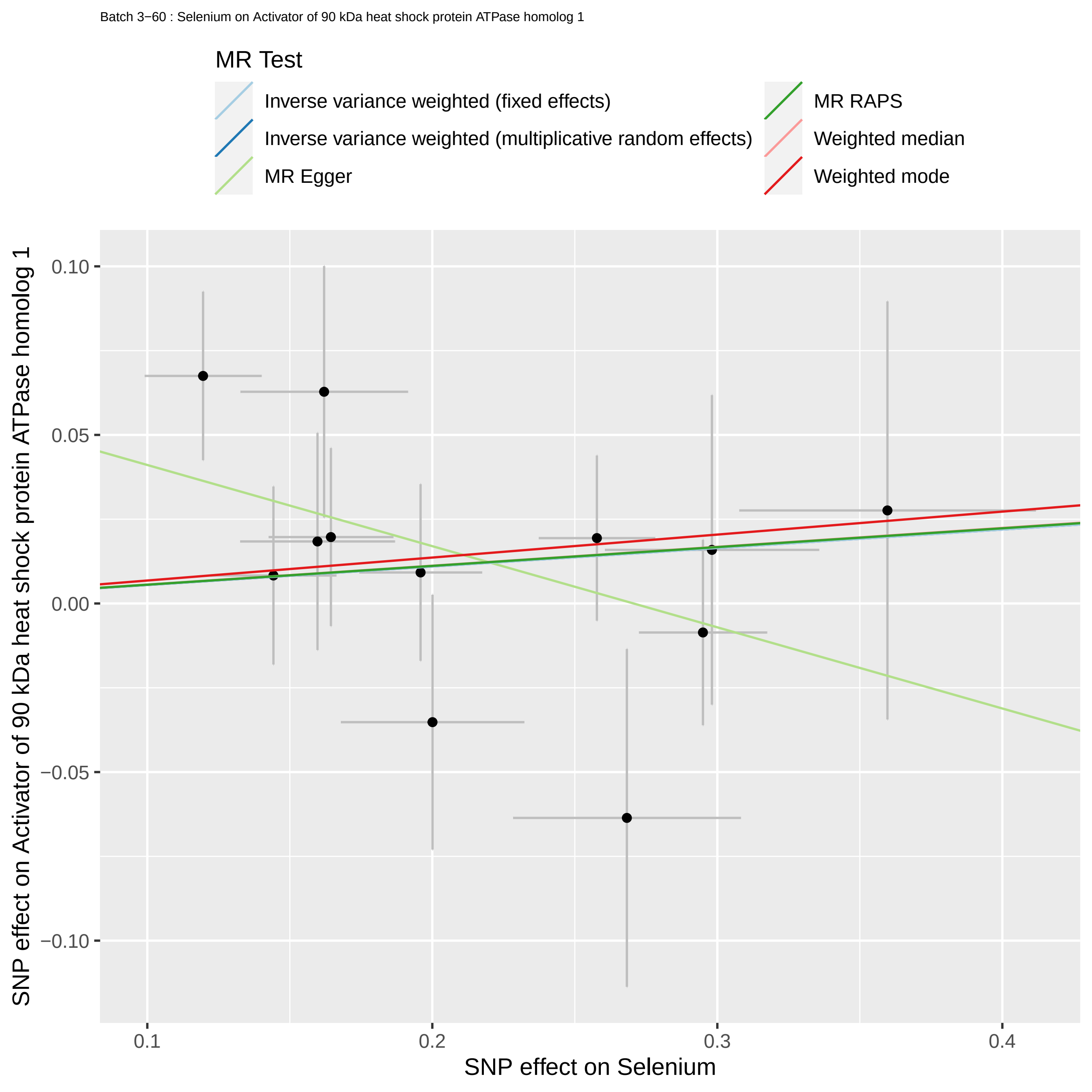

Batch 3−60 : Selenium on Activator of 90 kDa heat shock protein ATPase homolog 1
MR Test
Inverse variance weighted (fixed effects)
Inverse variance weighted (multiplicative random effects)
MR Egger
MR RAPS
Weighted median
Weighted mode
SNP effect on Activator of 90 kDa heat shock protein ATPase homolog 1
0.10
0.05
0.00
−0.05
−0.10
0.1
0.2
0.3
0.4
SNP effect on Selenium

Batch 3−61 : Selenium on Alpha−2−HS−glycoprotein

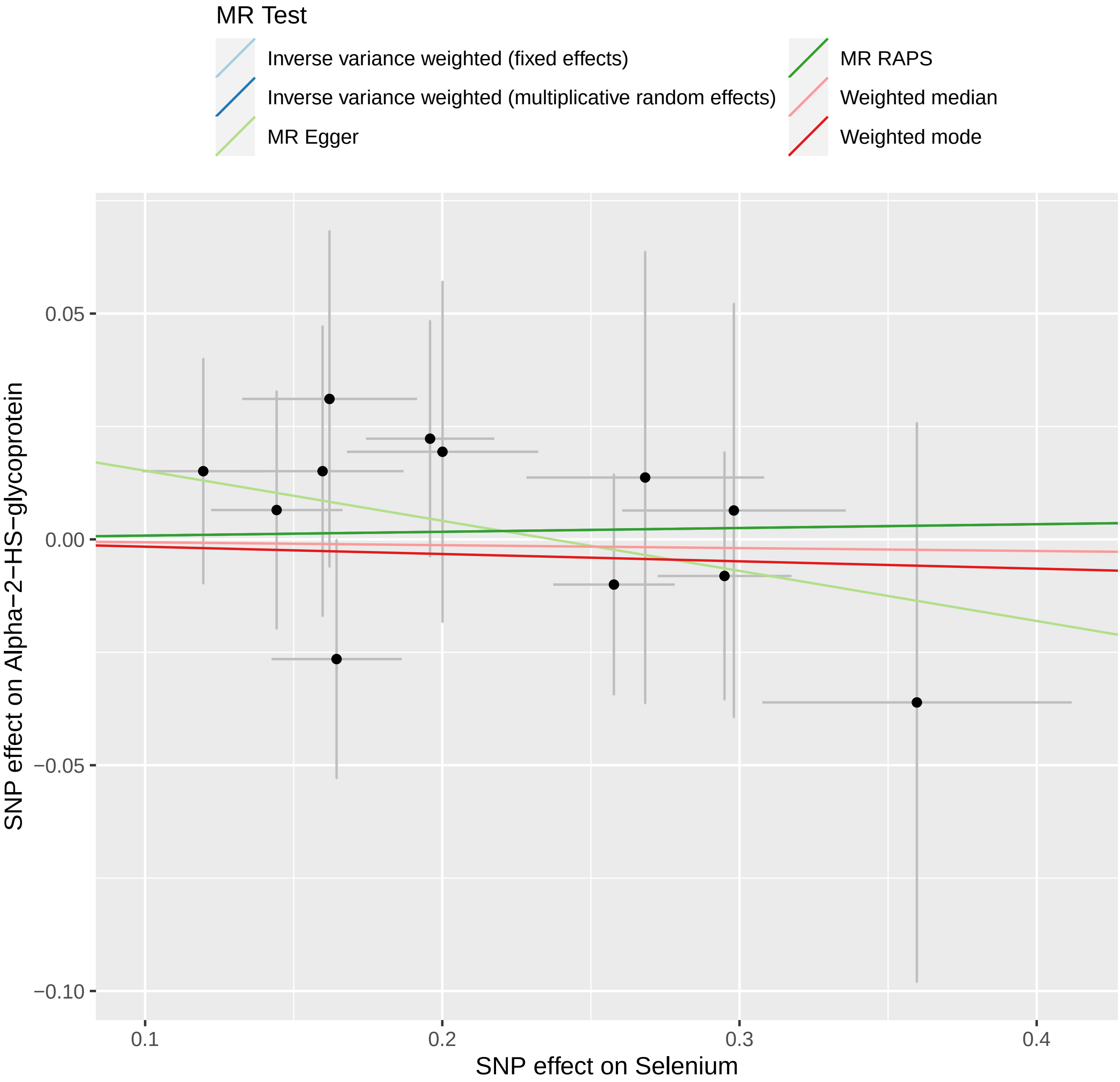

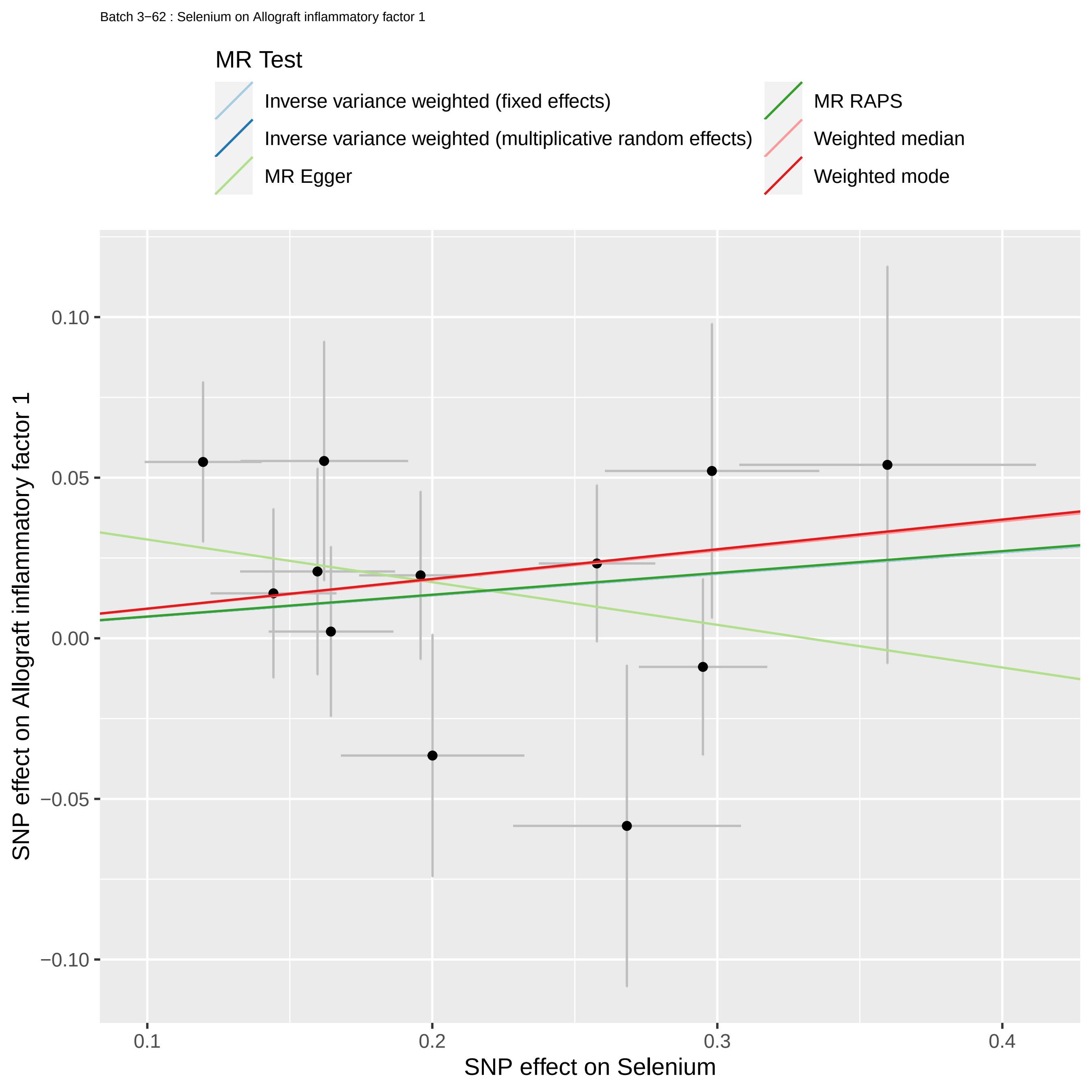

Batch 3−62 : Selenium on Allograft inflammatory factor 1
MR Test
Inverse variance weighted (fixed effects)
Inverse variance weighted (multiplicative random effects)
MR Egger
MR RAPS
Weighted median
Weighted mode
0.10
0.05
0.00
−0.05
−0.10
0.1
0.2
0.3
0.4
SNP effect on Allograft inflammatory factor 1
SNP effect on Selenium

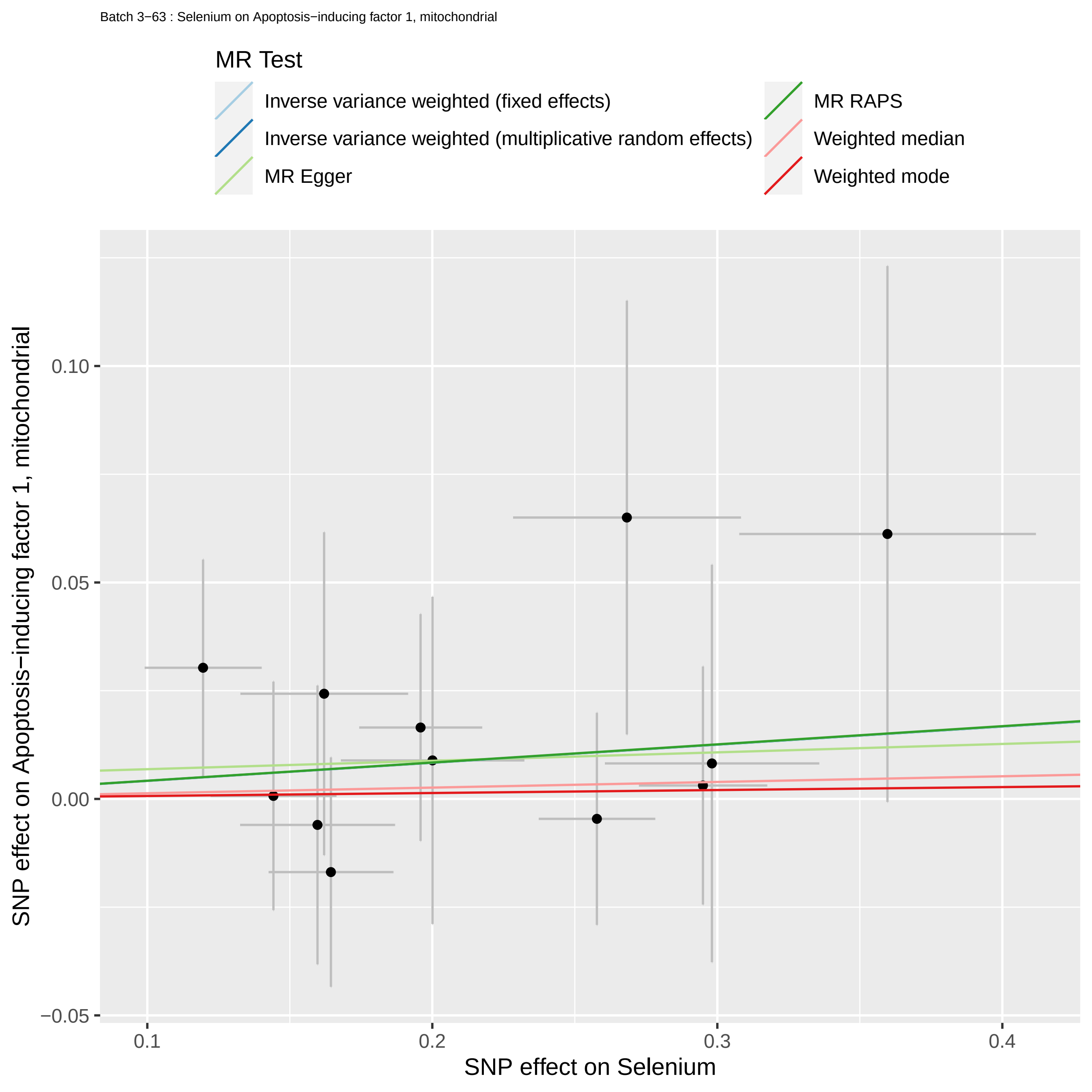
Batch 3−63 : Selenium on Apoptosis−inducing factor 1, mitochondrial
MR Test
Inverse variance weighted (fixed effects)
Inverse variance weighted (multiplicative random effects)
MR Egger
MR RAPS
Weighted median
Weighted mode
SNP effect on Apoptosis−inducing factor 1, mitochondrial
0.10
0.05
0.00
−0.05
0.1
0.2
0.3
0.4
SNP effect on Selenium

Batch 3−64 : Selenium on Apoptosis−inducing factor 1, mitochondrial

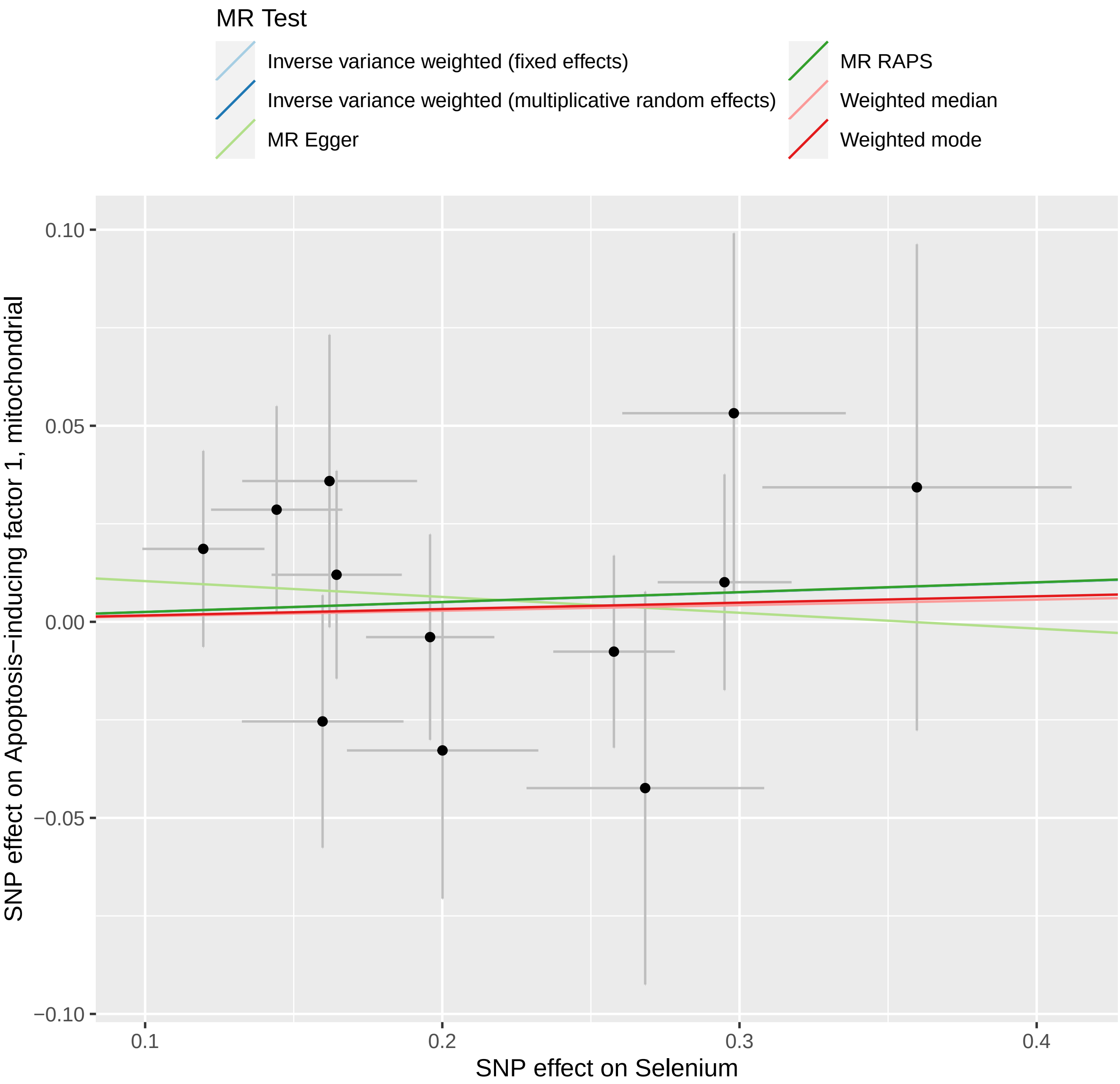

Batch 3−65 : Selenium on Endothelial monocyte−activating polypeptide 2

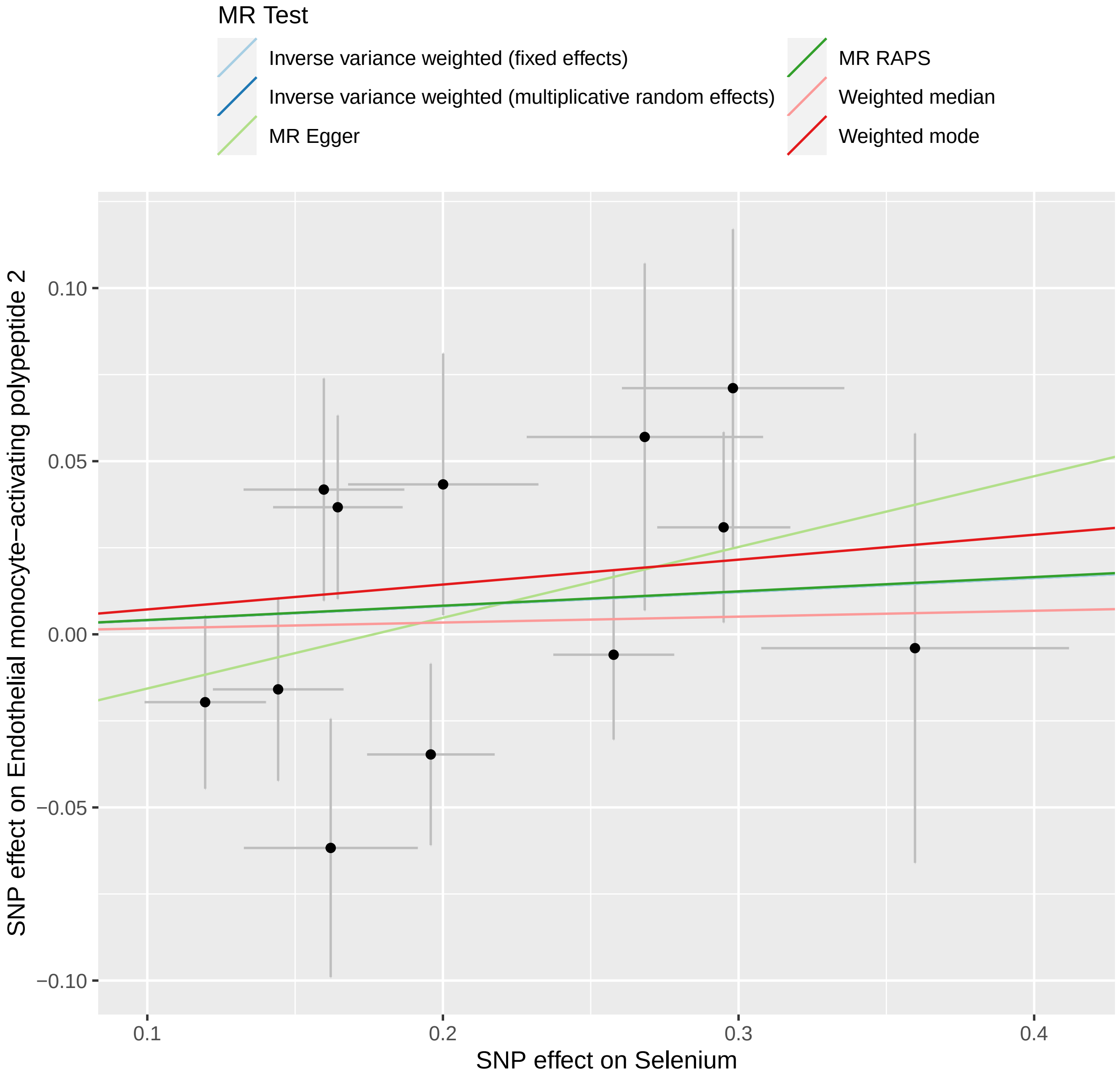

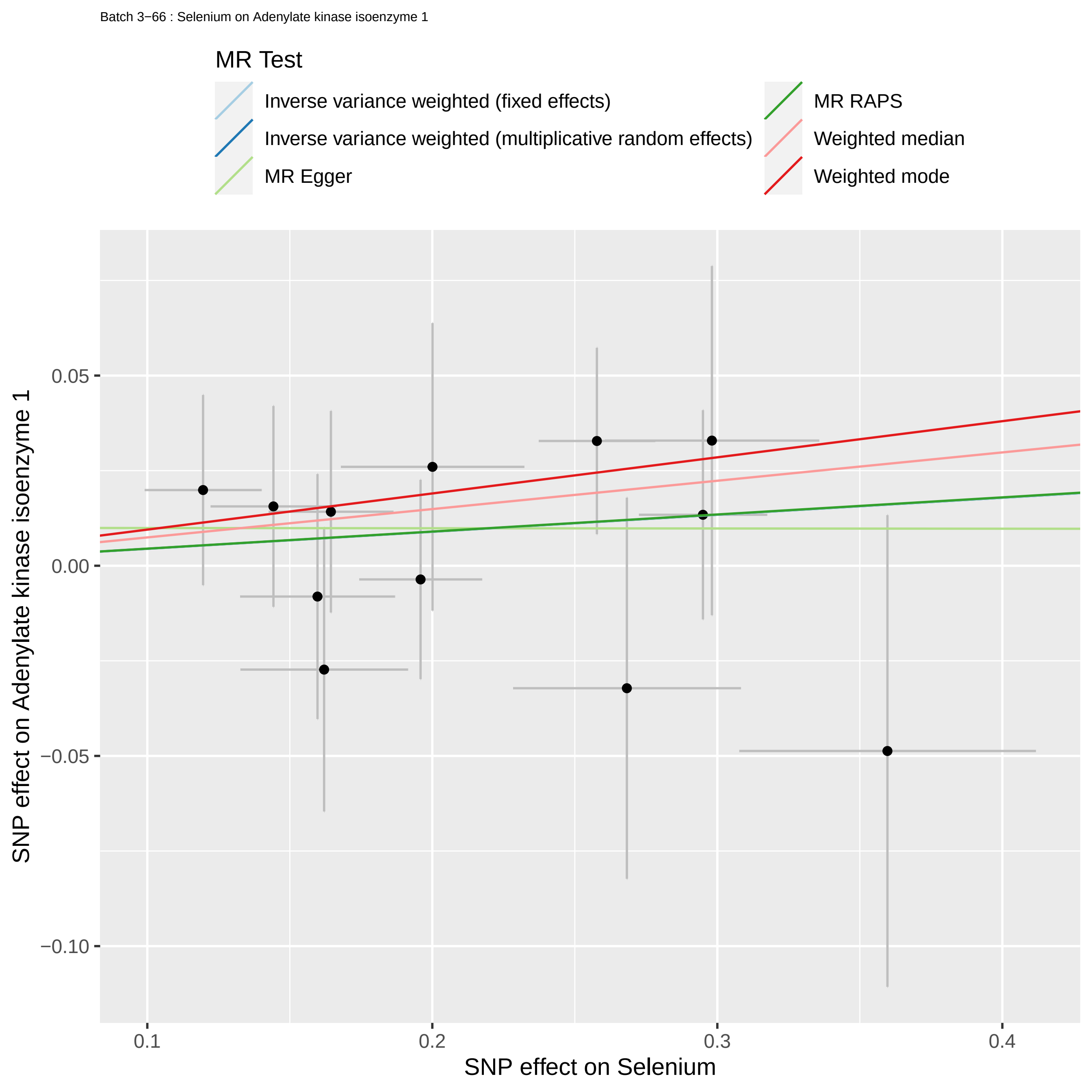

Batch 3−66 : Selenium on Adenylate kinase isoenzyme 1
MR Test
Inverse variance weighted (fixed effects)
Inverse variance weighted (multiplicative random effects)
MR Egger
MR RAPS
Weighted median
Weighted mode
0.05
0.00
−0.05
−0.10
0.1
0.2
0.3
0.4
SNP effect on Adenylate kinase isoenzyme 1
SNP effect on Selenium

Batch 3−67 : Selenium on Alcohol dehydrogenase [NADP(+)]

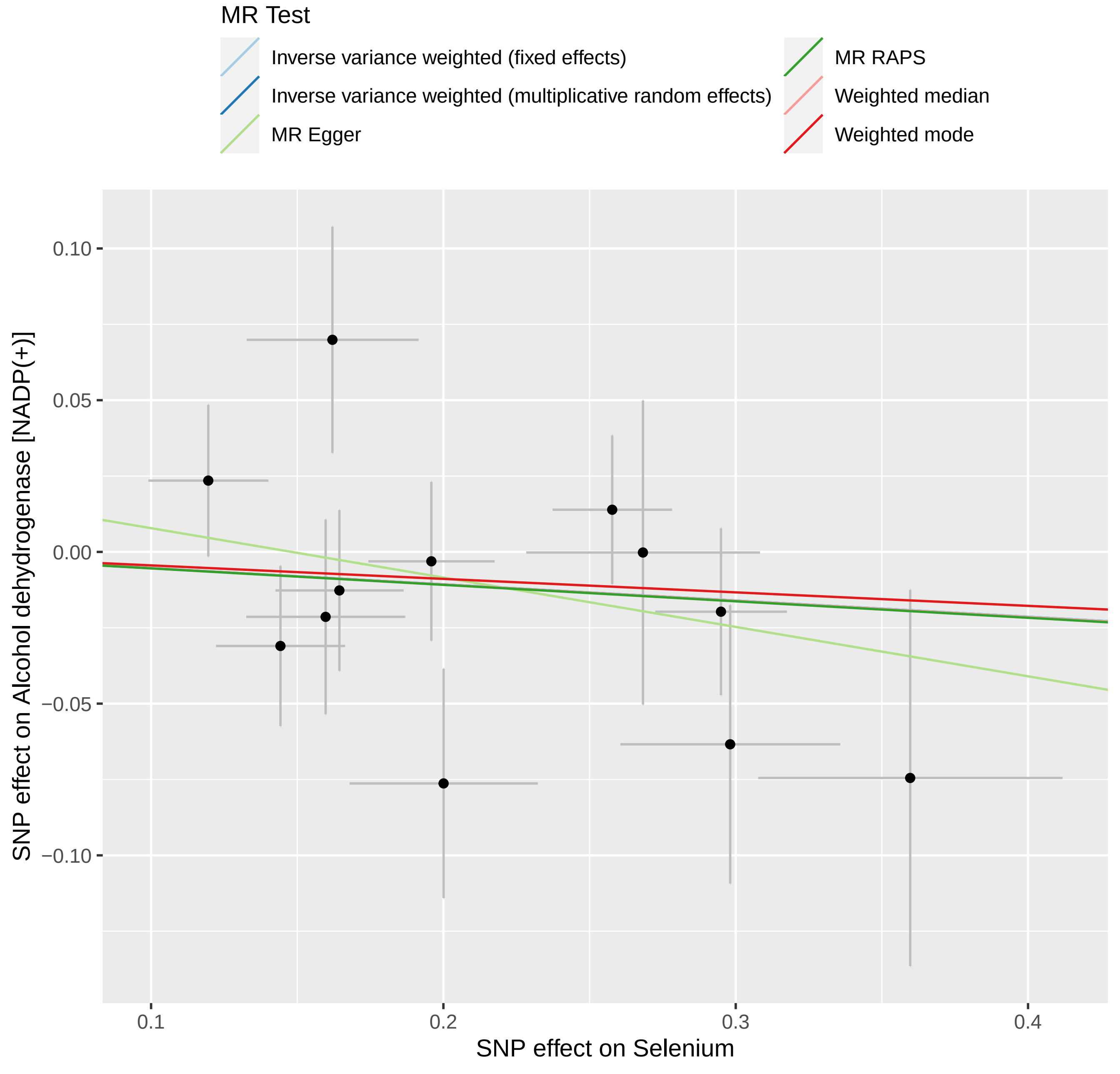

Batch 3−68 : Selenium on Aldose reductase

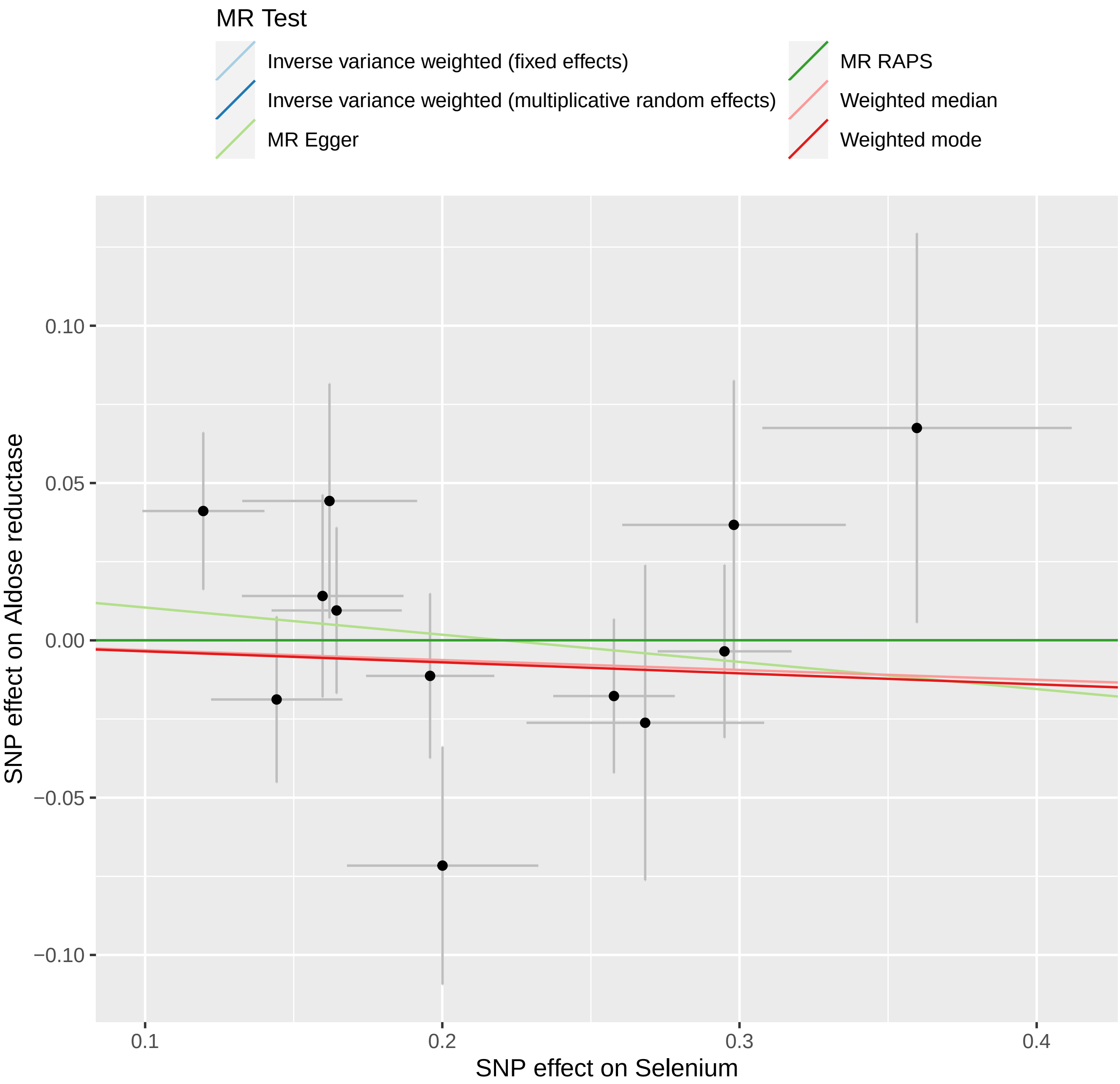

Batch 3−69 : Selenium on Aldo−keto reductase family 1 member C1

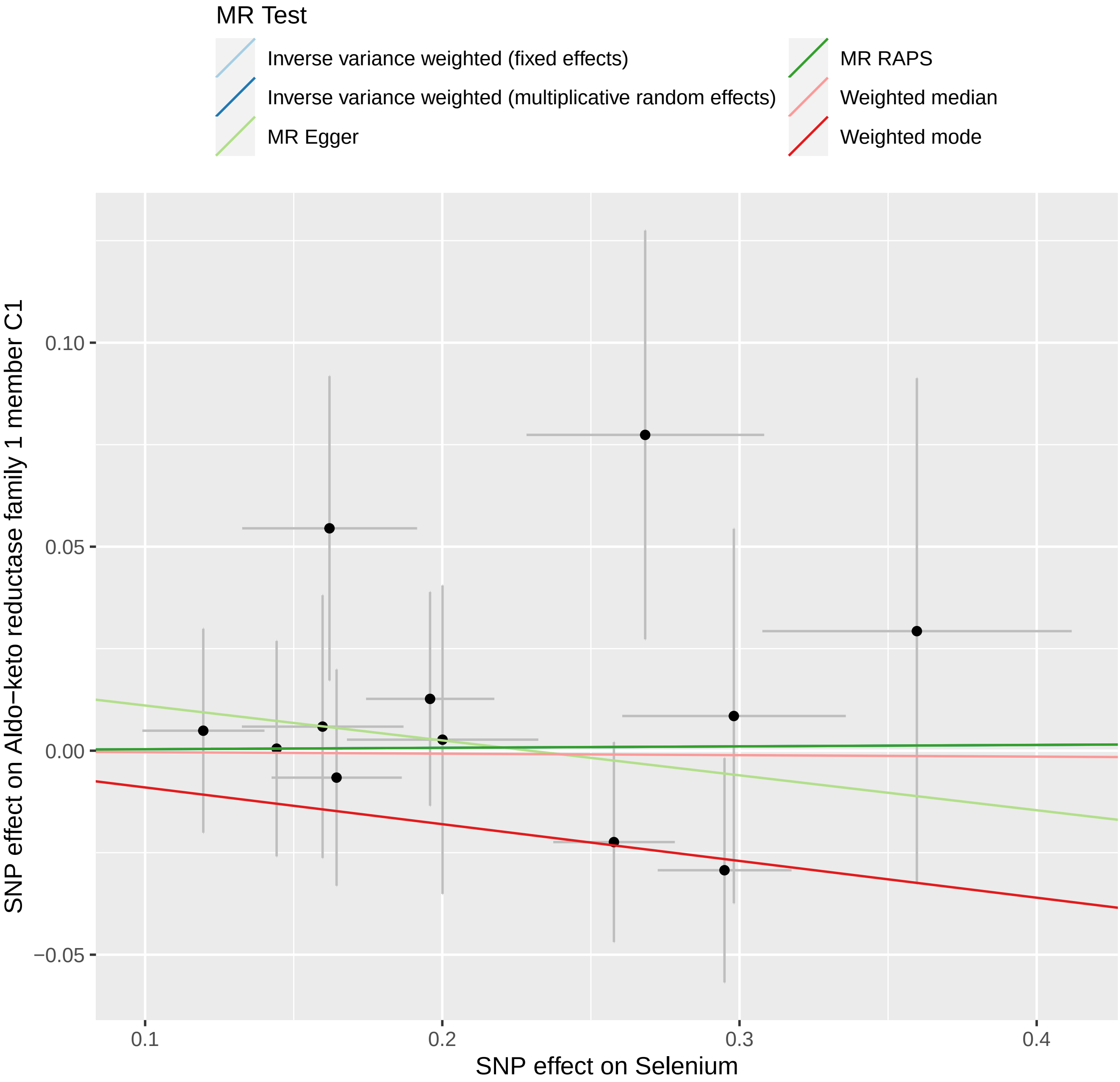

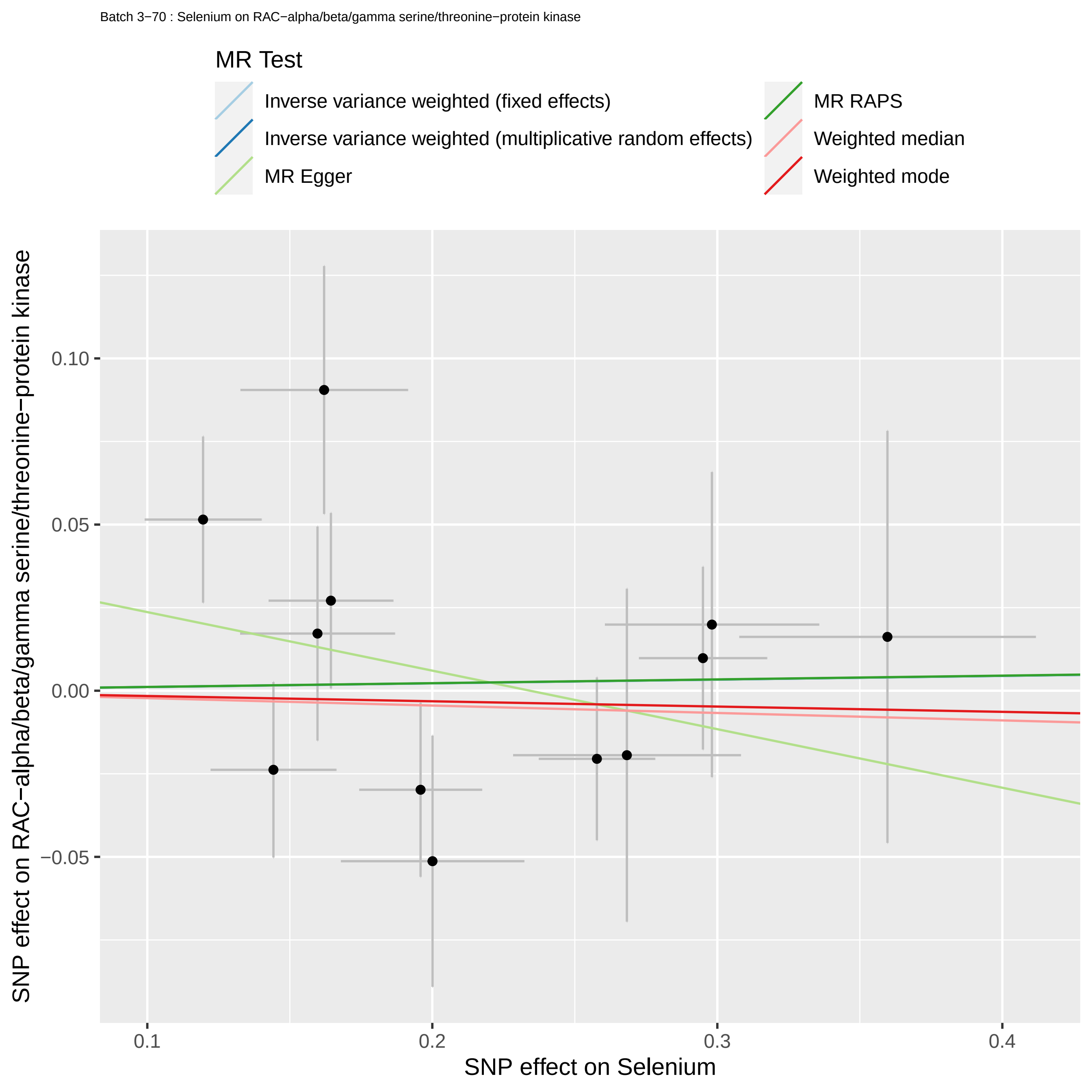

Batch 3−70 : Selenium on RAC−alpha/beta/gamma serine/threonine−protein kinase
MR Test
Inverse variance weighted (fixed effects)
Inverse variance weighted (multiplicative random effects)
MR Egger
MR RAPS
Weighted median
Weighted mode
SNP effect on RAC−alpha/beta/gamma serine/threonine−protein kinase
0.10
0.05
0.00
−0.05
0.1
0.2
0.3
0.4
SNP effect on Selenium

Batch 3−71 : Selenium on RAC−alpha/beta/gamma serine/threonine−protein kinase

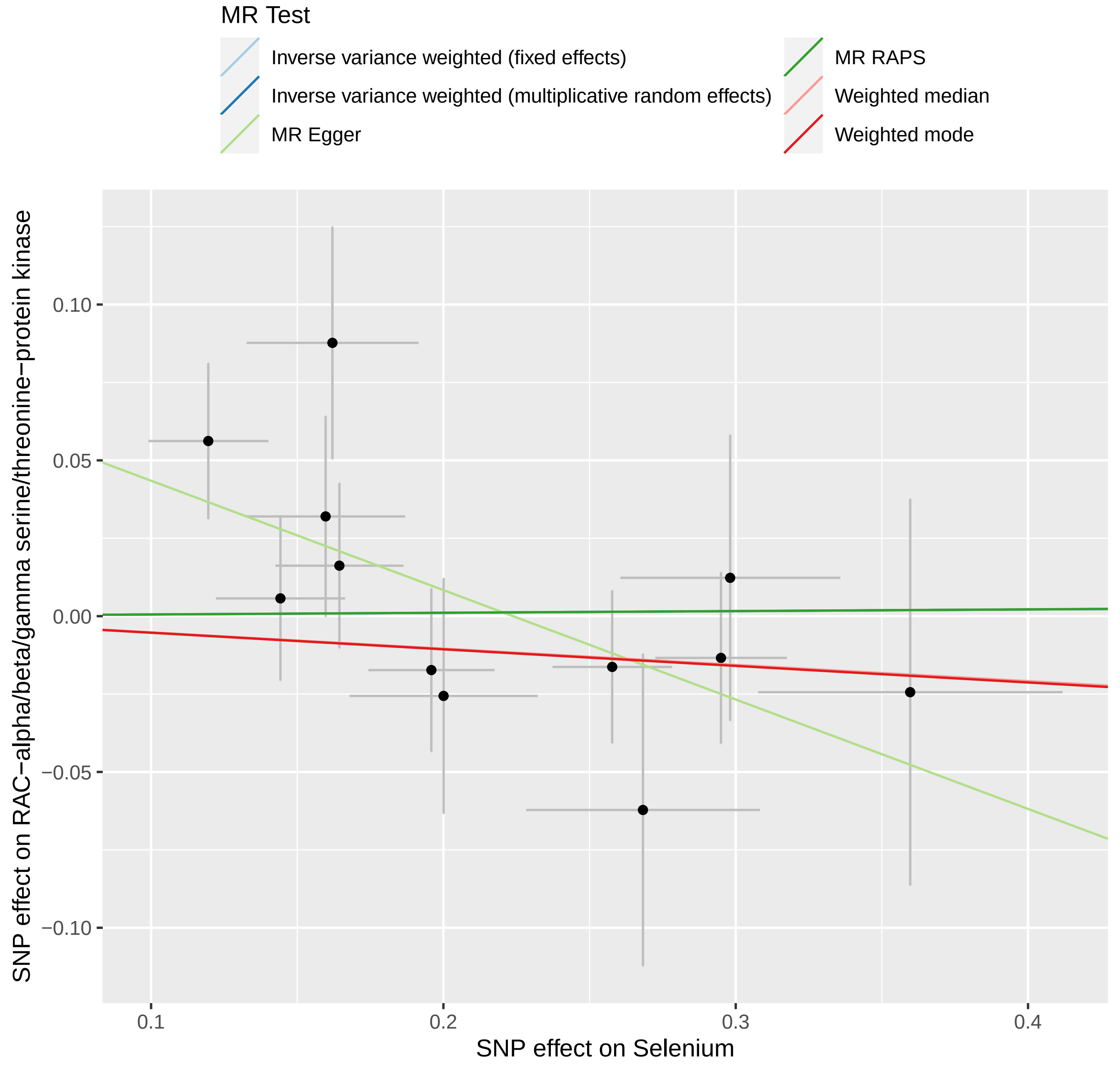

Batch 3−72 : Selenium on RAC−beta serine/threonine−protein kinase

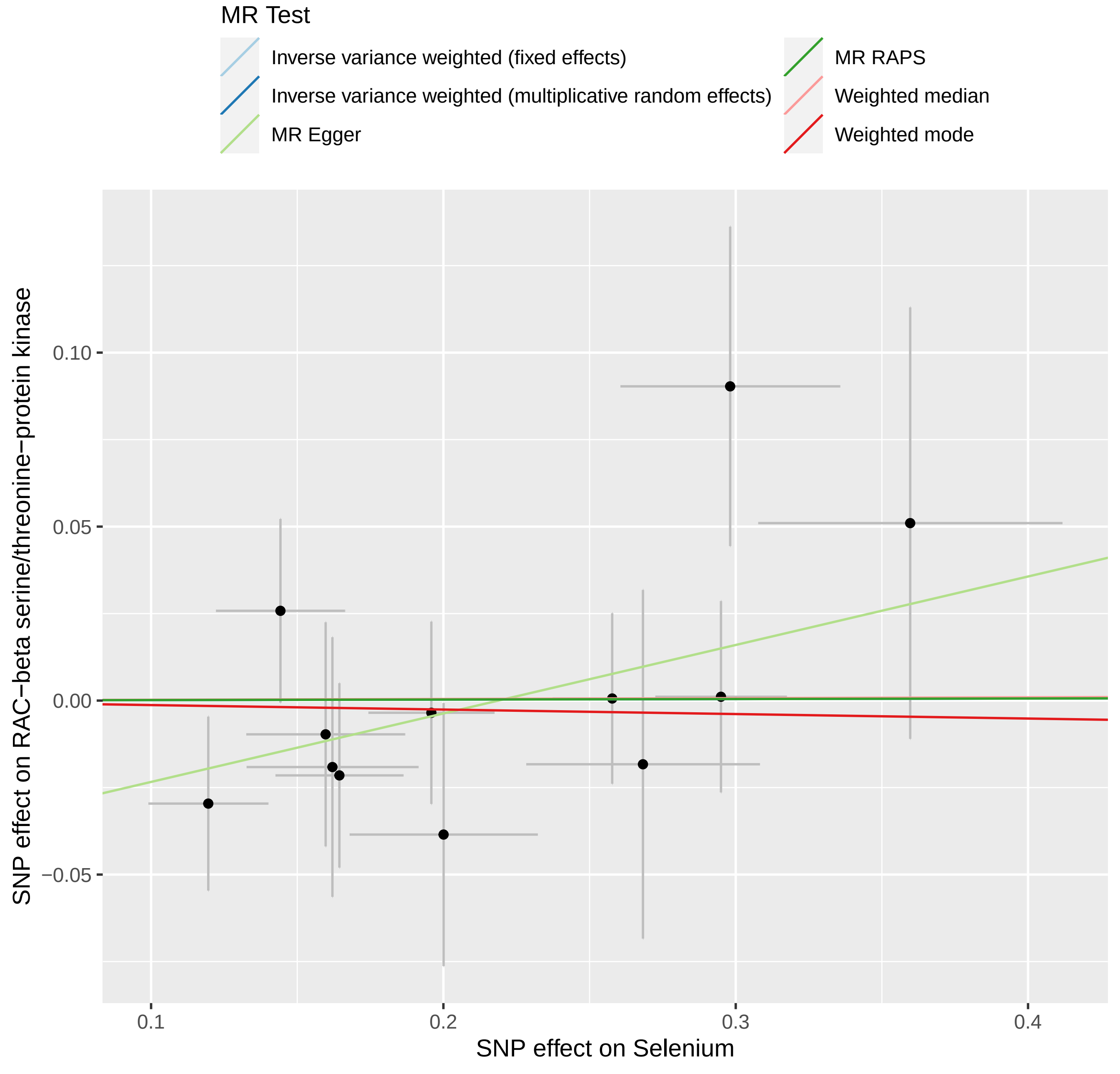

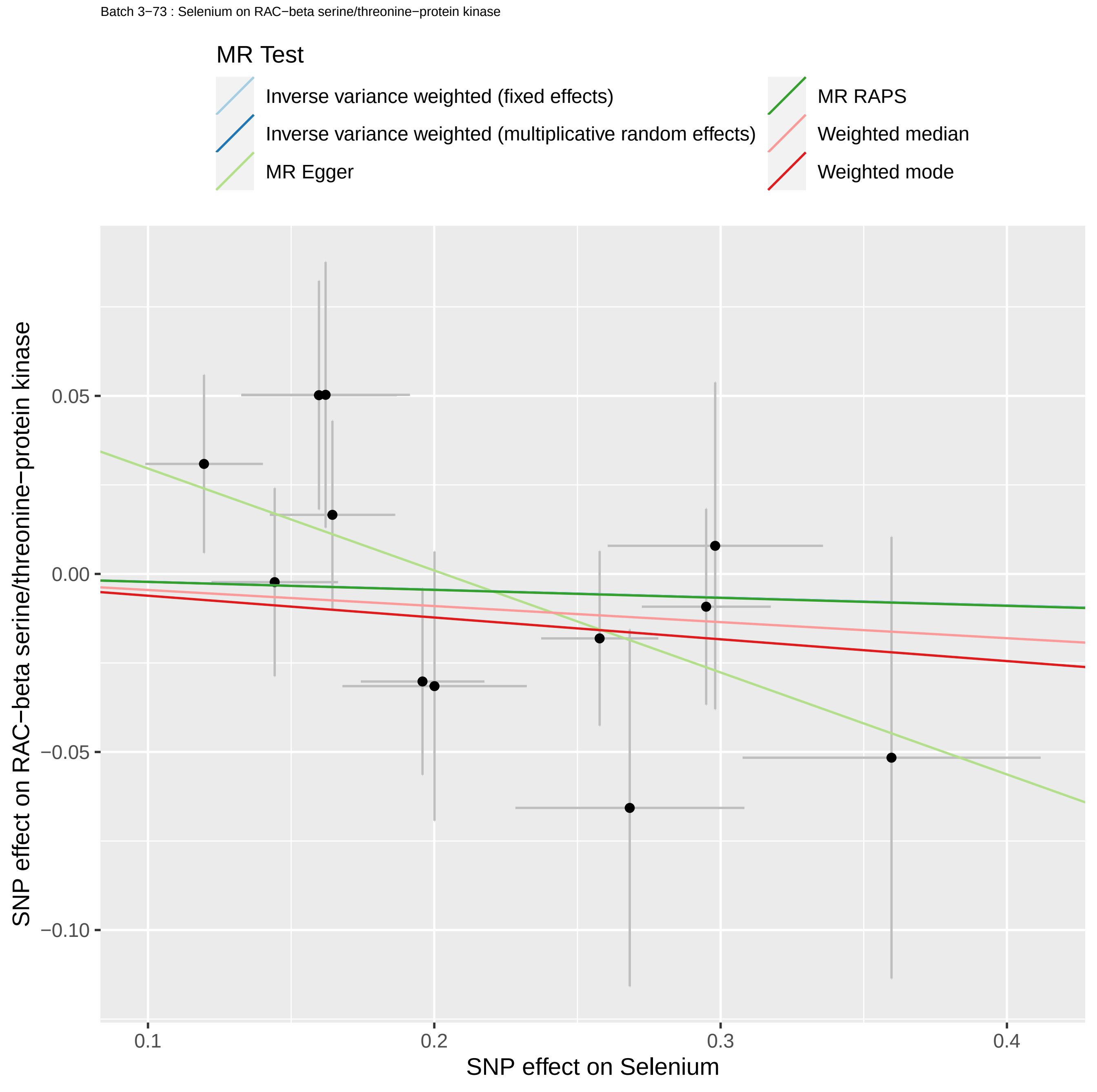

Batch 3−73 : Selenium on RAC−beta serine/threonine−protein kinase
MR Test
Inverse variance weighted (fixed effects)
Inverse variance weighted (multiplicative random effects)
MR Egger
MR RAPS
Weighted median
Weighted mode
SNP effect on RAC−beta serine/threonine−protein kinase
0.05
0.00
−0.05
−0.10
0.1
0.2
0.3
0.4
SNP effect on Selenium

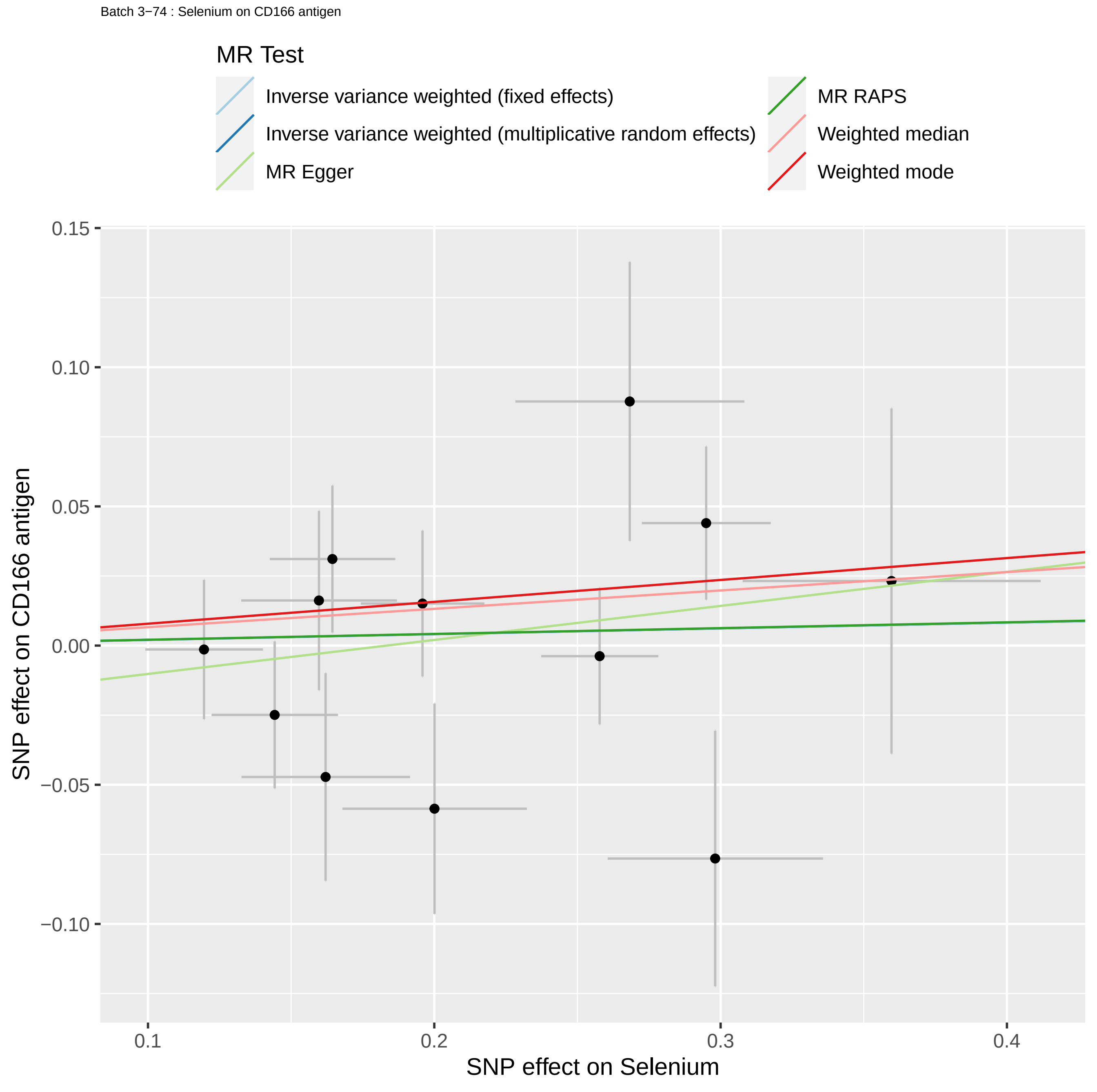
Batch 3−74 : Selenium on CD166 antigen
MR Test
Inverse variance weighted (fixed effects)
Inverse variance weighted (multiplicative random effects)
MR Egger
MR RAPS
Weighted median
Weighted mode
0.15
0.10
0.05
0.00
−0.05
−0.10
0.1
0.2
0.3
0.4
SNP effect on CD166 antigen
SNP effect on Selenium

Batch 3−75 : Selenium on Aldehyde dehydrogenase, dimeric NADP−preferring

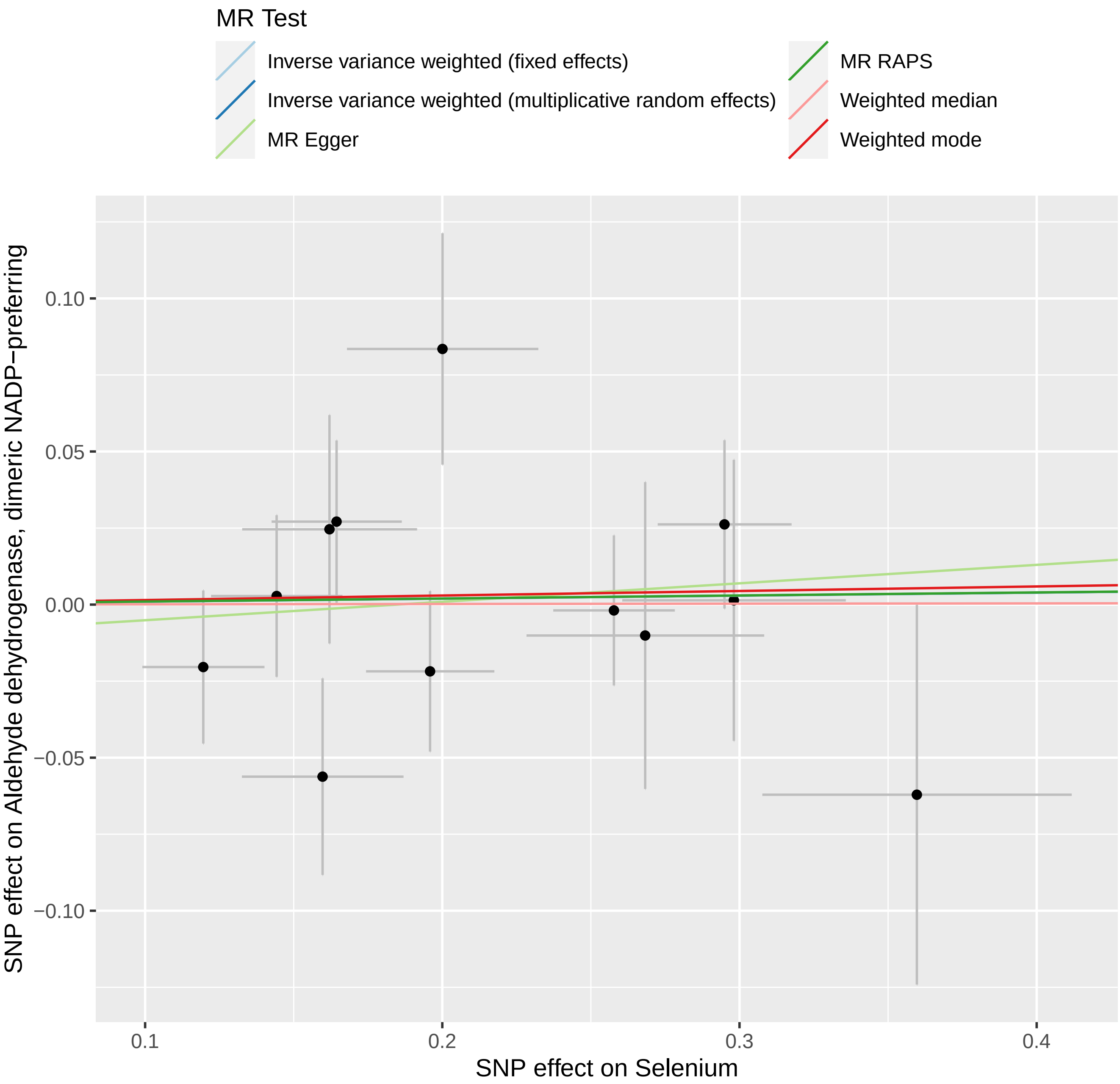

Batch 3−76 : Selenium on Aldehyde dehydrogenase family 3 member B1

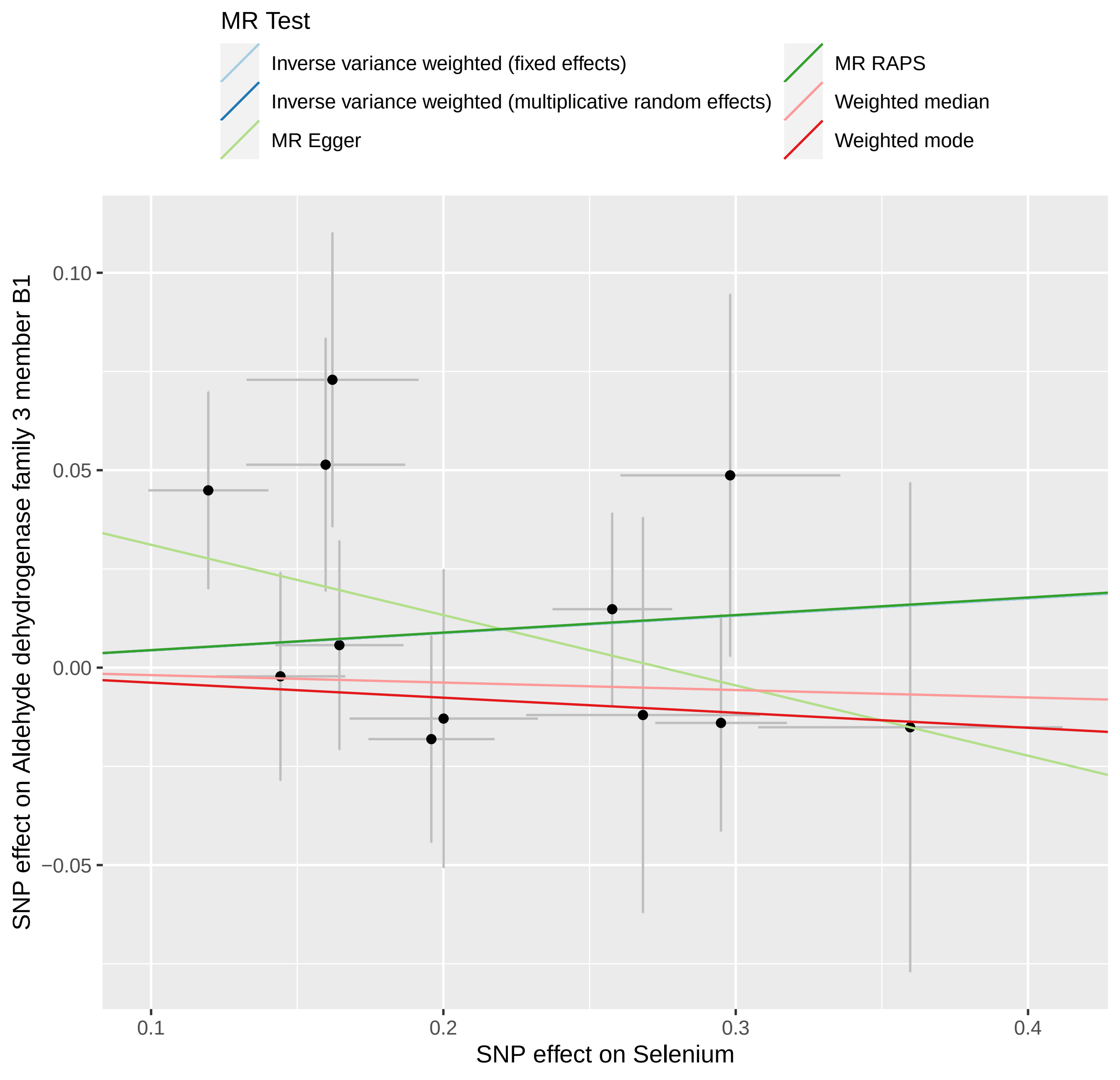

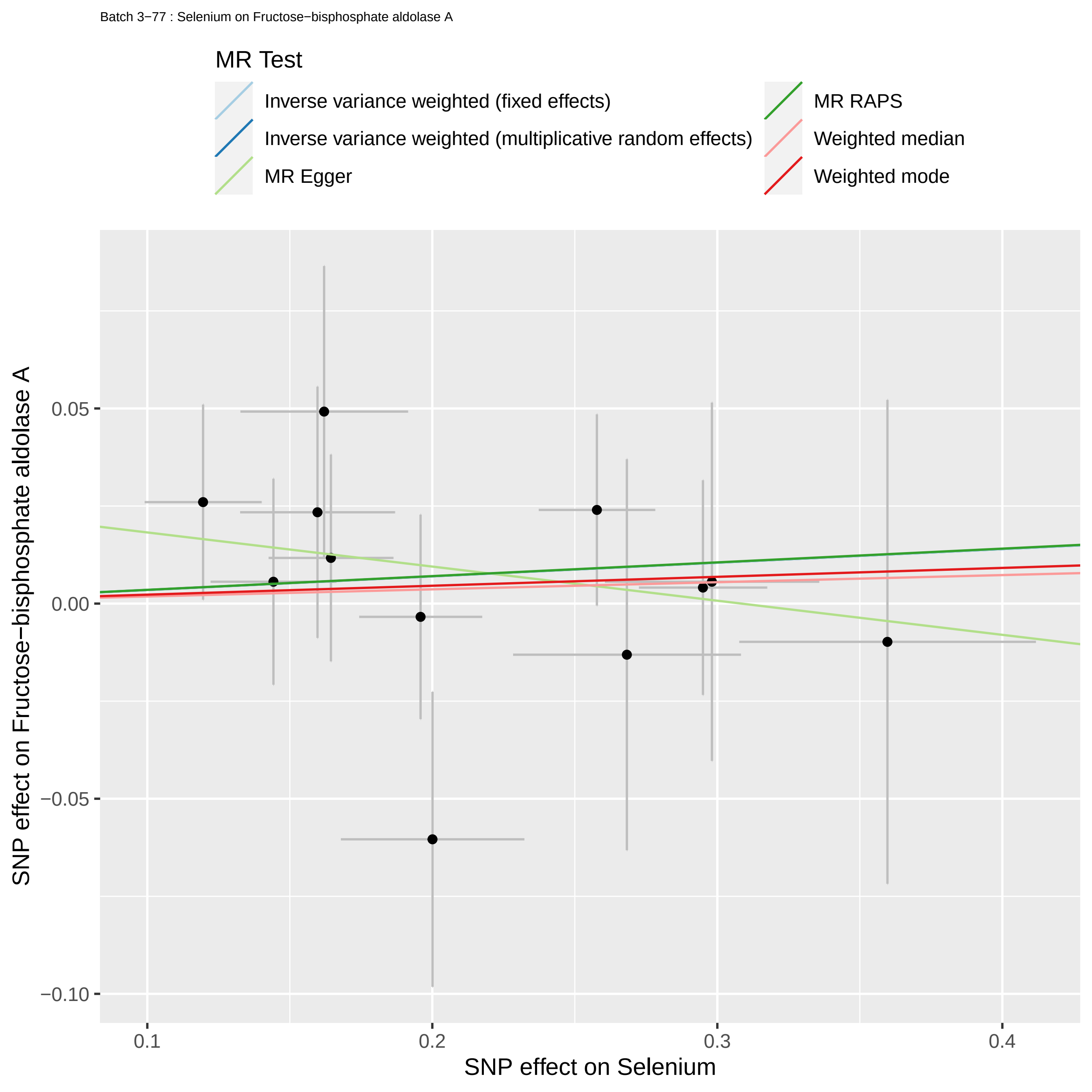
Batch 3−77 : Selenium on Fructose−bisphosphate aldolase A
MR Test
Inverse variance weighted (fixed effects)
Inverse variance weighted (multiplicative random effects)
MR Egger
MR RAPS
Weighted median
Weighted mode
SNP effect on Fructose−bisphosphate aldolase A
0.05
0.00
−0.05
−0.10
0.1
0.2
0.3
0.4
SNP effect on Selenium

Batch 3−78 : Selenium on Fructose−bisphosphate aldolase C

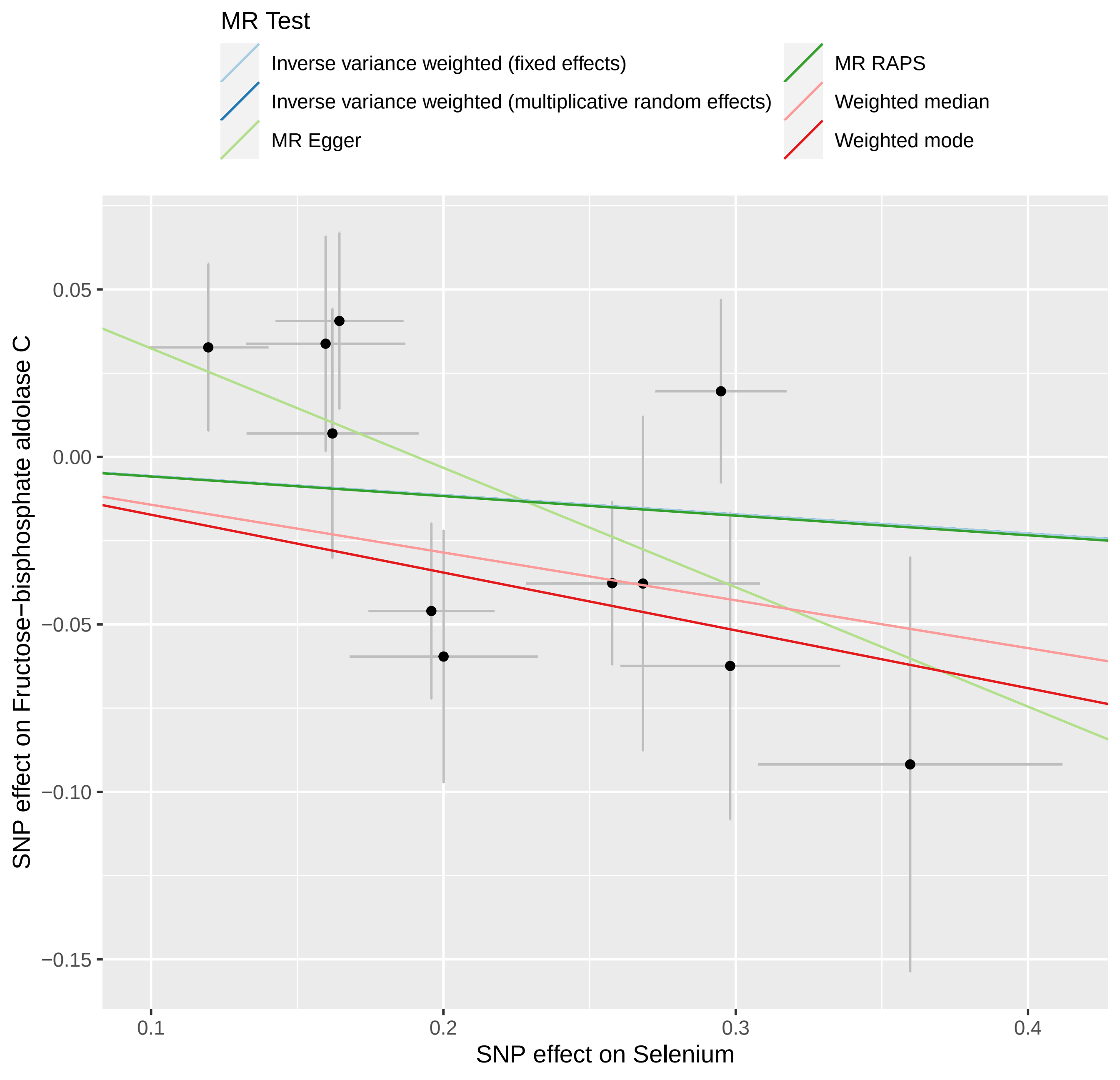

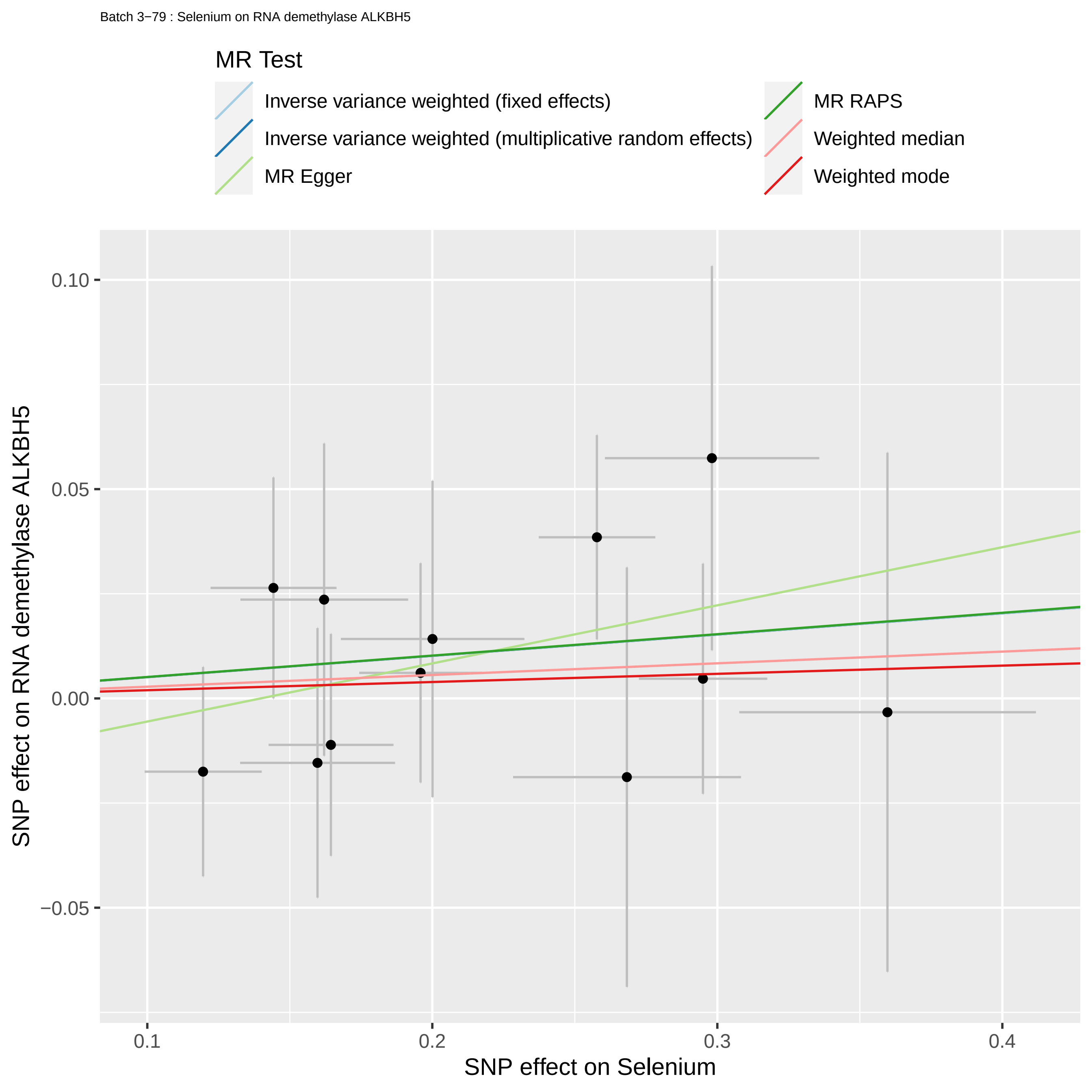
Batch 3−79 : Selenium on RNA demethylase ALKBH5
MR Test
Inverse variance weighted (fixed effects)
Inverse variance weighted (multiplicative random effects)
MR Egger
MR RAPS
Weighted median
Weighted mode
0.10
0.05
0.00
−0.05
0.1
0.2
0.3
0.4
SNP effect on RNA demethylase ALKBH5
SNP effect on Selenium

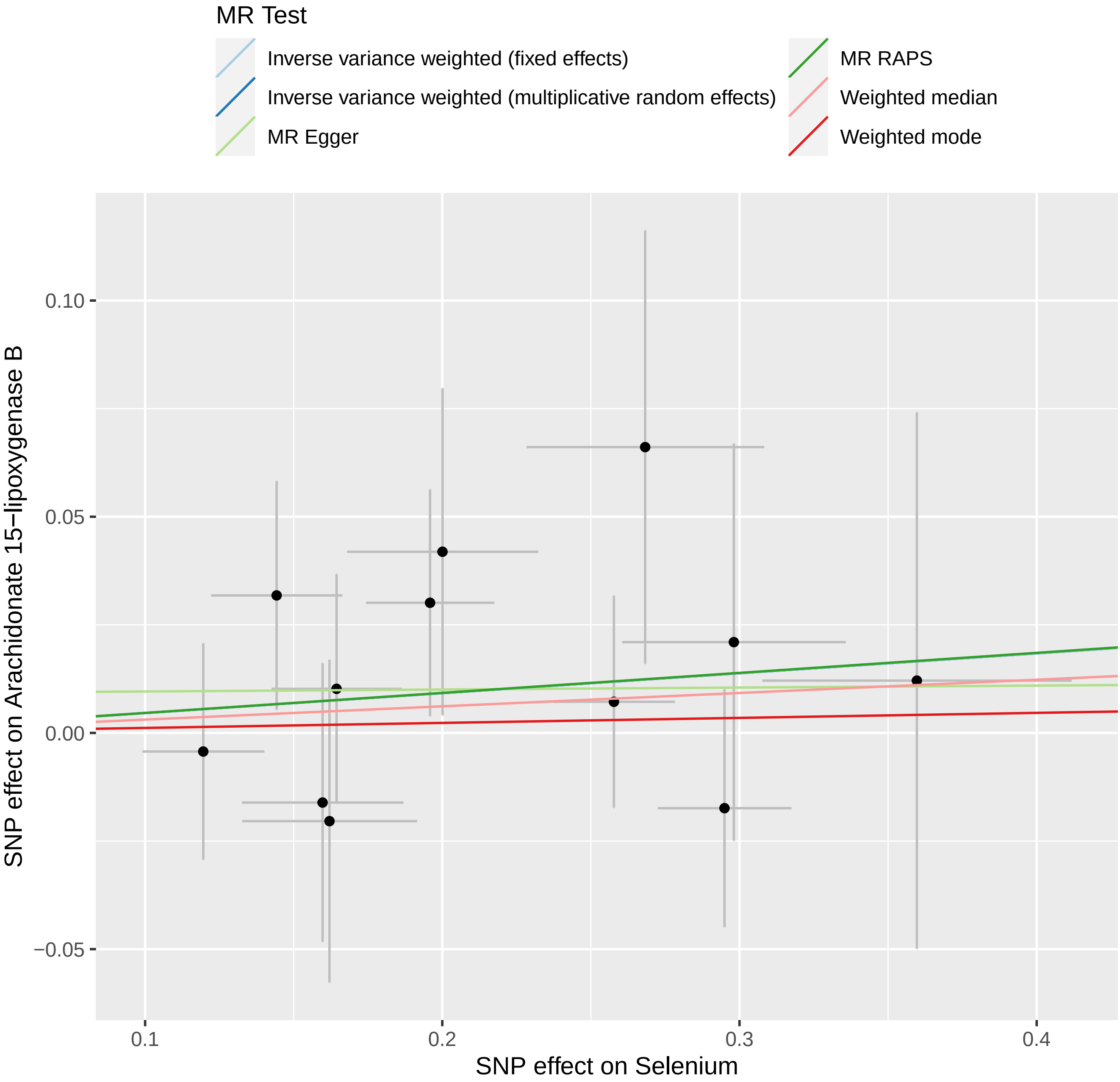
MR Test
Inverse variance weighted (fixed effects)
Inverse variance weighted (multiplicative random effects)
MR Egger
MR RAPS
Weighted median
Weighted mode
0.10
0.05
0.00
−0.05
0.1
0.2
0.3
0.4
SNP effect on Arachidonate 15−lipoxygenase B
SNP effect on Selenium

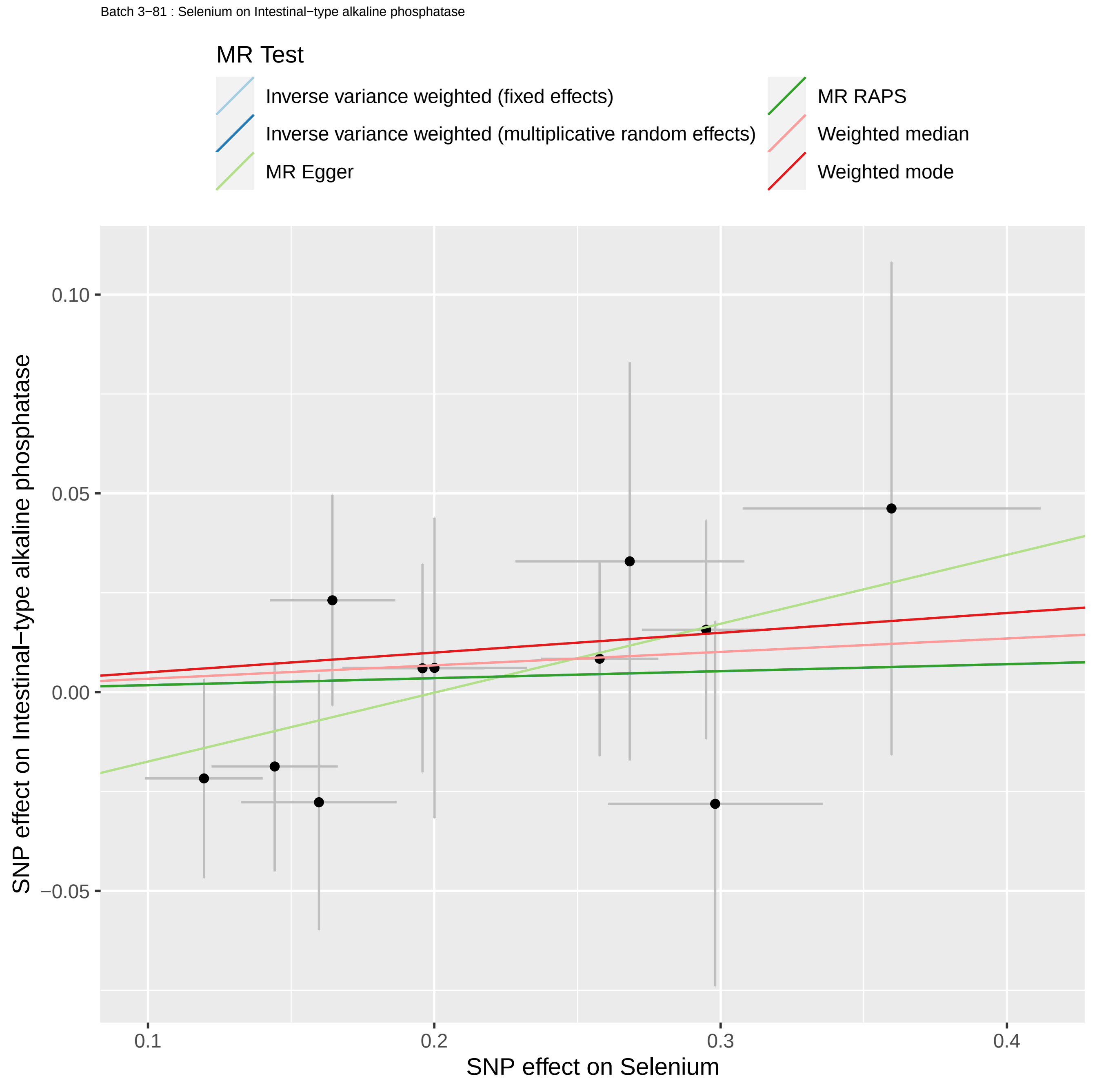
Batch 3−81 : Selenium on Intestinal−type alkaline phosphatase
MR Test
Inverse variance weighted (fixed effects)
Inverse variance weighted (multiplicative random effects)
MR Egger
MR RAPS
Weighted median
Weighted mode
0.10
0.05
0.00
−0.05
0.1
0.2
0.3
0.4
SNP effect on Intestinal−type alkaline phosphatase
SNP effect on Selenium

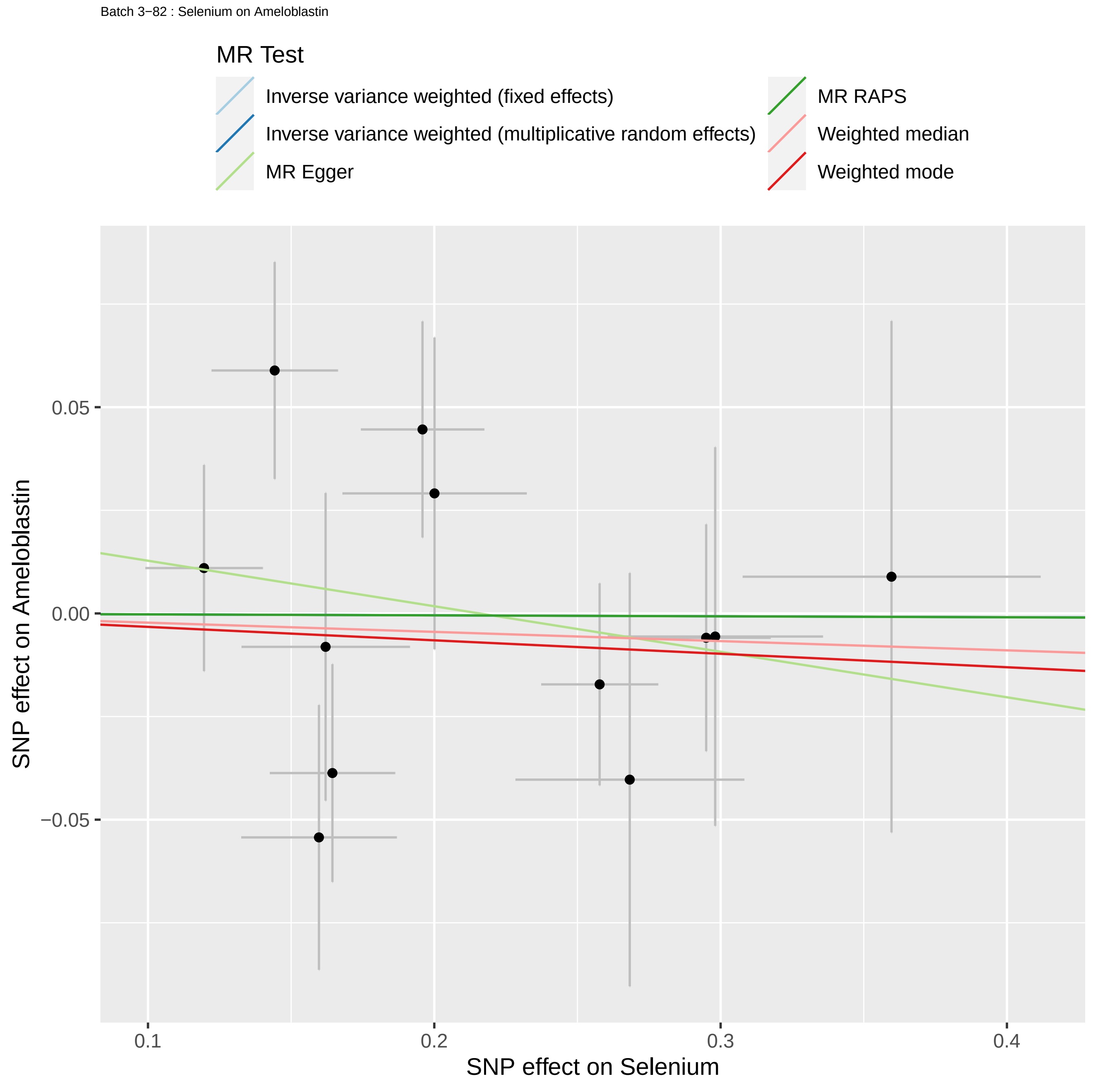

Batch 3−82 : Selenium on Ameloblastin
MR Test
Inverse variance weighted (fixed effects)
Inverse variance weighted (multiplicative random effects)
MR Egger
MR RAPS
Weighted median
Weighted mode
0.05
0.00
−0.05
0.1
0.2
0.3
0.4
SNP effect on Ameloblastin
SNP effect on Selenium

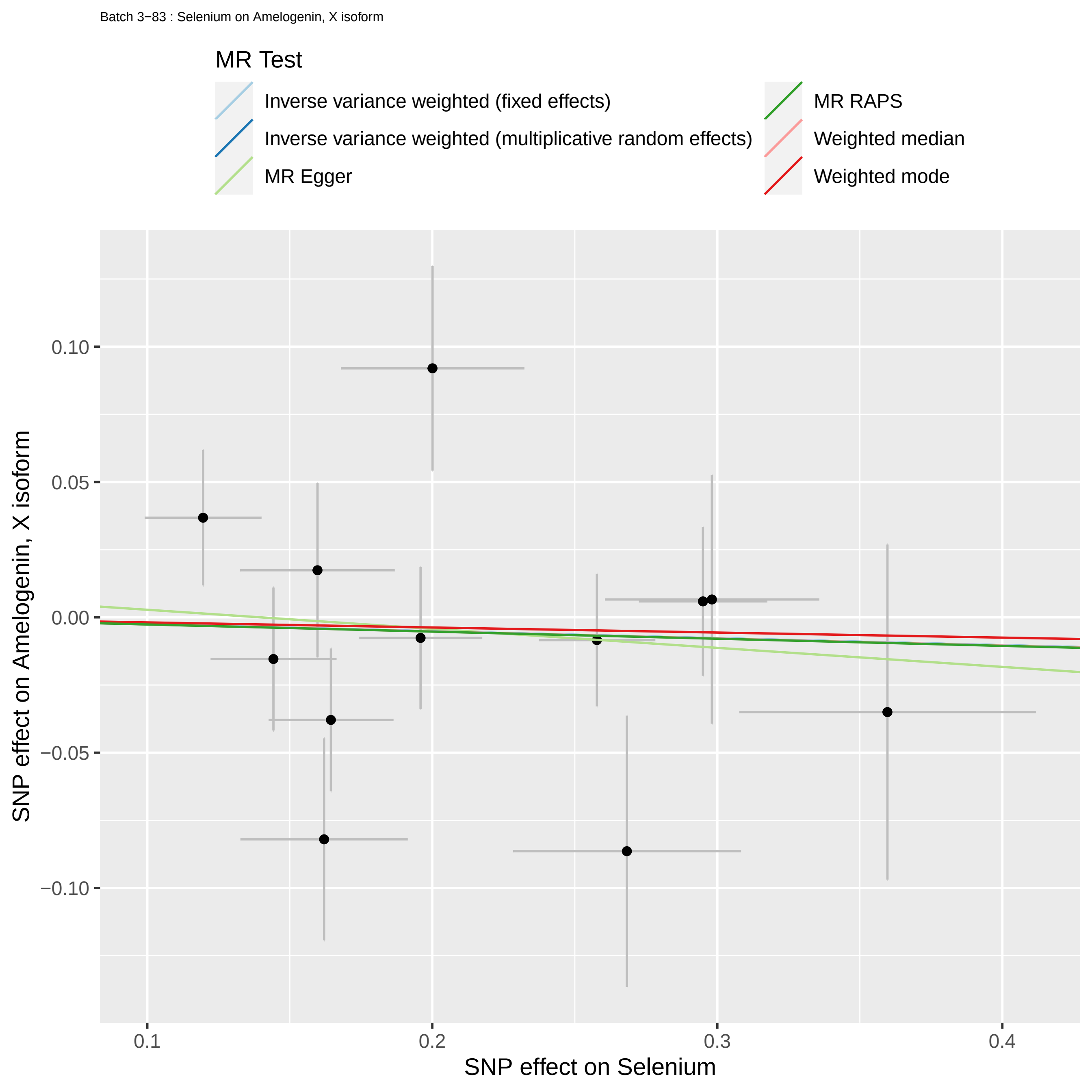
Batch 3−83 : Selenium on Amelogenin, X isoform
MR Test
Inverse variance weighted (fixed effects)
Inverse variance weighted (multiplicative random effects)
MR Egger
MR RAPS
Weighted median
Weighted mode
0.10
0.05
0.00
−0.05
−0.10
0.1
0.2
0.3
0.4
SNP effect on Amelogenin, X isoform
SNP effect on Selenium

Batch 3−84 : Selenium on Muellerian−inhibiting factor

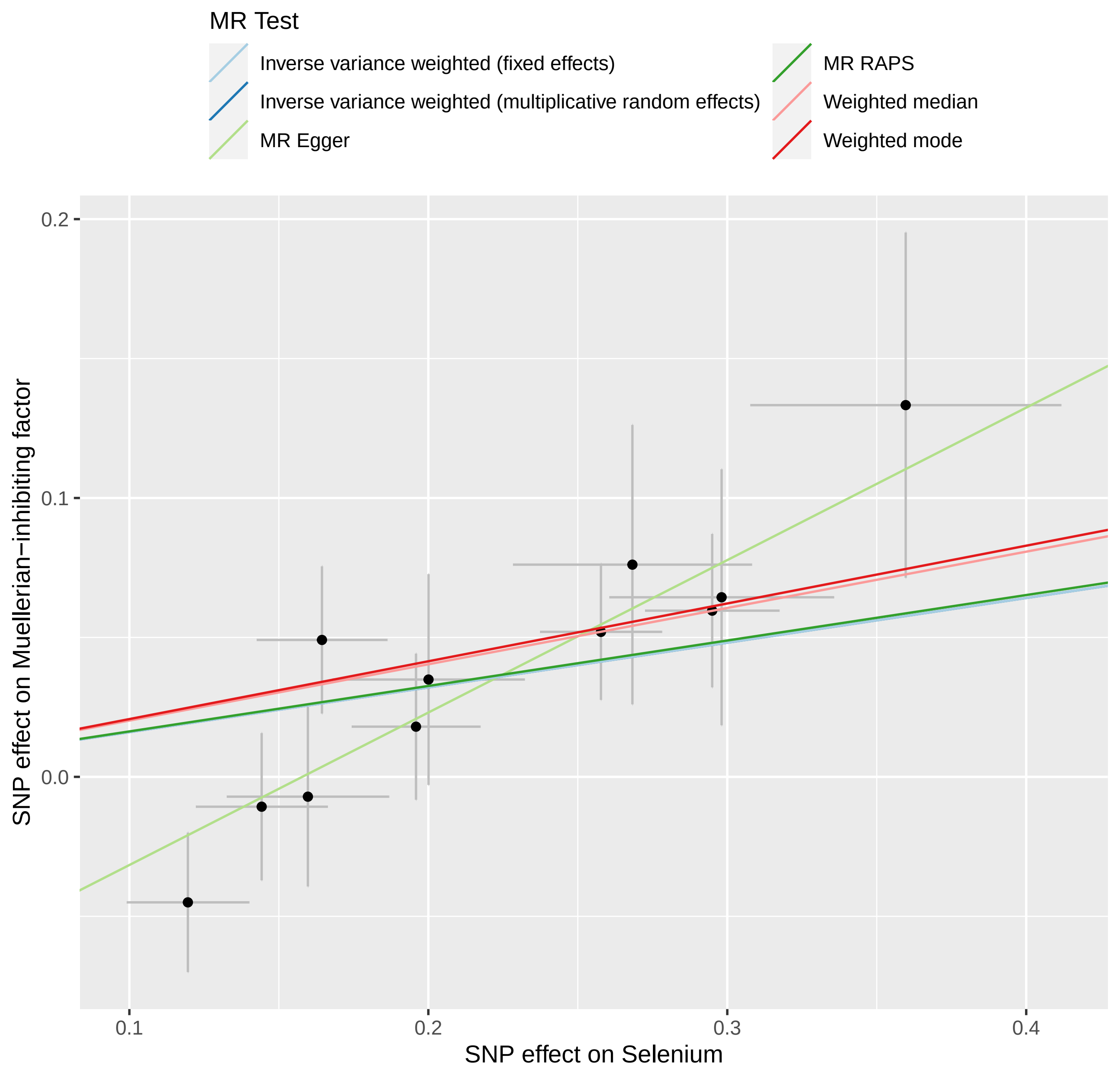

Batch 3−85 : Selenium on Junctional adhesion molecule−like

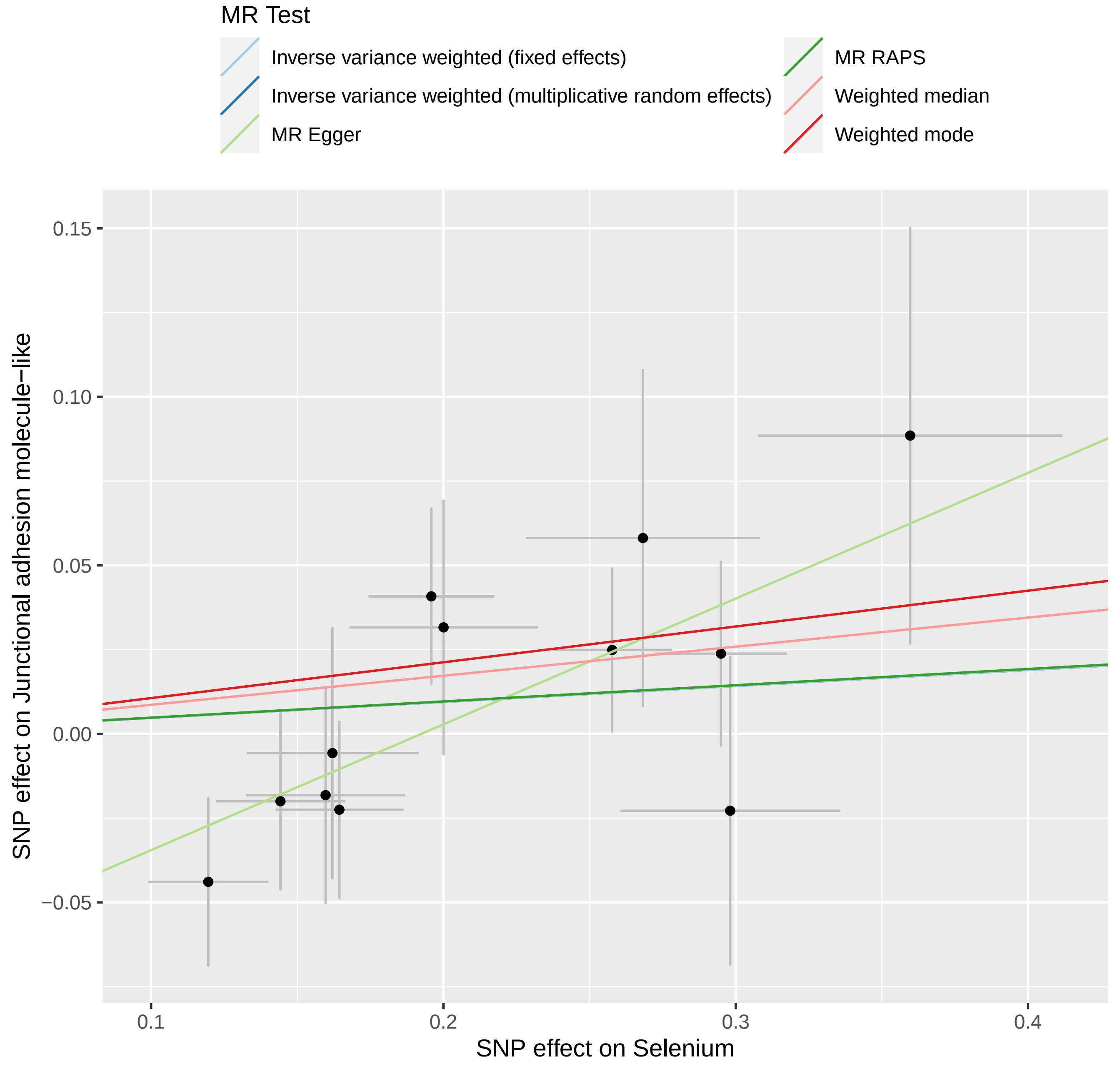

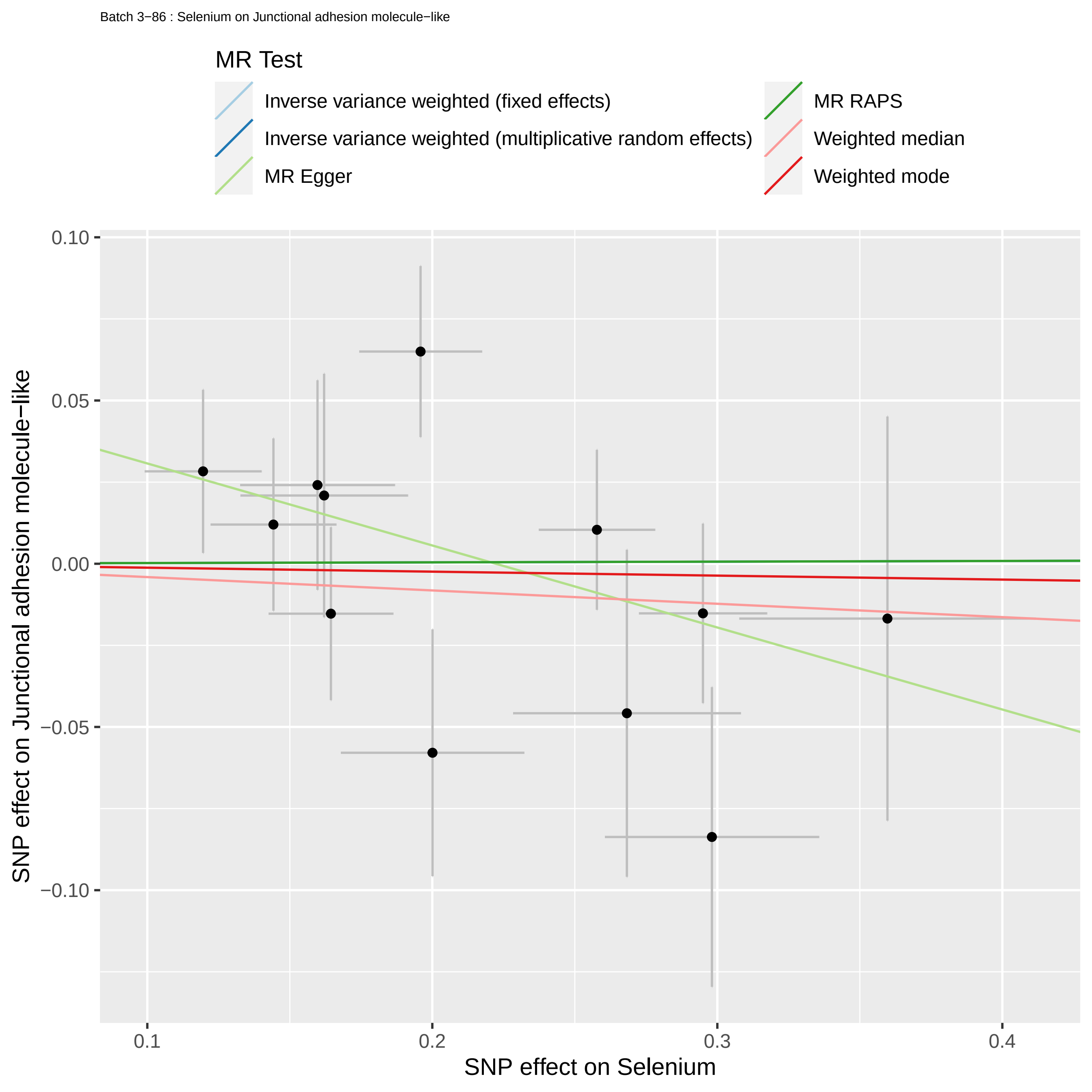

Batch 3−86 : Selenium on Junctional adhesion molecule−like
MR Test
Inverse variance weighted (fixed effects)
Inverse variance weighted (multiplicative random effects)
MR Egger
MR RAPS
Weighted median
Weighted mode
0.10
0.05
0.00
−0.05
−0.10
0.1
0.2
0.3
0.4
SNP effect on Junctional adhesion molecule−like
SNP effect on Selenium

Batch 3−87 : Selenium on Amphoterin−induced protein 1

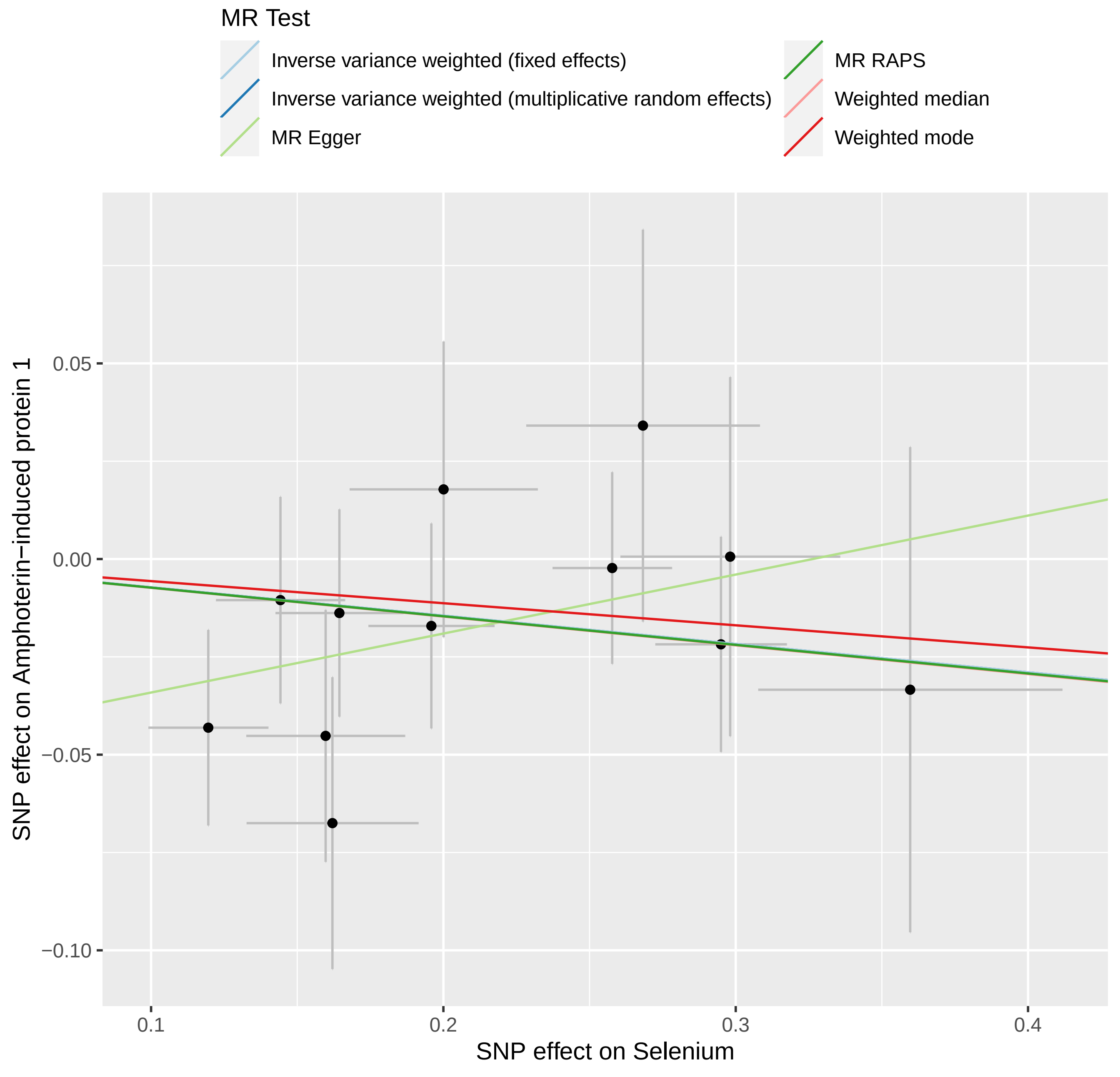

Batch 3−88 : Selenium on Protein amnionless

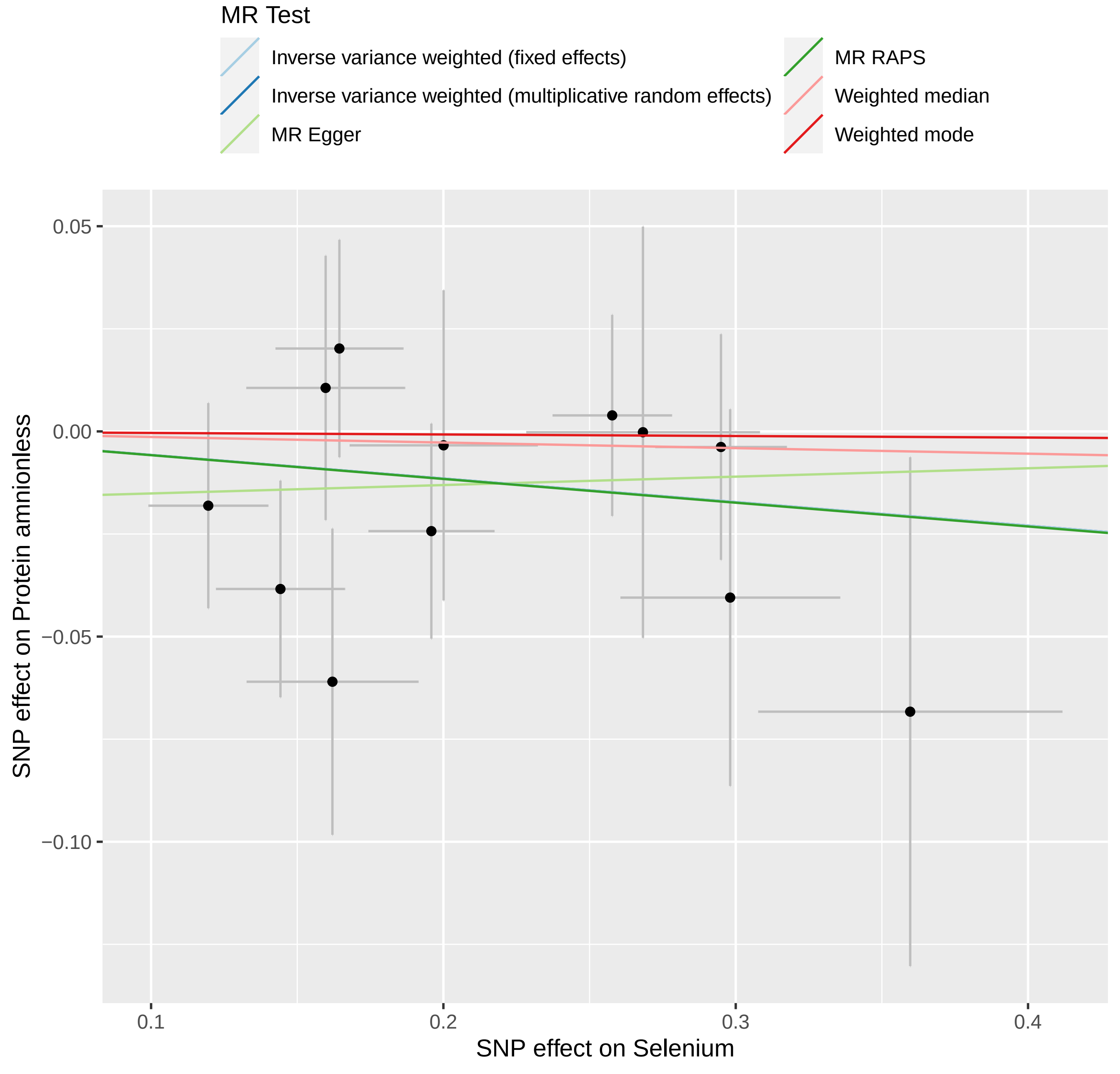

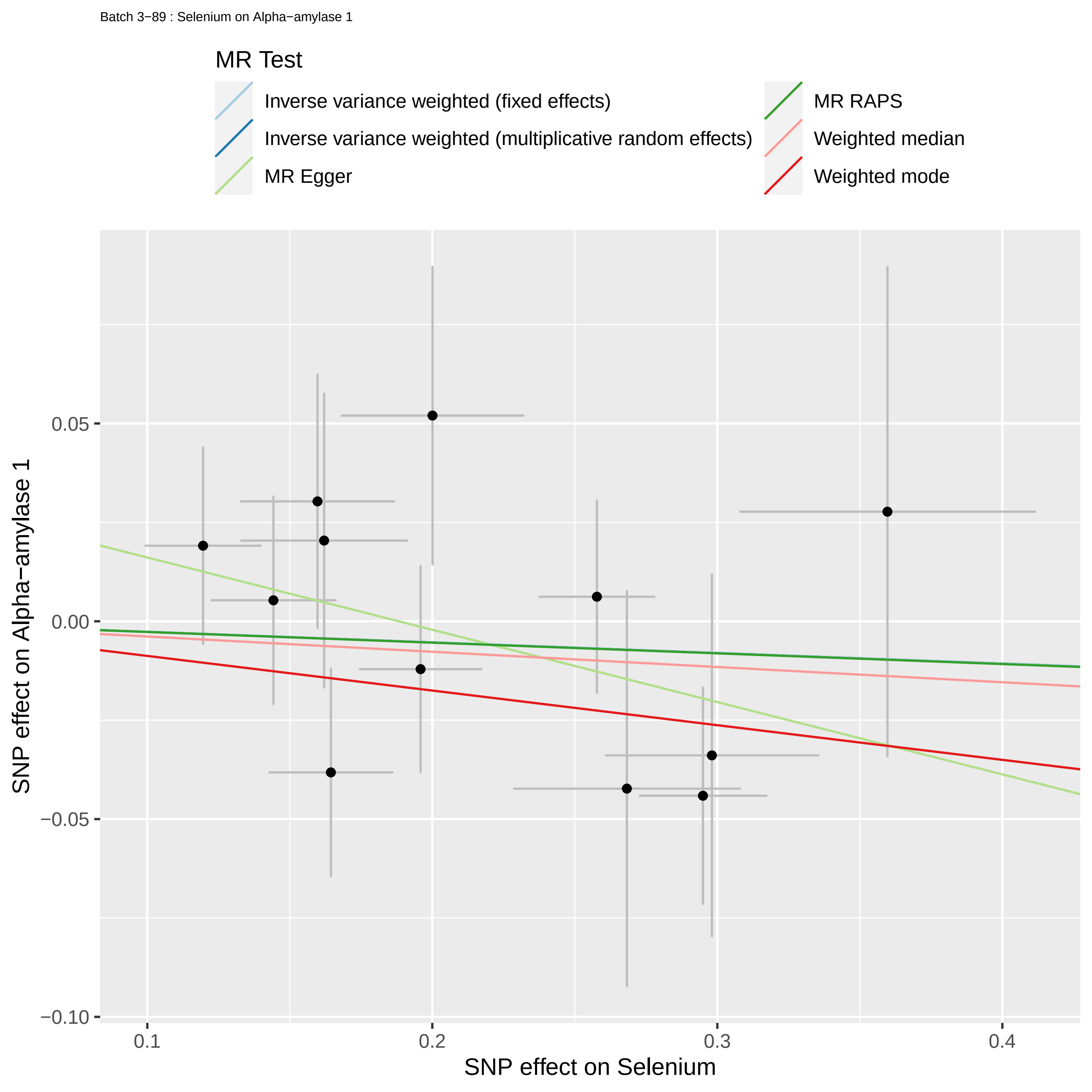

Batch 3−89 : Selenium on Alpha−amylase 1
MR Test
Inverse variance weighted (fixed effects)
Inverse variance weighted (multiplicative random effects)
MR Egger
MR RAPS
Weighted median
Weighted mode
SNP effect on Alpha−amylase 1
0.05
0.00
−0.05
−0.10
0.1
0.2
0.3
0.4
SNP effect on Selenium

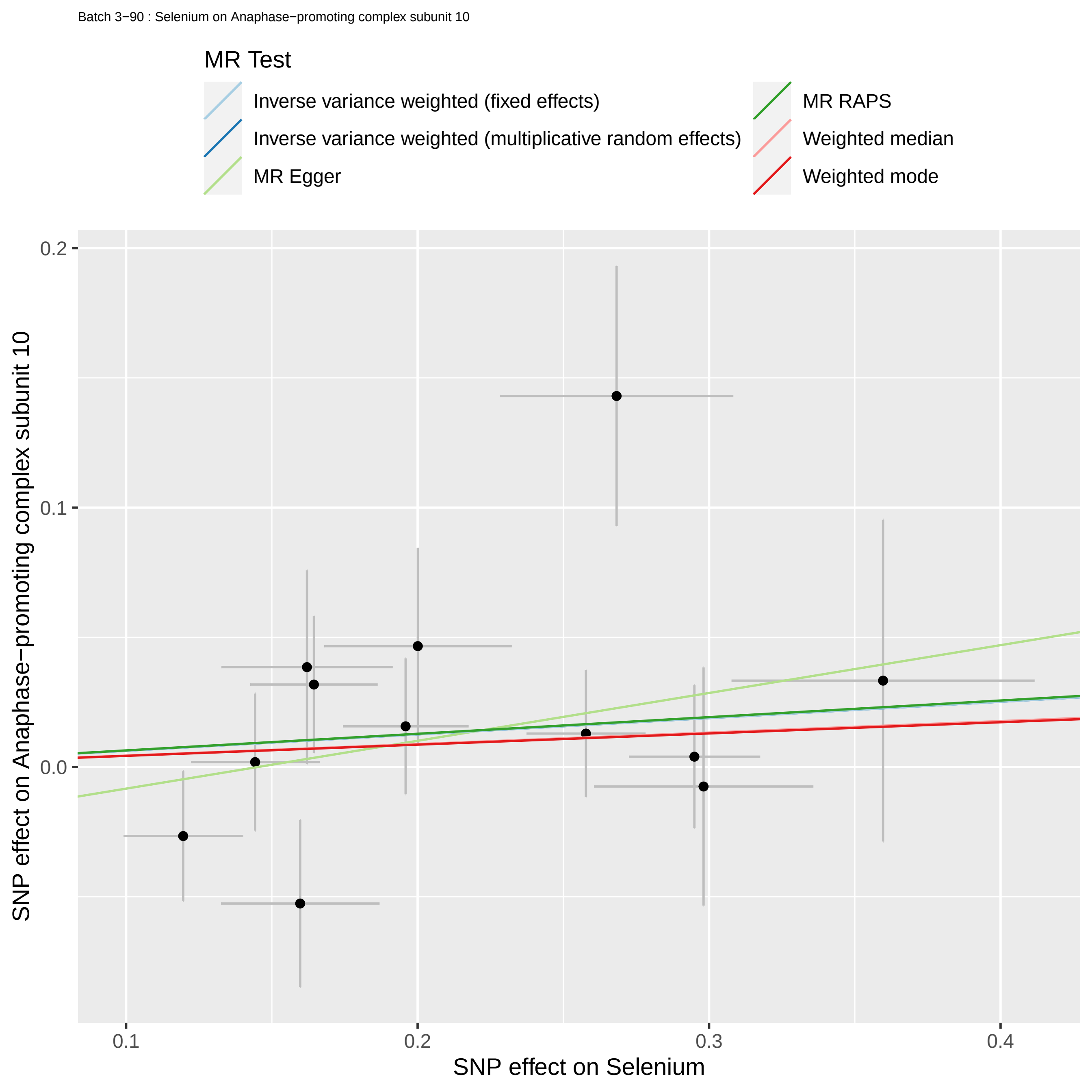

Batch 3−90 : Selenium on Anaphase−promoting complex subunit 10
MR Test
Inverse variance weighted (fixed effects)
Inverse variance weighted (multiplicative random effects)
MR Egger
MR RAPS
Weighted median
Weighted mode
0.2
0.1
0.0
0.1
0.2
0.3
0.4
SNP effect on Anaphase−promoting complex subunit 10
SNP effect on Selenium

Batch 3−91 : Selenium on Anaphase−promoting complex subunit 7

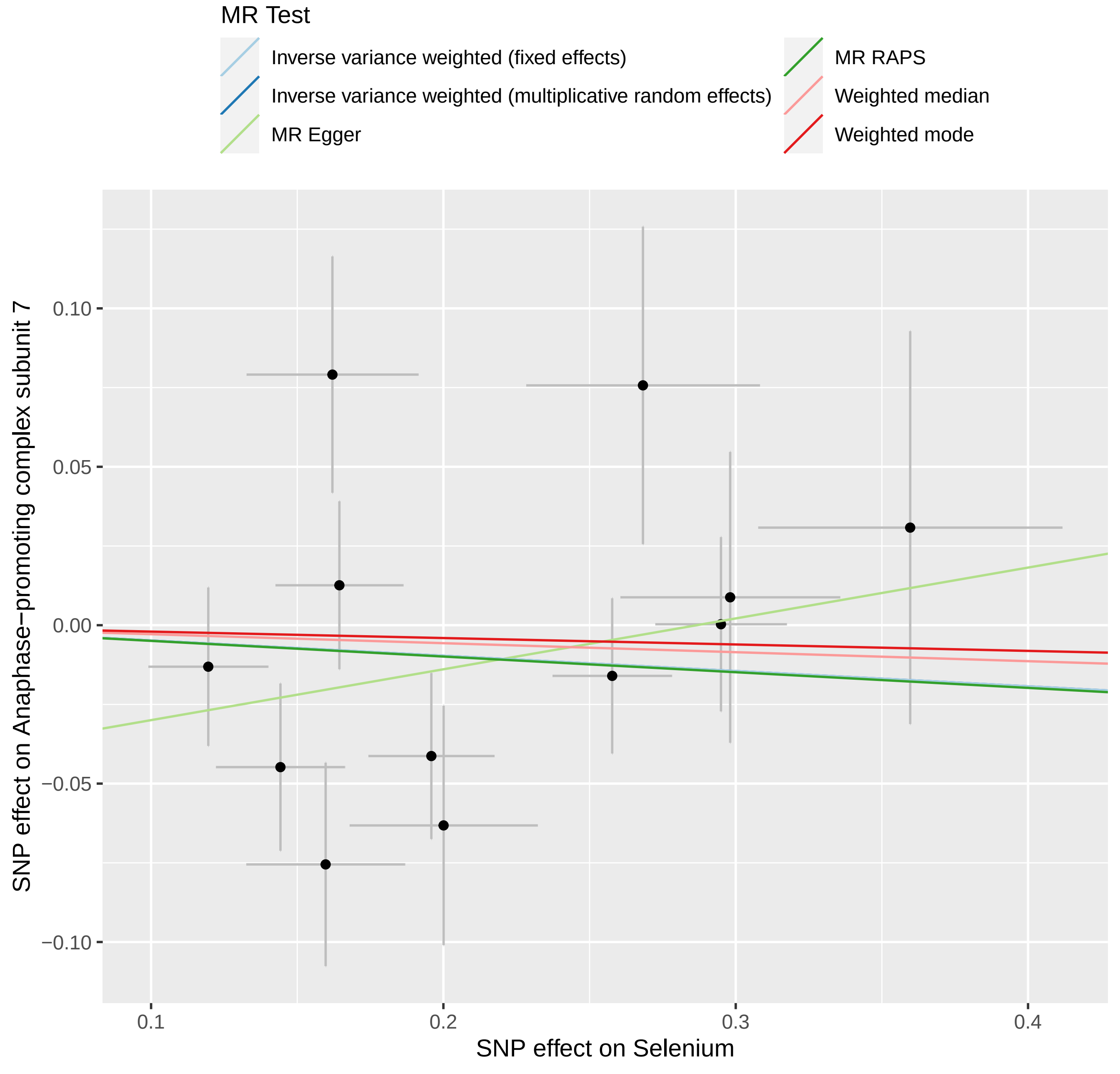

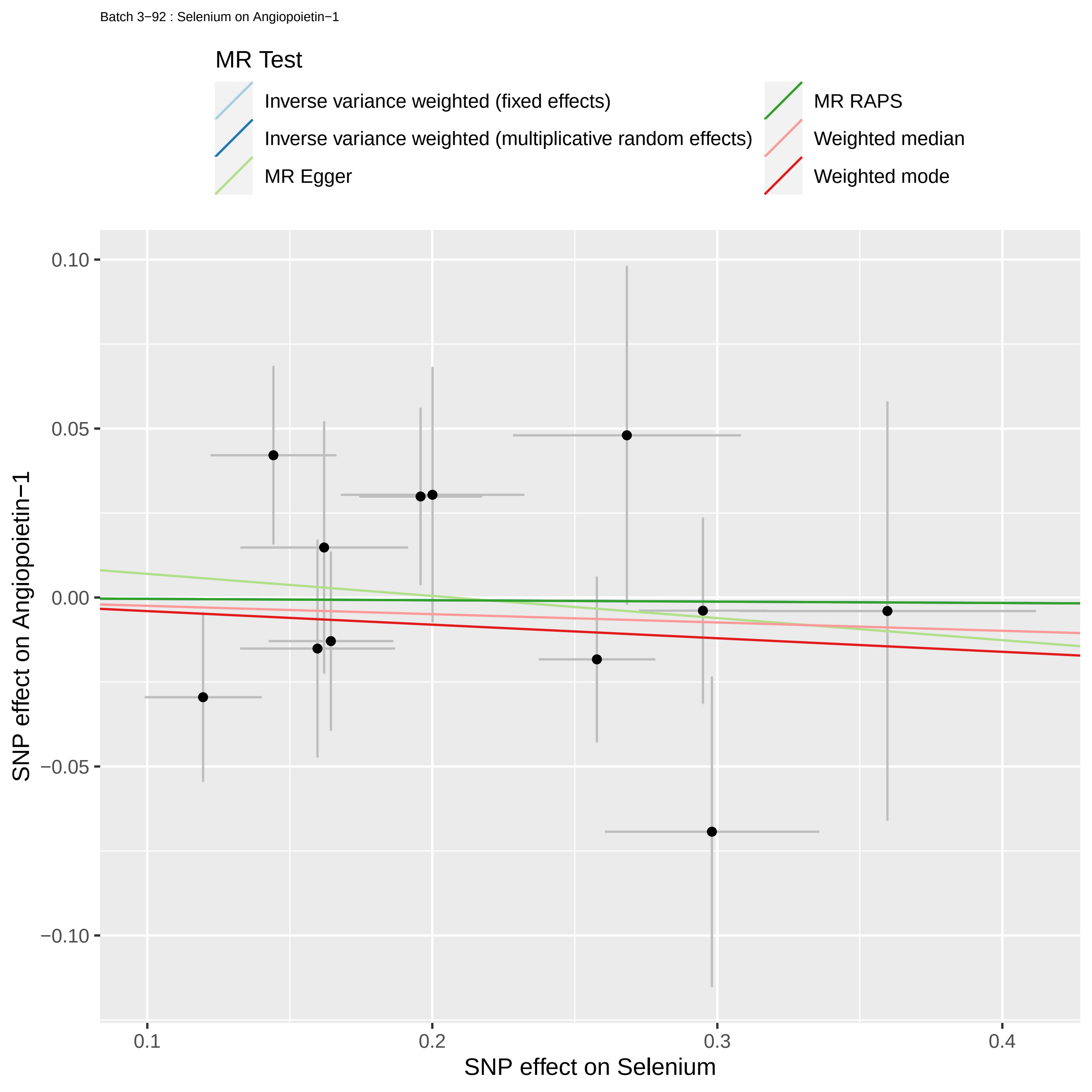
Batch 3−92 : Selenium on Angiopoietin−1
MR Test
Inverse variance weighted (fixed effects)
Inverse variance weighted (multiplicative random effects)
MR Egger
MR RAPS
Weighted median
Weighted mode
0.10
0.05
0.00
−0.05
−0.10
SNP effect on Angiopoietin−1
0.1
0.2
0.3
0.4
SNP effect on Selenium

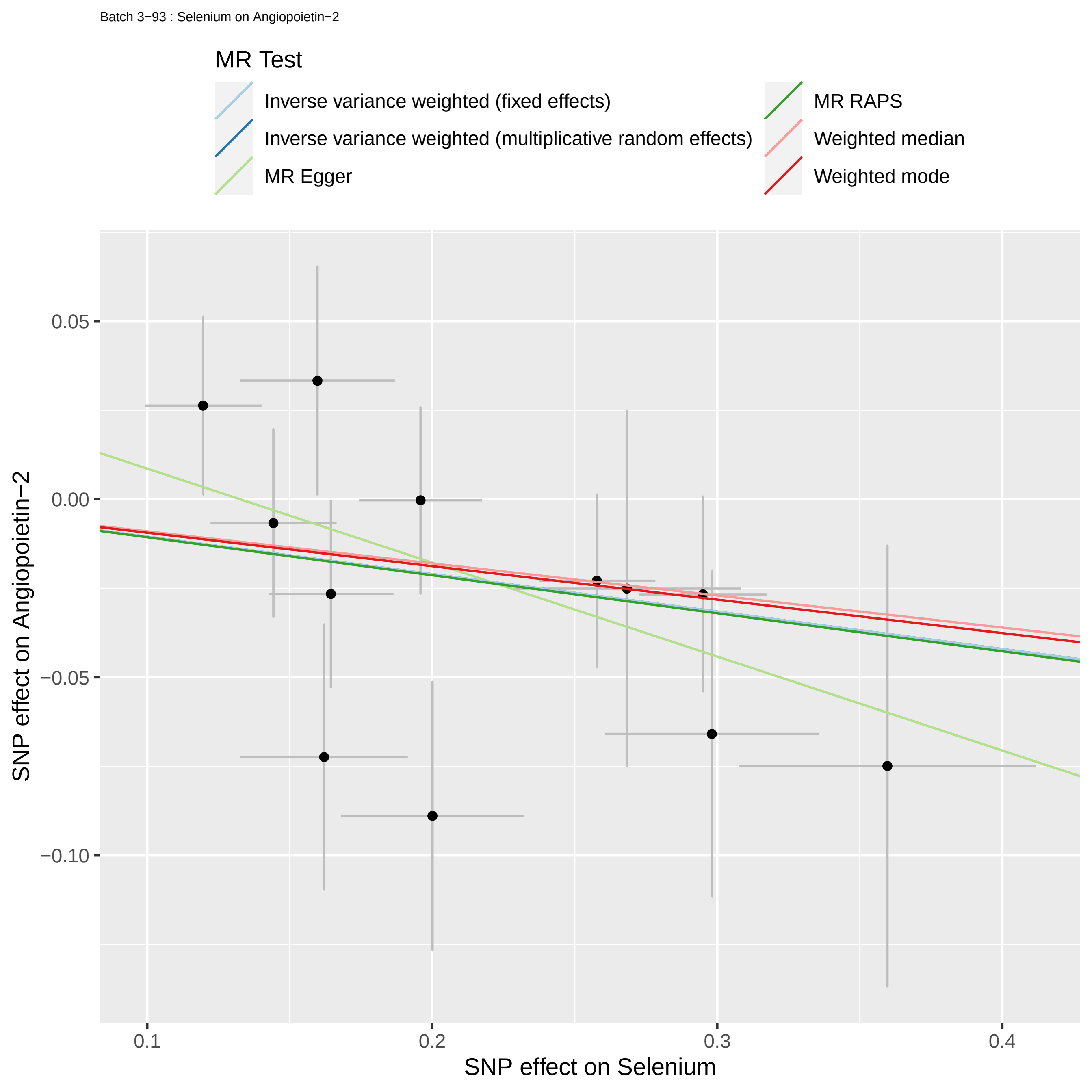

Batch 3−93 : Selenium on Angiopoietin−2
MR Test
Inverse variance weighted (fixed effects)
Inverse variance weighted (multiplicative random effects)
MR Egger
MR RAPS
Weighted median
Weighted mode
0.05
0.00
−0.05
−0.10
0.1
0.2
0.3
0.4
SNP effect on Angiopoietin−2
SNP effect on Selenium

Batch 3−94 : Selenium on Angiopoietin−2

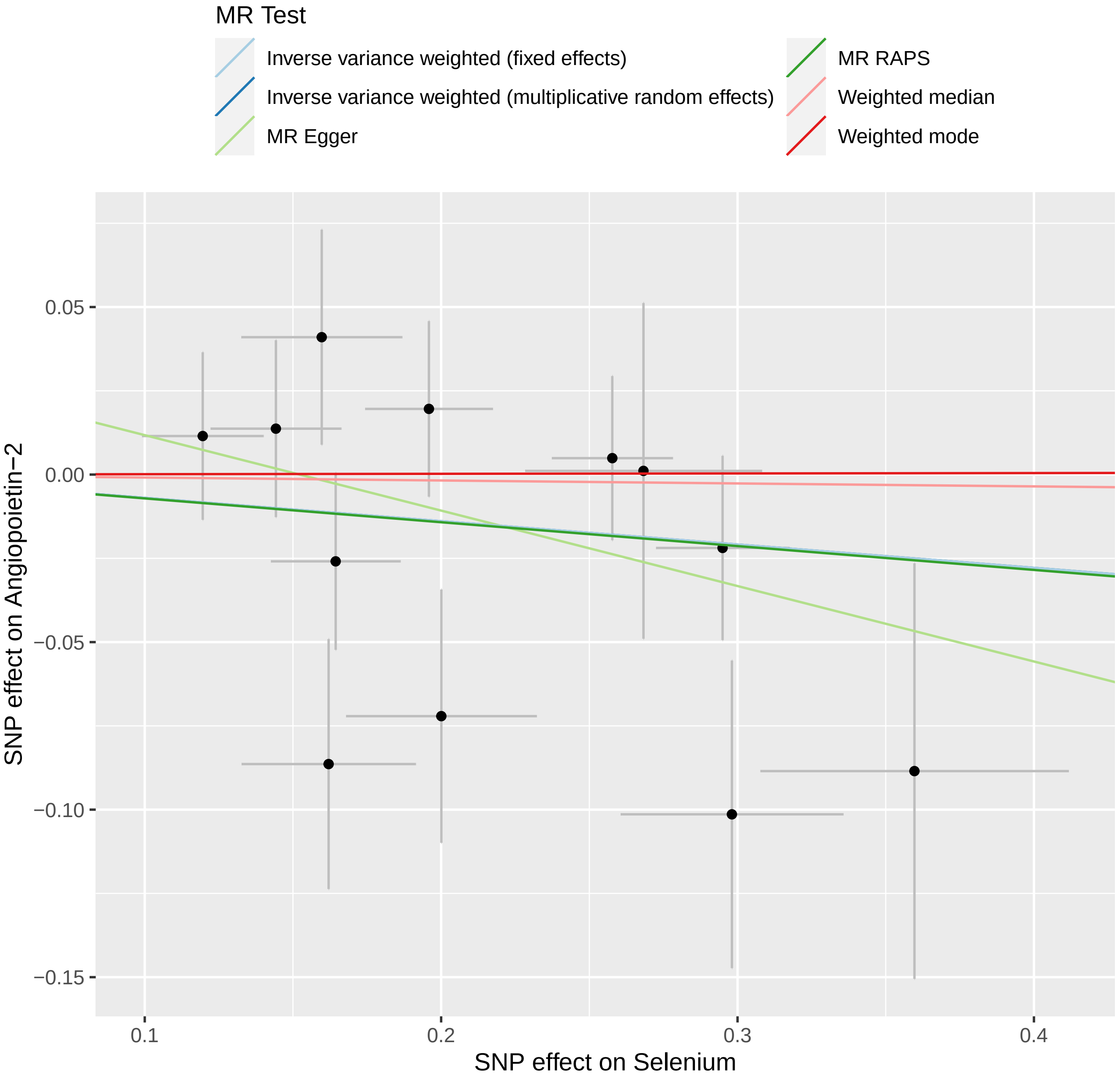

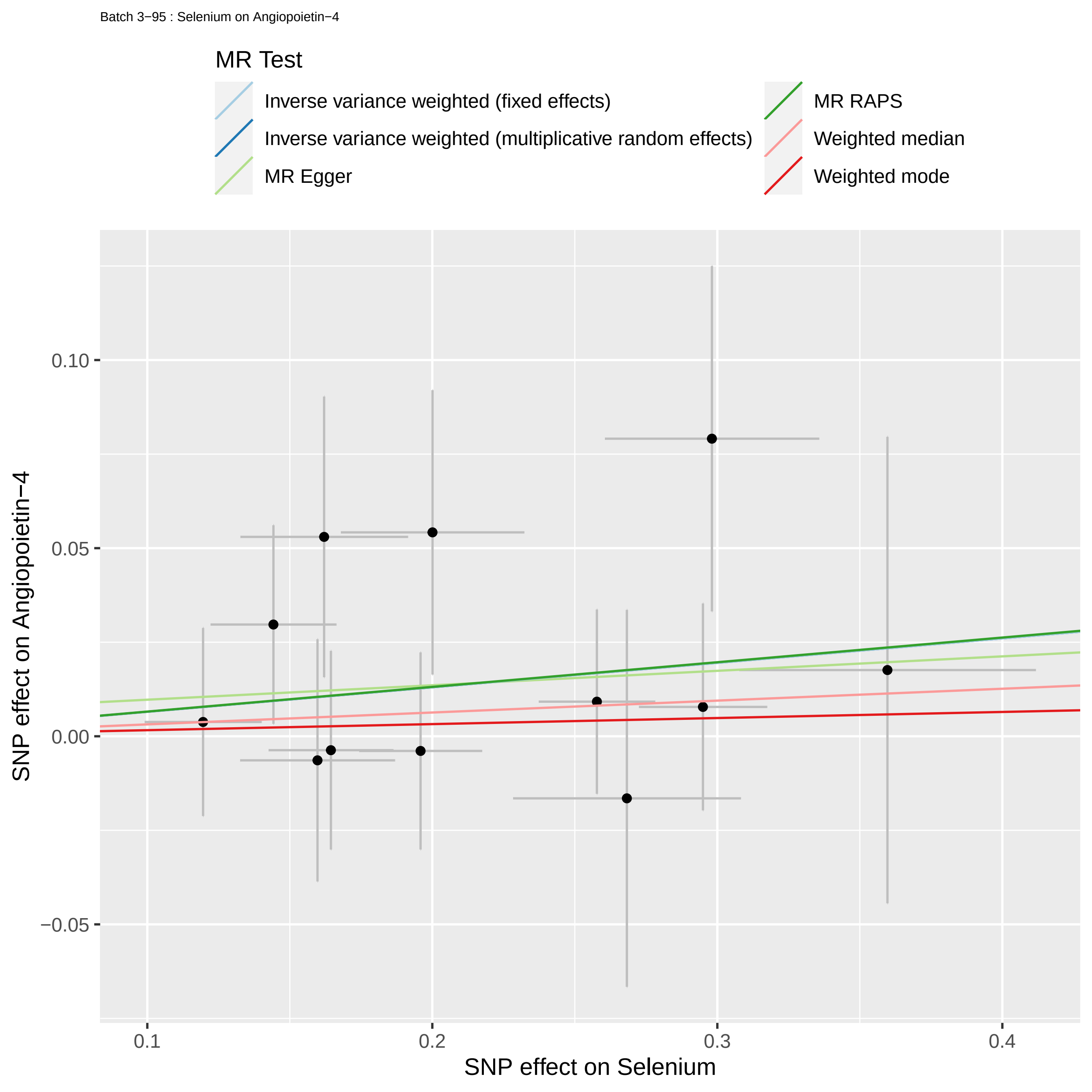
Batch 3−95 : Selenium on Angiopoietin−4
MR Test
Inverse variance weighted (fixed effects)
Inverse variance weighted (multiplicative random effects)
MR Egger
MR RAPS
Weighted median
Weighted mode
0.10
0.05
0.00
−0.05
0.1
0.2
0.3
0.4
SNP effect on Angiopoietin−4
SNP effect on Selenium

Batch 3−96 : Selenium on Angiopoietin−related protein 1

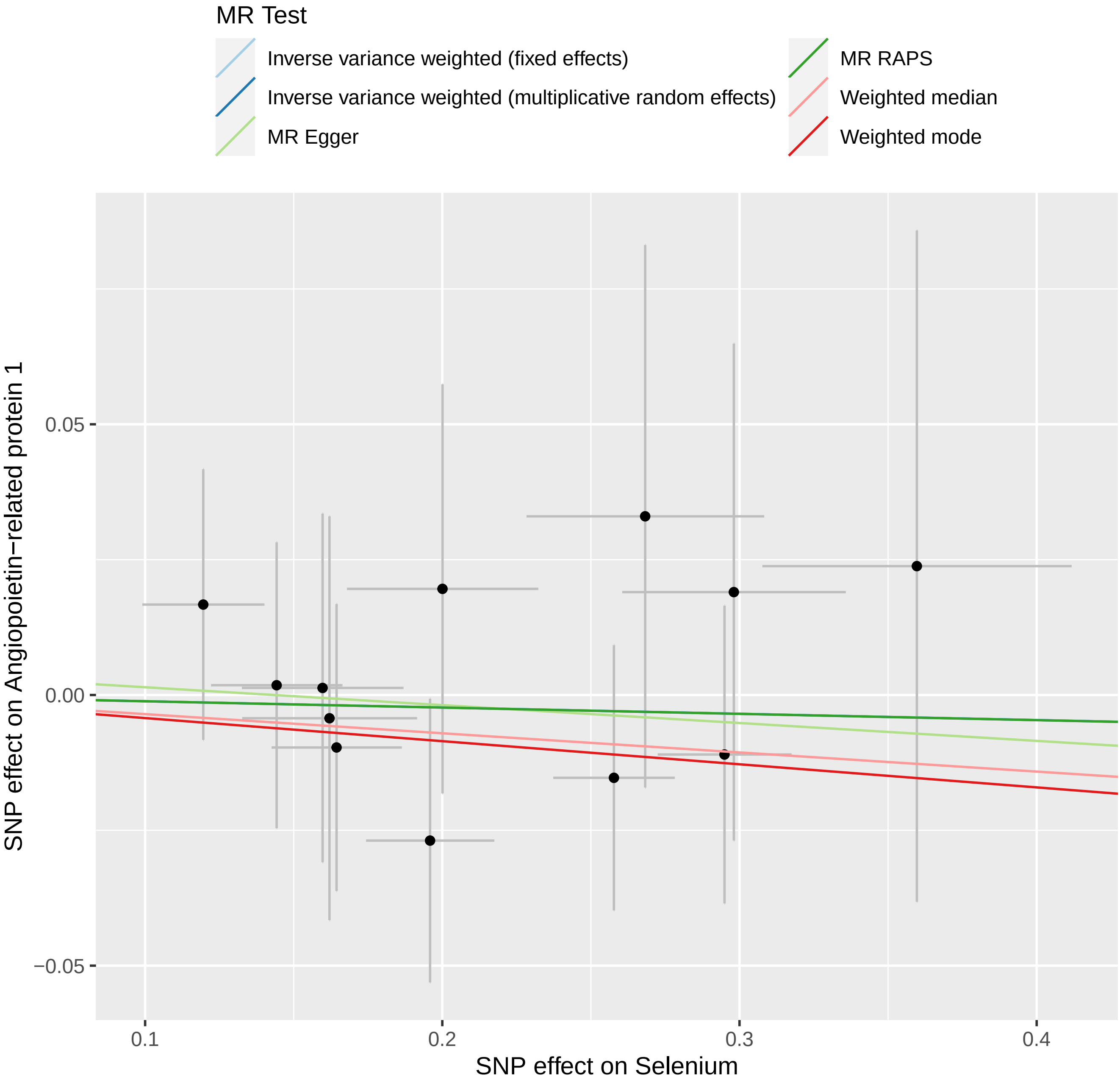

Batch 3−97 : Selenium on Angiopoietin−related protein 1

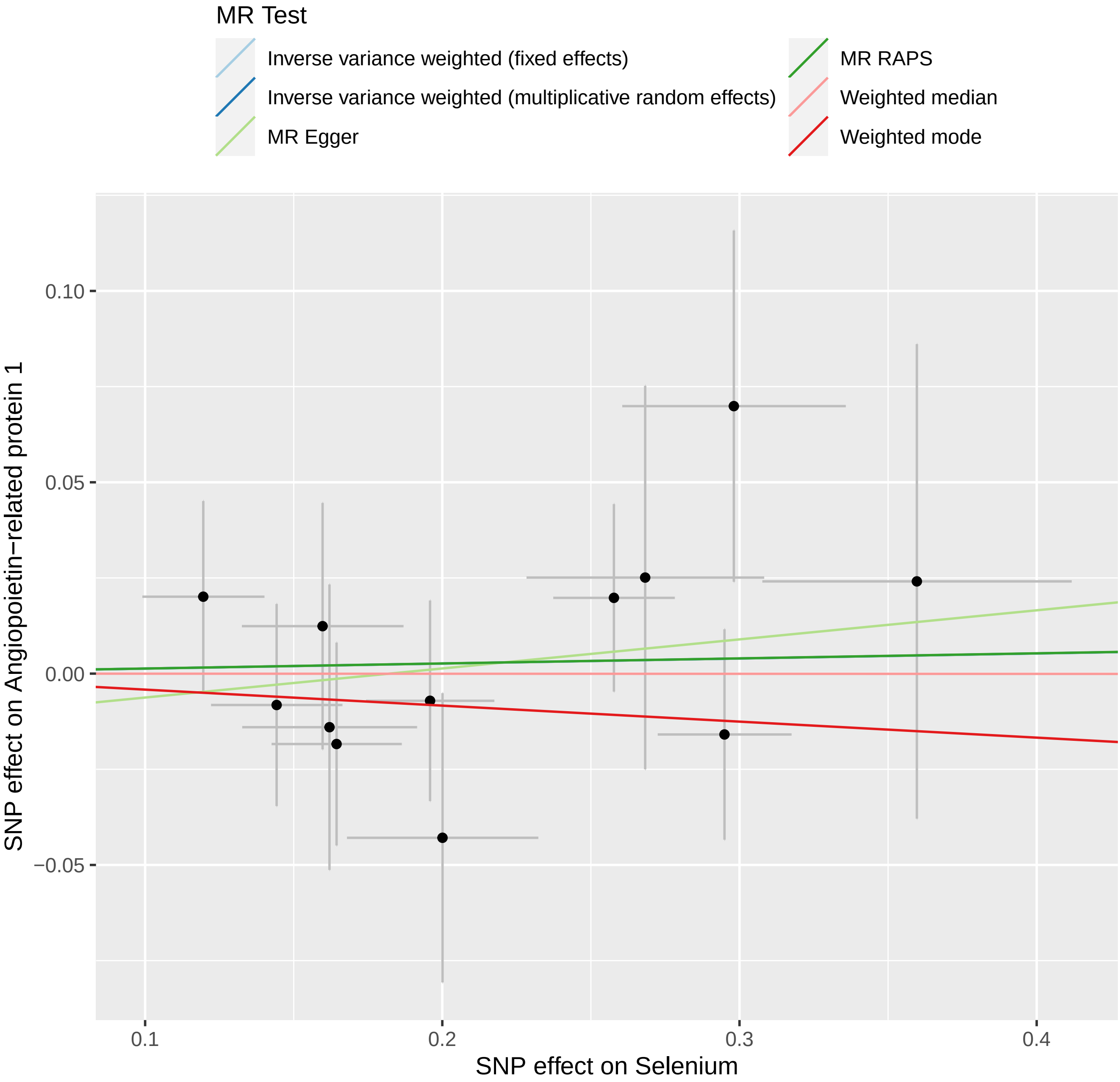

Batch 3−98 : Selenium on Angiopoietin−related protein 3

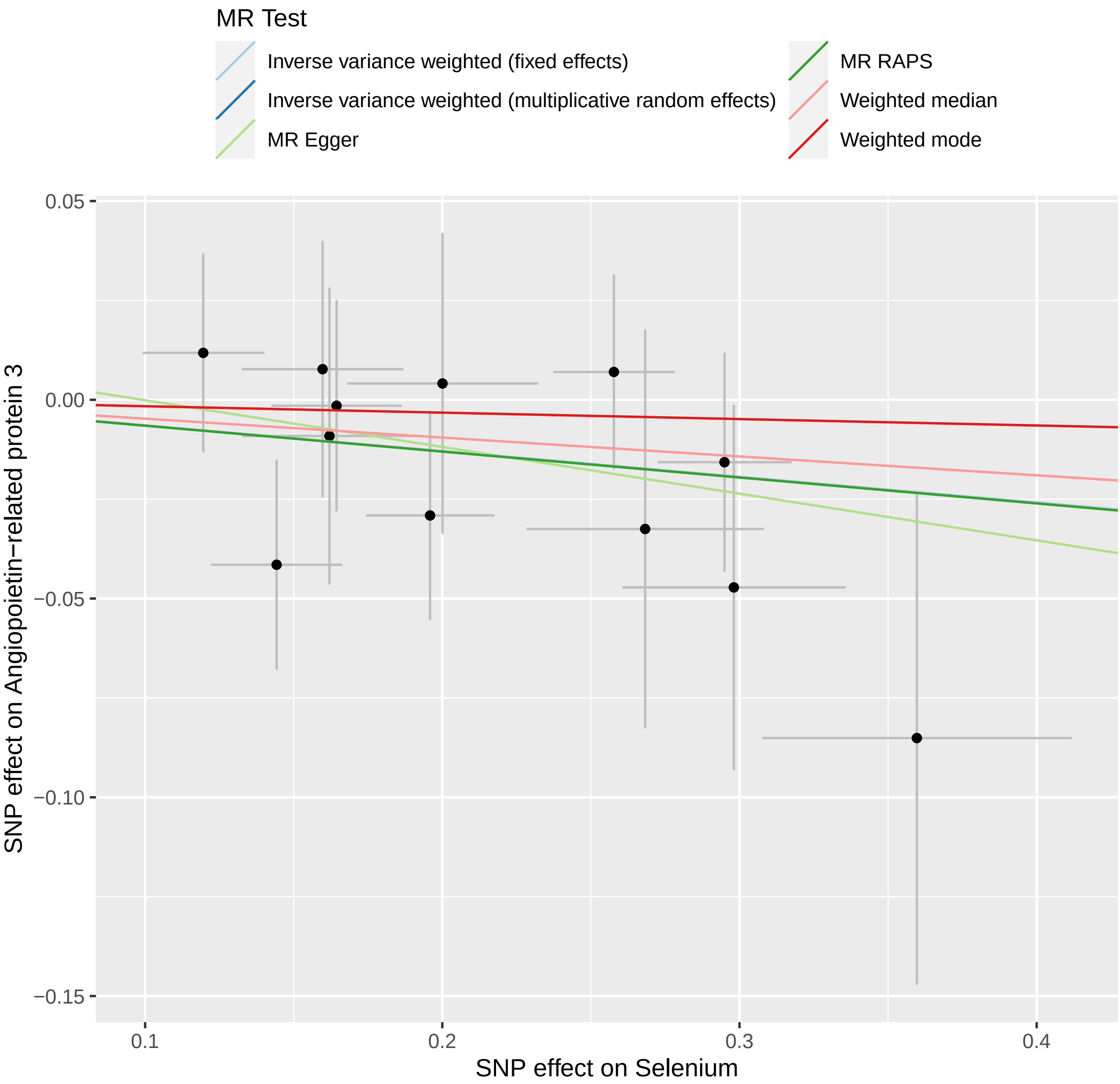

Batch 3−99 : Selenium on Angiopoietin−related protein 4

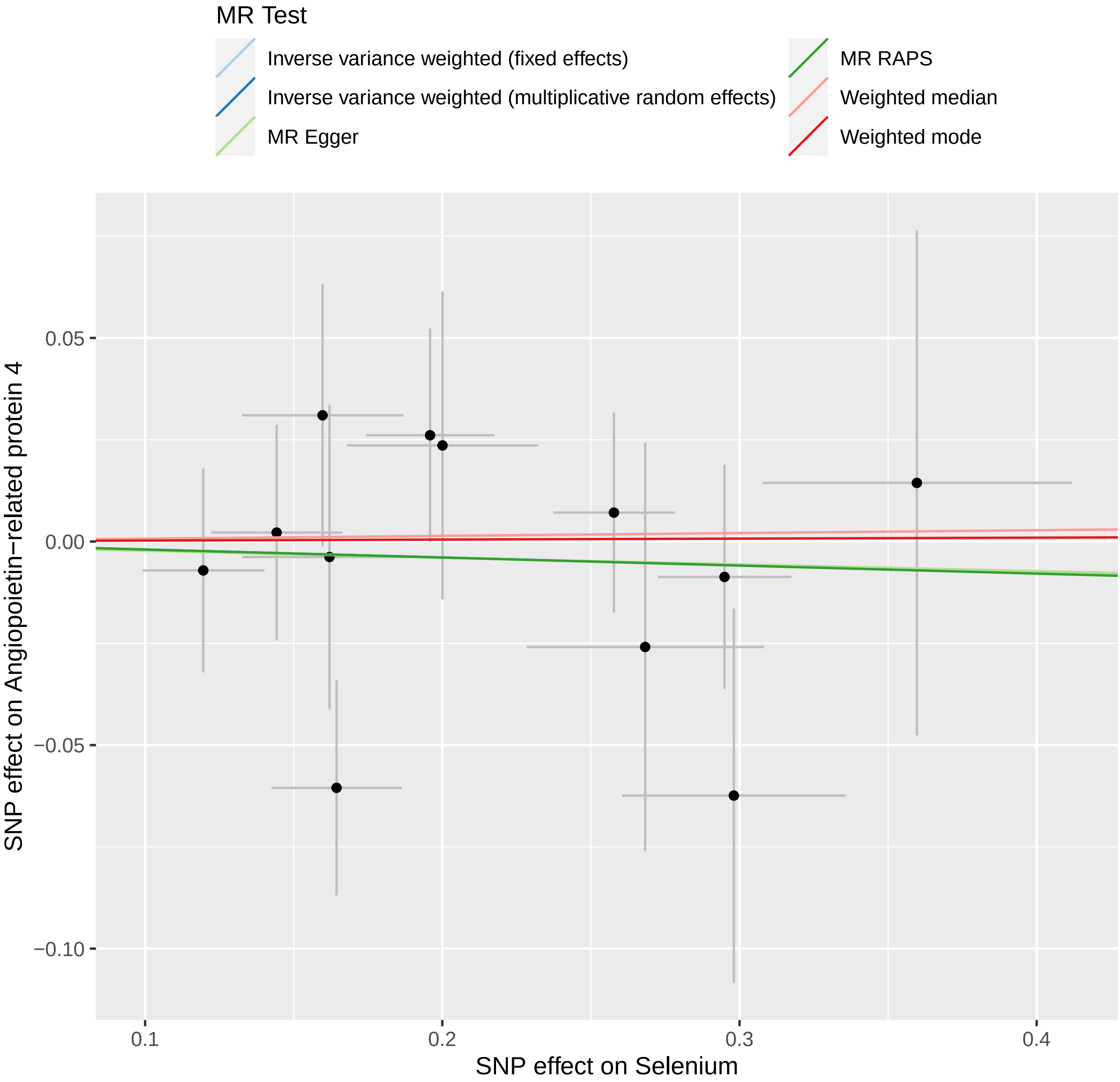

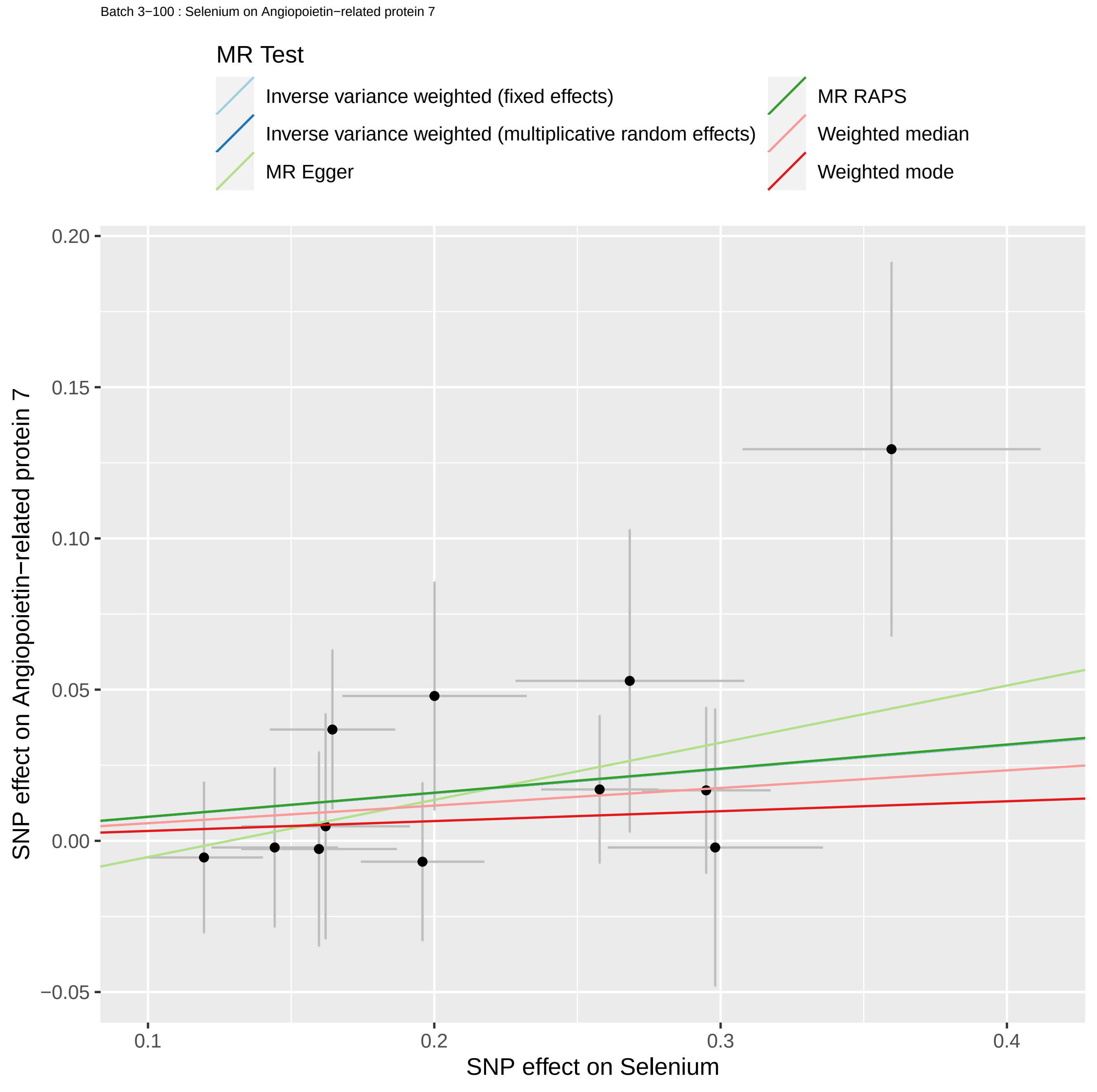

Batch 3−100 : Selenium on Angiopoietin−related protein 7
MR Test
Inverse variance weighted (fixed effects)
Inverse variance weighted (multiplicative random effects)
MR Egger
MR RAPS
Weighted median
Weighted mode
0.20
0.15
0.10
0.05
0.00
−0.05
0.1
0.2
0.3
0.4
SNP effect on Selenium
SNP effect on Angiopoietin−related protein 7

Batch 3−101 : Selenium on Ankyrin−2

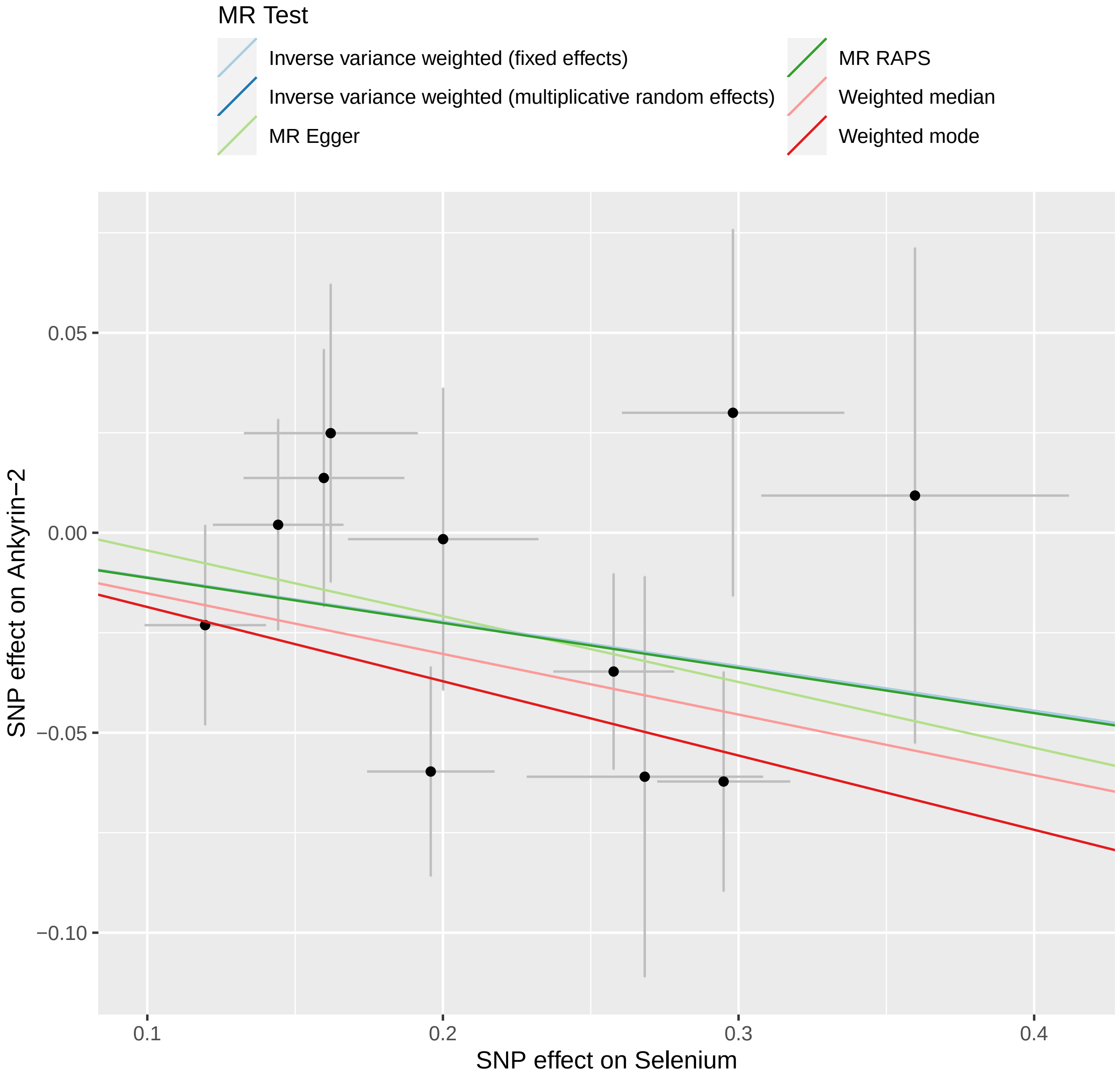

Batch 3−102 : Selenium on Ankyrin repeat domain−containing protein 27

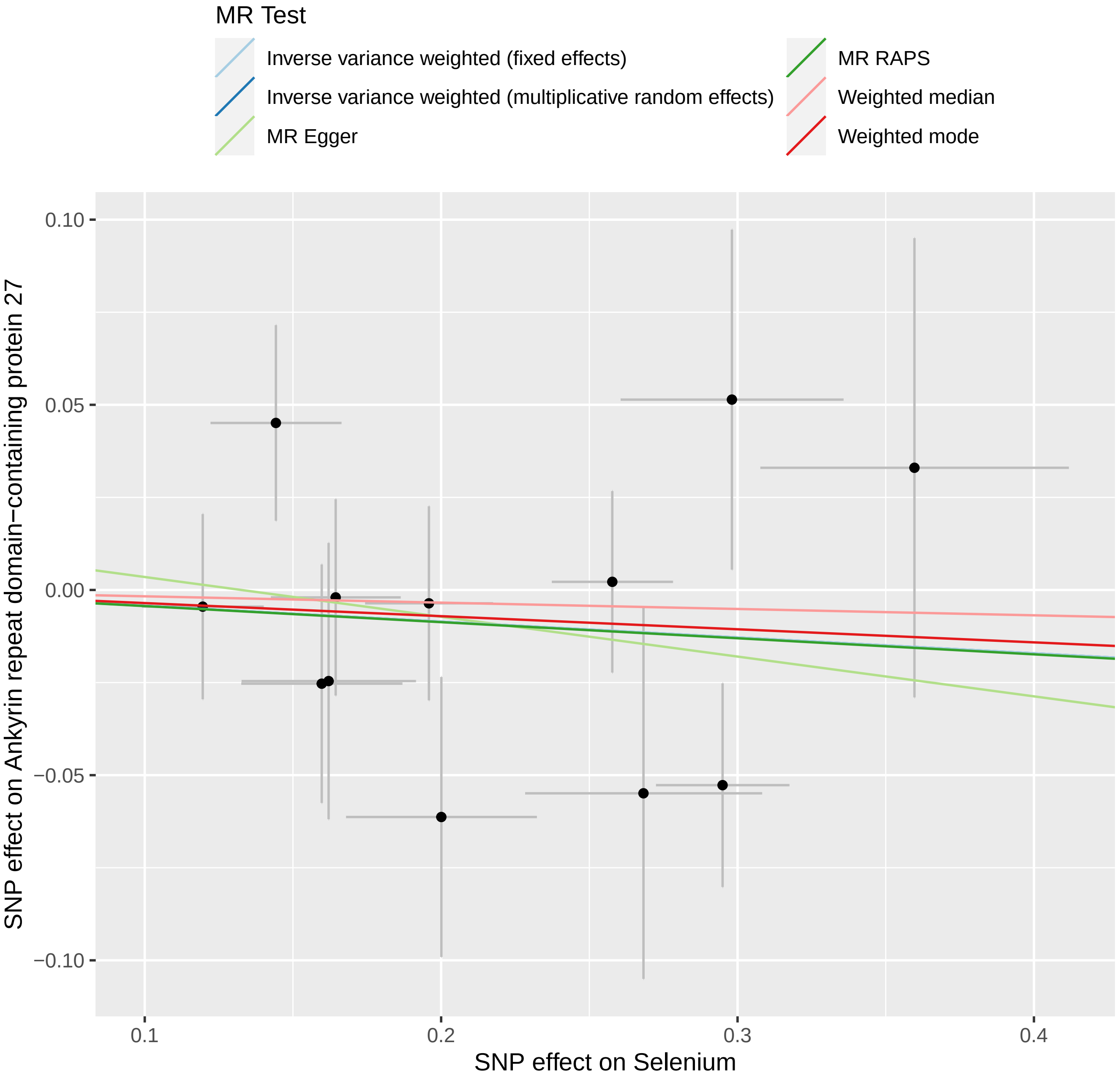

Batch 3−103 : Selenium on Ankyrin repeat domain−containing protein 46

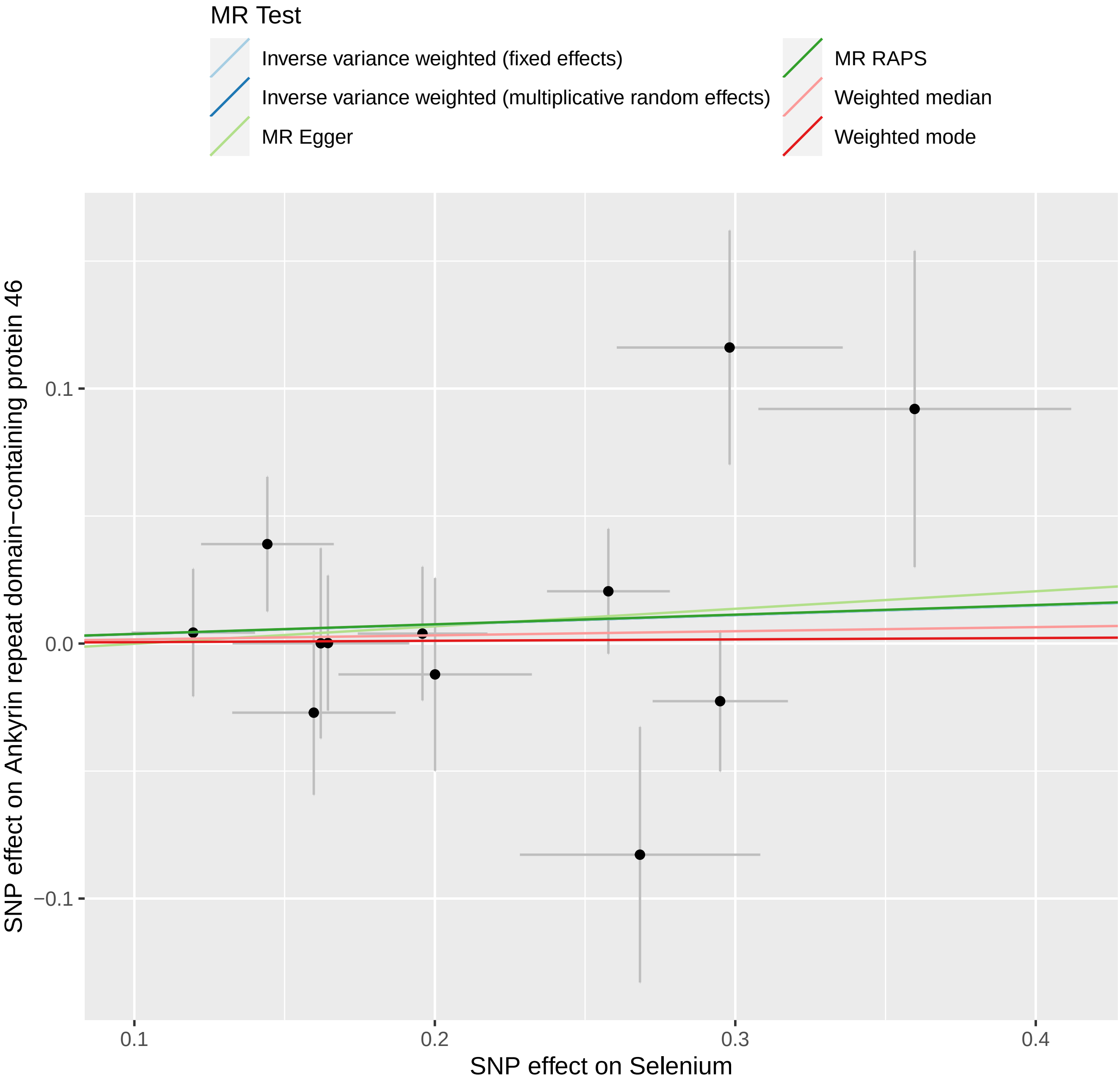

Batch 3−104 : Selenium on Acidic leucine−rich nuclear phosphoprotein 32 family member A

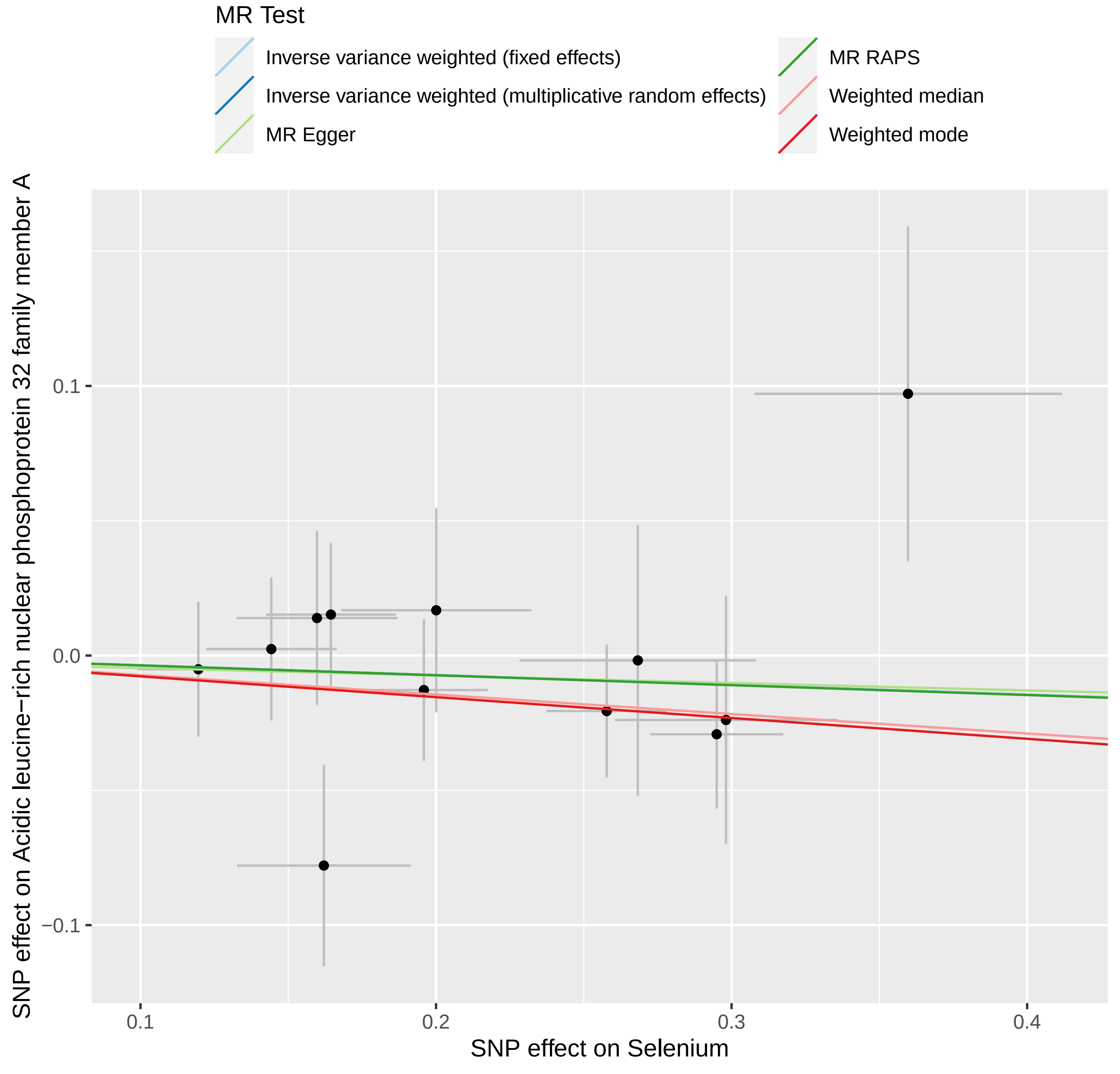

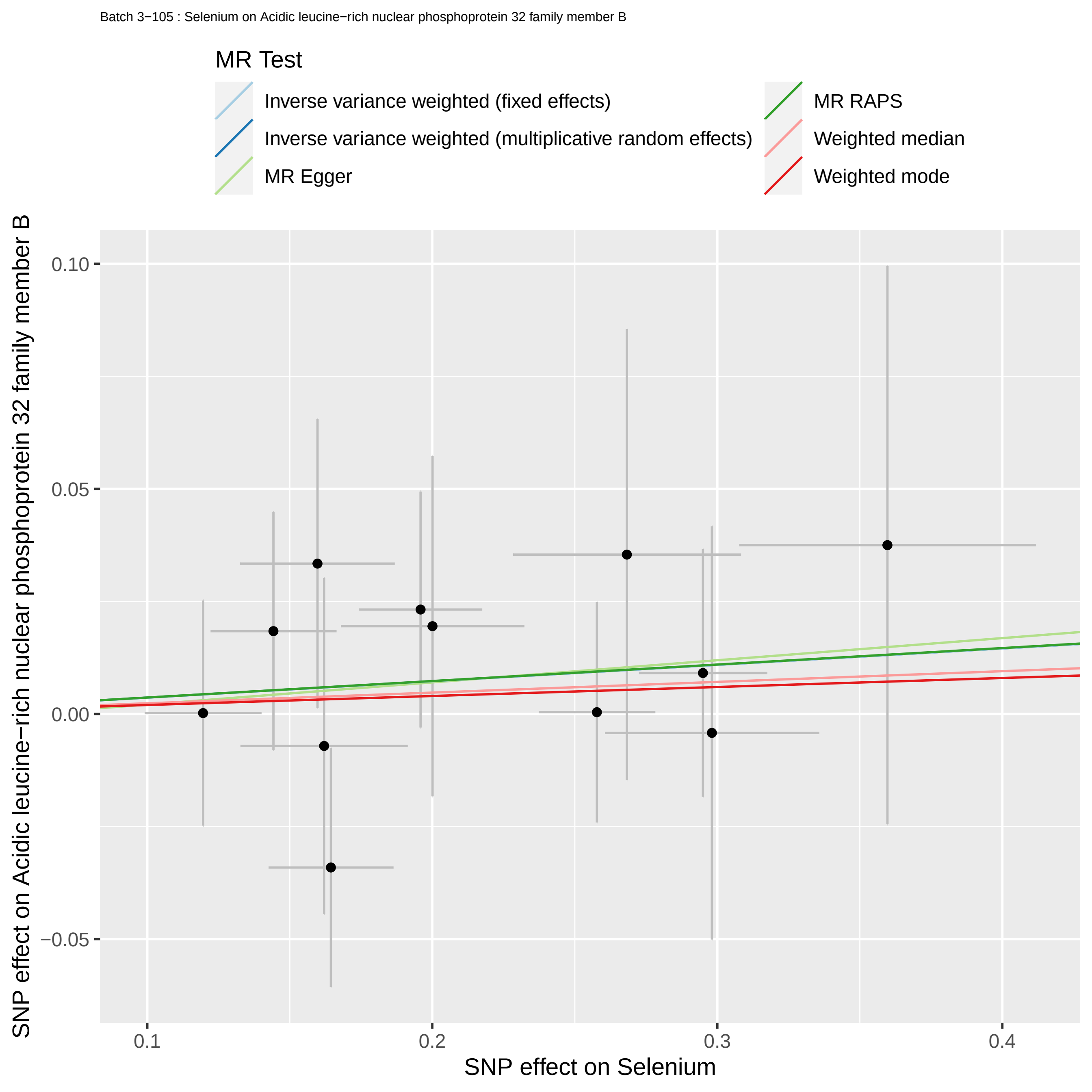
Batch 3−105 : Selenium on Acidic leucine−rich nuclear phosphoprotein 32 family member B
MR Test
Inverse variance weighted (fixed effects)
Inverse variance weighted (multiplicative random effects)
MR Egger
MR RAPS
Weighted median
Weighted mode
SNP effect on Acidic leucine−rich nuclear phosphoprotein 32 family member B
0.10
0.05
0.00
−0.05
0.1
0.2
0.3
0.4
SNP effect on Selenium

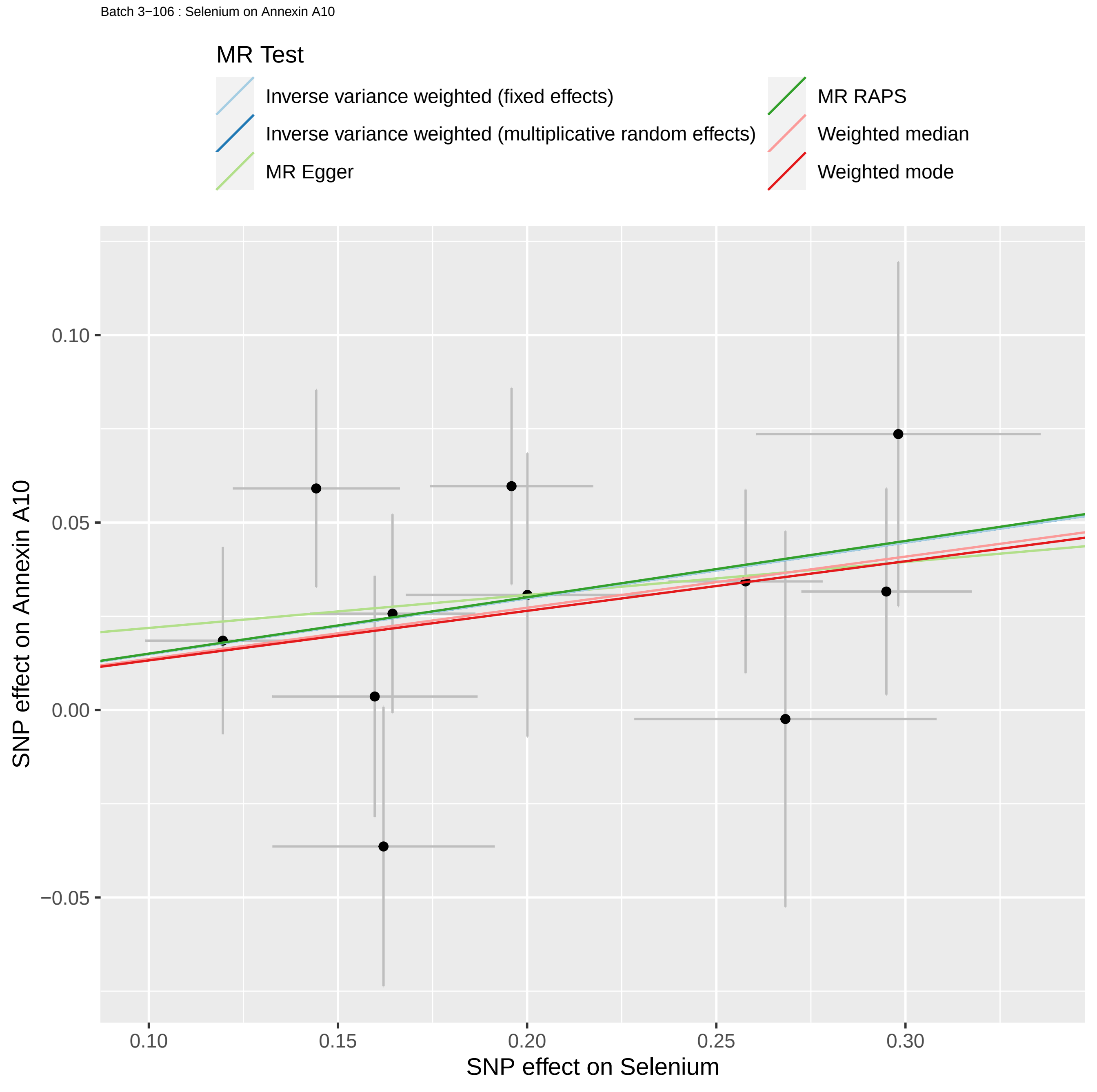

Batch 3−106 : Selenium on Annexin A10
MR Test
Inverse variance weighted (fixed effects)
Inverse variance weighted (multiplicative random effects)
MR Egger
MR RAPS
Weighted median
Weighted mode
0.10
0.05
0.00
−0.05
0.10
0.15
0.20
0.25
0.30
SNP effect on Annexin A10
SNP effect on Selenium

Batch 3−107 : Selenium on Annexin A1

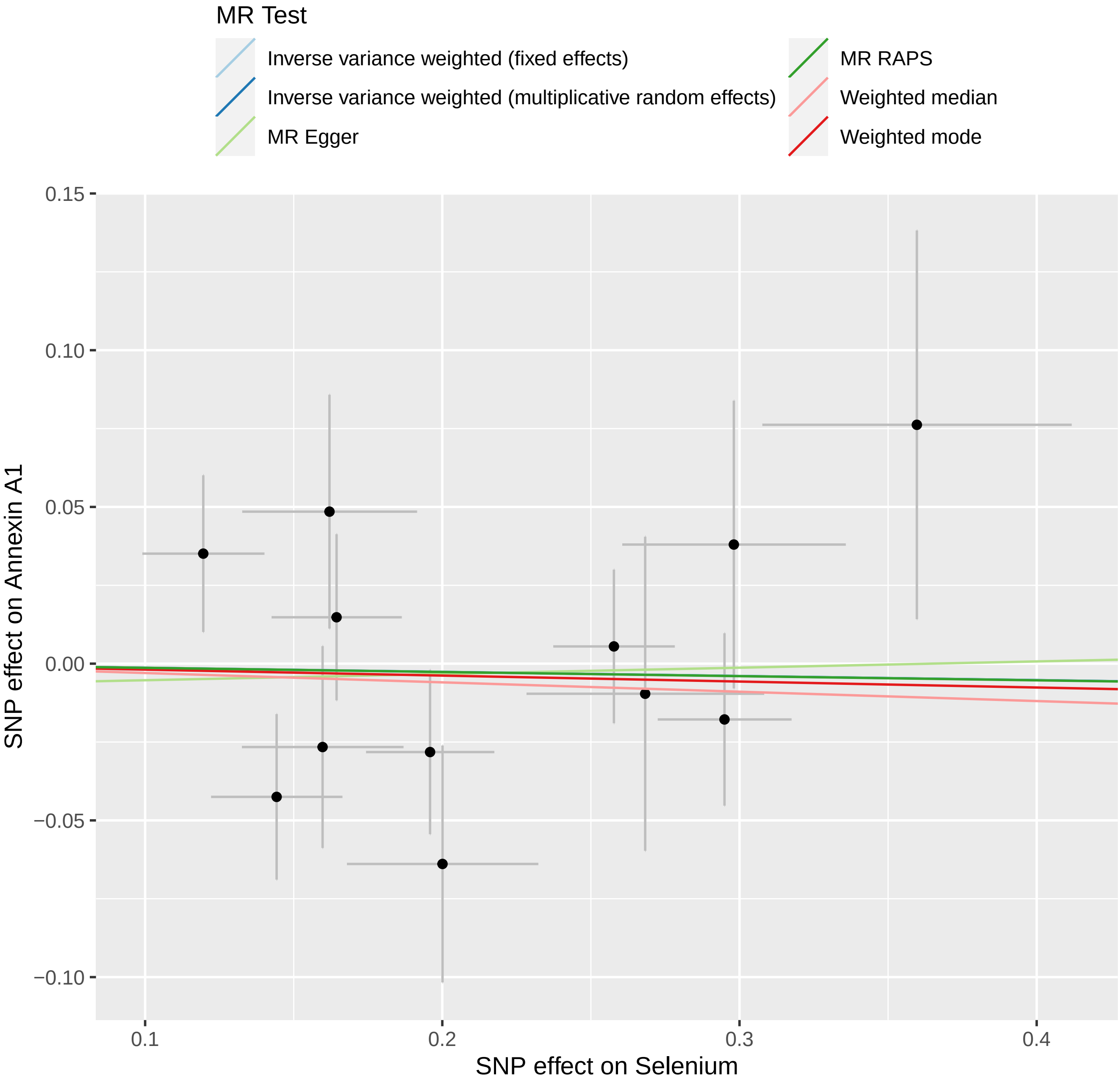

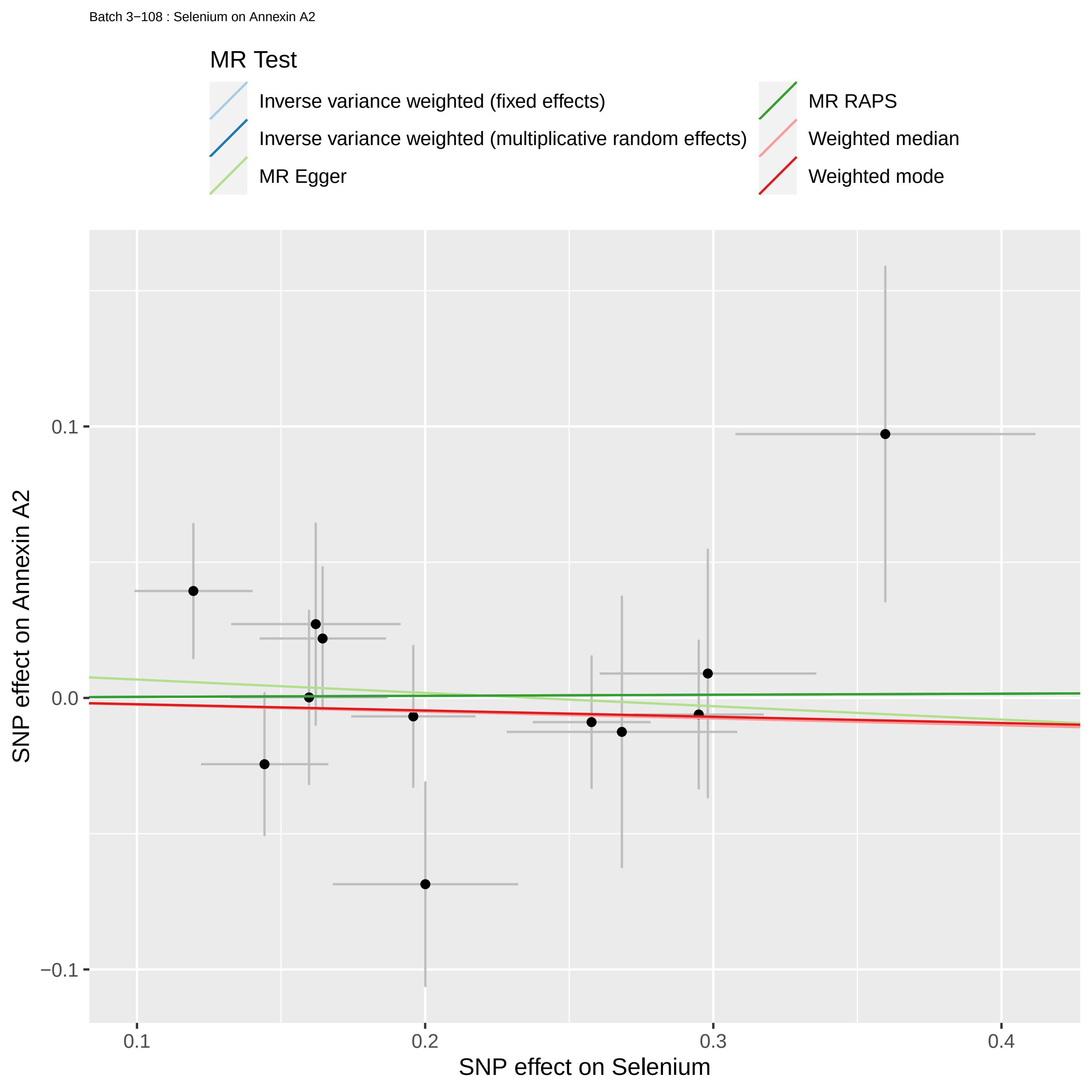

Batch 3−108 : Selenium on Annexin A2
MR Test
Inverse variance weighted (fixed effects)
Inverse variance weighted (multiplicative random effects)
MR Egger
MR RAPS
Weighted median
Weighted mode
SNP effect on Annexin A2
0.1
0.0
−0.1
0.1
0.2
0.3
0.4
SNP effect on Selenium

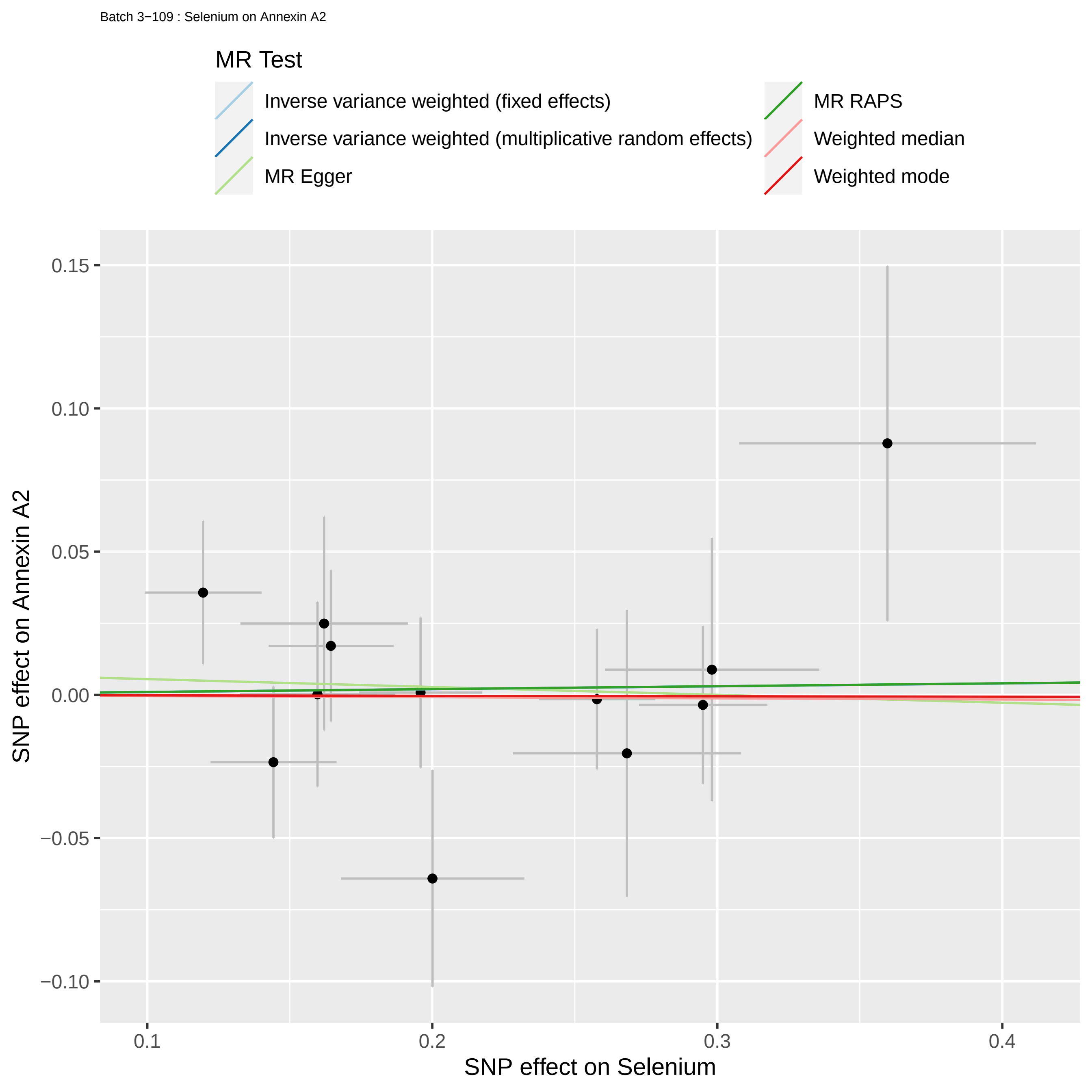

Batch 3−109 : Selenium on Annexin A2
MR Test
Inverse variance weighted (fixed effects)
Inverse variance weighted (multiplicative random effects)
MR Egger
MR RAPS
Weighted median
Weighted mode
0.15
0.10
0.05
0.00
−0.05
−0.10
0.1
0.2
0.3
0.4
SNP effect on Annexin A2
SNP effect on Selenium

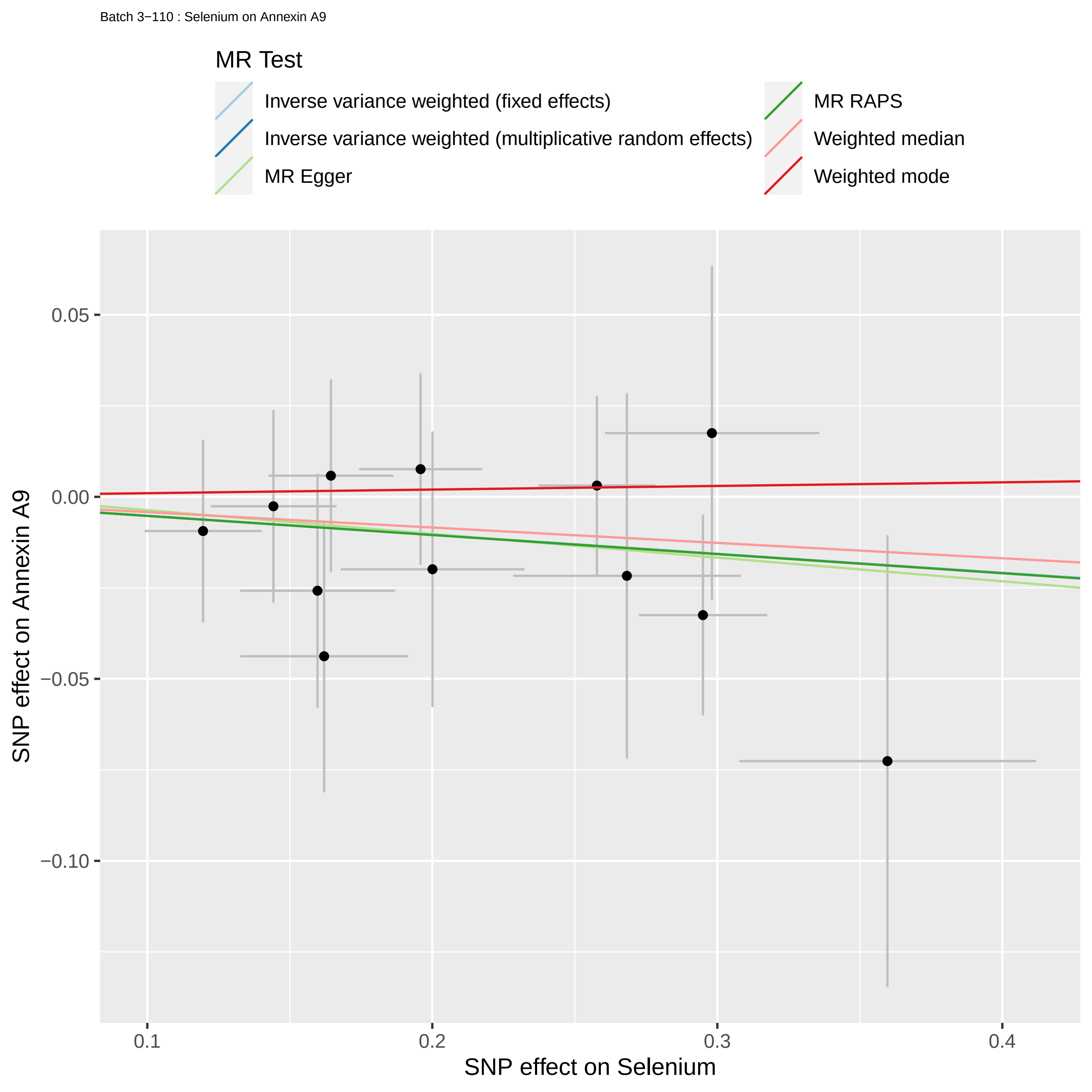
Batch 3−110 : Selenium on Annexin A9
MR Test
Inverse variance weighted (fixed effects)
Inverse variance weighted (multiplicative random effects)
MR Egger
MR RAPS
Weighted median
Weighted mode
0.05
0.00
−0.05
−0.10
0.1
0.2
0.3
0.4
SNP effect on Annexin A9
SNP effect on Selenium

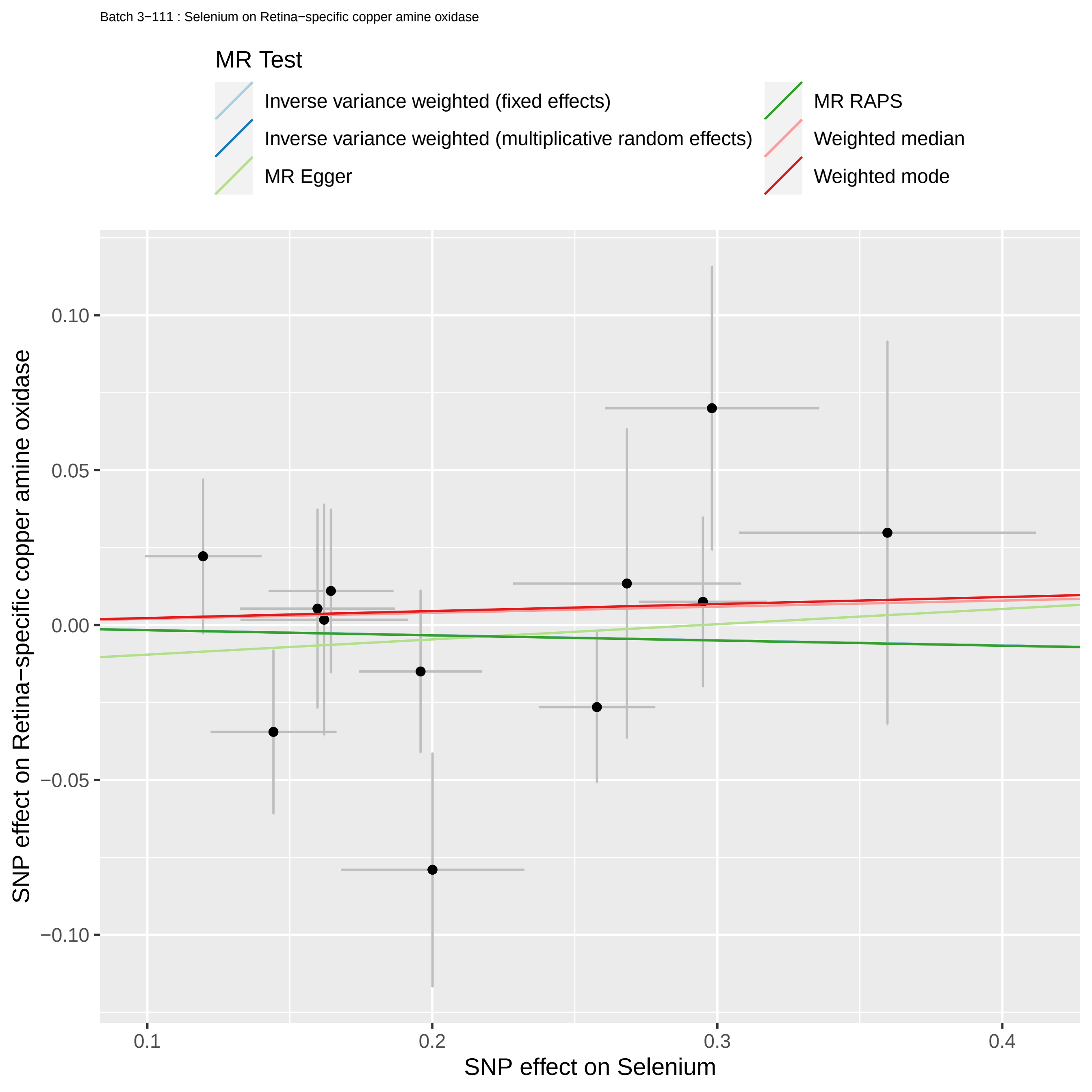
Batch 3−111 : Selenium on Retina−specific copper amine oxidase
MR Test
Inverse variance weighted (fixed effects)
Inverse variance weighted (multiplicative random effects)
MR Egger
MR RAPS
Weighted median
Weighted mode
0.10
0.05
0.00
−0.05
−0.10
0.1
0.2
0.3
0.4
SNP effect on Retina−specific copper amine oxidase
SNP effect on Selenium

Batch 3−112 : Selenium on AP−4 complex subunit mu−1

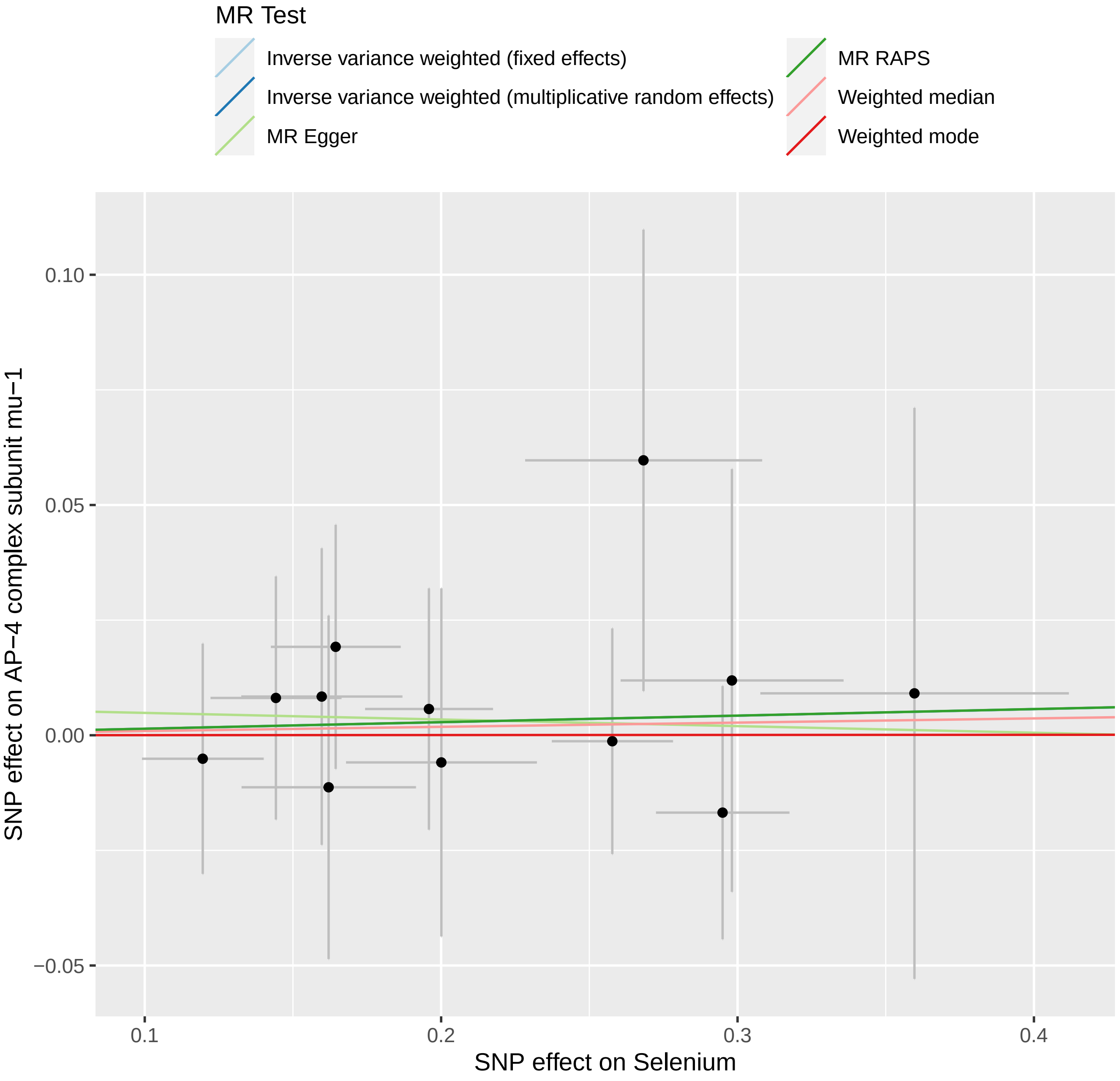

Batch 3−113 : Selenium on Apoptotic protease−activating factor 1

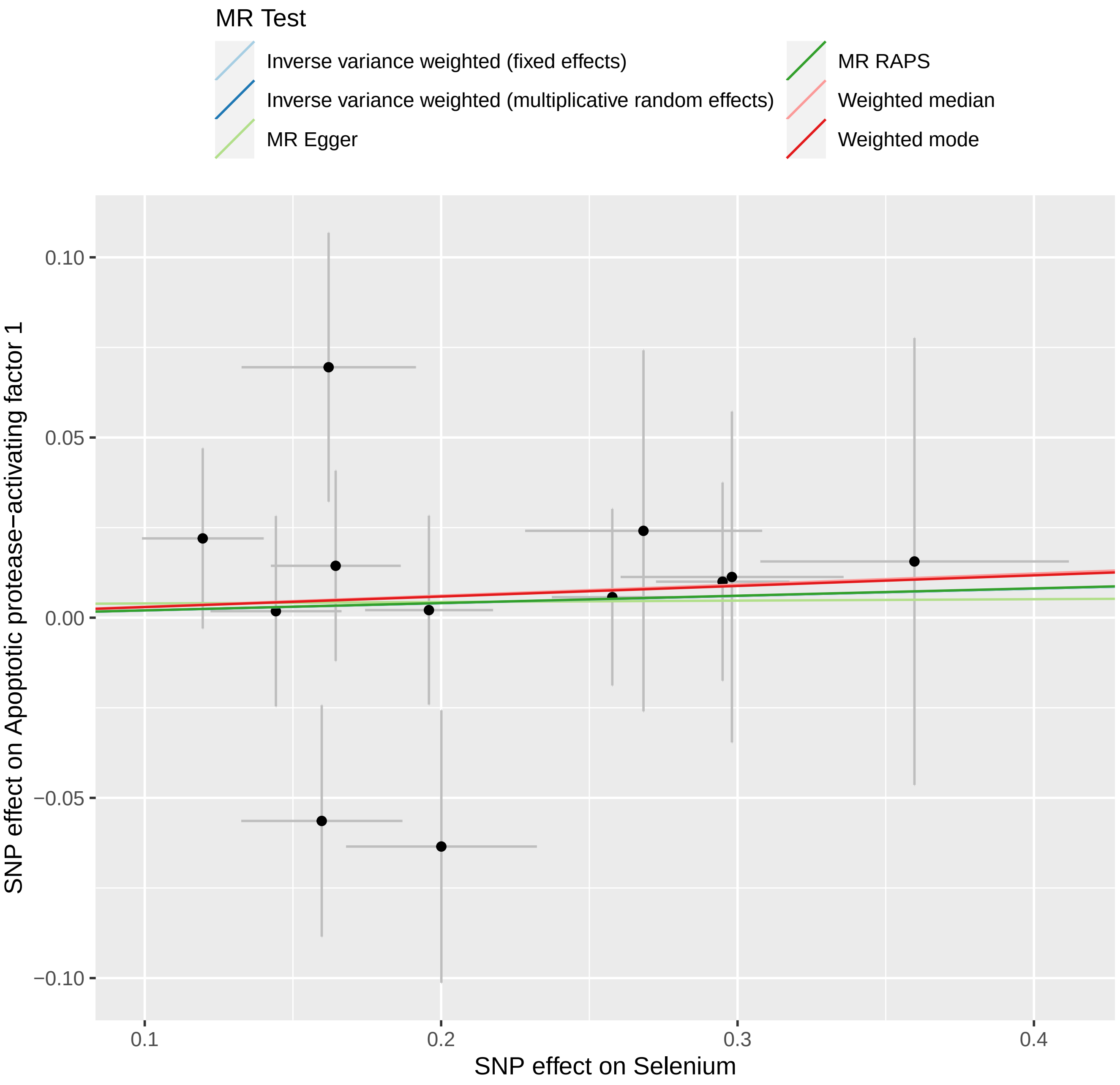

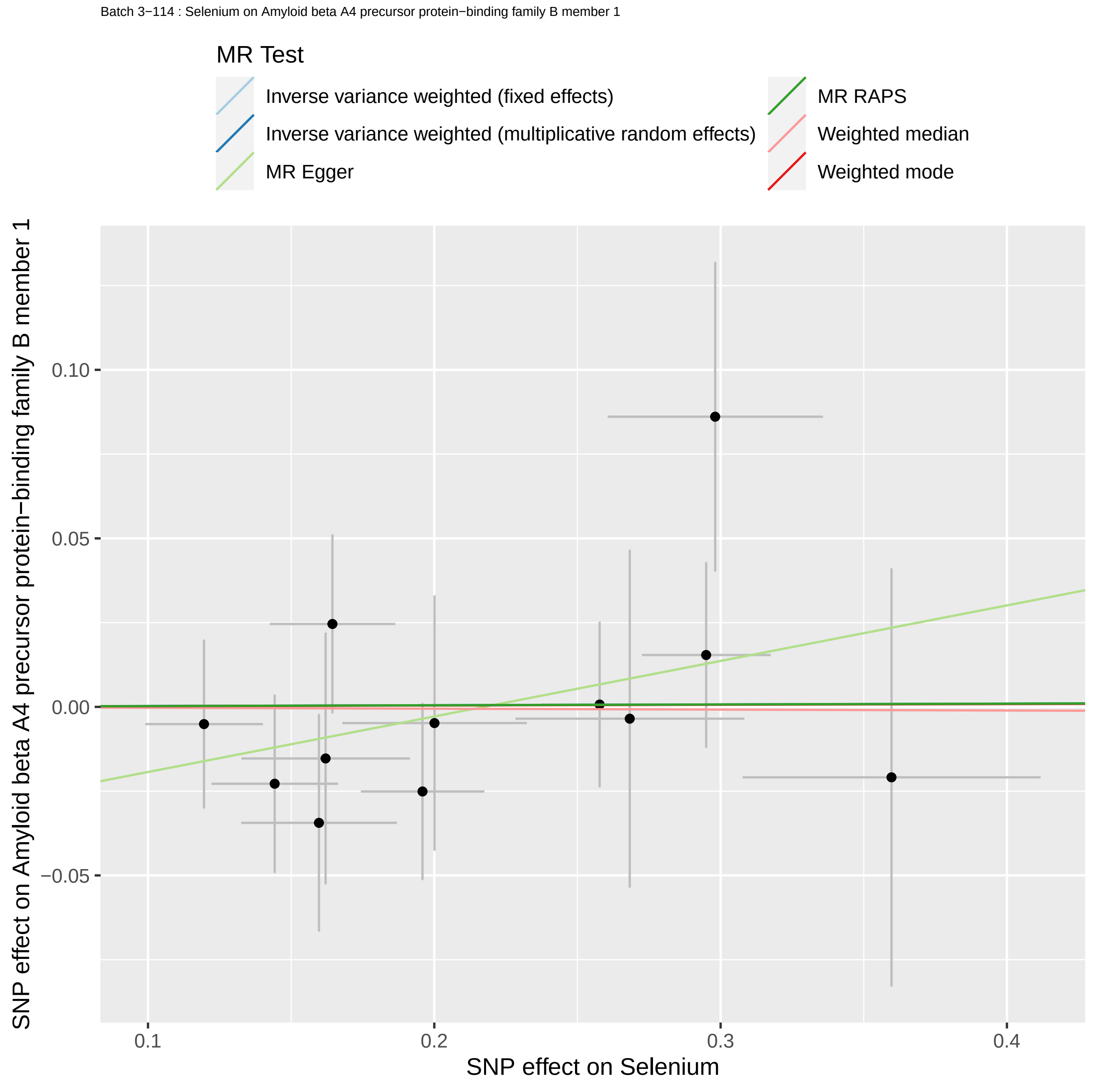

Batch 3−114 : Selenium on Amyloid beta A4 precursor protein−binding family B member 1
MR Test
Inverse variance weighted (fixed effects)
Inverse variance weighted (multiplicative random effects)
MR Egger
MR RAPS
Weighted median
Weighted mode
SNP effect on Amyloid beta A4 precursor protein−binding family B member 1
0.10
0.05
0.00
−0.05
0.1
0.2
0.3
0.4
SNP effect on Selenium

Batch 3−115 : Selenium on Amyloid beta A4 precursor protein−binding family B member 1

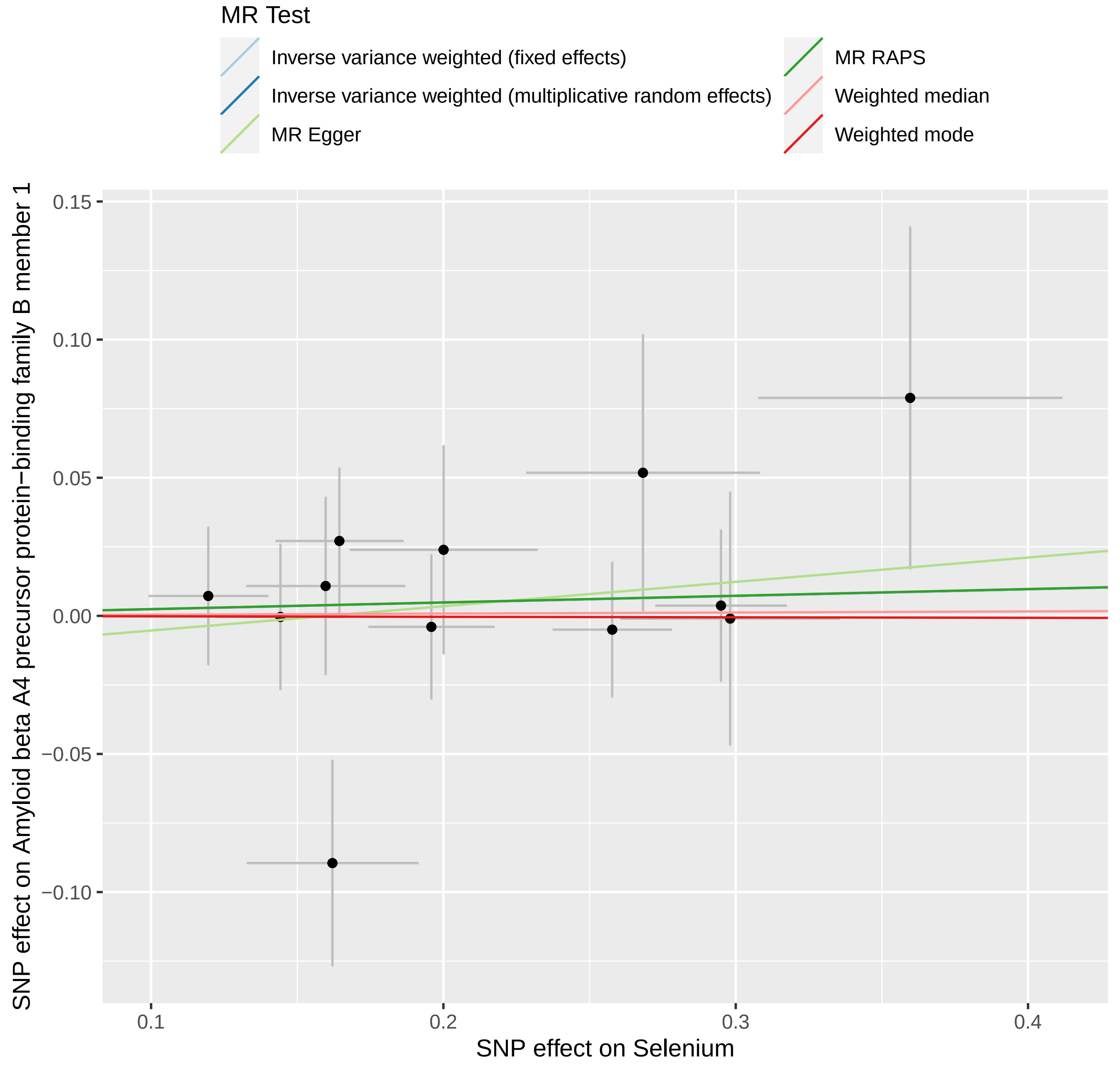

Batch 3−116 : Selenium on Amyloid beta A4 precursor protein−binding family B member 2

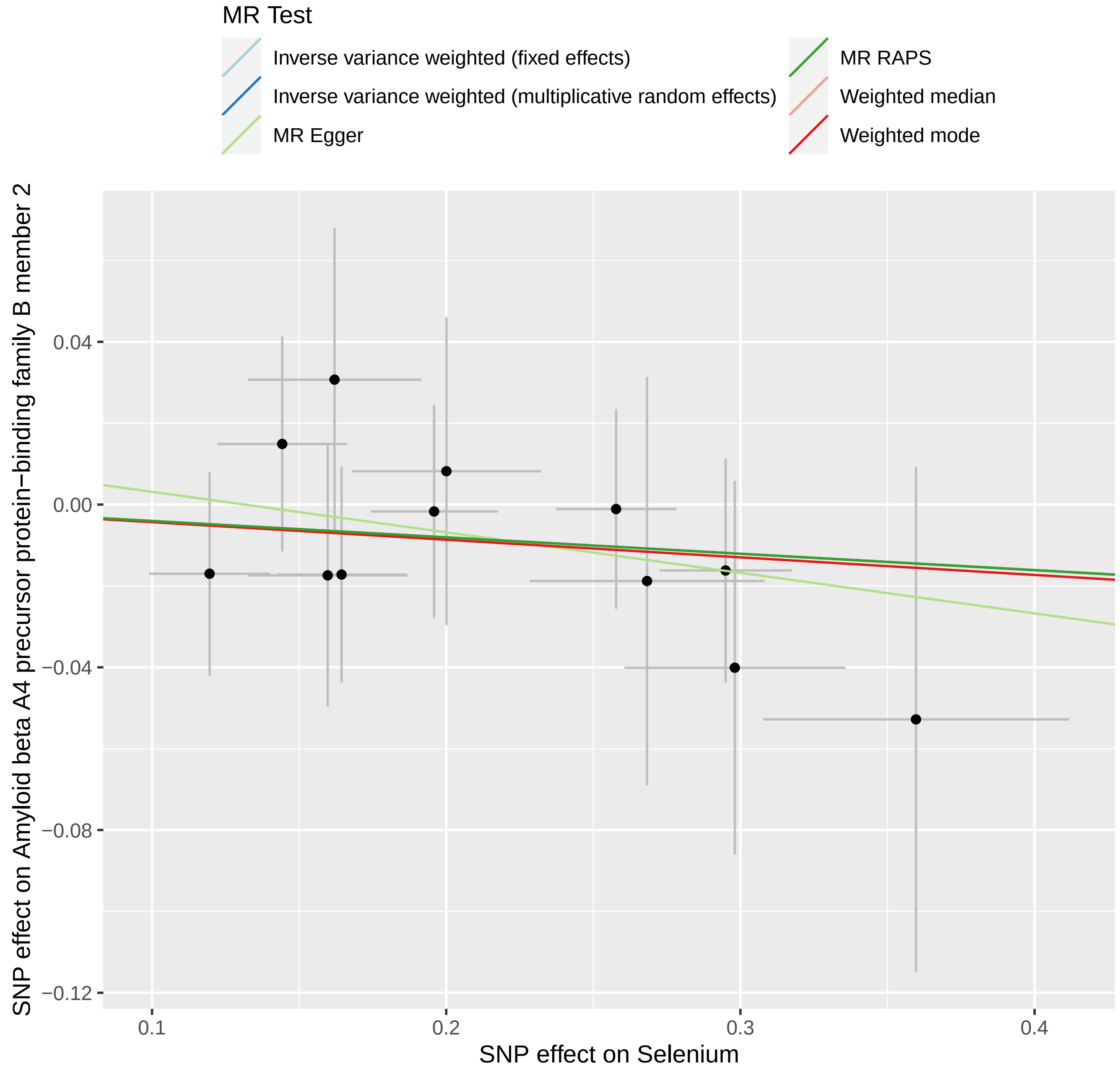

Batch 3−117 : Selenium on Amyloid beta A4 precursor protein−binding family B member 2

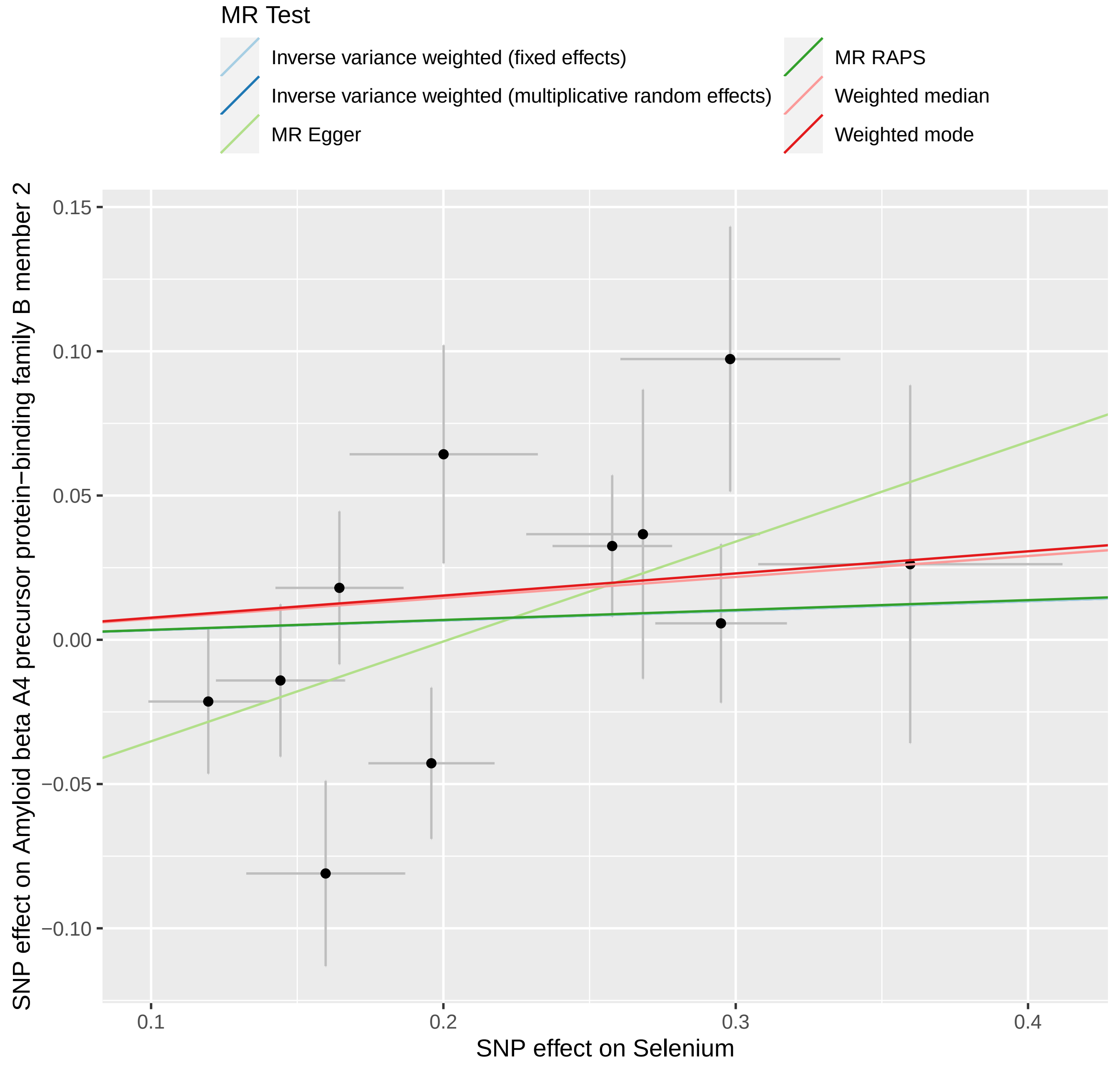

Batch 3−118 : Selenium on Amyloid beta A4 precursor protein−binding family B member 3

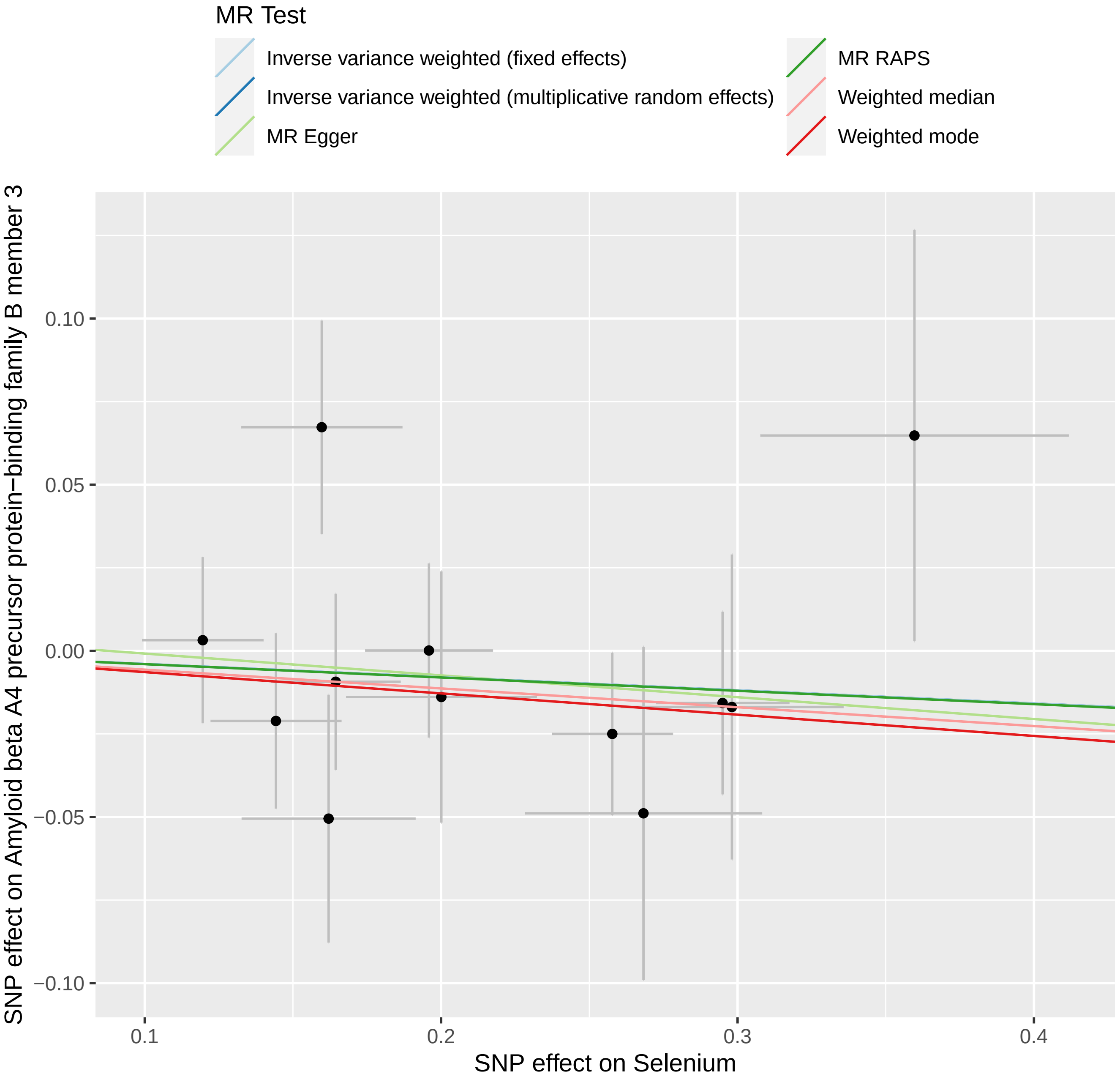

Batch 3−119 : Selenium on Protein APCDD1

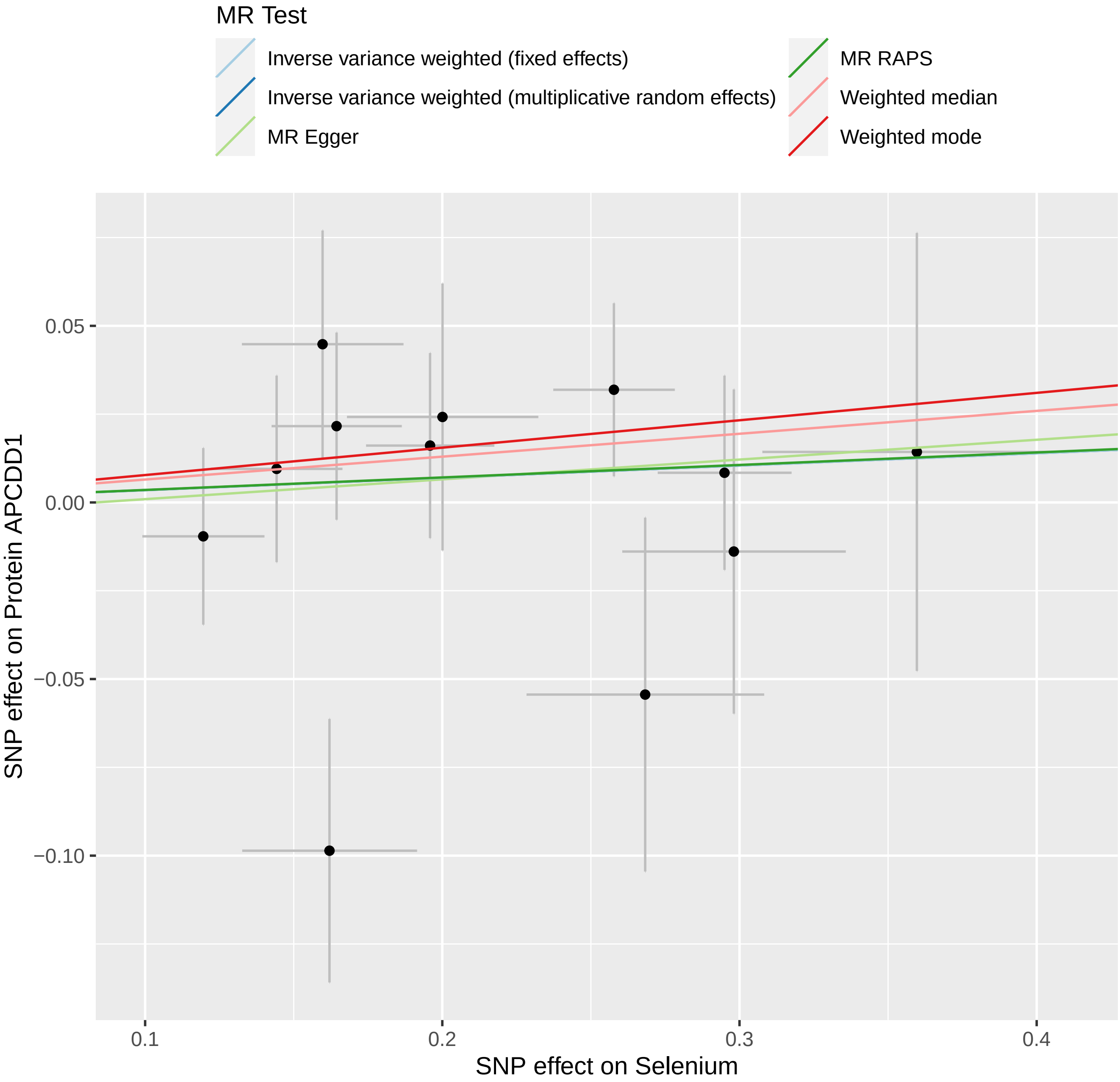

Batch 3−120 : Selenium on Serum amyloid P−component

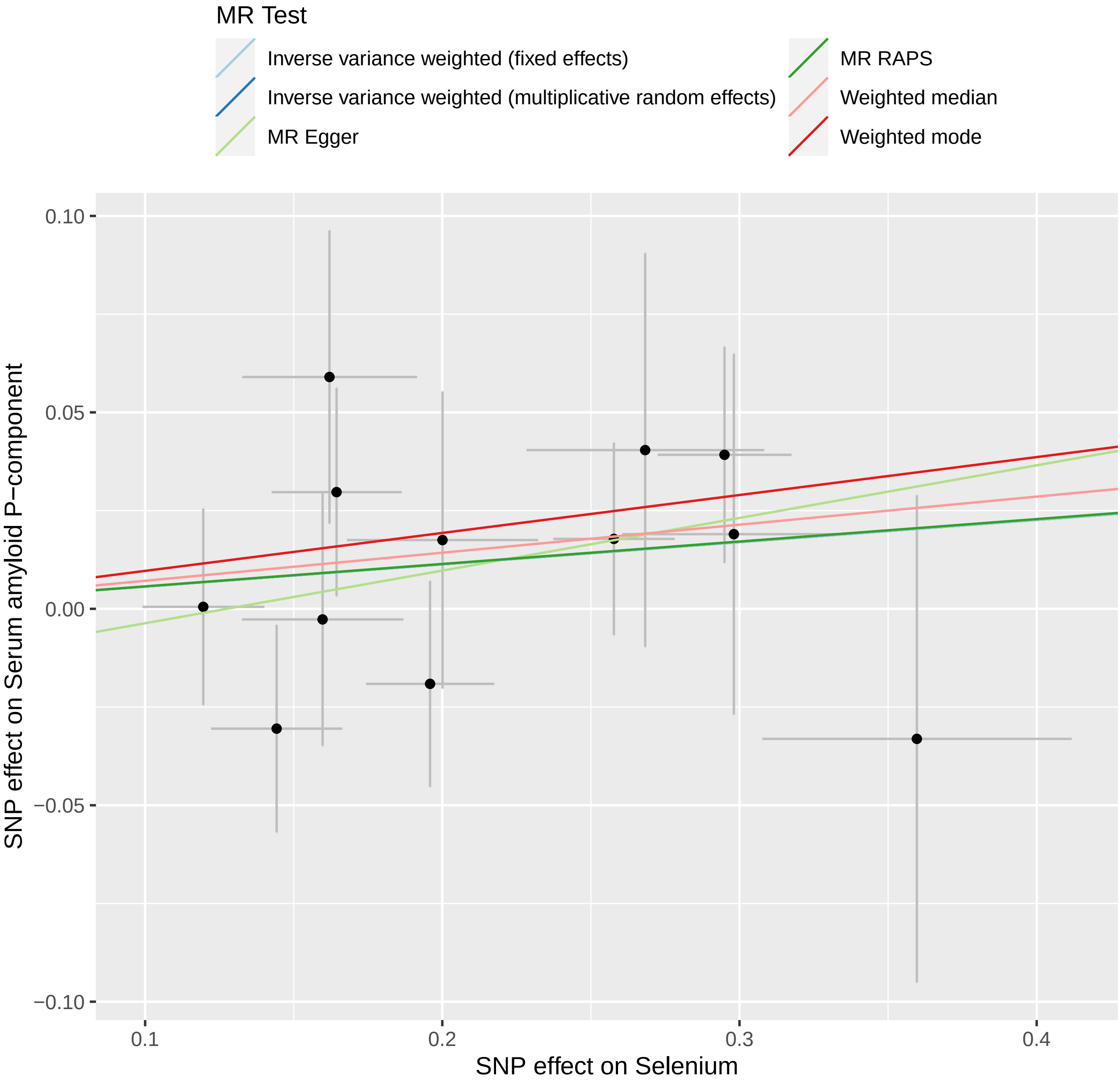

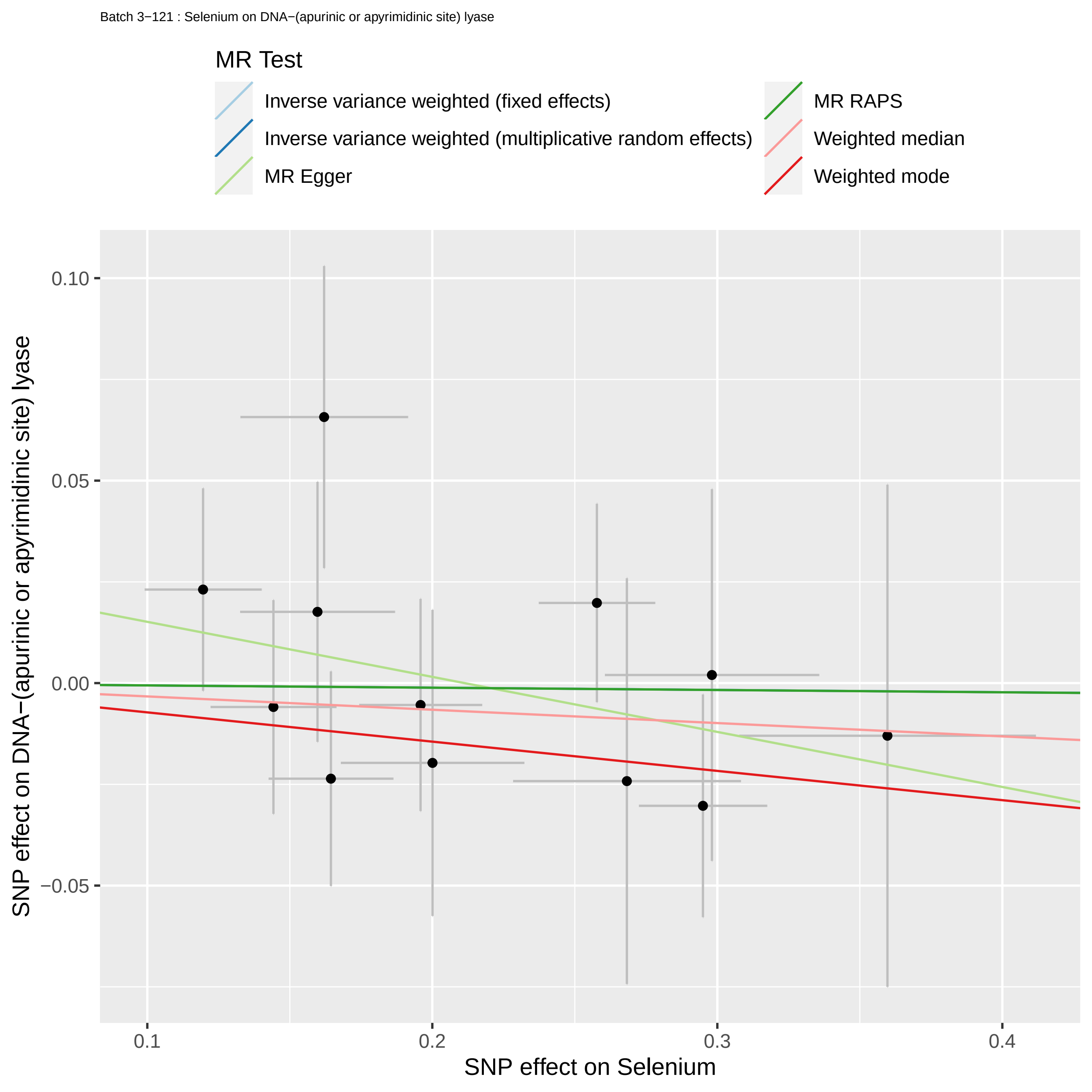
Batch 3−121 : Selenium on DNA−(apurinic or apyrimidinic site) lyase
MR Test
Inverse variance weighted (fixed effects)
Inverse variance weighted (multiplicative random effects)
MR Egger
MR RAPS
Weighted median
Weighted mode
0.10
0.05
0.00
−0.05
0.1
0.2
0.3
0.4
SNP effect on DNA−(apurinic or apyrimidinic site) lyase
SNP effect on Selenium

Batch 3−122 : Selenium on Amyloid−like protein 2

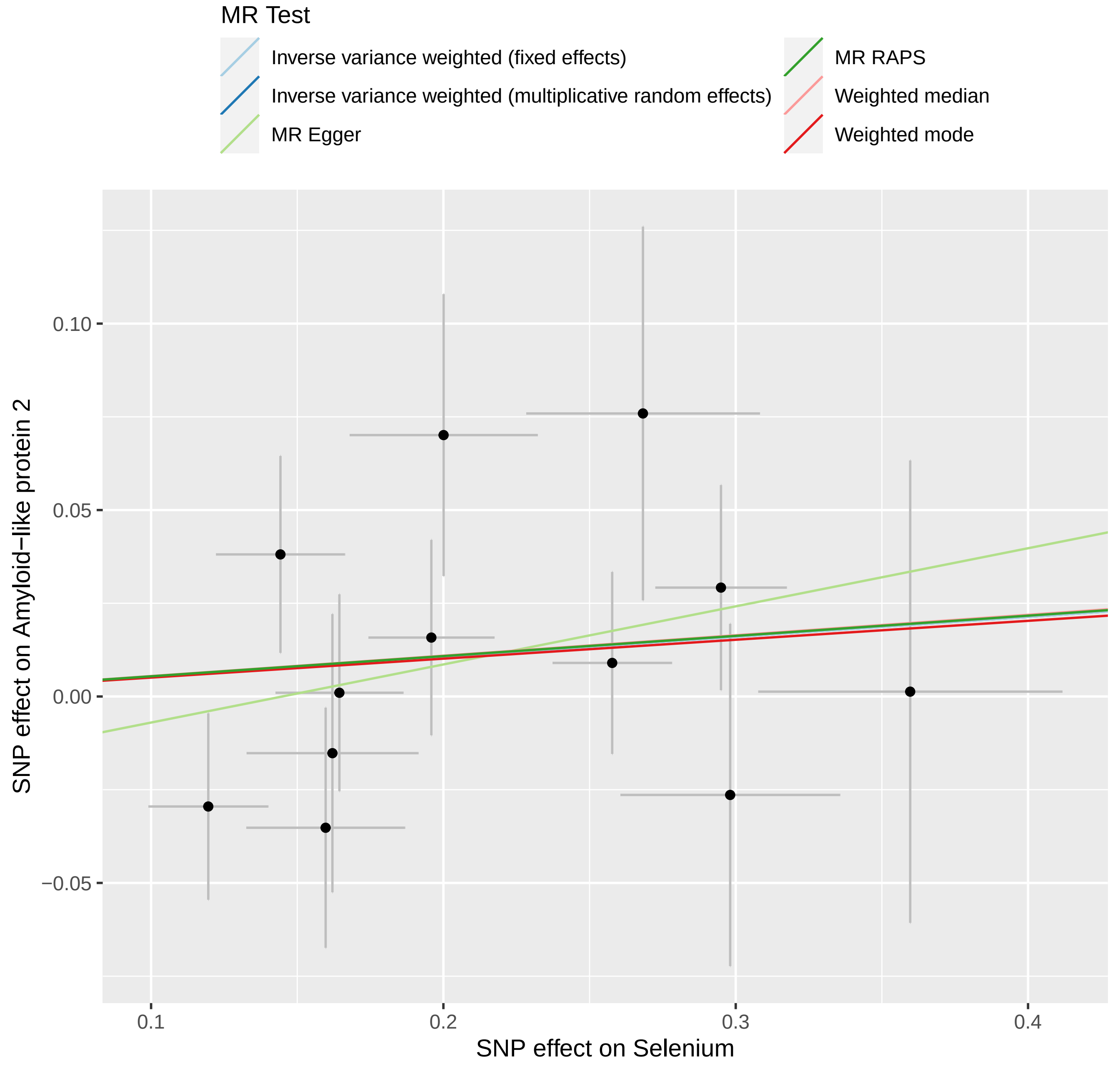

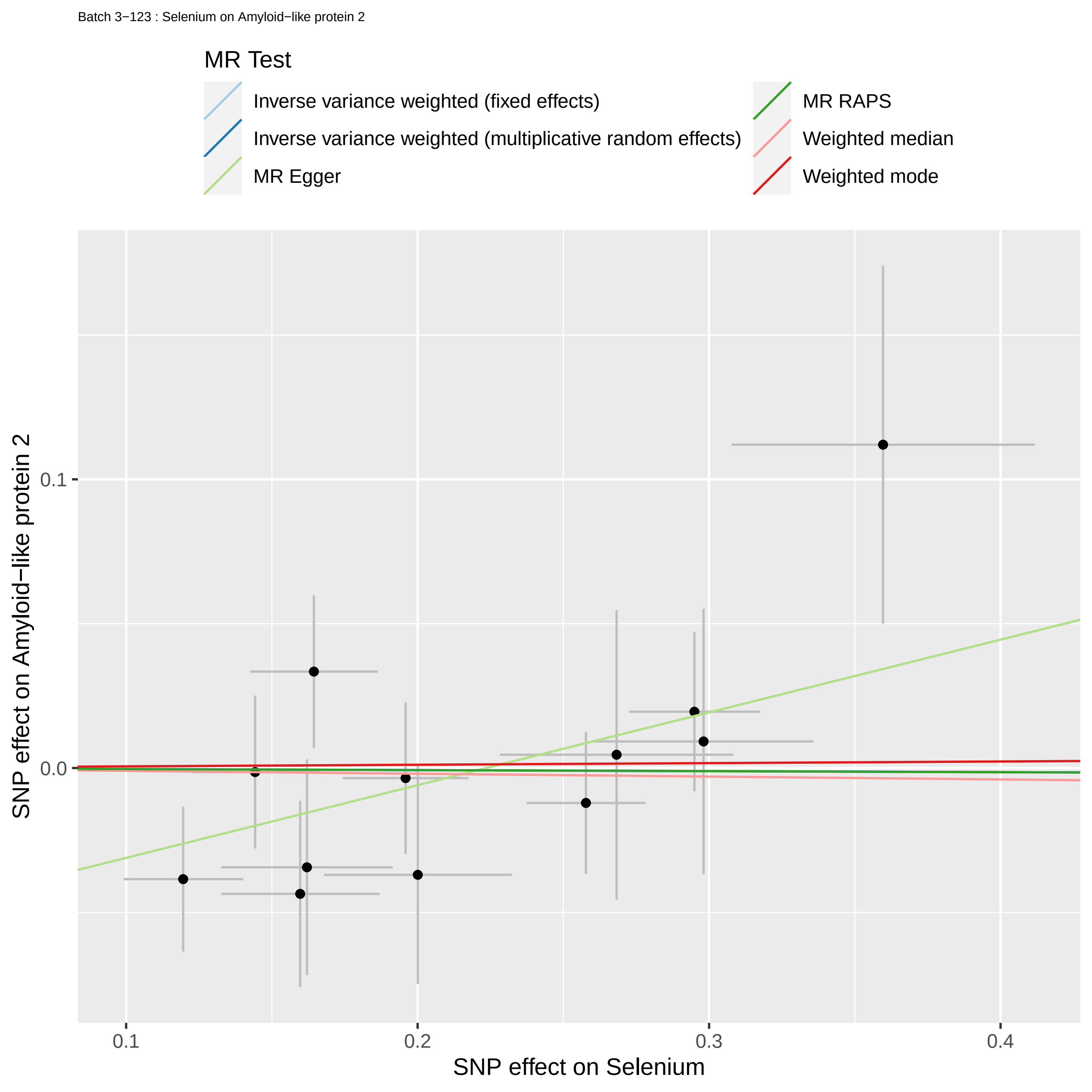

Batch 3−123 : Selenium on Amyloid−like protein 2
MR Test
Inverse variance weighted (fixed effects)
Inverse variance weighted (multiplicative random effects)
MR Egger
MR RAPS
Weighted median
Weighted mode
0.1
0.0
0.1
0.2
0.3
0.4
SNP effect on Selenium
SNP effect on Amyloid−like protein 2

Batch 3−124 : Selenium on Adipocyte plasma membrane−associated protein

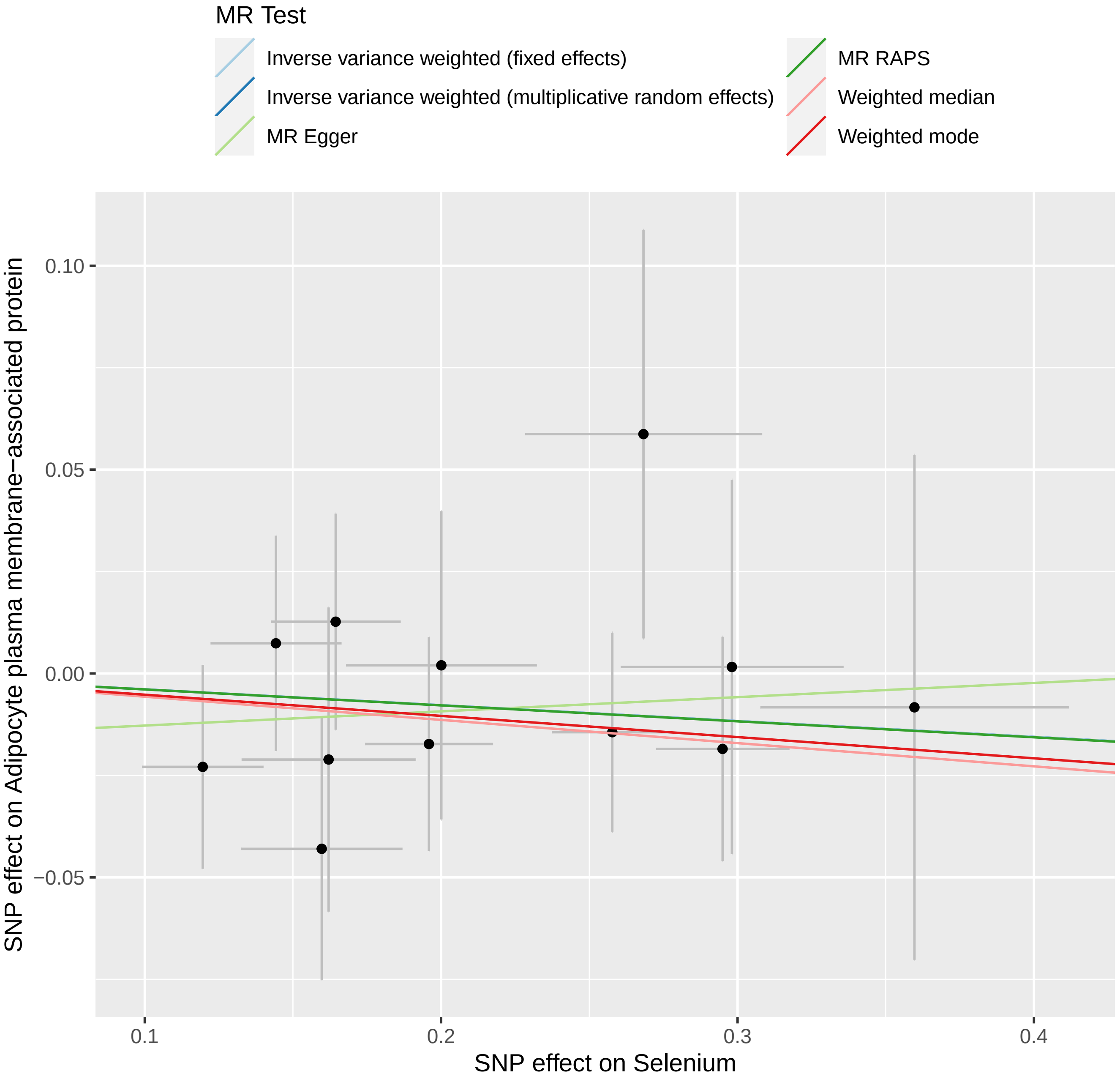

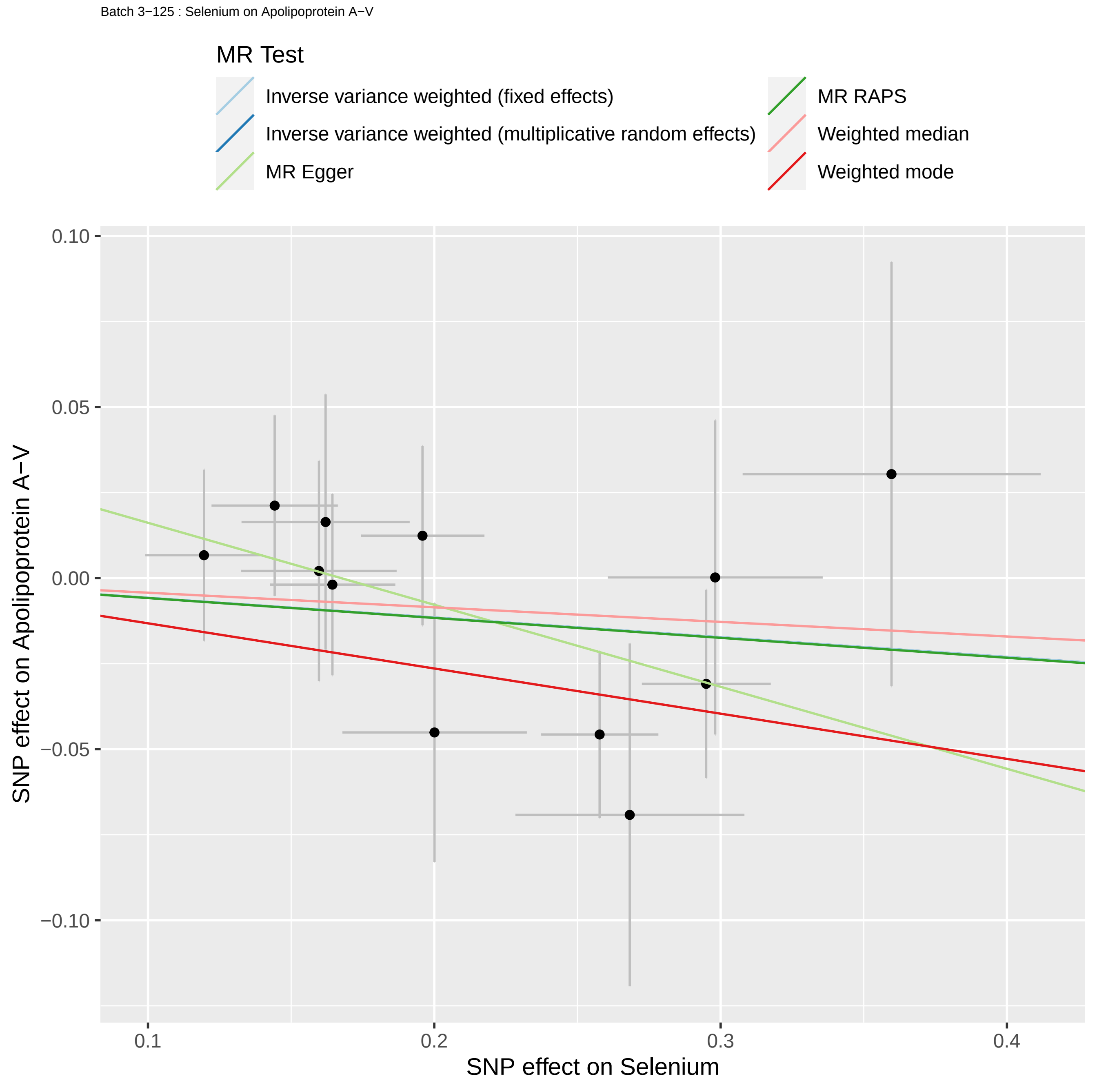
Batch 3−125 : Selenium on Apolipoprotein A−V
MR Test
Inverse variance weighted (fixed effects)
Inverse variance weighted (multiplicative random effects)
MR Egger
MR RAPS
Weighted median
Weighted mode
0.10
0.05
0.00
−0.05
−0.10
0.1
0.2
0.3
0.4
SNP effect on Apolipoprotein A−V
SNP effect on Selenium

Batch 3−126 : Selenium on Apolipoprotein A−V

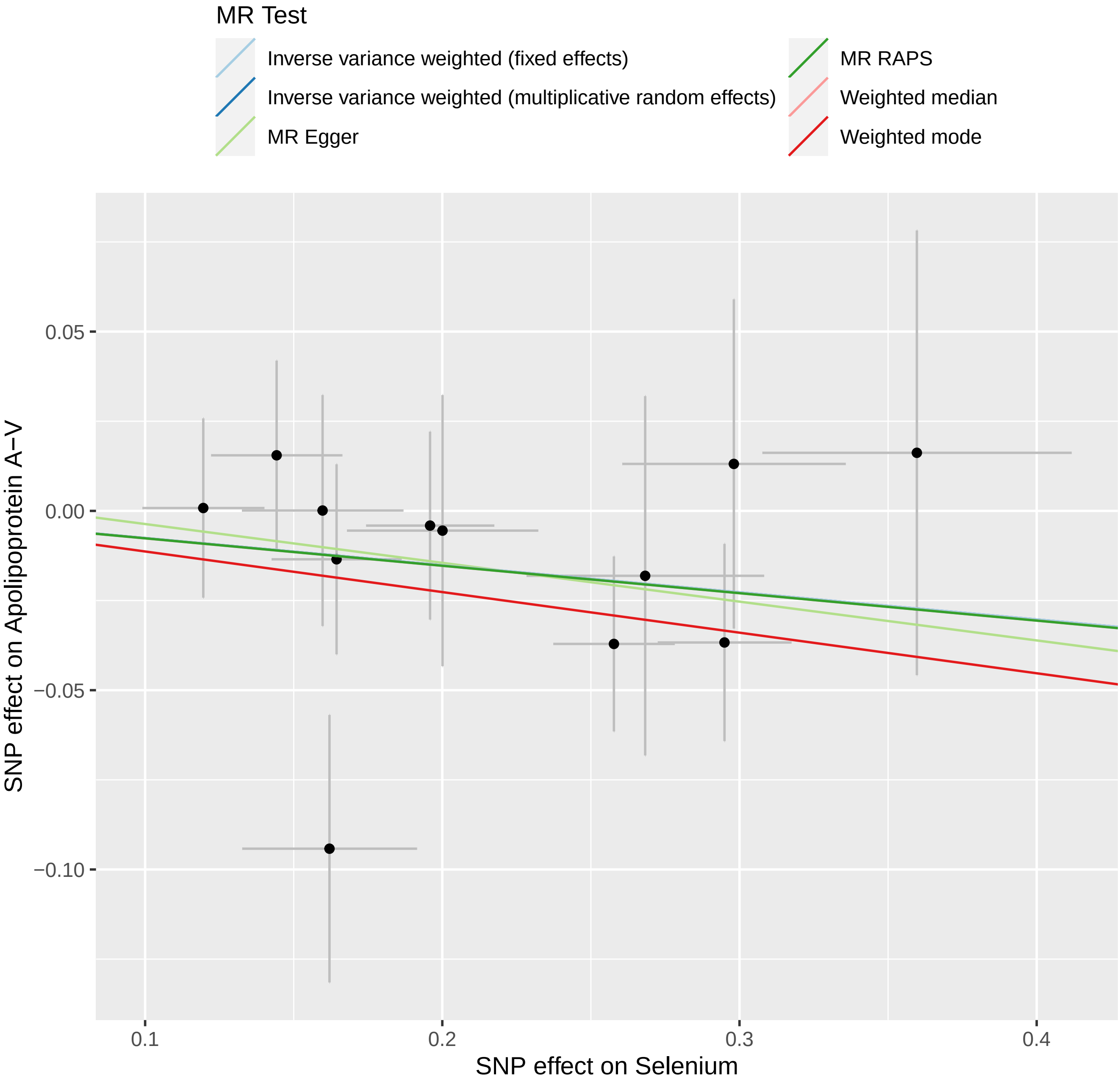

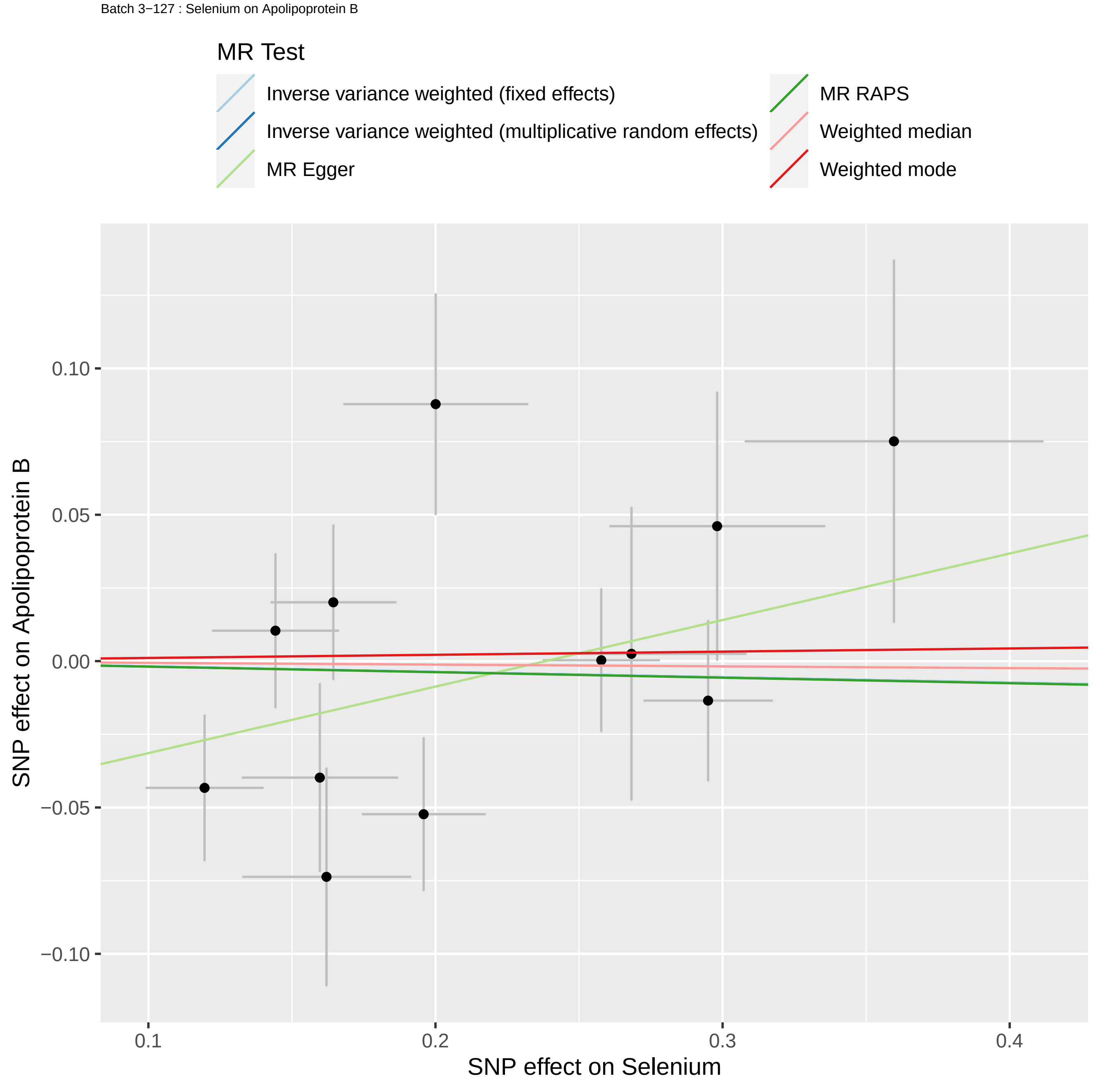
Batch 3−127 : Selenium on Apolipoprotein B
MR Test
Inverse variance weighted (fixed effects)
Inverse variance weighted (multiplicative random effects)
MR Egger
MR RAPS
Weighted median
Weighted mode
SNP effect on Apolipoprotein B
0.10
0.05
0.00
−0.05
−0.10
0.1
0.2
0.3
0.4
SNP effect on Selenium

Batch 3−128 : Selenium on DNA dC−>dU−editing enzyme APOBEC−3G

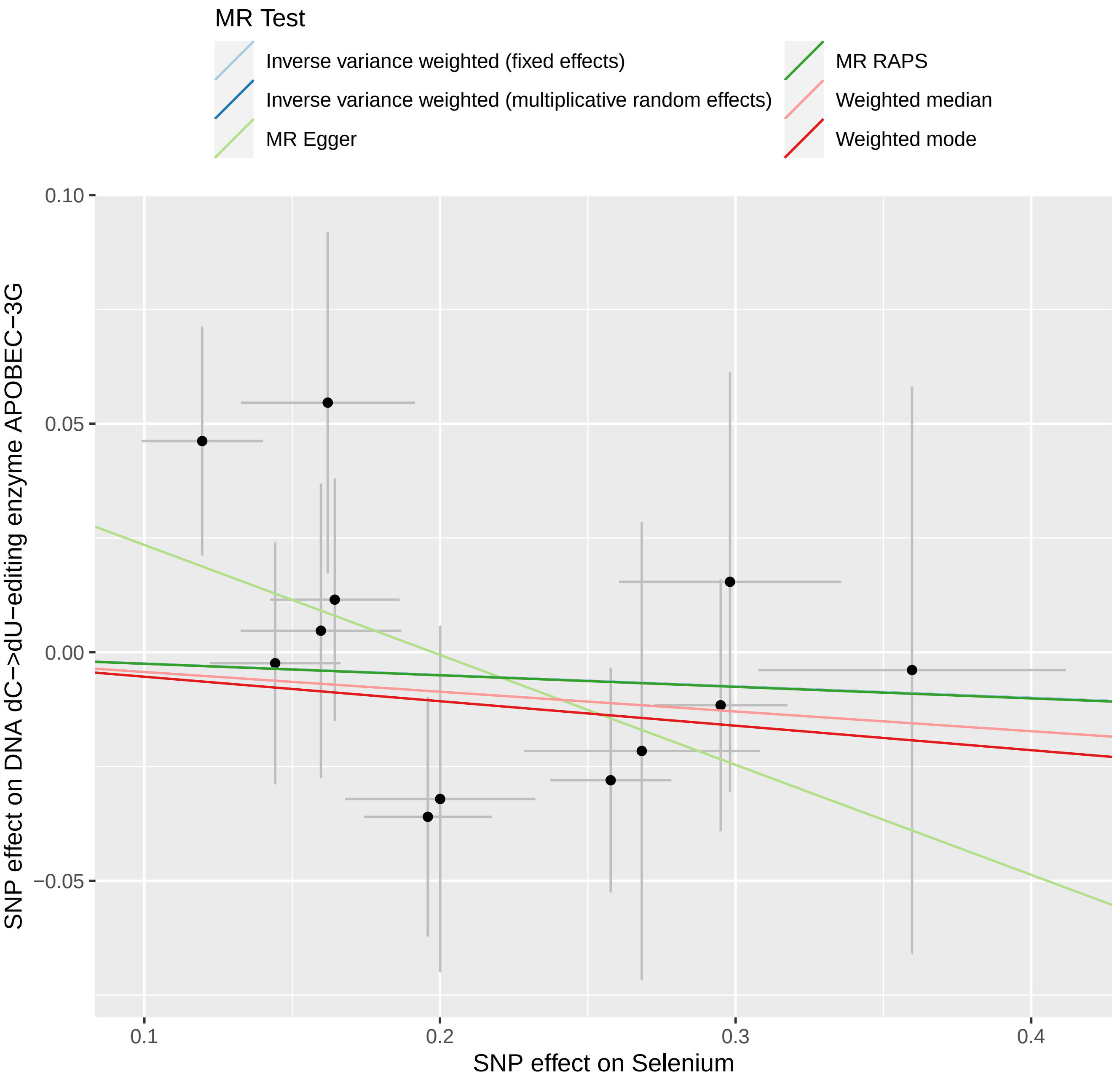

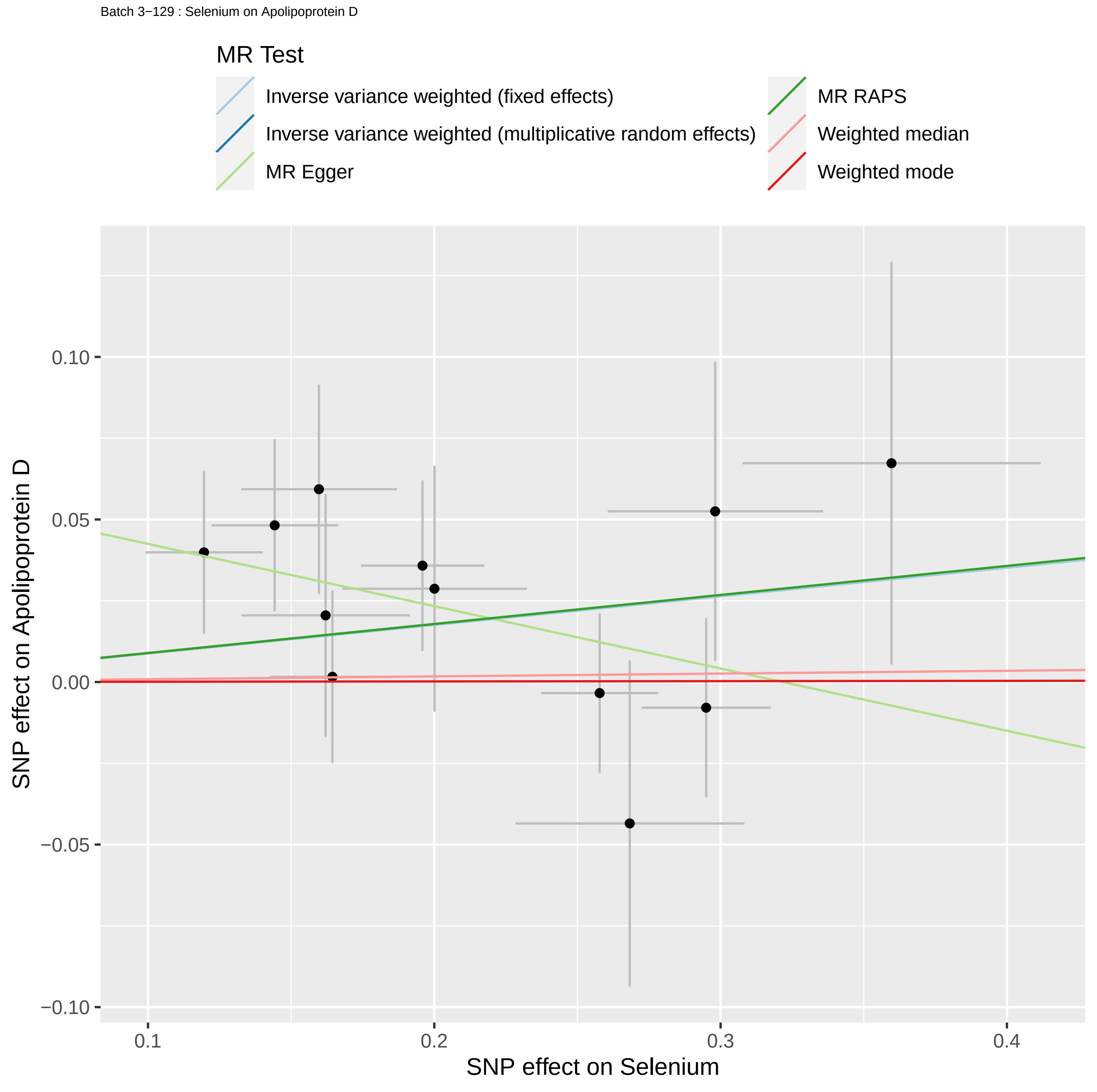

Batch 3−129 : Selenium on Apolipoprotein D
MR Test
Inverse variance weighted (fixed effects)
Inverse variance weighted (multiplicative random effects)
MR Egger
MR RAPS
Weighted median
Weighted mode
0.10
0.05
0.00
−0.05
−0.10
0.1
0.2
0.3
0.4
SNP effect on Apolipoprotein D
SNP effect on Selenium

Batch 3−130 : Selenium on Apolipoprotein D

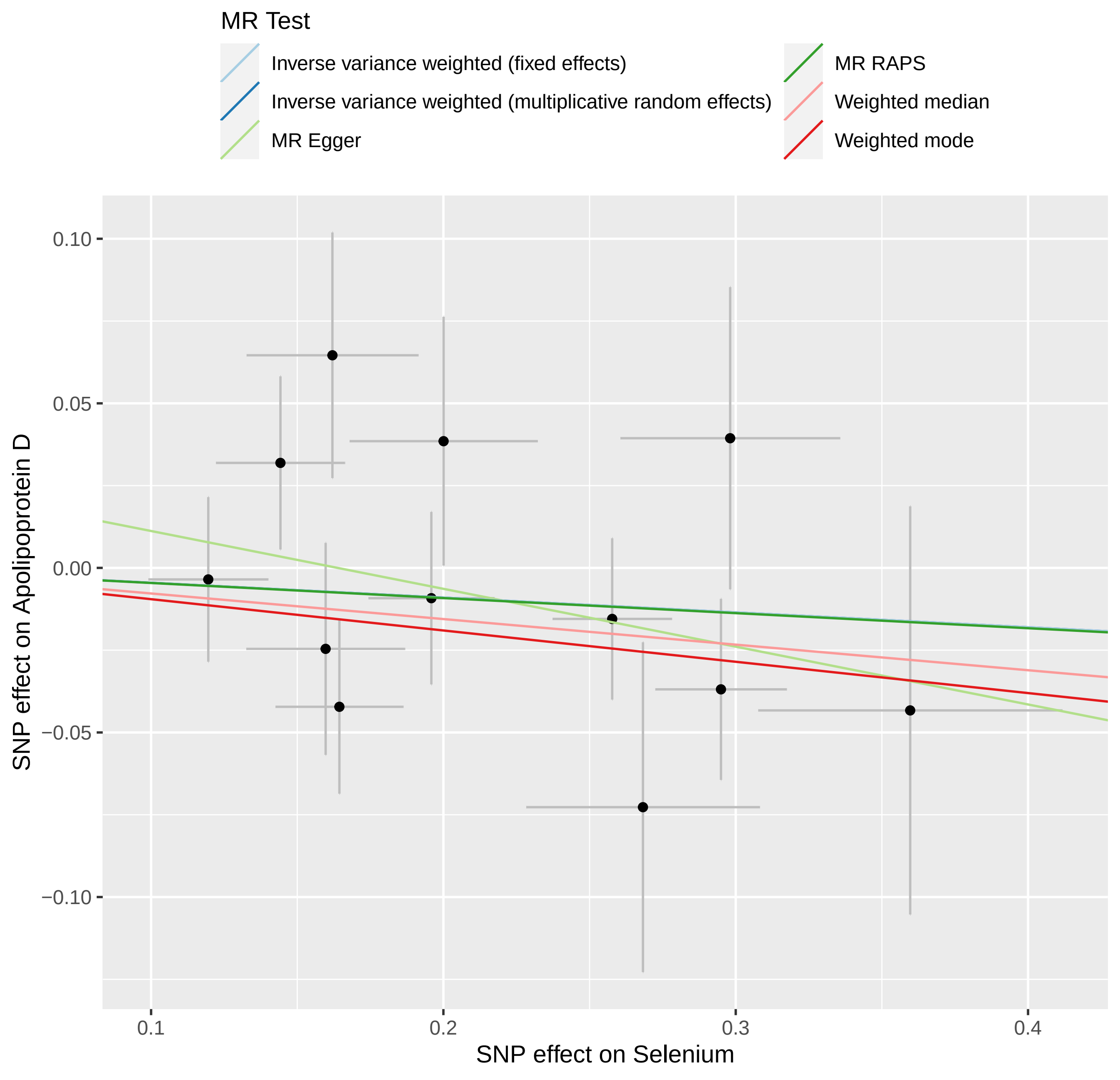

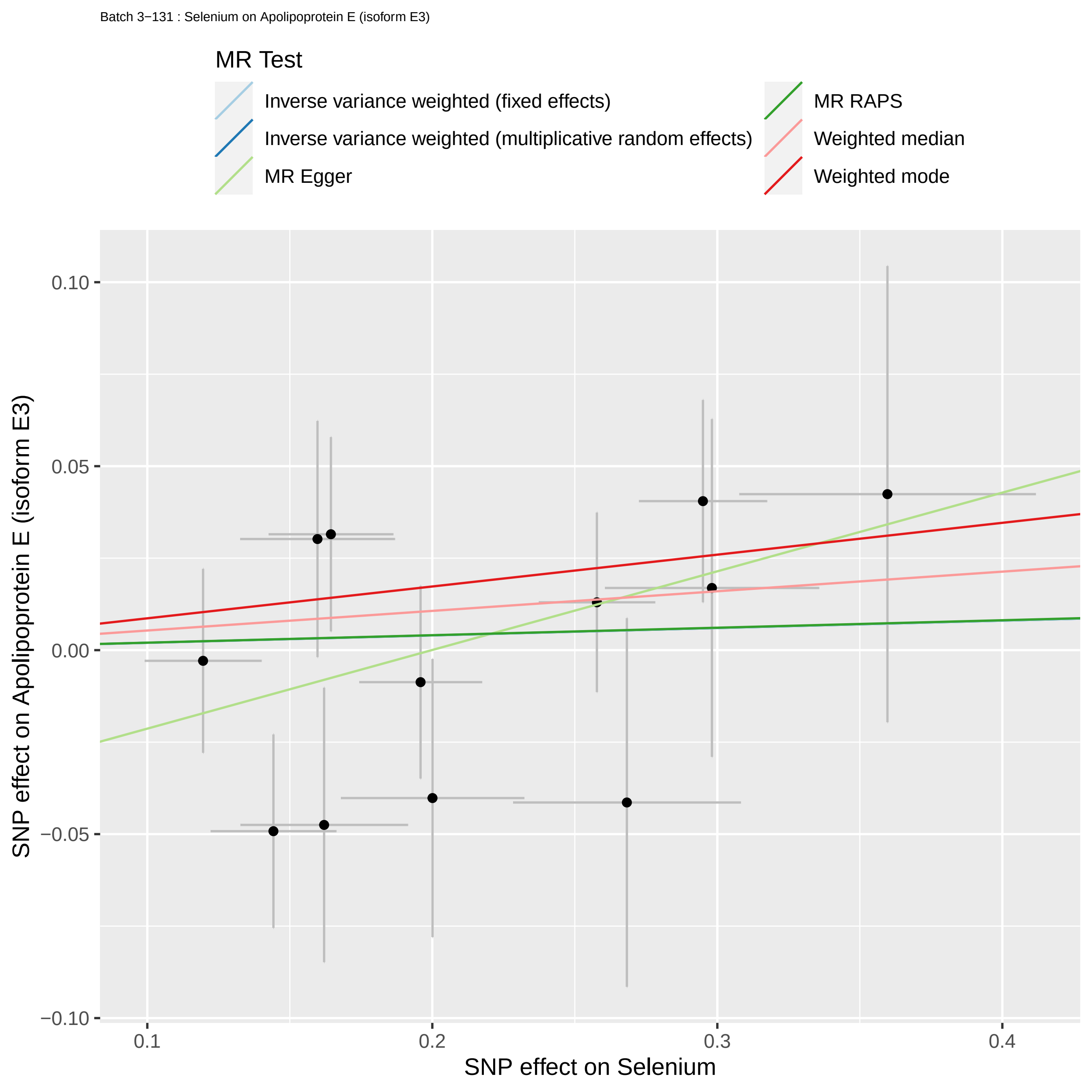
Batch 3−131 : Selenium on Apolipoprotein E (isoform E3)
MR Test
Inverse variance weighted (fixed effects)
Inverse variance weighted (multiplicative random effects)
MR Egger
MR RAPS
Weighted median
Weighted mode
0.10
0.05
0.00
−0.05
−0.10
0.1
0.2
0.3
0.4
SNP effect on Apolipoprotein E (isoform E3)
SNP effect on Selenium

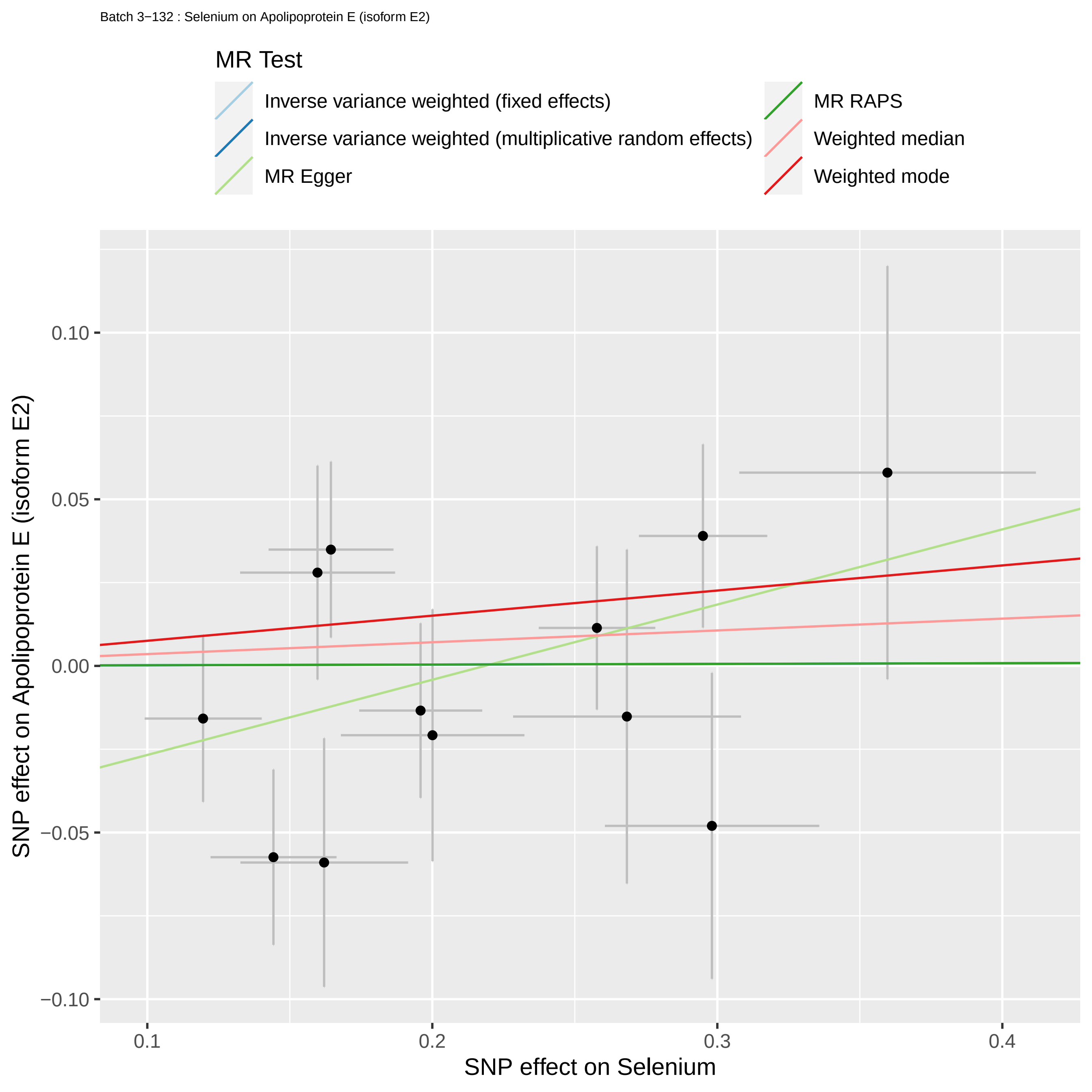
Batch 3−132 : Selenium on Apolipoprotein E (isoform E2)
MR Test
Inverse variance weighted (fixed effects)
Inverse variance weighted (multiplicative random effects)
MR Egger
MR RAPS
Weighted median
Weighted mode
0.10
0.05
0.00
−0.05
−0.10
0.1
0.2
0.3
0.4
SNP effect on Apolipoprotein E (isoform E2)
SNP effect on Selenium

Batch 3−133 : Selenium on Apolipoprotein F

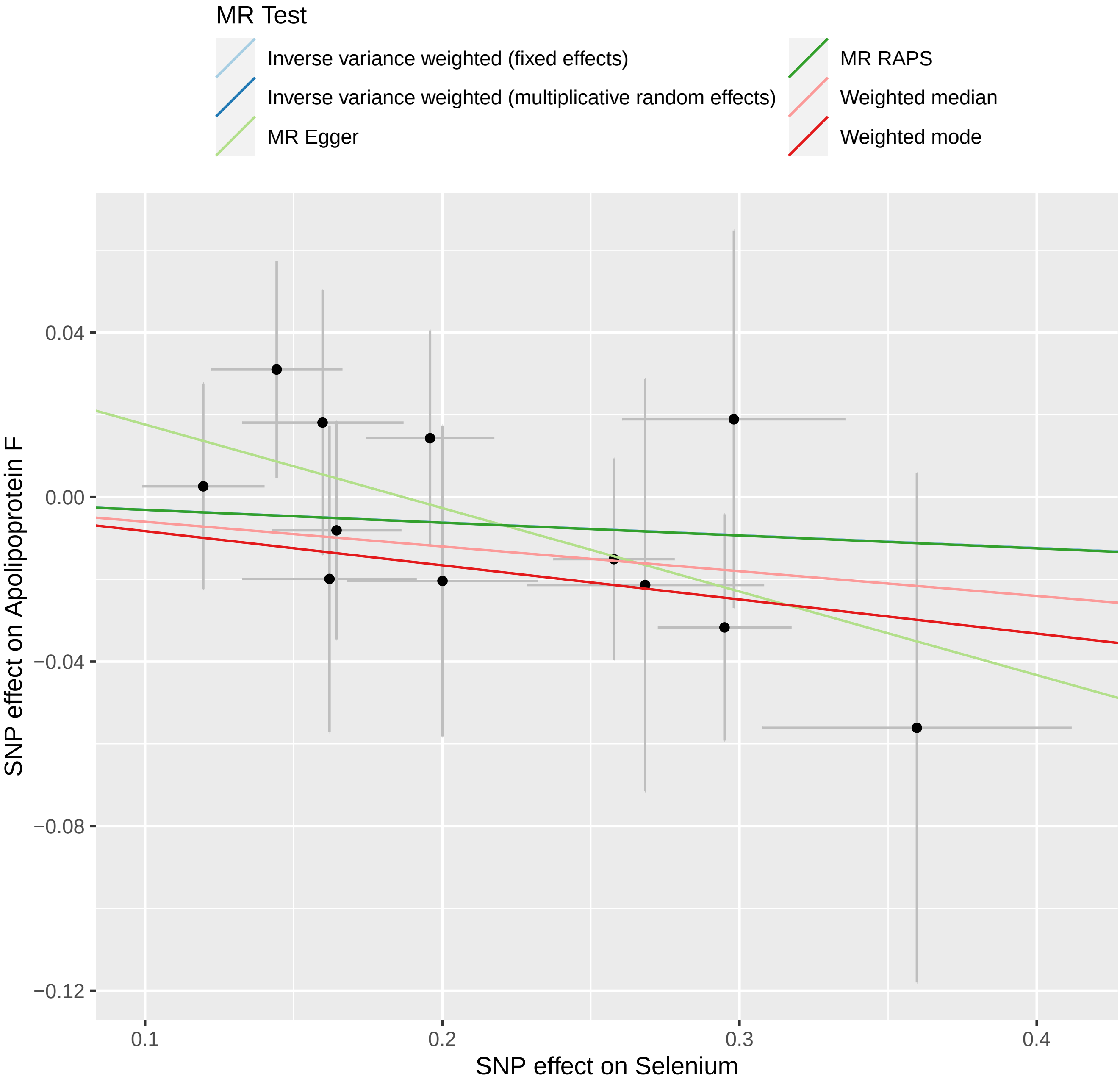

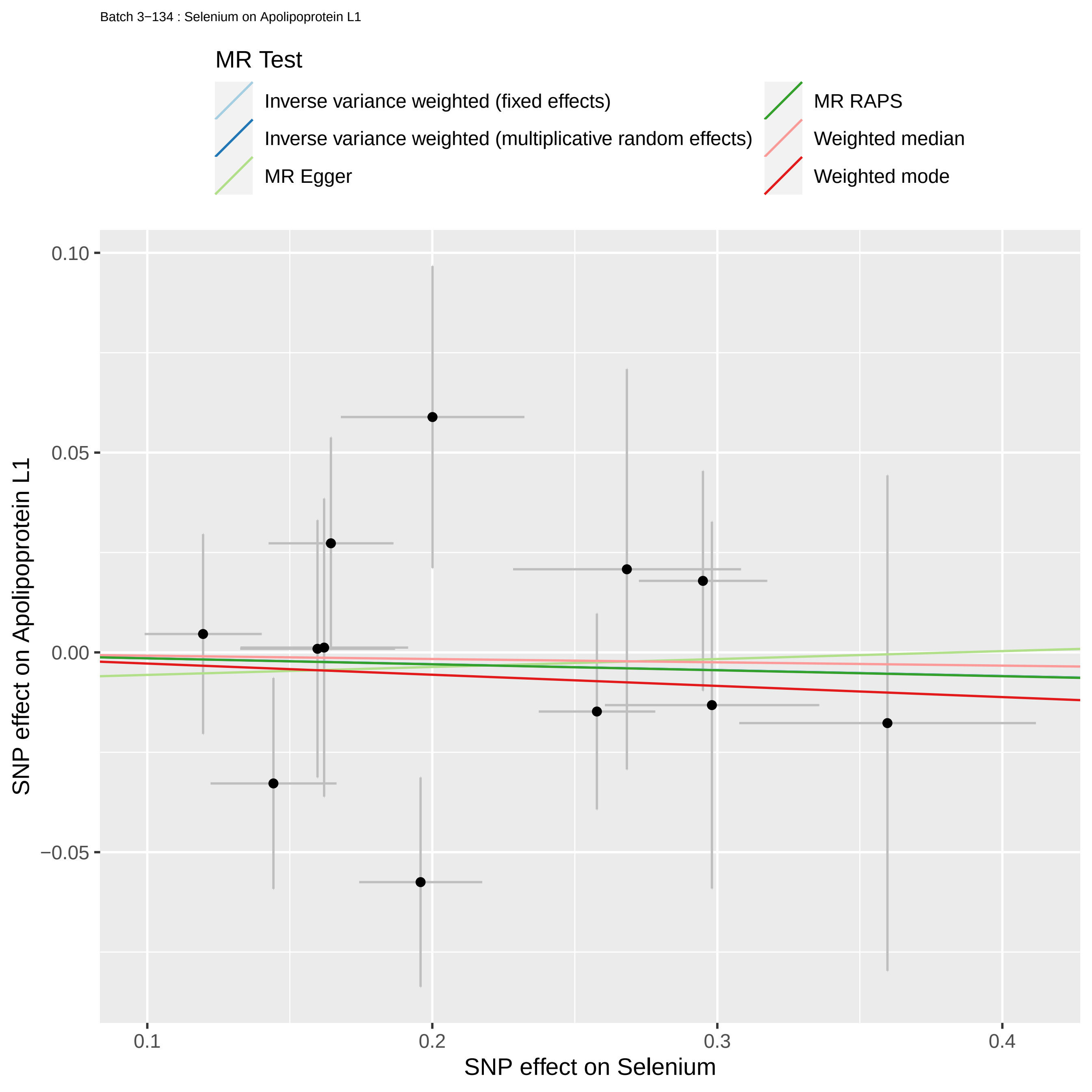

Batch 3−134 : Selenium on Apolipoprotein L1
MR Test
Inverse variance weighted (fixed effects)
Inverse variance weighted (multiplicative random effects)
MR Egger
MR RAPS
Weighted median
Weighted mode
0.10
0.05
0.00
−0.05
0.1
0.2
0.3
0.4
SNP effect on Apolipoprotein L1
SNP effect on Selenium

Batch 3−135 : Selenium on Apolipoprotein L1

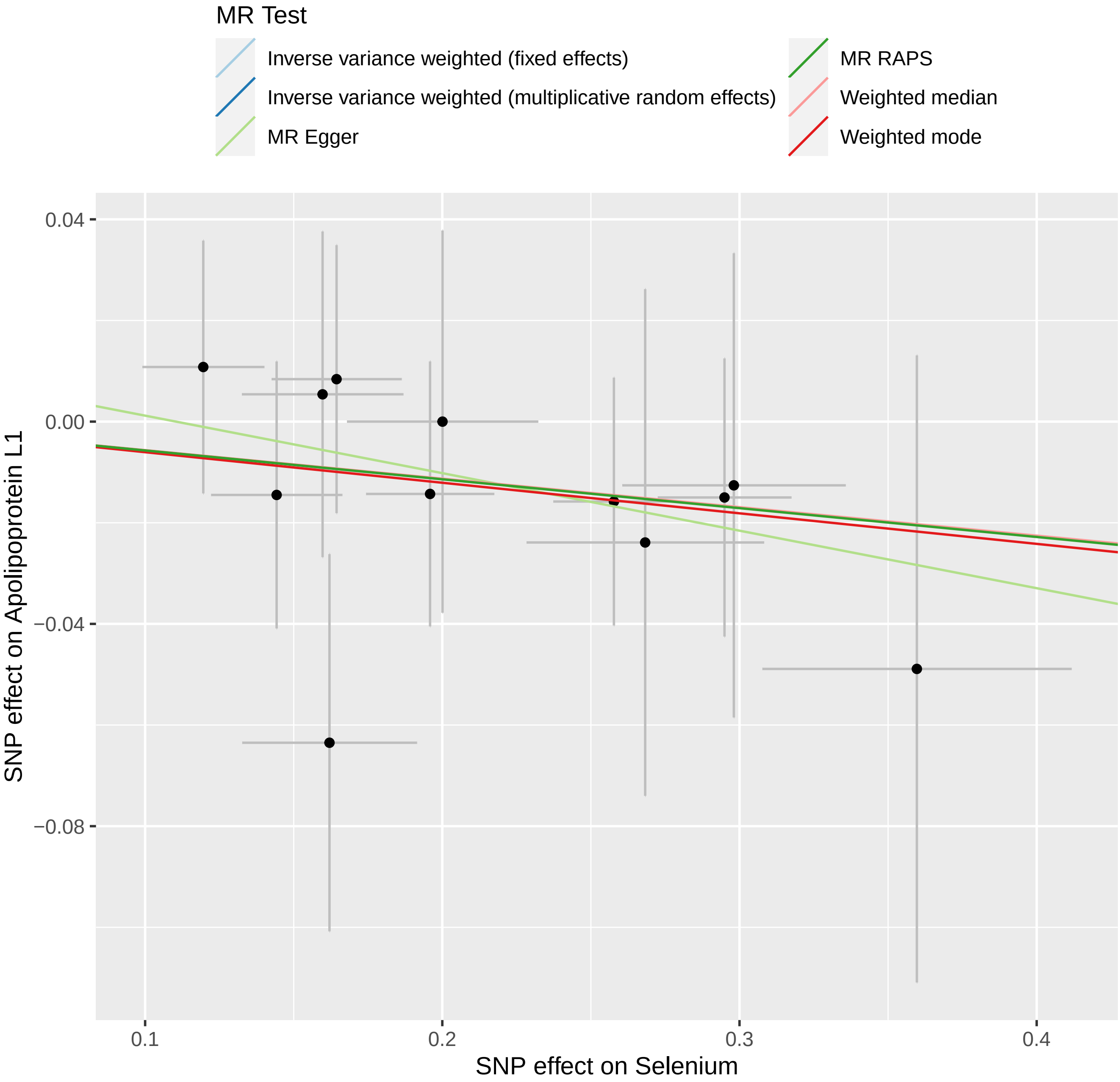

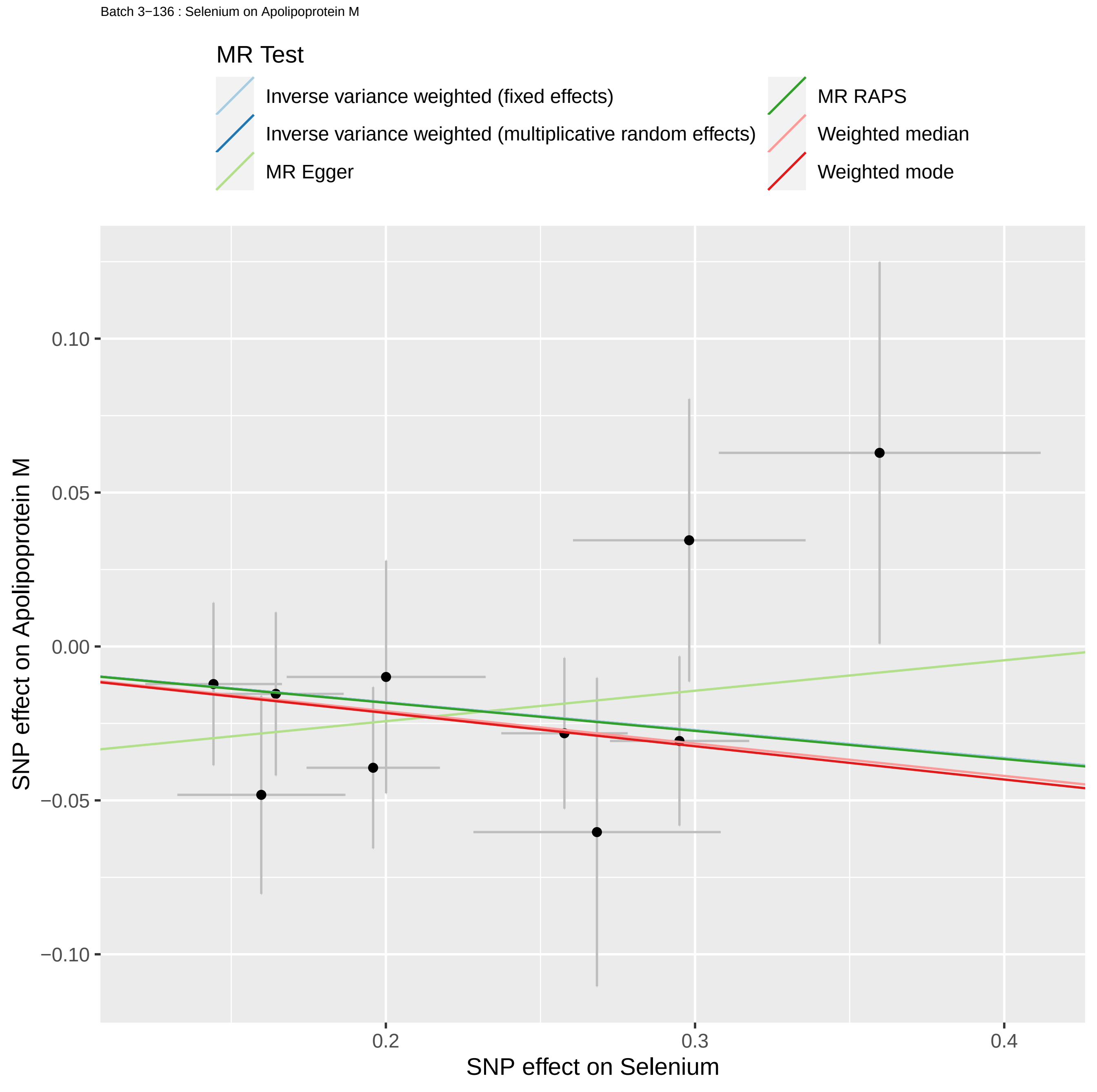

Batch 3−136 : Selenium on Apolipoprotein M
MR Test
Inverse variance weighted (fixed effects)
Inverse variance weighted (multiplicative random effects)
MR Egger
MR RAPS
Weighted median
Weighted mode
0.10
0.05
0.00
−0.05
−0.10
SNP effect on Apolipoprotein M
0.2
0.3
0.4
SNP effect on Selenium

Batch 3−137 : Selenium on Apolipoprotein M

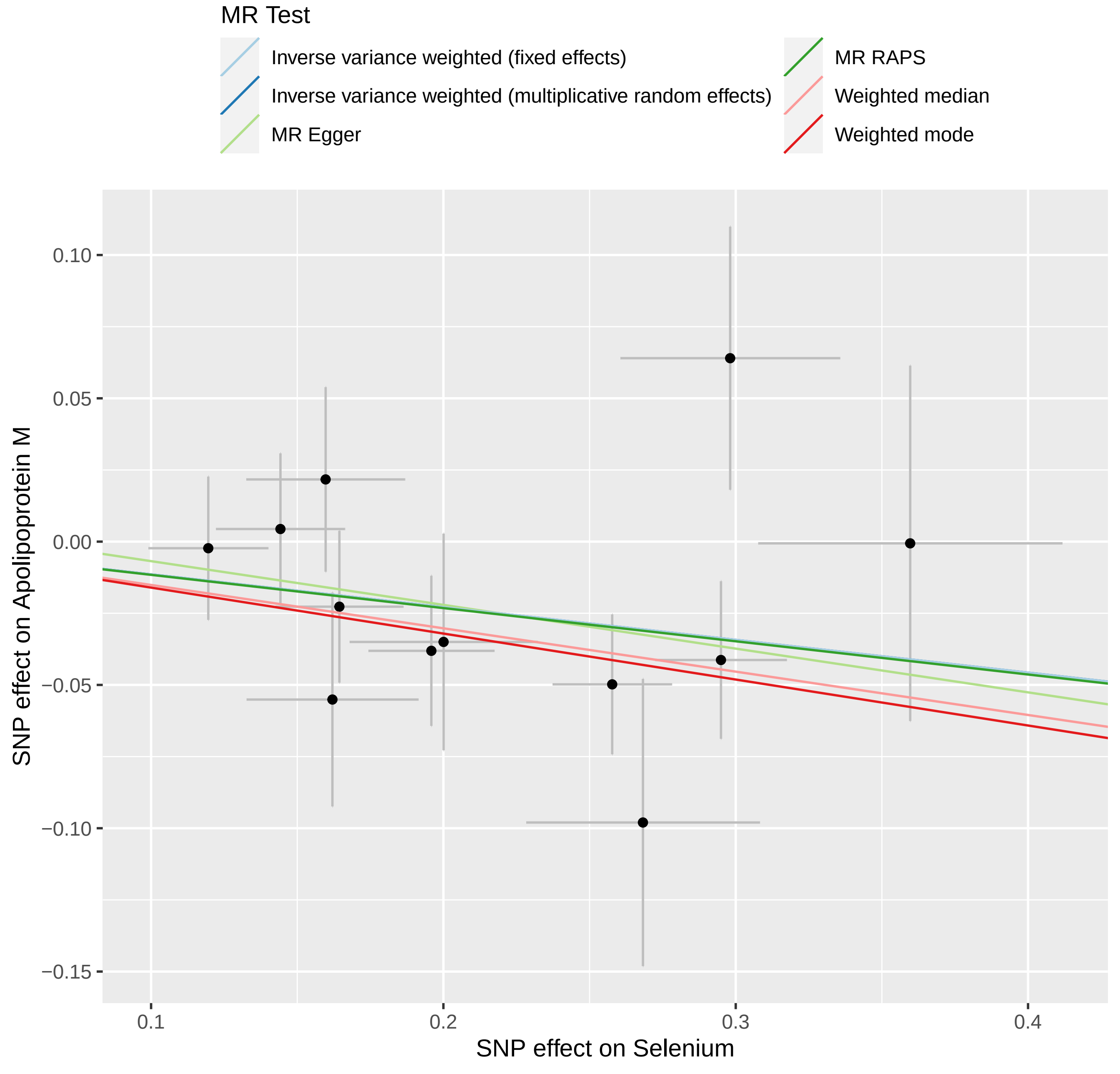

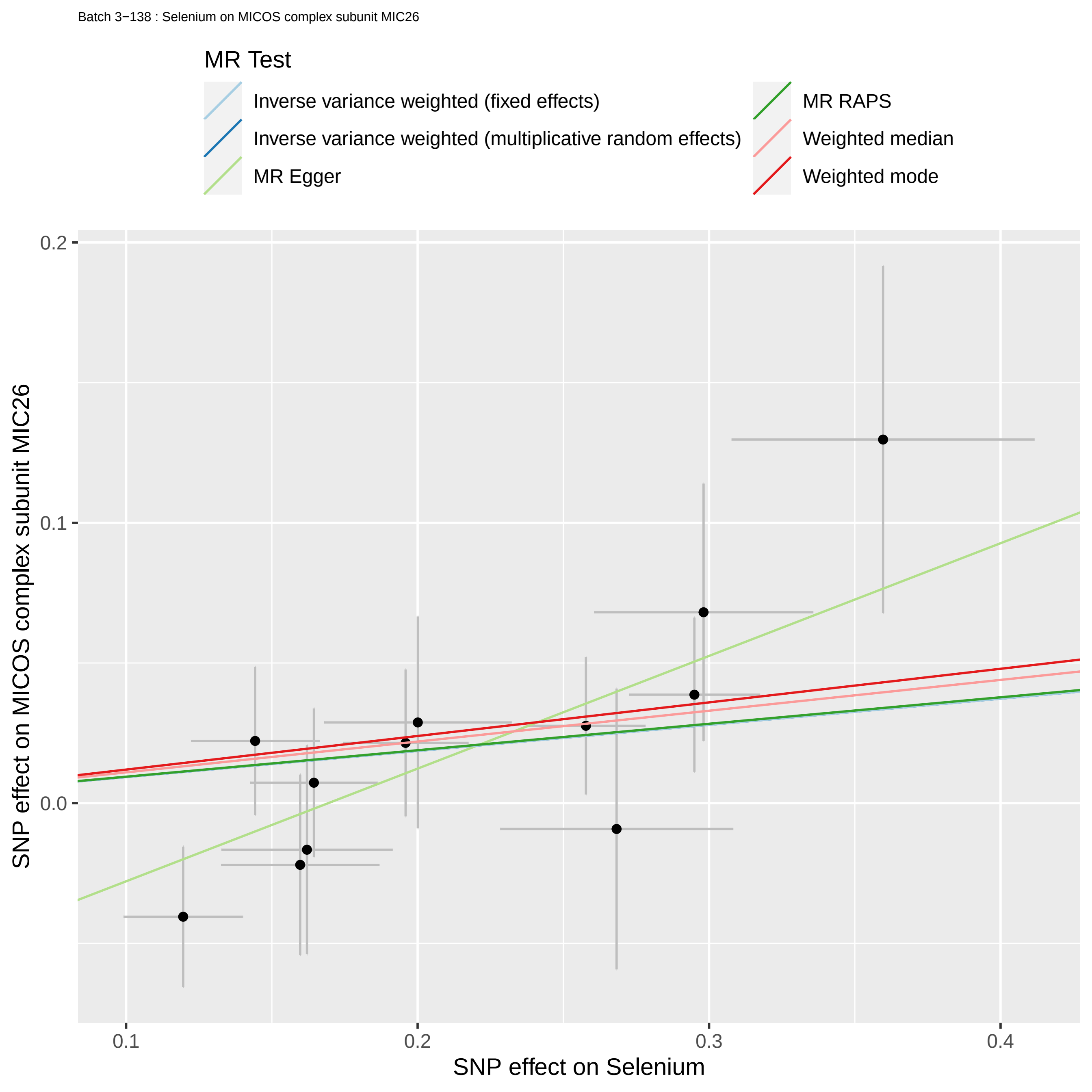

Batch 3−138 : Selenium on MICOS complex subunit MIC26
MR Test
Inverse variance weighted (fixed effects)
Inverse variance weighted (multiplicative random effects)
MR Egger
MR RAPS
Weighted median
Weighted mode
0.2
0.1
0.0
0.1
0.2
0.3
0.4
SNP effect on MICOS complex subunit MIC26
SNP effect on Selenium

Batch 3−139 : Selenium on Amyloid beta A4 protein

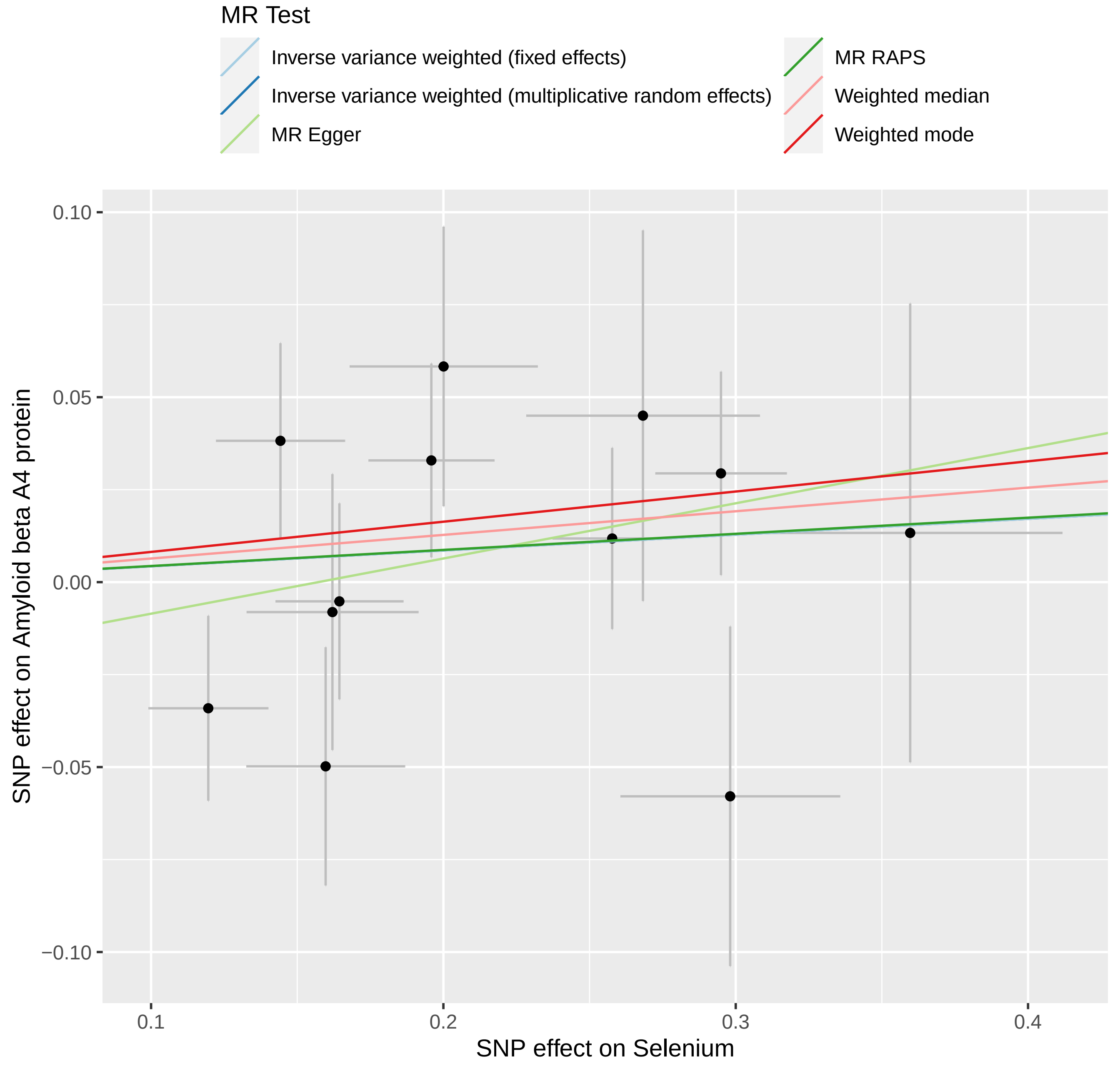

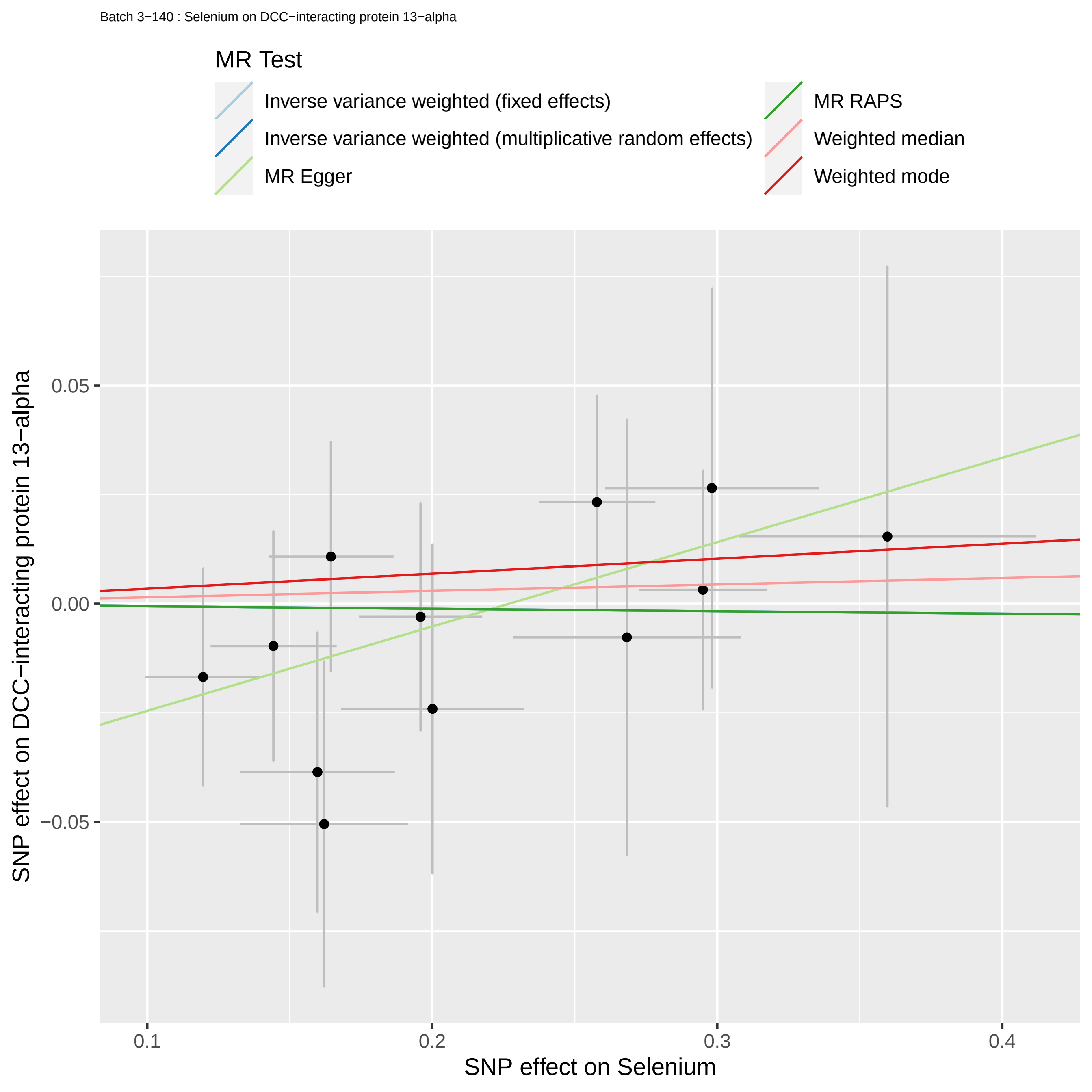

Batch 3−140 : Selenium on DCC−interacting protein 13−alpha
MR Test
Inverse variance weighted (fixed effects)
Inverse variance weighted (multiplicative random effects)
MR Egger
MR RAPS
Weighted median
Weighted mode
SNP effect on DCC−interacting protein 13−alpha
0.05
0.00
−0.05
0.1
0.2
0.3
0.4
SNP effect on Selenium

Batch 3−141 : Selenium on Amphiregulin

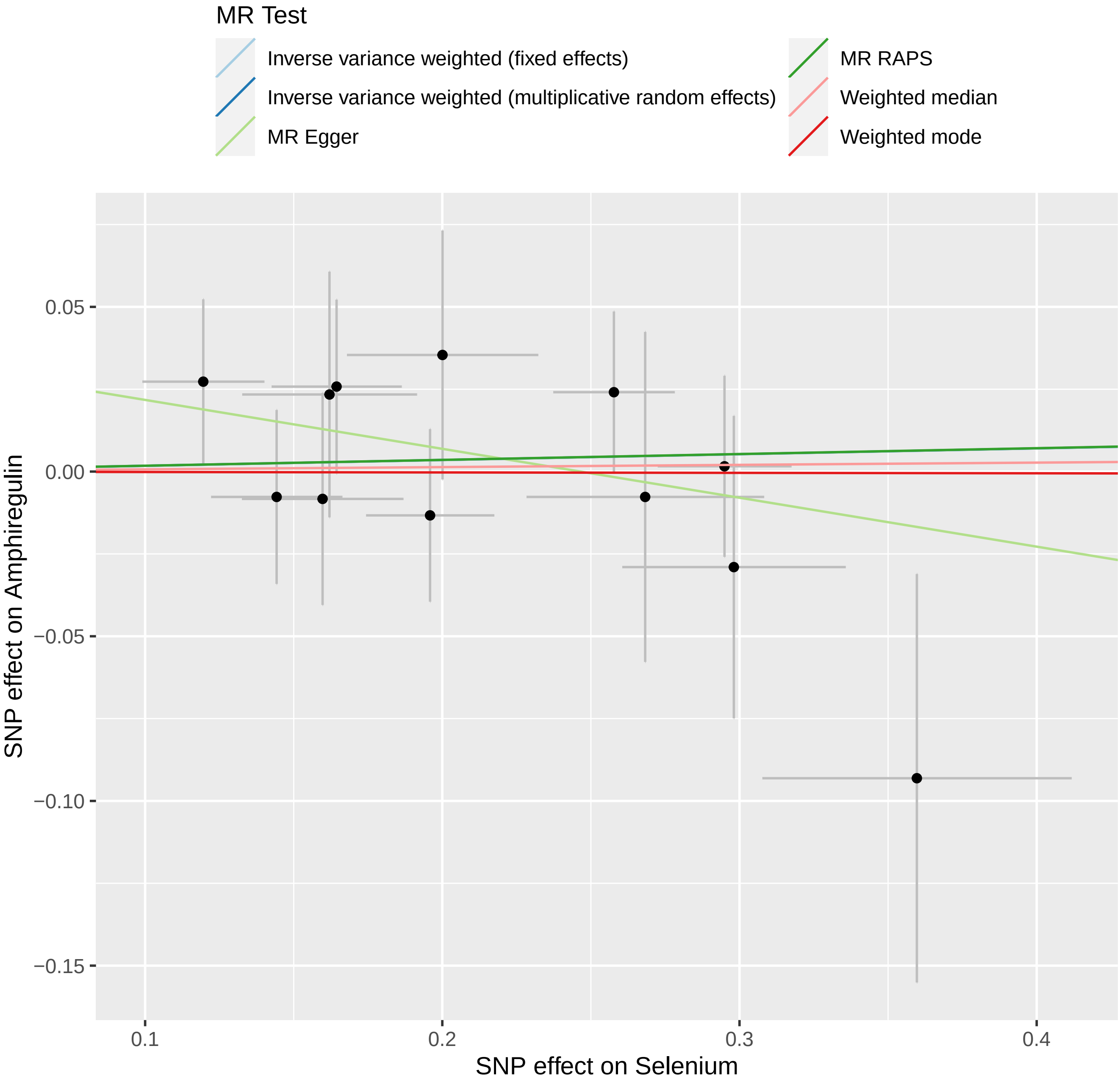

Batch 3−142 : Selenium on ADP−ribosylation factor 3

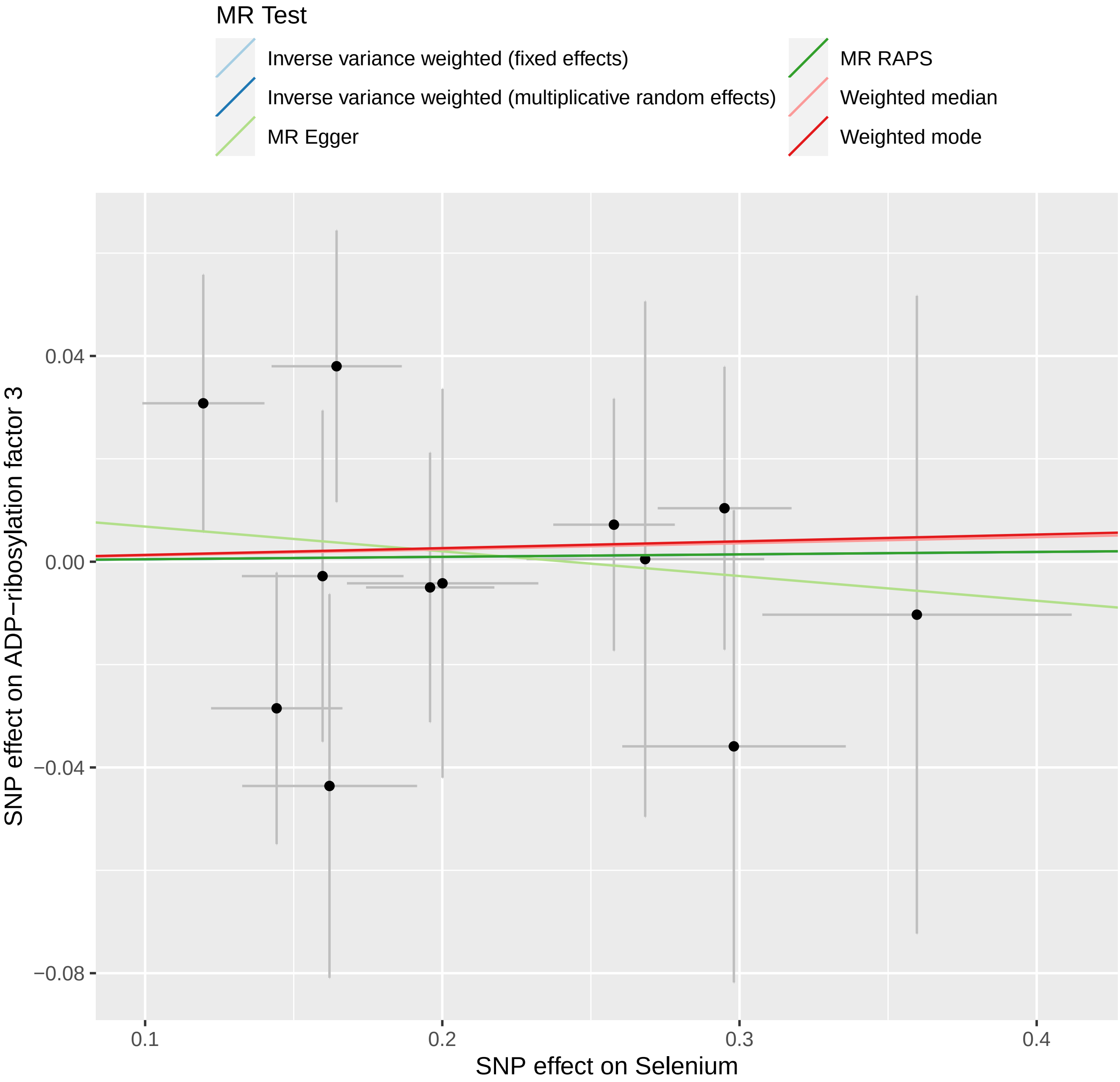

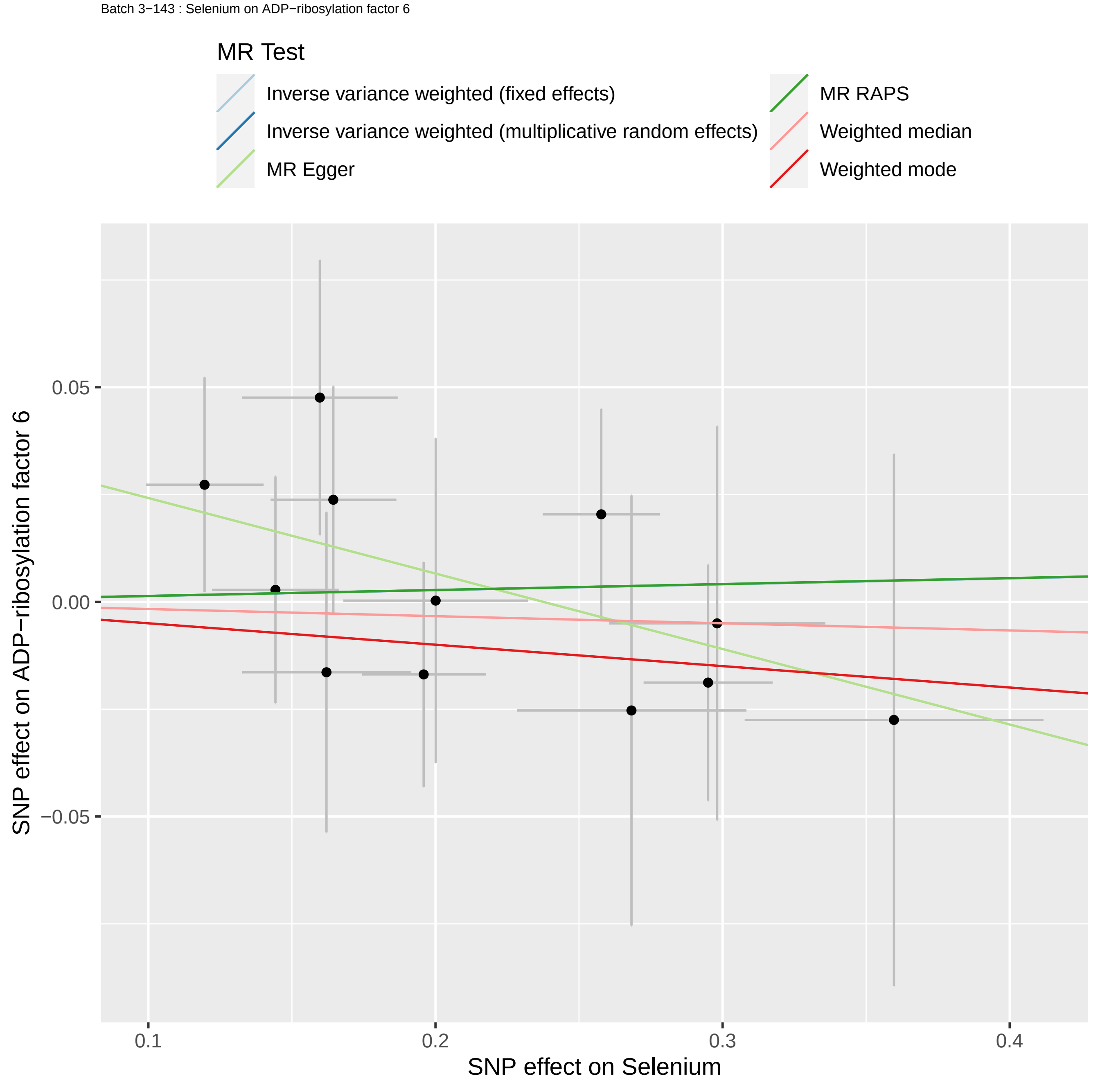

Batch 3−143 : Selenium on ADP−ribosylation factor 6
MR Test
Inverse variance weighted (fixed effects)
Inverse variance weighted (multiplicative random effects)
MR Egger
MR RAPS
Weighted median
Weighted mode
0.05
0.00
−0.05
0.1
0.2
0.3
0.4
SNP effect on ADP−ribosylation factor 6
SNP effect on Selenium

Batch 3−144 : Selenium on ADP−ribosylation factor GTPase−activating protein 2

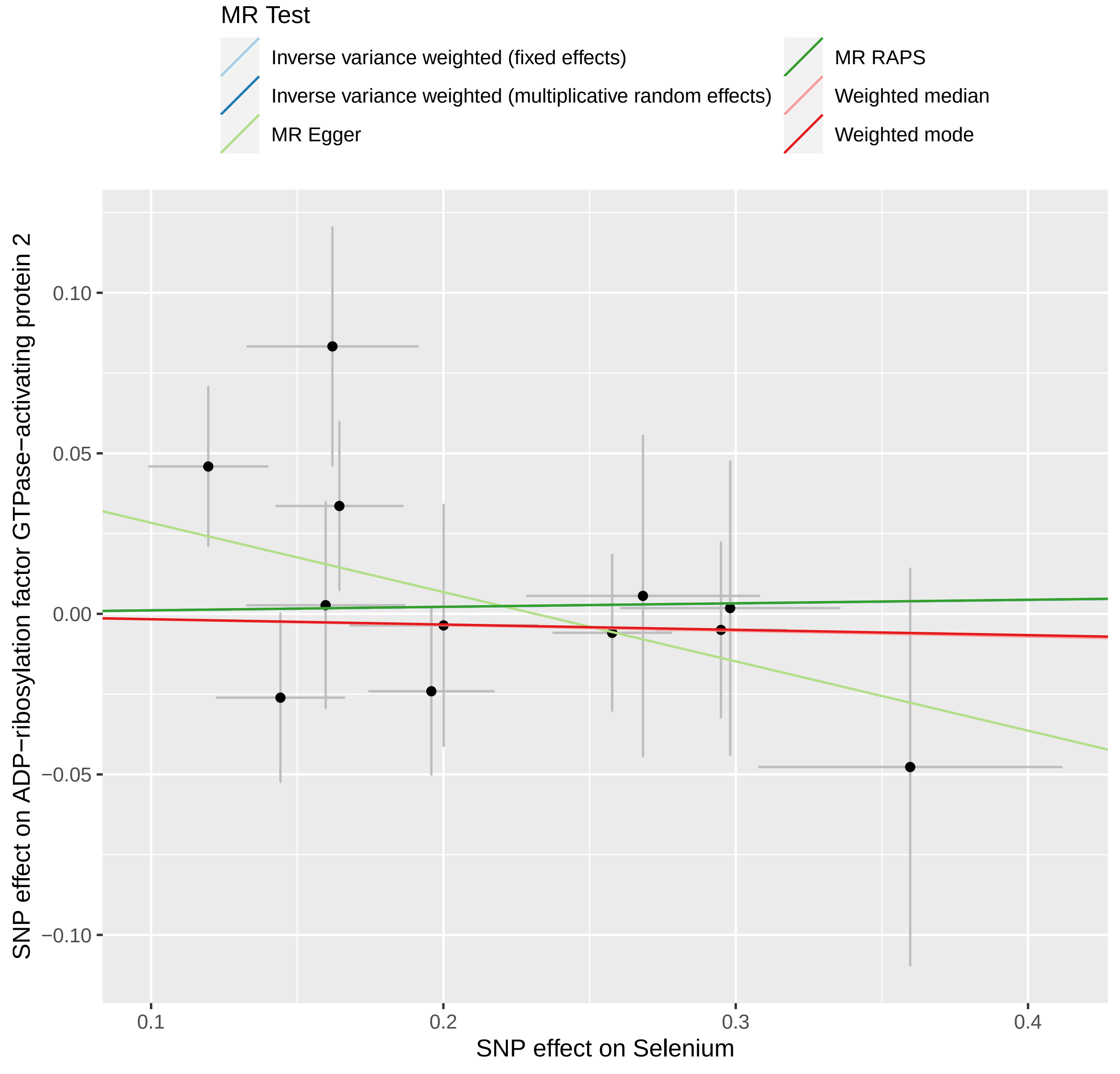

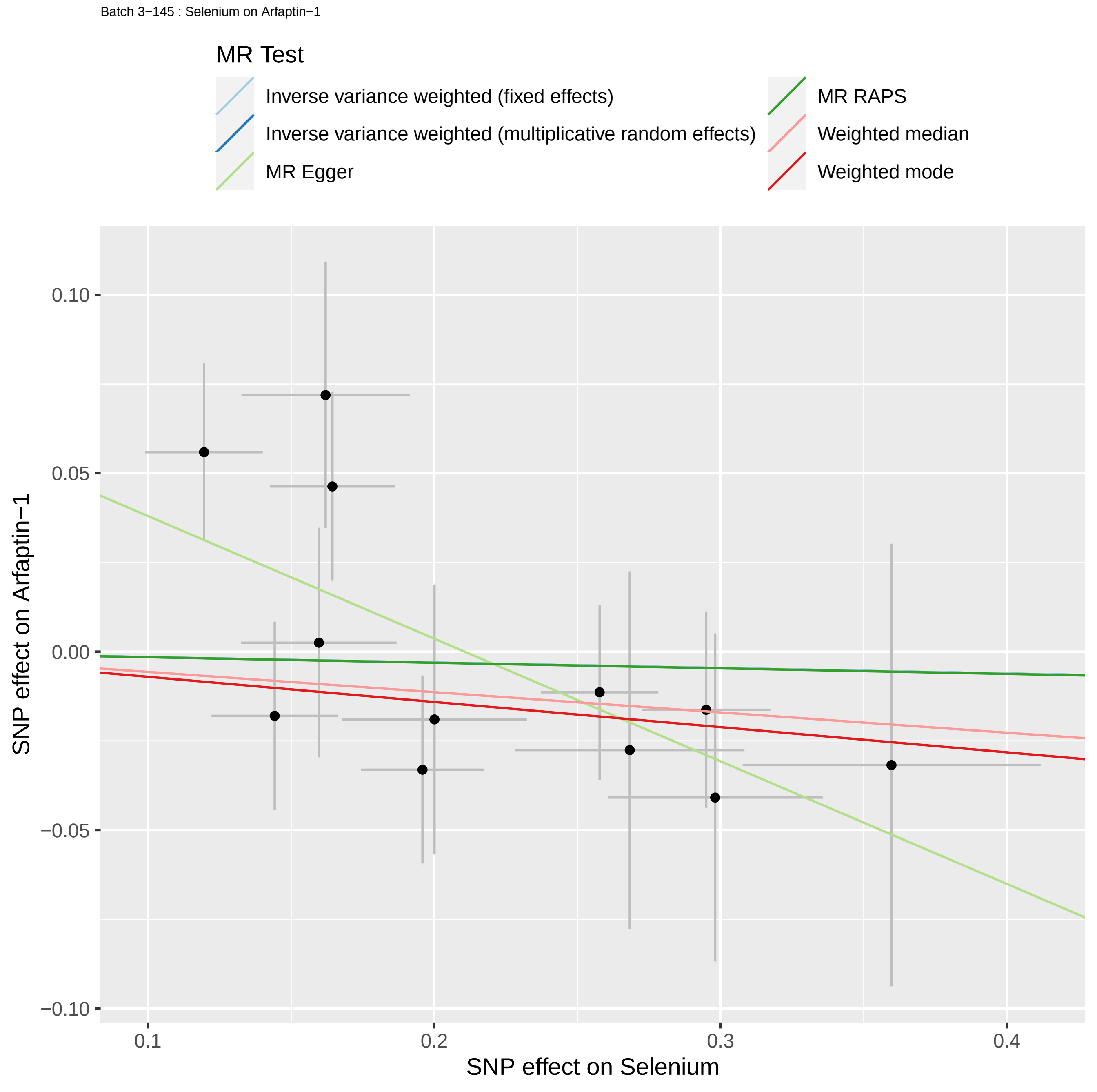

Batch 3−145 : Selenium on Arfaptin−1
MR Test
Inverse variance weighted (fixed effects)
Inverse variance weighted (multiplicative random effects)
MR Egger
MR RAPS
Weighted median
Weighted mode
0.10
0.05
0.00
−0.05
−0.10
0.1
0.2
0.3
0.4
SNP effect on Arfaptin−1
SNP effect on Selenium

Batch 3−146 : Selenium on Arfaptin−2

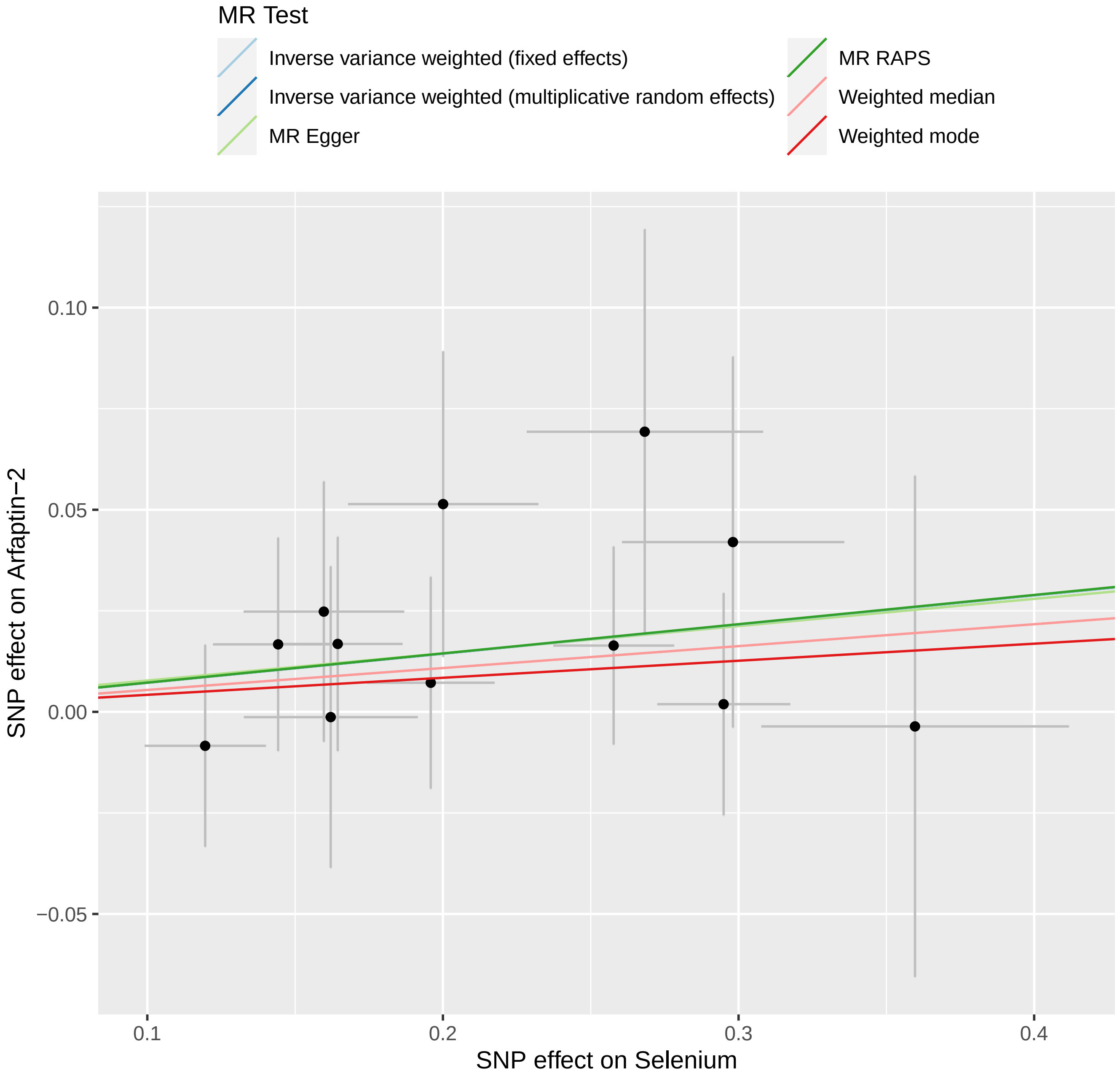

Batch 3−147 : Selenium on Rho GTPase−activating protein 1

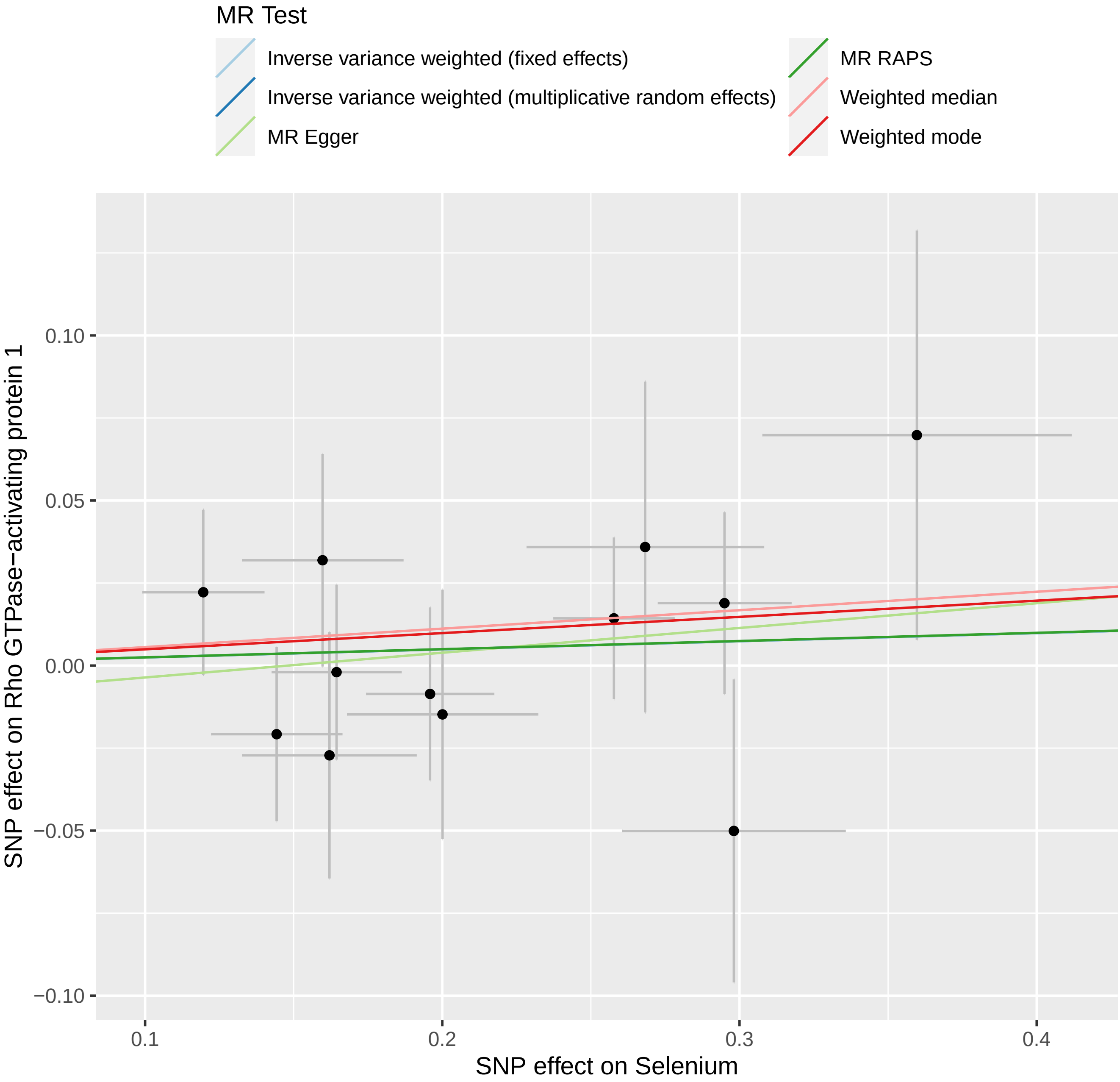

Batch 3−148 : Selenium on Rho GTPase−activating protein 25

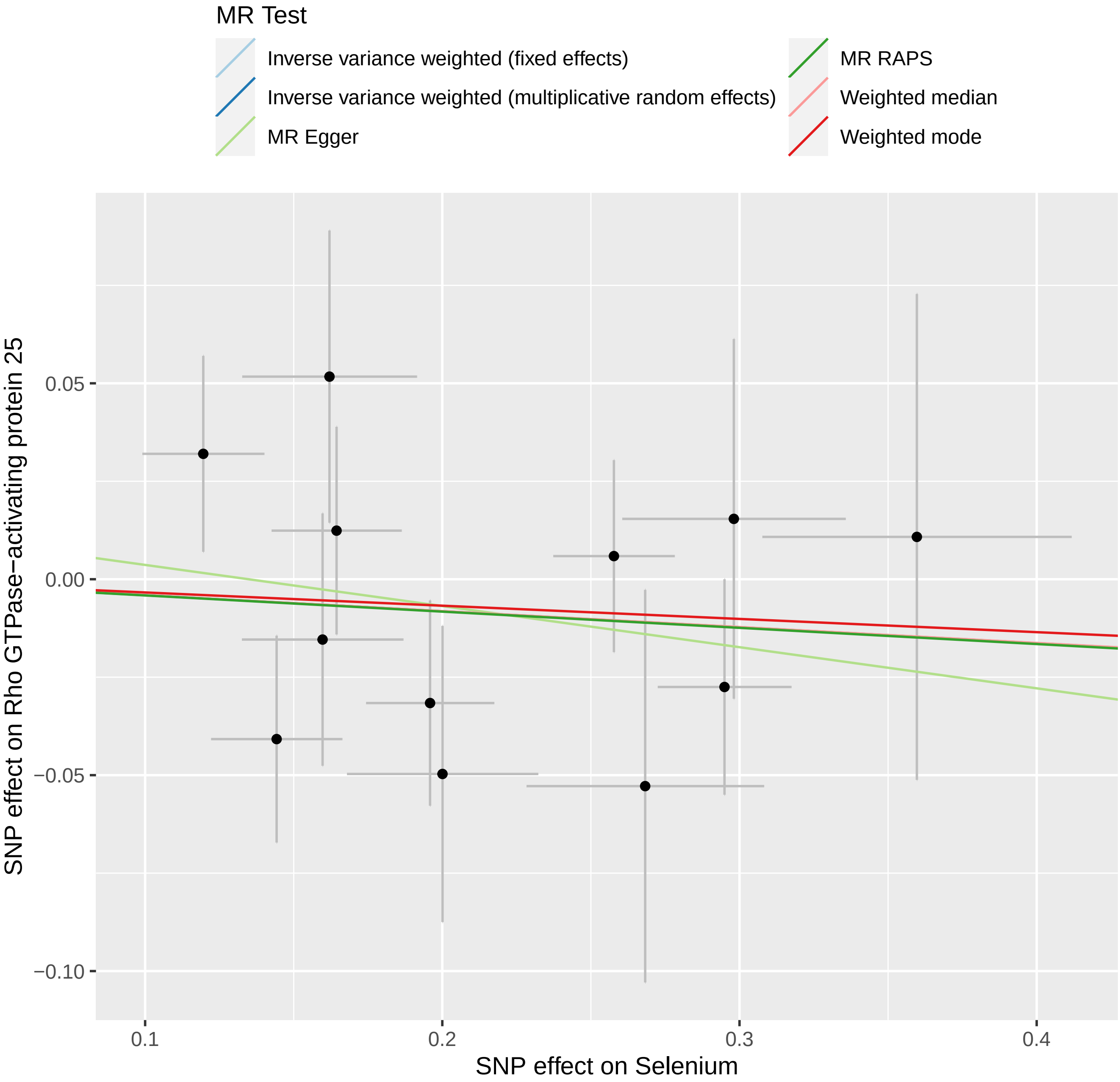

Batch 3−149 : Selenium on Rho GTPase−activating protein 30

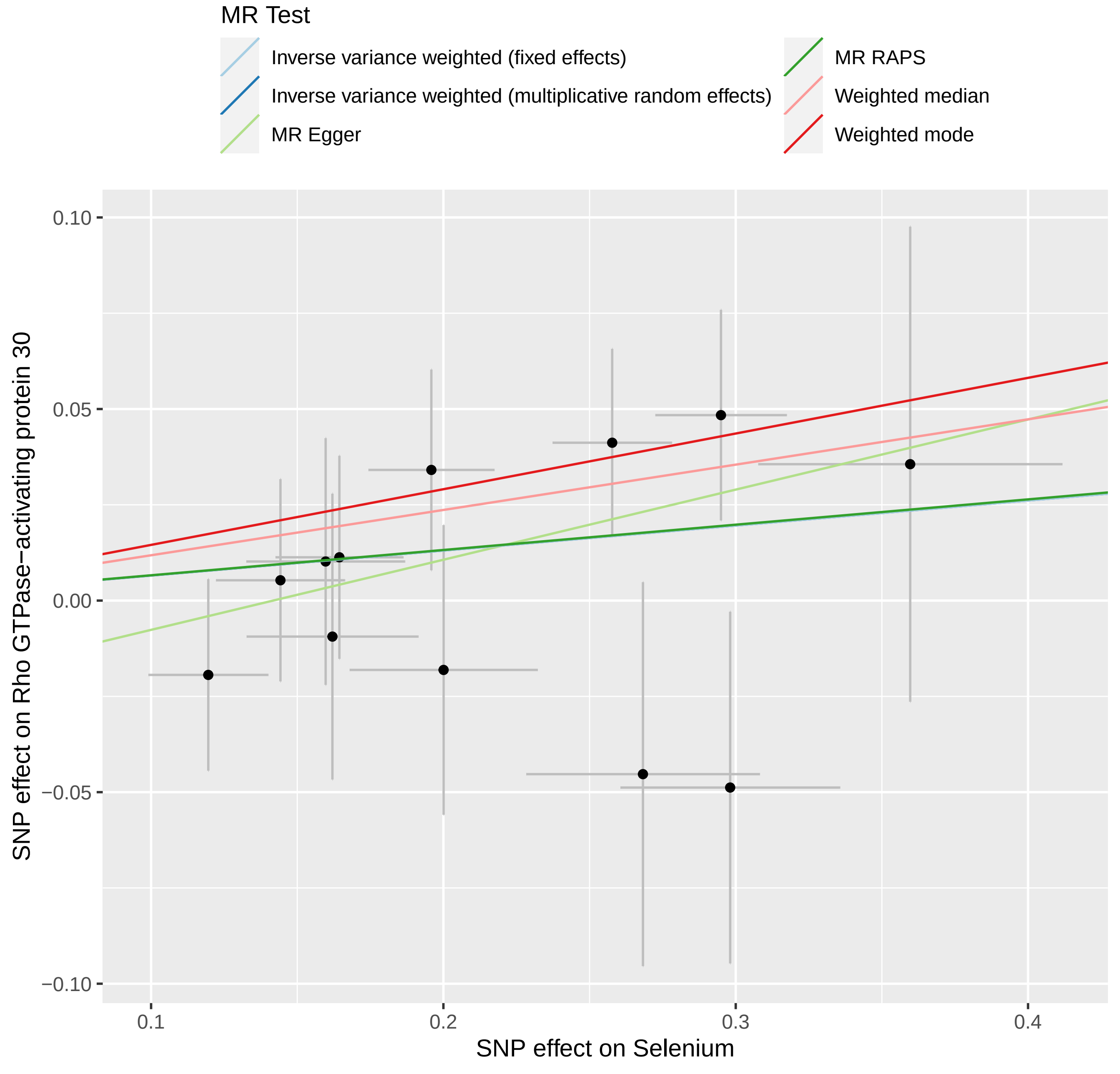

Batch 3−150 : Selenium on Rho GTPase−activating protein 36

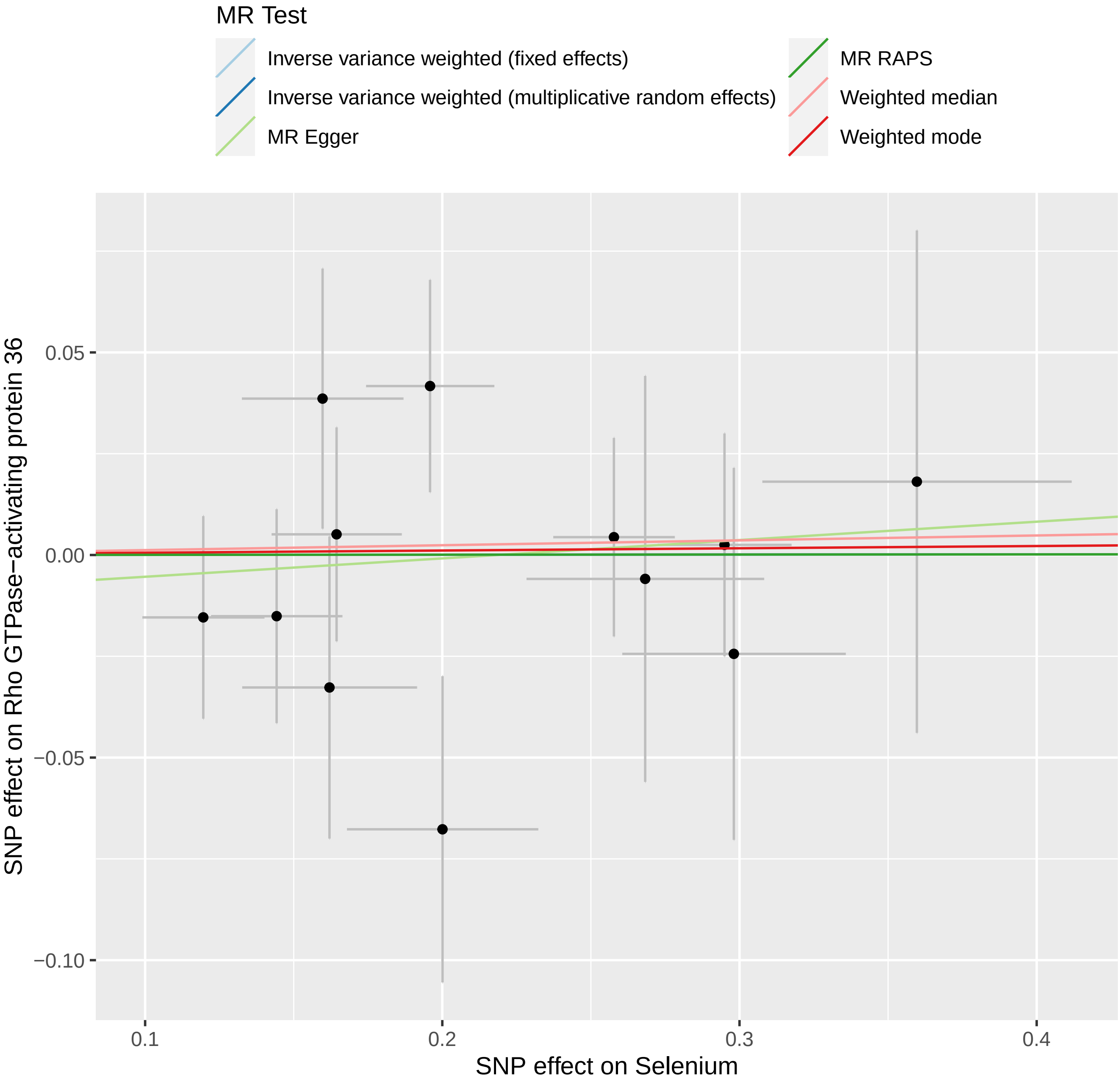

Batch 3−151 : Selenium on Rho GTPase−activating protein 5

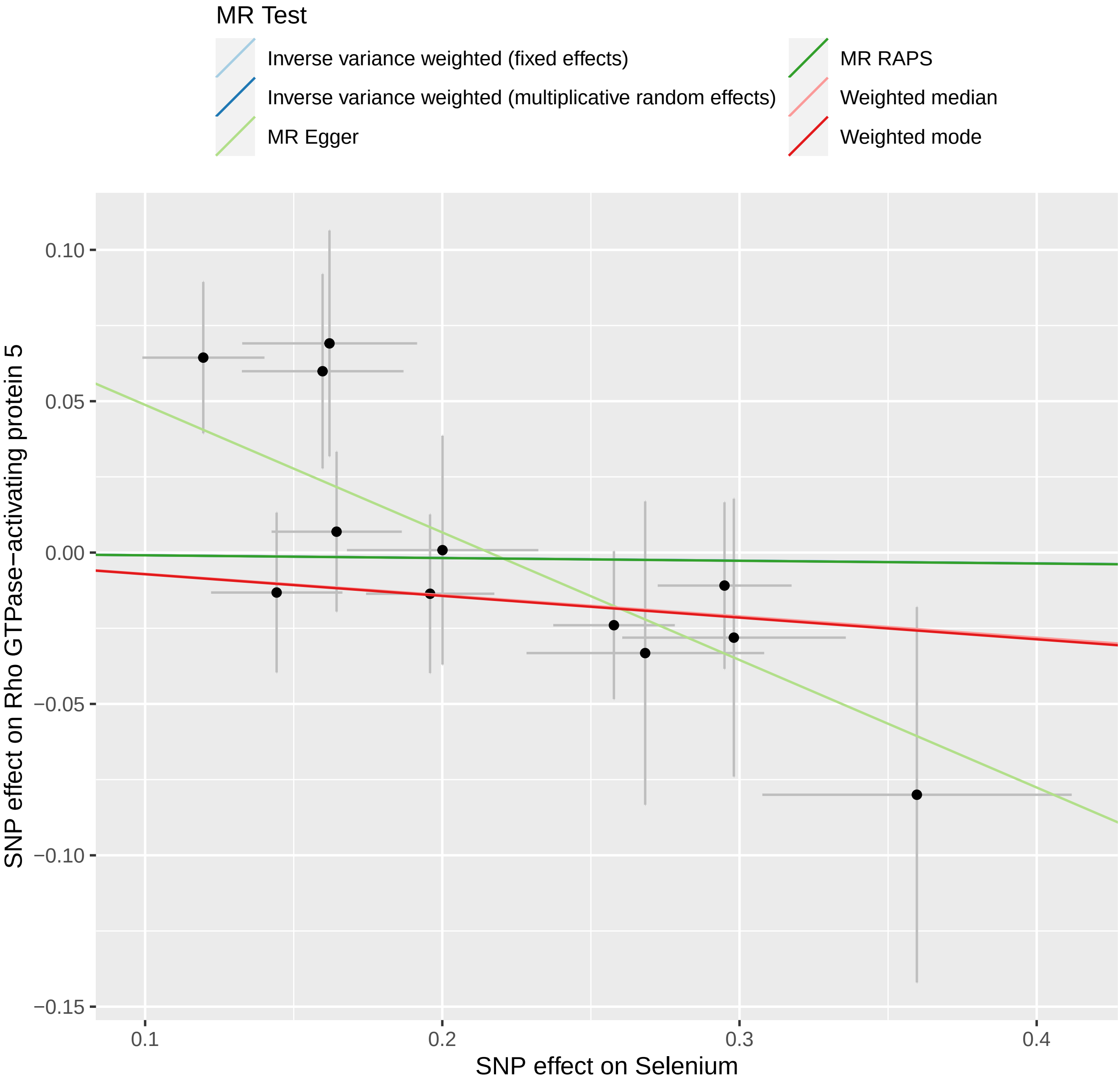

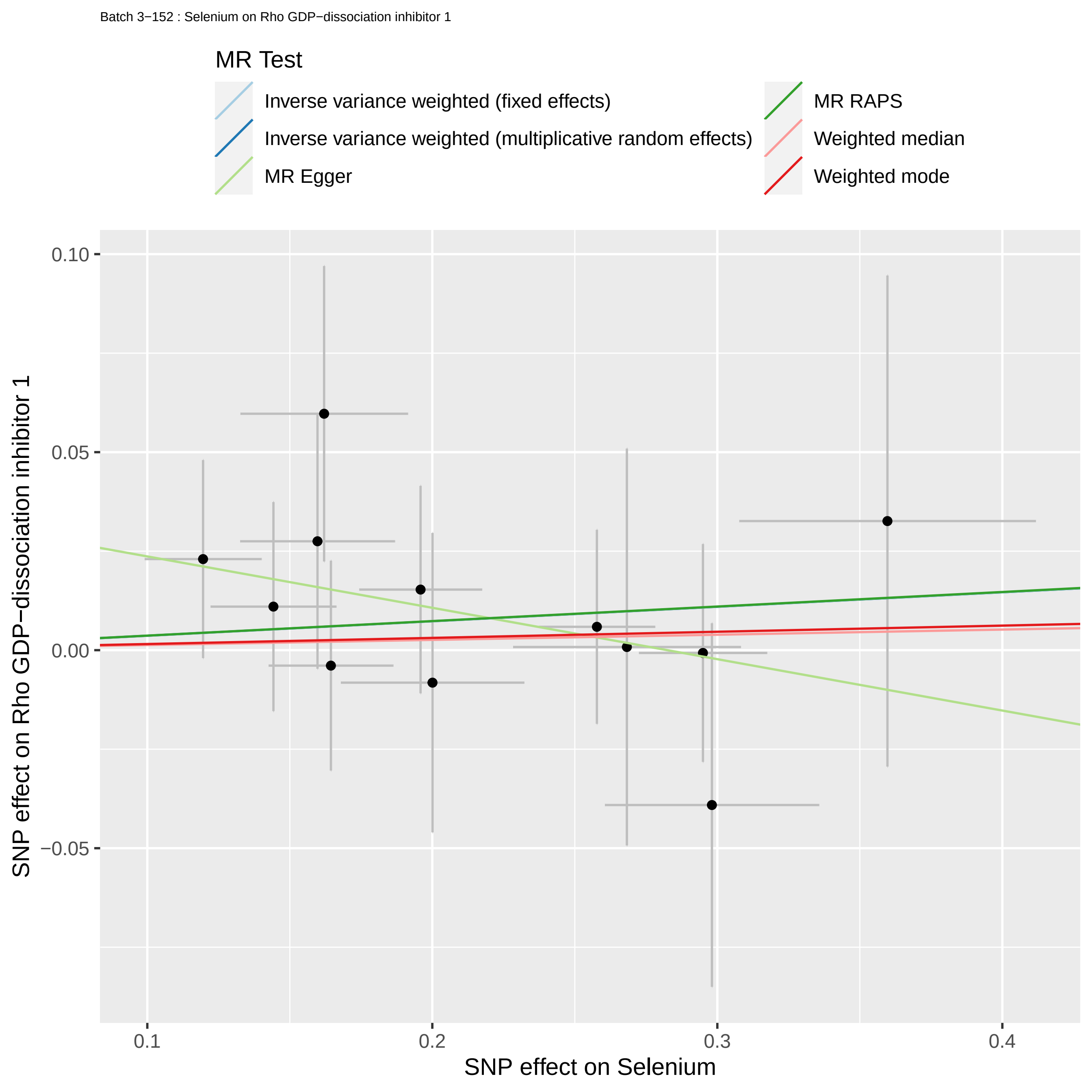

Batch 3−152 : Selenium on Rho GDP−dissociation inhibitor 1
MR Test
Inverse variance weighted (fixed effects)
Inverse variance weighted (multiplicative random effects)
MR Egger
MR RAPS
Weighted median
Weighted mode
0.10
0.05
0.00
−0.05
0.1
0.2
0.3
0.4
SNP effect on Rho GDP−dissociation inhibitor 1
SNP effect on Selenium

Batch 3−153 : Selenium on Rho GDP−dissociation inhibitor 2

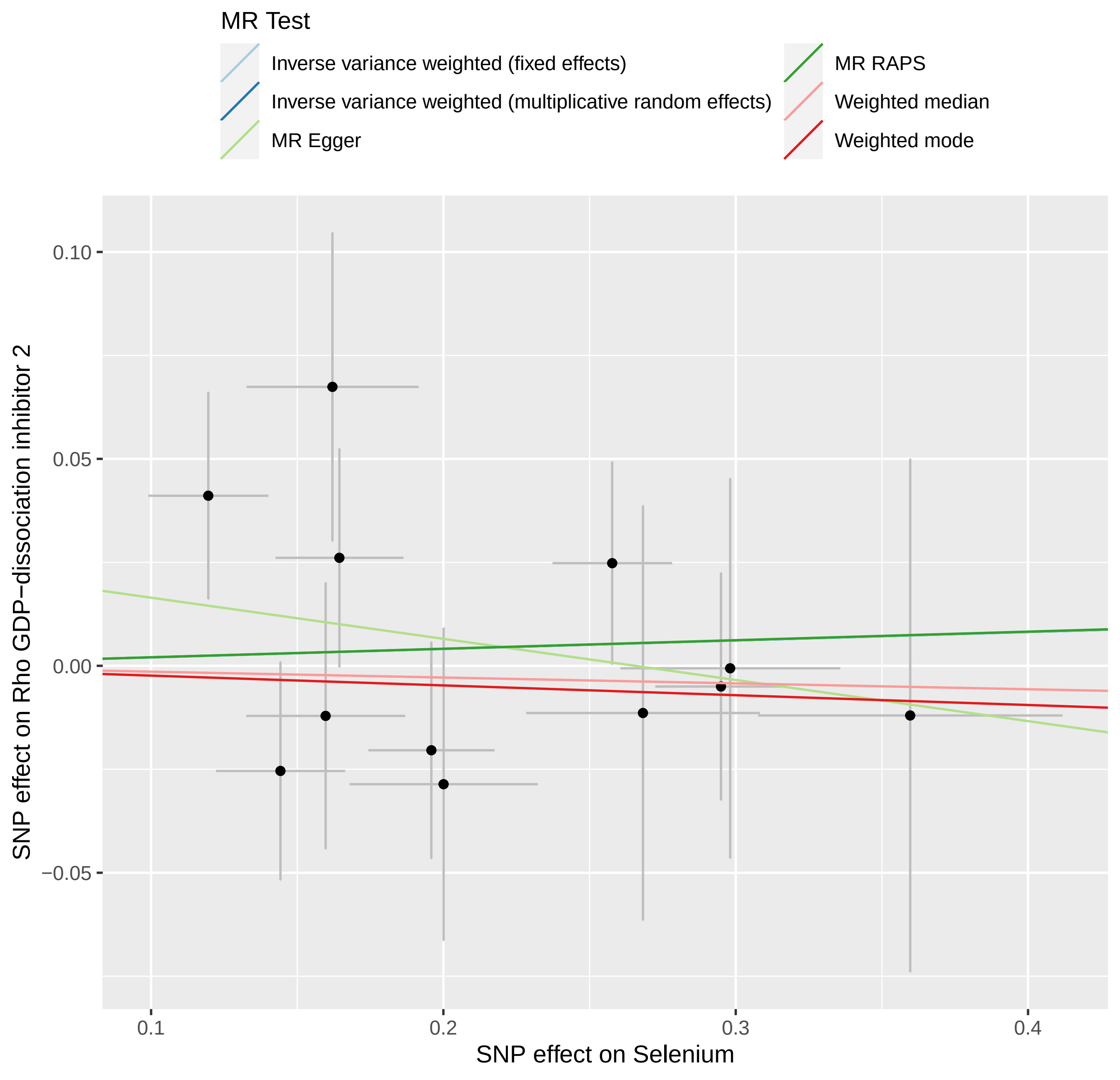

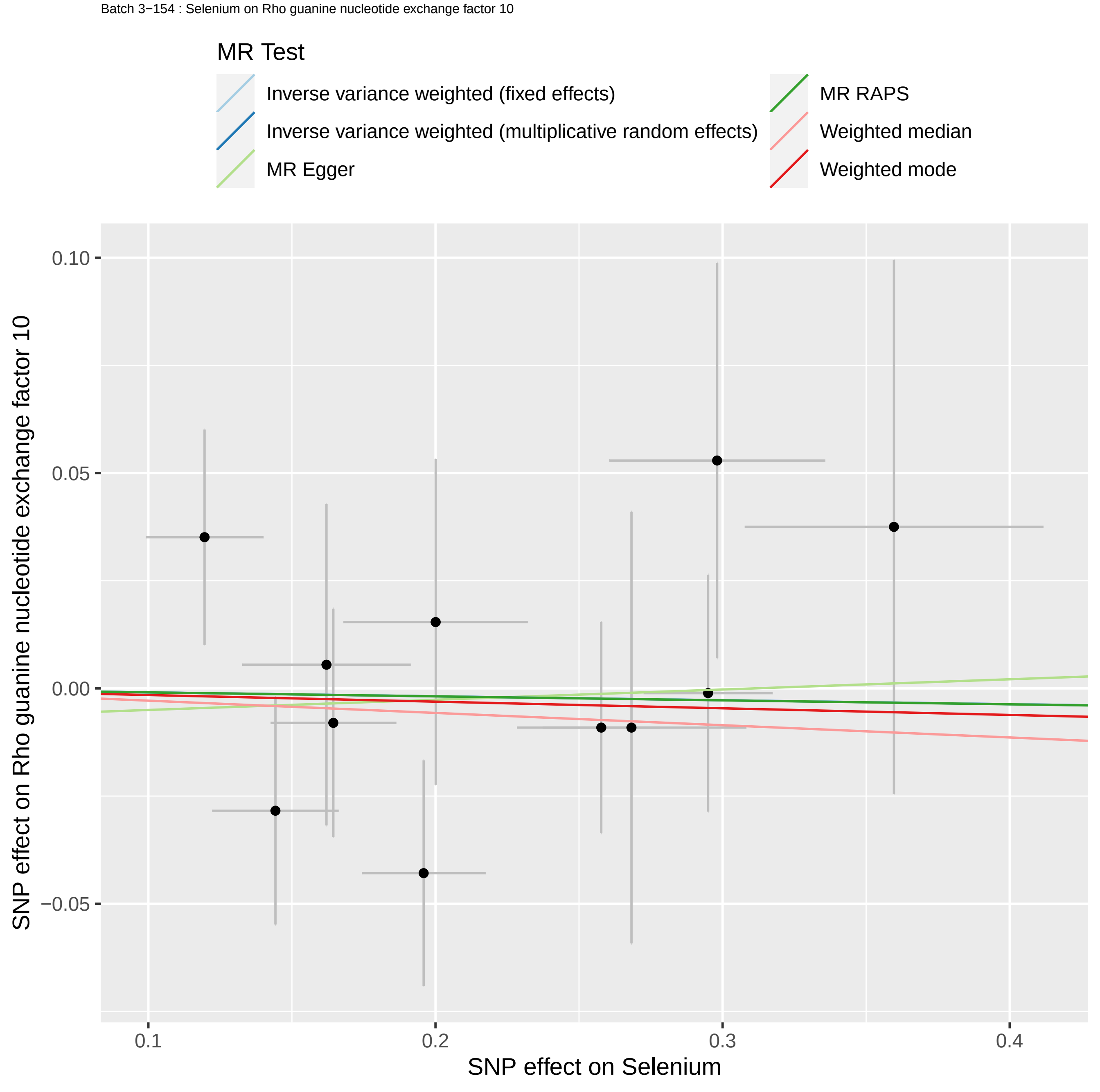

Batch 3−154 : Selenium on Rho guanine nucleotide exchange factor 10
MR Test
Inverse variance weighted (fixed effects)
Inverse variance weighted (multiplicative random effects)
MR Egger
MR RAPS
Weighted median
Weighted mode
0.10
0.05
0.00
−0.05
0.1
0.2
0.3
0.4
SNP effect on Rho guanine nucleotide exchange factor 10
SNP effect on Selenium

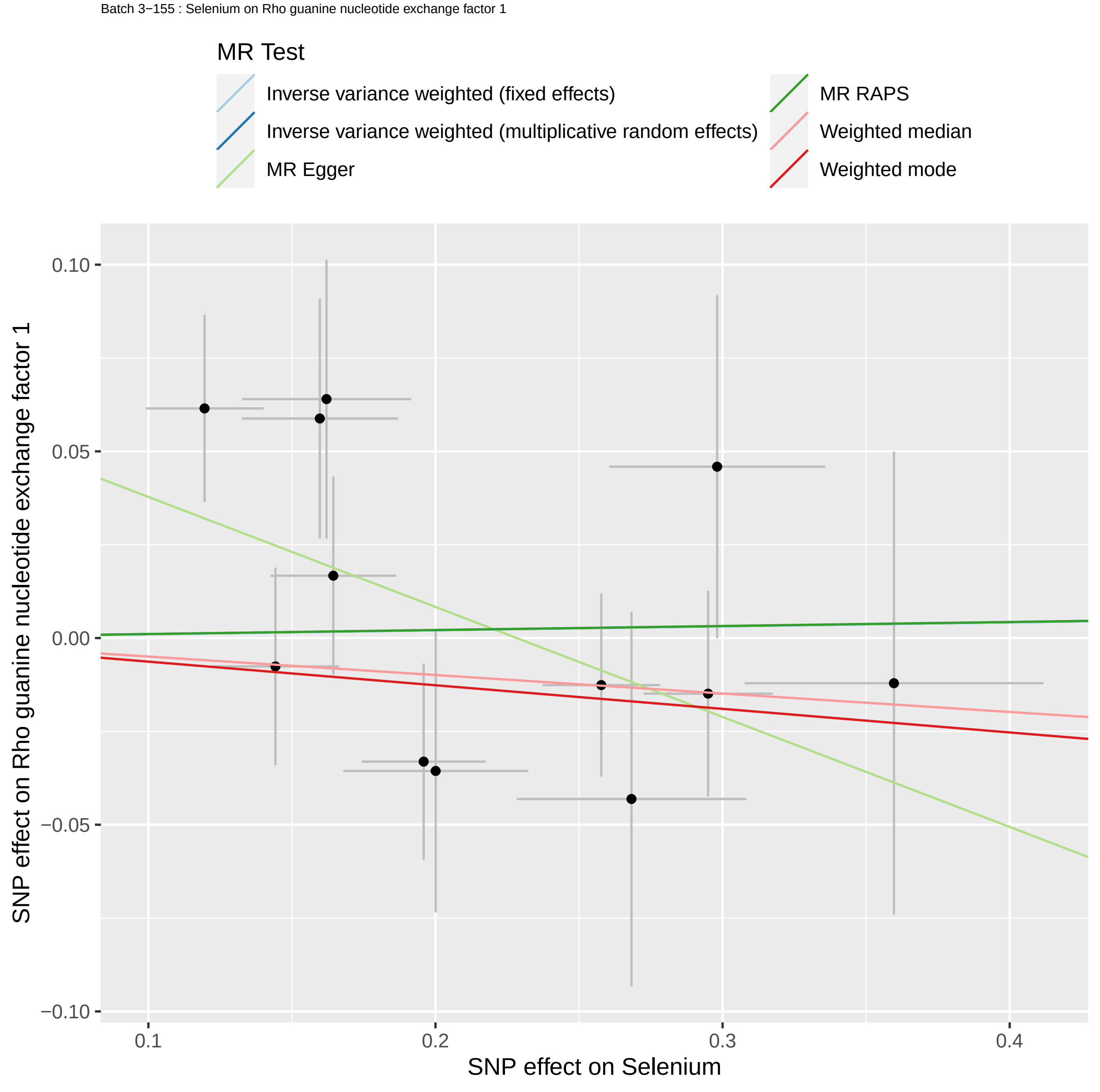

Batch 3−155 : Selenium on Rho guanine nucleotide exchange factor 1
MR Test
Inverse variance weighted (fixed effects)
Inverse variance weighted (multiplicative random effects)
MR Egger
MR RAPS
Weighted median
Weighted mode
0.10
0.05
0.00
−0.05
−0.10
0.1
0.2
0.3
0.4
SNP effect on Selenium
SNP effect on Rho guanine nucleotide exchange factor 1

Batch 3−156 : Selenium on Rho guanine nucleotide exchange factor 2

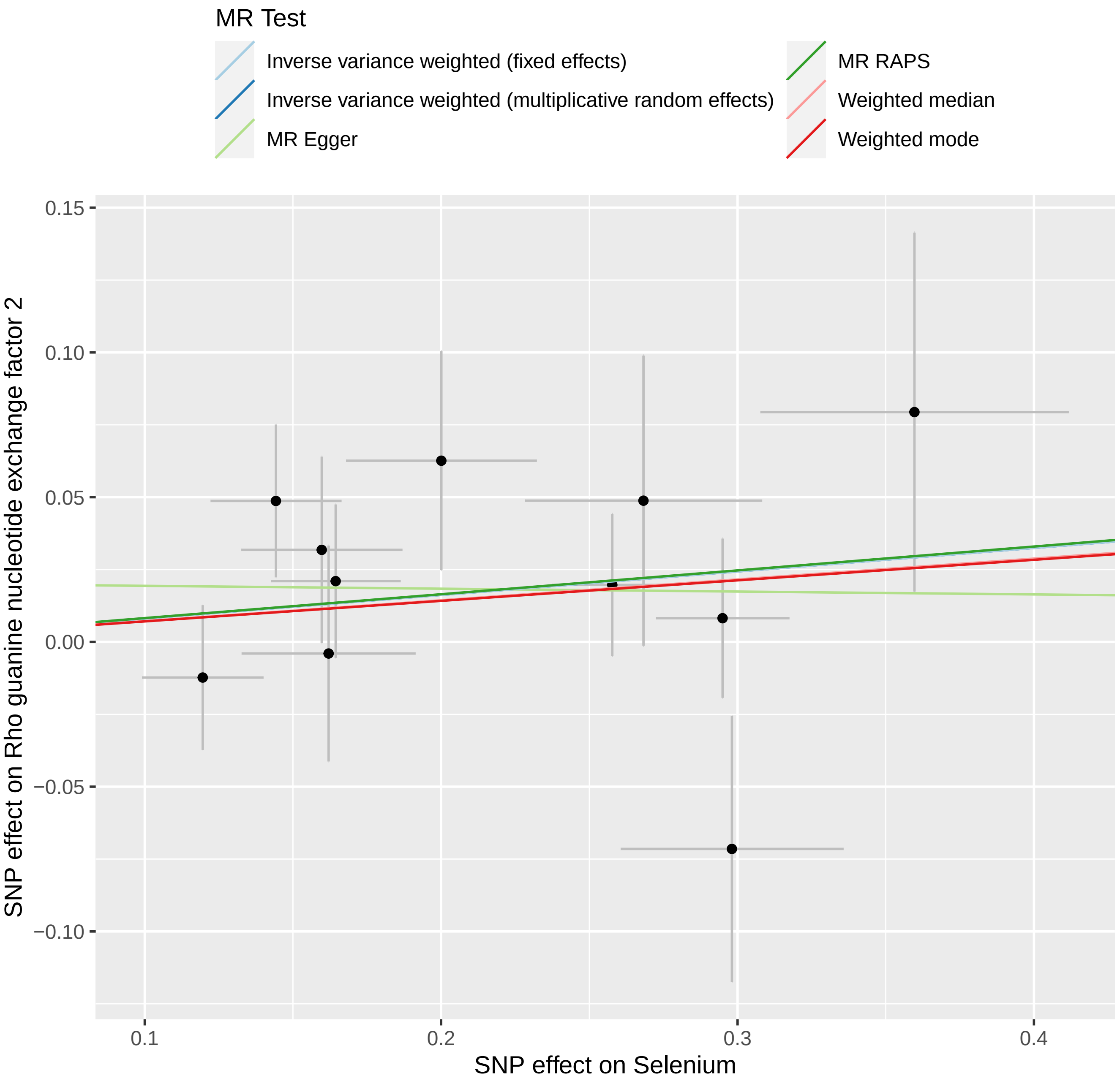

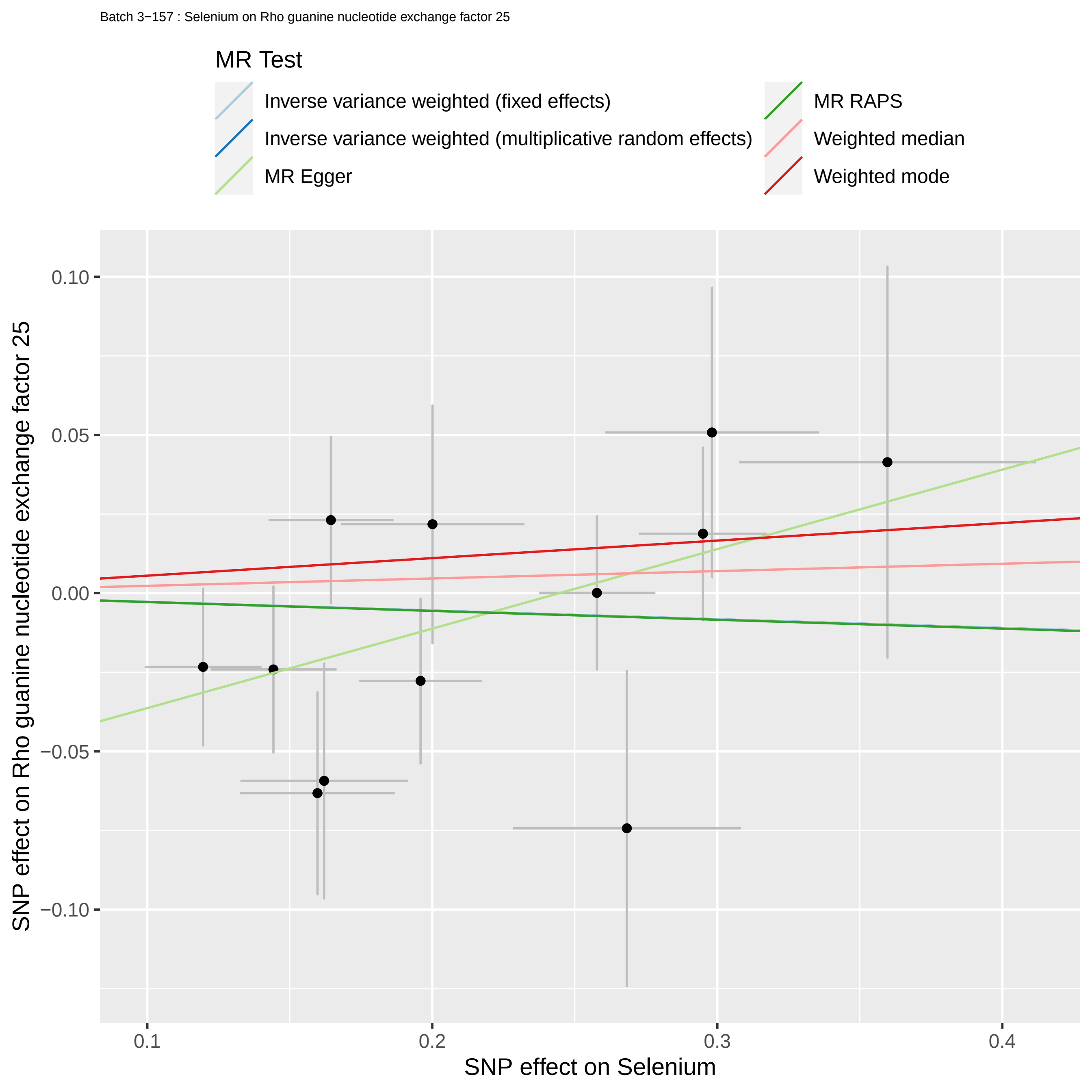

Batch 3−157 : Selenium on Rho guanine nucleotide exchange factor 25
MR Test
Inverse variance weighted (fixed effects)
Inverse variance weighted (multiplicative random effects)
MR Egger
MR RAPS
Weighted median
Weighted mode
0.10
0.05
0.00
−0.05
−0.10
0.1
0.2
0.3
0.4
SNP effect on Rho guanine nucleotide exchange factor 25
SNP effect on Selenium

Batch 3−158 : Selenium on Rho guanine nucleotide exchange factor 7

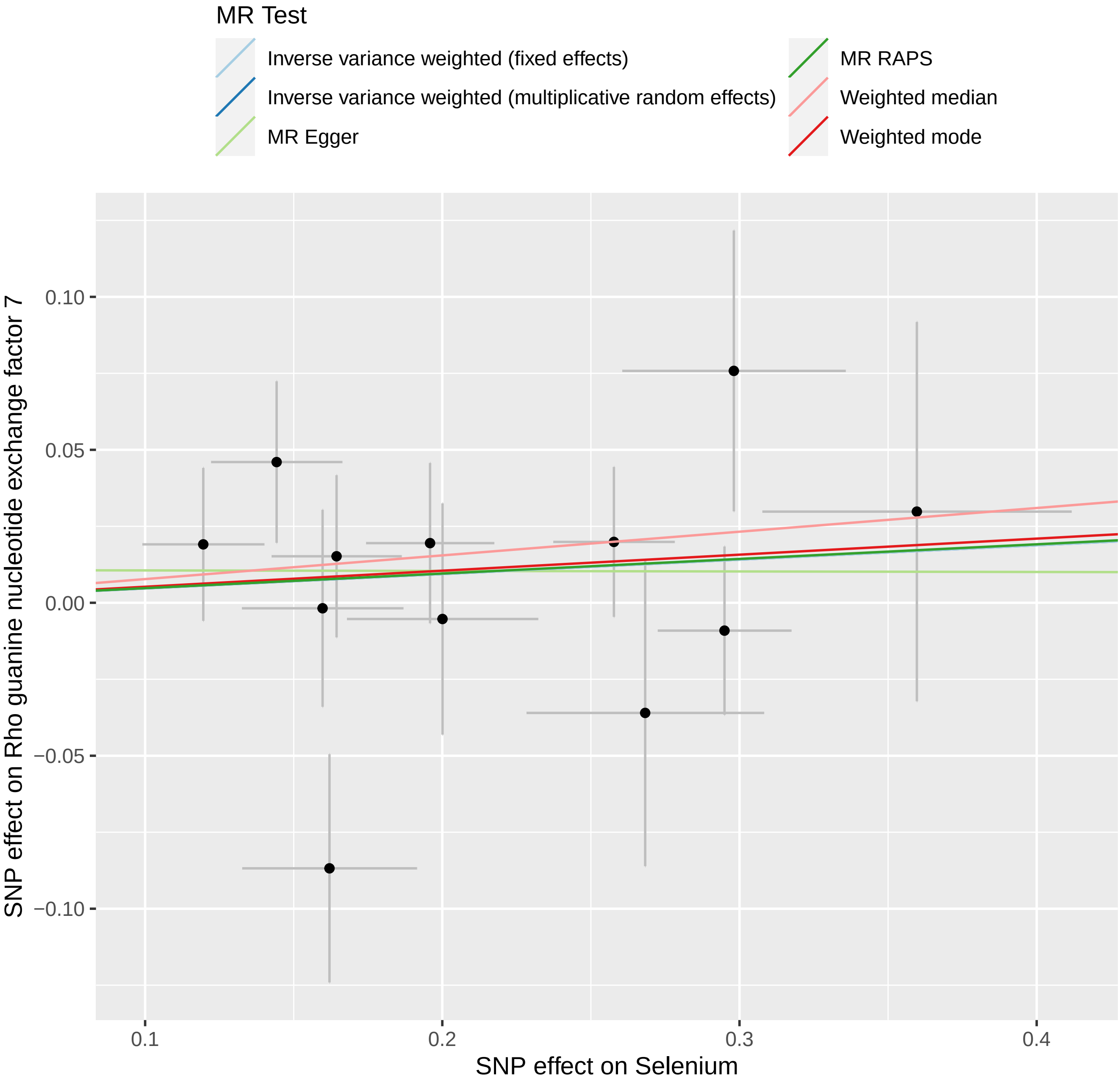

Batch 3−159 : Selenium on AT−rich interactive domain−containing protein 3A

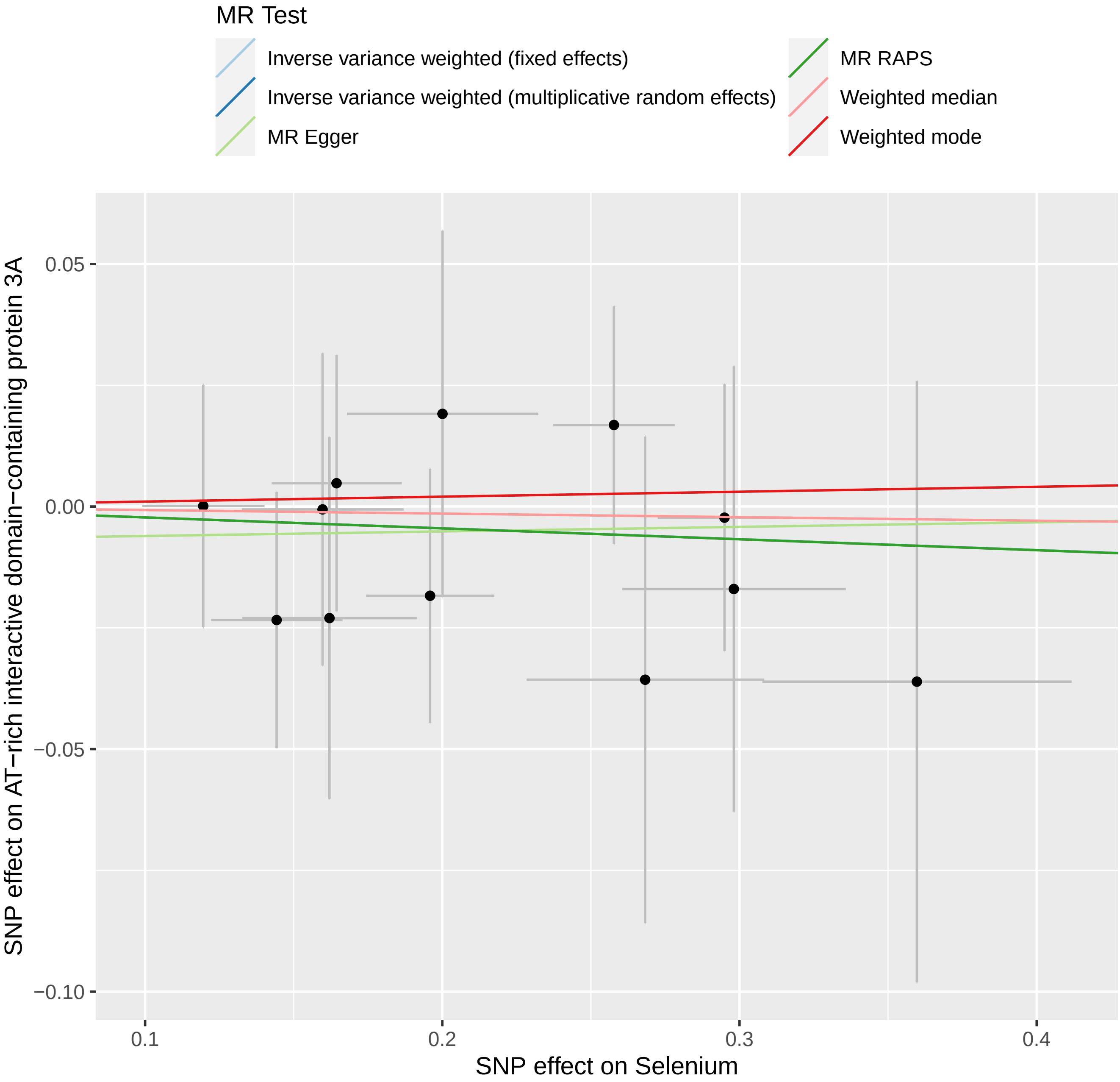

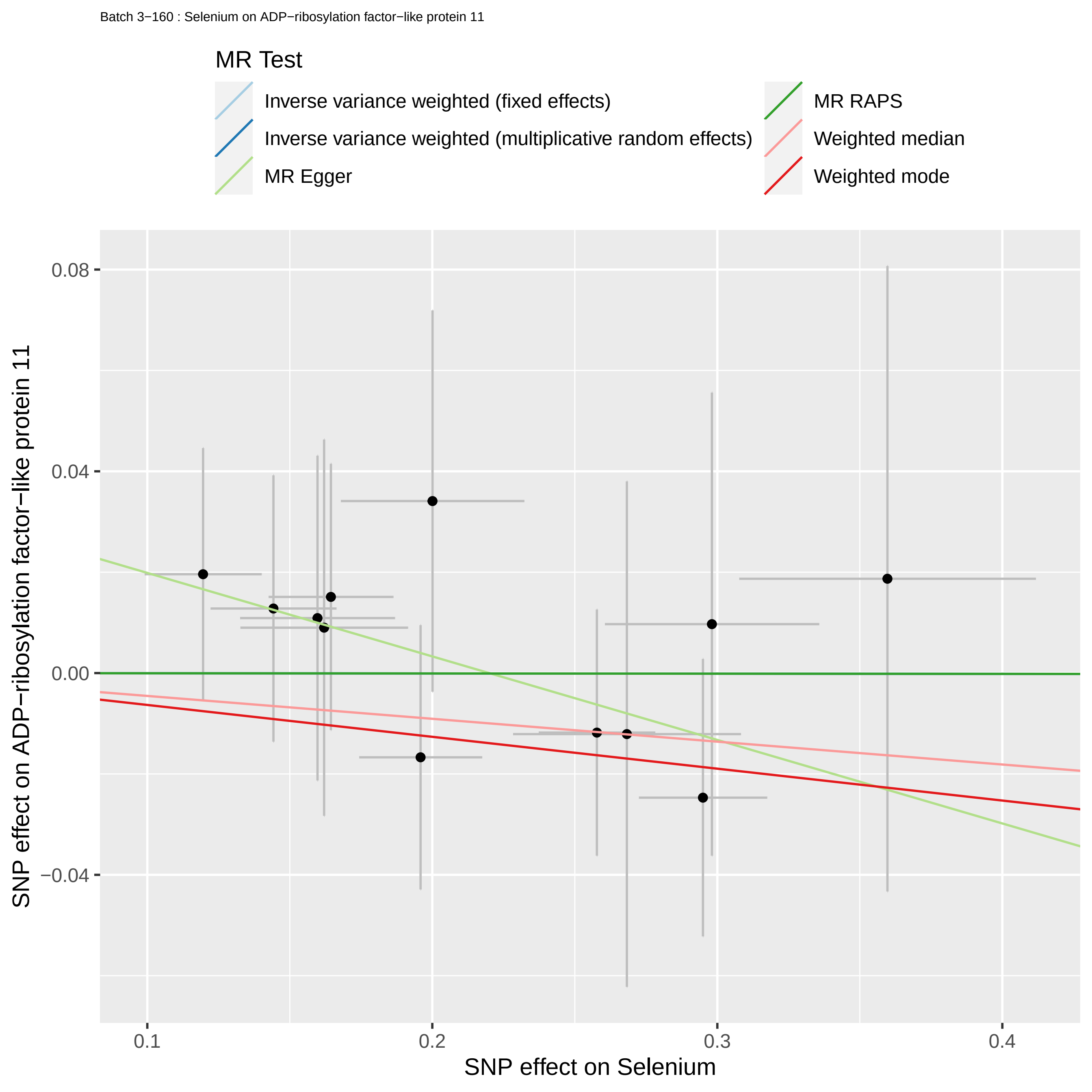

Batch 3−160 : Selenium on ADP−ribosylation factor−like protein 11
MR Test
Inverse variance weighted (fixed effects)
Inverse variance weighted (multiplicative random effects)
MR Egger
MR RAPS
Weighted median
Weighted mode
0.08
0.04
0.00
−0.04
0.1
0.2
0.3
0.4
SNP effect on Selenium
SNP effect on ADP−ribosylation factor−like protein 11

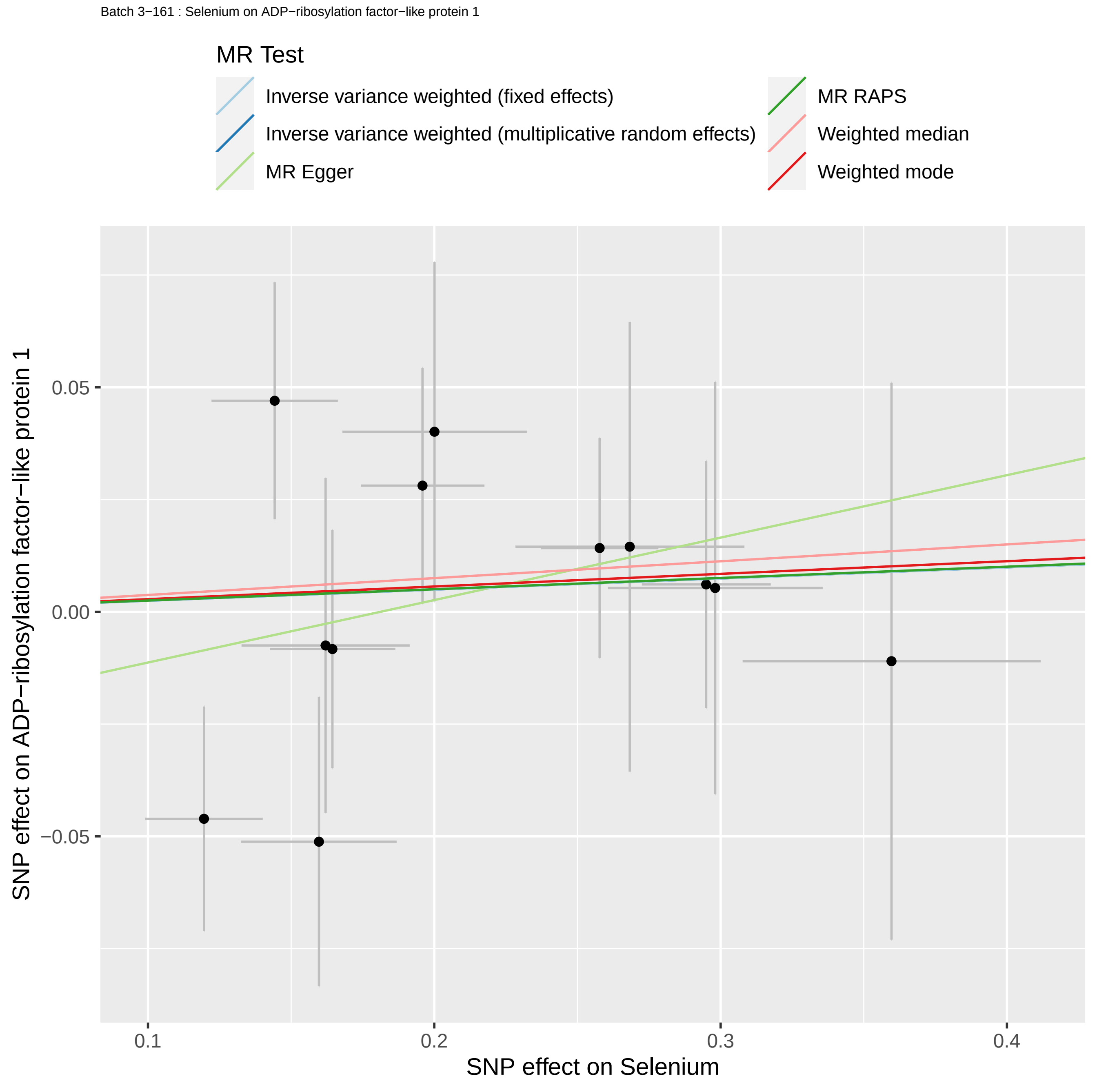

Batch 3−161 : Selenium on ADP−ribosylation factor−like protein 1
MR Test
Inverse variance weighted (fixed effects)
Inverse variance weighted (multiplicative random effects)
MR Egger
MR RAPS
Weighted median
Weighted mode
0.05
0.00
−0.05
0.1
0.2
0.3
0.4
SNP effect on Selenium
SNP effect on ADP−ribosylation factor−like protein 1

Batch 3−162 : Selenium on ADP−ribosylation factor−like protein 3

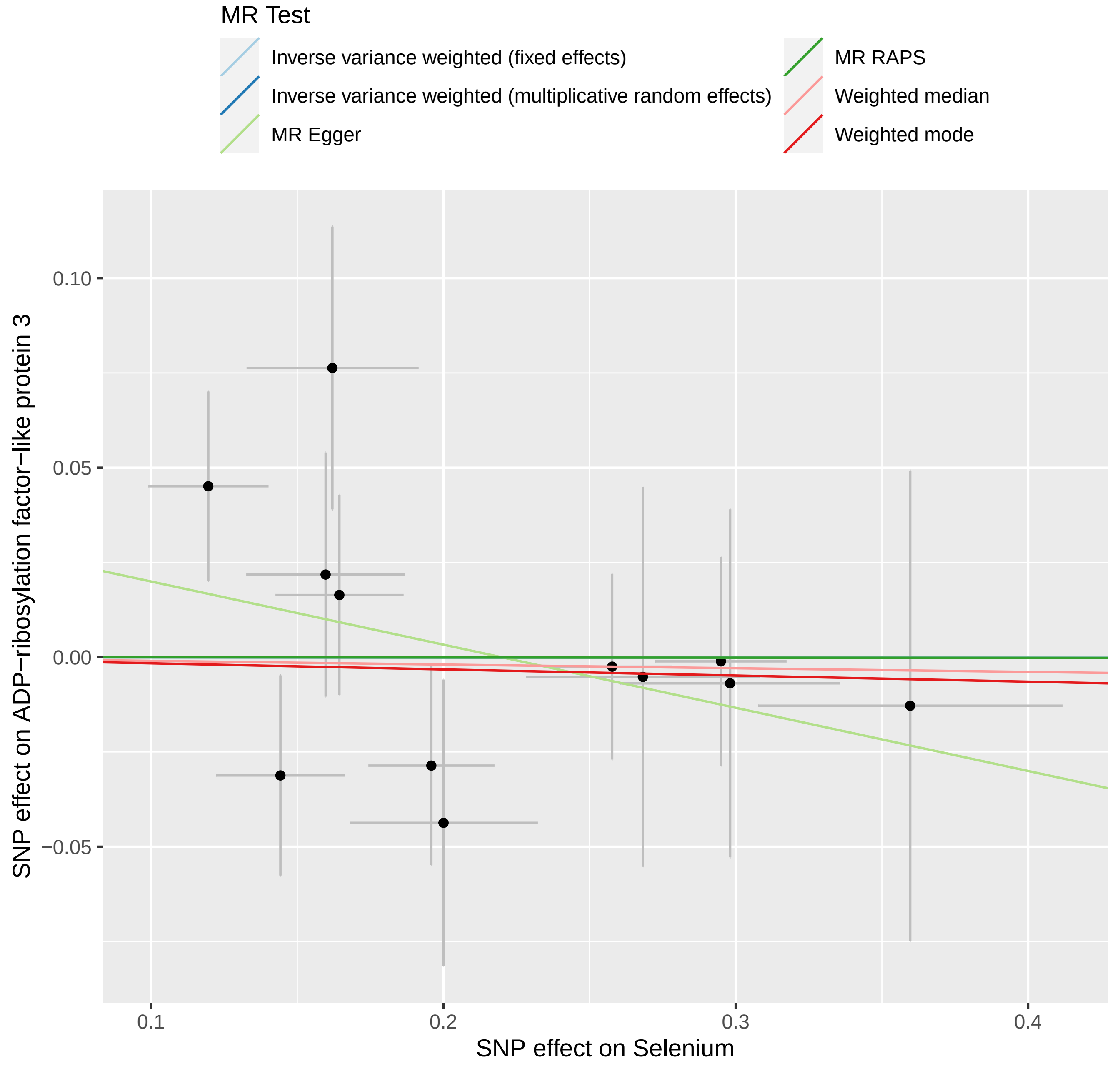

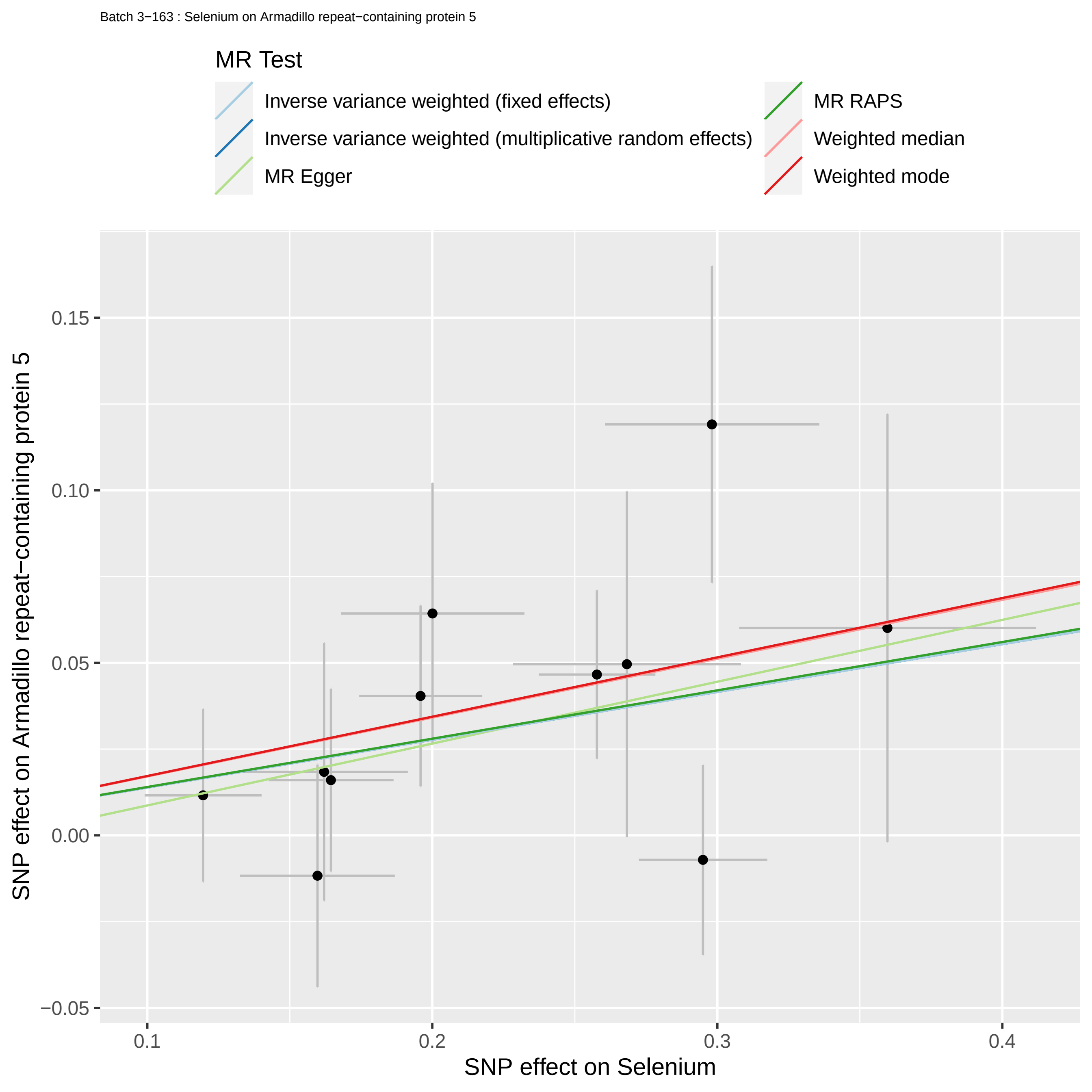
Batch 3−163 : Selenium on Armadillo repeat−containing protein 5
MR Test
Inverse variance weighted (fixed effects)
Inverse variance weighted (multiplicative random effects)
MR Egger
MR RAPS
Weighted median
Weighted mode
SNP effect on Armadillo repeat−containing protein 5
0.15
0.10
0.05
0.00
−0.05
0.1
0.2
0.3
0.4
SNP effect on Selenium

Batch 3−164 : Selenium on Armadillo repeat−containing protein 5

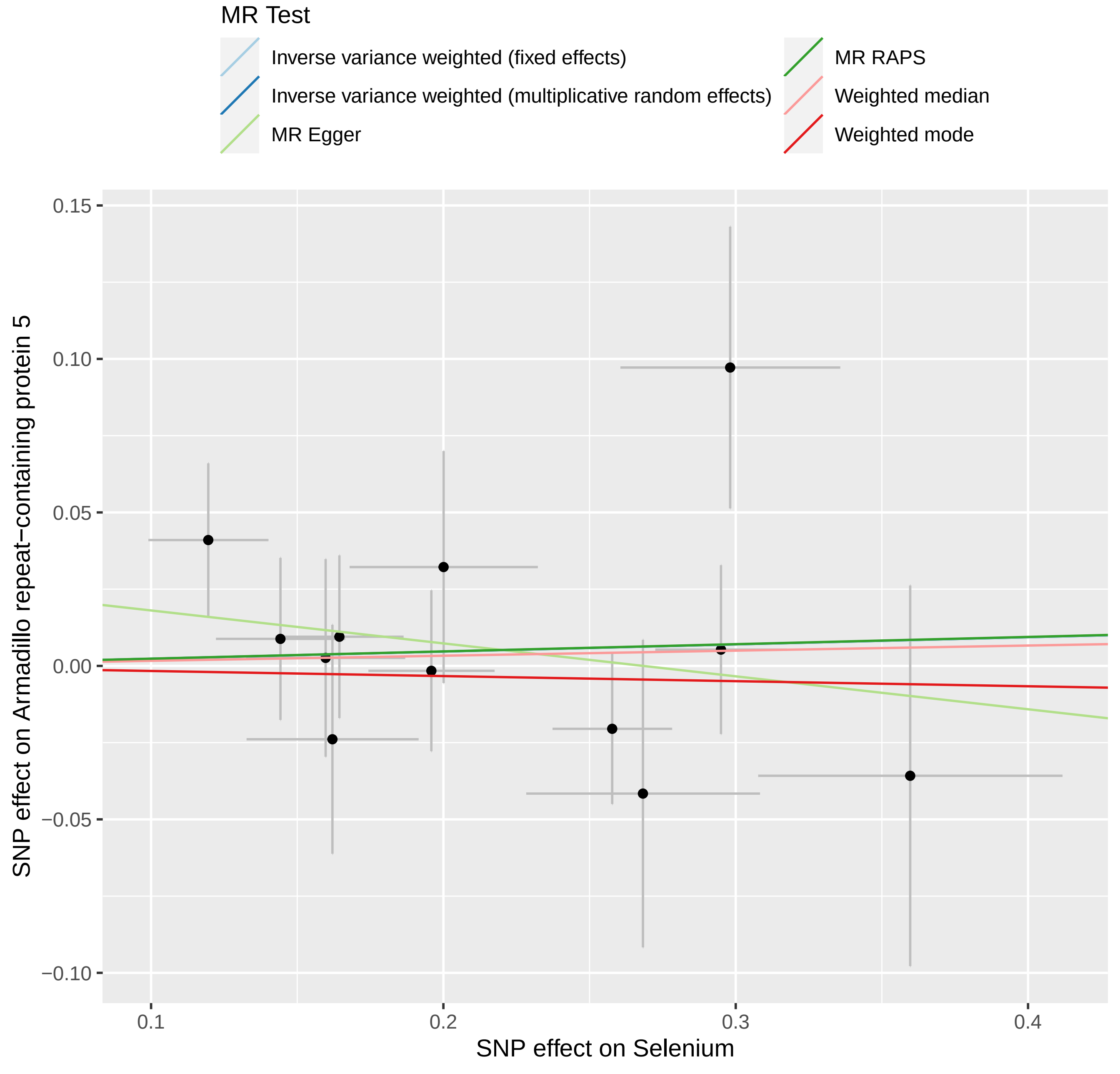

Batch 3−165 : Selenium on Armadillo repeat−containing protein 5

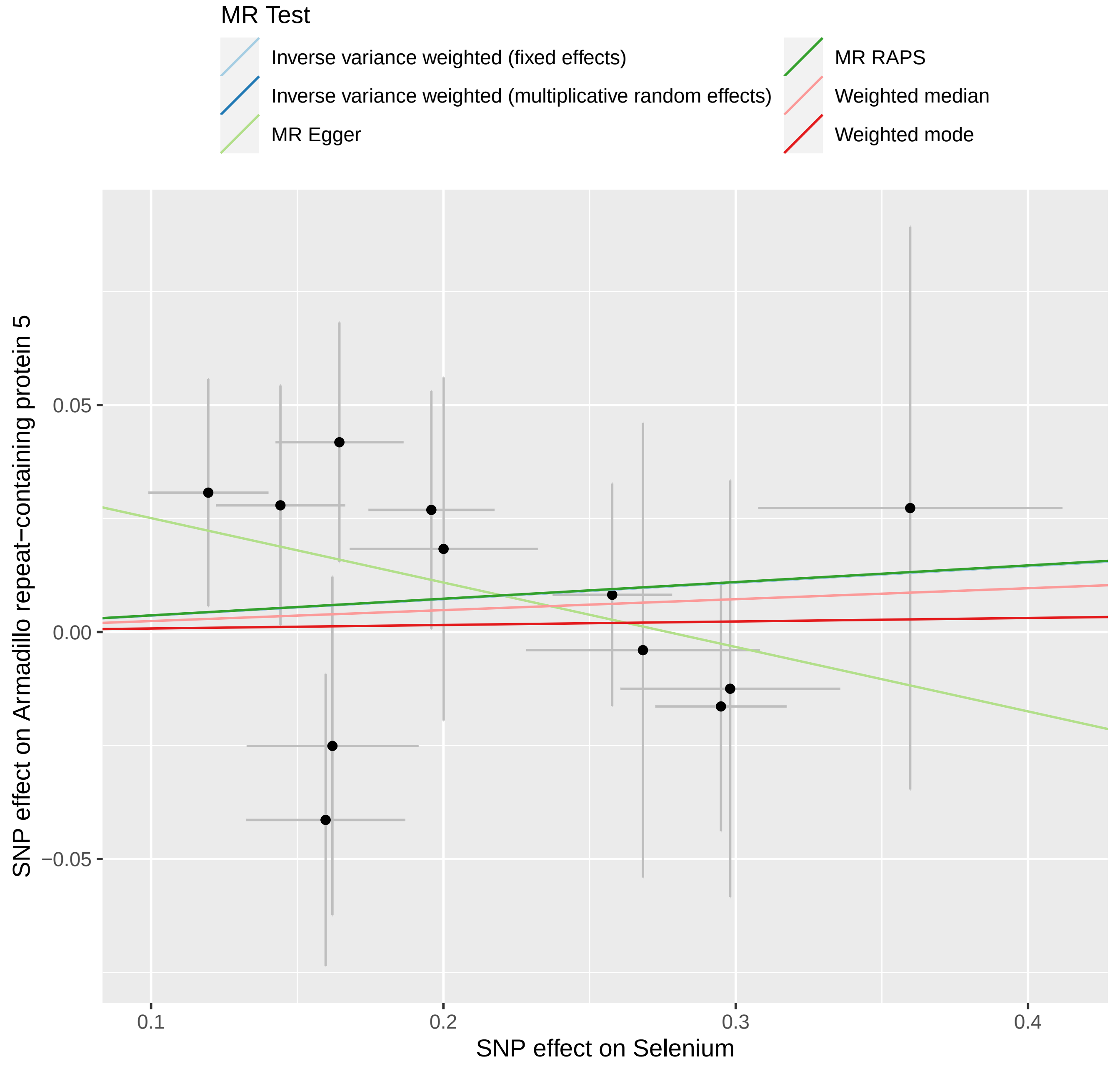

Batch 3−166 : Selenium on Actin−related protein 2/3 complex subunit 3

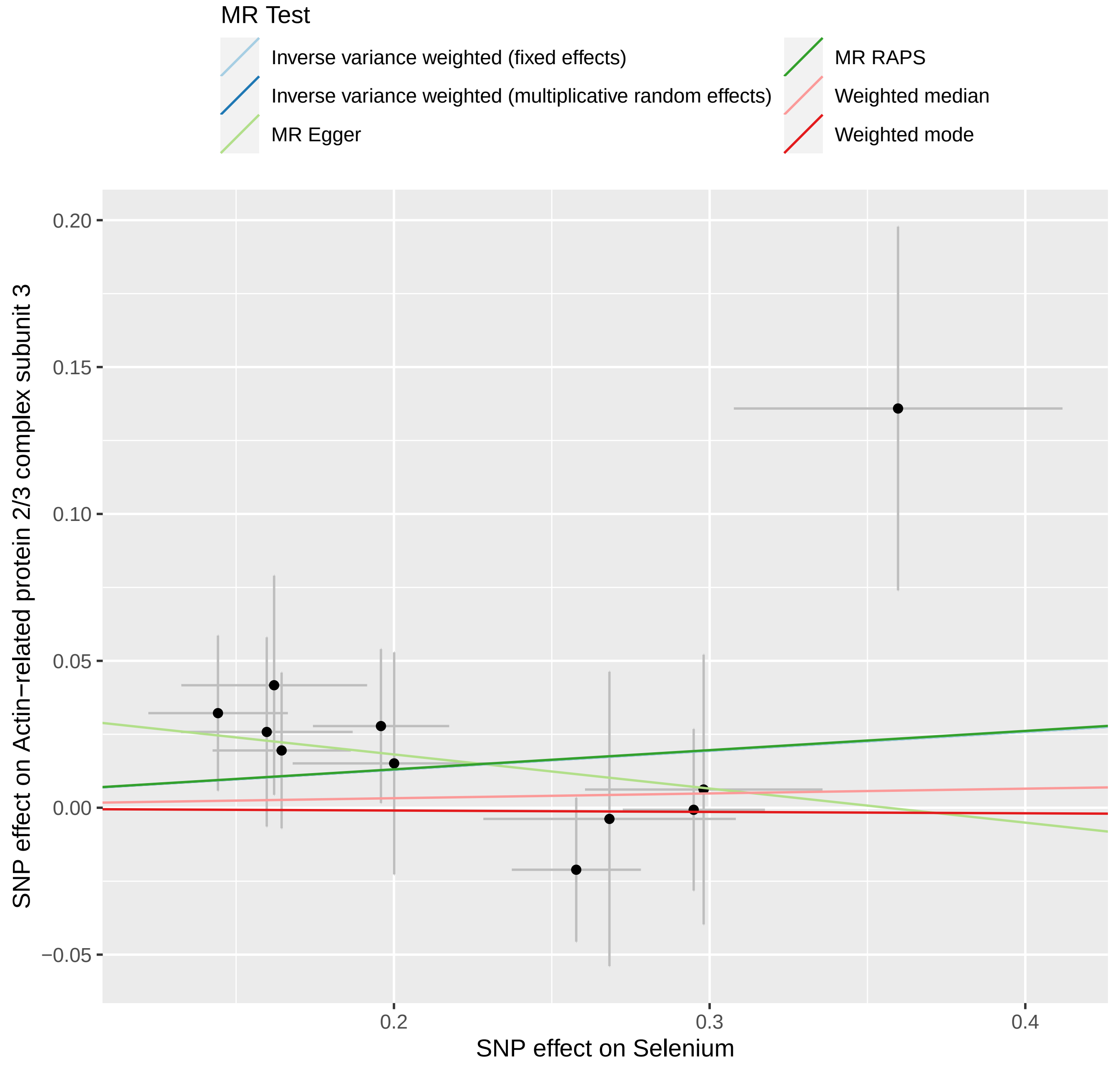

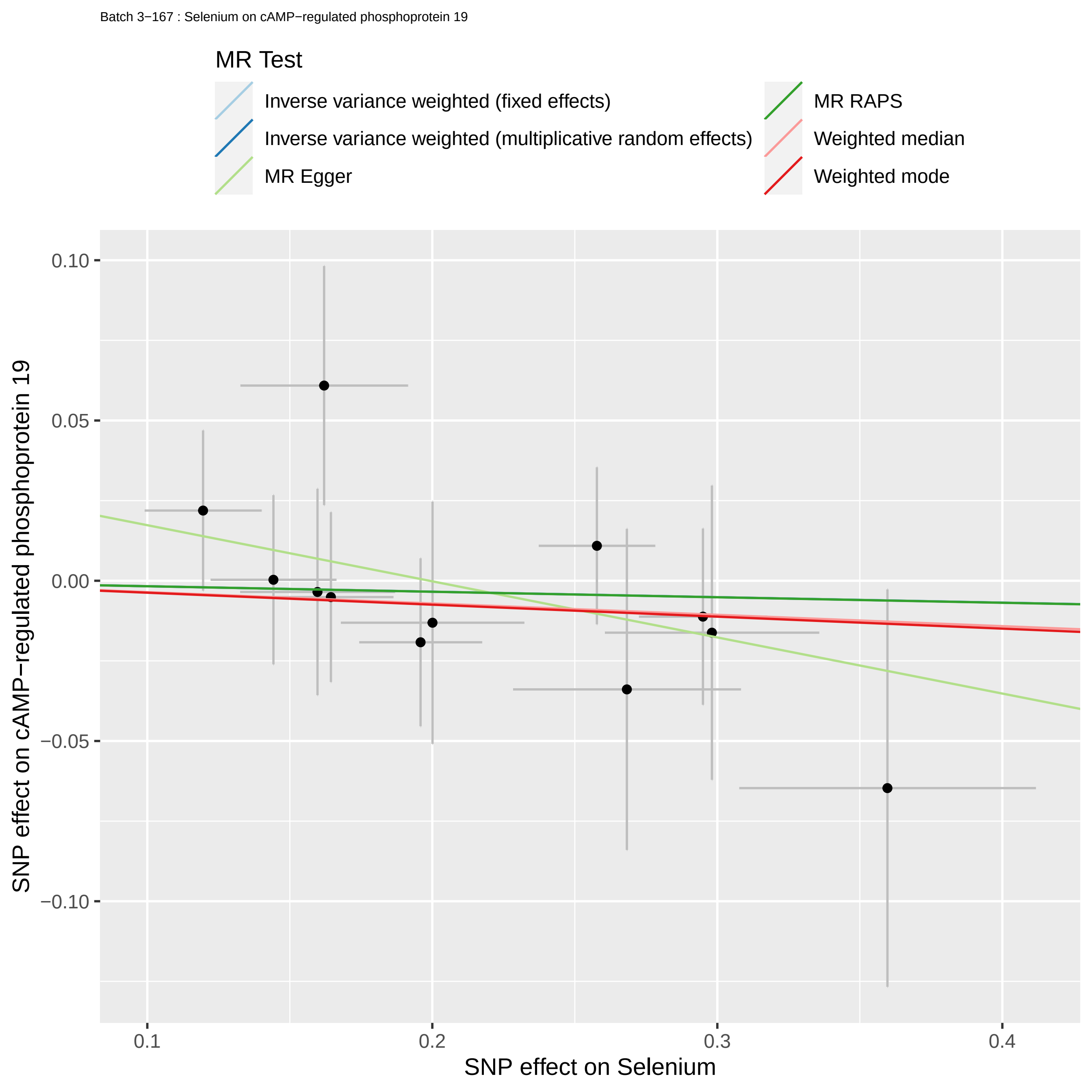
Batch 3−167 : Selenium on cAMP−regulated phosphoprotein 19
MR Test
Inverse variance weighted (fixed effects)
Inverse variance weighted (multiplicative random effects)
MR Egger
MR RAPS
Weighted median
Weighted mode
0.10
0.05
0.00
−0.05
−0.10
SNP effect on cAMP−regulated phosphoprotein 19
0.1
0.2
0.3
0.4
SNP effect on Selenium

Batch 3−168 : Selenium on cAMP−regulated phosphoprotein 21

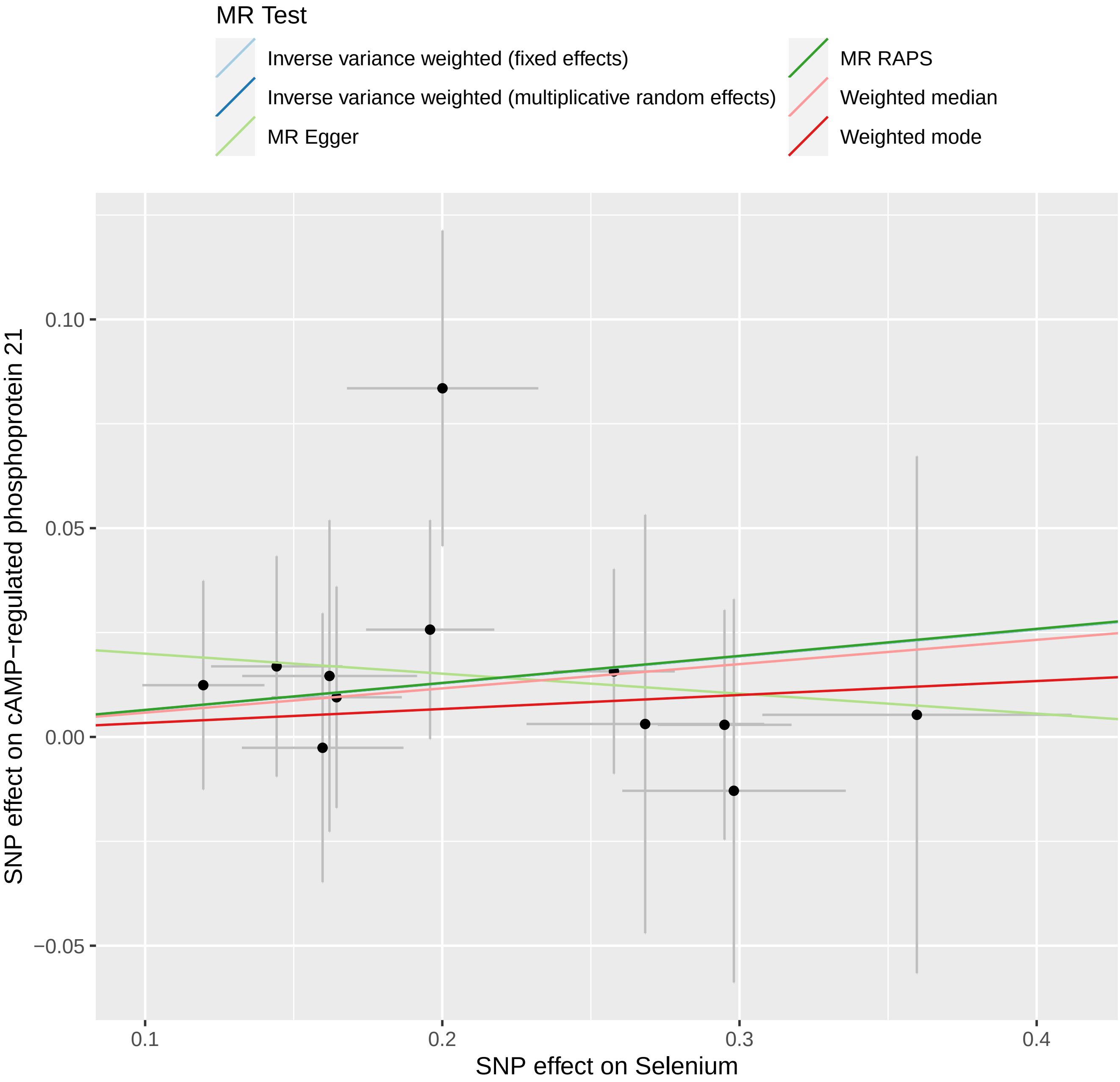

Batch 3−169 : Selenium on Beta−arrestin−1

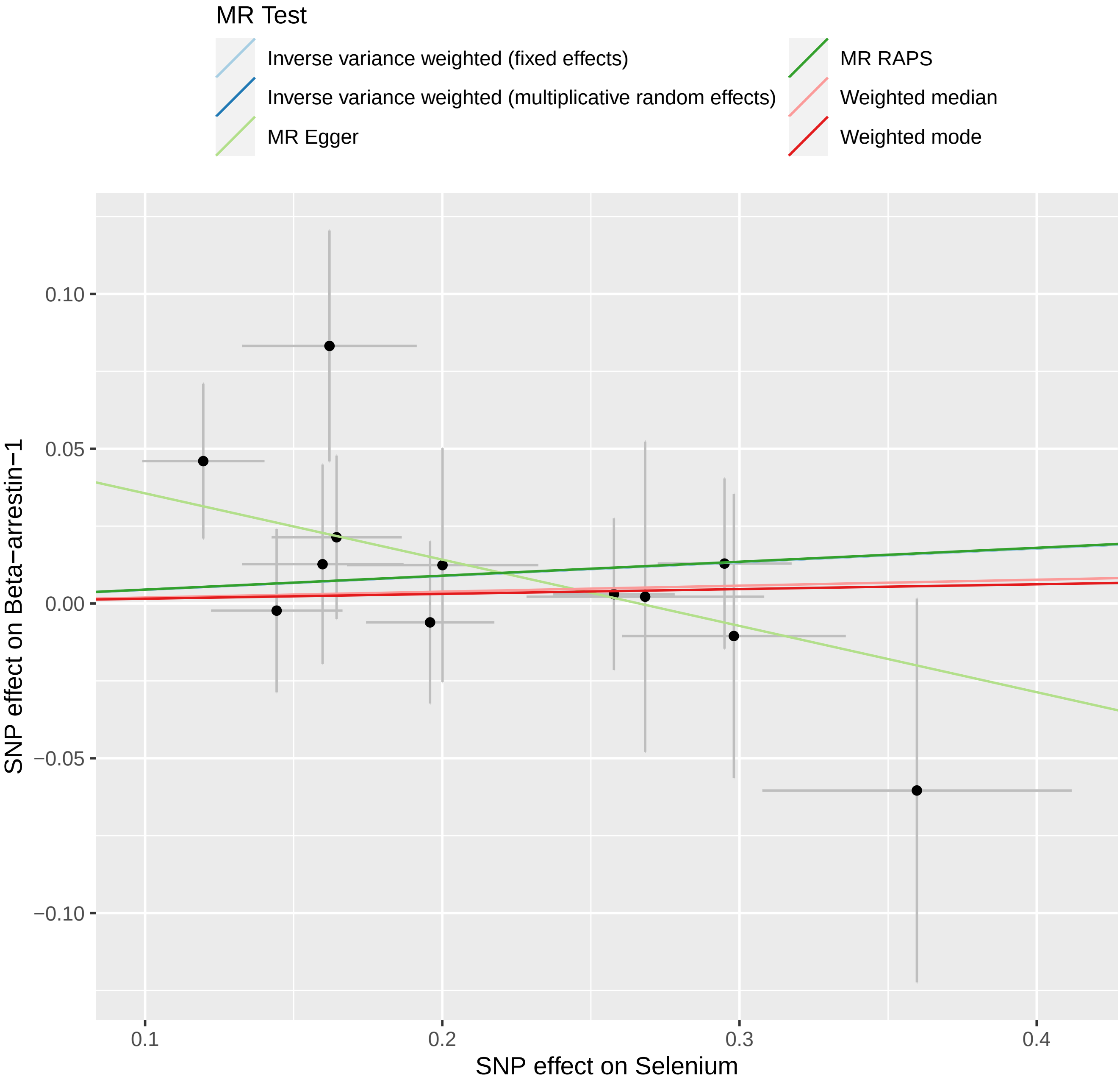

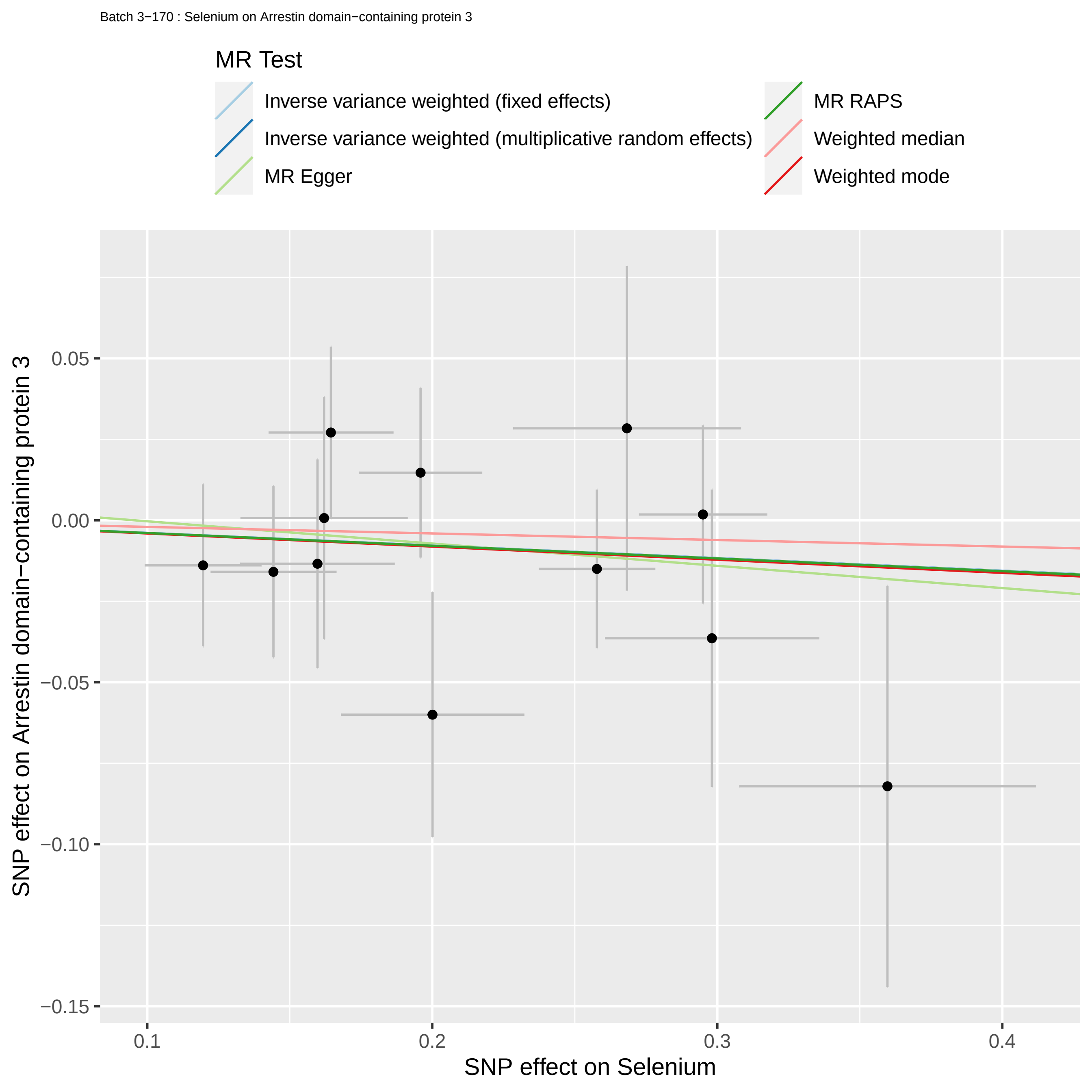

Batch 3−170 : Selenium on Arrestin domain−containing protein 3
MR Test
Inverse variance weighted (fixed effects)
Inverse variance weighted (multiplicative random effects)
MR Egger
MR RAPS
Weighted median
Weighted mode
0.05
0.00
−0.05
−0.10
−0.15
0.1
0.2
0.3
0.4
SNP effect on Arrestin domain−containing protein 3
SNP effect on Selenium

Batch 3−171 : Selenium on Arylsulfatase A

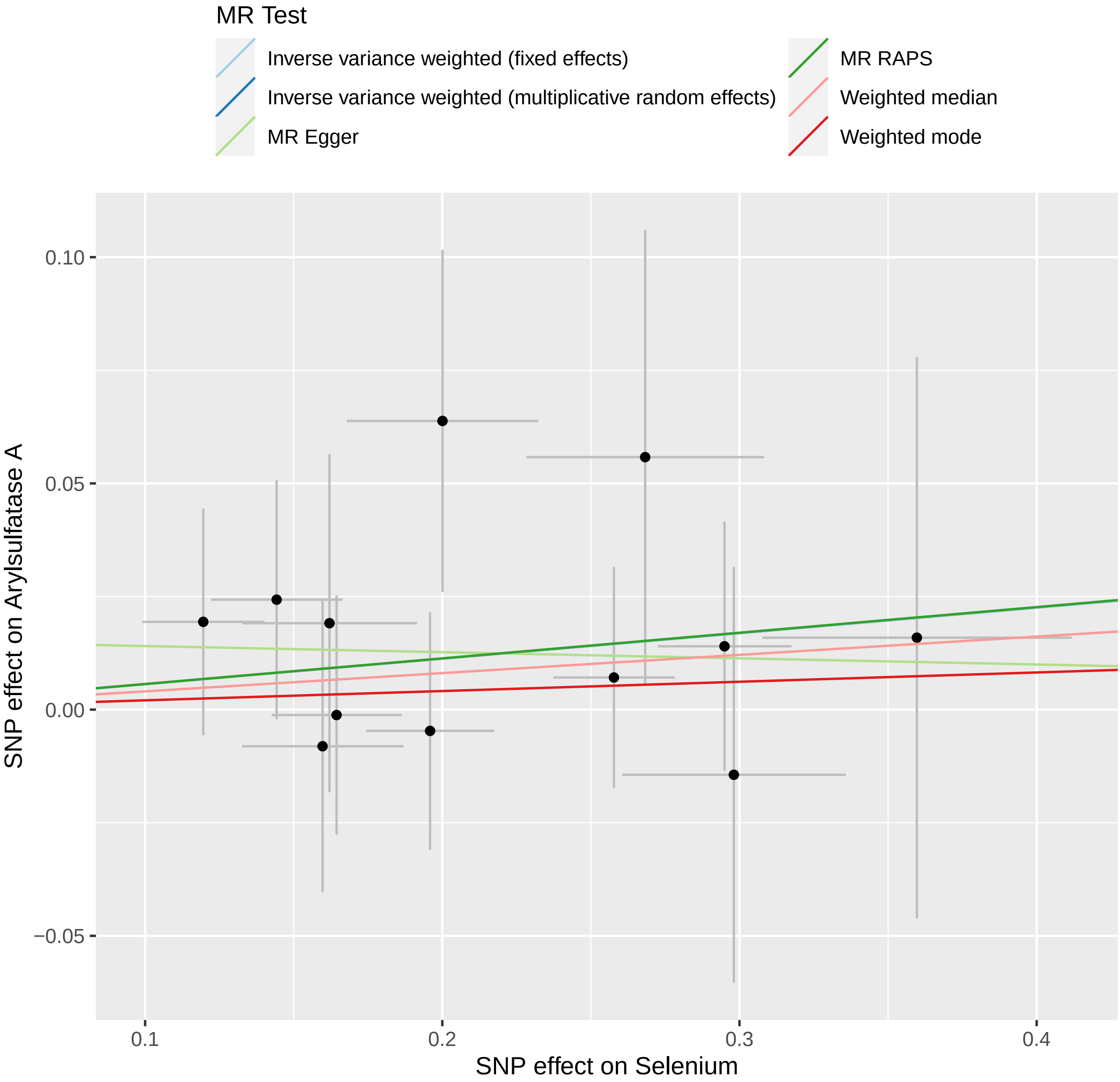

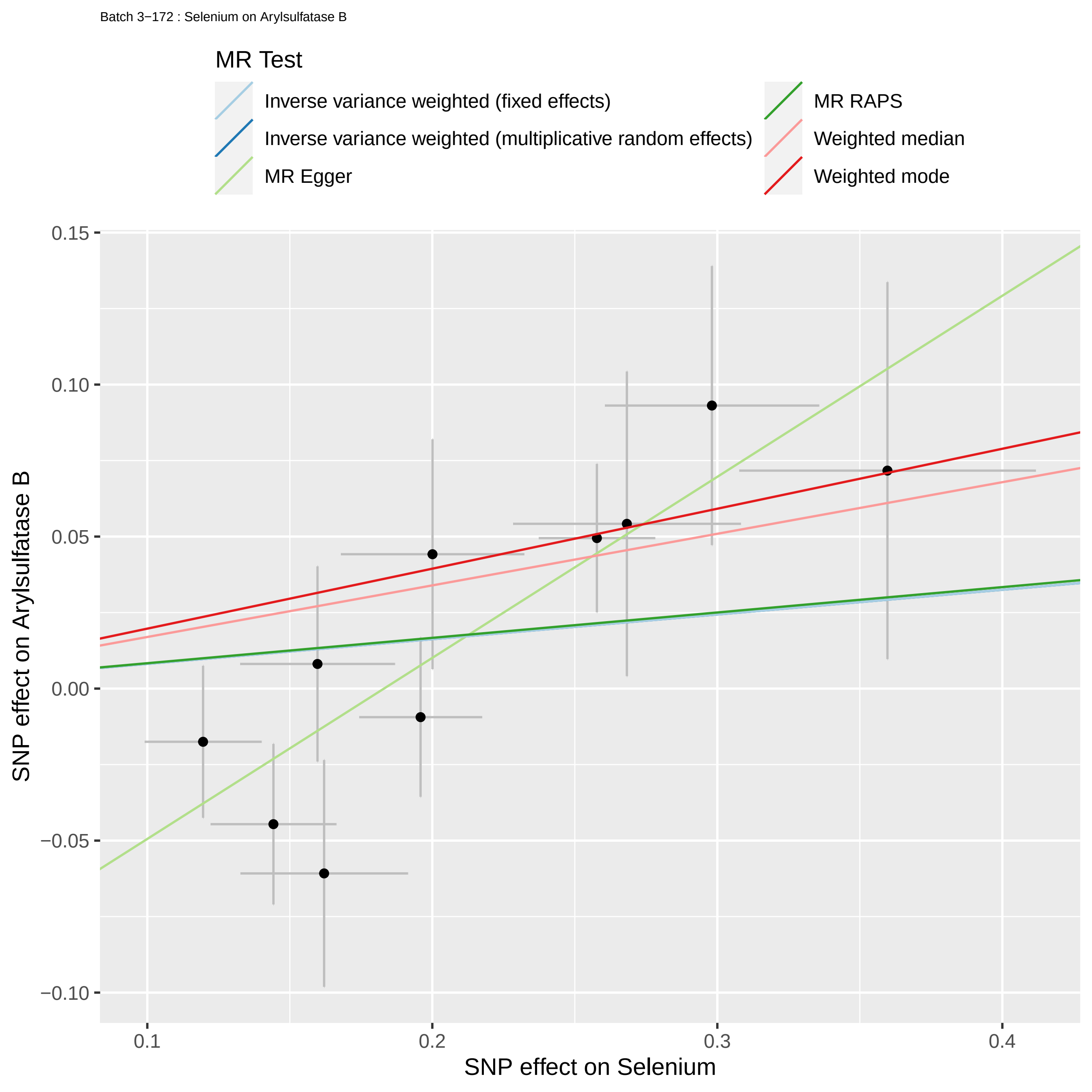
Batch 3−172 : Selenium on Arylsulfatase B
MR Test
Inverse variance weighted (fixed effects)
Inverse variance weighted (multiplicative random effects)
MR Egger
MR RAPS
Weighted median
Weighted mode
0.15
0.10
0.05
0.00
−0.05
−0.10
0.1
0.2
0.3
0.4
SNP effect on Arylsulfatase B
SNP effect on Selenium

Batch 3−173 : Selenium on Arylsulfatase K

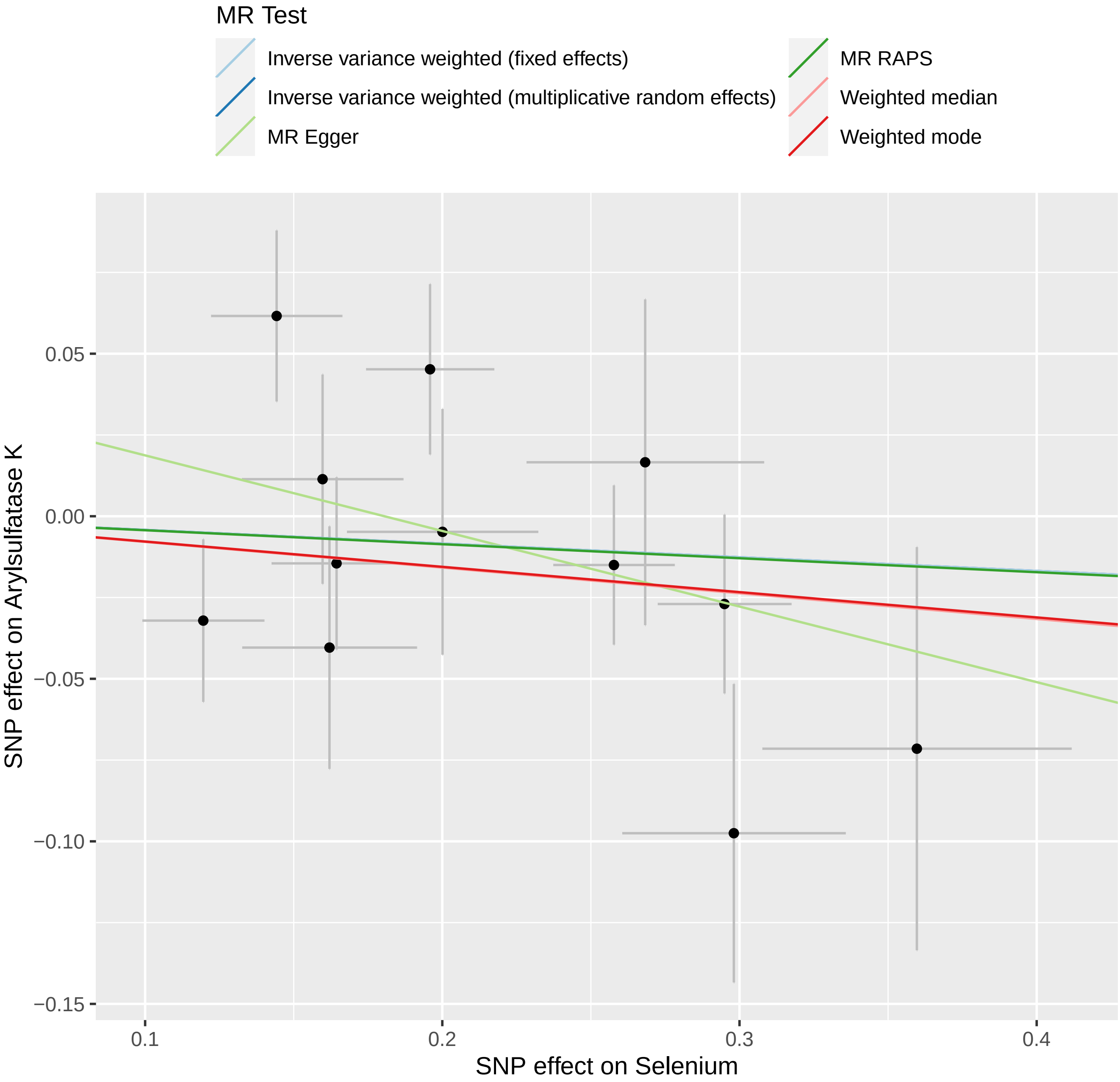

Batch 3−174 : Selenium on Ecto−ADP−ribosyltransferase 3

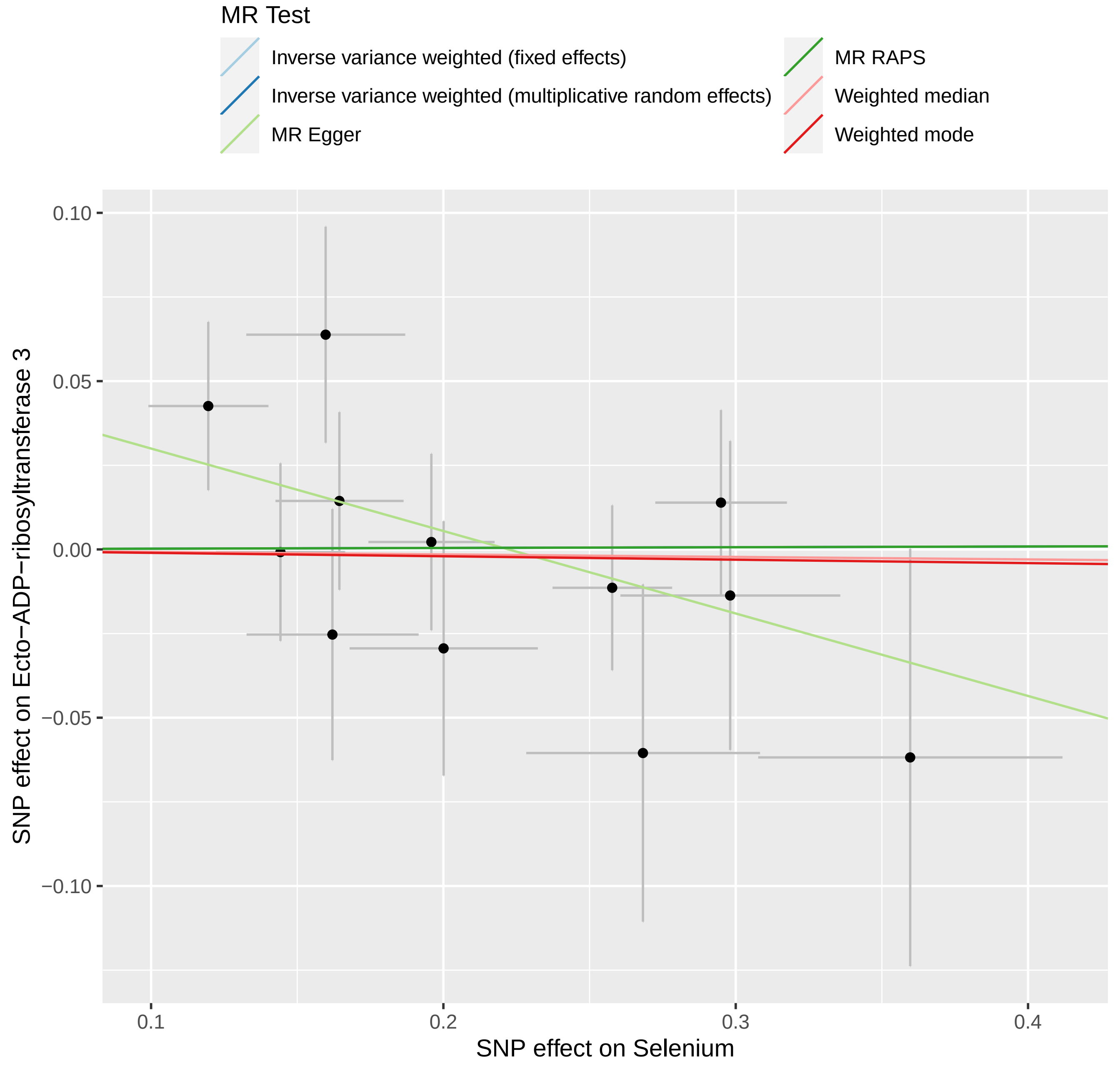

Batch 3−175 : Selenium on Ecto−ADP−ribosyltransferase 3

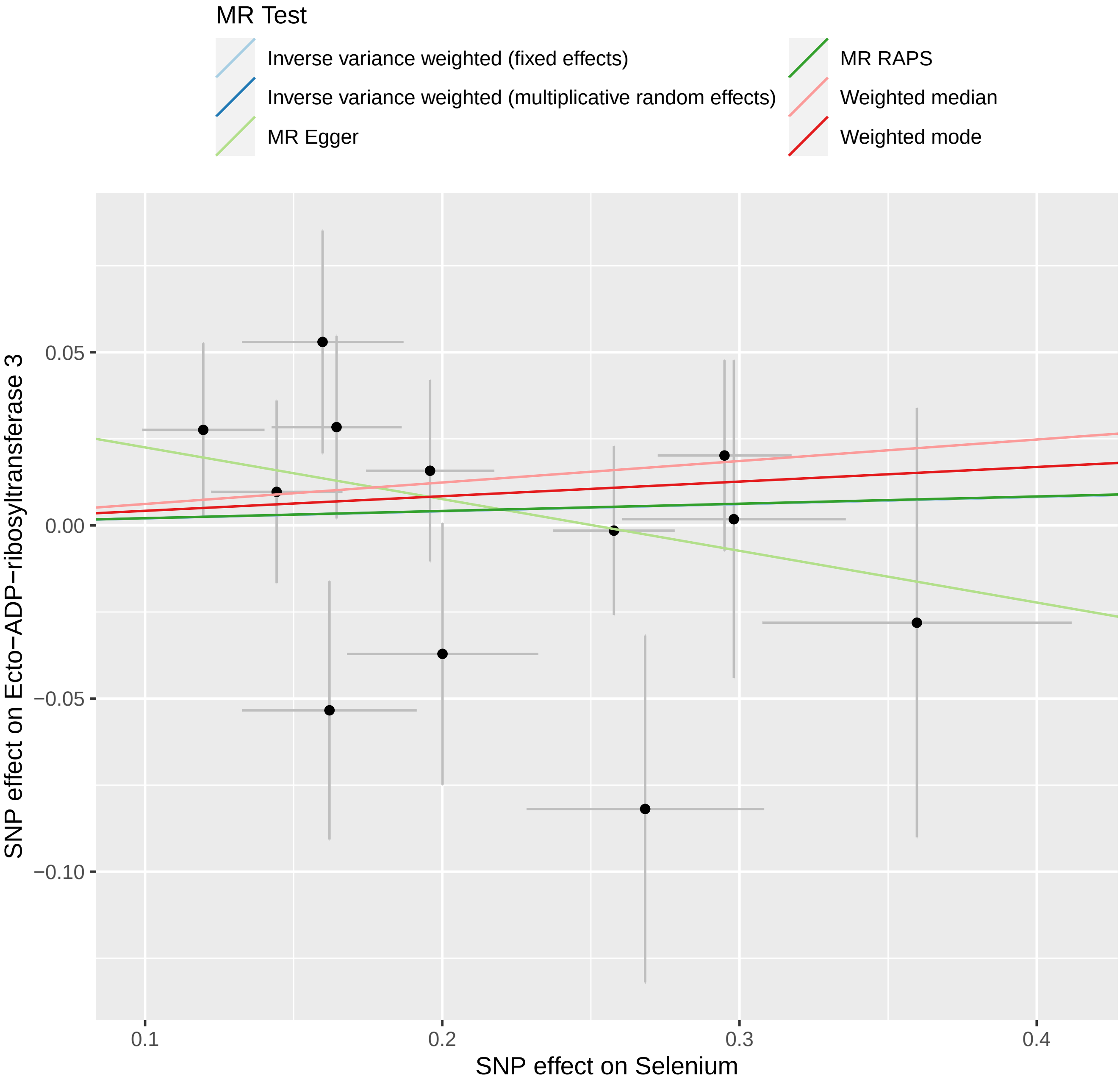

Batch 3−176 : Selenium on Ecto−ADP−ribosyltransferase 4

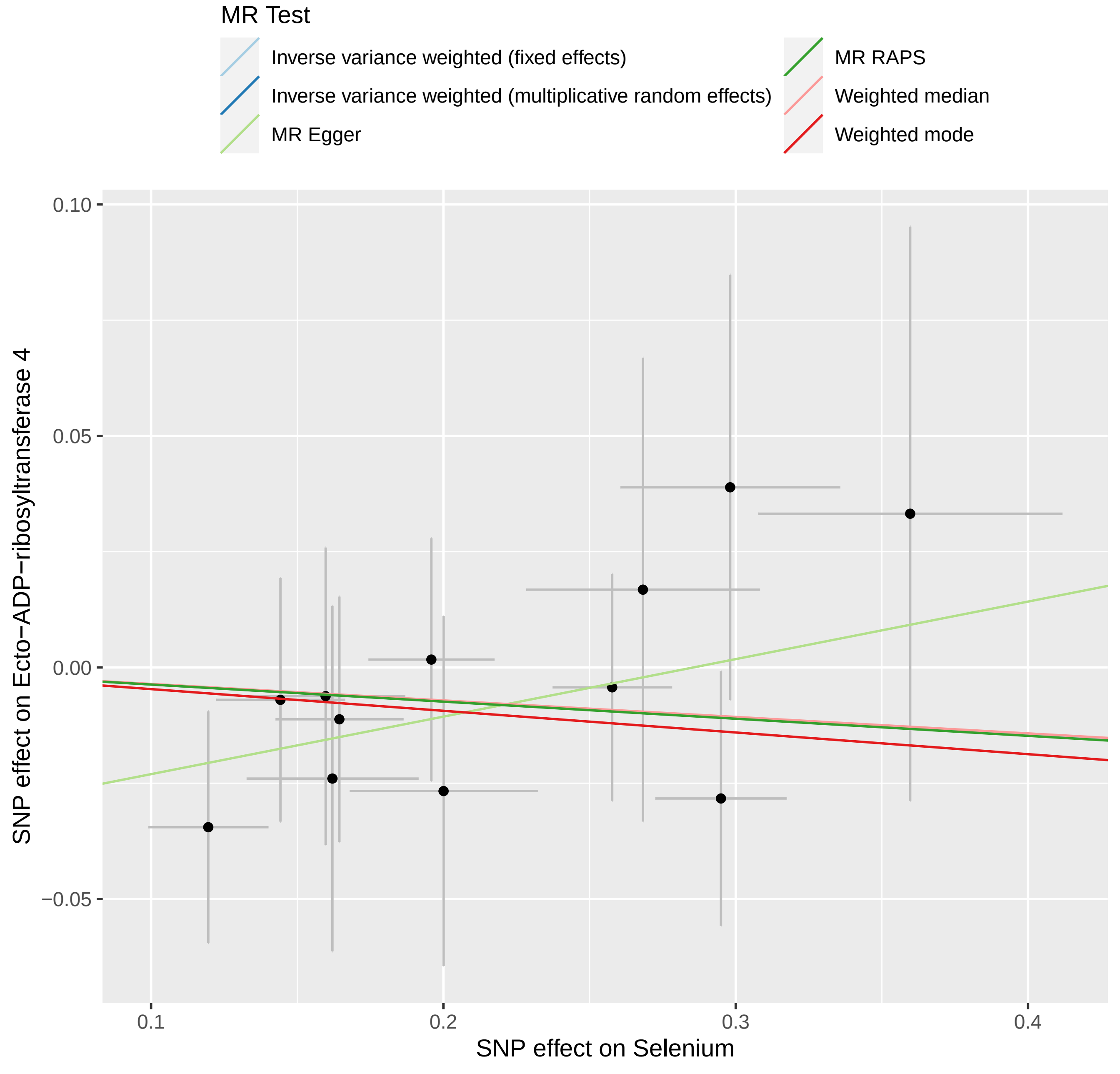

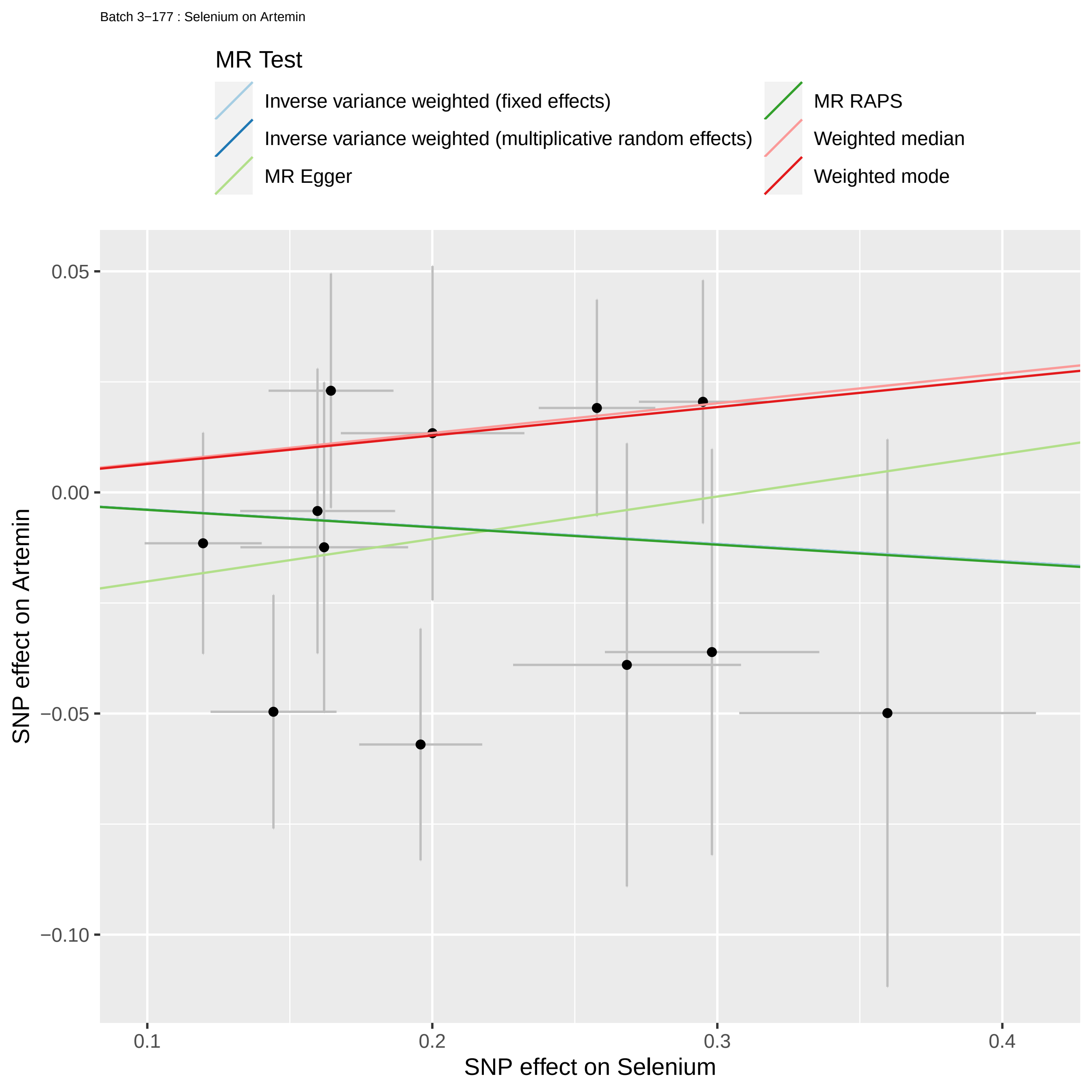

Batch 3−177 : Selenium on Artemin
MR Test
Inverse variance weighted (fixed effects)
Inverse variance weighted (multiplicative random effects)
MR Egger
MR RAPS
Weighted median
Weighted mode
0.05
0.00
−0.05
−0.10
0.1
0.2
0.3
0.4
SNP effect on Artemin
SNP effect on Selenium

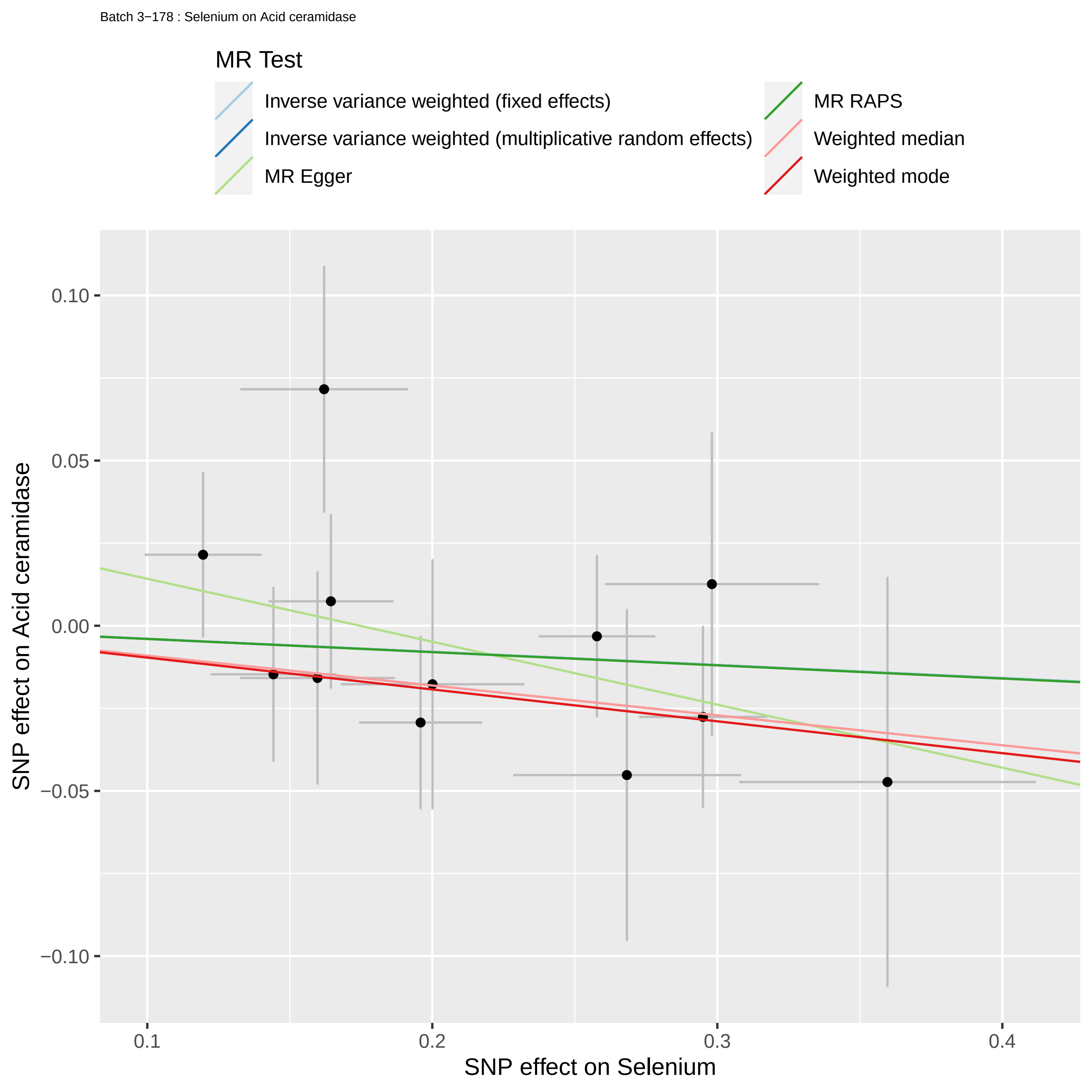
Batch 3−178 : Selenium on Acid ceramidase
MR Test
Inverse variance weighted (fixed effects)
Inverse variance weighted (multiplicative random effects)
MR Egger
MR RAPS
Weighted median
Weighted mode
0.10
0.05
0.00
−0.05
−0.10
0.1
0.2
0.3
0.4
SNP effect on Selenium
SNP effect on Acid ceramidase

Batch 3−179 : Selenium on Neutral ceramidase

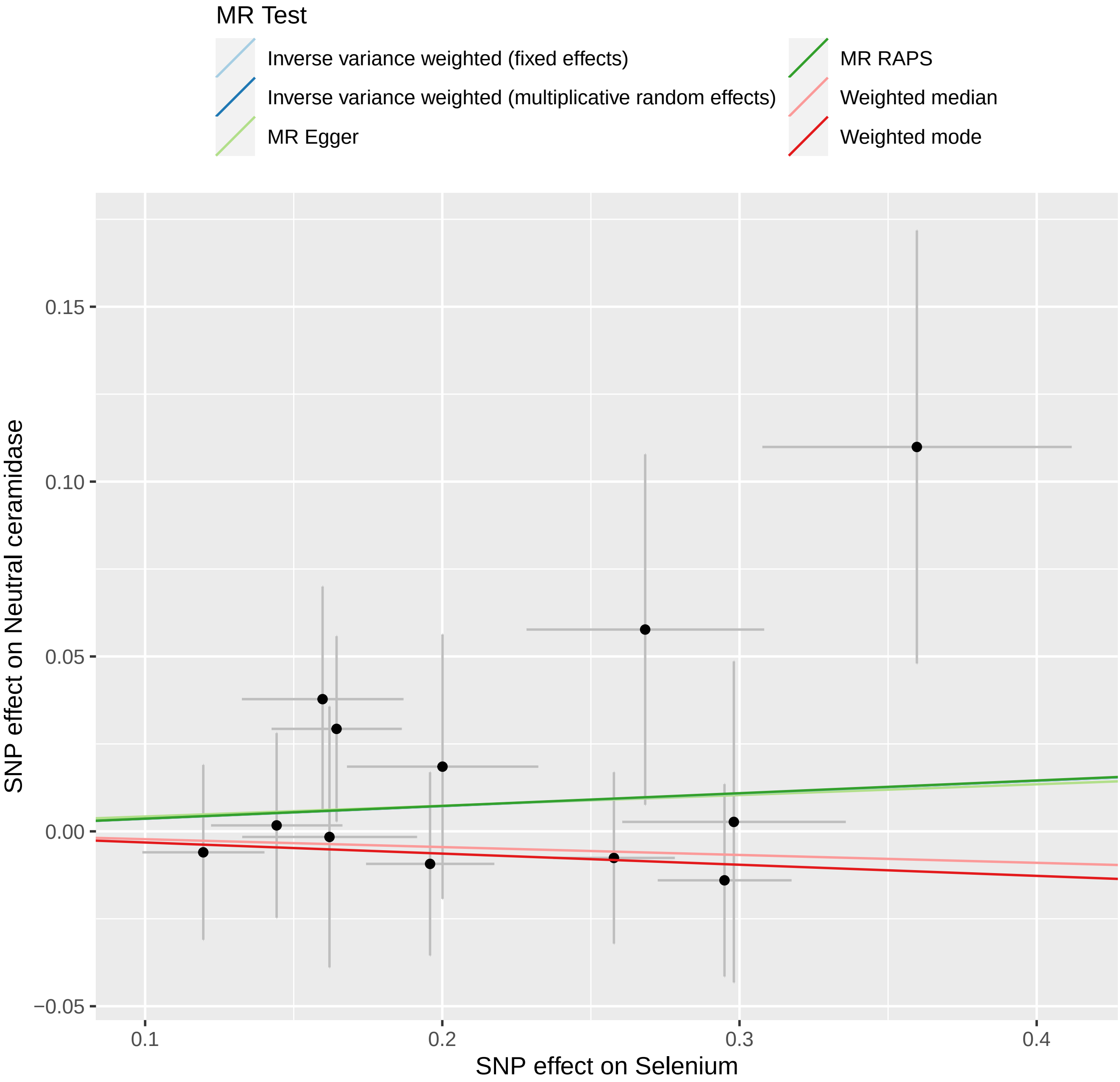

Batch 3−180 : Selenium on Arf−GAP with SH3 domain, ANK repeat and PH domain−containing protein 2

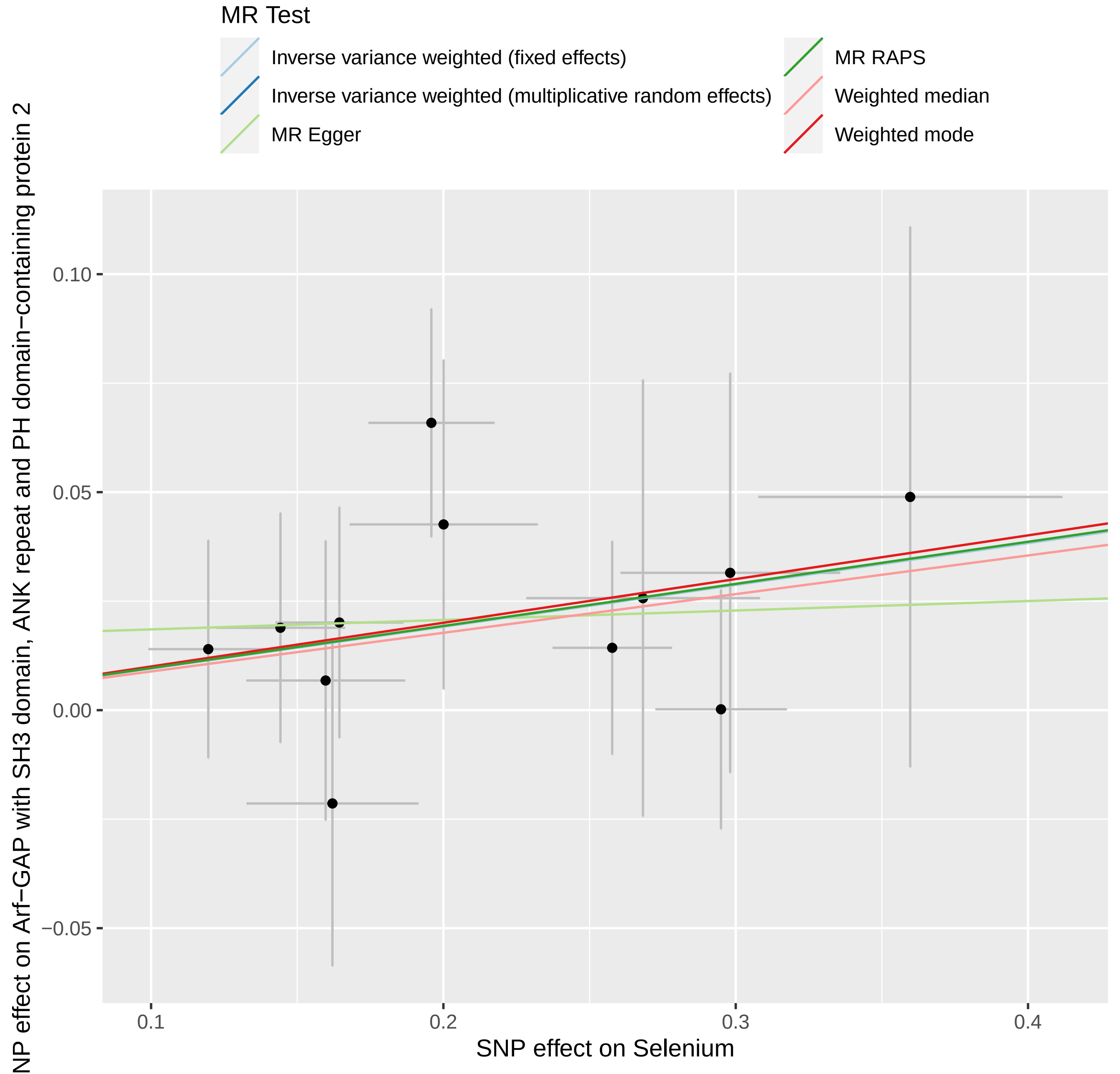

Batch 3−181 : Selenium on Activating signal cointegrator 1 complex subunit 1

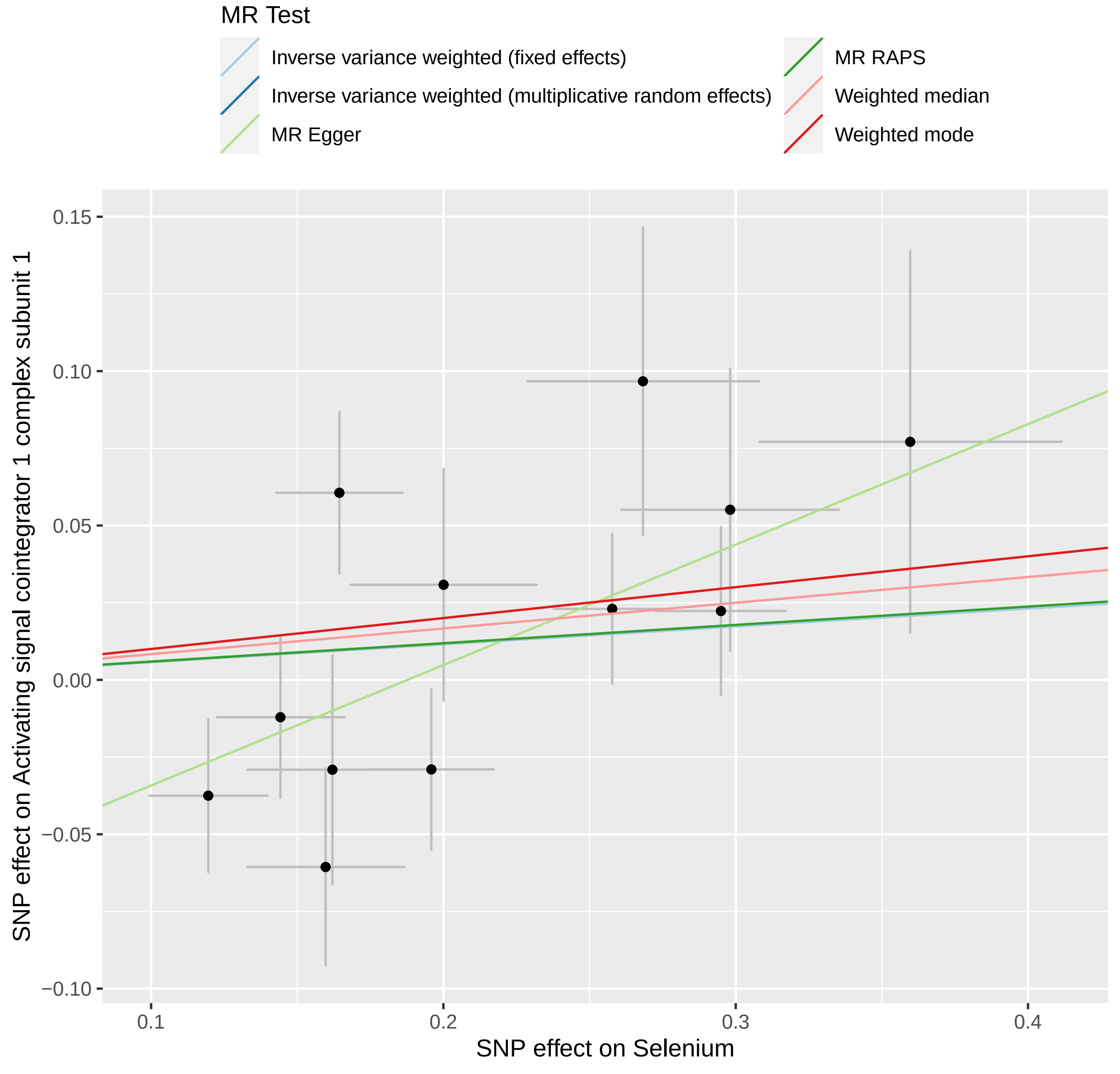

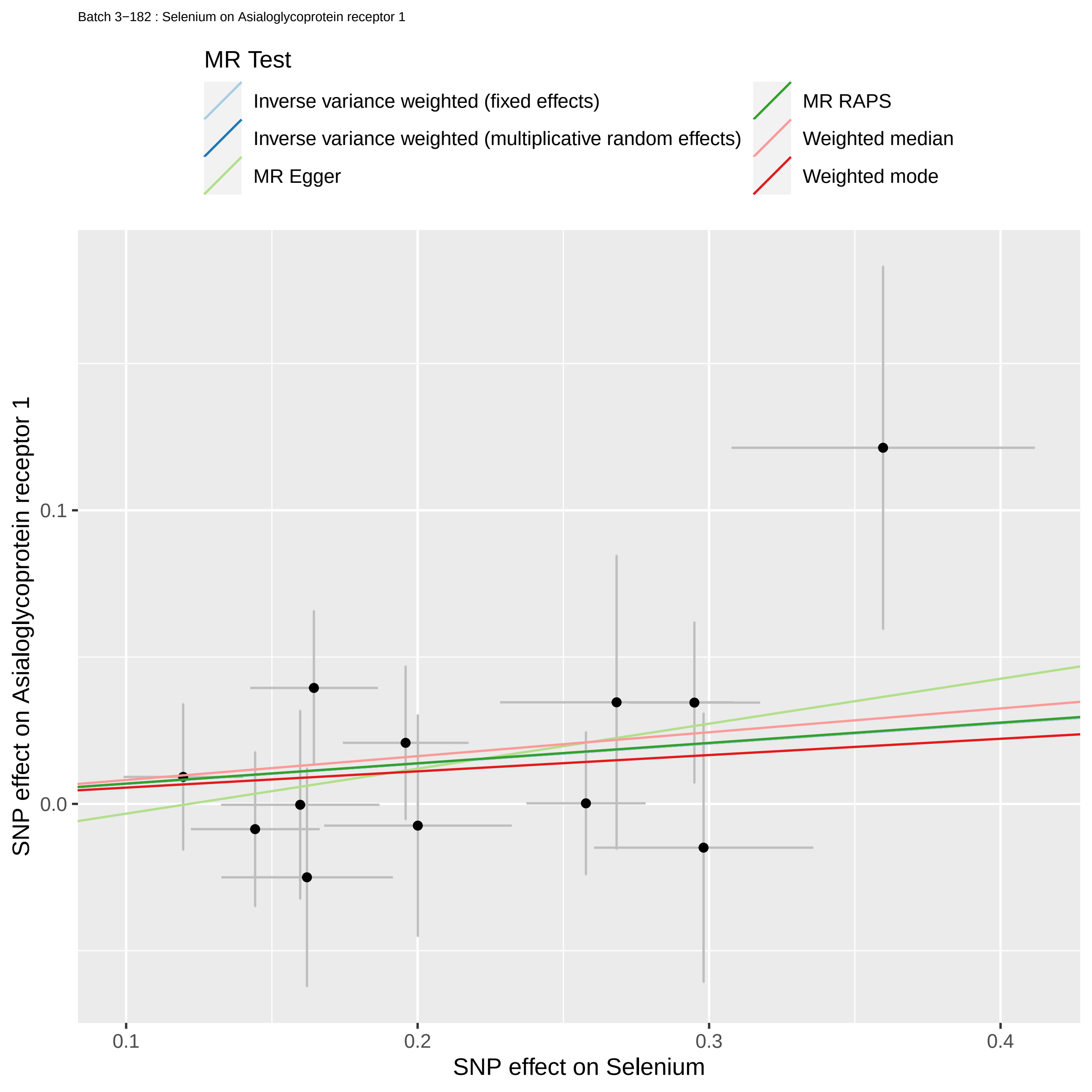

Batch 3−182 : Selenium on Asialoglycoprotein receptor 1
MR Test
Inverse variance weighted (fixed effects)
Inverse variance weighted (multiplicative random effects)
MR Egger
MR RAPS
Weighted median
Weighted mode
SNP effect on Asialoglycoprotein receptor 1
0.1
0.0
0.1
0.2
0.3
0.4
SNP effect on Selenium

Batch 3−183 : Selenium on Asialoglycoprotein receptor 2

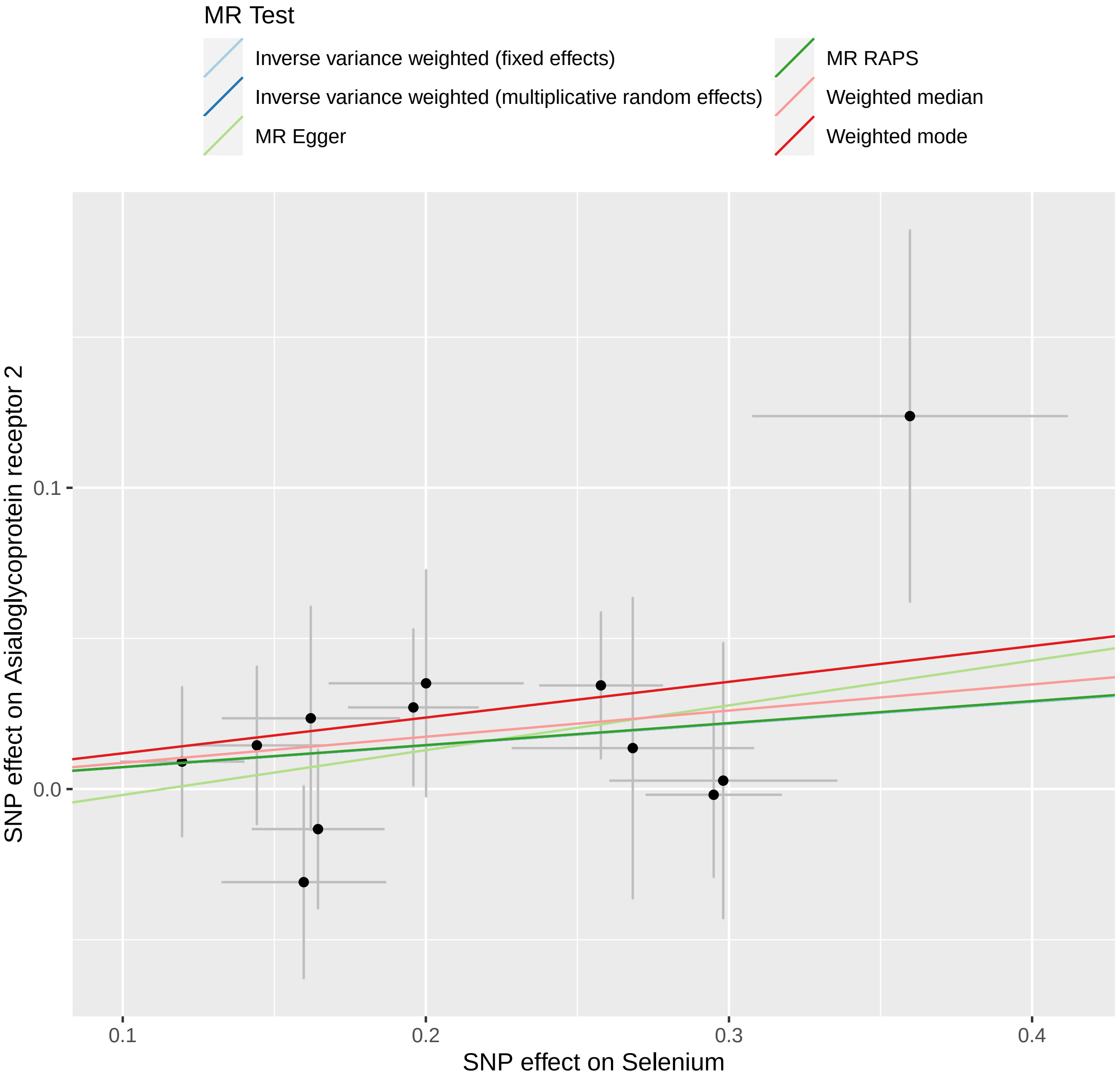

Batch 3−184 : Selenium on Histone−lysine N−methyltransferase ASH1L

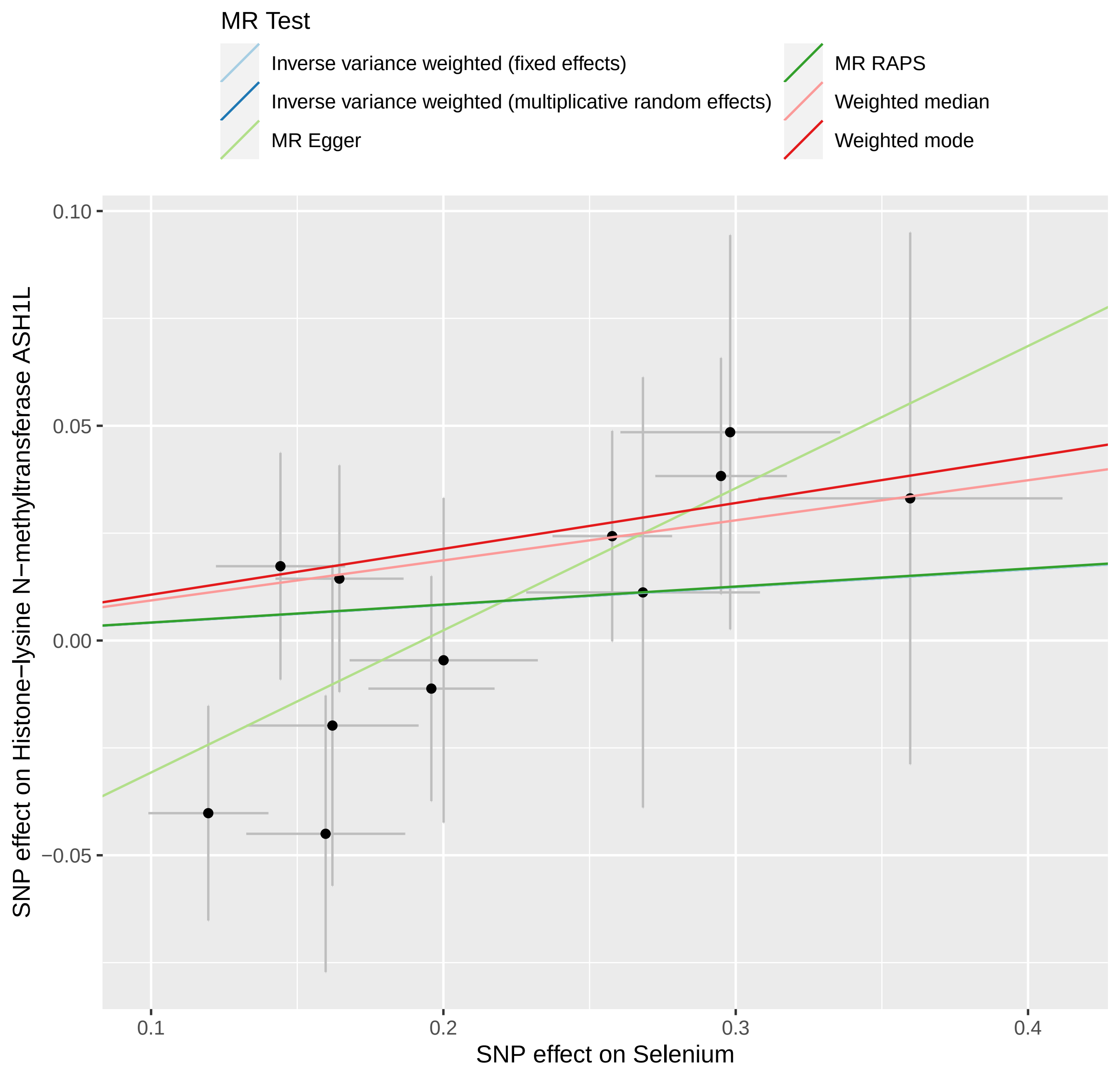

Batch 3−185 : Selenium on Set1/Ash2 histone methyltransferase complex subunit ASH2

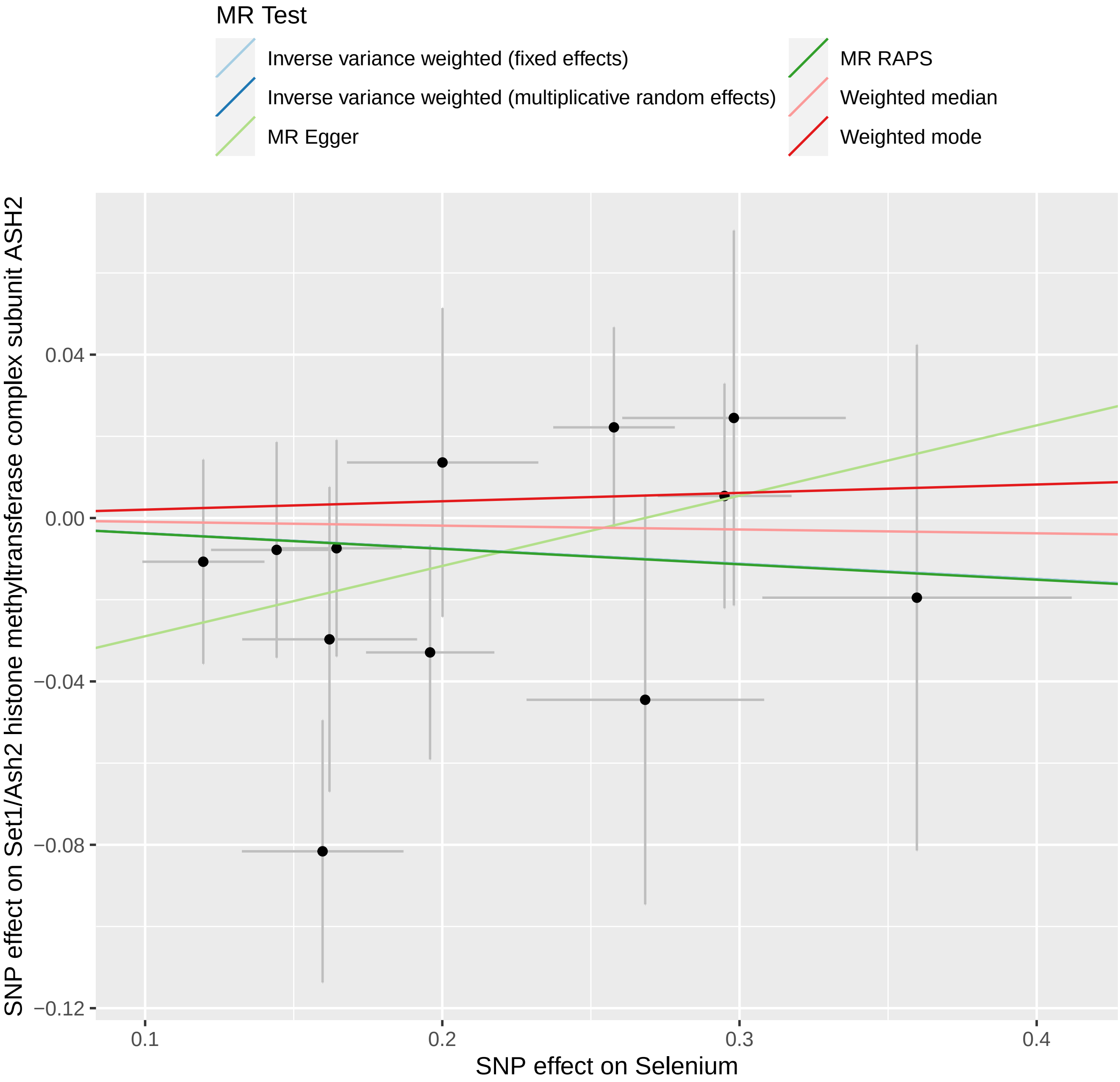

Batch 3−186 : Selenium on Acid−sensing ion channel 4

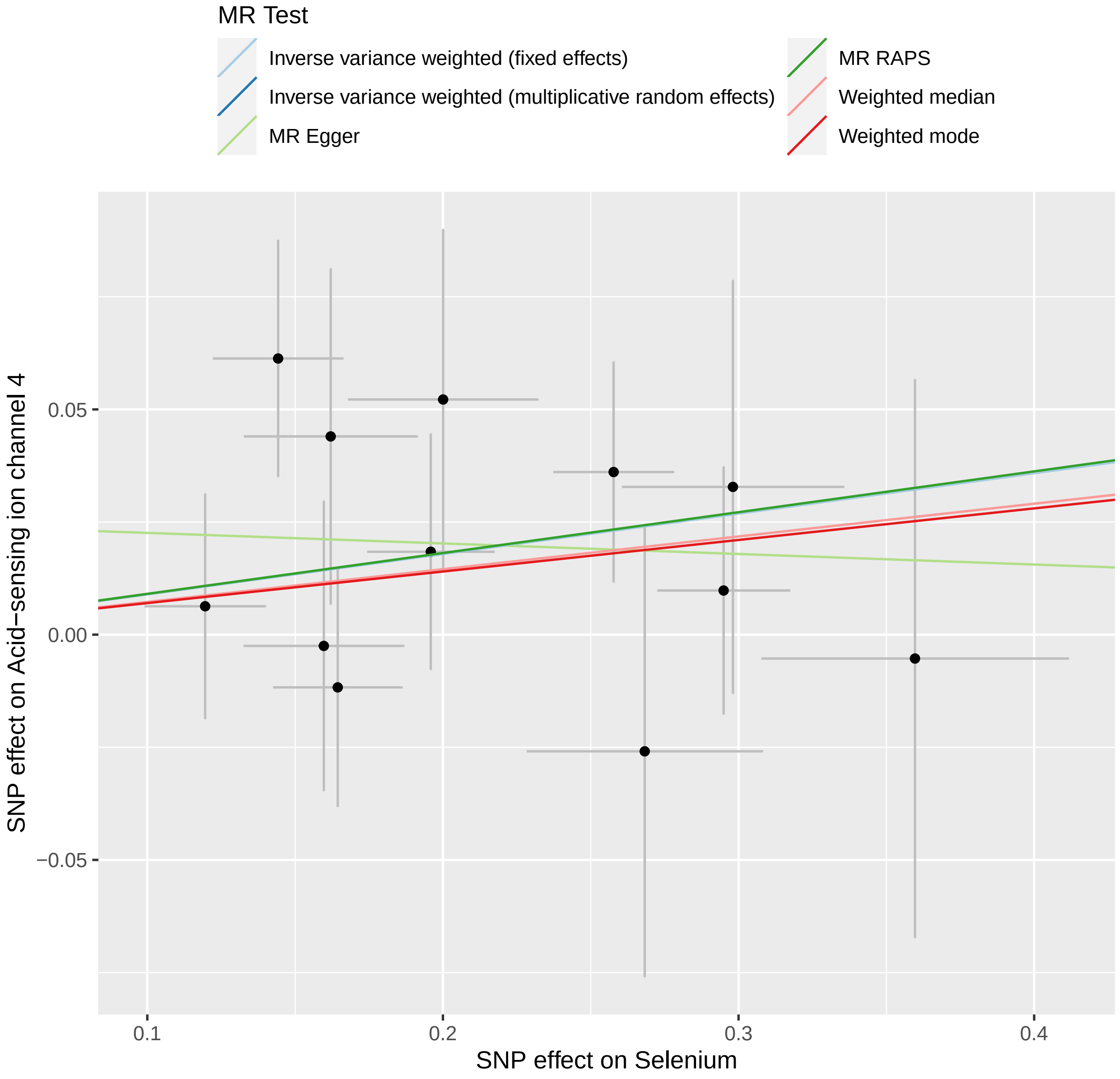

Batch 3−187 : Selenium on Agouti−signaling protein

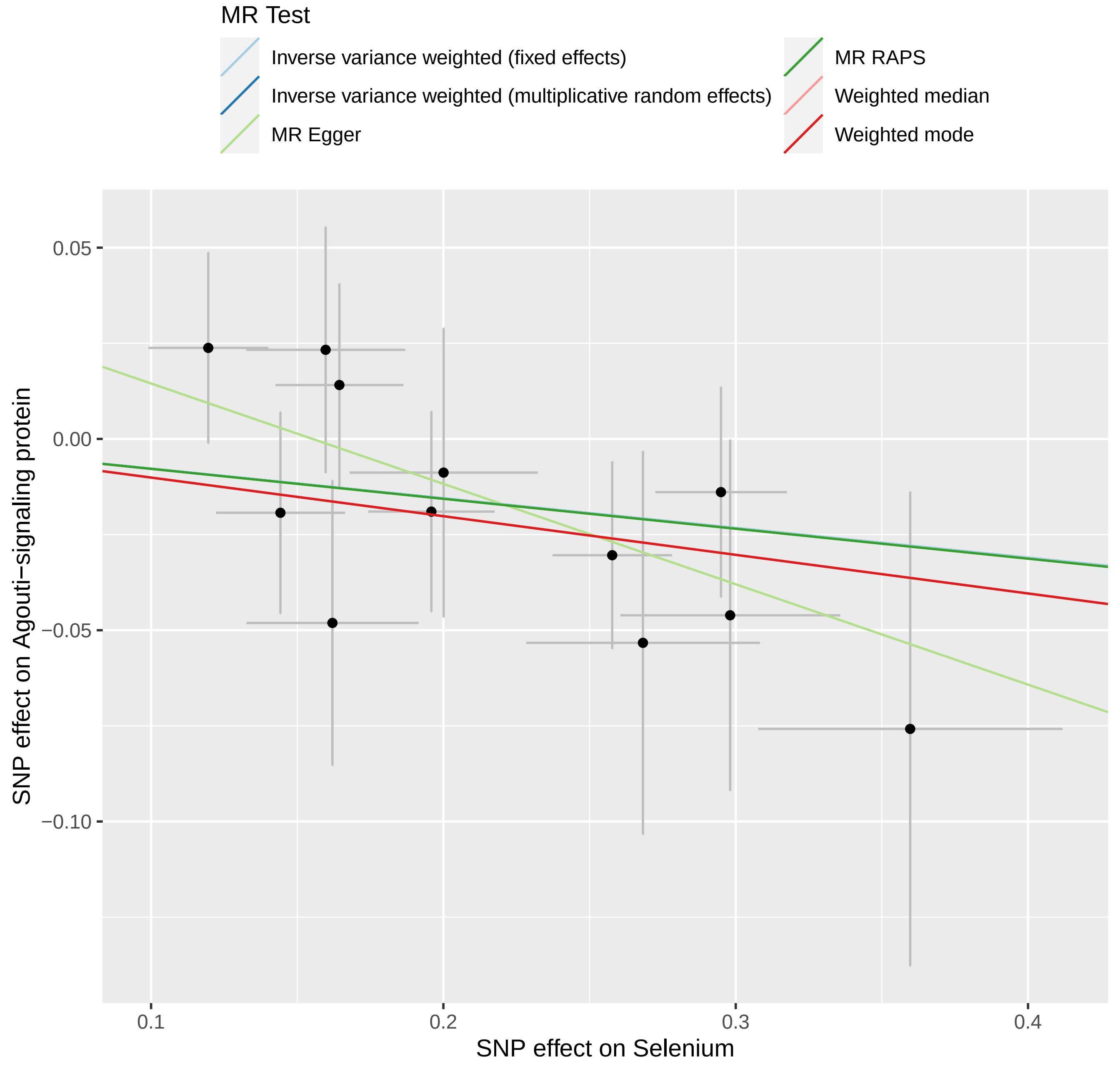

Batch 3−188 : Selenium on Argininosuccinate lyase

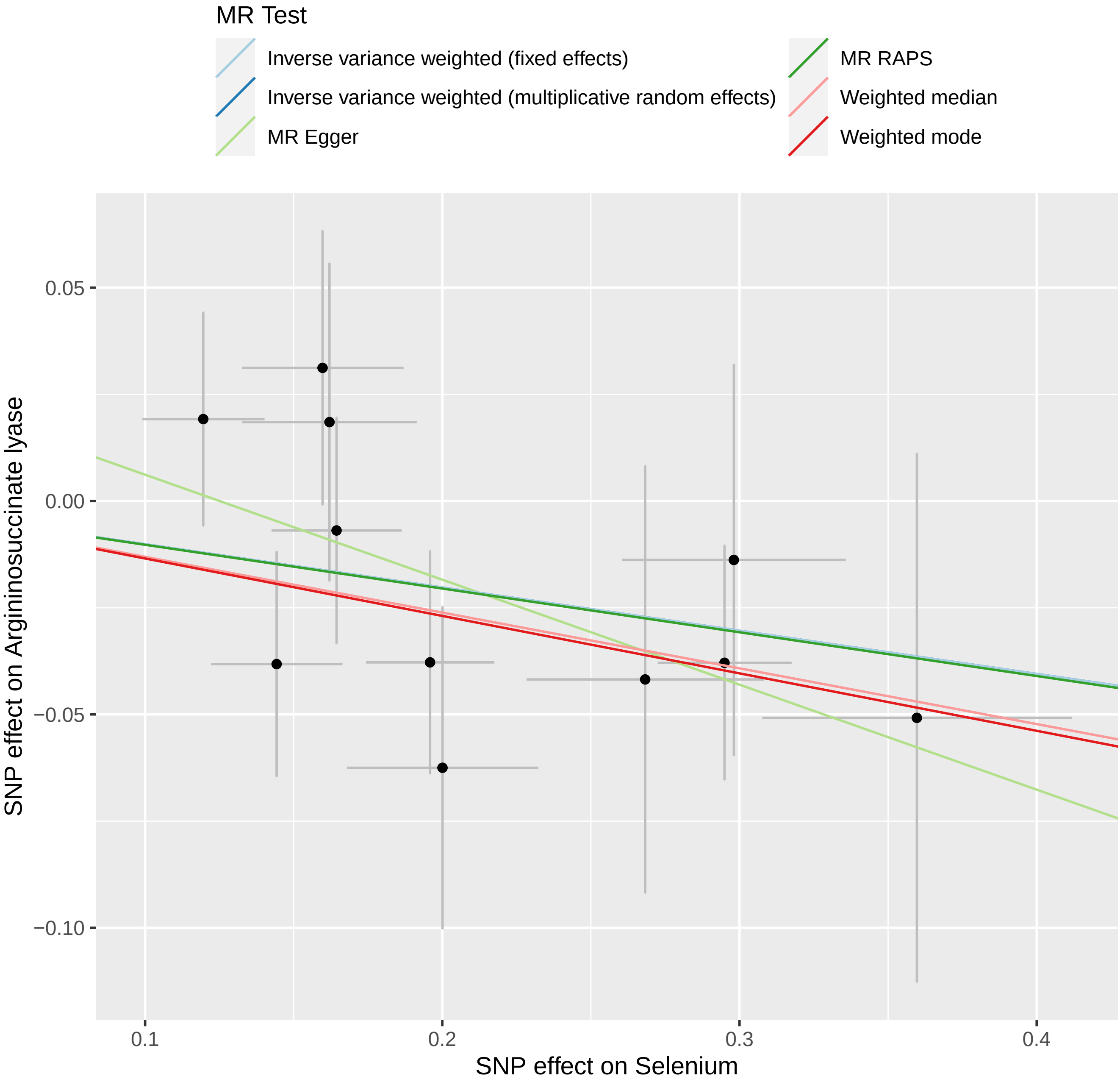

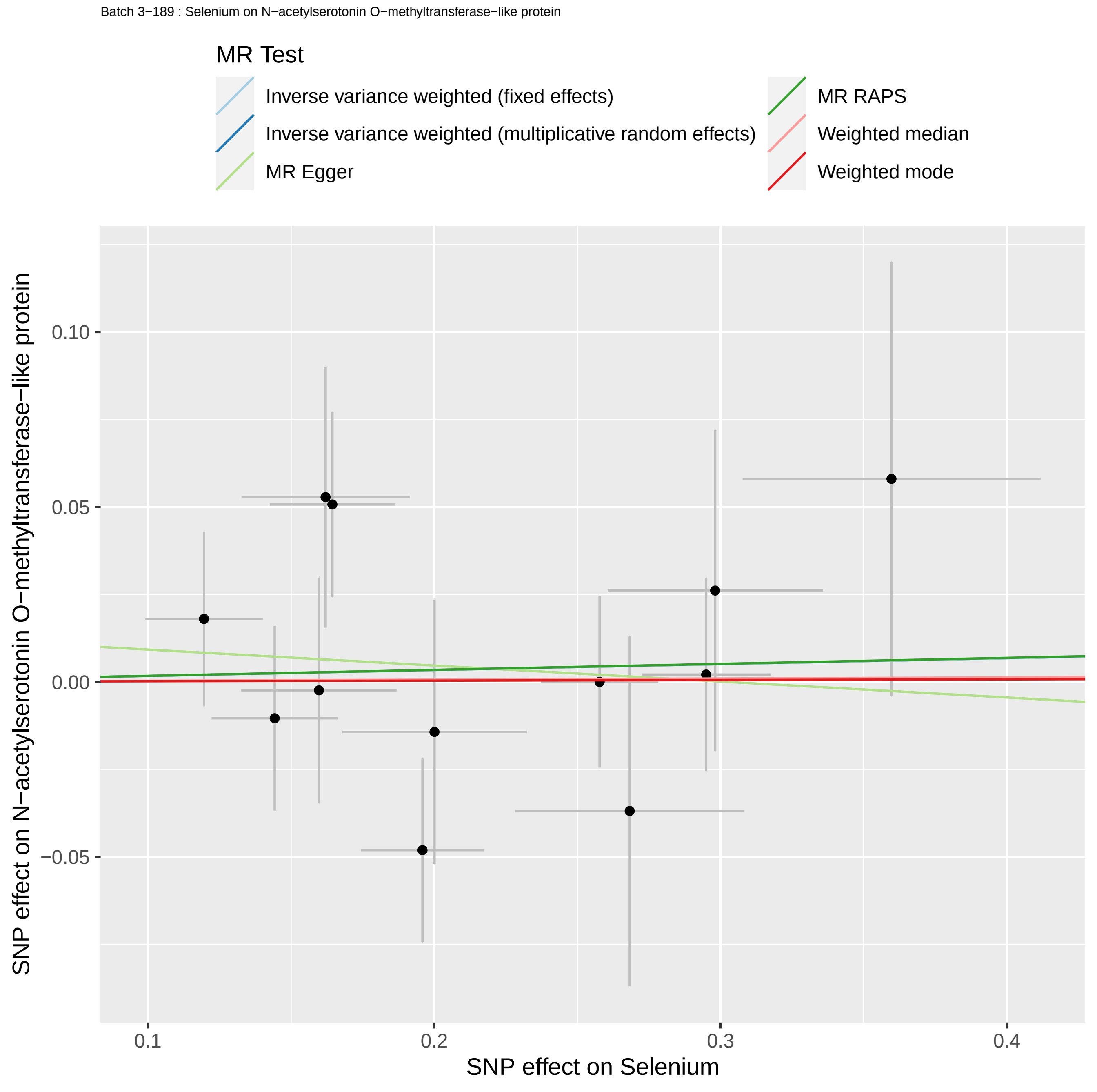

Batch 3−189 : Selenium on N−acetylserotonin O−methyltransferase−like protein
MR Test
Inverse variance weighted (fixed effects)
Inverse variance weighted (multiplicative random effects)
MR Egger
MR RAPS
Weighted median
Weighted mode
0.10
0.05
0.00
−0.05
0.1
0.2
0.3
0.4
SNP effect on N−acetylserotonin O−methyltransferase−like protein
SNP effect on Selenium

Batch 3−190 : Selenium on ATPase ASNA1

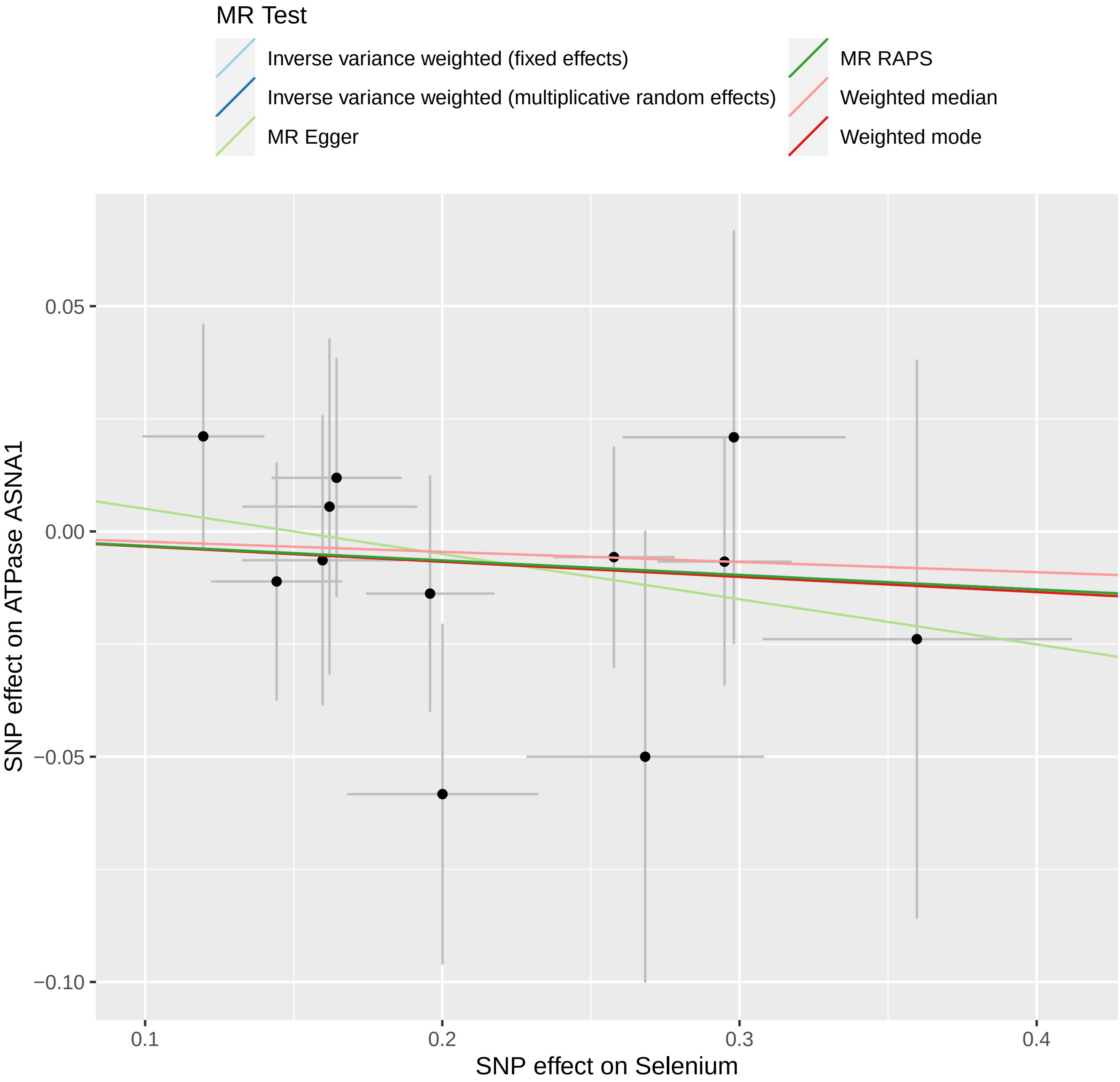

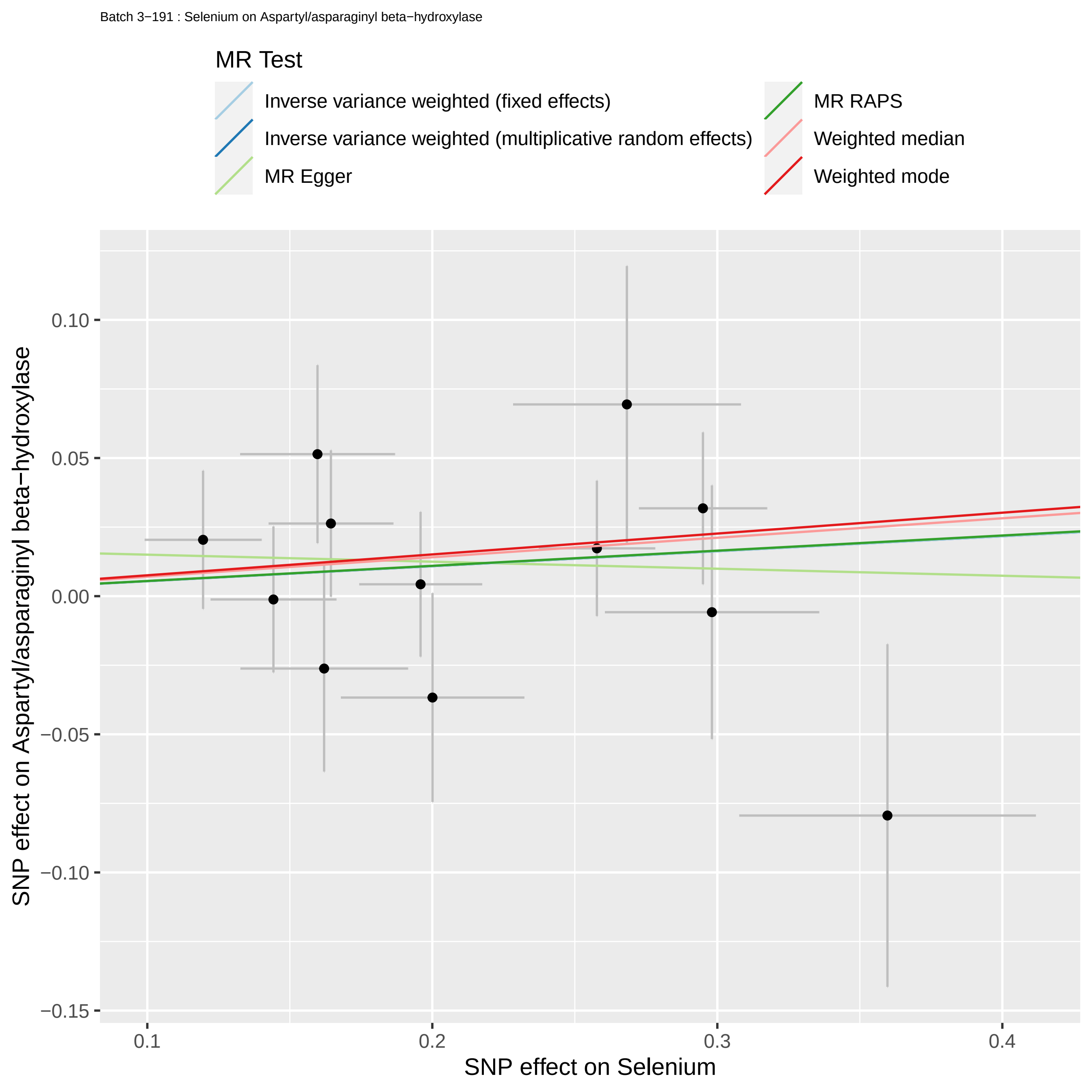

Batch 3−191 : Selenium on Aspartyl/asparaginyl beta−hydroxylase
MR Test
Inverse variance weighted (fixed effects)
Inverse variance weighted (multiplicative random effects)
MR Egger
MR RAPS
Weighted median
Weighted mode
0.10
0.05
0.00
−0.05
−0.10
−0.15
0.1
0.2
0.3
0.4
SNP effect on Aspartyl/asparaginyl beta−hydroxylase
SNP effect on Selenium

Batch 3−192 : Selenium on Asporin

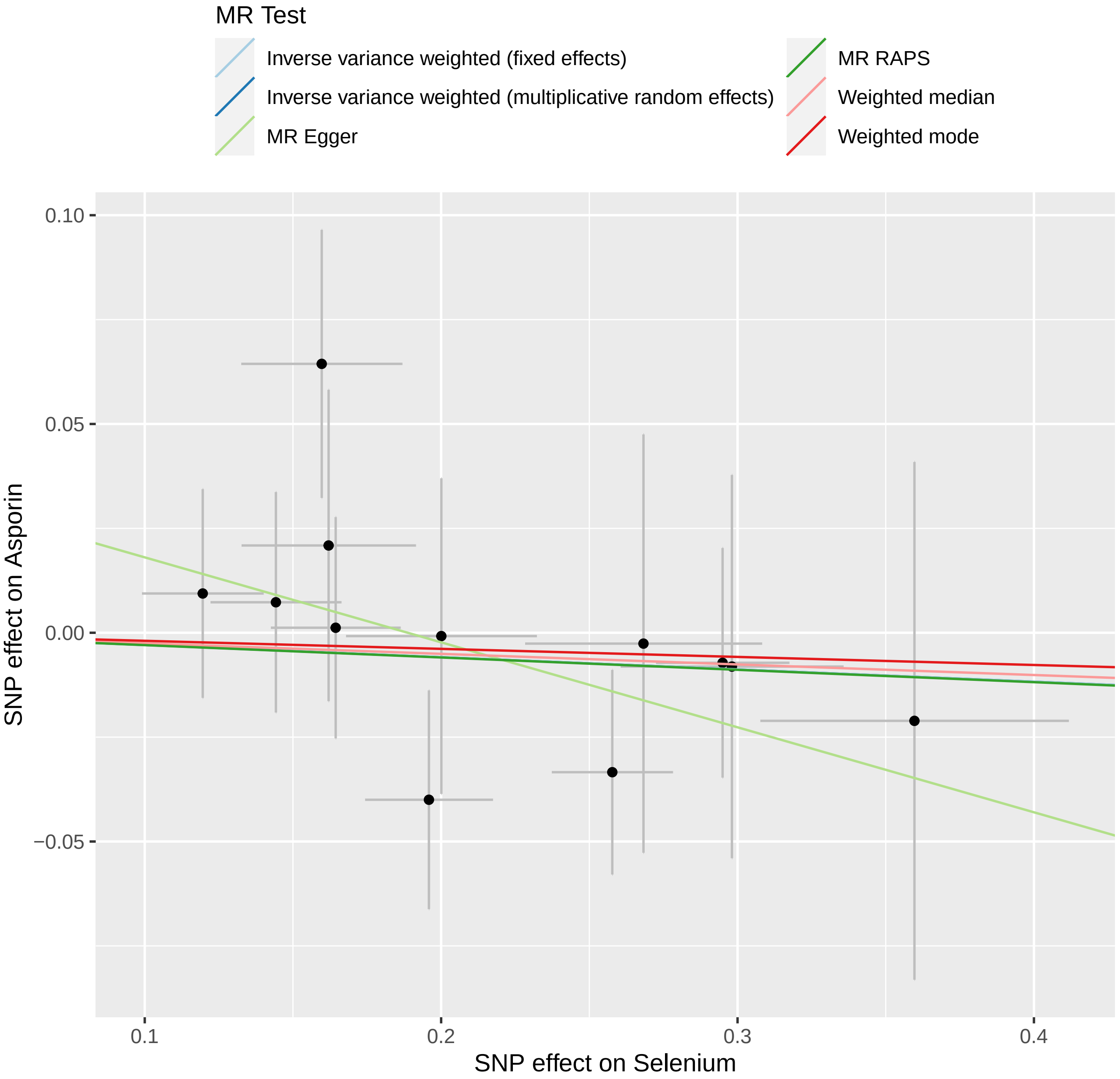

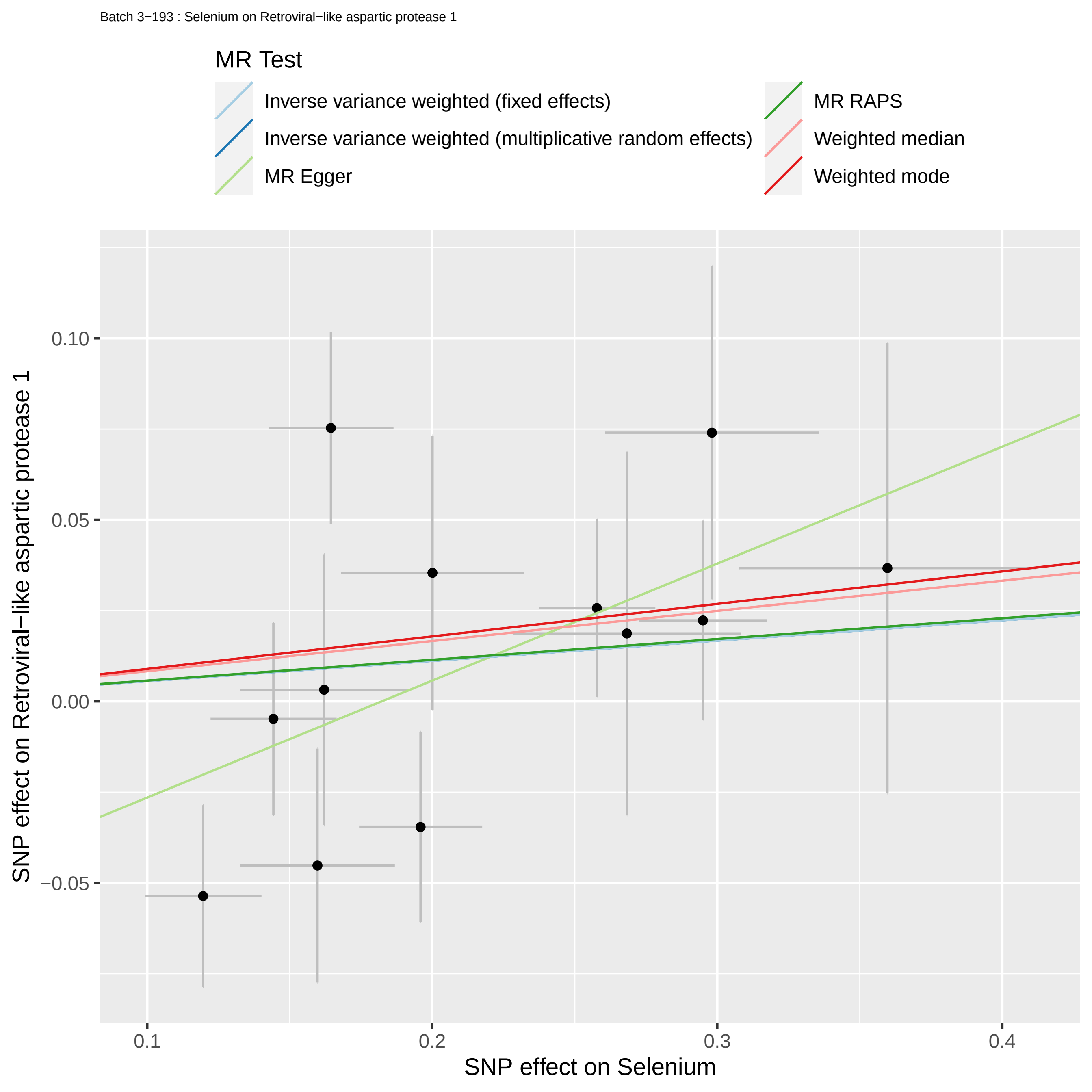

Batch 3−193 : Selenium on Retroviral−like aspartic protease 1
MR Test
Inverse variance weighted (fixed effects)
Inverse variance weighted (multiplicative random effects)
MR Egger
MR RAPS
Weighted median
Weighted mode
SNP effect on Retroviral−like aspartic protease 1
0.10
0.05
0.00
−0.05
0.1
0.2
0.3
0.4
SNP effect on Selenium

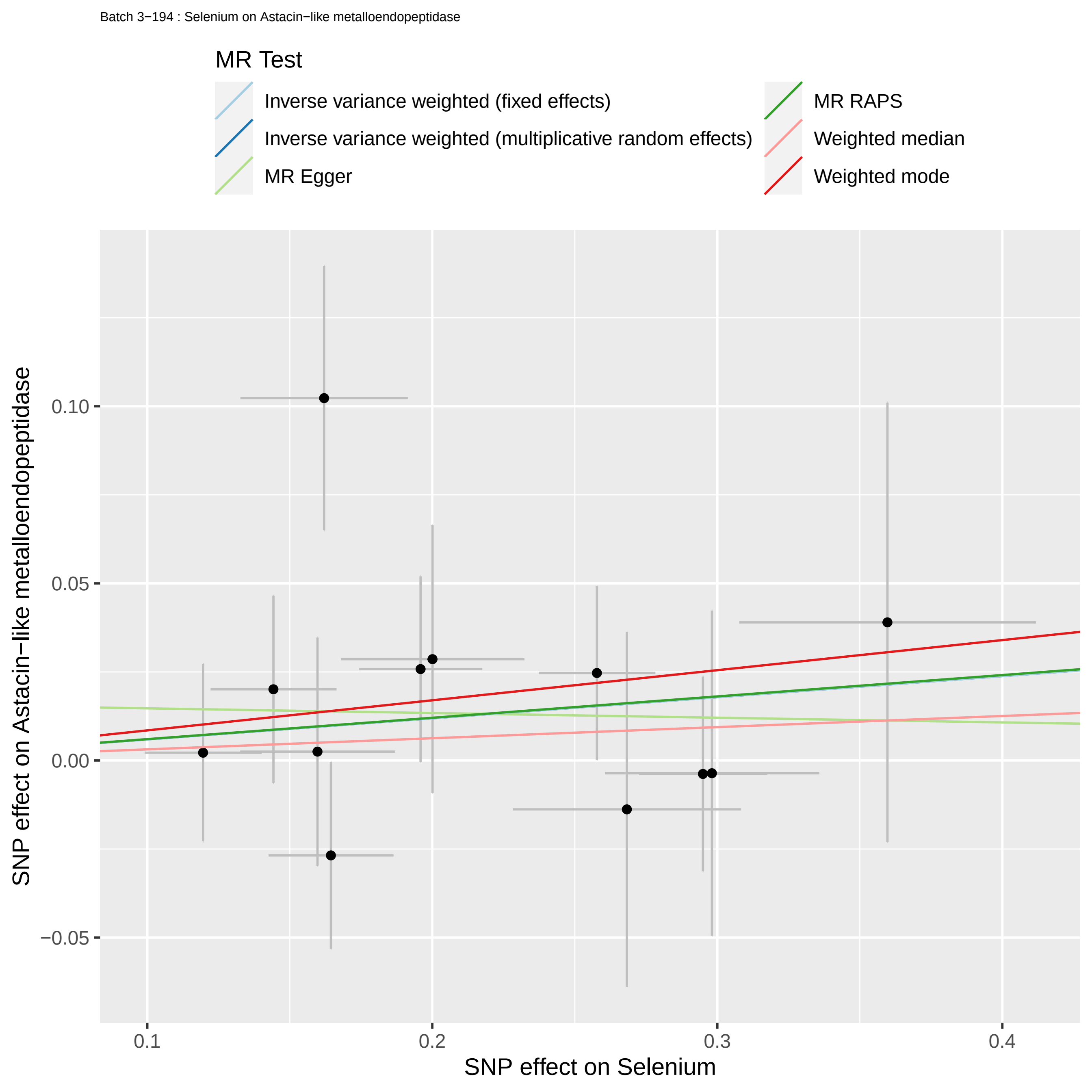
Batch 3−194 : Selenium on Astacin−like metalloendopeptidase
MR Test
Inverse variance weighted (fixed effects)
Inverse variance weighted (multiplicative random effects)
MR Egger
MR RAPS
Weighted median
Weighted mode
SNP effect on Astacin−like metalloendopeptidase
0.10
0.05
0.00
−0.05
0.1
0.2
0.3
0.4
SNP effect on Selenium

Batch 3−195 : Selenium on ATPase family AAA domain−containing protein 1

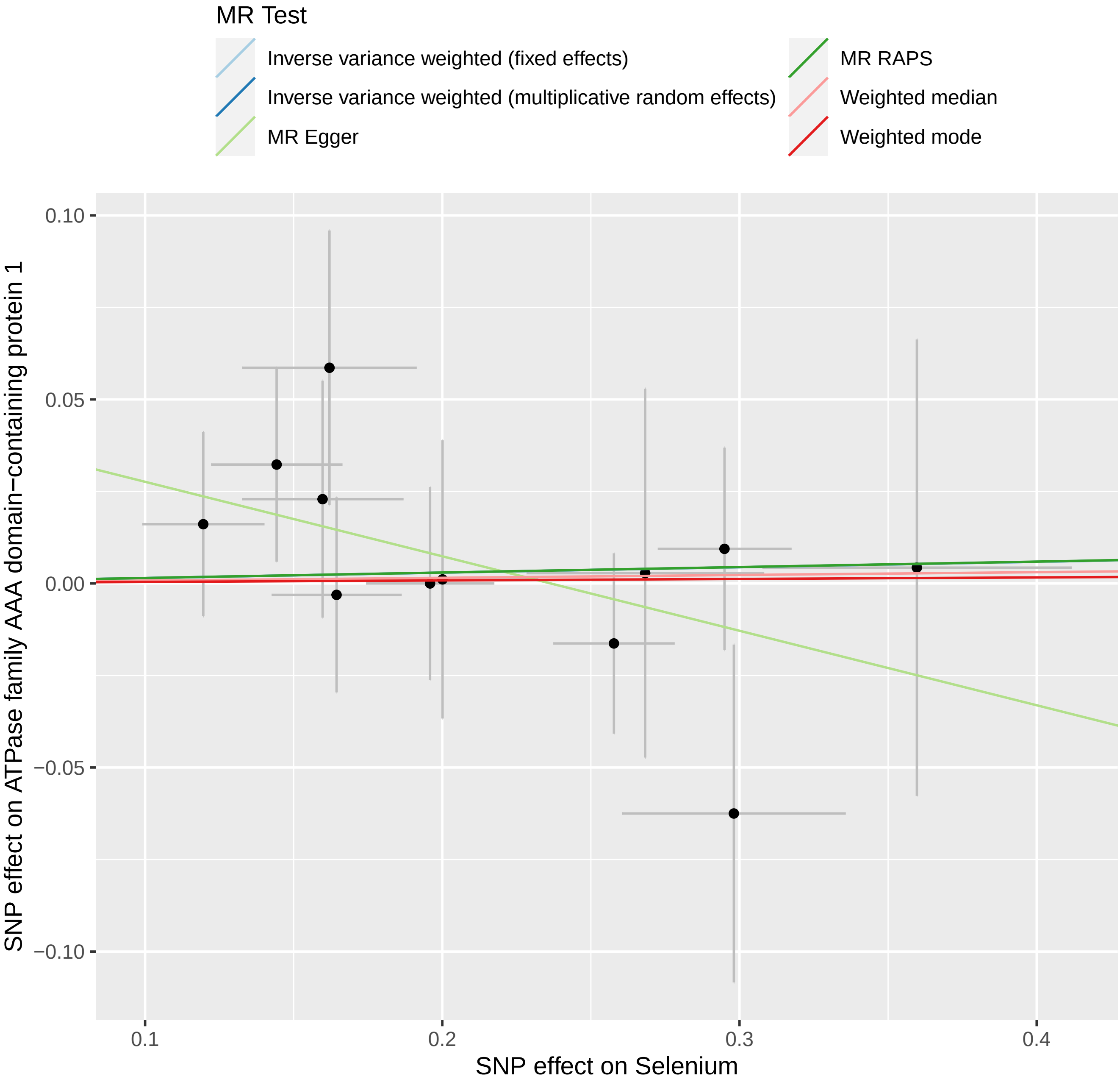

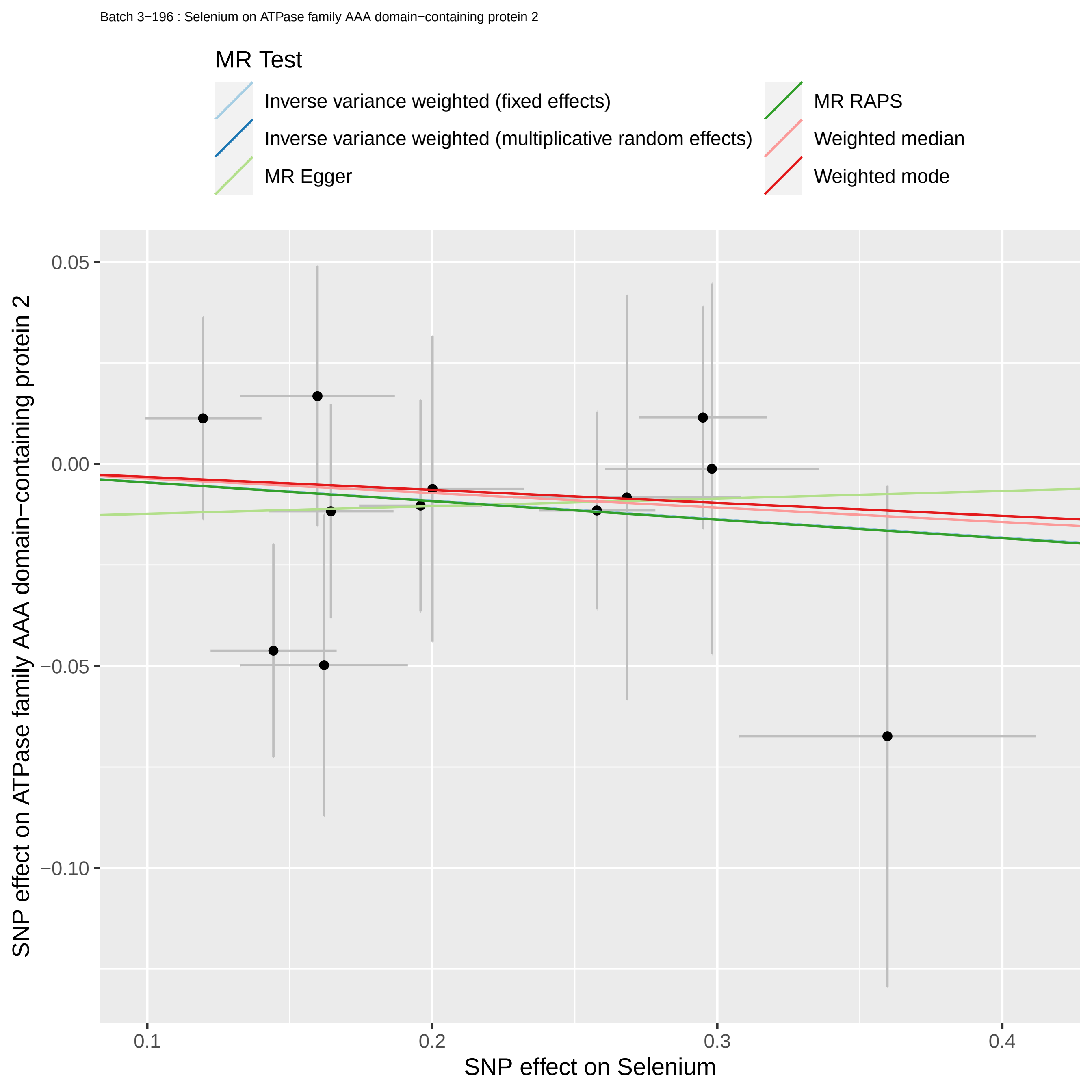
Batch 3−196 : Selenium on ATPase family AAA domain−containing protein 2
MR Test
Inverse variance weighted (fixed effects)
Inverse variance weighted (multiplicative random effects)
MR Egger
MR RAPS
Weighted median
Weighted mode
0.05
0.00
−0.05
−0.10
0.1
0.2
0.3
0.4
SNP effect on Selenium
SNP effect on ATPase family AAA domain−containing protein 2

Batch 3−197 : Selenium on Cyclic AMP−dependent transcription factor ATF−6 alpha

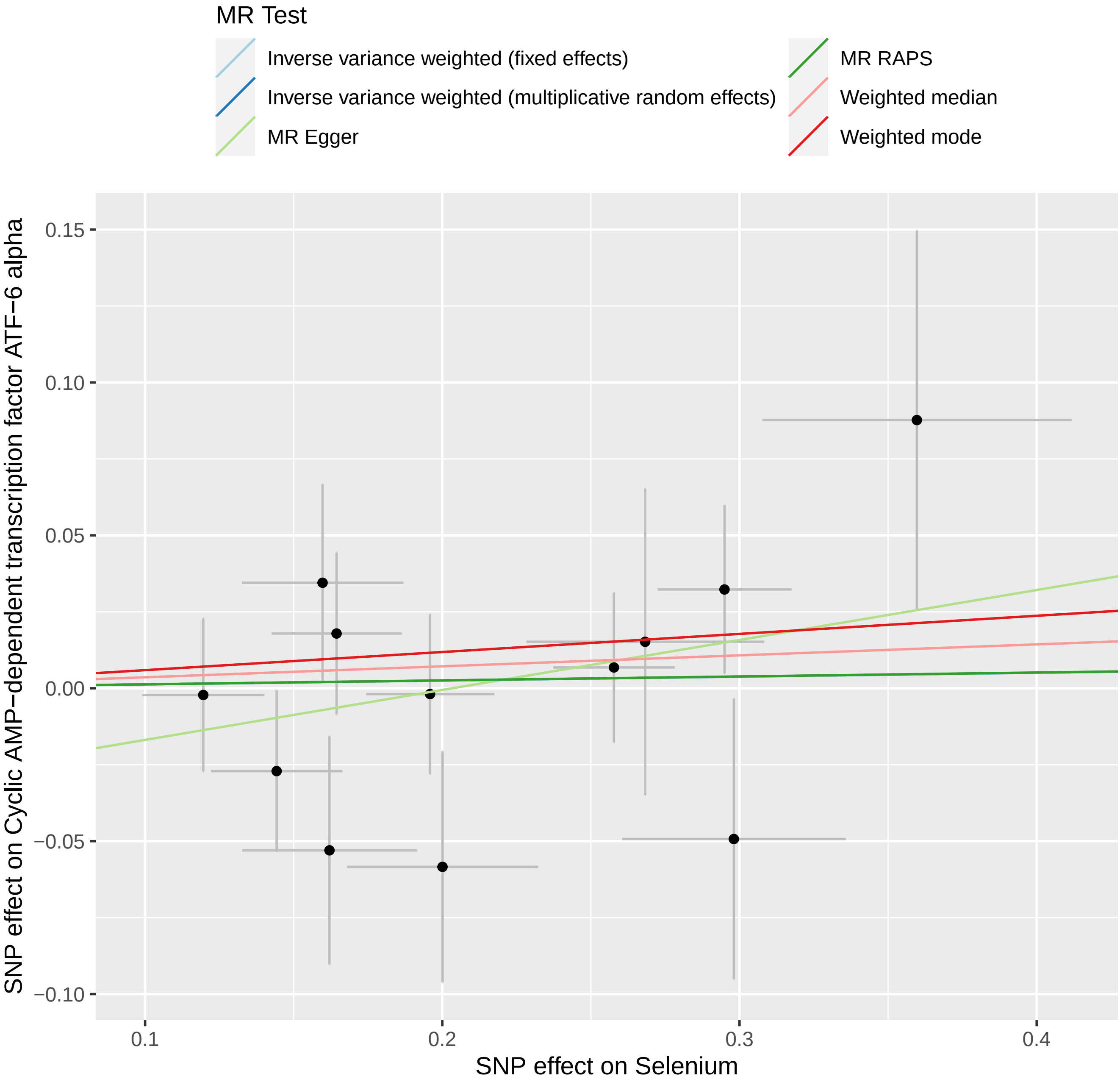

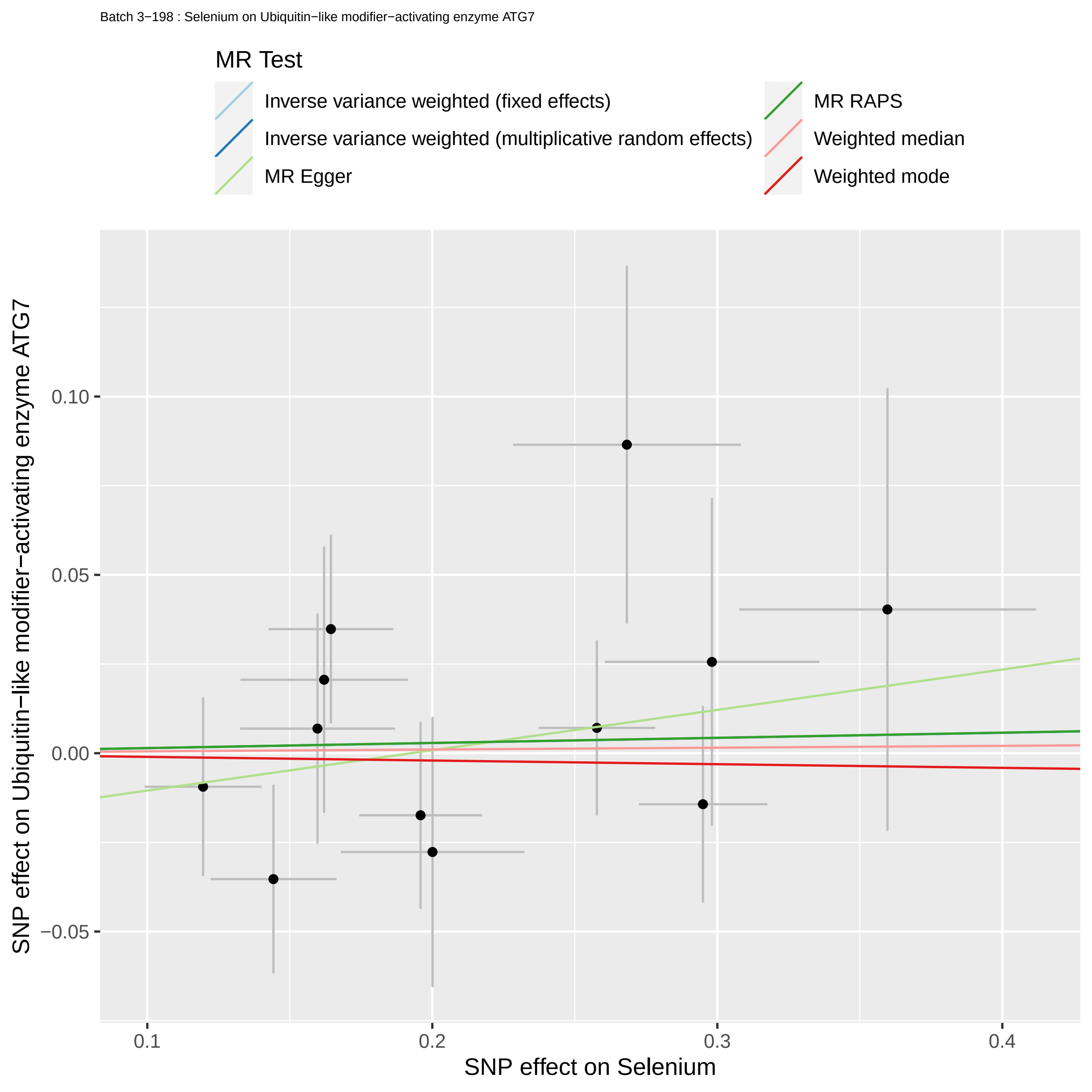

Batch 3−198 : Selenium on Ubiquitin−like modifier−activating enzyme ATG7
MR Test
Inverse variance weighted (fixed effects)
Inverse variance weighted (multiplicative random effects)
MR Egger
MR RAPS
Weighted median
Weighted mode
0.10
0.05
0.00
−0.05
0.1
0.2
0.3
0.4
SNP effect on Ubiquitin−like modifier−activating enzyme ATG7
SNP effect on Selenium

Batch 3−199 : Selenium on Sodium/potassium−transporting ATPase subunit alpha−1

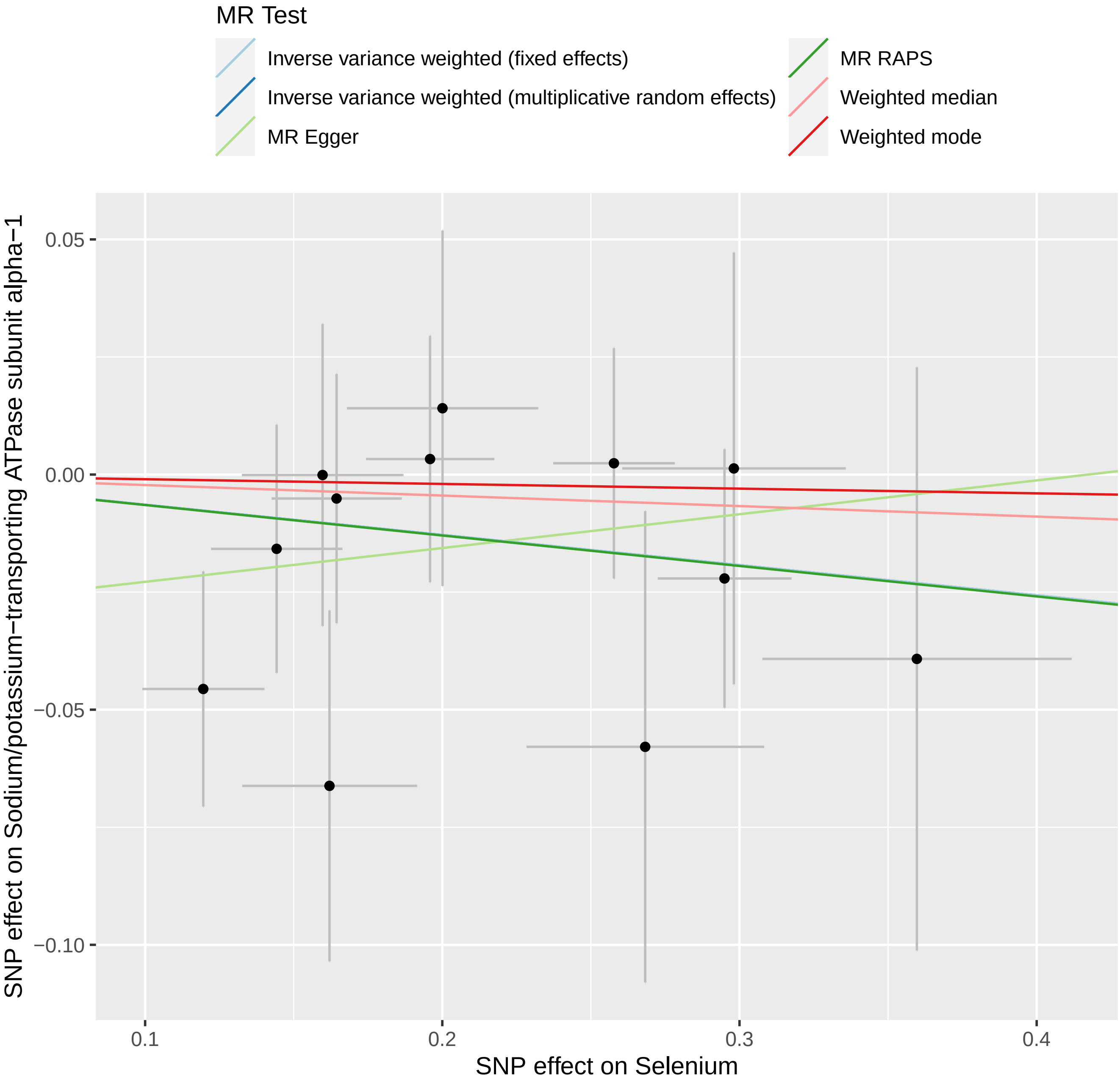

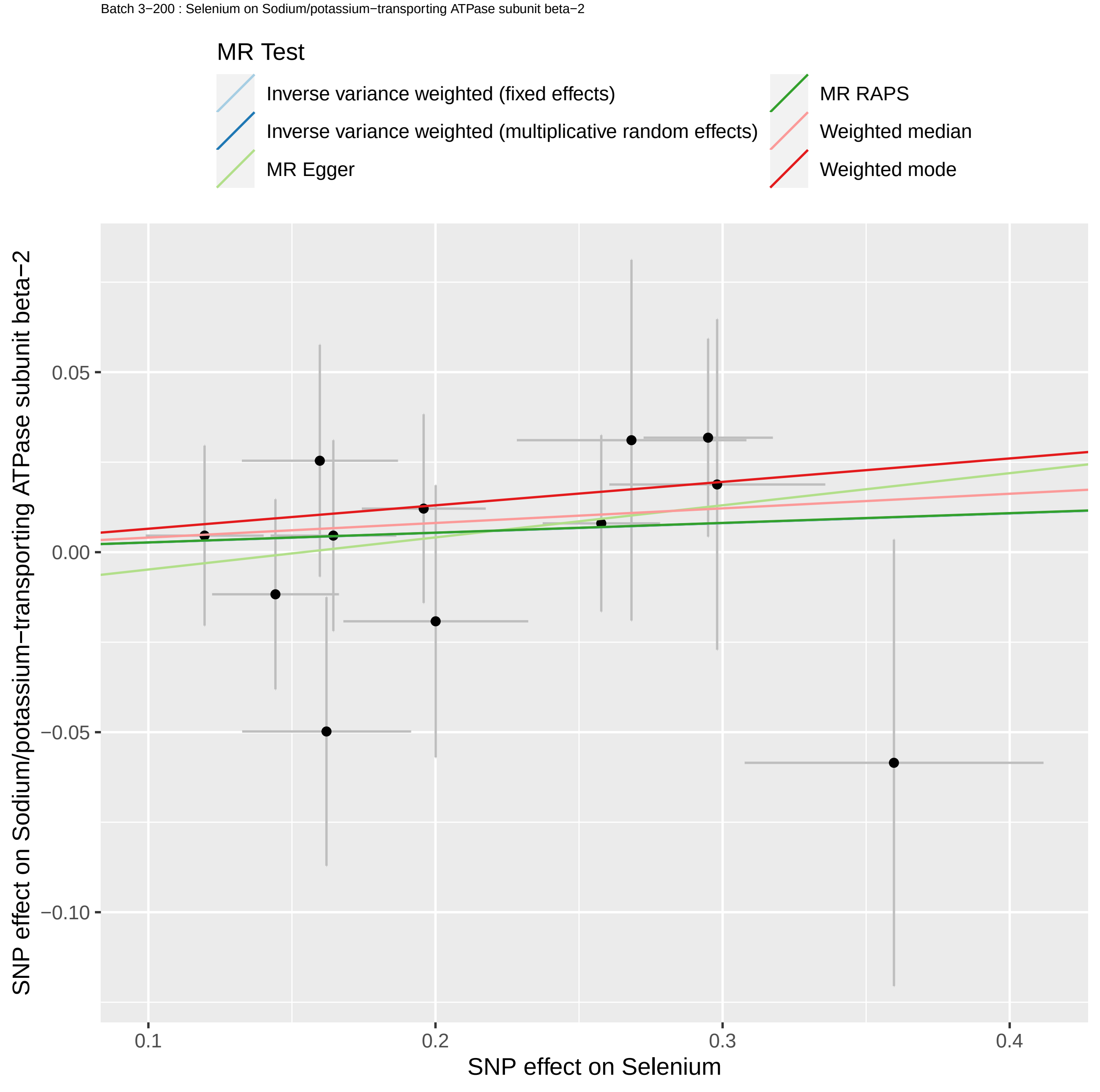
Batch 3−200 : Selenium on Sodium/potassium−transporting ATPase subunit beta−2
MR Test
Inverse variance weighted (fixed effects)
Inverse variance weighted (multiplicative random effects)
MR Egger
MR RAPS
Weighted median
Weighted mode
SNP effect on Sodium/potassium−transporting ATPase subunit beta−2
0.05
0.00
−0.05
−0.10
0.1
0.2
0.3
0.4
SNP effect on Selenium

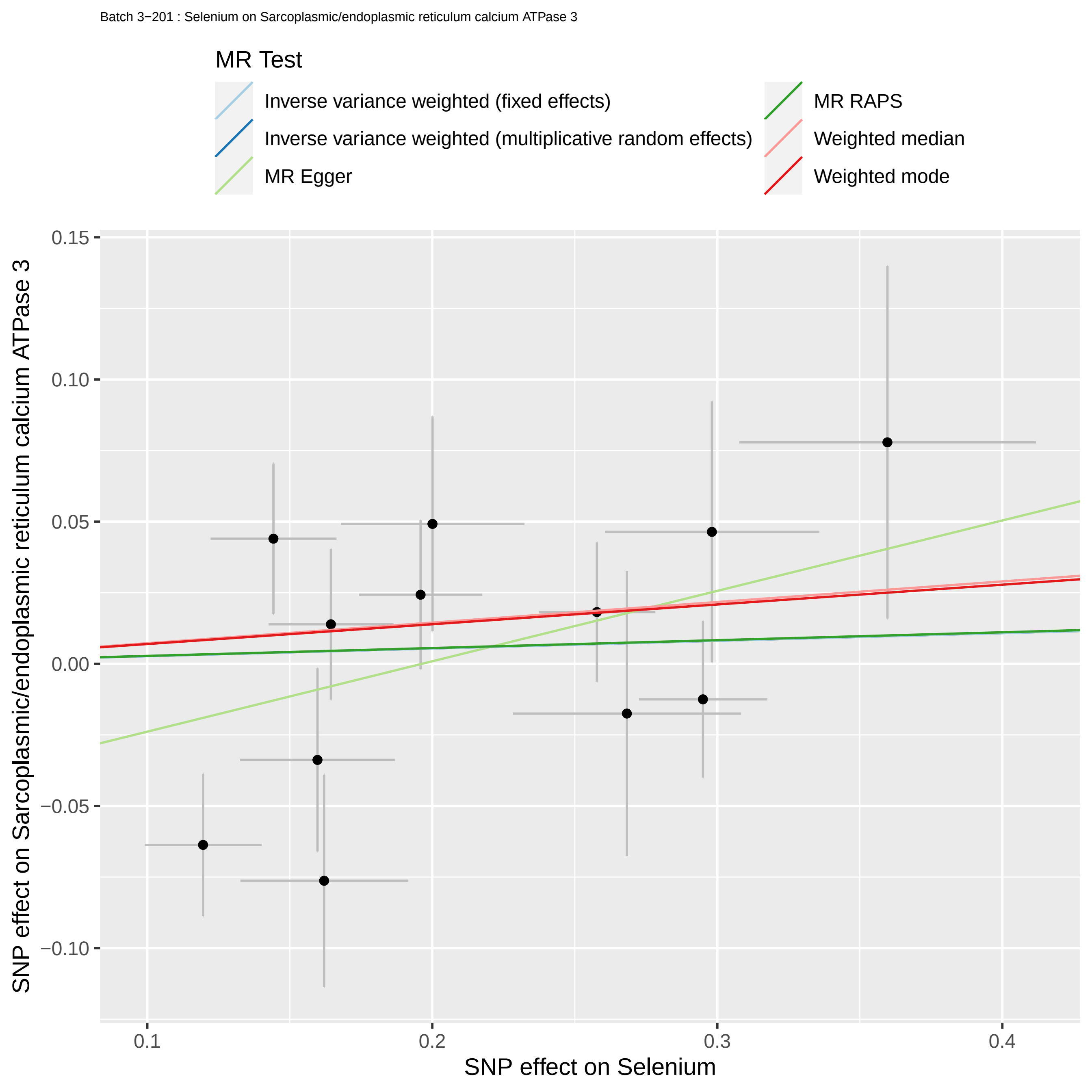

Batch 3−201 : Selenium on Sarcoplasmic/endoplasmic reticulum calcium ATPase 3
MR Test
Inverse variance weighted (fixed effects)
Inverse variance weighted (multiplicative random effects)
MR Egger
MR RAPS
Weighted median
Weighted mode
0.15
0.10
0.05
0.00
−0.05
−0.10
0.1
0.2
0.3
0.4
SNP effect on Sarcoplasmic/endoplasmic reticulum calcium ATPase 3
SNP effect on Selenium

Batch 3−202 : Selenium on Potassium−transporting ATPase subunit beta

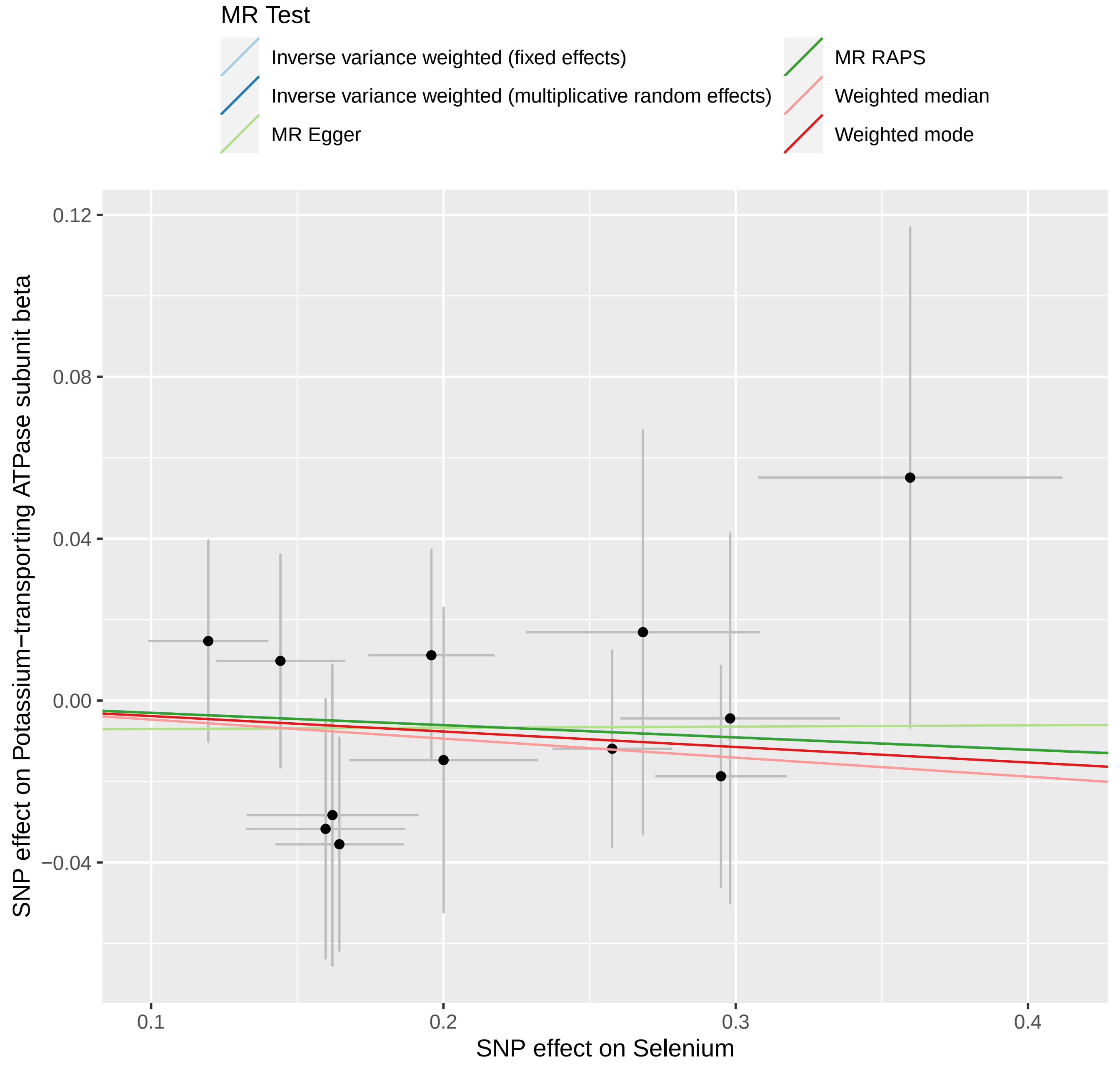

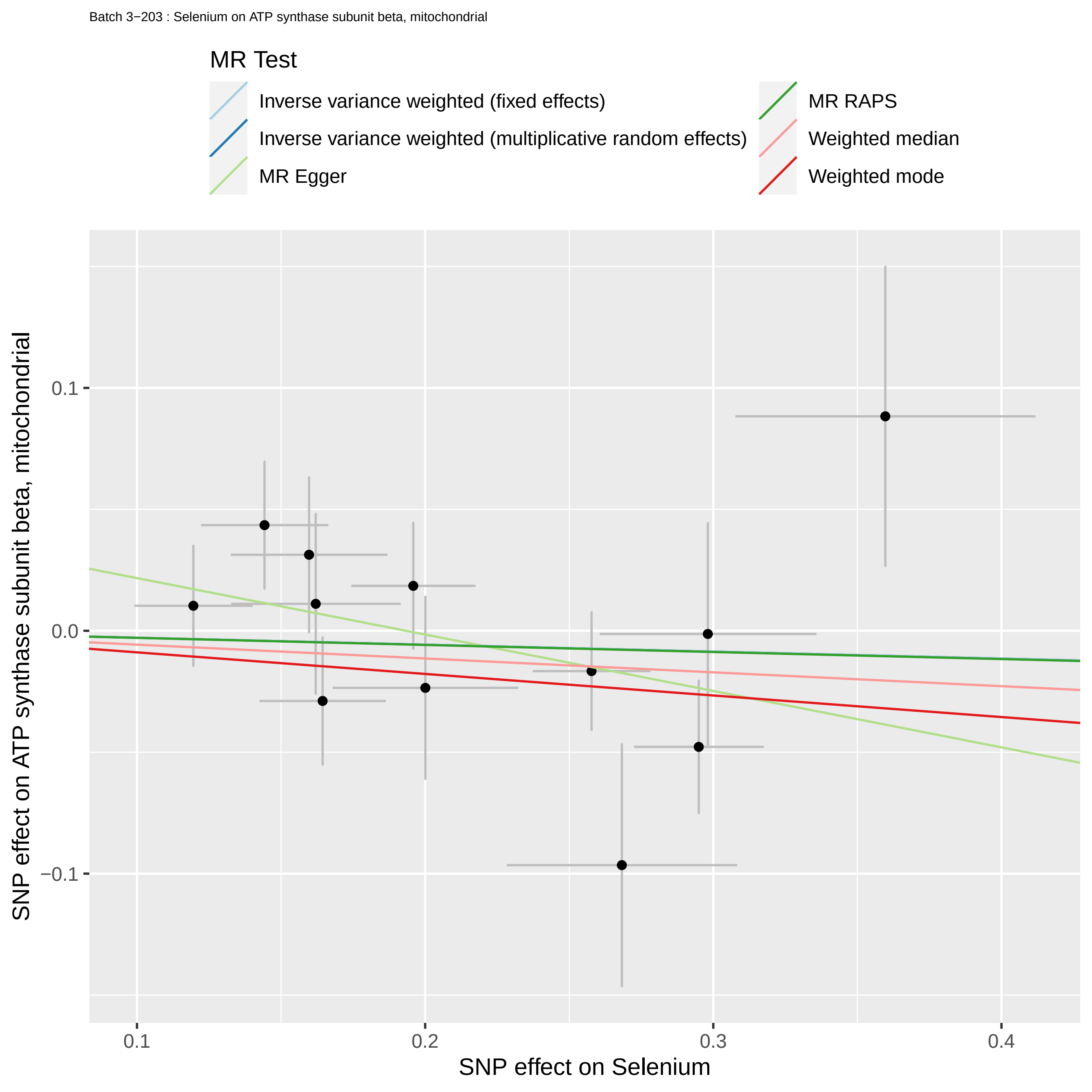

Batch 3−203 : Selenium on ATP synthase subunit beta, mitochondrial
MR Test
Inverse variance weighted (fixed effects)
Inverse variance weighted (multiplicative random effects)
MR Egger
MR RAPS
Weighted median
Weighted mode
SNP effect on ATP synthase subunit beta, mitochondrial
0.1
0.0
−0.1
0.1
0.2
0.3
0.4
SNP effect on Selenium

Batch 3−204 : Selenium on Aurora kinase A

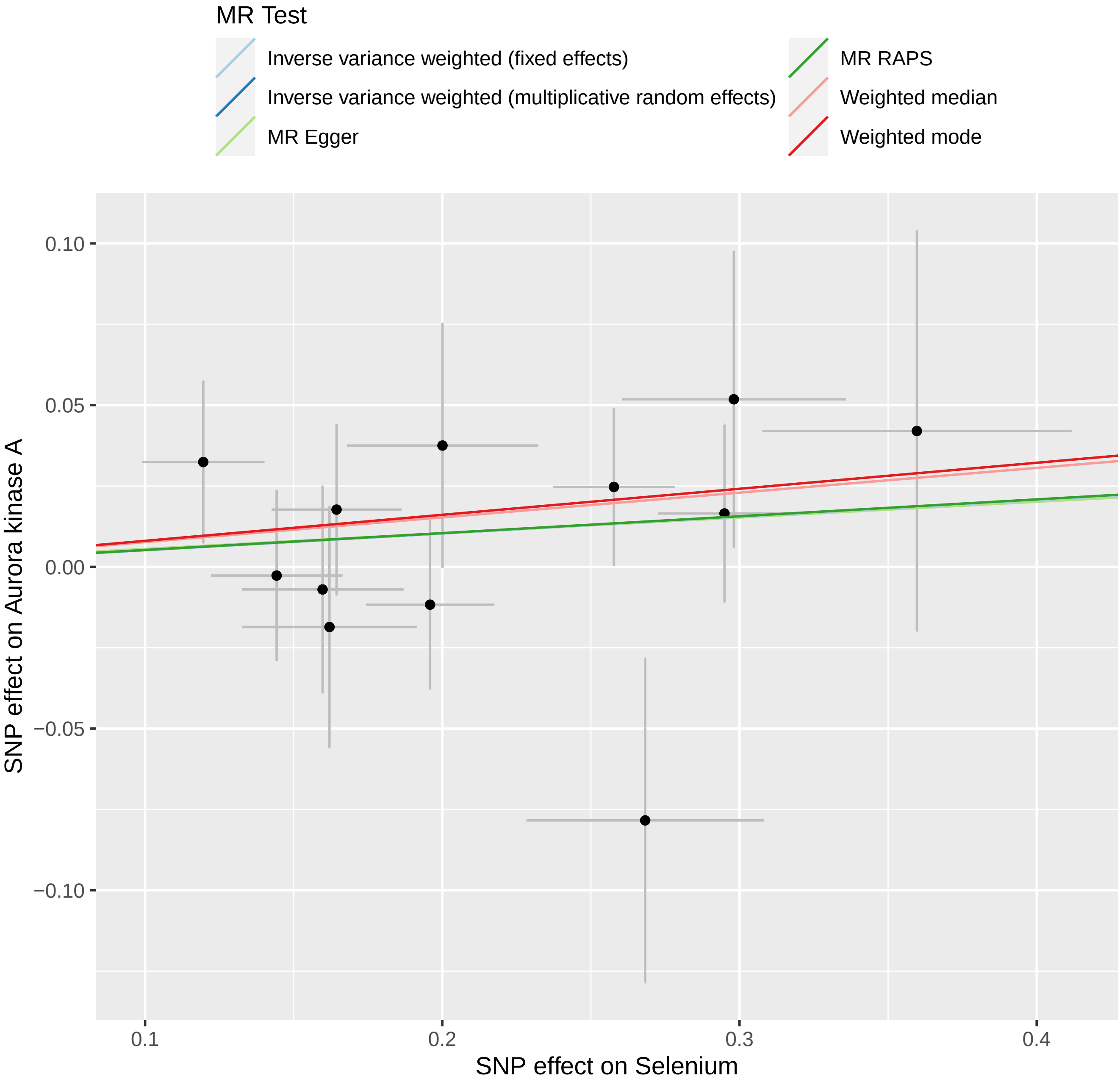

Batch 3−205 : Selenium on Aurora kinase B

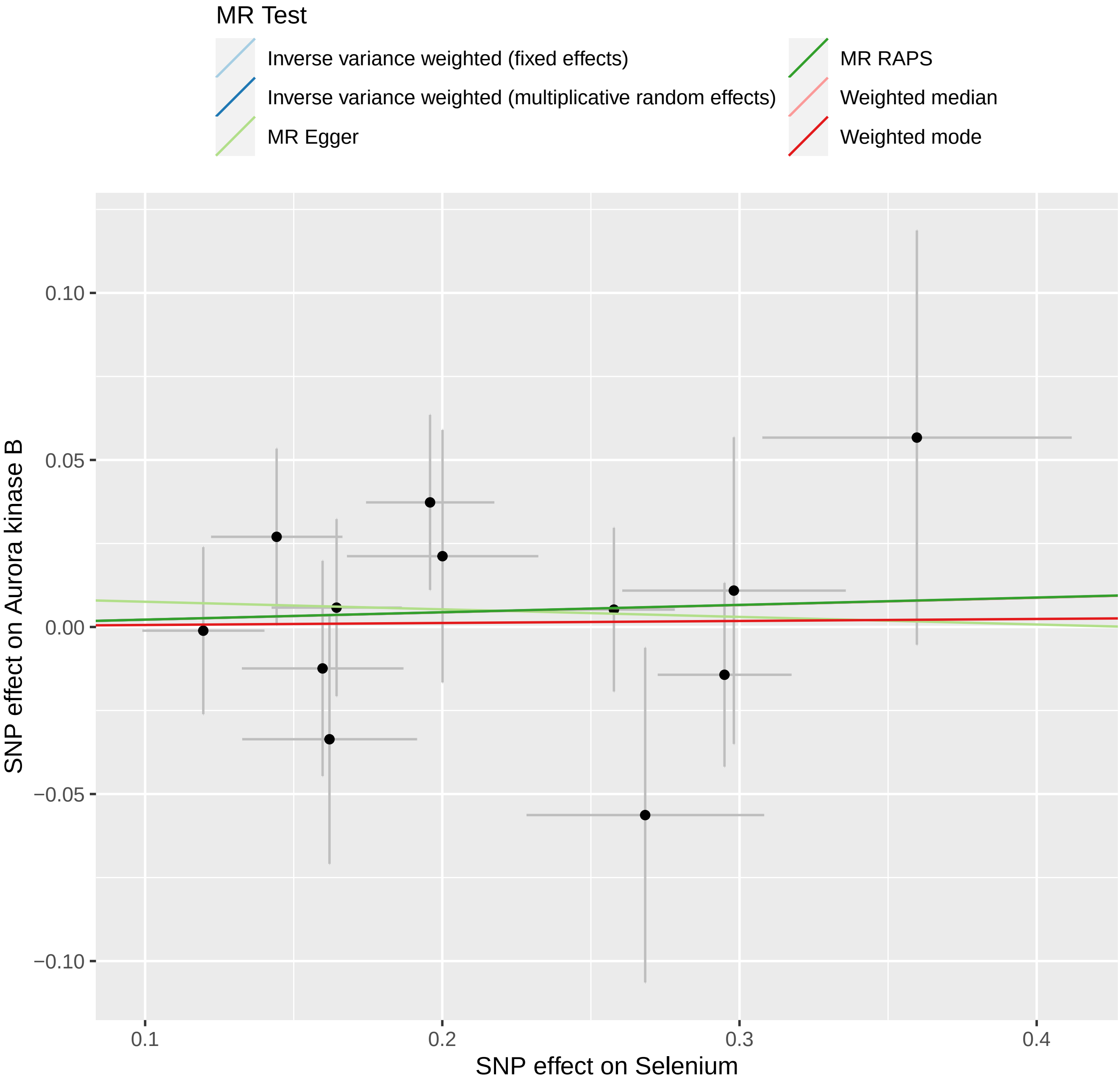

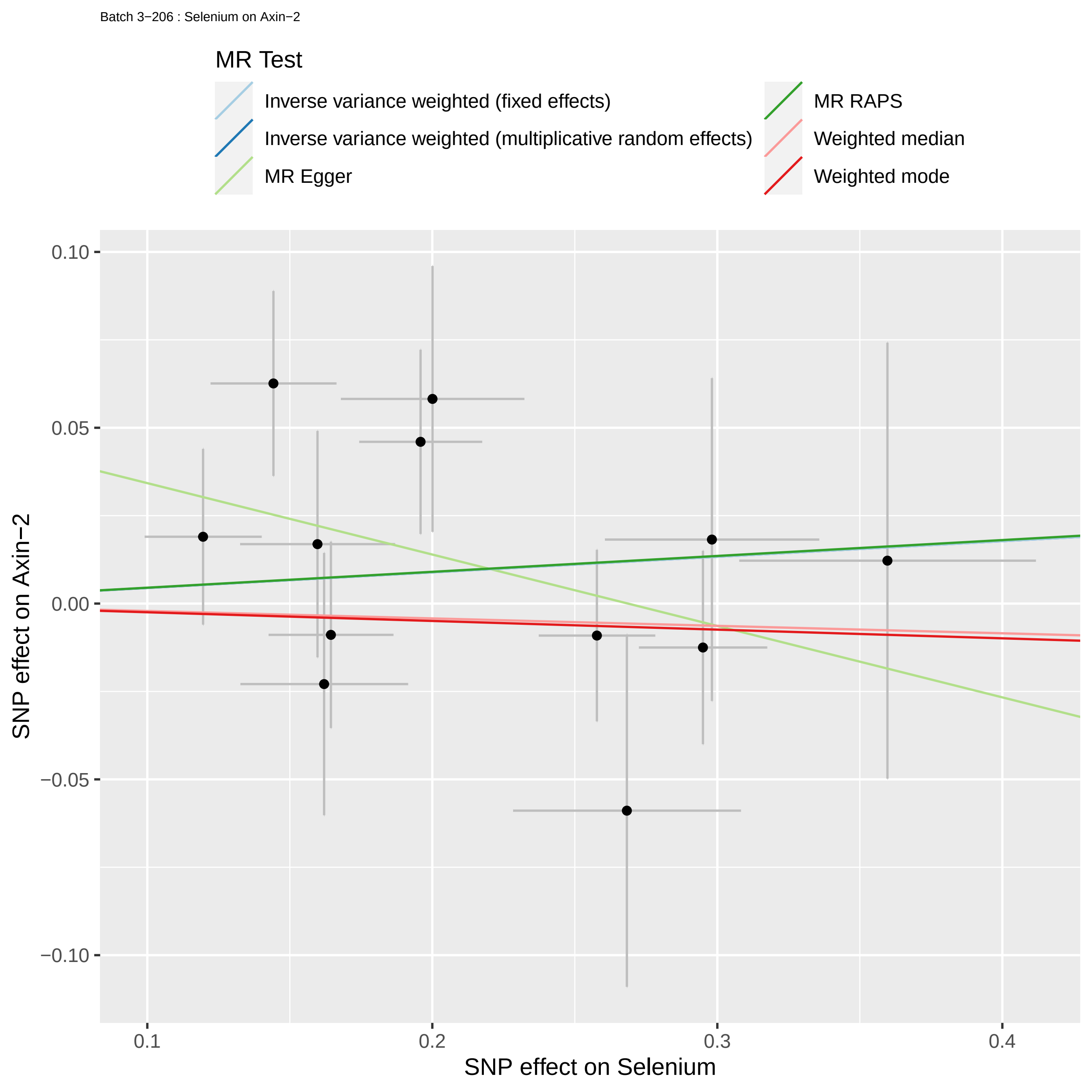

Batch 3−206 : Selenium on Axin−2
MR Test
Inverse variance weighted (fixed effects)
Inverse variance weighted (multiplicative random effects)
MR Egger
MR RAPS
Weighted median
Weighted mode
0.10
0.05
0.00
−0.05
−0.10
SNP effect on Axin−2
0.1
0.2
0.3
0.4
SNP effect on Selenium

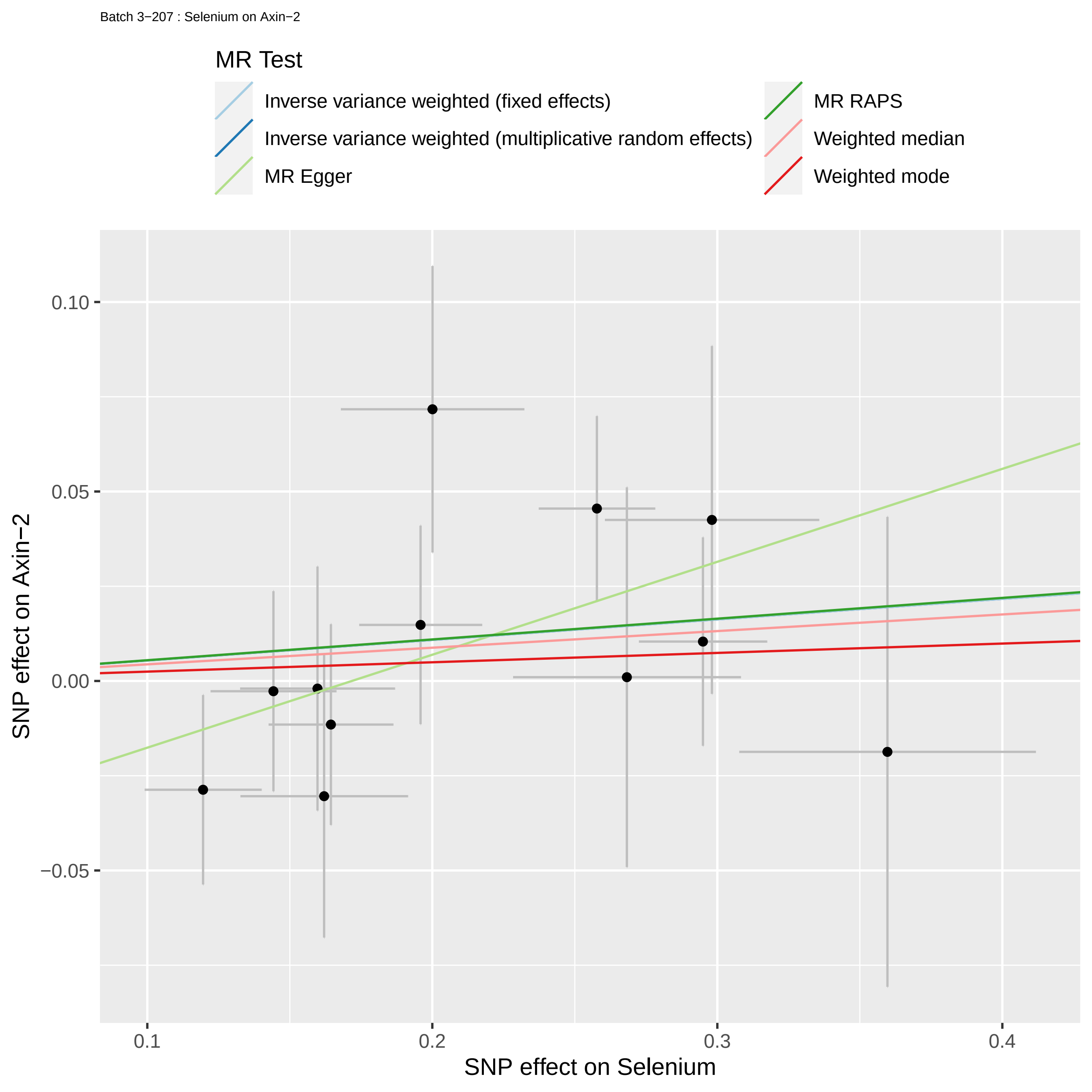
Batch 3−207 : Selenium on Axin−2
MR Test
Inverse variance weighted (fixed effects)
Inverse variance weighted (multiplicative random effects)
MR Egger
MR RAPS
Weighted median
Weighted mode
0.10
0.05
0.00
−0.05
SNP effect on Axin−2
0.1
0.2
0.3
0.4
SNP effect on Selenium

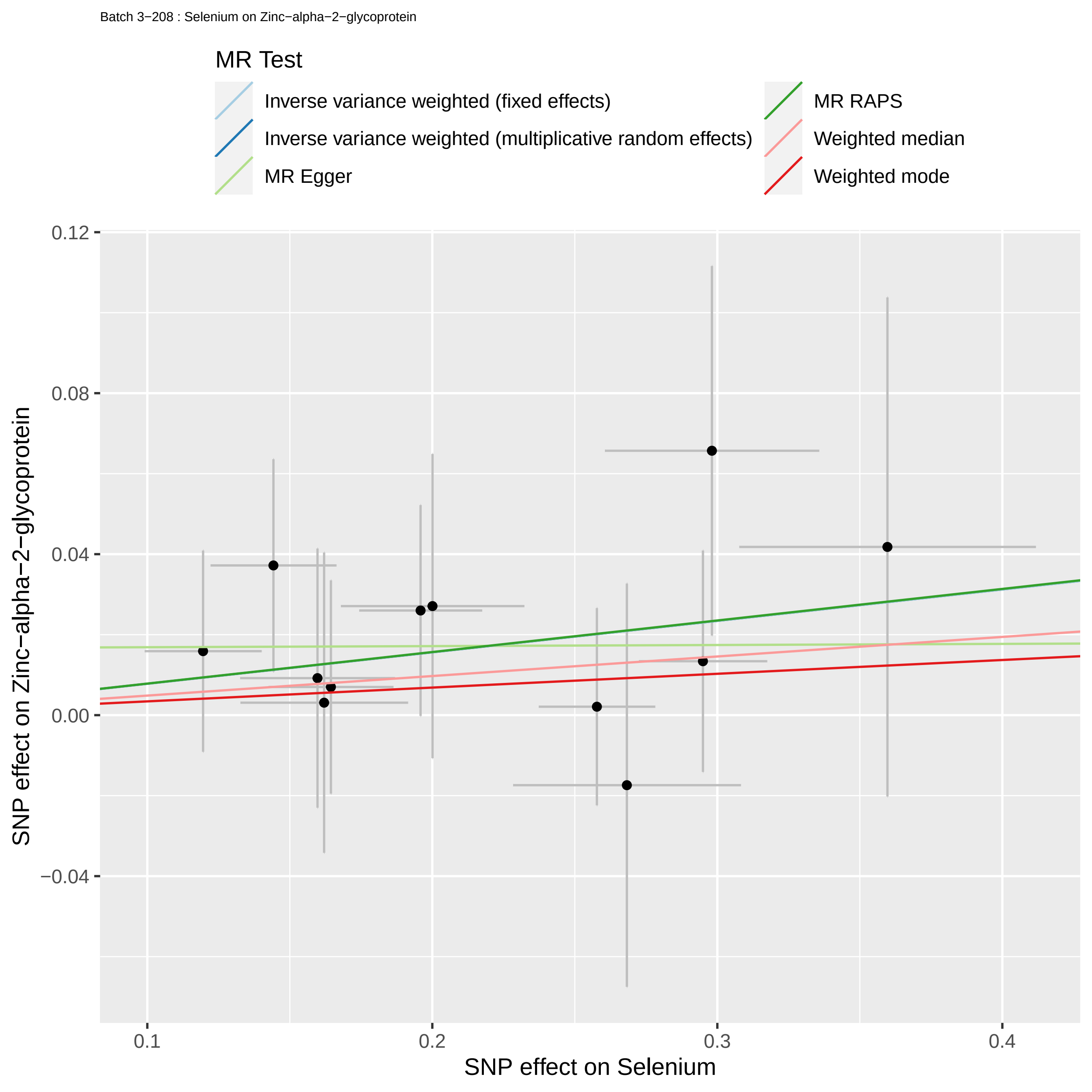
Batch 3−208 : Selenium on Zinc−alpha−2−glycoprotein
MR Test
Inverse variance weighted (fixed effects)
Inverse variance weighted (multiplicative random effects)
MR Egger
MR RAPS
Weighted median
Weighted mode
0.12
0.08
0.04
0.00
−0.04
0.1
0.2
0.3
0.4
SNP effect on Selenium
SNP effect on Zinc−alpha−2−glycoprotein

Batch 3−209 : Selenium on Beta−1,3−galactosyltransferase 6

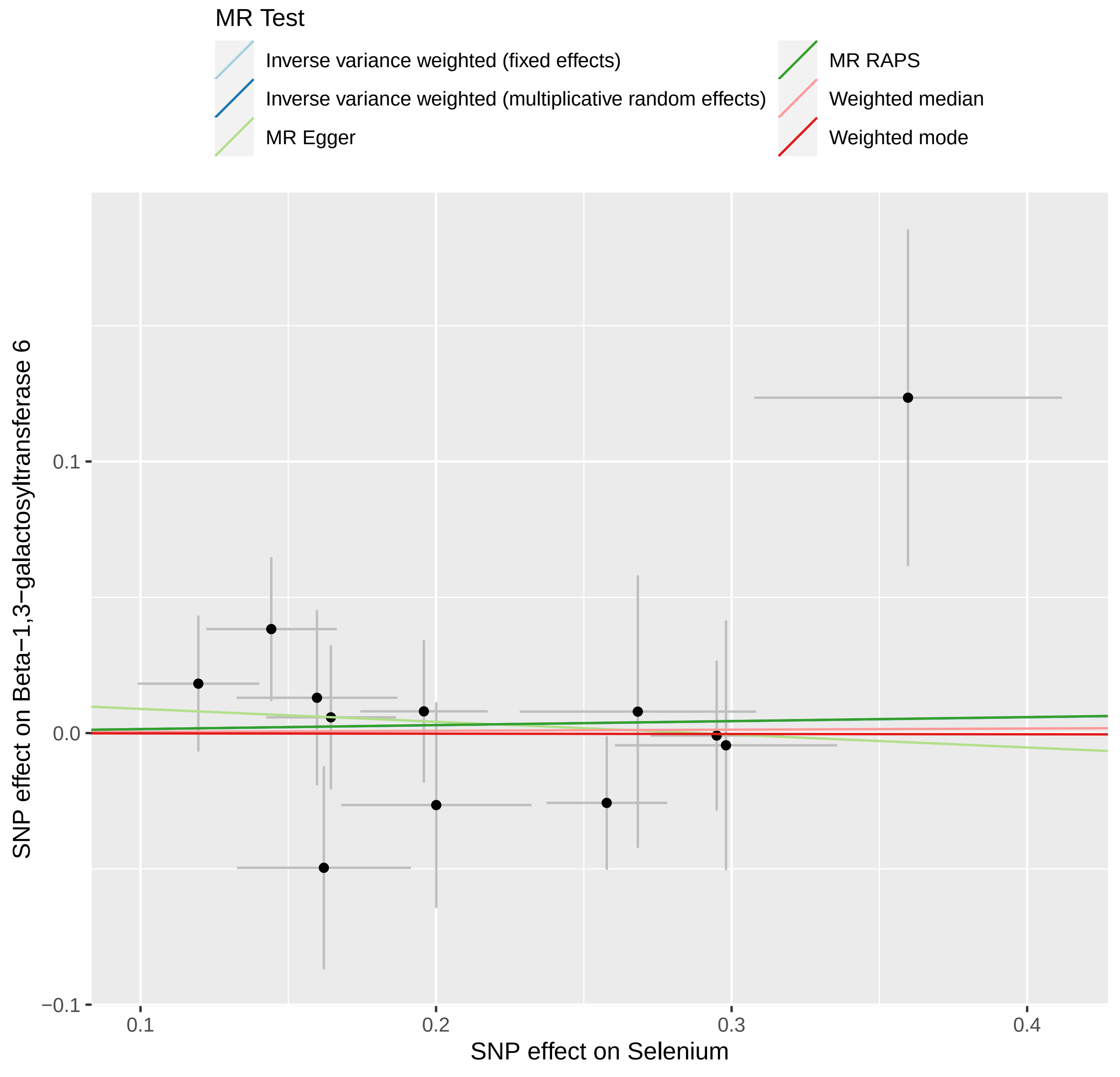

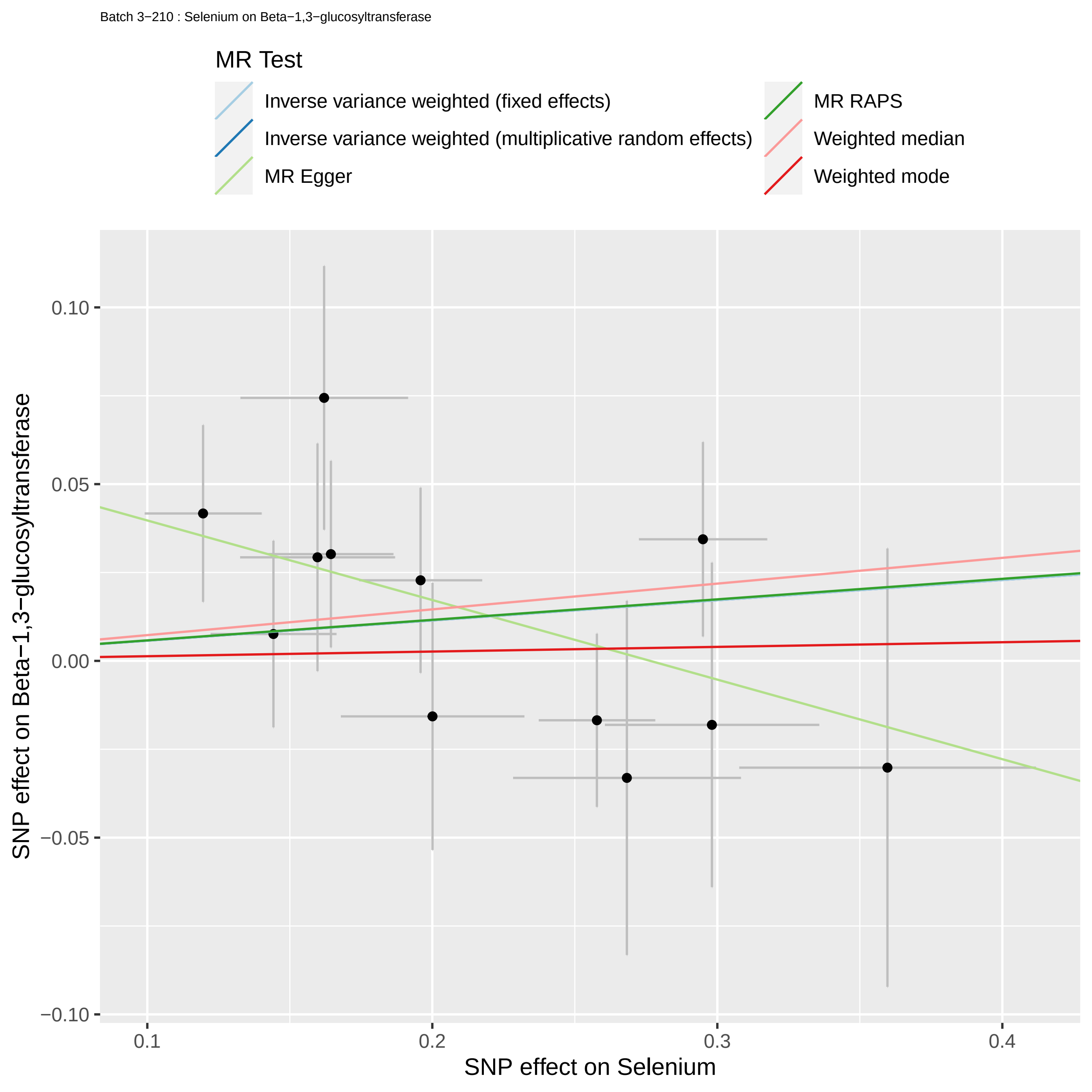

Batch 3−210 : Selenium on Beta−1,3−glucosyltransferase
MR Test
Inverse variance weighted (fixed effects)
Inverse variance weighted (multiplicative random effects)
MR Egger
MR RAPS
Weighted median
Weighted mode
0.10
0.05
0.00
−0.05
−0.10
0.1
0.2
0.3
0.4
SNP effect on Beta−1,3−glucosyltransferase
SNP effect on Selenium

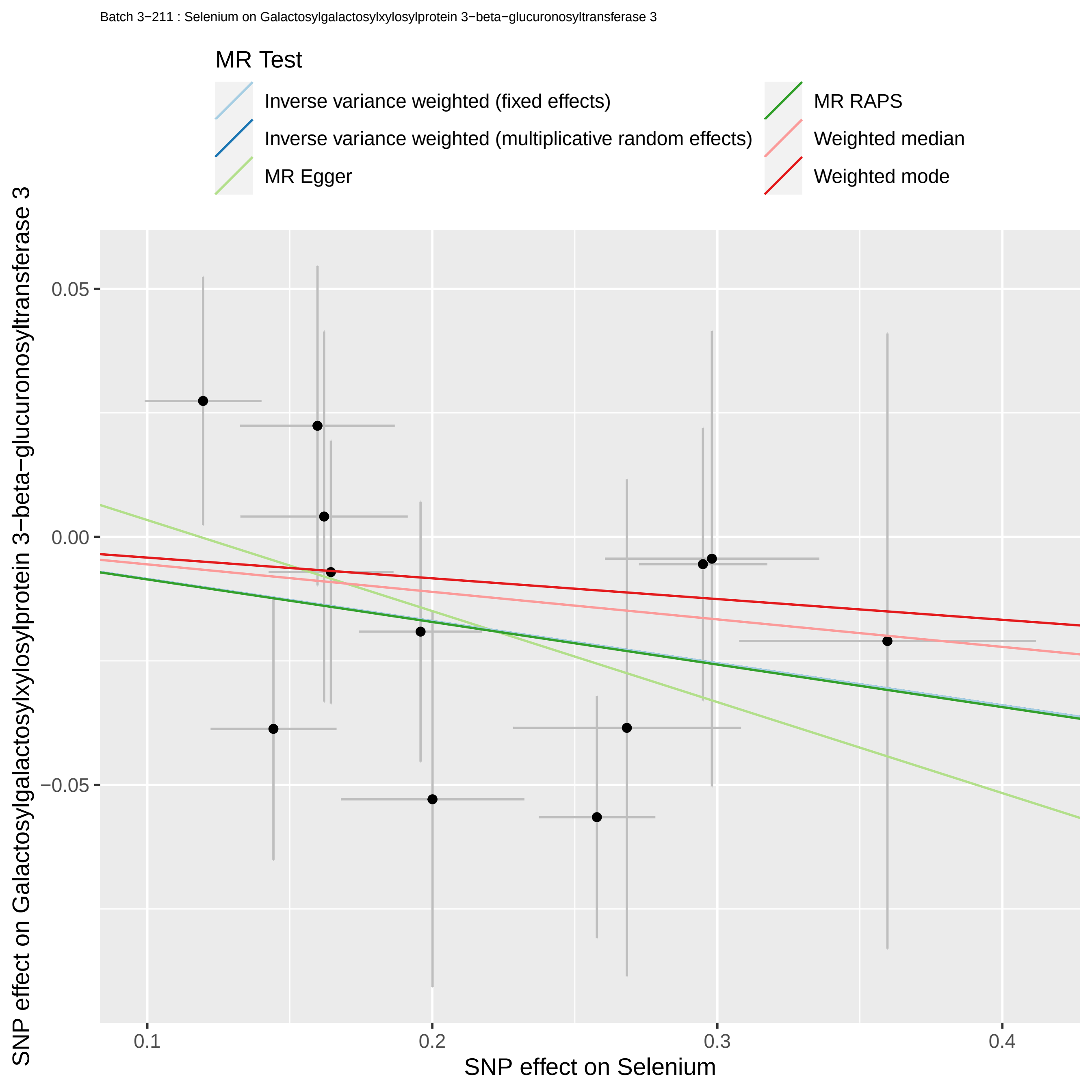
Batch 3−211 : Selenium on Galactosylgalactosylxylosylprotein 3−beta−glucuronosyltransferase 3
MR Test
Inverse variance weighted (fixed effects)
Inverse variance weighted (multiplicative random effects)
MR Egger
MR RAPS
Weighted median
Weighted mode
0.05
0.00
−0.05
0.1
0.2
0.3
0.4
SNP effect on Galactosylgalactosylxylosylprotein 3−beta−glucuronosyltransferase 3
SNP effect on Selenium

Batch 3−212 : Selenium on Beta−1,4−glucuronyltransferase 1

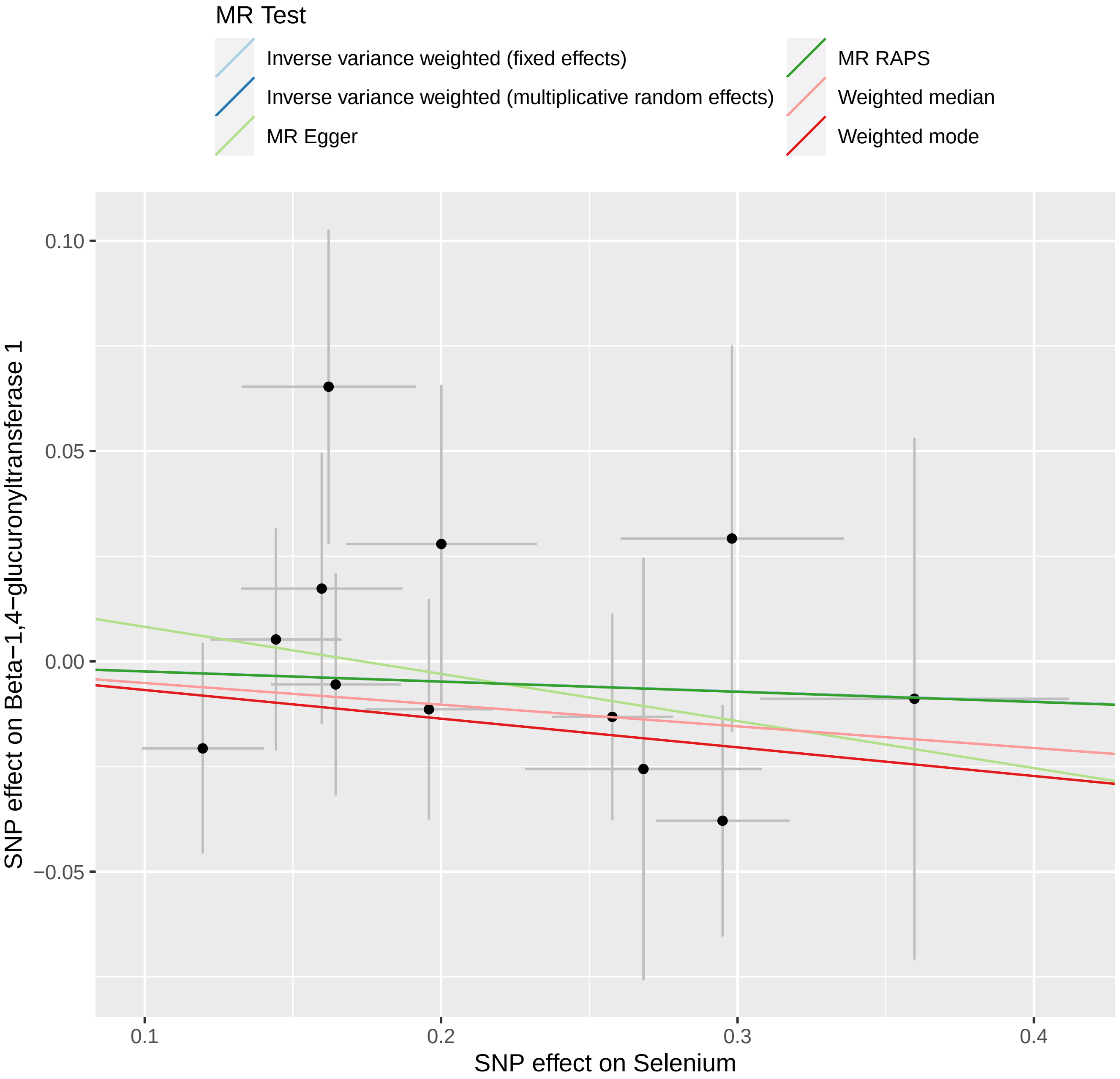

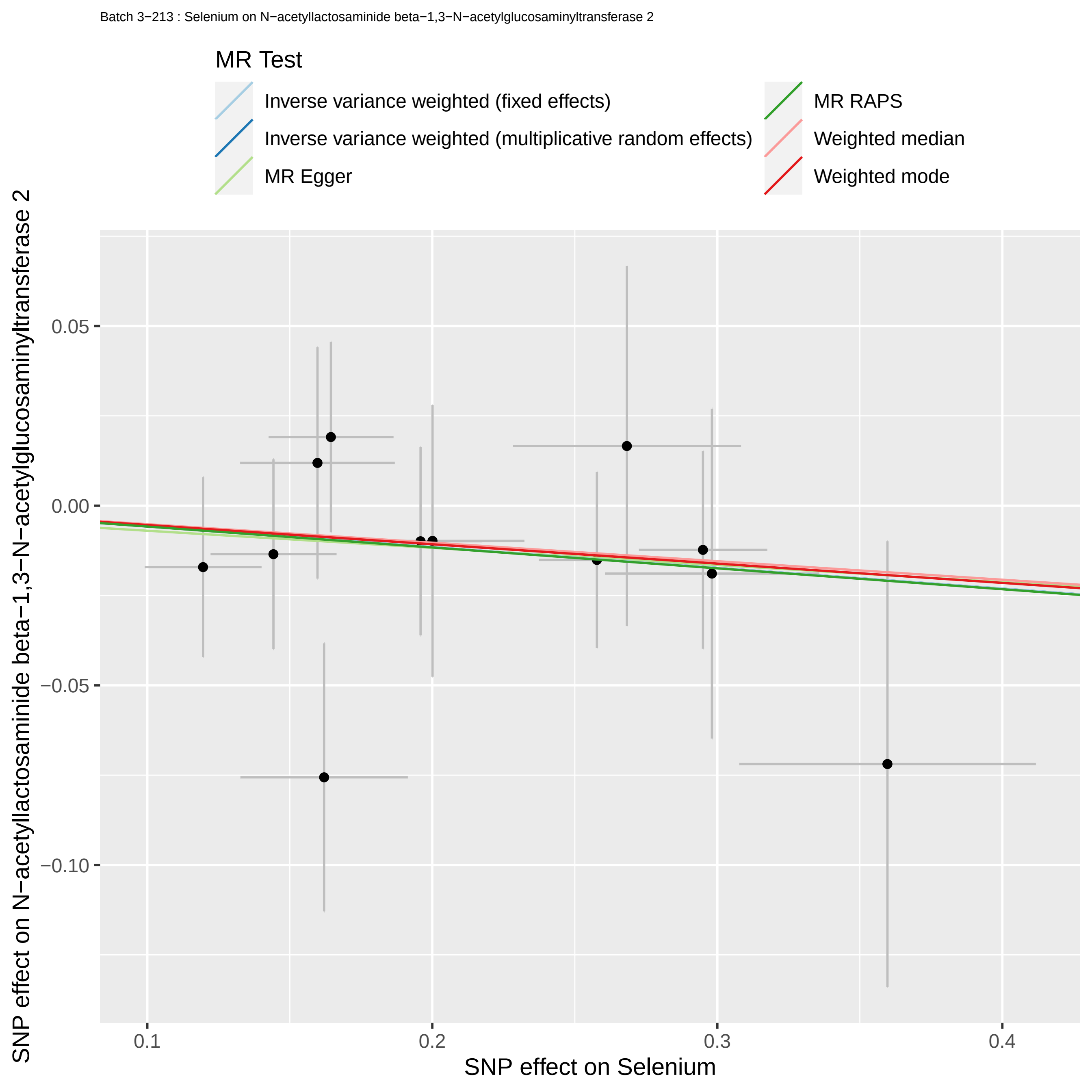

Batch 3−213 : Selenium on N−acetyllactosaminide beta−1,3−N−acetylglucosaminyltransferase 2
MR Test
Inverse variance weighted (fixed effects)
Inverse variance weighted (multiplicative random effects)
MR Egger
MR RAPS
Weighted median
Weighted mode
SNP effect on N−acetyllactosaminide beta−1,3−N−acetylglucosaminyltransferase 2
0.05
0.00
−0.05
−0.10
0.1
0.2
0.3
0.4
SNP effect on Selenium

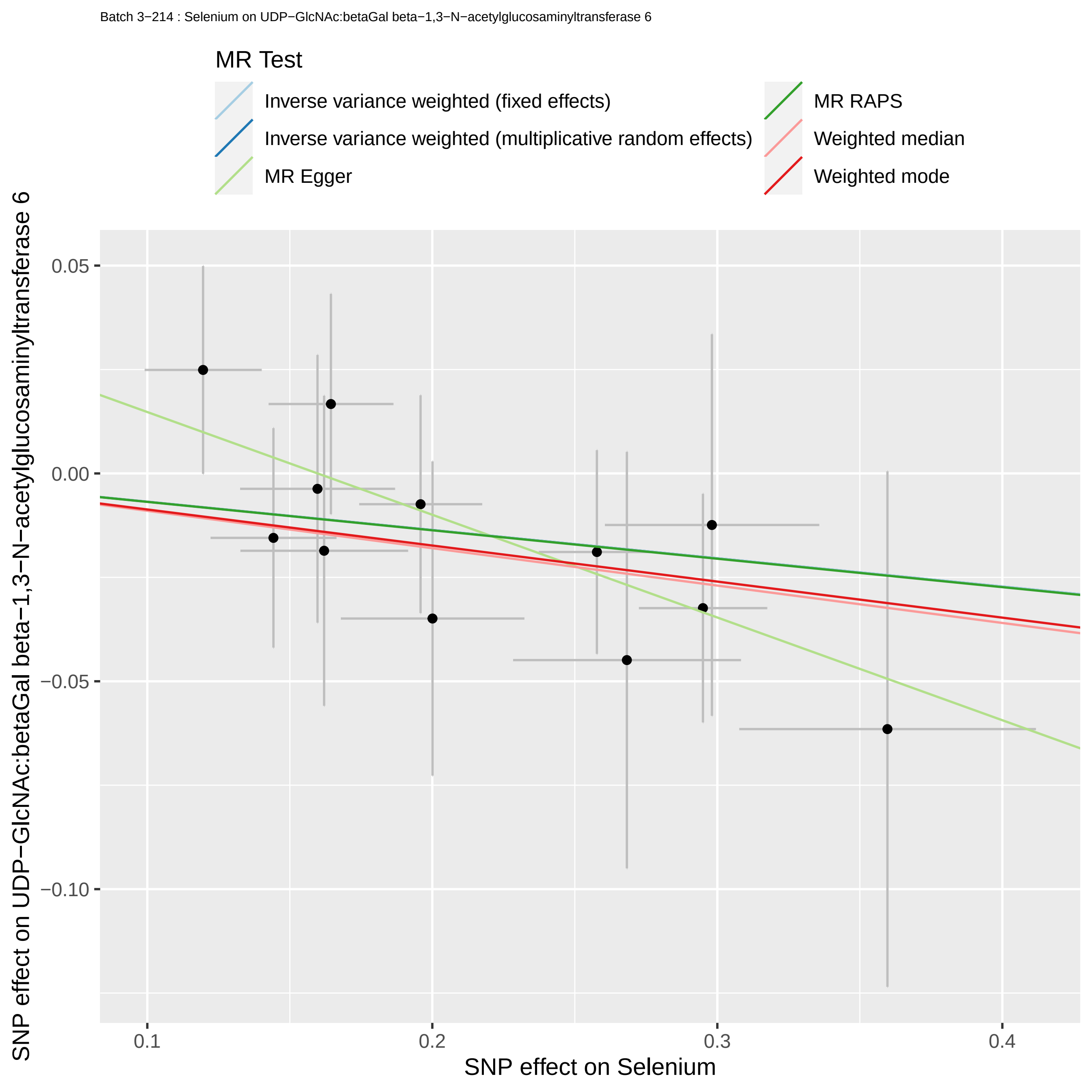
Batch 3−214 : Selenium on UDP−GlcNAc:betaGal beta−1,3−N−acetylglucosaminyltransferase 6
MR Test
Inverse variance weighted (fixed effects)
Inverse variance weighted (multiplicative random effects)
MR Egger
MR RAPS
Weighted median
Weighted mode
SNP effect on UDP−GlcNAc:betaGal beta−1,3−N−acetylglucosaminyltransferase 6
0.05
0.00
−0.05
−0.10
0.1
0.2
0.3
0.4
SNP effect on Selenium

Batch 3−215 : Selenium on UDP−GlcNAc:betaGal beta−1,3−N−acetylglucosaminyltransferase 8

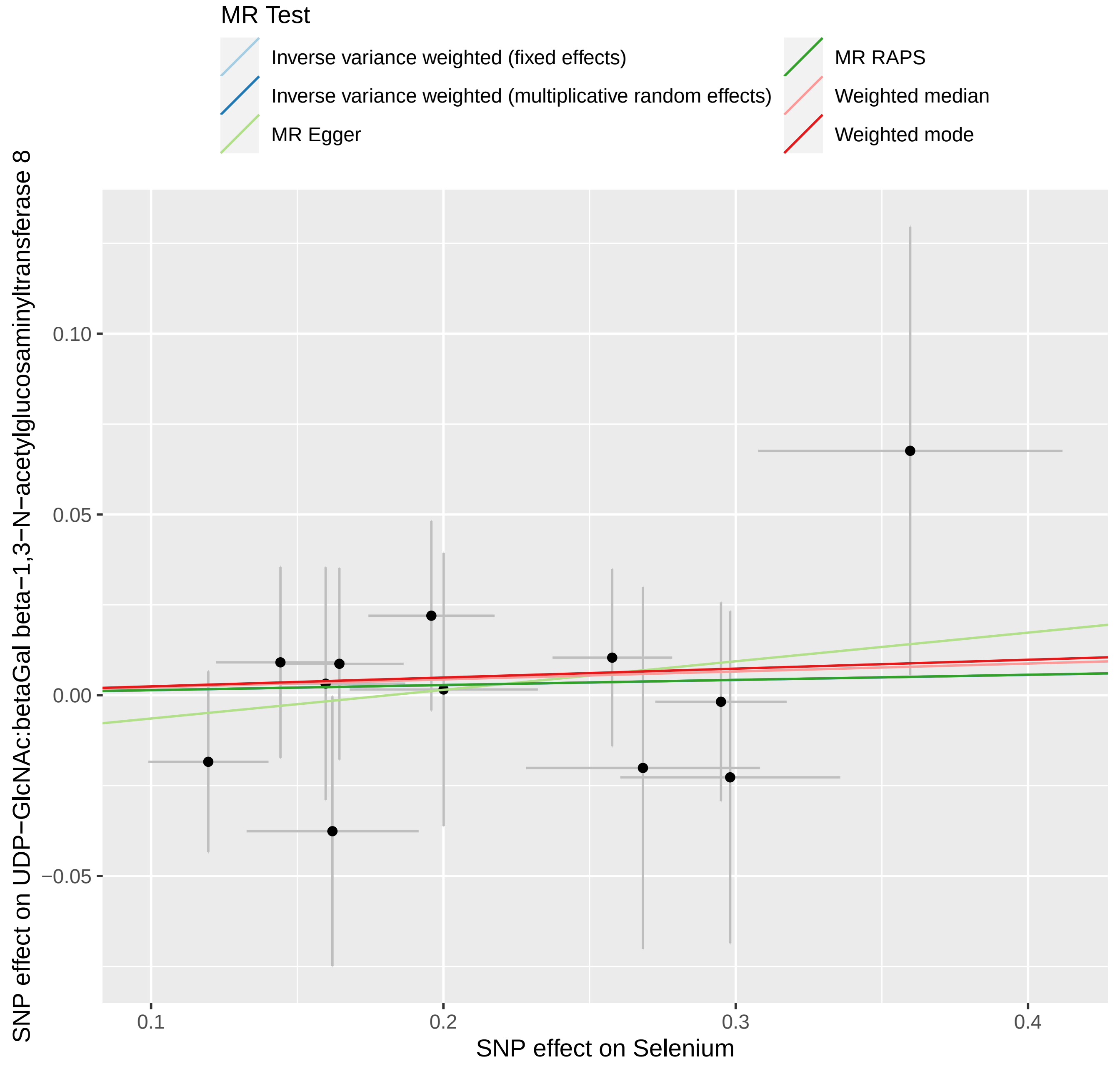

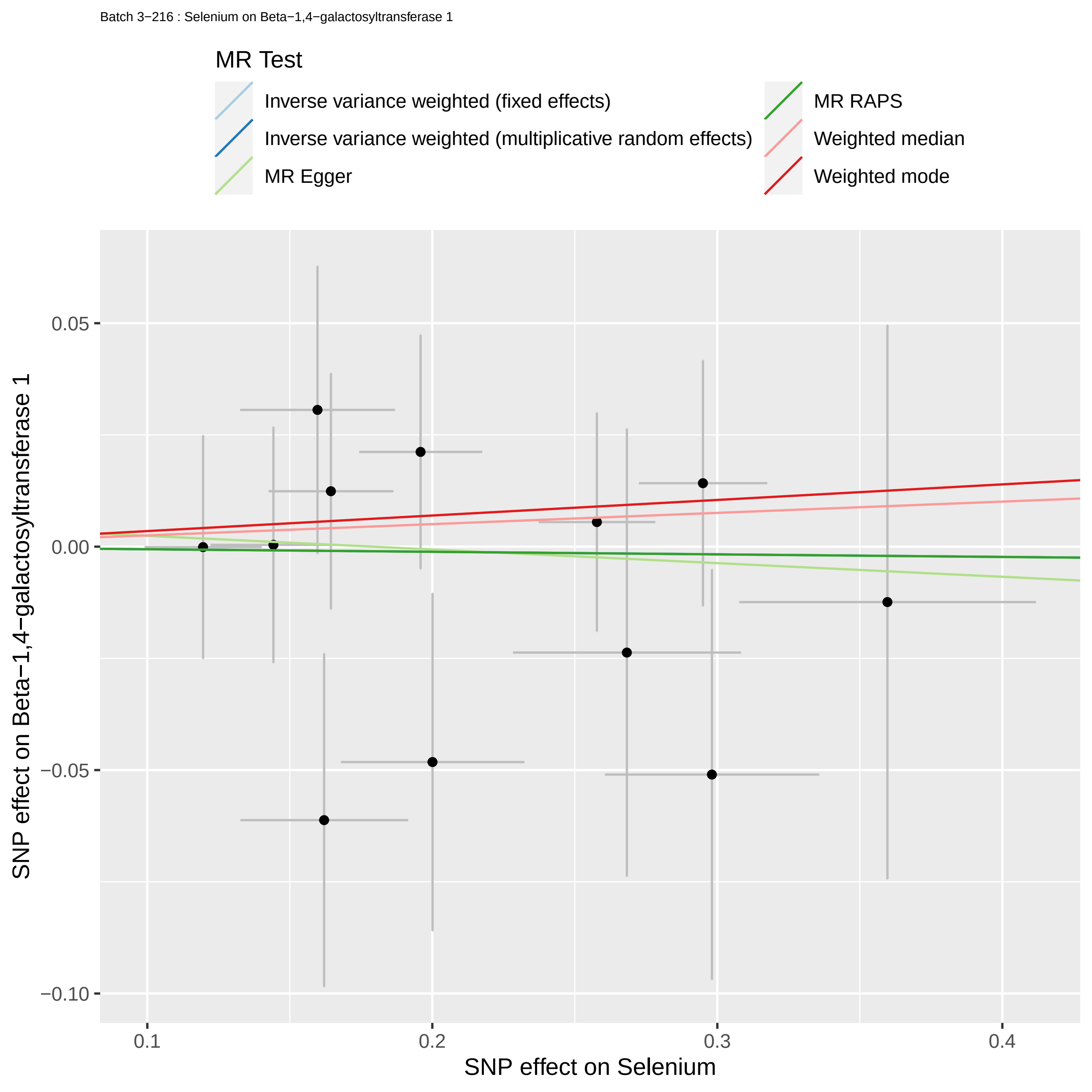

Batch 3−216 : Selenium on Beta−1,4−galactosyltransferase 1
MR Test
Inverse variance weighted (fixed effects)
Inverse variance weighted (multiplicative random effects)
MR Egger
MR RAPS
Weighted median
Weighted mode
0.05
0.00
−0.05
−0.10
0.1
0.2
0.3
0.4
SNP effect on Beta−1,4−galactosyltransferase 1
SNP effect on Selenium

Batch 3−217 : Selenium on Beta−1,4−galactosyltransferase 2

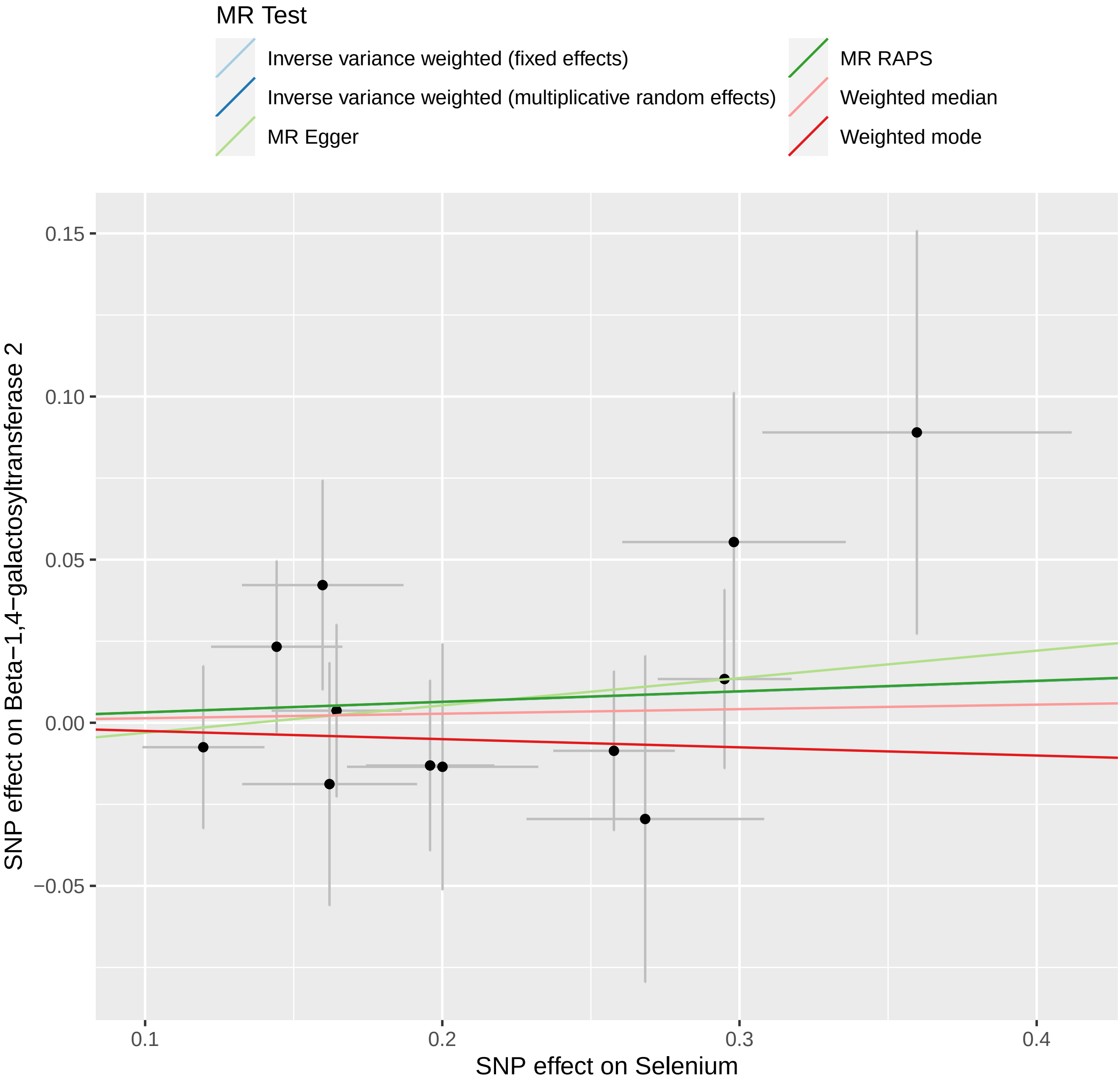

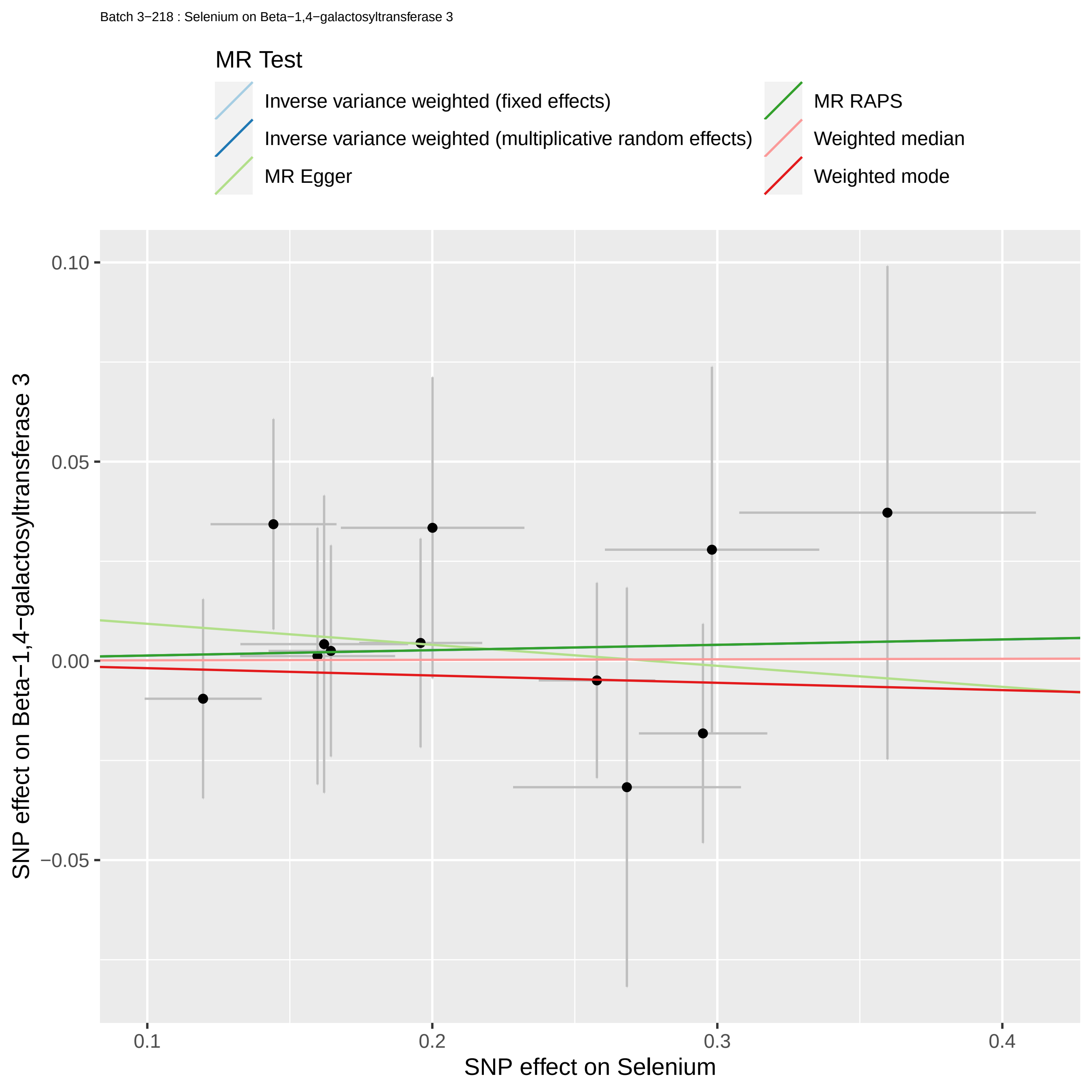

Batch 3−218 : Selenium on Beta−1,4−galactosyltransferase 3
MR Test
Inverse variance weighted (fixed effects)
Inverse variance weighted (multiplicative random effects)
MR Egger
MR RAPS
Weighted median
Weighted mode
0.10
0.05
0.00
−0.05
0.1
0.2
0.3
0.4
SNP effect on Selenium
SNP effect on Beta−1,4−galactosyltransferase 3

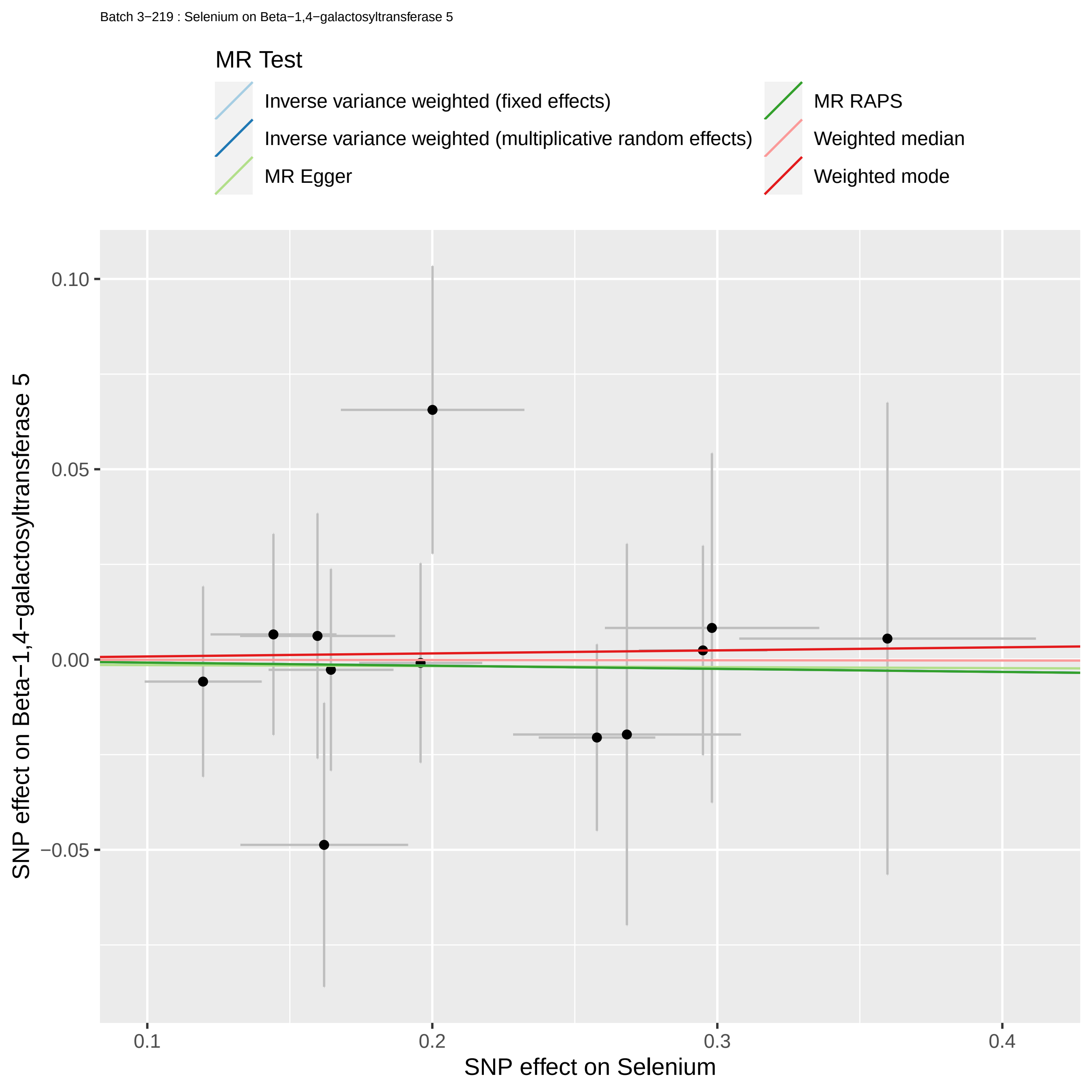

Batch 3−219 : Selenium on Beta−1,4−galactosyltransferase 5
MR Test
Inverse variance weighted (fixed effects)
Inverse variance weighted (multiplicative random effects)
MR Egger
MR RAPS
Weighted median
Weighted mode
0.10
0.05
0.00
−0.05
0.1
0.2
0.3
0.4
SNP effect on Selenium
SNP effect on Beta−1,4−galactosyltransferase 5

Batch 3−220 : Selenium on Beta−1,4−galactosyltransferase 6

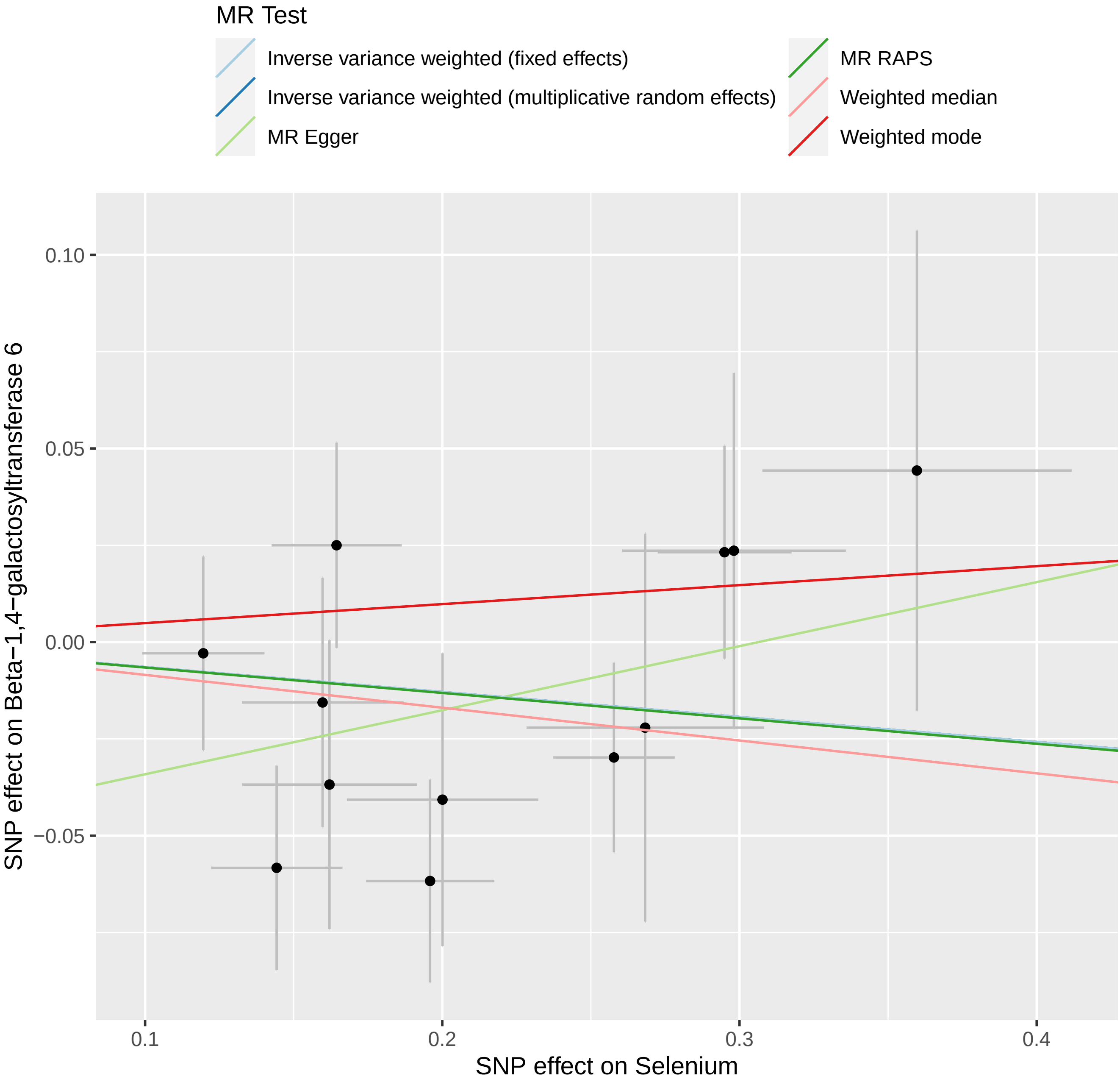

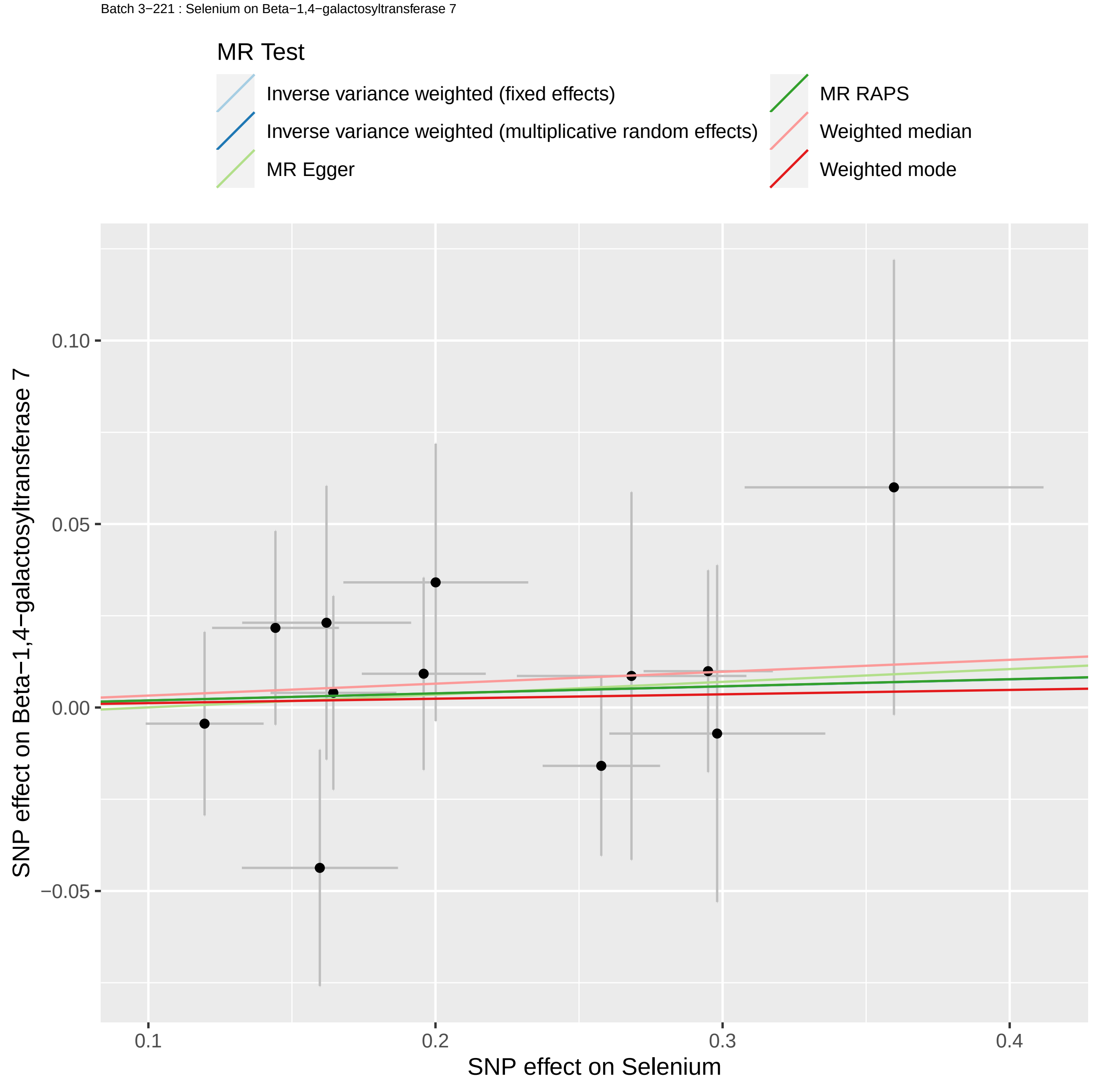

Batch 3−221 : Selenium on Beta−1,4−galactosyltransferase 7
MR Test
Inverse variance weighted (fixed effects)
Inverse variance weighted (multiplicative random effects)
MR Egger
MR RAPS
Weighted median
Weighted mode
SNP effect on Beta−1,4−galactosyltransferase 7
0.10
0.05
0.00
−0.05
0.1
0.2
0.3
0.4
SNP effect on Selenium

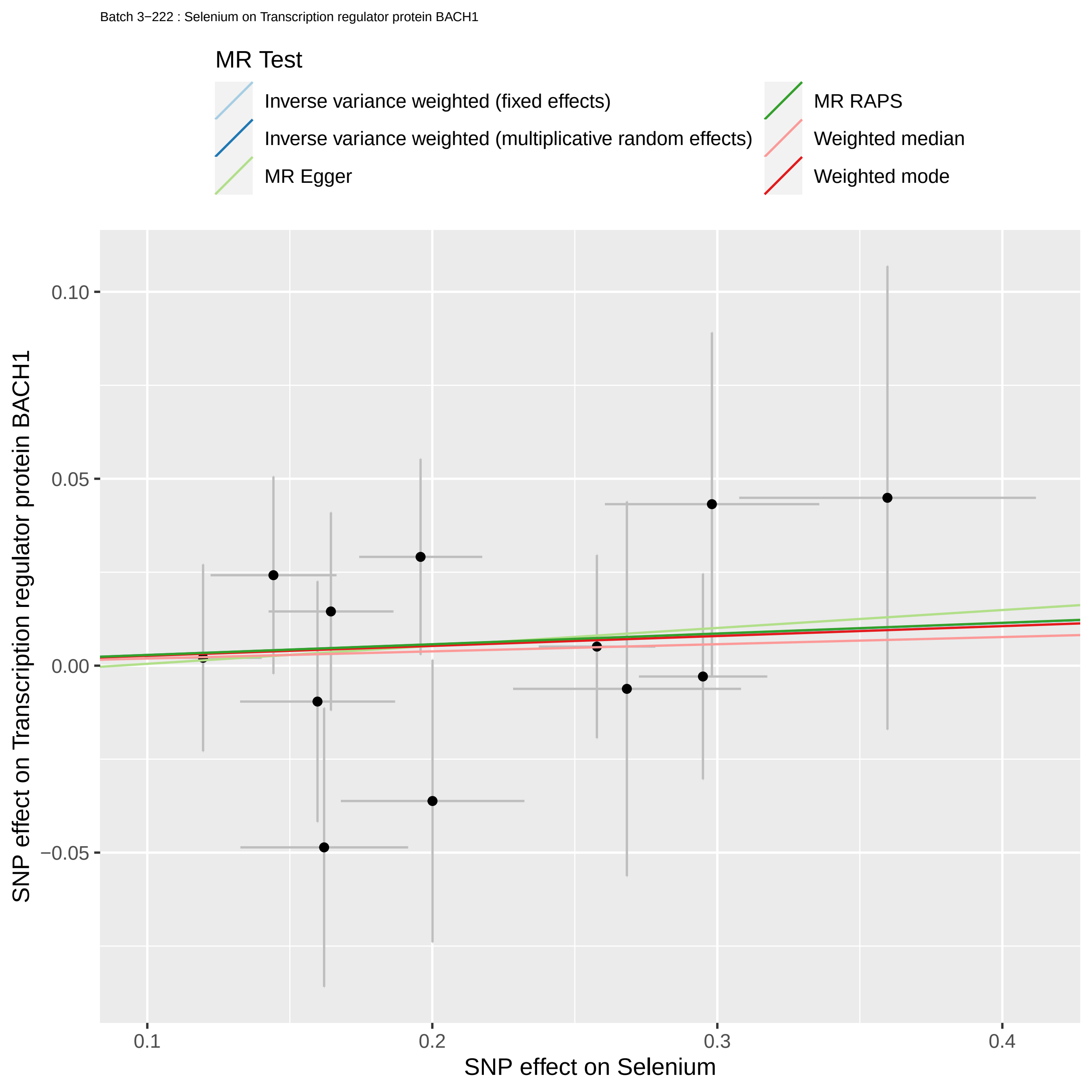

Batch 3−222 : Selenium on Transcription regulator protein BACH1
MR Test
Inverse variance weighted (fixed effects)
Inverse variance weighted (multiplicative random effects)
MR Egger
MR RAPS
Weighted median
Weighted mode
0.10
0.05
0.00
−0.05
0.1
0.2
0.3
0.4
SNP effect on Selenium
SNP effect on Transcription regulator protein BACH1

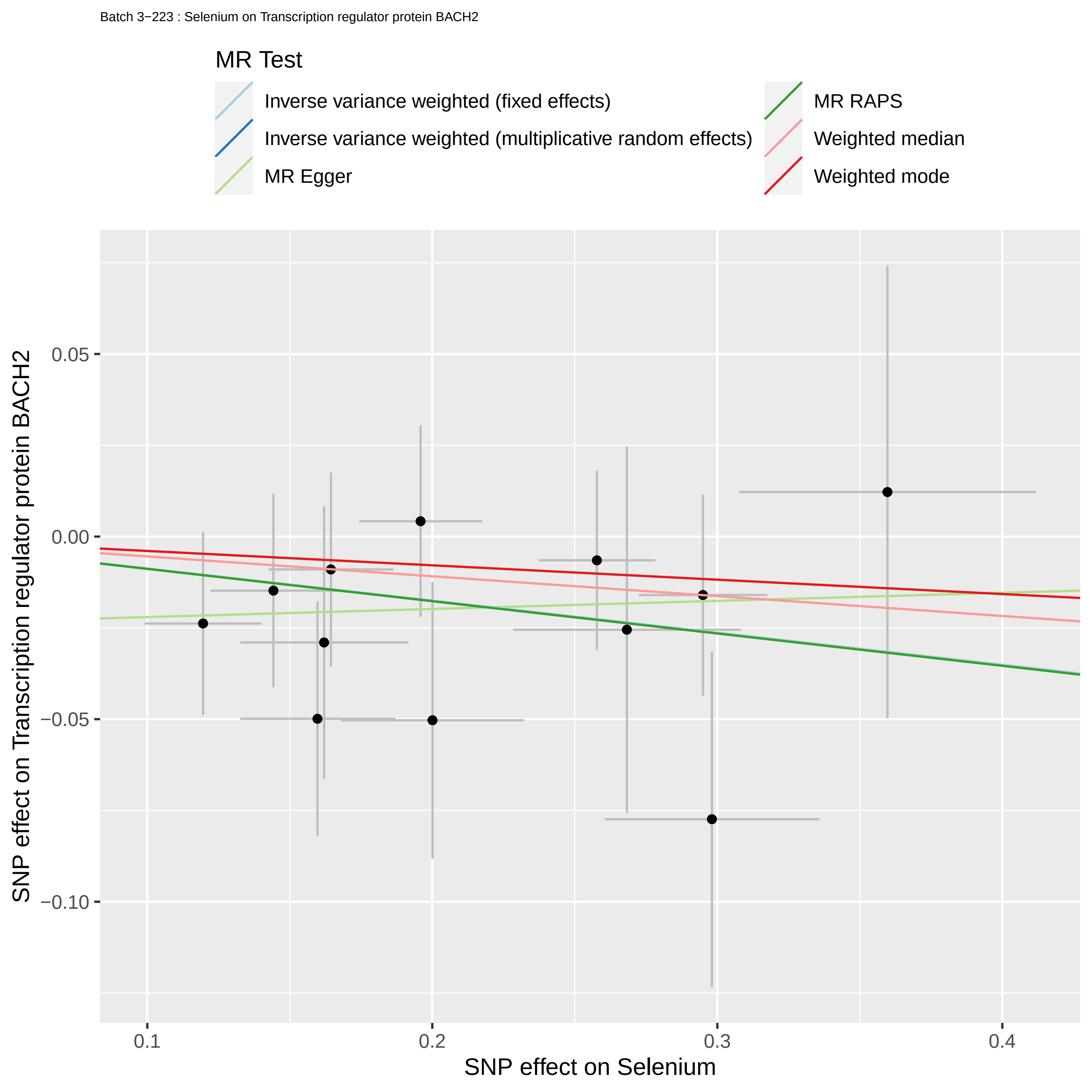

Batch 3−223 : Selenium on Transcription regulator protein BACH2
MR Test
Inverse variance weighted (fixed effects)
Inverse variance weighted (multiplicative random effects)
MR Egger
MR RAPS
Weighted median
Weighted mode
0.05
0.00
−0.05
−0.10
0.1
0.2
0.3
0.4
SNP effect on Selenium
SNP effect on Transcription regulator protein BACH2

Batch 3−224 : Selenium on Bcl2−associated agonist of cell death

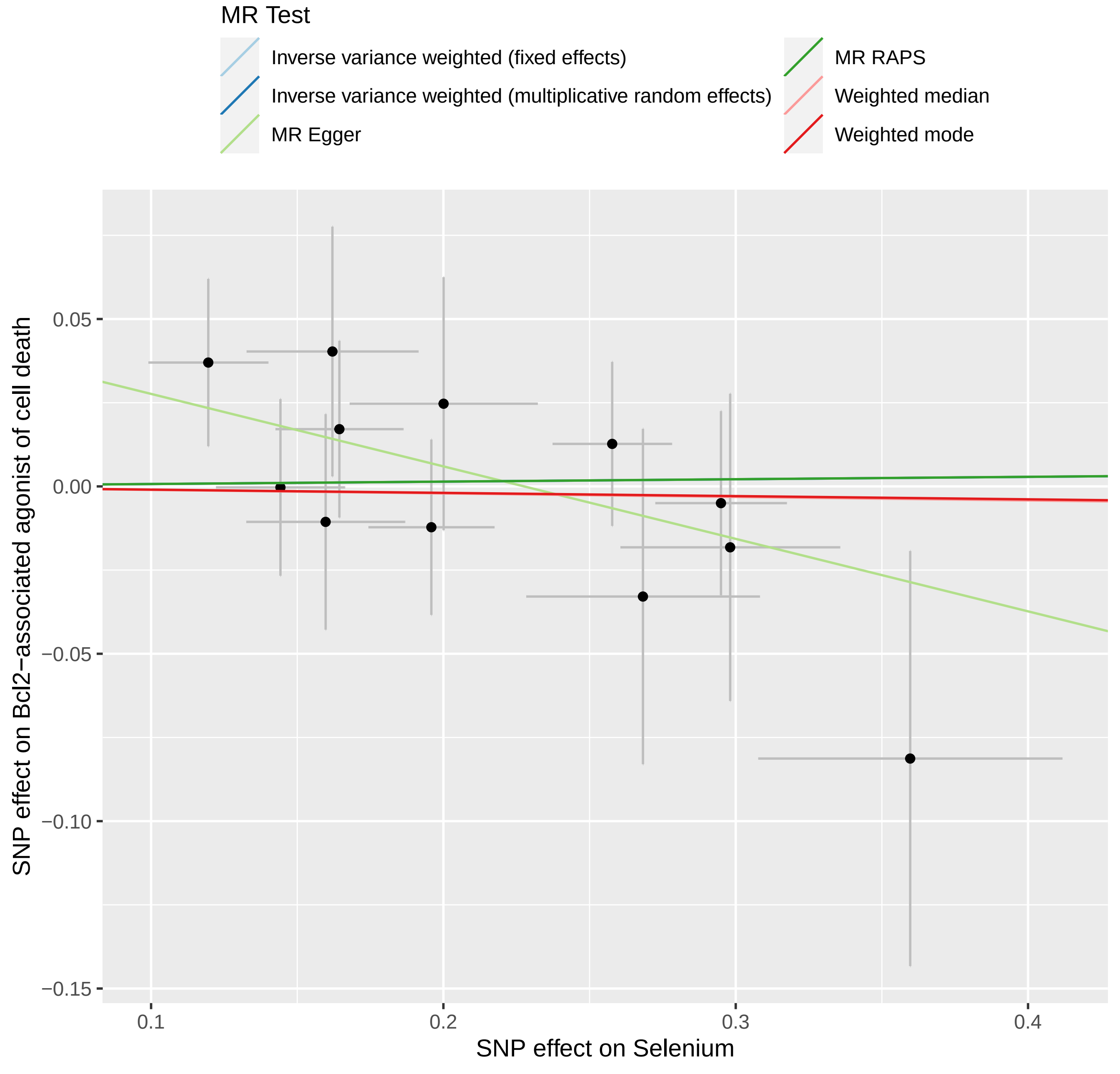

Batch 3−225 : Selenium on BAG family molecular chaperone regulator 3

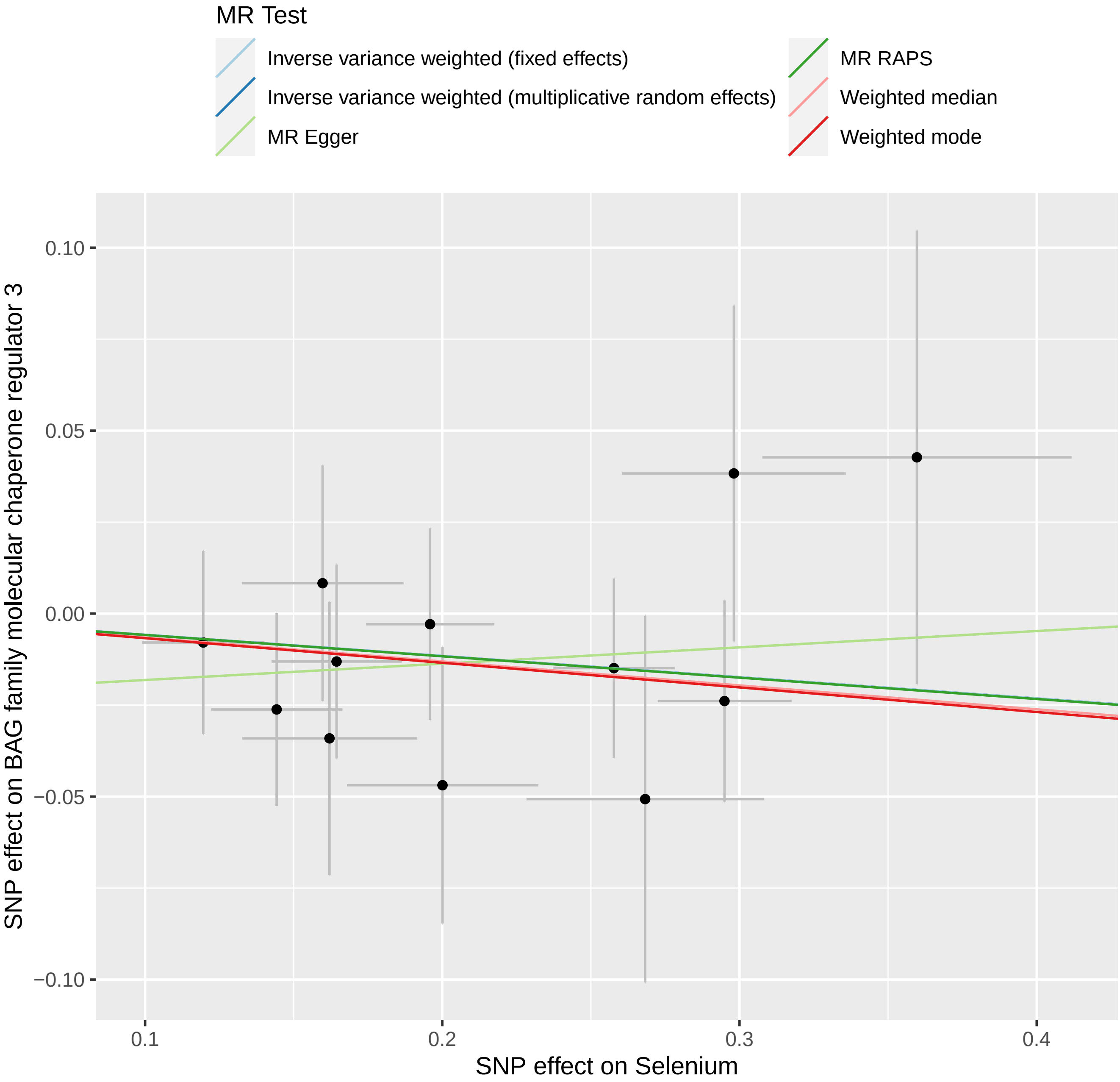

Batch 3−226 : Selenium on BAG family molecular chaperone regulator 4

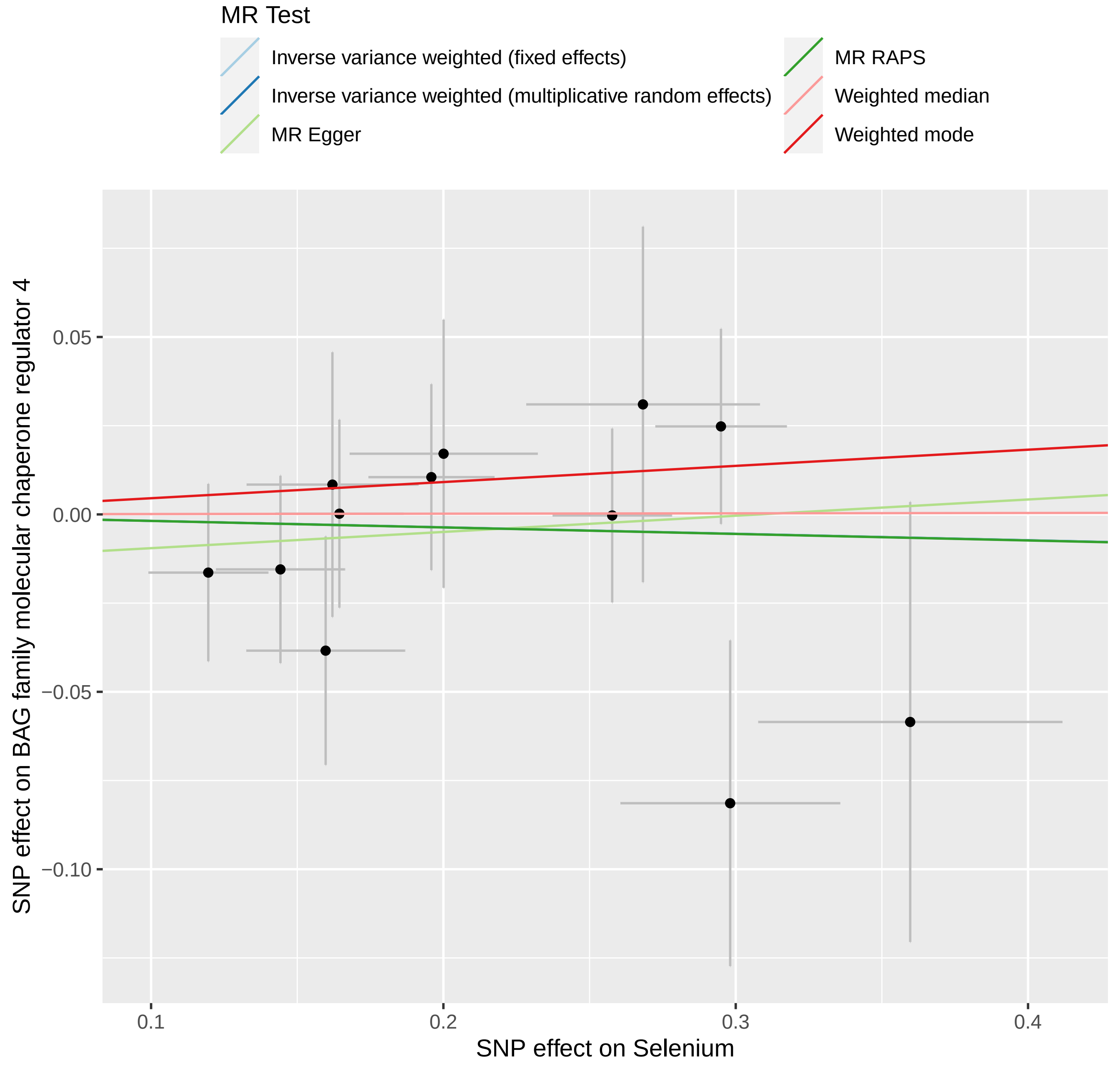

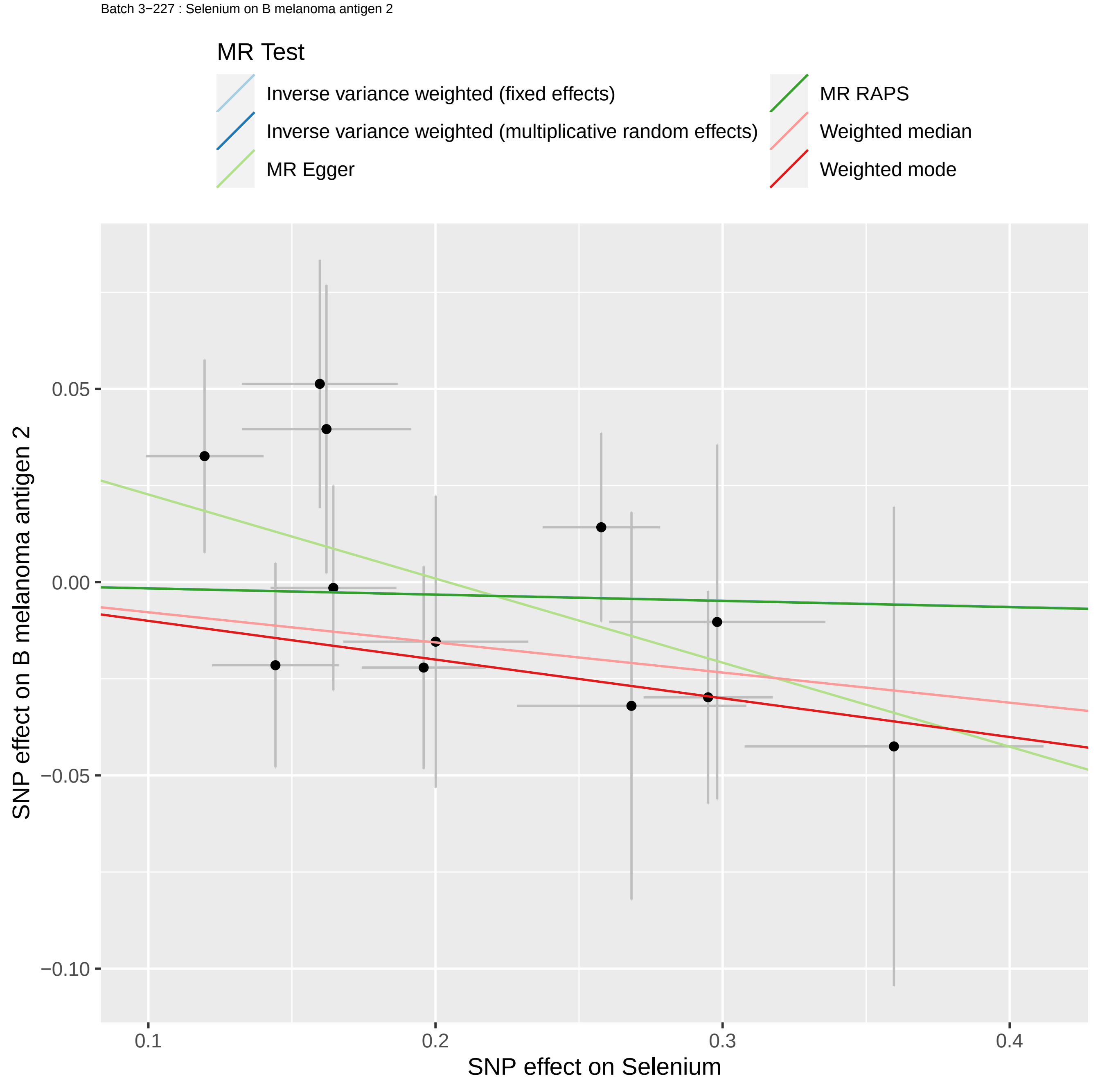

Batch 3−227 : Selenium on B melanoma antigen 2
MR Test
Inverse variance weighted (fixed effects)
Inverse variance weighted (multiplicative random effects)
MR Egger
MR RAPS
Weighted median
Weighted mode
0.05
0.00
−0.05
−0.10
0.1
0.2
0.3
0.4
SNP effect on B melanoma antigen 2
SNP effect on Selenium

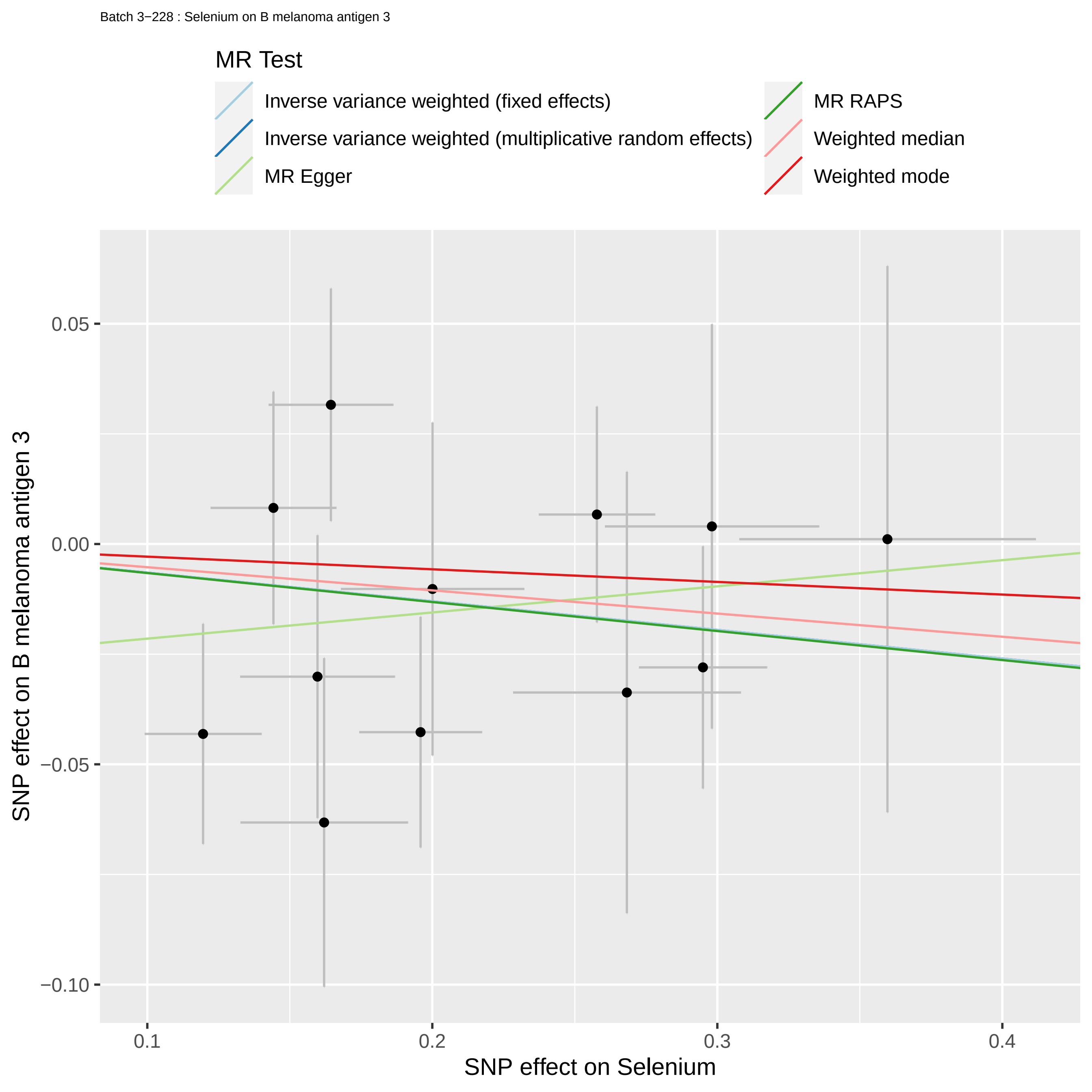

Batch 3−228 : Selenium on B melanoma antigen 3
MR Test
Inverse variance weighted (fixed effects)
Inverse variance weighted (multiplicative random effects)
MR Egger
MR RAPS
Weighted median
Weighted mode
0.05
0.00
−0.05
−0.10
0.1
0.2
0.3
0.4
SNP effect on B melanoma antigen 3
SNP effect on Selenium

Batch 3−229 : Selenium on BRCA1−associated RING domain protein 1

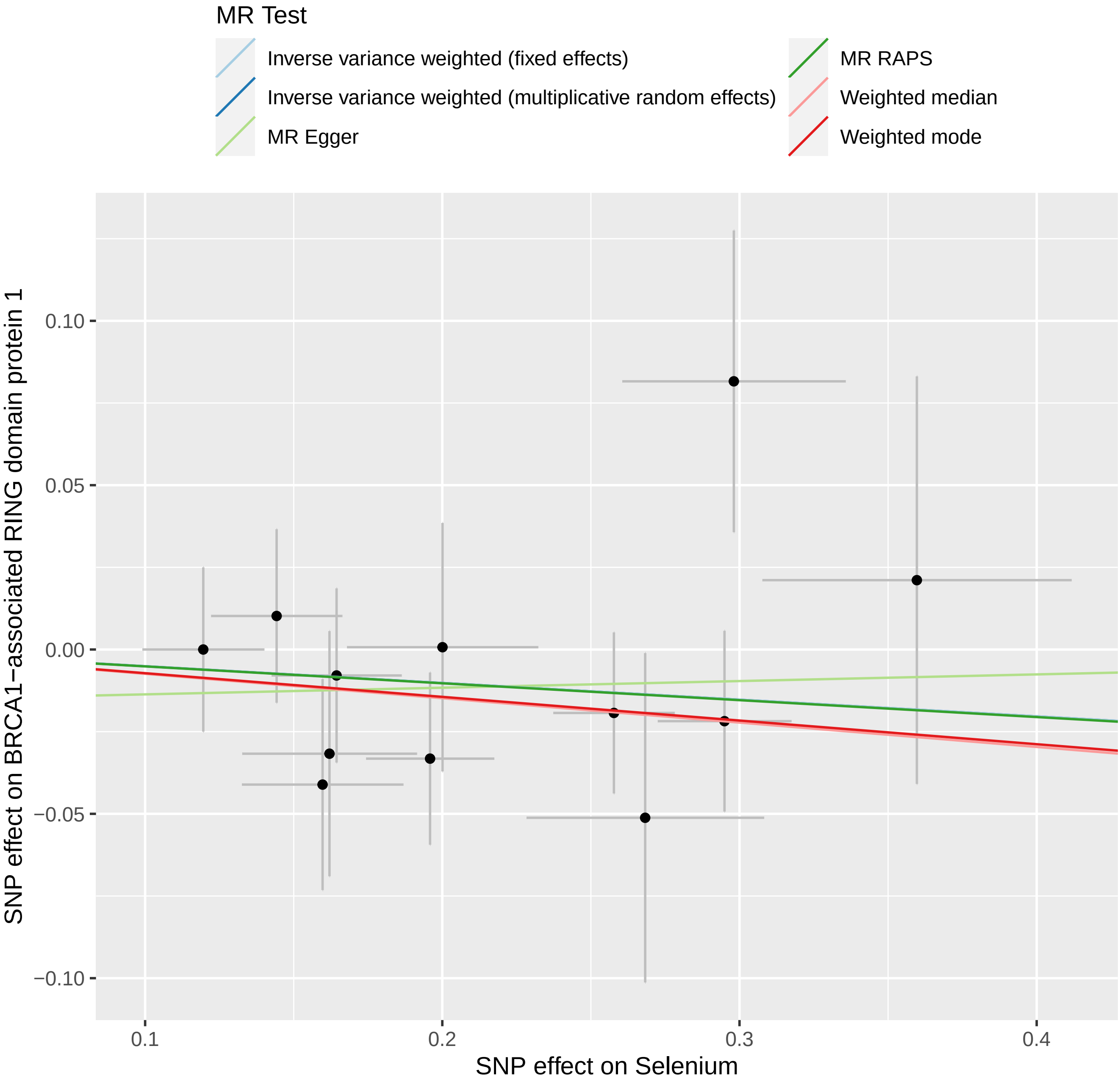

Batch 3−230 : Selenium on Basic leucine zipper transcriptional factor ATF−like 3

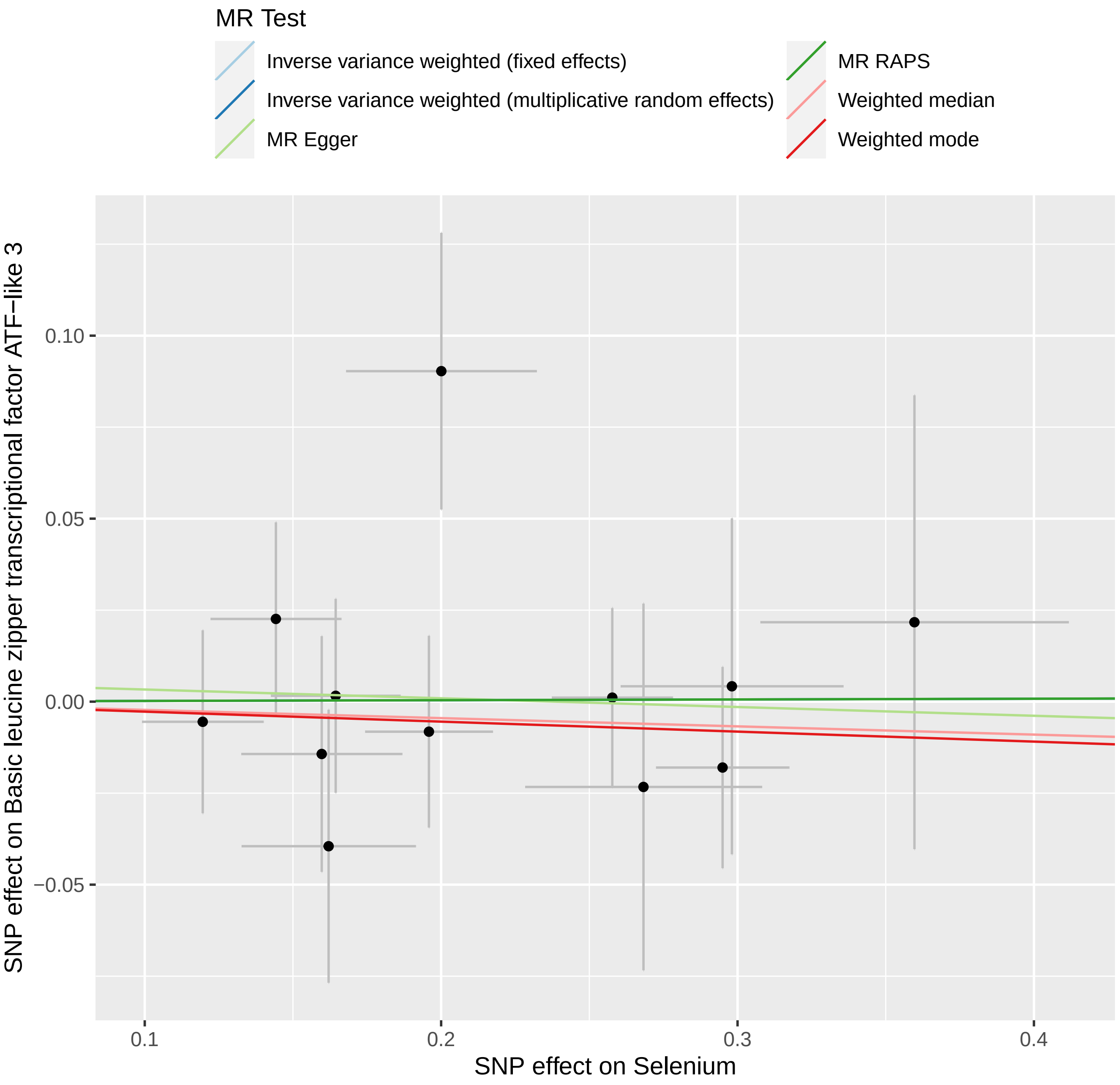

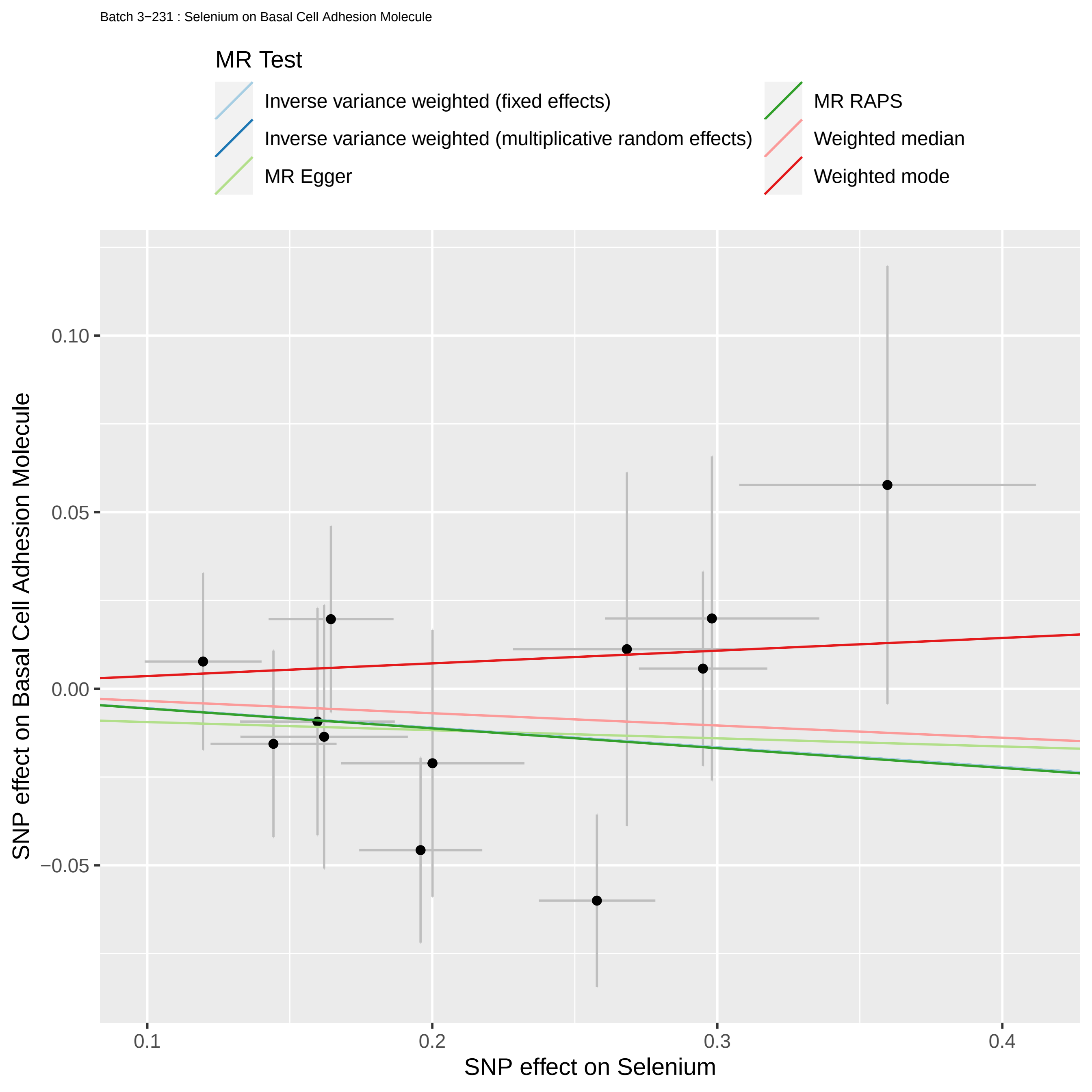
Batch 3−231 : Selenium on Basal Cell Adhesion Molecule
MR Test
Inverse variance weighted (fixed effects)
Inverse variance weighted (multiplicative random effects)
MR Egger
MR RAPS
Weighted median
Weighted mode
0.10
0.05
0.00
−0.05
0.1
0.2
0.3
0.4
SNP effect on Basal Cell Adhesion Molecule
SNP effect on Selenium

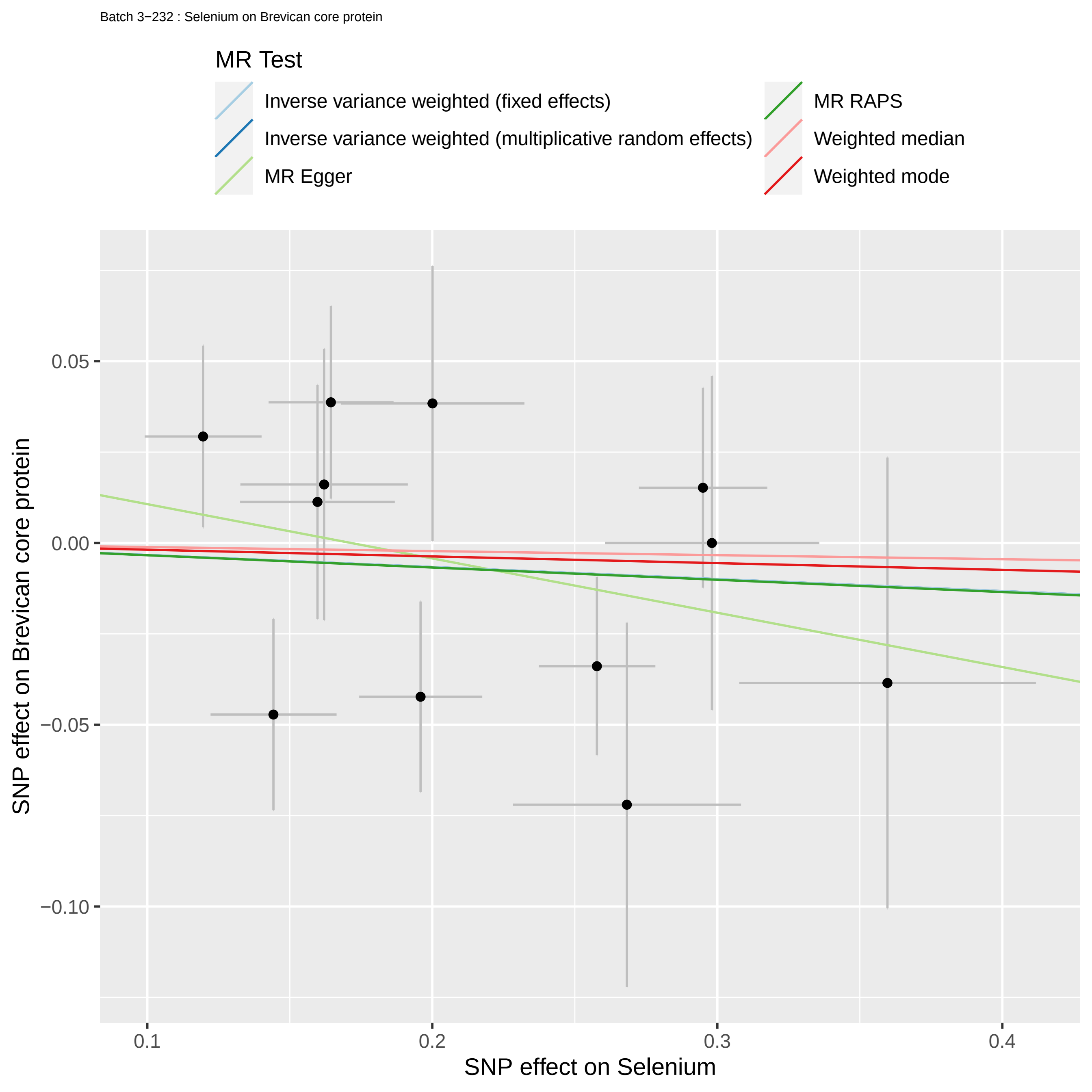
Batch 3−232 : Selenium on Brevican core protein
MR Test
Inverse variance weighted (fixed effects)
Inverse variance weighted (multiplicative random effects)
MR Egger
MR RAPS
Weighted median
Weighted mode
0.05
0.00
−0.05
−0.10
0.1
0.2
0.3
0.4
SNP effect on Brevican core protein
SNP effect on Selenium

Batch 3−233 : Selenium on B−cell receptor−associated protein 29

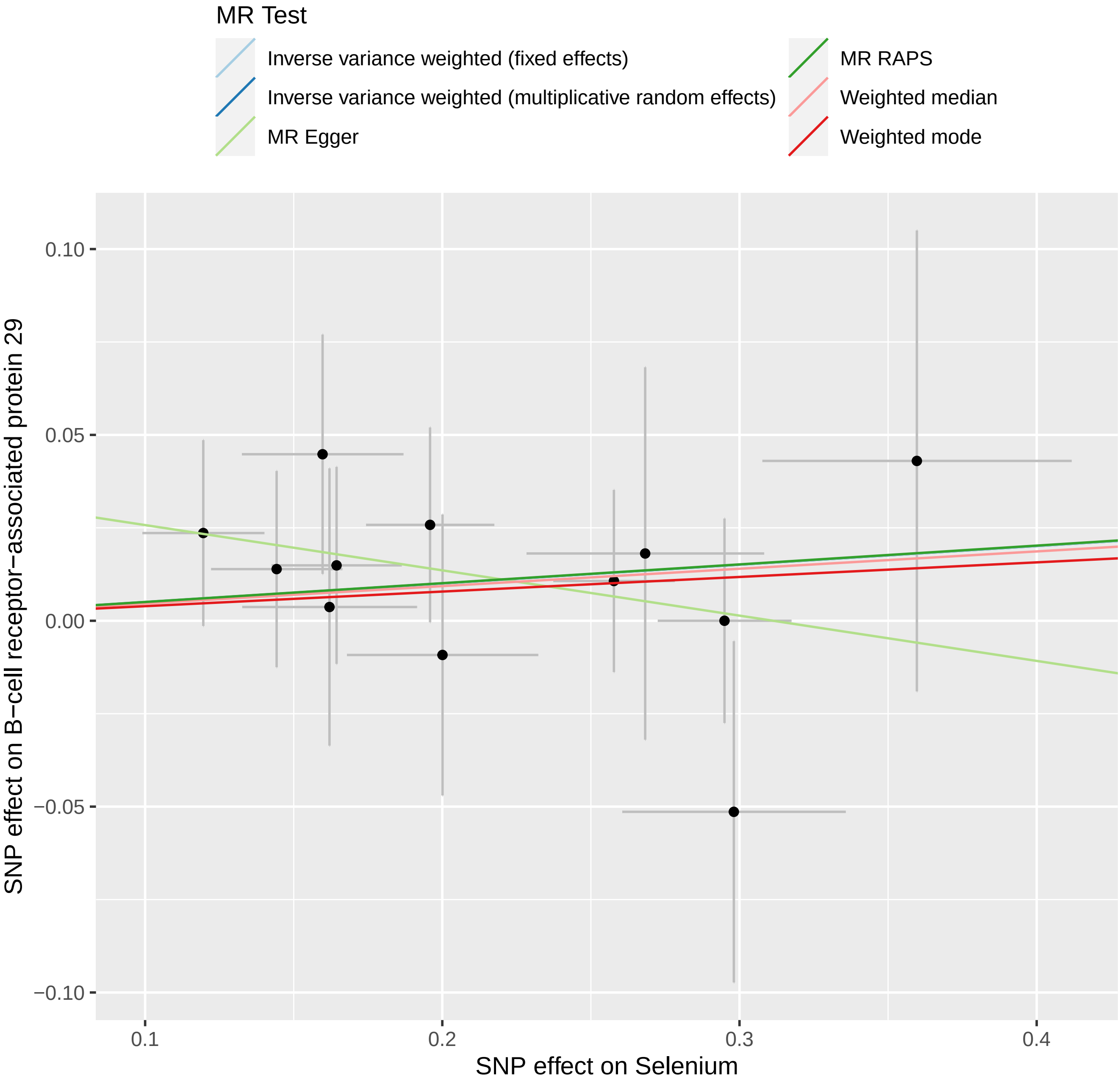

Batch 3−234 : Selenium on Breast cancer anti−estrogen resistance protein 3

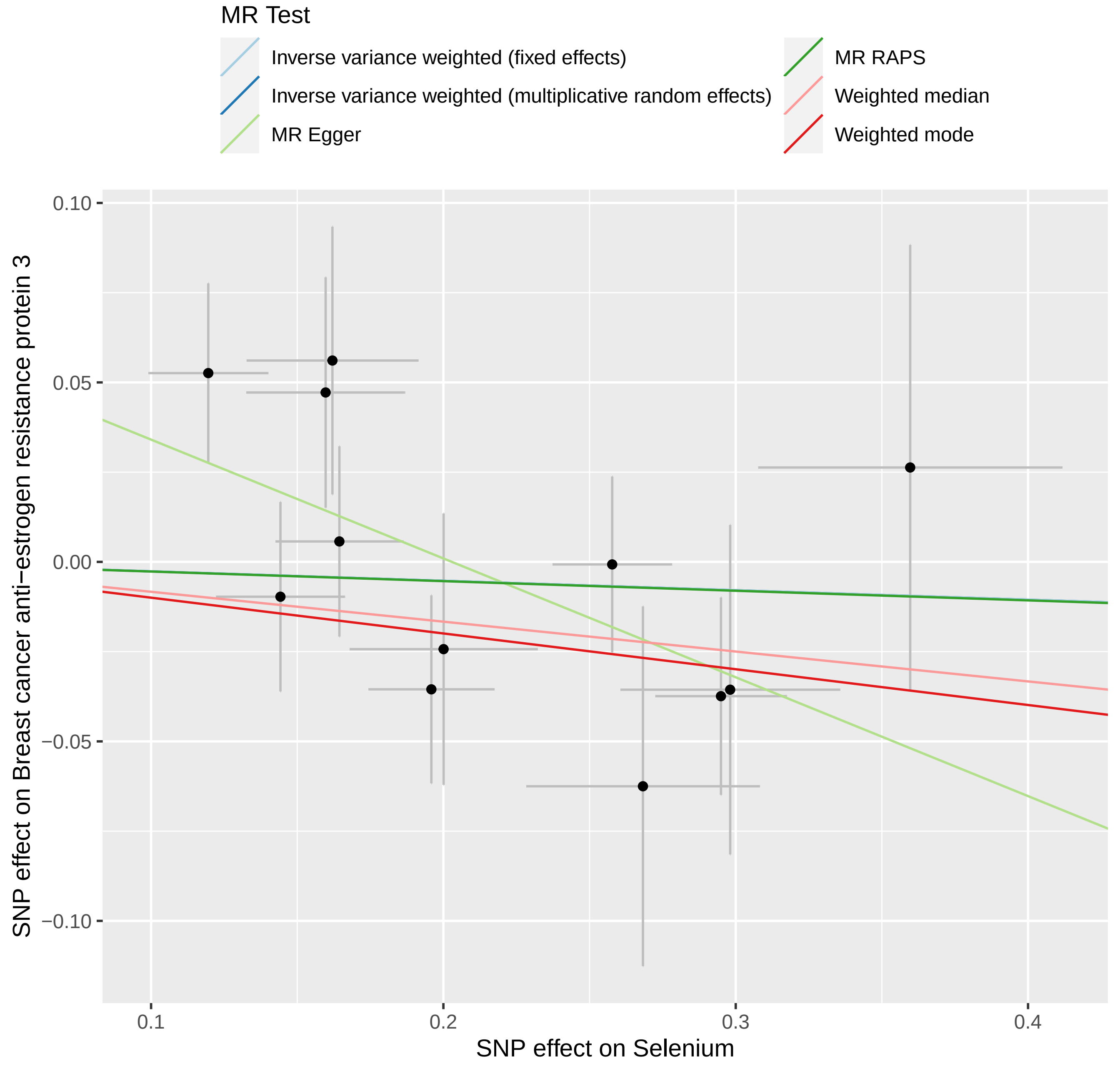

Batch 3−235 : Selenium on B−cell lymphoma/leukemia 10

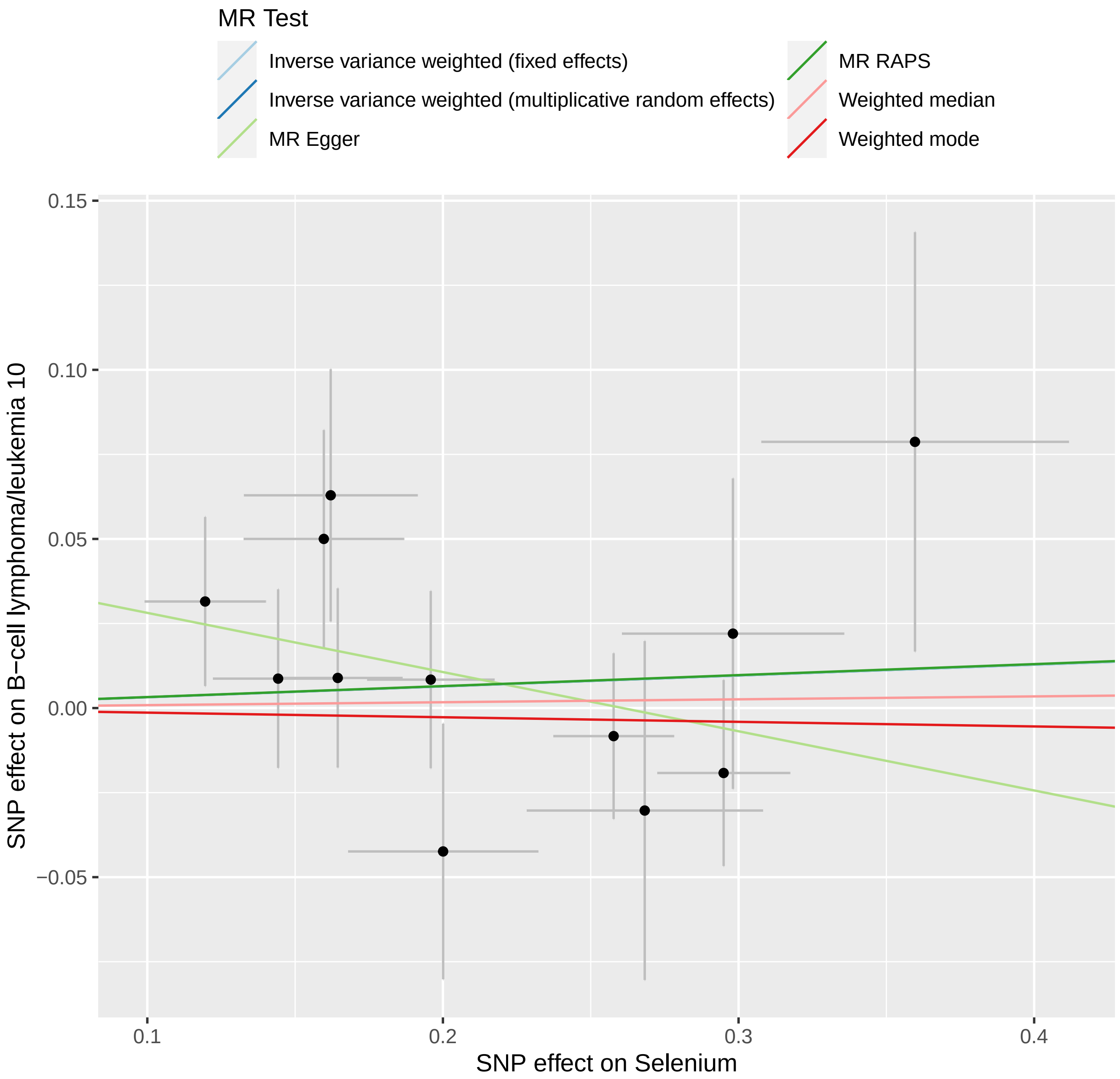

Batch 3−236 : Selenium on Apoptosis regulator Bcl−2

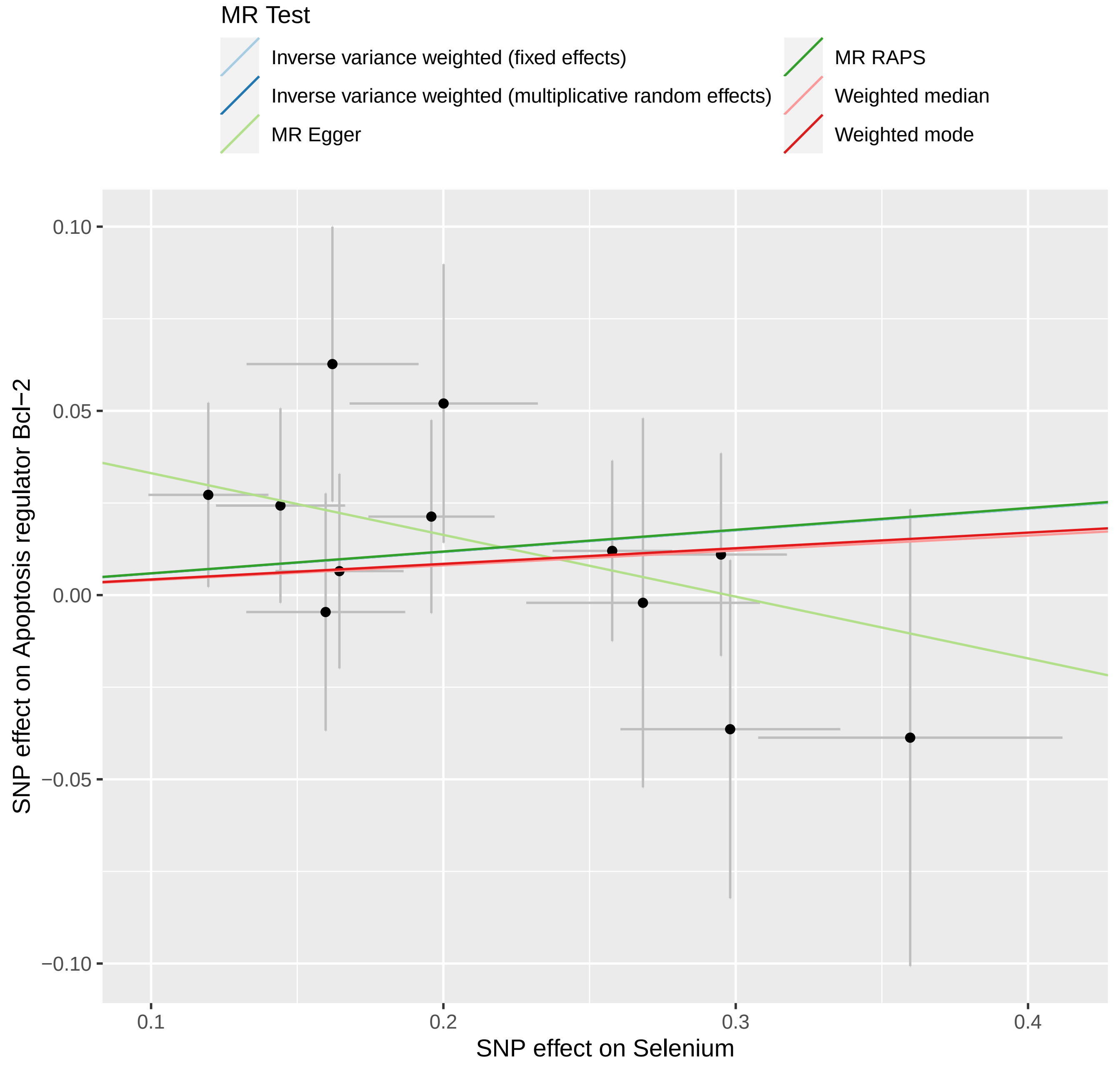

Batch 3−237 : Selenium on Bcl−2−related protein A1

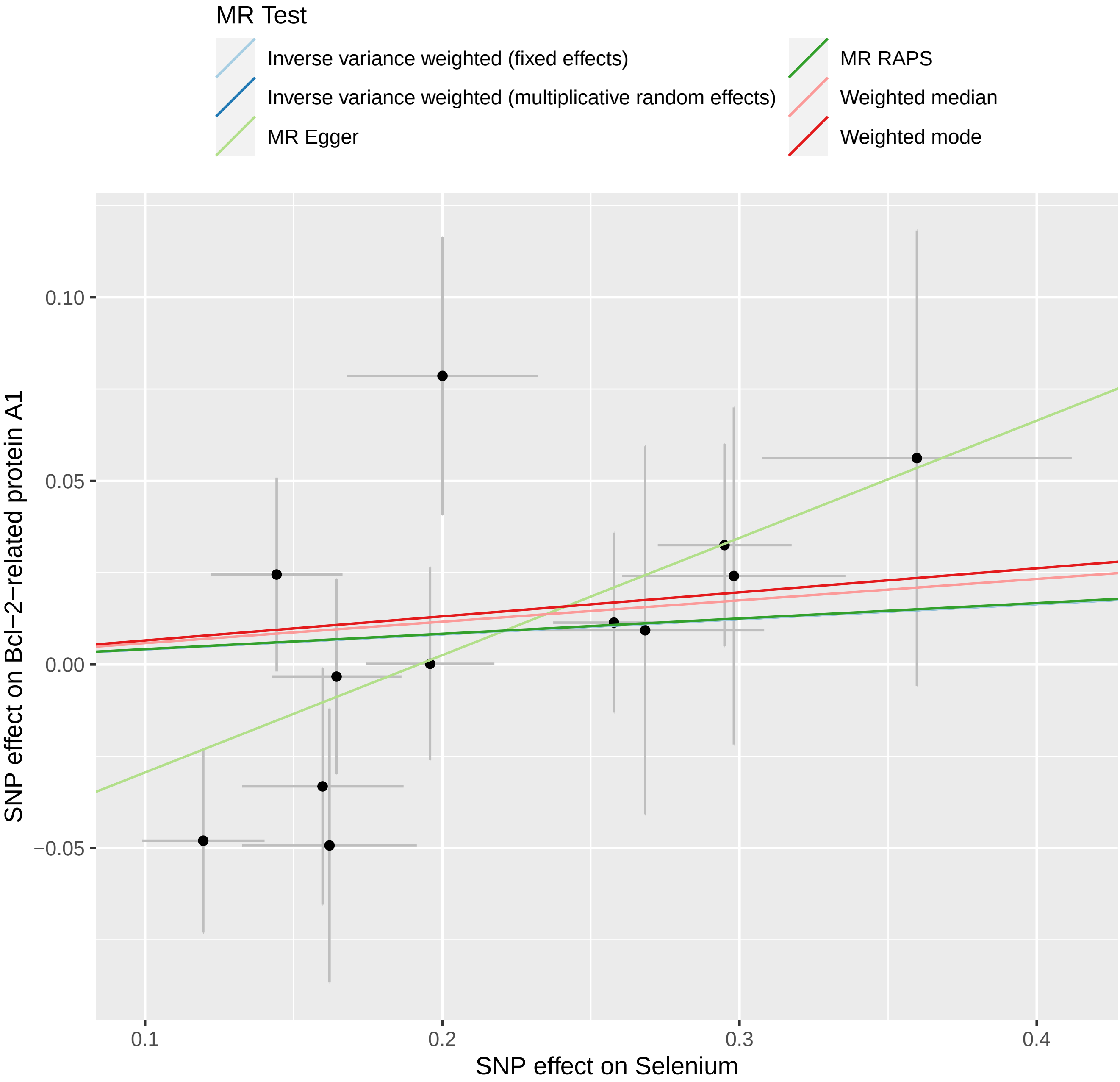

Batch 3−238 : Selenium on Bcl−2−like protein 1

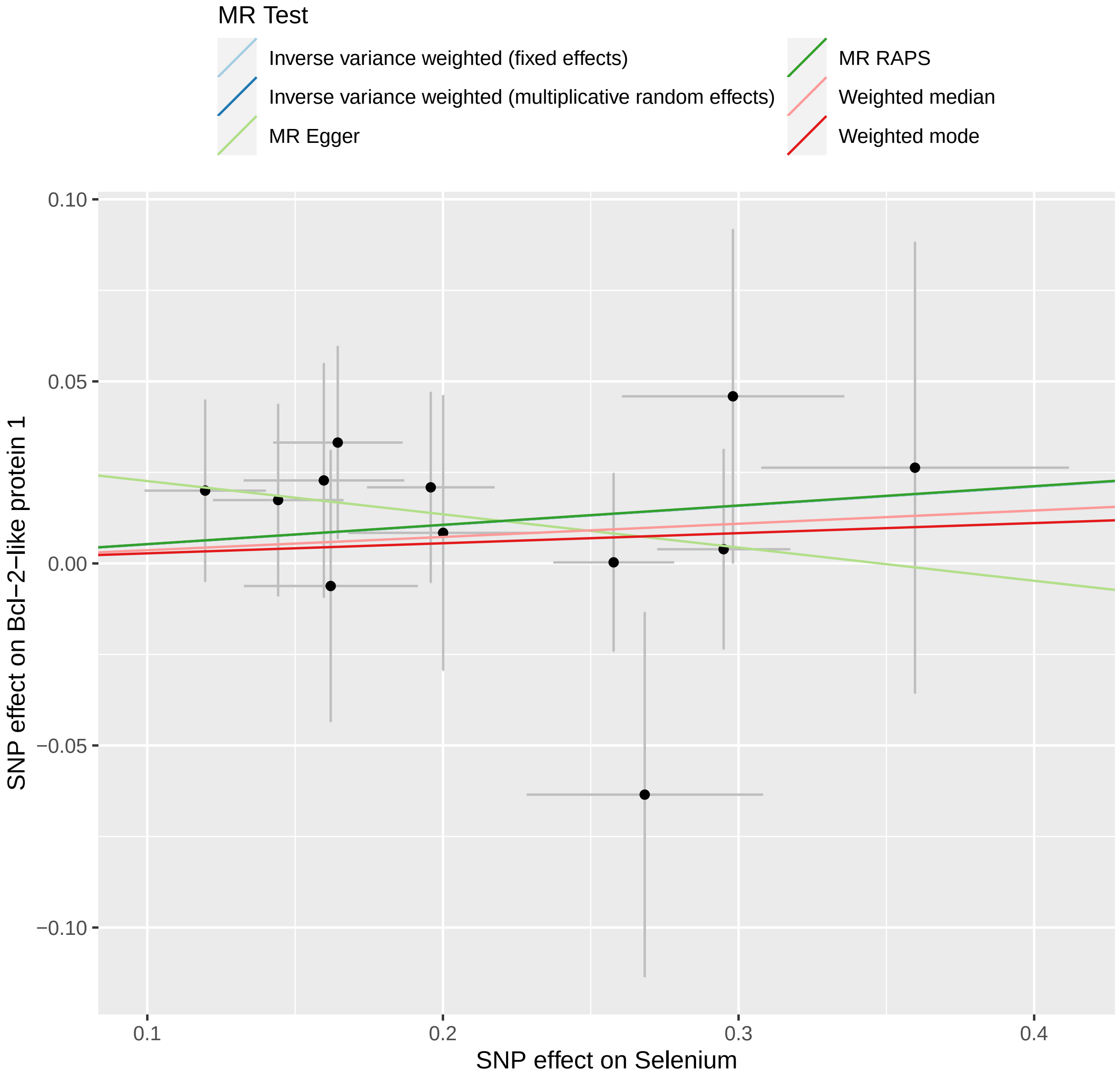

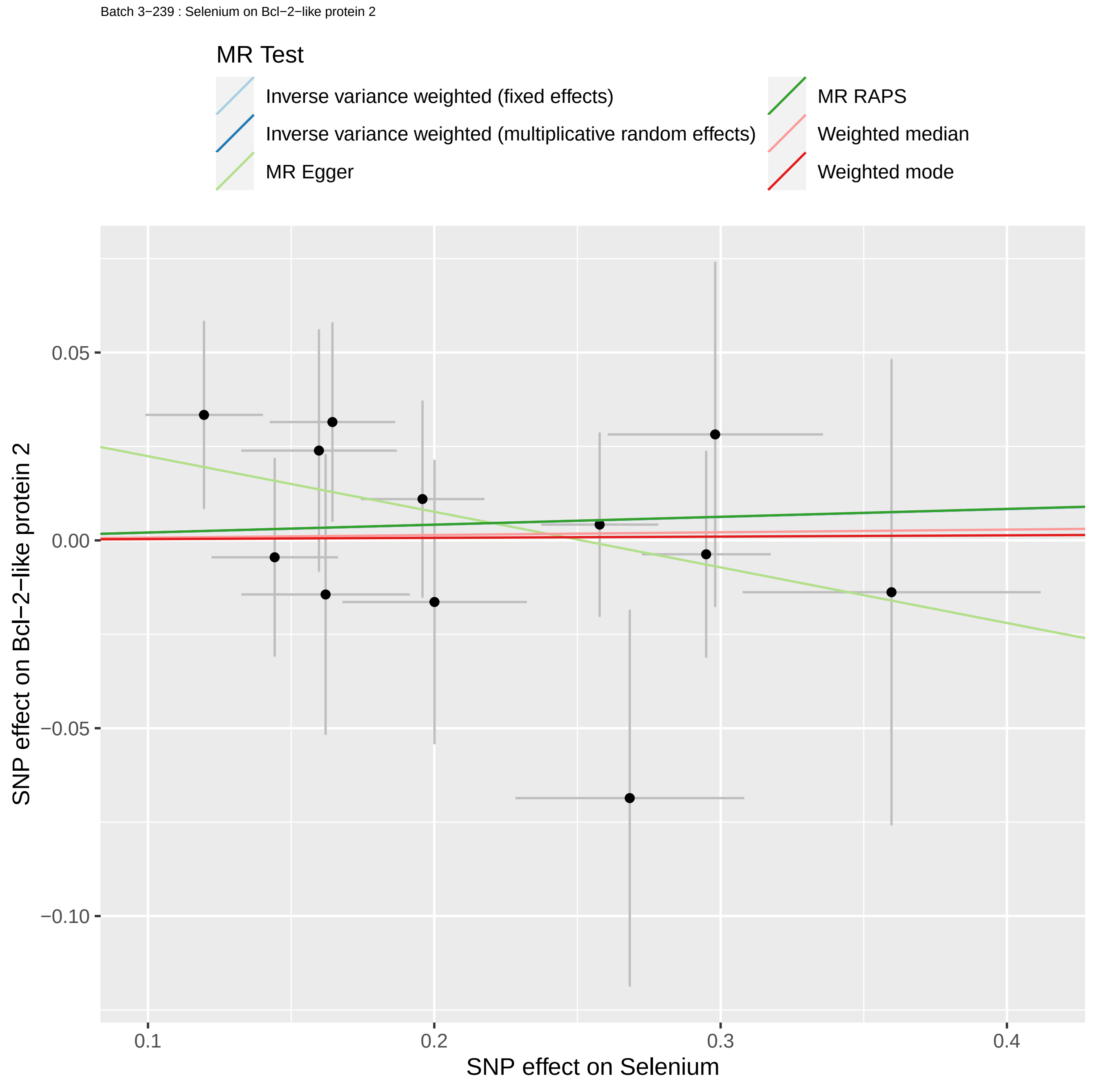

Batch 3−239 : Selenium on Bcl−2−like protein 2
MR Test
Inverse variance weighted (fixed effects)
Inverse variance weighted (multiplicative random effects)
MR Egger
MR RAPS
Weighted median
Weighted mode
0.05
0.00
−0.05
−0.10
SNP effect on Bcl−2−like protein 2
0.1
0.2
0.3
0.4
SNP effect on Selenium

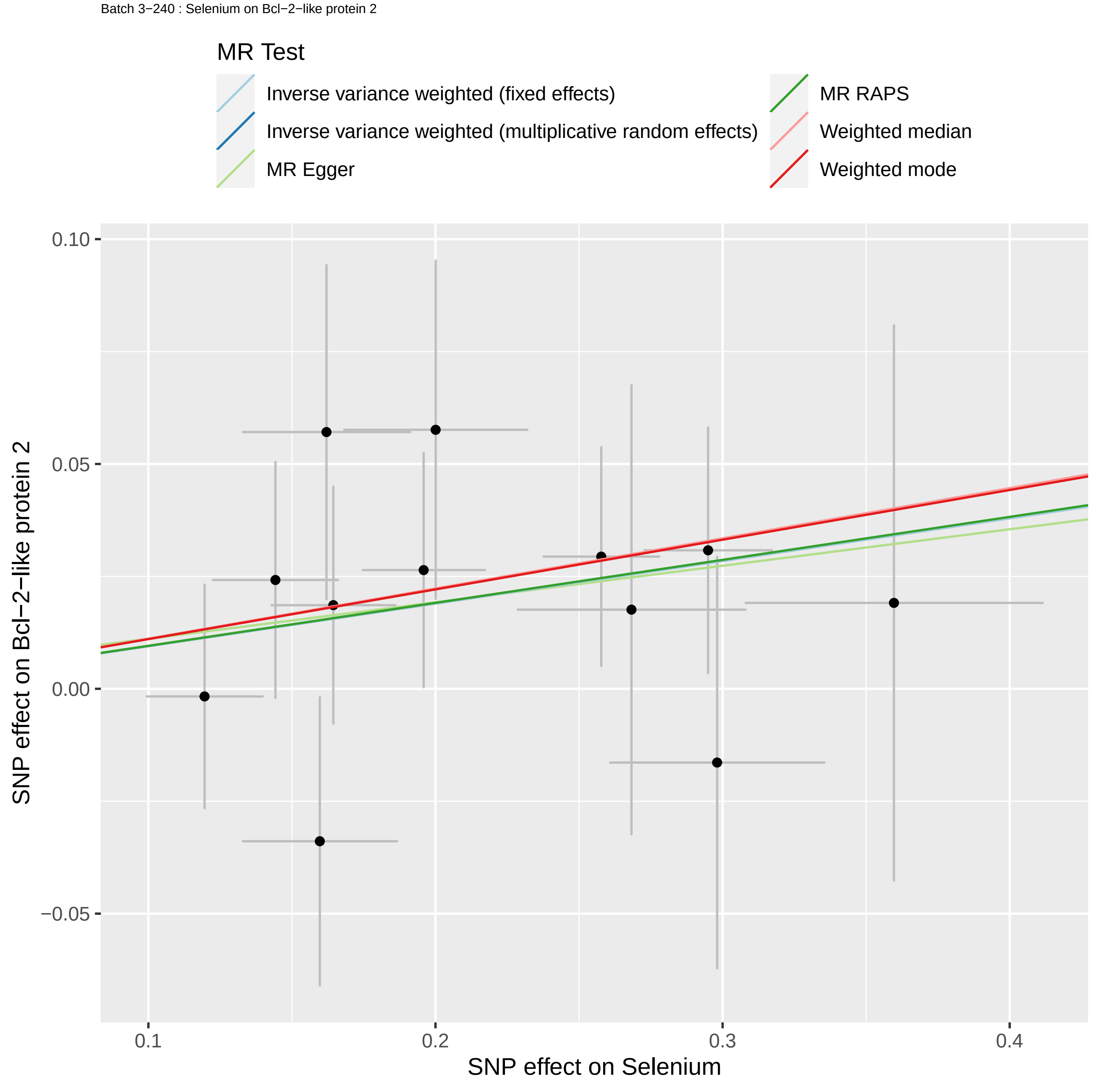

Batch 3−240 : Selenium on Bcl−2−like protein 2
MR Test
Inverse variance weighted (fixed effects)
Inverse variance weighted (multiplicative random effects)
MR Egger
MR RAPS
Weighted median
Weighted mode
0.10
0.05
0.00
−0.05
0.1
0.2
0.3
0.4
SNP effect on Bcl−2−like protein 2
SNP effect on Selenium

Batch 3−241 : Selenium on B−cell lymphoma 6 protein

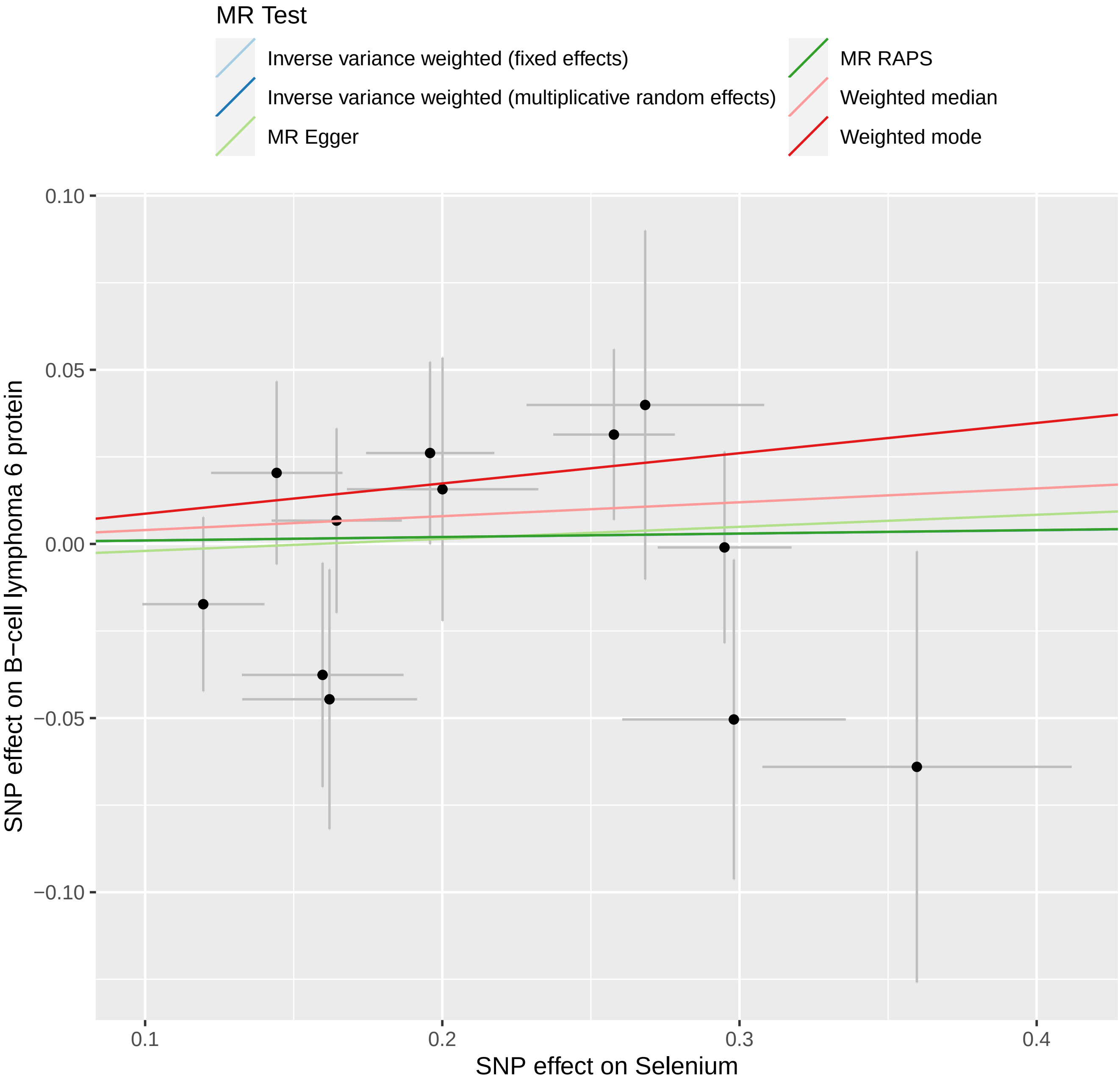

Batch 3−242 : Selenium on Brain−derived neurotrophic factor

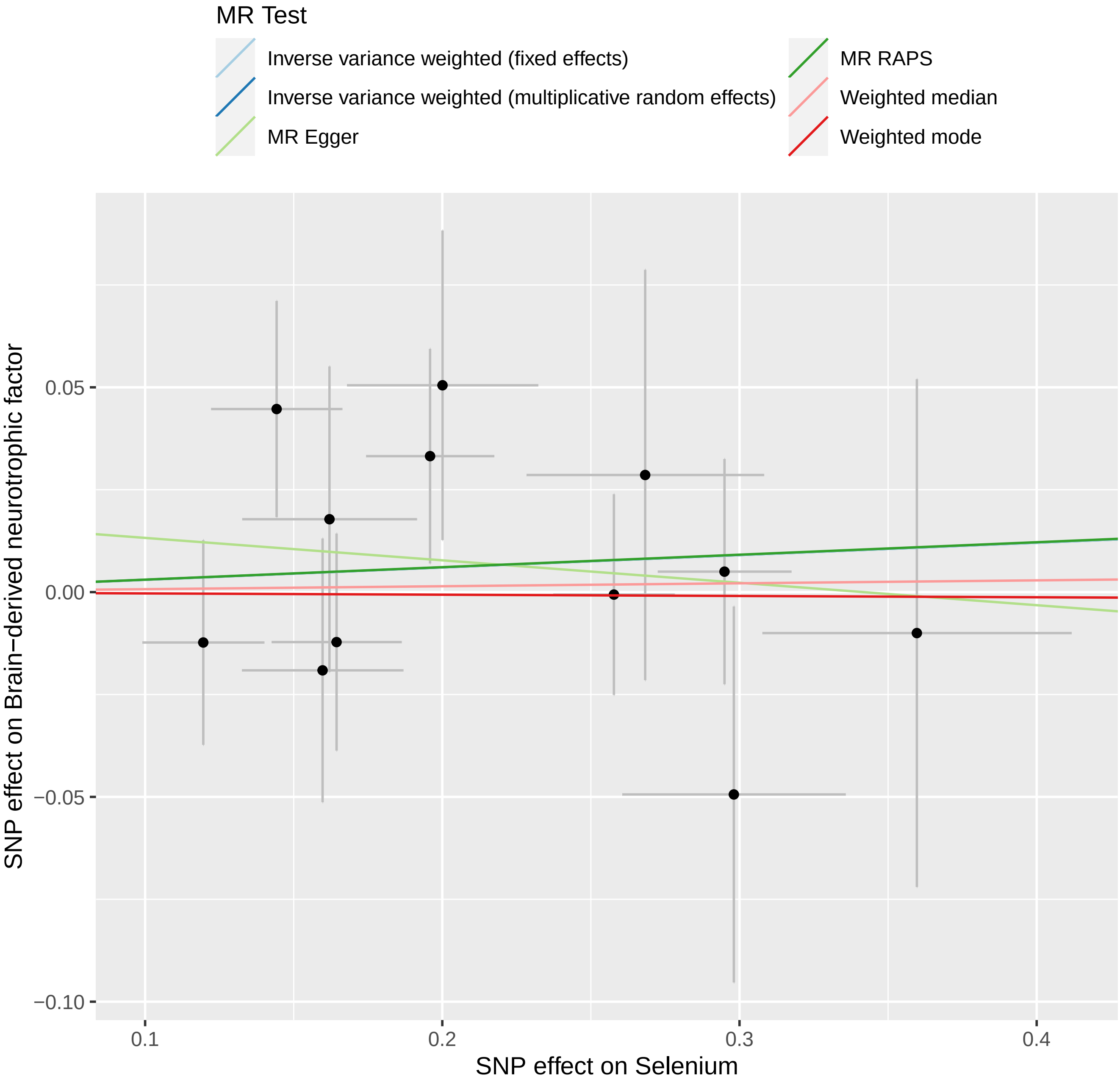

Batch 3−243 : Selenium on Transcription factor TFIIIB component B" homolog

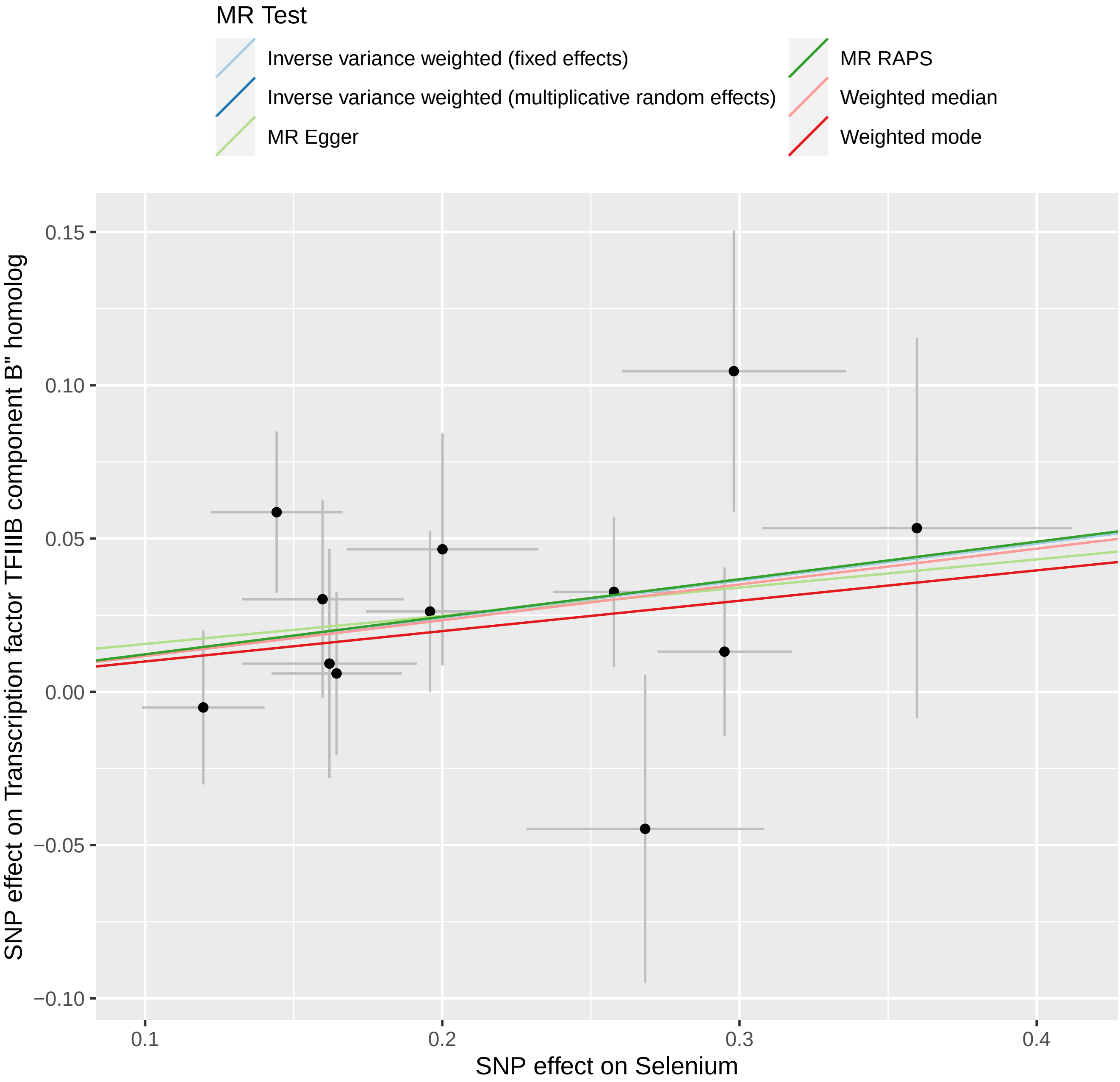

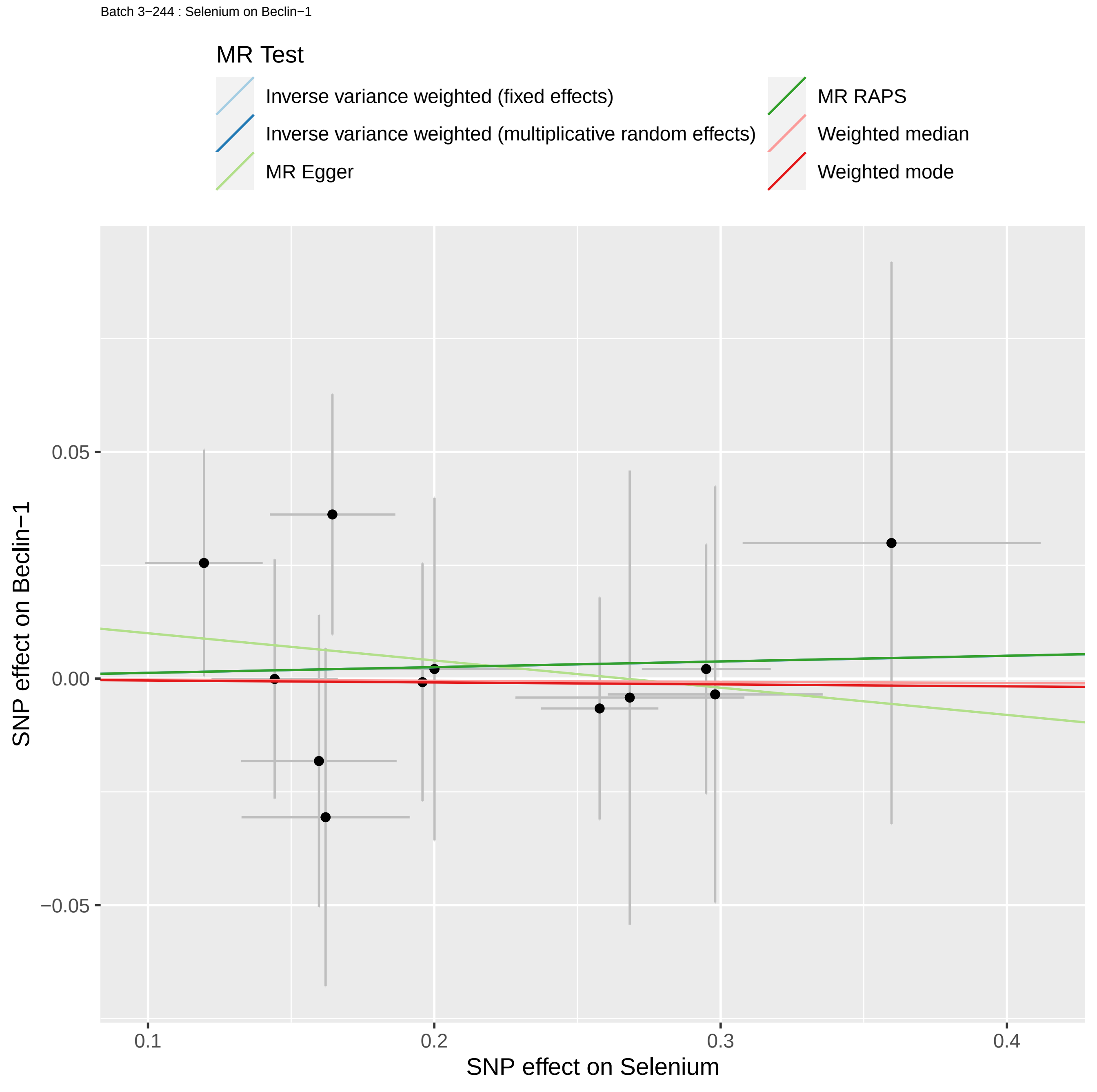

Batch 3−244 : Selenium on Beclin−1
MR Test
Inverse variance weighted (fixed effects)
Inverse variance weighted (multiplicative random effects)
MR Egger
MR RAPS
Weighted median
Weighted mode
0.05
0.00
−0.05
0.1
0.2
0.3
0.4
SNP effect on Selenium
SNP effect on Beclin−1

Batch 3−245 : Selenium on BET1−like protein

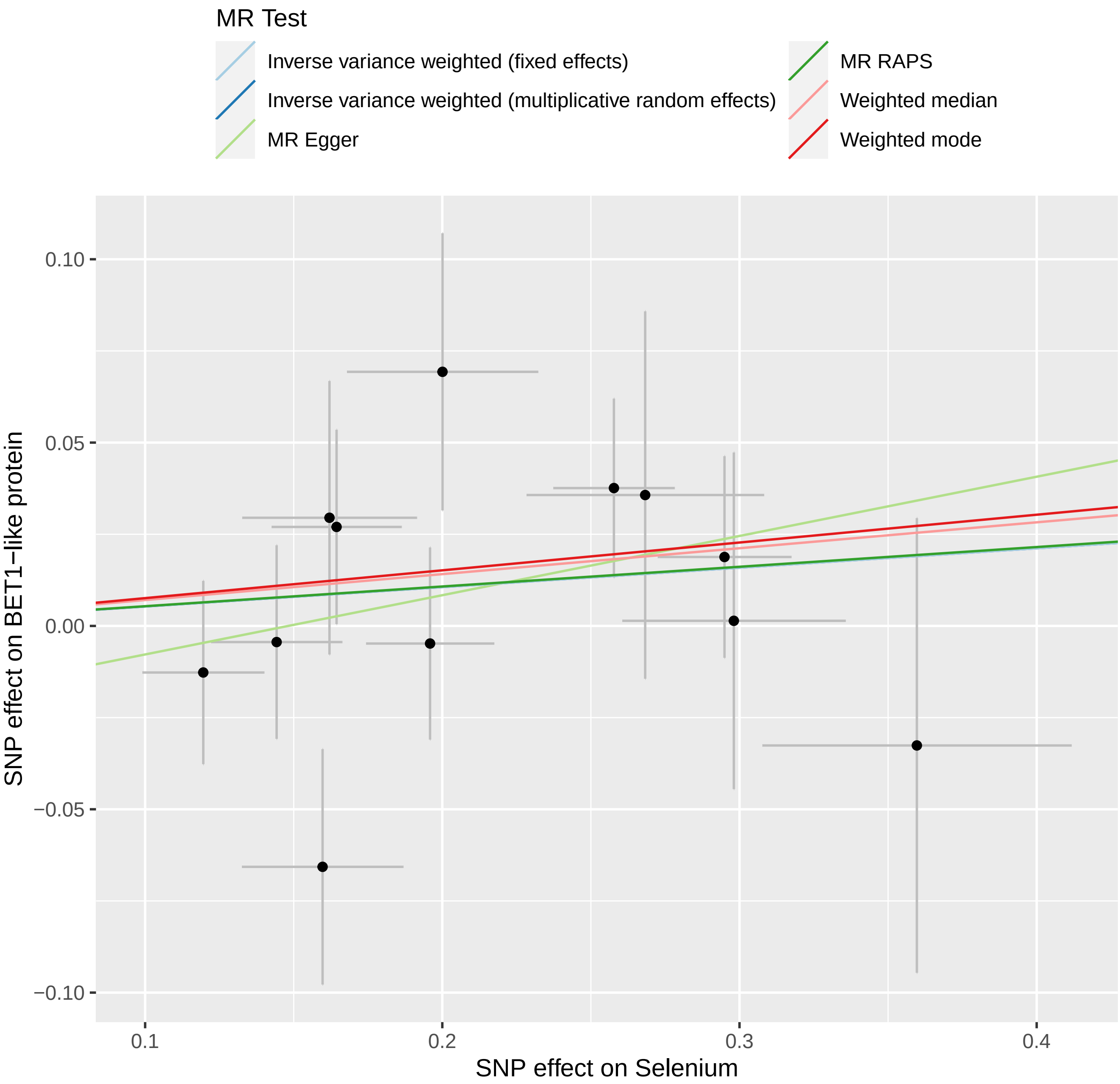

Batch 3−246 : Selenium on Osteocalcin

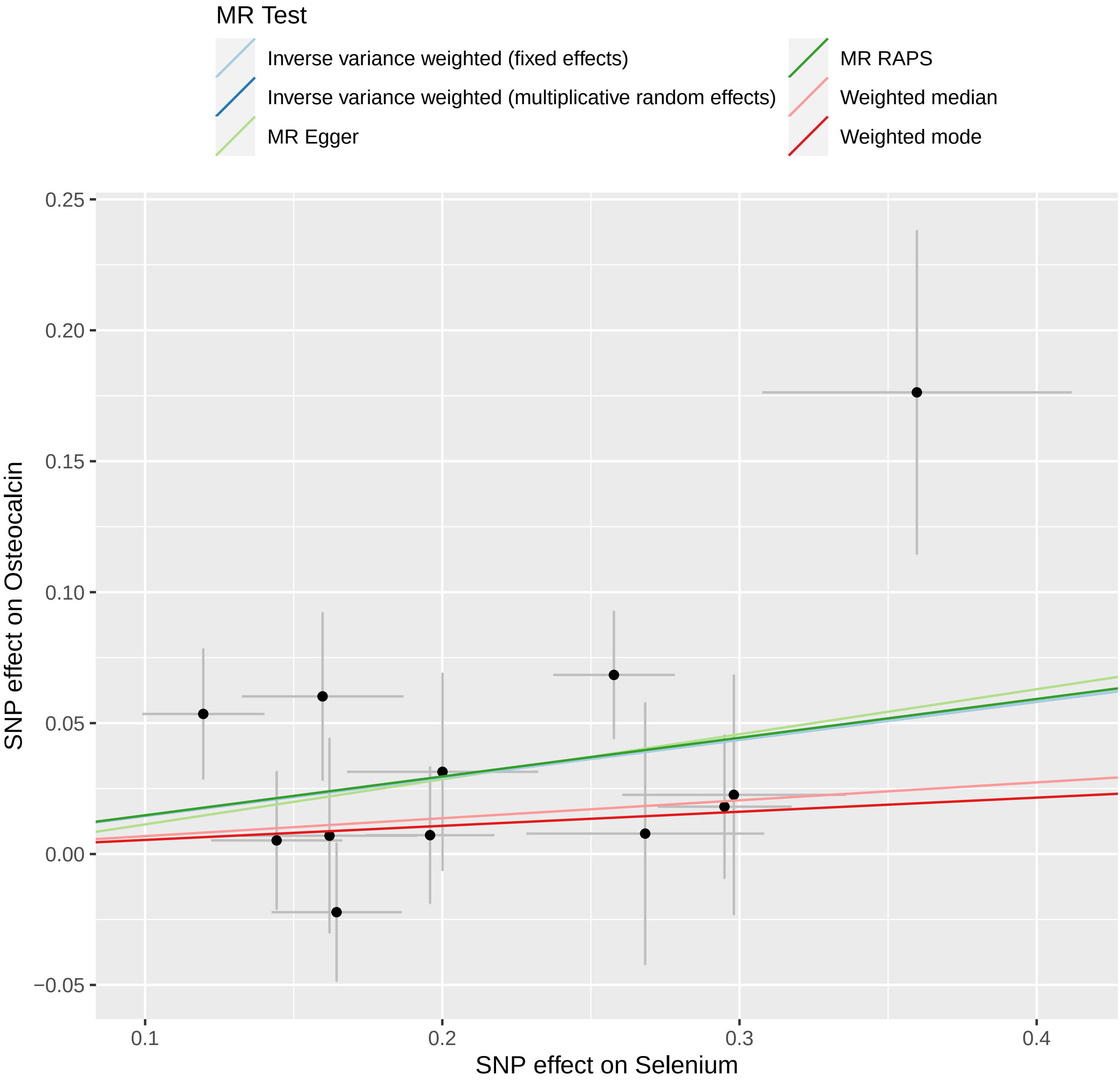

Batch 3−247 : Selenium on Biglycan

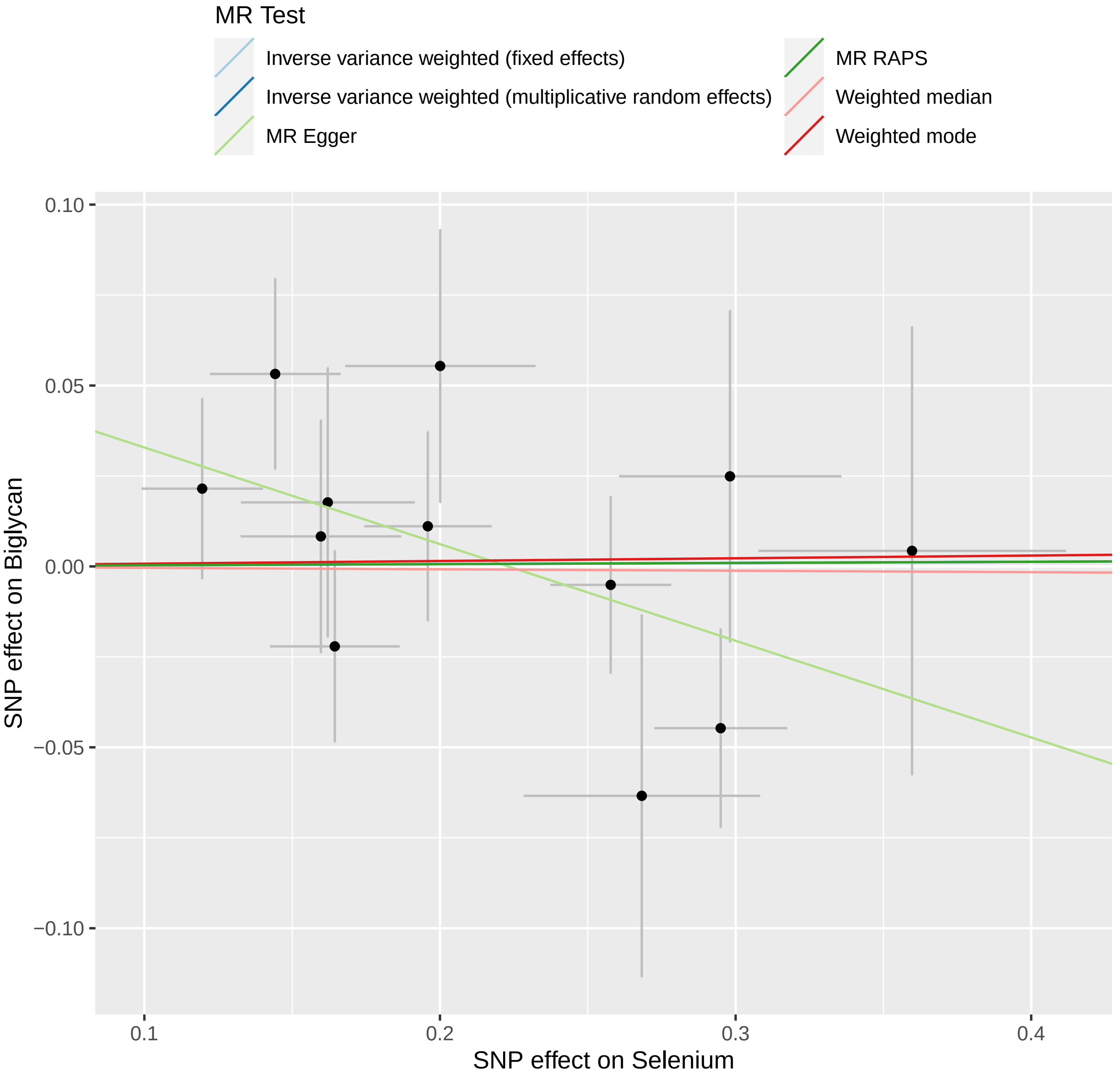

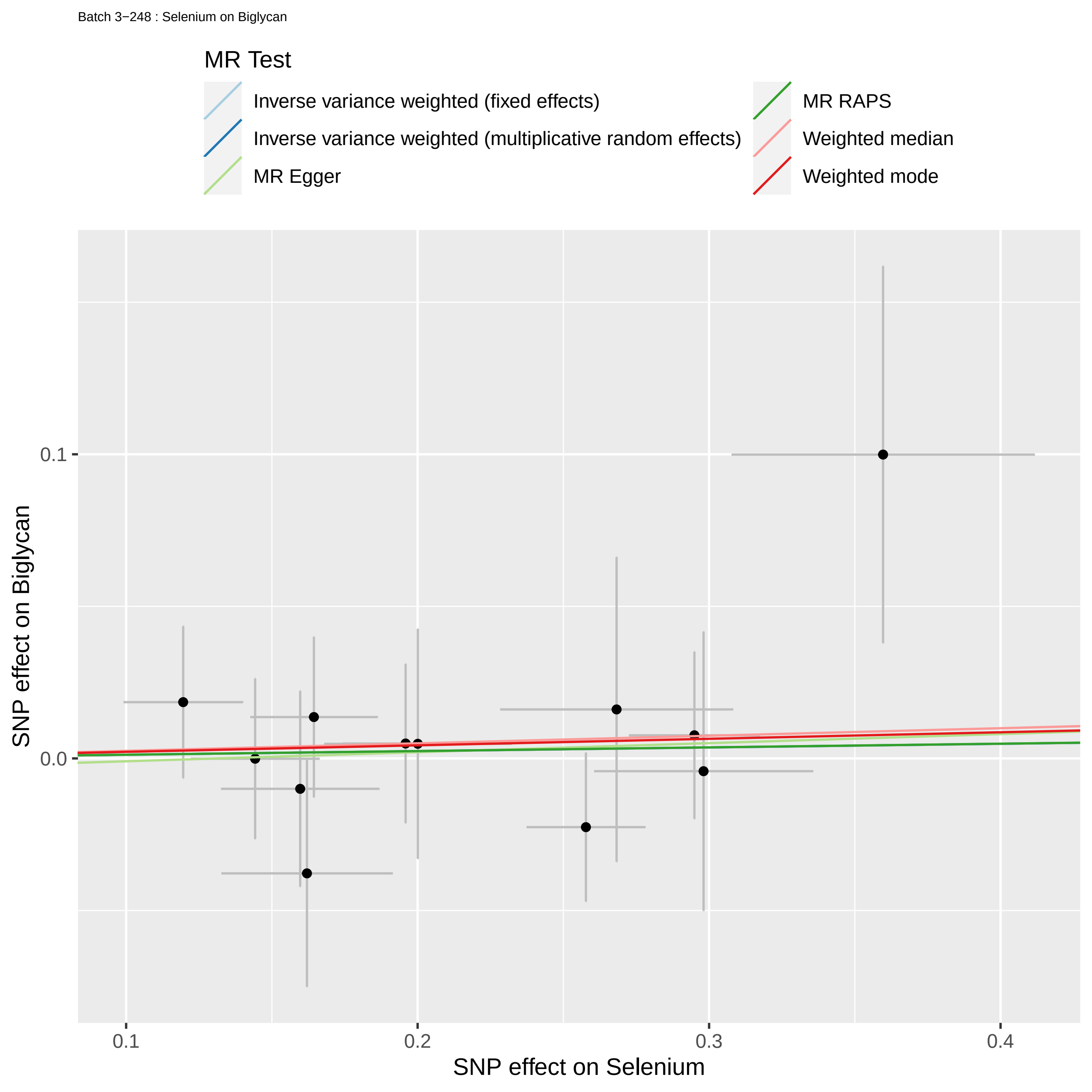
Batch 3−248 : Selenium on Biglycan
MR Test
Inverse variance weighted (fixed effects)
Inverse variance weighted (multiplicative random effects)
MR Egger
MR RAPS
Weighted median
Weighted mode
0.1
0.0
SNP effect on Biglycan
0.1
0.2
0.3
0.4
SNP effect on Selenium

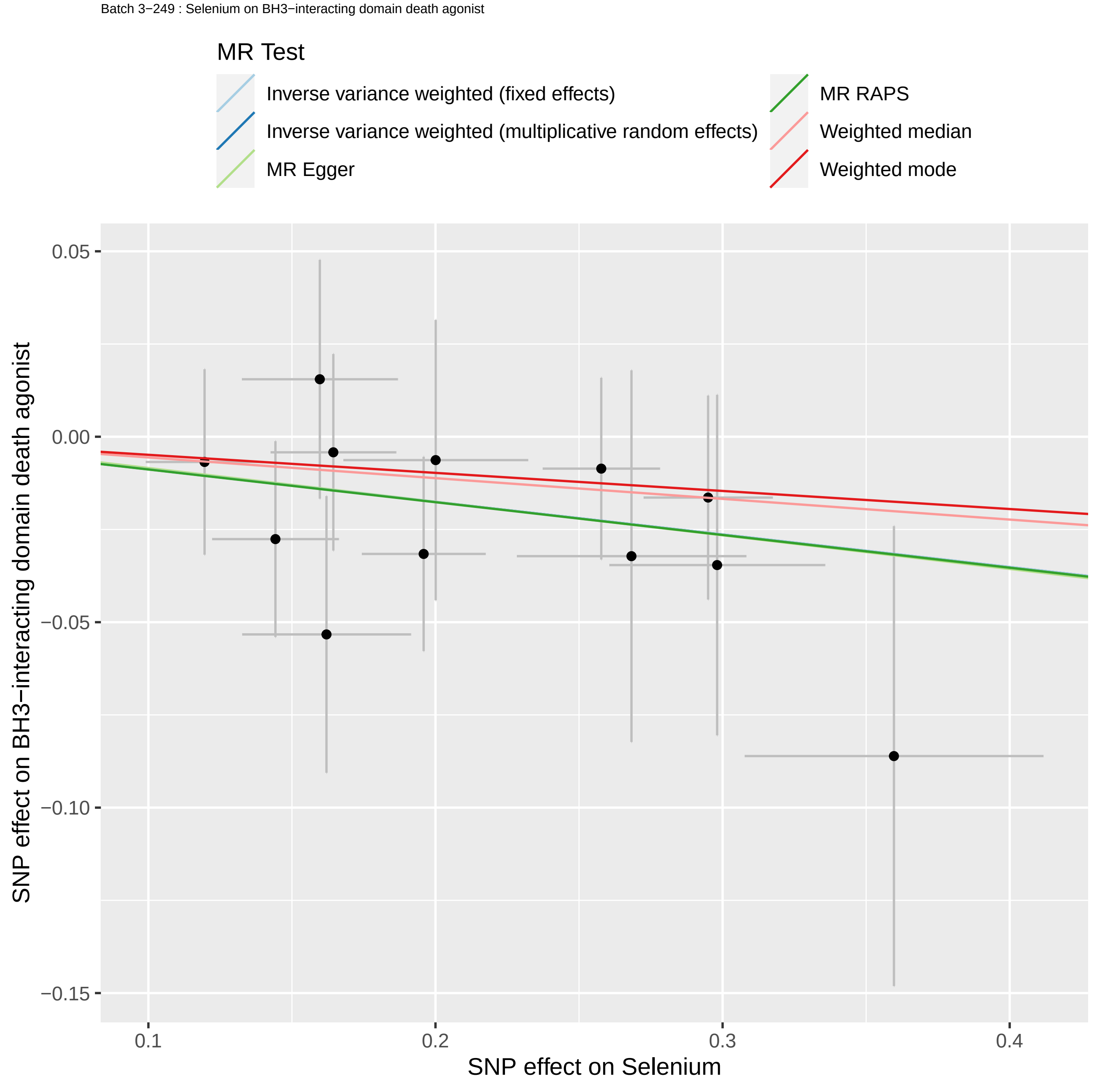

Batch 3−249 : Selenium on BH3−interacting domain death agonist
MR Test
Inverse variance weighted (fixed effects)
Inverse variance weighted (multiplicative random effects)
MR Egger
MR RAPS
Weighted median
Weighted mode
0.05
0.00
−0.05
−0.10
−0.15
0.1
0.2
0.3
0.4
SNP effect on BH3−interacting domain death agonist
SNP effect on Selenium

Batch 3−250 : Selenium on Myc box−dependent−interacting protein 1

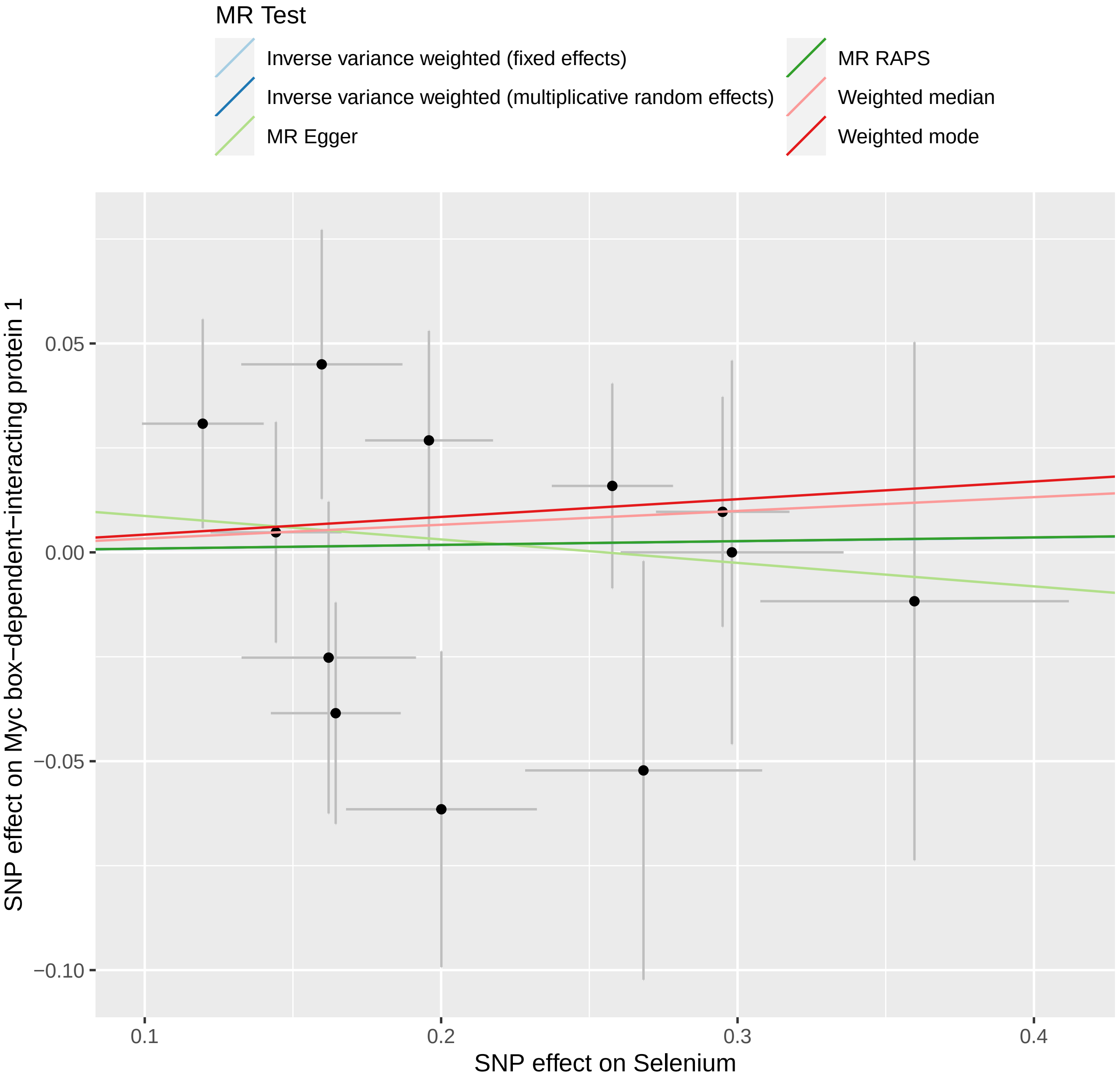

Batch 3−251 : Selenium on Baculoviral IAP repeat−containing protein 3

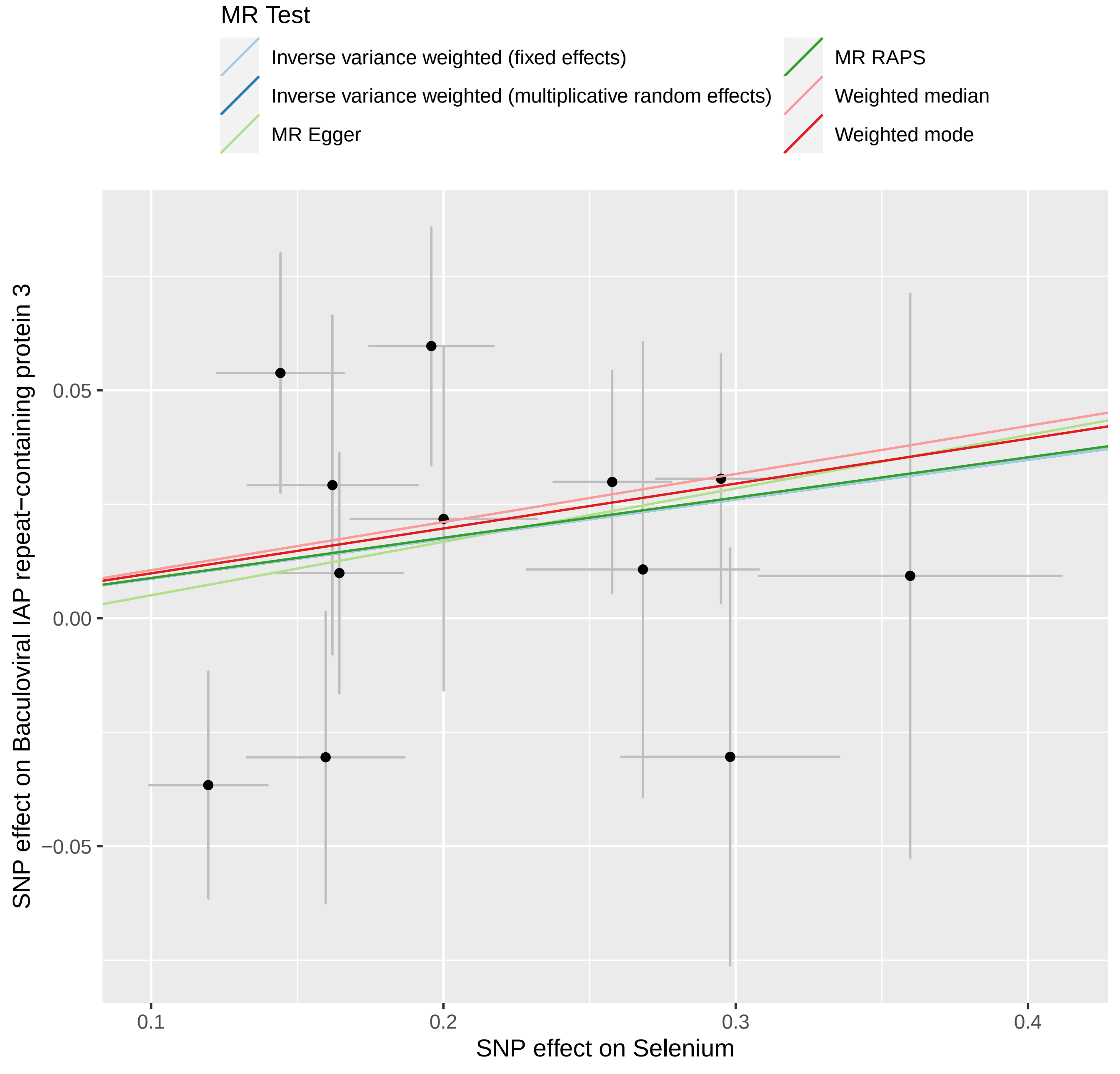

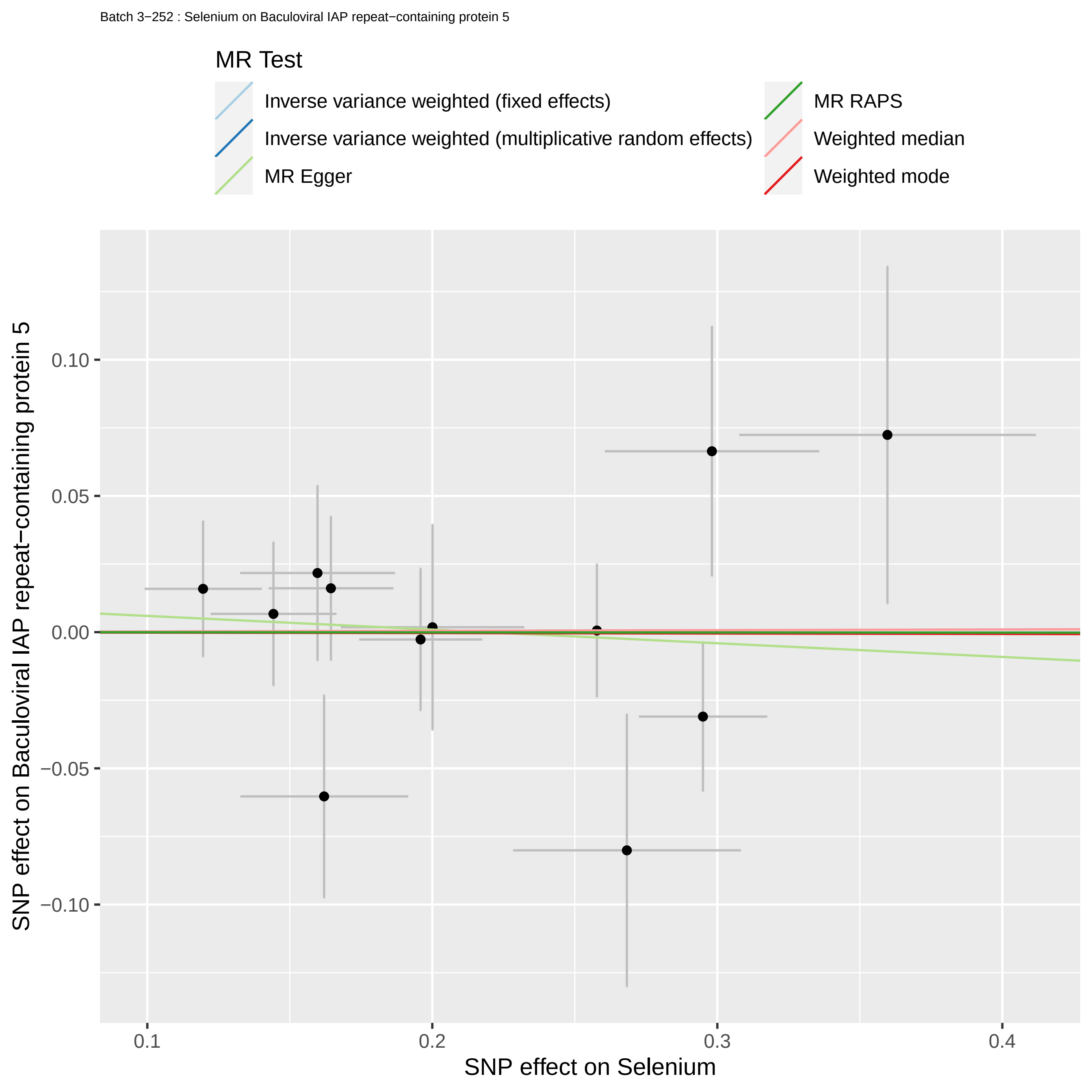
Batch 3−252 : Selenium on Baculoviral IAP repeat−containing protein 5
MR Test
Inverse variance weighted (fixed effects)
Inverse variance weighted (multiplicative random effects)
MR Egger
MR RAPS
Weighted median
Weighted mode
SNP effect on Baculoviral IAP repeat−containing protein 5
0.10
0.05
0.00
−0.05
−0.10
0.1
0.2
0.3
0.4
SNP effect on Selenium

Batch 3−253 : Selenium on Biliverdin reductase A

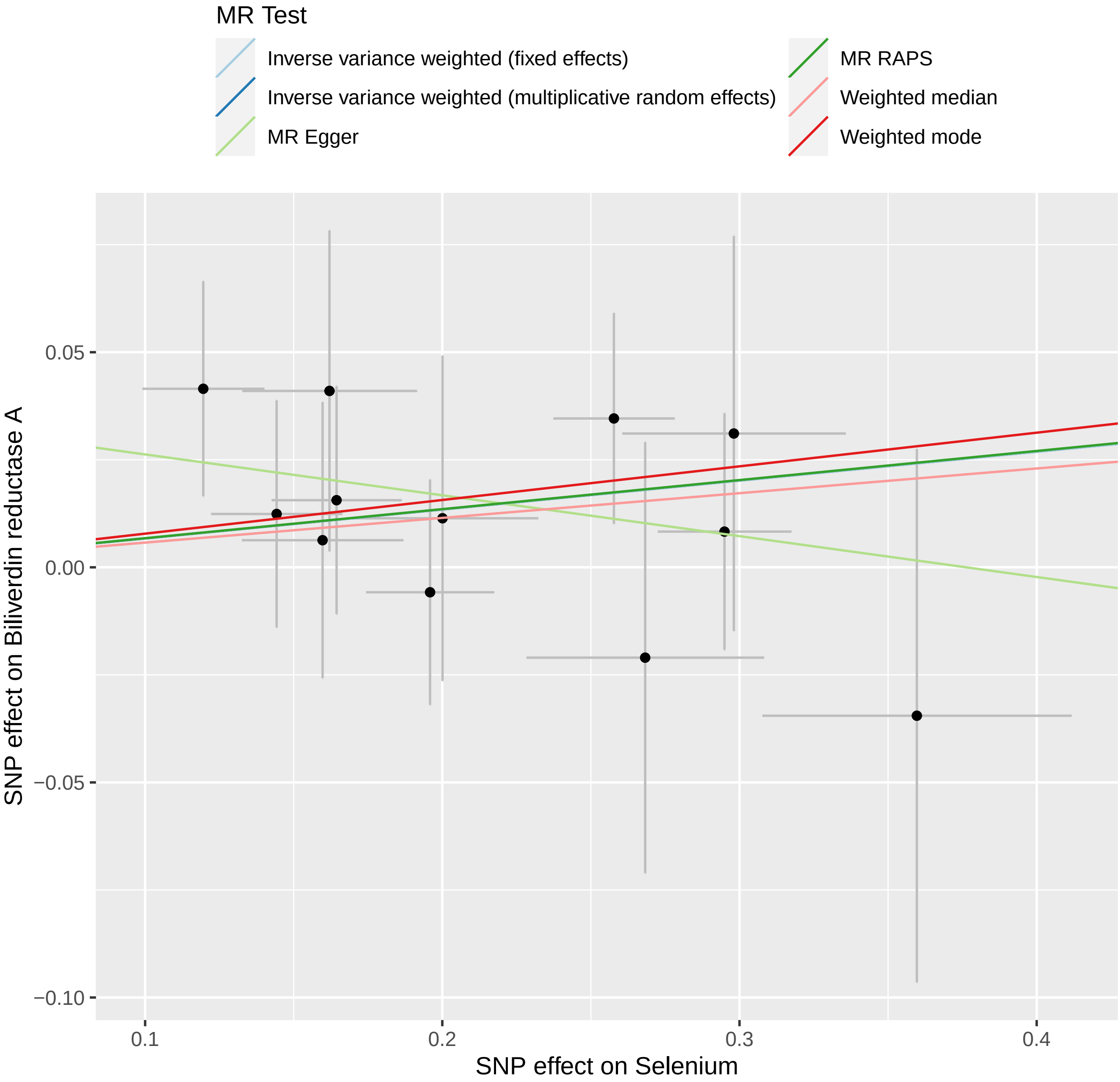

Batch 3−254 : Selenium on Bone morphogenetic protein 10

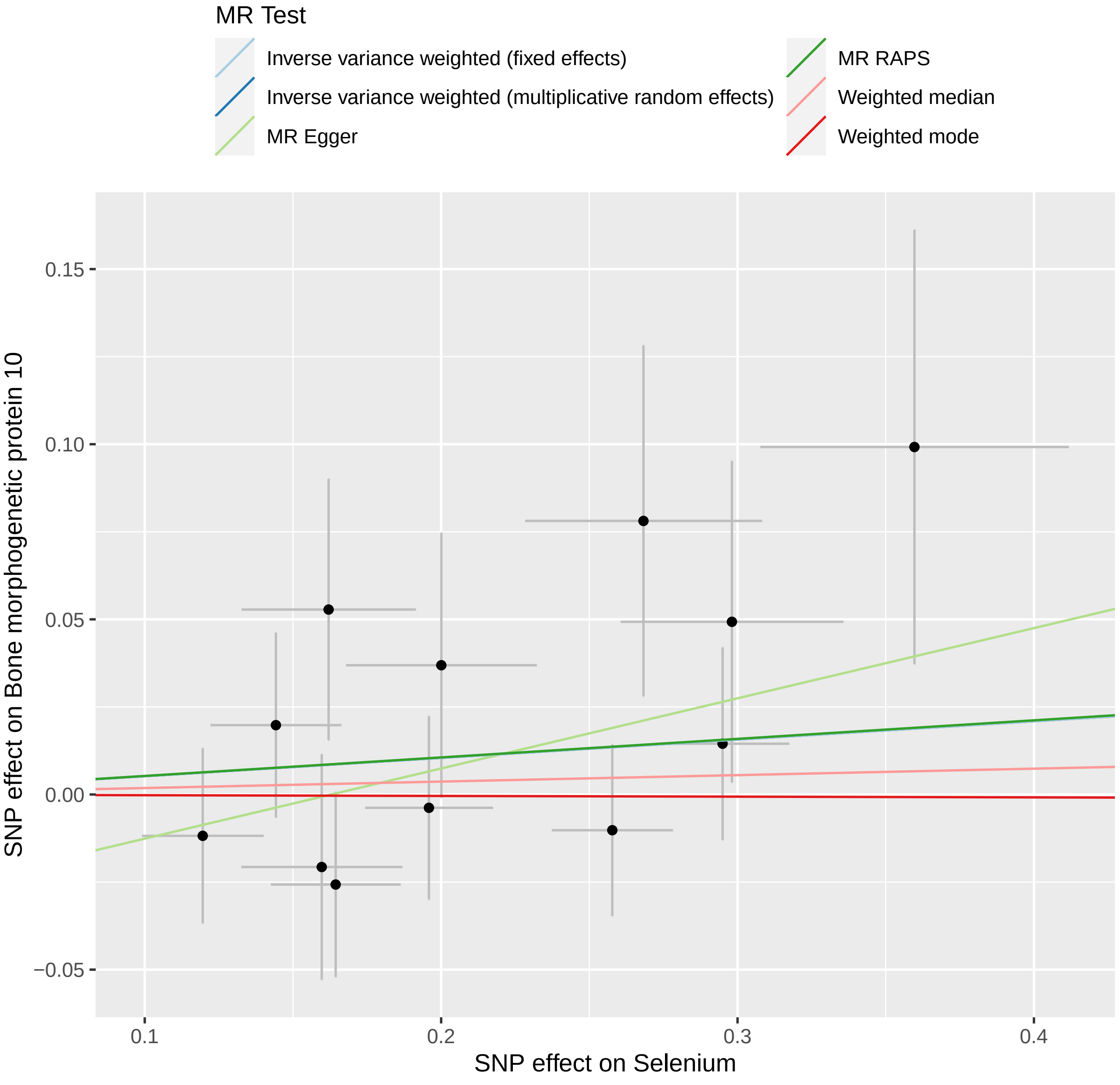

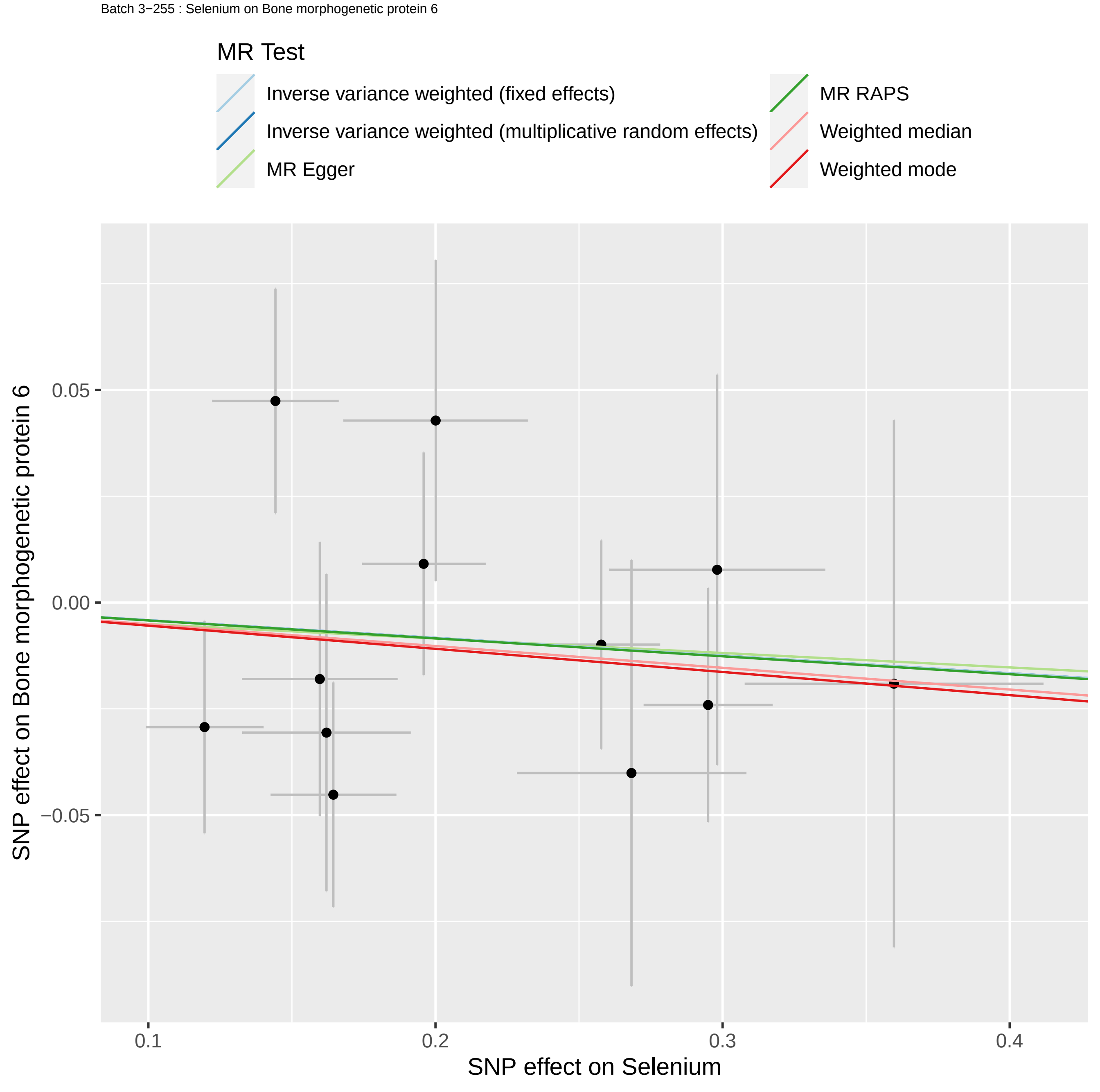
Batch 3−255 : Selenium on Bone morphogenetic protein 6
MR Test
Inverse variance weighted (fixed effects)
Inverse variance weighted (multiplicative random effects)
MR Egger
MR RAPS
Weighted median
Weighted mode
SNP effect on Bone morphogenetic protein 6
0.05
0.00
−0.05
0.1
0.2
0.3
0.4
SNP effect on Selenium

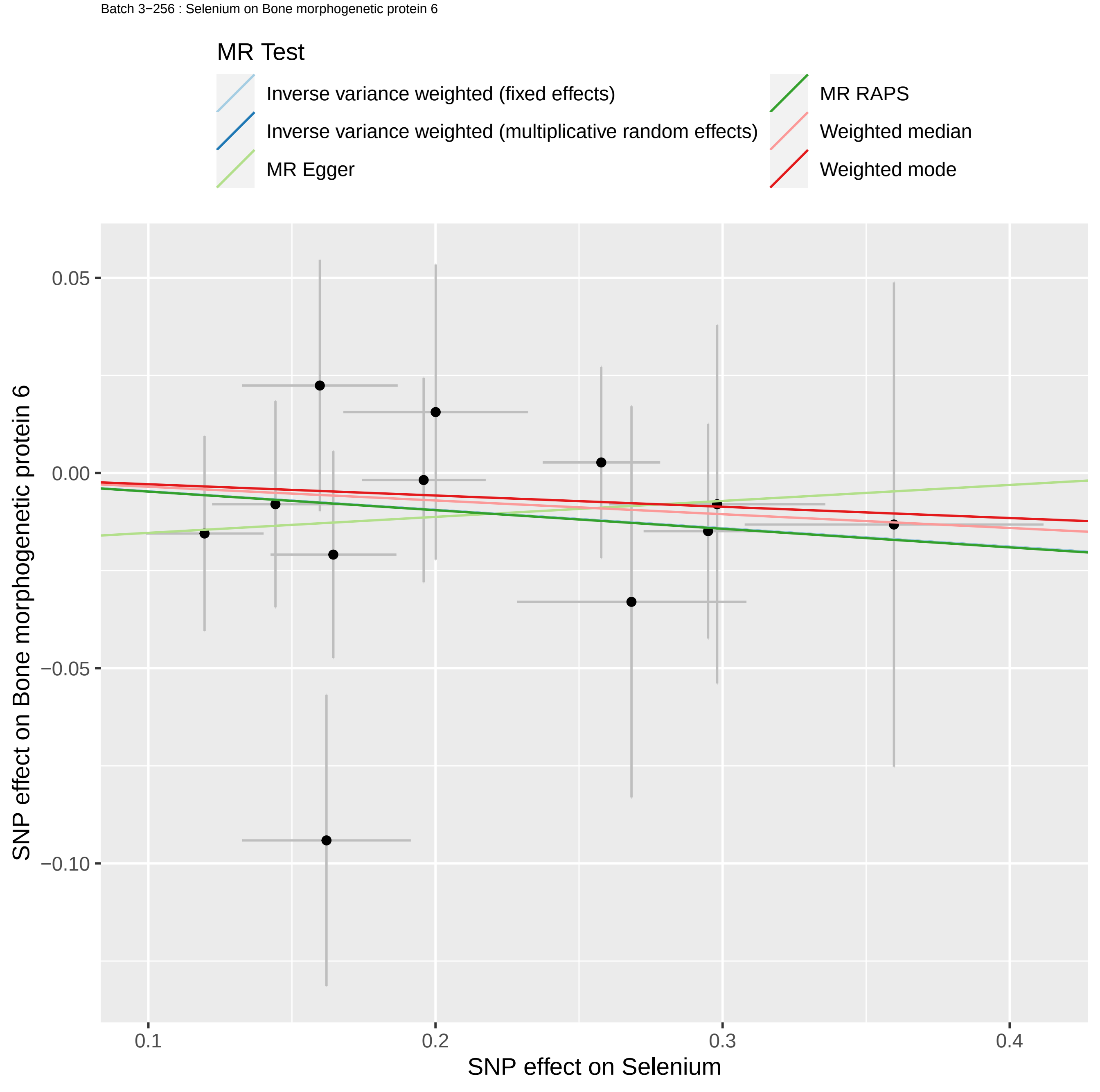

Batch 3−256 : Selenium on Bone morphogenetic protein 6
MR Test
Inverse variance weighted (fixed effects)
Inverse variance weighted (multiplicative random effects)
MR Egger
MR RAPS
Weighted median
Weighted mode
0.05
0.00
−0.05
−0.10
0.1
0.2
0.3
0.4
SNP effect on Bone morphogenetic protein 6
SNP effect on Selenium

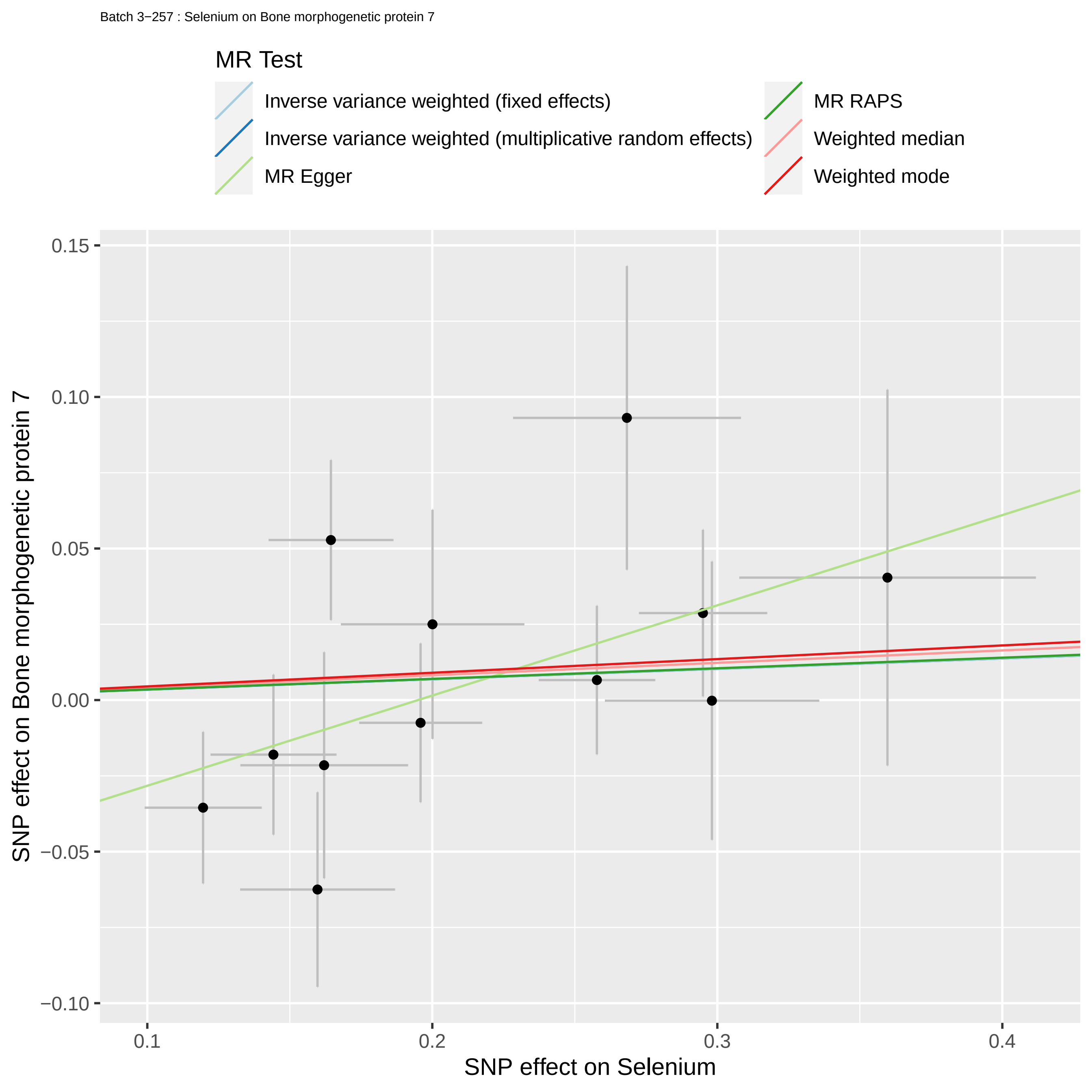

Batch 3−257 : Selenium on Bone morphogenetic protein 7
MR Test
Inverse variance weighted (fixed effects)
Inverse variance weighted (multiplicative random effects)
MR Egger
MR RAPS
Weighted median
Weighted mode
0.15
0.10
0.05
0.00
−0.05
−0.10
0.1
0.2
0.3
0.4
SNP effect on Bone morphogenetic protein 7
SNP effect on Selenium

Batch 3−258 : Selenium on BMP−binding endothelial regulator protein

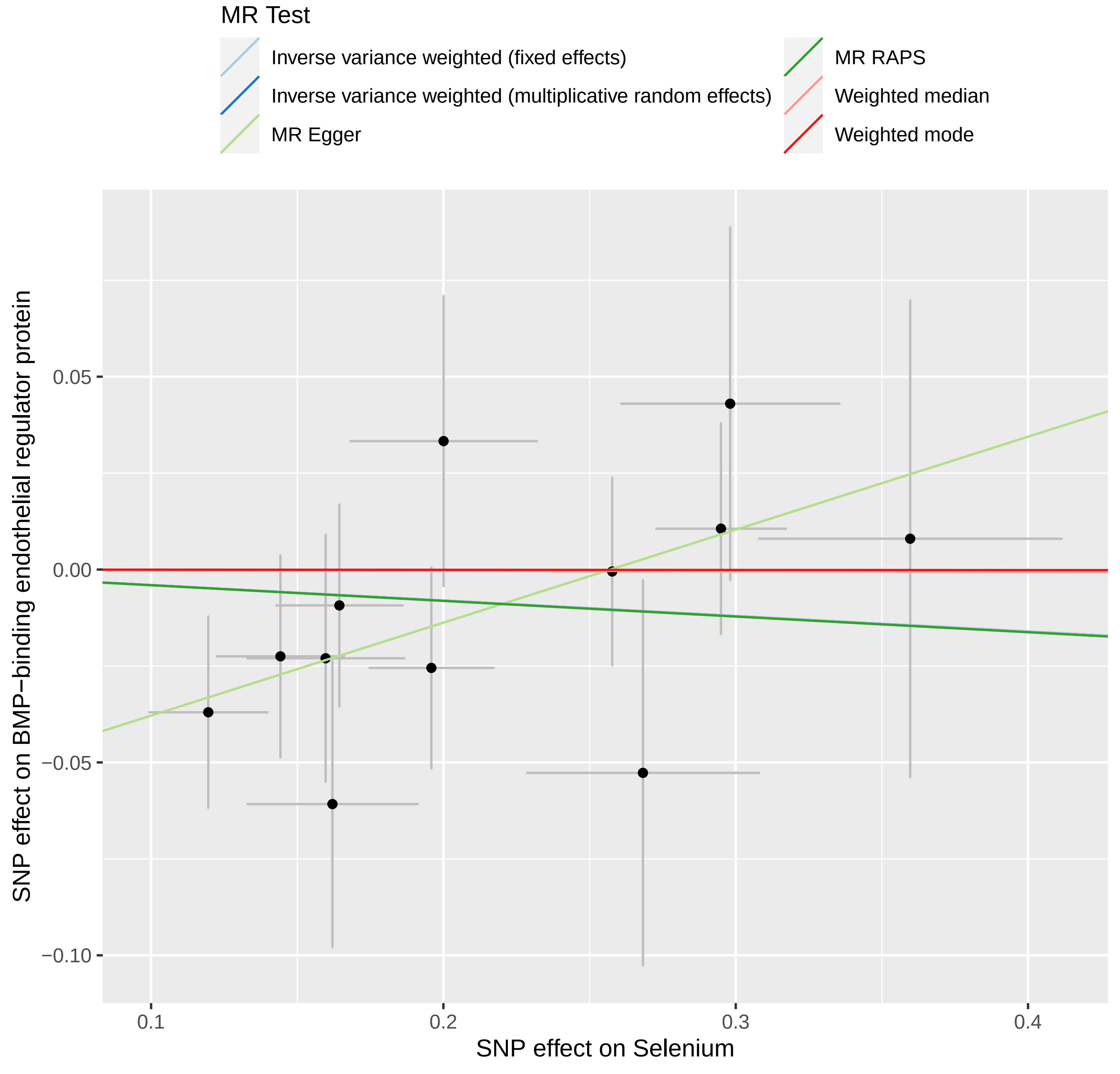

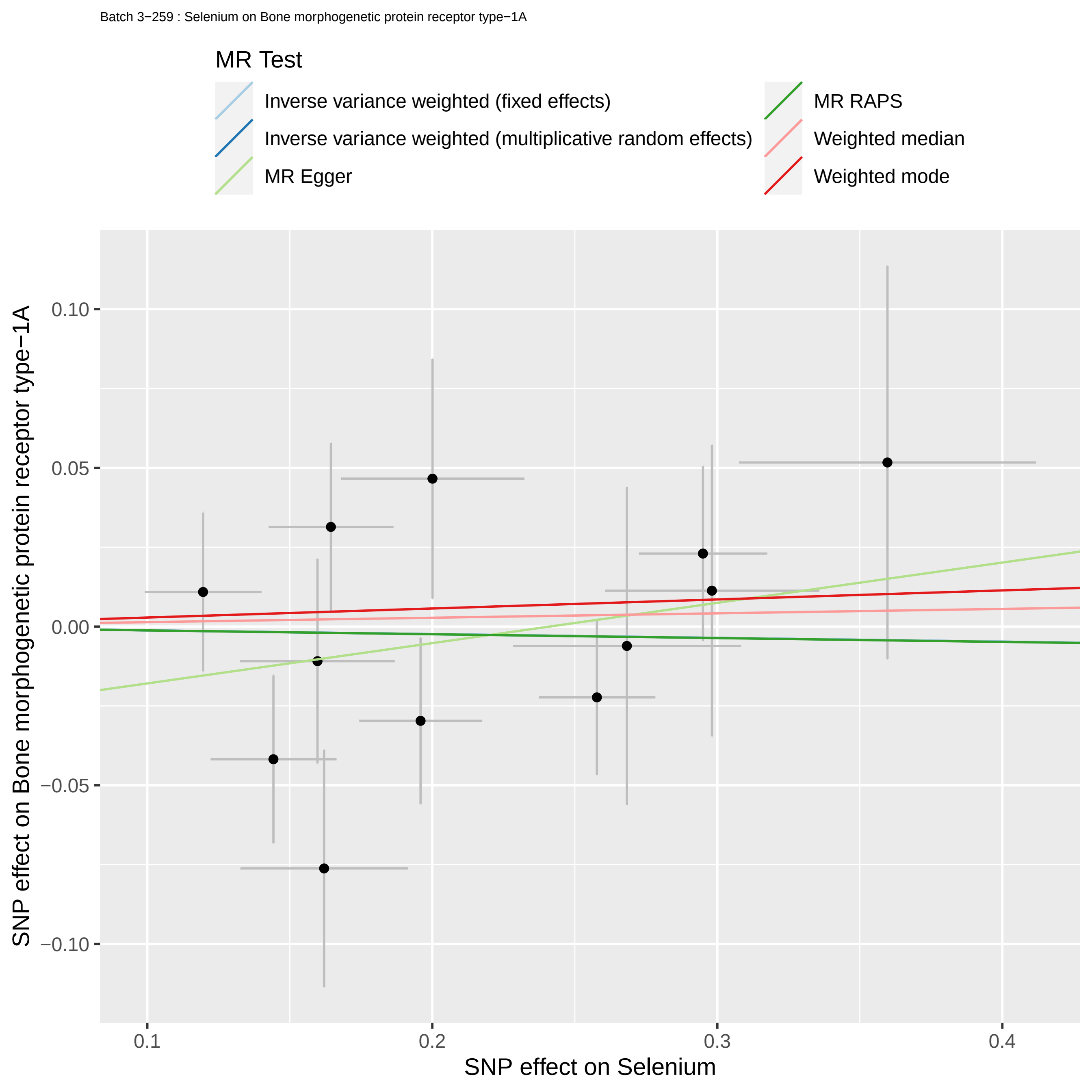

Batch 3−259 : Selenium on Bone morphogenetic protein receptor type−1A
MR Test
Inverse variance weighted (fixed effects)
Inverse variance weighted (multiplicative random effects)
MR Egger
MR RAPS
Weighted median
Weighted mode
0.10
0.05
0.00
−0.05
−0.10
0.1
0.2
0.3
0.4
SNP effect on Selenium
SNP effect on Bone morphogenetic protein receptor type−1A

Batch 3−260 : Selenium on Cytoplasmic tyrosine−protein kinase BMX

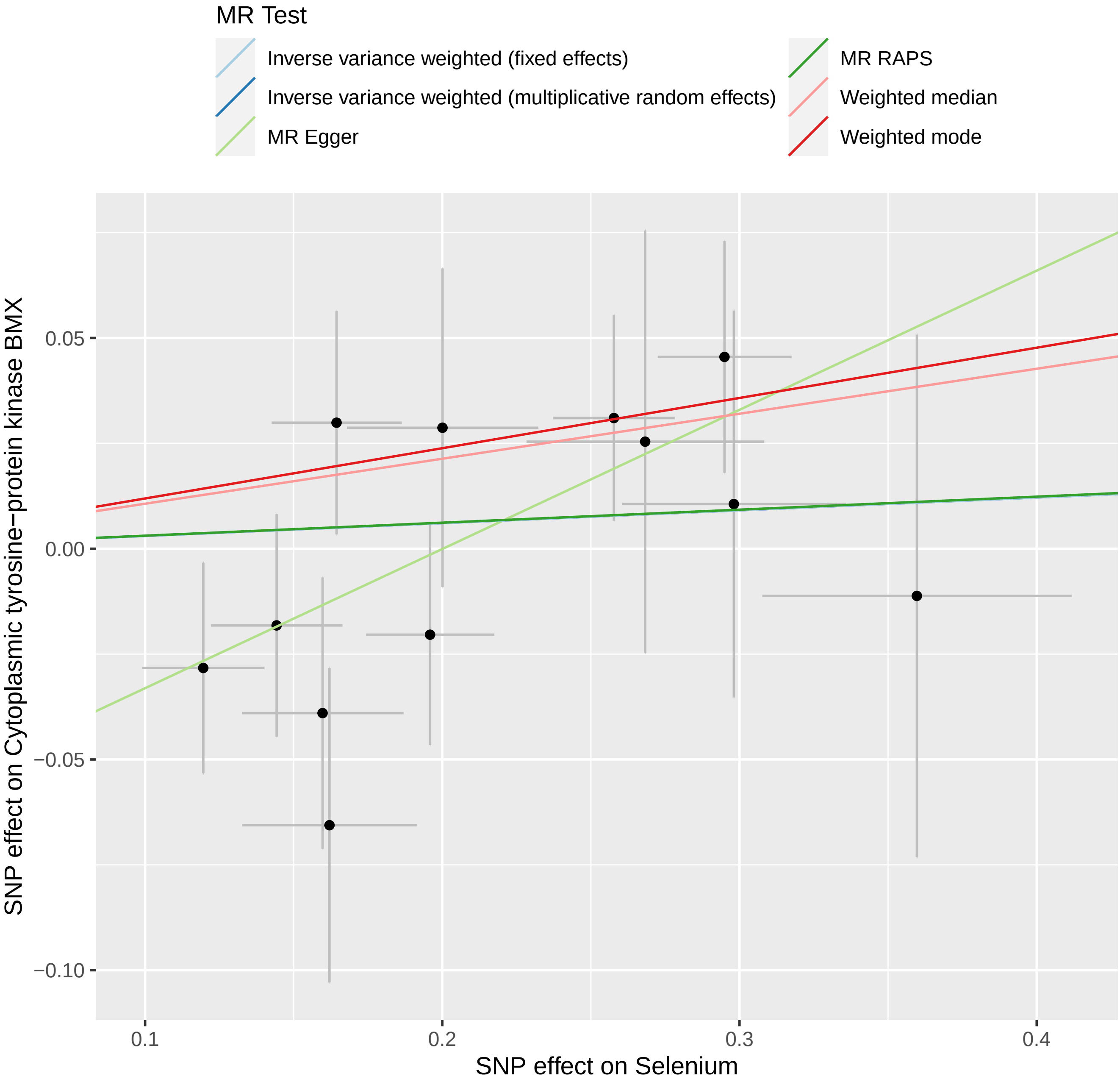

Batch 3−261 : Selenium on BCL2/adenovirus E1B 19 kDa protein−interacting protein 3

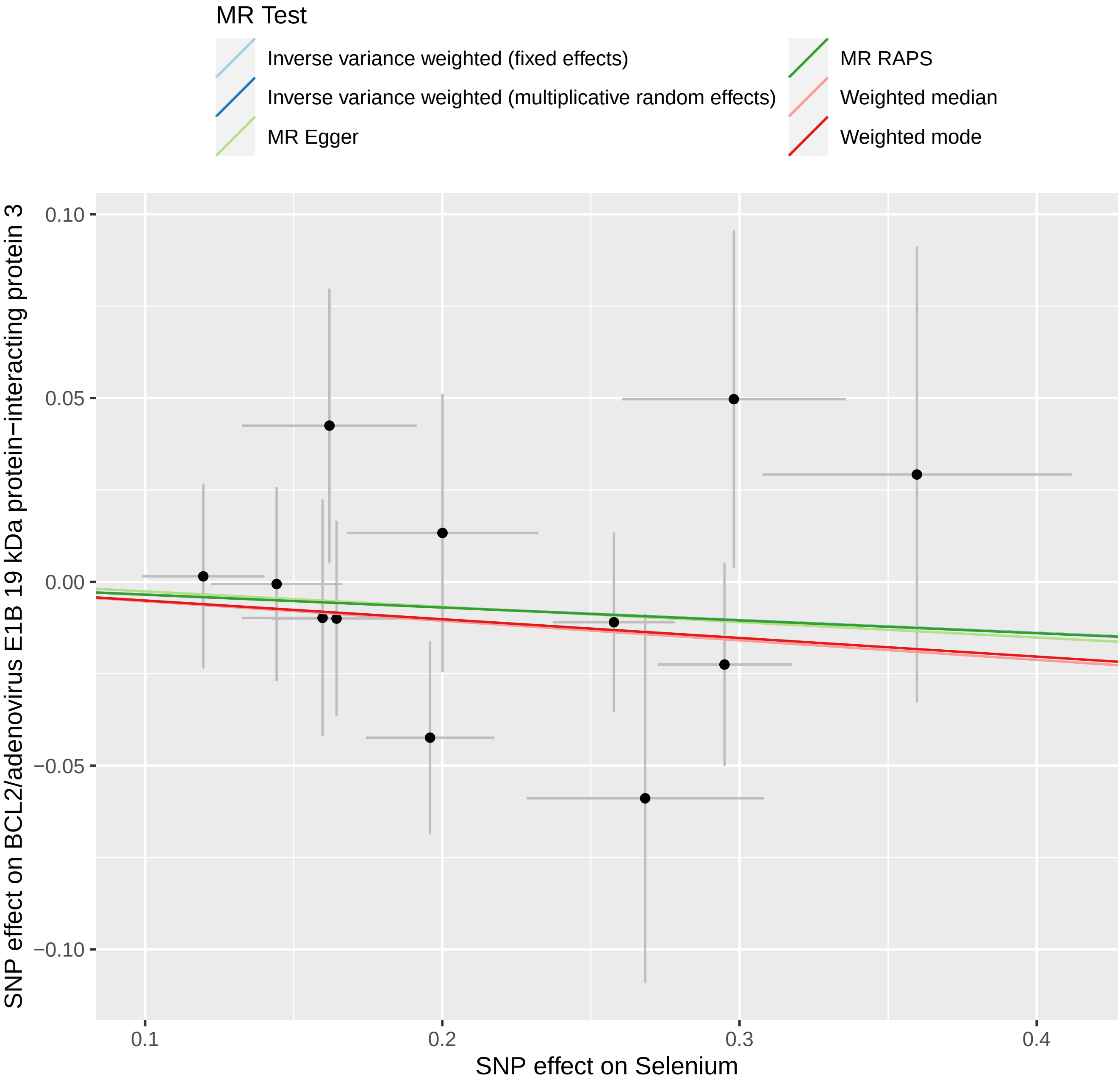

Batch 3−262 : Selenium on BCL2/adenovirus E1B 19 kDa protein−interacting protein 3−like

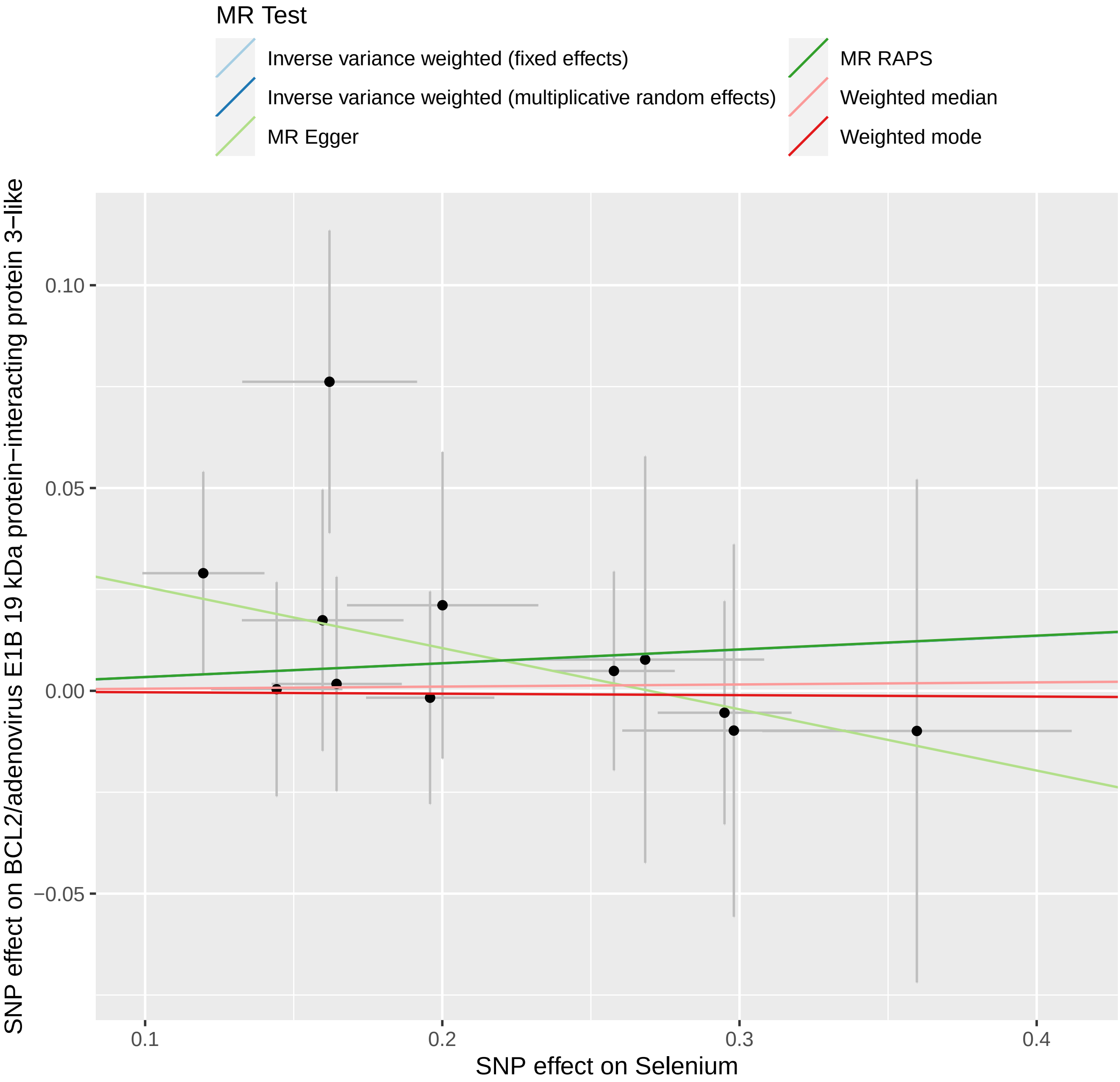

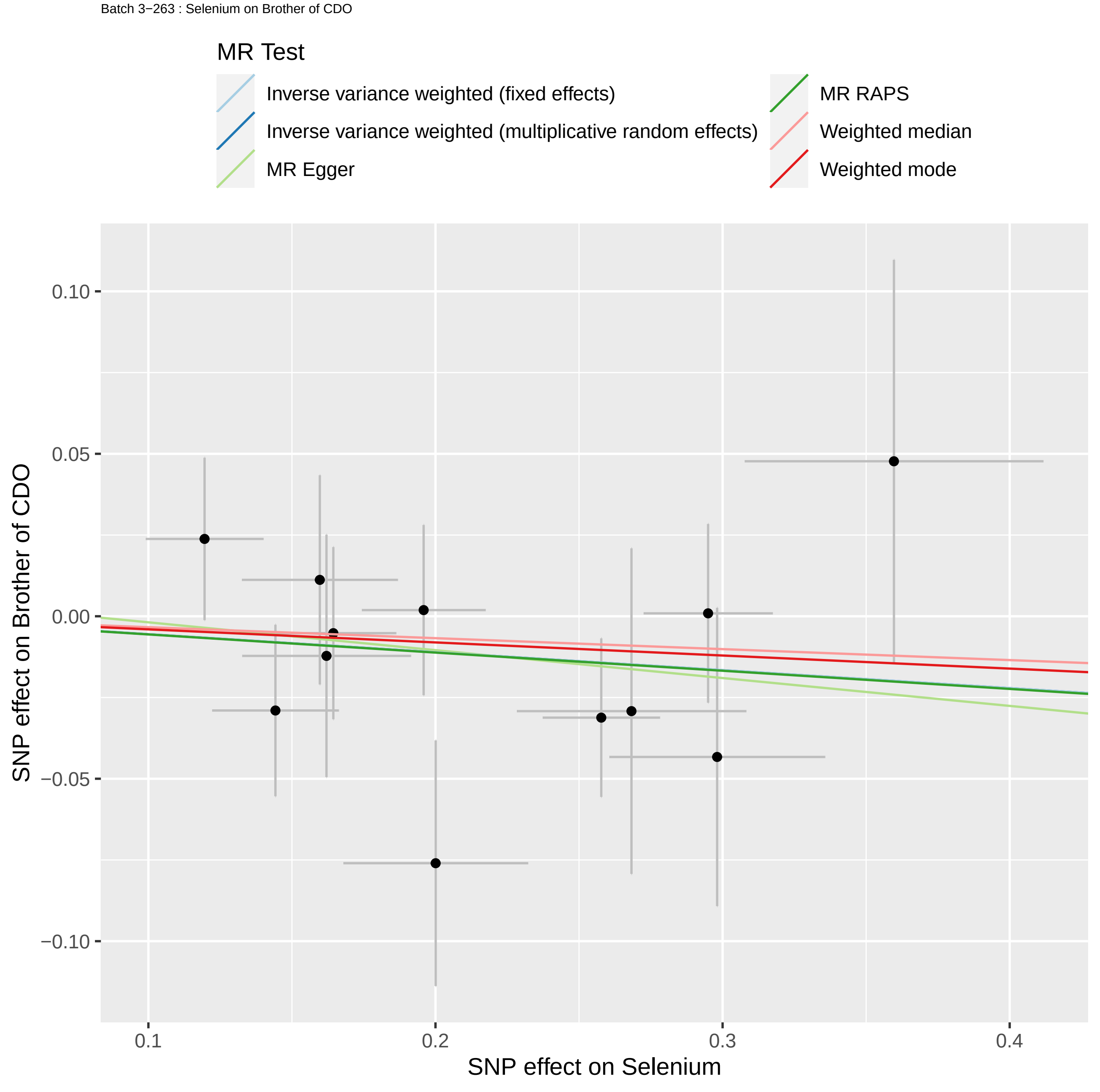
Batch 3−263 : Selenium on Brother of CDO
MR Test
Inverse variance weighted (fixed effects)
Inverse variance weighted (multiplicative random effects)
MR Egger
MR RAPS
Weighted median
Weighted mode
0.10
0.05
0.00
−0.05
−0.10
SNP effect on Brother of CDO
0.1
0.2
0.3
0.4
SNP effect on Selenium

Batch 3−264 : Selenium on BolA−like protein 2

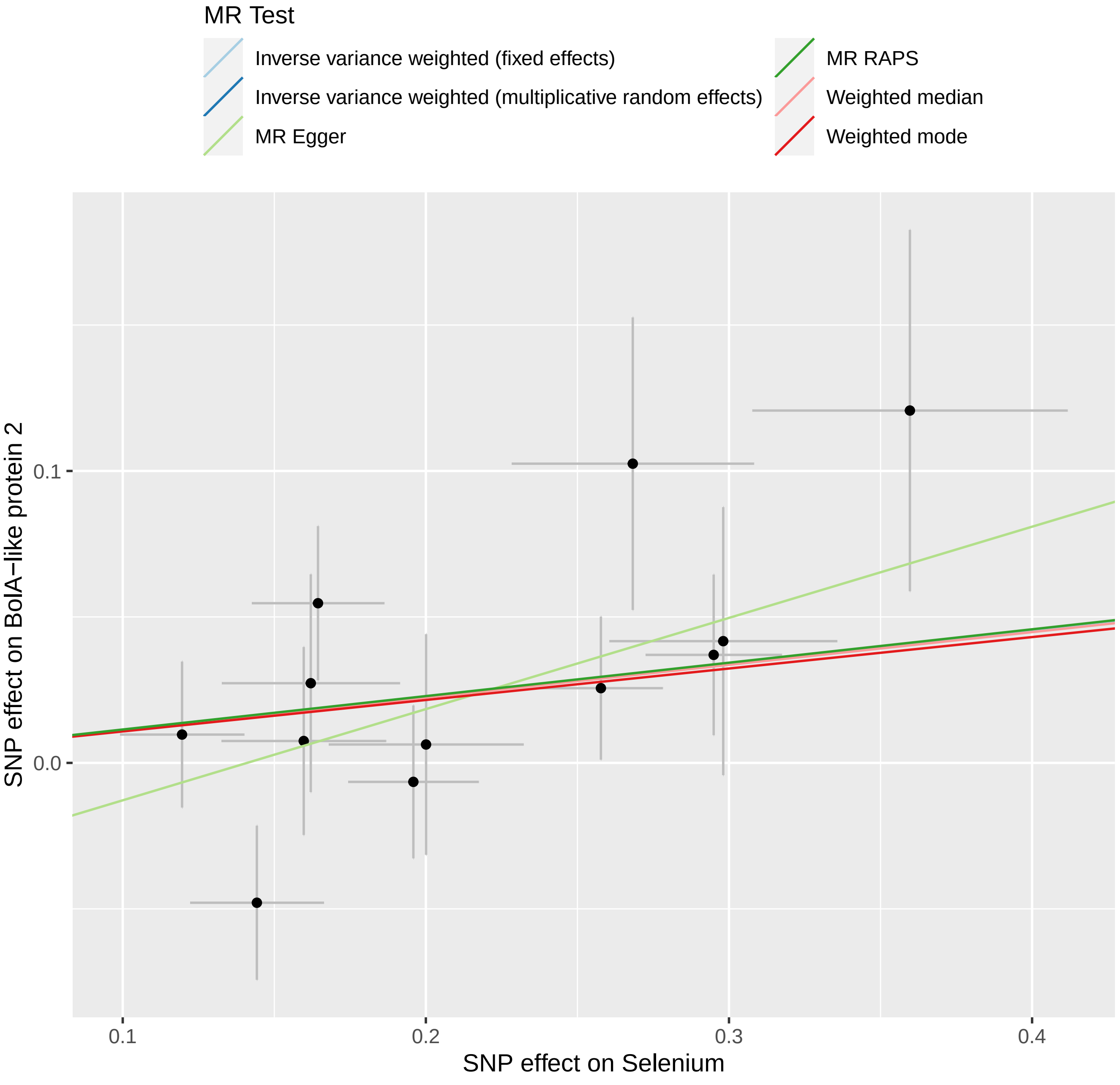

Batch 3−265 : Selenium on Bisphosphoglycerate mutase

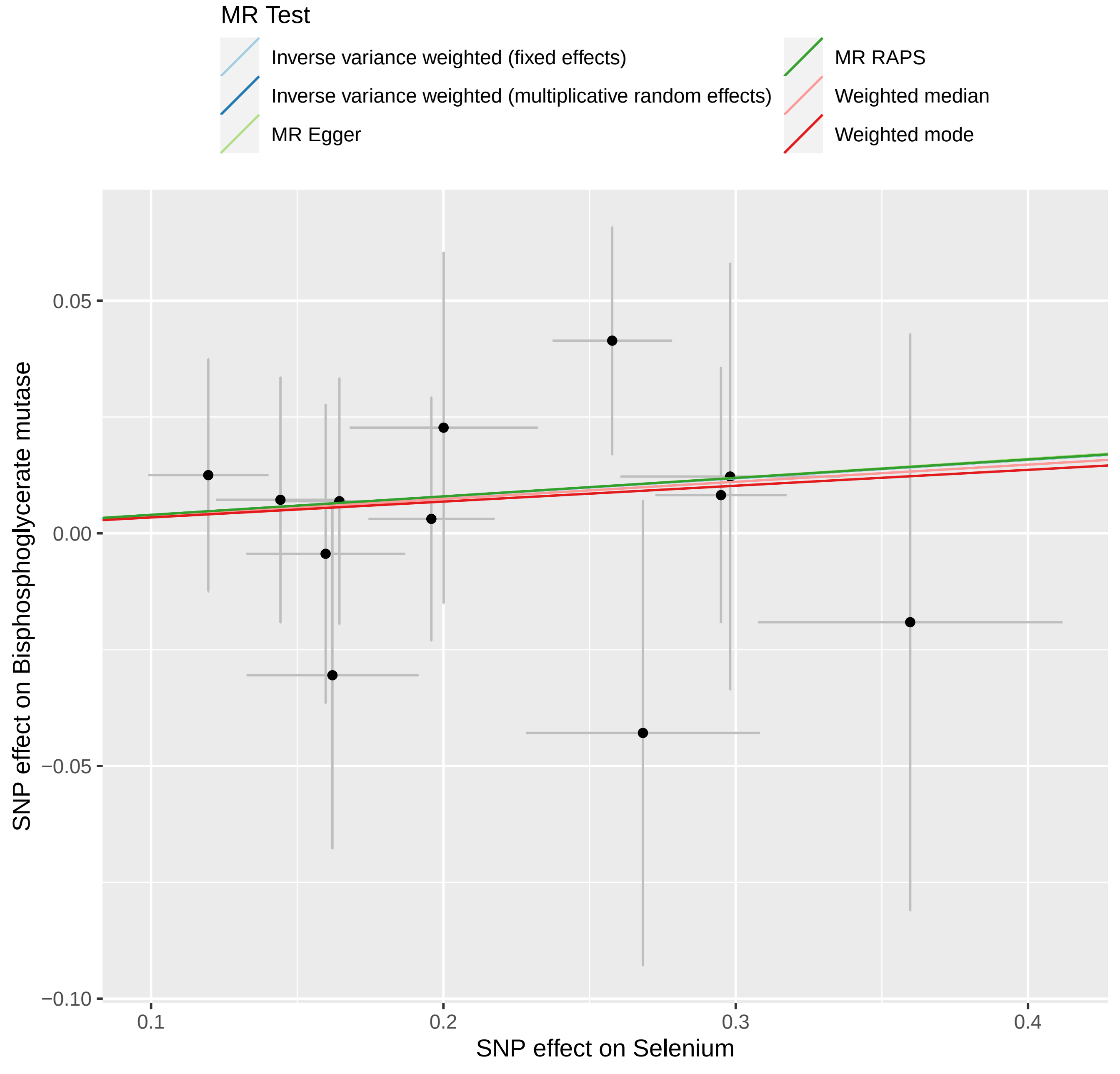

Batch 3−266 : Selenium on Bactericidal permeability−increasing protein

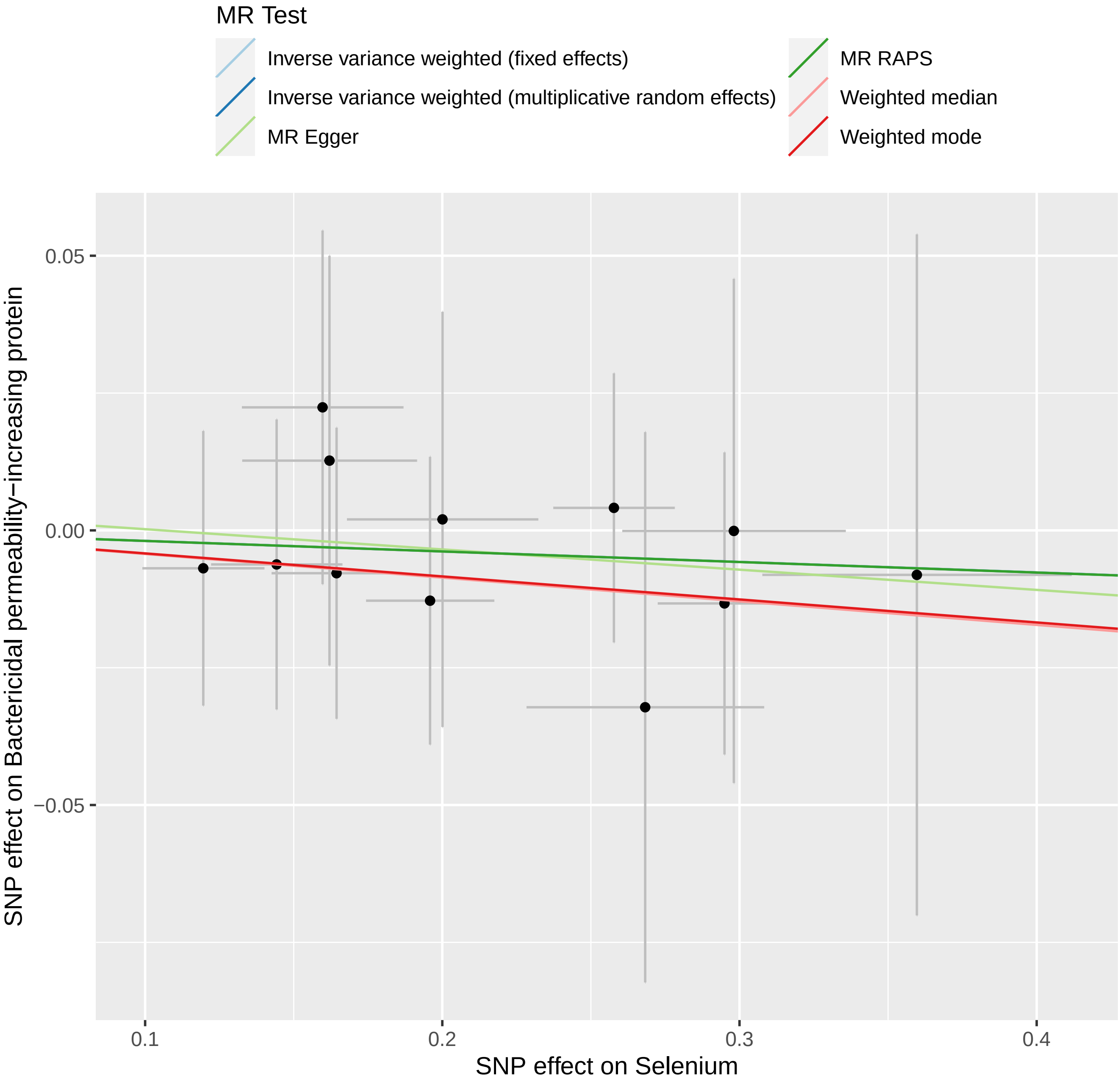

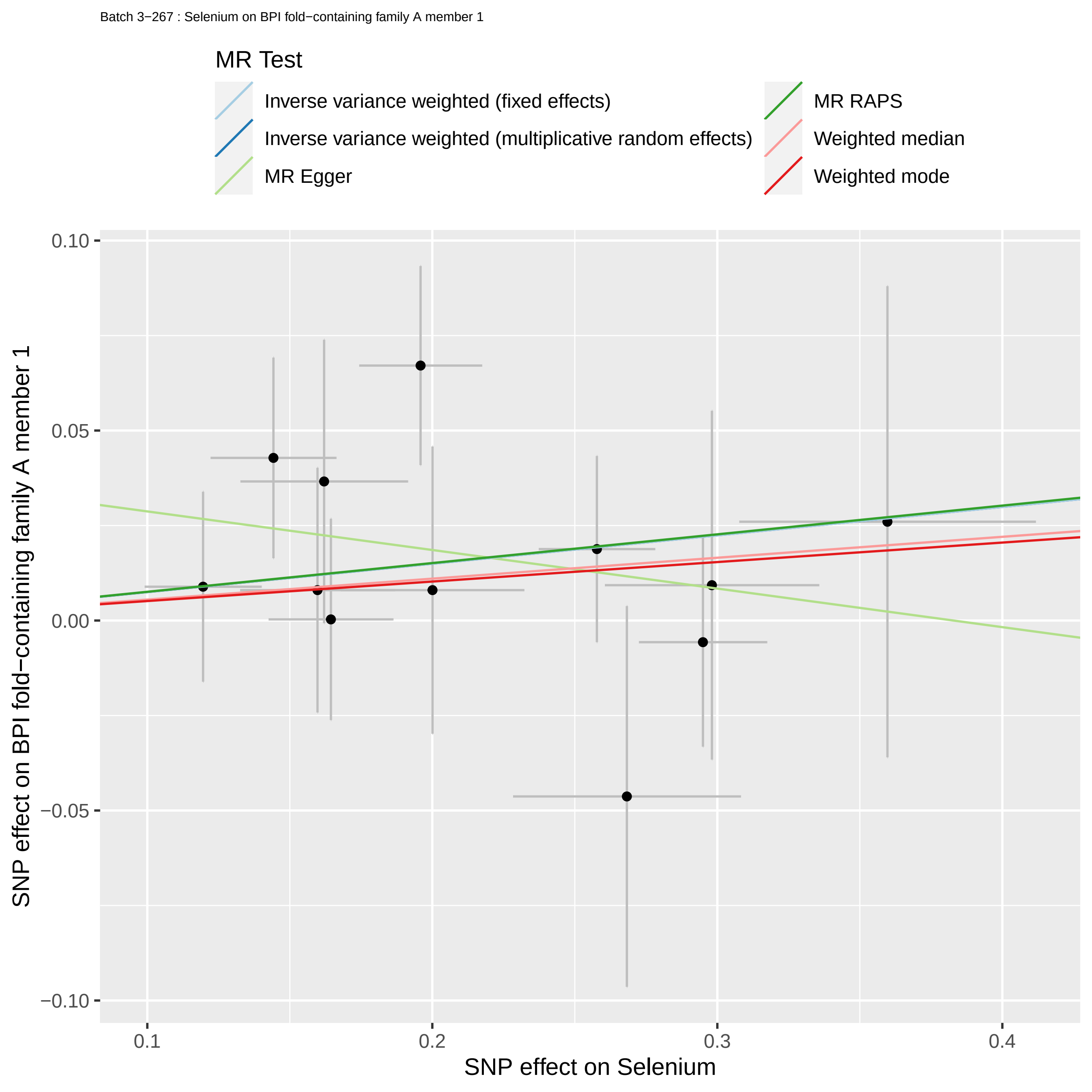
Batch 3−267 : Selenium on BPI fold−containing family A member 1
MR Test
Inverse variance weighted (fixed effects)
Inverse variance weighted (multiplicative random effects)
MR Egger
MR RAPS
Weighted median
Weighted mode
0.10
0.05
0.00
−0.05
−0.10
0.1
0.2
0.3
0.4
SNP effect on BPI fold−containing family A member 1
SNP effect on Selenium

Batch 3−268 : Selenium on BPI fold−containing family B member 1

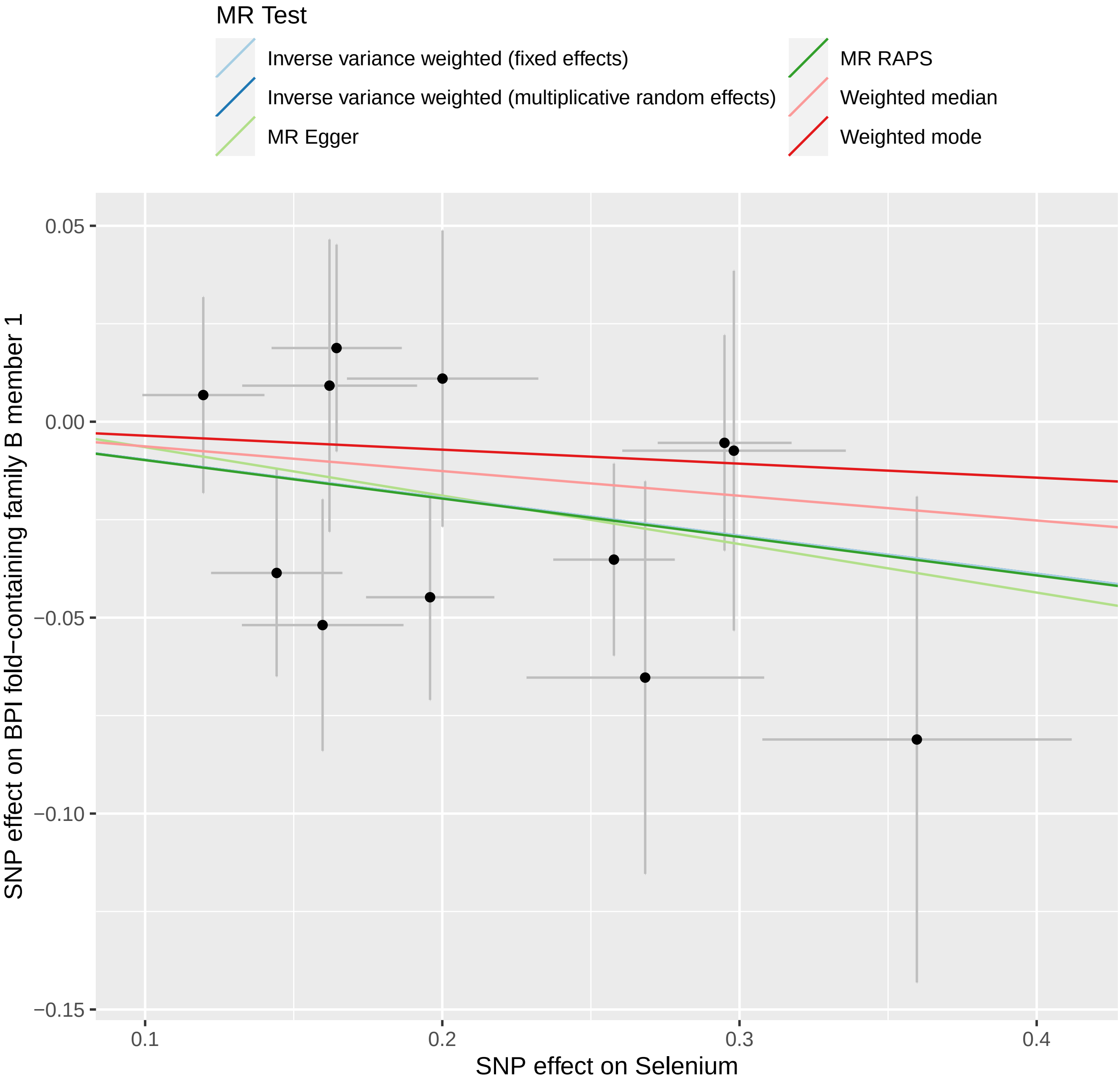

Batch 3−269 : Selenium on Bromodomain−containing protein 1

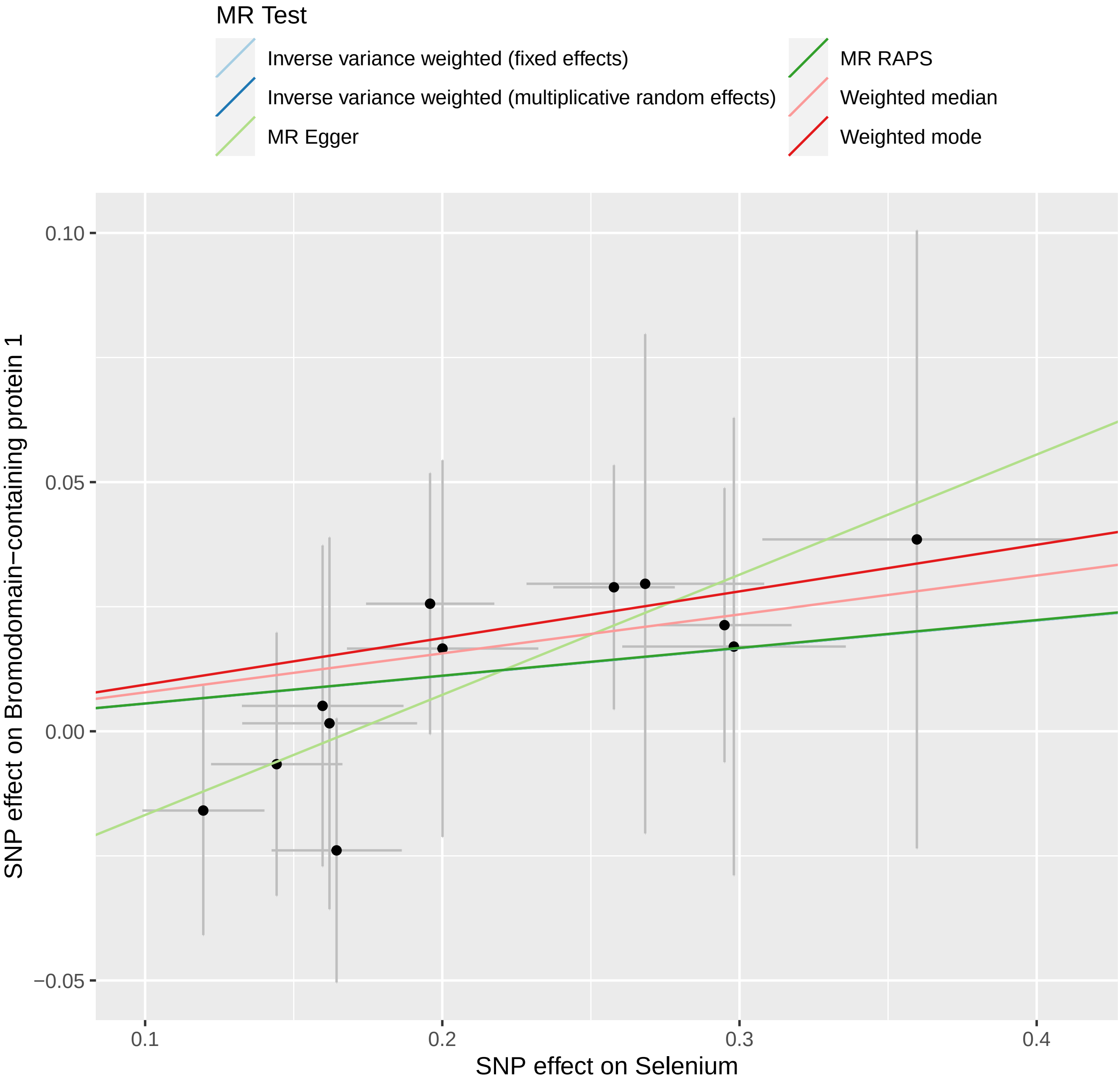

Batch 3−270 : Selenium on Bromodomain testis−specific protein

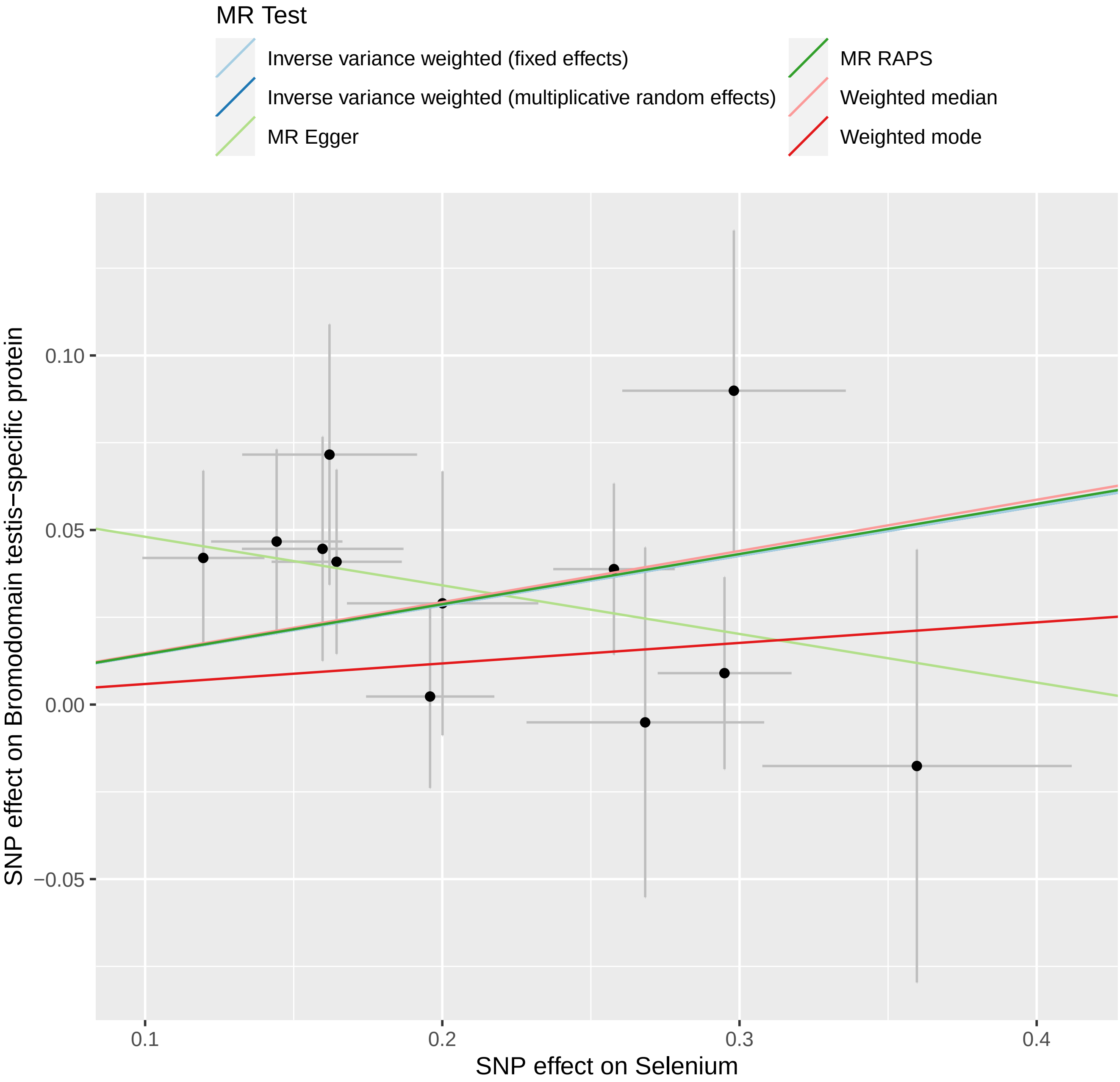

Batch 3−271 : Selenium on Transcription factor IIIB 90 kDa subunit

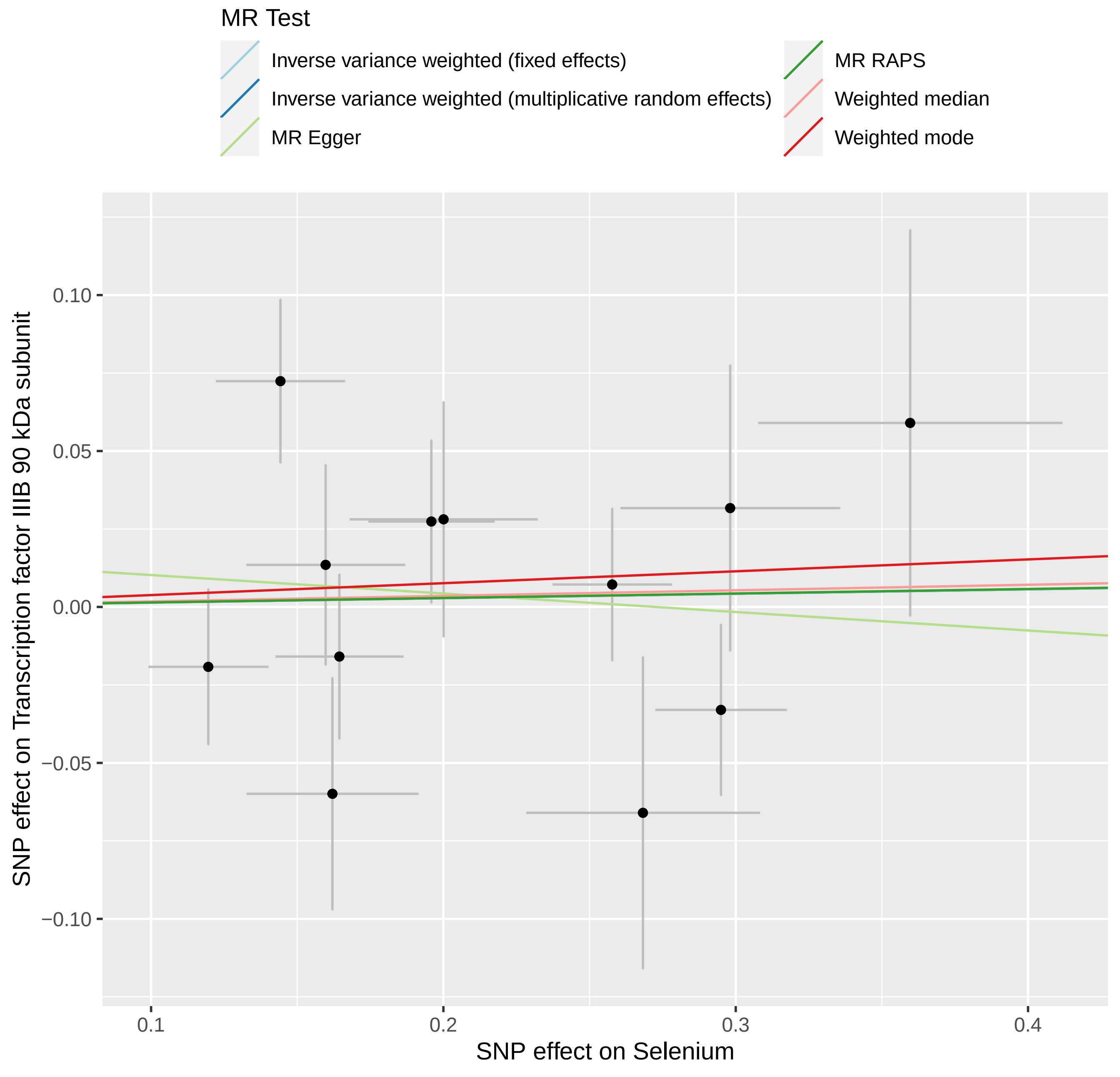

Batch 3−272 : Selenium on BRICHOS domain−containing protein 5

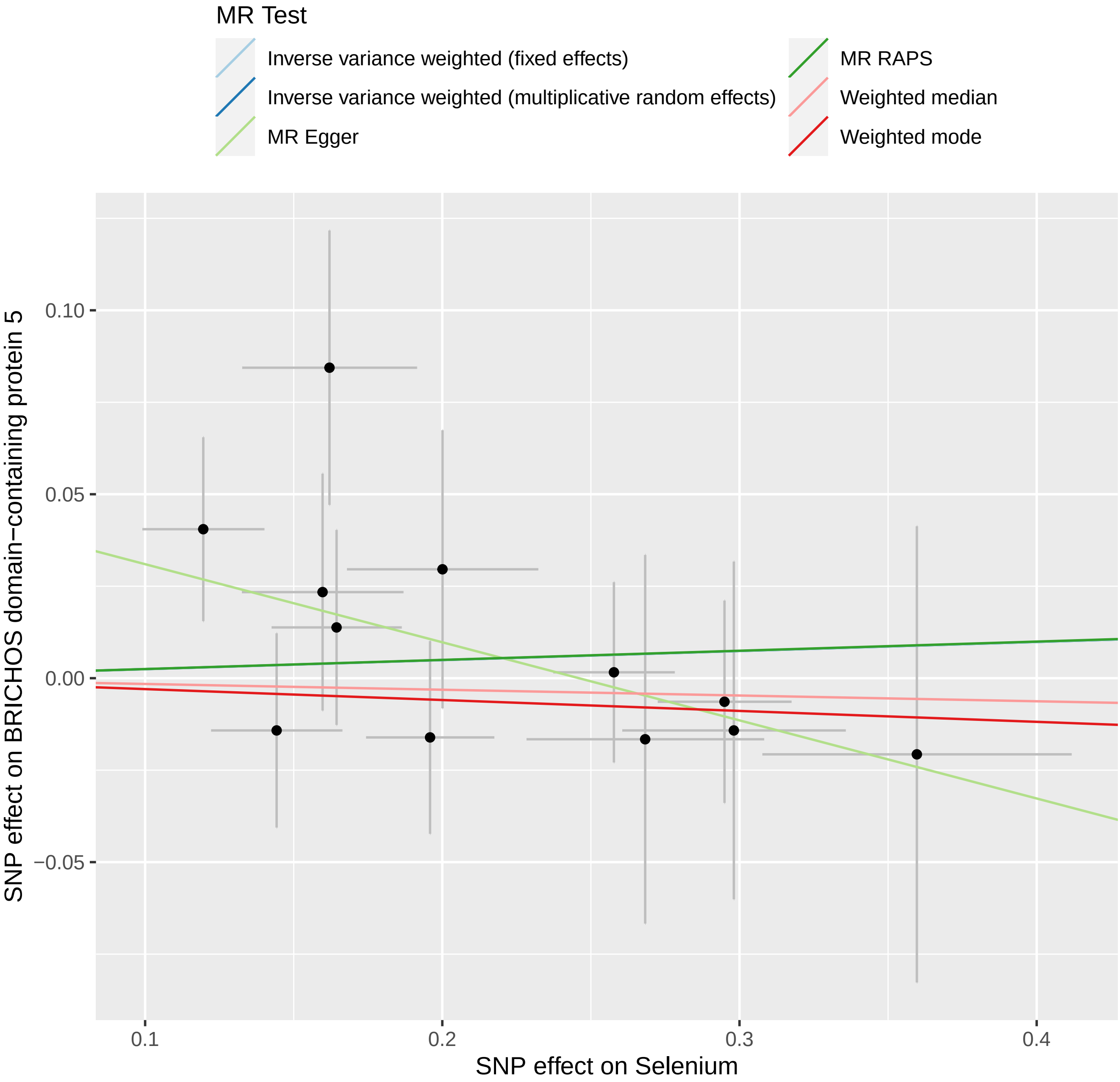

Batch 3−273 : Selenium on Peregrin

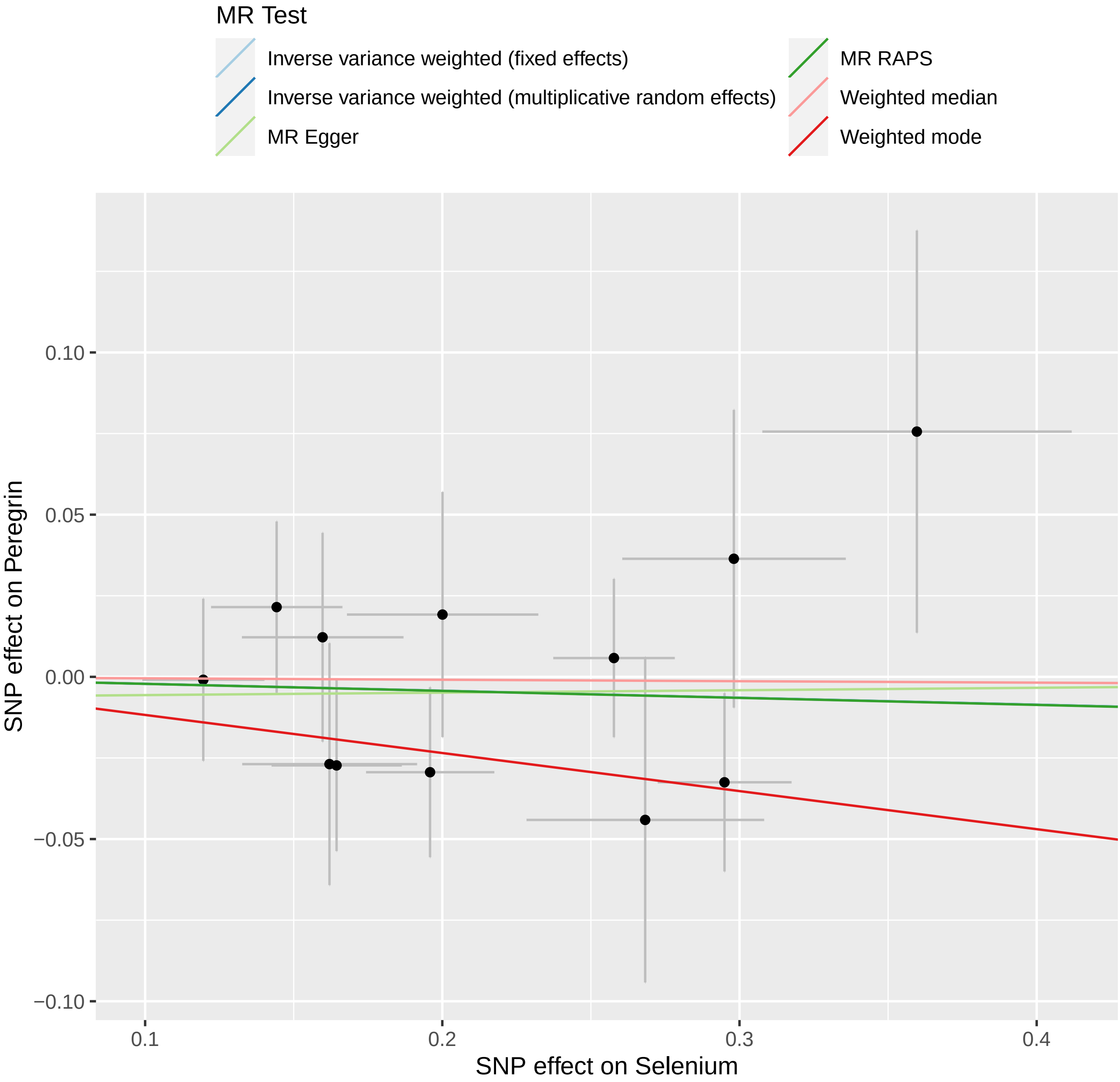

Batch 3−274 : Selenium on Serine/threonine−protein kinase BRSK2

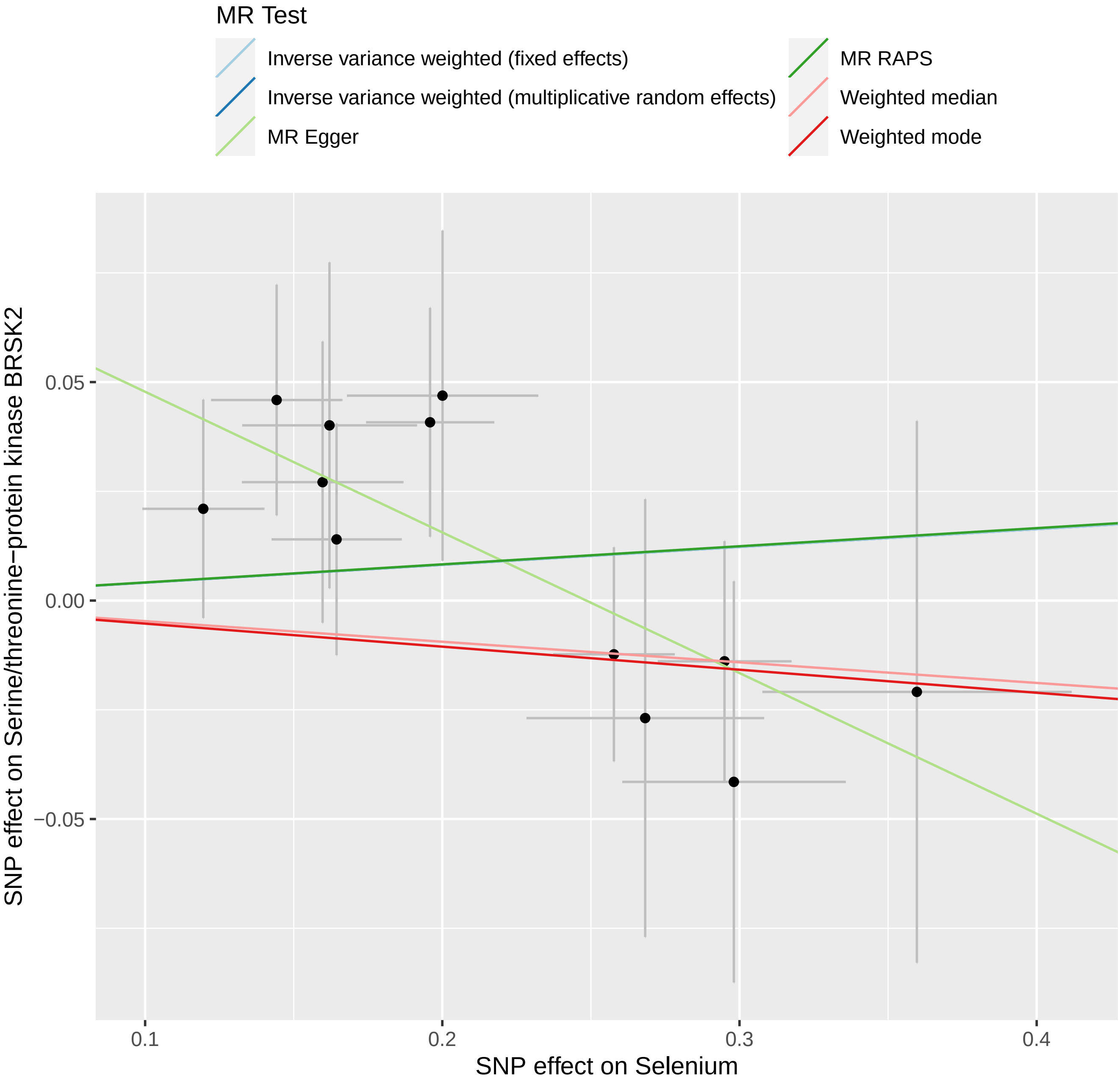

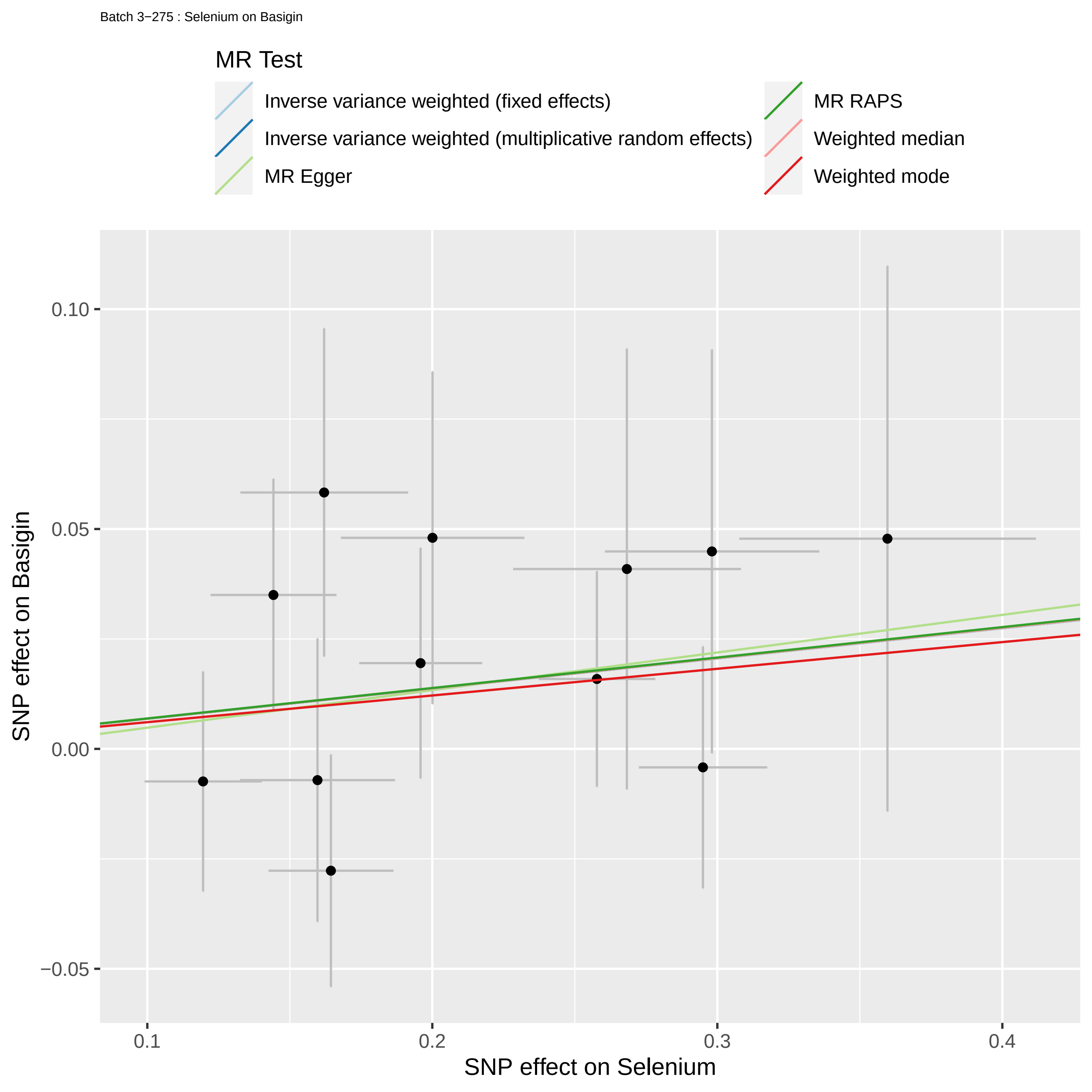

Batch 3−275 : Selenium on Basigin
MR Test
Inverse variance weighted (fixed effects)
Inverse variance weighted (multiplicative random effects)
MR Egger
MR RAPS
Weighted median
Weighted mode
0.10
0.05
0.00
−0.05
0.1
0.2
0.3
0.4
SNP effect on Basigin
SNP effect on Selenium

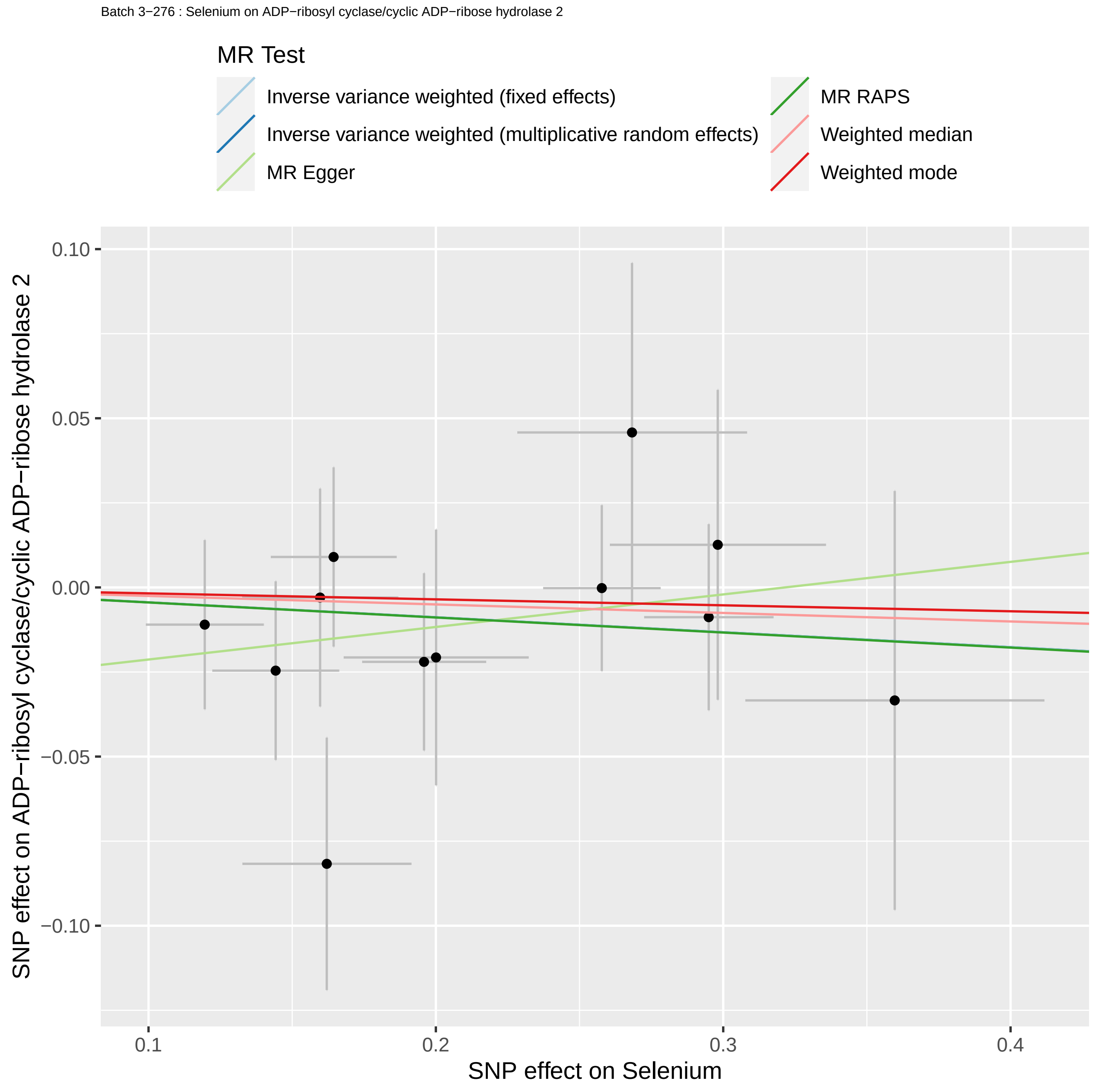
Batch 3−276 : Selenium on ADP−ribosyl cyclase/cyclic ADP−ribose hydrolase 2
MR Test
Inverse variance weighted (fixed effects)
Inverse variance weighted (multiplicative random effects)
MR Egger
MR RAPS
Weighted median
Weighted mode
0.10
0.05
0.00
−0.05
−0.10
SNP effect on ADP−ribosyl cyclase/cyclic ADP−ribose hydrolase 2
0.1
0.2
0.3
0.4
SNP effect on Selenium

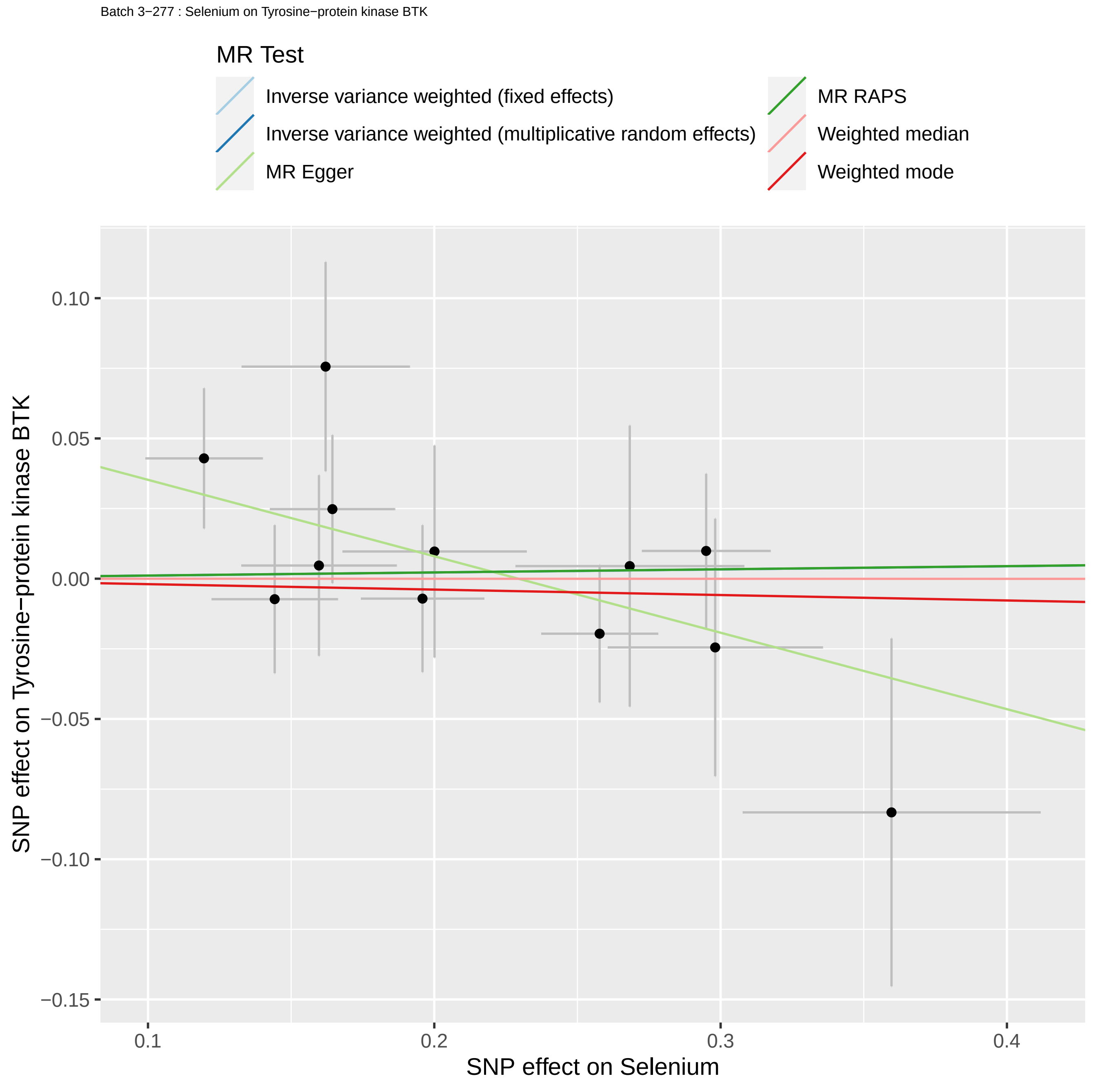
Batch 3−277 : Selenium on Tyrosine−protein kinase BTK
MR Test
Inverse variance weighted (fixed effects)
Inverse variance weighted (multiplicative random effects)
MR Egger
MR RAPS
Weighted median
Weighted mode
SNP effect on Tyrosine−protein kinase BTK
0.10
0.05
0.00
−0.05
−0.10
−0.15
0.1
0.2
0.3
0.4
SNP effect on Selenium

Batch 3−278 : Selenium on Butyrophilin−like protein 9

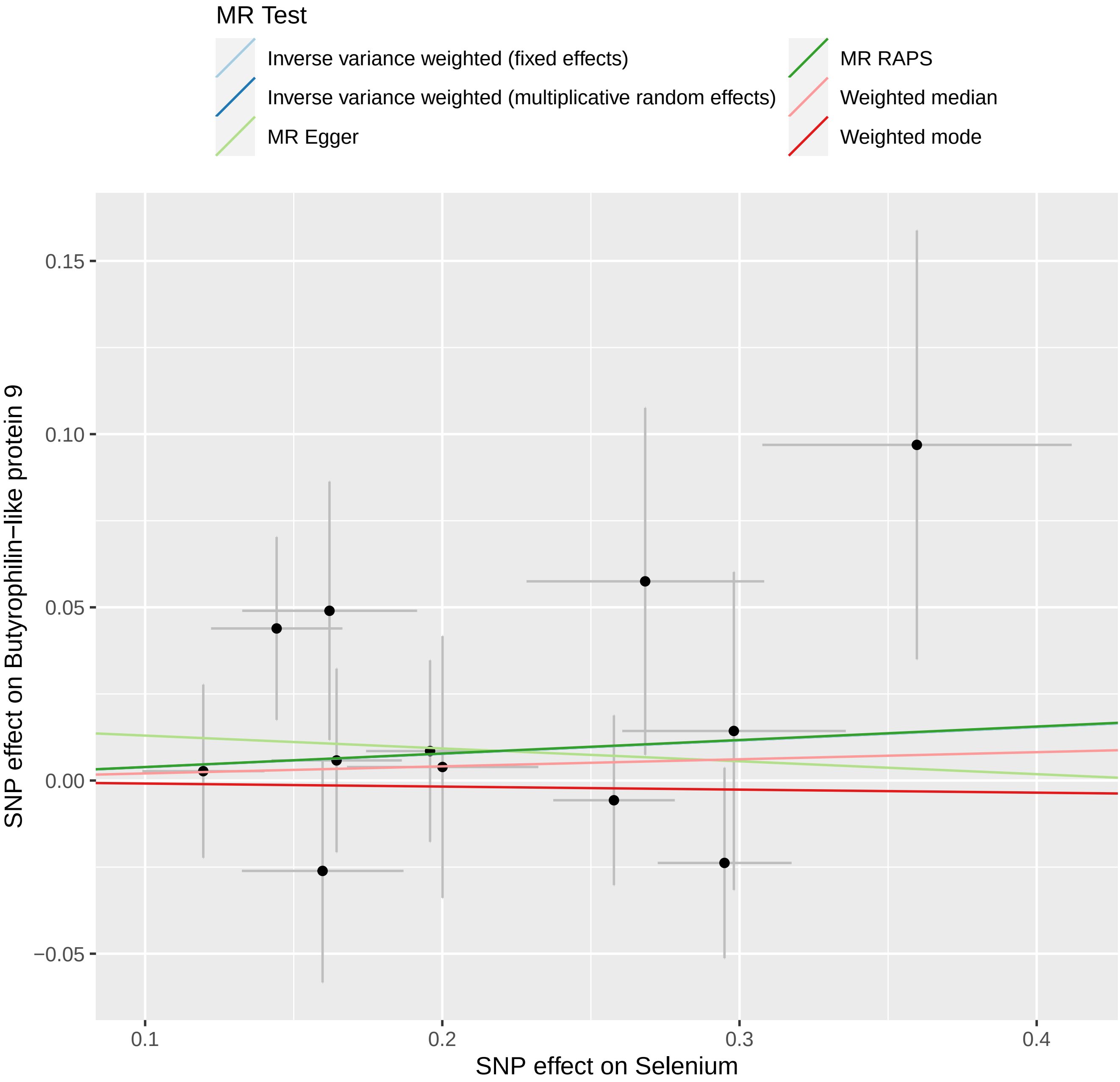

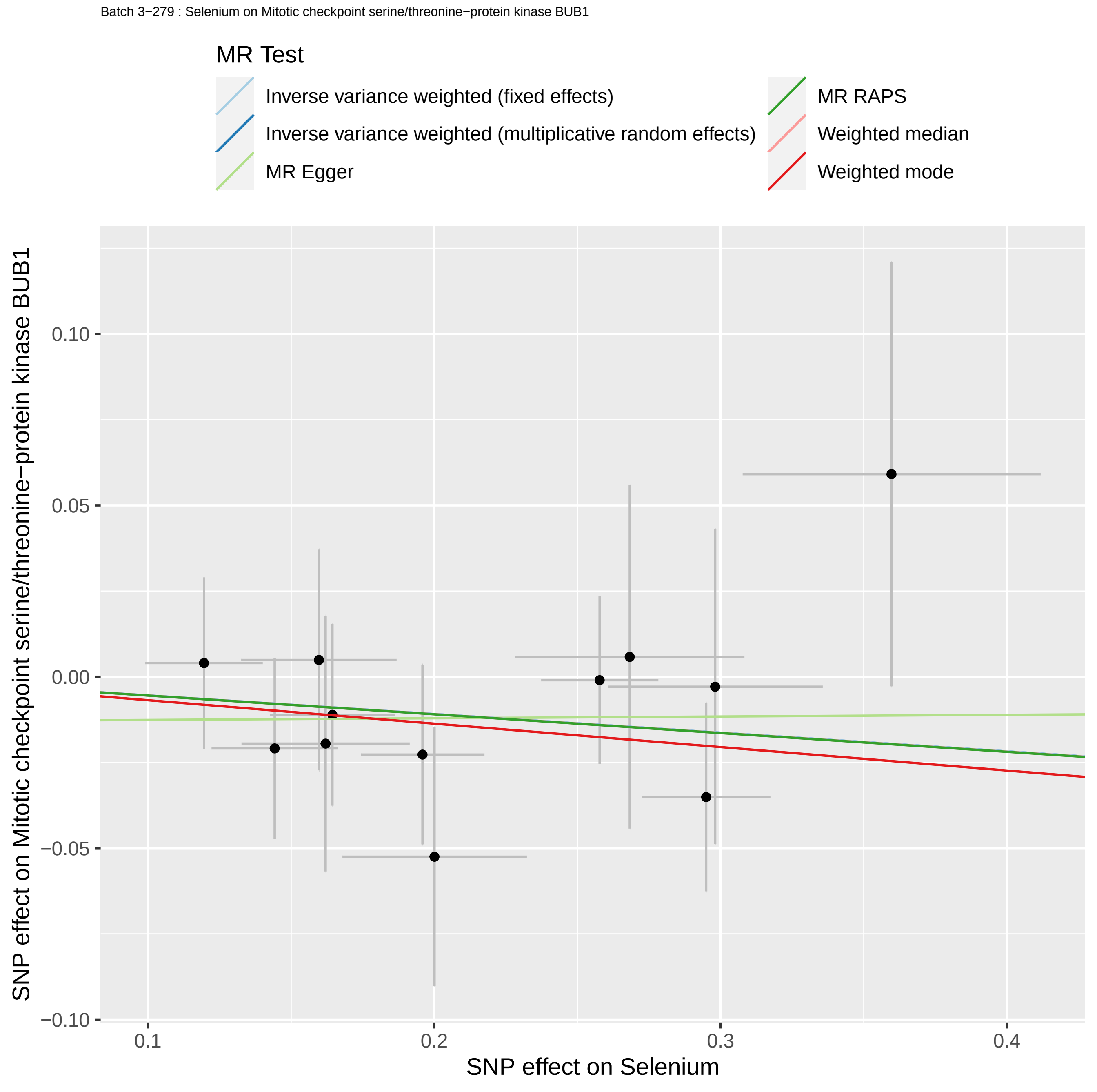

Batch 3−279 : Selenium on Mitotic checkpoint serine/threonine−protein kinase BUB1
MR Test
Inverse variance weighted (fixed effects)
Inverse variance weighted (multiplicative random effects)
MR Egger
MR RAPS
Weighted median
Weighted mode
SNP effect on Mitotic checkpoint serine/threonine−protein kinase BUB1
0.10
0.05
0.00
−0.05
−0.10
0.1
0.2
0.3
0.4
SNP effect on Selenium

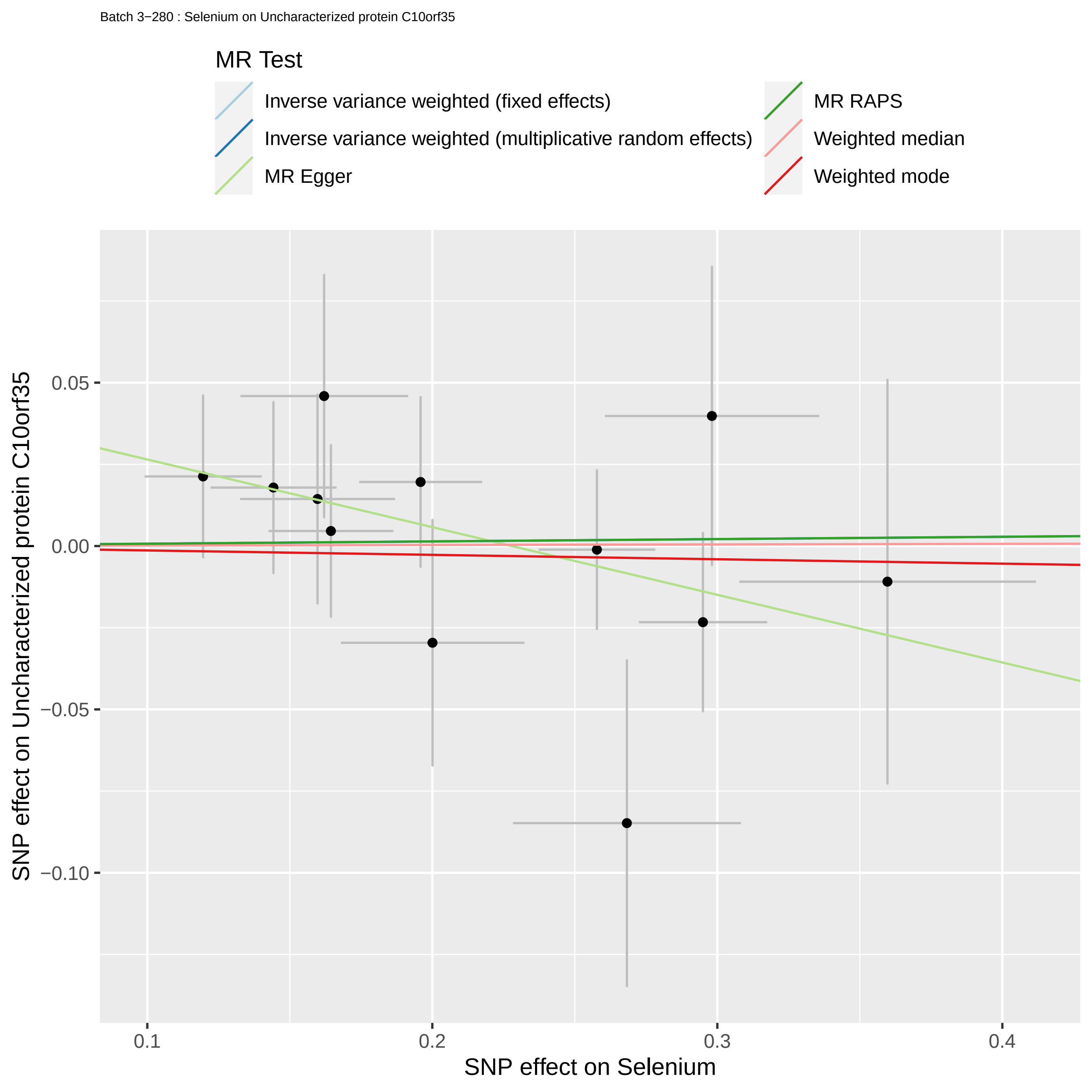

Batch 3−280 : Selenium on Uncharacterized protein C10orf35
MR Test
Inverse variance weighted (fixed effects)
Inverse variance weighted (multiplicative random effects)
MR Egger
MR RAPS
Weighted median
Weighted mode
0.05
0.00
−0.05
−0.10
0.1
0.2
0.3
0.4
SNP effect on Uncharacterized protein C10orf35
SNP effect on Selenium

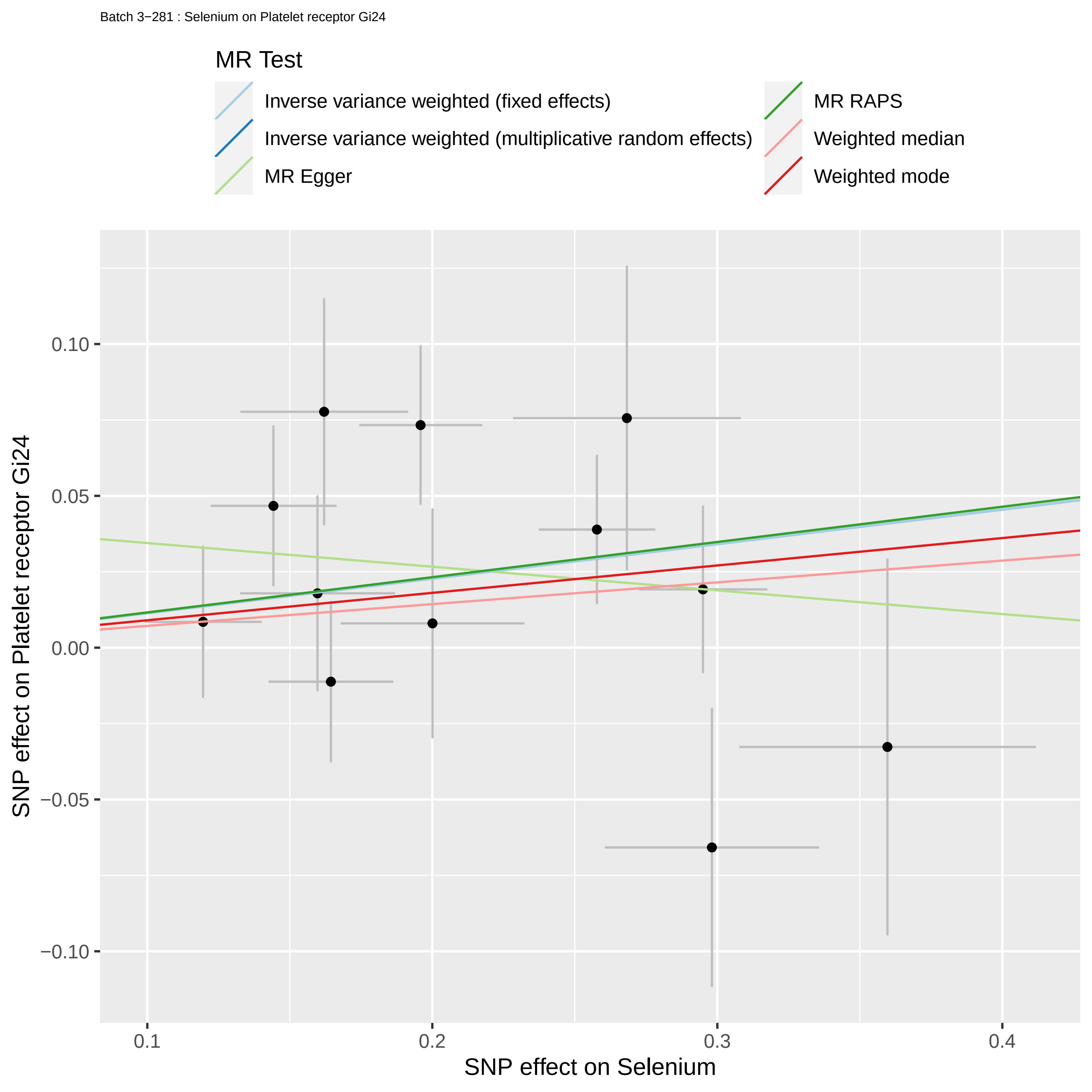

Batch 3−281 : Selenium on Platelet receptor Gi24
MR Test
Inverse variance weighted (fixed effects)
Inverse variance weighted (multiplicative random effects)
MR Egger
MR RAPS
Weighted median
Weighted mode
0.10
0.05
0.00
−0.05
−0.10
0.1
0.2
0.3
0.4
SNP effect on Platelet receptor Gi24
SNP effect on Selenium

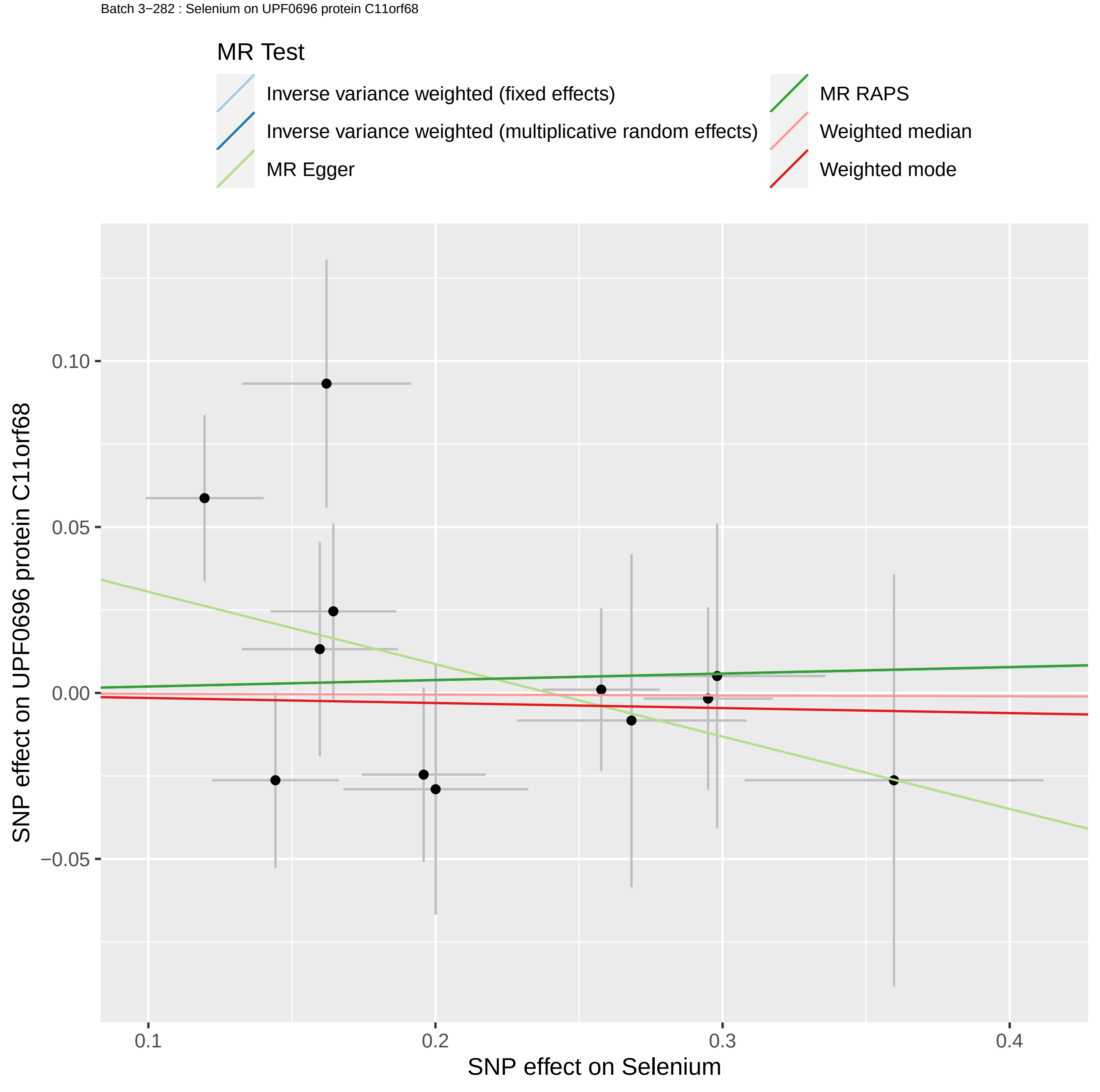

Batch 3−282 : Selenium on UPF0696 protein C11orf68
MR Test
Inverse variance weighted (fixed effects)
Inverse variance weighted (multiplicative random effects)
MR Egger
MR RAPS
Weighted median
Weighted mode
0.10
0.05
0.00
−0.05
0.1
0.2
0.3
0.4
SNP effect on UPF0696 protein C11orf68
SNP effect on Selenium

Batch 3−283 : Selenium on UPF0454 protein C12orf49

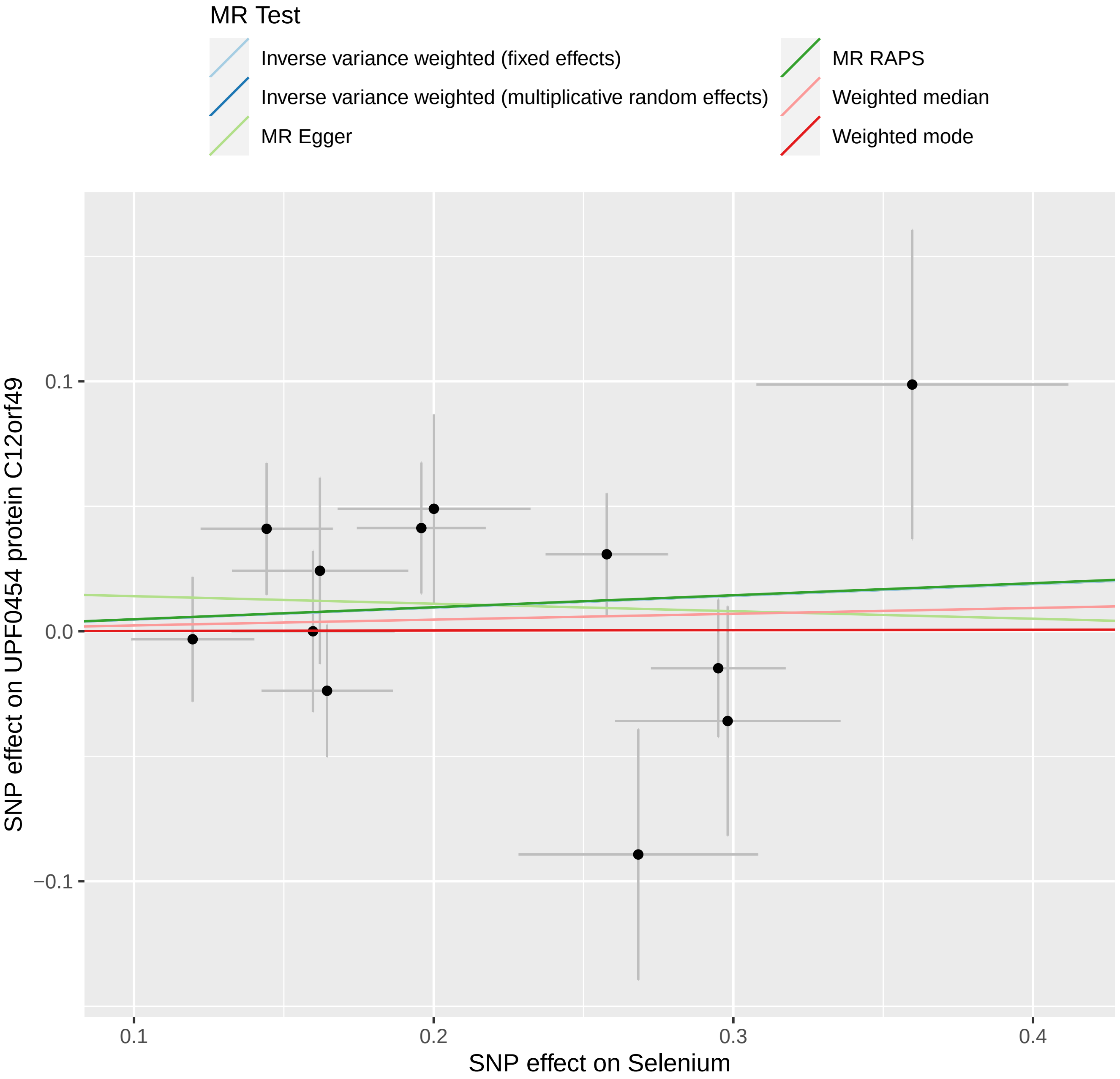

Batch 3−284 : Selenium on Uncharacterized protein C14orf93

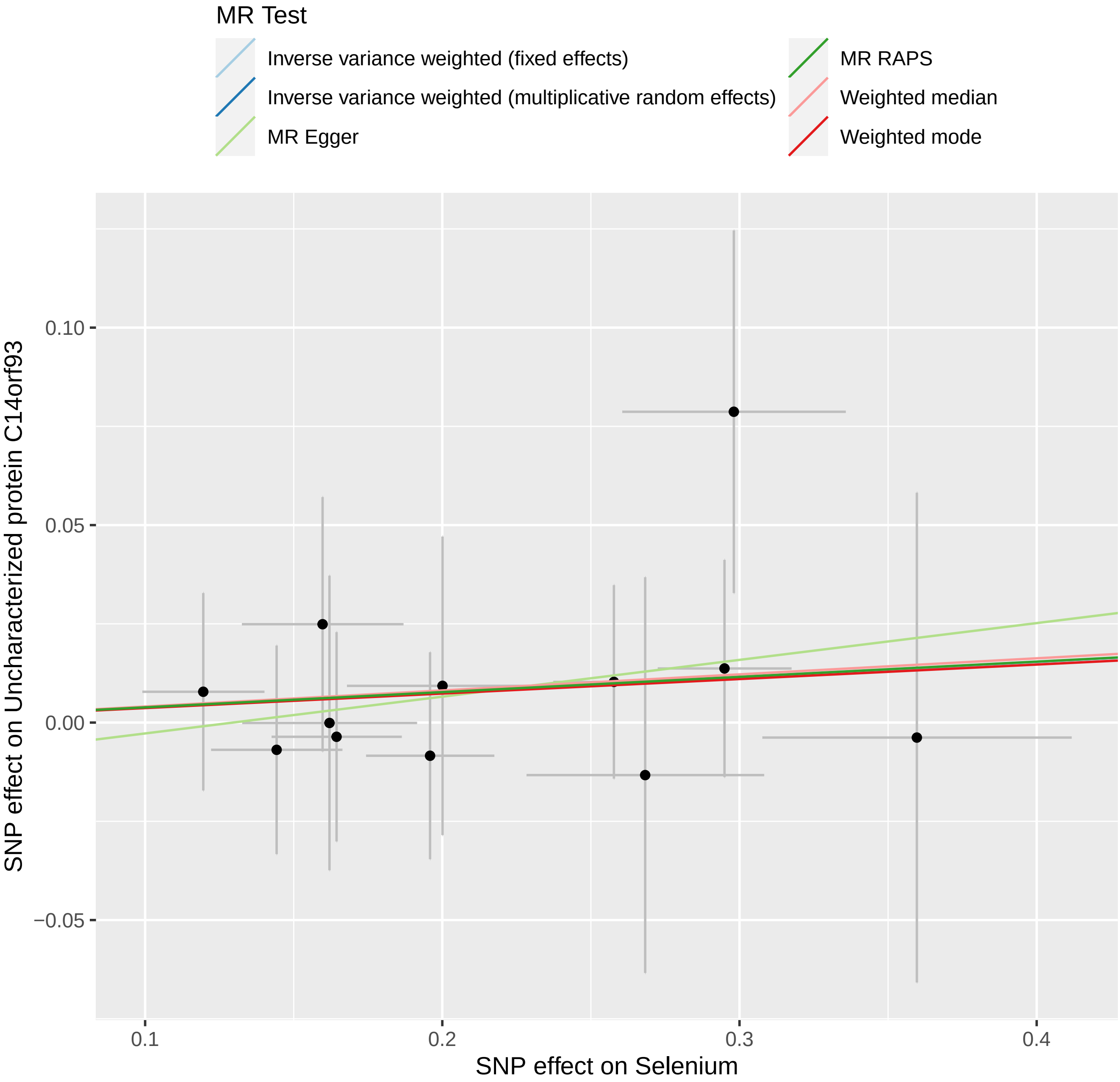

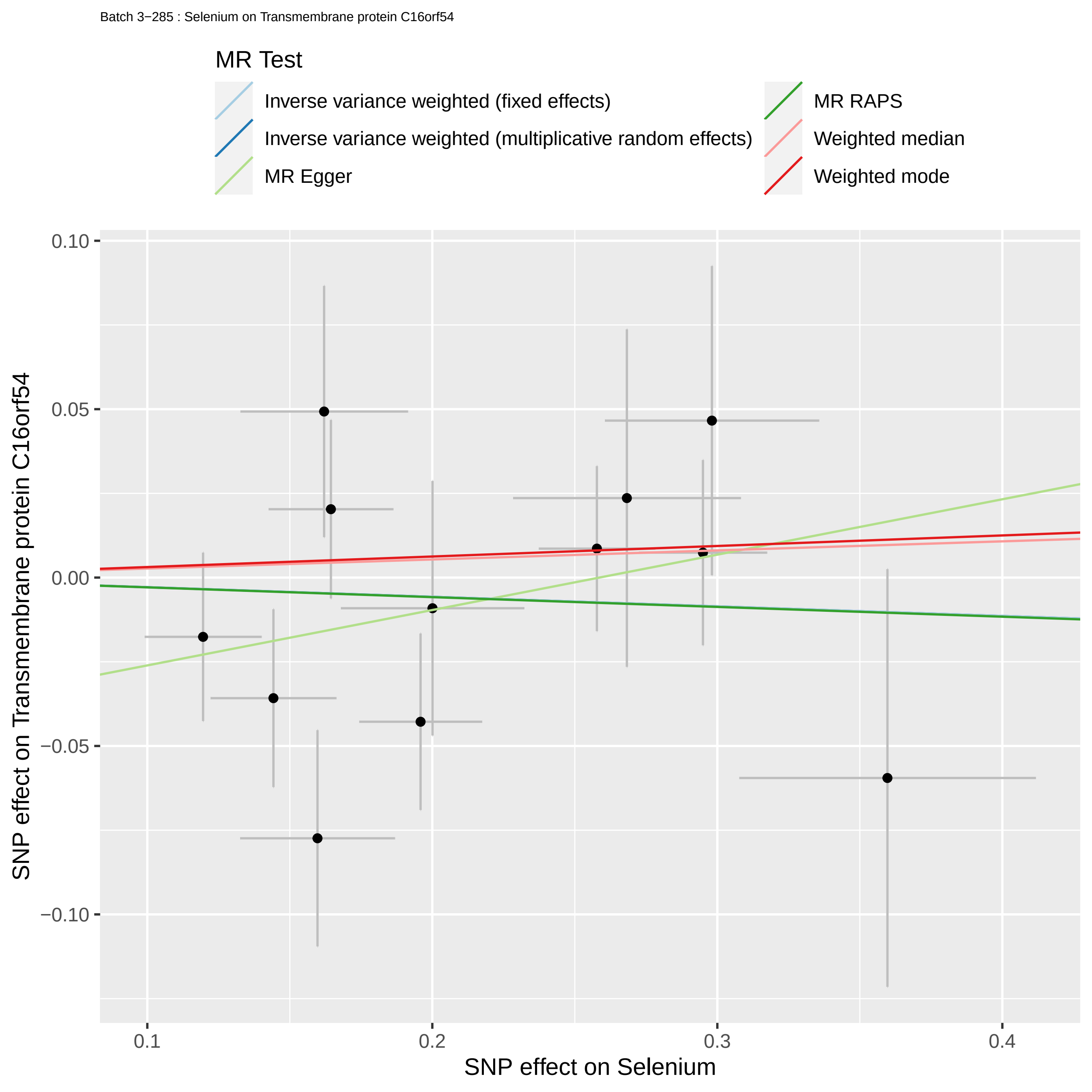

Batch 3−285 : Selenium on Transmembrane protein C16orf54
MR Test
Inverse variance weighted (fixed effects)
Inverse variance weighted (multiplicative random effects)
MR Egger
MR RAPS
Weighted median
Weighted mode
0.10
0.05
0.00
−0.05
−0.10
0.1
0.2
0.3
0.4
SNP effect on Transmembrane protein C16orf54
SNP effect on Selenium

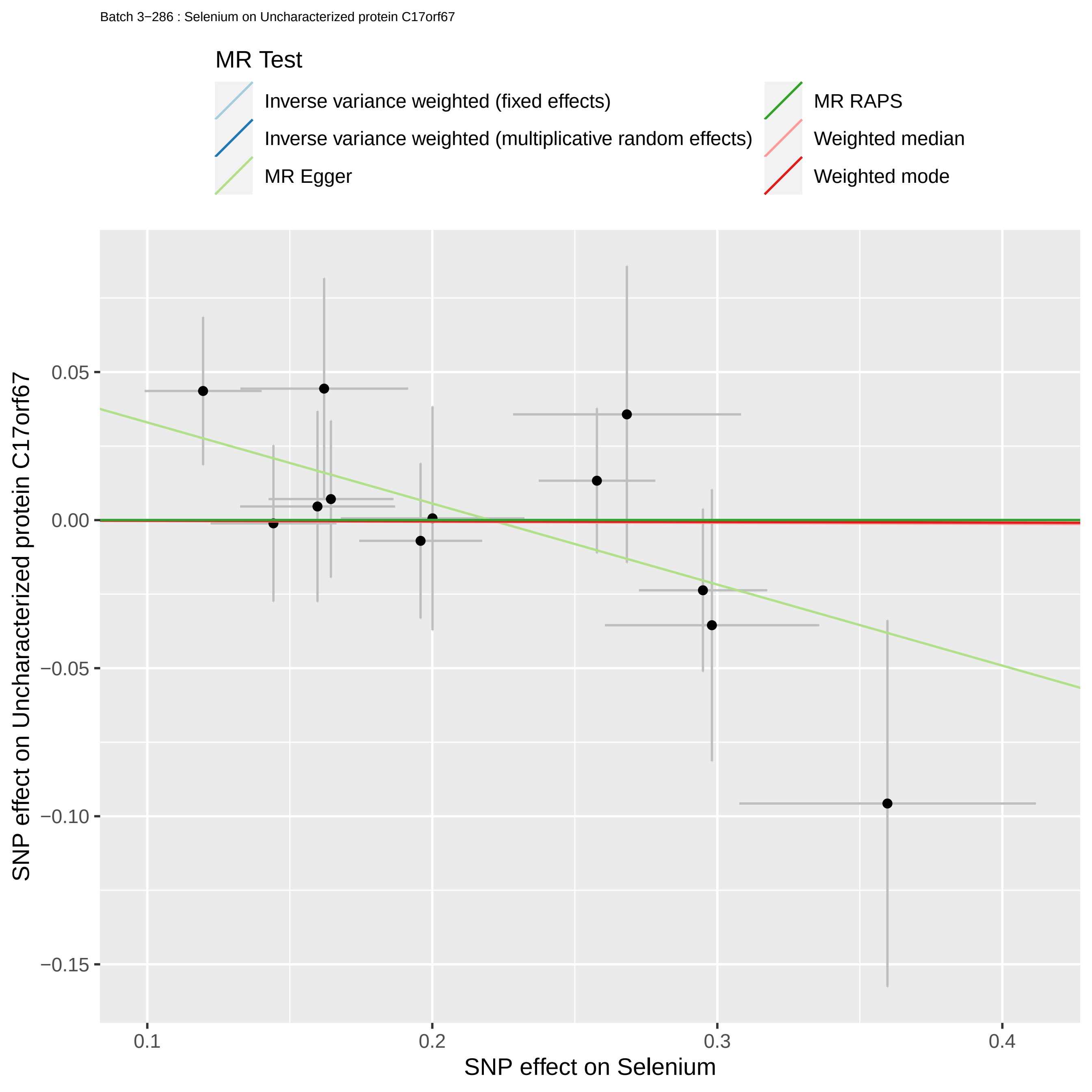
Batch 3−286 : Selenium on Uncharacterized protein C17orf67
MR Test
Inverse variance weighted (fixed effects)
Inverse variance weighted (multiplicative random effects)
MR Egger
MR RAPS
Weighted median
Weighted mode
0.05
0.00
−0.05
−0.10
−0.15
0.1
0.2
0.3
0.4
SNP effect on Uncharacterized protein C17orf67
SNP effect on Selenium

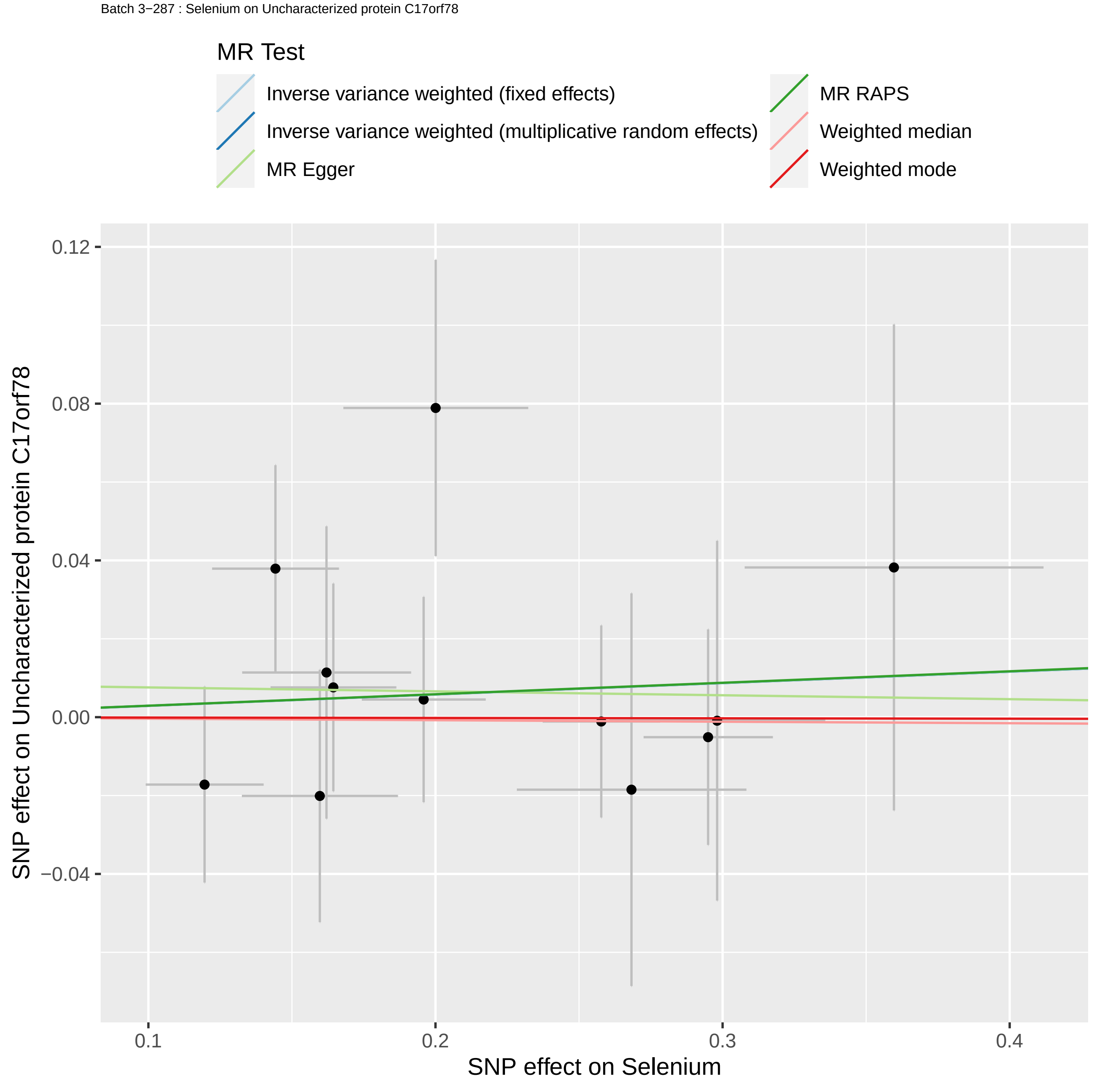
Batch 3−287 : Selenium on Uncharacterized protein C17orf78
MR Test
Inverse variance weighted (fixed effects)
Inverse variance weighted (multiplicative random effects)
MR Egger
MR RAPS
Weighted median
Weighted mode
0.12
0.08
0.04
0.00
−0.04
0.1
0.2
0.3
0.4
SNP effect on Uncharacterized protein C17orf78
SNP effect on Selenium

Batch 3−288 : Selenium on Uncharacterized protein C17orf78

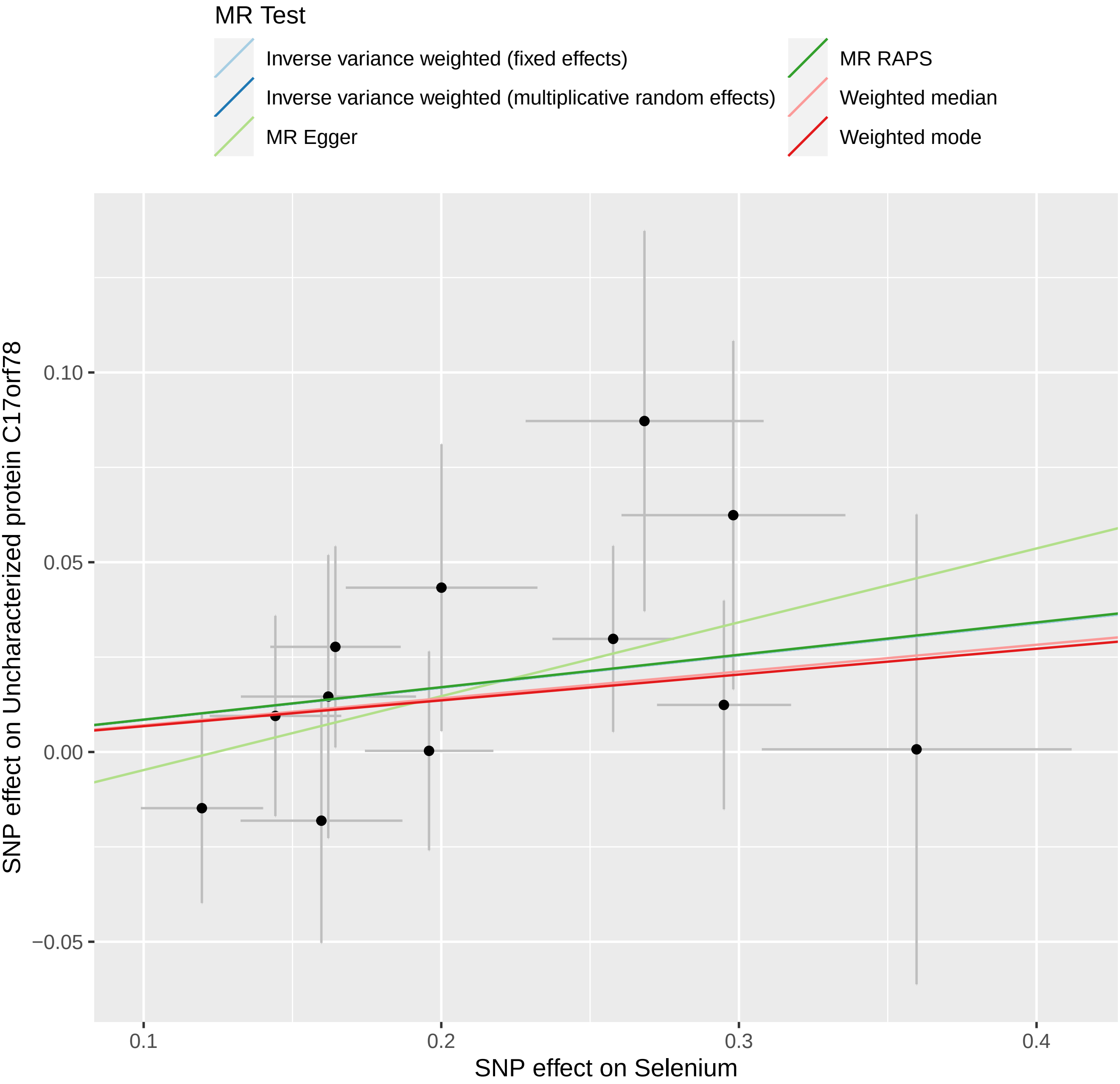

Batch 3−289 : Selenium on Uncharacterized protein C17orf89

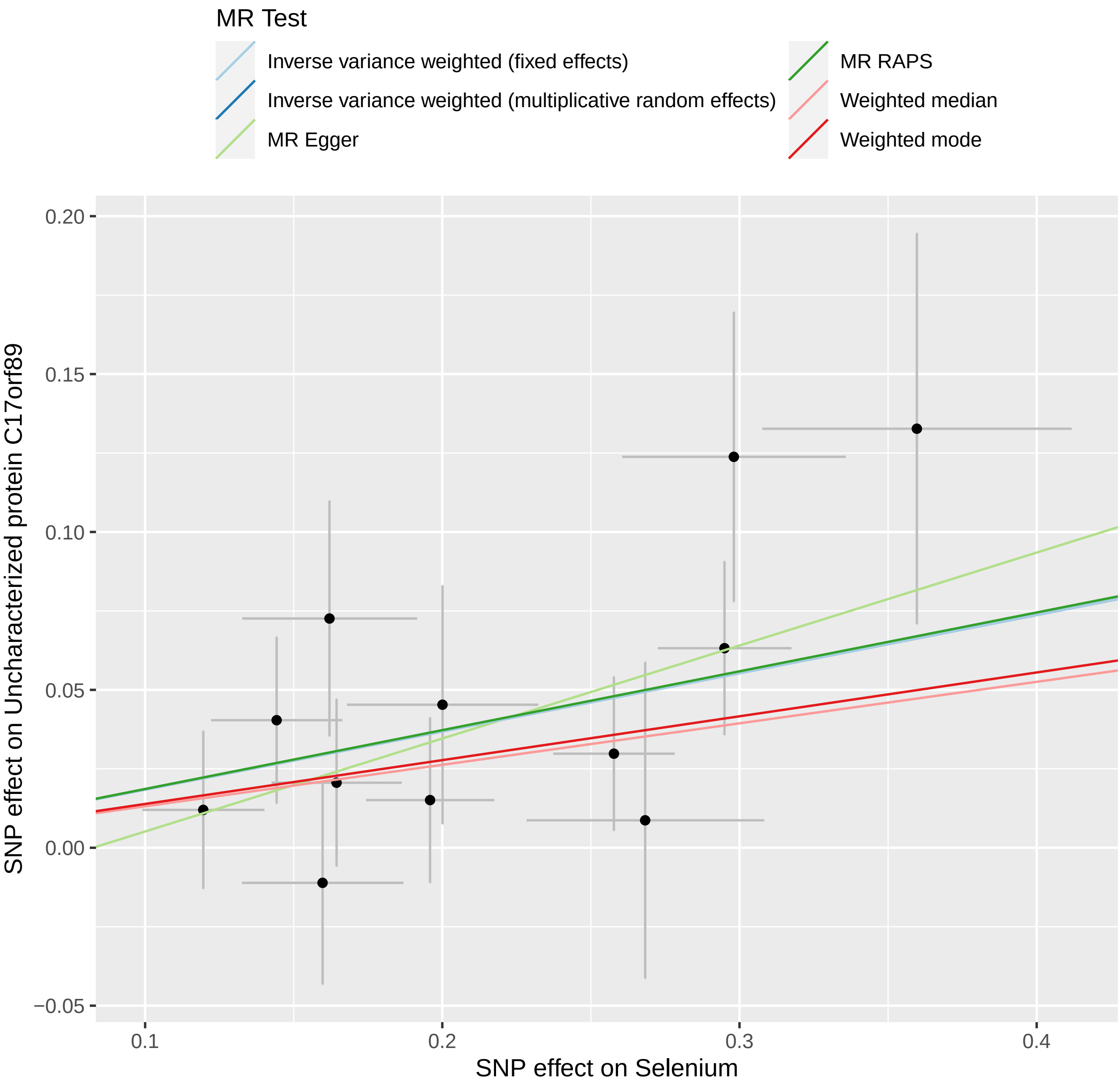

Batch 3−290 : Selenium on UPF0729 protein C18orf32

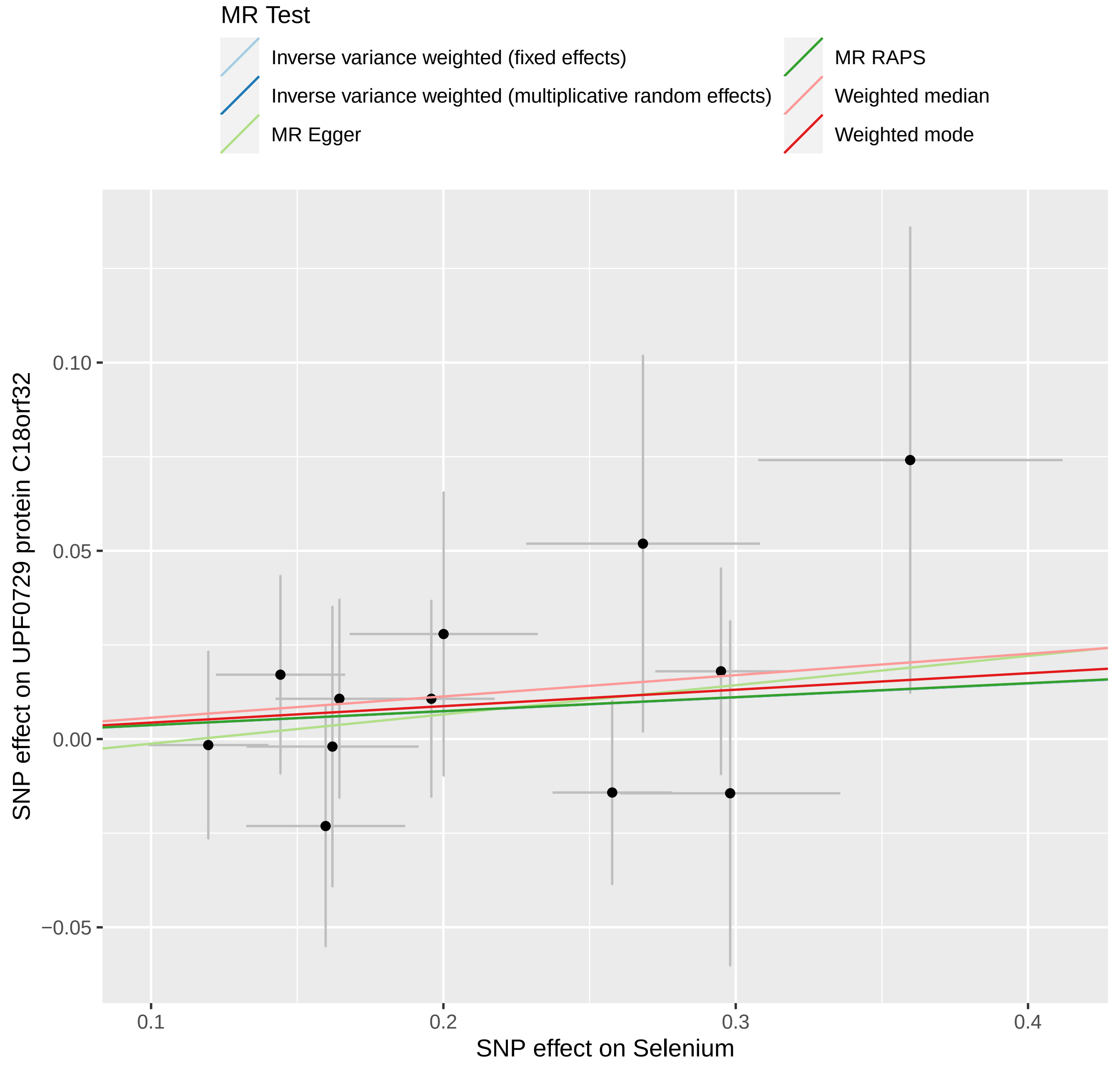

Batch 3−291 : Selenium on Uncharacterized protein C19orf18

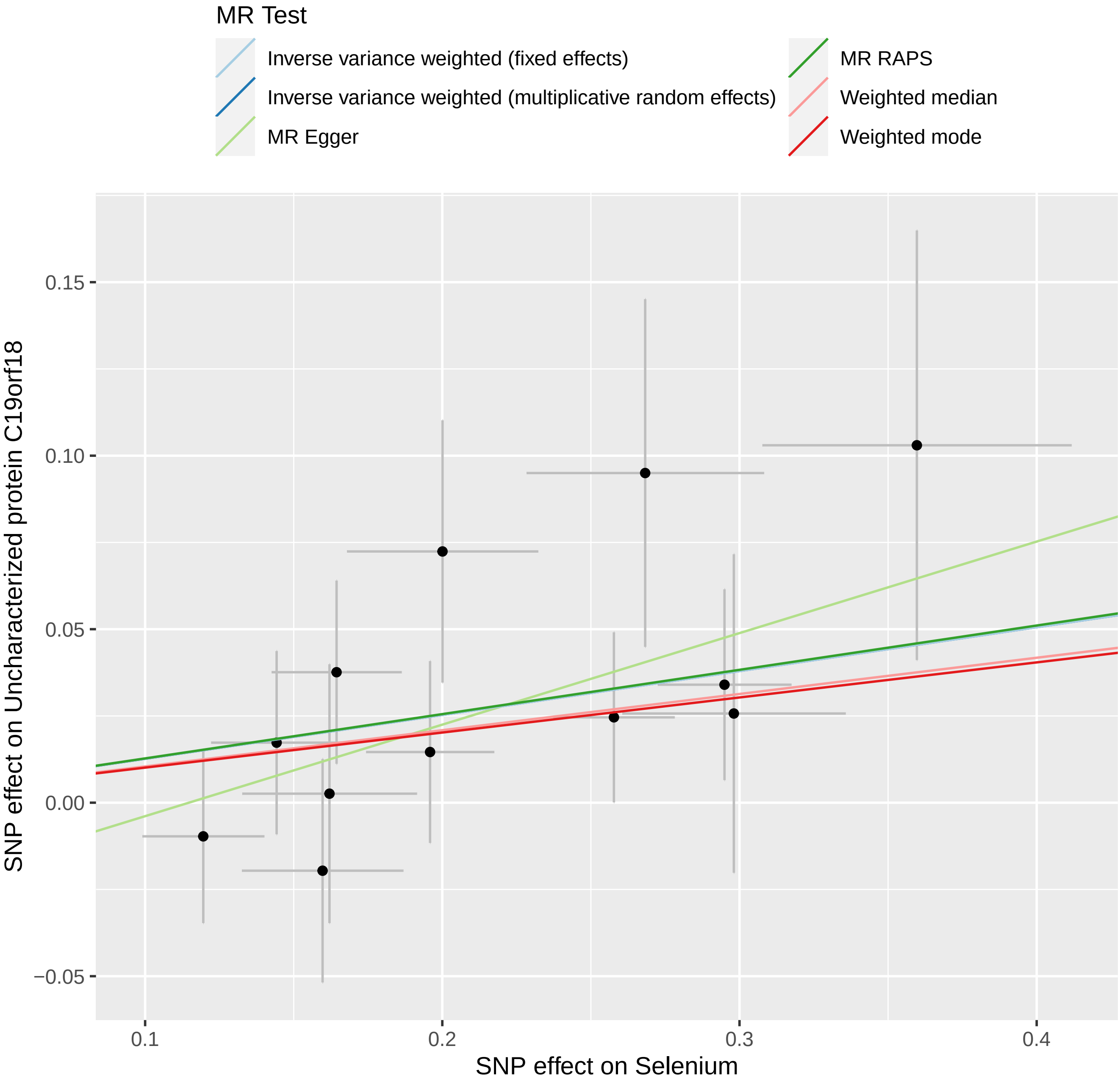

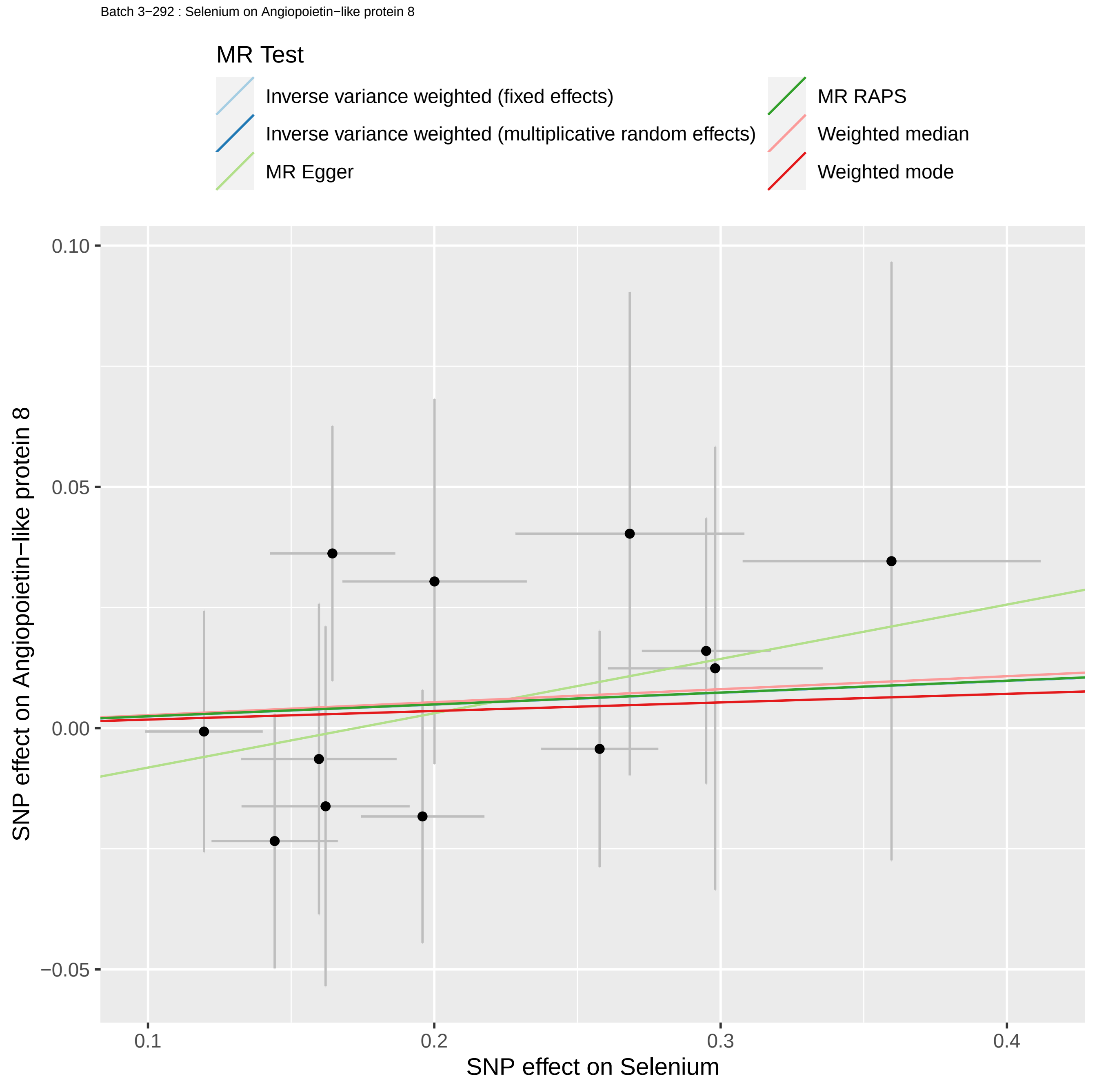

Batch 3−292 : Selenium on Angiopoietin−like protein 8
MR Test
Inverse variance weighted (fixed effects)
Inverse variance weighted (multiplicative random effects)
MR Egger
MR RAPS
Weighted median
Weighted mode
0.10
0.05
0.00
−0.05
0.1
0.2
0.3
0.4
SNP effect on Selenium
SNP effect on Angiopoietin−like protein 8

Batch 3−293 : Selenium on Nuclear nucleic acid−binding protein C1D

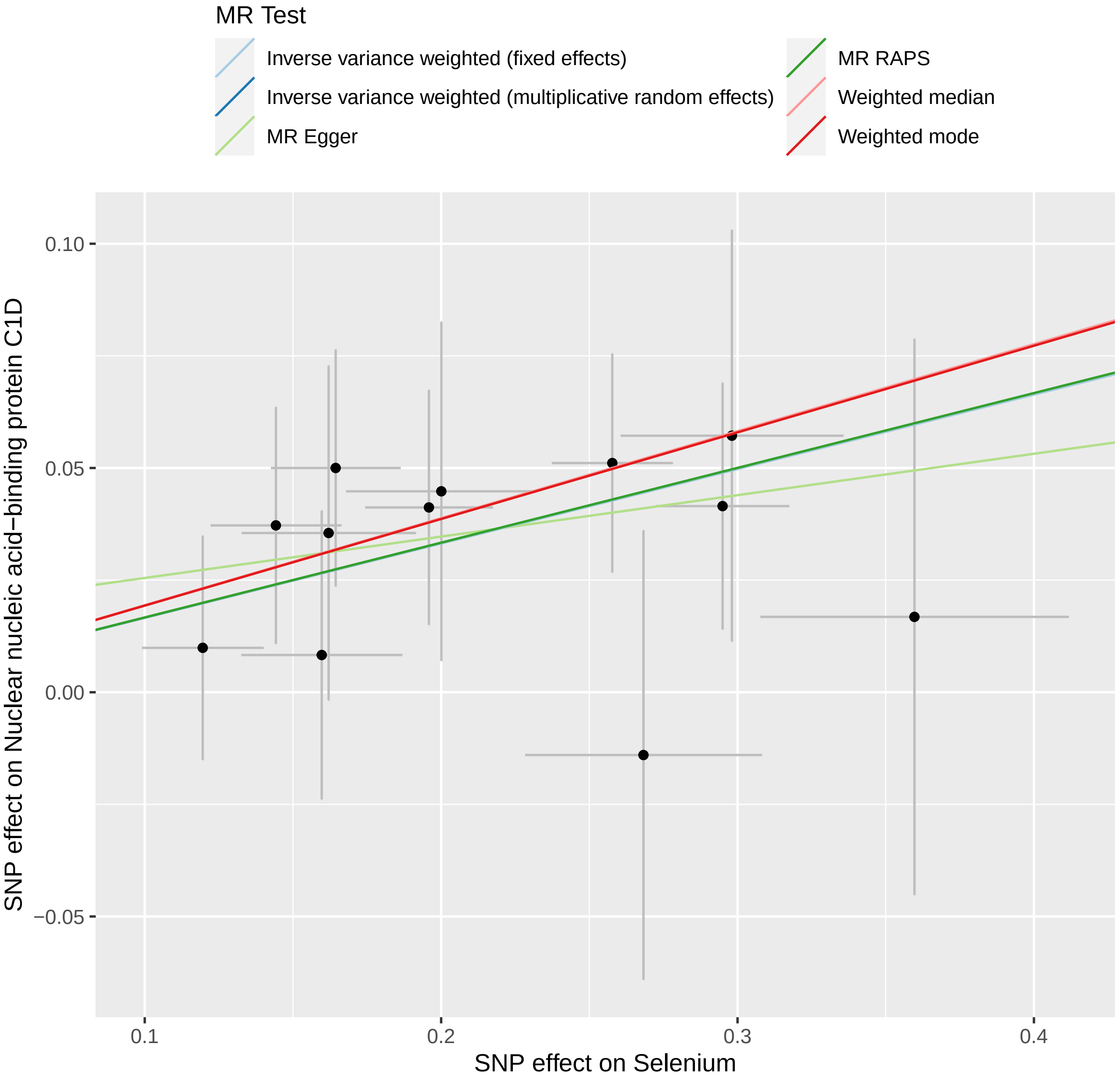

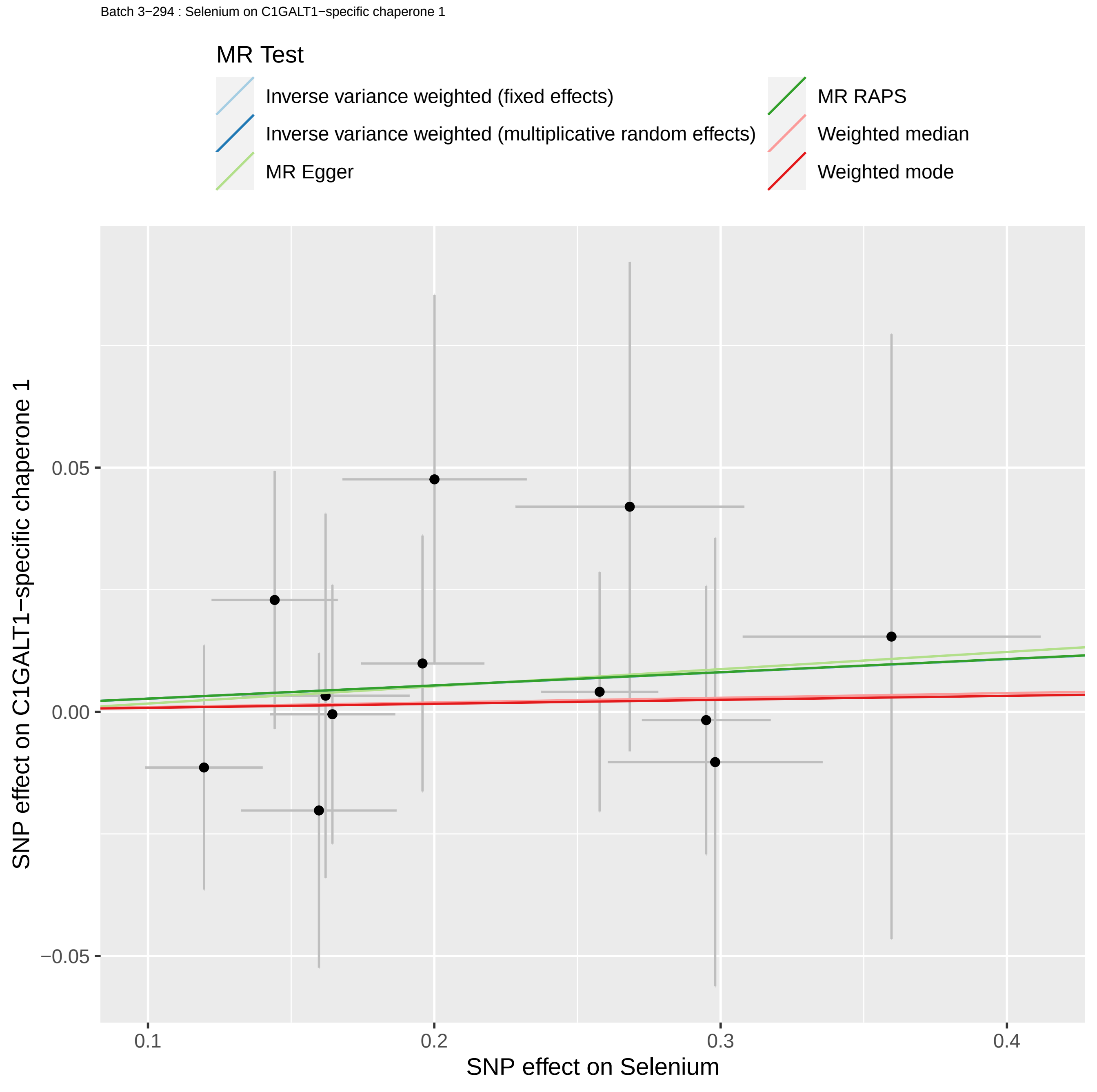
Batch 3−294 : Selenium on C1GALT1−specific chaperone 1
MR Test
Inverse variance weighted (fixed effects)
Inverse variance weighted (multiplicative random effects)
MR Egger
MR RAPS
Weighted median
Weighted mode
SNP effect on C1GALT1−specific chaperone 1
0.05
0.00
−0.05
0.1
0.2
0.3
0.4
SNP effect on Selenium

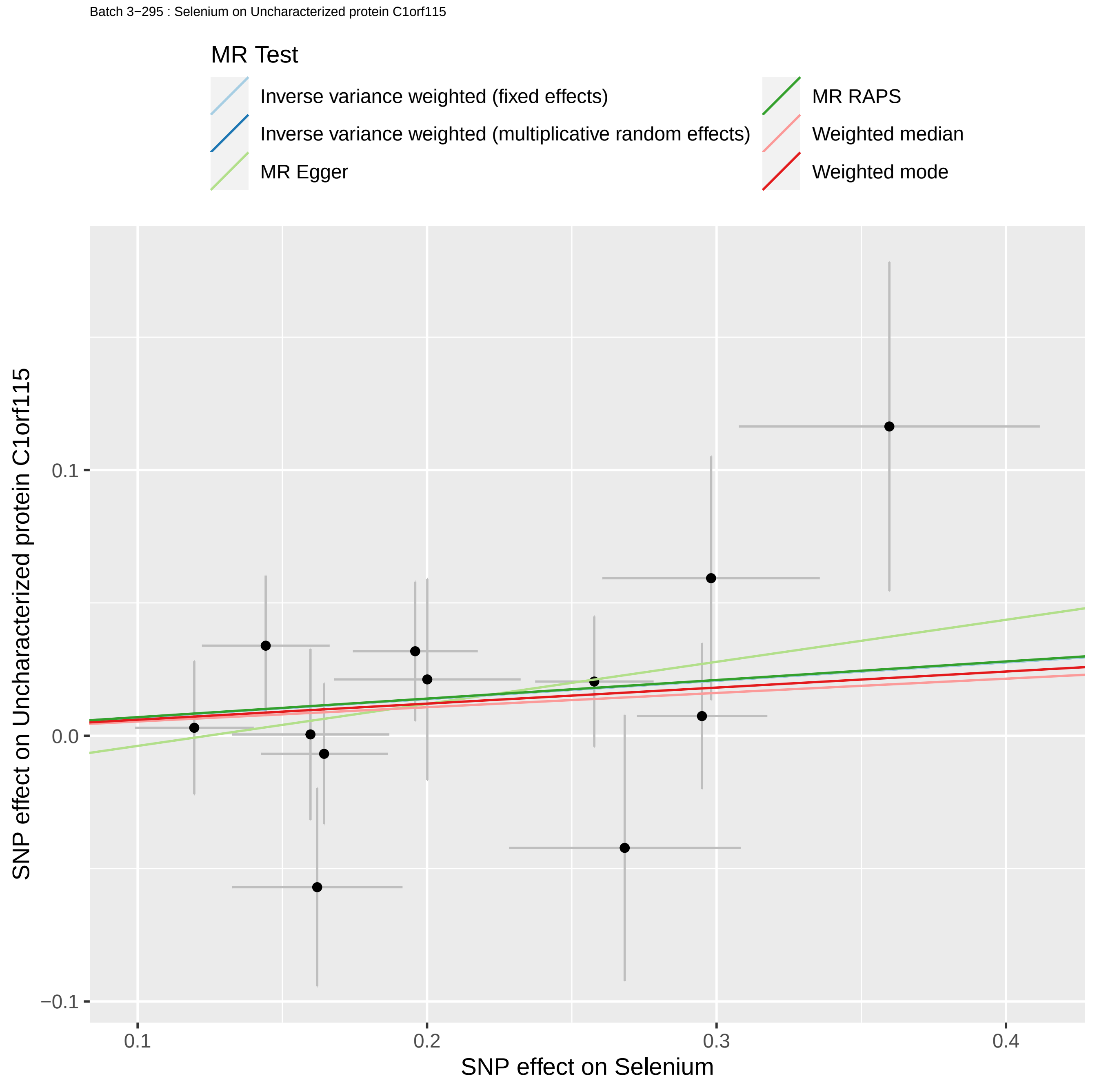

Batch 3−295 : Selenium on Uncharacterized protein C1orf115
MR Test
Inverse variance weighted (fixed effects)
Inverse variance weighted (multiplicative random effects)
MR Egger
MR RAPS
Weighted median
Weighted mode
SNP effect on Uncharacterized protein C1orf115
0.1
0.0
−0.1
0.1
0.2
0.3
0.4
SNP effect on Selenium

Batch 3−296 : Selenium on Uncharacterized protein C1orf185

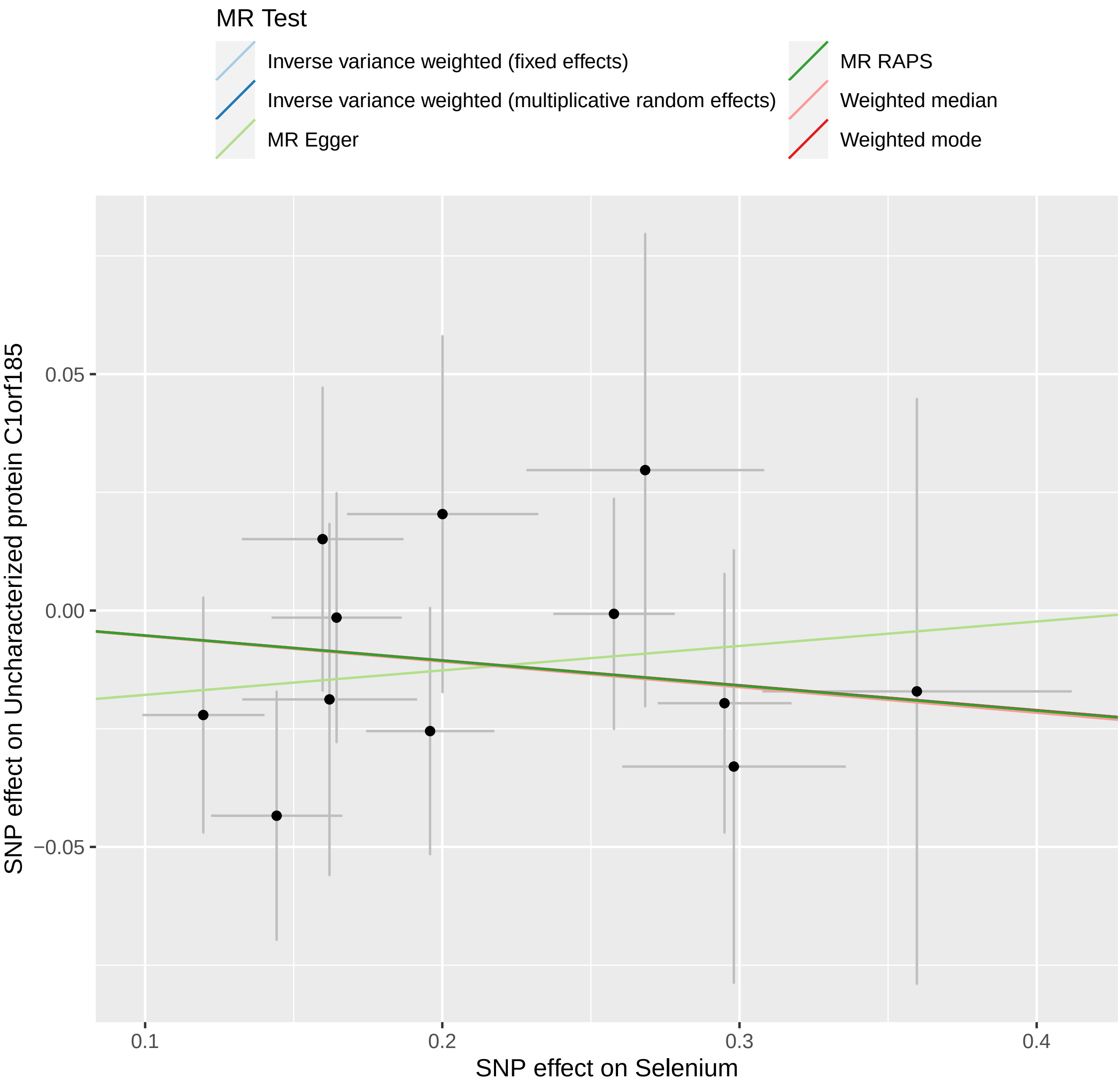

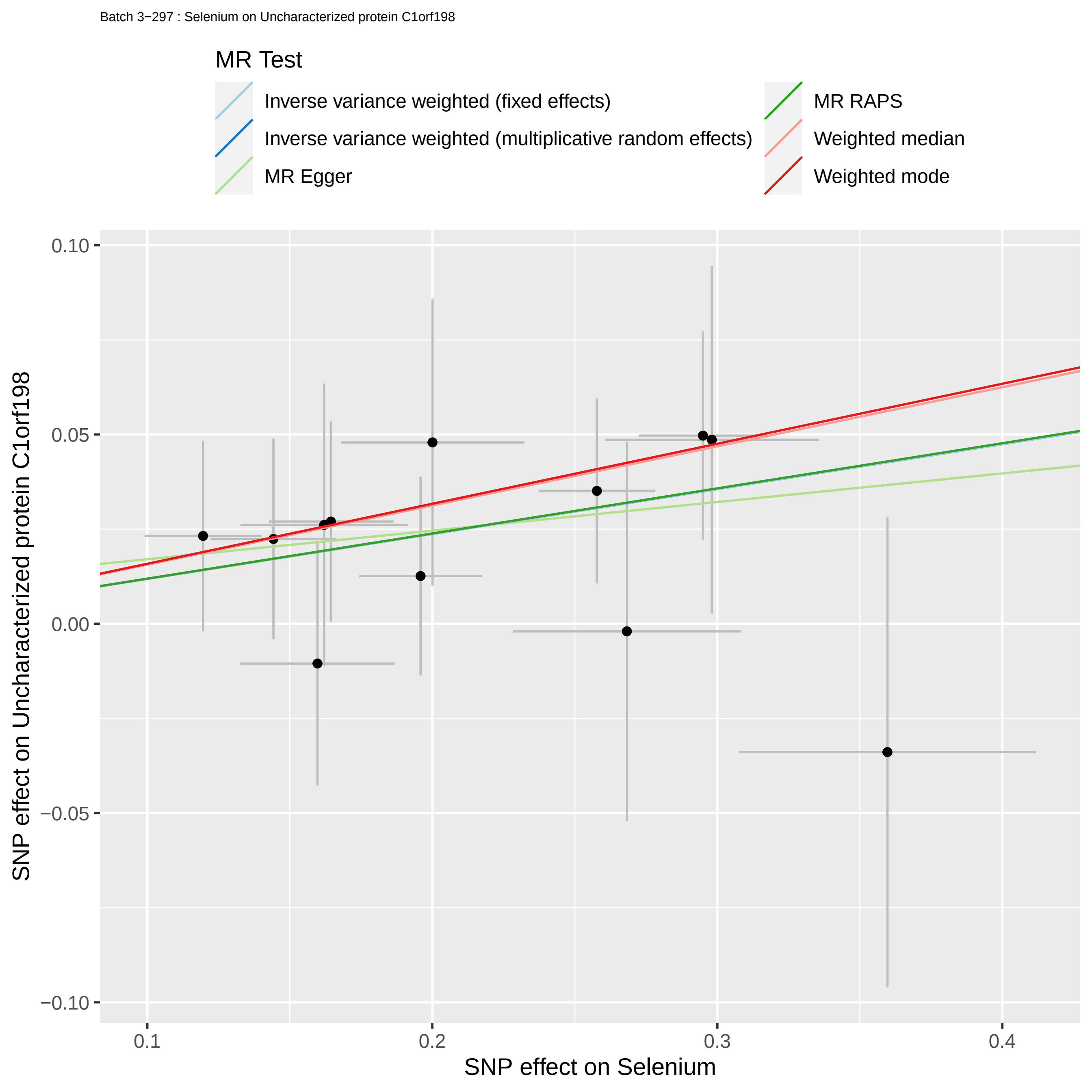
Batch 3−297 : Selenium on Uncharacterized protein C1orf198
MR Test
Inverse variance weighted (fixed effects)
Inverse variance weighted (multiplicative random effects)
MR Egger
MR RAPS
Weighted median
Weighted mode
SNP effect on Uncharacterized protein C1orf198
0.10
0.05
0.00
−0.05
−0.10
0.1
0.2
0.3
0.4
SNP effect on Selenium

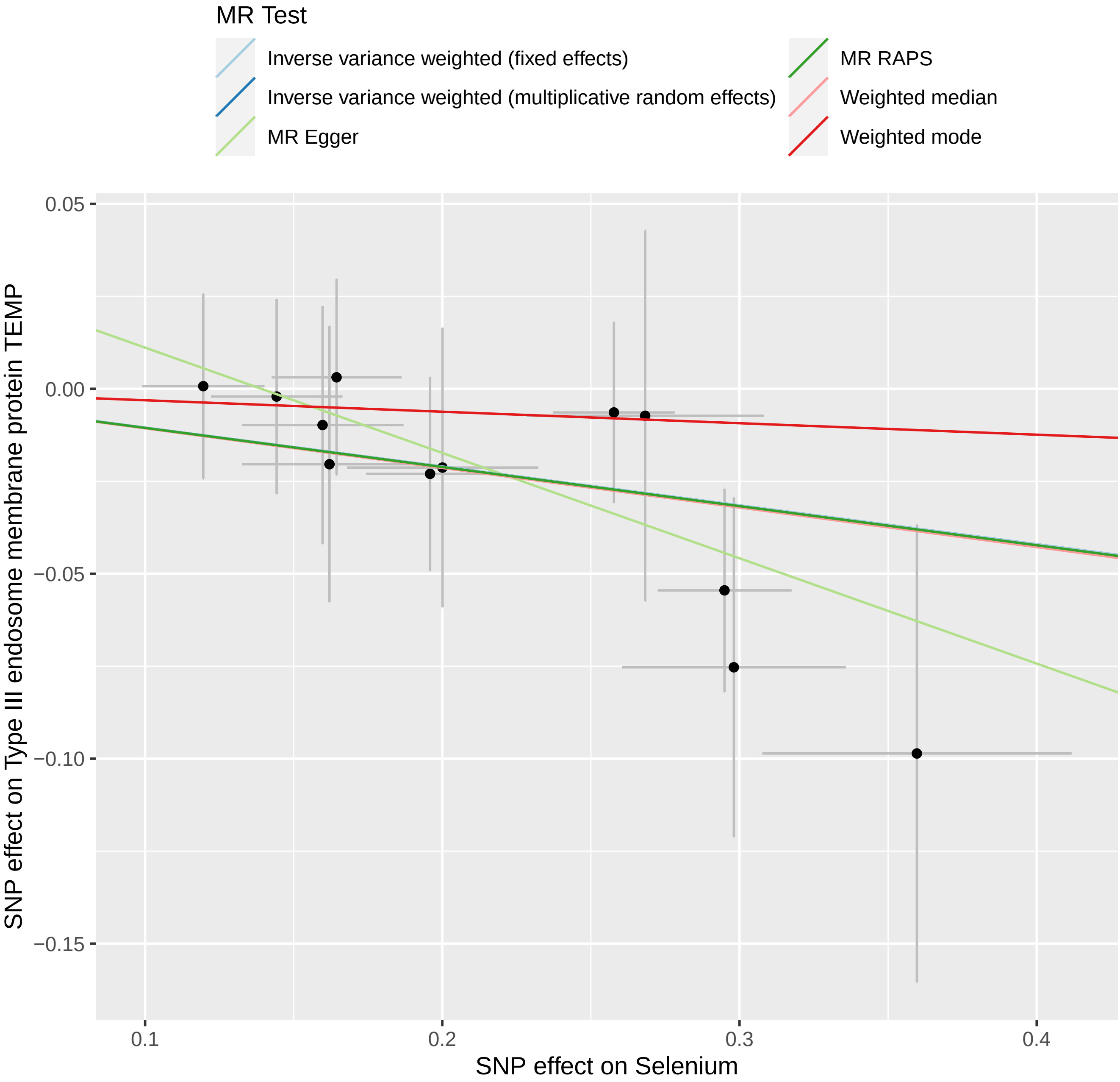
MR Test
Inverse variance weighted (fixed effects)
Inverse variance weighted (multiplicative random effects)
MR Egger
MR RAPS
Weighted median
Weighted mode
0.05
0.00
−0.05
−0.10
−0.15
0.1
0.2
0.3
0.4
SNP effect on Type III endosome membrane protein TEMP
SNP effect on Selenium

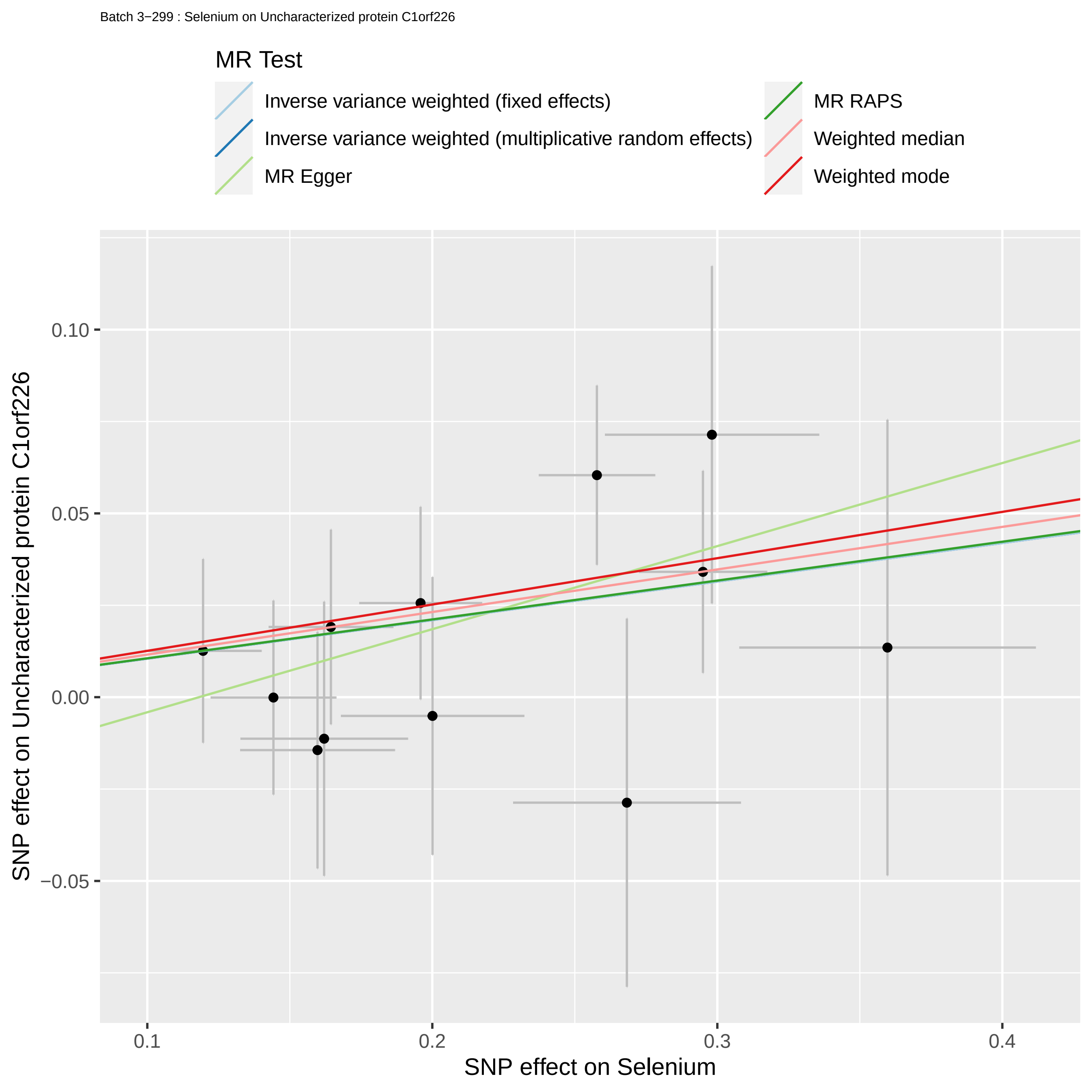

Batch 3−299 : Selenium on Uncharacterized protein C1orf226
MR Test
Inverse variance weighted (fixed effects)
Inverse variance weighted (multiplicative random effects)
MR Egger
MR RAPS
Weighted median
Weighted mode
0.10
0.05
0.00
−0.05
0.1
0.2
0.3
0.4
SNP effect on Uncharacterized protein C1orf226
SNP effect on Selenium

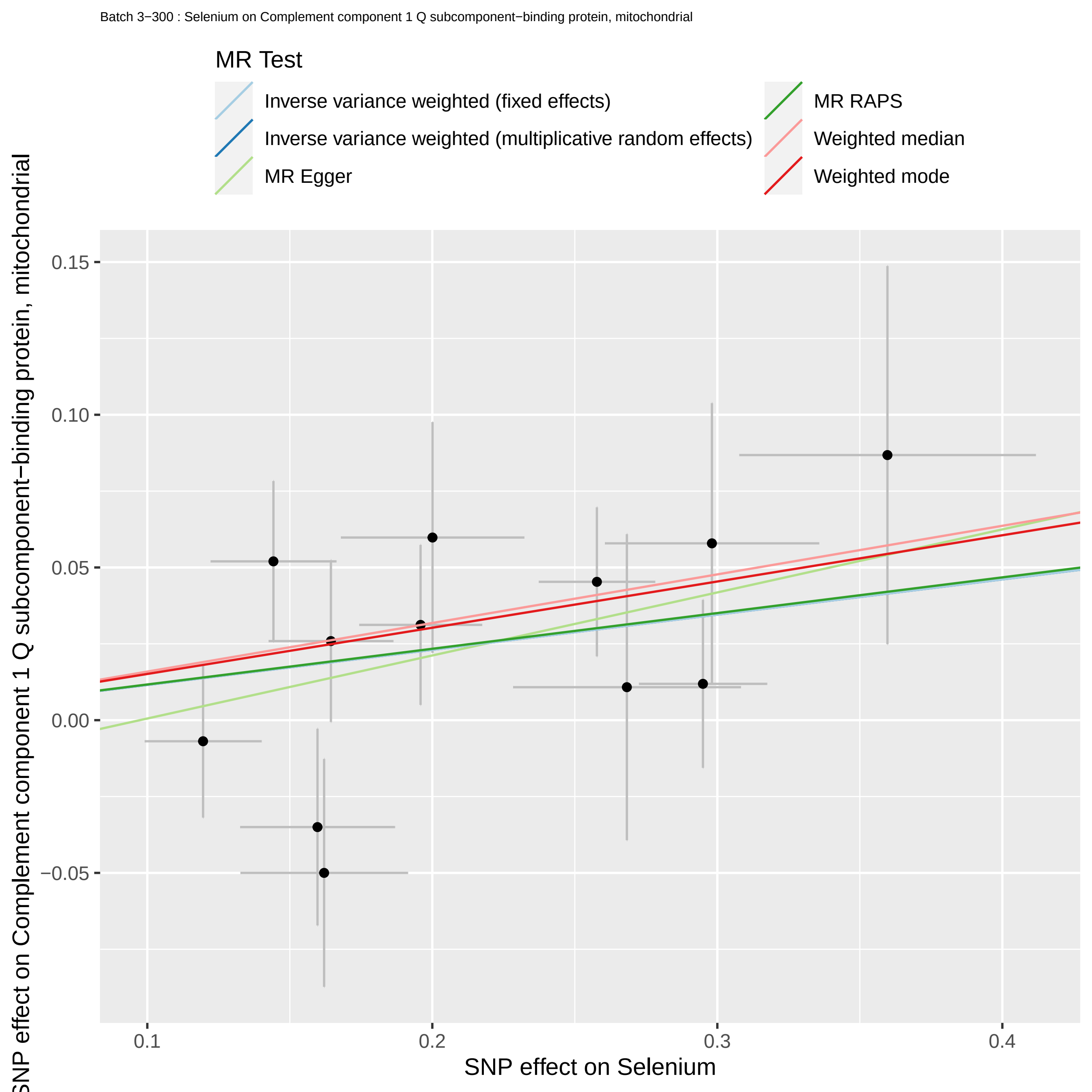

Batch 3−300 : Selenium on Complement component 1 Q subcomponent−binding protein, mitochondrial
MR Test
Inverse variance weighted (fixed effects)
Inverse variance weighted (multiplicative random effects)
MR Egger
MR RAPS
Weighted median
Weighted mode
0.15
0.10
0.05
0.00
−0.05
0.1
0.2
0.3
0.4
SNP effect on Selenium
SNP effect on Complement component 1 Q subcomponent−binding protein, mitochondrial

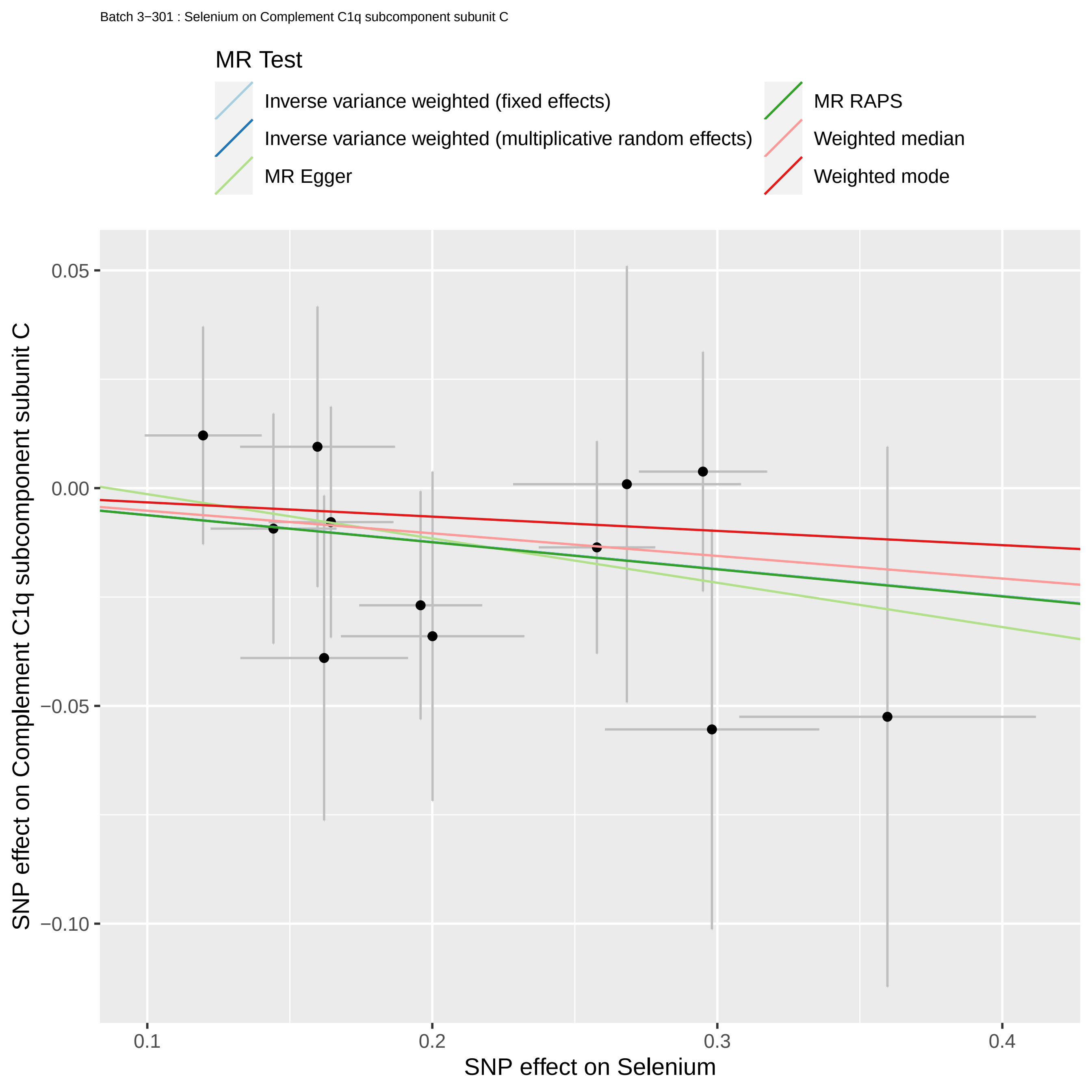
Batch 3−301 : Selenium on Complement C1q subcomponent subunit C
MR Test
Inverse variance weighted (fixed effects)
Inverse variance weighted (multiplicative random effects)
MR Egger
MR RAPS
Weighted median
Weighted mode
0.05
0.00
−0.05
−0.10
0.1
0.2
0.3
0.4
SNP effect on Complement C1q subcomponent subunit C
SNP effect on Selenium

Batch 3−302 : Selenium on Complement C1q−like protein 4

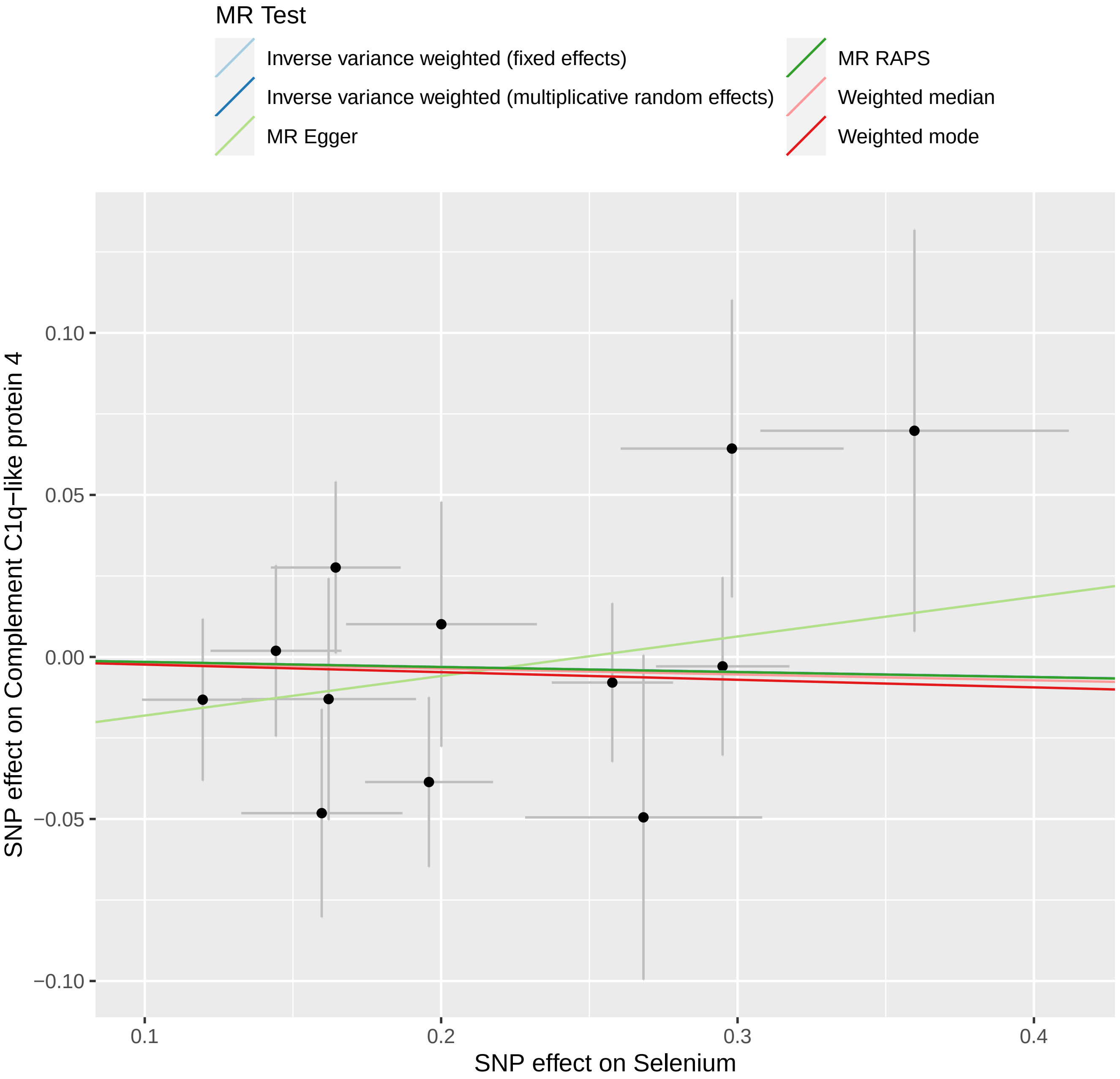

Batch 3−303 : Selenium on Complement C1q tumor necrosis factor−related protein 1

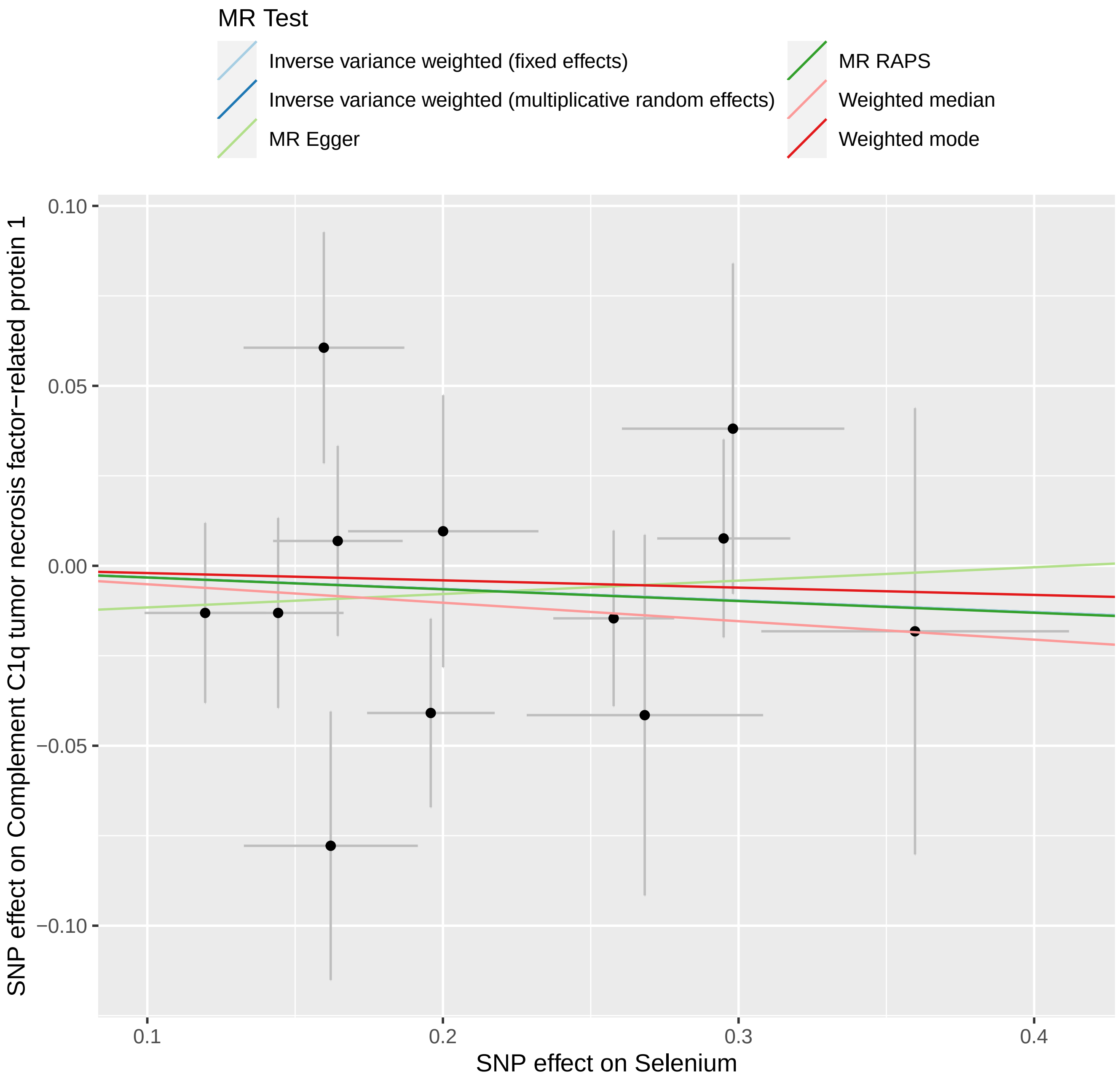

Batch 3−304 : Selenium on Complement C1q tumor necrosis factor−related protein 3

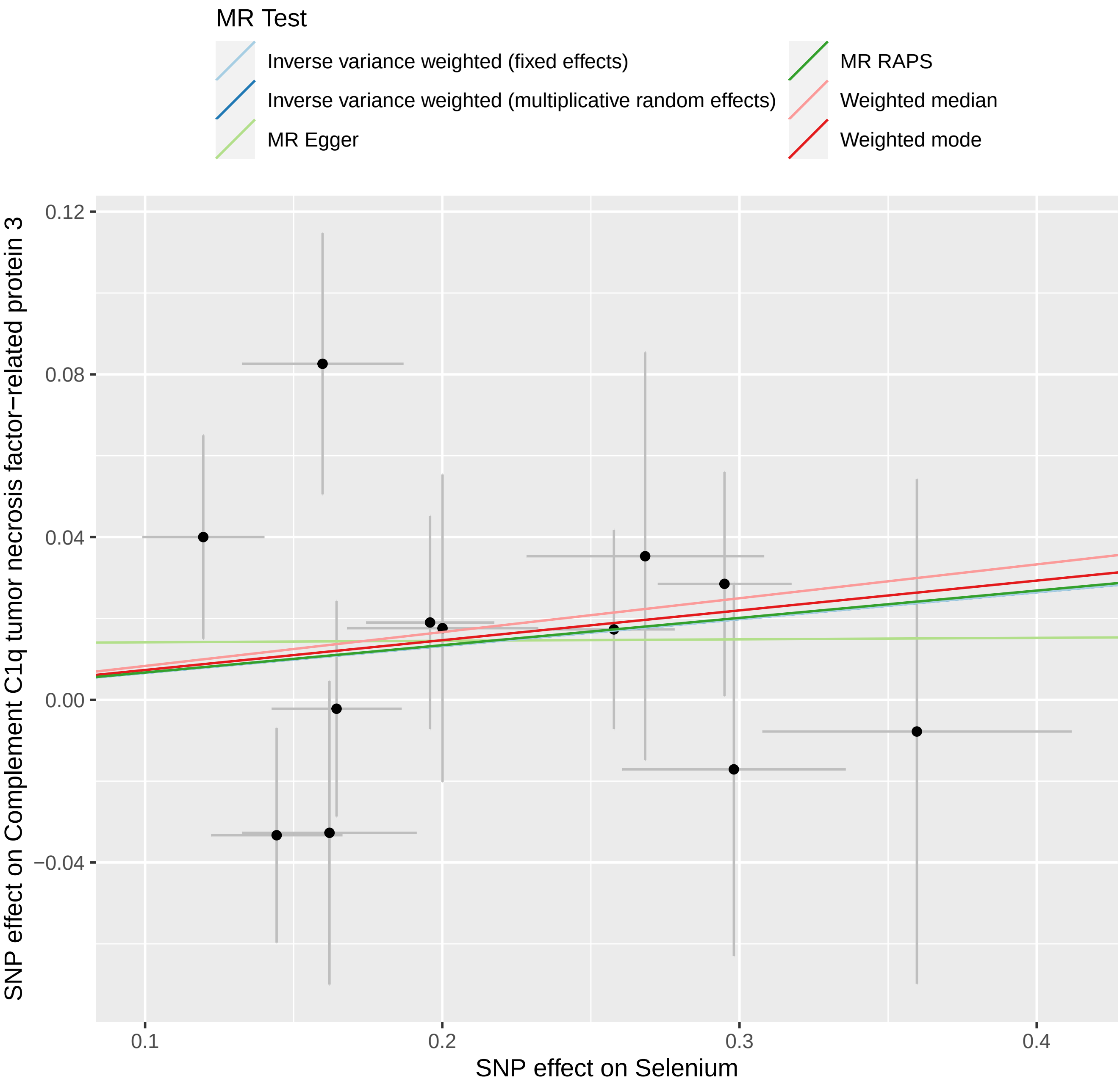

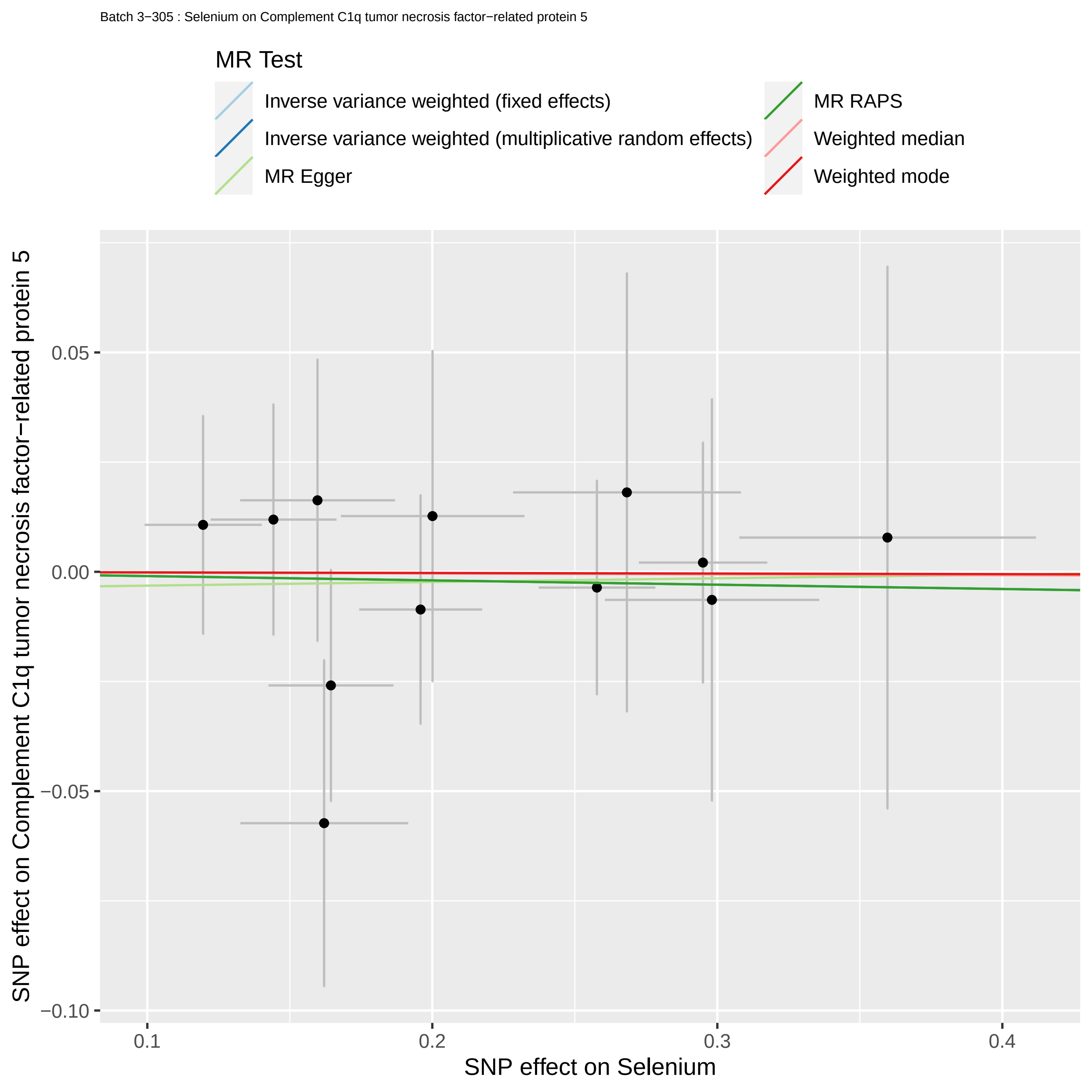

Batch 3−305 : Selenium on Complement C1q tumor necrosis factor−related protein 5
MR Test
Inverse variance weighted (fixed effects)
Inverse variance weighted (multiplicative random effects)
MR Egger
MR RAPS
Weighted median
Weighted mode
SNP effect on Complement C1q tumor necrosis factor−related protein 5
0.05
0.00
−0.05
−0.10
0.1
0.2
0.3
0.4
SNP effect on Selenium

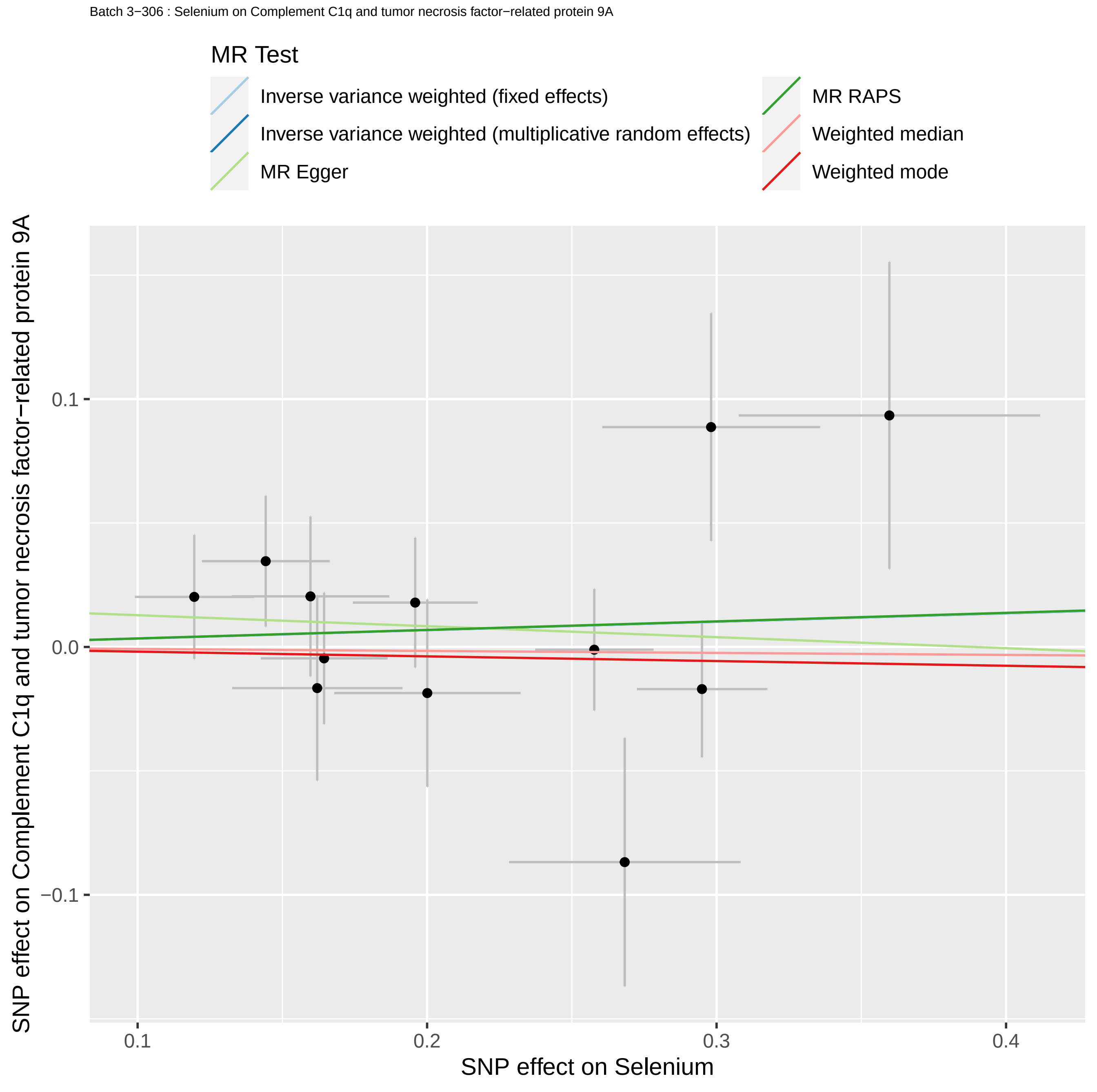

Batch 3−306 : Selenium on Complement C1q and tumor necrosis factor−related protein 9A
MR Test
Inverse variance weighted (fixed effects)
Inverse variance weighted (multiplicative random effects)
MR Egger
MR RAPS
Weighted median
Weighted mode
SNP effect on Complement C1q and tumor necrosis factor−related protein 9A
0.1
0.0
−0.1
0.1
0.2
0.3
0.4
SNP effect on Selenium

Batch 3−307 : Selenium on Complement C1r subcomponent−like protein

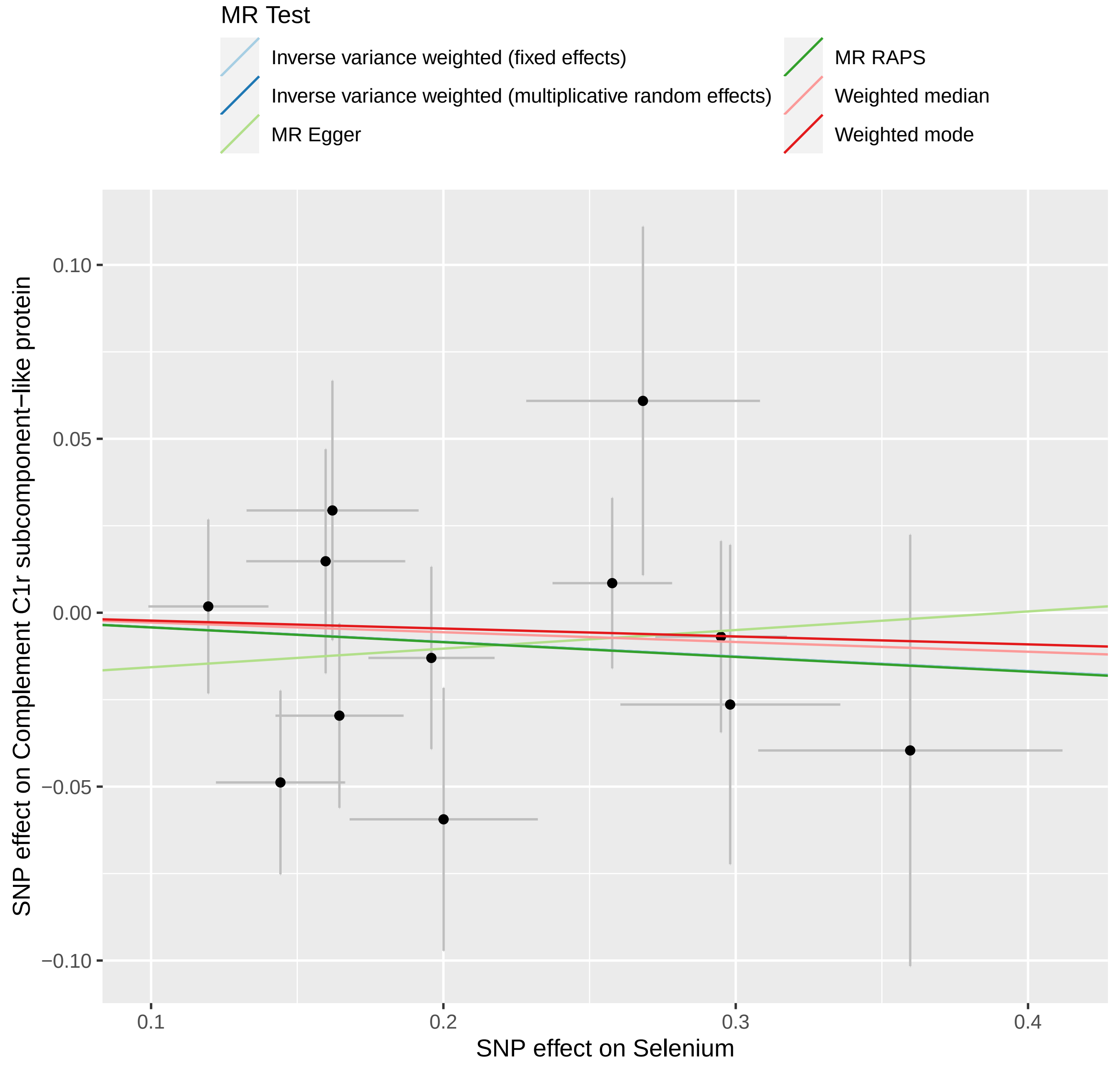

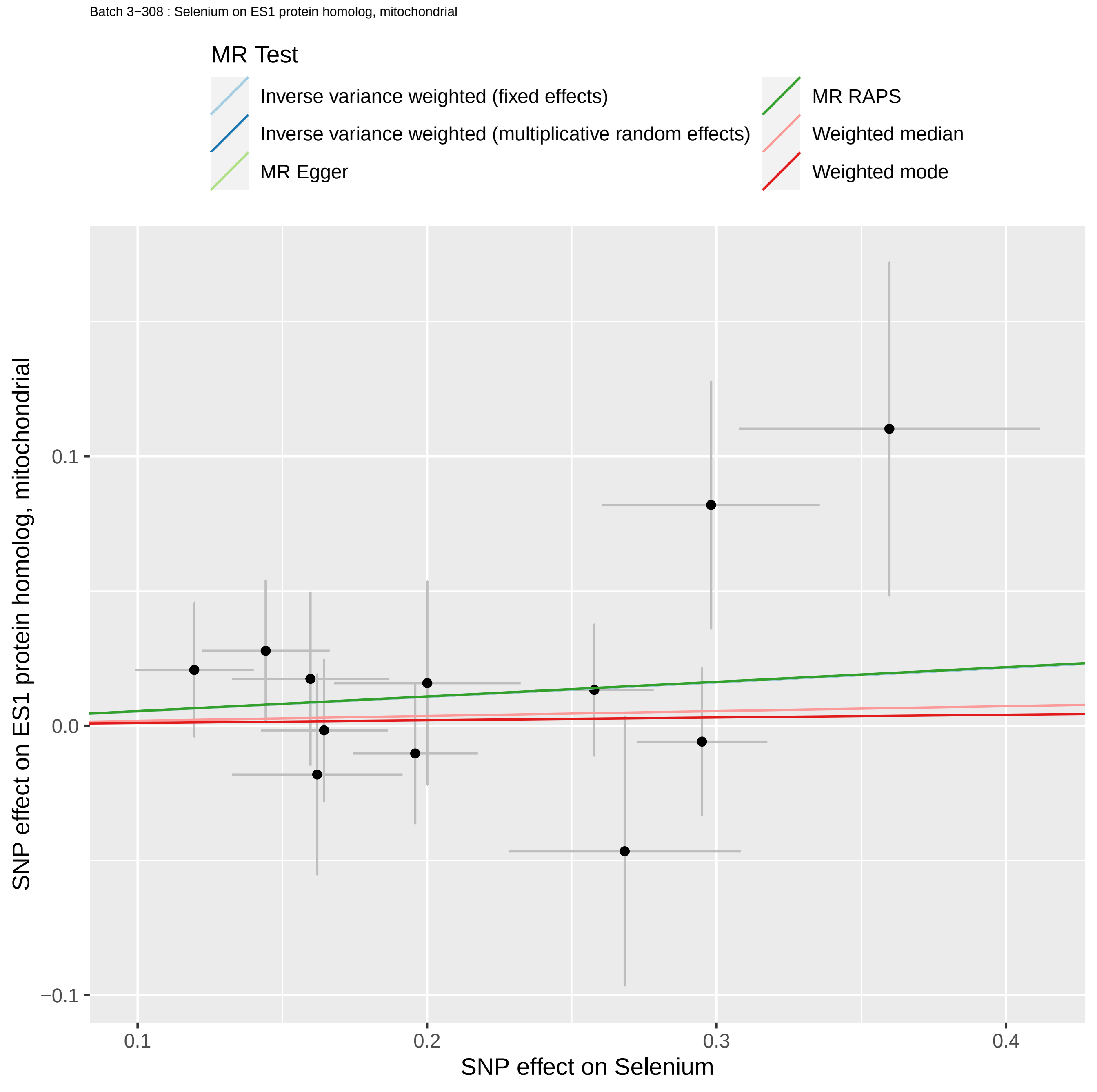

Batch 3−308 : Selenium on ES1 protein homolog, mitochondrial
MR Test
Inverse variance weighted (fixed effects)
Inverse variance weighted (multiplicative random effects)
MR Egger
MR RAPS
Weighted median
Weighted mode
SNP effect on ES1 protein homolog, mitochondrial
0.1
0.0
−0.1
0.1
0.2
0.3
0.4
SNP effect on Selenium

Batch 3−309 : Selenium on Uncharacterized protein C22orf15

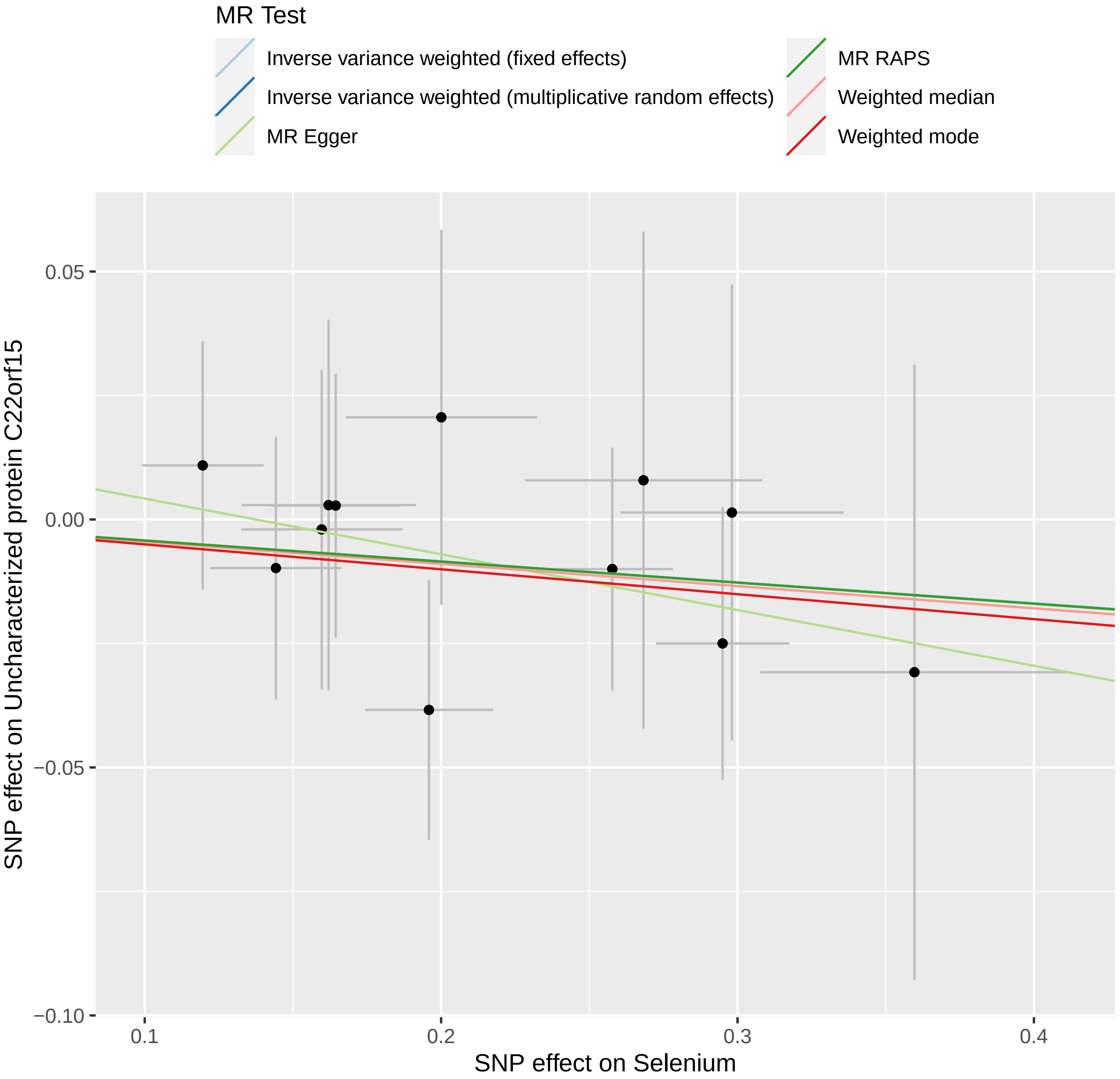

Batch 3−310 : Selenium on Augurin

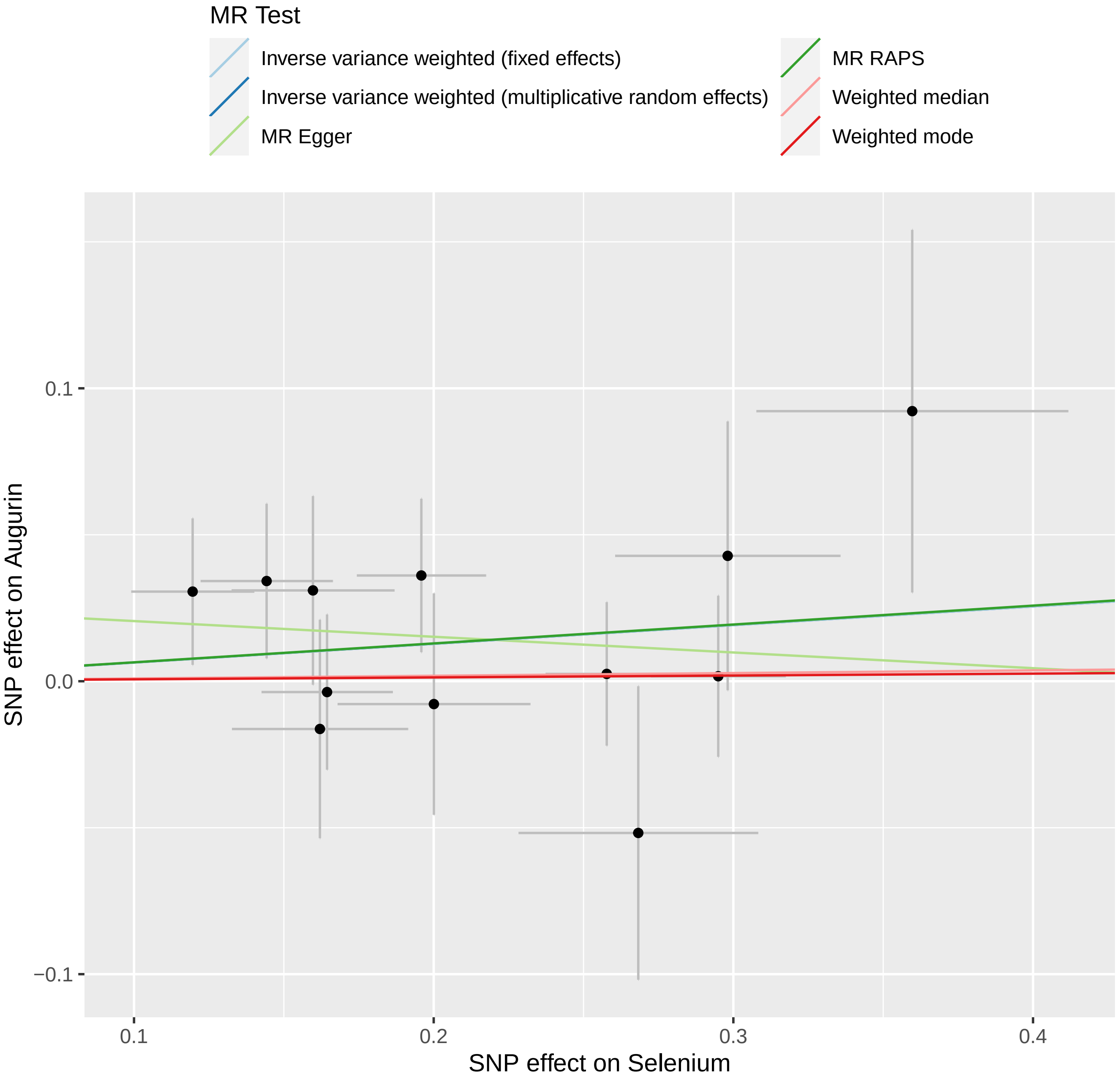

Batch 3−311 : Selenium on Uncharacterized protein C2orf66

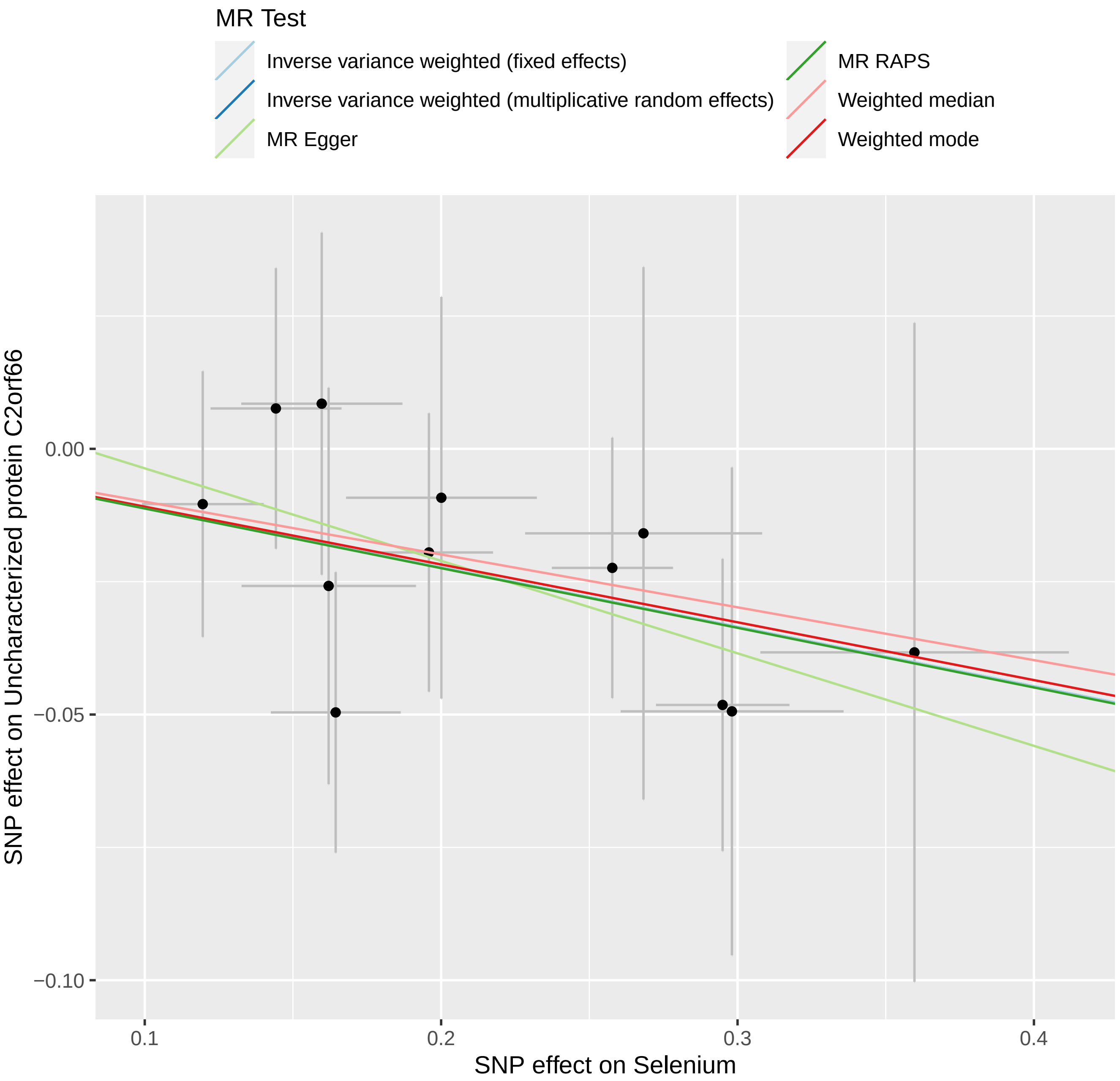

Batch 3−312 : Selenium on Uncharacterized protein C2orf82

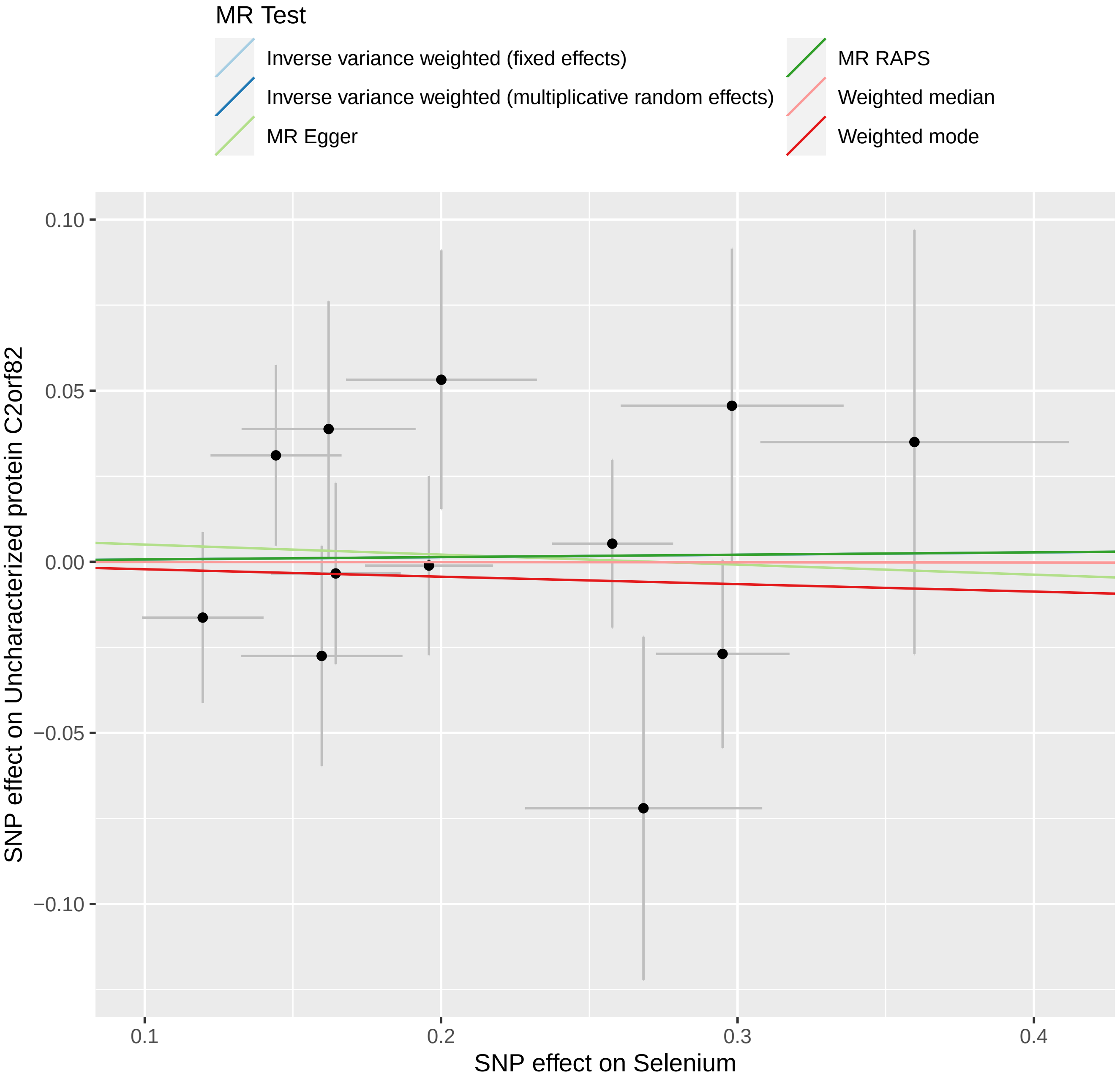

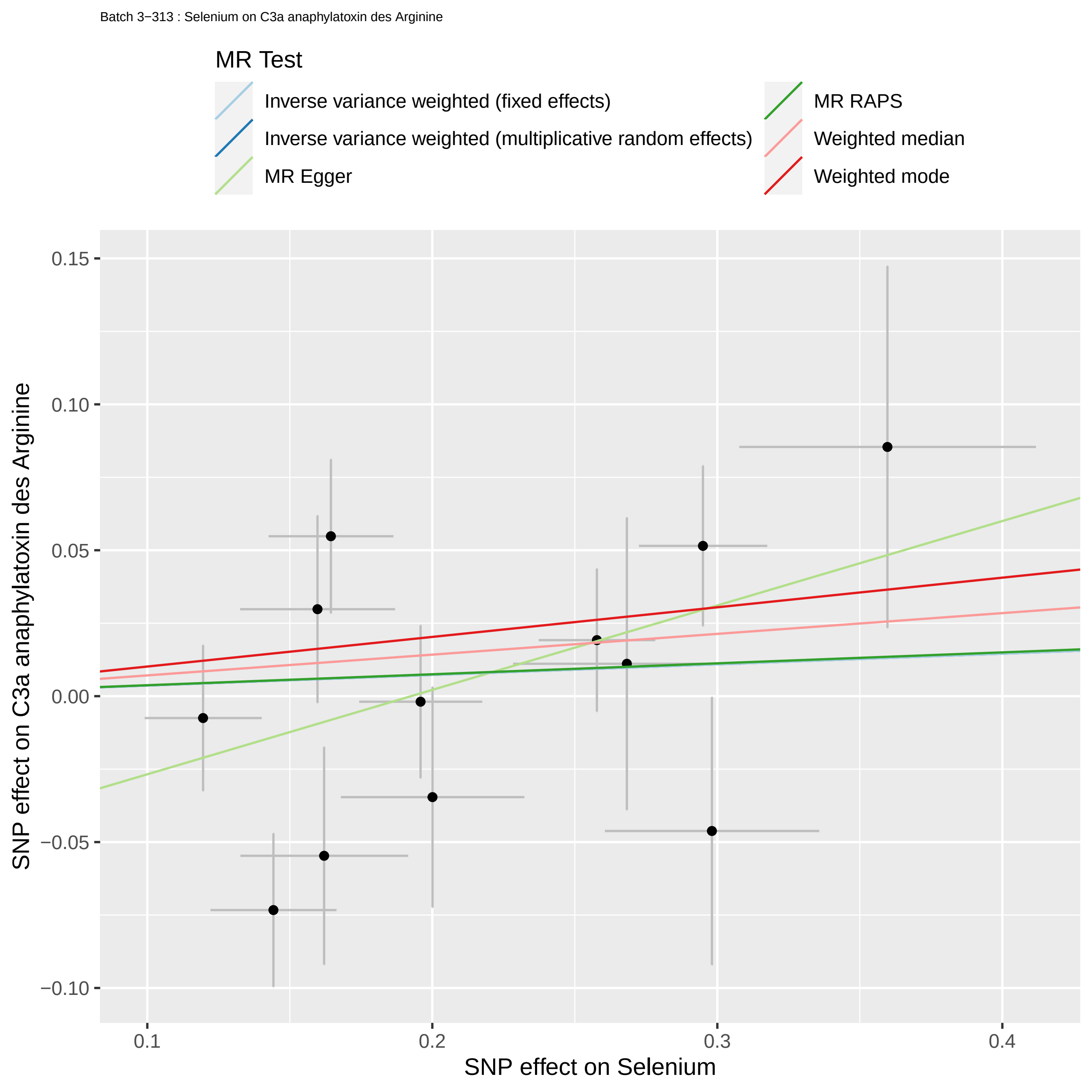
Batch 3−313 : Selenium on C3a anaphylatoxin des Arginine
MR Test
Inverse variance weighted (fixed effects)
Inverse variance weighted (multiplicative random effects)
MR Egger
MR RAPS
Weighted median
Weighted mode
0.15
0.10
0.05
0.00
−0.05
−0.10
0.1
0.2
0.3
0.4
SNP effect on C3a anaphylatoxin des Arginine
SNP effect on Selenium

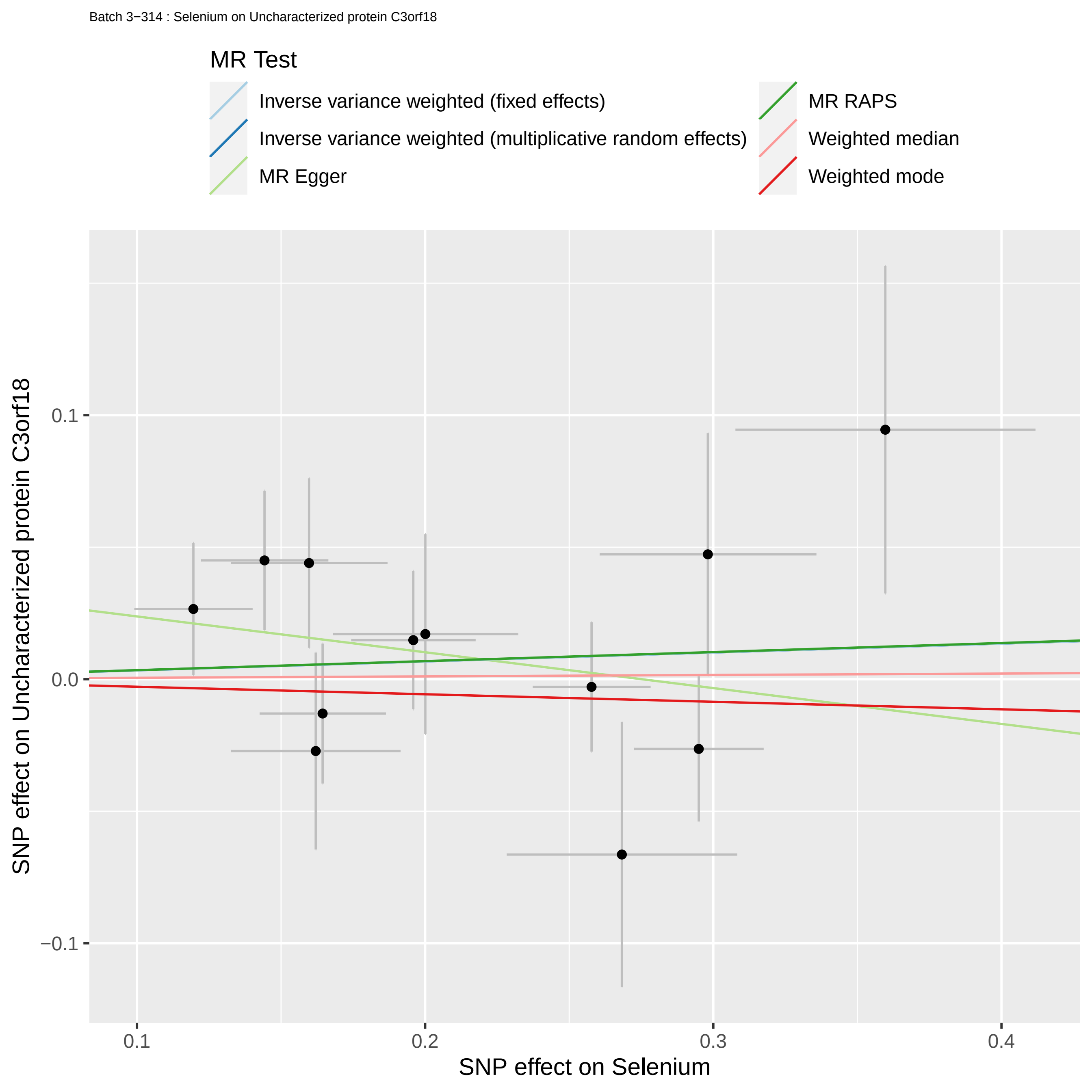
Batch 3−314 : Selenium on Uncharacterized protein C3orf18
MR Test
Inverse variance weighted (fixed effects)
Inverse variance weighted (multiplicative random effects)
MR Egger
MR RAPS
Weighted median
Weighted mode
0.1
0.0
−0.1
0.1
0.2
0.3
0.4
SNP effect on Uncharacterized protein C3orf18
SNP effect on Selenium

Batch 3−315 : Selenium on Complement C4

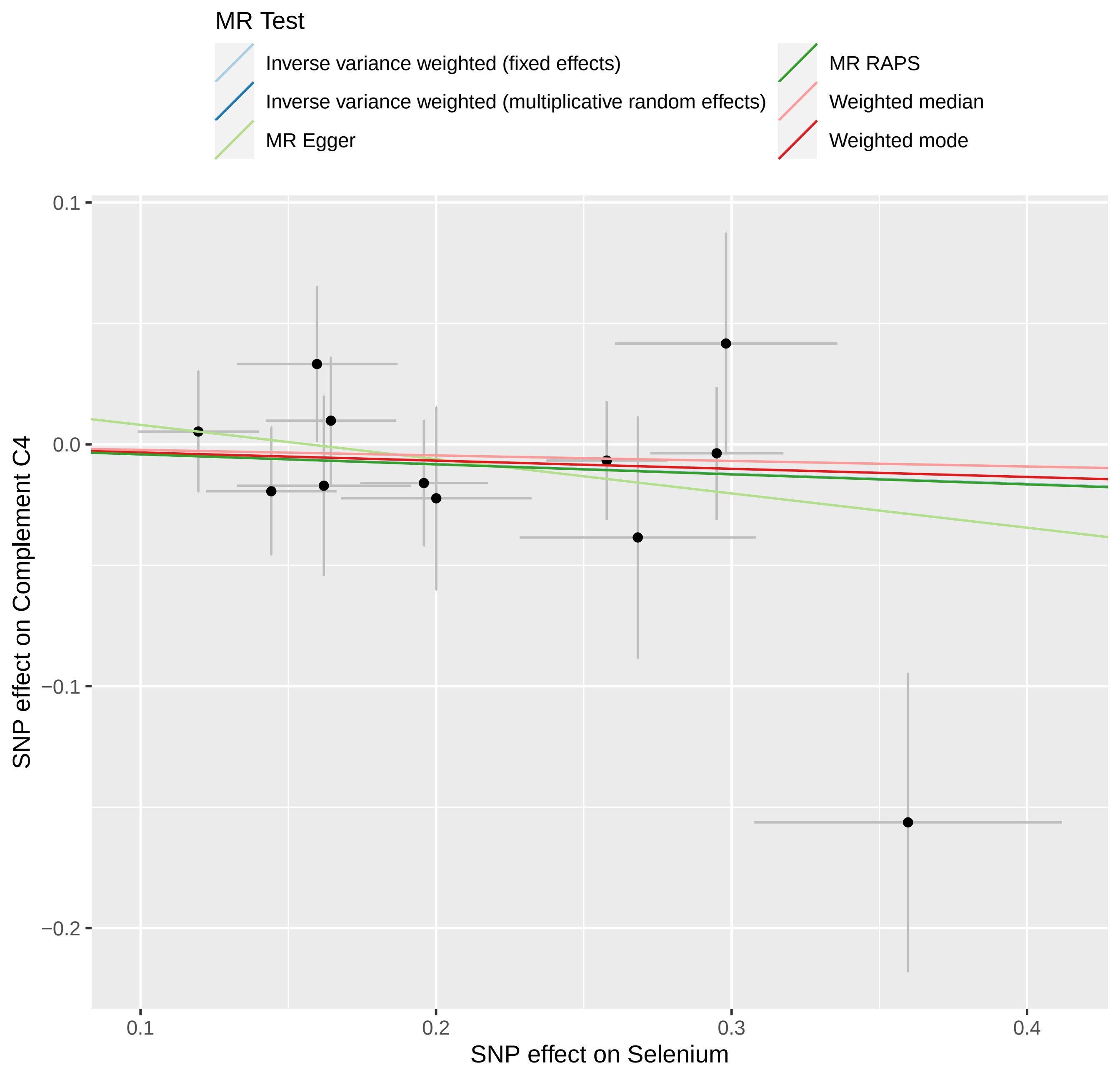

Batch 3−316 : Selenium on Protein CEI

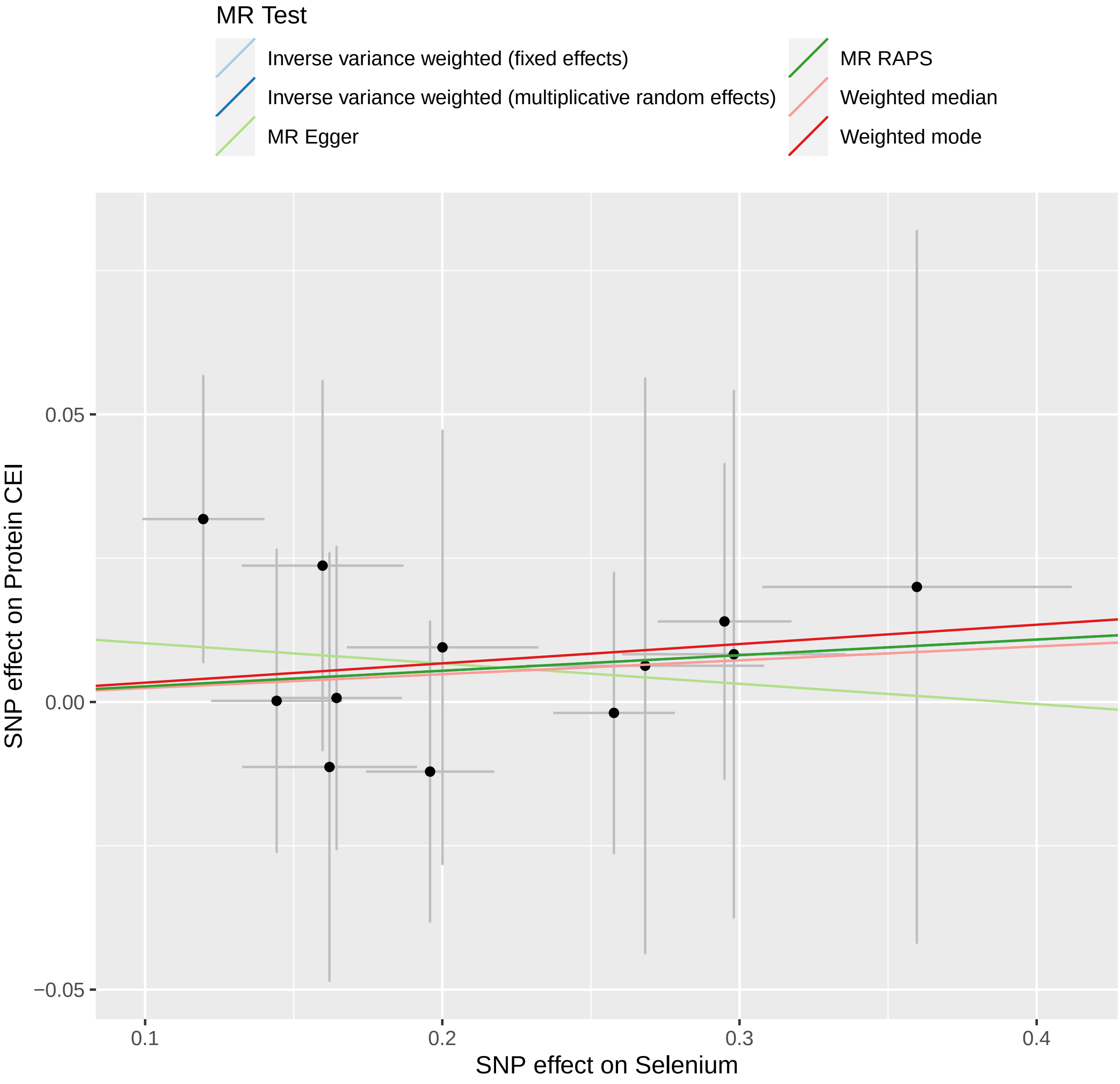

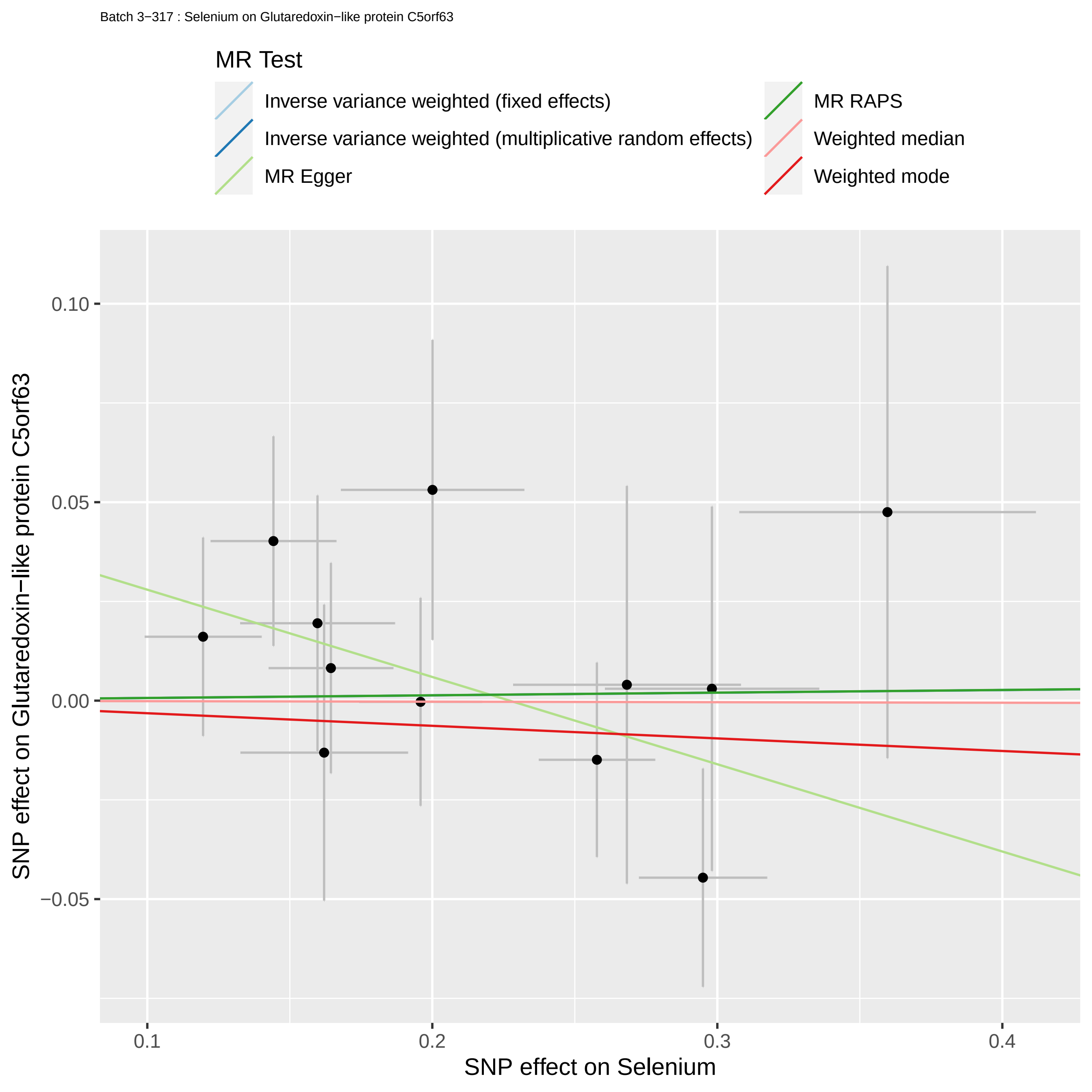

Batch 3−317 : Selenium on Glutaredoxin−like protein C5orf63
MR Test
Inverse variance weighted (fixed effects)
Inverse variance weighted (multiplicative random effects)
MR Egger
MR RAPS
Weighted median
Weighted mode
SNP effect on Glutaredoxin−like protein C5orf63
0.10
0.05
0.00
−0.05
0.1
0.2
0.3
0.4
SNP effect on Selenium

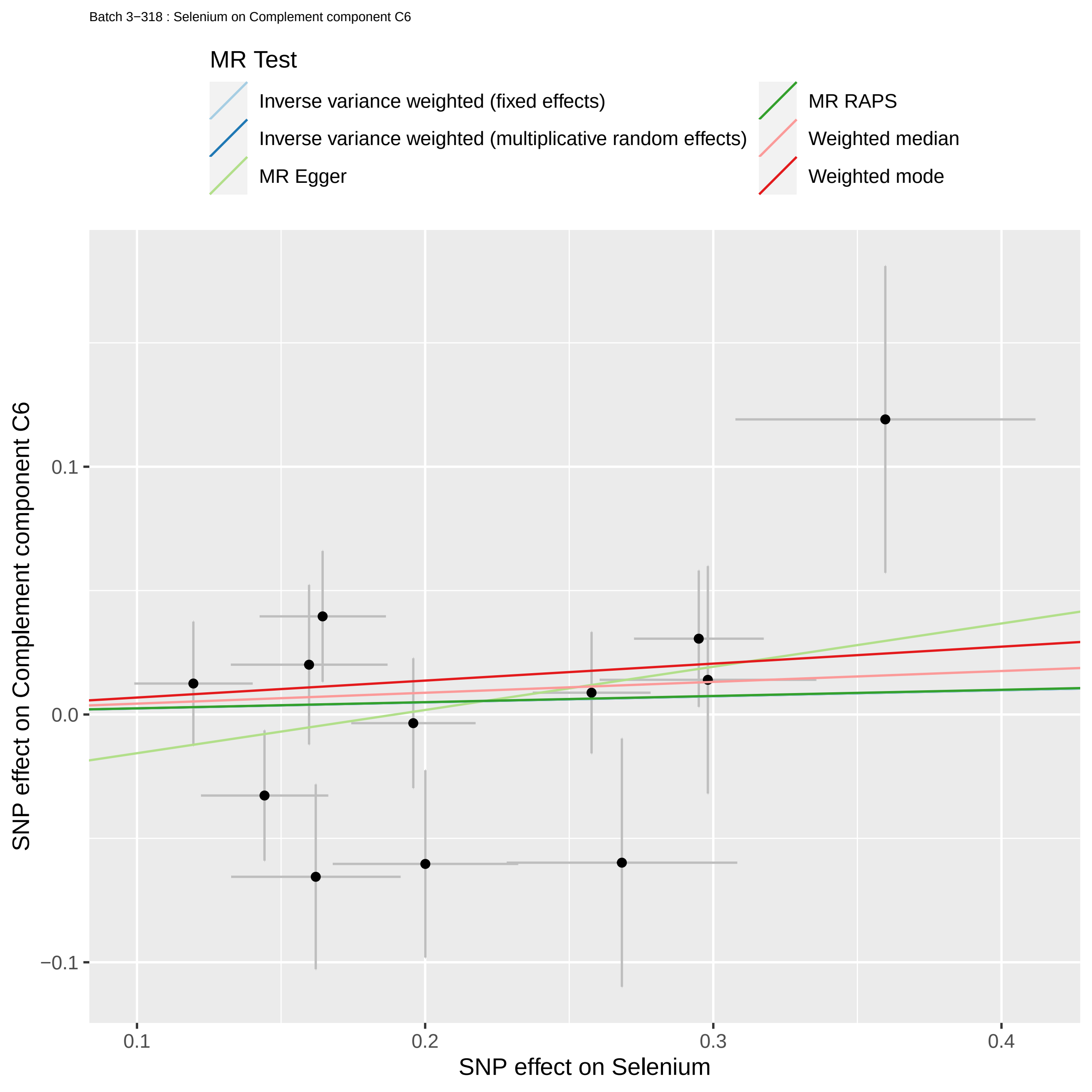
Batch 3−318 : Selenium on Complement component C6
MR Test
Inverse variance weighted (fixed effects)
Inverse variance weighted (multiplicative random effects)
MR Egger
MR RAPS
Weighted median
Weighted mode
SNP effect on Complement component C6
0.1
0.0
−0.1
0.1
0.2
0.3
0.4
SNP effect on Selenium

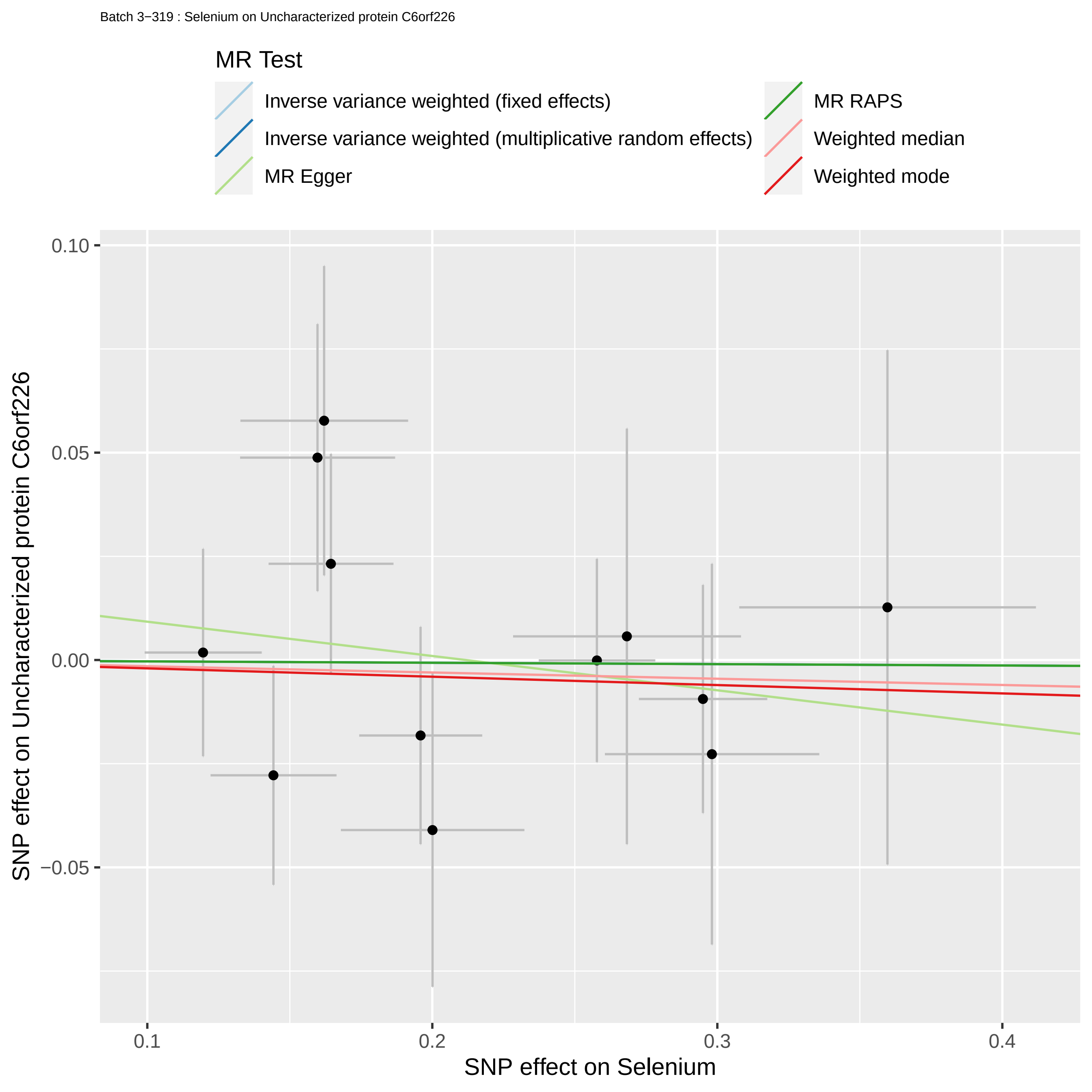

Batch 3−319 : Selenium on Uncharacterized protein C6orf226
MR Test
Inverse variance weighted (fixed effects)
Inverse variance weighted (multiplicative random effects)
MR Egger
MR RAPS
Weighted median
Weighted mode
0.10
0.05
0.00
−0.05
0.1
0.2
0.3
0.4
SNP effect on Selenium
SNP effect on Uncharacterized protein C6orf226

Batch 3−320 : Selenium on Bombesin receptor−activated protein C6orf89

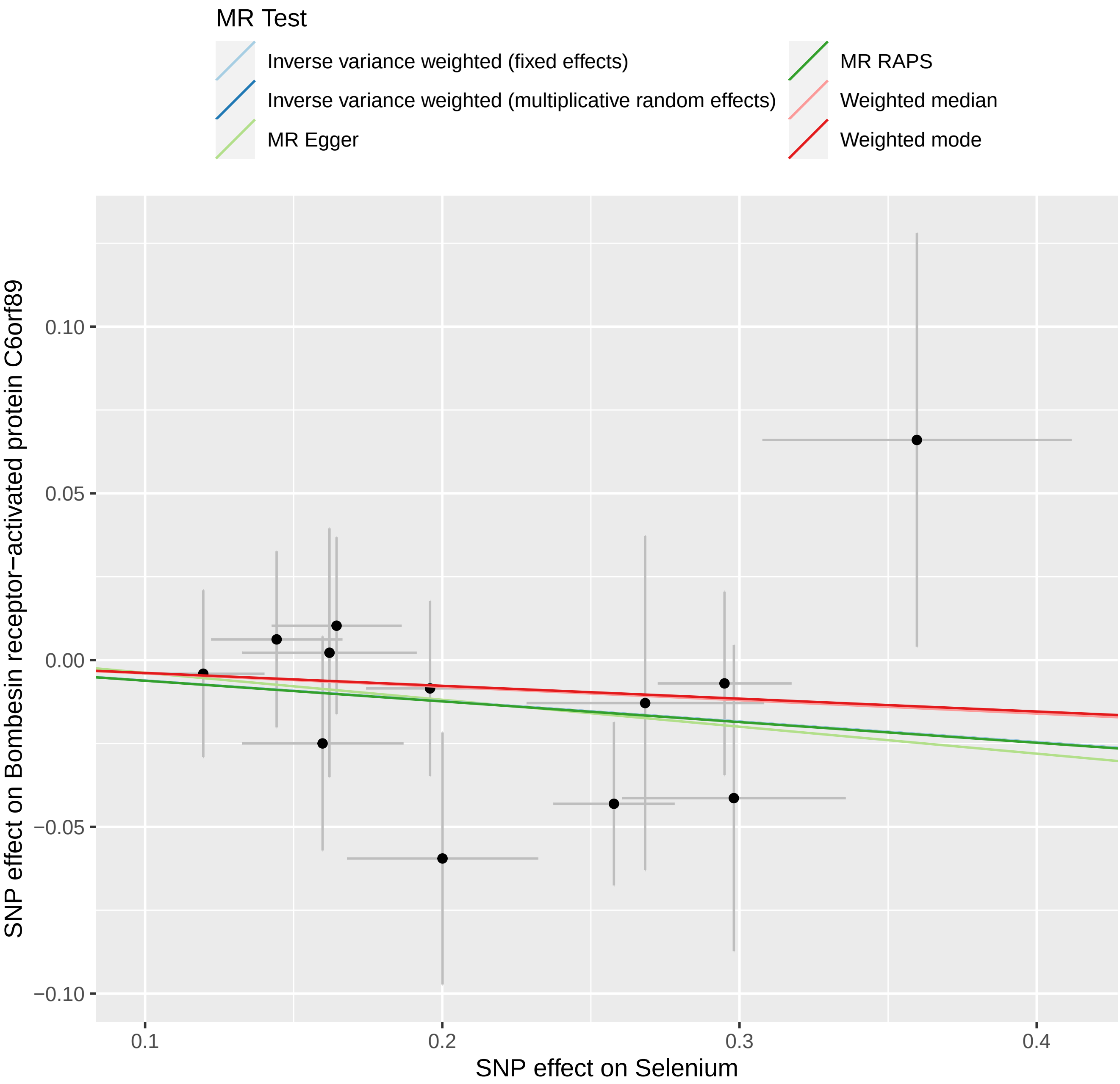

Batch 3−321 : Selenium on Uncharacterized protein C7orf69

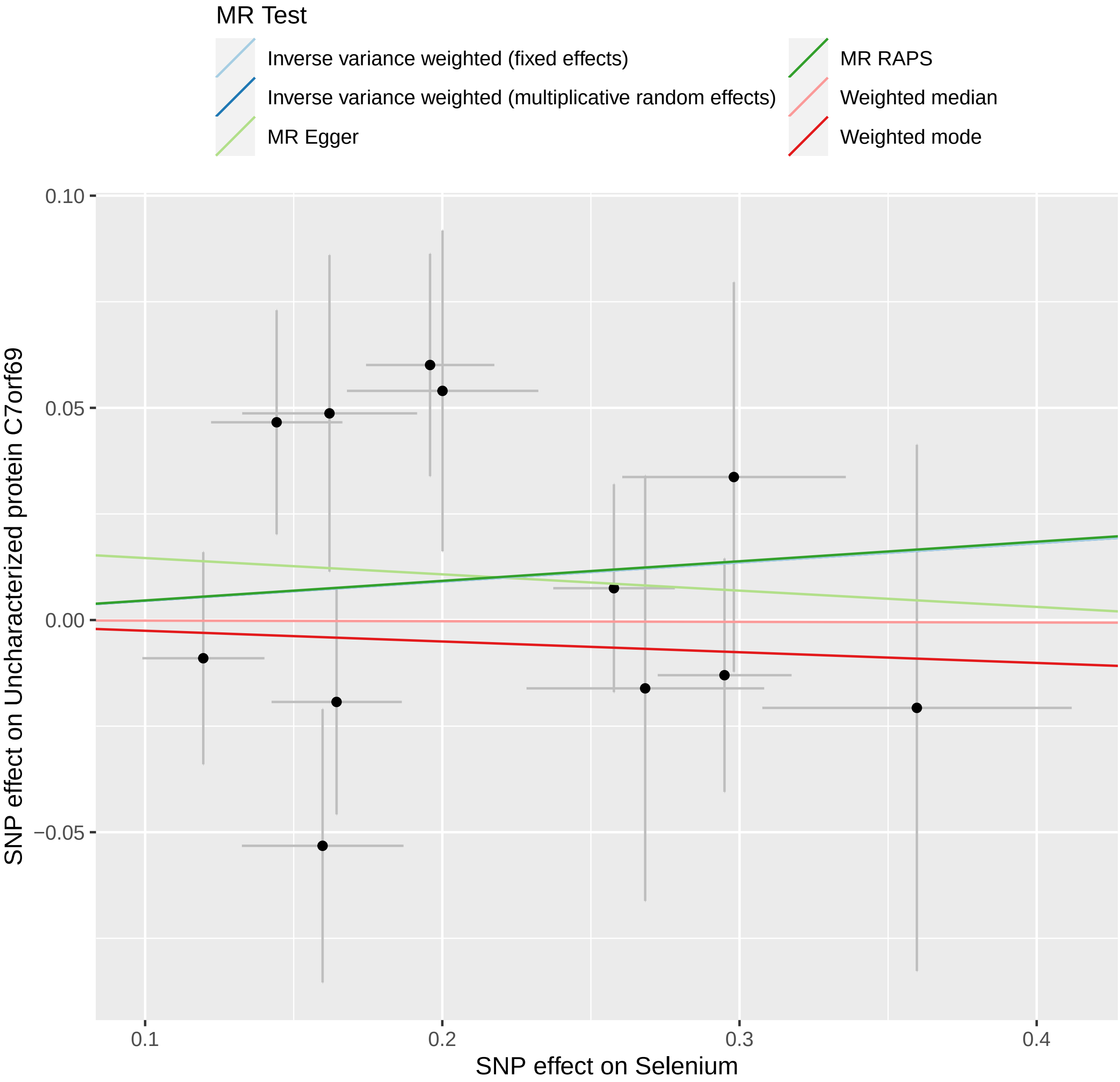

Batch 3−322 : Selenium on Complement component C8

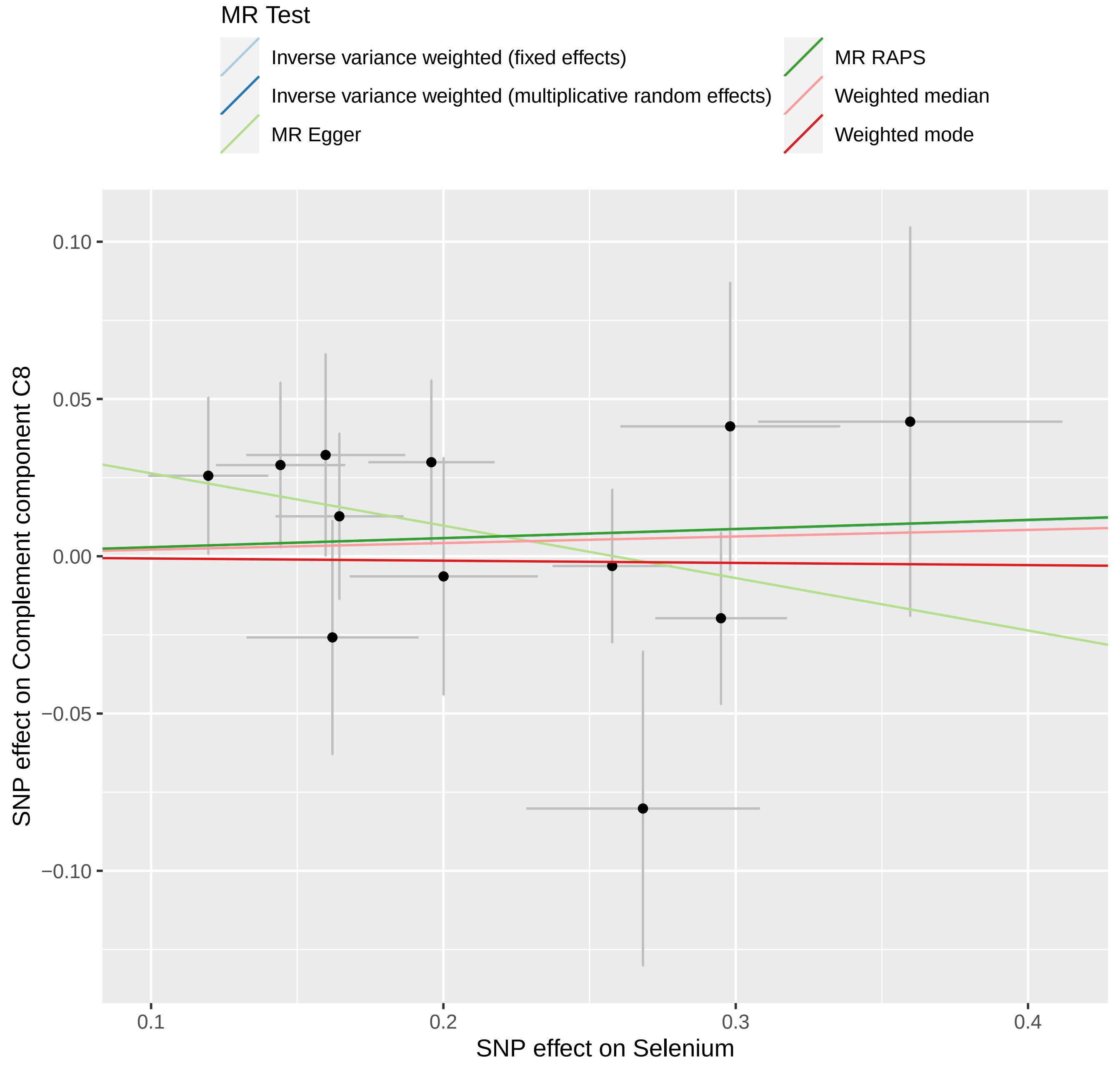

Batch 3−323 : Selenium on UPF0488 protein C8orf33

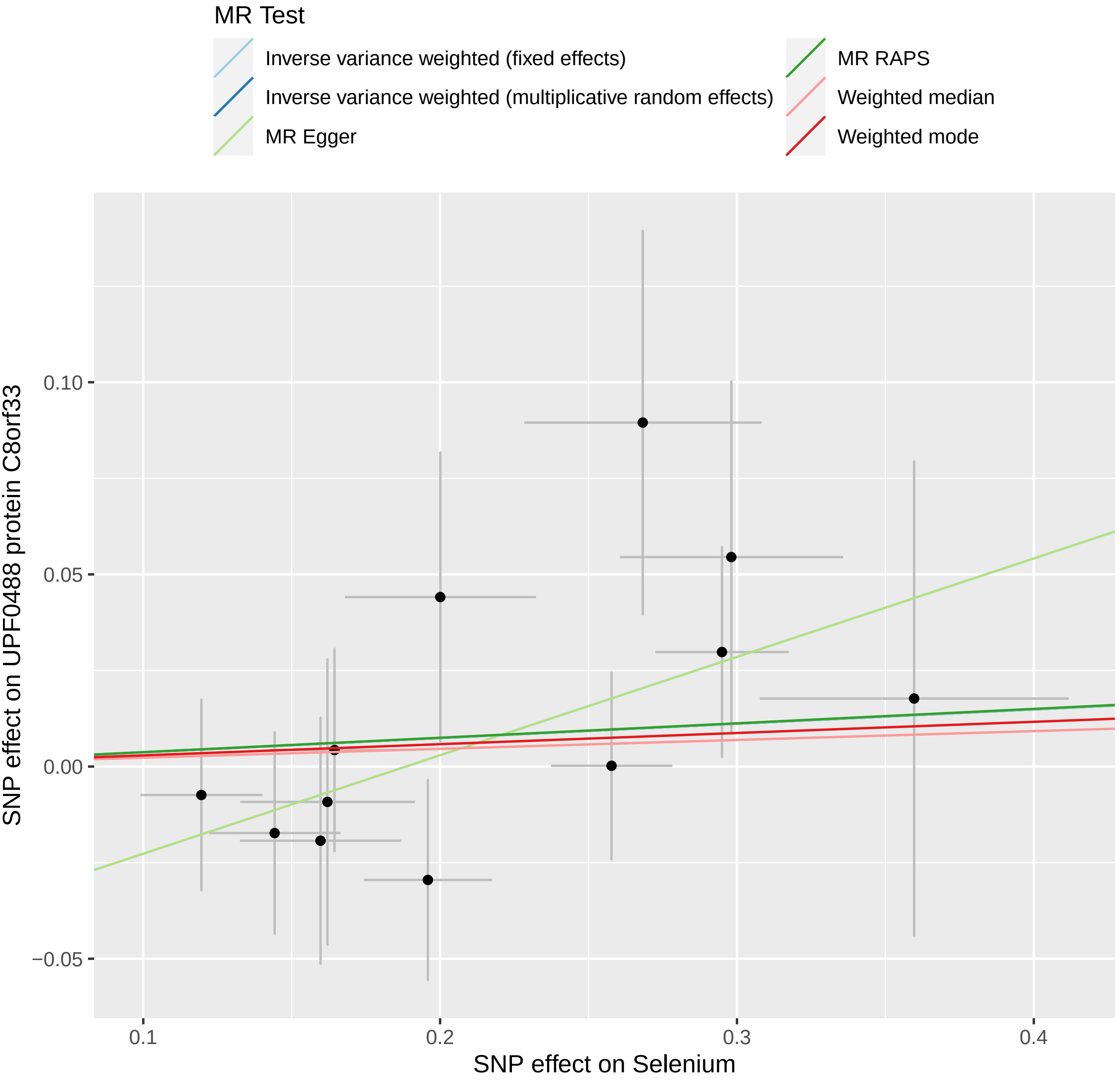

Batch 3−324 : Selenium on Complement component C9

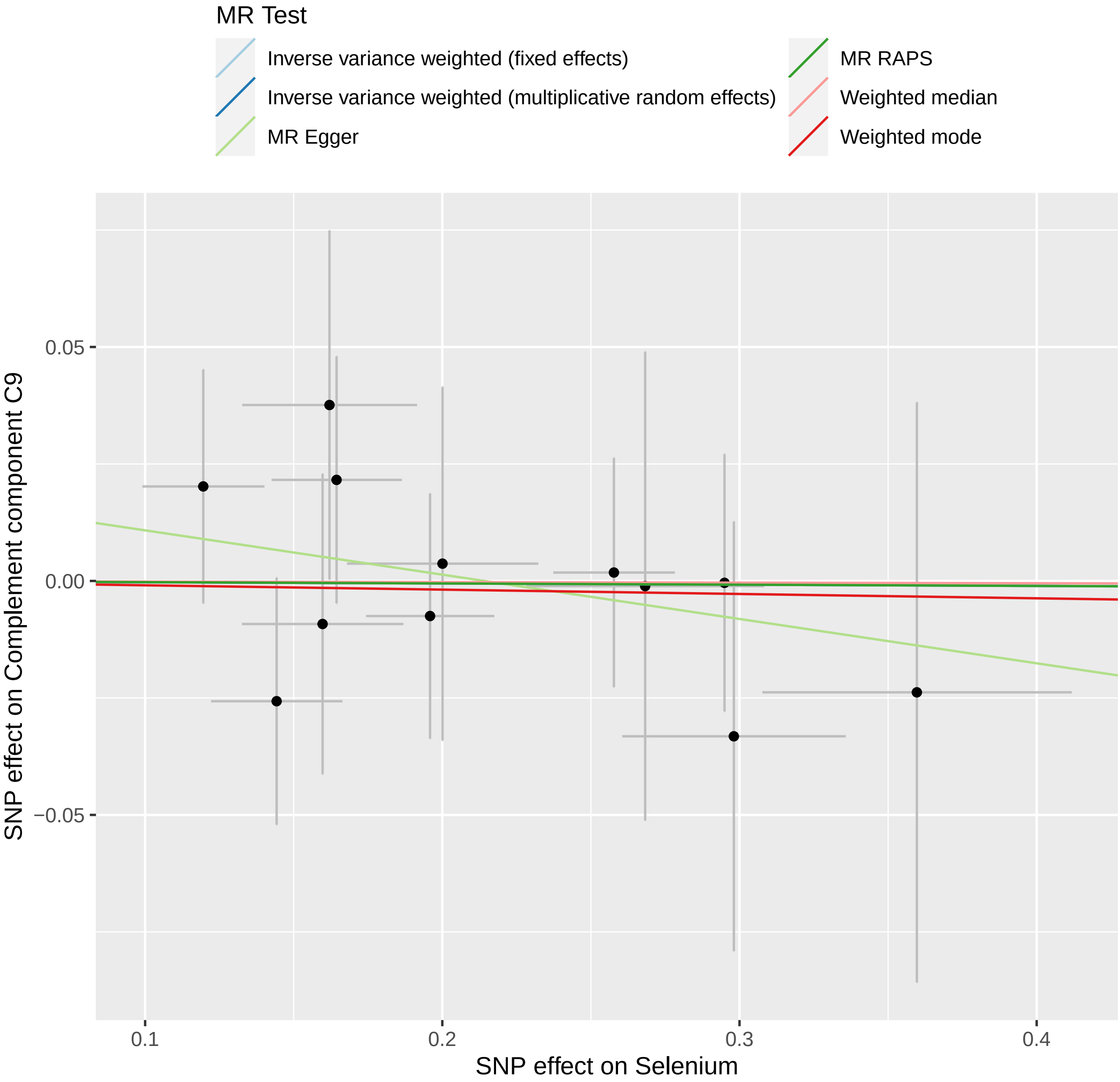

Batch 3−325 : Selenium on Bcl10−interacting CARD protein

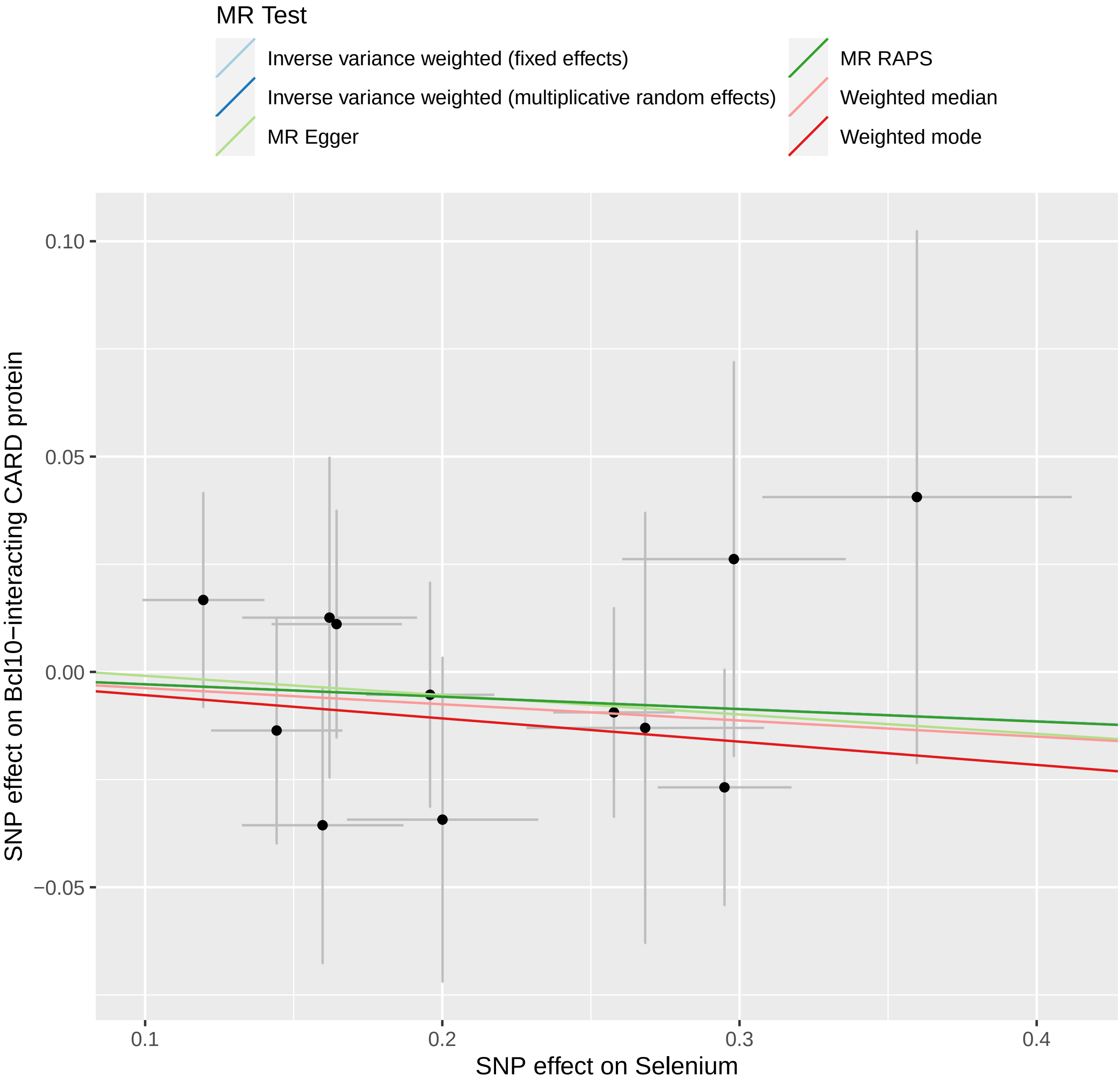

Batch 3−326 : Selenium on Carbonic anhydrase−related protein 10

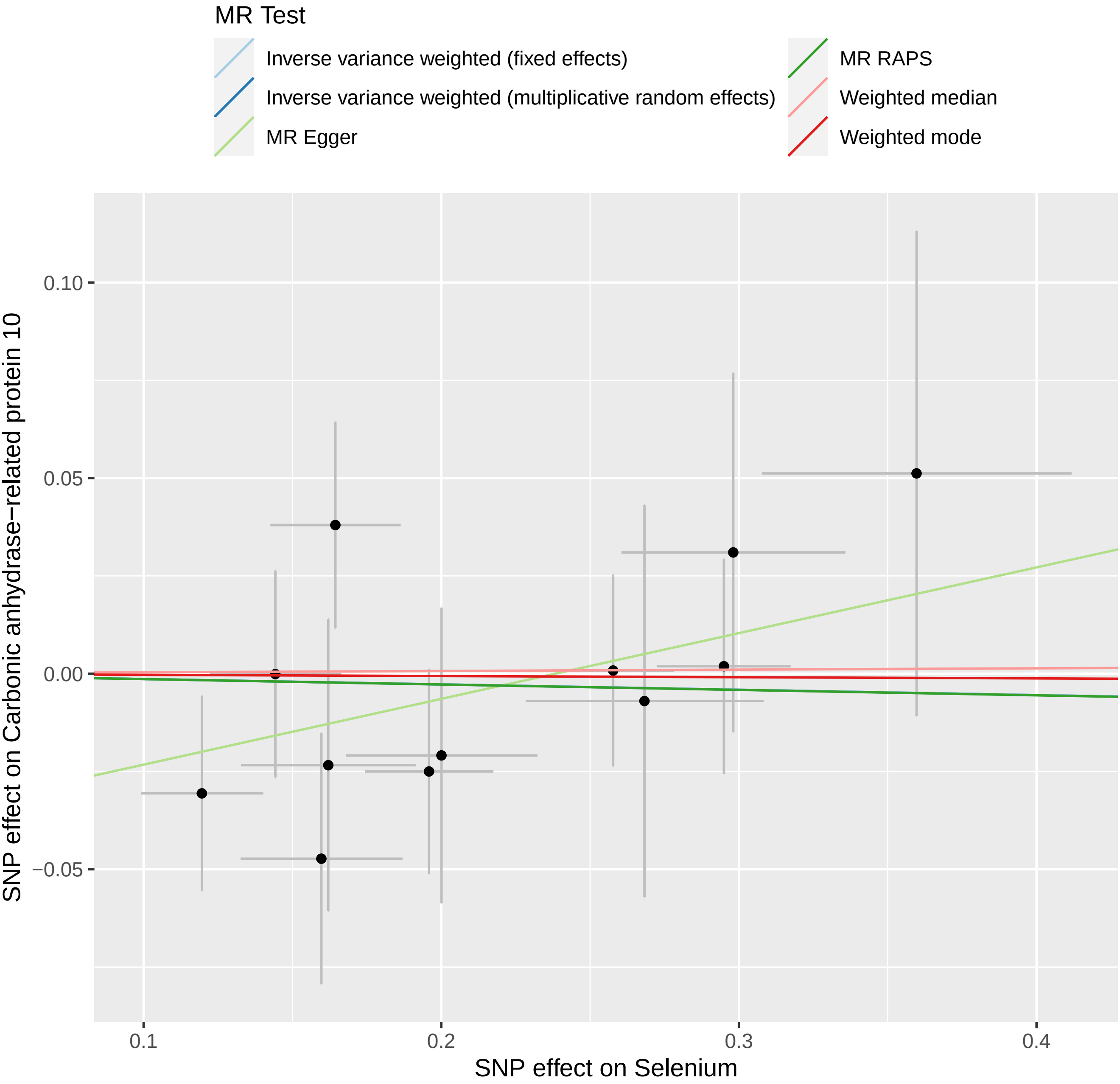

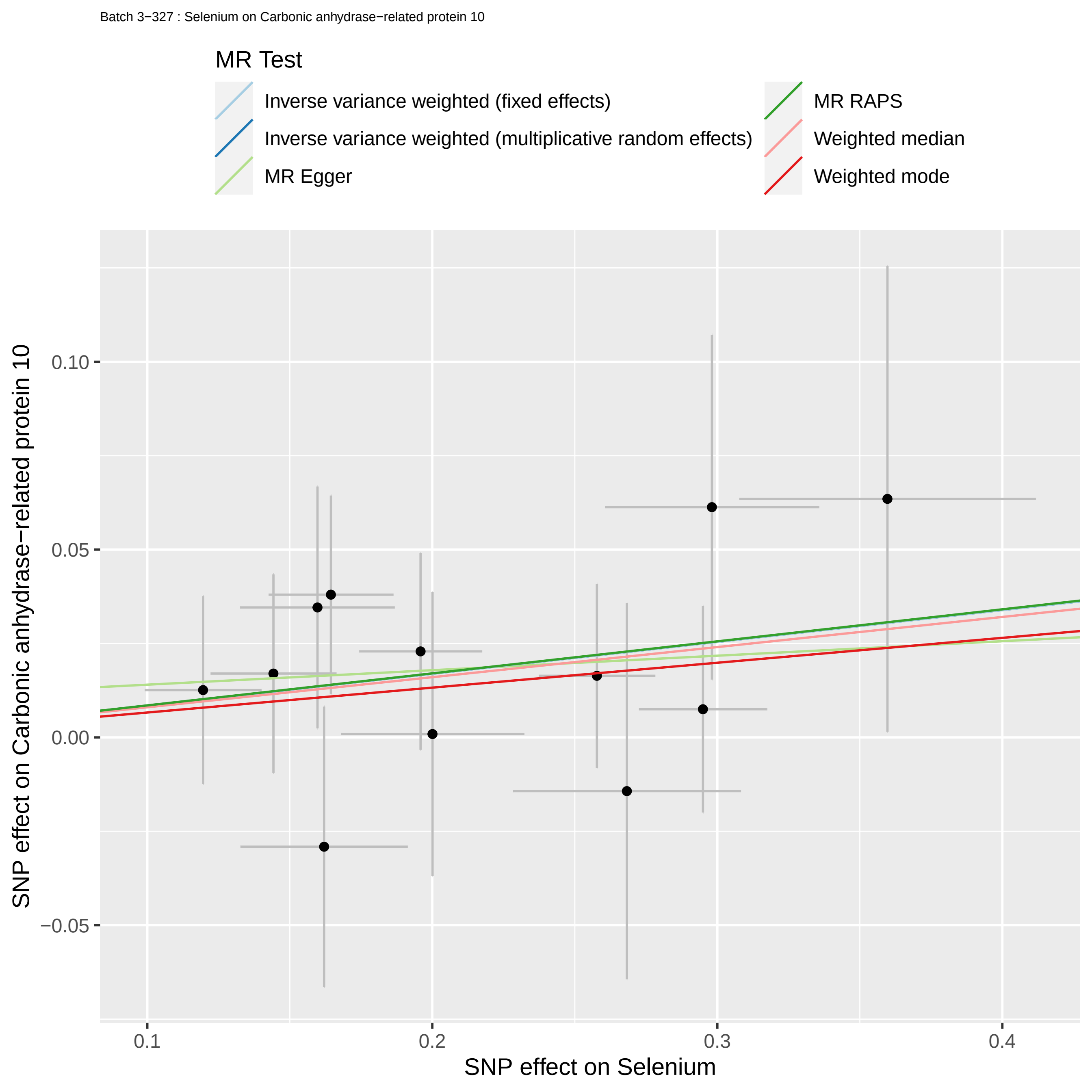
Batch 3−327 : Selenium on Carbonic anhydrase−related protein 10
MR Test
Inverse variance weighted (fixed effects)
Inverse variance weighted (multiplicative random effects)
MR Egger
MR RAPS
Weighted median
Weighted mode
SNP effect on Carbonic anhydrase−related protein 10
0.10
0.05
0.00
−0.05
0.1
0.2
0.3
0.4
SNP effect on Selenium

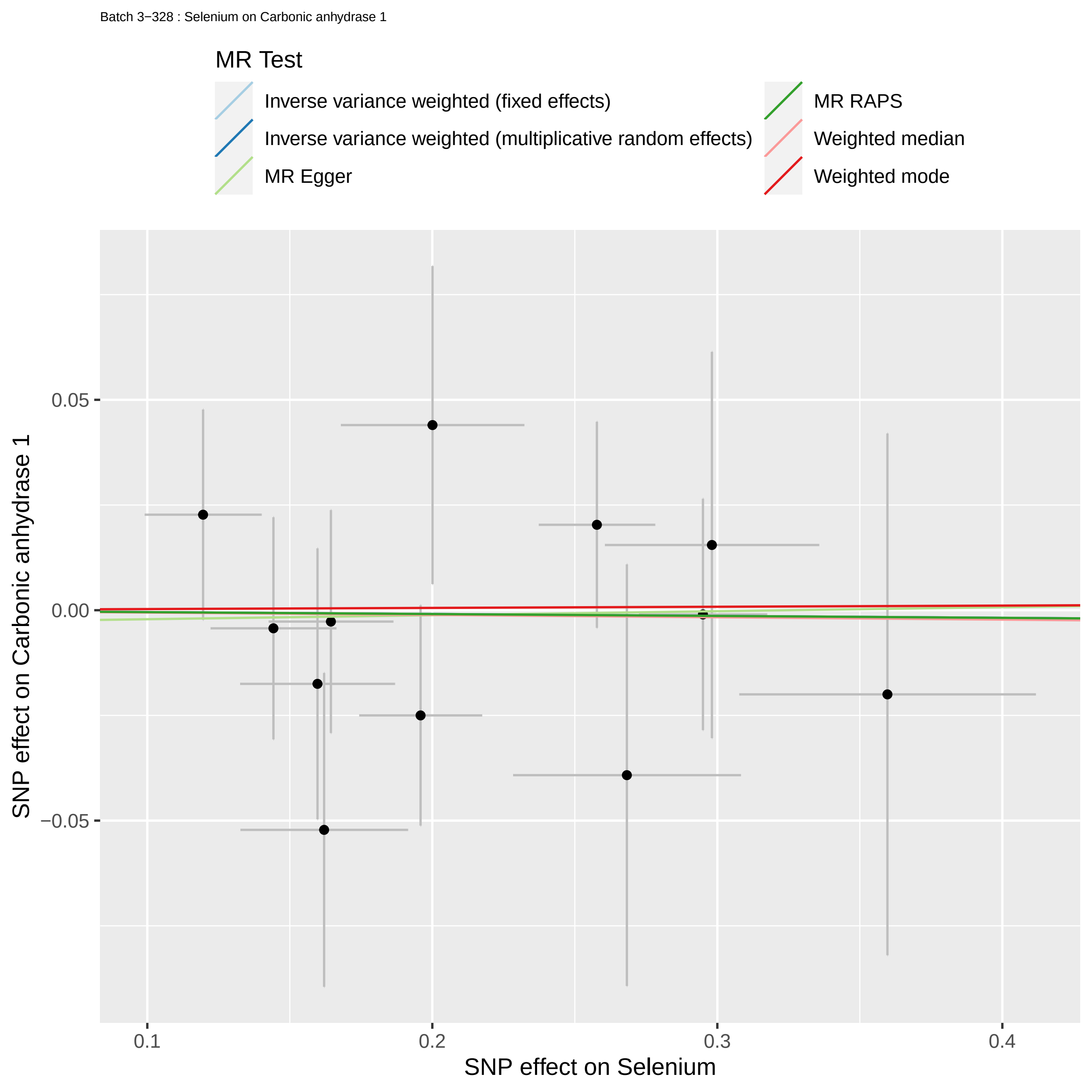

Batch 3−328 : Selenium on Carbonic anhydrase 1
MR Test
Inverse variance weighted (fixed effects)
Inverse variance weighted (multiplicative random effects)
MR Egger
MR RAPS
Weighted median
Weighted mode
0.05
0.00
−0.05
SNP effect on Carbonic anhydrase 1
0.1
0.2
0.3
0.4
SNP effect on Selenium

Batch 3−329 : Selenium on Carbonic anhydrase 2

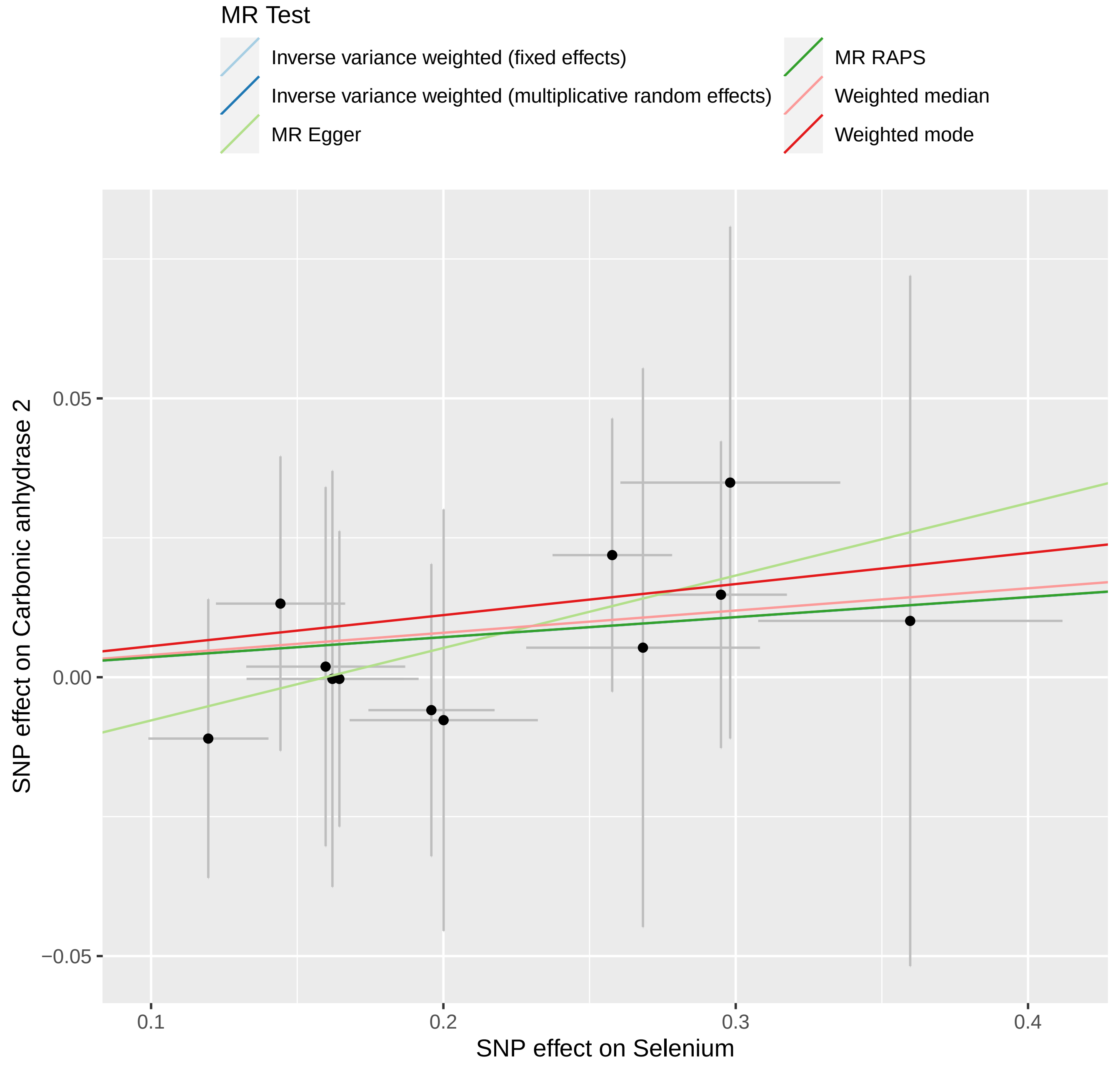

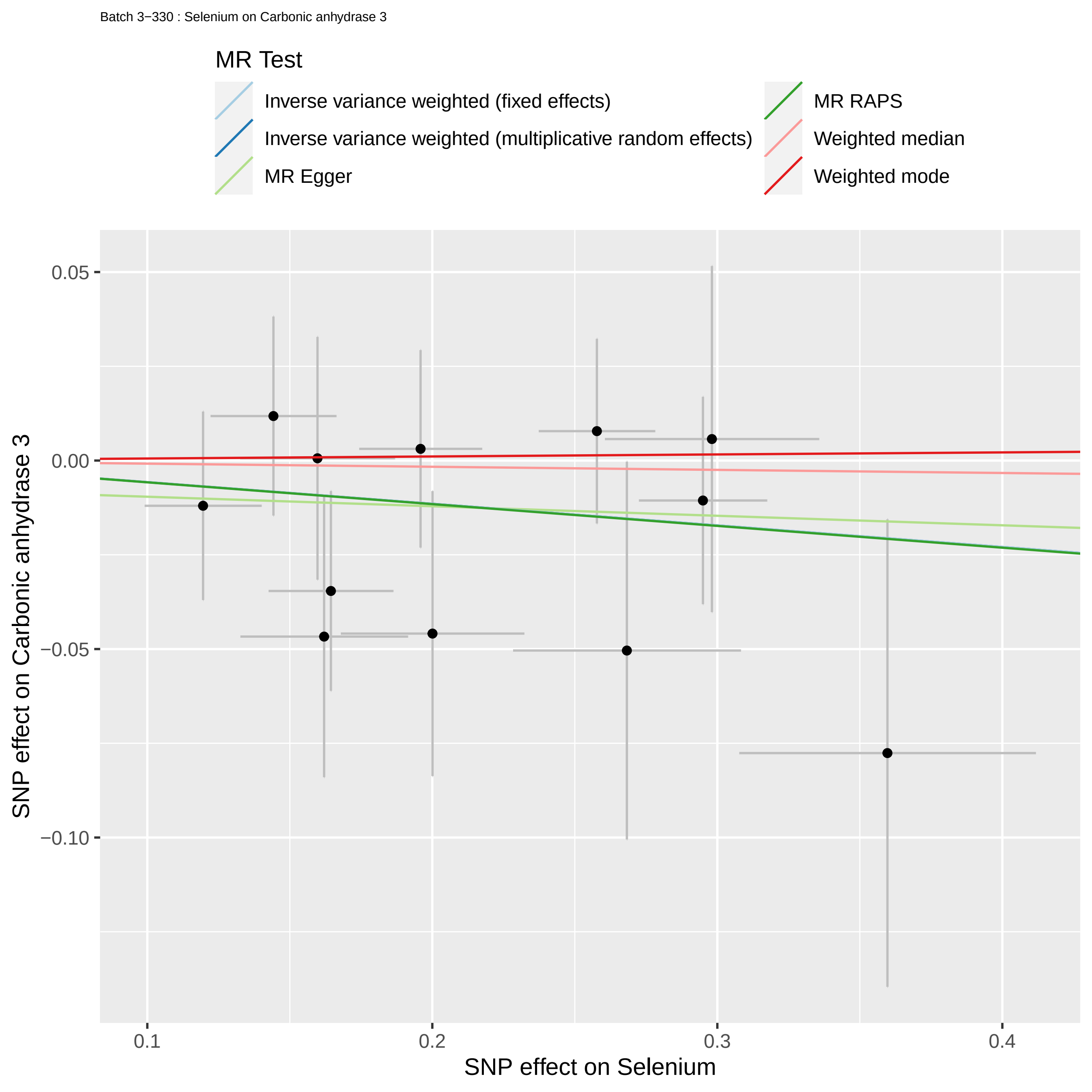
Batch 3−330 : Selenium on Carbonic anhydrase 3
MR Test
Inverse variance weighted (fixed effects)
Inverse variance weighted (multiplicative random effects)
MR Egger
MR RAPS
Weighted median
Weighted mode
0.05
0.00
−0.05
−0.10
0.1
0.2
0.3
0.4
SNP effect on Carbonic anhydrase 3
SNP effect on Selenium

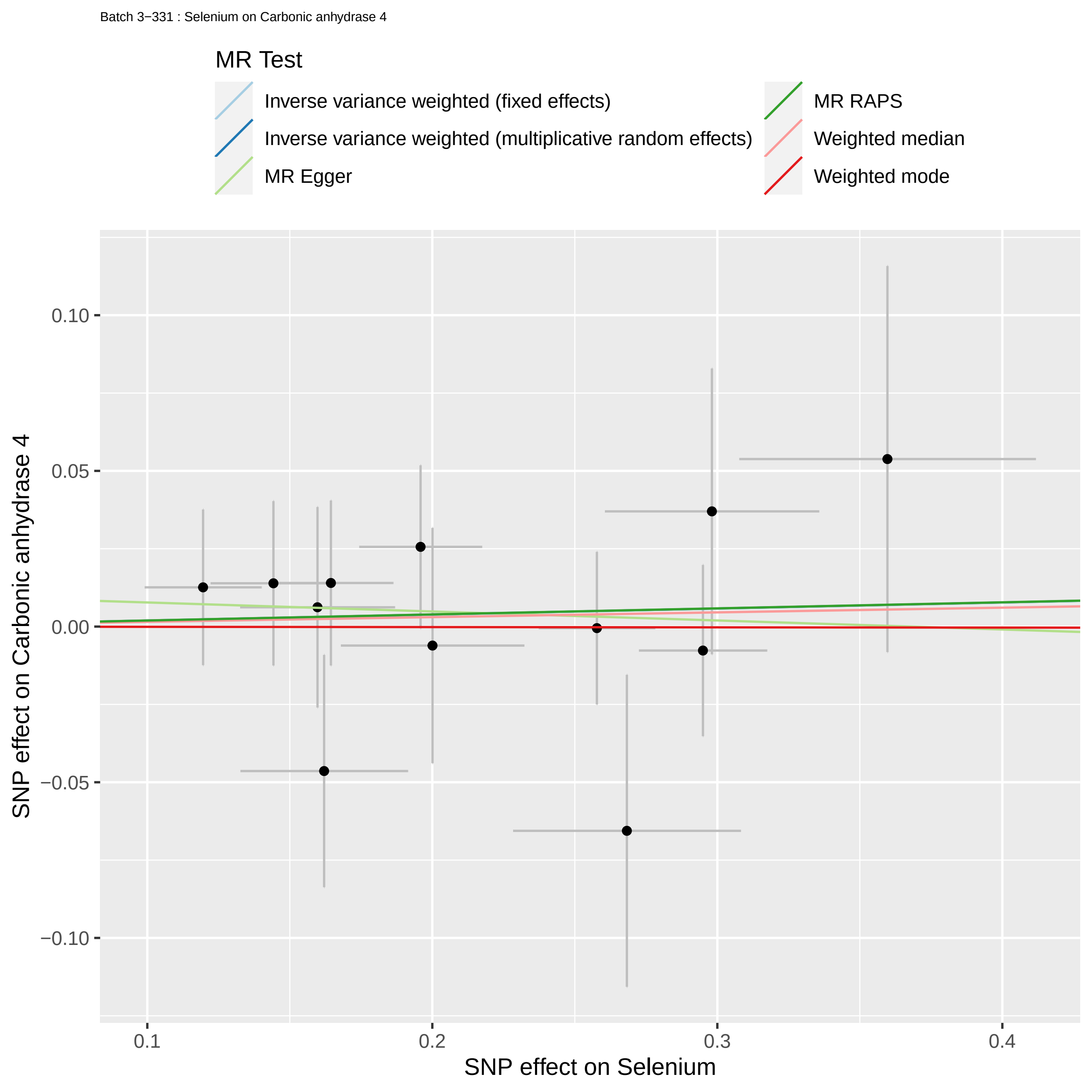
Batch 3−331 : Selenium on Carbonic anhydrase 4
MR Test
Inverse variance weighted (fixed effects)
Inverse variance weighted (multiplicative random effects)
MR Egger
MR RAPS
Weighted median
Weighted mode
0.10
0.05
0.00
−0.05
−0.10
0.1
0.2
0.3
0.4
SNP effect on Carbonic anhydrase 4
SNP effect on Selenium

Batch 3−332 : Selenium on Carbonic anhydrase 5A, mitochondrial

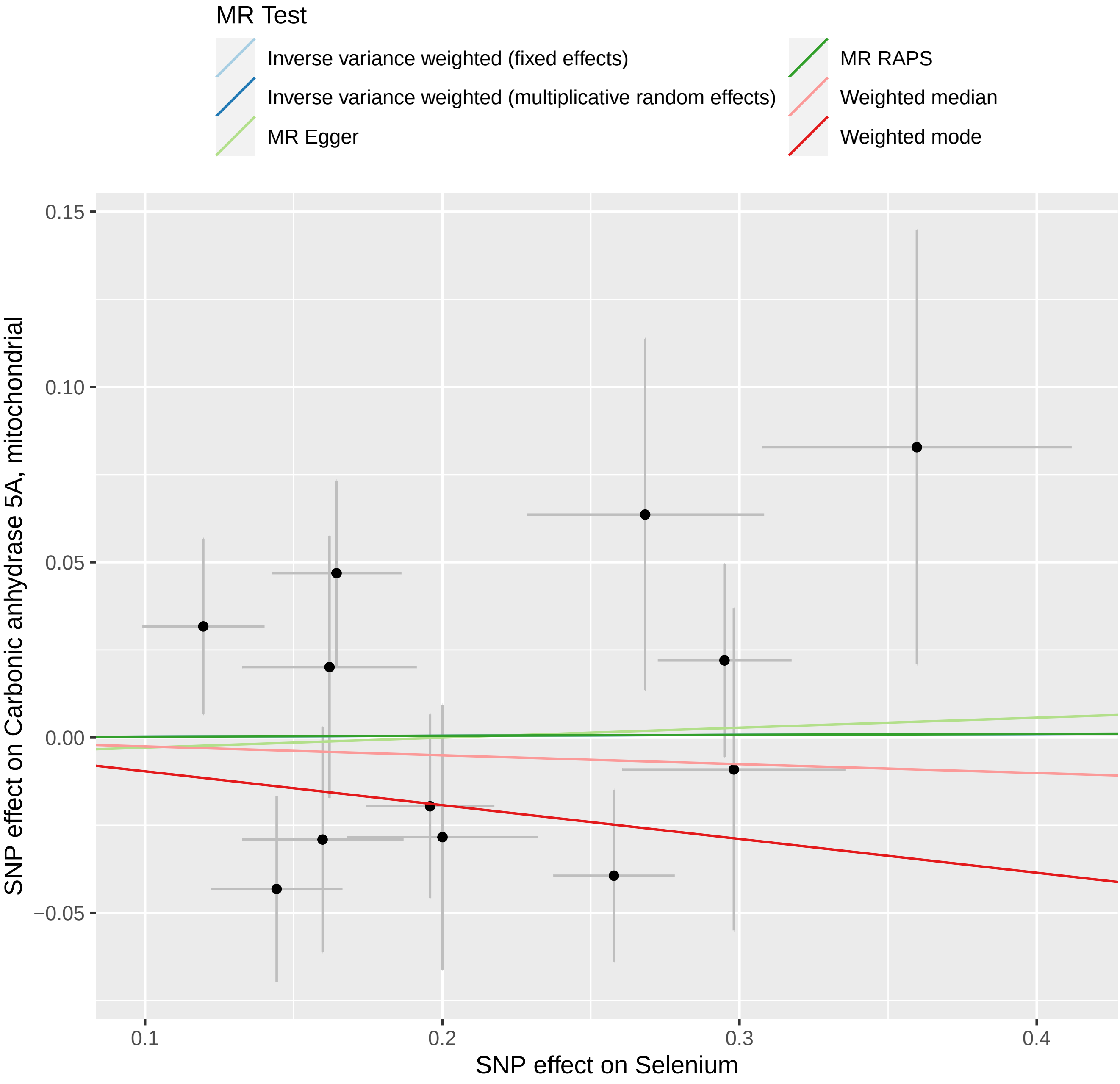

Batch 3−333 : Selenium on Carbonic anhydrase 6

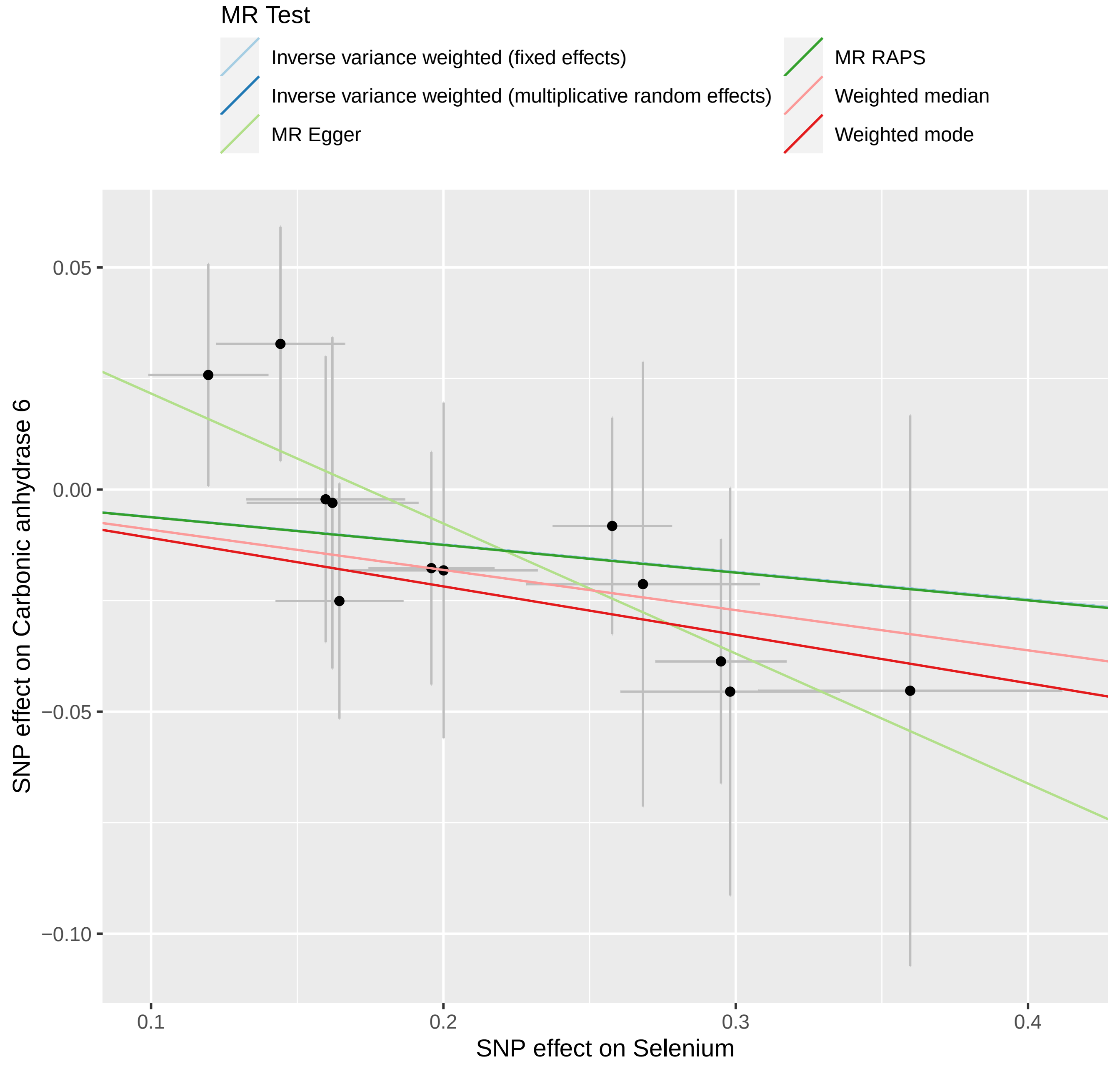

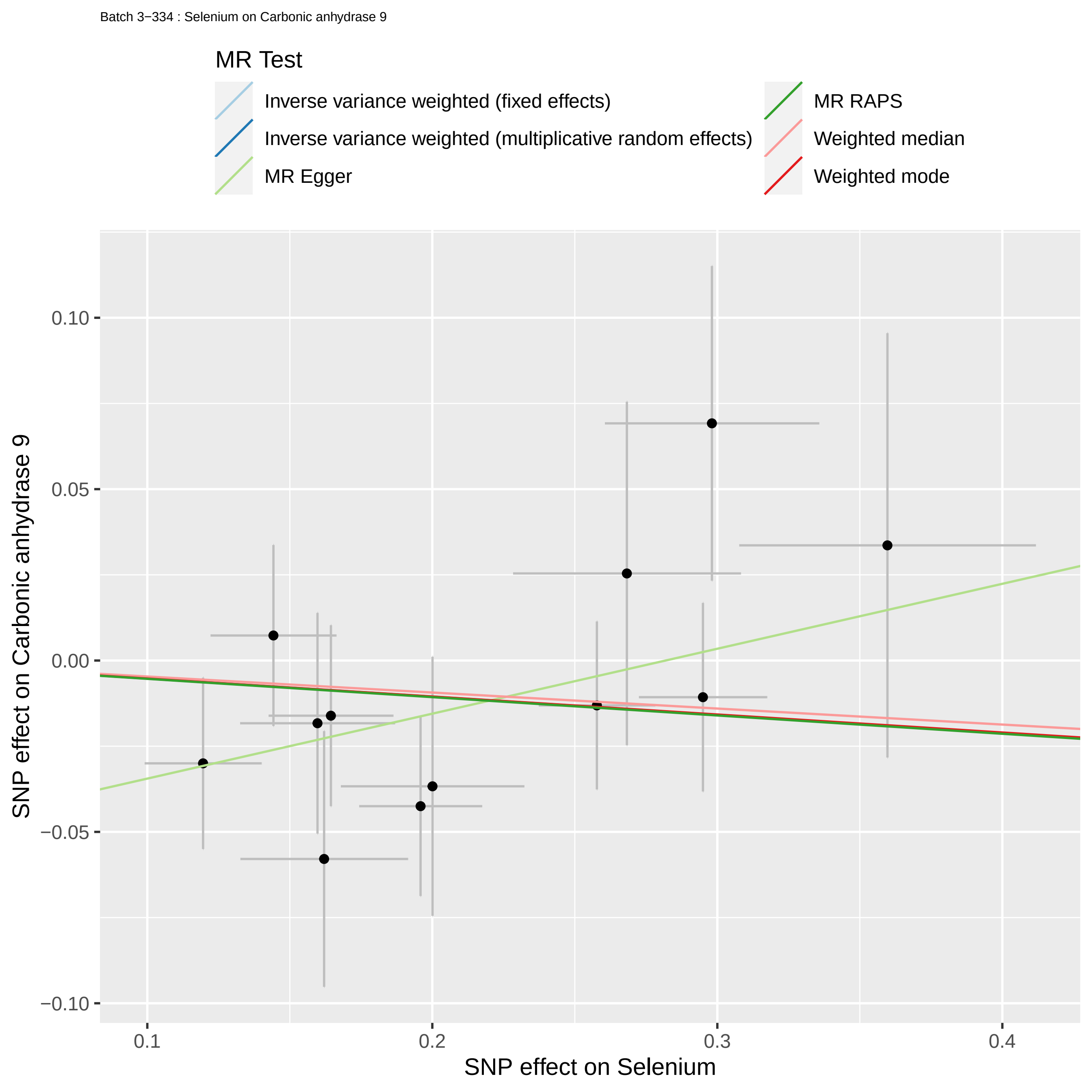

Batch 3−334 : Selenium on Carbonic anhydrase 9
MR Test
Inverse variance weighted (fixed effects)
Inverse variance weighted (multiplicative random effects)
MR Egger
MR RAPS
Weighted median
Weighted mode
SNP effect on Carbonic anhydrase 9
0.10
0.05
0.00
−0.05
−0.10
0.1
0.2
0.3
0.4
SNP effect on Selenium

Batch 3−335 : Selenium on Voltage−dependent calcium channel subunit alpha−2/delta−3

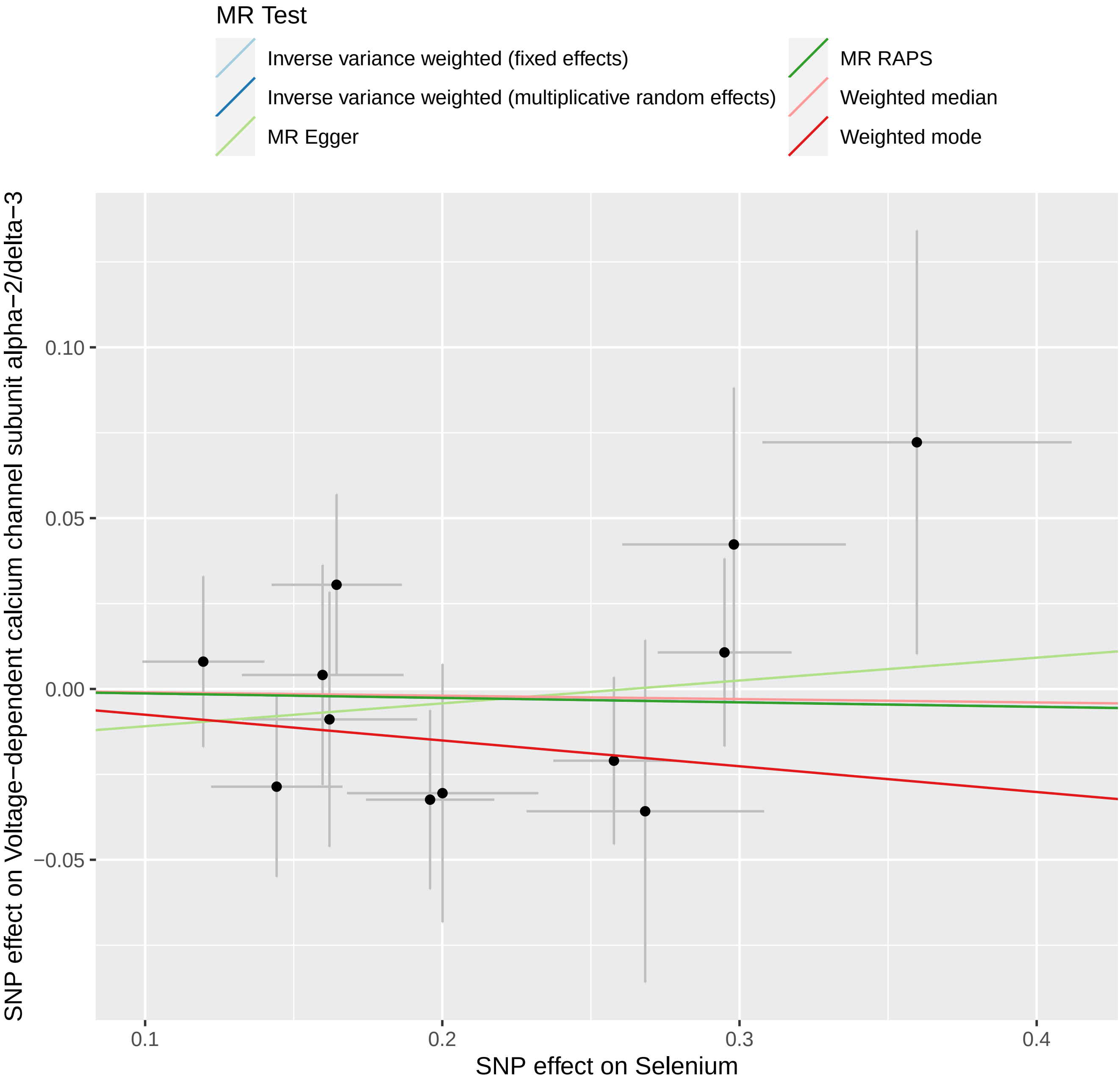

Batch 3−336 : Selenium on Calcyclin−binding protein

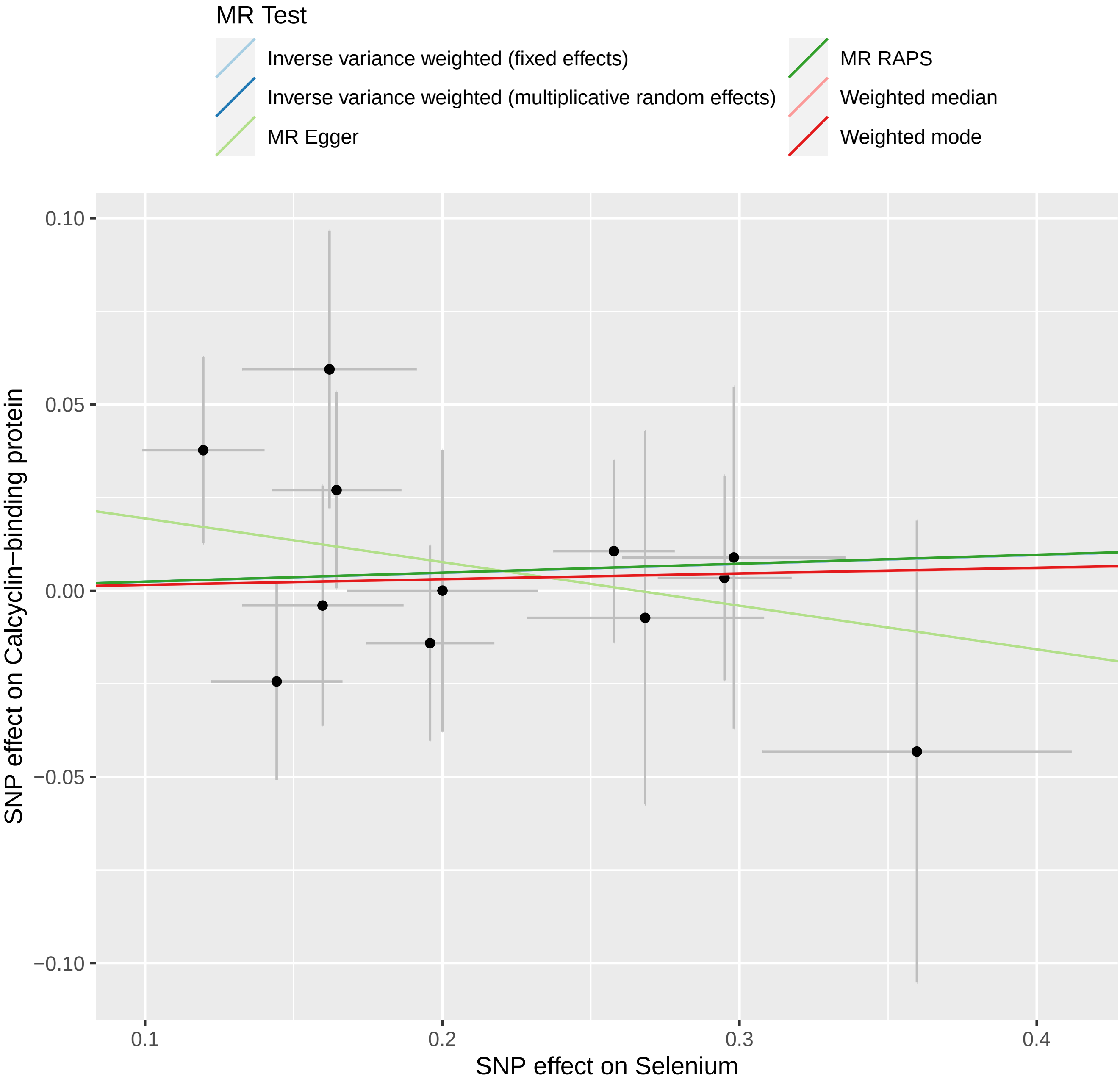

Batch 3−337 : Selenium on Cell adhesion molecule 1

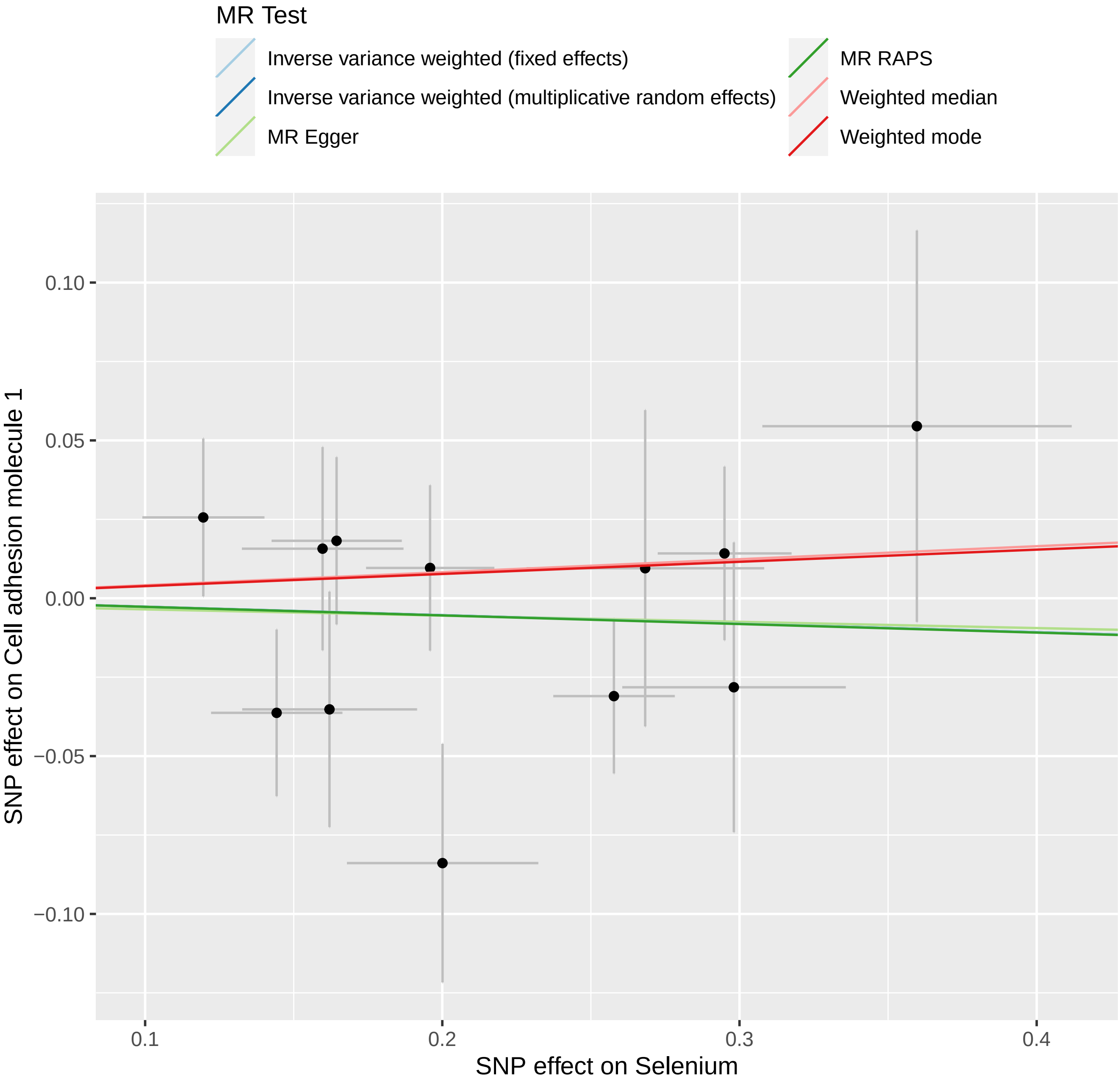

Batch 3−338 : Selenium on Cell adhesion molecule 3

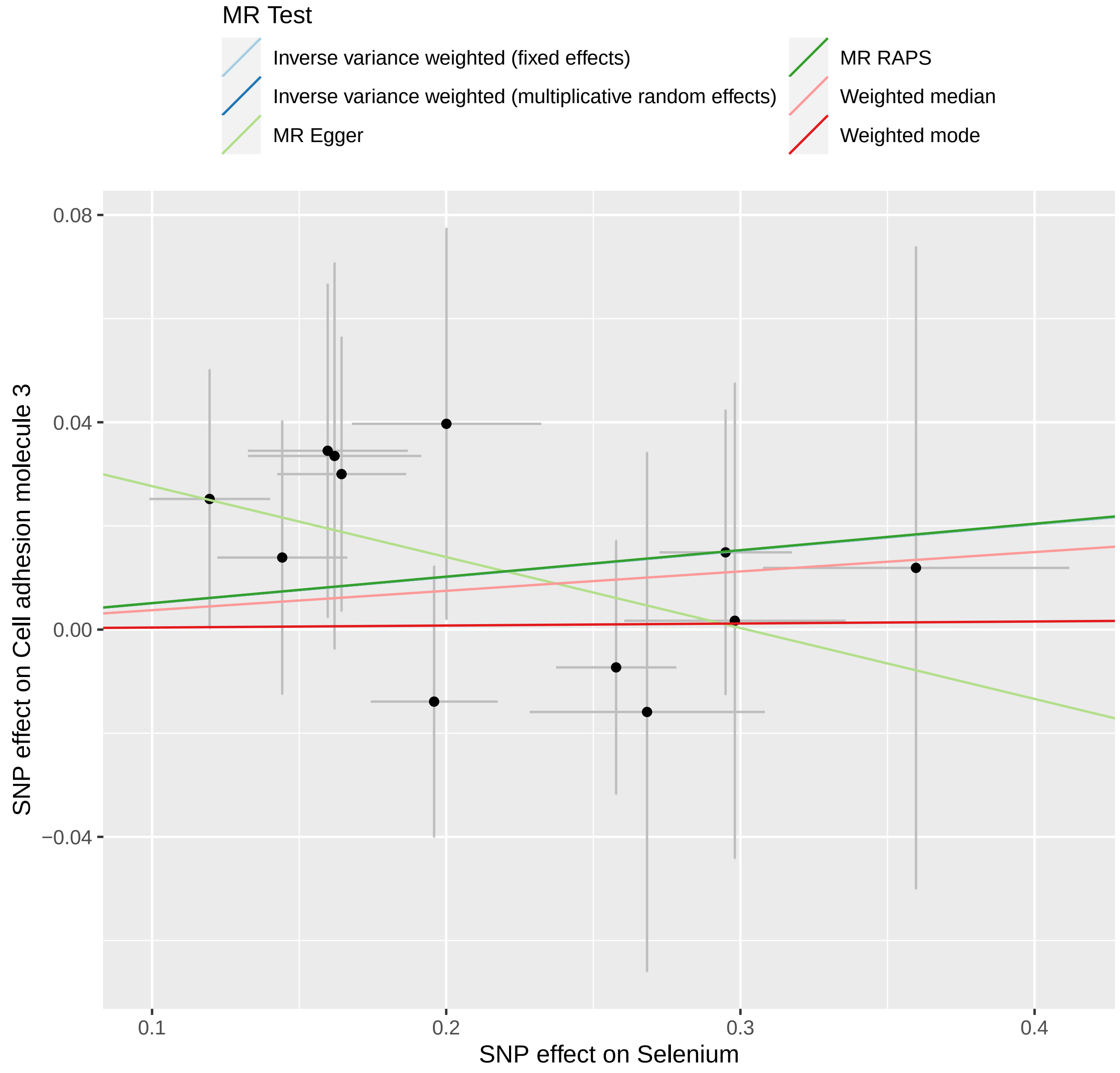

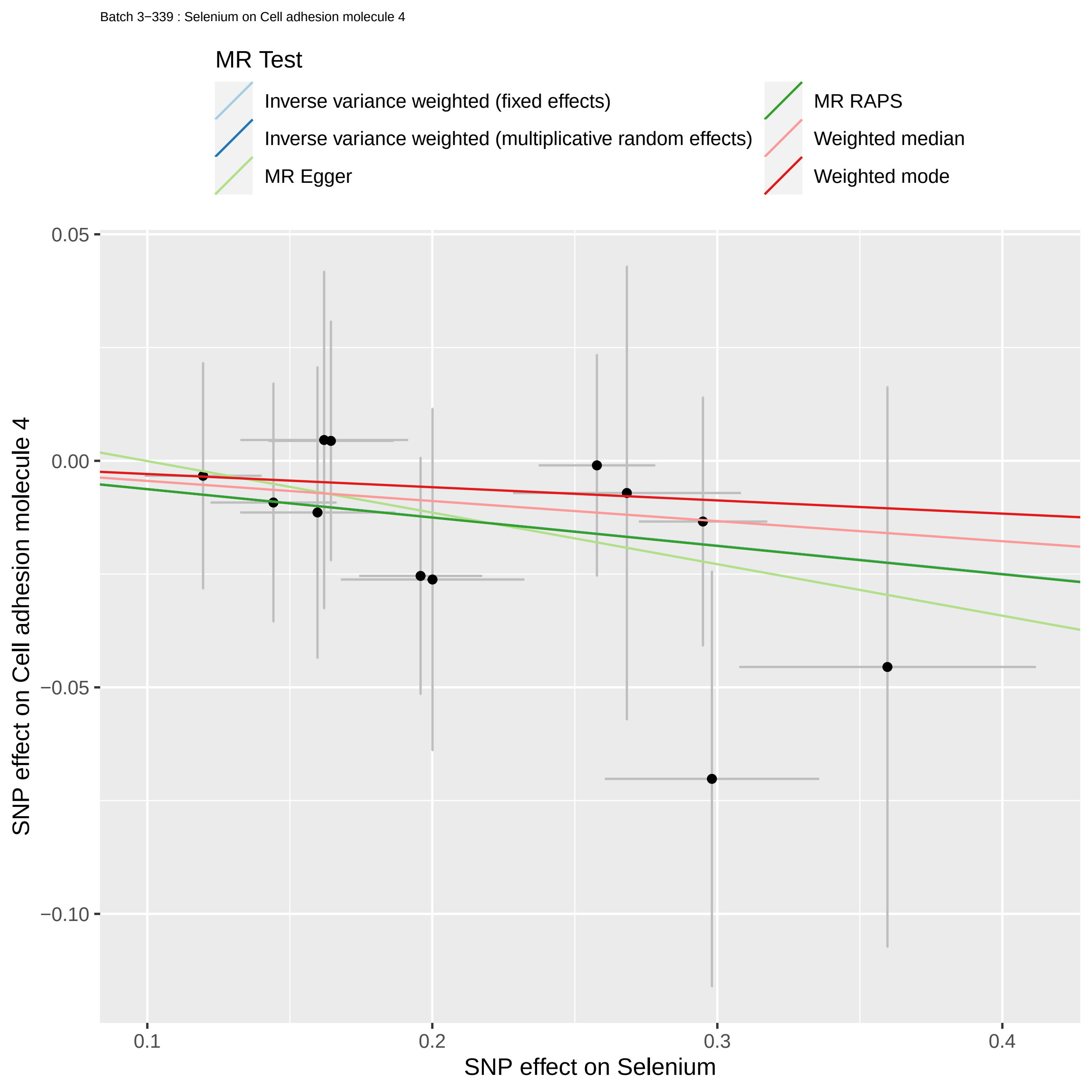
Batch 3−339 : Selenium on Cell adhesion molecule 4
MR Test
Inverse variance weighted (fixed effects)
Inverse variance weighted (multiplicative random effects)
MR Egger
MR RAPS
Weighted median
Weighted mode
0.05
0.00
−0.05
−0.10
0.1
0.2
0.3
0.4
SNP effect on Selenium
SNP effect on Cell adhesion molecule 4

Batch 3−340 : Selenium on Calbindin

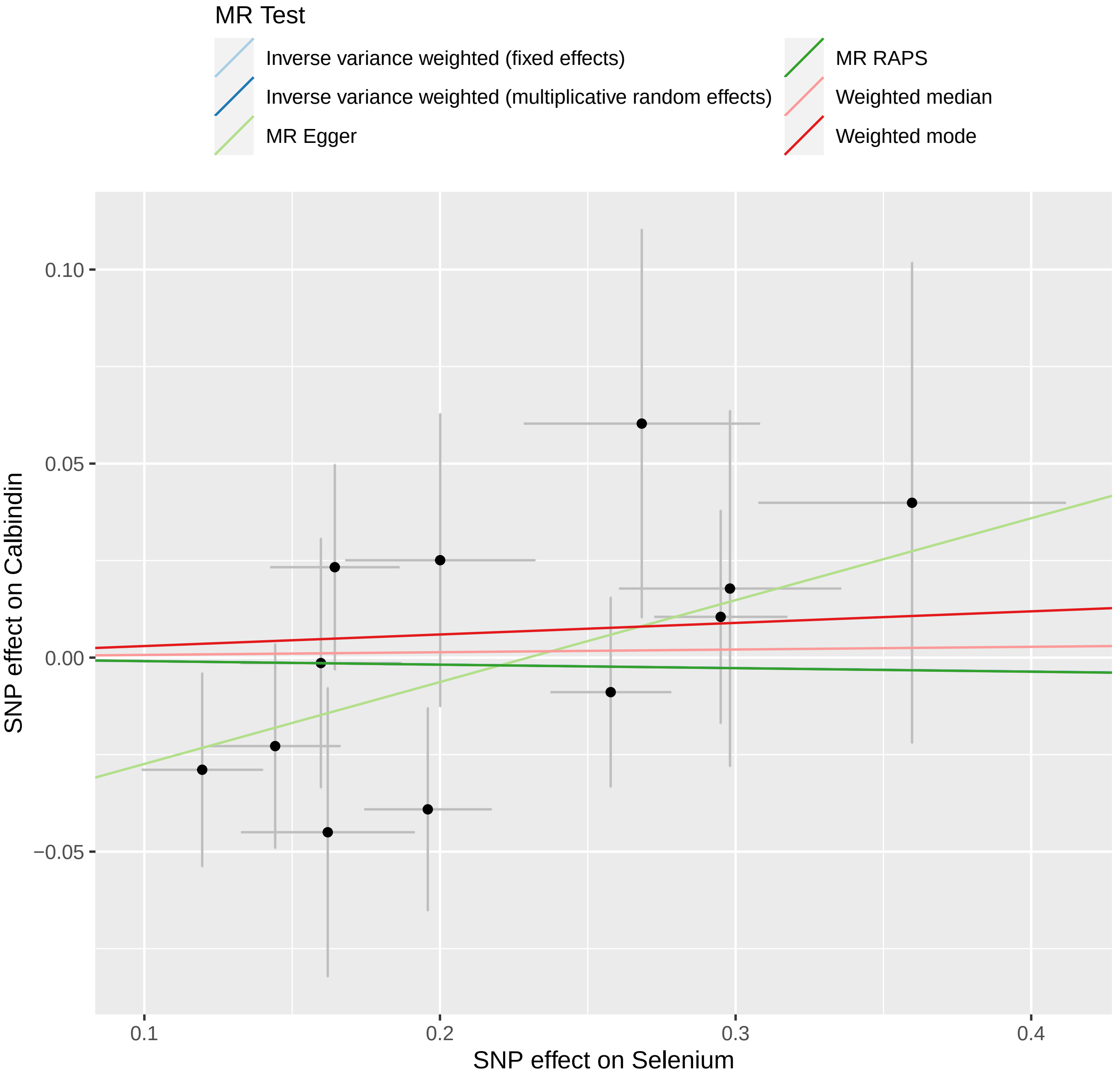

Batch 3−341 : Selenium on Procalcitonin

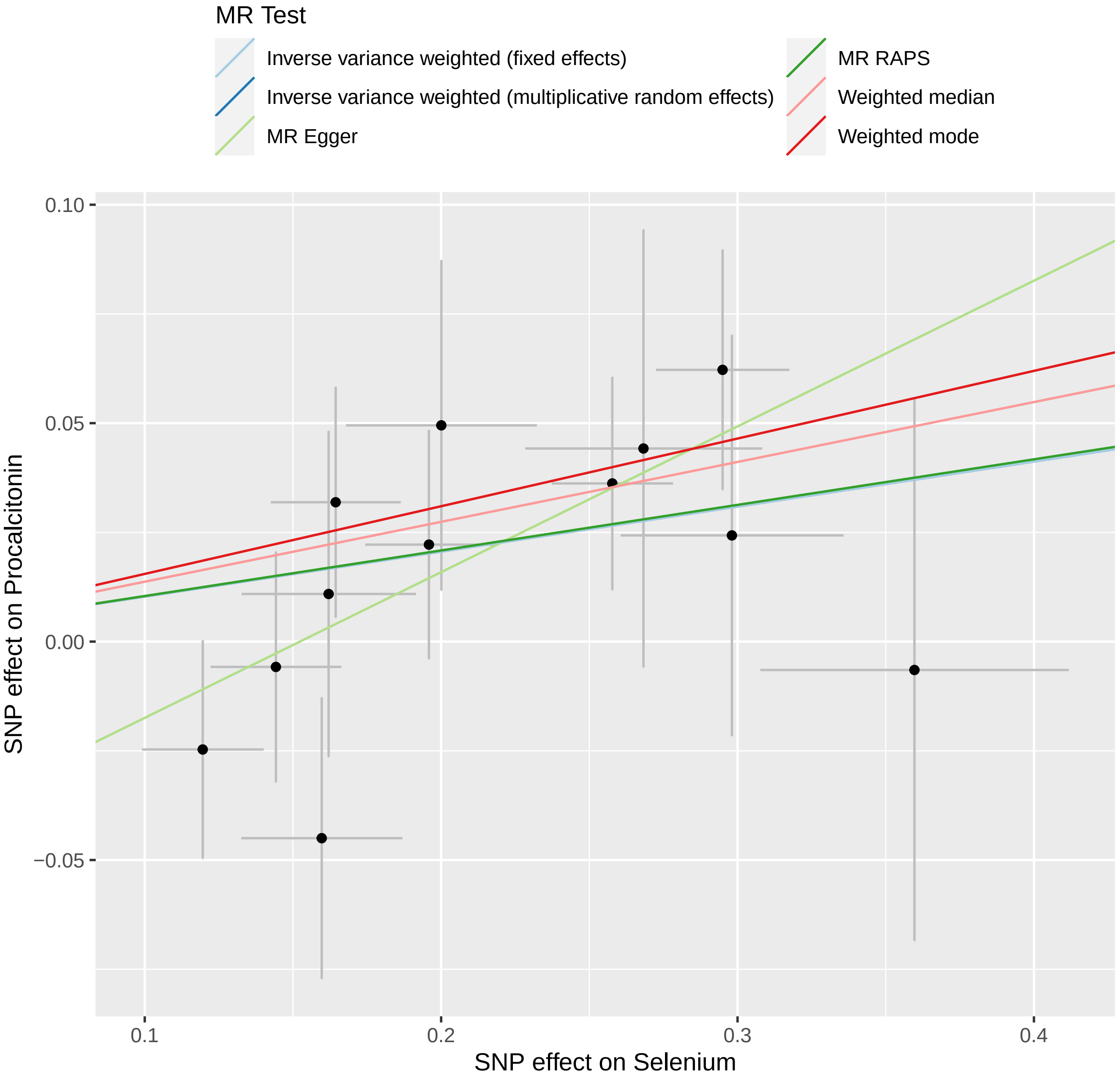

Batch 3−342 : Selenium on Calcium−binding and coiled−coil domain−containing protein 2

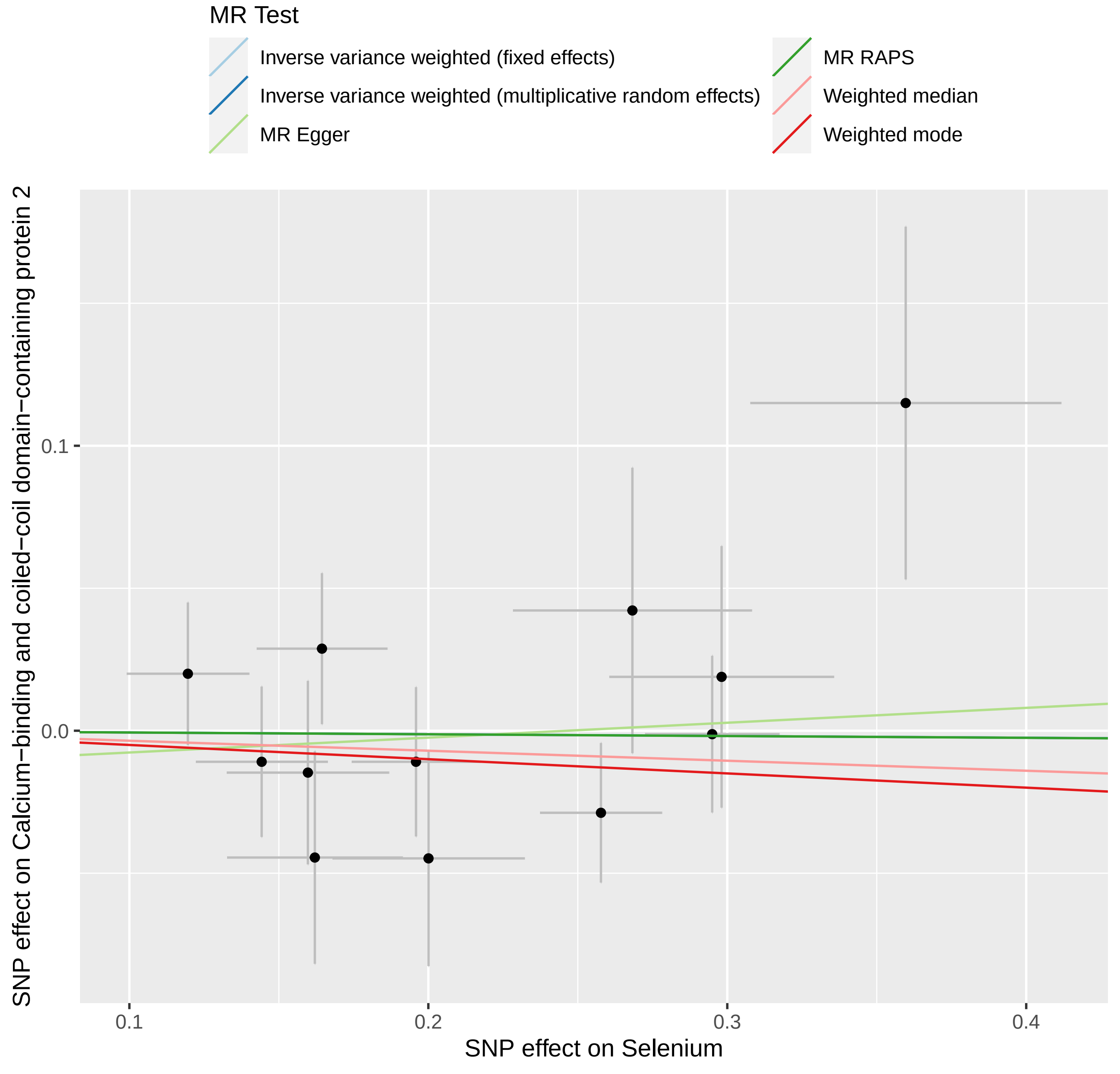

Batch 3−343 : Selenium on Calcium−binding protein 8

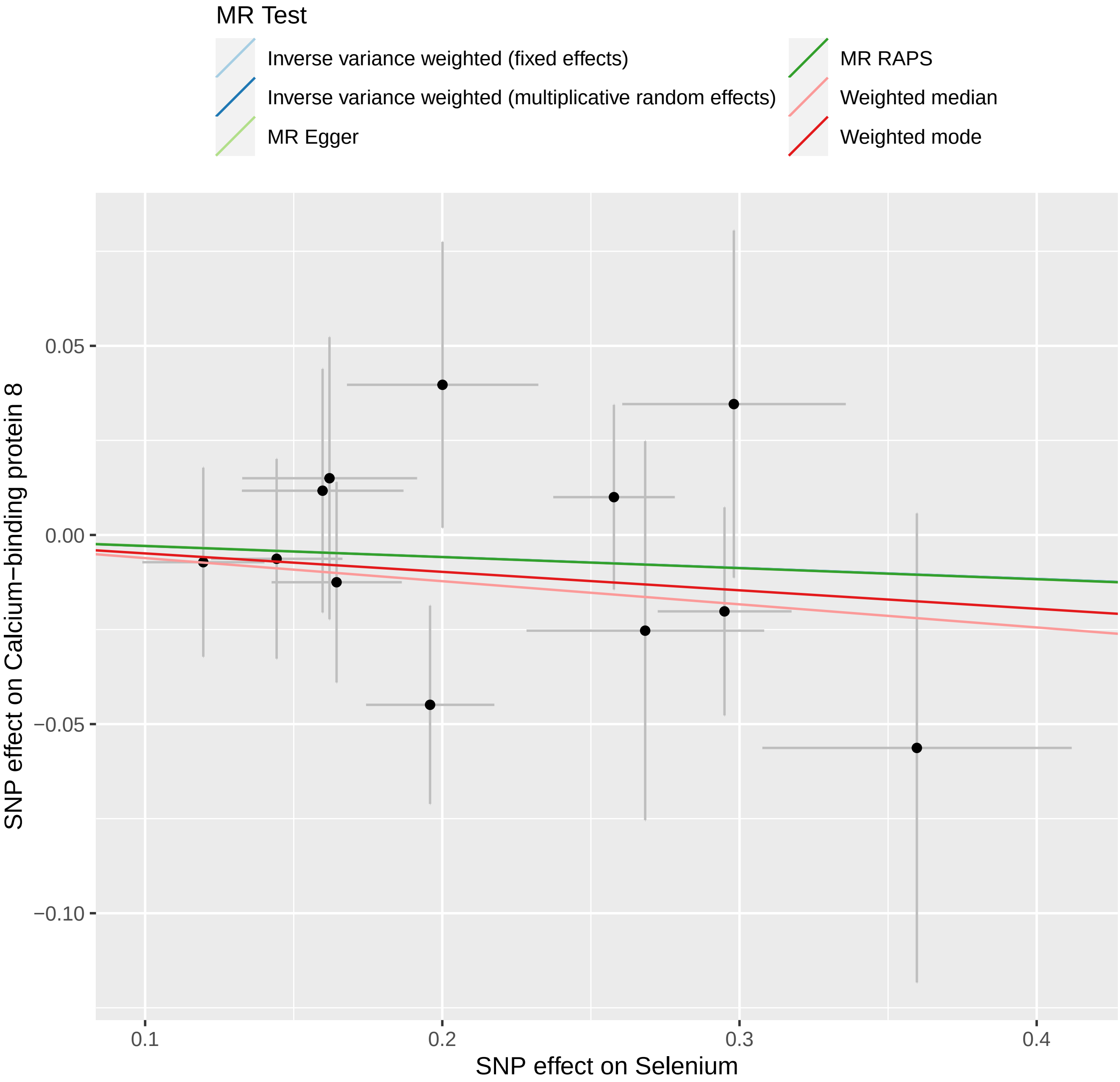

Batch 3−344 : Selenium on Calreticulin

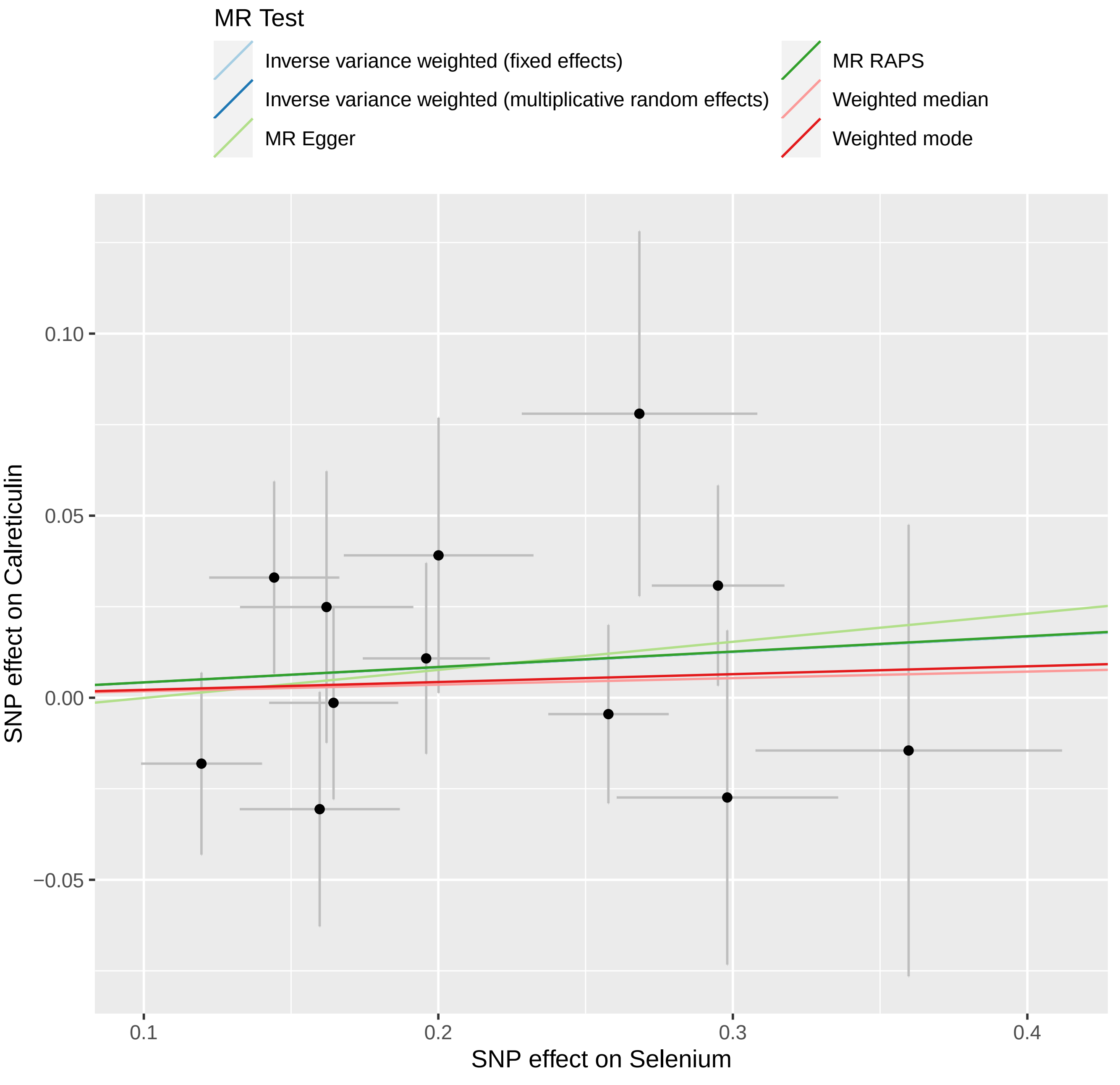

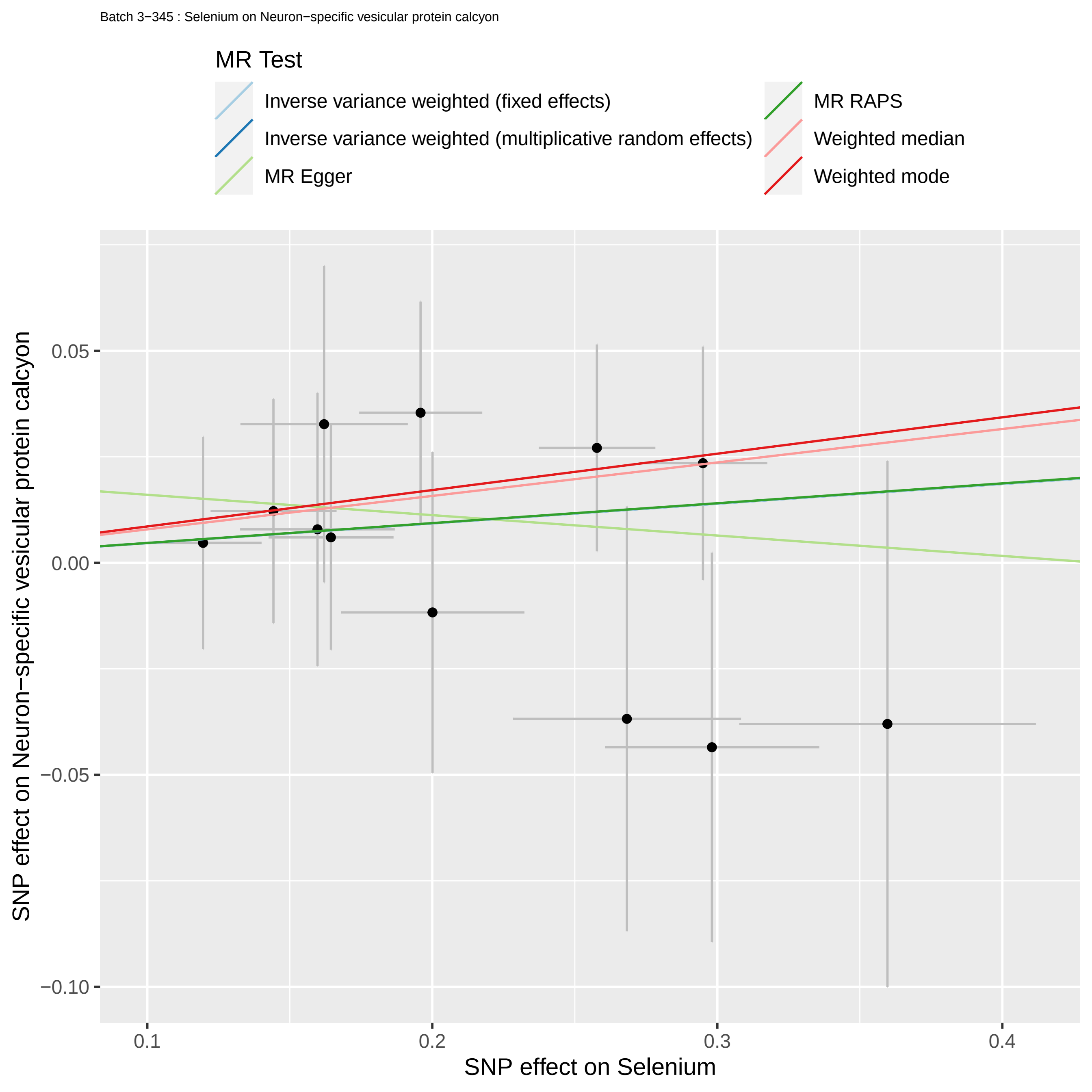
Batch 3−345 : Selenium on Neuron−specific vesicular protein calcyon
MR Test
Inverse variance weighted (fixed effects)
Inverse variance weighted (multiplicative random effects)
MR Egger
MR RAPS
Weighted median
Weighted mode
0.05
0.00
−0.05
−0.10
0.1
0.2
0.3
0.4
SNP effect on Selenium
SNP effect on Neuron−specific vesicular protein calcyon

Batch 3−346 : Selenium on Calcium/calmodulin−dependent protein kinase type 1

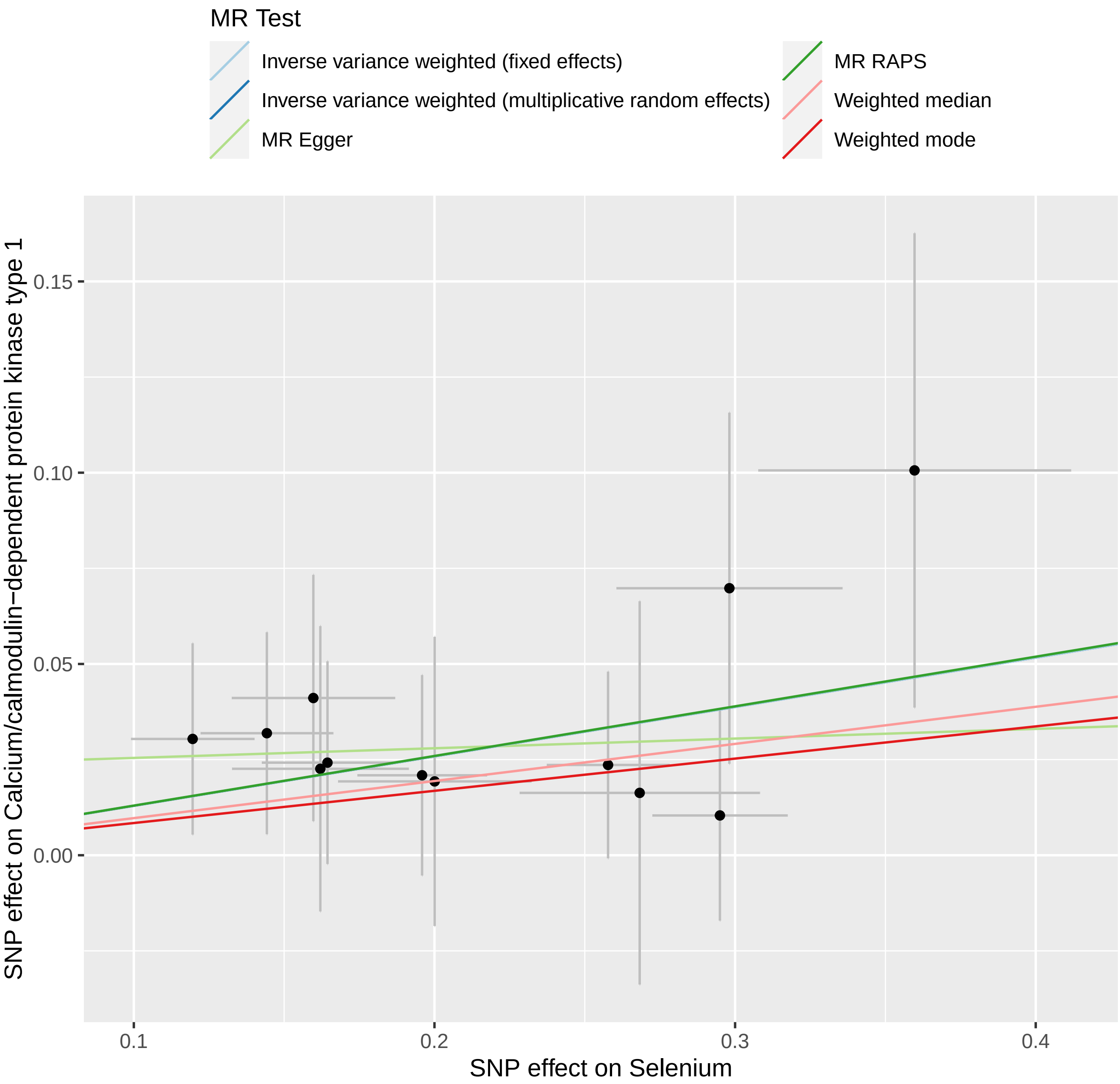

Batch 3−347 : Selenium on Calcium/calmodulin−dependent protein kinase type 1D

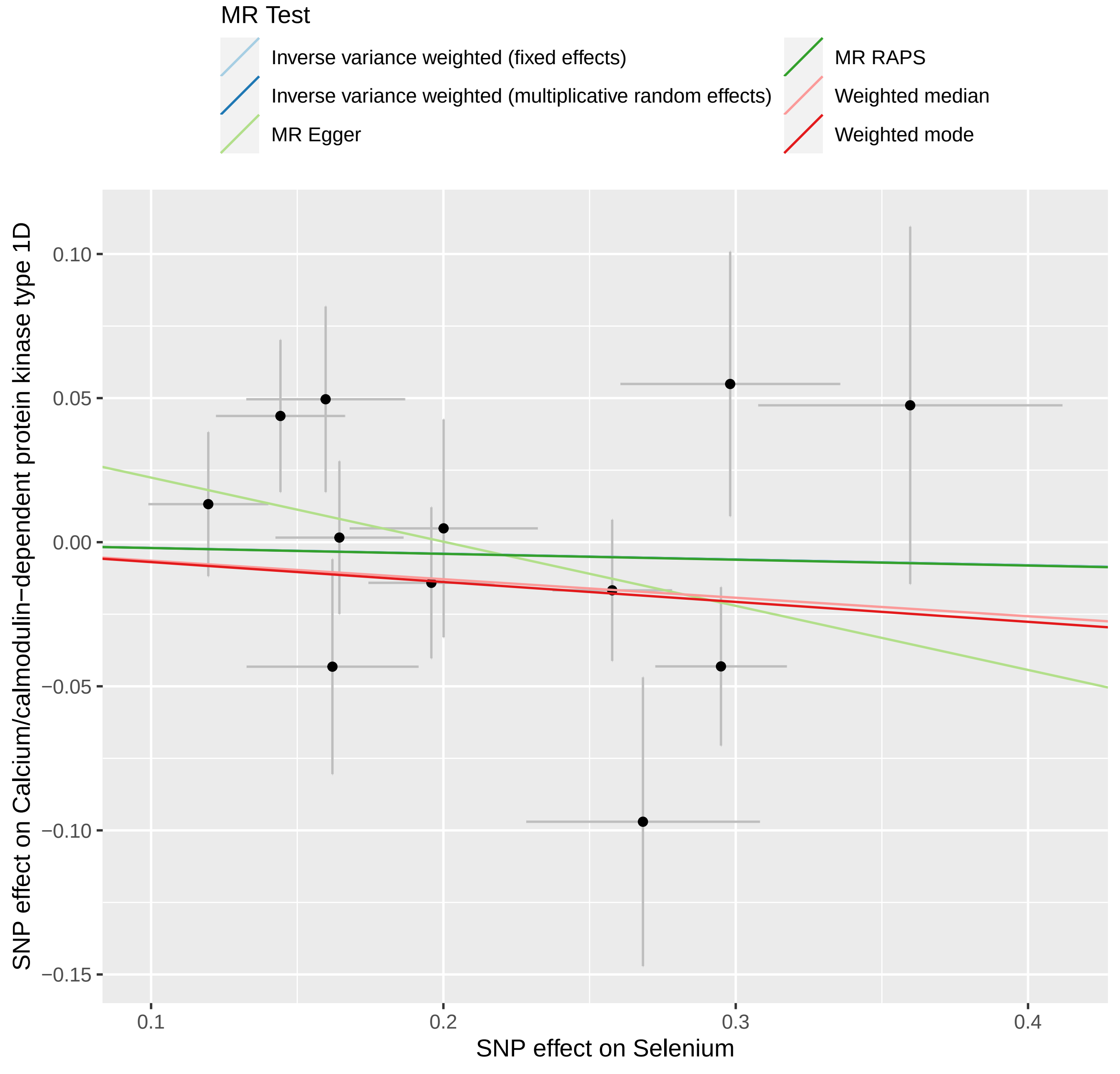

Batch 3−348 : Selenium on Calcium/calmodulin−dependent protein kinase type II subunit alpha

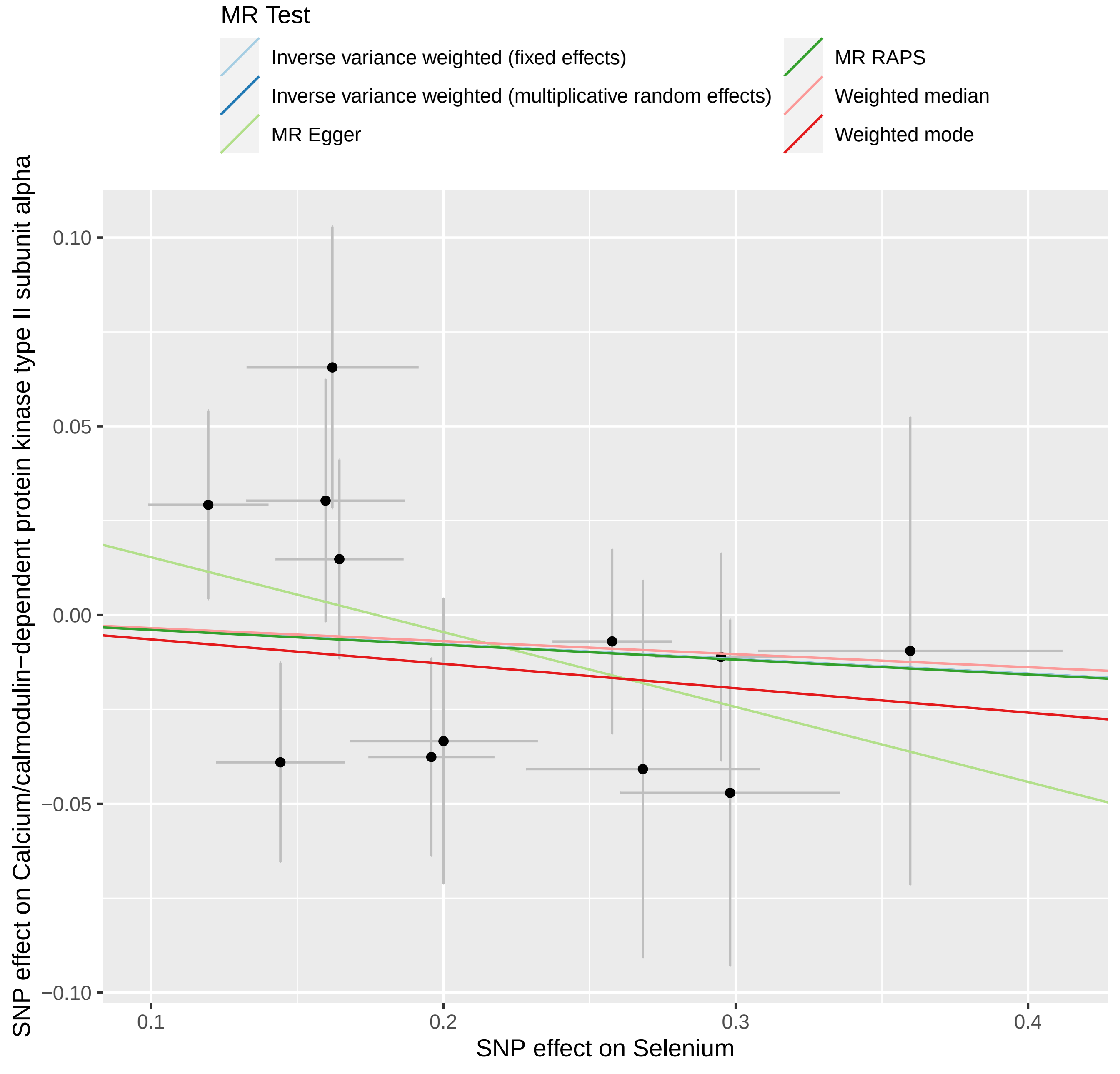

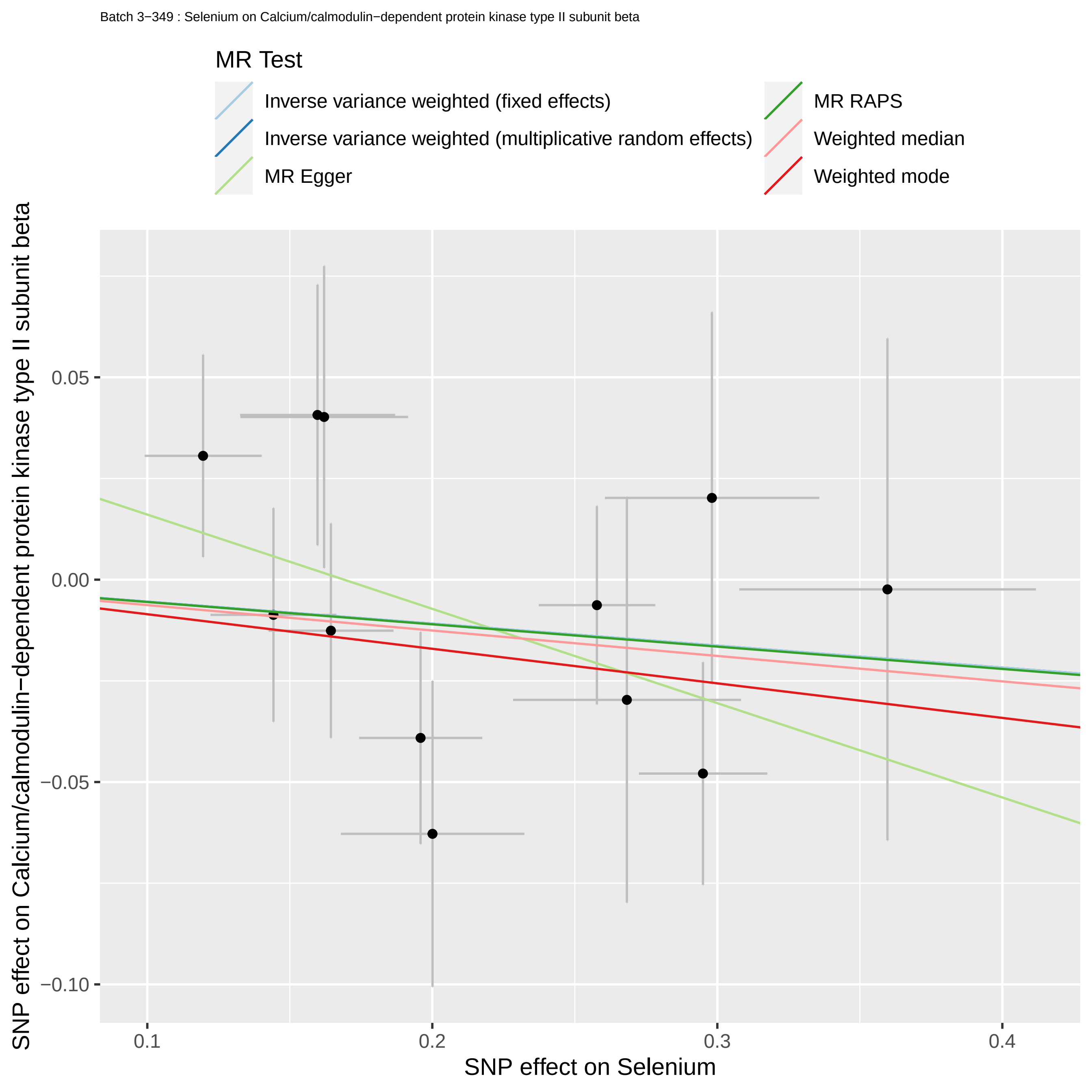

Batch 3−349 : Selenium on Calcium/calmodulin−dependent protein kinase type II subunit beta
MR Test
Inverse variance weighted (fixed effects)
Inverse variance weighted (multiplicative random effects)
MR Egger
MR RAPS
Weighted median
Weighted mode
SNP effect on Calcium/calmodulin−dependent protein kinase type II subunit beta
0.05
0.00
−0.05
−0.10
0.1
0.2
0.3
0.4
SNP effect on Selenium

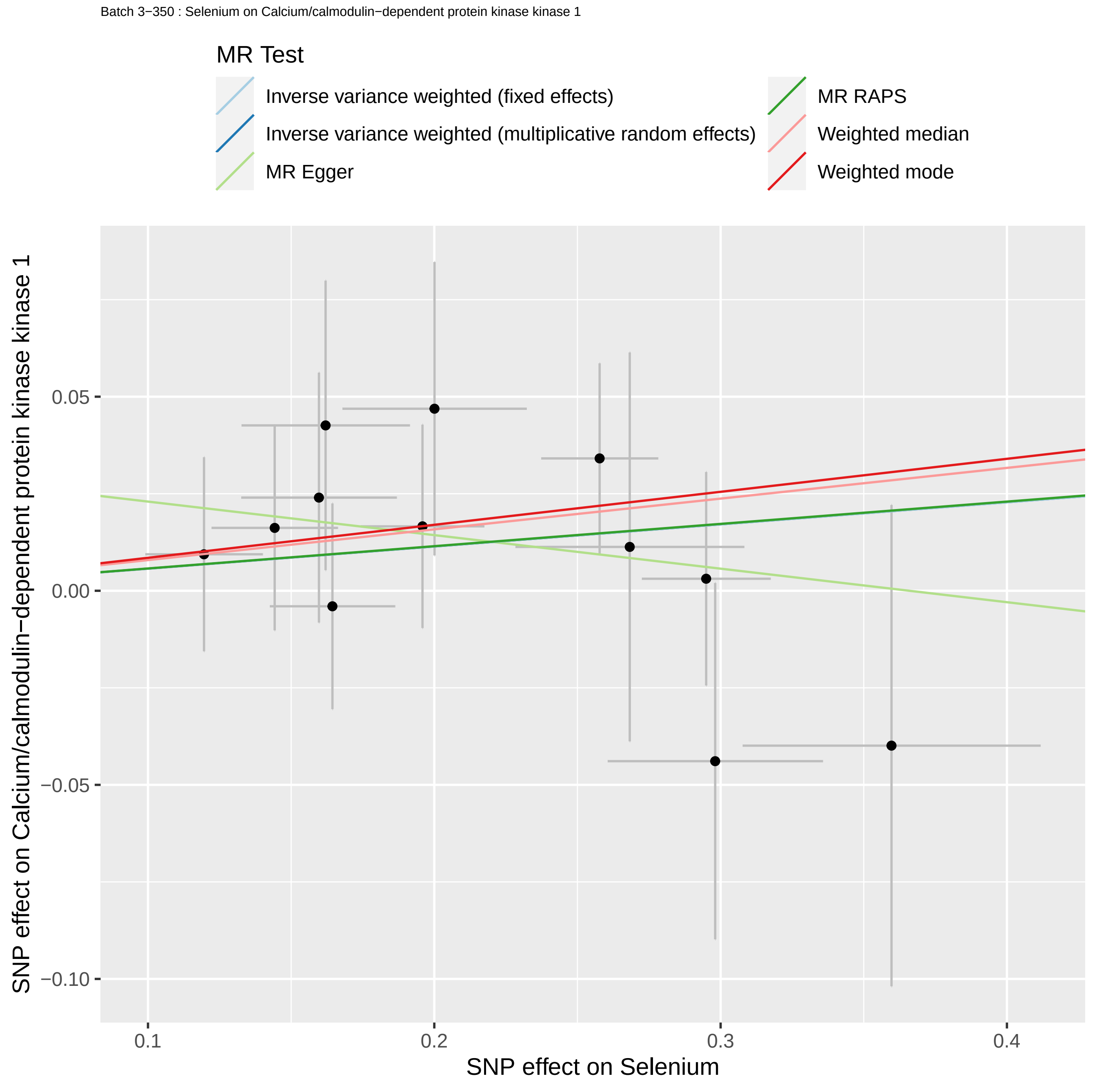
Batch 3−350 : Selenium on Calcium/calmodulin−dependent protein kinase kinase 1
MR Test
Inverse variance weighted (fixed effects)
Inverse variance weighted (multiplicative random effects)
MR Egger
MR RAPS
Weighted median
Weighted mode
0.05
0.00
−0.05
−0.10
0.1
0.2
0.3
0.4
SNP effect on Selenium
SNP effect on Calcium/calmodulin−dependent protein kinase kinase 1

Batch 3−351 : Selenium on Cullin−associated NEDD8−dissociated protein 1

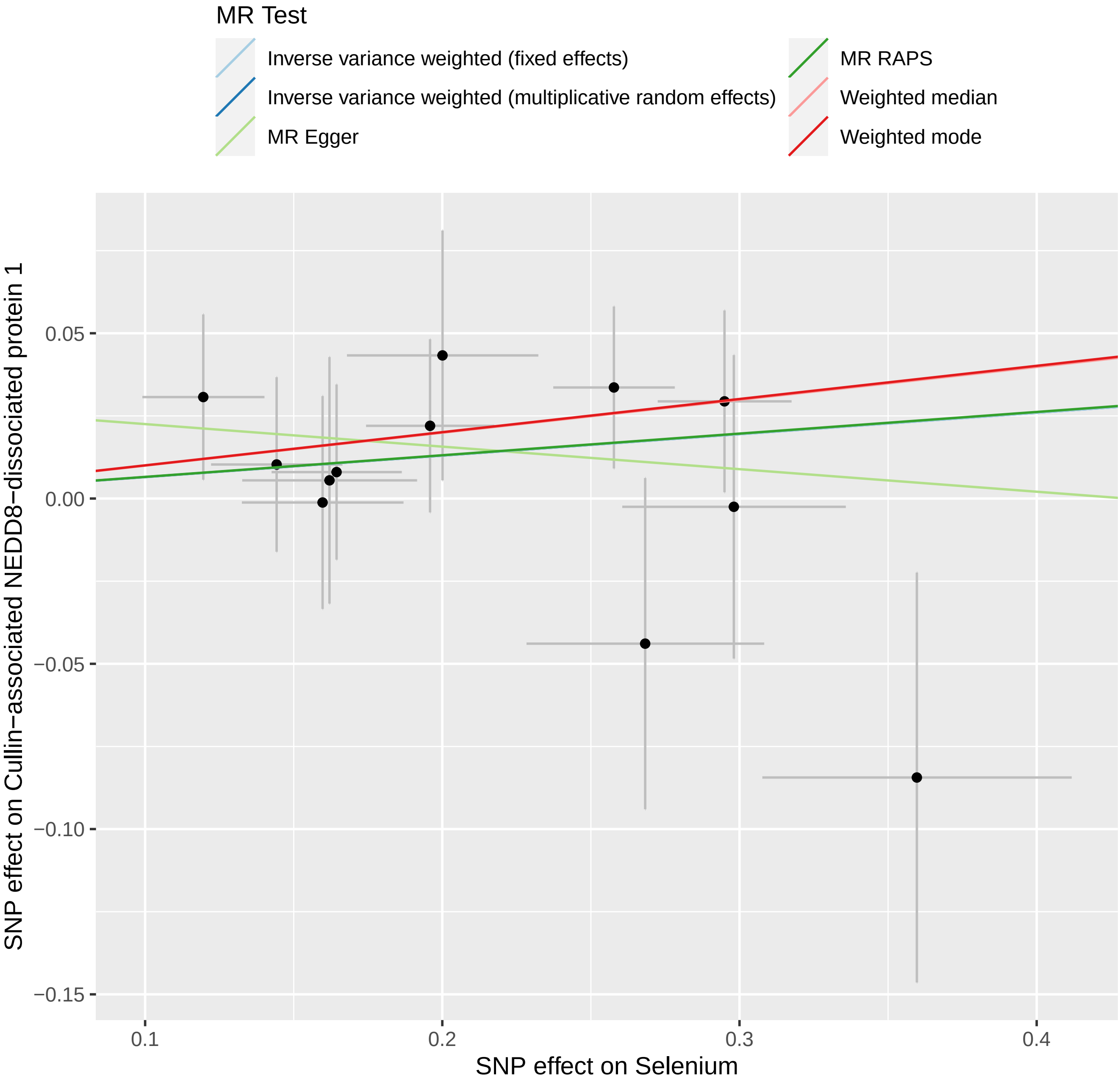

Batch 3−352 : Selenium on Soluble calcium−activated nucleotidase 1

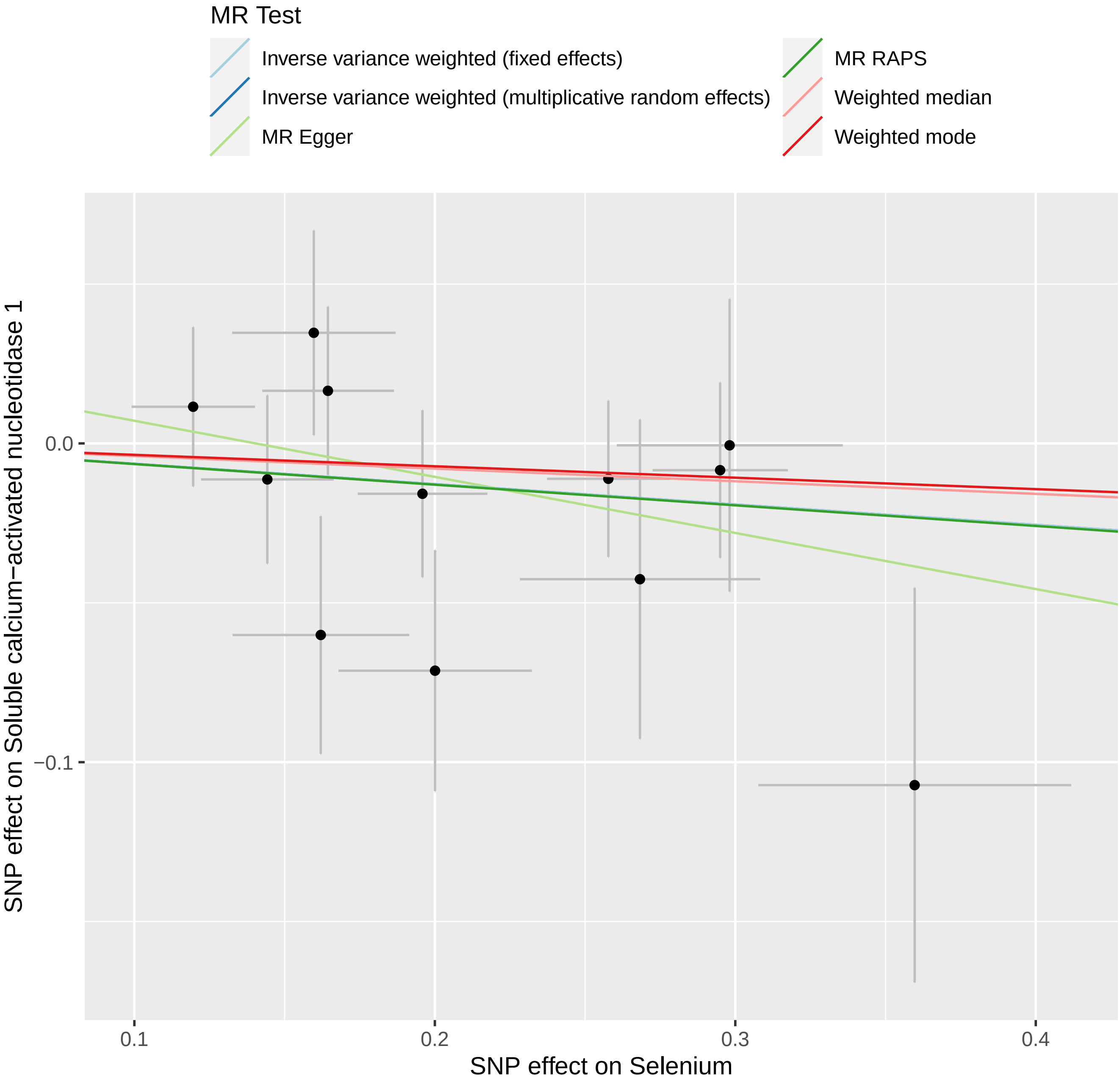

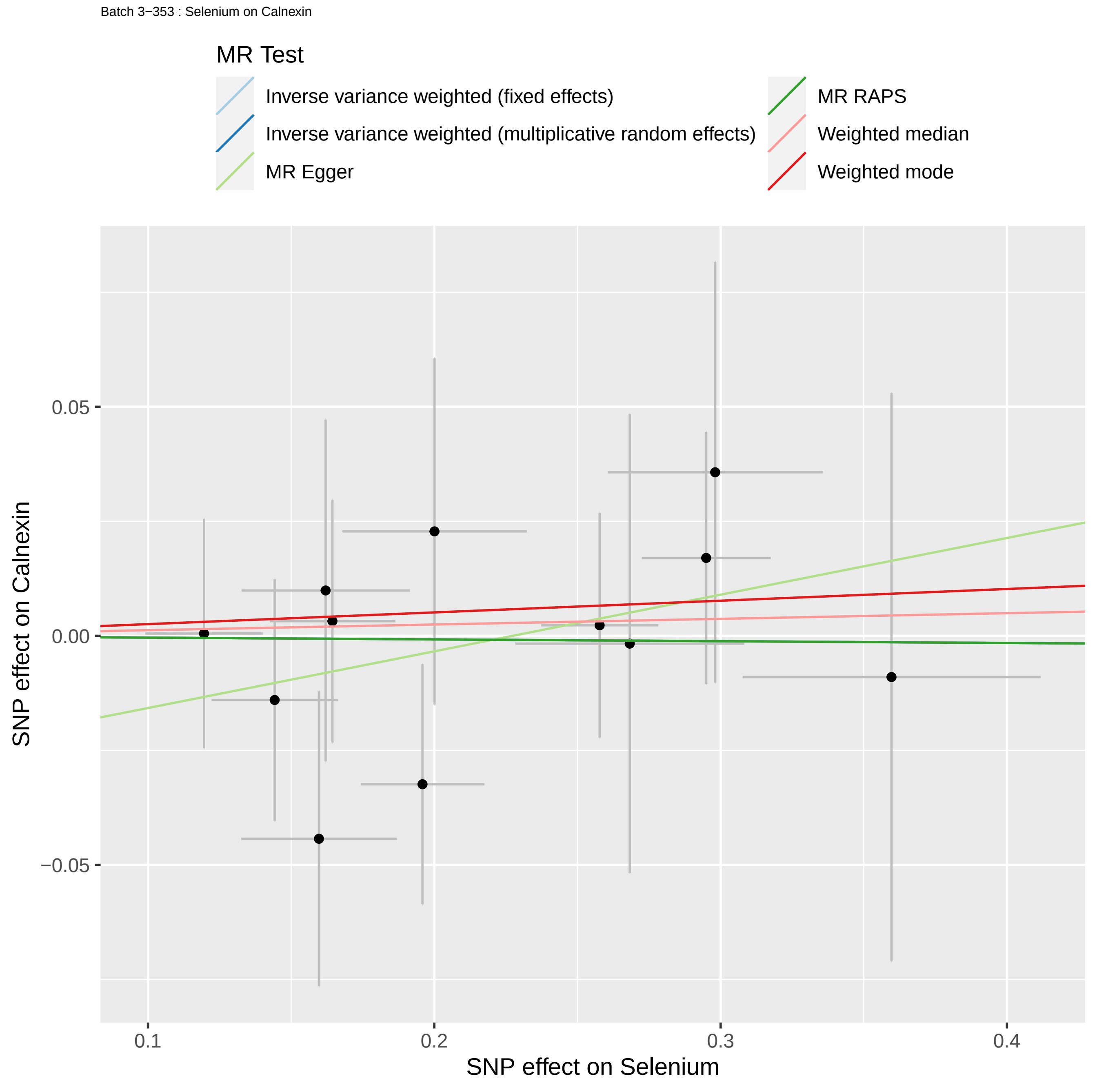

Batch 3−353 : Selenium on Calnexin
MR Test
Inverse variance weighted (fixed effects)
Inverse variance weighted (multiplicative random effects)
MR Egger
MR RAPS
Weighted median
Weighted mode
0.05
0.00
−0.05
0.1
0.2
0.3
0.4
SNP effect on Calnexin
SNP effect on Selenium

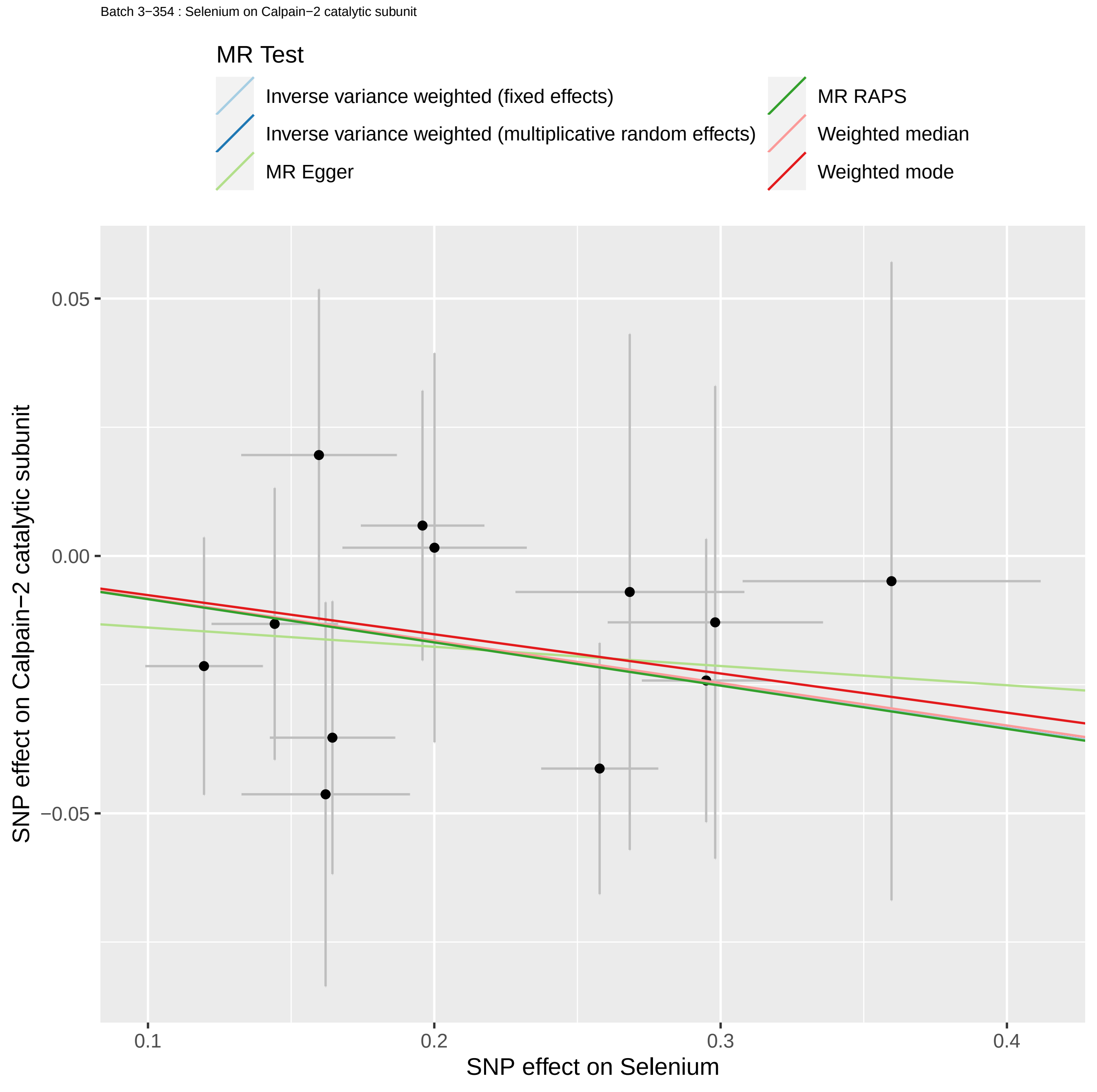

Batch 3−354 : Selenium on Calpain−2 catalytic subunit
MR Test
Inverse variance weighted (fixed effects)
Inverse variance weighted (multiplicative random effects)
MR Egger
MR RAPS
Weighted median
Weighted mode
0.05
0.00
−0.05
0.1
0.2
0.3
0.4
SNP effect on Selenium
SNP effect on Calpain−2 catalytic subunit

Batch 3−355 : Selenium on Calpain−3

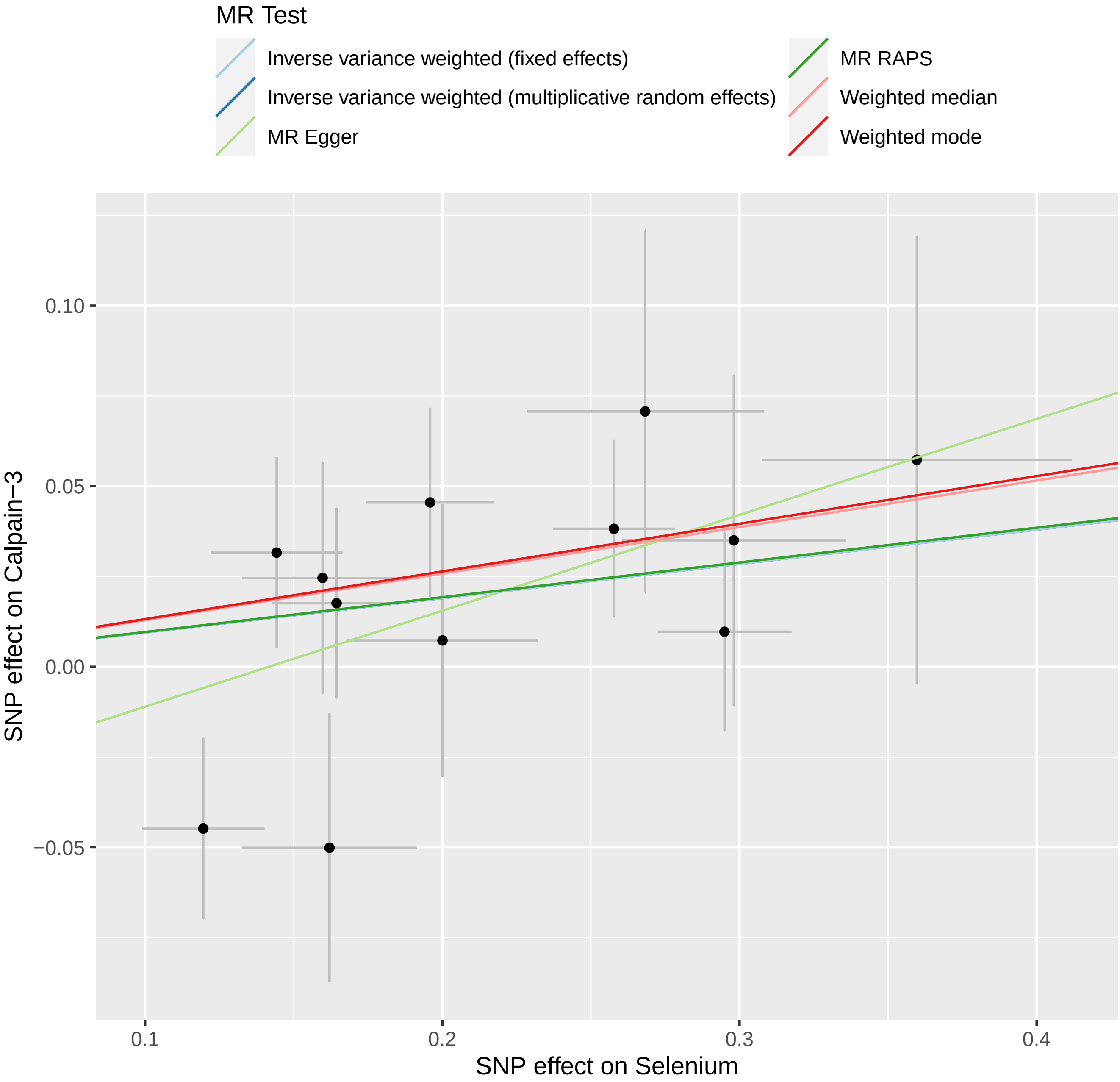

Batch 3−356 : Selenium on Calcyphosin−like protein

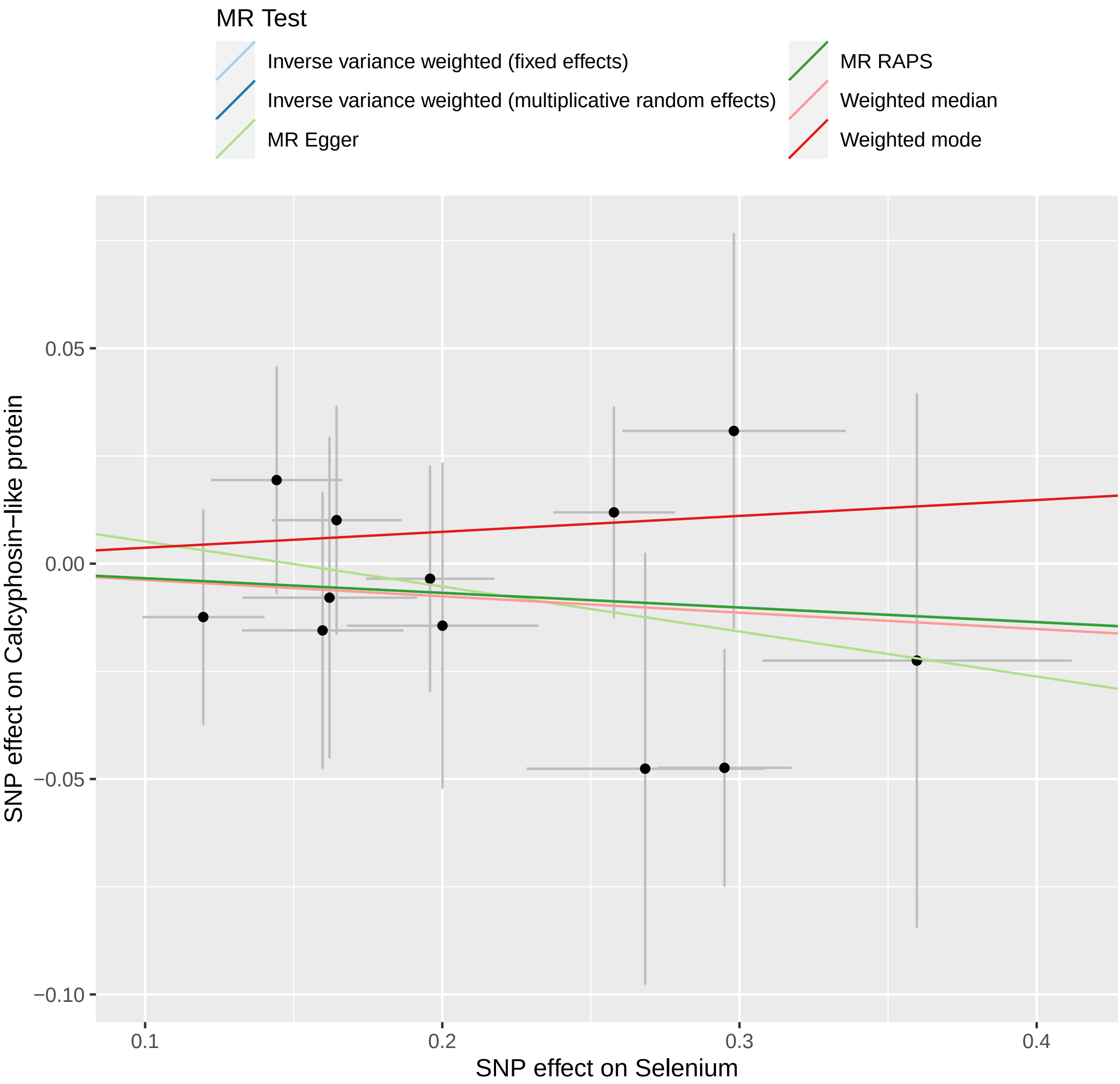

Batch 3−357 : Selenium on Calcium−regulated heat stable protein 1

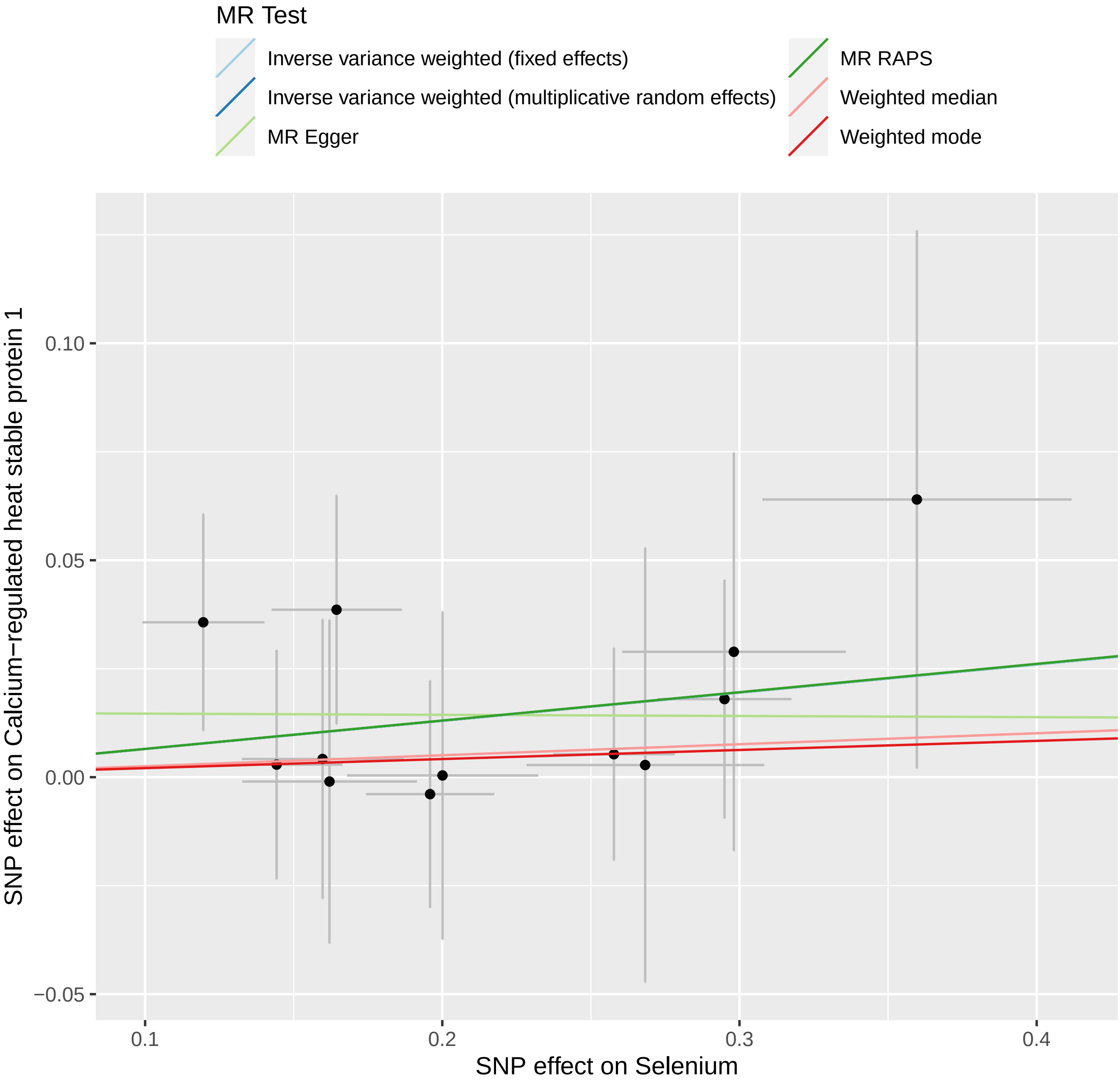

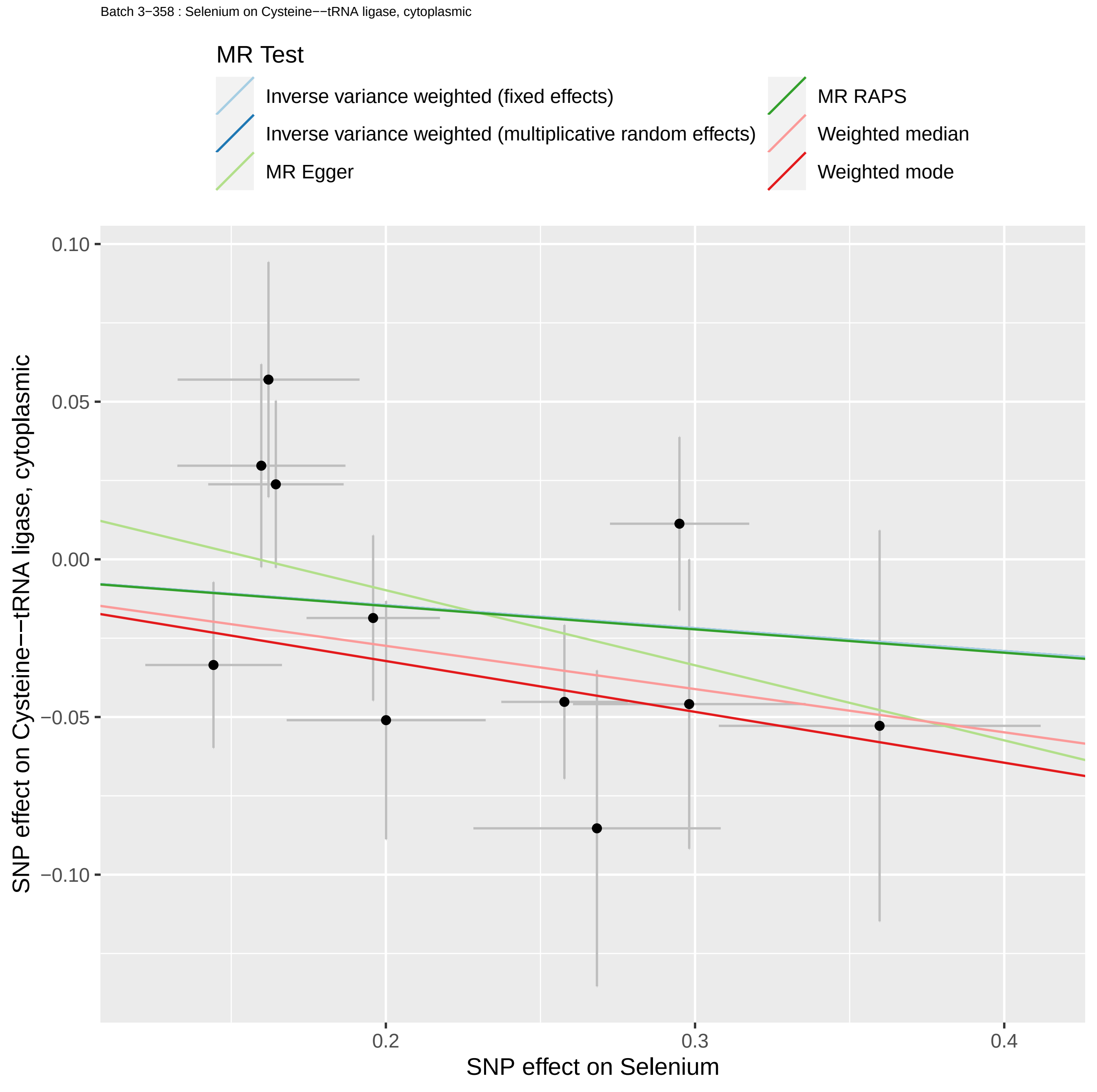

Batch 3−358 : Selenium on Cysteine−−tRNA ligase, cytoplasmic
MR Test
Inverse variance weighted (fixed effects)
Inverse variance weighted (multiplicative random effects)
MR Egger
MR RAPS
Weighted median
Weighted mode
0.10
0.05
0.00
−0.05
−0.10
0.2
0.3
0.4
SNP effect on Selenium
SNP effect on Cysteine−−tRNA ligase, cytoplasmic

Batch 3−359 : Selenium on Cocaine− and amphetamine−regulated transcript protein

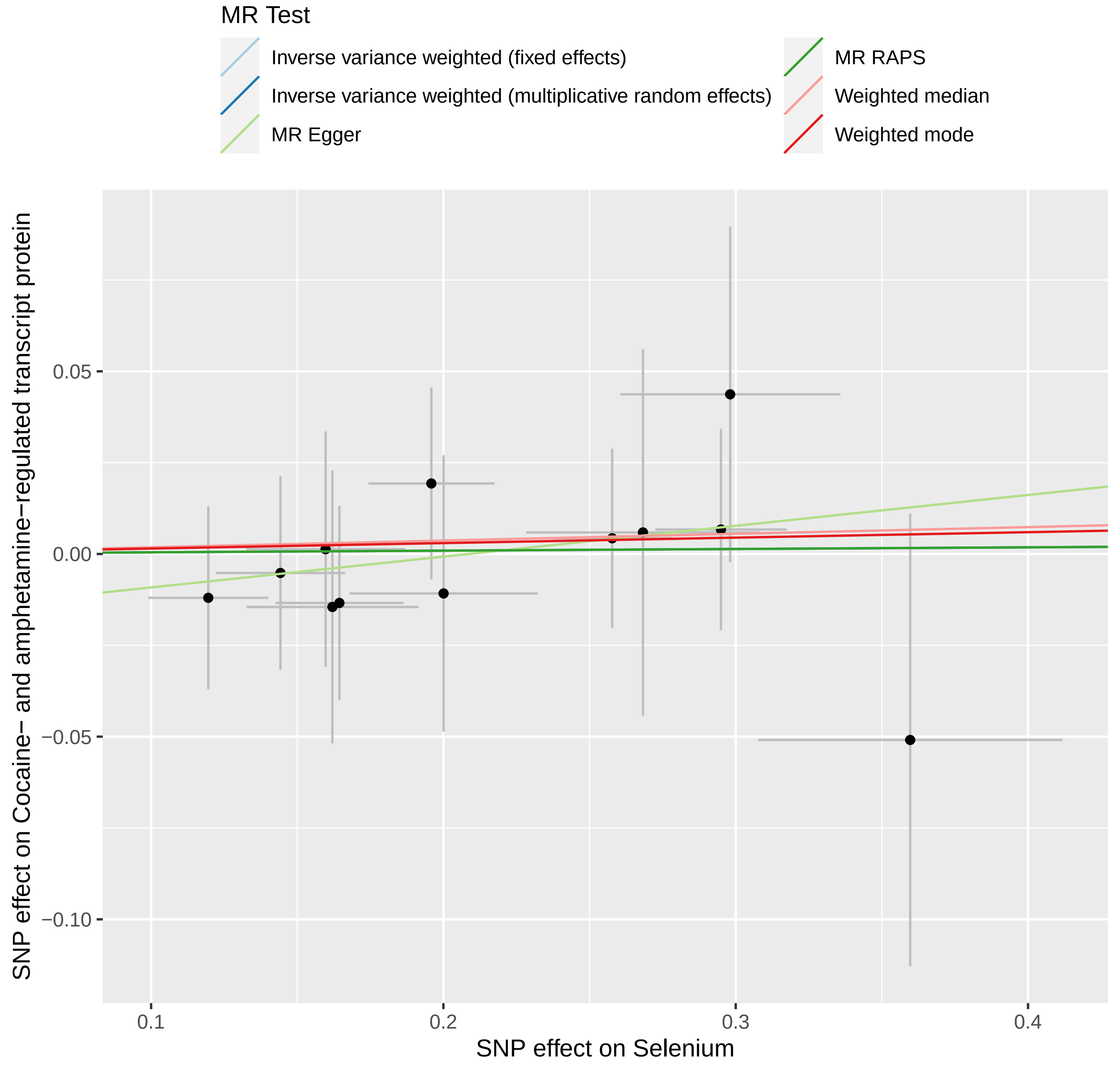

Batch 3−360 : Selenium on Protein CASC4

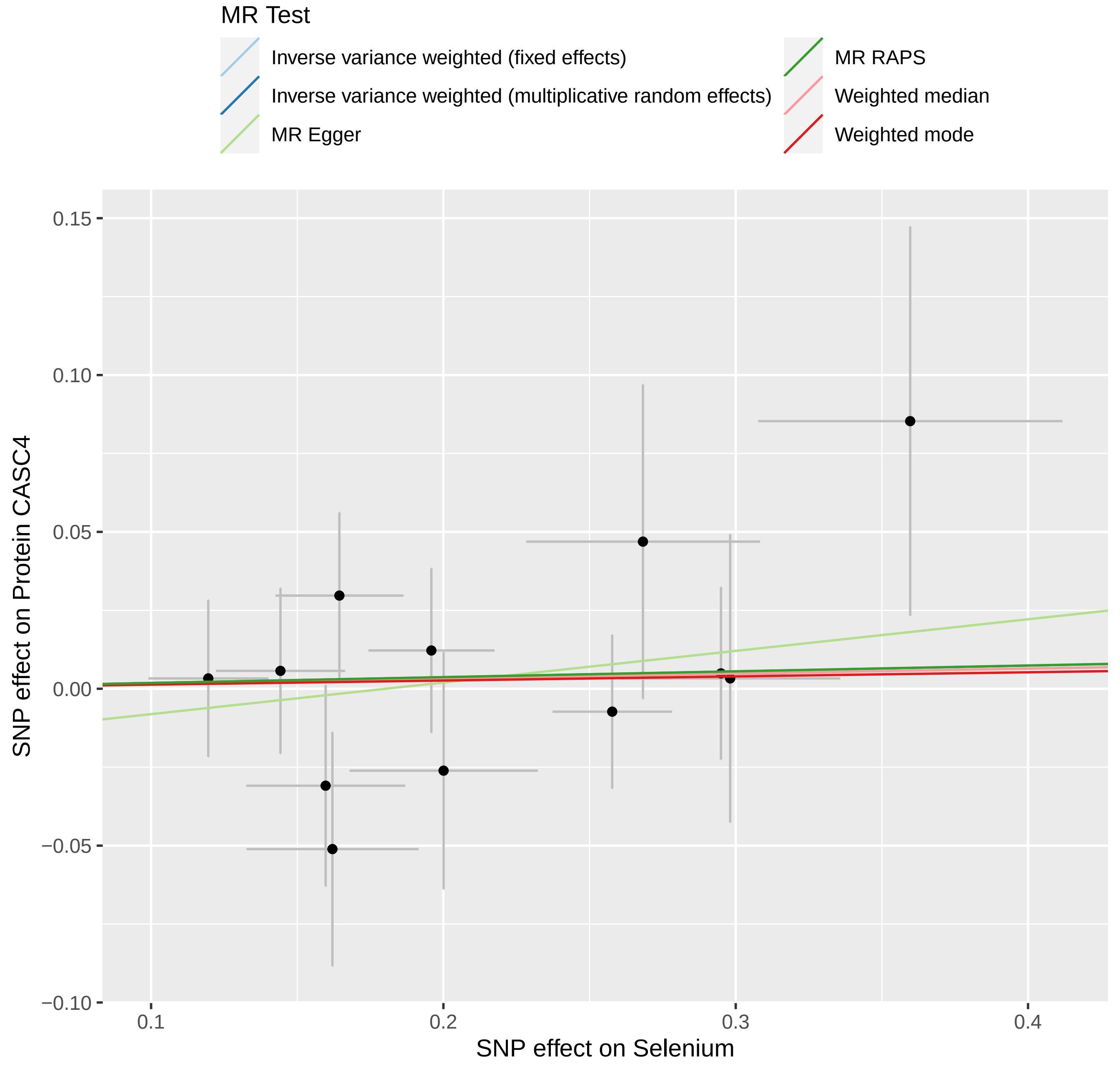

Batch 3−361 : Selenium on Protein CASC4

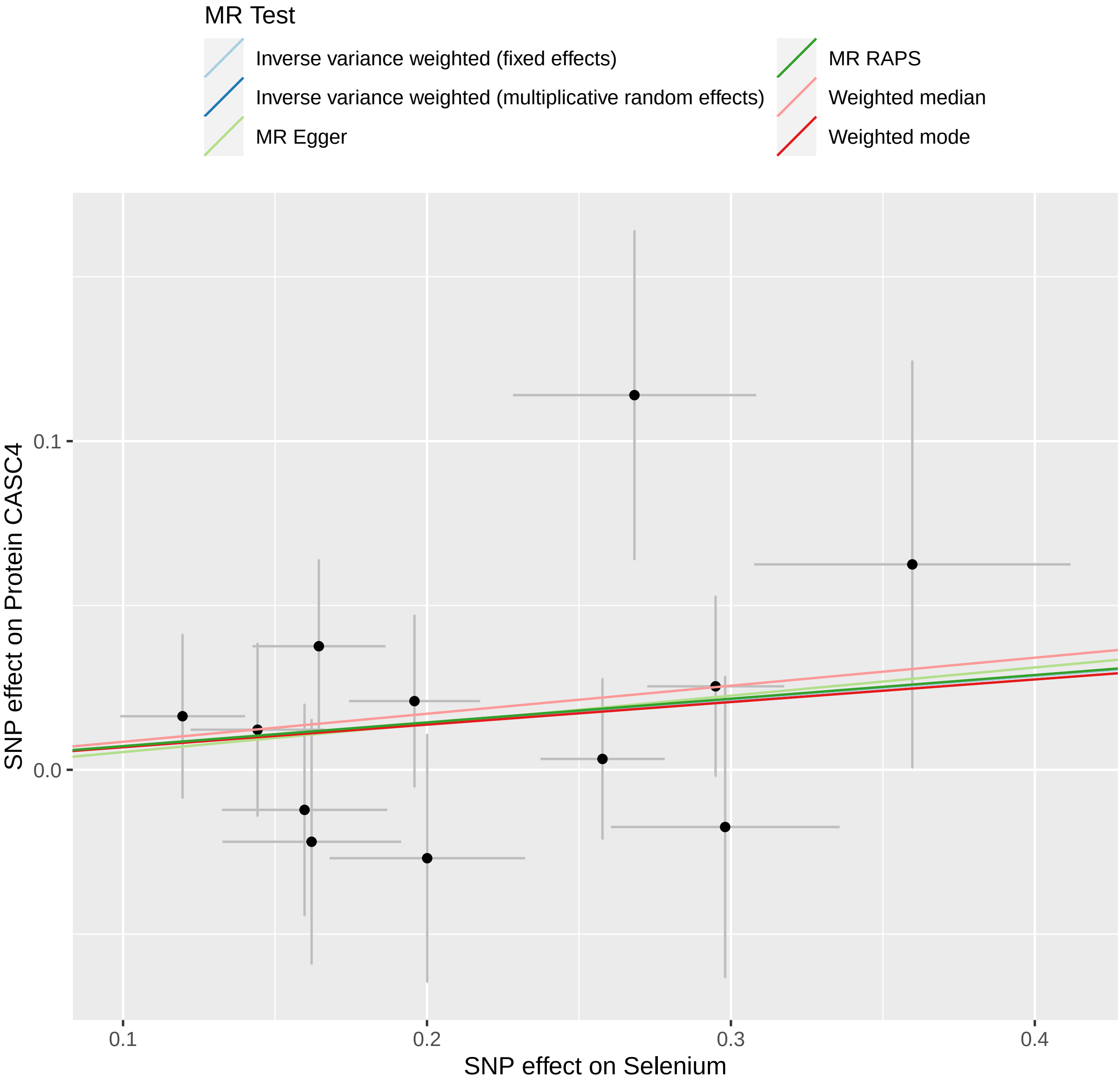

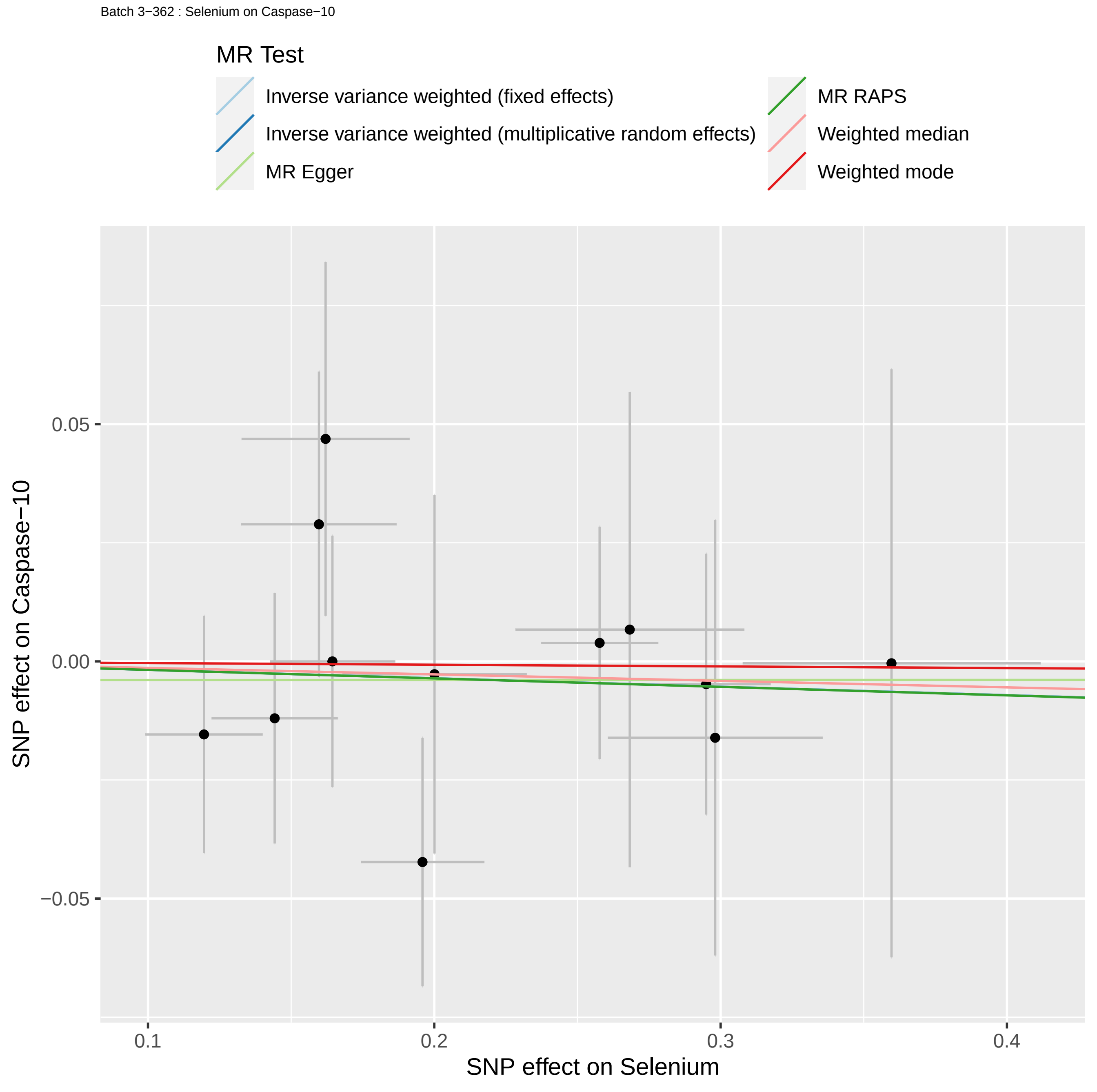

Batch 3−362 : Selenium on Caspase−10
MR Test
Inverse variance weighted (fixed effects)
Inverse variance weighted (multiplicative random effects)
MR Egger
MR RAPS
Weighted median
Weighted mode
0.05
0.00
−0.05
0.1
0.2
0.3
0.4
SNP effect on Caspase−10
SNP effect on Selenium

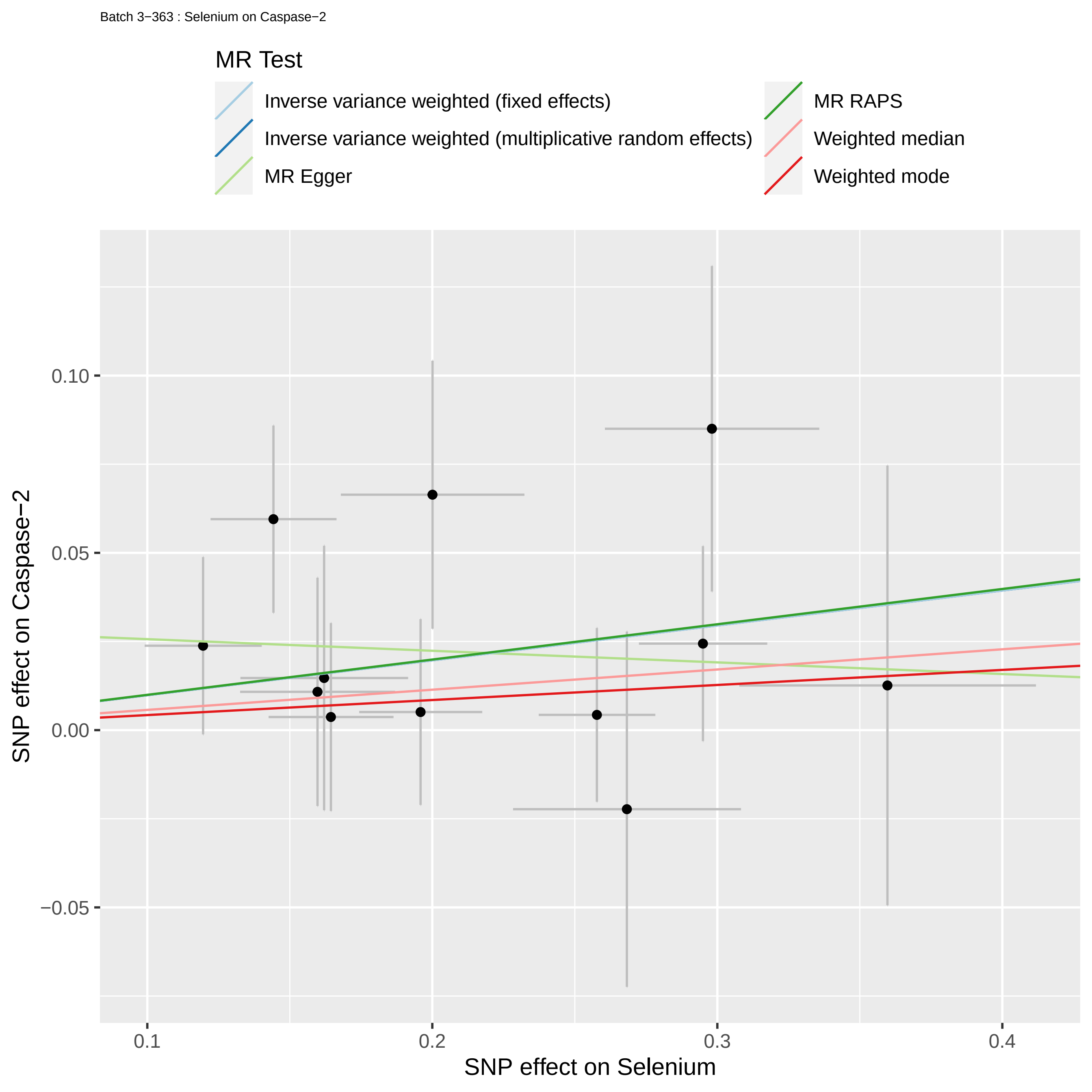
Batch 3−363 : Selenium on Caspase−2
MR Test
Inverse variance weighted (fixed effects)
Inverse variance weighted (multiplicative random effects)
MR Egger
MR RAPS
Weighted median
Weighted mode
SNP effect on Caspase−2
0.10
0.05
0.00
−0.05
0.1
0.2
0.3
0.4
SNP effect on Selenium

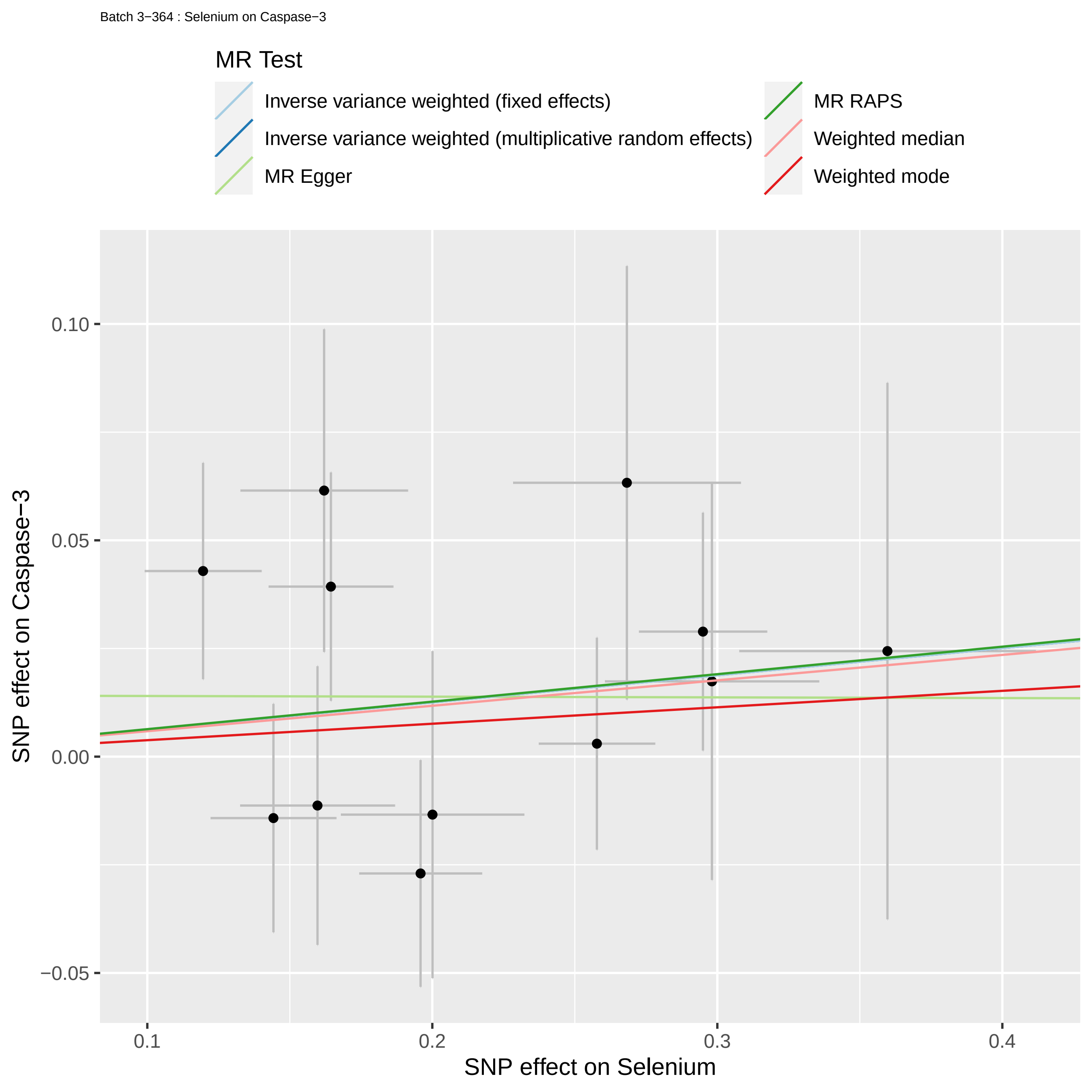

Batch 3−364 : Selenium on Caspase−3
MR Test
Inverse variance weighted (fixed effects)
Inverse variance weighted (multiplicative random effects)
MR Egger
MR RAPS
Weighted median
Weighted mode
0.10
0.05
0.00
−0.05
0.1
0.2
0.3
0.4
SNP effect on Caspase−3
SNP effect on Selenium

Batch 3−365 : Selenium on Cas scaffolding protein family member 4

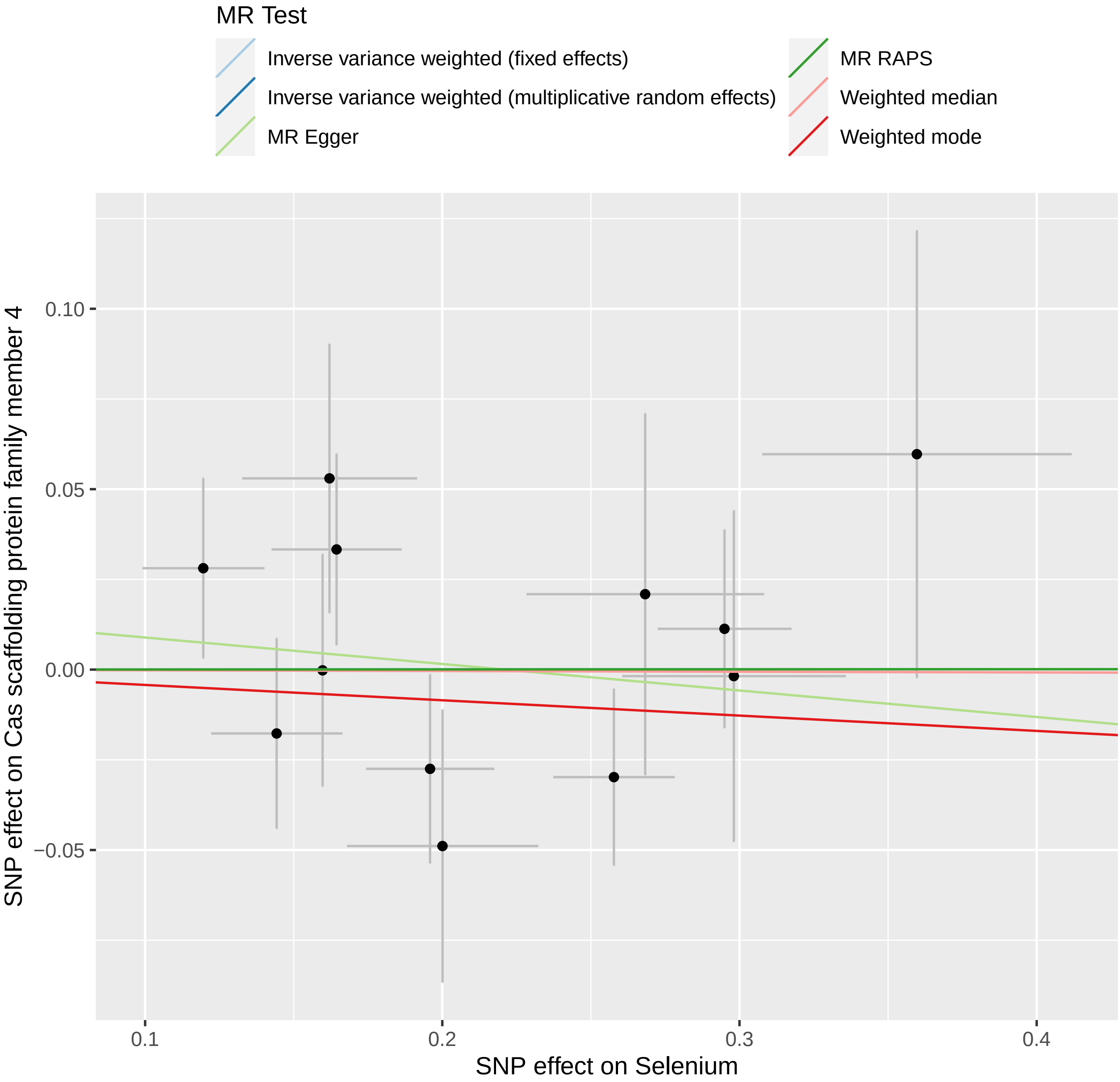

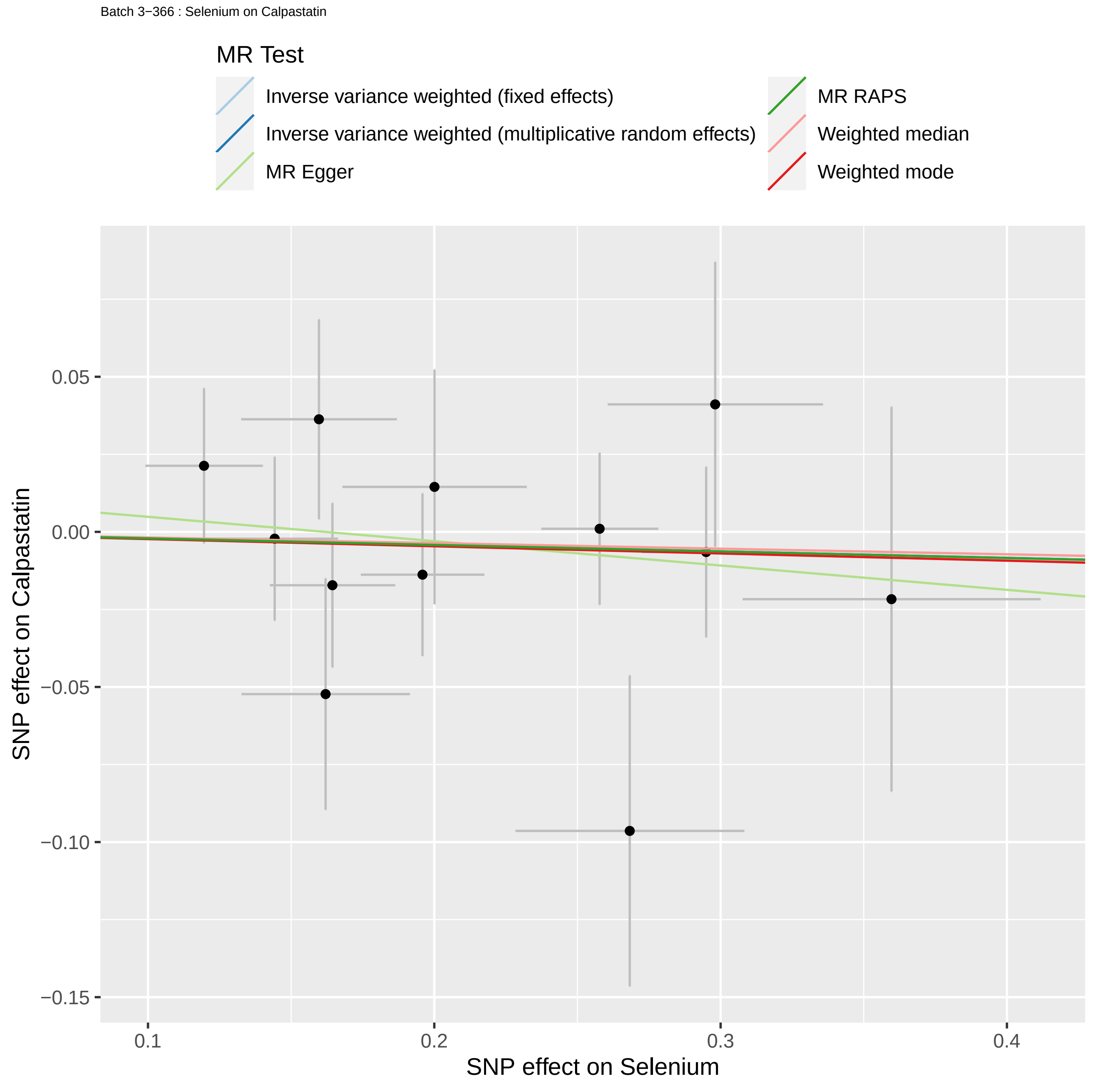

Batch 3−366 : Selenium on Calpastatin
MR Test
Inverse variance weighted (fixed effects)
Inverse variance weighted (multiplicative random effects)
MR Egger
MR RAPS
Weighted median
Weighted mode
0.05
0.00
−0.05
−0.10
−0.15
0.1
0.2
0.3
0.4
SNP effect on Calpastatin
SNP effect on Selenium

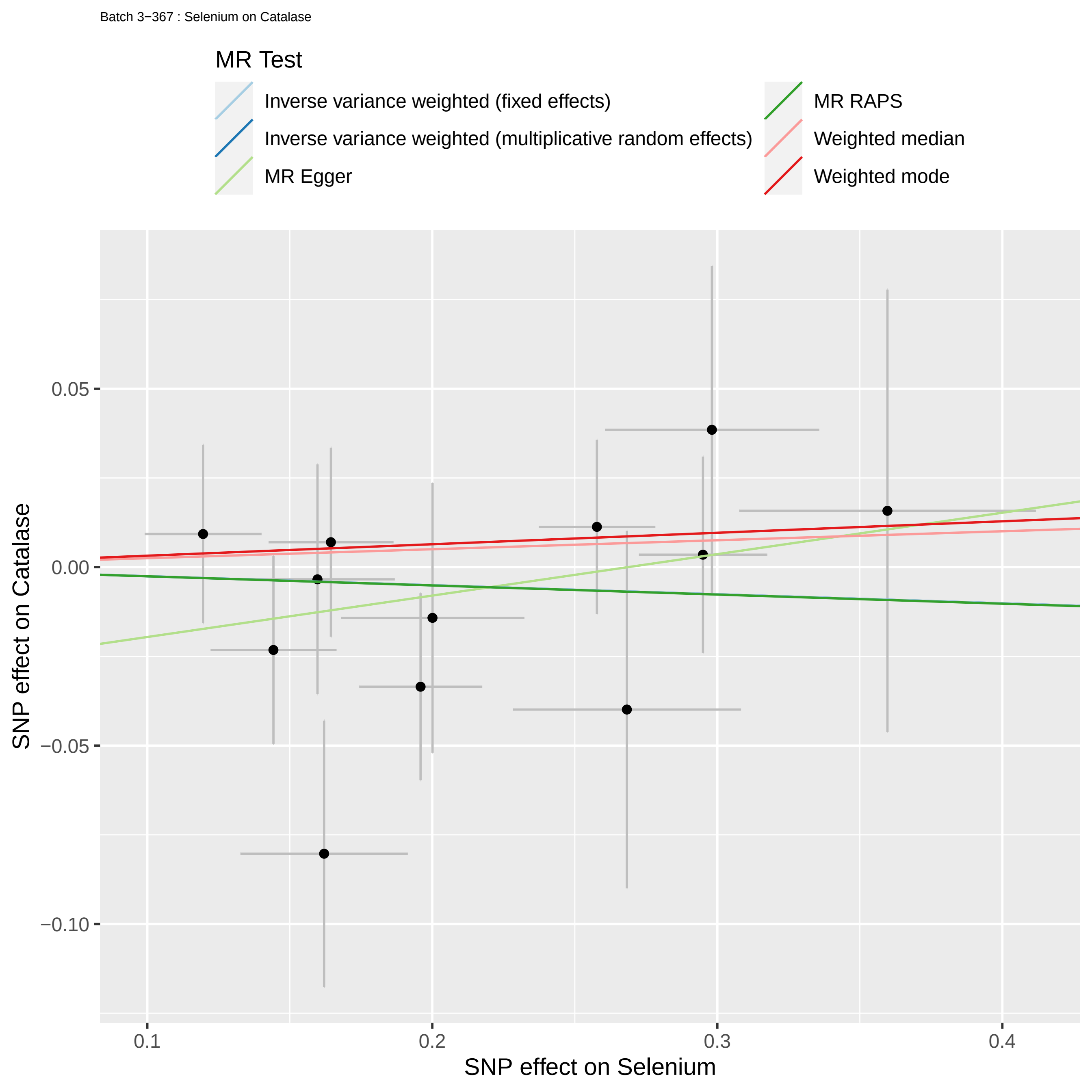

Batch 3−367 : Selenium on Catalase
MR Test
Inverse variance weighted (fixed effects)
Inverse variance weighted (multiplicative random effects)
MR Egger
MR RAPS
Weighted median
Weighted mode
0.05
0.00
−0.05
−0.10
0.1
0.2
0.3
0.4
SNP effect on Selenium
SNP effect on Catalase

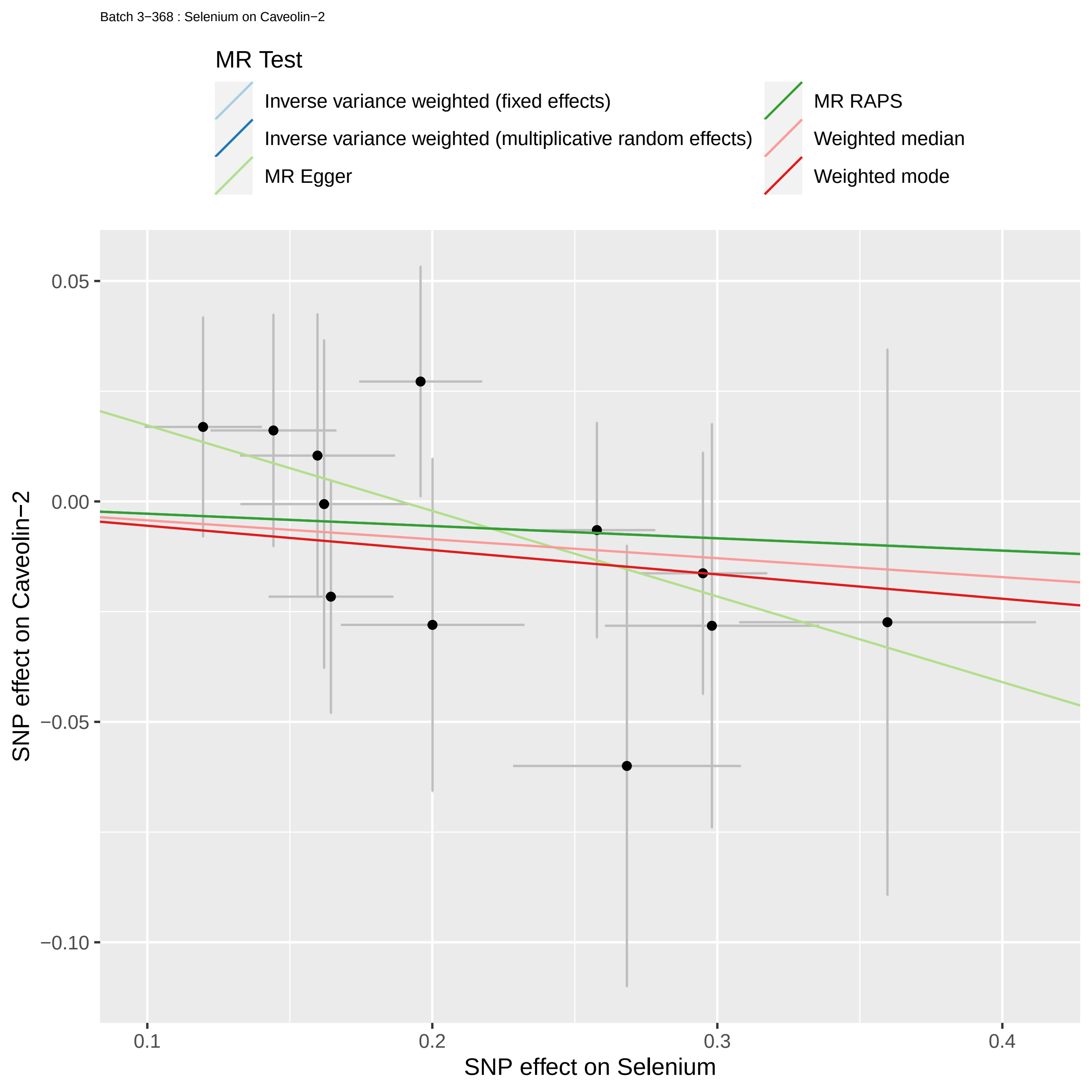
Batch 3−368 : Selenium on Caveolin−2
MR Test
Inverse variance weighted (fixed effects)
Inverse variance weighted (multiplicative random effects)
MR Egger
MR RAPS
Weighted median
Weighted mode
0.05
0.00
−0.05
−0.10
SNP effect on Caveolin−2
0.1
0.2
0.3
0.4
SNP effect on Selenium

Batch 3−369 : Selenium on Core−binding factor subunit beta

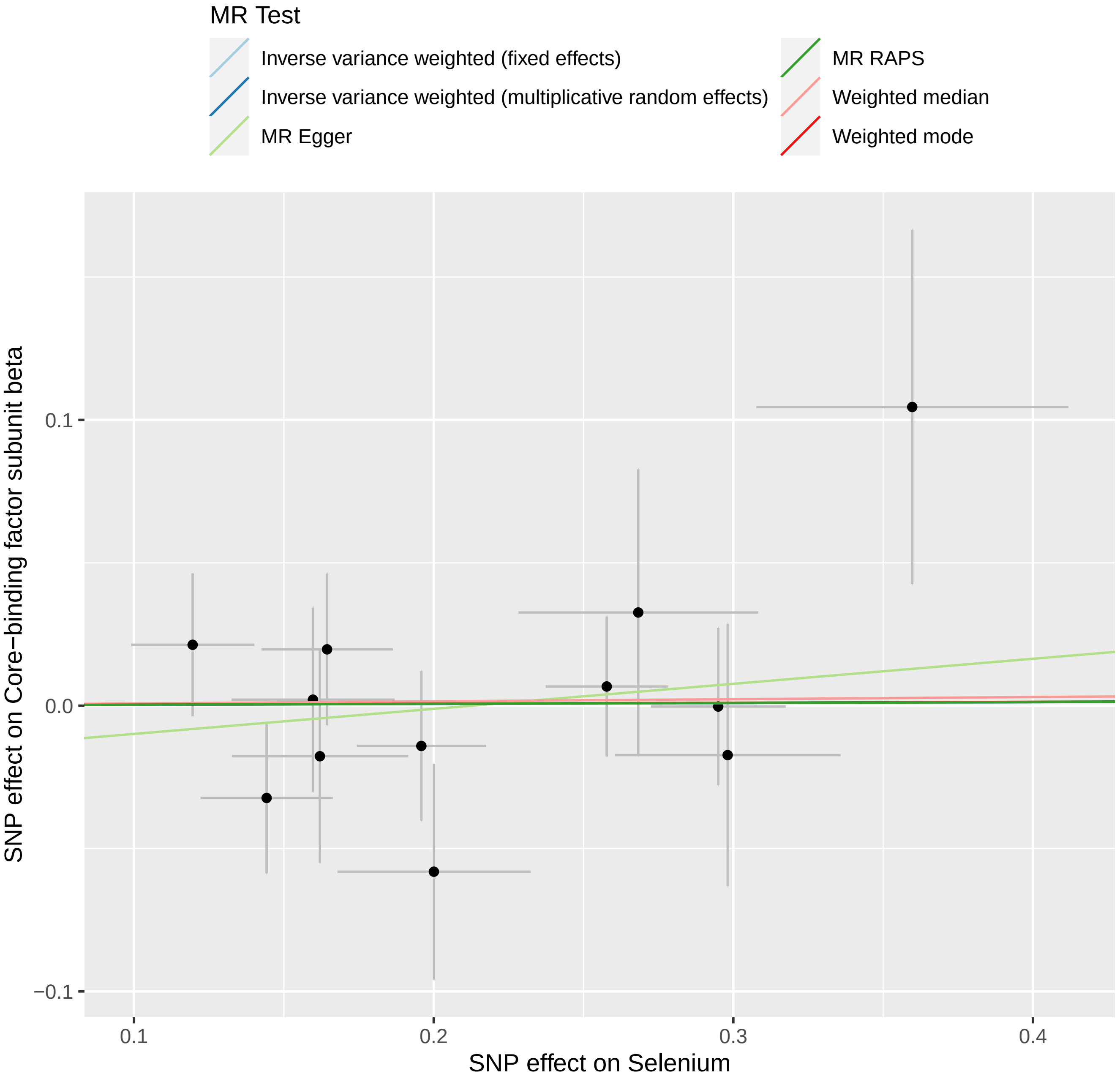

Batch 3−370 : Selenium on E3 ubiquitin−protein ligase CBL

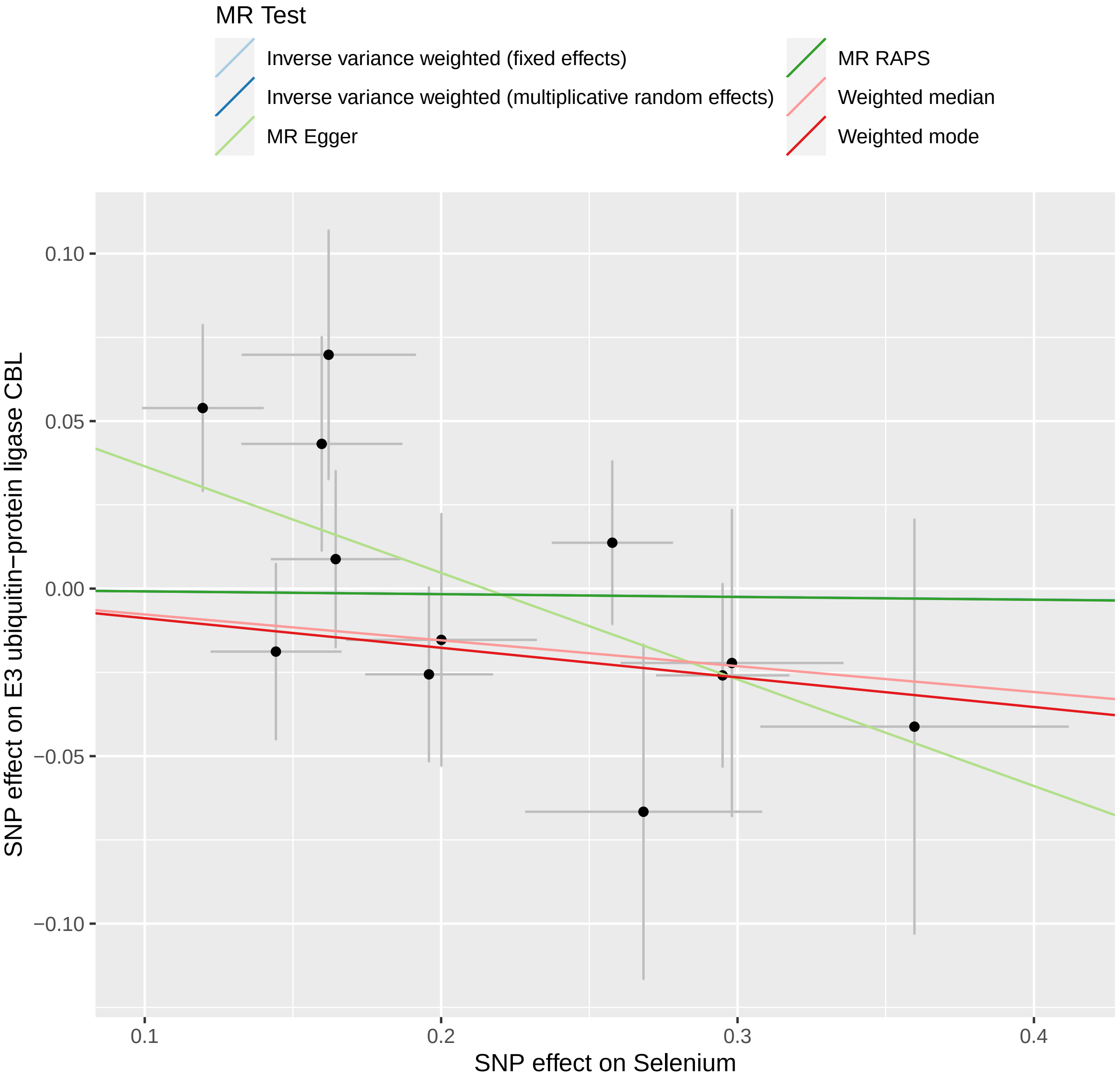

Batch 3−371 : Selenium on Cerebellin−1

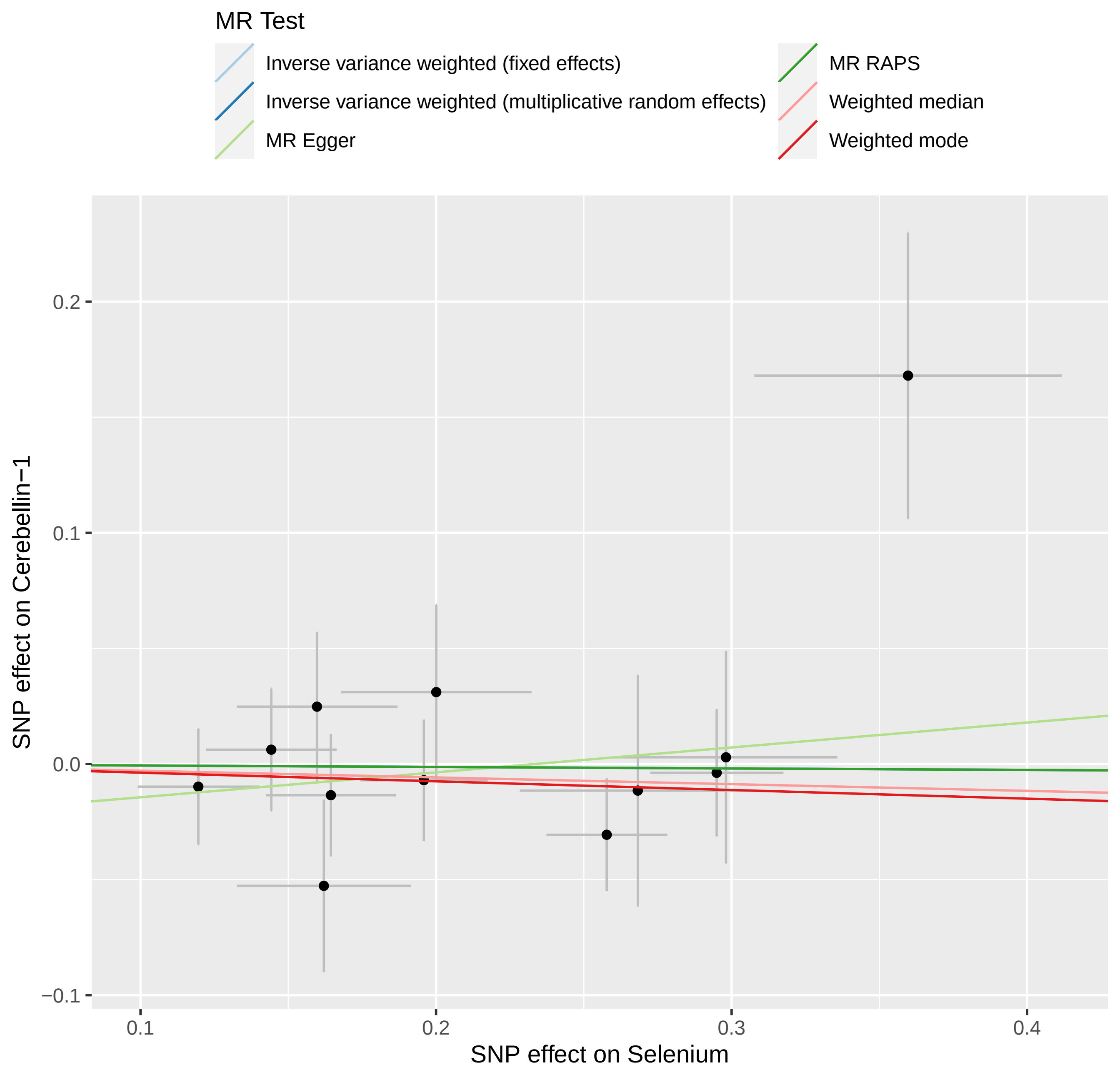

Batch 3−372 : Selenium on Cerebellin−4

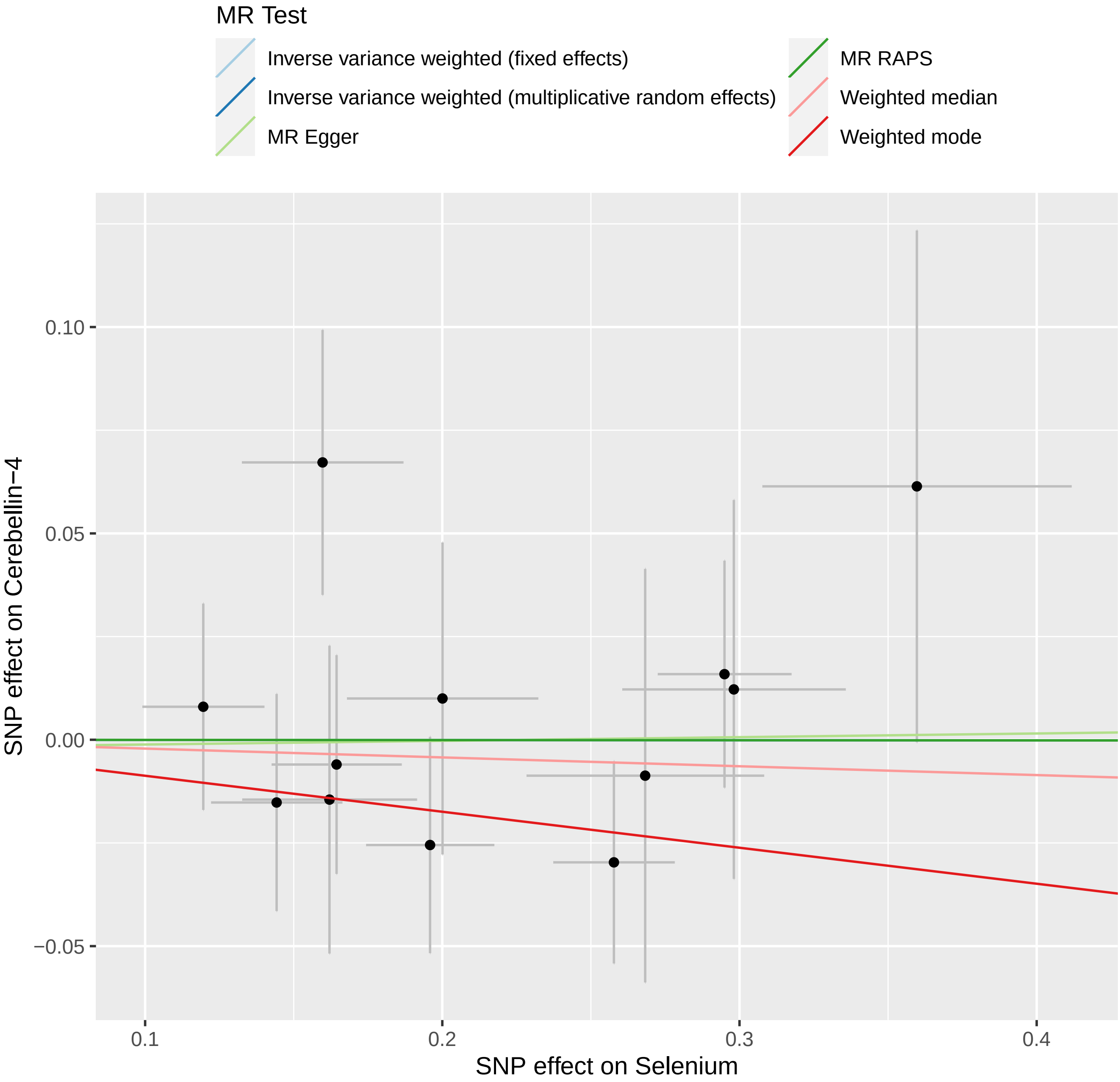

Batch 3−373 : Selenium on Carbonyl reductase [NADPH] 1

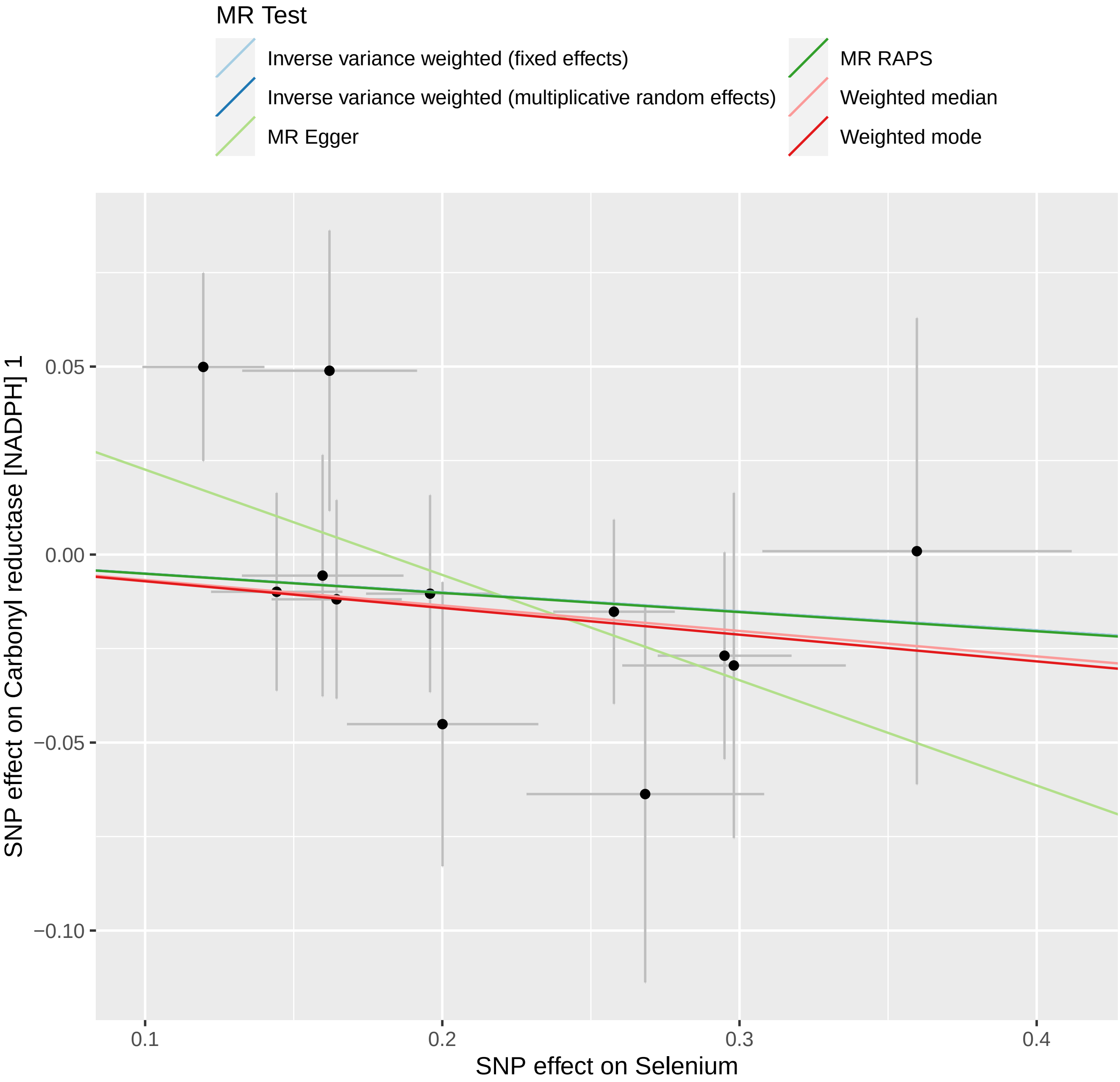

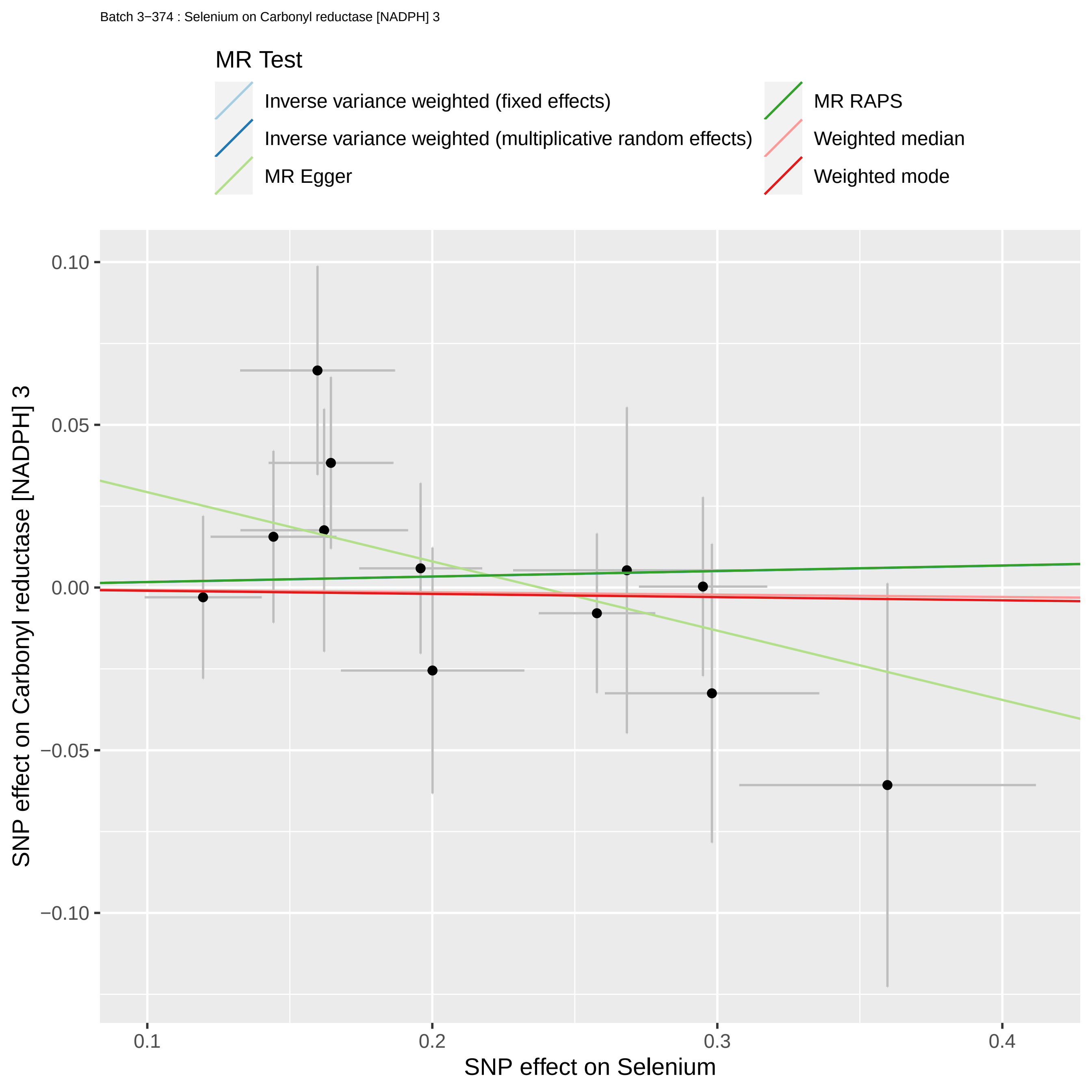
Batch 3−374 : Selenium on Carbonyl reductase [NADPH] 3
MR Test
Inverse variance weighted (fixed effects)
Inverse variance weighted (multiplicative random effects)
MR Egger
MR RAPS
Weighted median
Weighted mode
0.10
0.05
0.00
−0.05
−0.10
0.1
0.2
0.3
0.4
SNP effect on Carbonyl reductase [NADPH] 3
SNP effect on Selenium

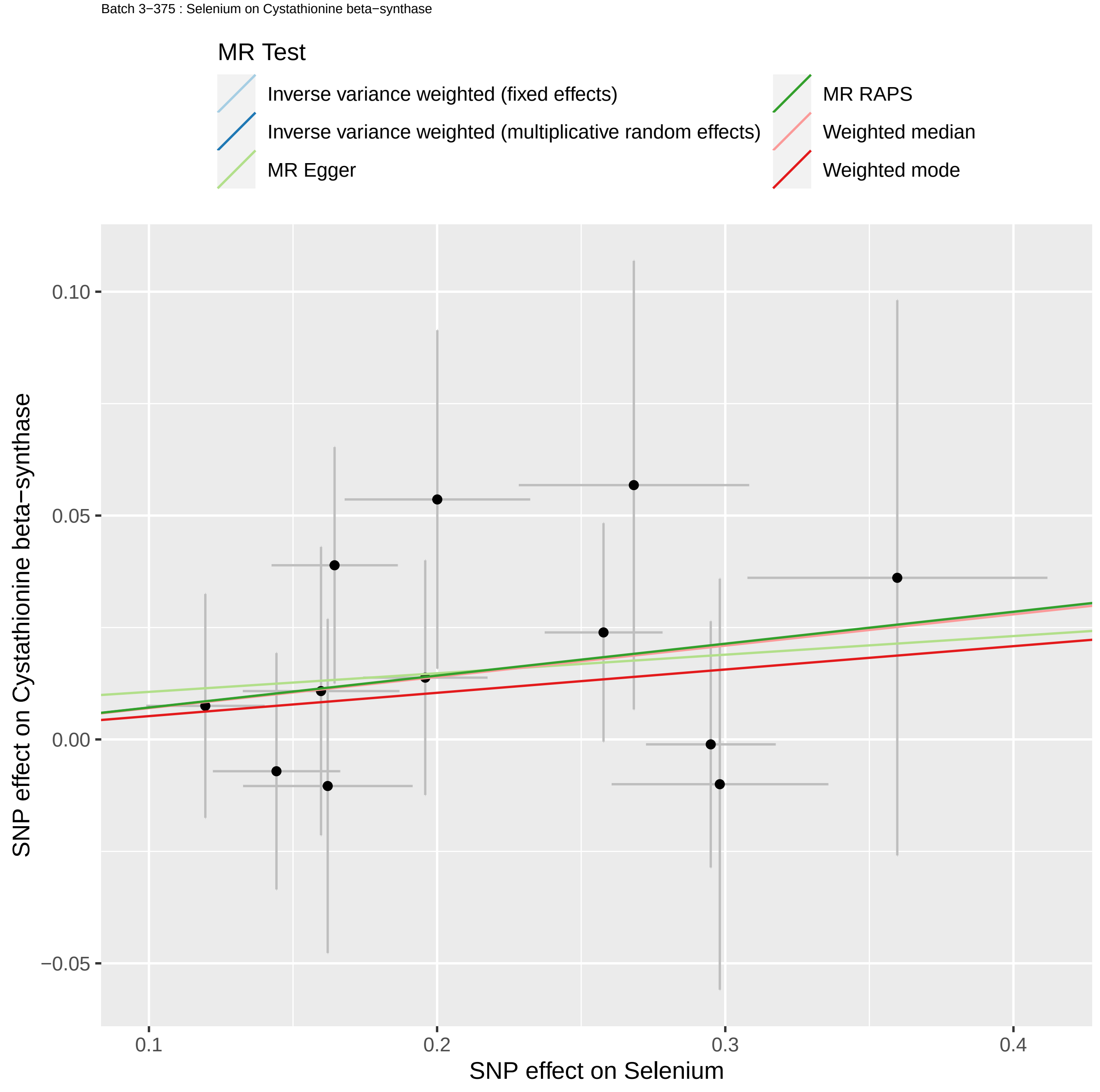
Batch 3−375 : Selenium on Cystathionine beta−synthase
MR Test
Inverse variance weighted (fixed effects)
Inverse variance weighted (multiplicative random effects)
MR Egger
MR RAPS
Weighted median
Weighted mode
0.10
0.05
0.00
−0.05
0.1
0.2
0.3
0.4
SNP effect on Cystathionine beta−synthase
SNP effect on Selenium

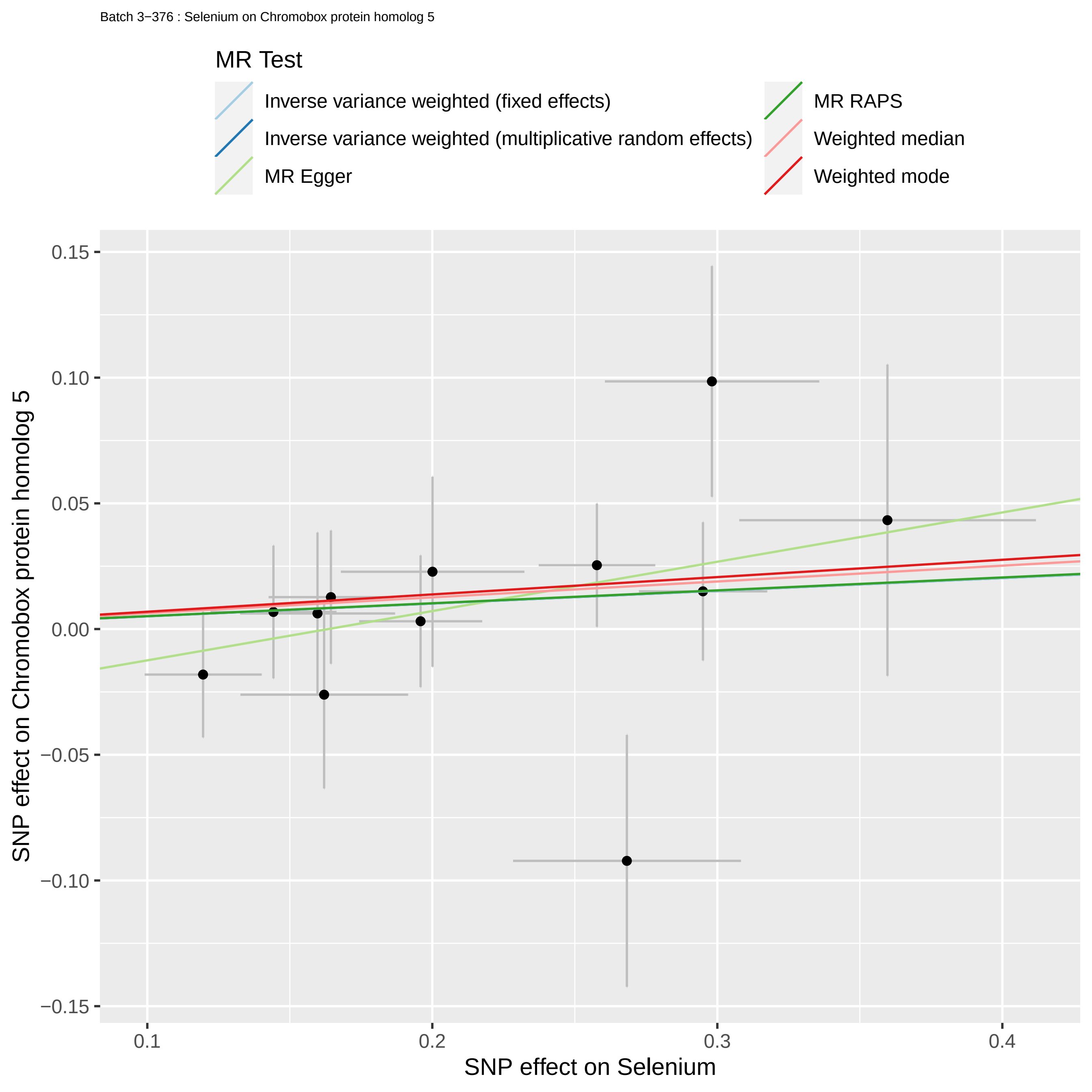

Batch 3−376 : Selenium on Chromobox protein homolog 5
MR Test
Inverse variance weighted (fixed effects)
Inverse variance weighted (multiplicative random effects)
MR Egger
MR RAPS
Weighted median
Weighted mode
SNP effect on Chromobox protein homolog 5
0.15
0.10
0.05
0.00
−0.05
−0.10
−0.15
0.1
0.2
0.3
0.4
SNP effect on Selenium

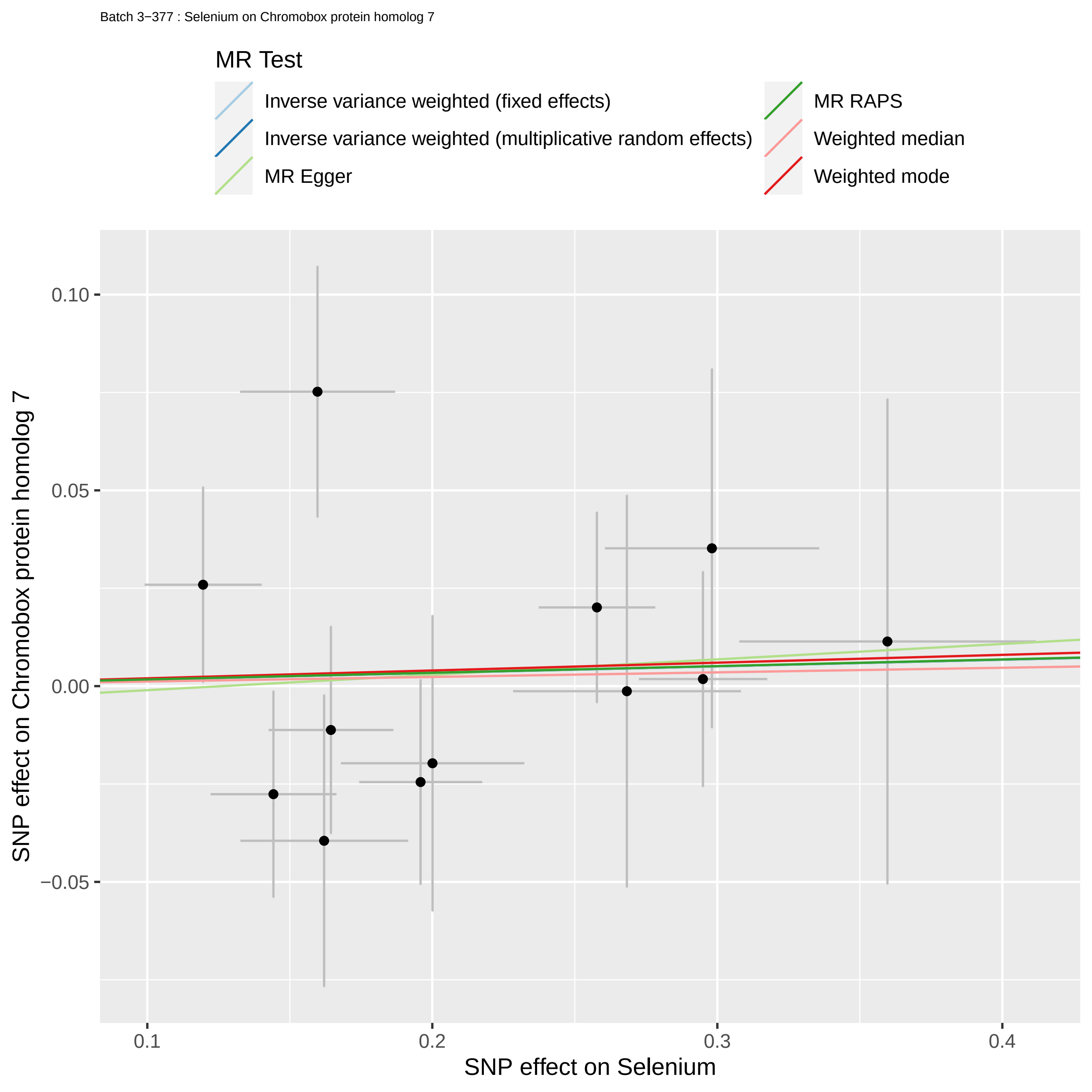
Batch 3−377 : Selenium on Chromobox protein homolog 7
MR Test
Inverse variance weighted (fixed effects)
Inverse variance weighted (multiplicative random effects)
MR Egger
MR RAPS
Weighted median
Weighted mode
SNP effect on Chromobox protein homolog 7
0.10
0.05
0.00
−0.05
0.1
0.2
0.3
0.4
SNP effect on Selenium

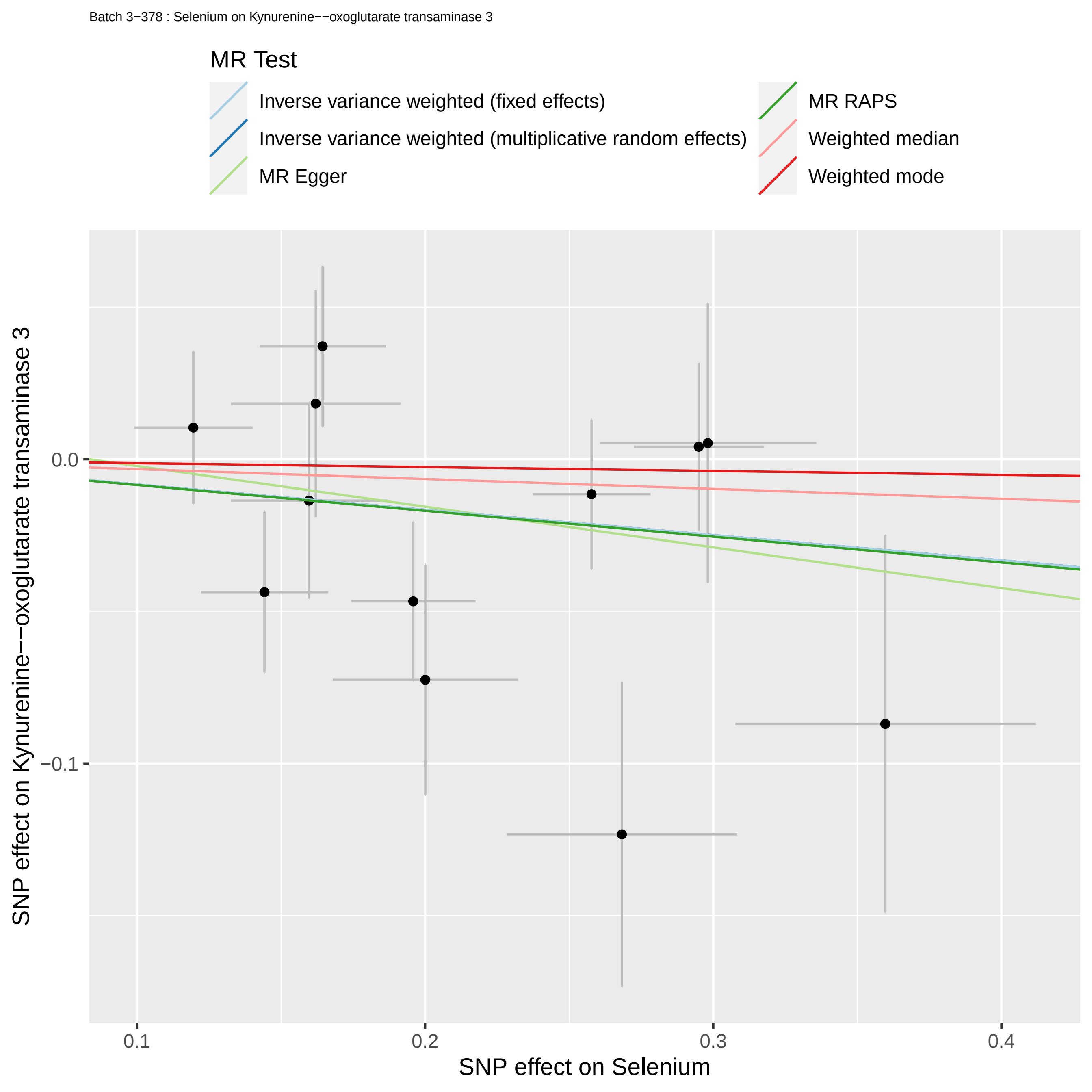

Batch 3−378 : Selenium on Kynurenine−−oxoglutarate transaminase 3
MR Test
Inverse variance weighted (fixed effects)
Inverse variance weighted (multiplicative random effects)
MR Egger
MR RAPS
Weighted median
Weighted mode
0.0
−0.1
0.1
0.2
0.3
0.4
SNP effect on Selenium
SNP effect on Kynurenine−−oxoglutarate transaminase 3

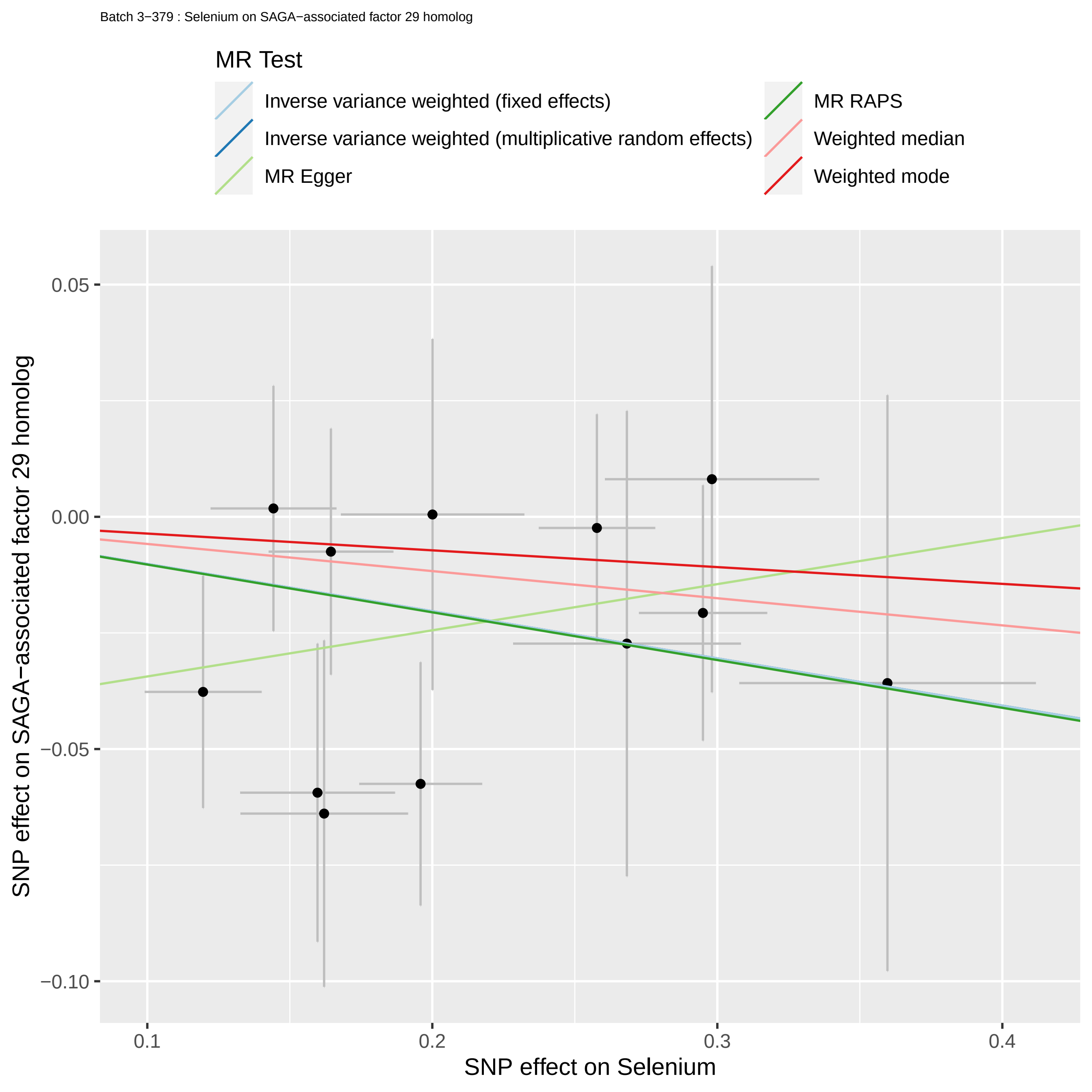
Batch 3−379 : Selenium on SAGA−associated factor 29 homolog
MR Test
Inverse variance weighted (fixed effects)
Inverse variance weighted (multiplicative random effects)
MR Egger
MR RAPS
Weighted median
Weighted mode
0.05
0.00
−0.05
−0.10
SNP effect on SAGA−associated factor 29 homolog
0.1
0.2
0.3
0.4
SNP effect on Selenium

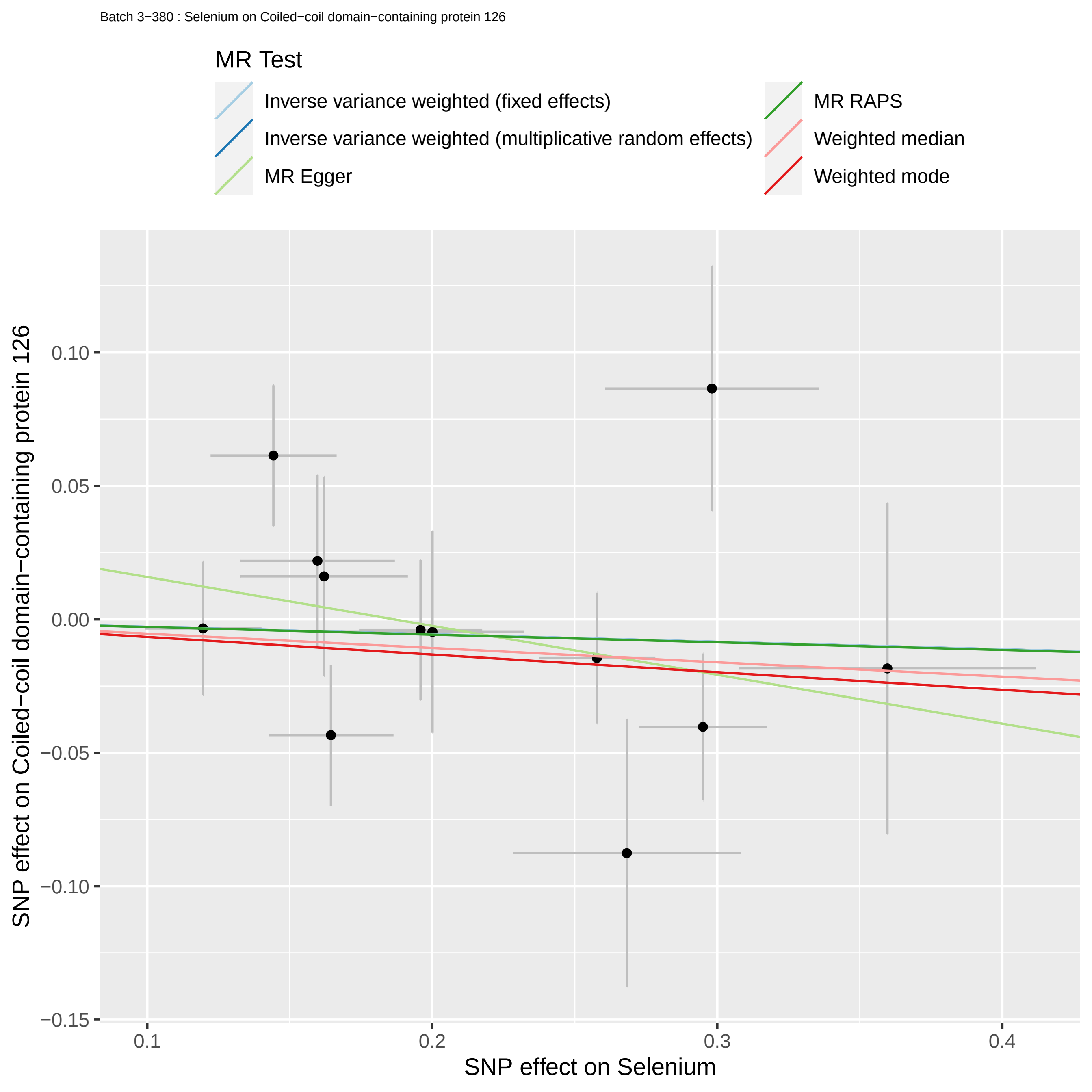

Batch 3−380 : Selenium on Coiled−coil domain−containing protein 126
MR Test
Inverse variance weighted (fixed effects)
Inverse variance weighted (multiplicative random effects)
MR Egger
MR RAPS
Weighted median
Weighted mode
SNP effect on Coiled−coil domain−containing protein 126
0.10
0.05
0.00
−0.05
−0.10
−0.15
0.1
0.2
0.3
0.4
SNP effect on Selenium

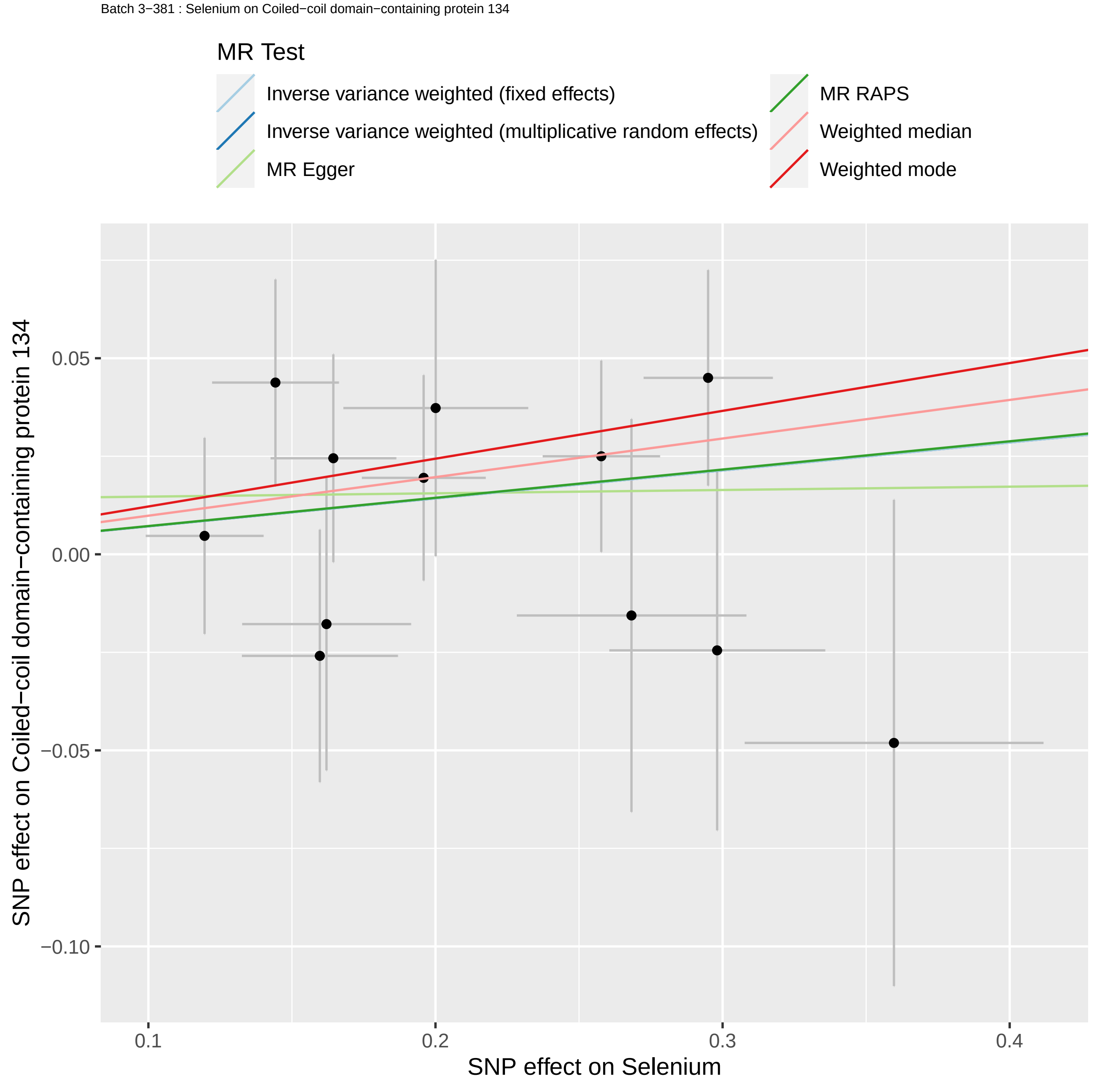

Batch 3−381 : Selenium on Coiled−coil domain−containing protein 134
MR Test
Inverse variance weighted (fixed effects)
Inverse variance weighted (multiplicative random effects)
MR Egger
MR RAPS
Weighted median
Weighted mode
SNP effect on Coiled−coil domain−containing protein 134
0.05
0.00
−0.05
−0.10
0.1
0.2
0.3
0.4
SNP effect on Selenium

Batch 3−382 : Selenium on Coiled−coil domain−containing protein 167

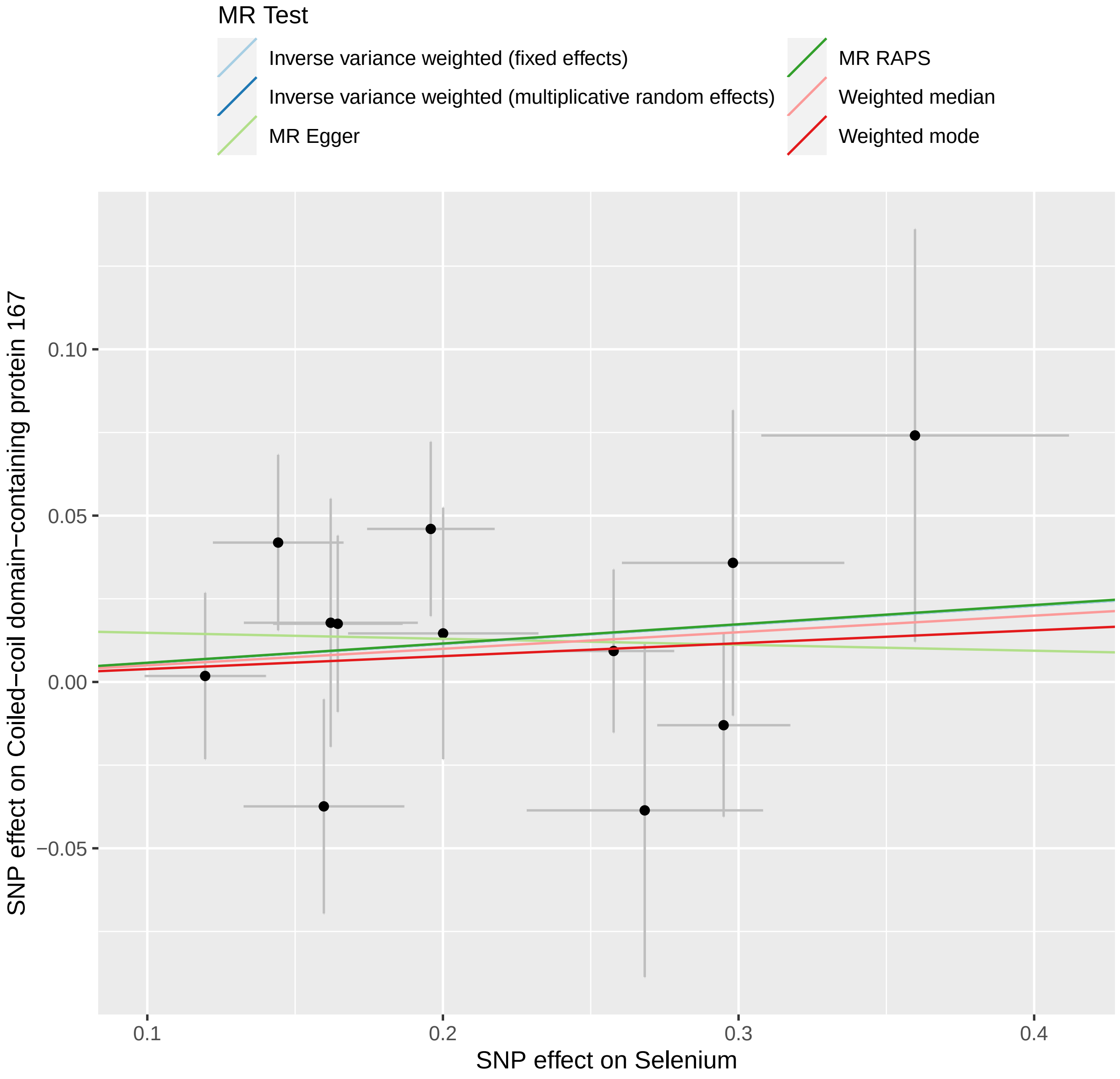

Batch 3−383 : Selenium on Coiled−coil domain−containing protein 51

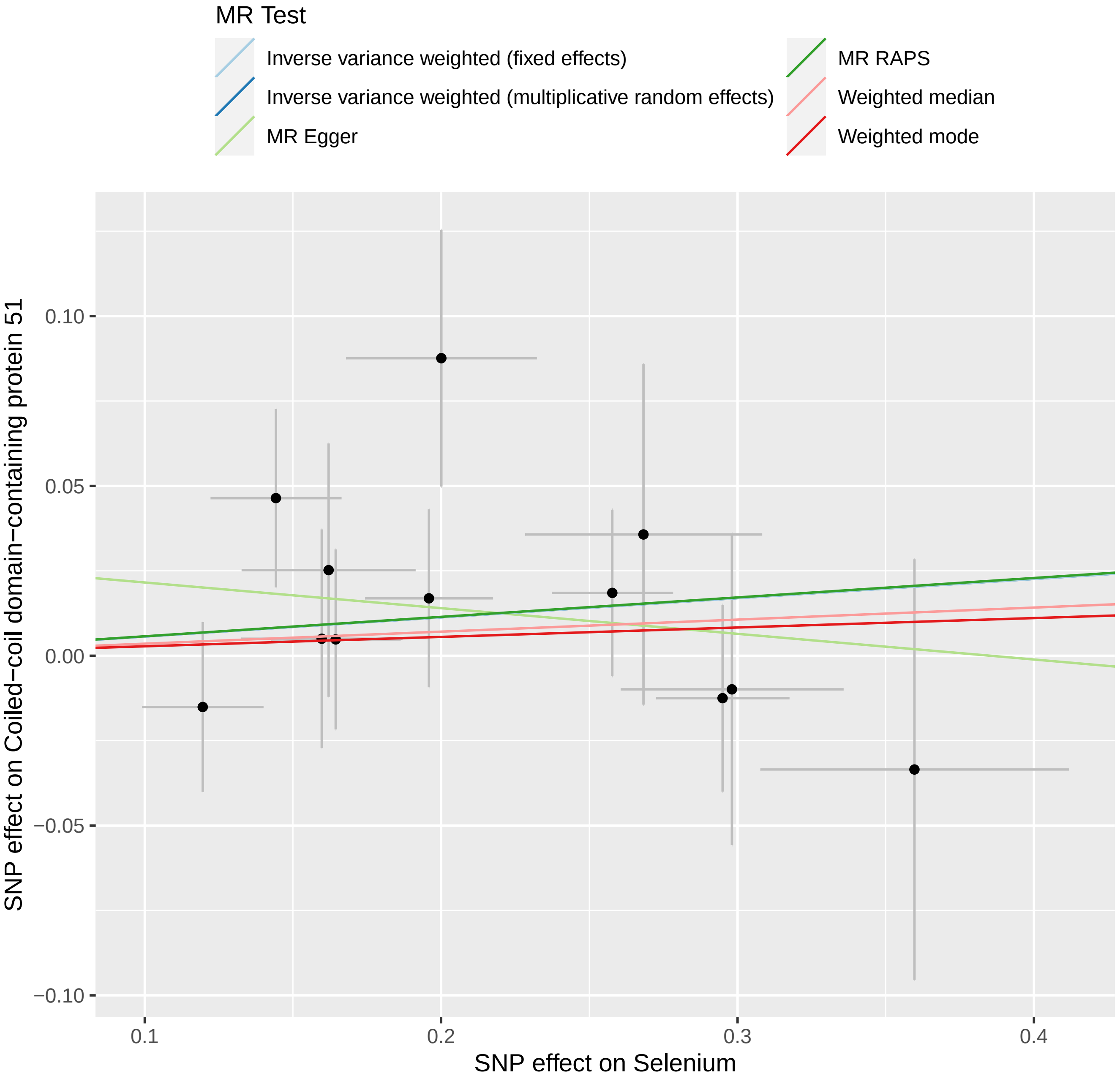

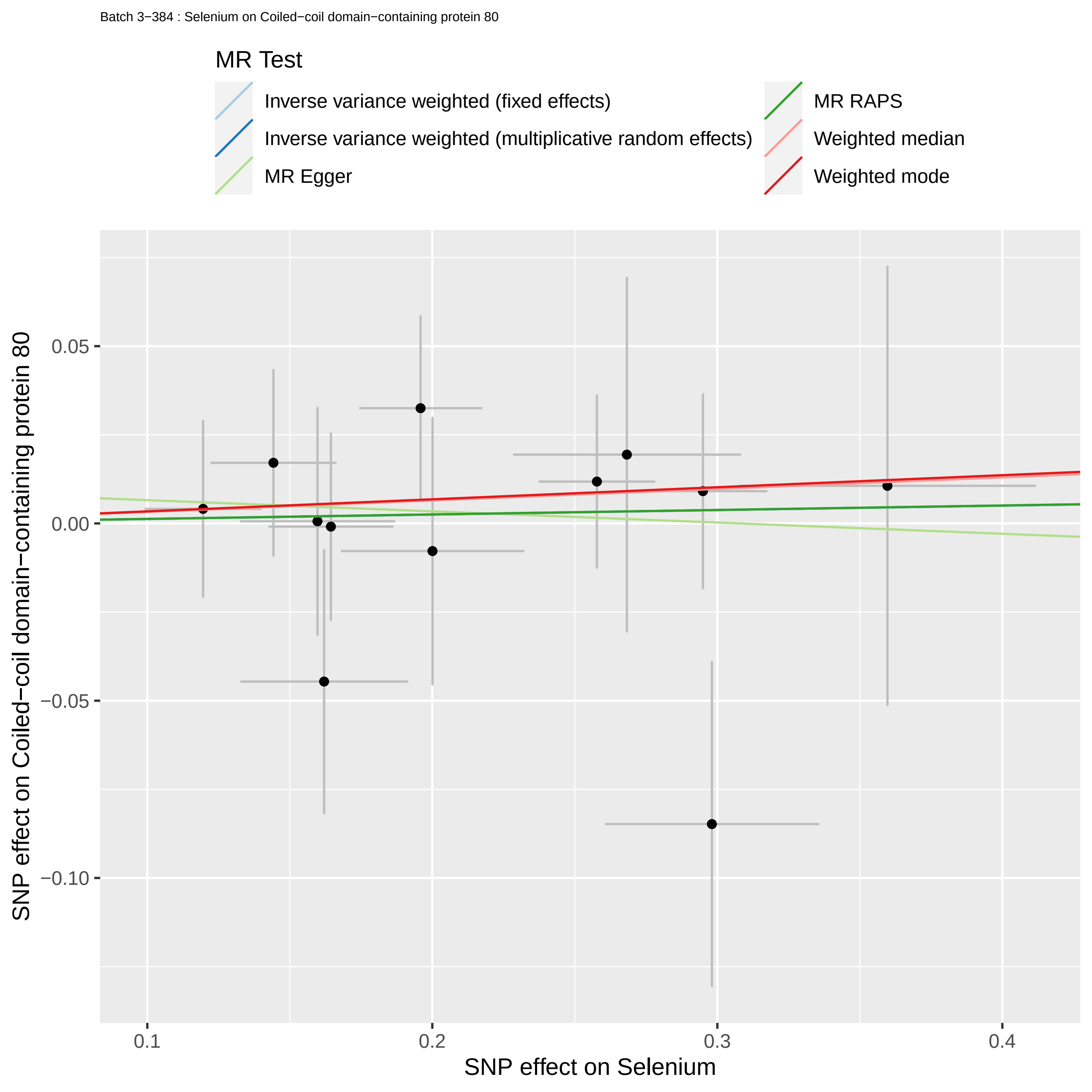
Batch 3−384 : Selenium on Coiled−coil domain−containing protein 80
MR Test
Inverse variance weighted (fixed effects)
Inverse variance weighted (multiplicative random effects)
MR Egger
MR RAPS
Weighted median
Weighted mode
SNP effect on Coiled−coil domain−containing protein 80
0.05
0.00
−0.05
−0.10
0.1
0.2
0.3
0.4
SNP effect on Selenium

Batch 3−385 : Selenium on Coiled−coil domain−containing protein 90B, mitochondrial

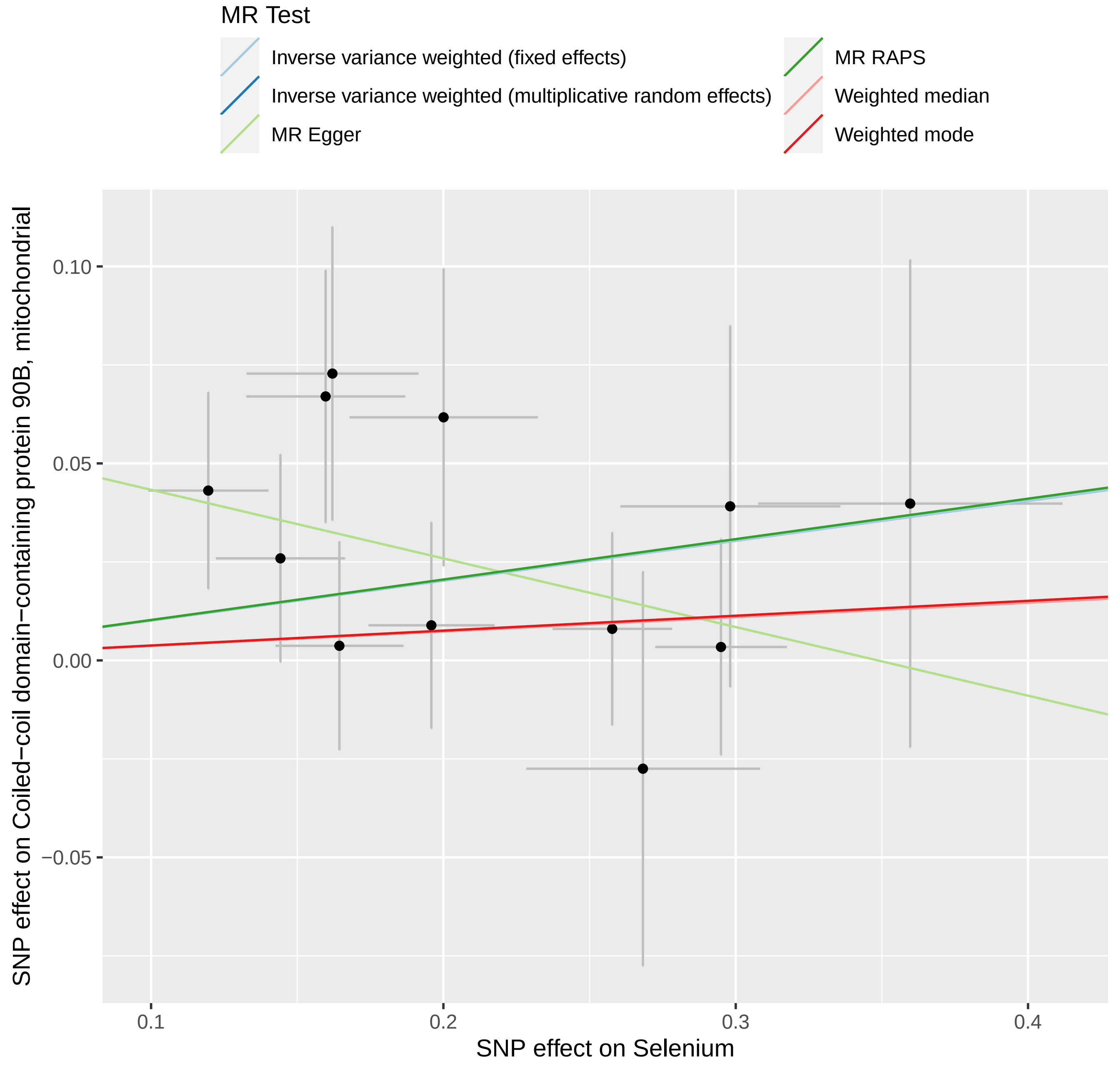

Batch 3−386 : Selenium on C−C motif chemokine 1

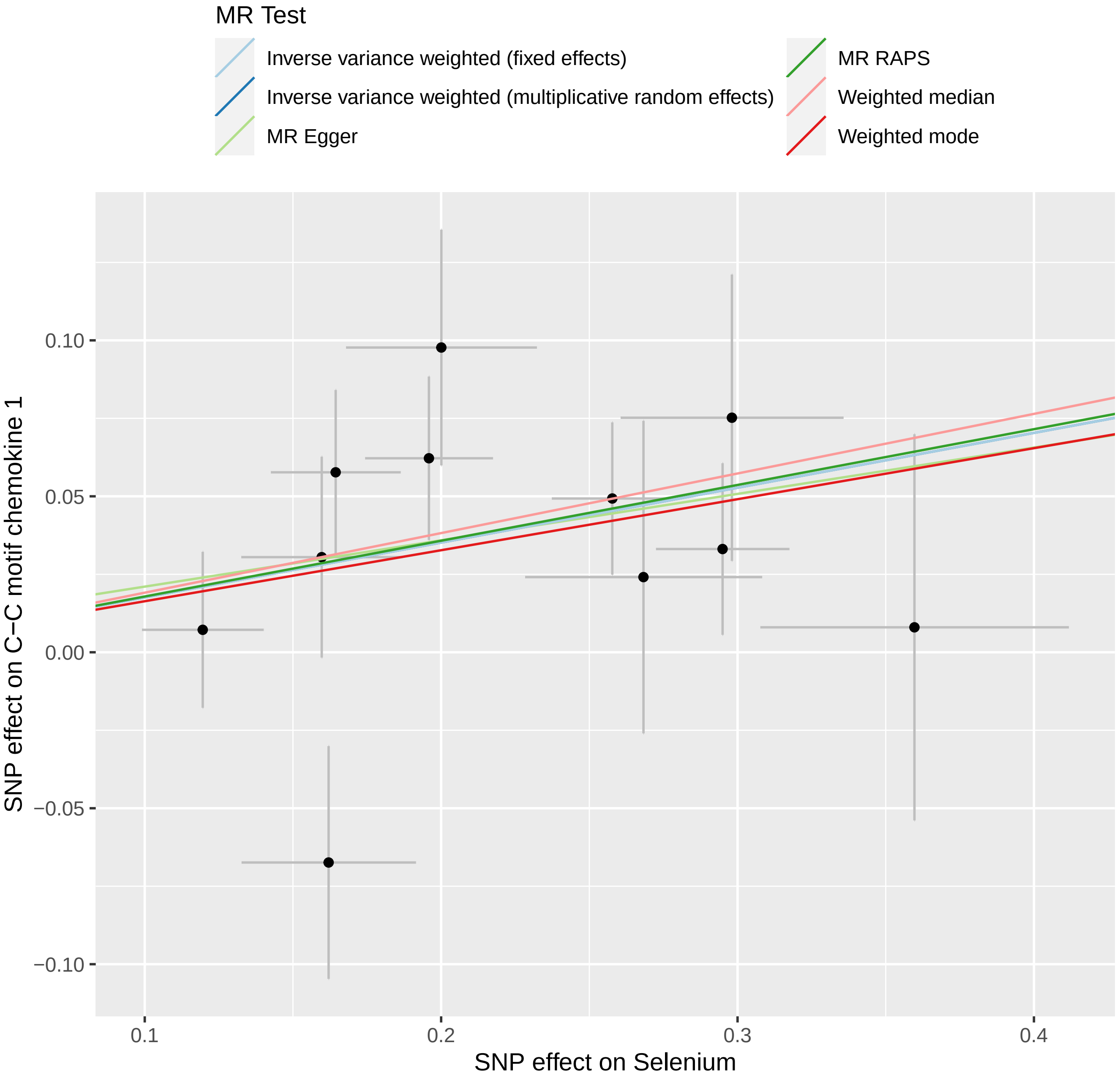

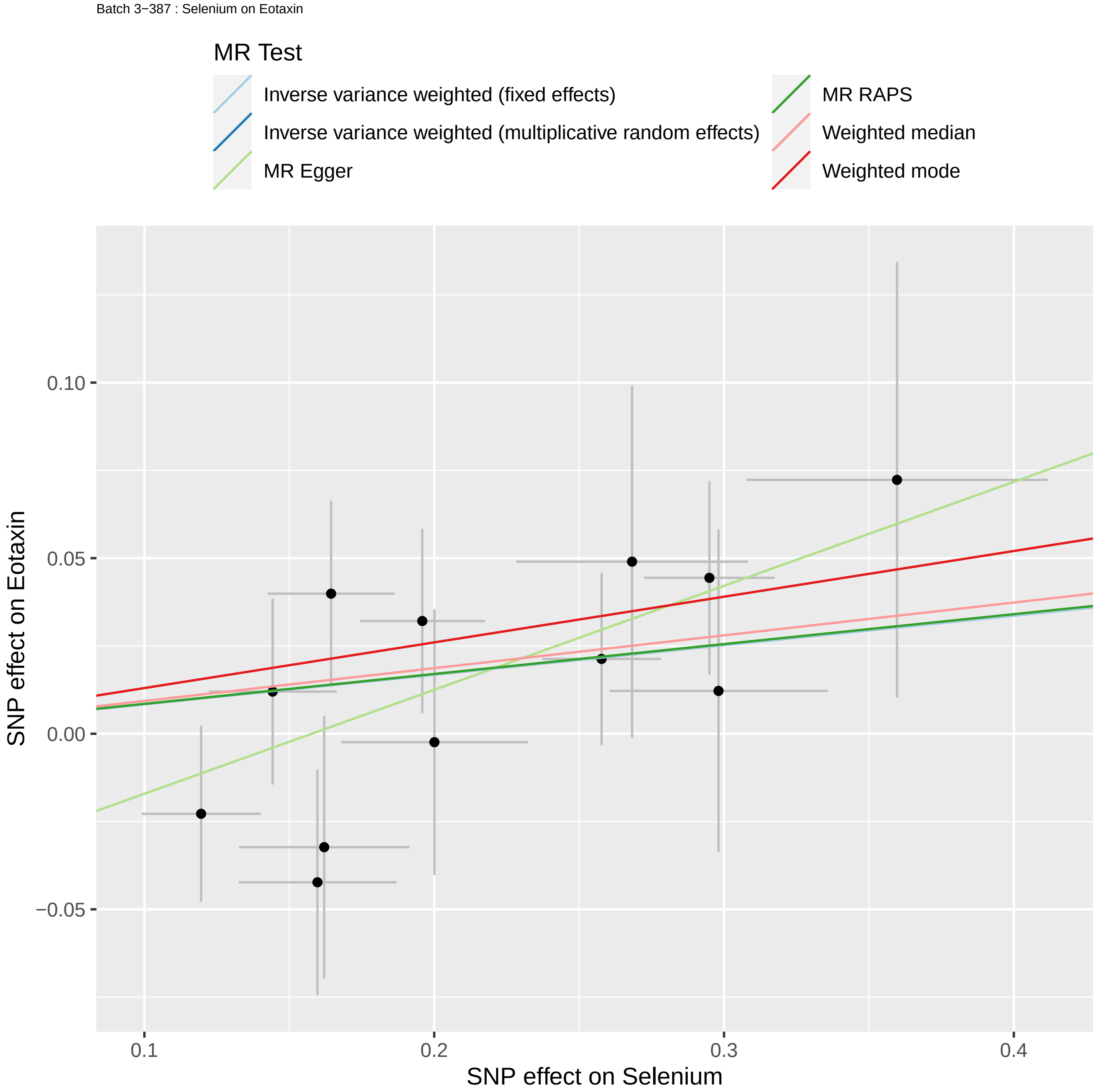

Batch 3−387 : Selenium on Eotaxin
MR Test
Inverse variance weighted (fixed effects)
Inverse variance weighted (multiplicative random effects)
MR Egger
MR RAPS
Weighted median
Weighted mode
0.10
0.05
0.00
−0.05
0.1
0.2
0.3
0.4
SNP effect on Eotaxin
SNP effect on Selenium

Batch 3−388 : Selenium on C−C motif chemokine 1

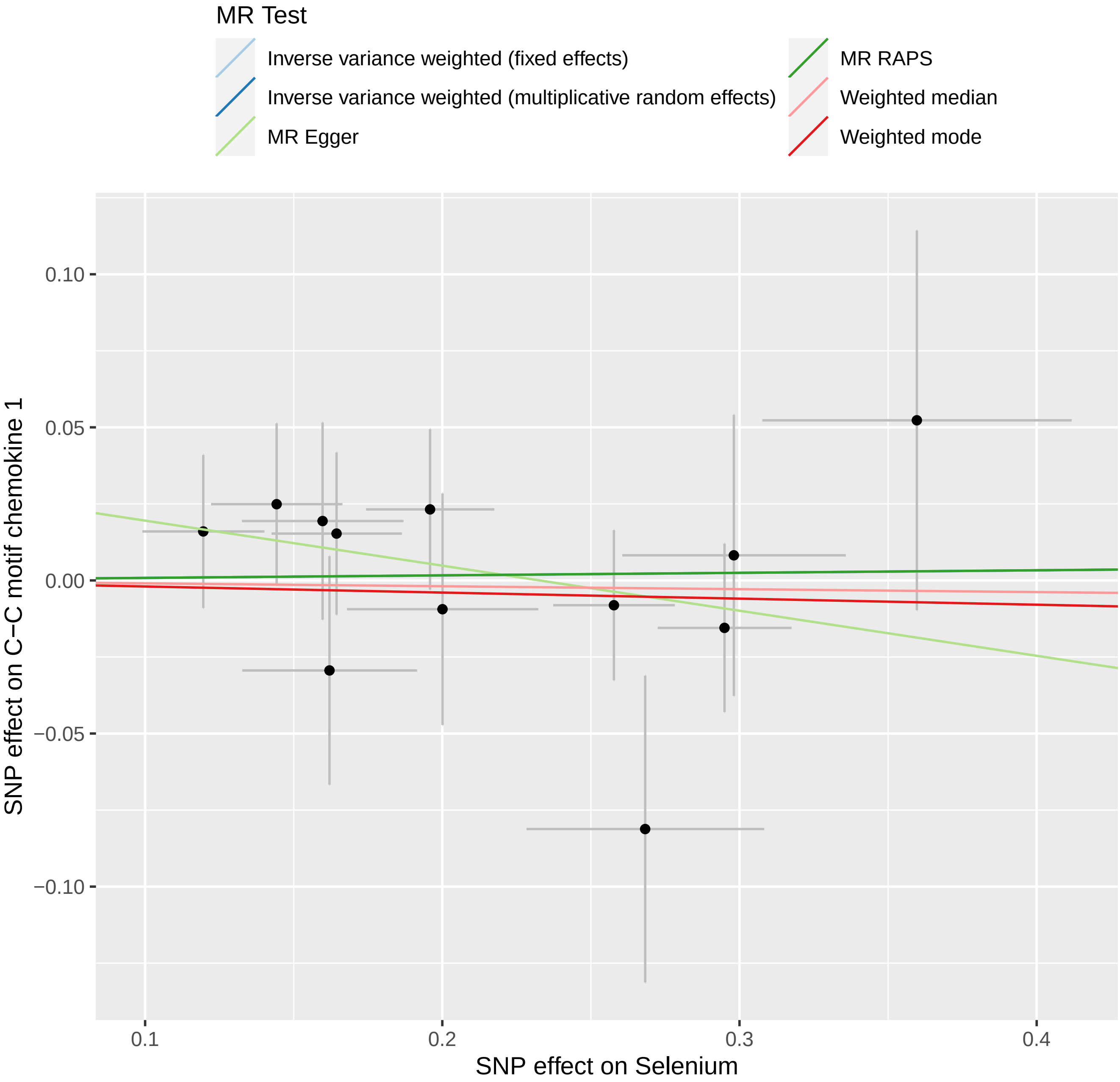

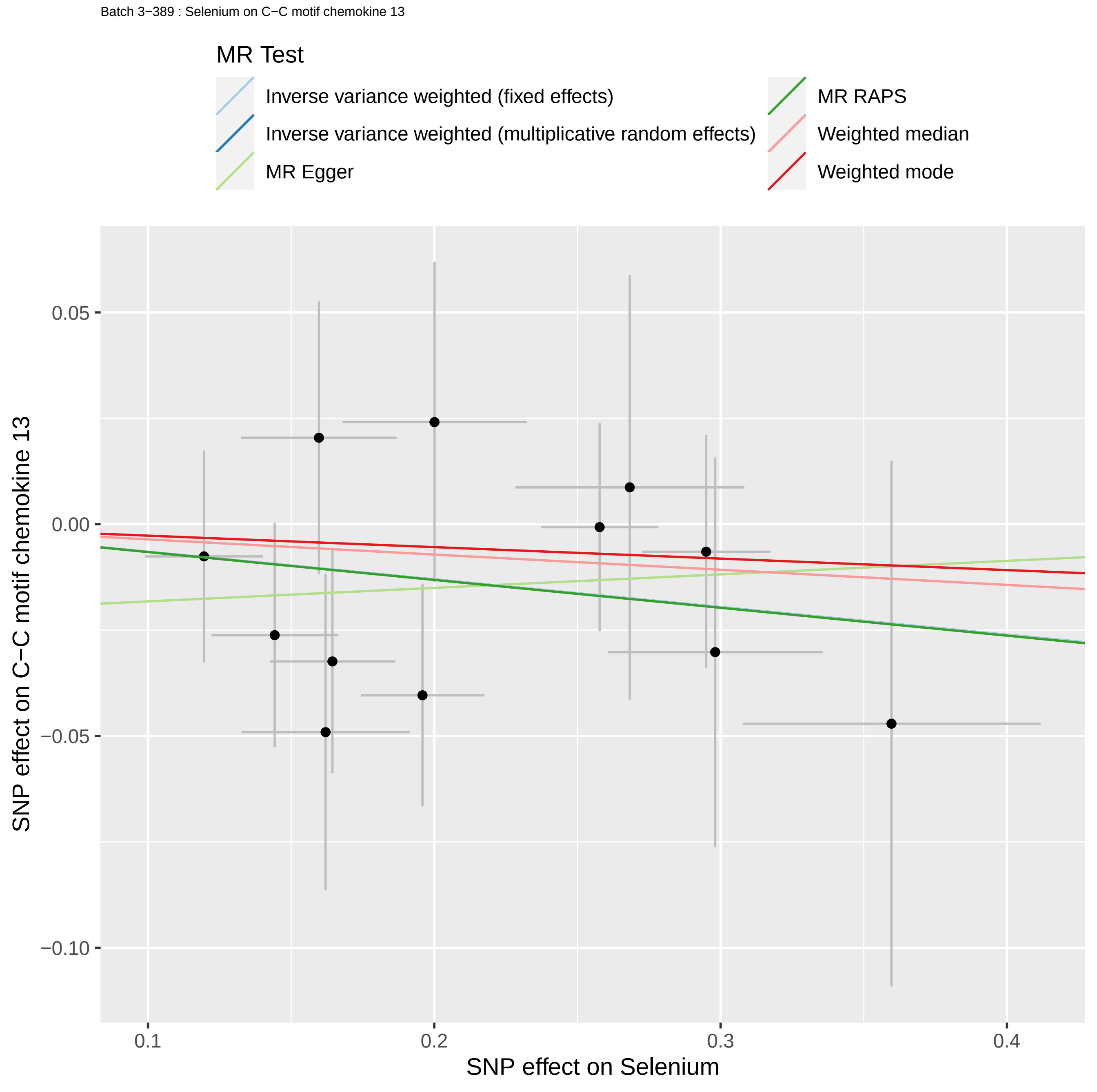

Batch 3−389 : Selenium on C−C motif chemokine 13
MR Test
Inverse variance weighted (fixed effects)
Inverse variance weighted (multiplicative random effects)
MR Egger
MR RAPS
Weighted median
Weighted mode
0.05
0.00
−0.05
−0.10
0.1
0.2
0.3
0.4
SNP effect on C−C motif chemokine 13
SNP effect on Selenium

Batch 3−390 : Selenium on C−C motif chemokine 14

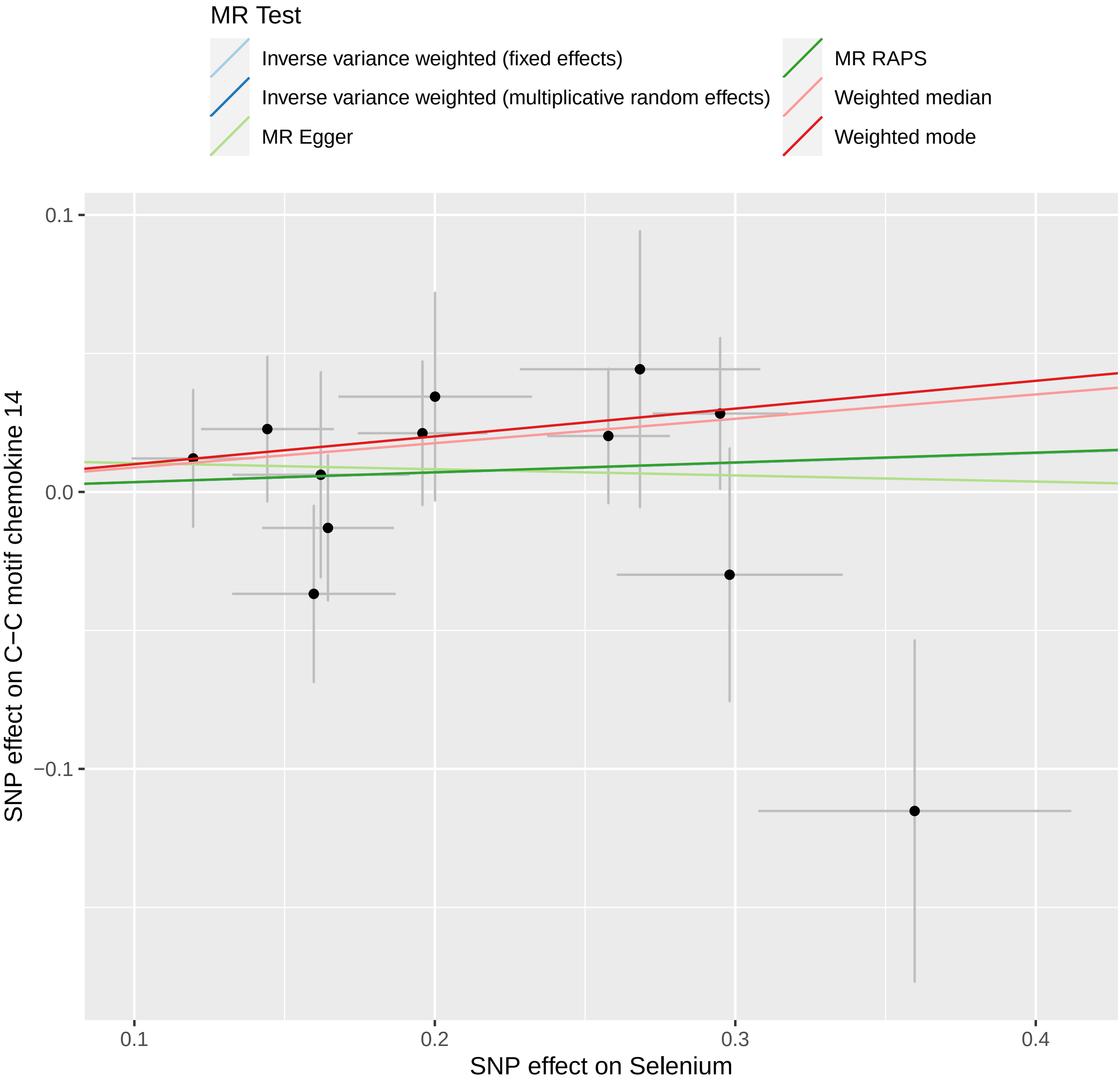

Batch 3−391 : Selenium on C−C motif chemokine 15

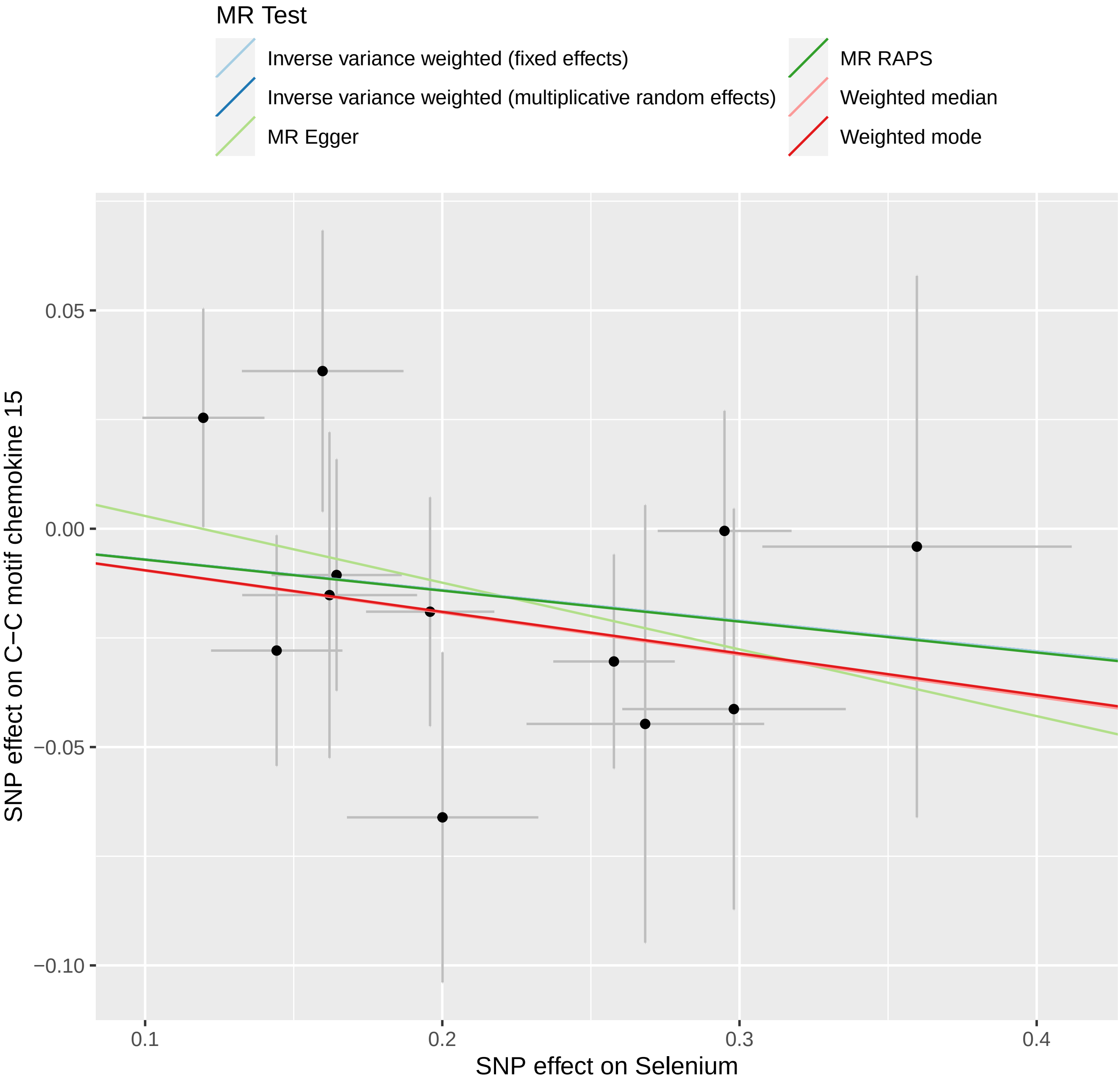

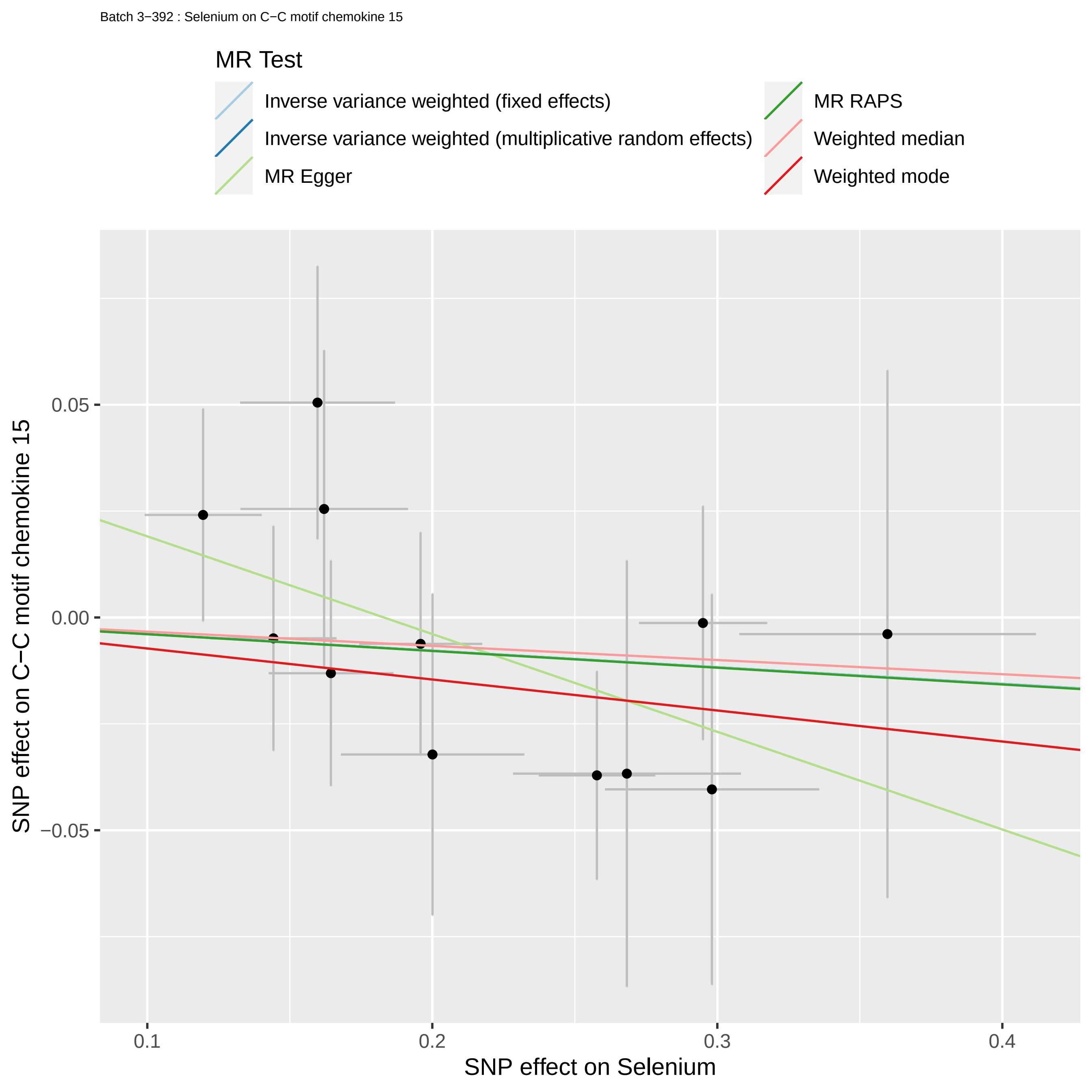

Batch 3−392 : Selenium on C−C motif chemokine 15
MR Test
Inverse variance weighted (fixed effects)
Inverse variance weighted (multiplicative random effects)
MR Egger
MR RAPS
Weighted median
Weighted mode
0.05
0.00
−0.05
0.1
0.2
0.3
0.4
SNP effect on C−C motif chemokine 15
SNP effect on Selenium

Batch 3−393 : Selenium on C−C motif chemokine 16

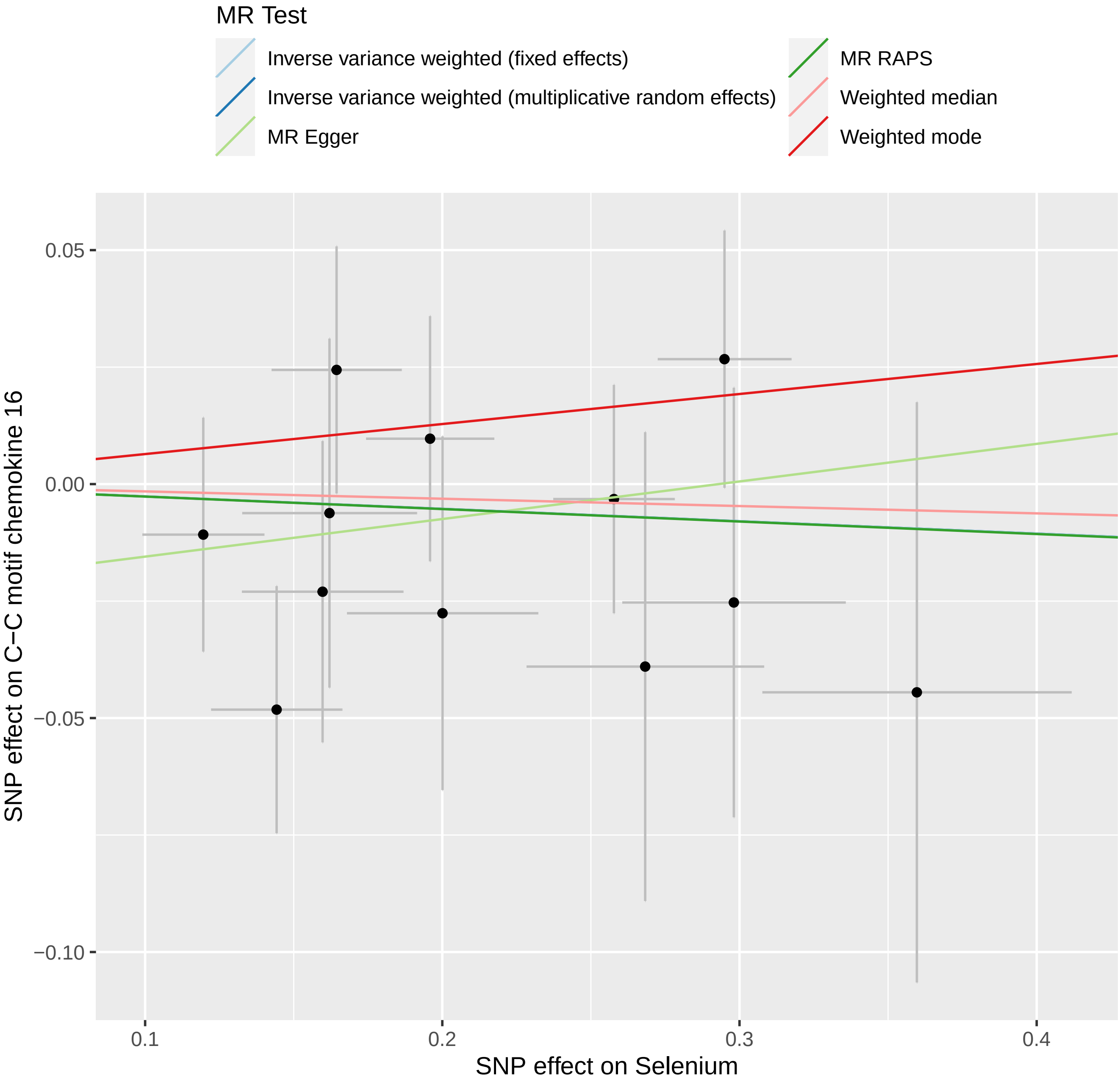

Batch 3−394 : Selenium on C−C motif chemokine 17

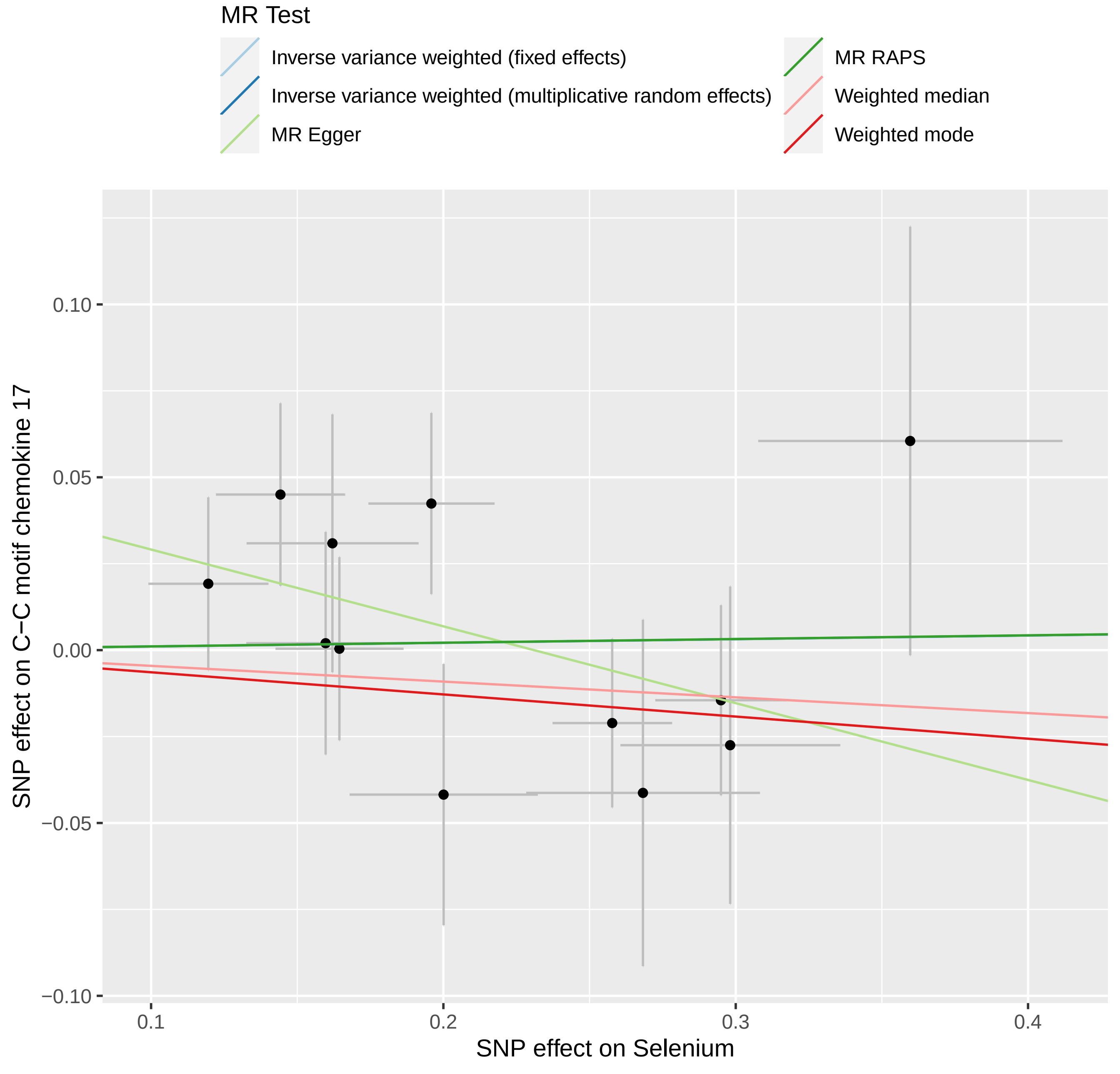

Batch 3−395 : Selenium on C−C motif chemokine 19

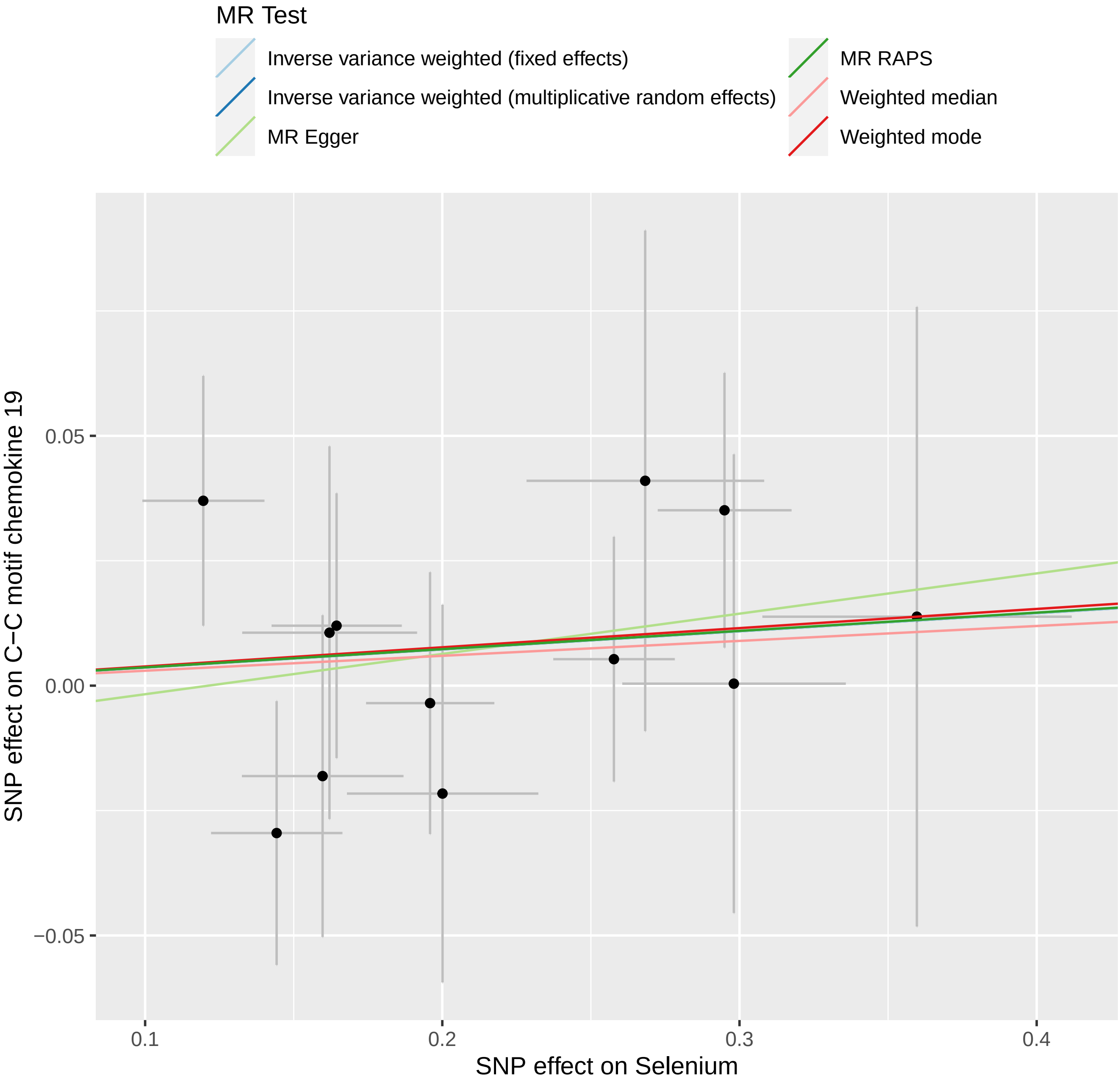

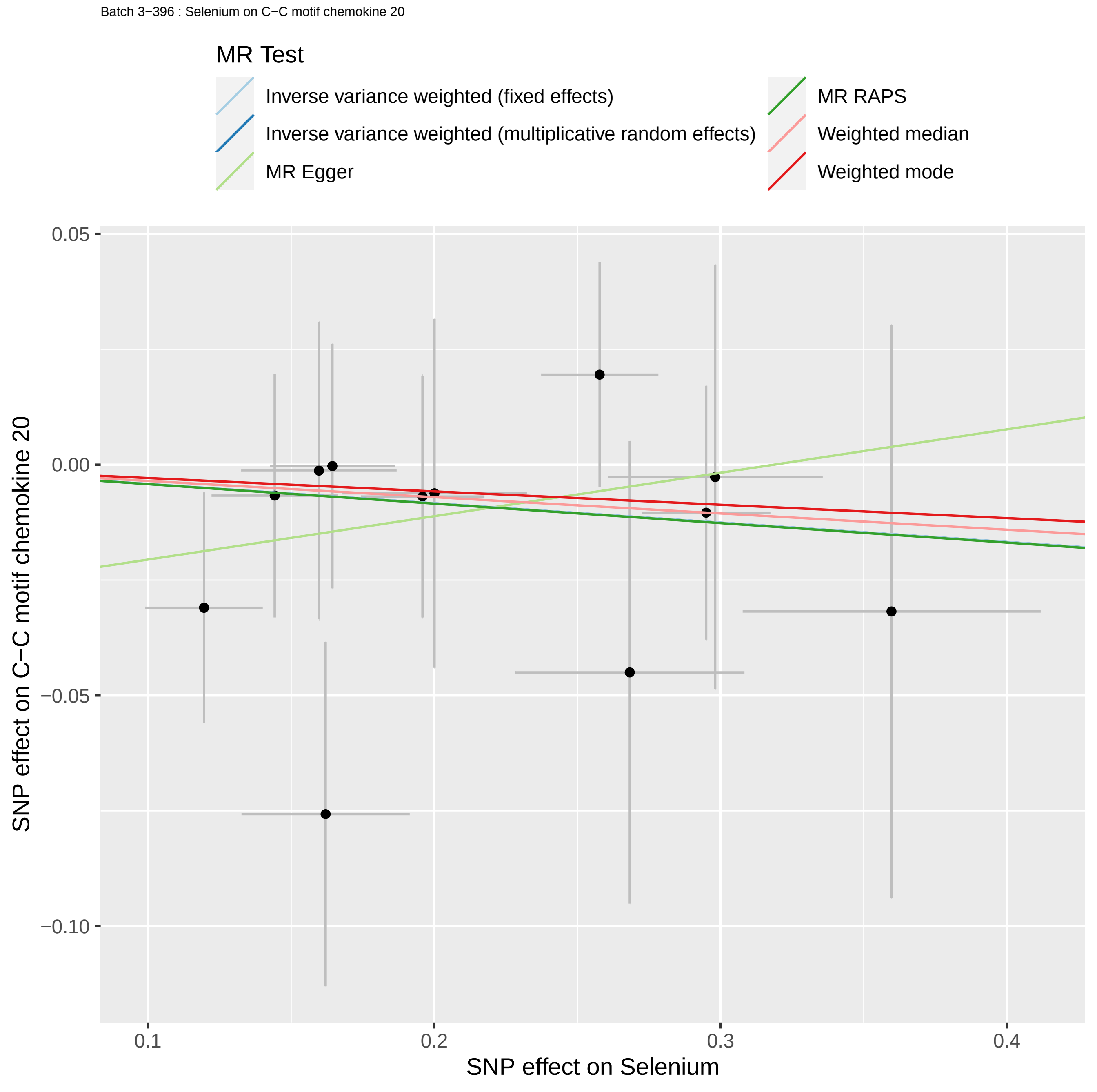
Batch 3−396 : Selenium on C−C motif chemokine 20
MR Test
Inverse variance weighted (fixed effects)
Inverse variance weighted (multiplicative random effects)
MR Egger
MR RAPS
Weighted median
Weighted mode
0.05
0.00
−0.05
−0.10
0.1
0.2
0.3
0.4
SNP effect on C−C motif chemokine 20
SNP effect on Selenium

Batch 3−397 : Selenium on C−C motif chemokine 21

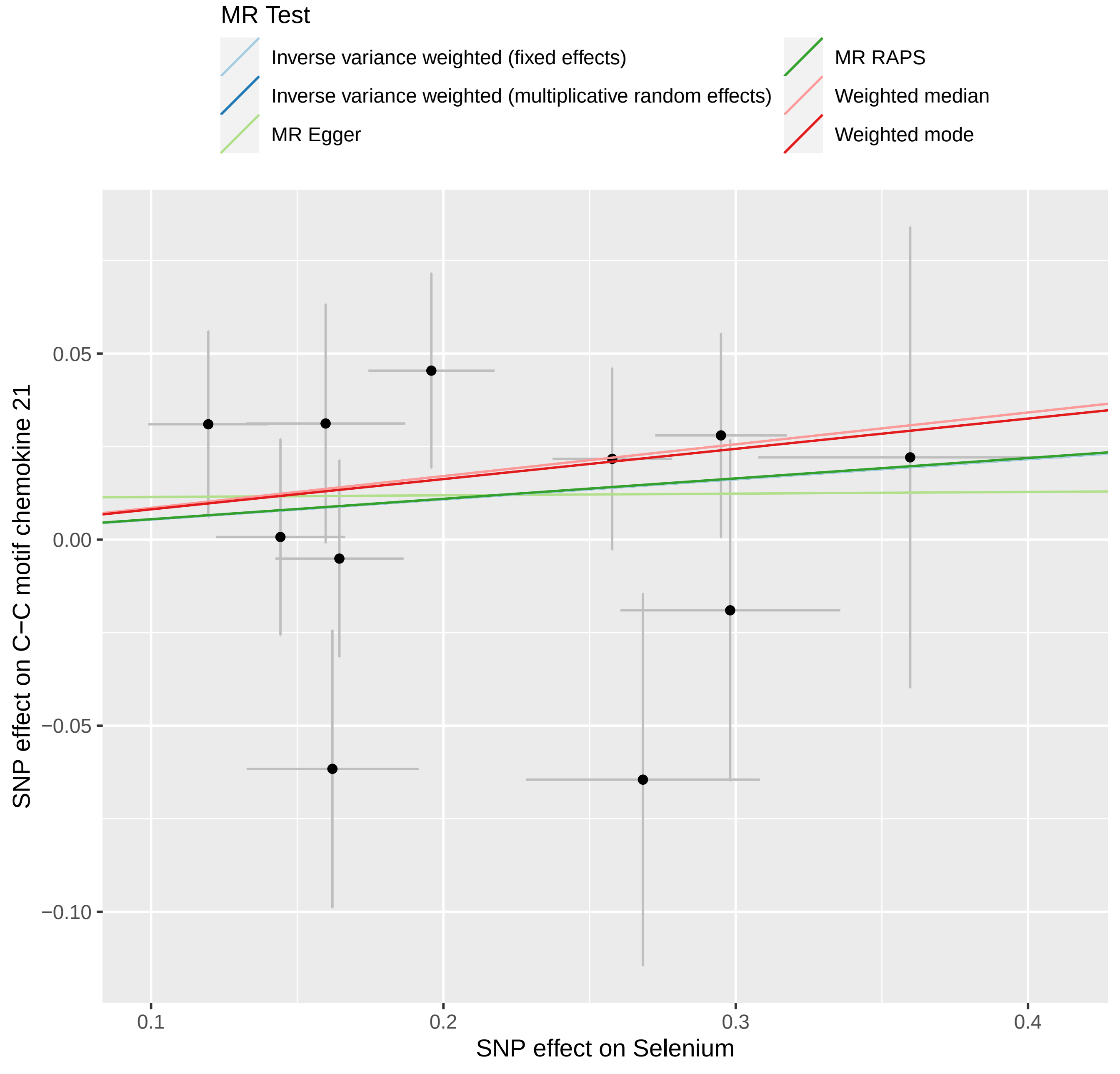

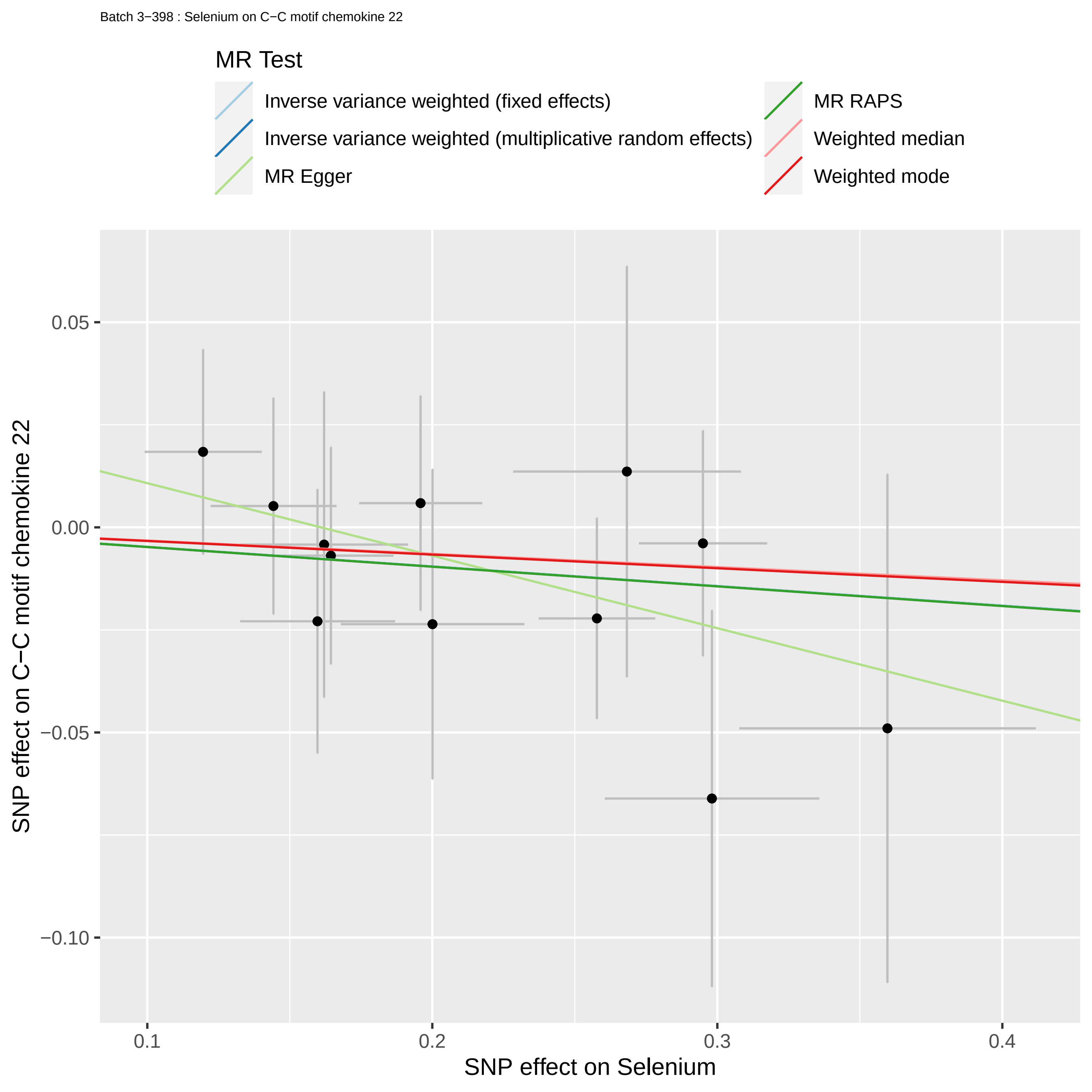
Batch 3−398 : Selenium on C−C motif chemokine 22
MR Test
Inverse variance weighted (fixed effects)
Inverse variance weighted (multiplicative random effects)
MR Egger
MR RAPS
Weighted median
Weighted mode
0.05
0.00
−0.05
−0.10
0.1
0.2
0.3
0.4
SNP effect on C−C motif chemokine 22
SNP effect on Selenium

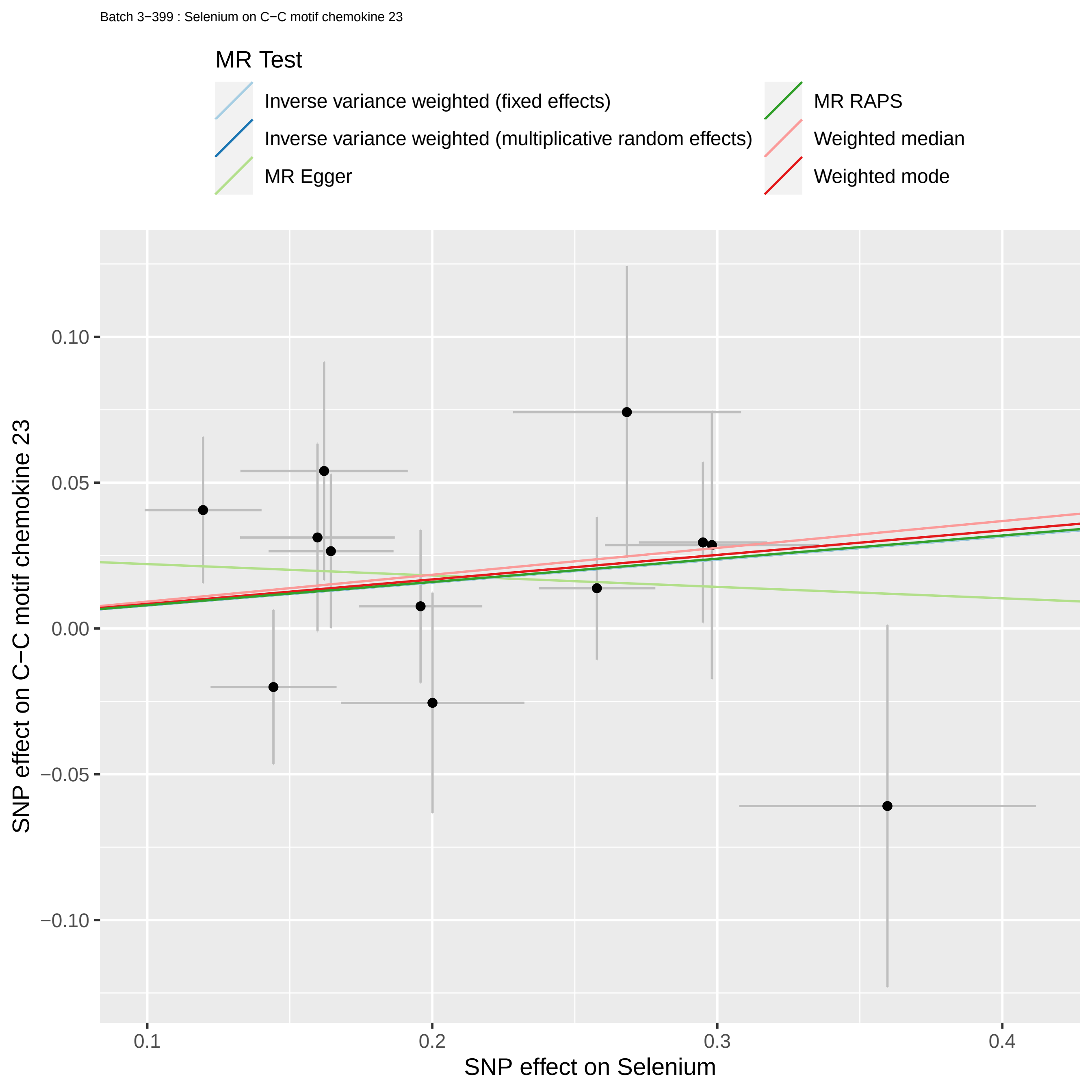
Batch 3−399 : Selenium on C−C motif chemokine 23
MR Test
Inverse variance weighted (fixed effects)
Inverse variance weighted (multiplicative random effects)
MR Egger
MR RAPS
Weighted median
Weighted mode
0.10
0.05
0.00
−0.05
−0.10
0.1
0.2
0.3
0.4
SNP effect on C−C motif chemokine 23
SNP effect on Selenium

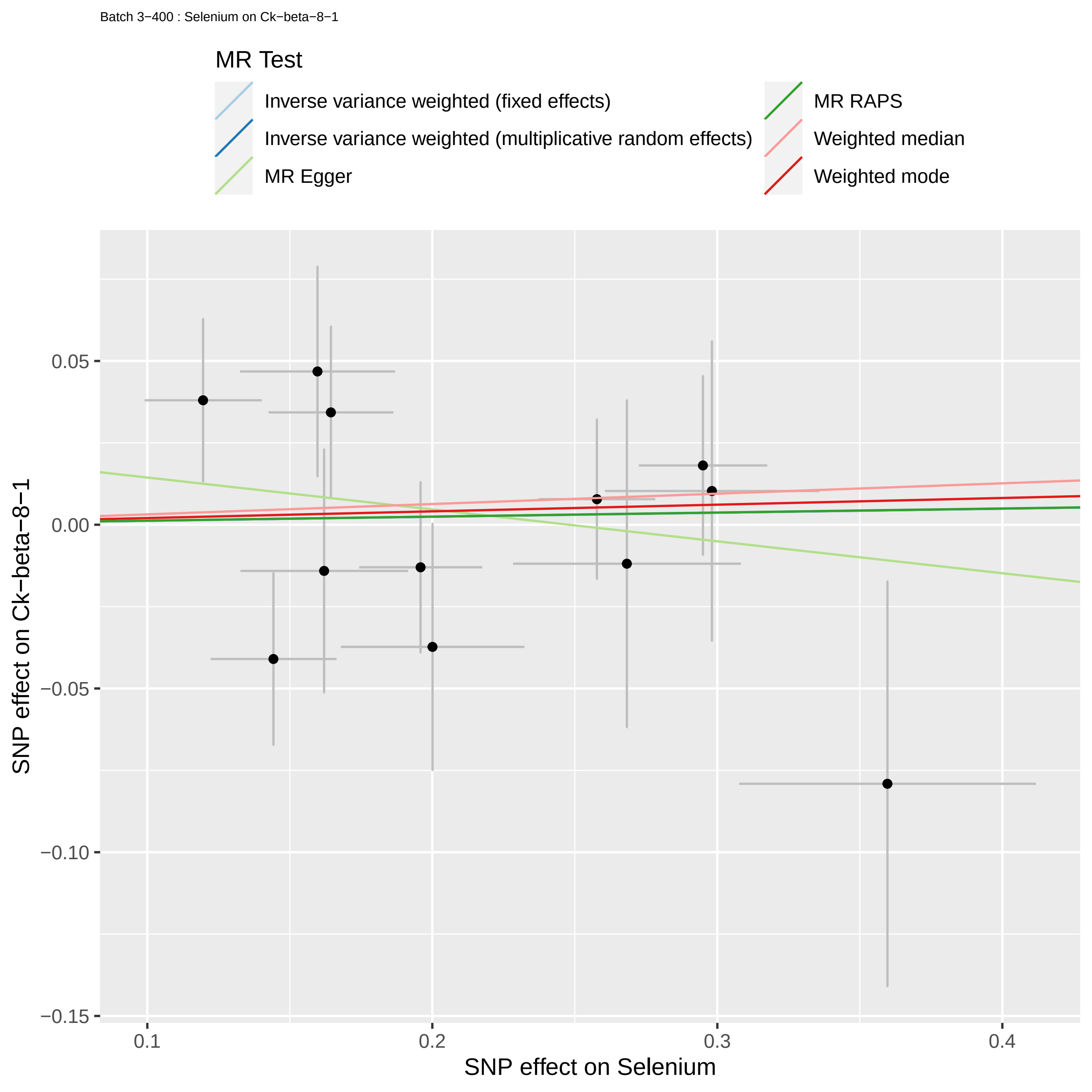

Batch 3−400 : Selenium on Ck−beta−8−1
MR Test
Inverse variance weighted (fixed effects)
Inverse variance weighted (multiplicative random effects)
MR Egger
MR RAPS
Weighted median
Weighted mode
SNP effect on Ck−beta−8−1
0.05
0.00
−0.05
−0.10
−0.15
0.1
0.2
0.3
0.4
SNP effect on Selenium

Batch 3−401 : Selenium on C−C motif chemokine 24

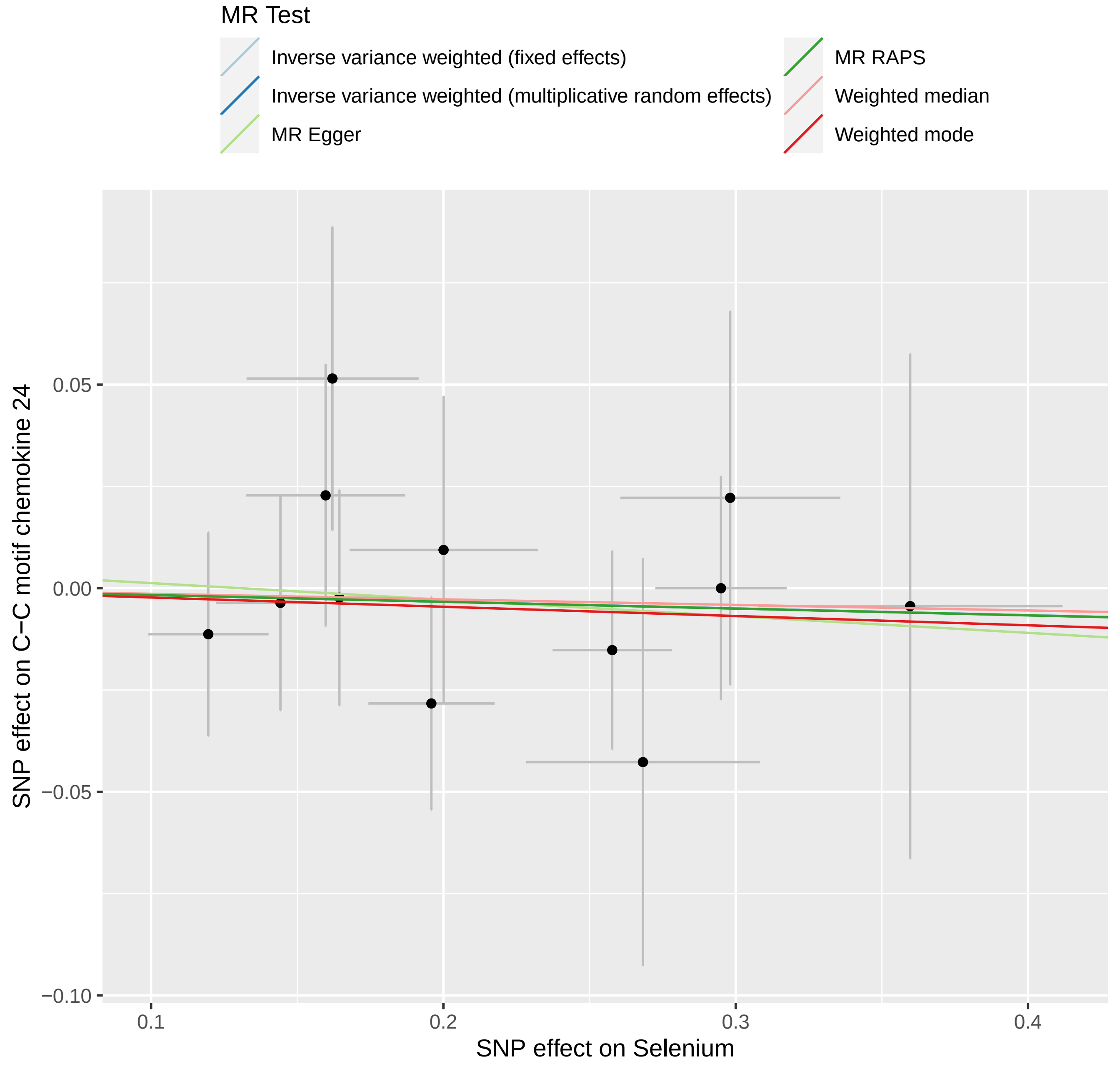

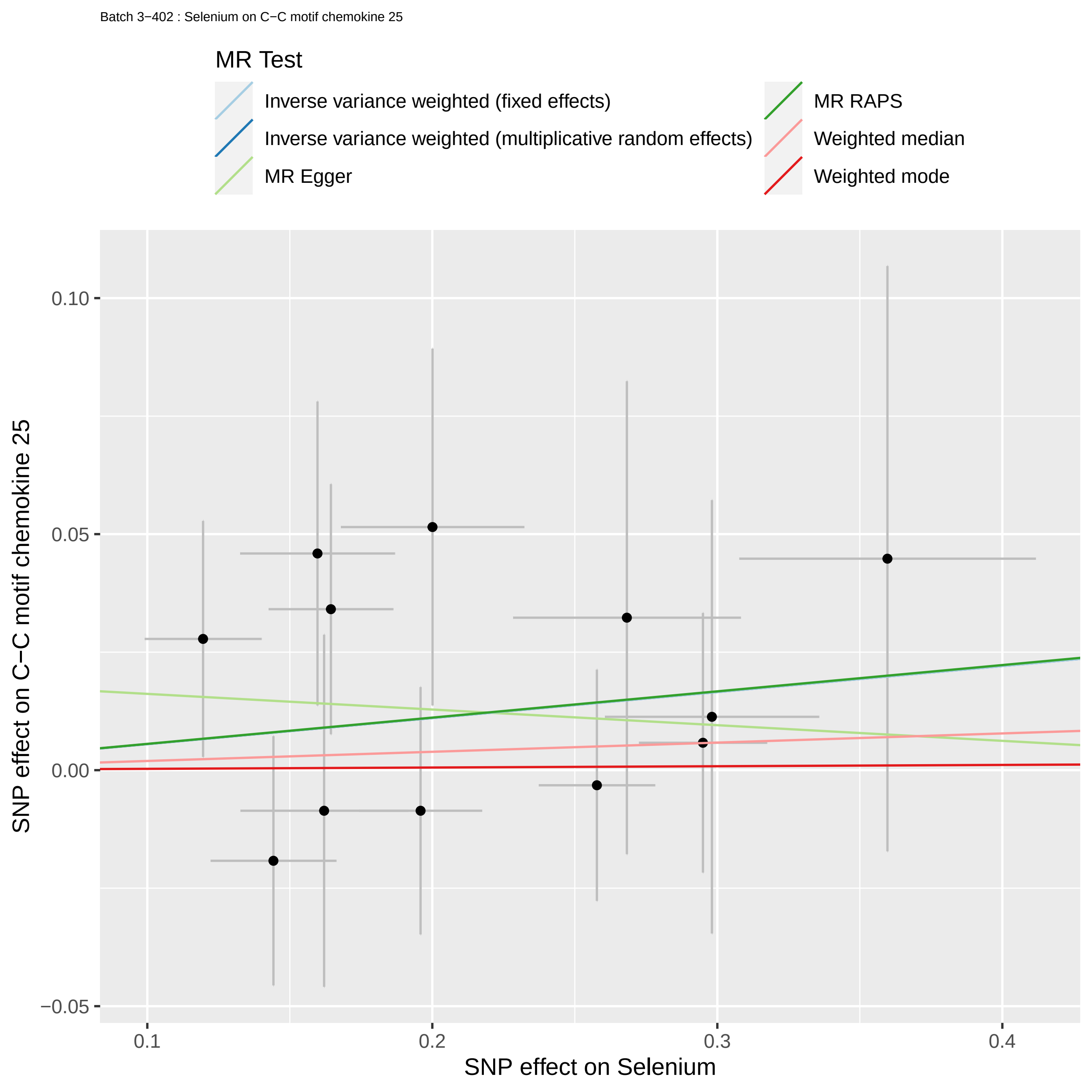

Batch 3−402 : Selenium on C−C motif chemokine 25
MR Test
Inverse variance weighted (fixed effects)
Inverse variance weighted (multiplicative random effects)
MR Egger
MR RAPS
Weighted median
Weighted mode
0.10
0.05
0.00
−0.05
0.1
0.2
0.3
0.4
SNP effect on C−C motif chemokine 25
SNP effect on Selenium

Batch 3−403 : Selenium on C−C motif chemokine 25

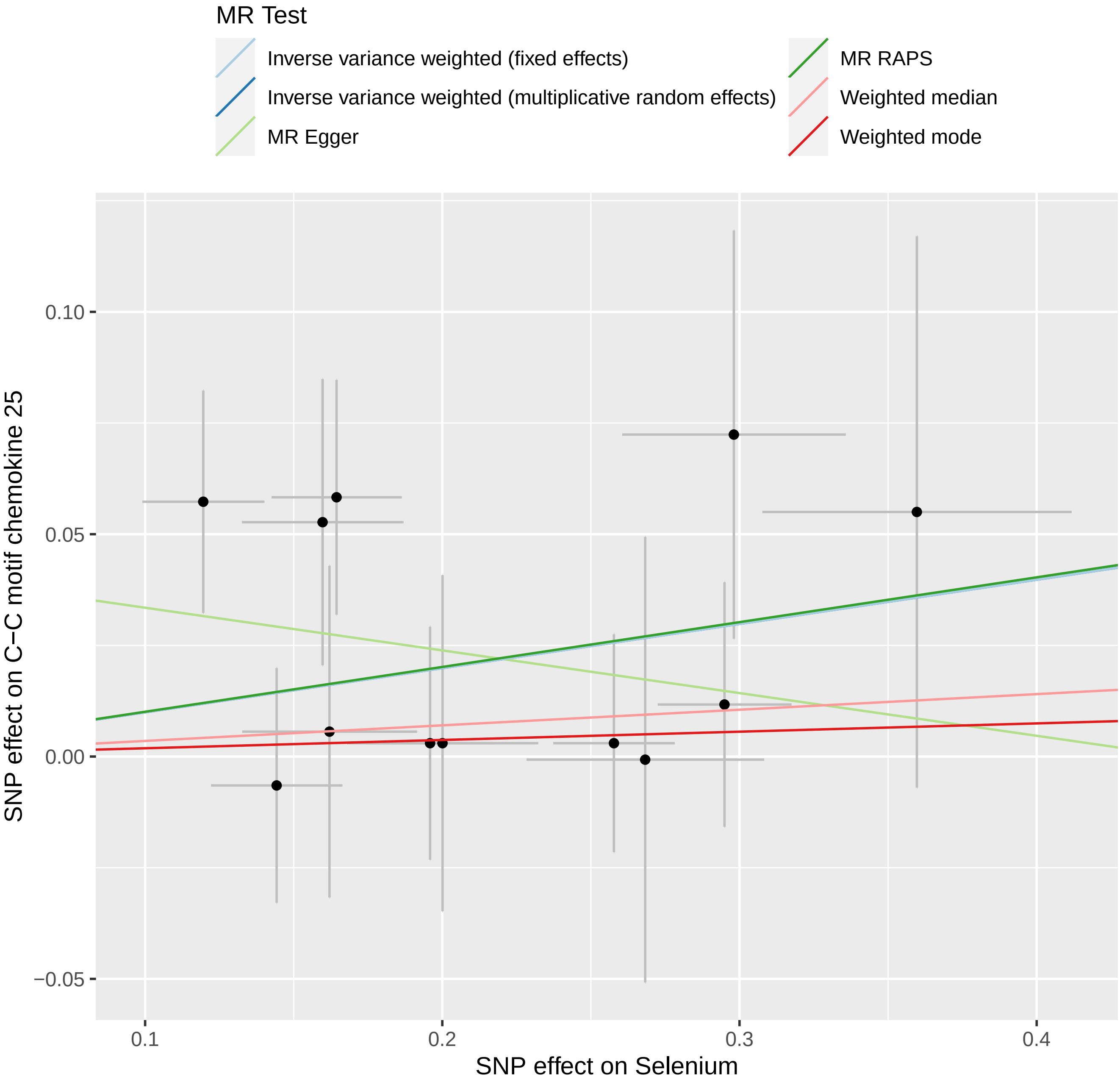

Batch 3−404 : Selenium on C−C motif chemokine 26

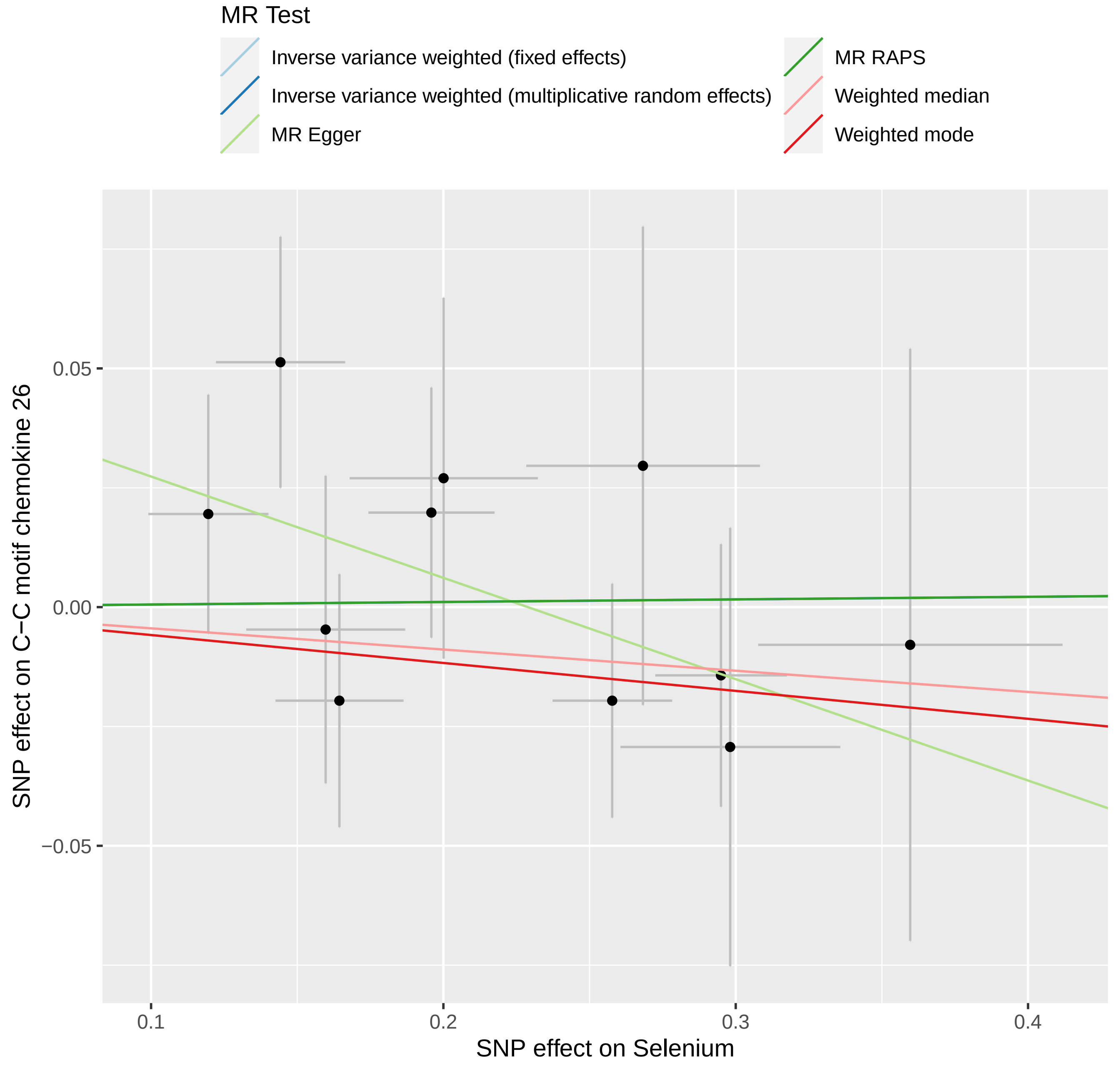

Batch 3−405 : Selenium on C−C motif chemokine 27

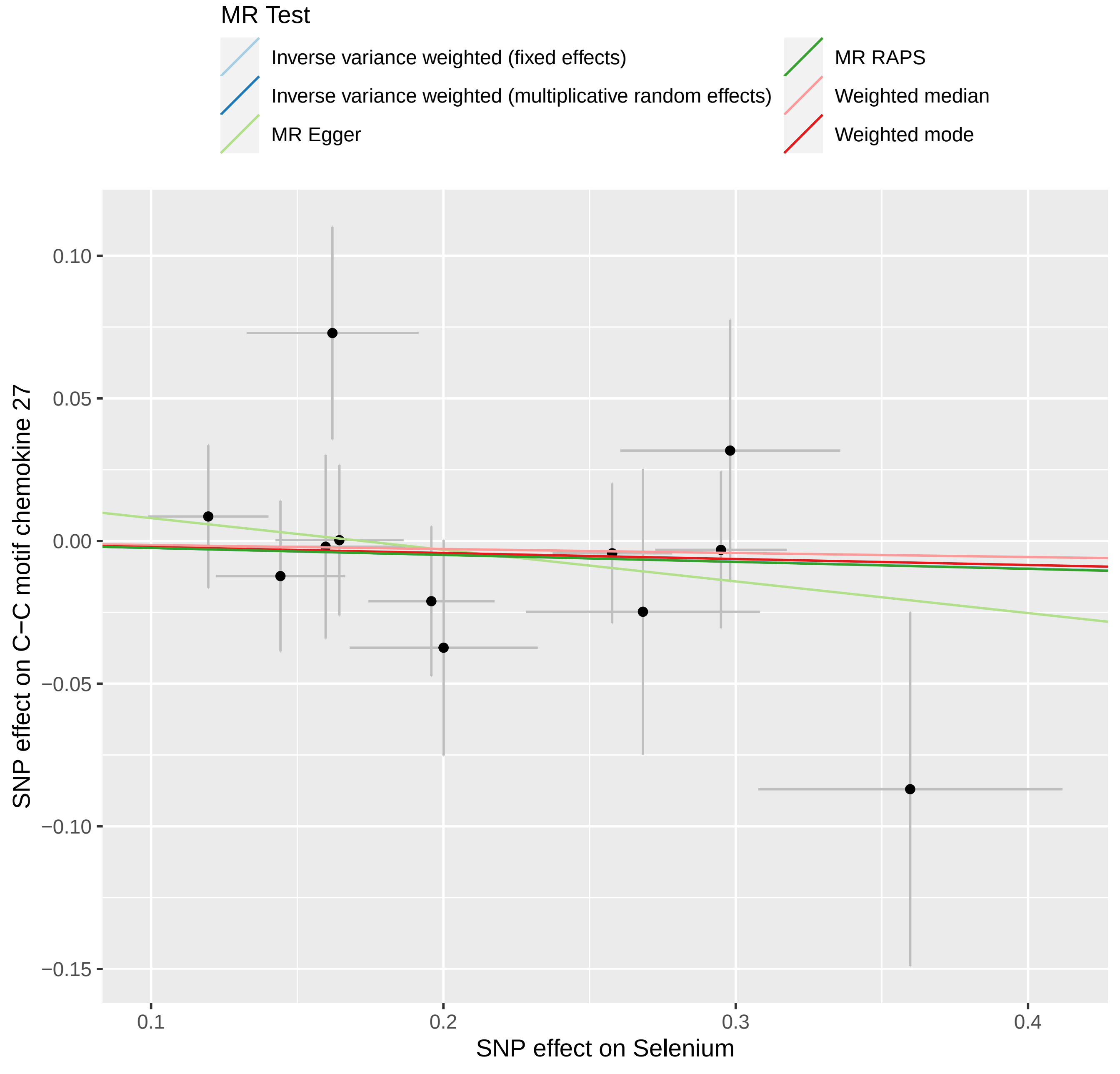

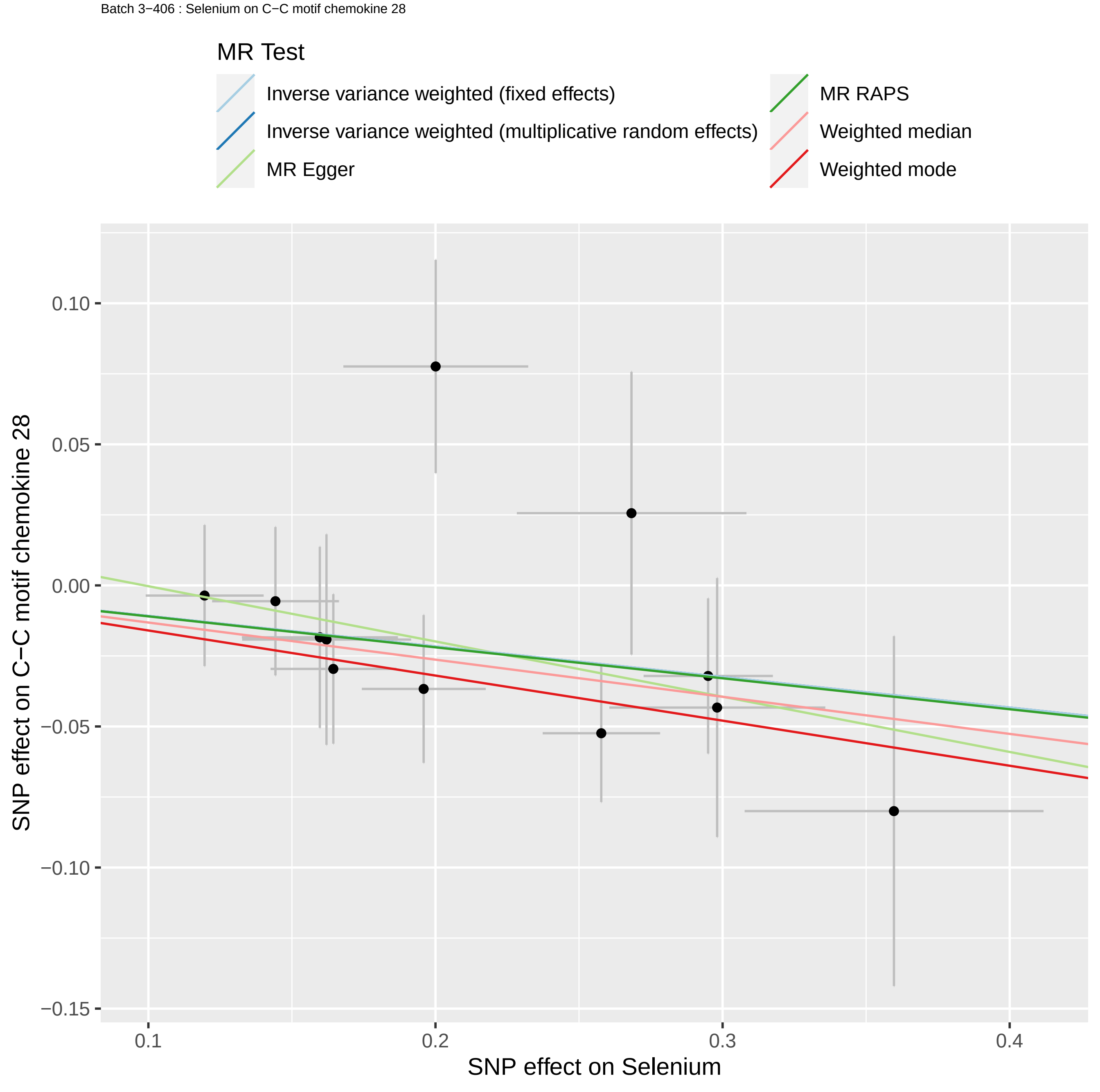
Batch 3−406 : Selenium on C−C motif chemokine 28
MR Test
Inverse variance weighted (fixed effects)
Inverse variance weighted (multiplicative random effects)
MR Egger
MR RAPS
Weighted median
Weighted mode
0.10
0.05
0.00
−0.05
−0.10
−0.15
0.1
0.2
0.3
0.4
SNP effect on C−C motif chemokine 28
SNP effect on Selenium

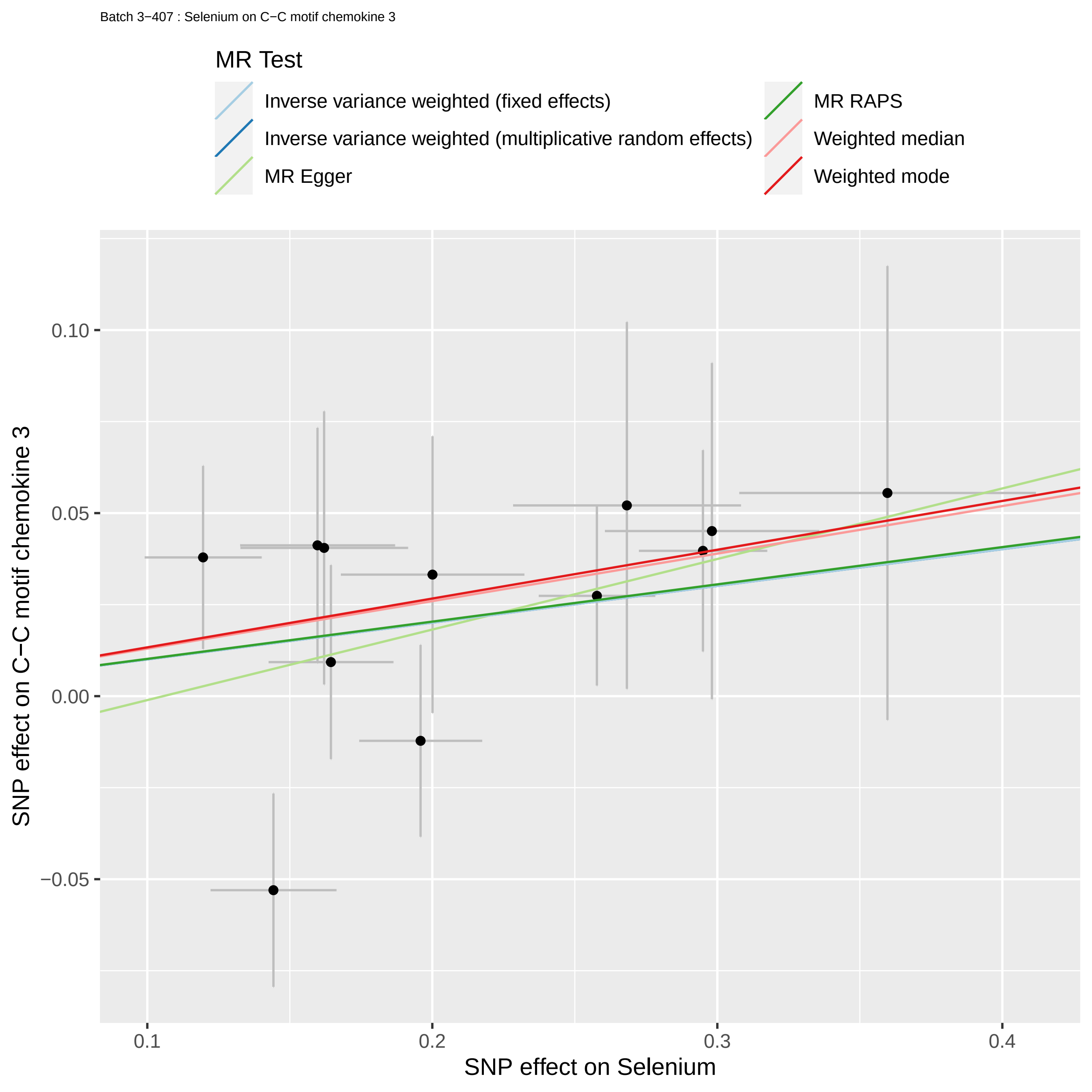

Batch 3−407 : Selenium on C−C motif chemokine 3
MR Test
Inverse variance weighted (fixed effects)
Inverse variance weighted (multiplicative random effects)
MR Egger
MR RAPS
Weighted median
Weighted mode
SNP effect on C−C motif chemokine 3
0.10
0.05
0.00
−0.05
0.1
0.2
0.3
0.4
SNP effect on Selenium

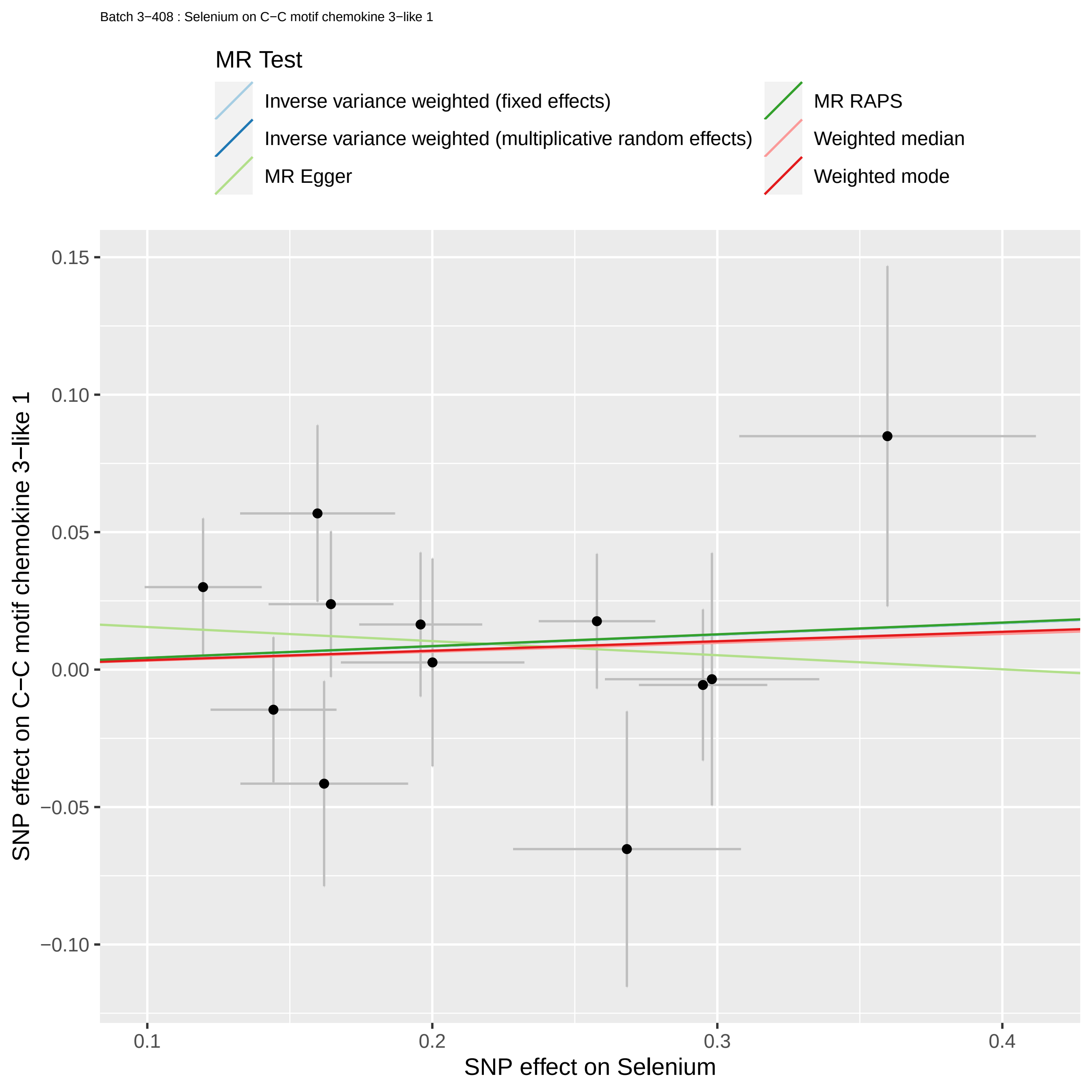

Batch 3−408 : Selenium on C−C motif chemokine 3−like 1
MR Test
Inverse variance weighted (fixed effects)
Inverse variance weighted (multiplicative random effects)
MR Egger
MR RAPS
Weighted median
Weighted mode
SNP effect on C−C motif chemokine 3−like 1
0.15
0.10
0.05
0.00
−0.05
−0.10
0.1
0.2
0.3
0.4
SNP effect on Selenium

Batch 3−409 : Selenium on C−C motif chemokine 5

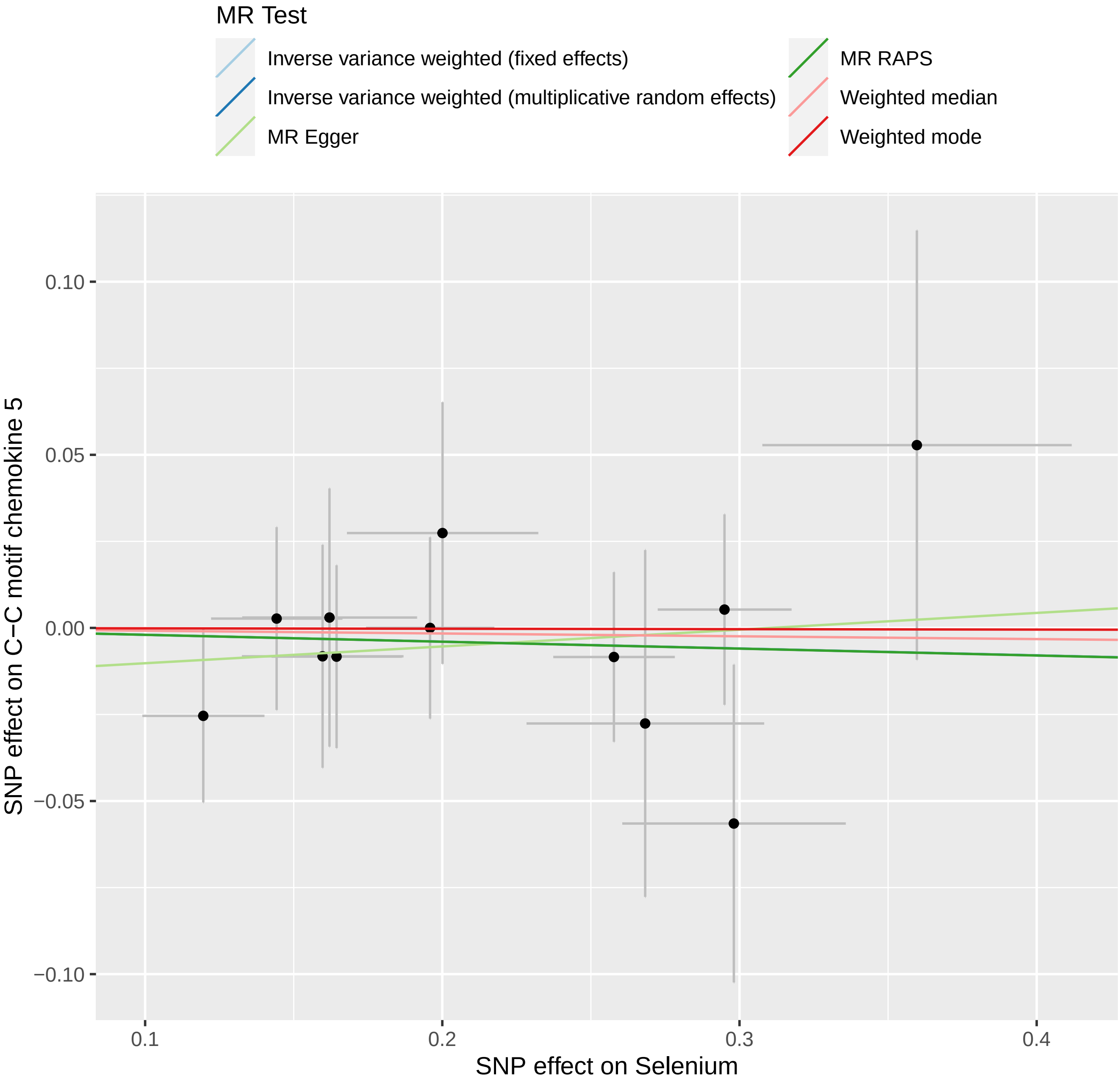

Batch 3−410 : Selenium on C−C motif chemokine 7

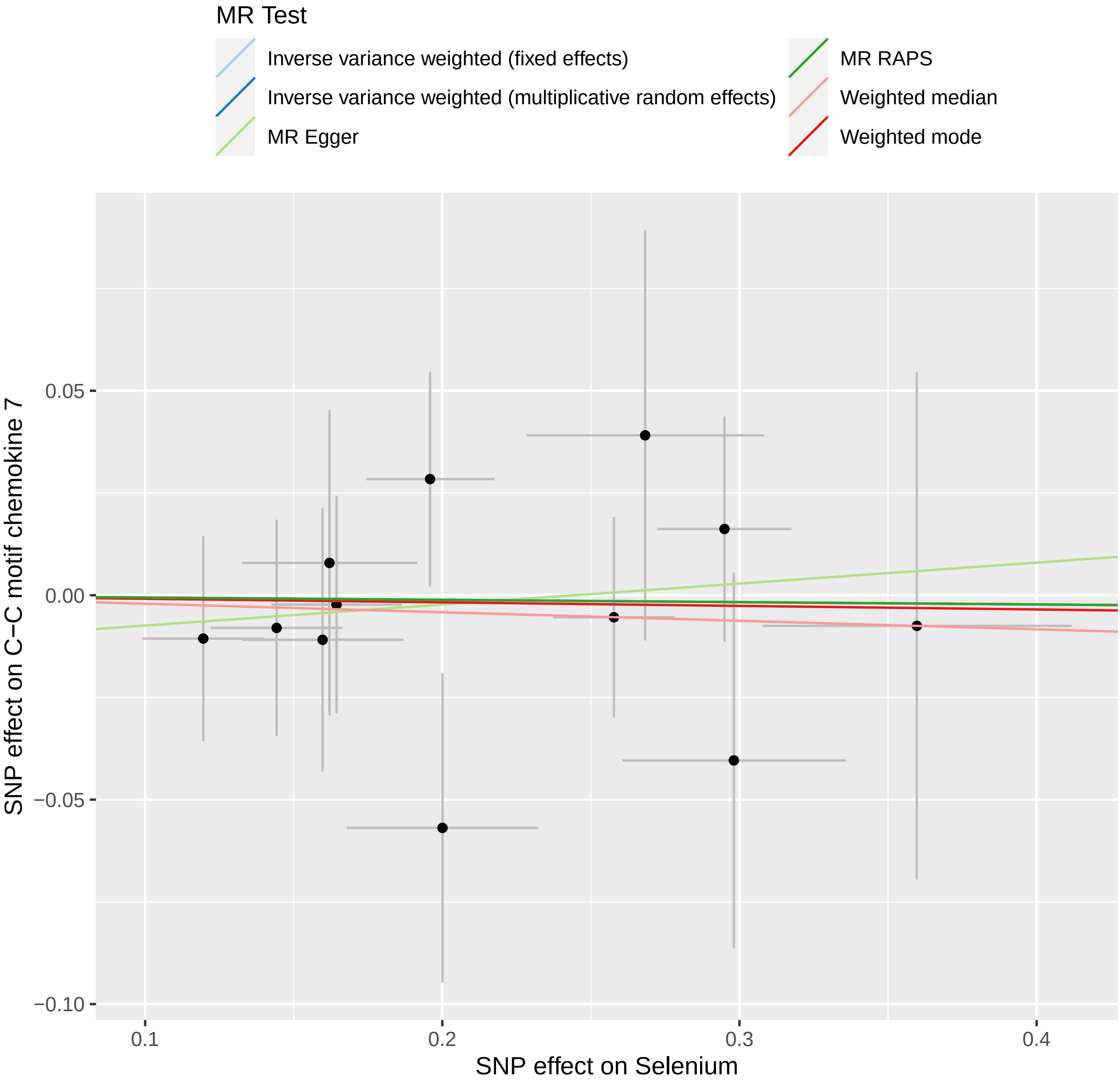

Batch 3−411 : Selenium on C−C motif chemokine 8

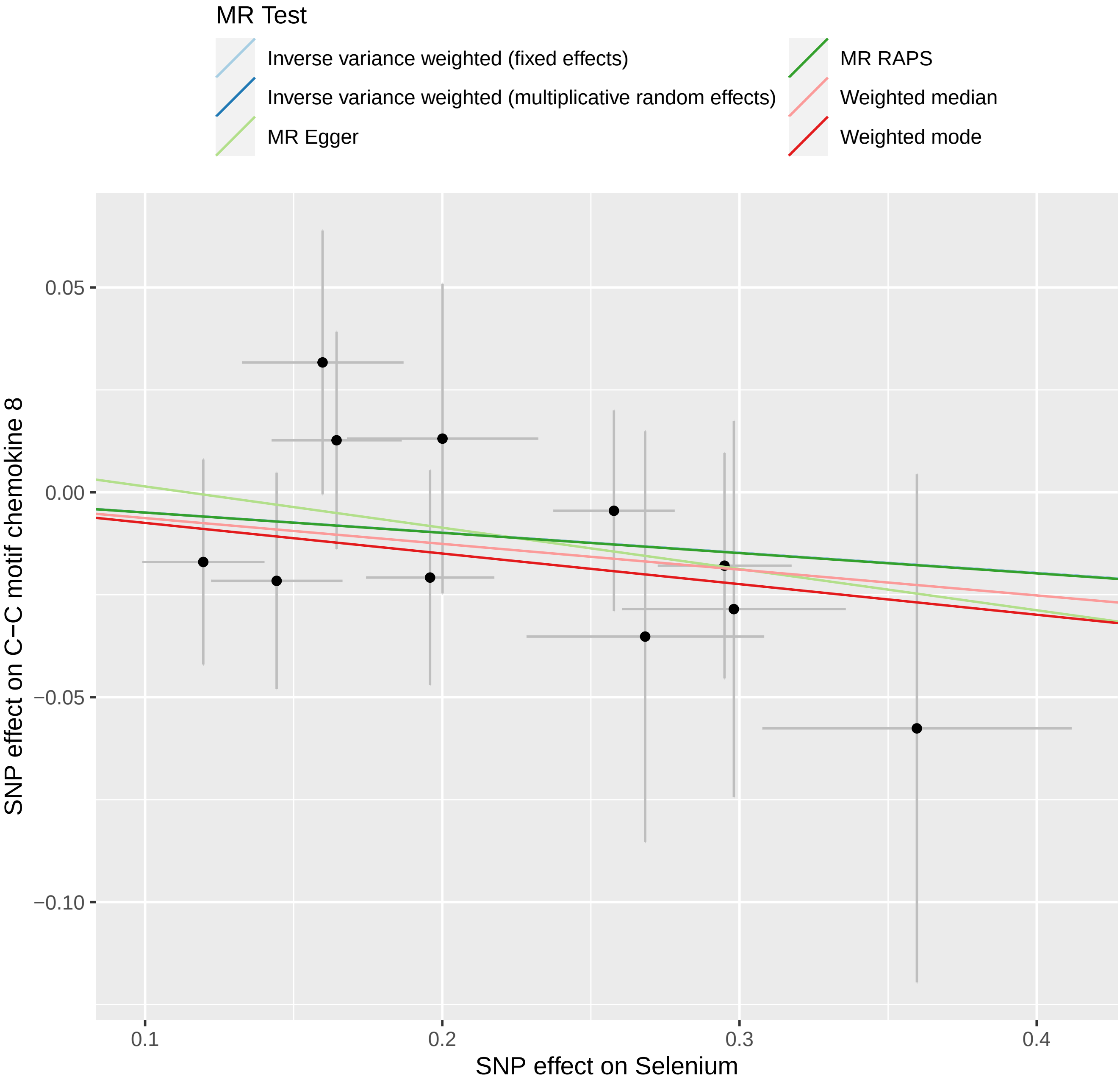

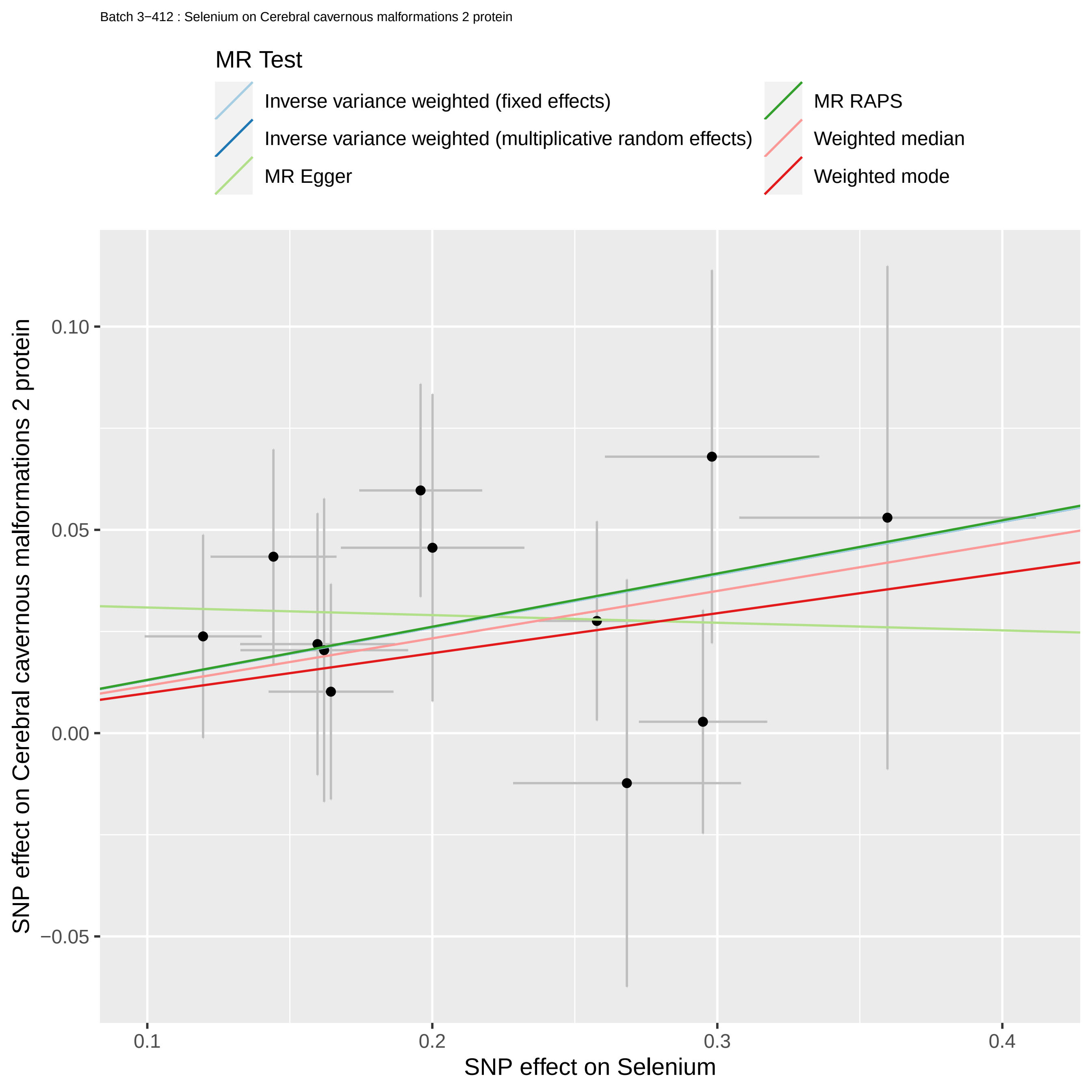
Batch 3−412 : Selenium on Cerebral cavernous malformations 2 protein
MR Test
Inverse variance weighted (fixed effects)
Inverse variance weighted (multiplicative random effects)
MR Egger
MR RAPS
Weighted median
Weighted mode
SNP effect on Cerebral cavernous malformations 2 protein
0.10
0.05
0.00
−0.05
0.1
0.2
0.3
0.4
SNP effect on Selenium

Batch 3−413 : Selenium on G2/mitotic−specific cyclin−B1

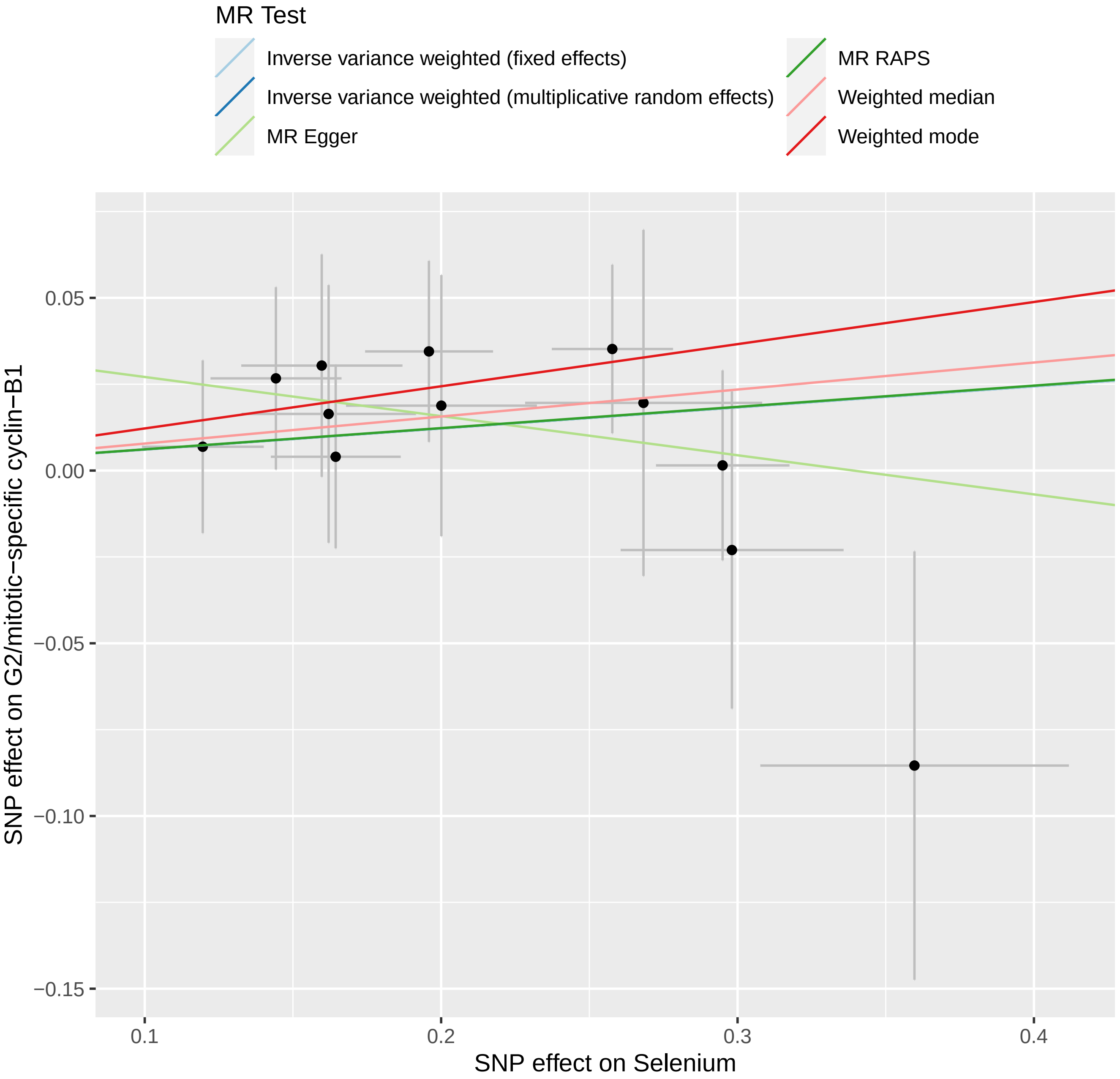

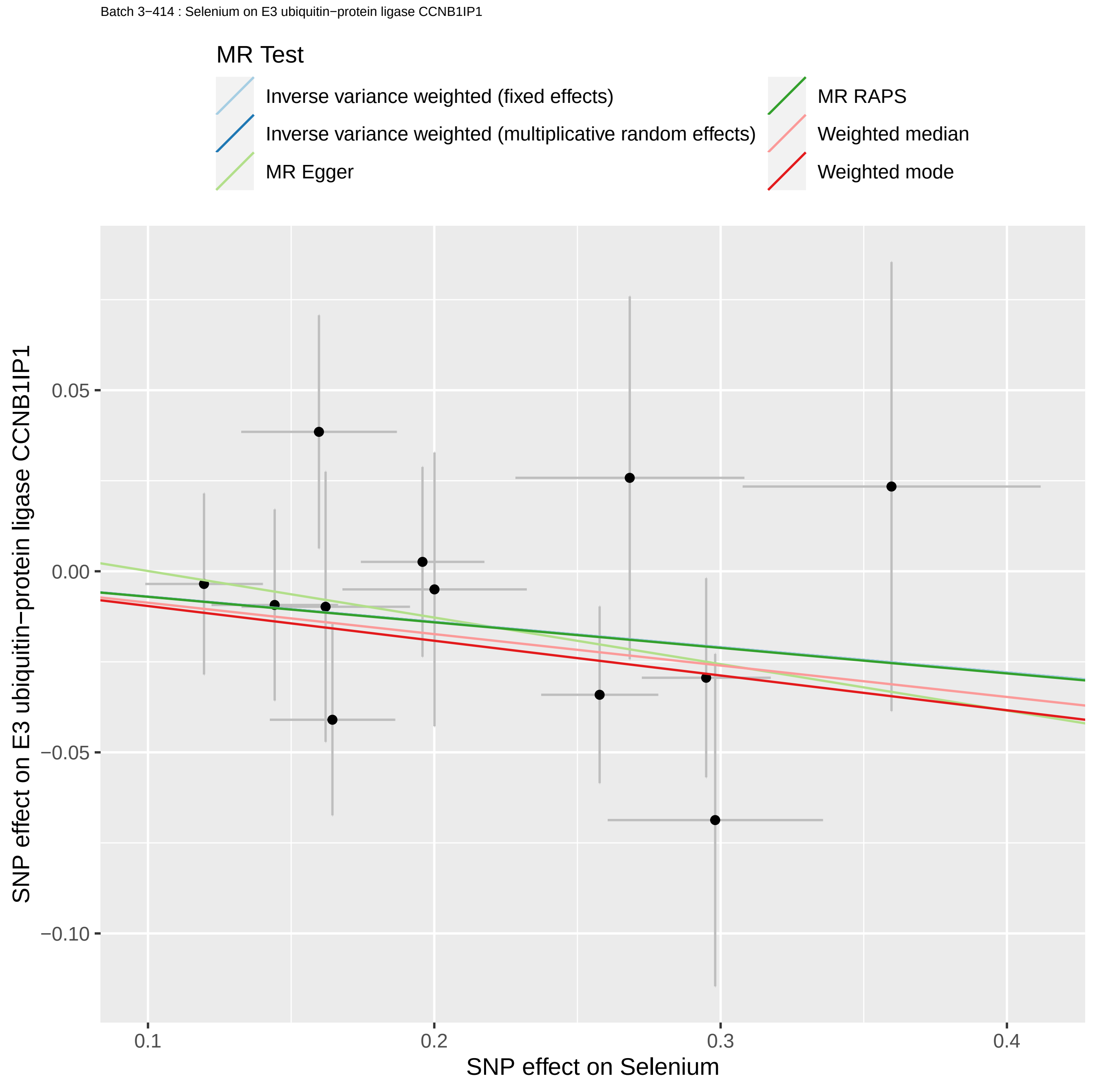
Batch 3−414 : Selenium on E3 ubiquitin−protein ligase CCNB1IP1
MR Test
Inverse variance weighted (fixed effects)
Inverse variance weighted (multiplicative random effects)
MR Egger
MR RAPS
Weighted median
Weighted mode
SNP effect on E3 ubiquitin−protein ligase CCNB1IP1
0.05
0.00
−0.05
−0.10
0.1
0.2
0.3
0.4
SNP effect on Selenium

Batch 3−415 : Selenium on Cyclin−C

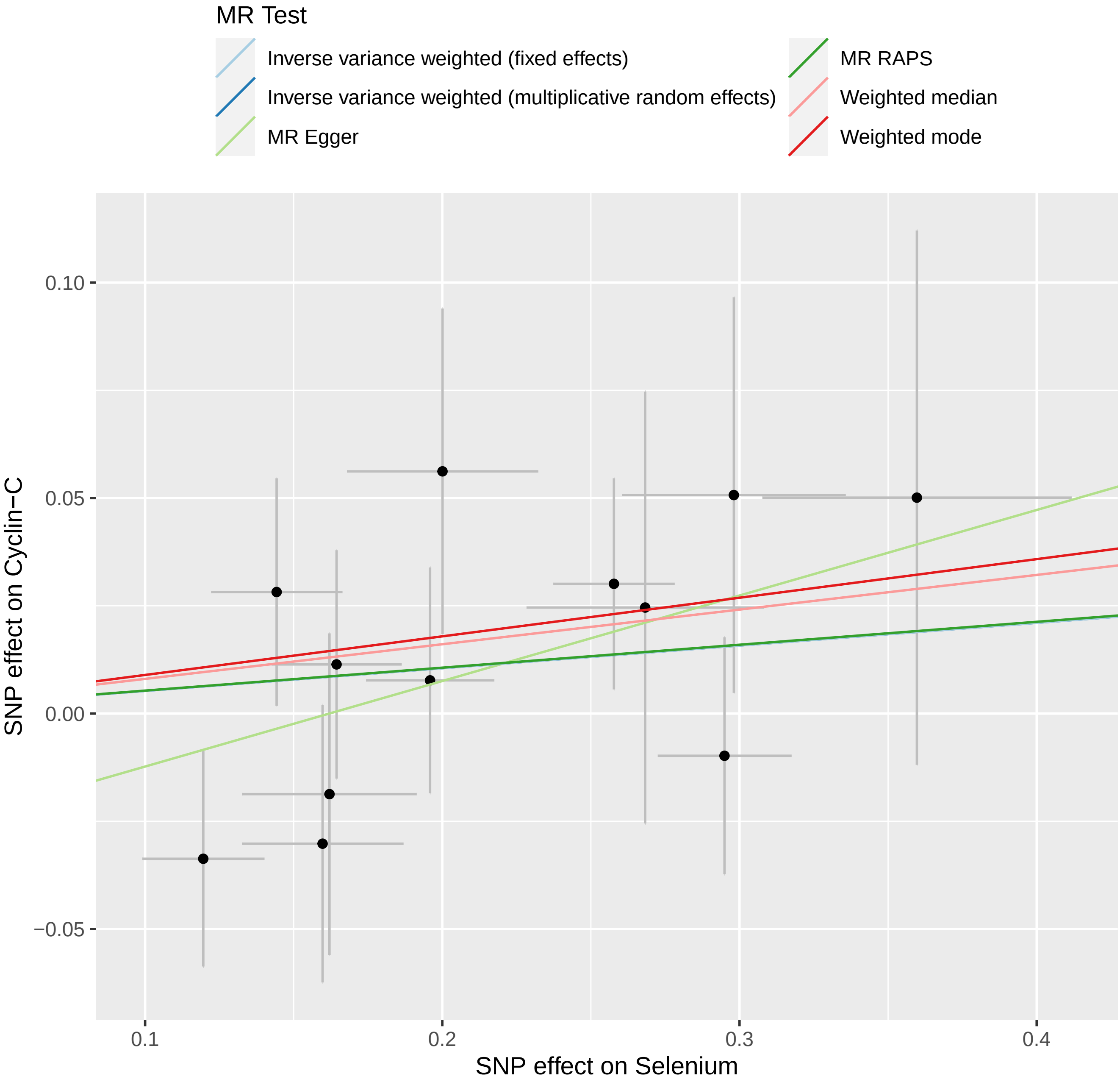

Batch 3−416 : Selenium on Cyclin−H

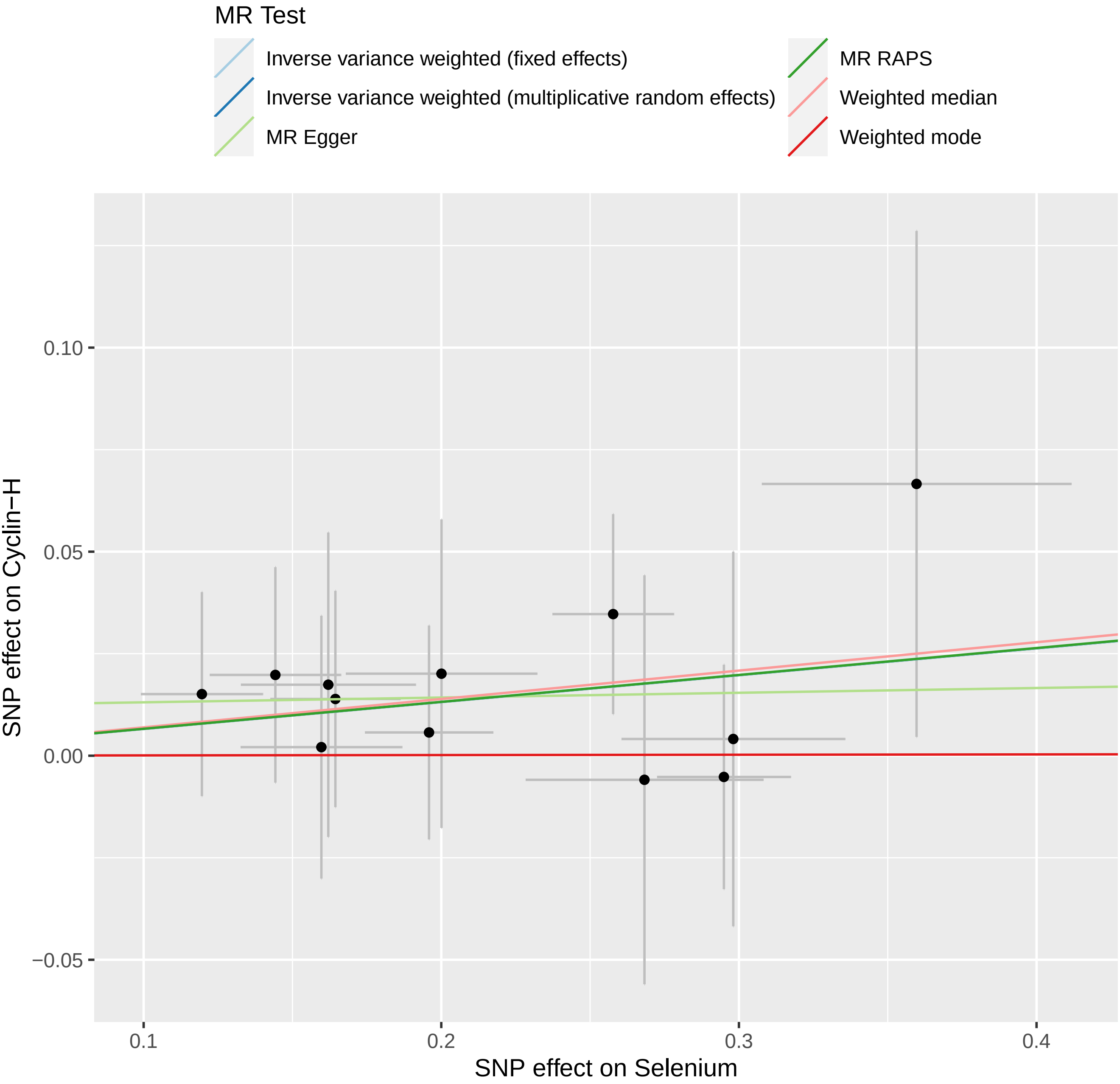

Batch 3−417 : Selenium on Cell cycle progression protein 1

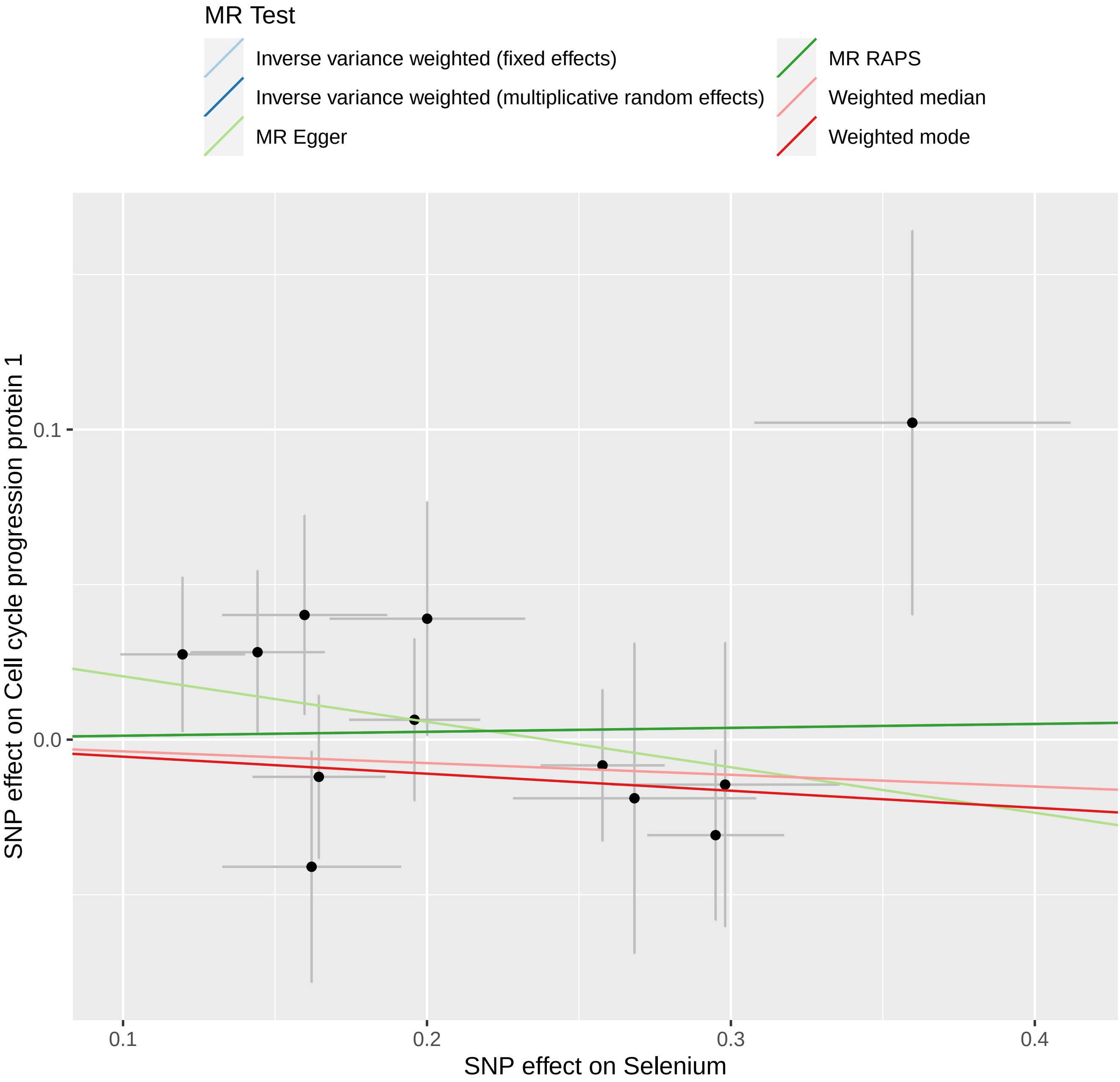

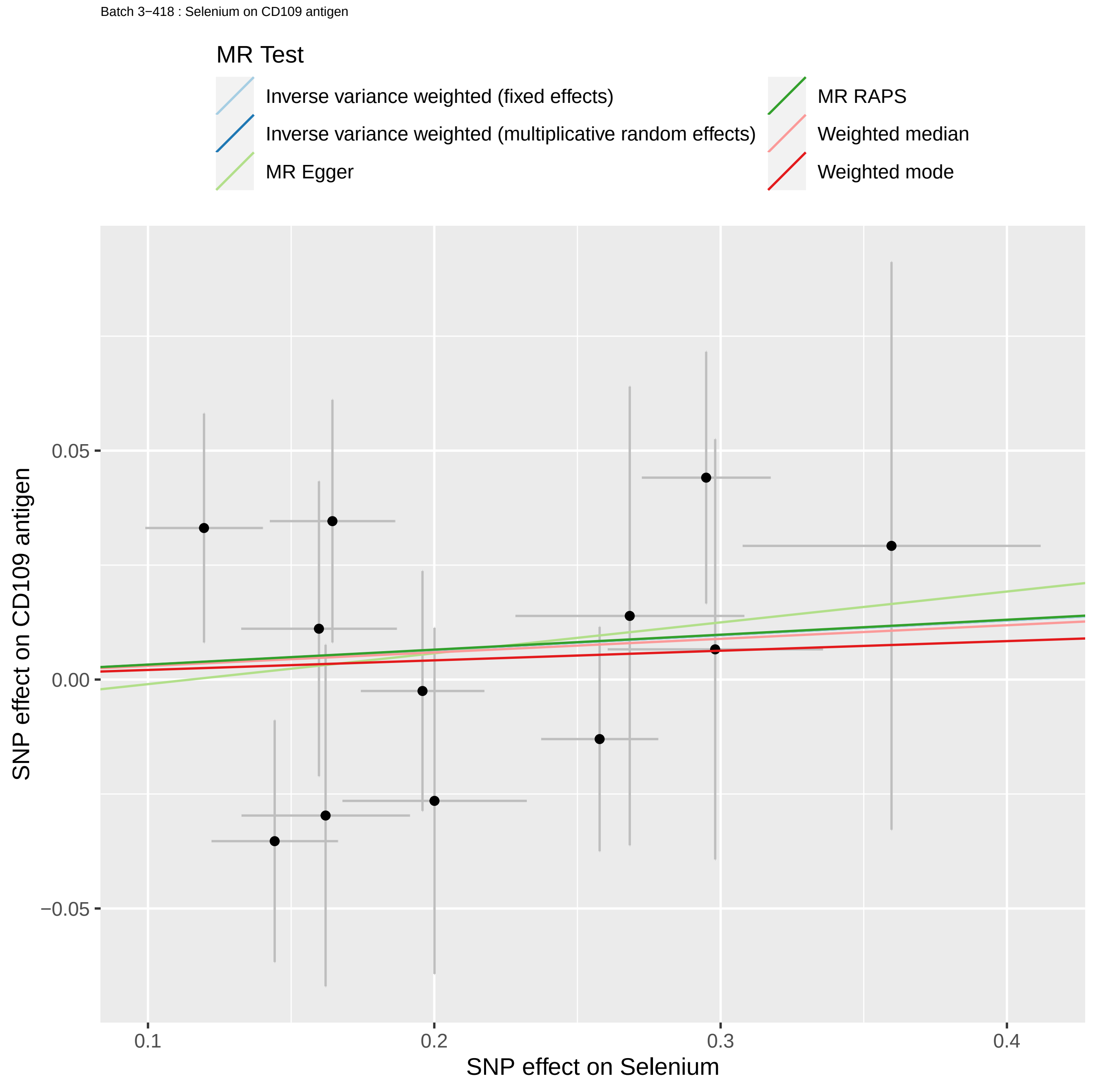
Batch 3−418 : Selenium on CD109 antigen
MR Test
Inverse variance weighted (fixed effects)
Inverse variance weighted (multiplicative random effects)
MR Egger
MR RAPS
Weighted median
Weighted mode
SNP effect on CD109 antigen
0.05
0.00
−0.05
SNP effect on Selenium
0.1
0.2
0.3
0.4

Batch 3−419 : Selenium on Scavenger receptor cysteine−rich type 1 protein M130

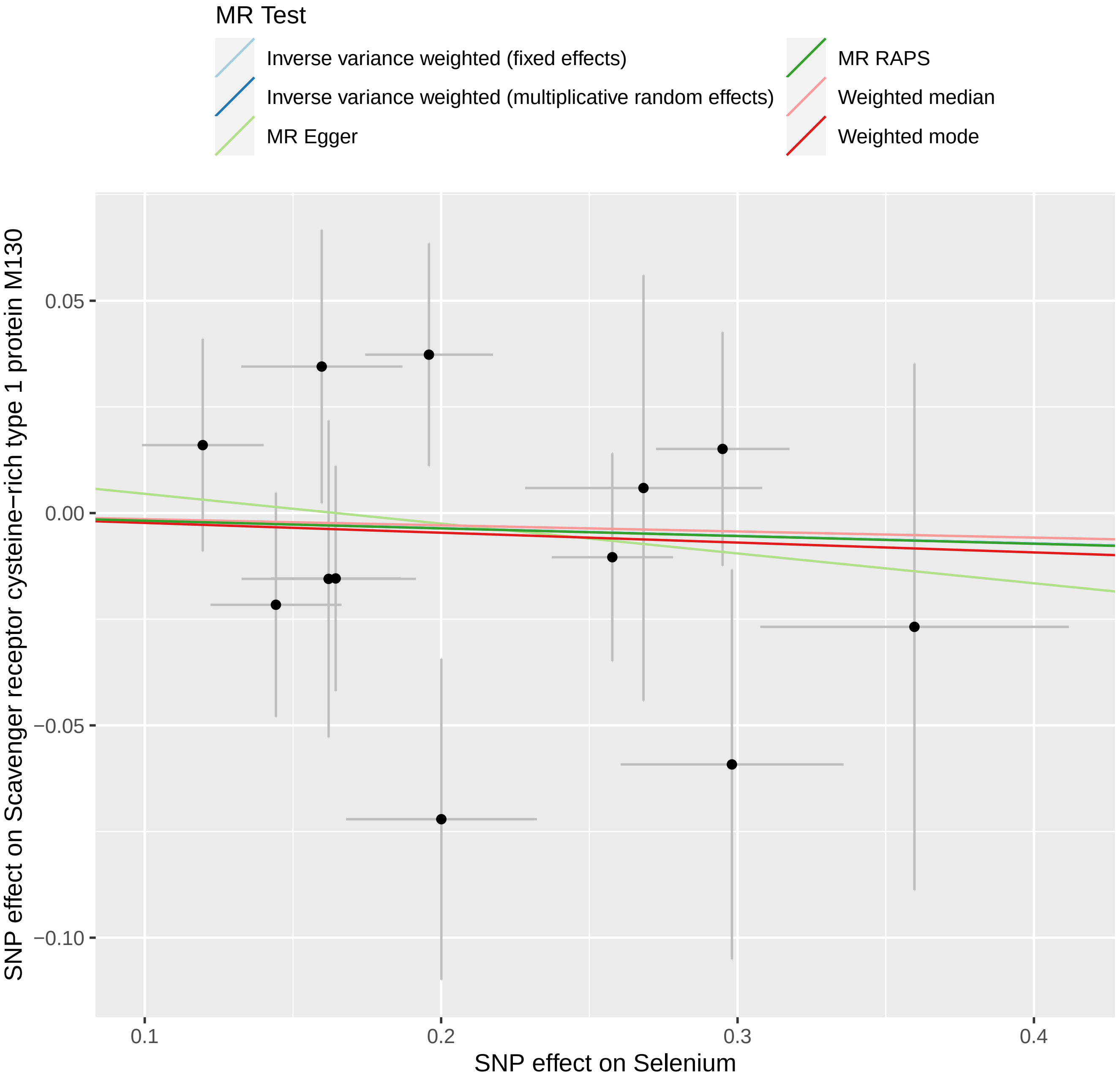

Batch 3−420 : Selenium on CD177 antigen

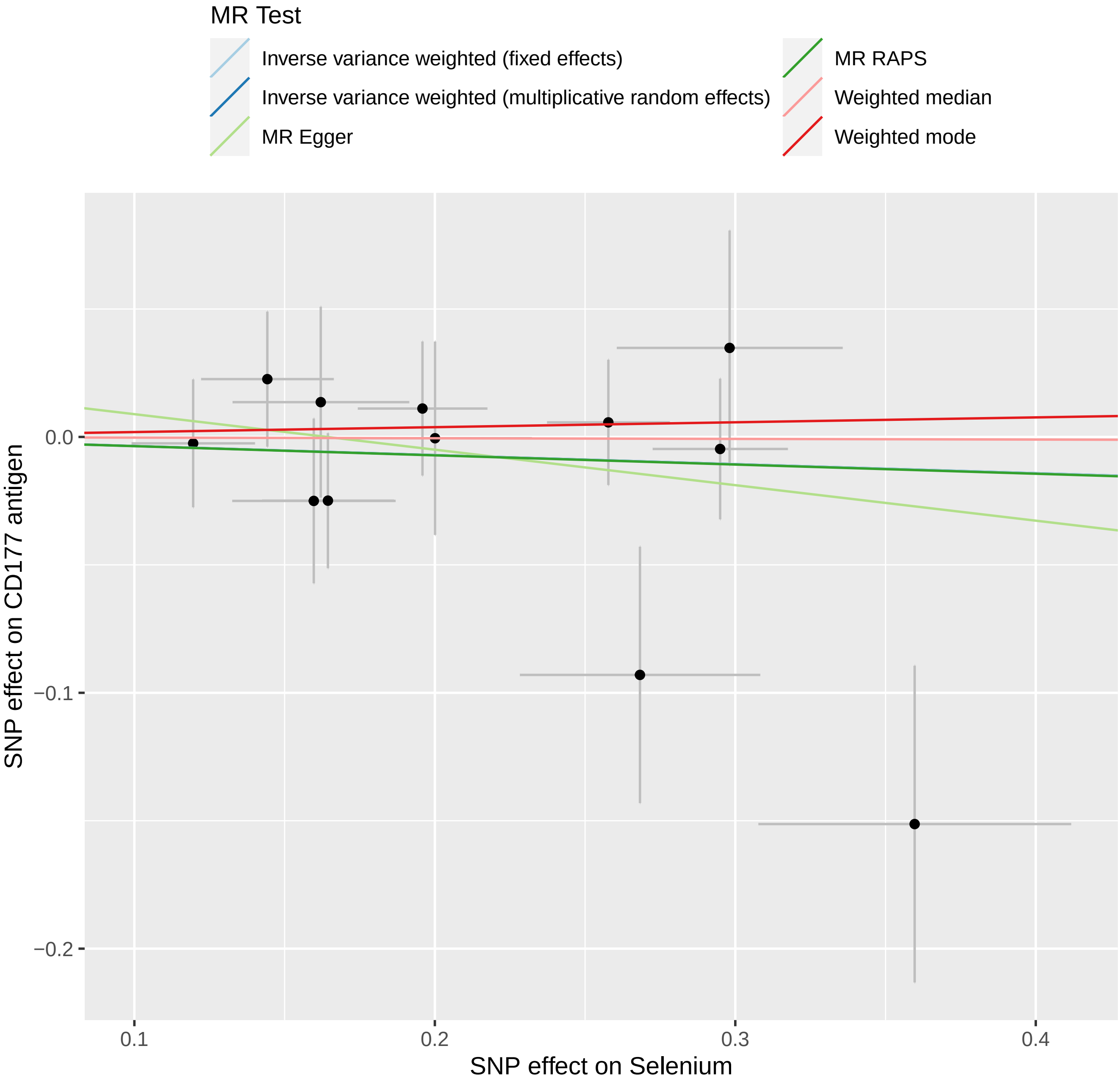

Batch 3−421 : Selenium on Antigen−presenting glycoprotein CD1d

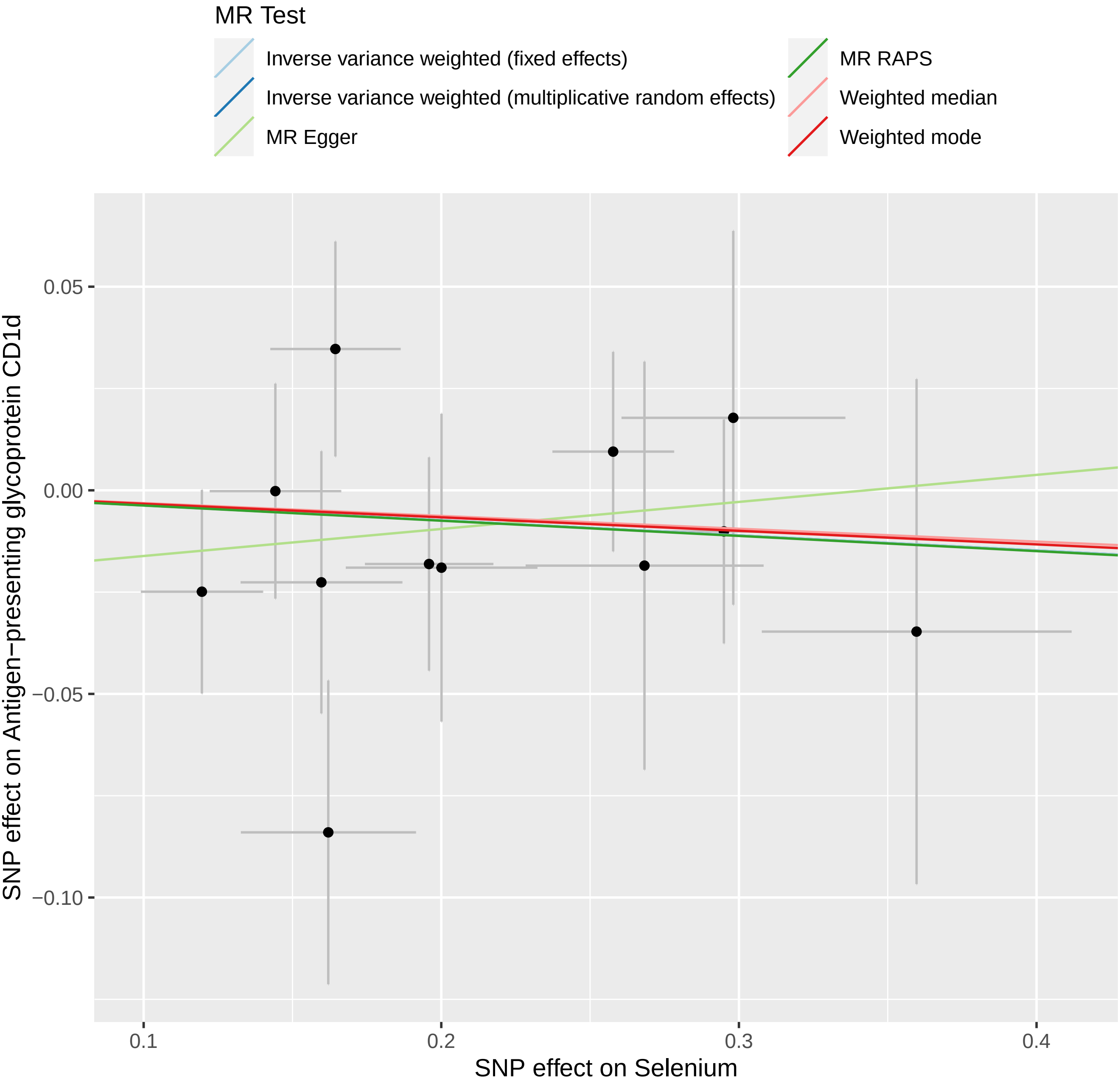

Batch 3−422 : Selenium on OX−2 membrane glycoprotein

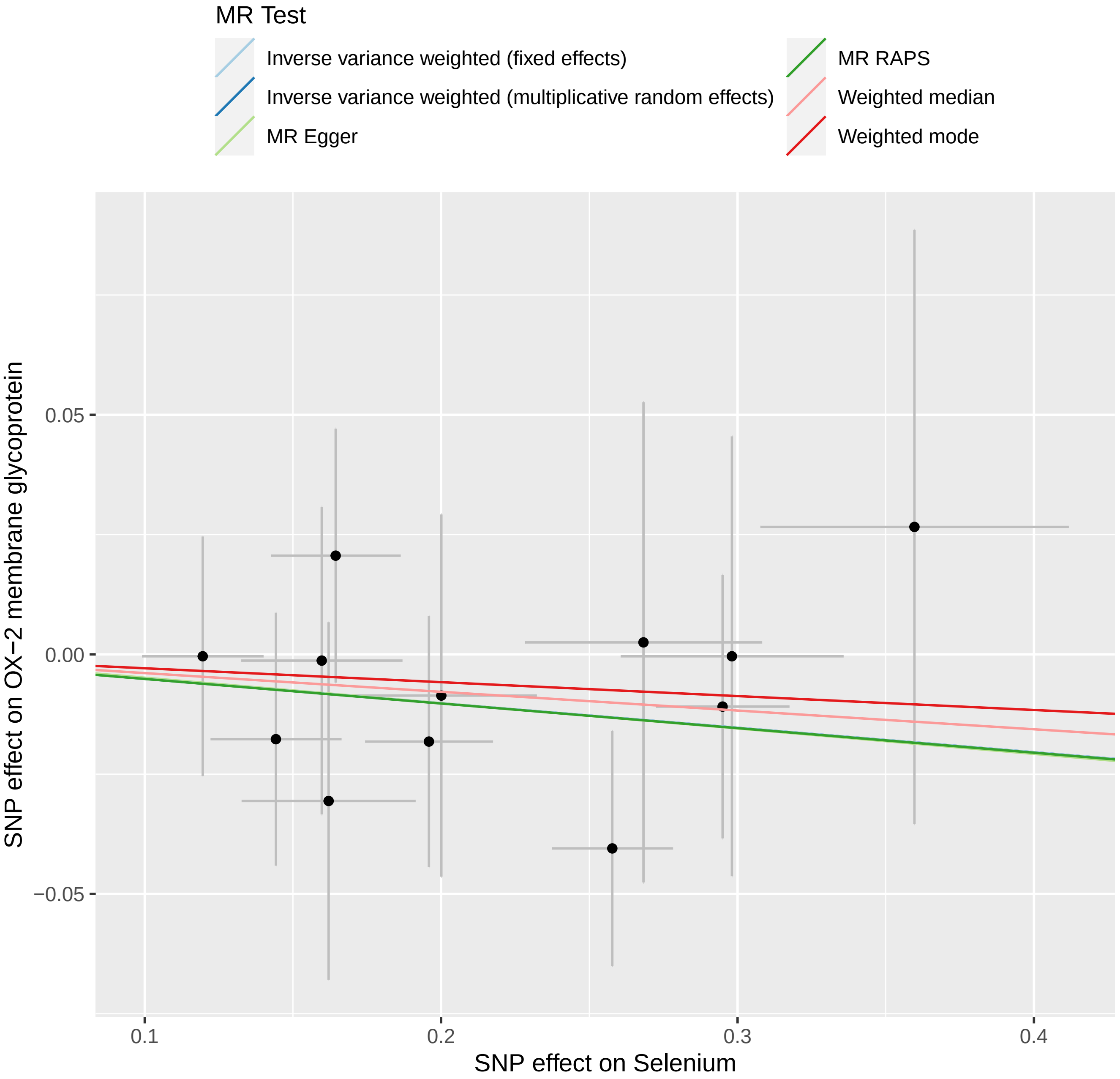

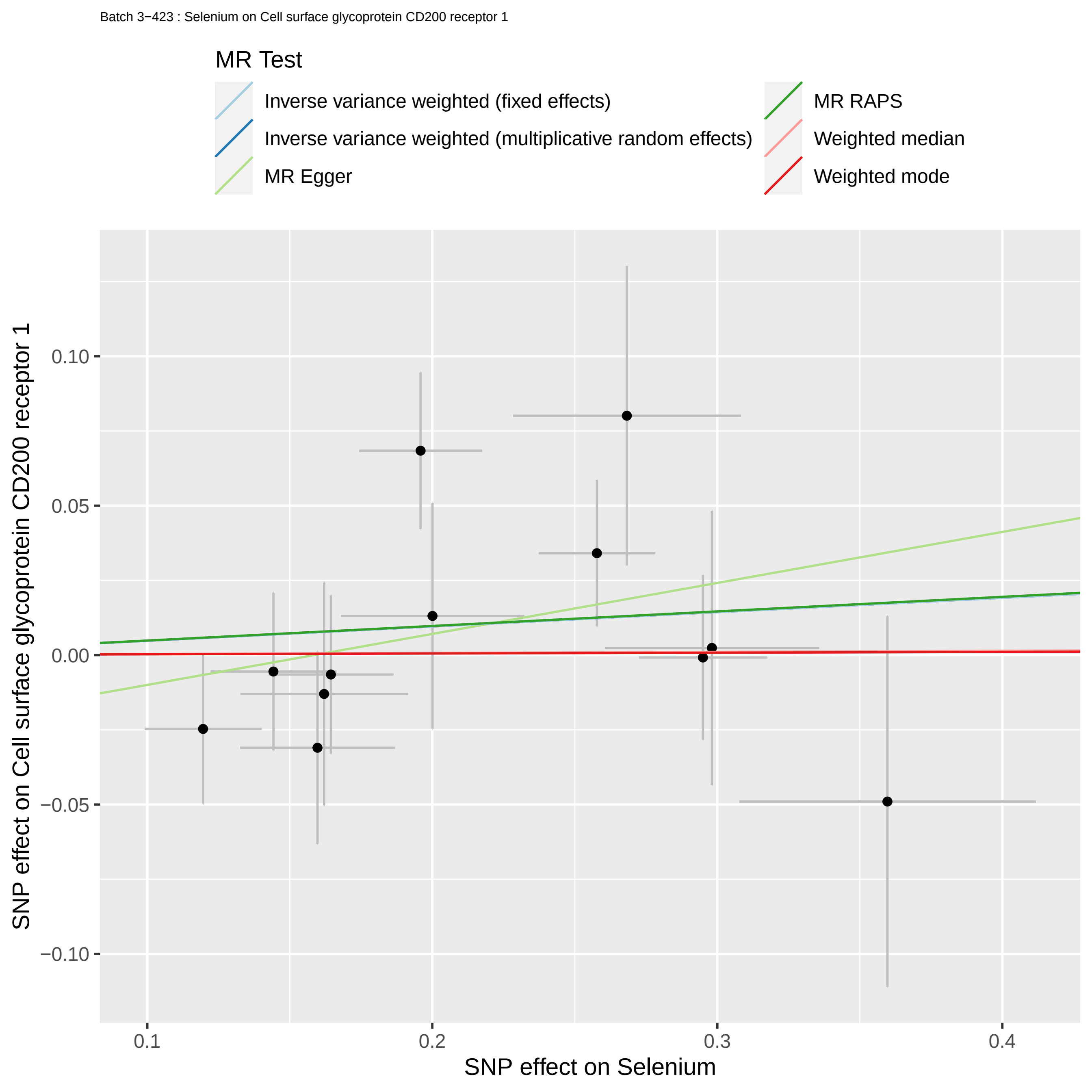

Batch 3−423 : Selenium on Cell surface glycoprotein CD200 receptor 1
MR Test
Inverse variance weighted (fixed effects)
Inverse variance weighted (multiplicative random effects)
MR Egger
MR RAPS
Weighted median
Weighted mode
0.10
0.05
0.00
−0.05
−0.10
0.1
0.2
0.3
0.4
SNP effect on Selenium
SNP effect on Cell surface glycoprotein CD200 receptor 1

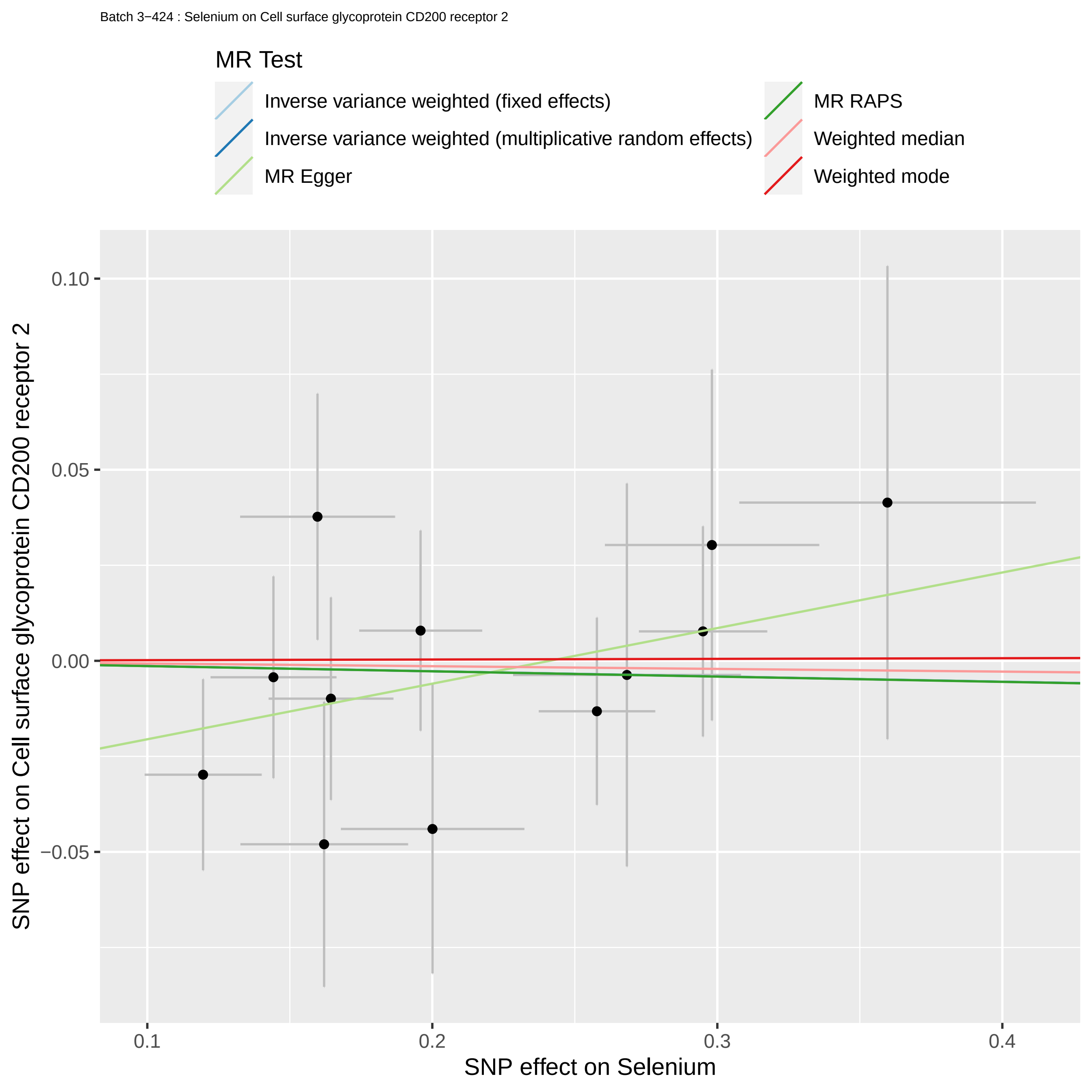
Batch 3−424 : Selenium on Cell surface glycoprotein CD200 receptor 2
MR Test
Inverse variance weighted (fixed effects)
Inverse variance weighted (multiplicative random effects)
MR Egger
MR RAPS
Weighted median
Weighted mode
SNP effect on Cell surface glycoprotein CD200 receptor 2
0.10
0.05
0.00
−0.05
0.1
0.2
0.3
0.4
SNP effect on Selenium

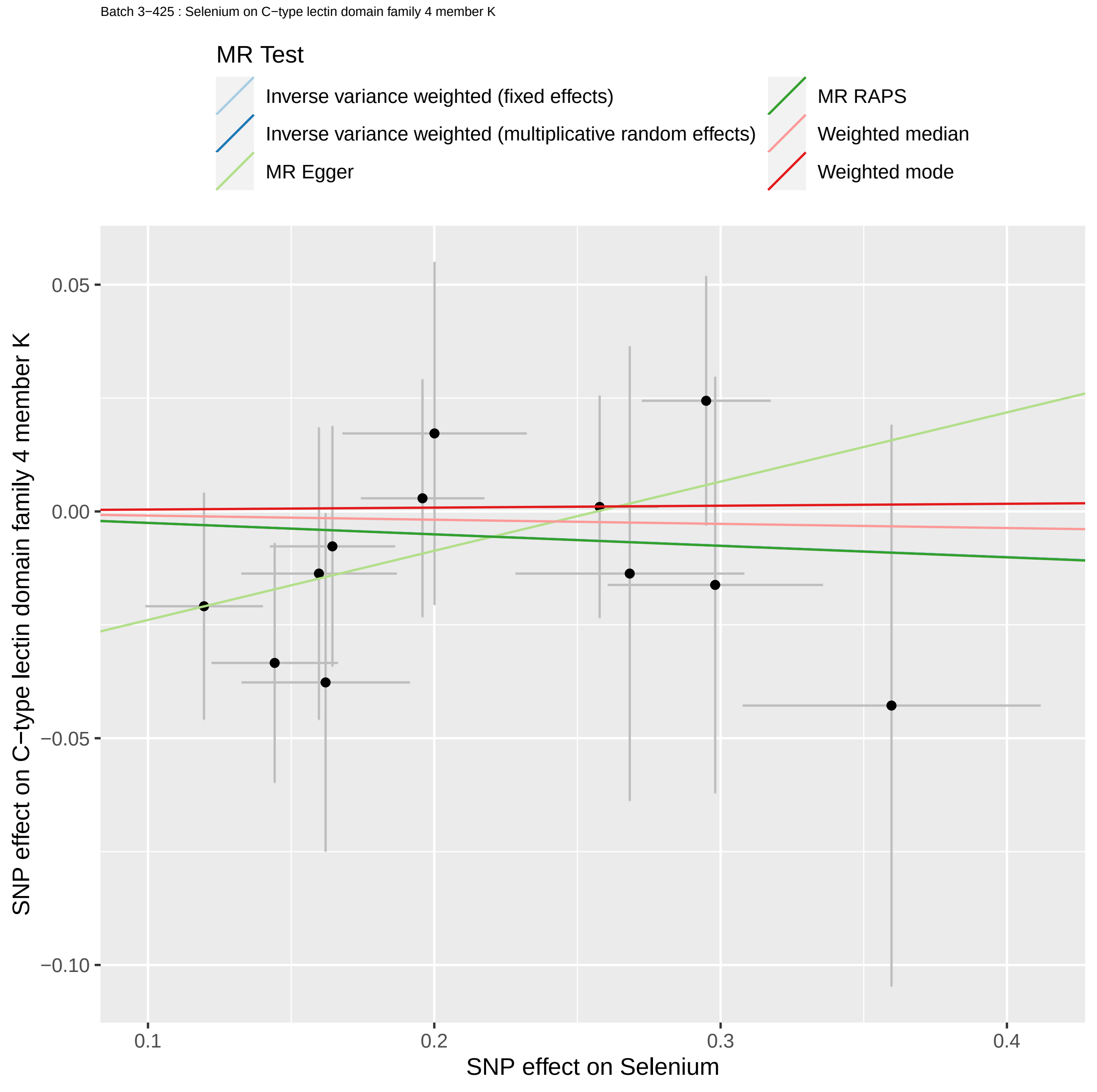
Batch 3−425 : Selenium on C−type lectin domain family 4 member K
MR Test
Inverse variance weighted (fixed effects)
Inverse variance weighted (multiplicative random effects)
MR Egger
MR RAPS
Weighted median
Weighted mode
0.05
0.00
−0.05
−0.10
0.1
0.2
0.3
0.4
SNP effect on C−type lectin domain family 4 member K
SNP effect on Selenium

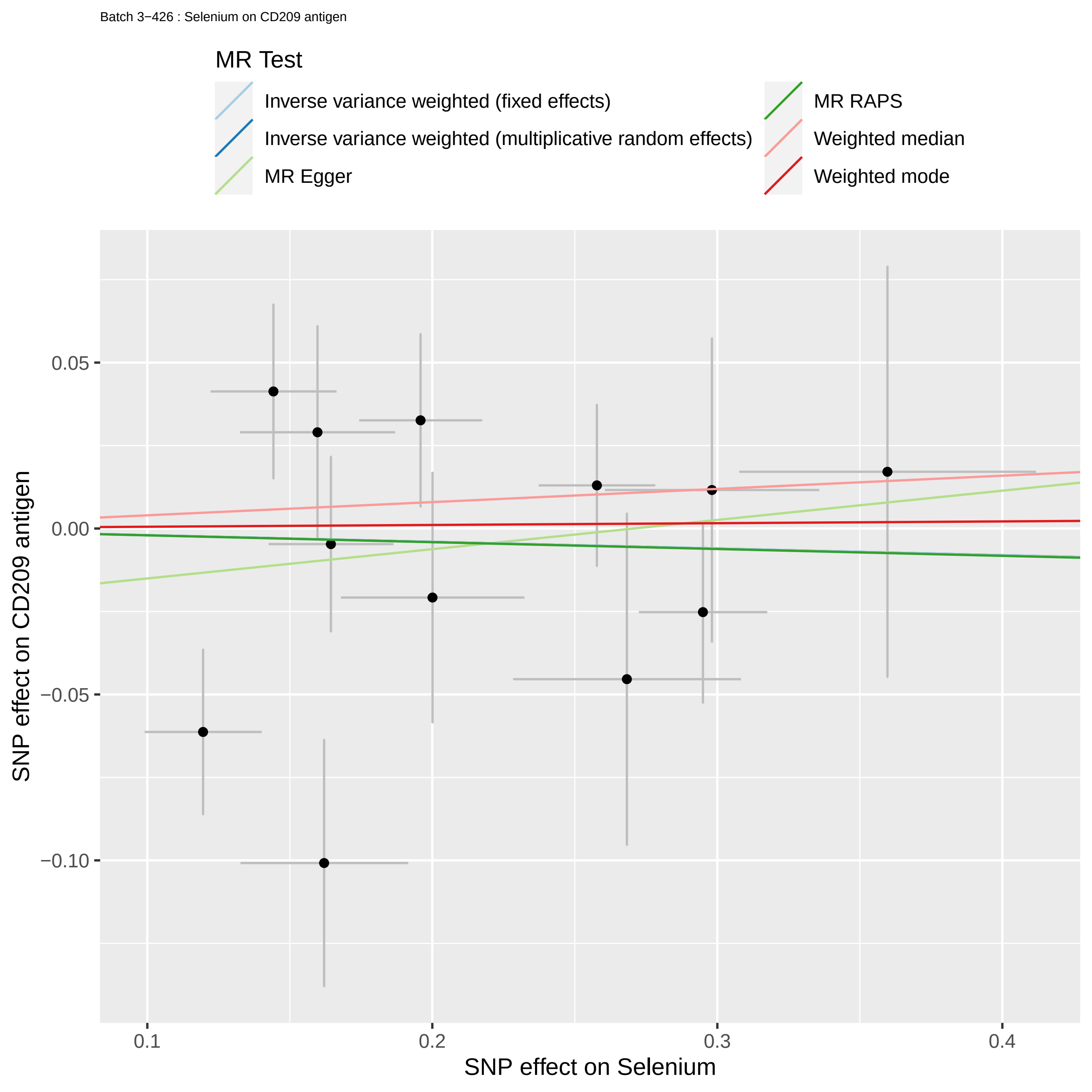

Batch 3−426 : Selenium on CD209 antigen
MR Test
Inverse variance weighted (fixed effects)
Inverse variance weighted (multiplicative random effects)
MR Egger
MR RAPS
Weighted median
Weighted mode
0.05
0.00
−0.05
−0.10
0.1
0.2
0.3
0.4
SNP effect on CD209 antigen
SNP effect on Selenium

Batch 3−427 : Selenium on B−cell receptor CD22

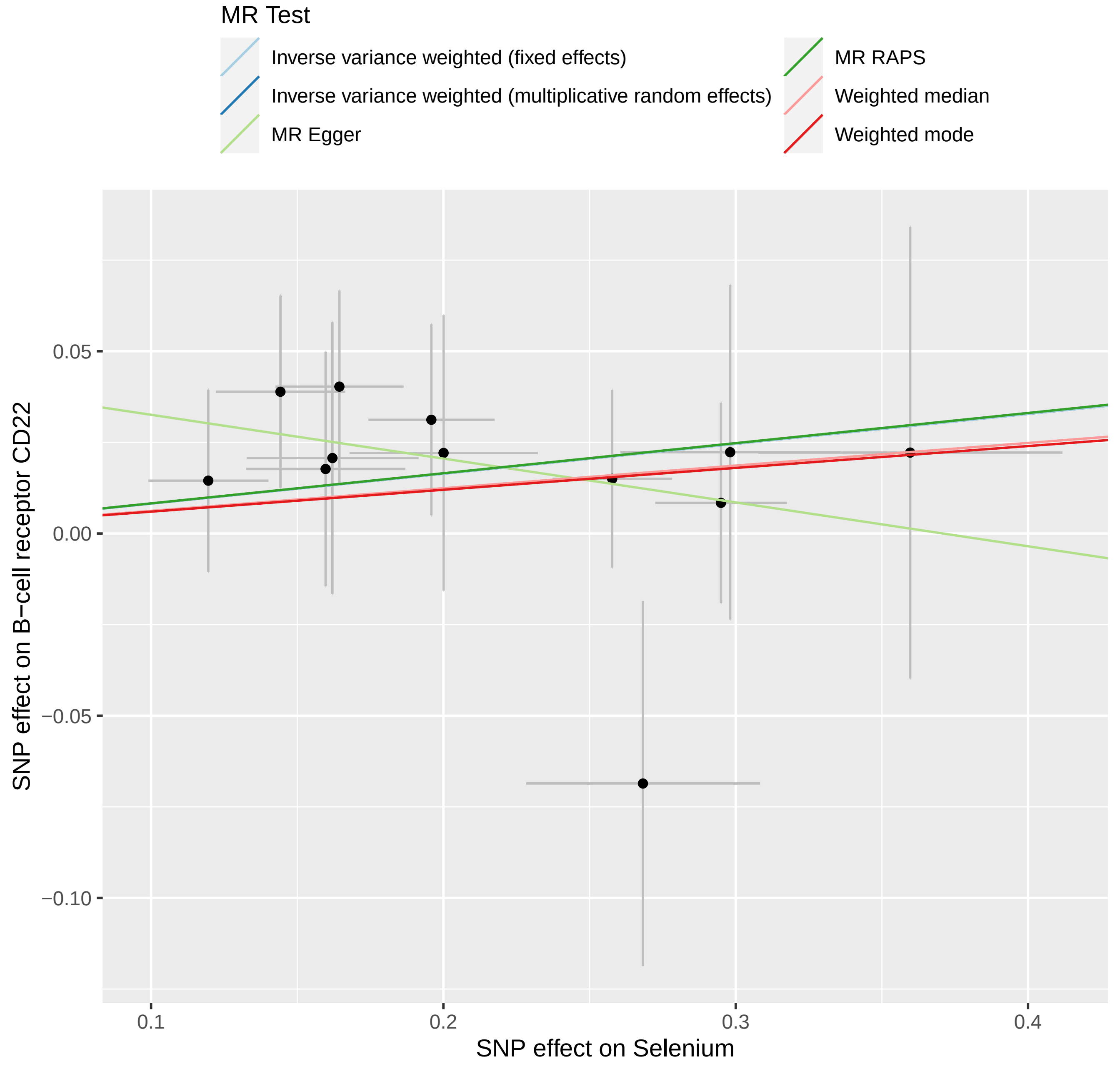

Batch 3−428 : Selenium on CD226 antigen

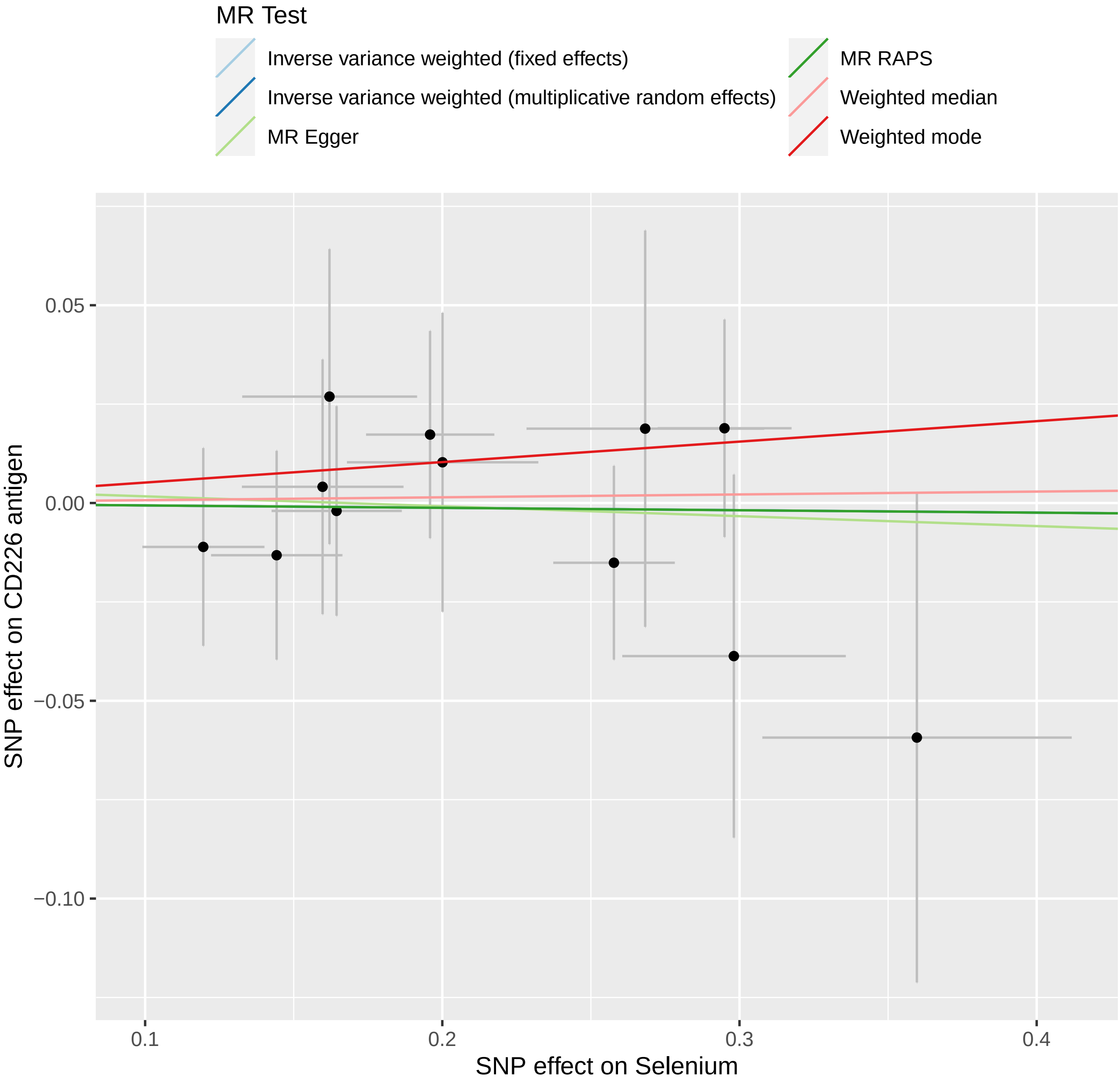

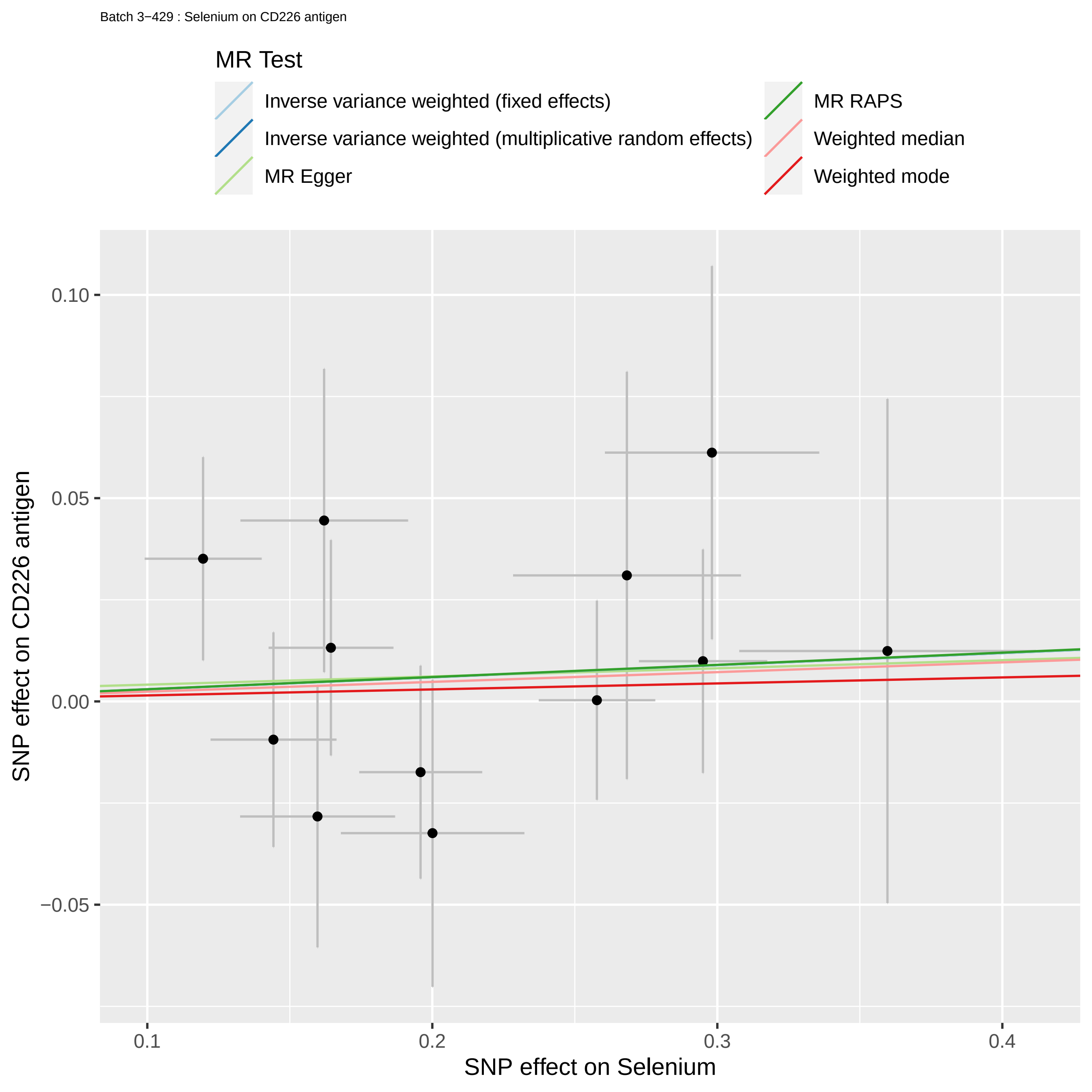

Batch 3−429 : Selenium on CD226 antigen
MR Test
Inverse variance weighted (fixed effects)
Inverse variance weighted (multiplicative random effects)
MR Egger
MR RAPS
Weighted median
Weighted mode
0.10
0.05
0.00
−0.05
0.1
0.2
0.3
0.4
SNP effect on CD226 antigen
SNP effect on Selenium

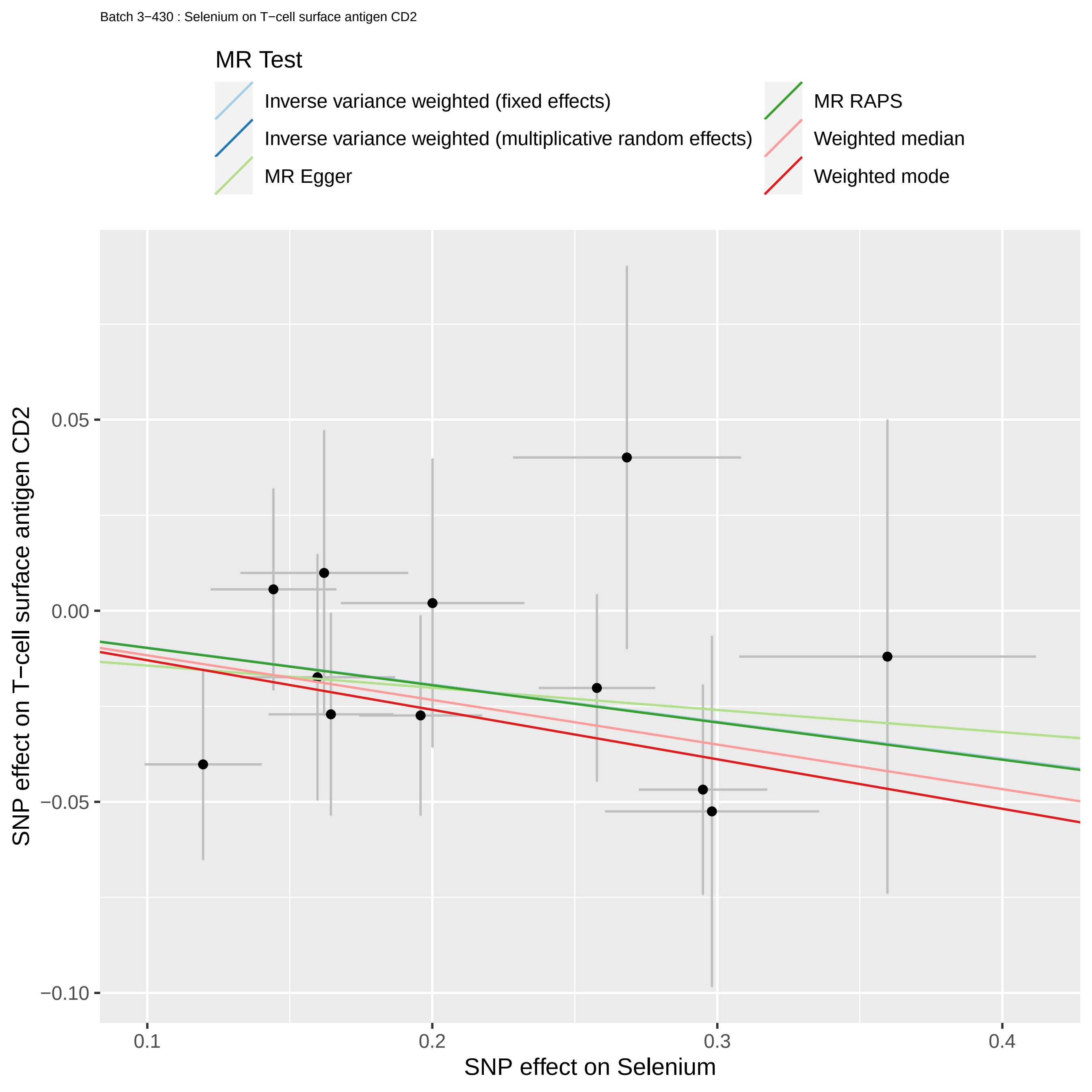
Batch 3−430 : Selenium on T−cell surface antigen CD2
MR Test
Inverse variance weighted (fixed effects)
Inverse variance weighted (multiplicative random effects)
MR Egger
MR RAPS
Weighted median
Weighted mode
0.05
0.00
−0.05
−0.10
0.1
0.2
0.3
0.4
SNP effect on T−cell surface antigen CD2
SNP effect on Selenium

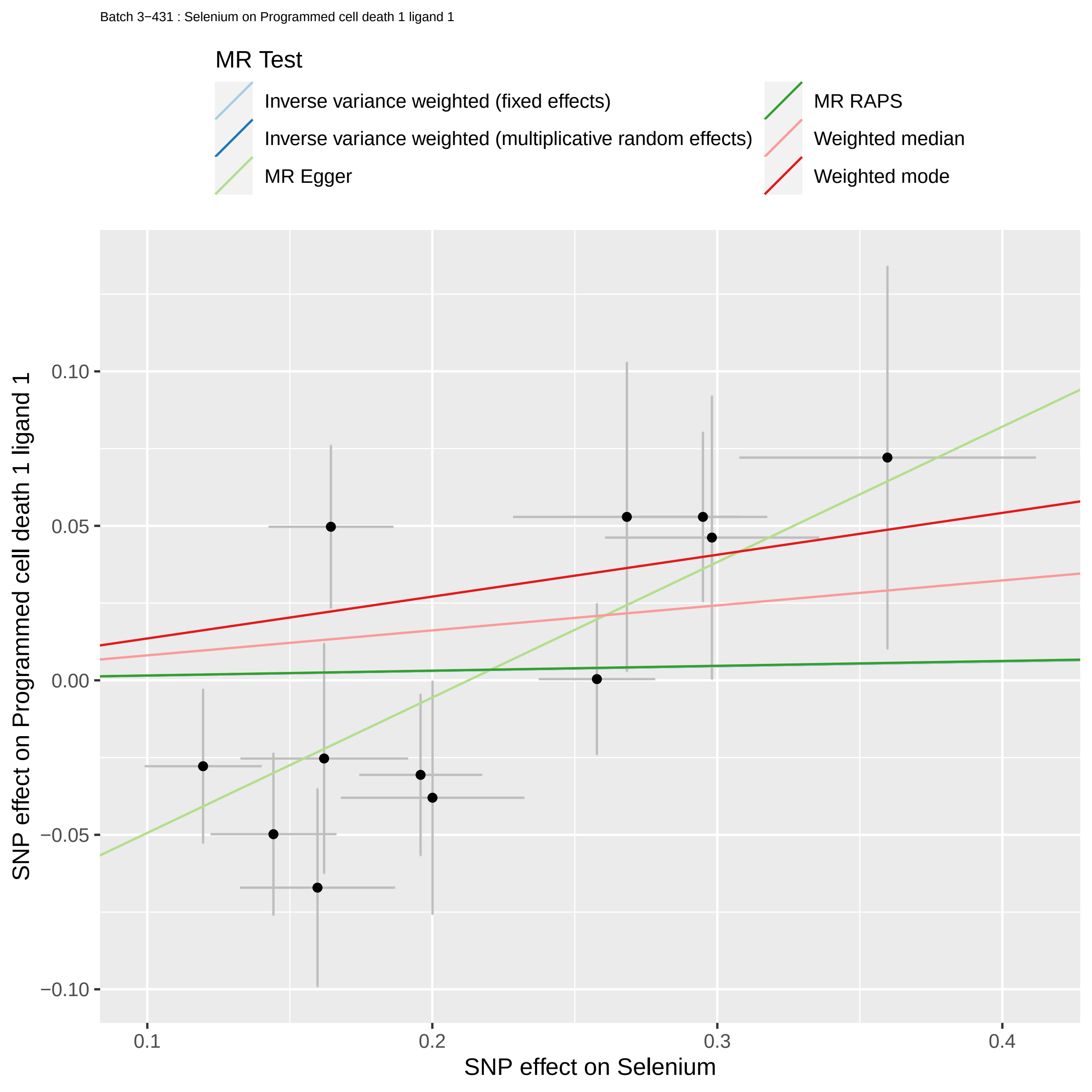

Batch 3−431 : Selenium on Programmed cell death 1 ligand 1
MR Test
Inverse variance weighted (fixed effects)
Inverse variance weighted (multiplicative random effects)
MR Egger
MR RAPS
Weighted median
Weighted mode
SNP effect on Programmed cell death 1 ligand 1
0.10
0.05
0.00
−0.05
−0.10
0.1
0.2
0.3
0.4
SNP effect on Selenium

Batch 3−432 : Selenium on CMRF35−like molecule 8

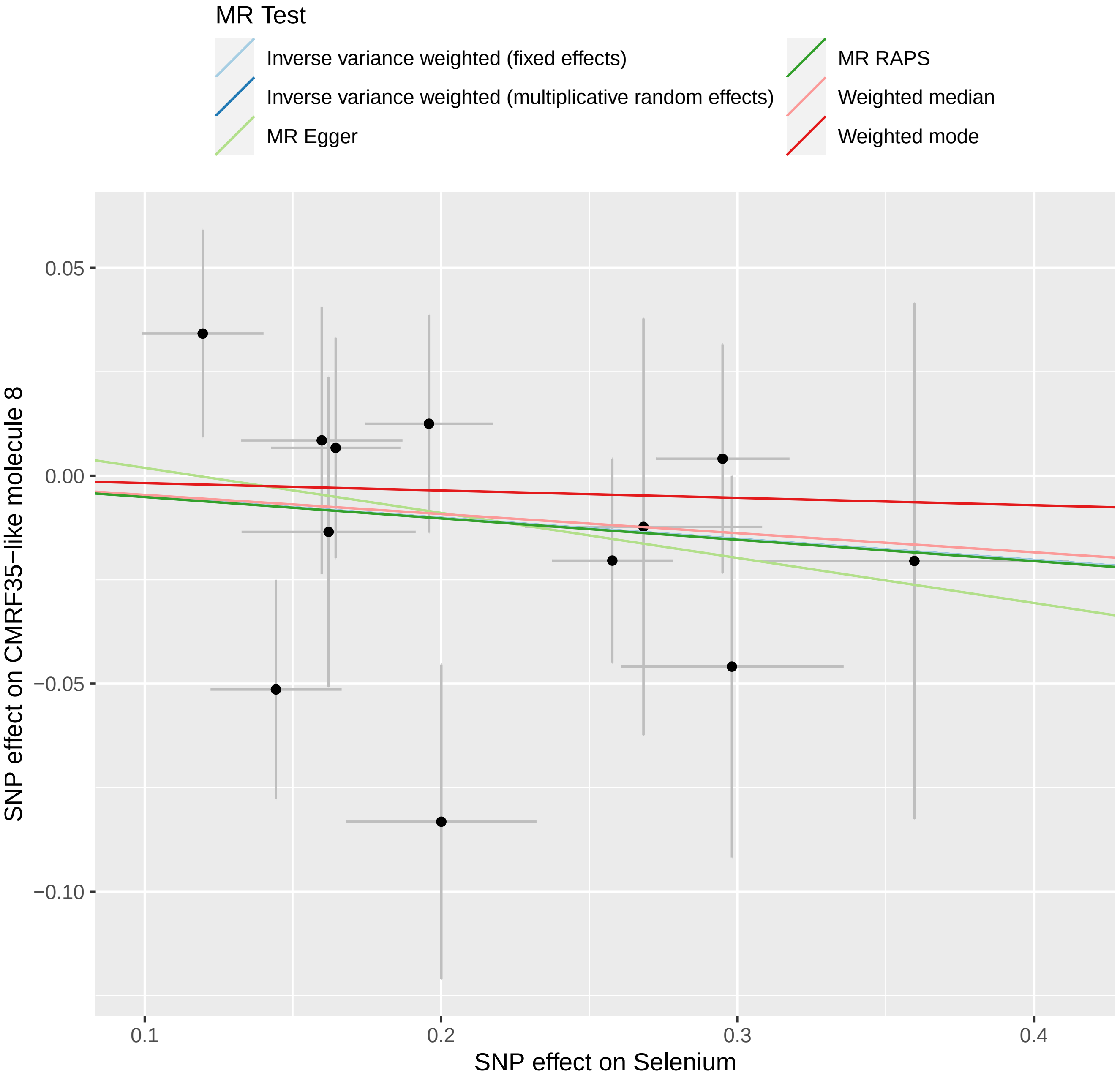

Batch 3−433 : Selenium on CMRF35−like molecule 6

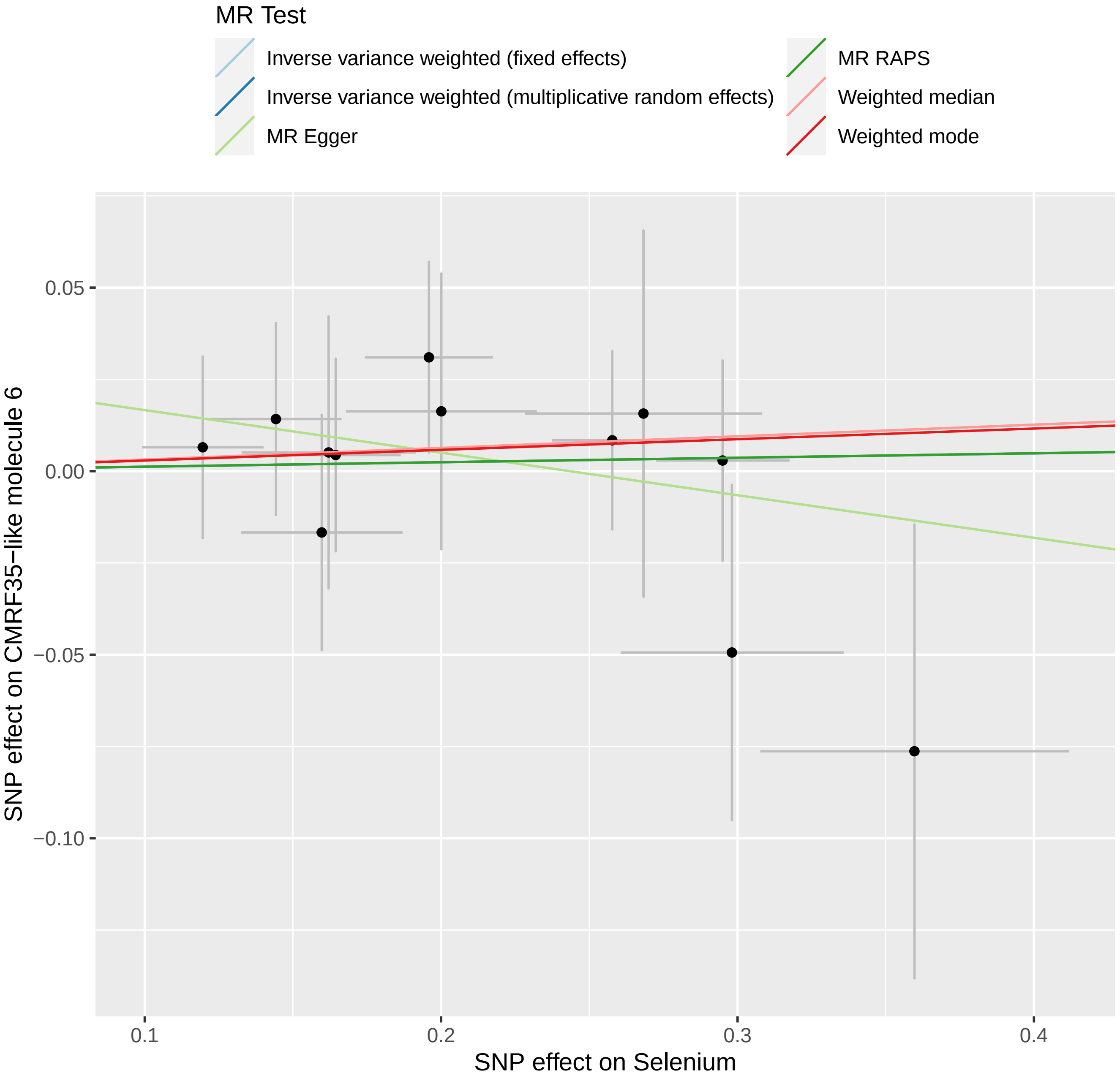

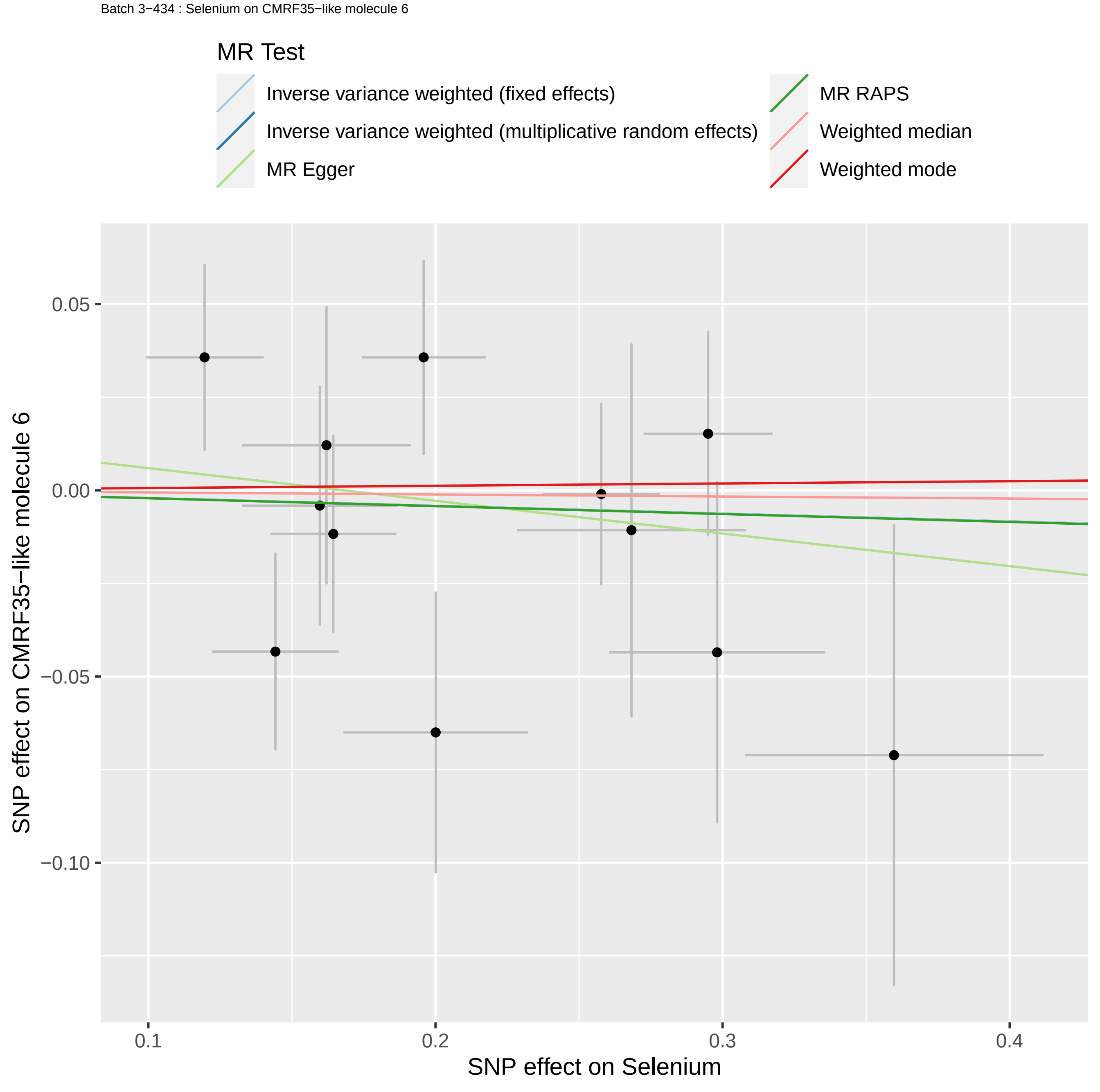
Batch 3−434 : Selenium on CMRF35−like molecule 6
MR Test
Inverse variance weighted (fixed effects)
Inverse variance weighted (multiplicative random effects)
MR Egger
MR RAPS
Weighted median
Weighted mode
SNP effect on CMRF35−like molecule 6
0.05
0.00
−0.05
−0.10
0.1
0.2
0.3
0.4
SNP effect on Selenium

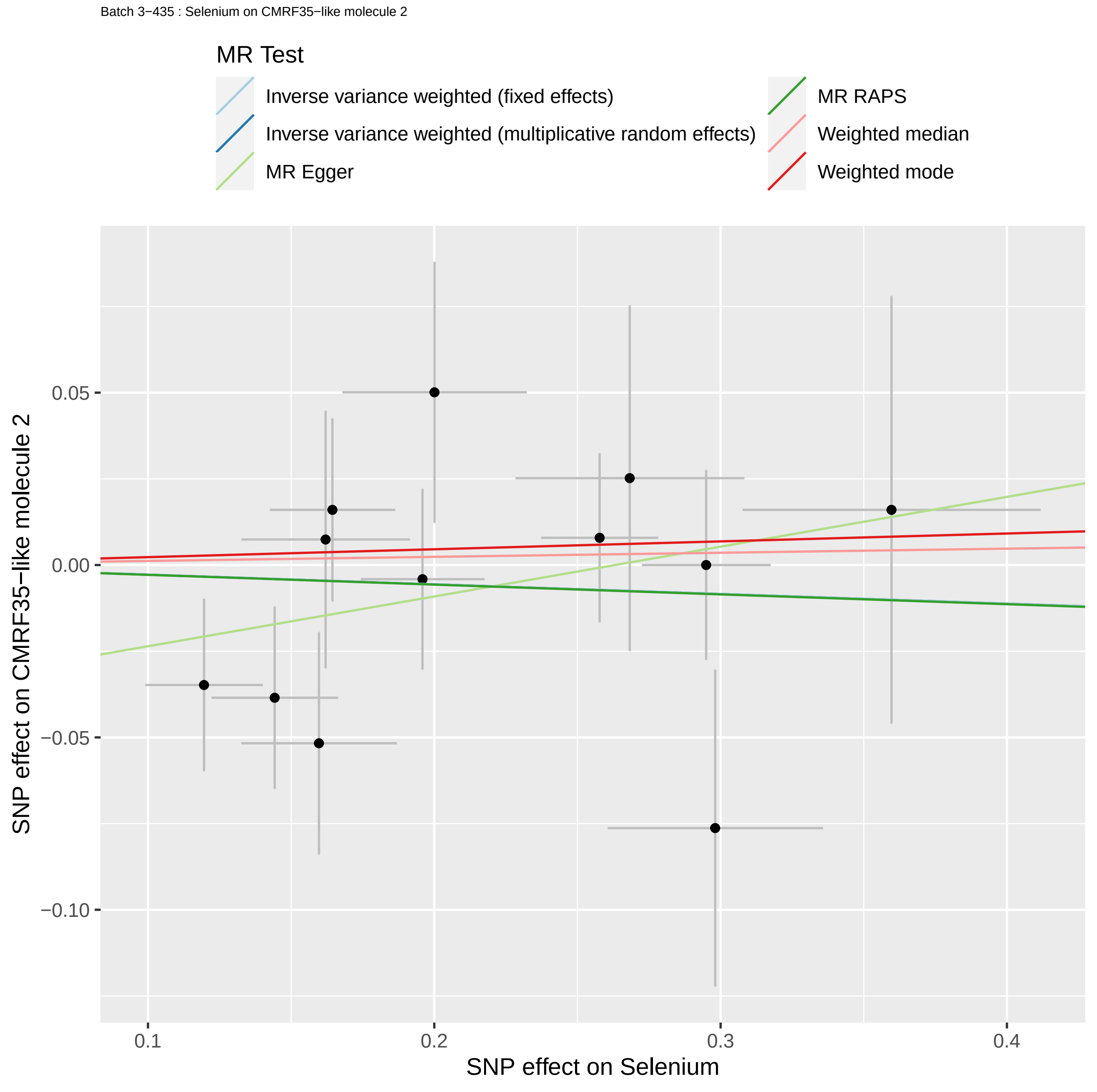
Batch 3−435 : Selenium on CMRF35−like molecule 2
MR Test
Inverse variance weighted (fixed effects)
Inverse variance weighted (multiplicative random effects)
MR Egger
MR RAPS
Weighted median
Weighted mode
0.05
0.00
−0.05
−0.10
0.1
0.2
0.3
0.4
SNP effect on CMRF35−like molecule 2
SNP effect on Selenium

Batch 3−436 : Selenium on CMRF35−like molecule 7

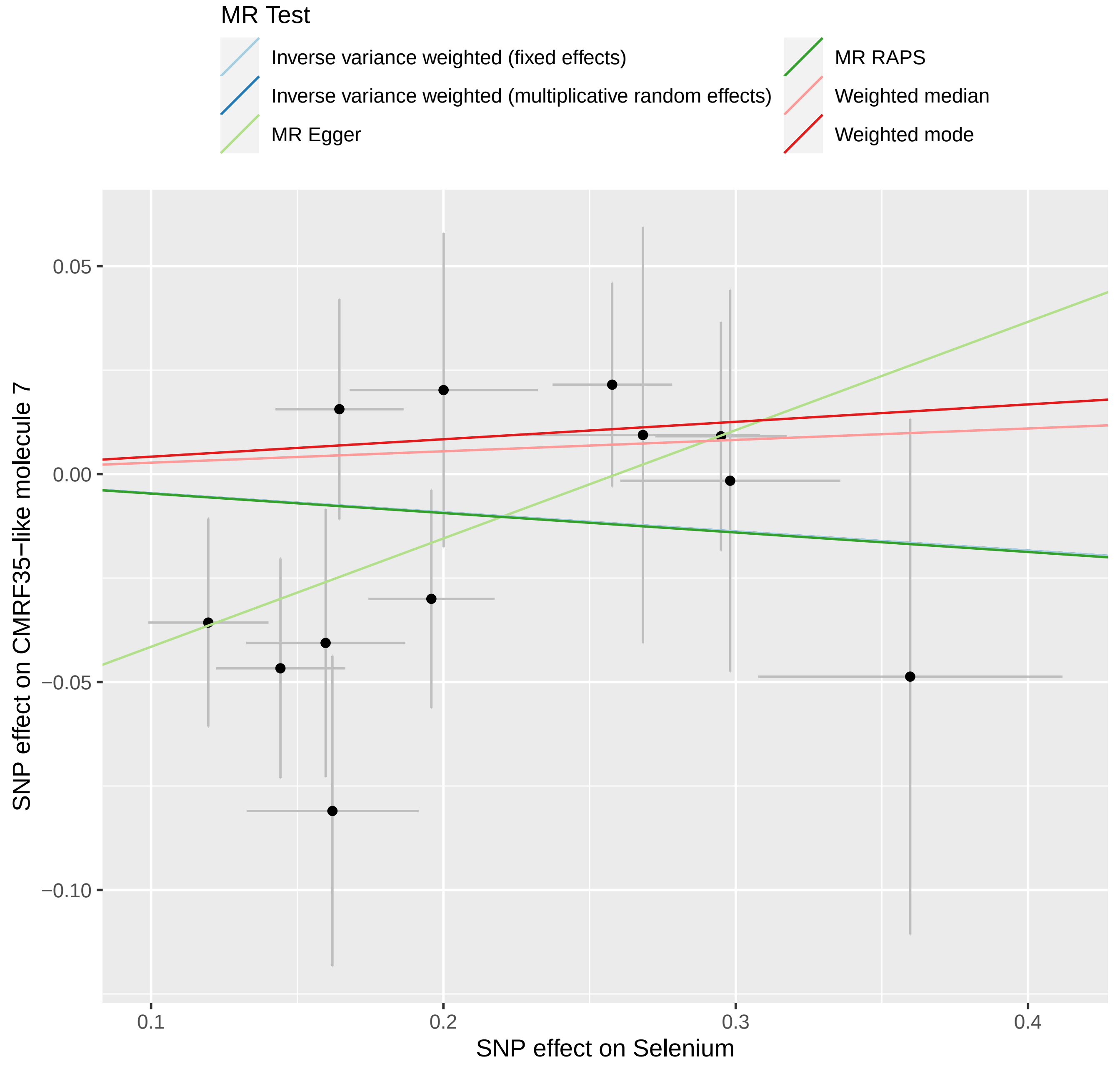

Batch 3−437 : Selenium on CMRF35−like molecule 1

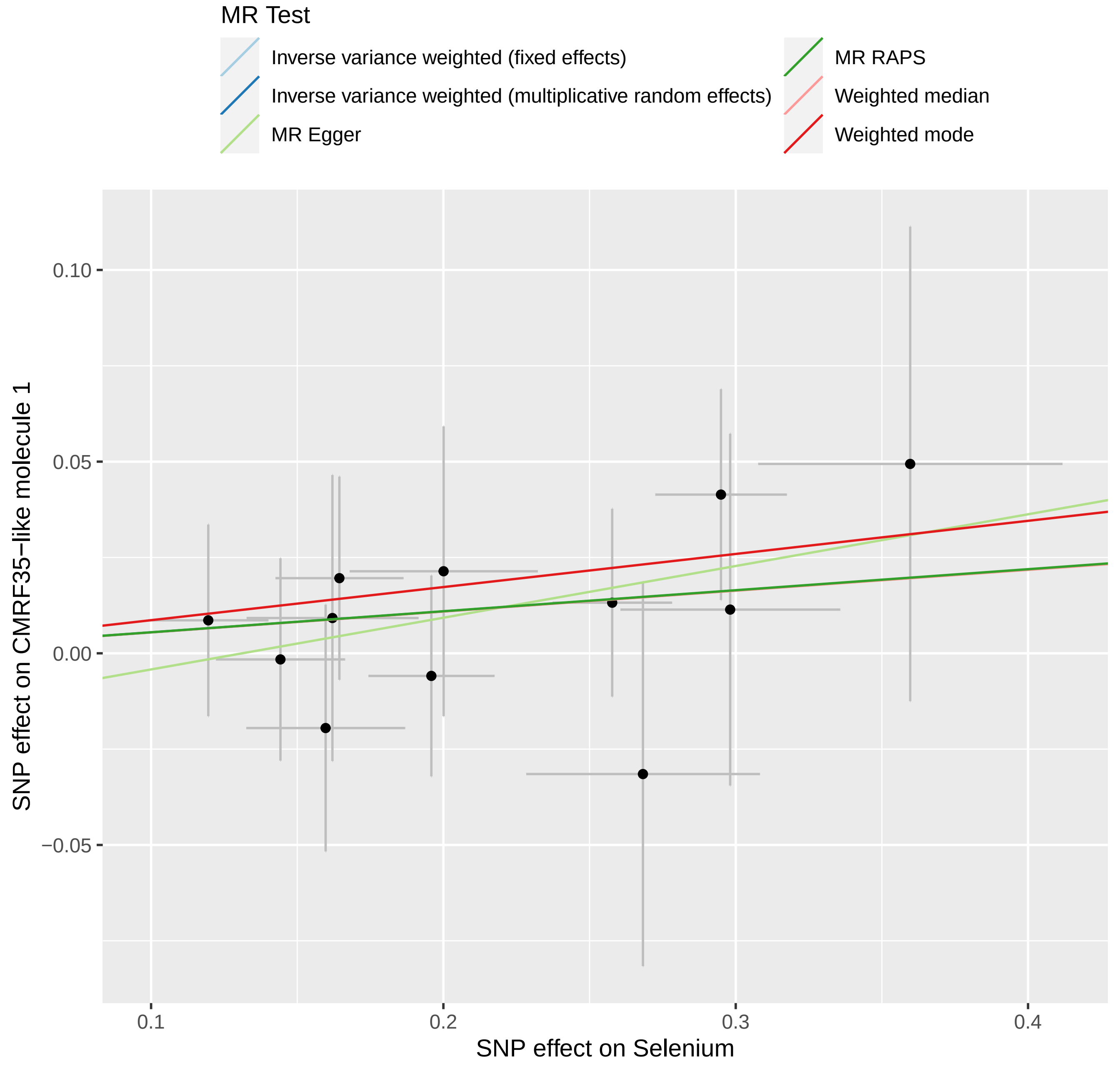

Batch 3−438 : Selenium on CMRF35−like molecule 9

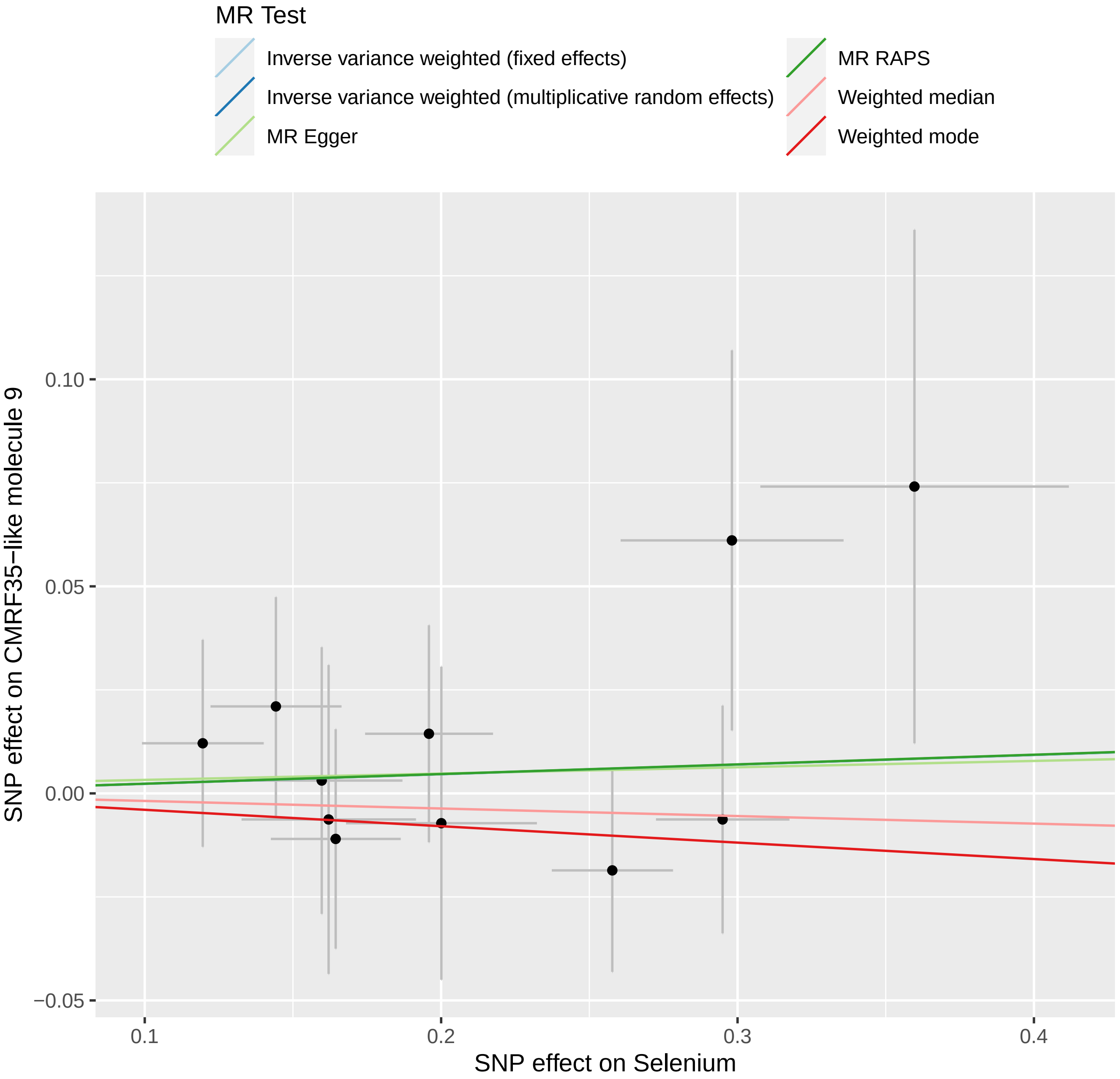

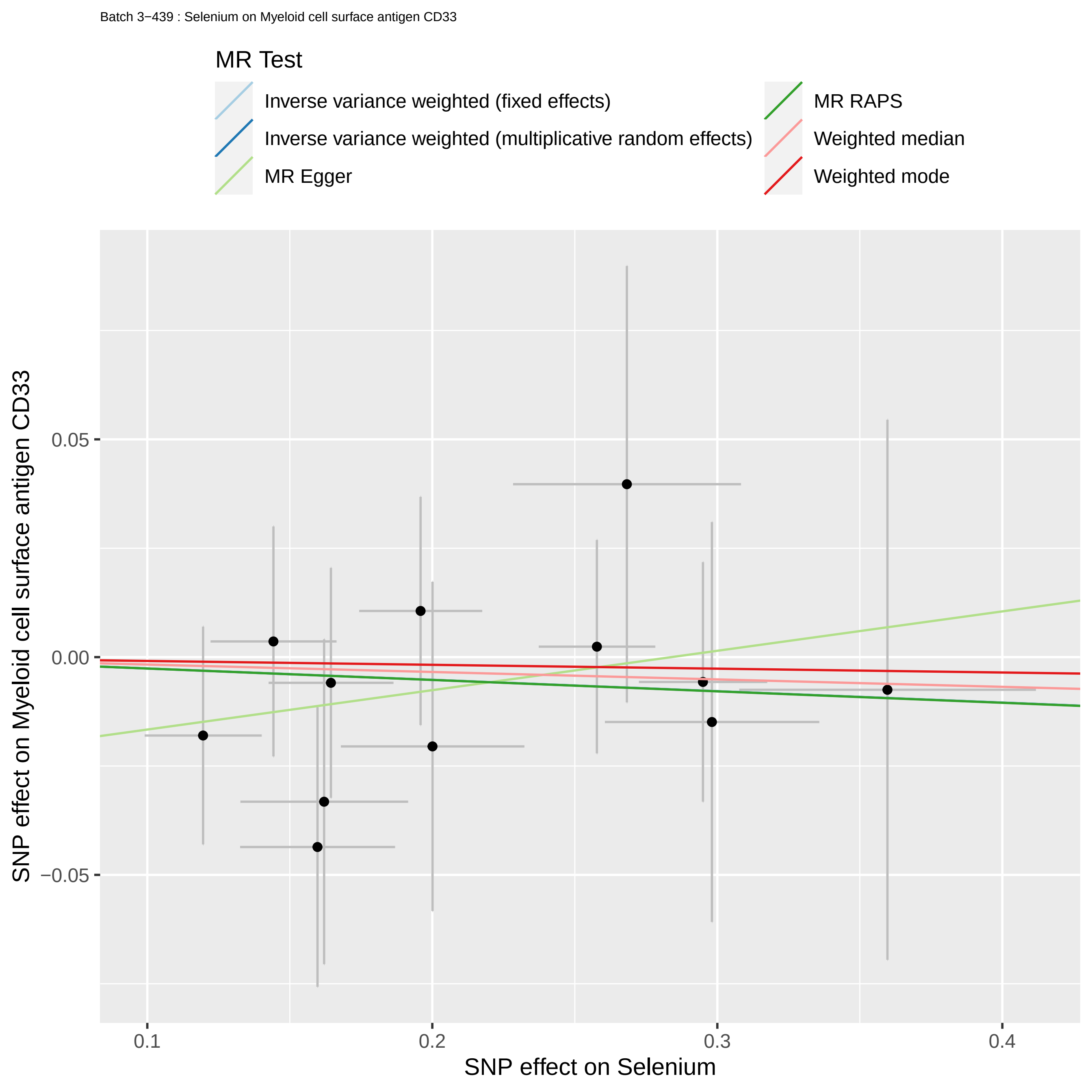
Batch 3−439 : Selenium on Myeloid cell surface antigen CD33
MR Test
Inverse variance weighted (fixed effects)
Inverse variance weighted (multiplicative random effects)
MR Egger
MR RAPS
Weighted median
Weighted mode
SNP effect on Myeloid cell surface antigen CD33
0.05
0.00
−0.05
0.1
0.2
0.3
0.4
SNP effect on Selenium

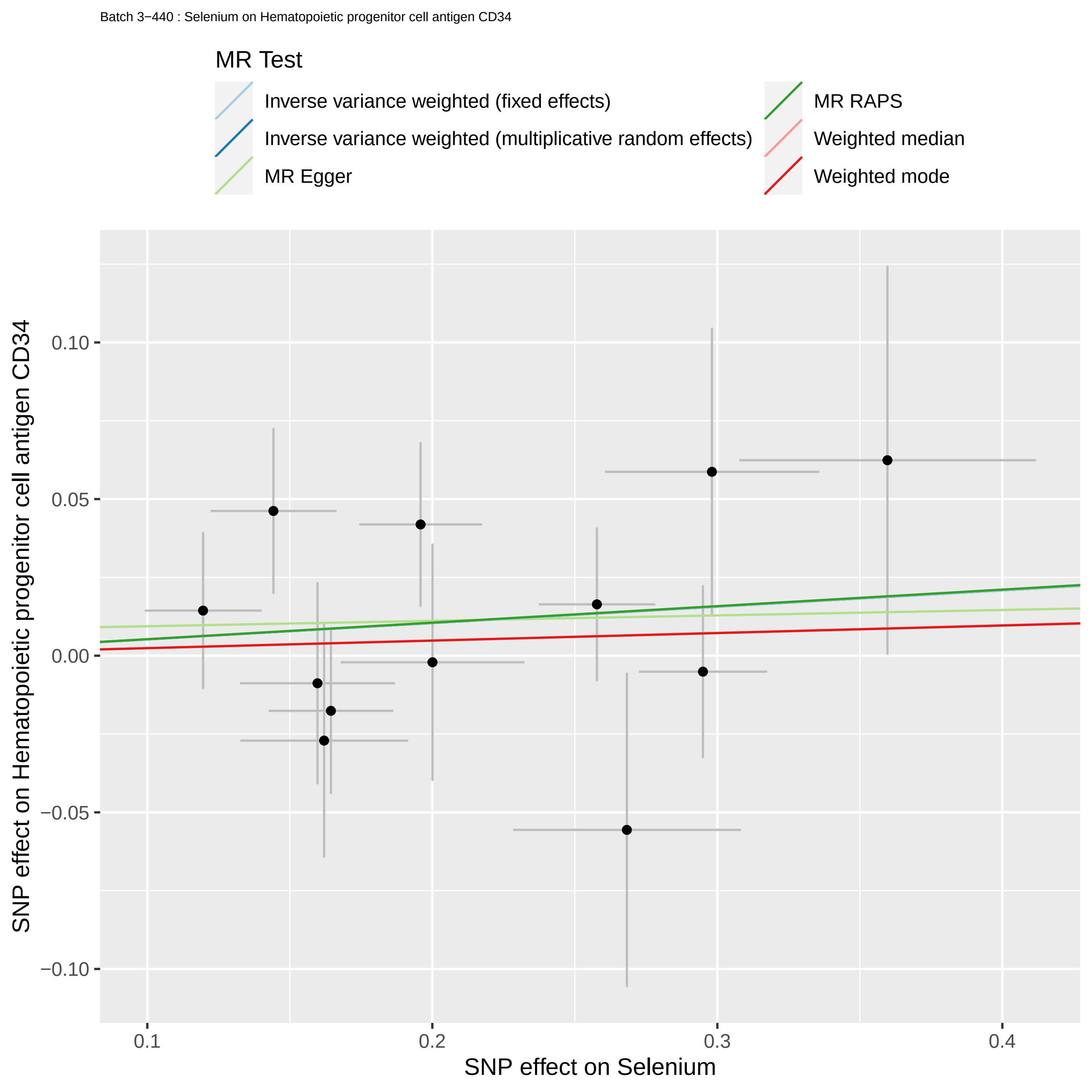
Batch 3−440 : Selenium on Hematopoietic progenitor cell antigen CD34
MR Test
Inverse variance weighted (fixed effects)
Inverse variance weighted (multiplicative random effects)
MR Egger
MR RAPS
Weighted median
Weighted mode
0.10
0.05
0.00
−0.05
−0.10
0.1
0.2
0.3
0.4
SNP effect on Hematopoietic progenitor cell antigen CD34
SNP effect on Selenium

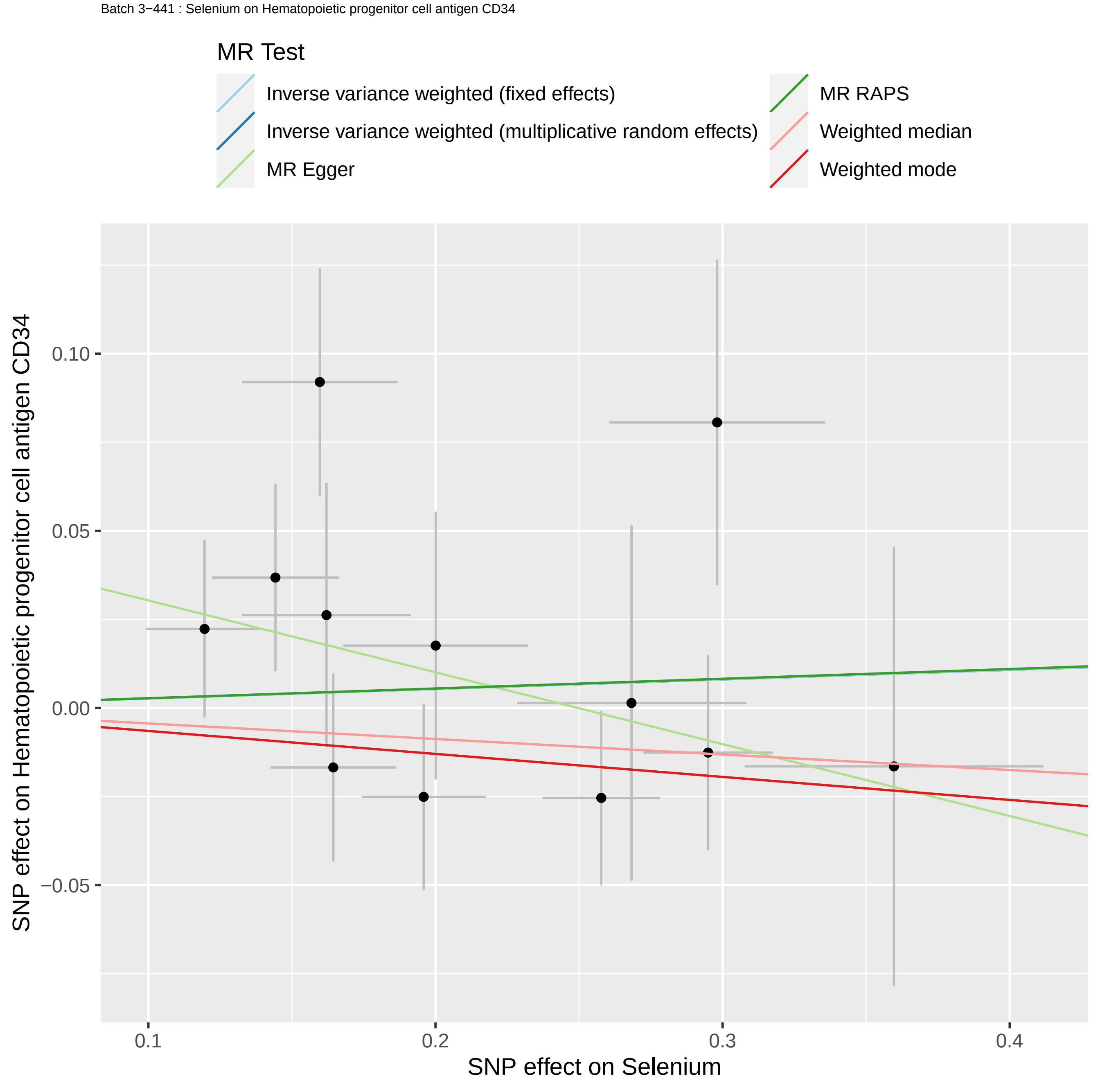
Batch 3−441 : Selenium on Hematopoietic progenitor cell antigen CD34
MR Test
Inverse variance weighted (fixed effects)
Inverse variance weighted (multiplicative random effects)
MR Egger
MR RAPS
Weighted median
Weighted mode
SNP effect on Hematopoietic progenitor cell antigen CD34
0.10
0.05
0.00
−0.05
0.1
0.2
0.3
0.4
SNP effect on Selenium

Batch 3−442 : Selenium on Platelet glycoprotein 4

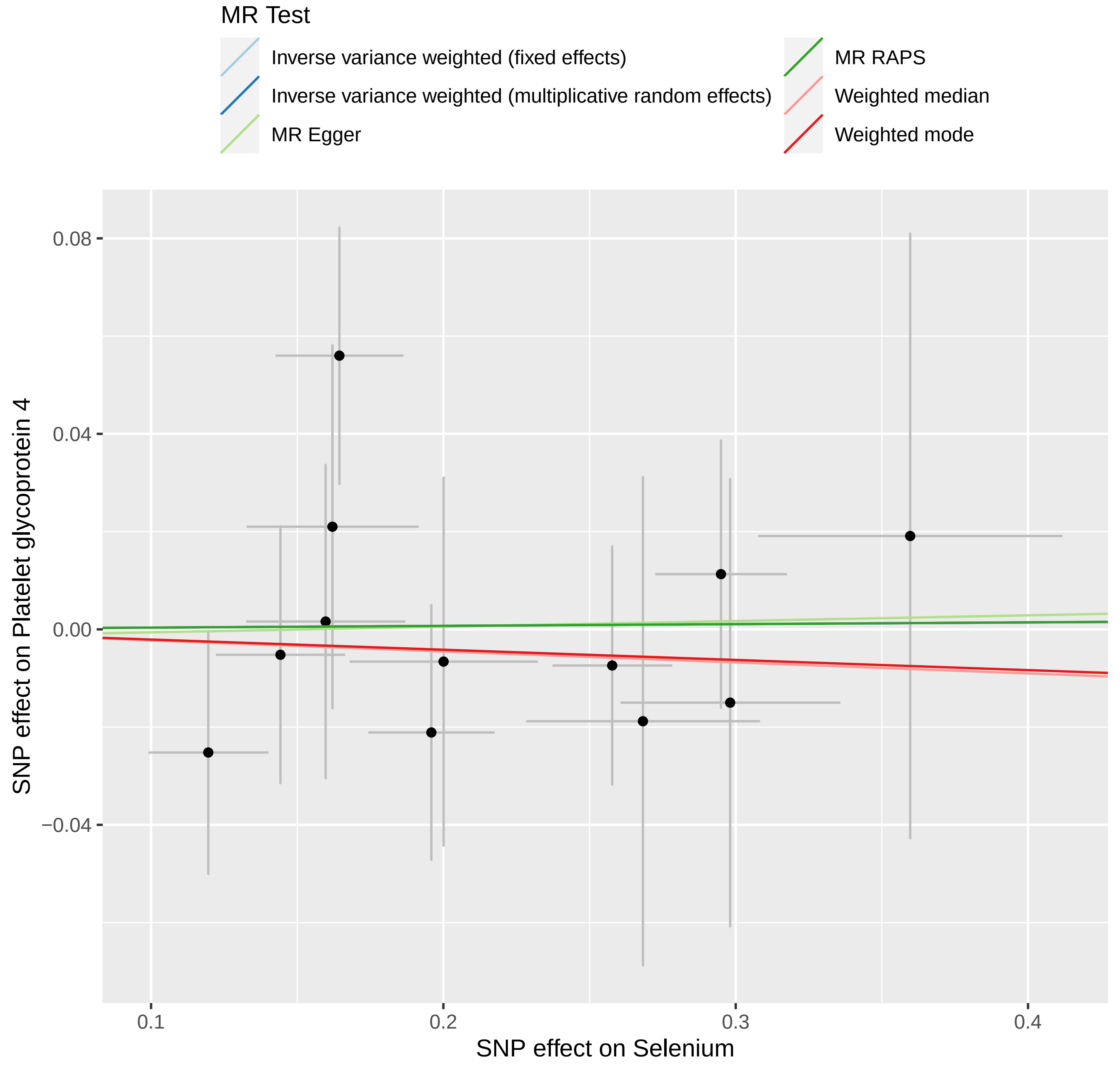

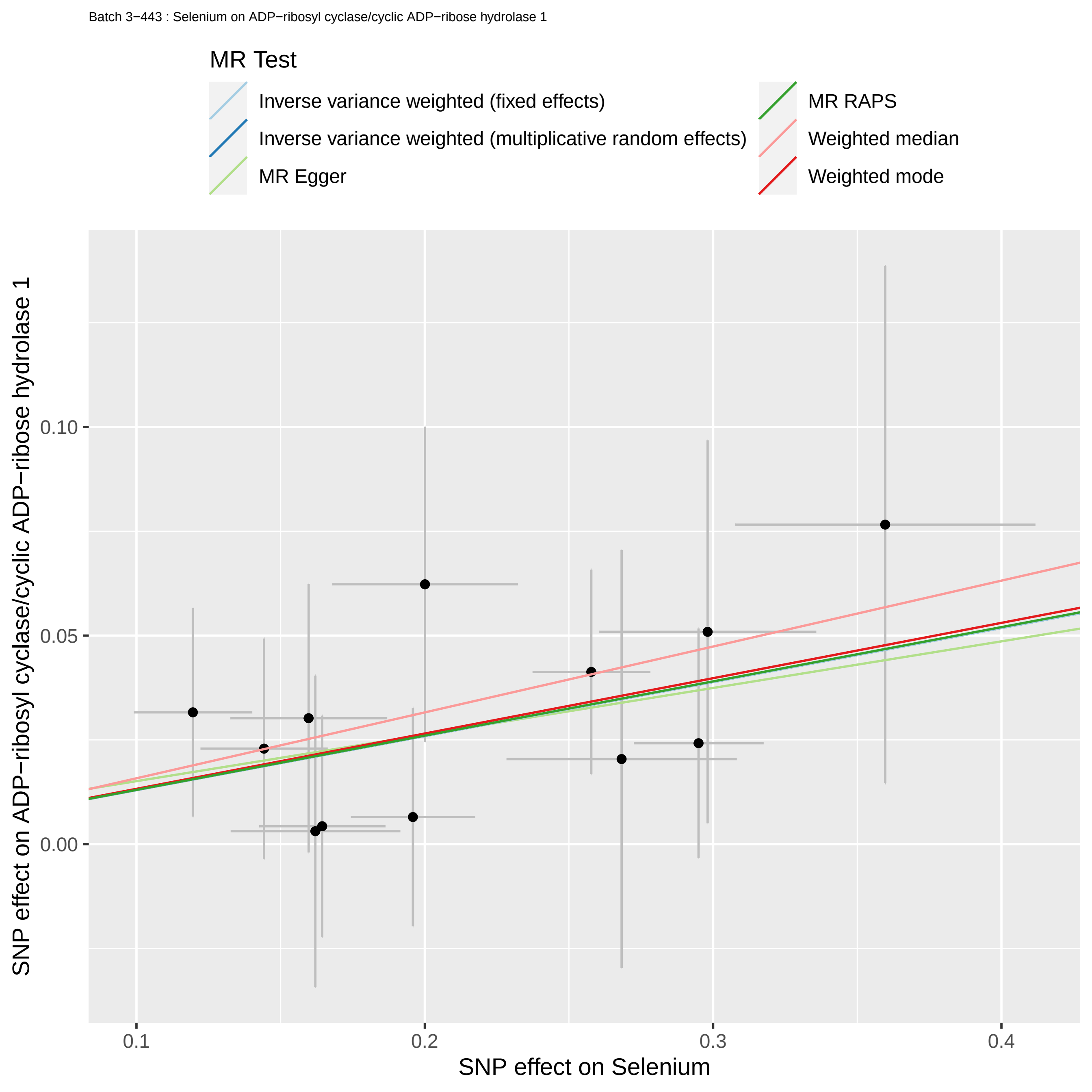
Batch 3−443 : Selenium on ADP−ribosyl cyclase/cyclic ADP−ribose hydrolase 1
MR Test
Inverse variance weighted (fixed effects)
Inverse variance weighted (multiplicative random effects)
MR Egger
MR RAPS
Weighted median
Weighted mode
SNP effect on ADP−ribosyl cyclase/cyclic ADP−ribose hydrolase 1
0.10
0.05
0.00
0.1
0.2
0.3
0.4
SNP effect on Selenium

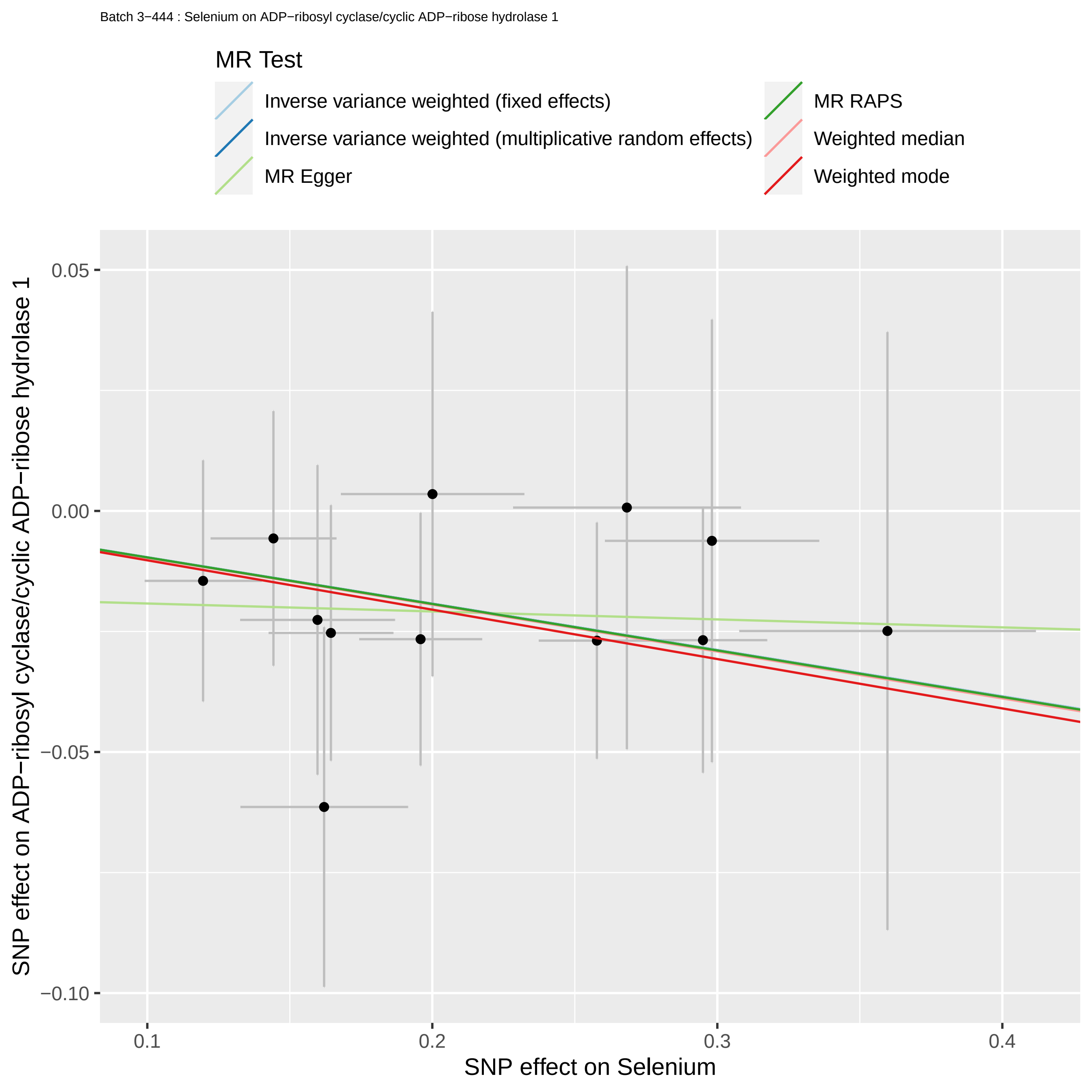

Batch 3−444 : Selenium on ADP−ribosyl cyclase/cyclic ADP−ribose hydrolase 1
MR Test
Inverse variance weighted (fixed effects)
Inverse variance weighted (multiplicative random effects)
MR Egger
MR RAPS
Weighted median
Weighted mode
SNP effect on ADP−ribosyl cyclase/cyclic ADP−ribose hydrolase 1
0.05
0.00
−0.05
−0.10
0.1
0.2
0.3
0.4
SNP effect on Selenium

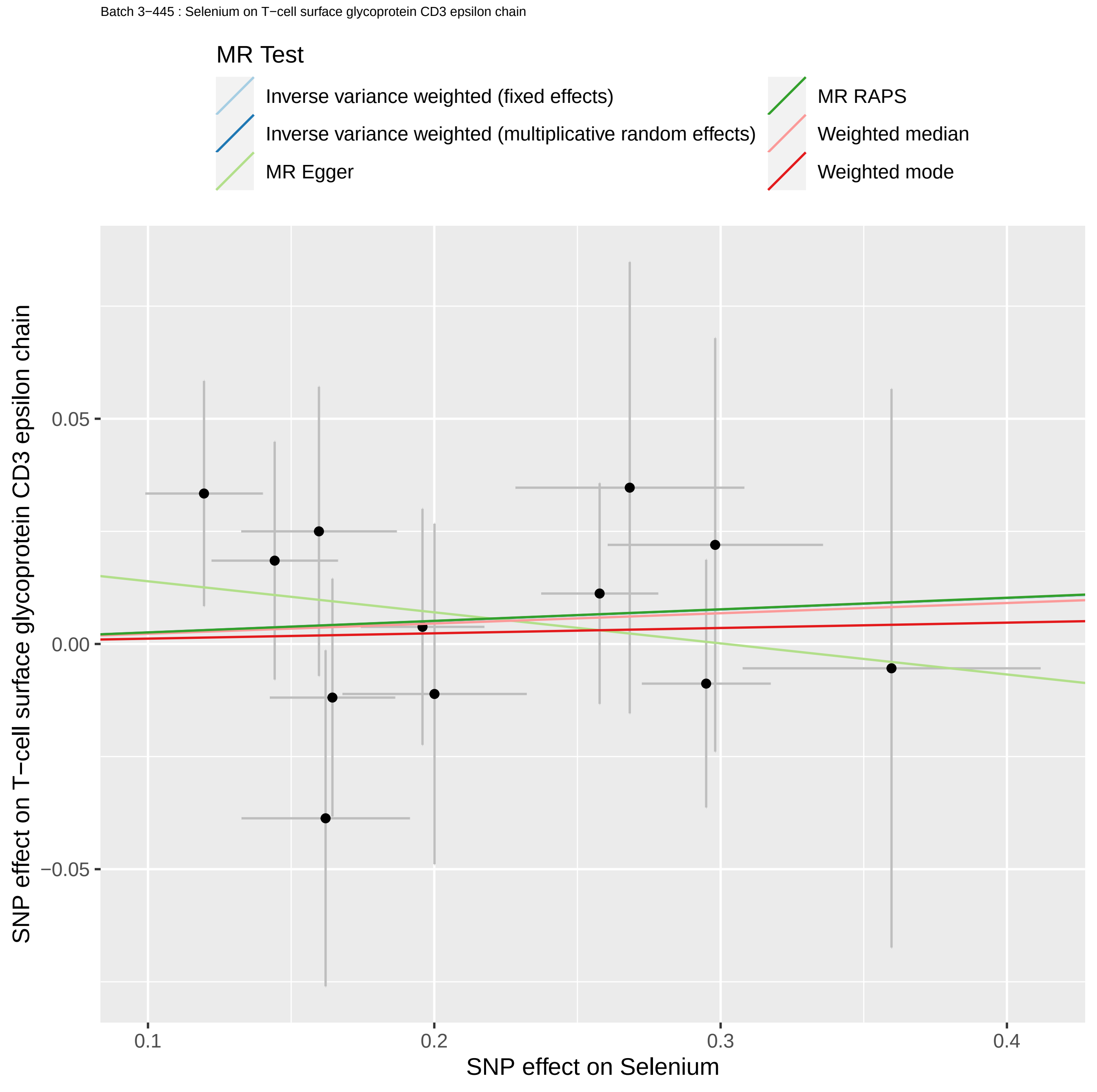

Batch 3−445 : Selenium on T−cell surface glycoprotein CD3 epsilon chain
MR Test
Inverse variance weighted (fixed effects)
Inverse variance weighted (multiplicative random effects)
MR Egger
MR RAPS
Weighted median
Weighted mode
SNP effect on T−cell surface glycoprotein CD3 epsilon chain
0.05
0.00
−0.05
0.1
0.2
0.3
0.4
SNP effect on Selenium

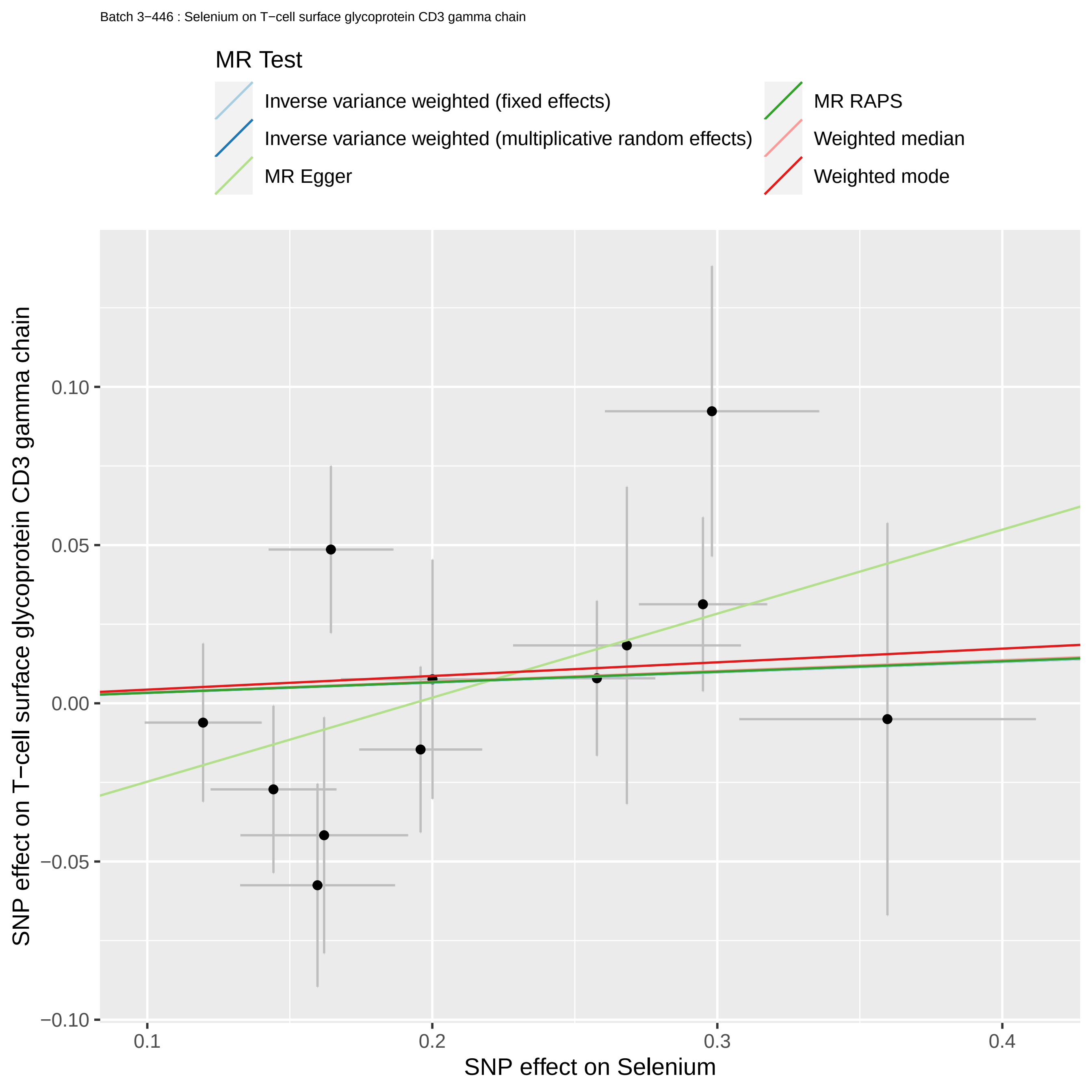

Batch 3−446 : Selenium on T−cell surface glycoprotein CD3 gamma chain
MR Test
Inverse variance weighted (fixed effects)
Inverse variance weighted (multiplicative random effects)
MR Egger
MR RAPS
Weighted median
Weighted mode
SNP effect on T−cell surface glycoprotein CD3 gamma chain
0.10
0.05
0.00
−0.05
−0.10
0.1
0.2
0.3
0.4
SNP effect on Selenium

Batch 3−447 : Selenium on CD40 ligand

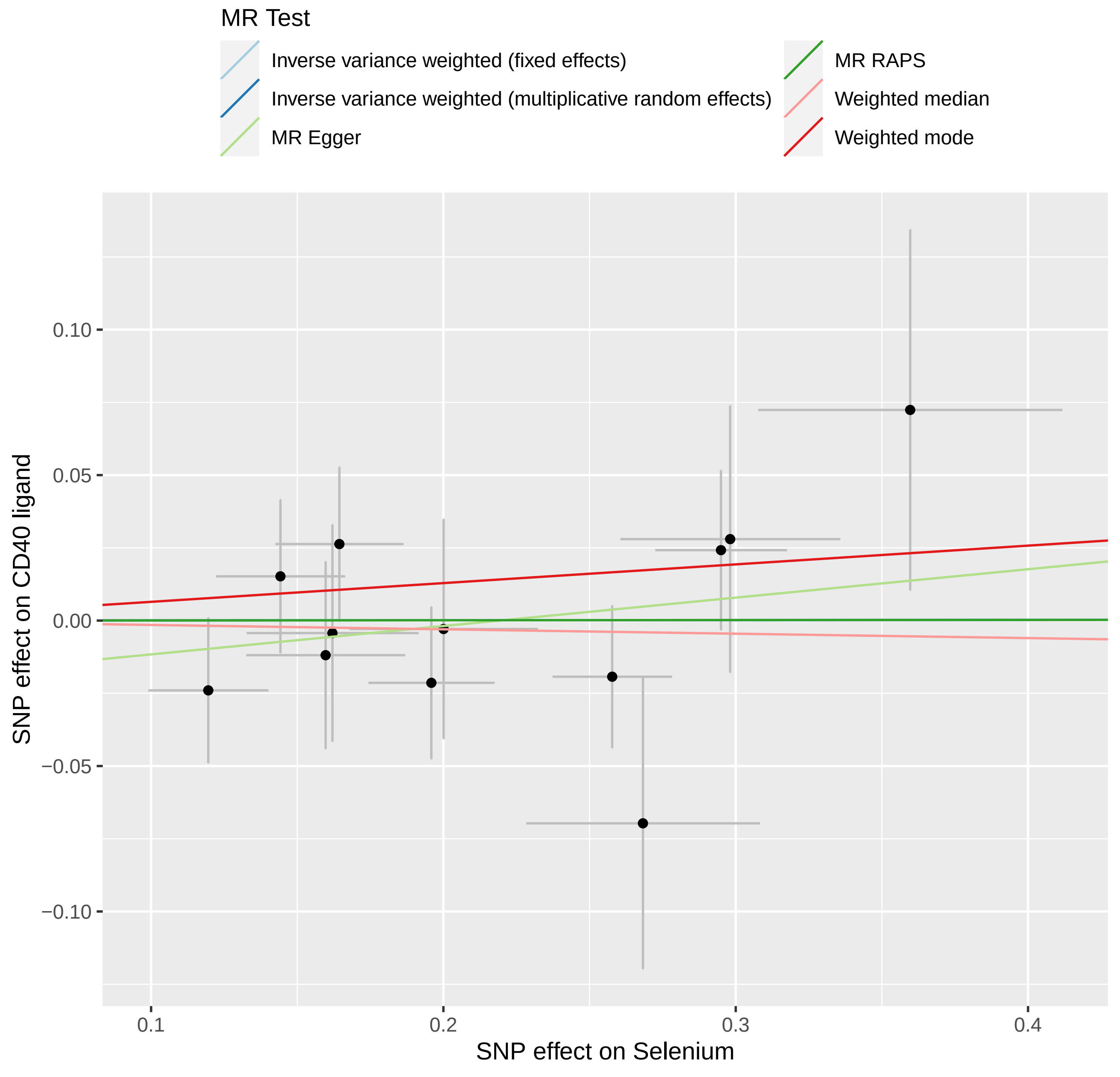

Batch 3−448 : Selenium on T−cell surface glycoprotein CD4

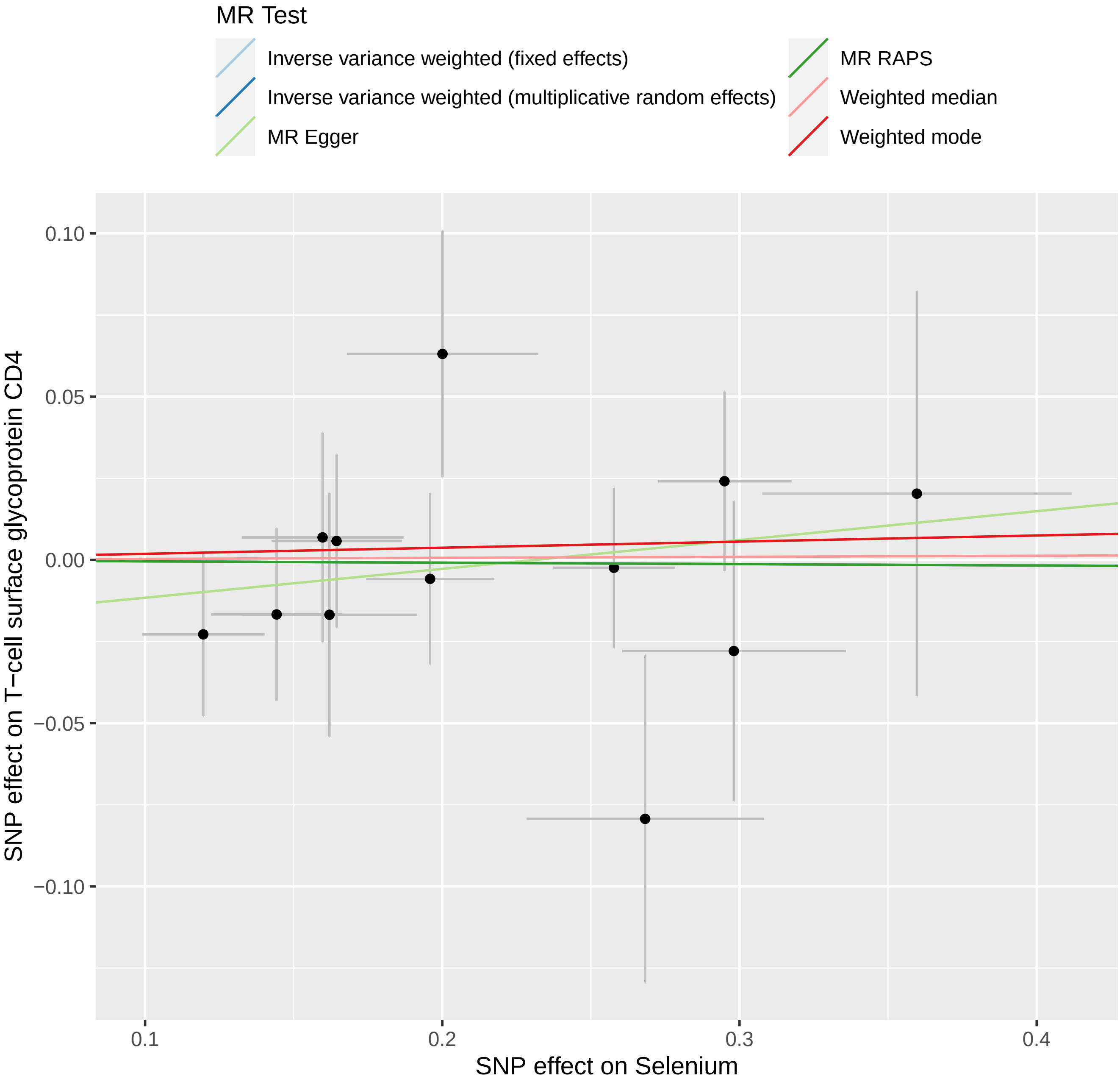

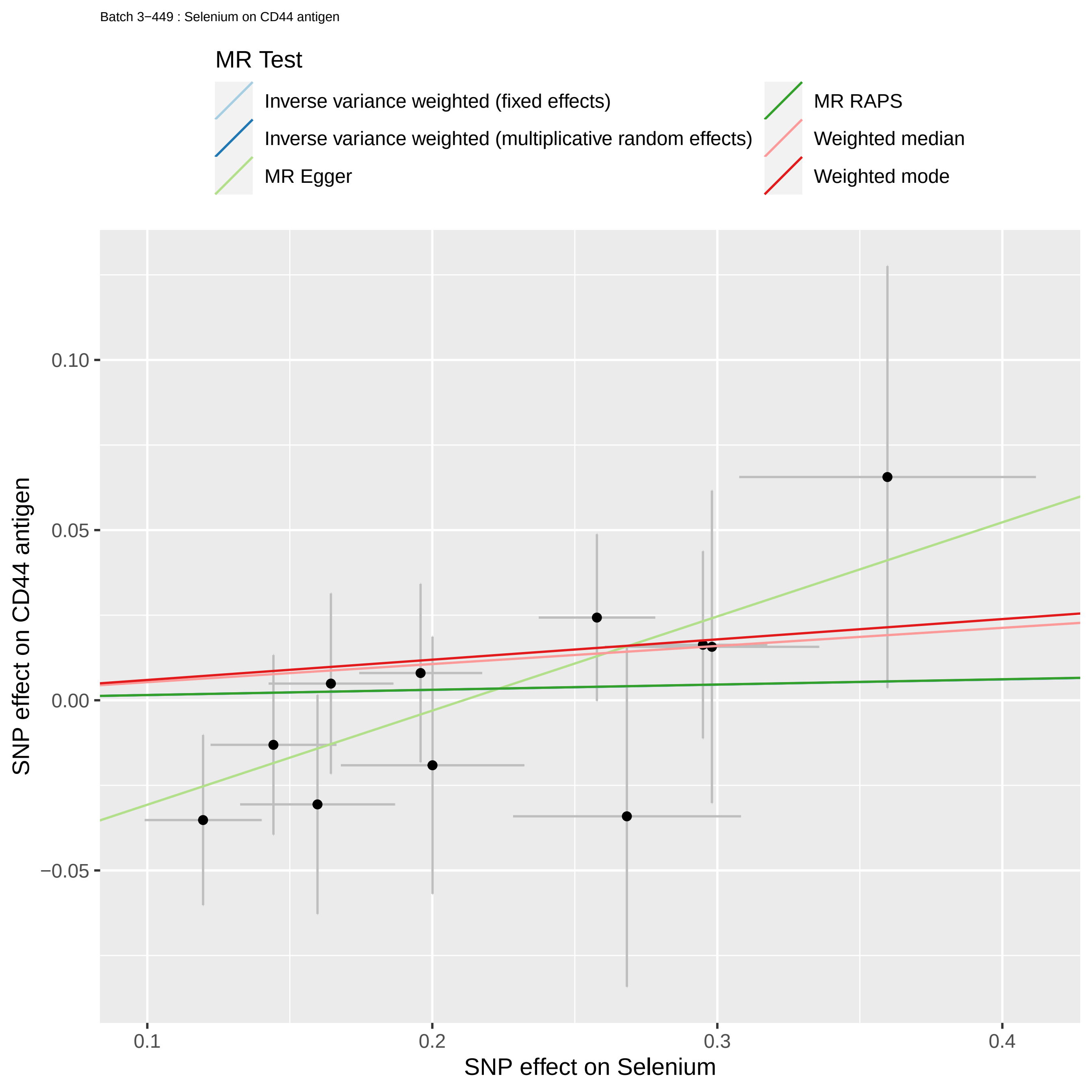
Batch 3−449 : Selenium on CD44 antigen
MR Test
Inverse variance weighted (fixed effects)
Inverse variance weighted (multiplicative random effects)
MR Egger
MR RAPS
Weighted median
Weighted mode
SNP effect on CD44 antigen
0.10
0.05
0.00
−0.05
0.1
0.2
0.3
0.4
SNP effect on Selenium

Batch 3−450 : Selenium on Leukocyte surface antigen CD47

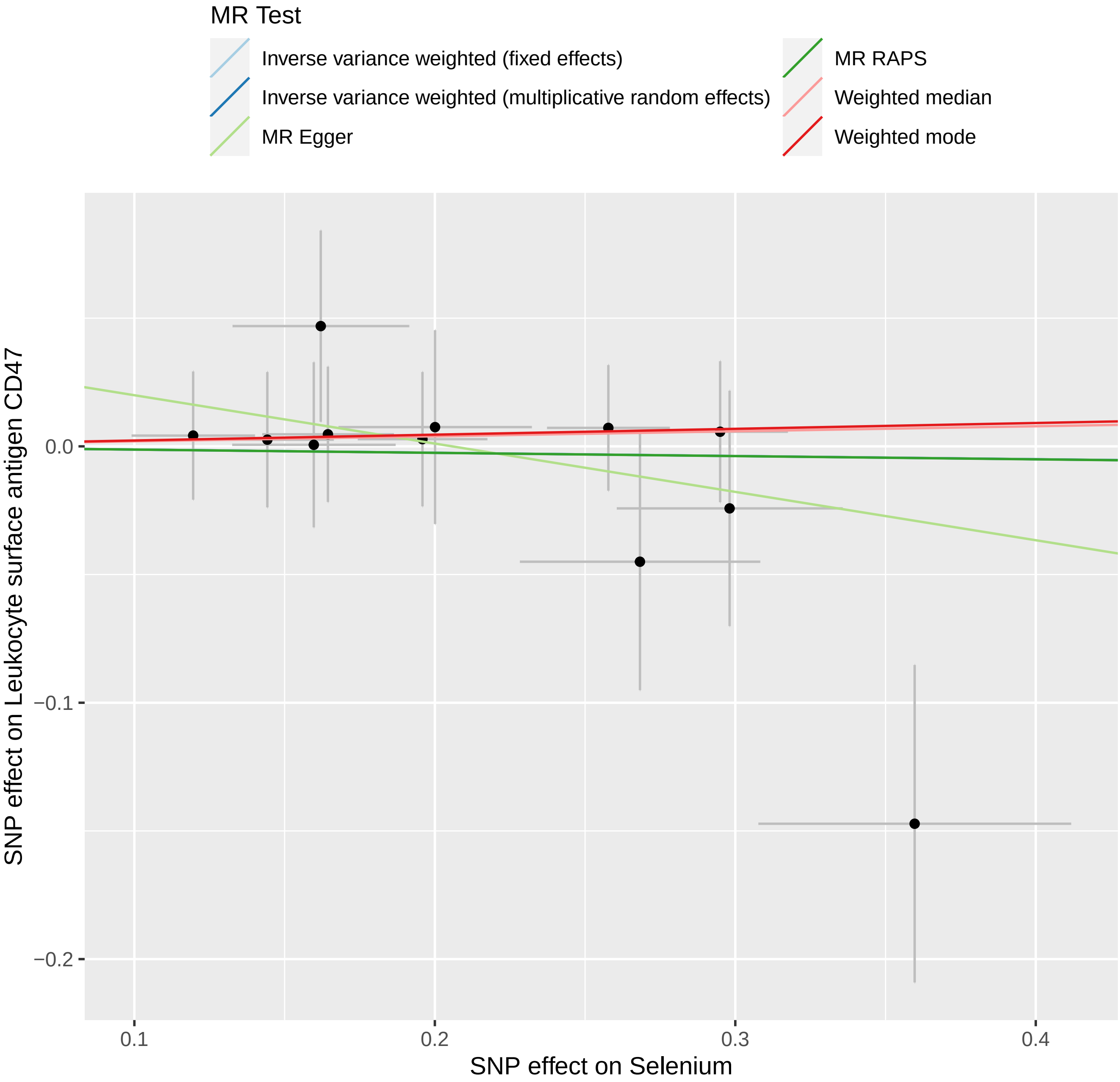

Batch 3−451 : Selenium on CD48 antigen

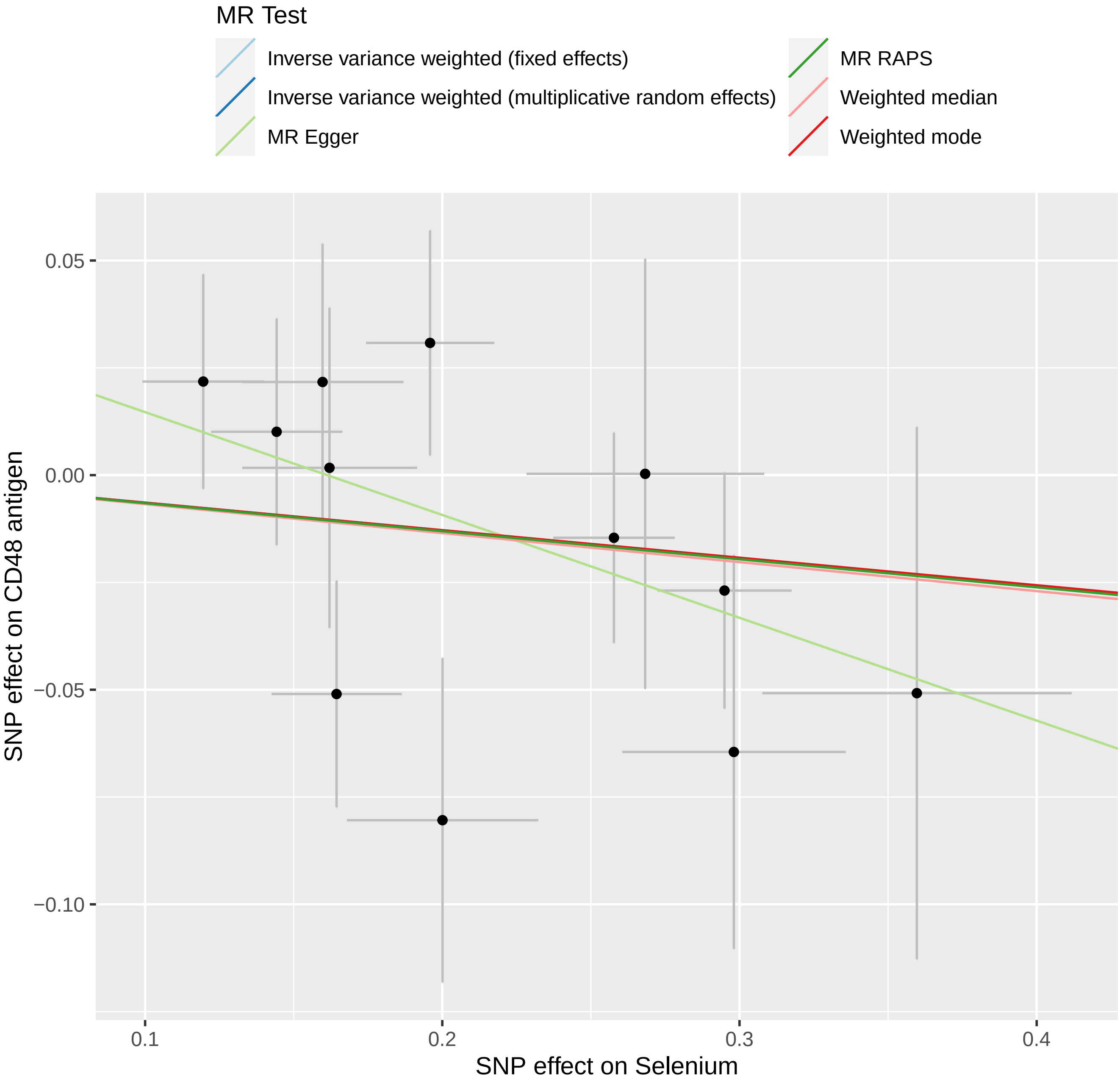

Batch 3−452 : Selenium on T−cell surface glycoprotein CD5

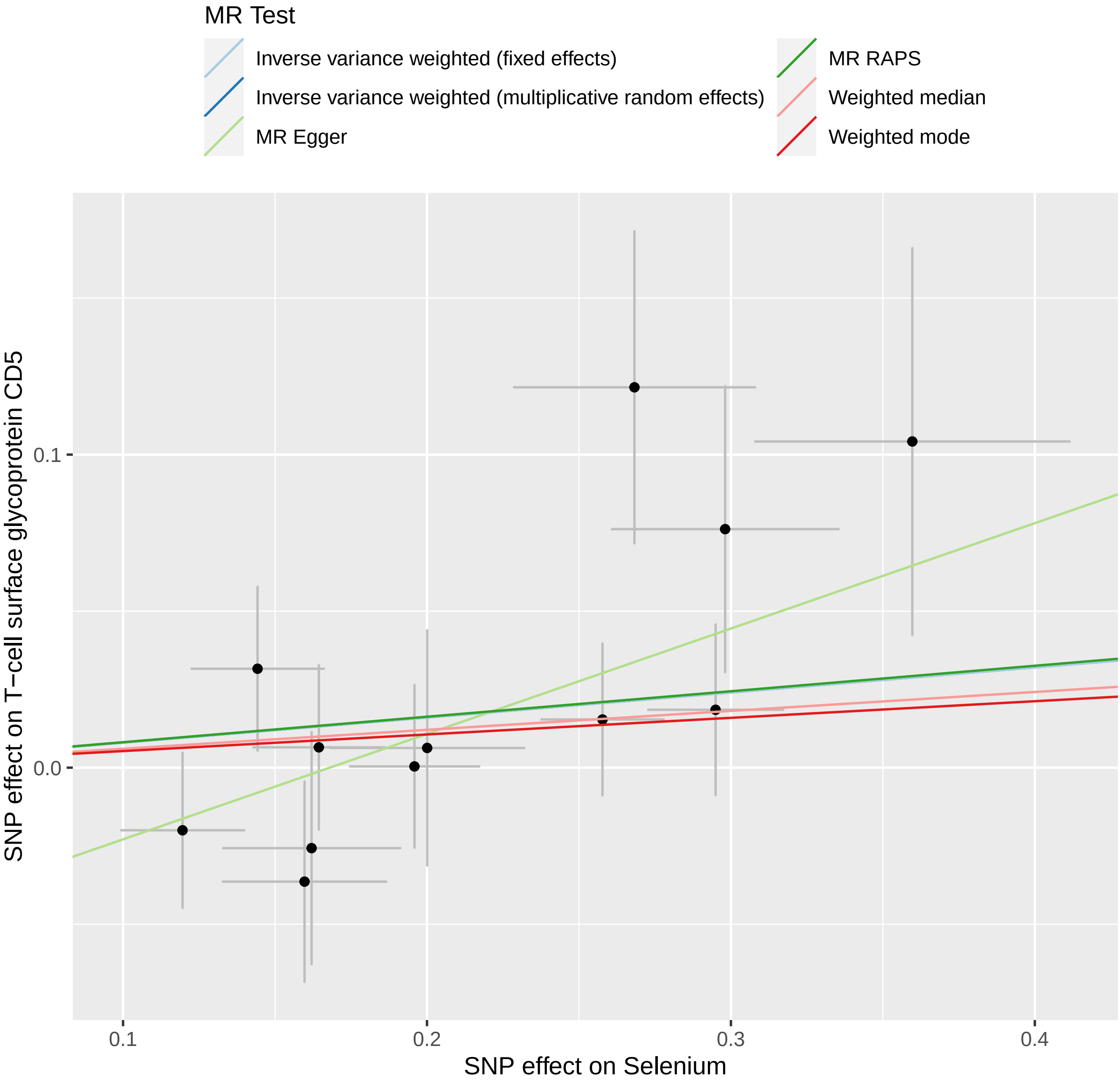

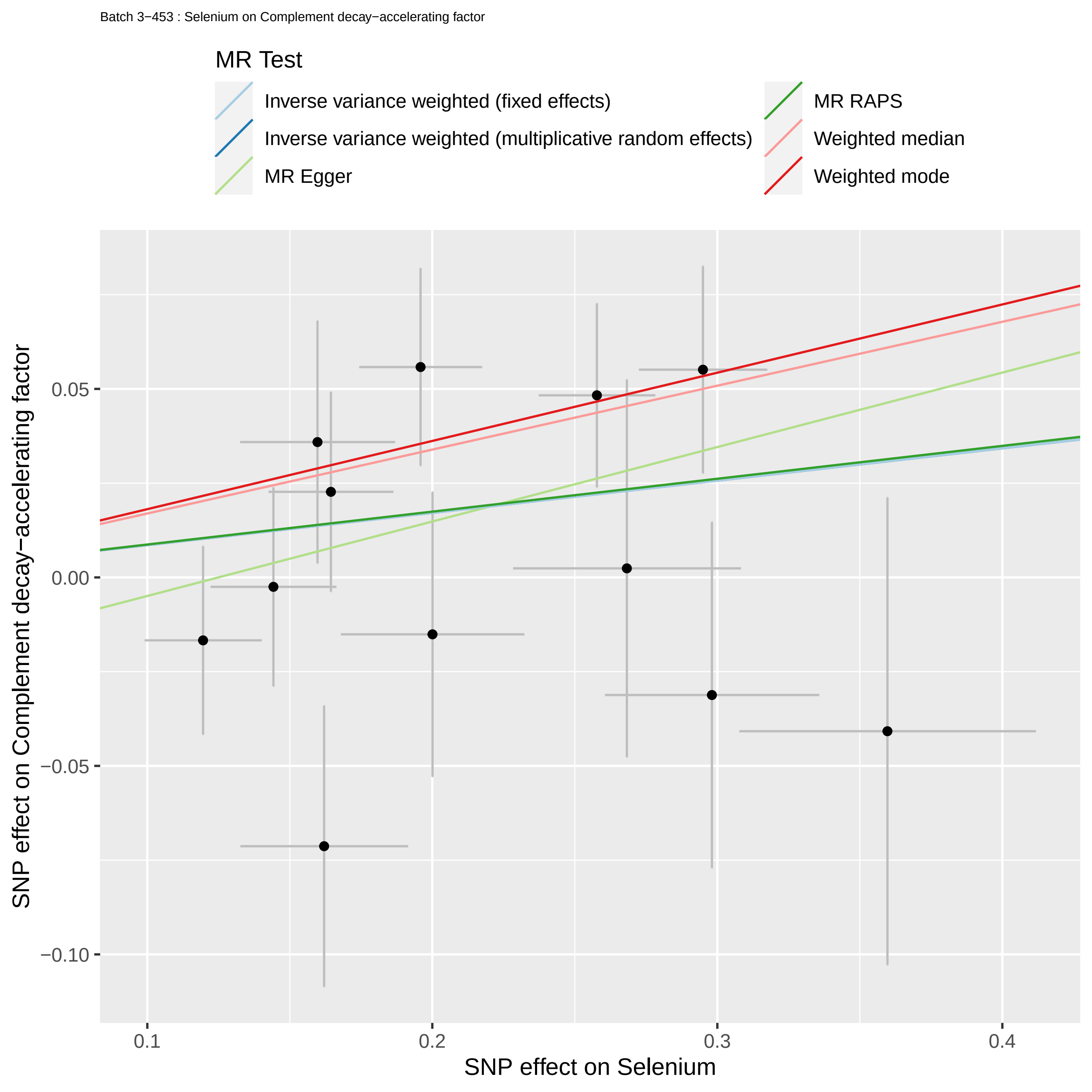
Batch 3−453 : Selenium on Complement decay−accelerating factor
MR Test
Inverse variance weighted (fixed effects)
Inverse variance weighted (multiplicative random effects)
MR Egger
MR RAPS
Weighted median
Weighted mode
SNP effect on Complement decay−accelerating factor
0.05
0.00
−0.05
−0.10
0.1
0.2
0.3
0.4
SNP effect on Selenium

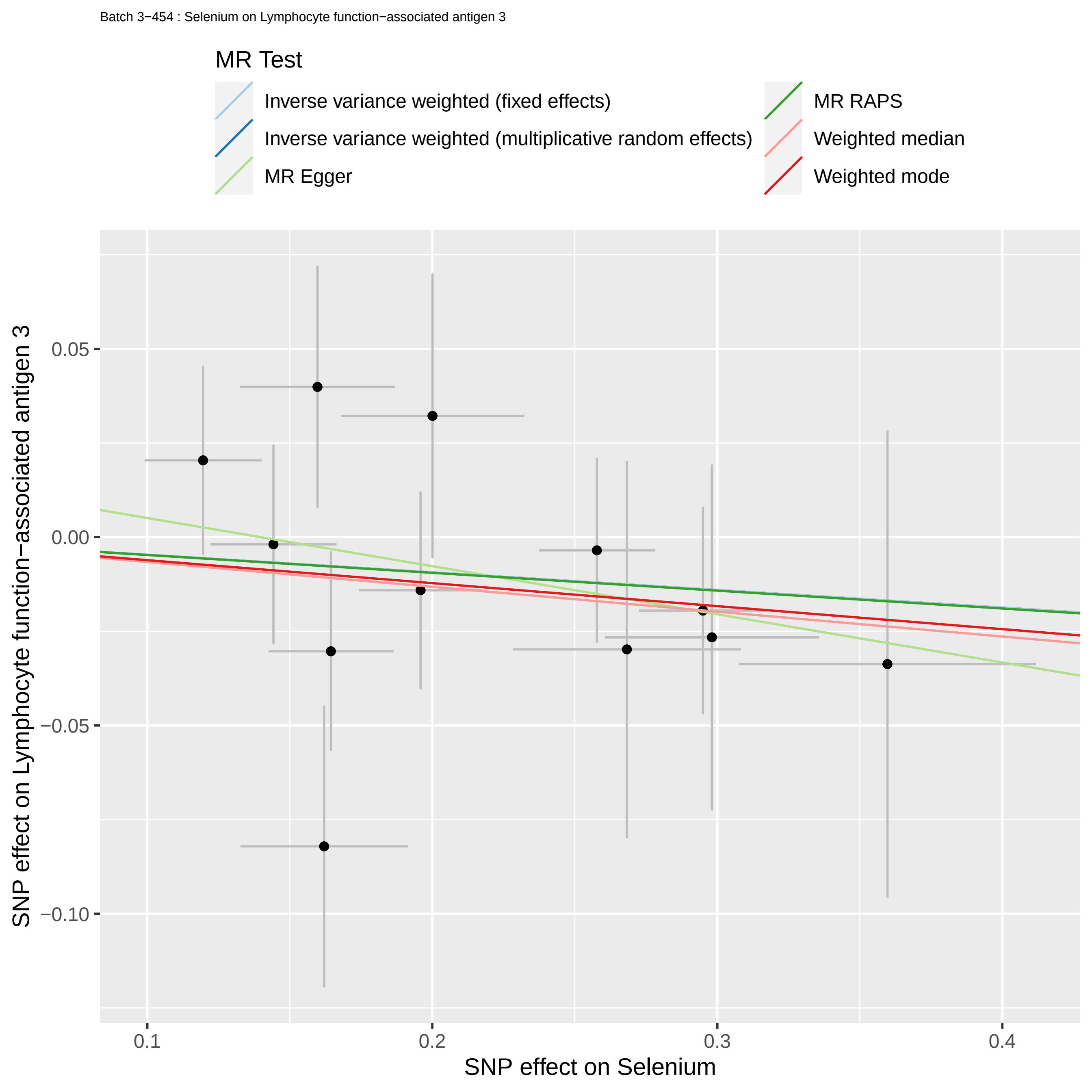

Batch 3−454 : Selenium on Lymphocyte function−associated antigen 3
MR Test
Inverse variance weighted (fixed effects)
Inverse variance weighted (multiplicative random effects)
MR Egger
MR RAPS
Weighted median
Weighted mode
SNP effect on Lymphocyte function−associated antigen 3
0.05
0.00
−0.05
−0.10
0.1
0.2
0.3
0.4
SNP effect on Selenium

Batch 3−455 : Selenium on CD59 glycoprotein

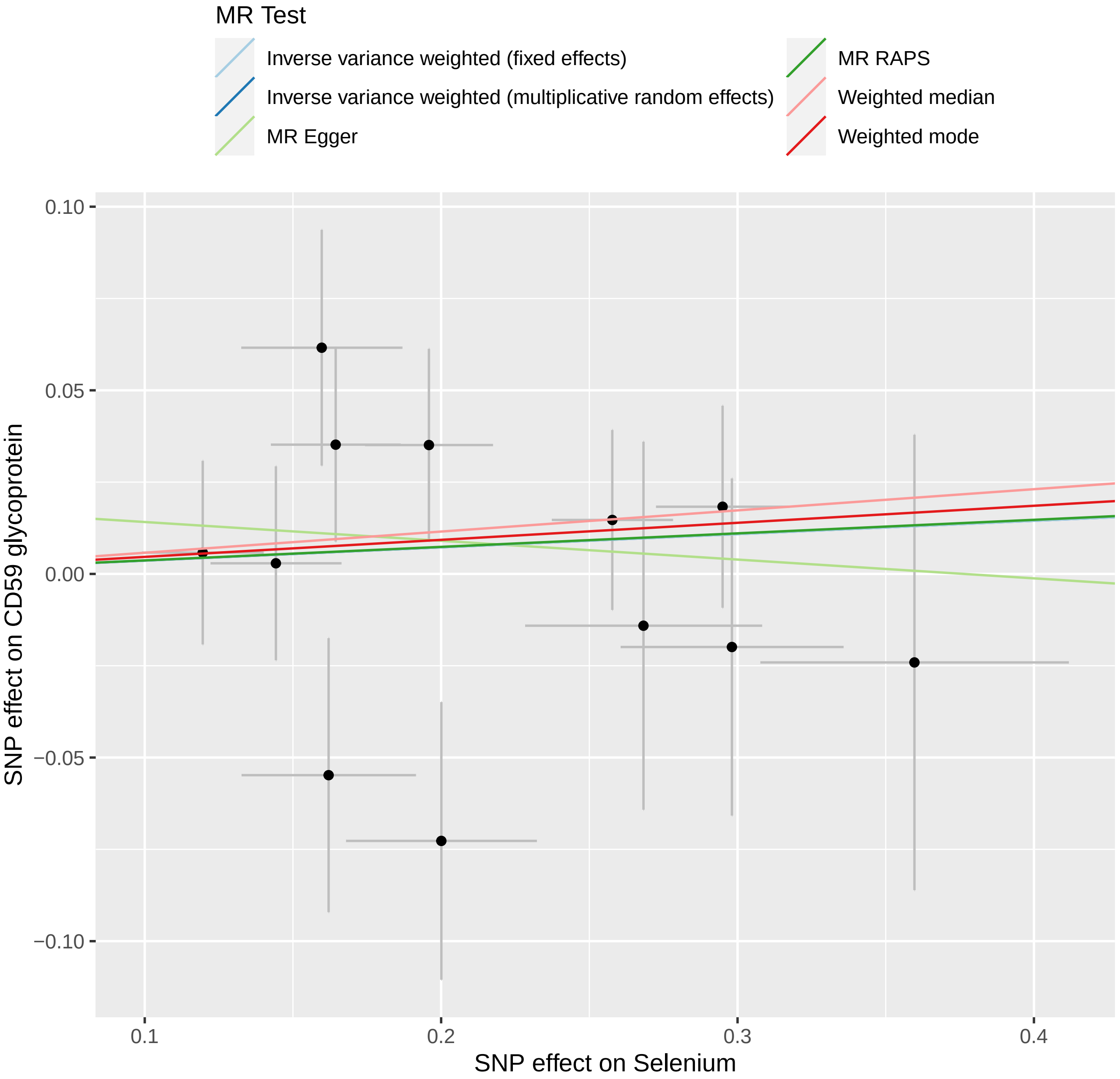

Batch 3−456 : Selenium on CD63 antigen

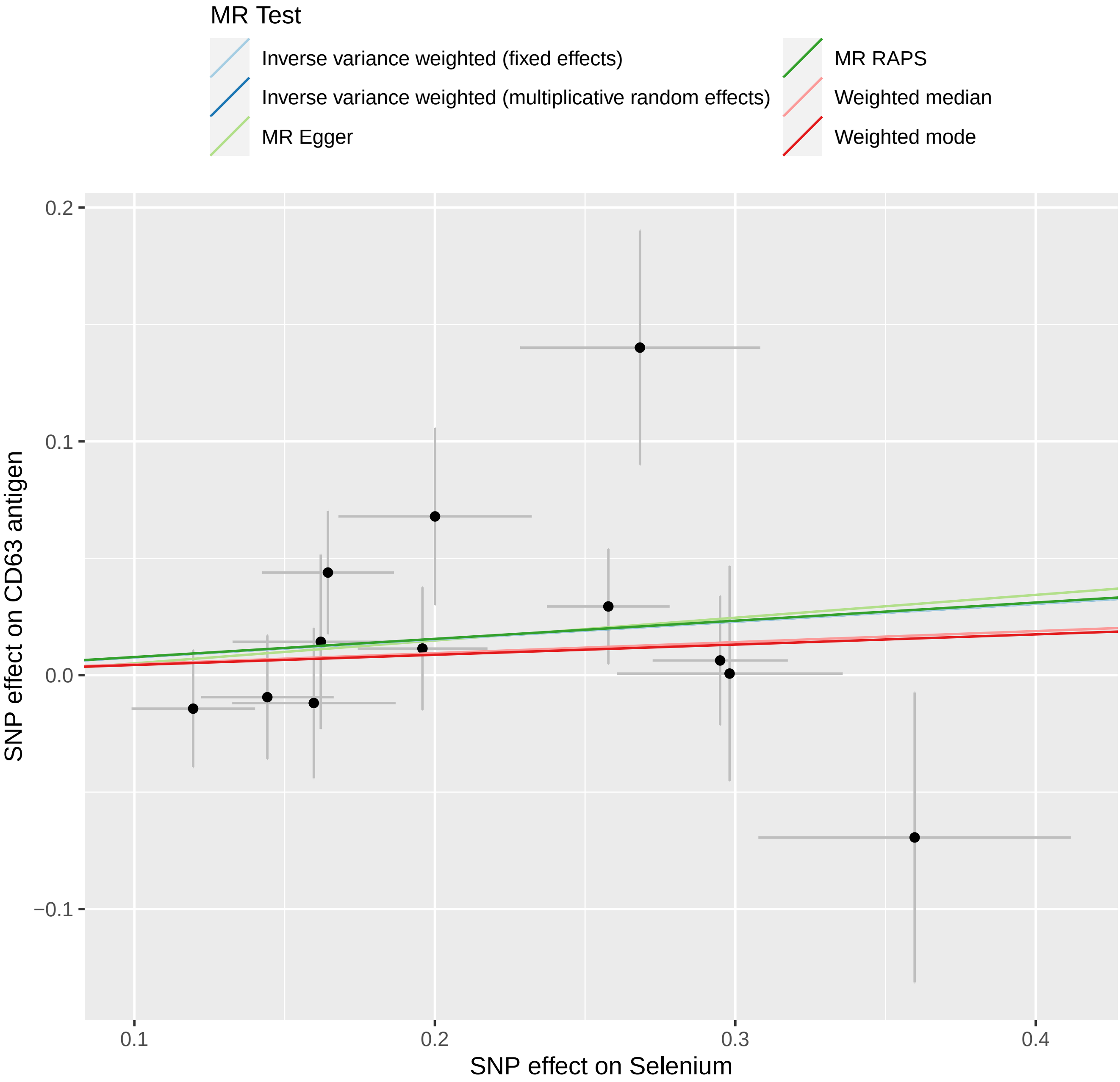

Batch 3−457 : Selenium on CD70 antigen

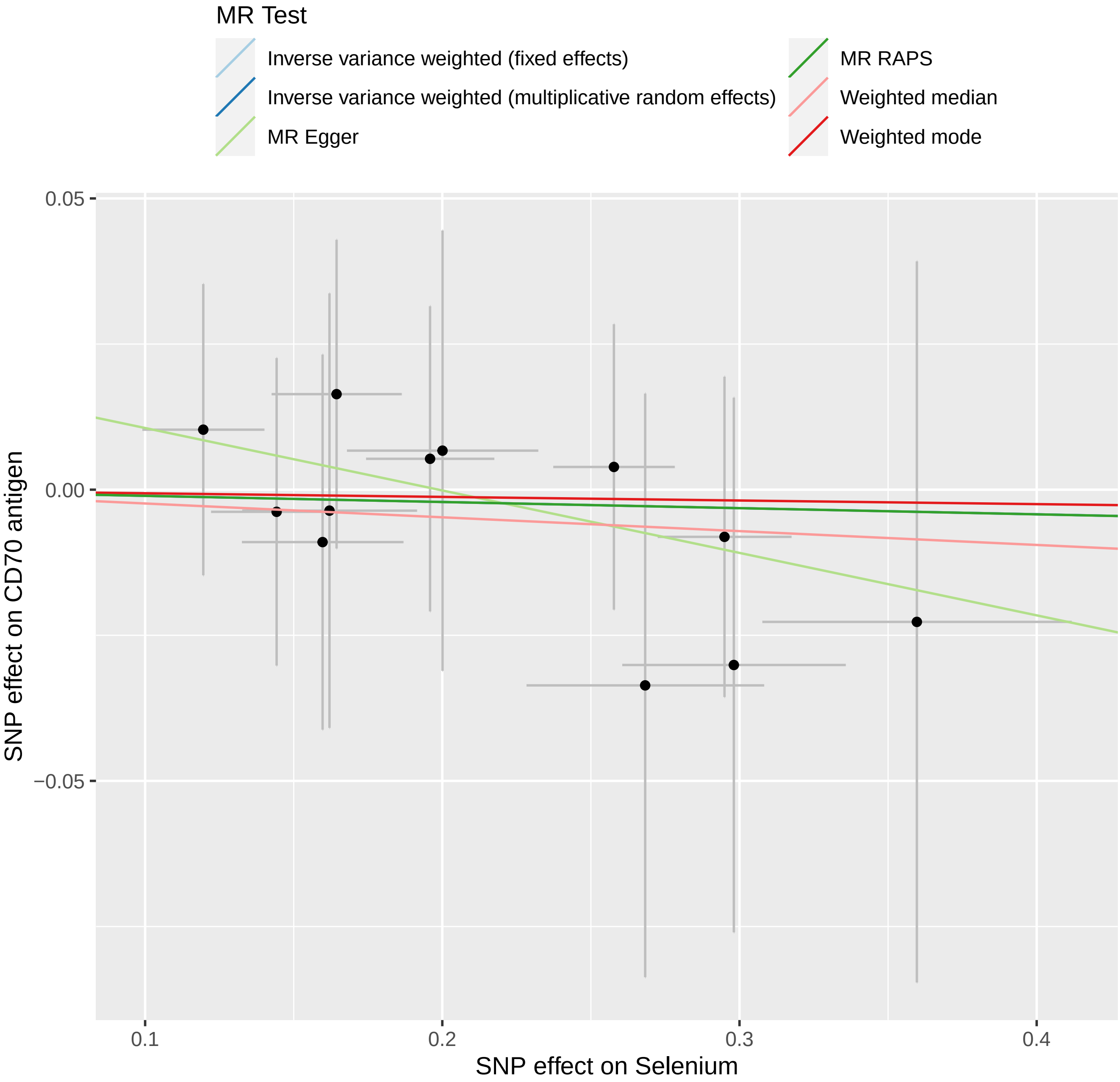

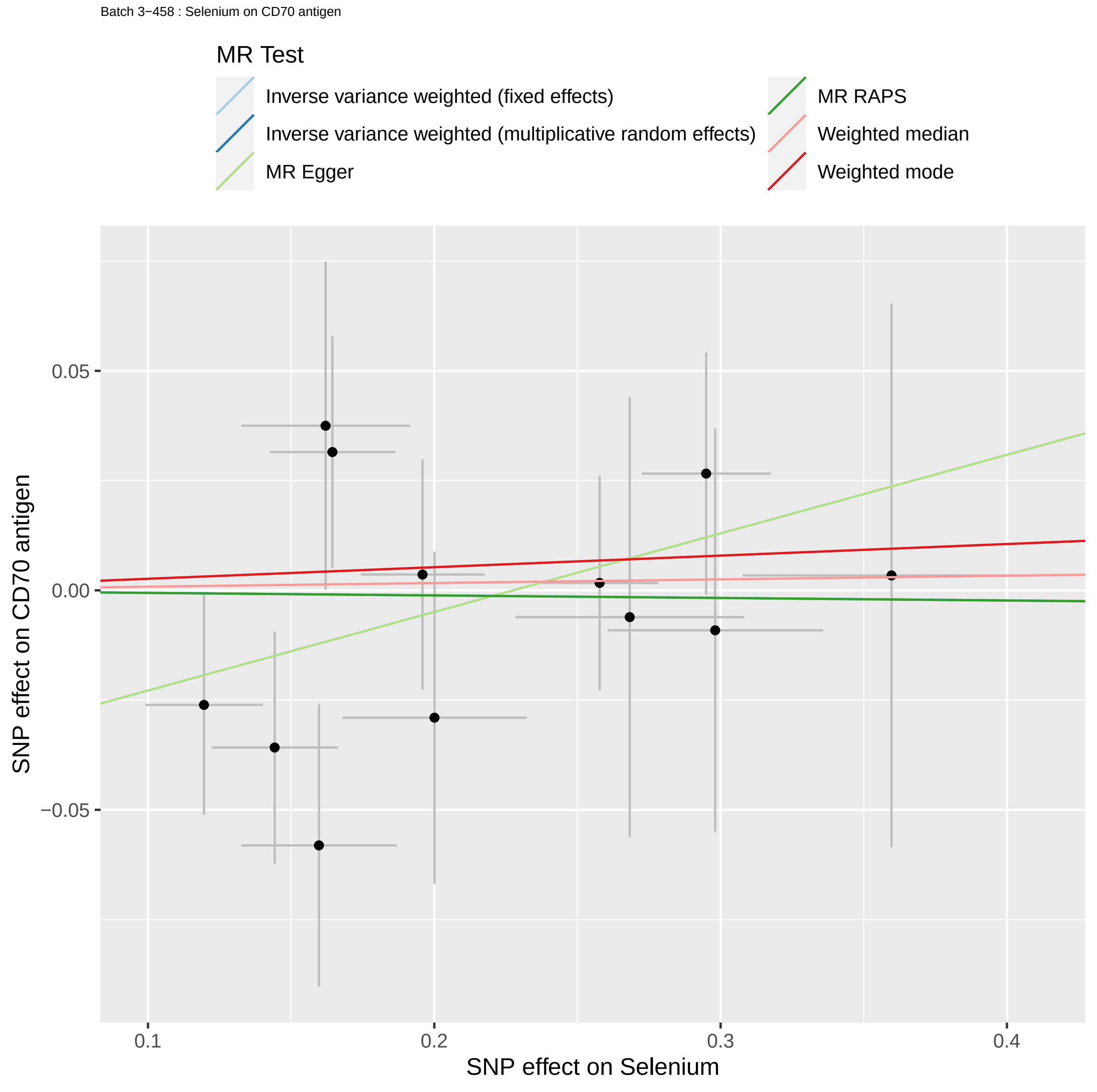
Batch 3−458 : Selenium on CD70 antigen
MR Test
Inverse variance weighted (fixed effects)
Inverse variance weighted (multiplicative random effects)
MR Egger
MR RAPS
Weighted median
Weighted mode
0.05
0.00
−0.05
0.1
0.2
0.3
0.4
SNP effect on Selenium
SNP effect on CD70 antigen

Batch 3−459 : Selenium on HLA class II histocompatibility antigen gamma chain

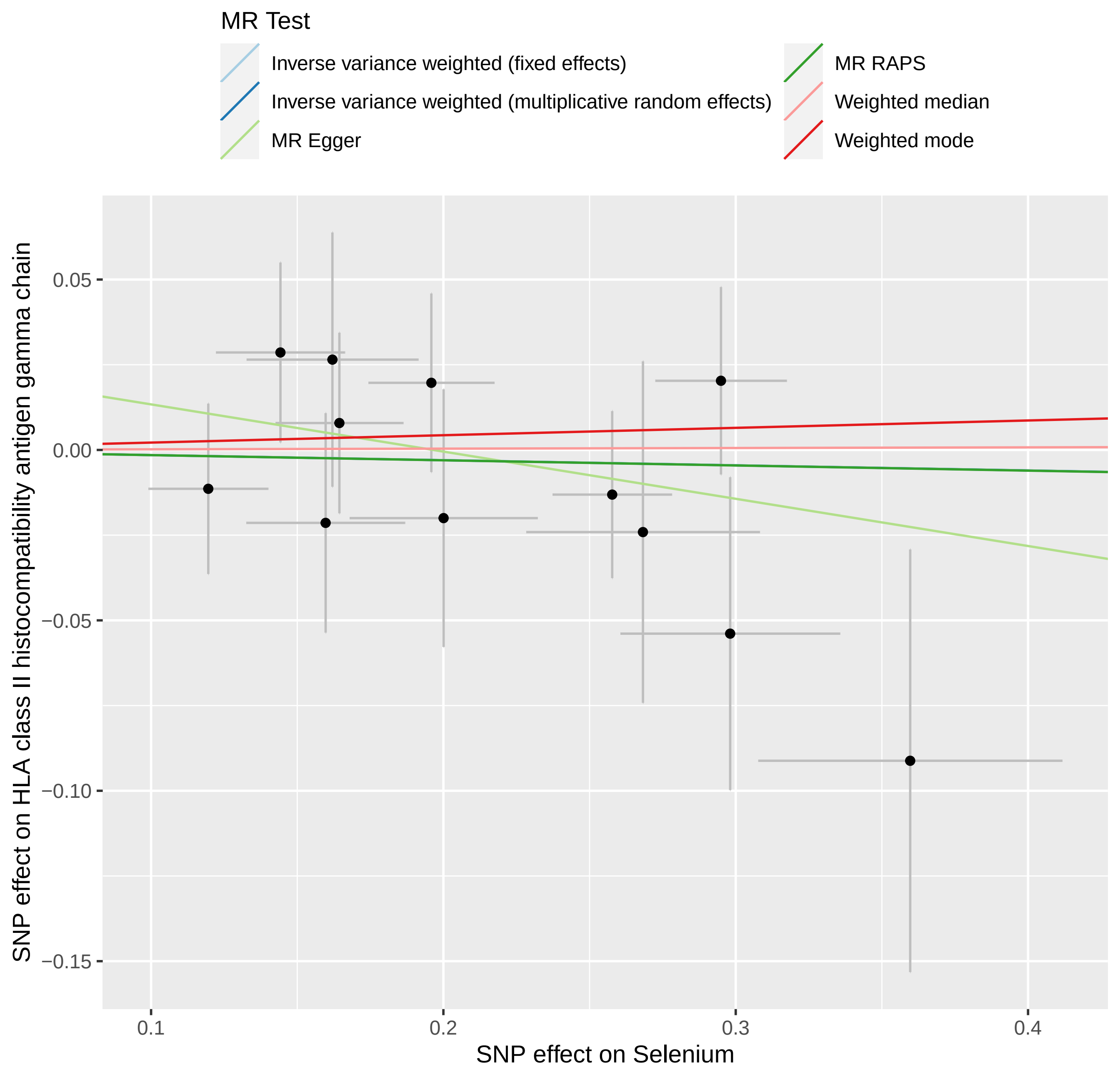

Batch 3−460 : Selenium on HLA class II histocompatibility antigen gamma chain

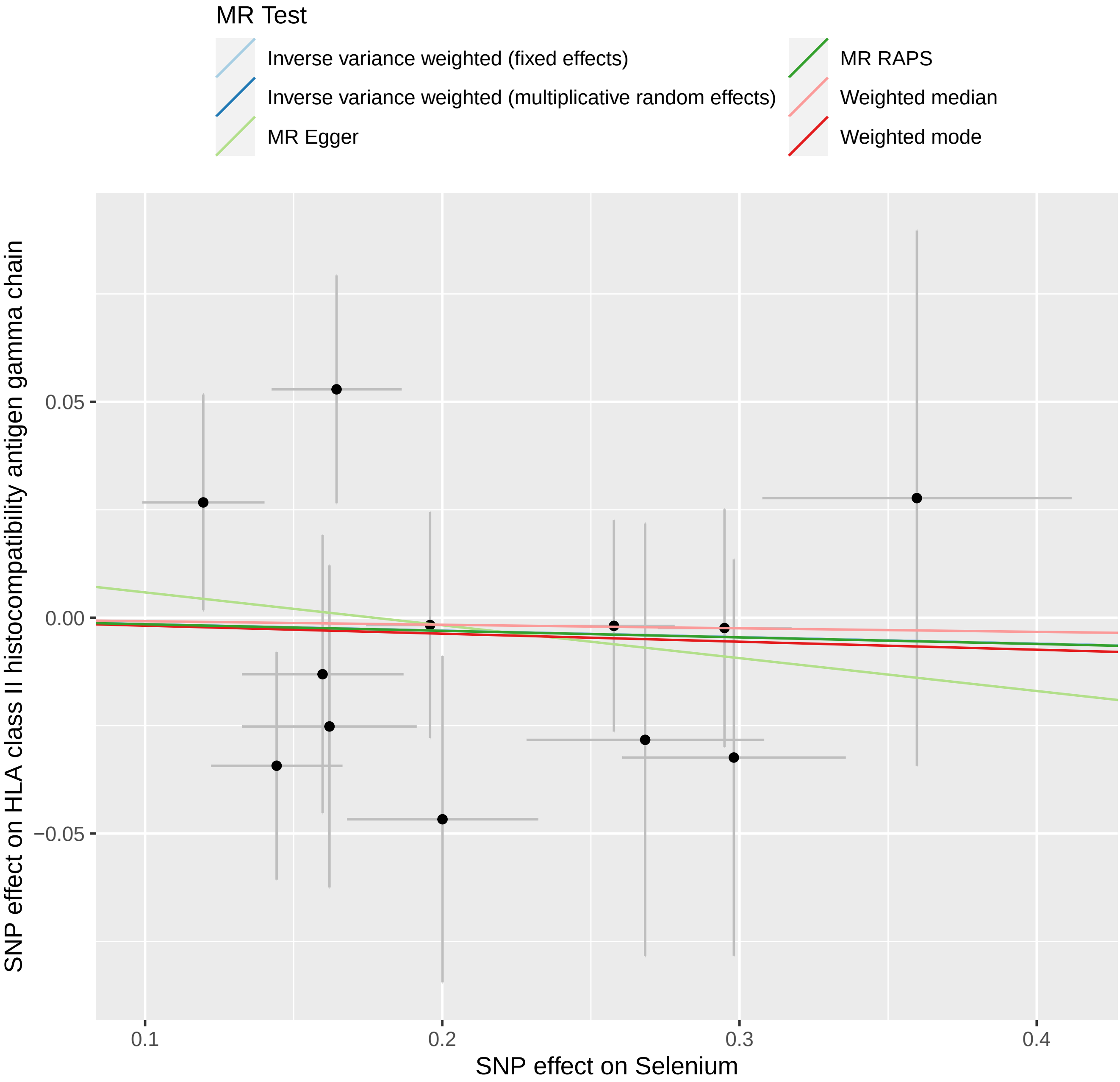

Batch 3−461 : Selenium on B−cell antigen receptor complex−associated protein alpha chain

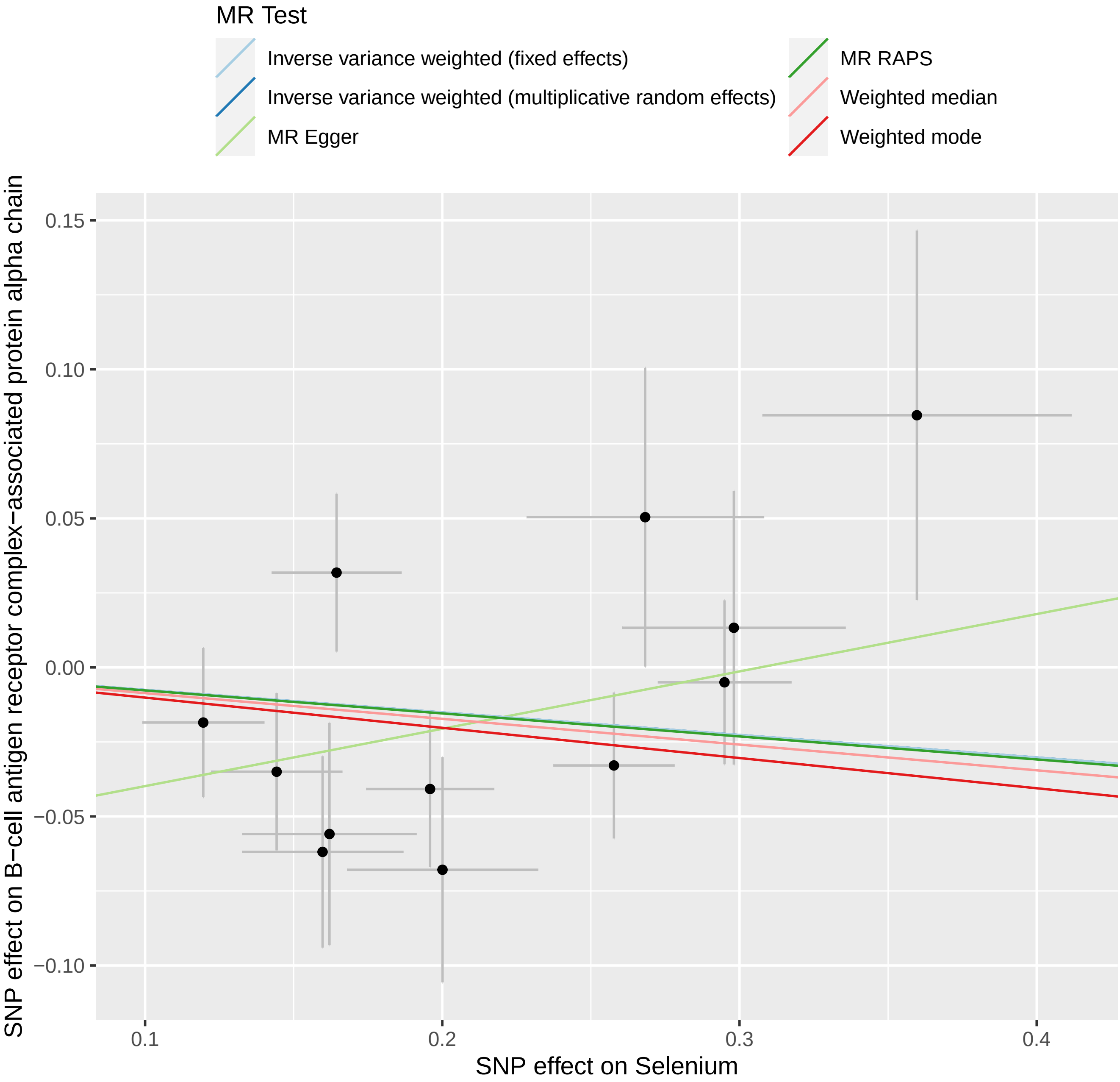

Batch 3−462 : Selenium on B−cell antigen receptor complex−associated protein beta chain

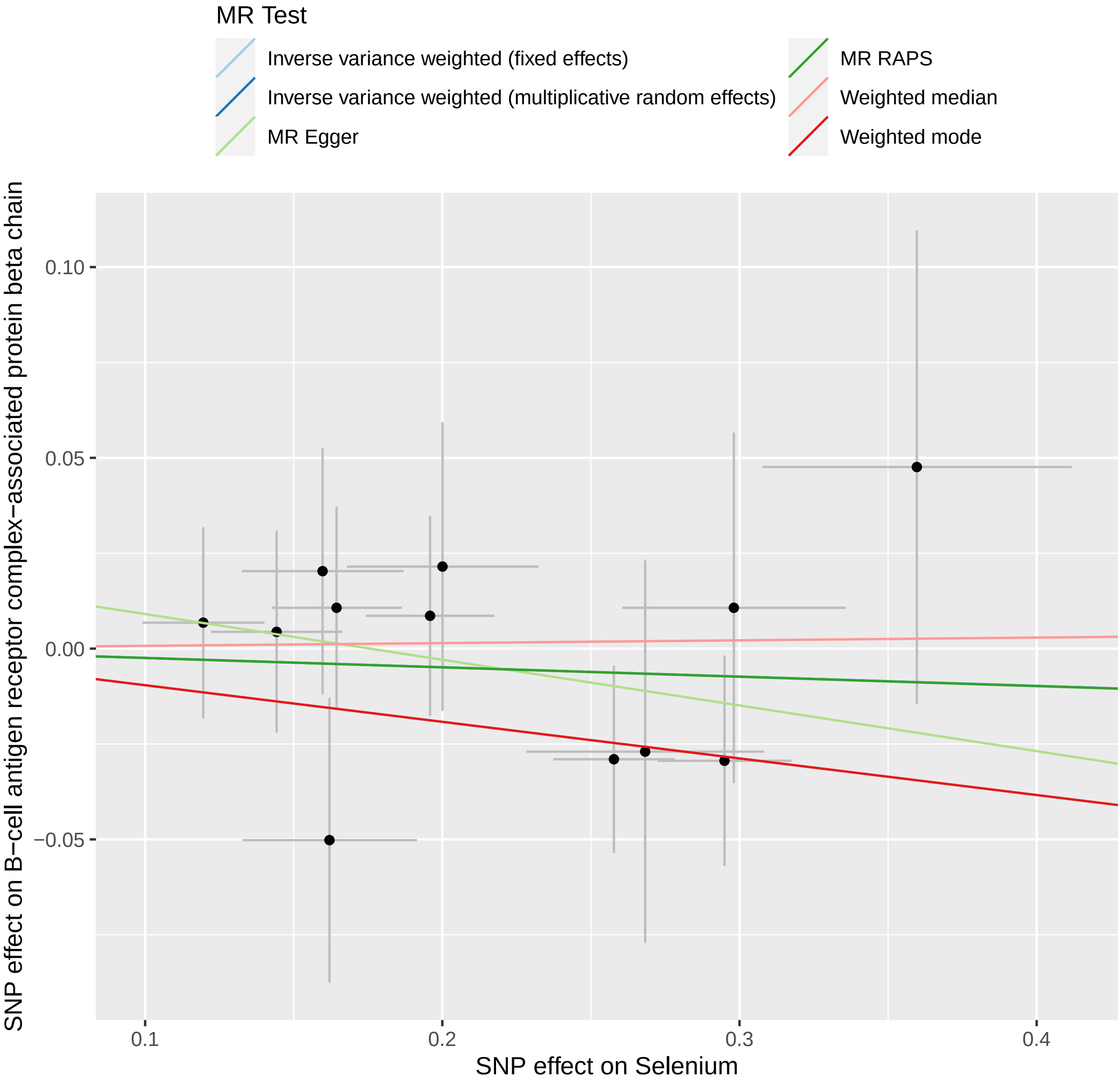

Batch 3−463 : Selenium on T−lymphocyte activation antigen CD80

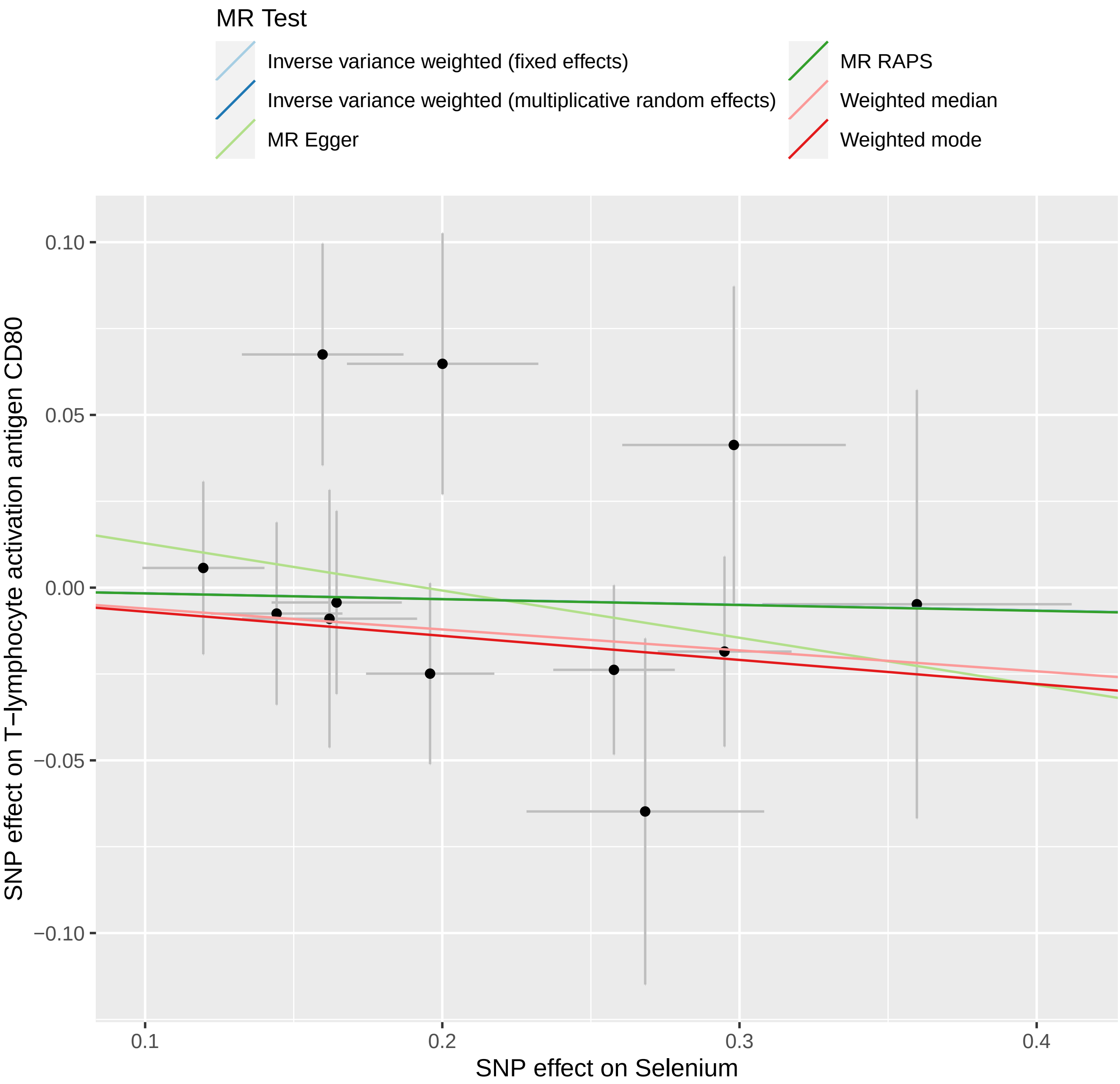

Batch 3−464 : Selenium on SLAM family member 5

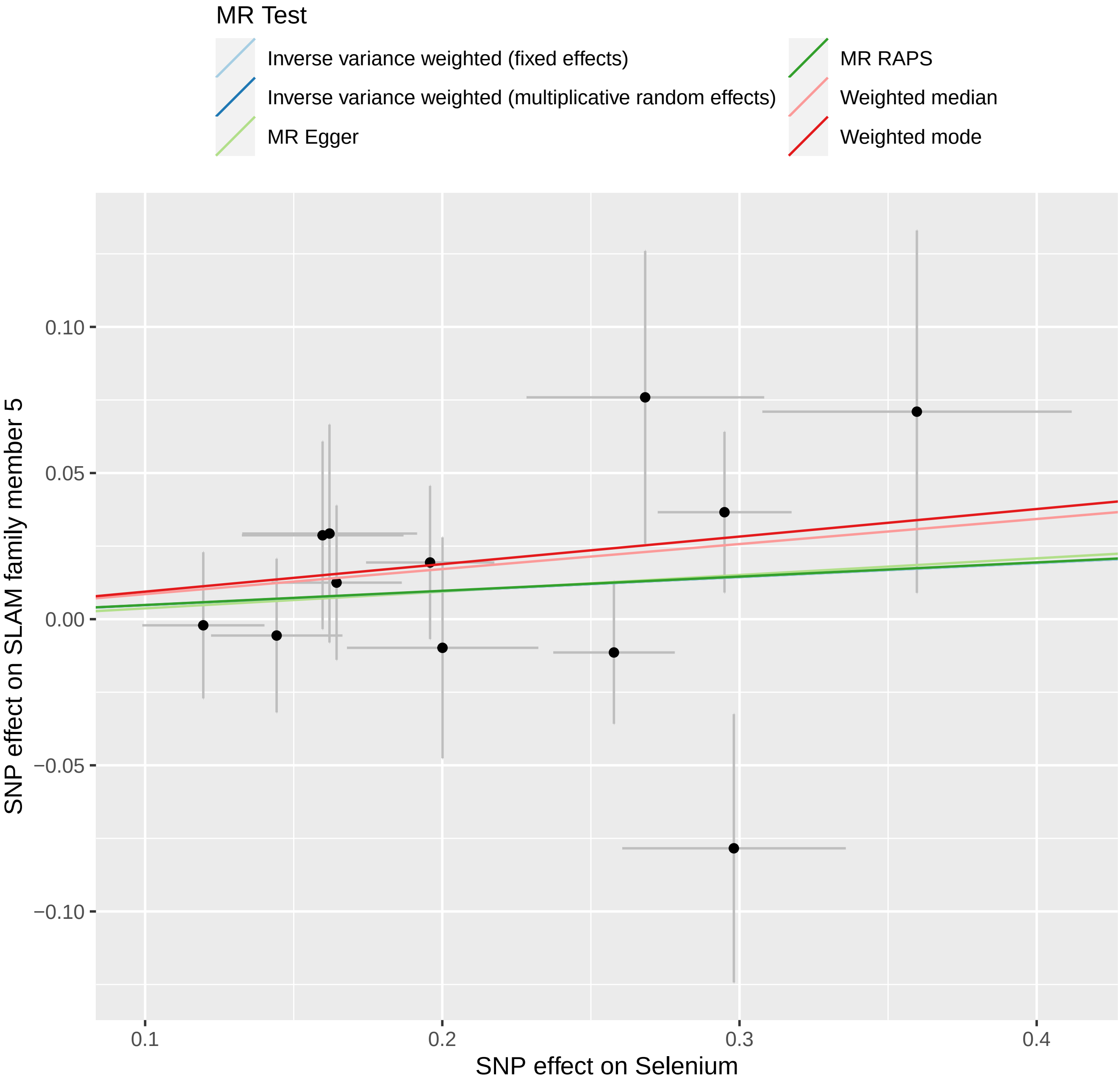

Batch 3−465 : Selenium on SLAM family member 5

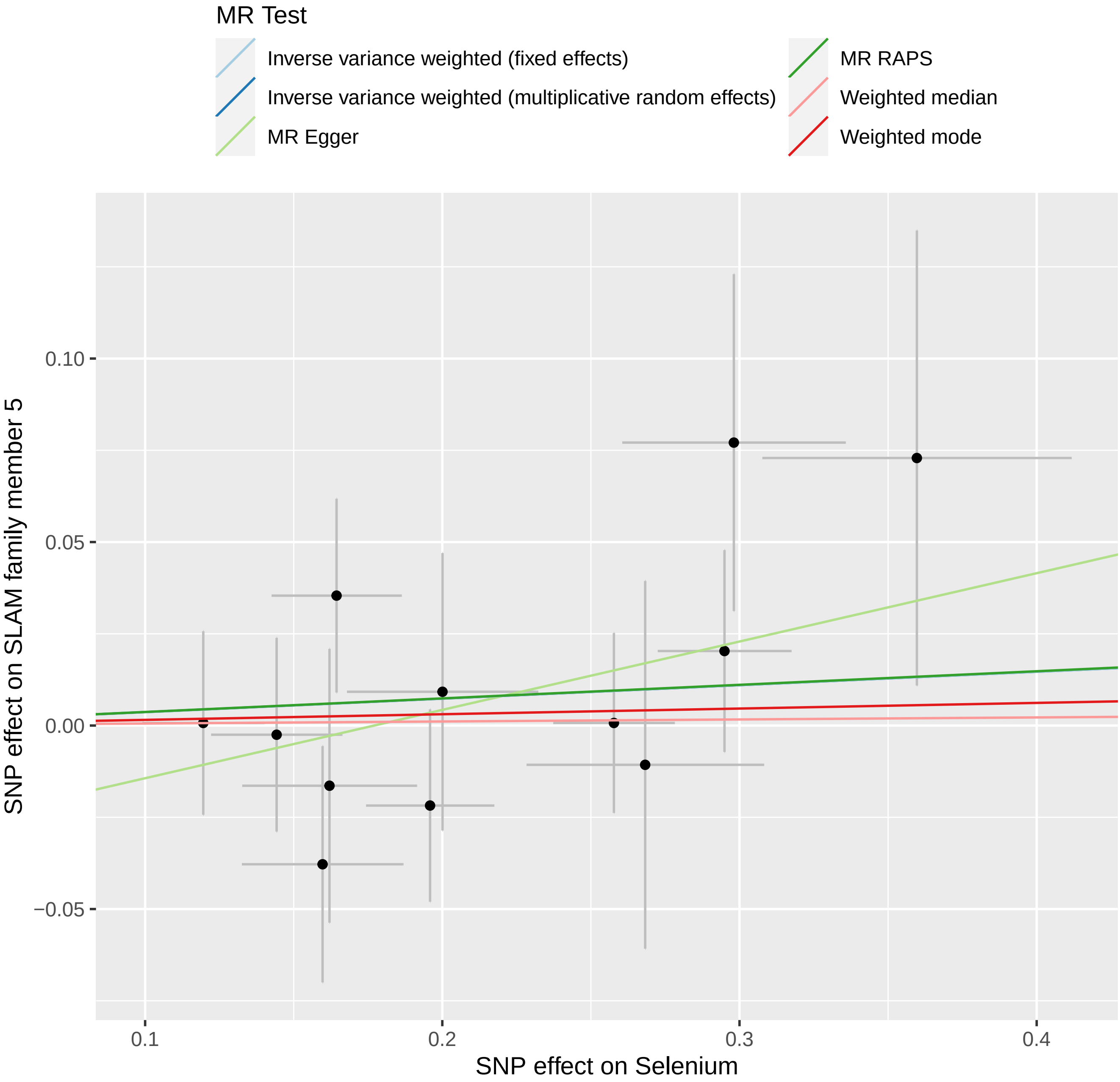

Batch 3−466 : Selenium on T−lymphocyte activation antigen CD86

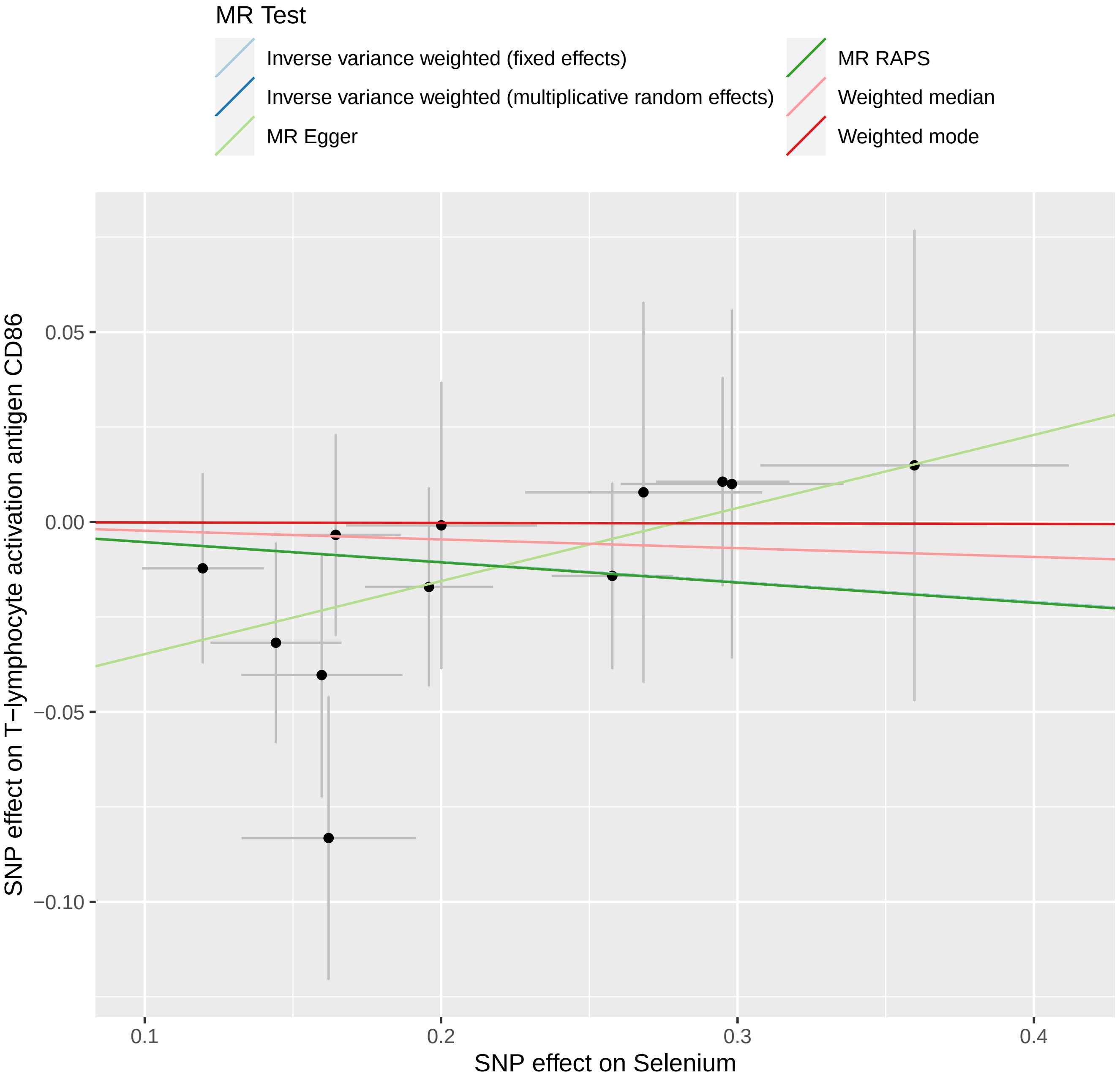

Batch 3−467 : Selenium on T−cell surface glycoprotein CD8 beta chain

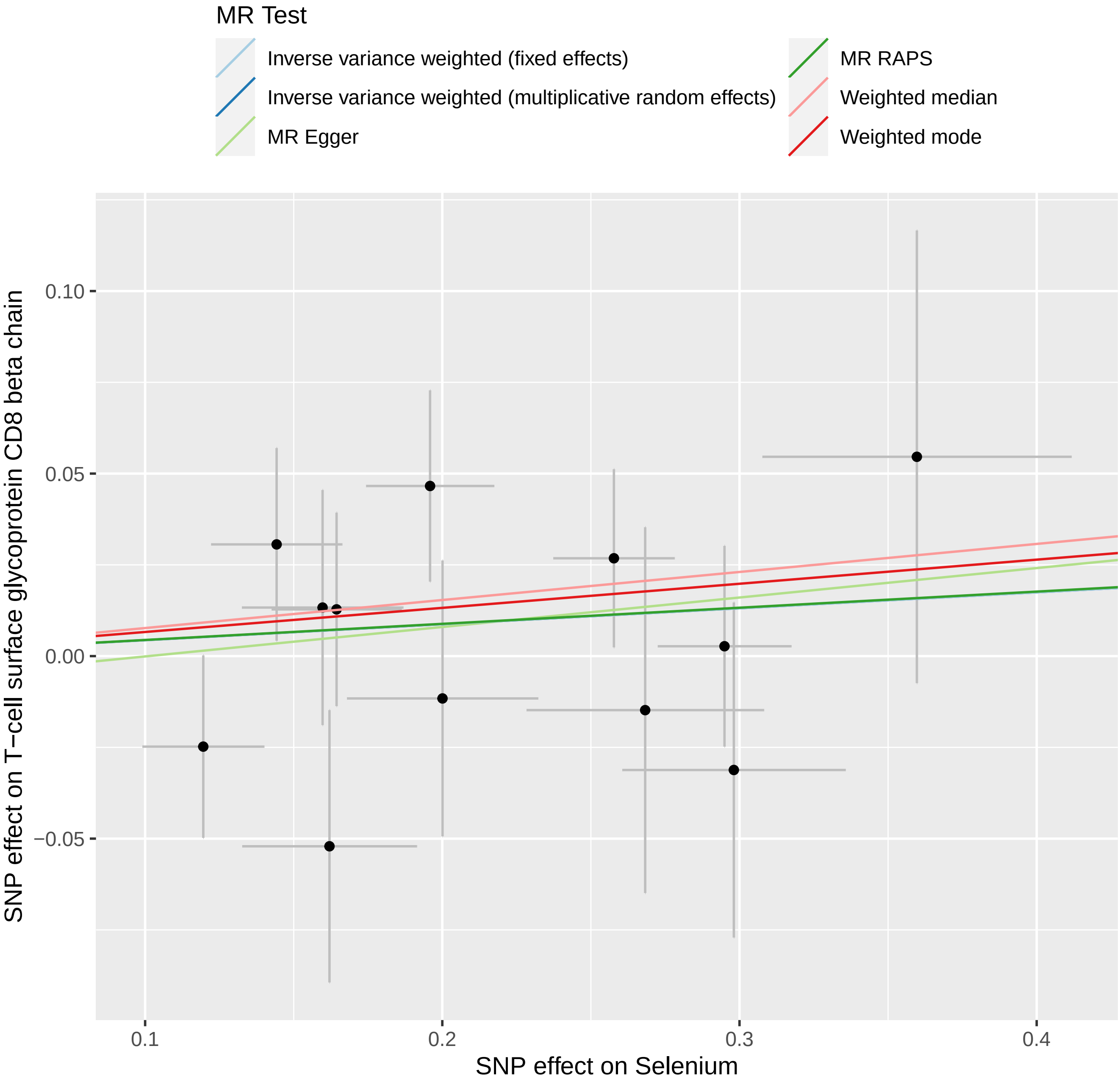

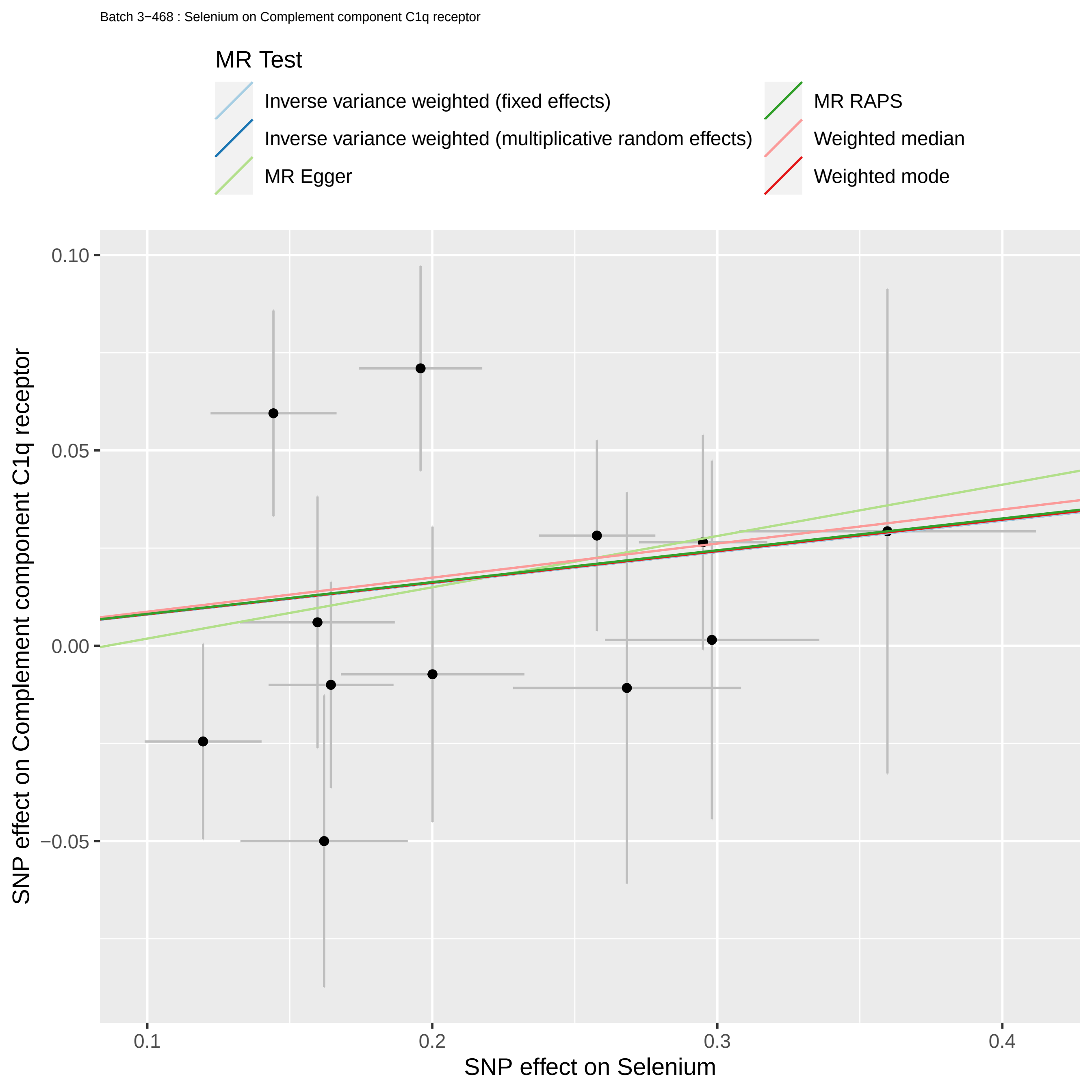
Batch 3−468 : Selenium on Complement component C1q receptor
MR Test
Inverse variance weighted (fixed effects)
Inverse variance weighted (multiplicative random effects)
MR Egger
MR RAPS
Weighted median
Weighted mode
0.10
0.05
0.00
−0.05
0.1
0.2
0.3
0.4
SNP effect on Selenium
SNP effect on Complement component C1q receptor

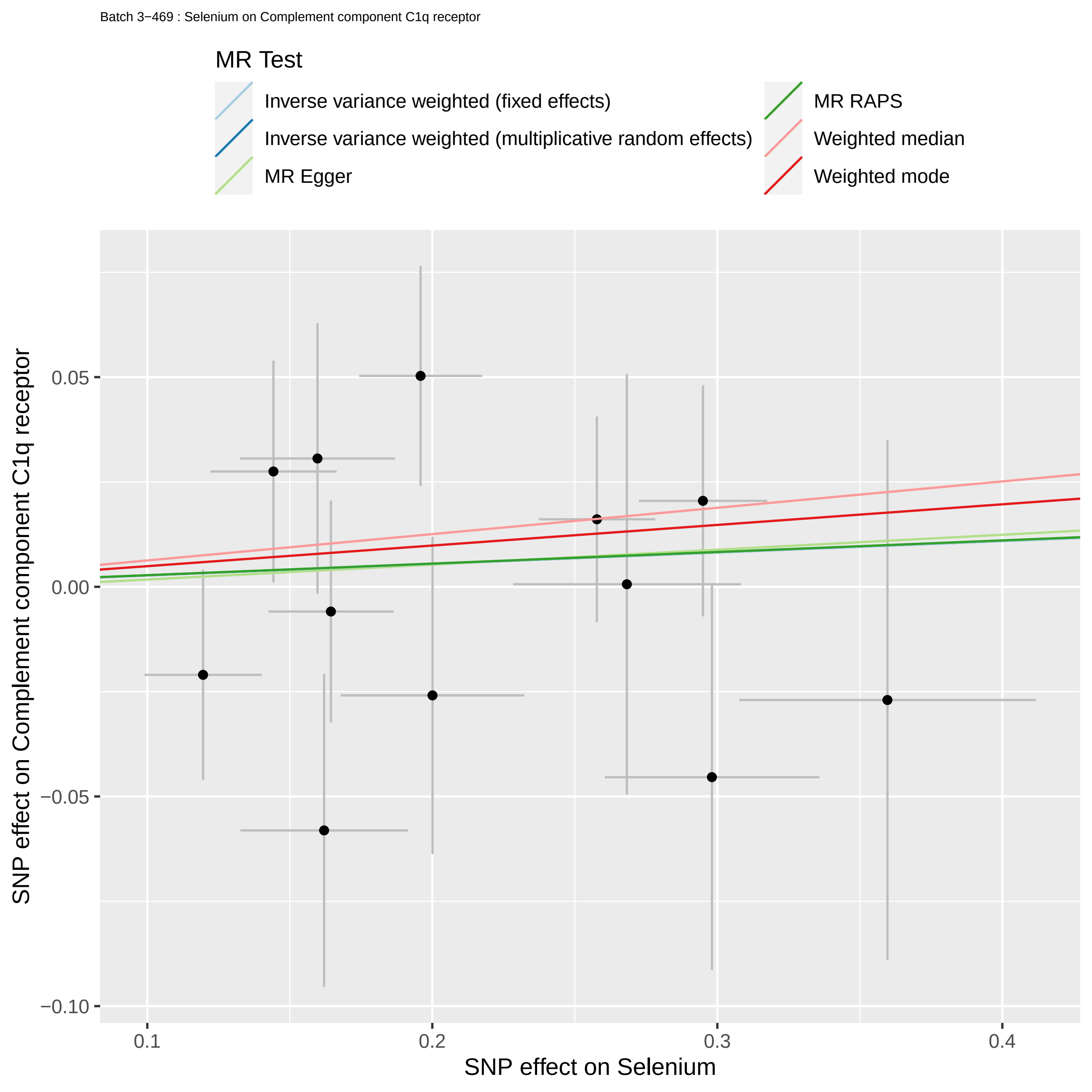
Batch 3−469 : Selenium on Complement component C1q receptor
MR Test
Inverse variance weighted (fixed effects)
Inverse variance weighted (multiplicative random effects)
MR Egger
MR RAPS
Weighted median
Weighted mode
SNP effect on Complement component C1q receptor
0.05
0.00
−0.05
−0.10
0.1
0.2
0.3
0.4
SNP effect on Selenium

Batch 3−470 : Selenium on T−cell surface protein tactile

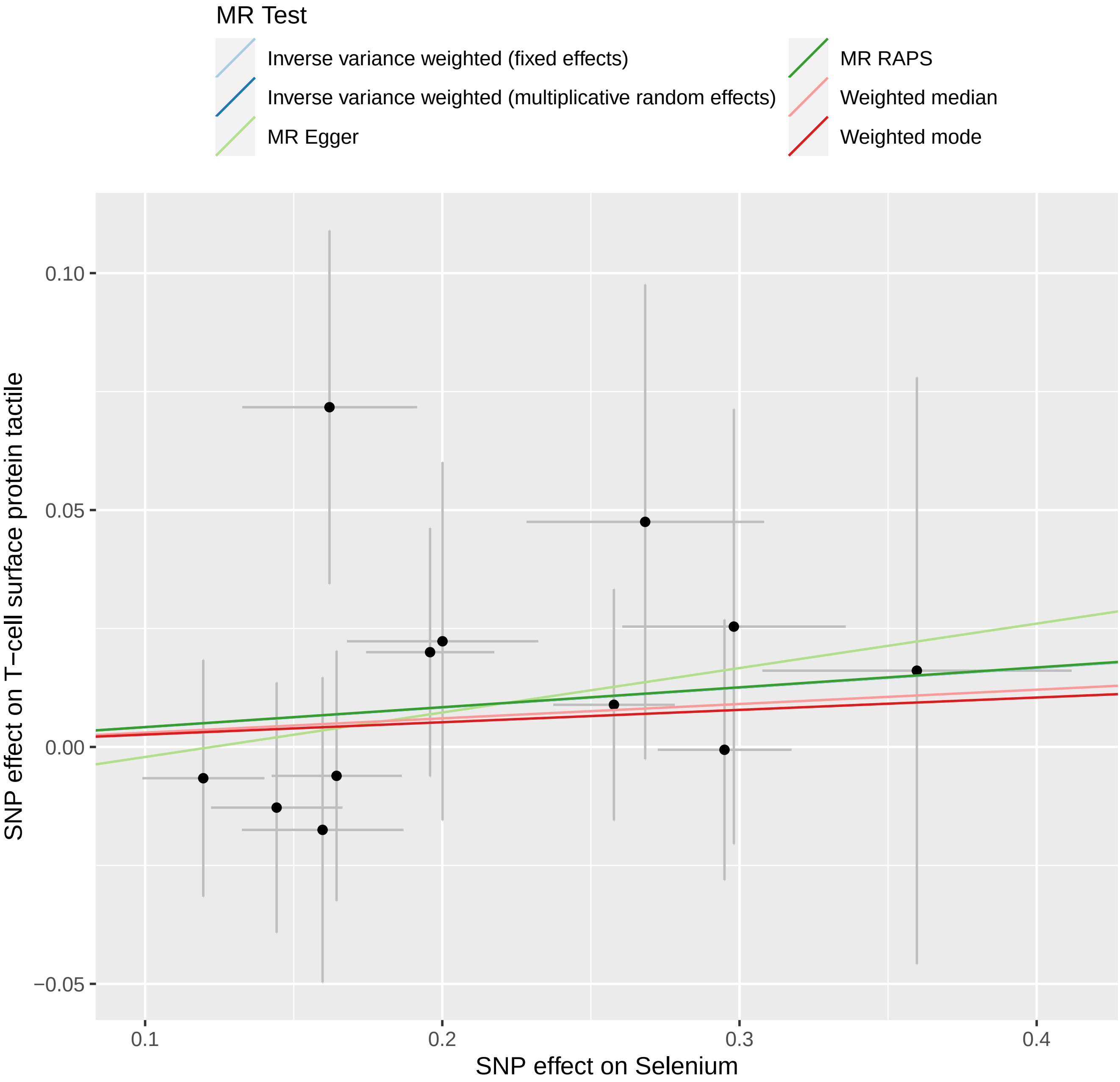

Batch 3−471 : Selenium on CD97 antigen

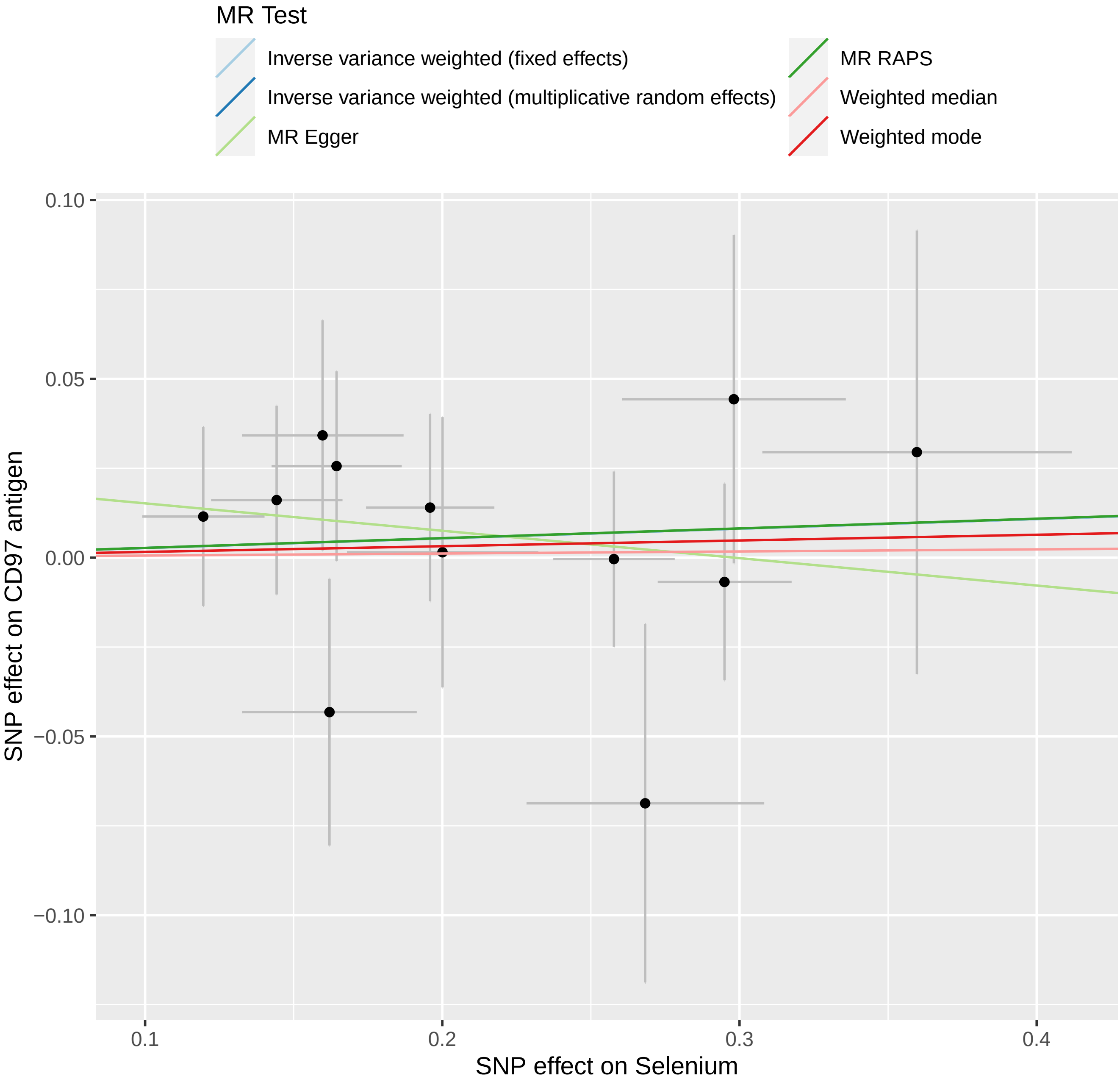

Batch 3−472 : Selenium on M−phase inducer phosphatase 2

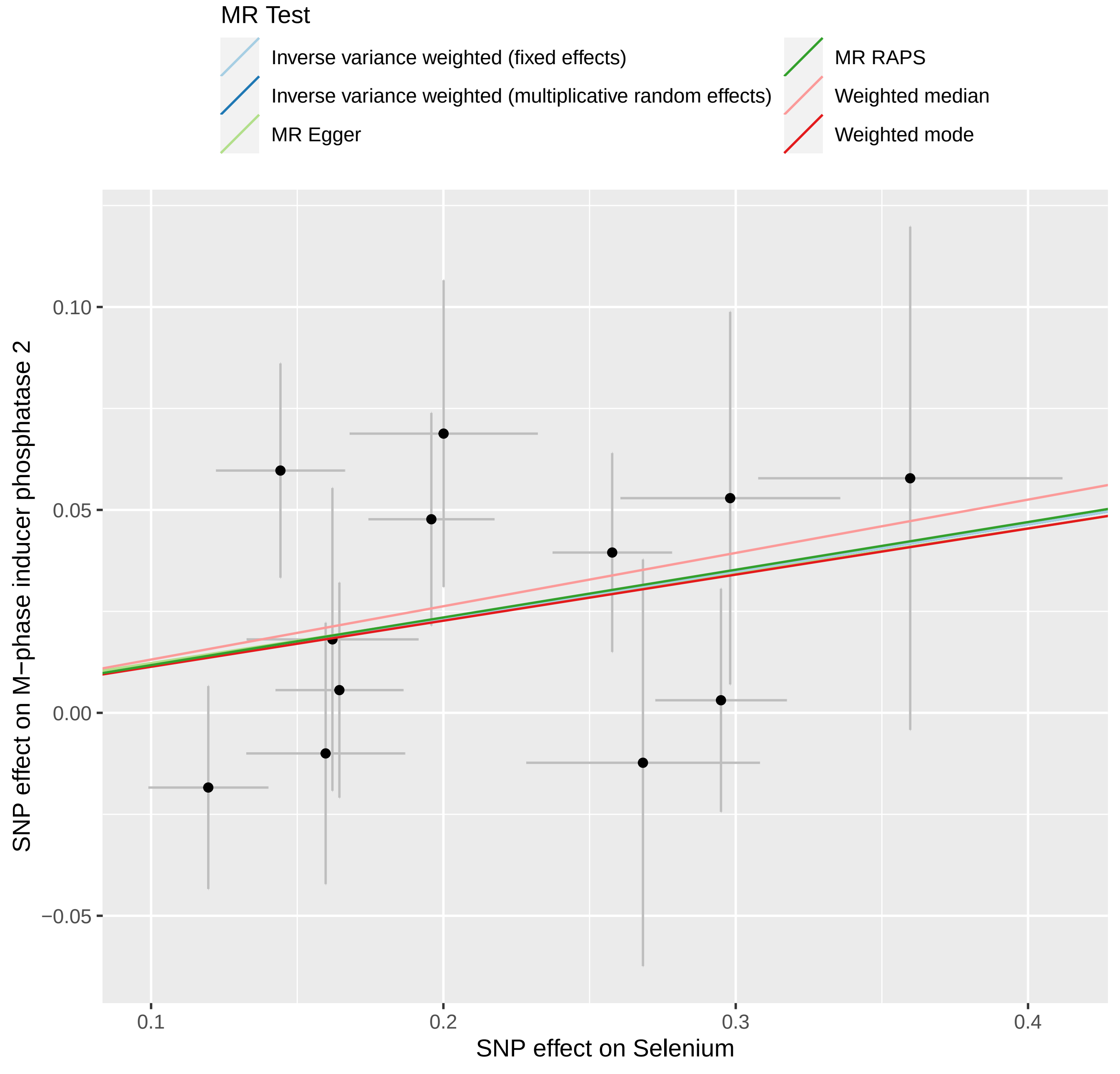

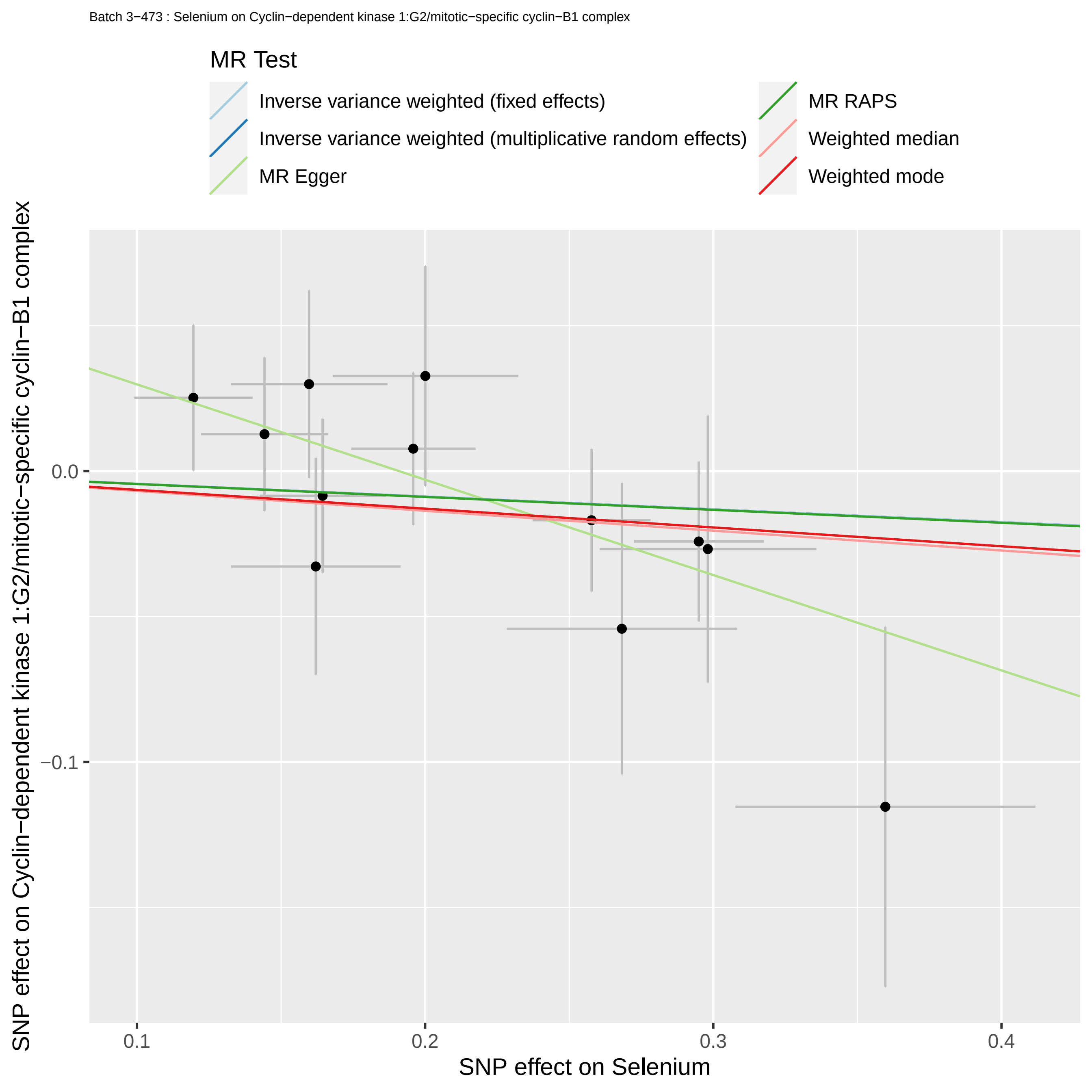

Batch 3−473 : Selenium on Cyclin−dependent kinase 1:G2/mitotic−specific cyclin−B1 complex
MR Test
Inverse variance weighted (fixed effects)
Inverse variance weighted (multiplicative random effects)
MR Egger
MR RAPS
Weighted median
Weighted mode
0.0
−0.1
0.1
0.2
0.3
0.4
SNP effect on Selenium
SNP effect on Cyclin−dependent kinase 1:G2/mitotic−specific cyclin−B1 complex

Batch 3−474 : Selenium on Cell division control protein 42 homolog

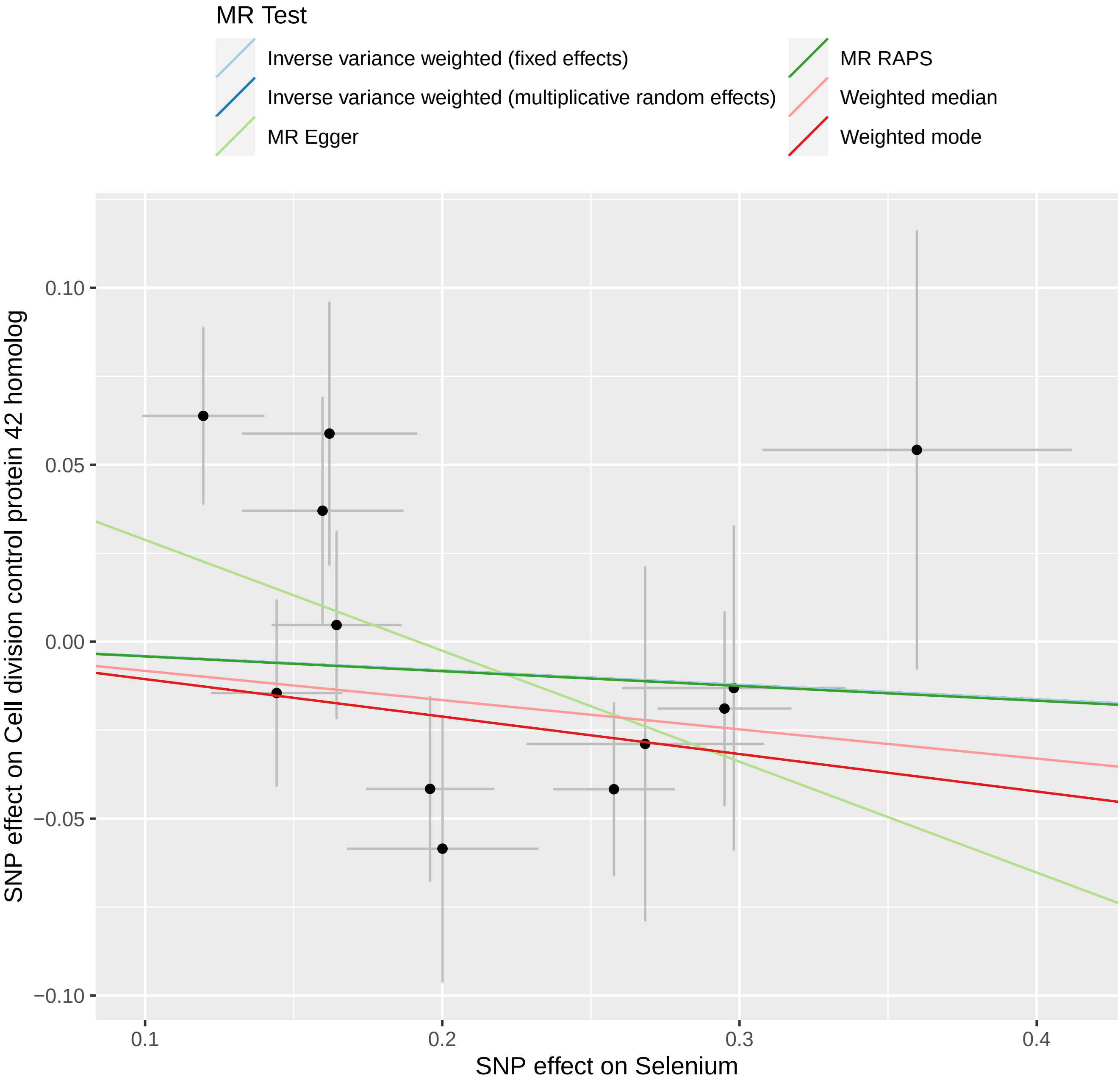

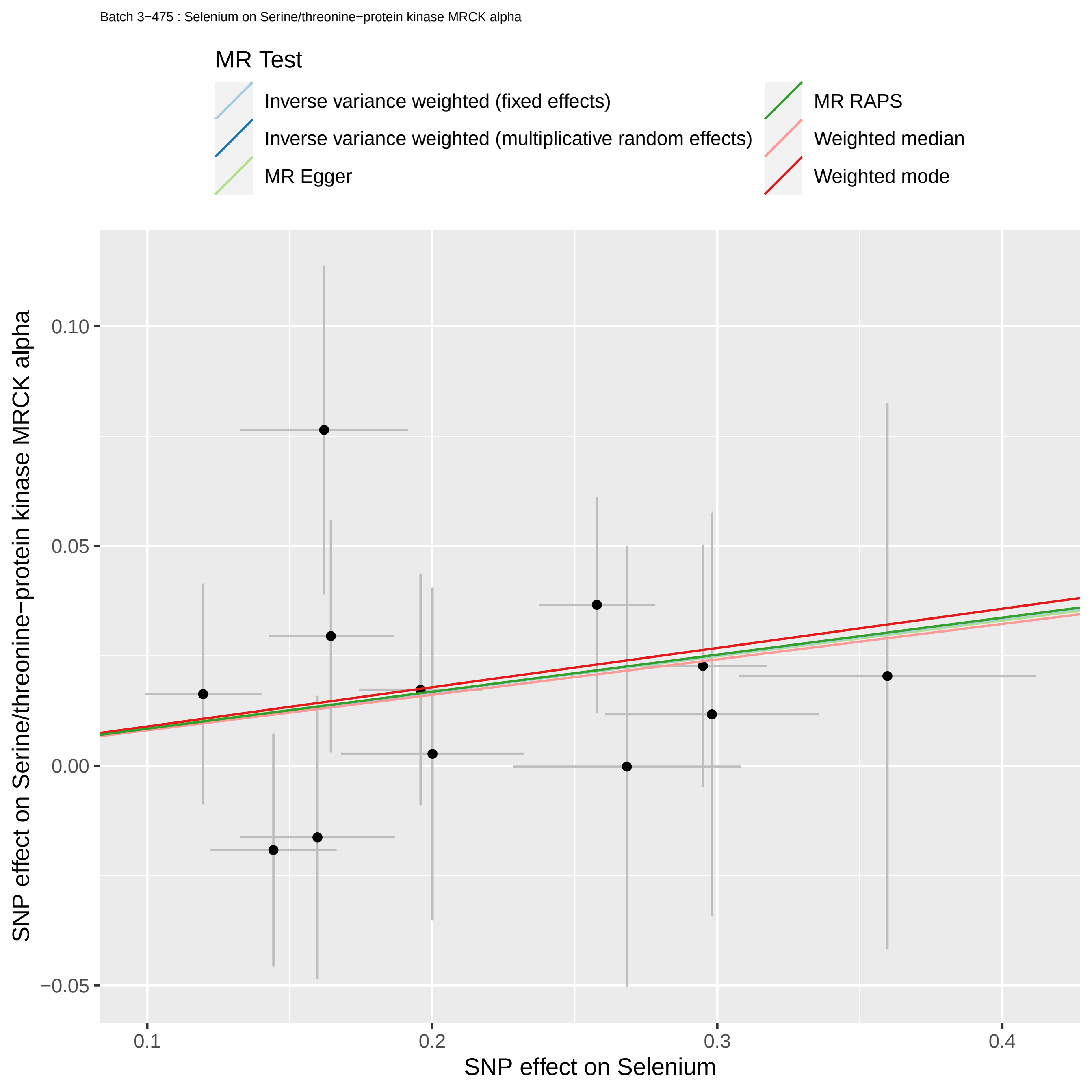

Batch 3−475 : Selenium on Serine/threonine−protein kinase MRCK alpha
MR Test
Inverse variance weighted (fixed effects)
Inverse variance weighted (multiplicative random effects)
MR Egger
MR RAPS
Weighted median
Weighted mode
0.10
0.05
0.00
−0.05
0.1
0.2
0.3
0.4
SNP effect on Serine/threonine−protein kinase MRCK alpha
SNP effect on Selenium

Batch 3−476 : Selenium on Serine/threonine−protein kinase MRCK beta

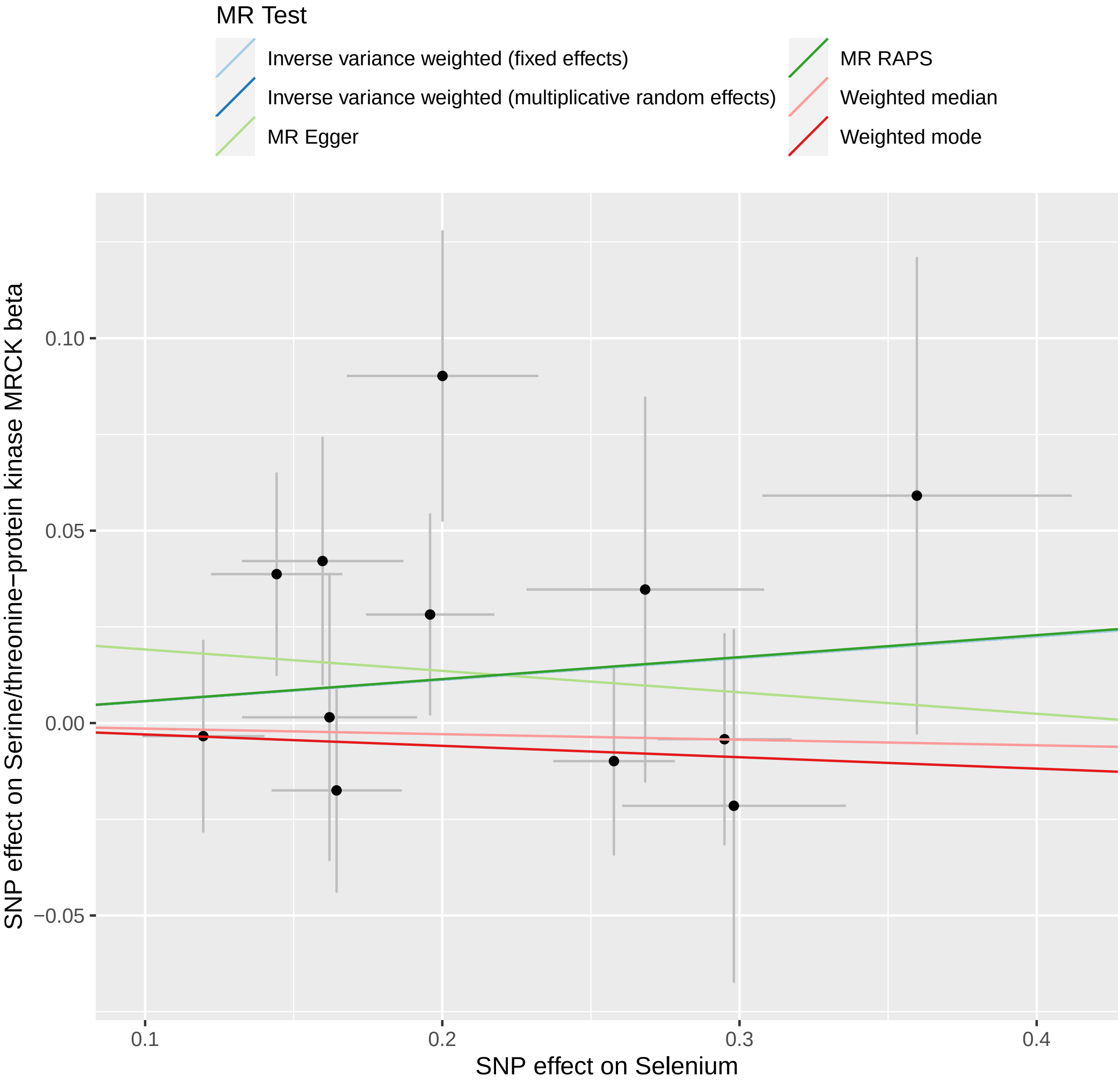

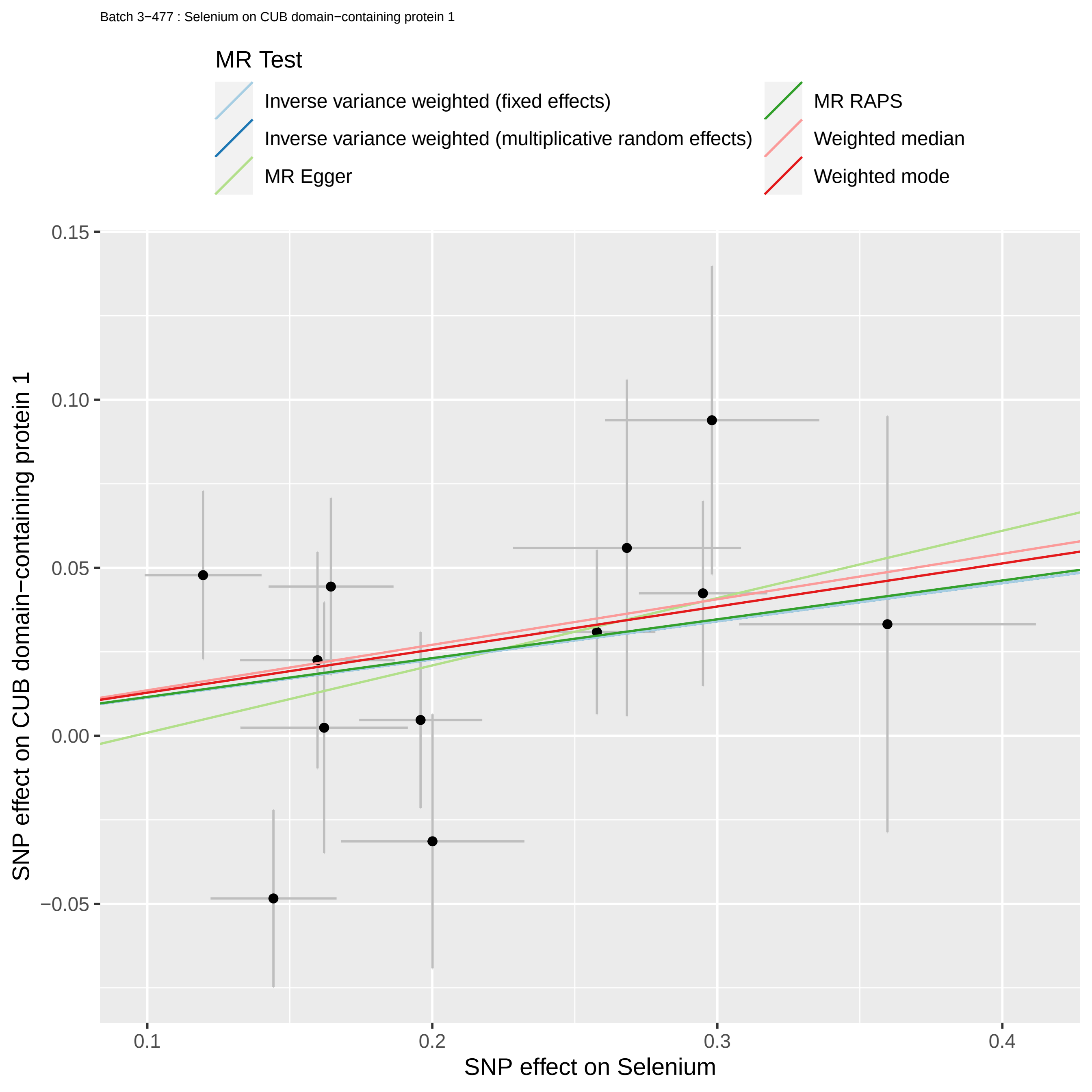

Batch 3−477 : Selenium on CUB domain−containing protein 1
MR Test
Inverse variance weighted (fixed effects)
Inverse variance weighted (multiplicative random effects)
MR Egger
MR RAPS
Weighted median
Weighted mode
0.15
0.10
0.05
0.00
−0.05
0.1
0.2
0.3
0.4
SNP effect on CUB domain−containing protein 1
SNP effect on Selenium

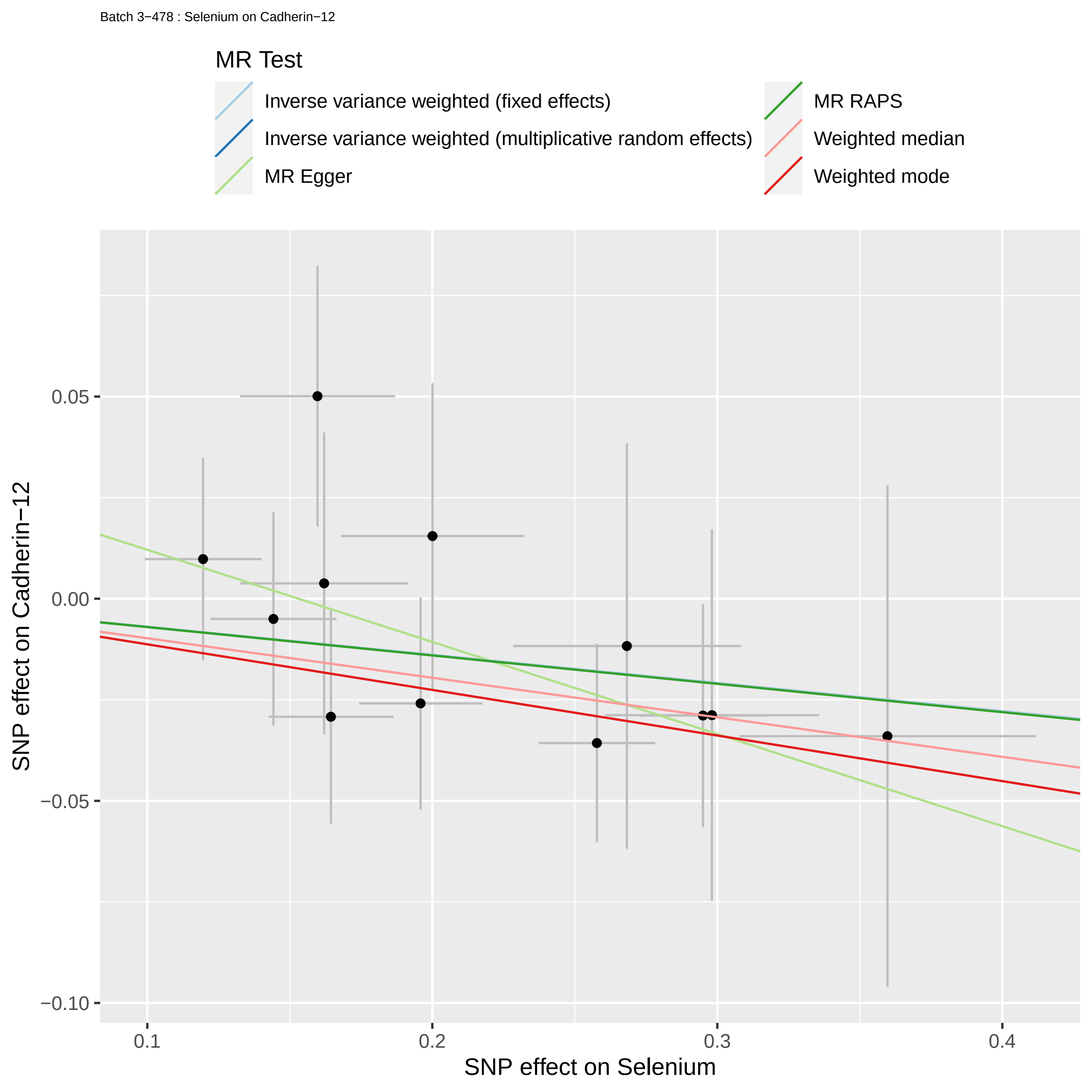

Batch 3−478 : Selenium on Cadherin−12
MR Test
Inverse variance weighted (fixed effects)
Inverse variance weighted (multiplicative random effects)
MR Egger
MR RAPS
Weighted median
Weighted mode
SNP effect on Cadherin−12
0.05
0.00
−0.05
−0.10
0.1
0.2
0.3
0.4
SNP effect on Selenium

Batch 3−479 : Selenium on Cadherin−12

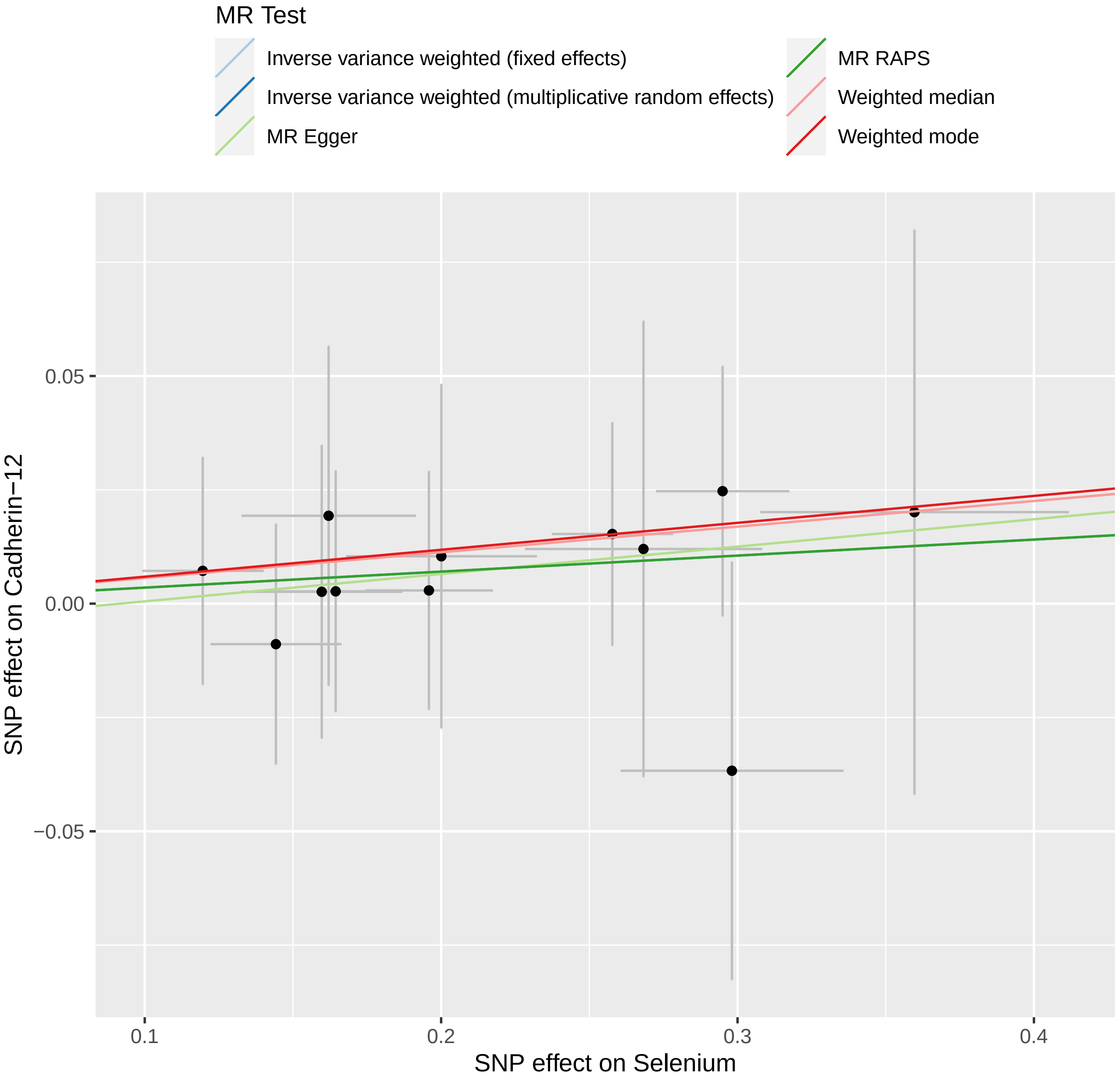

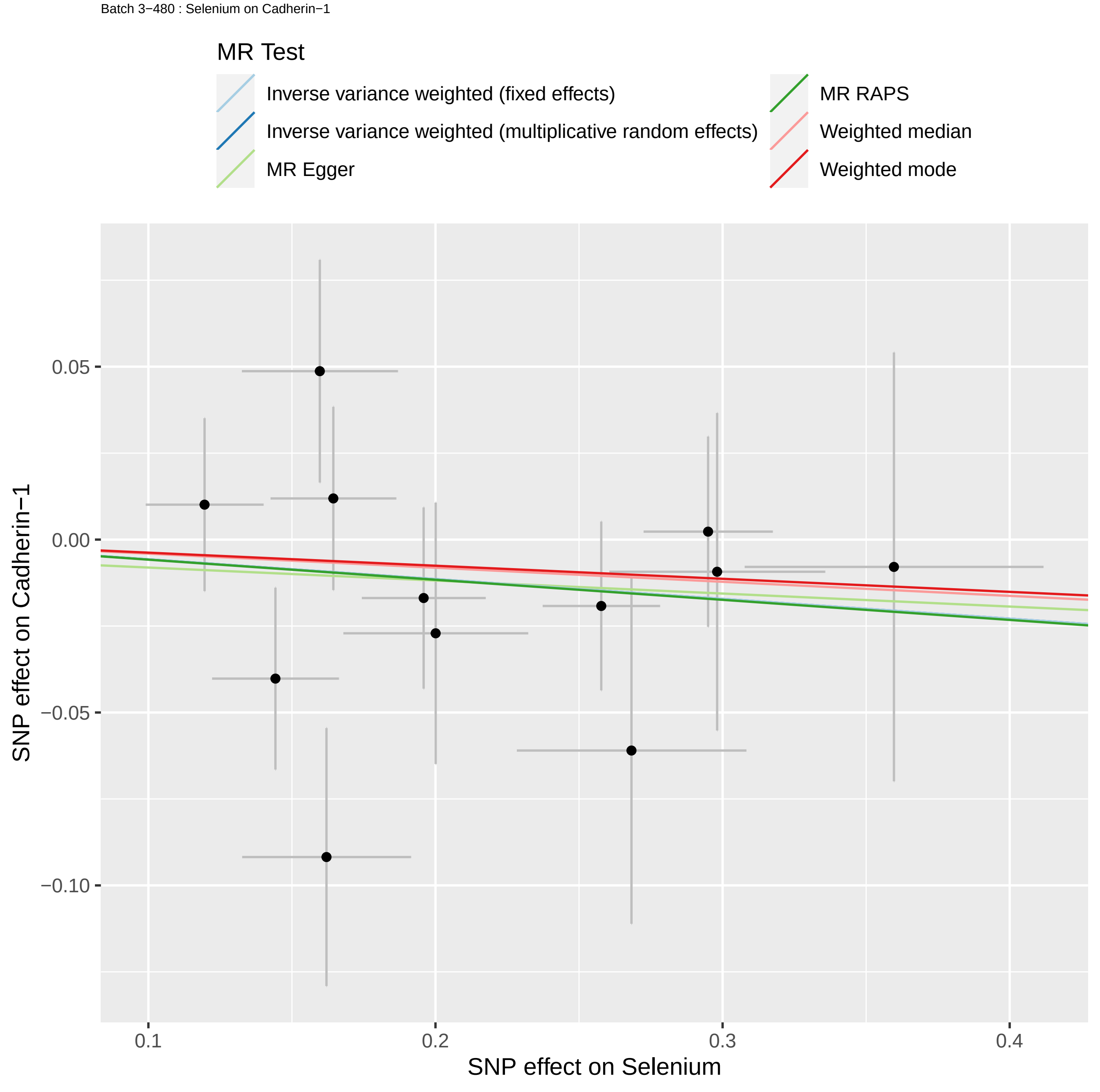
Batch 3−480 : Selenium on Cadherin−1
MR Test
Inverse variance weighted (fixed effects)
Inverse variance weighted (multiplicative random effects)
MR Egger
MR RAPS
Weighted median
Weighted mode
0.05
0.00
−0.05
−0.10
0.1
0.2
0.3
0.4
SNP effect on Cadherin−1
SNP effect on Selenium

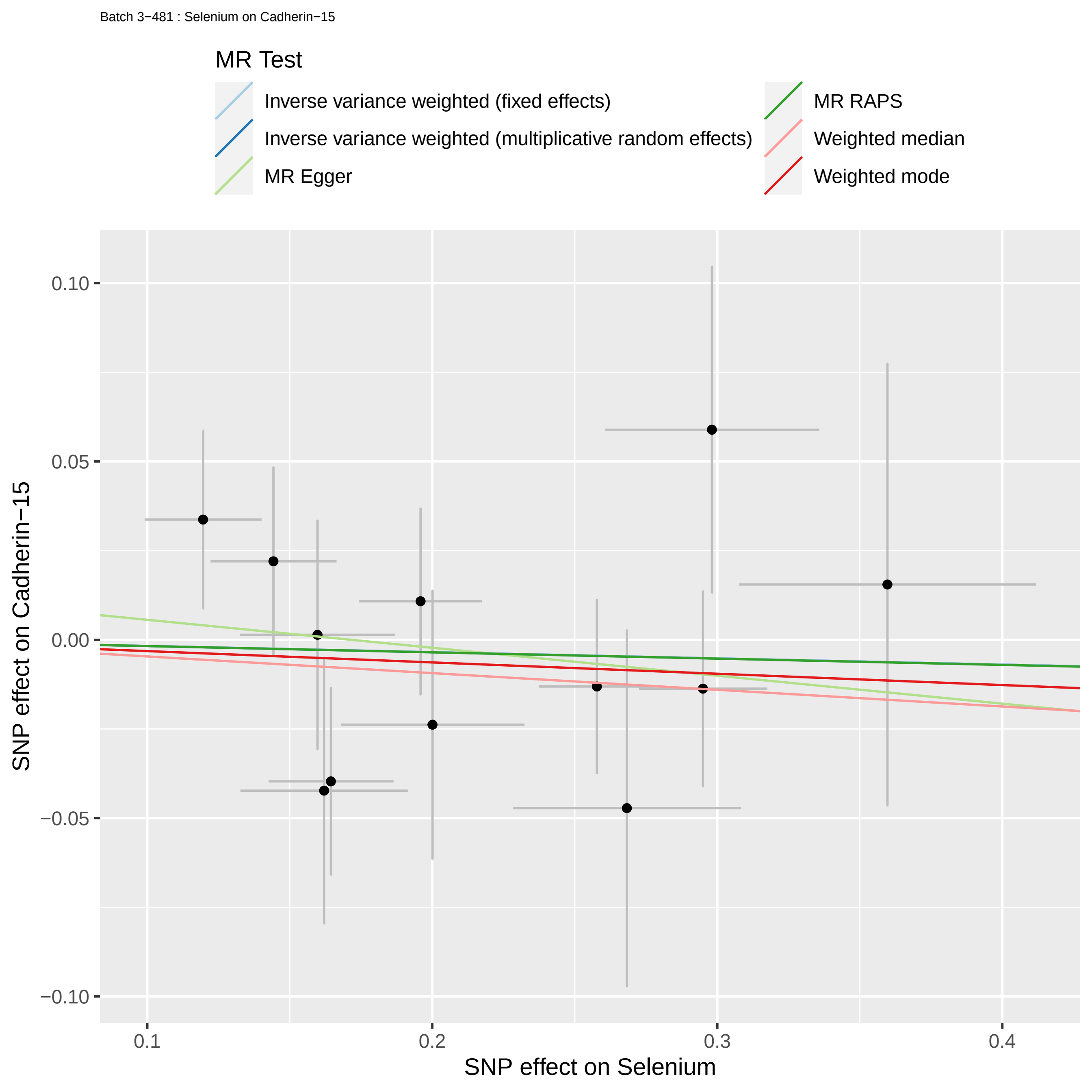
Batch 3−481 : Selenium on Cadherin−15
MR Test
Inverse variance weighted (fixed effects)
Inverse variance weighted (multiplicative random effects)
MR Egger
MR RAPS
Weighted median
Weighted mode
0.10
0.05
0.00
−0.05
−0.10
0.1
0.2
0.3
0.4
SNP effect on Cadherin−15
SNP effect on Selenium

Batch 3−482 : Selenium on Cadherin−15

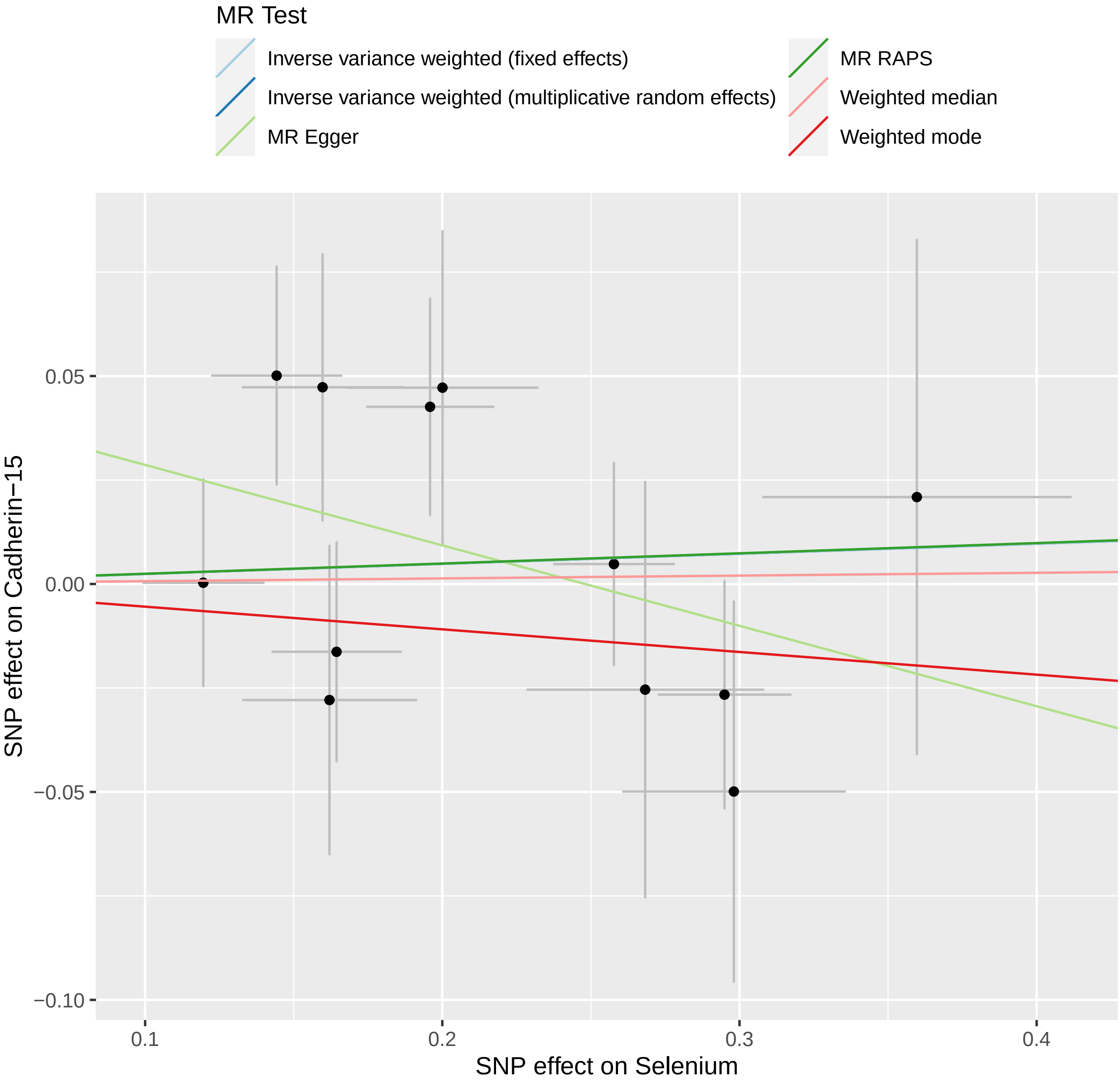

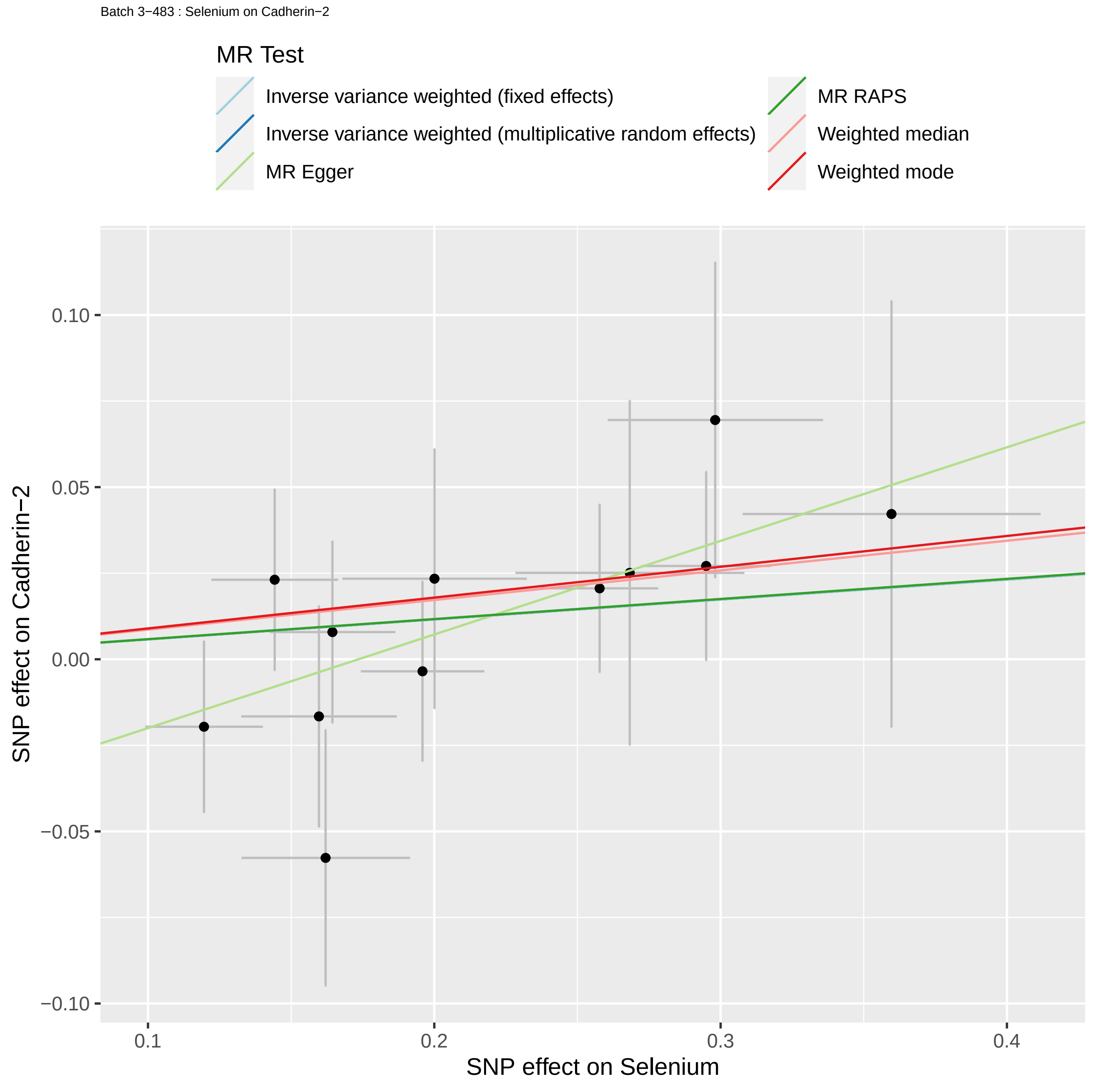

Batch 3−483 : Selenium on Cadherin−2
MR Test
Inverse variance weighted (fixed effects)
Inverse variance weighted (multiplicative random effects)
MR Egger
MR RAPS
Weighted median
Weighted mode
SNP effect on Cadherin−2
0.10
0.05
0.00
−0.05
−0.10
0.1
0.2
0.3
0.4
SNP effect on Selenium

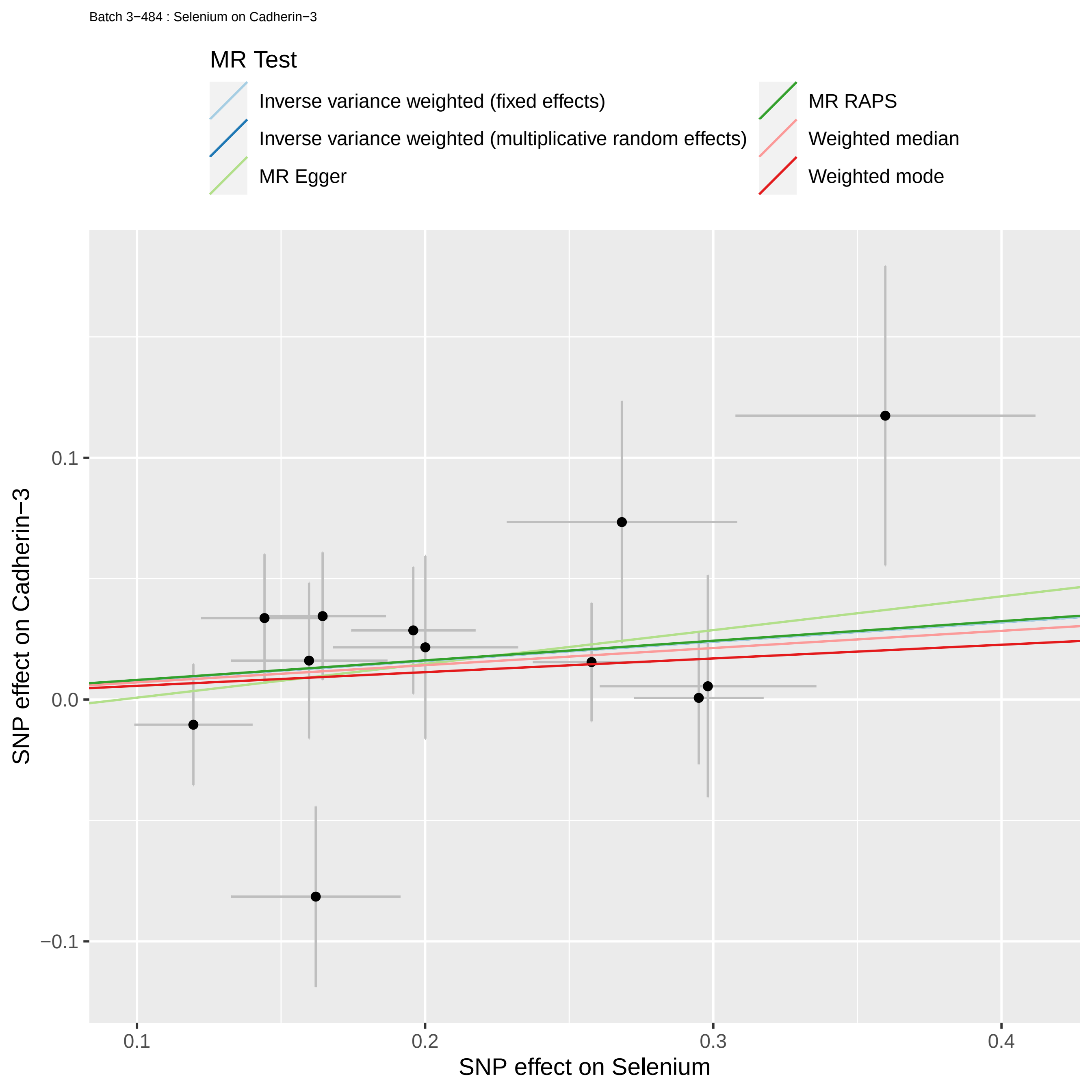
Batch 3−484 : Selenium on Cadherin−3
MR Test
Inverse variance weighted (fixed effects)
Inverse variance weighted (multiplicative random effects)
MR Egger
MR RAPS
Weighted median
Weighted mode
0.1
0.0
−0.1
0.1
0.2
0.3
0.4
SNP effect on Cadherin−3
SNP effect on Selenium

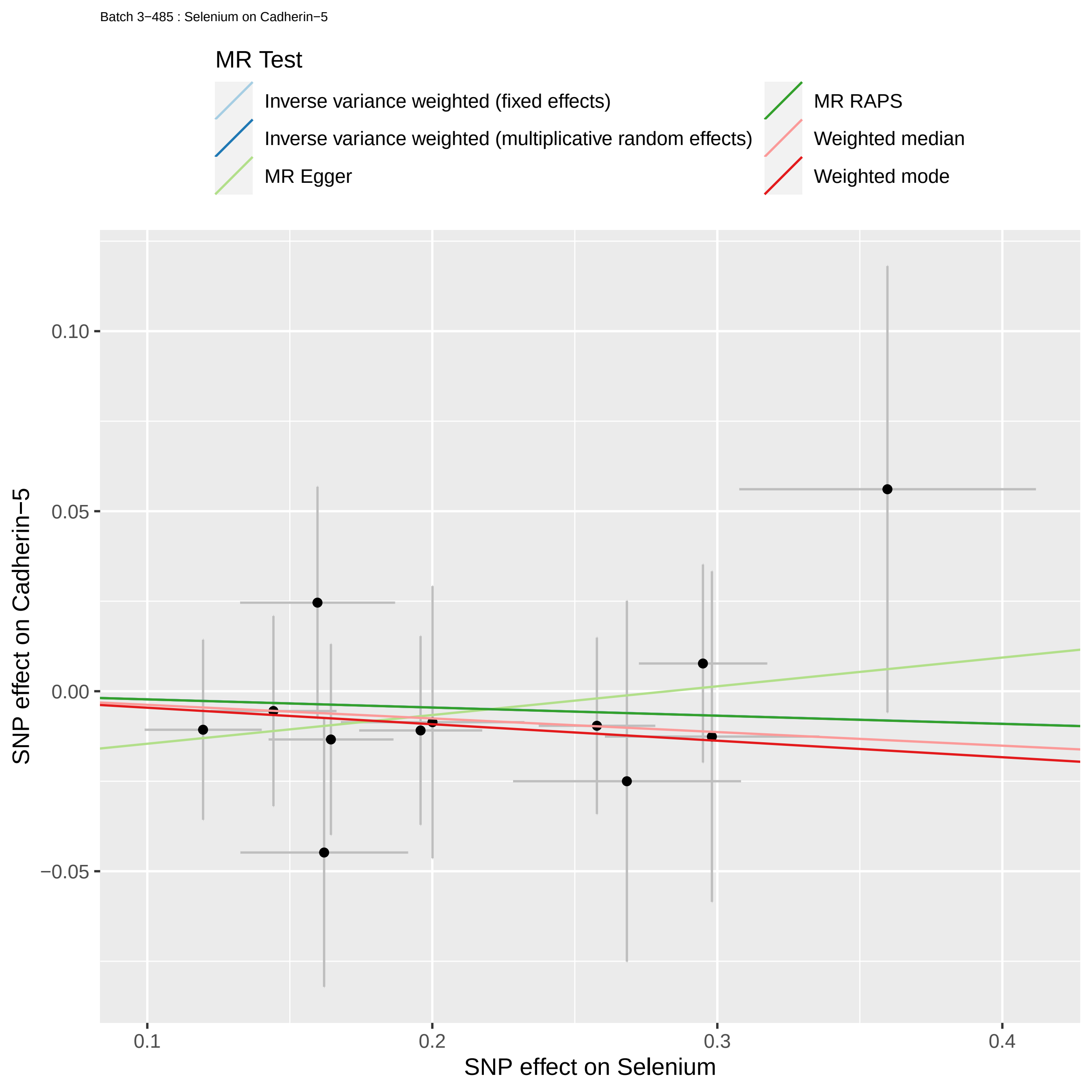
Batch 3−485 : Selenium on Cadherin−5
MR Test
Inverse variance weighted (fixed effects)
Inverse variance weighted (multiplicative random effects)
MR Egger
MR RAPS
Weighted median
Weighted mode
SNP effect on Cadherin−5
0.10
0.05
0.00
−0.05
0.1
0.2
0.3
0.4
SNP effect on Selenium

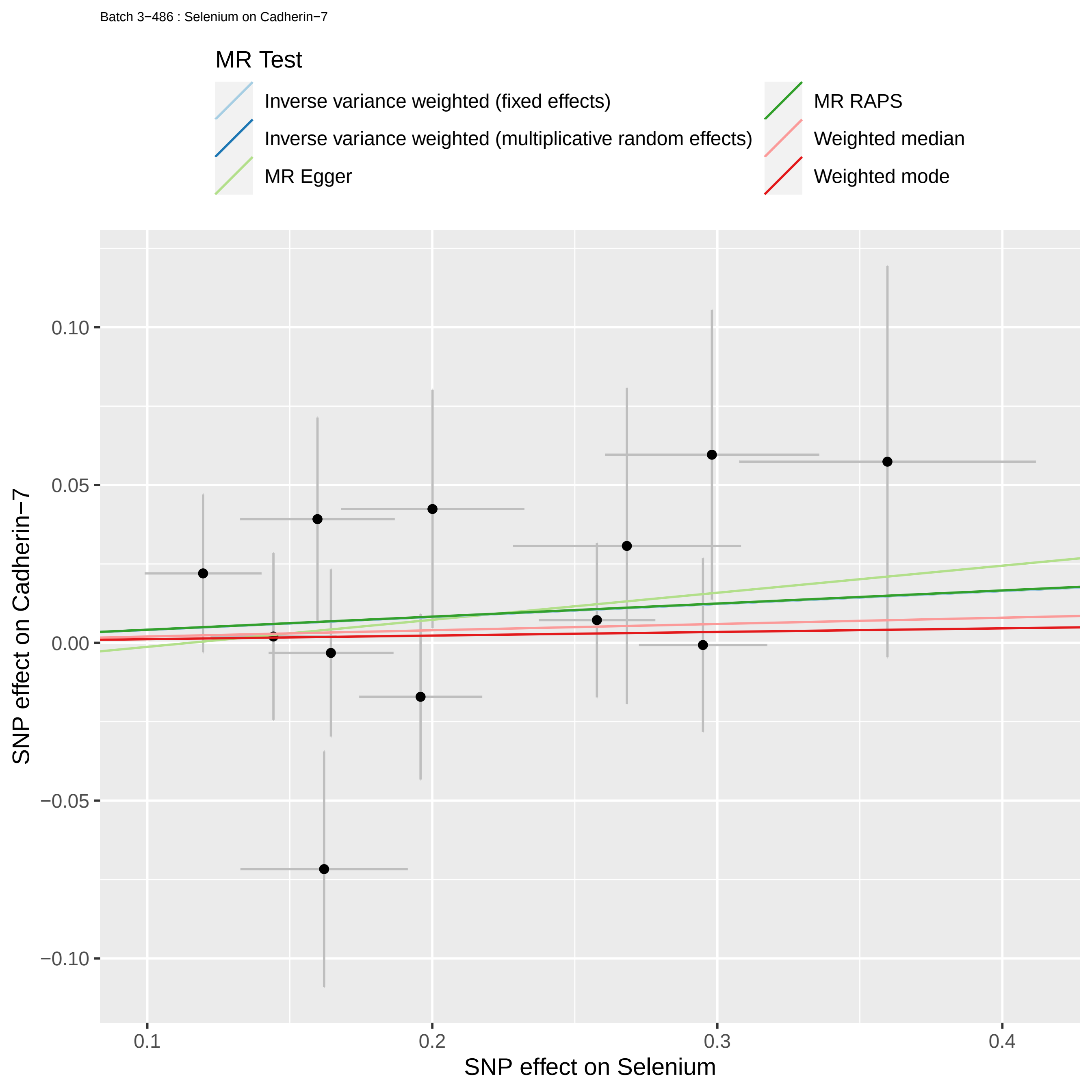

Batch 3−486 : Selenium on Cadherin−7
MR Test
Inverse variance weighted (fixed effects)
Inverse variance weighted (multiplicative random effects)
MR Egger
MR RAPS
Weighted median
Weighted mode
SNP effect on Cadherin−7
0.10
0.05
0.00
−0.05
−0.10
0.1
0.2
0.3
0.4
SNP effect on Selenium

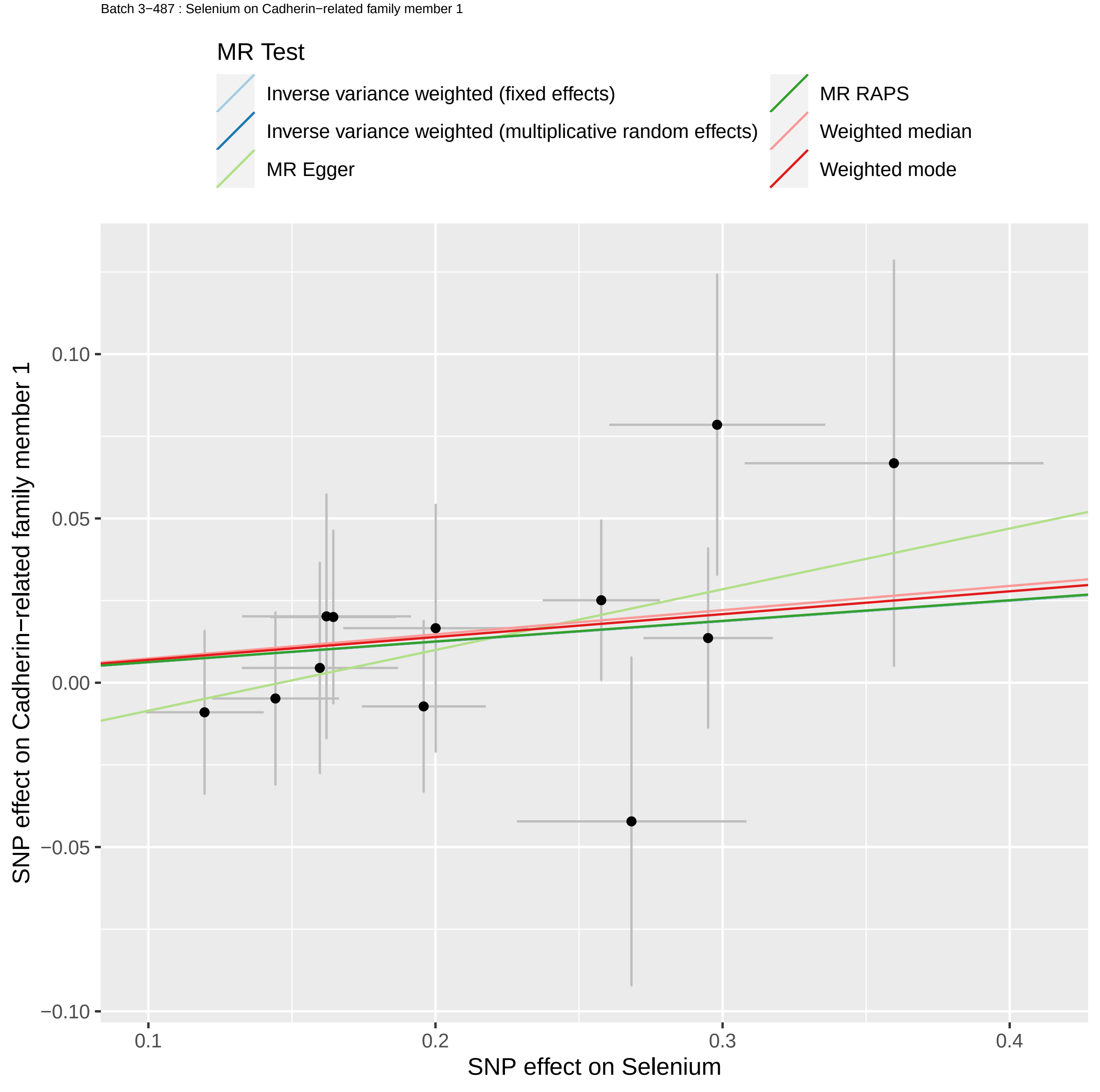
Batch 3−487 : Selenium on Cadherin−related family member 1
MR Test
Inverse variance weighted (fixed effects)
Inverse variance weighted (multiplicative random effects)
MR Egger
MR RAPS
Weighted median
Weighted mode
0.10
0.05
0.00
−0.05
−0.10
0.1
0.2
0.3
0.4
SNP effect on Cadherin−related family member 1
SNP effect on Selenium

Batch 3−488 : Selenium on Cadherin−related family member 3

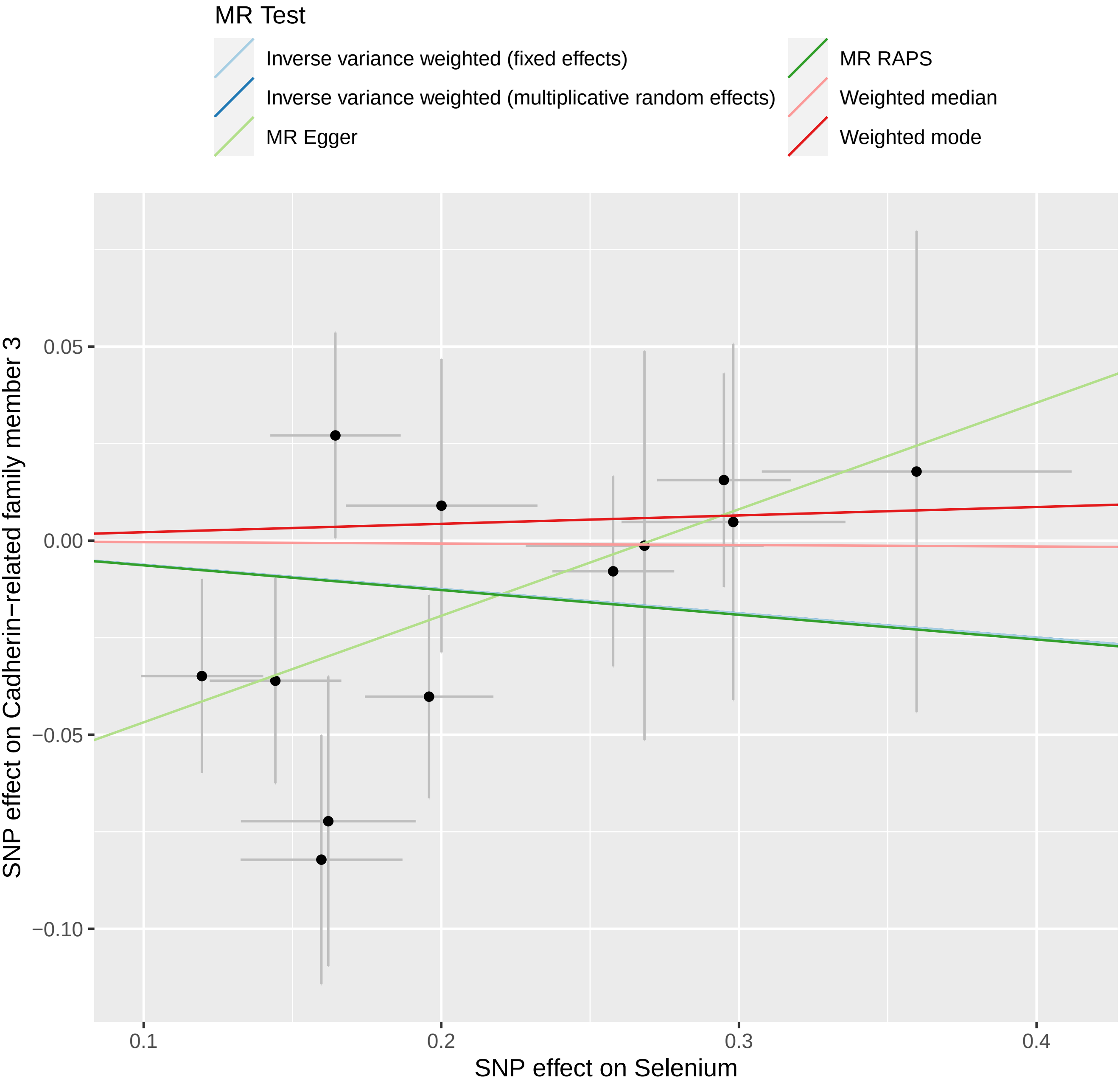

Batch 3−489 : Selenium on Cadherin−related family member 3

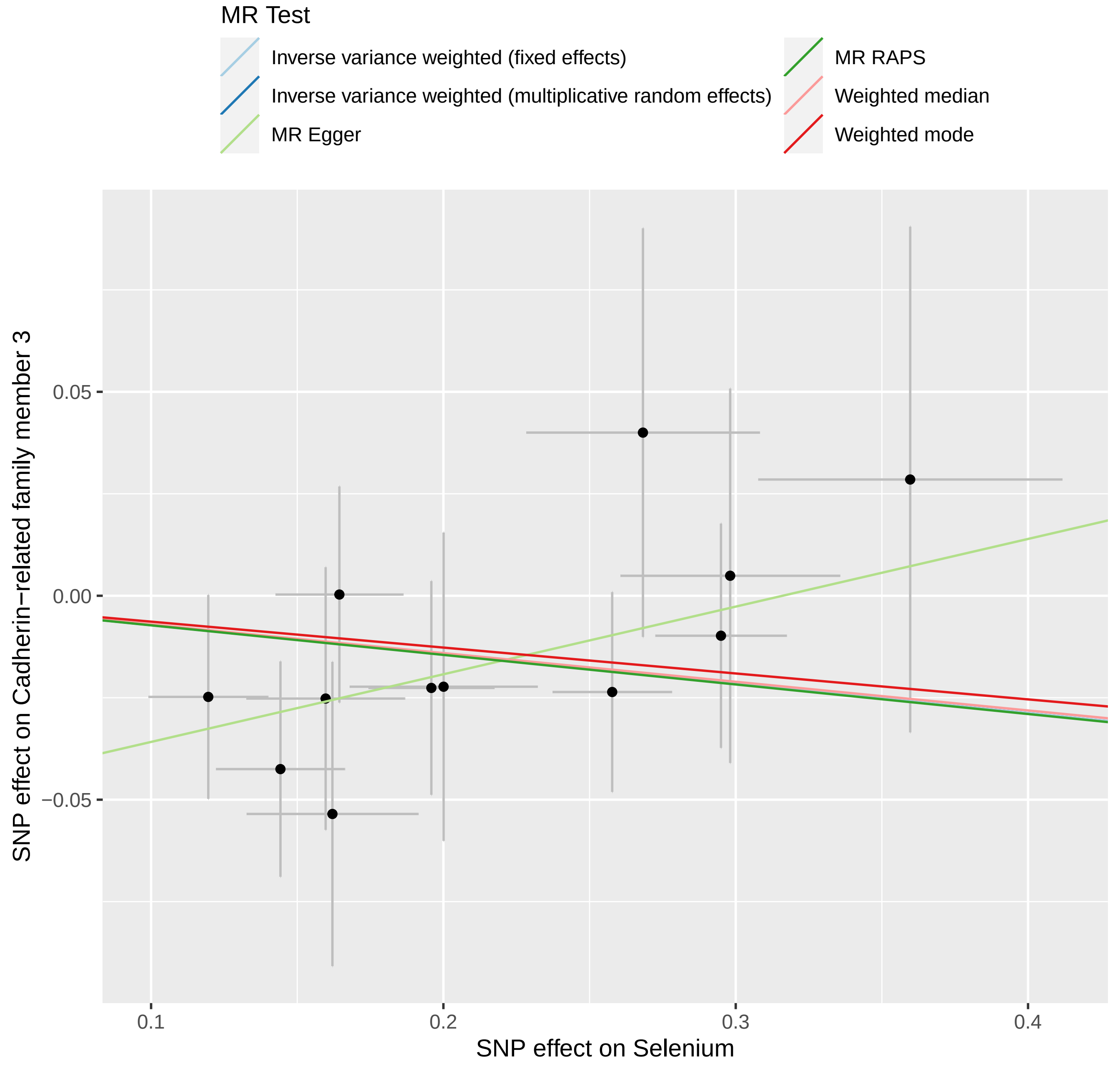

Batch 3−490 : Selenium on Cadherin−related family member 5

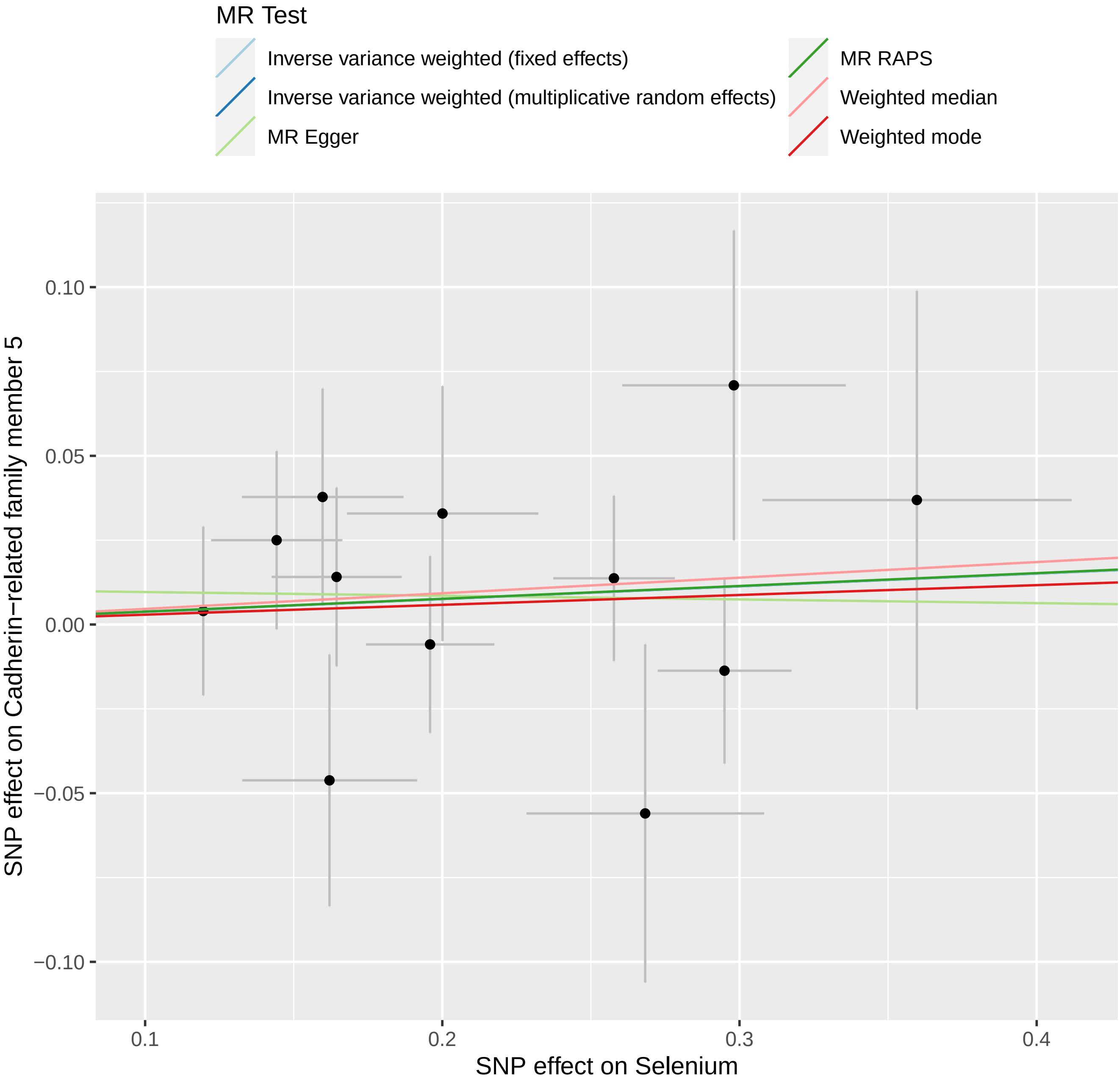

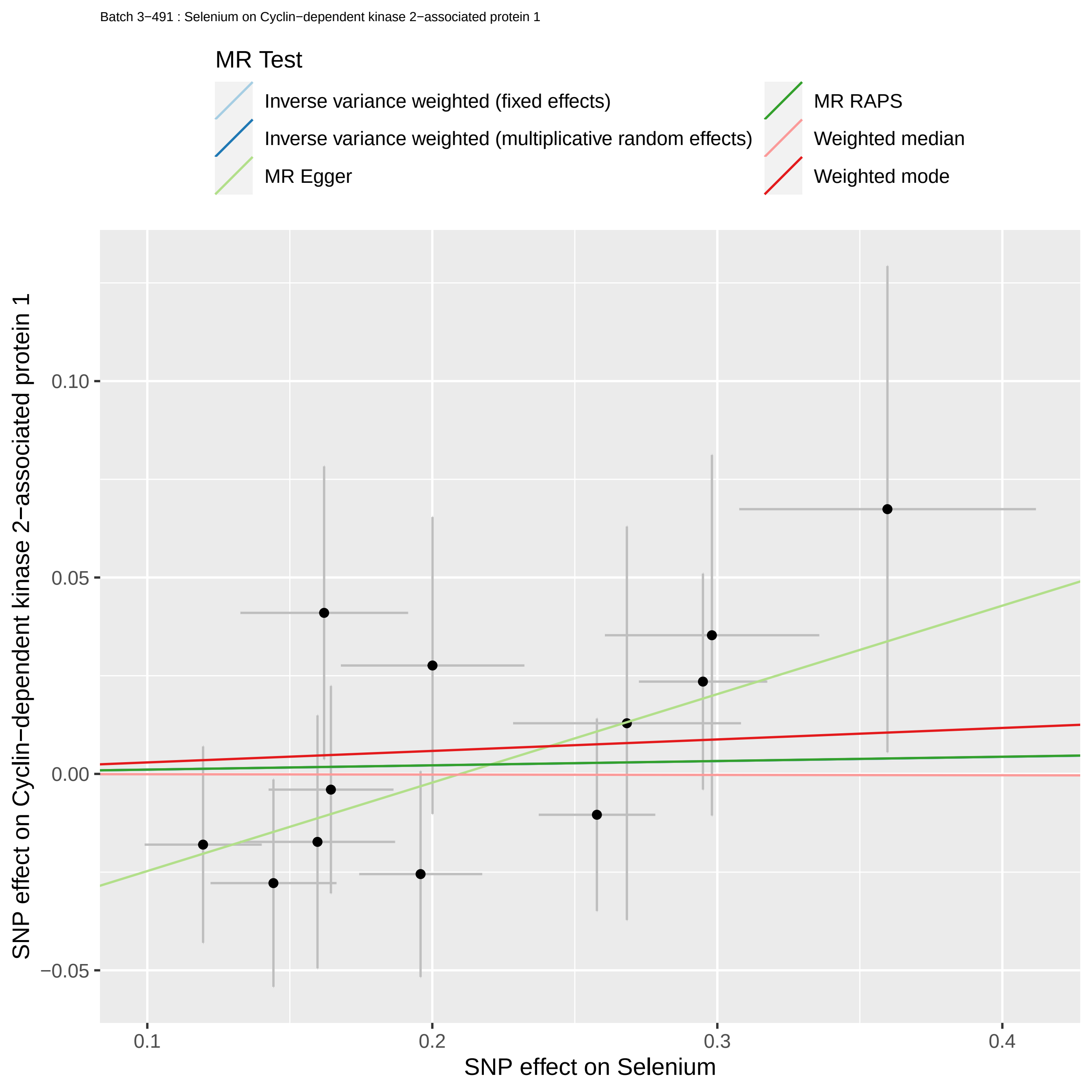
Batch 3−491 : Selenium on Cyclin−dependent kinase 2−associated protein 1
MR Test
Inverse variance weighted (fixed effects)
Inverse variance weighted (multiplicative random effects)
MR Egger
MR RAPS
Weighted median
Weighted mode
0.10
0.05
0.00
−0.05
0.1
0.2
0.3
0.4
SNP effect on Cyclin−dependent kinase 2−associated protein 1
SNP effect on Selenium

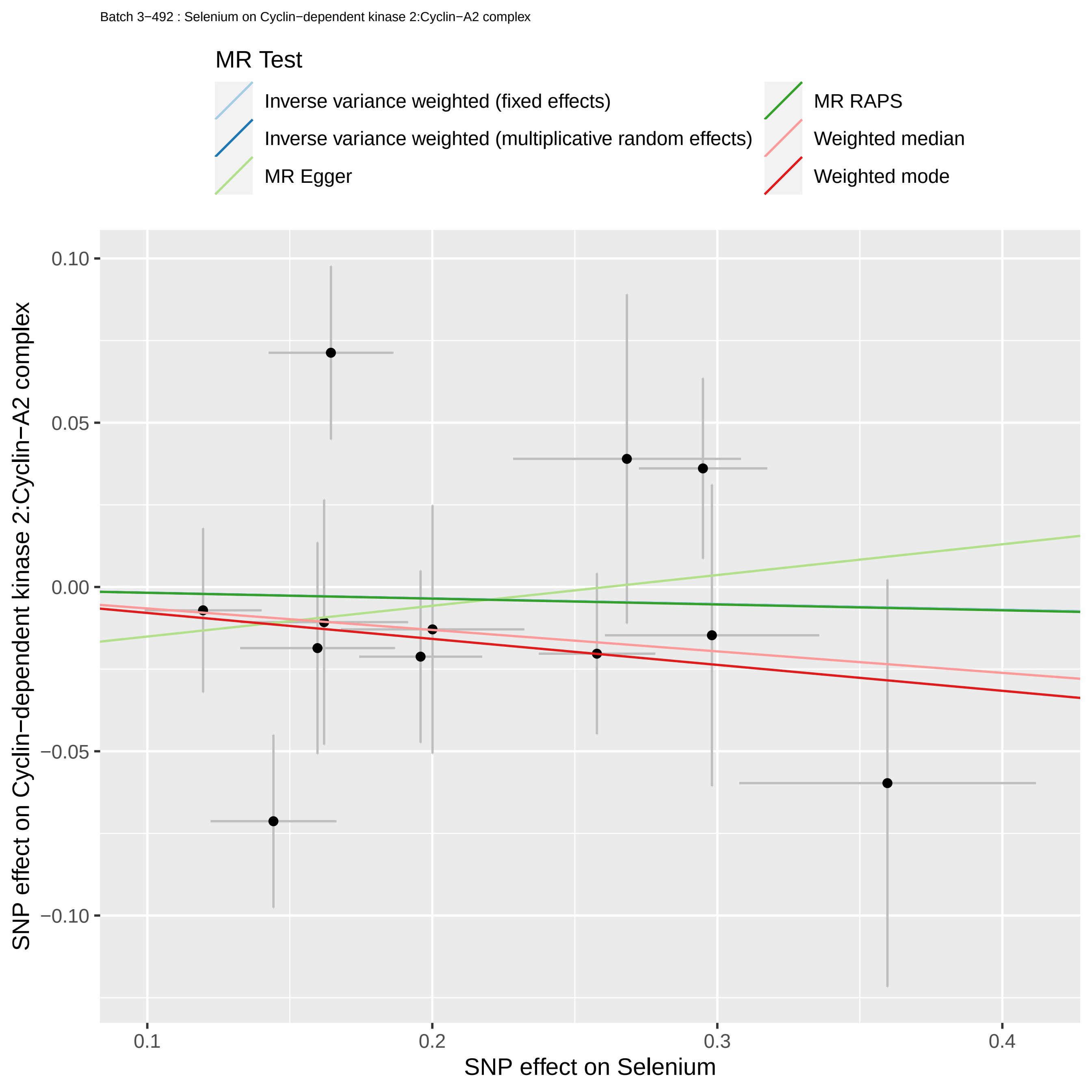
Batch 3−492 : Selenium on Cyclin−dependent kinase 2:Cyclin−A2 complex
MR Test
Inverse variance weighted (fixed effects)
Inverse variance weighted (multiplicative random effects)
MR Egger
MR RAPS
Weighted median
Weighted mode
SNP effect on Cyclin−dependent kinase 2:Cyclin−A2 complex
0.10
0.05
0.00
−0.05
−0.10
0.1
0.2
0.3
0.4
SNP effect on Selenium

Batch 3−493 : Selenium on Cyclin−dependent kinase 5:Cyclin−dependent kinase 5 activator 1 complex

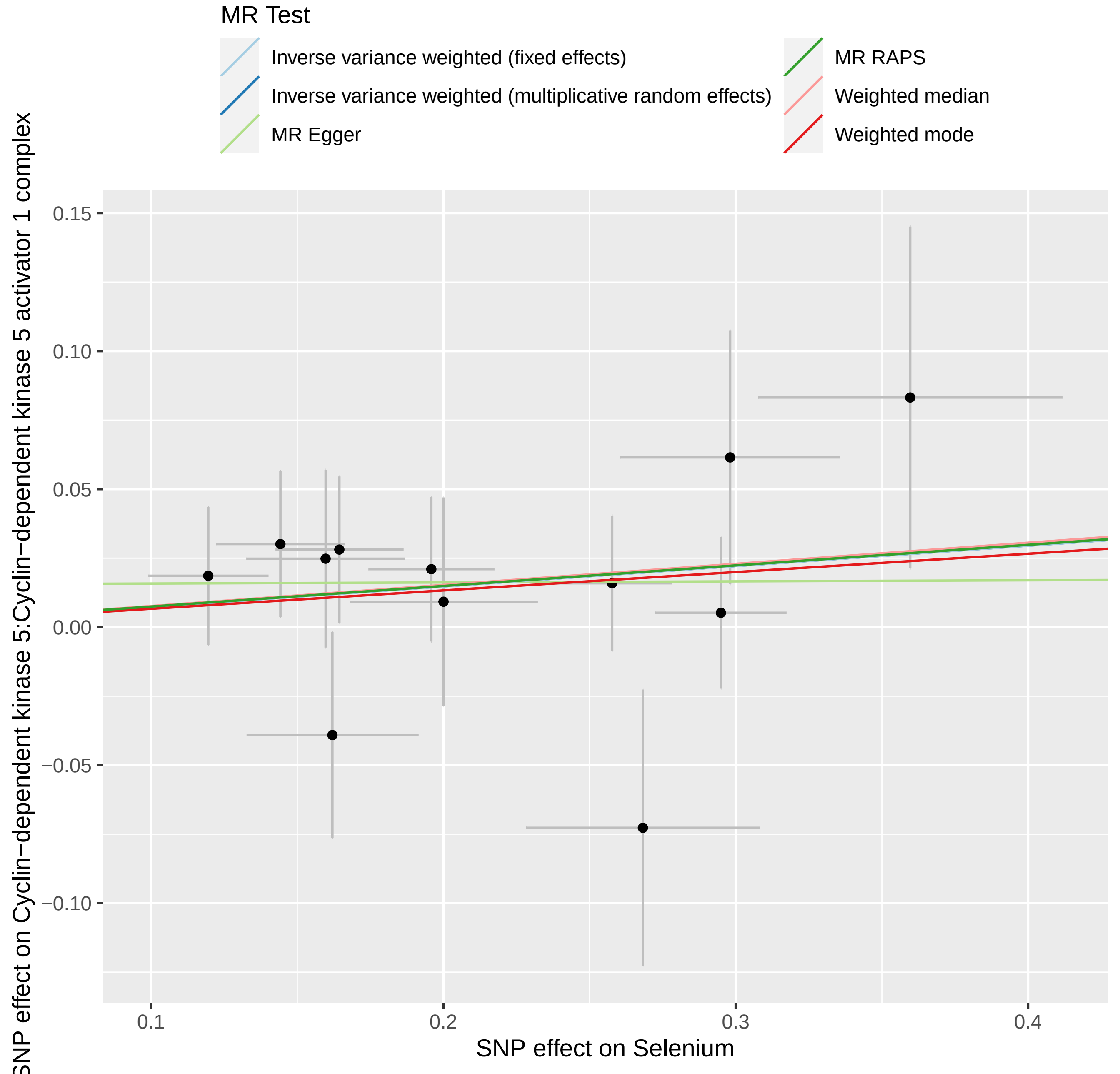

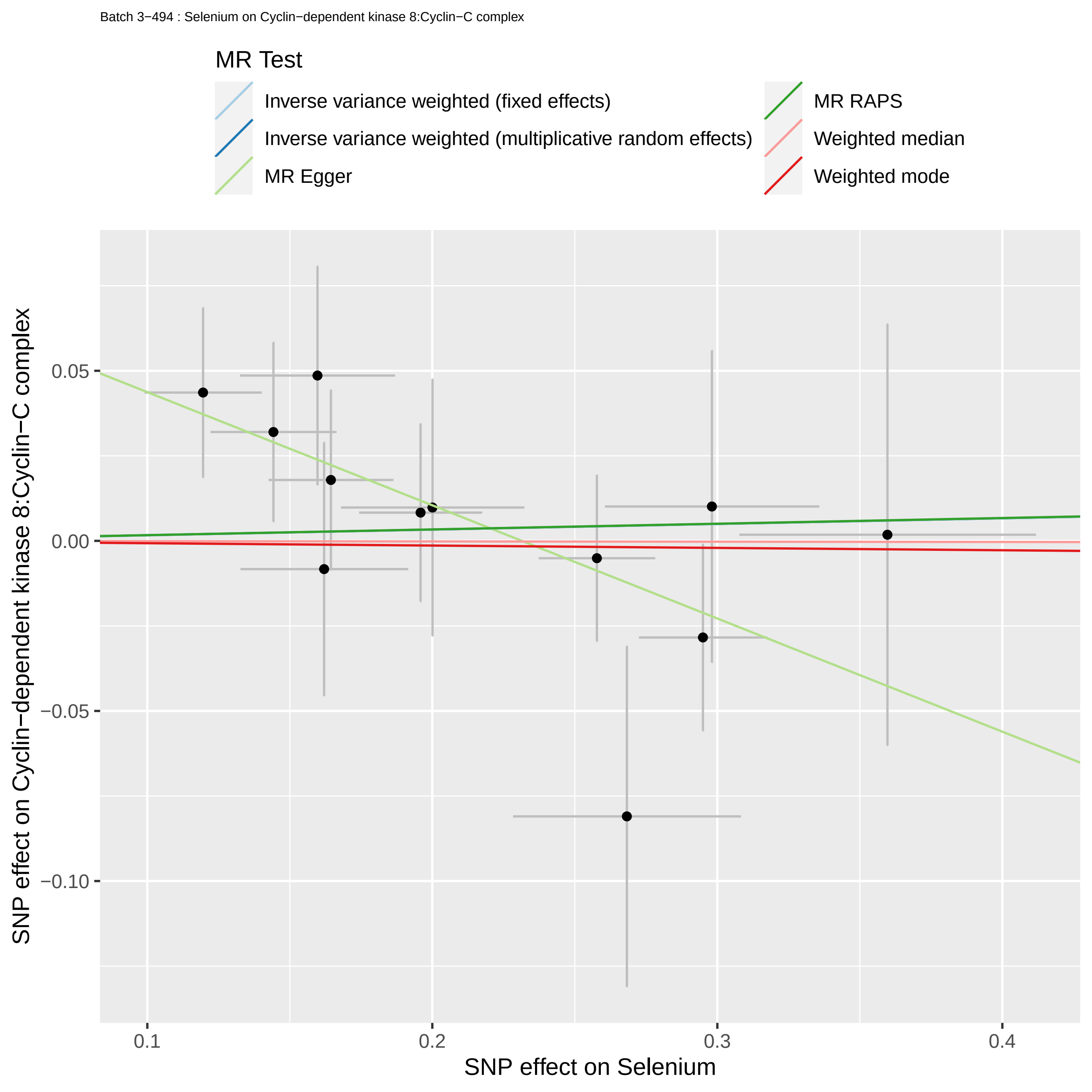

Batch 3−494 : Selenium on Cyclin−dependent kinase 8:Cyclin−C complex
MR Test
Inverse variance weighted (fixed effects)
Inverse variance weighted (multiplicative random effects)
MR Egger
MR RAPS
Weighted median
Weighted mode
SNP effect on Cyclin−dependent kinase 8:Cyclin−C complex
0.05
0.00
−0.05
−0.10
0.1
0.2
0.3
0.4
SNP effect on Selenium

Batch 3−495 : Selenium on Cyclin−dependent kinase inhibitor 1B

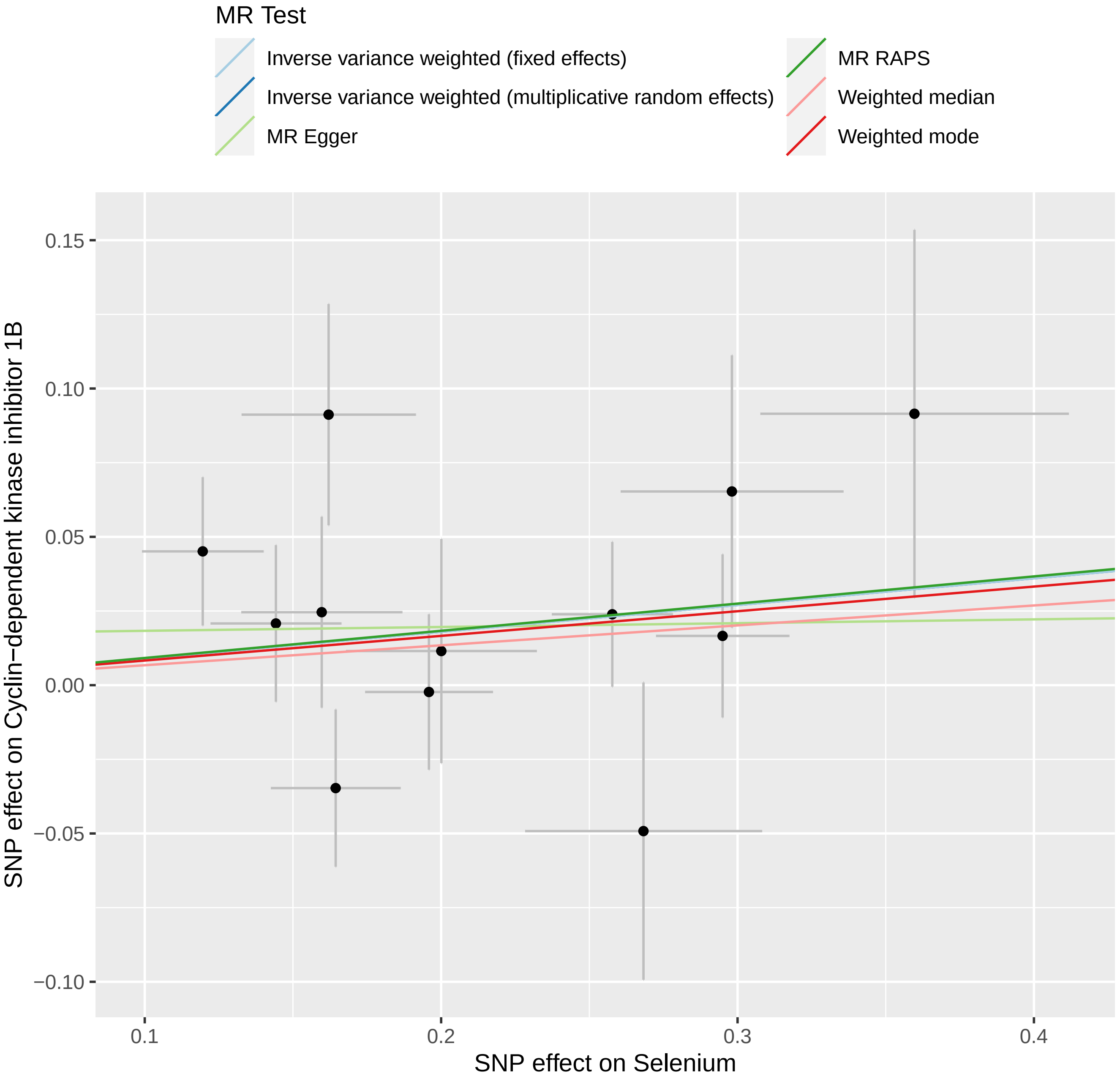

Batch 3−496 : Selenium on Cyclin−dependent kinase 4 inhibitor B

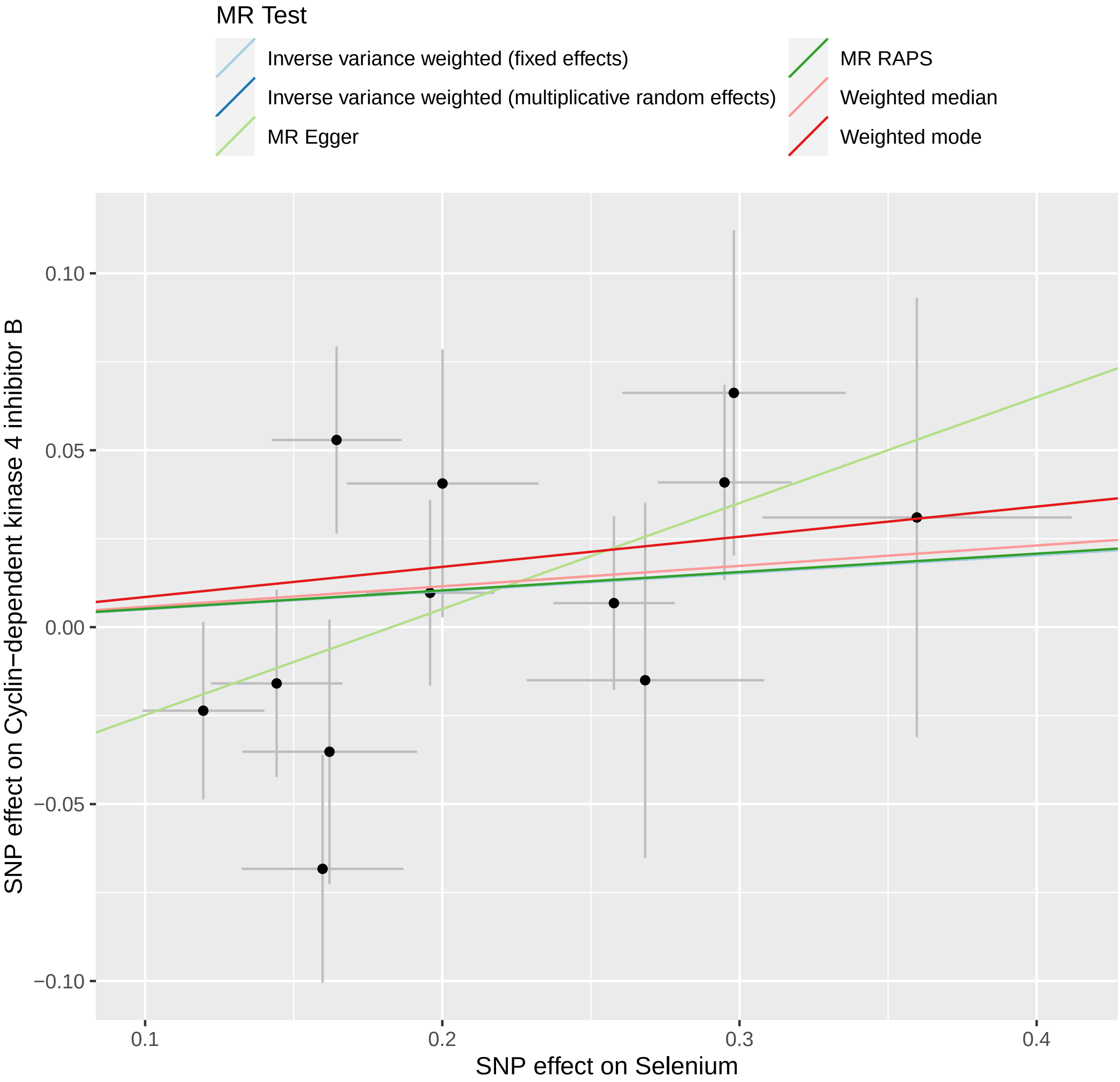

Batch 3−497 : Selenium on Cyclin−dependent kinase inhibitor 3

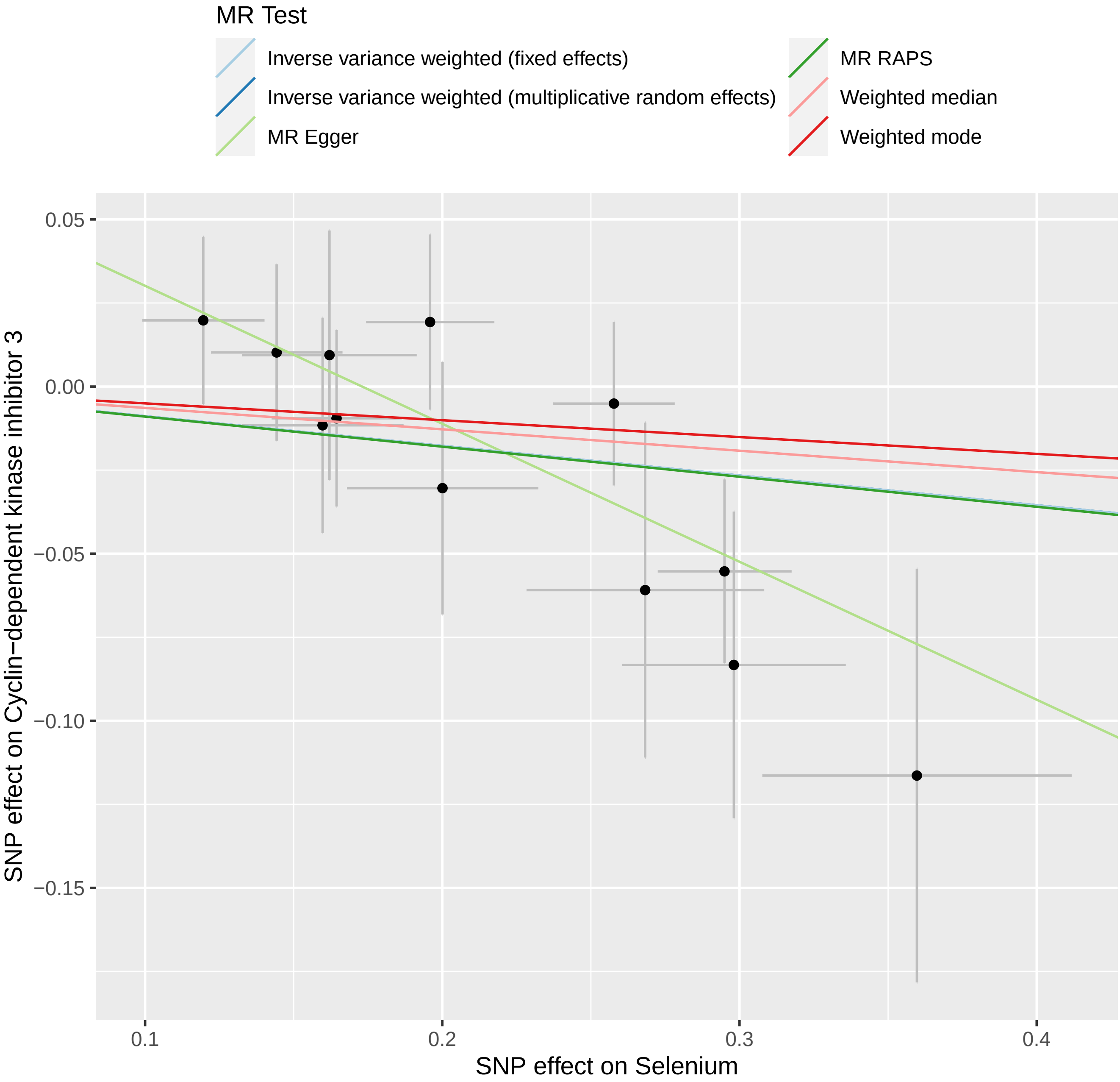

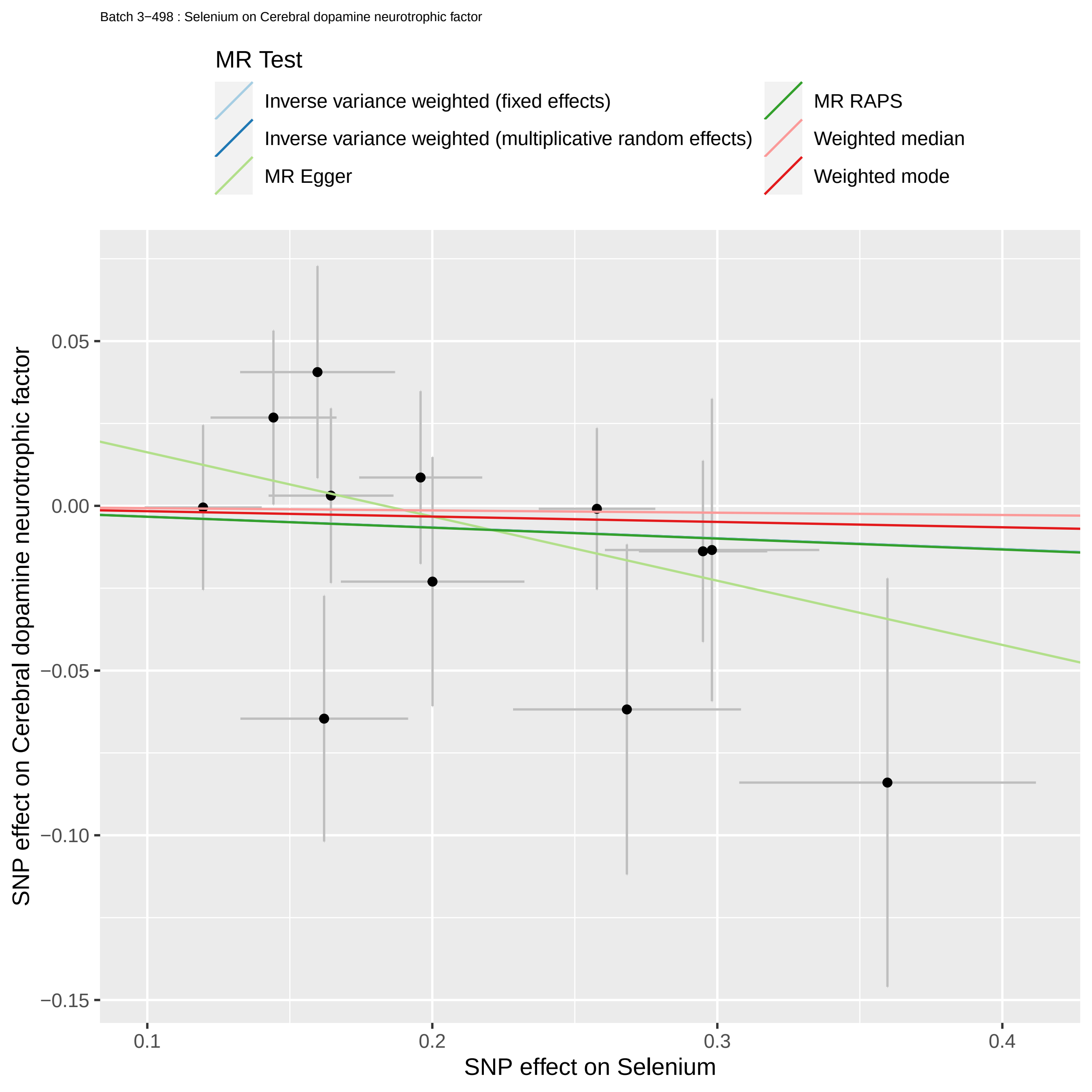
Batch 3−498 : Selenium on Cerebral dopamine neurotrophic factor
MR Test
Inverse variance weighted (fixed effects)
Inverse variance weighted (multiplicative random effects)
MR Egger
MR RAPS
Weighted median
Weighted mode
SNP effect on Cerebral dopamine neurotrophic factor
0.05
0.00
−0.05
−0.10
−0.15
0.1
0.2
0.3
0.4
SNP effect on Selenium

Batch 3−499 : Selenium on Cell adhesion molecule−related/down−regulated by oncogenes

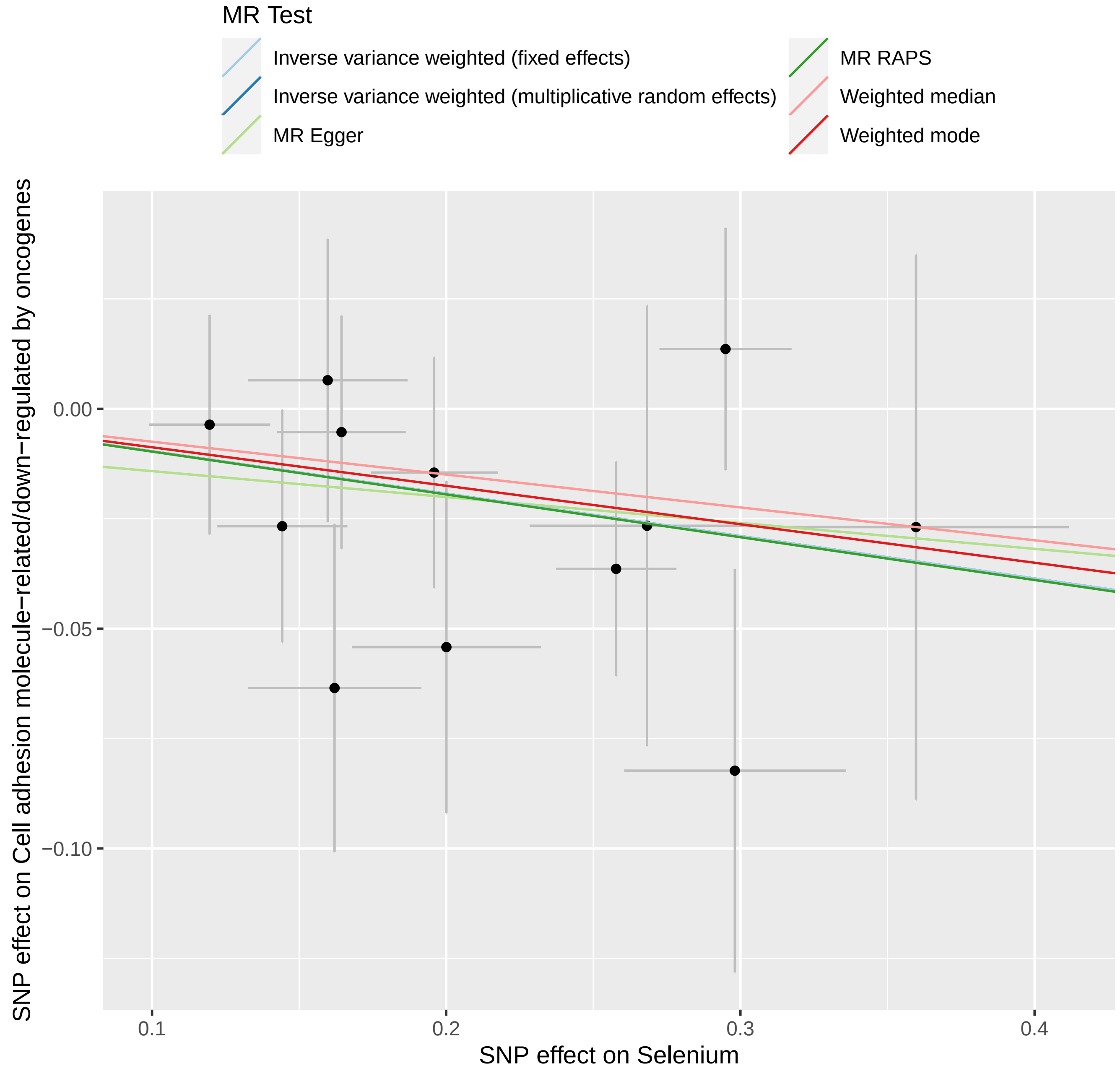

Batch 3−500 : Selenium on Corneodesmosin

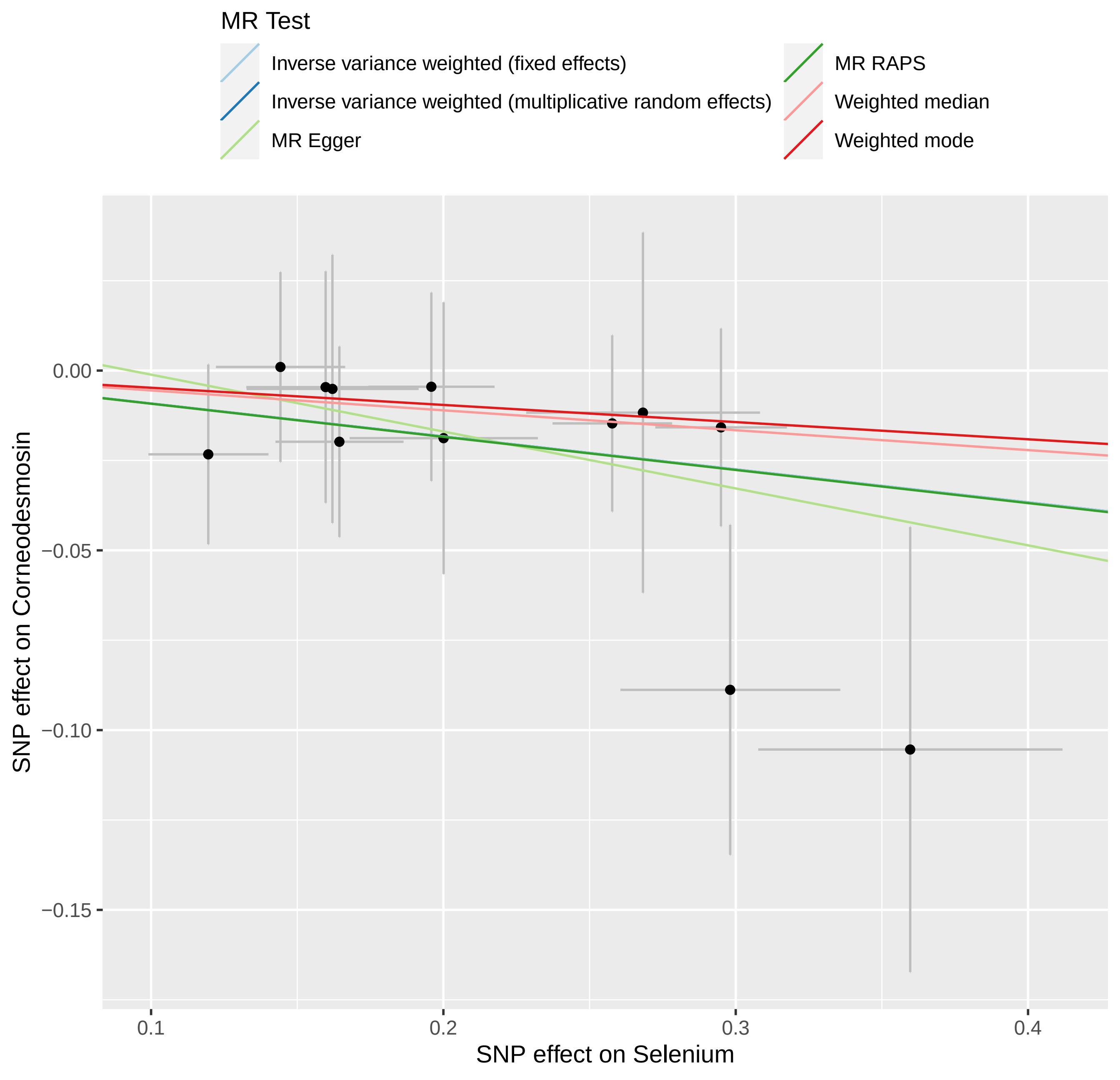

Batch 3−501 : Selenium on Testis−specific chromodomain protein Y 1

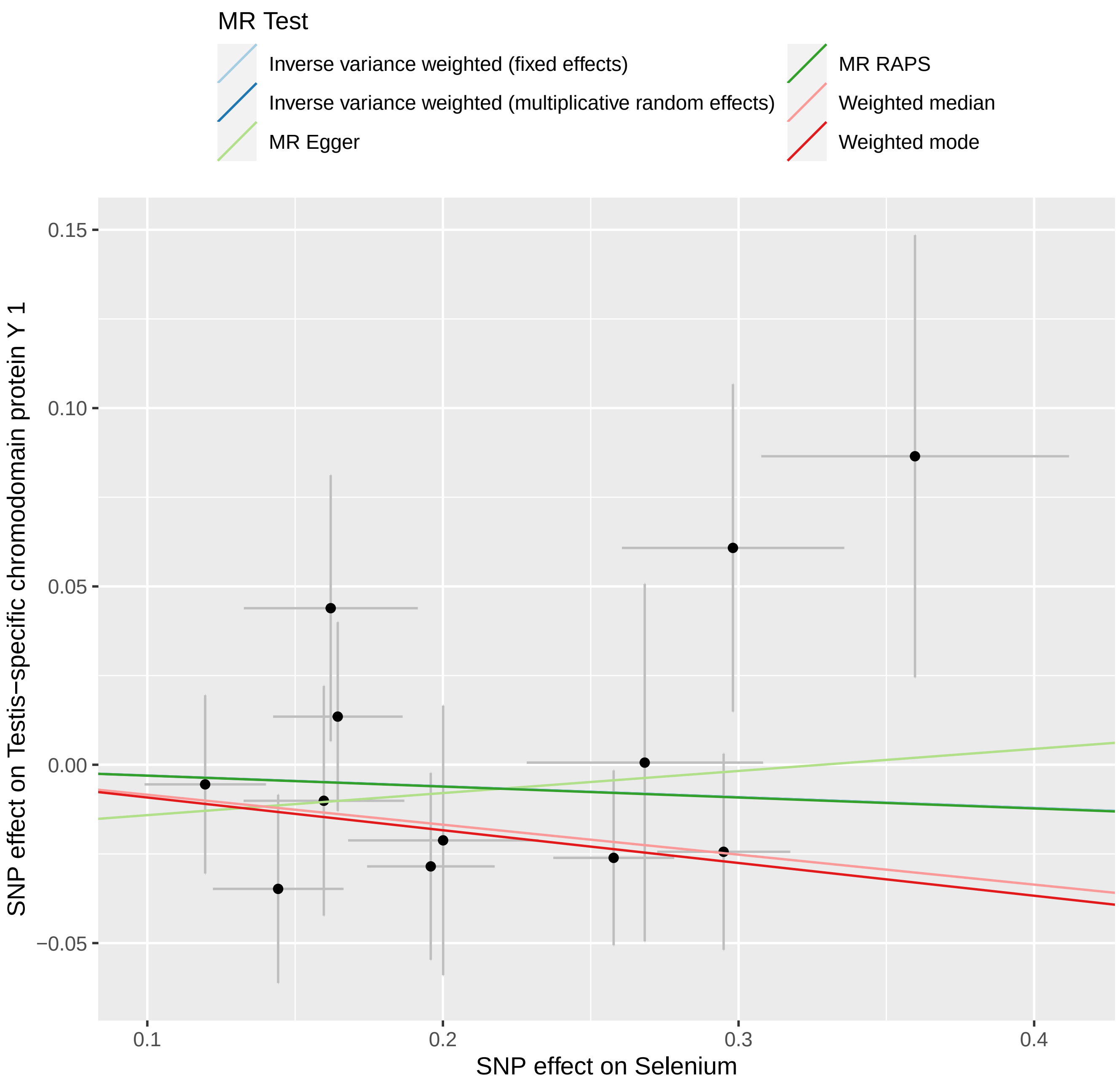

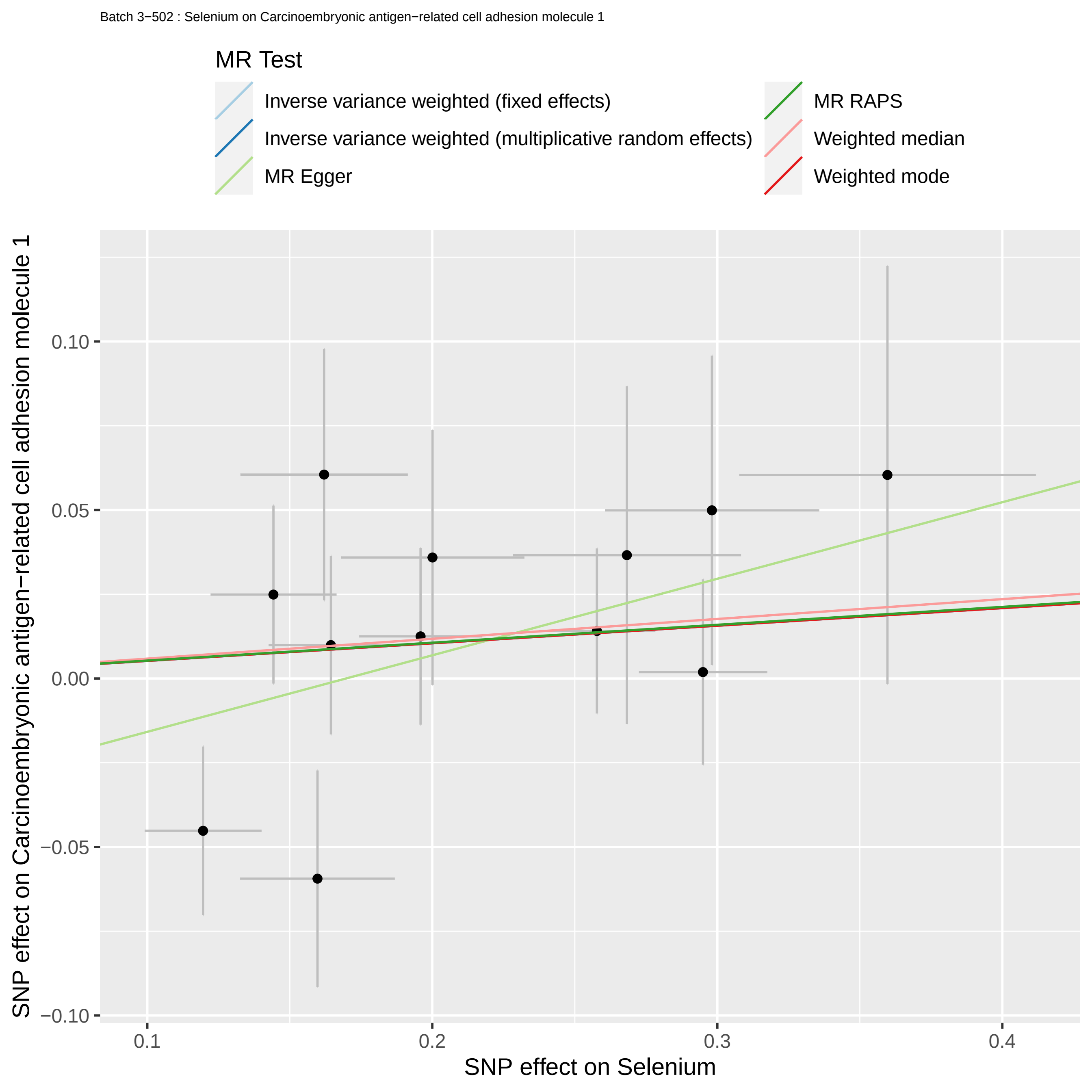

Batch 3−502 : Selenium on Carcinoembryonic antigen−related cell adhesion molecule 1
MR Test
Inverse variance weighted (fixed effects)
Inverse variance weighted (multiplicative random effects)
MR Egger
MR RAPS
Weighted median
Weighted mode
SNP effect on Carcinoembryonic antigen−related cell adhesion molecule 1
0.10
0.05
0.00
−0.05
−0.10
0.1
0.2
0.3
0.4
SNP effect on Selenium

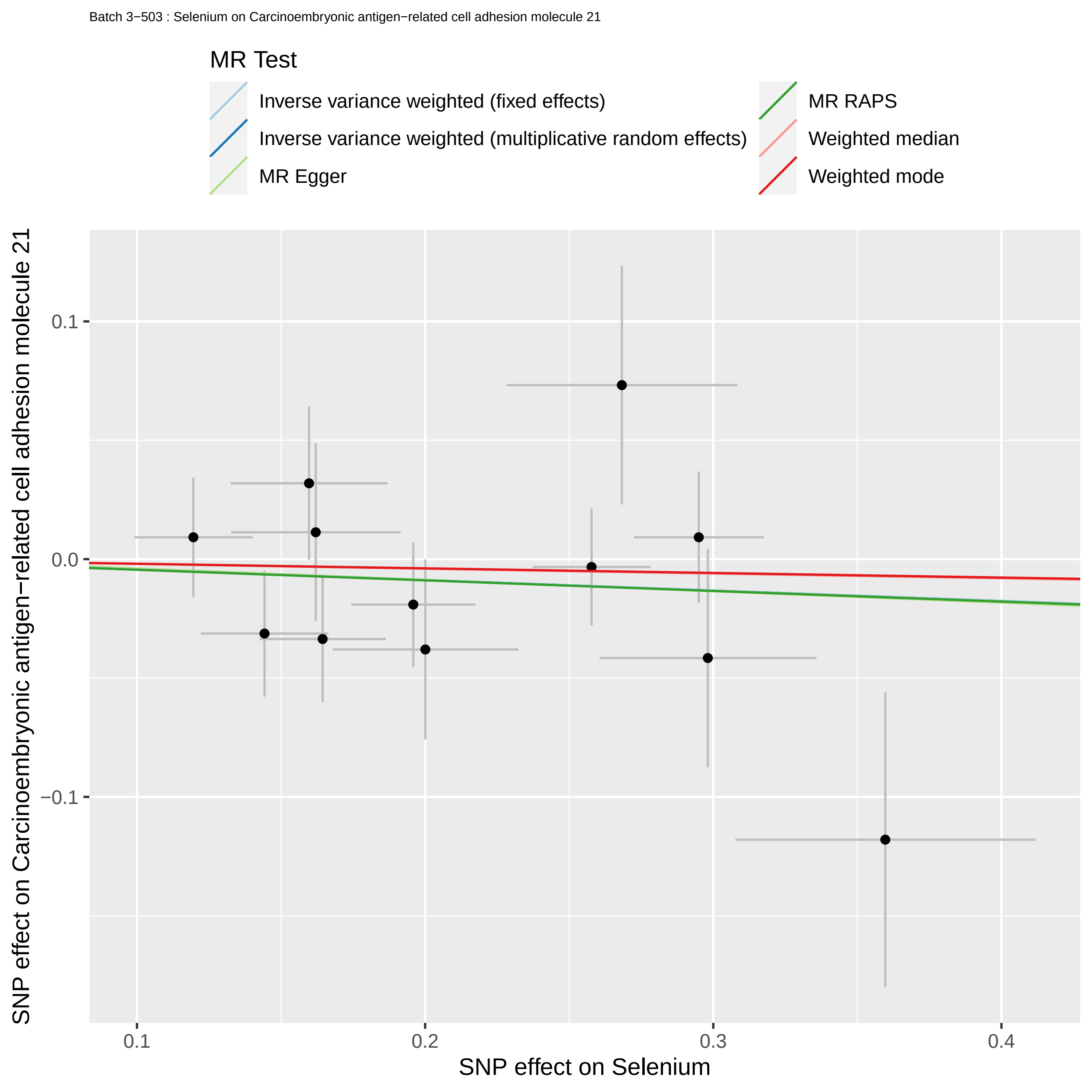

Batch 3−503 : Selenium on Carcinoembryonic antigen−related cell adhesion molecule 21
MR Test
Inverse variance weighted (fixed effects)
Inverse variance weighted (multiplicative random effects)
MR Egger
MR RAPS
Weighted median
Weighted mode
SNP effect on Carcinoembryonic antigen−related cell adhesion molecule 21
0.1
0.0
−0.1
0.1
0.2
0.3
0.4
SNP effect on Selenium

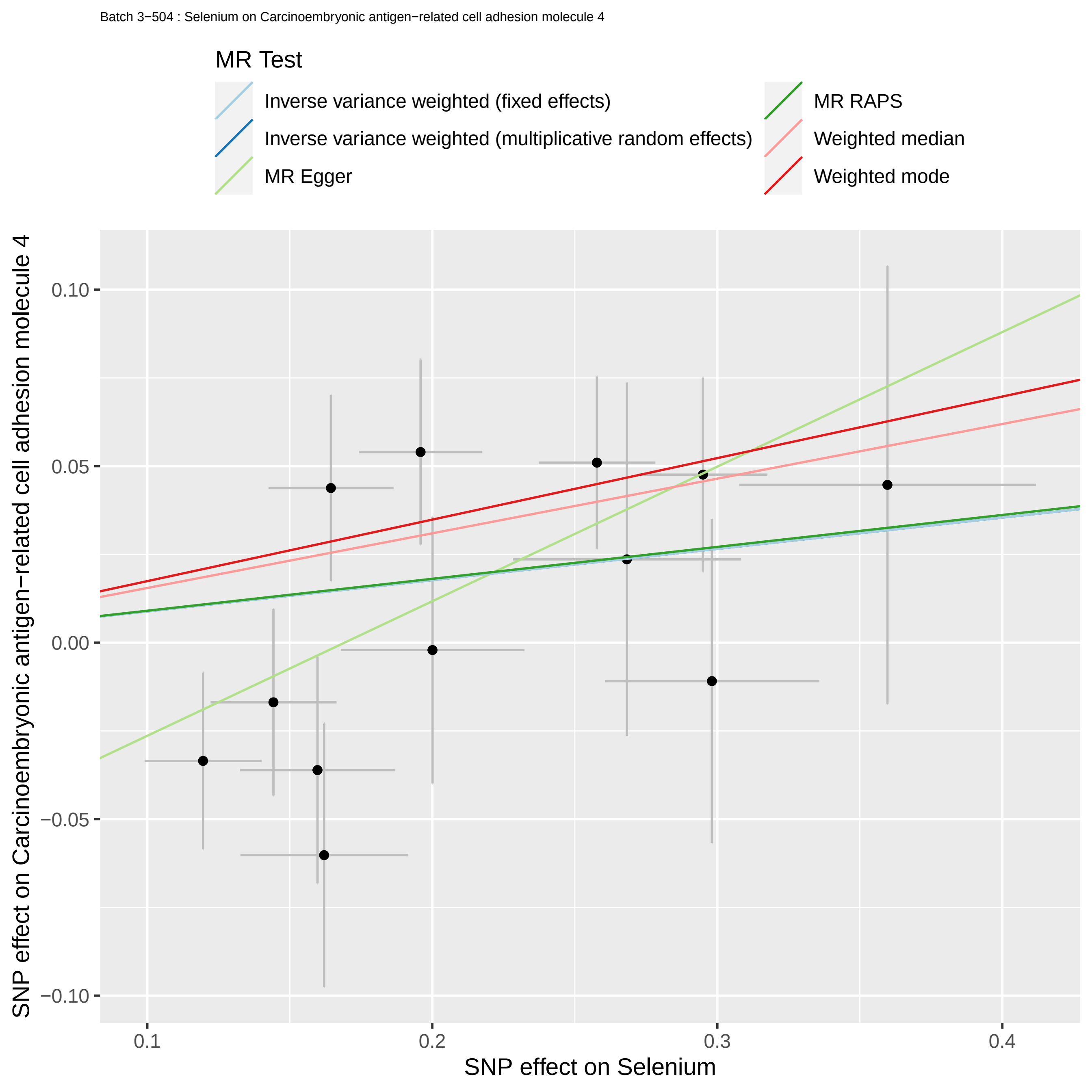

Batch 3−504 : Selenium on Carcinoembryonic antigen−related cell adhesion molecule 4
MR Test
Inverse variance weighted (fixed effects)
Inverse variance weighted (multiplicative random effects)
MR Egger
MR RAPS
Weighted median
Weighted mode
SNP effect on Carcinoembryonic antigen−related cell adhesion molecule 4
0.10
0.05
0.00
−0.05
−0.10
0.1
0.2
0.3
0.4
SNP effect on Selenium

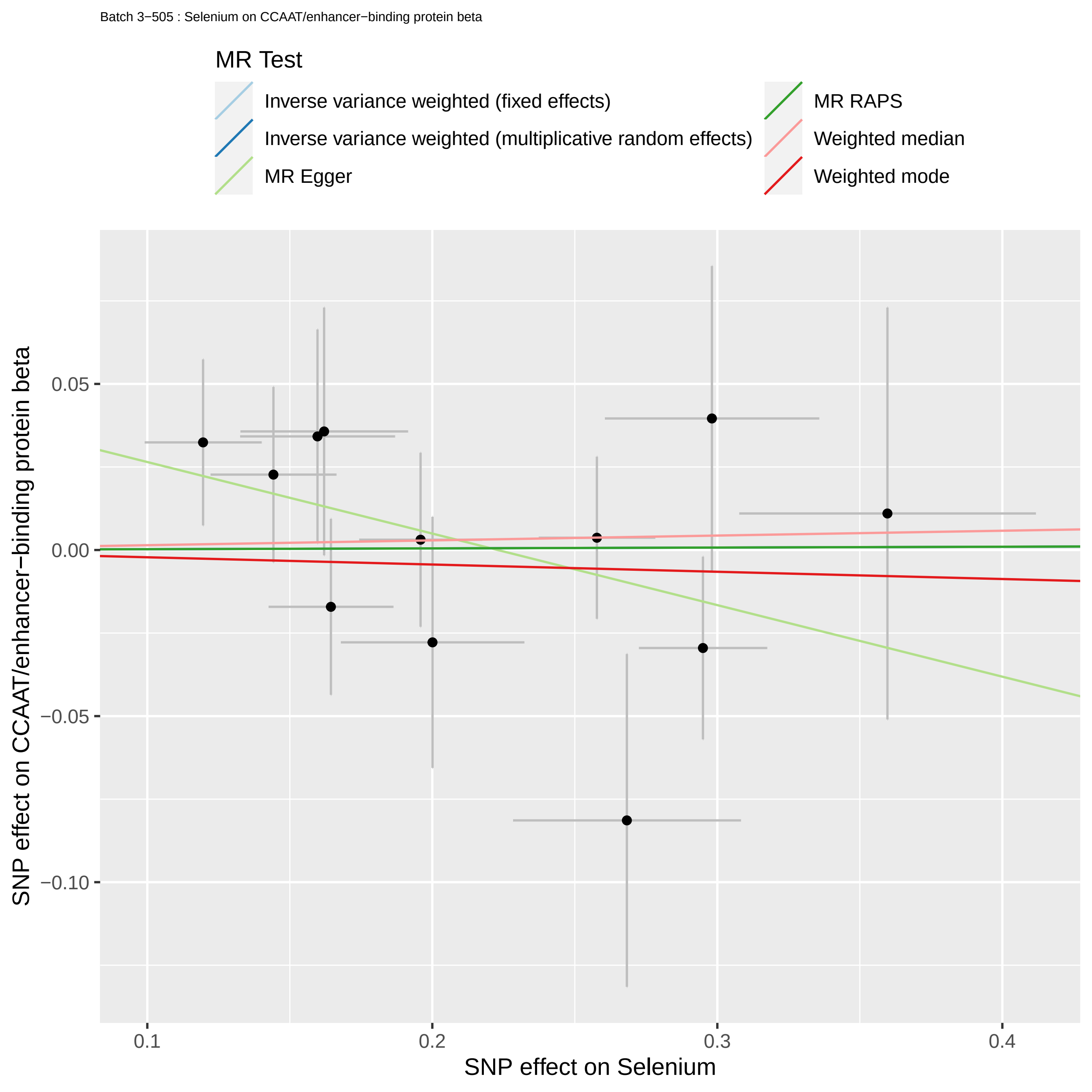

Batch 3−505 : Selenium on CCAAT/enhancer−binding protein beta
MR Test
Inverse variance weighted (fixed effects)
Inverse variance weighted (multiplicative random effects)
MR Egger
MR RAPS
Weighted median
Weighted mode
SNP effect on CCAAT/enhancer−binding protein beta
0.05
0.00
−0.05
−0.10
0.1
0.2
0.3
0.4
SNP effect on Selenium

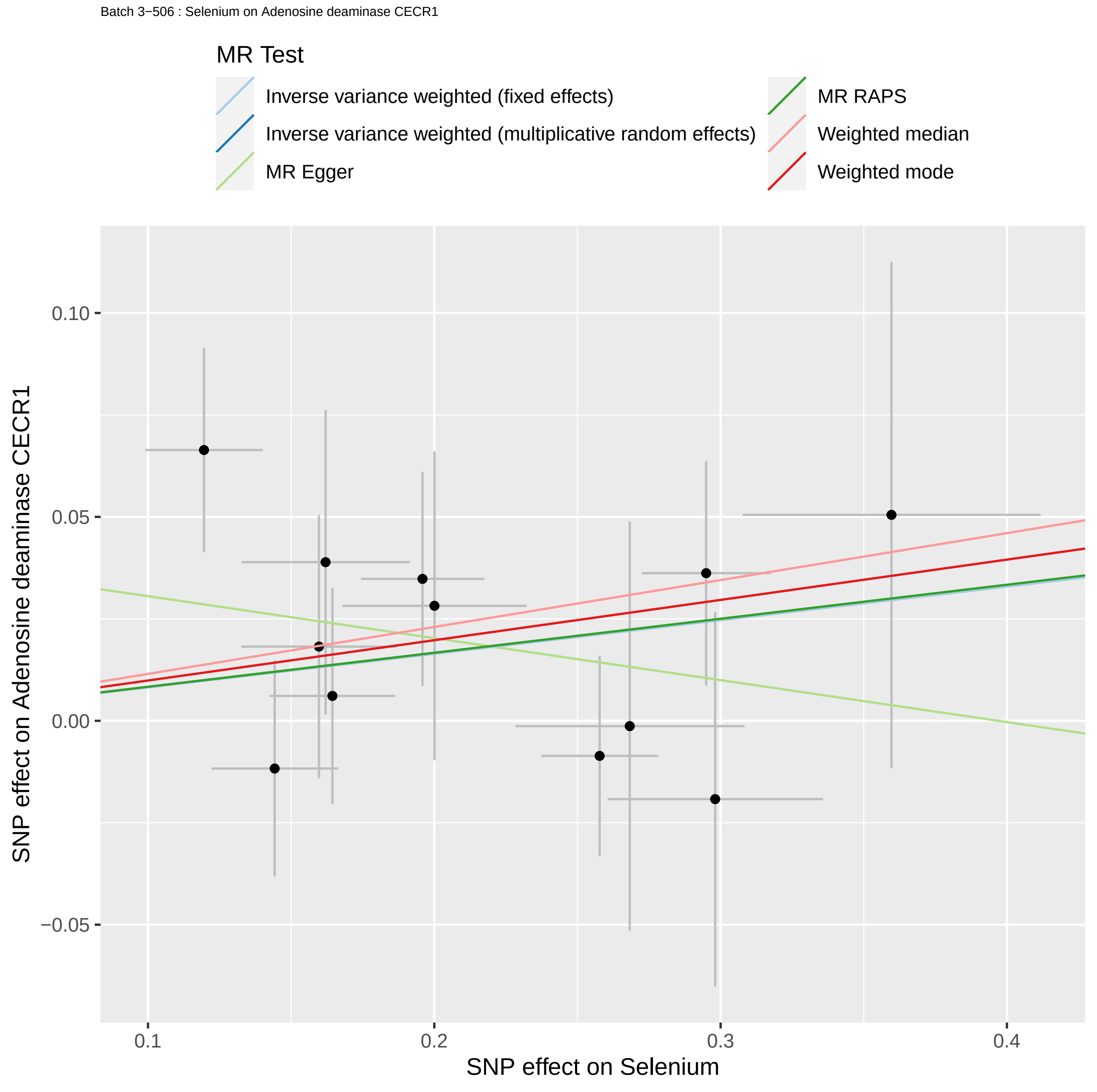

Batch 3−506 : Selenium on Adenosine deaminase CECR1
MR Test
Inverse variance weighted (fixed effects)
Inverse variance weighted (multiplicative random effects)
MR Egger
MR RAPS
Weighted median
Weighted mode
0.10
0.05
0.00
−0.05
0.1
0.2
0.3
0.4
SNP effect on Adenosine deaminase CECR1
SNP effect on Selenium

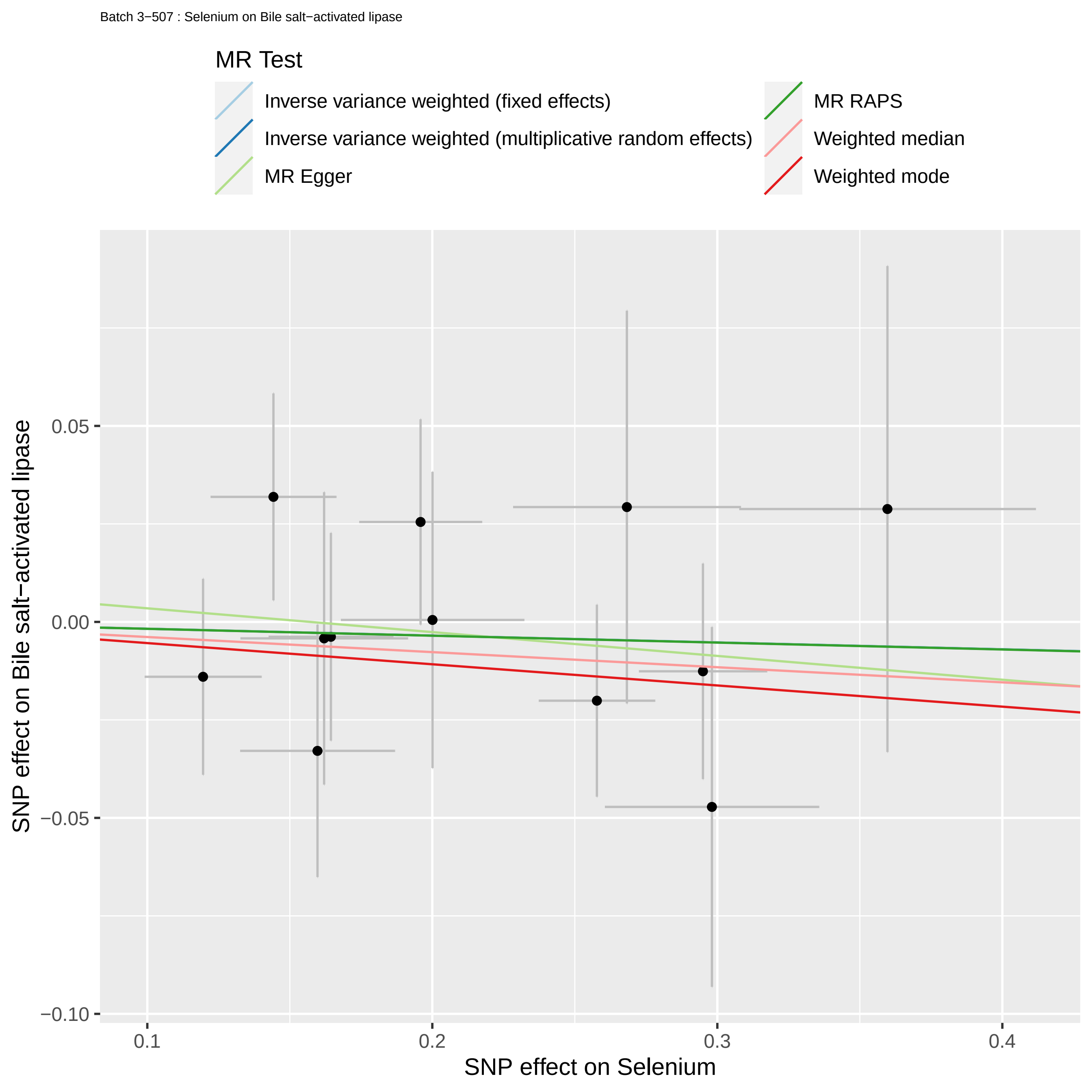
Batch 3−507 : Selenium on Bile salt−activated lipase
MR Test
Inverse variance weighted (fixed effects)
Inverse variance weighted (multiplicative random effects)
MR Egger
MR RAPS
Weighted median
Weighted mode
SNP effect on Bile salt−activated lipase
0.05
0.00
−0.05
−0.10
0.1
0.2
0.3
0.4
SNP effect on Selenium

Batch 3−508 : Selenium on Chymotrypsin−like elastase family member 2A

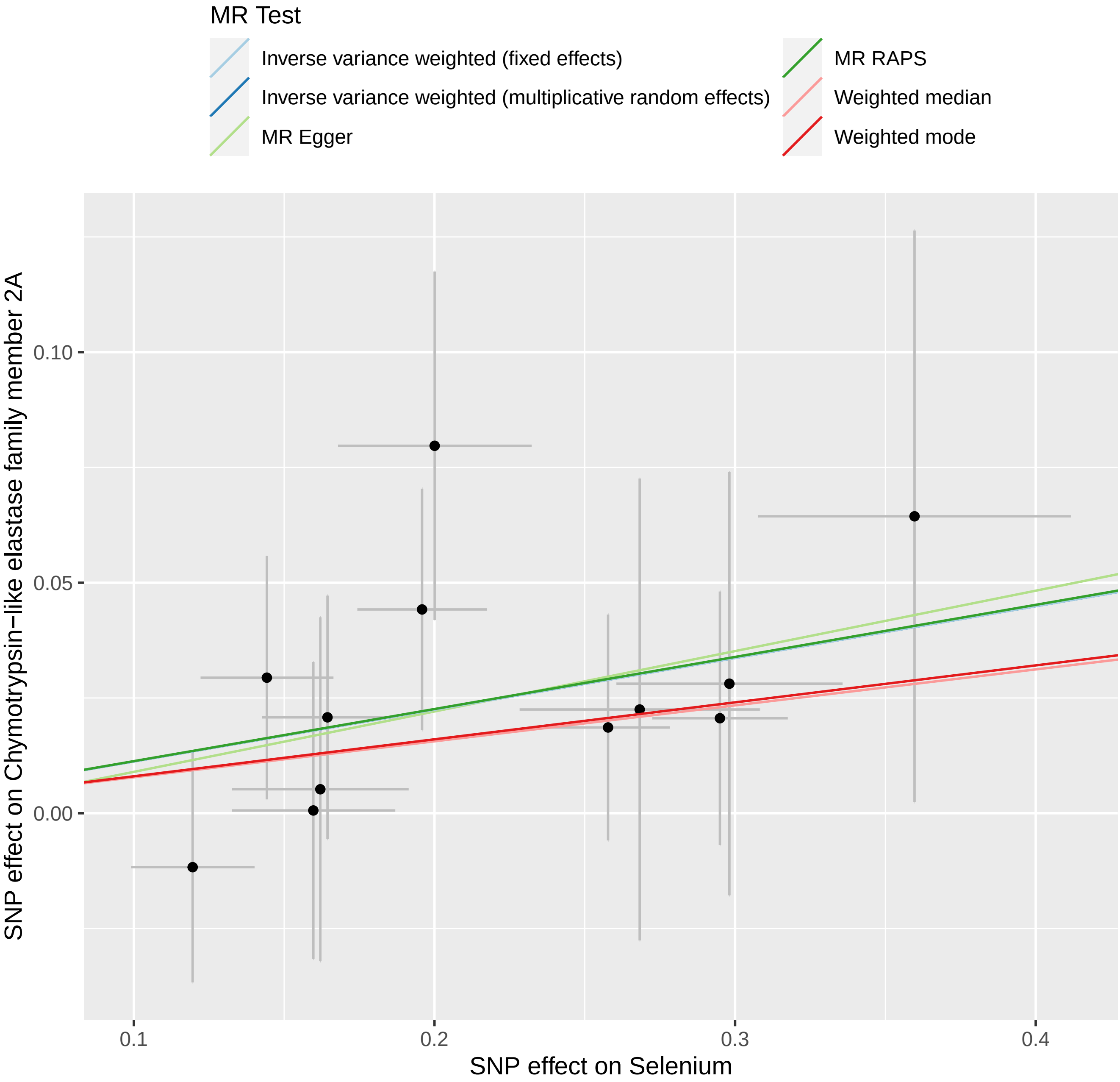

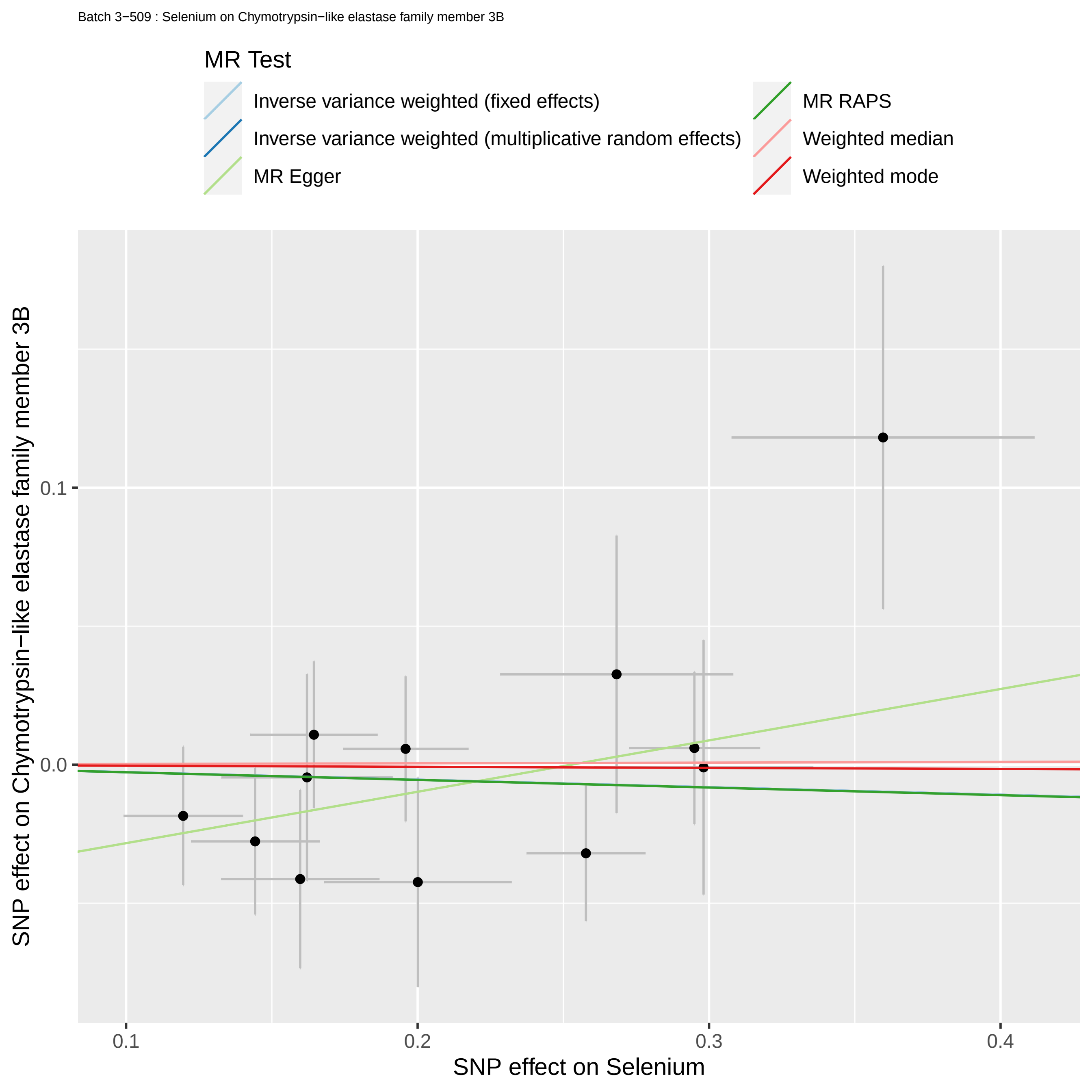

Batch 3−509 : Selenium on Chymotrypsin−like elastase family member 3B
MR Test
Inverse variance weighted (fixed effects)
Inverse variance weighted (multiplicative random effects)
MR Egger
MR RAPS
Weighted median
Weighted mode
0.1
0.0
0.1
0.2
0.3
0.4
SNP effect on Selenium
SNP effect on Chymotrypsin−like elastase family member 3B

Batch 3−510 : Selenium on CUGBP Elav−like family member 2

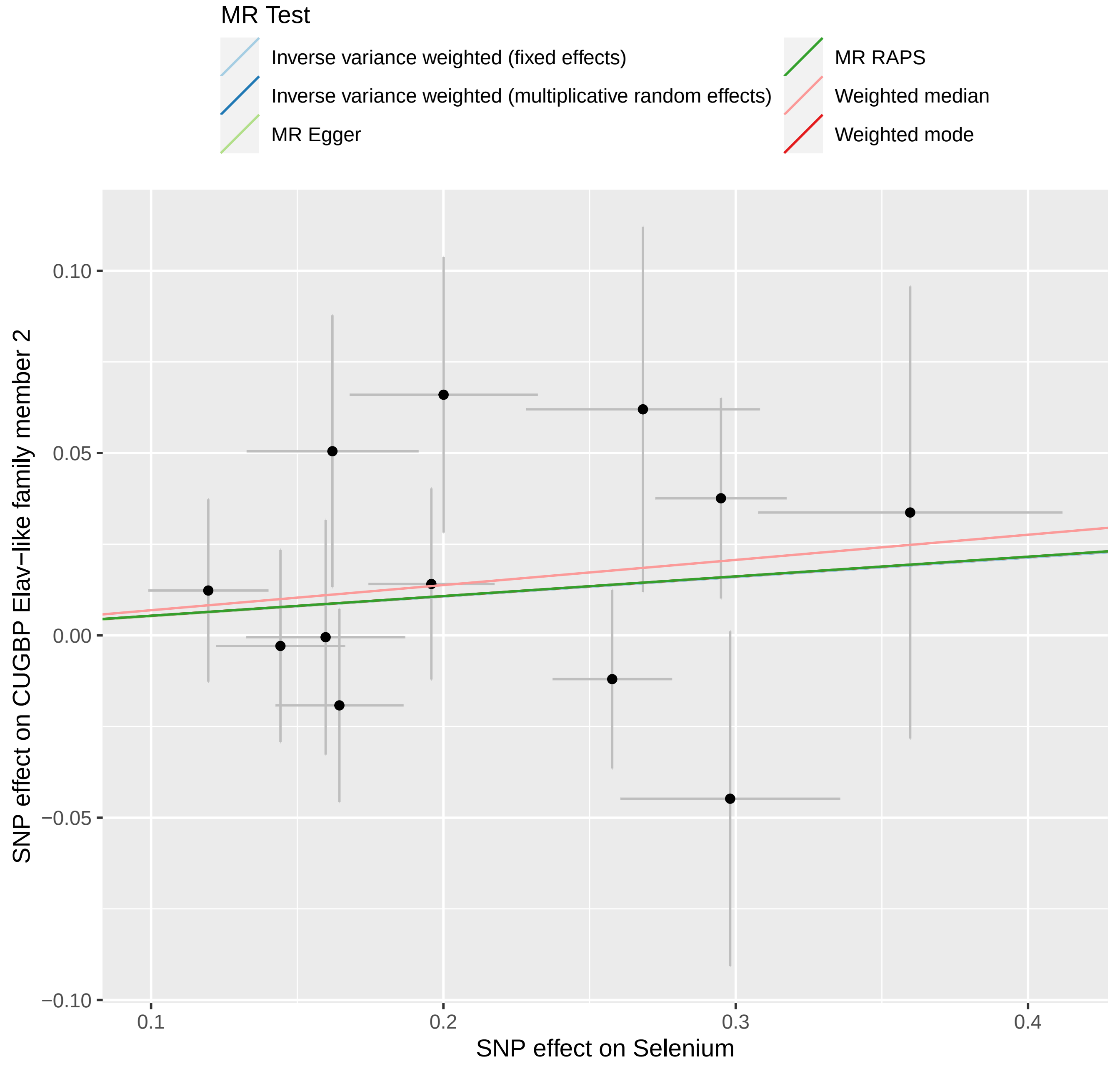

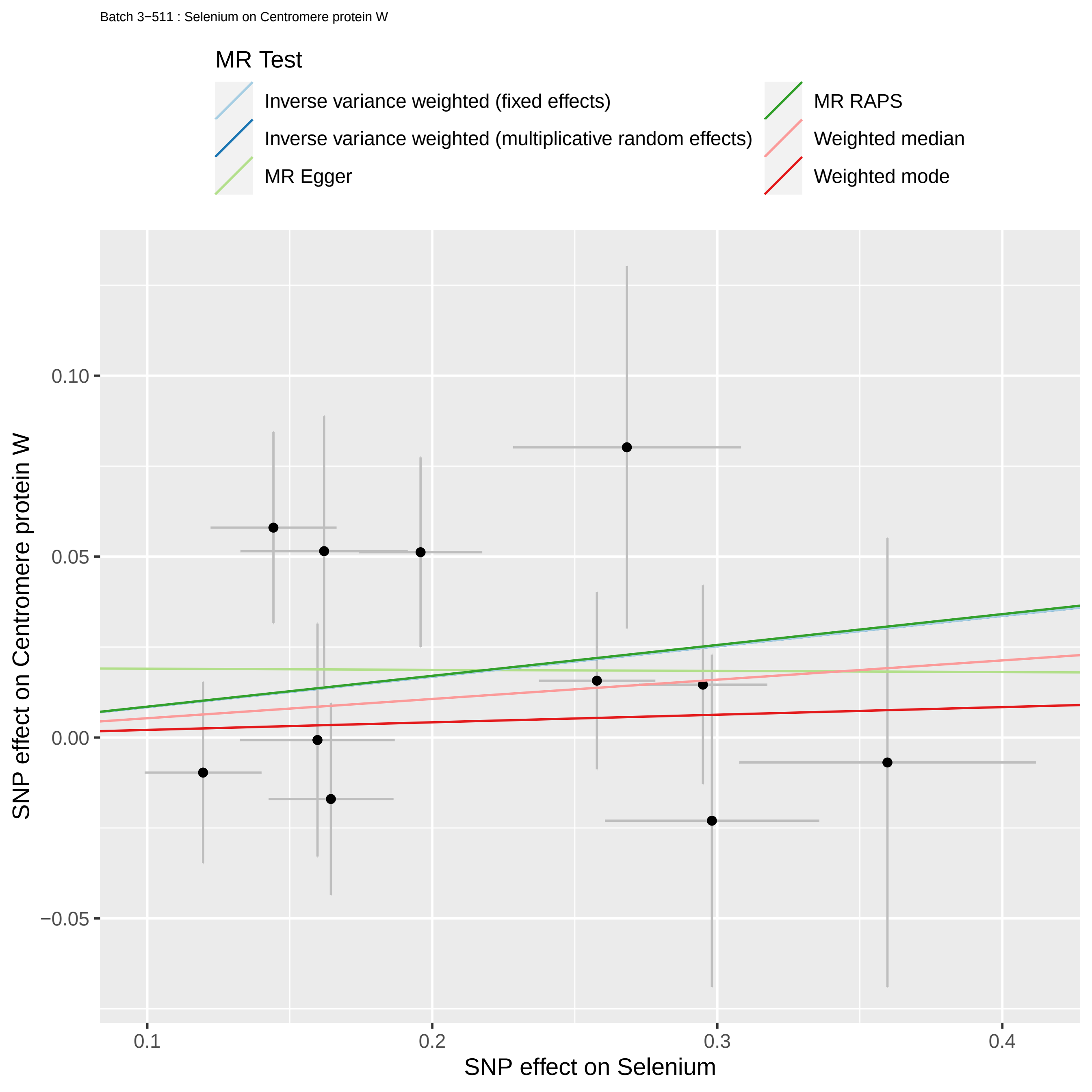

Batch 3−511 : Selenium on Centromere protein W
MR Test
Inverse variance weighted (fixed effects)
Inverse variance weighted (multiplicative random effects)
MR Egger
MR RAPS
Weighted median
Weighted mode
SNP effect on Centromere protein W
0.10
0.05
0.00
−0.05
0.1
0.2
0.3
0.4
SNP effect on Selenium

Batch 3−512 : Selenium on Centrosomal protein of 57 kDa

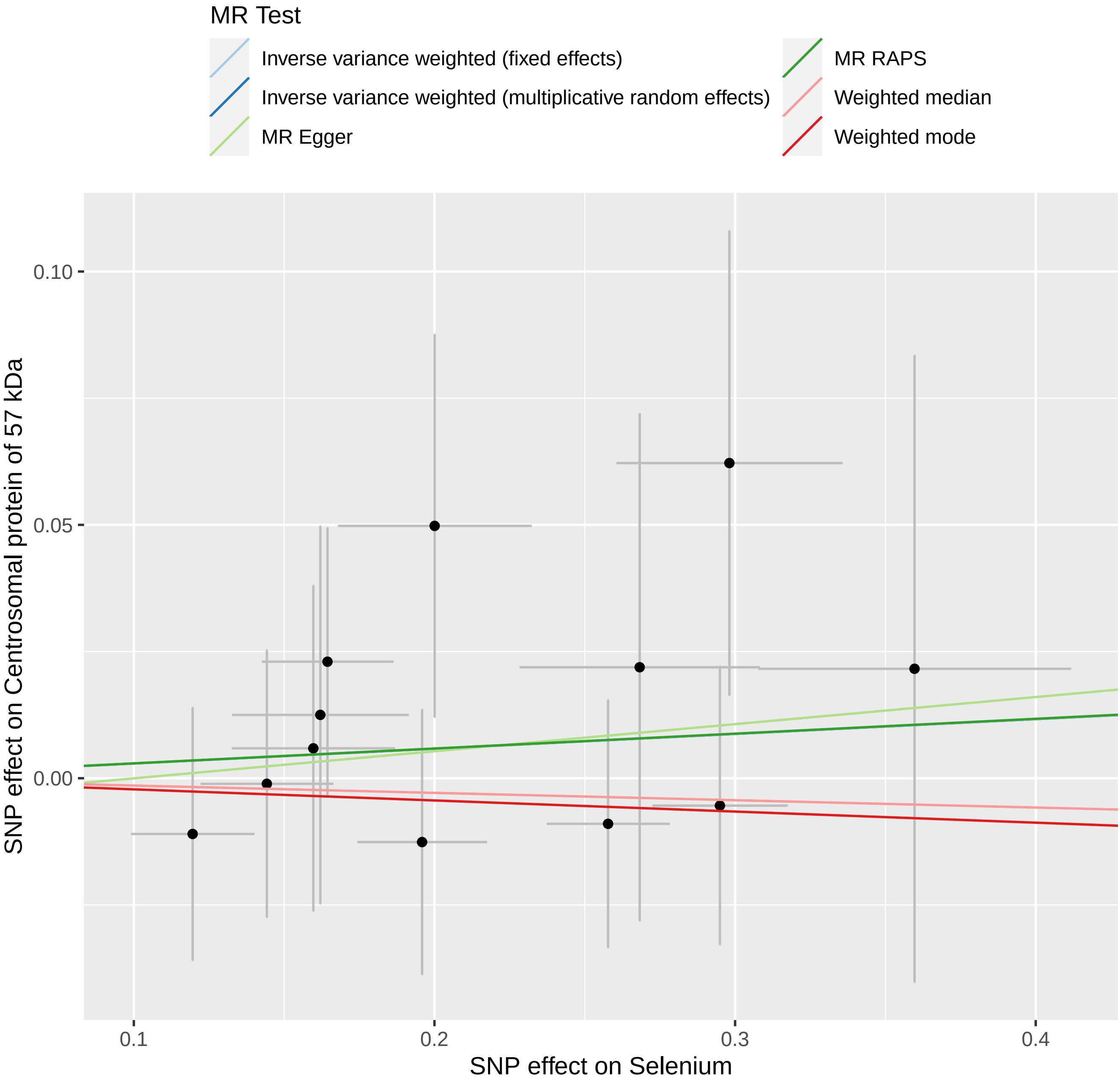

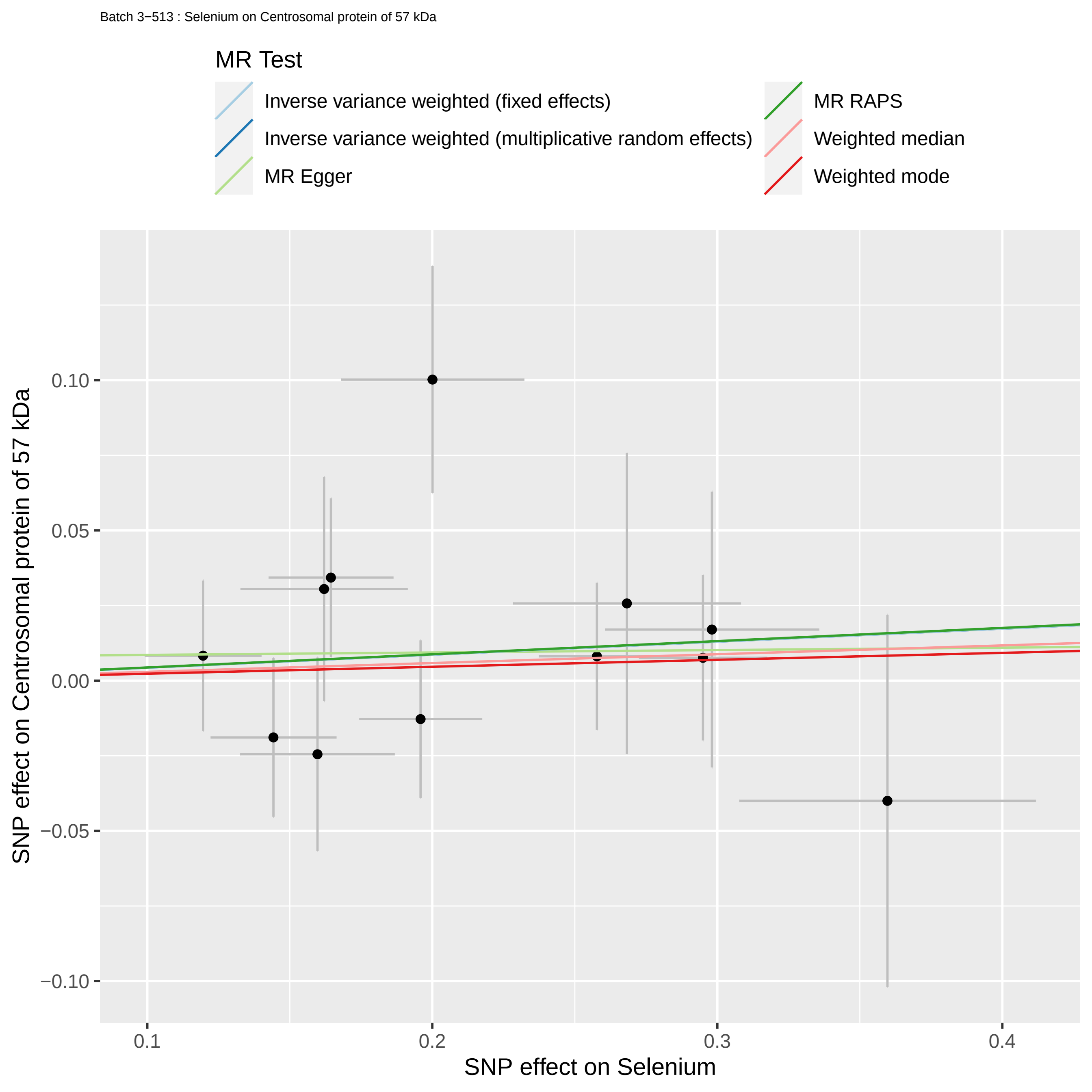

Batch 3−513 : Selenium on Centrosomal protein of 57 kDa
MR Test
Inverse variance weighted (fixed effects)
Inverse variance weighted (multiplicative random effects)
MR Egger
MR RAPS
Weighted median
Weighted mode
0.10
0.05
0.00
−0.05
−0.10
0.1
0.2
0.3
0.4
SNP effect on Centrosomal protein of 57 kDa
SNP effect on Selenium

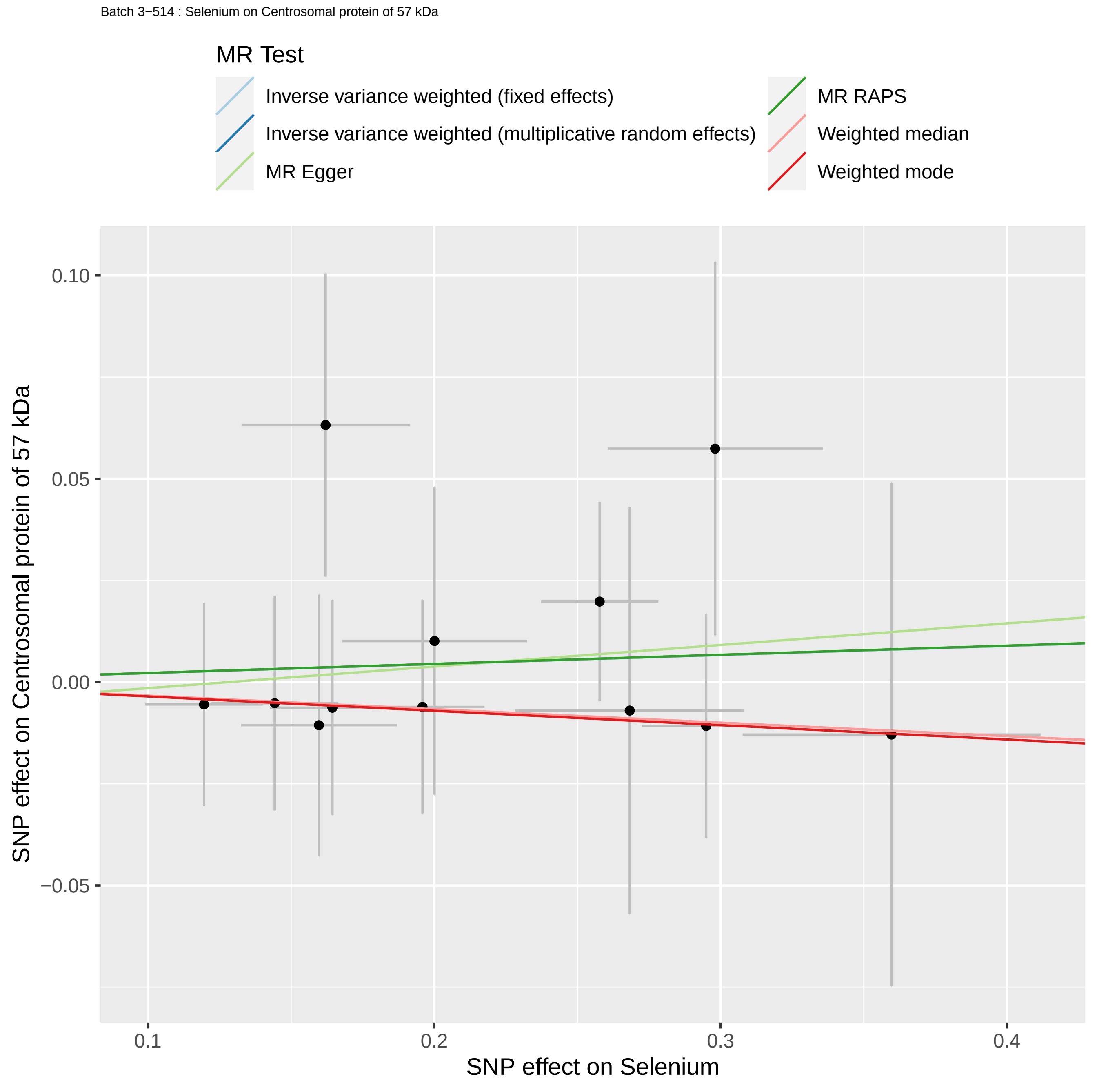

Batch 3−514 : Selenium on Centrosomal protein of 57 kDa
MR Test
Inverse variance weighted (fixed effects)
Inverse variance weighted (multiplicative random effects)
MR Egger
MR RAPS
Weighted median
Weighted mode
0.10
0.05
0.00
−0.05
0.1
0.2
0.3
0.4
SNP effect on Centrosomal protein of 57 kDa
SNP effect on Selenium

Batch 3−515 : Selenium on Ceramide synthase 5

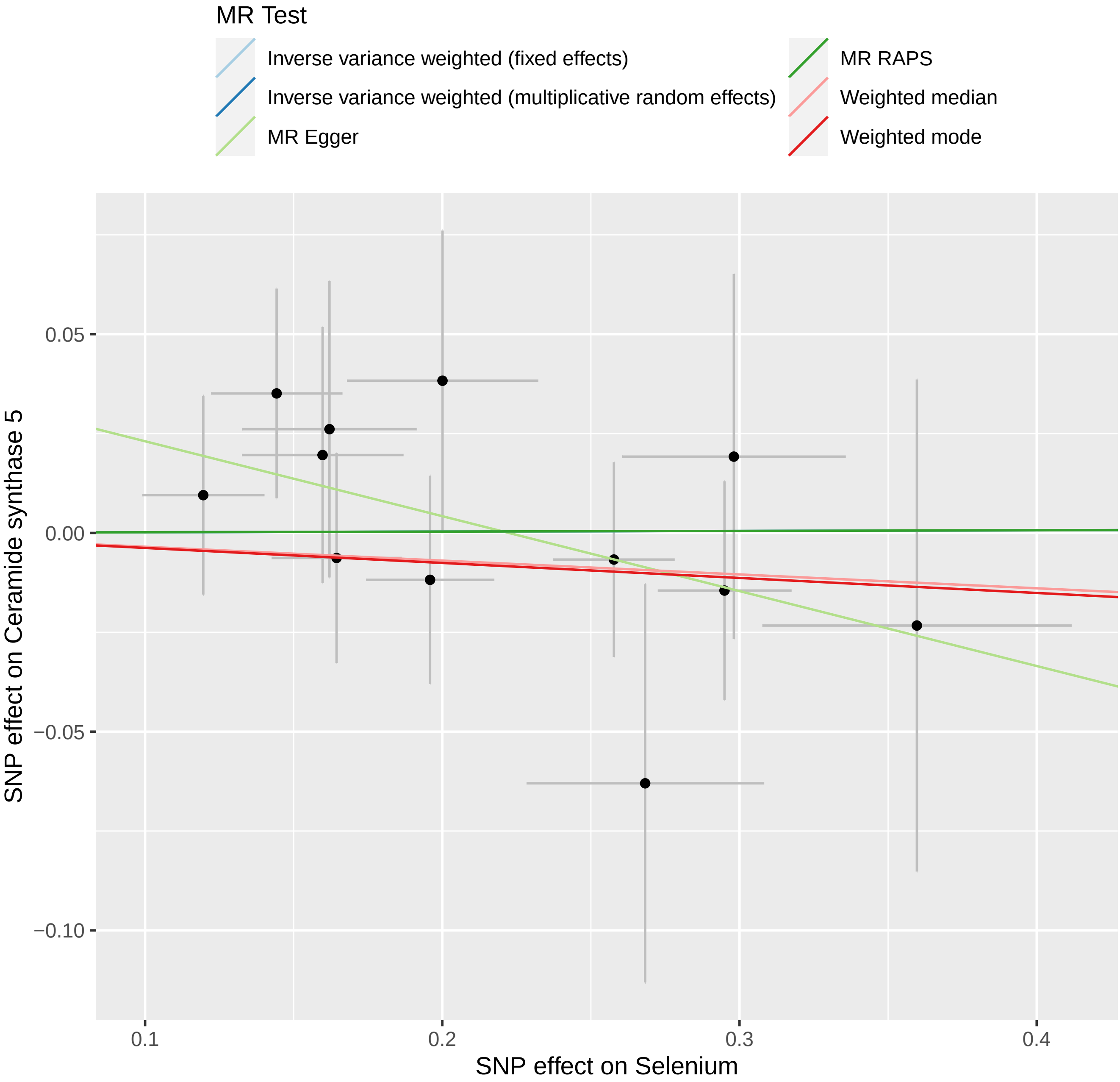

Batch 3−516 : Selenium on Centrin−2

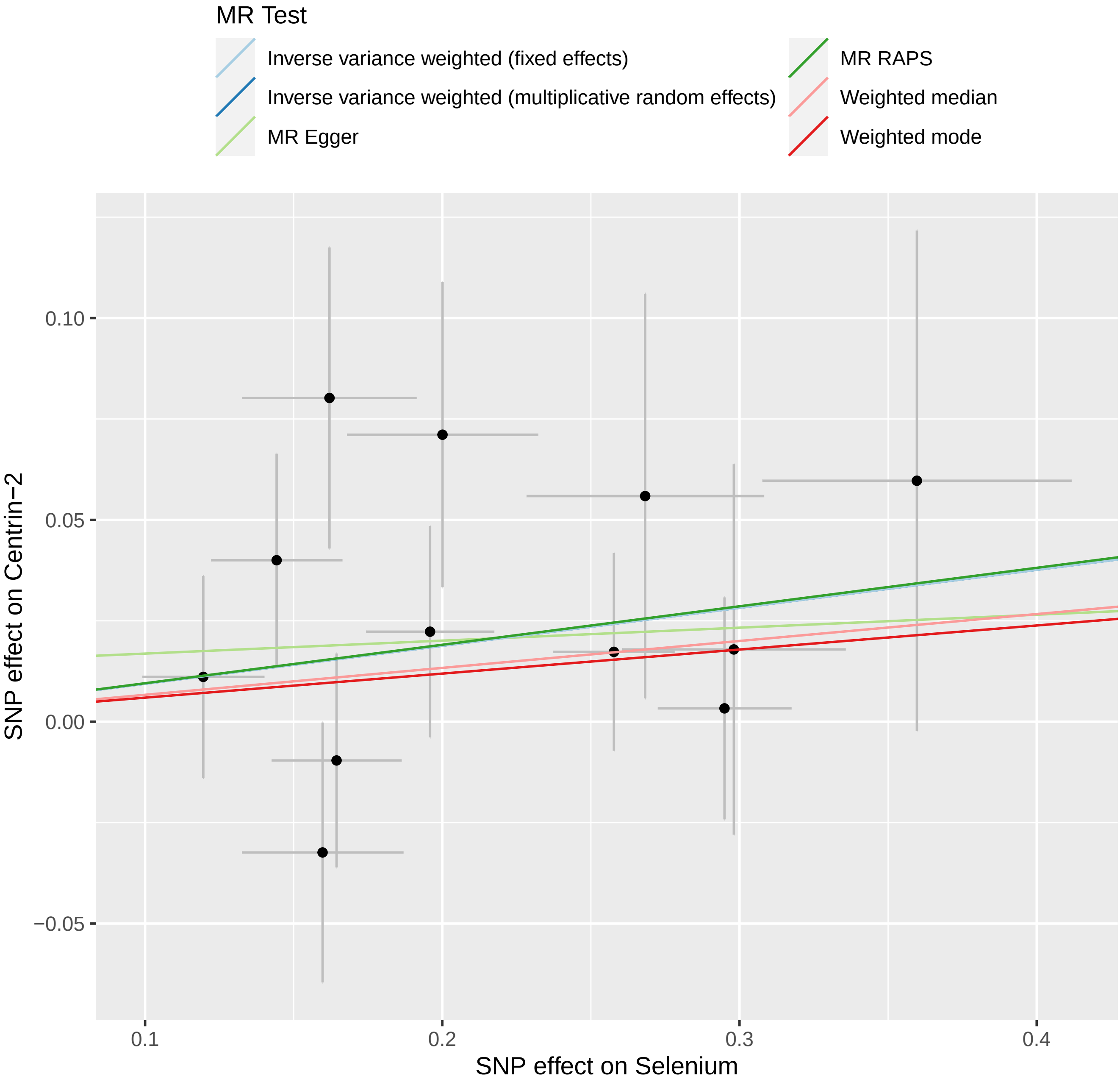

Batch 3−517 : Selenium on Cilia− and flagella−associated protein 45

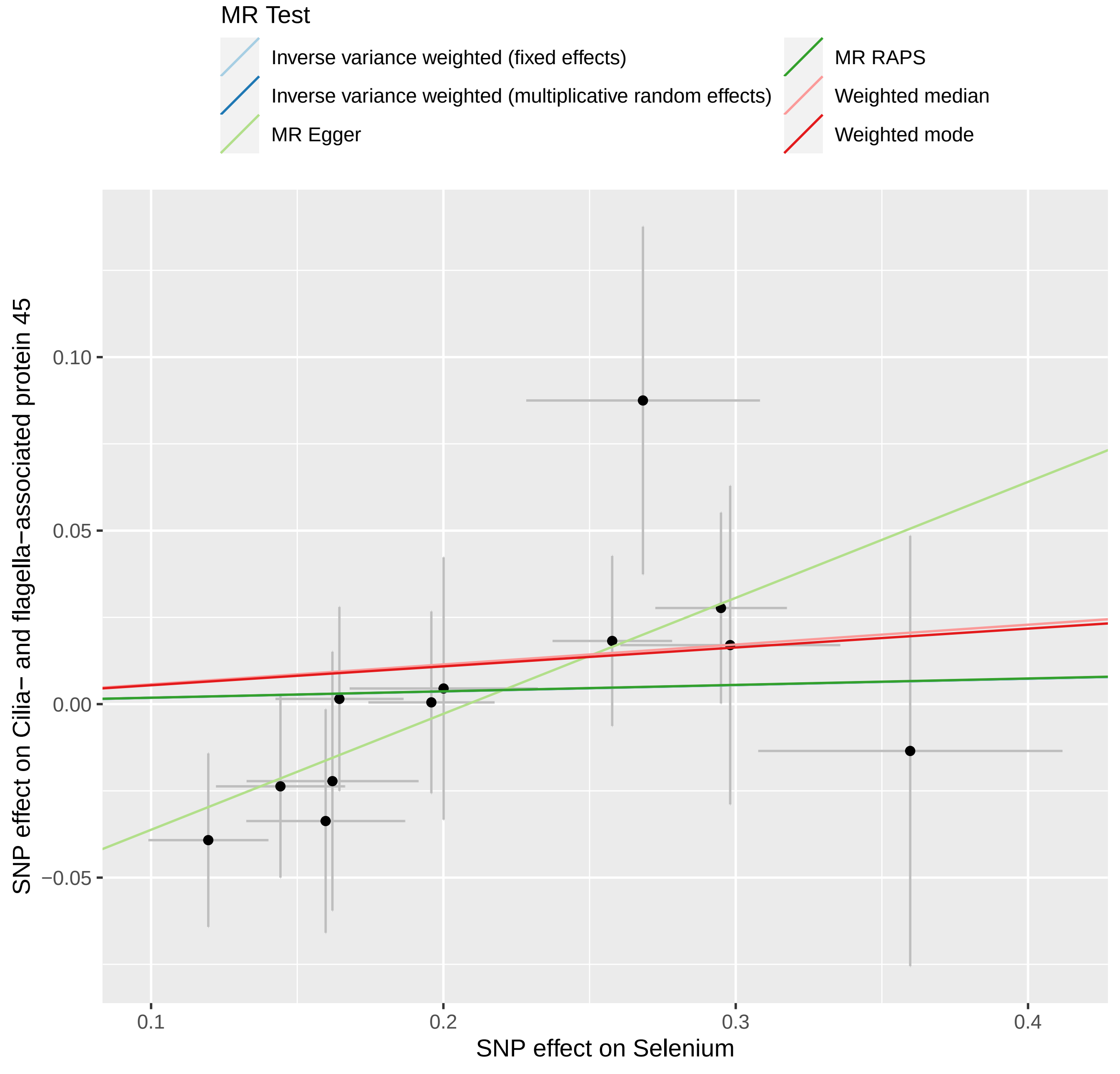

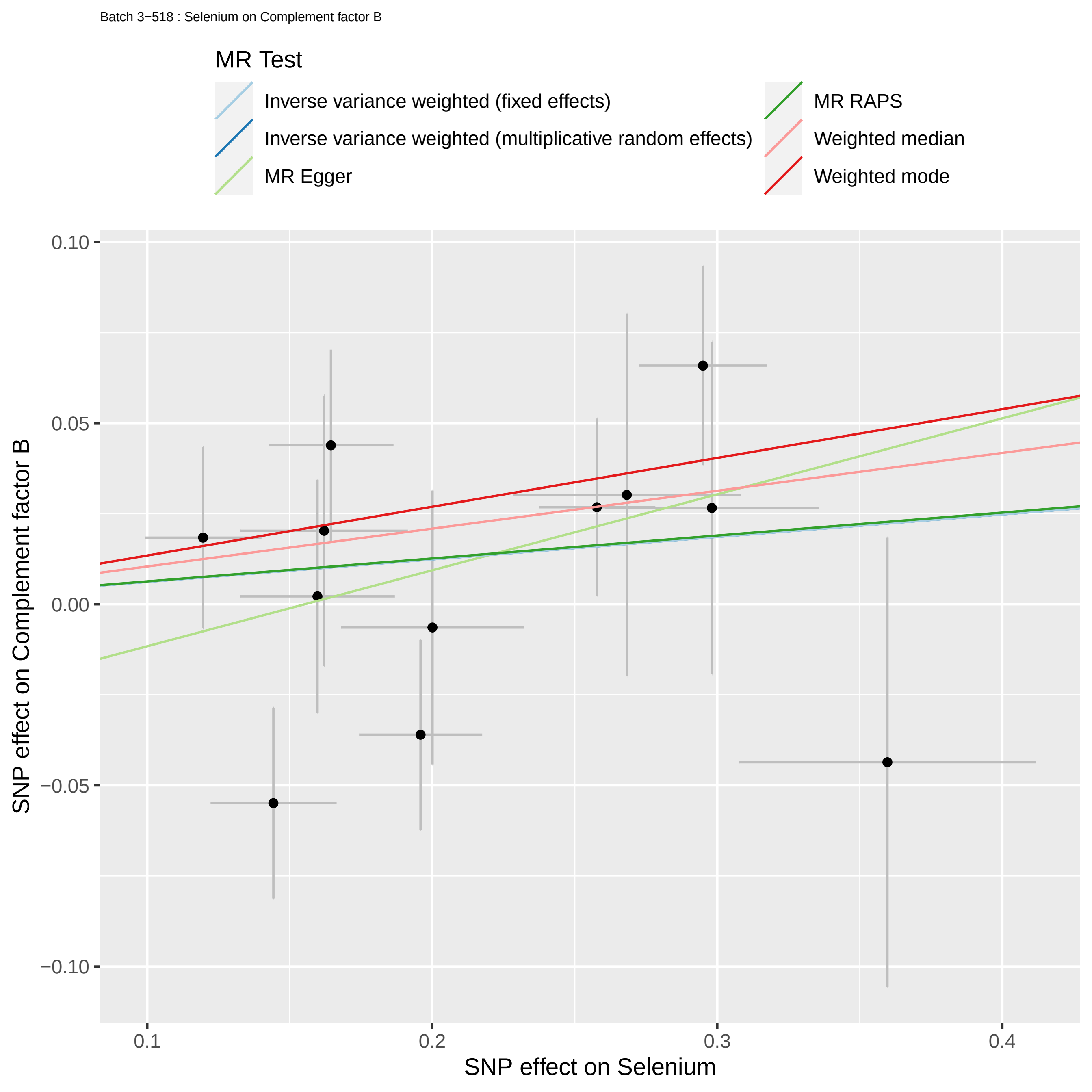
Batch 3−518 : Selenium on Complement factor B
MR Test
Inverse variance weighted (fixed effects)
Inverse variance weighted (multiplicative random effects)
MR Egger
MR RAPS
Weighted median
Weighted mode
0.10
0.05
0.00
−0.05
−0.10
0.1
0.2
0.3
0.4
SNP effect on Complement factor B
SNP effect on Selenium

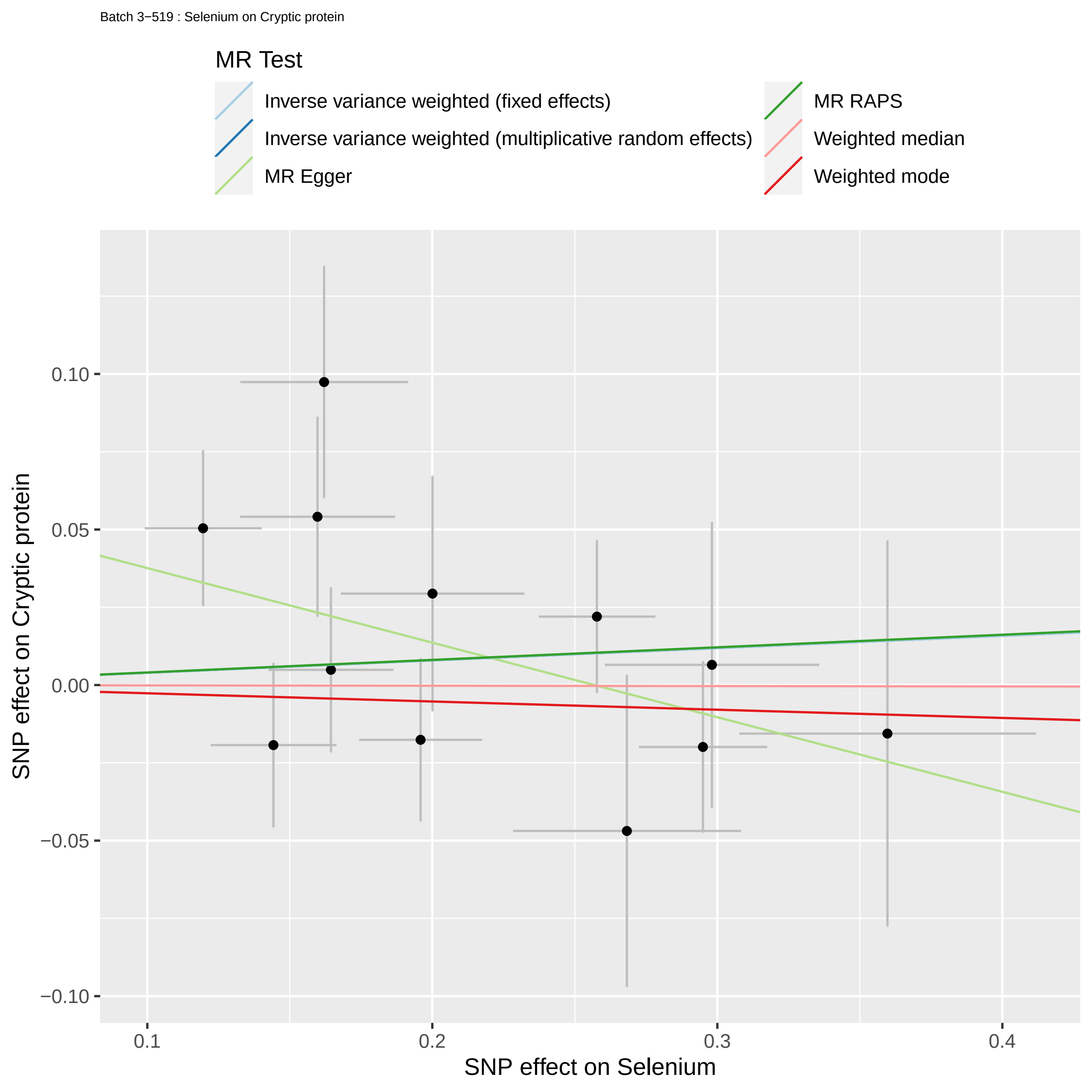
Batch 3−519 : Selenium on Cryptic protein
MR Test
Inverse variance weighted (fixed effects)
Inverse variance weighted (multiplicative random effects)
MR Egger
MR RAPS
Weighted median
Weighted mode
0.10
0.05
0.00
−0.05
−0.10
0.1
0.2
0.3
0.4
SNP effect on Cryptic protein
SNP effect on Selenium

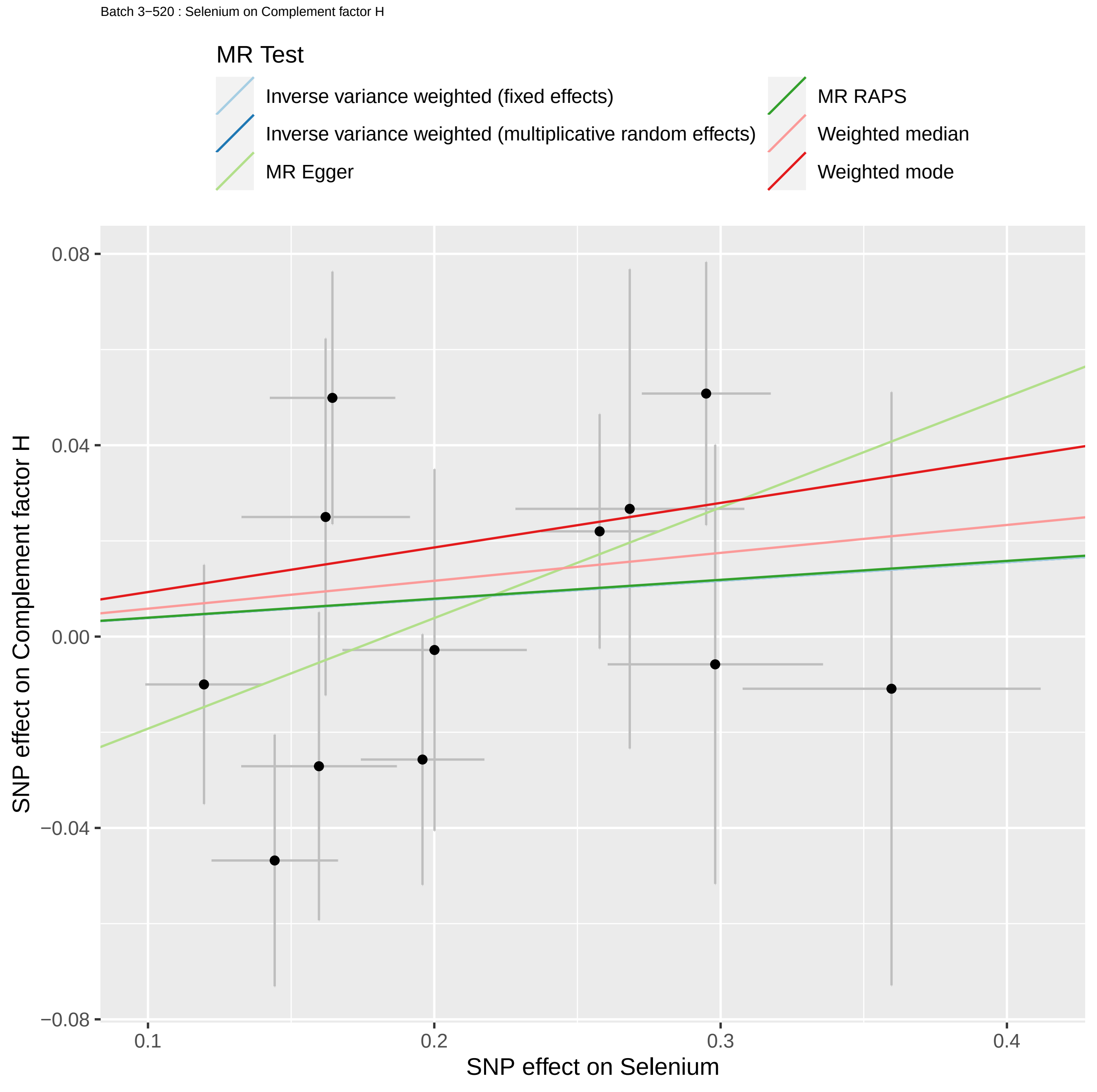
Batch 3−520 : Selenium on Complement factor H
MR Test
Inverse variance weighted (fixed effects)
Inverse variance weighted (multiplicative random effects)
MR Egger
MR RAPS
Weighted median
Weighted mode
0.08
0.04
0.00
−0.04
−0.08
0.1
0.2
0.3
0.4
SNP effect on Complement factor H
SNP effect on Selenium

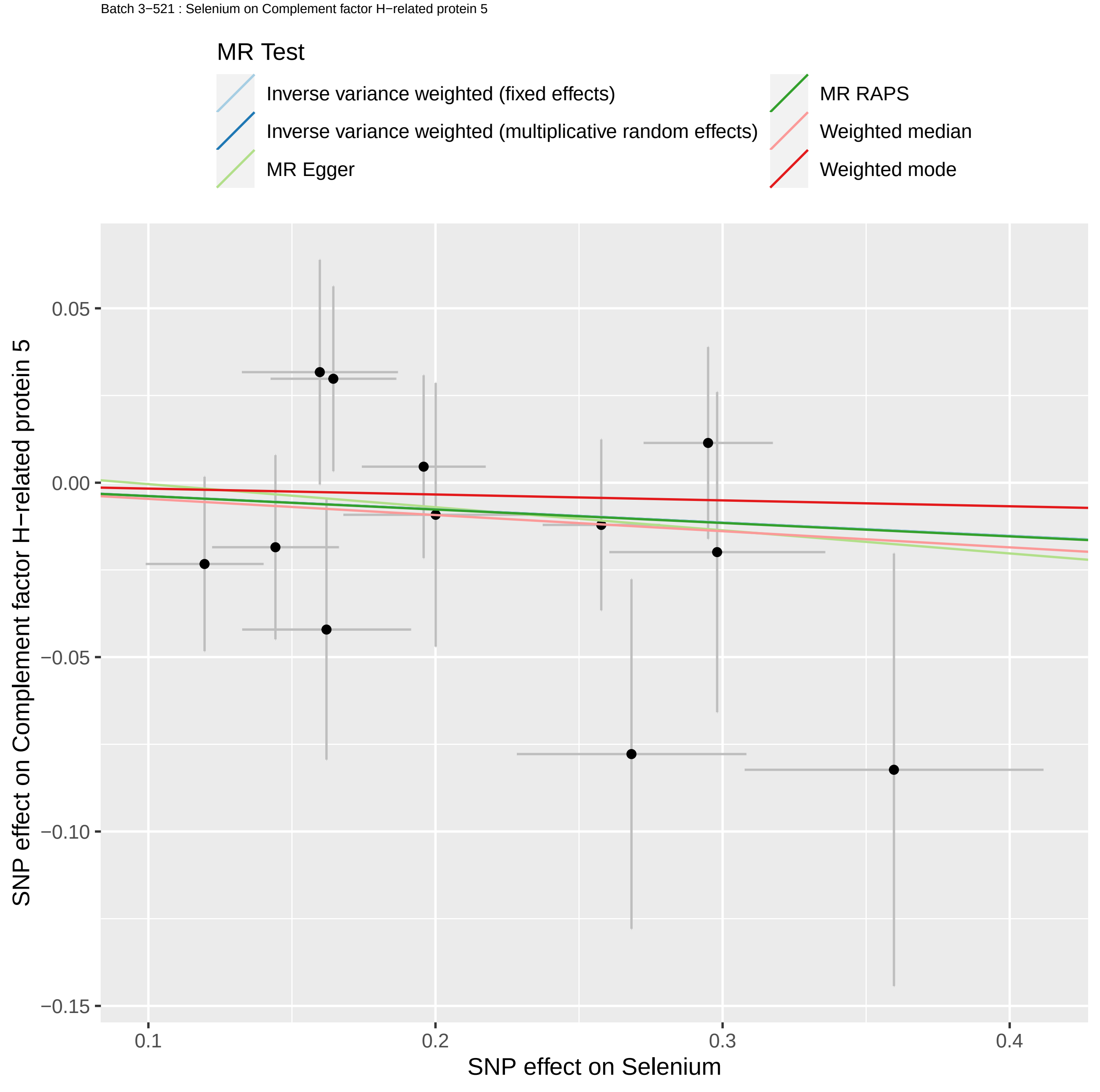

Batch 3−521 : Selenium on Complement factor H−related protein 5
MR Test
Inverse variance weighted (fixed effects)
Inverse variance weighted (multiplicative random effects)
MR Egger
MR RAPS
Weighted median
Weighted mode
SNP effect on Complement factor H−related protein 5
0.05
0.00
−0.05
−0.10
−0.15
0.1
0.2
0.3
0.4
SNP effect on Selenium

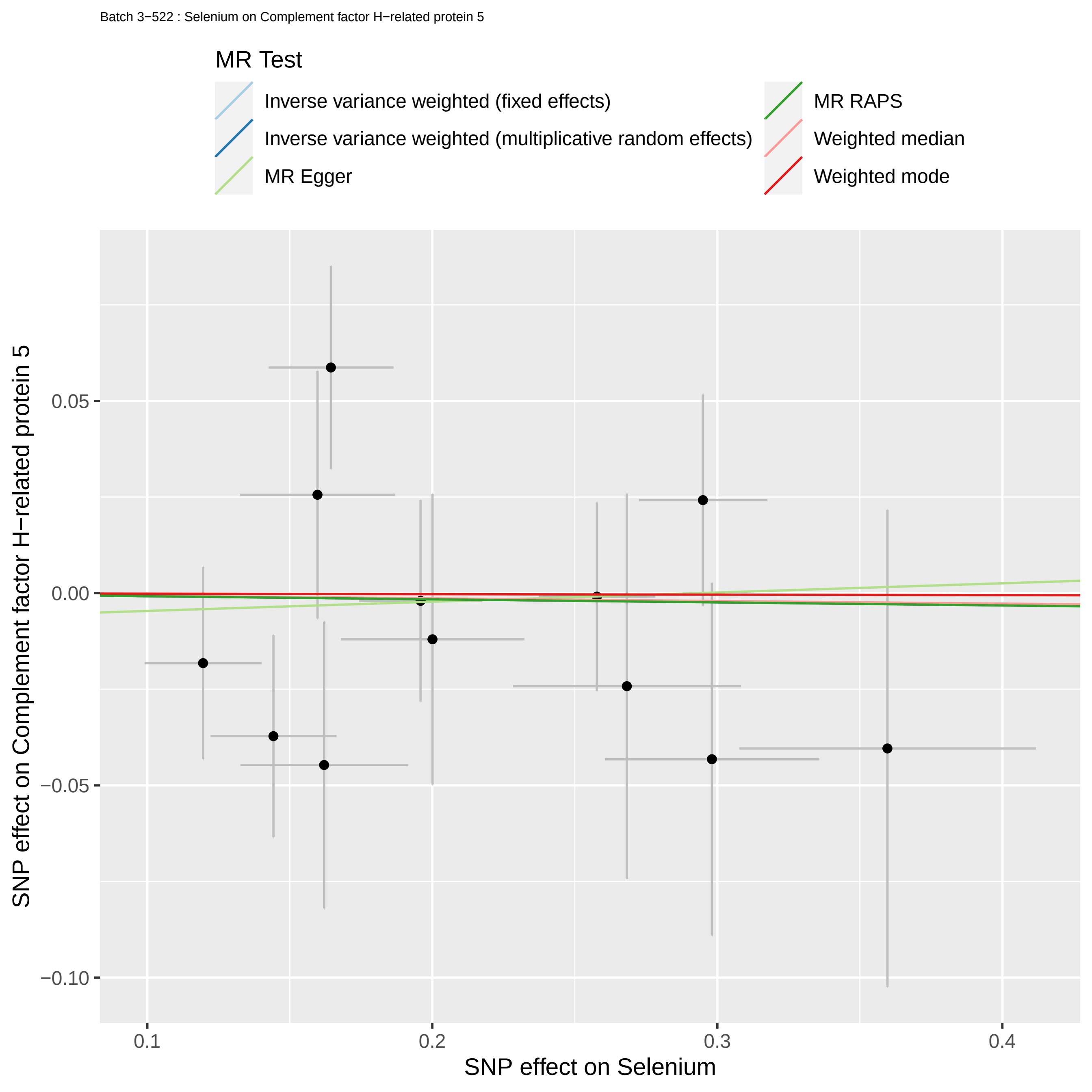

Batch 3−522 : Selenium on Complement factor H−related protein 5
MR Test
Inverse variance weighted (fixed effects)
Inverse variance weighted (multiplicative random effects)
MR Egger
MR RAPS
Weighted median
Weighted mode
0.05
0.00
−0.05
−0.10
0.1
0.2
0.3
0.4
SNP effect on Complement factor H−related protein 5
SNP effect on Selenium

Batch 3−523 : Selenium on Complement factor I

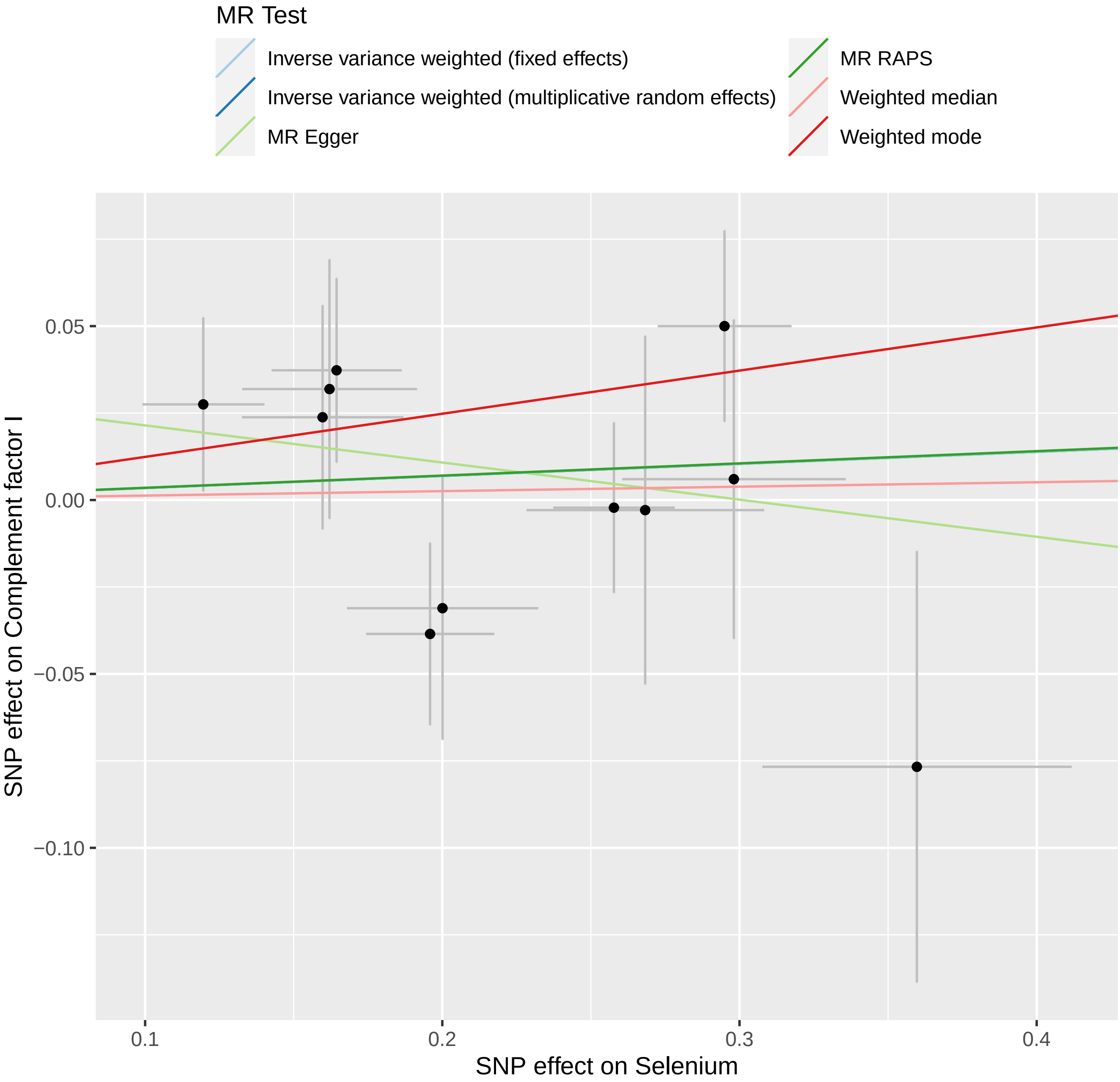

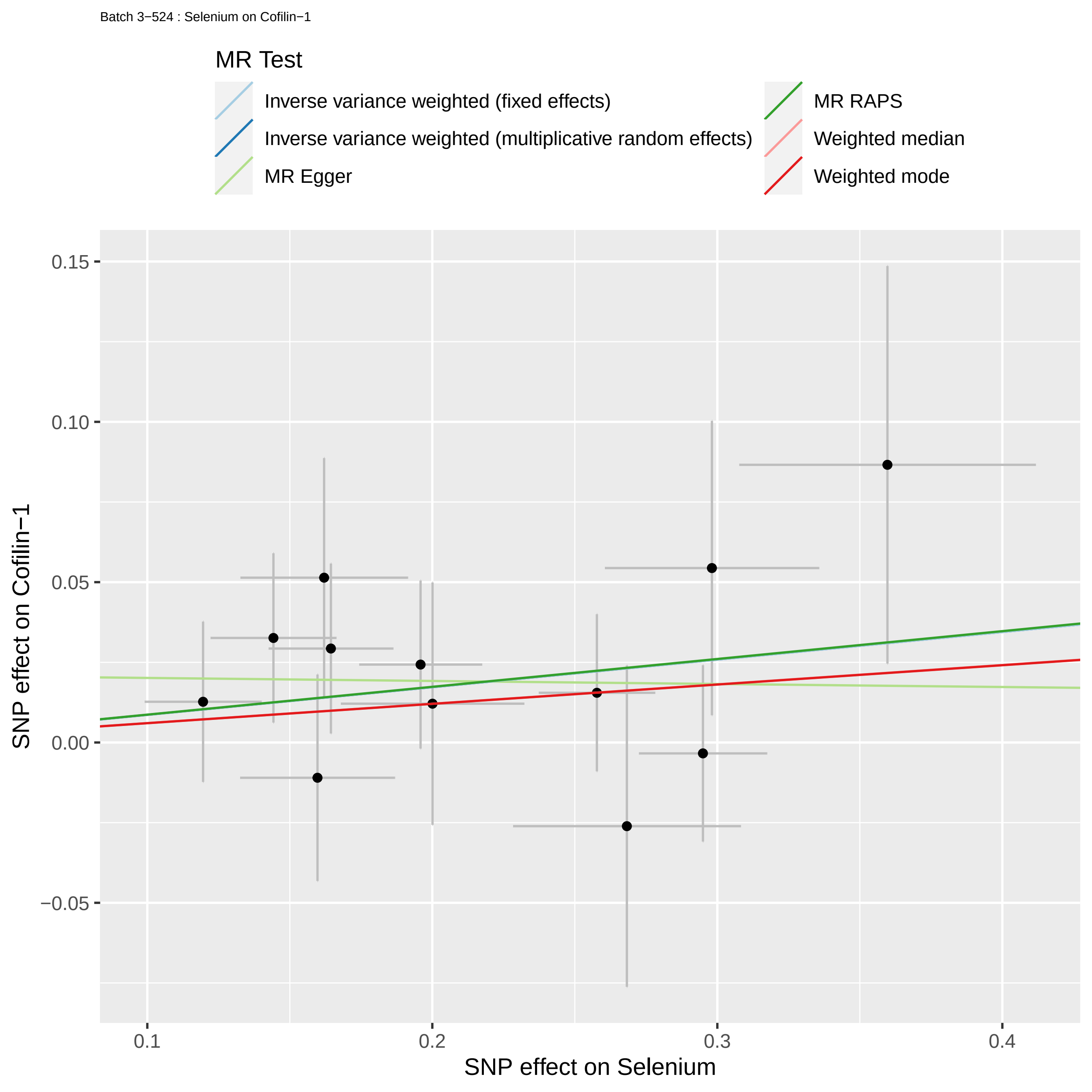
Batch 3−524 : Selenium on Cofilin−1
MR Test
Inverse variance weighted (fixed effects)
Inverse variance weighted (multiplicative random effects)
MR Egger
MR RAPS
Weighted median
Weighted mode
0.15
0.10
0.05
0.00
−0.05
0.1
0.2
0.3
0.4
SNP effect on Cofilin−1
SNP effect on Selenium

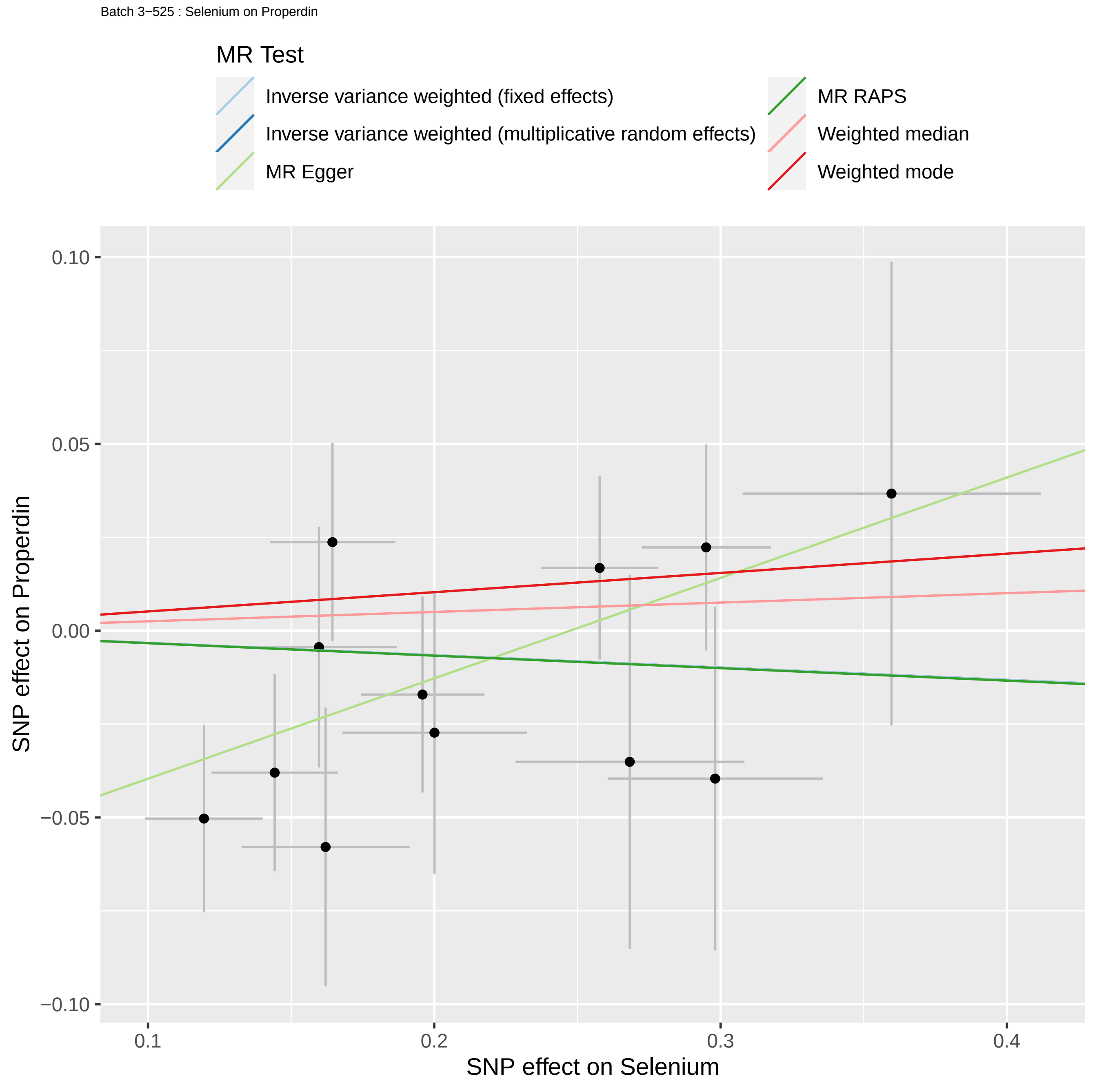

Batch 3−525 : Selenium on Properdin
MR Test
Inverse variance weighted (fixed effects)
Inverse variance weighted (multiplicative random effects)
MR Egger
MR RAPS
Weighted median
Weighted mode
0.10
0.05
0.00
−0.05
−0.10
0.1
0.2
0.3
0.4
SNP effect on Properdin
SNP effect on Selenium

Batch 3−526 : Selenium on Glycoprotein hormones alpha chain

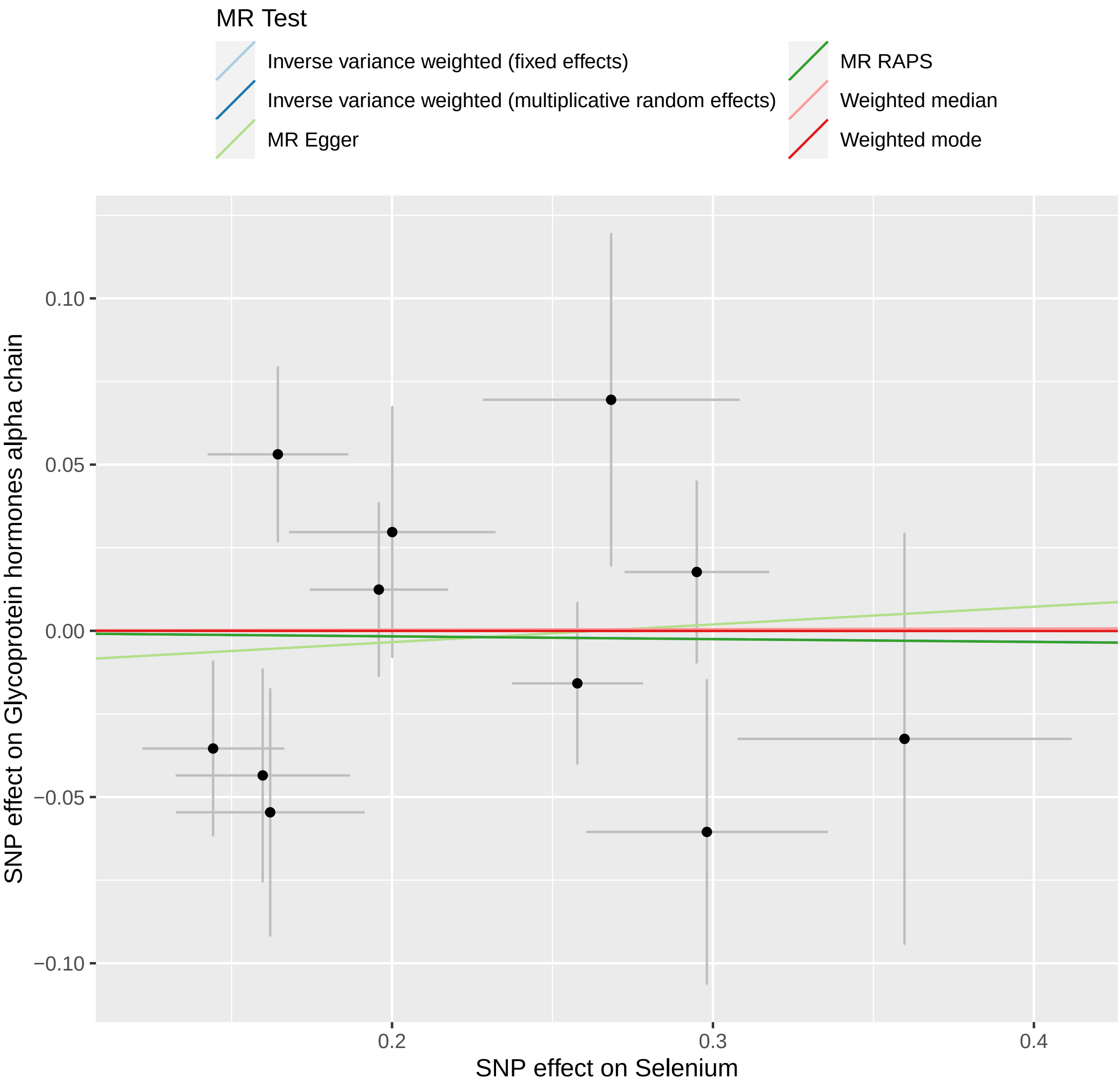

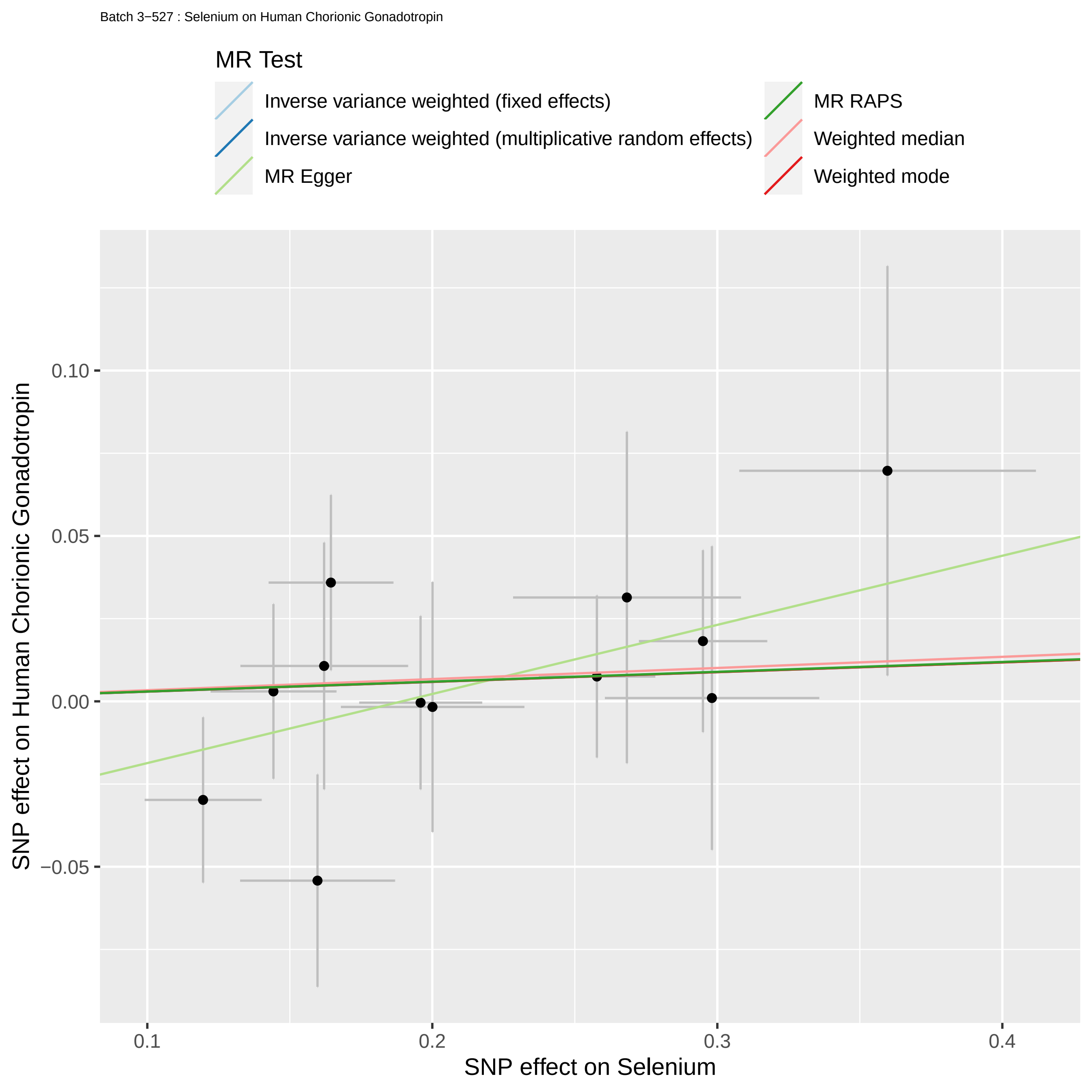

Batch 3−527 : Selenium on Human Chorionic Gonadotropin
MR Test
Inverse variance weighted (fixed effects)
Inverse variance weighted (multiplicative random effects)
MR Egger
MR RAPS
Weighted median
Weighted mode
SNP effect on Human Chorionic Gonadotropin
0.10
0.05
0.00
−0.05
0.1
0.2
0.3
0.4
SNP effect on Selenium

Batch 3−528 : Selenium on Follicle stimulating hormone

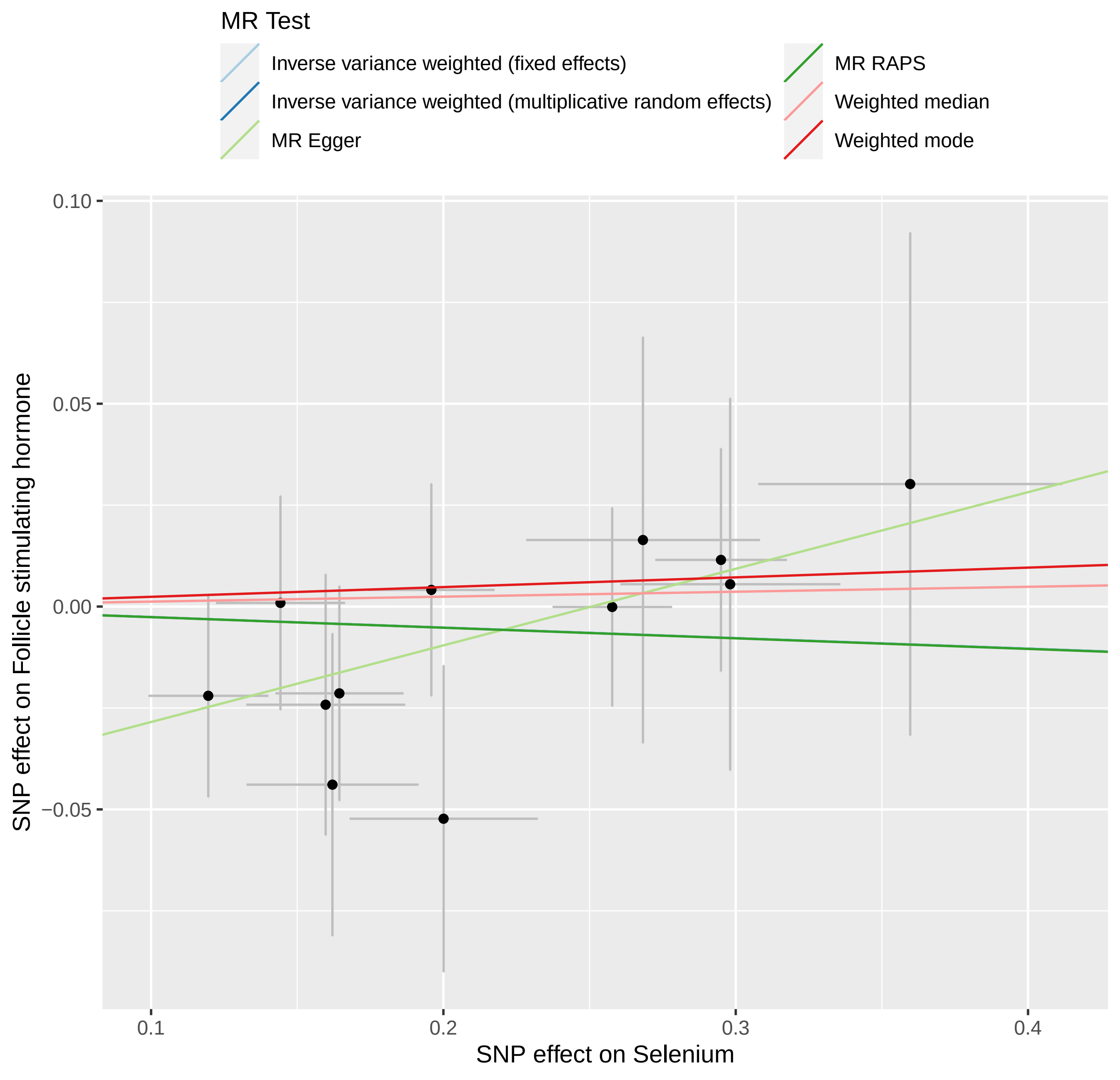

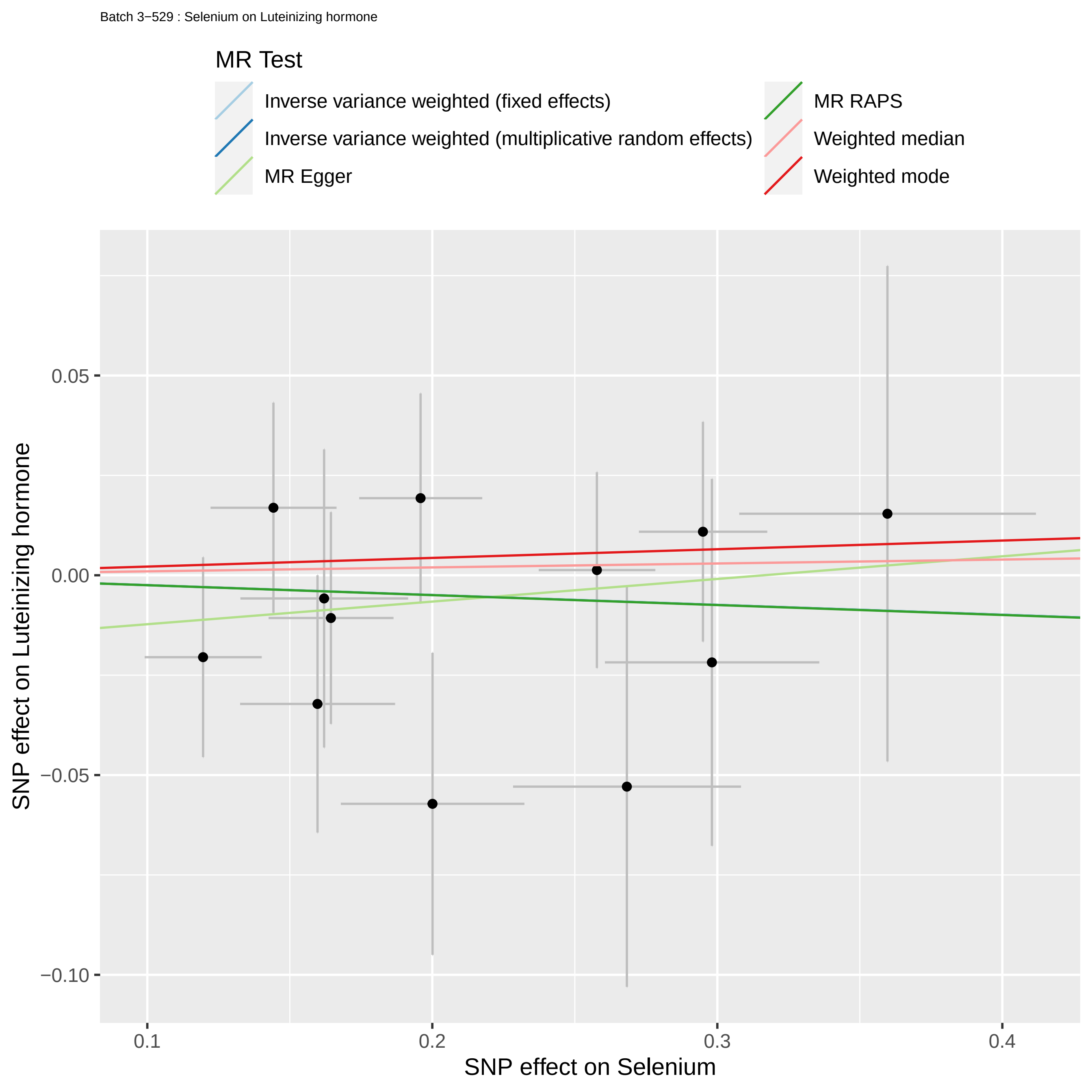

Batch 3−529 : Selenium on Luteinizing hormone
MR Test
Inverse variance weighted (fixed effects)
Inverse variance weighted (multiplicative random effects)
MR Egger
MR RAPS
Weighted median
Weighted mode
0.05
0.00
−0.05
−0.10
0.1
0.2
0.3
0.4
SNP effect on Luteinizing hormone
SNP effect on Selenium

Batch 3−530 : Selenium on Thyroid Stimulating Hormone

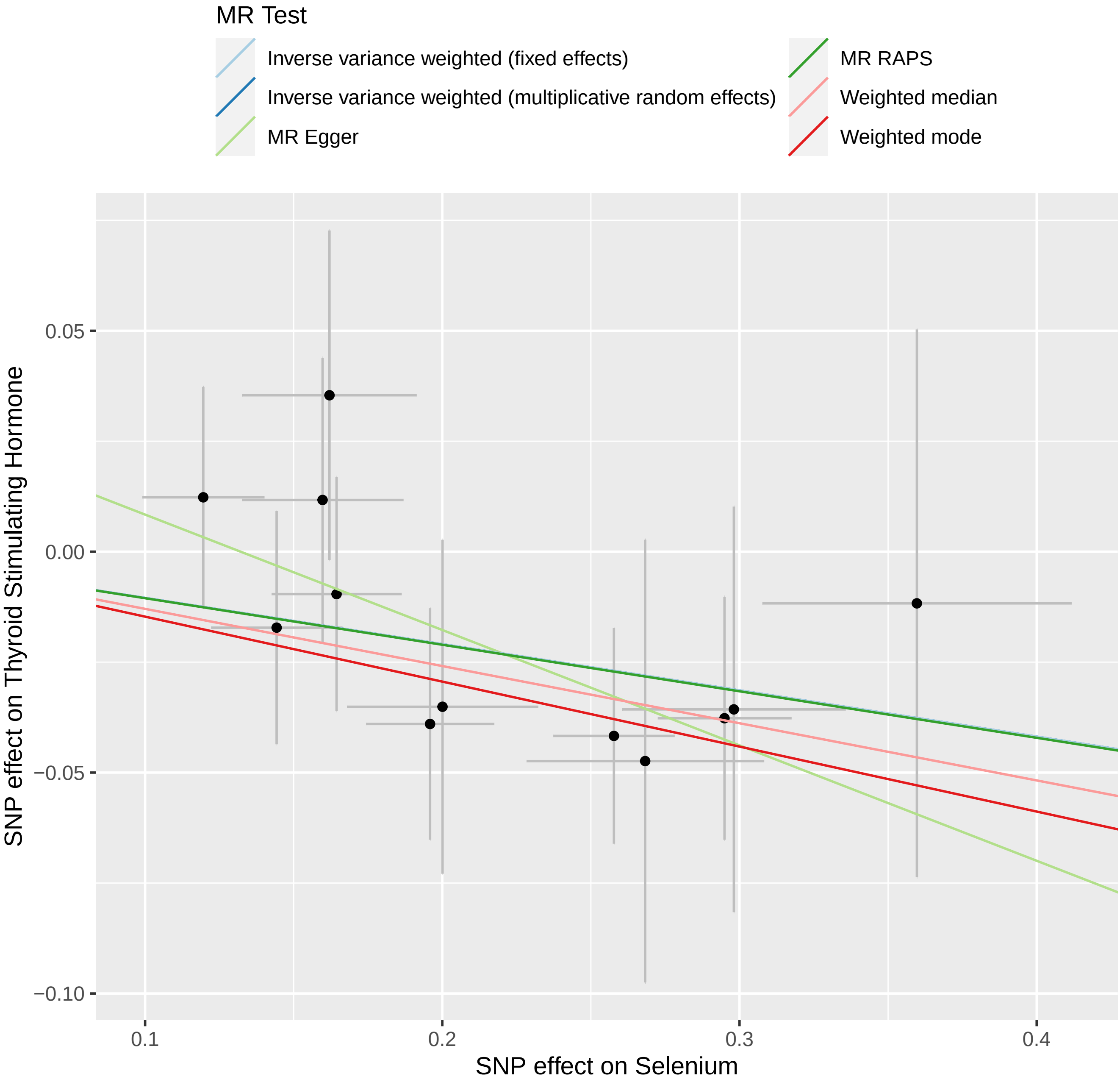

Batch 3−531 : Selenium on Choriogonadotropin subunit beta variant 2

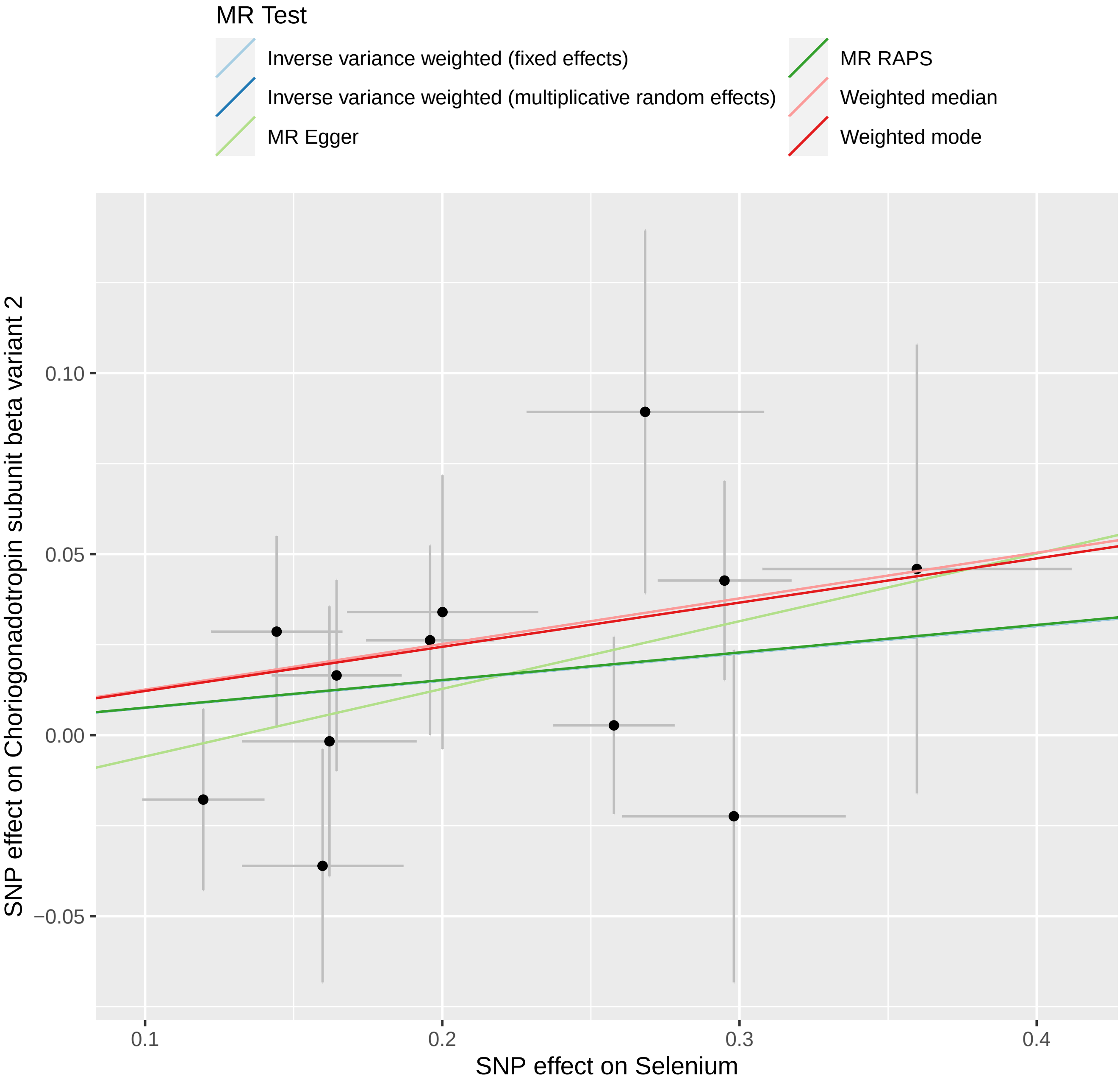

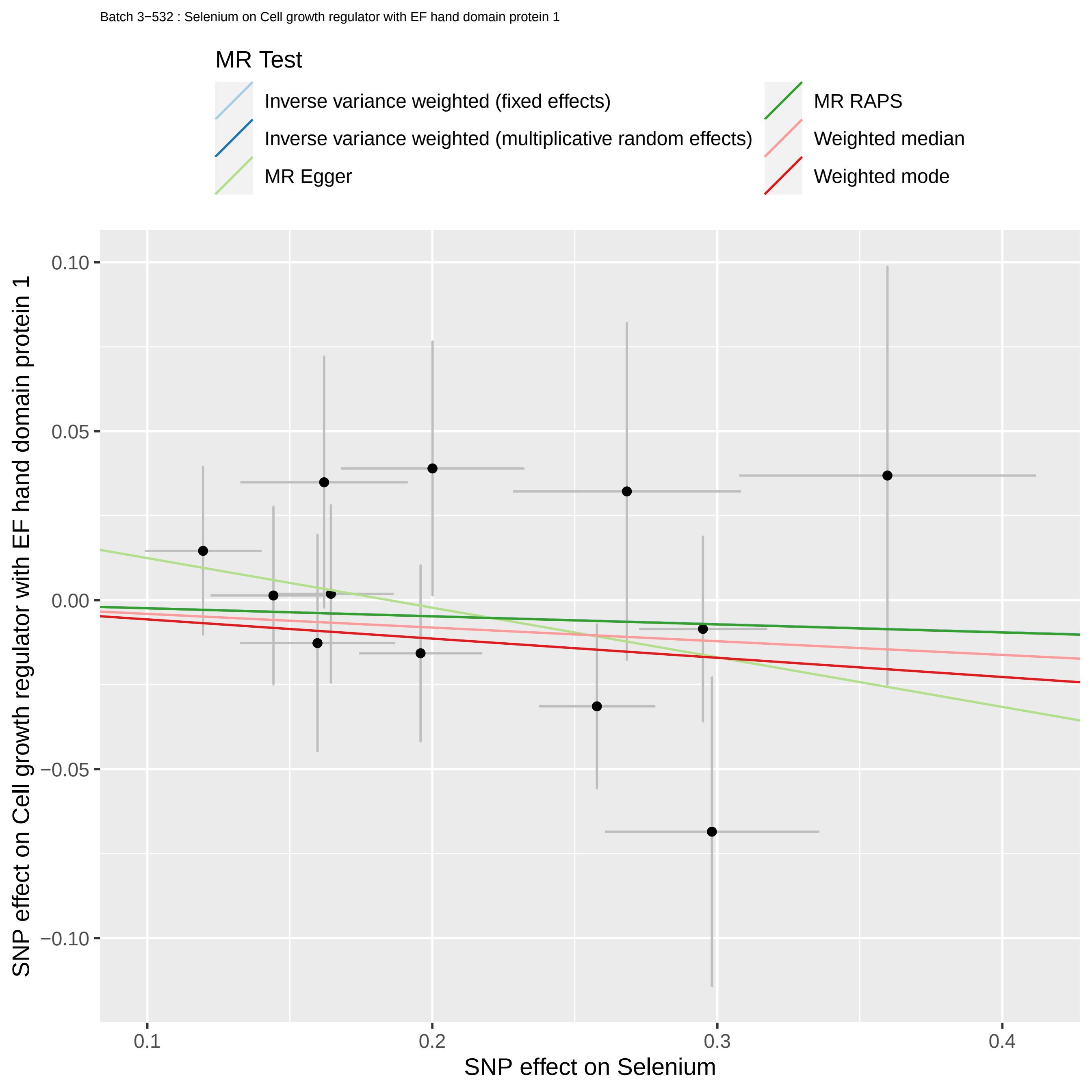

Batch 3−532 : Selenium on Cell growth regulator with EF hand domain protein 1
MR Test
Inverse variance weighted (fixed effects)
Inverse variance weighted (multiplicative random effects)
MR Egger
MR RAPS
Weighted median
Weighted mode
0.10
0.05
0.00
−0.05
−0.10
0.1
0.2
0.3
0.4
SNP effect on Cell growth regulator with EF hand domain protein 1
SNP effect on Selenium

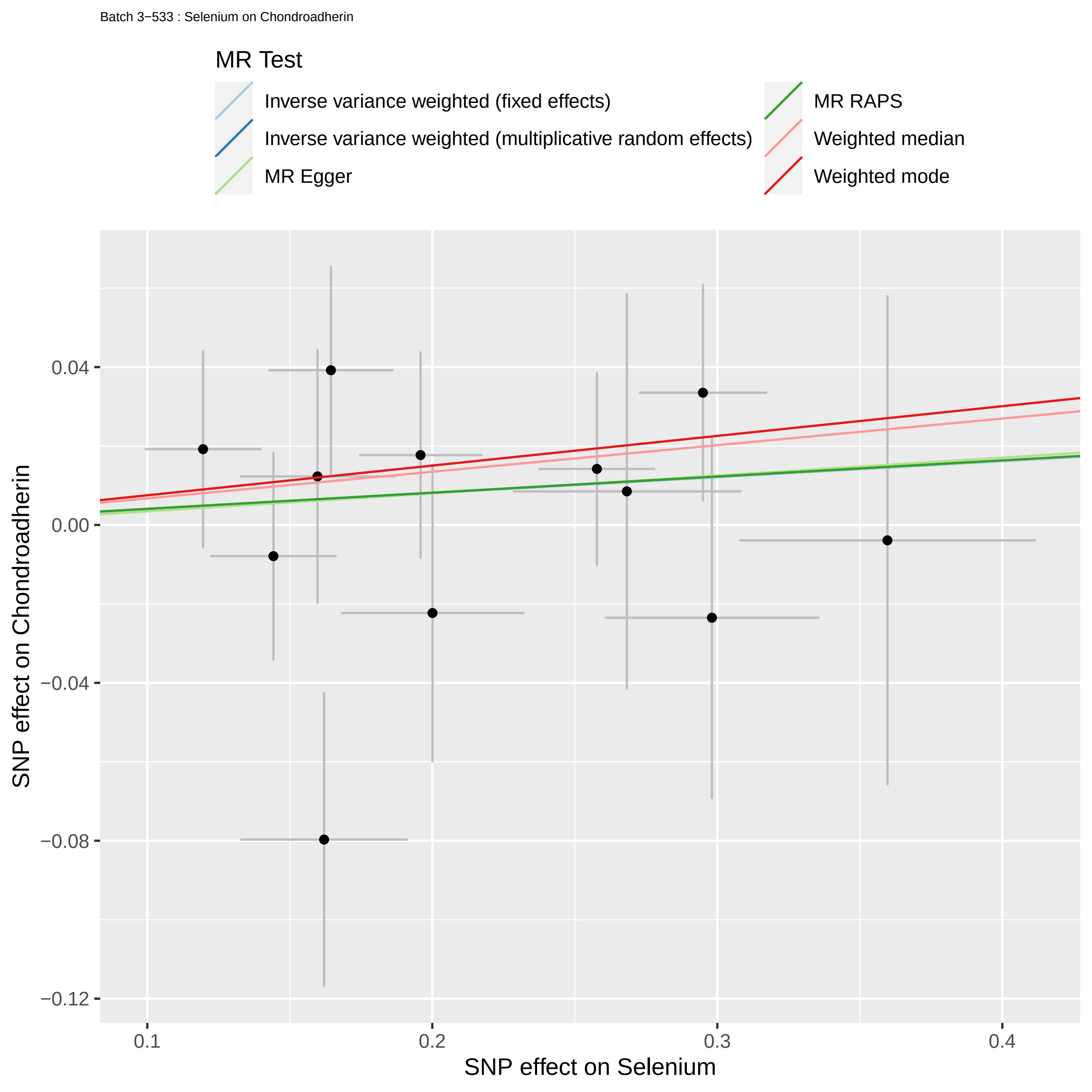

Batch 3−533 : Selenium on Chondroadherin
MR Test
Inverse variance weighted (fixed effects)
Inverse variance weighted (multiplicative random effects)
MR Egger
MR RAPS
Weighted median
Weighted mode
0.04
0.00
−0.04
−0.08
−0.12
0.1
0.2
0.3
0.4
SNP effect on Chondroadherin
SNP effect on Selenium

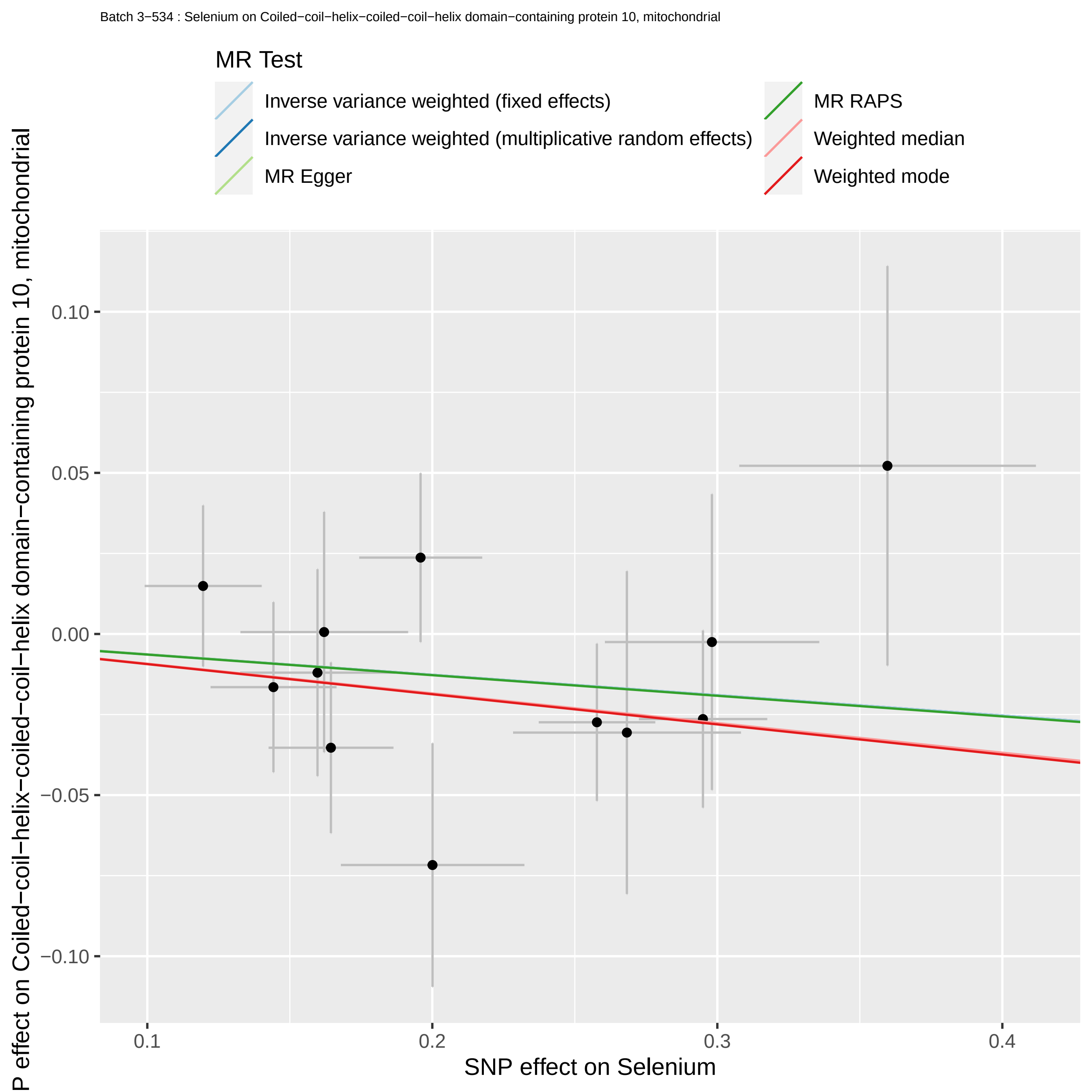

Batch 3−534 : Selenium on Coiled−coil−helix−coiled−coil−helix domain−containing protein 10, mitochondrial
MR Test
Inverse variance weighted (fixed effects)
Inverse variance weighted (multiplicative random effects)
MR Egger
MR RAPS
Weighted median
Weighted mode
0.10
0.05
0.00
−0.05
−0.10
0.1
0.2
0.3
0.4
SNP effect on Selenium
P effect on Coiled−coil−helix−coiled−coil−helix domain−containing protein 10, mitochondrial

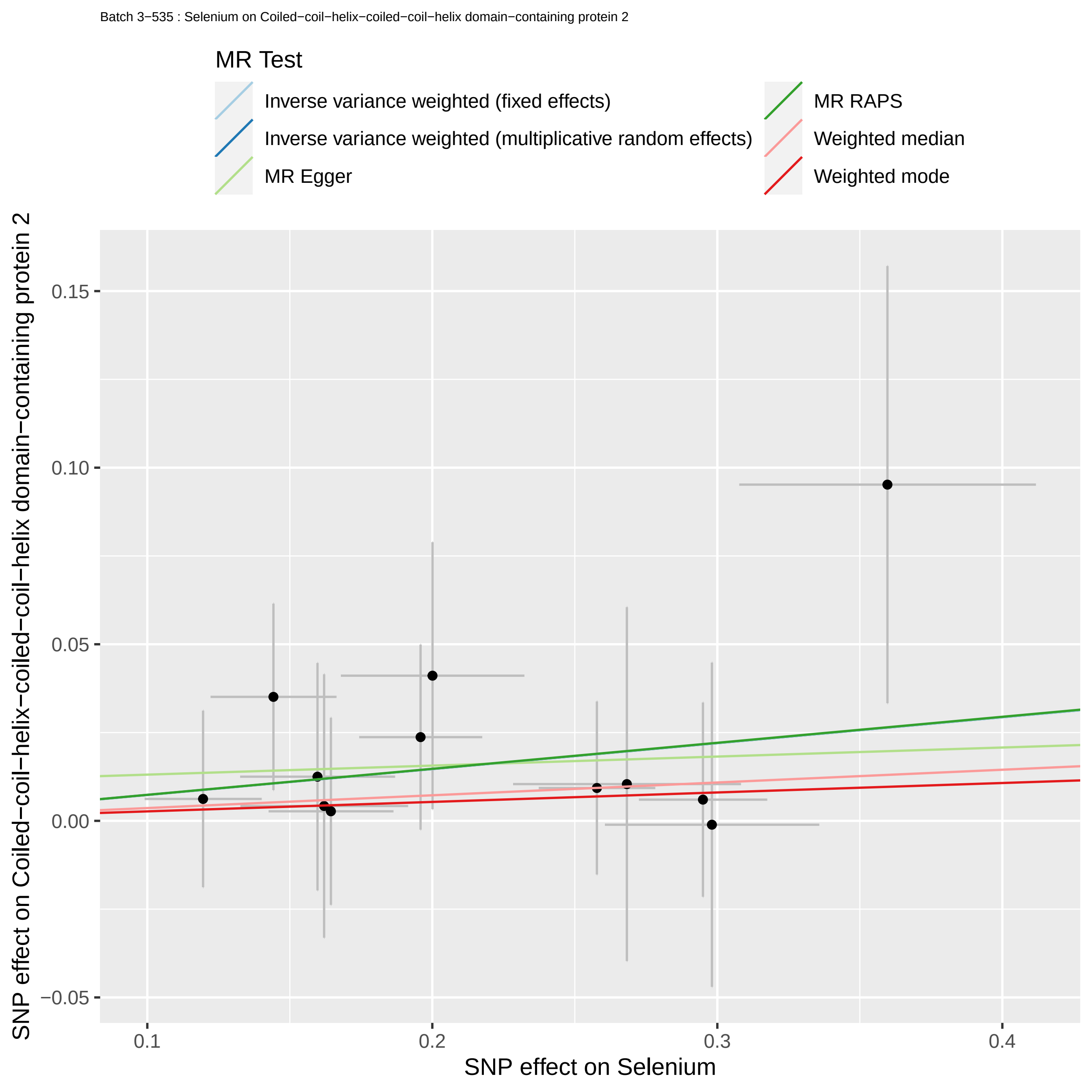

Batch 3−535 : Selenium on Coiled−coil−helix−coiled−coil−helix domain−containing protein 2
MR Test
Inverse variance weighted (fixed effects)
Inverse variance weighted (multiplicative random effects)
MR Egger
MR RAPS
Weighted median
Weighted mode
SNP effect on Coiled−coil−helix−coiled−coil−helix domain−containing protein 2
0.15
0.10
0.05
0.00
−0.05
0.1
0.2
0.3
0.4
SNP effect on Selenium

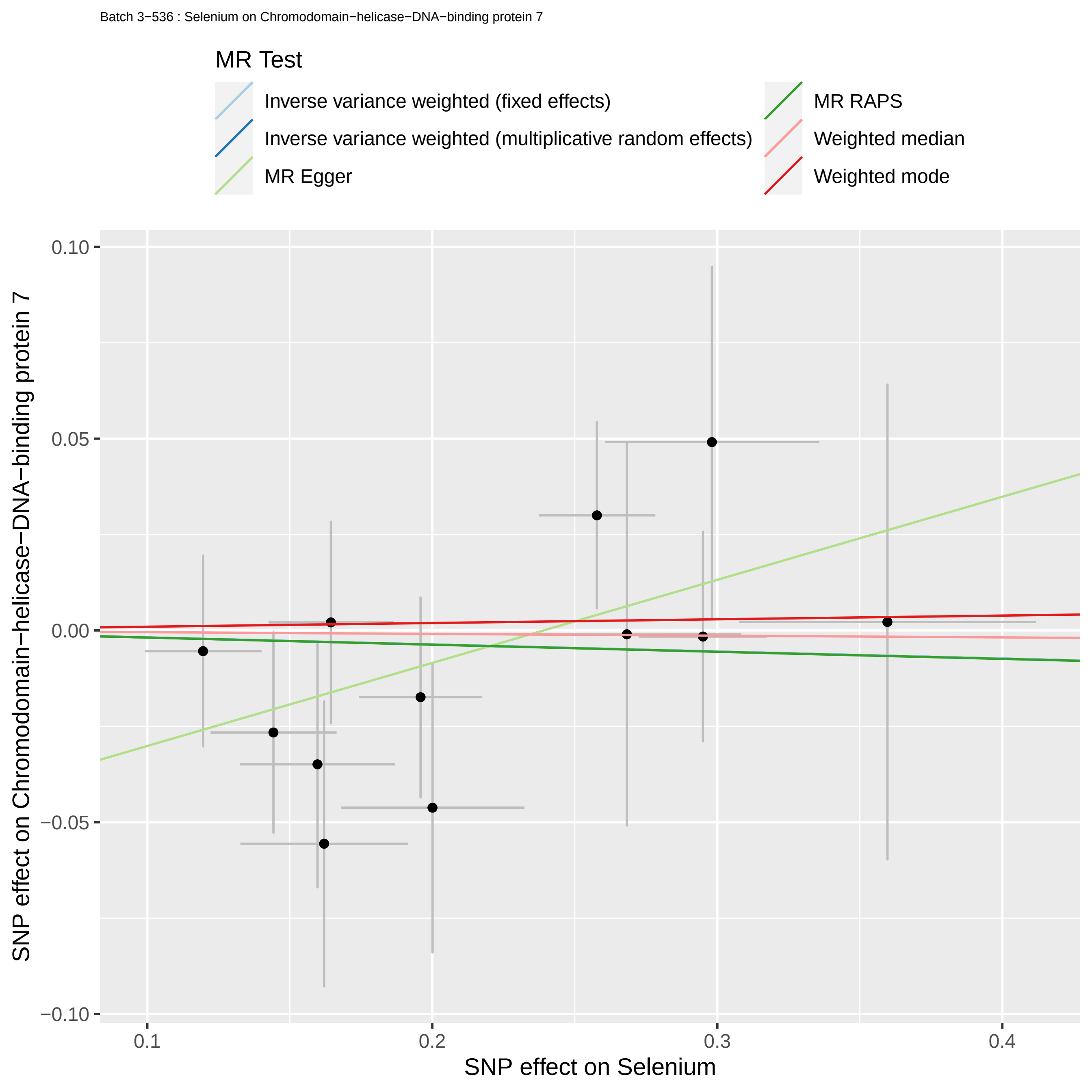
Batch 3−536 : Selenium on Chromodomain−helicase−DNA−binding protein 7
MR Test
Inverse variance weighted (fixed effects)
Inverse variance weighted (multiplicative random effects)
MR Egger
MR RAPS
Weighted median
Weighted mode
SNP effect on Chromodomain−helicase−DNA−binding protein 7
0.10
0.05
0.00
−0.05
−0.10
0.1
0.2
0.3
0.4
SNP effect on Selenium

Batch 3−537 : Selenium on Serine/threonine−protein kinase Chk1

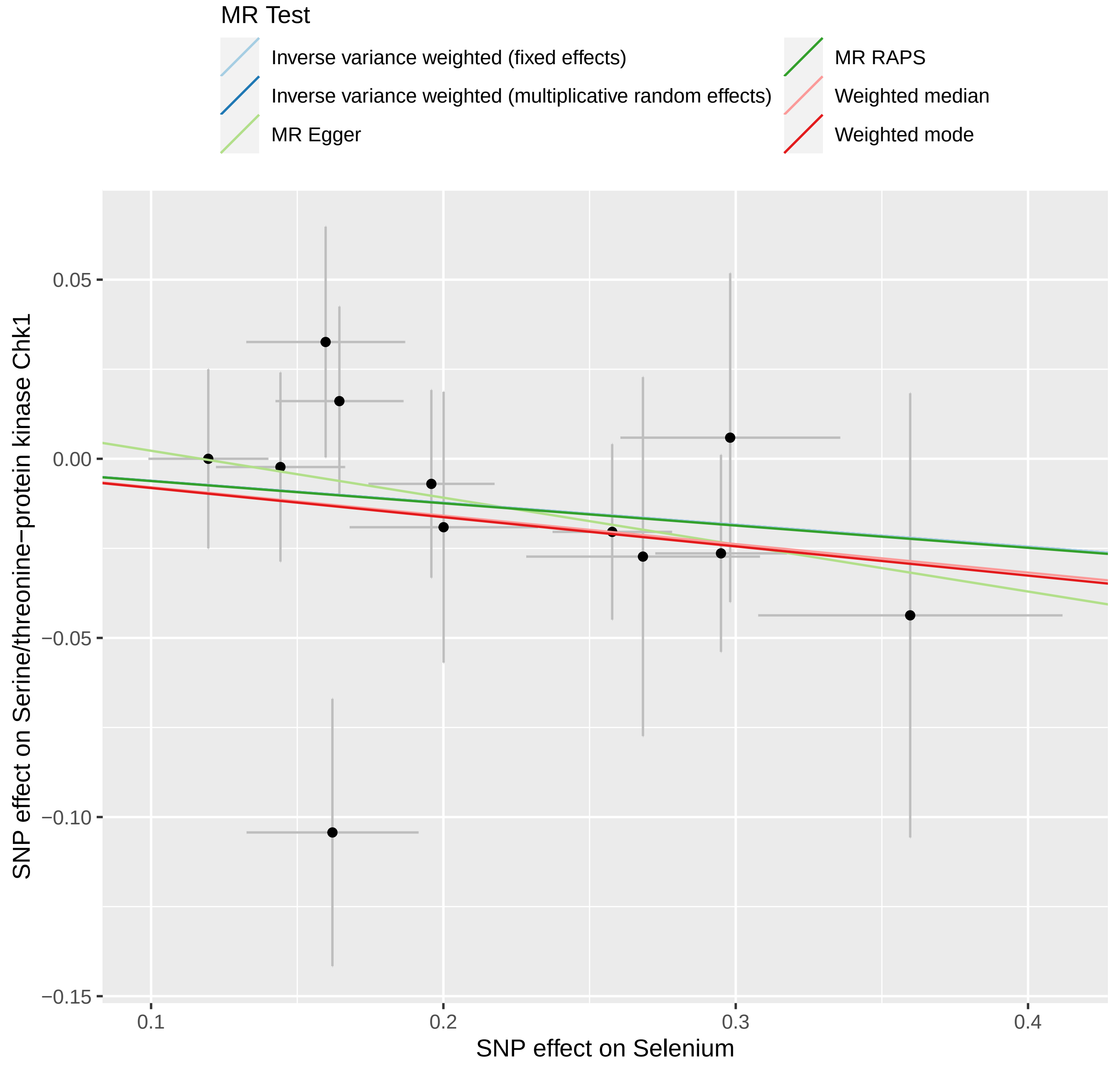

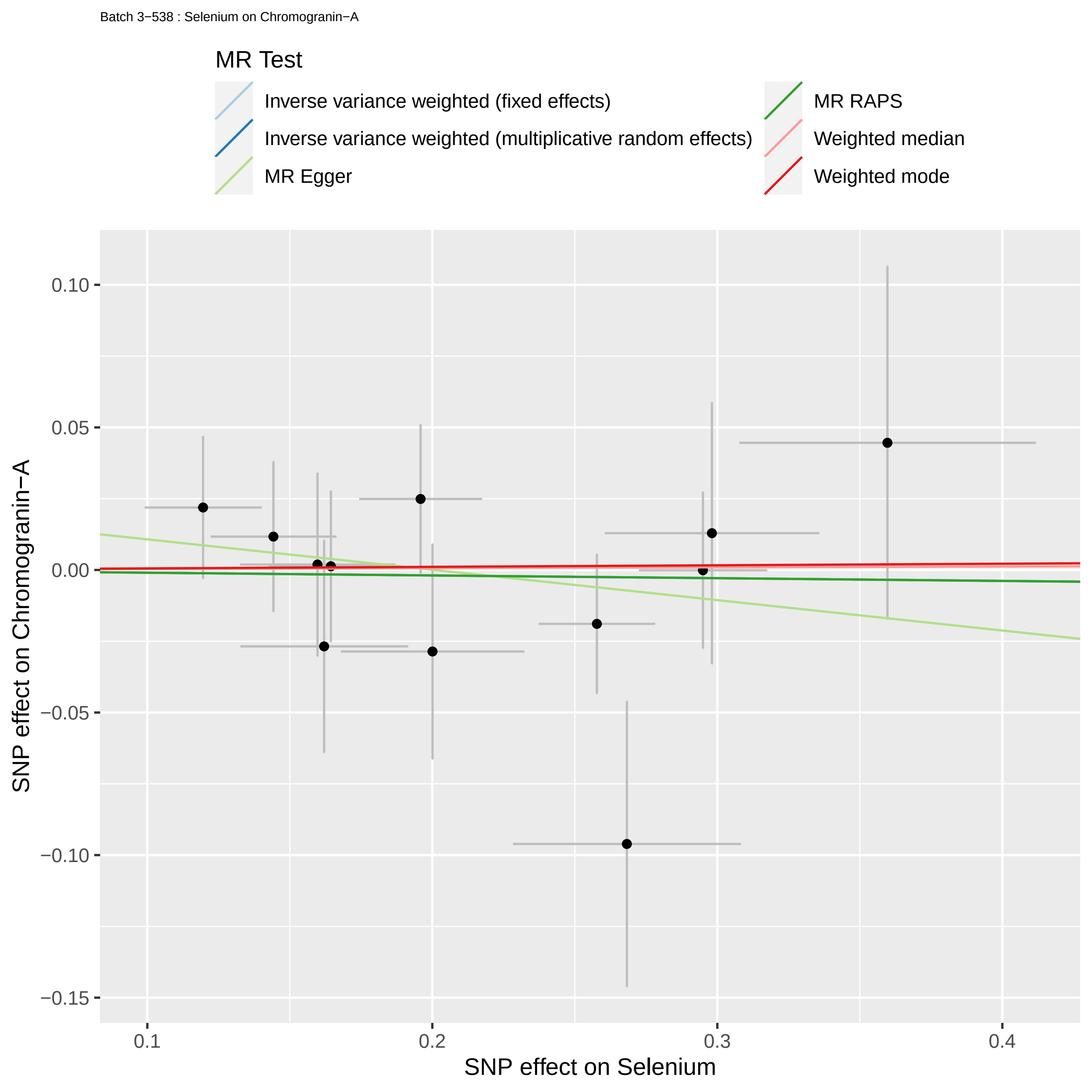

Batch 3−538 : Selenium on Chromogranin−A
MR Test
Inverse variance weighted (fixed effects)
Inverse variance weighted (multiplicative random effects)
MR Egger
MR RAPS
Weighted median
Weighted mode
0.10
0.05
0.00
−0.05
−0.10
−0.15
SNP effect on Chromogranin−A
0.1
0.2
0.3
0.4
SNP effect on Selenium

Batch 3−539 : Selenium on Secretogranin−1

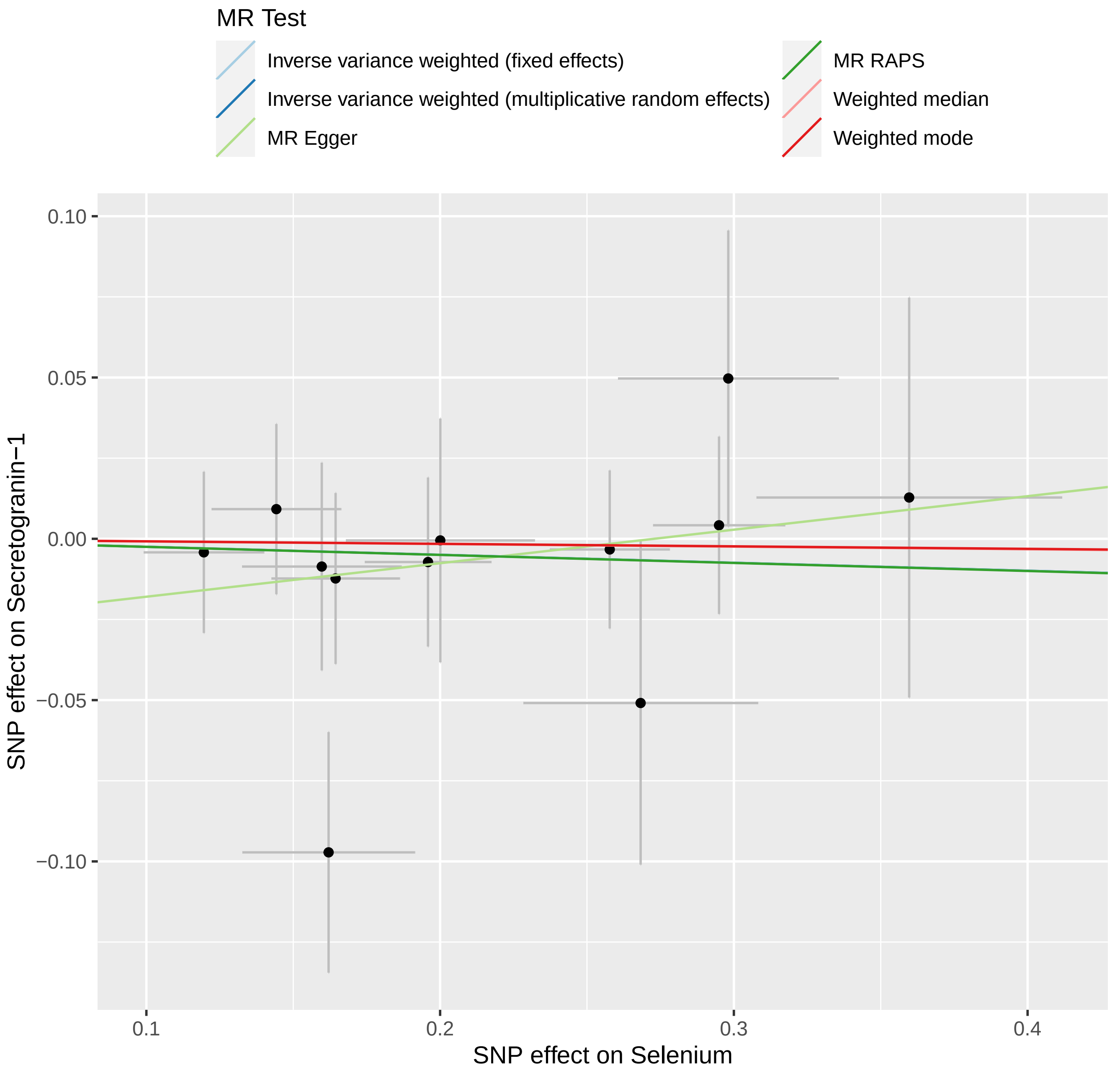

Batch 3−540 : Selenium on Chitinase−3−like protein 1

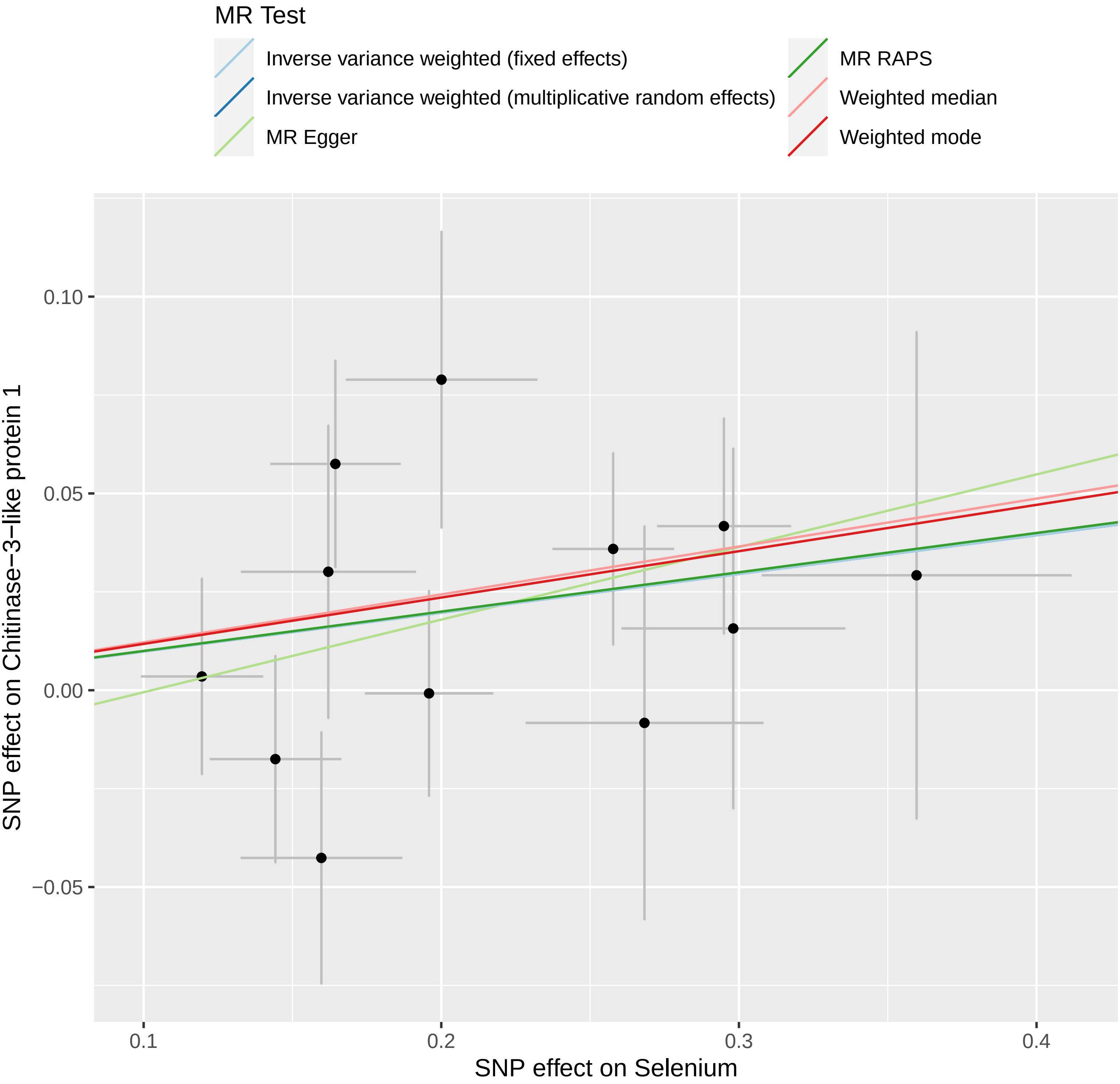

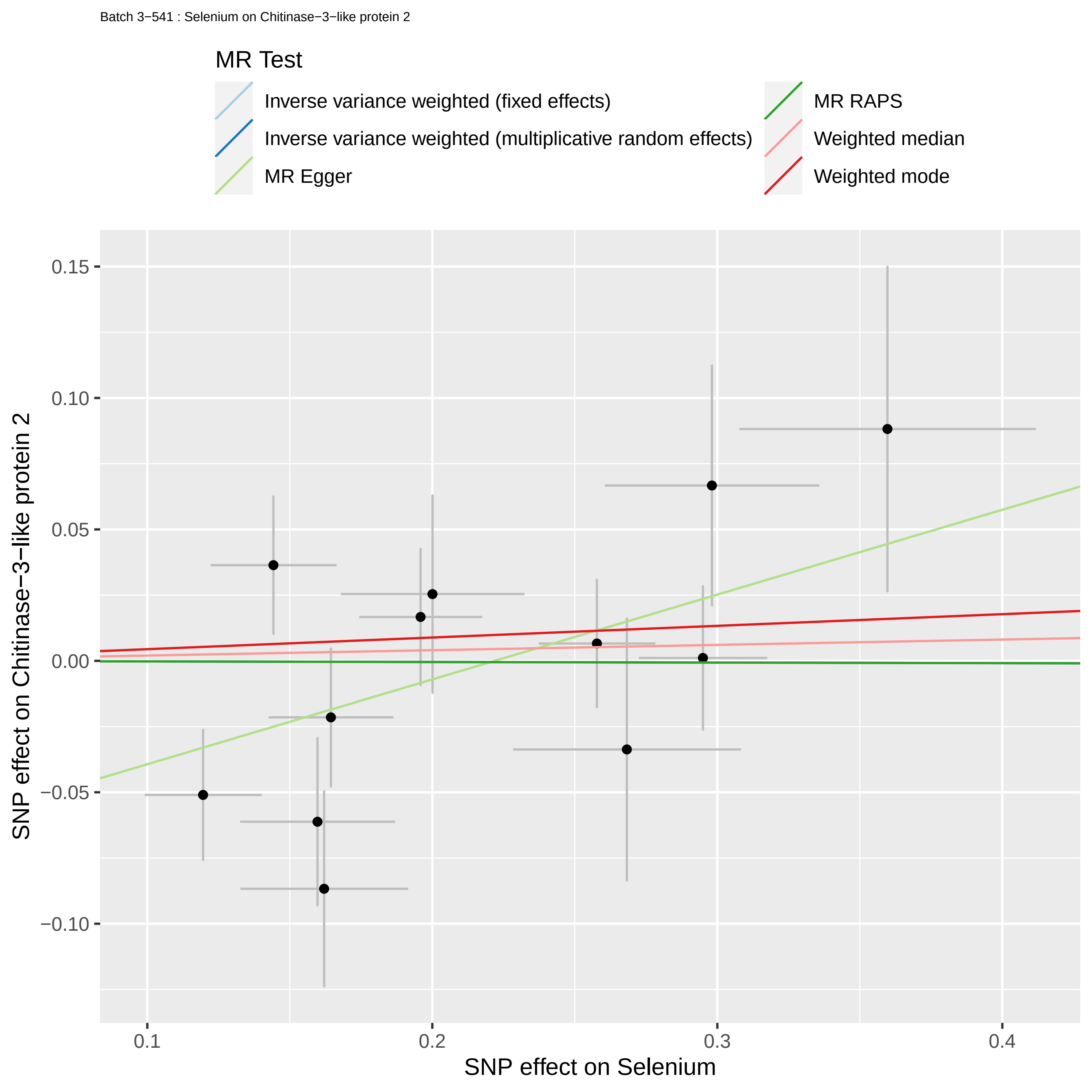

Batch 3−541 : Selenium on Chitinase−3−like protein 2
MR Test
Inverse variance weighted (fixed effects)
Inverse variance weighted (multiplicative random effects)
MR Egger
MR RAPS
Weighted median
Weighted mode
0.15
0.10
0.05
0.00
−0.05
−0.10
0.1
0.2
0.3
0.4
SNP effect on Chitinase−3−like protein 2
SNP effect on Selenium

Batch 3−542 : Selenium on Cysteine−rich hydrophobic domain−containing protein 2

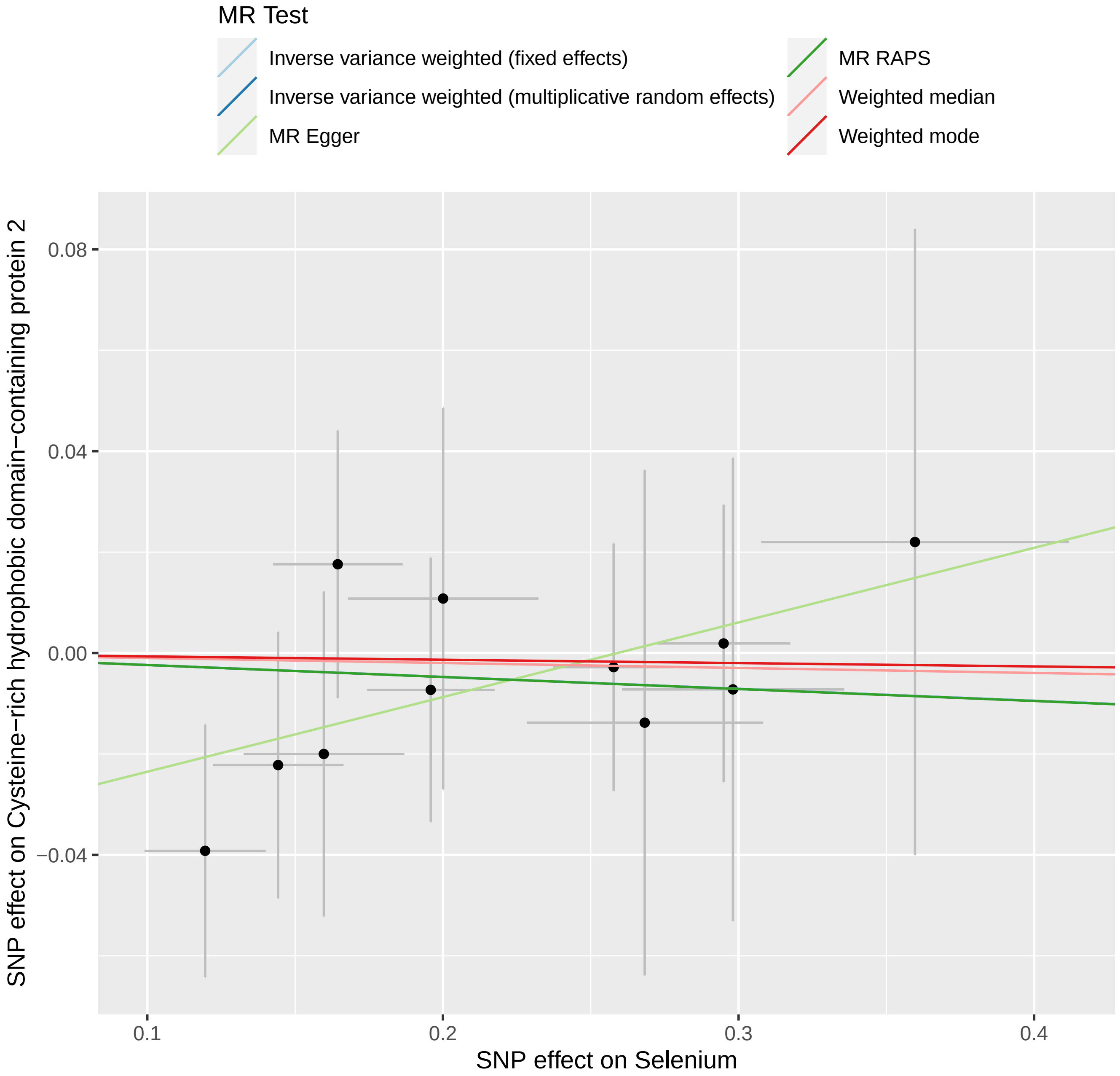

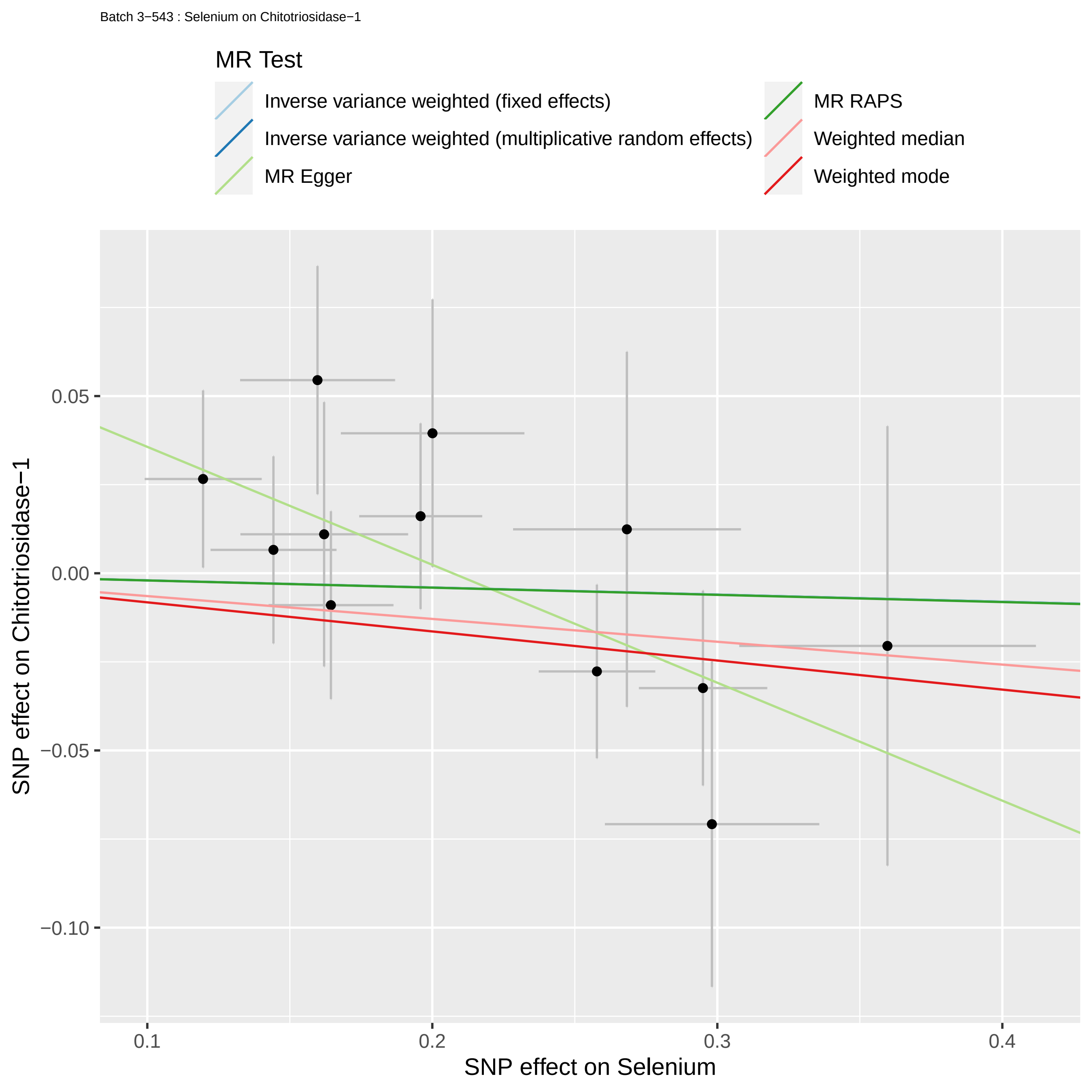

Batch 3−543 : Selenium on Chitotriosidase−1
MR Test
Inverse variance weighted (fixed effects)
Inverse variance weighted (multiplicative random effects)
MR Egger
MR RAPS
Weighted median
Weighted mode
0.05
0.00
−0.05
−0.10
0.1
0.2
0.3
0.4
SNP effect on Chitotriosidase−1
SNP effect on Selenium

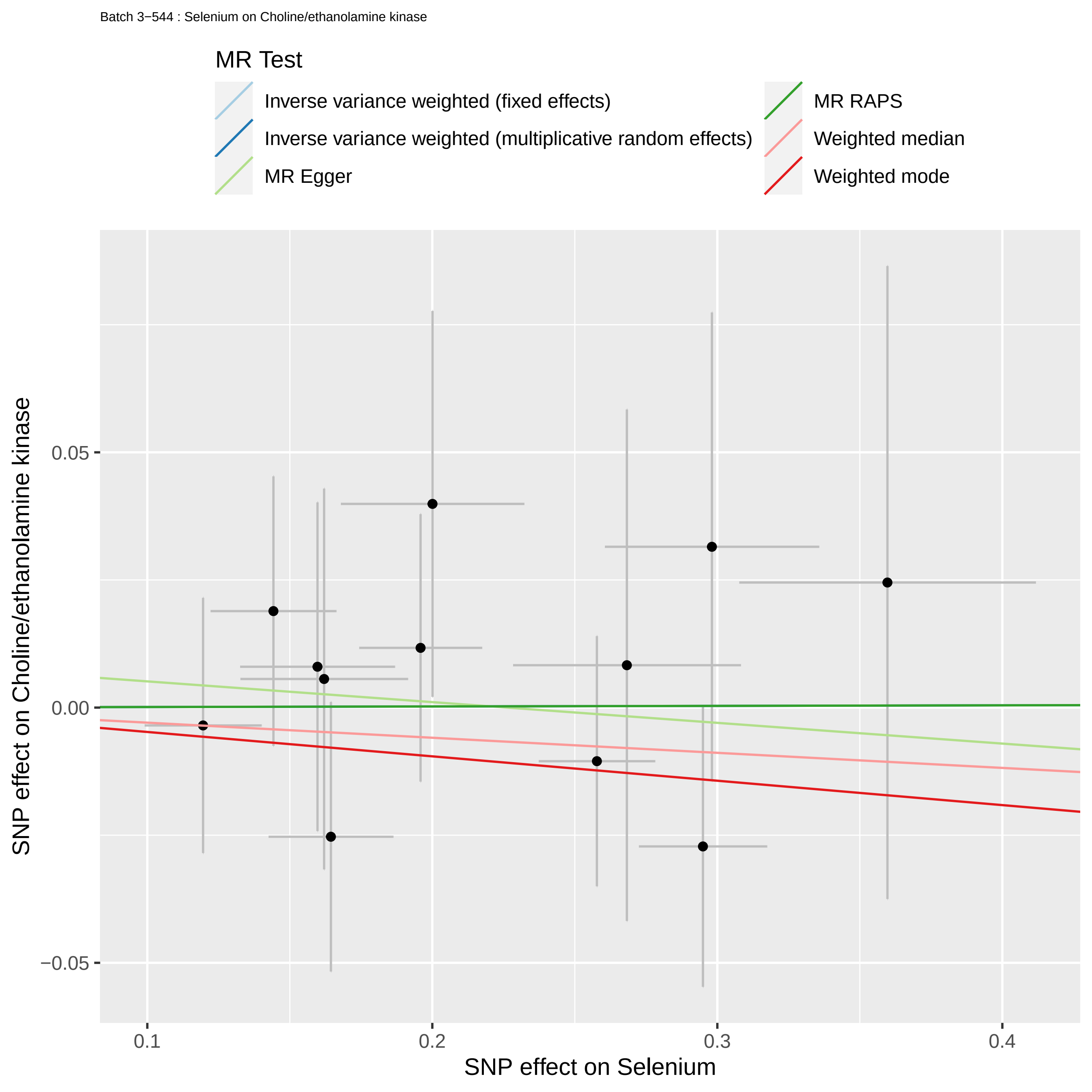
Batch 3−544 : Selenium on Choline/ethanolamine kinase
MR Test
Inverse variance weighted (fixed effects)
Inverse variance weighted (multiplicative random effects)
MR Egger
MR RAPS
Weighted median
Weighted mode
SNP effect on Choline/ethanolamine kinase
0.05
0.00
−0.05
0.1
0.2
0.3
0.4
SNP effect on Selenium

Batch 3−545 : Selenium on Neural cell adhesion molecule L1−like protein

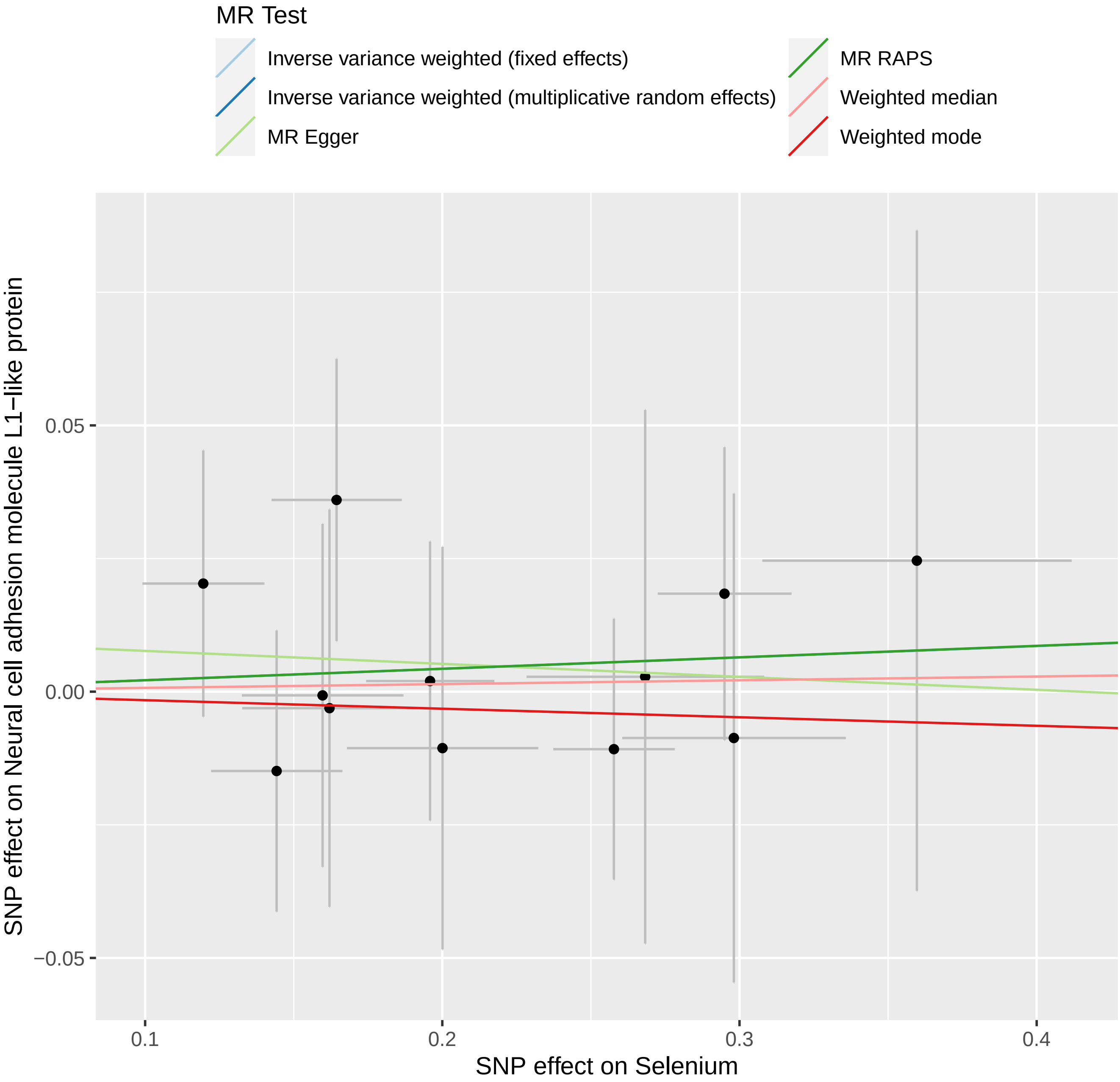

Batch 3−546 : Selenium on Calcineurin B homologous protein 1

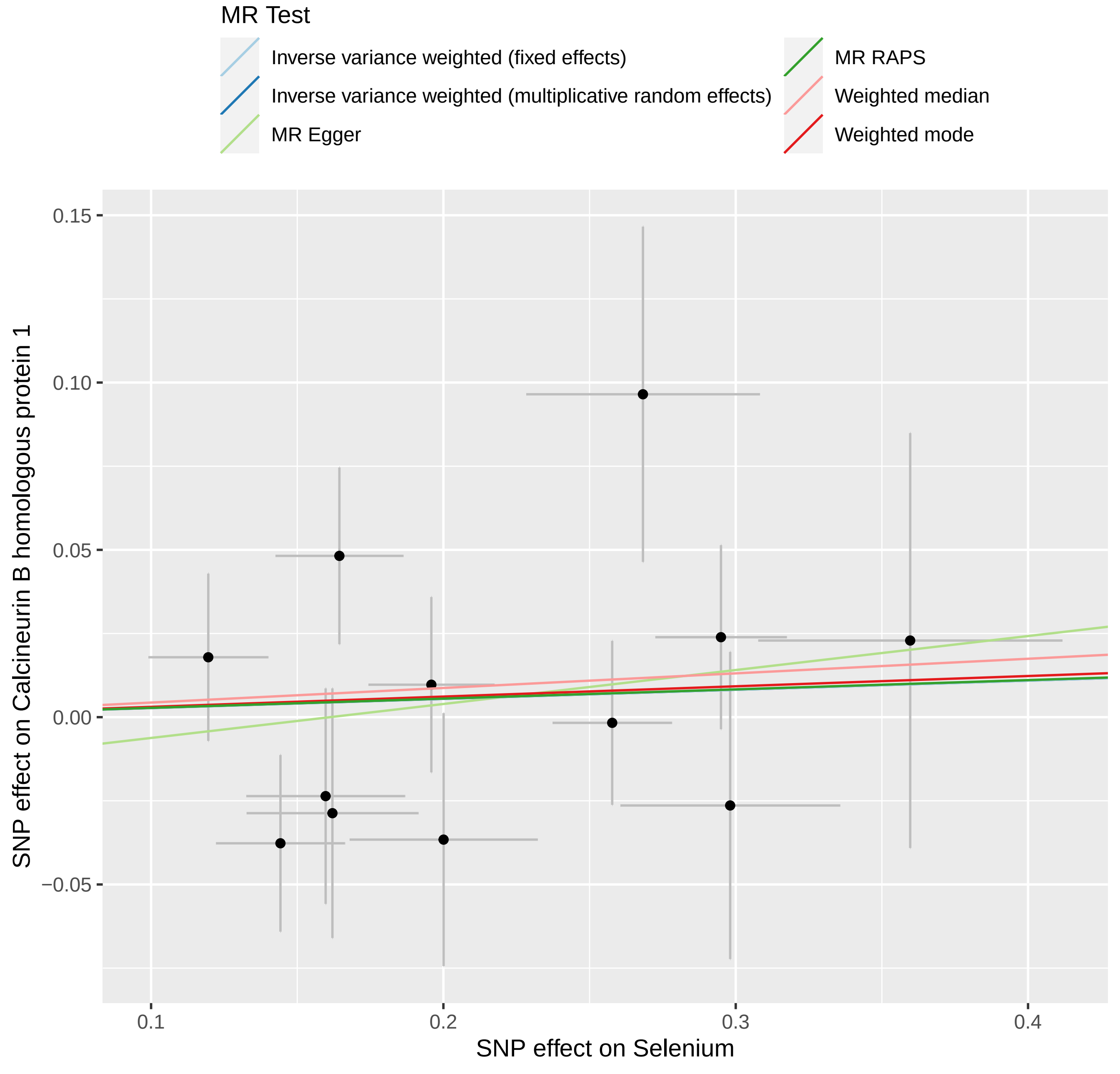

Batch 3−547 : Selenium on Chordin

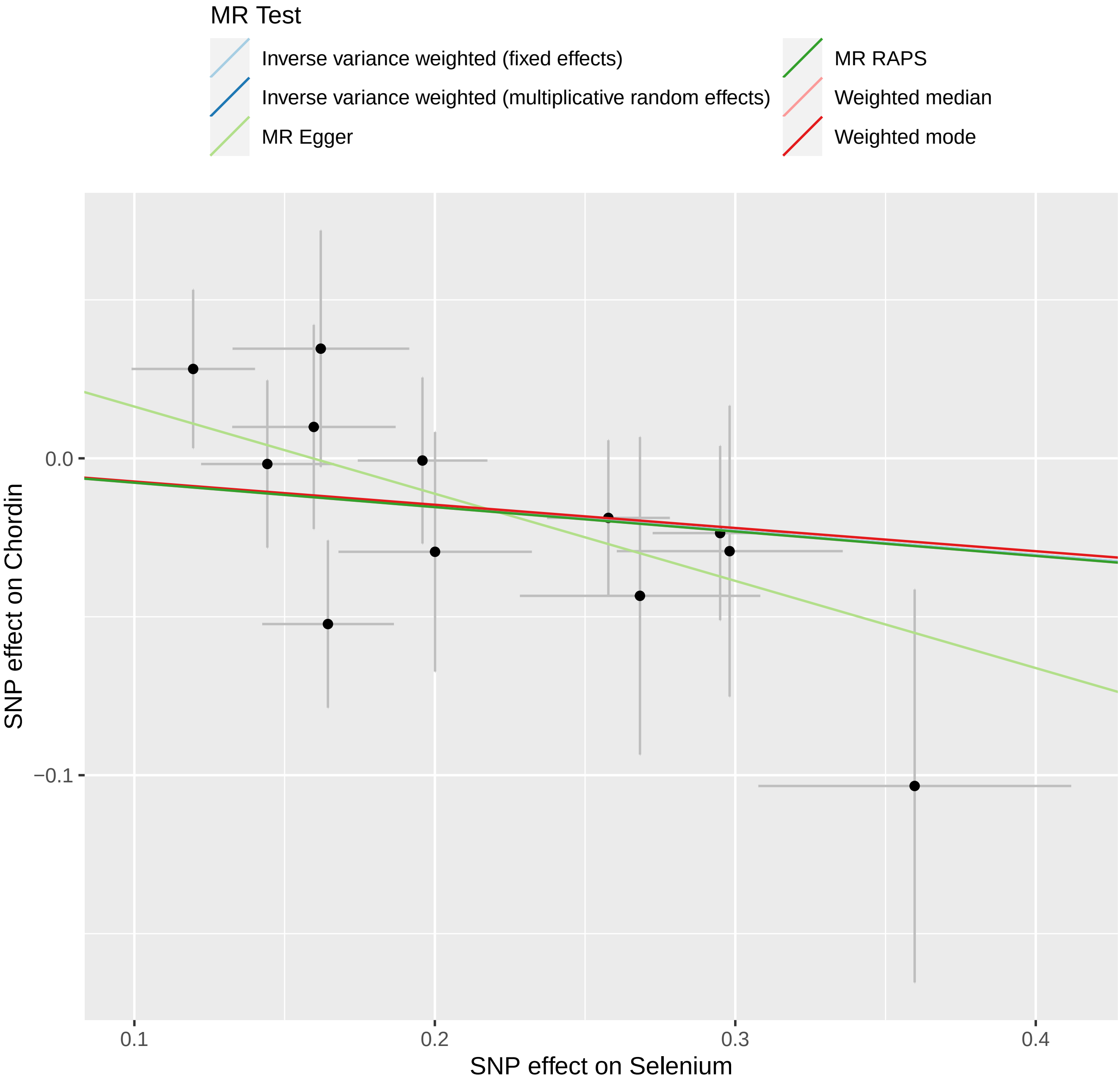

Batch 3−548 : Selenium on Chordin−like protein 1

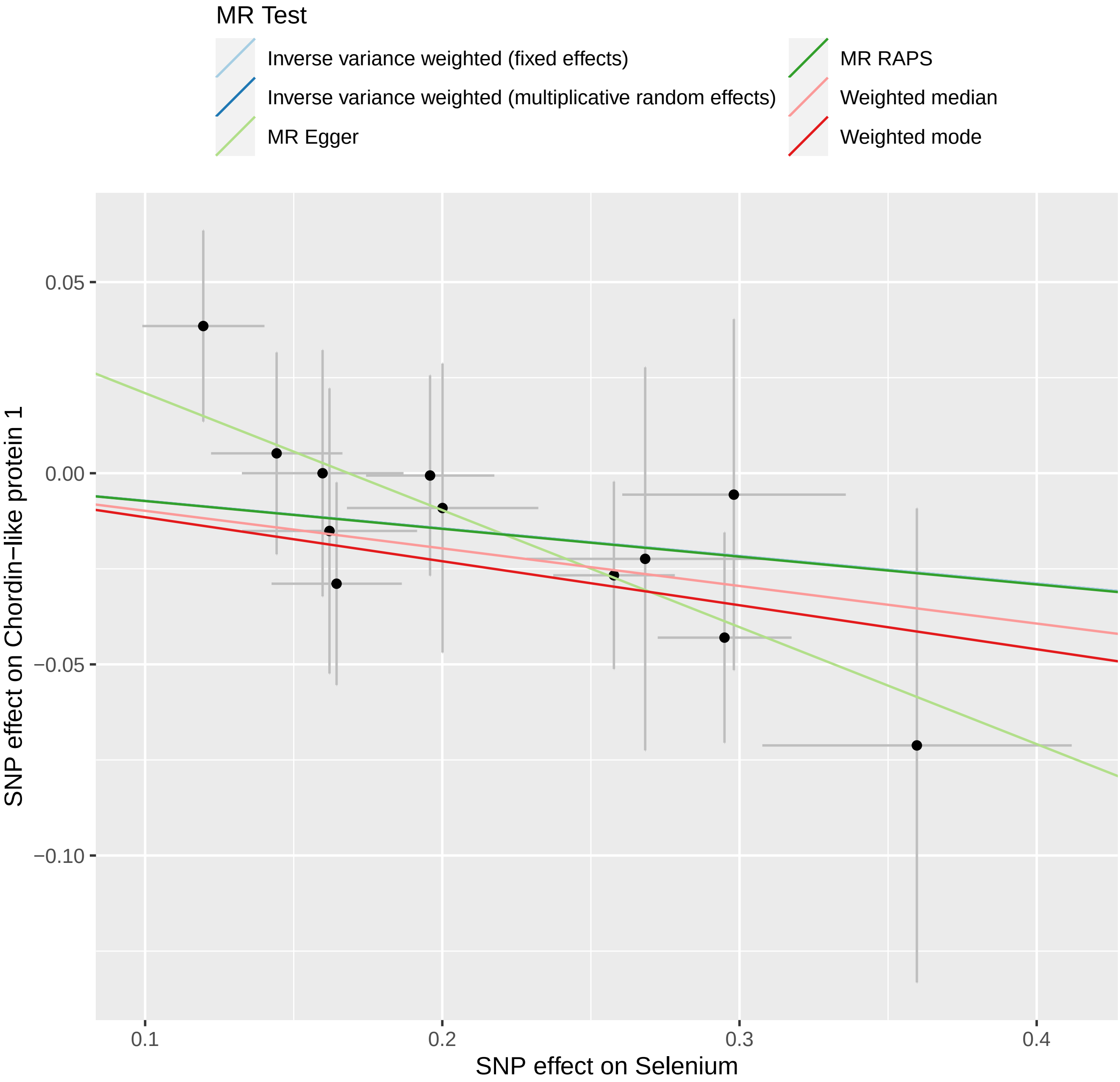

Batch 3−549 : Selenium on Chordin−like protein 2

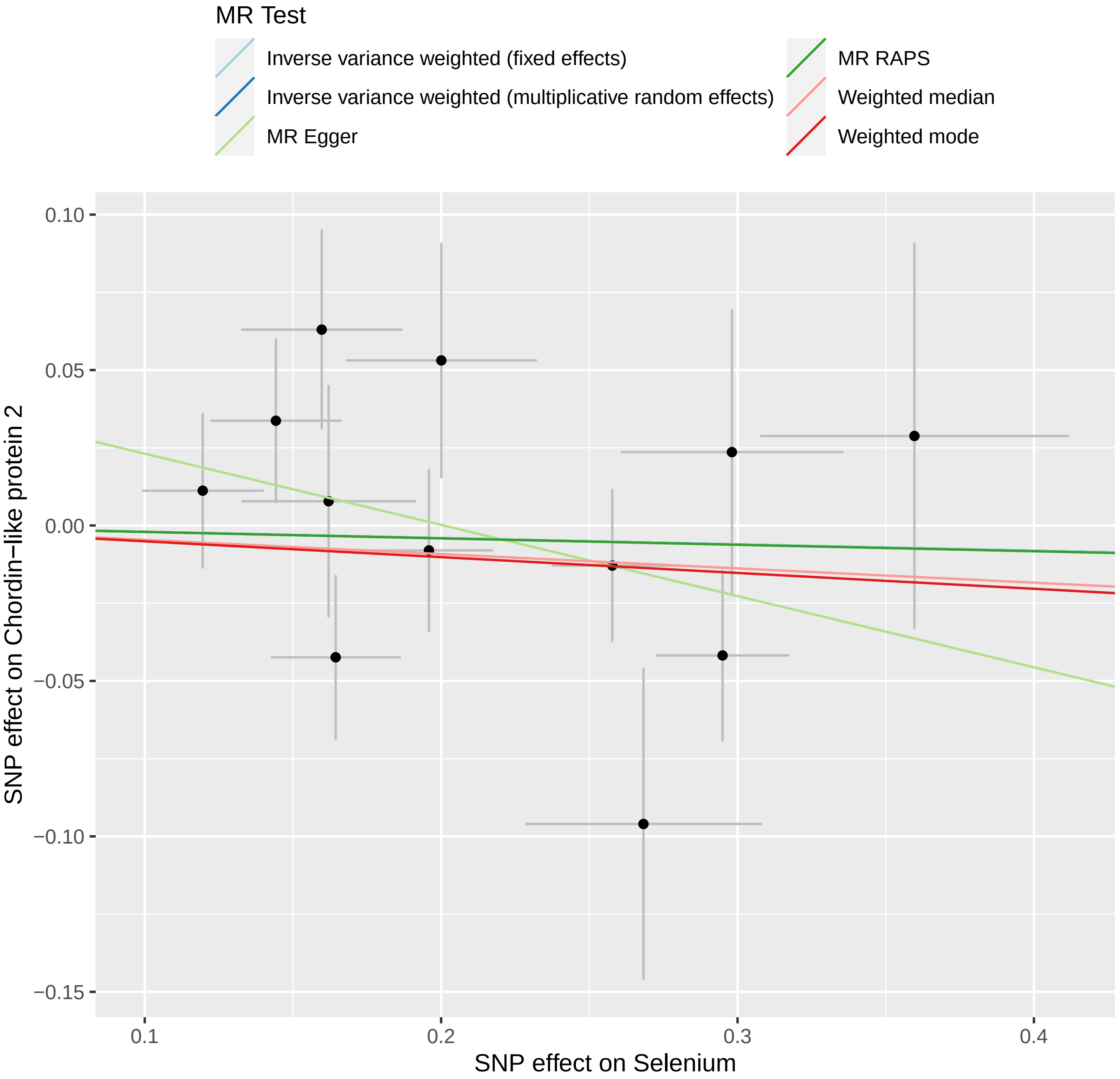

Batch 3−550 : Selenium on Carbohydrate sulfotransferase 11

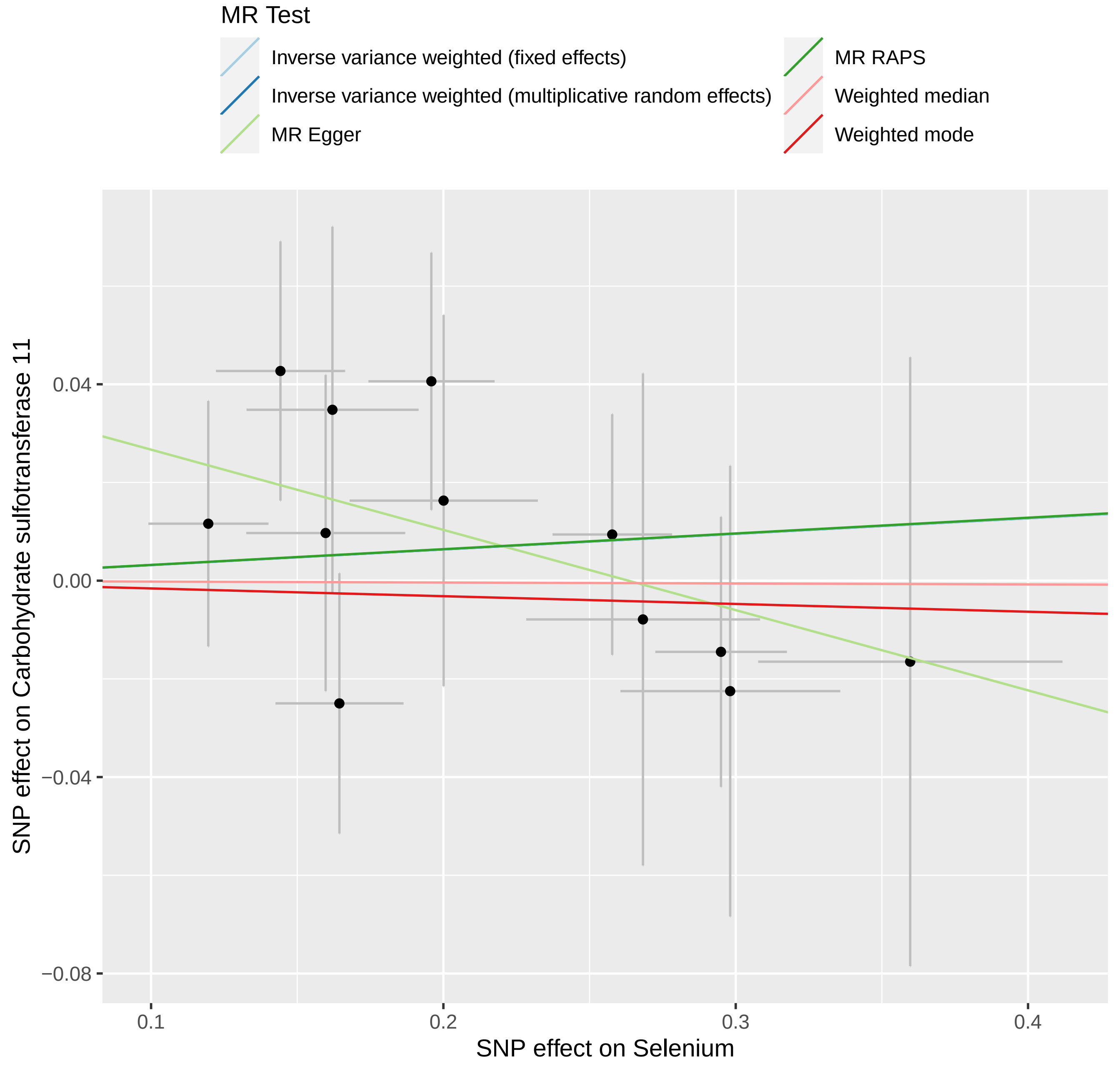

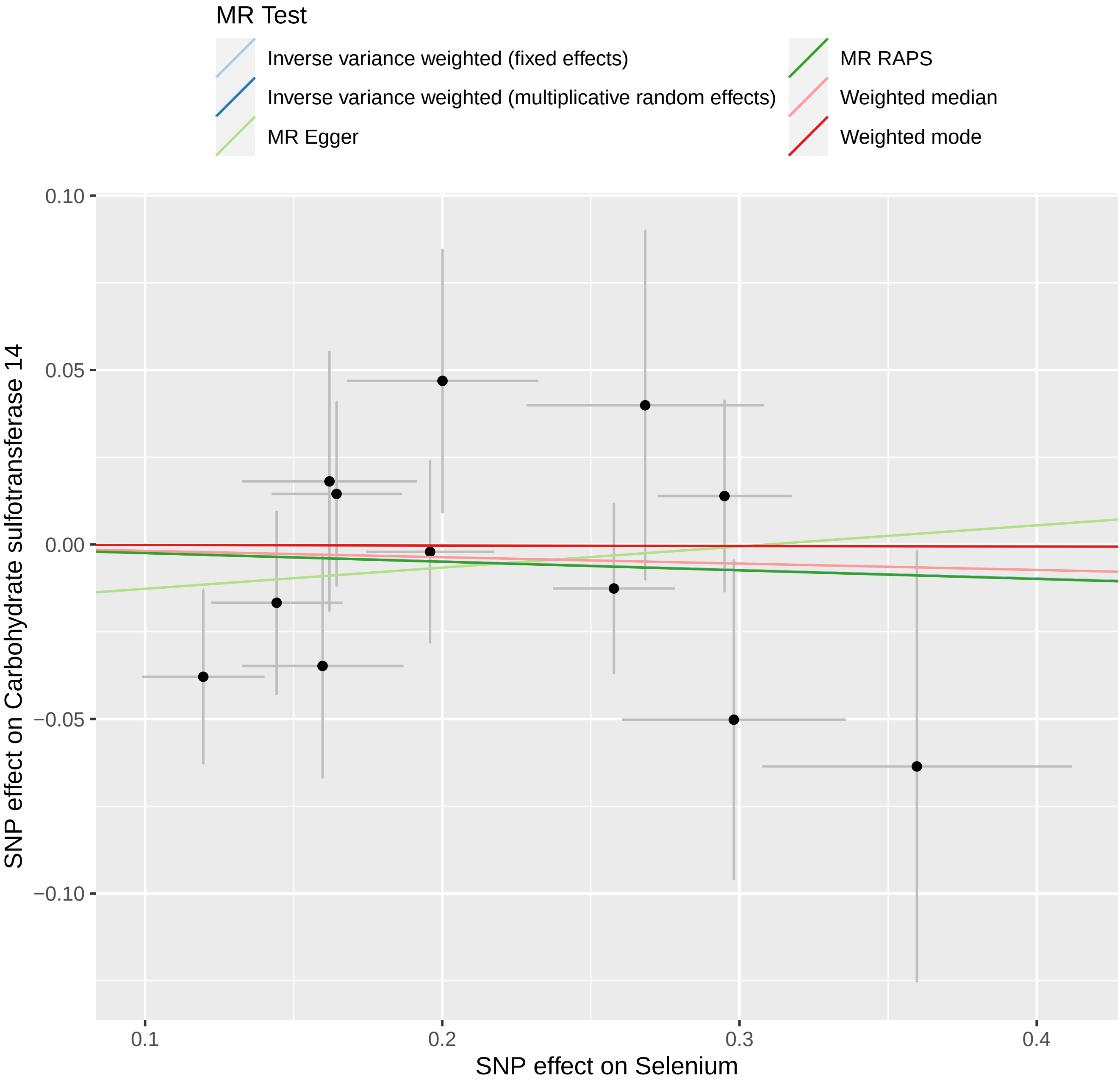
MR Test
Inverse variance weighted (fixed effects)
Inverse variance weighted (multiplicative random effects)
MR Egger
MR RAPS
Weighted median
Weighted mode
0.10
0.05
0.00
−0.05
−0.10
0.1
0.2
0.3
0.4
SNP effect on Selenium
SNP effect on Carbohydrate sulfotransferase 14

Batch 3−552 : Selenium on Carbohydrate sulfotransferase 15

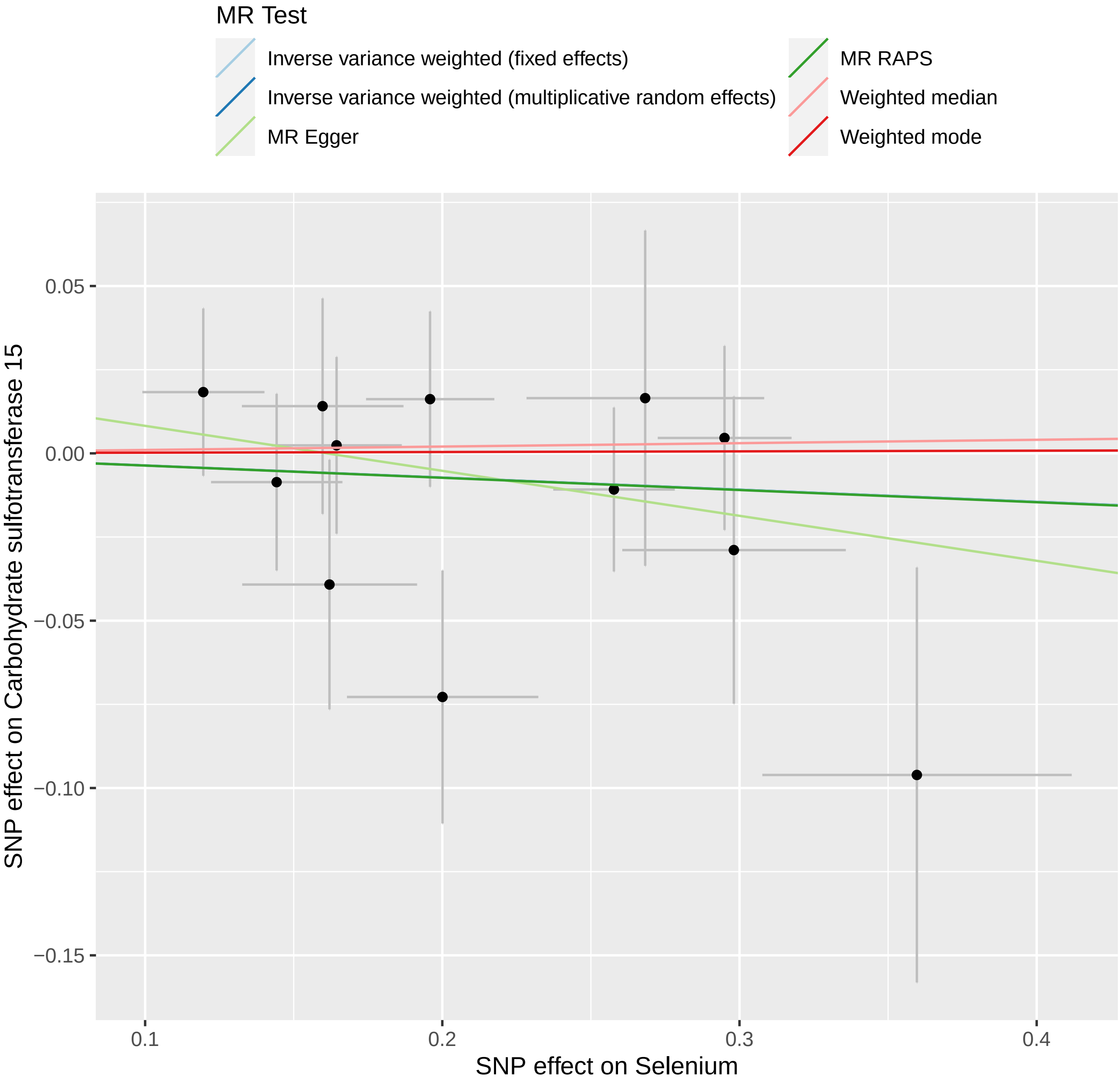

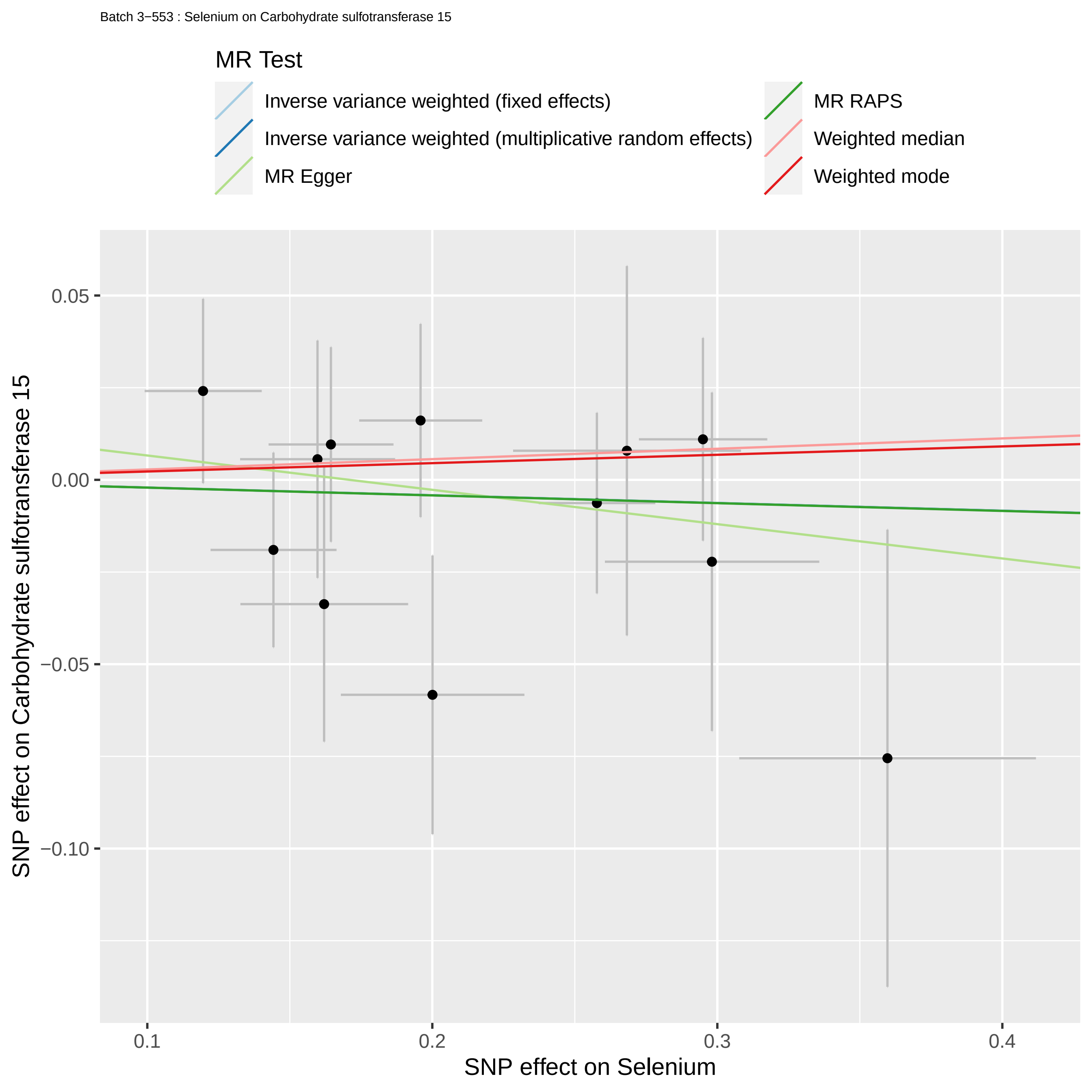
Batch 3−553 : Selenium on Carbohydrate sulfotransferase 15
MR Test
Inverse variance weighted (fixed effects)
Inverse variance weighted (multiplicative random effects)
MR Egger
MR RAPS
Weighted median
Weighted mode
0.05
0.00
−0.05
−0.10
0.1
0.2
0.3
0.4
SNP effect on Carbohydrate sulfotransferase 15
SNP effect on Selenium

Batch 3−554 : Selenium on Carbohydrate sulfotransferase 1

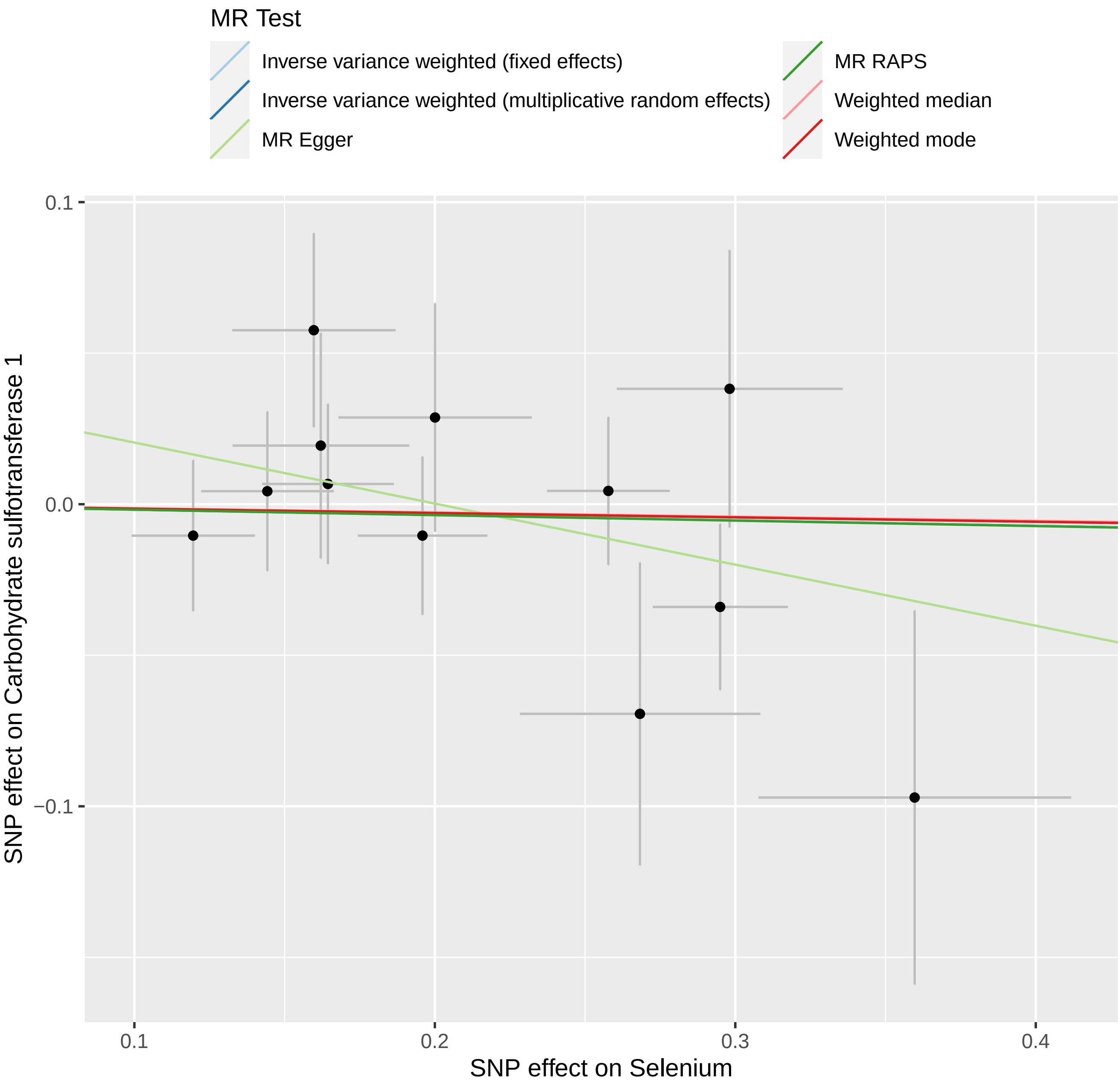

Batch 3−555 : Selenium on Carbohydrate sulfotransferase 2

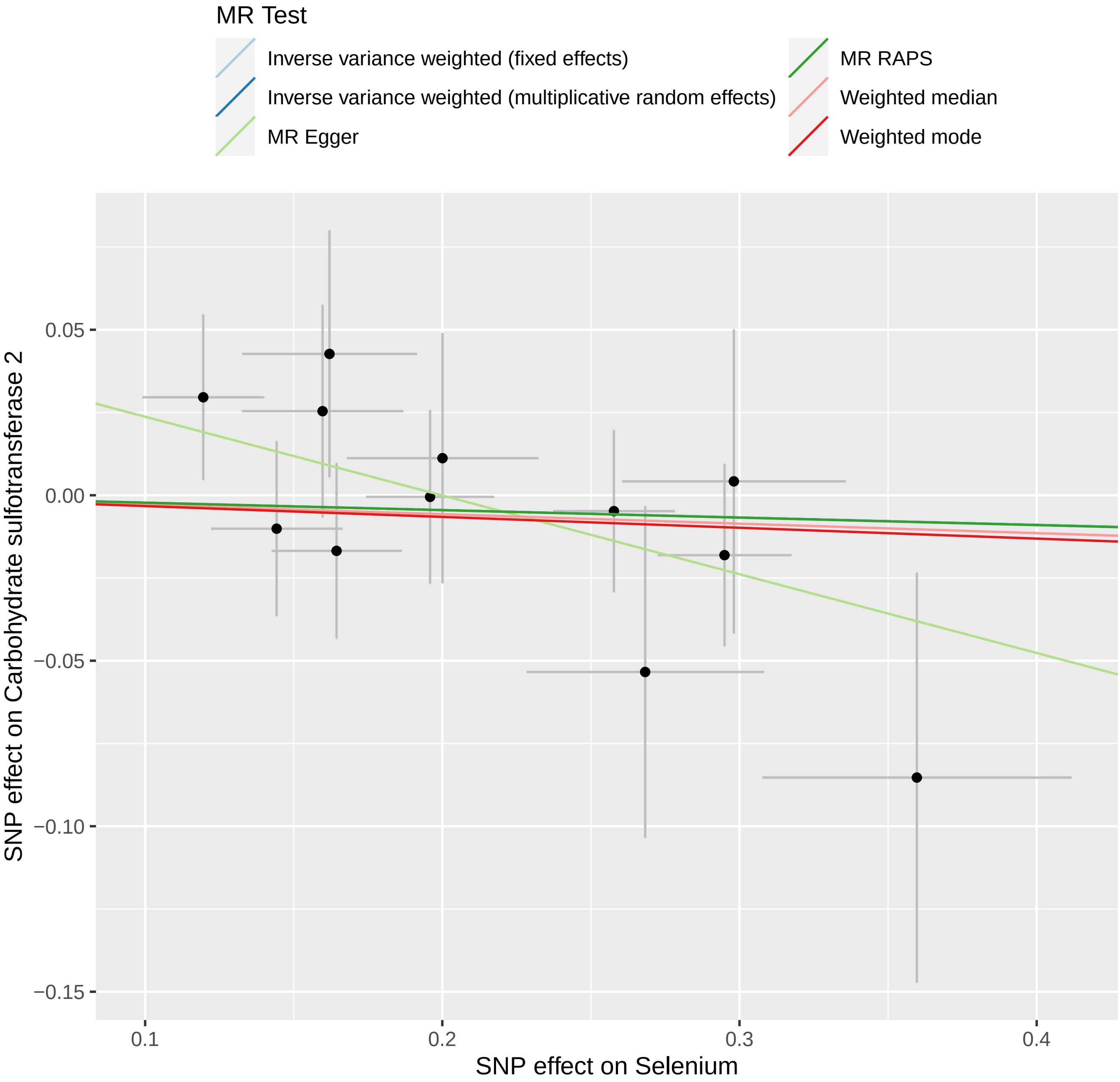

Batch 3−556 : Selenium on Carbohydrate sulfotransferase 3

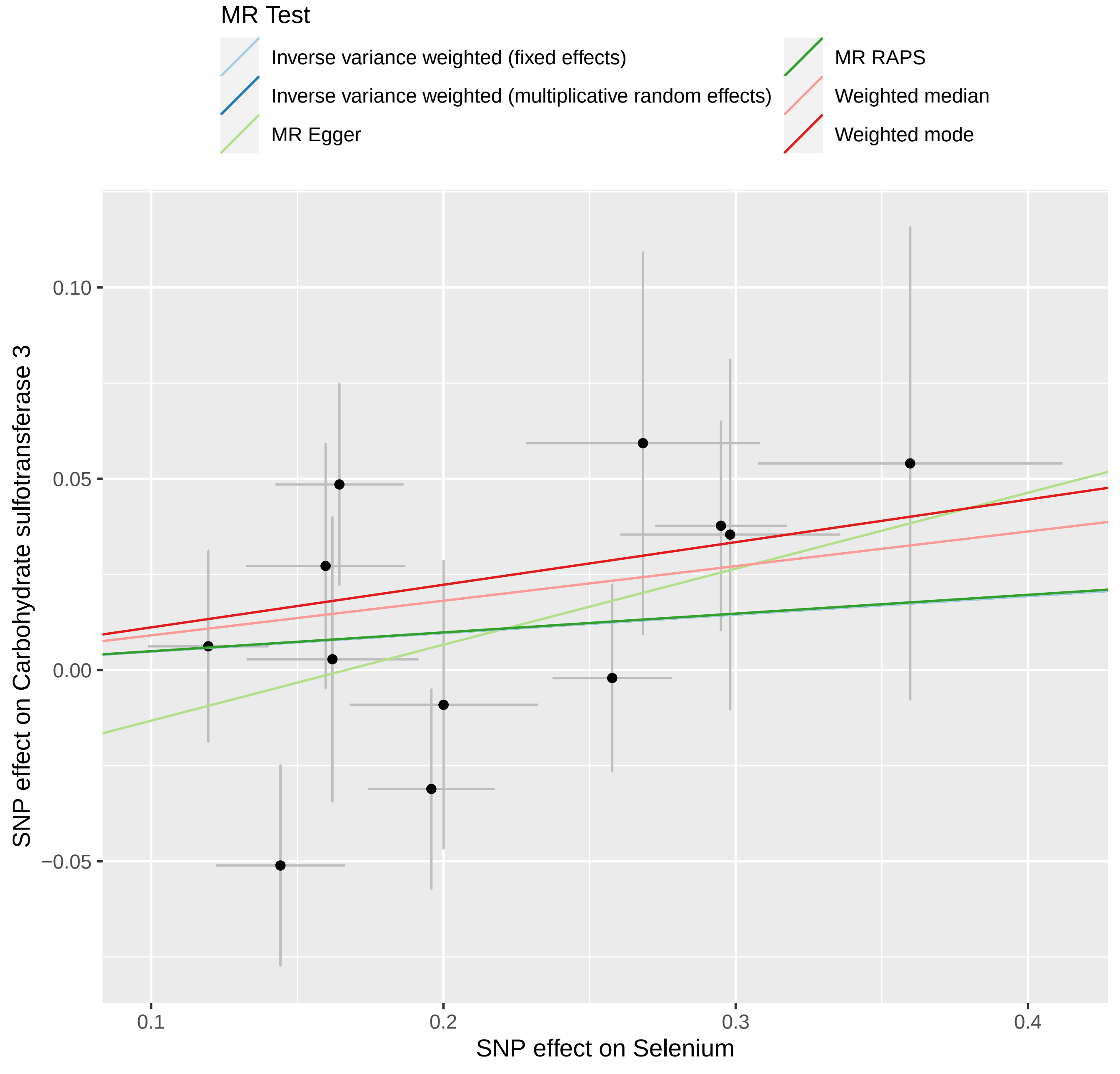

Batch 3−557 : Selenium on Carbohydrate sulfotransferase 5

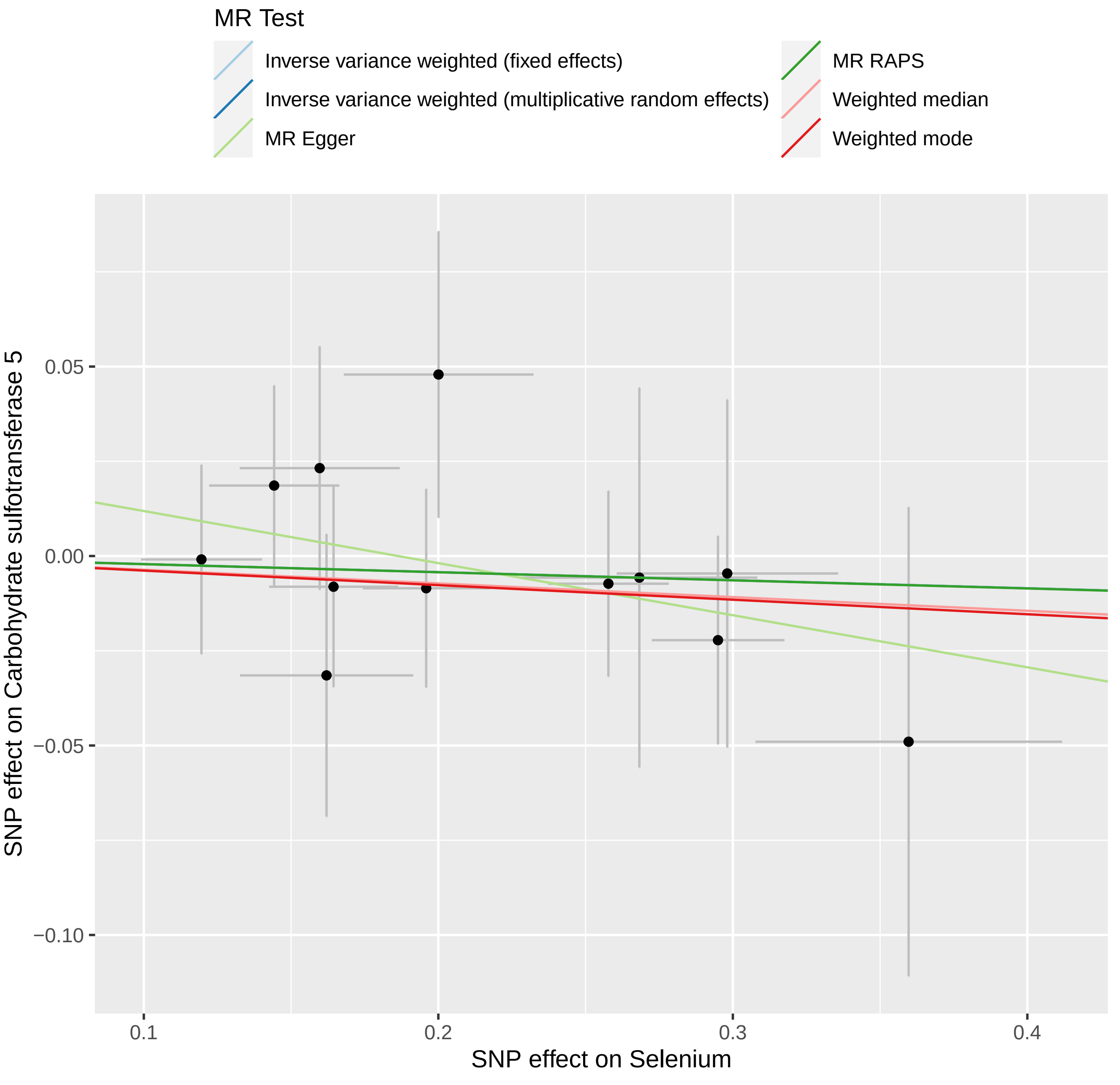

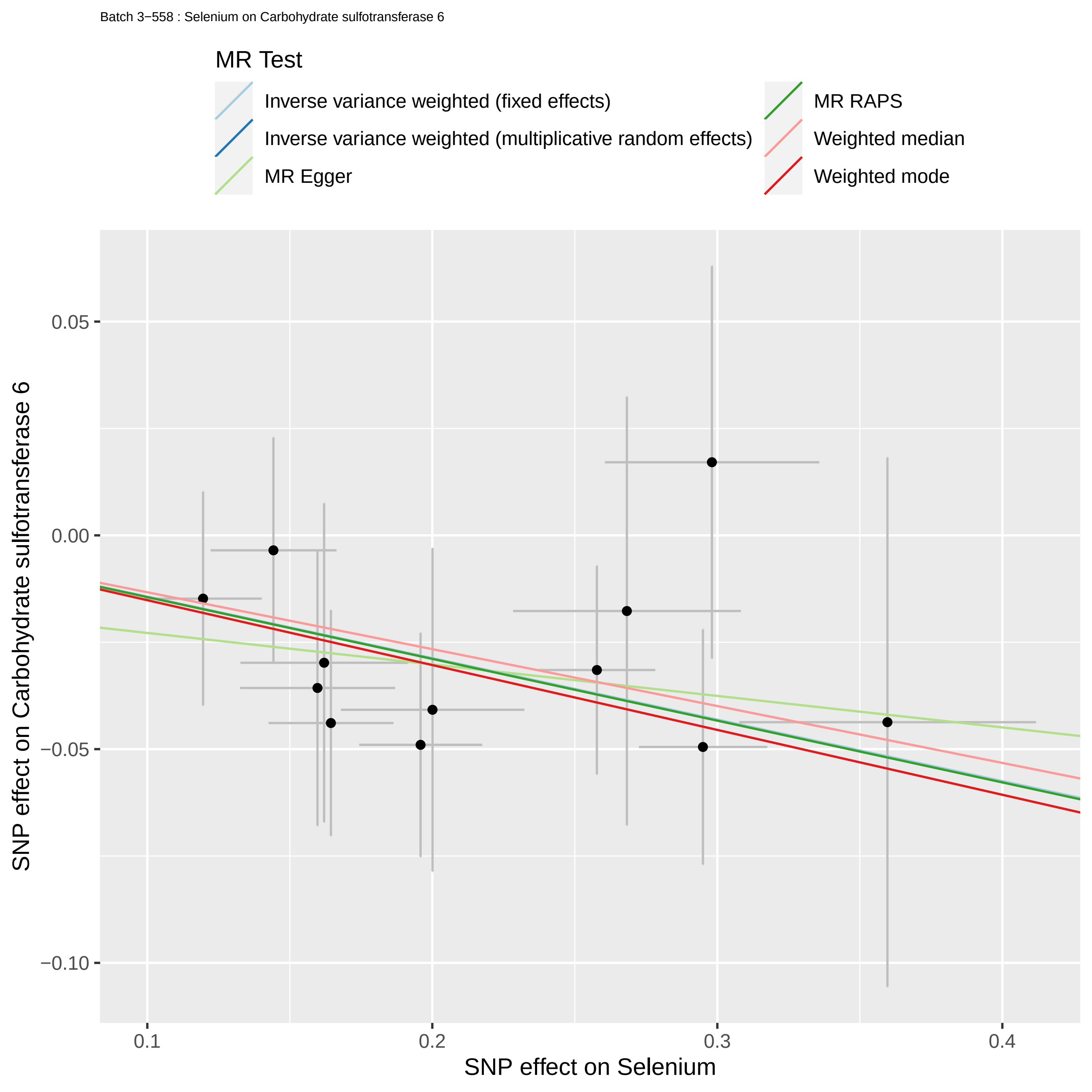

Batch 3−558 : Selenium on Carbohydrate sulfotransferase 6
MR Test
Inverse variance weighted (fixed effects)
Inverse variance weighted (multiplicative random effects)
MR Egger
MR RAPS
Weighted median
Weighted mode
0.05
0.00
−0.05
−0.10
0.1
0.2
0.3
0.4
SNP effect on Carbohydrate sulfotransferase 6
SNP effect on Selenium

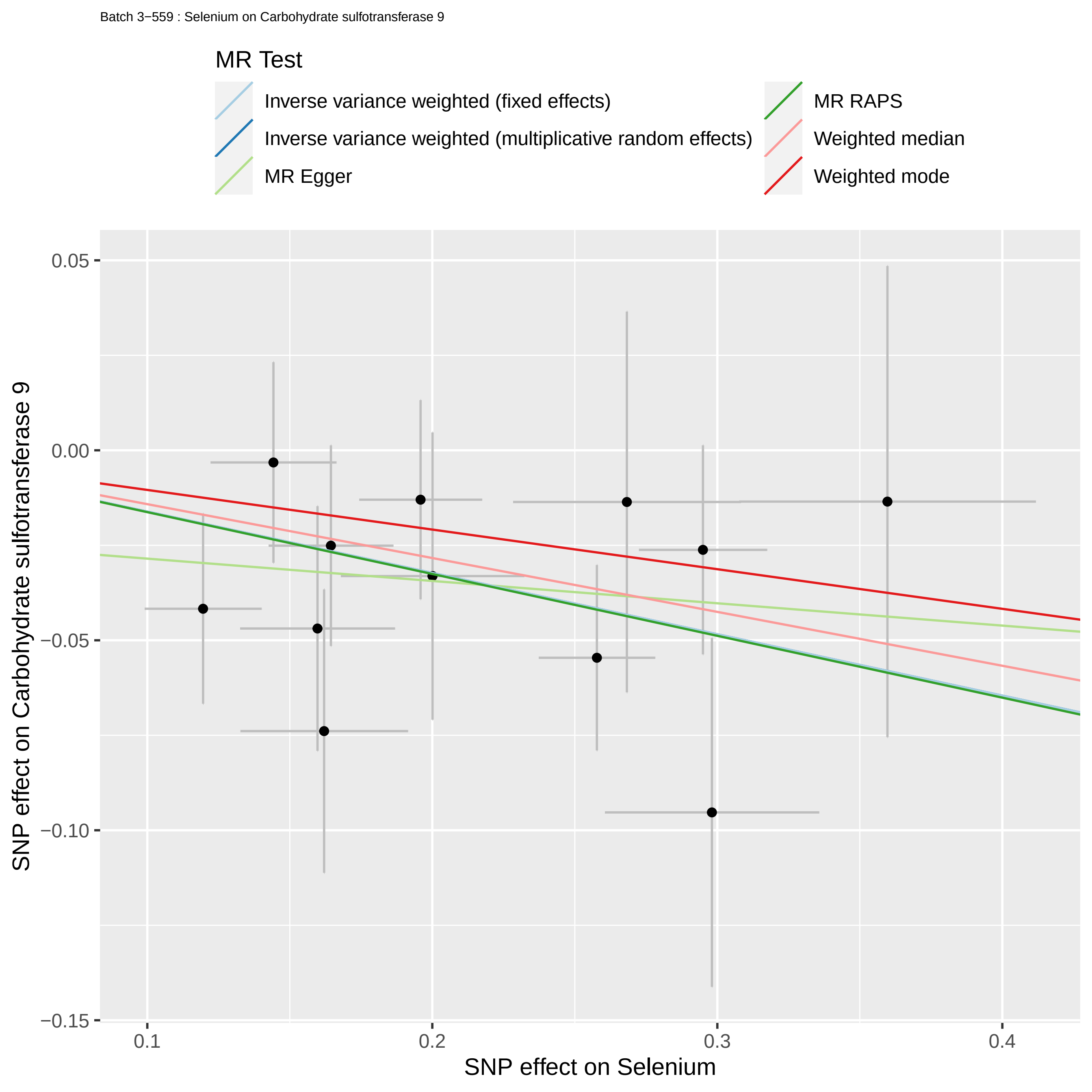

Batch 3−559 : Selenium on Carbohydrate sulfotransferase 9
MR Test
Inverse variance weighted (fixed effects)
Inverse variance weighted (multiplicative random effects)
MR Egger
MR RAPS
Weighted median
Weighted mode
0.05
0.00
−0.05
−0.10
−0.15
0.1
0.2
0.3
0.4
SNP effect on Selenium
SNP effect on Carbohydrate sulfotransferase 9

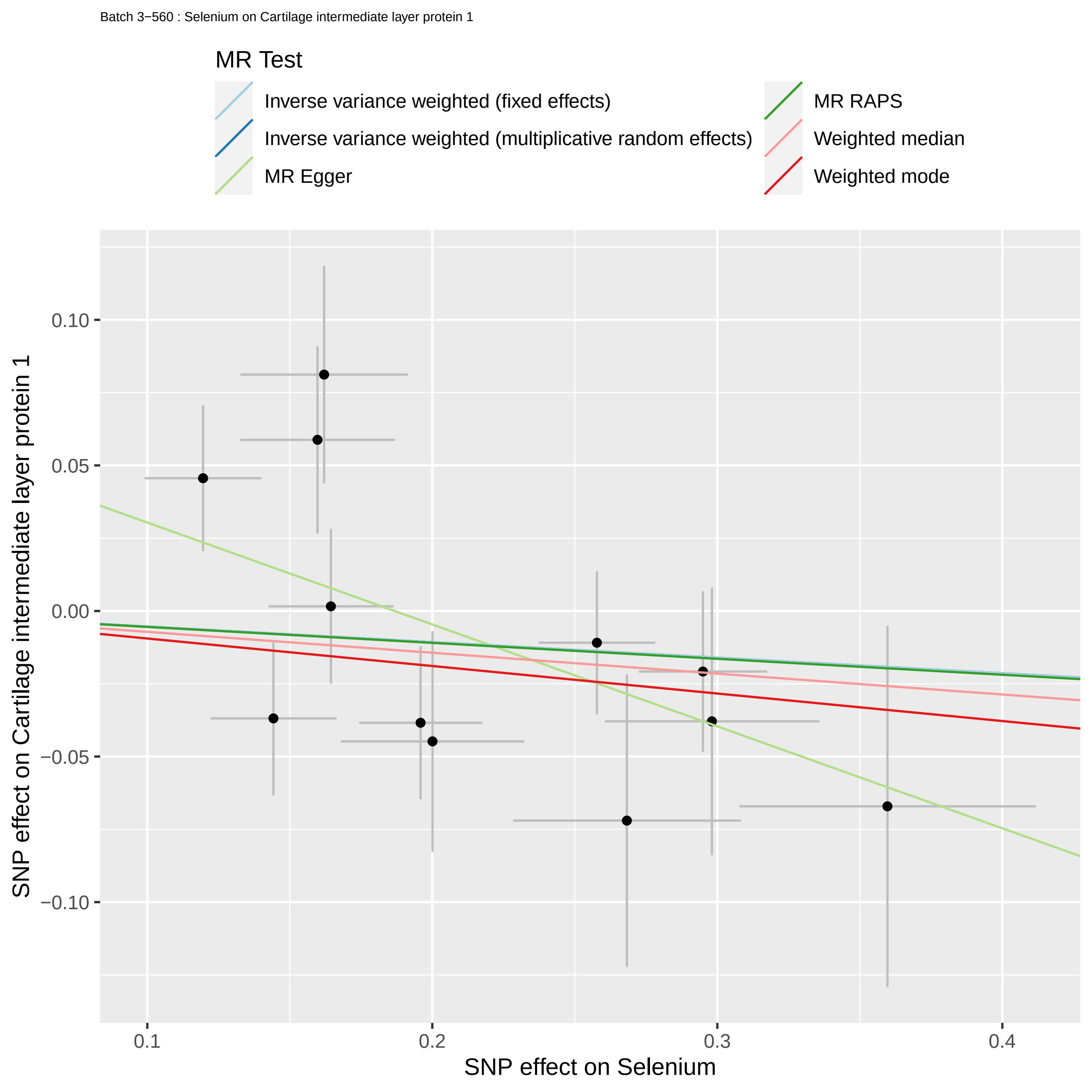
Batch 3−560 : Selenium on Cartilage intermediate layer protein 1
MR Test
Inverse variance weighted (fixed effects)
Inverse variance weighted (multiplicative random effects)
MR Egger
MR RAPS
Weighted median
Weighted mode
SNP effect on Cartilage intermediate layer protein 1
0.10
0.05
0.00
−0.05
−0.10
0.1
0.2
0.3
0.4
SNP effect on Selenium

Batch 3−561 : Selenium on Cold−inducible RNA−binding protein

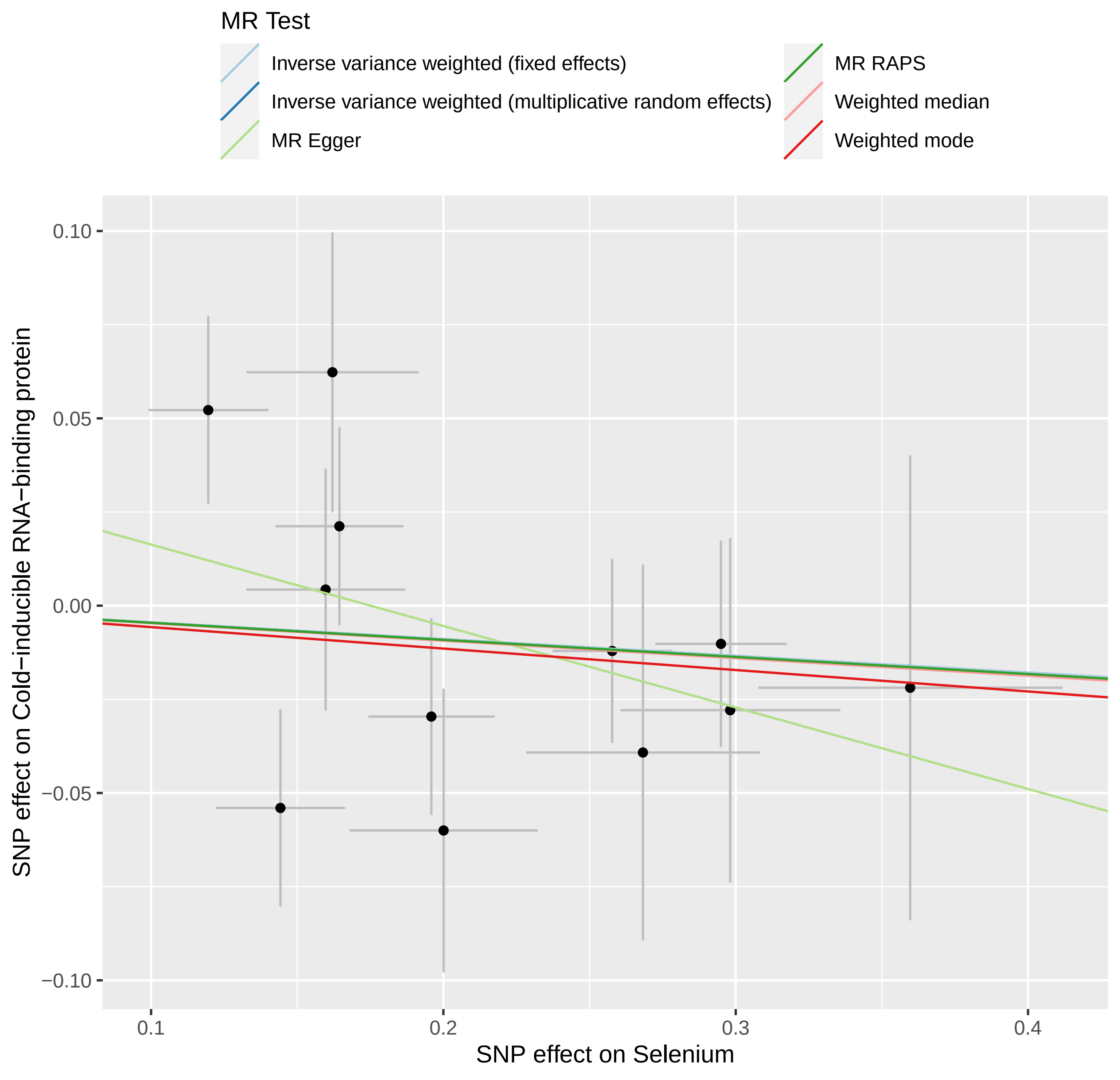

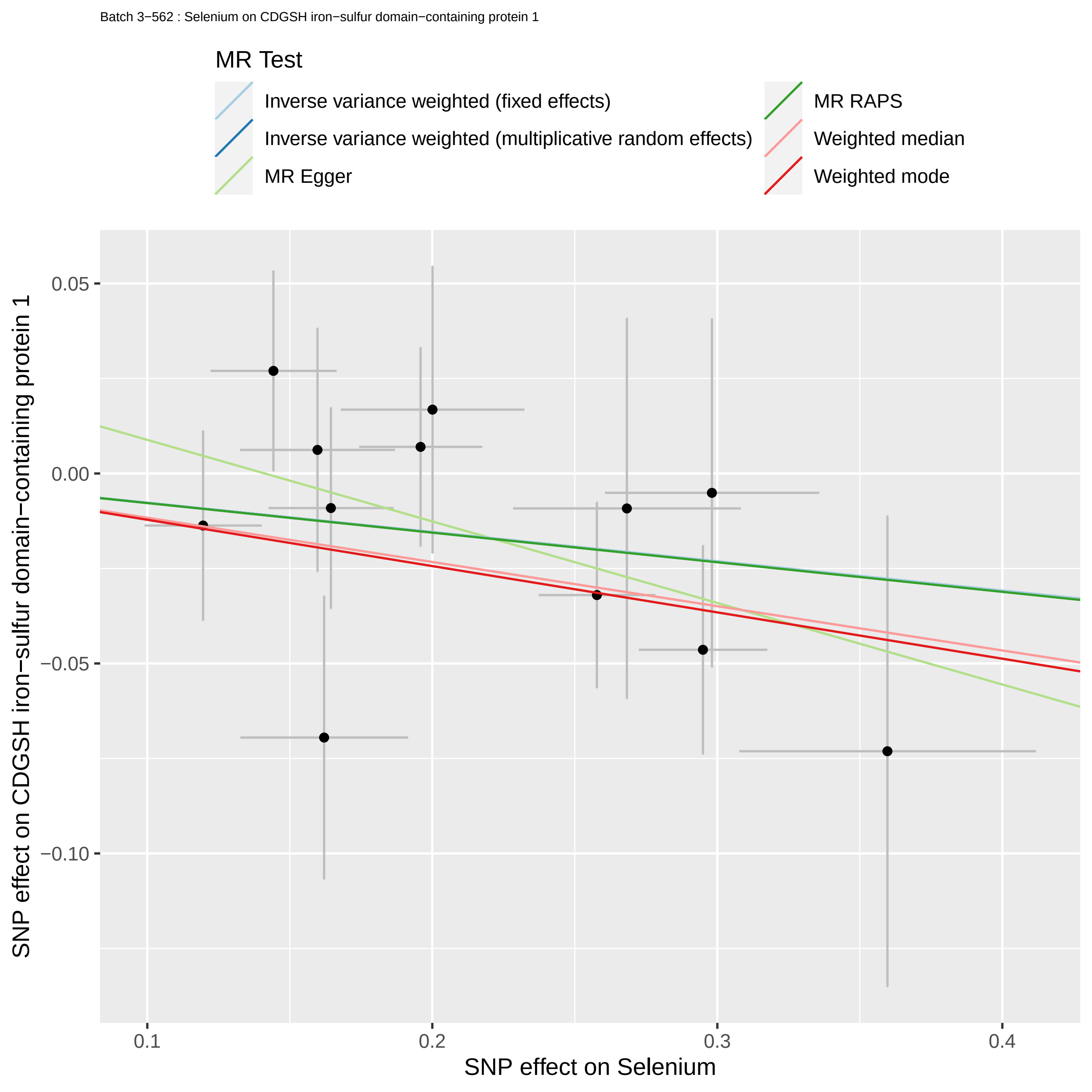

Batch 3−562 : Selenium on CDGSH iron−sulfur domain−containing protein 1
MR Test
Inverse variance weighted (fixed effects)
Inverse variance weighted (multiplicative random effects)
MR Egger
MR RAPS
Weighted median
Weighted mode
0.05
0.00
−0.05
−0.10
0.1
0.2
0.3
0.4
SNP effect on Selenium
SNP effect on CDGSH iron−sulfur domain−containing protein 1

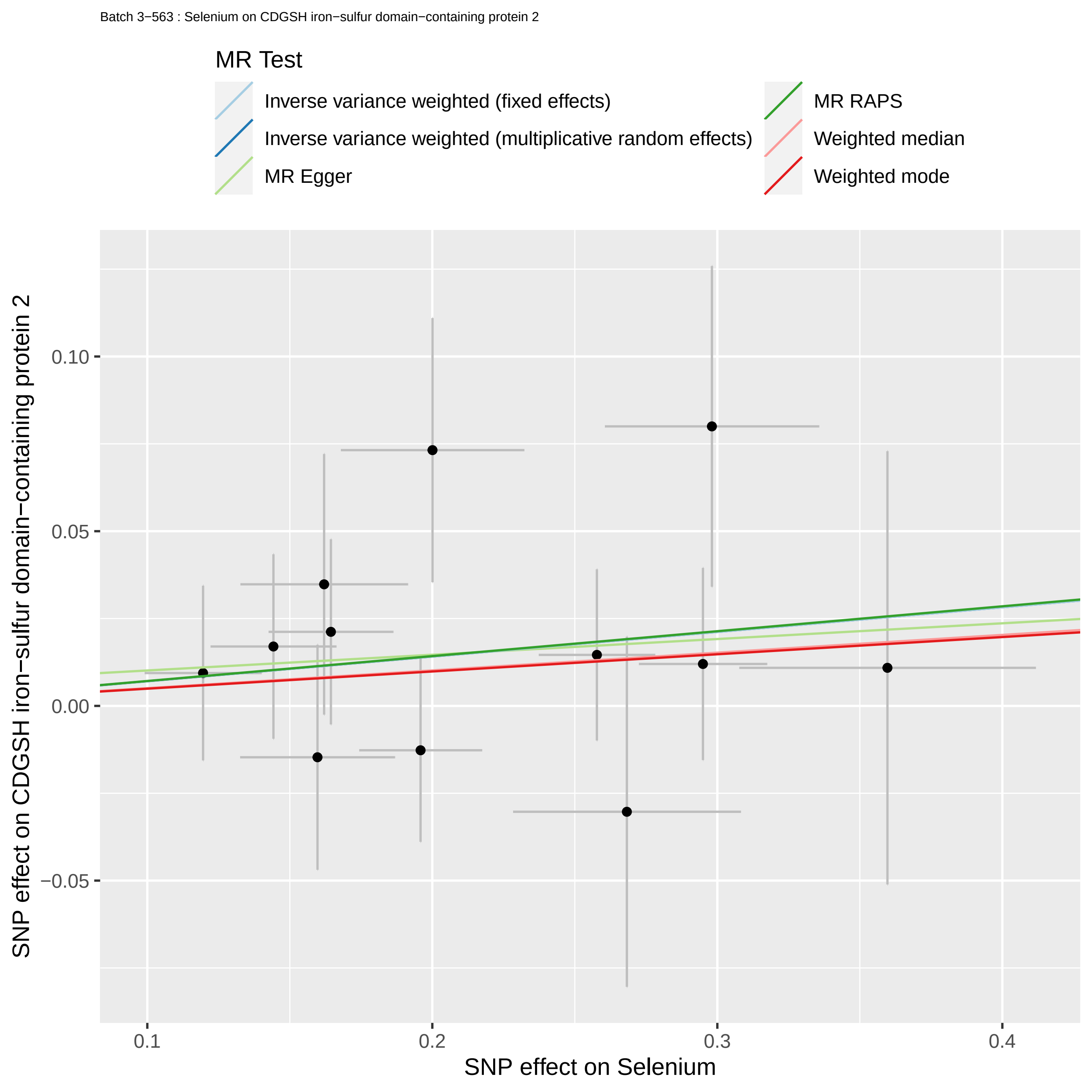
Batch 3−563 : Selenium on CDGSH iron−sulfur domain−containing protein 2
MR Test
Inverse variance weighted (fixed effects)
Inverse variance weighted (multiplicative random effects)
MR Egger
MR RAPS
Weighted median
Weighted mode
SNP effect on CDGSH iron−sulfur domain−containing protein 2
0.10
0.05
0.00
−0.05
0.1
0.2
0.3
0.4
SNP effect on Selenium

Batch 3−564 : Selenium on Cytoskeleton−associated protein 2

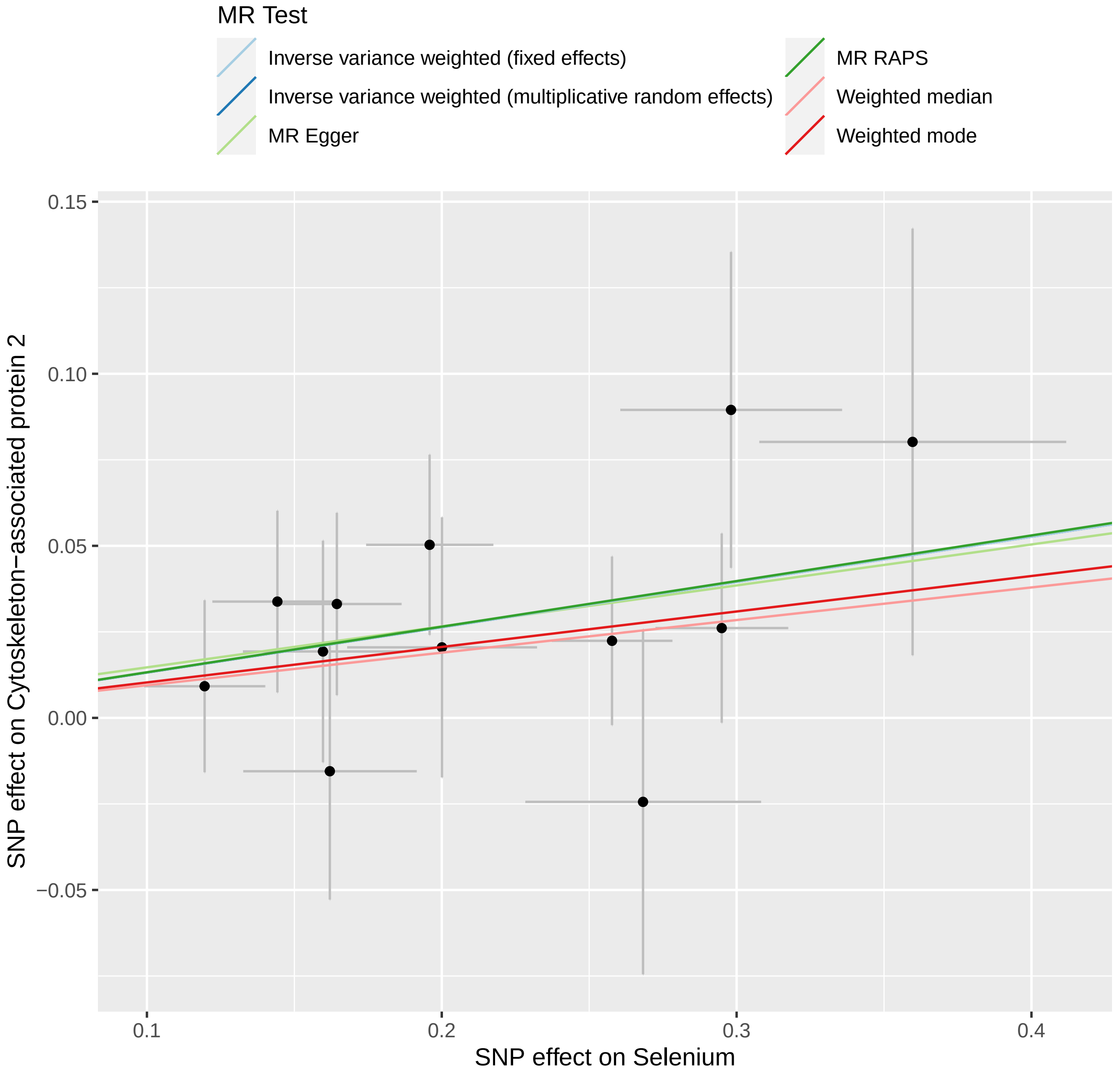

Batch 3−565 : Selenium on Creatine kinase B−type

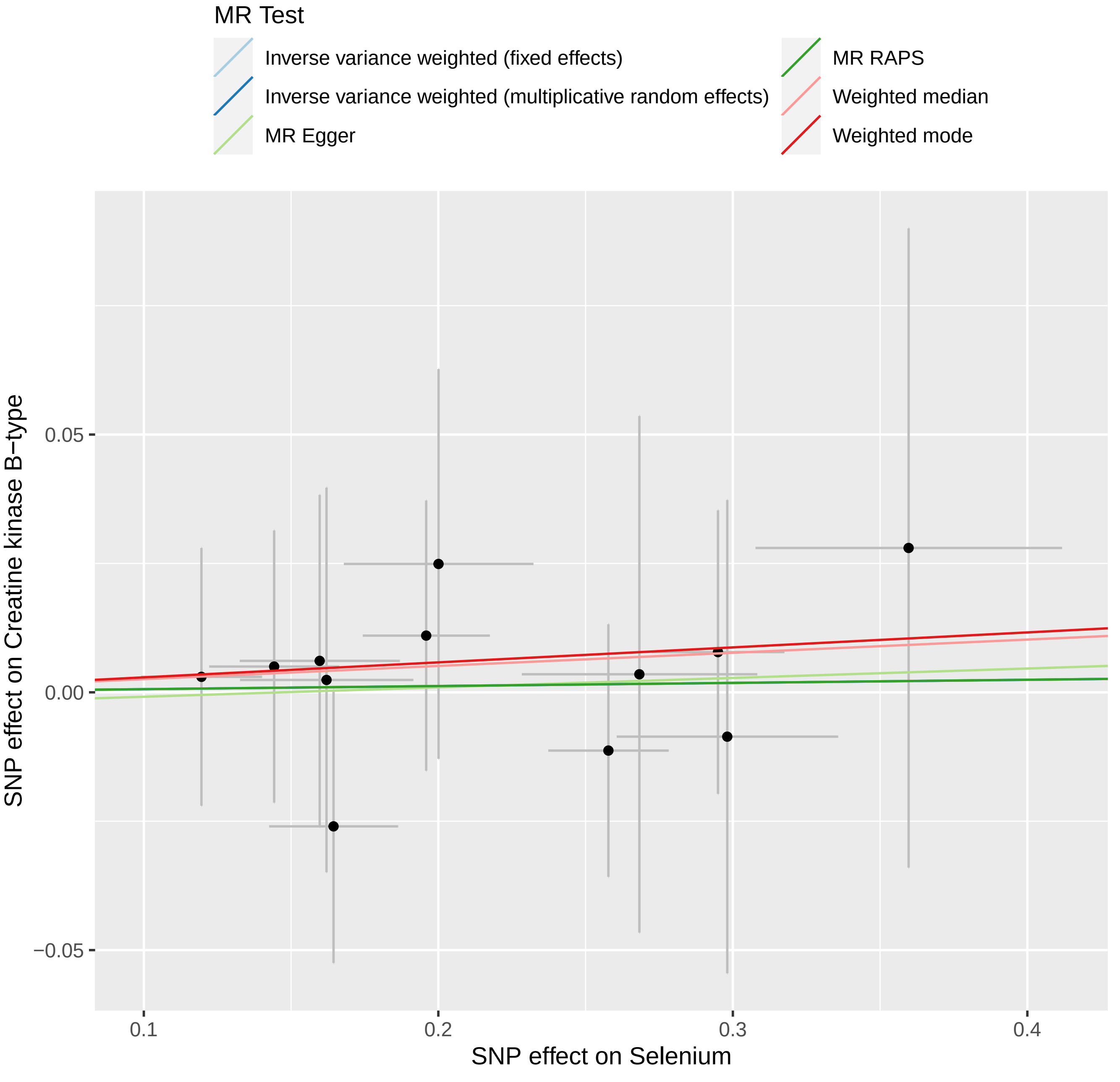

Batch 3−566 : Selenium on Creatine kinase M−type

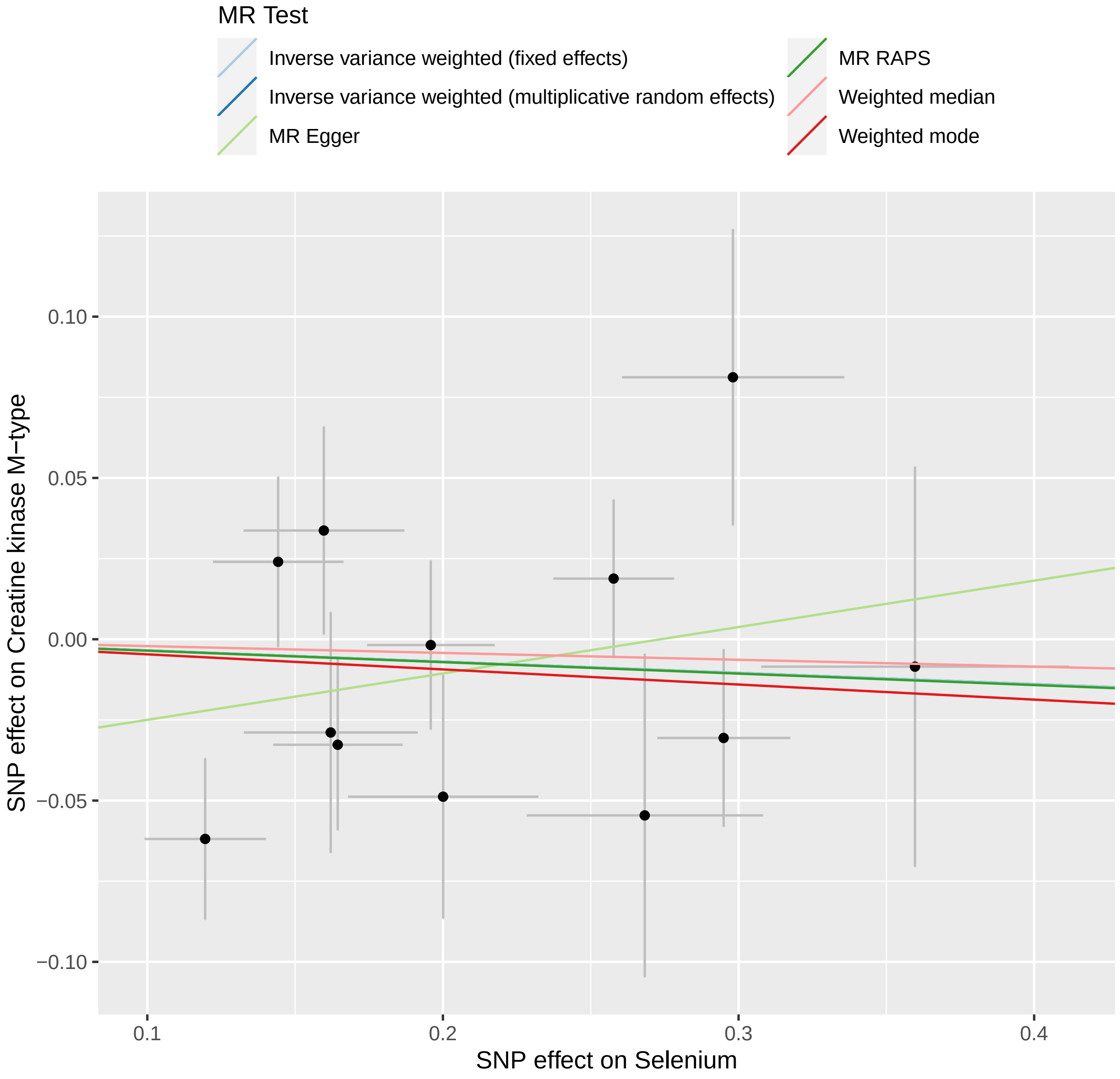

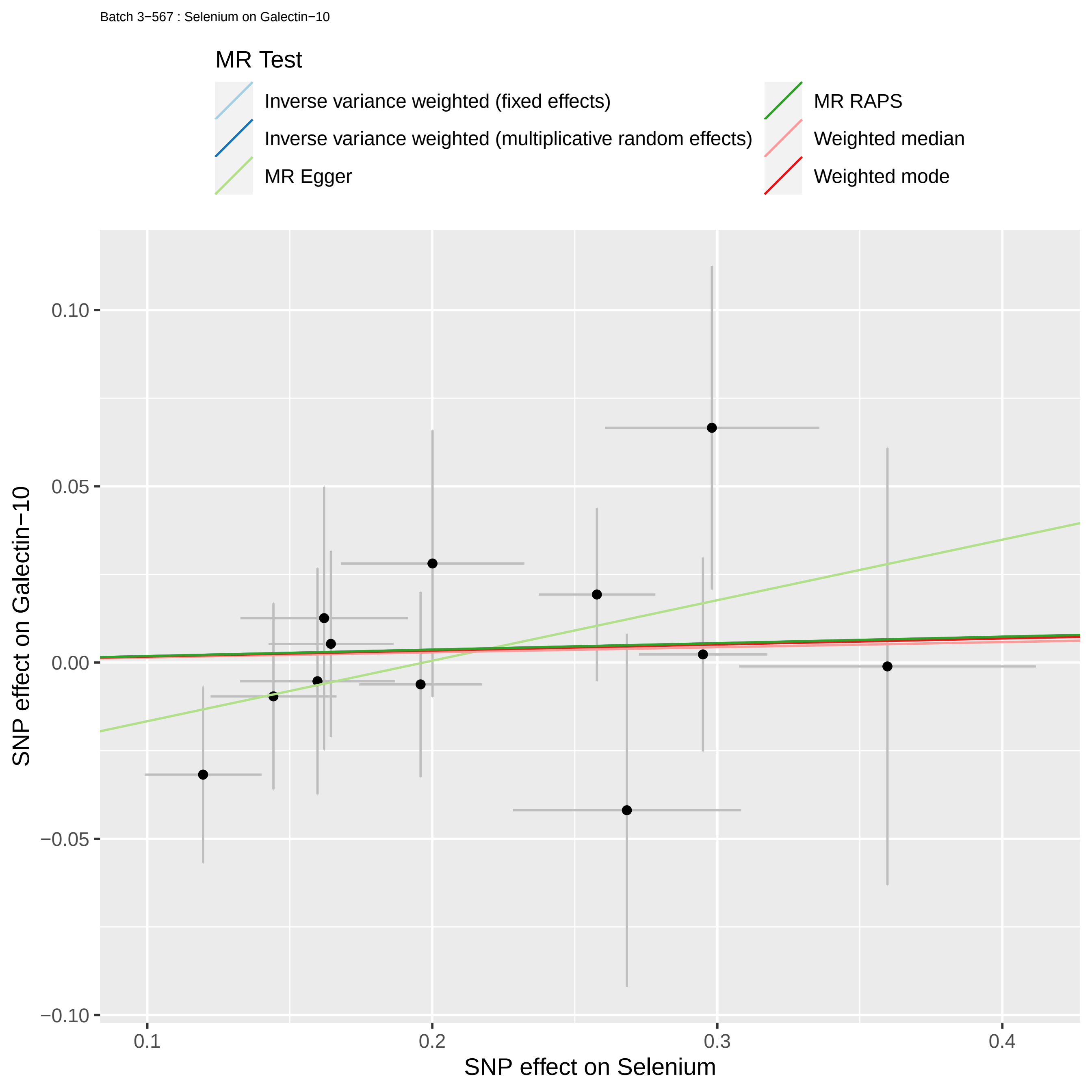
Batch 3−567 : Selenium on Galectin−10
MR Test
Inverse variance weighted (fixed effects)
Inverse variance weighted (multiplicative random effects)
MR Egger
MR RAPS
Weighted median
Weighted mode
0.10
0.05
0.00
−0.05
−0.10
0.1
0.2
0.3
0.4
SNP effect on Galectin−10
SNP effect on Selenium

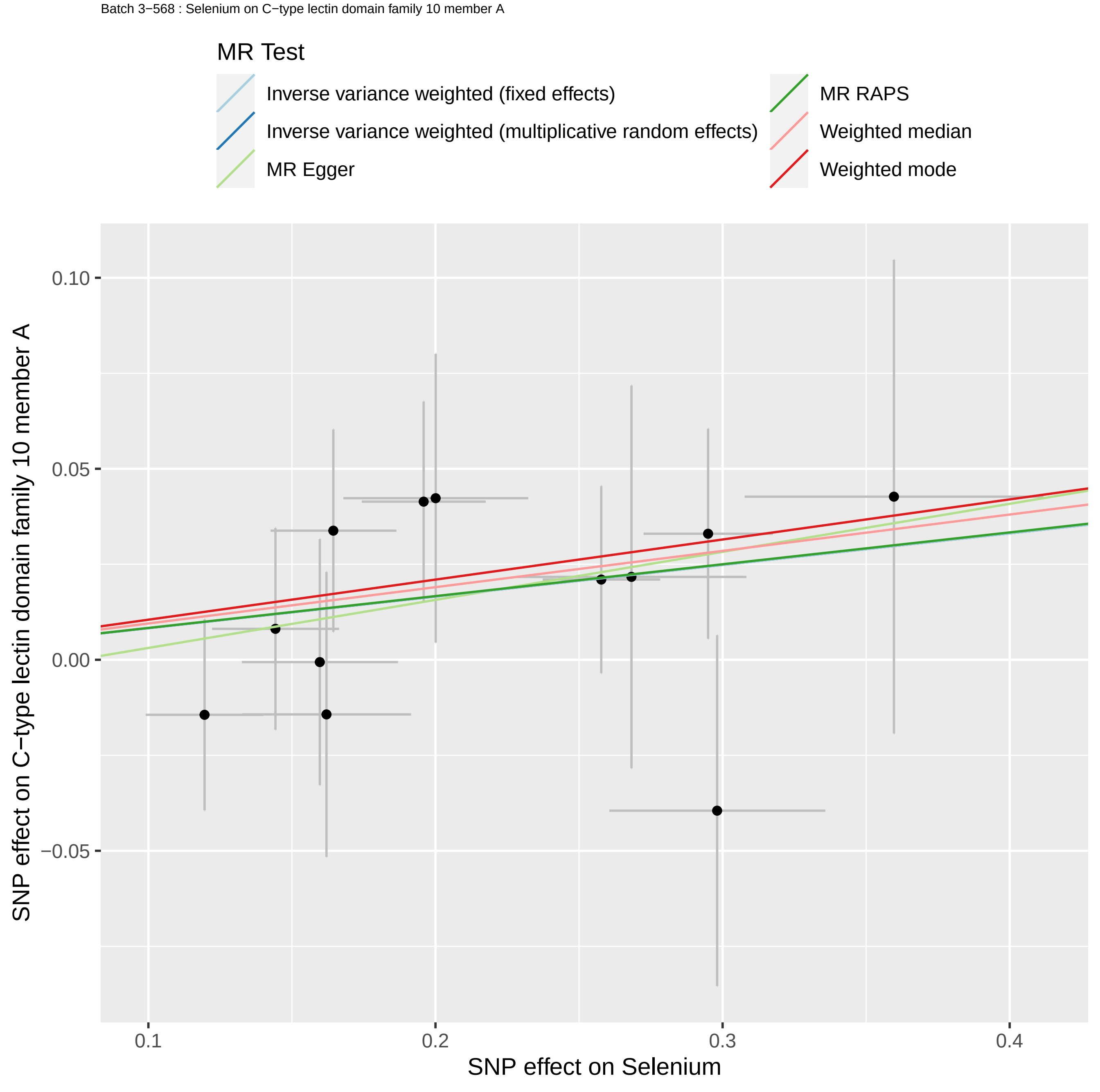

Batch 3−568 : Selenium on C−type lectin domain family 10 member A
MR Test
Inverse variance weighted (fixed effects)
Inverse variance weighted (multiplicative random effects)
MR Egger
MR RAPS
Weighted median
Weighted mode
SNP effect on C−type lectin domain family 10 member A
0.10
0.05
0.00
−0.05
0.1
0.2
0.3
0.4
SNP effect on Selenium

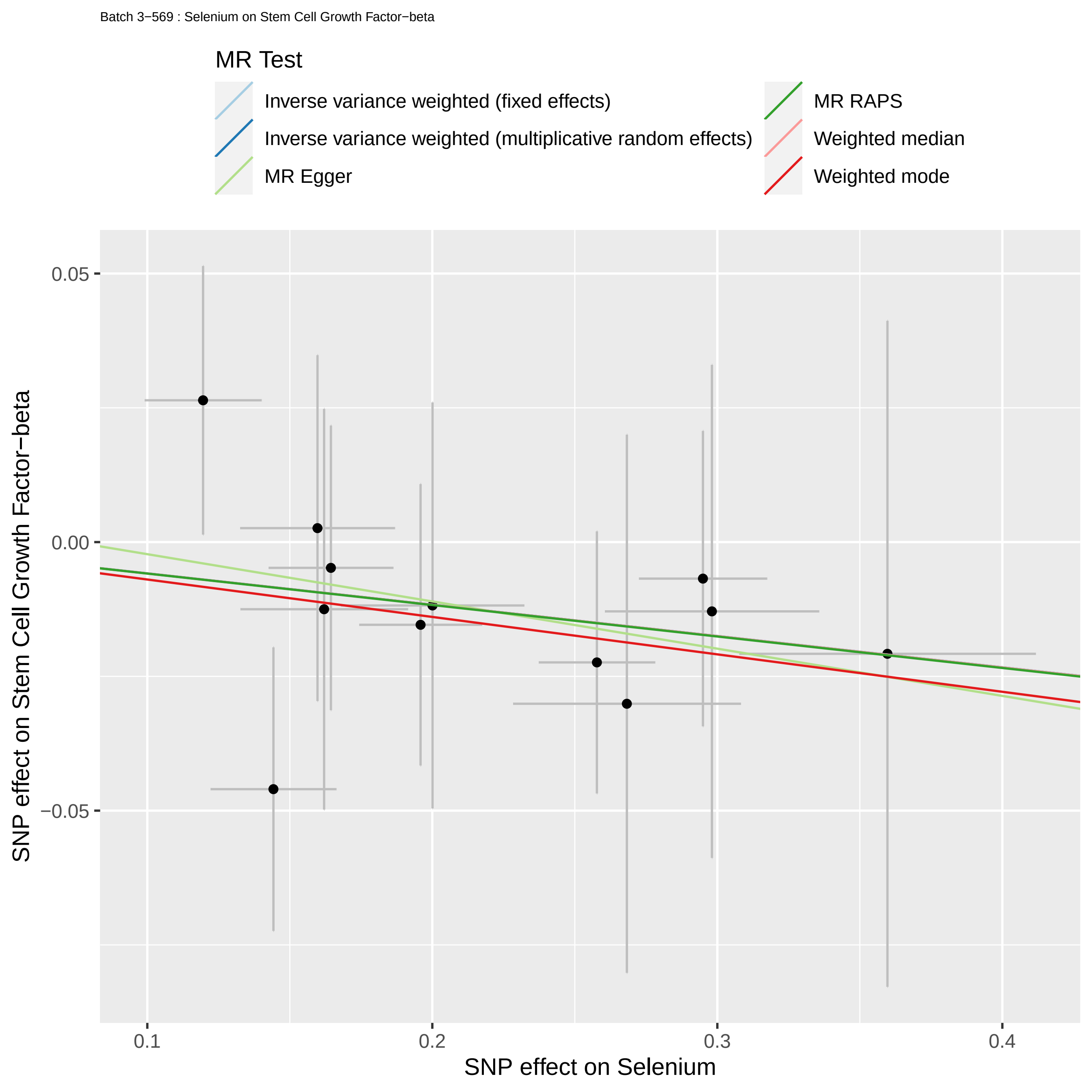
Batch 3−569 : Selenium on Stem Cell Growth Factor−beta
MR Test
Inverse variance weighted (fixed effects)
Inverse variance weighted (multiplicative random effects)
MR Egger
MR RAPS
Weighted median
Weighted mode
0.05
0.00
−0.05
0.1
0.2
0.3
0.4
SNP effect on Stem Cell Growth Factor−beta
SNP effect on Selenium

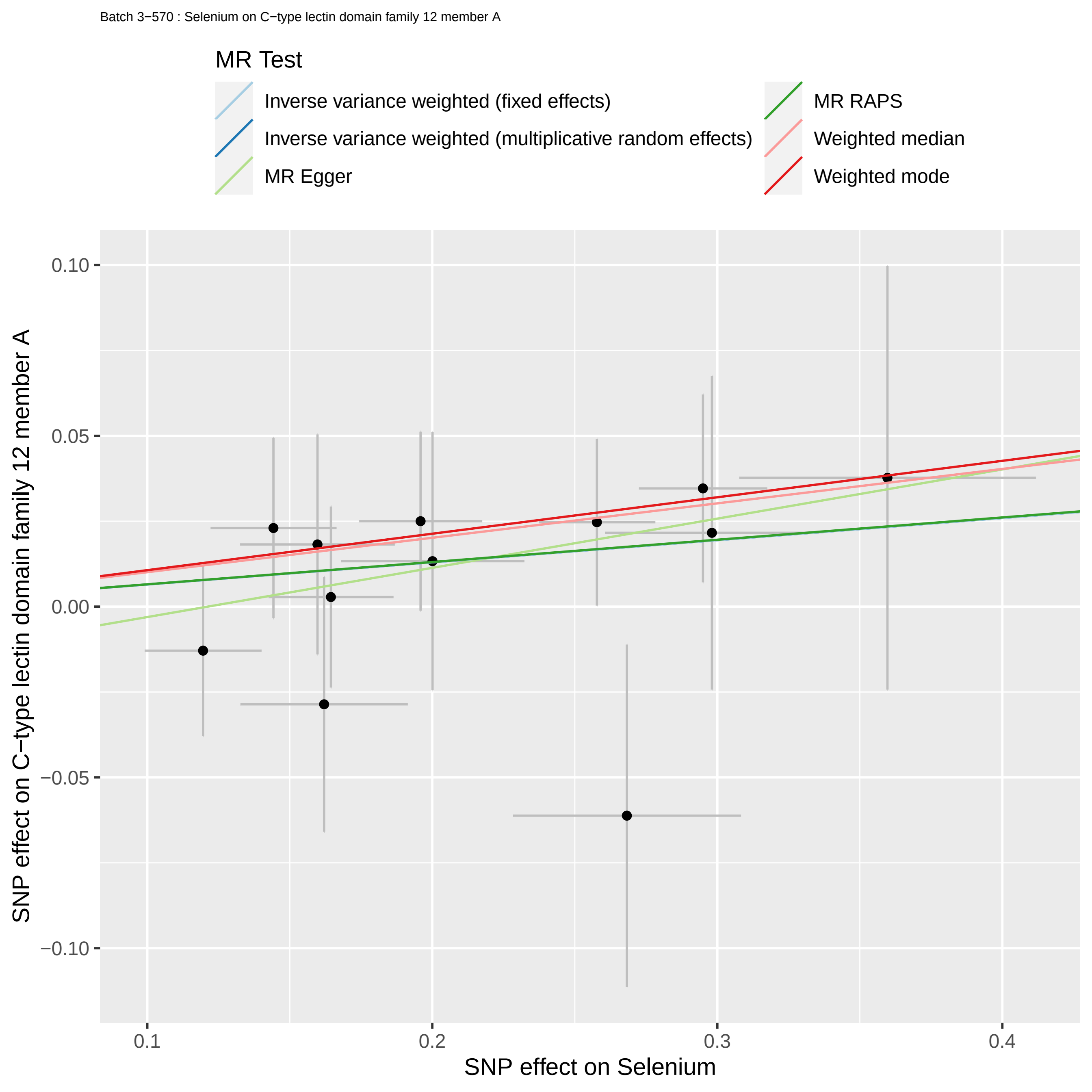

Batch 3−570 : Selenium on C−type lectin domain family 12 member A
MR Test
Inverse variance weighted (fixed effects)
Inverse variance weighted (multiplicative random effects)
MR Egger
MR RAPS
Weighted median
Weighted mode
0.10
0.05
0.00
−0.05
−0.10
0.1
0.2
0.3
0.4
SNP effect on C−type lectin domain family 12 member A
SNP effect on Selenium

Batch 3−571 : Selenium on C−type lectin domain family 14 member A

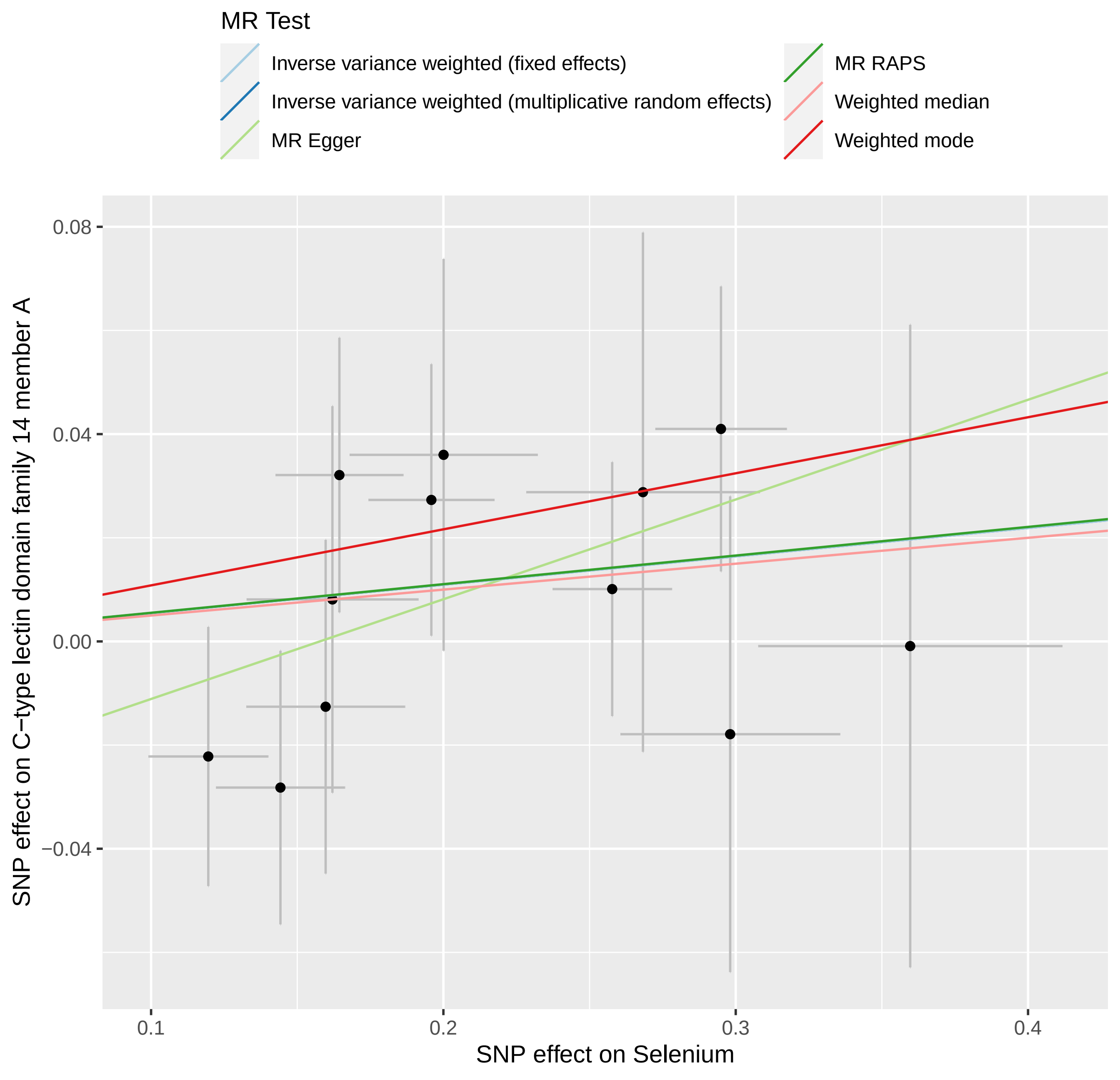

Batch 3−572 : Selenium on C−type lectin domain family 2 member A

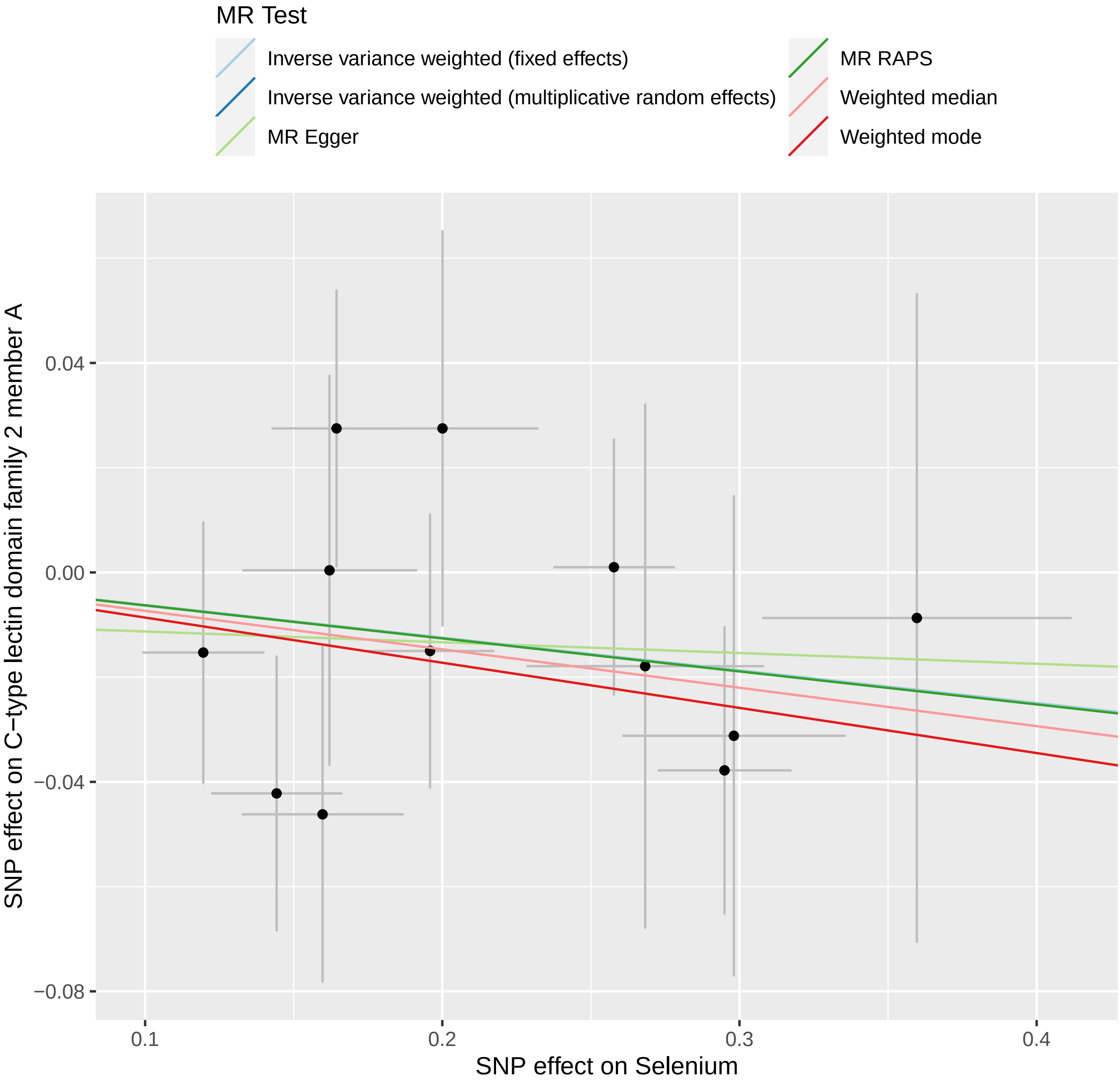

Batch 3−573 : Selenium on C−type lectin domain family 2 member B

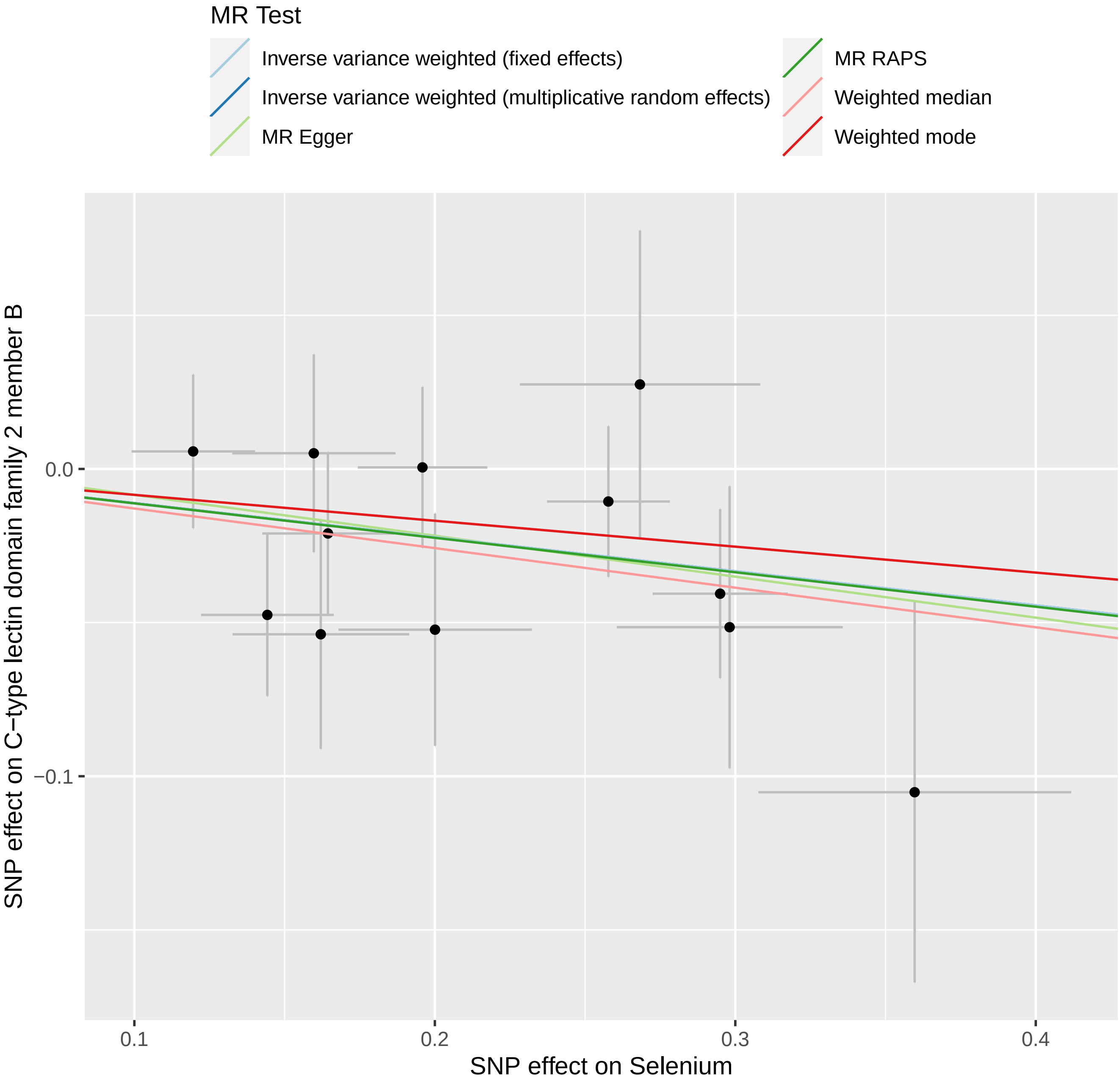

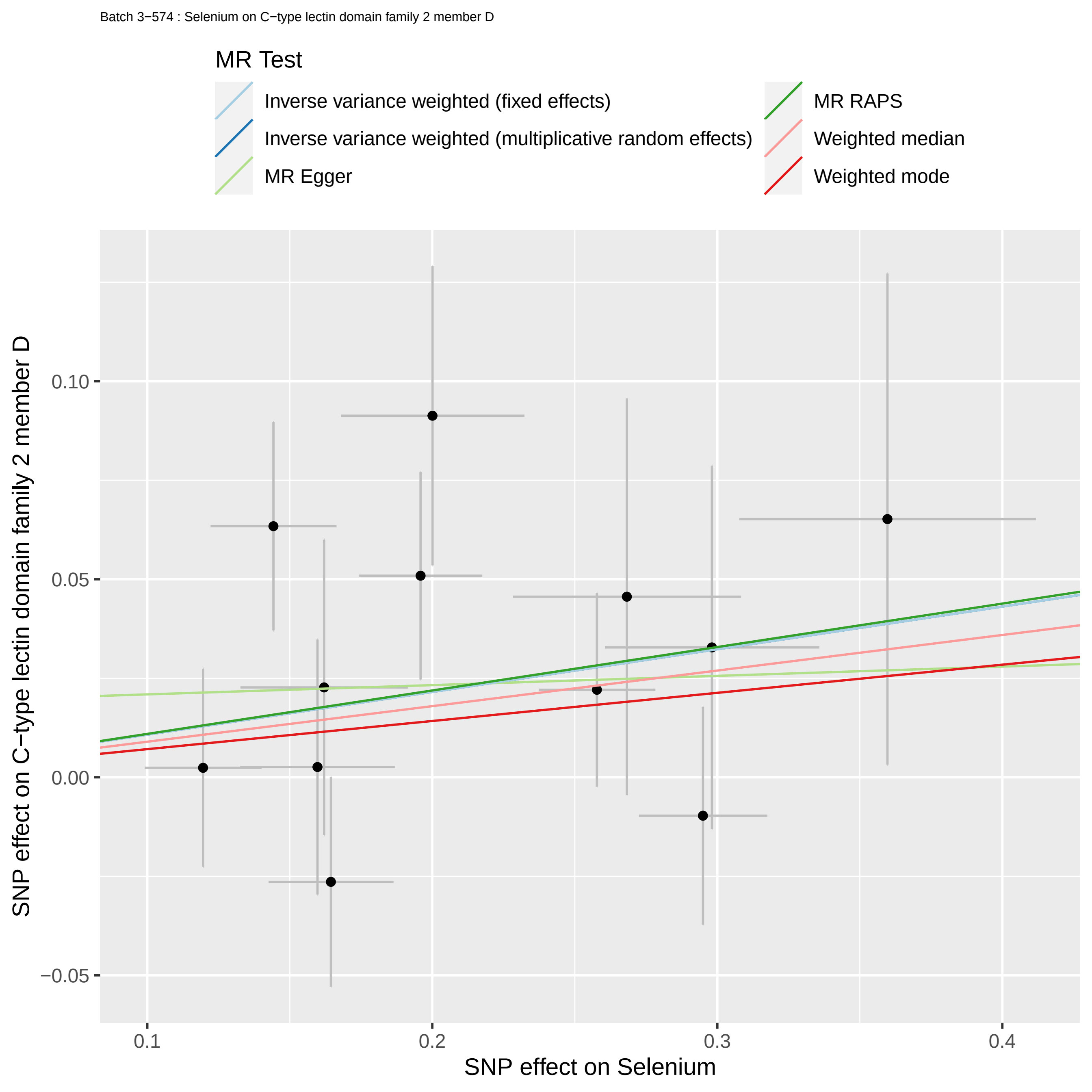

Batch 3−574 : Selenium on C−type lectin domain family 2 member D
MR Test
Inverse variance weighted (fixed effects)
Inverse variance weighted (multiplicative random effects)
MR Egger
MR RAPS
Weighted median
Weighted mode
0.10
0.05
0.00
−0.05
0.1
0.2
0.3
0.4
SNP effect on C−type lectin domain family 2 member D
SNP effect on Selenium

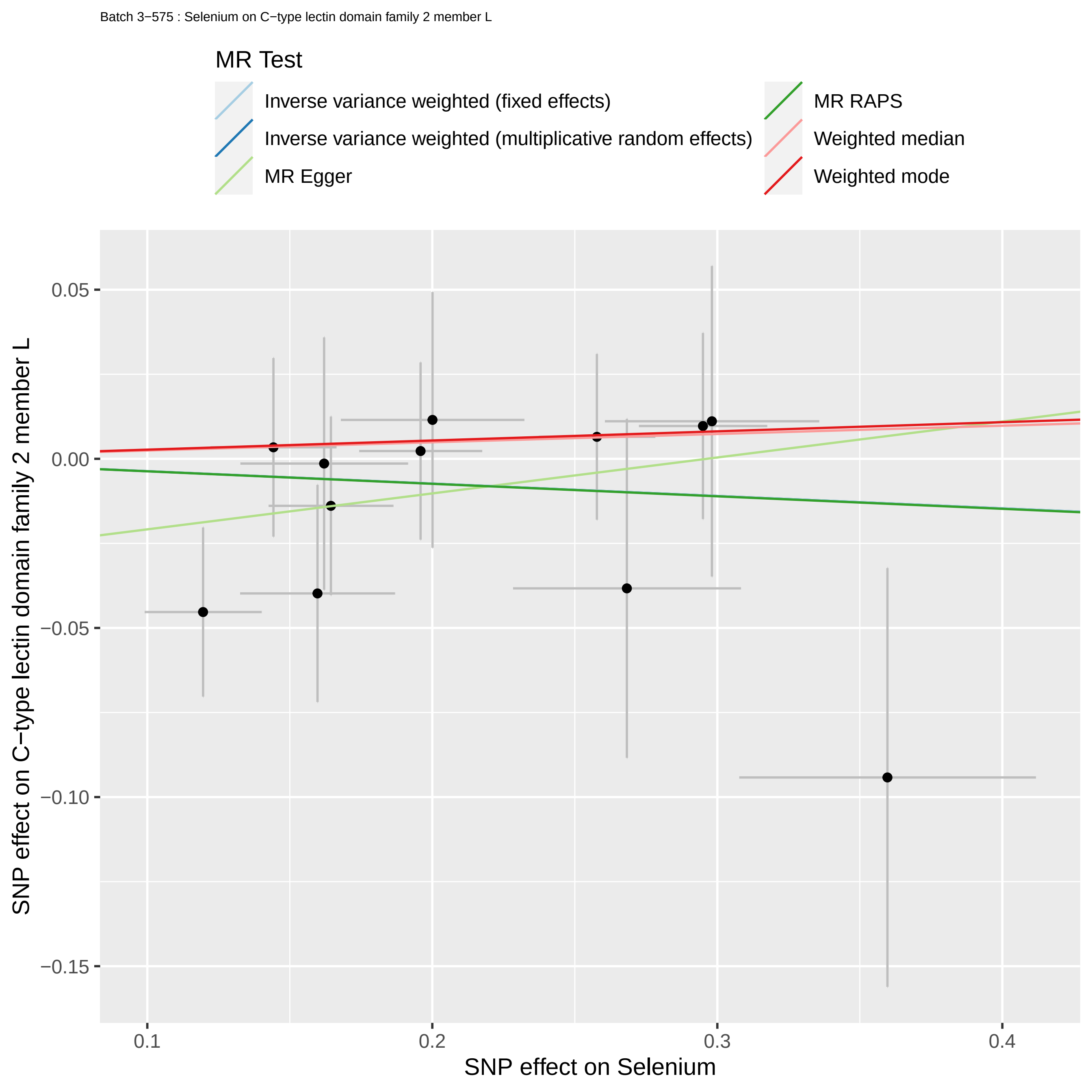

Batch 3−575 : Selenium on C−type lectin domain family 2 member L
MR Test
Inverse variance weighted (fixed effects)
Inverse variance weighted (multiplicative random effects)
MR Egger
MR RAPS
Weighted median
Weighted mode
SNP effect on C−type lectin domain family 2 member L
0.05
0.00
−0.05
−0.10
−0.15
0.1
0.2
0.3
0.4
SNP effect on Selenium

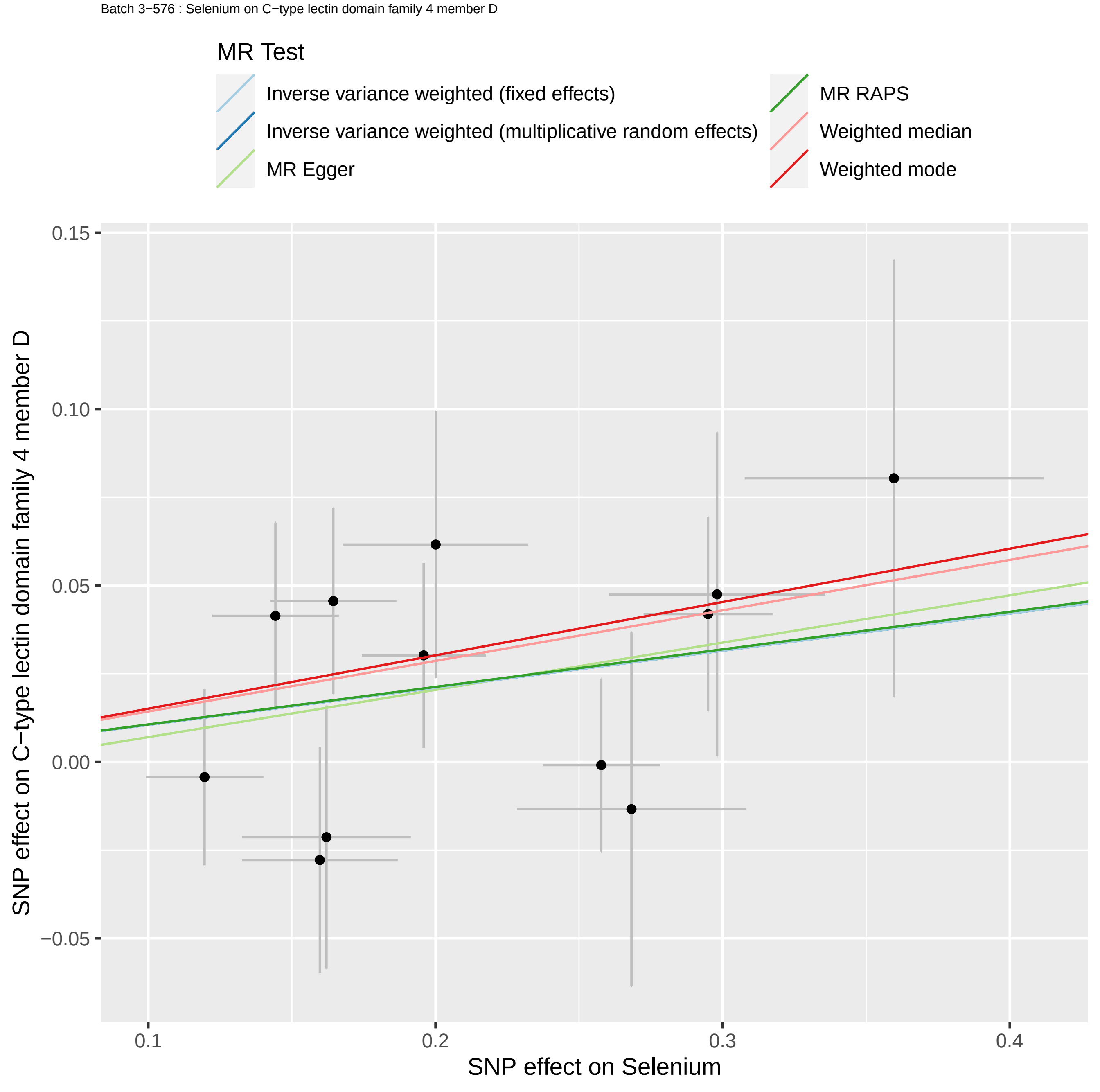
Batch 3−576 : Selenium on C−type lectin domain family 4 member D
MR Test
Inverse variance weighted (fixed effects)
Inverse variance weighted (multiplicative random effects)
MR Egger
MR RAPS
Weighted median
Weighted mode
0.15
0.10
0.05
0.00
−0.05
0.1
0.2
0.3
0.4
SNP effect on C−type lectin domain family 4 member D
SNP effect on Selenium

Batch 3−577 : Selenium on C−type lectin domain family 4 member E

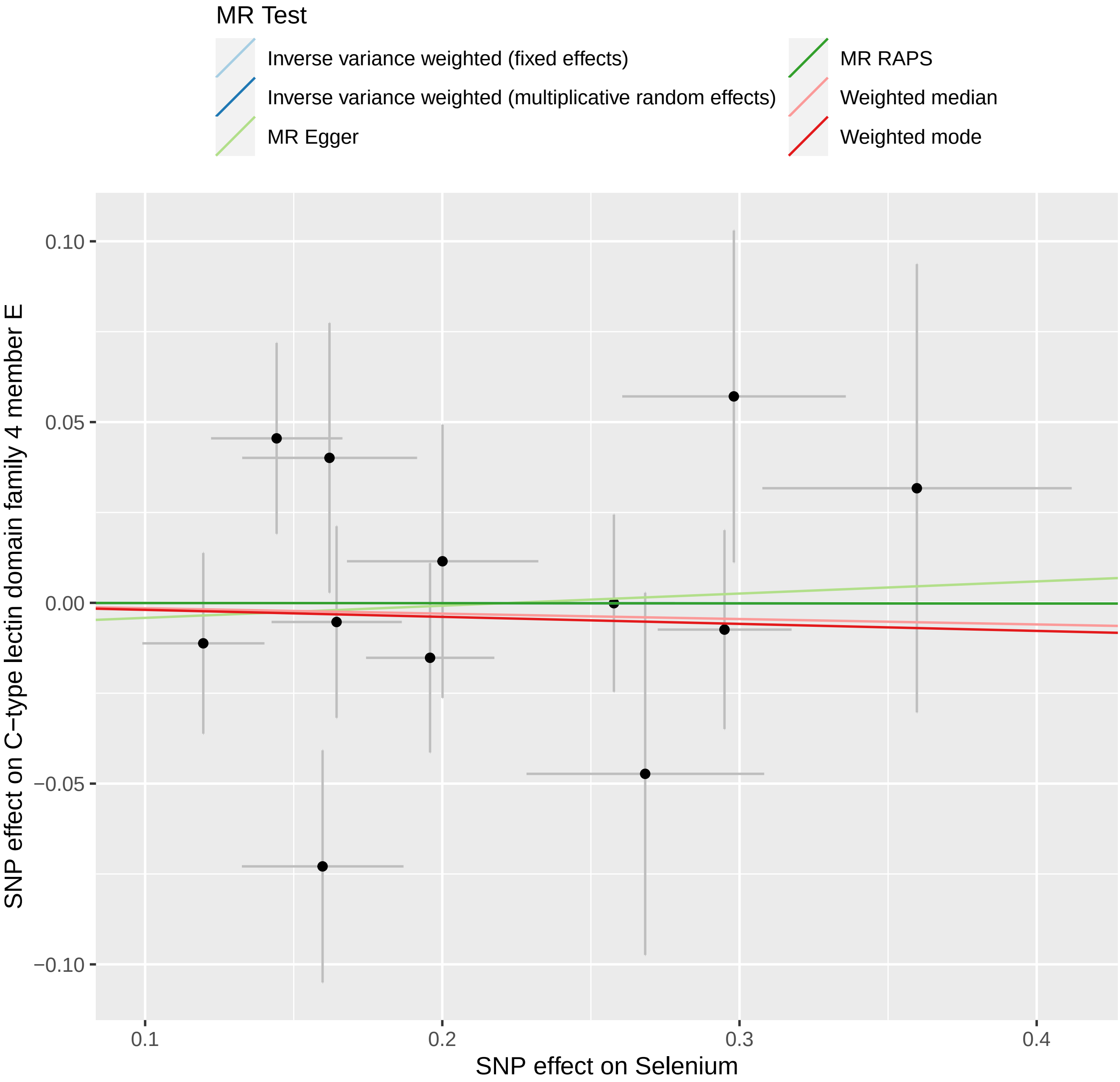

Batch 3−578 : Selenium on C−type lectin domain family 4 member M

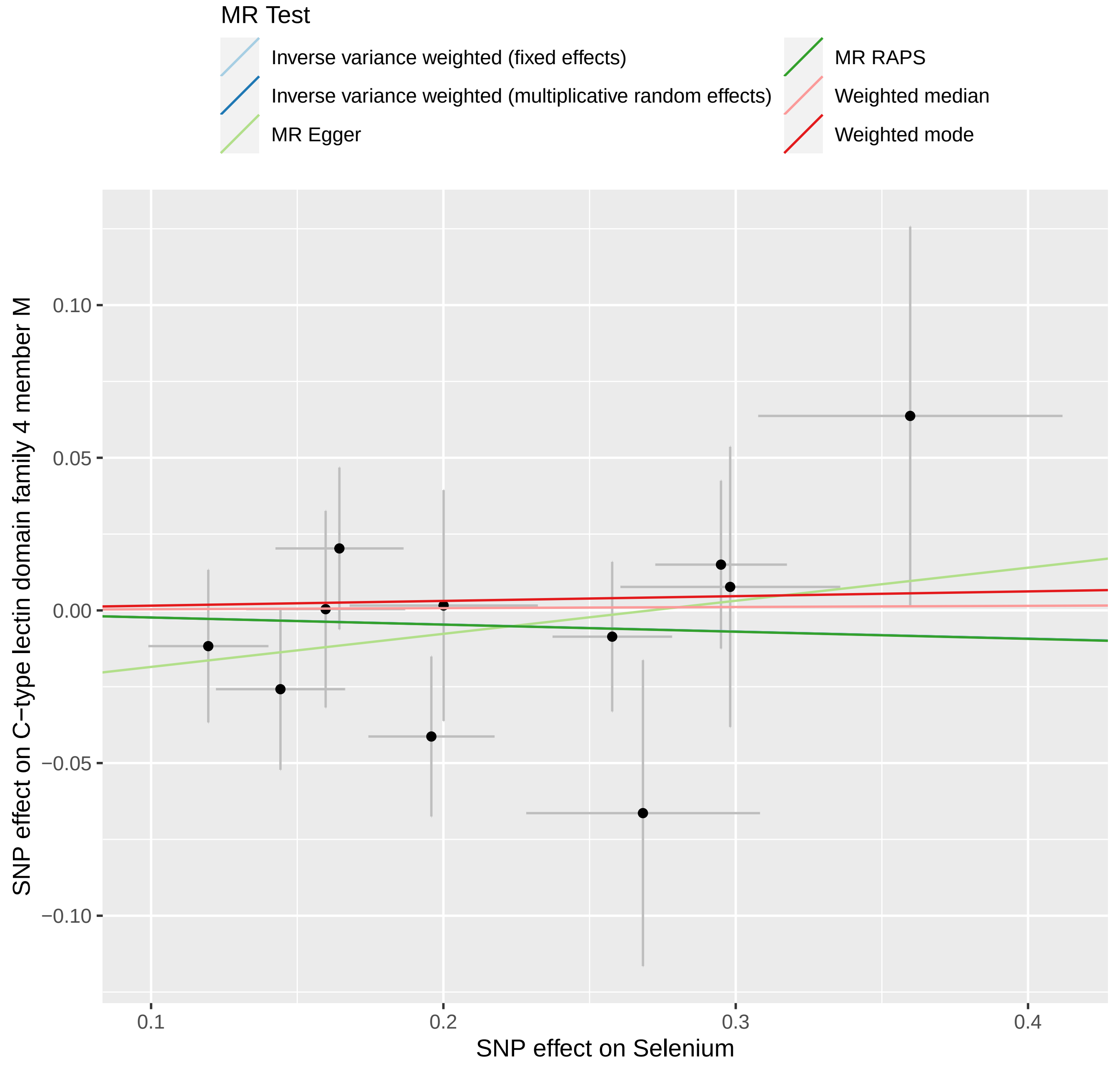

Batch 3−579 : Selenium on C−type lectin domain family 5 member A

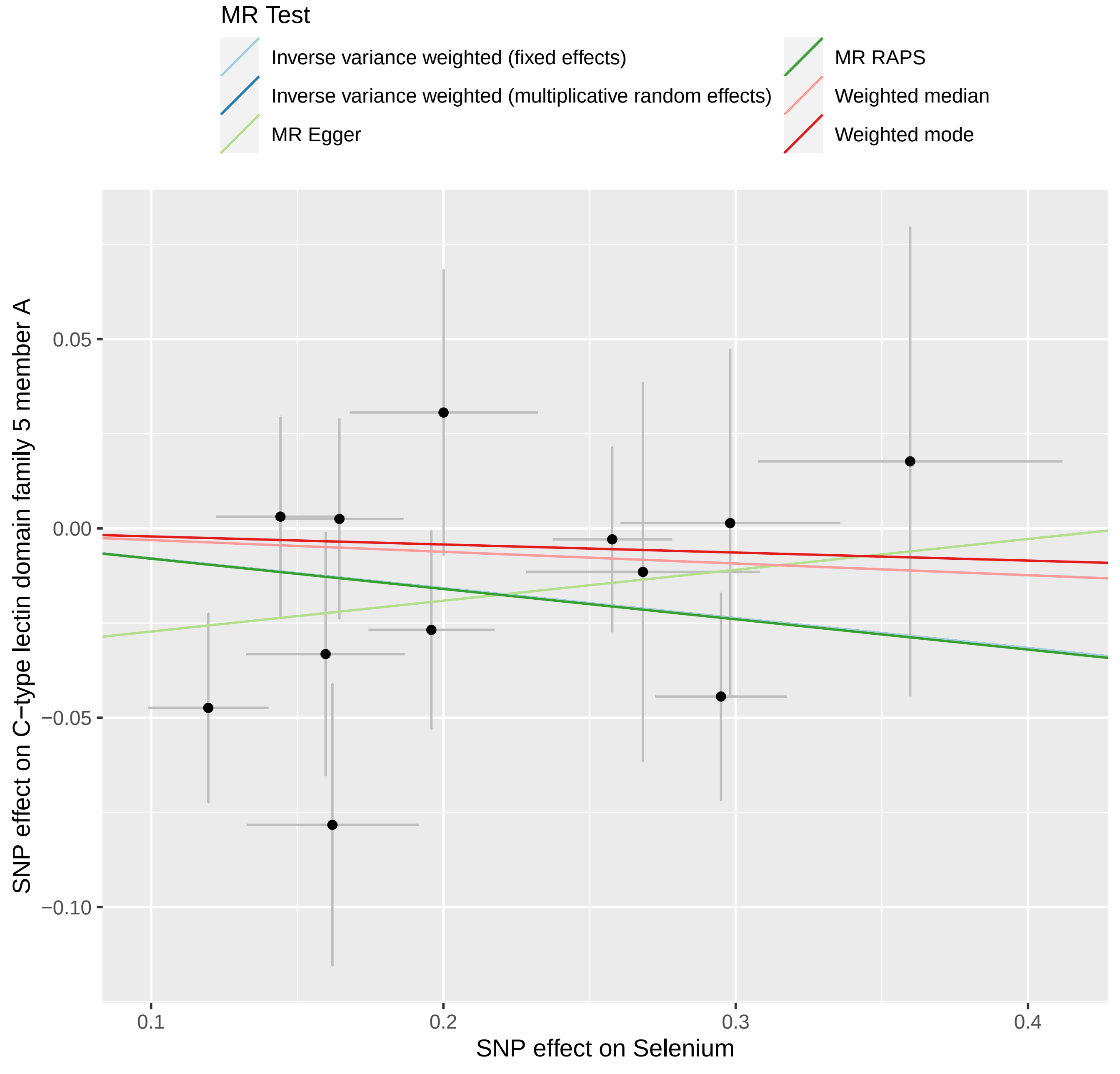

Batch 3−580 : Selenium on C−type lectin domain family 6 member A

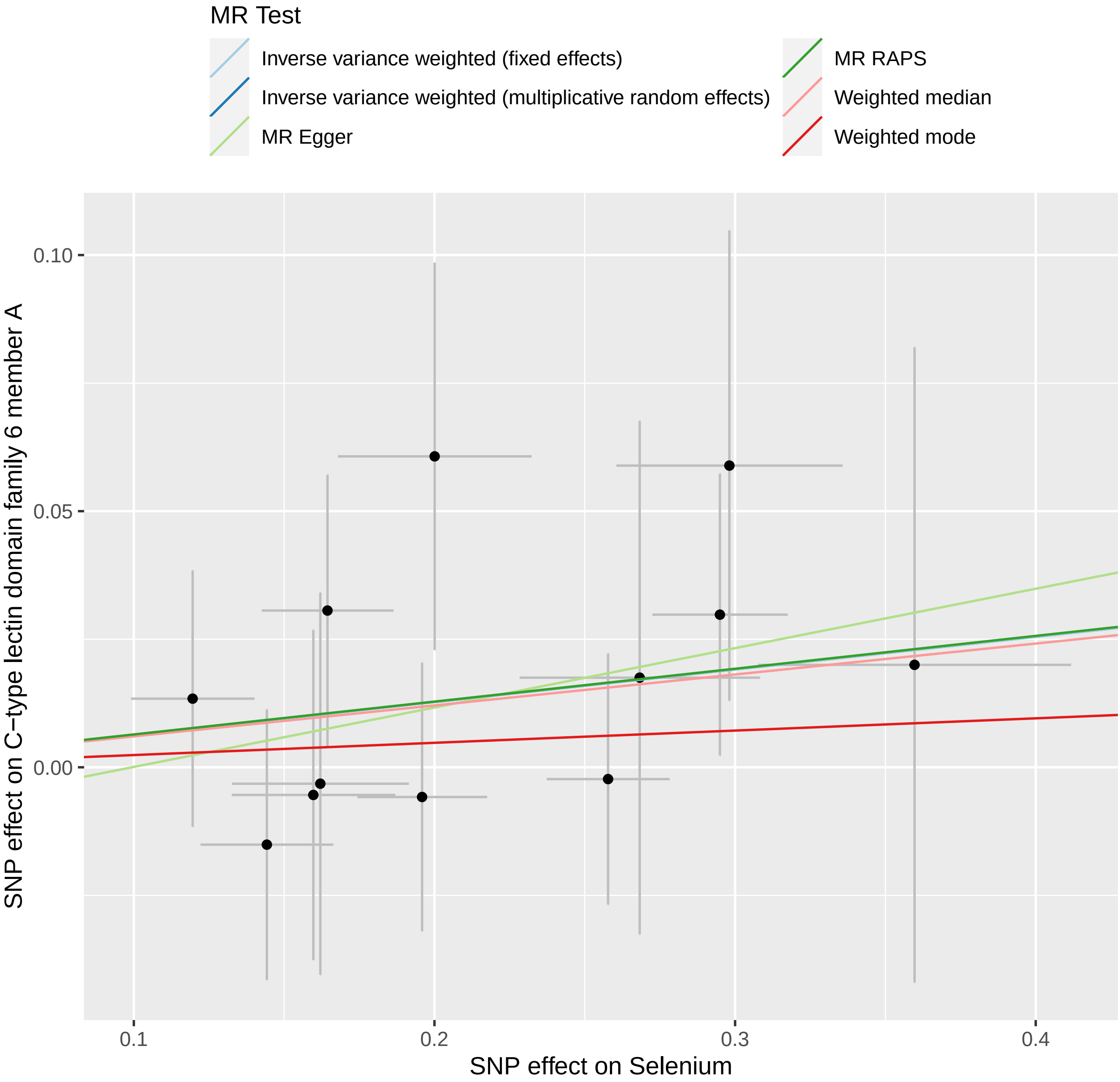

Batch 3−581 : Selenium on C−type lectin domain family 7 member A

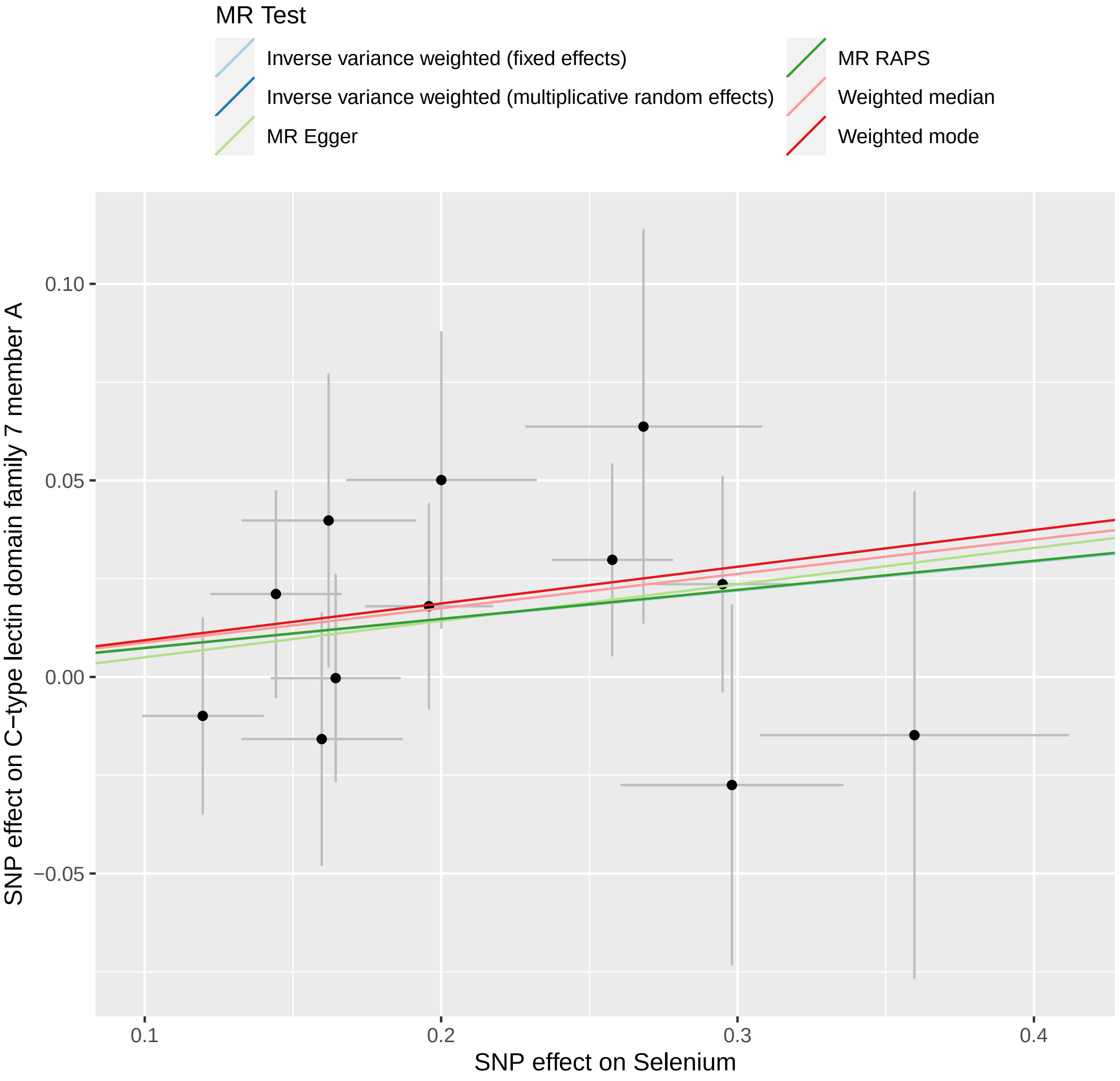

Batch 3−582 : Selenium on C−type lectin−like domain family 1

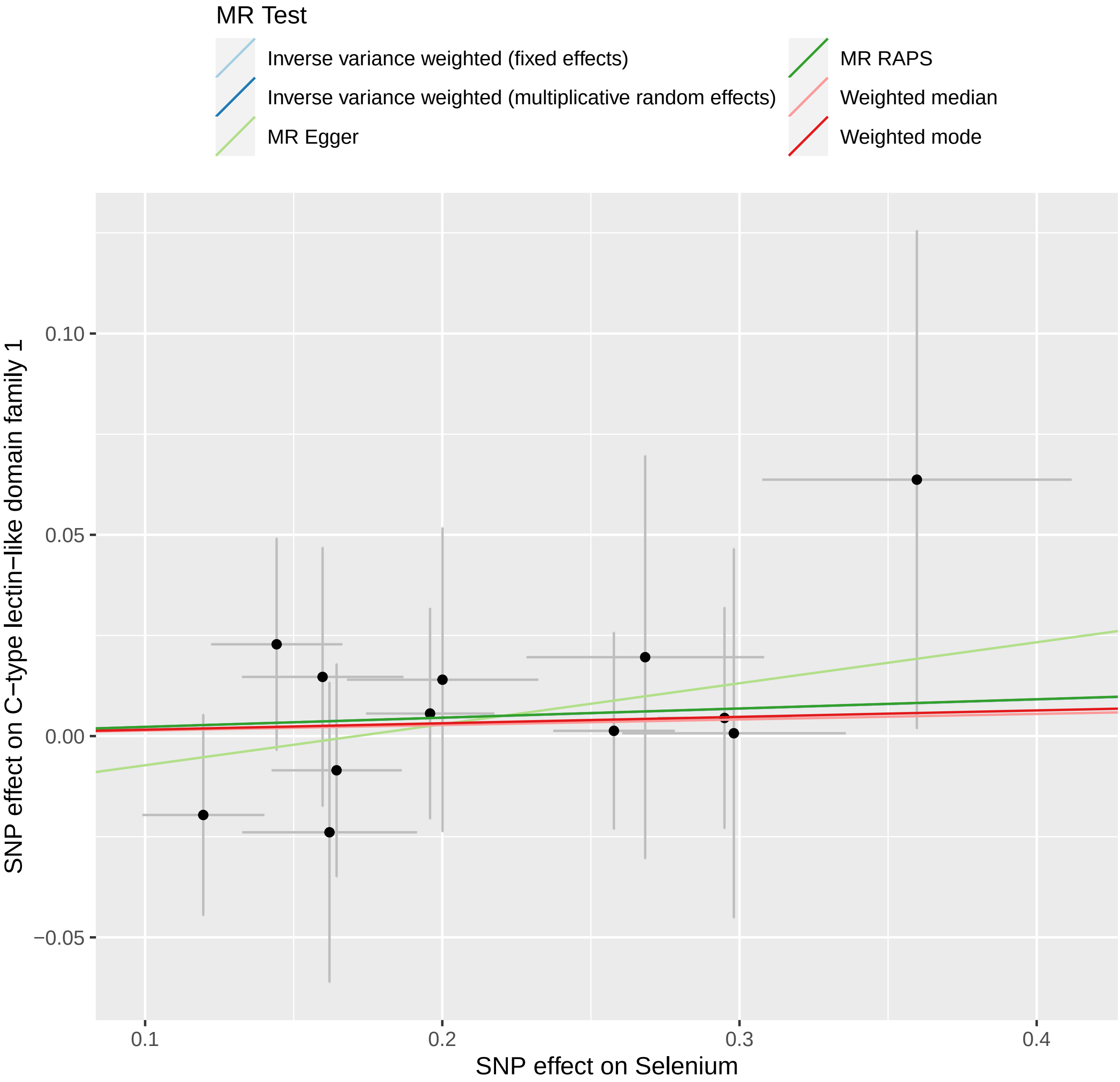

Batch 3−583 : Selenium on Chloride intracellular channel protein 4

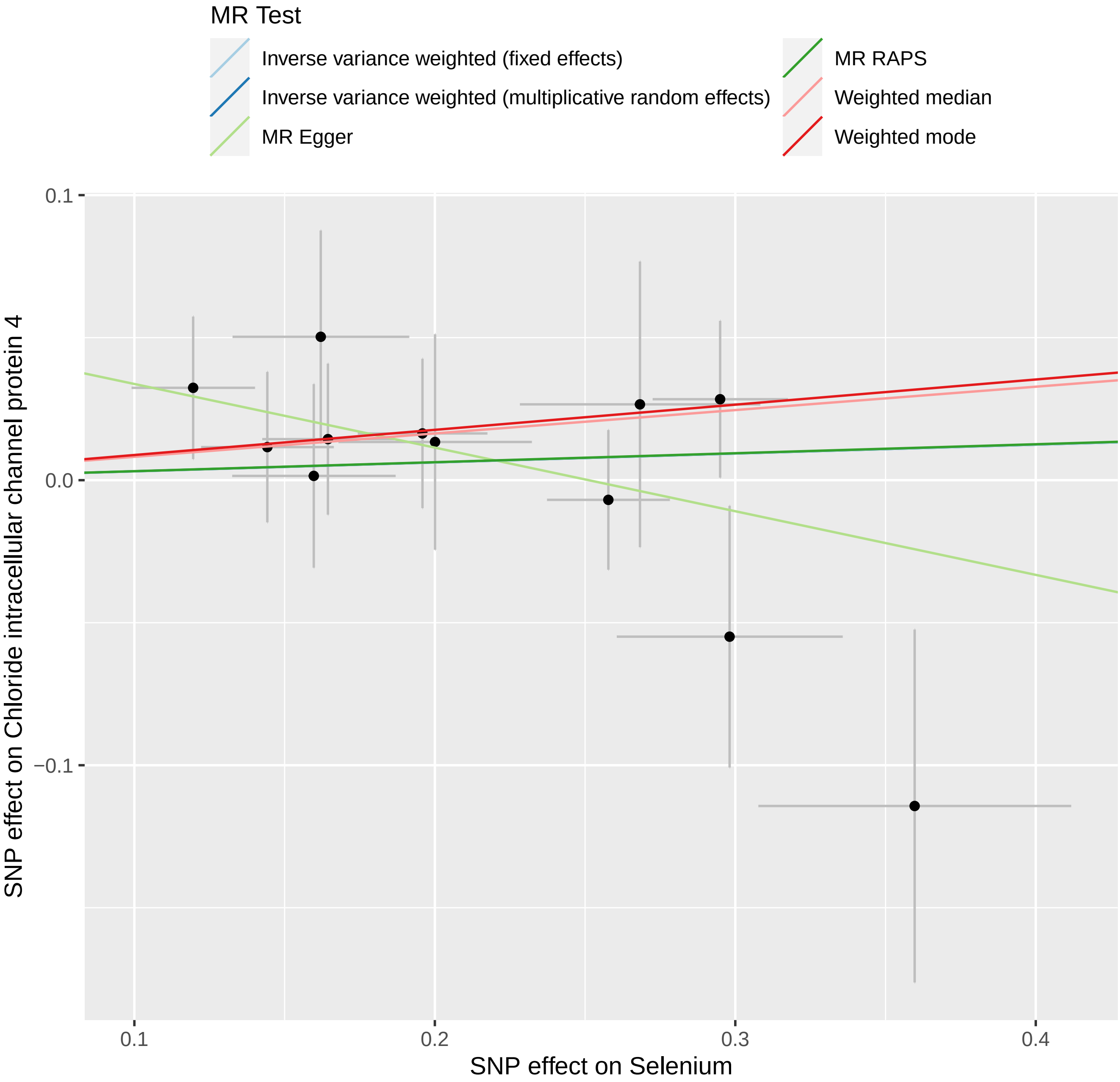

Batch 3−584 : Selenium on Chloride intracellular channel protein 5

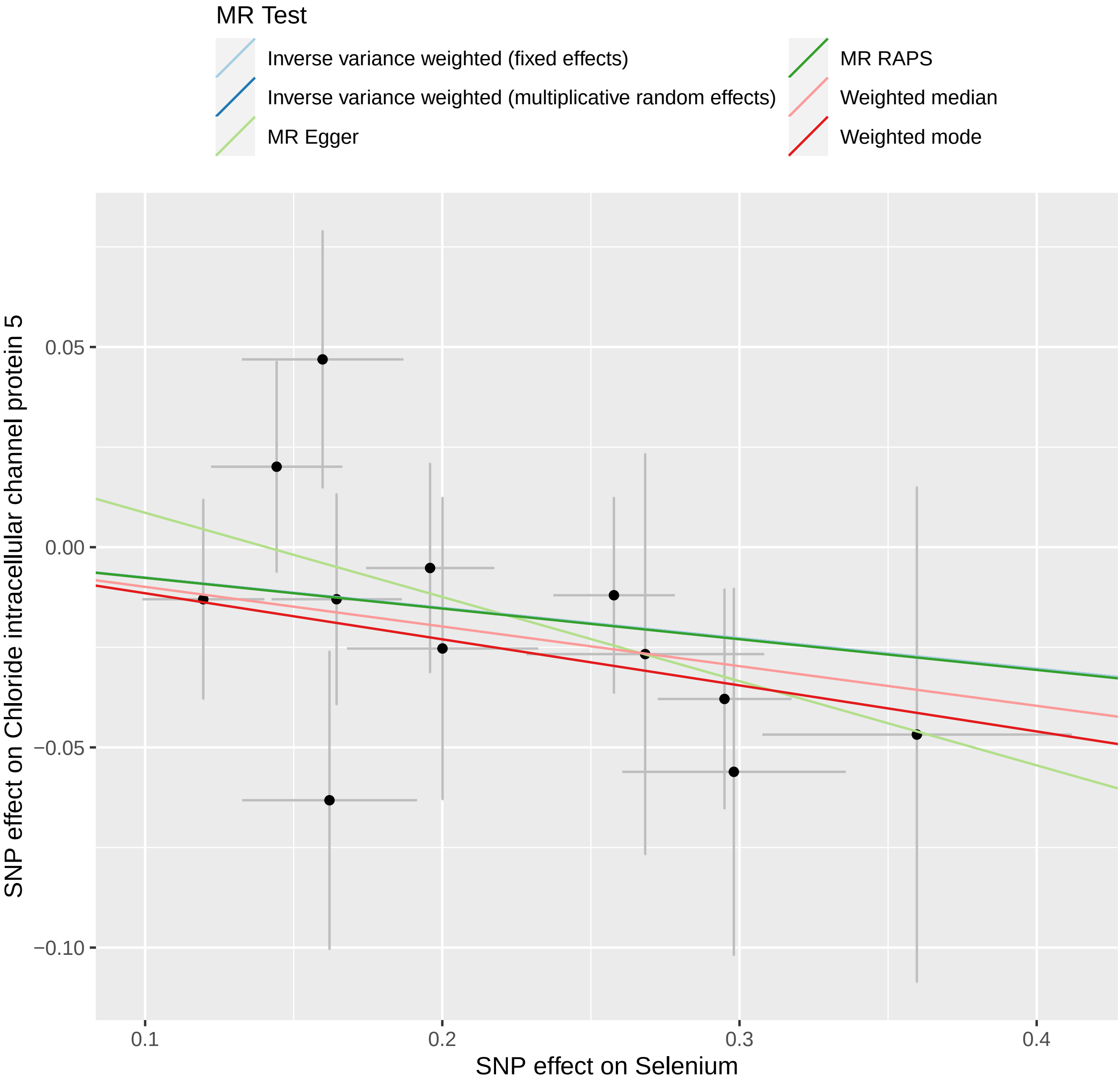

Batch 3−585 : Selenium on Clathrin interactor 1

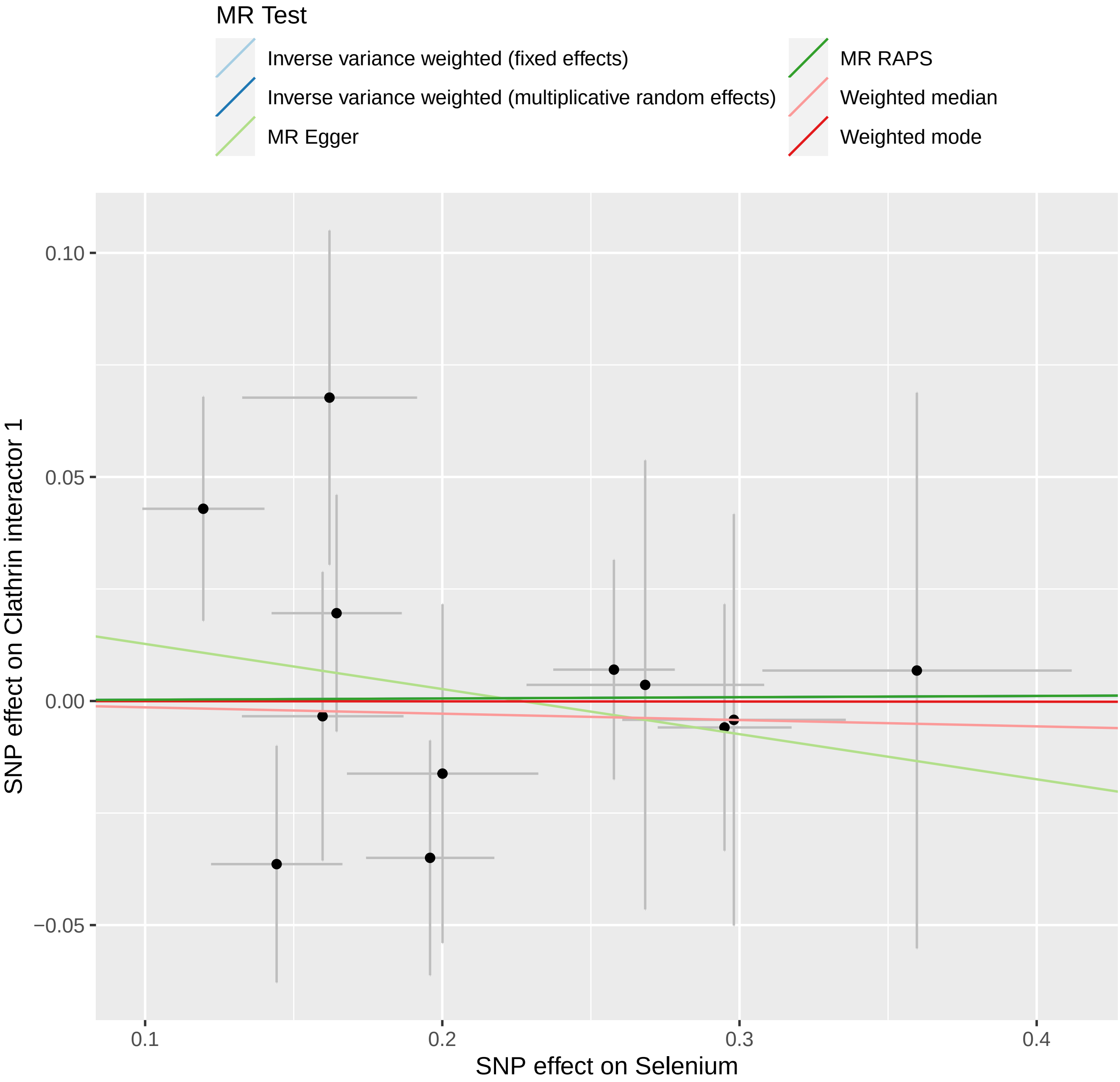

Batch 3−586 : Selenium on Dual specificity protein kinase CLK2

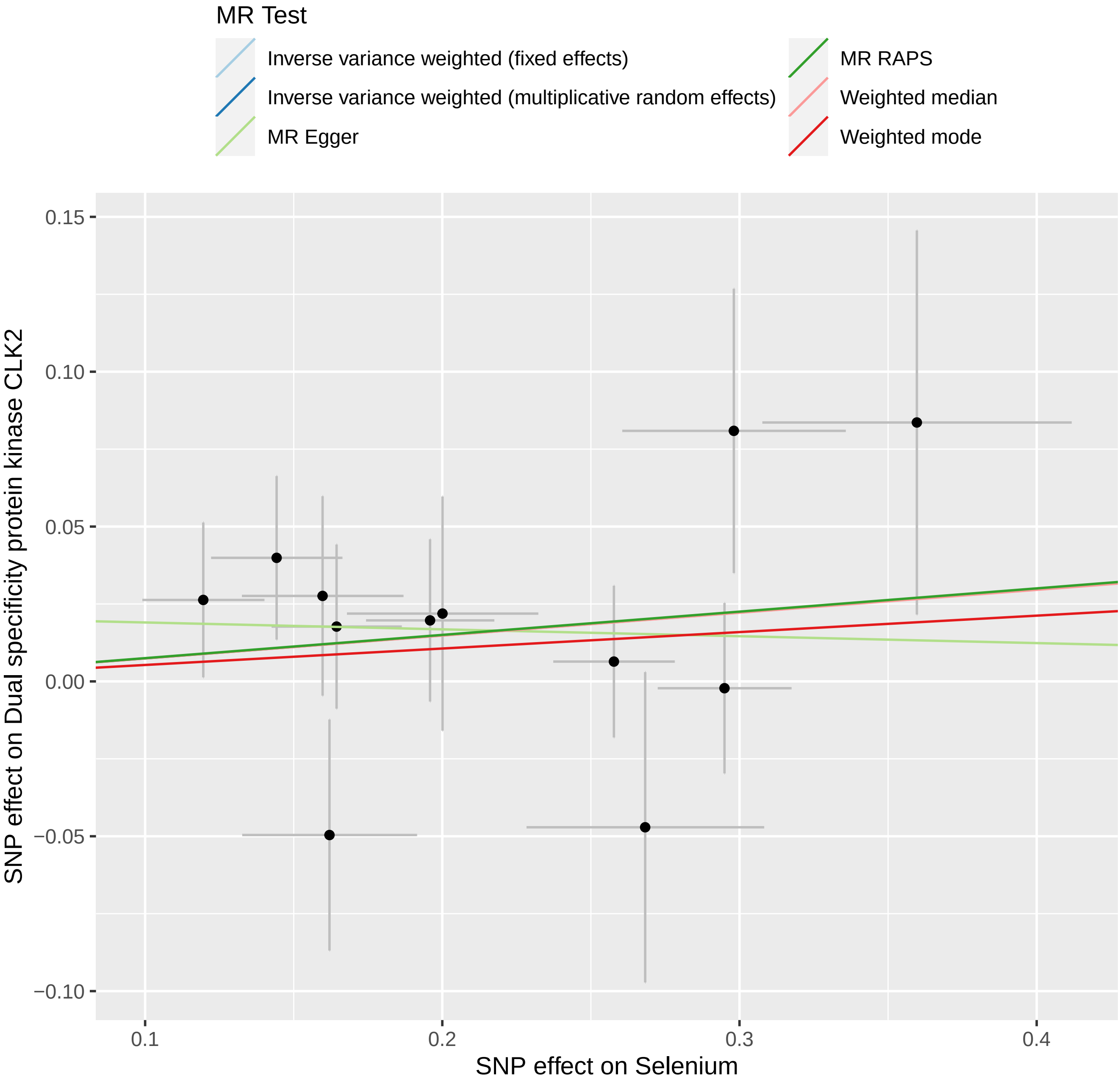

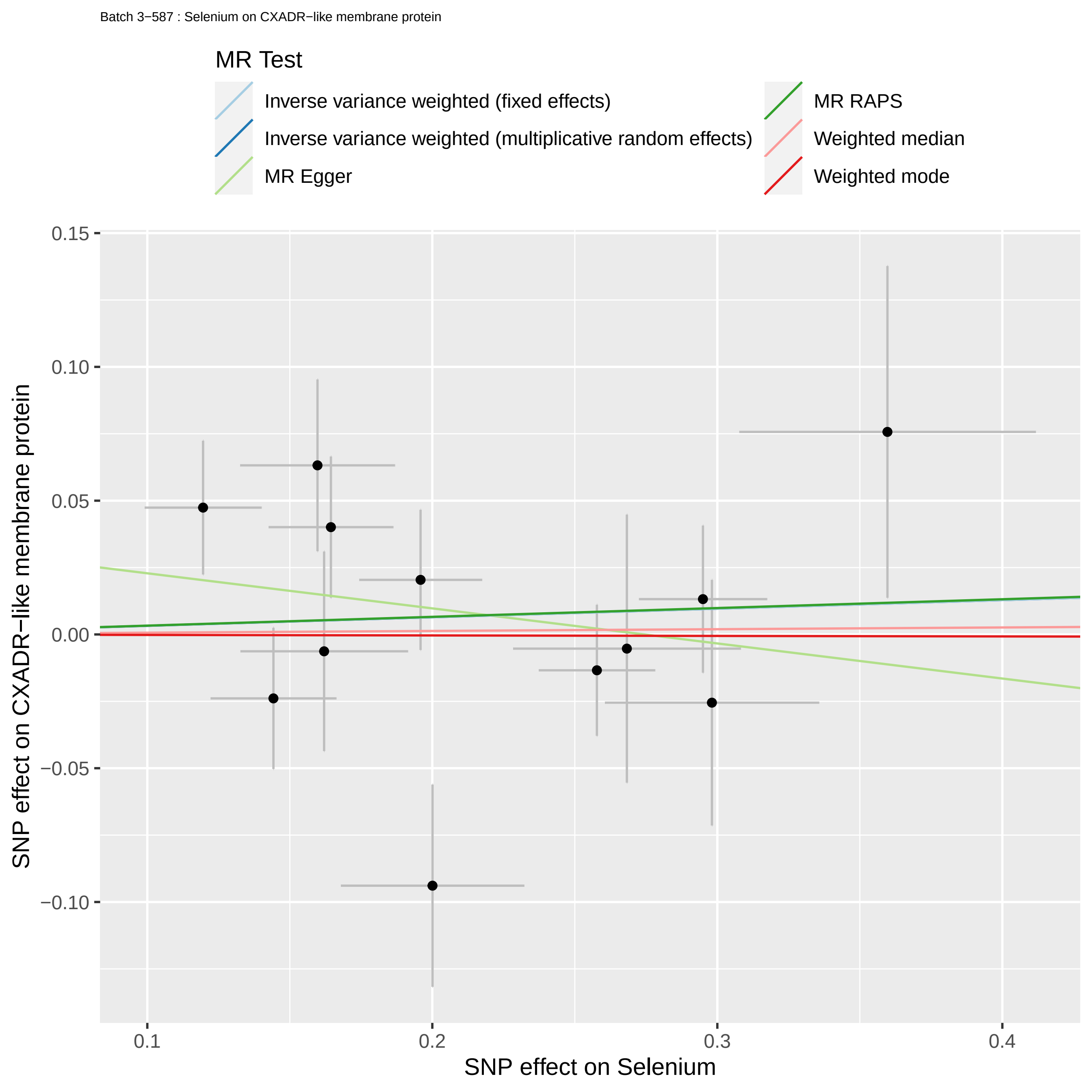

Batch 3−587 : Selenium on CXADR−like membrane protein
MR Test
Inverse variance weighted (fixed effects)
Inverse variance weighted (multiplicative random effects)
MR Egger
MR RAPS
Weighted median
Weighted mode
0.15
0.10
0.05
0.00
−0.05
−0.10
0.1
0.2
0.3
0.4
SNP effect on CXADR−like membrane protein
SNP effect on Selenium

Batch 3−588 : Selenium on CXADR−like membrane protein

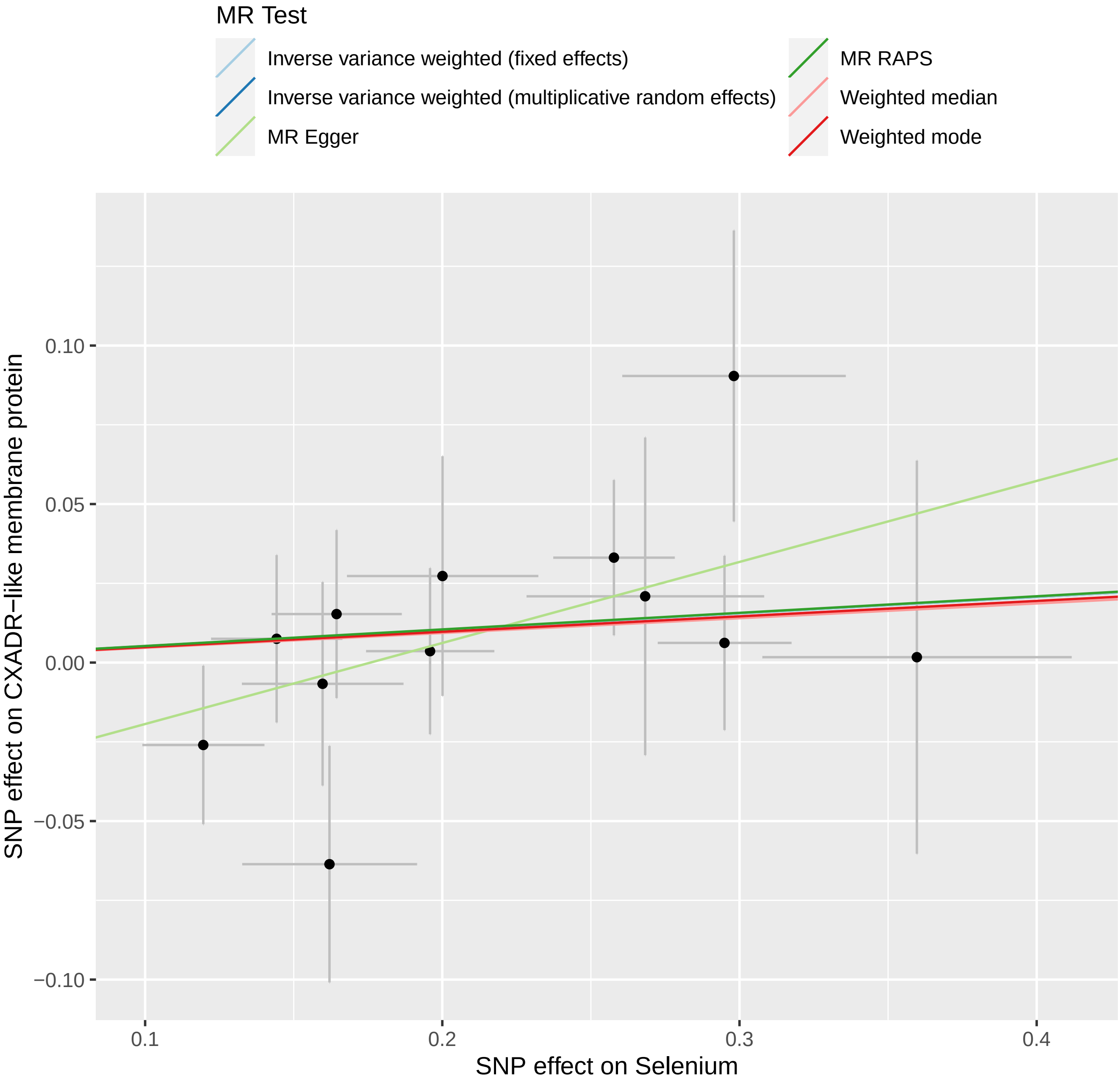

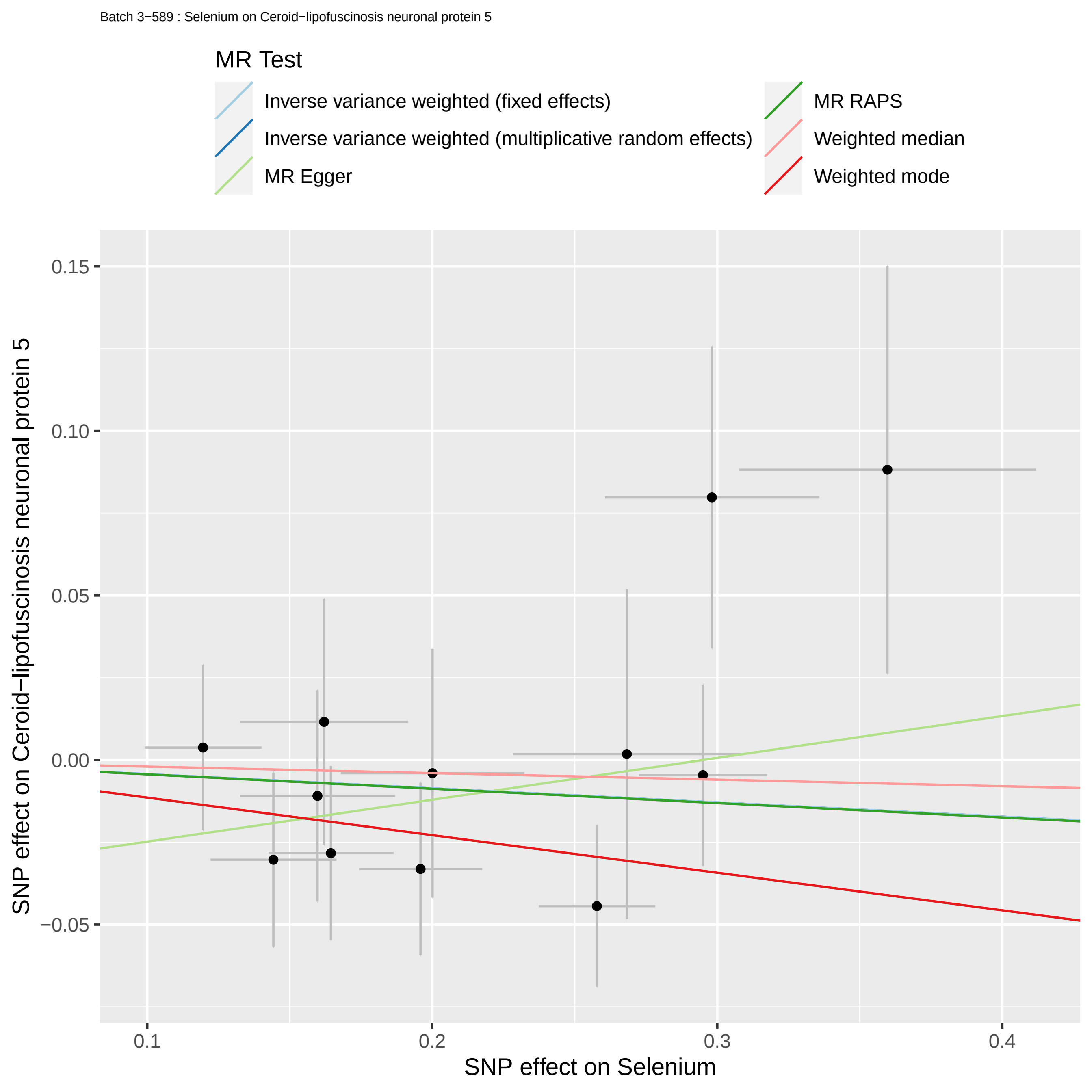

Batch 3−589 : Selenium on Ceroid−lipofuscinosis neuronal protein 5
MR Test
Inverse variance weighted (fixed effects)
Inverse variance weighted (multiplicative random effects)
MR Egger
MR RAPS
Weighted median
Weighted mode
0.15
0.10
0.05
0.00
−0.05
0.1
0.2
0.3
0.4
SNP effect on Ceroid−lipofuscinosis neuronal protein 5
SNP effect on Selenium

Batch 3−590 : Selenium on Ceroid−lipofuscinosis neuronal protein 5

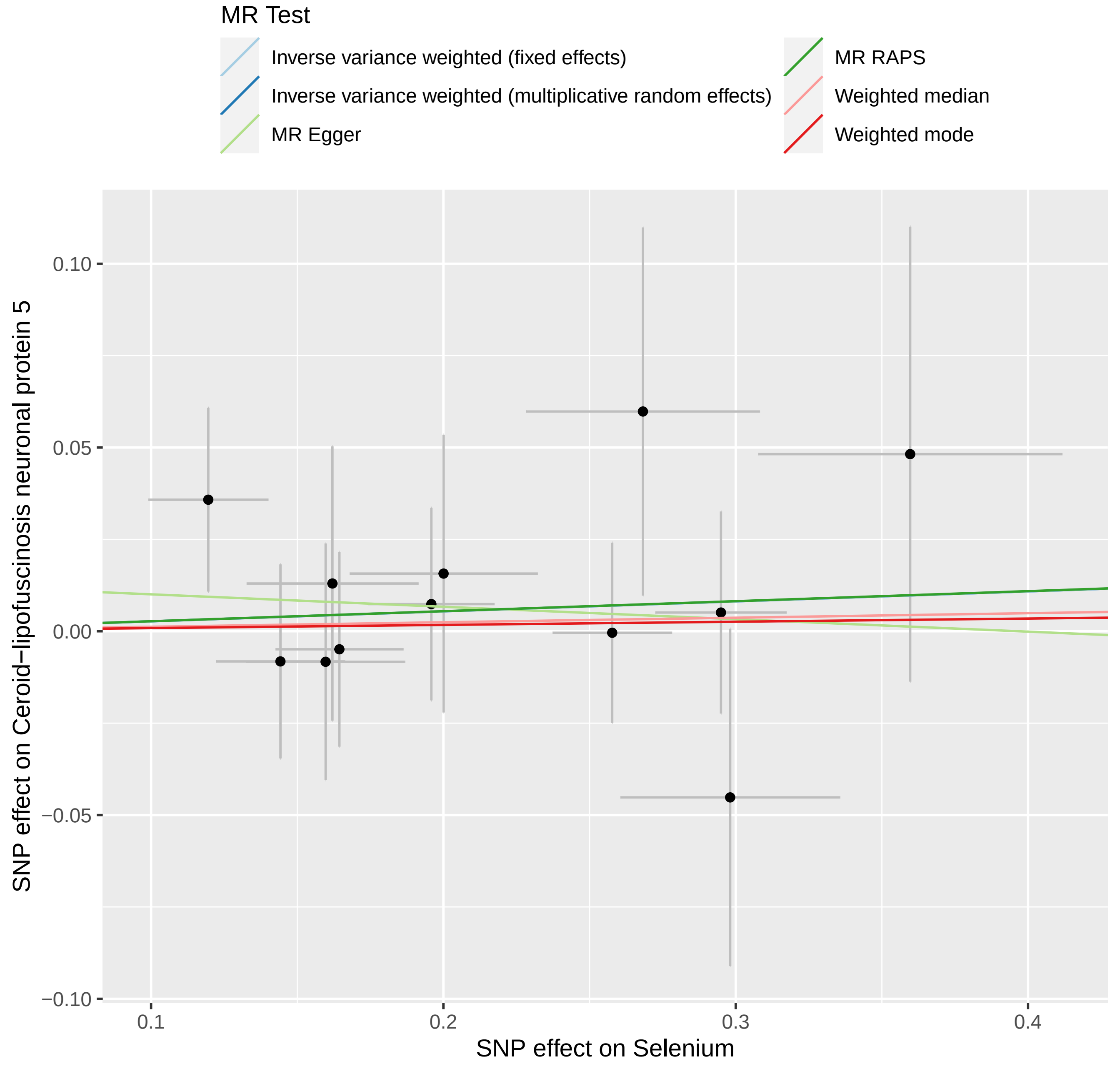

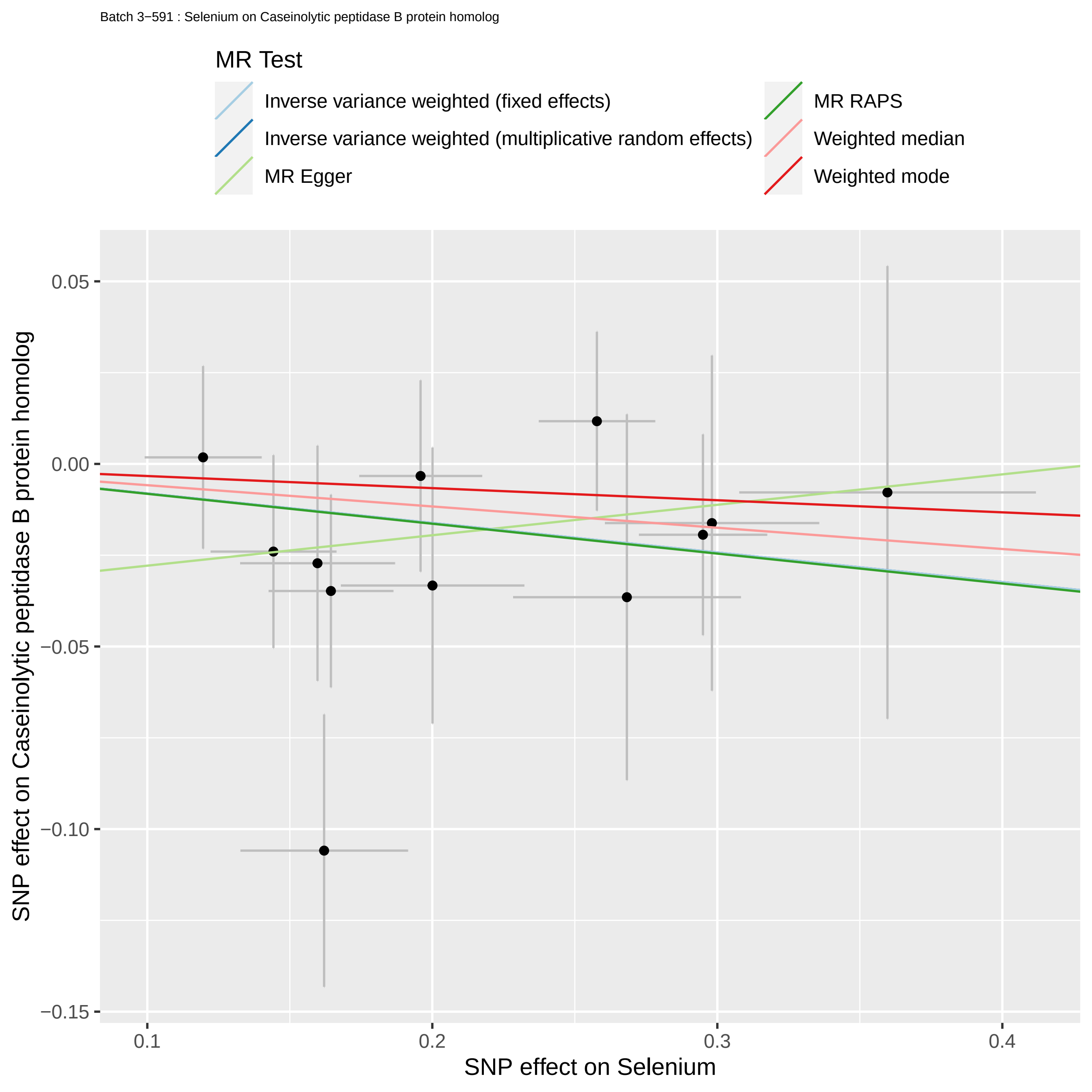
Batch 3−591 : Selenium on Caseinolytic peptidase B protein homolog
MR Test
Inverse variance weighted (fixed effects)
Inverse variance weighted (multiplicative random effects)
MR Egger
MR RAPS
Weighted median
Weighted mode
0.05
0.00
−0.05
−0.10
−0.15
0.1
0.2
0.3
0.4
SNP effect on Caseinolytic peptidase B protein homolog
SNP effect on Selenium

Batch 3−592 : Selenium on Colipase

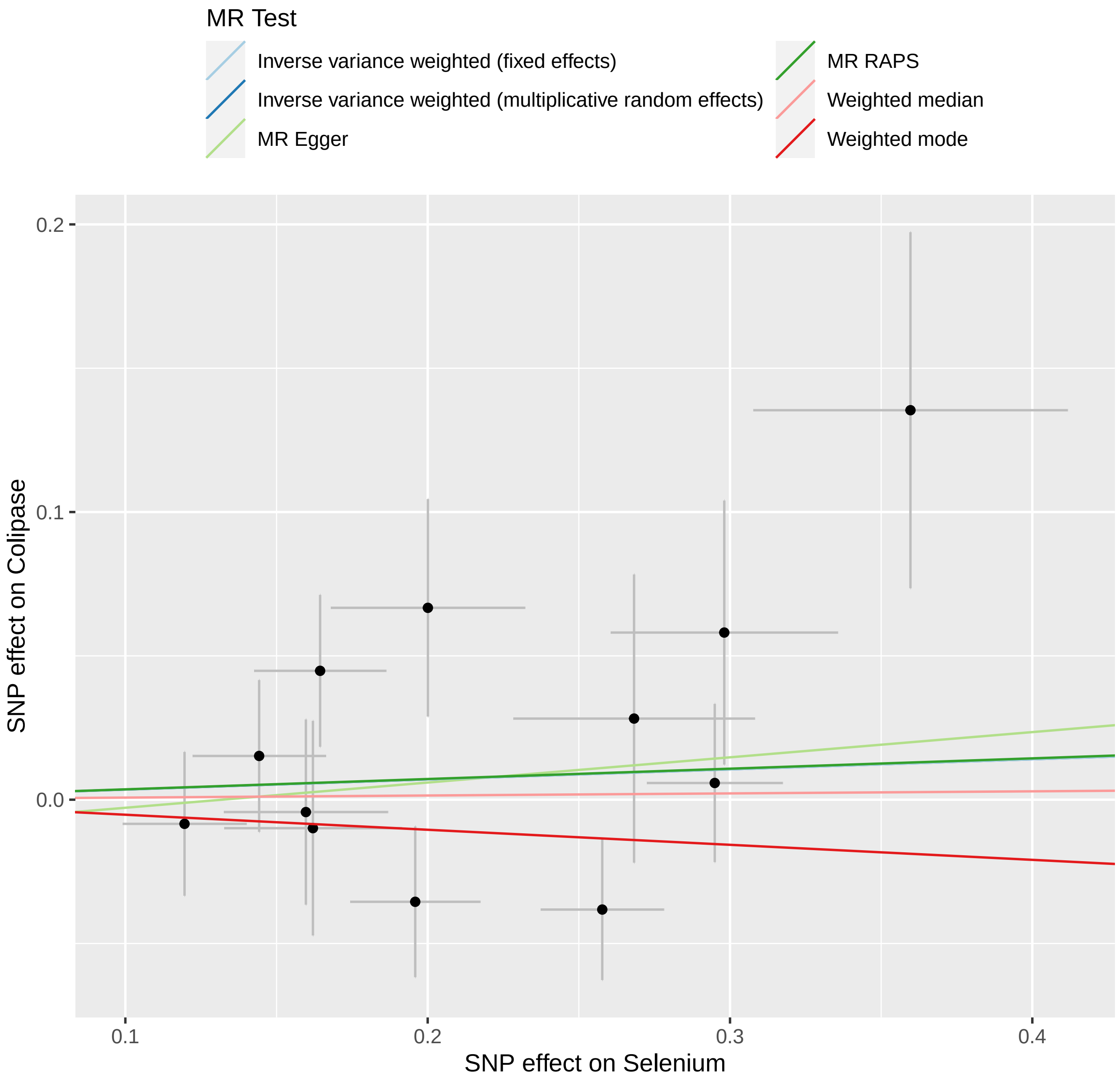

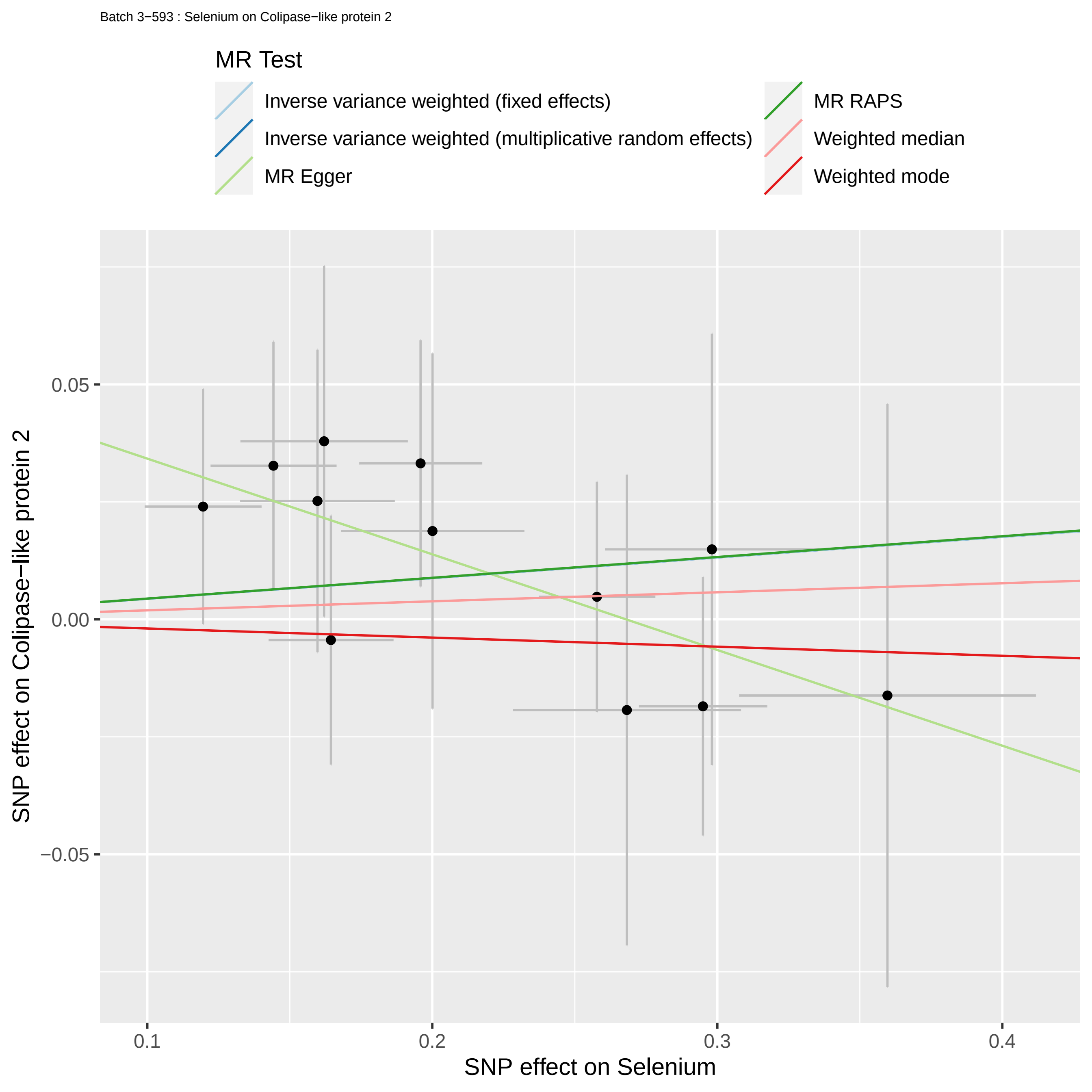

Batch 3−593 : Selenium on Colipase−like protein 2
MR Test
Inverse variance weighted (fixed effects)
Inverse variance weighted (multiplicative random effects)
MR Egger
MR RAPS
Weighted median
Weighted mode
0.05
0.00
−0.05
0.1
0.2
0.3
0.4
SNP effect on Colipase−like protein 2
SNP effect on Selenium

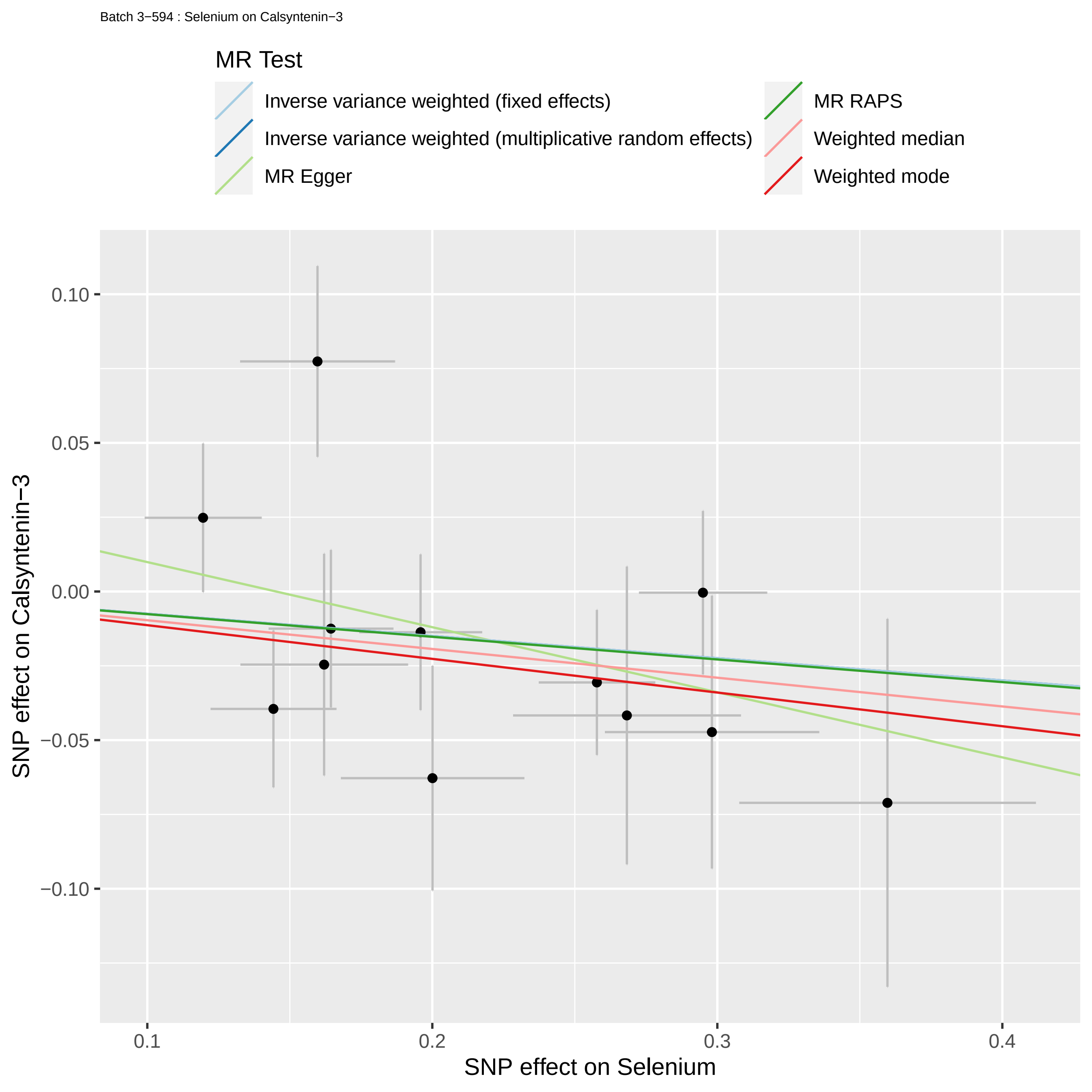
Batch 3−594 : Selenium on Calsyntenin−3
MR Test
Inverse variance weighted (fixed effects)
Inverse variance weighted (multiplicative random effects)
MR Egger
MR RAPS
Weighted median
Weighted mode
0.10
0.05
0.00
−0.05
−0.10
SNP effect on Calsyntenin−3
0.1
0.2
0.3
0.4
SNP effect on Selenium

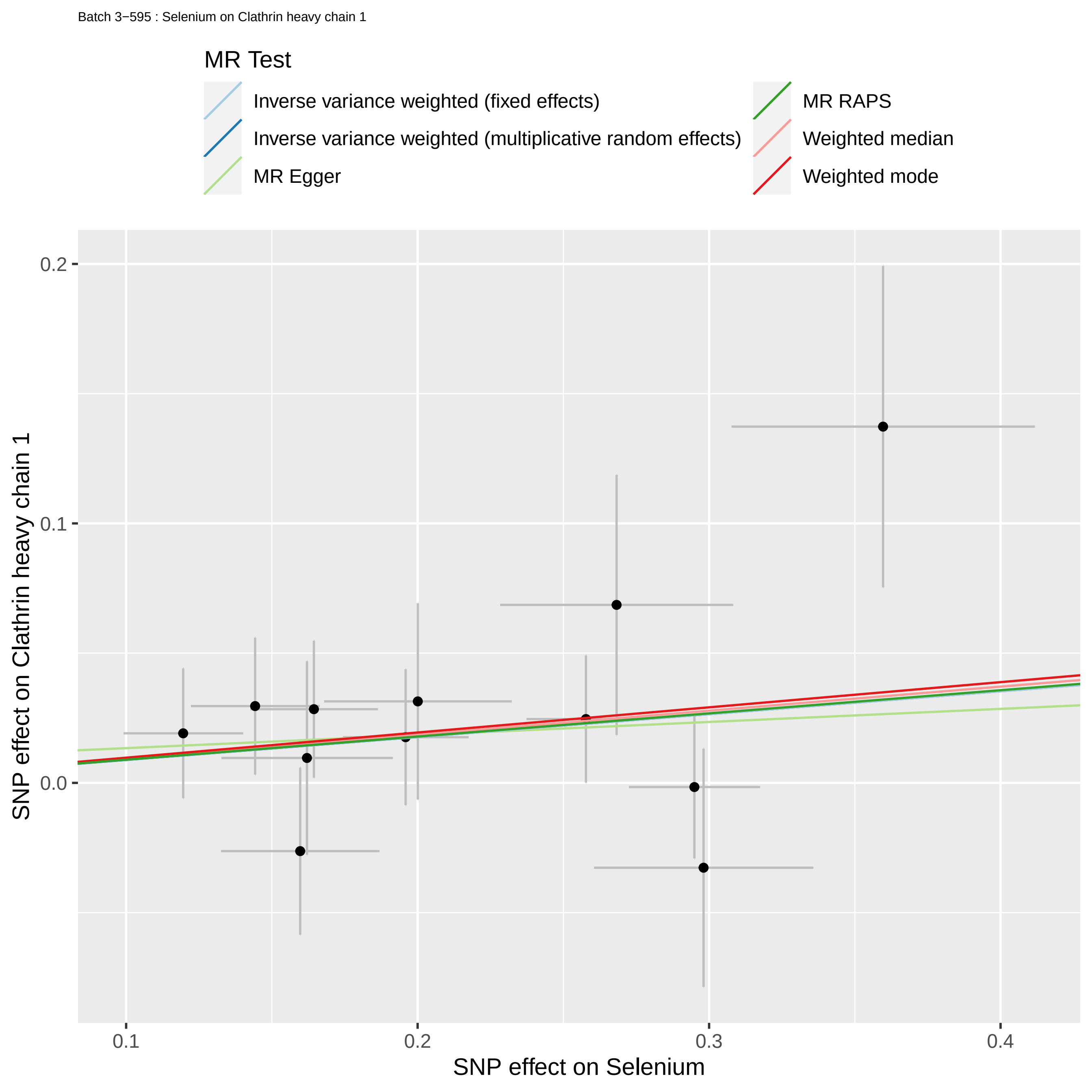

Batch 3−595 : Selenium on Clathrin heavy chain 1
MR Test
Inverse variance weighted (fixed effects)
Inverse variance weighted (multiplicative random effects)
MR Egger
MR RAPS
Weighted median
Weighted mode
0.2
0.1
0.0
0.1
0.2
0.3
0.4
SNP effect on Clathrin heavy chain 1
SNP effect on Selenium

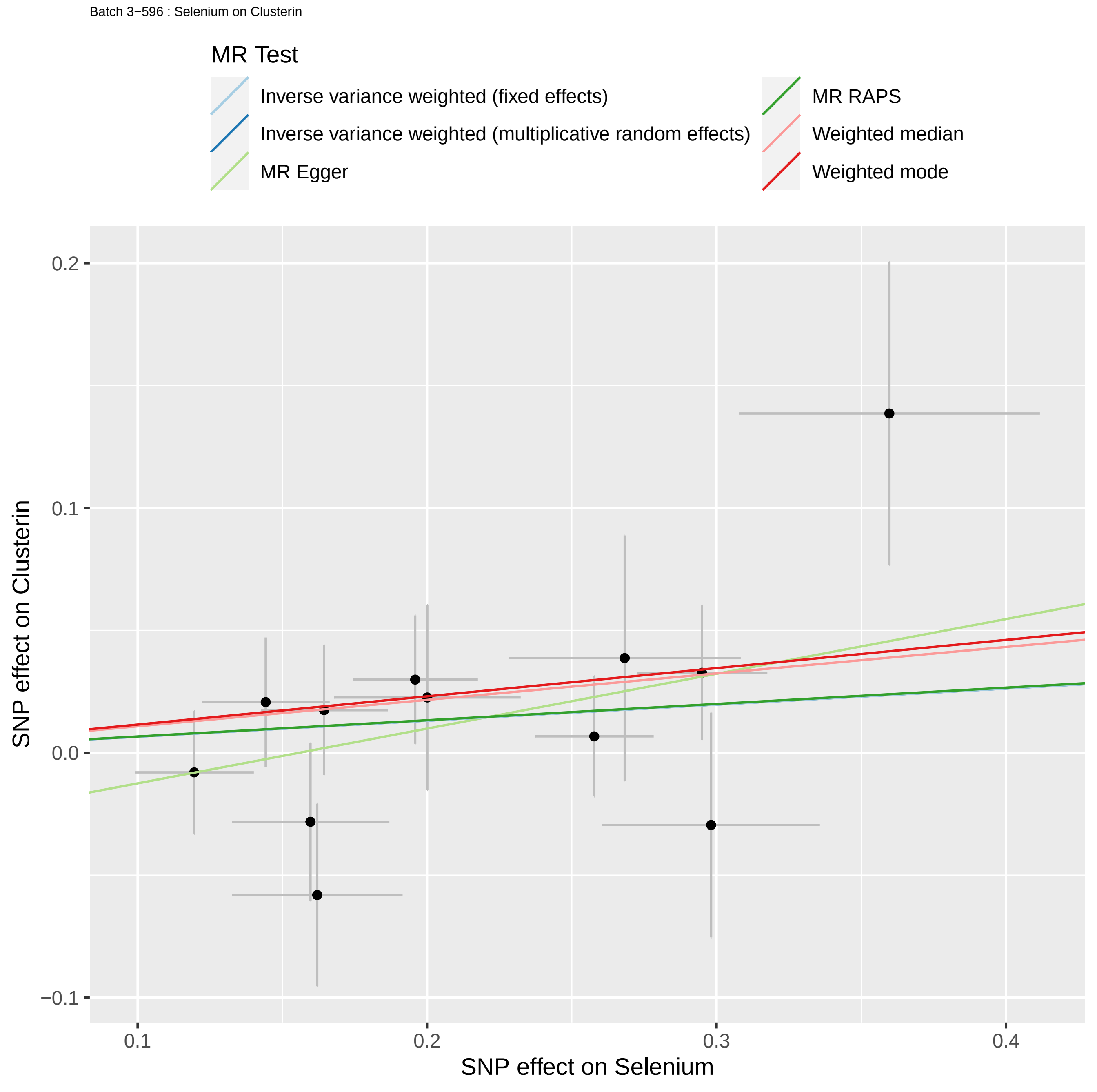

Batch 3−596 : Selenium on Clusterin
MR Test
Inverse variance weighted (fixed effects)
Inverse variance weighted (multiplicative random effects)
MR Egger
MR RAPS
Weighted median
Weighted mode
0.2
0.1
0.0
−0.1
0.1
0.2
0.3
0.4
SNP effect on Clusterin
SNP effect on Selenium

Batch 3−597 : Selenium on Chymase

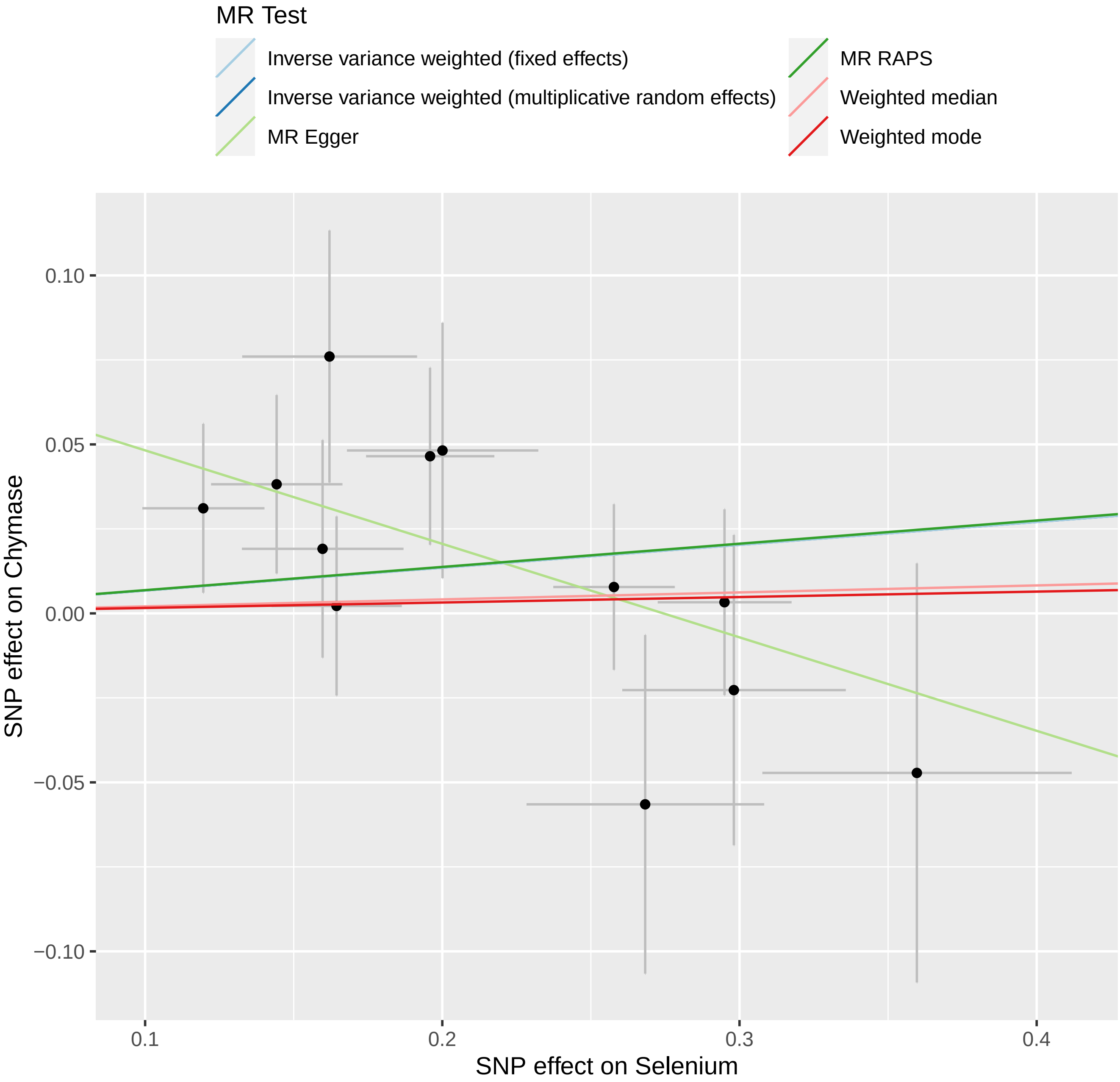

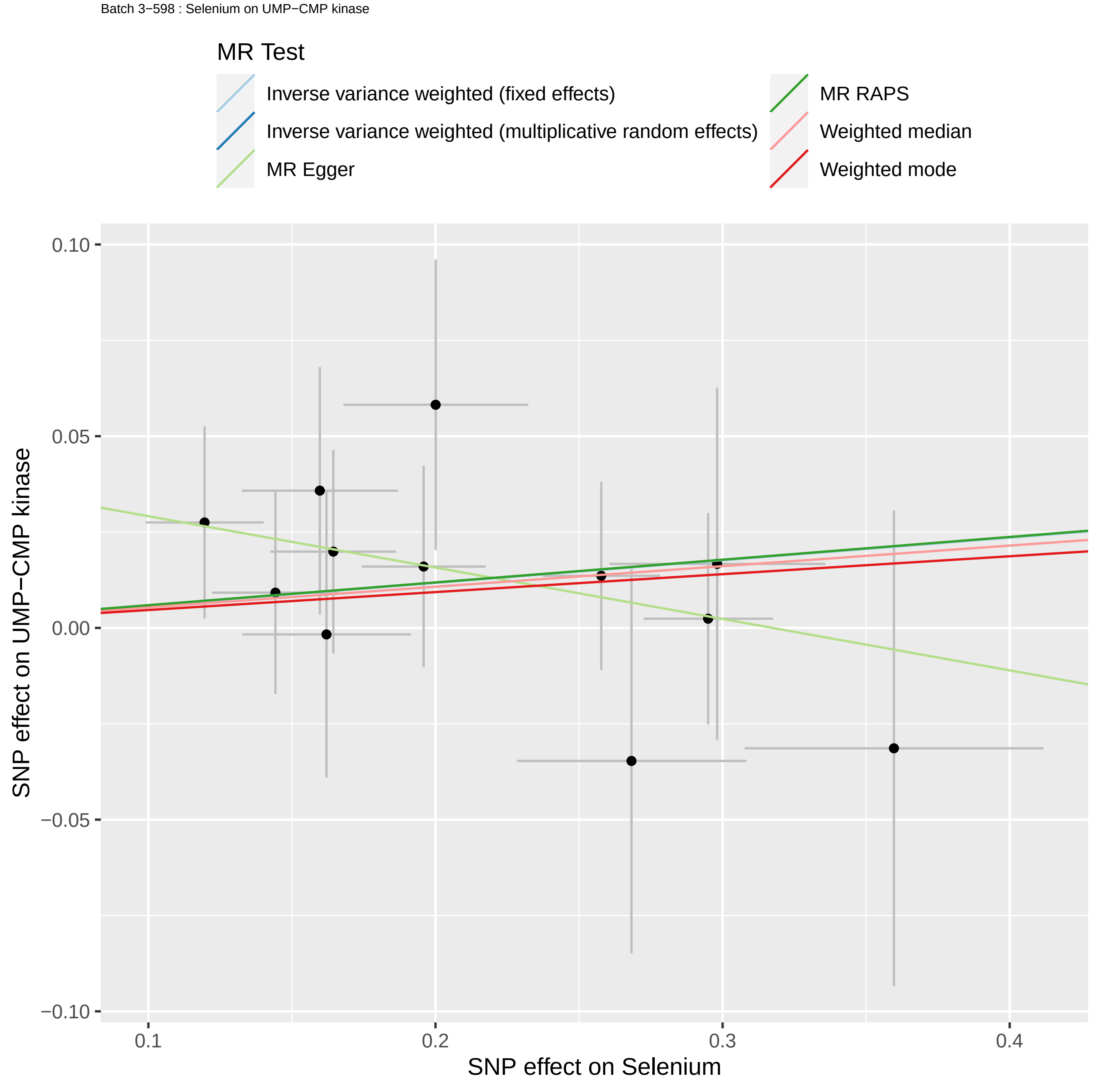
Batch 3−598 : Selenium on UMP−CMP kinase
MR Test
Inverse variance weighted (fixed effects)
Inverse variance weighted (multiplicative random effects)
MR Egger
MR RAPS
Weighted median
Weighted mode
0.10
0.05
0.00
−0.05
−0.10
SNP effect on UMP−CMP kinase
0.1
0.2
0.3
0.4
SNP effect on Selenium

Batch 3−599 : Selenium on UMP−CMP kinase

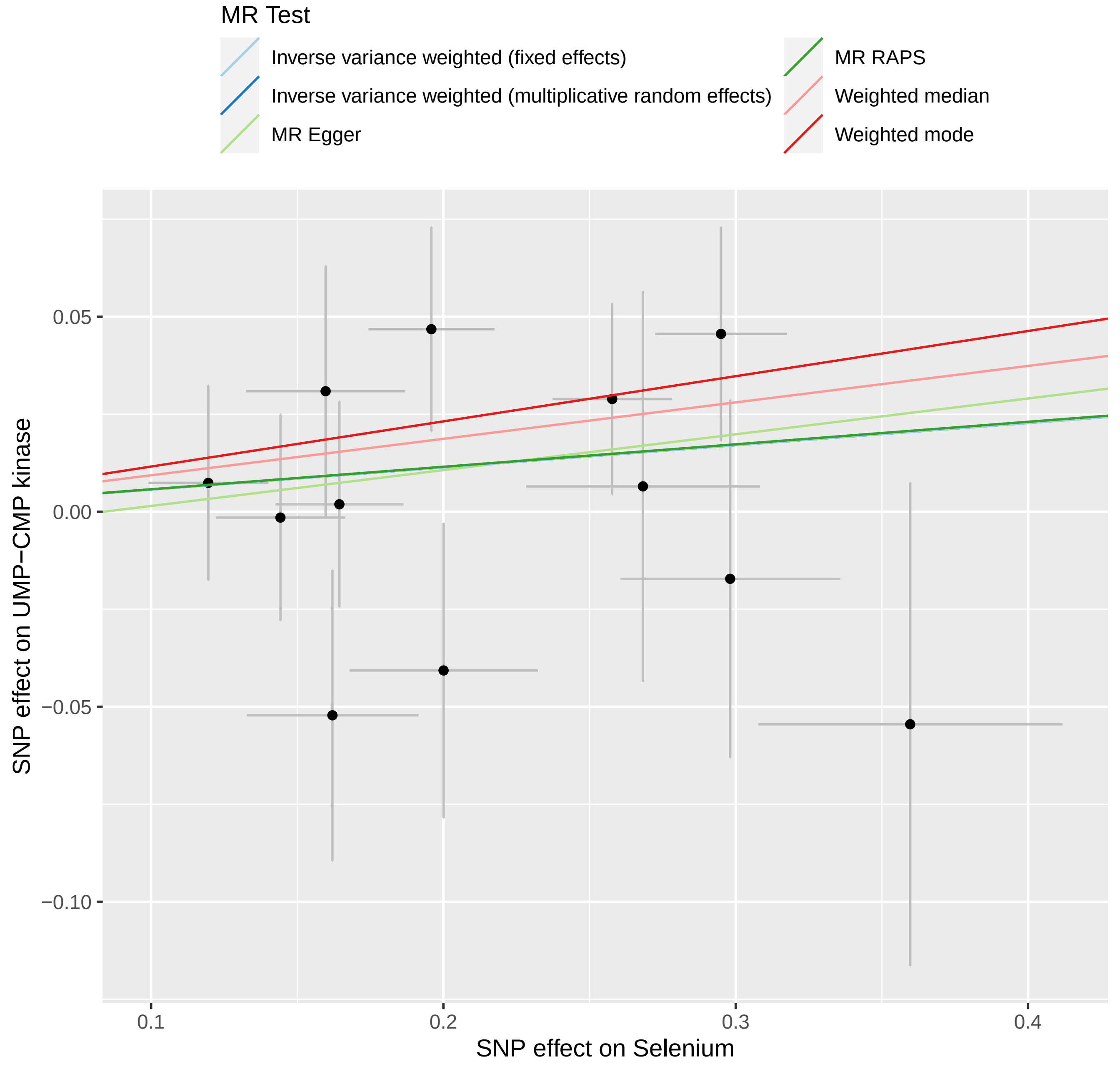

Batch 3−600 : Selenium on Cytosolic non−specific dipeptidase

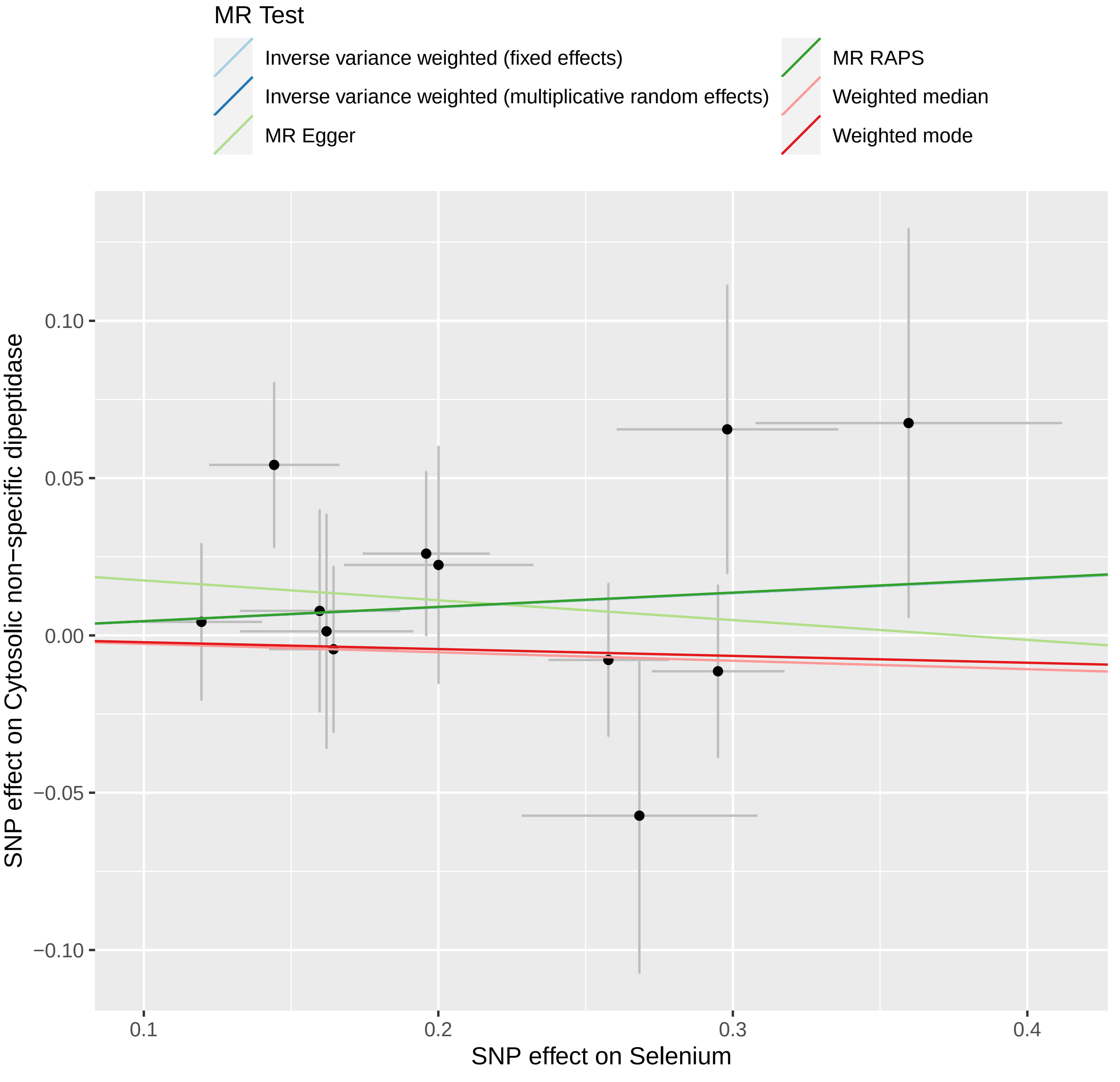

Batch 3−601 : Selenium on Nuclear envelope phosphatase−regulatory subunit 1

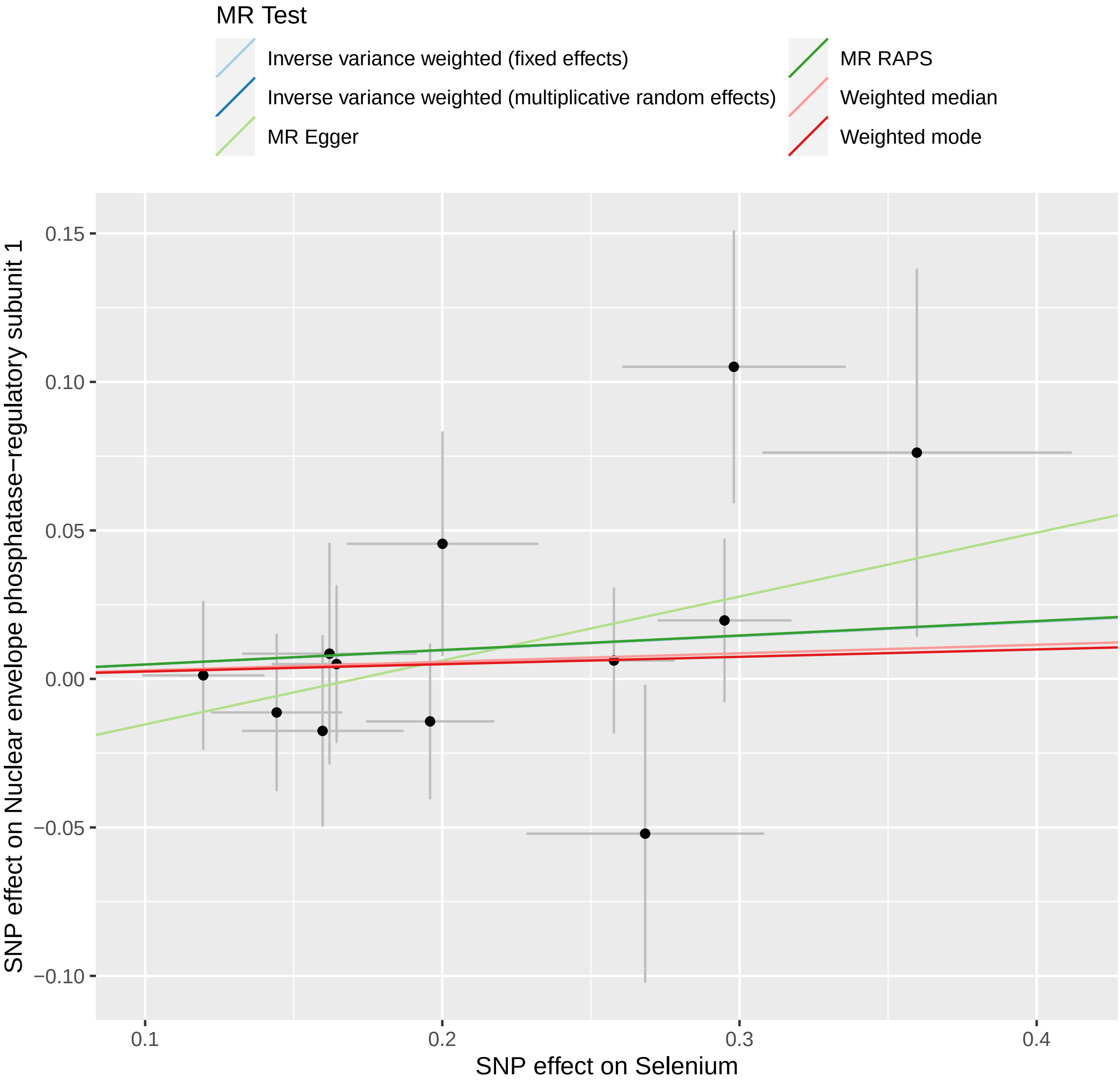

Batch 3−602 : Selenium on CCR4−NOT transcription complex subunit 1

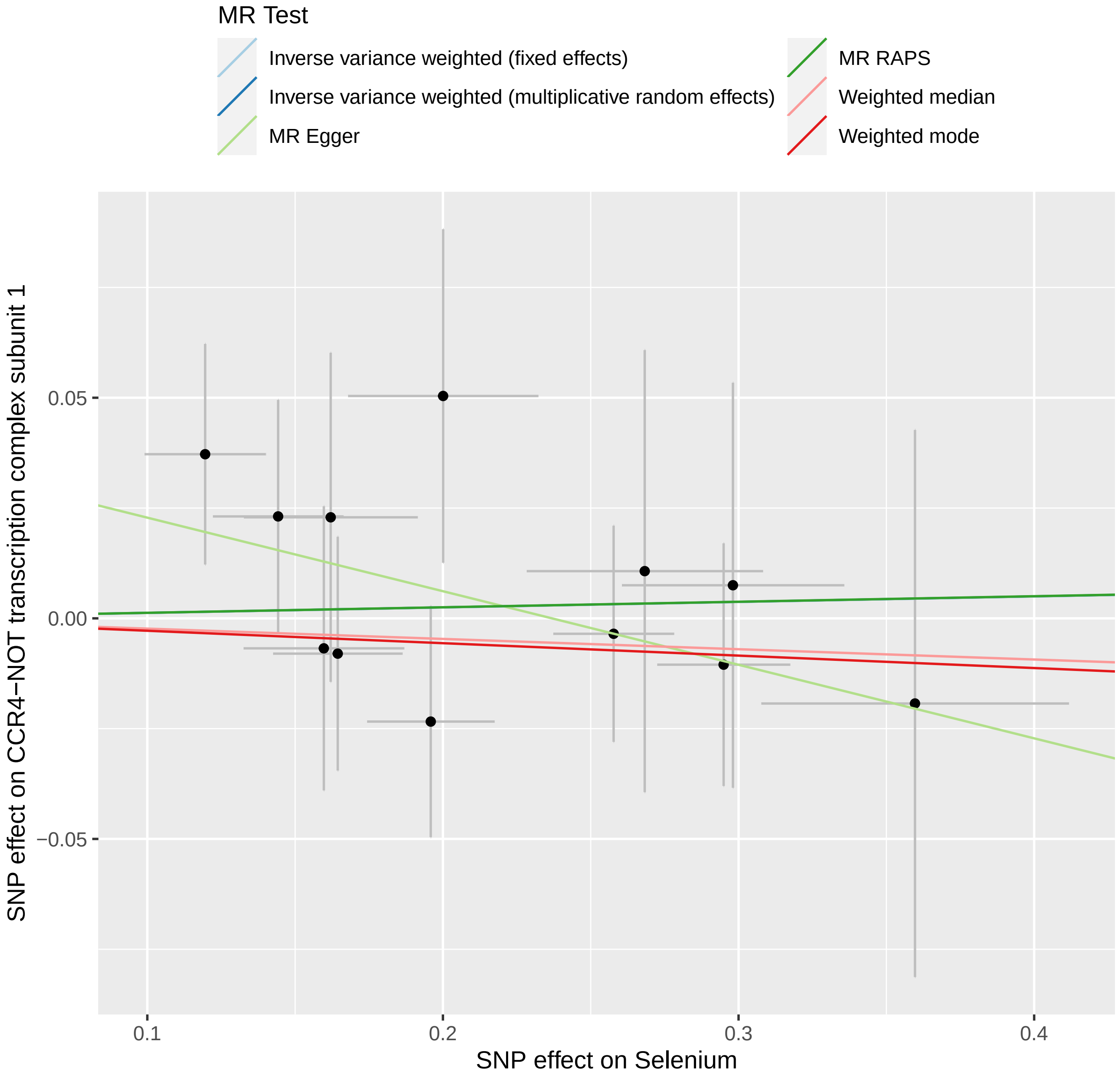

Batch 3−603 : Selenium on 2',3'−cyclic−nucleotide 3'−phosphodiesterase

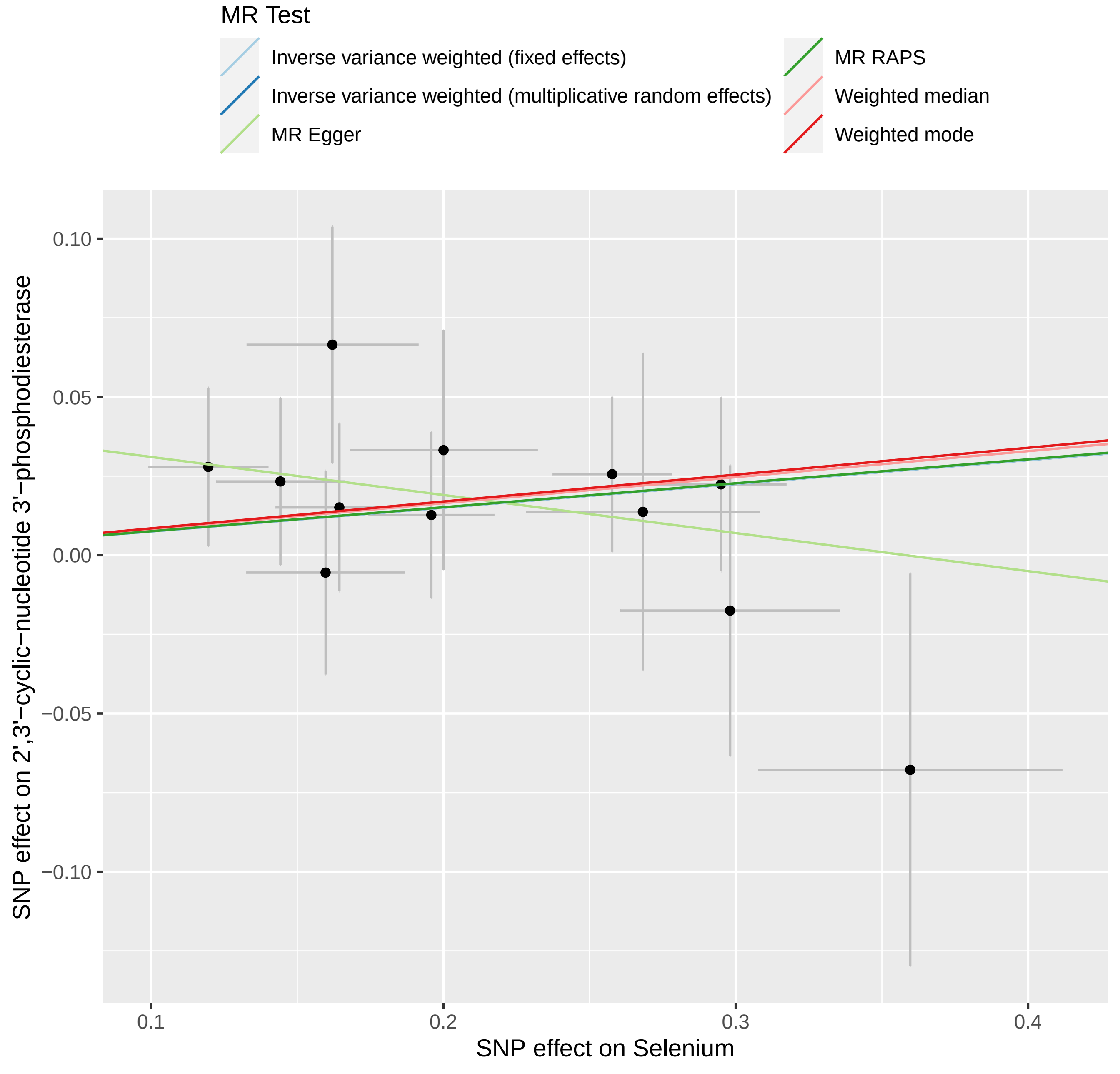

Batch 3−604 : Selenium on Ciliary neurotrophic factor

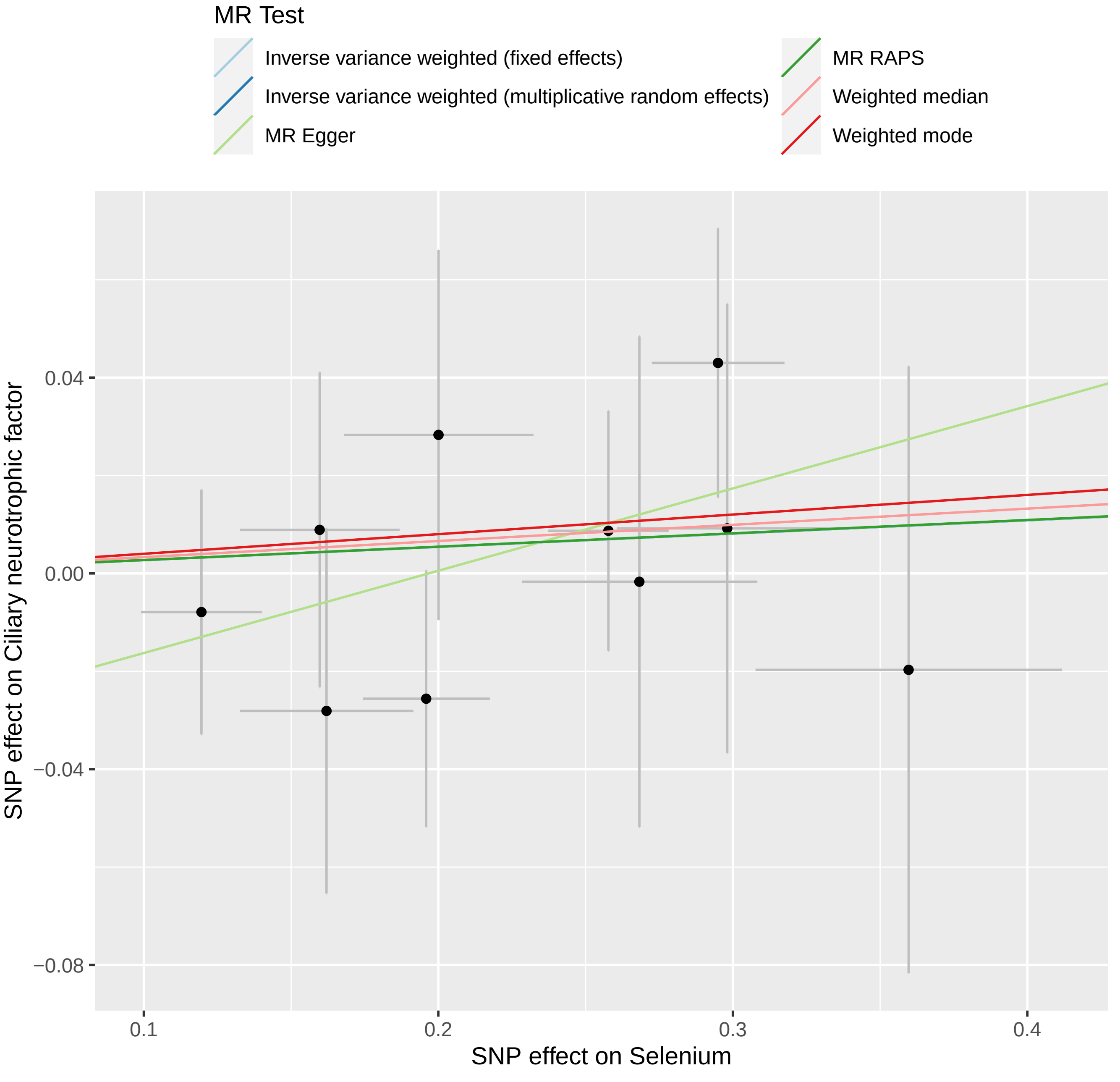

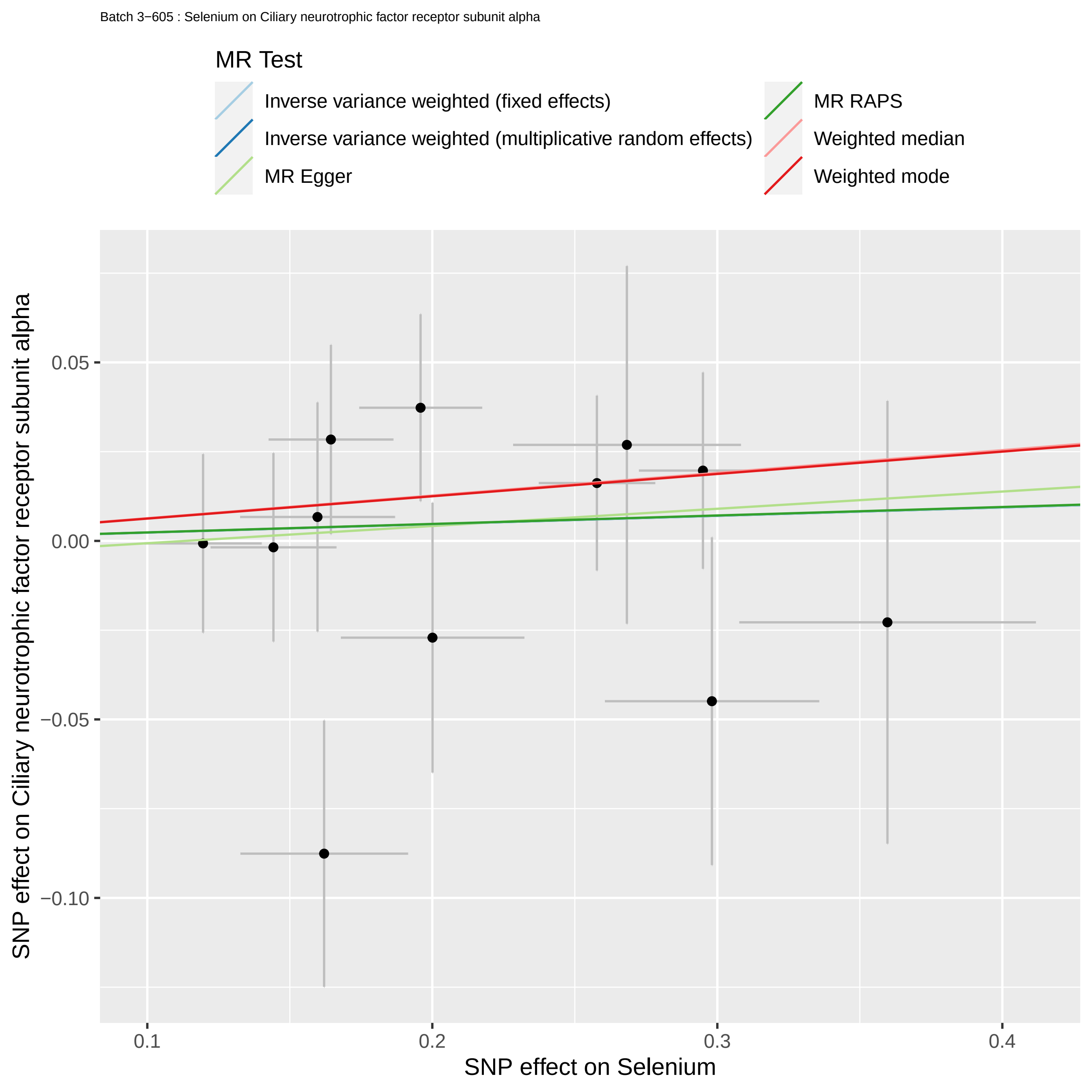

Batch 3−605 : Selenium on Ciliary neurotrophic factor receptor subunit alpha
MR Test
Inverse variance weighted (fixed effects)
Inverse variance weighted (multiplicative random effects)
MR Egger
MR RAPS
Weighted median
Weighted mode
SNP effect on Ciliary neurotrophic factor receptor subunit alpha
0.05
0.00
−0.05
−0.10
0.1
0.2
0.3
0.4
SNP effect on Selenium

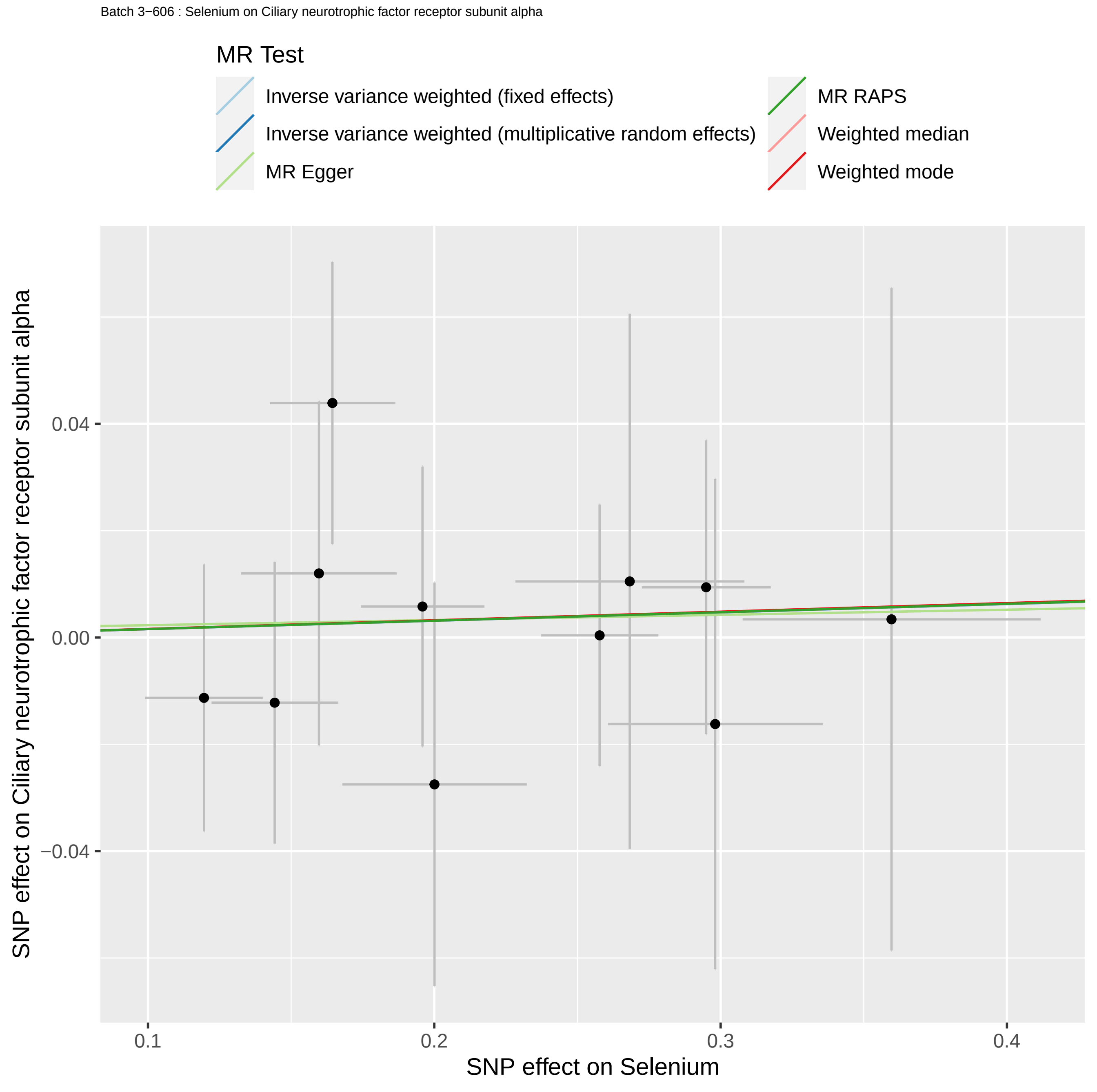

Batch 3−606 : Selenium on Ciliary neurotrophic factor receptor subunit alpha
MR Test
Inverse variance weighted (fixed effects)
Inverse variance weighted (multiplicative random effects)
MR Egger
MR RAPS
Weighted median
Weighted mode
0.04
0.00
−0.04
0.1
0.2
0.3
0.4
SNP effect on Selenium
SNP effect on Ciliary neurotrophic factor receptor subunit alpha

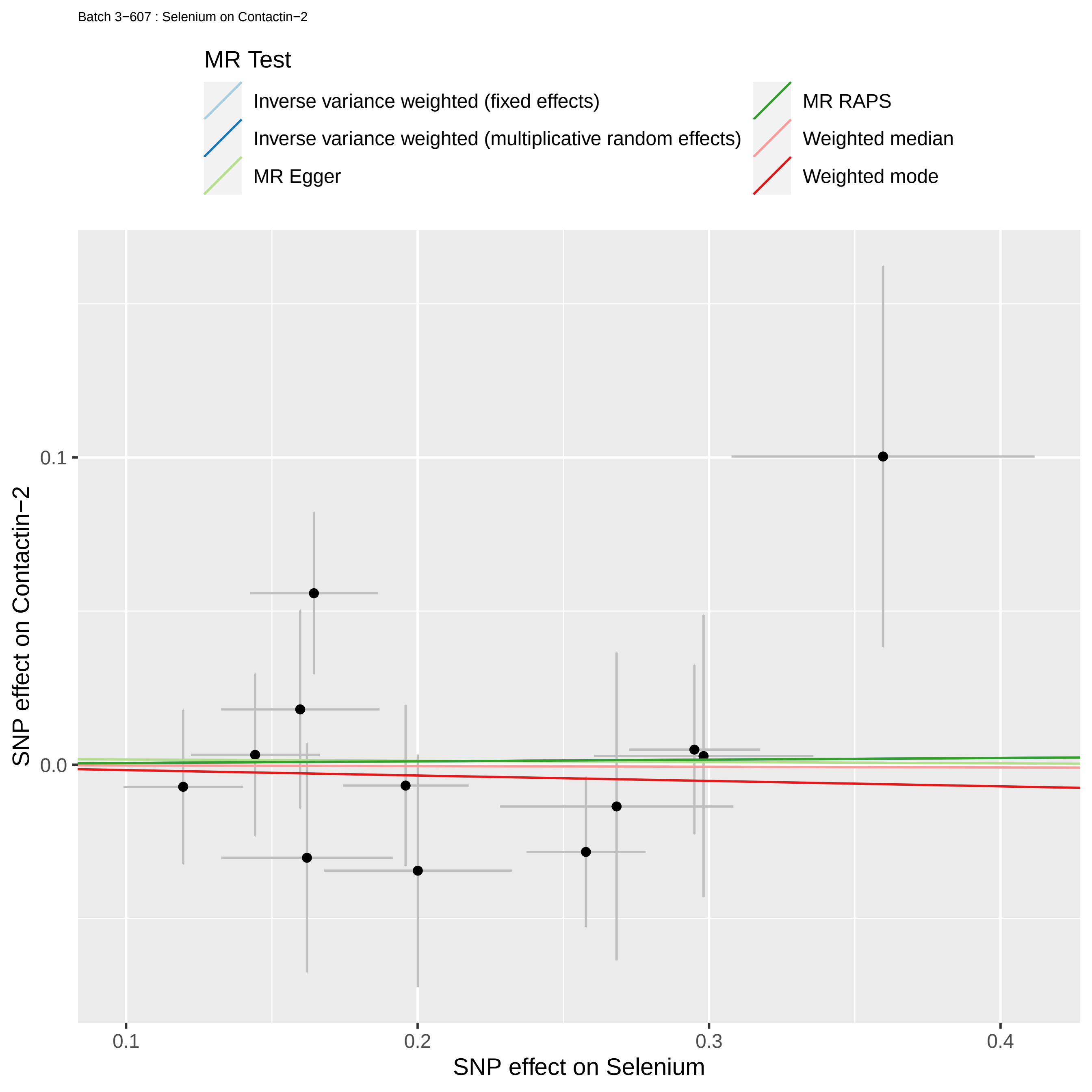

Batch 3−607 : Selenium on Contactin−2
MR Test
Inverse variance weighted (fixed effects)
Inverse variance weighted (multiplicative random effects)
MR Egger
MR RAPS
Weighted median
Weighted mode
SNP effect on Contactin−2
0.1
0.0
0.1
0.2
0.3
0.4
SNP effect on Selenium

Batch 3−608 : Selenium on Contactin−4

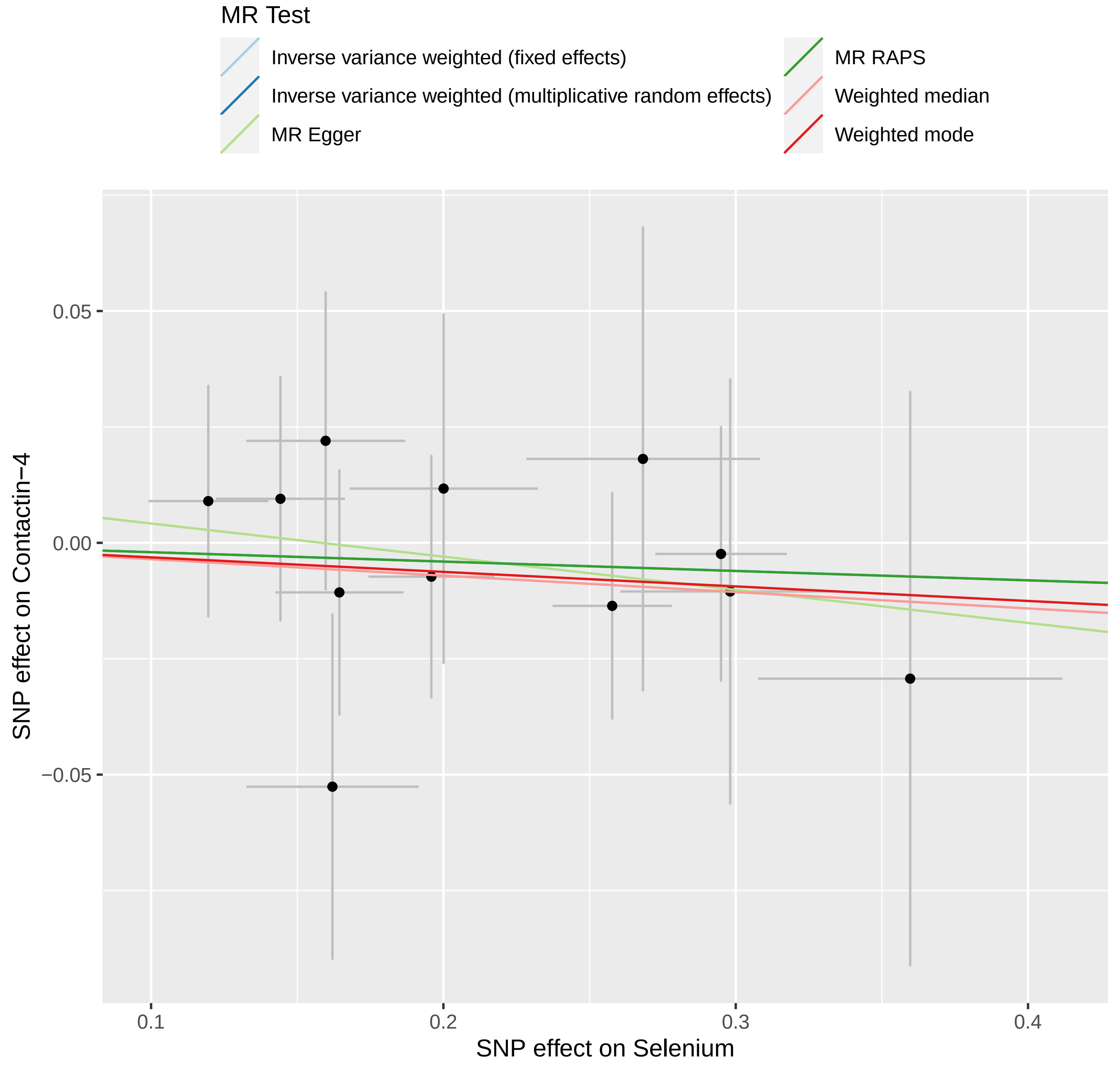

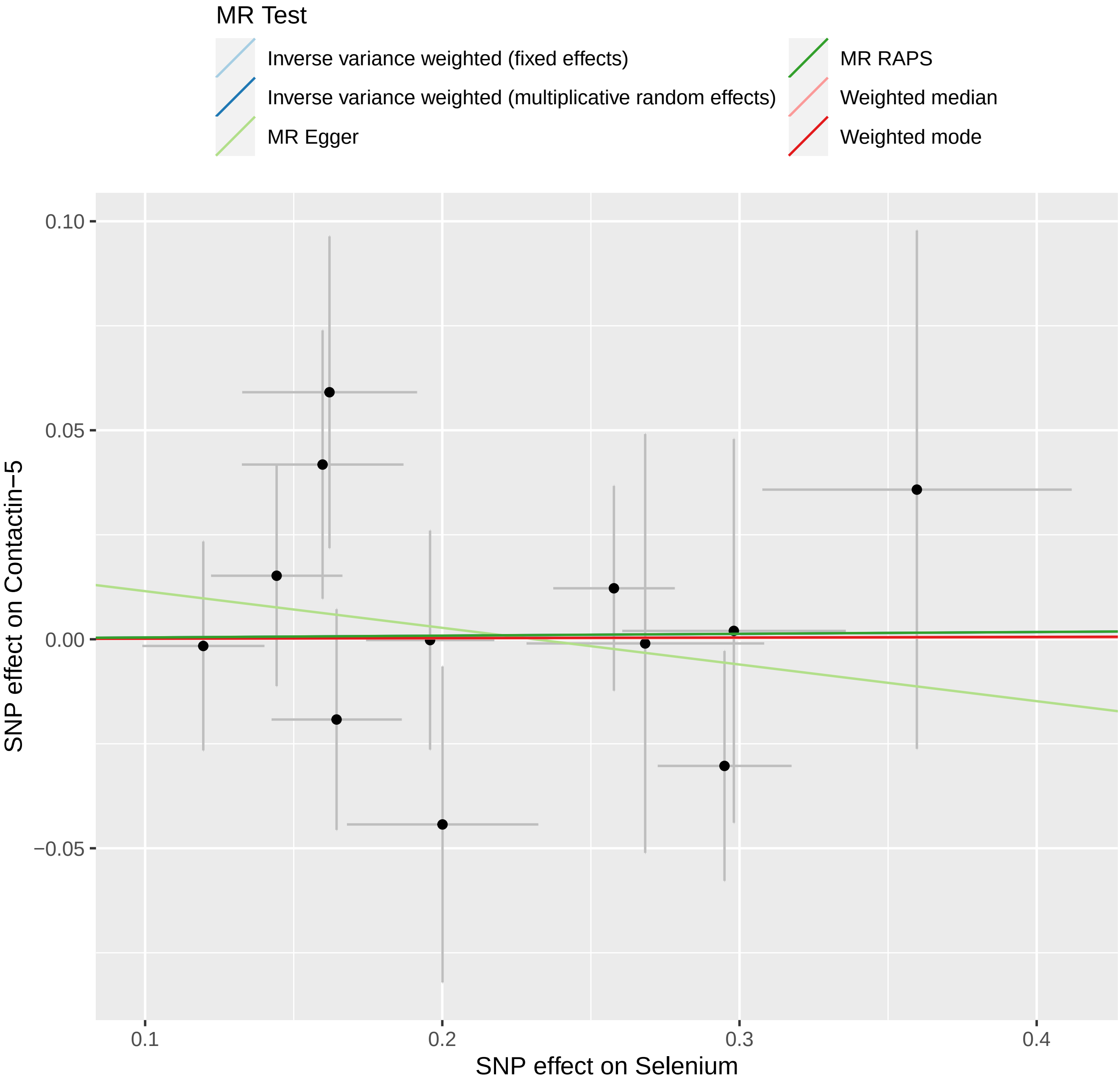
MR Test
Inverse variance weighted (fixed effects)
Inverse variance weighted (multiplicative random effects)
MR Egger
MR RAPS
Weighted median
Weighted mode
0.10
0.05
0.00
−0.05
0.1
0.2
0.3
0.4
SNP effect on Contactin−5
SNP effect on Selenium

Batch 3−610 : Selenium on Contactin−associated protein 1

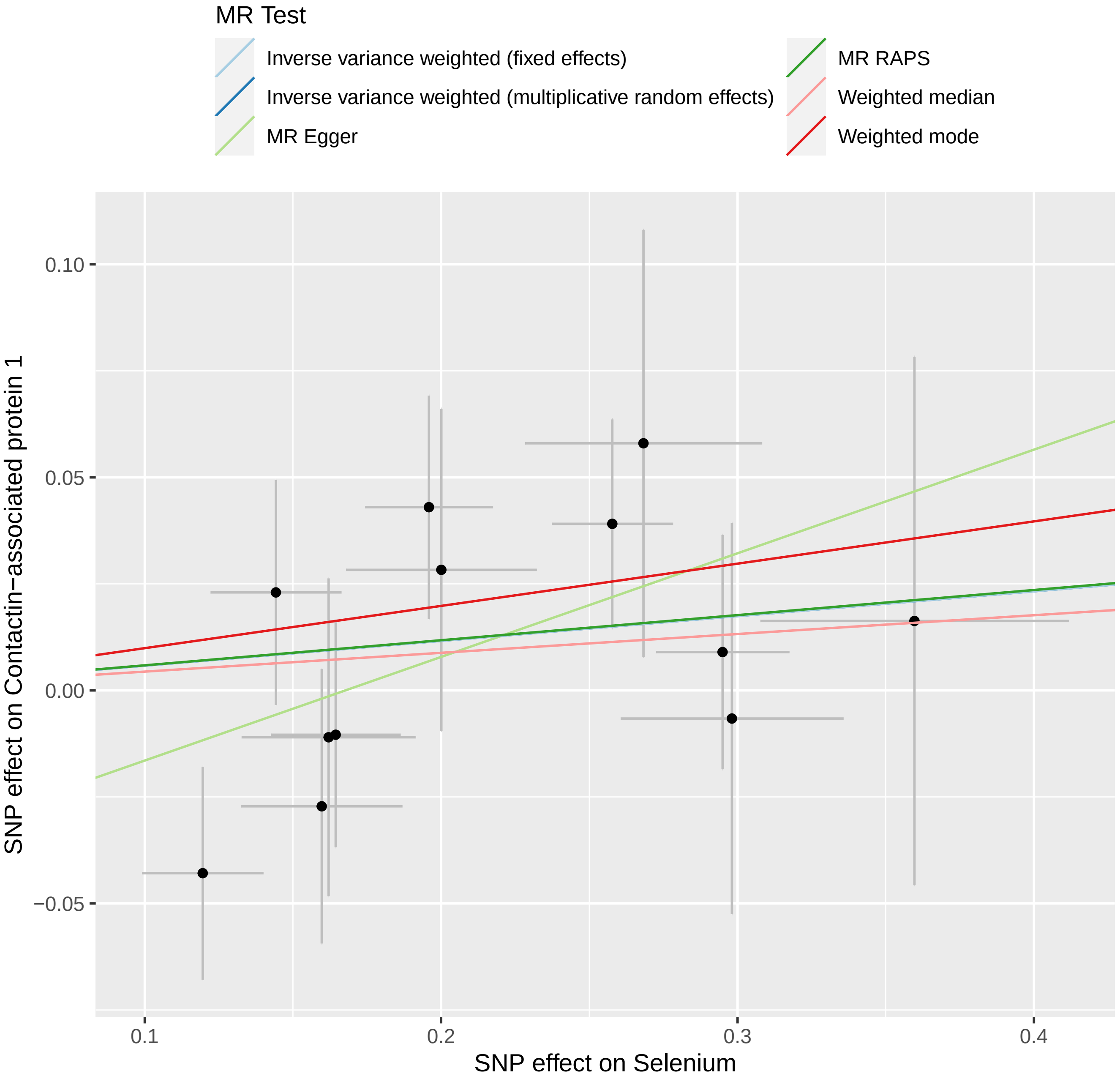

Batch 3−611 : Selenium on Contactin−associated protein−like 2

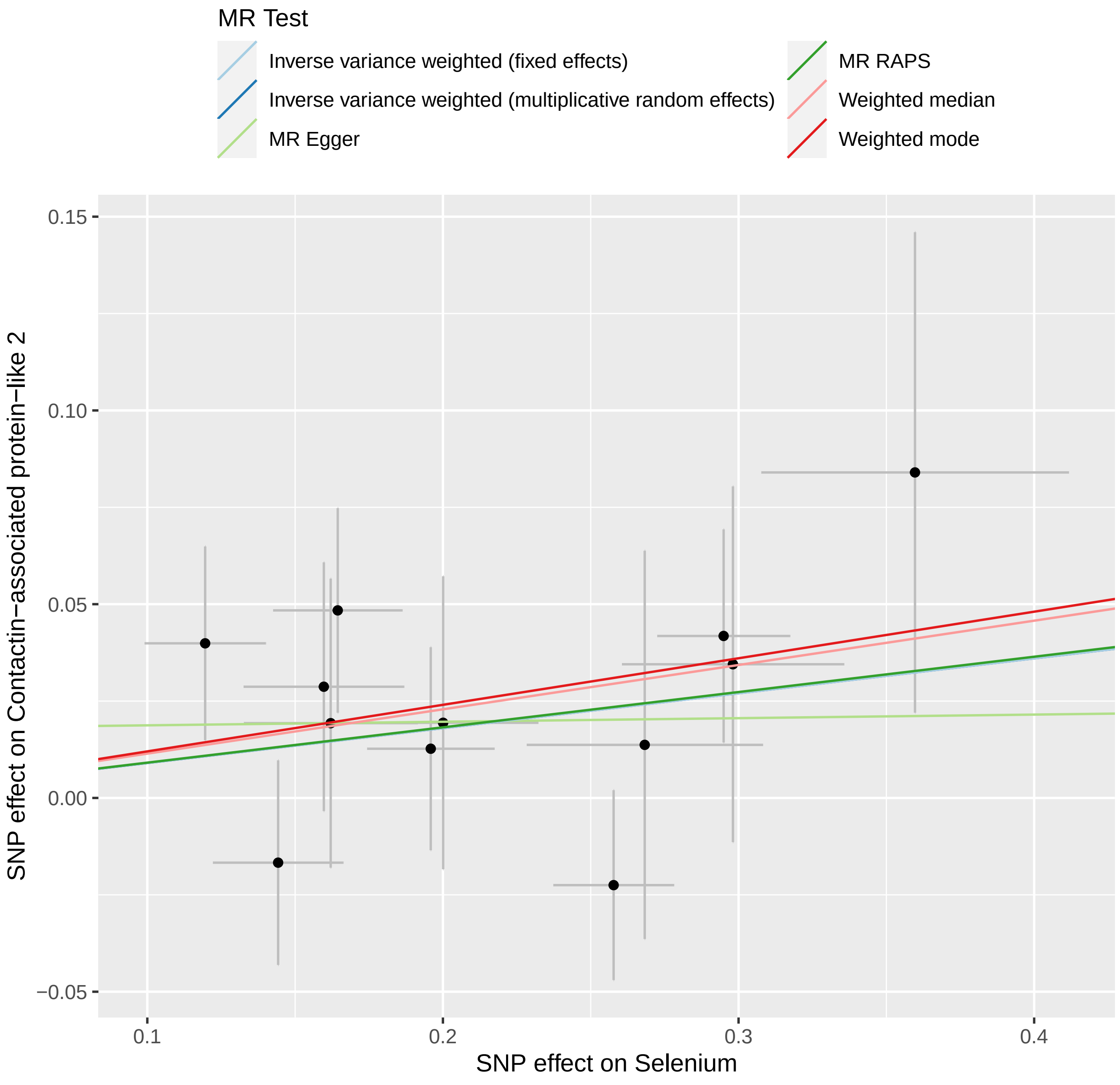

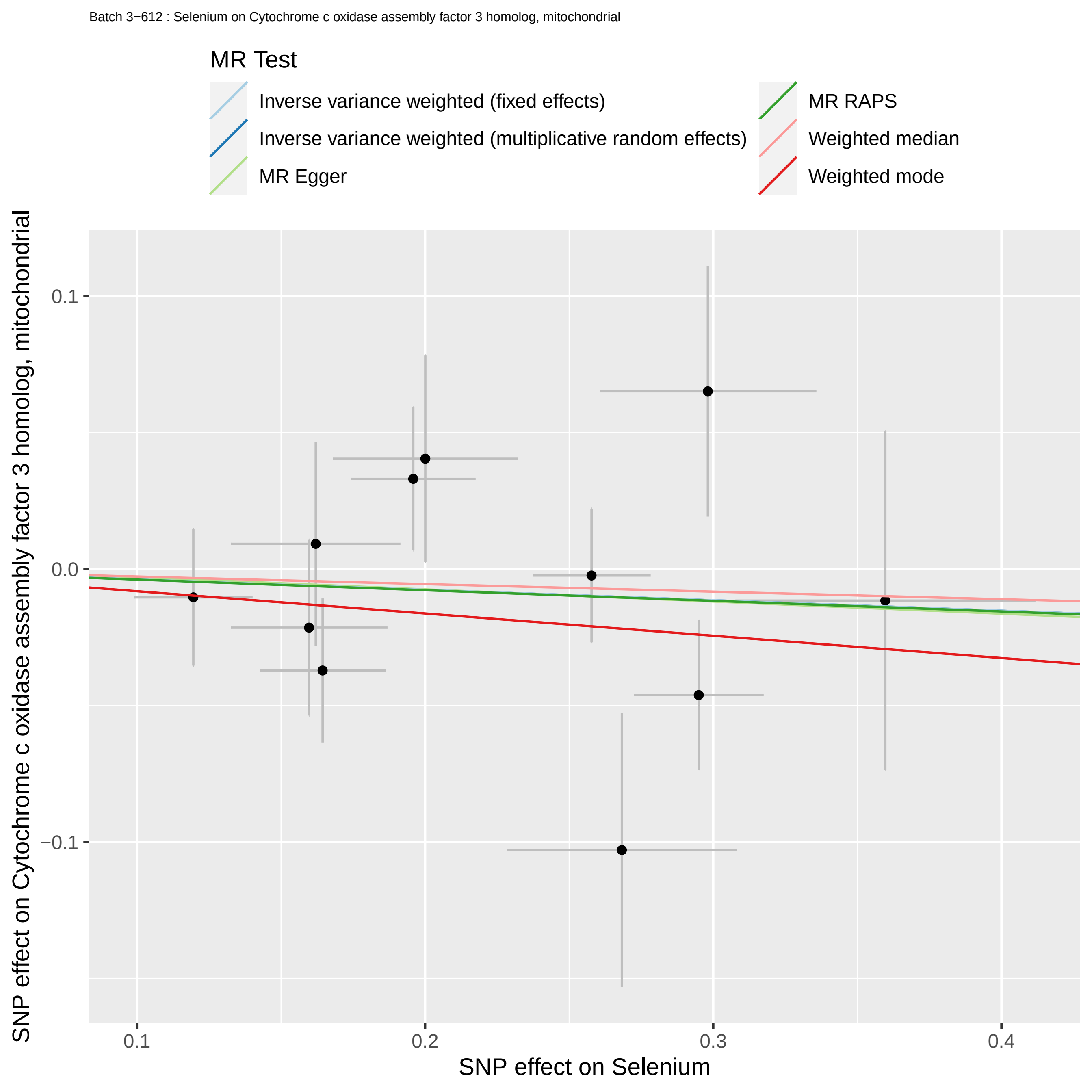
Batch 3−612 : Selenium on Cytochrome c oxidase assembly factor 3 homolog, mitochondrial
MR Test
Inverse variance weighted (fixed effects)
Inverse variance weighted (multiplicative random effects)
MR Egger
MR RAPS
Weighted median
Weighted mode
SNP effect on Cytochrome c oxidase assembly factor 3 homolog, mitochondrial
0.1
0.0
−0.1
0.1
0.2
0.3
0.4
SNP effect on Selenium

Batch 3−613 : Selenium on Cochlin

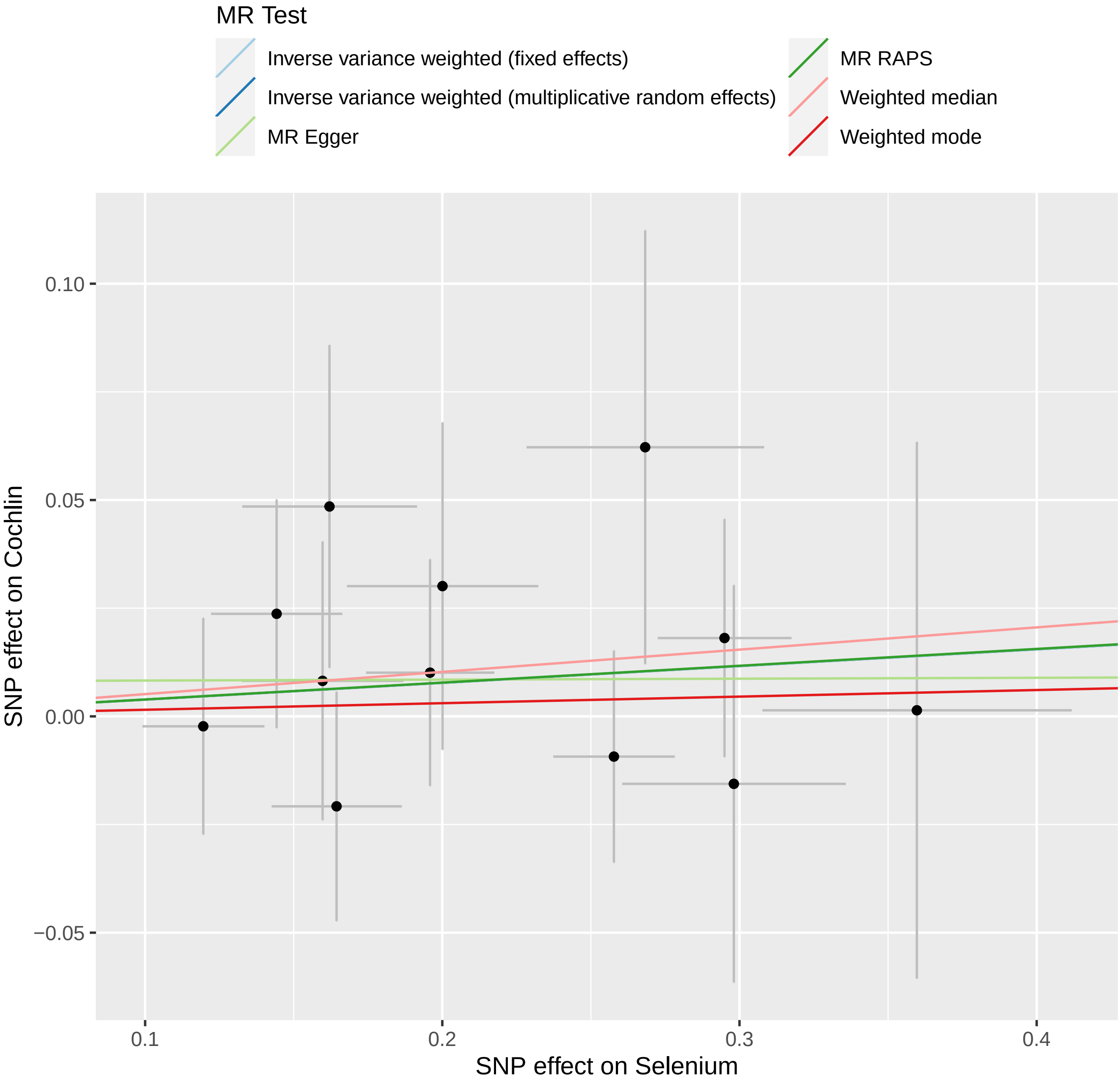

Batch 3−614 : Selenium on Conserved oligomeric Golgi complex subunit 8

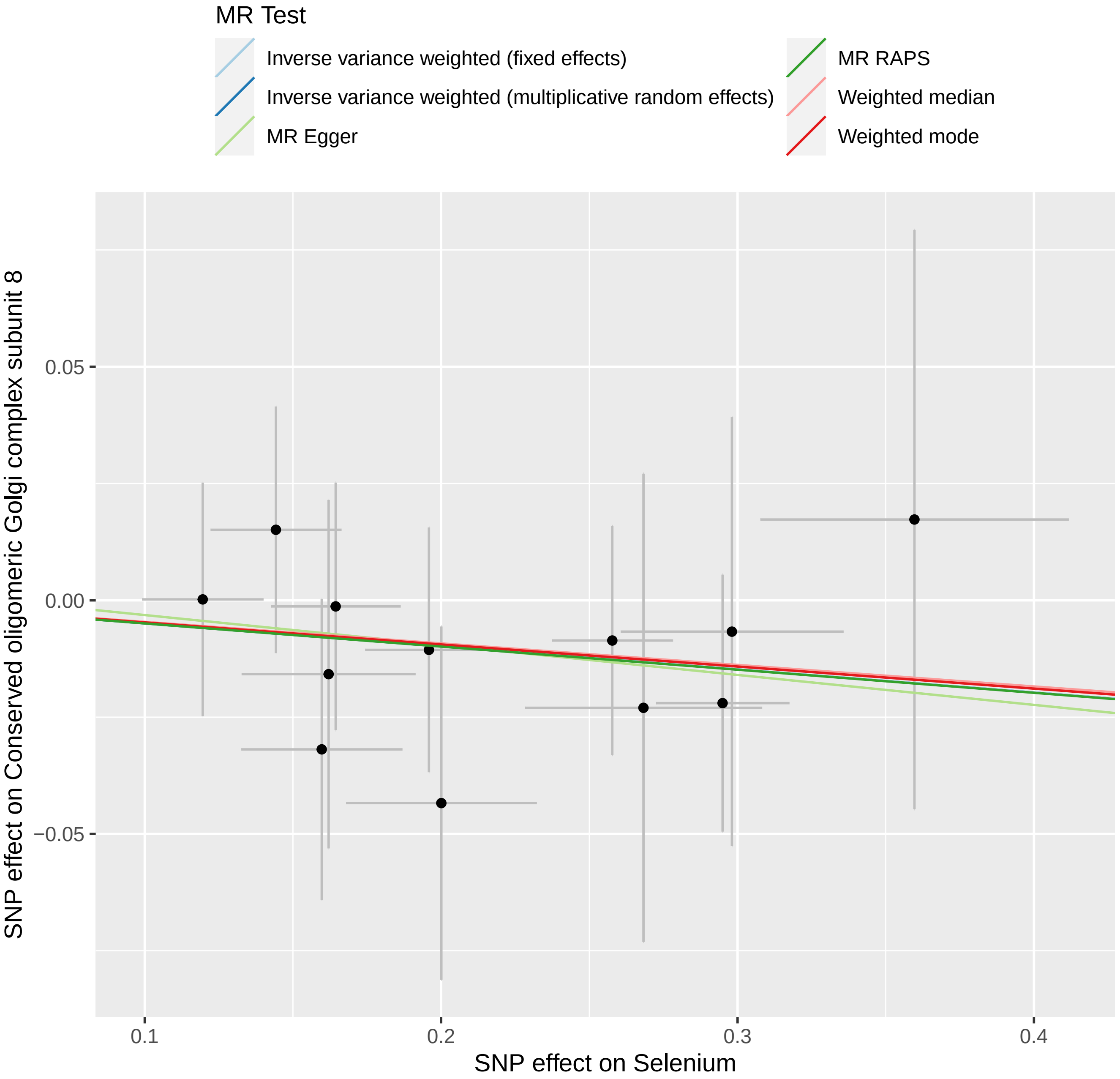

Batch 3−615 : Selenium on Collagen alpha−2(XI) chain

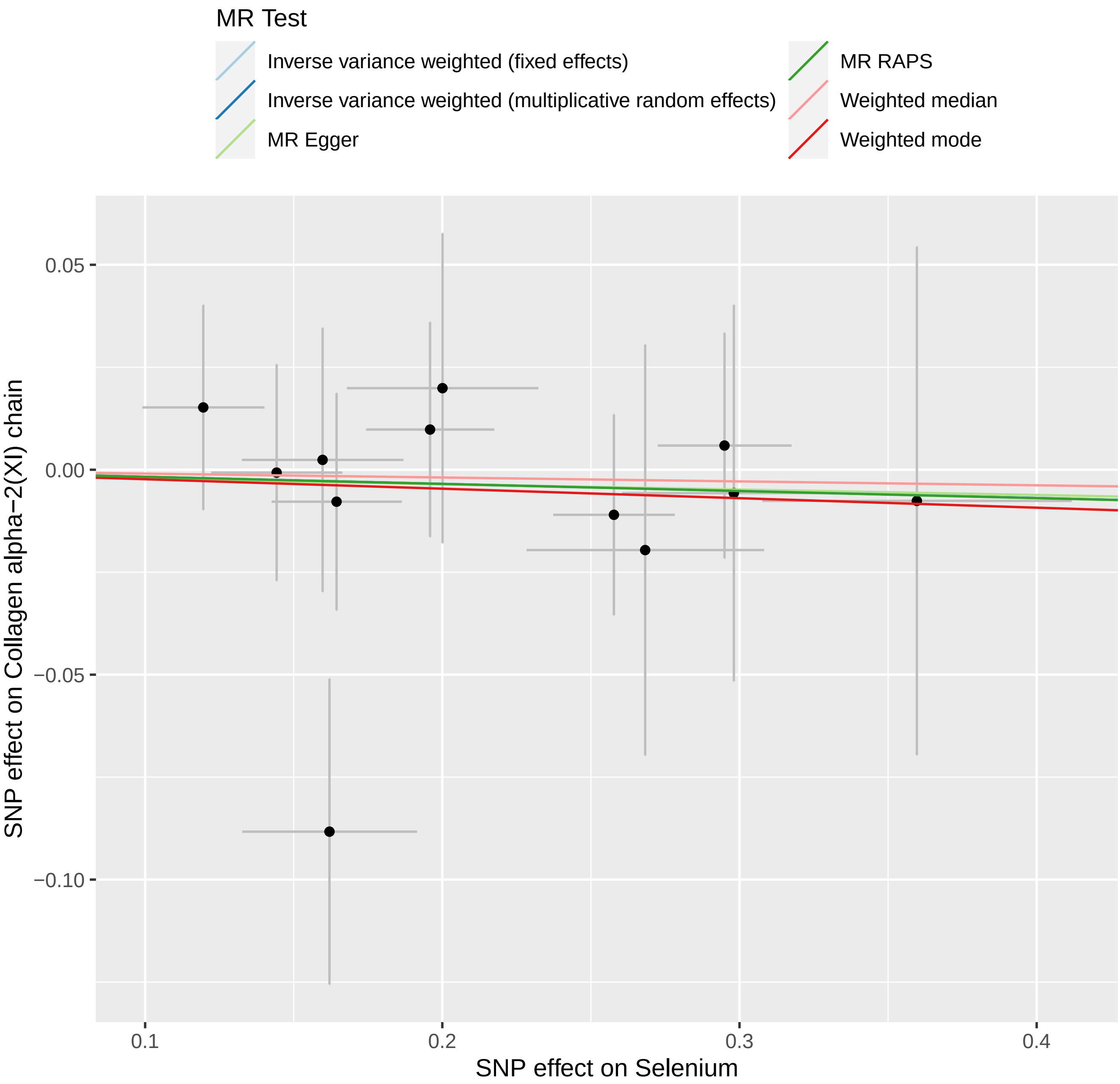

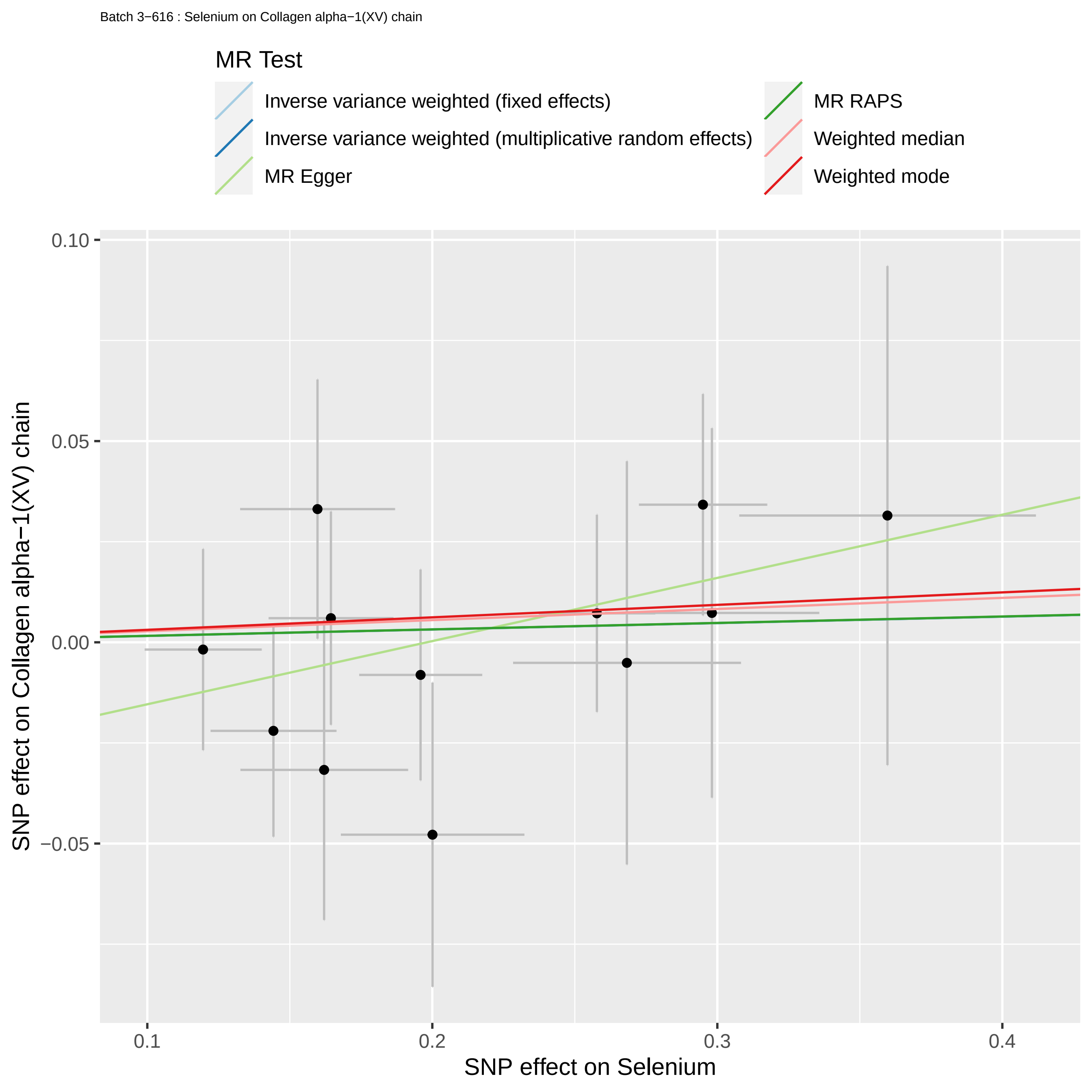

Batch 3−616 : Selenium on Collagen alpha−1(XV) chain
MR Test
Inverse variance weighted (fixed effects)
Inverse variance weighted (multiplicative random effects)
MR Egger
MR RAPS
Weighted median
Weighted mode
0.10
0.05
0.00
−0.05
0.1
0.2
0.3
0.4
SNP effect on Collagen alpha−1(XV) chain
SNP effect on Selenium

Batch 3−617 : Selenium on Endostatin

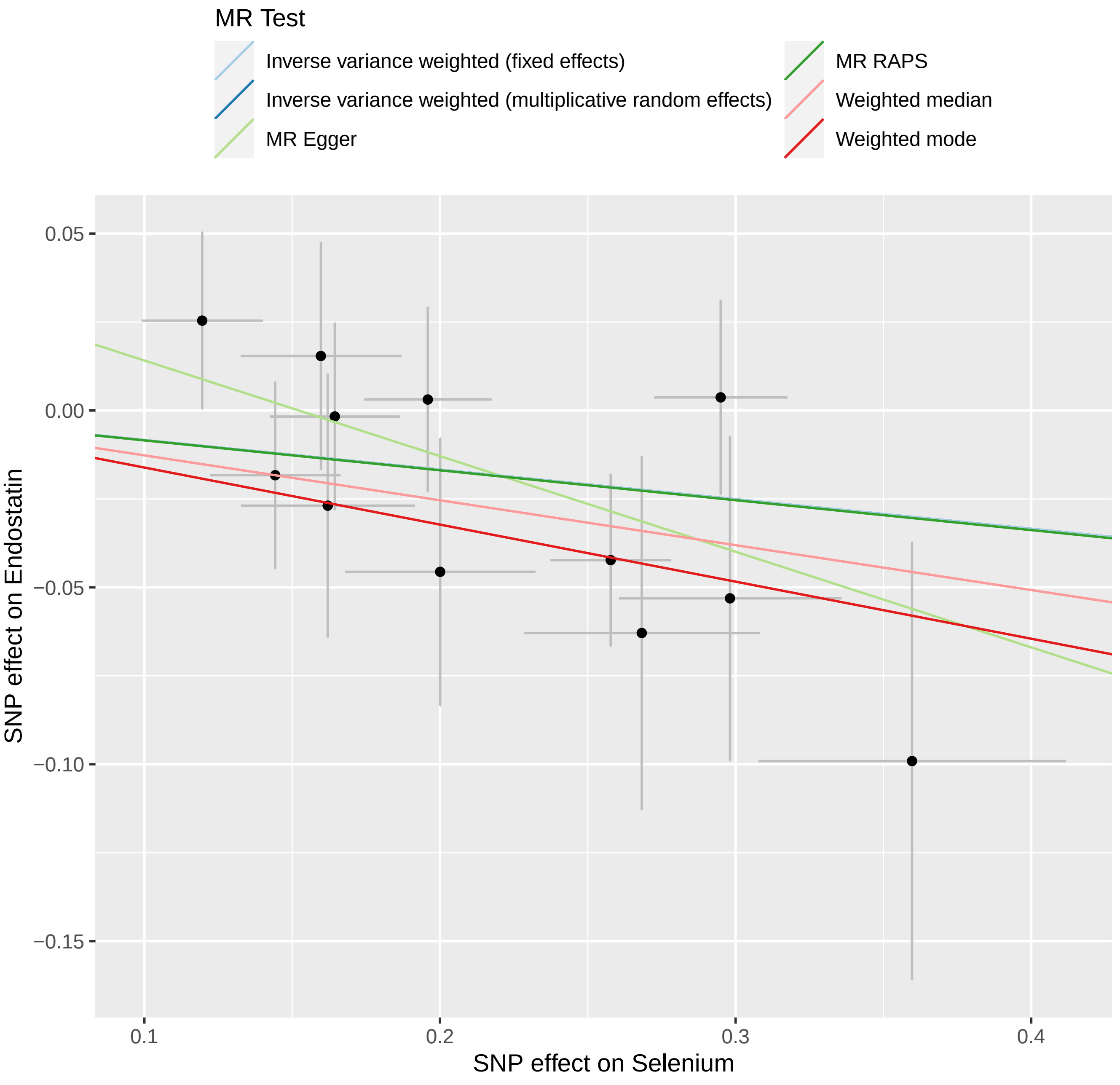

Batch 3−618 : Selenium on Collagen alpha−1(I) chain

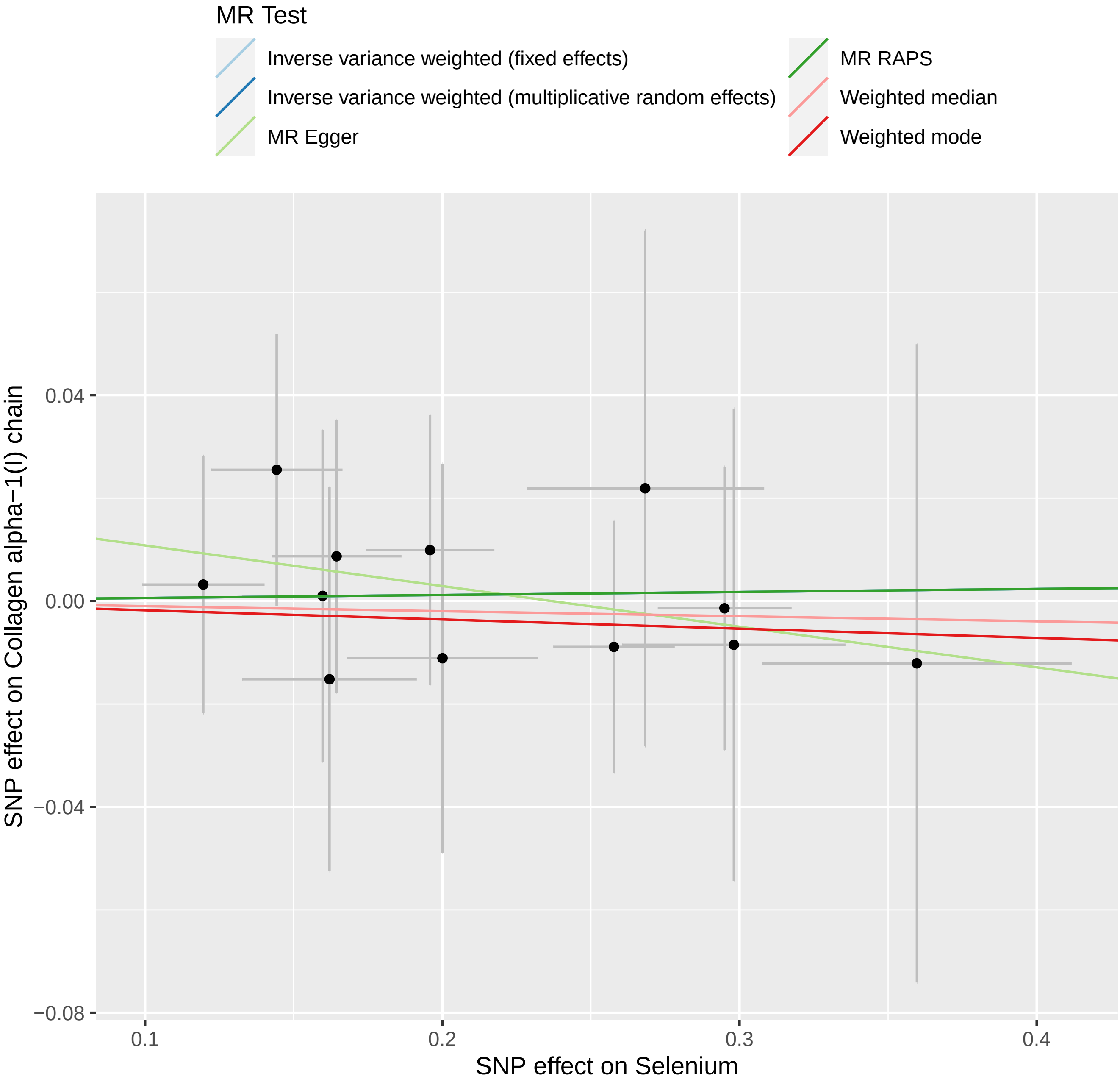

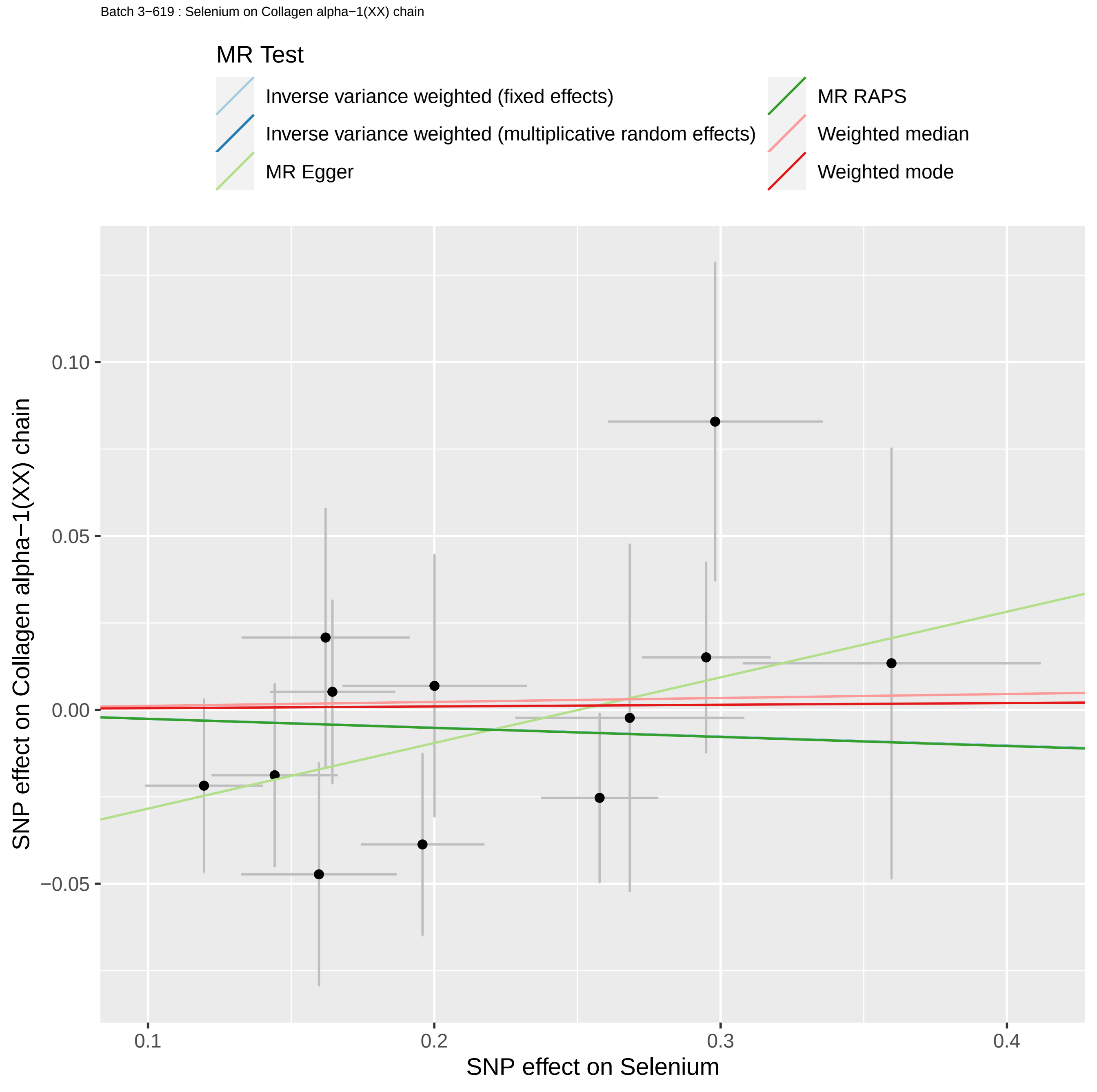
Batch 3−619 : Selenium on Collagen alpha−1(XX) chain
MR Test
Inverse variance weighted (fixed effects)
Inverse variance weighted (multiplicative random effects)
MR Egger
MR RAPS
Weighted median
Weighted mode
0.10
0.05
0.00
−0.05
0.1
0.2
0.3
0.4
SNP effect on Collagen alpha−1(XX) chain
SNP effect on Selenium

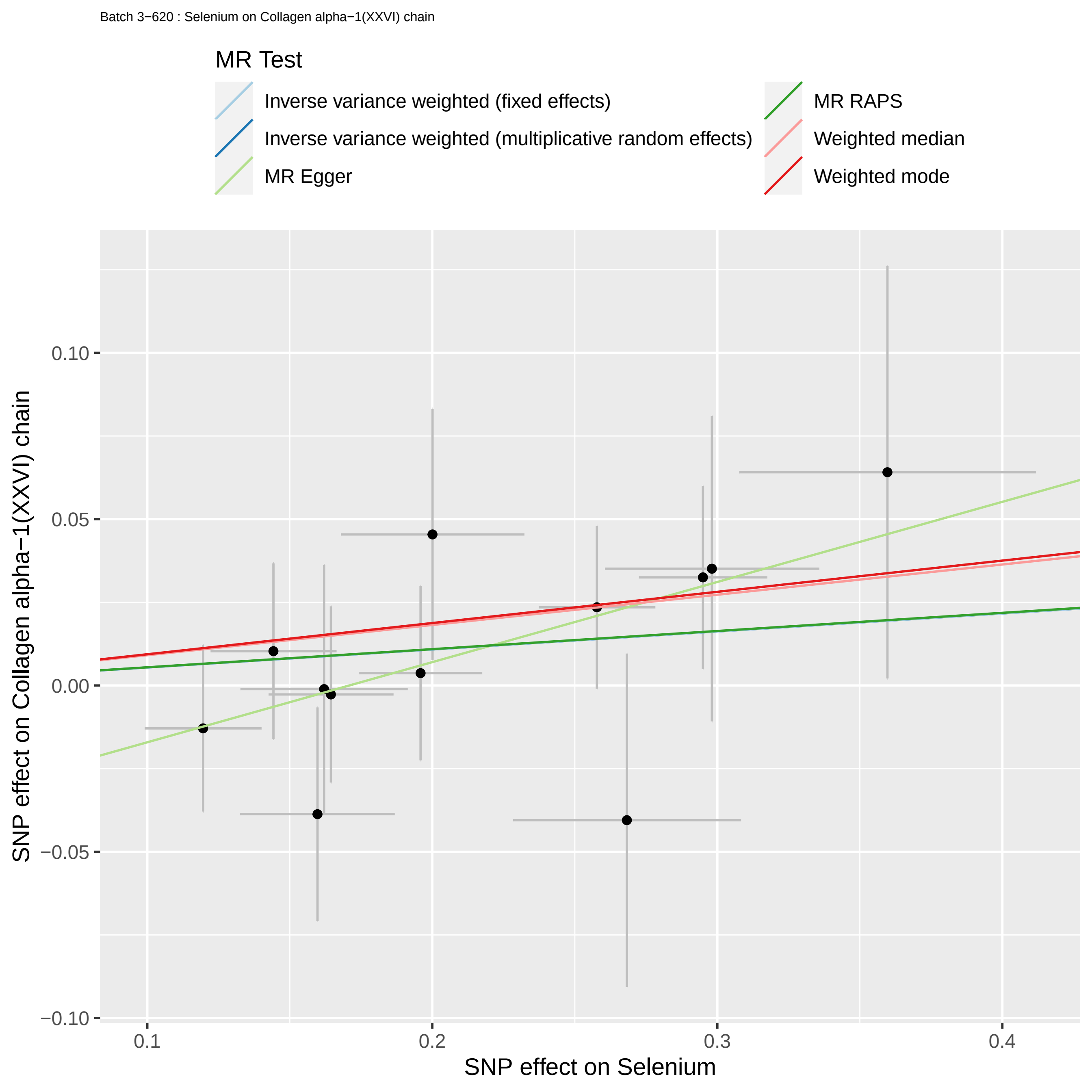
Batch 3−620 : Selenium on Collagen alpha−1(XXVI) chain
MR Test
Inverse variance weighted (fixed effects)
Inverse variance weighted (multiplicative random effects)
MR Egger
MR RAPS
Weighted median
Weighted mode
0.10
0.05
0.00
−0.05
−0.10
0.1
0.2
0.3
0.4
SNP effect on Collagen alpha−1(XXVI) chain
SNP effect on Selenium

Batch 3−621 : Selenium on Collagen alpha−1(XXVIII) chain

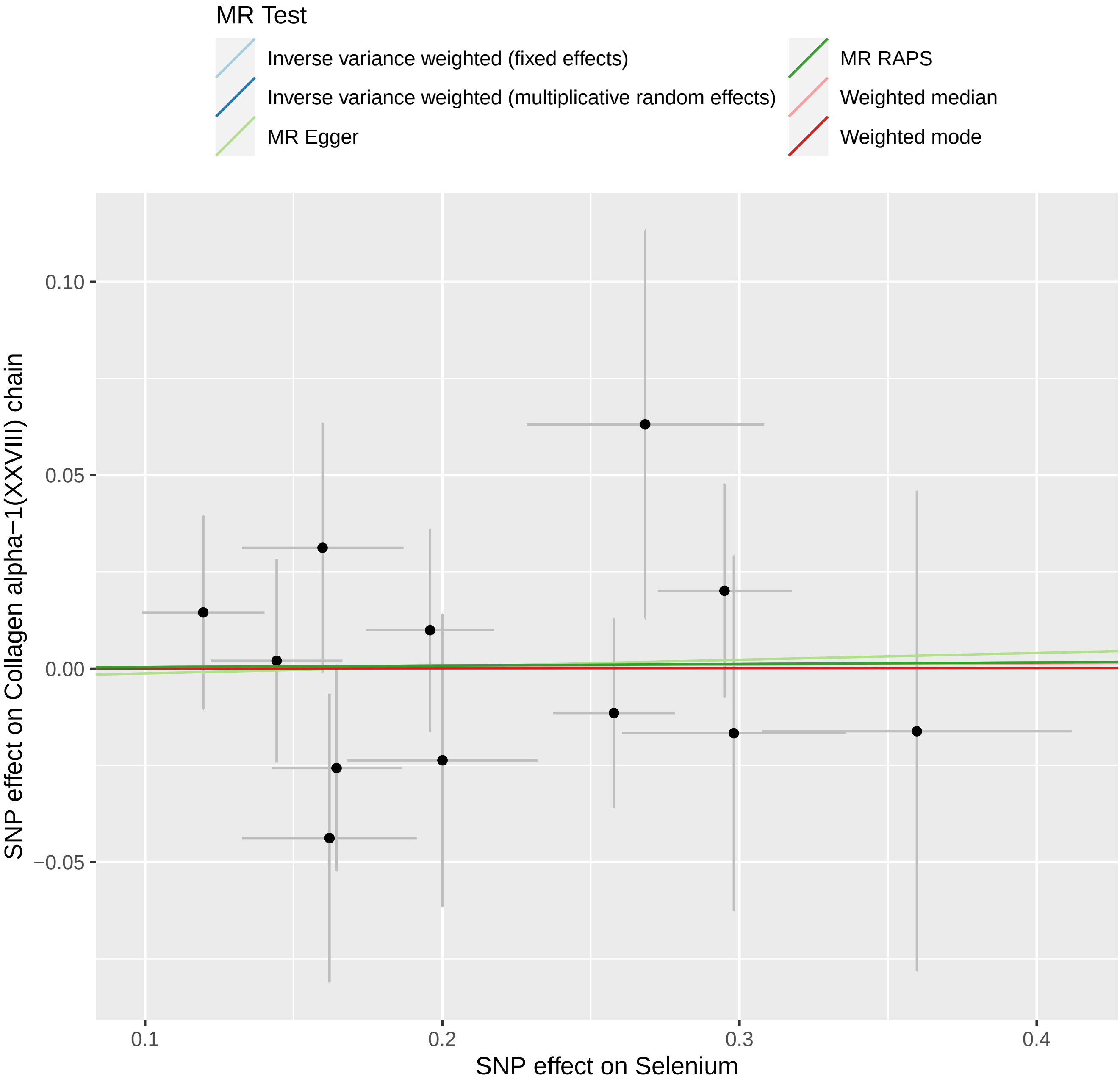

Batch 3−622 : Selenium on Collagen alpha−1(VI) chain

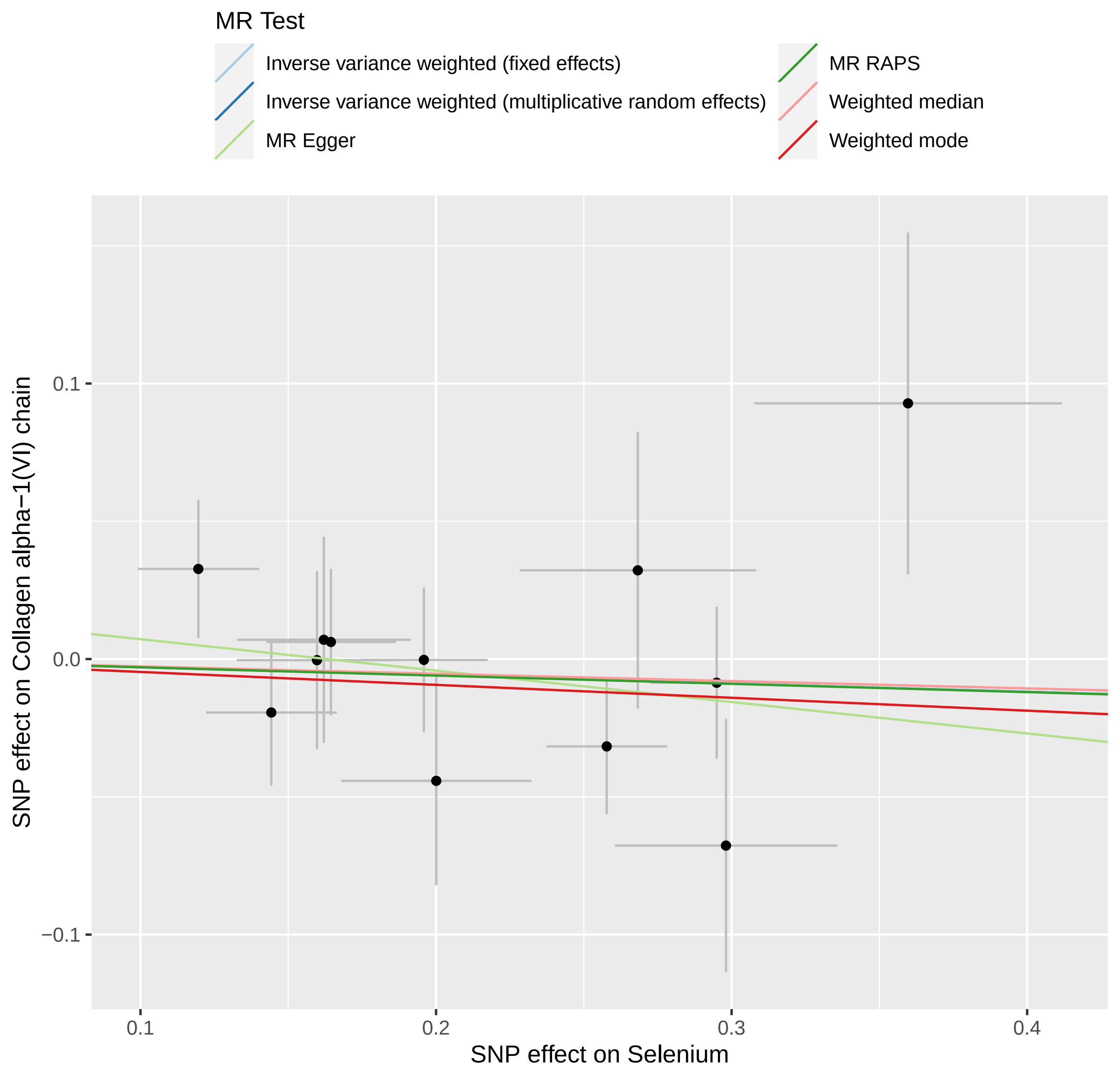

Batch 3−623 : Selenium on Collagen alpha−3(VI) chain

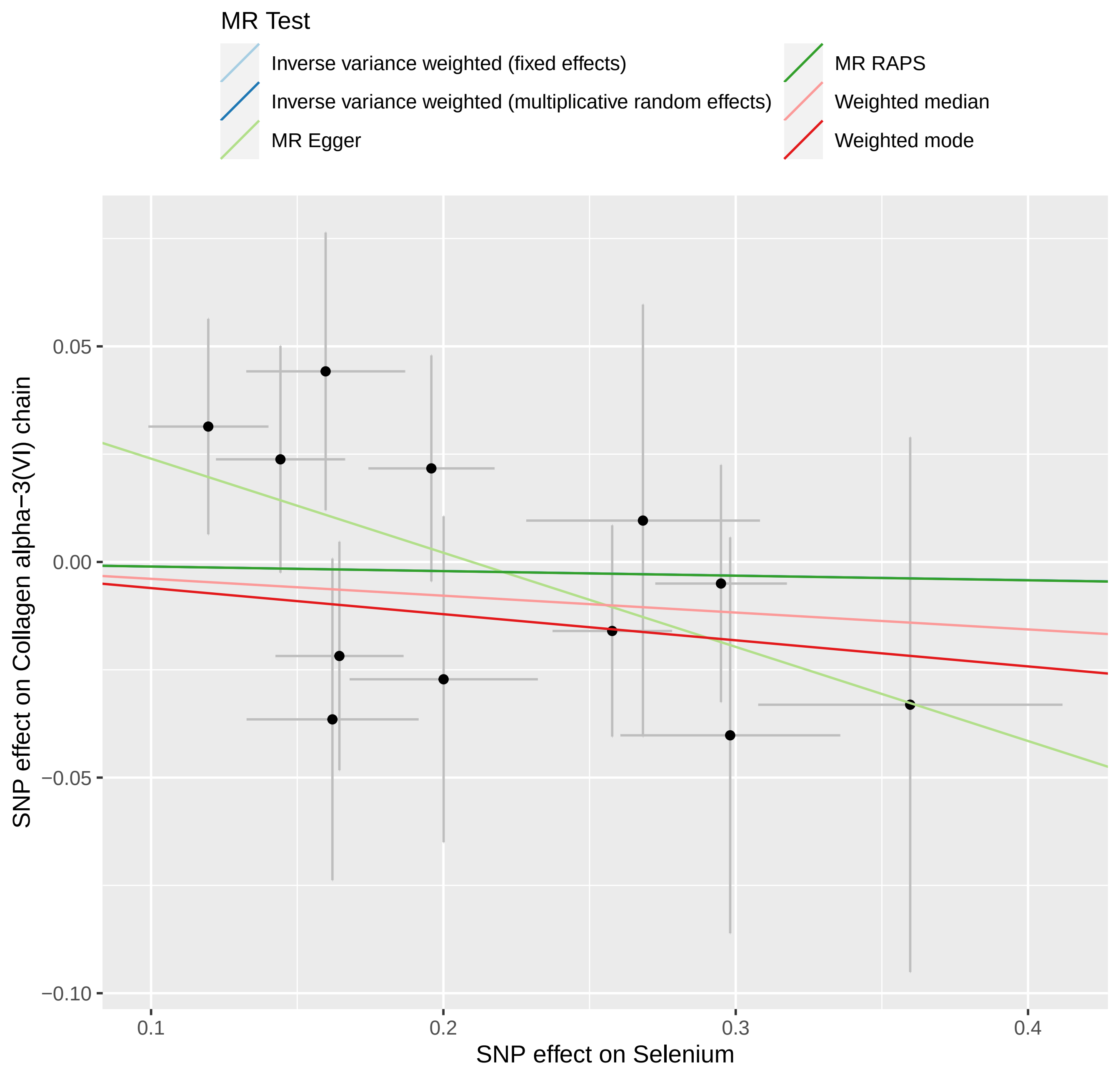

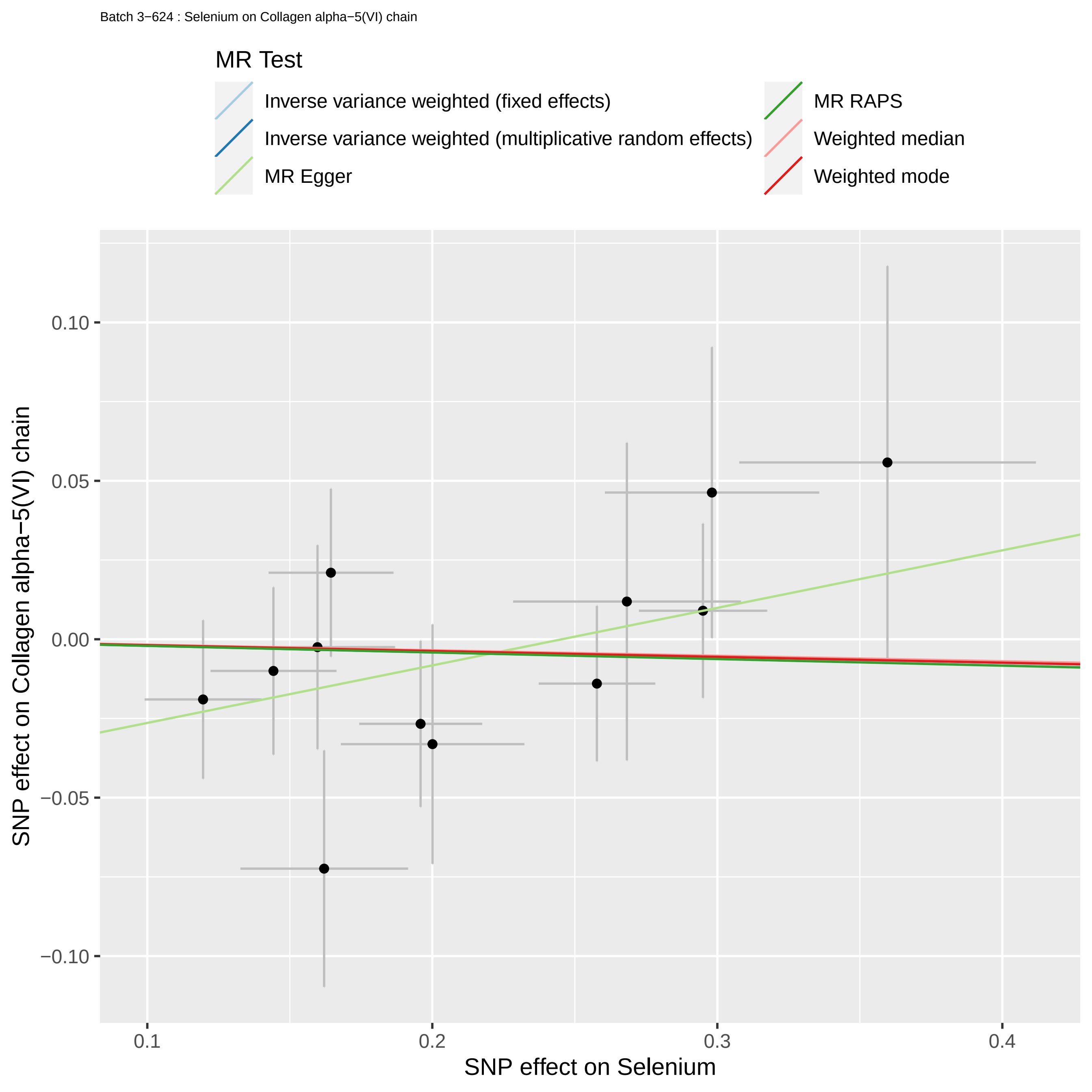

Batch 3−624 : Selenium on Collagen alpha−5(VI) chain
MR Test
Inverse variance weighted (fixed effects)
Inverse variance weighted (multiplicative random effects)
MR Egger
MR RAPS
Weighted median
Weighted mode
0.10
0.05
0.00
−0.05
−0.10
0.1
0.2
0.3
0.4
SNP effect on Collagen alpha−5(VI) chain
SNP effect on Selenium

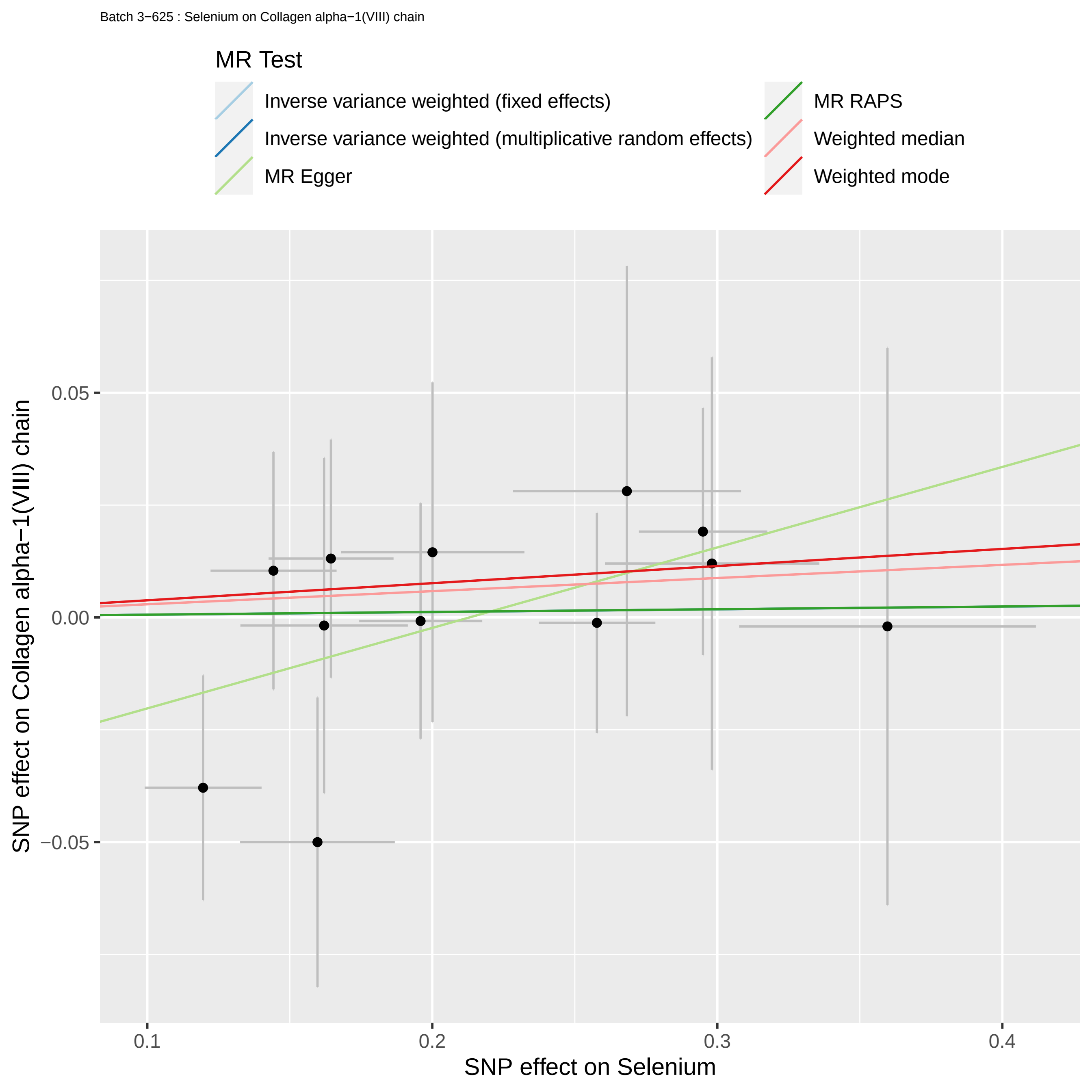

Batch 3−625 : Selenium on Collagen alpha−1(VIII) chain
MR Test
Inverse variance weighted (fixed effects)
Inverse variance weighted (multiplicative random effects)
MR Egger
MR RAPS
Weighted median
Weighted mode
0.05
0.00
−0.05
0.1
0.2
0.3
0.4
SNP effect on Selenium
SNP effect on Collagen alpha−1(VIII) chain

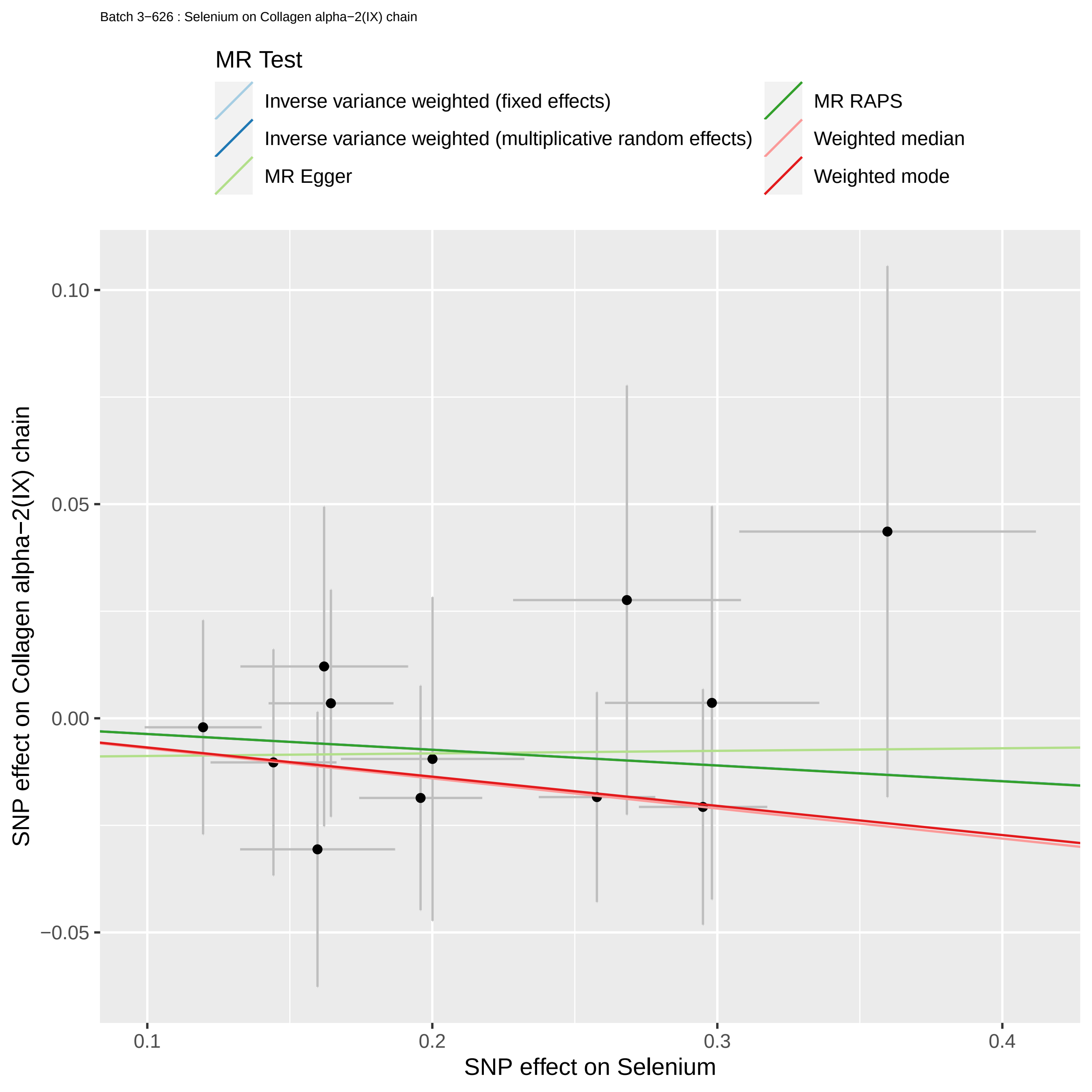

Batch 3−626 : Selenium on Collagen alpha−2(IX) chain
MR Test
Inverse variance weighted (fixed effects)
Inverse variance weighted (multiplicative random effects)
MR Egger
MR RAPS
Weighted median
Weighted mode
0.10
0.05
0.00
−0.05
0.1
0.2
0.3
0.4
SNP effect on Selenium
SNP effect on Collagen alpha−2(IX) chain

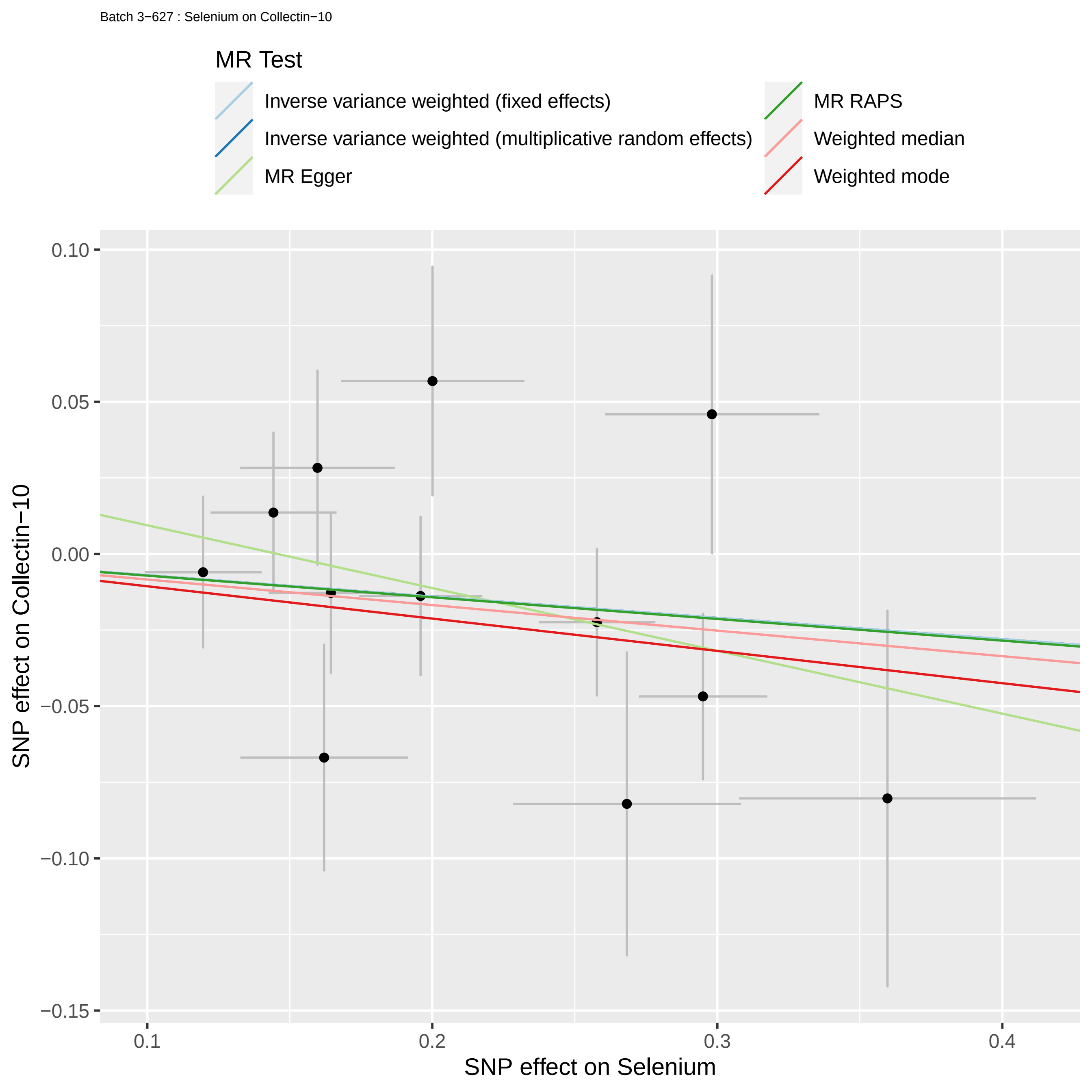

Batch 3−627 : Selenium on Collectin−10
MR Test
Inverse variance weighted (fixed effects)
Inverse variance weighted (multiplicative random effects)
MR Egger
MR RAPS
Weighted median
Weighted mode
0.10
0.05
0.00
−0.05
−0.10
−0.15
0.1
0.2
0.3
0.4
SNP effect on Collectin−10
SNP effect on Selenium

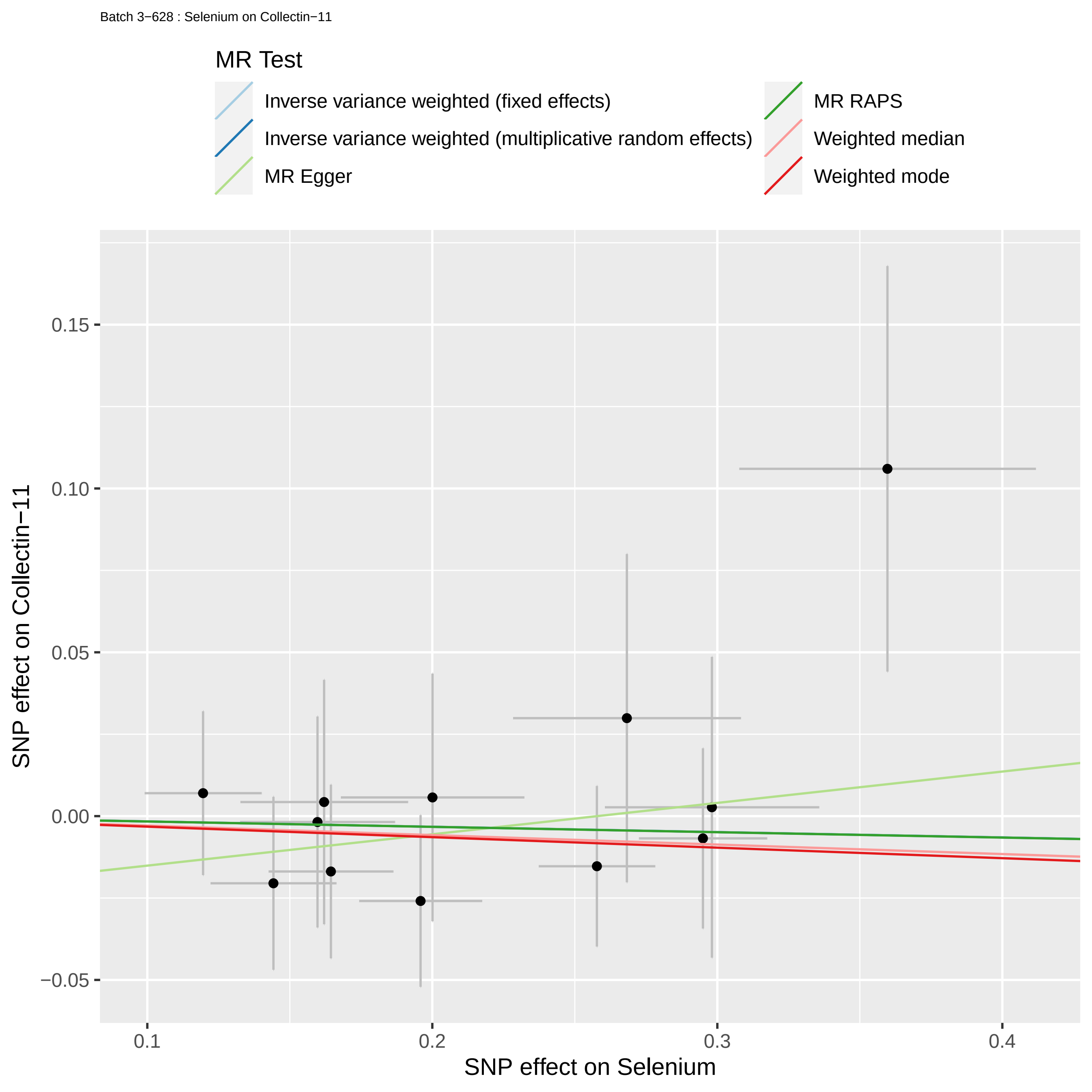
Batch 3−628 : Selenium on Collectin−11
MR Test
Inverse variance weighted (fixed effects)
Inverse variance weighted (multiplicative random effects)
MR Egger
MR RAPS
Weighted median
Weighted mode
0.15
0.10
0.05
0.00
−0.05
0.1
0.2
0.3
0.4
SNP effect on Collectin−11
SNP effect on Selenium

Batch 3−629 : Selenium on Collectin−12

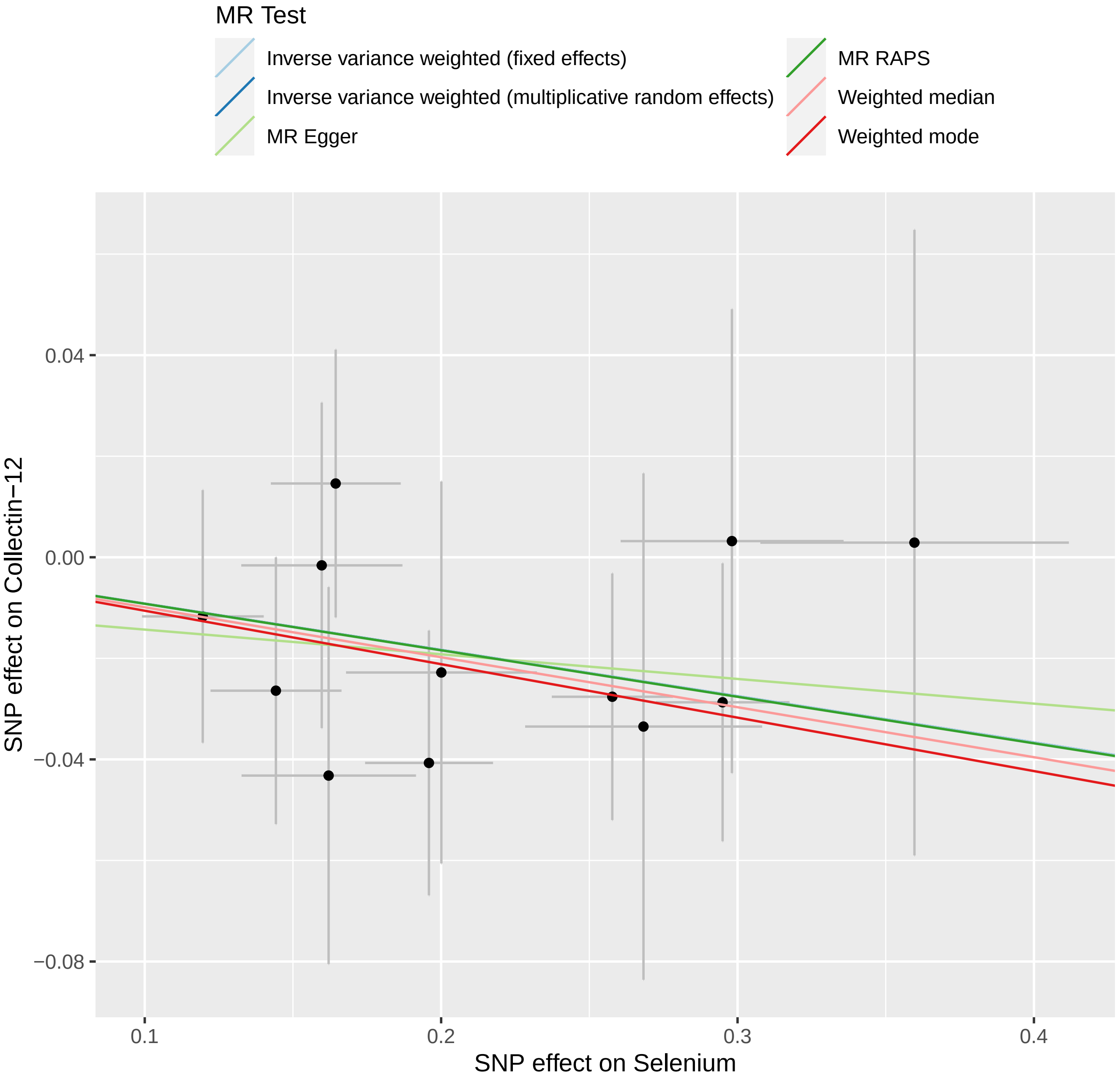

Batch 3−630 : Selenium on Collectin−12

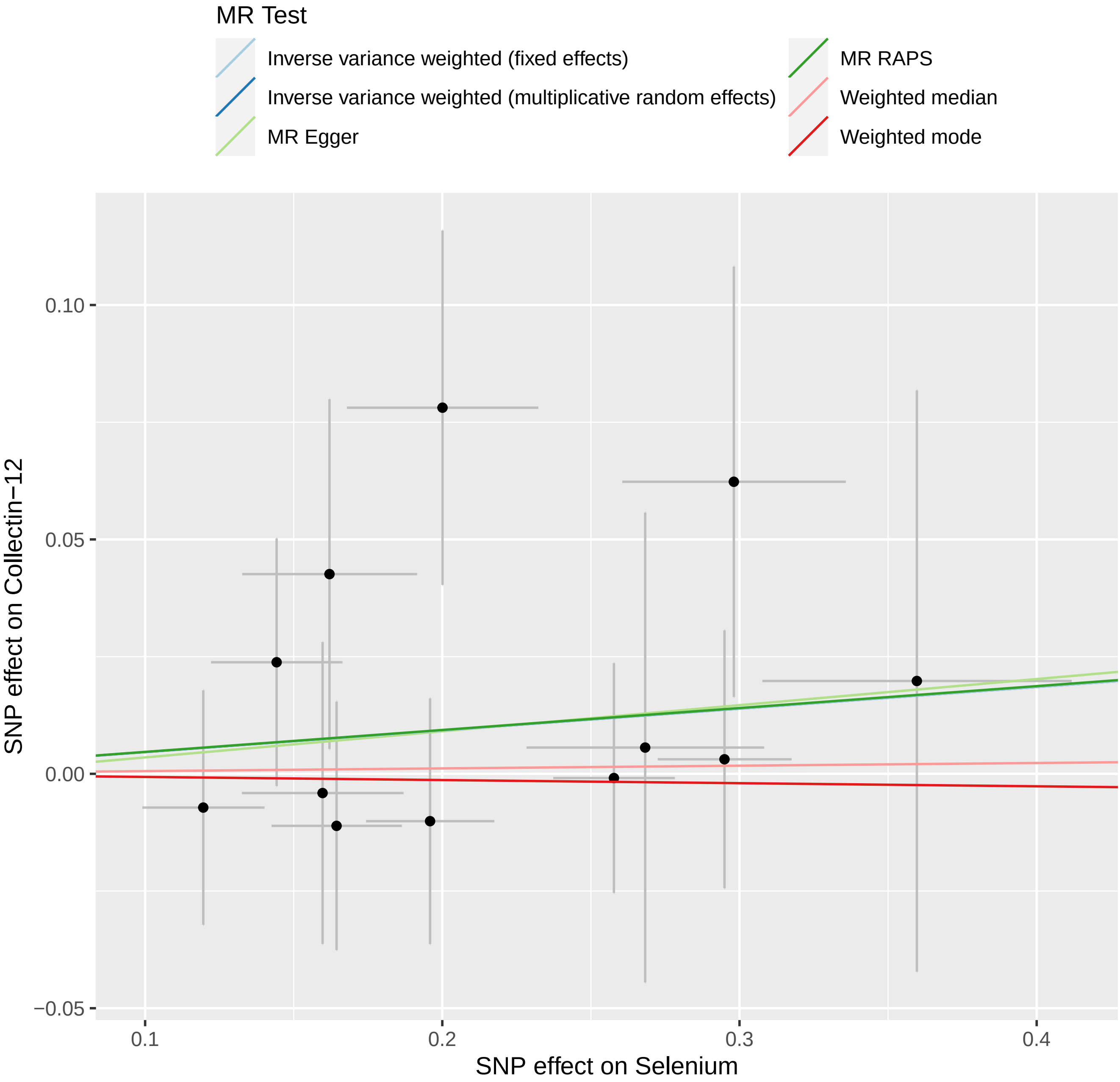

Batch 3−631 : Selenium on Procollagen galactosyltransferase 1

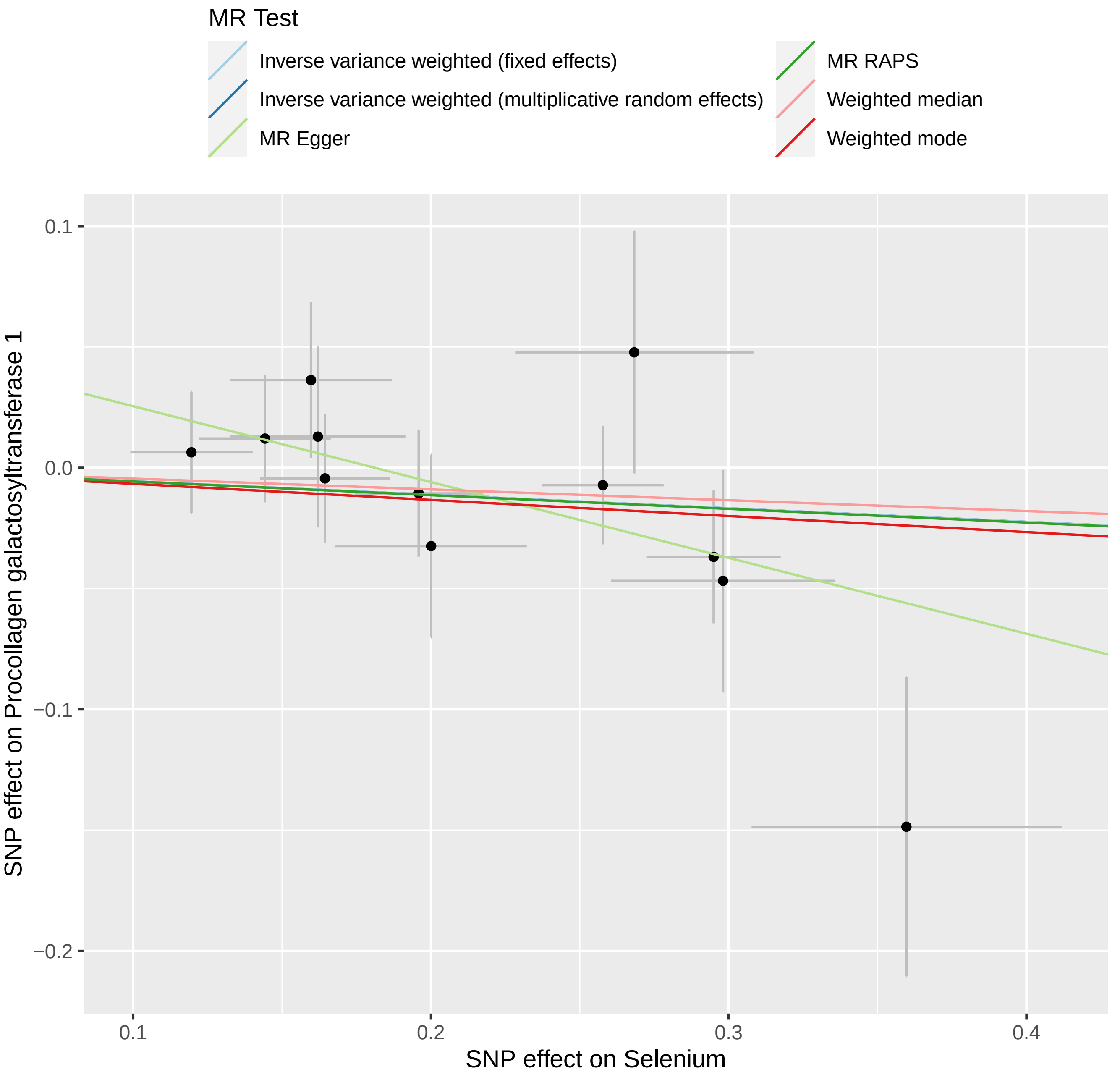

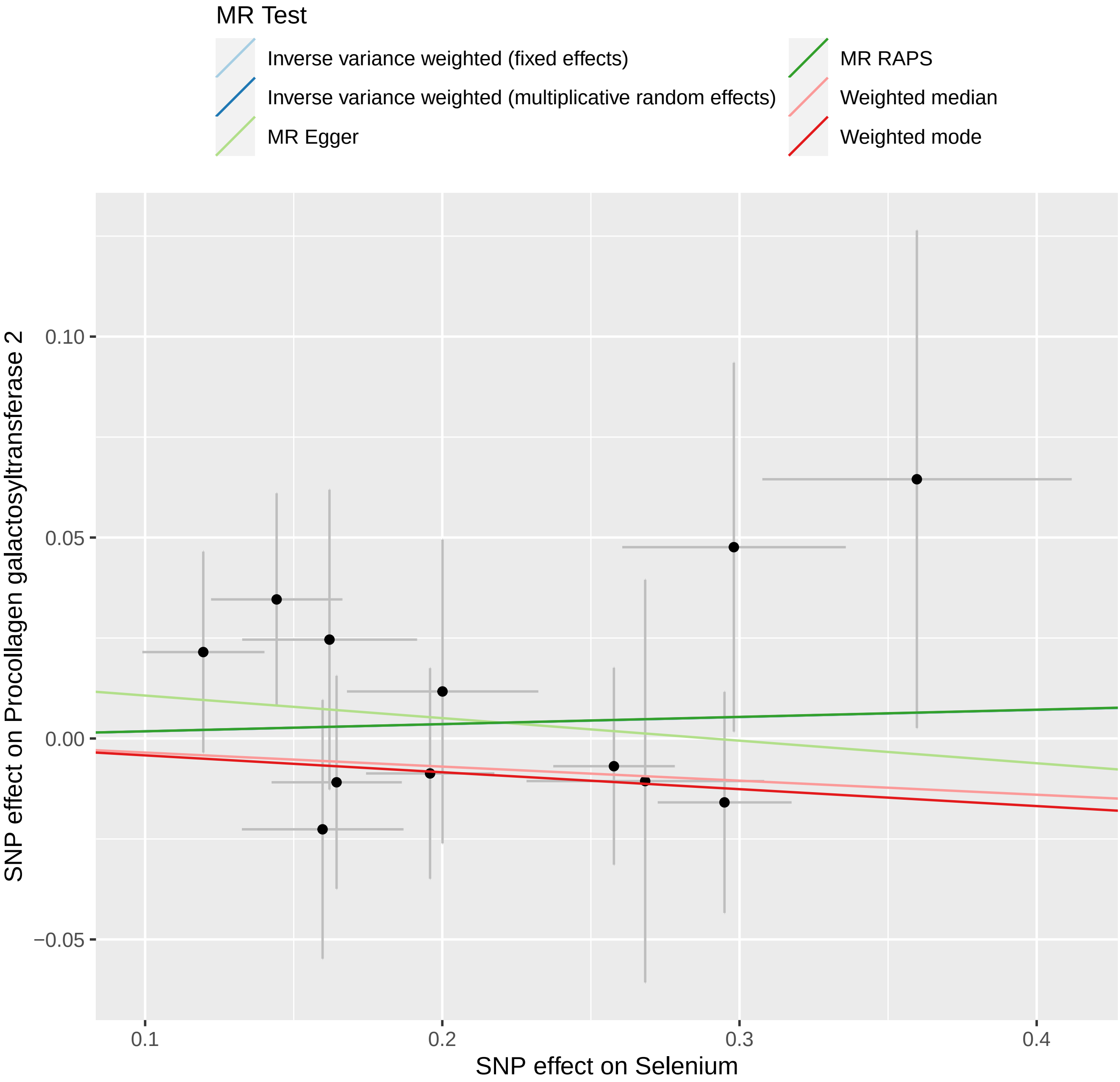
MR Test
Inverse variance weighted (fixed effects)
Inverse variance weighted (multiplicative random effects)
MR Egger
MR RAPS
Weighted median
Weighted mode
0.10
0.05
0.00
−0.05
0.1
0.2
0.3
0.4
SNP effect on Procollagen galactosyltransferase 2
SNP effect on Selenium

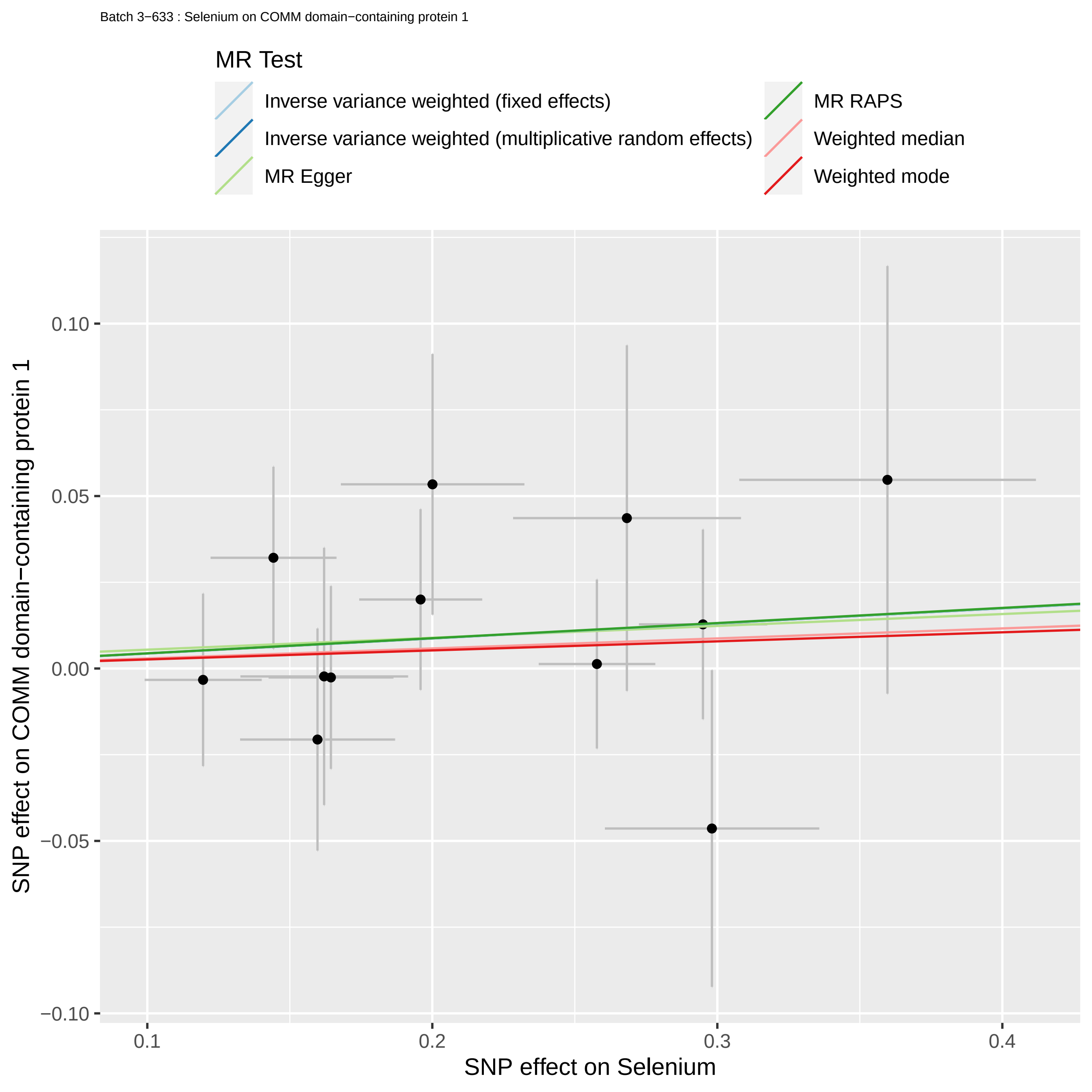

Batch 3−633 : Selenium on COMM domain−containing protein 1
MR Test
Inverse variance weighted (fixed effects)
Inverse variance weighted (multiplicative random effects)
MR Egger
MR RAPS
Weighted median
Weighted mode
SNP effect on COMM domain−containing protein 1
0.10
0.05
0.00
−0.05
−0.10
0.1
0.2
0.3
0.4
SNP effect on Selenium

Batch 3−634 : Selenium on COMM domain−containing protein 7

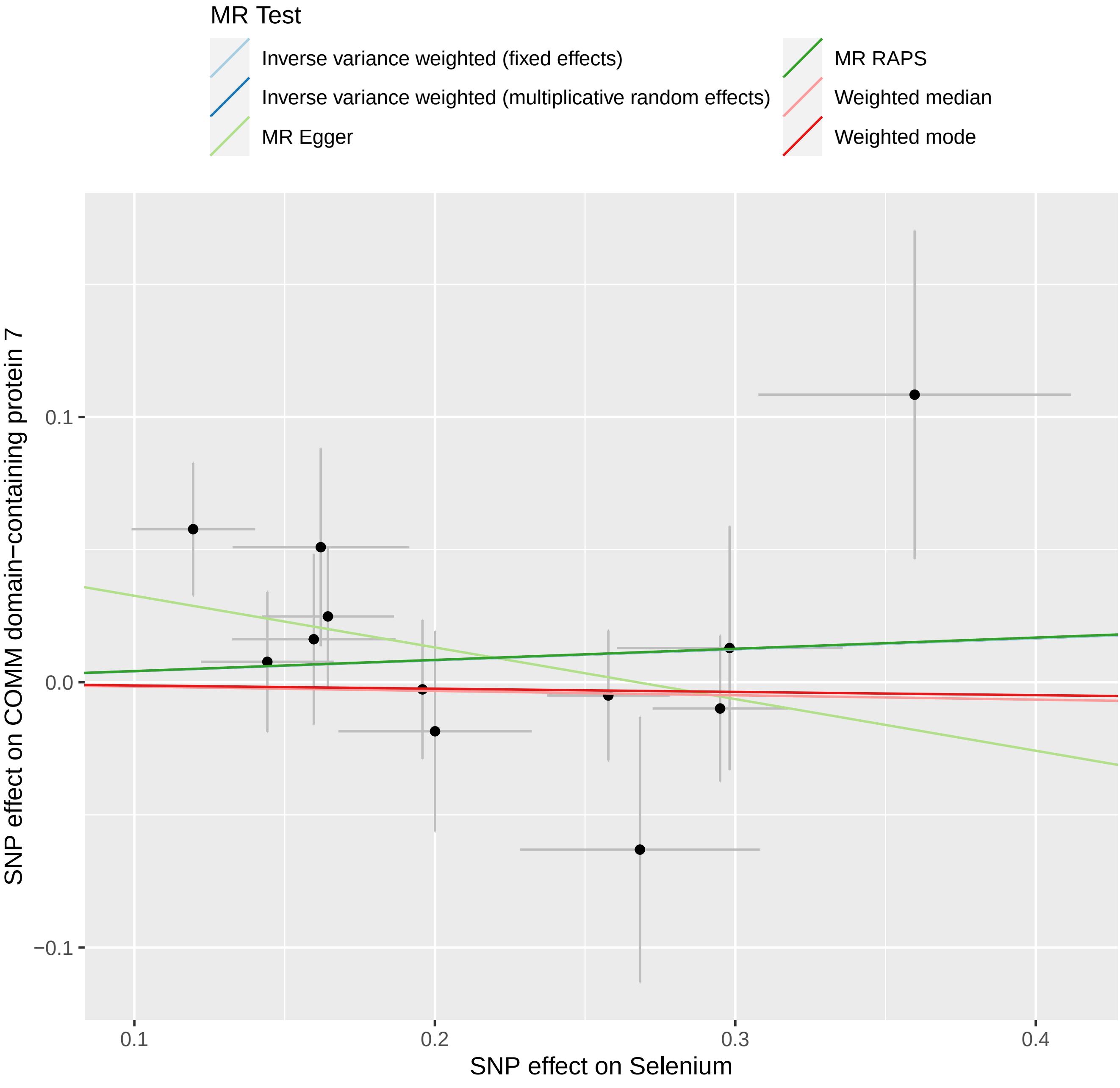

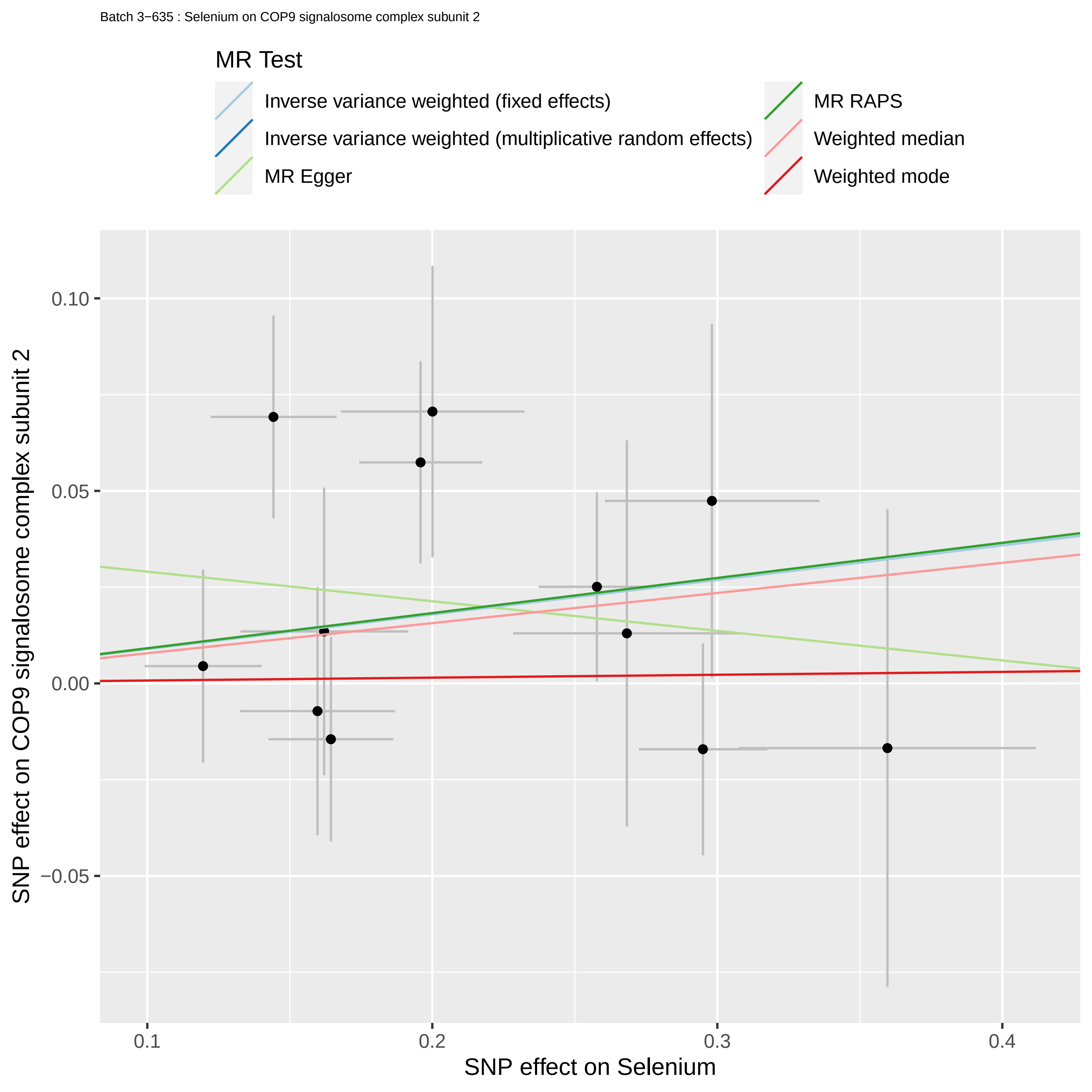

Batch 3−635 : Selenium on COP9 signalosome complex subunit 2
MR Test
Inverse variance weighted (fixed effects)
Inverse variance weighted (multiplicative random effects)
MR Egger
MR RAPS
Weighted median
Weighted mode
SNP effect on COP9 signalosome complex subunit 2
0.10
0.05
0.00
−0.05
0.1
0.2
0.3
0.4
SNP effect on Selenium

Batch 3−636 : Selenium on Coactosin−like protein

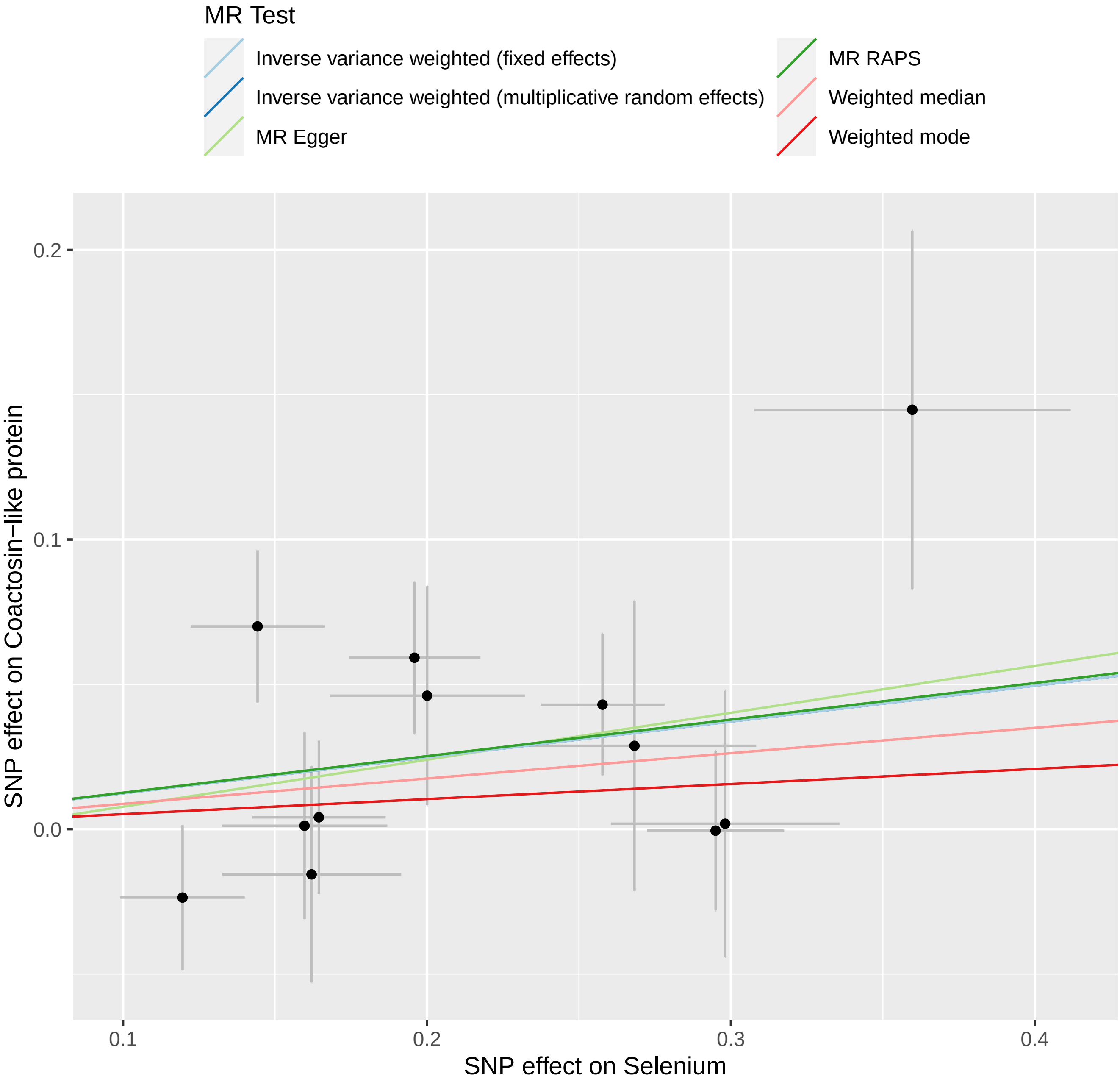

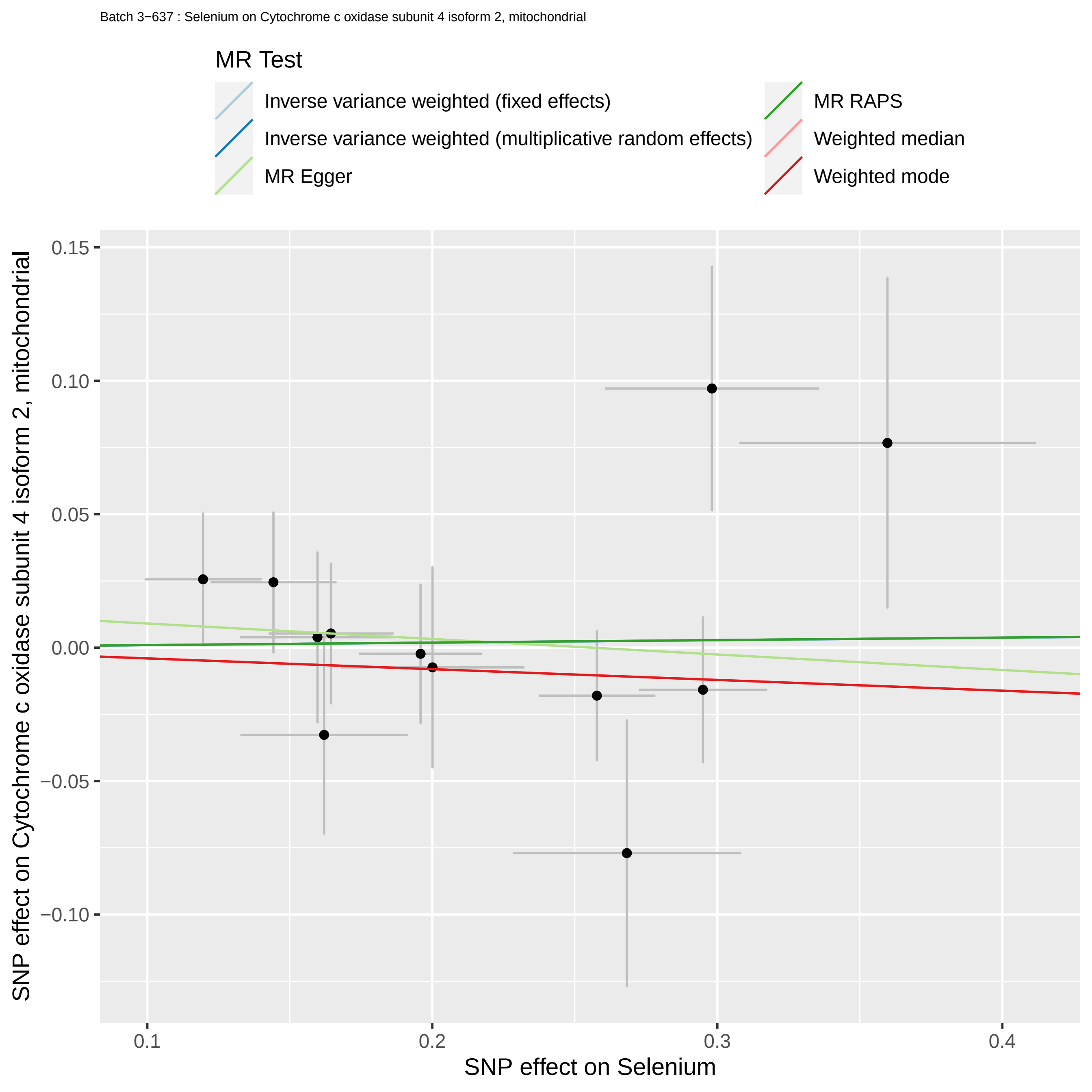
Batch 3−637 : Selenium on Cytochrome c oxidase subunit 4 isoform 2, mitochondrial
MR Test
Inverse variance weighted (fixed effects)
Inverse variance weighted (multiplicative random effects)
MR Egger
MR RAPS
Weighted median
Weighted mode
SNP effect on Cytochrome c oxidase subunit 4 isoform 2, mitochondrial
0.15
0.10
0.05
0.00
−0.05
−0.10
0.1
0.2
0.3
0.4
SNP effect on Selenium

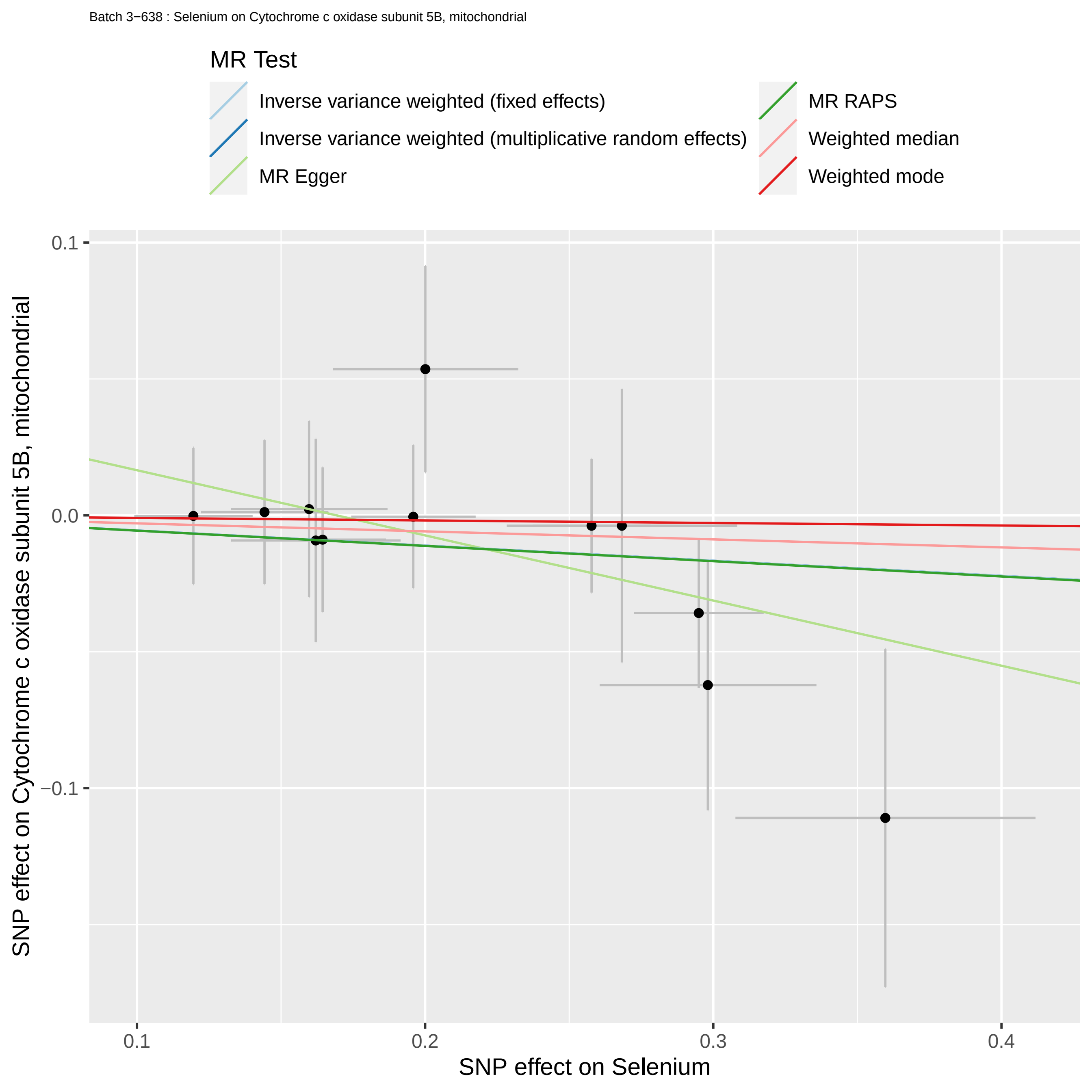
Batch 3−638 : Selenium on Cytochrome c oxidase subunit 5B, mitochondrial
MR Test
Inverse variance weighted (fixed effects)
Inverse variance weighted (multiplicative random effects)
MR Egger
MR RAPS
Weighted median
Weighted mode
0.1
0.0
−0.1
0.1
0.2
0.3
0.4
SNP effect on Cytochrome c oxidase subunit 5B, mitochondrial
SNP effect on Selenium

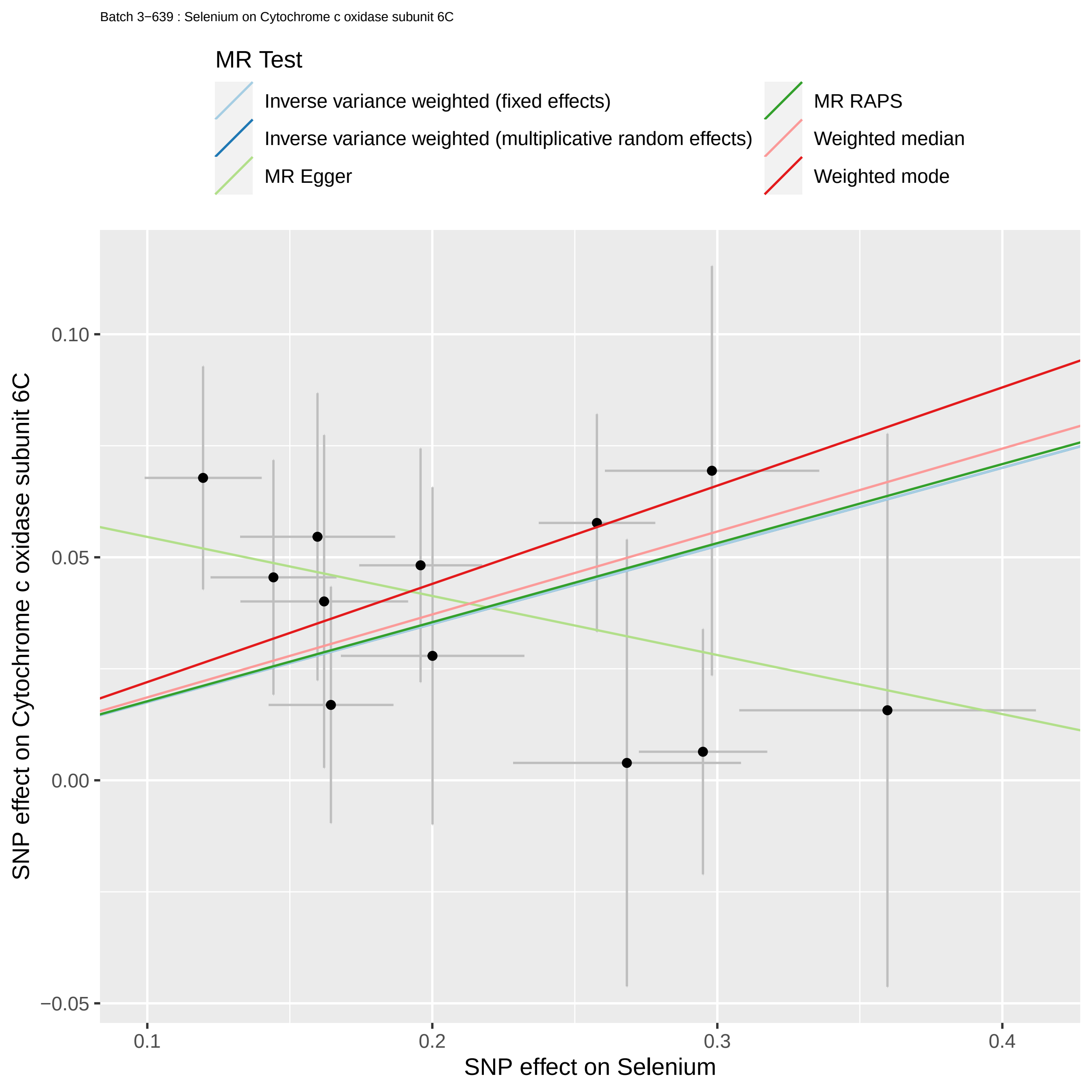

Batch 3−639 : Selenium on Cytochrome c oxidase subunit 6C
MR Test
Inverse variance weighted (fixed effects)
Inverse variance weighted (multiplicative random effects)
MR Egger
MR RAPS
Weighted median
Weighted mode
0.10
0.05
0.00
−0.05
0.1
0.2
0.3
0.4
SNP effect on Cytochrome c oxidase subunit 6C
SNP effect on Selenium

Batch 3−640 : Selenium on Cytochrome c oxidase subunit 7A1, mitochondrial

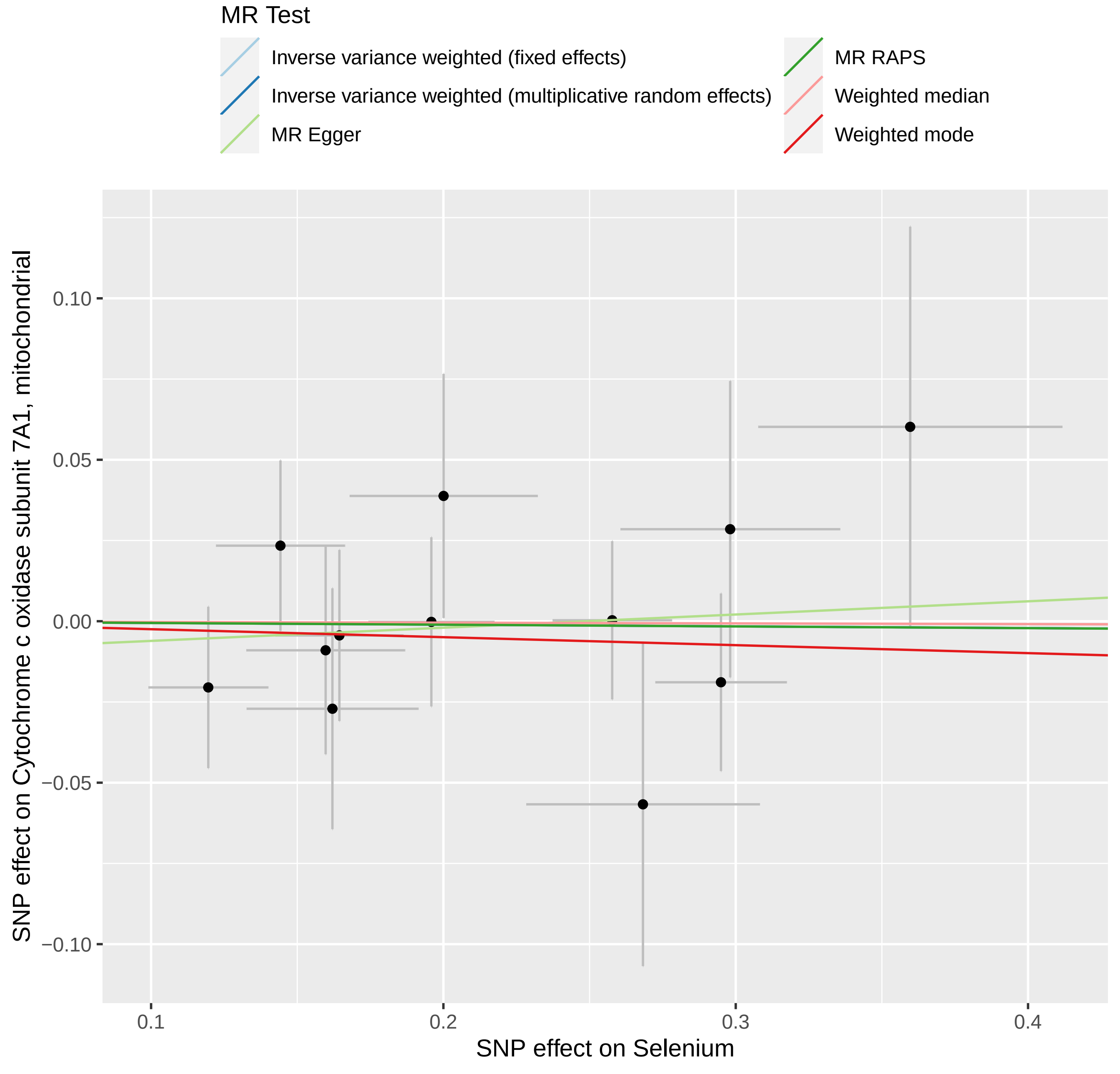

Batch 3−641 : Selenium on Cytochrome c oxidase subunit 8A, mitochondrial

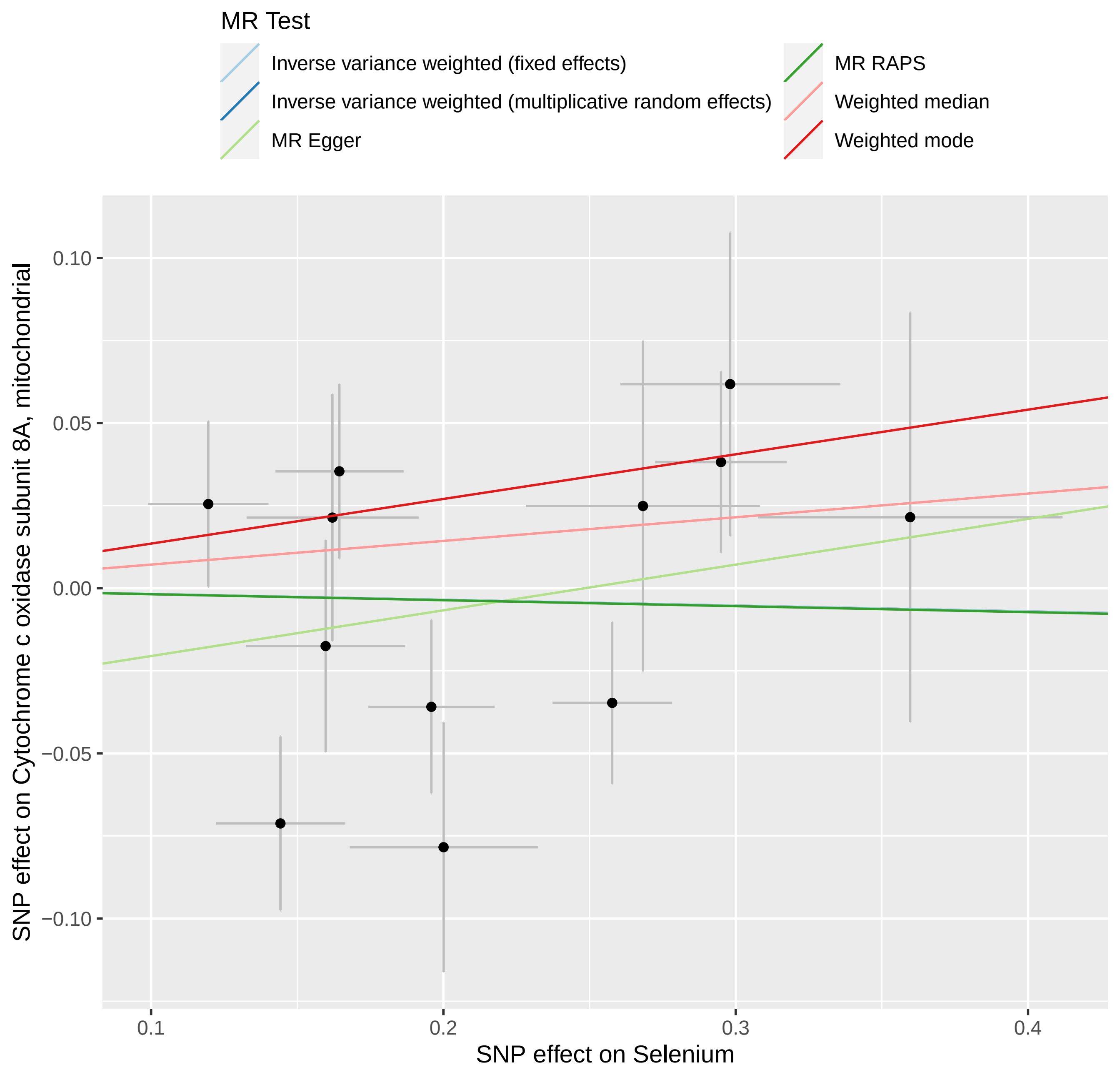

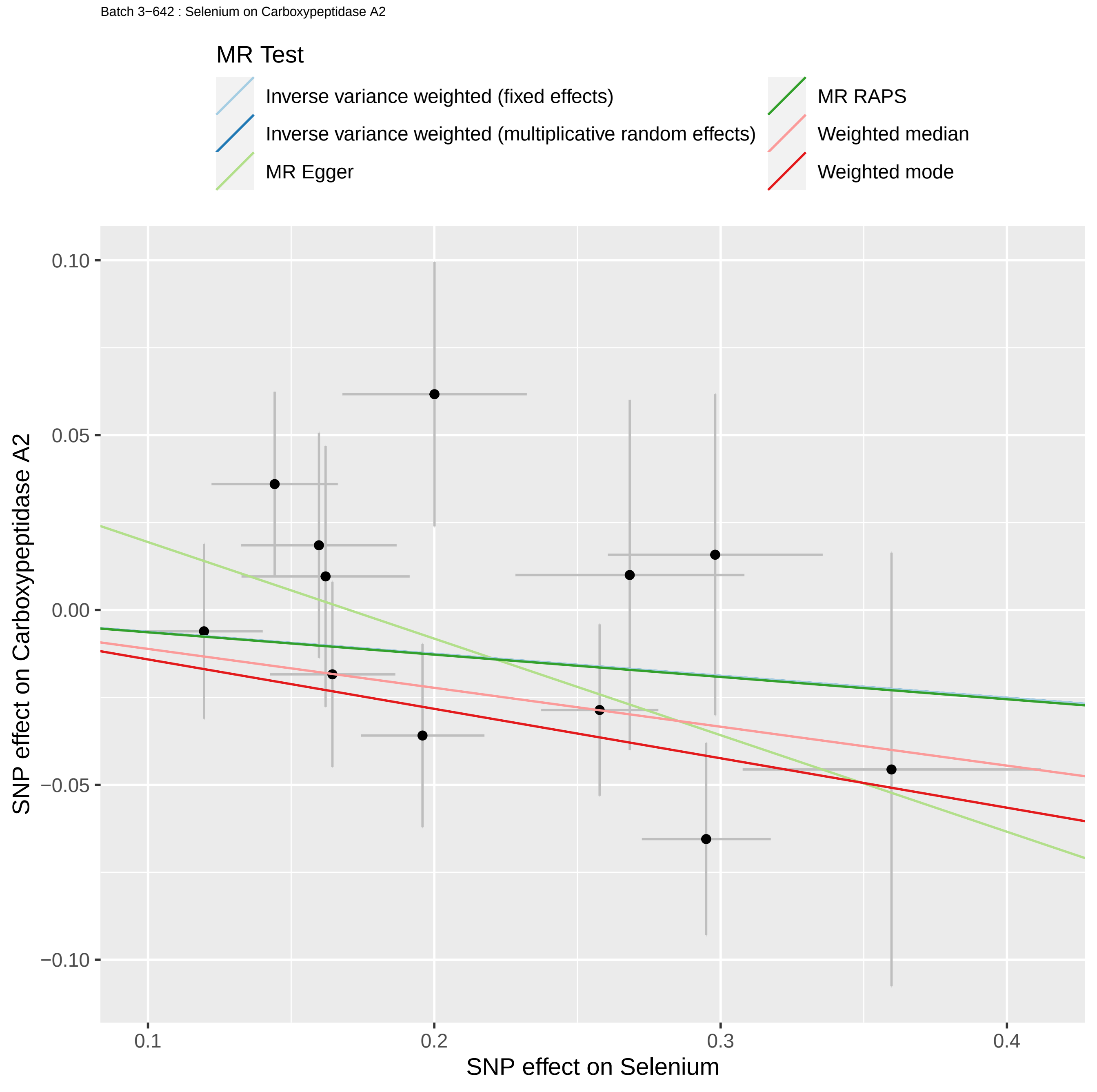

Batch 3−642 : Selenium on Carboxypeptidase A2
MR Test
Inverse variance weighted (fixed effects)
Inverse variance weighted (multiplicative random effects)
MR Egger
MR RAPS
Weighted median
Weighted mode
SNP effect on Carboxypeptidase A2
0.10
0.05
0.00
−0.05
−0.10
0.1
0.2
0.3
0.4
SNP effect on Selenium

Batch 3−643 : Selenium on Carboxypeptidase A4

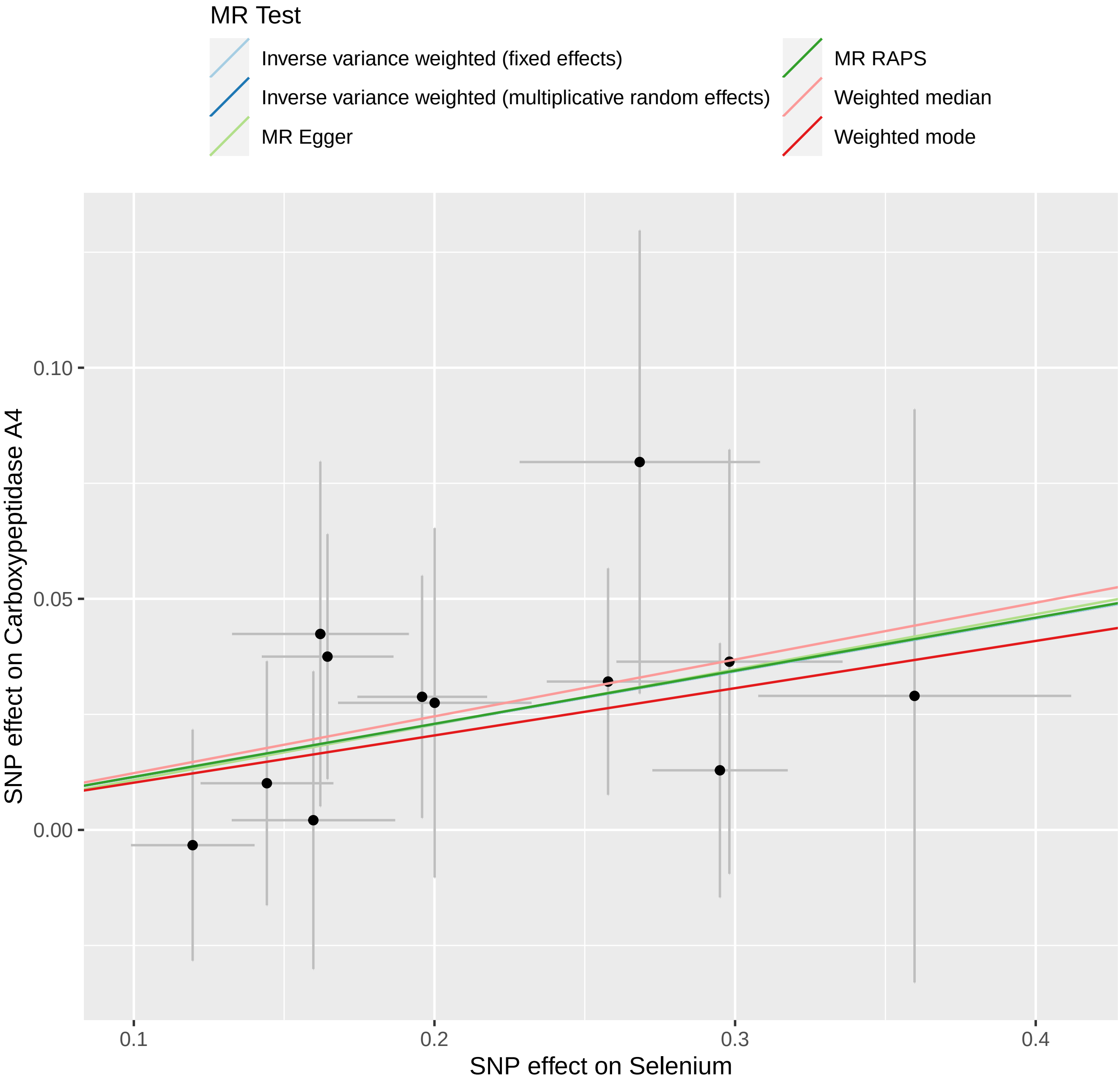

Batch 3−644 : Selenium on Carboxypeptidase B

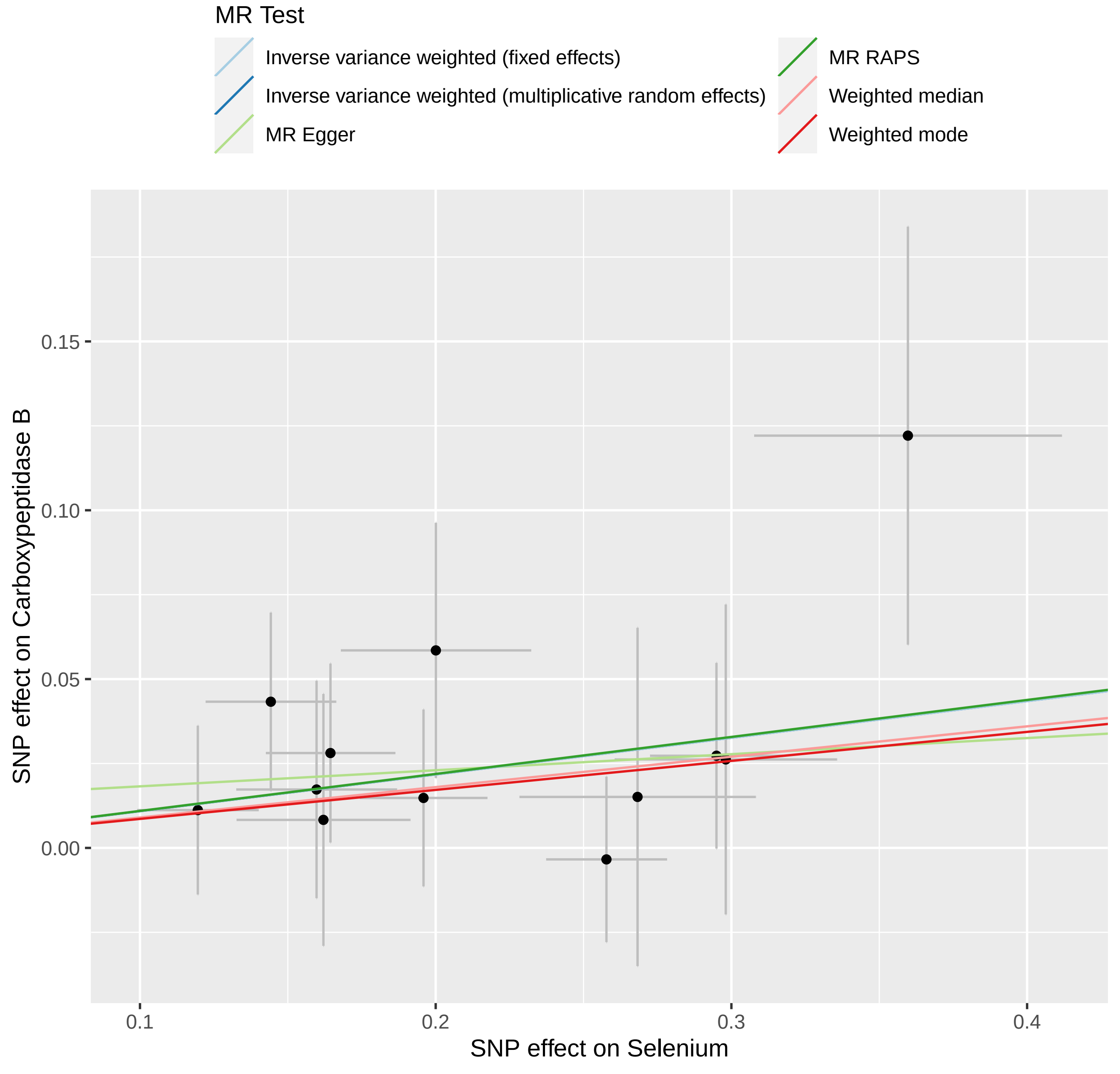

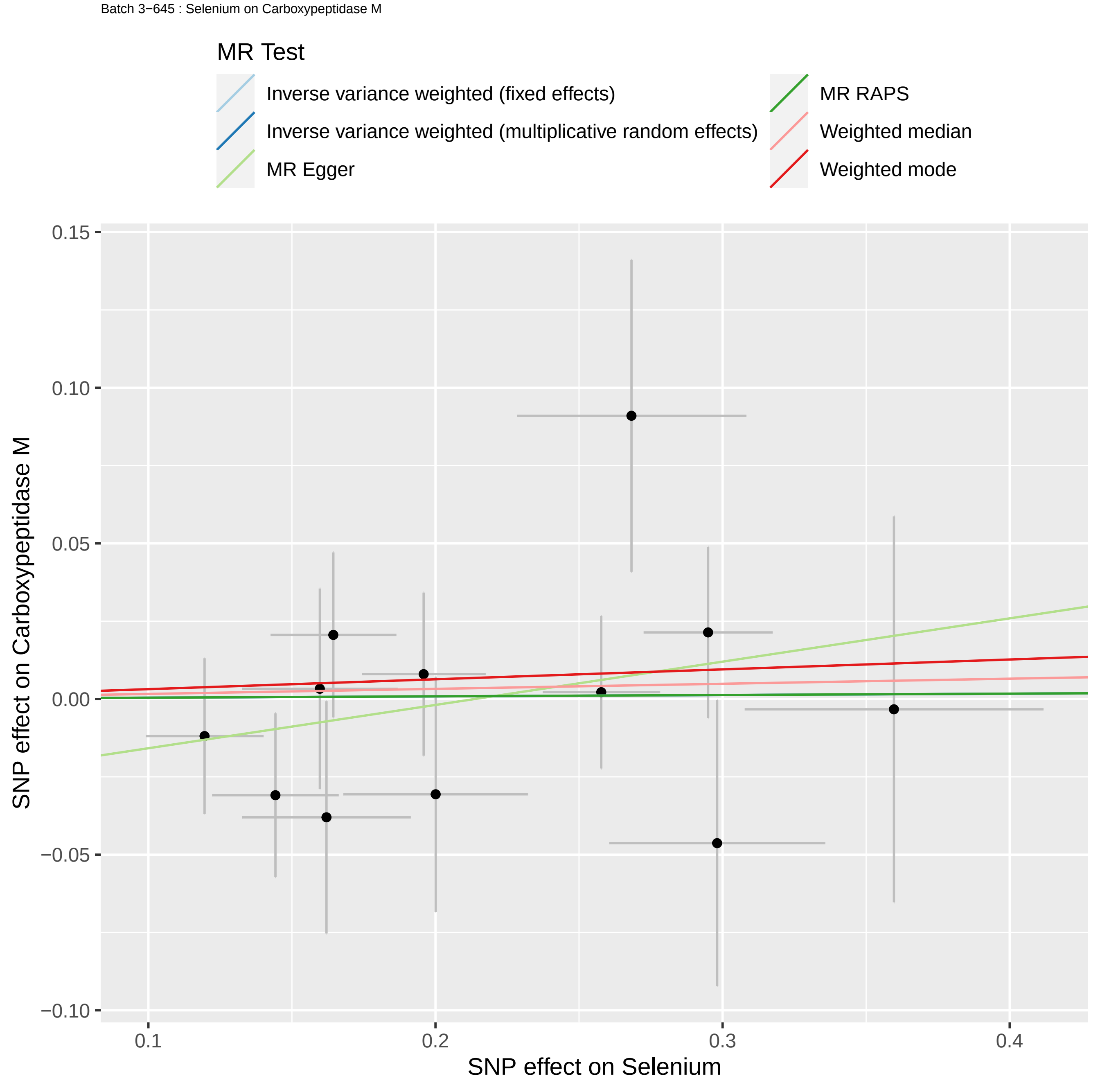
Batch 3−645 : Selenium on Carboxypeptidase M
MR Test
Inverse variance weighted (fixed effects)
Inverse variance weighted (multiplicative random effects)
MR Egger
MR RAPS
Weighted median
Weighted mode
0.15
0.10
0.05
0.00
−0.05
−0.10
0.1
0.2
0.3
0.4
SNP effect on Carboxypeptidase M
SNP effect on Selenium

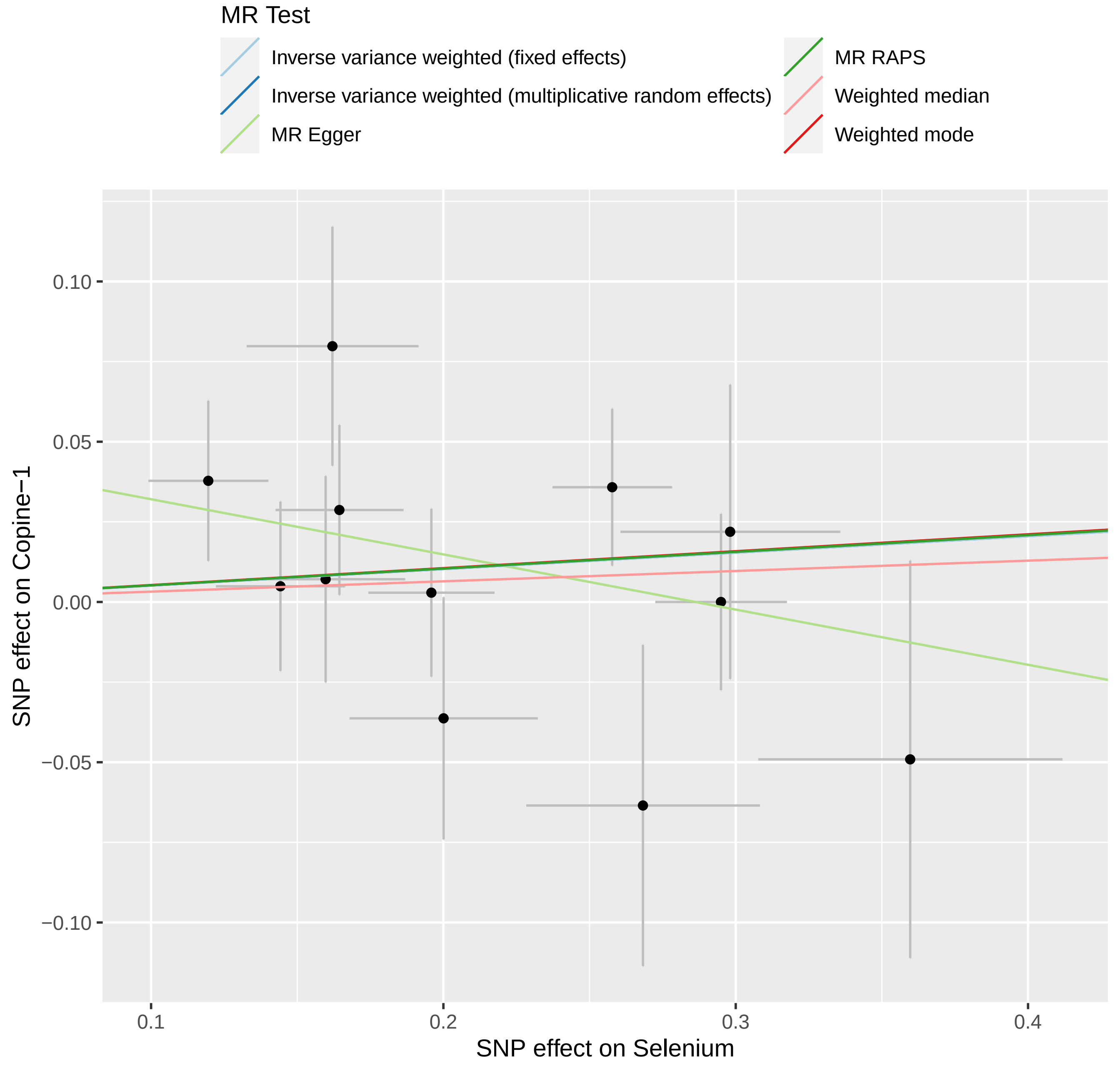
MR Test
Inverse variance weighted (fixed effects)
Inverse variance weighted (multiplicative random effects)
MR Egger
MR RAPS
Weighted median
Weighted mode
0.10
0.05
0.00
−0.05
−0.10
SNP effect on Copine−1
0.1
0.2
0.3
0.4
SNP effect on Selenium

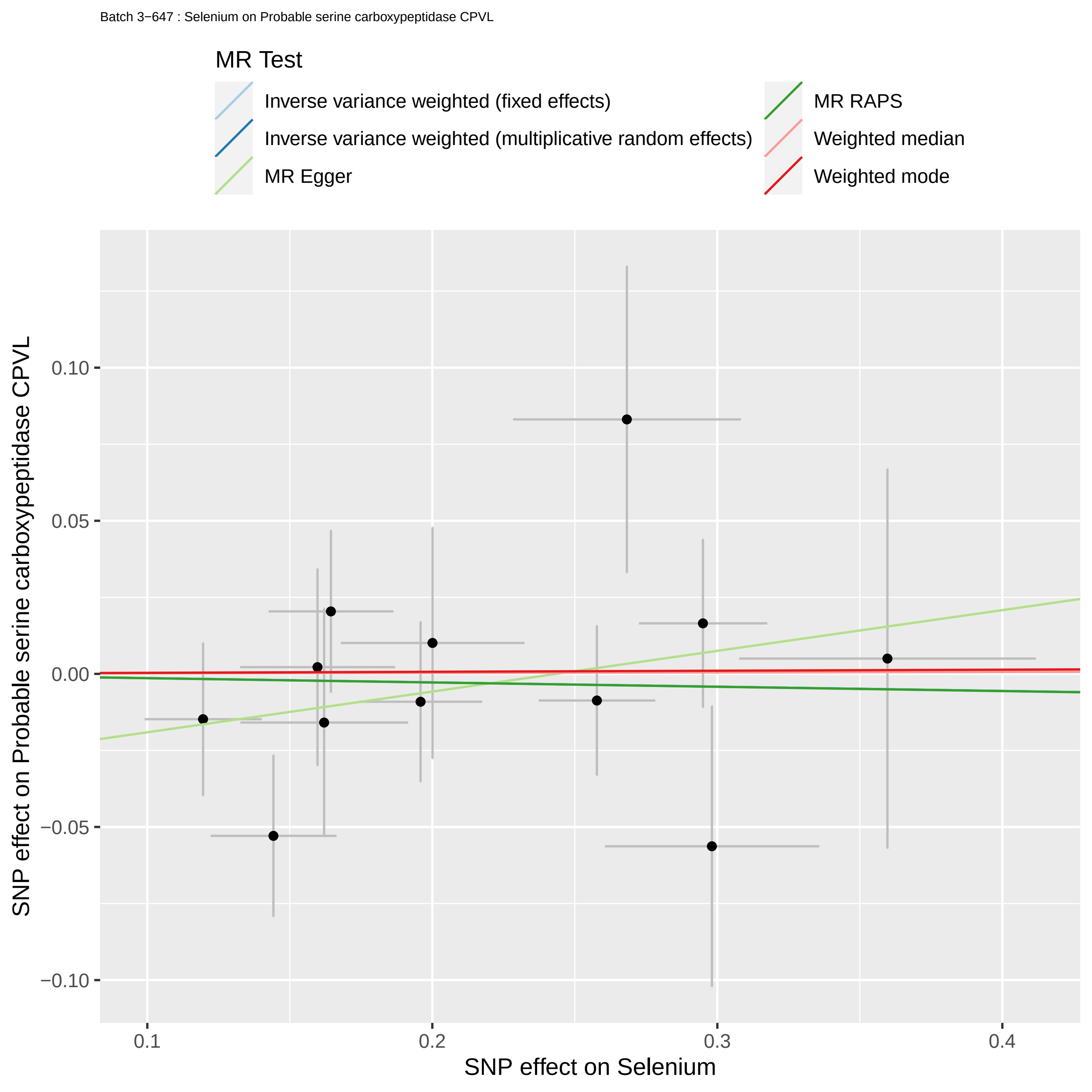

Batch 3−647 : Selenium on Probable serine carboxypeptidase CPVL
MR Test
Inverse variance weighted (fixed effects)
Inverse variance weighted (multiplicative random effects)
MR Egger
MR RAPS
Weighted median
Weighted mode
SNP effect on Probable serine carboxypeptidase CPVL
0.10
0.05
0.00
−0.05
−0.10
0.1
0.2
0.3
0.4
SNP effect on Selenium

Batch 3−648 : Selenium on Probable carboxypeptidase X1

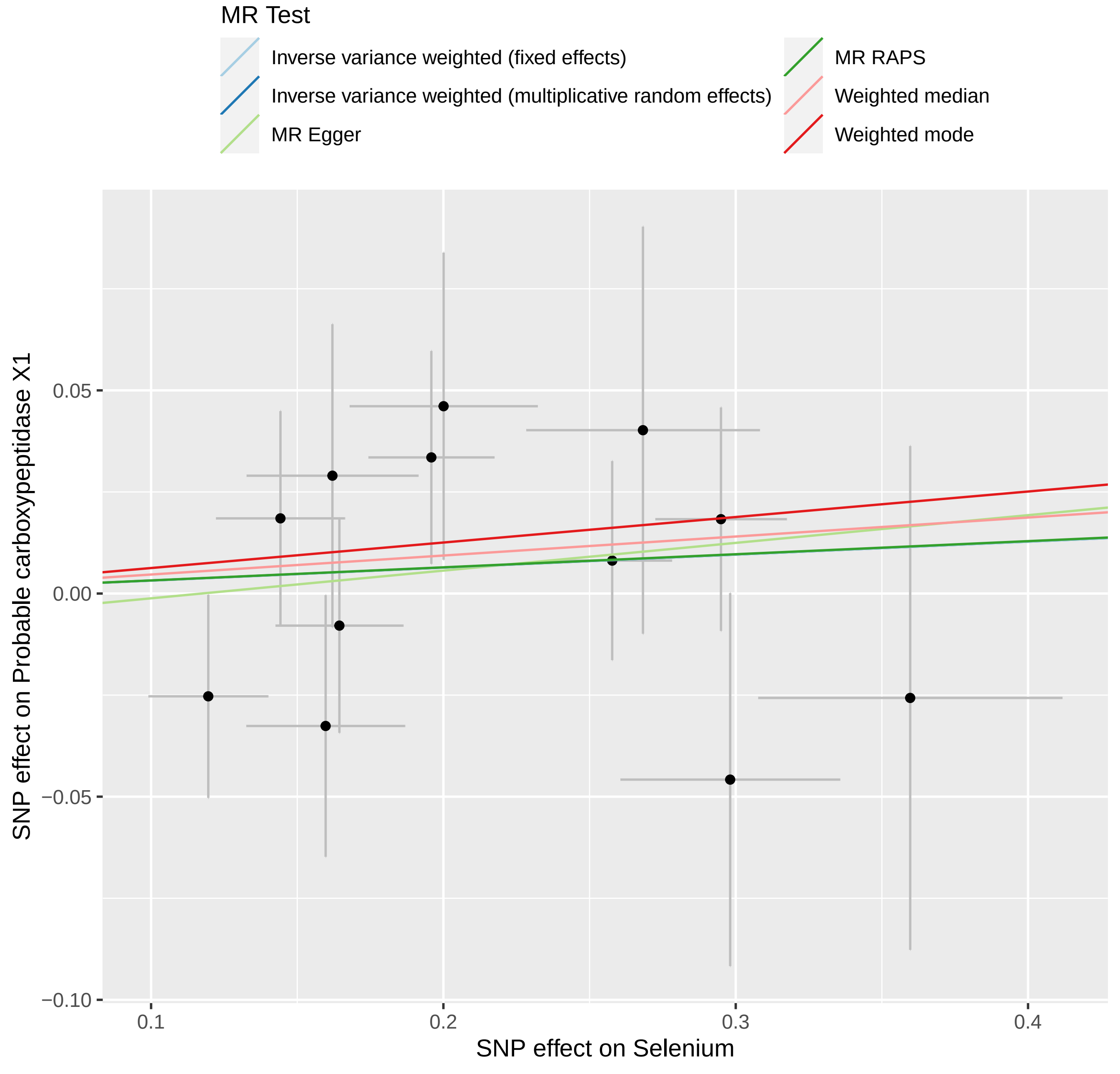

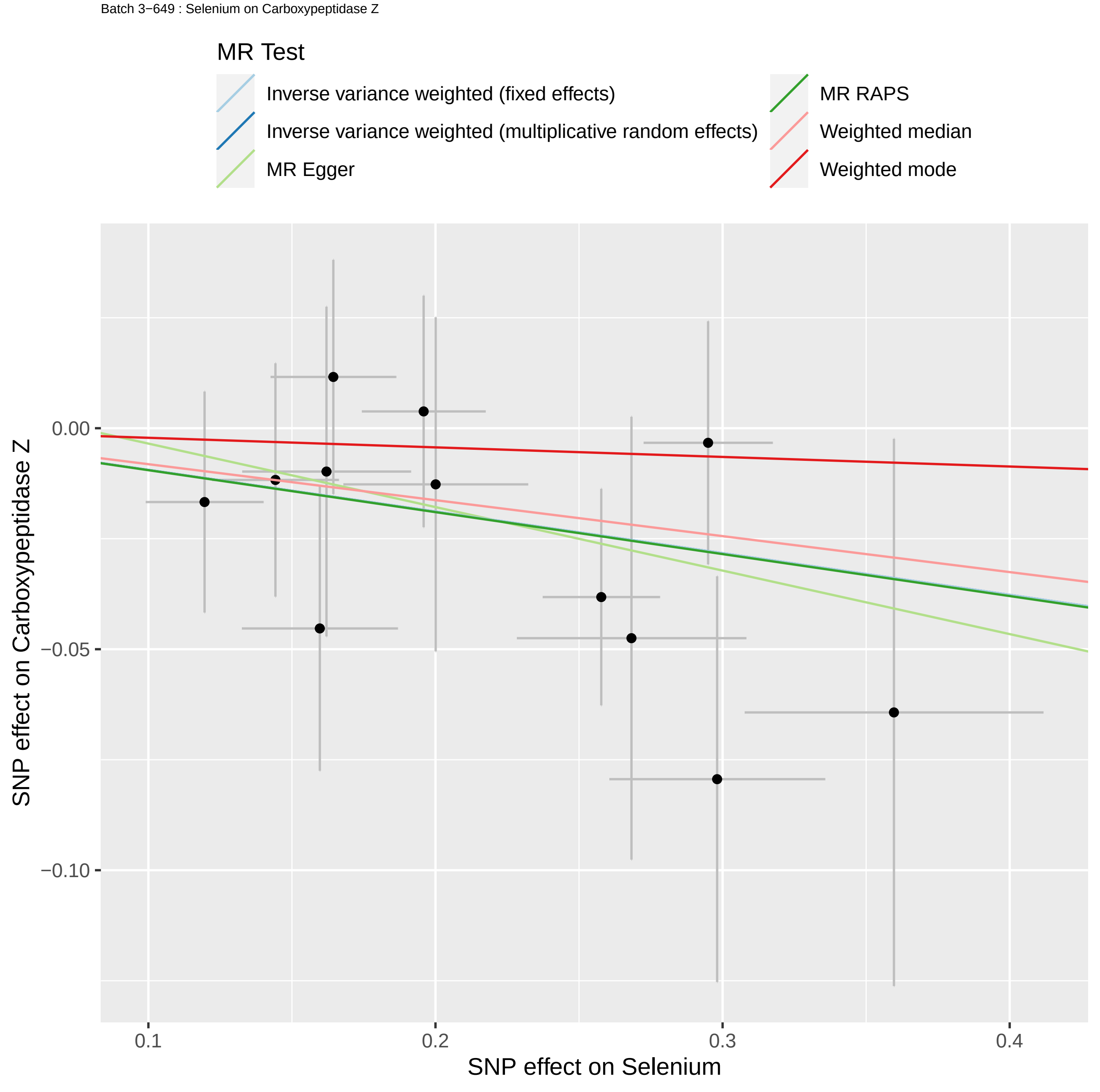
Batch 3−649 : Selenium on Carboxypeptidase Z
MR Test
Inverse variance weighted (fixed effects)
Inverse variance weighted (multiplicative random effects)
MR Egger
MR RAPS
Weighted median
Weighted mode
0.00
−0.05
−0.10
0.1
0.2
0.3
0.4
SNP effect on Carboxypeptidase Z
SNP effect on Selenium

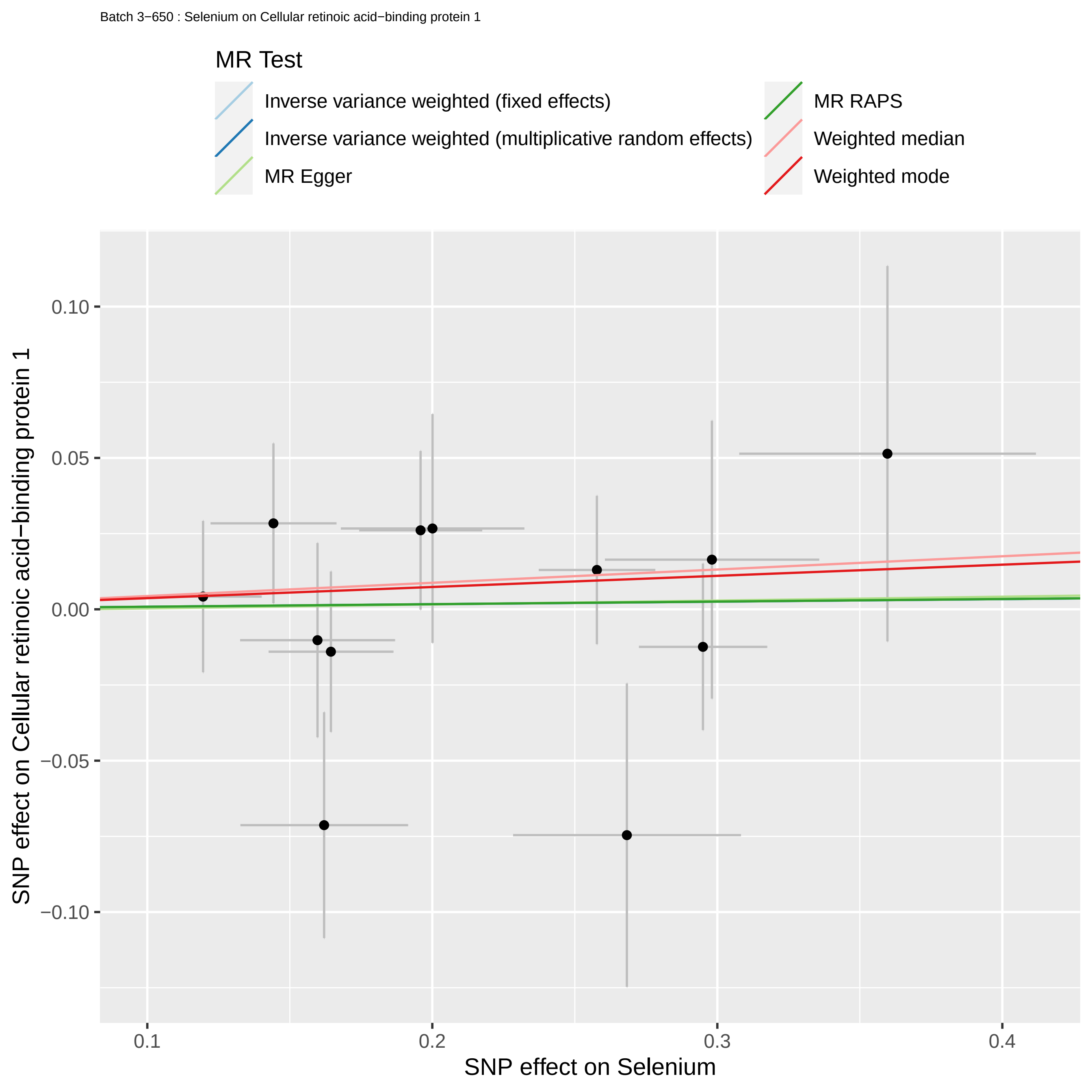

Batch 3−650 : Selenium on Cellular retinoic acid−binding protein 1
MR Test
Inverse variance weighted (fixed effects)
Inverse variance weighted (multiplicative random effects)
MR Egger
MR RAPS
Weighted median
Weighted mode
0.10
0.05
0.00
−0.05
−0.10
0.1
0.2
0.3
0.4
SNP effect on Cellular retinoic acid−binding protein 1
SNP effect on Selenium

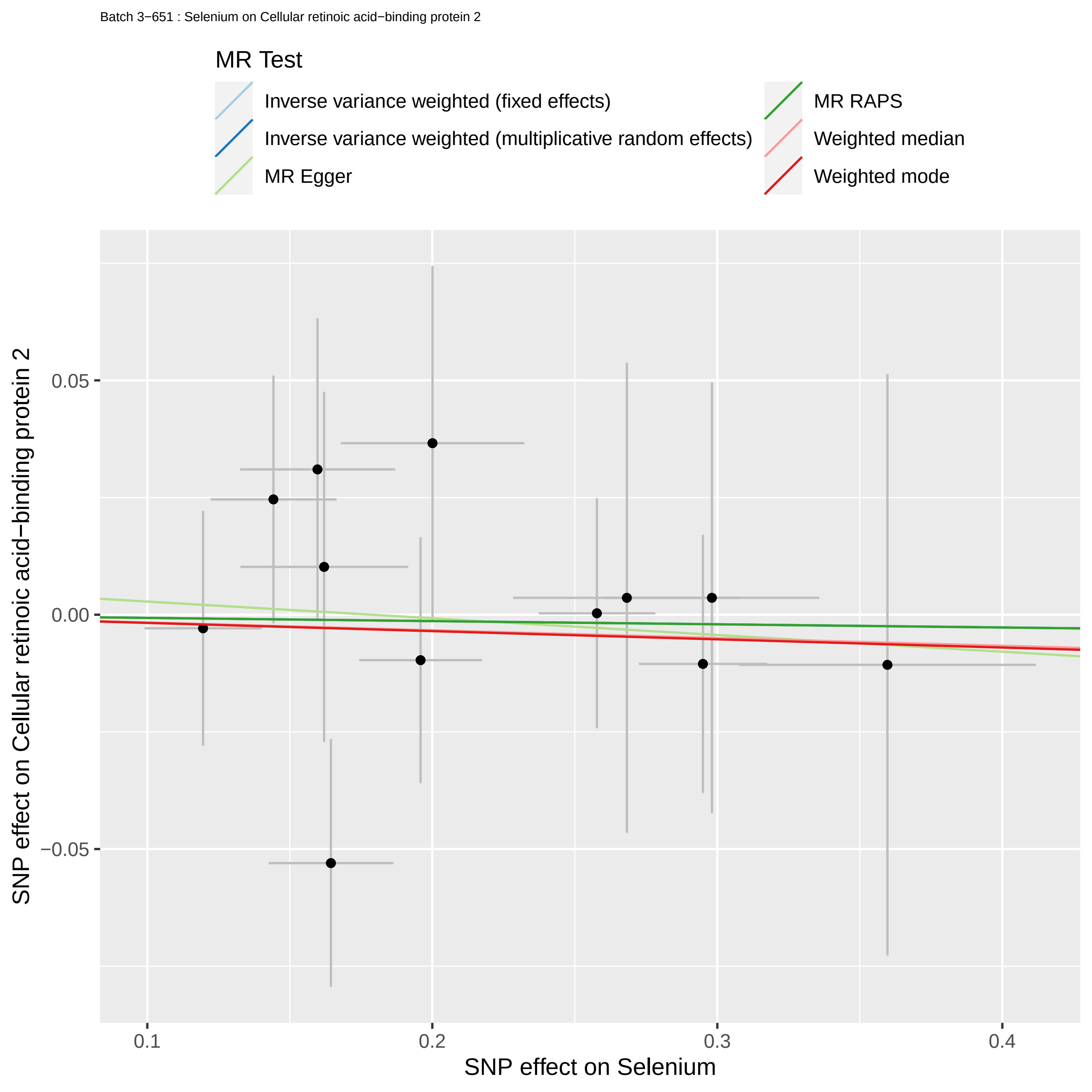

Batch 3−651 : Selenium on Cellular retinoic acid−binding protein 2
MR Test
Inverse variance weighted (fixed effects)
Inverse variance weighted (multiplicative random effects)
MR Egger
MR RAPS
Weighted median
Weighted mode
SNP effect on Cellular retinoic acid−binding protein 2
0.05
0.00
−0.05
0.1
0.2
0.3
0.4
SNP effect on Selenium

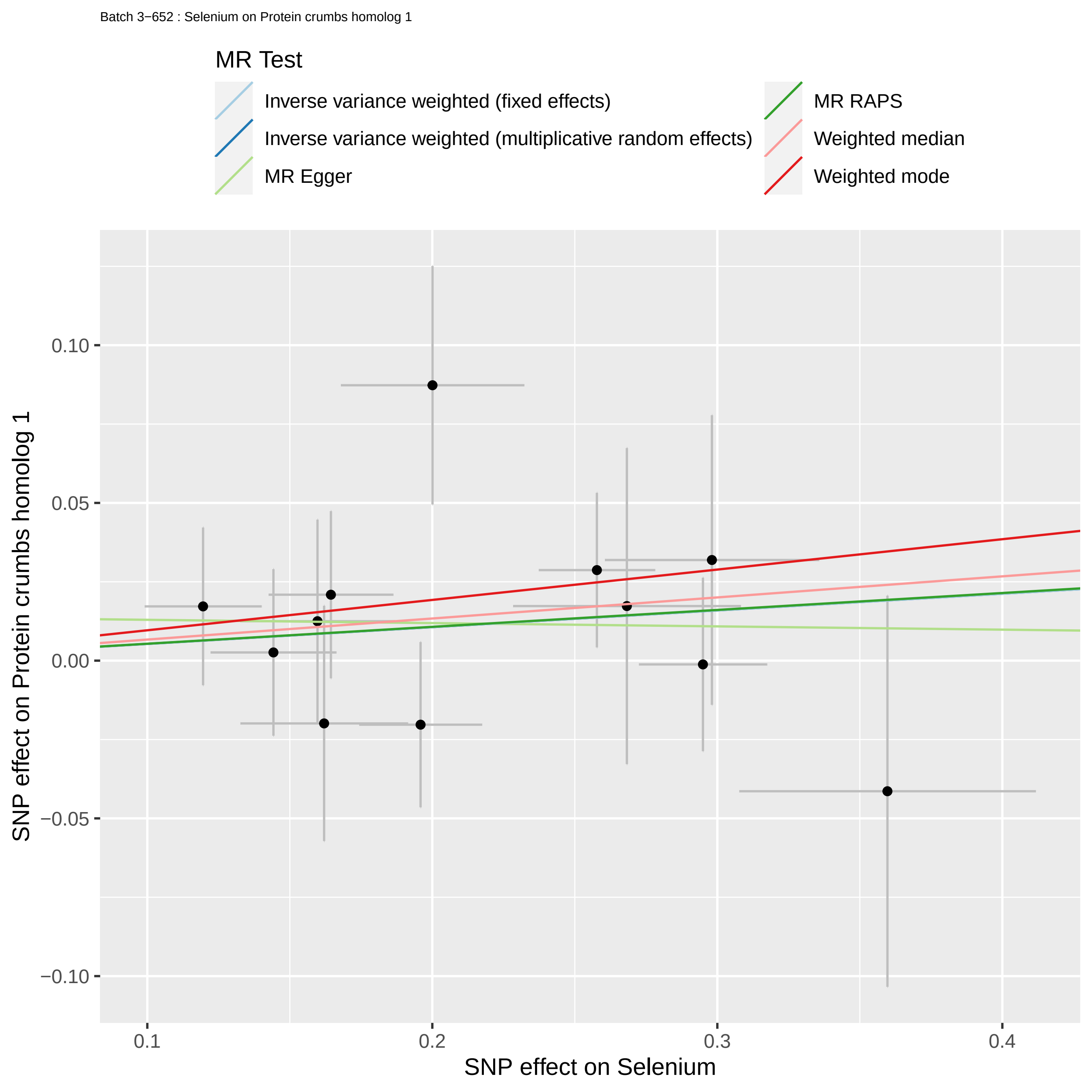

Batch 3−652 : Selenium on Protein crumbs homolog 1
MR Test
Inverse variance weighted (fixed effects)
Inverse variance weighted (multiplicative random effects)
MR Egger
MR RAPS
Weighted median
Weighted mode
0.10
0.05
0.00
−0.05
−0.10
0.1
0.2
0.3
0.4
SNP effect on Selenium
SNP effect on Protein crumbs homolog 1

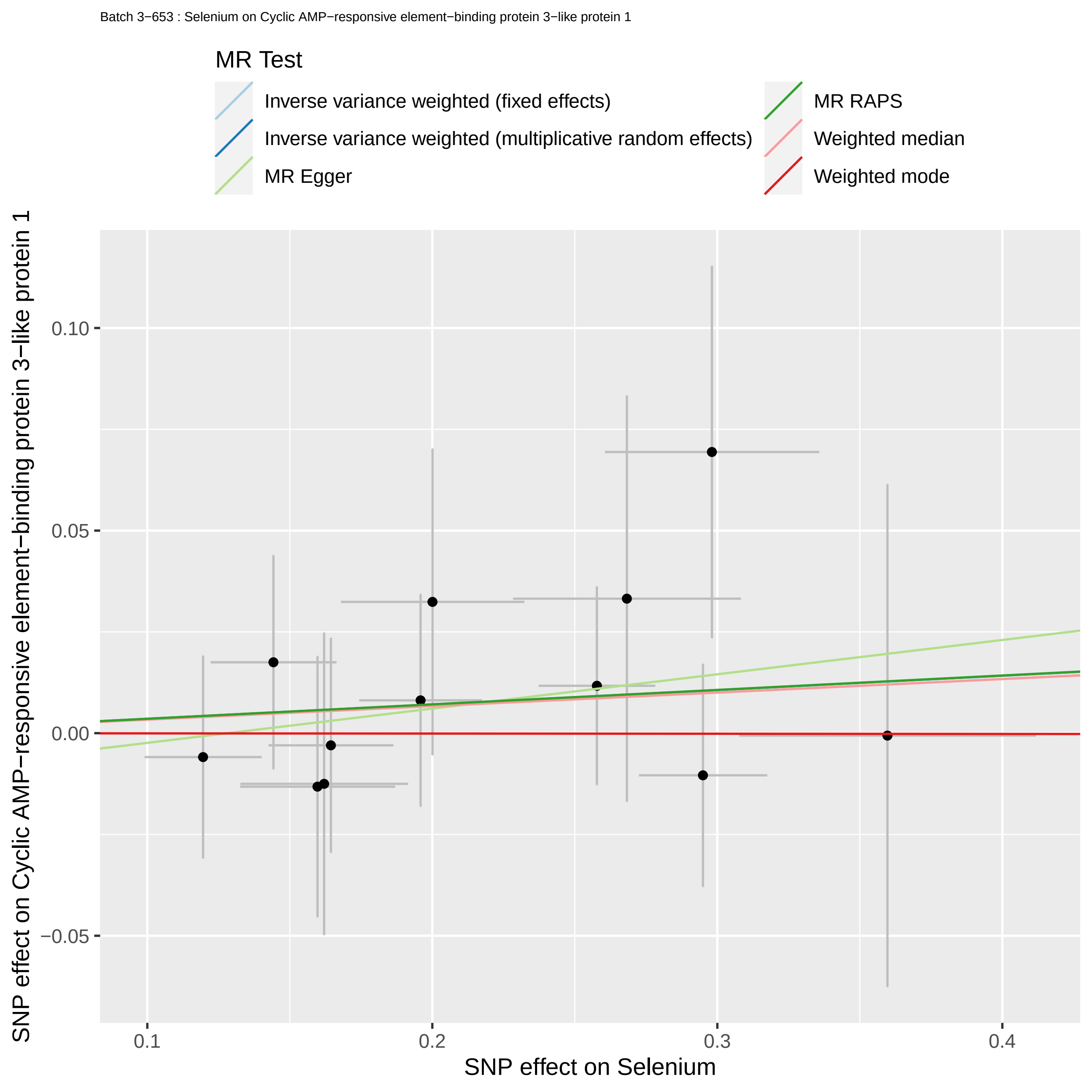

Batch 3−653 : Selenium on Cyclic AMP−responsive element−binding protein 3−like protein 1
MR Test
Inverse variance weighted (fixed effects)
Inverse variance weighted (multiplicative random effects)
MR Egger
MR RAPS
Weighted median
Weighted mode
SNP effect on Cyclic AMP−responsive element−binding protein 3−like protein 1
0.10
0.05
0.00
−0.05
0.1
0.2
0.3
0.4
SNP effect on Selenium

Batch 3−654 : Selenium on Cyclic AMP−responsive element−binding protein 3−like protein 4

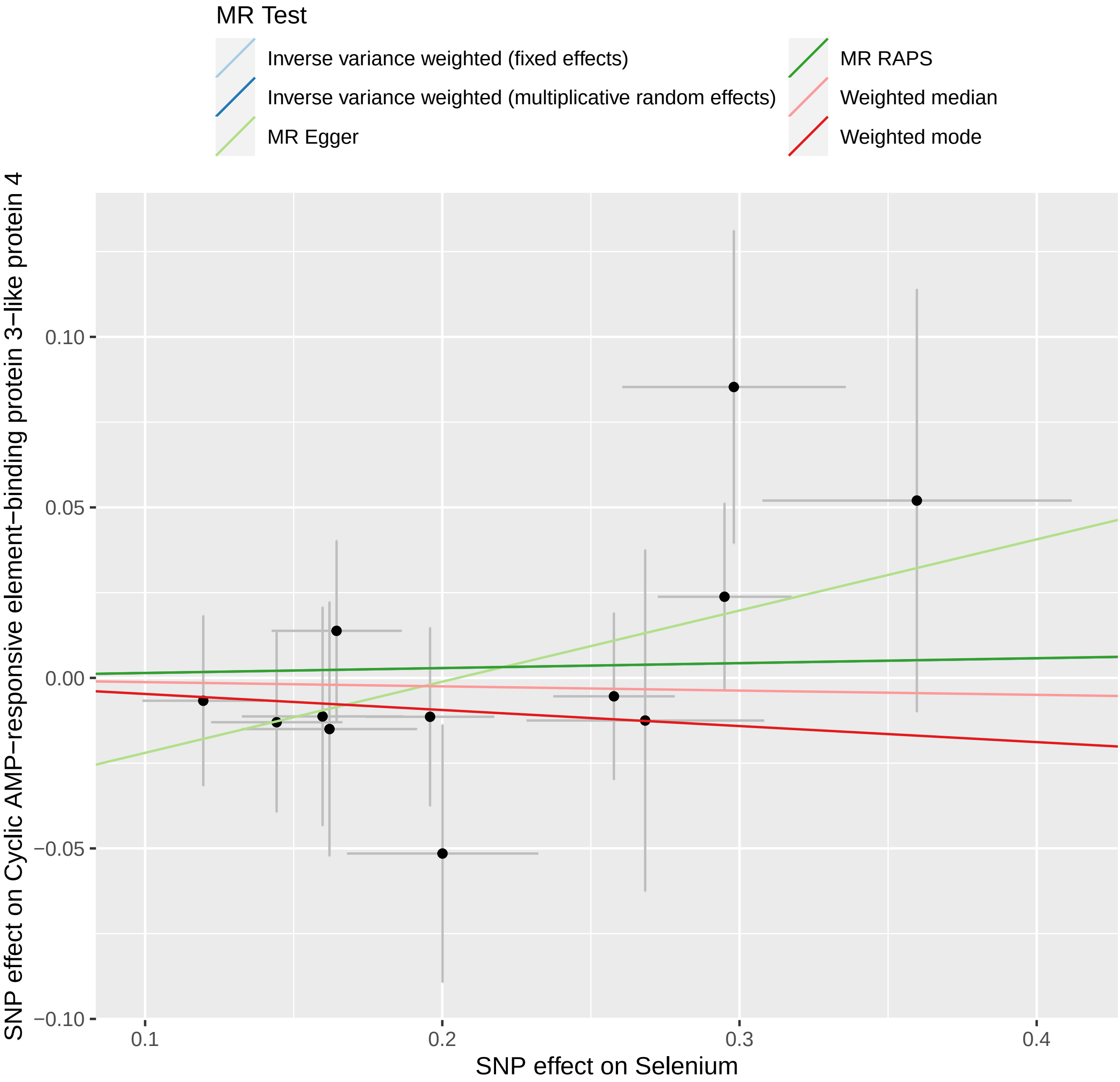

Batch 3−655 : Selenium on CREB−binding protein

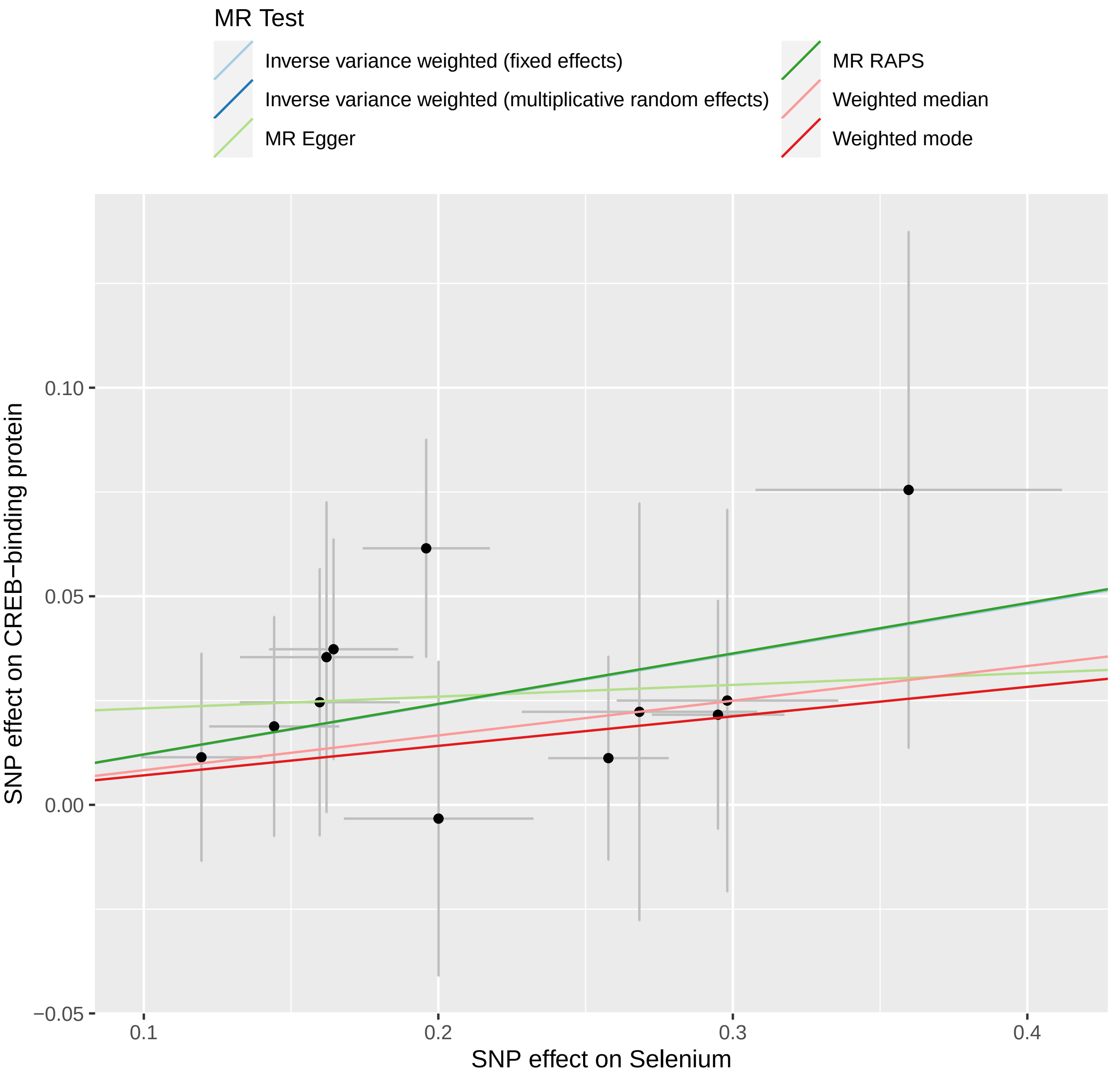

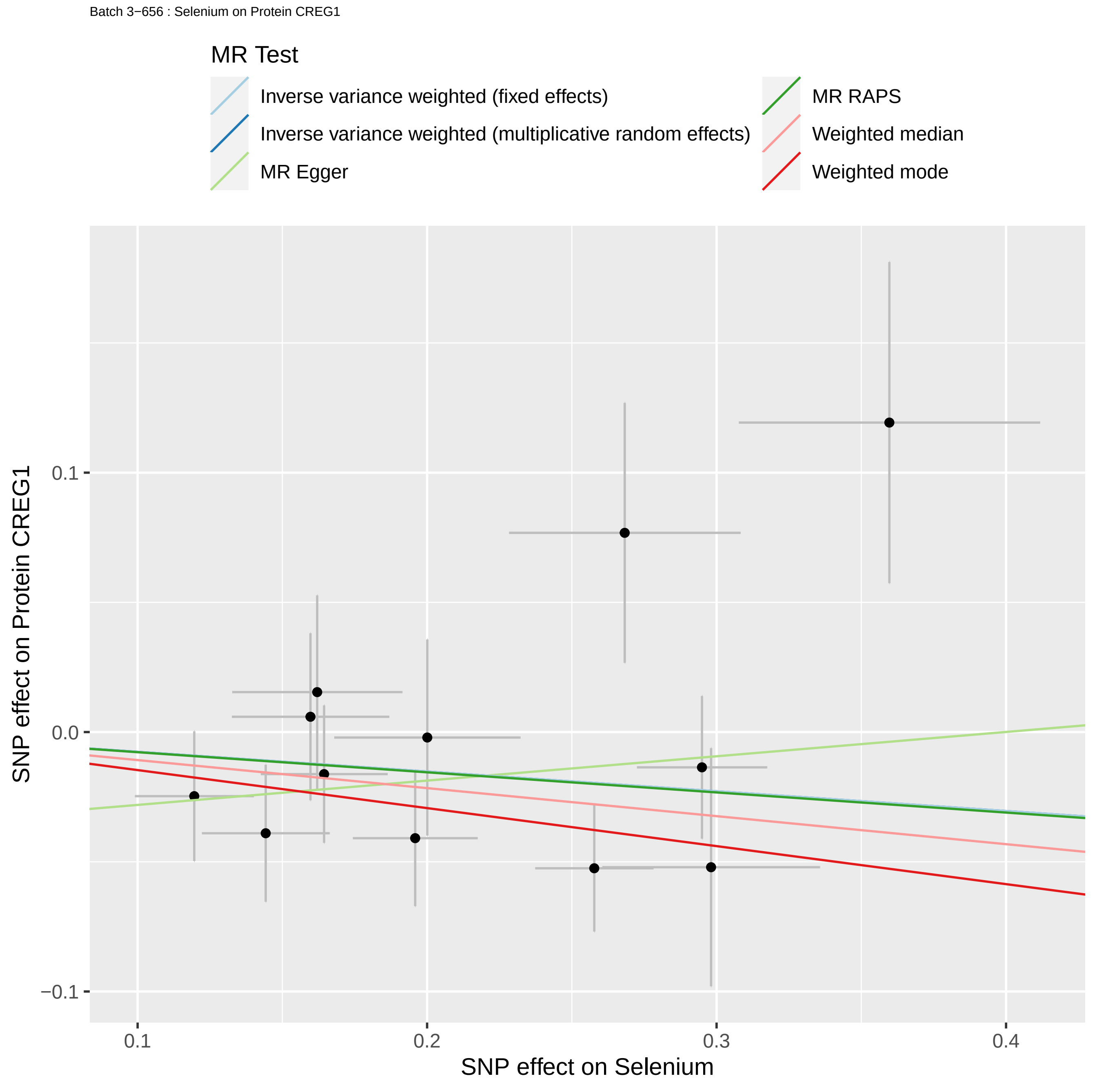
Batch 3−656 : Selenium on Protein CREG1
MR Test
Inverse variance weighted (fixed effects)
Inverse variance weighted (multiplicative random effects)
MR Egger
MR RAPS
Weighted median
Weighted mode
0.1
0.0
−0.1
0.1
0.2
0.3
0.4
SNP effect on Protein CREG1
SNP effect on Selenium

Batch 3−657 : Selenium on Cysteine−rich with EGF−like domain protein 1

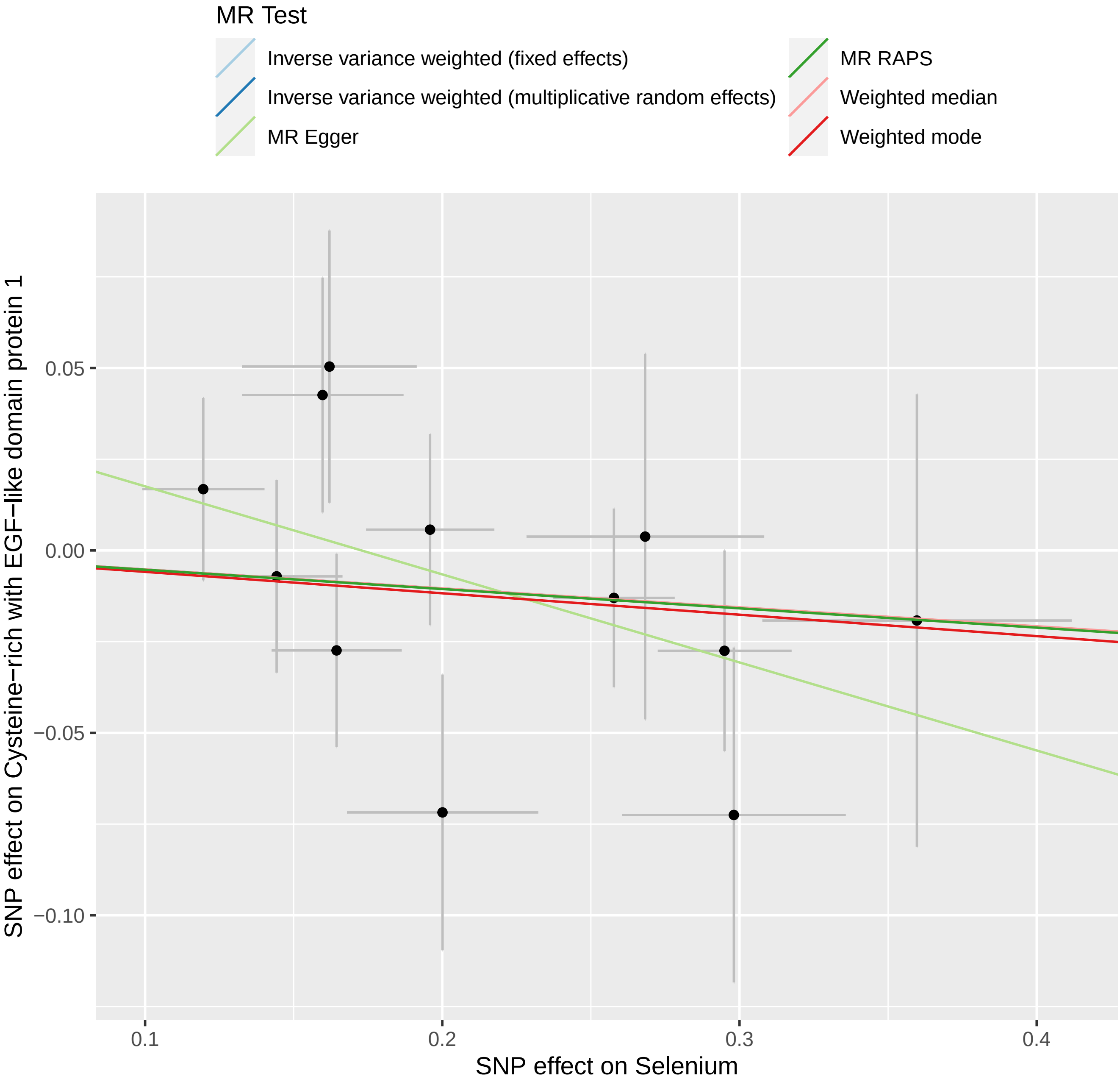

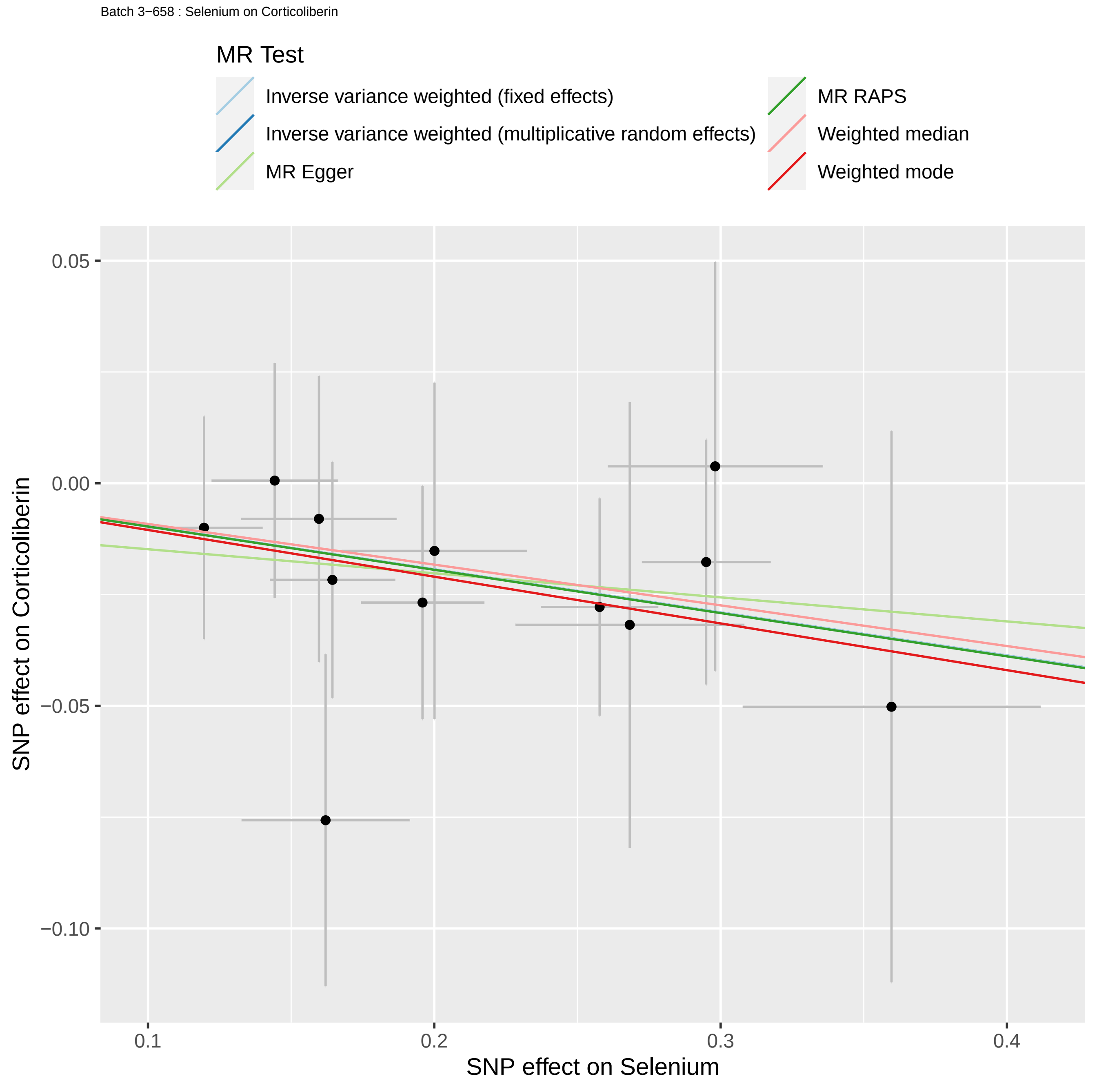
Batch 3−658 : Selenium on Corticoliberin
MR Test
Inverse variance weighted (fixed effects)
Inverse variance weighted (multiplicative random effects)
MR Egger
MR RAPS
Weighted median
Weighted mode
0.05
0.00
−0.05
−0.10
0.1
0.2
0.3
0.4
SNP effect on Corticoliberin
SNP effect on Selenium

Batch 3−659 : Selenium on Corticotropin−releasing factor−binding protein

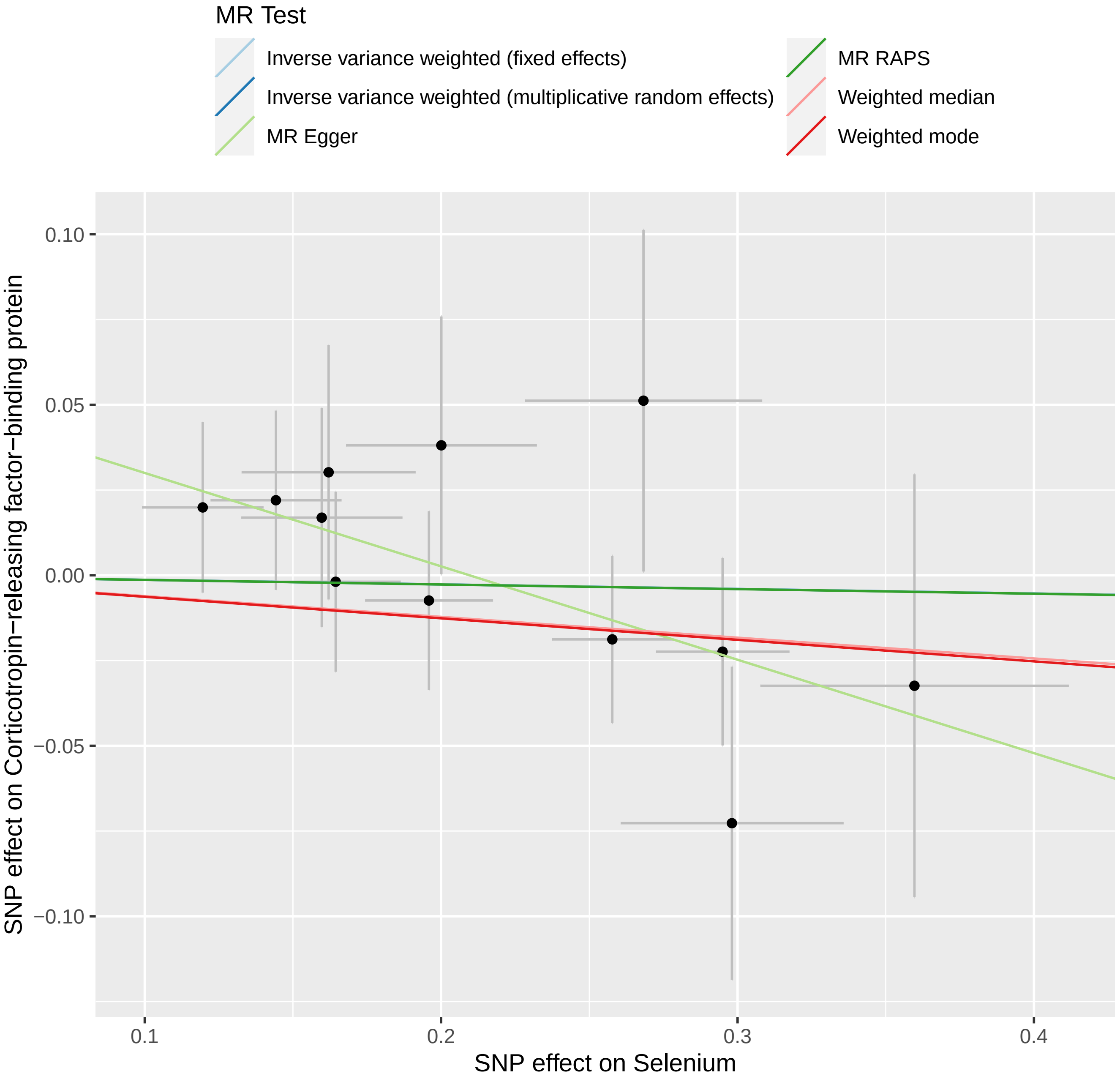

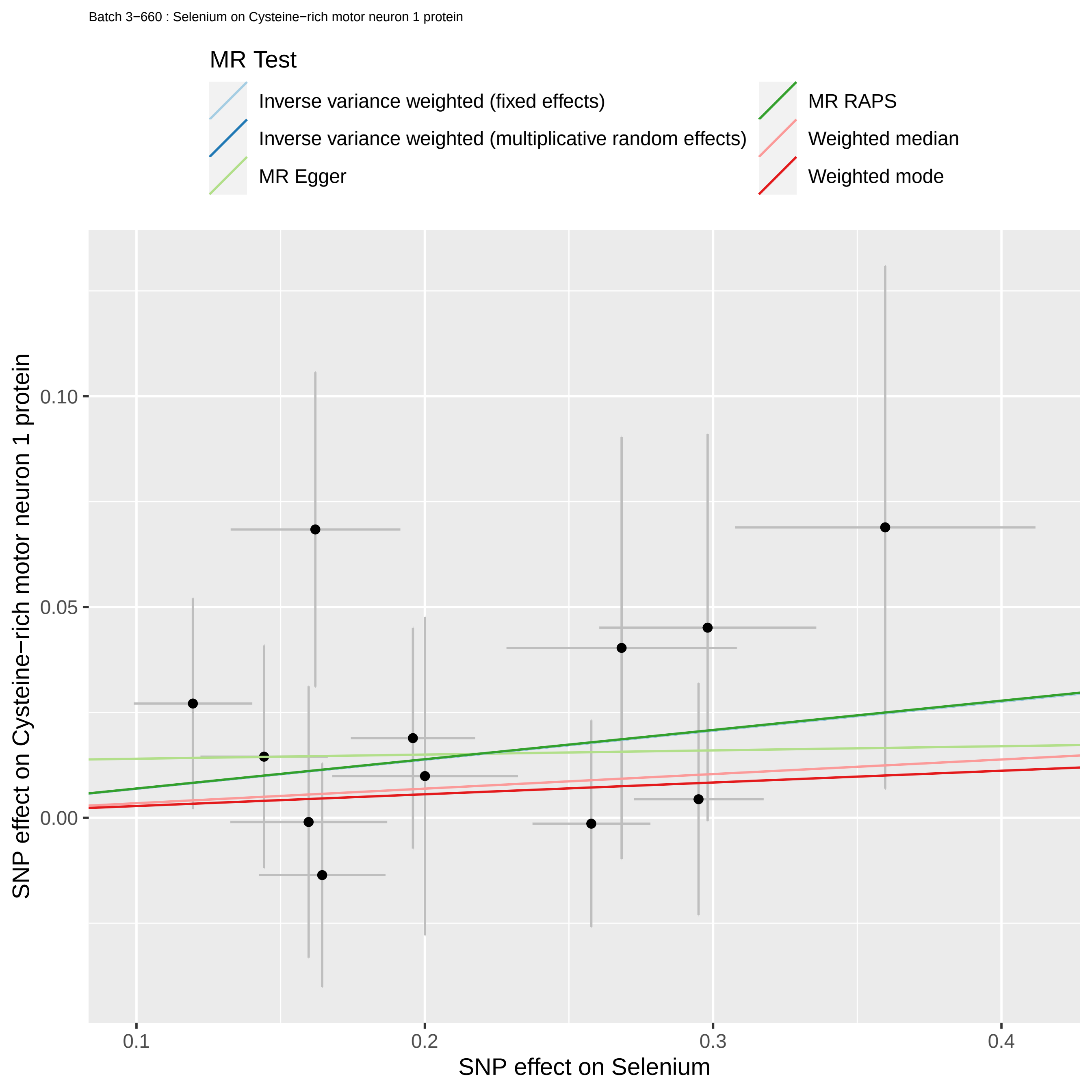

Batch 3−660 : Selenium on Cysteine−rich motor neuron 1 protein
MR Test
Inverse variance weighted (fixed effects)
Inverse variance weighted (multiplicative random effects)
MR Egger
MR RAPS
Weighted median
Weighted mode
SNP effect on Cysteine−rich motor neuron 1 protein
0.10
0.05
0.00
0.1
0.2
0.3
0.4
SNP effect on Selenium

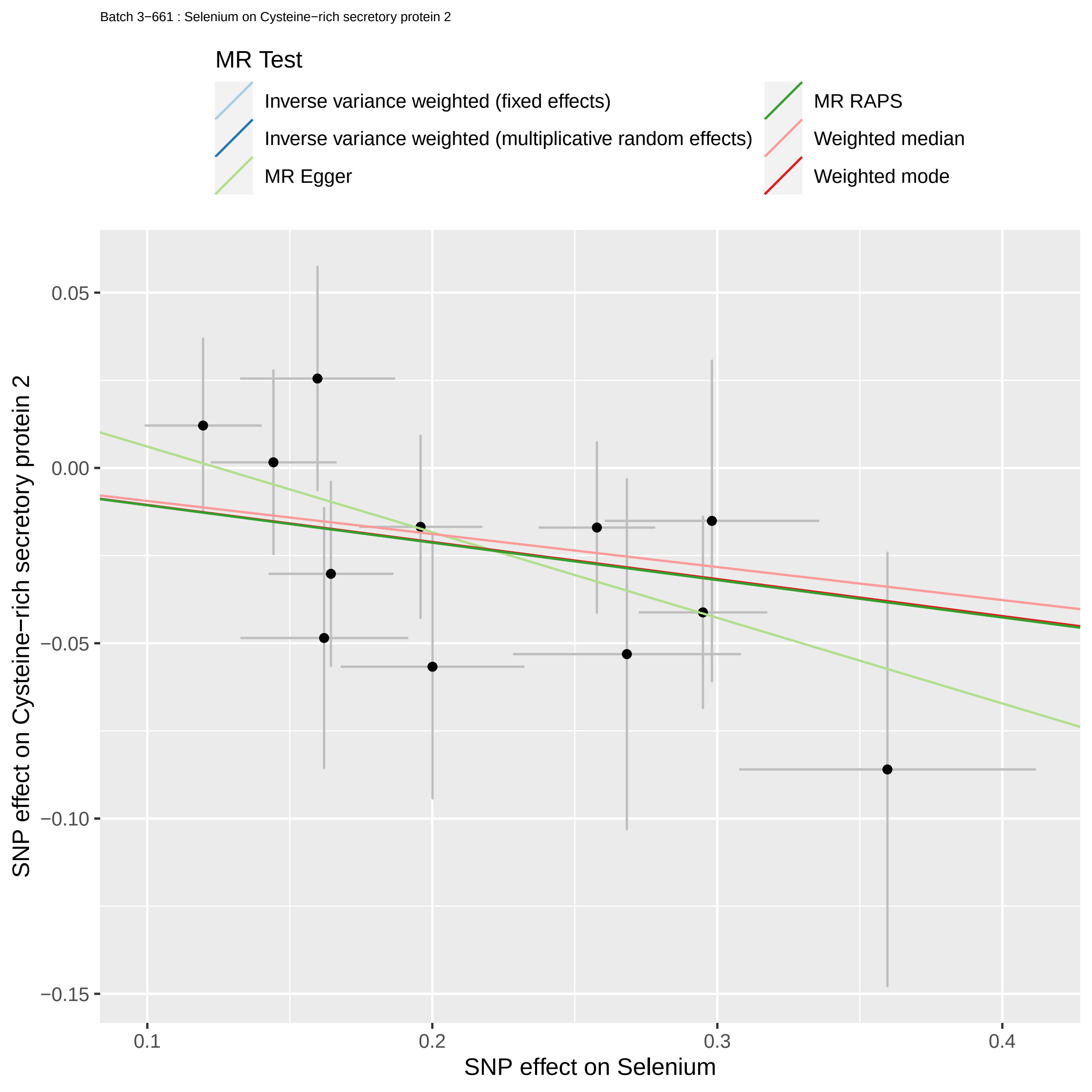

Batch 3−661 : Selenium on Cysteine−rich secretory protein 2
MR Test
Inverse variance weighted (fixed effects)
Inverse variance weighted (multiplicative random effects)
MR Egger
MR RAPS
Weighted median
Weighted mode
0.05
0.00
−0.05
−0.10
−0.15
0.1
0.2
0.3
0.4
SNP effect on Cysteine−rich secretory protein 2
SNP effect on Selenium

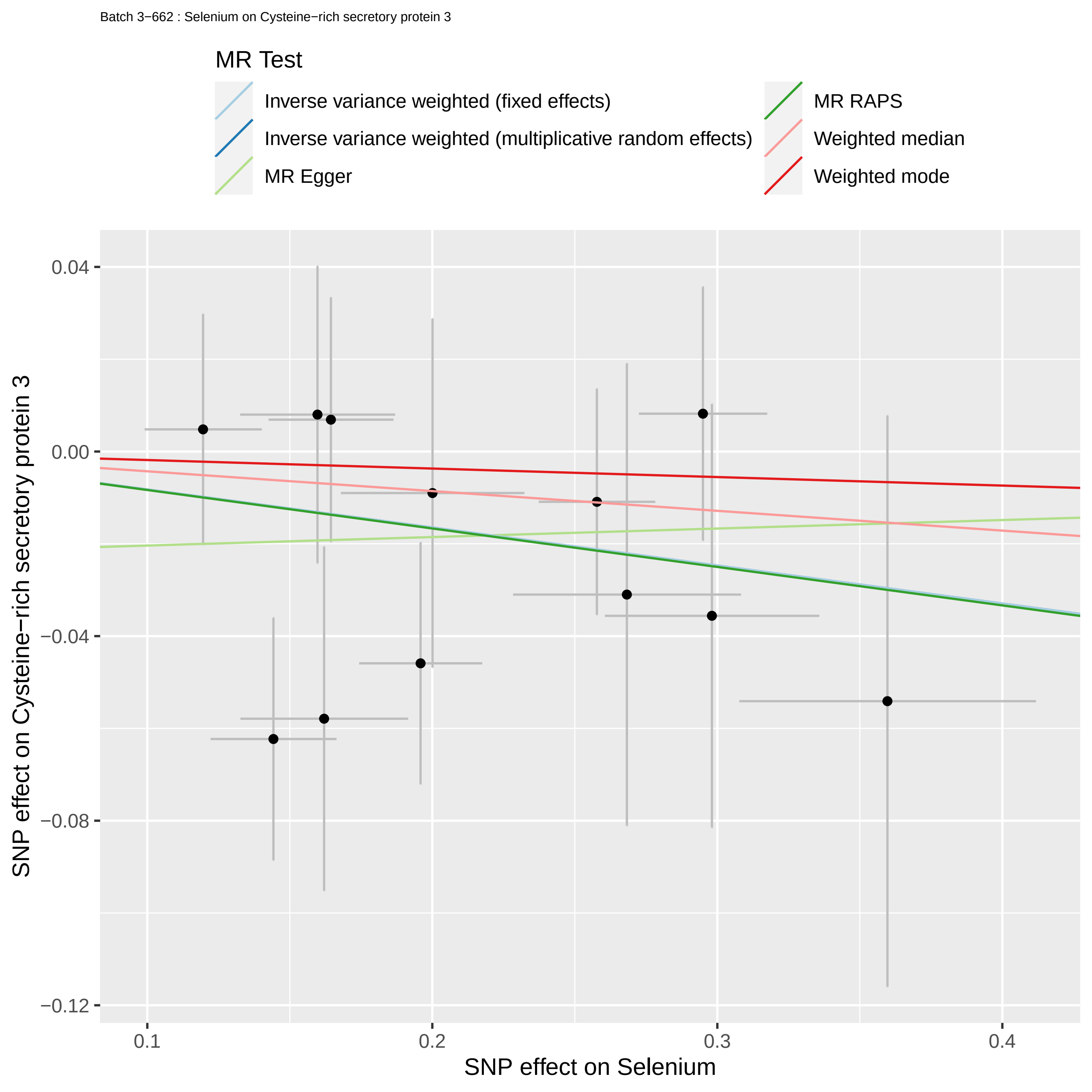
Batch 3−662 : Selenium on Cysteine−rich secretory protein 3
MR Test
Inverse variance weighted (fixed effects)
Inverse variance weighted (multiplicative random effects)
MR Egger
MR RAPS
Weighted median
Weighted mode
0.04
0.00
−0.04
−0.08
−0.12
0.1
0.2
0.3
0.4
SNP effect on Cysteine−rich secretory protein 3
SNP effect on Selenium

Batch 3−663 : Selenium on Cysteine−rich secretory protein LCCL domain−containing 2

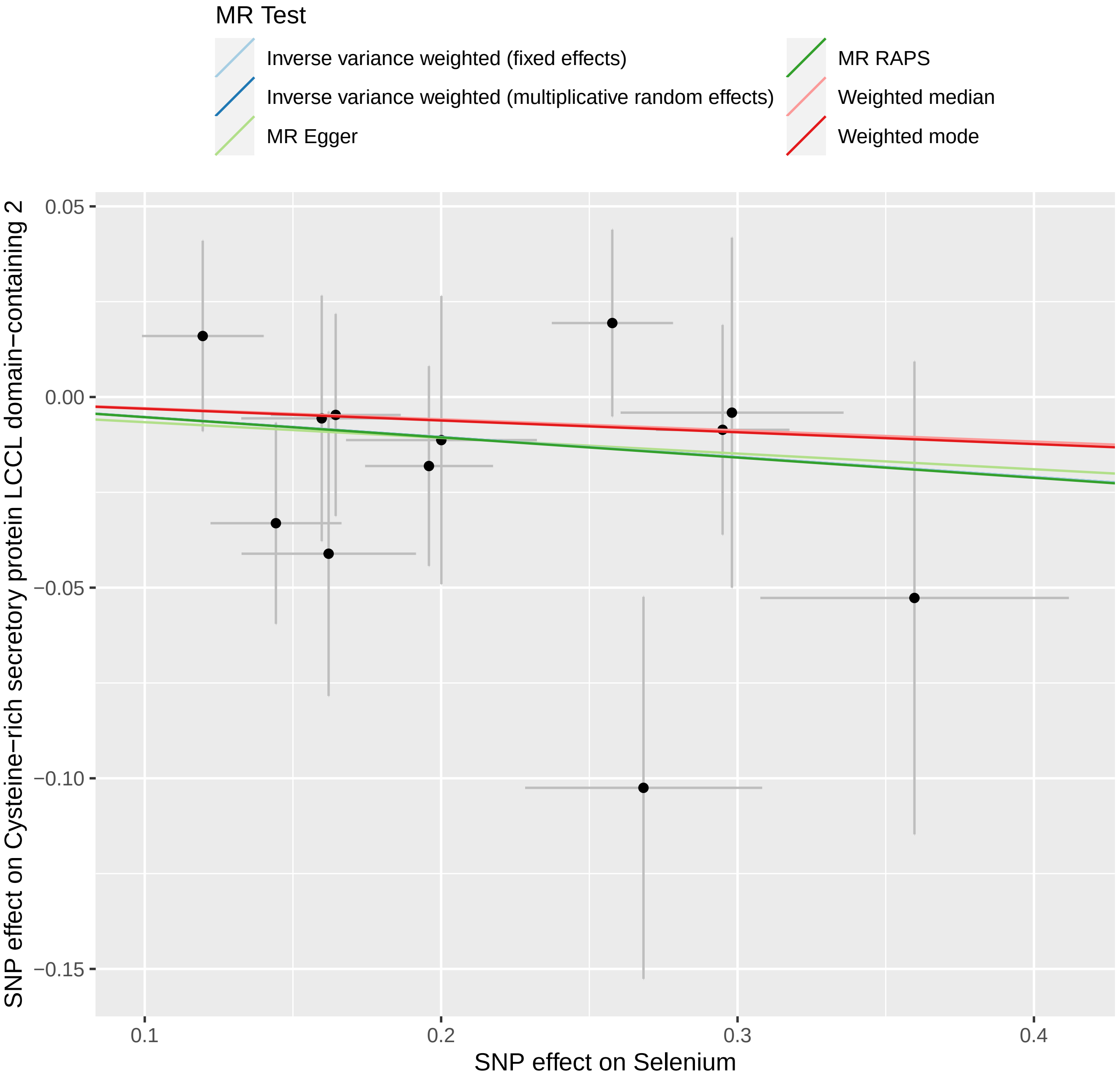

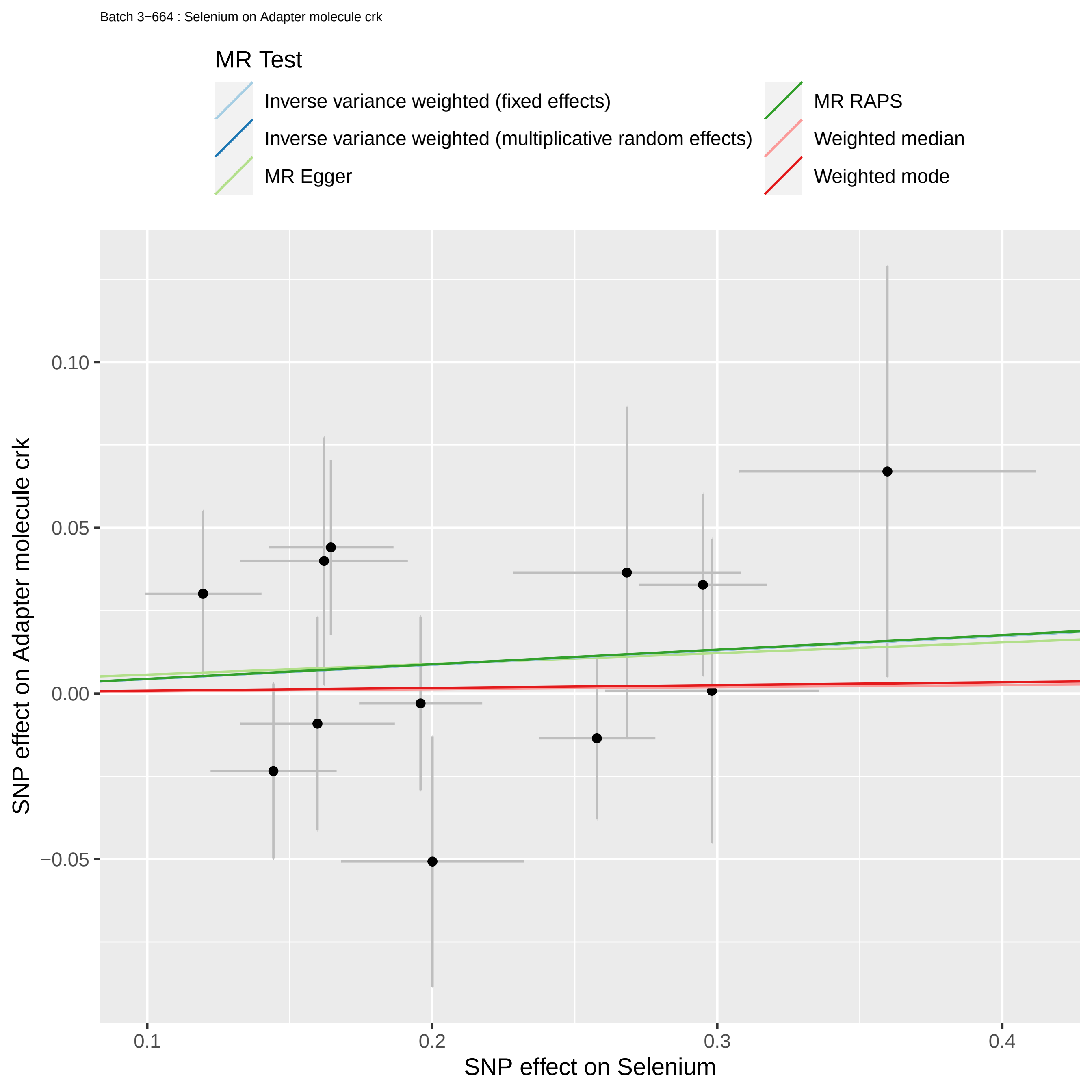
Batch 3−664 : Selenium on Adapter molecule crk
MR Test
Inverse variance weighted (fixed effects)
Inverse variance weighted (multiplicative random effects)
MR Egger
MR RAPS
Weighted median
Weighted mode
SNP effect on Adapter molecule crk
0.10
0.05
0.00
−0.05
0.1
0.2
0.3
0.4
SNP effect on Selenium

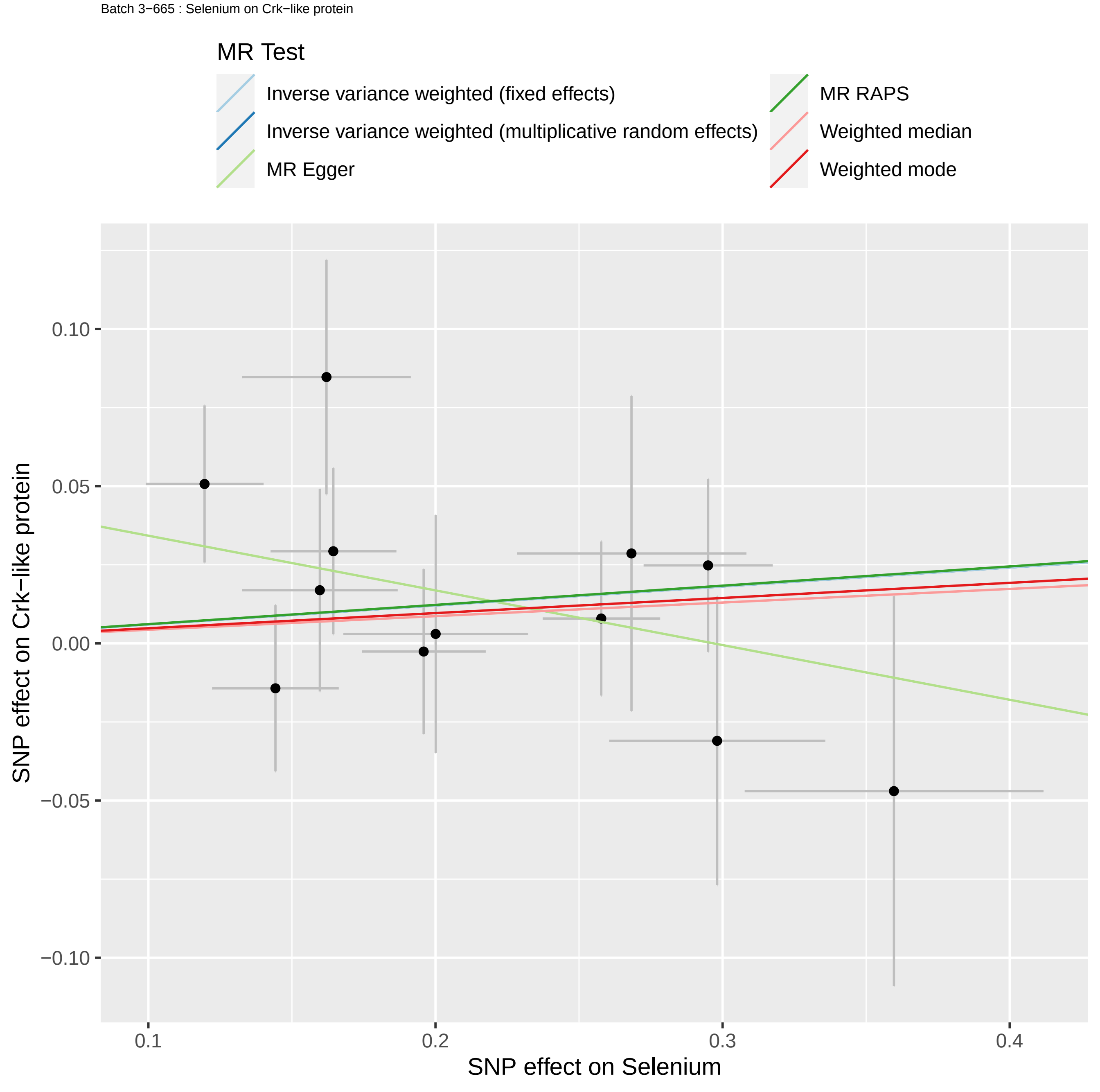

Batch 3−665 : Selenium on Crk−like protein
MR Test
Inverse variance weighted (fixed effects)
Inverse variance weighted (multiplicative random effects)
MR Egger
MR RAPS
Weighted median
Weighted mode
0.10
0.05
0.00
−0.05
−0.10
0.1
0.2
0.3
0.4
SNP effect on Crk−like protein
SNP effect on Selenium

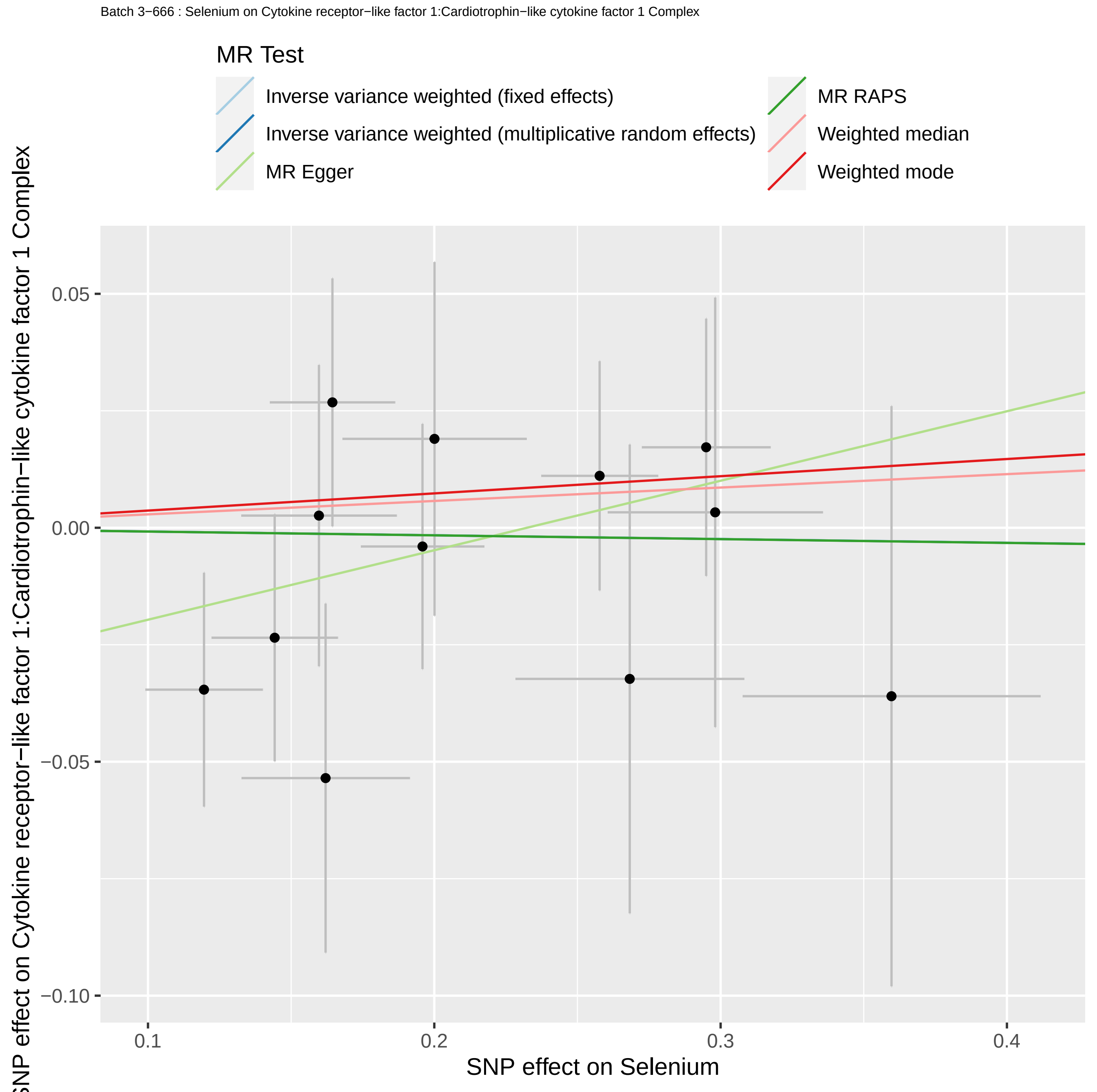

Batch 3−666 : Selenium on Cytokine receptor−like factor 1:Cardiotrophin−like cytokine factor 1 Complex
MR Test
Inverse variance weighted (fixed effects)
Inverse variance weighted (multiplicative random effects)
MR Egger
MR RAPS
Weighted median
Weighted mode
0.05
0.00
−0.05
−0.10
0.1
0.2
0.3
0.4
SNP effect on Selenium
SNP effect on Cytokine receptor−like factor 1:Cardiotrophin−like cytokine factor 1 Complex

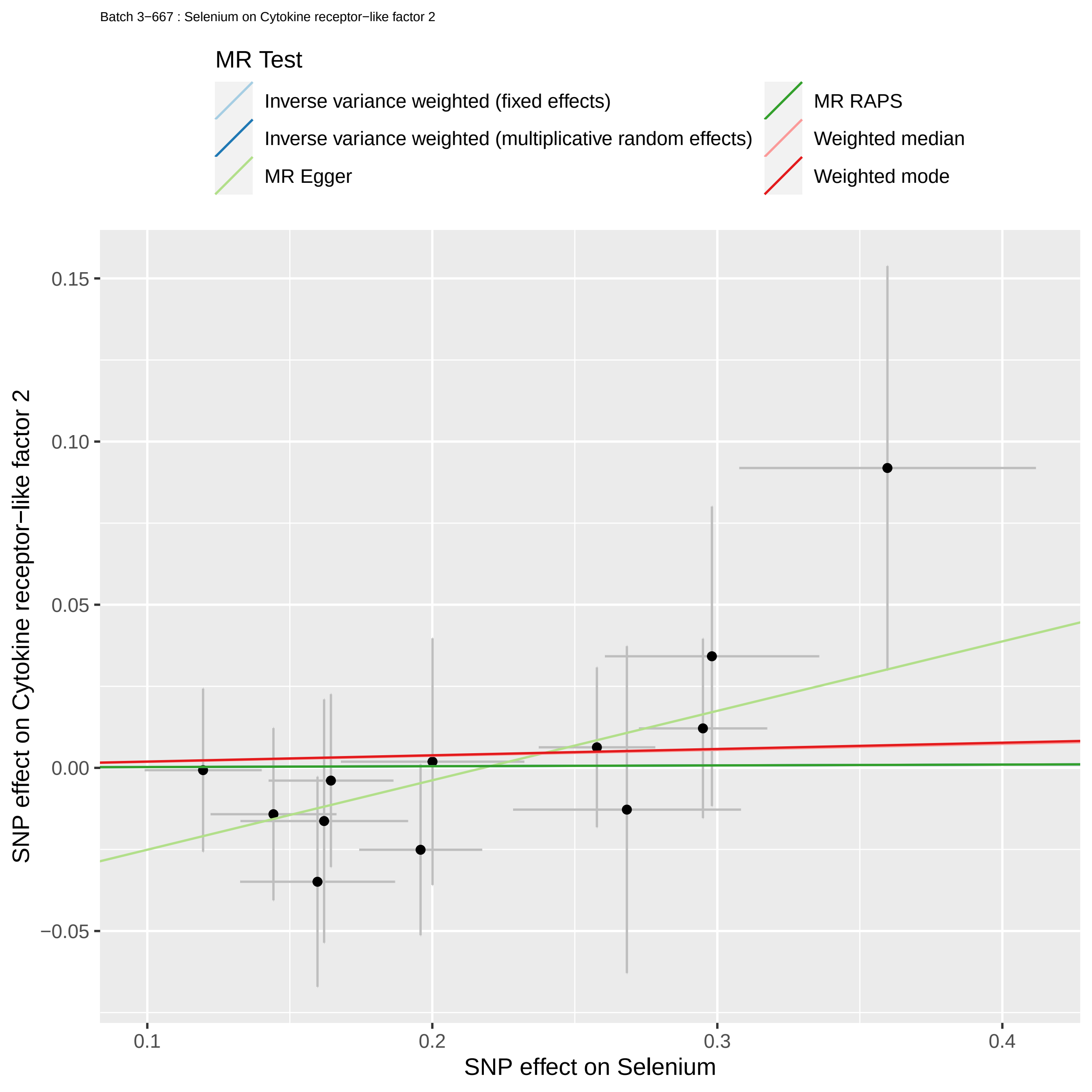

Batch 3−667 : Selenium on Cytokine receptor−like factor 2
MR Test
Inverse variance weighted (fixed effects)
Inverse variance weighted (multiplicative random effects)
MR Egger
MR RAPS
Weighted median
Weighted mode
SNP effect on Cytokine receptor−like factor 2
0.15
0.10
0.05
0.00
−0.05
0.1
0.2
0.3
0.4
SNP effect on Selenium

Batch 3−668 : Selenium on Cytokine receptor−like factor 2

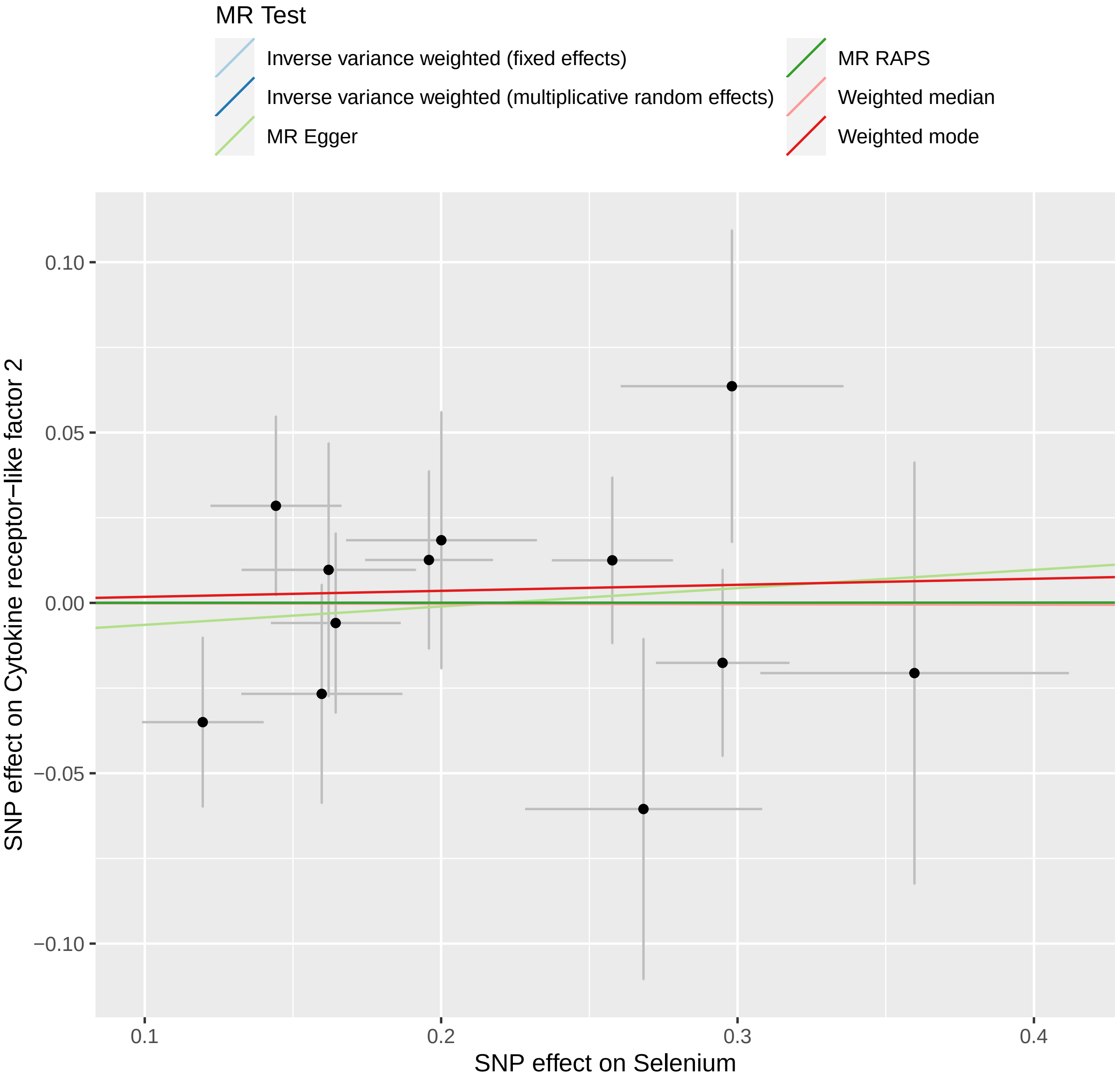

Batch 3−669 : Selenium on Peroxisomal carnitine O−octanoyltransferase

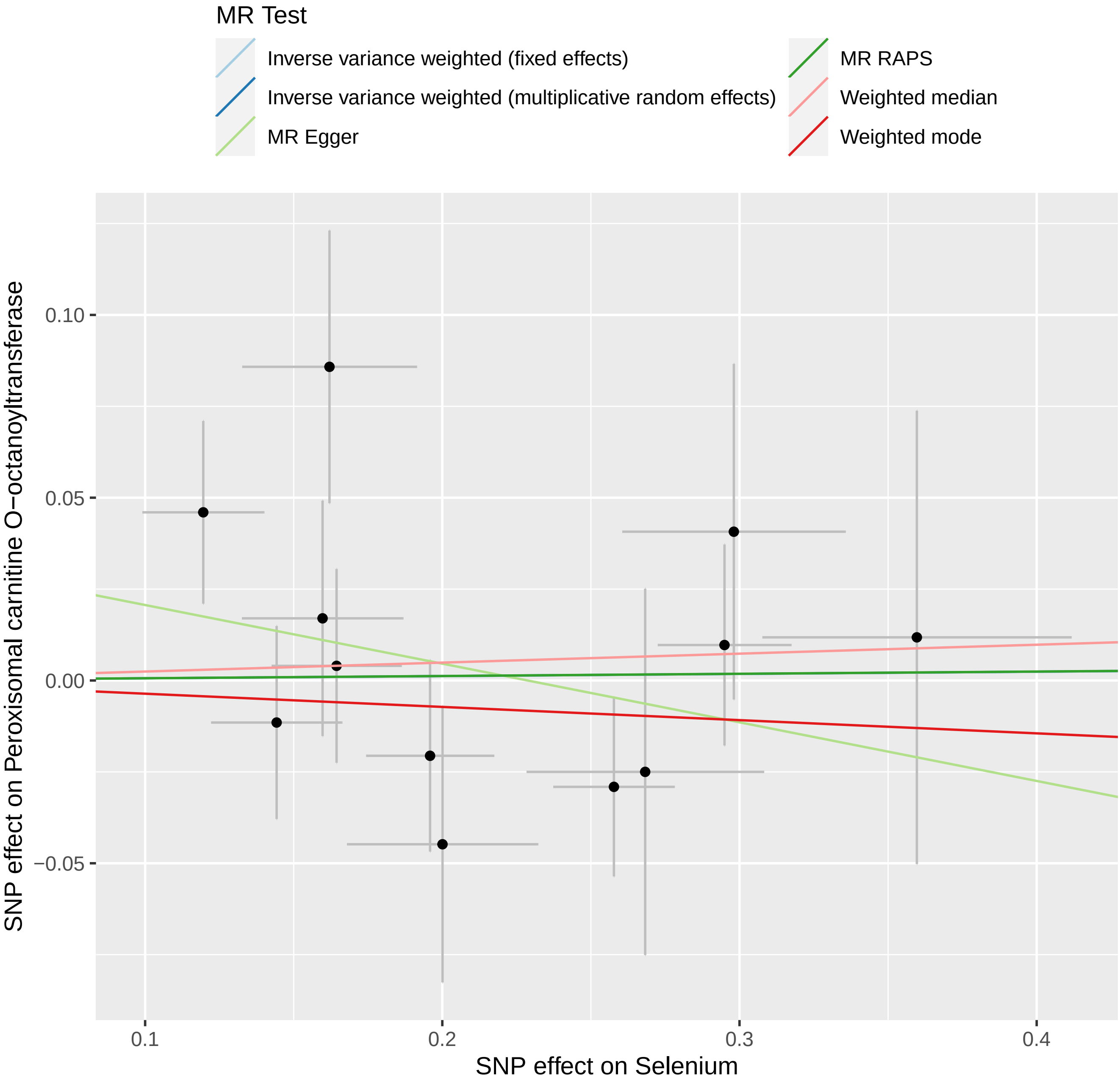

Batch 3−670 : Selenium on C−reactive protein

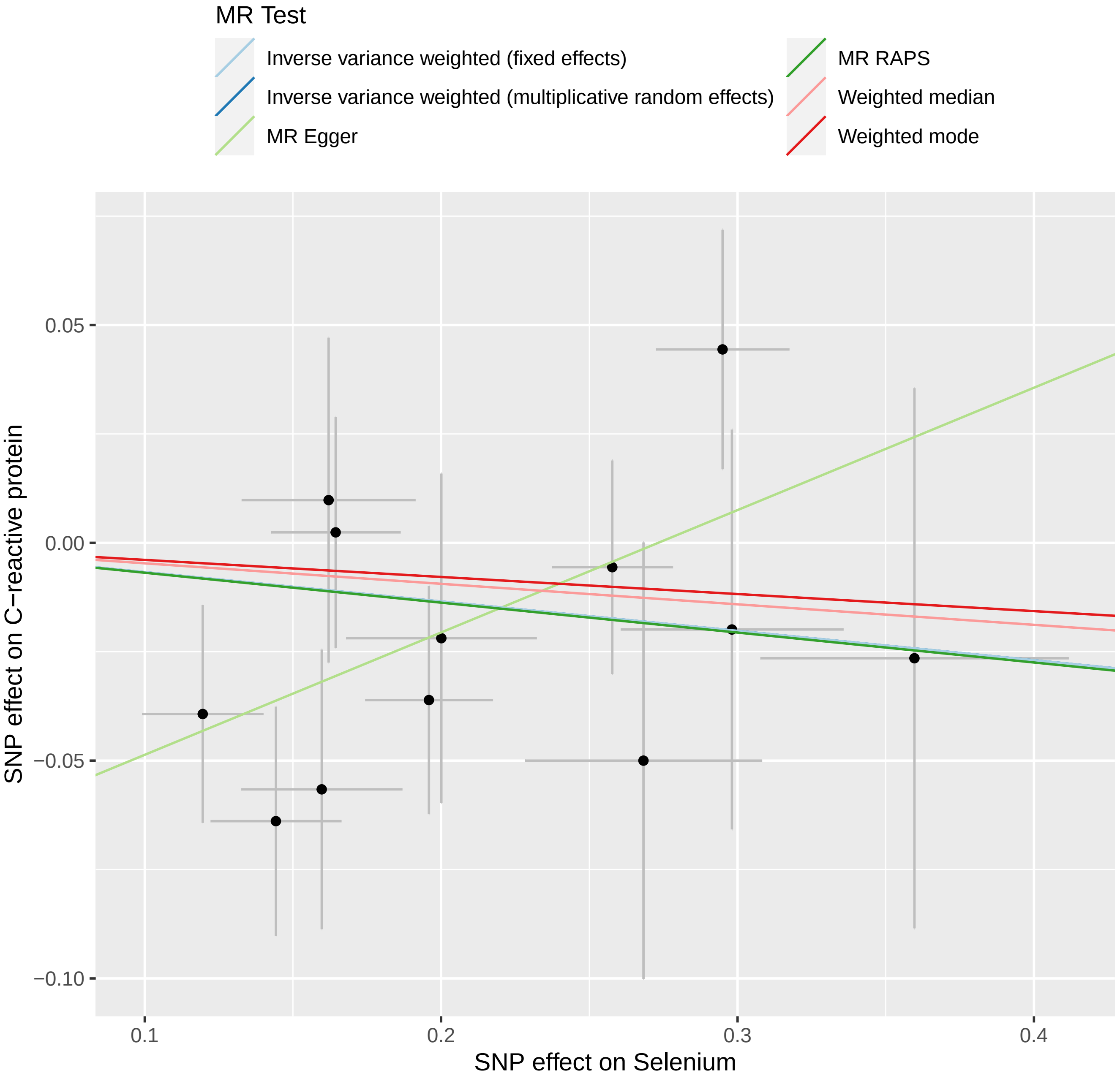

Batch 3−671 : Selenium on Cytotoxic and regulatory T−cell molecule

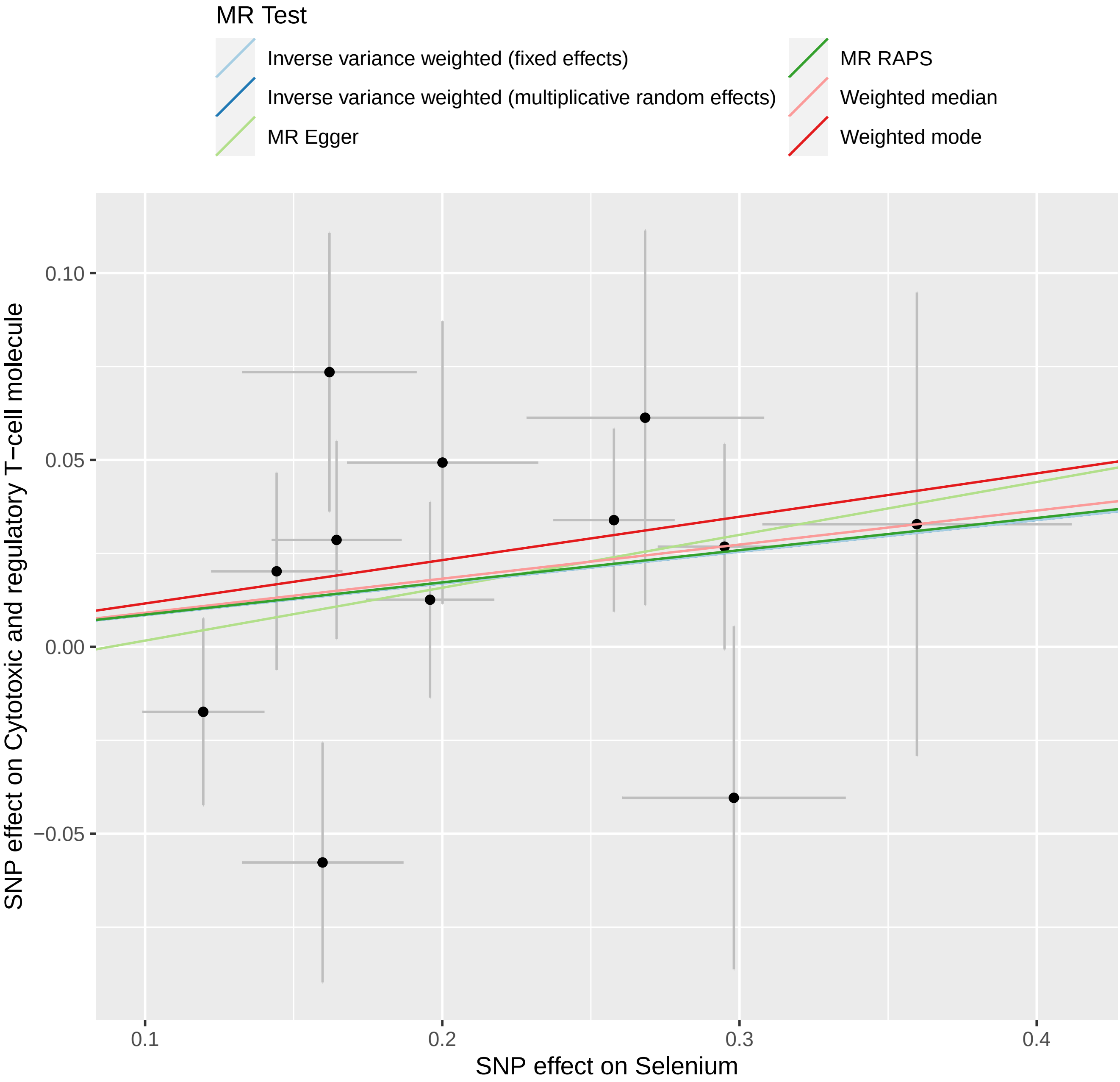

Batch 3−672 : Selenium on Cytotoxic and regulatory T−cell molecule

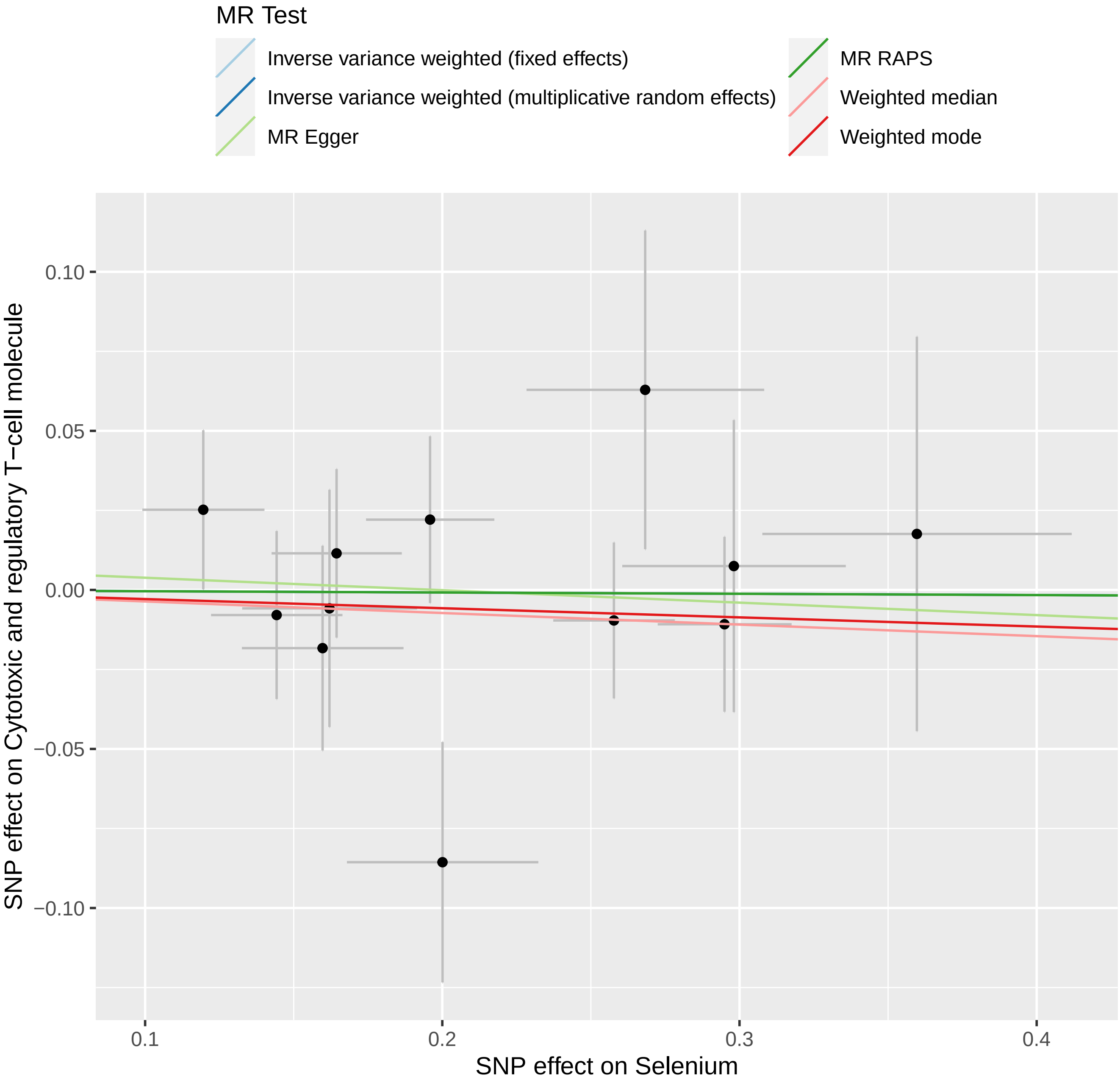

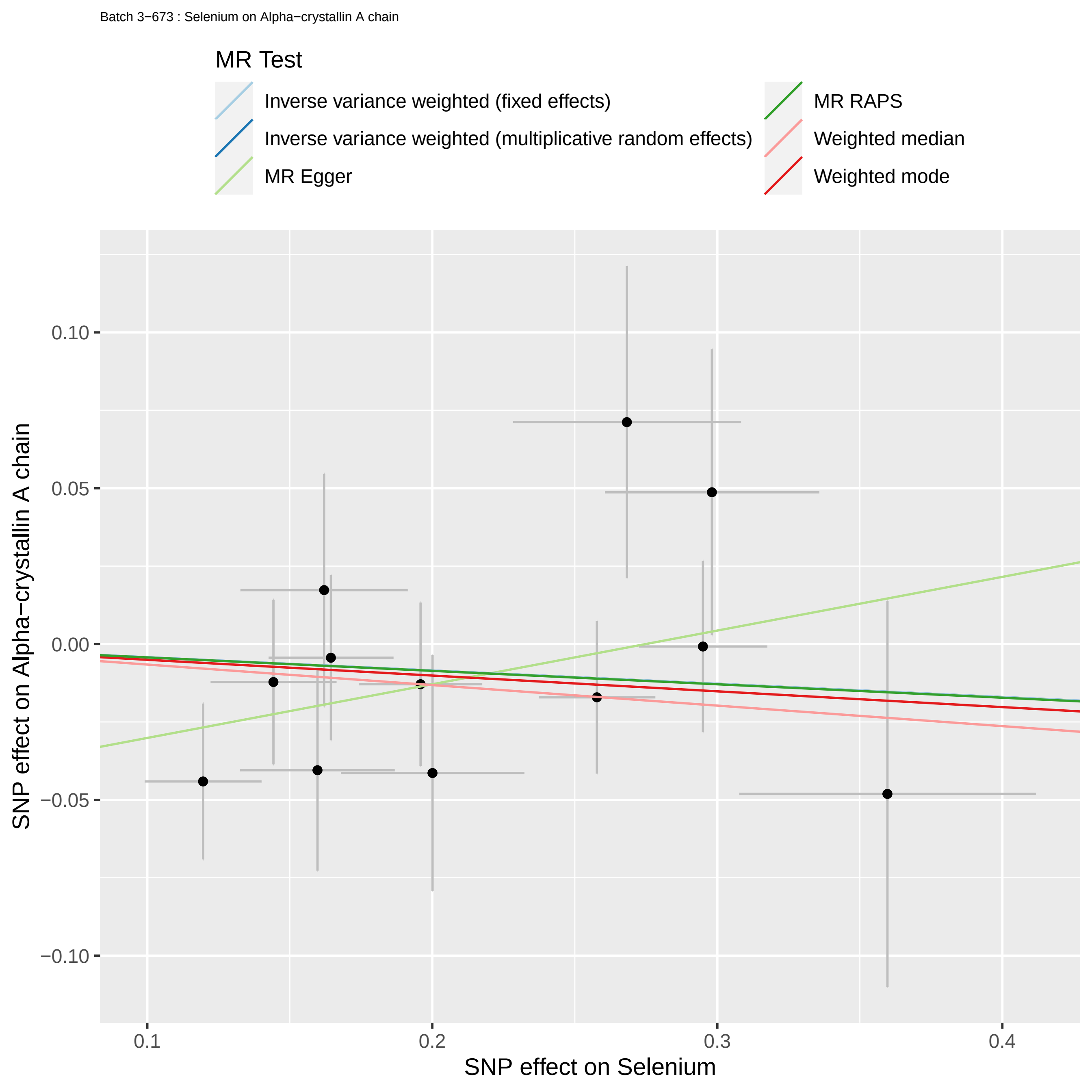

Batch 3−673 : Selenium on Alpha−crystallin A chain
MR Test
Inverse variance weighted (fixed effects)
Inverse variance weighted (multiplicative random effects)
MR Egger
MR RAPS
Weighted median
Weighted mode
SNP effect on Alpha−crystallin A chain
0.10
0.05
0.00
−0.05
−0.10
0.1
0.2
0.3
0.4
SNP effect on Selenium

Batch 3−674 : Selenium on Beta−crystallin B2

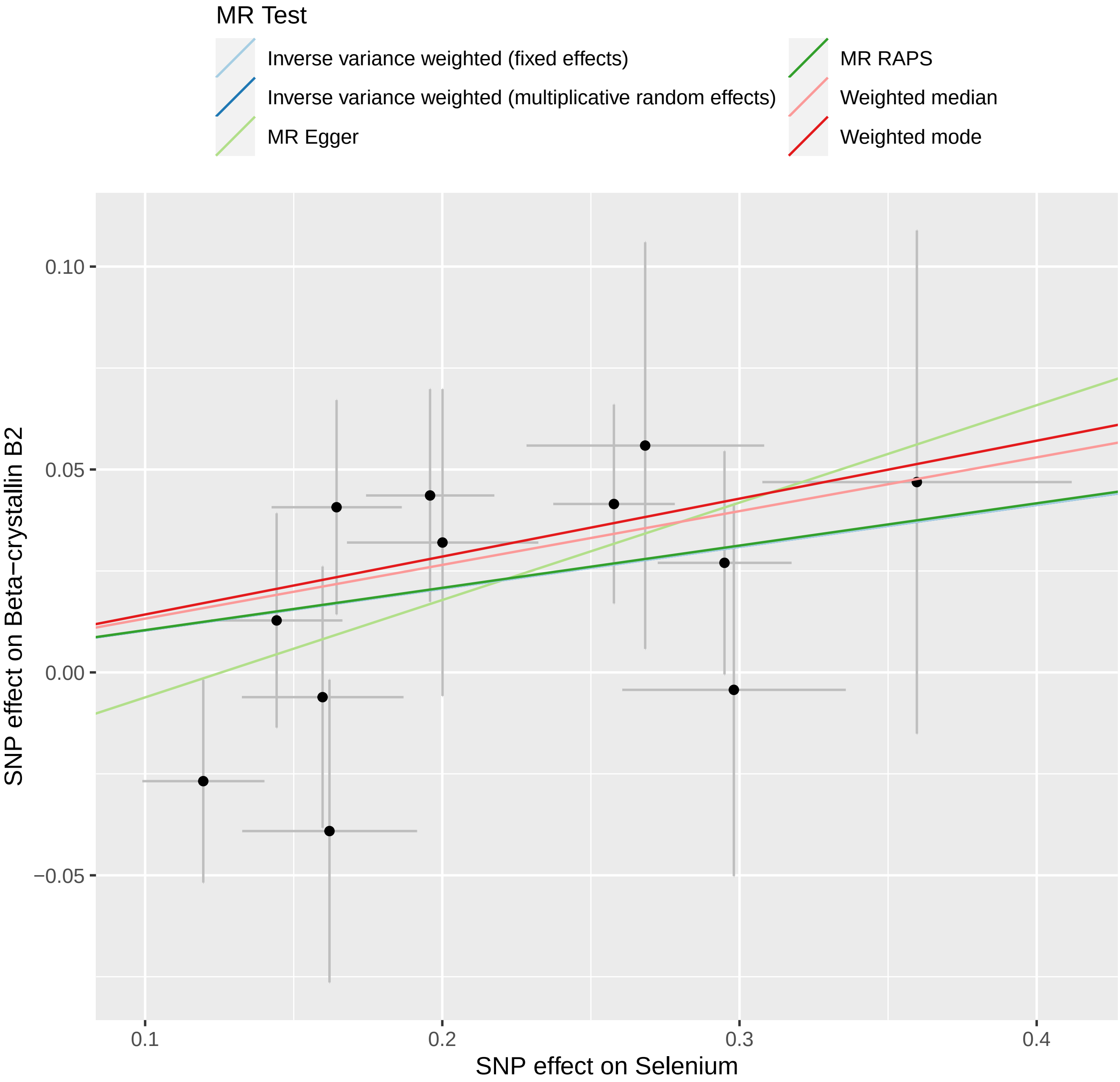

Batch 3−675 : Selenium on Gamma−crystallin D

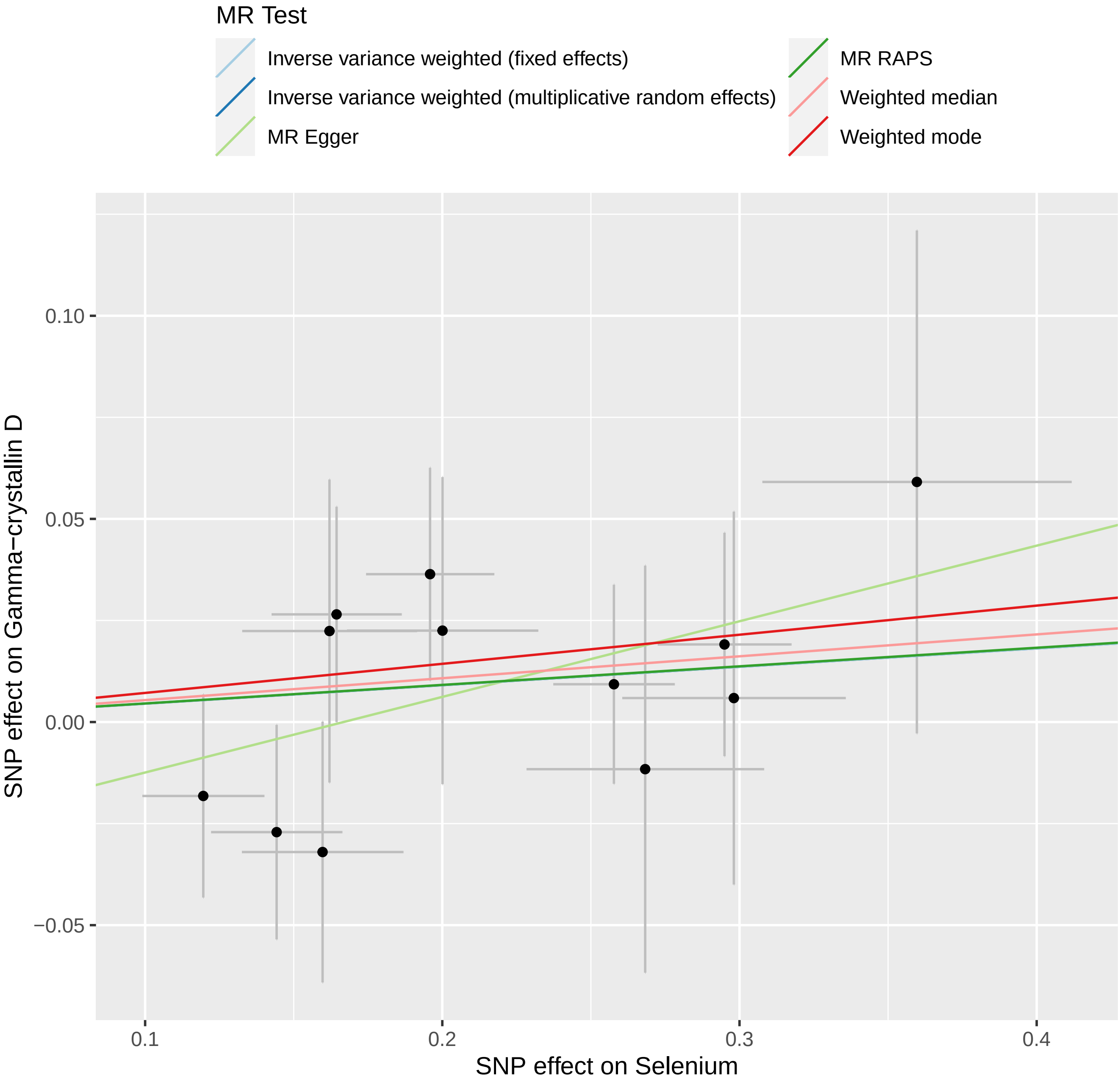

Batch 3−676 : Selenium on Quinone oxidoreductase−like protein 1

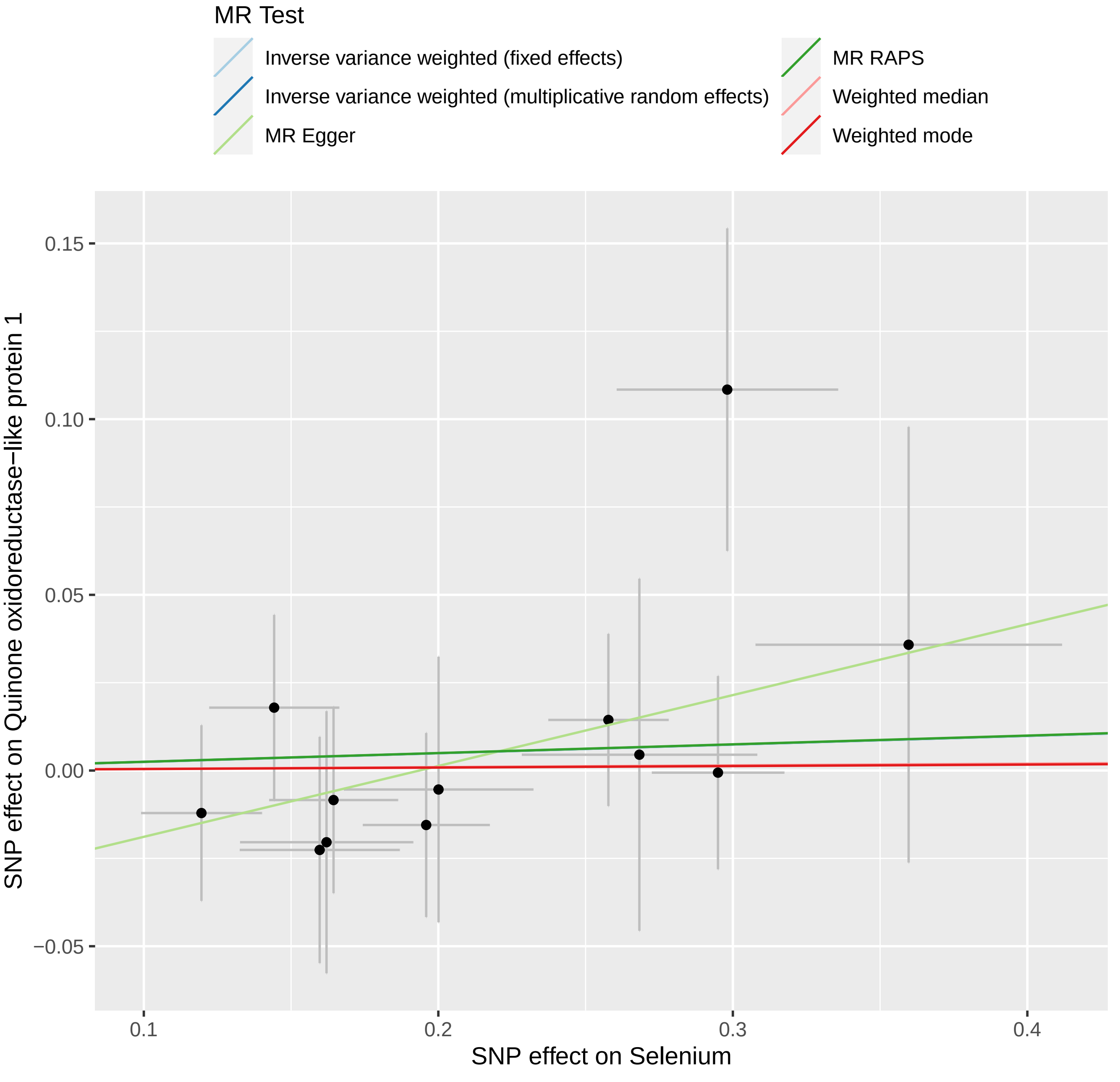

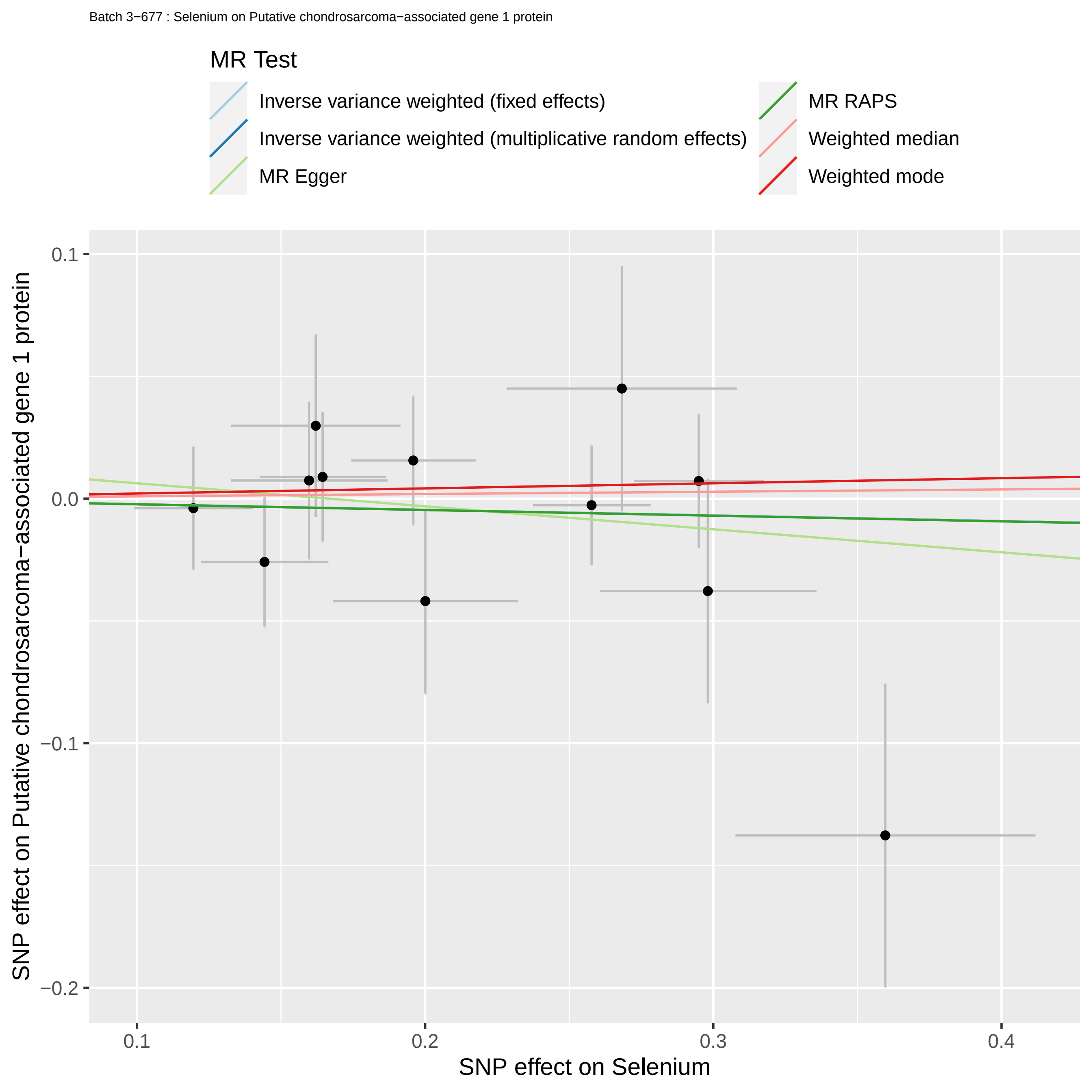

Batch 3−677 : Selenium on Putative chondrosarcoma−associated gene 1 protein
MR Test
Inverse variance weighted (fixed effects)
Inverse variance weighted (multiplicative random effects)
MR Egger
MR RAPS
Weighted median
Weighted mode
0.1
0.0
−0.1
−0.2
0.1
0.2
0.3
0.4
SNP effect on Putative chondrosarcoma−associated gene 1 protein
SNP effect on Selenium

Batch 3−678 : Selenium on Cold shock domain−containing protein C2

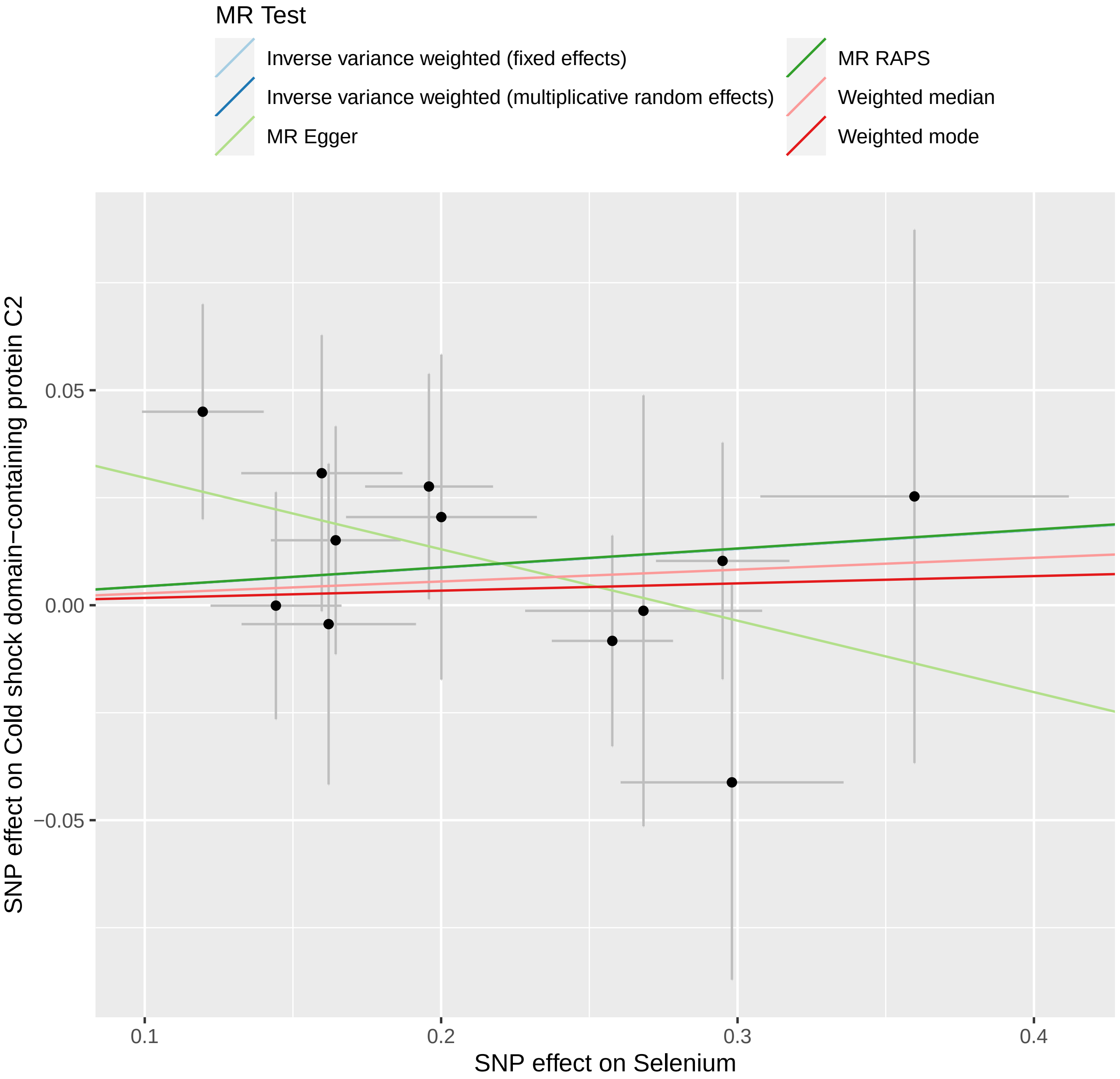

Batch 3−679 : Selenium on Cold shock domain−containing protein E1

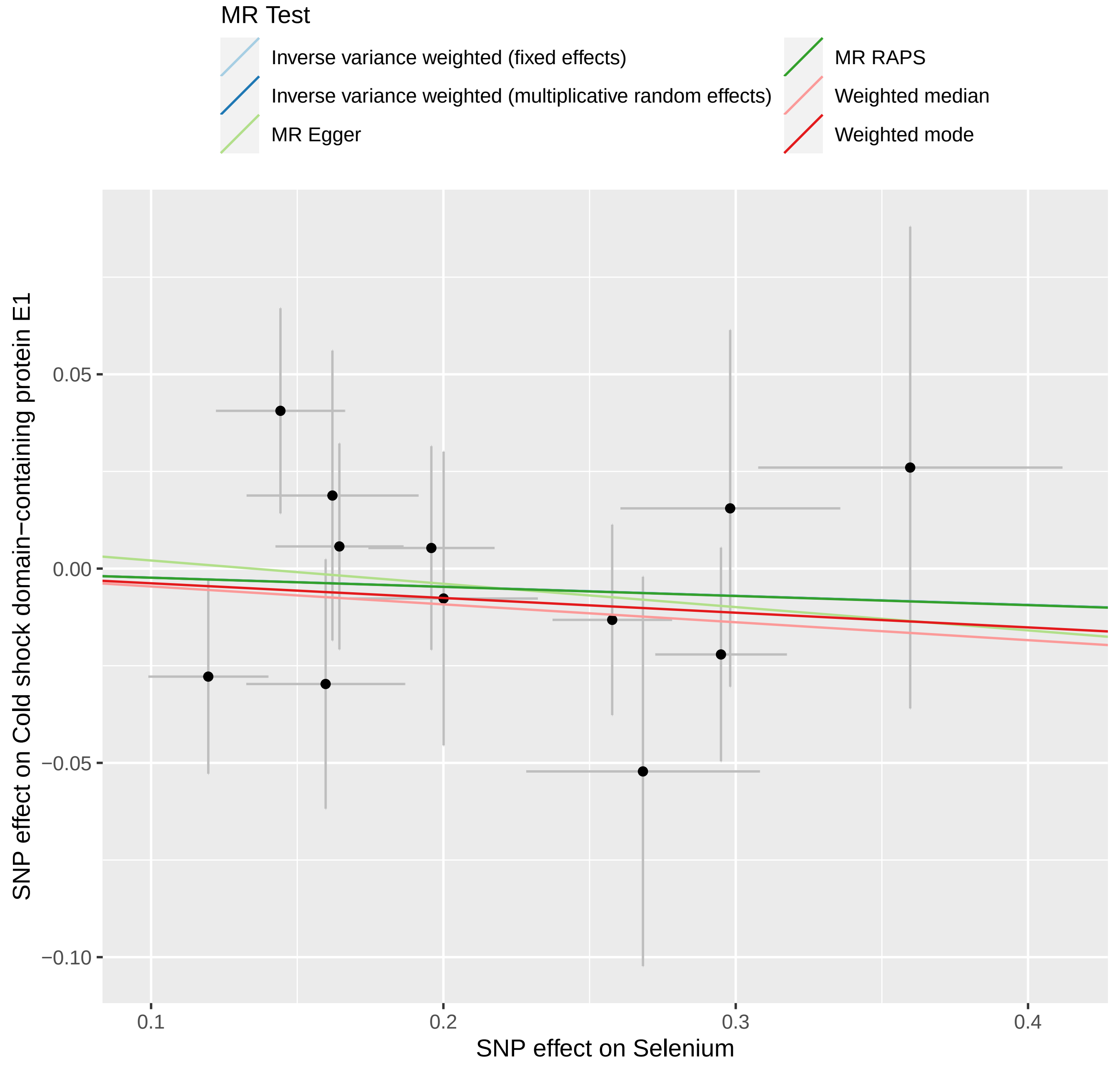

Batch 3−680 : Selenium on Macrophage colony−stimulating factor 1

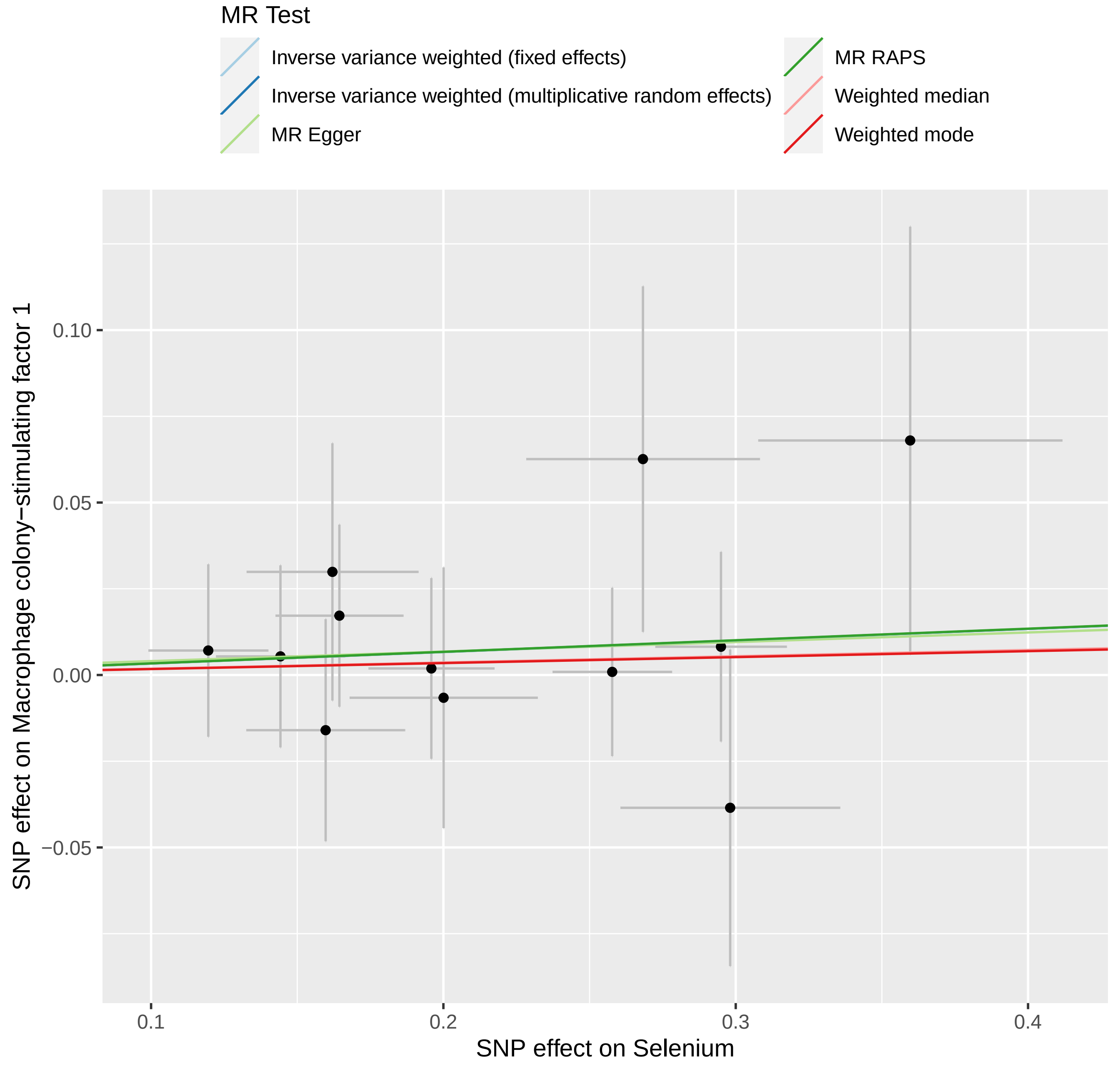

Batch 3−681 : Selenium on Granulocyte−macrophage colony−stimulating factor

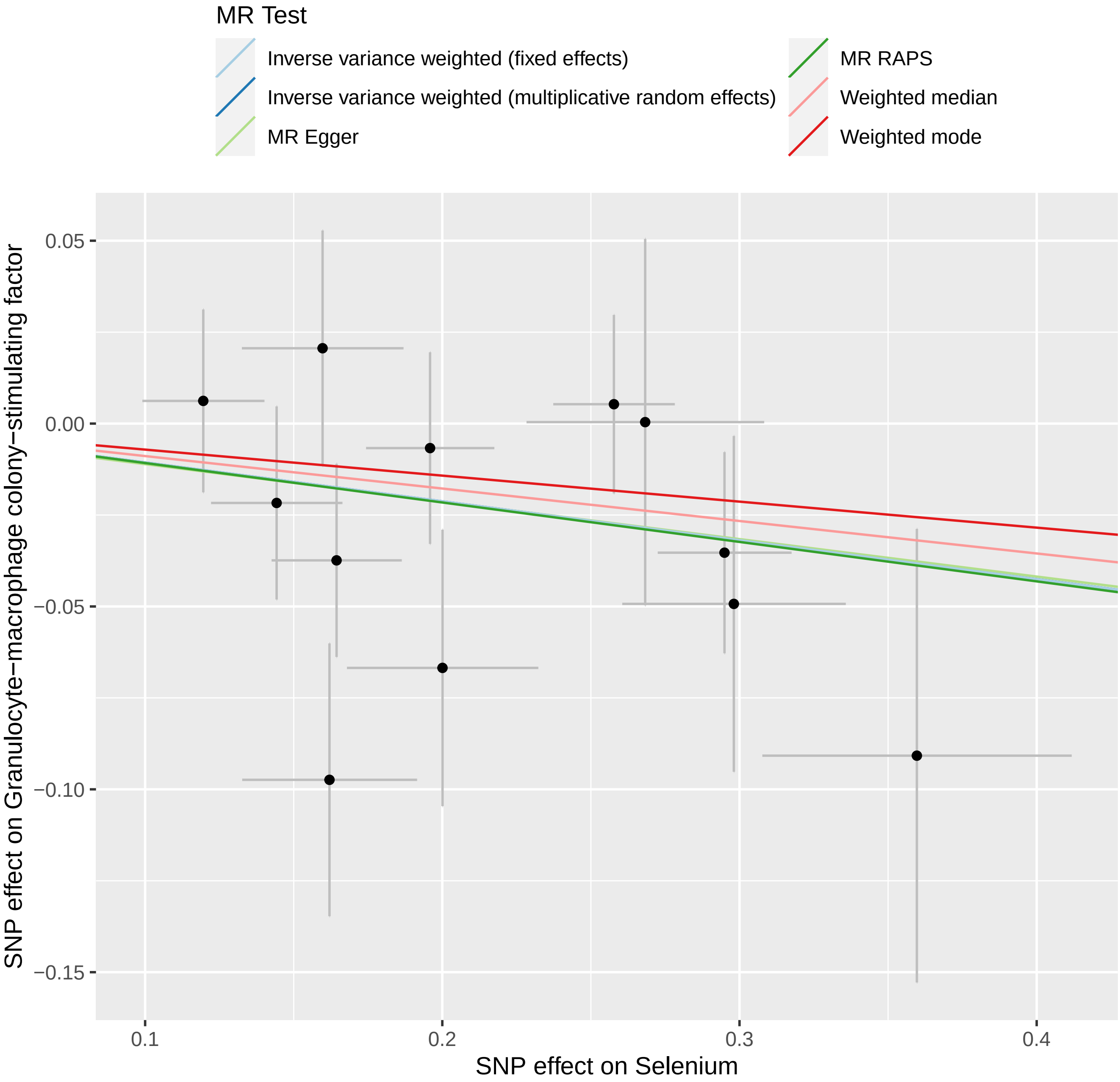

Batch 3−682 : Selenium on Granulocyte−macrophage colony−stimulating factor receptor subunit alpha

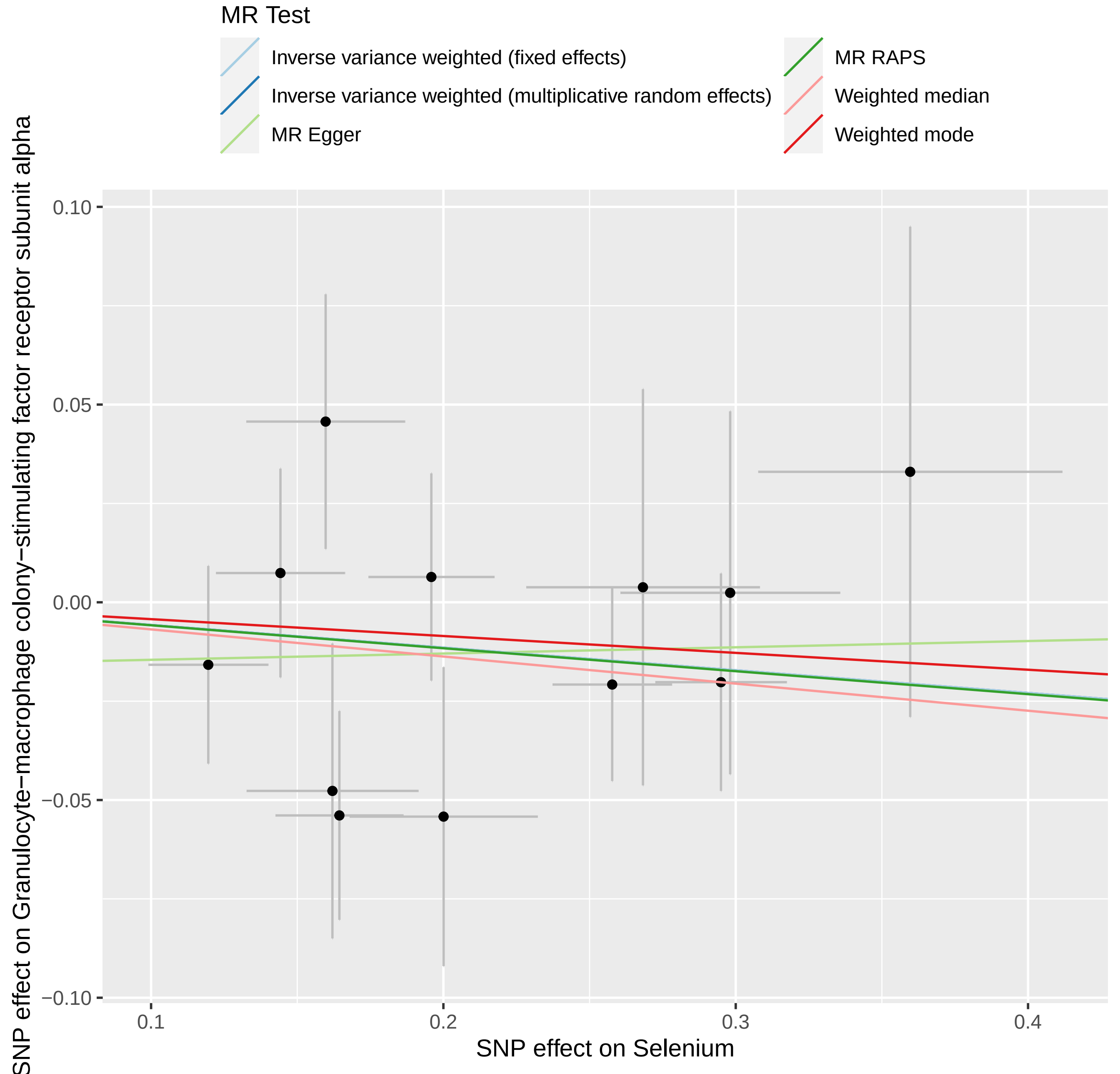

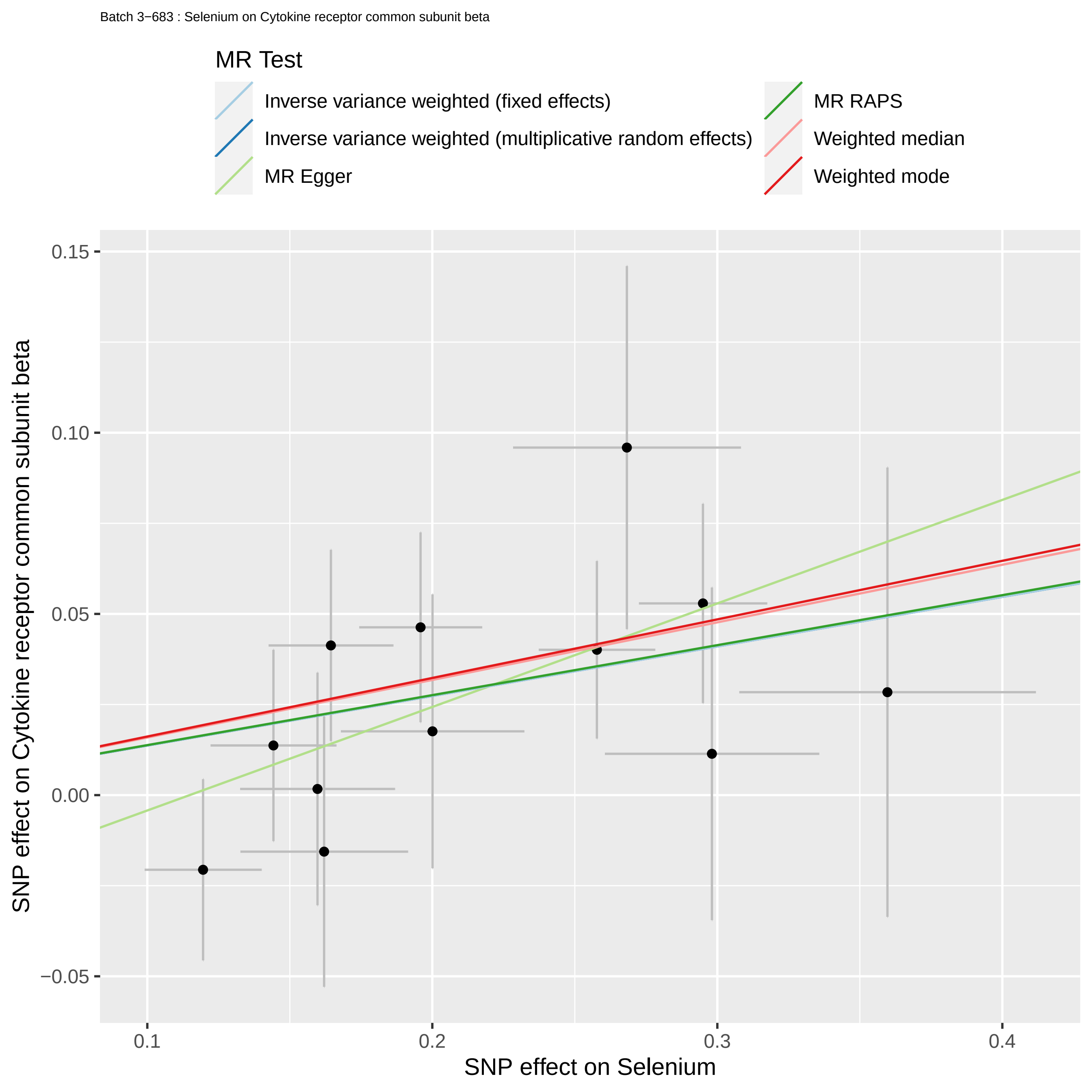
Batch 3−683 : Selenium on Cytokine receptor common subunit beta
MR Test
Inverse variance weighted (fixed effects)
Inverse variance weighted (multiplicative random effects)
MR Egger
MR RAPS
Weighted median
Weighted mode
SNP effect on Cytokine receptor common subunit beta
0.15
0.10
0.05
0.00
−0.05
0.1
0.2
0.3
0.4
SNP effect on Selenium

Batch 3−684 : Selenium on Cytokine receptor common subunit beta

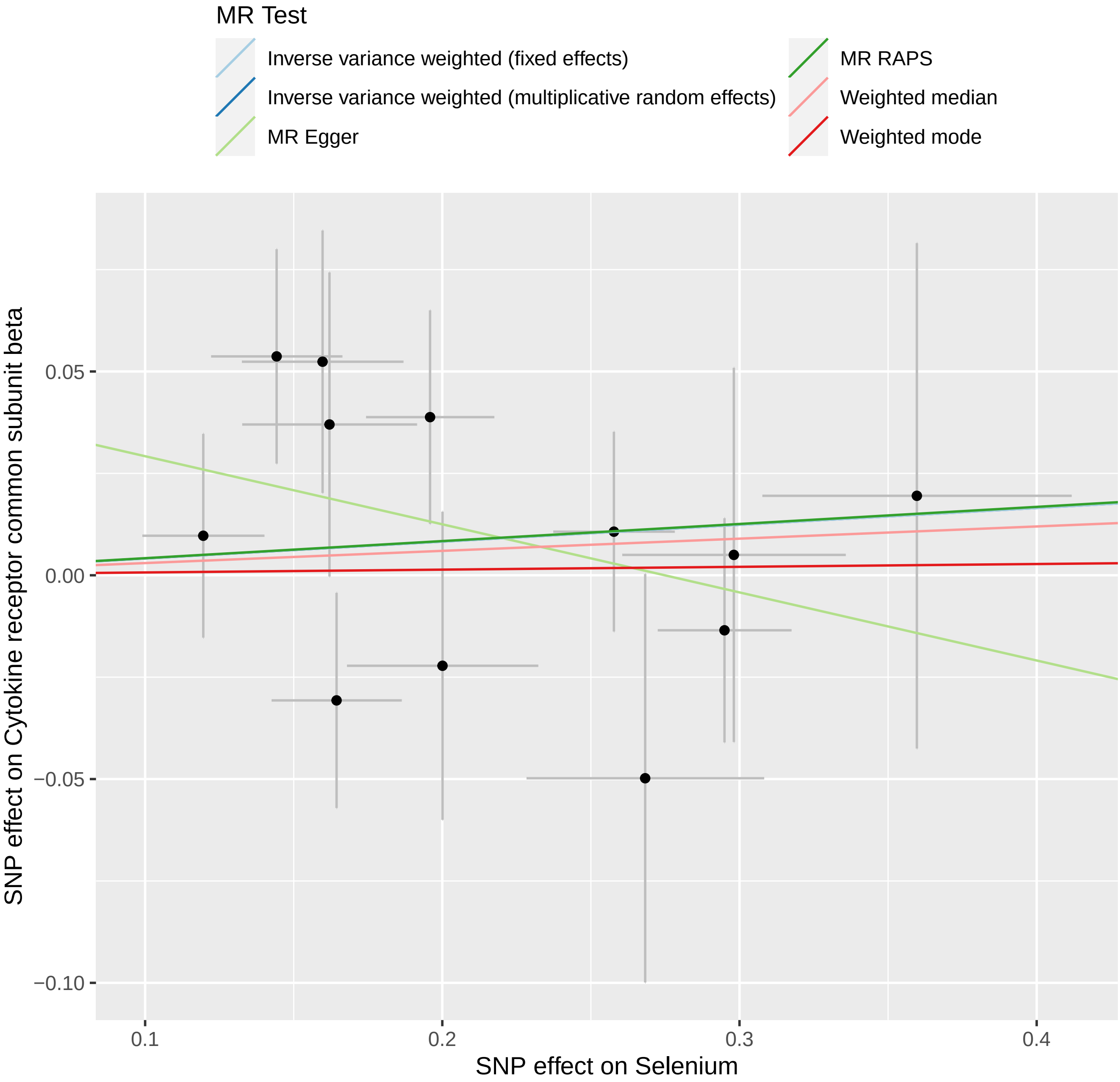

Batch 3−685 : Selenium on Granulocyte colony−stimulating factor

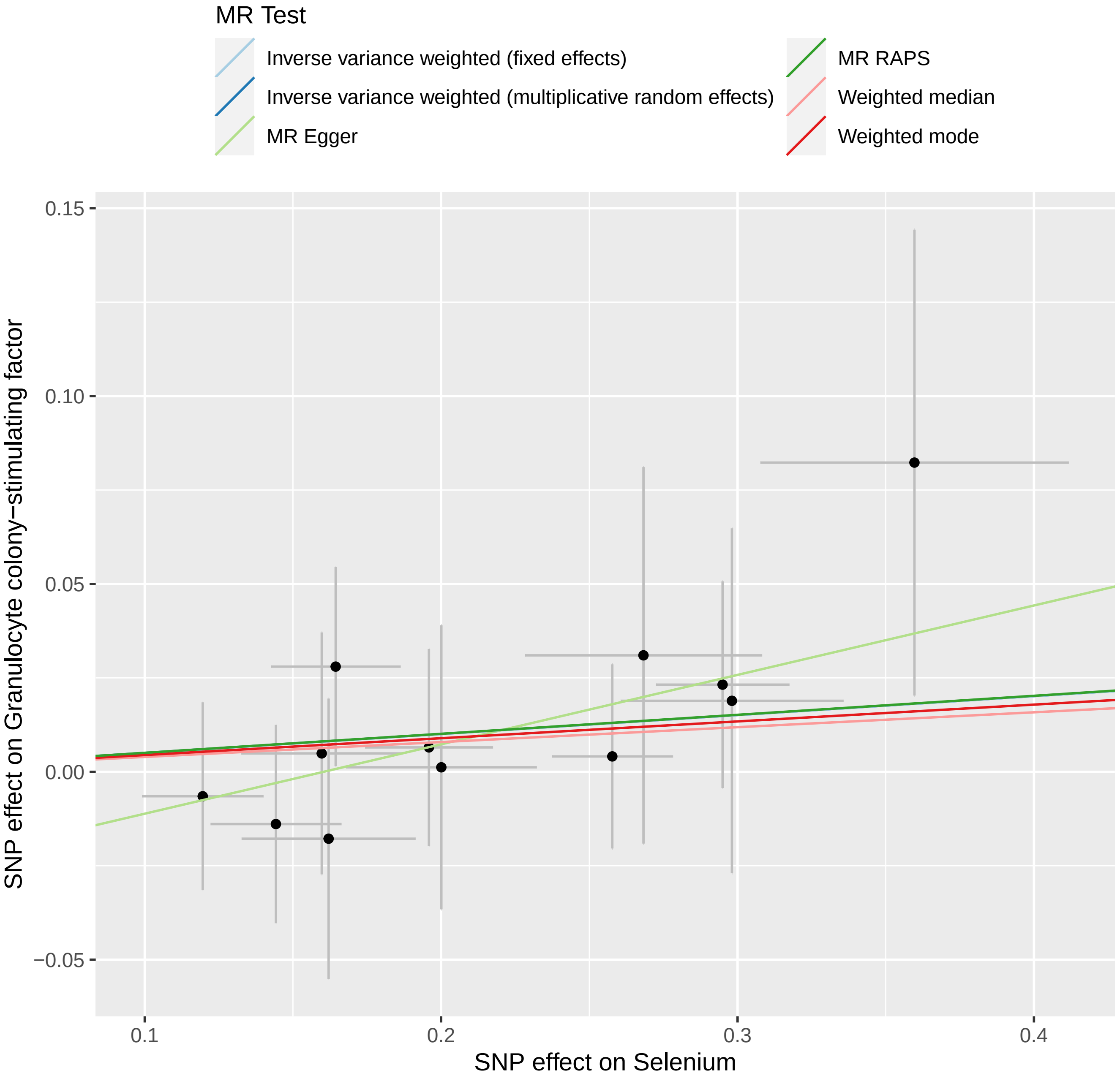

Batch 3−686 : Selenium on Granulocyte colony−stimulating factor receptor

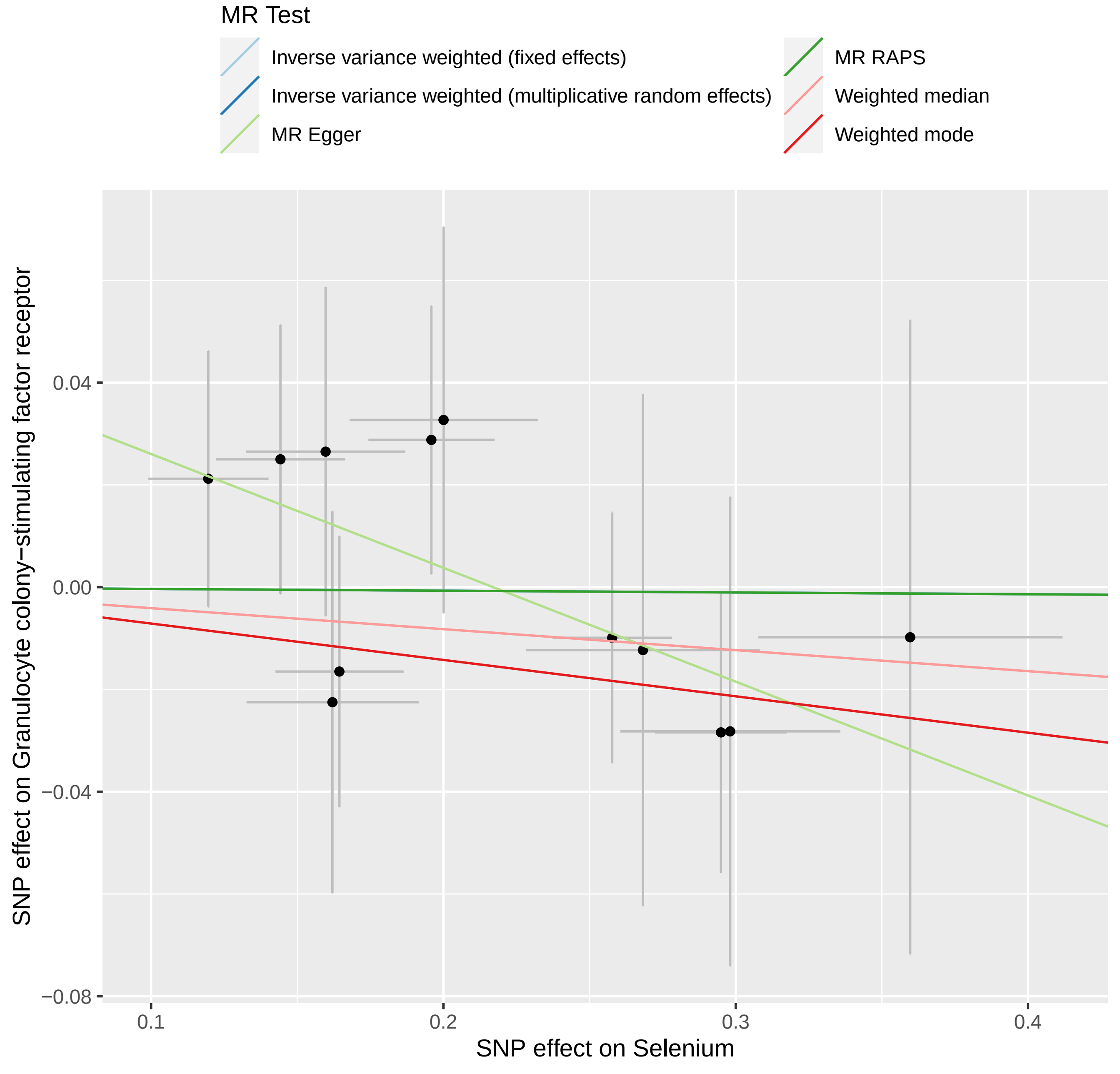

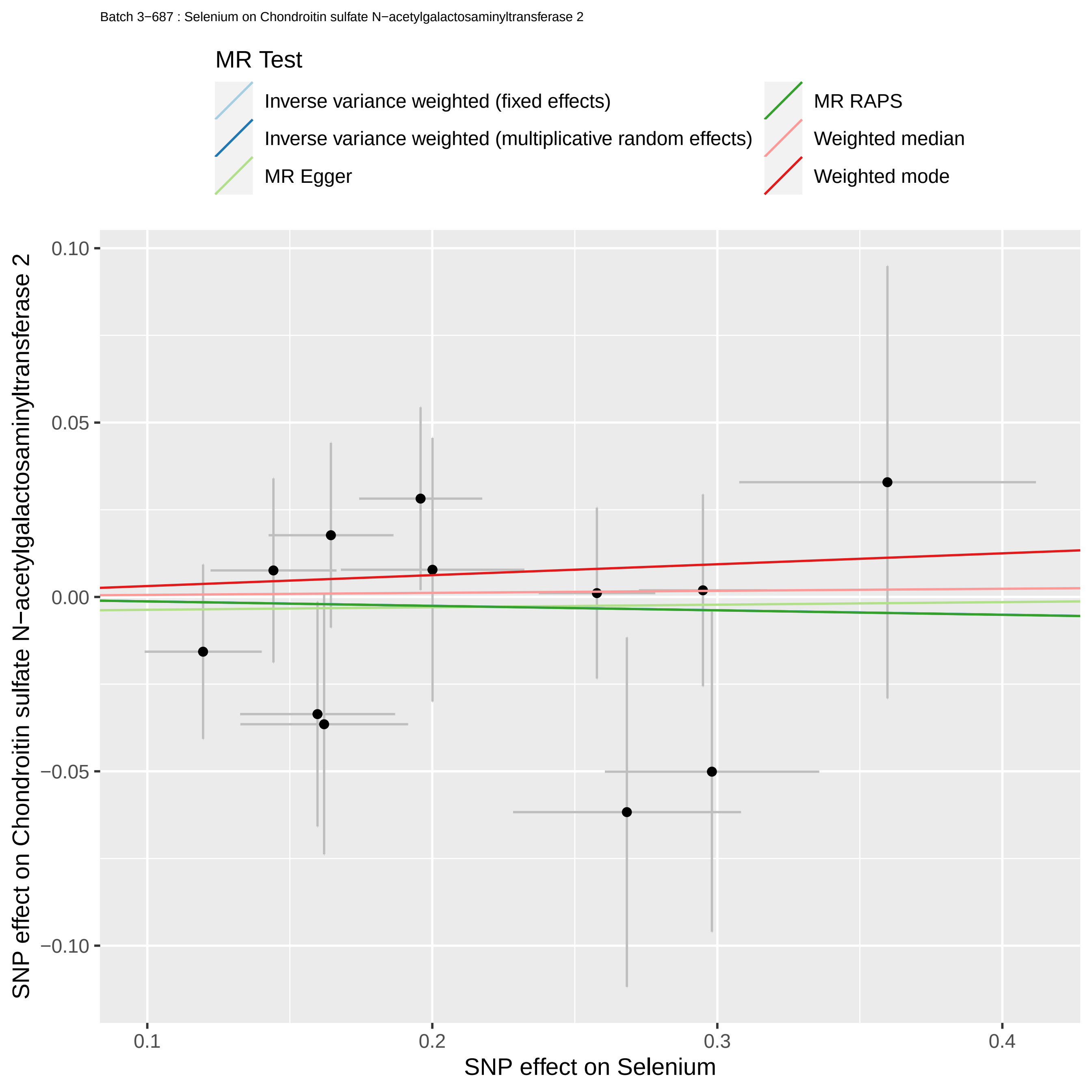

Batch 3−687 : Selenium on Chondroitin sulfate N−acetylgalactosaminyltransferase 2
MR Test
Inverse variance weighted (fixed effects)
Inverse variance weighted (multiplicative random effects)
MR Egger
MR RAPS
Weighted median
Weighted mode
0.10
0.05
0.00
−0.05
−0.10
0.1
0.2
0.3
0.4
SNP effect on Chondroitin sulfate N−acetylgalactosaminyltransferase 2
SNP effect on Selenium

Batch 3−688 : Selenium on Chorionic somatomammotropin hormone

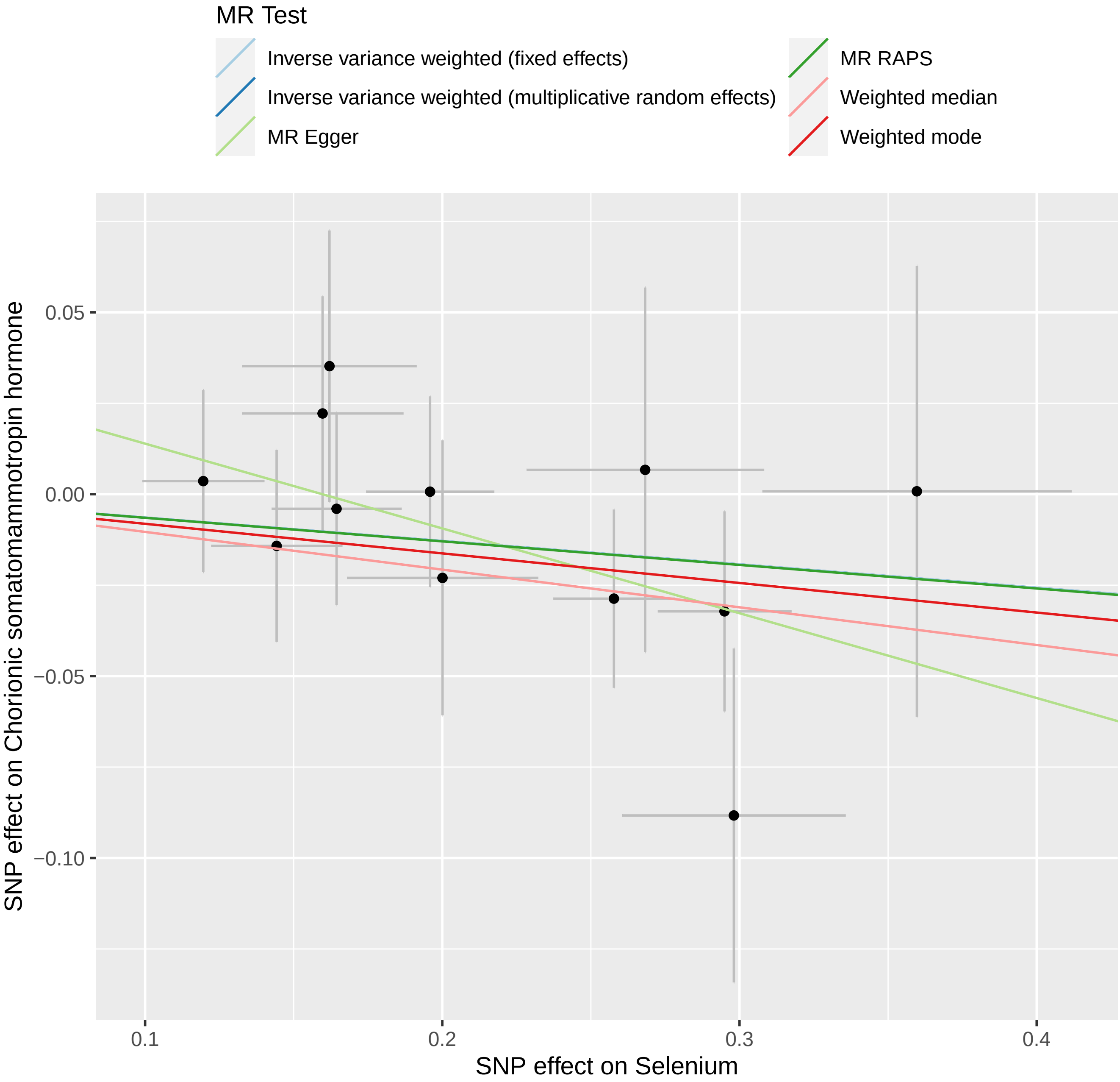

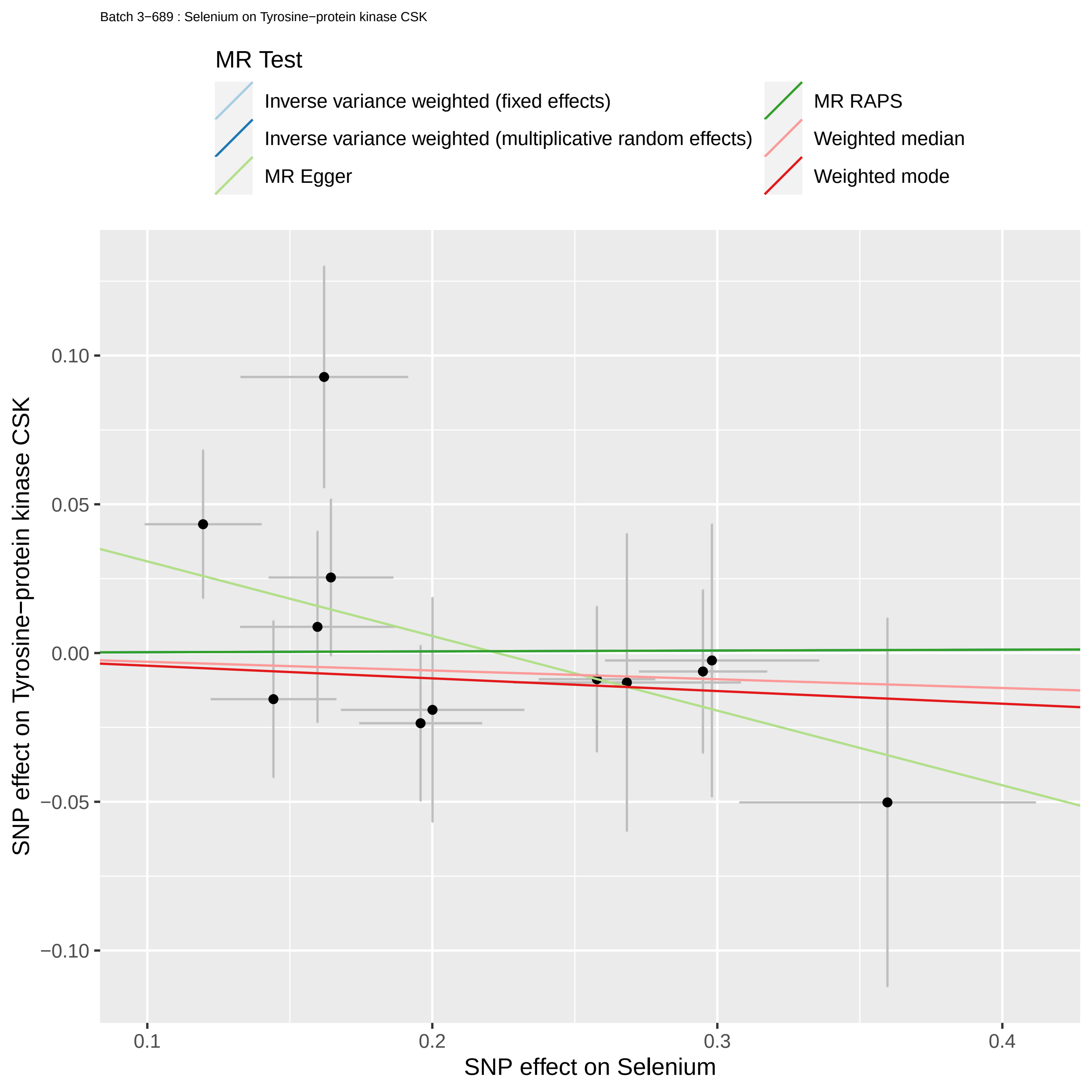

Batch 3−689 : Selenium on Tyrosine−protein kinase CSK
MR Test
Inverse variance weighted (fixed effects)
Inverse variance weighted (multiplicative random effects)
MR Egger
MR RAPS
Weighted median
Weighted mode
0.10
0.05
0.00
−0.05
−0.10
0.1
0.2
0.3
0.4
SNP effect on Tyrosine−protein kinase CSK
SNP effect on Selenium

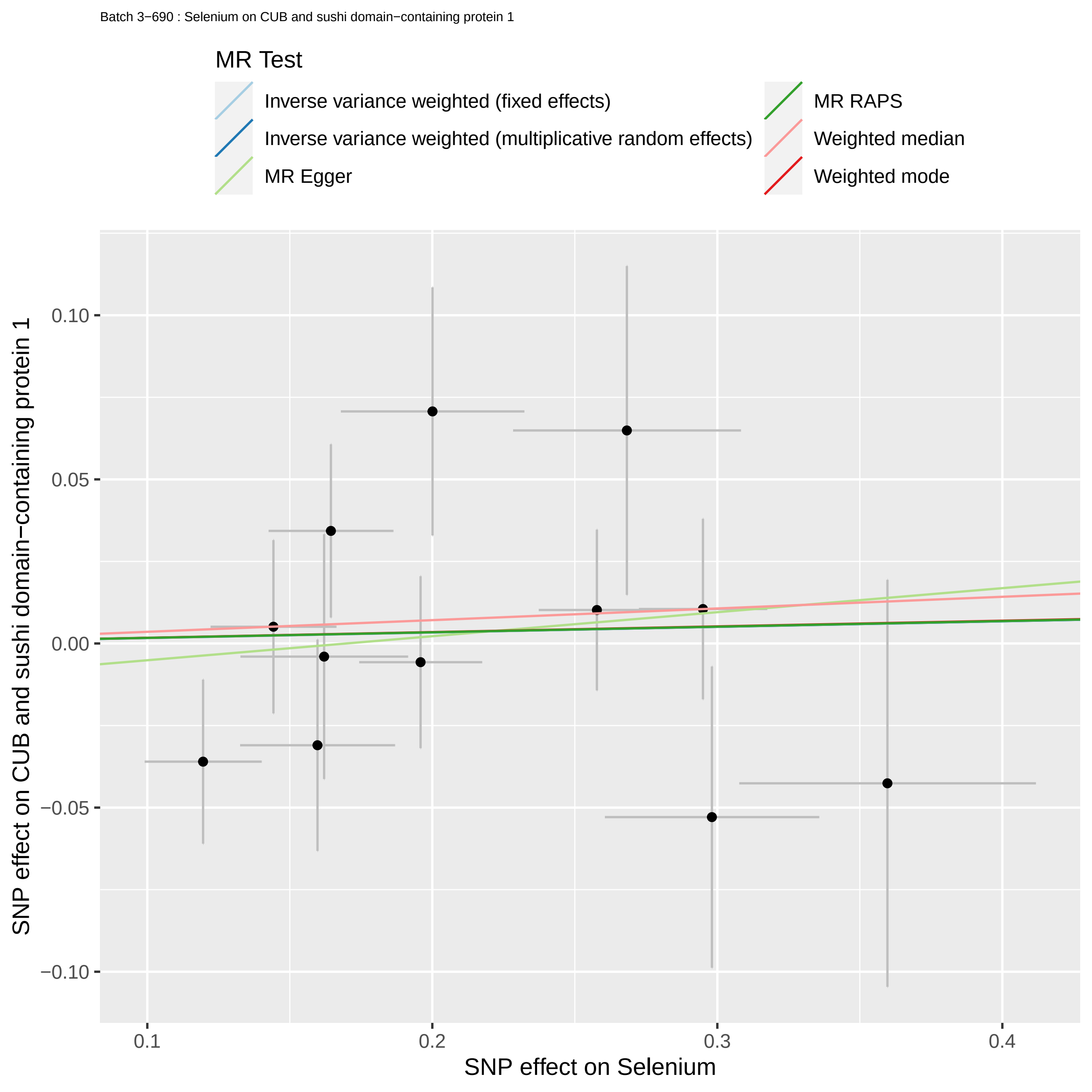
Batch 3−690 : Selenium on CUB and sushi domain−containing protein 1
MR Test
Inverse variance weighted (fixed effects)
Inverse variance weighted (multiplicative random effects)
MR Egger
MR RAPS
Weighted median
Weighted mode
0.10
0.05
0.00
−0.05
−0.10
0.1
0.2
0.3
0.4
SNP effect on Selenium
SNP effect on CUB and sushi domain−containing protein 1

Batch 3−691 : Selenium on CUB and sushi domain−containing protein 2

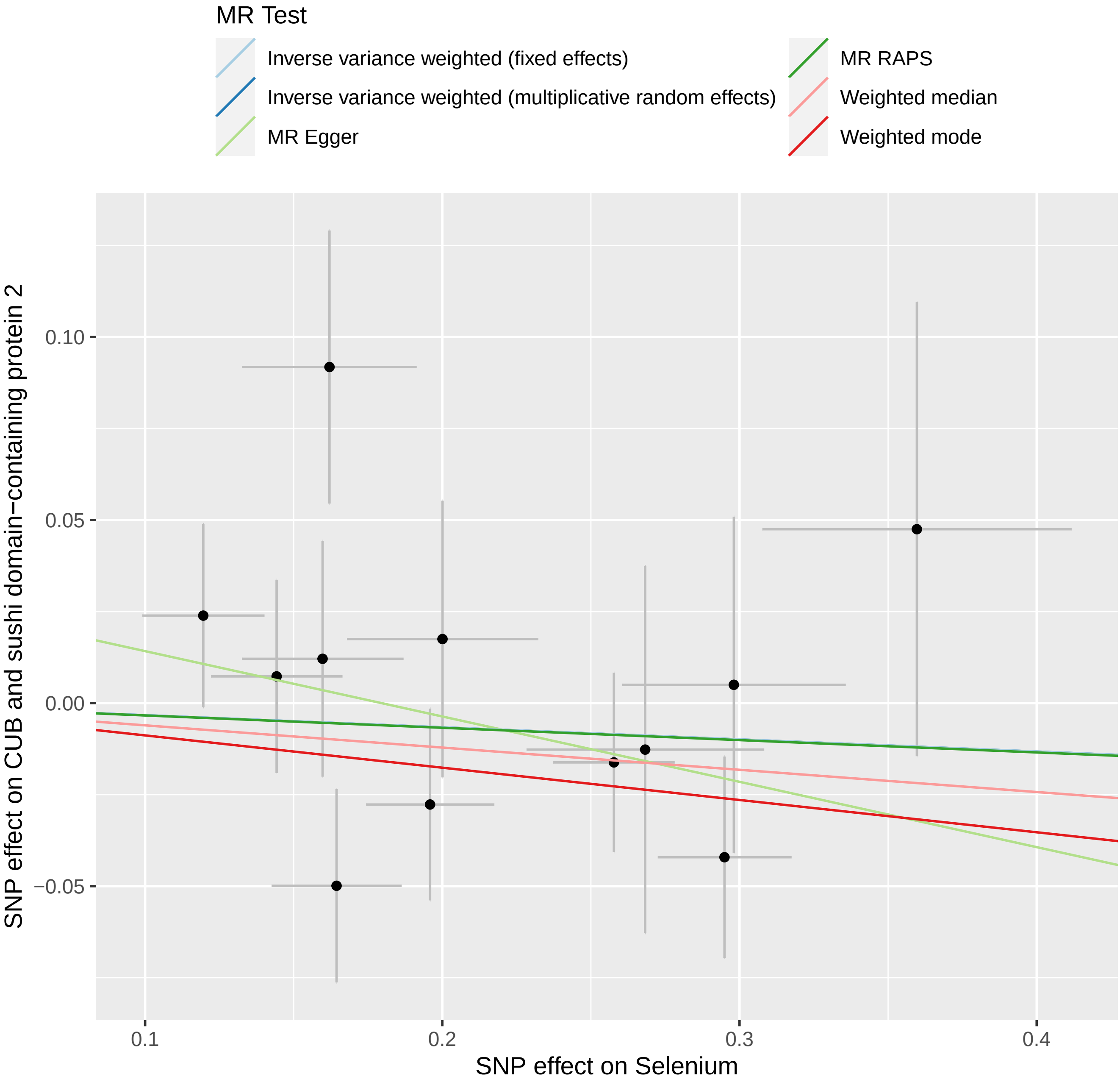

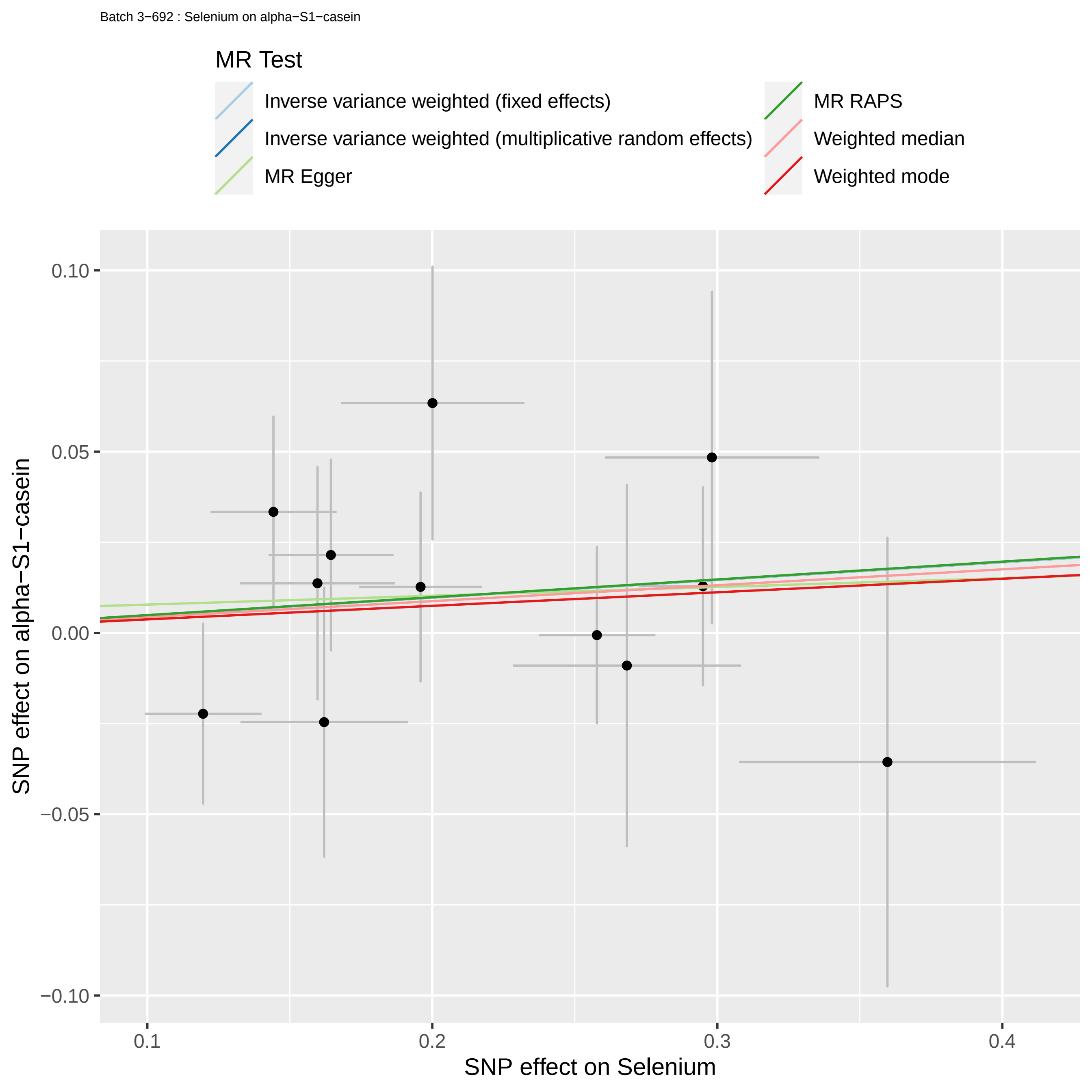
Batch 3−692 : Selenium on alpha−S1−casein
MR Test
Inverse variance weighted (fixed effects)
Inverse variance weighted (multiplicative random effects)
MR Egger
MR RAPS
Weighted median
Weighted mode
0.10
0.05
0.00
−0.05
−0.10
0.1
0.2
0.3
0.4
SNP effect on Selenium
SNP effect on alpha−S1−casein

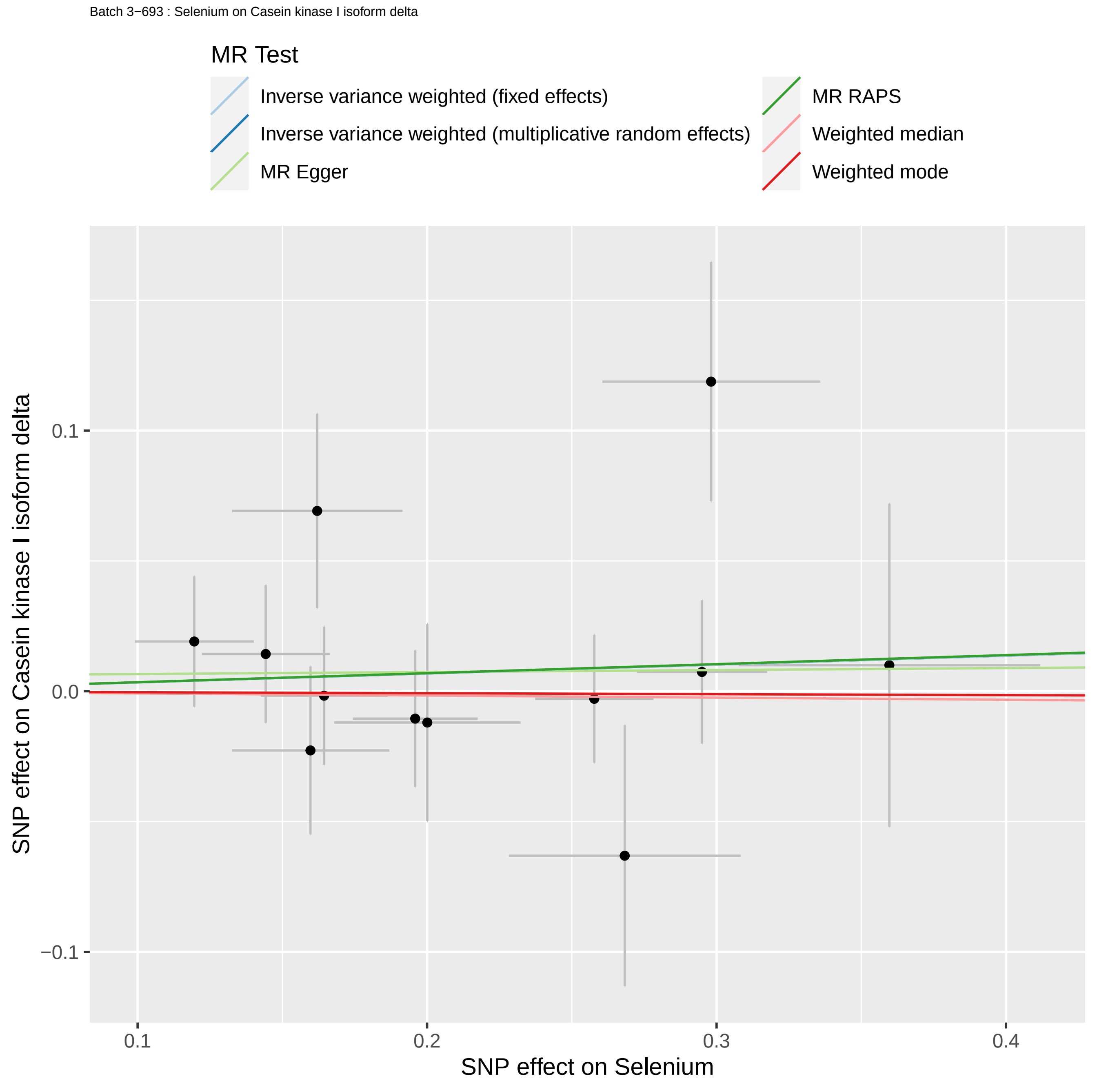

Batch 3−693 : Selenium on Casein kinase I isoform delta
MR Test
Inverse variance weighted (fixed effects)
Inverse variance weighted (multiplicative random effects)
MR Egger
MR RAPS
Weighted median
Weighted mode
SNP effect on Casein kinase I isoform delta
0.1
0.0
−0.1
0.1
0.2
0.3
0.4
SNP effect on Selenium

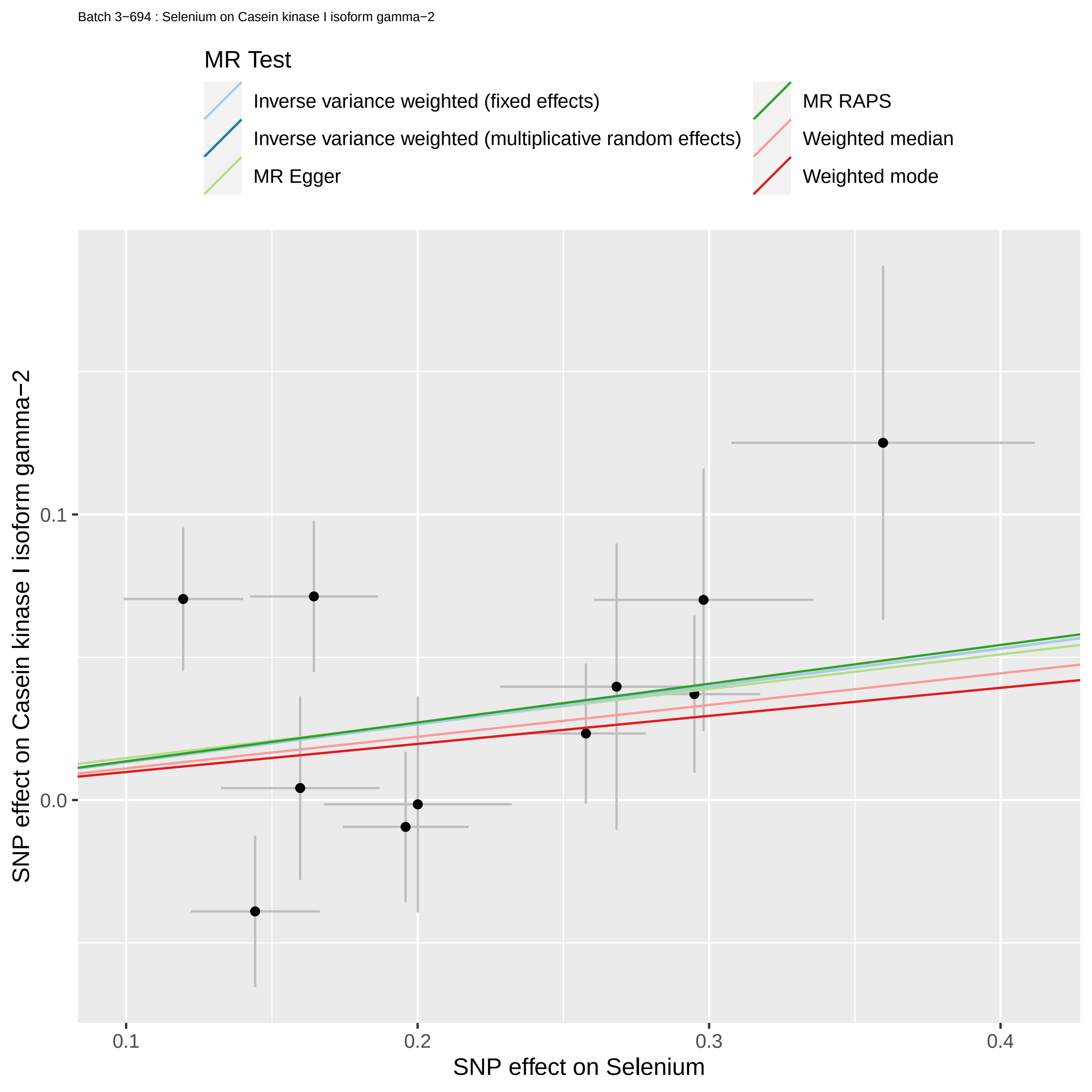

Batch 3−694 : Selenium on Casein kinase I isoform gamma−2
MR Test
Inverse variance weighted (fixed effects)
Inverse variance weighted (multiplicative random effects)
MR Egger
MR RAPS
Weighted median
Weighted mode
SNP effect on Casein kinase I isoform gamma−2
0.1
0.0
0.1
0.2
0.3
0.4
SNP effect on Selenium

Batch 3−695 : Selenium on Casein kinase II subunit alpha

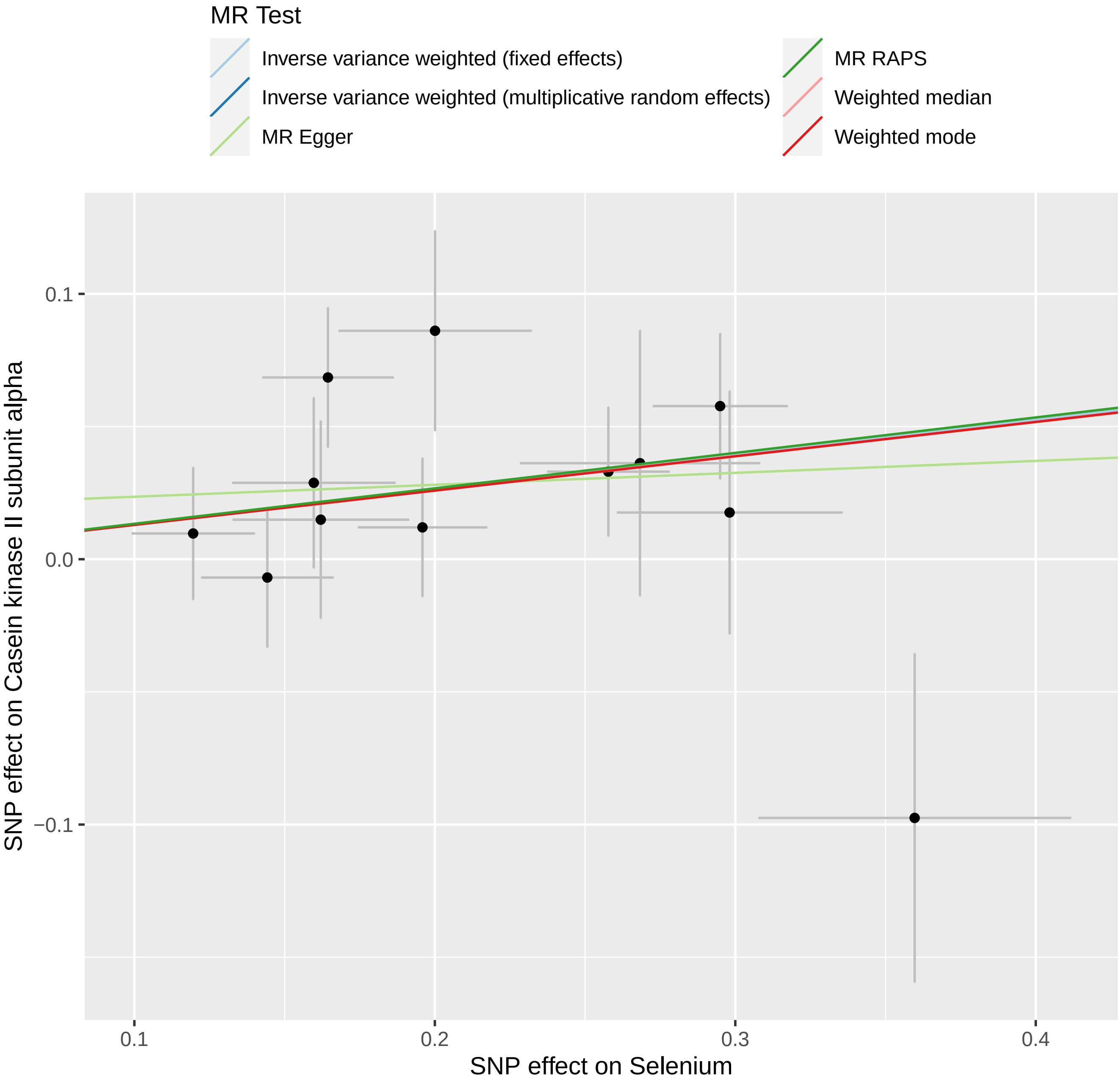

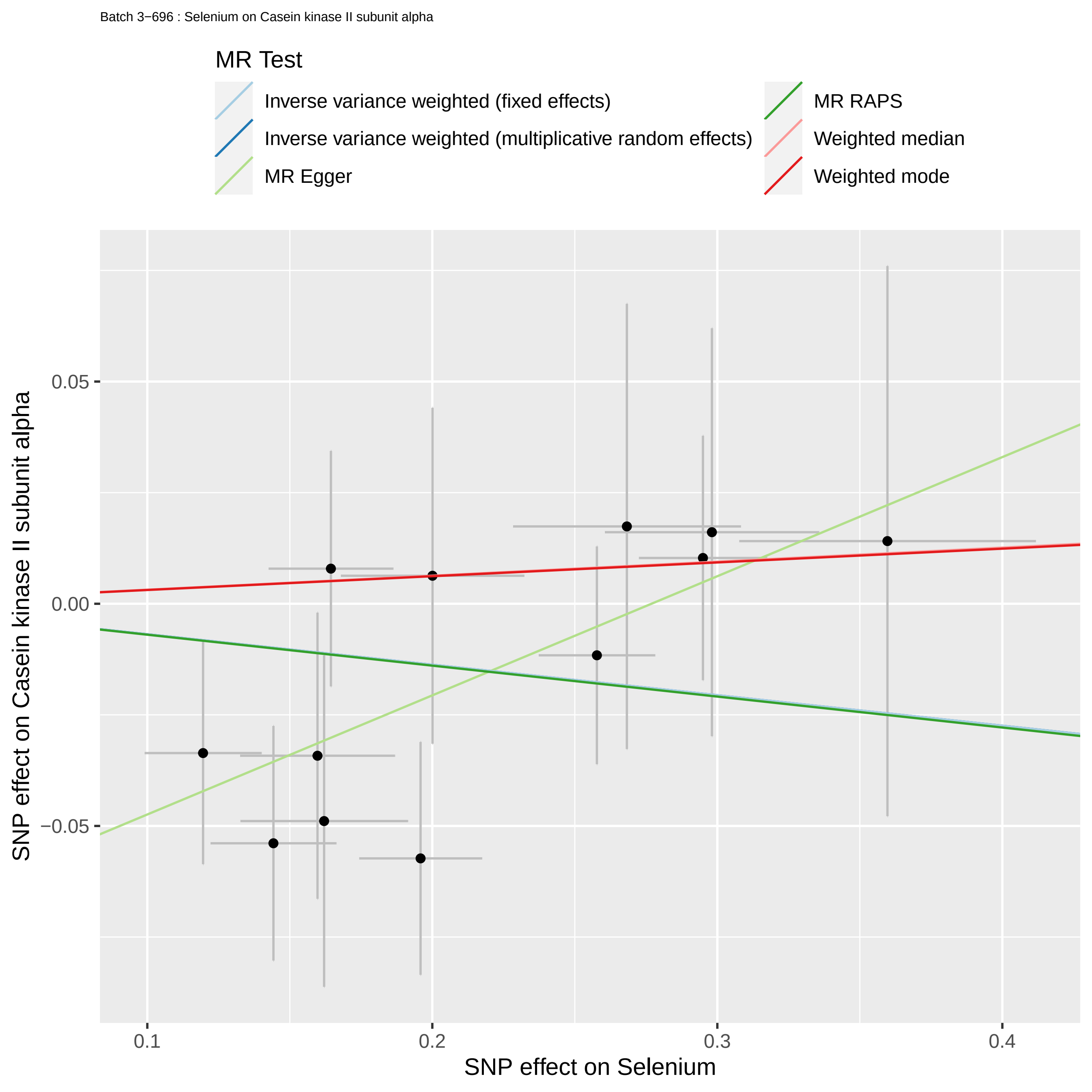

Batch 3−696 : Selenium on Casein kinase II subunit alpha
MR Test
Inverse variance weighted (fixed effects)
Inverse variance weighted (multiplicative random effects)
MR Egger
MR RAPS
Weighted median
Weighted mode
SNP effect on Casein kinase II subunit alpha
0.05
0.00
−0.05
0.1
0.2
0.3
0.4
SNP effect on Selenium

Batch 3−697 : Selenium on Casein kinase II 2−alpha:2−beta heterotetramer

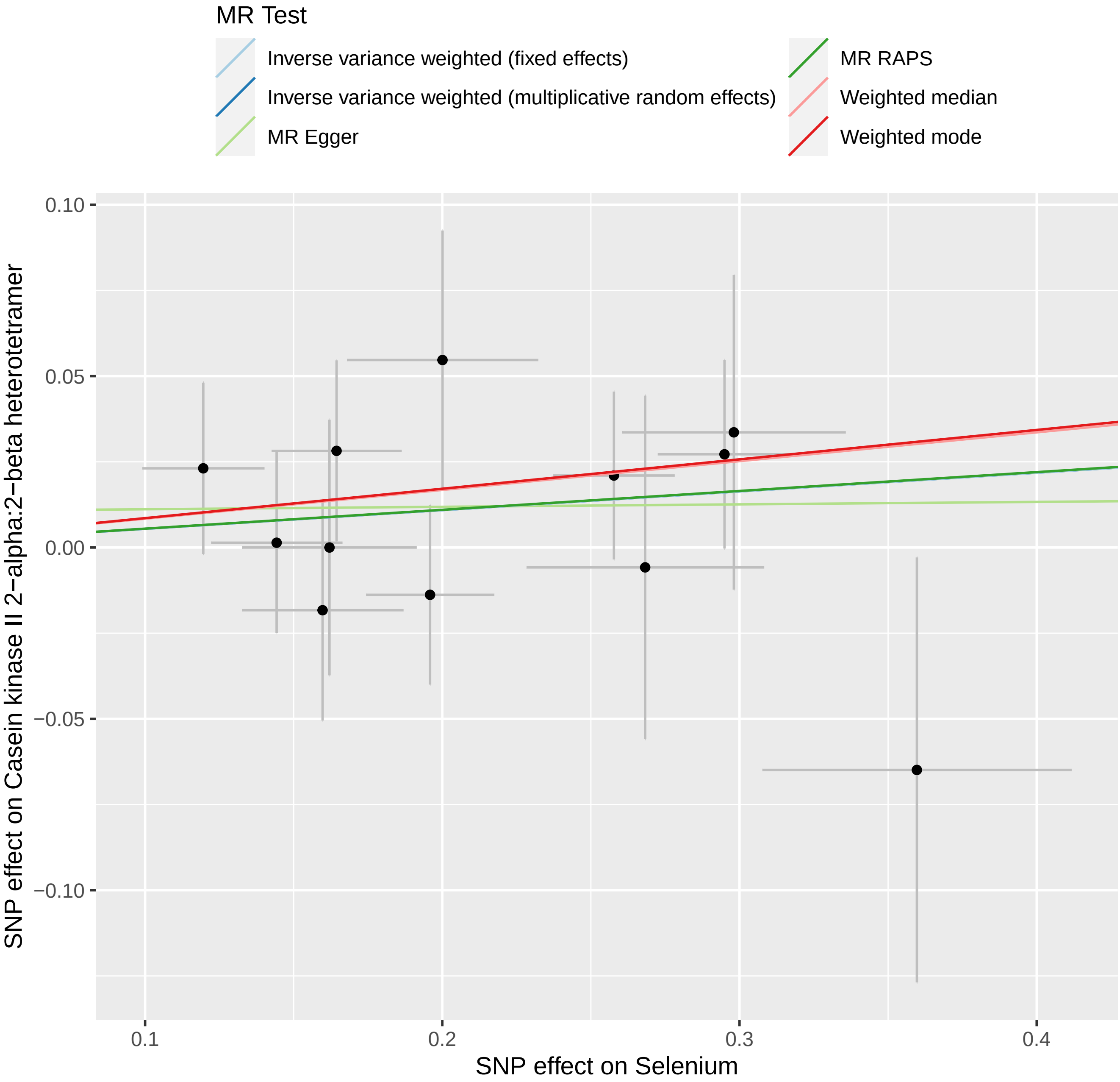

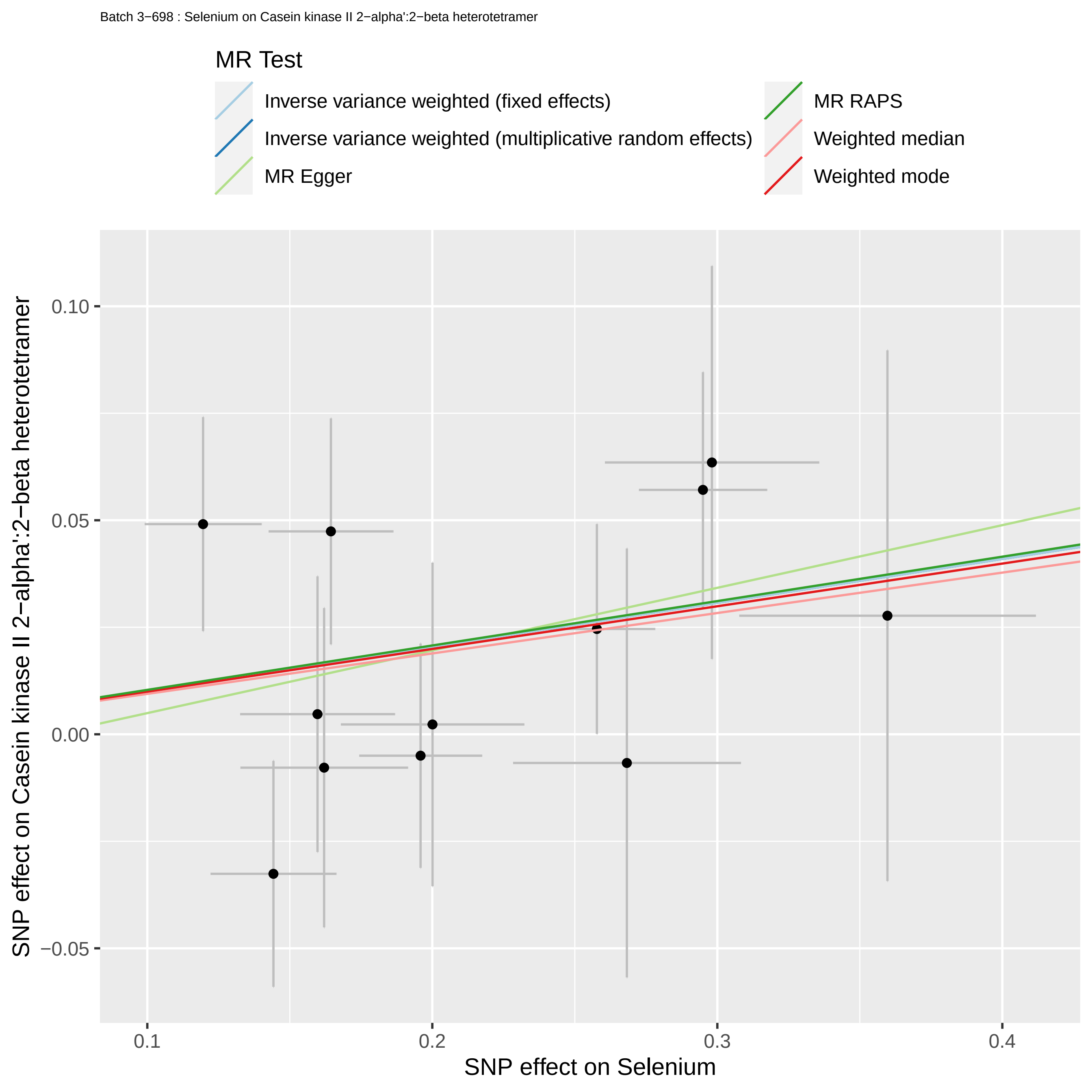

Batch 3−698 : Selenium on Casein kinase II 2−alpha':2−beta heterotetramer
MR Test
Inverse variance weighted (fixed effects)
Inverse variance weighted (multiplicative random effects)
MR Egger
MR RAPS
Weighted median
Weighted mode
0.10
0.05
0.00
−0.05
0.1
0.2
0.3
0.4
SNP effect on Casein kinase II 2−alpha':2−beta heterotetramer
SNP effect on Selenium

Batch 3−699 : Selenium on Chondroitin sulfate proteoglycan 4

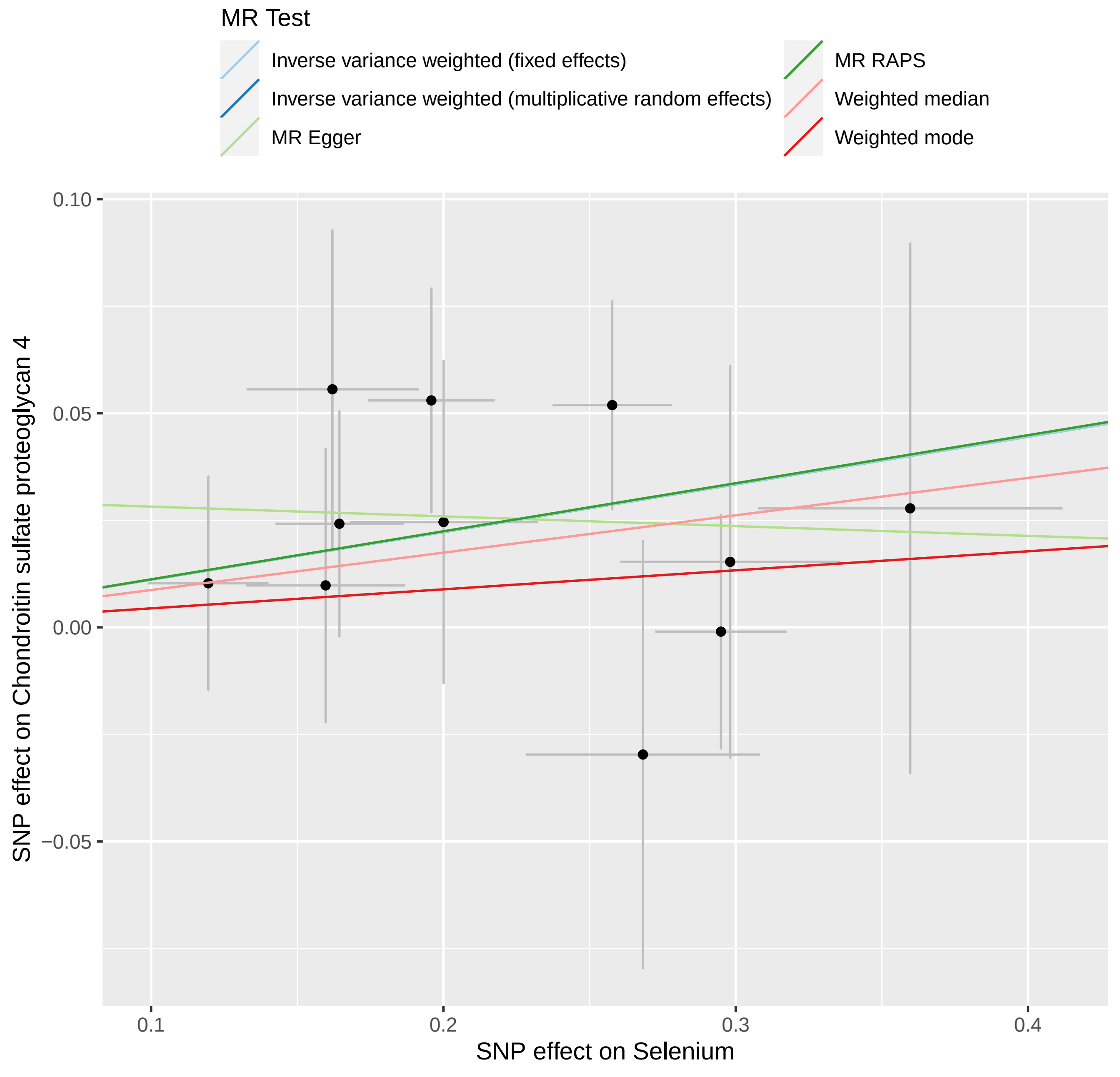

Batch 3−700 : Selenium on Cysteine and glycine−rich protein 2

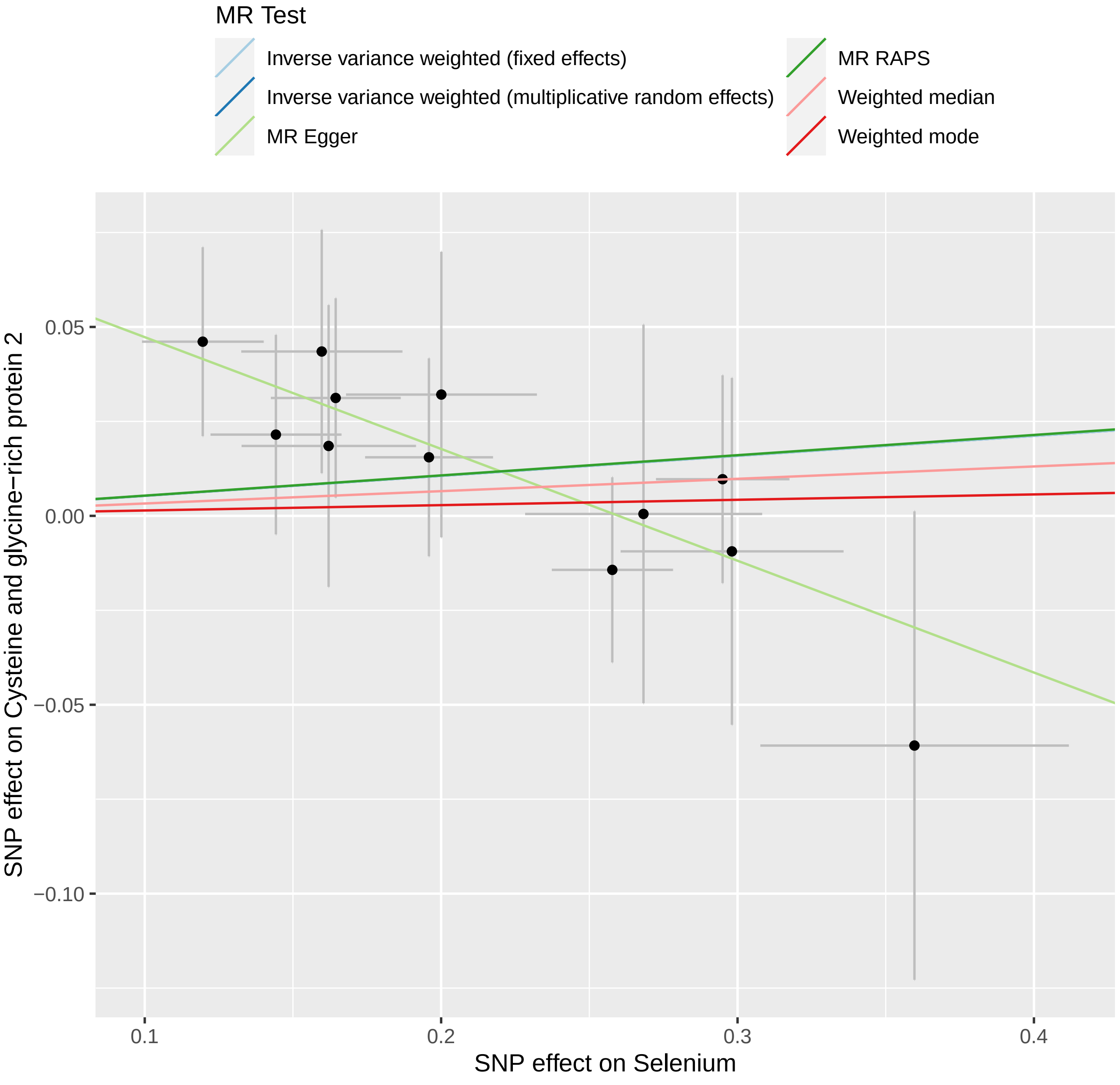

Batch 3−701 : Selenium on Cysteine and glycine−rich protein 3

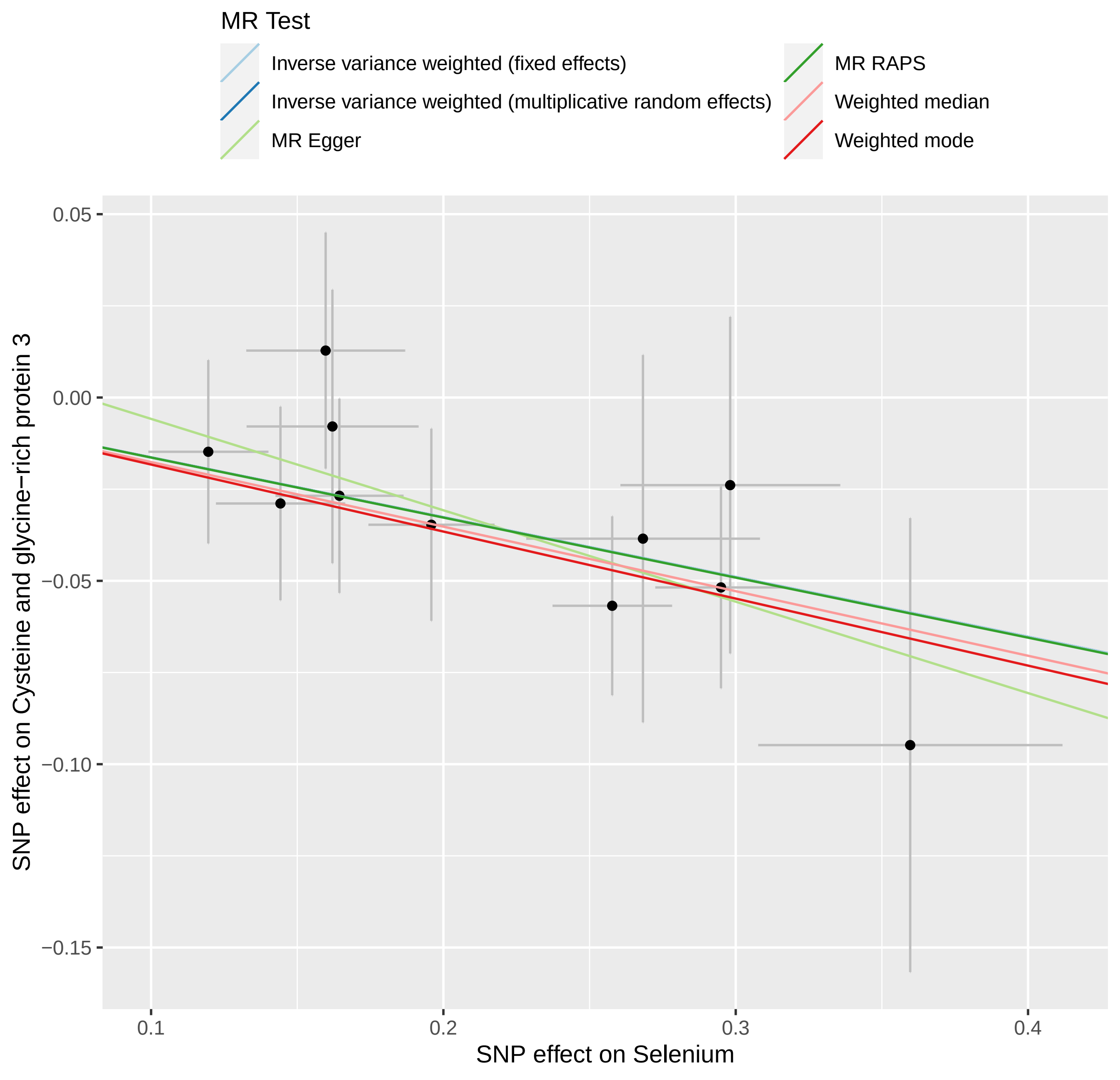

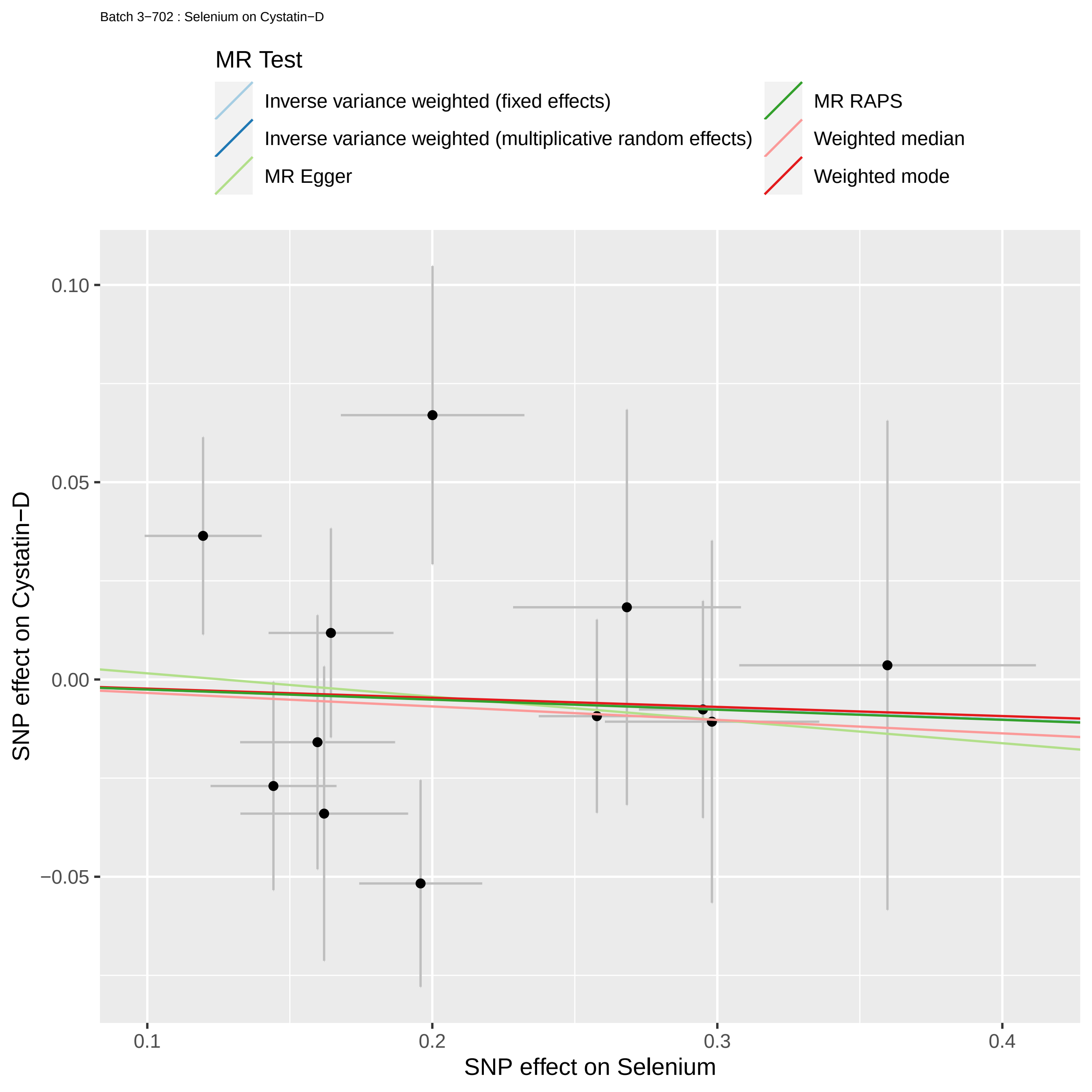
Batch 3−702 : Selenium on Cystatin−D
MR Test
Inverse variance weighted (fixed effects)
Inverse variance weighted (multiplicative random effects)
MR Egger
MR RAPS
Weighted median
Weighted mode
0.10
0.05
0.00
−0.05
0.1
0.2
0.3
0.4
SNP effect on Cystatin−D
SNP effect on Selenium

Batch 3−703 : Selenium on Cystatin−M

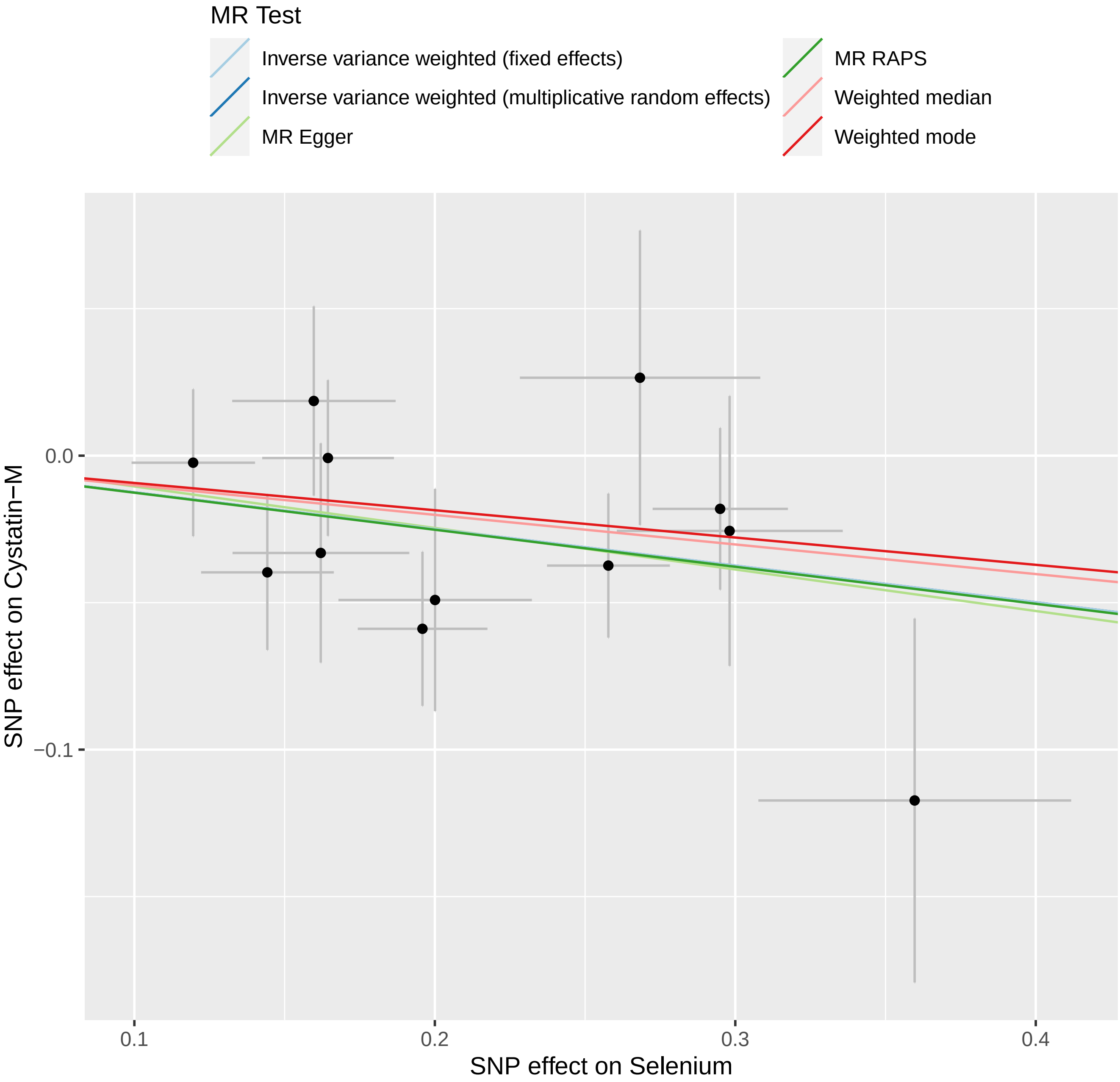

Batch 3−704 : Selenium on Cystatin−M

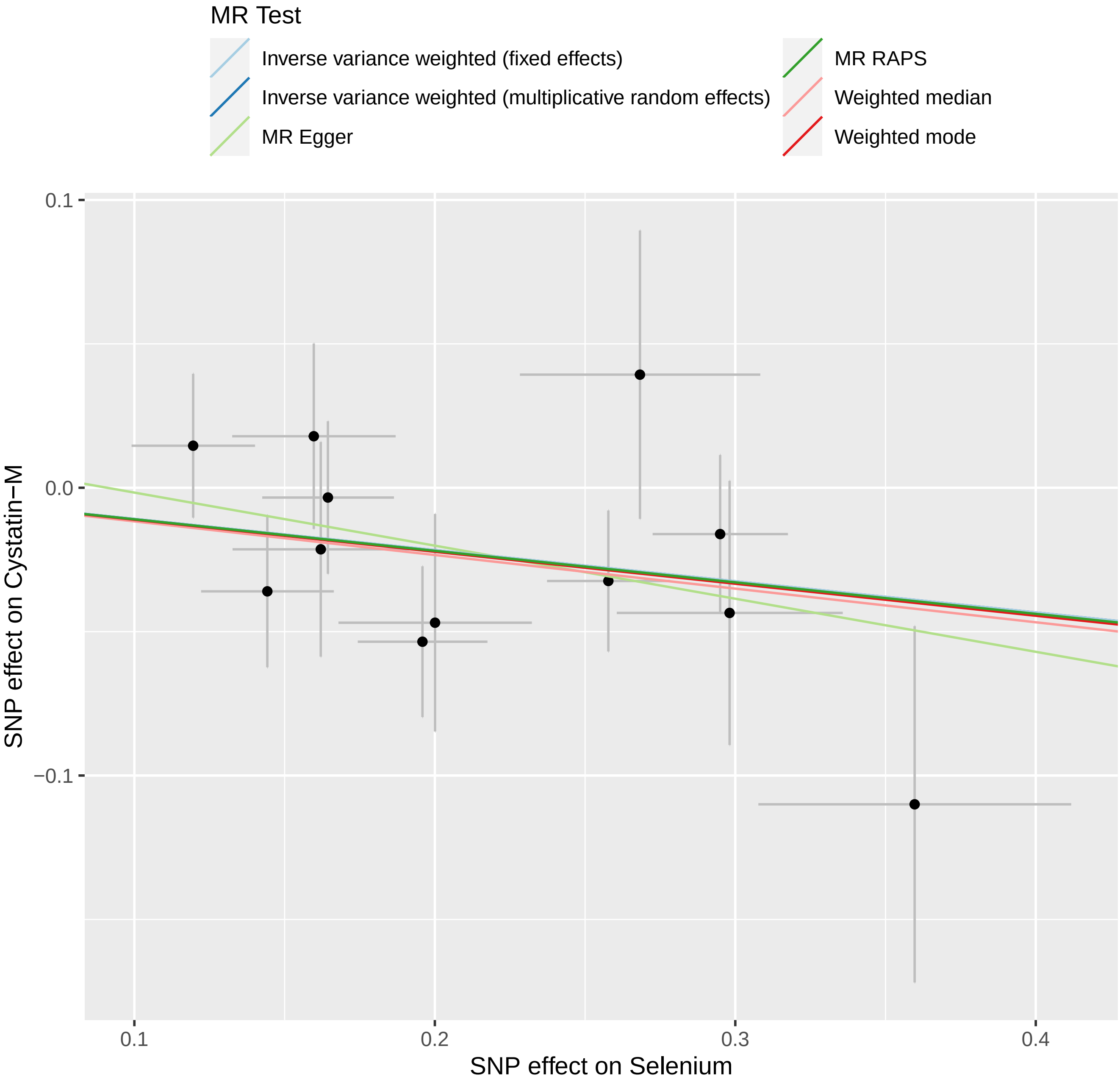

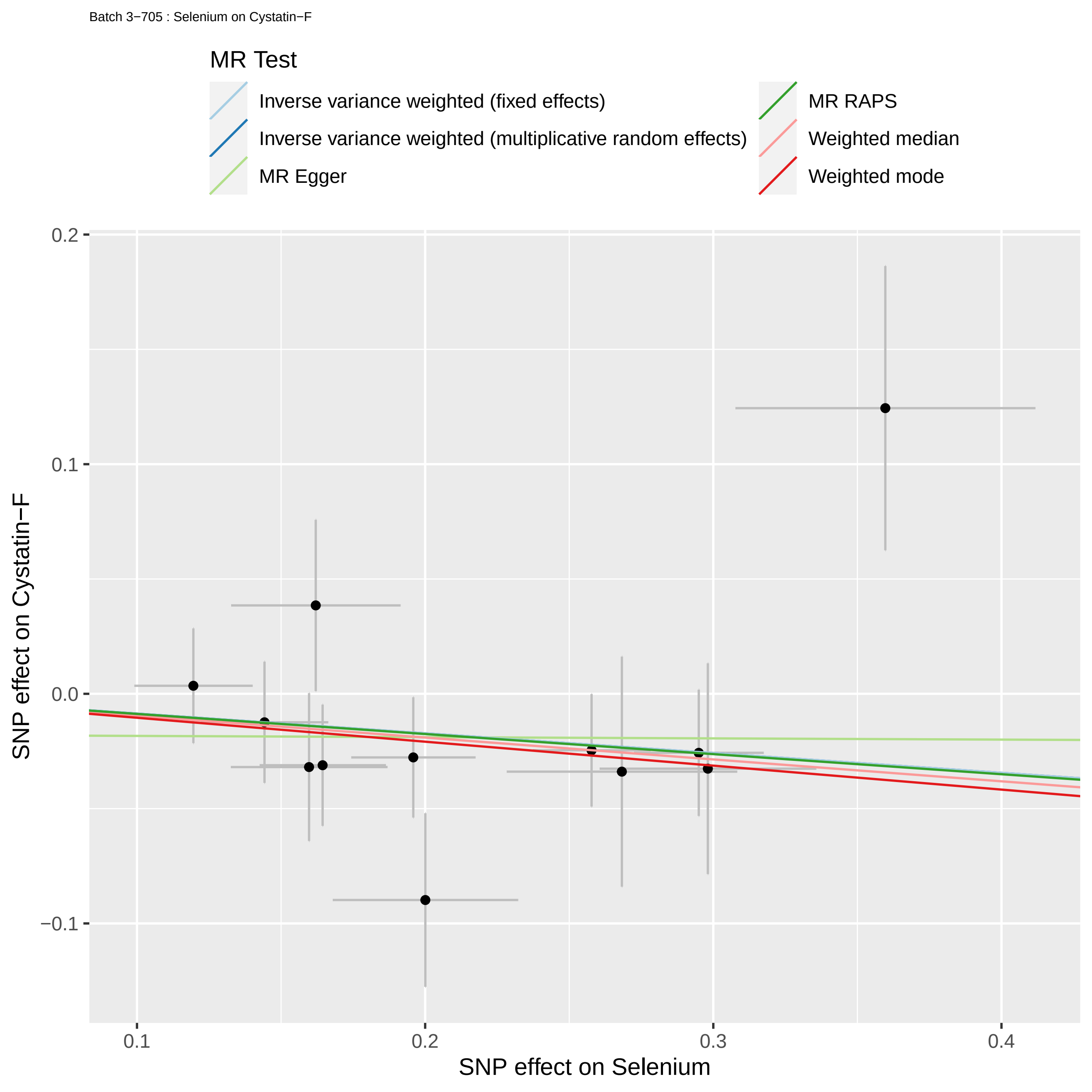
Batch 3−705 : Selenium on Cystatin−F
MR Test
Inverse variance weighted (fixed effects)
Inverse variance weighted (multiplicative random effects)
MR Egger
MR RAPS
Weighted median
Weighted mode
0.2
0.1
0.0
−0.1
SNP effect on Cystatin−F
0.1
0.2
0.3
0.4
SNP effect on Selenium

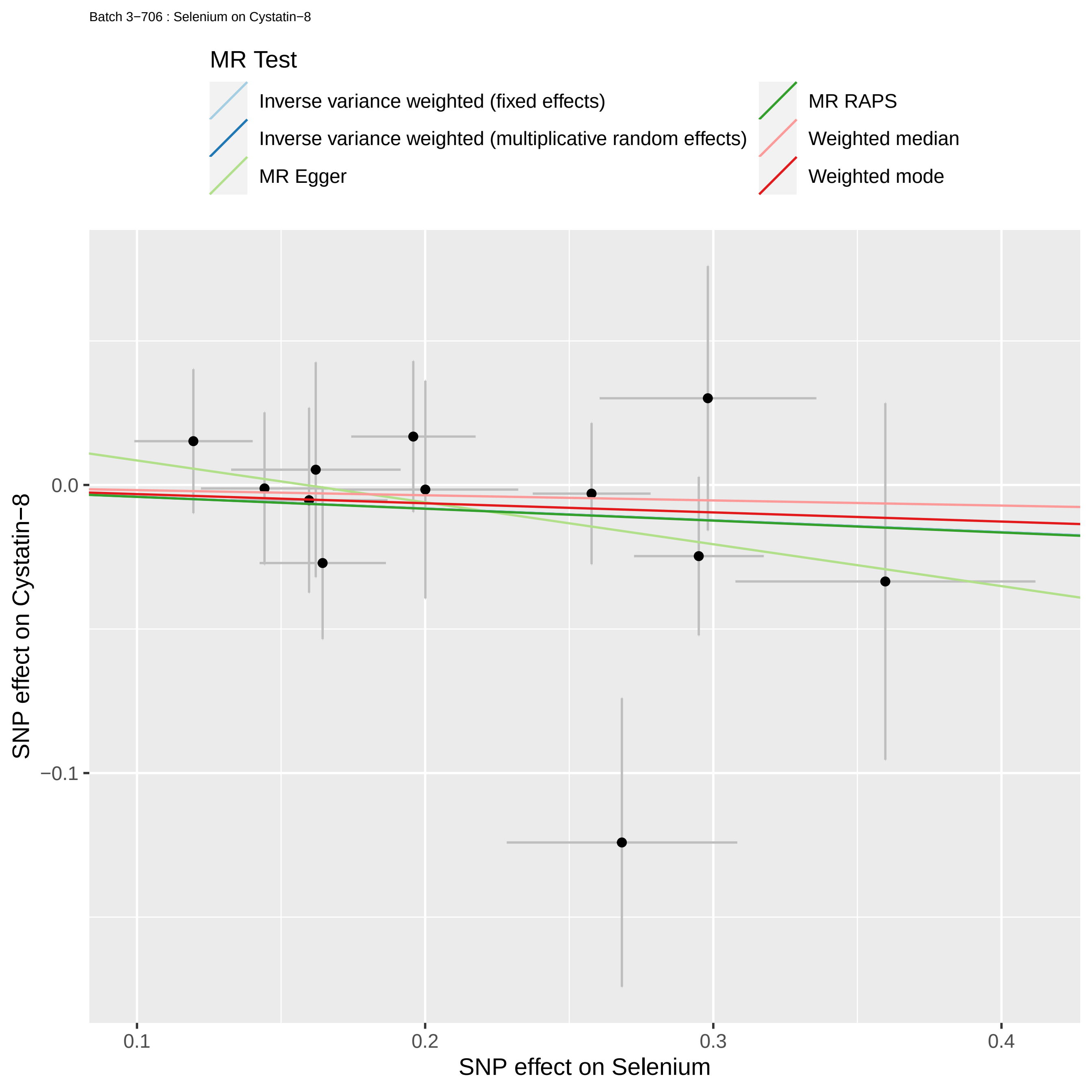

Batch 3−706 : Selenium on Cystatin−8
MR Test
Inverse variance weighted (fixed effects)
Inverse variance weighted (multiplicative random effects)
MR Egger
MR RAPS
Weighted median
Weighted mode
SNP effect on Cystatin−8
0.0
−0.1
0.1
0.2
0.3
0.4
SNP effect on Selenium

Batch 3−707 : Selenium on Cancer/testis antigen 55

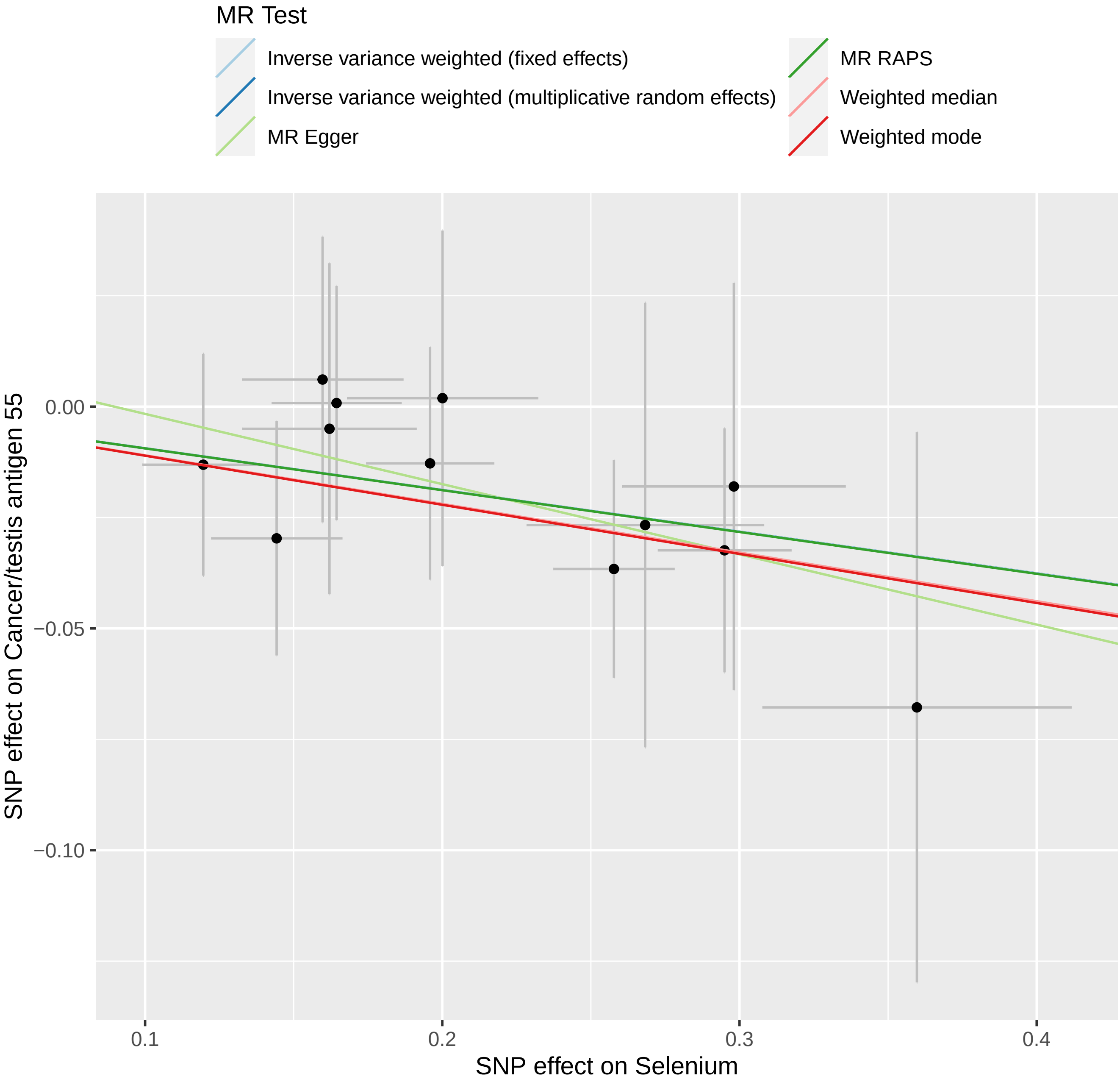

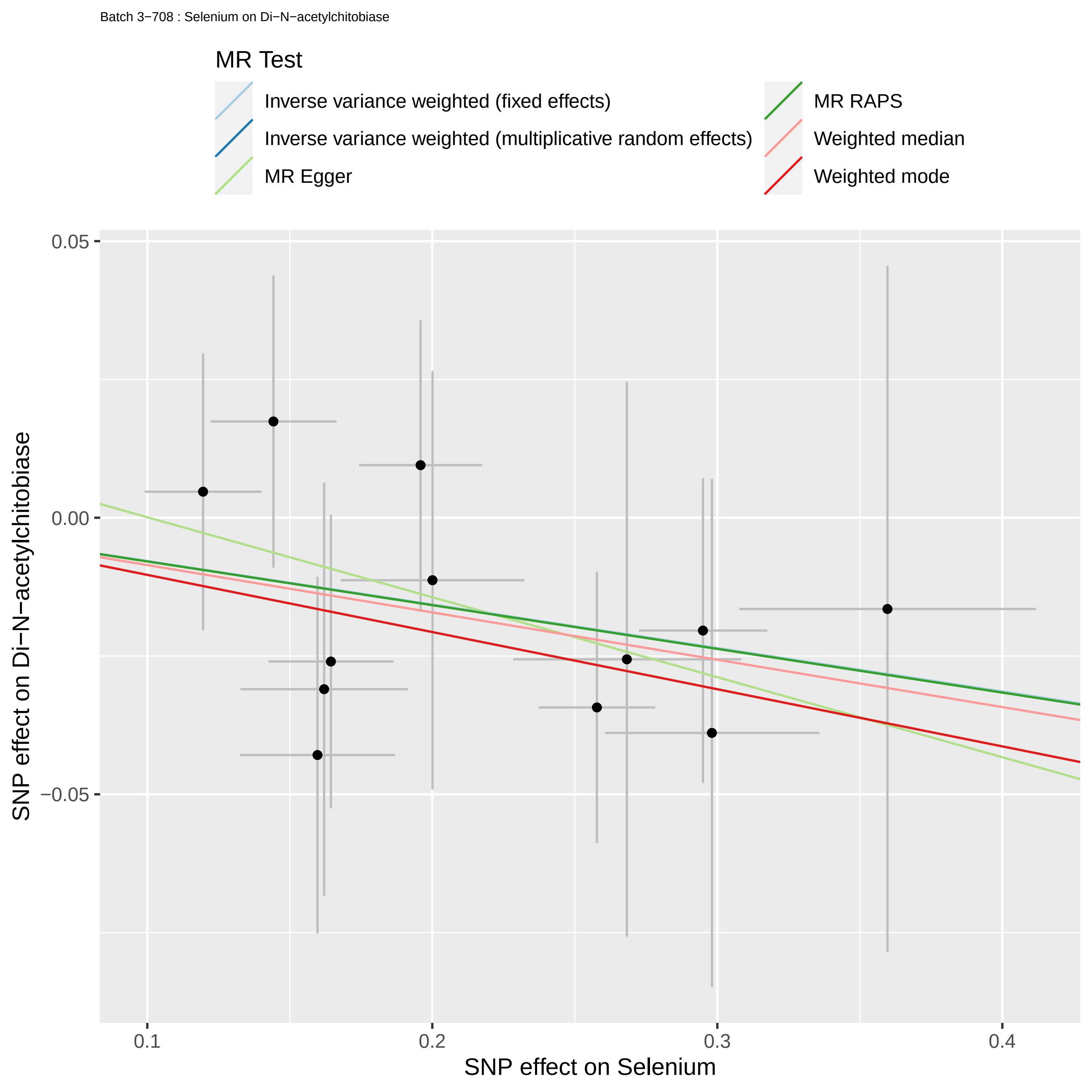
Batch 3−708 : Selenium on Di−N−acetylchitobiase
MR Test
Inverse variance weighted (fixed effects)
Inverse variance weighted (multiplicative random effects)
MR Egger
MR RAPS
Weighted median
Weighted mode
0.05
0.00
−0.05
0.1
0.2
0.3
0.4
SNP effect on Di−N−acetylchitobiase
SNP effect on Selenium

Batch 3−709 : Selenium on Transcriptional repressor CTCF

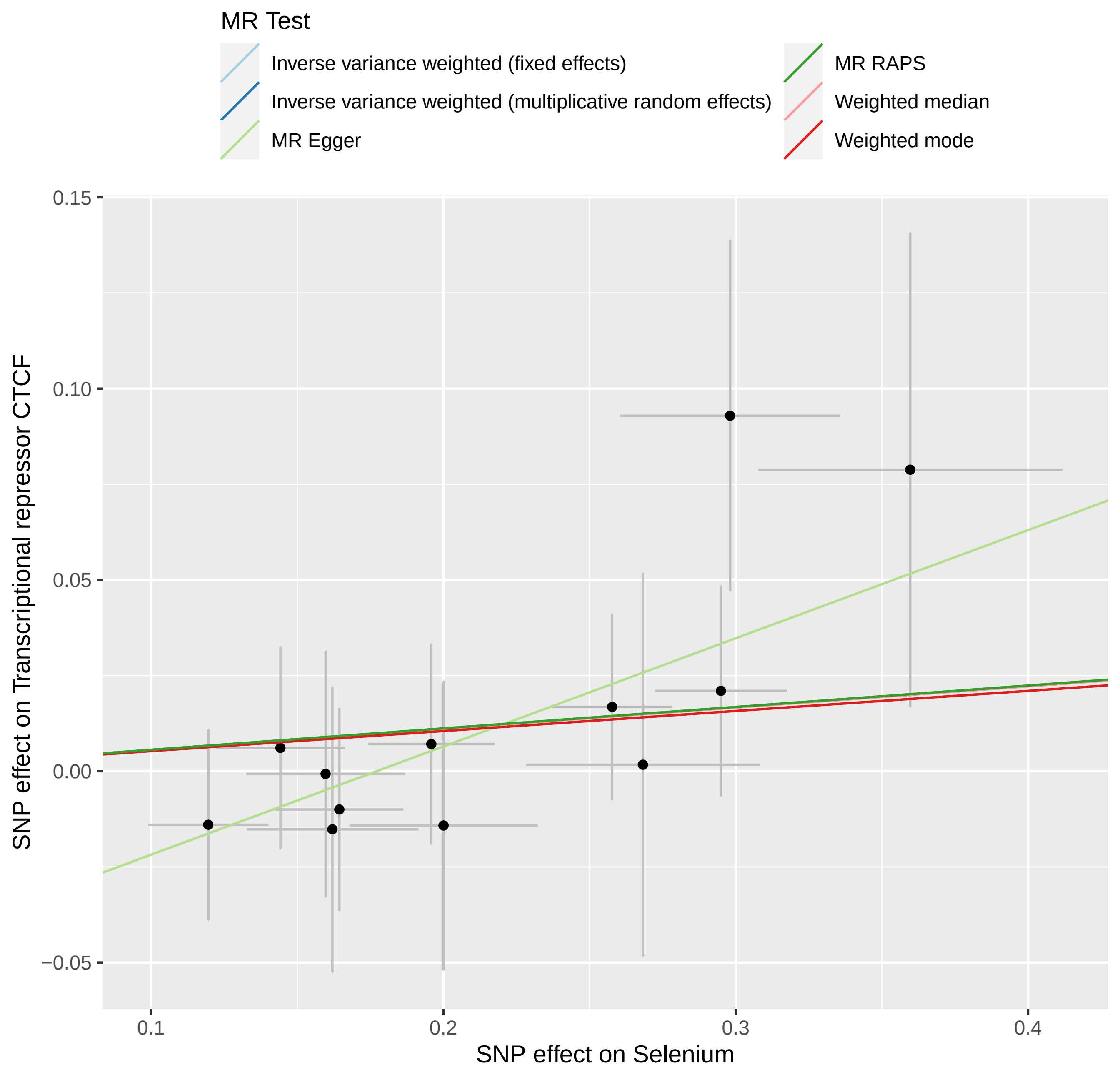

Batch 3−710 : Selenium on Cardiotrophin−1

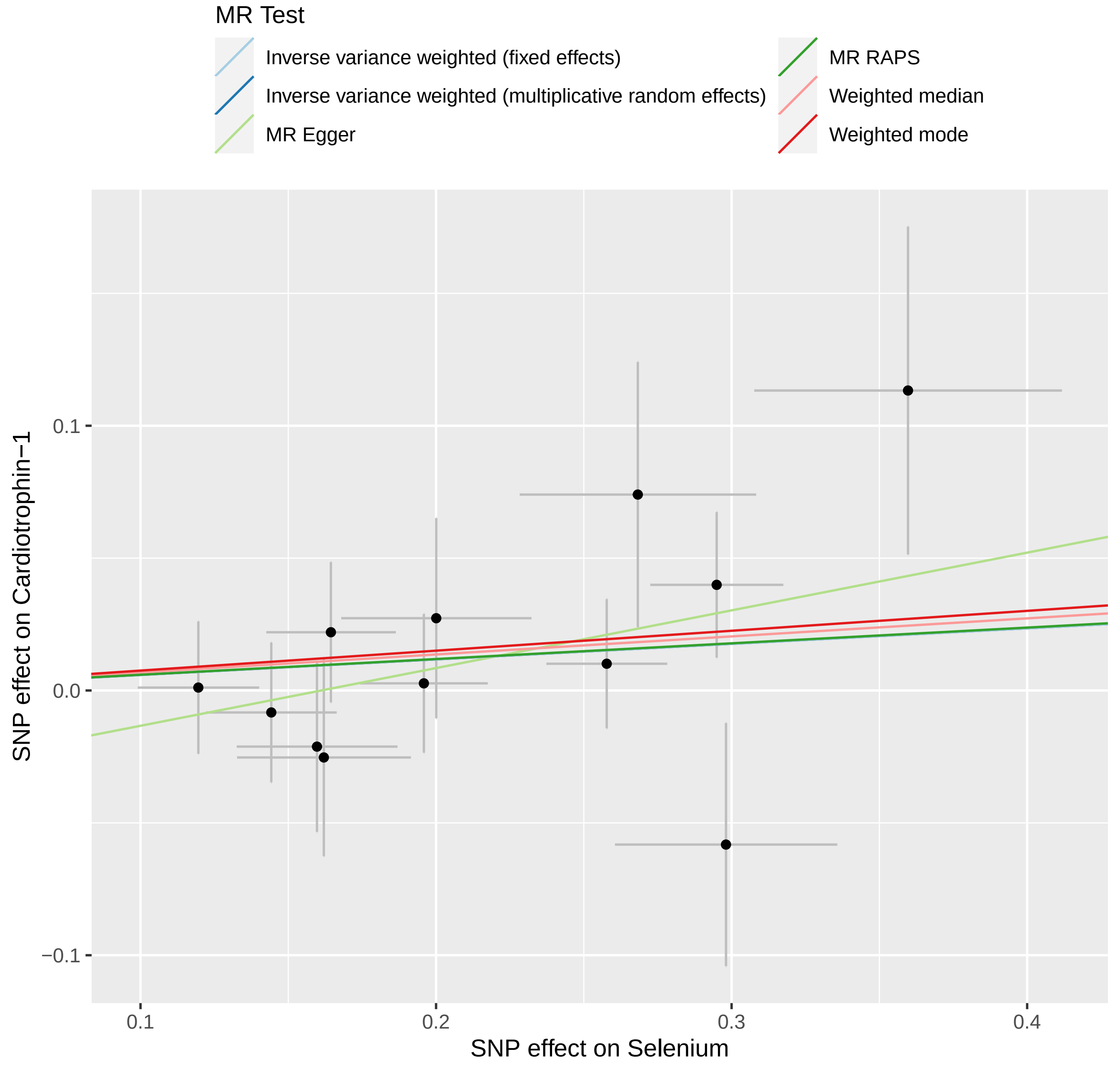

Batch 3−711 : Selenium on Cardiotrophin−1

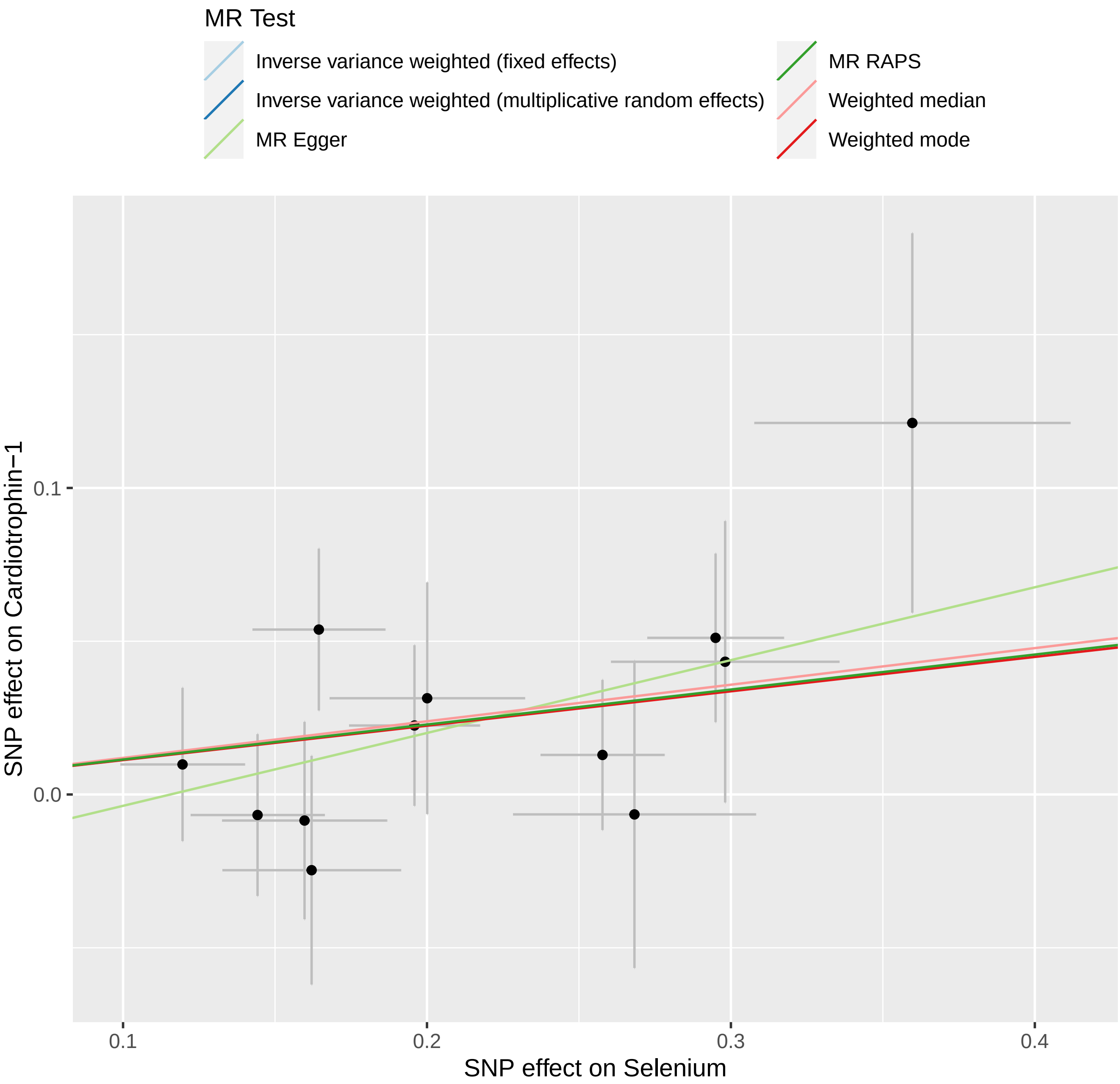

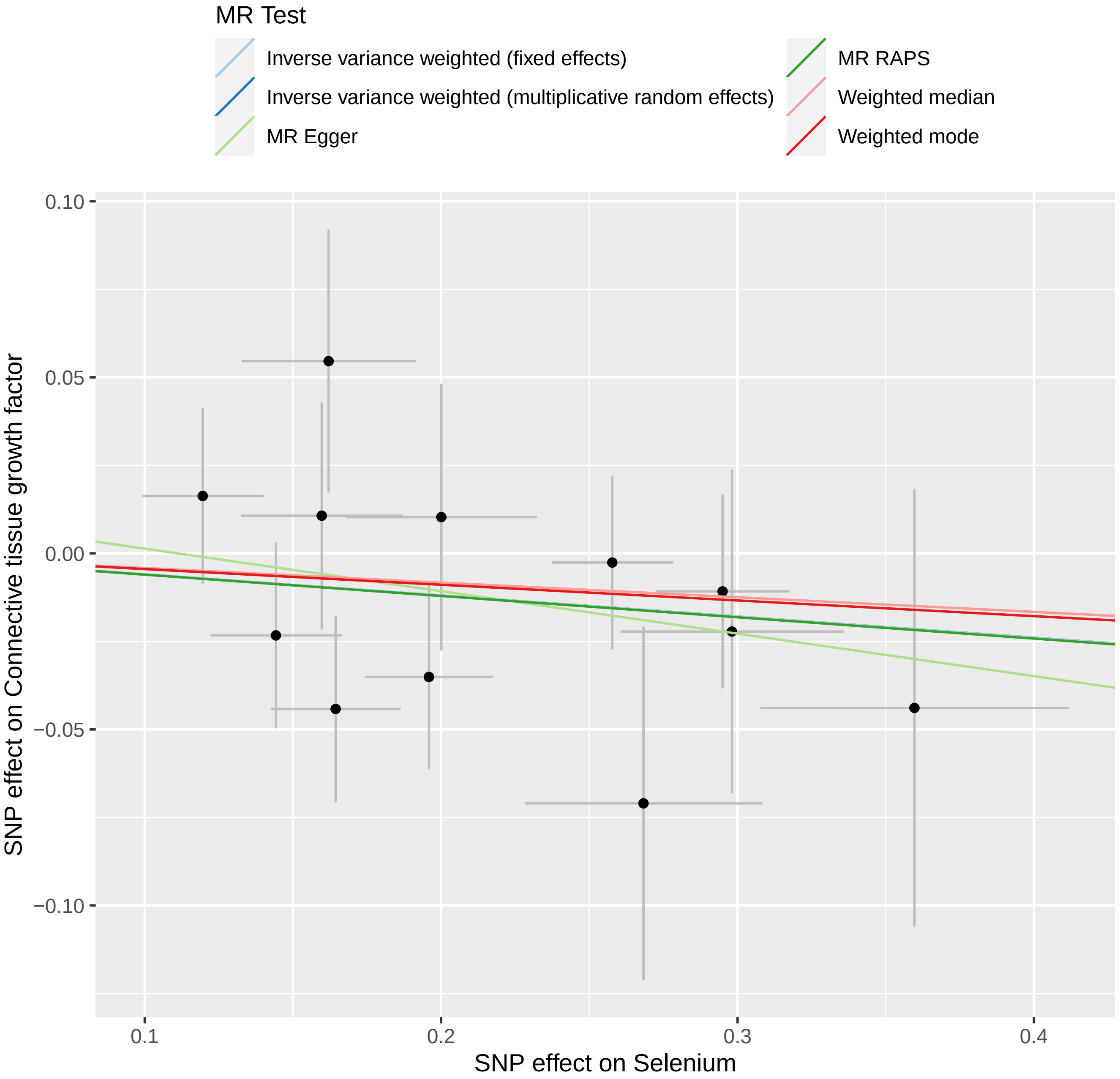

MR Test
Inverse variance weighted (fixed effects)
Inverse variance weighted (multiplicative random effects)
MR Egger
MR RAPS
Weighted median
Weighted mode
0.10
0.05
0.00
−0.05
−0.10
0.1
0.2
0.3
0.4
SNP effect on Connective tissue growth factor
SNP effect on Selenium

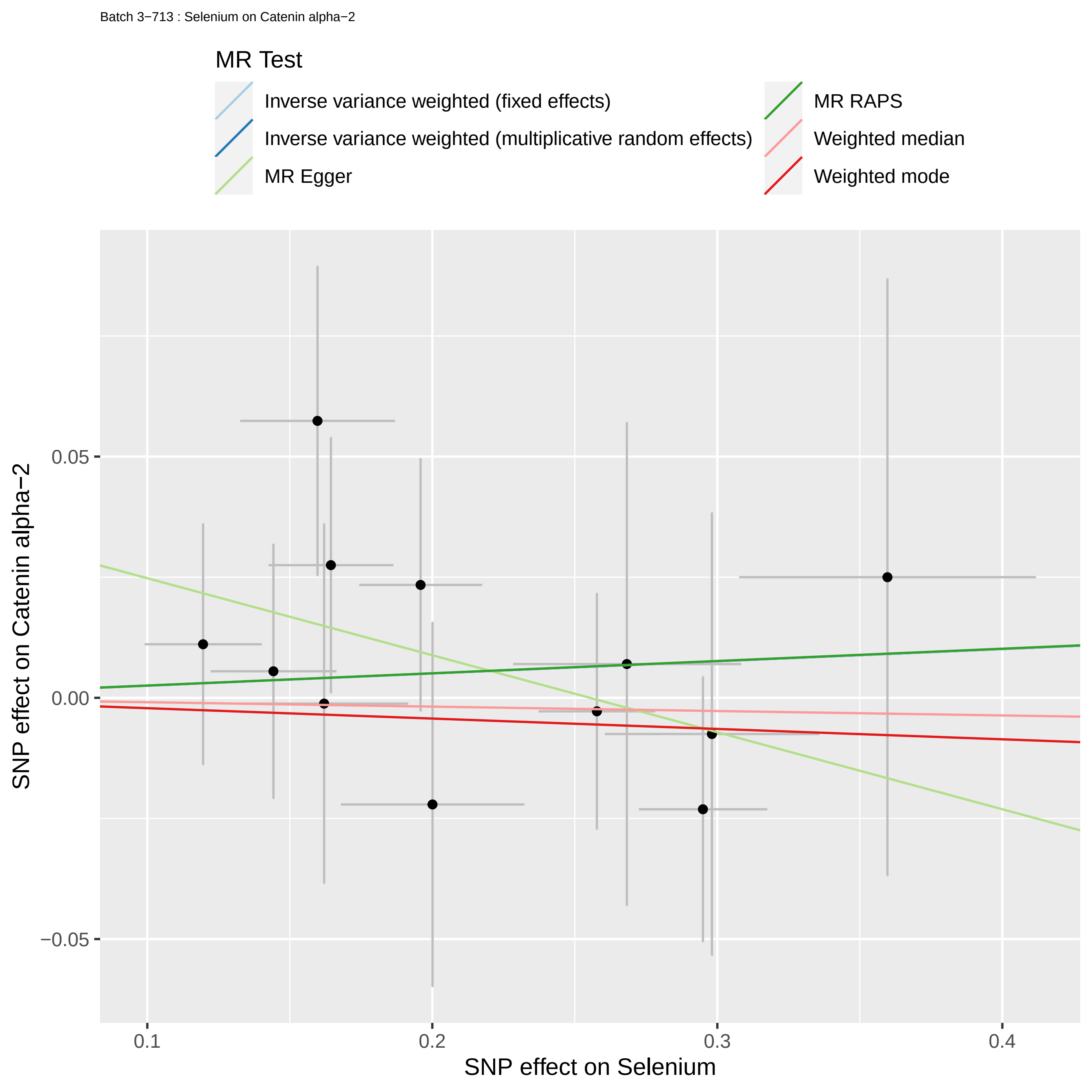

Batch 3−713 : Selenium on Catenin alpha−2
MR Test
Inverse variance weighted (fixed effects)
Inverse variance weighted (multiplicative random effects)
MR Egger
MR RAPS
Weighted median
Weighted mode
SNP effect on Catenin alpha−2
0.05
0.00
−0.05
0.1
0.2
0.3
0.4
SNP effect on Selenium

Batch 3−714 : Selenium on Chymotrypsinogen B

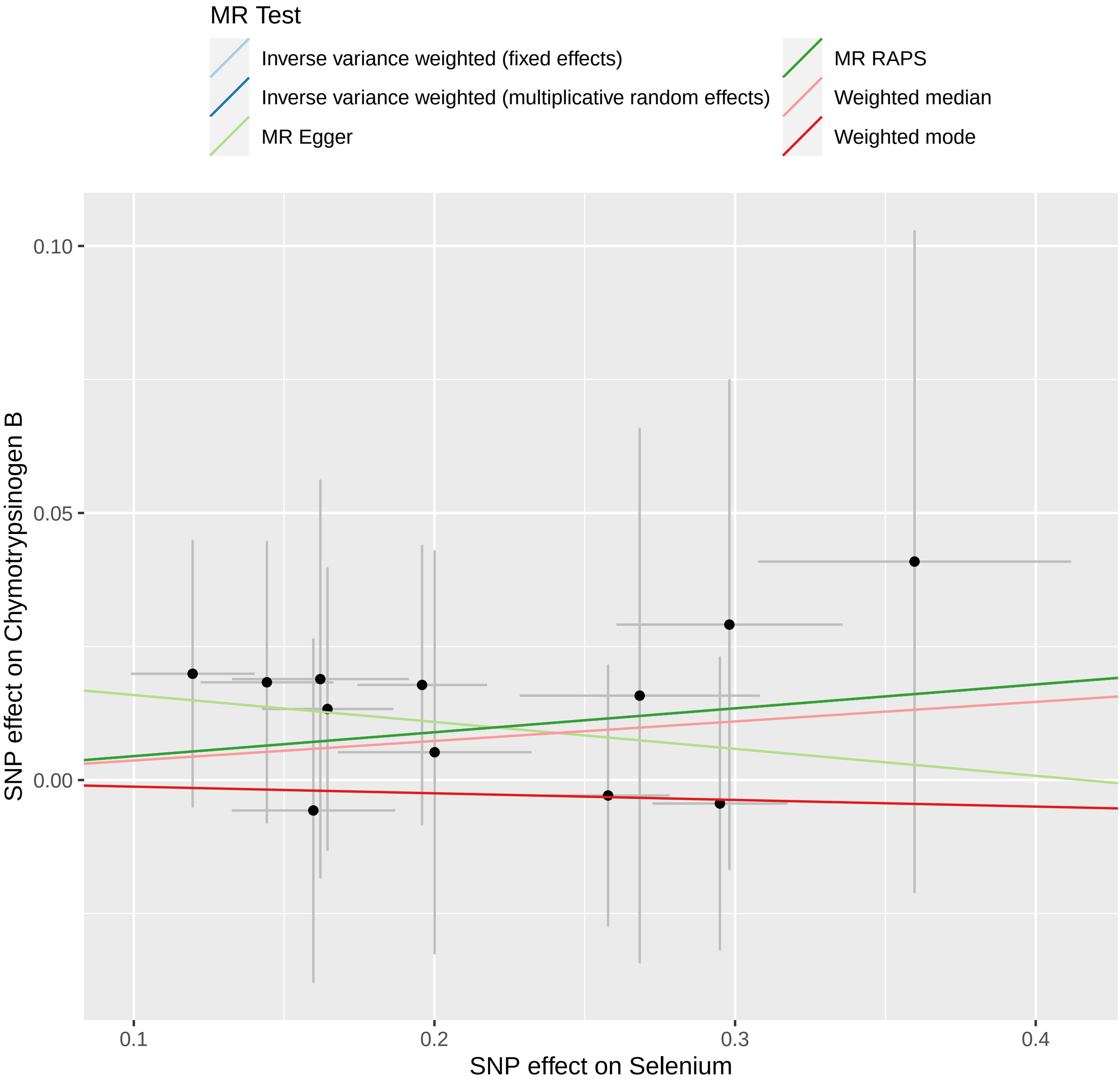

Batch 3−715 : Selenium on Chymotrypsin−C

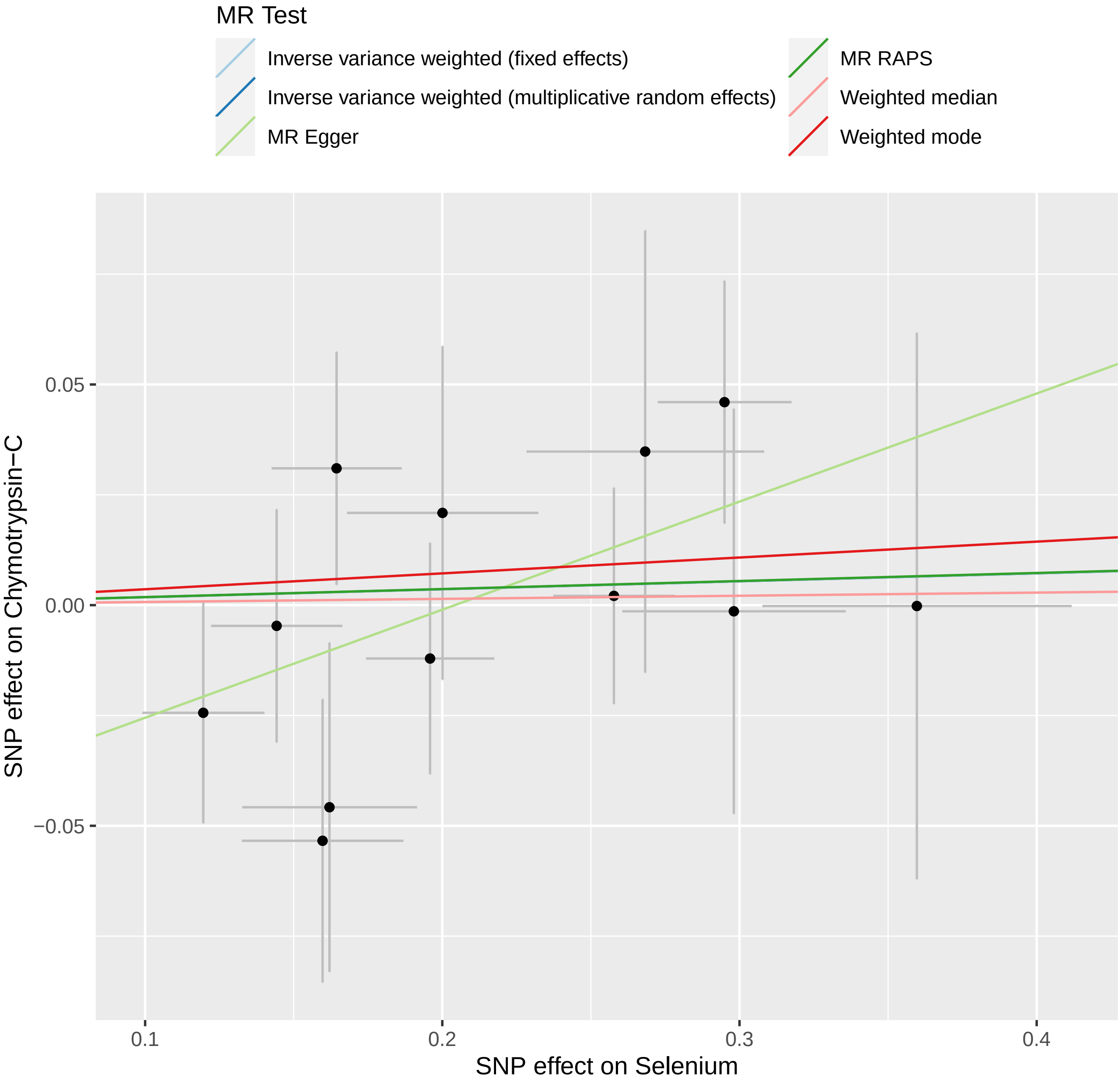

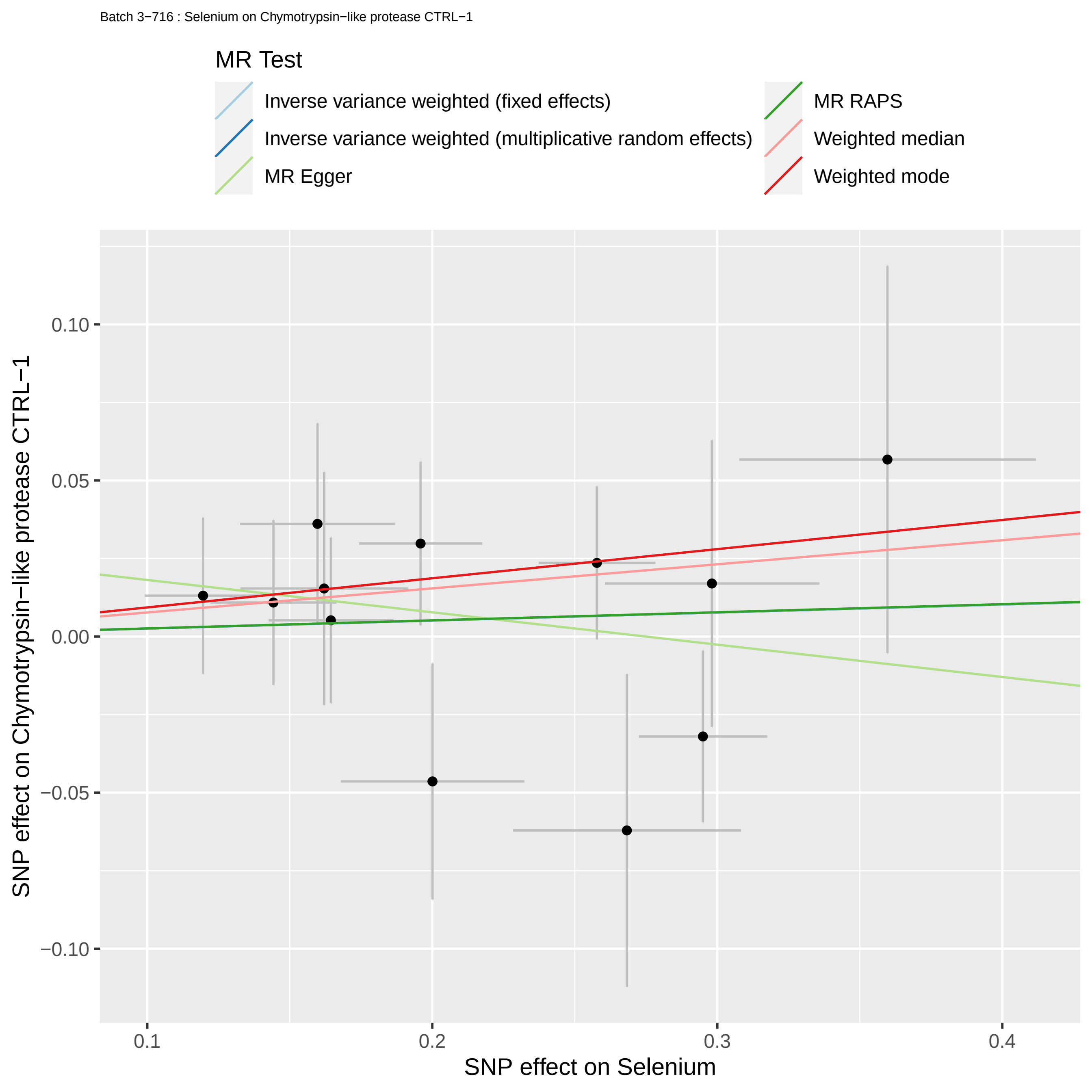

Batch 3−716 : Selenium on Chymotrypsin−like protease CTRL−1
MR Test
Inverse variance weighted (fixed effects)
Inverse variance weighted (multiplicative random effects)
MR Egger
MR RAPS
Weighted median
Weighted mode
0.10
0.05
0.00
−0.05
−0.10
0.1
0.2
0.3
0.4
SNP effect on Selenium
SNP effect on Chymotrypsin−like protease CTRL−1

Batch 3−717 : Selenium on Lysosomal protective protein

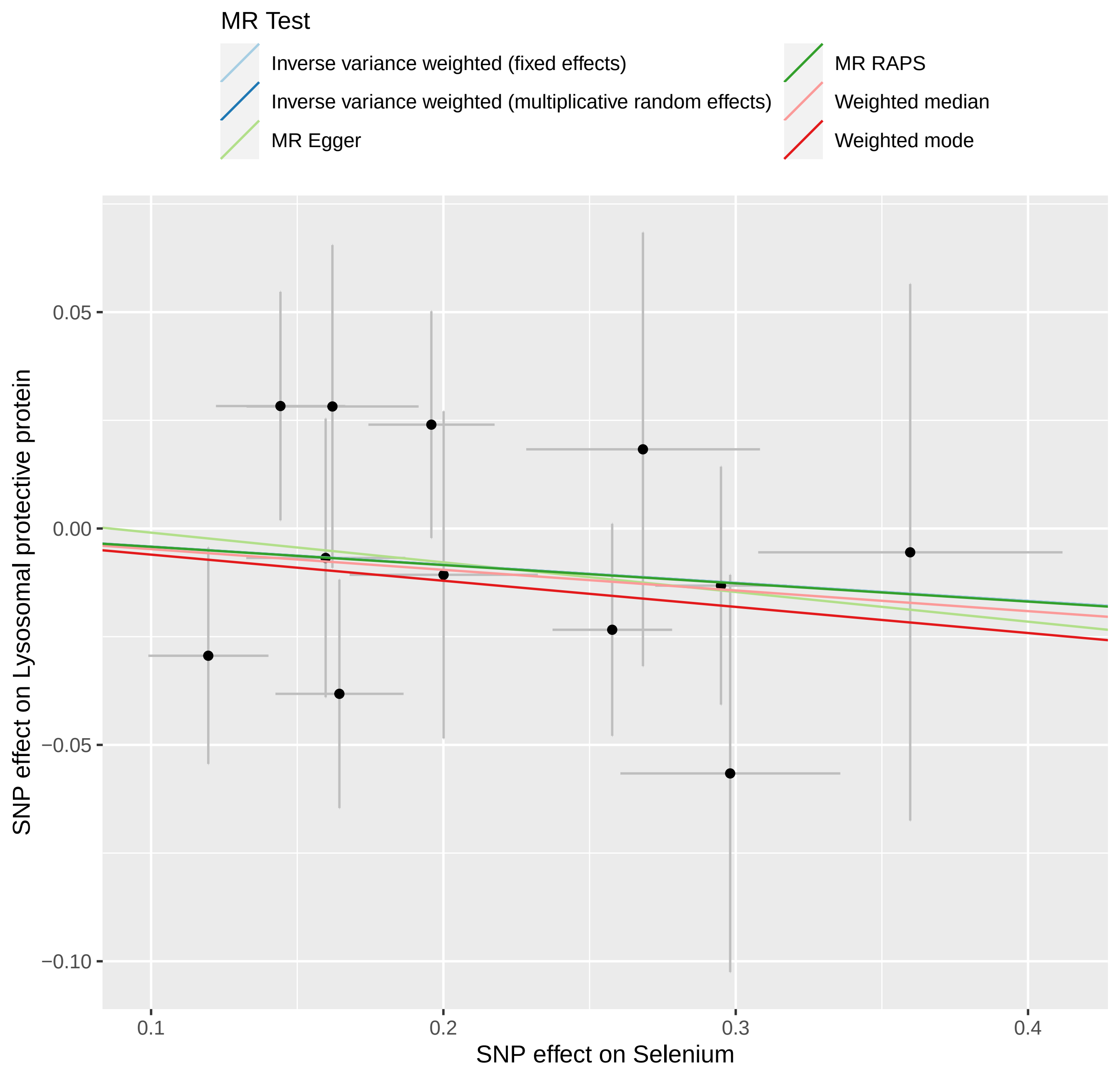

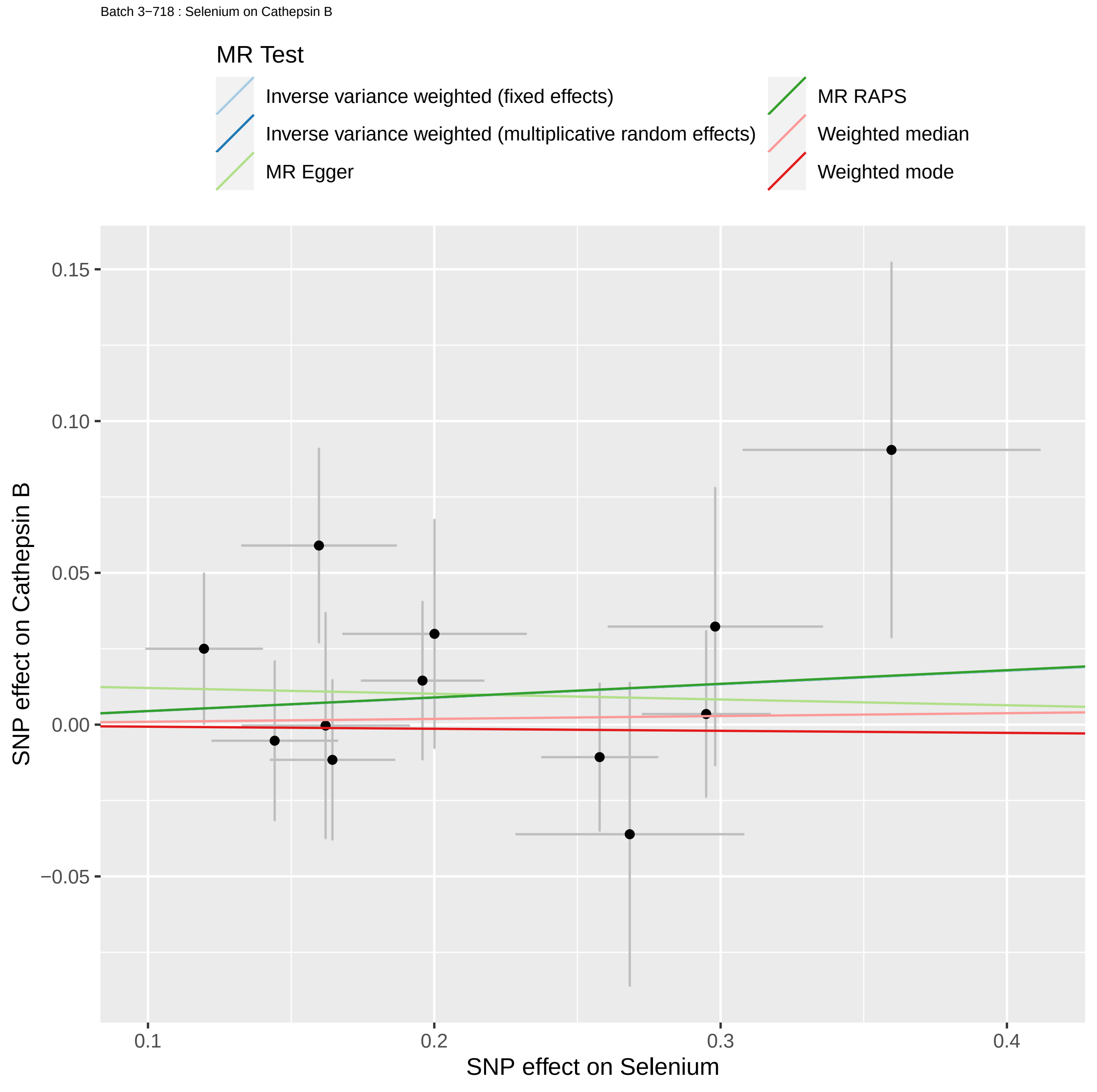
Batch 3−718 : Selenium on Cathepsin B
MR Test
Inverse variance weighted (fixed effects)
Inverse variance weighted (multiplicative random effects)
MR Egger
MR RAPS
Weighted median
Weighted mode
0.15
0.10
0.05
0.00
−0.05
0.1
0.2
0.3
0.4
SNP effect on Cathepsin B
SNP effect on Selenium

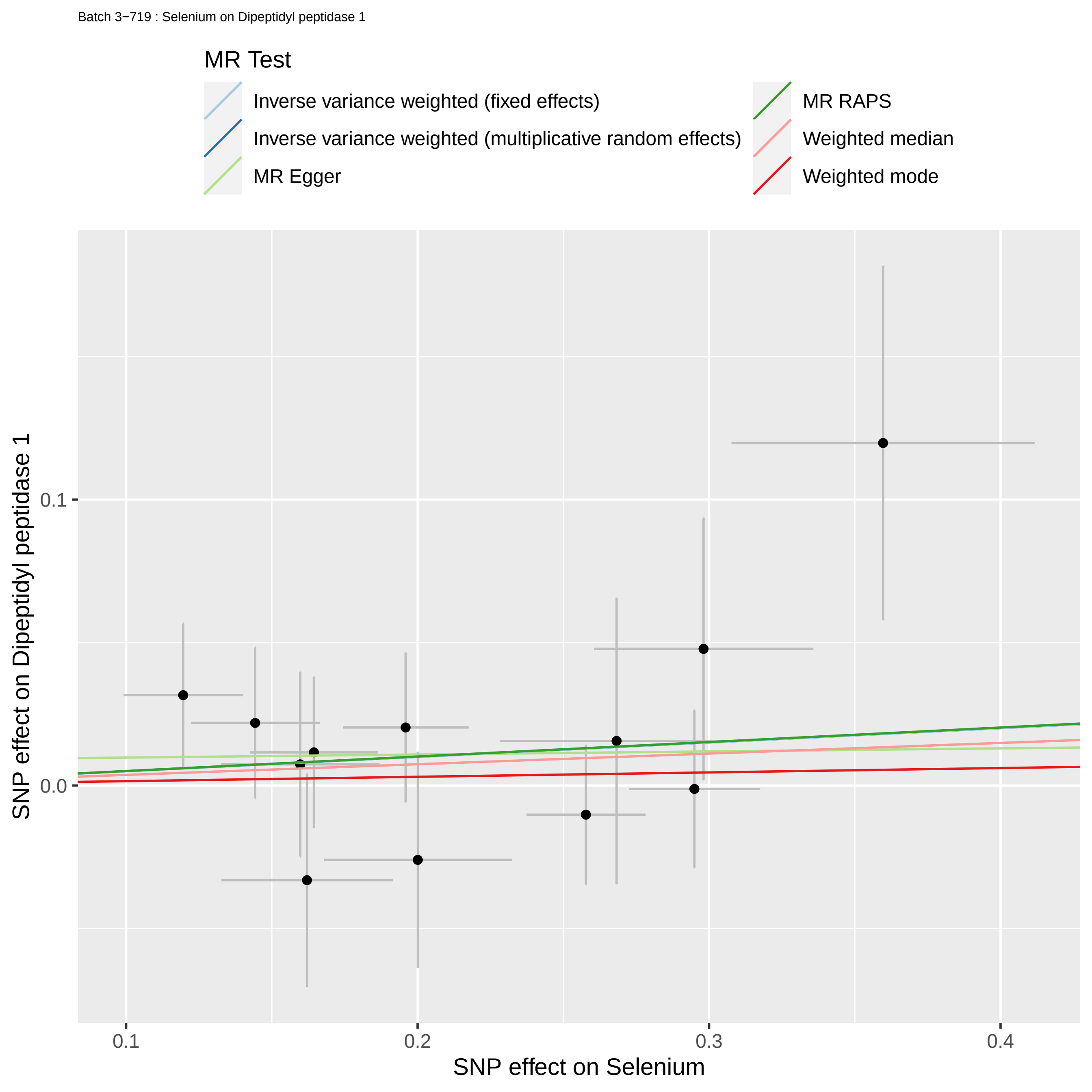

Batch 3−719 : Selenium on Dipeptidyl peptidase 1
MR Test
Inverse variance weighted (fixed effects)
Inverse variance weighted (multiplicative random effects)
MR Egger
MR RAPS
Weighted median
Weighted mode
SNP effect on Dipeptidyl peptidase 1
0.1
0.0
0.1
0.2
0.3
0.4
SNP effect on Selenium

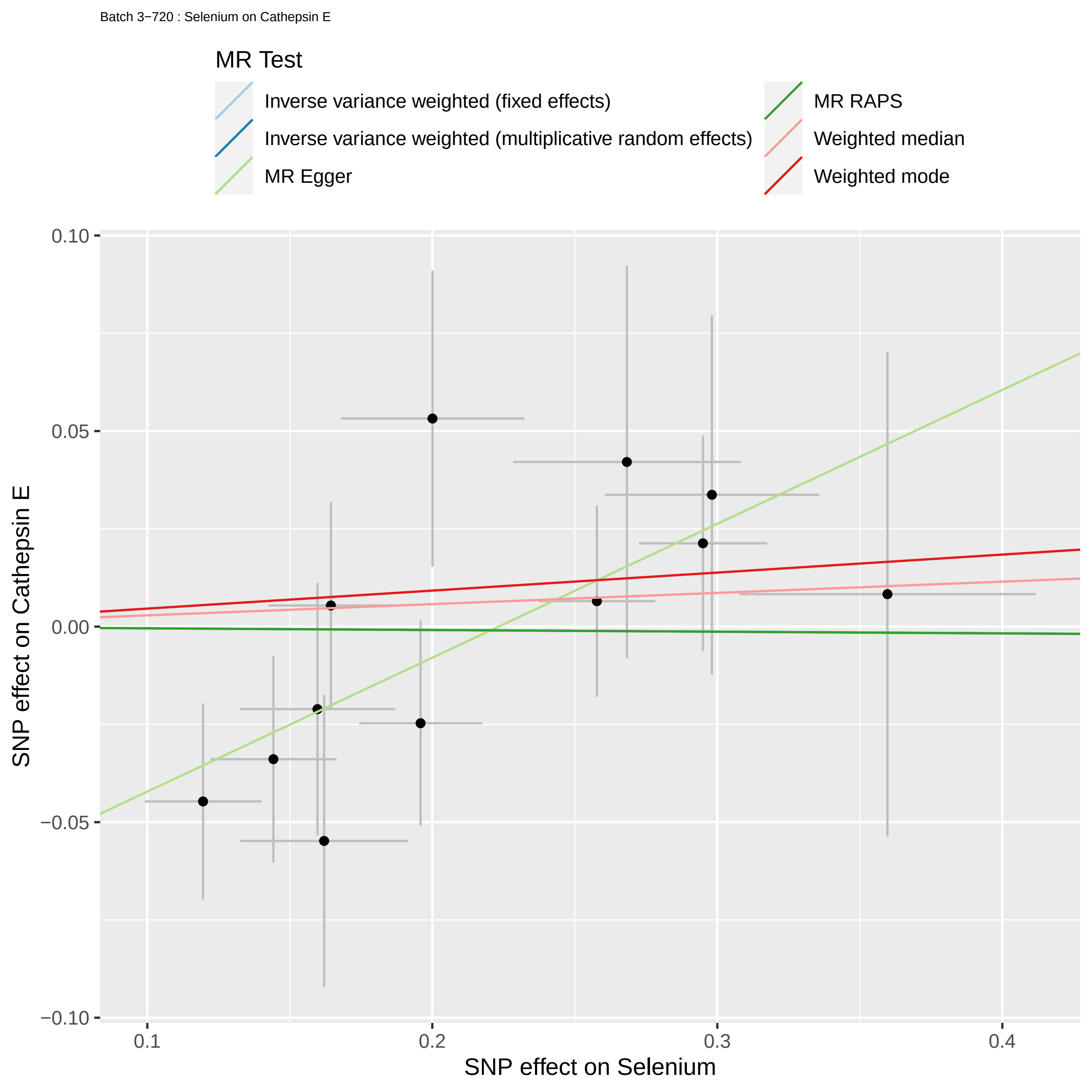
Batch 3−720 : Selenium on Cathepsin E
MR Test
Inverse variance weighted (fixed effects)
Inverse variance weighted (multiplicative random effects)
MR Egger
MR RAPS
Weighted median
Weighted mode
0.10
0.05
0.00
−0.05
−0.10
0.1
0.2
0.3
0.4
SNP effect on Cathepsin E
SNP effect on Selenium

Batch 3−721 : Selenium on Cathepsin F

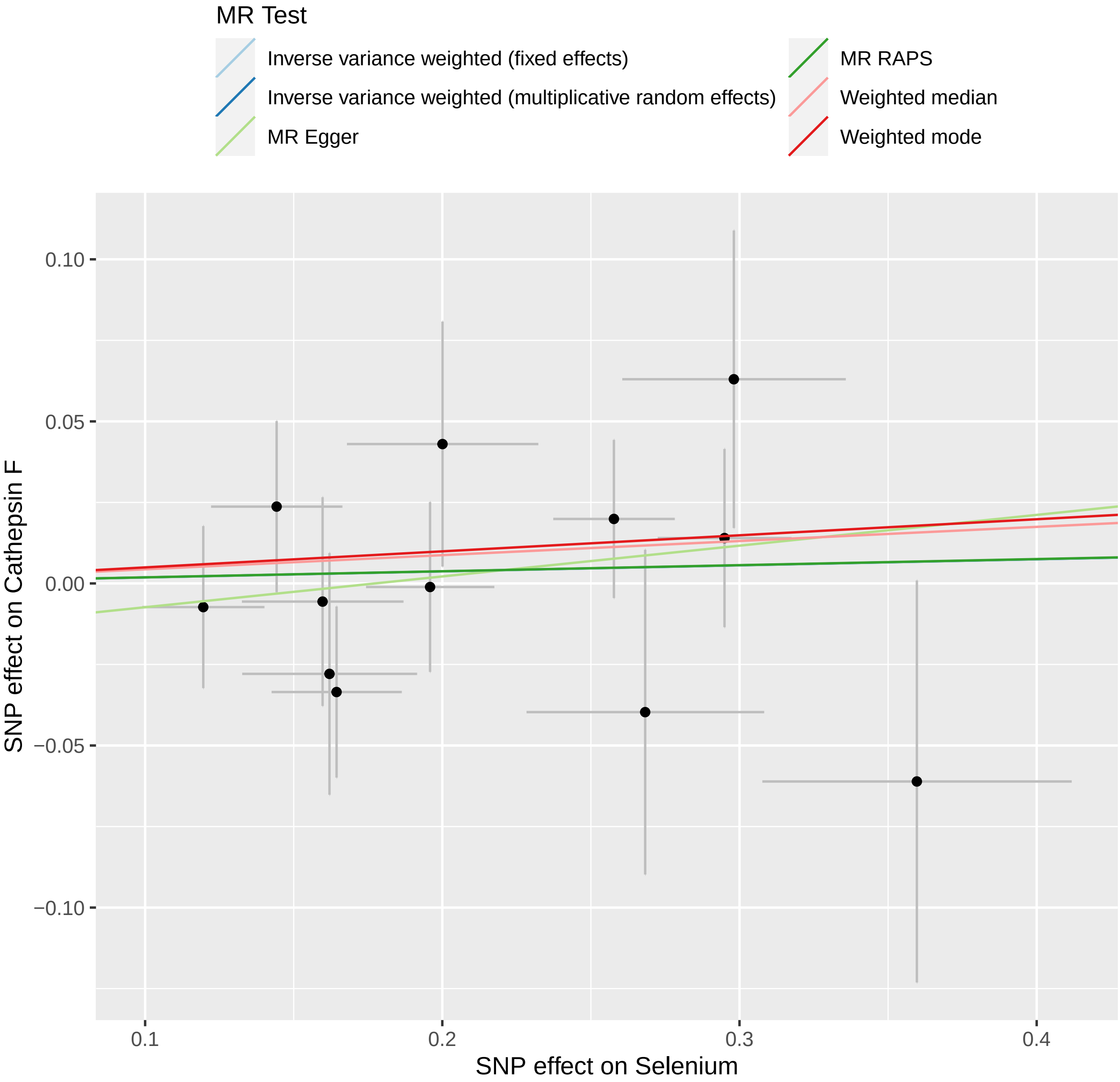

Batch 3−722 : Selenium on Cathepsin F

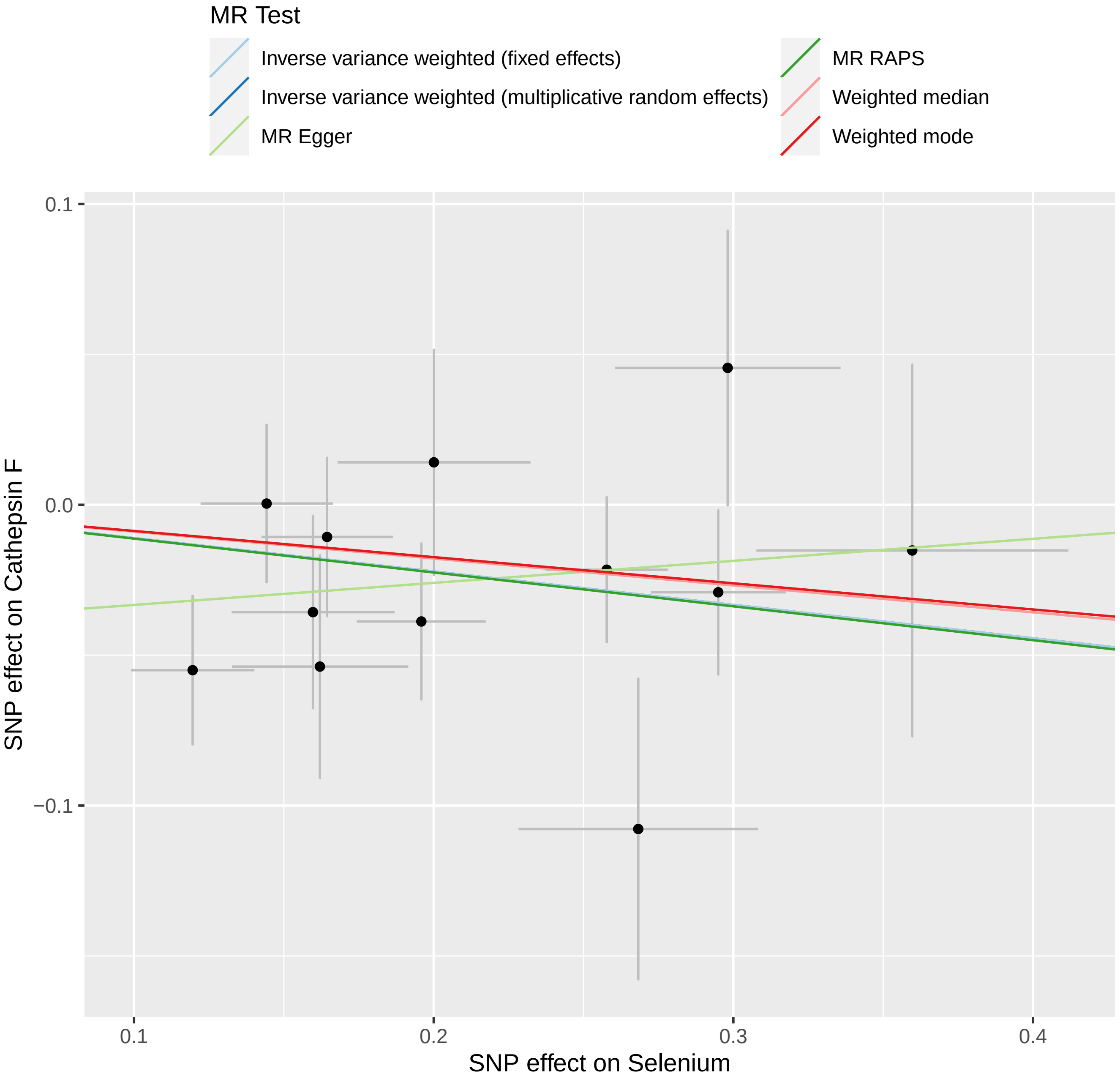

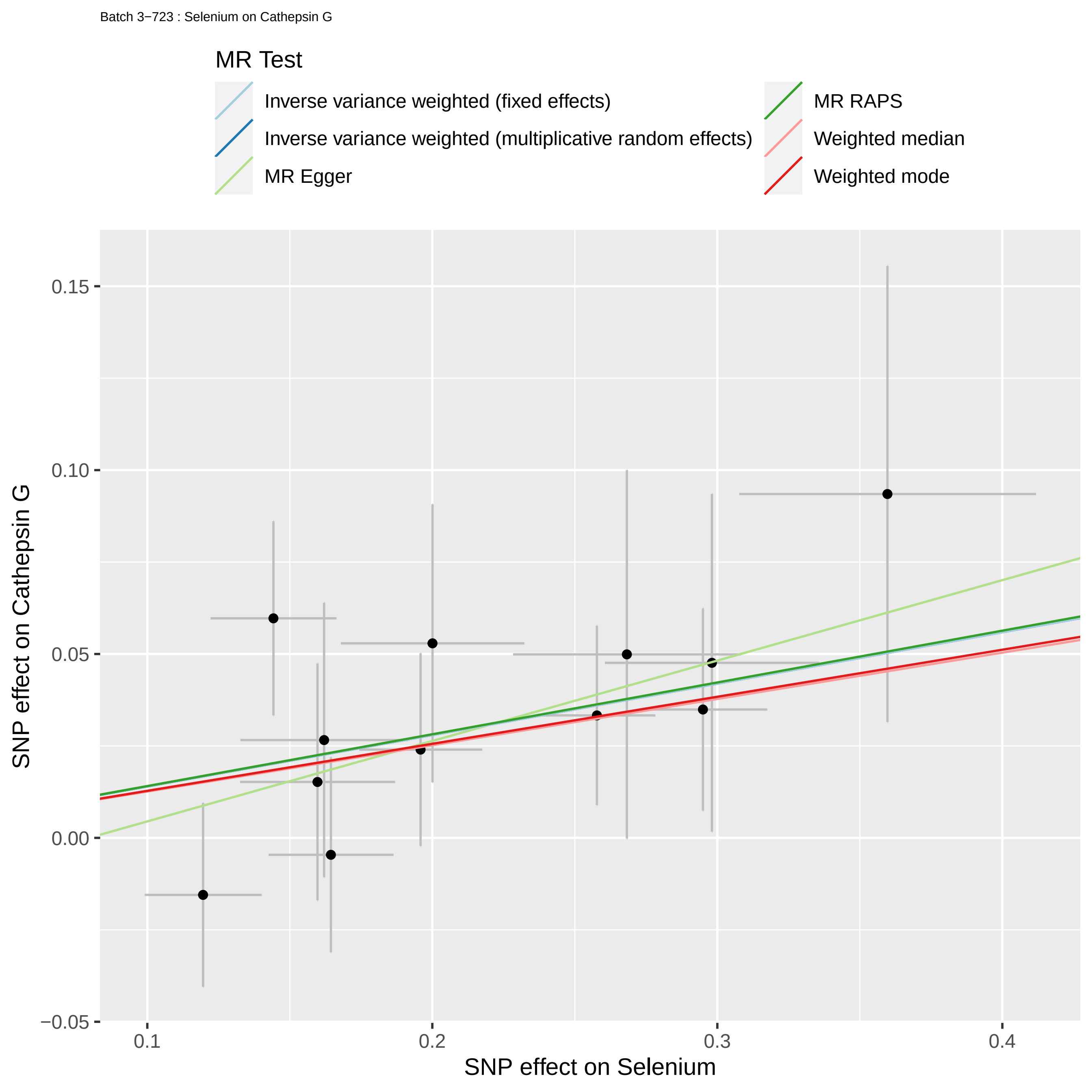

Batch 3−723 : Selenium on Cathepsin G
MR Test
Inverse variance weighted (fixed effects)
Inverse variance weighted (multiplicative random effects)
MR Egger
MR RAPS
Weighted median
Weighted mode
0.15
0.10
0.05
0.00
−0.05
0.1
0.2
0.3
0.4
SNP effect on Cathepsin G
SNP effect on Selenium

Batch 3−724 : Selenium on Cathepsin H

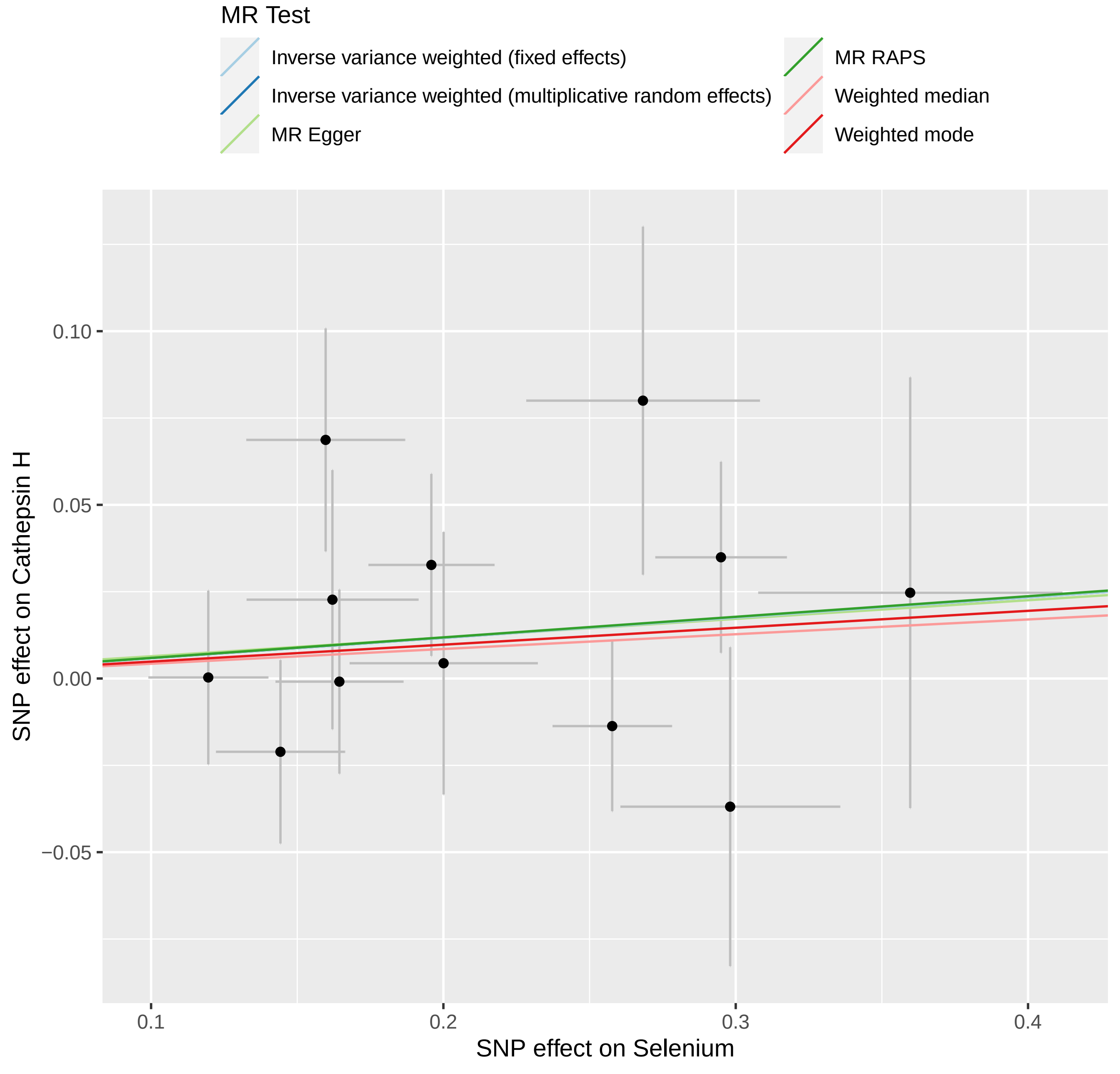

Batch 3−725 : Selenium on Cathepsin H

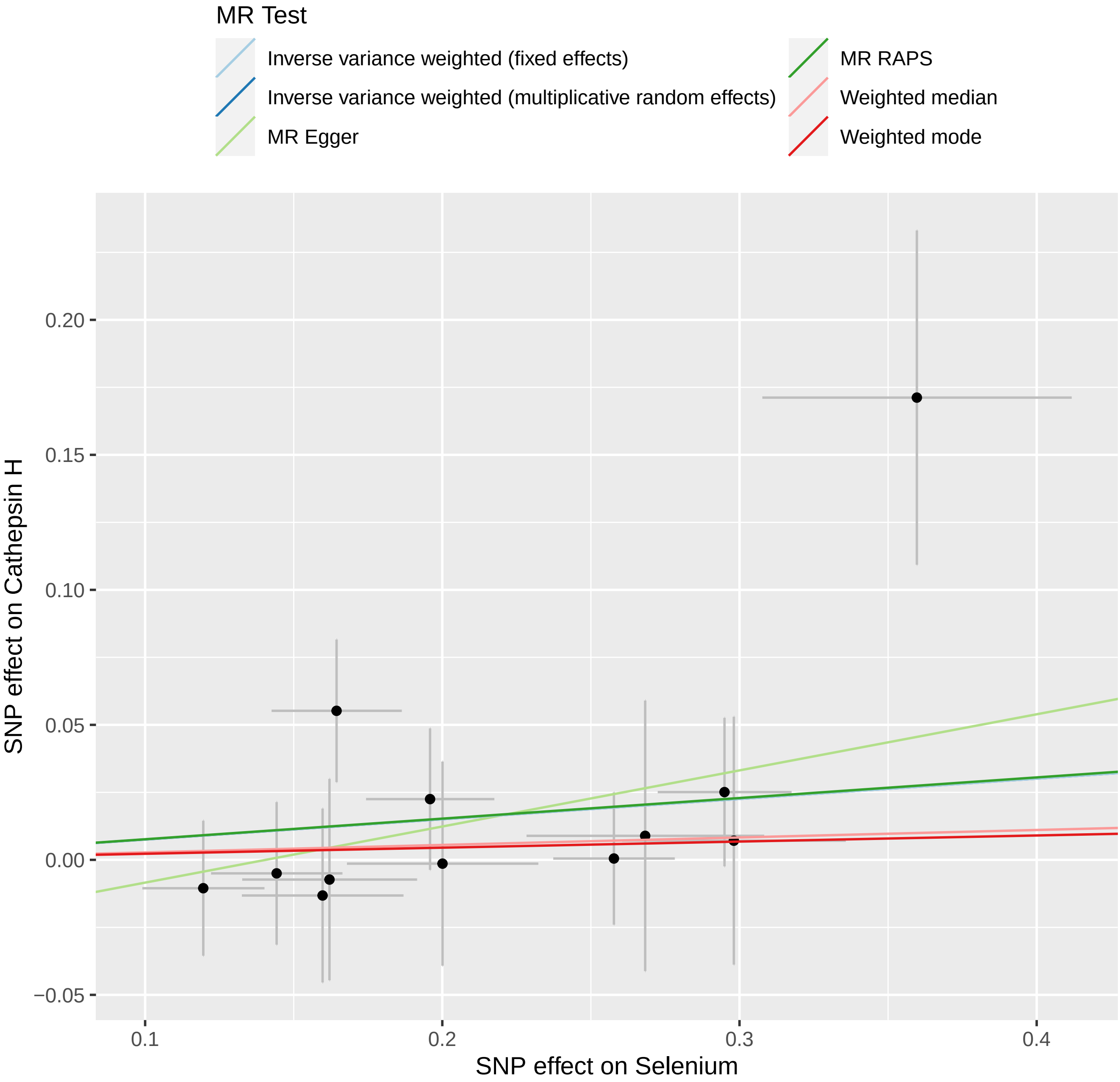

Batch 3−726 : Selenium on Cathepsin O

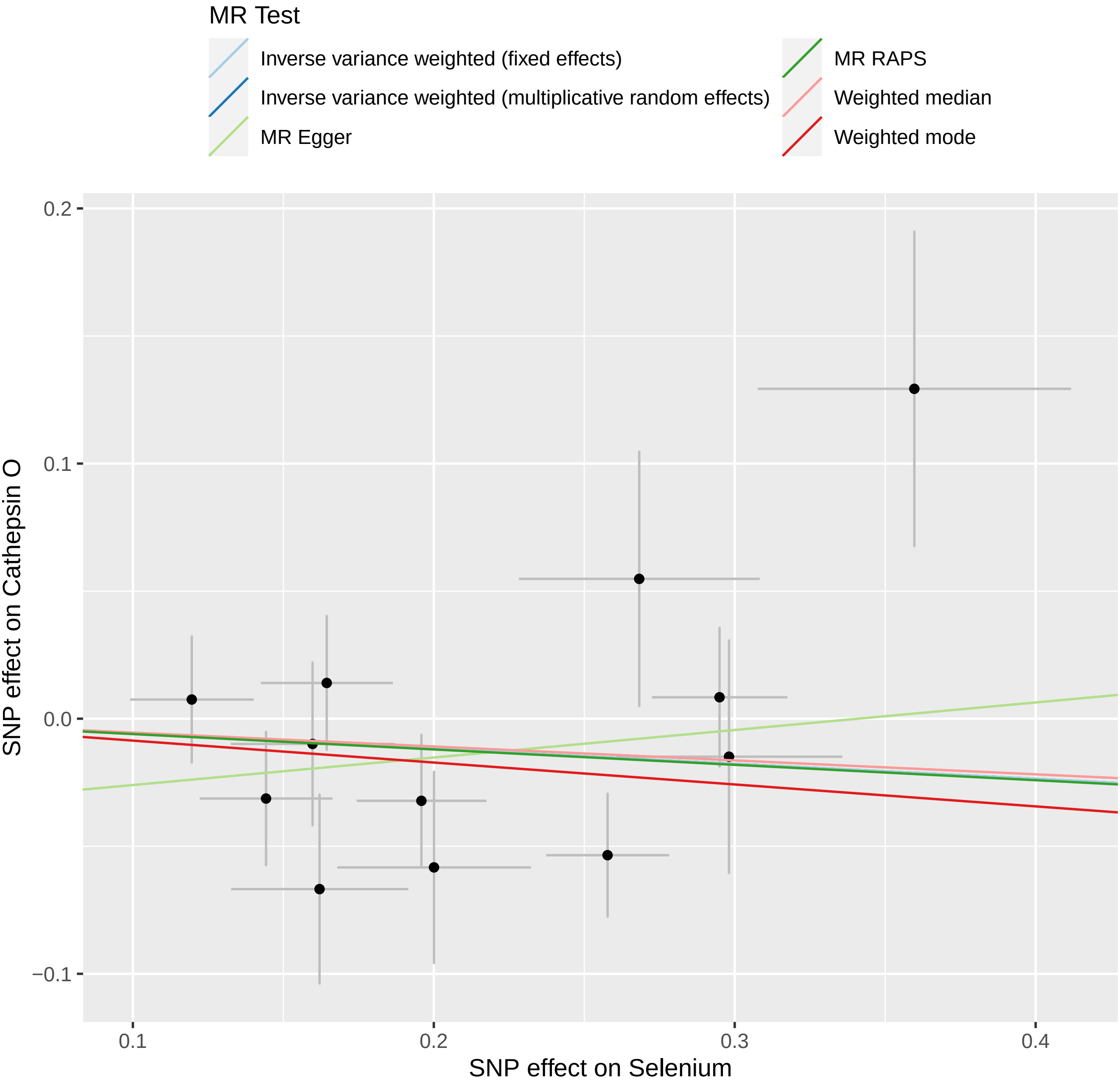

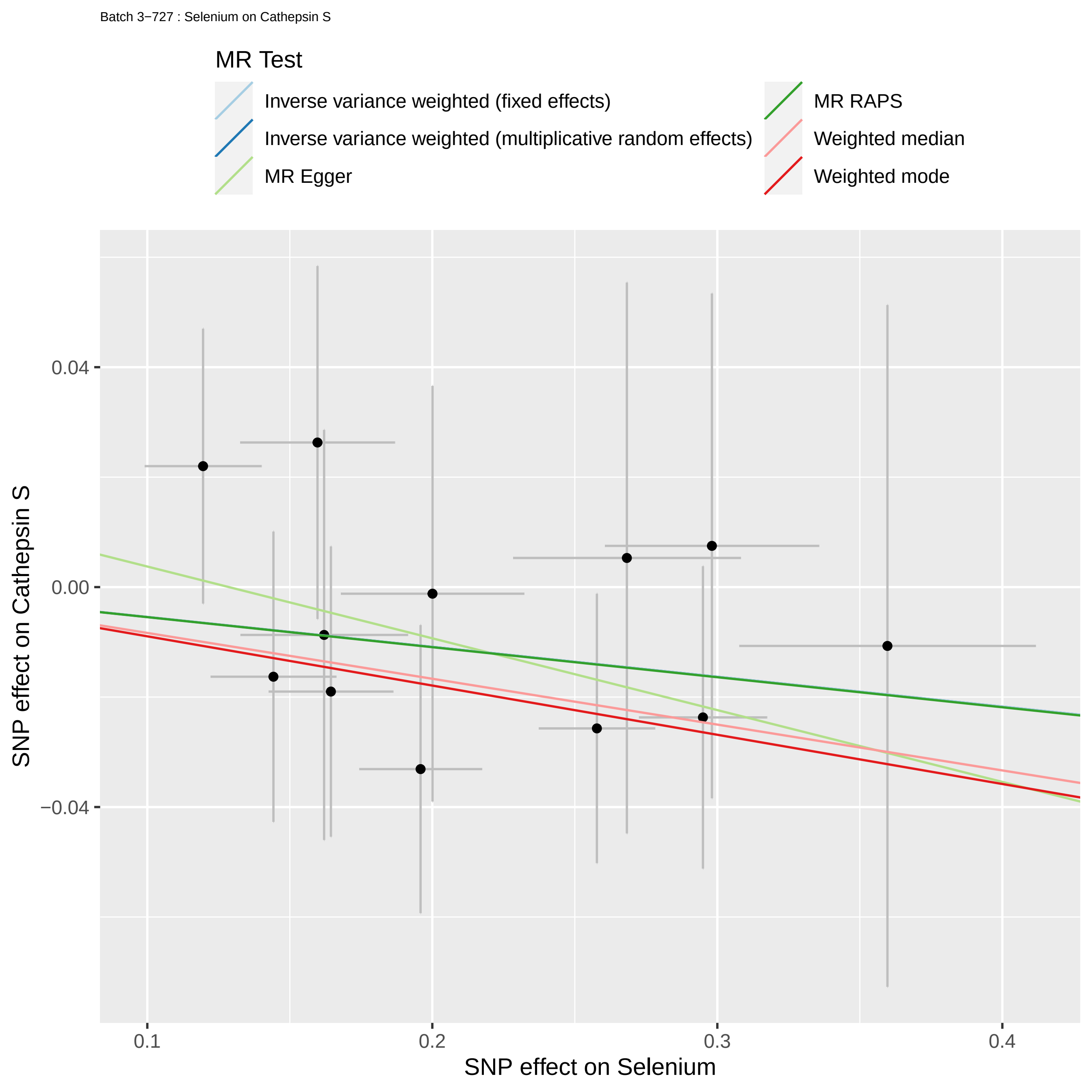
Batch 3−727 : Selenium on Cathepsin S
MR Test
Inverse variance weighted (fixed effects)
Inverse variance weighted (multiplicative random effects)
MR Egger
MR RAPS
Weighted median
Weighted mode
0.04
0.00
−0.04
0.1
0.2
0.3
0.4
SNP effect on Cathepsin S
SNP effect on Selenium

Batch 3−728 : Selenium on Cathepsin L2

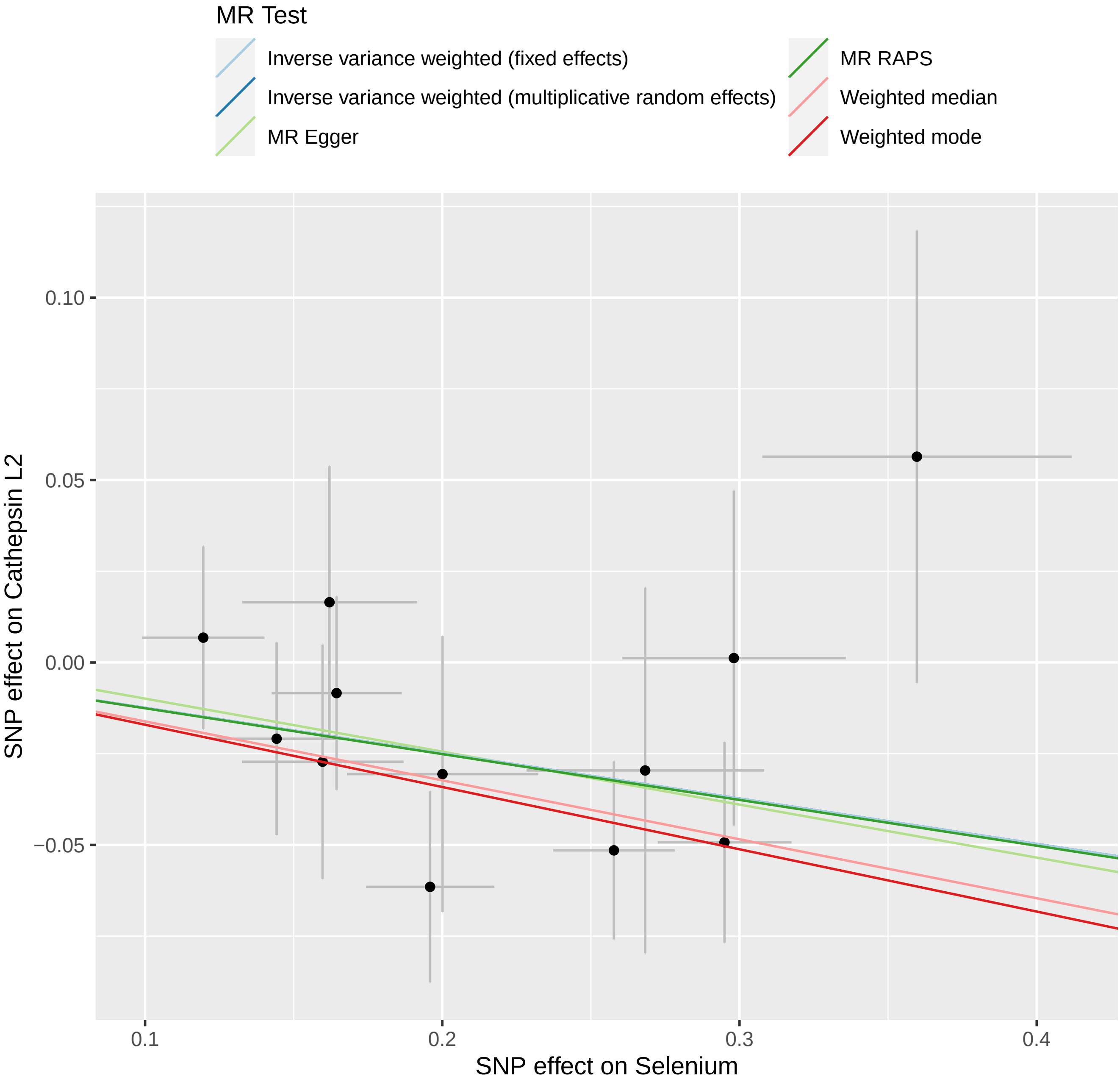

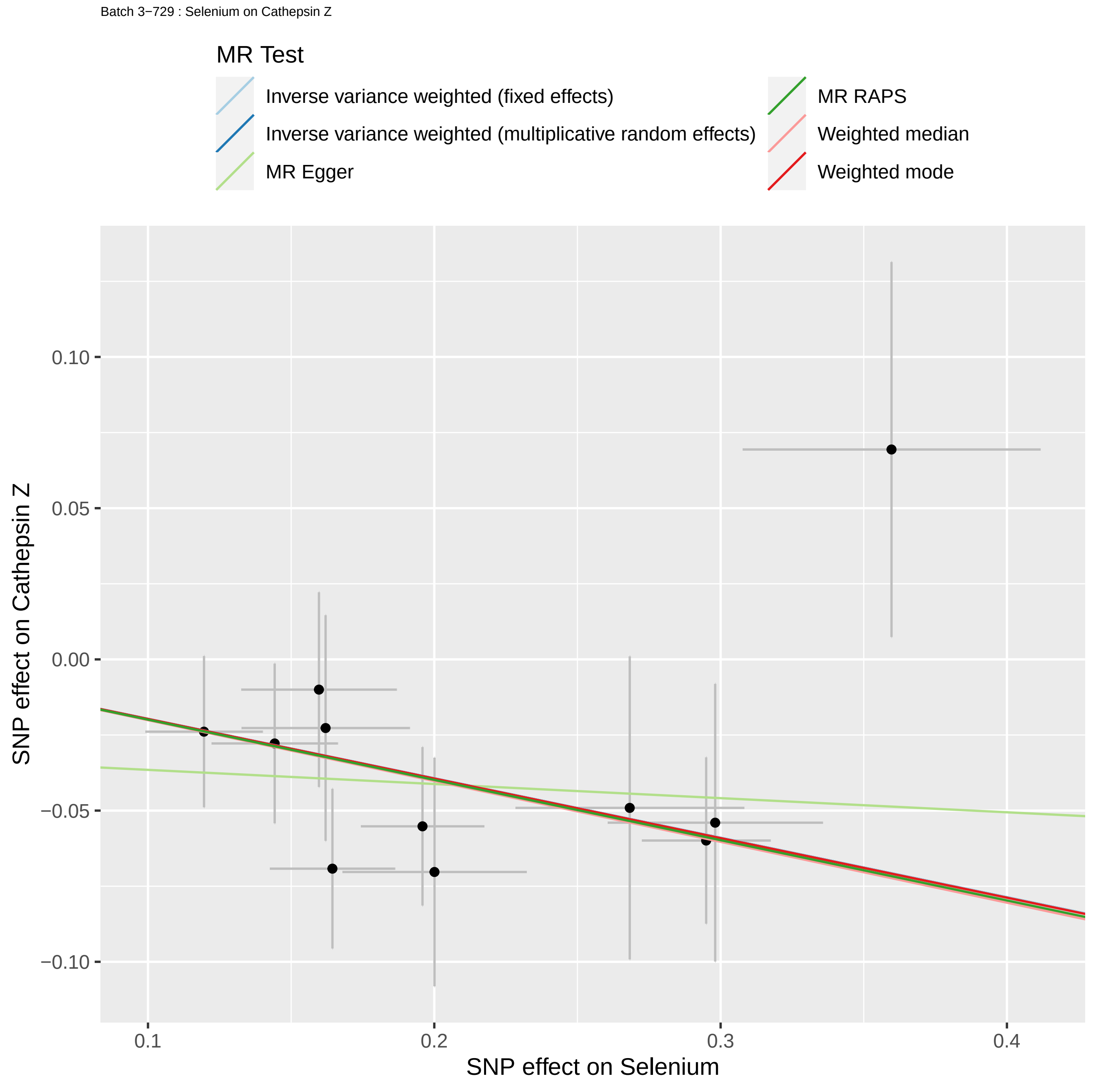
Batch 3−729 : Selenium on Cathepsin Z
MR Test
Inverse variance weighted (fixed effects)
Inverse variance weighted (multiplicative random effects)
MR Egger
MR RAPS
Weighted median
Weighted mode
0.10
0.05
0.00
−0.05
−0.10
0.1
0.2
0.3
0.4
SNP effect on Cathepsin Z
SNP effect on Selenium

Batch 3−730 : Selenium on Cortexin−3

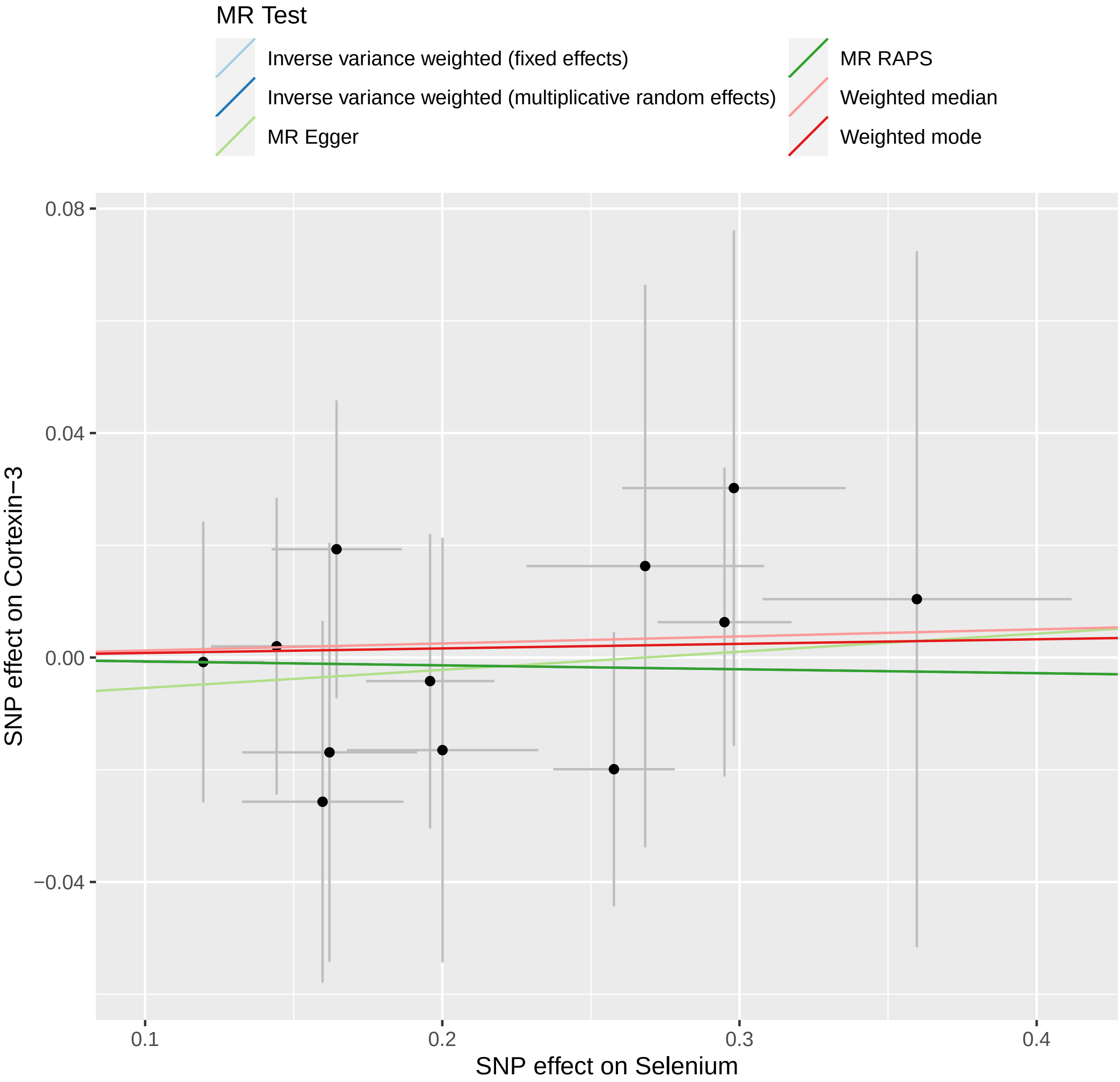

Batch 3−731 : Selenium on cubilin

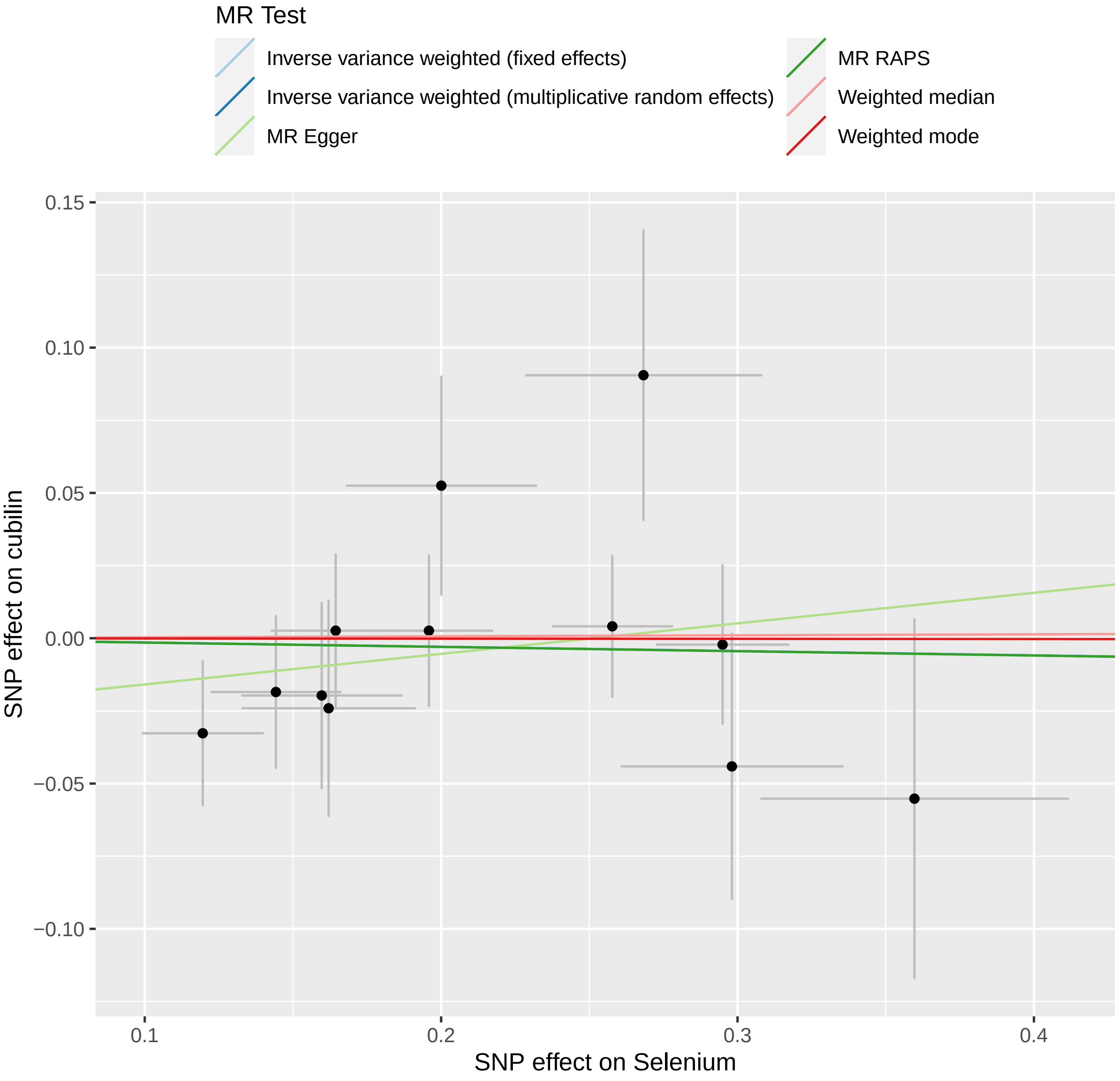

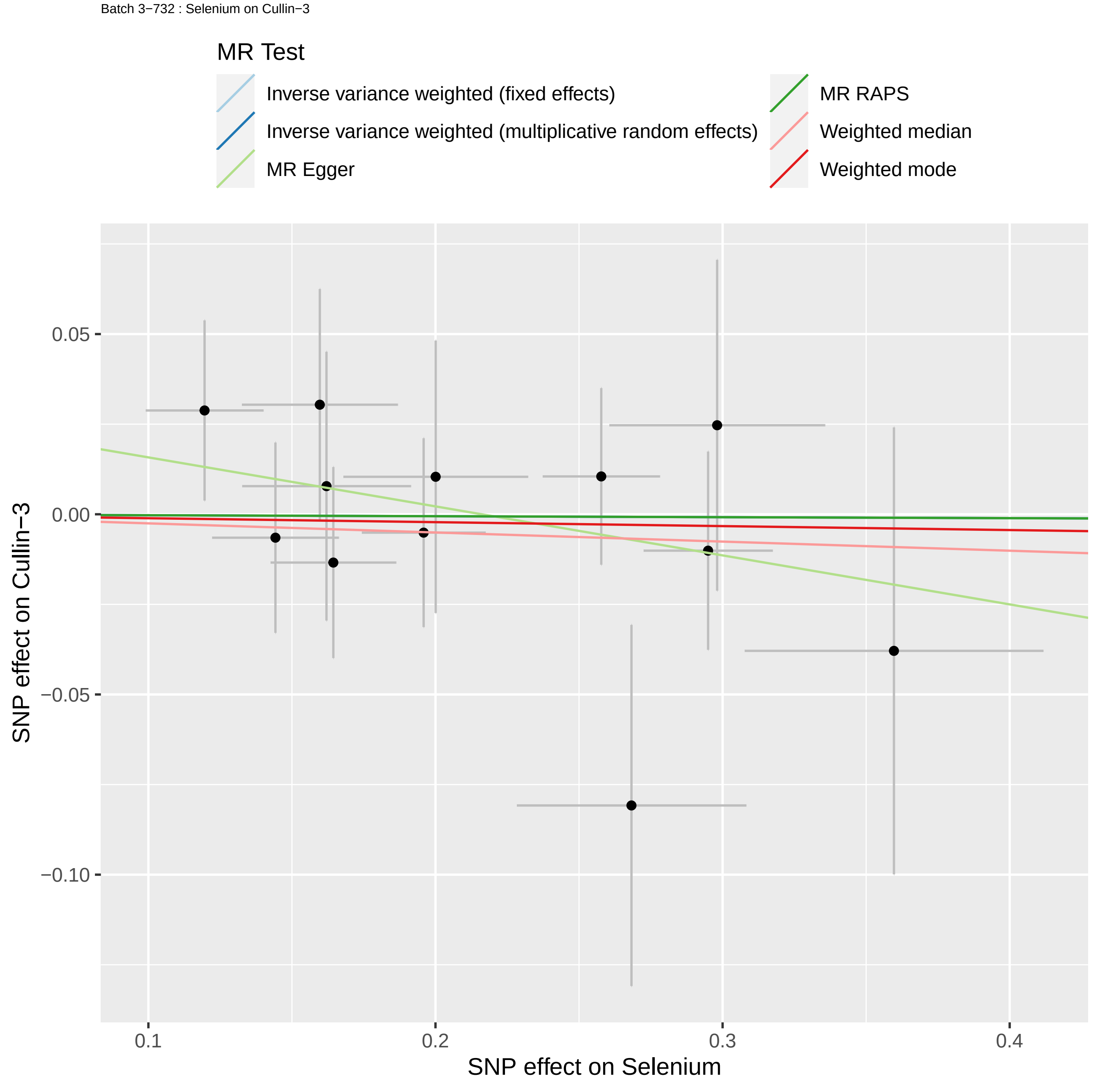

Batch 3−732 : Selenium on Cullin−3
MR Test
Inverse variance weighted (fixed effects)
Inverse variance weighted (multiplicative random effects)
MR Egger
MR RAPS
Weighted median
Weighted mode
0.05
0.00
−0.05
−0.10
SNP effect on Cullin−3
0.1
0.2
0.3
0.4
SNP effect on Selenium

Batch 3−733 : Selenium on Cullin−4B

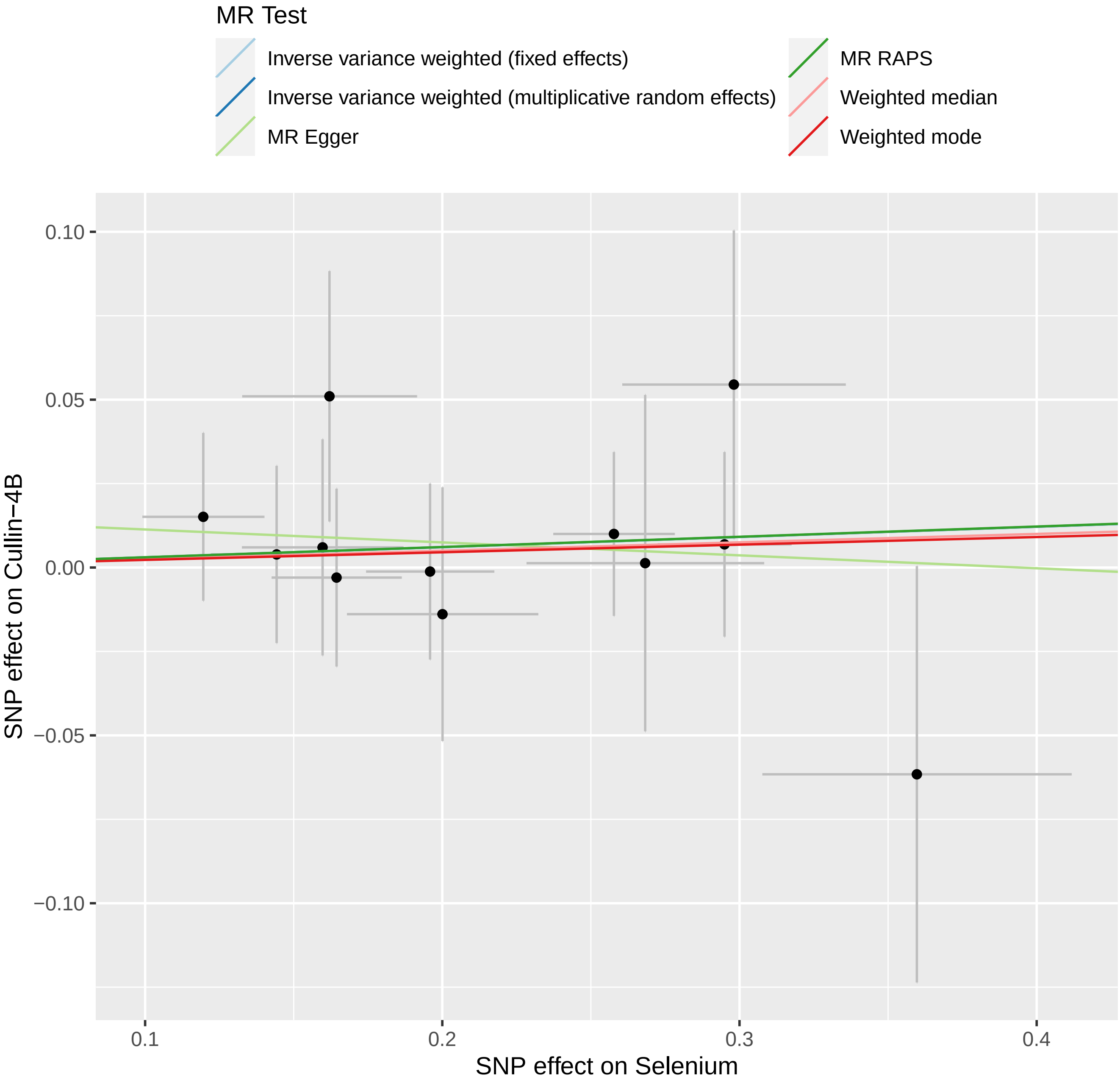

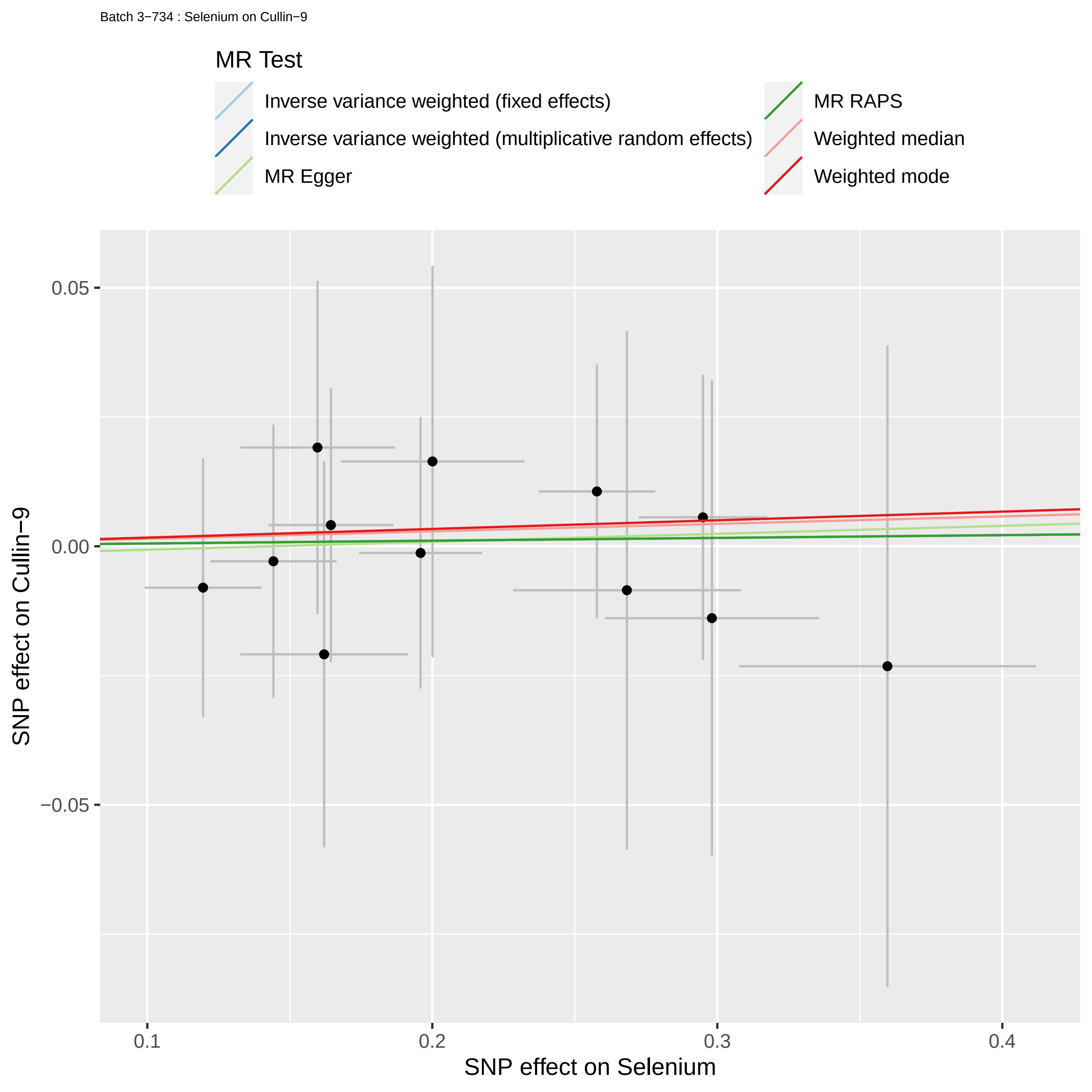
Batch 3−734 : Selenium on Cullin−9
MR Test
Inverse variance weighted (fixed effects)
Inverse variance weighted (multiplicative random effects)
MR Egger
MR RAPS
Weighted median
Weighted mode
0.05
0.00
−0.05
0.1
0.2
0.3
0.4
SNP effect on Cullin−9
SNP effect on Selenium

Batch 3−735 : Selenium on CUB and zona pellucida−like domain−containing protein 1

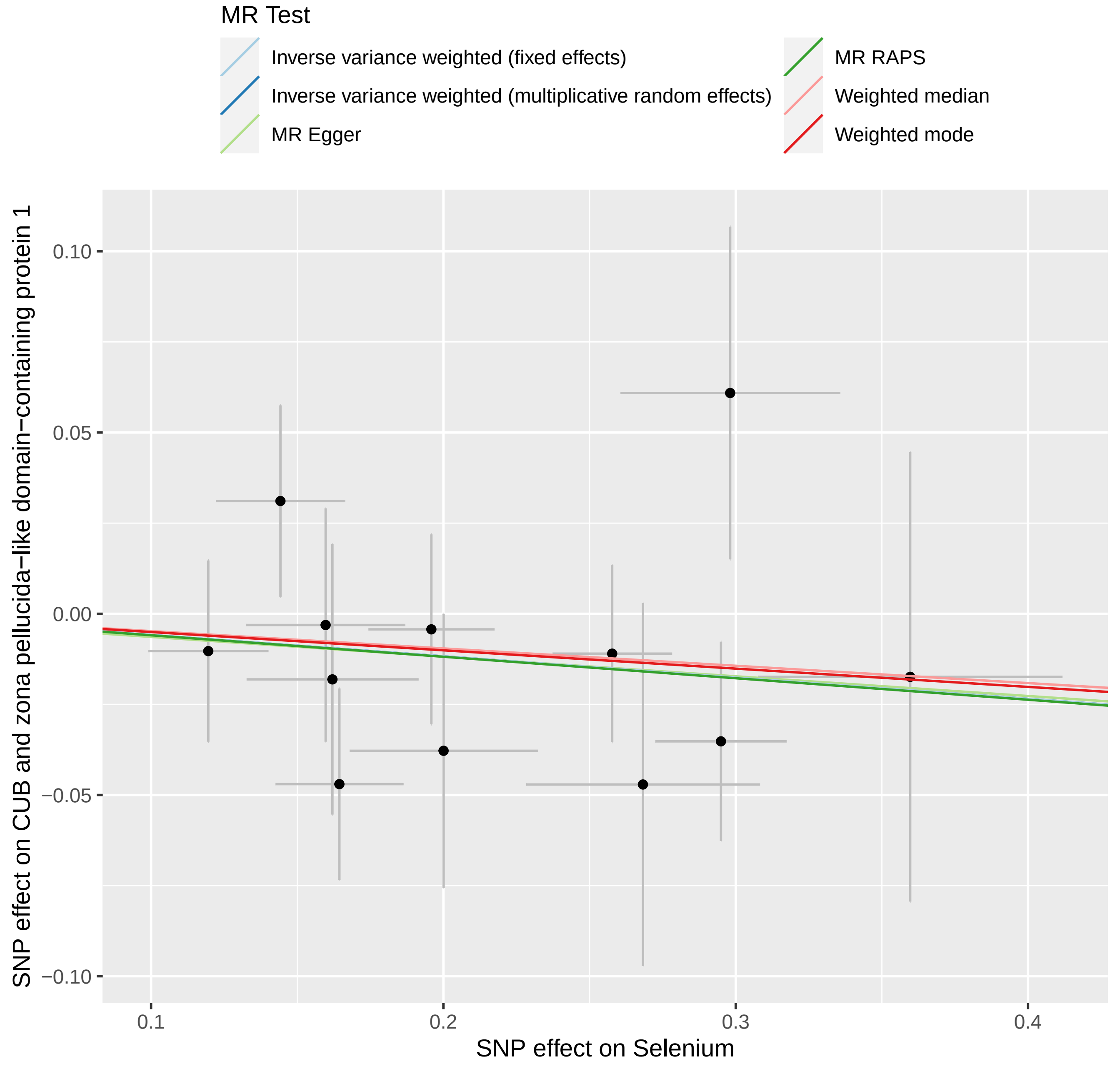

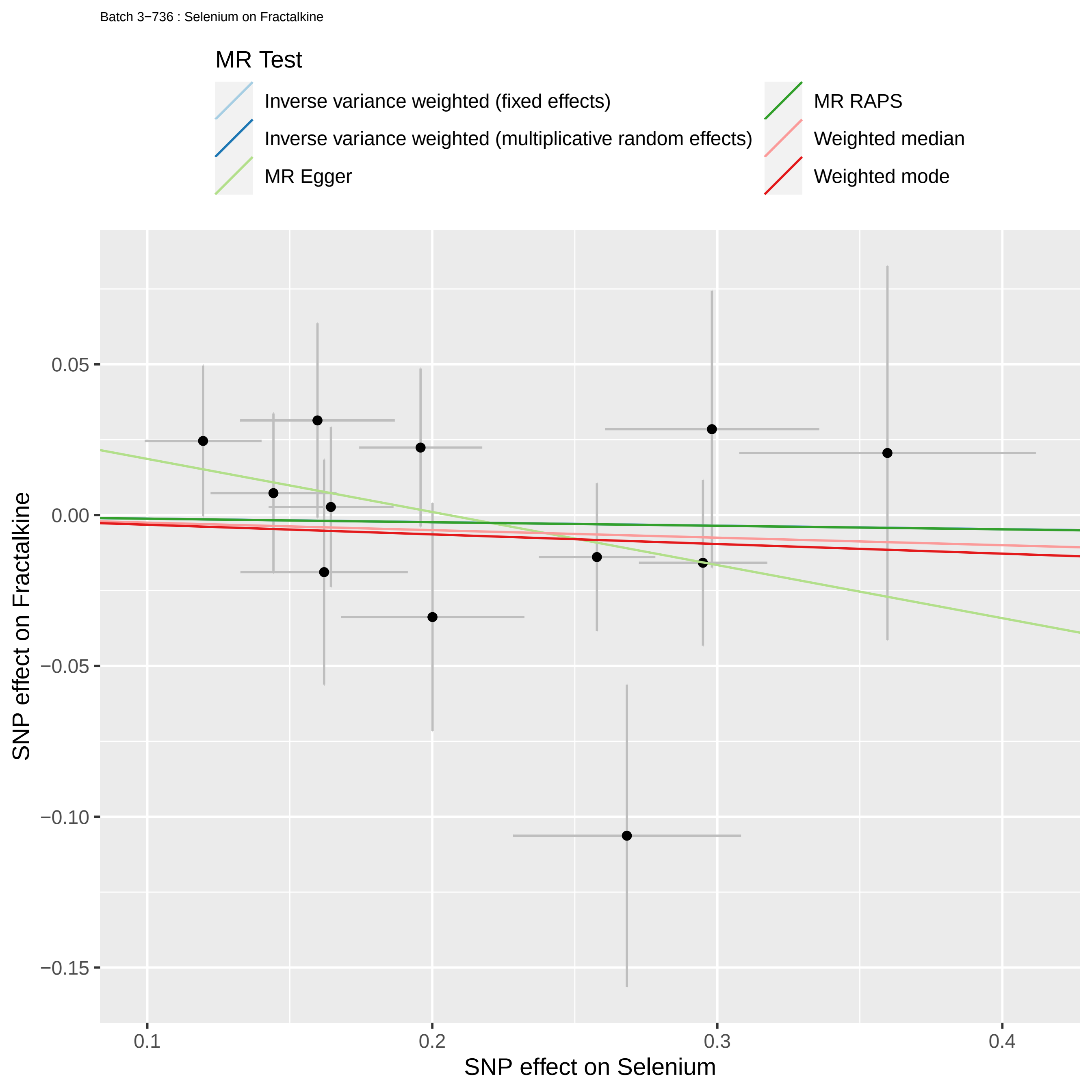
Batch 3−736 : Selenium on Fractalkine
MR Test
Inverse variance weighted (fixed effects)
Inverse variance weighted (multiplicative random effects)
MR Egger
MR RAPS
Weighted median
Weighted mode
0.05
0.00
−0.05
−0.10
−0.15
0.1
0.2
0.3
0.4
SNP effect on Fractalkine
SNP effect on Selenium

Batch 3−737 : Selenium on Coxsackievirus and adenovirus receptor

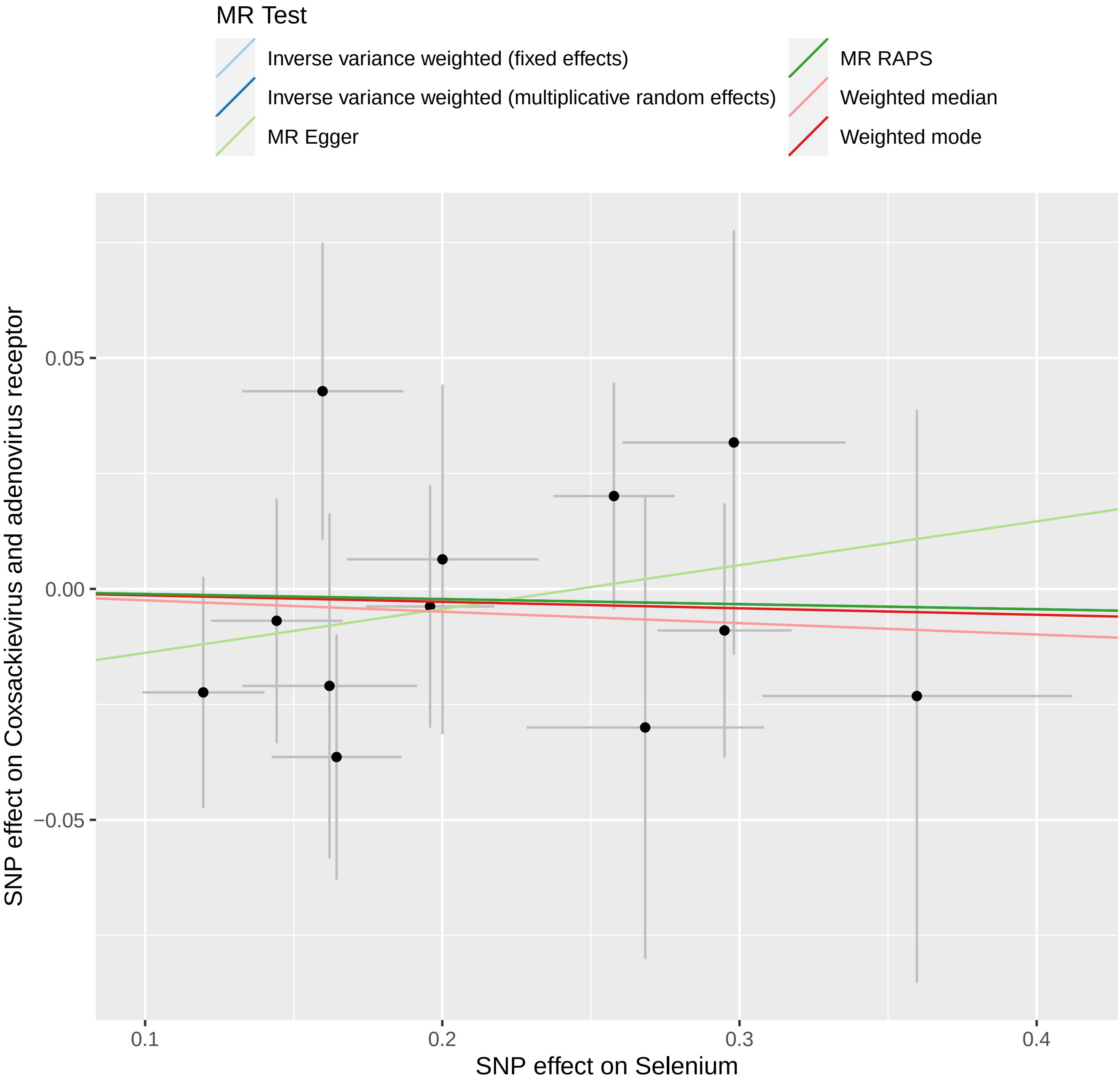

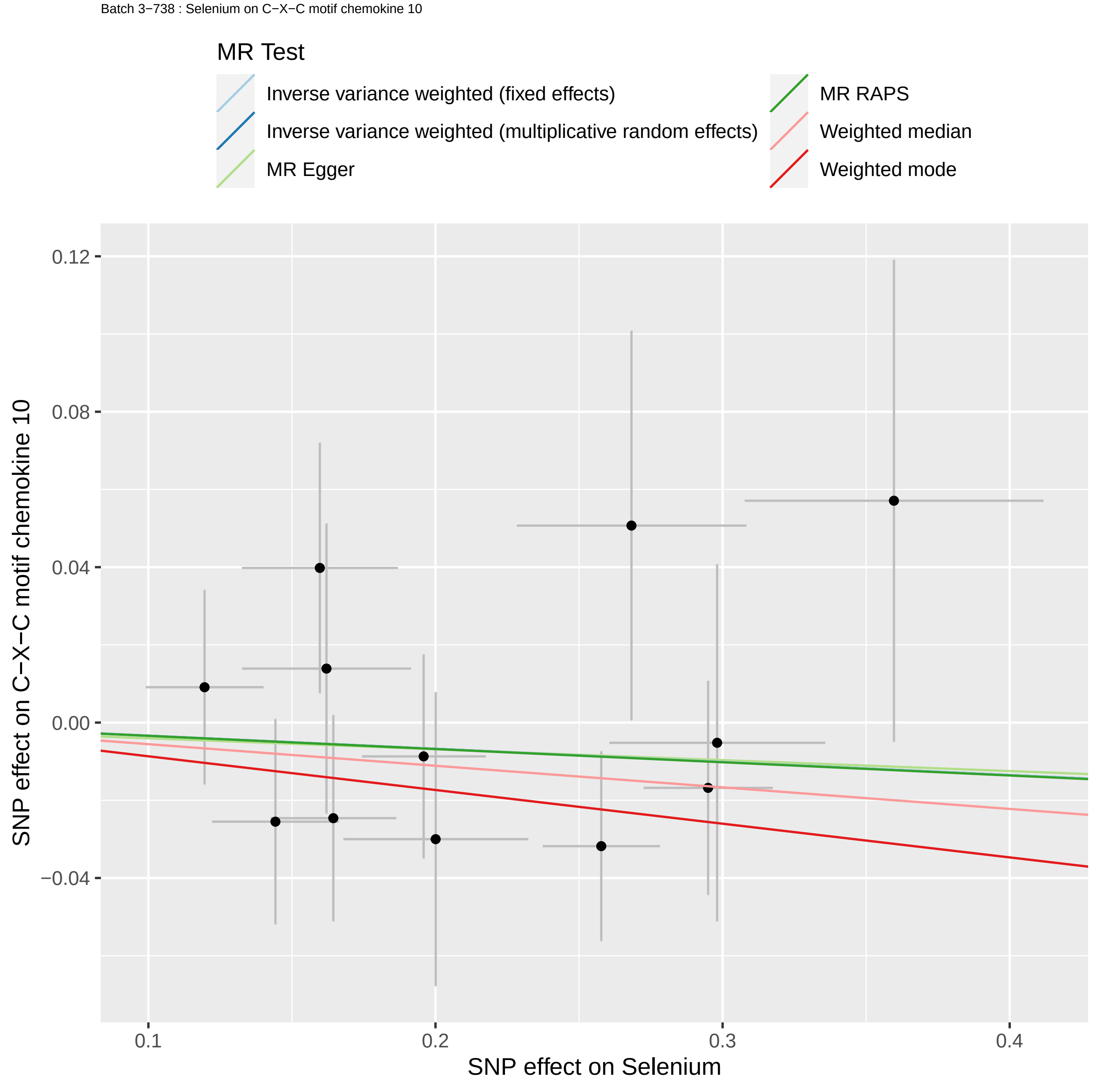
Batch 3−738 : Selenium on C−X−C motif chemokine 10
MR Test
Inverse variance weighted (fixed effects)
Inverse variance weighted (multiplicative random effects)
MR Egger
MR RAPS
Weighted median
Weighted mode
0.12
0.08
0.04
0.00
−0.04
0.1
0.2
0.3
0.4
SNP effect on C−X−C motif chemokine 10
SNP effect on Selenium

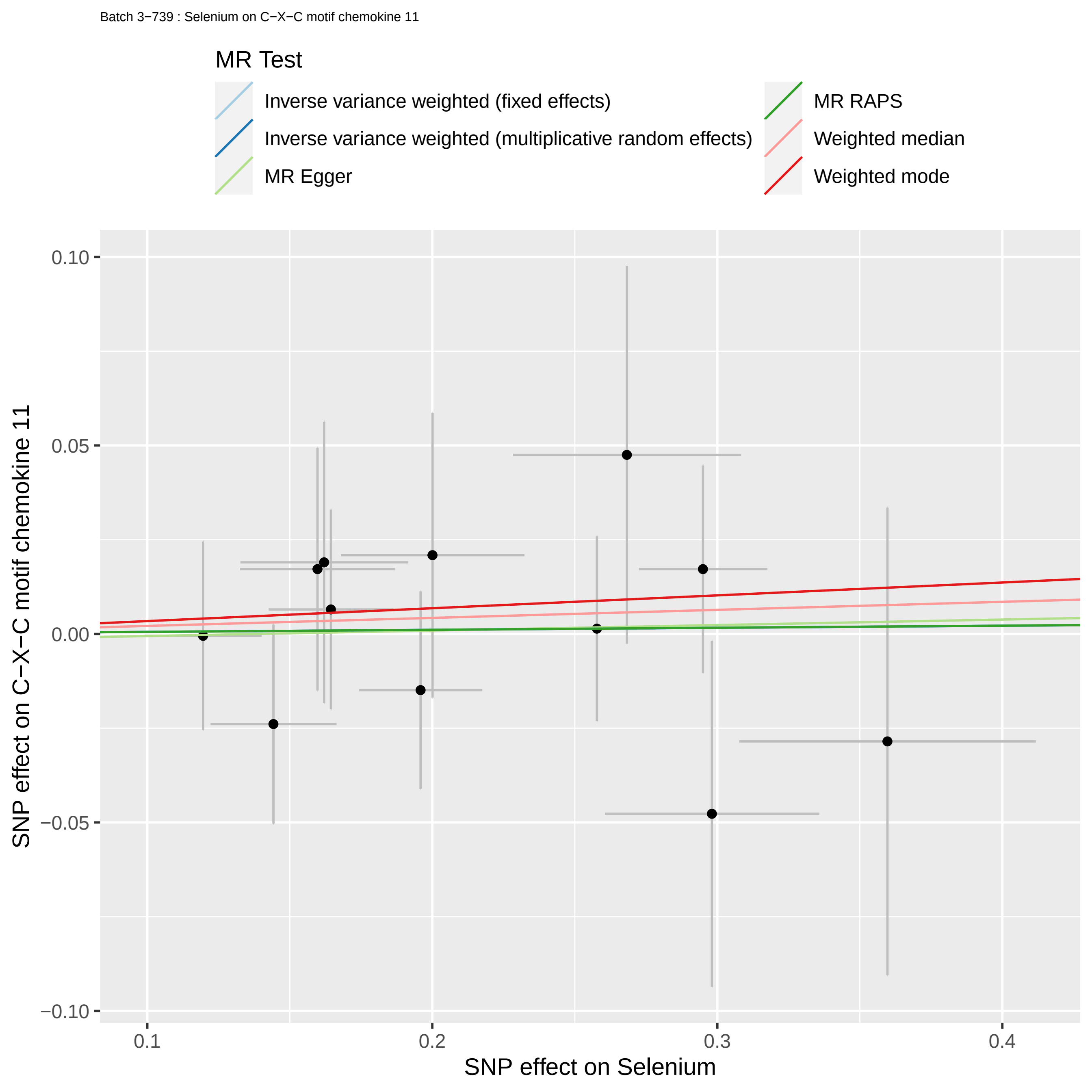

Batch 3−739 : Selenium on C−X−C motif chemokine 11
MR Test
Inverse variance weighted (fixed effects)
Inverse variance weighted (multiplicative random effects)
MR Egger
MR RAPS
Weighted median
Weighted mode
SNP effect on C−X−C motif chemokine 11
0.10
0.05
0.00
−0.05
−0.10
0.1
0.2
0.3
0.4
SNP effect on Selenium

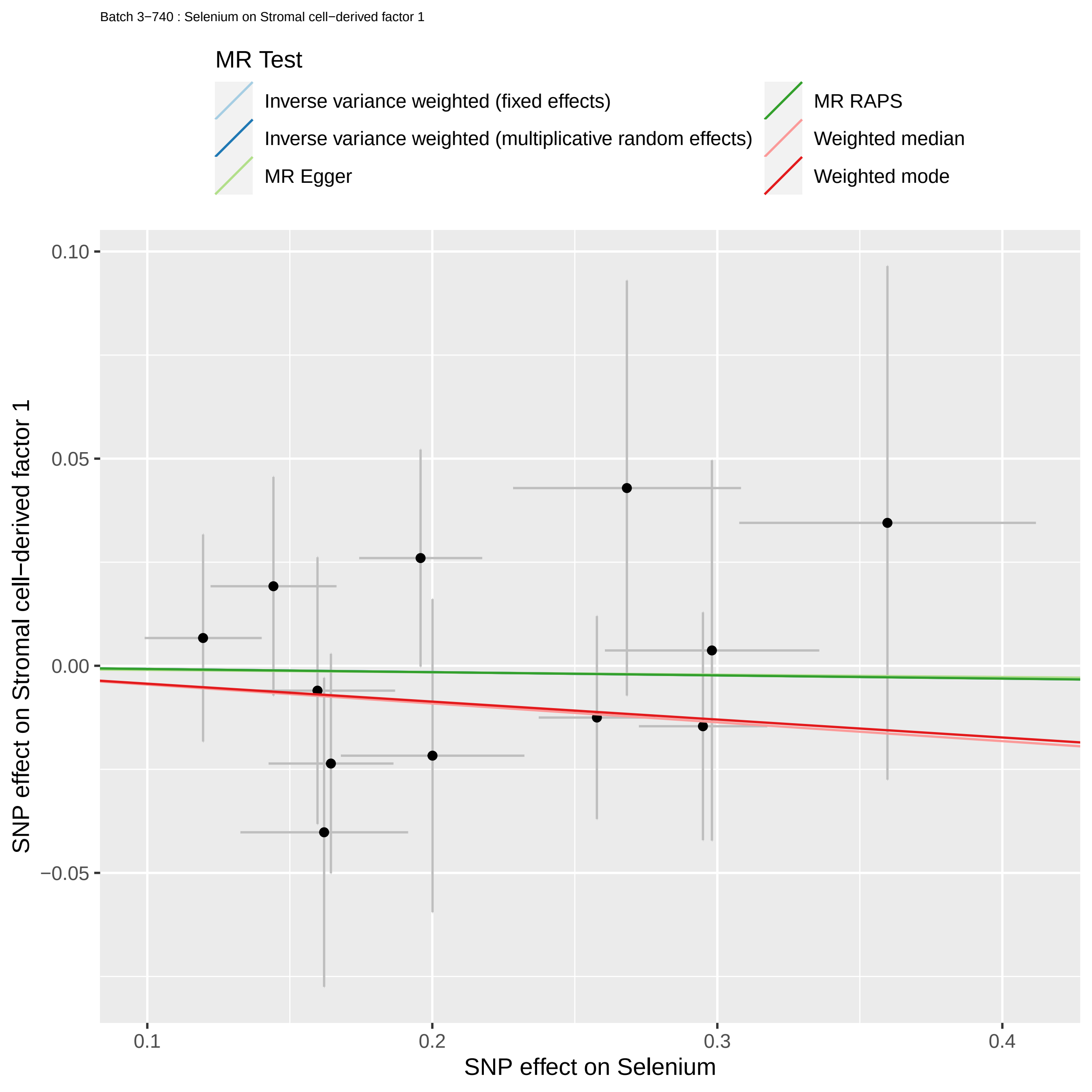
Batch 3−740 : Selenium on Stromal cell−derived factor 1
MR Test
Inverse variance weighted (fixed effects)
Inverse variance weighted (multiplicative random effects)
MR Egger
MR RAPS
Weighted median
Weighted mode
0.10
0.05
0.00
−0.05
0.1
0.2
0.3
0.4
SNP effect on Stromal cell−derived factor 1
SNP effect on Selenium

Batch 3−741 : Selenium on Stromal cell−derived factor 1

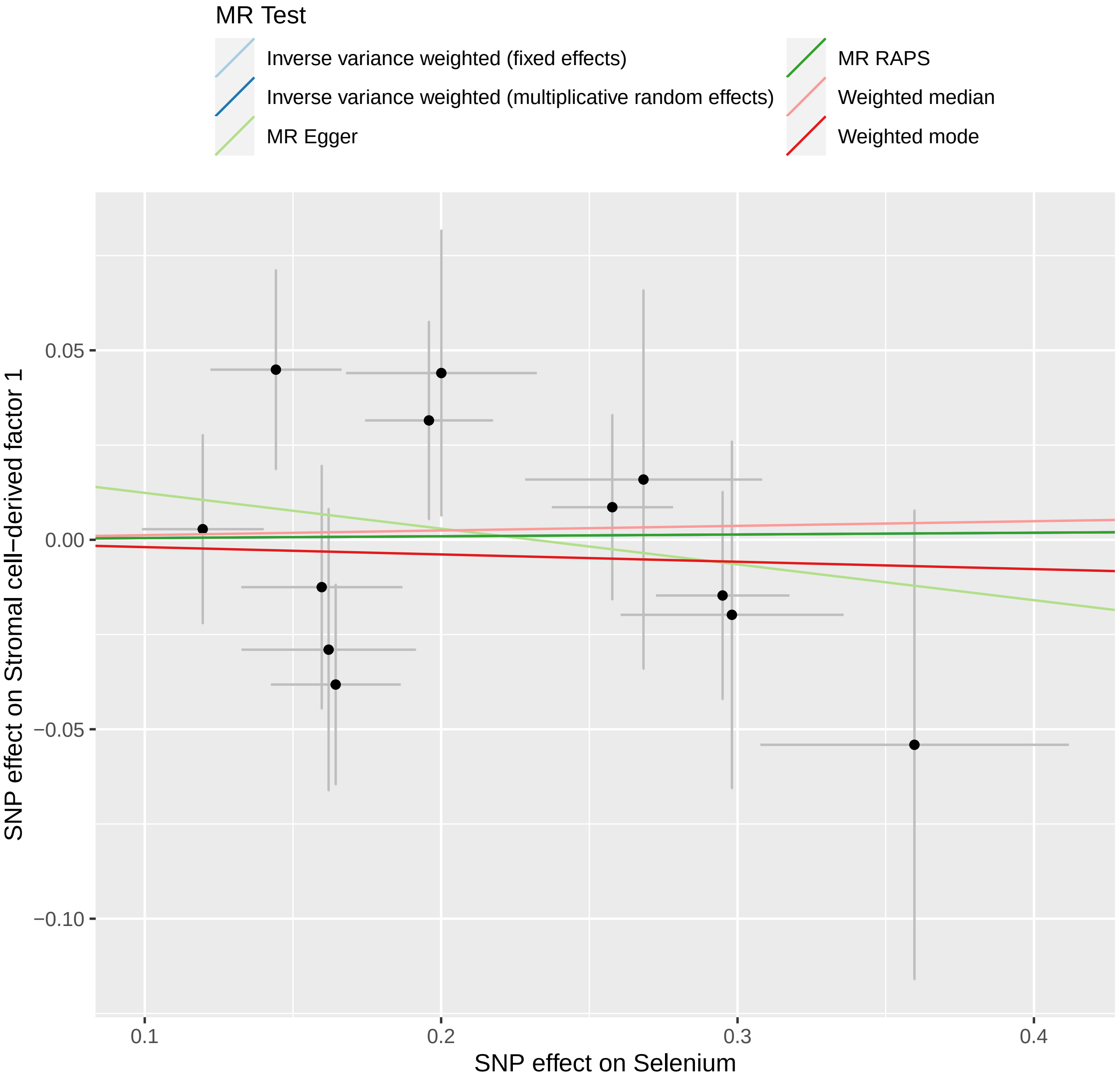

Batch 3−742 : Selenium on Growth−regulated alpha protein

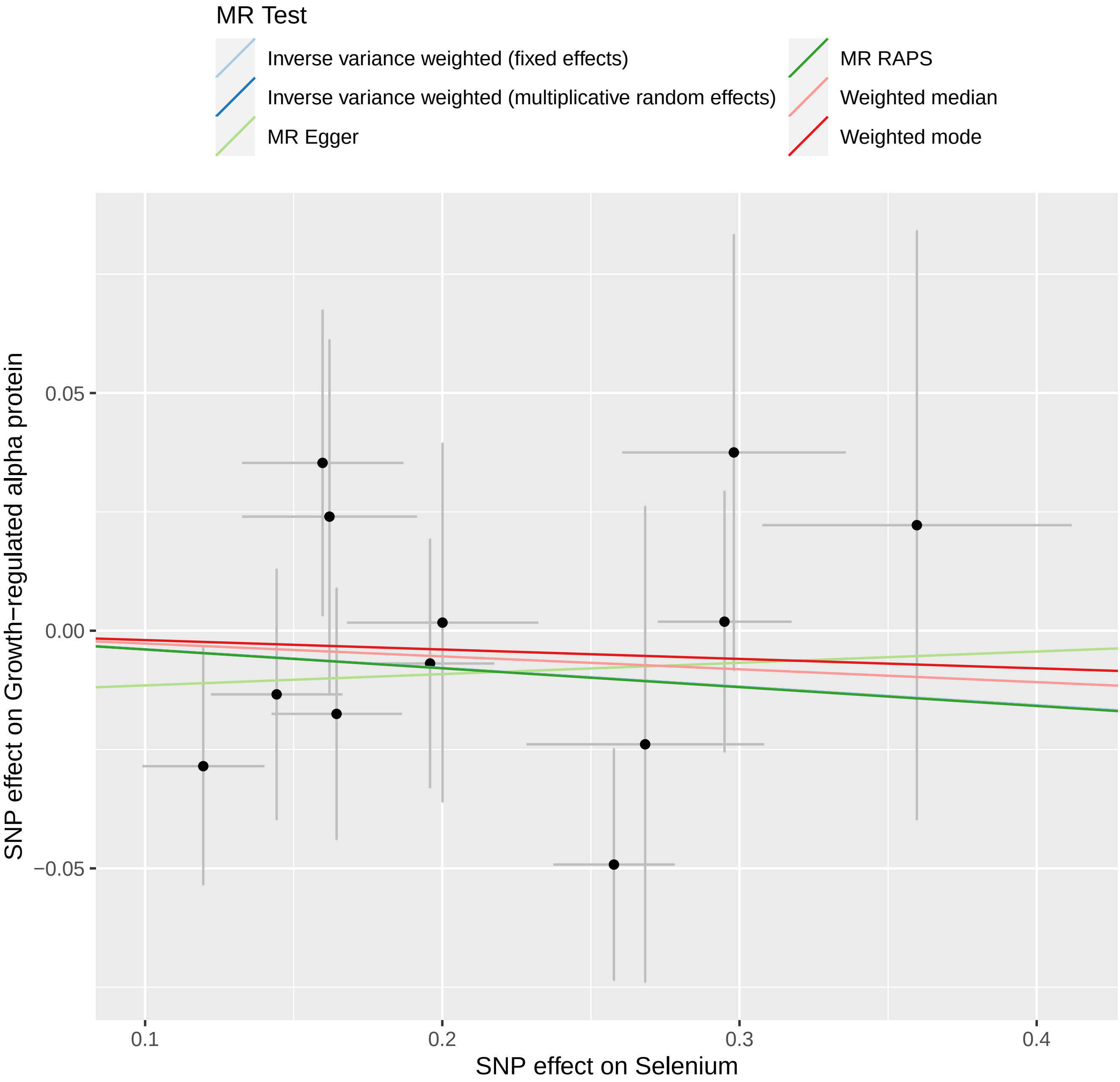

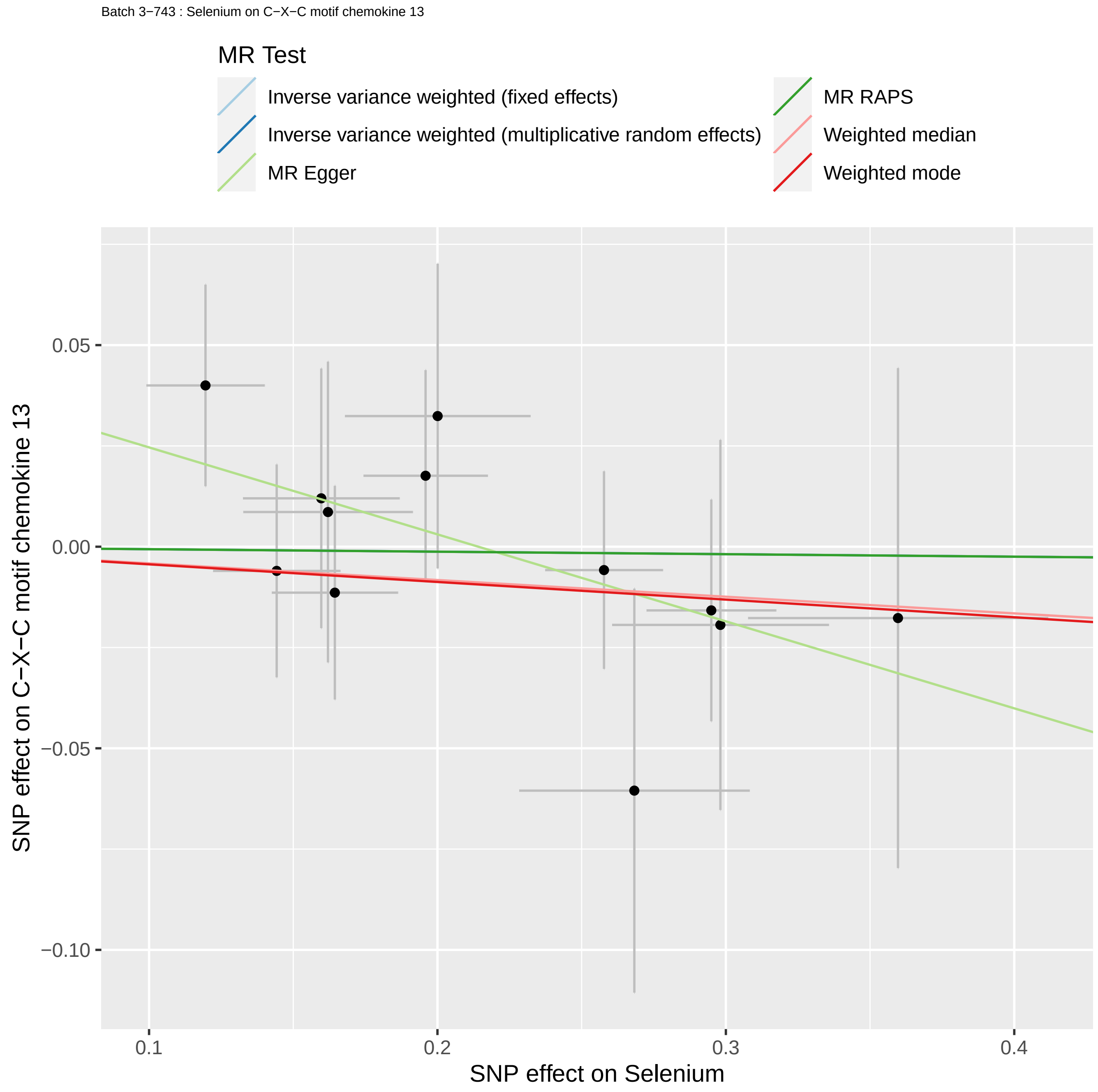
Batch 3−743 : Selenium on C−X−C motif chemokine 13
MR Test
Inverse variance weighted (fixed effects)
Inverse variance weighted (multiplicative random effects)
MR Egger
MR RAPS
Weighted median
Weighted mode
0.05
0.00
−0.05
−0.10
0.1
0.2
0.3
0.4
SNP effect on C−X−C motif chemokine 13
SNP effect on Selenium

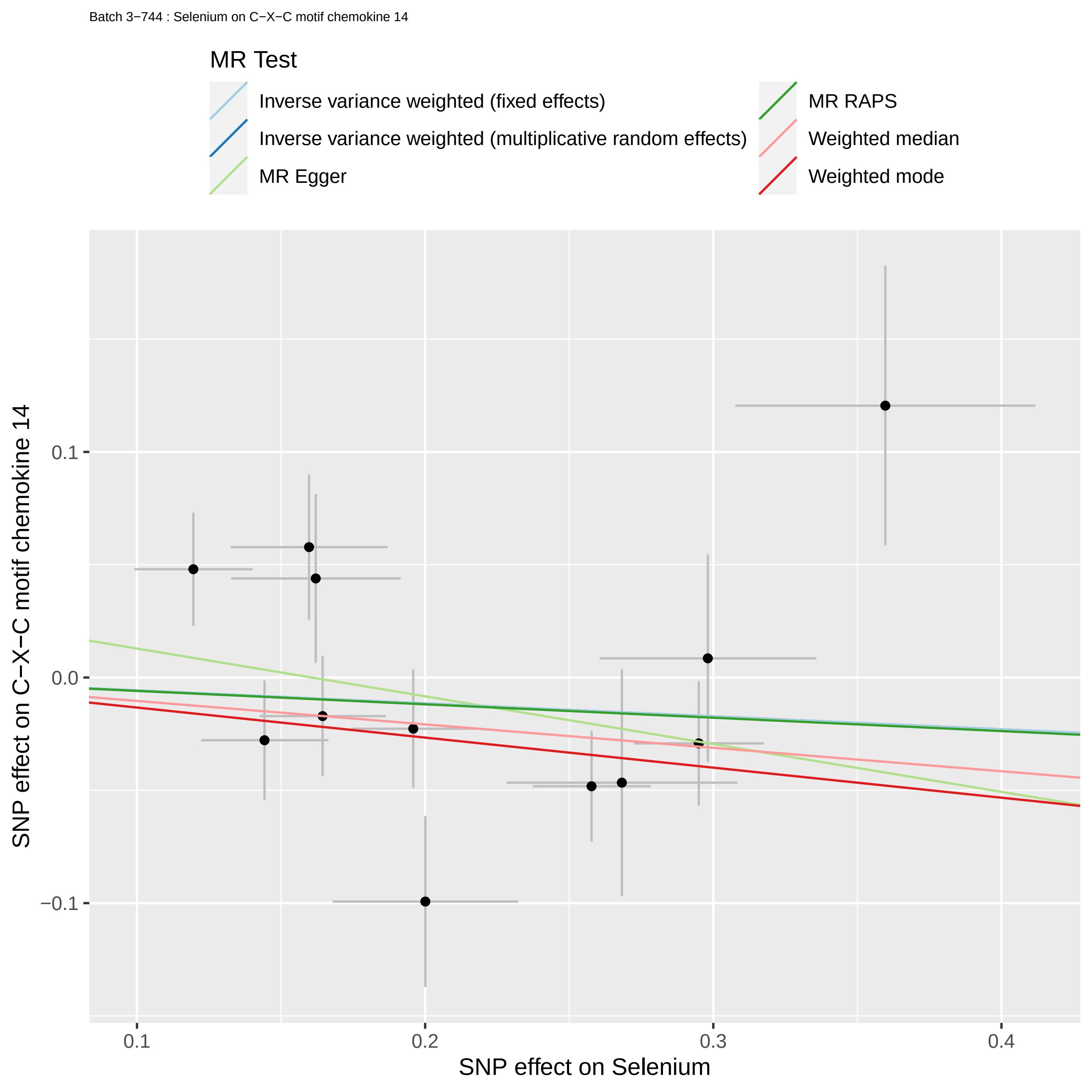

Batch 3−744 : Selenium on C−X−C motif chemokine 14
MR Test
Inverse variance weighted (fixed effects)
Inverse variance weighted (multiplicative random effects)
MR Egger
MR RAPS
Weighted median
Weighted mode
SNP effect on C−X−C motif chemokine 14
0.1
0.0
−0.1
0.1
0.2
0.3
0.4
SNP effect on Selenium

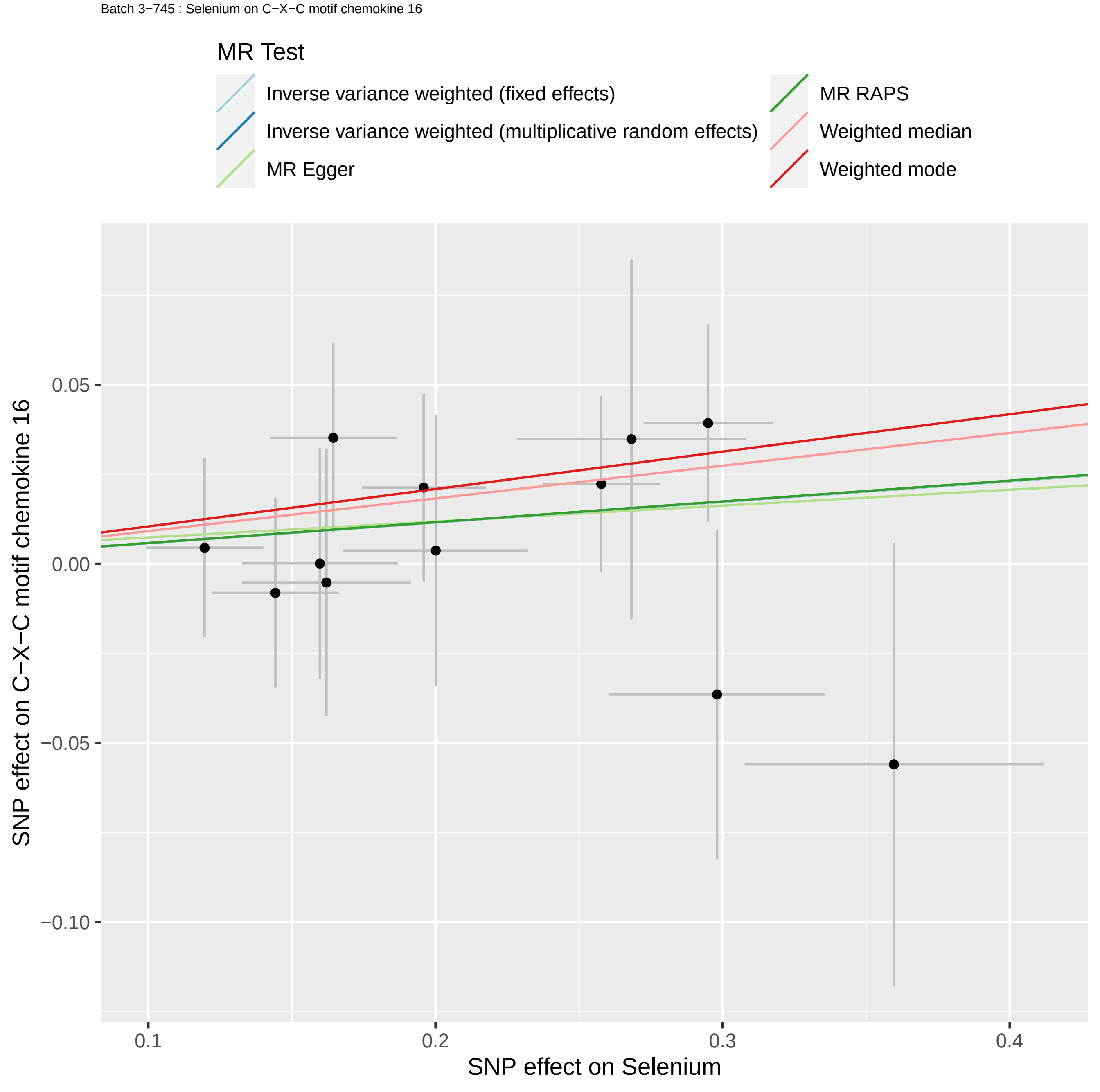

Batch 3−745 : Selenium on C−X−C motif chemokine 16
MR Test
Inverse variance weighted (fixed effects)
Inverse variance weighted (multiplicative random effects)
MR Egger
MR RAPS
Weighted median
Weighted mode
0.05
0.00
−0.05
−0.10
0.1
0.2
0.3
0.4
SNP effect on C−X−C motif chemokine 16
SNP effect on Selenium

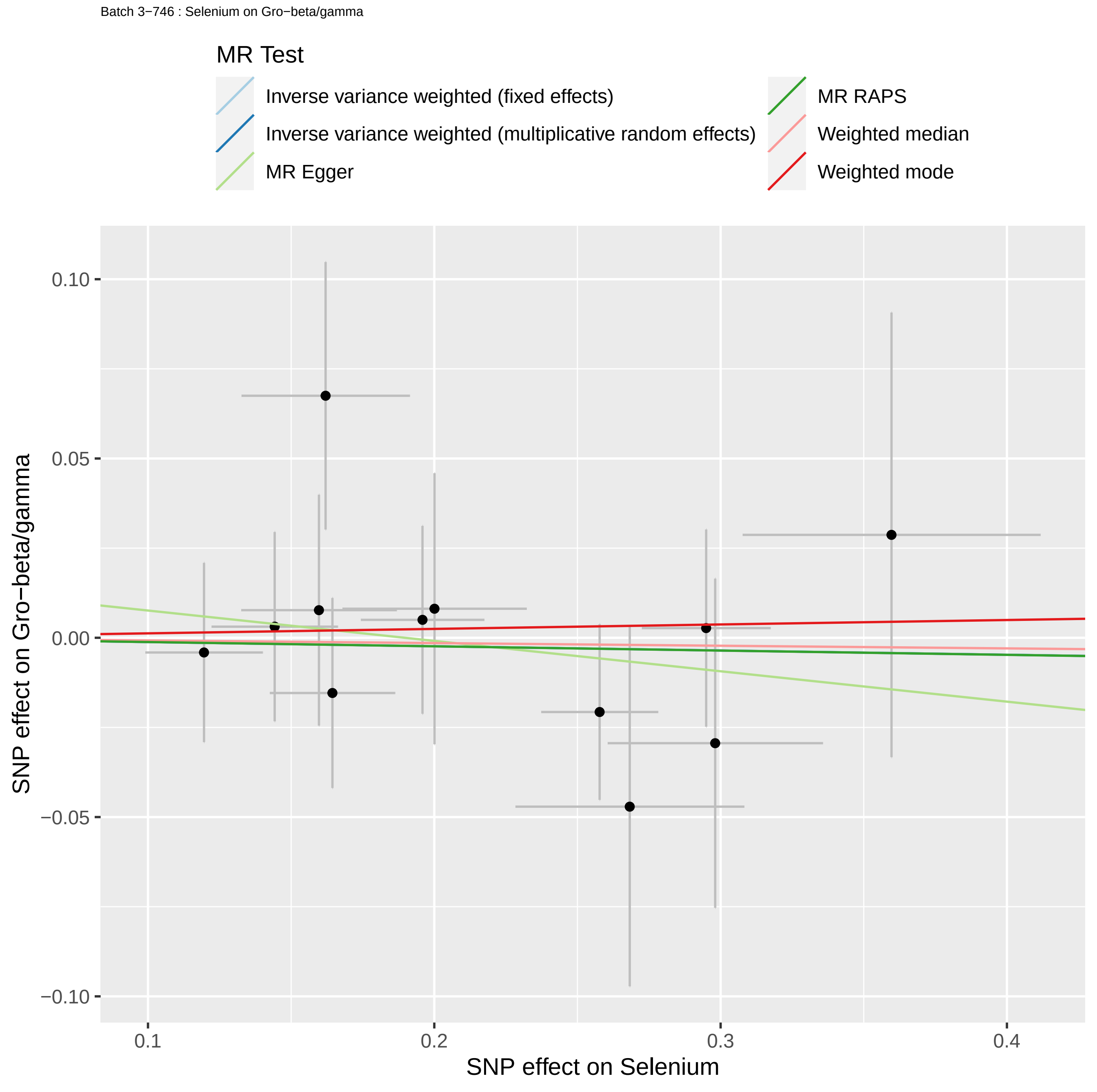

Batch 3−746 : Selenium on Gro−beta/gamma
MR Test
Inverse variance weighted (fixed effects)
Inverse variance weighted (multiplicative random effects)
MR Egger
MR RAPS
Weighted median
Weighted mode
SNP effect on Gro−beta/gamma
0.10
0.05
0.00
−0.05
−0.10
SNP effect on Selenium
0.1
0.2
0.3
0.4

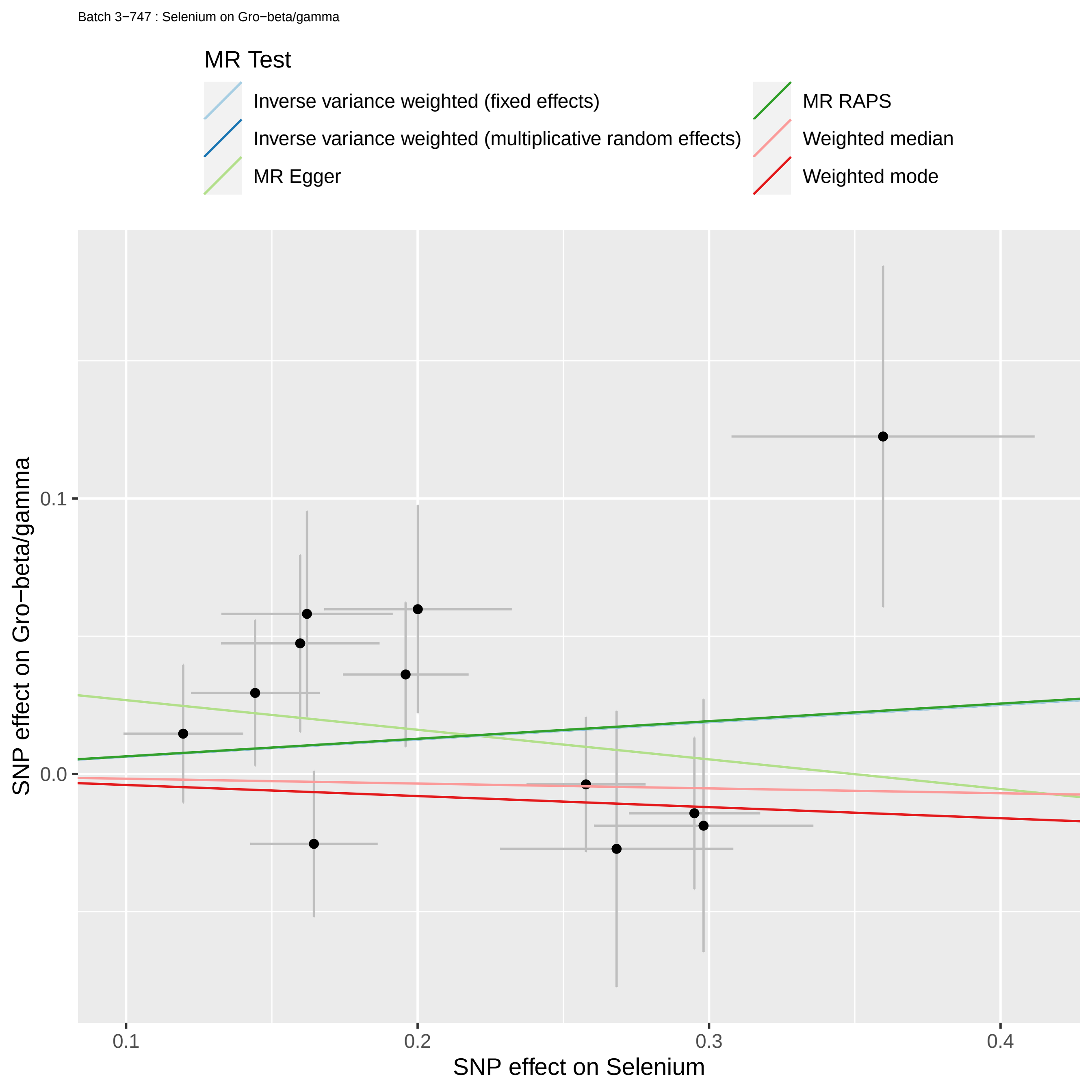

Batch 3−747 : Selenium on Gro−beta/gamma
MR Test
Inverse variance weighted (fixed effects)
Inverse variance weighted (multiplicative random effects)
MR Egger
MR RAPS
Weighted median
Weighted mode
SNP effect on Gro−beta/gamma
0.1
0.0
0.1
0.2
0.3
0.4
SNP effect on Selenium

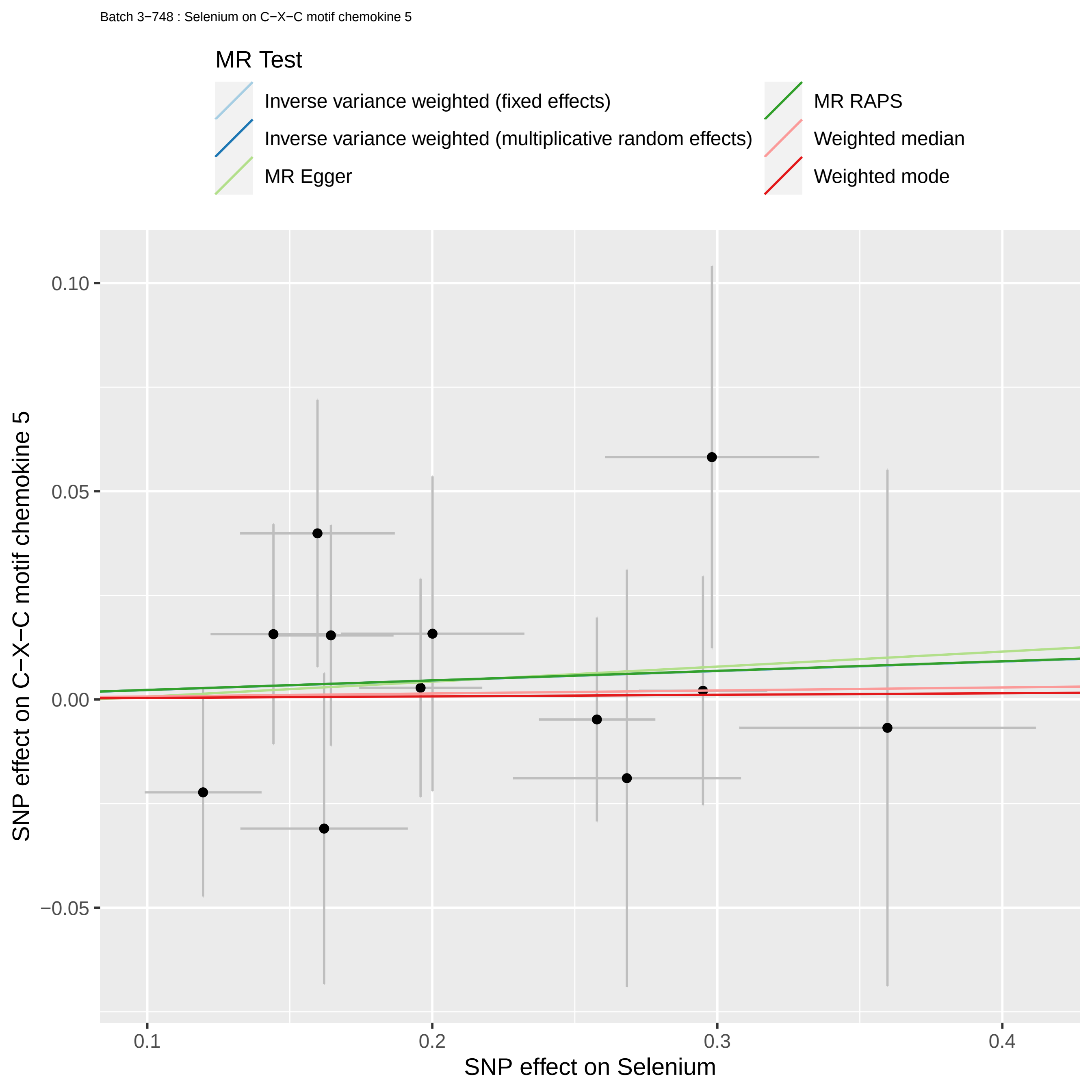

Batch 3−748 : Selenium on C−X−C motif chemokine 5
MR Test
Inverse variance weighted (fixed effects)
Inverse variance weighted (multiplicative random effects)
MR Egger
MR RAPS
Weighted median
Weighted mode
0.10
0.05
0.00
−0.05
0.1
0.2
0.3
0.4
SNP effect on C−X−C motif chemokine 5
SNP effect on Selenium

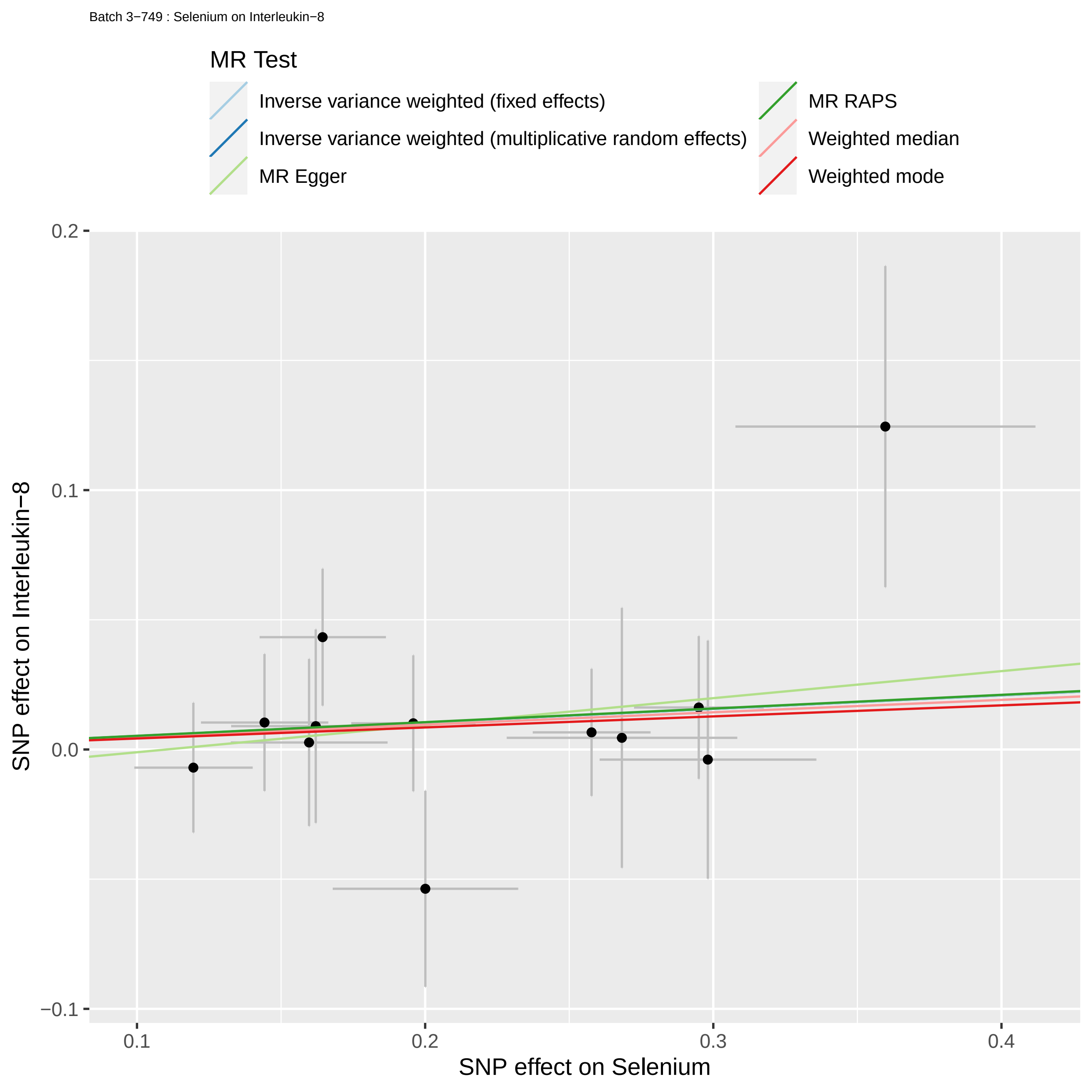
Batch 3−749 : Selenium on Interleukin−8
MR Test
Inverse variance weighted (fixed effects)
Inverse variance weighted (multiplicative random effects)
MR Egger
MR RAPS
Weighted median
Weighted mode
0.2
0.1
0.0
−0.1
0.1
0.2
0.3
0.4
SNP effect on Selenium
SNP effect on Interleukin−8

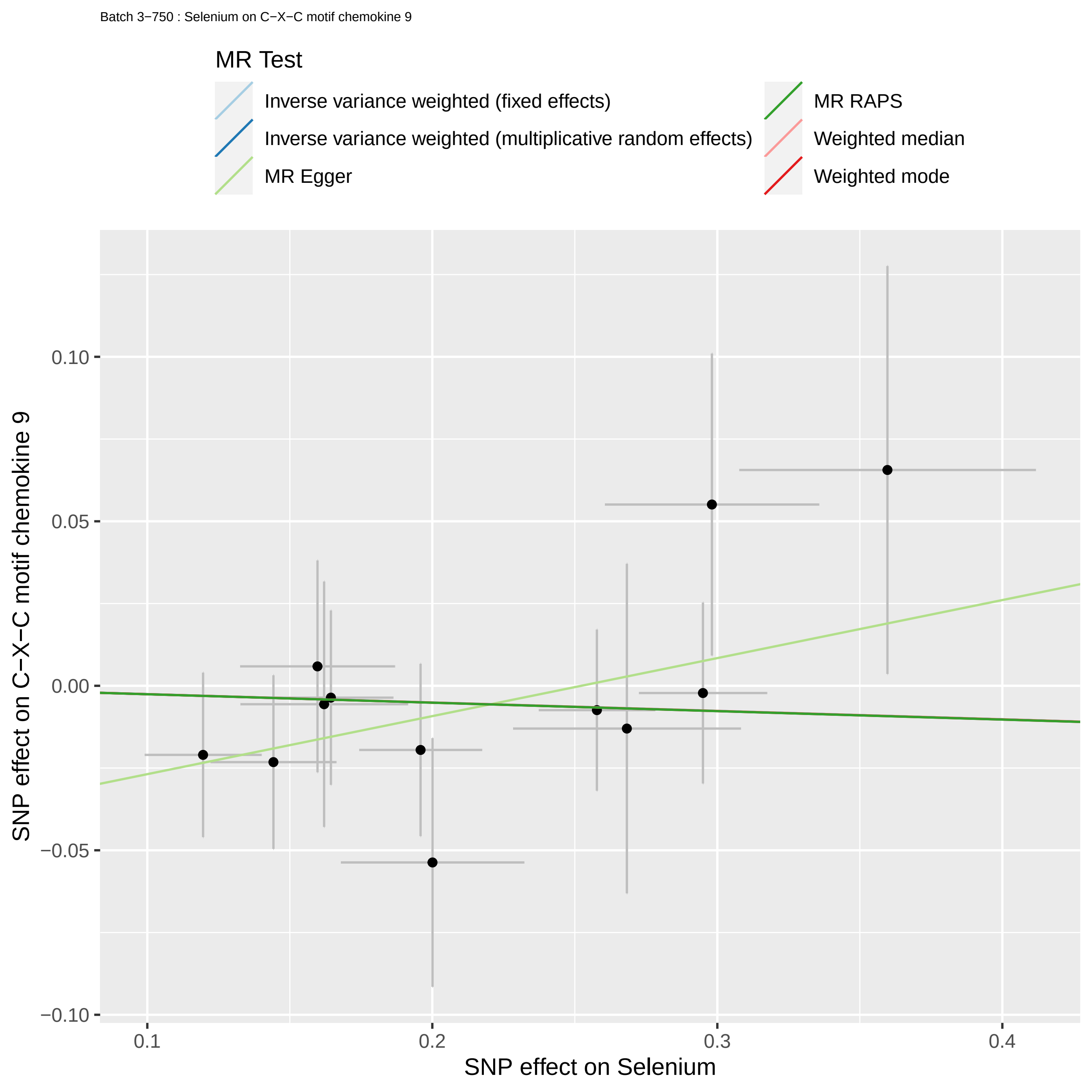
Batch 3−750 : Selenium on C−X−C motif chemokine 9
MR Test
Inverse variance weighted (fixed effects)
Inverse variance weighted (multiplicative random effects)
MR Egger
MR RAPS
Weighted median
Weighted mode
SNP effect on C−X−C motif chemokine 9
0.10
0.05
0.00
−0.05
−0.10
0.1
0.2
0.3
0.4
SNP effect on Selenium

Batch 3−751 : Selenium on C−X−C motif chemokine 9

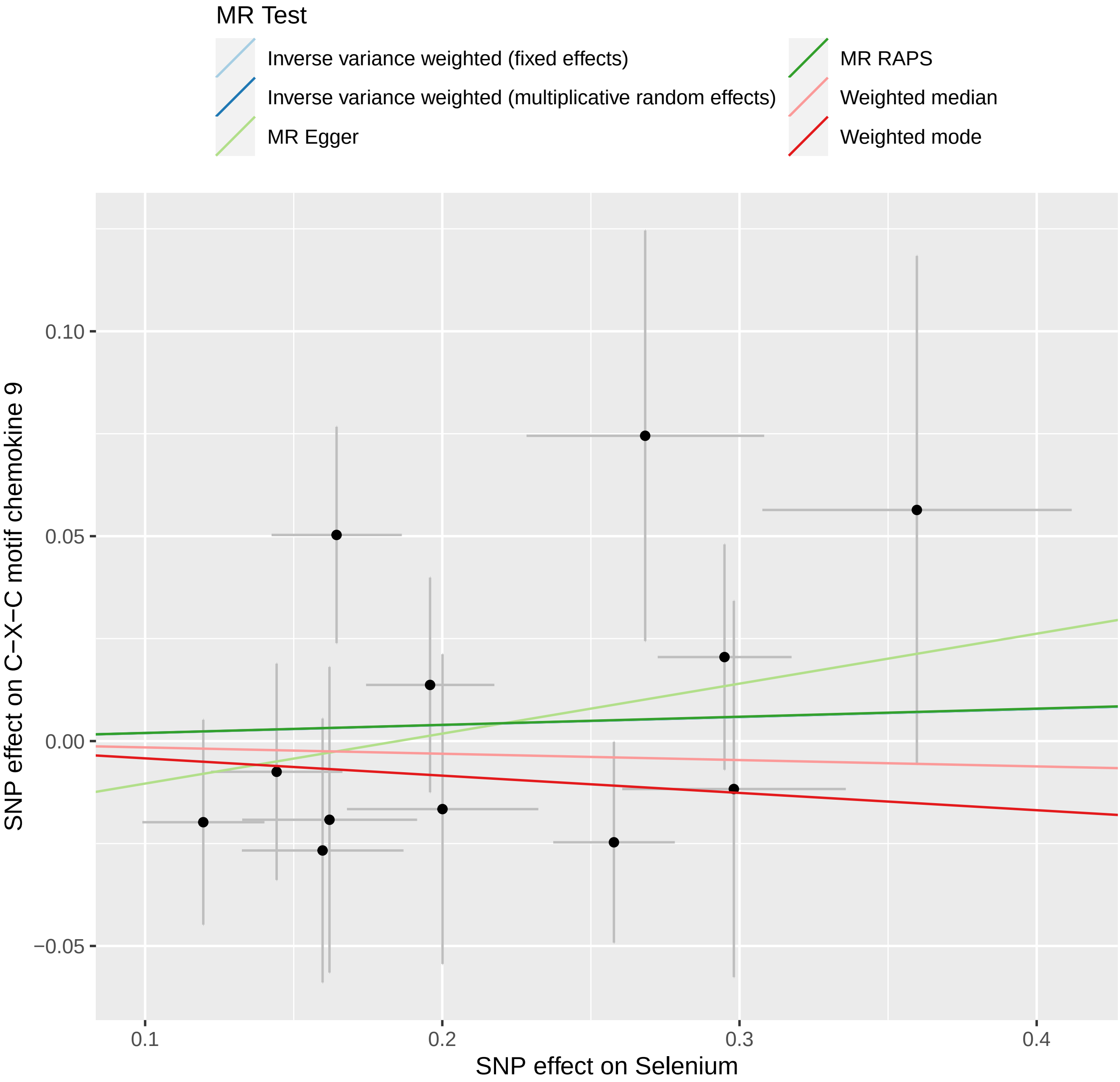

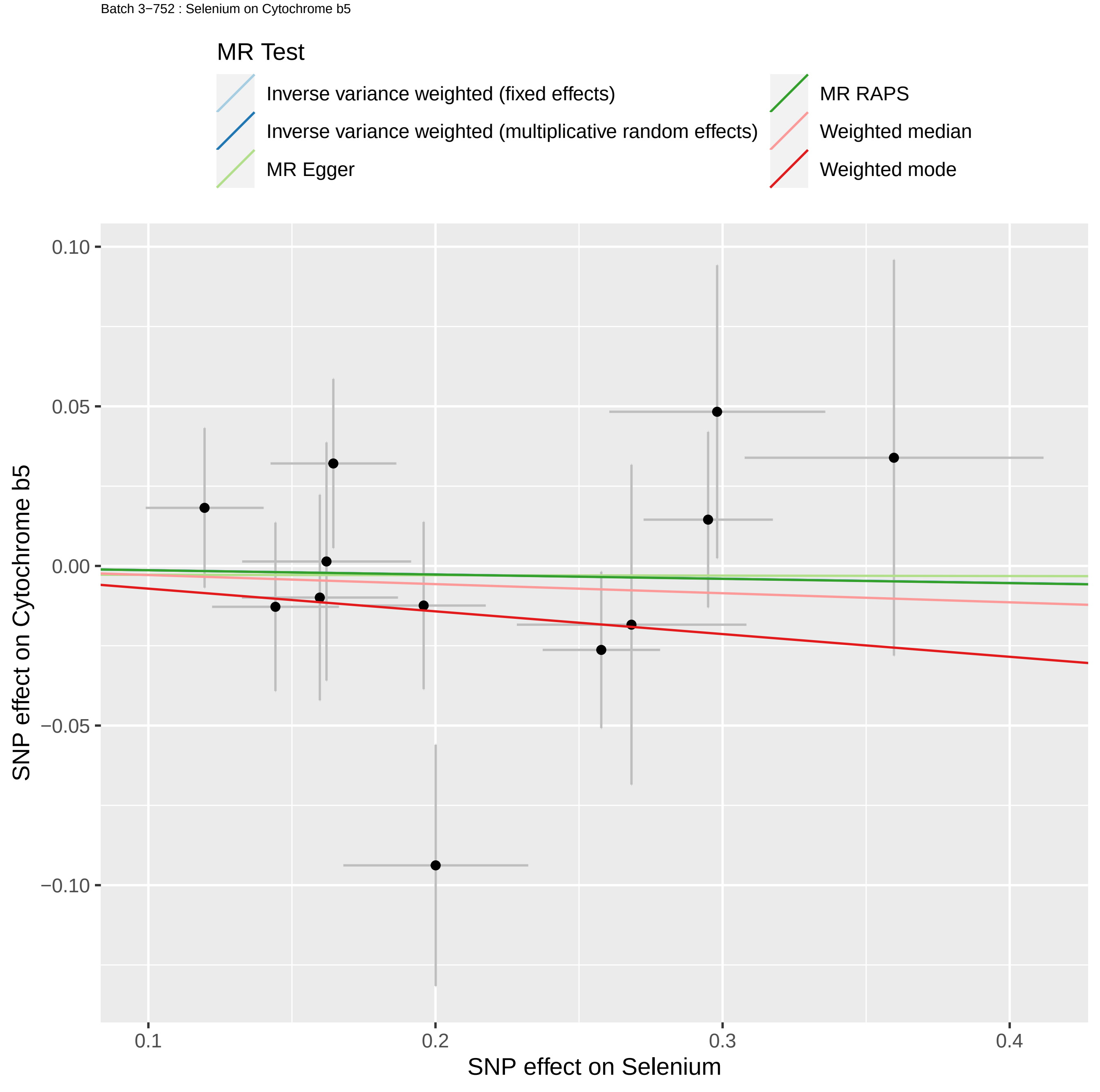

Batch 3−752 : Selenium on Cytochrome b5
MR Test
Inverse variance weighted (fixed effects)
Inverse variance weighted (multiplicative random effects)
MR Egger
MR RAPS
Weighted median
Weighted mode
0.10
0.05
0.00
−0.05
−0.10
0.1
0.2
0.3
0.4
SNP effect on Cytochrome b5
SNP effect on Selenium

Batch 3−753 : Selenium on NADH−cytochrome b5 reductase 3

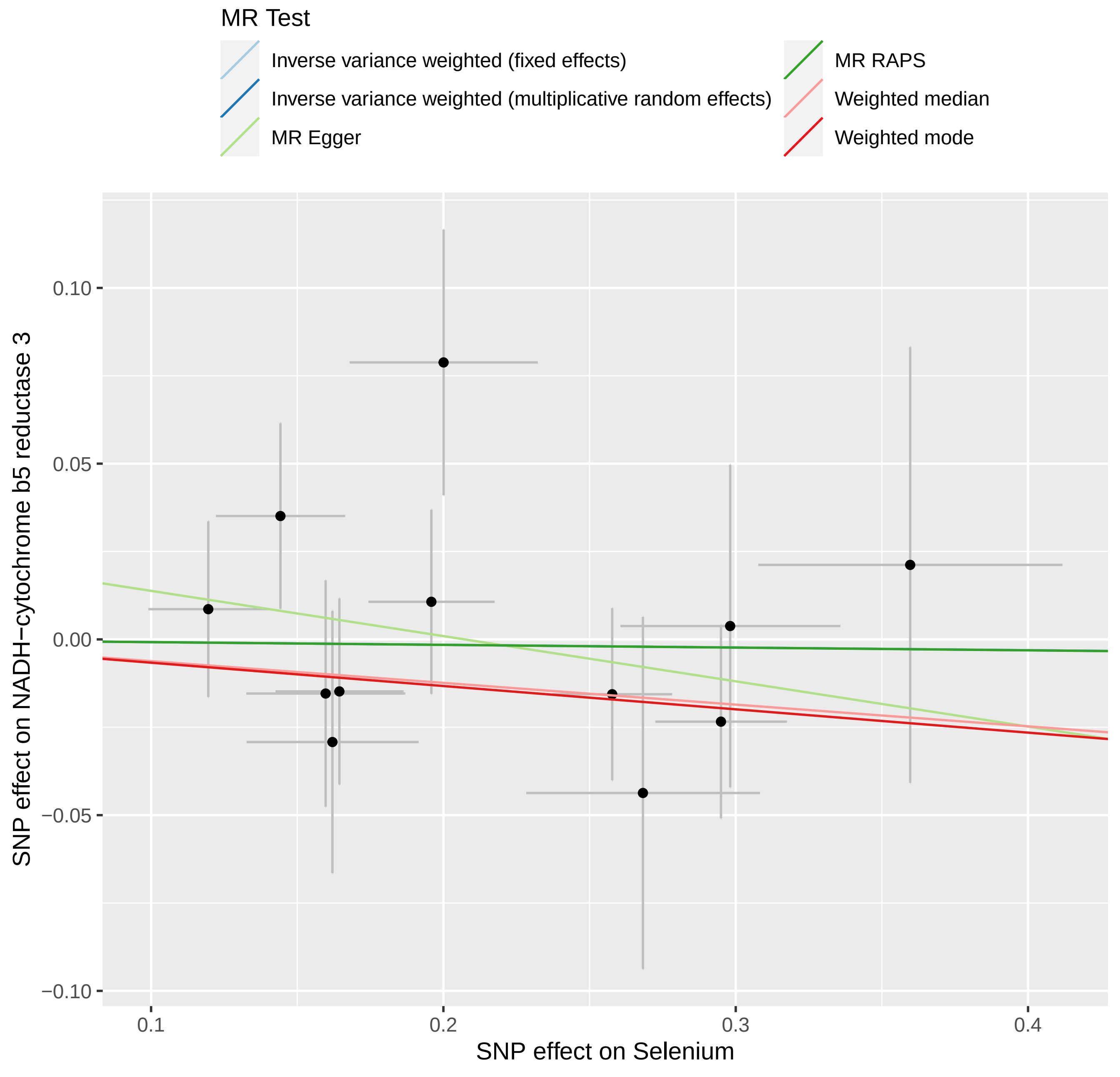

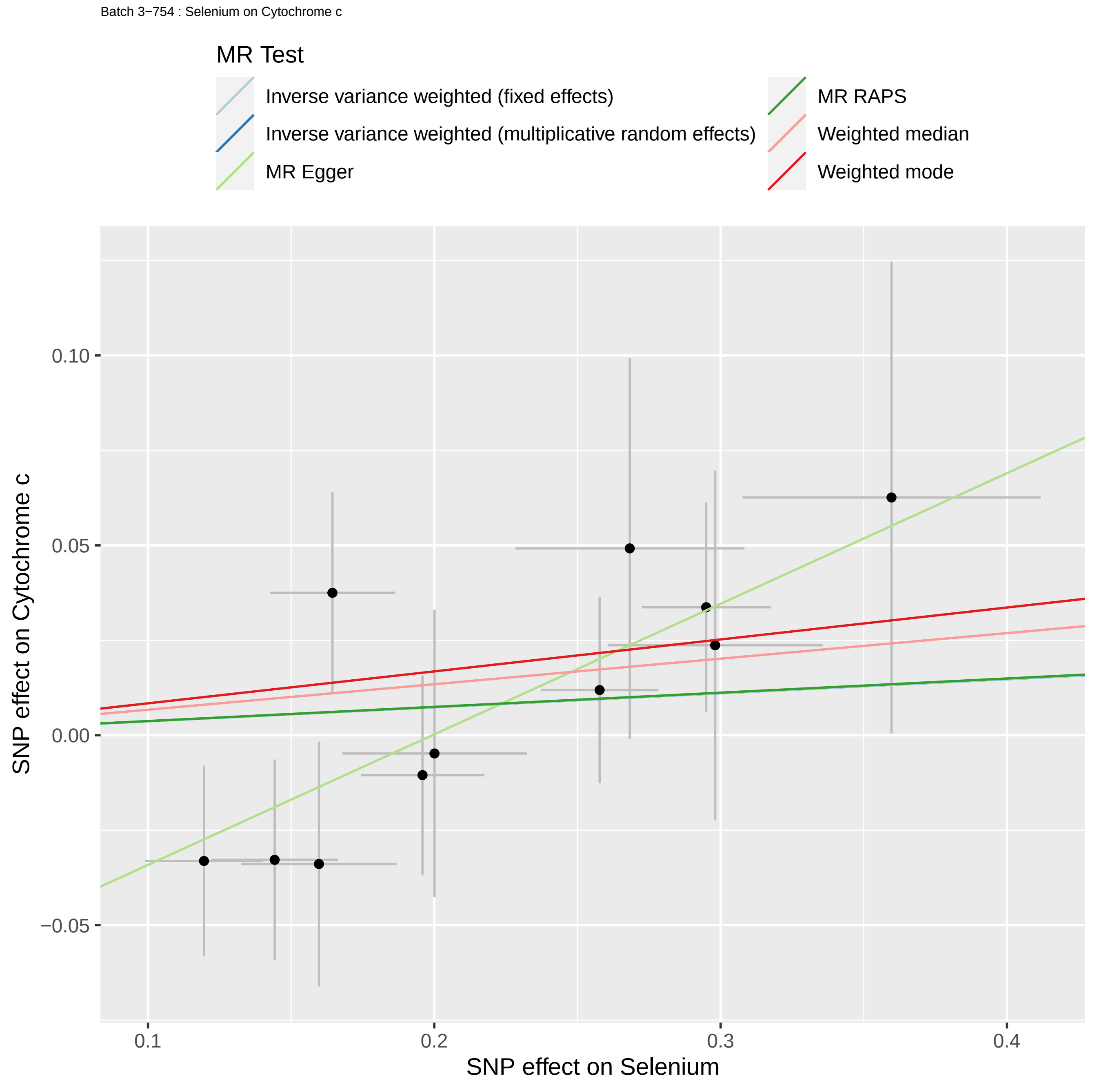
Batch 3−754 : Selenium on Cytochrome c
MR Test
Inverse variance weighted (fixed effects)
Inverse variance weighted (multiplicative random effects)
MR Egger
MR RAPS
Weighted median
Weighted mode
SNP effect on Cytochrome c
0.10
0.05
0.00
−0.05
0.1
0.2
0.3
0.4
SNP effect on Selenium

Batch 3−755 : Selenium on Cytoglobin

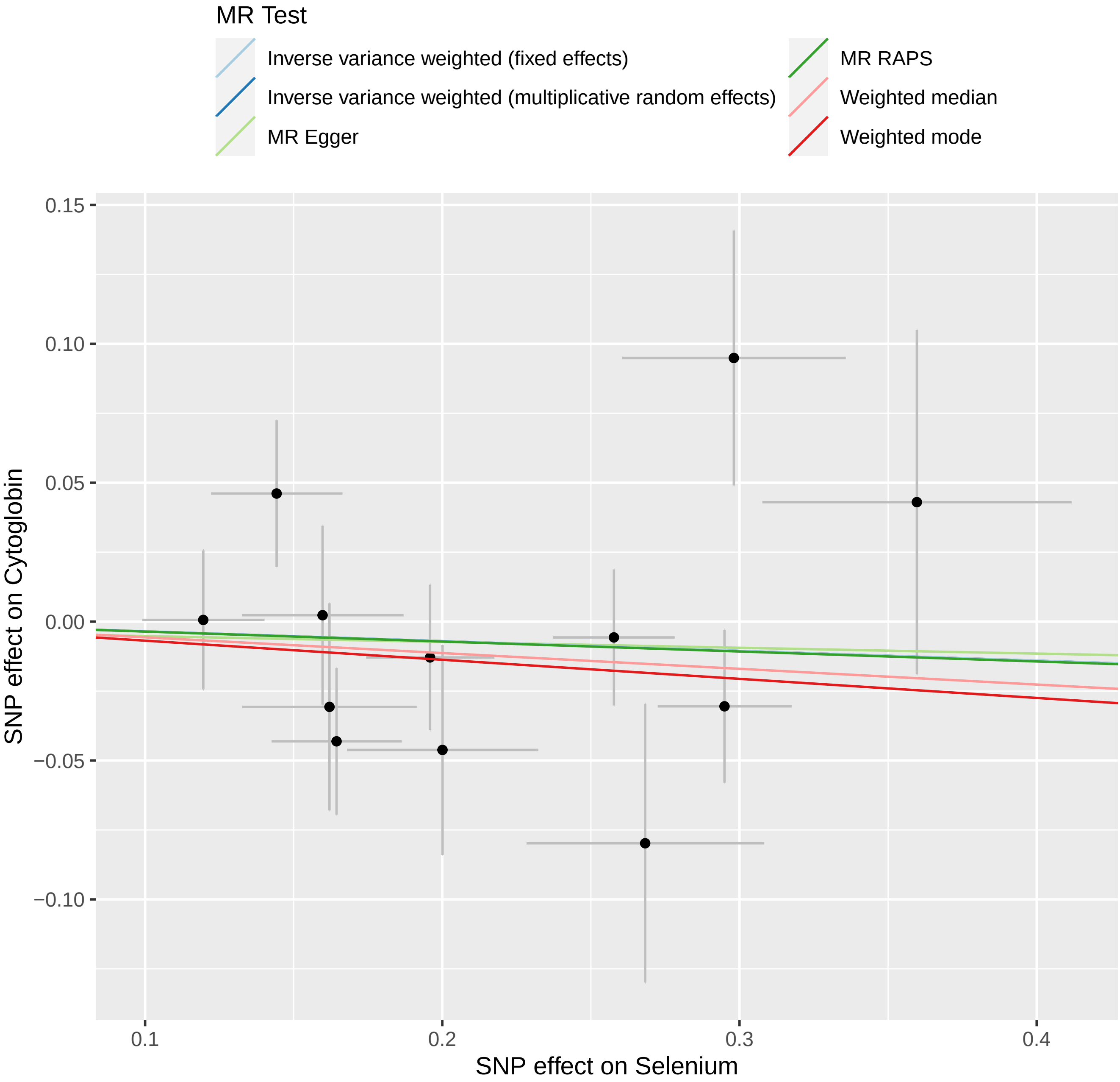

Batch 3−756 : Selenium on Cytochrome P450 3A4

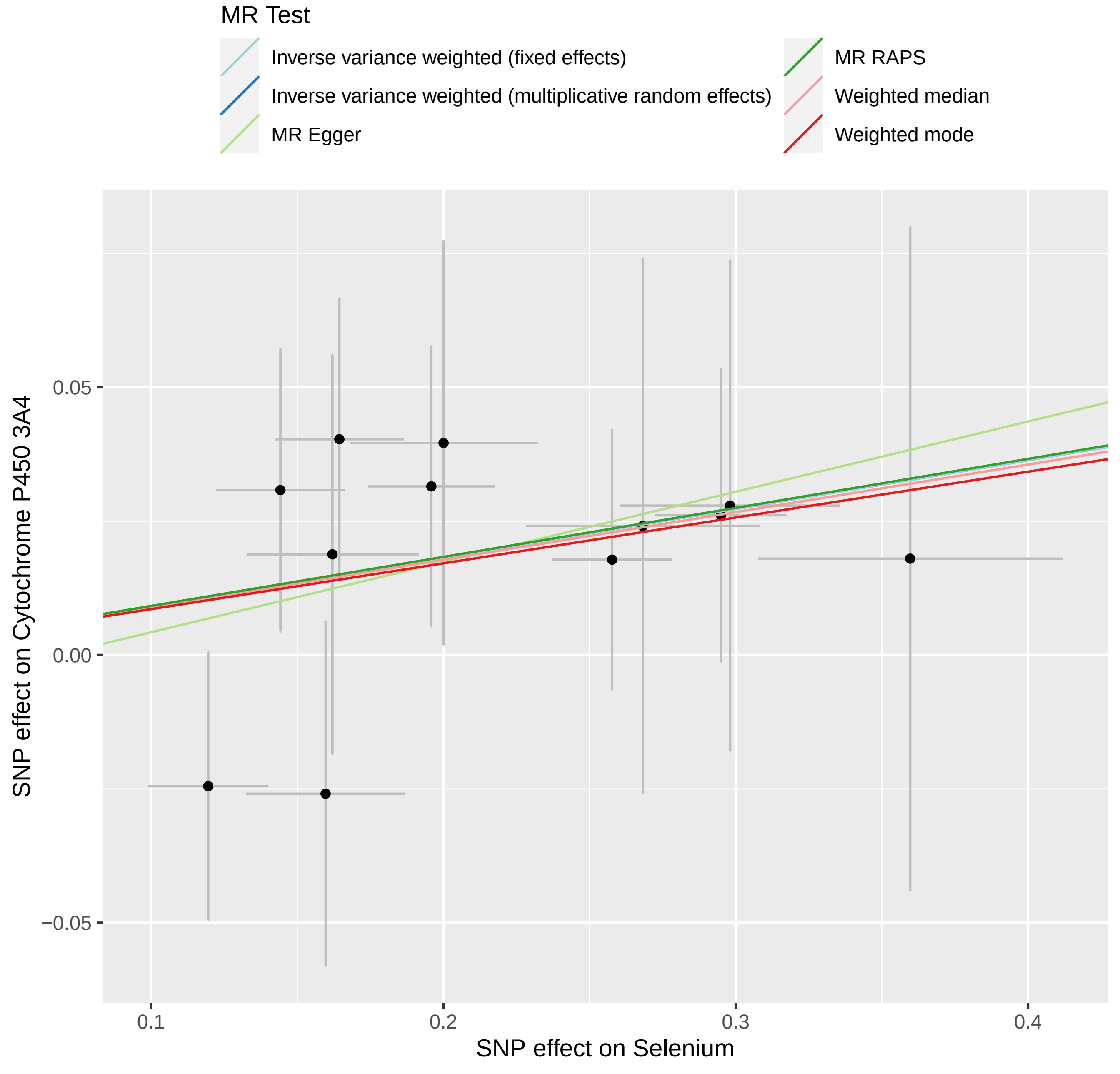

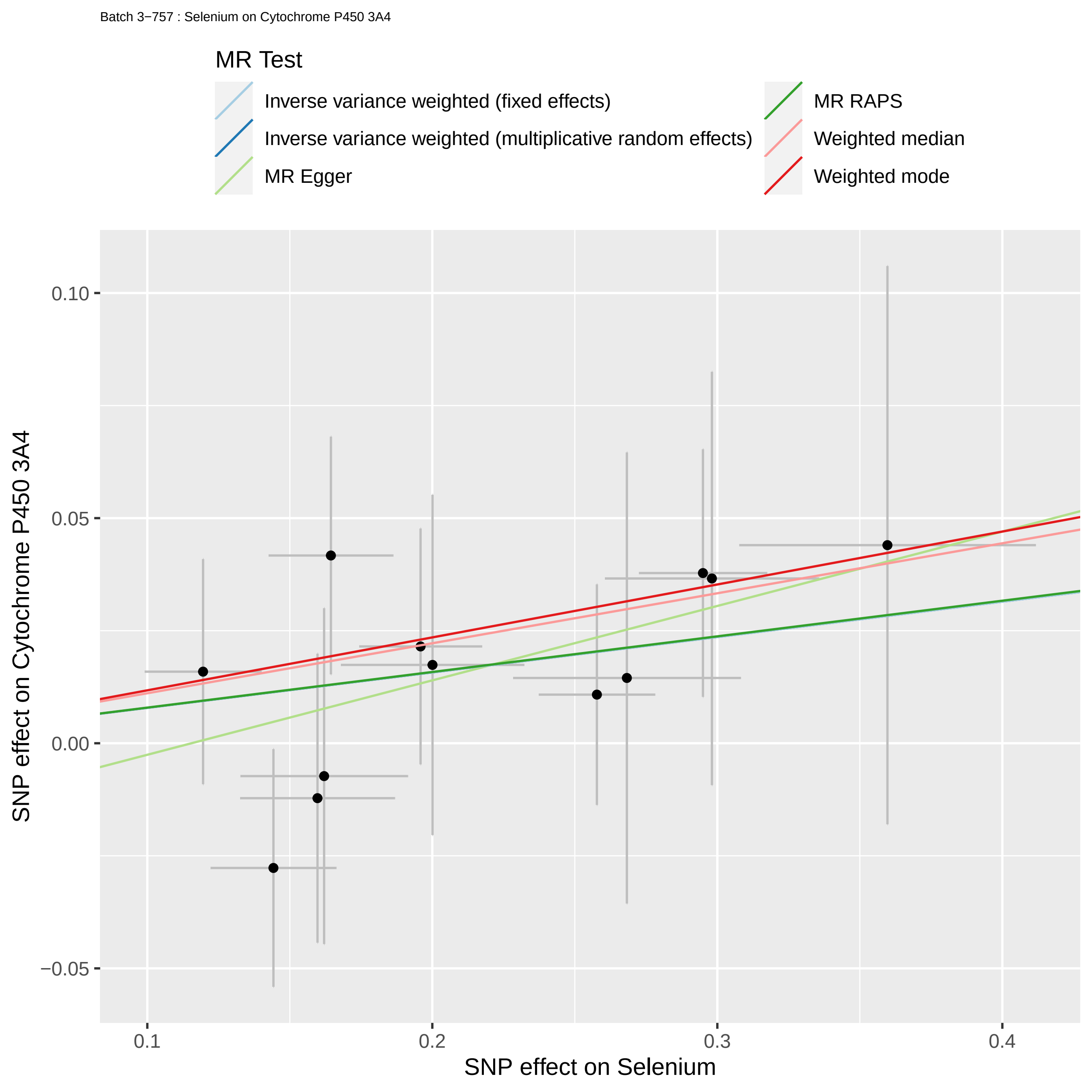

Batch 3−757 : Selenium on Cytochrome P450 3A4
MR Test
Inverse variance weighted (fixed effects)
Inverse variance weighted (multiplicative random effects)
MR Egger
MR RAPS
Weighted median
Weighted mode
0.10
0.05
0.00
−0.05
0.1
0.2
0.3
0.4
SNP effect on Cytochrome P450 3A4
SNP effect on Selenium

Batch 3−758 : Selenium on Protein CYR61

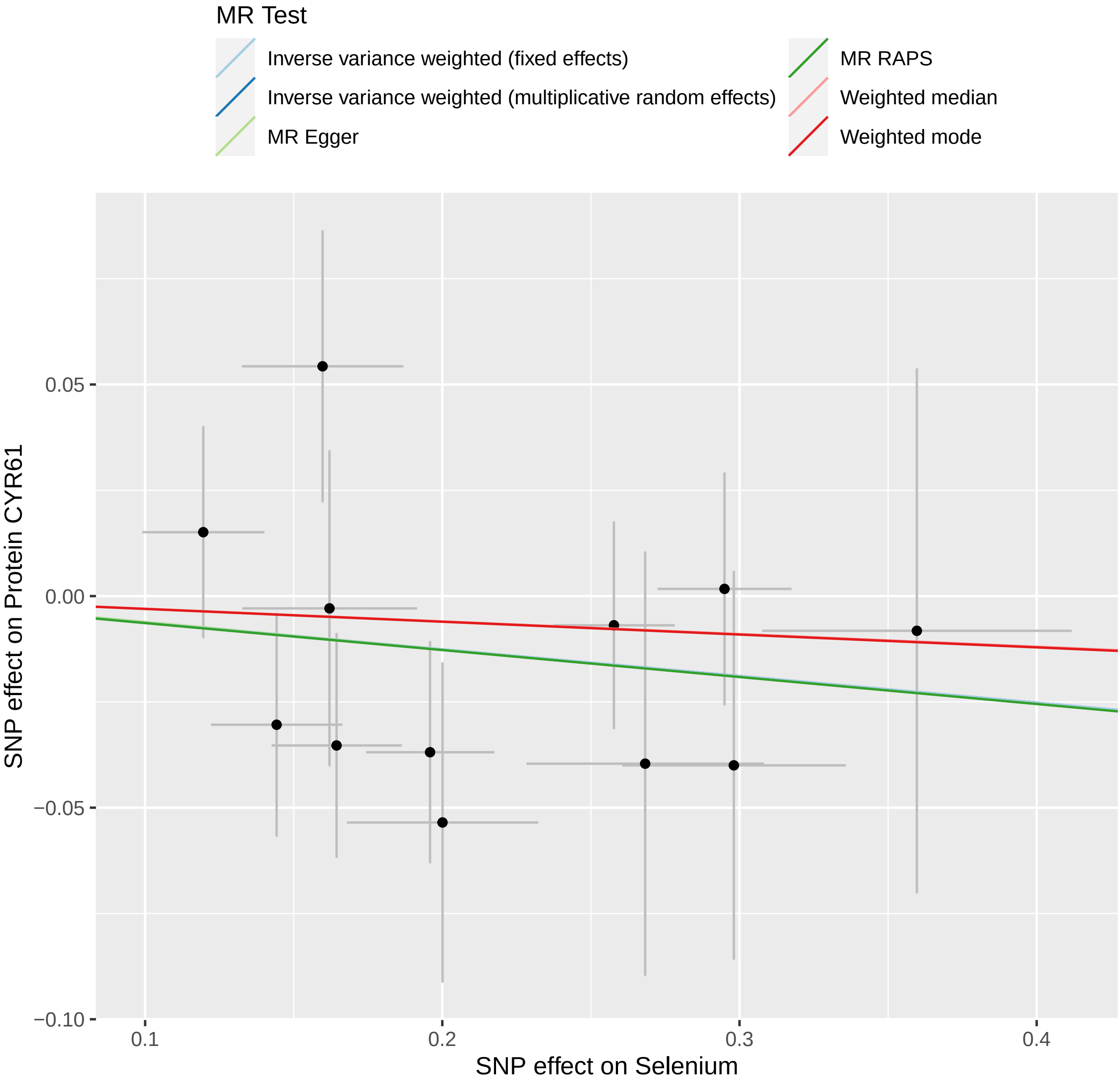

Batch 3−759 : Selenium on Cytohesin−2

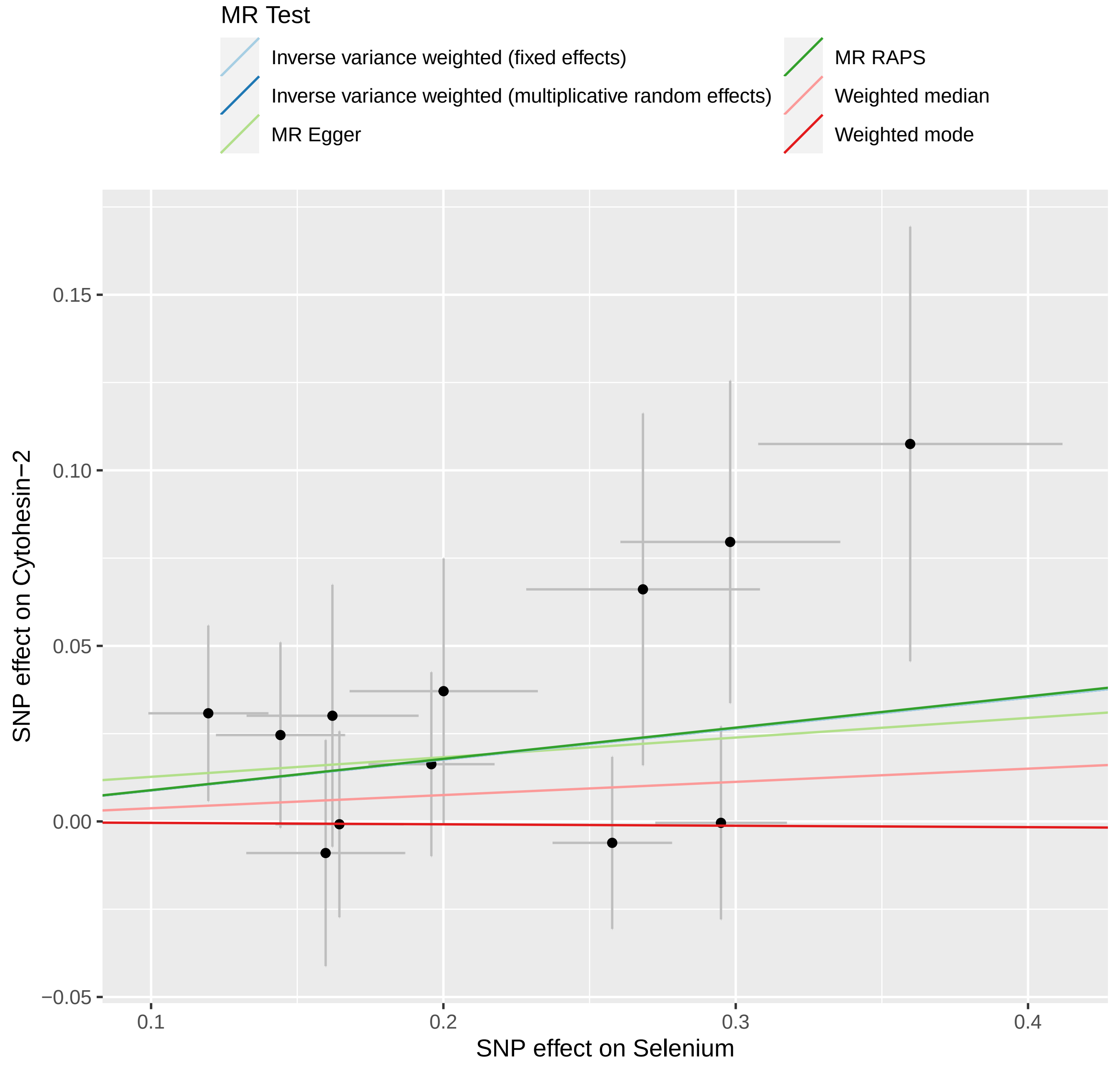

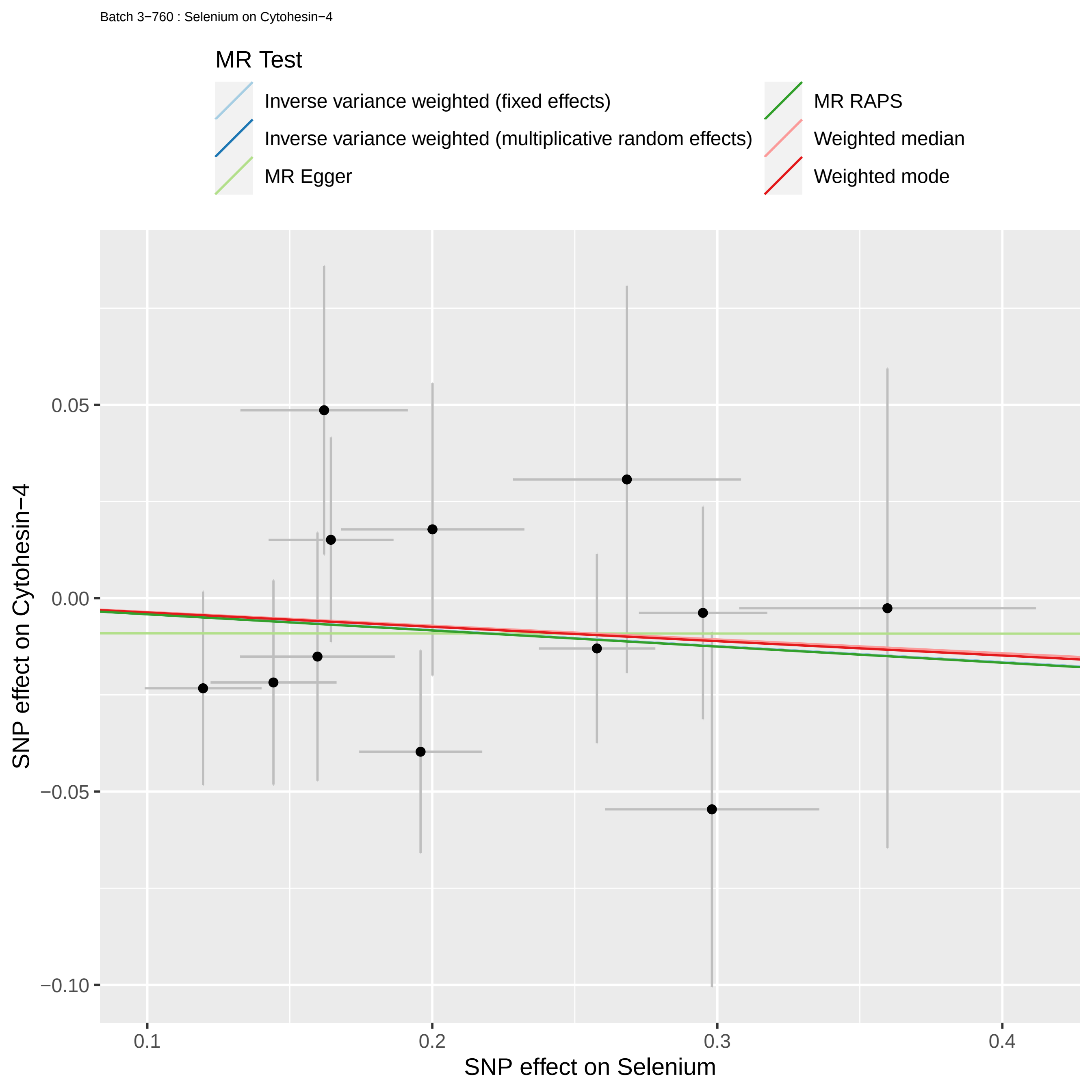
Batch 3−760 : Selenium on Cytohesin−4
MR Test
Inverse variance weighted (fixed effects)
Inverse variance weighted (multiplicative random effects)
MR Egger
MR RAPS
Weighted median
Weighted mode
SNP effect on Cytohesin−4
0.05
0.00
−0.05
−0.10
0.1
0.2
0.3
0.4
SNP effect on Selenium

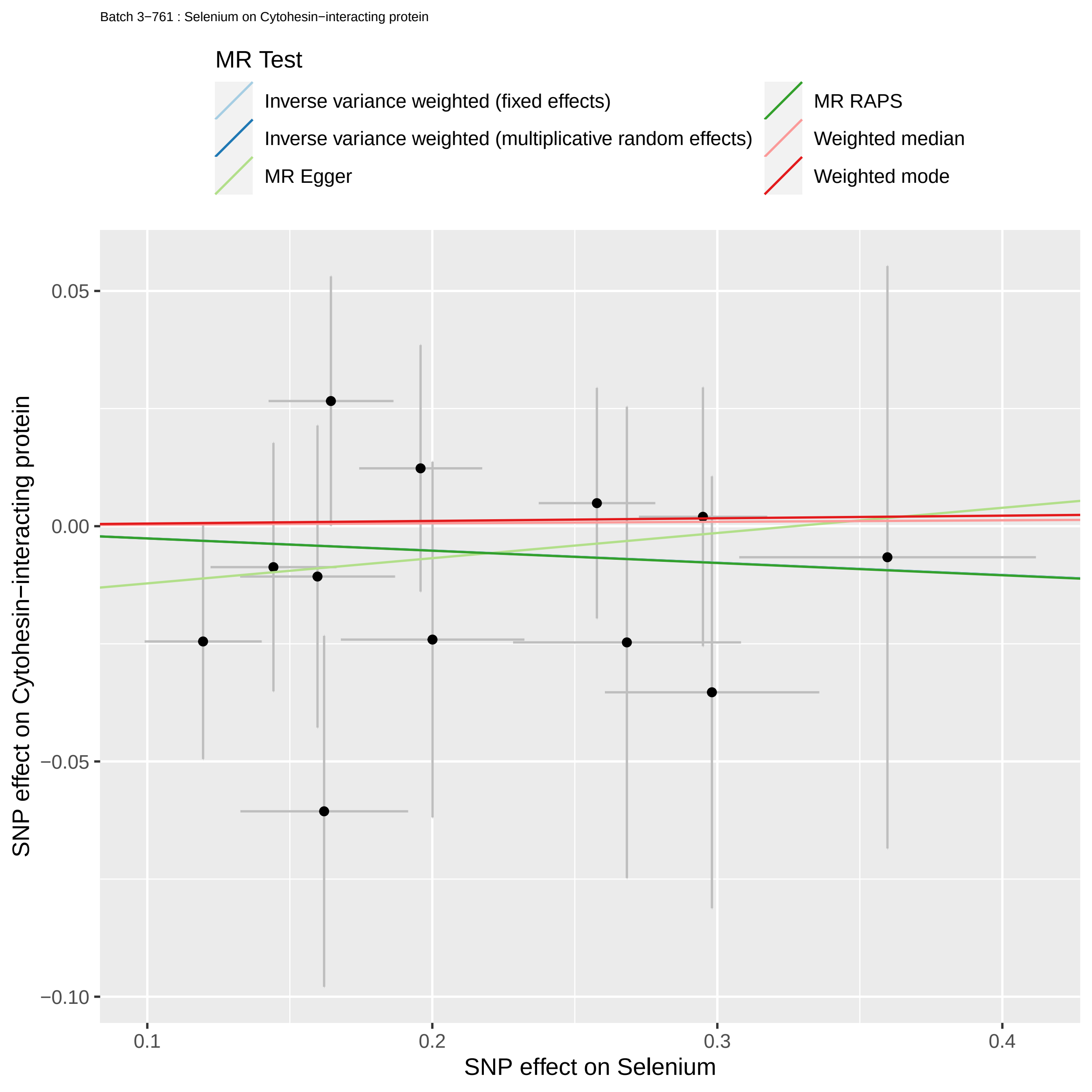

Batch 3−761 : Selenium on Cytohesin−interacting protein
MR Test
Inverse variance weighted (fixed effects)
Inverse variance weighted (multiplicative random effects)
MR Egger
MR RAPS
Weighted median
Weighted mode
0.05
0.00
−0.05
−0.10
0.1
0.2
0.3
0.4
SNP effect on Selenium
SNP effect on Cytohesin−interacting protein

Batch 3−762 : Selenium on Dystroglycan

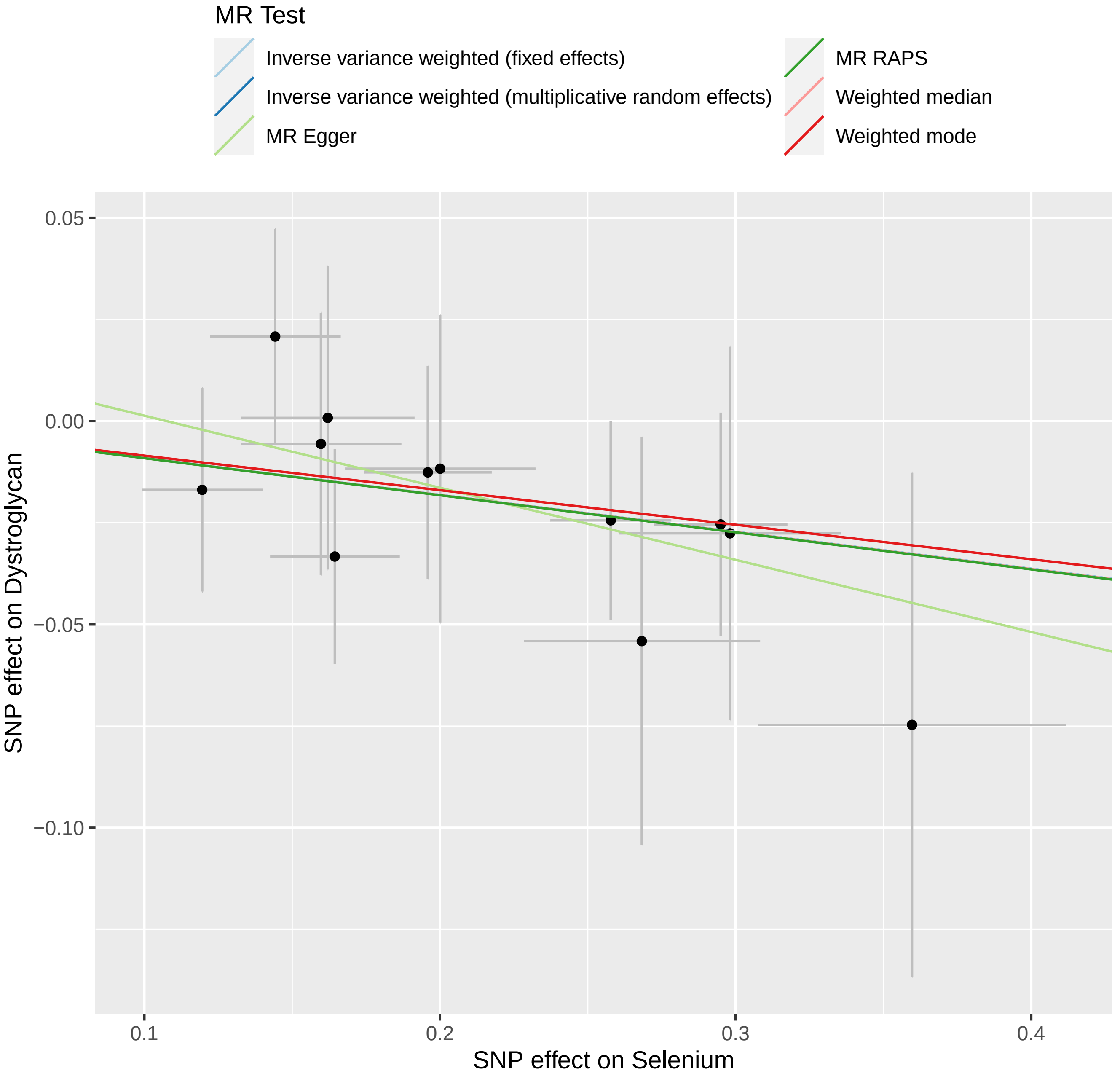

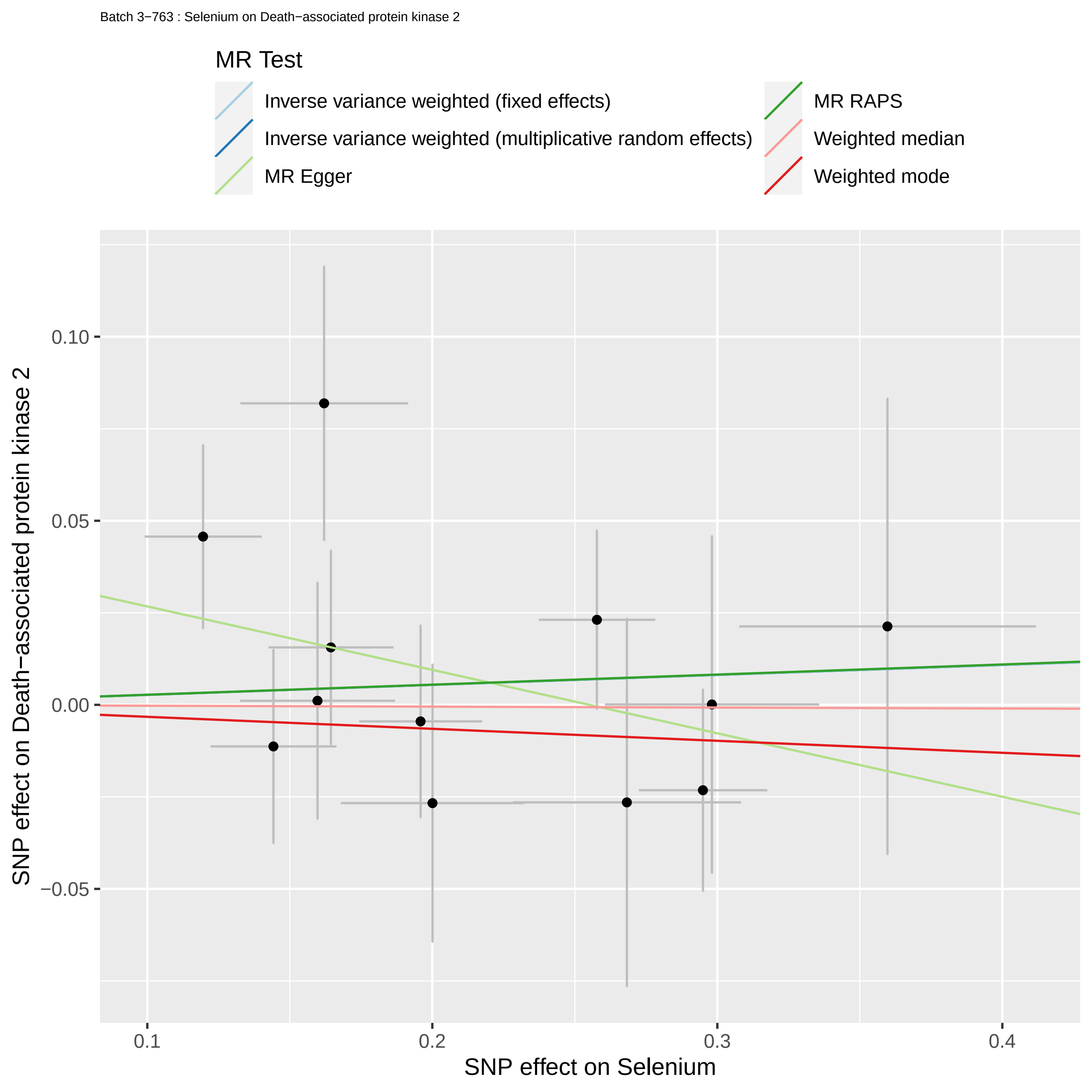
Batch 3−763 : Selenium on Death−associated protein kinase 2
MR Test
Inverse variance weighted (fixed effects)
Inverse variance weighted (multiplicative random effects)
MR Egger
MR RAPS
Weighted median
Weighted mode
0.10
0.05
0.00
−0.05
SNP effect on Death−associated protein kinase 2
0.1
0.2
0.3
0.4
SNP effect on Selenium

Batch 3−764 : Selenium on Drebrin−like protein

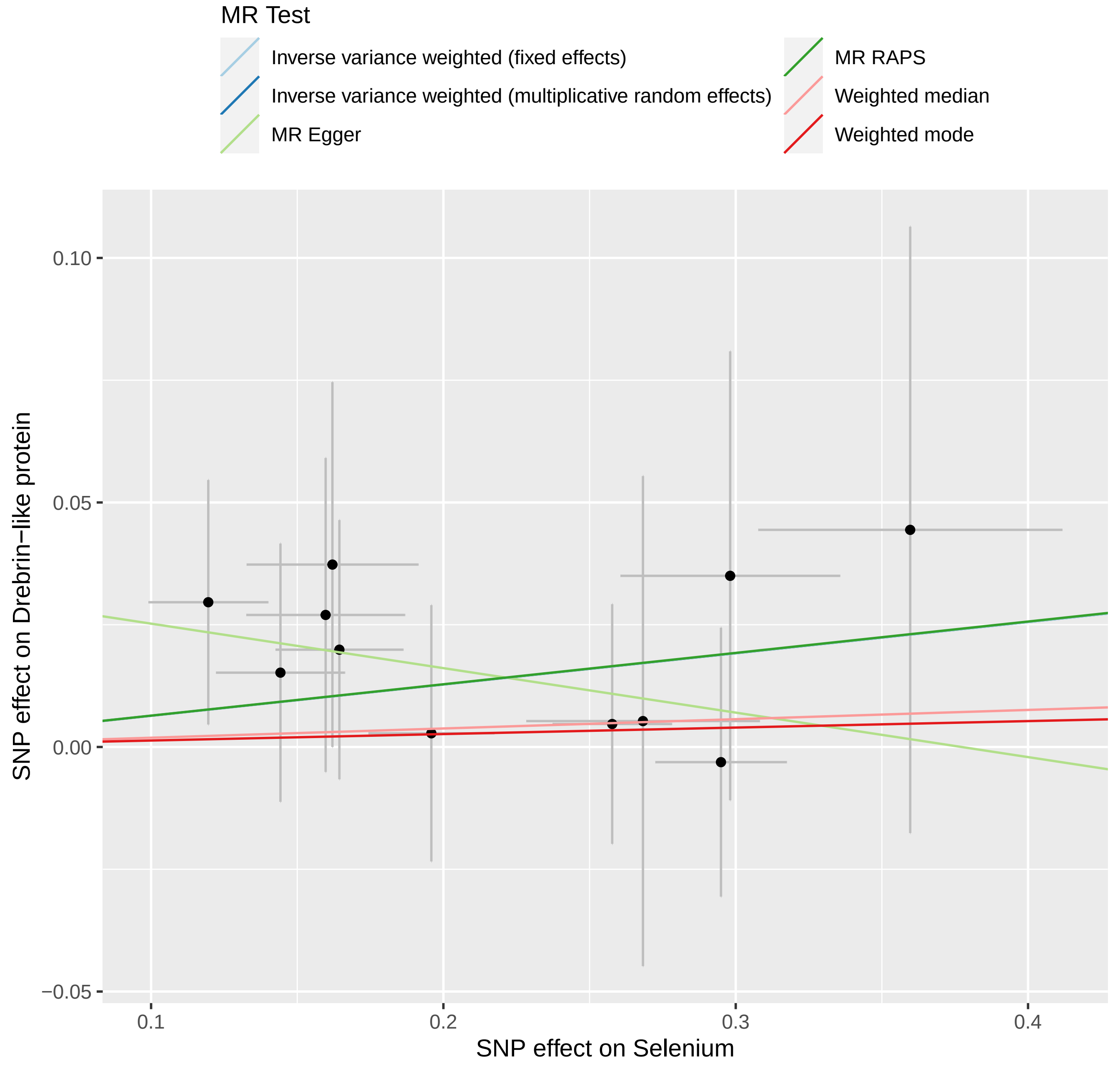

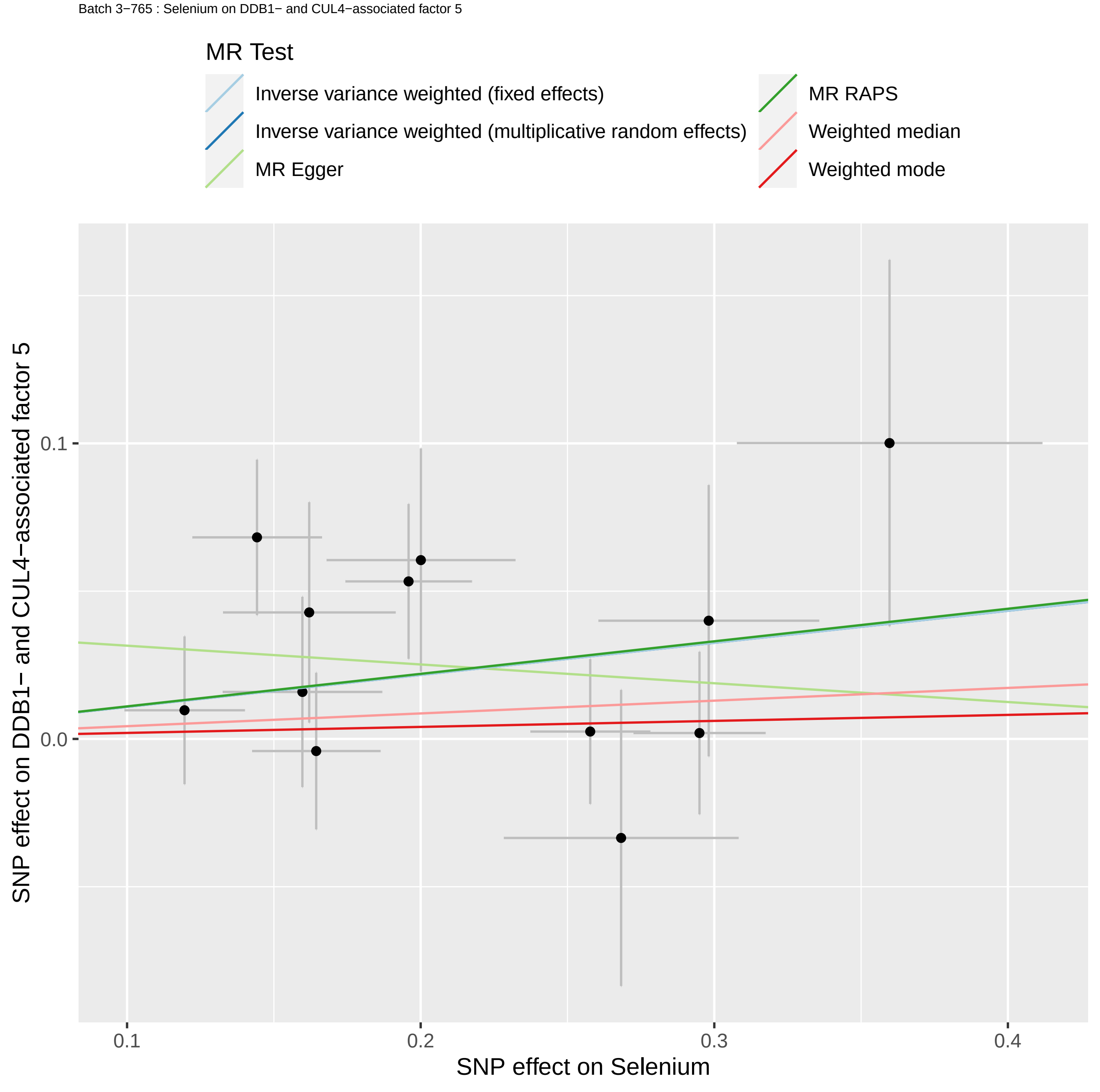

Batch 3−765 : Selenium on DDB1− and CUL4−associated factor 5
MR Test
Inverse variance weighted (fixed effects)
Inverse variance weighted (multiplicative random effects)
MR Egger
MR RAPS
Weighted median
Weighted mode
SNP effect on DDB1− and CUL4−associated factor 5
0.1
0.0
0.1
0.2
0.3
0.4
SNP effect on Selenium

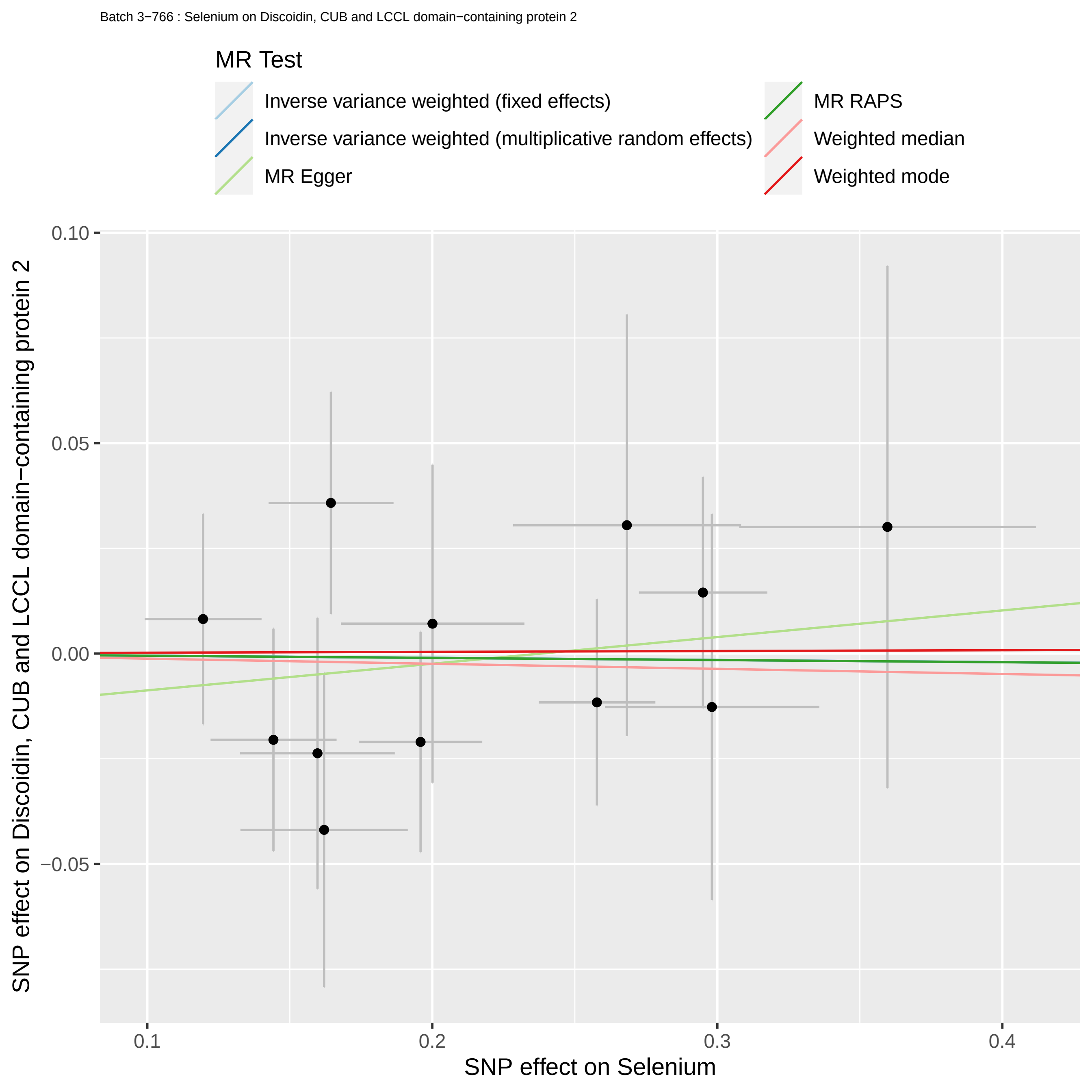
Batch 3−766 : Selenium on Discoidin, CUB and LCCL domain−containing protein 2
MR Test
Inverse variance weighted (fixed effects)
Inverse variance weighted (multiplicative random effects)
MR Egger
MR RAPS
Weighted median
Weighted mode
0.10
0.05
0.00
−0.05
0.1
0.2
0.3
0.4
SNP effect on Discoidin, CUB and LCCL domain−containing protein 2
SNP effect on Selenium

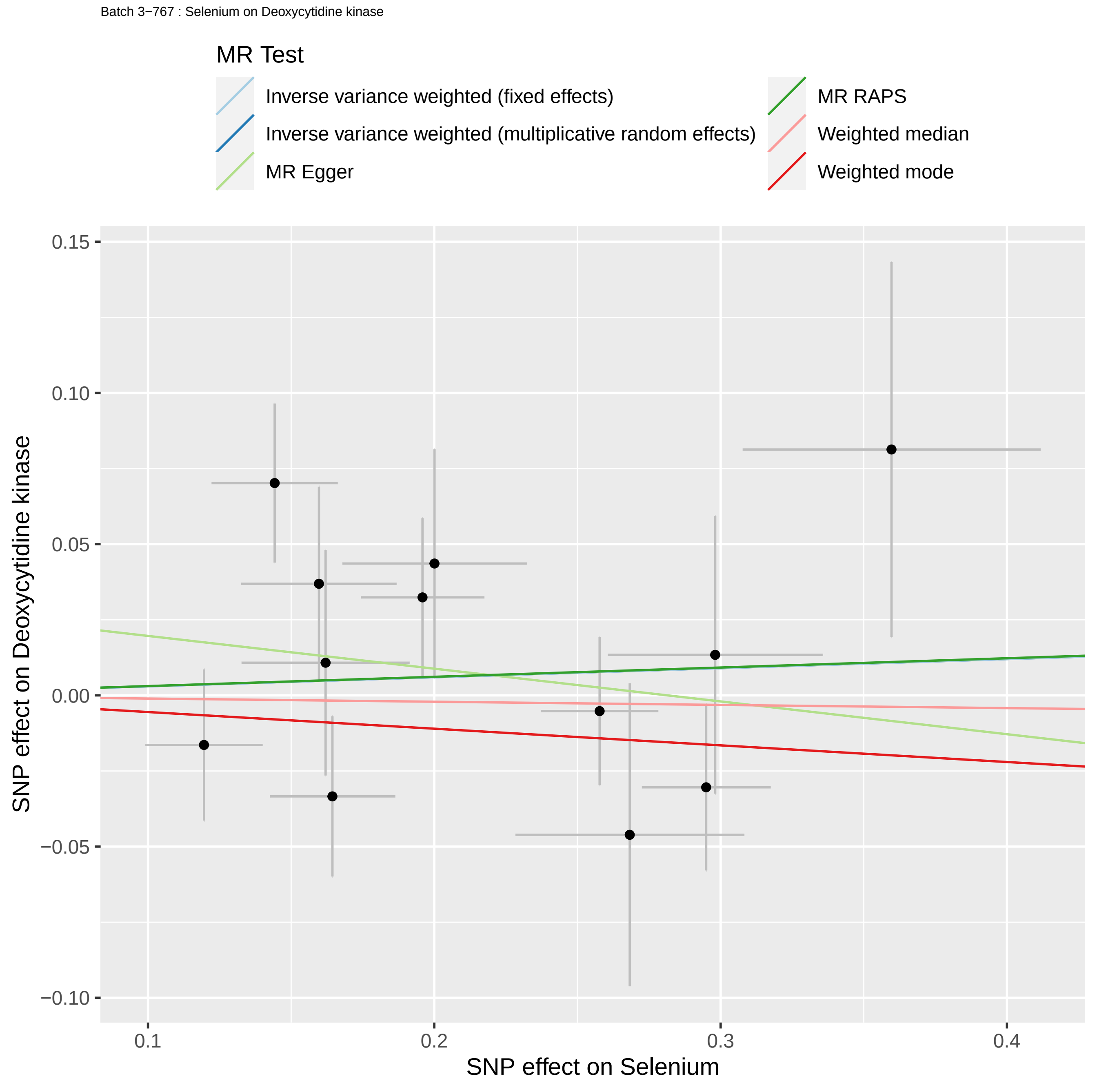
Batch 3−767 : Selenium on Deoxycytidine kinase
MR Test
Inverse variance weighted (fixed effects)
Inverse variance weighted (multiplicative random effects)
MR Egger
MR RAPS
Weighted median
Weighted mode
0.15
0.10
0.05
0.00
−0.05
−0.10
0.1
0.2
0.3
0.4
SNP effect on Deoxycytidine kinase
SNP effect on Selenium

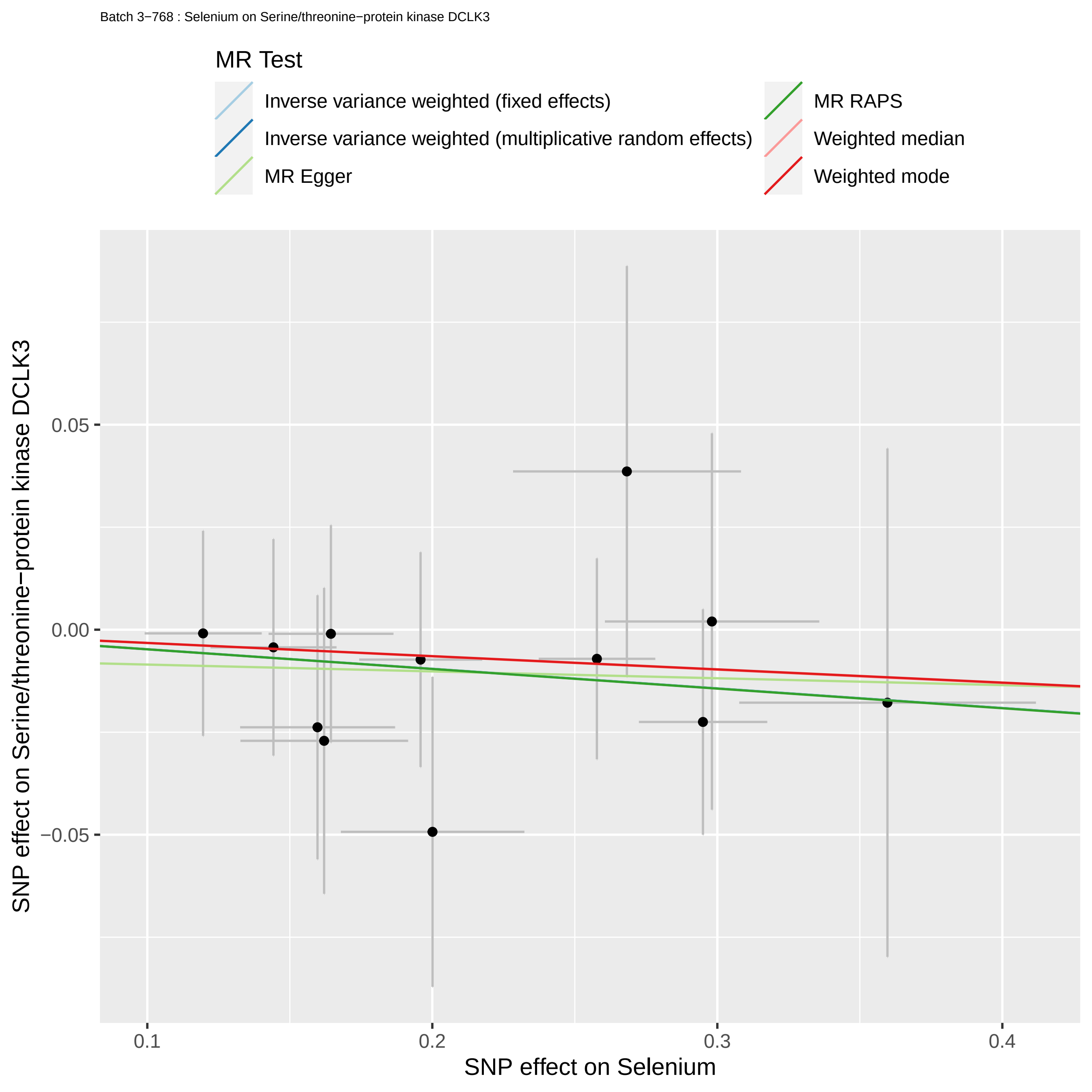
Batch 3−768 : Selenium on Serine/threonine−protein kinase DCLK3
MR Test
Inverse variance weighted (fixed effects)
Inverse variance weighted (multiplicative random effects)
MR Egger
MR RAPS
Weighted median
Weighted mode
SNP effect on Serine/threonine−protein kinase DCLK3
0.05
0.00
−0.05
0.1
0.2
0.3
0.4
SNP effect on Selenium

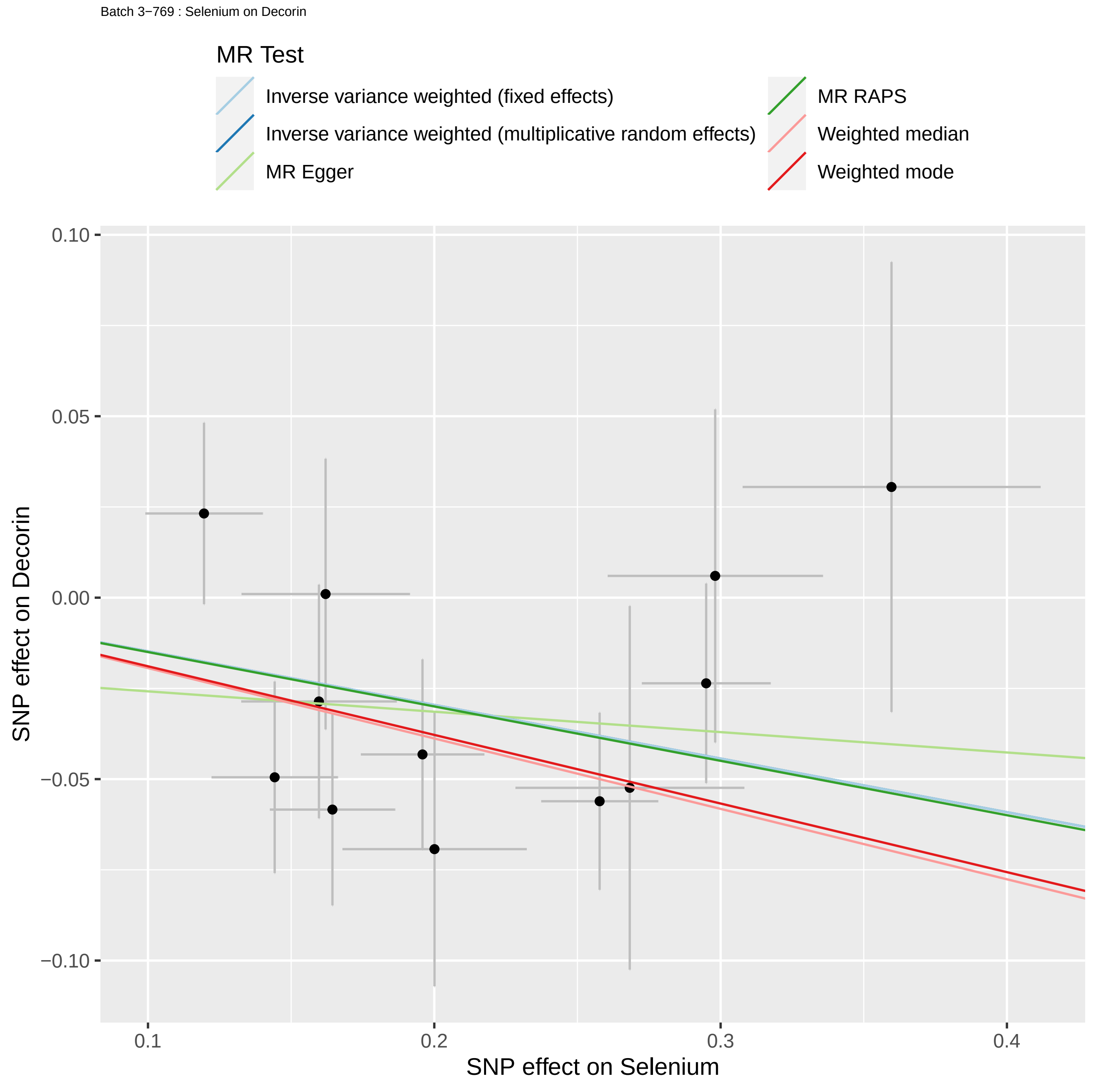
Batch 3−769 : Selenium on Decorin
MR Test
Inverse variance weighted (fixed effects)
Inverse variance weighted (multiplicative random effects)
MR Egger
MR RAPS
Weighted median
Weighted mode
0.10
0.05
0.00
−0.05
−0.10
SNP effect on Decorin
0.1
0.2
0.3
0.4
SNP effect on Selenium

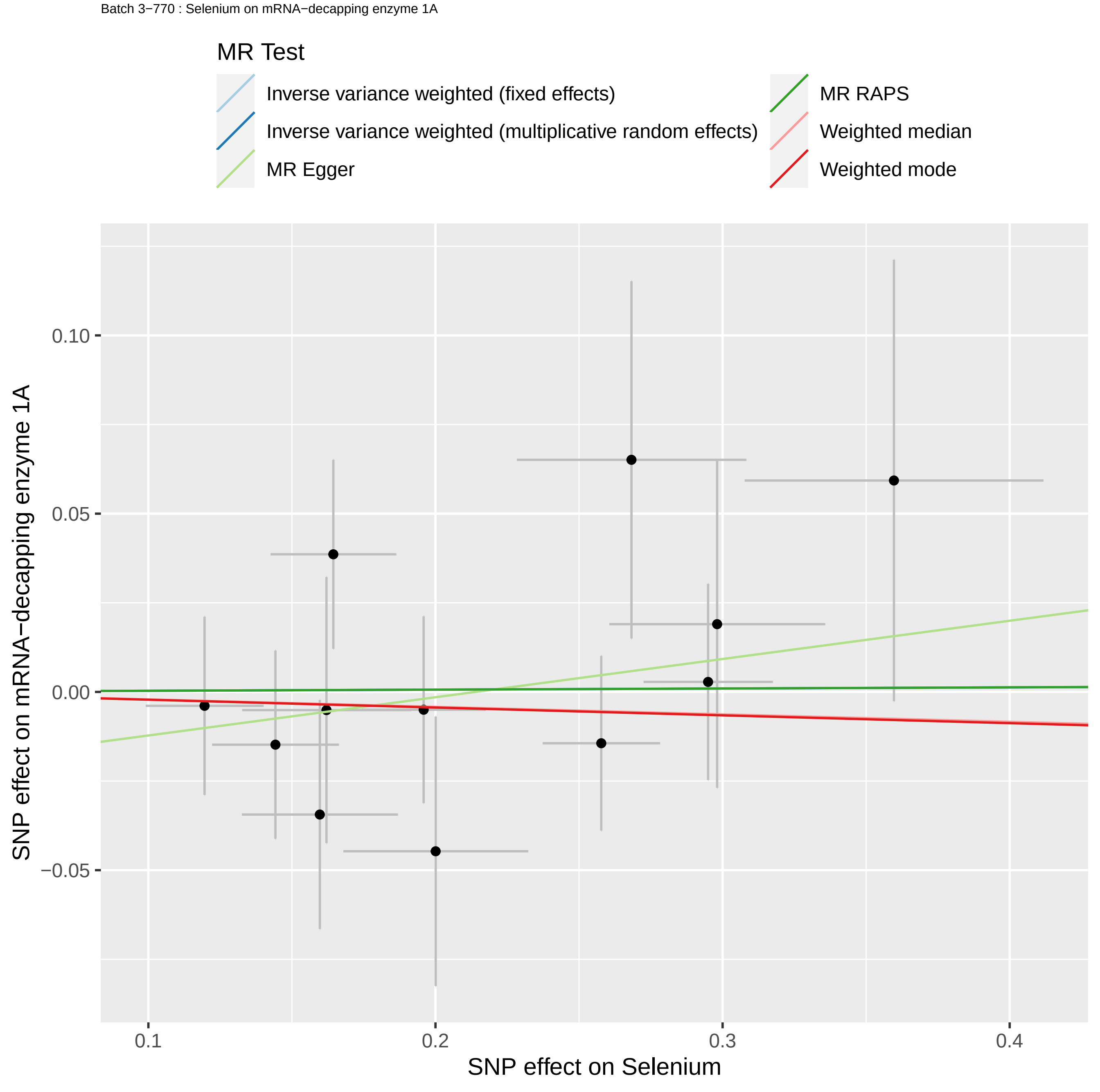

Batch 3−770 : Selenium on mRNA−decapping enzyme 1A
MR Test
Inverse variance weighted (fixed effects)
Inverse variance weighted (multiplicative random effects)
MR Egger
MR RAPS
Weighted median
Weighted mode
0.10
0.05
0.00
−0.05
0.1
0.2
0.3
0.4
SNP effect on mRNA−decapping enzyme 1A
SNP effect on Selenium

Batch 3−771 : Selenium on Dynactin subunit 2

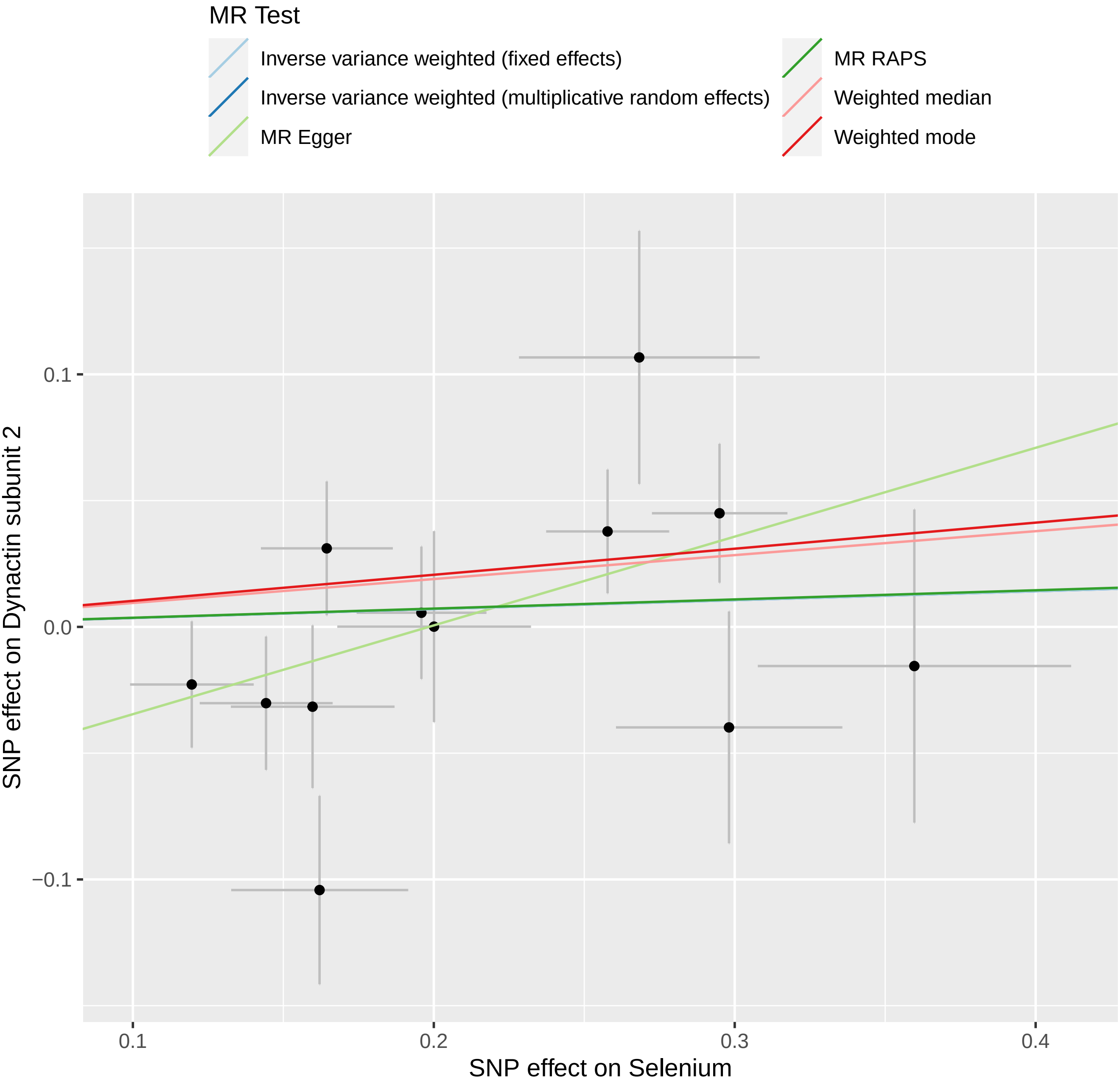

Batch 3−772 : Selenium on dCTP pyrophosphatase 1

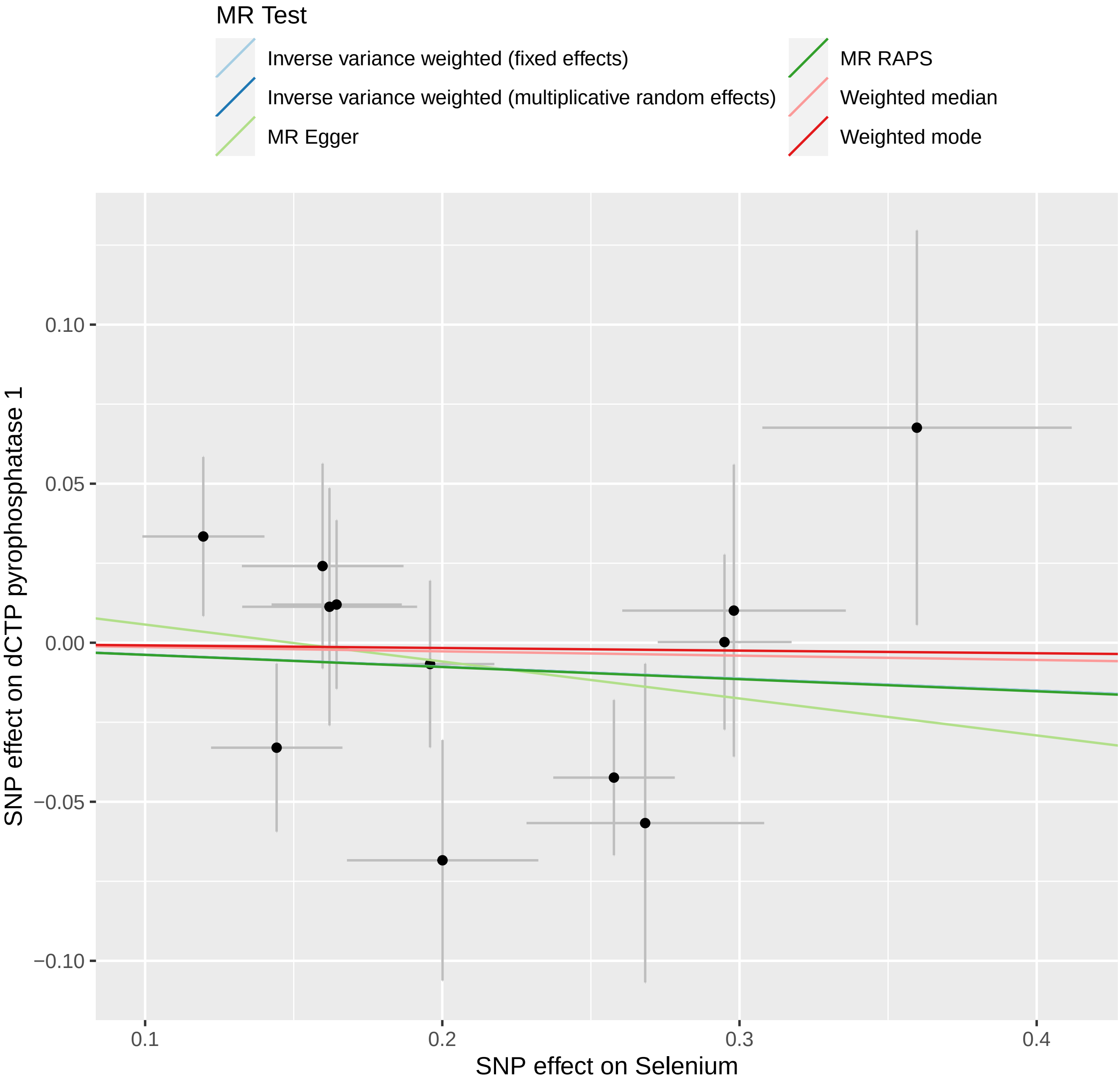

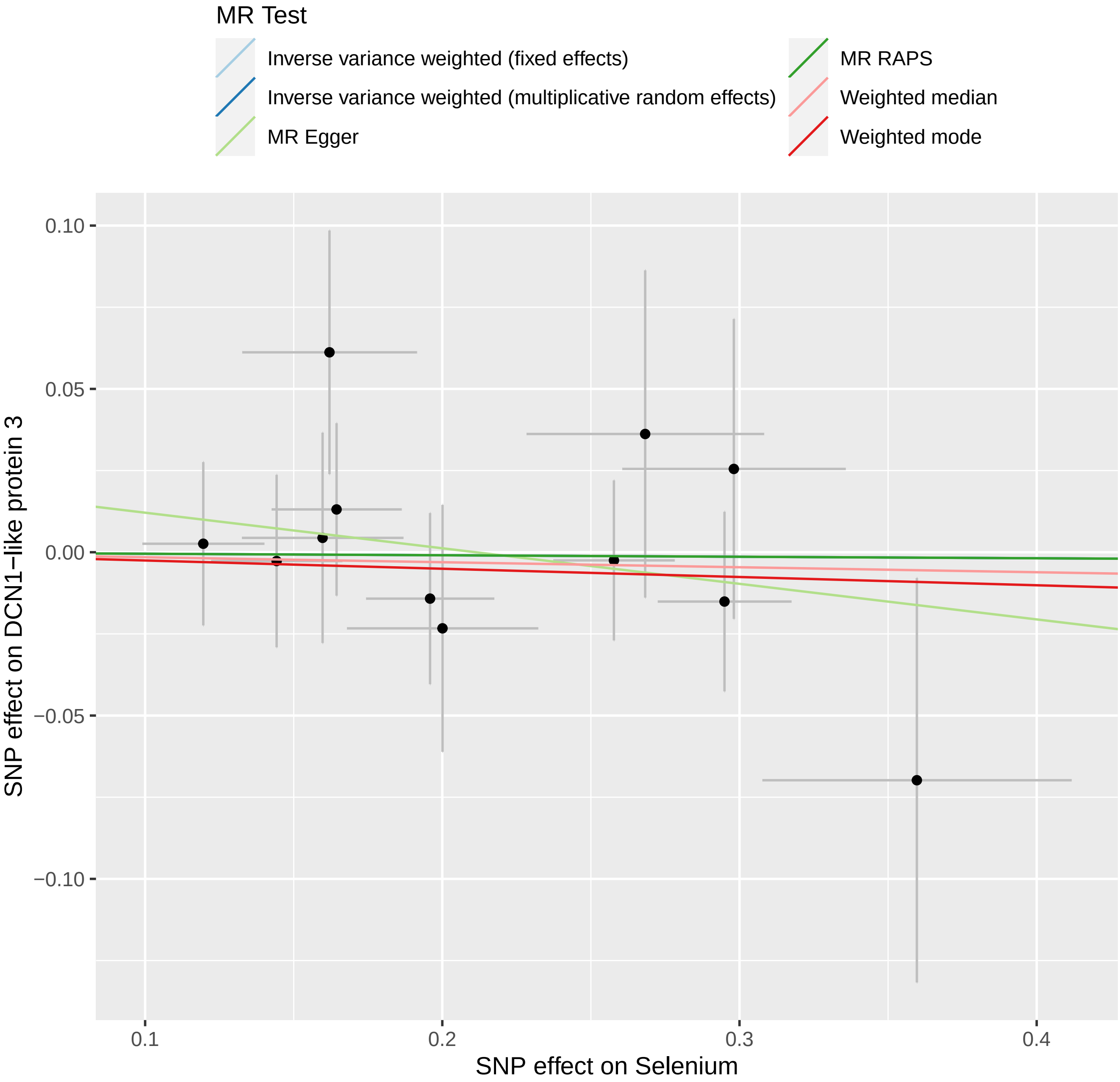
MR Test
Inverse variance weighted (fixed effects)
Inverse variance weighted (multiplicative random effects)
MR Egger
MR RAPS
Weighted median
Weighted mode
0.10
0.05
0.00
−0.05
−0.10
SNP effect on DCN1−like protein 3
0.1
0.2
0.3
0.4
SNP effect on Selenium

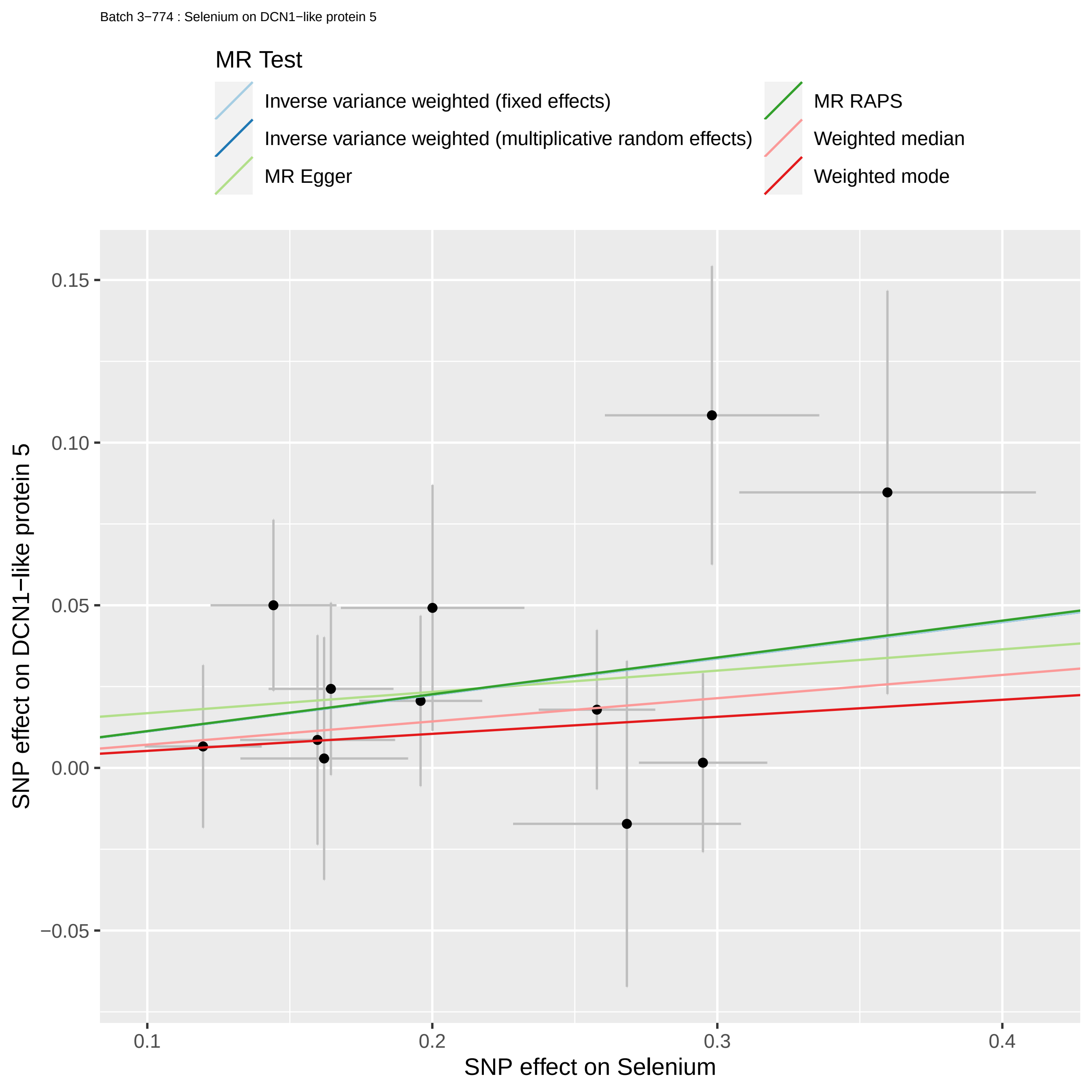
Batch 3−774 : Selenium on DCN1−like protein 5
MR Test
Inverse variance weighted (fixed effects)
Inverse variance weighted (multiplicative random effects)
MR Egger
MR RAPS
Weighted median
Weighted mode
0.15
0.10
0.05
0.00
−0.05
0.1
0.2
0.3
0.4
SNP effect on DCN1−like protein 5
SNP effect on Selenium

Batch 3−775 : Selenium on Aromatic−L−amino−acid decarboxylase

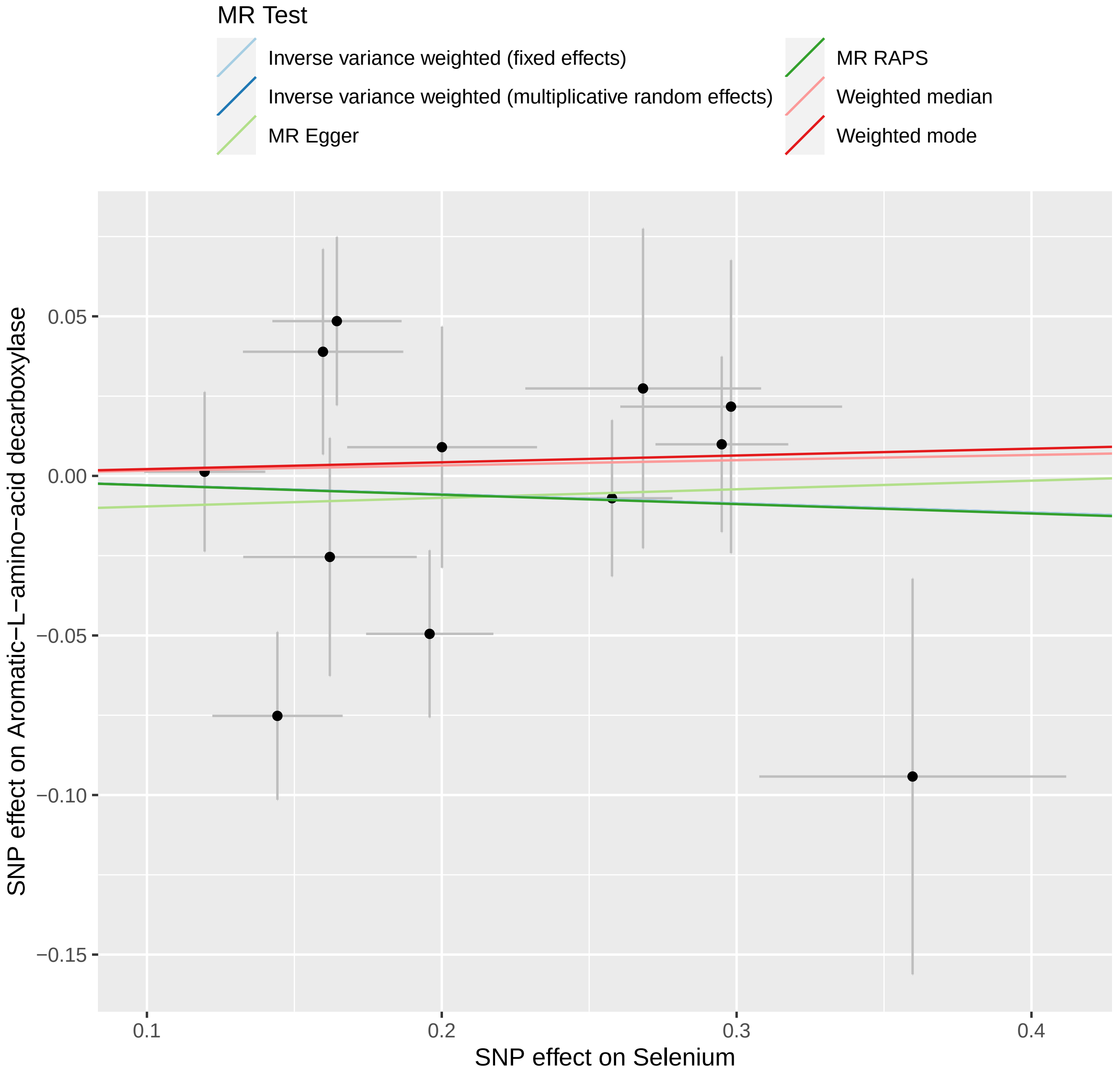

Batch 3−776 : Selenium on Discoidin domain−containing receptor 2

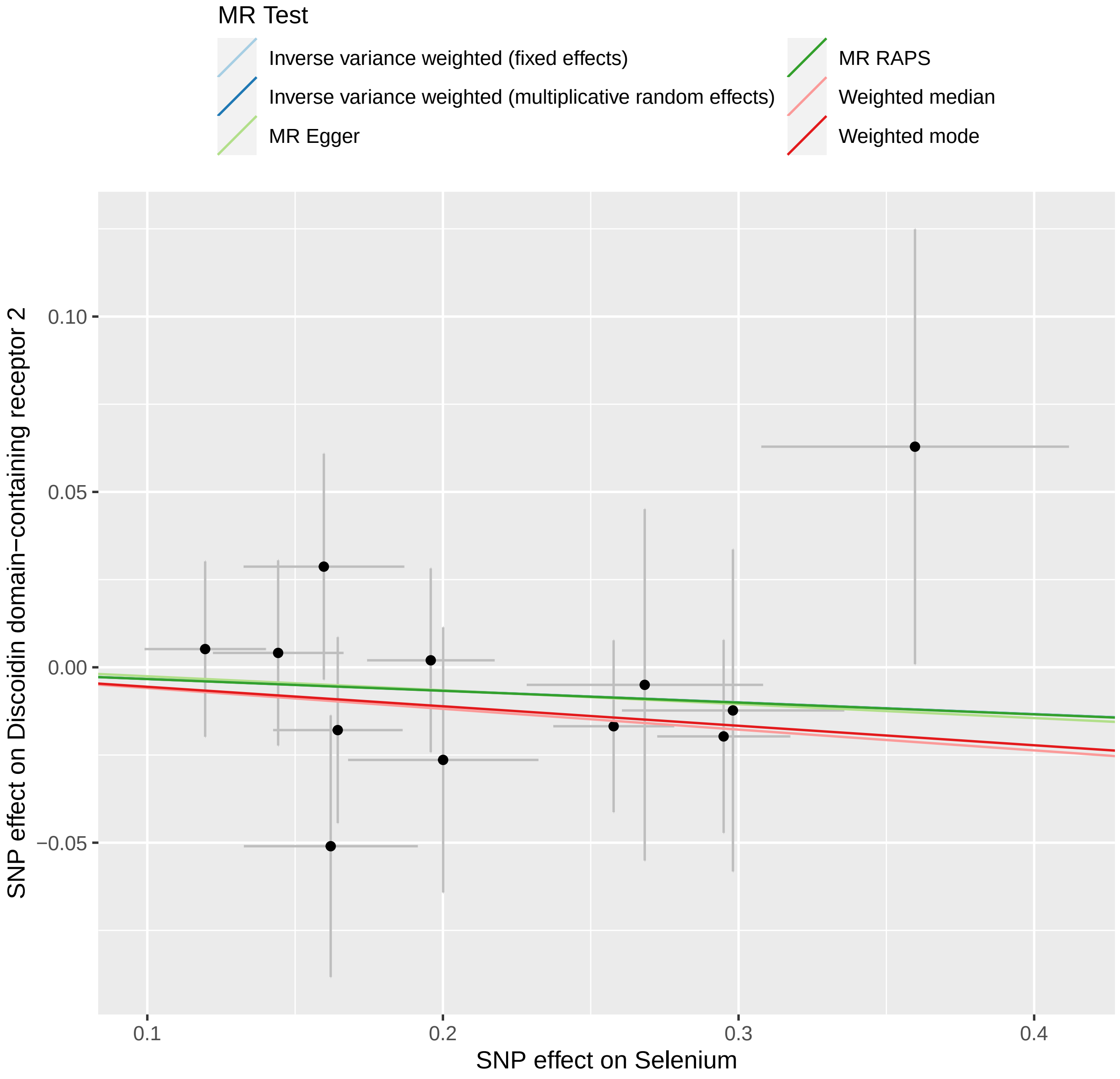

Batch 3−777 : Selenium on ATP−dependent RNA helicase DDX25

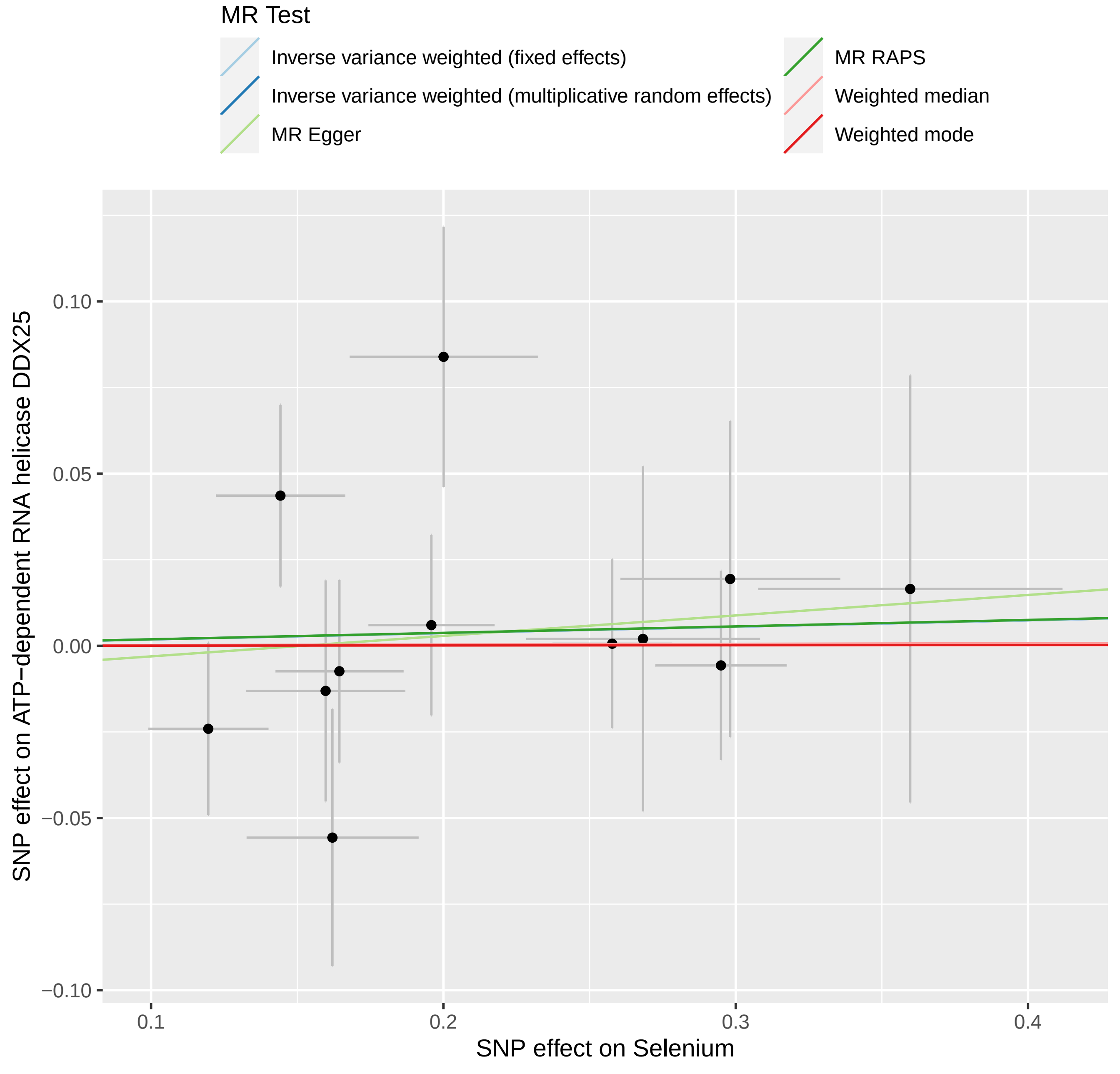

Batch 3−778 : Selenium on Spliceosome RNA helicase DDX39B

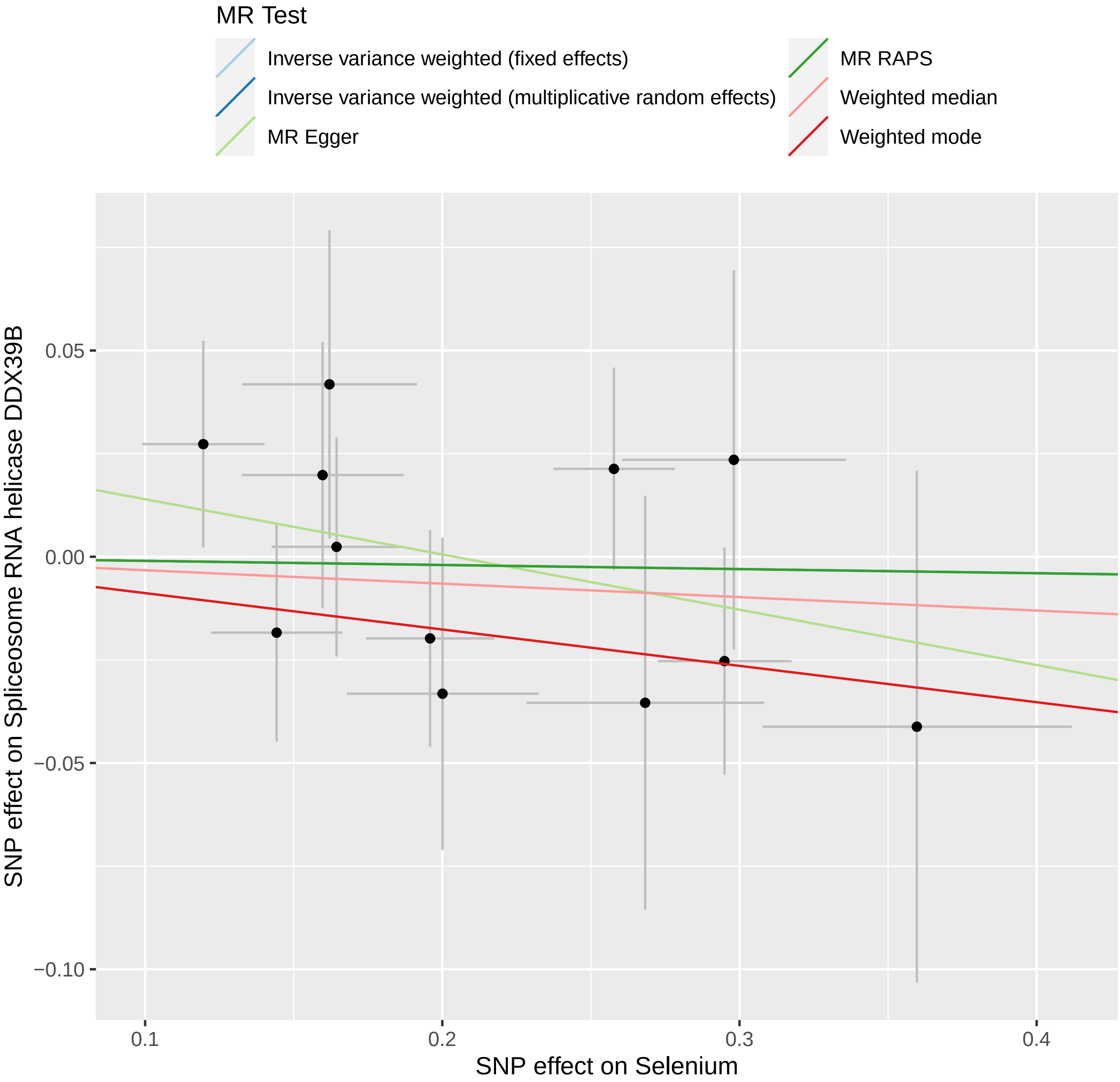

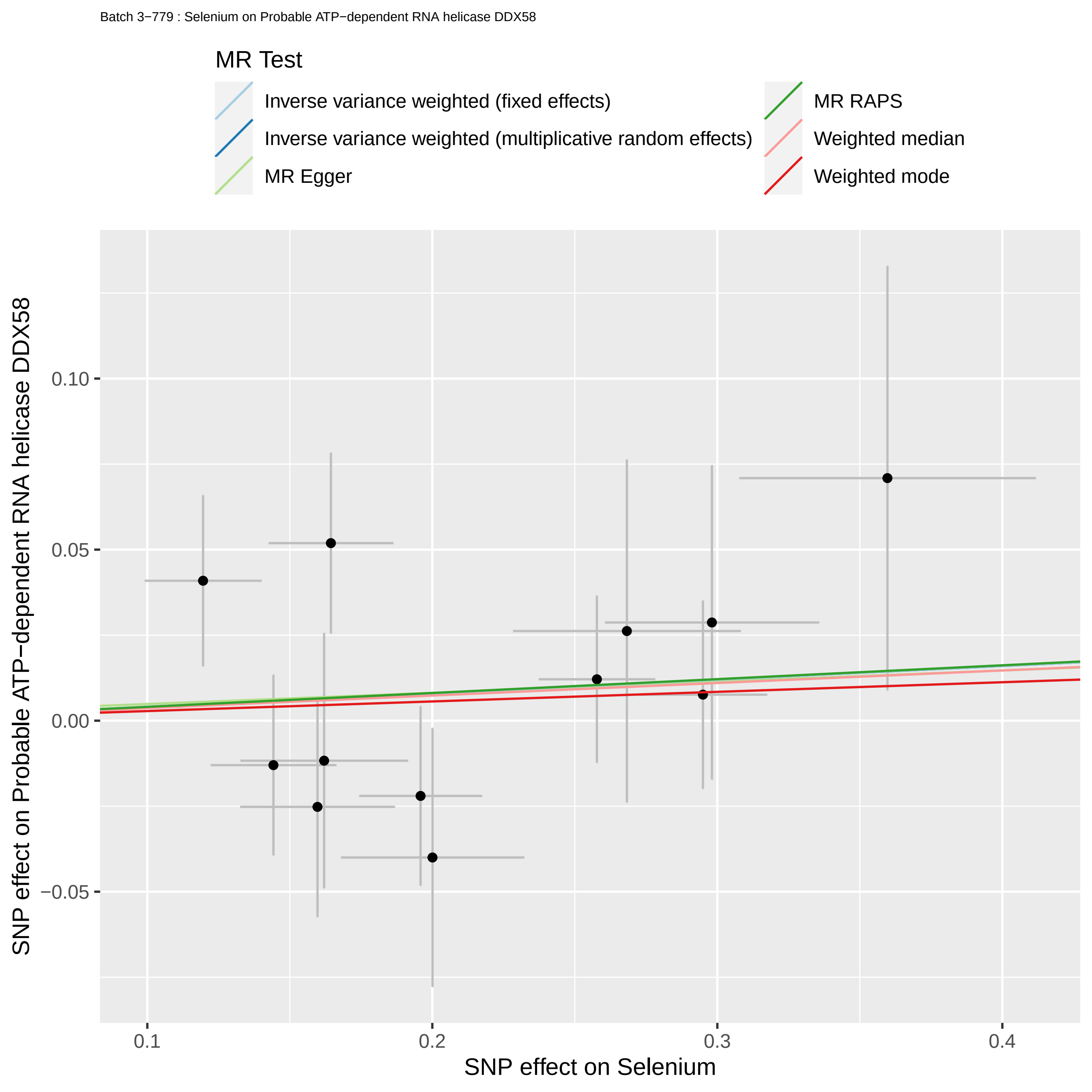
Batch 3−779 : Selenium on Probable ATP−dependent RNA helicase DDX58
MR Test
Inverse variance weighted (fixed effects)
Inverse variance weighted (multiplicative random effects)
MR Egger
MR RAPS
Weighted median
Weighted mode
SNP effect on Probable ATP−dependent RNA helicase DDX58
0.10
0.05
0.00
−0.05
0.1
0.2
0.3
0.4
SNP effect on Selenium

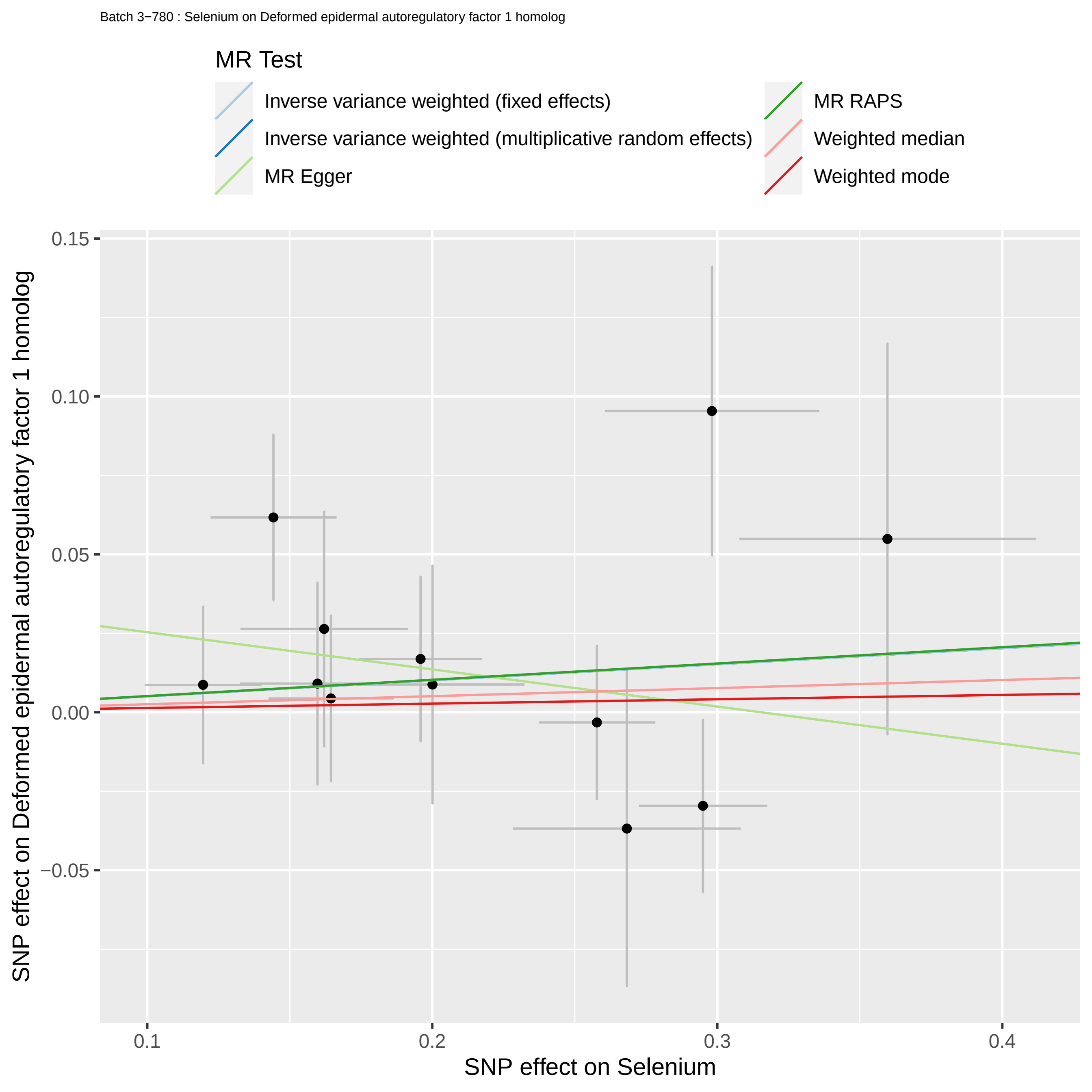

Batch 3−780 : Selenium on Deformed epidermal autoregulatory factor 1 homolog
MR Test
Inverse variance weighted (fixed effects)
Inverse variance weighted (multiplicative random effects)
MR Egger
MR RAPS
Weighted median
Weighted mode
SNP effect on Deformed epidermal autoregulatory factor 1 homolog
0.15
0.10
0.05
0.00
−0.05
0.1
0.2
0.3
0.4
SNP effect on Selenium

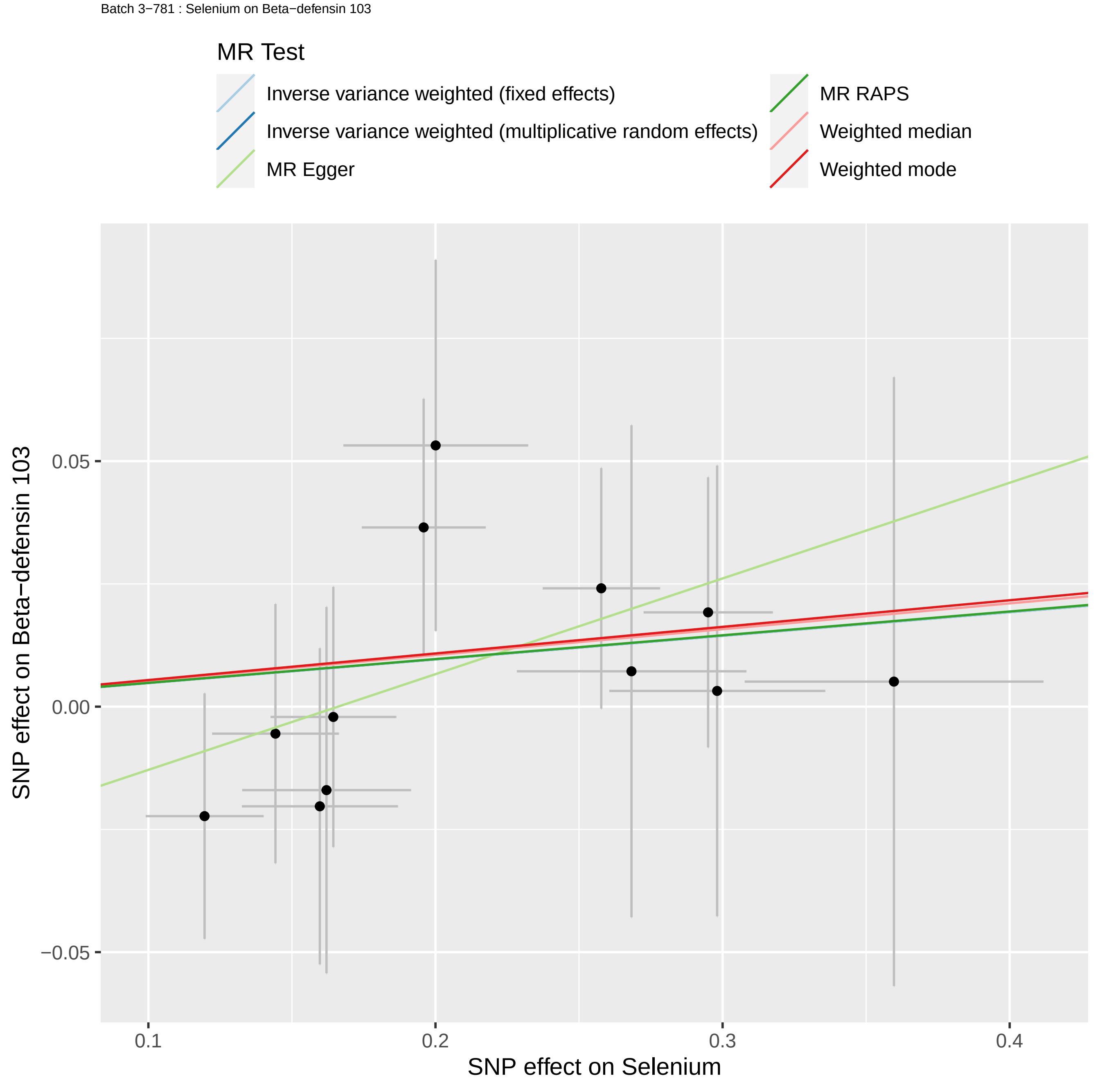
Batch 3−781 : Selenium on Beta−defensin 103
MR Test
Inverse variance weighted (fixed effects)
Inverse variance weighted (multiplicative random effects)
MR Egger
MR RAPS
Weighted median
Weighted mode
SNP effect on Beta−defensin 103
0.05
0.00
−0.05
0.1
0.2
0.3
0.4
SNP effect on Selenium

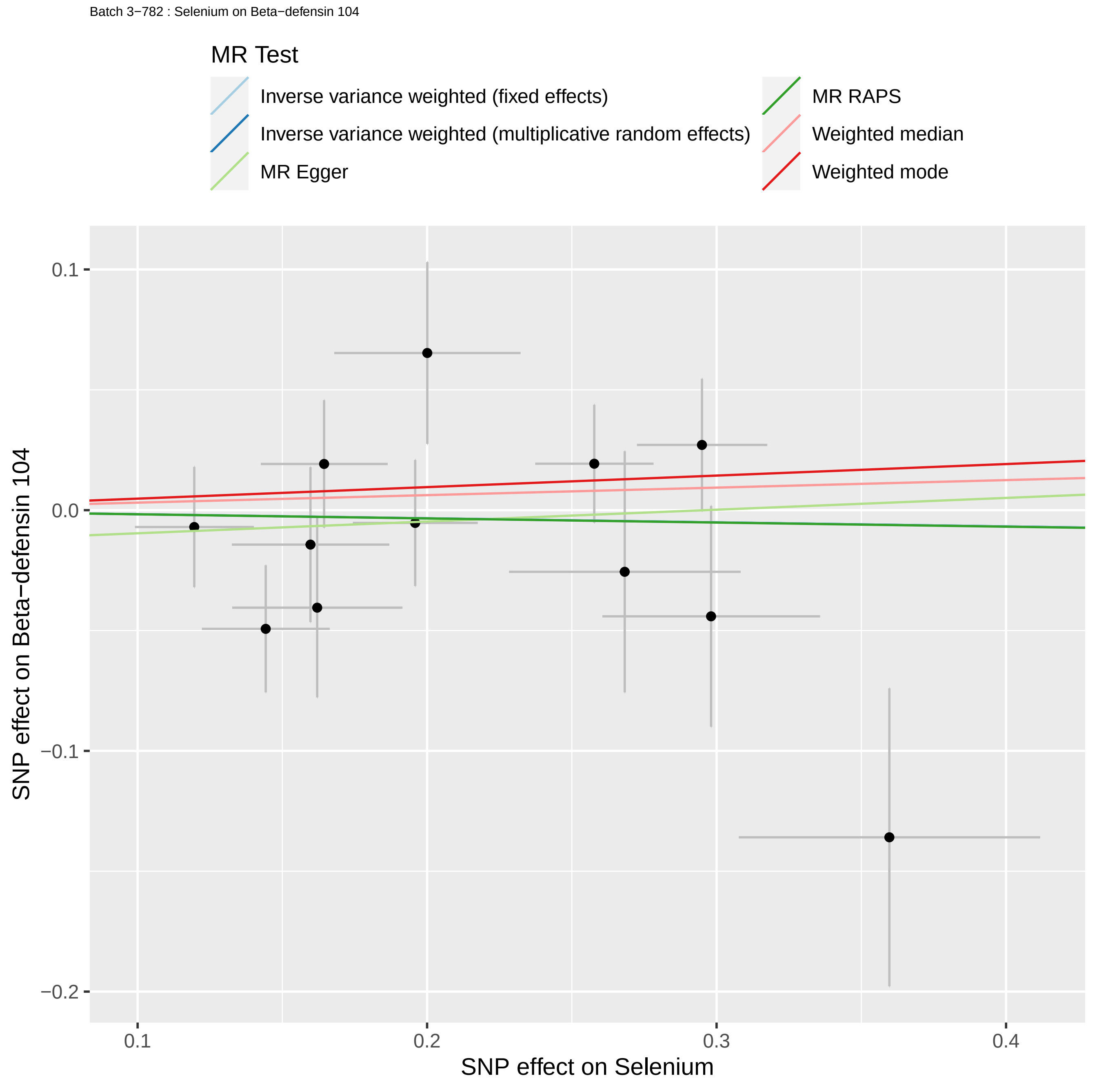
Batch 3−782 : Selenium on Beta−defensin 104
MR Test
Inverse variance weighted (fixed effects)
Inverse variance weighted (multiplicative random effects)
MR Egger
MR RAPS
Weighted median
Weighted mode
0.1
0.0
−0.1
−0.2
0.1
0.2
0.3
0.4
SNP effect on Beta−defensin 104
SNP effect on Selenium

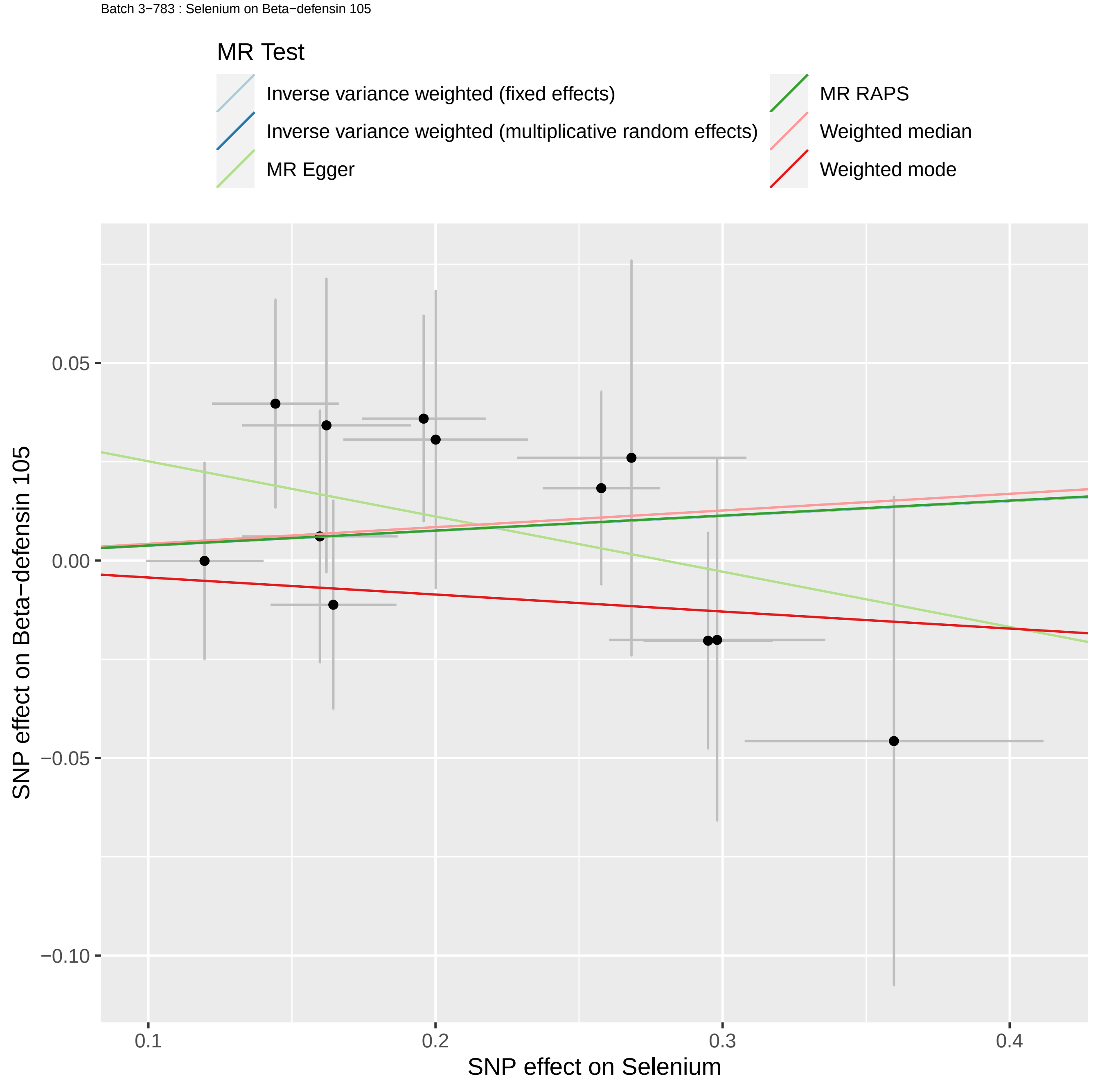
Batch 3−783 : Selenium on Beta−defensin 105
MR Test
Inverse variance weighted (fixed effects)
Inverse variance weighted (multiplicative random effects)
MR Egger
MR RAPS
Weighted median
Weighted mode
0.05
0.00
−0.05
−0.10
0.1
0.2
0.3
0.4
SNP effect on Beta−defensin 105
SNP effect on Selenium

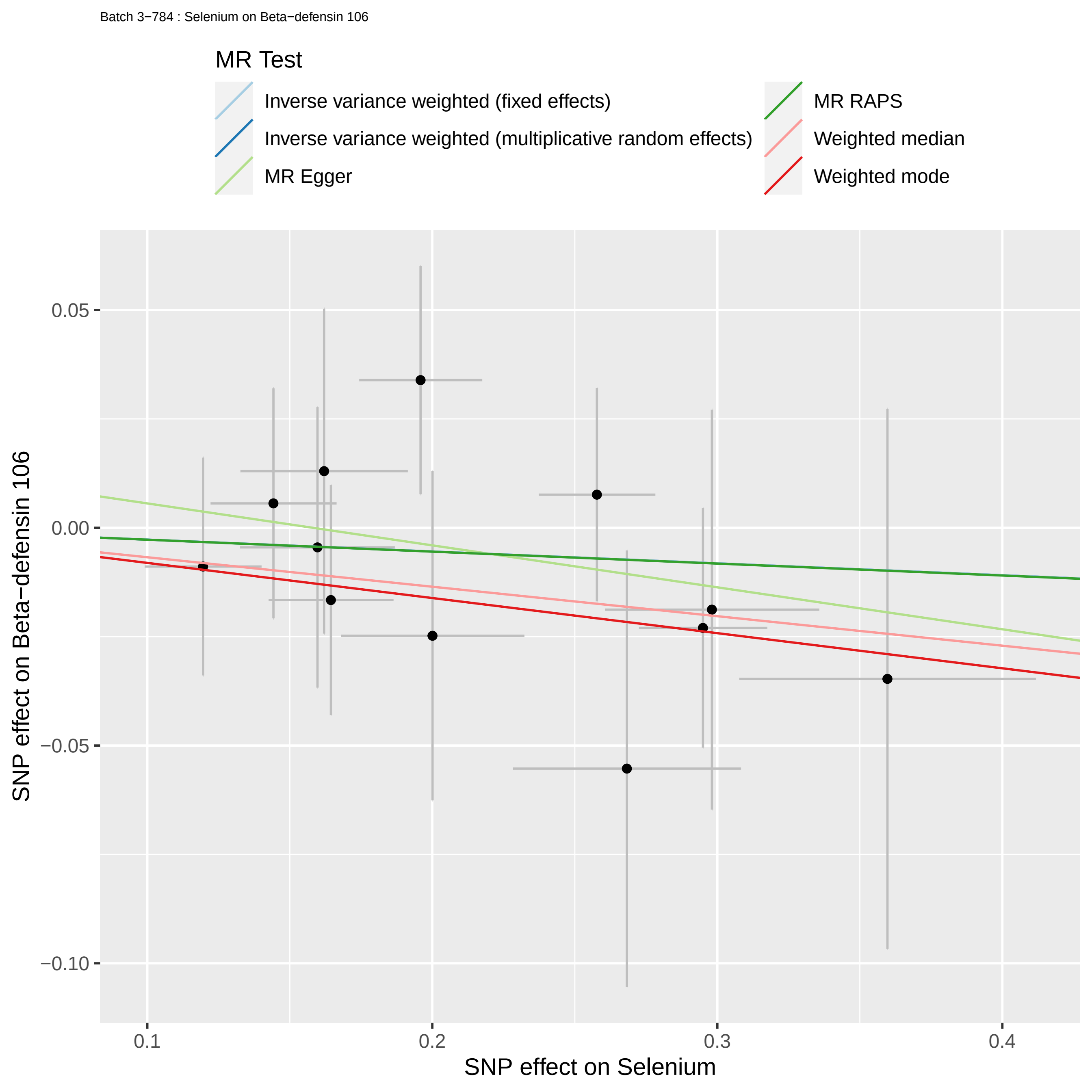

Batch 3−784 : Selenium on Beta−defensin 106
MR Test
Inverse variance weighted (fixed effects)
Inverse variance weighted (multiplicative random effects)
MR Egger
MR RAPS
Weighted median
Weighted mode
0.05
0.00
−0.05
−0.10
0.1
0.2
0.3
0.4
SNP effect on Beta−defensin 106
SNP effect on Selenium

Batch 3−785 : Selenium on Beta−defensin 107

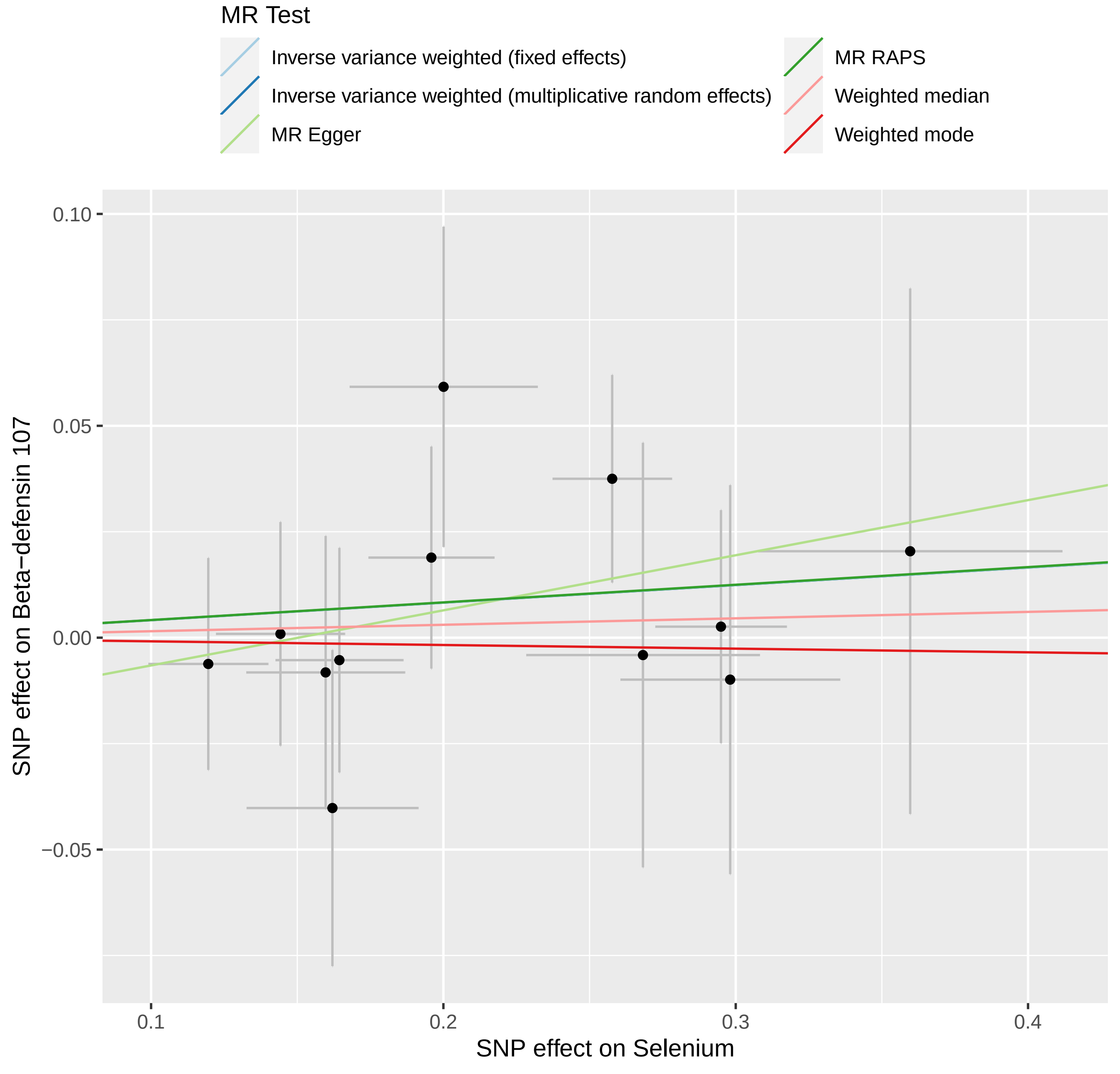

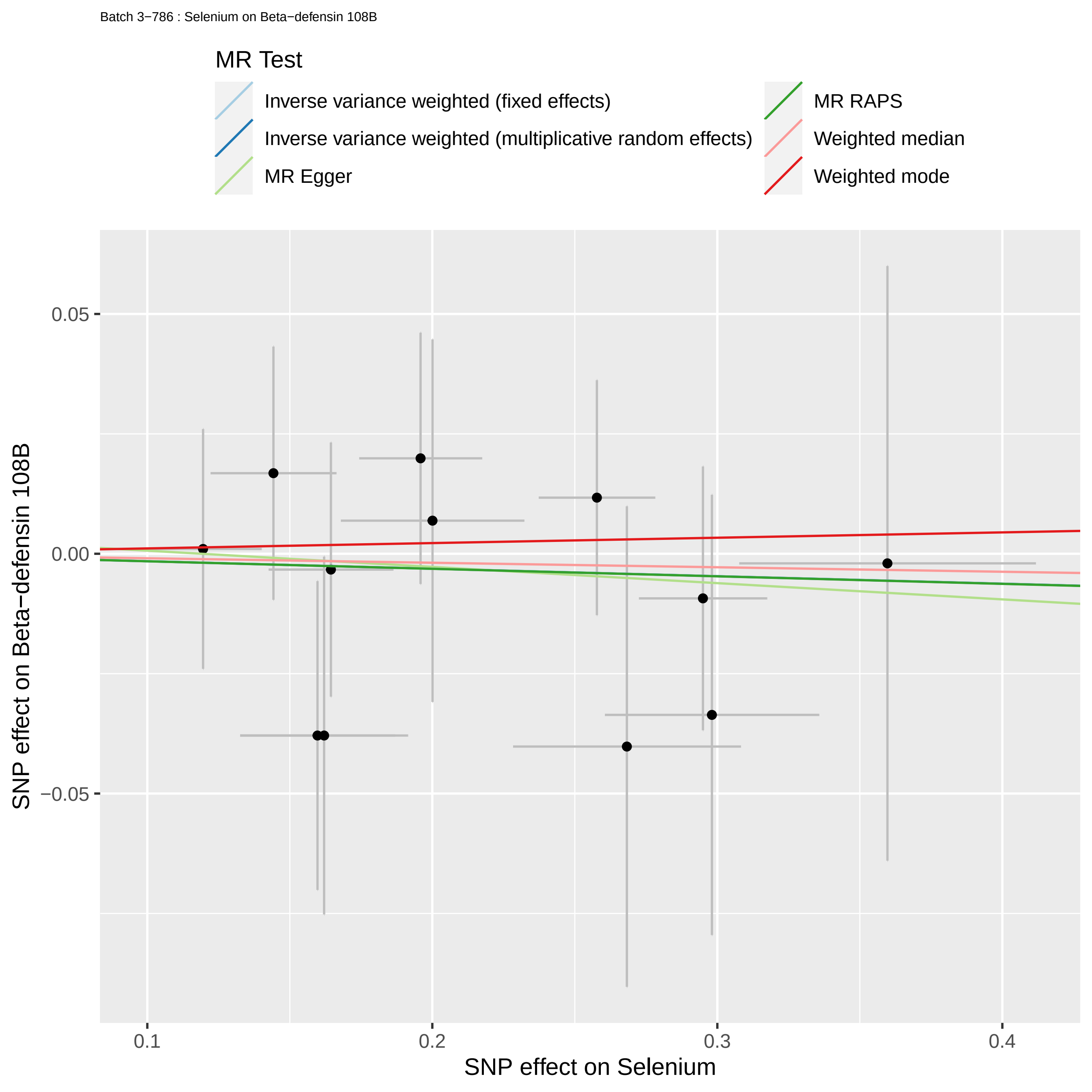

Batch 3−786 : Selenium on Beta−defensin 108B
MR Test
Inverse variance weighted (fixed effects)
Inverse variance weighted (multiplicative random effects)
MR Egger
MR RAPS
Weighted median
Weighted mode
0.05
0.00
−0.05
0.1
0.2
0.3
0.4
SNP effect on Beta−defensin 108B
SNP effect on Selenium

Batch 3−787 : Selenium on Beta−defensin 110

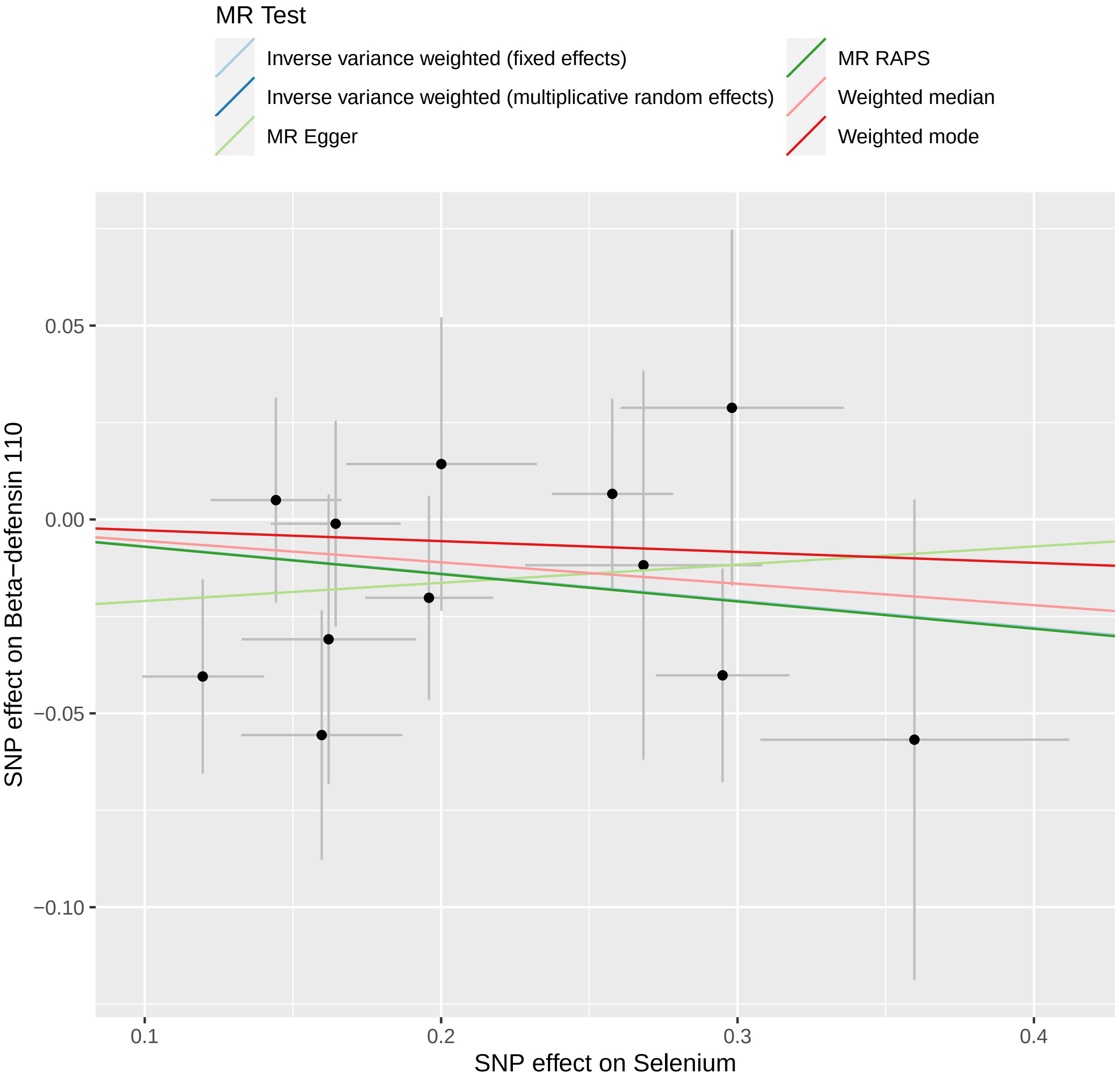

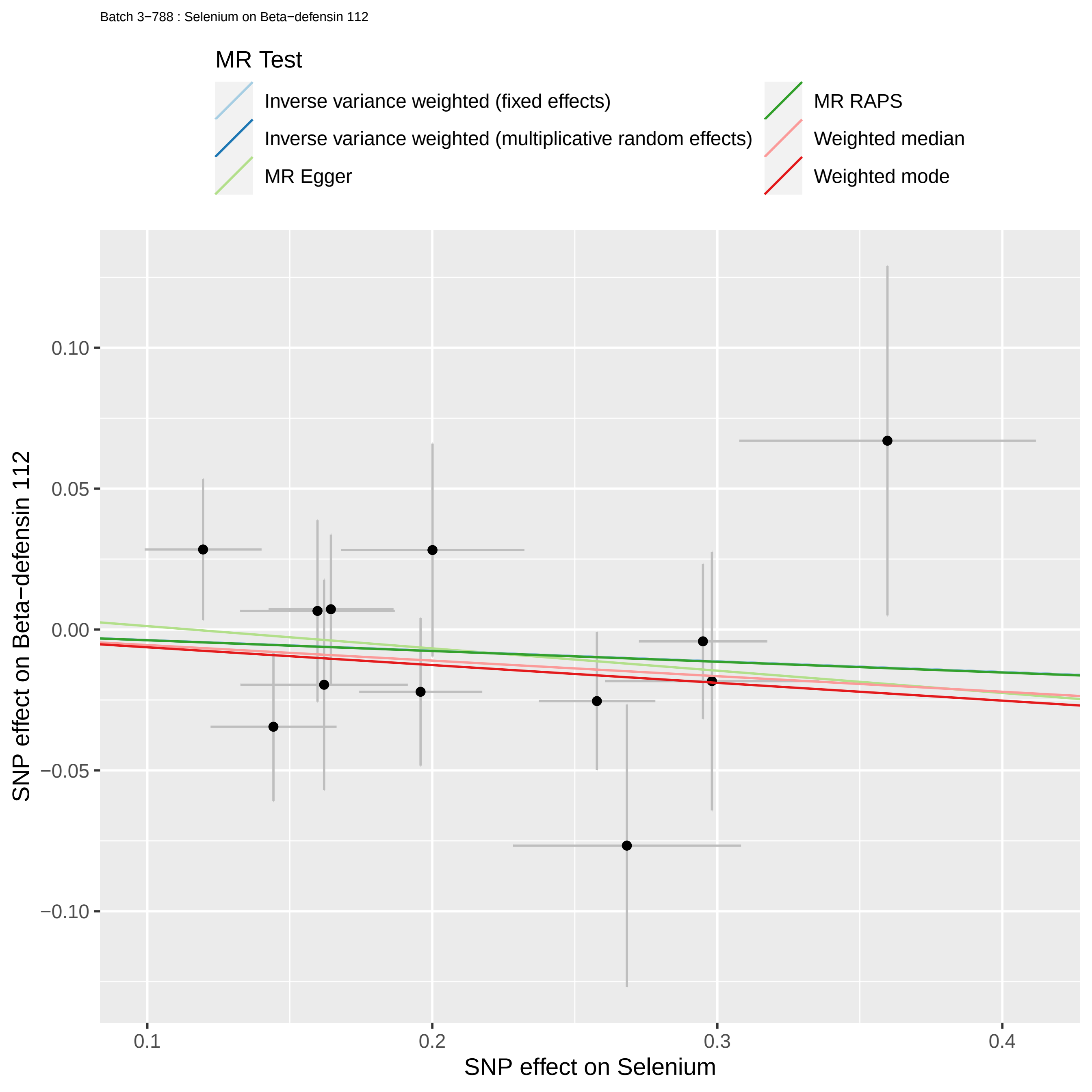

Batch 3−788 : Selenium on Beta−defensin 112
MR Test
Inverse variance weighted (fixed effects)
Inverse variance weighted (multiplicative random effects)
MR Egger
MR RAPS
Weighted median
Weighted mode
0.10
0.05
0.00
−0.05
−0.10
0.1
0.2
0.3
0.4
SNP effect on Beta−defensin 112
SNP effect on Selenium

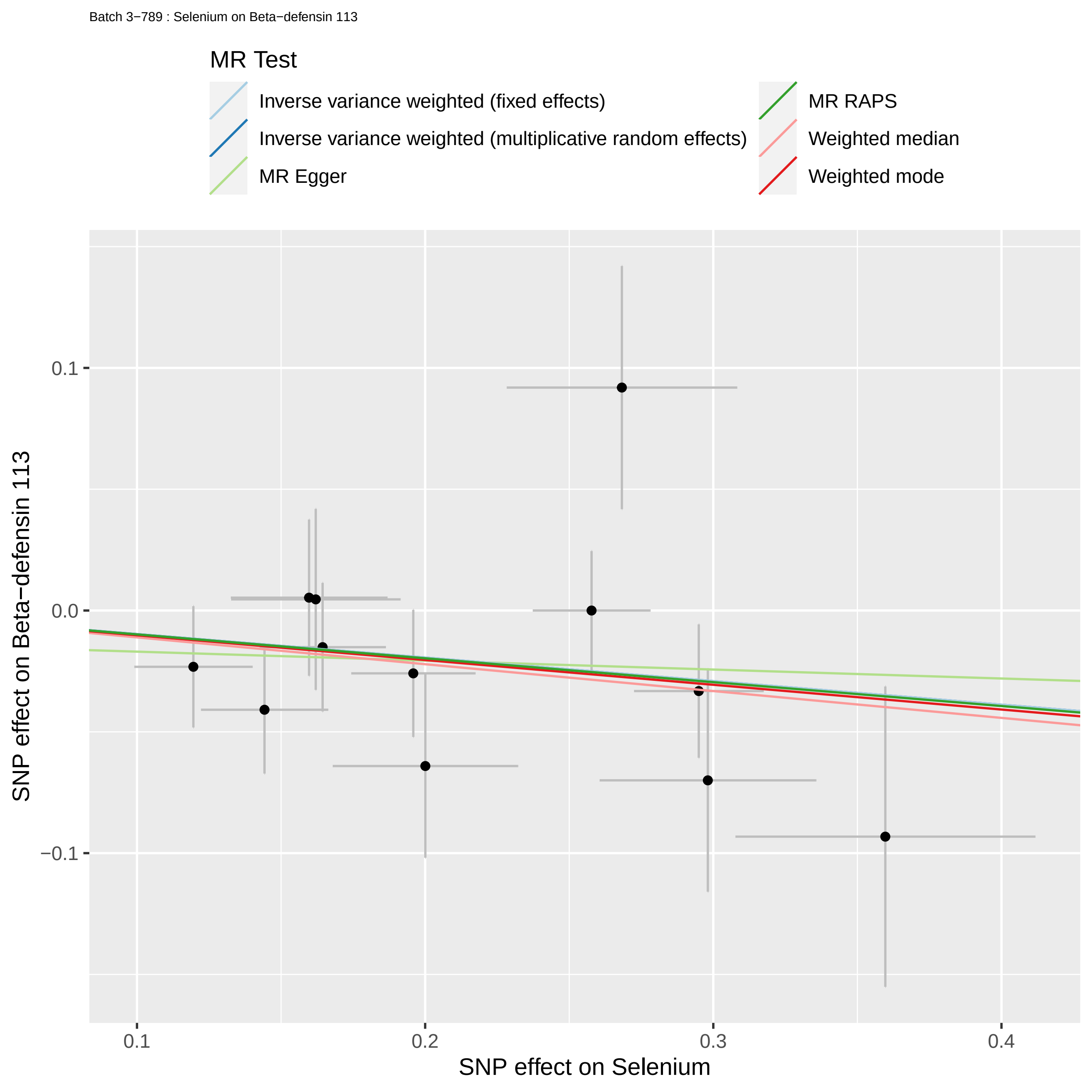

Batch 3−789 : Selenium on Beta−defensin 113
MR Test
Inverse variance weighted (fixed effects)
Inverse variance weighted (multiplicative random effects)
MR Egger
MR RAPS
Weighted median
Weighted mode
SNP effect on Beta−defensin 113
SNP effect on Selenium
0.1
0.0
−0.1
0.1
0.2
0.3
0.4

Batch 3−790 : Selenium on Beta−defensin 115

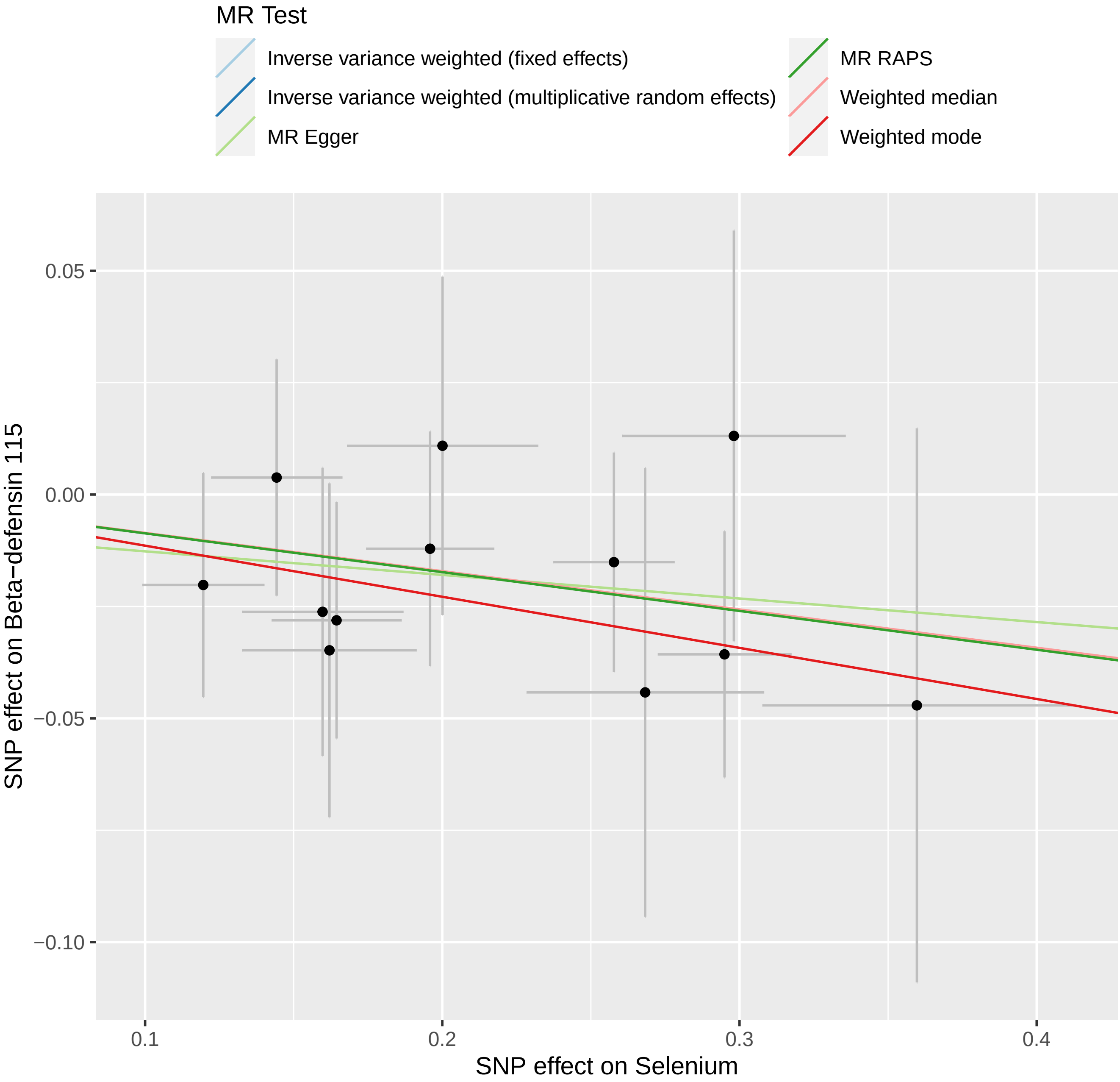

Batch 3−791 : Selenium on Beta−defensin 116

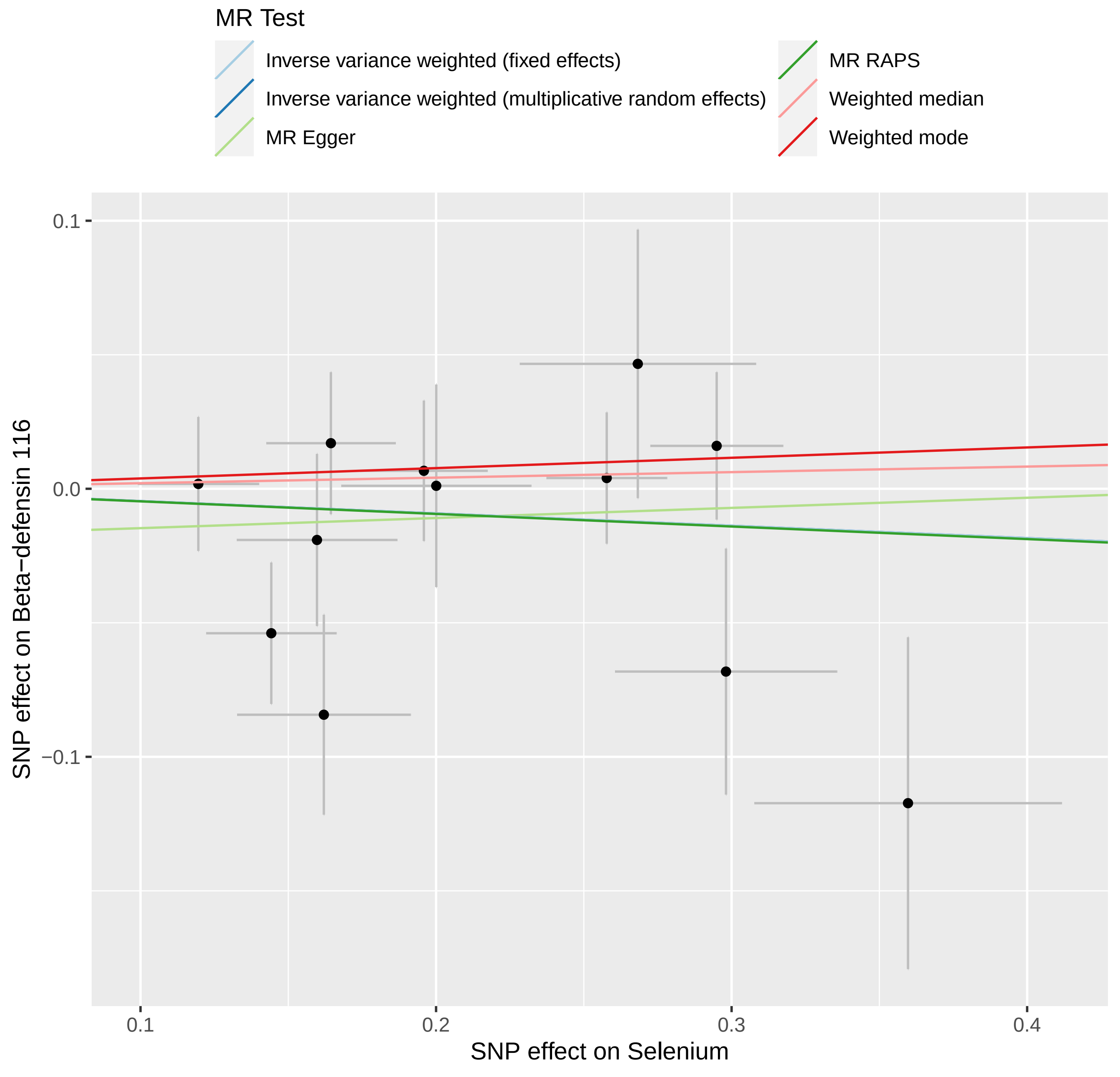

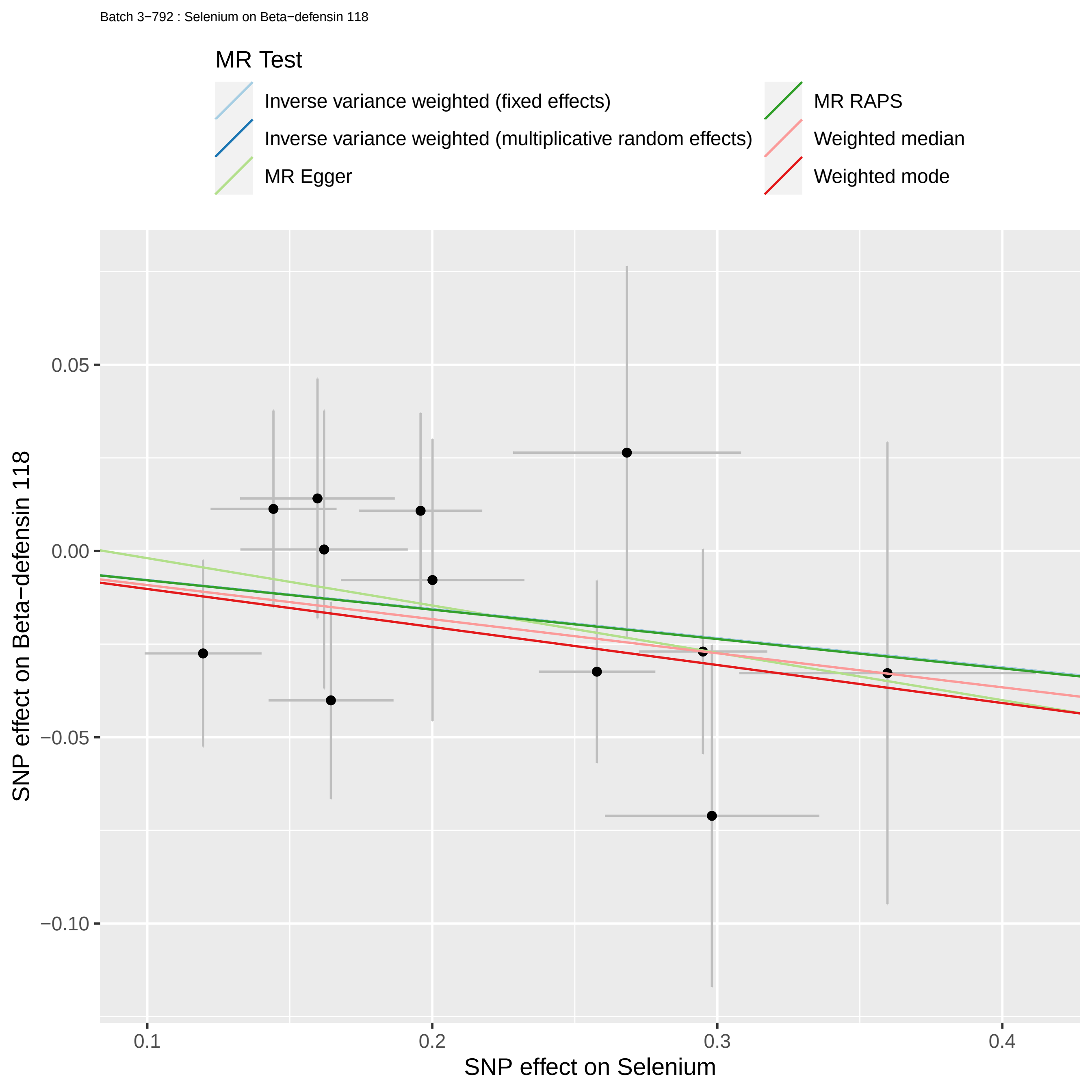

Batch 3−792 : Selenium on Beta−defensin 118
MR Test
Inverse variance weighted (fixed effects)
Inverse variance weighted (multiplicative random effects)
MR Egger
MR RAPS
Weighted median
Weighted mode
0.05
0.00
−0.05
−0.10
0.1
0.2
0.3
0.4
SNP effect on Beta−defensin 118
SNP effect on Selenium

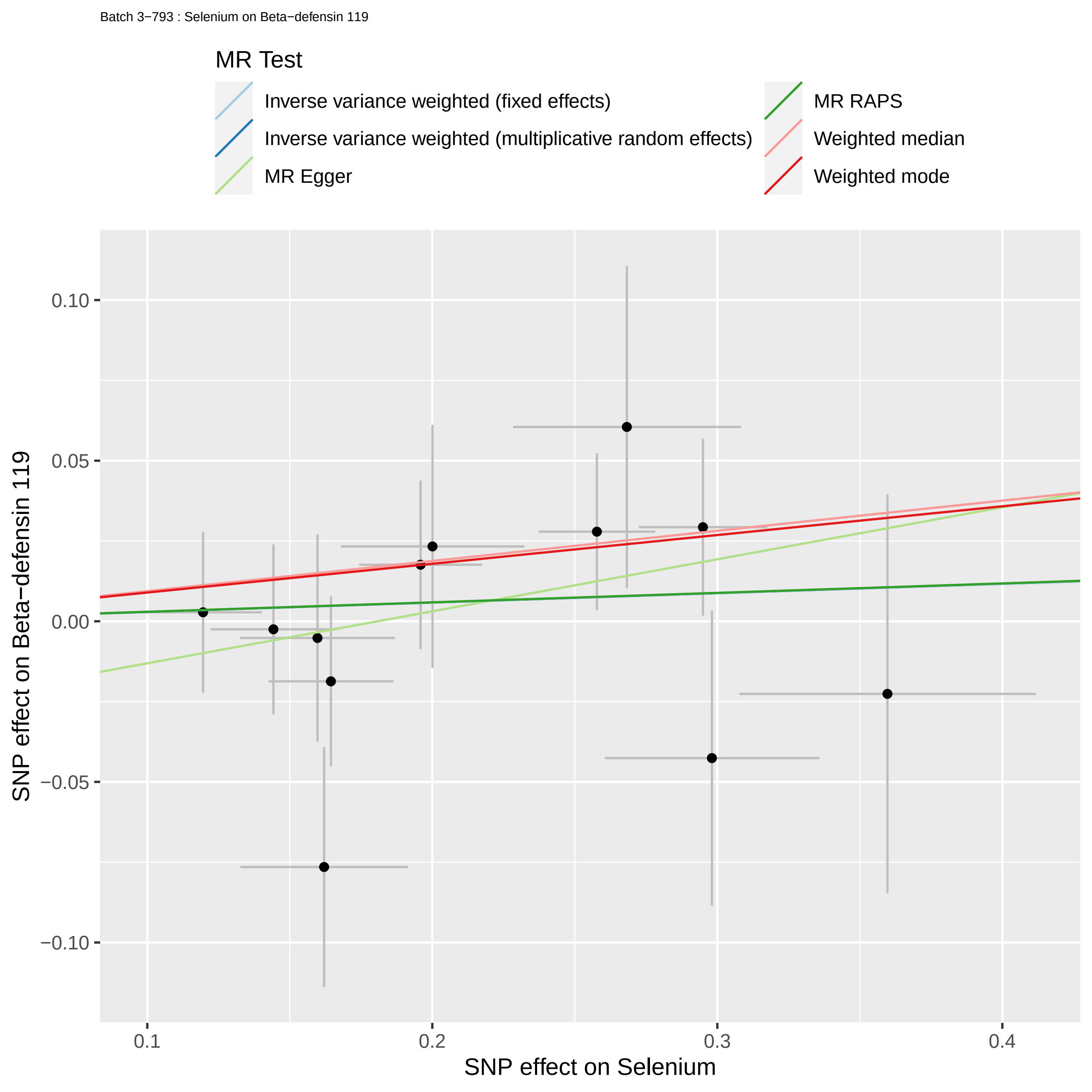

Batch 3−793 : Selenium on Beta−defensin 119
MR Test
Inverse variance weighted (fixed effects)
Inverse variance weighted (multiplicative random effects)
MR Egger
MR RAPS
Weighted median
Weighted mode
0.10
0.05
0.00
−0.05
−0.10
0.1
0.2
0.3
0.4
SNP effect on Beta−defensin 119
SNP effect on Selenium

Batch 3−794 : Selenium on Beta−defensin 119

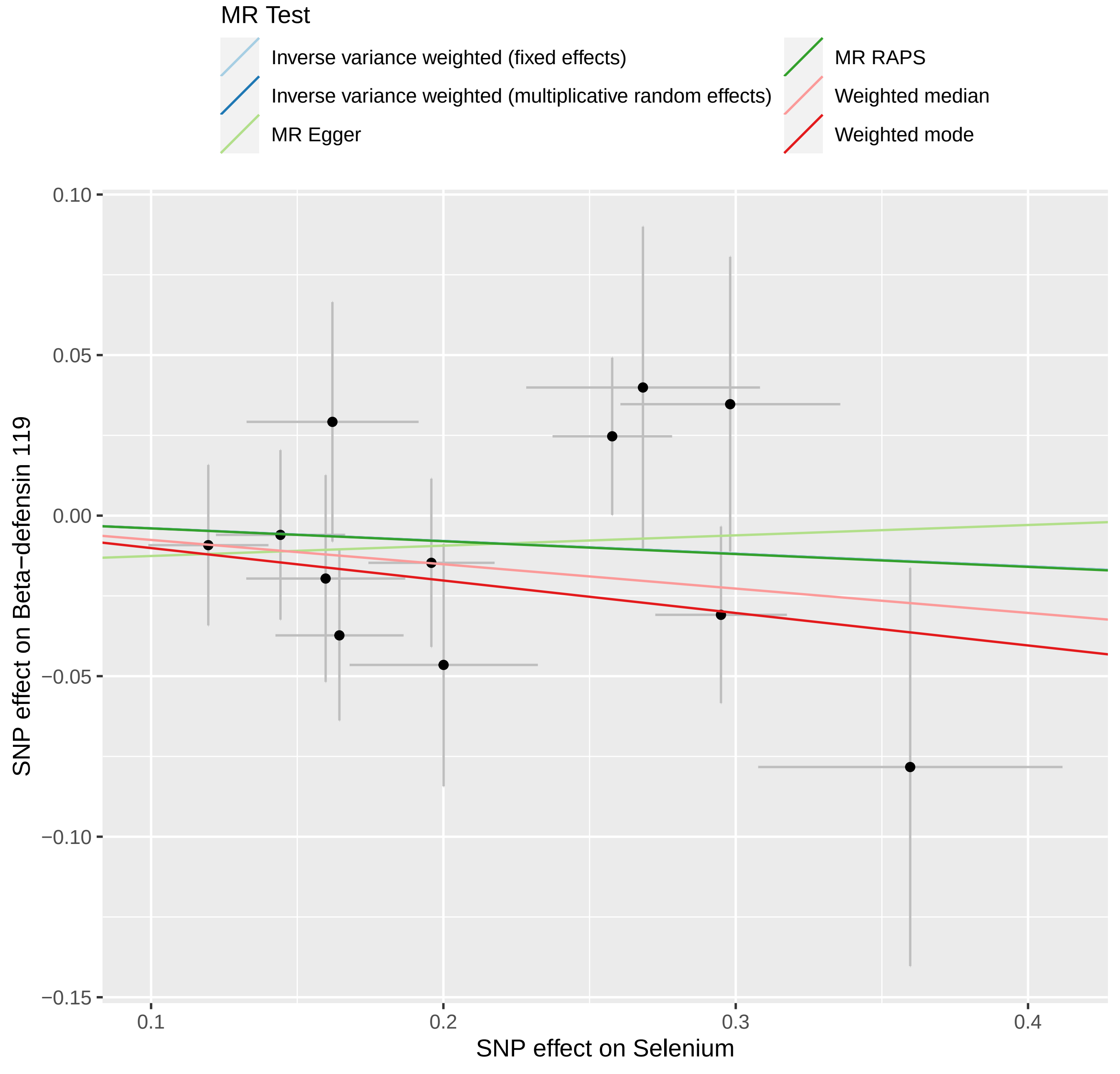

Batch 3−795 : Selenium on Beta−defensin 119

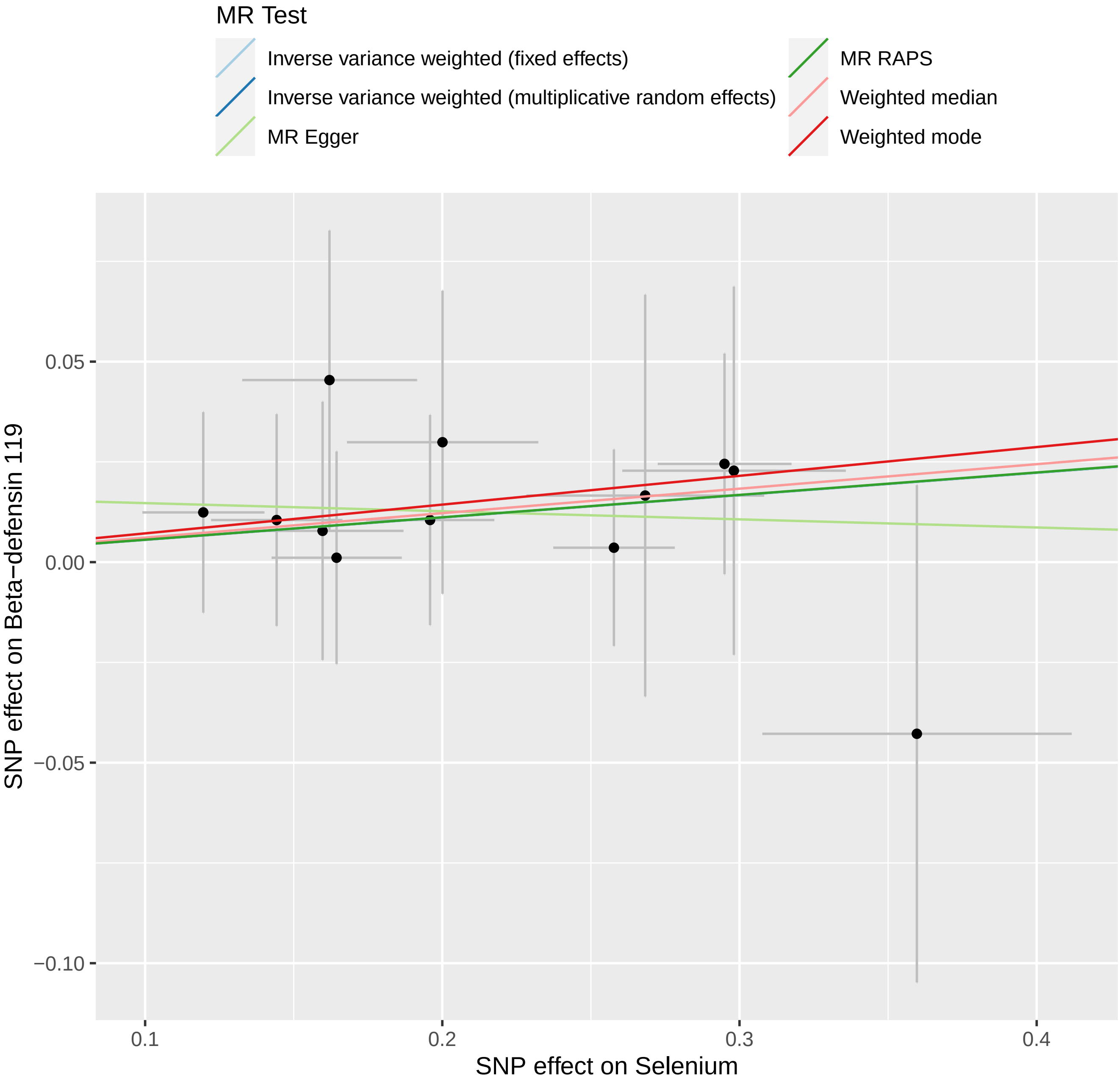

Batch 3−796 : Selenium on Beta−defensin 119

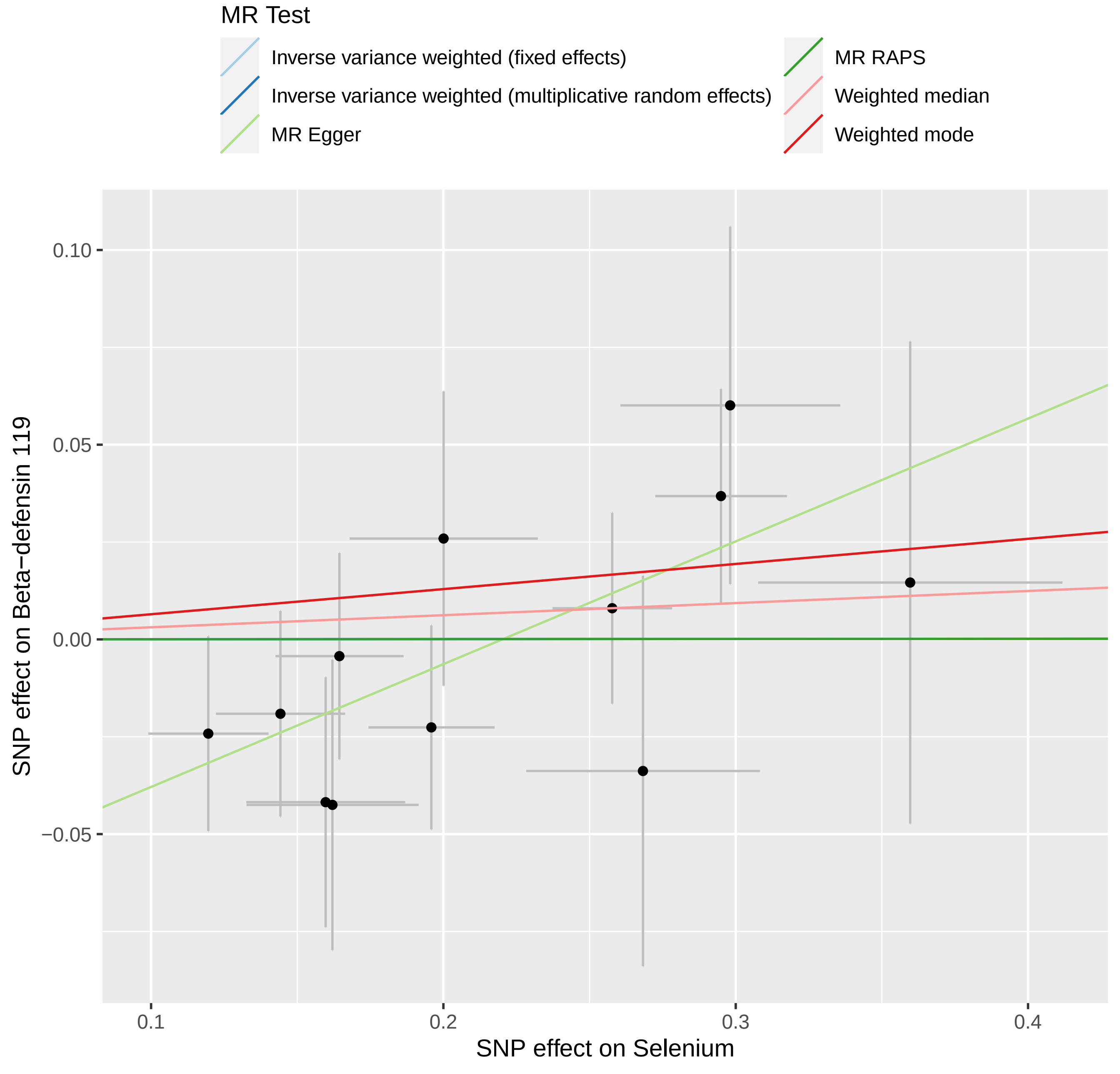

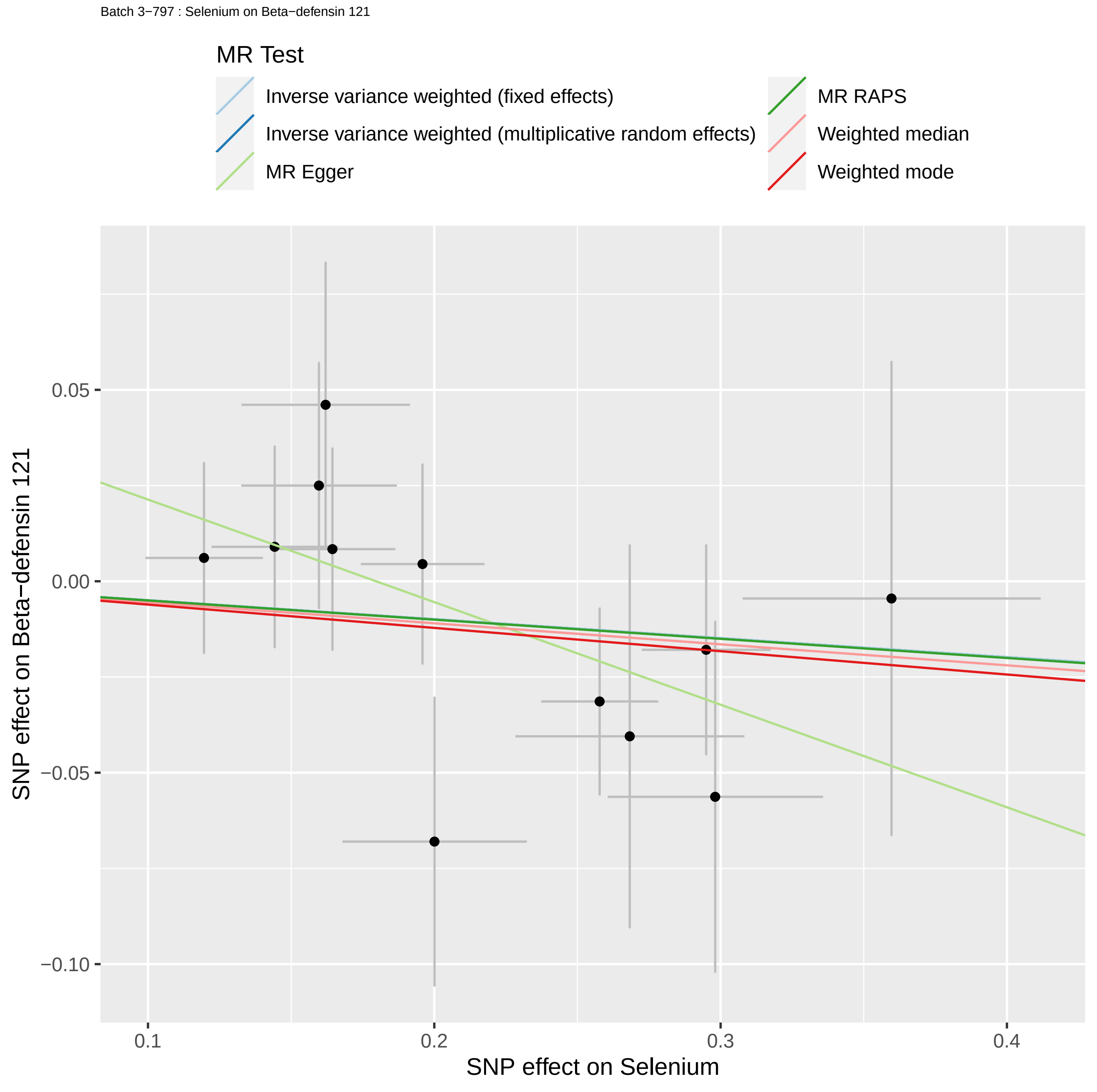
Batch 3−797 : Selenium on Beta−defensin 121
MR Test
Inverse variance weighted (fixed effects)
Inverse variance weighted (multiplicative random effects)
MR Egger
MR RAPS
Weighted median
Weighted mode
0.05
0.00
−0.05
−0.10
0.1
0.2
0.3
0.4
SNP effect on Beta−defensin 121
SNP effect on Selenium

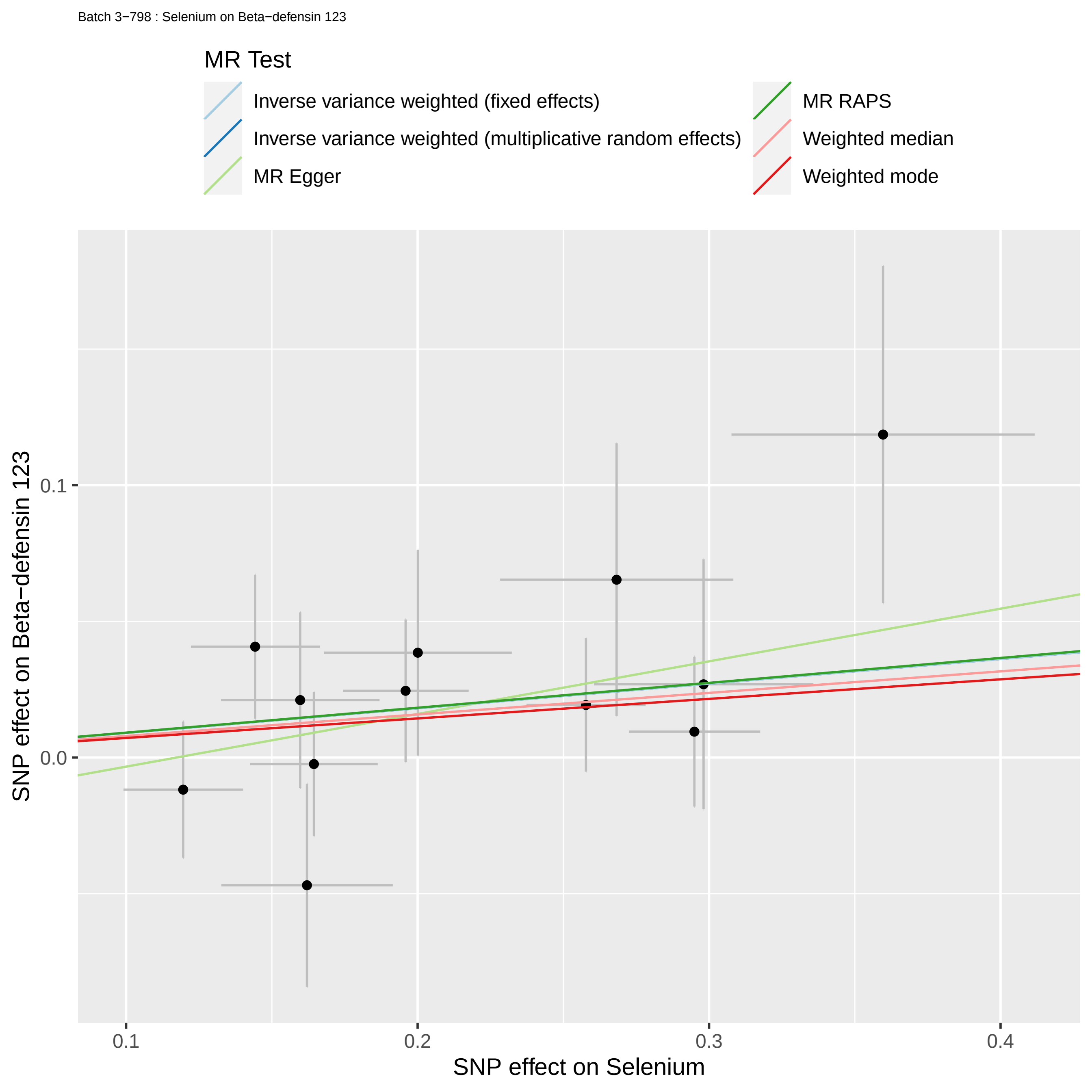

Batch 3−798 : Selenium on Beta−defensin 123
MR Test
Inverse variance weighted (fixed effects)
Inverse variance weighted (multiplicative random effects)
MR Egger
MR RAPS
Weighted median
Weighted mode
SNP effect on Beta−defensin 123
0.1
0.0
0.1
0.2
0.3
0.4
SNP effect on Selenium

Batch 3−799 : Selenium on Beta−defensin 125

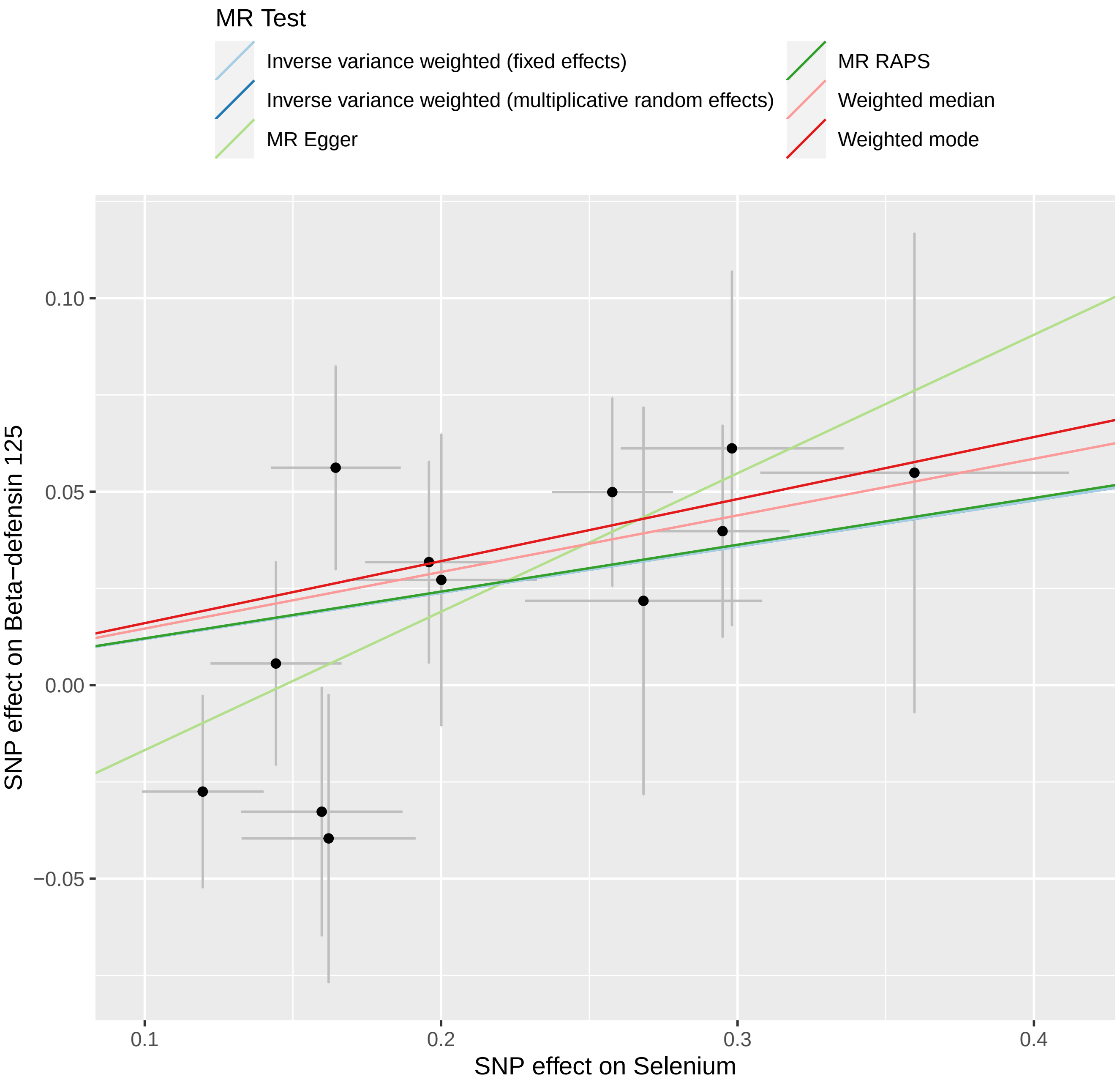

Batch 3−800 : Selenium on Beta−defensin 128

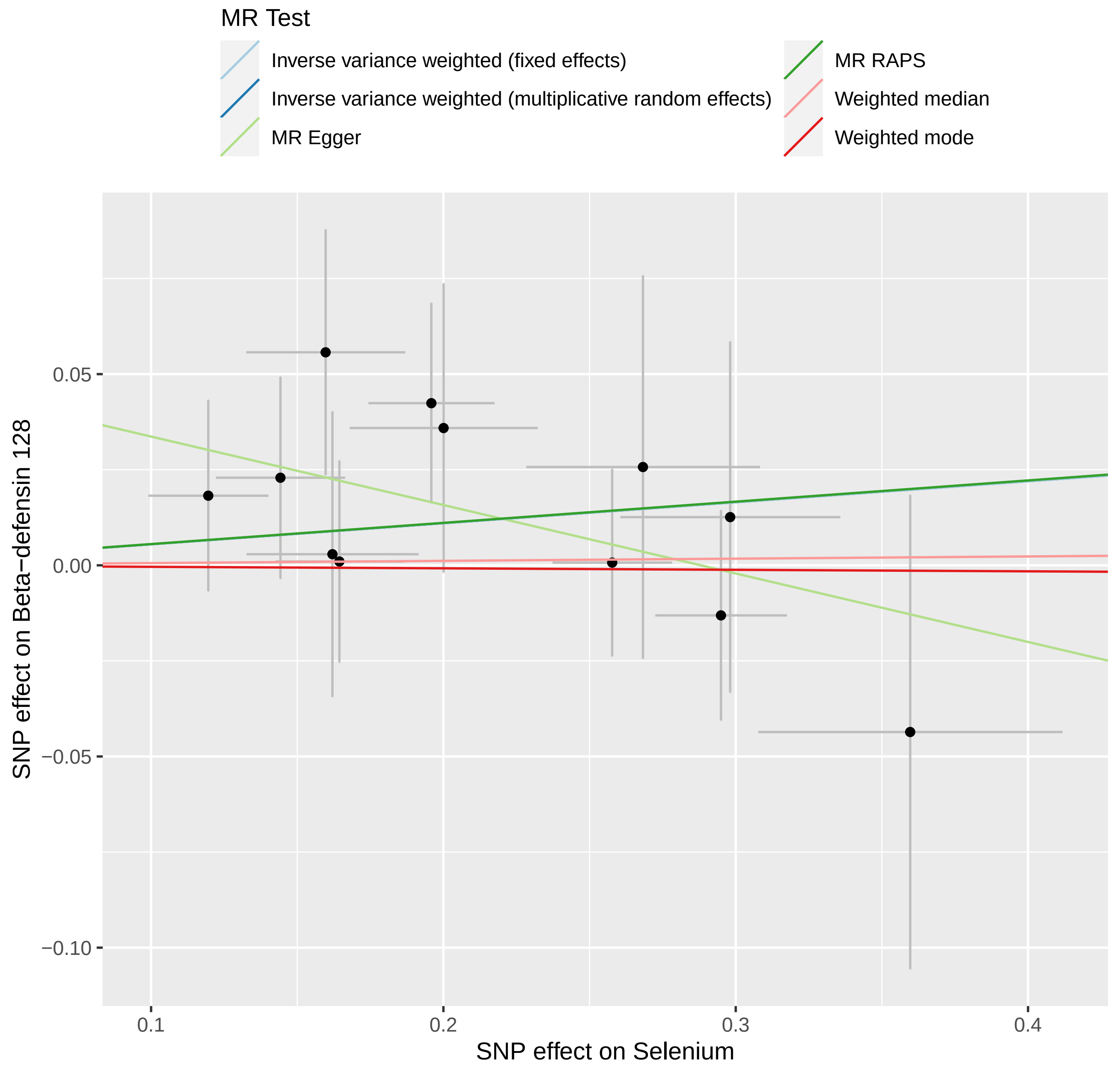

Batch 3−801 : Selenium on Beta−defensin 134

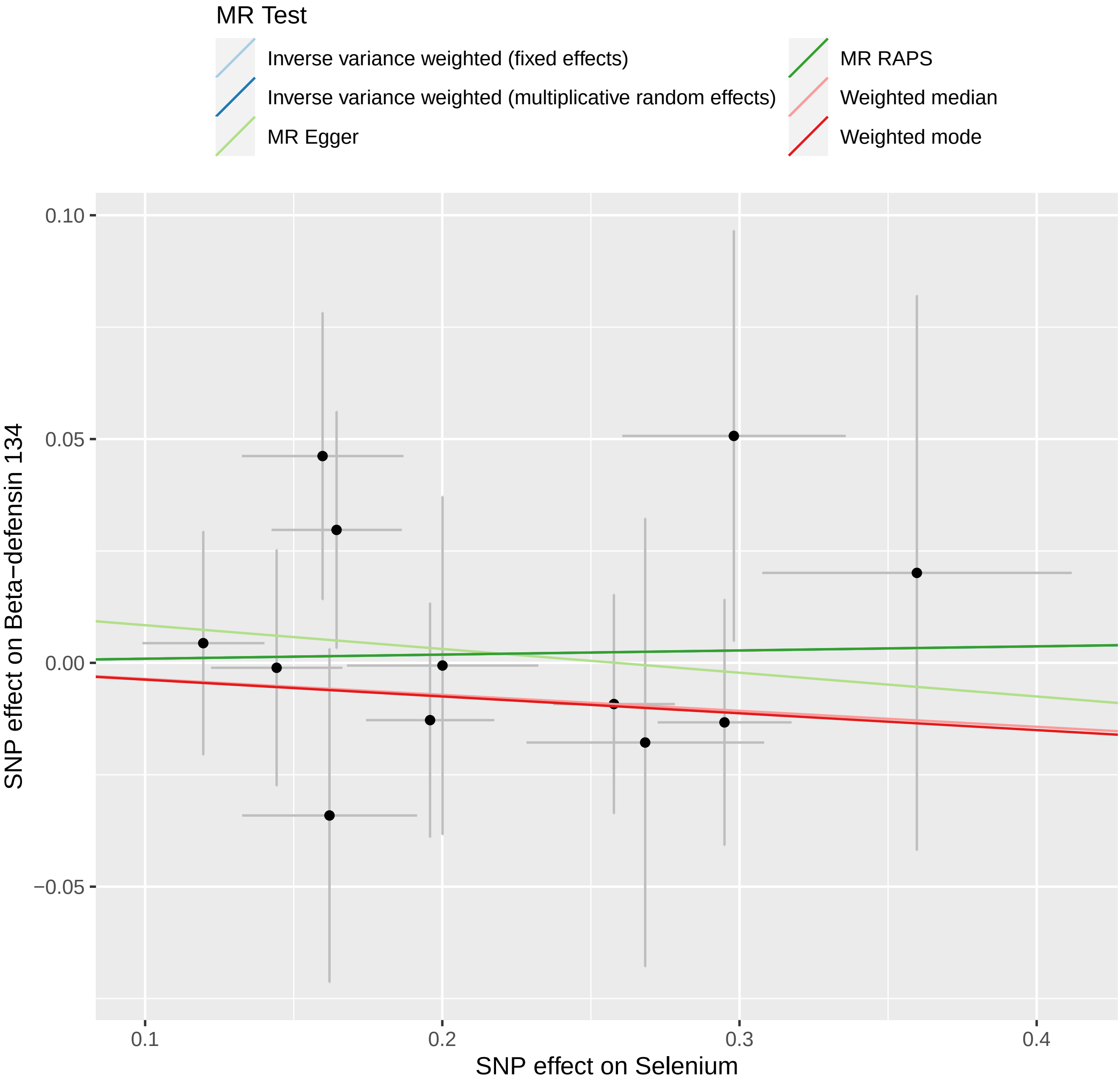

Batch 3−802 : Selenium on Beta−defensin 135

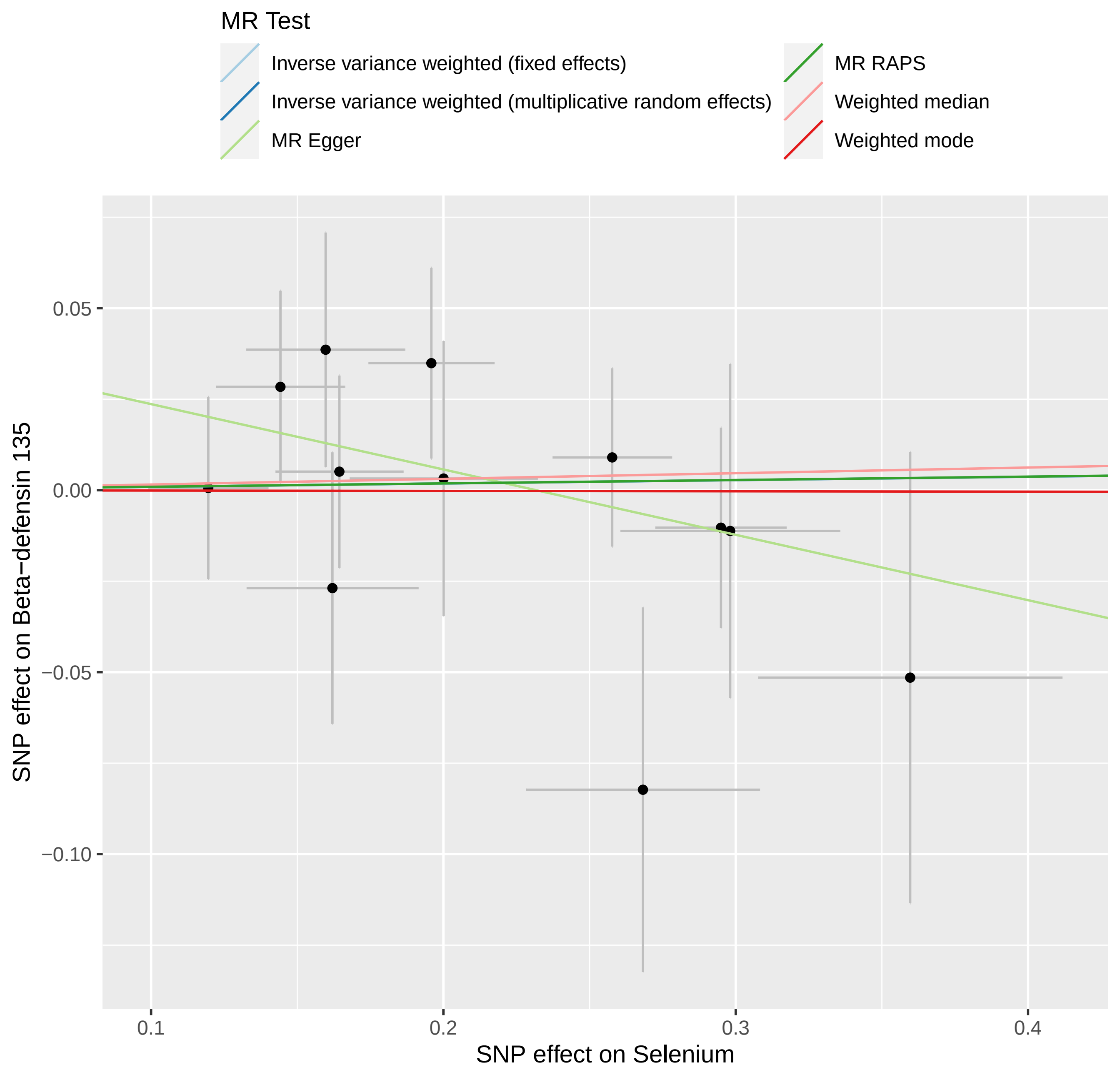

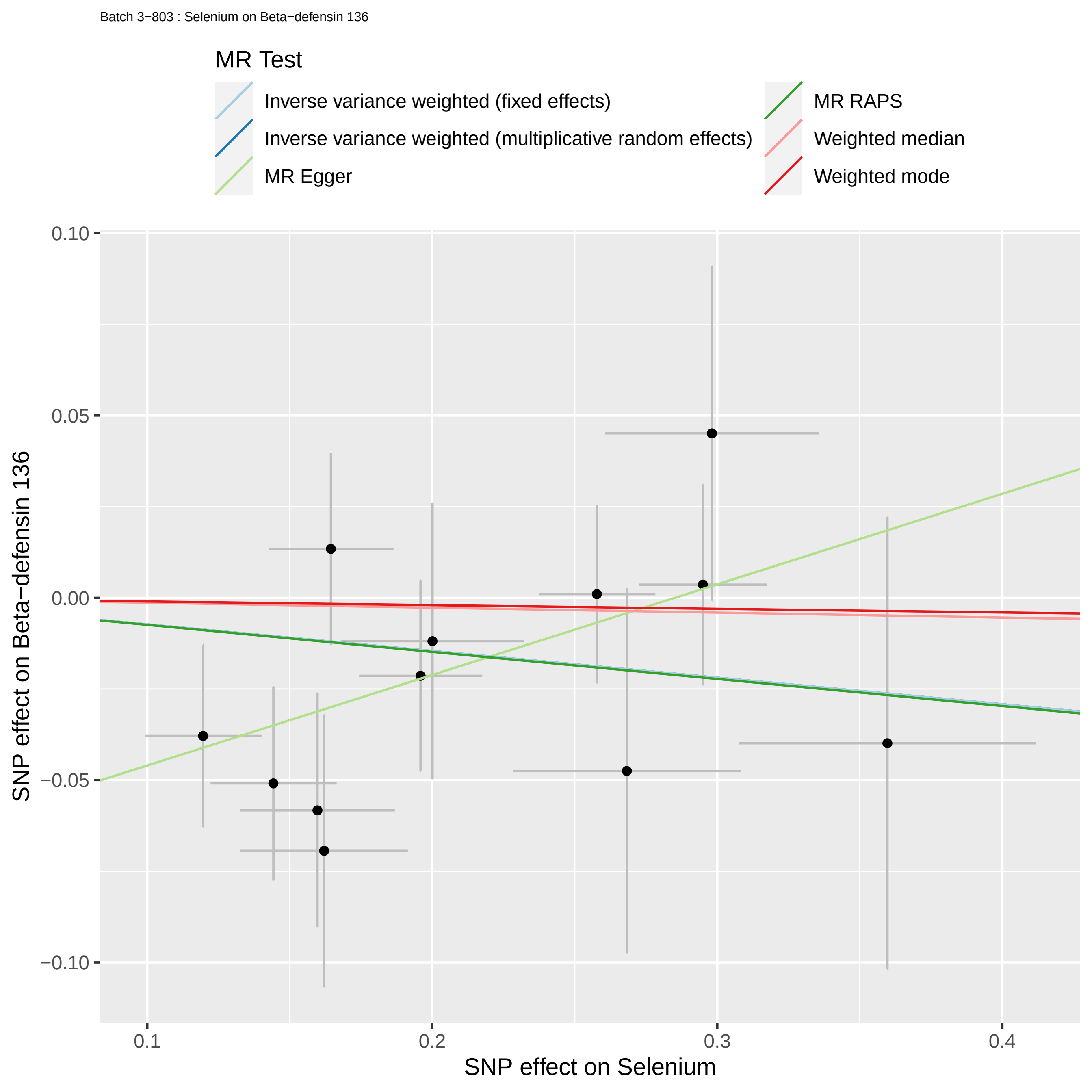
Batch 3−803 : Selenium on Beta−defensin 136
MR Test
Inverse variance weighted (fixed effects)
Inverse variance weighted (multiplicative random effects)
MR Egger
MR RAPS
Weighted median
Weighted mode
0.10
0.05
0.00
−0.05
−0.10
0.1
0.2
0.3
0.4
SNP effect on Selenium
SNP effect on Beta−defensin 136

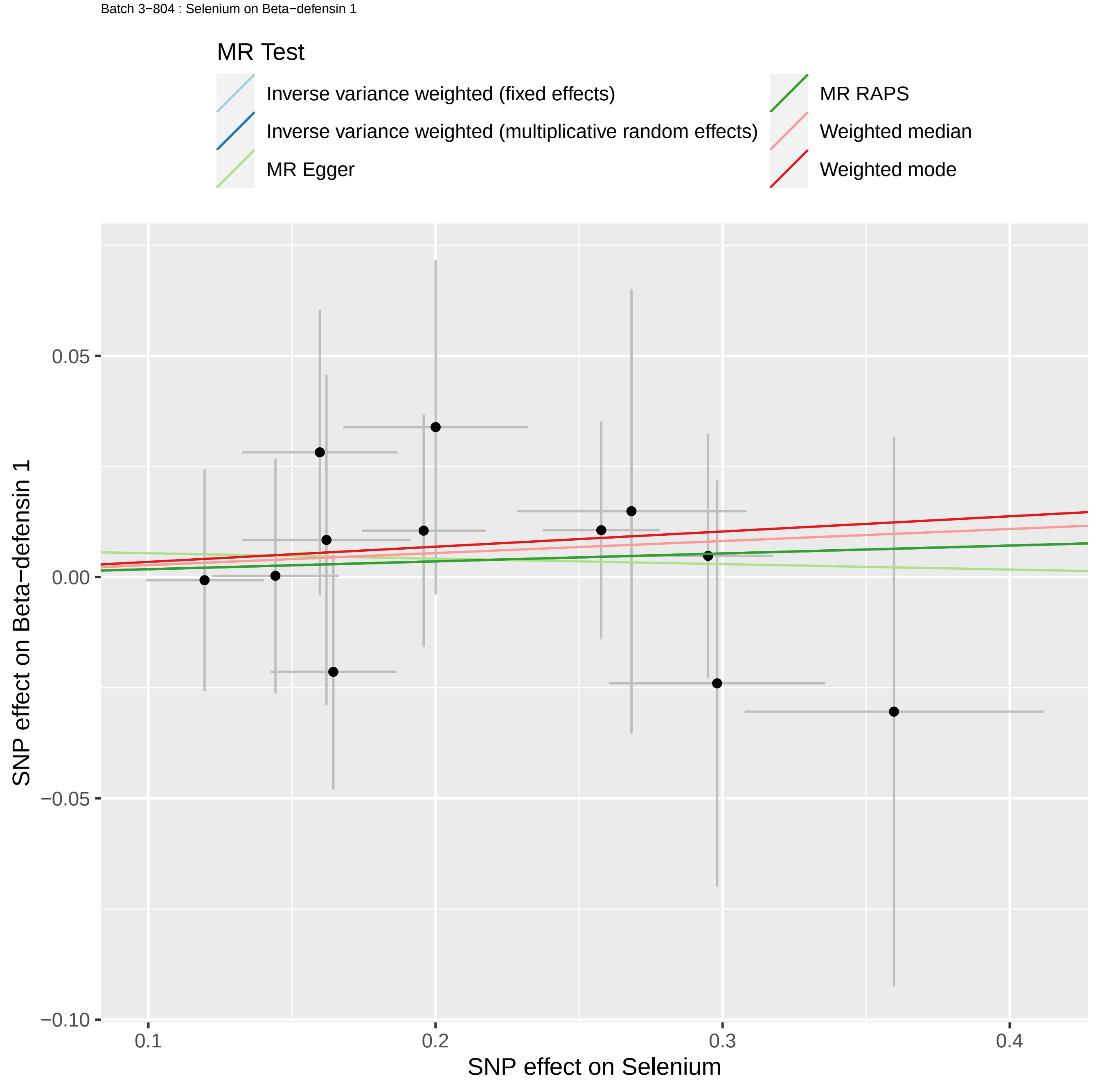

Batch 3−804 : Selenium on Beta−defensin 1
MR Test
Inverse variance weighted (fixed effects)
Inverse variance weighted (multiplicative random effects)
MR Egger
MR RAPS
Weighted median
Weighted mode
0.05
0.00
−0.05
−0.10
0.1
0.2
0.3
0.4
SNP effect on Beta−defensin 1
SNP effect on Selenium

Batch 3−805 : Selenium on Protein DEPP

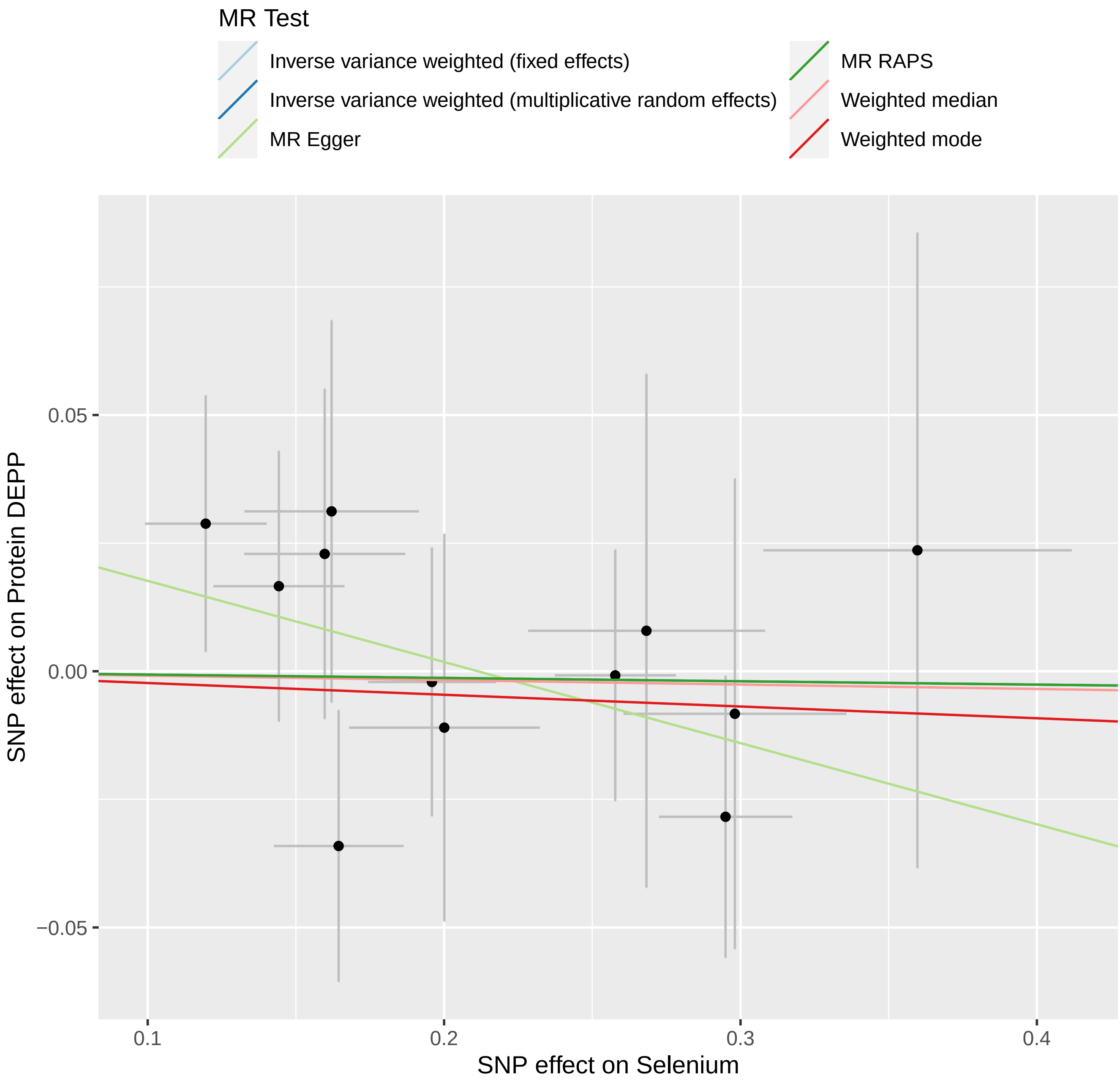

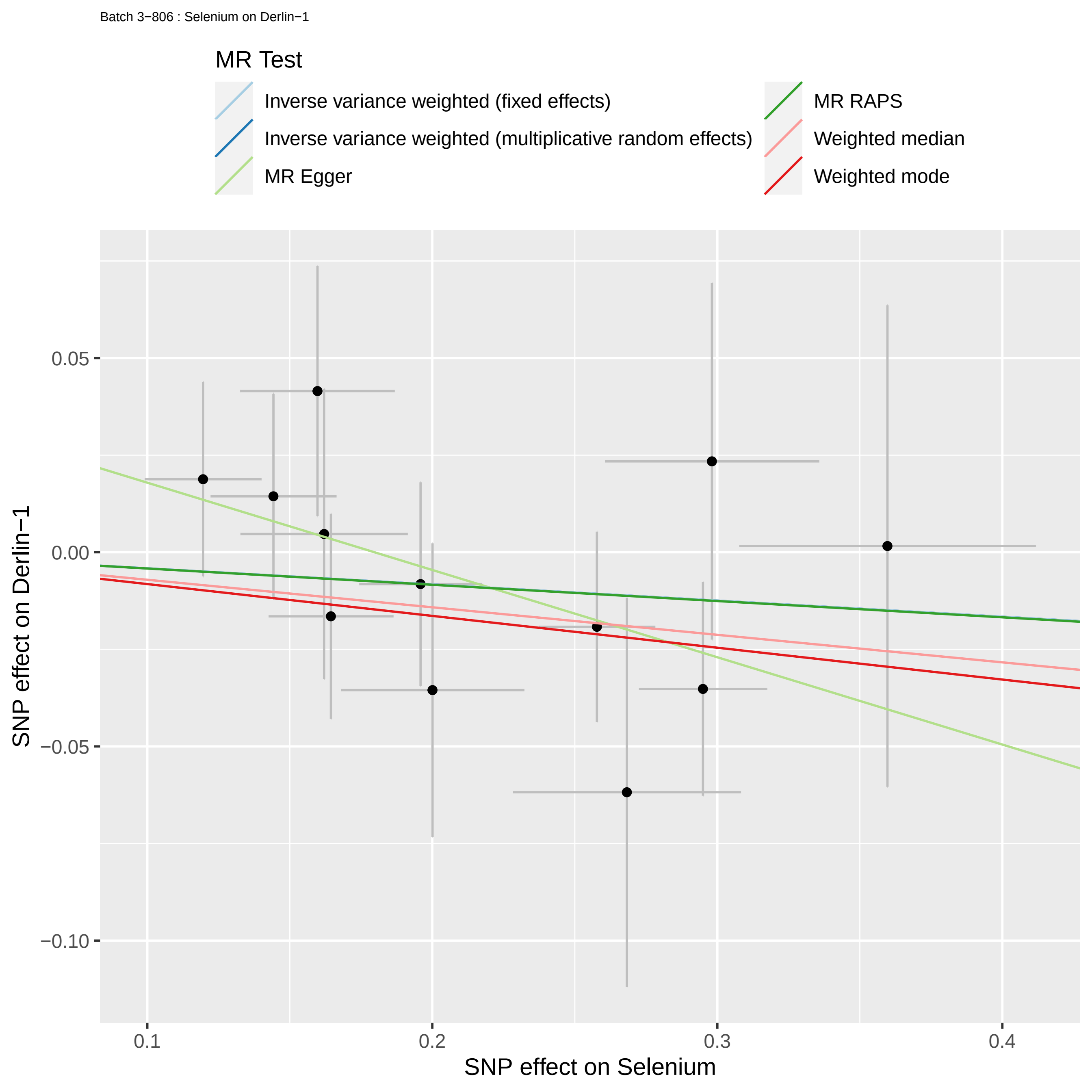

Batch 3−806 : Selenium on Derlin−1
MR Test
Inverse variance weighted (fixed effects)
Inverse variance weighted (multiplicative random effects)
MR Egger
MR RAPS
Weighted median
Weighted mode
0.05
0.00
−0.05
−0.10
0.1
0.2
0.3
0.4
SNP effect on Derlin−1
SNP effect on Selenium

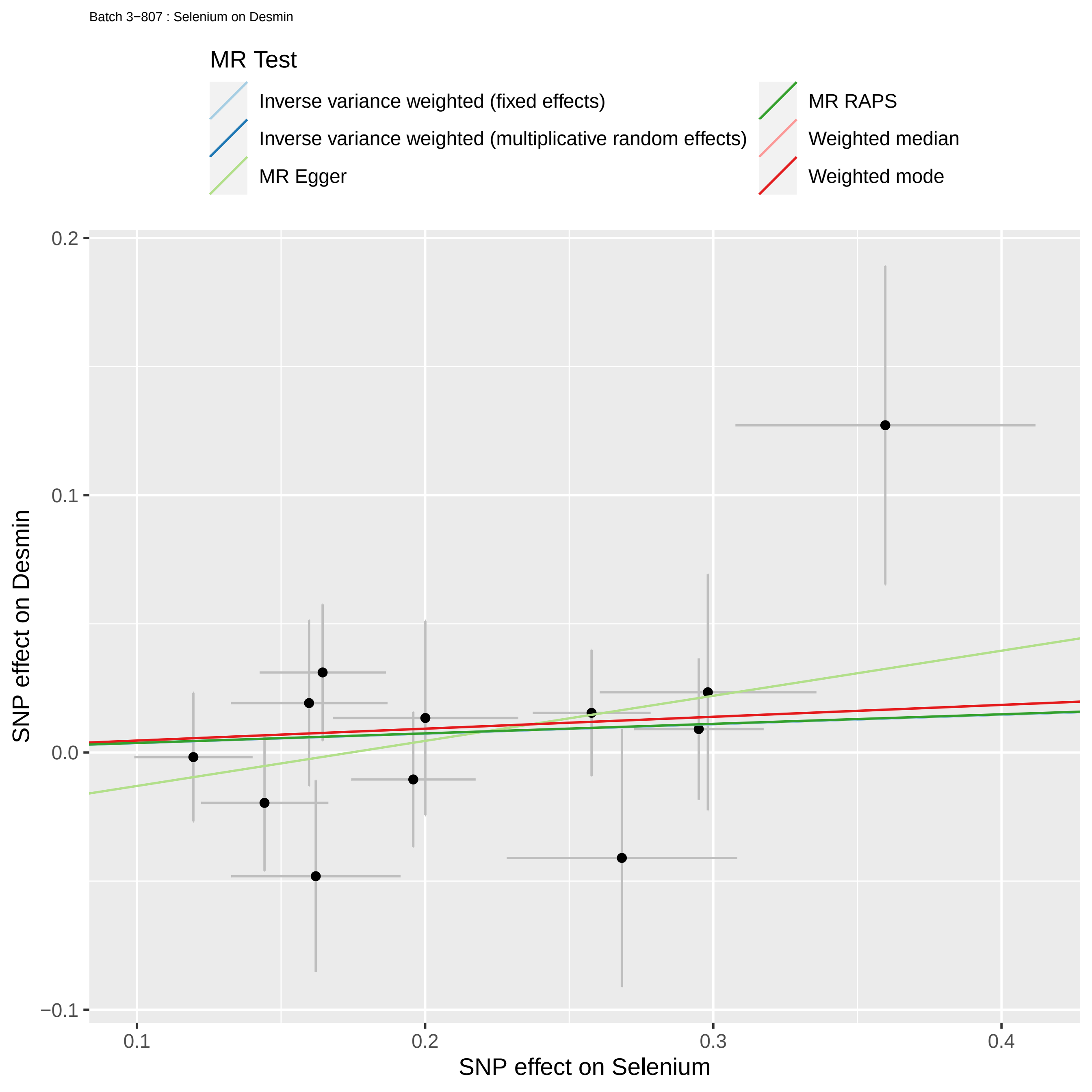
Batch 3−807 : Selenium on Desmin
MR Test
Inverse variance weighted (fixed effects)
Inverse variance weighted (multiplicative random effects)
MR Egger
MR RAPS
Weighted median
Weighted mode
SNP effect on Desmin
SNP effect on Selenium
0.2
0.1
0.0
−0.1
0.1
0.2
0.3
0.4

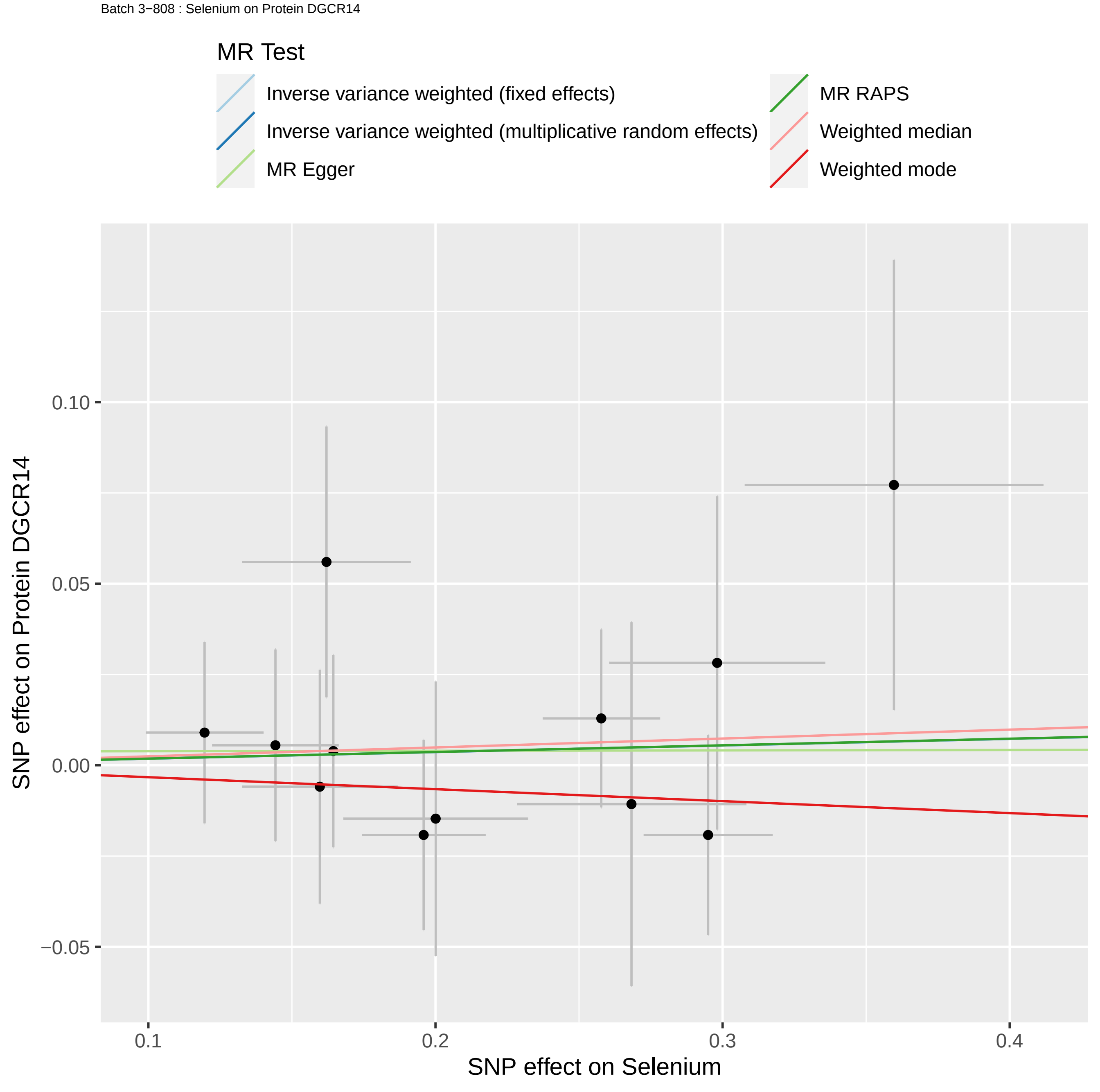
Batch 3−808 : Selenium on Protein DGCR14
MR Test
Inverse variance weighted (fixed effects)
Inverse variance weighted (multiplicative random effects)
MR Egger
MR RAPS
Weighted median
Weighted mode
SNP effect on Protein DGCR14
0.10
0.05
0.00
−0.05
0.1
0.2
0.3
0.4
SNP effect on Selenium

Batch 3−809 : Selenium on Integral membrane protein DGCR2/IDD

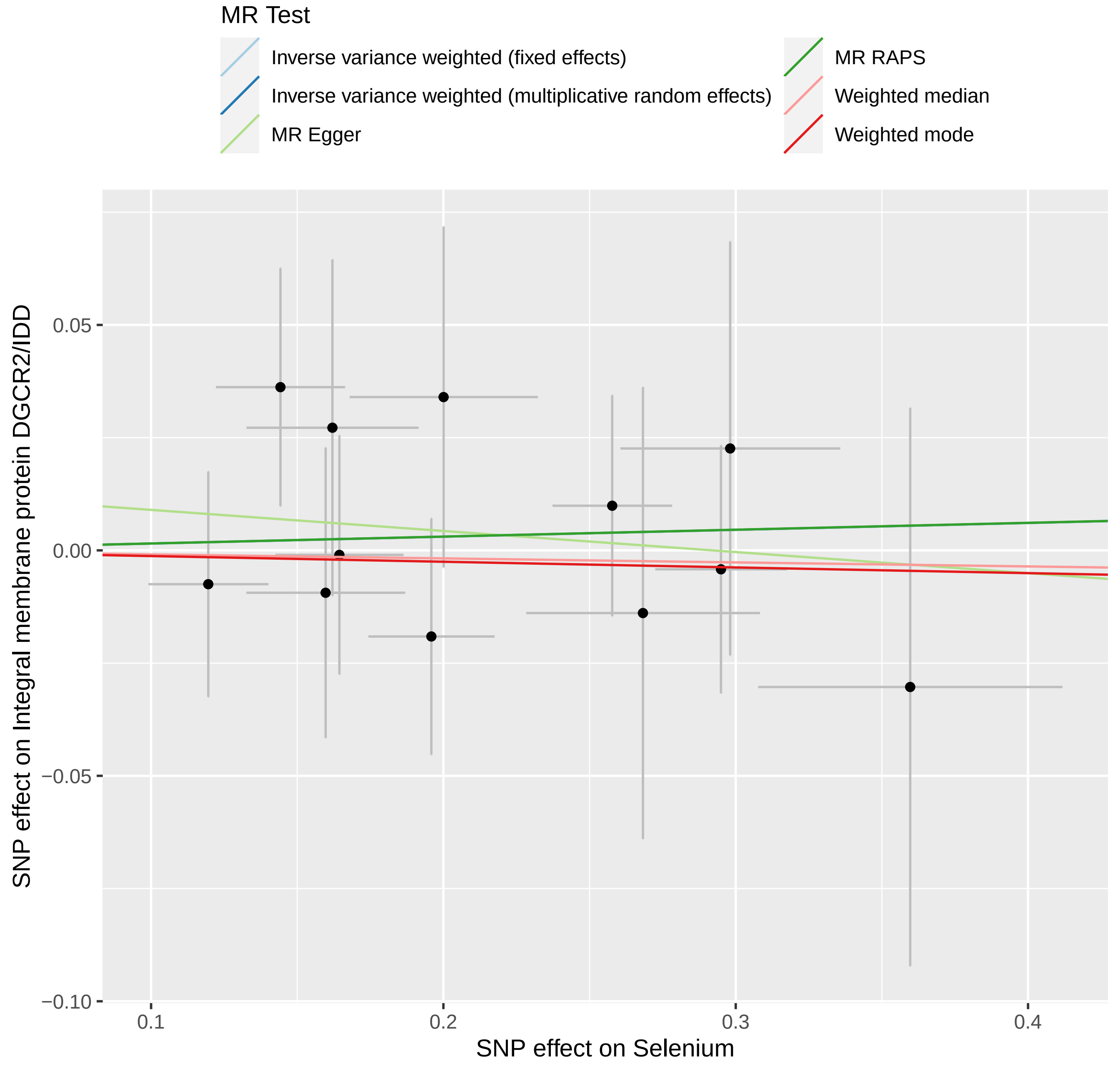

Batch 3−810 : Selenium on Protein DGCR6

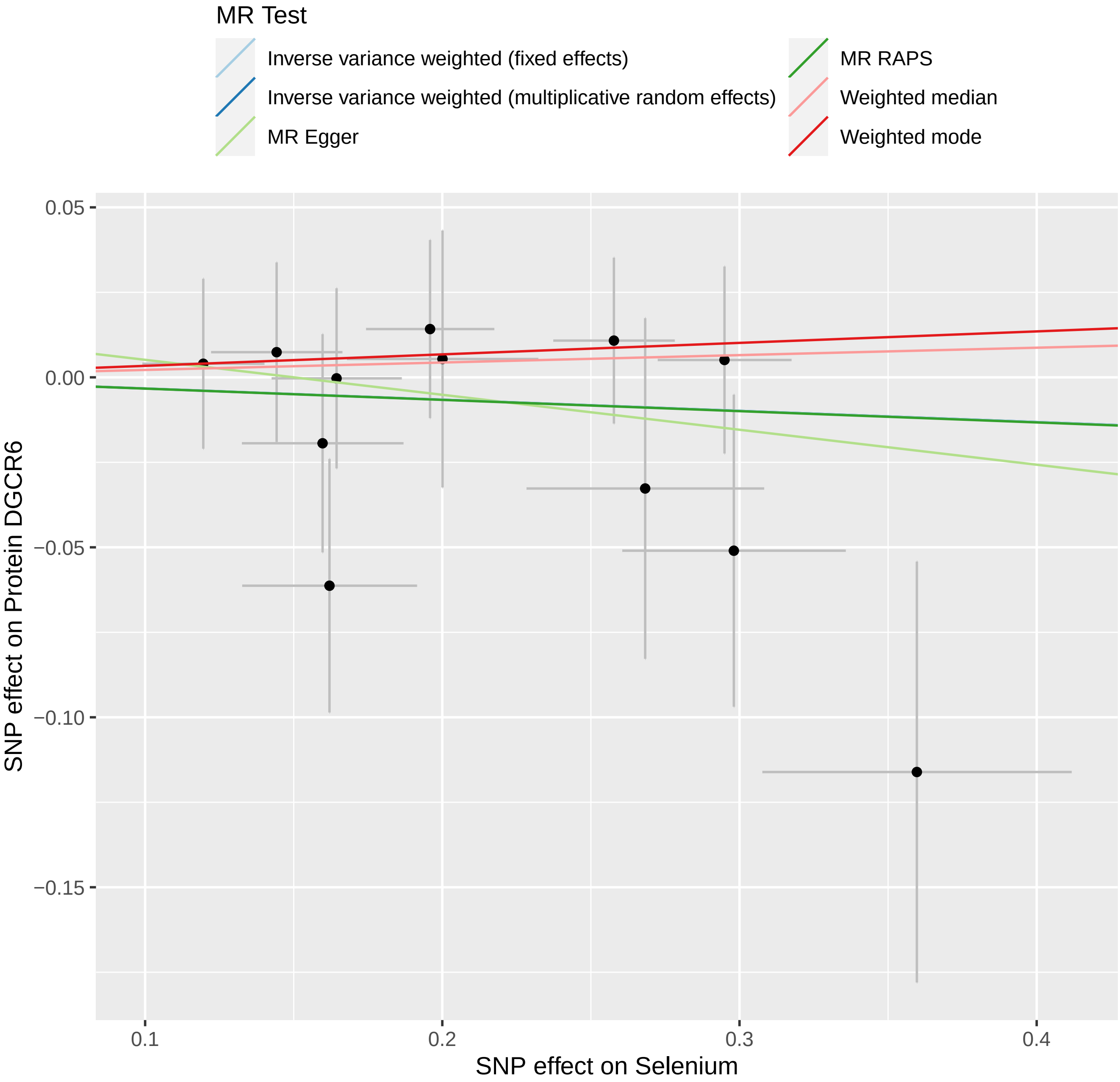

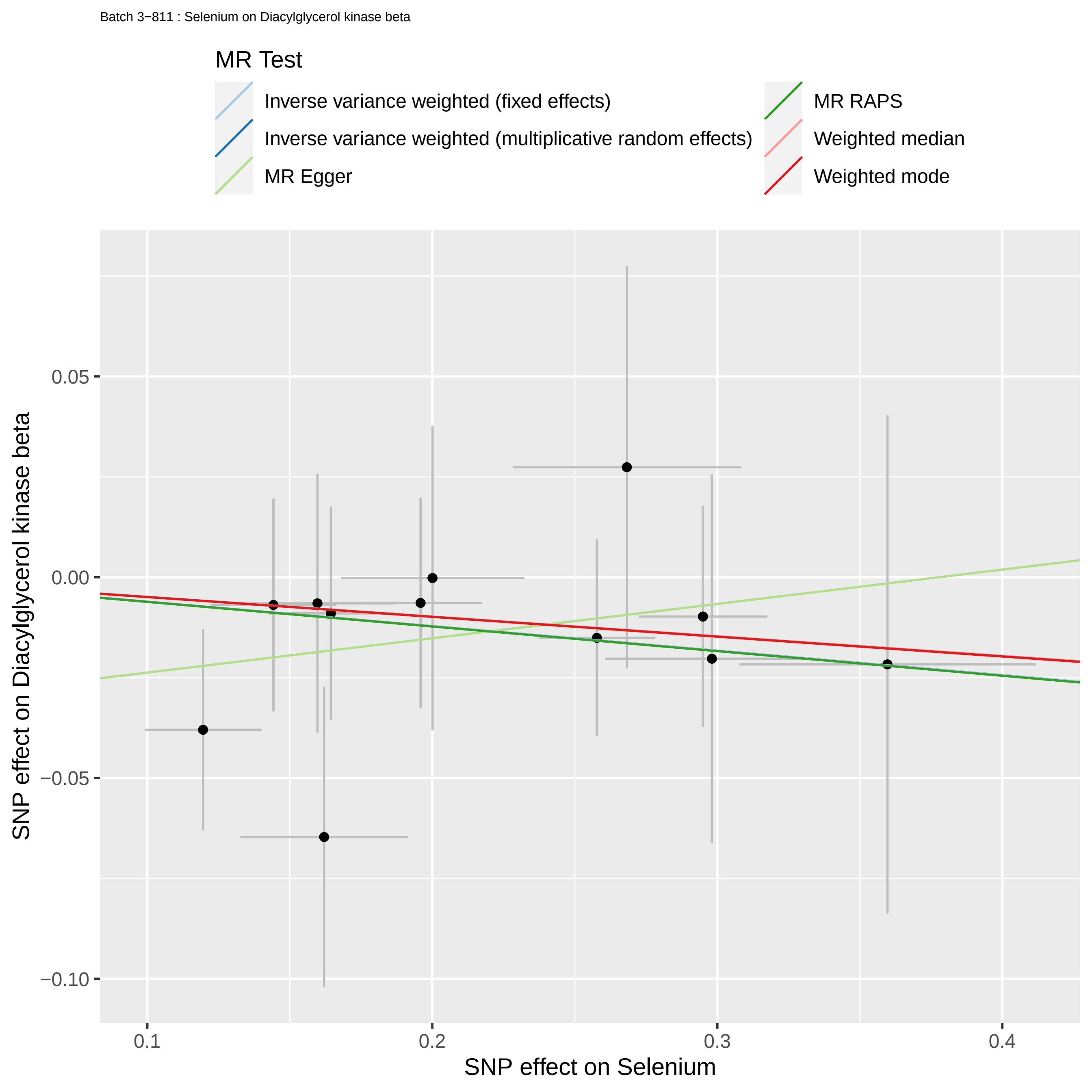
Batch 3−811 : Selenium on Diacylglycerol kinase beta
MR Test
Inverse variance weighted (fixed effects)
Inverse variance weighted (multiplicative random effects)
MR Egger
MR RAPS
Weighted median
Weighted mode
SNP effect on Diacylglycerol kinase beta
0.05
0.00
−0.05
−0.10
0.1
0.2
0.3
0.4
SNP effect on Selenium

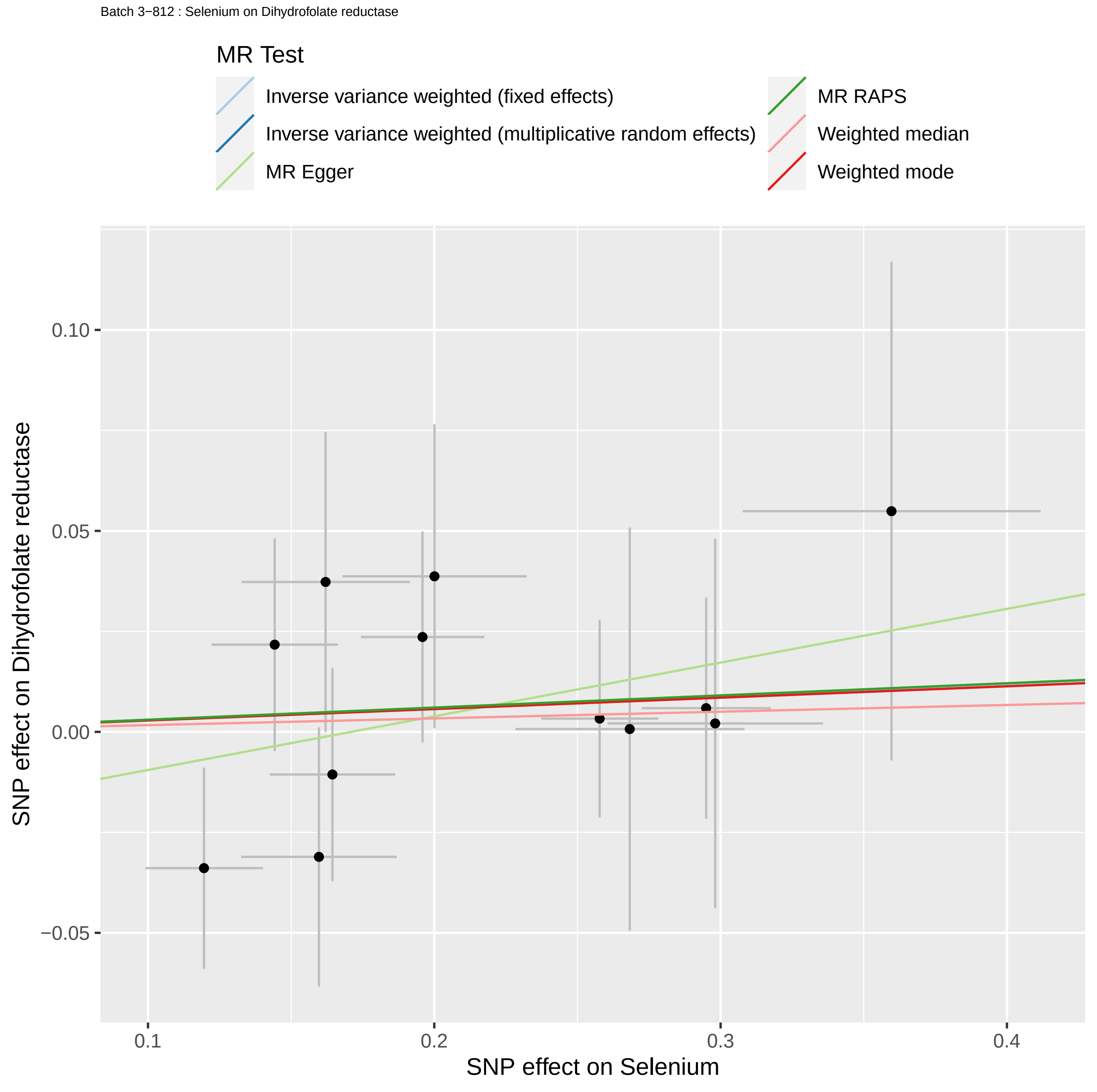

Batch 3−812 : Selenium on Dihydrofolate reductase
MR Test
Inverse variance weighted (fixed effects)
Inverse variance weighted (multiplicative random effects)
MR Egger
MR RAPS
Weighted median
Weighted mode
0.10
0.05
0.00
−0.05
0.1
0.2
0.3
0.4
SNP effect on Selenium
SNP effect on Dihydrofolate reductase

Batch 3−813 : Selenium on Desert hedgehog protein N−product

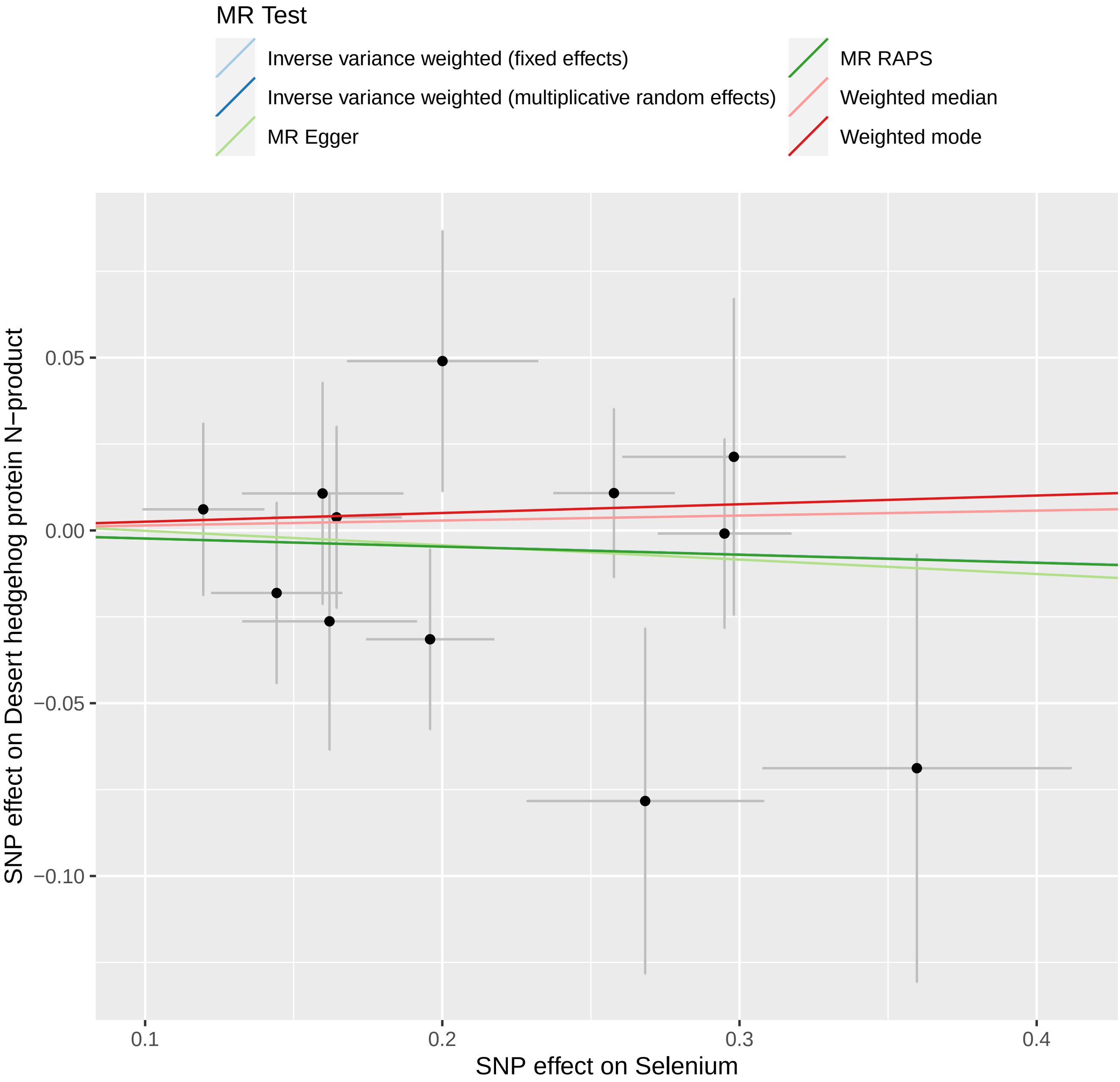

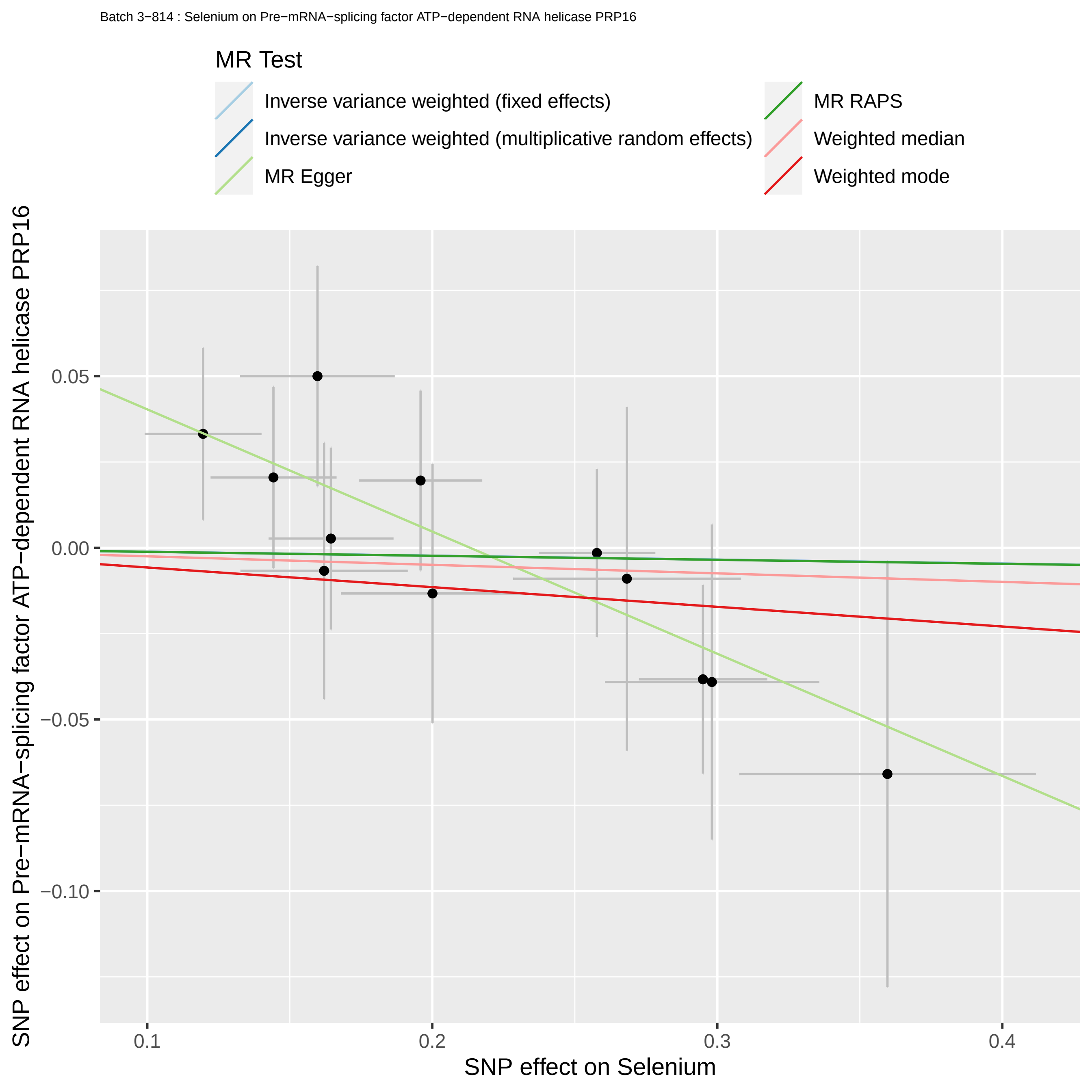
Batch 3−814 : Selenium on Pre−mRNA−splicing factor ATP−dependent RNA helicase PRP16
MR Test
Inverse variance weighted (fixed effects)
Inverse variance weighted (multiplicative random effects)
MR Egger
MR RAPS
Weighted median
Weighted mode
SNP effect on Pre−mRNA−splicing factor ATP−dependent RNA helicase PRP16
0.05
0.00
−0.05
−0.10
0.1
0.2
0.3
0.4
SNP effect on Selenium

Batch 3−815 : Selenium on Probable ATP−dependent RNA helicase DHX58

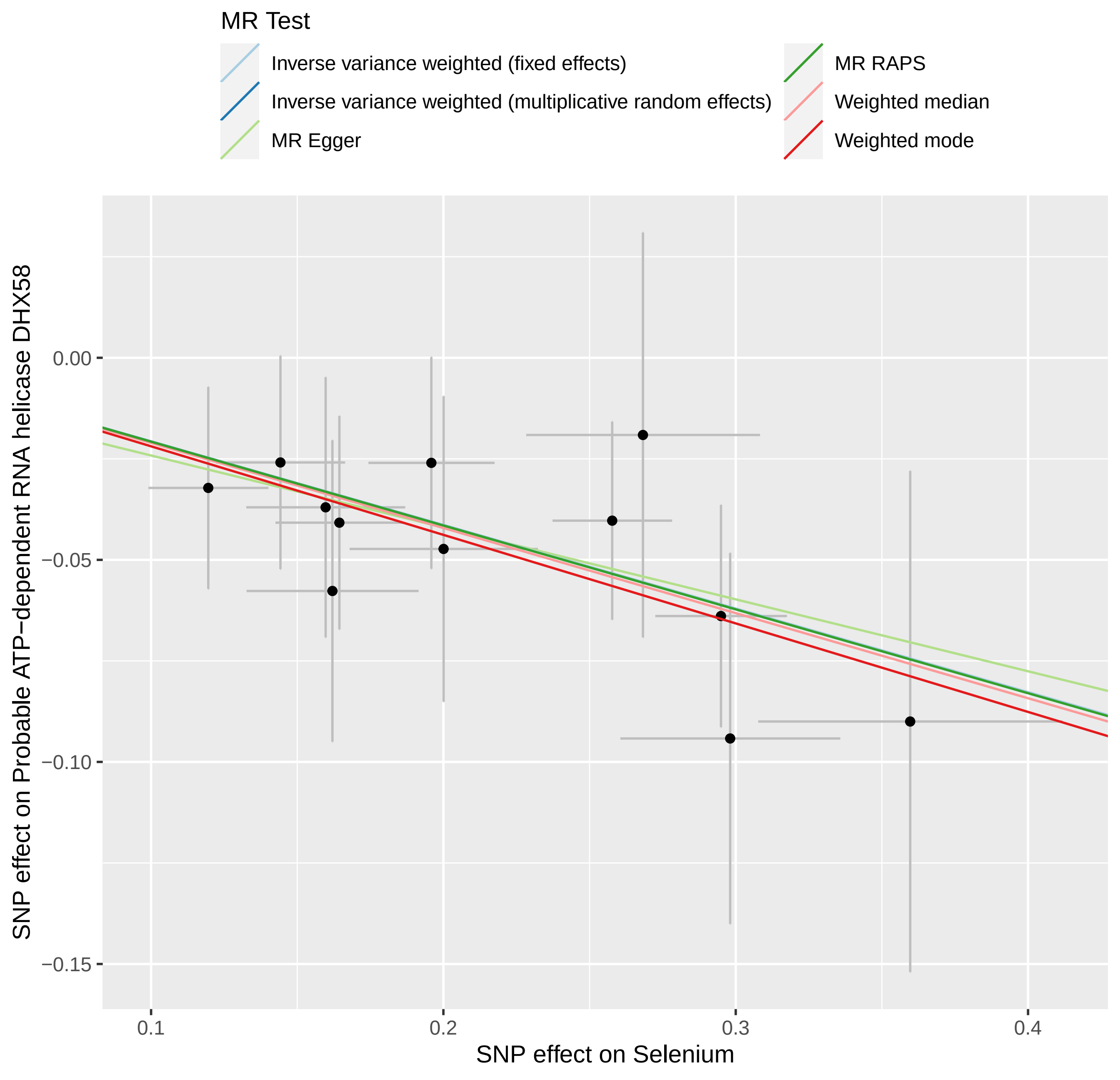

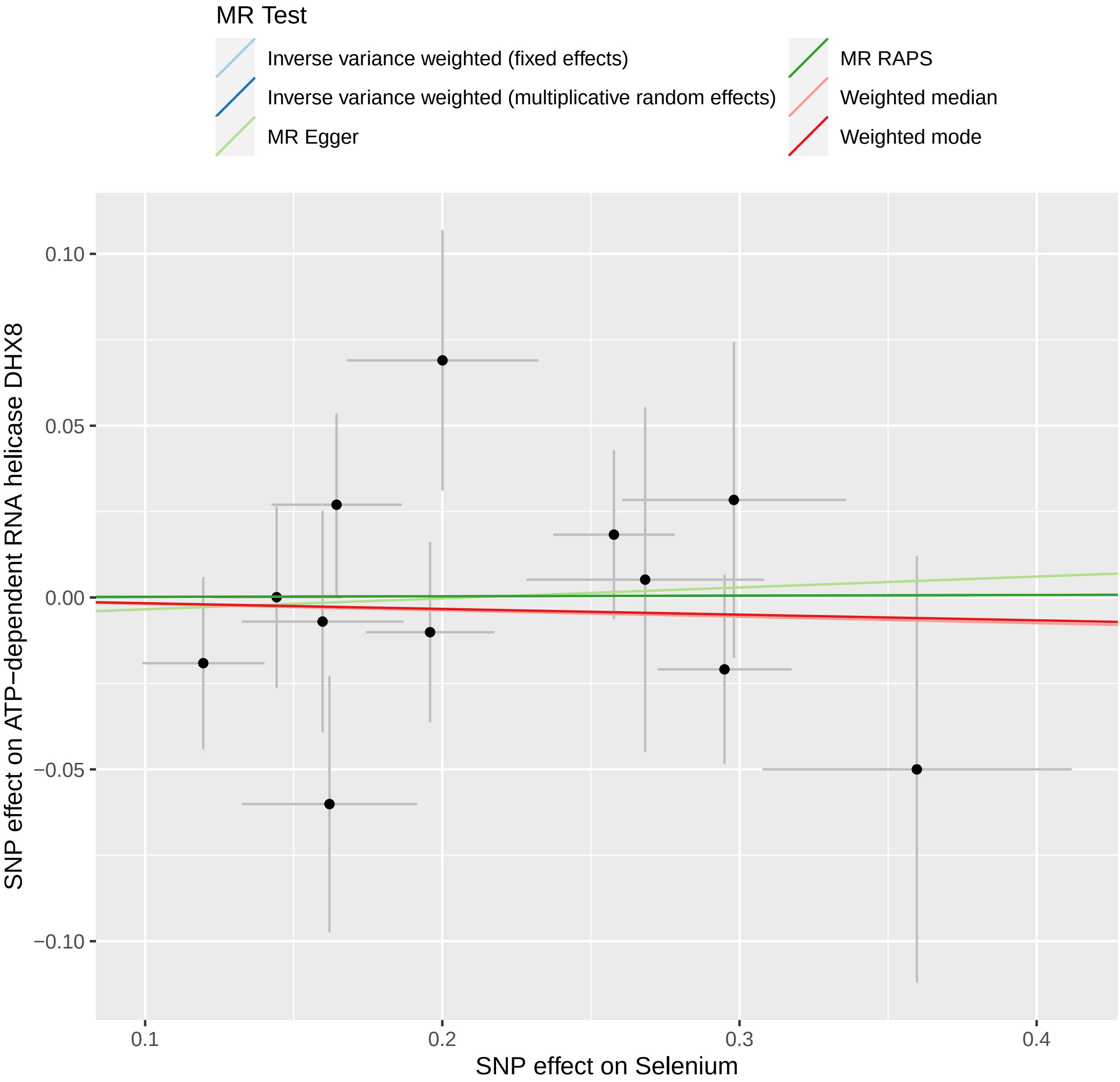
MR Test
Inverse variance weighted (fixed effects)
Inverse variance weighted (multiplicative random effects)
MR Egger
MR RAPS
Weighted median
Weighted mode
0.10
0.05
0.00
−0.05
−0.10
0.1
0.2
0.3
0.4
SNP effect on ATP−dependent RNA helicase DHX8
SNP effect on Selenium

Batch 3−817 : Selenium on ATP−dependent RNA helicase A

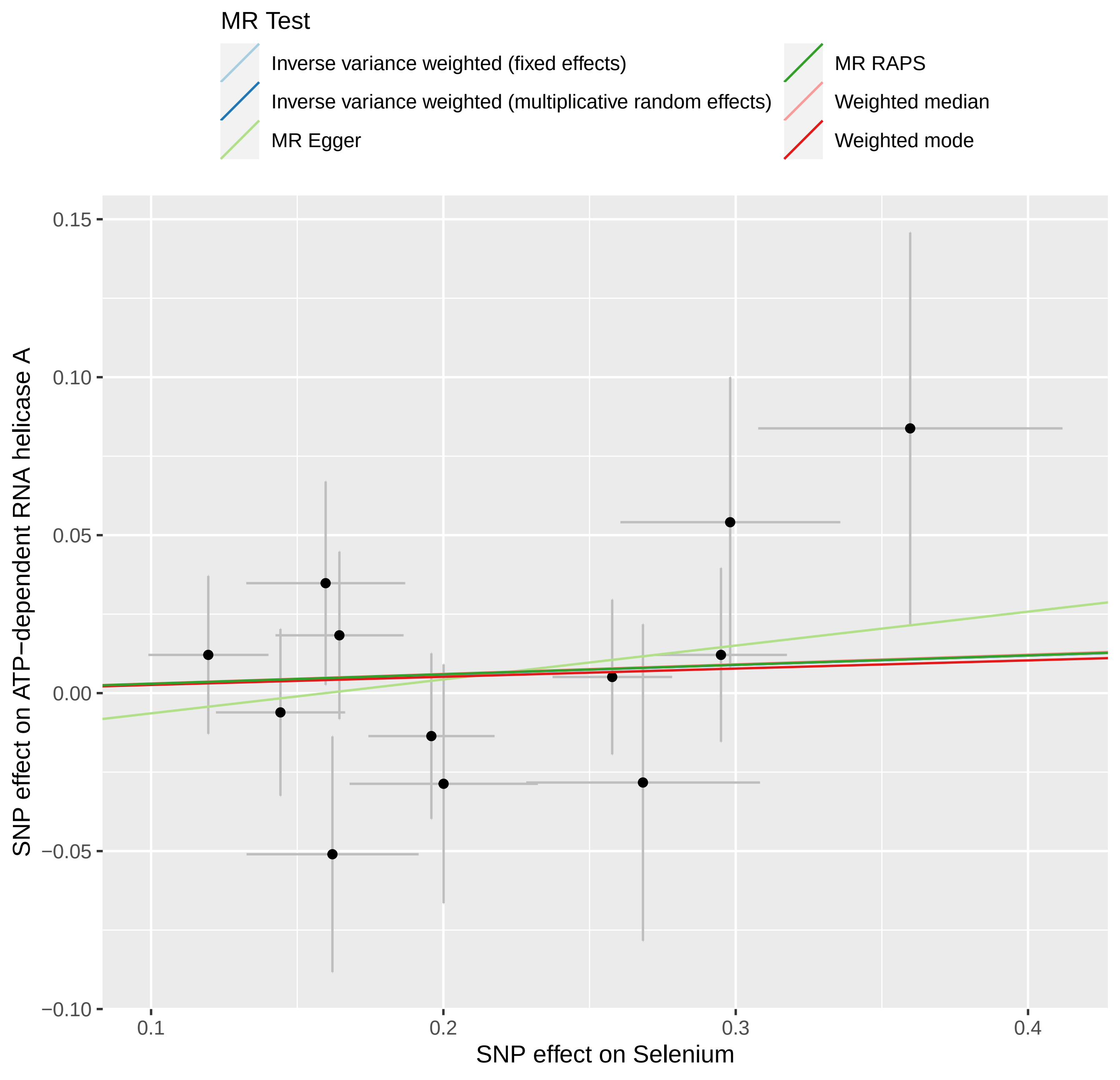

Batch 3−818 : Selenium on Diablo homolog, mitochondrial

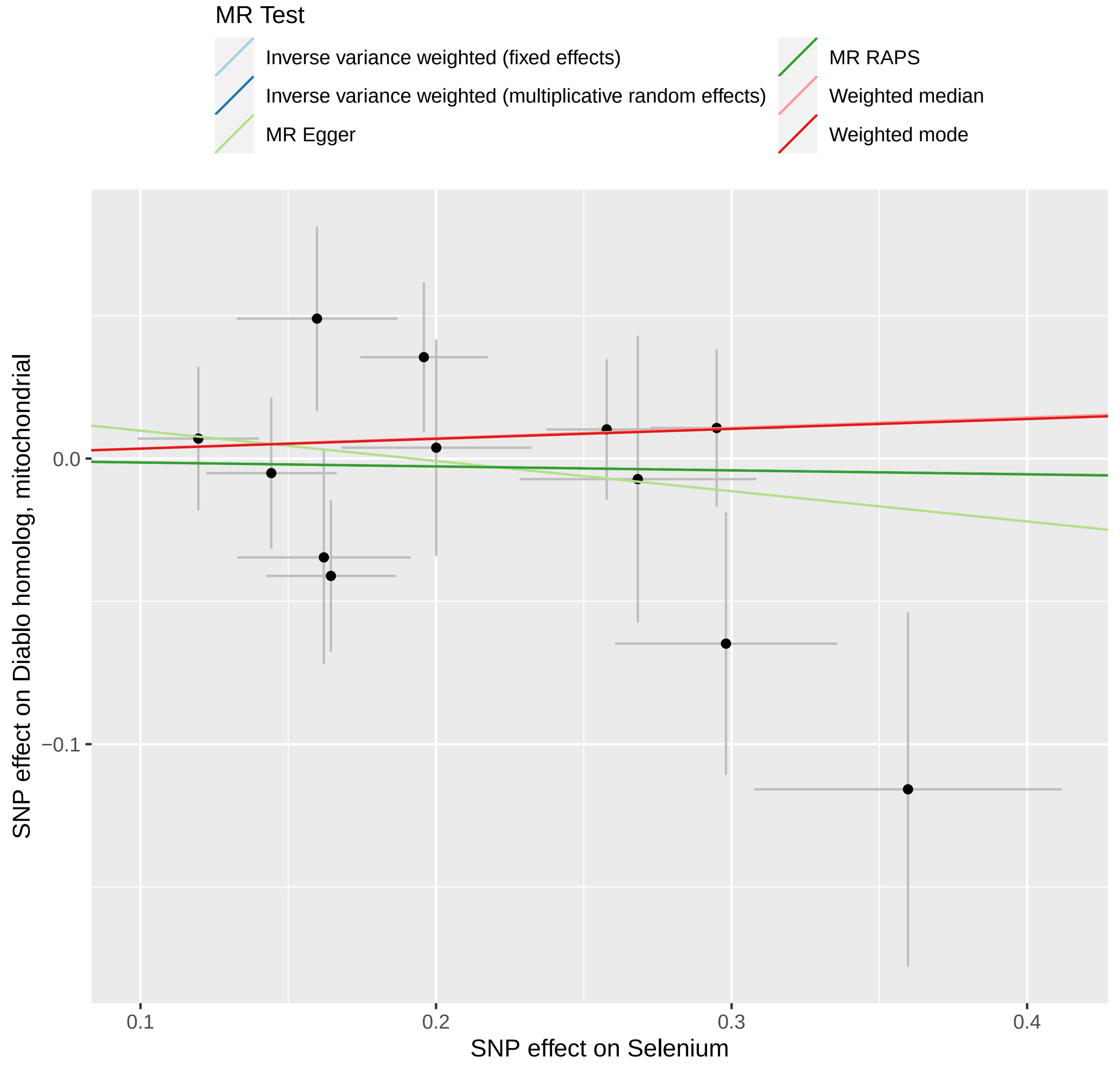

Batch 3−819 : Selenium on Probable dimethyladenosine transferase

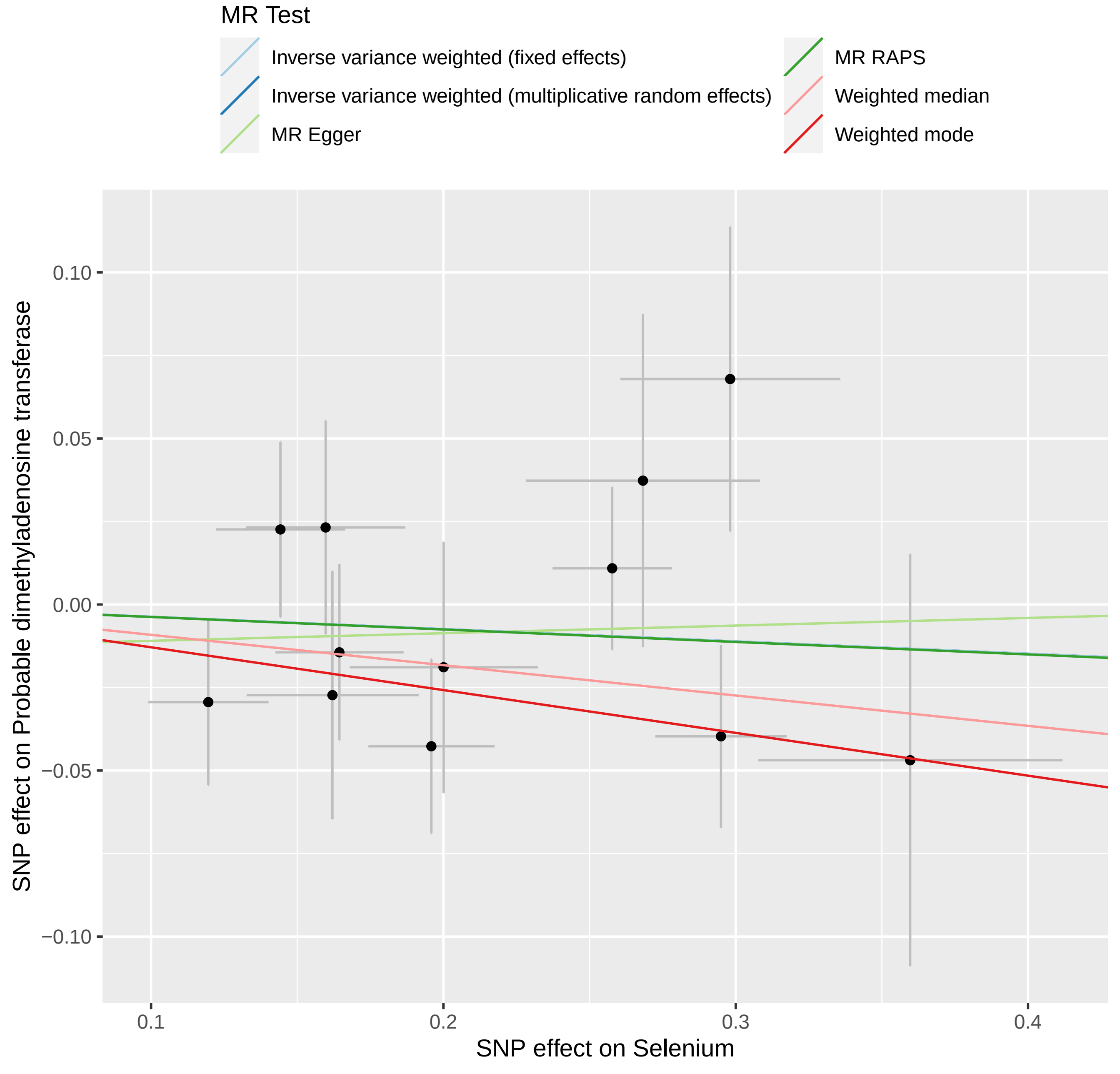

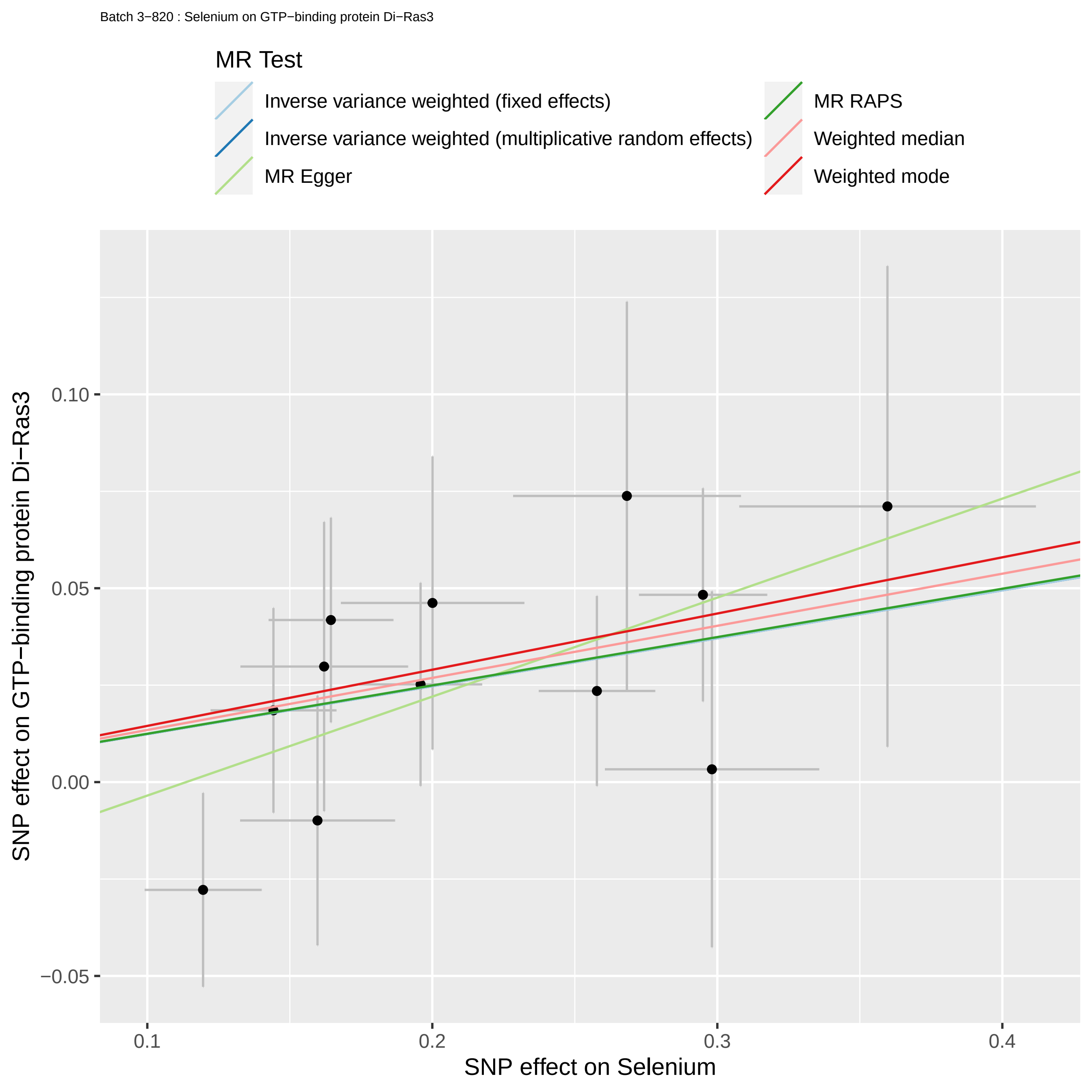

Batch 3−820 : Selenium on GTP−binding protein Di−Ras3
MR Test
Inverse variance weighted (fixed effects)
Inverse variance weighted (multiplicative random effects)
MR Egger
MR RAPS
Weighted median
Weighted mode
0.10
0.05
0.00
−0.05
0.1
0.2
0.3
0.4
SNP effect on GTP−binding protein Di−Ras3
SNP effect on Selenium

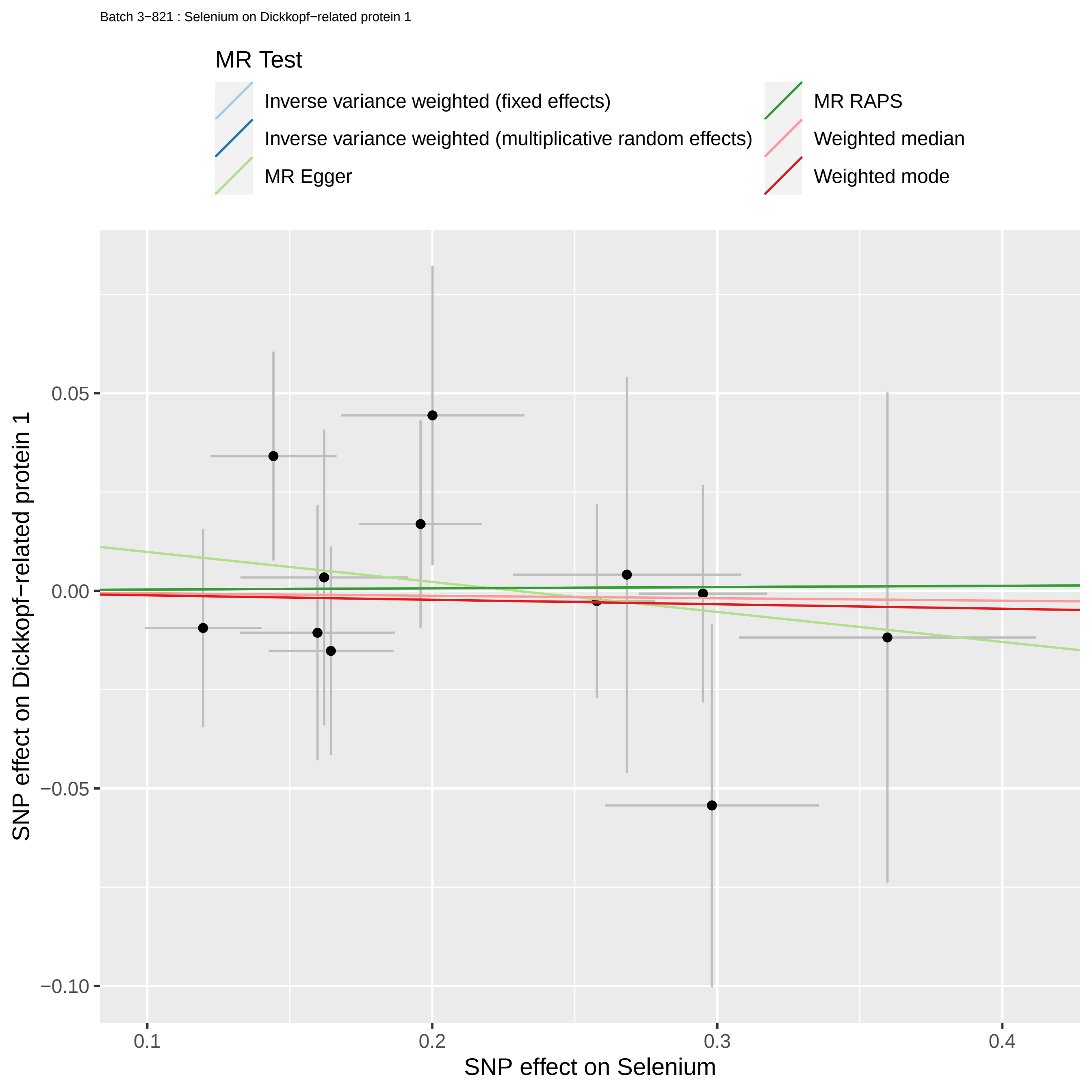
Batch 3−821 : Selenium on Dickkopf−related protein 1
MR Test
Inverse variance weighted (fixed effects)
Inverse variance weighted (multiplicative random effects)
MR Egger
MR RAPS
Weighted median
Weighted mode
SNP effect on Dickkopf−related protein 1
0.05
0.00
−0.05
−0.10
0.1
0.2
0.3
0.4
SNP effect on Selenium

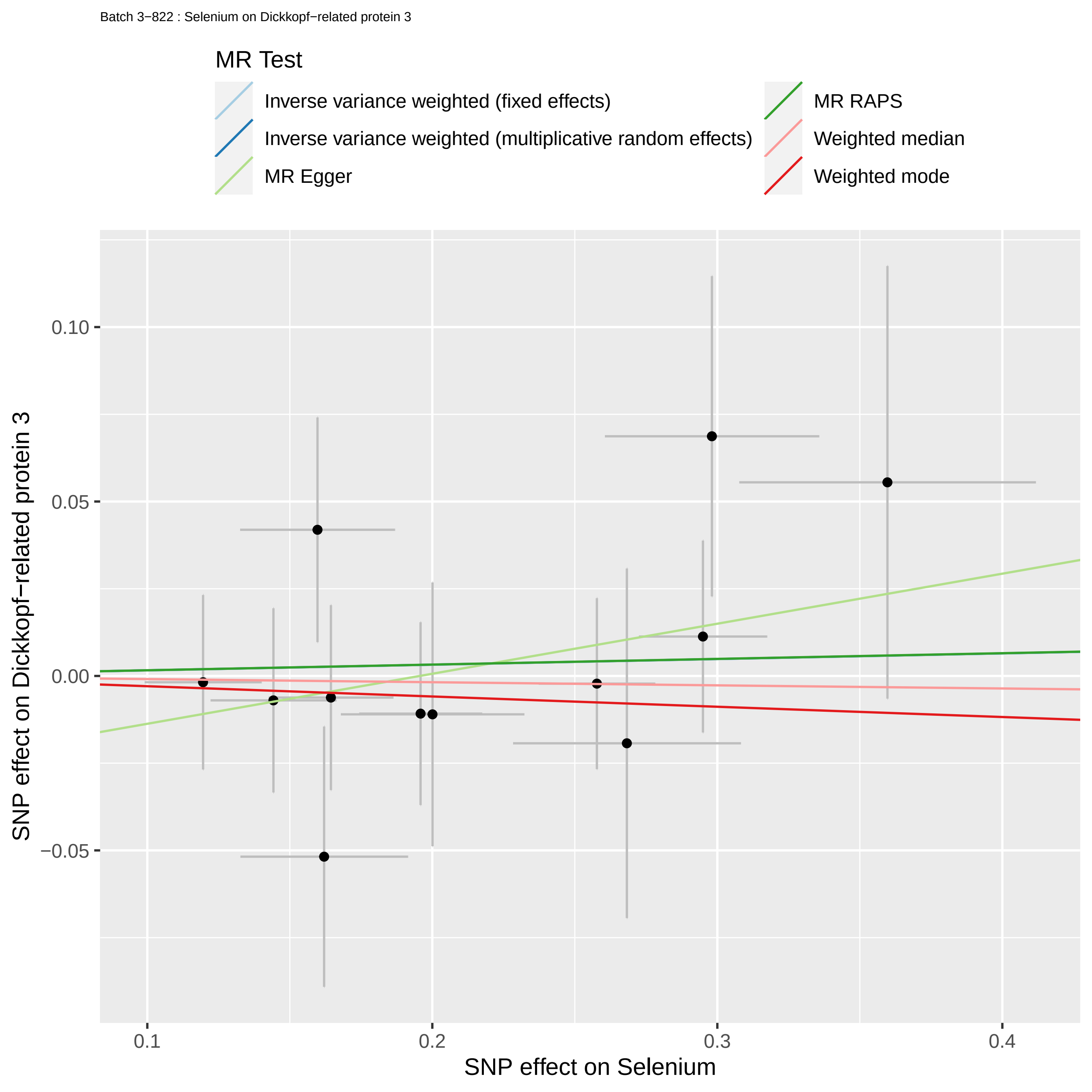

Batch 3−822 : Selenium on Dickkopf−related protein 3
MR Test
Inverse variance weighted (fixed effects)
Inverse variance weighted (multiplicative random effects)
MR Egger
MR RAPS
Weighted median
Weighted mode
0.10
0.05
0.00
−0.05
0.1
0.2
0.3
0.4
SNP effect on Dickkopf−related protein 3
SNP effect on Selenium

Batch 3−823 : Selenium on Dickkopf−related protein 4

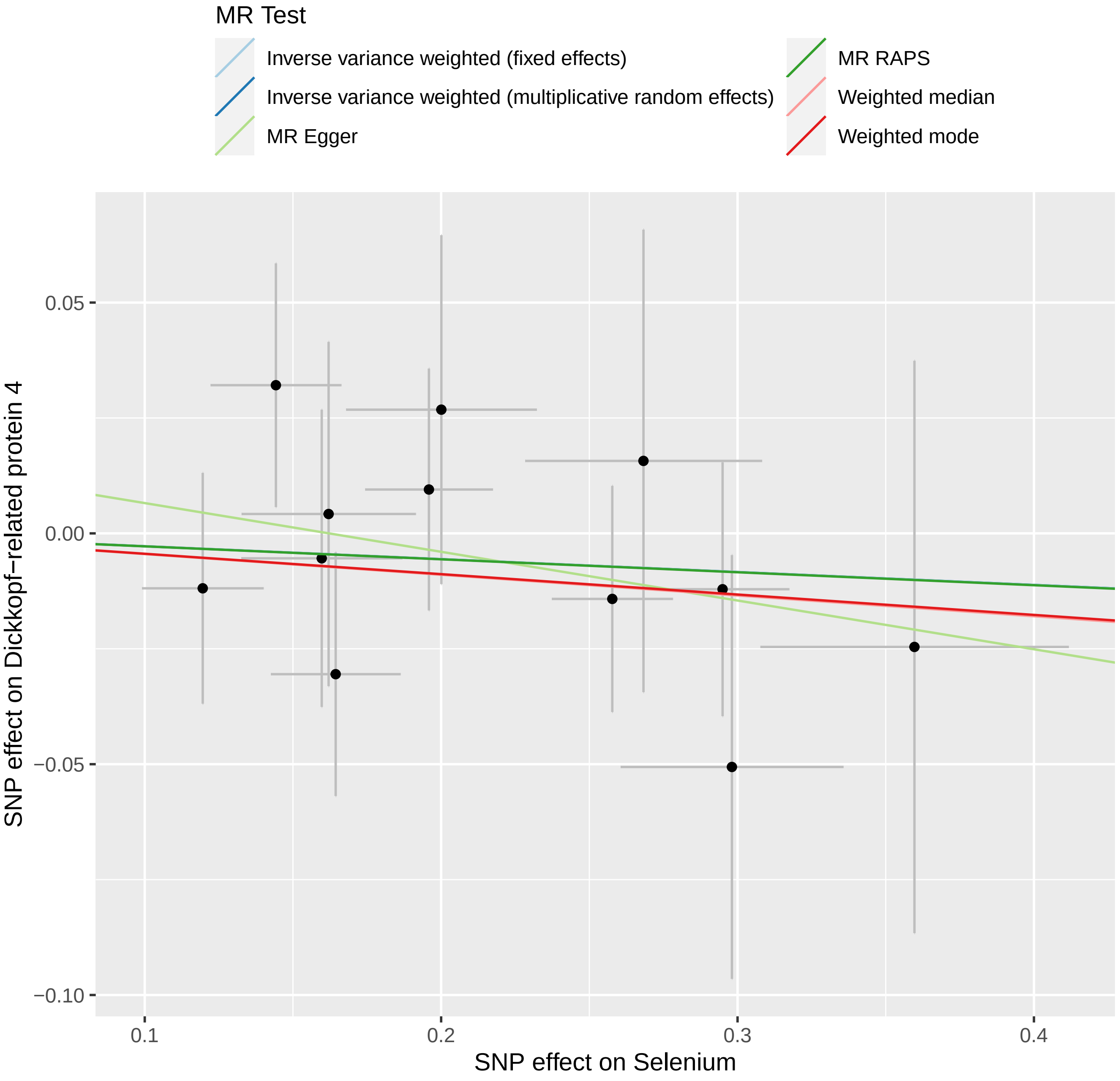

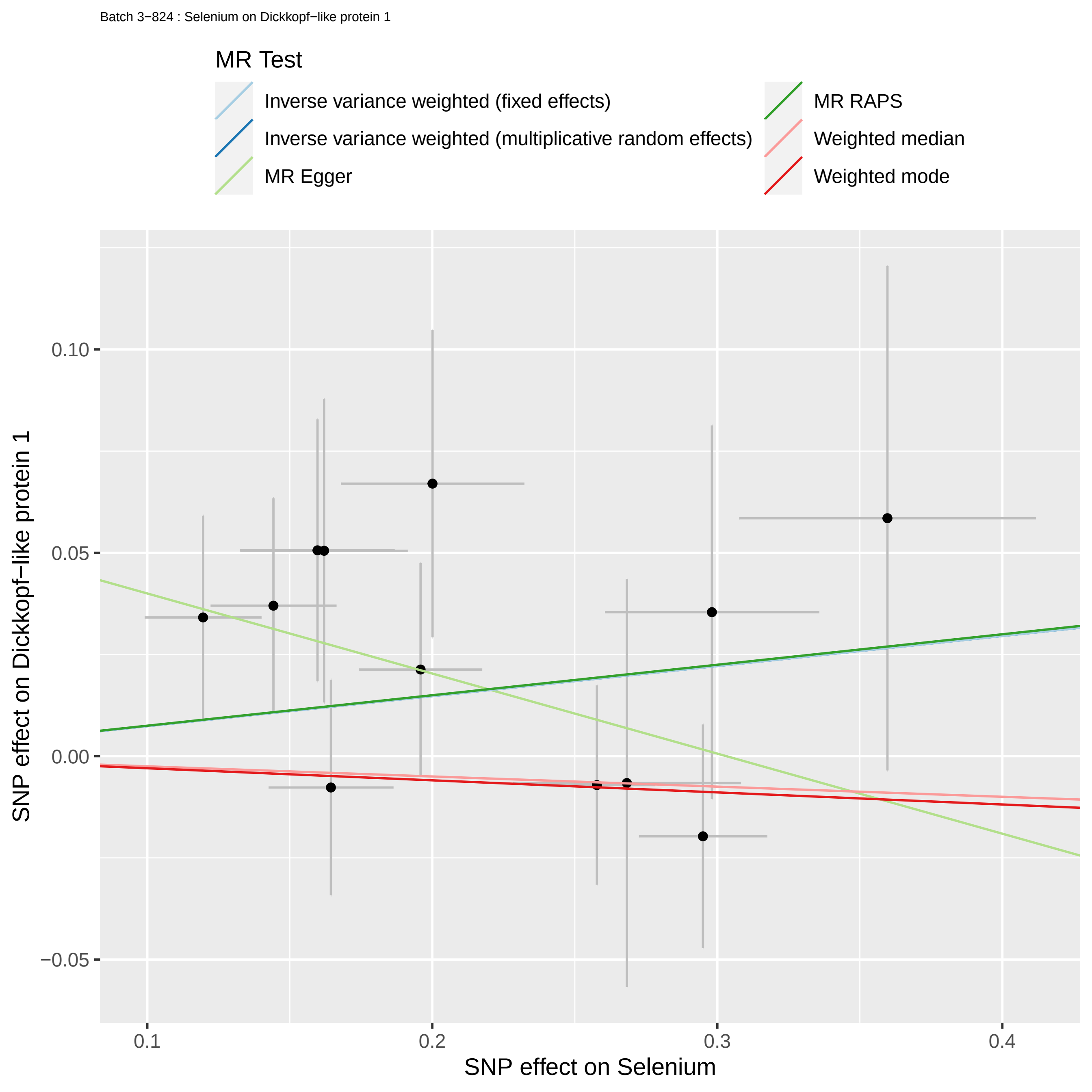

Batch 3−824 : Selenium on Dickkopf−like protein 1
MR Test
Inverse variance weighted (fixed effects)
Inverse variance weighted (multiplicative random effects)
MR Egger
MR RAPS
Weighted median
Weighted mode
0.10
0.05
0.00
−0.05
0.1
0.2
0.3
0.4
SNP effect on Dickkopf−like protein 1
SNP effect on Selenium

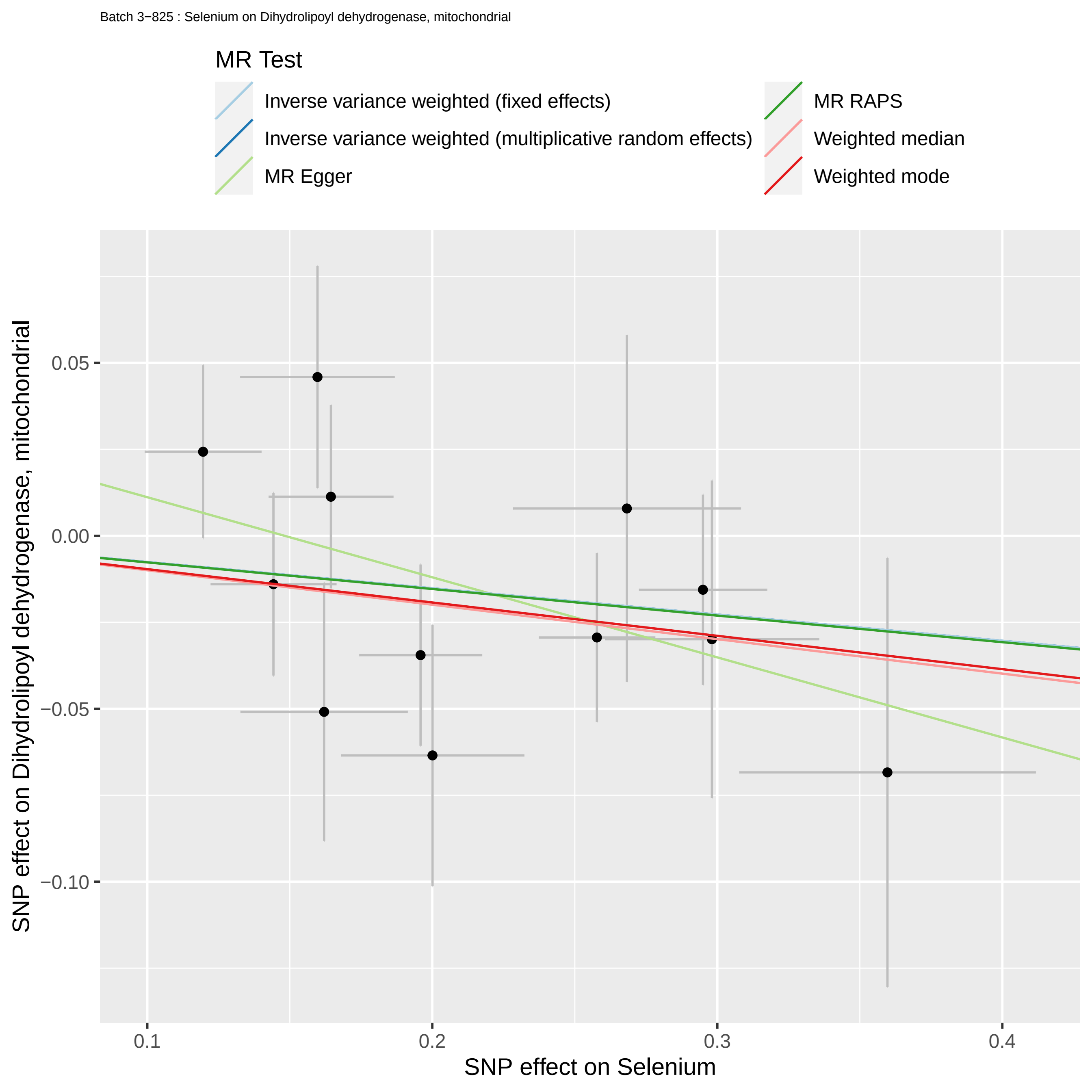
Batch 3−825 : Selenium on Dihydrolipoyl dehydrogenase, mitochondrial
MR Test
Inverse variance weighted (fixed effects)
Inverse variance weighted (multiplicative random effects)
MR Egger
MR RAPS
Weighted median
Weighted mode
SNP effect on Dihydrolipoyl dehydrogenase, mitochondrial
0.05
0.00
−0.05
−0.10
0.1
0.2
0.3
0.4
SNP effect on Selenium

Batch 3−826 : Selenium on Disks large homolog 3

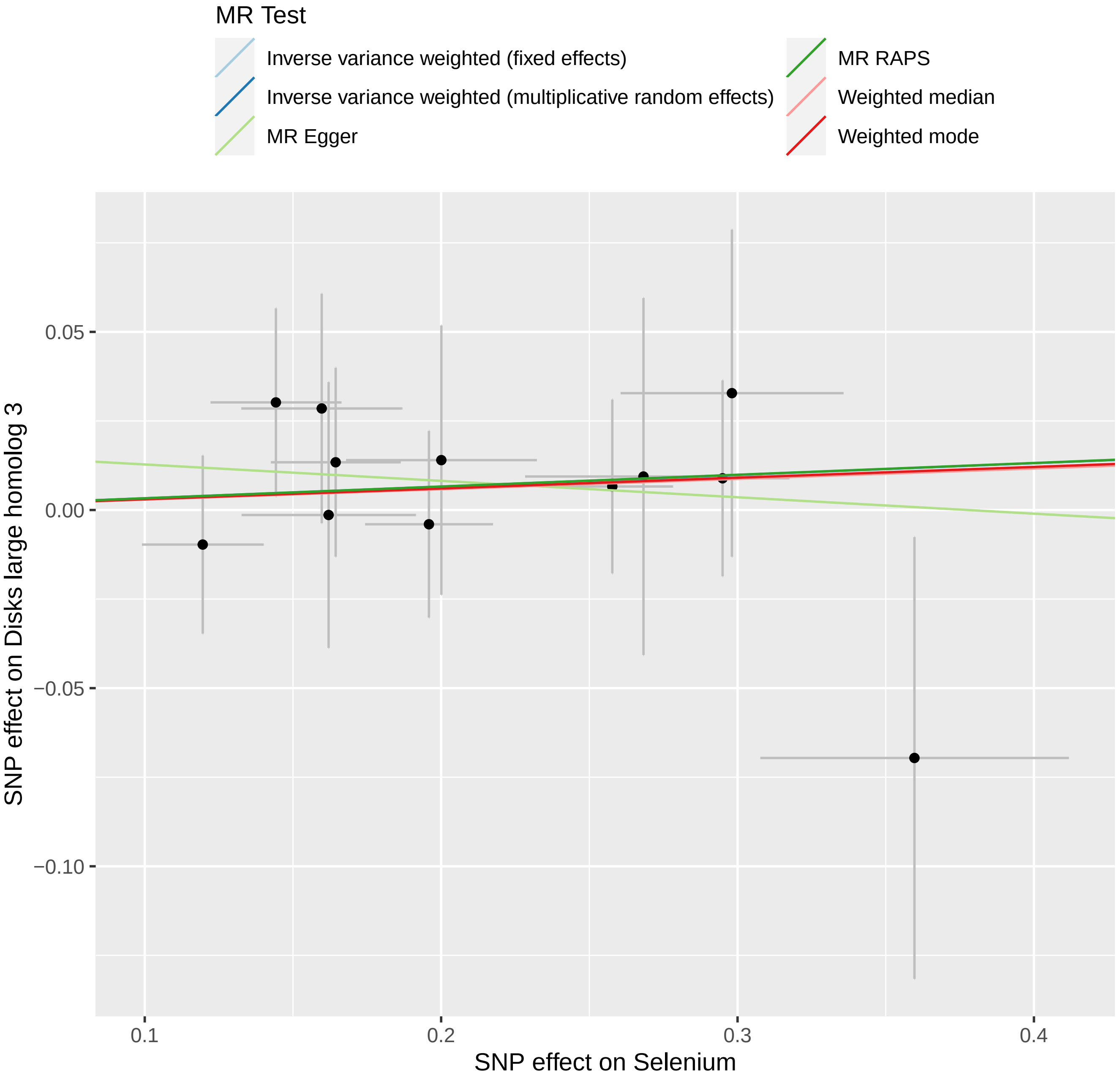

Batch 3−827 : Selenium on Protein delta homolog 1

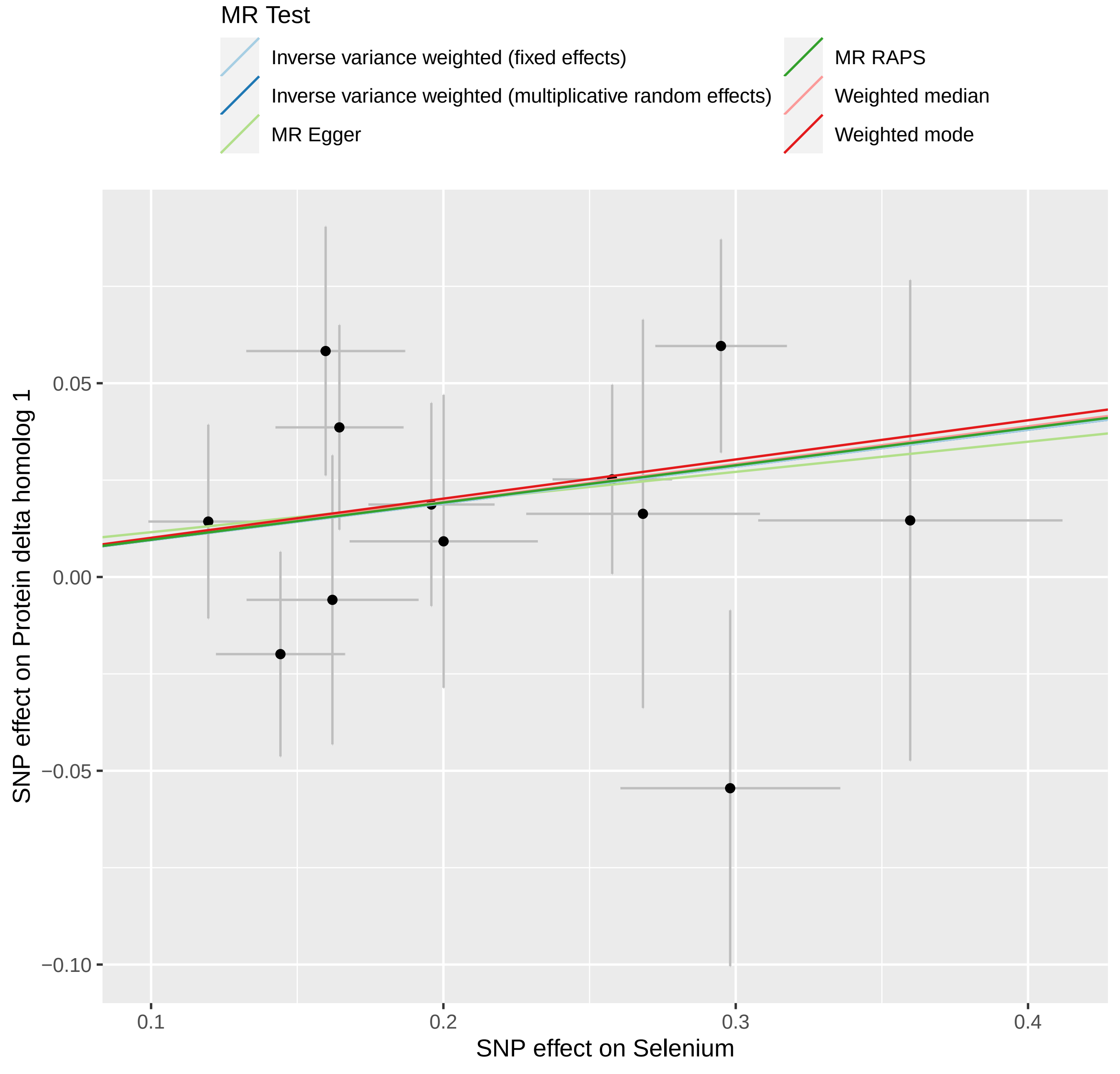

Batch 3−828 : Selenium on Protein delta homolog 1

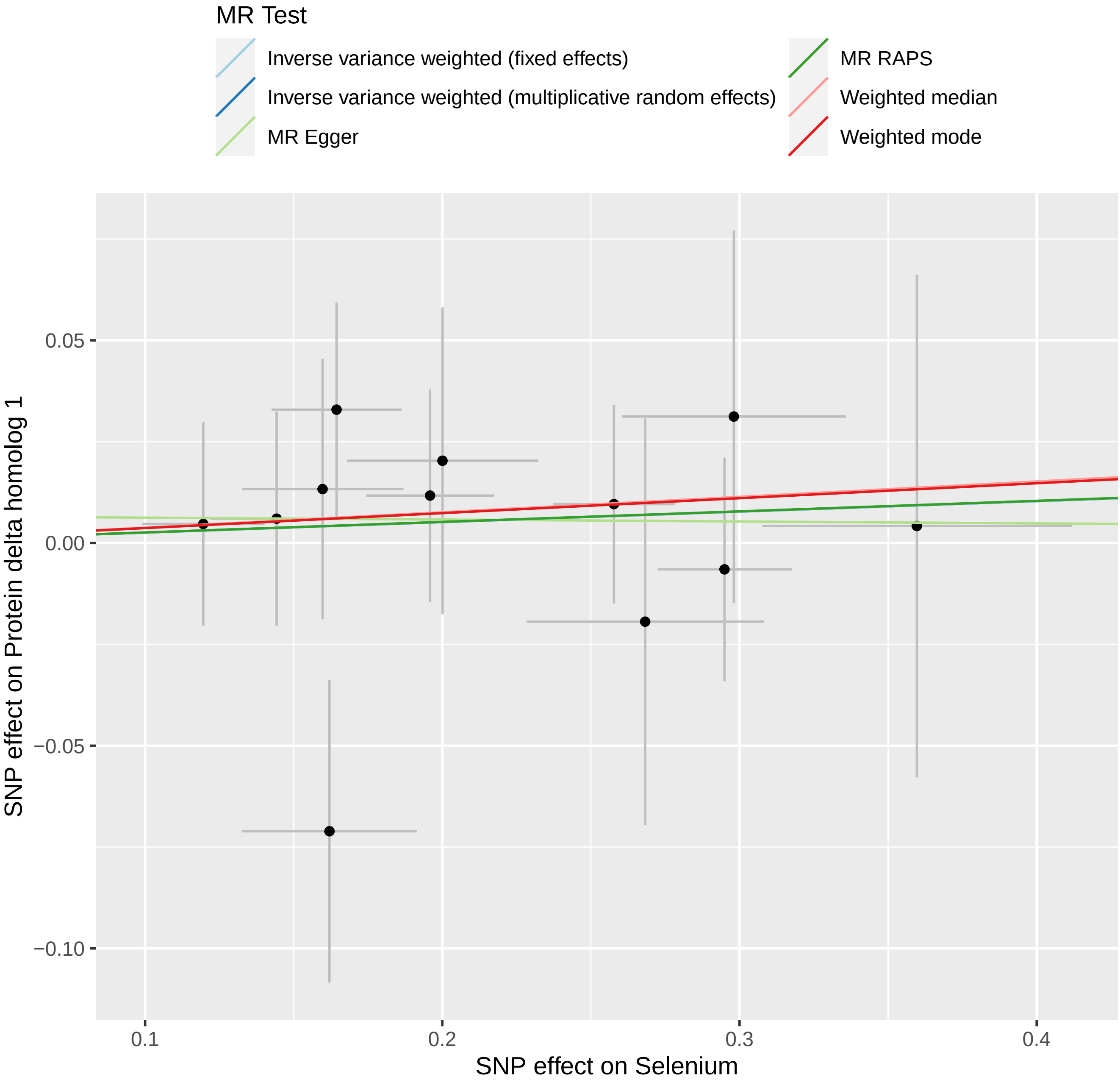

Batch 3−829 : Selenium on Protein delta homolog 2

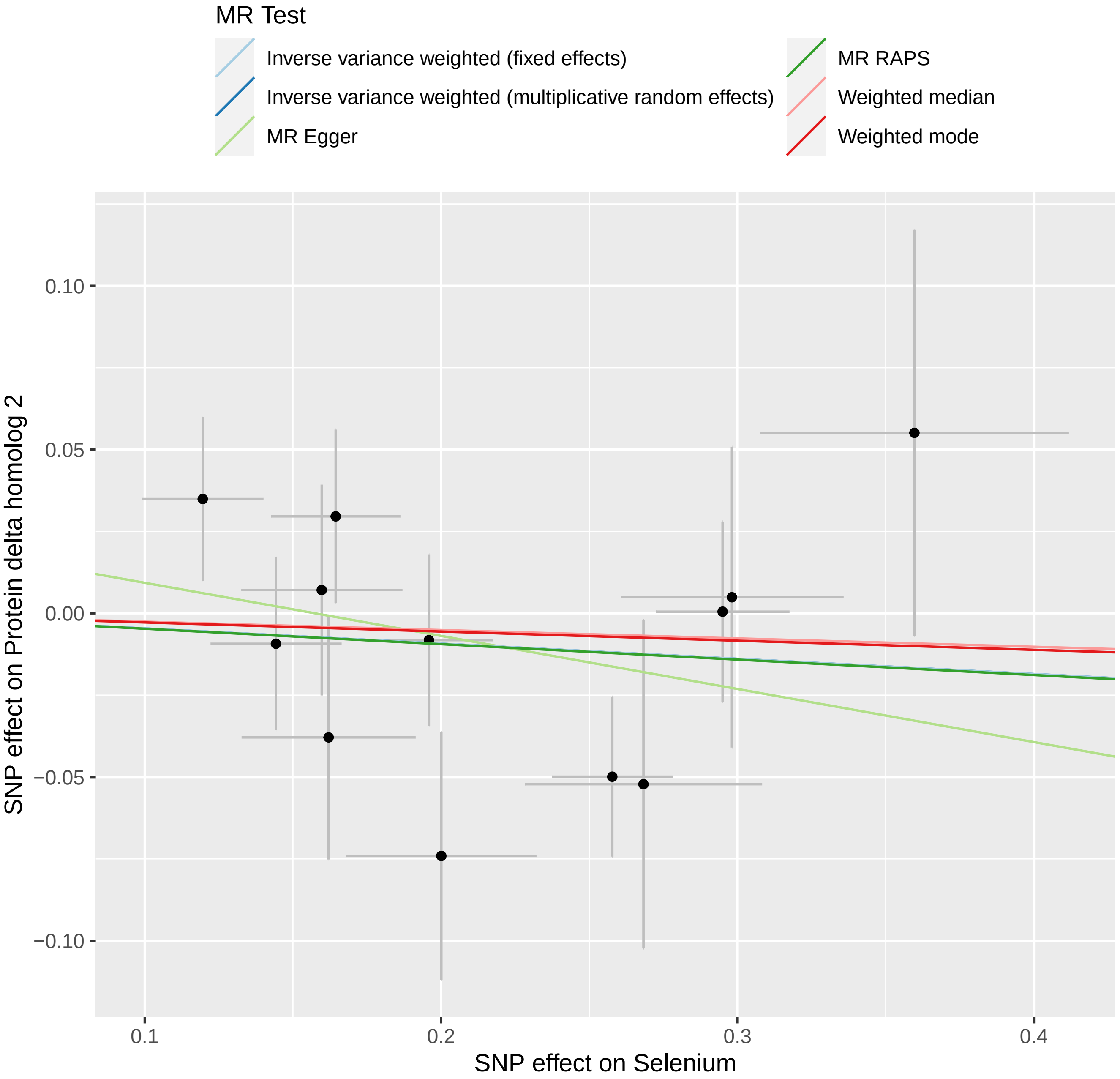

Batch 3−830 : Selenium on Delta−like protein 1

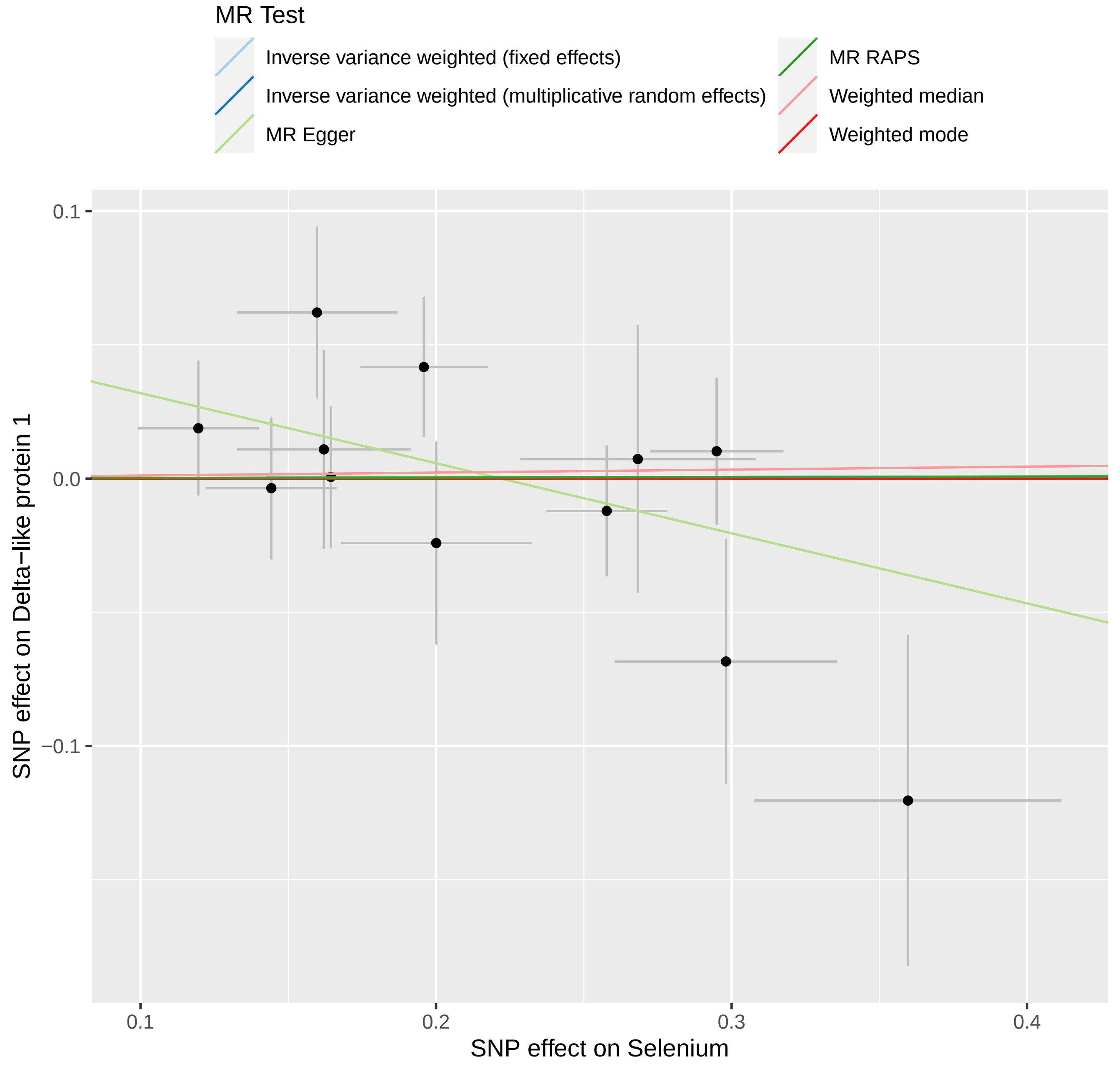

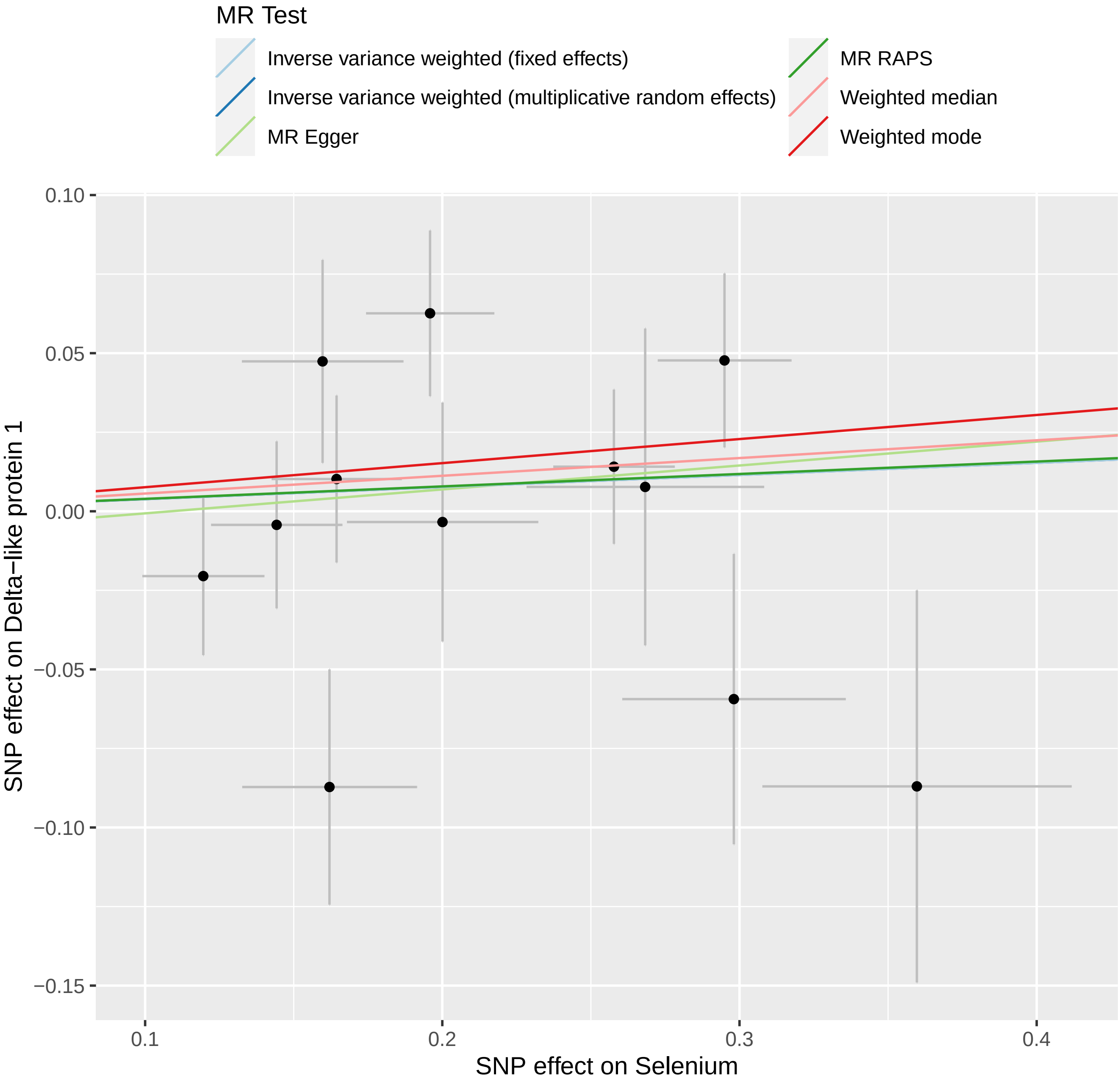
MR Test
Inverse variance weighted (fixed effects)
Inverse variance weighted (multiplicative random effects)
MR Egger
MR RAPS
Weighted median
Weighted mode
0.10
0.05
0.00
−0.05
−0.10
−0.15
0.1
0.2
0.3
0.4
SNP effect on Selenium
SNP effect on Delta−like protein 1

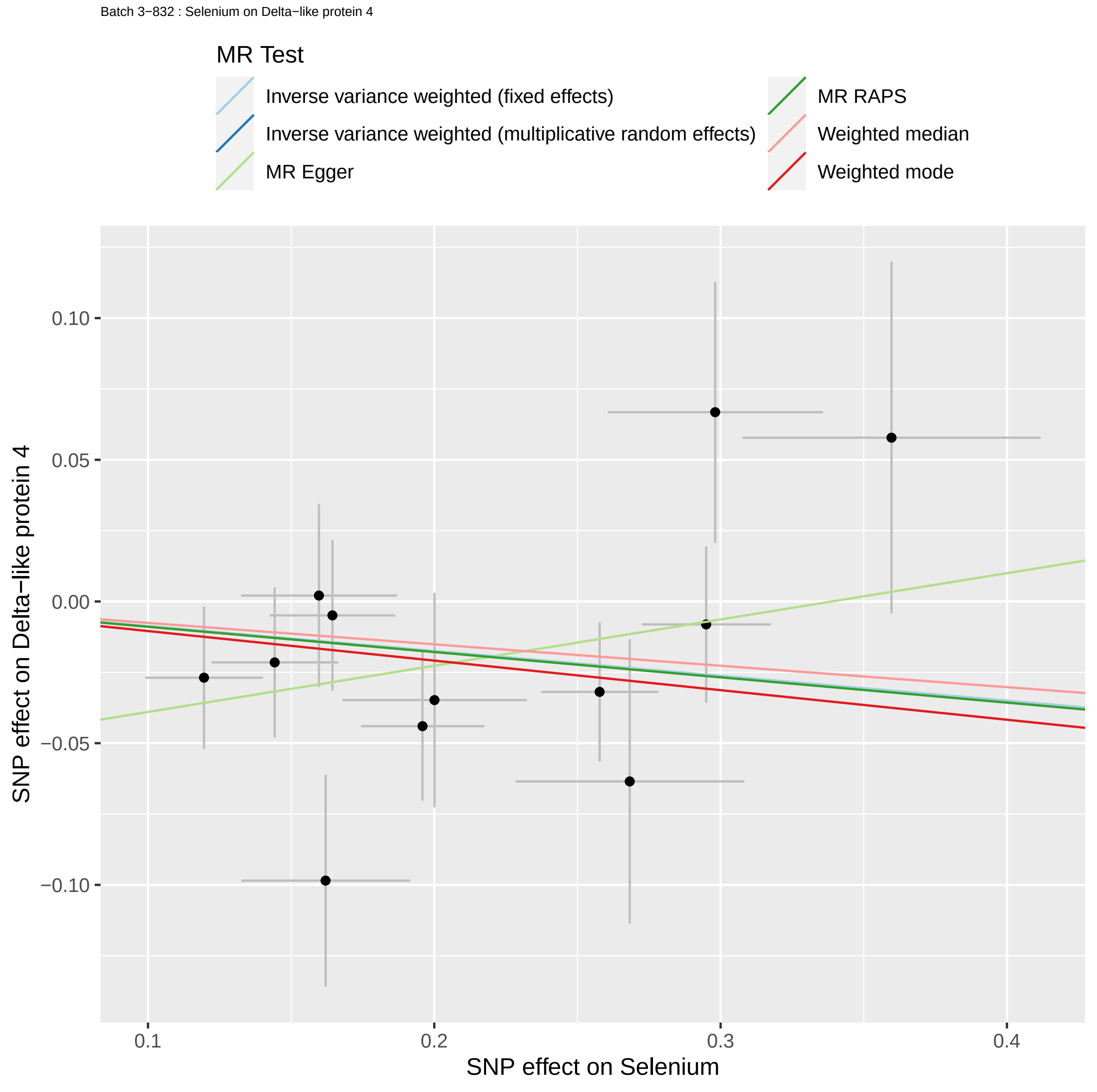
Batch 3−832 : Selenium on Delta−like protein 4
MR Test
Inverse variance weighted (fixed effects)
Inverse variance weighted (multiplicative random effects)
MR Egger
MR RAPS
Weighted median
Weighted mode
SNP effect on Delta−like protein 4
0.10
0.05
0.00
−0.05
−0.10
0.1
0.2
0.3
0.4
SNP effect on Selenium

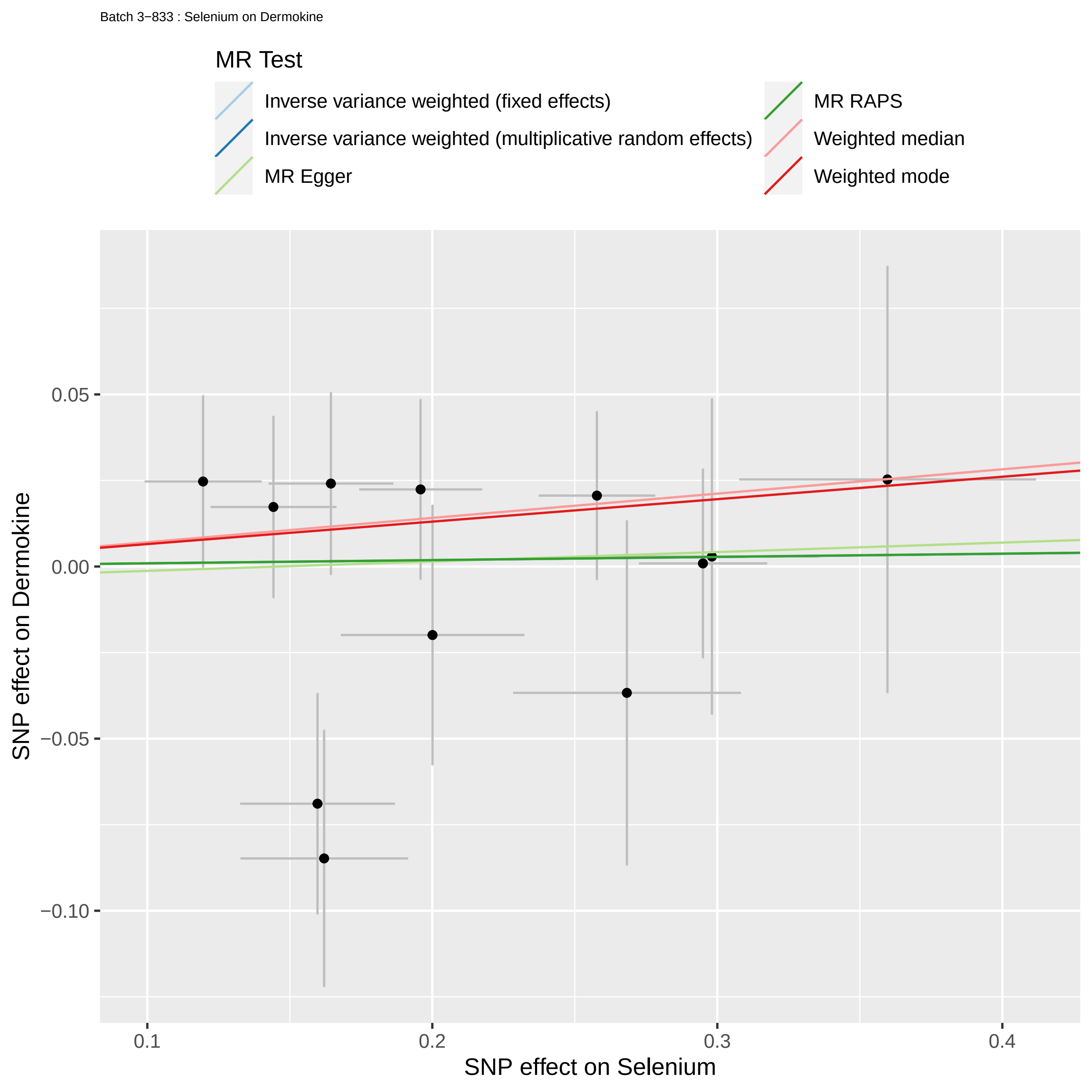
Batch 3−833 : Selenium on Dermokine
MR Test
Inverse variance weighted (fixed effects)
Inverse variance weighted (multiplicative random effects)
MR Egger
MR RAPS
Weighted median
Weighted mode
0.05
0.00
−0.05
−0.10
0.1
0.2
0.3
0.4
SNP effect on Dermokine
SNP effect on Selenium

Batch 3−834 : Selenium on Myotonin−protein kinase

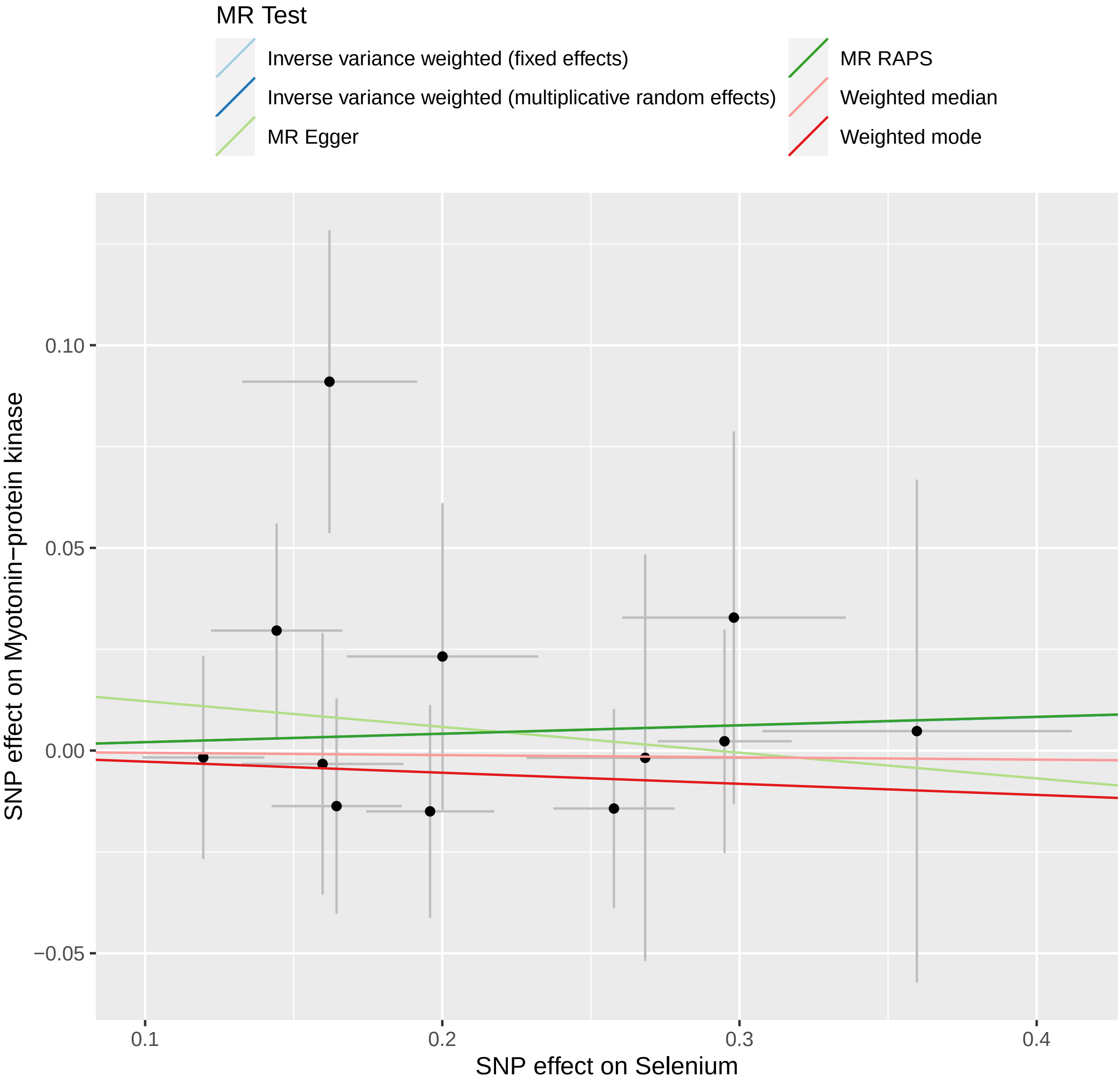

Batch 3−835 : Selenium on DnaJ homolog subfamily A member 4

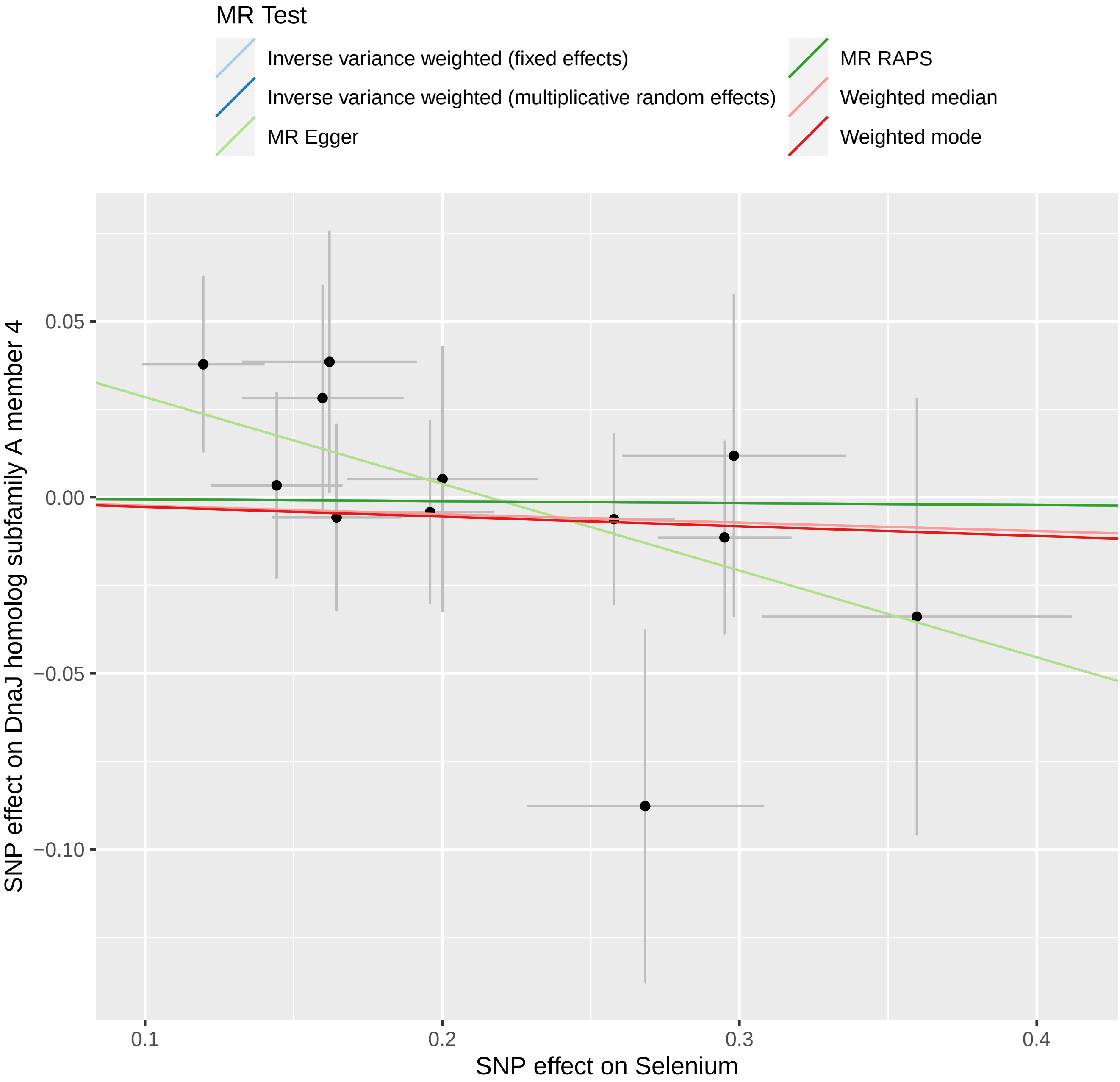

Batch 3−836 : Selenium on DnaJ homolog subfamily B member 11

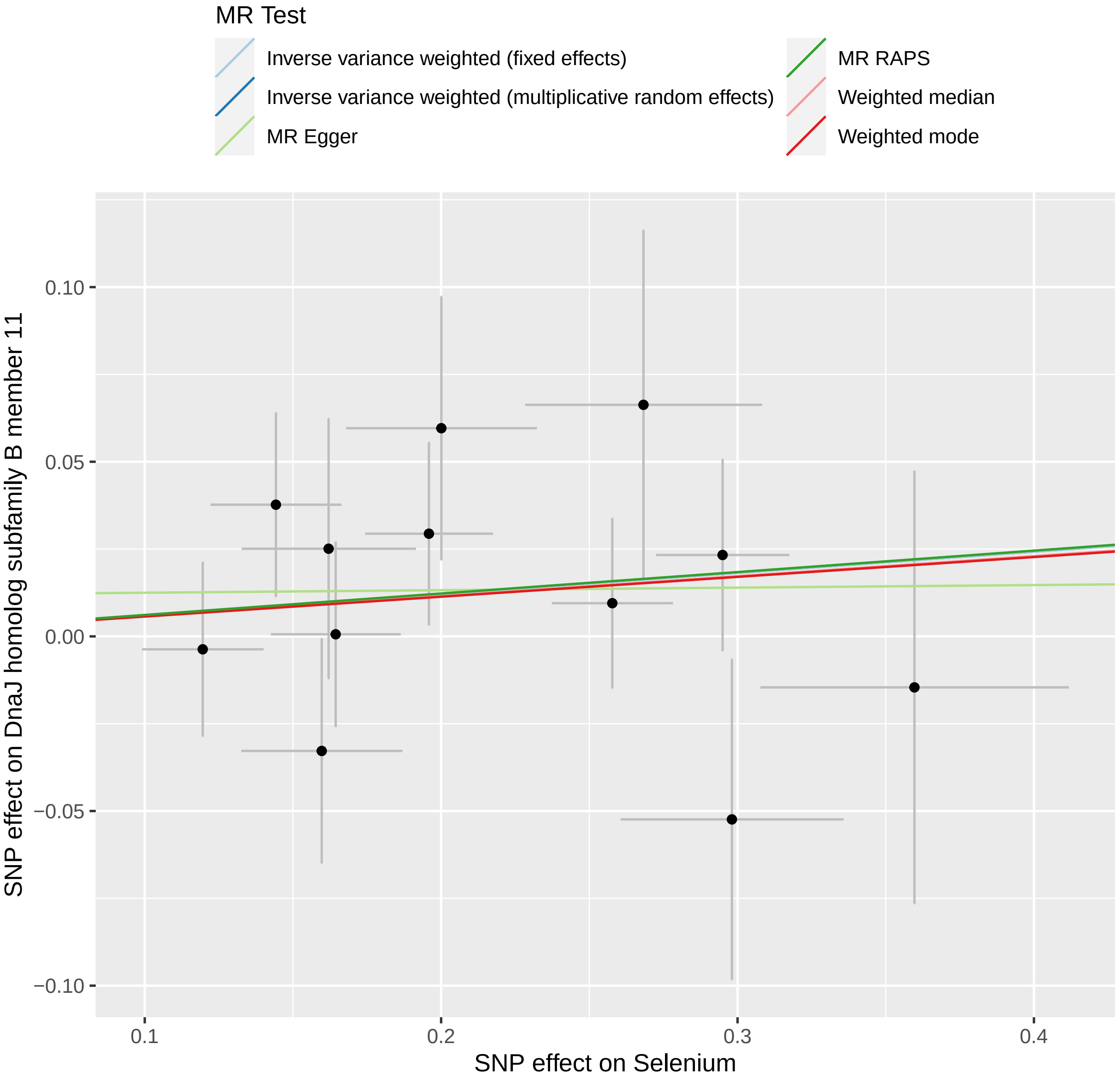

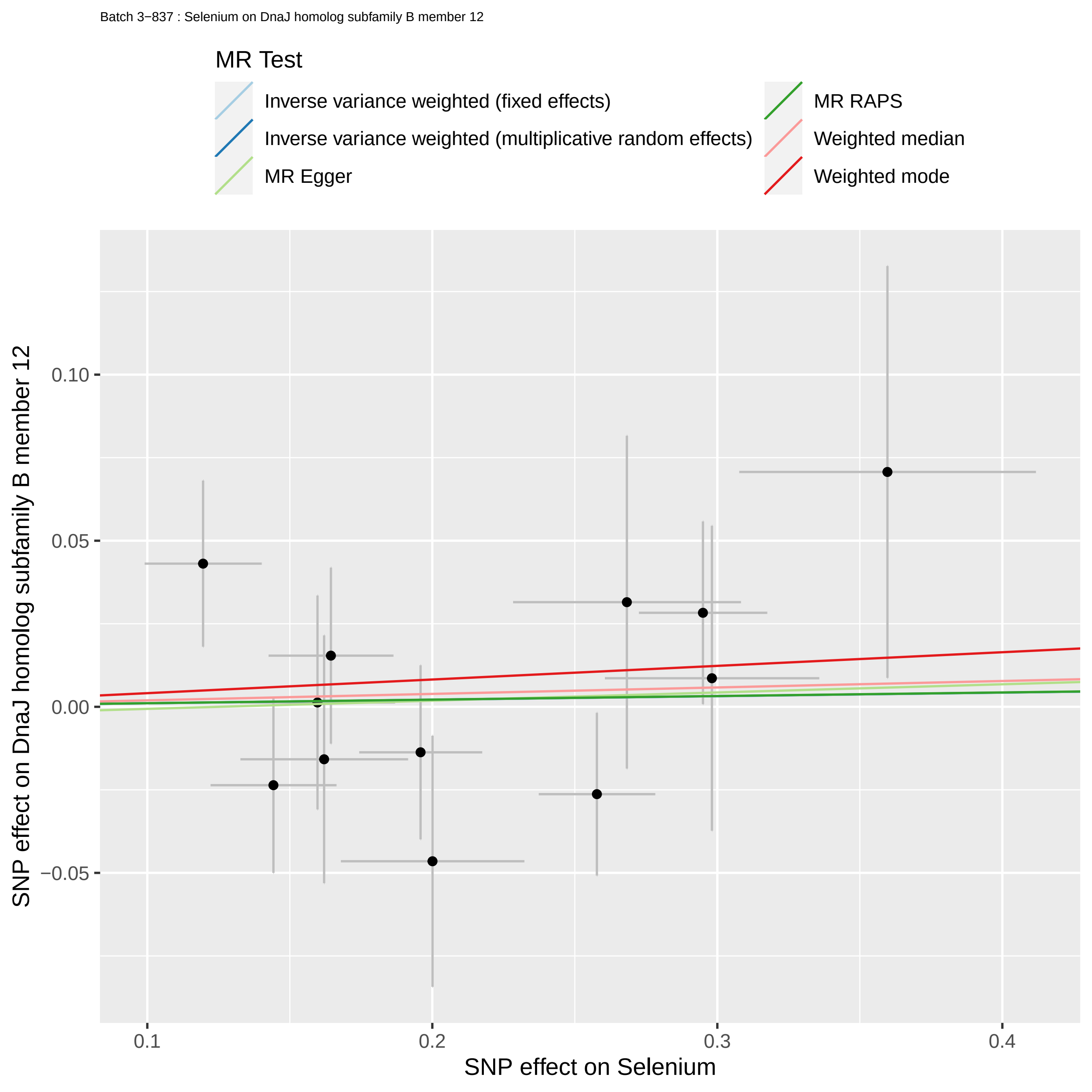

Batch 3−837 : Selenium on DnaJ homolog subfamily B member 12
MR Test
Inverse variance weighted (fixed effects)
Inverse variance weighted (multiplicative random effects)
MR Egger
MR RAPS
Weighted median
Weighted mode
0.10
0.05
0.00
−0.05
0.1
0.2
0.3
0.4
SNP effect on Selenium
SNP effect on DnaJ homolog subfamily B member 12

Batch 3−838 : Selenium on DnaJ homolog subfamily B member 1

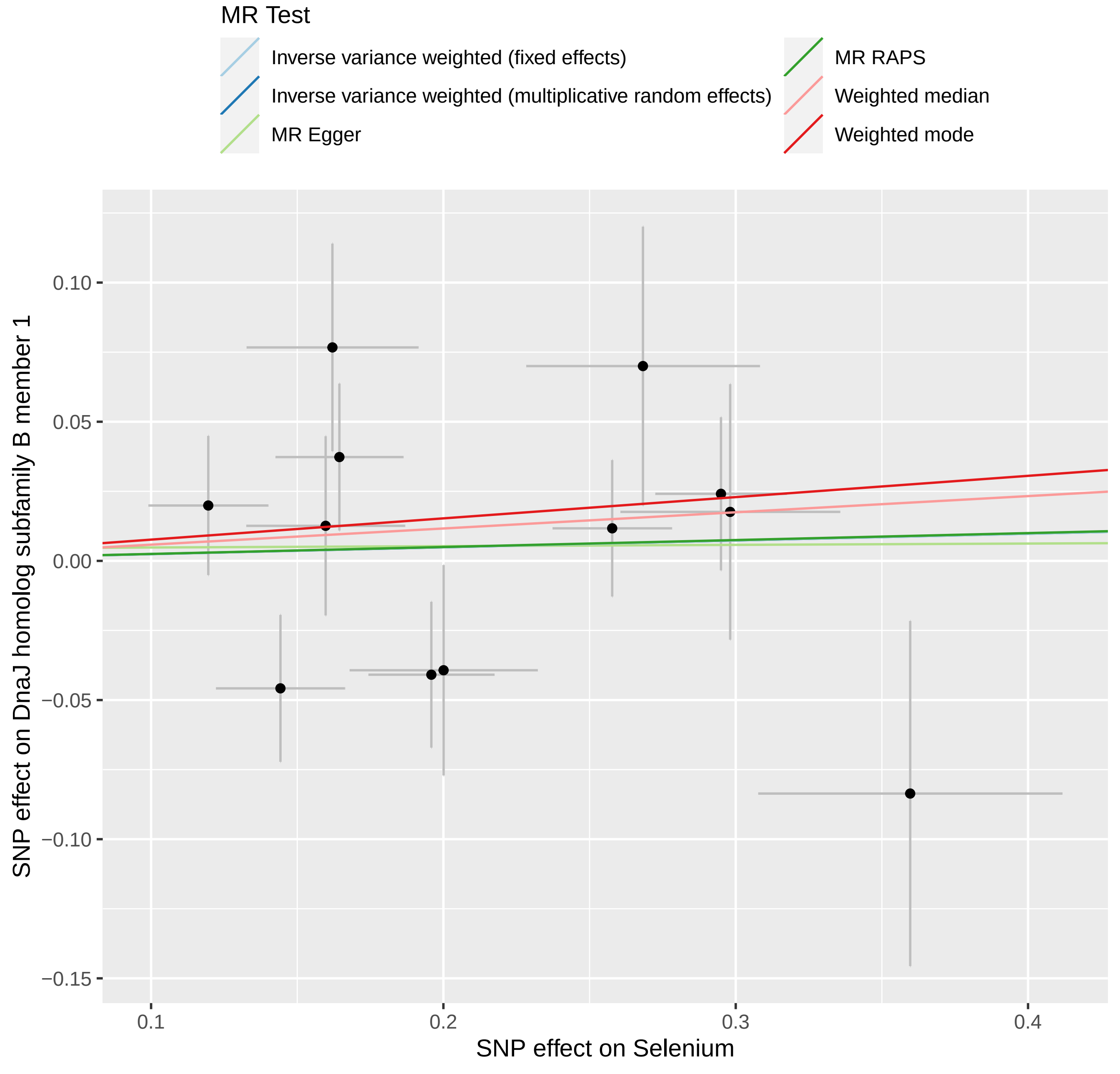

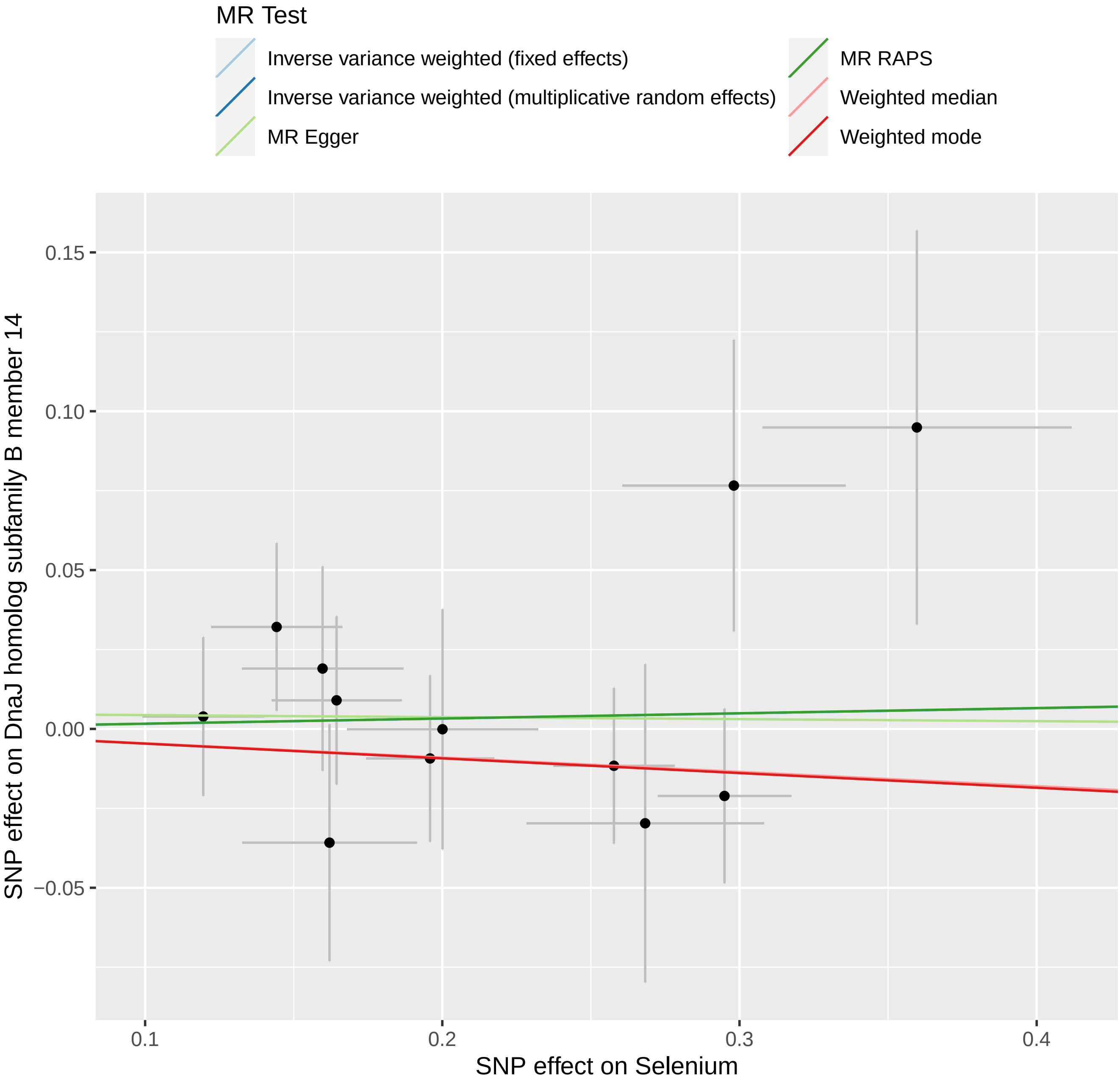
MR Test
Inverse variance weighted (fixed effects)
Inverse variance weighted (multiplicative random effects)
MR Egger
MR RAPS
Weighted median
Weighted mode
0.15
0.10
0.05
0.00
−0.05
0.1
0.2
0.3
0.4
SNP effect on DnaJ homolog subfamily B member 14
SNP effect on Selenium

Batch 3−840 : Selenium on DnaJ homolog subfamily B member 14

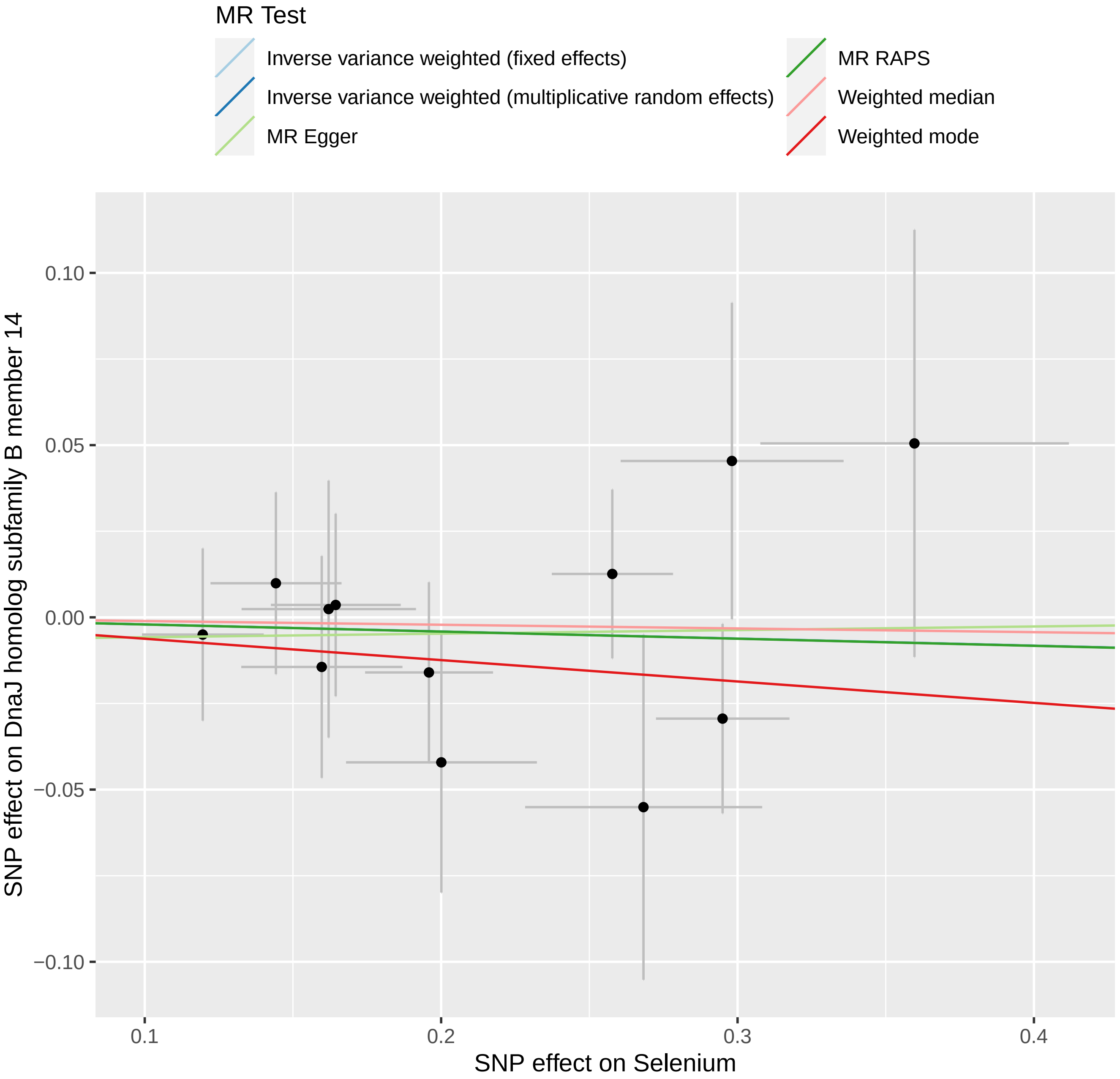

Batch 3−841 : Selenium on DnaJ homolog subfamily B member 2

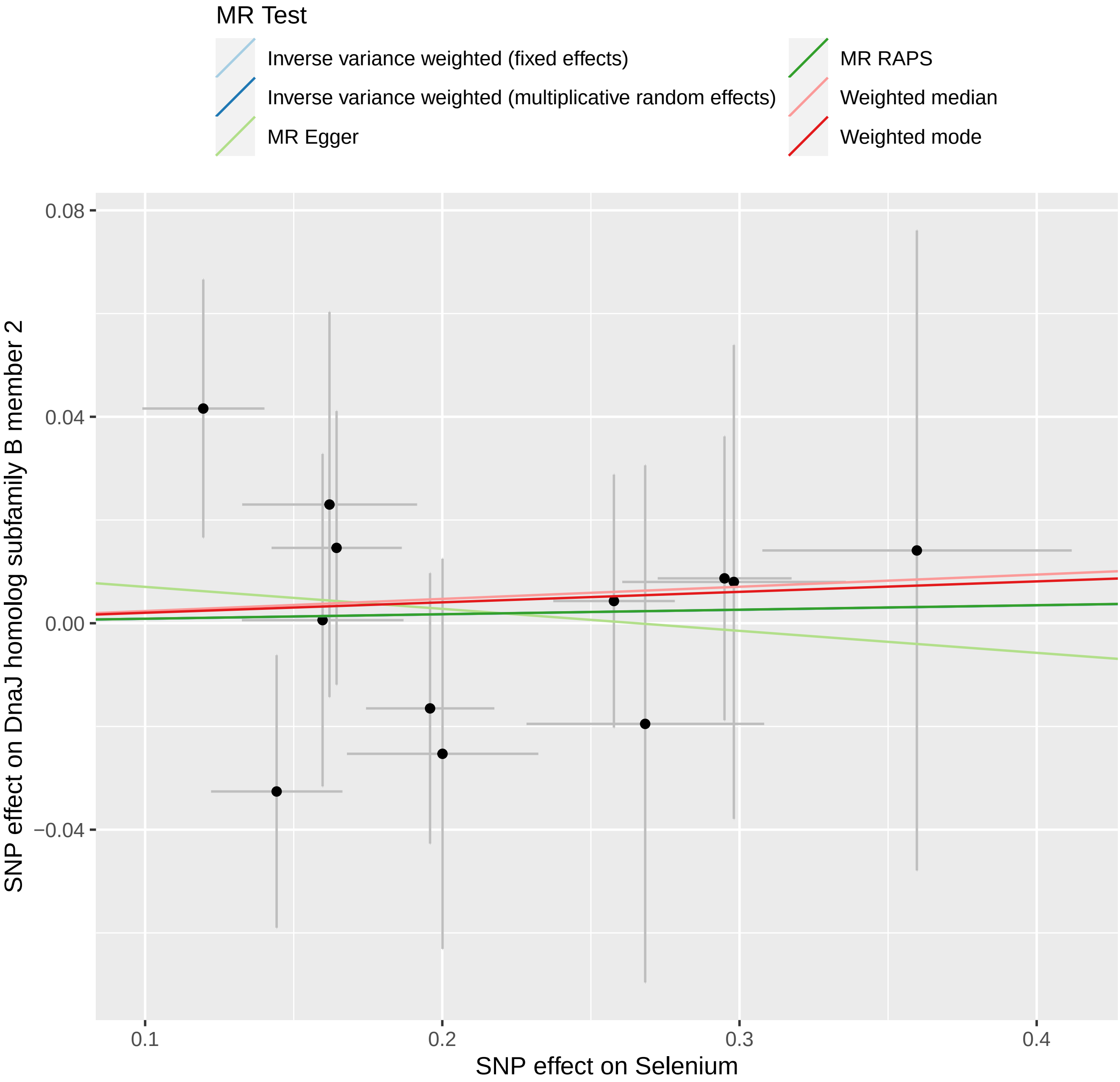

Batch 3−842 : Selenium on DnaJ homolog subfamily B member 9

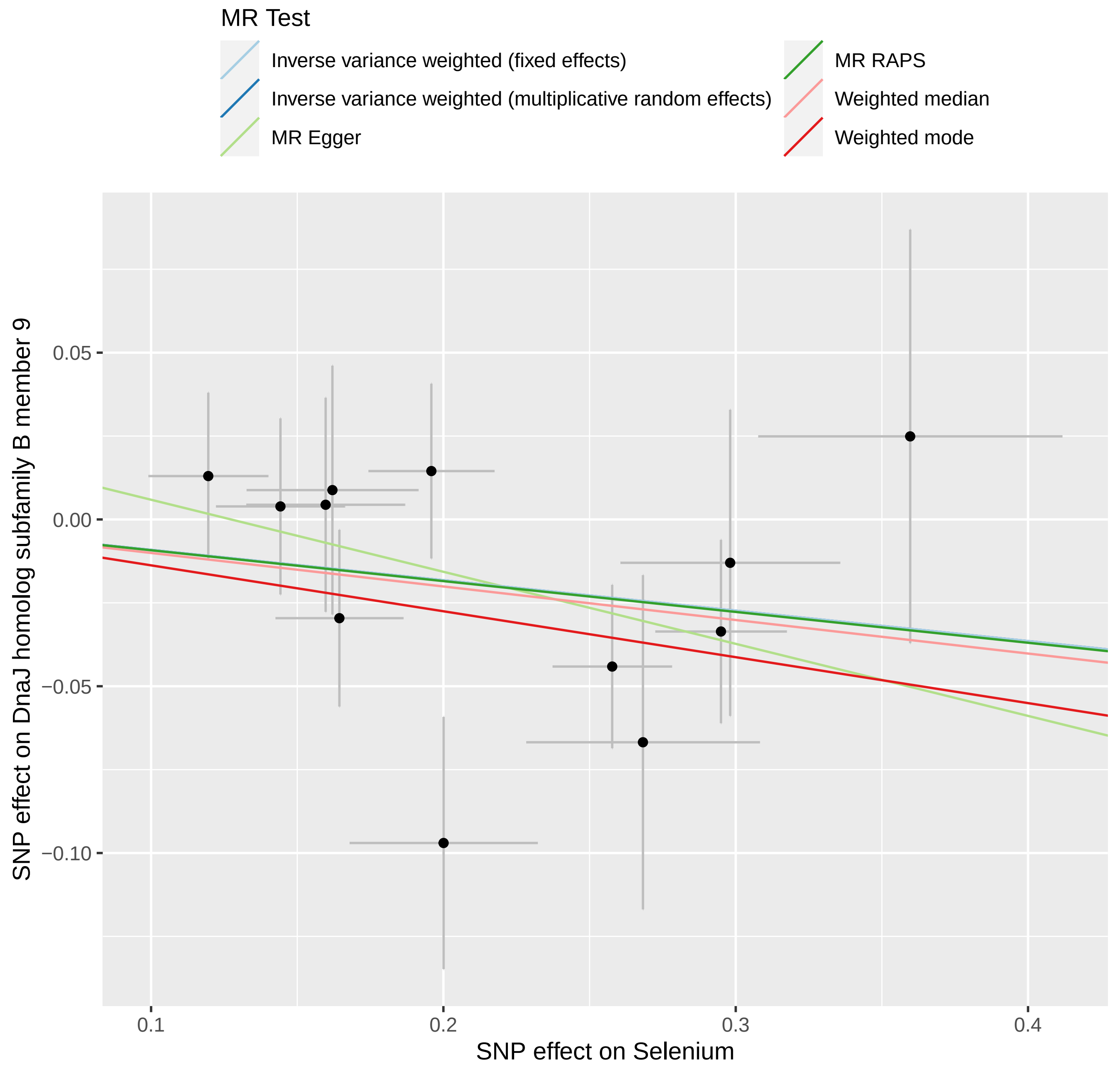

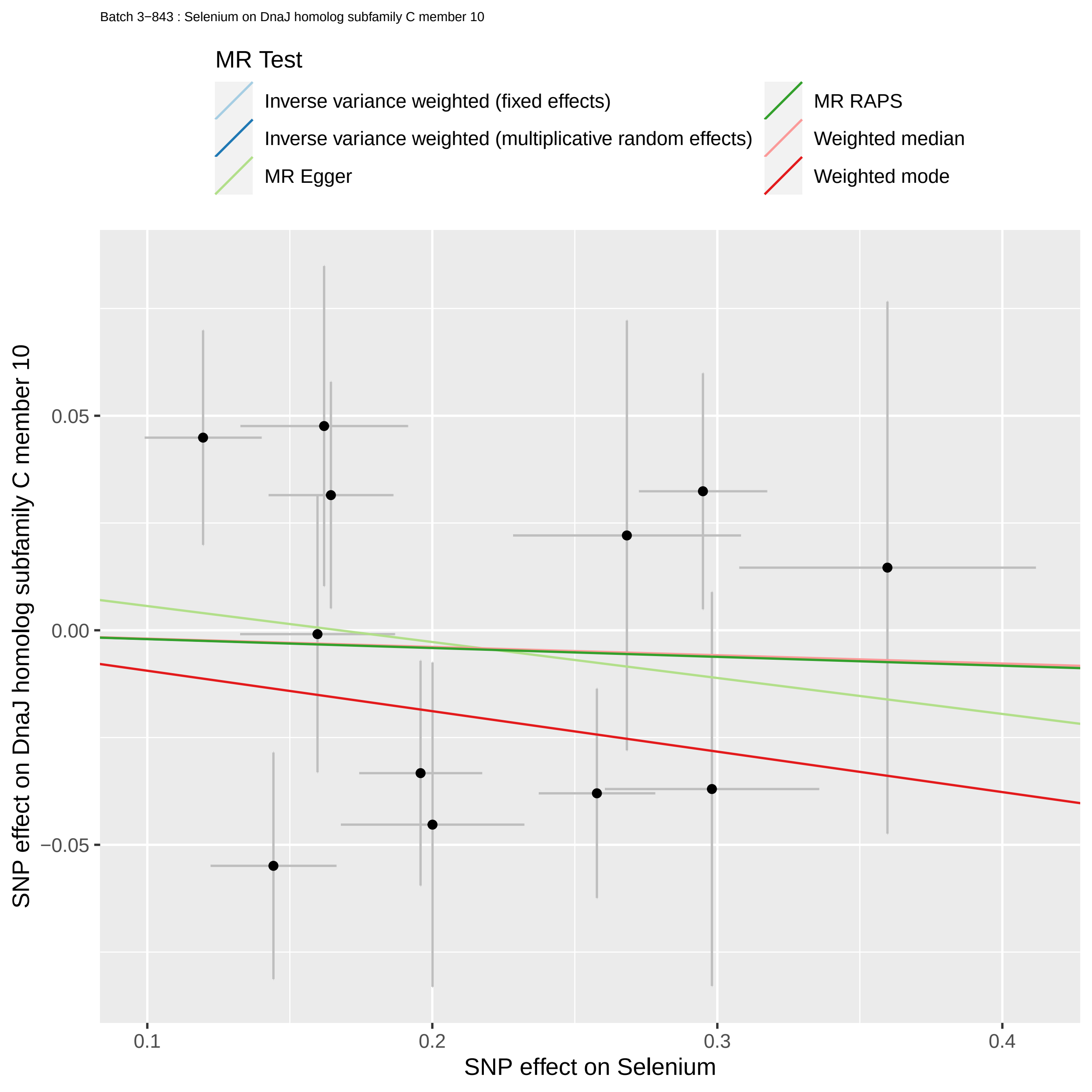

Batch 3−843 : Selenium on DnaJ homolog subfamily C member 10
MR Test
Inverse variance weighted (fixed effects)
Inverse variance weighted (multiplicative random effects)
MR Egger
MR RAPS
Weighted median
Weighted mode
SNP effect on DnaJ homolog subfamily C member 10
0.05
0.00
−0.05
0.1
0.2
0.3
0.4
SNP effect on Selenium

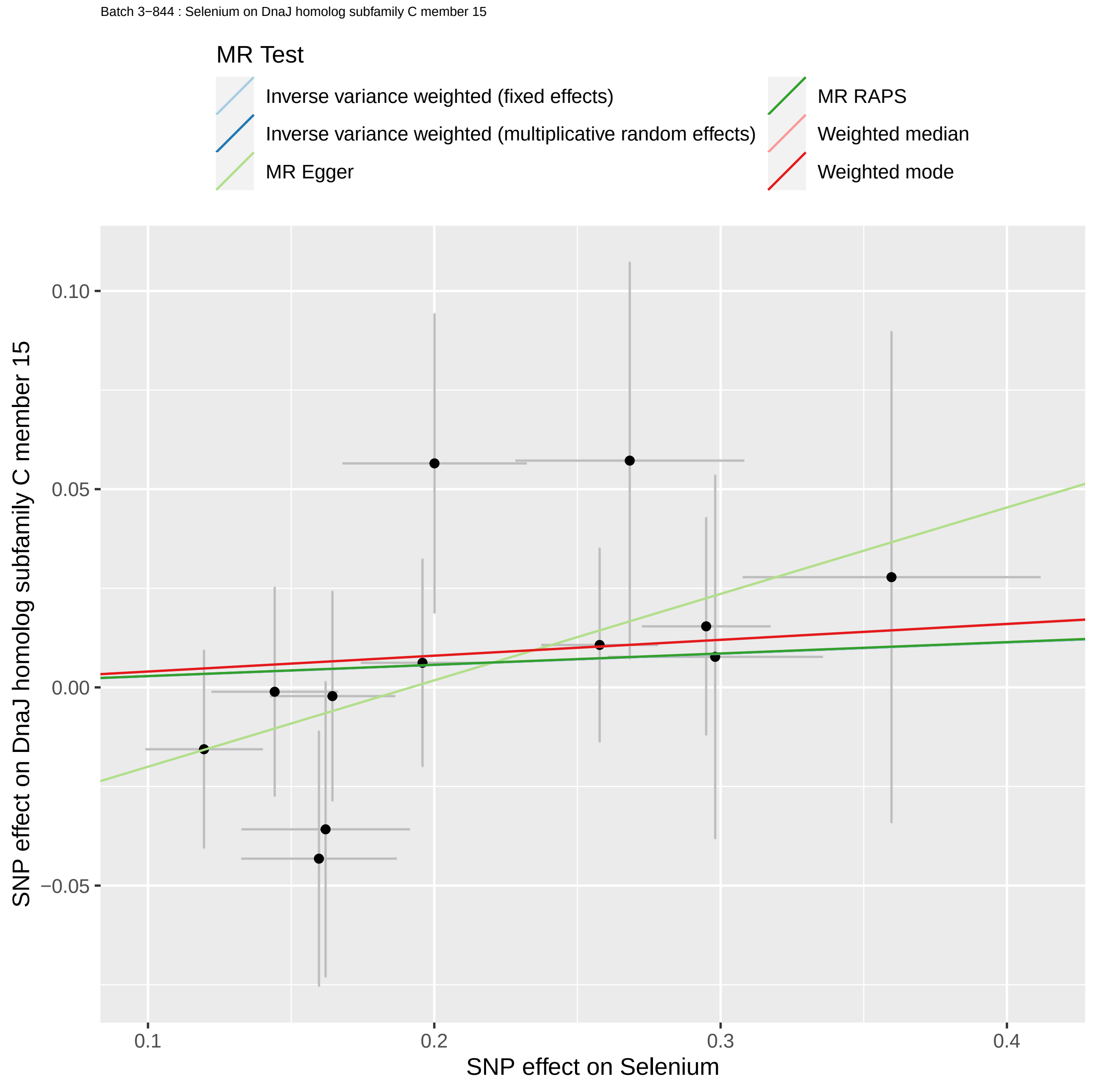

Batch 3−844 : Selenium on DnaJ homolog subfamily C member 15
MR Test
Inverse variance weighted (fixed effects)
Inverse variance weighted (multiplicative random effects)
MR Egger
MR RAPS
Weighted median
Weighted mode
0.10
0.05
0.00
−0.05
0.1
0.2
0.3
0.4
SNP effect on DnaJ homolog subfamily C member 15
SNP effect on Selenium

Batch 3−845 : Selenium on DnaJ homolog subfamily C member 17

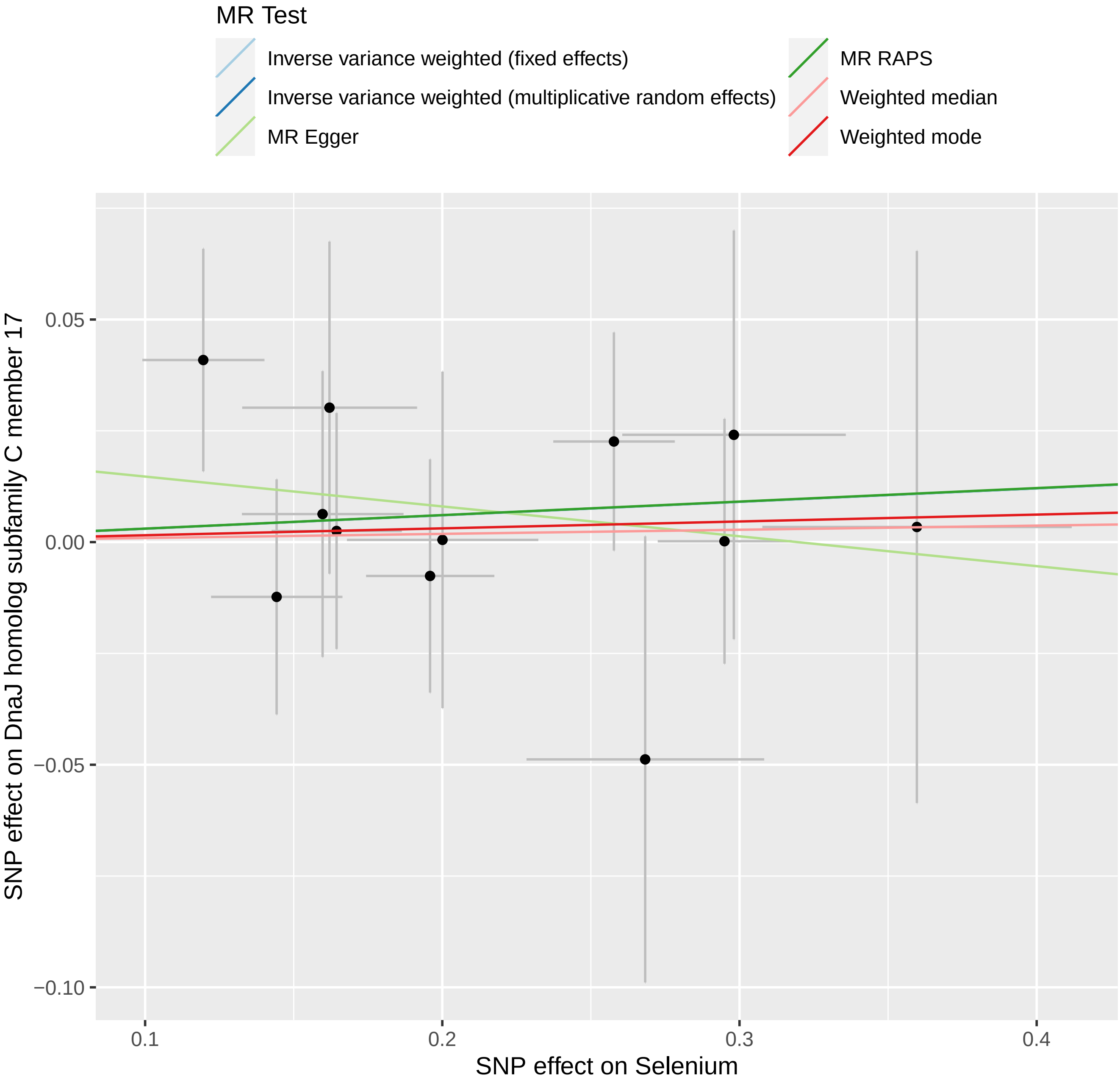

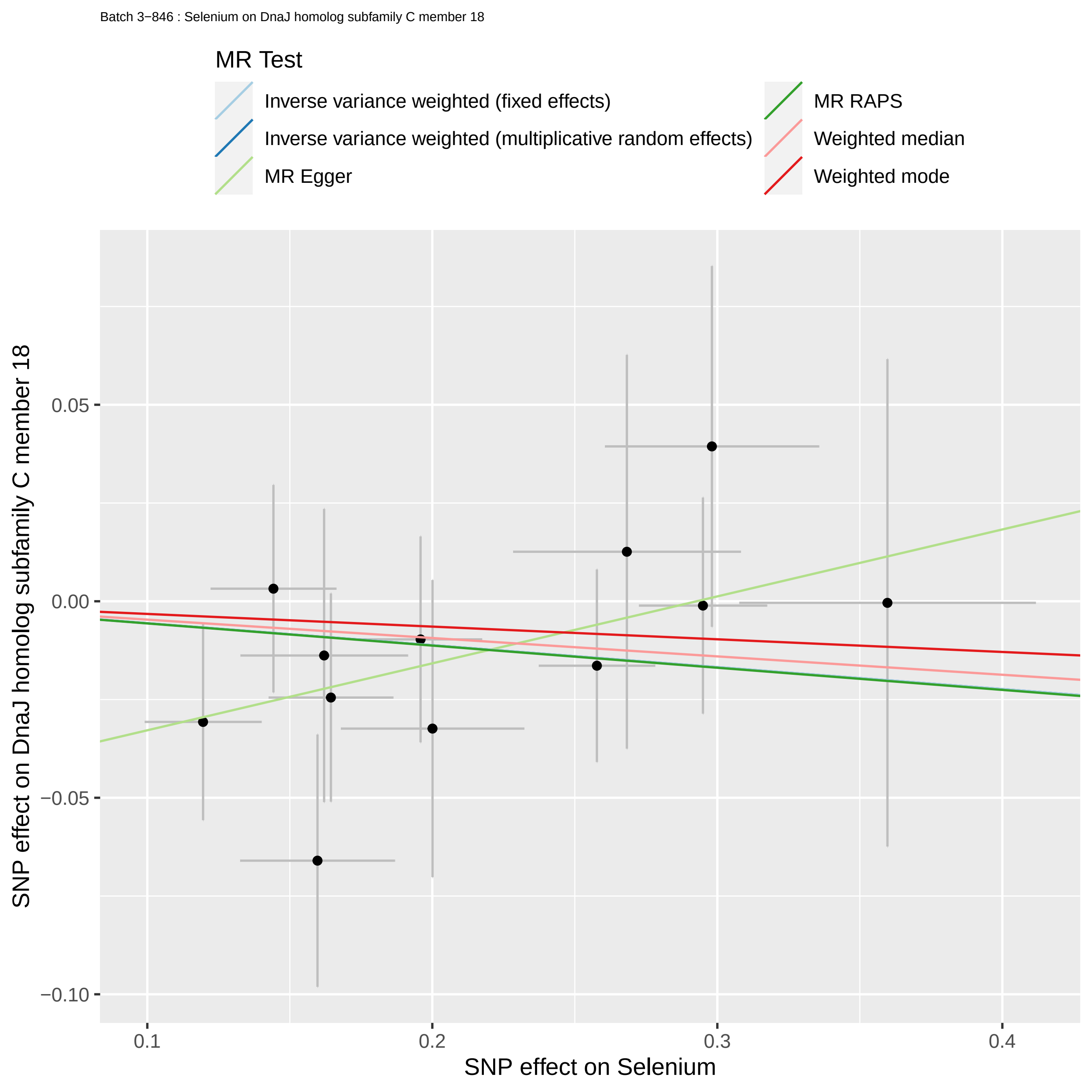

Batch 3−846 : Selenium on DnaJ homolog subfamily C member 18
MR Test
Inverse variance weighted (fixed effects)
Inverse variance weighted (multiplicative random effects)
MR Egger
MR RAPS
Weighted median
Weighted mode
0.05
0.00
−0.05
−0.10
0.1
0.2
0.3
0.4
SNP effect on Selenium
SNP effect on DnaJ homolog subfamily C member 18

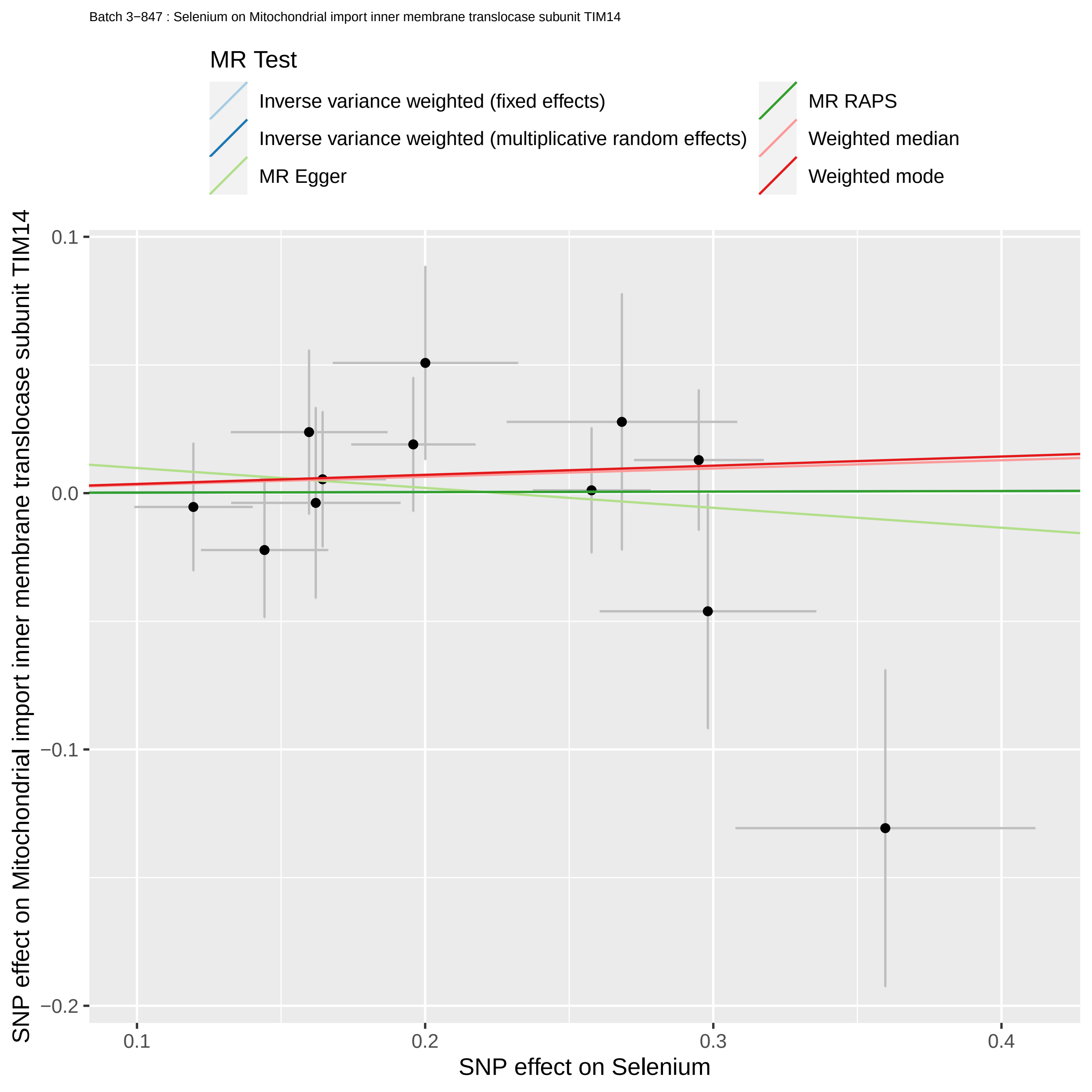

Batch 3−847 : Selenium on Mitochondrial import inner membrane translocase subunit TIM14
MR Test
Inverse variance weighted (fixed effects)
Inverse variance weighted (multiplicative random effects)
MR Egger
MR RAPS
Weighted median
Weighted mode
SNP effect on Mitochondrial import inner membrane translocase subunit TIM14
0.1
0.0
−0.1
−0.2
0.1
0.2
0.3
0.4
SNP effect on Selenium

Batch 3−848 : Selenium on DnaJ homolog subfamily C member 27

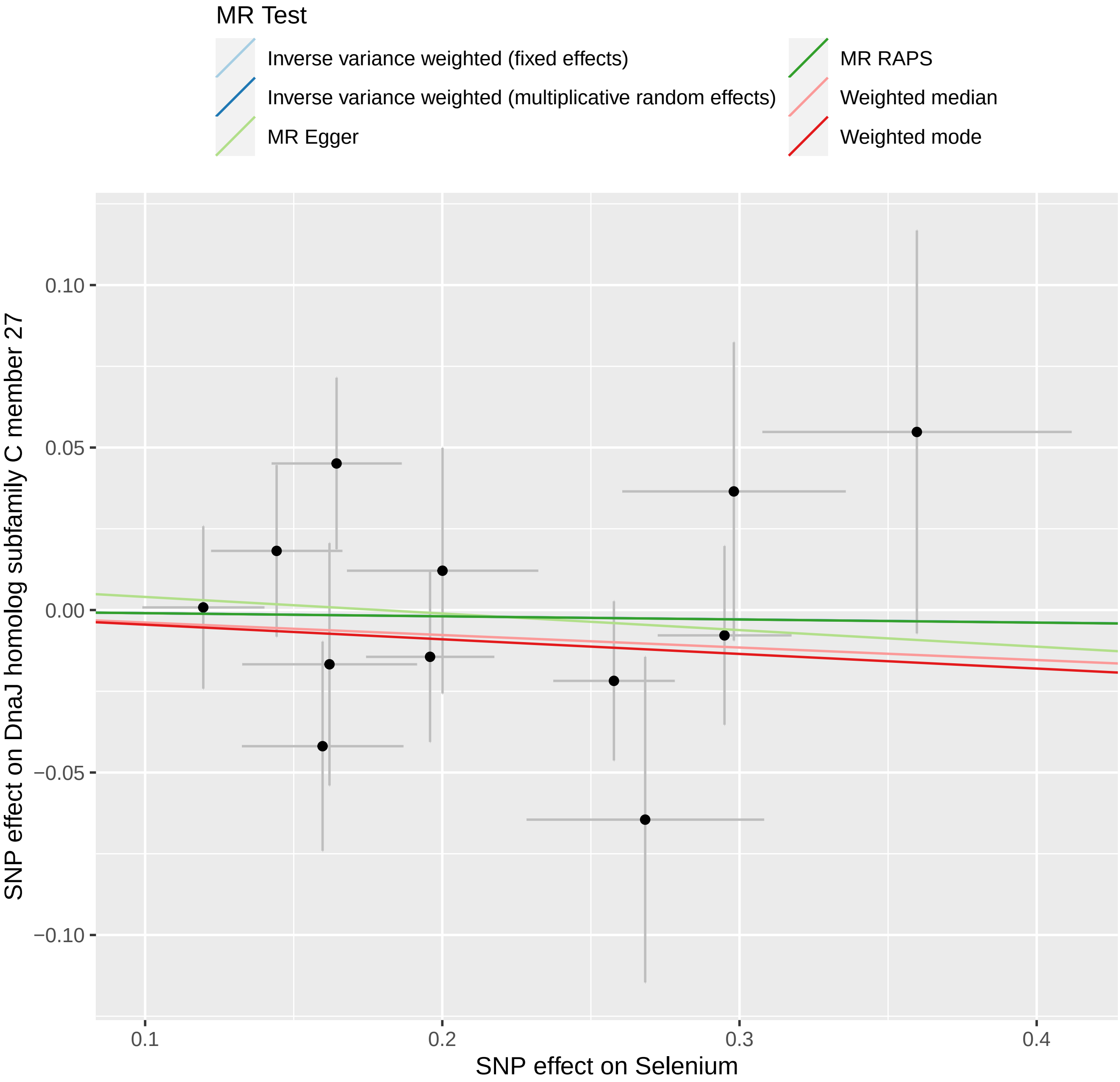

Batch 3−849 : Selenium on DnaJ homolog subfamily C member 30

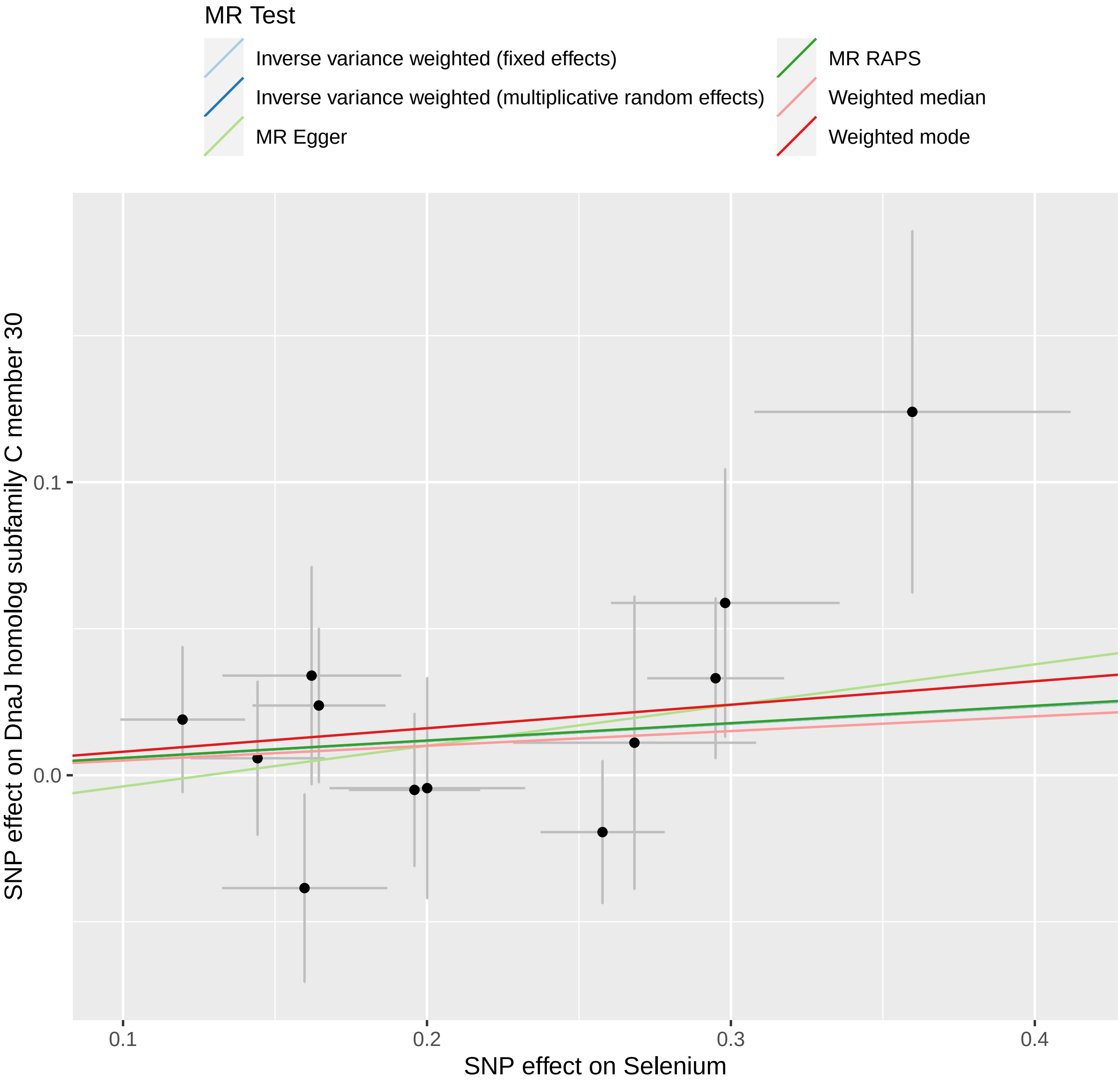

Batch 3−850 : Selenium on DnaJ homolog subfamily C member 4

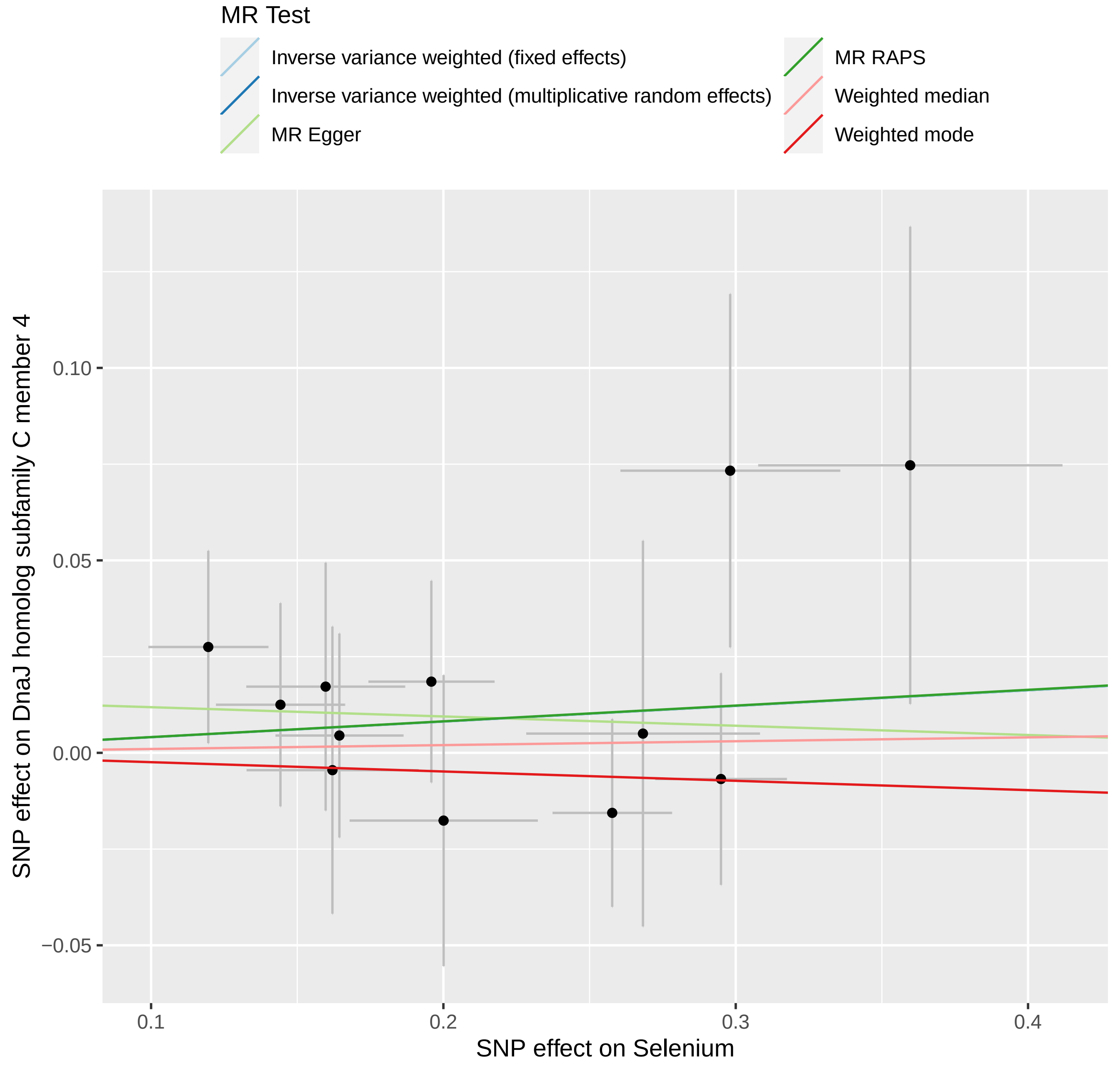

Batch 3−851 : Selenium on Deoxyribonuclease−2−beta

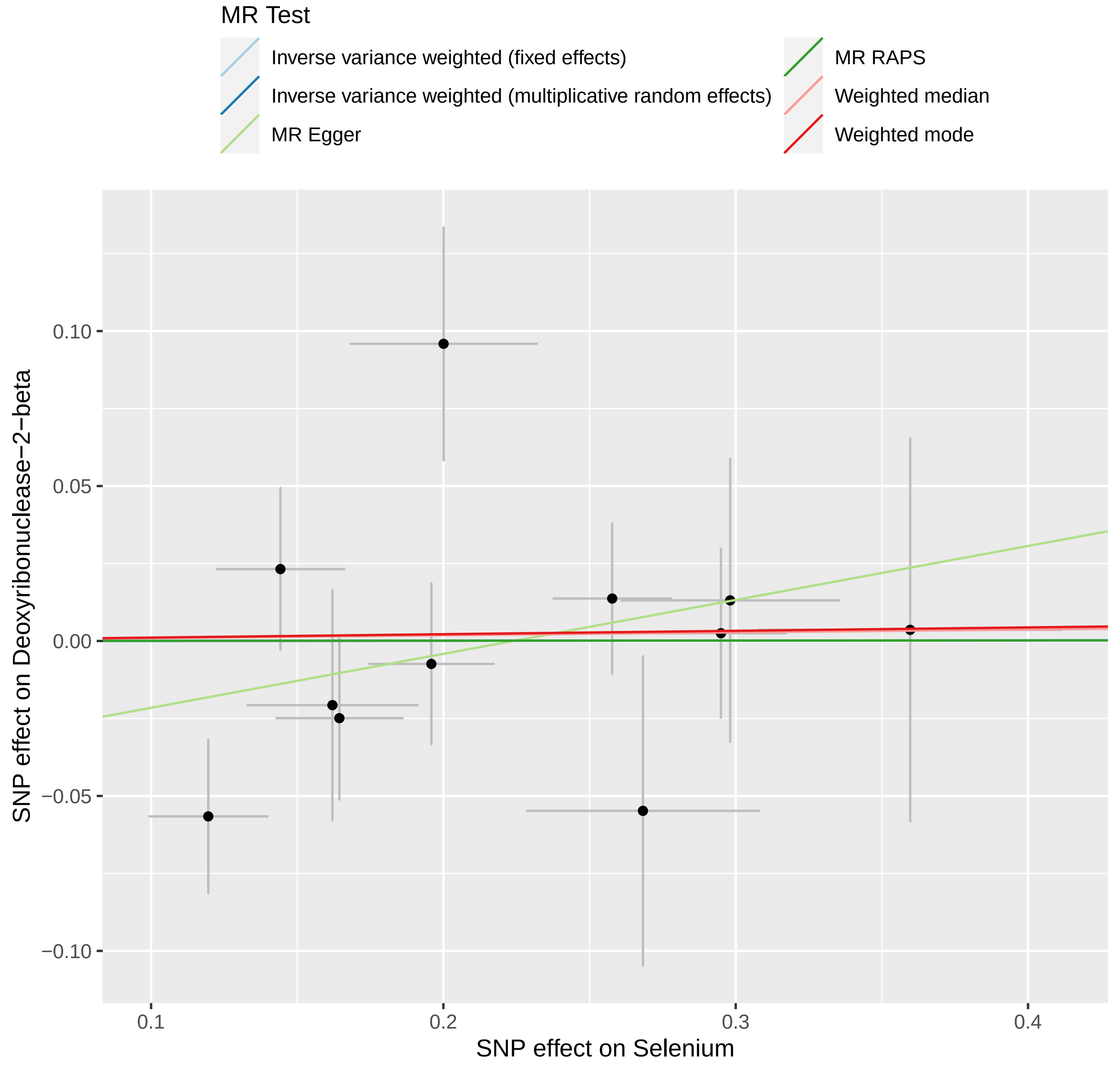

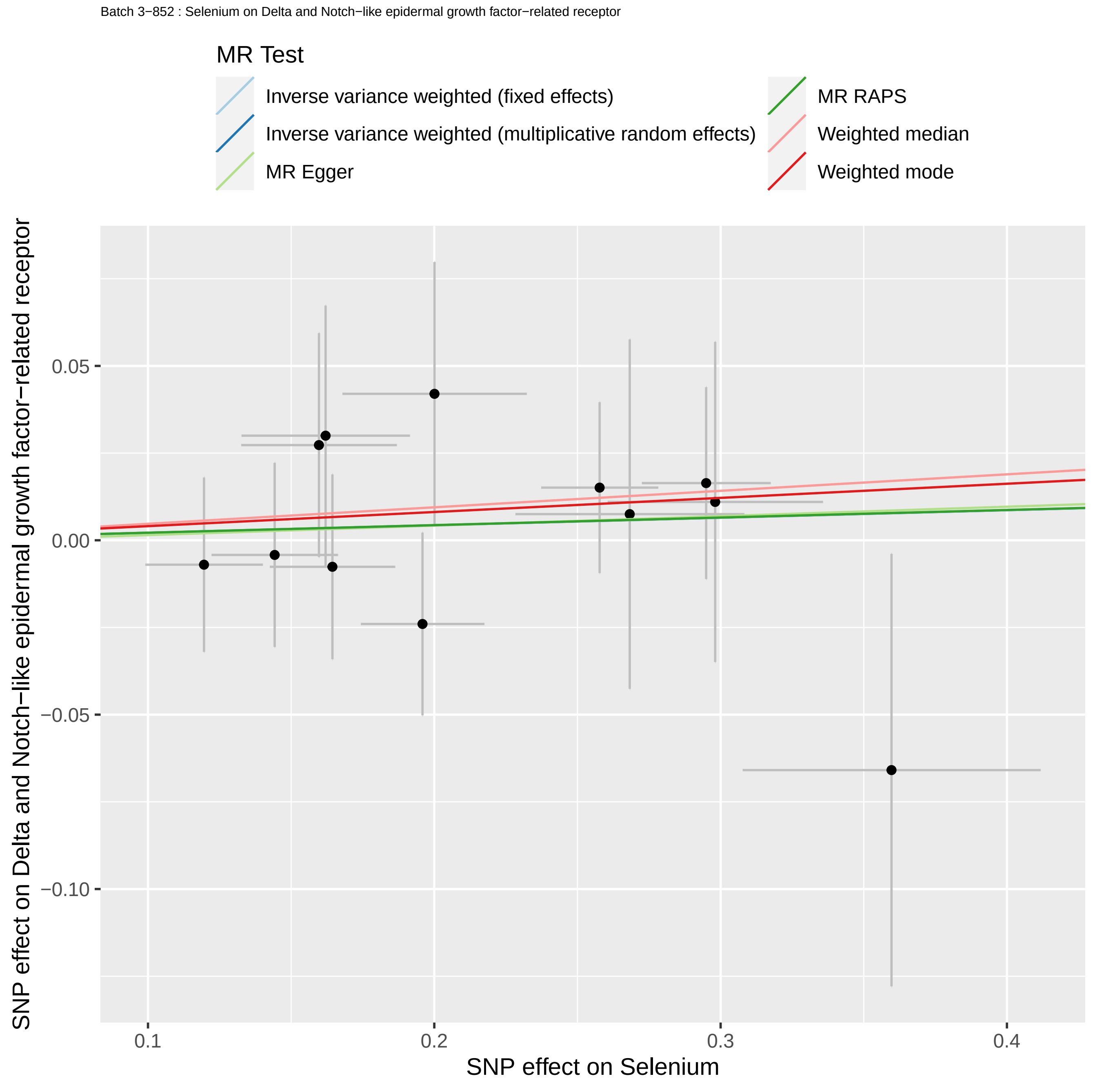

Batch 3−852 : Selenium on Delta and Notch−like epidermal growth factor−related receptor
MR Test
Inverse variance weighted (fixed effects)
Inverse variance weighted (multiplicative random effects)
MR Egger
MR RAPS
Weighted median
Weighted mode
SNP effect on Delta and Notch−like epidermal growth factor−related receptor
0.05
0.00
−0.05
−0.10
0.1
0.2
0.3
0.4
SNP effect on Selenium

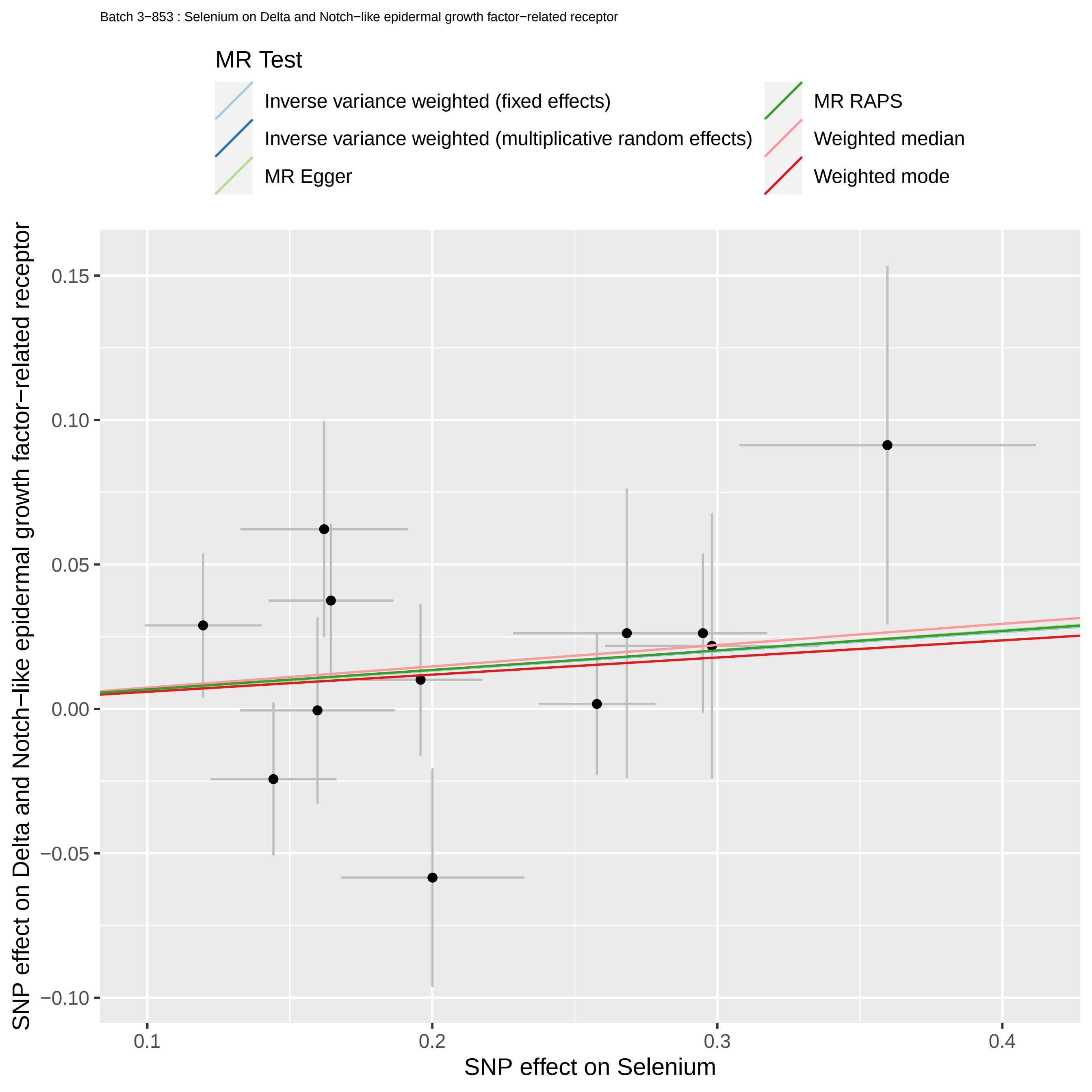
Batch 3−853 : Selenium on Delta and Notch−like epidermal growth factor−related receptor
MR Test
Inverse variance weighted (fixed effects)
Inverse variance weighted (multiplicative random effects)
MR Egger
MR RAPS
Weighted median
Weighted mode
SNP effect on Delta and Notch−like epidermal growth factor−related receptor
0.15
0.10
0.05
0.00
−0.05
−0.10
0.1
0.2
0.3
0.4
SNP effect on Selenium

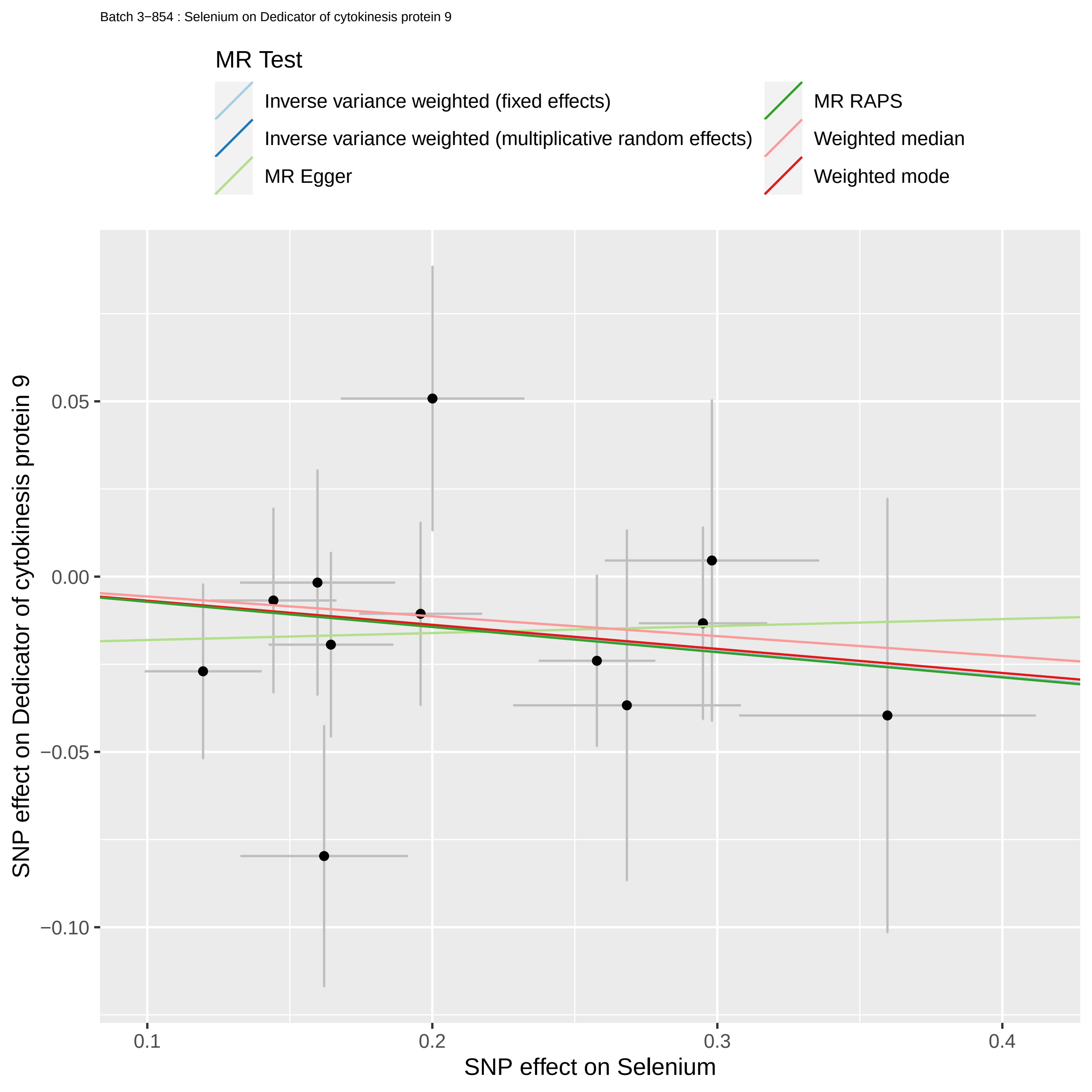

Batch 3−854 : Selenium on Dedicator of cytokinesis protein 9
MR Test
Inverse variance weighted (fixed effects)
Inverse variance weighted (multiplicative random effects)
MR Egger
MR RAPS
Weighted median
Weighted mode
SNP effect on Dedicator of cytokinesis protein 9
0.05
0.00
−0.05
−0.10
0.1
0.2
0.3
0.4
SNP effect on Selenium

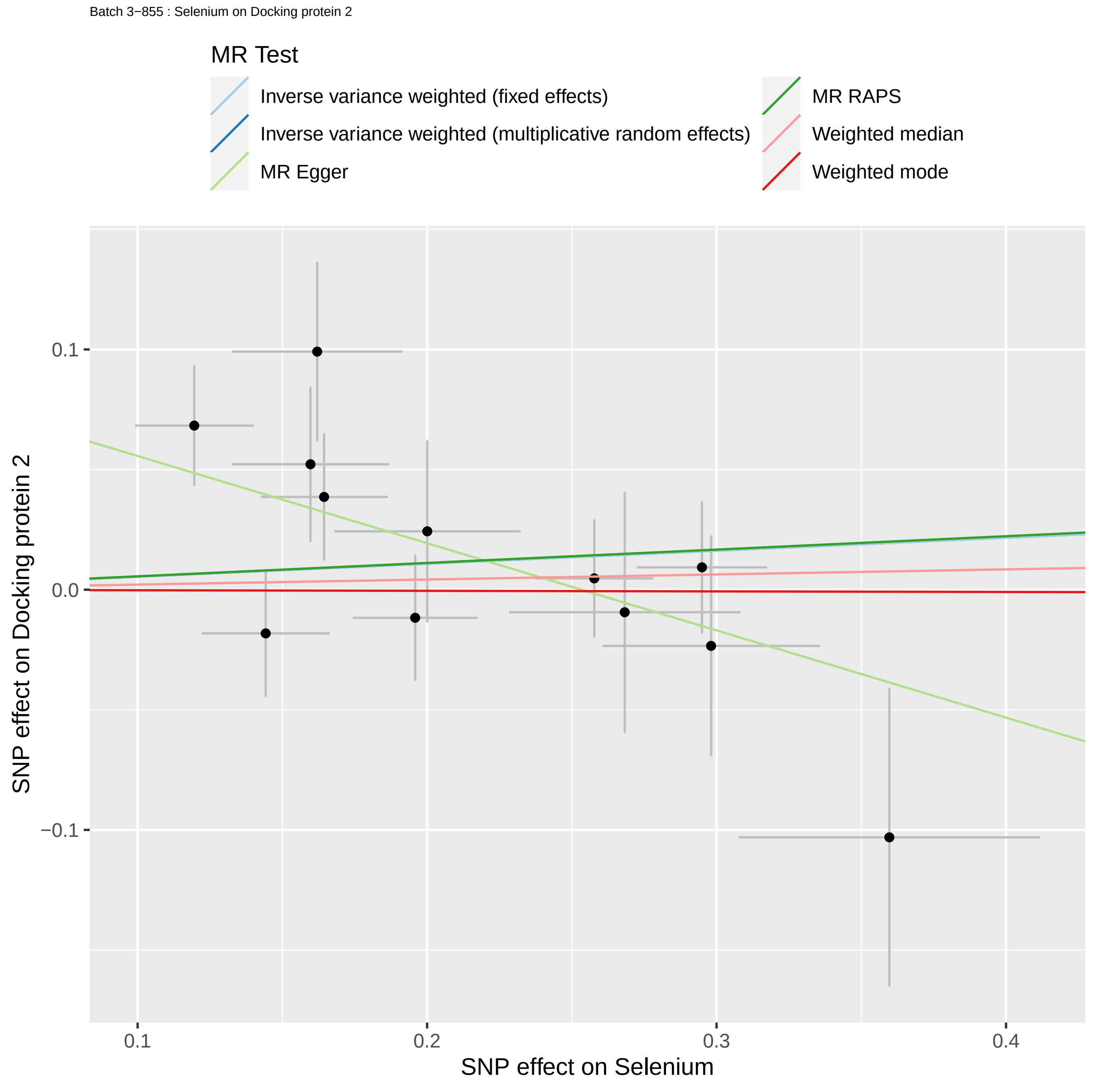
Batch 3−855 : Selenium on Docking protein 2
MR Test
Inverse variance weighted (fixed effects)
Inverse variance weighted (multiplicative random effects)
MR Egger
MR RAPS
Weighted median
Weighted mode
0.1
0.0
−0.1
0.1
0.2
0.3
0.4
SNP effect on Docking protein 2
SNP effect on Selenium

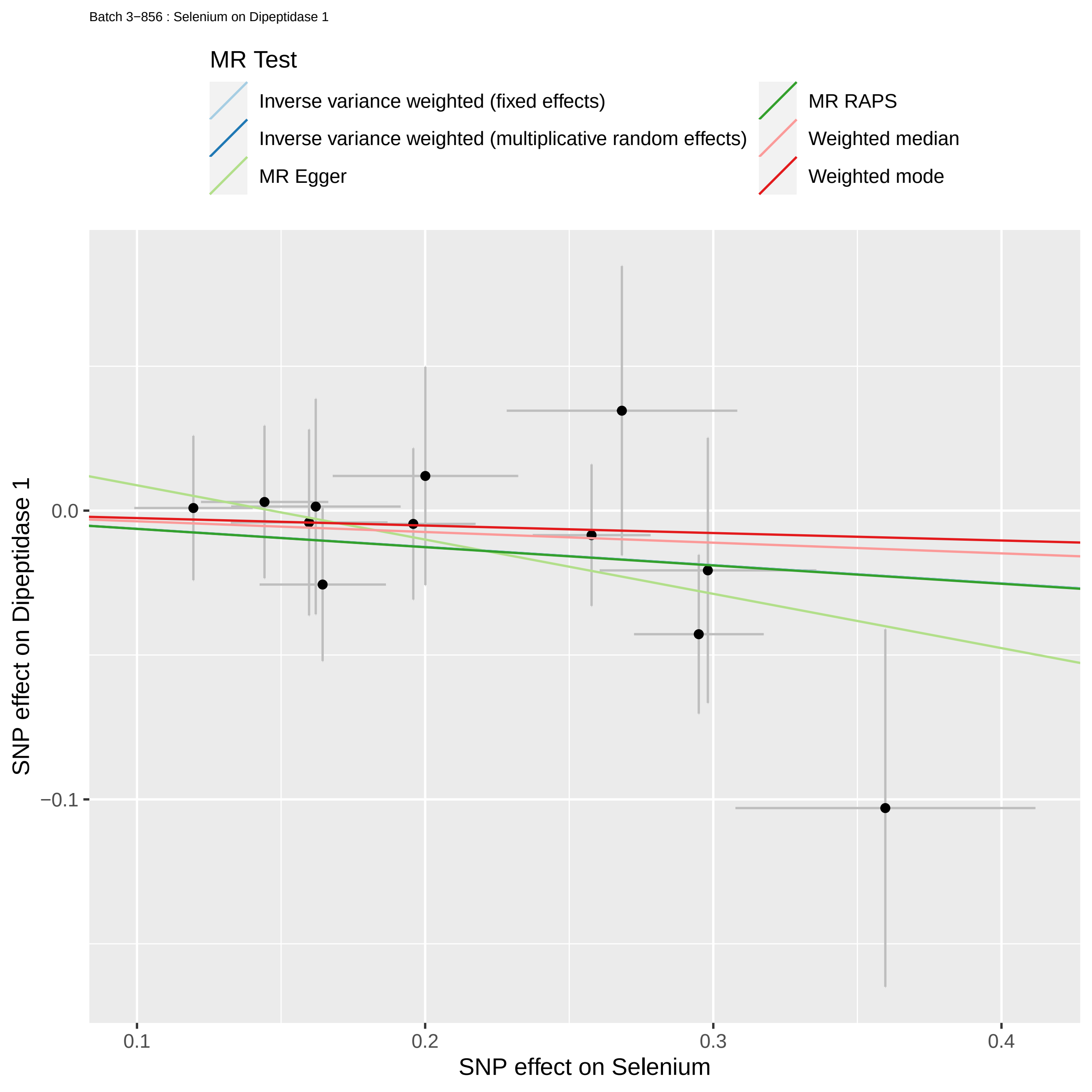
Batch 3−856 : Selenium on Dipeptidase 1
MR Test
Inverse variance weighted (fixed effects)
Inverse variance weighted (multiplicative random effects)
MR Egger
MR RAPS
Weighted median
Weighted mode
SNP effect on Dipeptidase 1
0.0
−0.1
0.1
0.2
0.3
0.4
SNP effect on Selenium

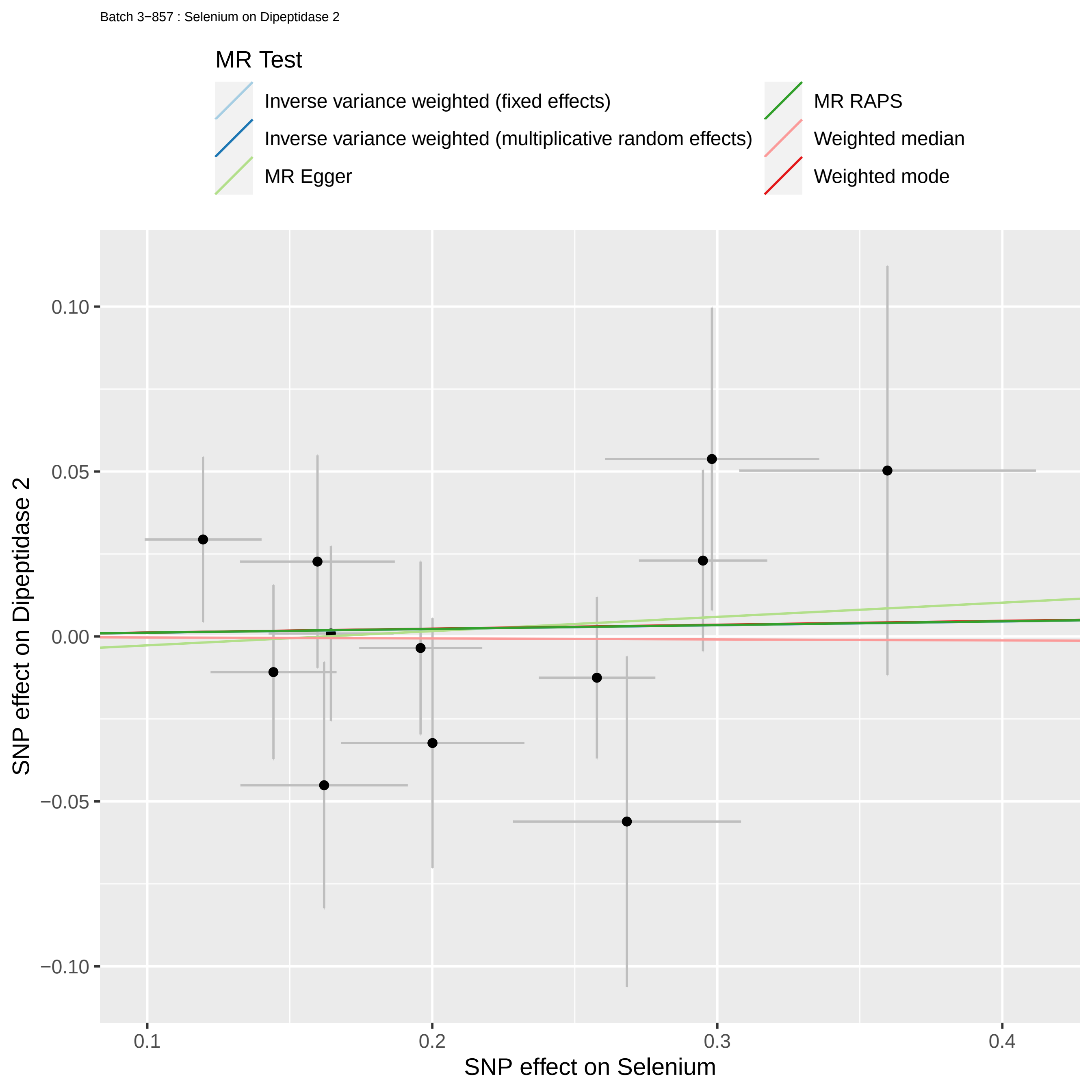

Batch 3−857 : Selenium on Dipeptidase 2
MR Test
Inverse variance weighted (fixed effects)
Inverse variance weighted (multiplicative random effects)
MR Egger
MR RAPS
Weighted median
Weighted mode
0.10
0.05
0.00
−0.05
−0.10
0.1
0.2
0.3
0.4
SNP effect on Selenium
SNP effect on Dipeptidase 2

Batch 3−858 : Selenium on Inactive dipeptidyl peptidase 10

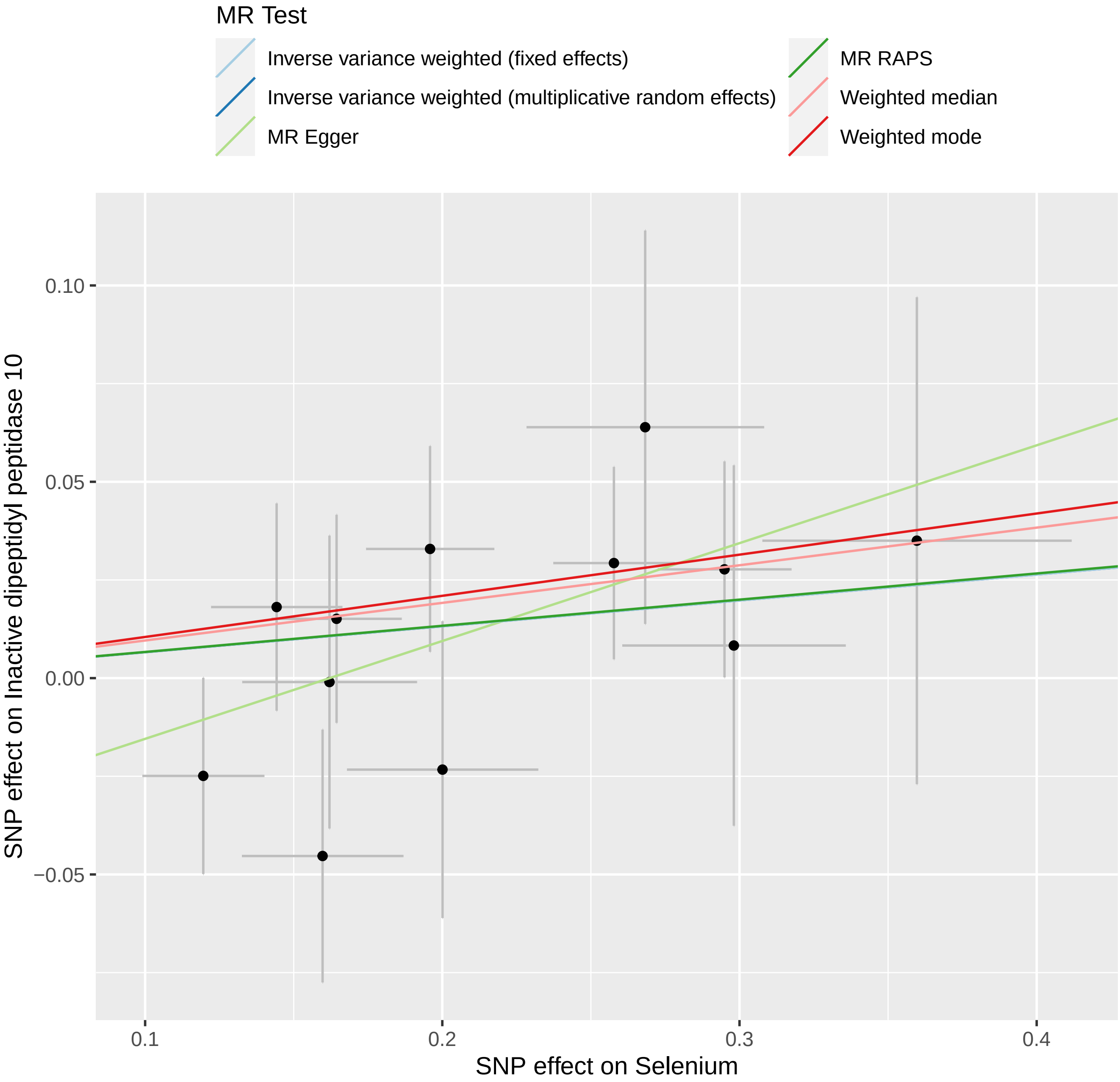

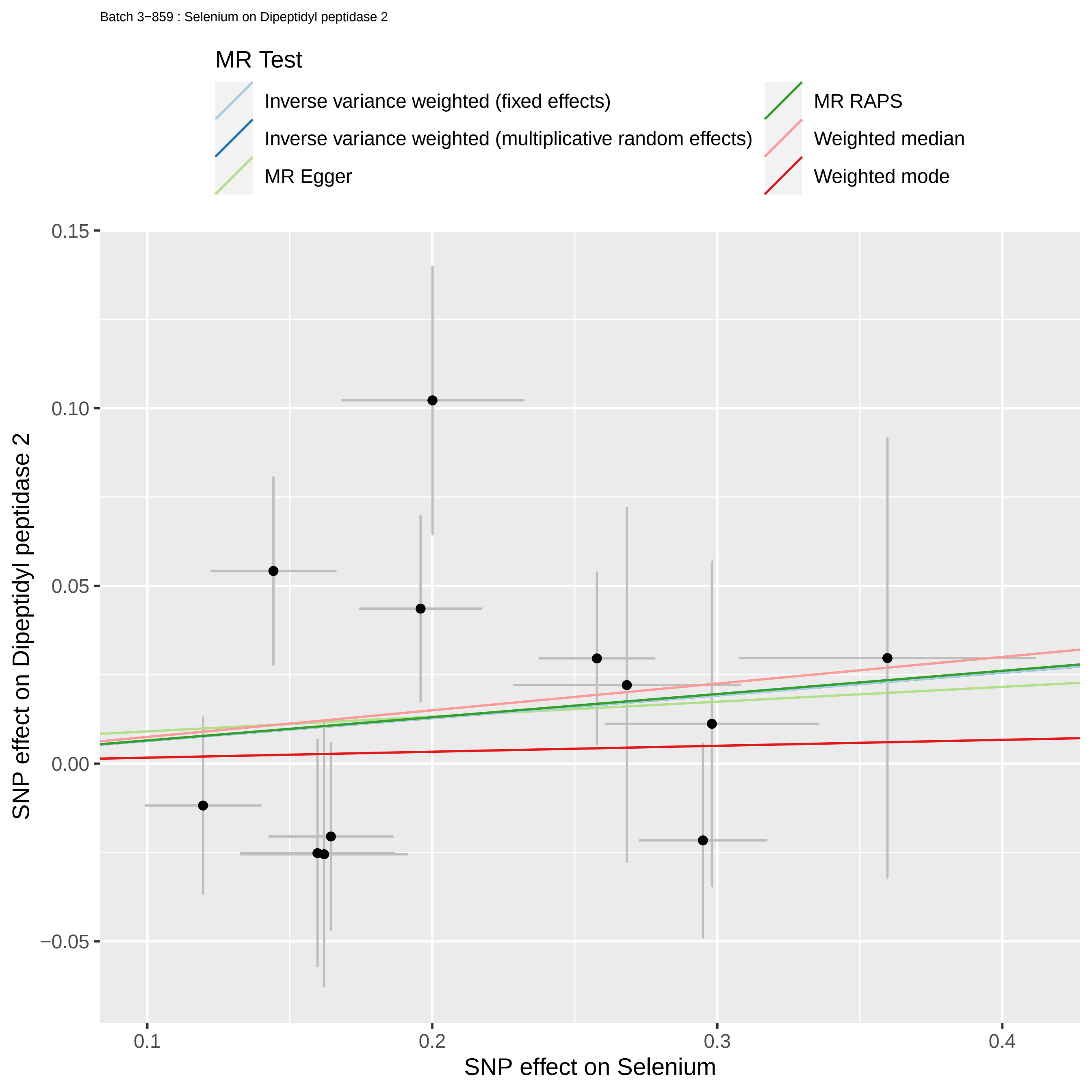

Batch 3−859 : Selenium on Dipeptidyl peptidase 2
MR Test
Inverse variance weighted (fixed effects)
Inverse variance weighted (multiplicative random effects)
MR Egger
MR RAPS
Weighted median
Weighted mode
0.15
0.10
0.05
0.00
−0.05
0.1
0.2
0.3
0.4
SNP effect on Dipeptidyl peptidase 2
SNP effect on Selenium

Batch 3−860 : Selenium on Dipeptidyl peptidase 2

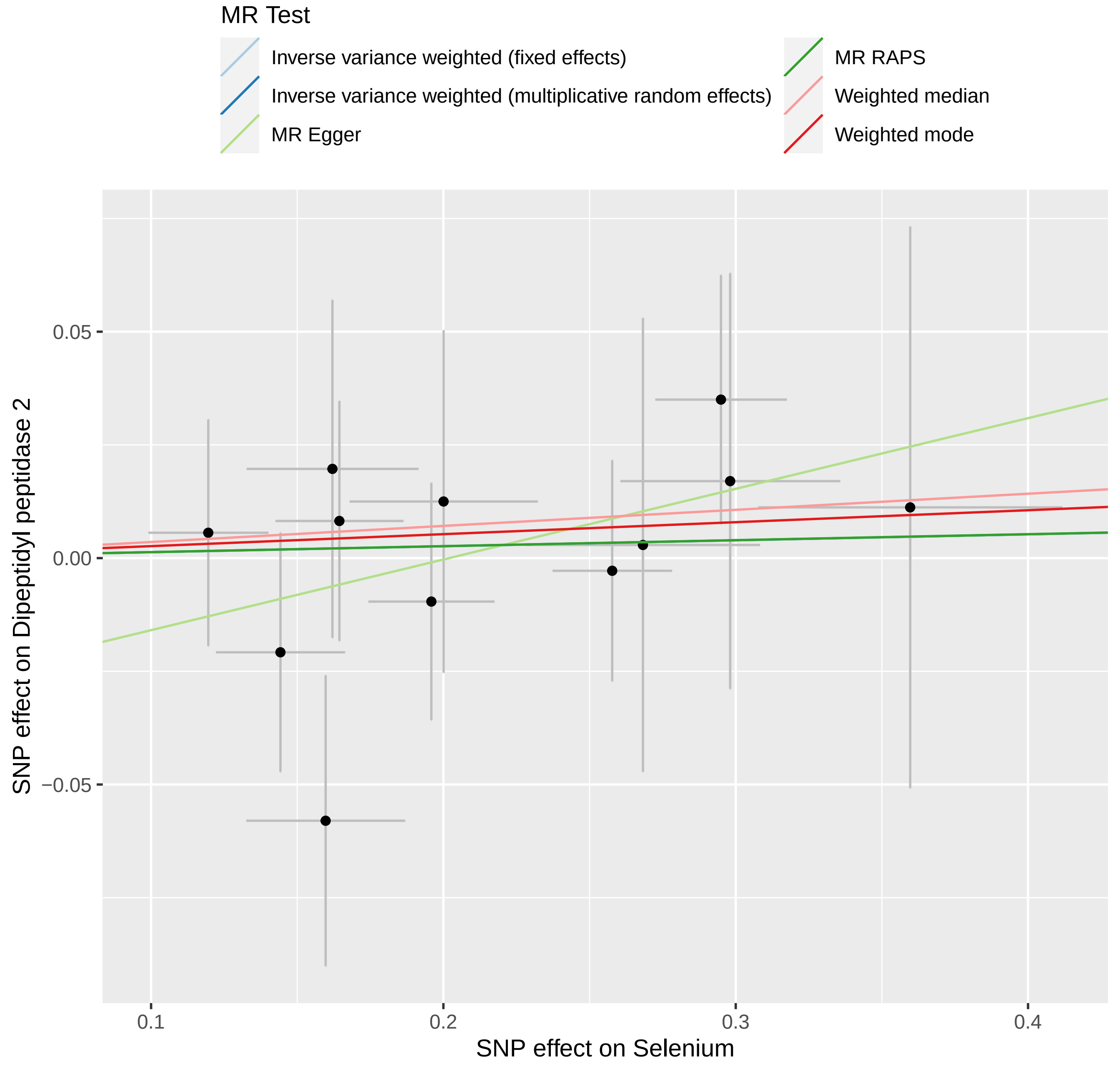

Batch 3−861 : Selenium on Dermatopontin

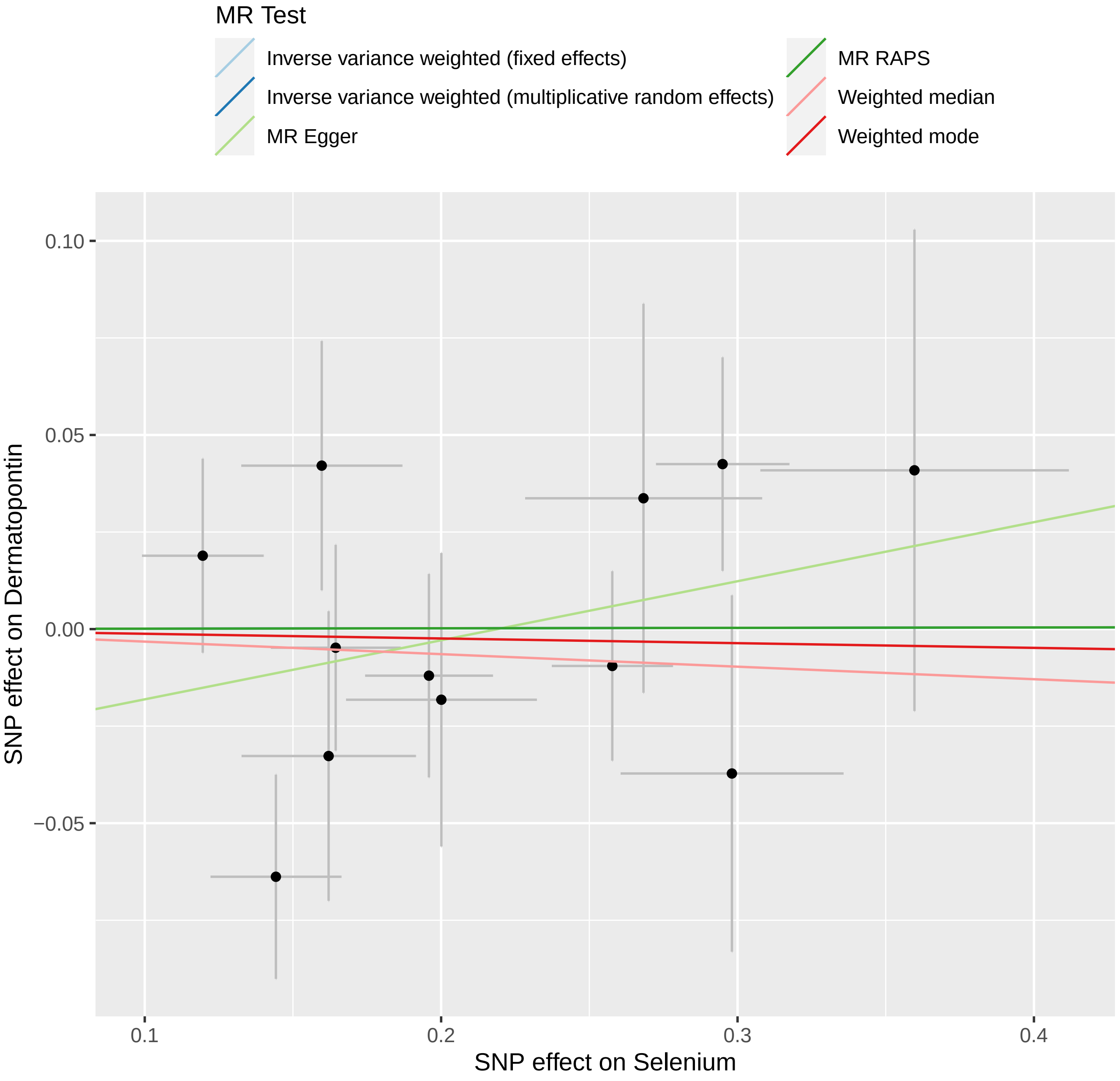

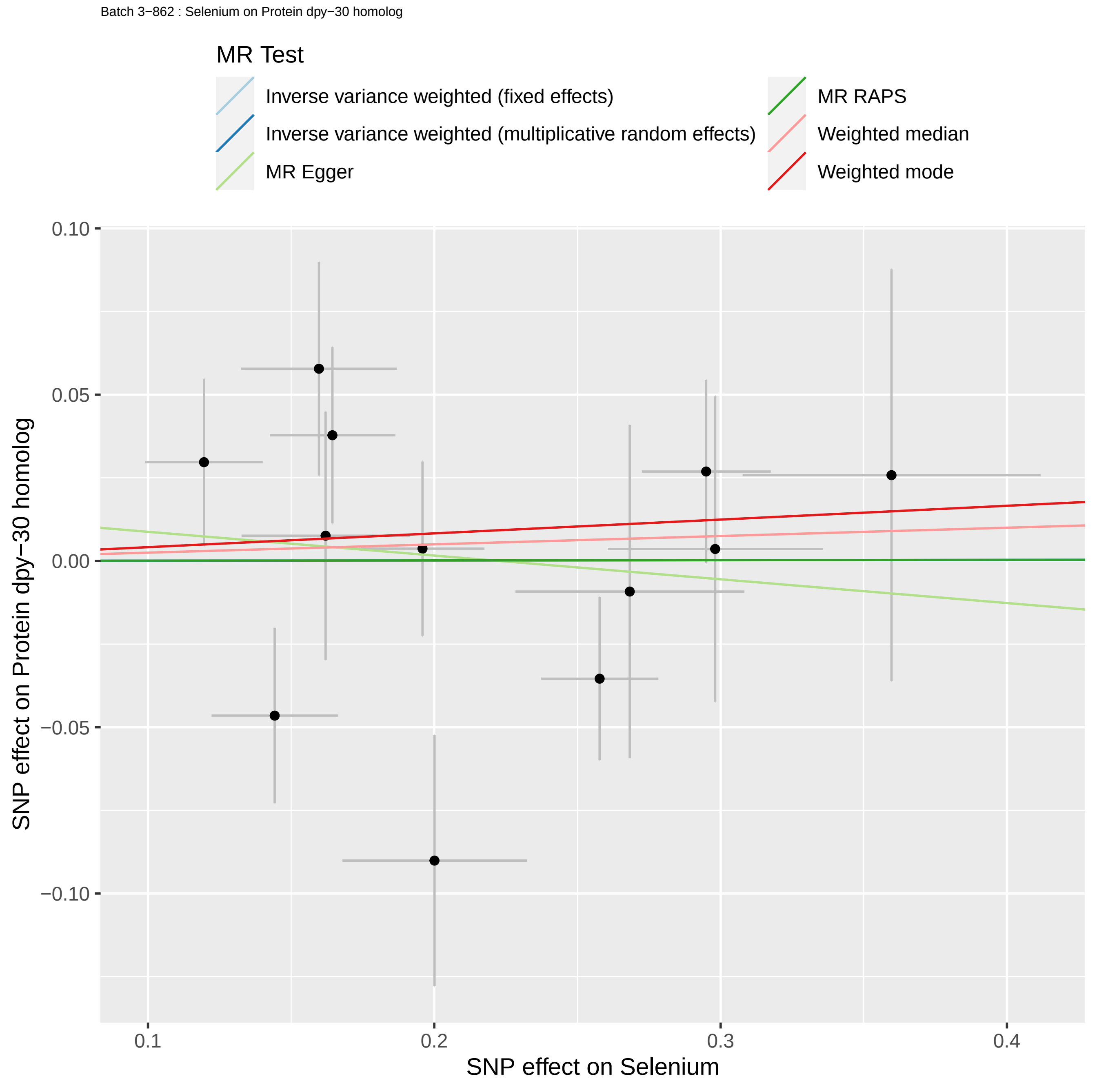
Batch 3−862 : Selenium on Protein dpy−30 homolog
MR Test
Inverse variance weighted (fixed effects)
Inverse variance weighted (multiplicative random effects)
MR Egger
MR RAPS
Weighted median
Weighted mode
0.10
0.05
0.00
−0.05
−0.10
0.1
0.2
0.3
0.4
SNP effect on Protein dpy−30 homolog
SNP effect on Selenium

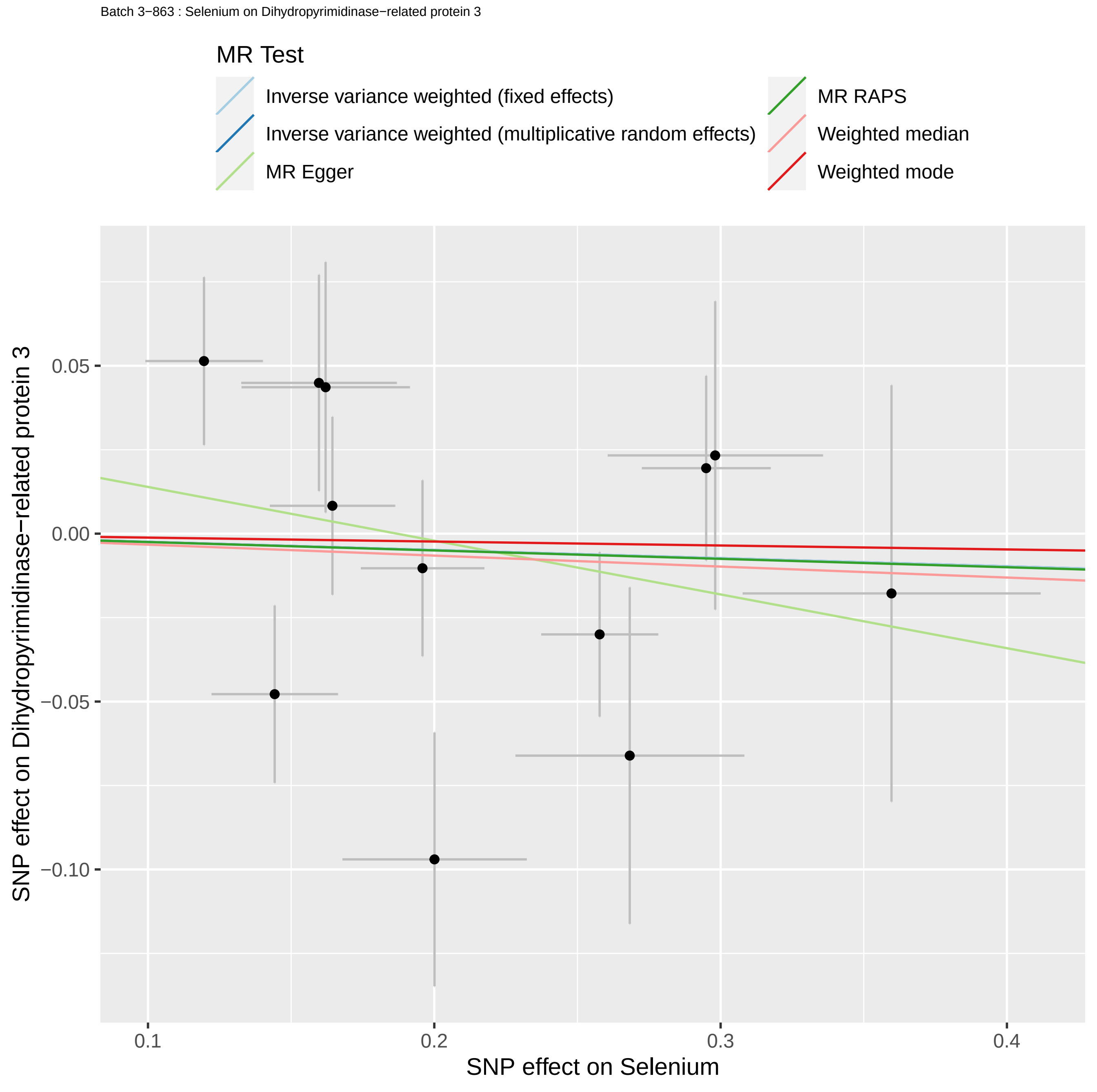
Batch 3−863 : Selenium on Dihydropyrimidinase−related protein 3
MR Test
Inverse variance weighted (fixed effects)
Inverse variance weighted (multiplicative random effects)
MR Egger
MR RAPS
Weighted median
Weighted mode
0.05
0.00
−0.05
−0.10
0.1
0.2
0.3
0.4
SNP effect on Selenium
SNP effect on Dihydropyrimidinase−related protein 3

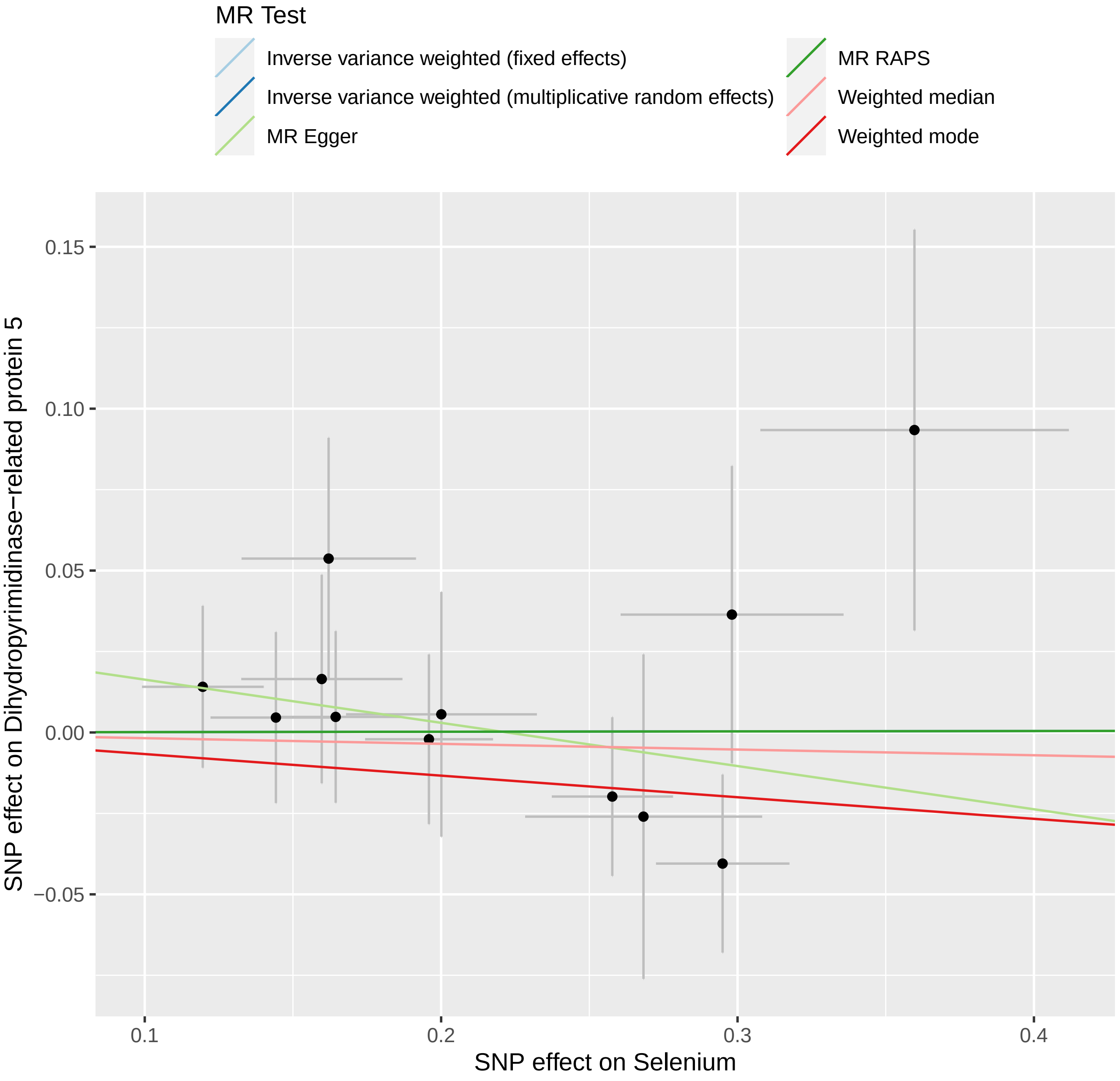
MR Test
Inverse variance weighted (fixed effects)
Inverse variance weighted (multiplicative random effects)
MR Egger
MR RAPS
Weighted median
Weighted mode
0.15
0.10
0.05
0.00
−0.05
0.1
0.2
0.3
0.4
SNP effect on Dihydropyrimidinase−related protein 5
SNP effect on Selenium

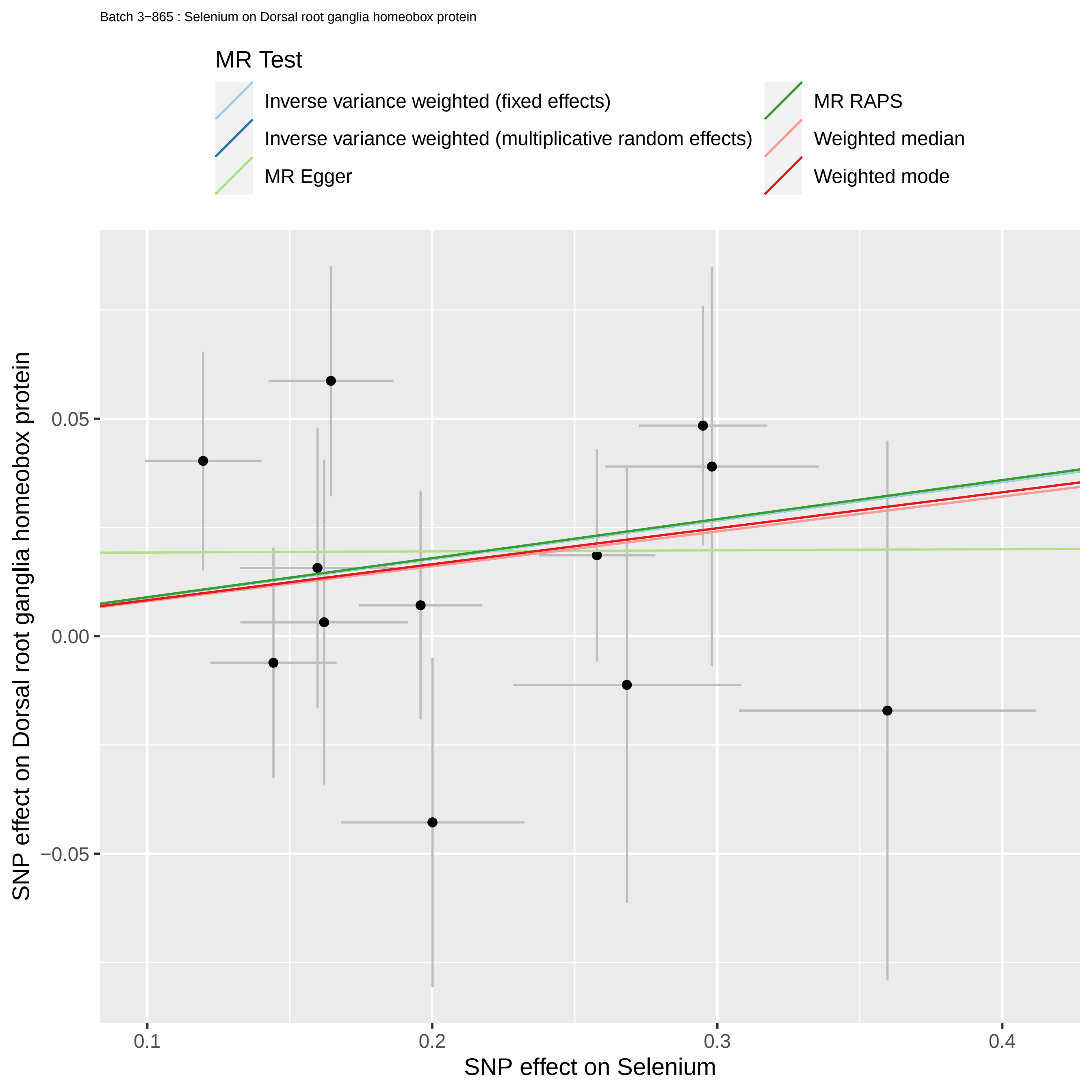
Batch 3−865 : Selenium on Dorsal root ganglia homeobox protein
MR Test
Inverse variance weighted (fixed effects)
Inverse variance weighted (multiplicative random effects)
MR Egger
MR RAPS
Weighted median
Weighted mode
SNP effect on Dorsal root ganglia homeobox protein
0.05
0.00
−0.05
0.1
0.2
0.3
0.4
SNP effect on Selenium

Batch 3−866 : Selenium on Desmocollin−2

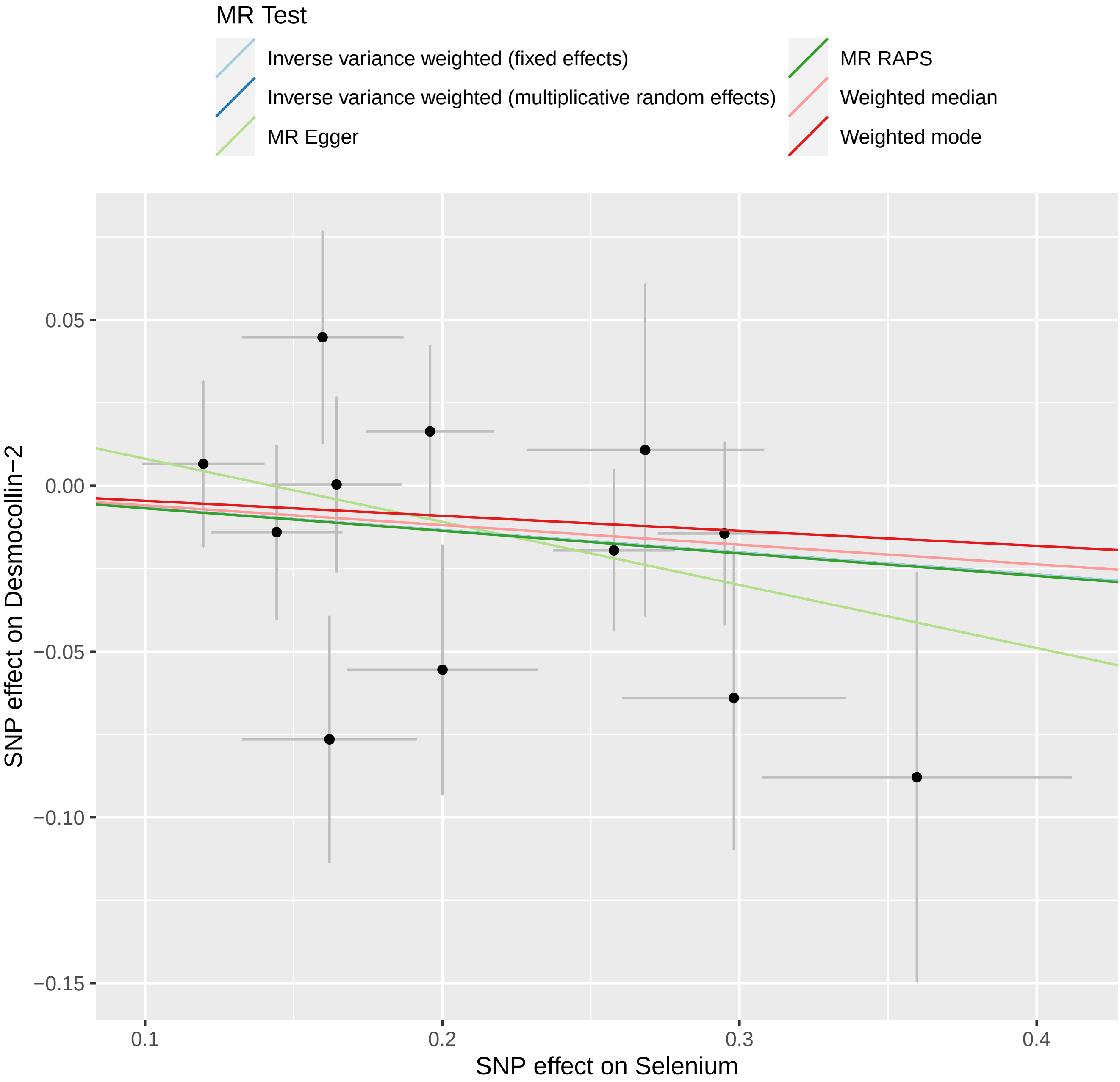

Batch 3−867 : Selenium on Desmocollin−3

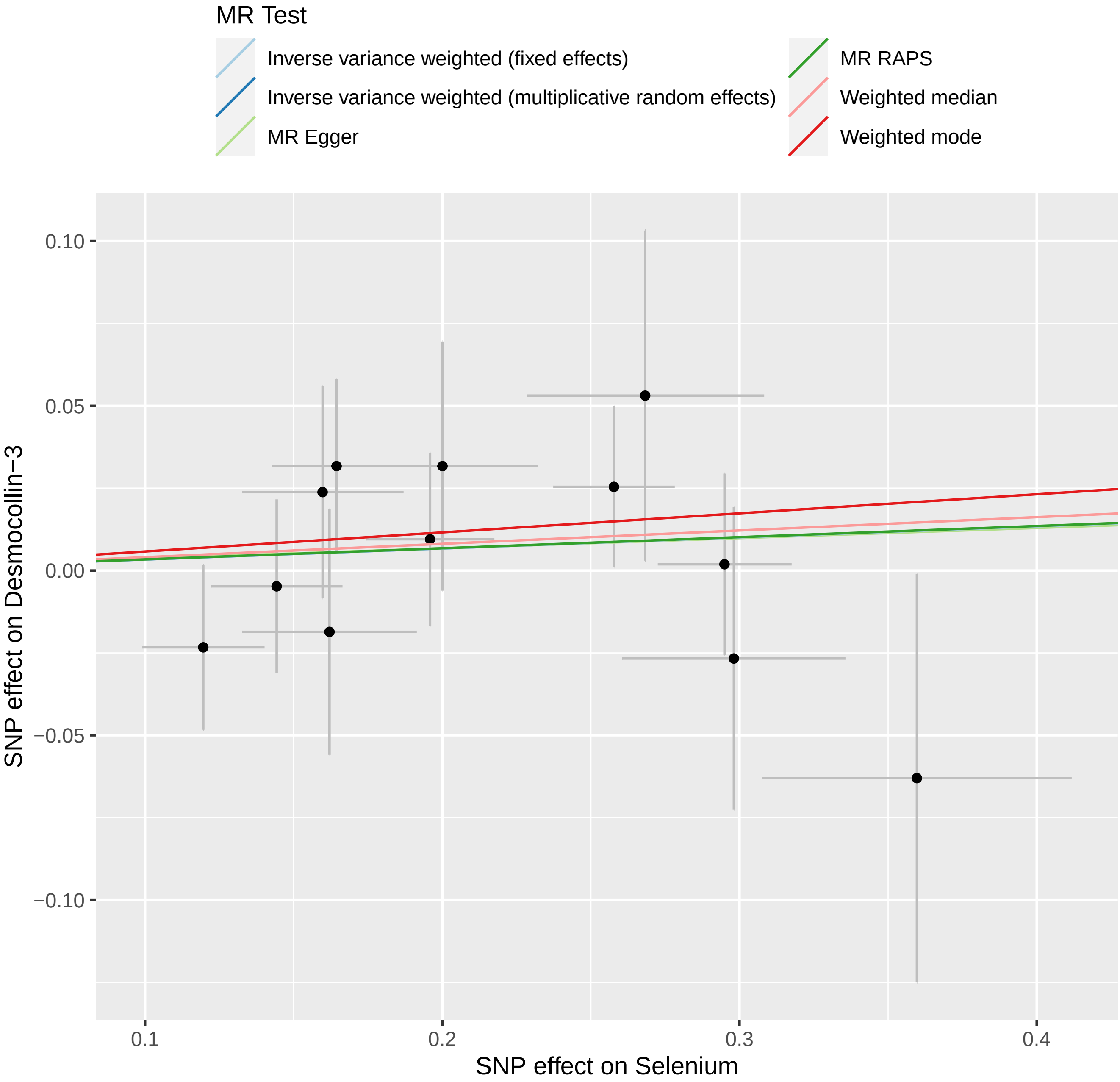

Batch 3−868 : Selenium on Down syndrome cell adhesion molecule

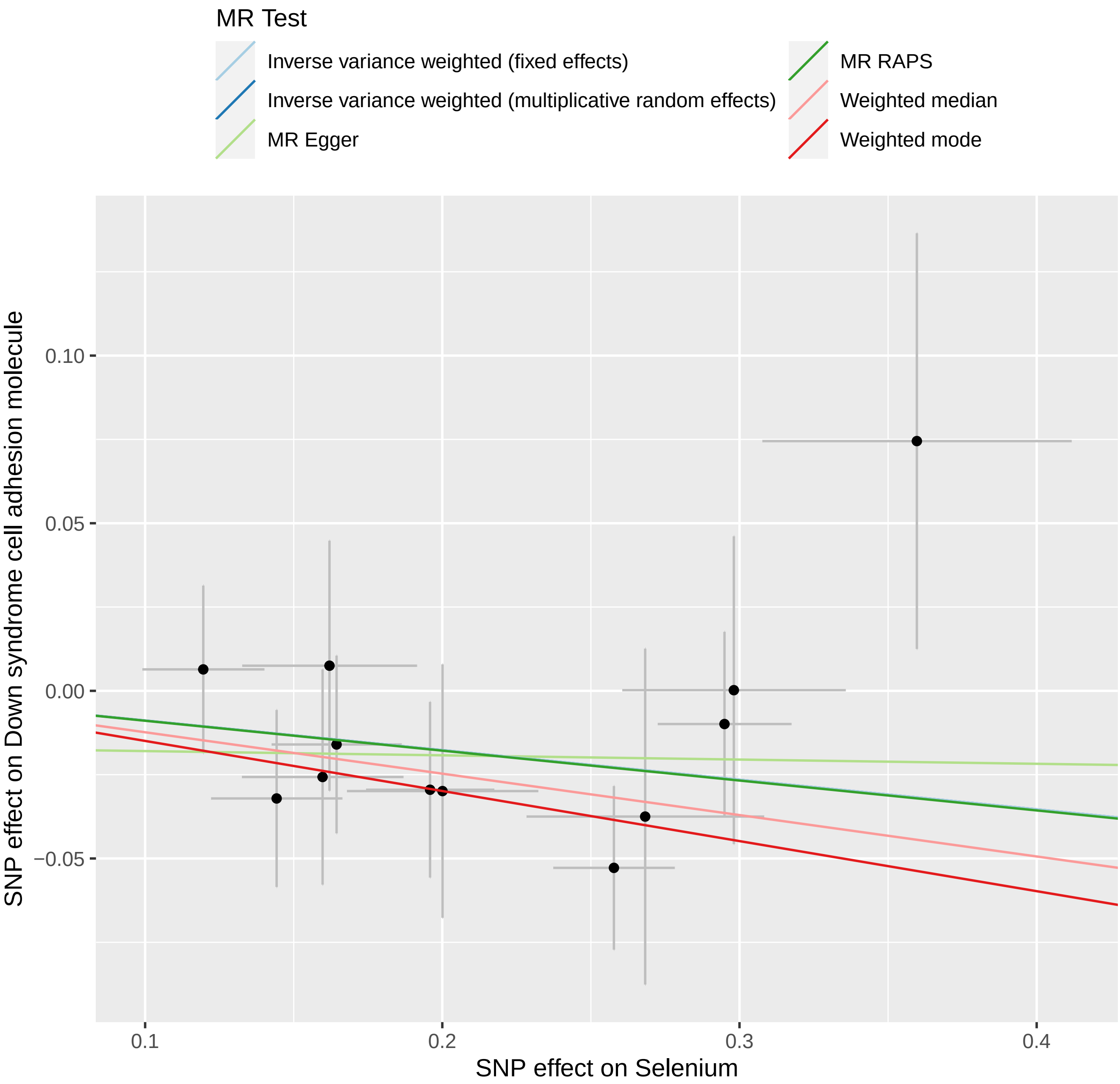

Batch 3−869 : Selenium on Desmoglein−1

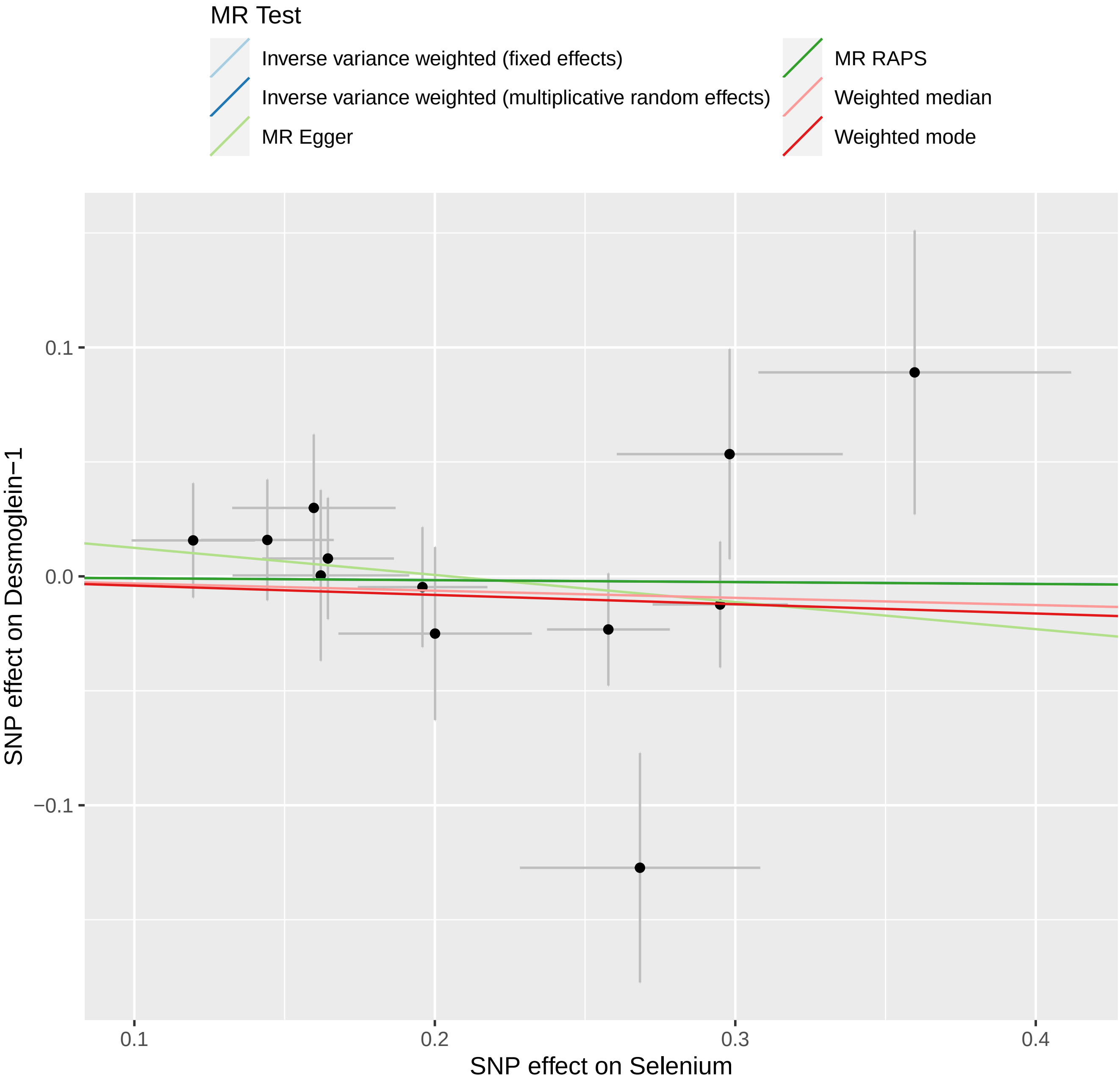

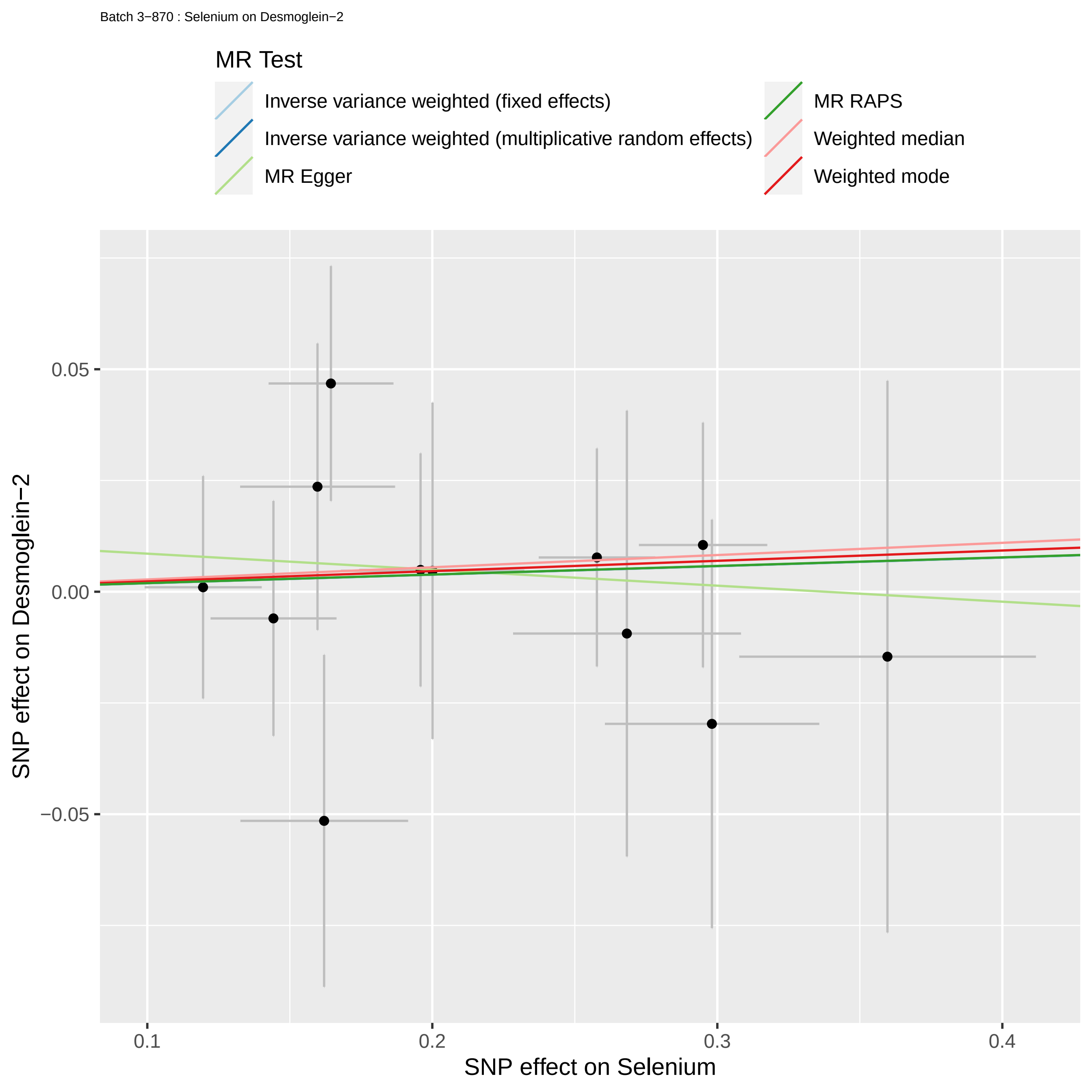
Batch 3−870 : Selenium on Desmoglein−2
MR Test
Inverse variance weighted (fixed effects)
Inverse variance weighted (multiplicative random effects)
MR Egger
MR RAPS
Weighted median
Weighted mode
0.05
0.00
−0.05
0.1
0.2
0.3
0.4
SNP effect on Desmoglein−2
SNP effect on Selenium

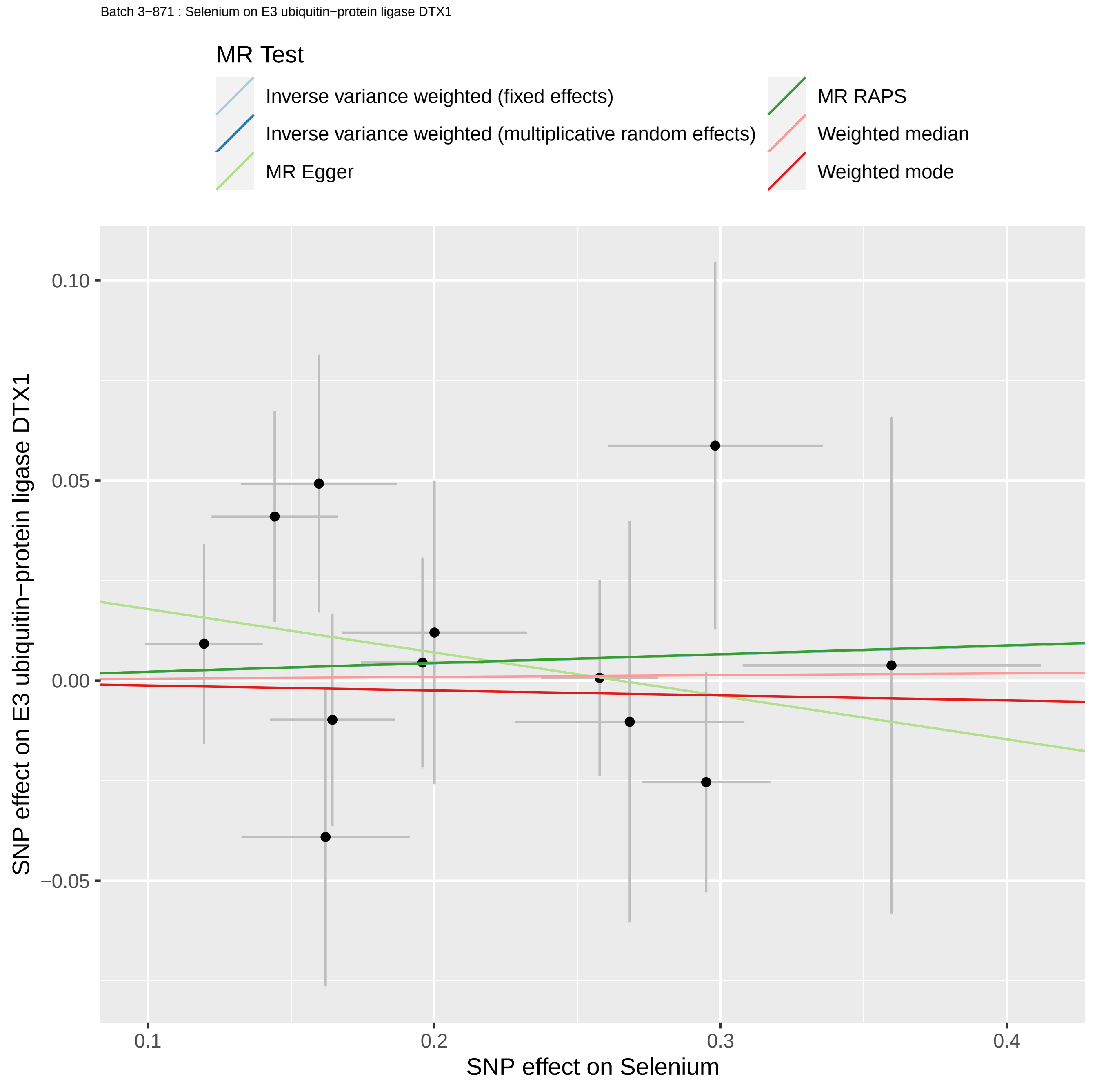

Batch 3−871 : Selenium on E3 ubiquitin−protein ligase DTX1
MR Test
Inverse variance weighted (fixed effects)
Inverse variance weighted (multiplicative random effects)
MR Egger
MR RAPS
Weighted median
Weighted mode
0.10
0.05
0.00
−0.05
0.1
0.2
0.3
0.4
SNP effect on E3 ubiquitin−protein ligase DTX1
SNP effect on Selenium

Batch 3−872 : Selenium on E3 ubiquitin−protein ligase DTX3L

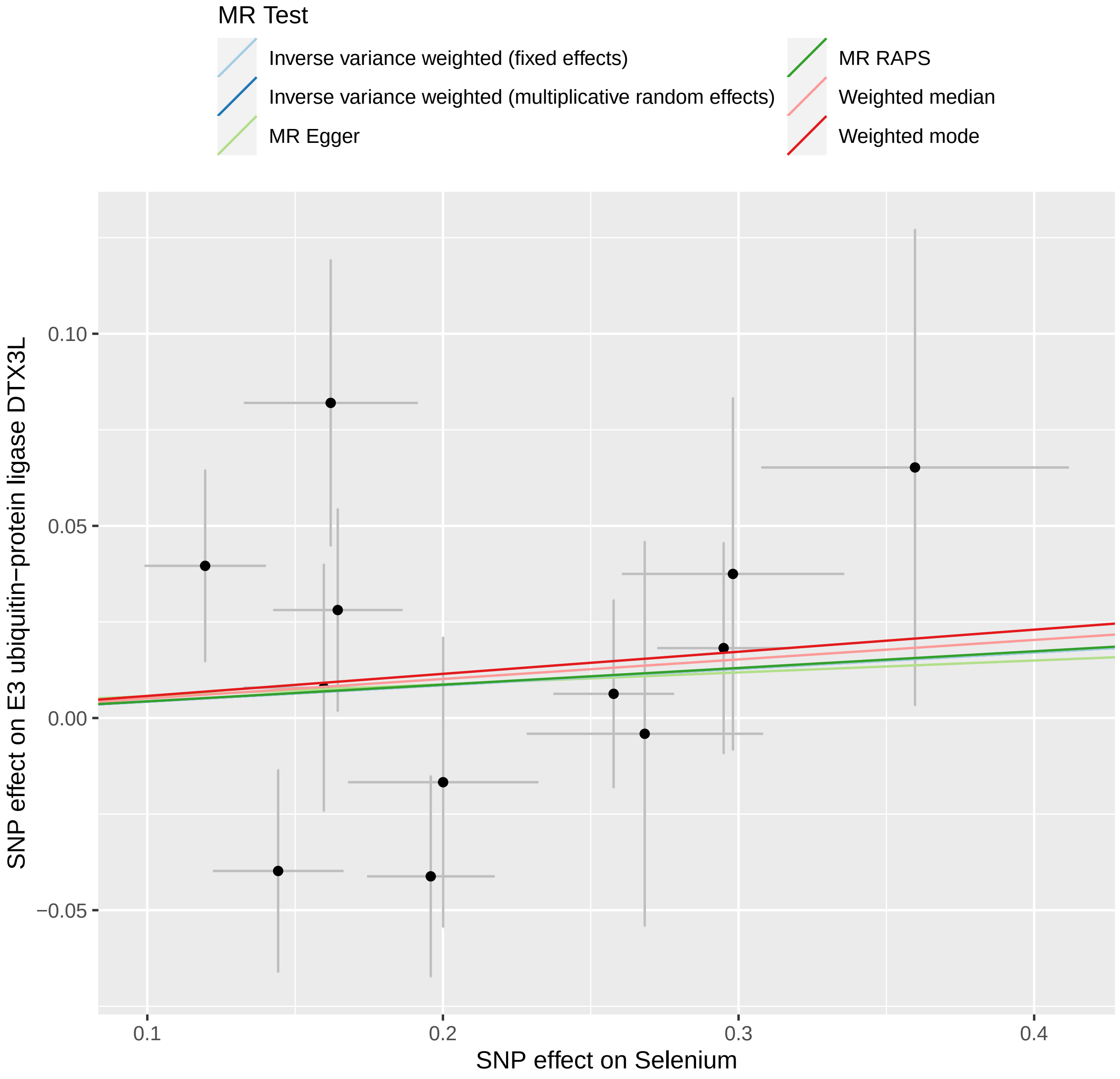

Batch 3−873 : Selenium on Dual specificity protein phosphatase 13 isoform A

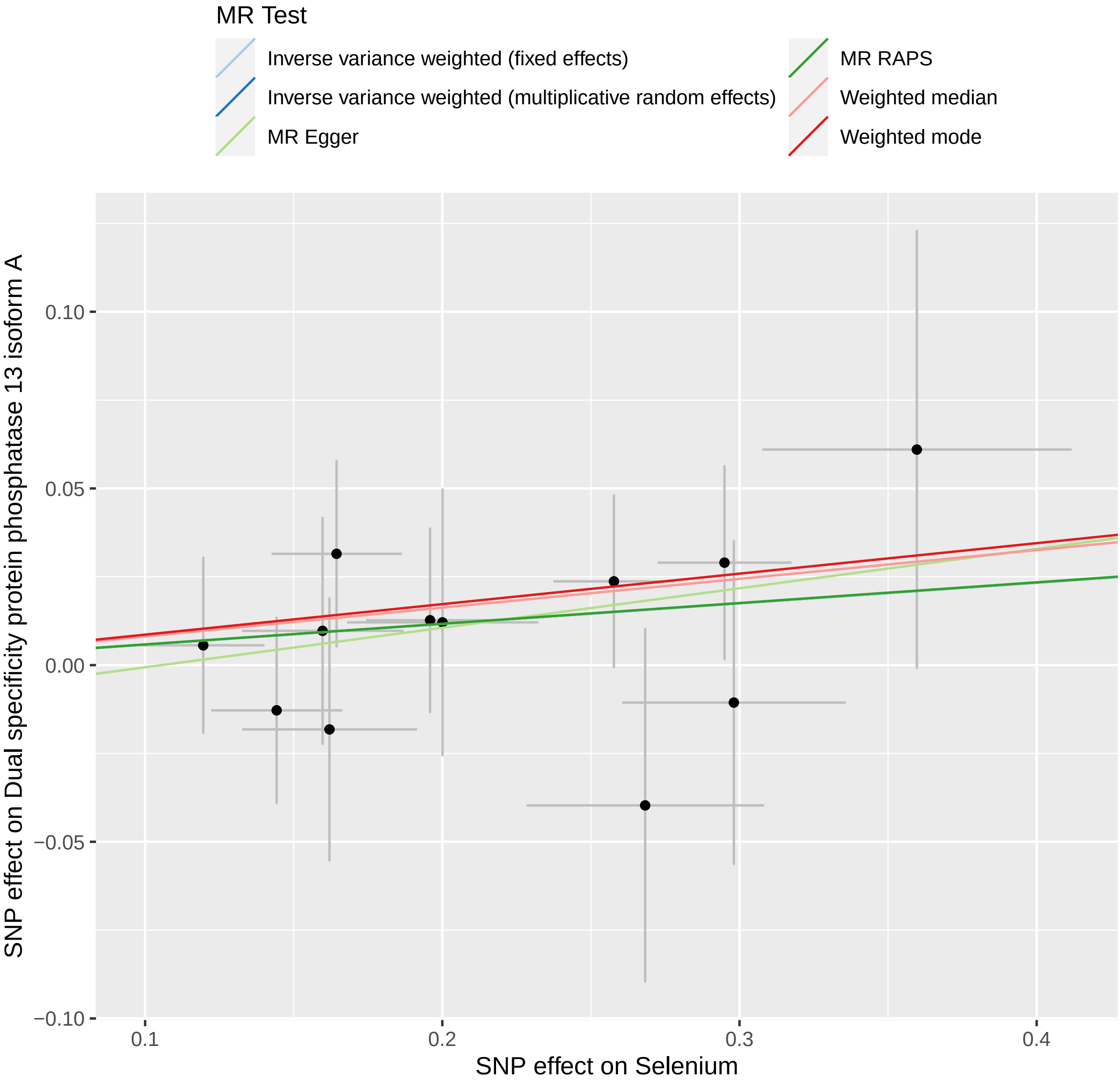

Batch 3−874 : Selenium on Dual specificity protein phosphatase 15

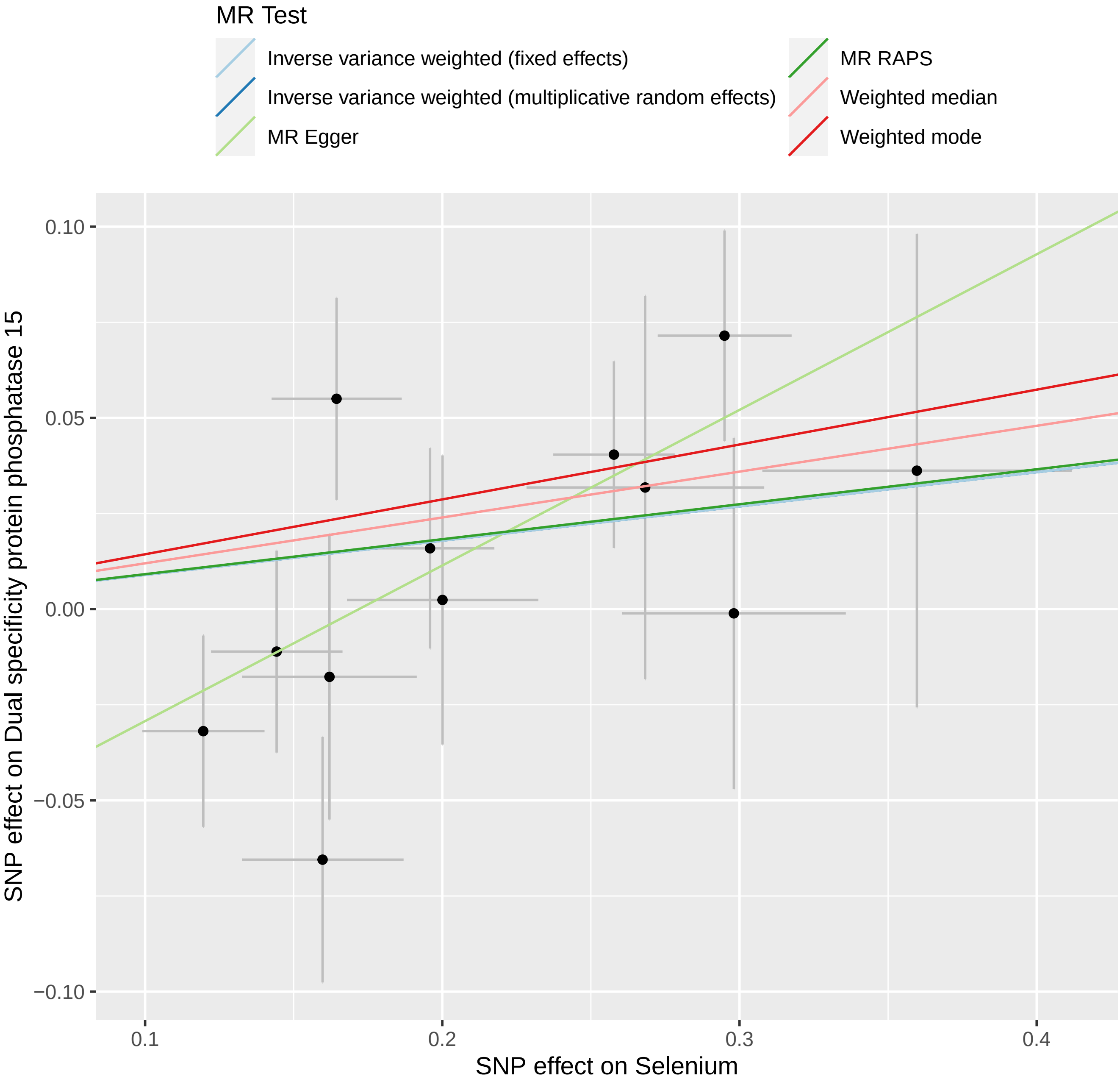

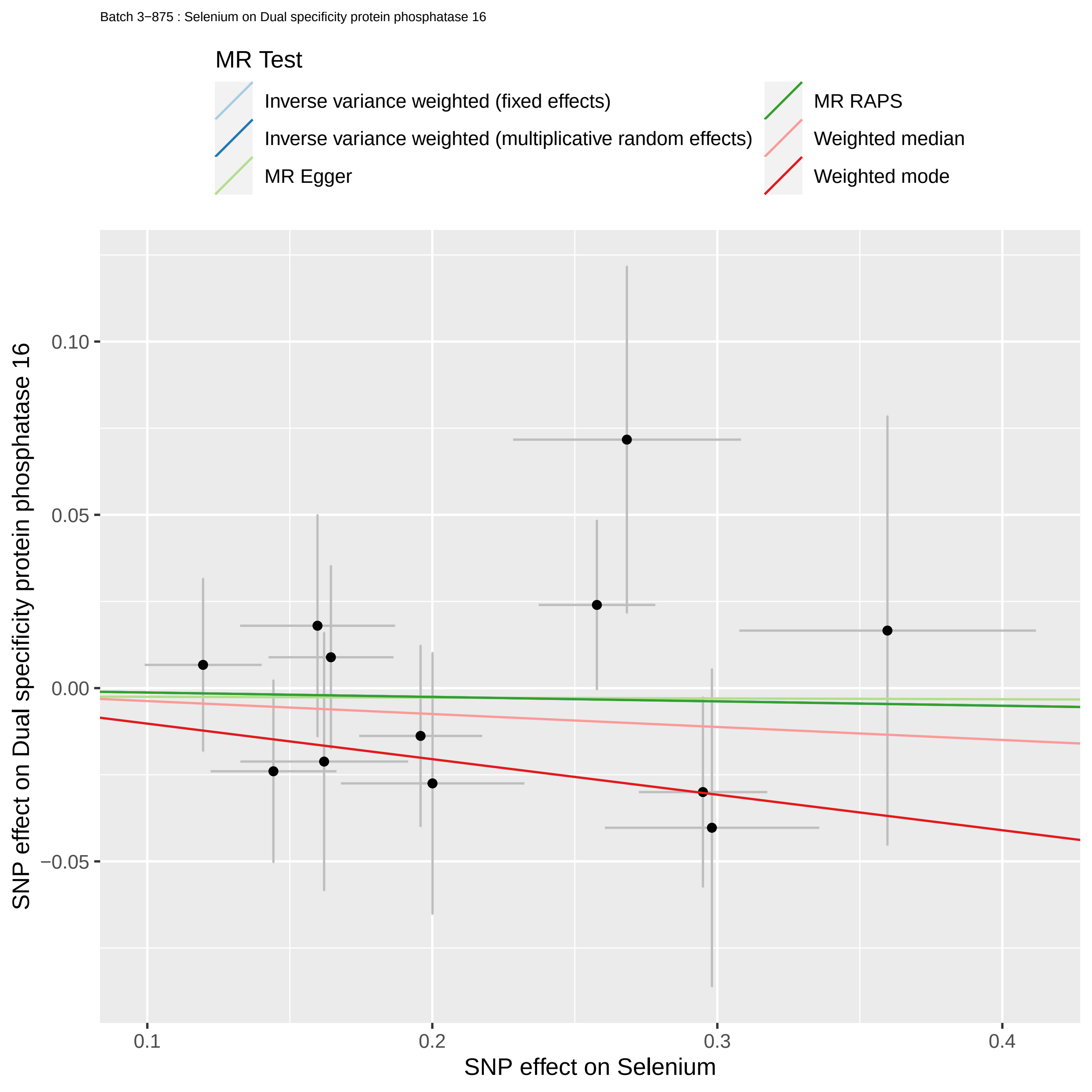
Batch 3−875 : Selenium on Dual specificity protein phosphatase 16
MR Test
Inverse variance weighted (fixed effects)
Inverse variance weighted (multiplicative random effects)
MR Egger
MR RAPS
Weighted median
Weighted mode
0.10
0.05
0.00
−0.05
0.1
0.2
0.3
0.4
SNP effect on Dual specificity protein phosphatase 16
SNP effect on Selenium

Batch 3−876 : Selenium on Dual specificity protein phosphatase 4

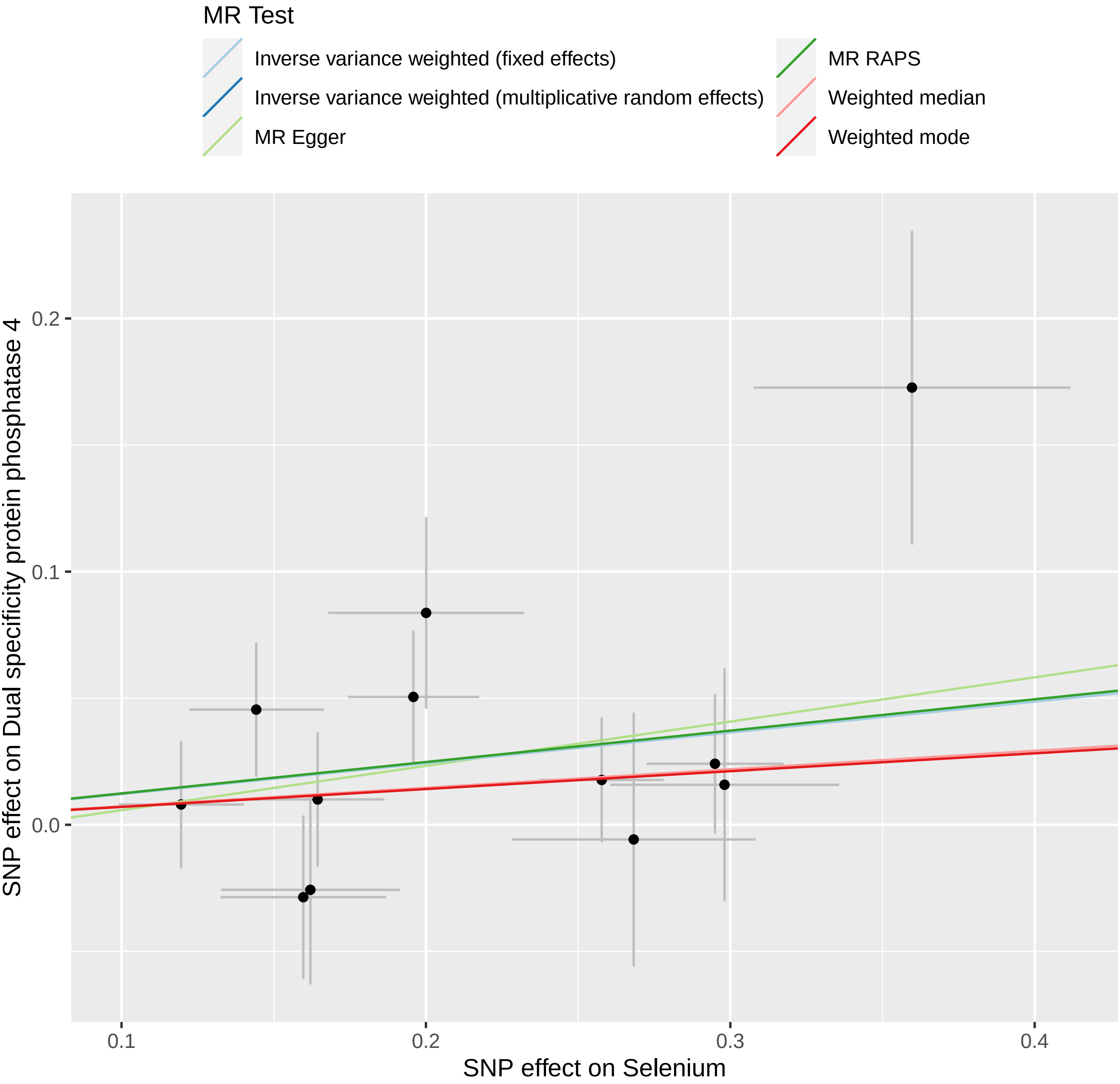

Batch 3−877 : Selenium on Dual specificity protein phosphatase 6

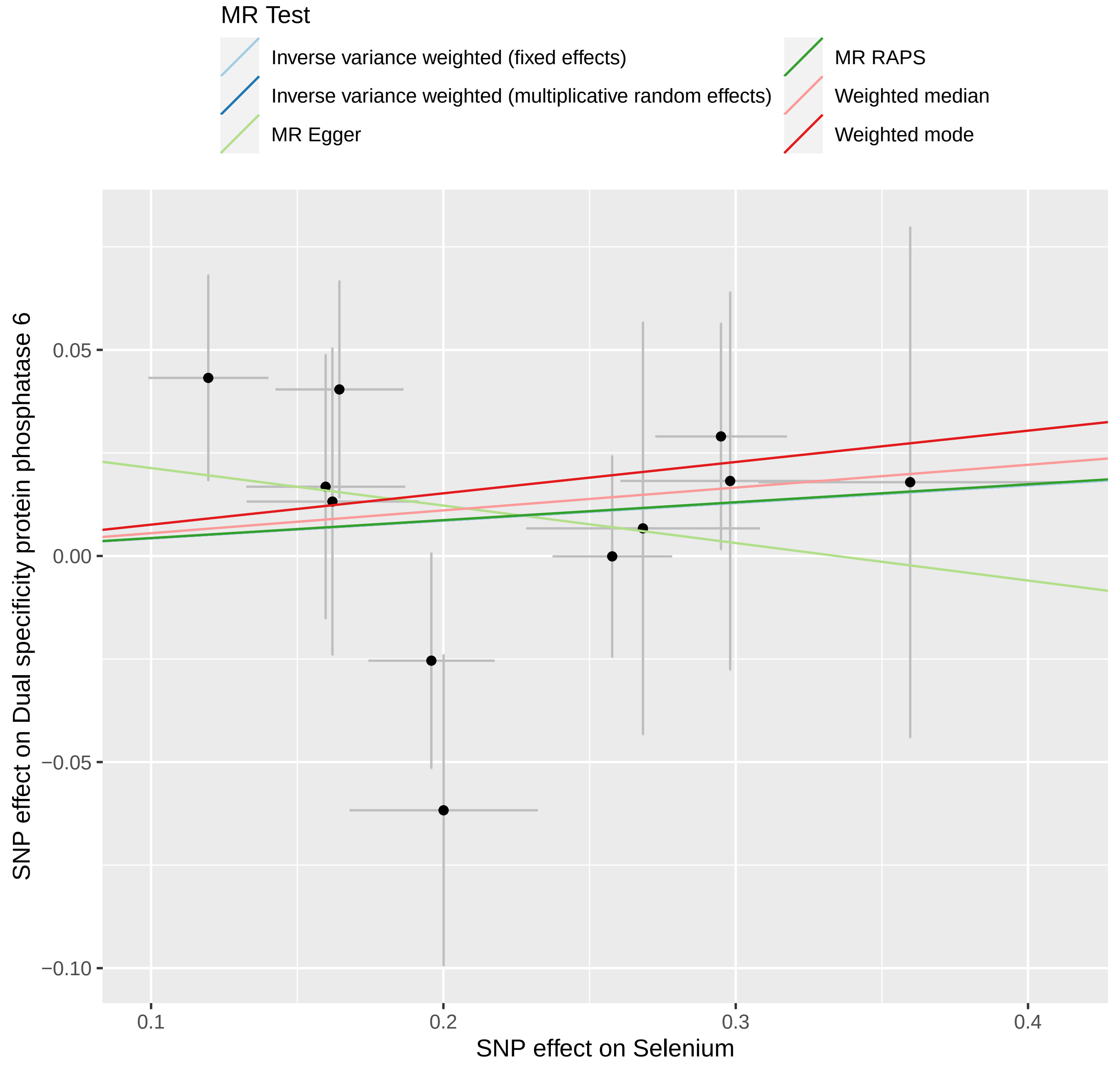

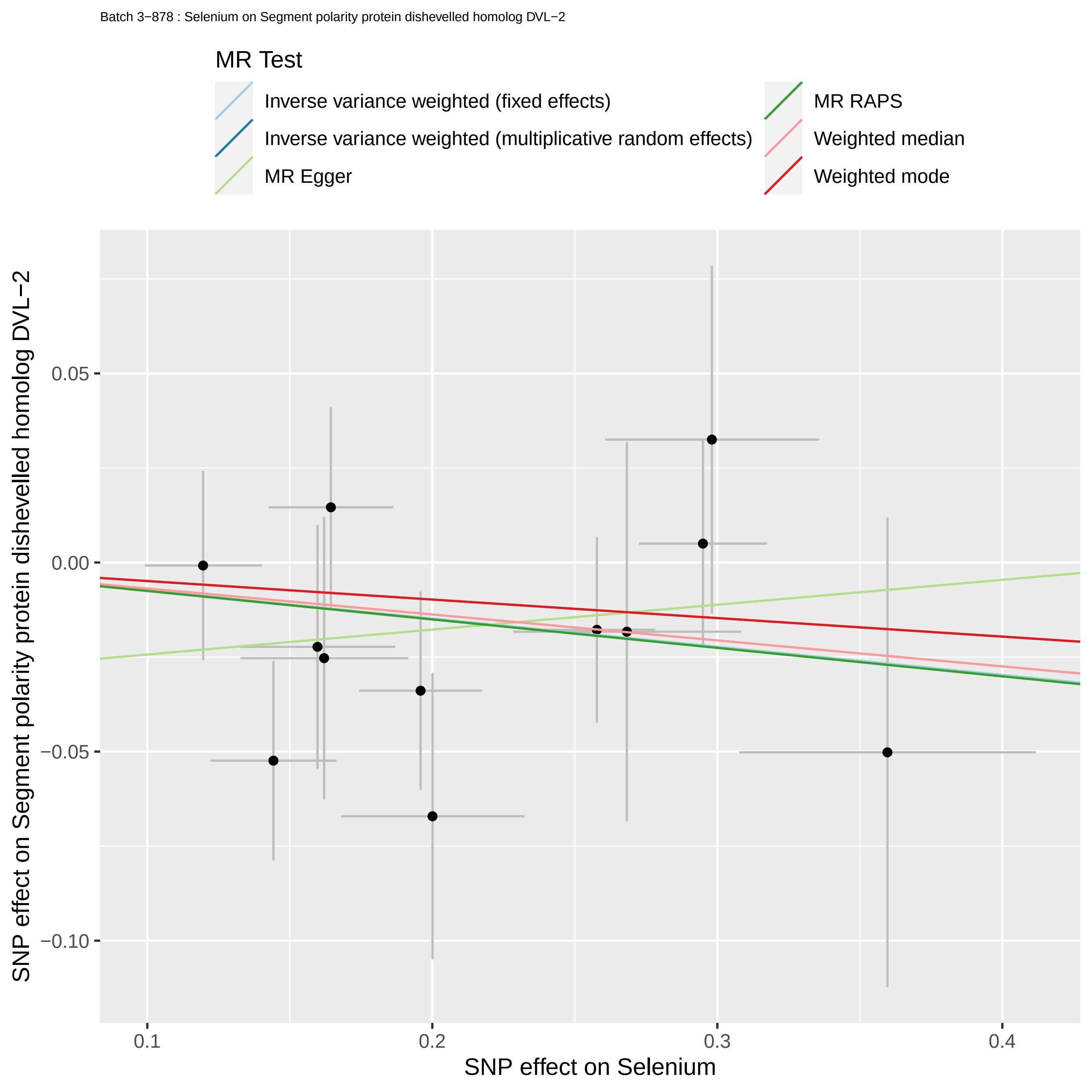

Batch 3−878 : Selenium on Segment polarity protein dishevelled homolog DVL−2
MR Test
Inverse variance weighted (fixed effects)
Inverse variance weighted (multiplicative random effects)
MR Egger
MR RAPS
Weighted median
Weighted mode
SNP effect on Segment polarity protein dishevelled homolog DVL−2
0.05
0.00
−0.05
−0.10
0.1
0.2
0.3
0.4
SNP effect on Selenium

Batch 3−879 : Selenium on Dynactin−associated protein

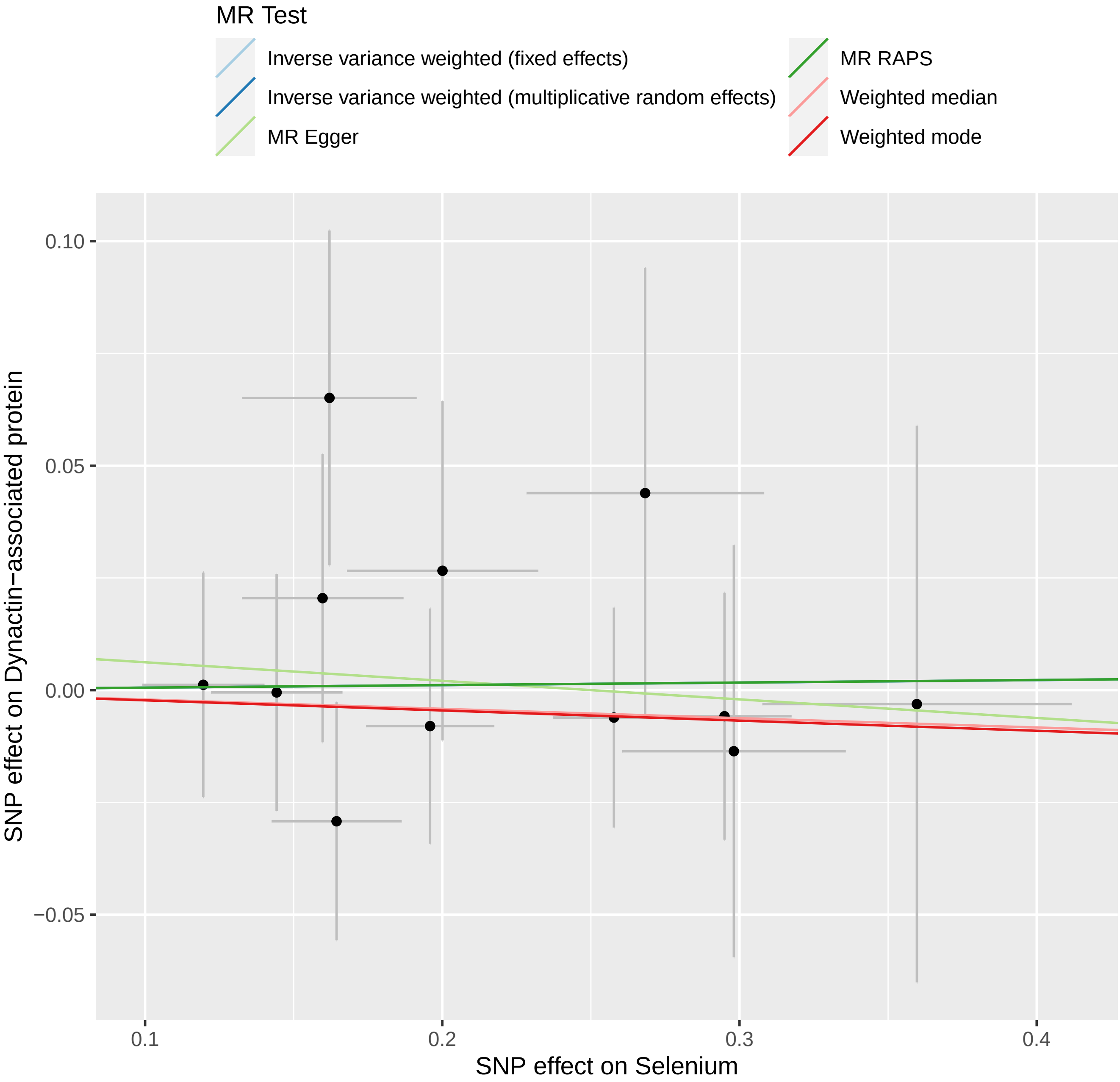

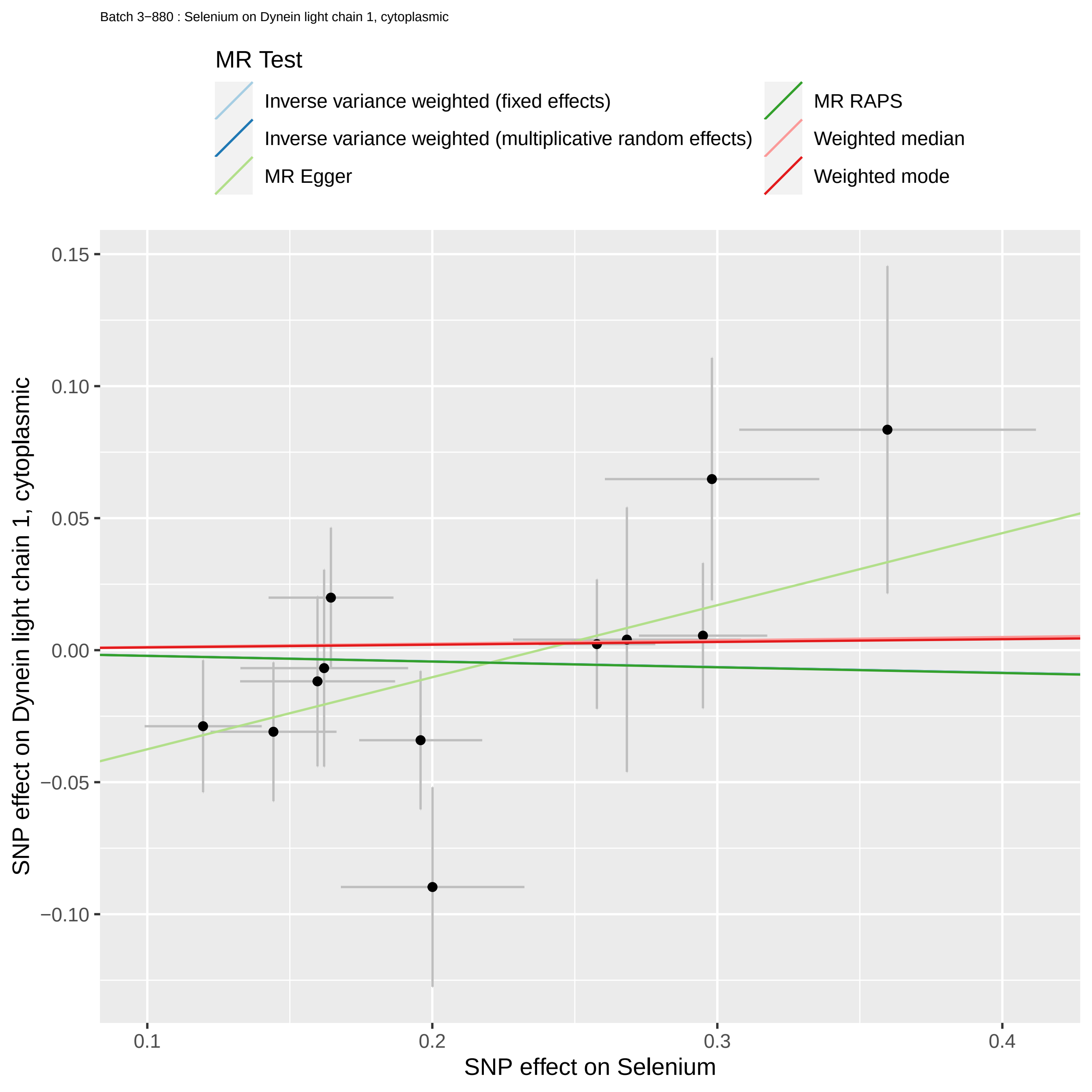

Batch 3−880 : Selenium on Dynein light chain 1, cytoplasmic
MR Test
Inverse variance weighted (fixed effects)
Inverse variance weighted (multiplicative random effects)
MR Egger
MR RAPS
Weighted median
Weighted mode
SNP effect on Dynein light chain 1, cytoplasmic
0.15
0.10
0.05
0.00
−0.05
−0.10
0.1
0.2
0.3
0.4
SNP effect on Selenium

Batch 3−881 : Selenium on Dynein light chain Tctex−type 3

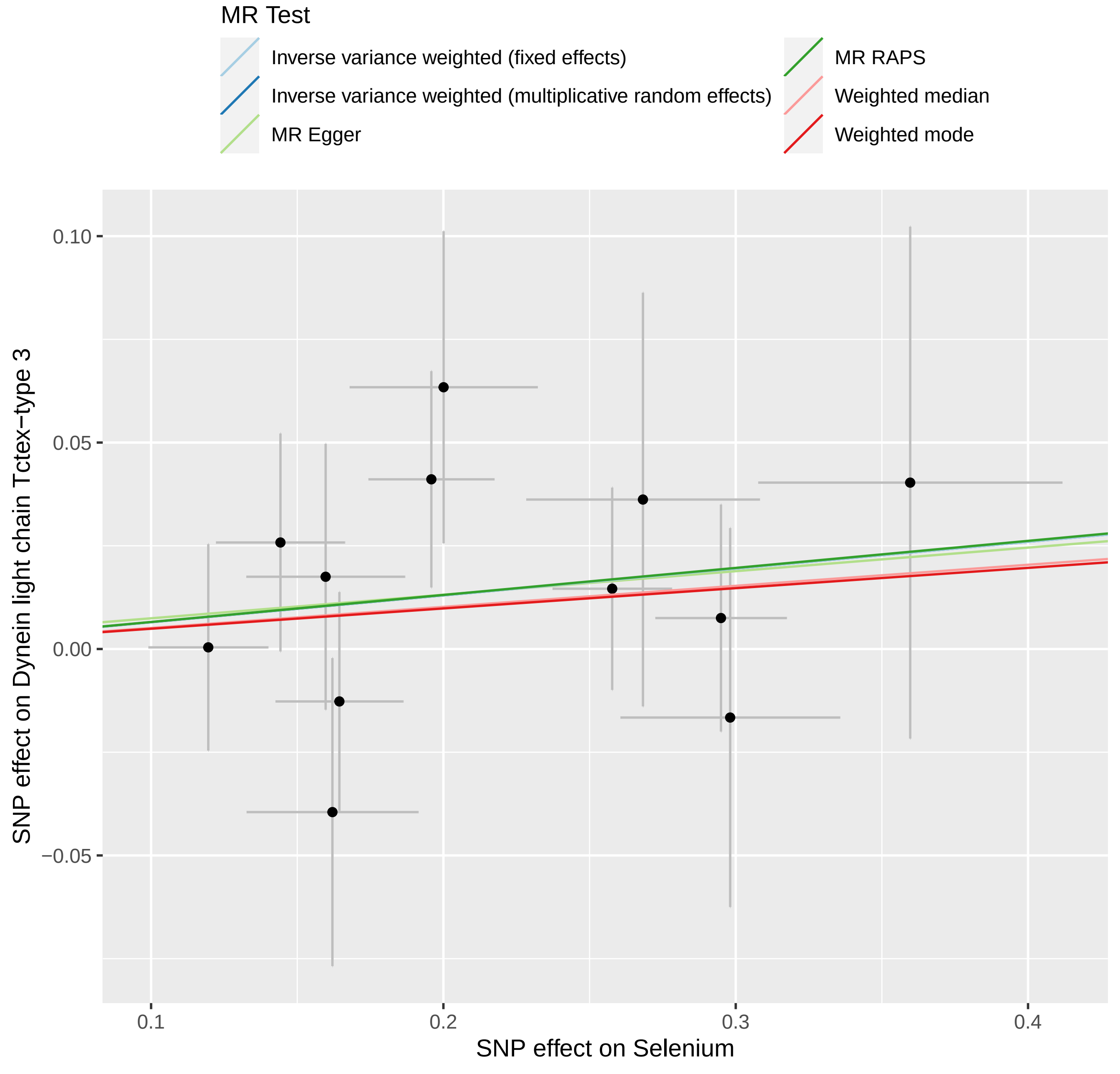

Batch 3−882 : Selenium on Dual specificity tyrosine−phosphorylation−regulated kinase 3

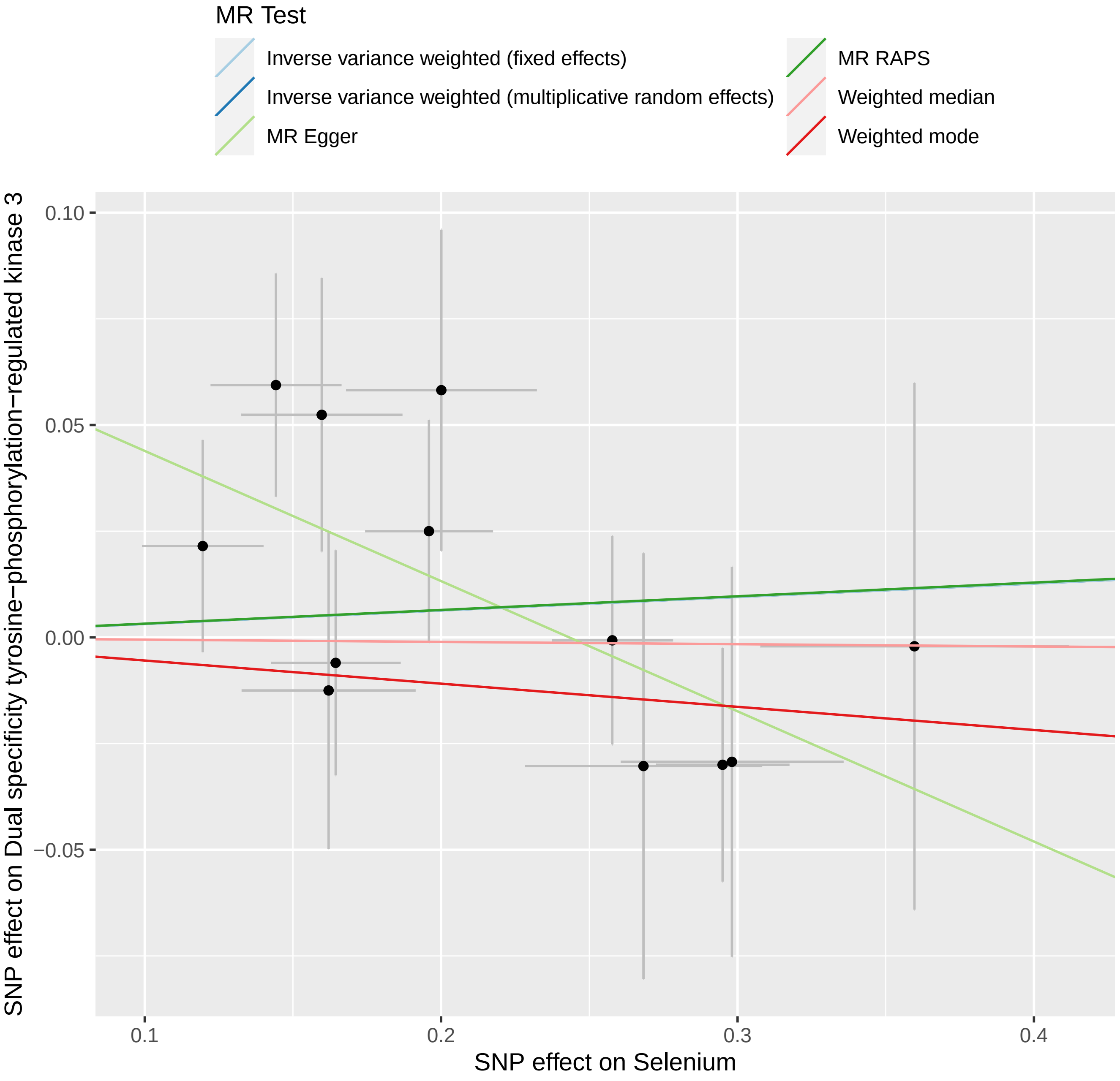

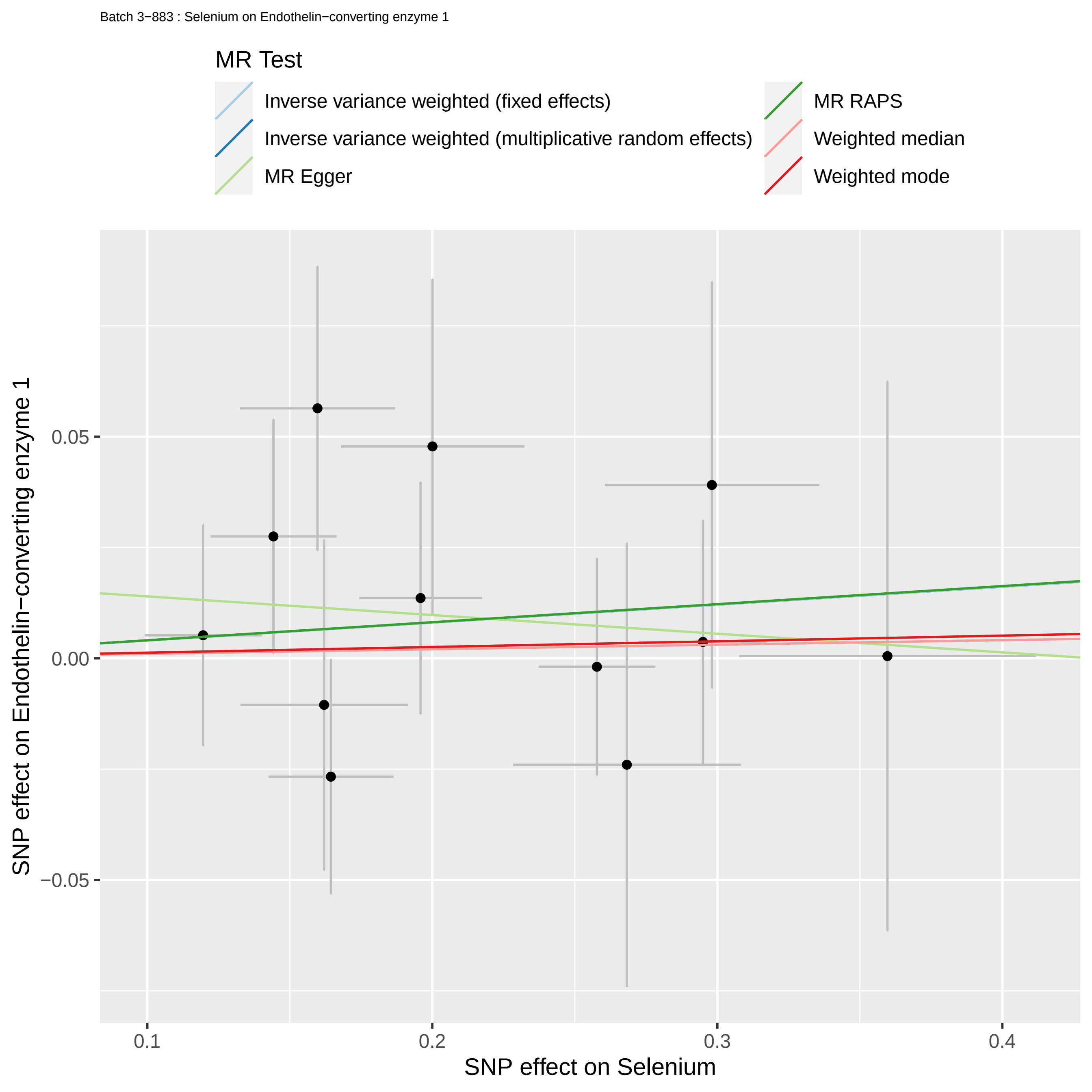

Batch 3−883 : Selenium on Endothelin−converting enzyme 1
MR Test
Inverse variance weighted (fixed effects)
Inverse variance weighted (multiplicative random effects)
MR Egger
MR RAPS
Weighted median
Weighted mode
SNP effect on Endothelin−converting enzyme 1
0.05
0.00
−0.05
0.1
0.2
0.3
0.4
SNP effect on Selenium

Batch 3−884 : Selenium on Endothelin−converting enzyme 1

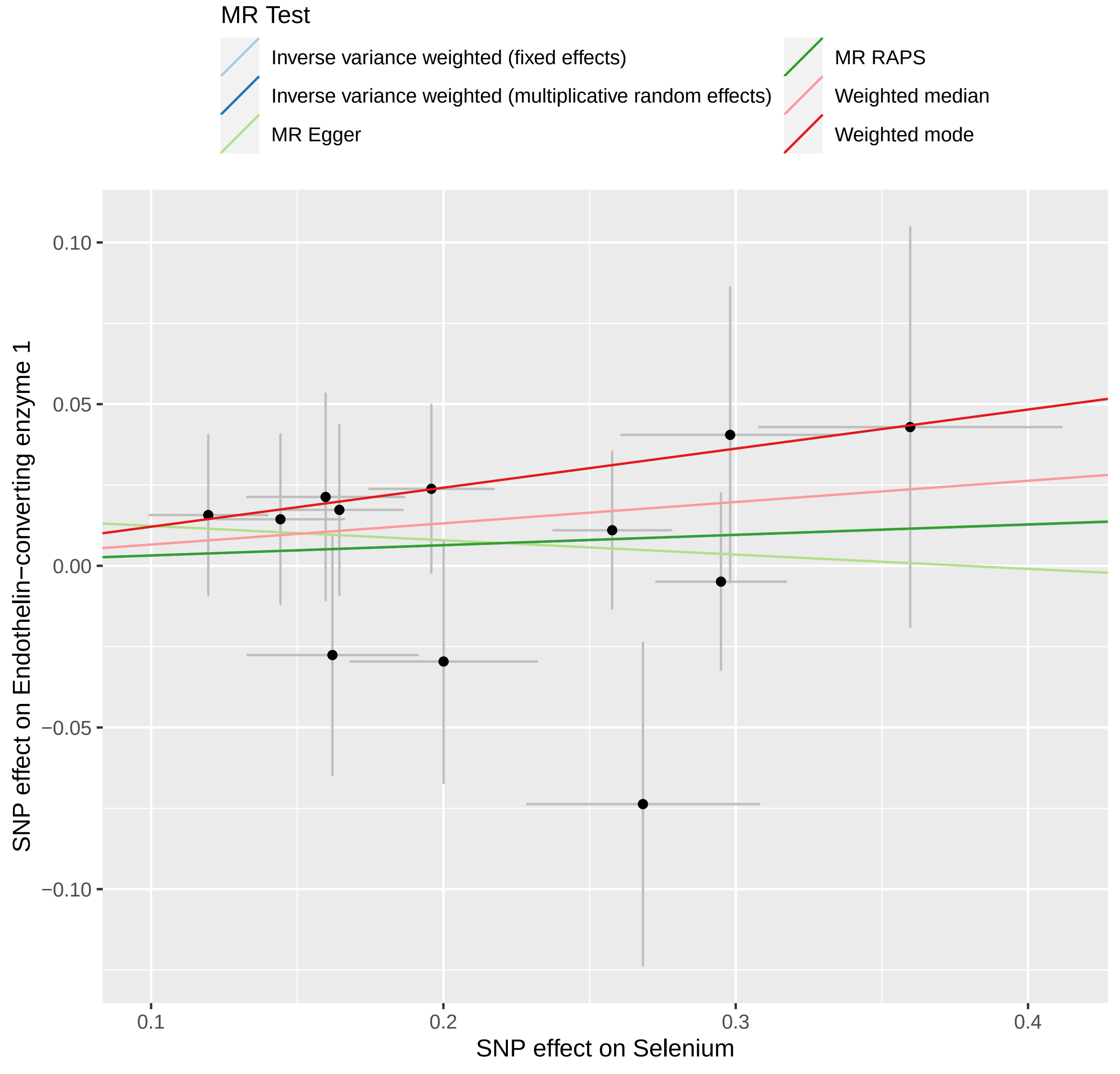

Batch 3−885 : Selenium on Ectodysplasin−A, secreted form

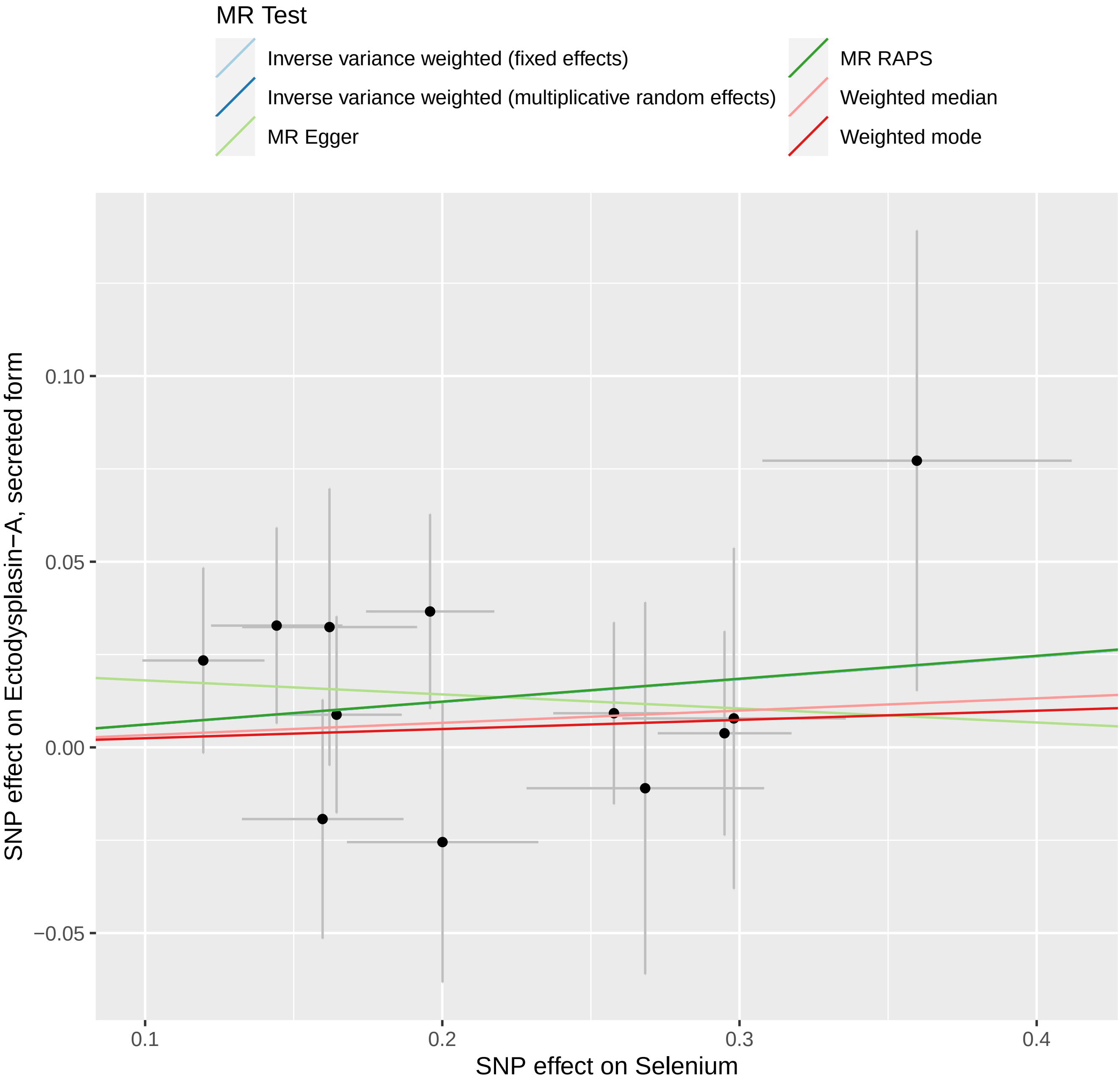

Batch 3−886 : Selenium on Ectodysplasin−A, secreted form

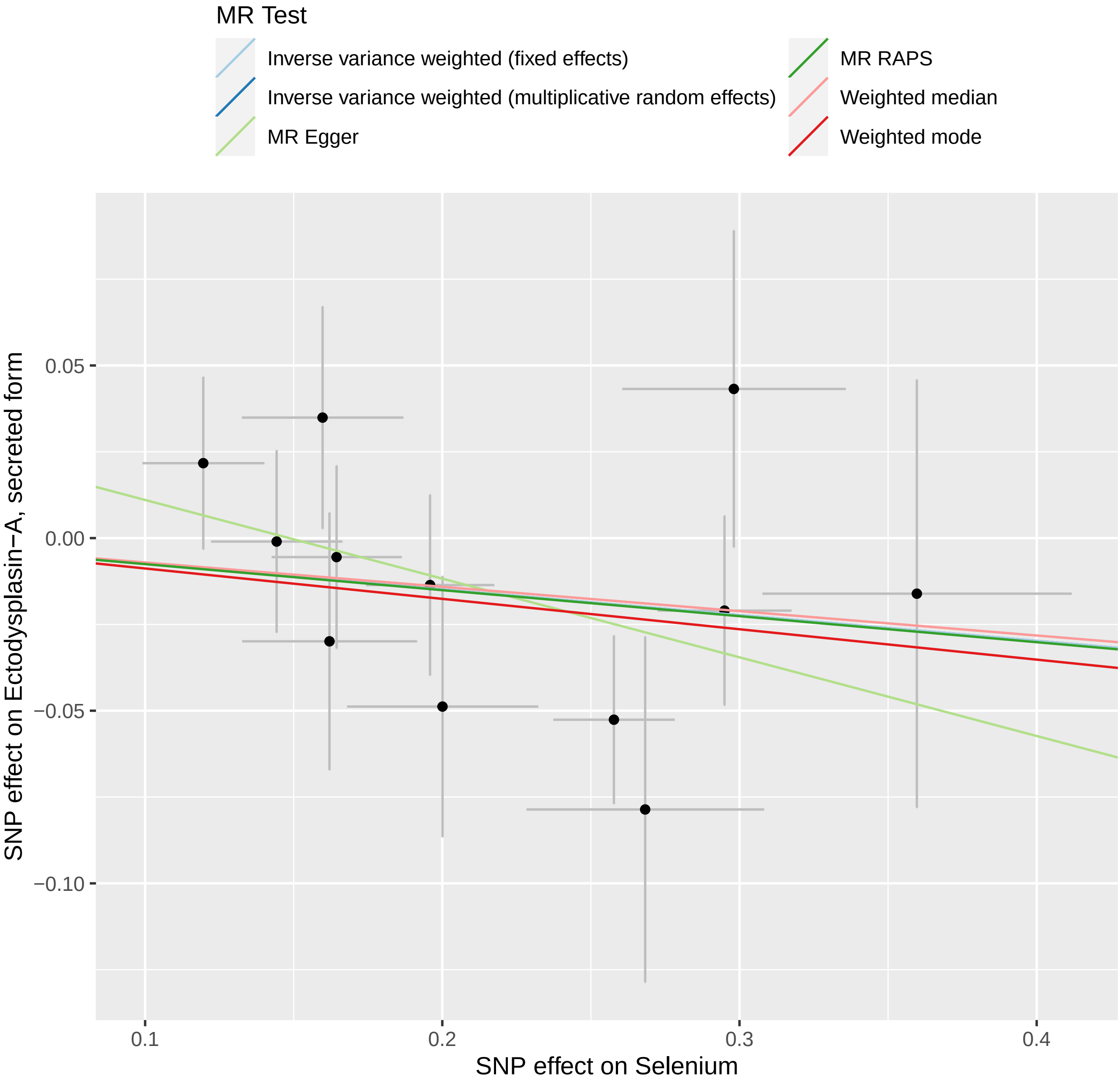

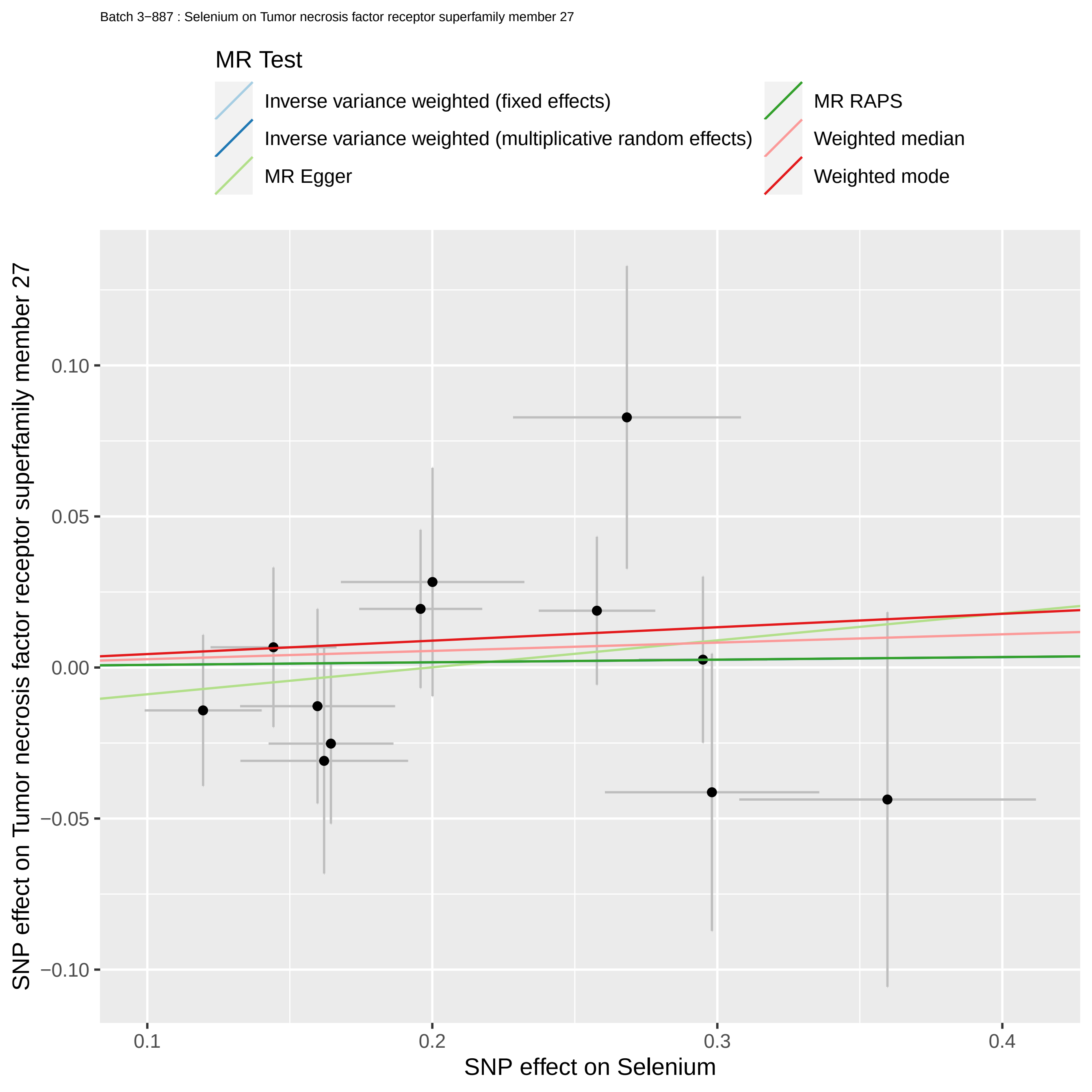
Batch 3−887 : Selenium on Tumor necrosis factor receptor superfamily member 27
MR Test
Inverse variance weighted (fixed effects)
Inverse variance weighted (multiplicative random effects)
MR Egger
MR RAPS
Weighted median
Weighted mode
SNP effect on Tumor necrosis factor receptor superfamily member 27
0.10
0.05
0.00
−0.05
−0.10
0.1
0.2
0.3
0.4
SNP effect on Selenium

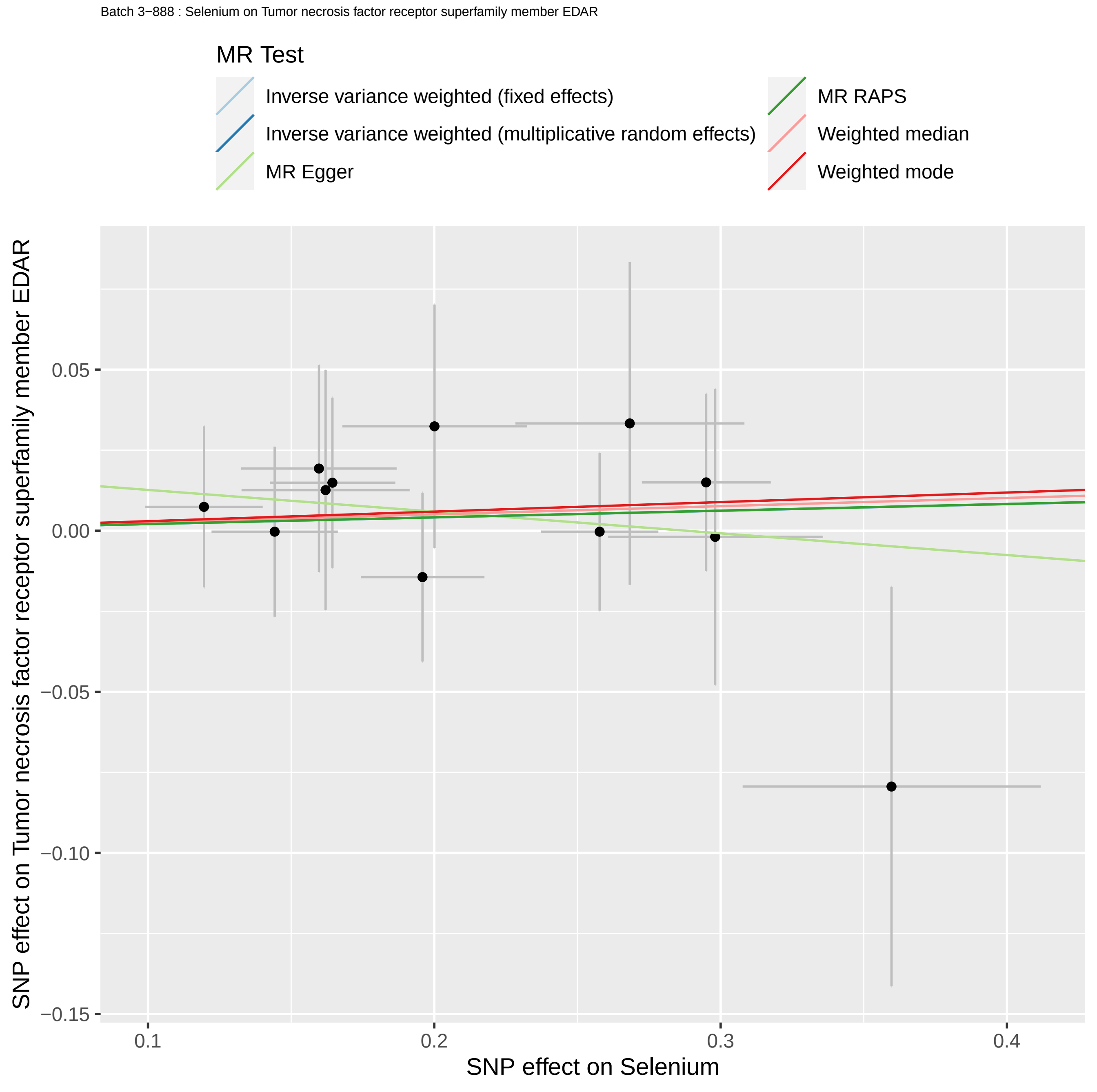

Batch 3−888 : Selenium on Tumor necrosis factor receptor superfamily member EDAR
MR Test
Inverse variance weighted (fixed effects)
Inverse variance weighted (multiplicative random effects)
MR Egger
MR RAPS
Weighted median
Weighted mode
0.05
0.00
−0.05
−0.10
−0.15
0.1
0.2
0.3
0.4
SNP effect on Tumor necrosis factor receptor superfamily member EDAR
SNP effect on Selenium

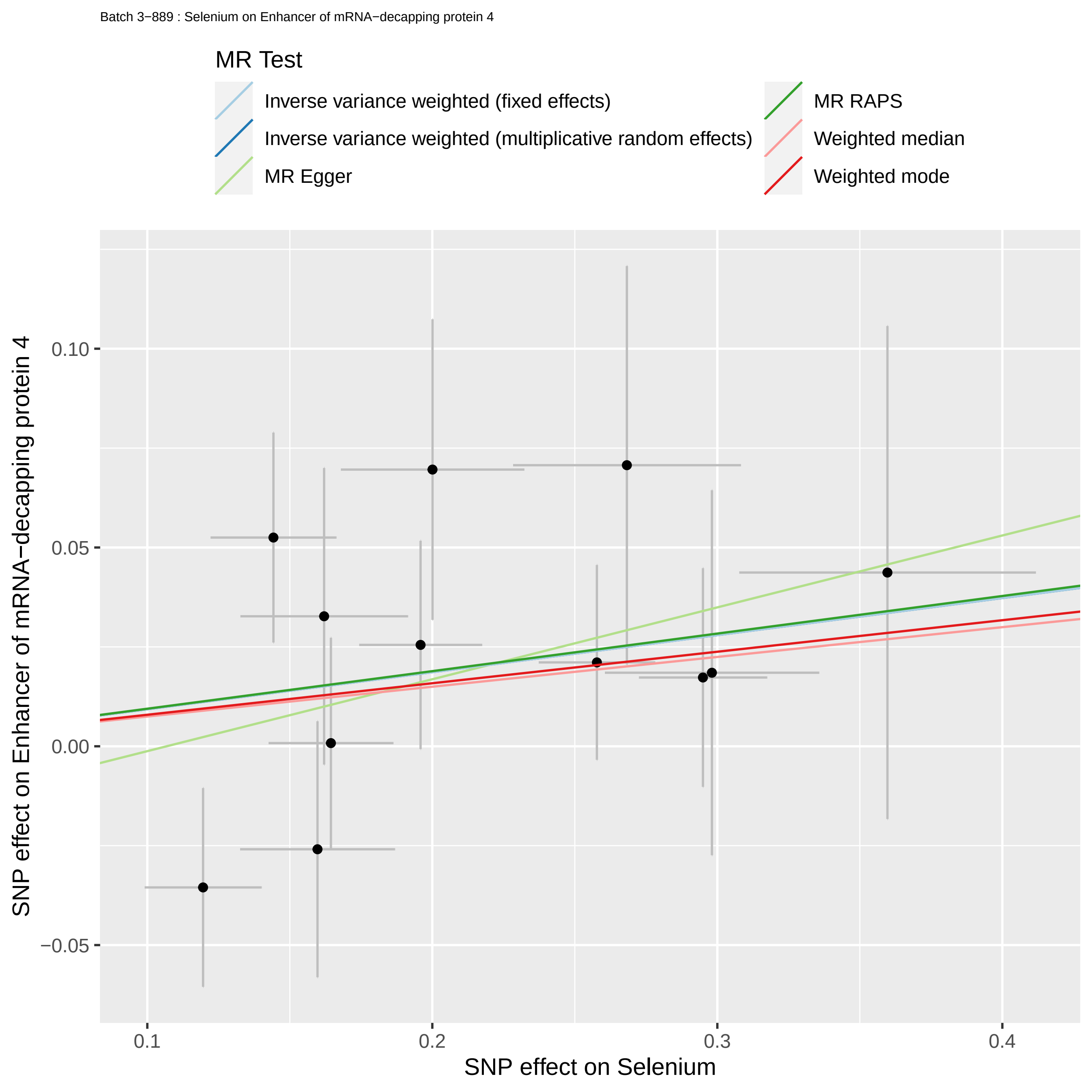
Batch 3−889 : Selenium on Enhancer of mRNA−decapping protein 4
MR Test
Inverse variance weighted (fixed effects)
Inverse variance weighted (multiplicative random effects)
MR Egger
MR RAPS
Weighted median
Weighted mode
SNP effect on Enhancer of mRNA−decapping protein 4
0.10
0.05
0.00
−0.05
0.1
0.2
0.3
0.4
SNP effect on Selenium

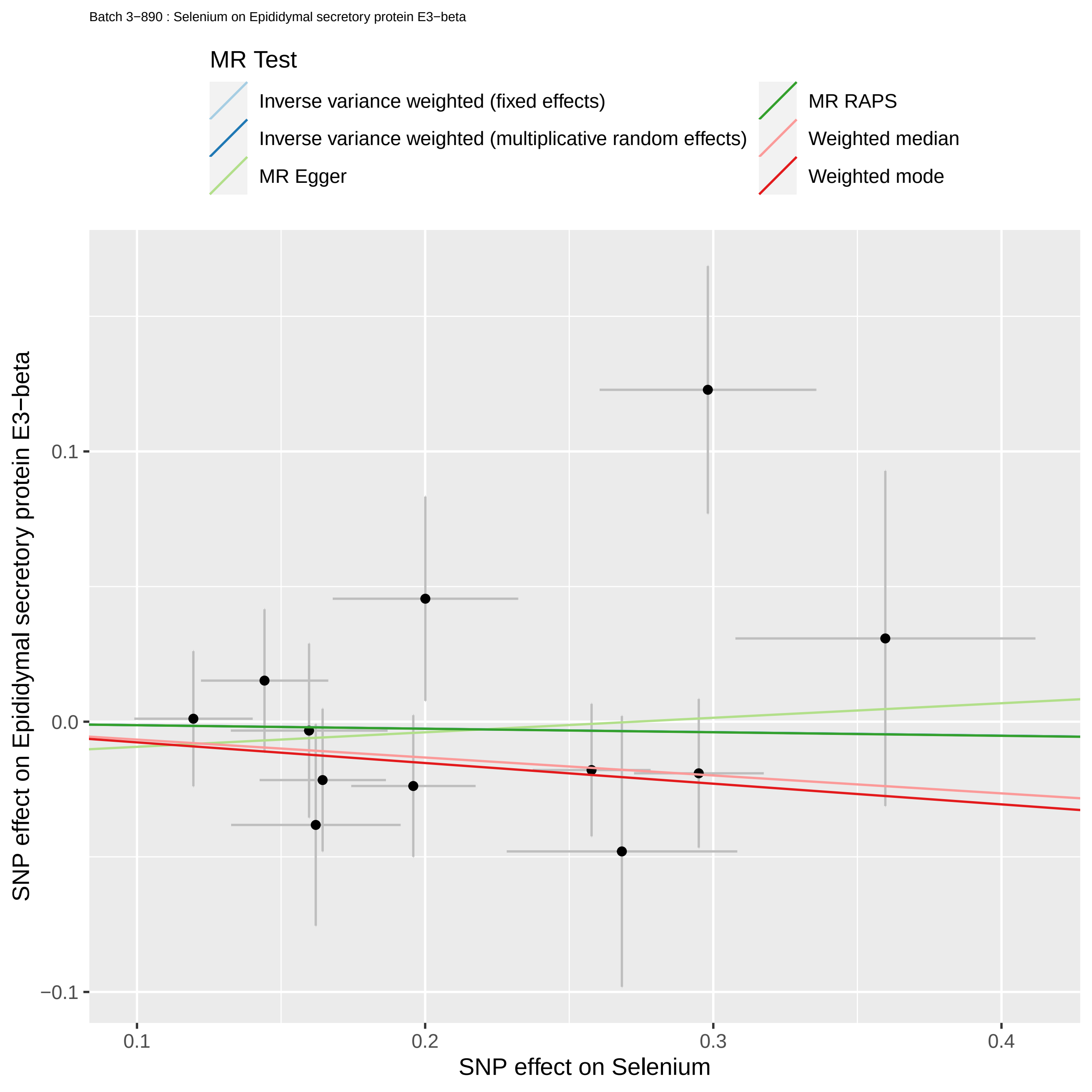

Batch 3−890 : Selenium on Epididymal secretory protein E3−beta
MR Test
Inverse variance weighted (fixed effects)
Inverse variance weighted (multiplicative random effects)
MR Egger
MR RAPS
Weighted median
Weighted mode
0.1
0.0
−0.1
0.1
0.2
0.3
0.4
SNP effect on Selenium
SNP effect on Epididymal secretory protein E3−beta

Batch 3−891 : Selenium on Endothelial differentiation−related factor 1

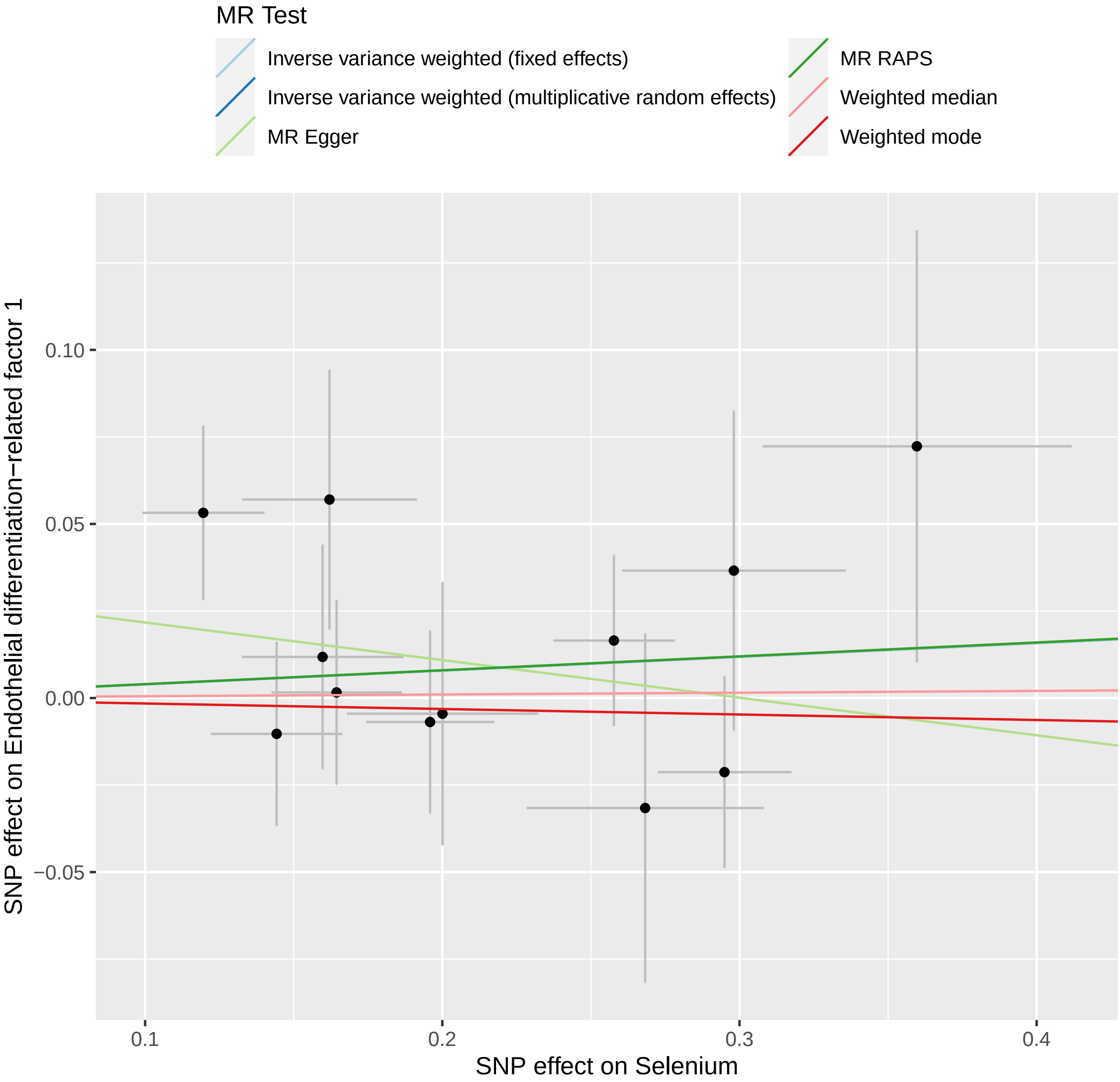

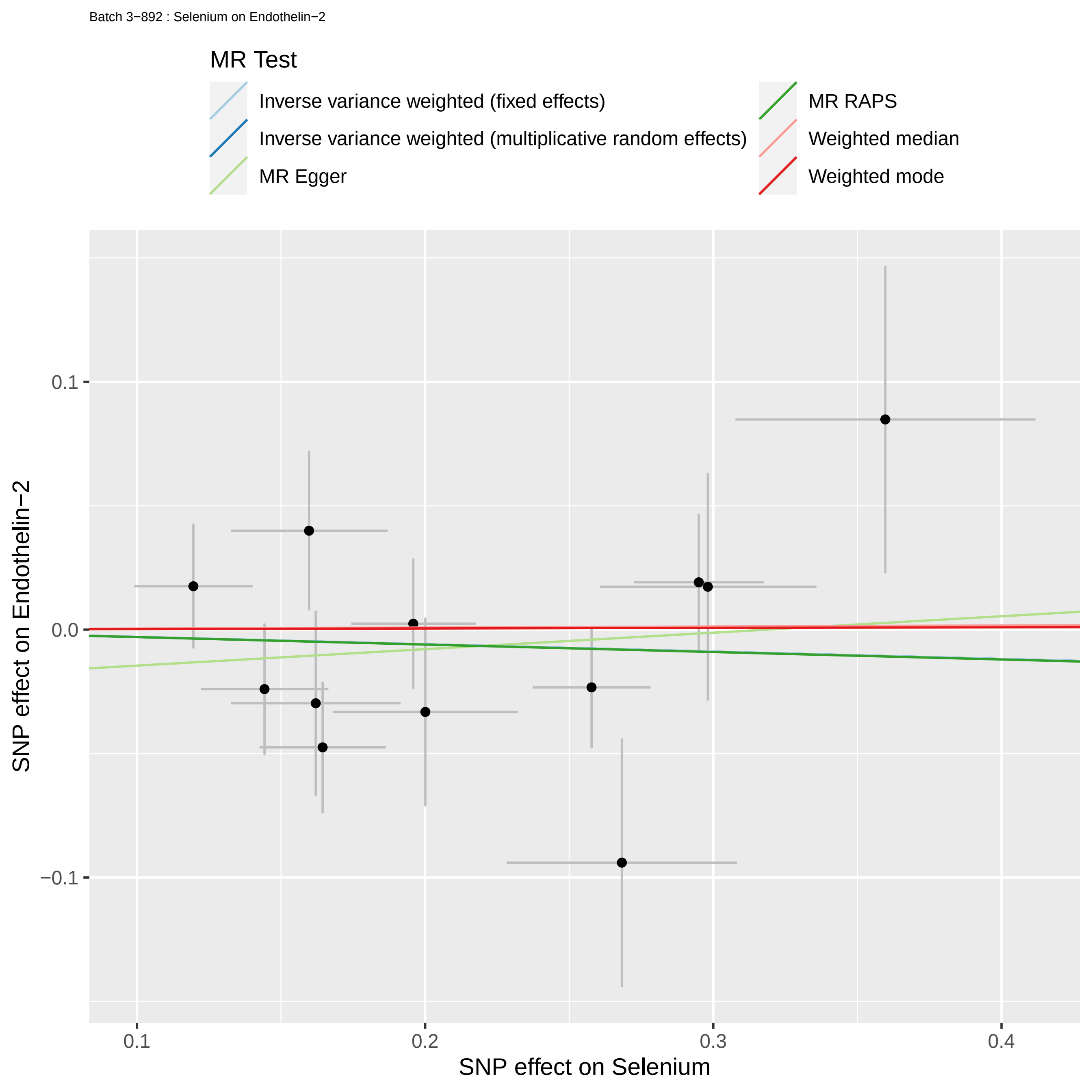
Batch 3−892 : Selenium on Endothelin−2
MR Test
Inverse variance weighted (fixed effects)
Inverse variance weighted (multiplicative random effects)
MR Egger
MR RAPS
Weighted median
Weighted mode
0.1
0.0
−0.1
0.1
0.2
0.3
0.4
SNP effect on Endothelin−2
SNP effect on Selenium

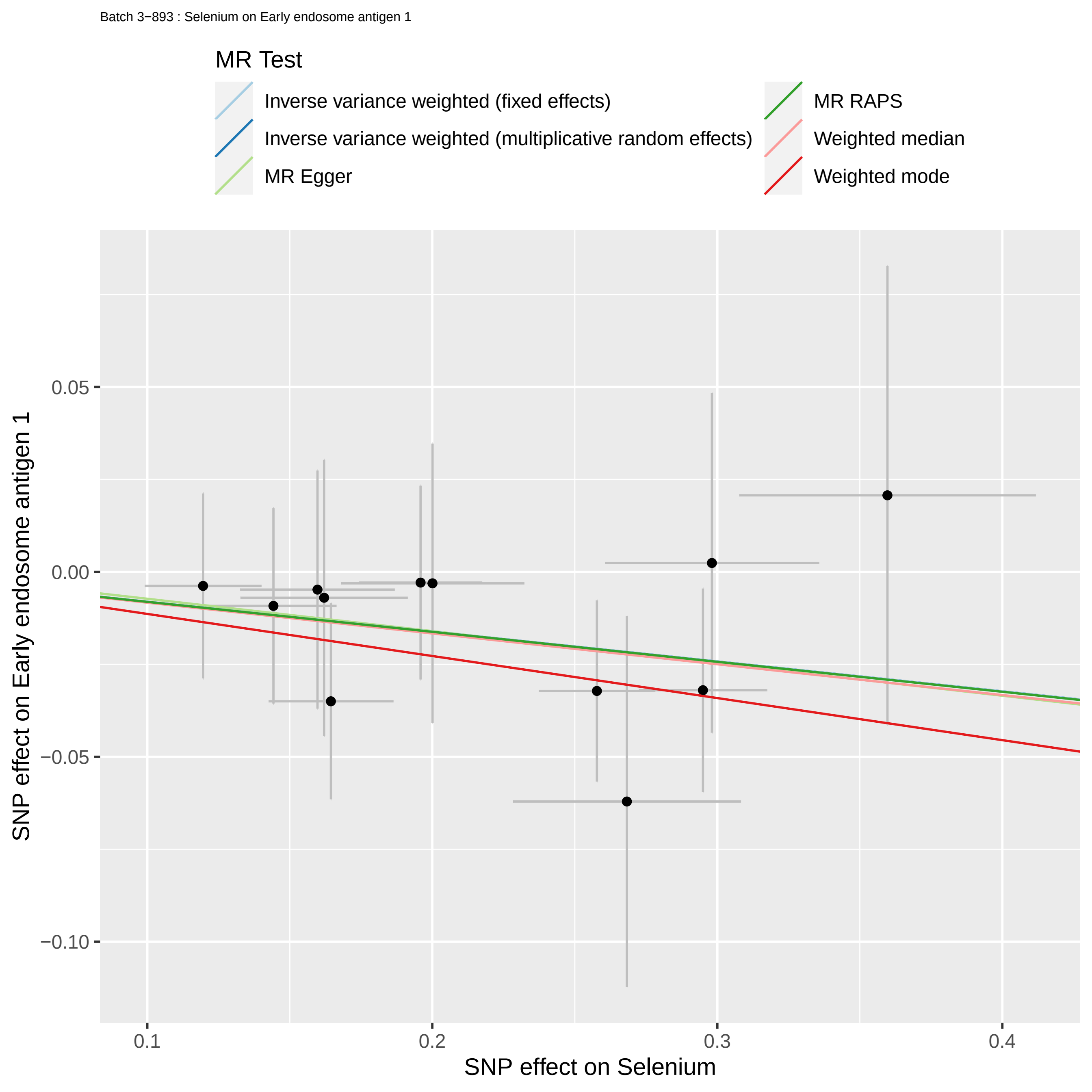
Batch 3−893 : Selenium on Early endosome antigen 1
MR Test
Inverse variance weighted (fixed effects)
Inverse variance weighted (multiplicative random effects)
MR Egger
MR RAPS
Weighted median
Weighted mode
0.05
0.00
−0.05
−0.10
0.1
0.2
0.3
0.4
SNP effect on Early endosome antigen 1
SNP effect on Selenium

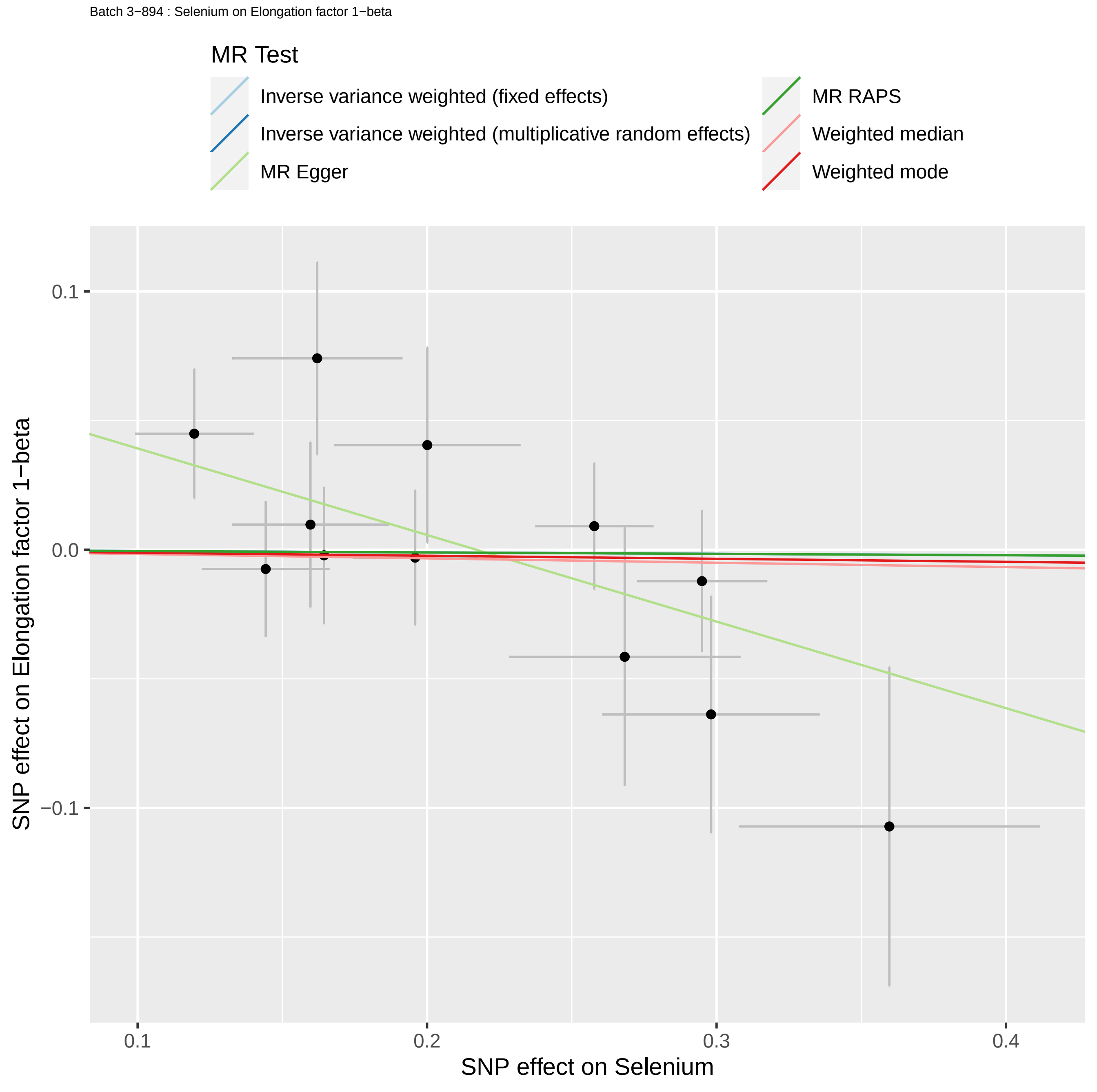

Batch 3−894 : Selenium on Elongation factor 1−beta
MR Test
Inverse variance weighted (fixed effects)
Inverse variance weighted (multiplicative random effects)
MR Egger
MR RAPS
Weighted median
Weighted mode
0.1
0.0
−0.1
0.1
0.2
0.3
0.4
SNP effect on Elongation factor 1−beta
SNP effect on Selenium

Batch 3−895 : Selenium on EF−hand calcium−binding domain−containing protein 14

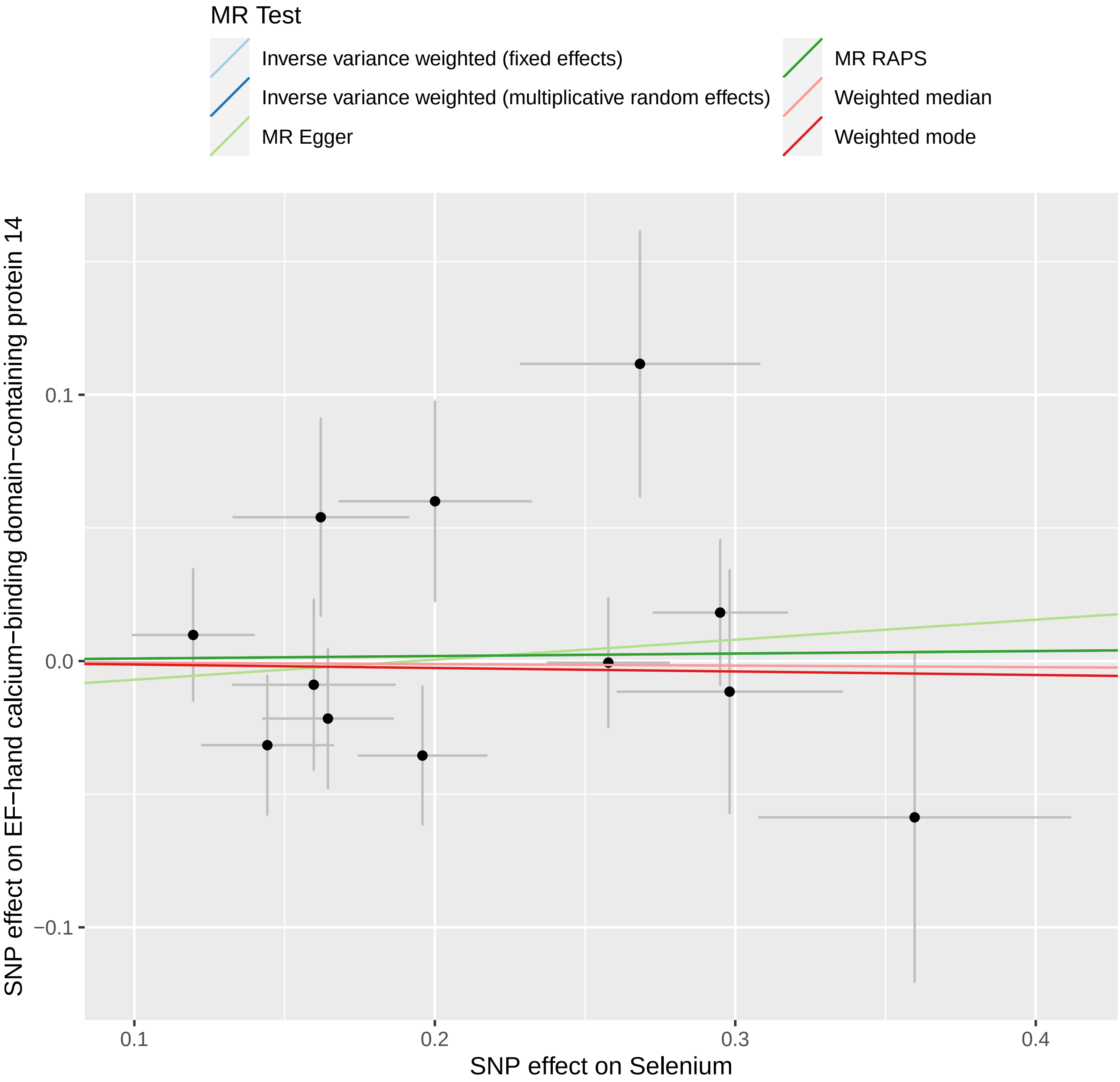

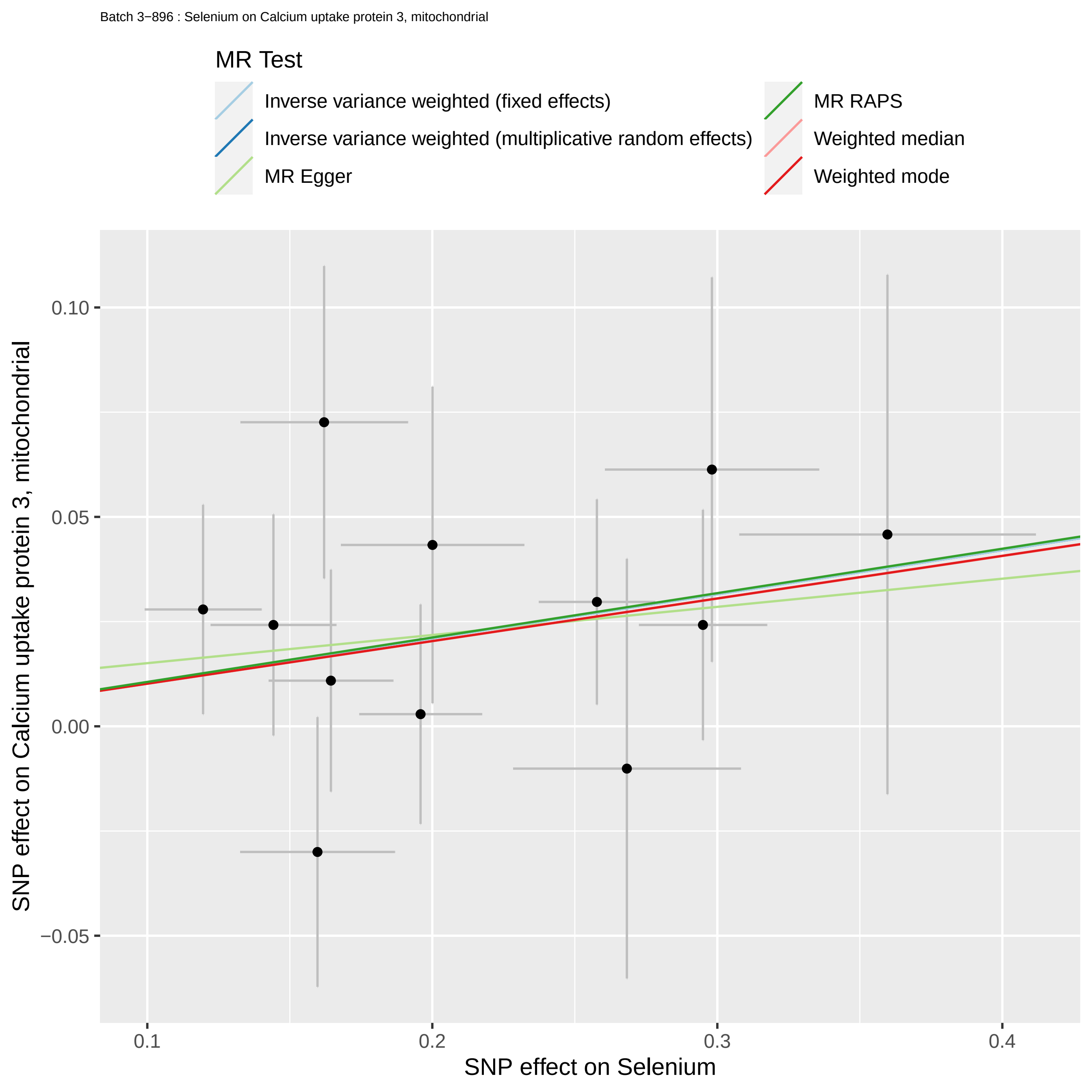
Batch 3−896 : Selenium on Calcium uptake protein 3, mitochondrial
MR Test
Inverse variance weighted (fixed effects)
Inverse variance weighted (multiplicative random effects)
MR Egger
MR RAPS
Weighted median
Weighted mode
SNP effect on Calcium uptake protein 3, mitochondrial
0.10
0.05
0.00
−0.05
0.1
0.2
0.3
0.4
SNP effect on Selenium

Batch 3−897 : Selenium on Ephrin−A2

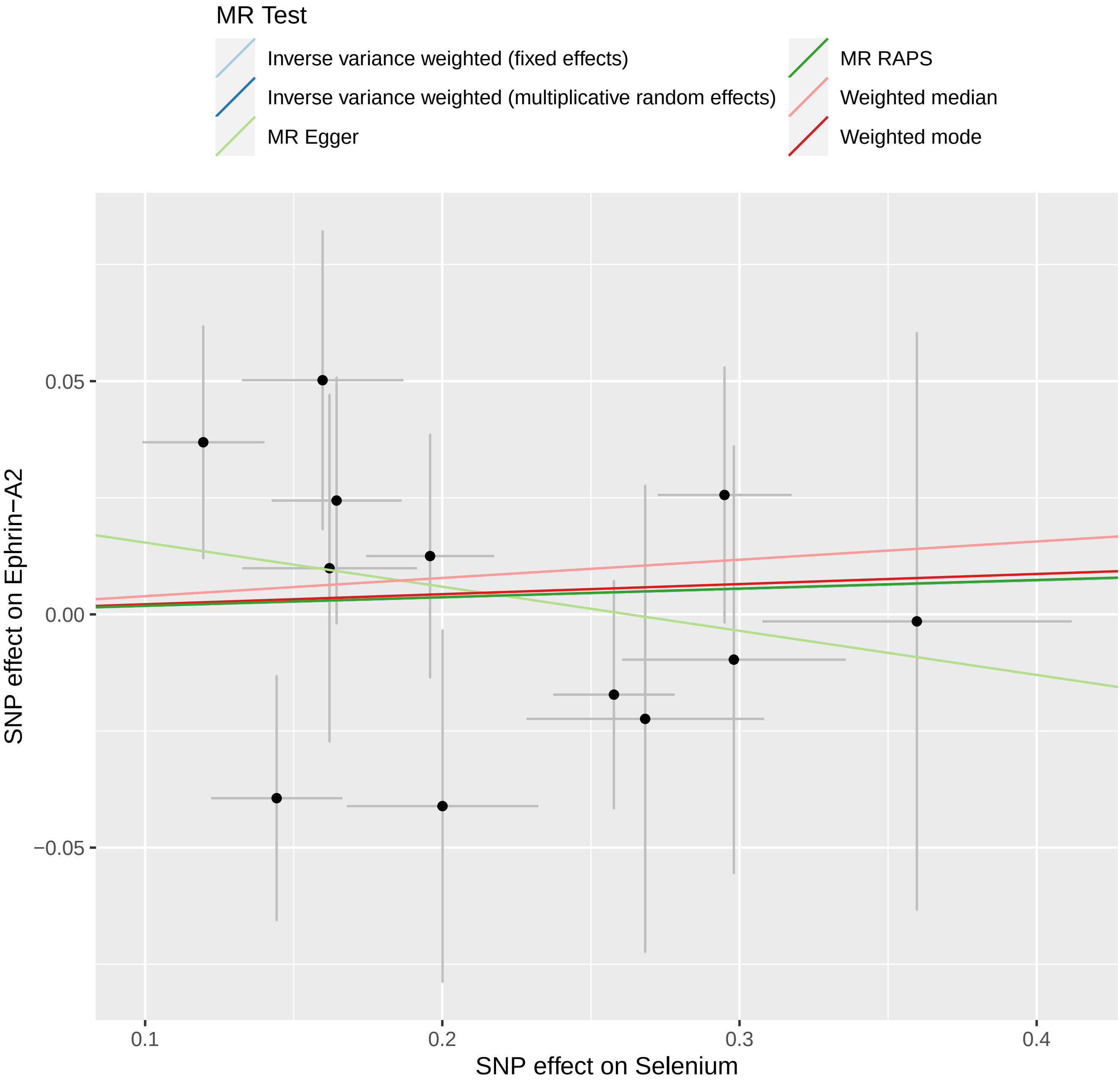

Batch 3−898 : Selenium on Ephrin−A3

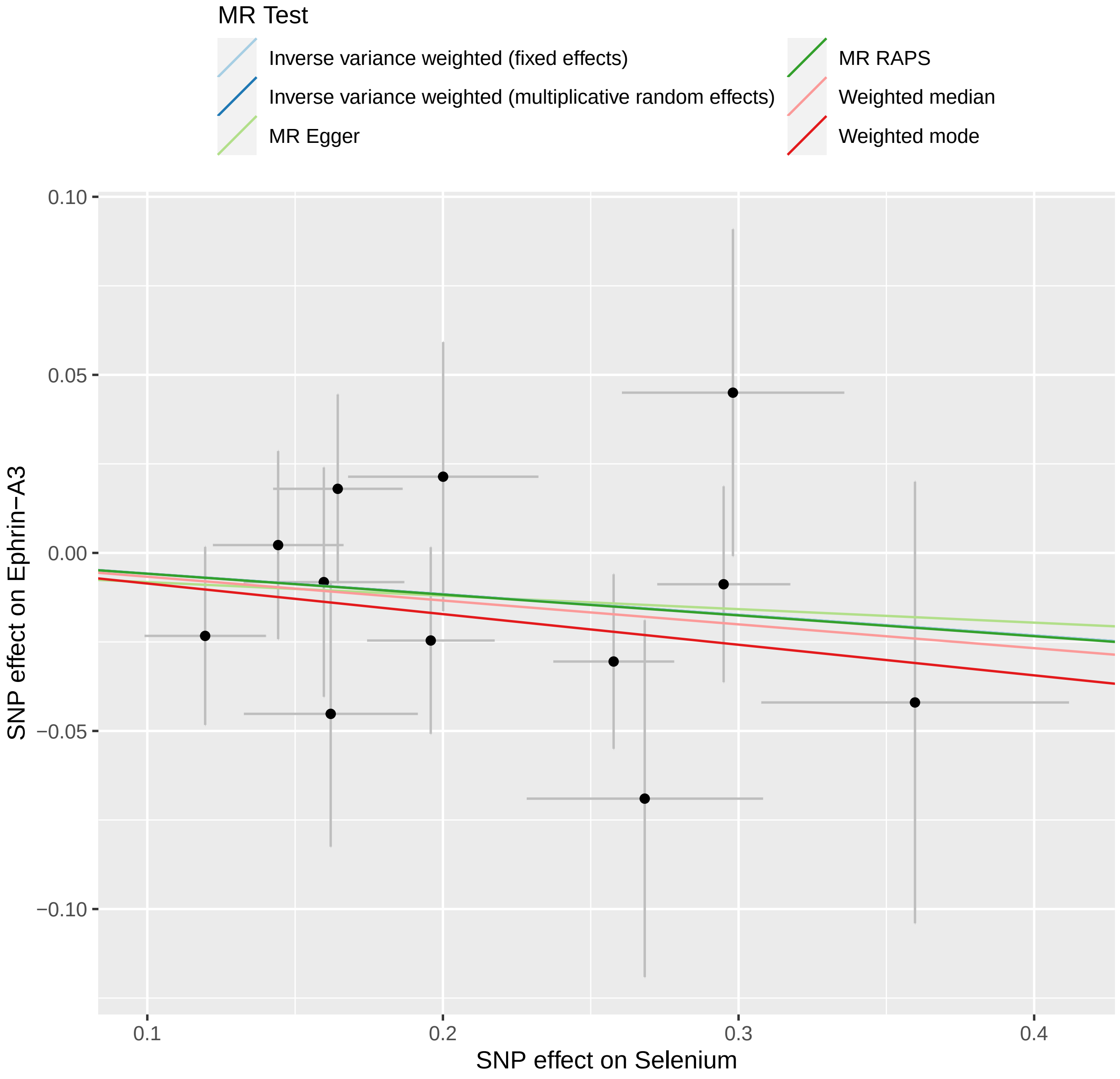

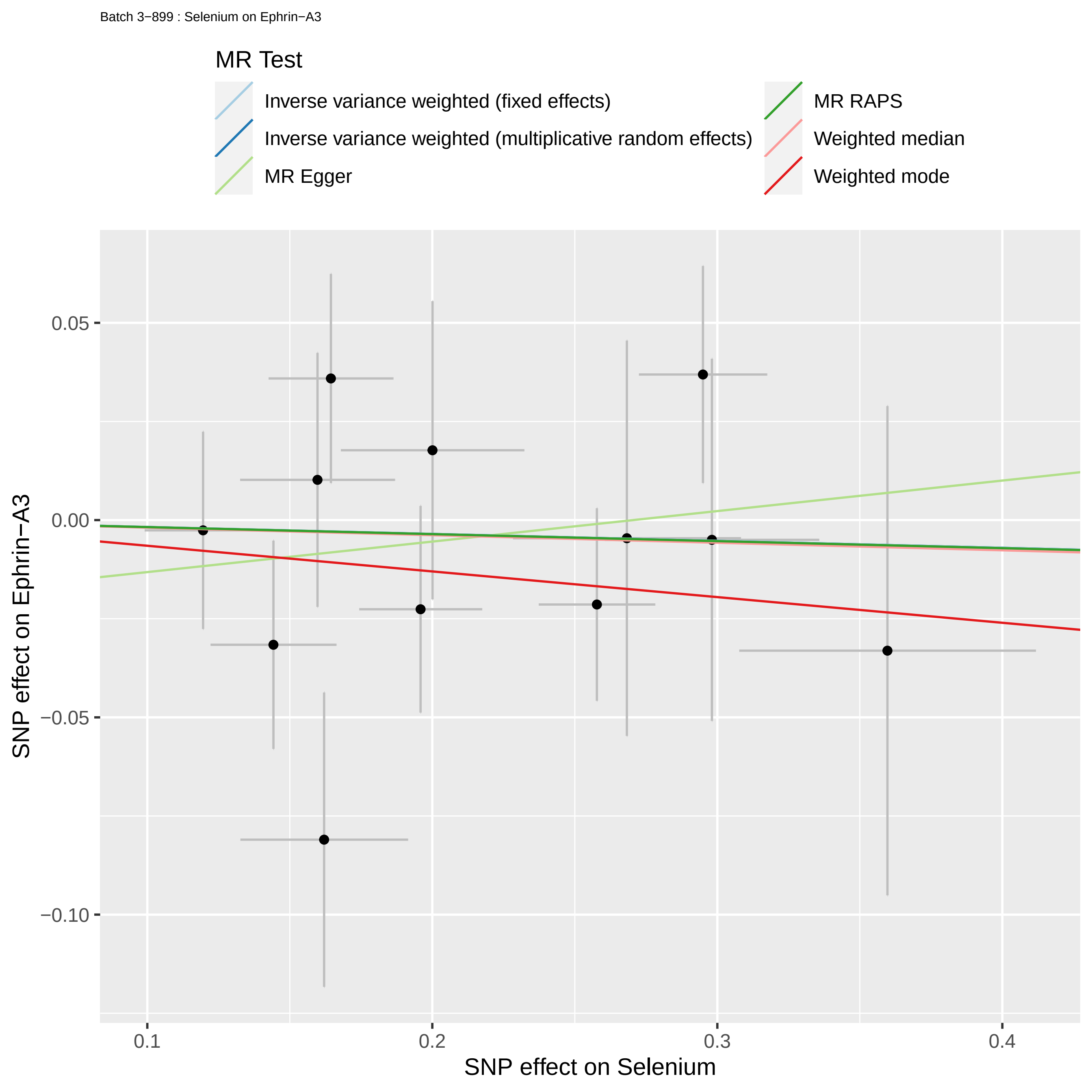

Batch 3−899 : Selenium on Ephrin−A3
MR Test
Inverse variance weighted (fixed effects)
Inverse variance weighted (multiplicative random effects)
MR Egger
MR RAPS
Weighted median
Weighted mode
0.05
0.00
−0.05
−0.10
SNP effect on Ephrin−A3
0.1
0.2
0.3
0.4
SNP effect on Selenium

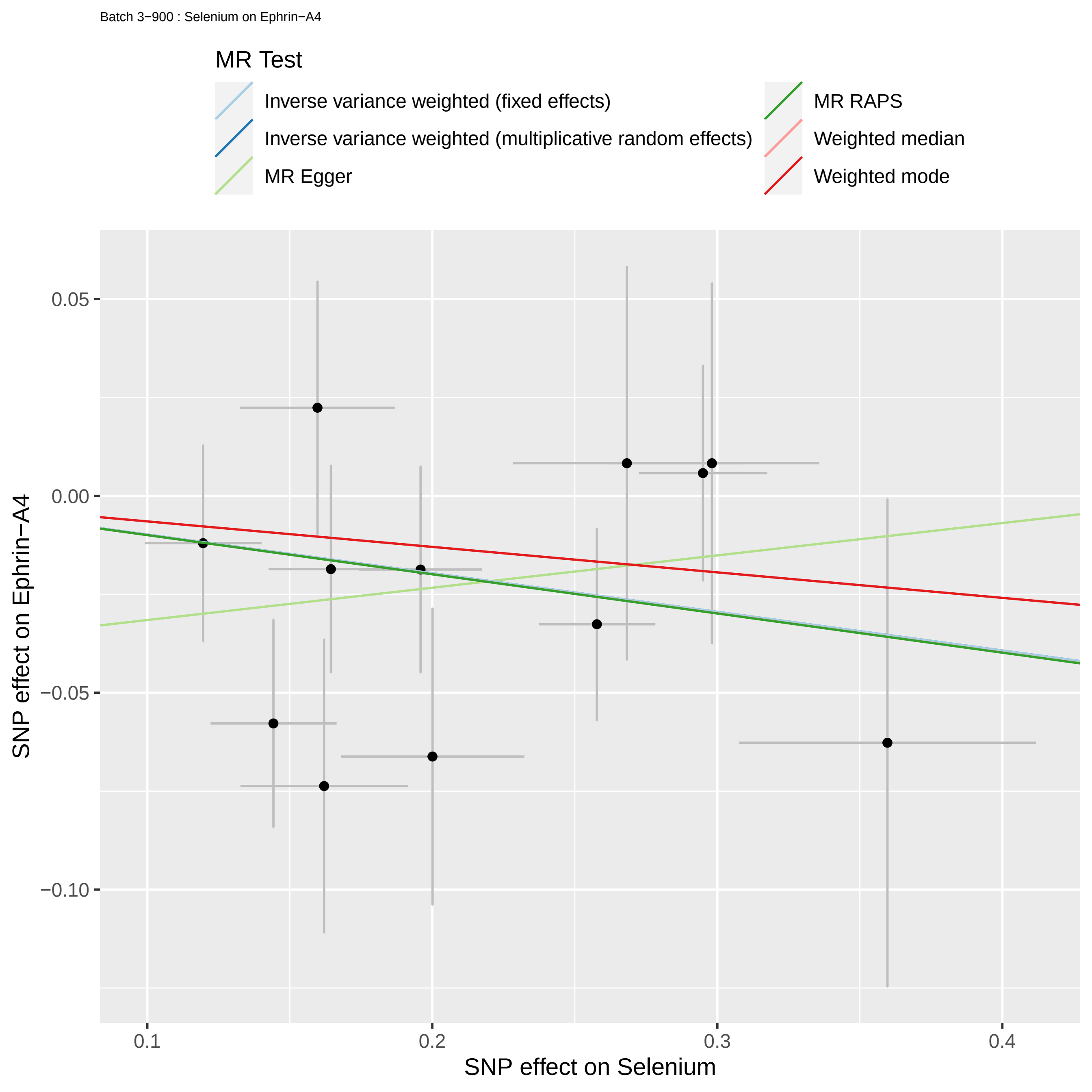

Batch 3−900 : Selenium on Ephrin−A4
MR Test
Inverse variance weighted (fixed effects)
Inverse variance weighted (multiplicative random effects)
MR Egger
MR RAPS
Weighted median
Weighted mode
0.05
0.00
−0.05
−0.10
SNP effect on Ephrin−A4
0.1
0.2
0.3
0.4
SNP effect on Selenium

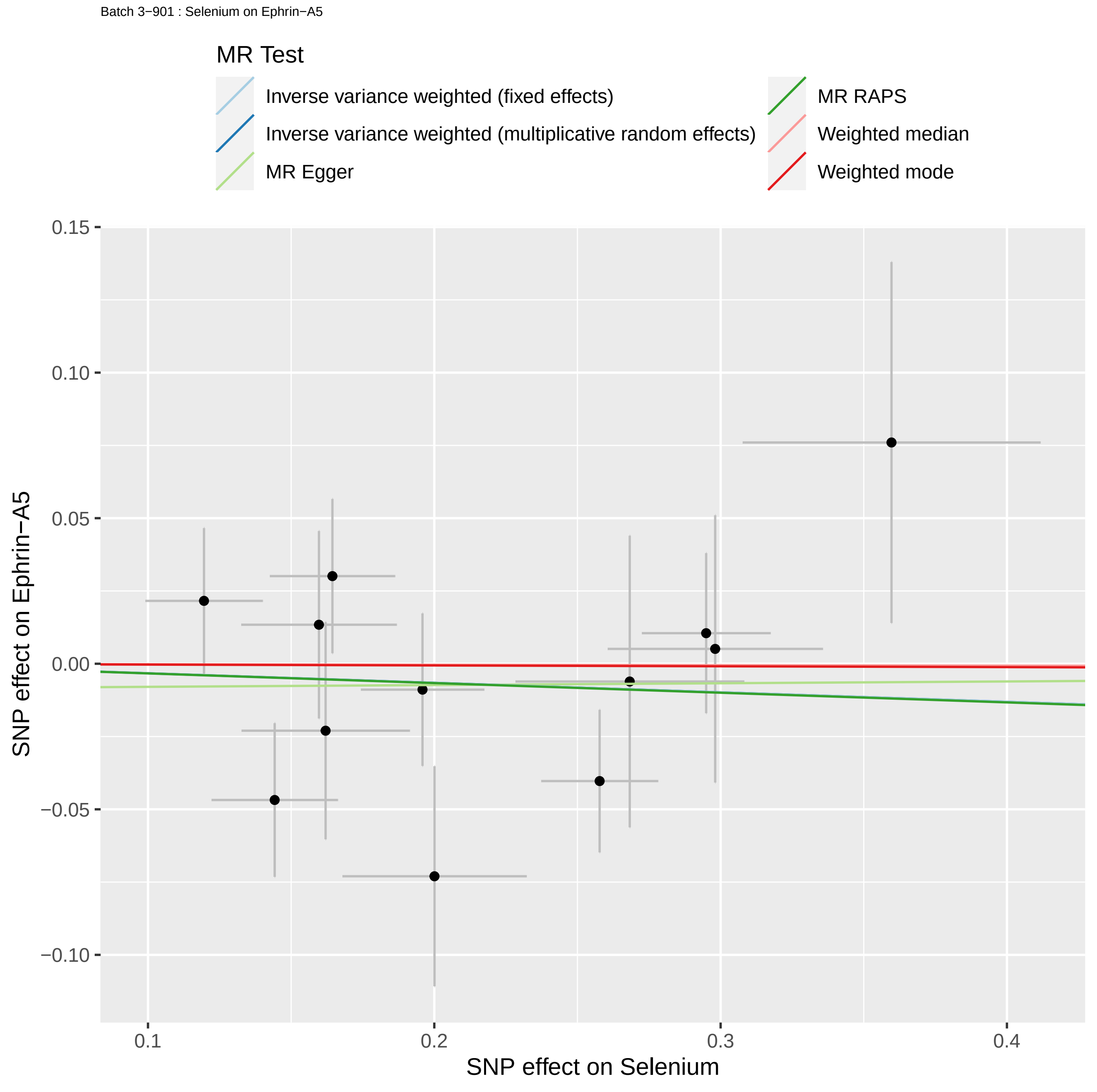
Batch 3−901 : Selenium on Ephrin−A5
MR Test
Inverse variance weighted (fixed effects)
Inverse variance weighted (multiplicative random effects)
MR Egger
MR RAPS
Weighted median
Weighted mode
0.15
0.10
0.05
0.00
−0.05
−0.10
0.1
0.2
0.3
0.4
SNP effect on Ephrin−A5
SNP effect on Selenium

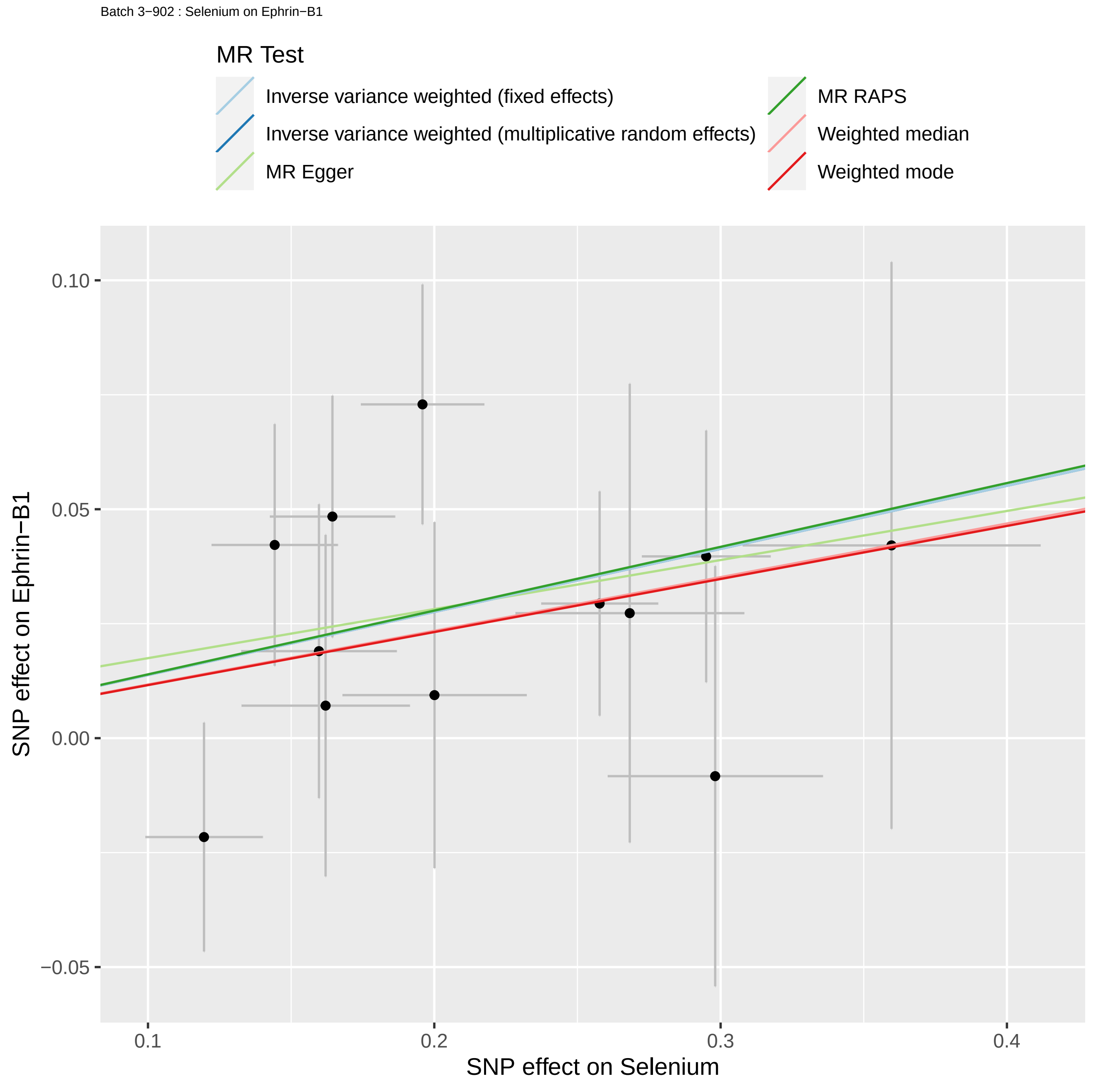
Batch 3−902 : Selenium on Ephrin−B1
MR Test
Inverse variance weighted (fixed effects)
Inverse variance weighted (multiplicative random effects)
MR Egger
MR RAPS
Weighted median
Weighted mode
SNP effect on Ephrin−B1
0.10
0.05
0.00
−0.05
0.1
0.2
0.3
0.4
SNP effect on Selenium

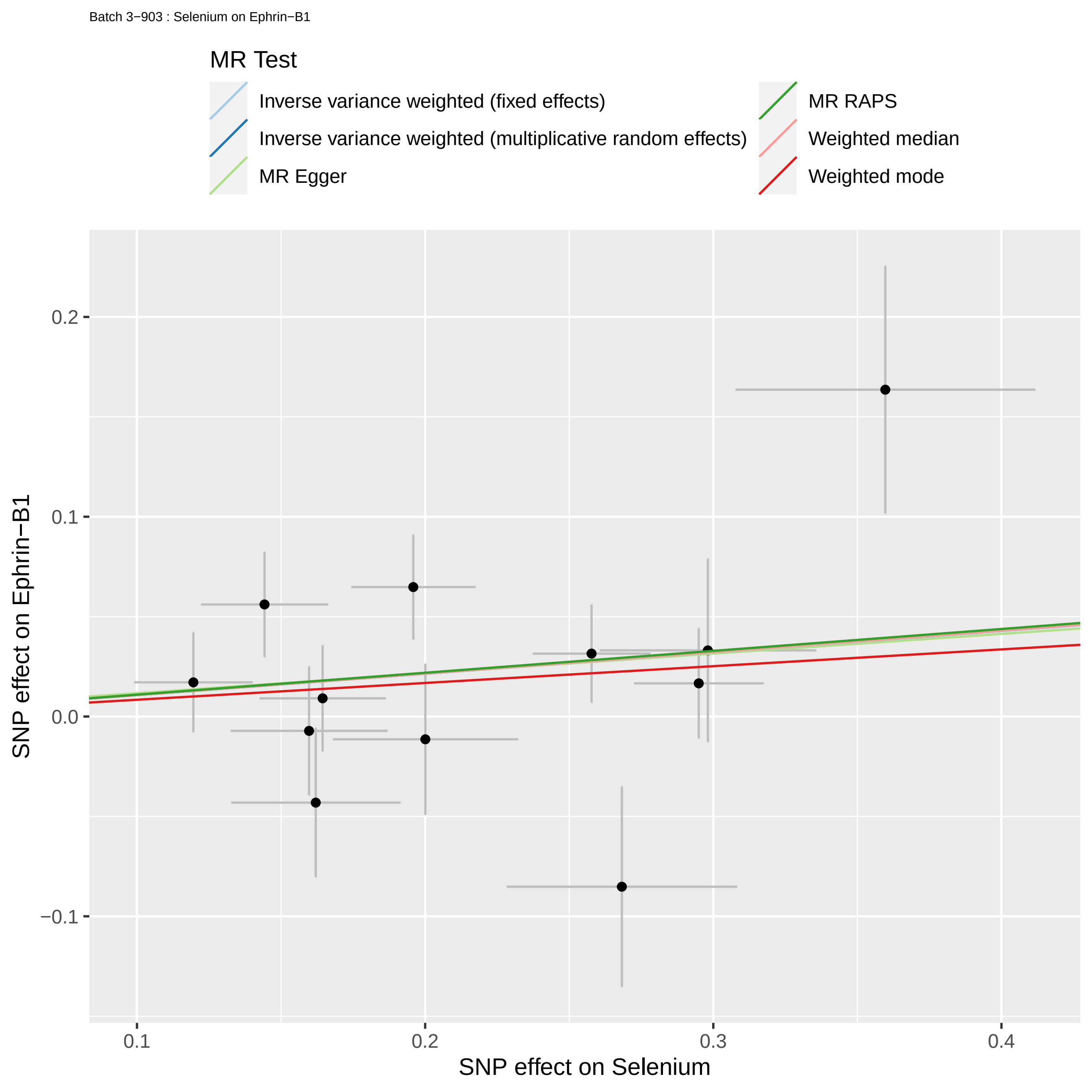
Batch 3−903 : Selenium on Ephrin−B1
MR Test
Inverse variance weighted (fixed effects)
Inverse variance weighted (multiplicative random effects)
MR Egger
MR RAPS
Weighted median
Weighted mode
0.2
0.1
0.0
−0.1
0.1
0.2
0.3
0.4
SNP effect on Ephrin−B1
SNP effect on Selenium

Batch 3−904 : Selenium on Ephrin−B2

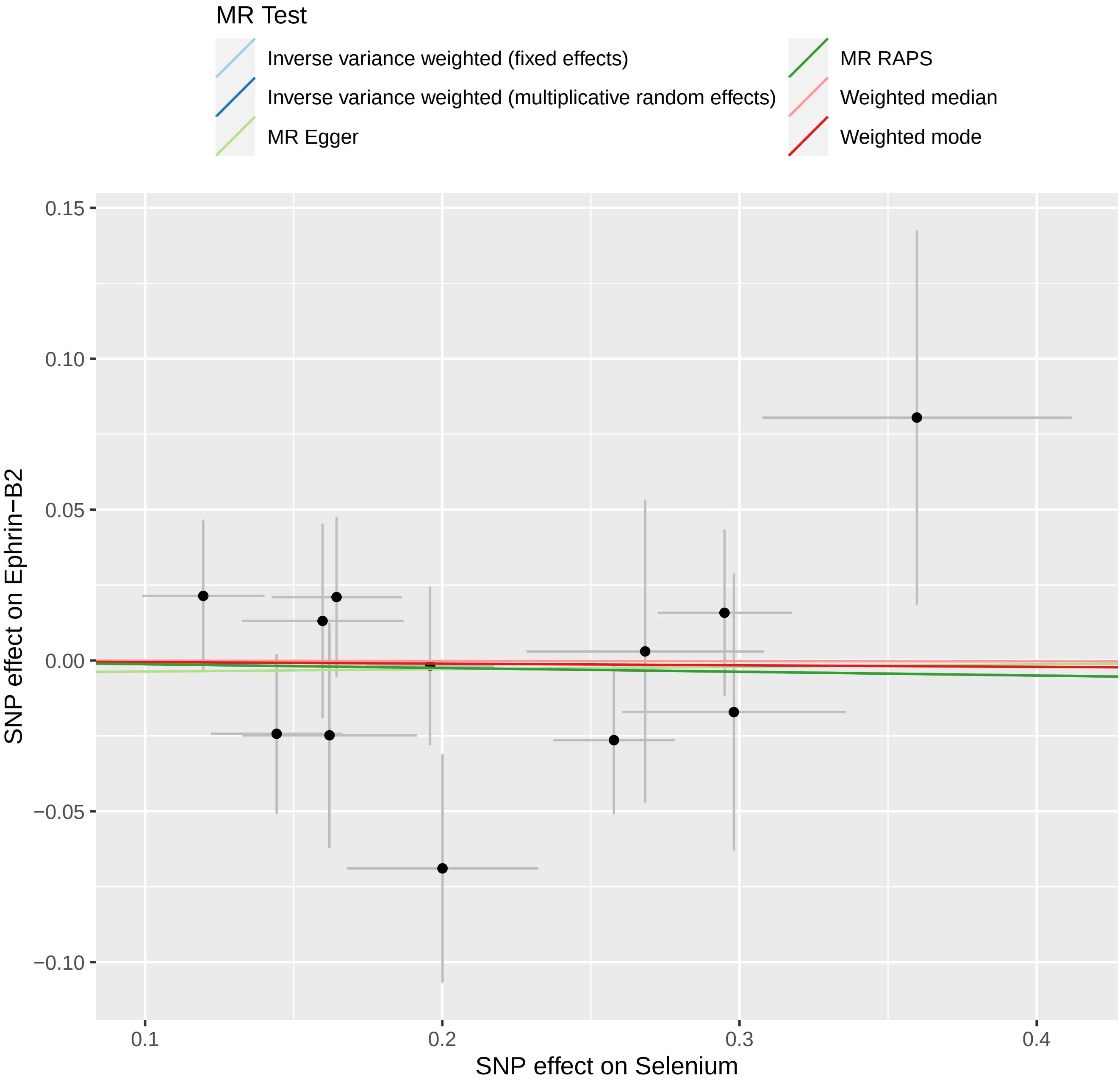

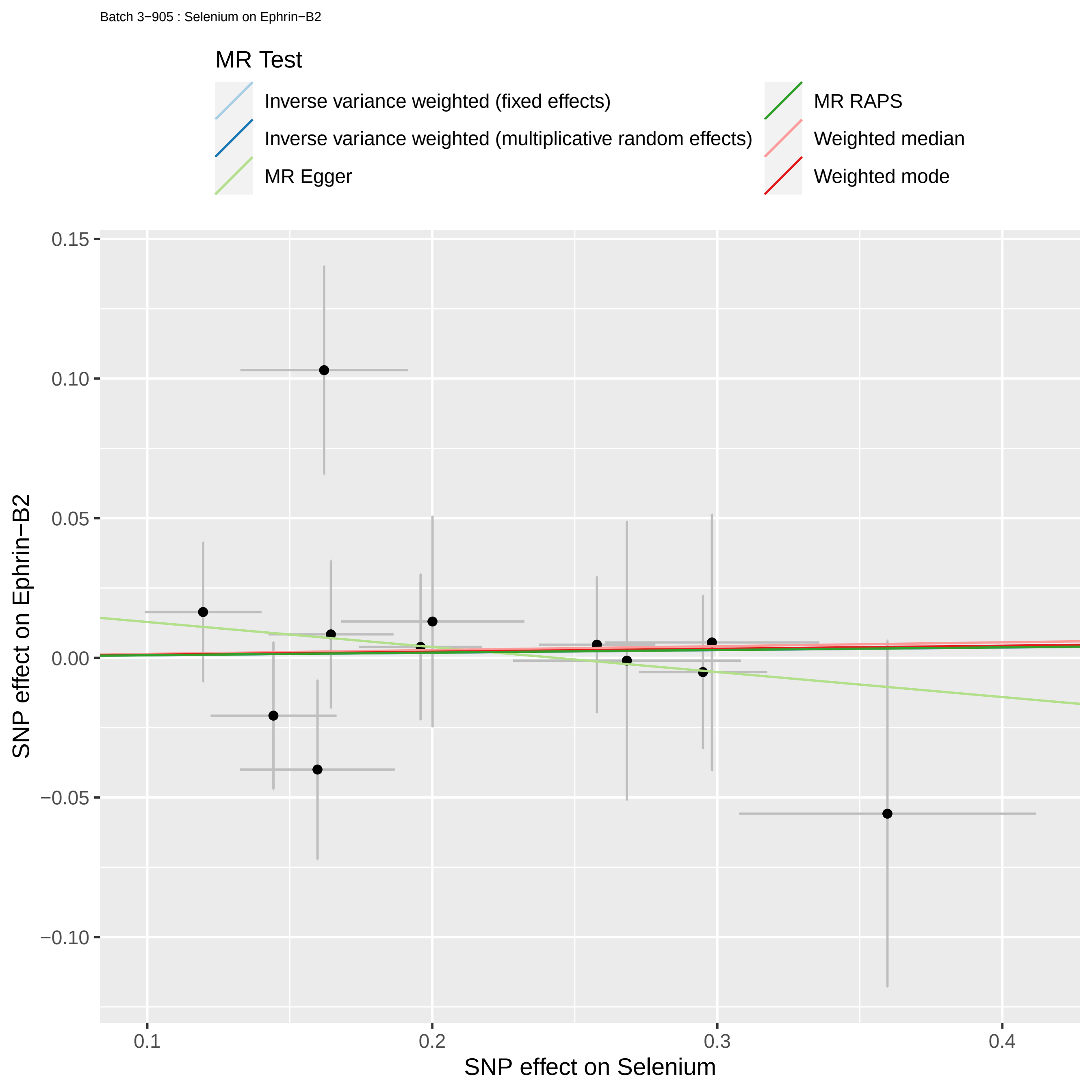
Batch 3−905 : Selenium on Ephrin−B2
MR Test
Inverse variance weighted (fixed effects)
Inverse variance weighted (multiplicative random effects)
MR Egger
MR RAPS
Weighted median
Weighted mode
0.15
0.10
0.05
0.00
−0.05
−0.10
SNP effect on Ephrin−B2
0.1
0.2
0.3
0.4
SNP effect on Selenium

Batch 3−906 : Selenium on Ephrin−B3

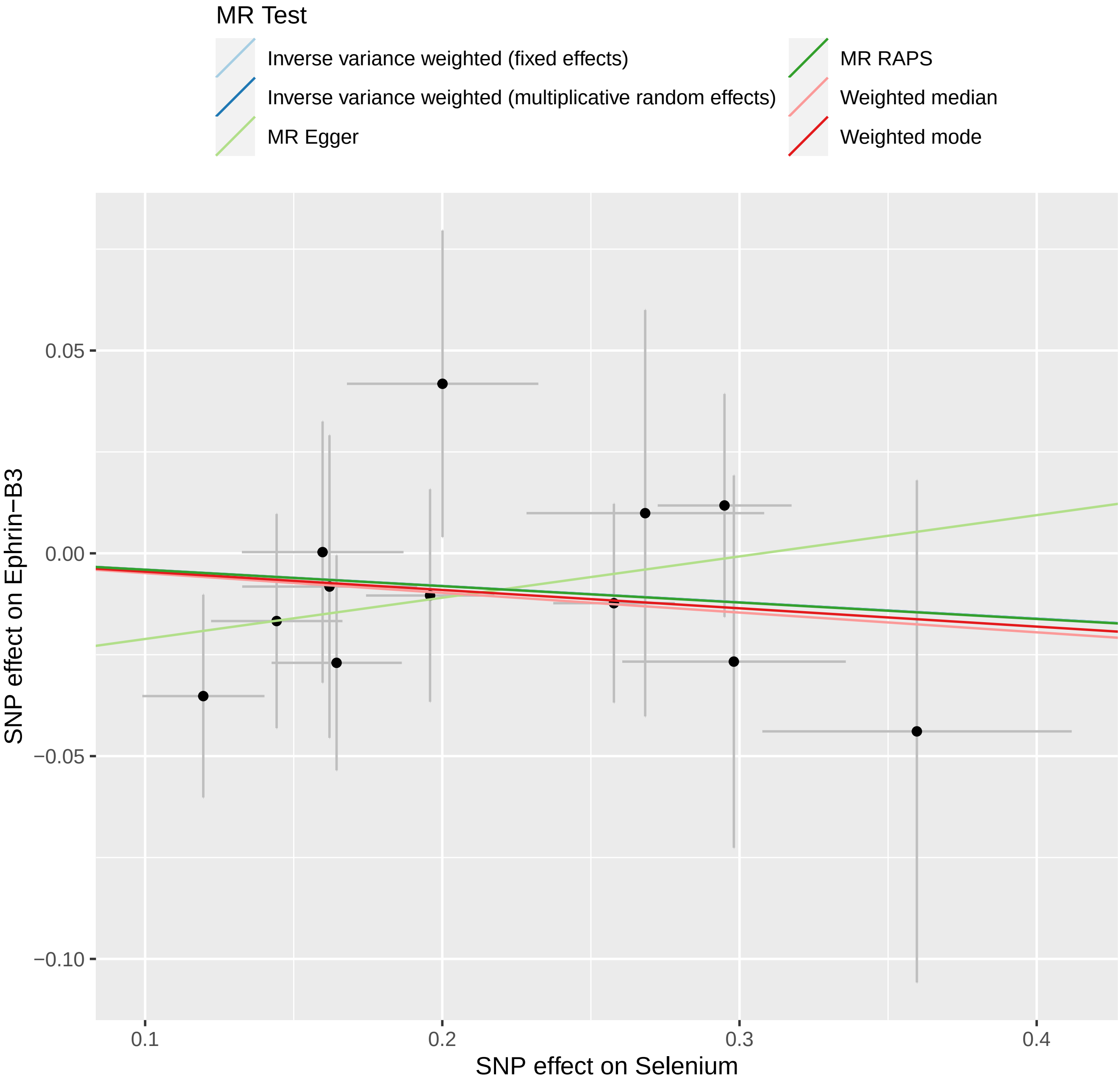

Batch 3−907 : Selenium on Ephrin−B3

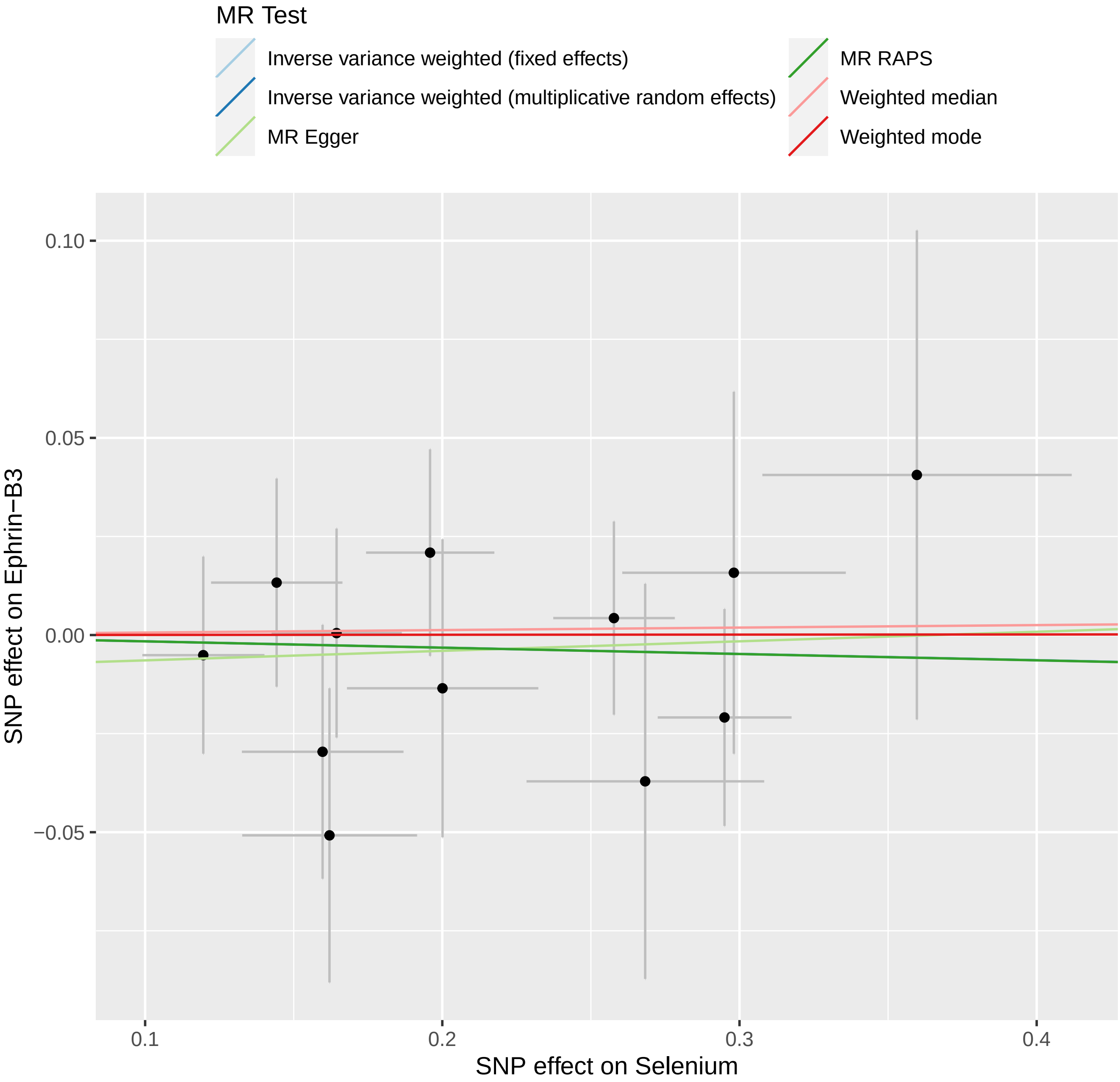

Batch 3−908 : Selenium on Epidermal growth factor

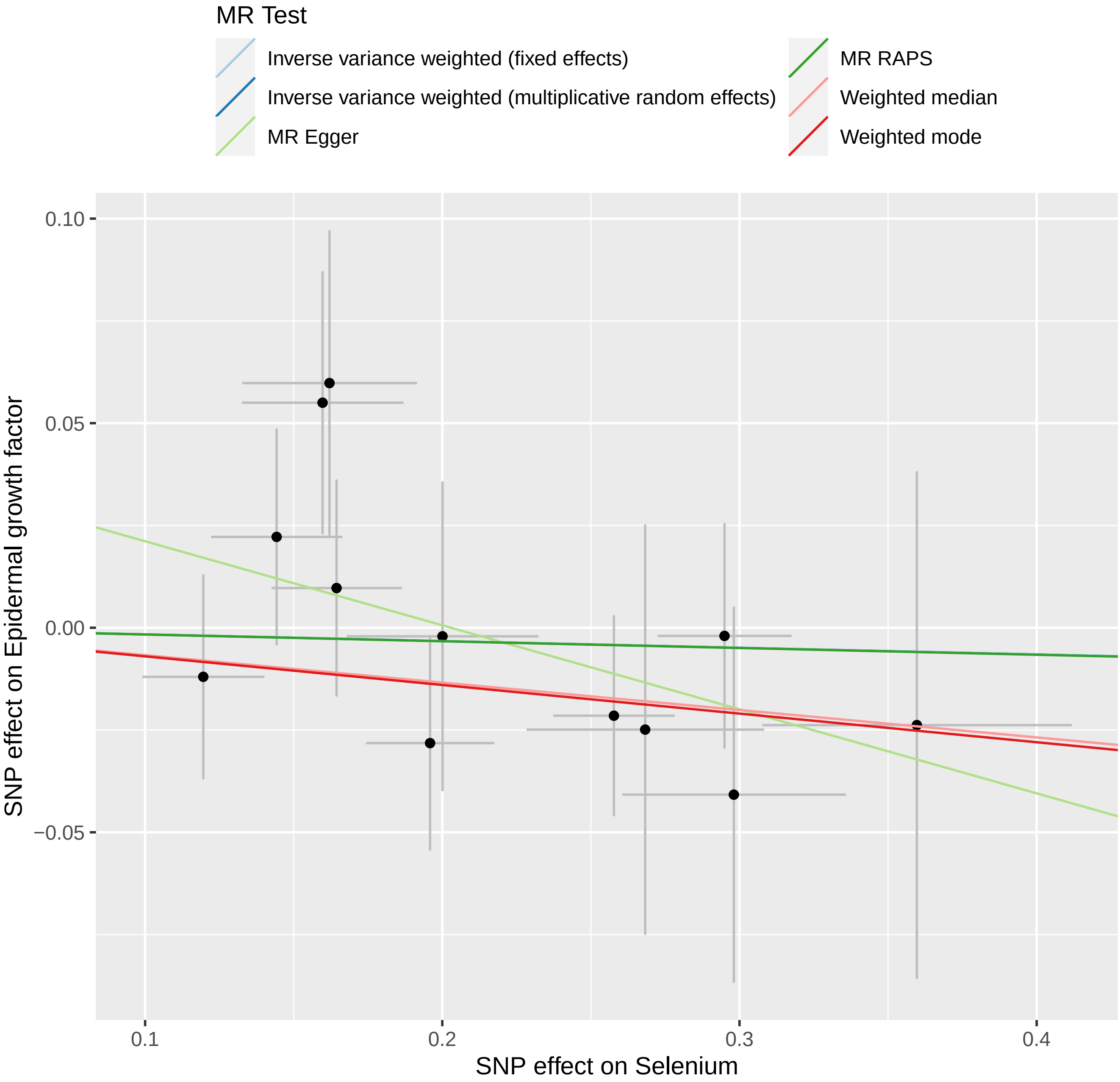

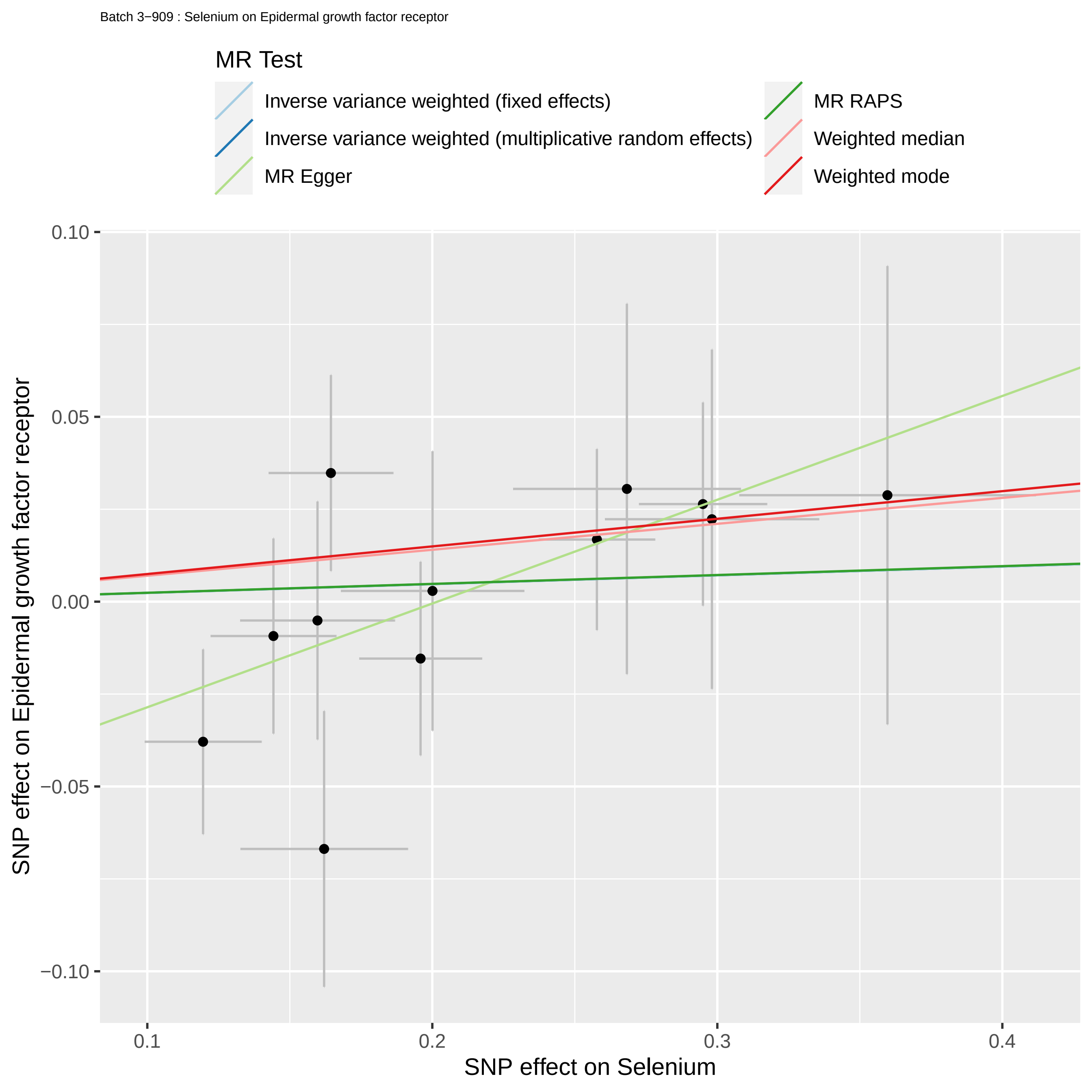
Batch 3−909 : Selenium on Epidermal growth factor receptor
MR Test
Inverse variance weighted (fixed effects)
Inverse variance weighted (multiplicative random effects)
MR Egger
MR RAPS
Weighted median
Weighted mode
0.10
0.05
0.00
−0.05
−0.10
SNP effect on Epidermal growth factor receptor
0.1
0.2
0.3
0.4
SNP effect on Selenium

Batch 3−910 : Selenium on Epidermal growth factor receptor variant III

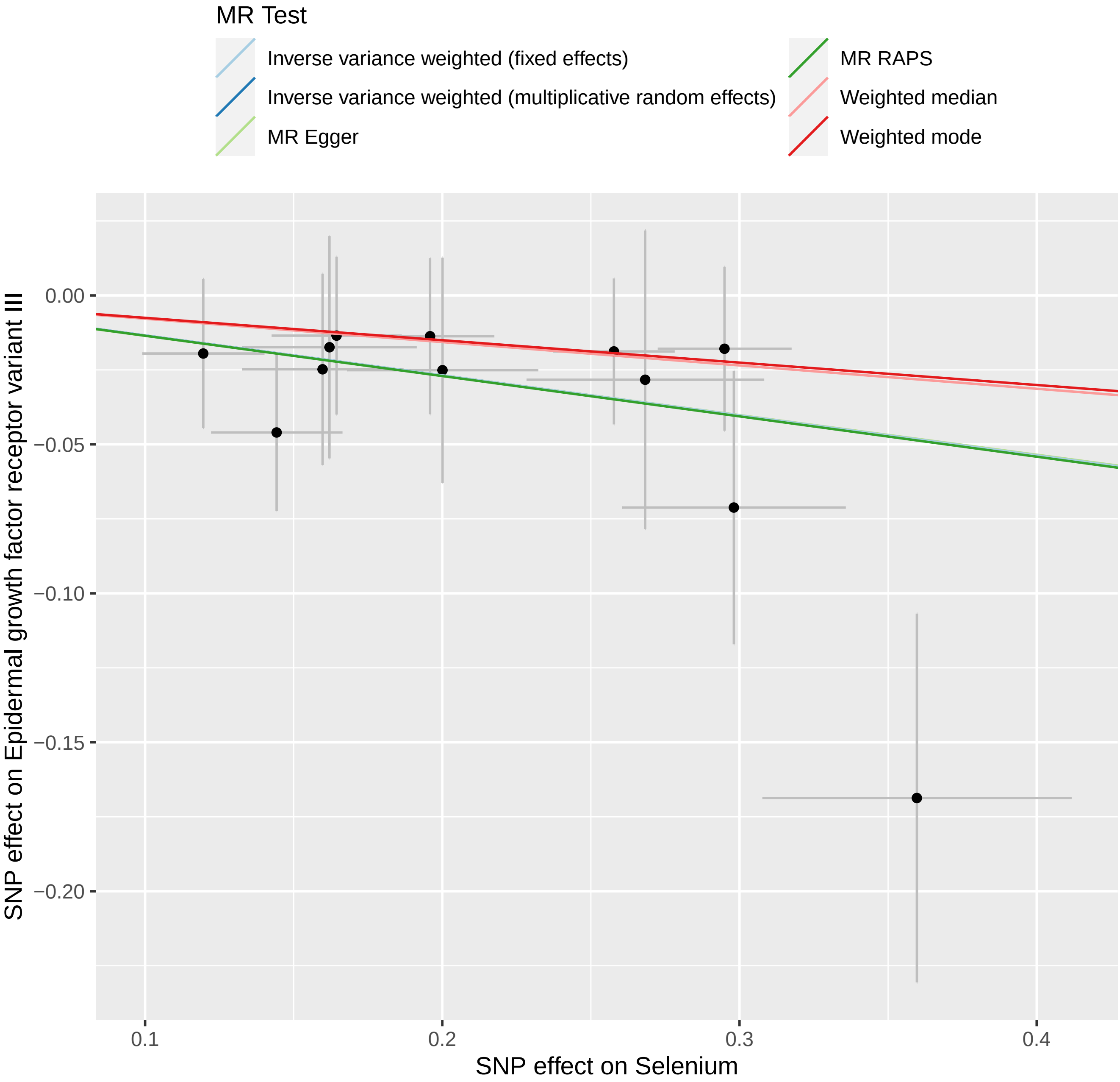

Batch 3−911 : Selenium on EH domain−binding protein 1

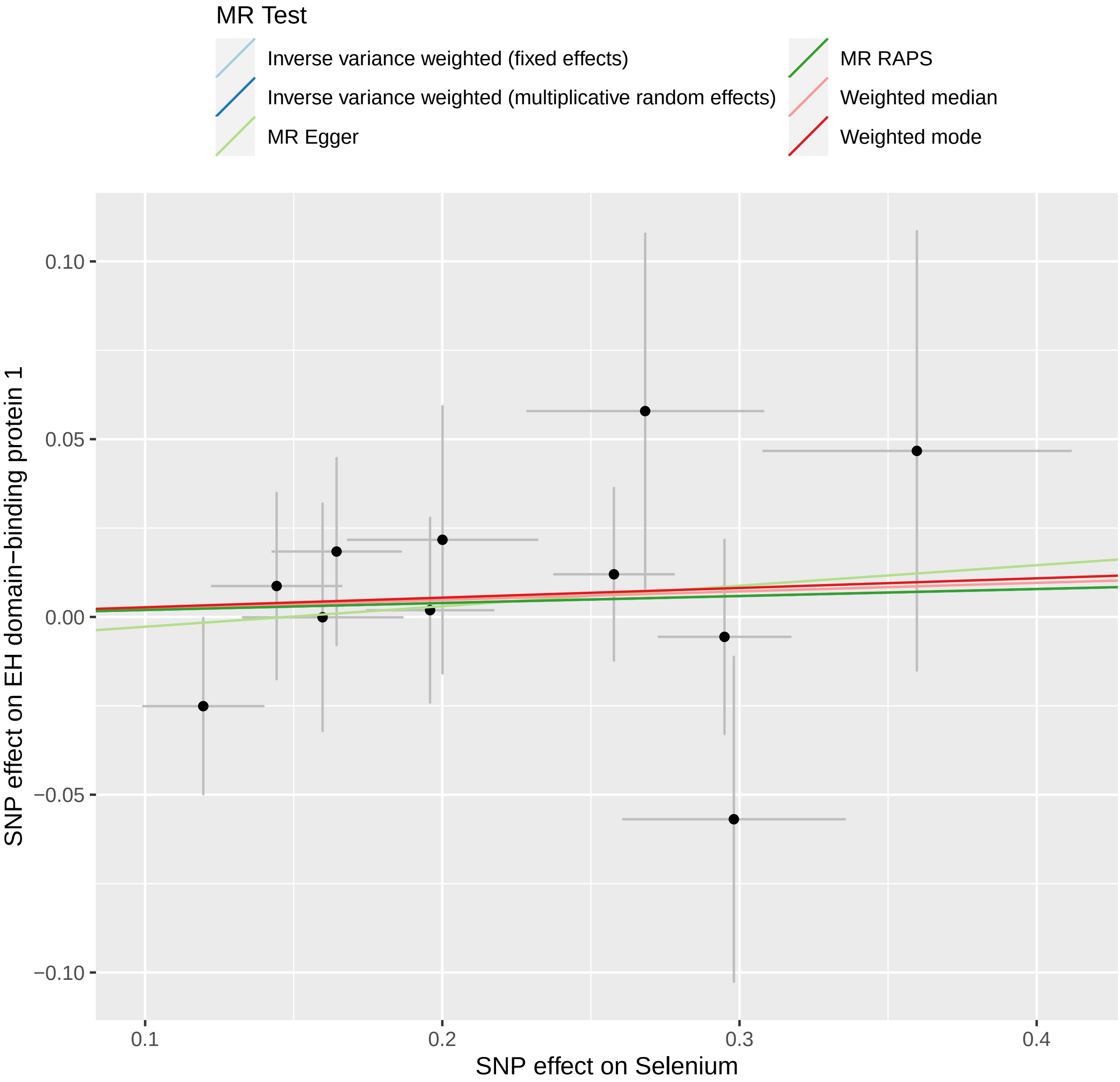

Batch 3−912 : Selenium on EH domain−containing protein 4

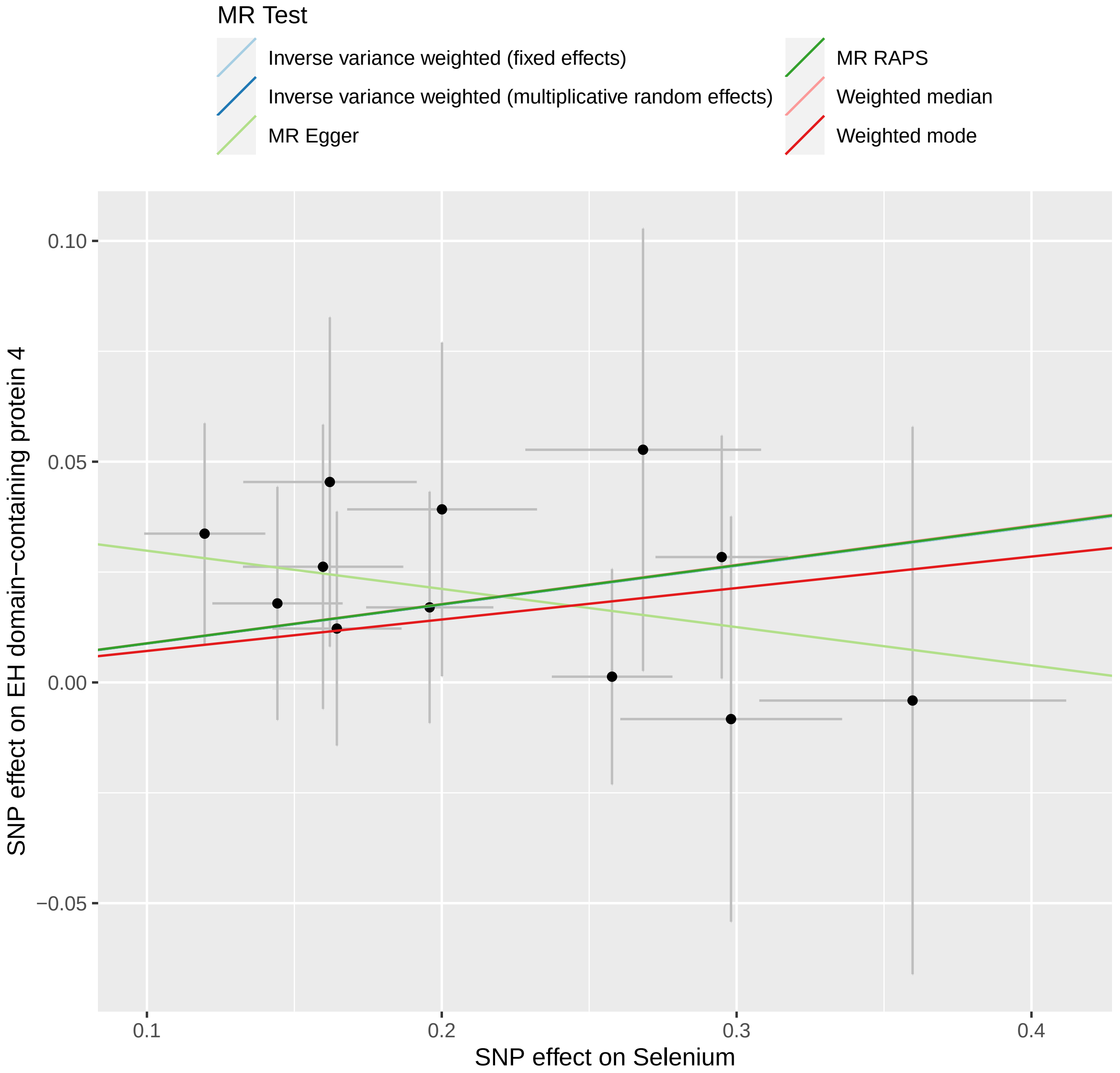

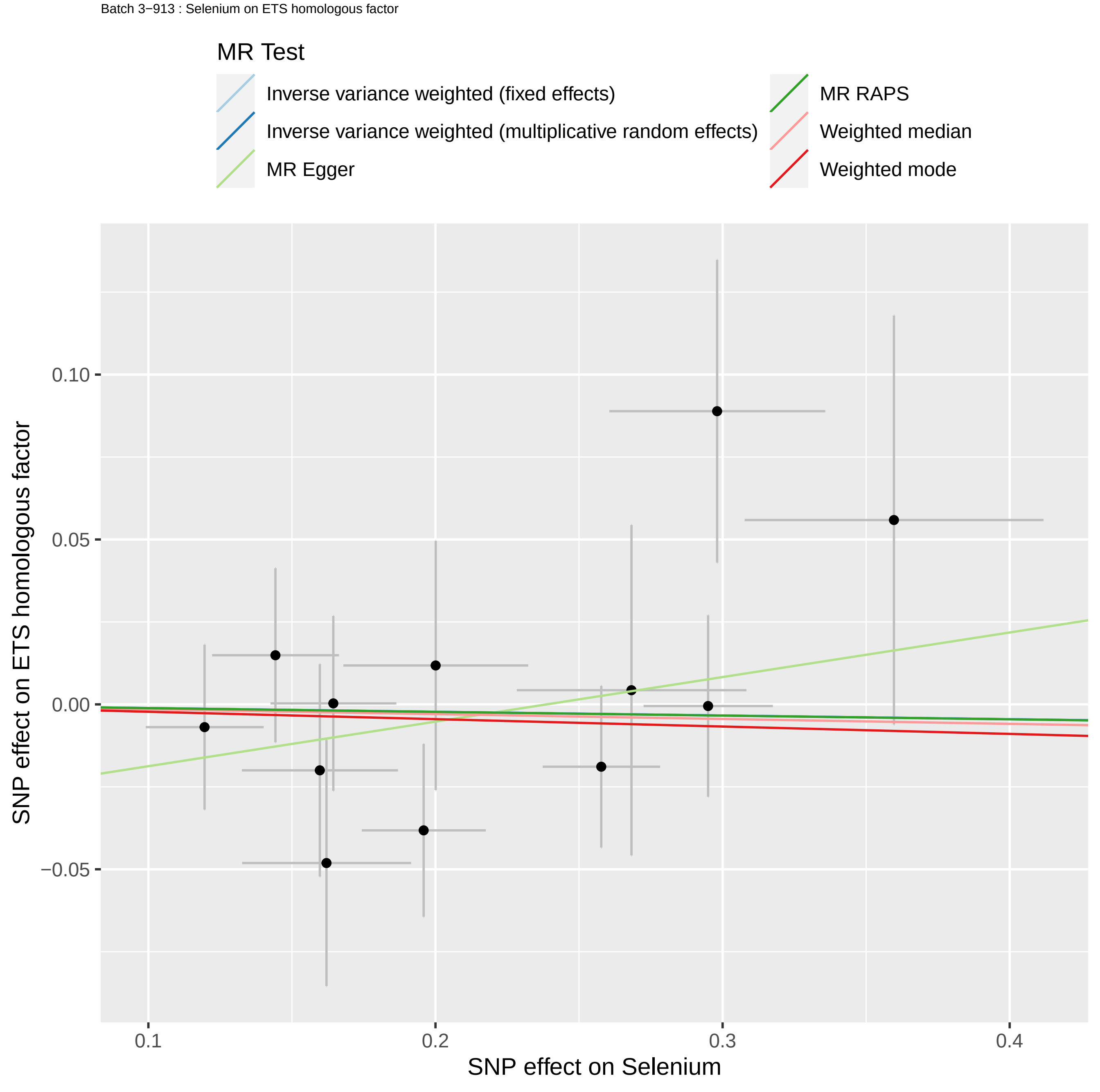

Batch 3−913 : Selenium on ETS homologous factor
MR Test
Inverse variance weighted (fixed effects)
Inverse variance weighted (multiplicative random effects)
MR Egger
MR RAPS
Weighted median
Weighted mode
0.10
0.05
0.00
−0.05
0.1
0.2
0.3
0.4
SNP effect on Selenium
SNP effect on ETS homologous factor

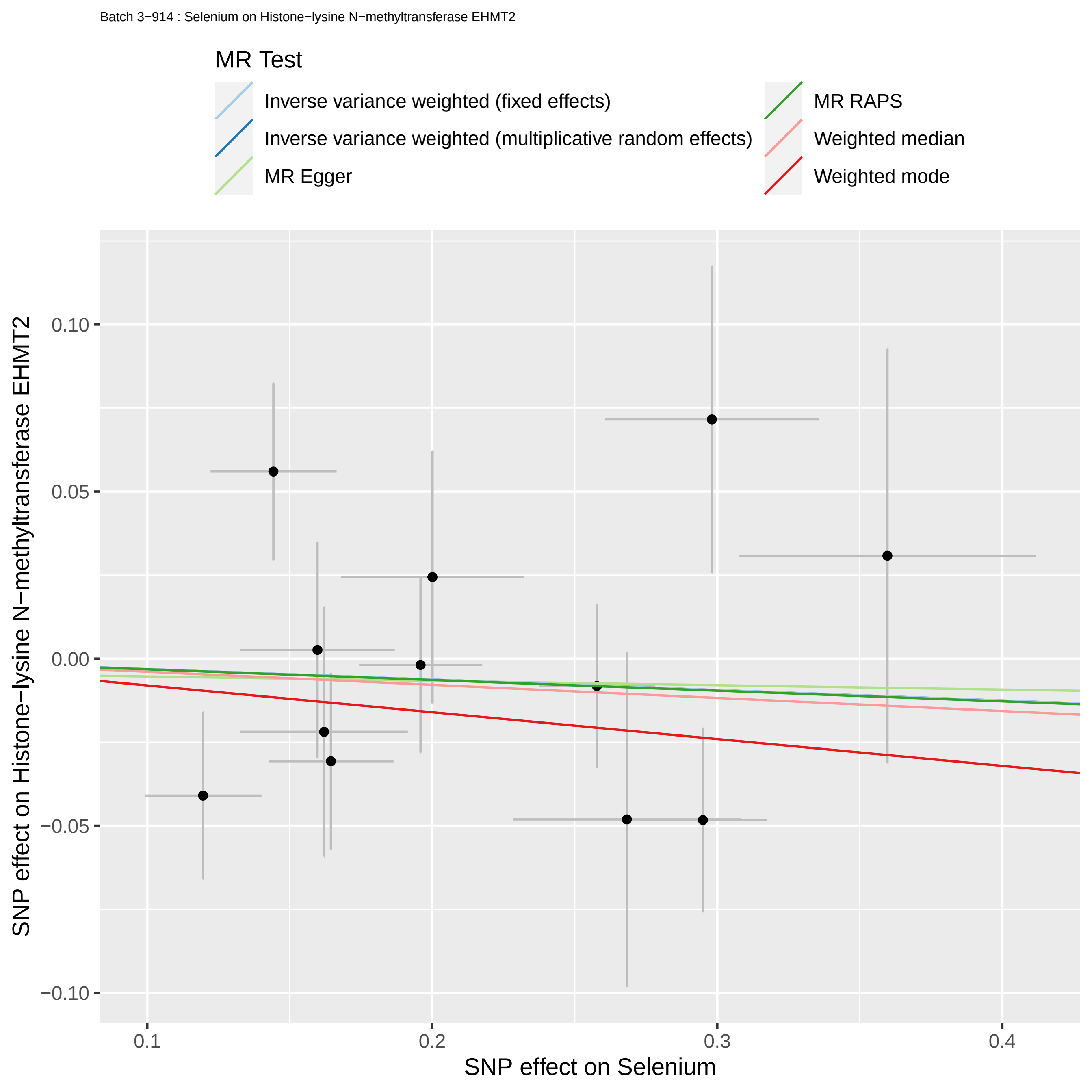

Batch 3−914 : Selenium on Histone−lysine N−methyltransferase EHMT2
MR Test
Inverse variance weighted (fixed effects)
Inverse variance weighted (multiplicative random effects)
MR Egger
MR RAPS
Weighted median
Weighted mode
SNP effect on Histone−lysine N−methyltransferase EHMT2
0.10
0.05
0.00
−0.05
−0.10
0.1
0.2
0.3
0.4
SNP effect on Selenium

Batch 3−915 : Selenium on EP300−interacting inhibitor of differentiation 3

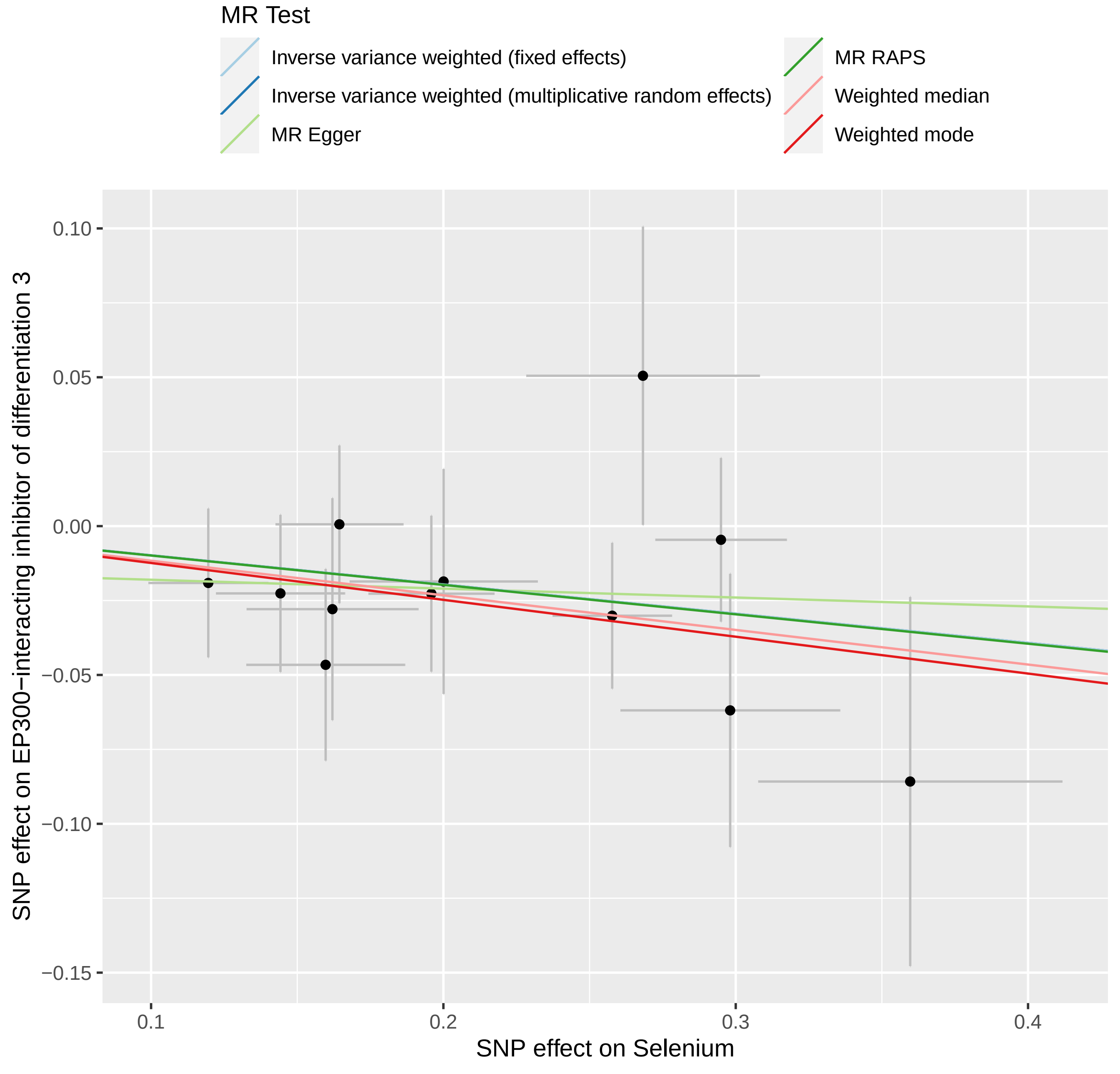

Batch 3−916 : Selenium on Probable RNA−binding protein EIF1AD

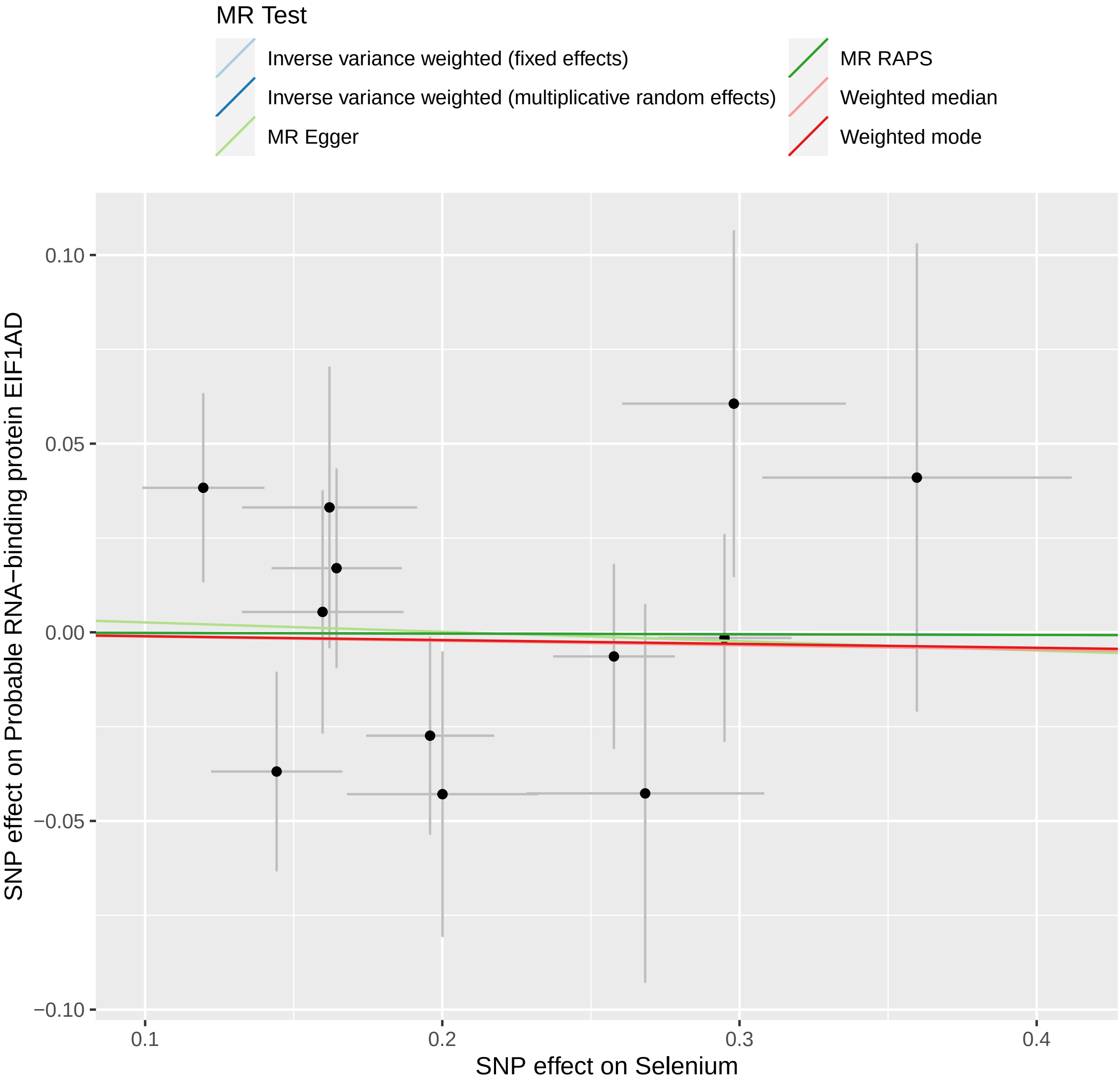

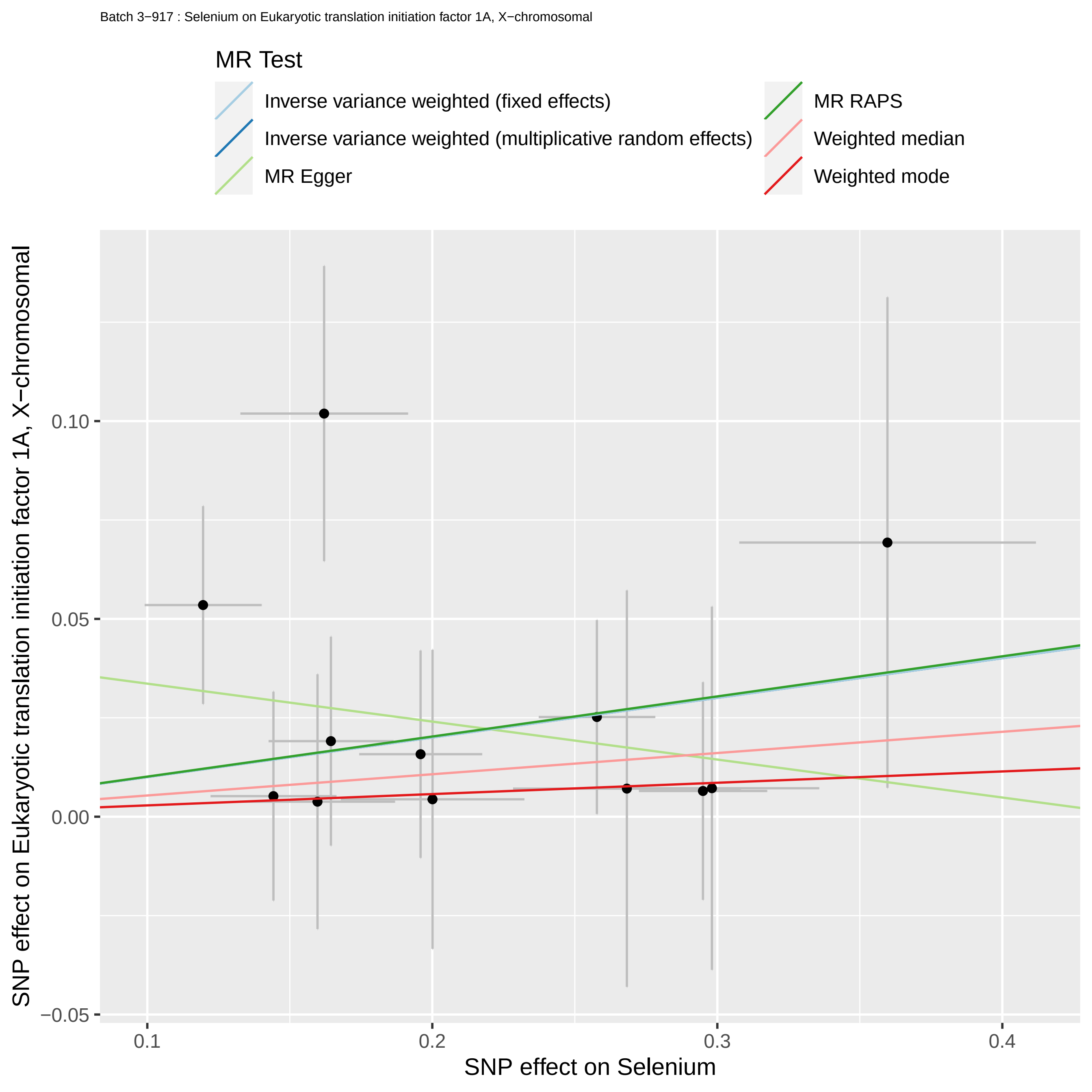
Batch 3−917 : Selenium on Eukaryotic translation initiation factor 1A, X−chromosomal
MR Test
Inverse variance weighted (fixed effects)
Inverse variance weighted (multiplicative random effects)
MR Egger
MR RAPS
Weighted median
Weighted mode
SNP effect on Eukaryotic translation initiation factor 1A, X−chromosomal
0.10
0.05
0.00
−0.05
0.1
0.2
0.3
0.4
SNP effect on Selenium

Batch 3−918 : Selenium on Translation initiation factor eIF−2B subunit alpha

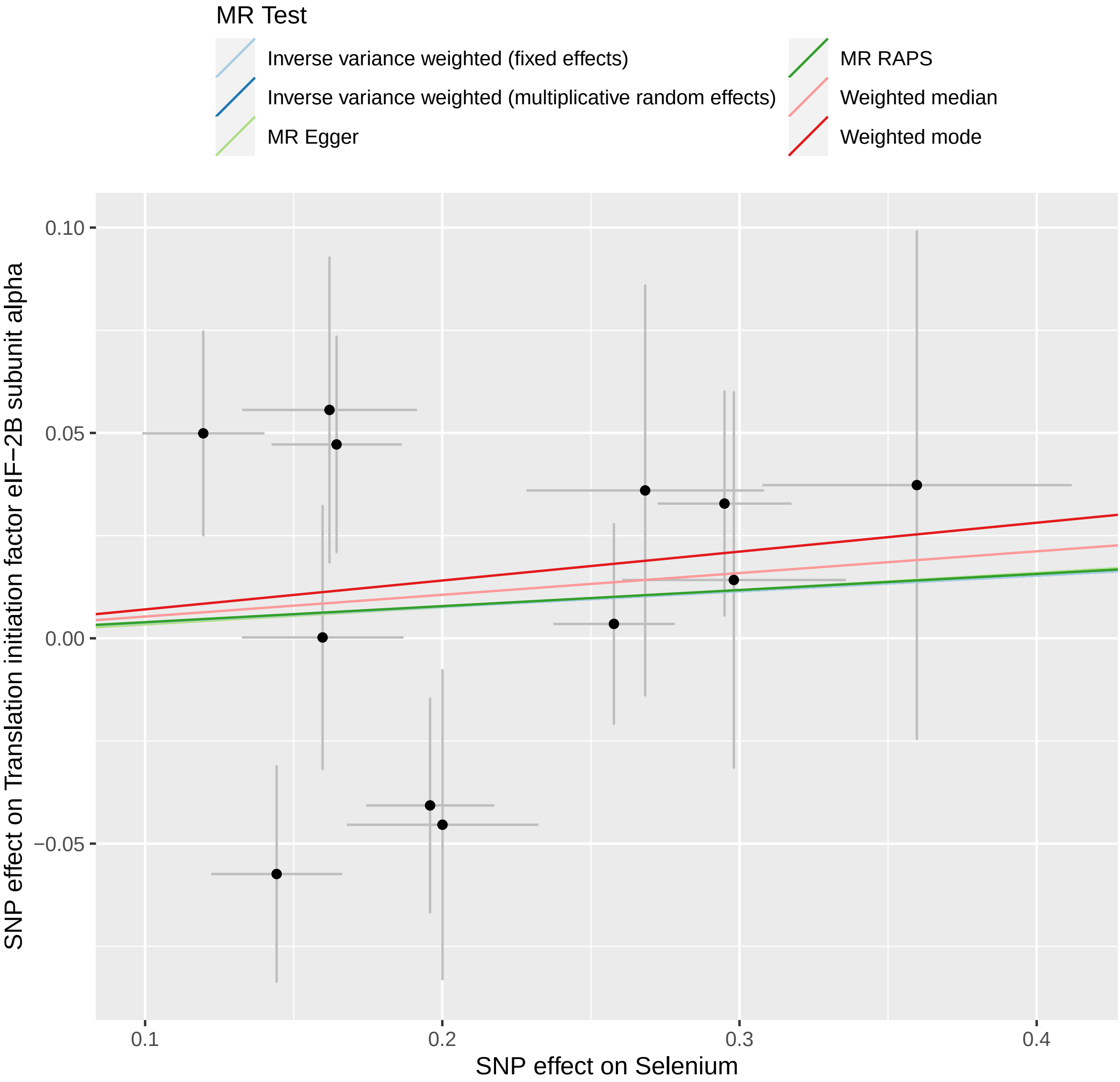

Batch 3−919 : Selenium on Eukaryotic translation initiation factor 3 subunit G

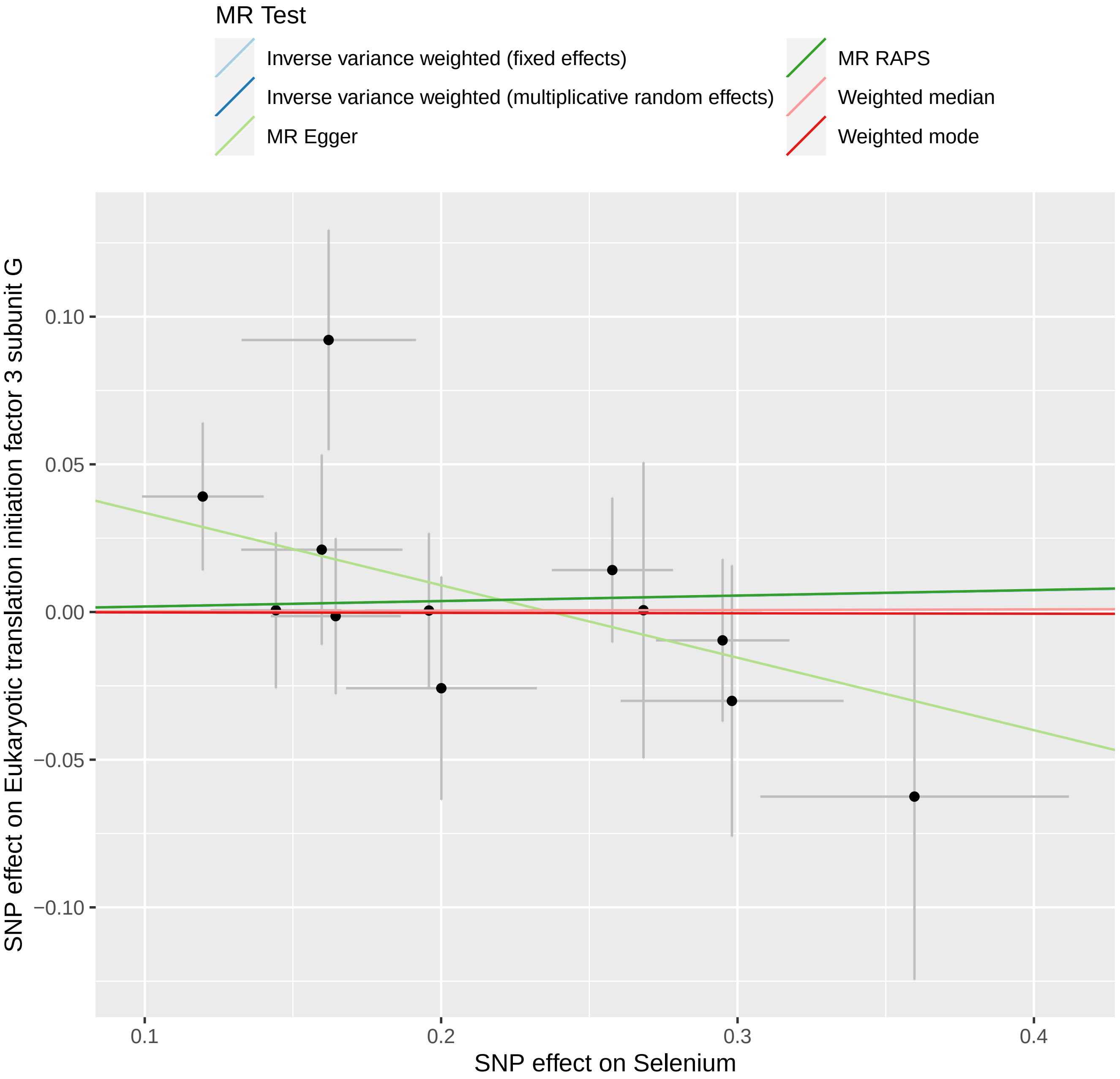

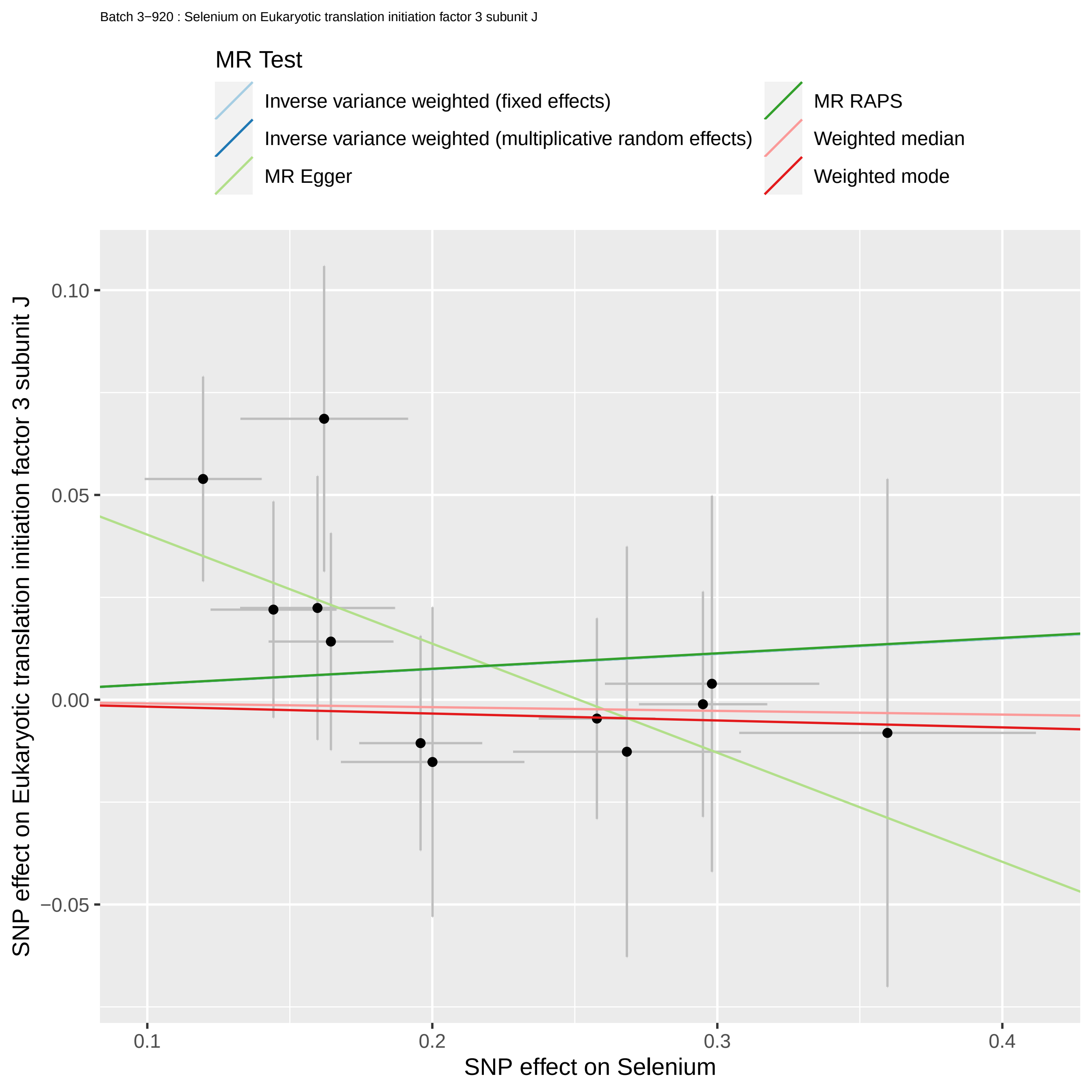

Batch 3−920 : Selenium on Eukaryotic translation initiation factor 3 subunit J
MR Test
Inverse variance weighted (fixed effects)
Inverse variance weighted (multiplicative random effects)
MR Egger
MR RAPS
Weighted median
Weighted mode
0.10
0.05
0.00
−0.05
0.1
0.2
0.3
0.4
SNP effect on Selenium
SNP effect on Eukaryotic translation initiation factor 3 subunit J

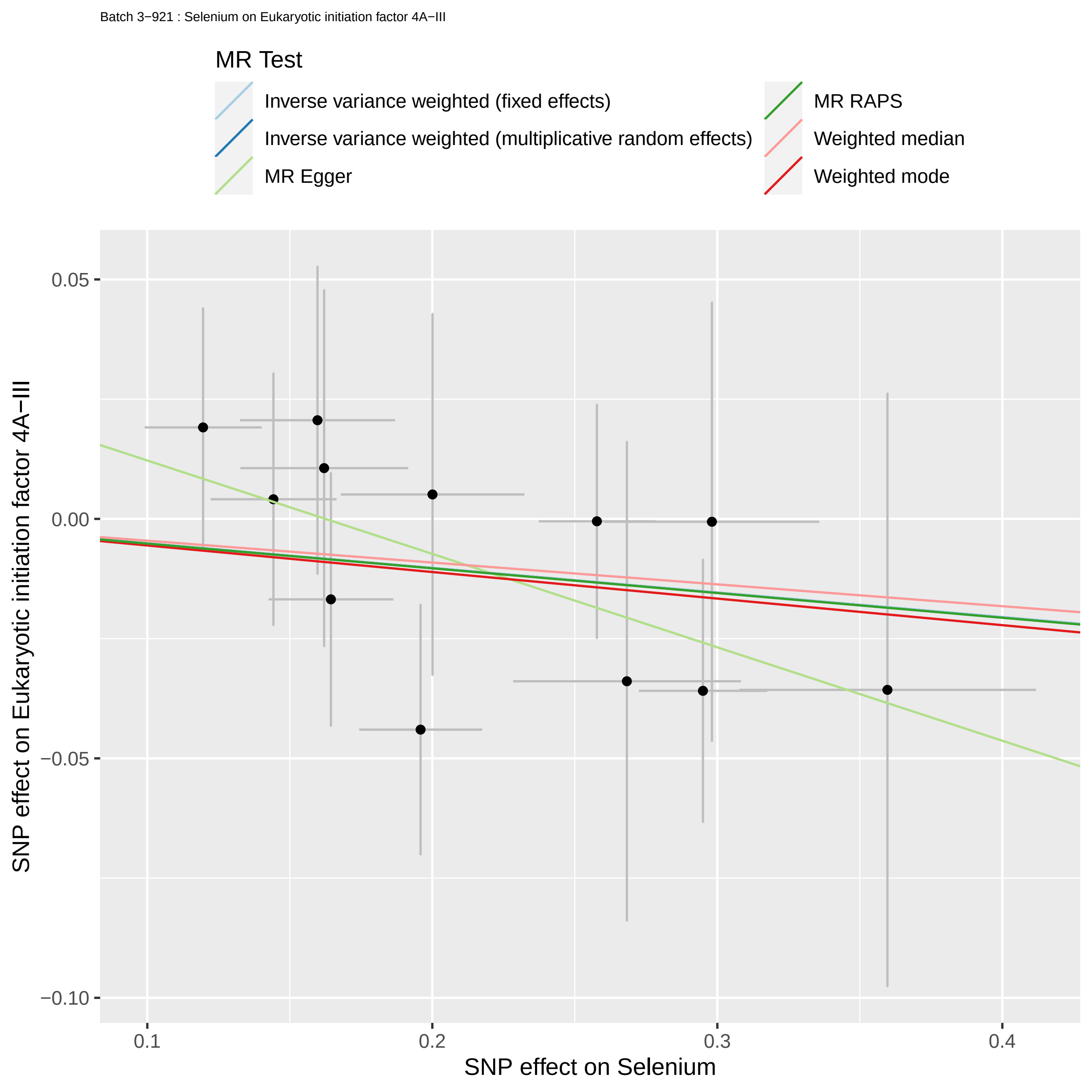
Batch 3−921 : Selenium on Eukaryotic initiation factor 4A−III
MR Test
Inverse variance weighted (fixed effects)
Inverse variance weighted (multiplicative random effects)
MR Egger
MR RAPS
Weighted median
Weighted mode
0.05
0.00
−0.05
−0.10
0.1
0.2
0.3
0.4
SNP effect on Eukaryotic initiation factor 4A−III
SNP effect on Selenium

Batch 3−922 : Selenium on Eukaryotic translation initiation factor 4B

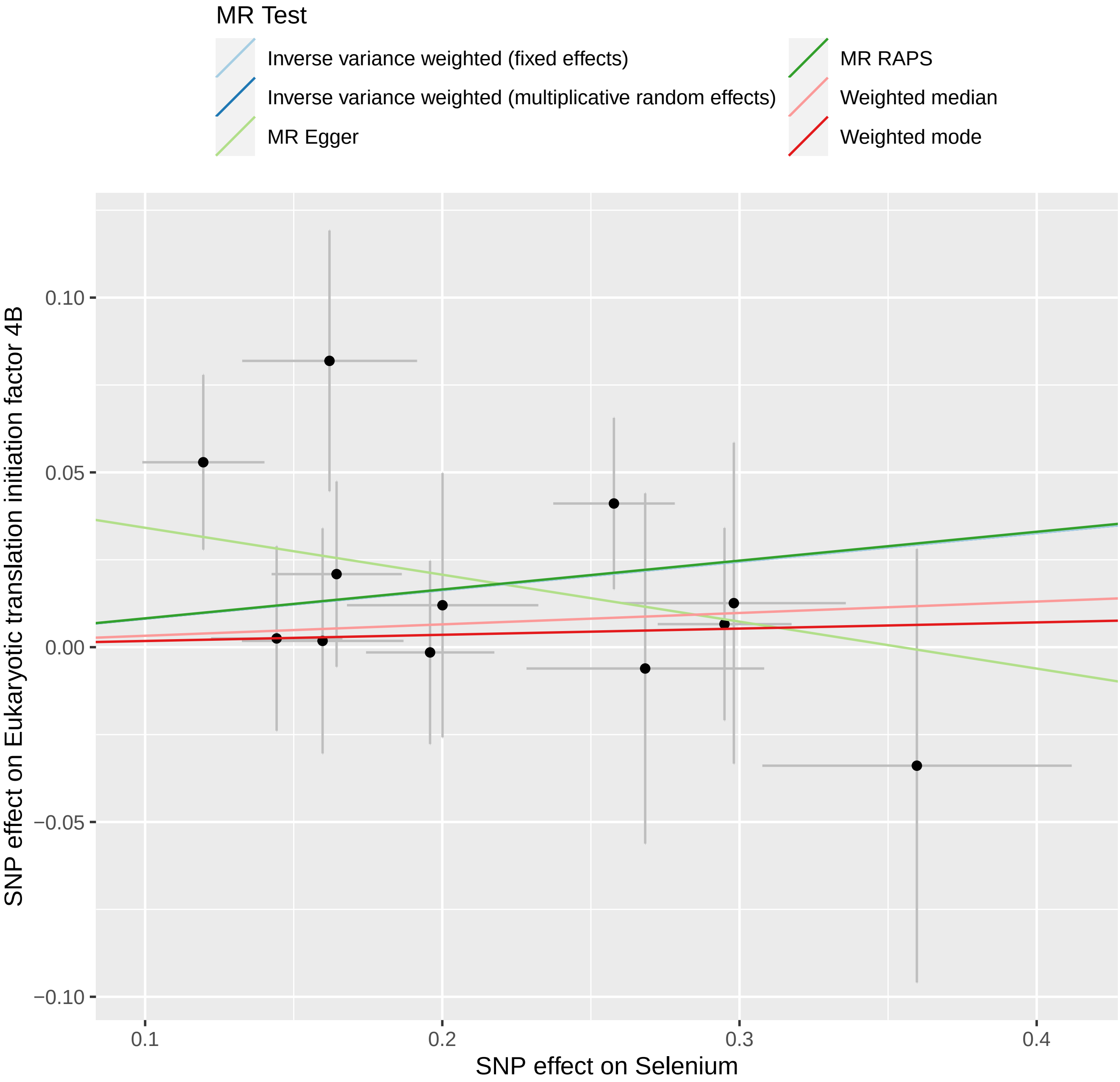

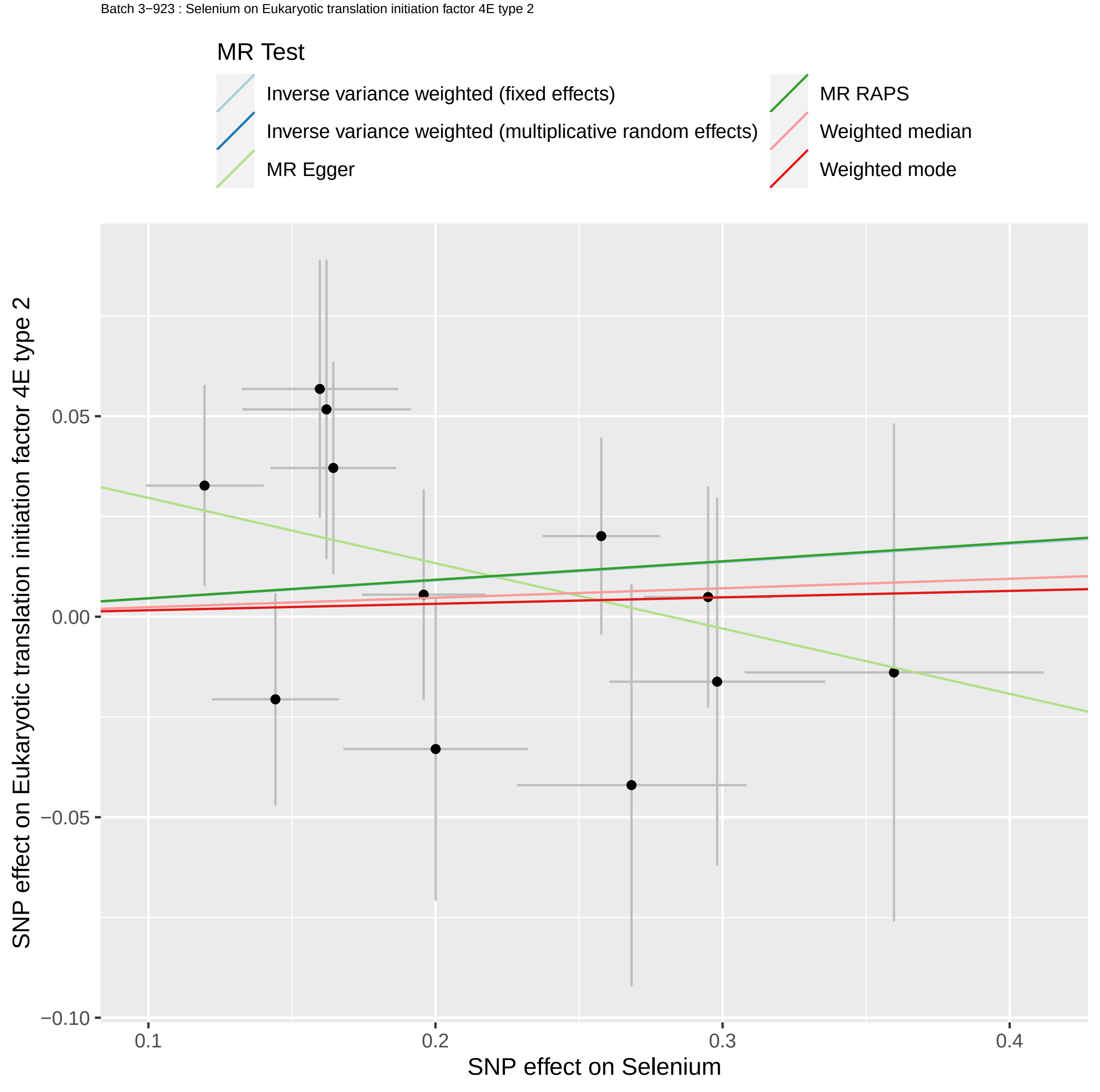
Batch 3−923 : Selenium on Eukaryotic translation initiation factor 4E type 2
MR Test
Inverse variance weighted (fixed effects)
Inverse variance weighted (multiplicative random effects)
MR Egger
MR RAPS
Weighted median
Weighted mode
SNP effect on Eukaryotic translation initiation factor 4E type 2
0.05
0.00
−0.05
−0.10
0.1
0.2
0.3
0.4
SNP effect on Selenium

Batch 3−924 : Selenium on Eukaryotic translation initiation factor 4E−binding protein 2

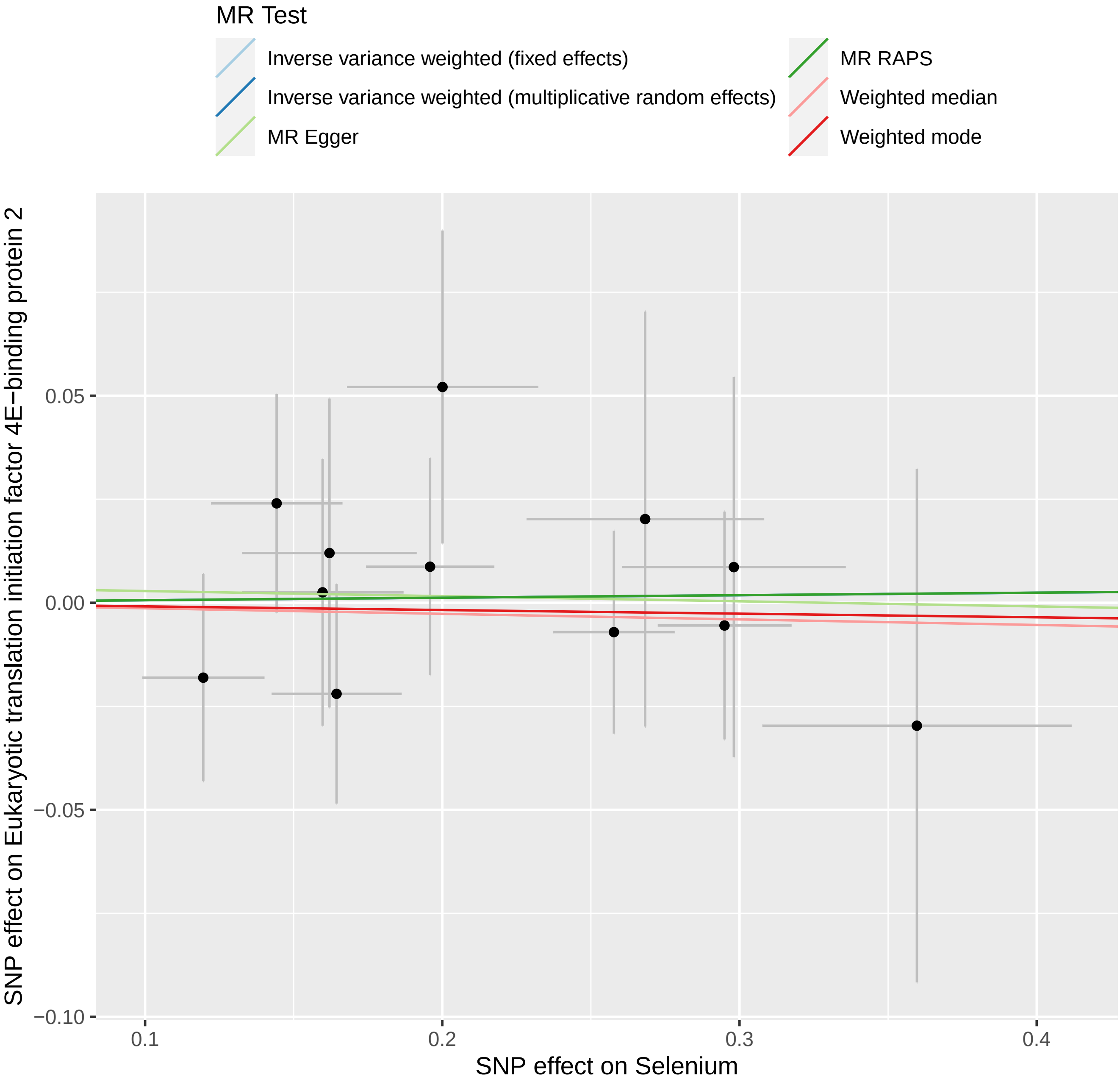

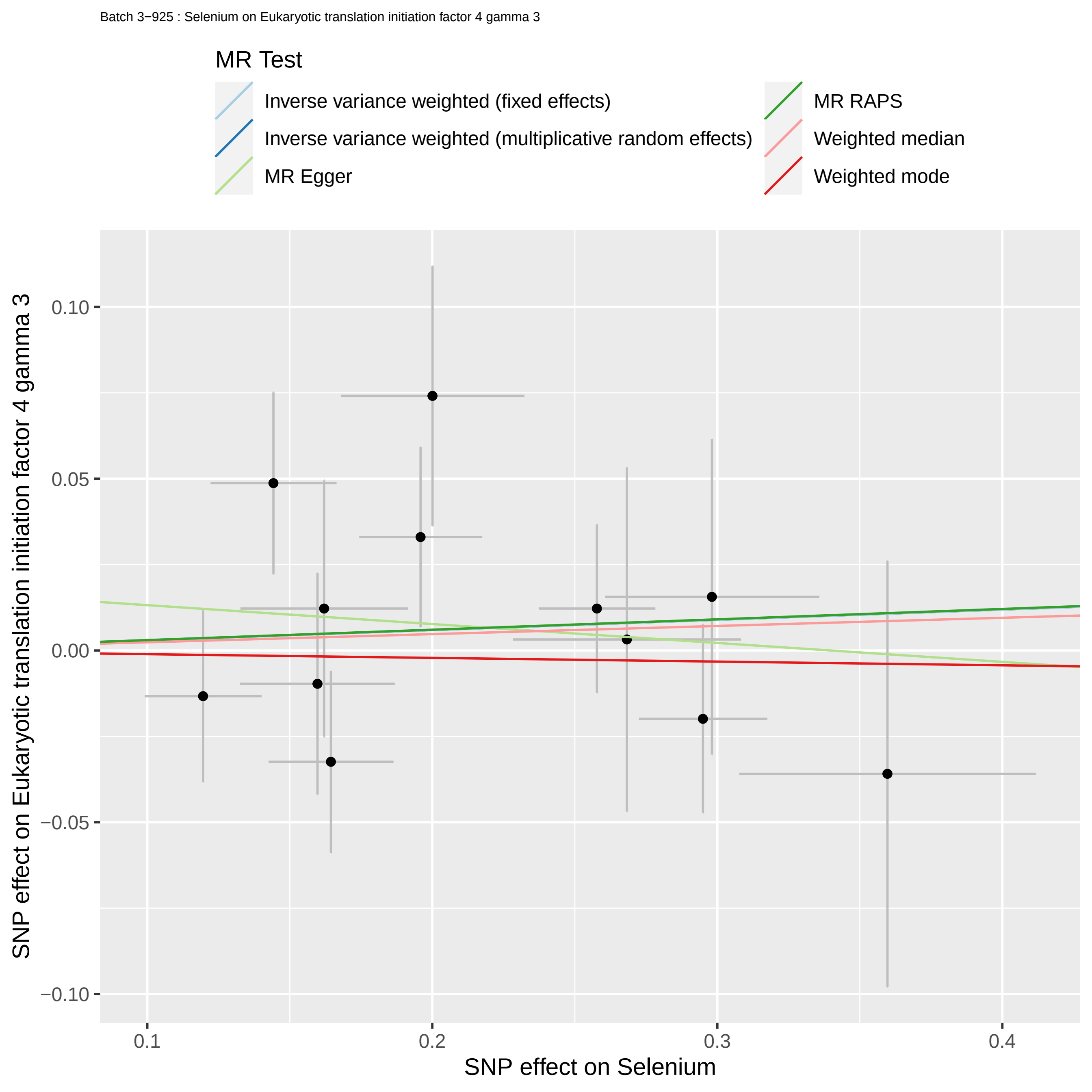
Batch 3−925 : Selenium on Eukaryotic translation initiation factor 4 gamma 3
MR Test
Inverse variance weighted (fixed effects)
Inverse variance weighted (multiplicative random effects)
MR Egger
MR RAPS
Weighted median
Weighted mode
SNP effect on Eukaryotic translation initiation factor 4 gamma 3
0.10
0.05
0.00
−0.05
−0.10
0.1
0.2
0.3
0.4
SNP effect on Selenium

Batch 3−926 : Selenium on Eukaryotic translation initiation factor 5

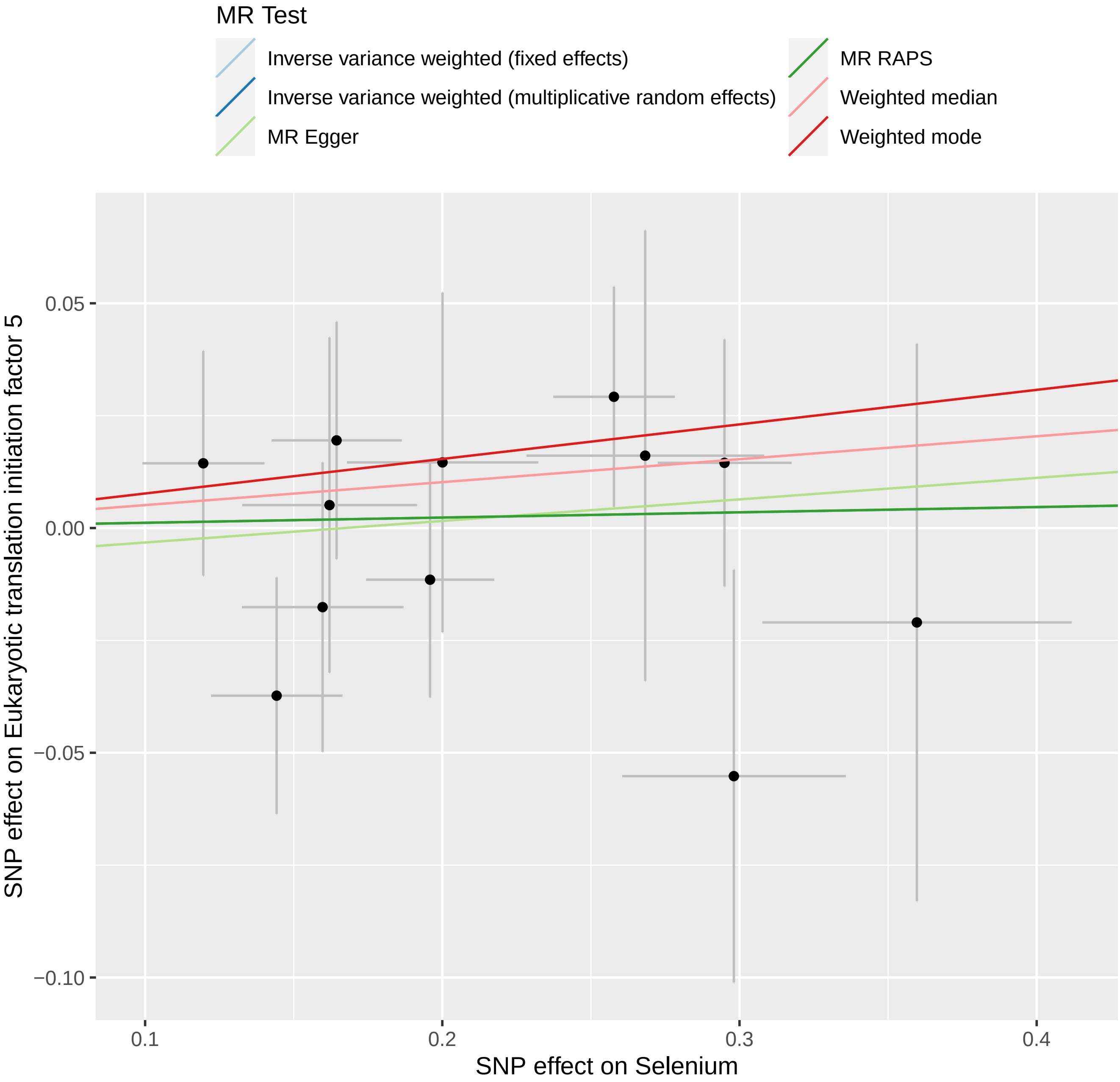

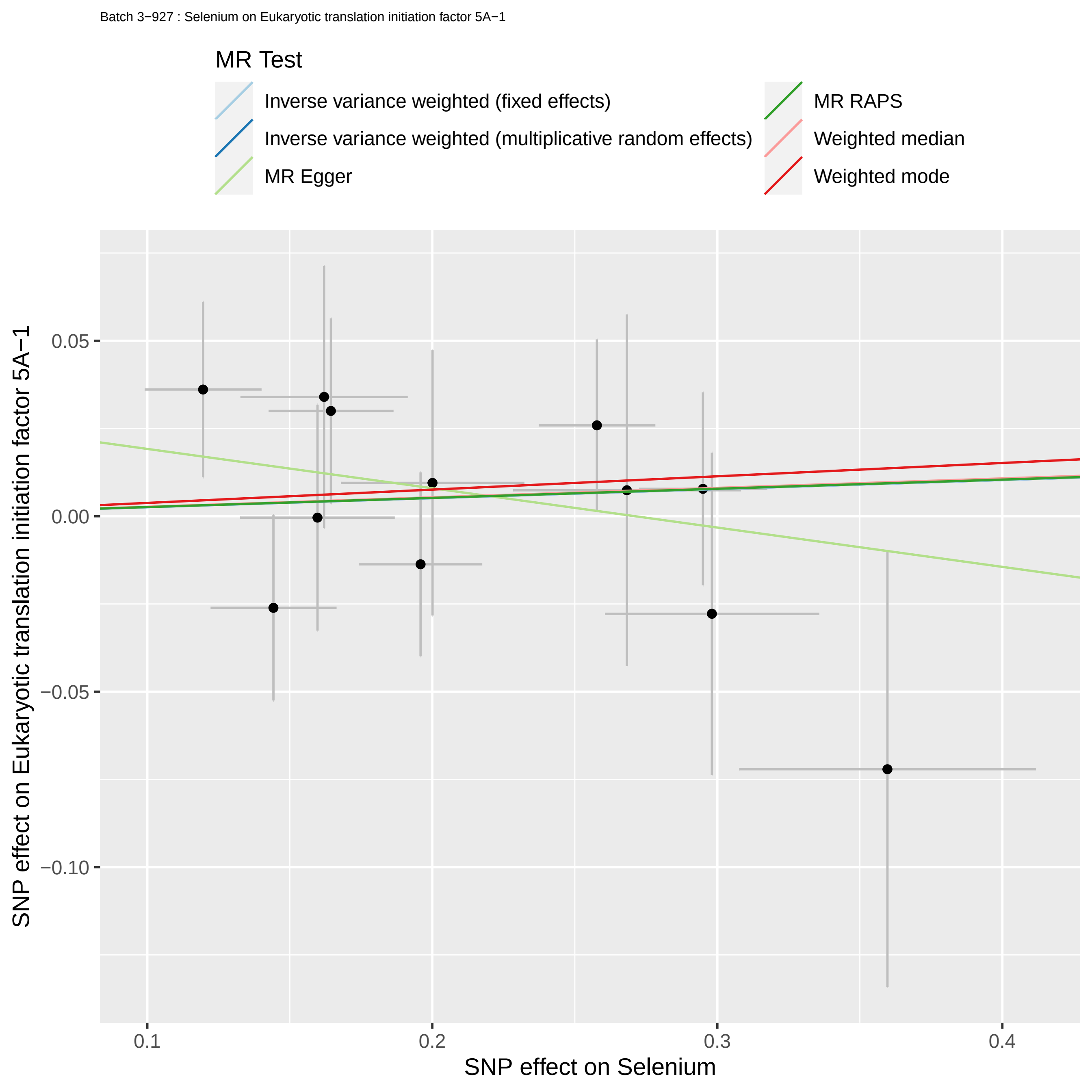

Batch 3−927 : Selenium on Eukaryotic translation initiation factor 5A−1
MR Test
Inverse variance weighted (fixed effects)
Inverse variance weighted (multiplicative random effects)
MR Egger
MR RAPS
Weighted median
Weighted mode
0.05
0.00
−0.05
−0.10
0.1
0.2
0.3
0.4
SNP effect on Eukaryotic translation initiation factor 5A−1
SNP effect on Selenium

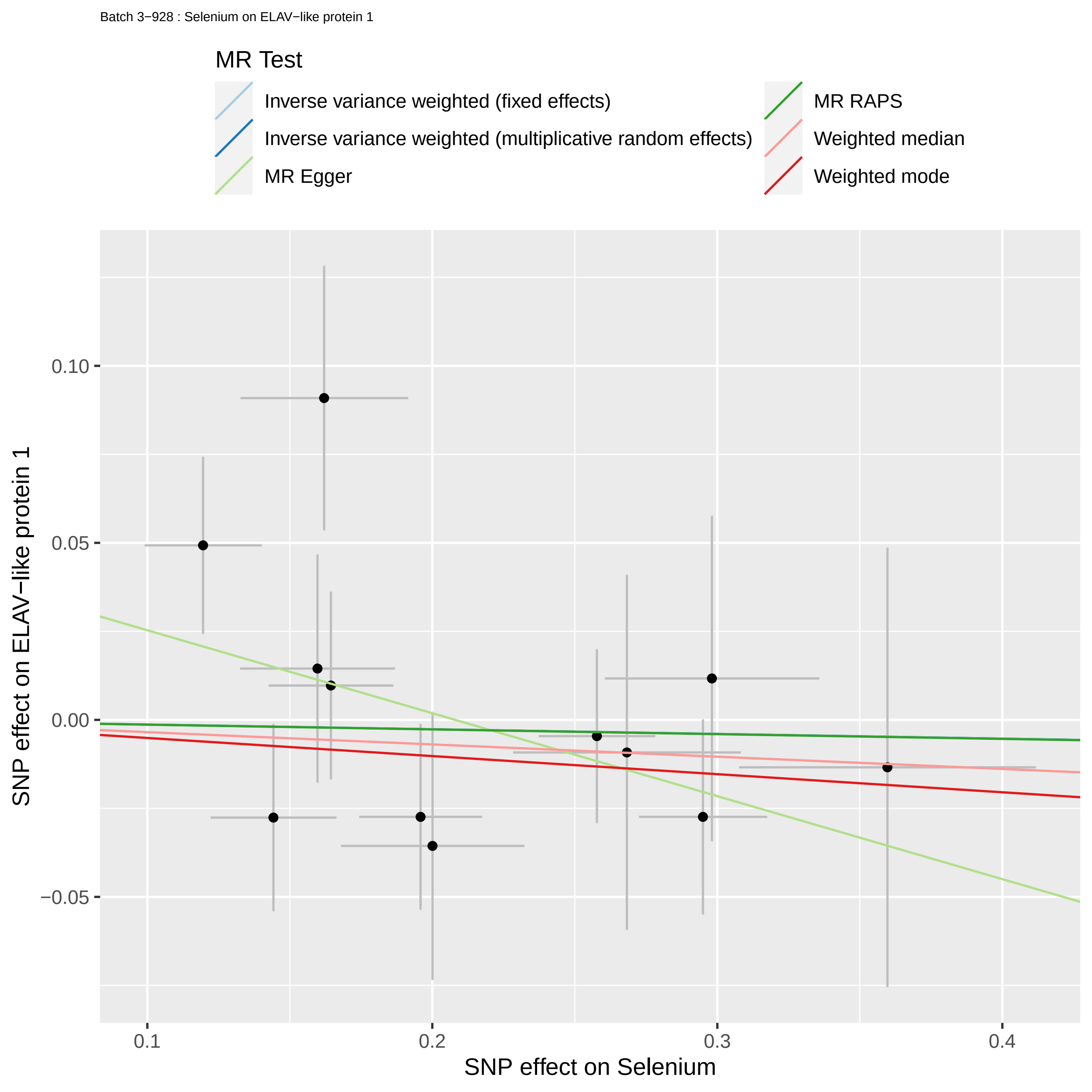

Batch 3−928 : Selenium on ELAV−like protein 1
MR Test
Inverse variance weighted (fixed effects)
Inverse variance weighted (multiplicative random effects)
MR Egger
MR RAPS
Weighted median
Weighted mode
0.10
0.05
0.00
−0.05
0.1
0.2
0.3
0.4
SNP effect on ELAV−like protein 1
SNP effect on Selenium

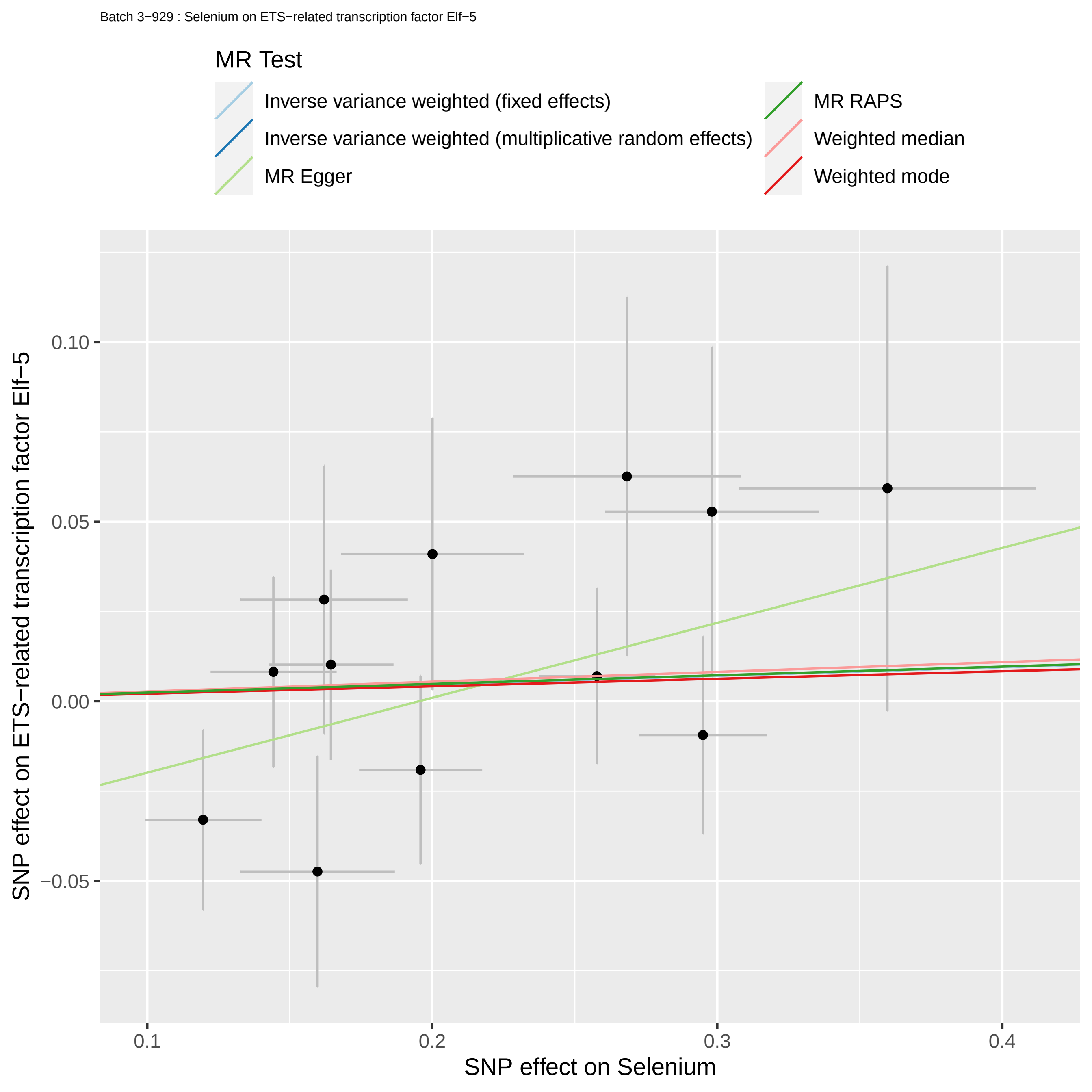
Batch 3−929 : Selenium on ETS−related transcription factor Elf−5
MR Test
Inverse variance weighted (fixed effects)
Inverse variance weighted (multiplicative random effects)
MR Egger
MR RAPS
Weighted median
Weighted mode
0.10
0.05
0.00
−0.05
0.1
0.2
0.3
0.4
SNP effect on ETS−related transcription factor Elf−5
SNP effect on Selenium

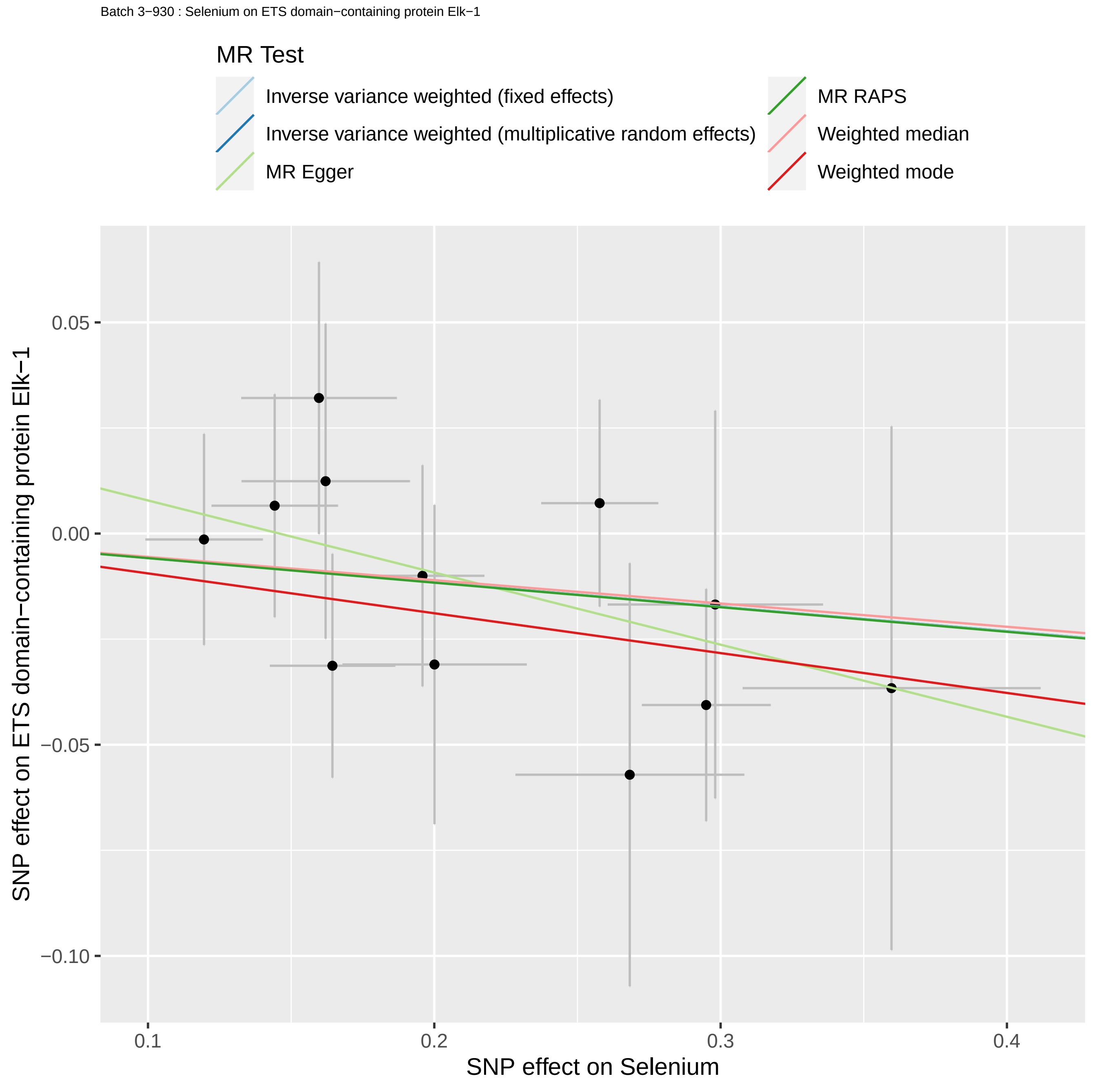

Batch 3−930 : Selenium on ETS domain−containing protein Elk−1
MR Test
Inverse variance weighted (fixed effects)
Inverse variance weighted (multiplicative random effects)
MR Egger
MR RAPS
Weighted median
Weighted mode
0.05
0.00
−0.05
−0.10
0.1
0.2
0.3
0.4
SNP effect on ETS domain−containing protein Elk−1
SNP effect on Selenium

Batch 3−931 : Selenium on ETS domain−containing protein Elk−3

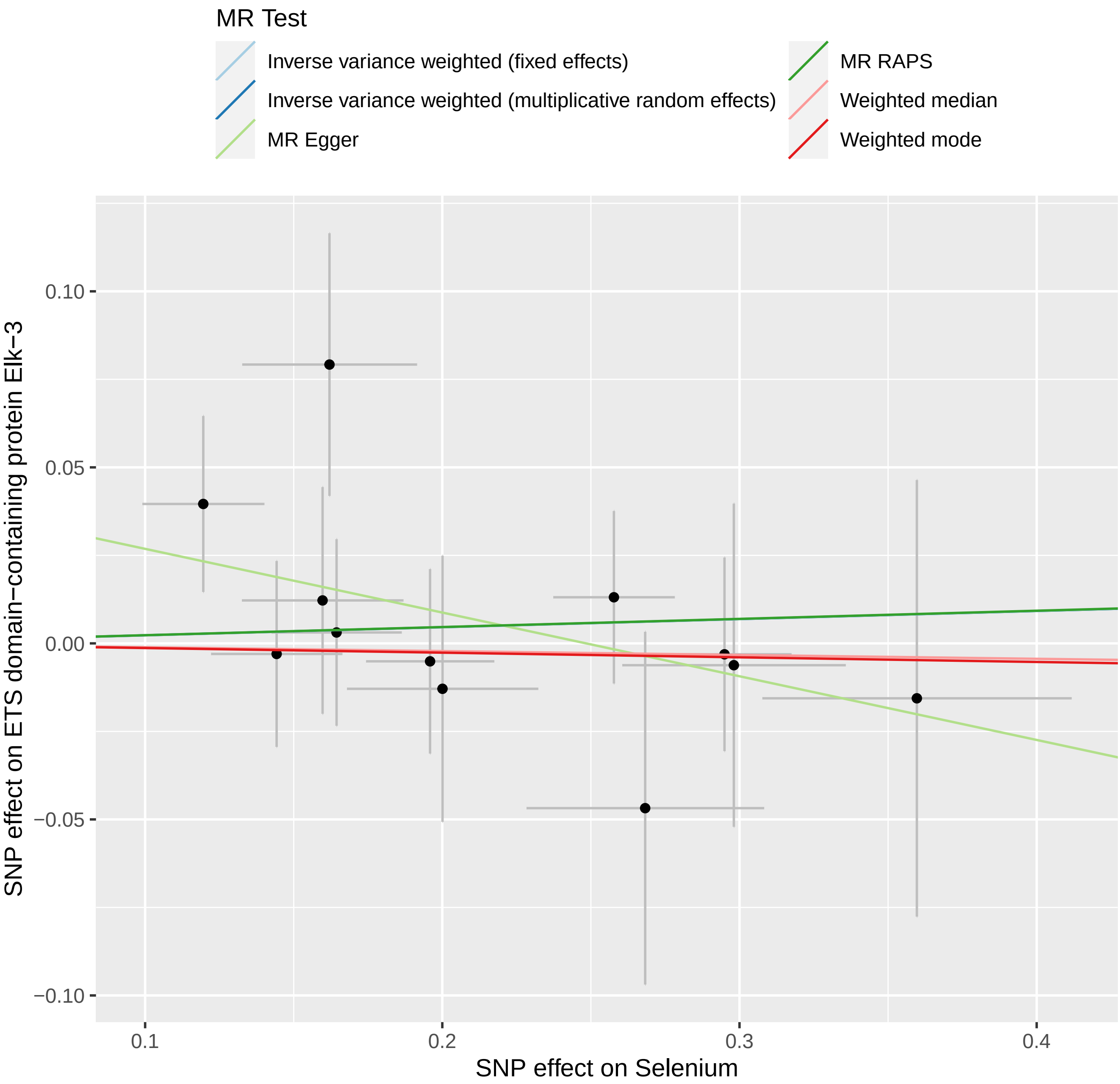

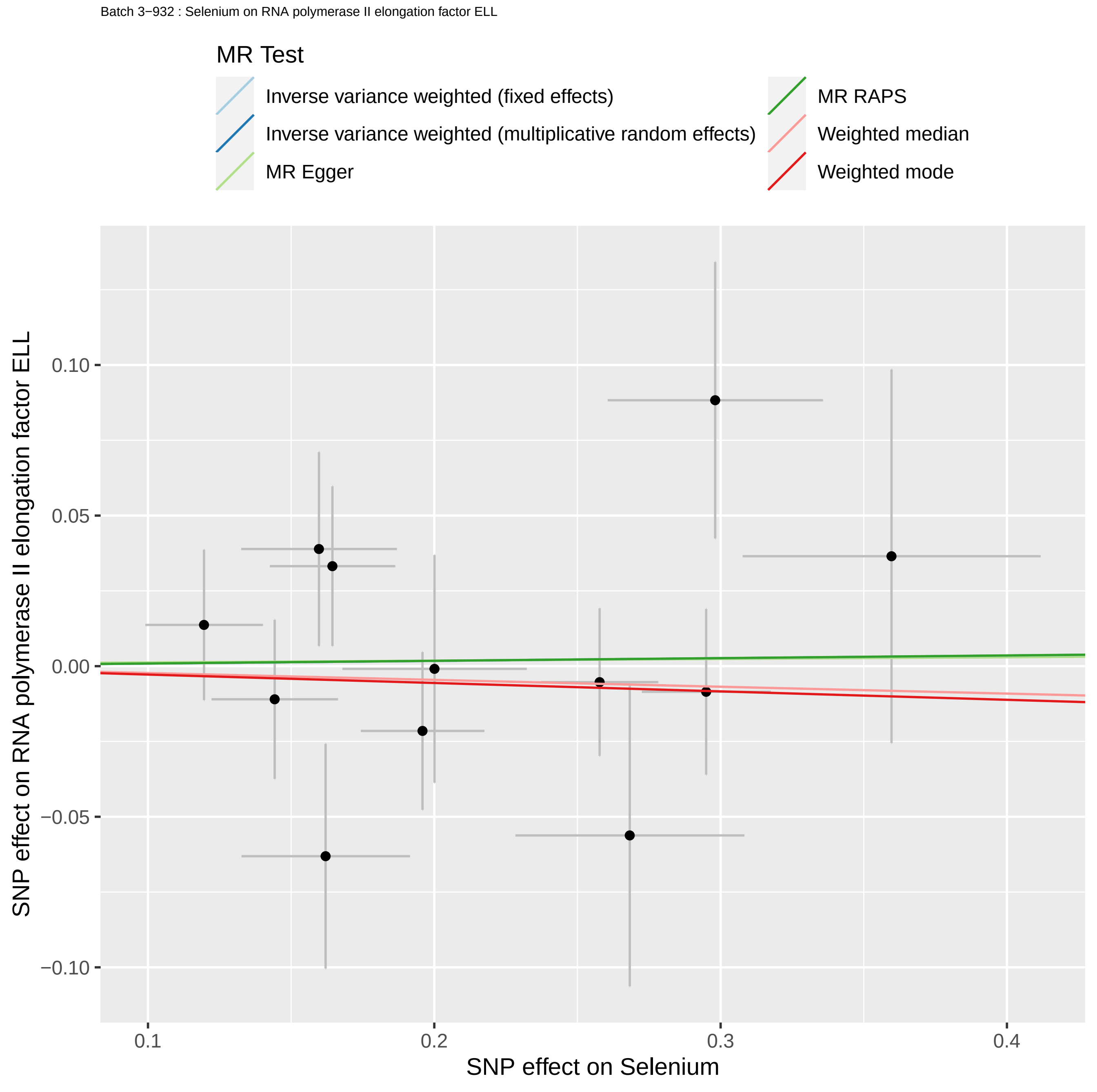

Batch 3−932 : Selenium on RNA polymerase II elongation factor ELL
MR Test
Inverse variance weighted (fixed effects)
Inverse variance weighted (multiplicative random effects)
MR Egger
MR RAPS
Weighted median
Weighted mode
SNP effect on RNA polymerase II elongation factor ELL
0.10
0.05
0.00
−0.05
−0.10
0.1
0.2
0.3
0.4
SNP effect on Selenium

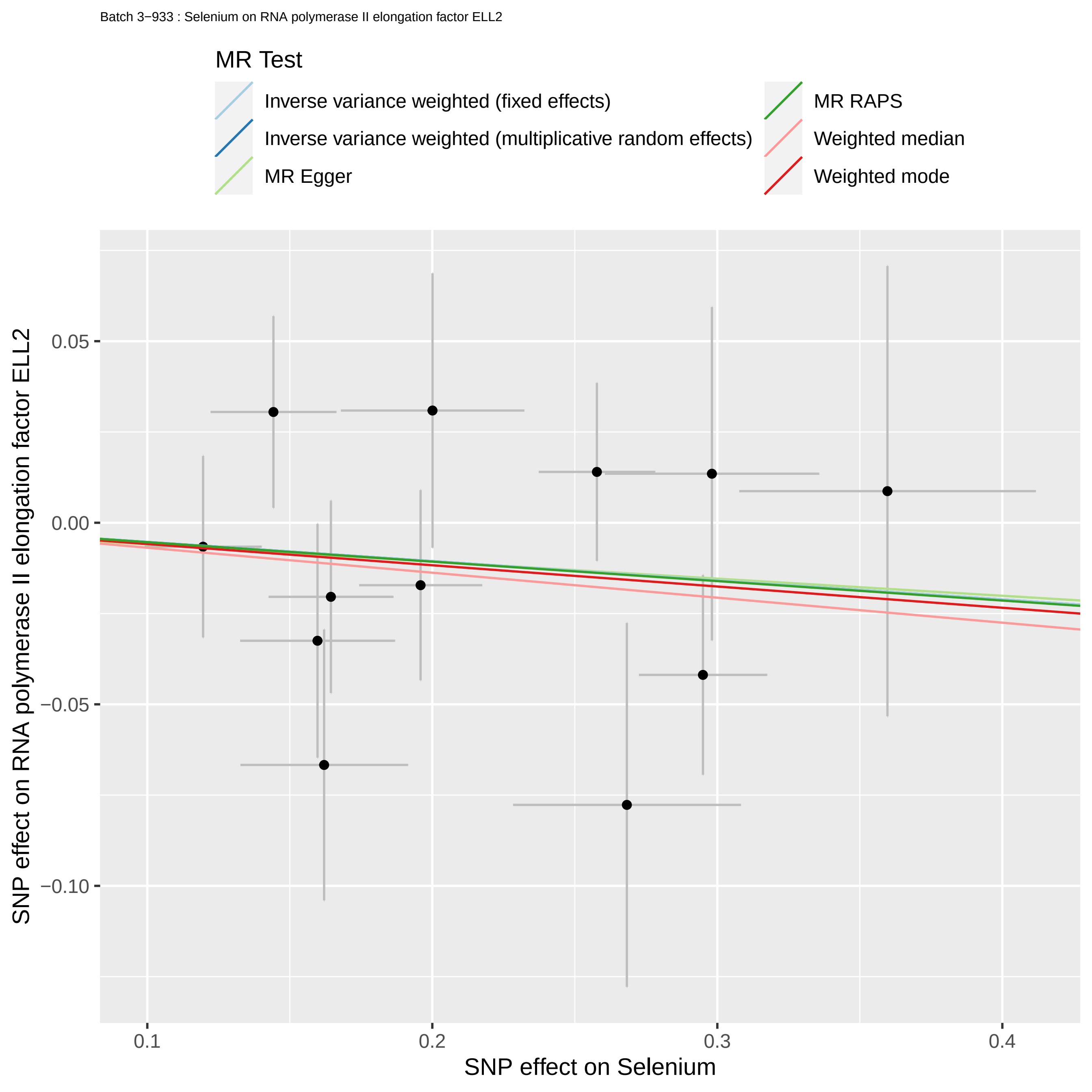
Batch 3−933 : Selenium on RNA polymerase II elongation factor ELL2
MR Test
Inverse variance weighted (fixed effects)
Inverse variance weighted (multiplicative random effects)
MR Egger
MR RAPS
Weighted median
Weighted mode
0.05
0.00
−0.05
−0.10
0.1
0.2
0.3
0.4
SNP effect on RNA polymerase II elongation factor ELL2
SNP effect on Selenium

Batch 3−934 : Selenium on Engulfment and cell motility protein 1

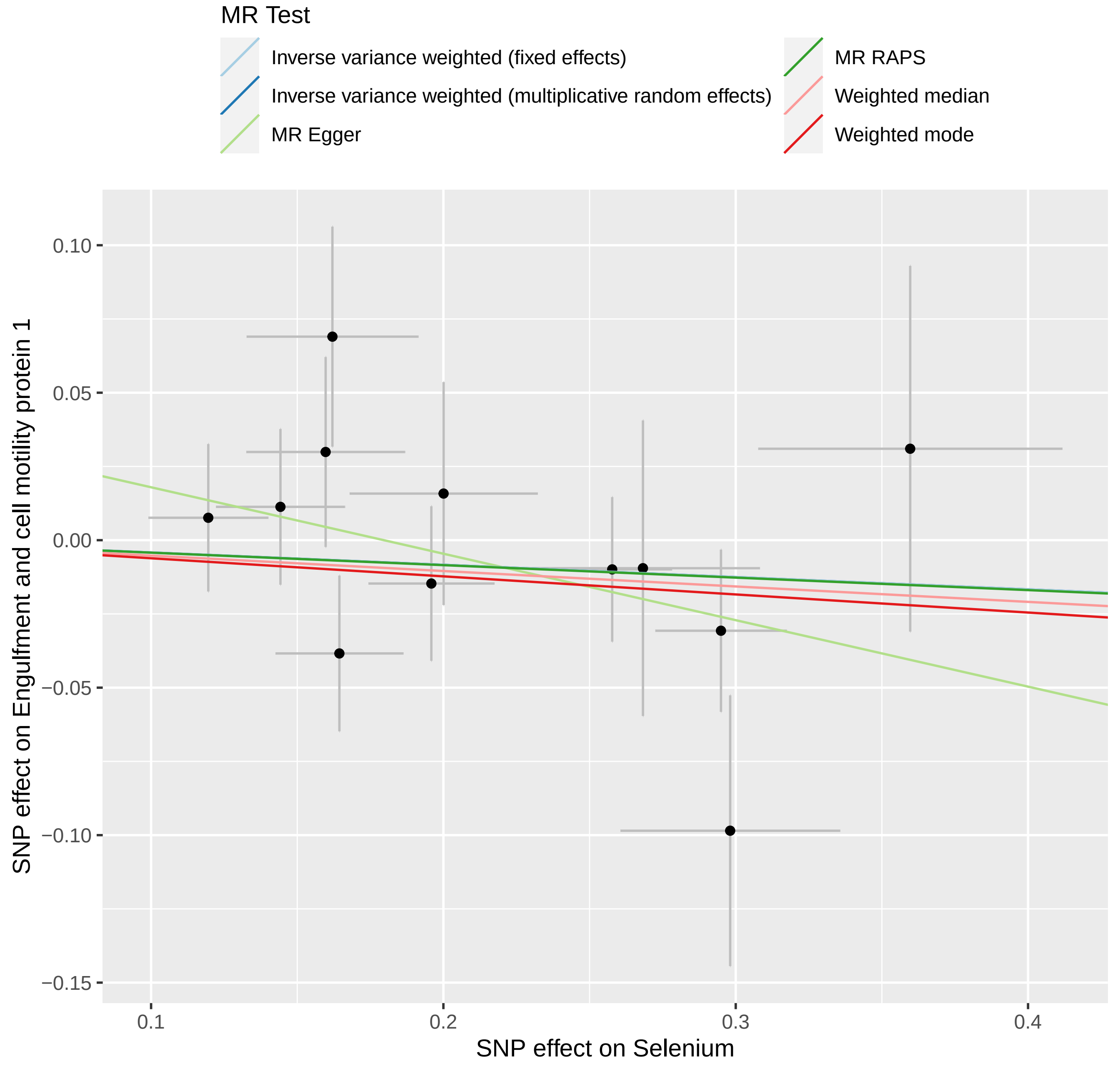

Batch 3−935 : Selenium on ER membrane protein complex subunit 1

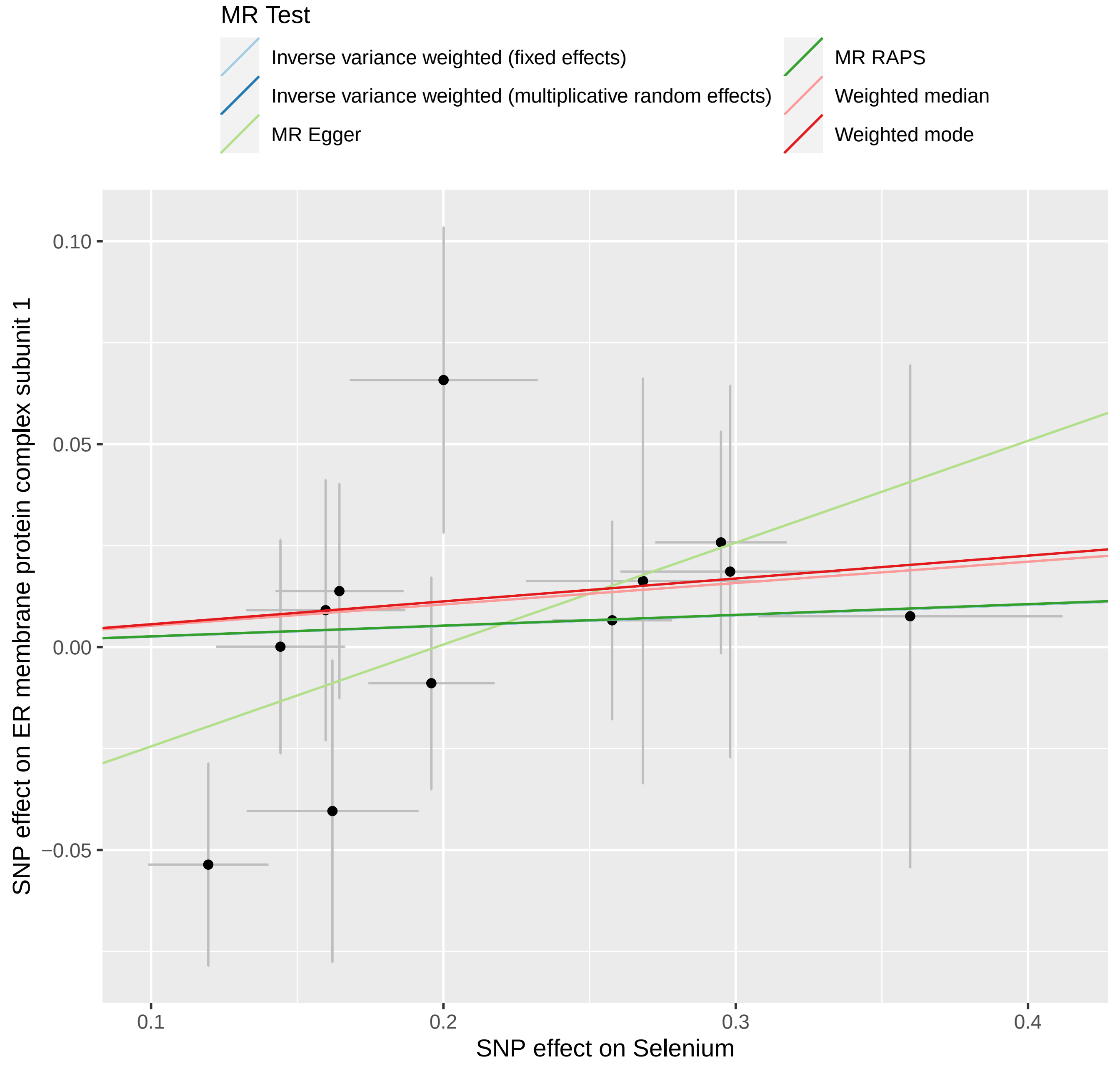

Batch 3−936 : Selenium on ER membrane protein complex subunit 4

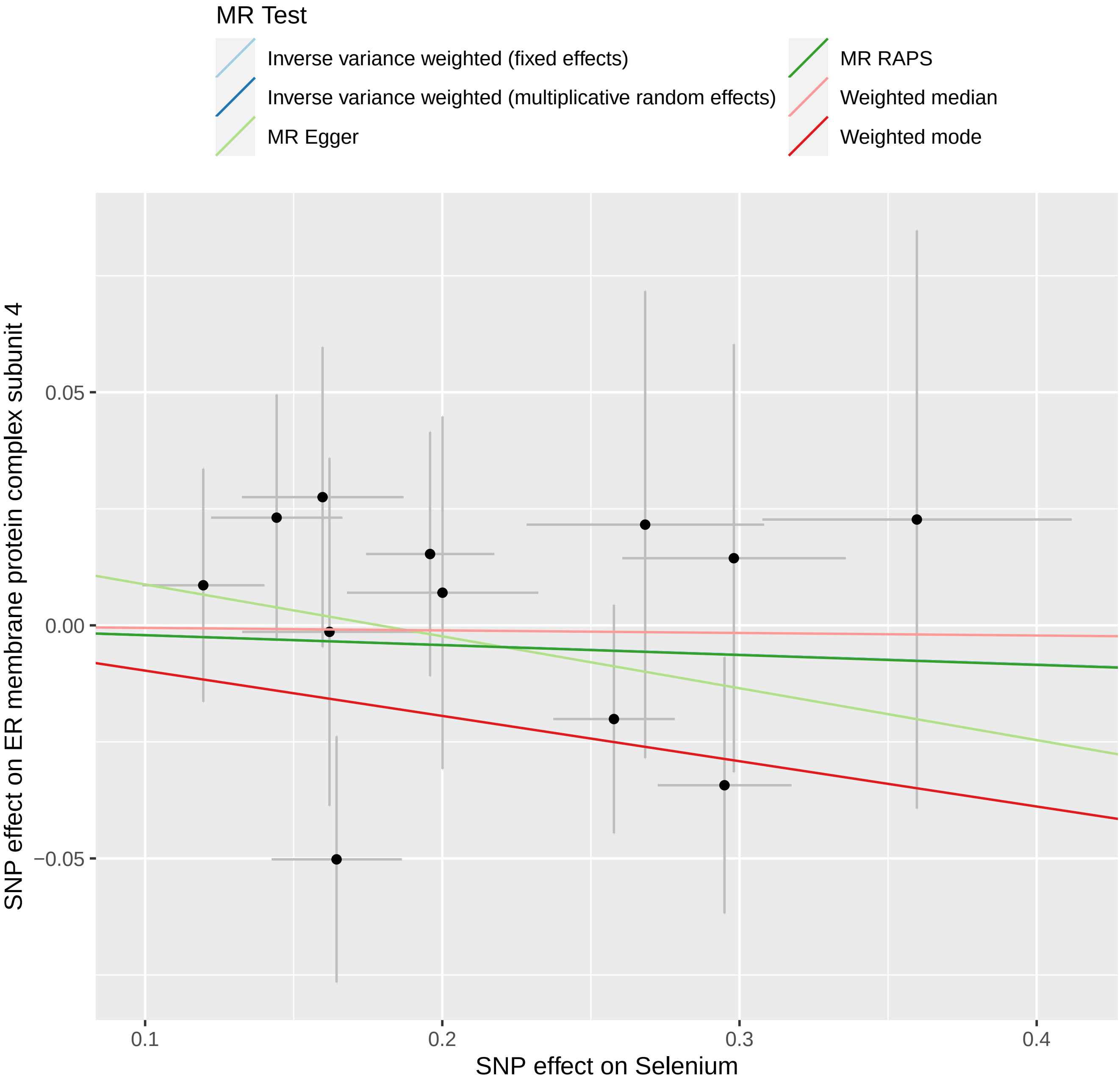

Batch 3−937 : Selenium on EMI domain−containing protein 1

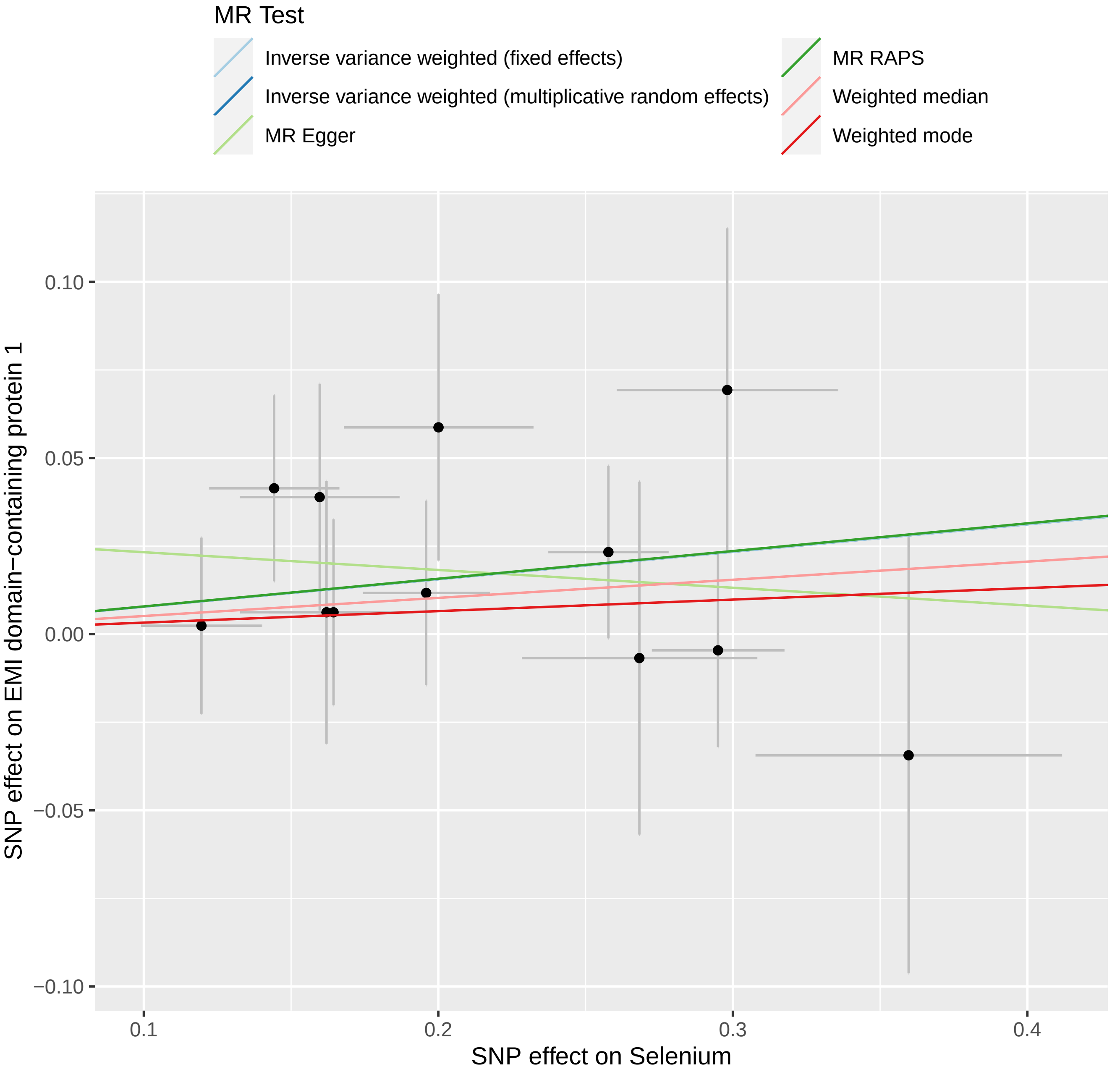

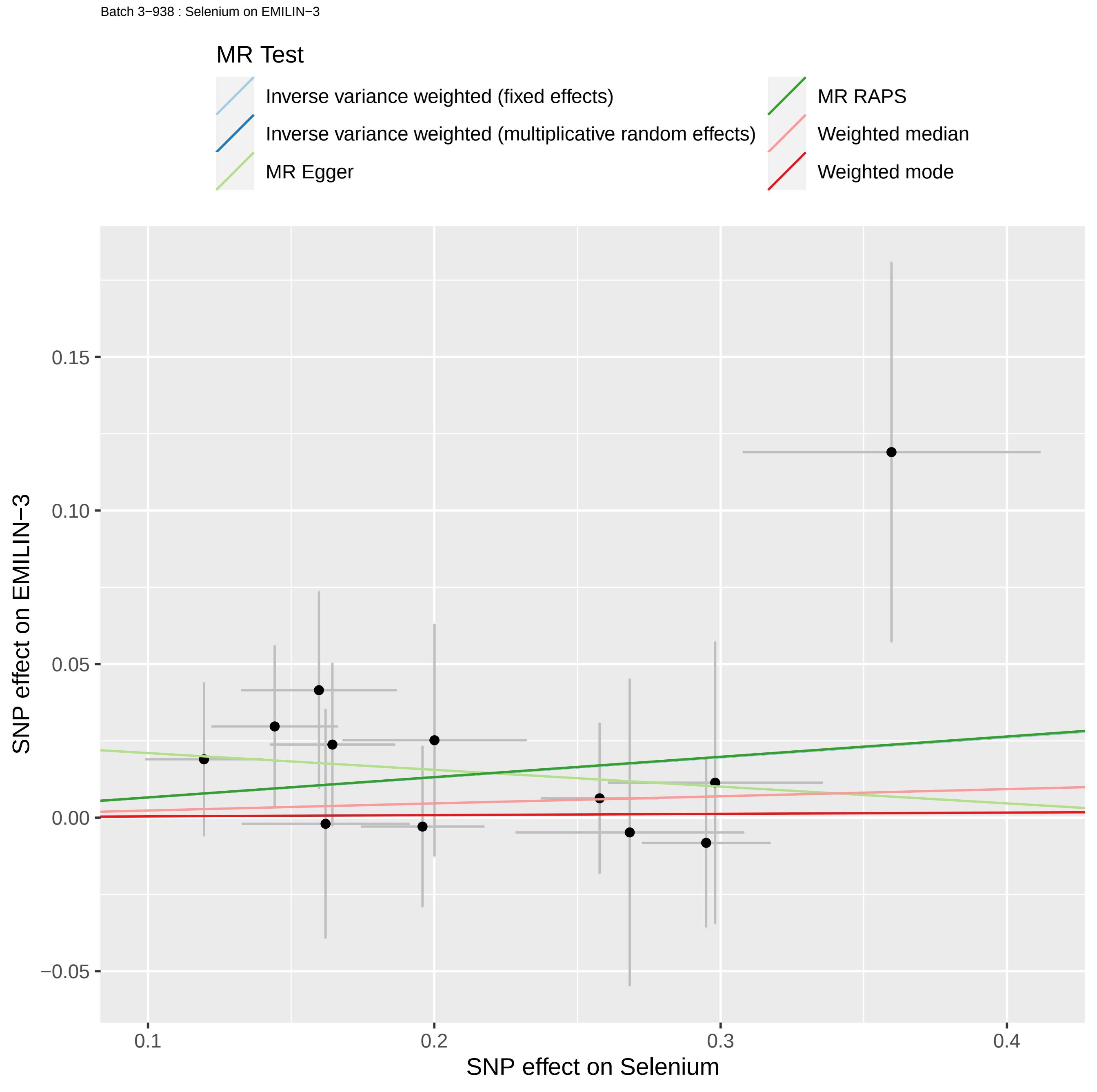

Batch 3−938 : Selenium on EMILIN−3
MR Test
Inverse variance weighted (fixed effects)
Inverse variance weighted (multiplicative random effects)
MR Egger
MR RAPS
Weighted median
Weighted mode
0.15
0.10
0.05
0.00
−0.05
0.1
0.2
0.3
0.4
SNP effect on EMILIN−3
SNP effect on Selenium

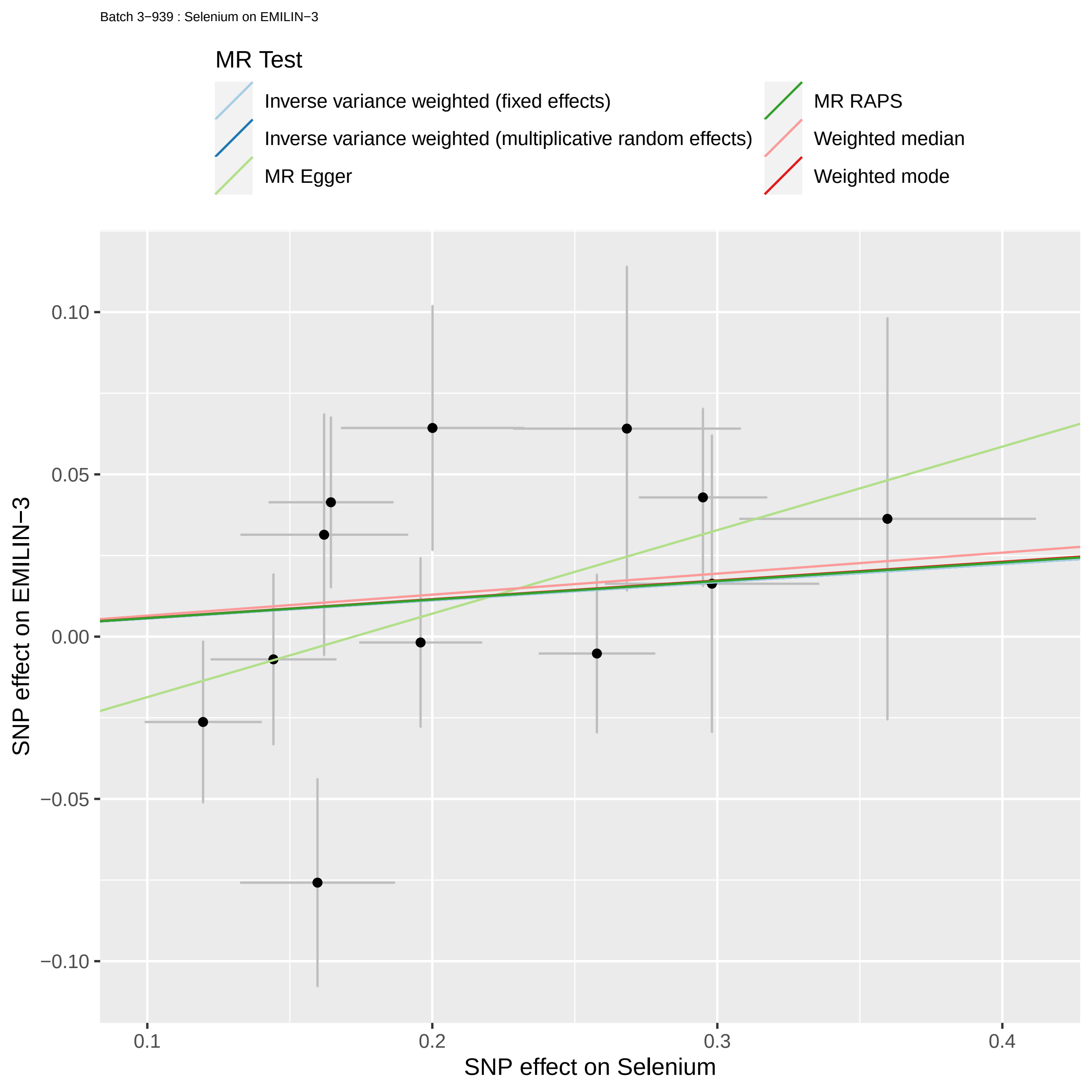
Batch 3−939 : Selenium on EMILIN−3
MR Test
Inverse variance weighted (fixed effects)
Inverse variance weighted (multiplicative random effects)
MR Egger
MR RAPS
Weighted median
Weighted mode
0.10
0.05
0.00
−0.05
−0.10
SNP effect on EMILIN−3
0.1
0.2
0.3
0.4
SNP effect on Selenium

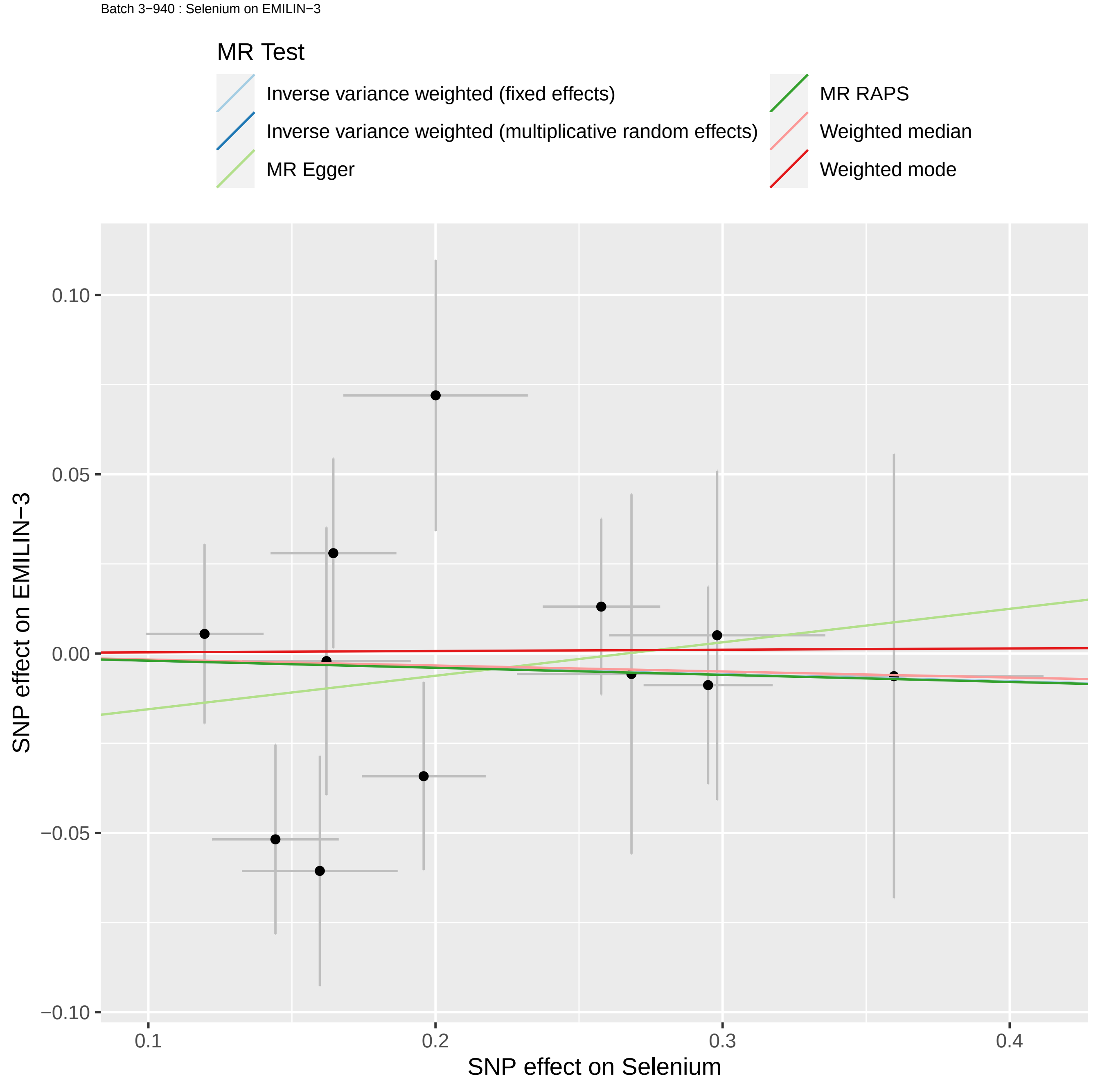

Batch 3−940 : Selenium on EMILIN−3
MR Test
Inverse variance weighted (fixed effects)
Inverse variance weighted (multiplicative random effects)
MR Egger
MR RAPS
Weighted median
Weighted mode
0.10
0.05
0.00
−0.05
−0.10
0.1
0.2
0.3
0.4
SNP effect on EMILIN−3
SNP effect on Selenium

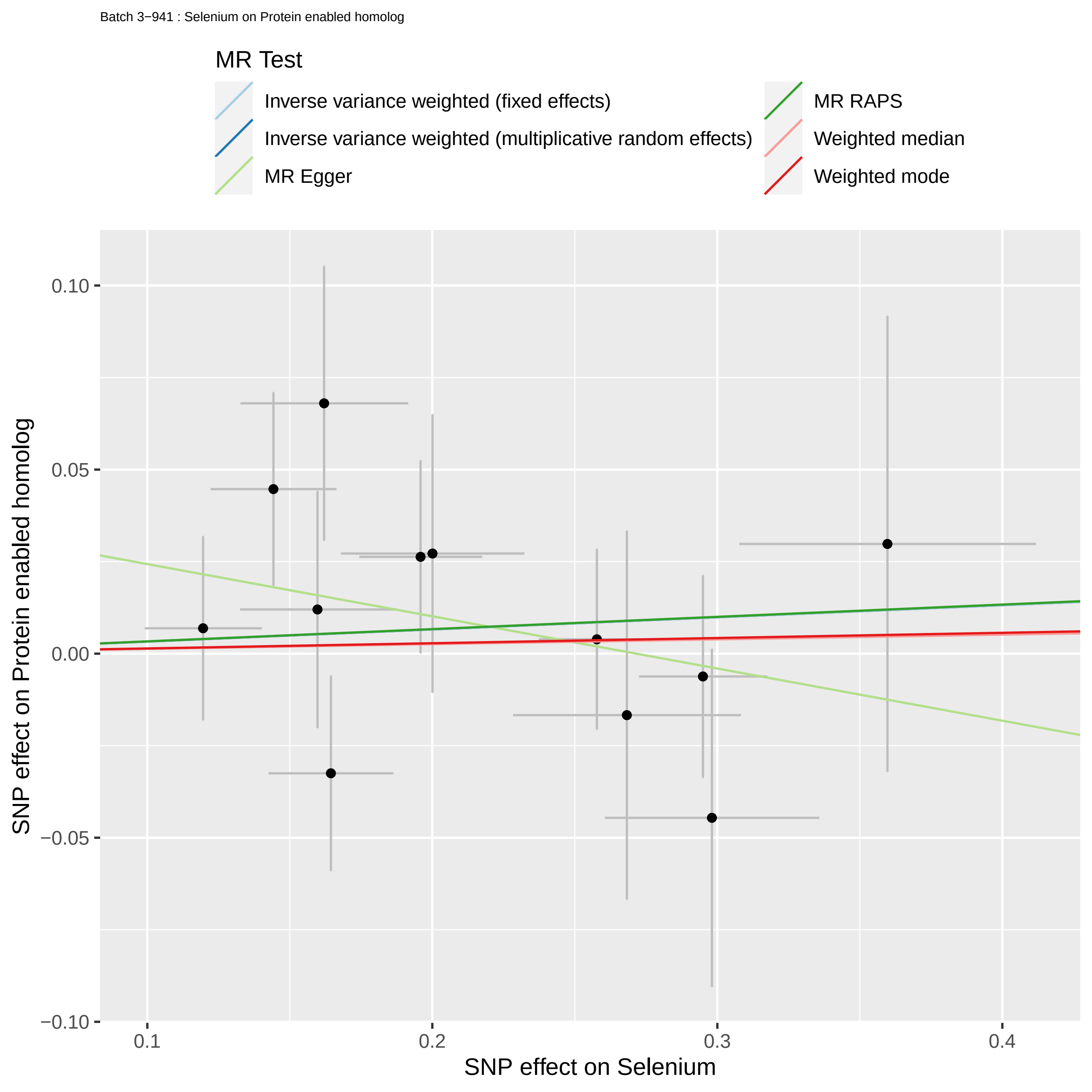
Batch 3−941 : Selenium on Protein enabled homolog
MR Test
Inverse variance weighted (fixed effects)
Inverse variance weighted (multiplicative random effects)
MR Egger
MR RAPS
Weighted median
Weighted mode
0.10
0.05
0.00
−0.05
−0.10
0.1
0.2
0.3
0.4
SNP effect on Protein enabled homolog
SNP effect on Selenium

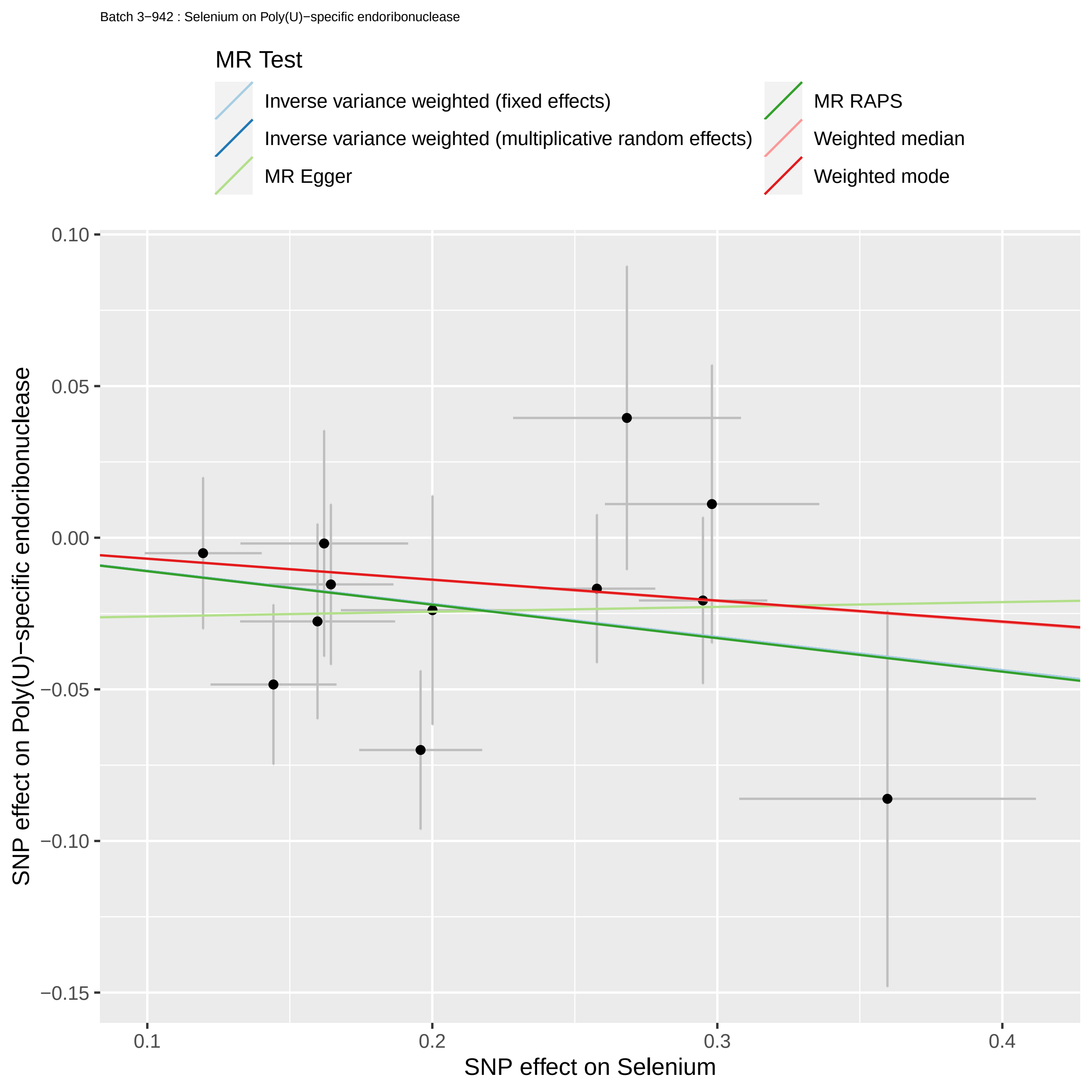
Batch 3−942 : Selenium on Poly(U)−specific endoribonuclease
MR Test
Inverse variance weighted (fixed effects)
Inverse variance weighted (multiplicative random effects)
MR Egger
MR RAPS
Weighted median
Weighted mode
0.10
0.05
0.00
−0.05
−0.10
−0.15
0.1
0.2
0.3
0.4
SNP effect on Selenium
SNP effect on Poly(U)−specific endoribonuclease

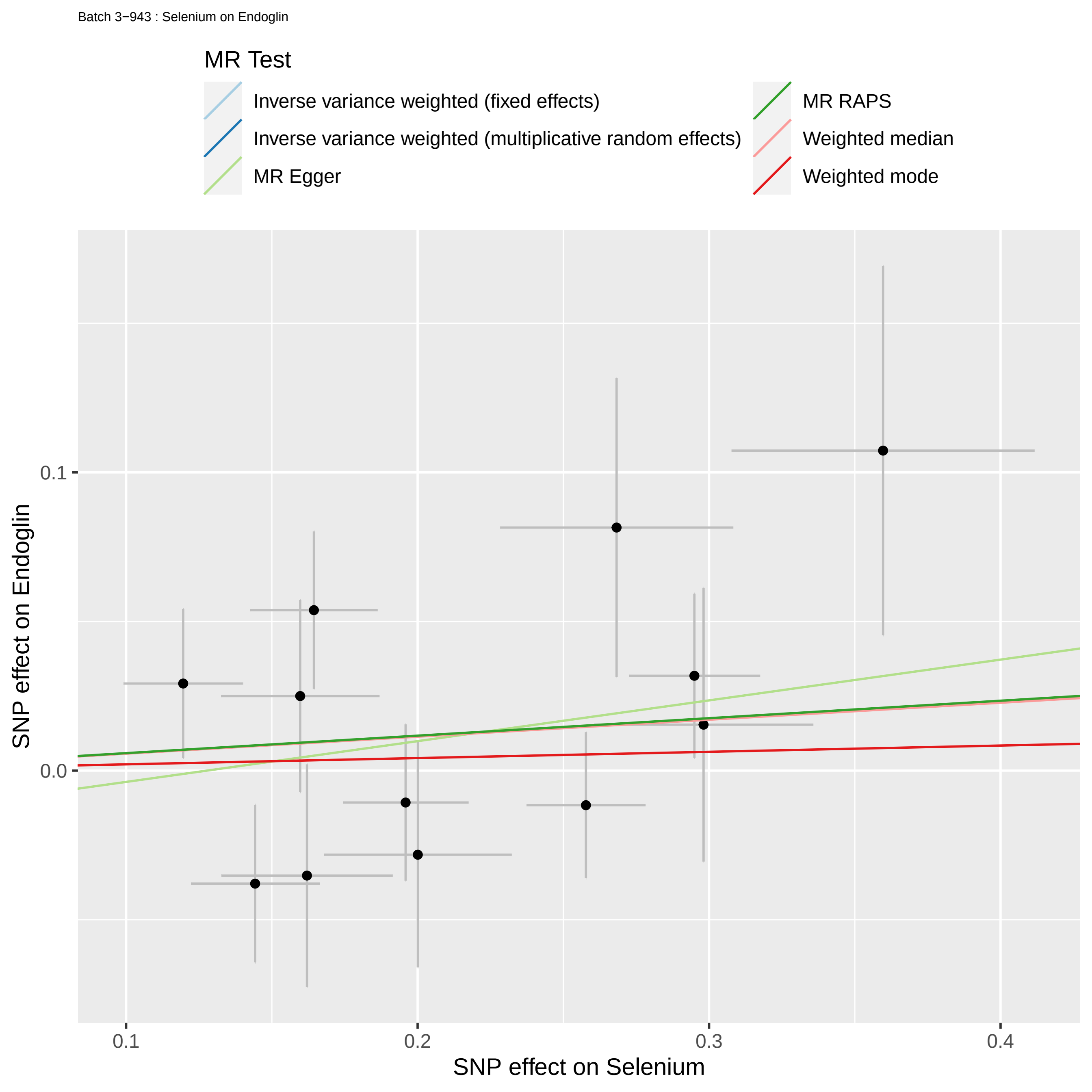
Batch 3−943 : Selenium on Endoglin
MR Test
Inverse variance weighted (fixed effects)
Inverse variance weighted (multiplicative random effects)
MR Egger
MR RAPS
Weighted median
Weighted mode
SNP effect on Endoglin
SNP effect on Selenium
0.1
0.0
0.1
0.2
0.3
0.4

Batch 3−944 : Selenium on Gamma−enolase

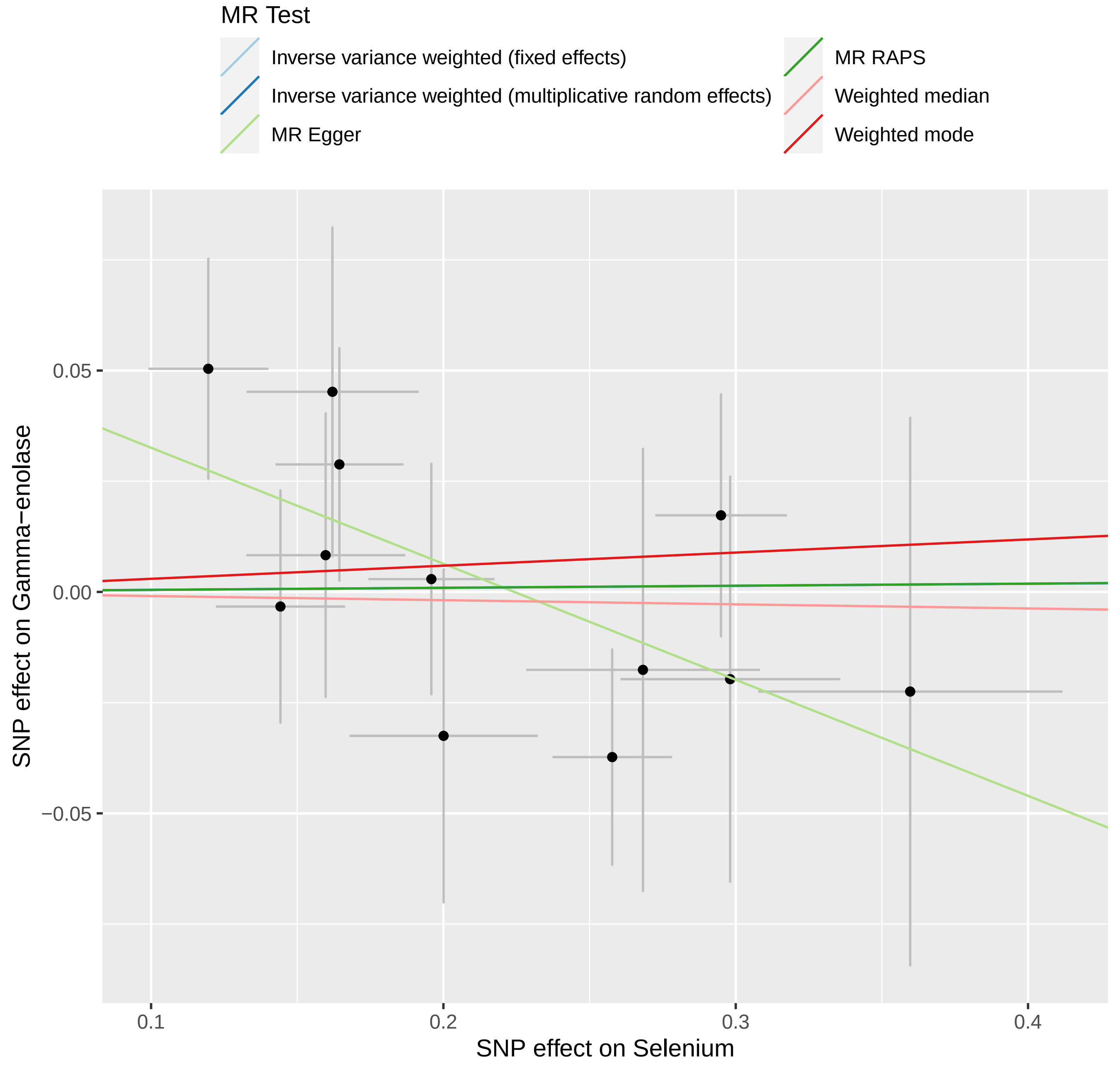

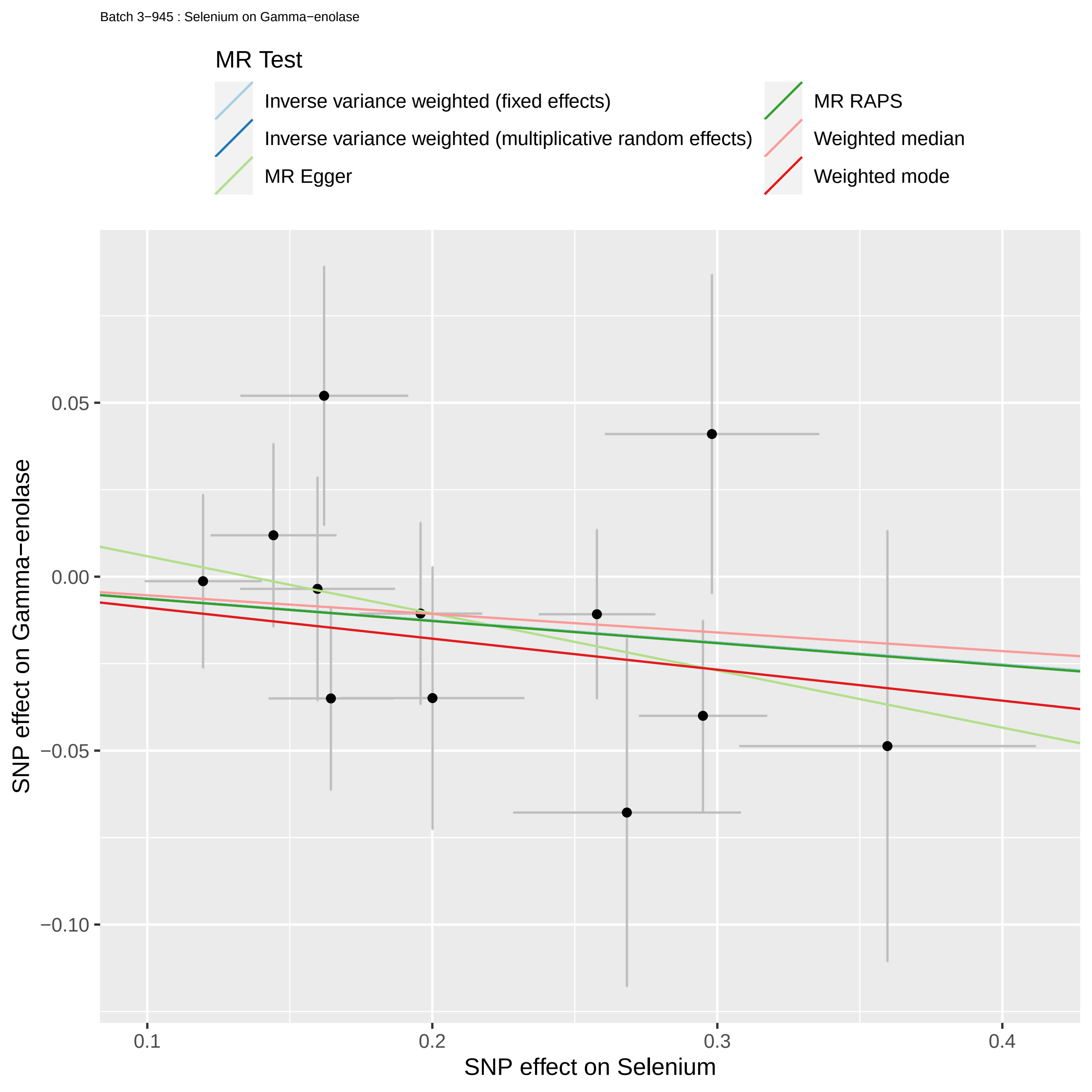
Batch 3−945 : Selenium on Gamma−enolase
MR Test
Inverse variance weighted (fixed effects)
Inverse variance weighted (multiplicative random effects)
MR Egger
MR RAPS
Weighted median
Weighted mode
0.05
0.00
−0.05
−0.10
0.1
0.2
0.3
0.4
SNP effect on Selenium
SNP effect on Gamma−enolase

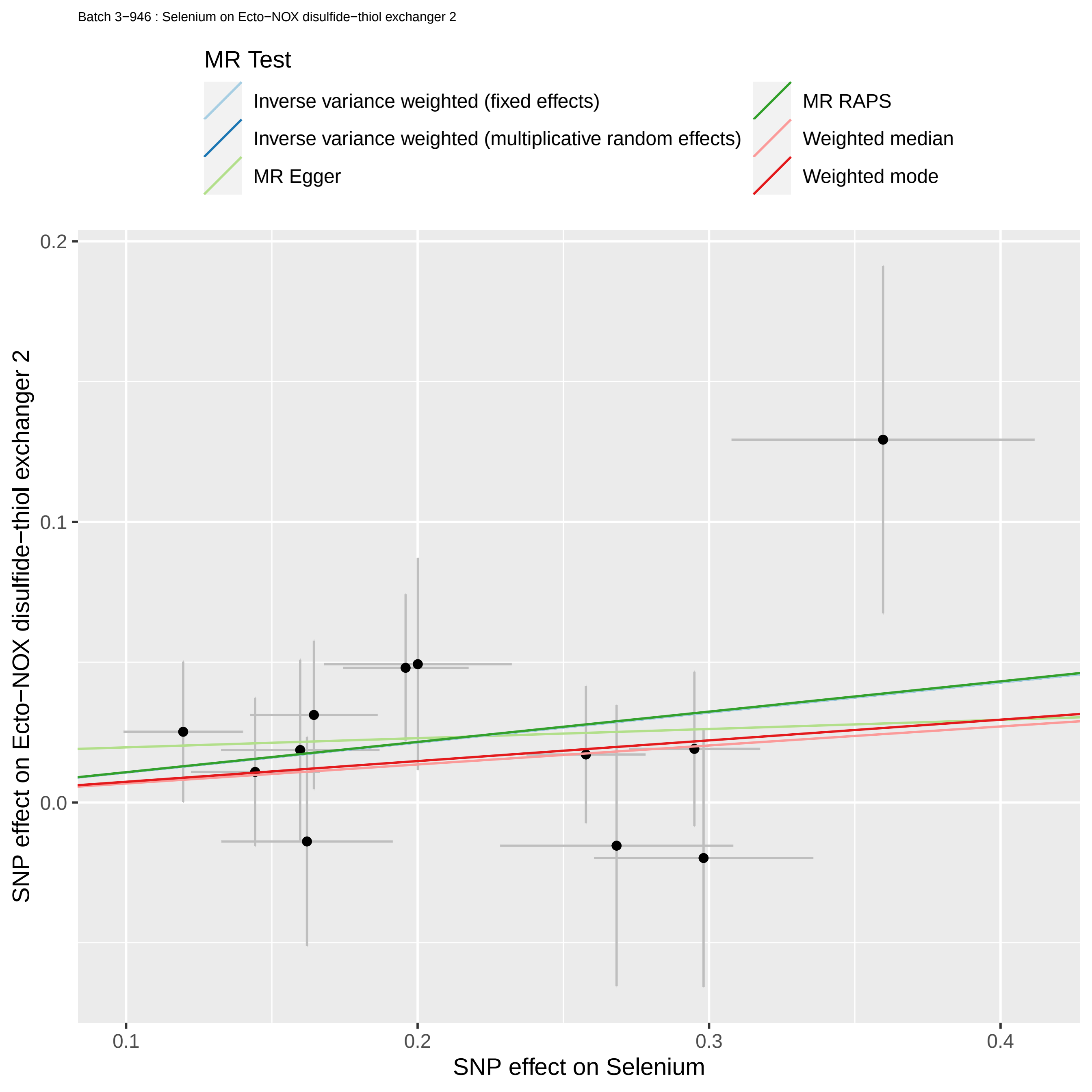

Batch 3−946 : Selenium on Ecto−NOX disulfide−thiol exchanger 2
MR Test
Inverse variance weighted (fixed effects)
Inverse variance weighted (multiplicative random effects)
MR Egger
MR RAPS
Weighted median
Weighted mode
SNP effect on Ecto−NOX disulfide−thiol exchanger 2
0.2
0.1
0.0
0.1
0.2
0.3
0.4
SNP effect on Selenium

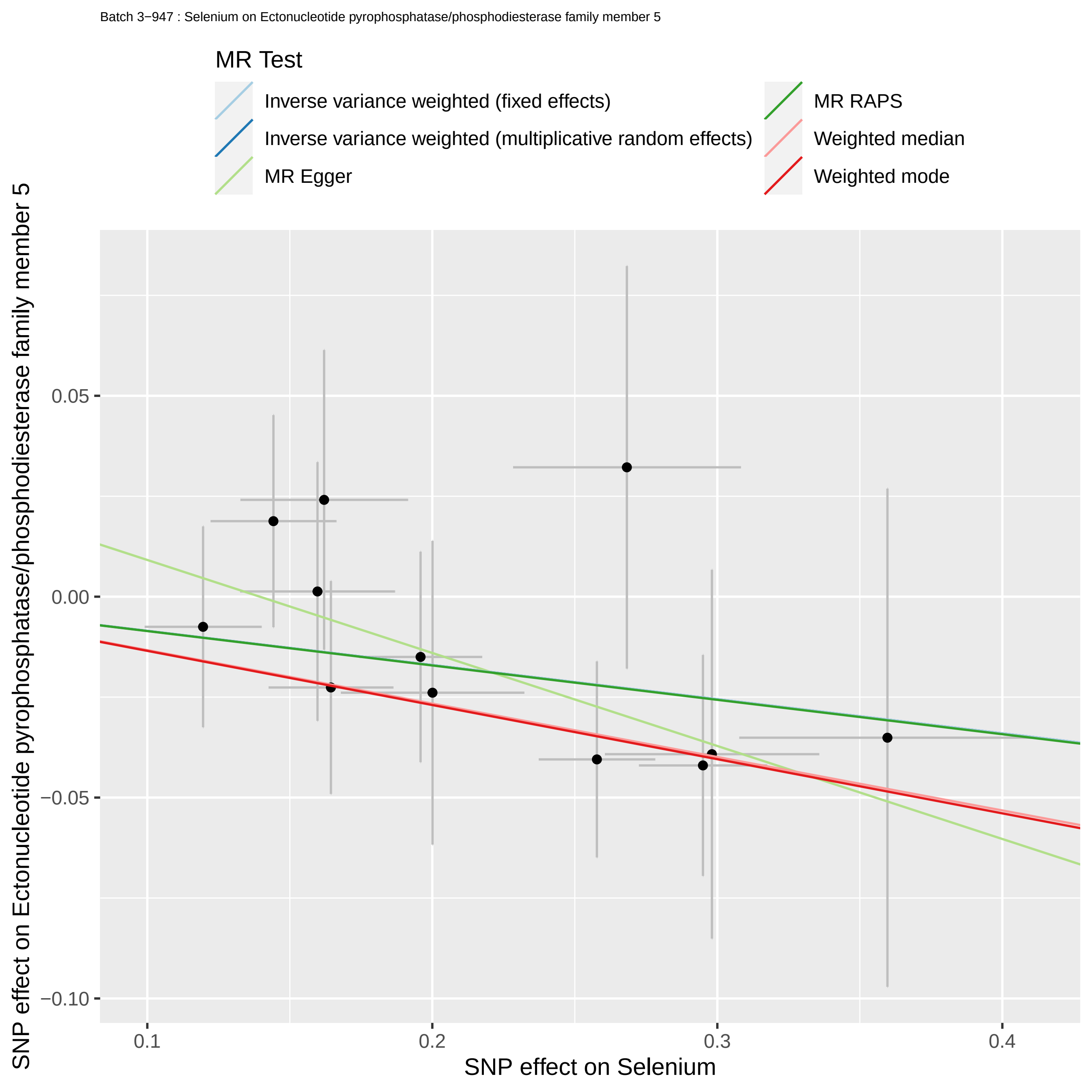

Batch 3−947 : Selenium on Ectonucleotide pyrophosphatase/phosphodiesterase family member 5
MR Test
Inverse variance weighted (fixed effects)
Inverse variance weighted (multiplicative random effects)
MR Egger
MR RAPS
Weighted median
Weighted mode
SNP effect on Ectonucleotide pyrophosphatase/phosphodiesterase family member 5
0.05
0.00
−0.05
−0.10
0.1
0.2
0.3
0.4
SNP effect on Selenium

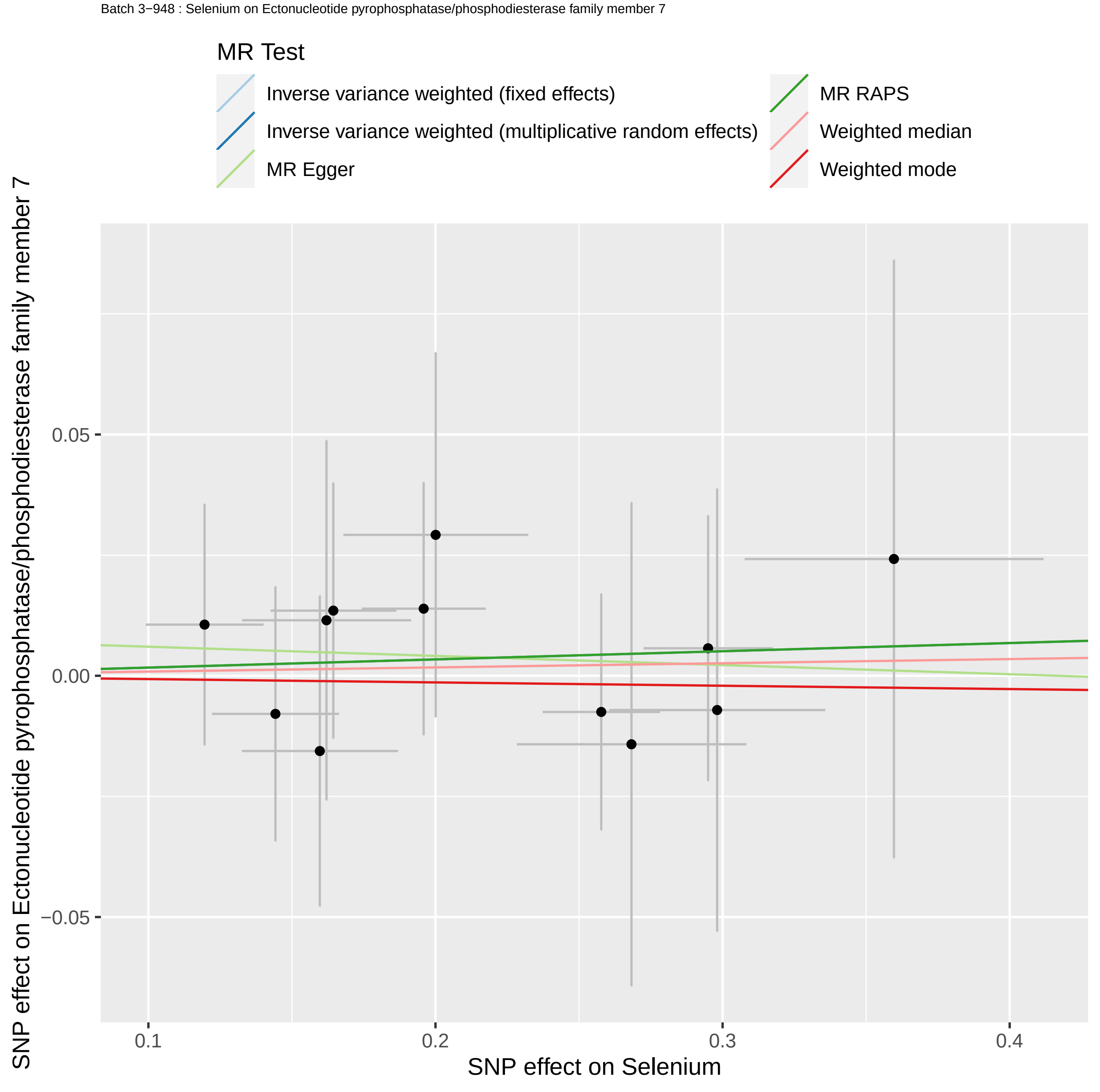

Batch 3−948 : Selenium on Ectonucleotide pyrophosphatase/phosphodiesterase family member 7
MR Test
Inverse variance weighted (fixed effects)
Inverse variance weighted (multiplicative random effects)
MR Egger
MR RAPS
Weighted median
Weighted mode
0.05
0.00
−0.05
0.1
0.2
0.3
0.4
SNP effect on Ectonucleotide pyrophosphatase/phosphodiesterase family member 7
SNP effect on Selenium

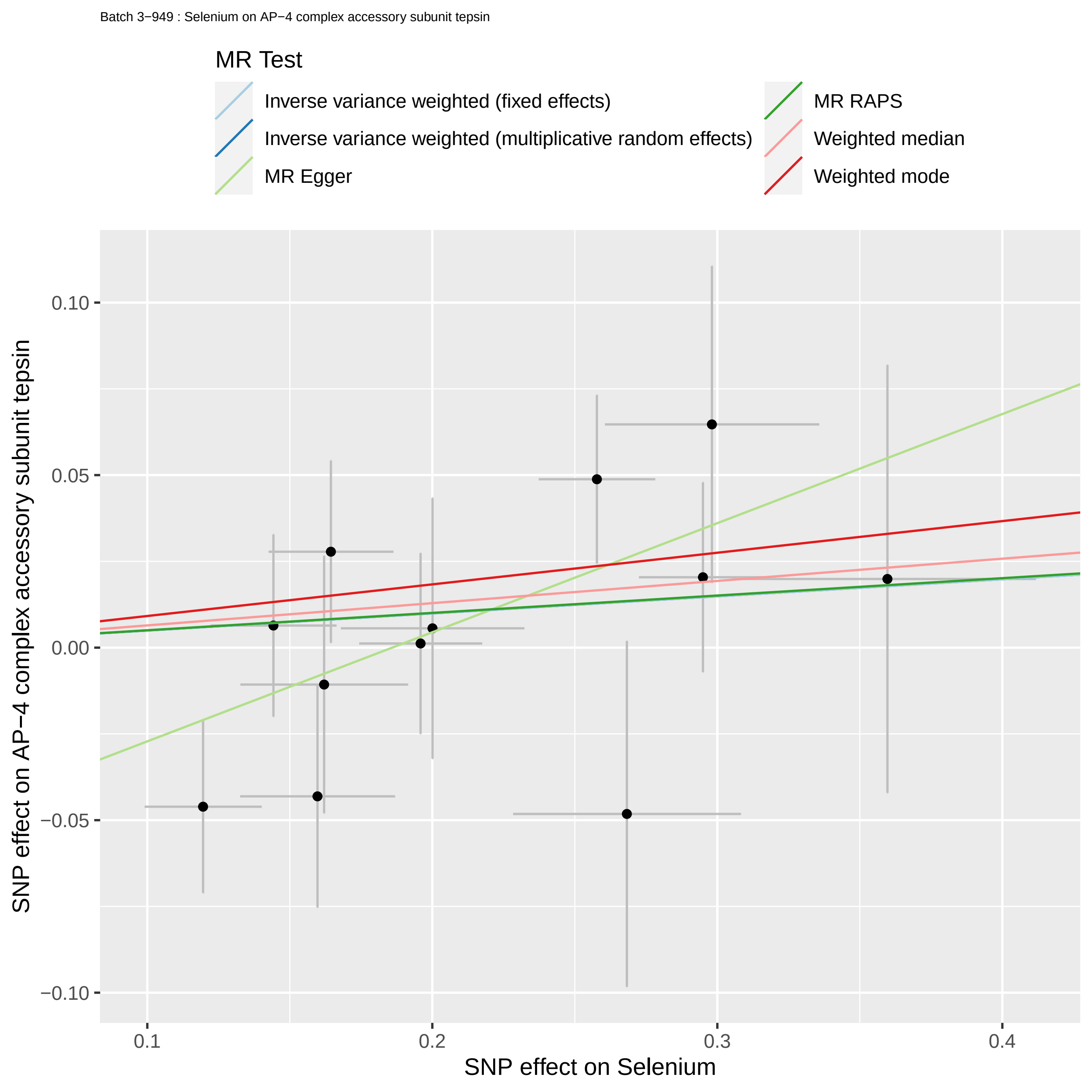

Batch 3−949 : Selenium on AP−4 complex accessory subunit tepsin
MR Test
Inverse variance weighted (fixed effects)
Inverse variance weighted (multiplicative random effects)
MR Egger
MR RAPS
Weighted median
Weighted mode
0.10
0.05
0.00
−0.05
−0.10
0.1
0.2
0.3
0.4
SNP effect on AP−4 complex accessory subunit tepsin
SNP effect on Selenium

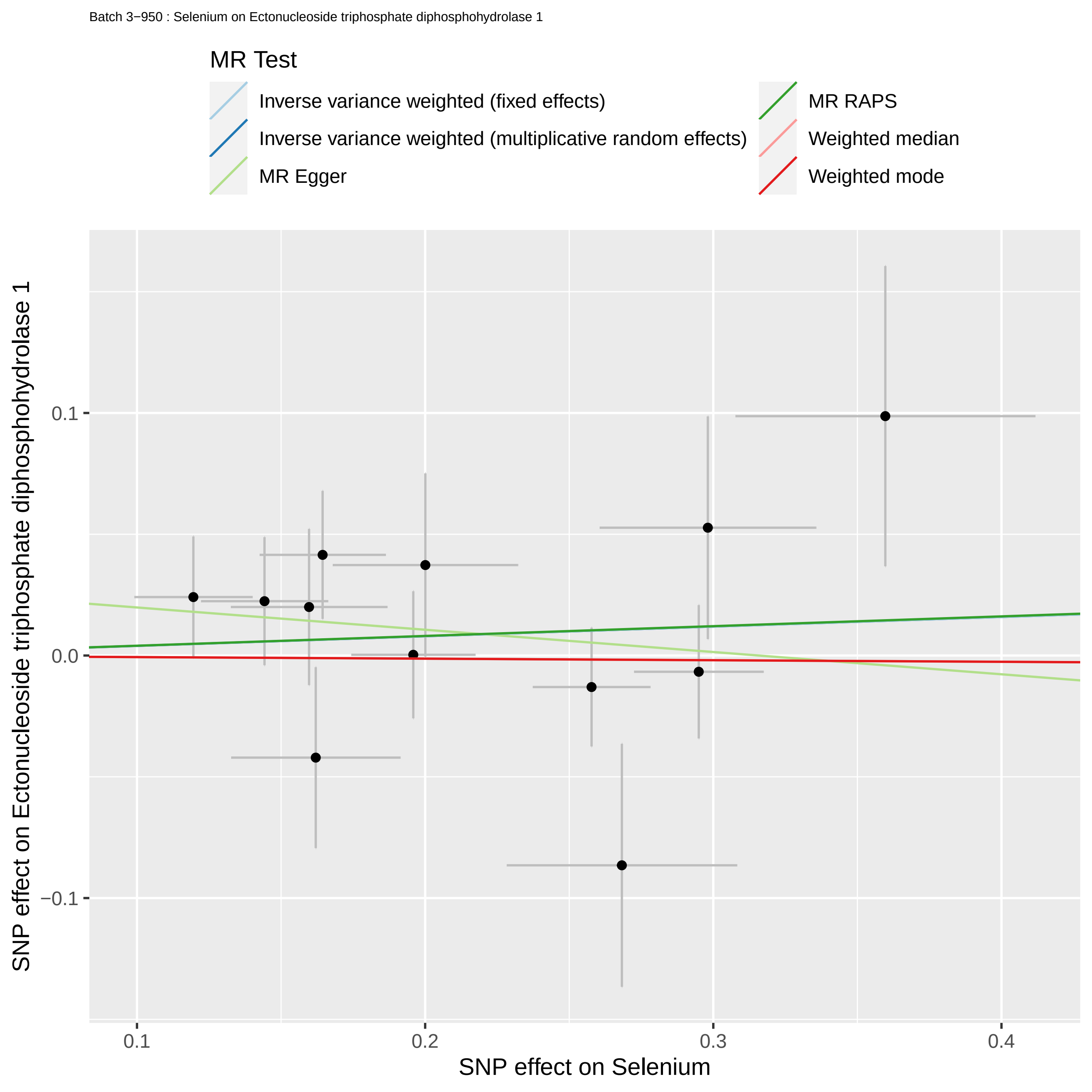
Batch 3−950 : Selenium on Ectonucleoside triphosphate diphosphohydrolase 1
MR Test
Inverse variance weighted (fixed effects)
Inverse variance weighted (multiplicative random effects)
MR Egger
MR RAPS
Weighted median
Weighted mode
SNP effect on Ectonucleoside triphosphate diphosphohydrolase 1
0.1
0.0
−0.1
0.1
0.2
0.3
0.4
SNP effect on Selenium

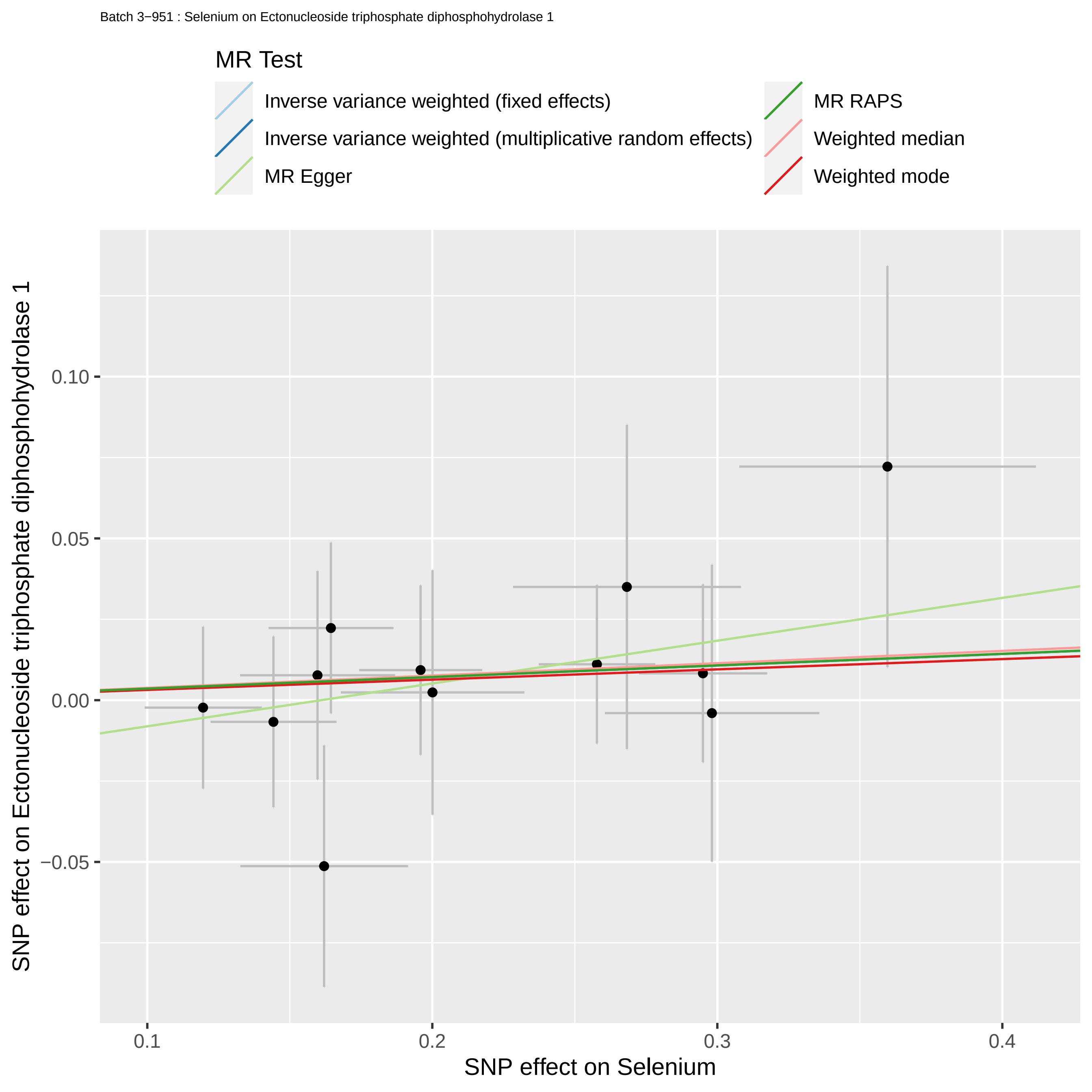

Batch 3−951 : Selenium on Ectonucleoside triphosphate diphosphohydrolase 1
MR Test
Inverse variance weighted (fixed effects)
Inverse variance weighted (multiplicative random effects)
MR Egger
MR RAPS
Weighted median
Weighted mode
SNP effect on Ectonucleoside triphosphate diphosphohydrolase 1
0.10
0.05
0.00
−0.05
0.1
0.2
0.3
0.4
SNP effect on Selenium

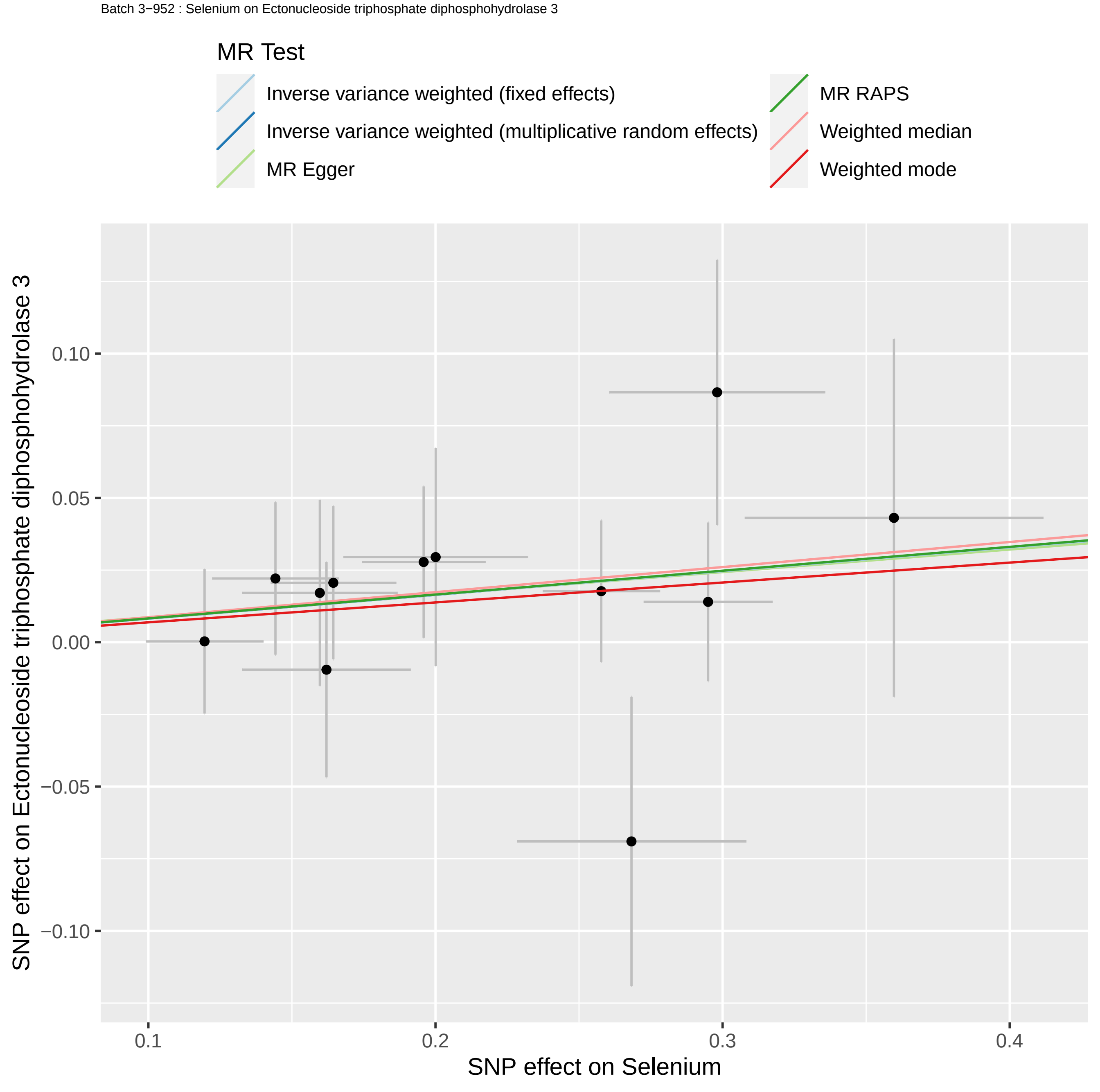

Batch 3−952 : Selenium on Ectonucleoside triphosphate diphosphohydrolase 3
MR Test
Inverse variance weighted (fixed effects)
Inverse variance weighted (multiplicative random effects)
MR Egger
MR RAPS
Weighted median
Weighted mode
SNP effect on Ectonucleoside triphosphate diphosphohydrolase 3
0.10
0.05
0.00
−0.05
−0.10
0.1
0.2
0.3
0.4
SNP effect on Selenium

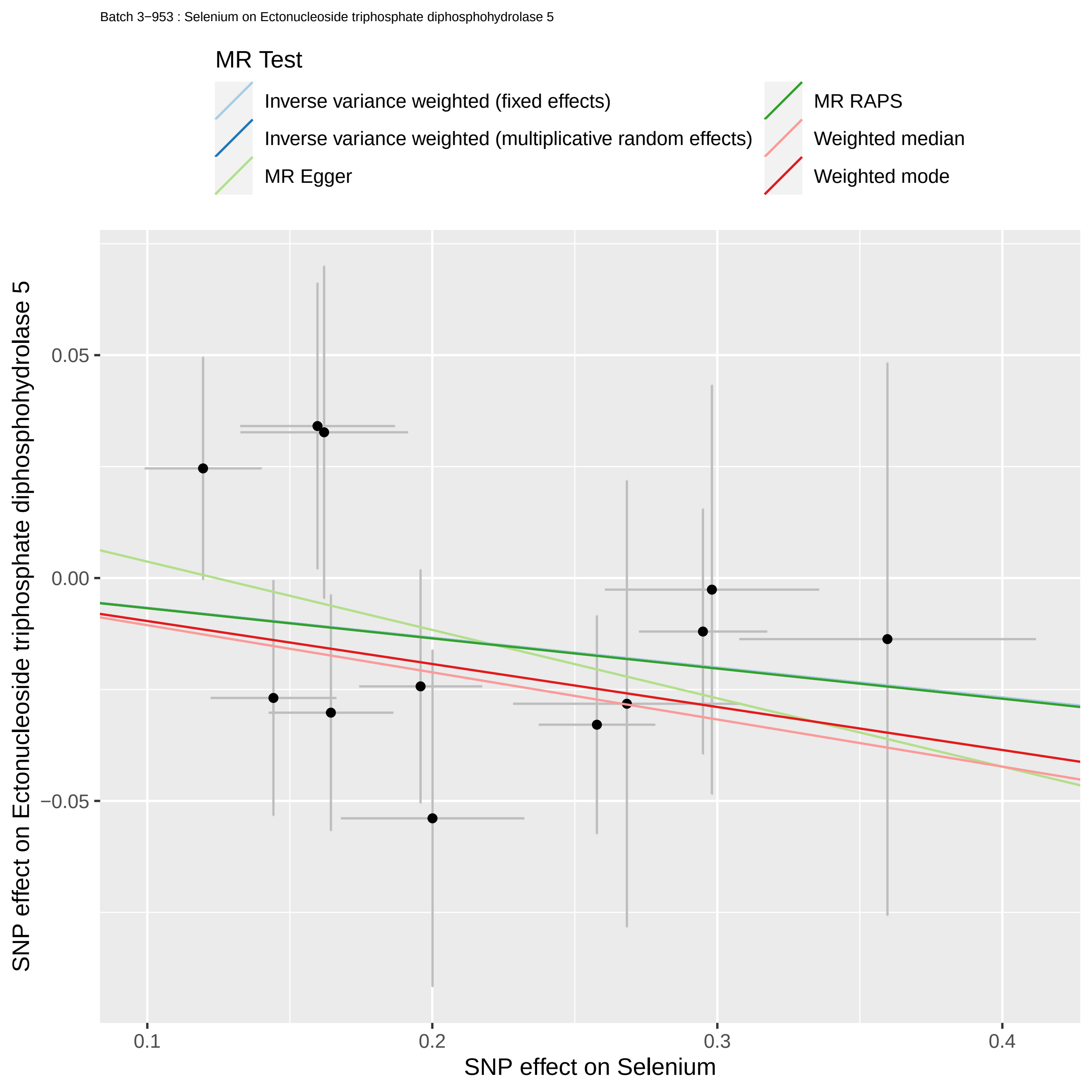
Batch 3−953 : Selenium on Ectonucleoside triphosphate diphosphohydrolase 5
MR Test
Inverse variance weighted (fixed effects)
Inverse variance weighted (multiplicative random effects)
MR Egger
MR RAPS
Weighted median
Weighted mode
0.05
0.00
−0.05
0.1
0.2
0.3
0.4
SNP effect on Ectonucleoside triphosphate diphosphohydrolase 5
SNP effect on Selenium

Batch 3−954 : Selenium on Ectonucleoside triphosphate diphosphohydrolase 6

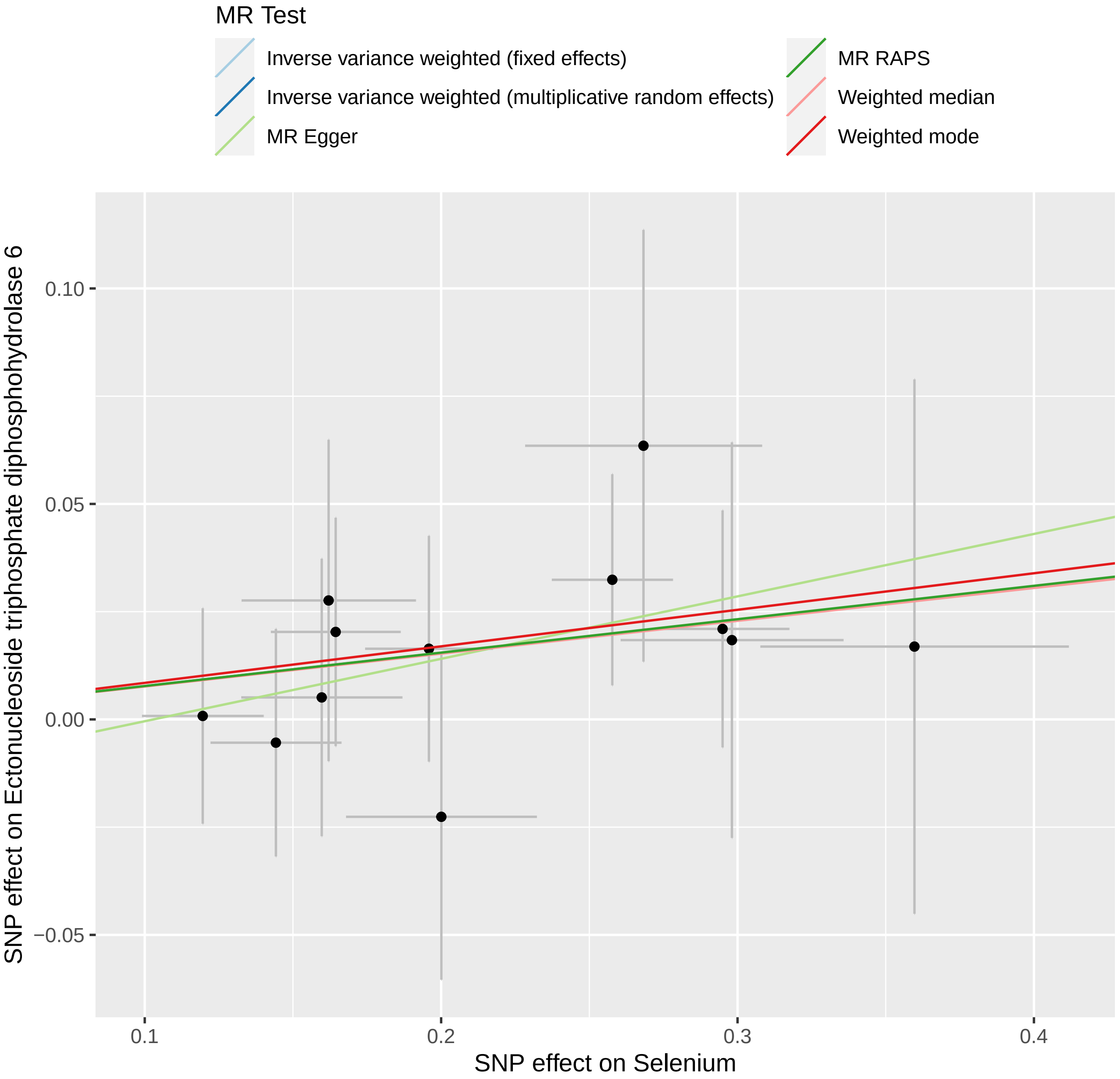

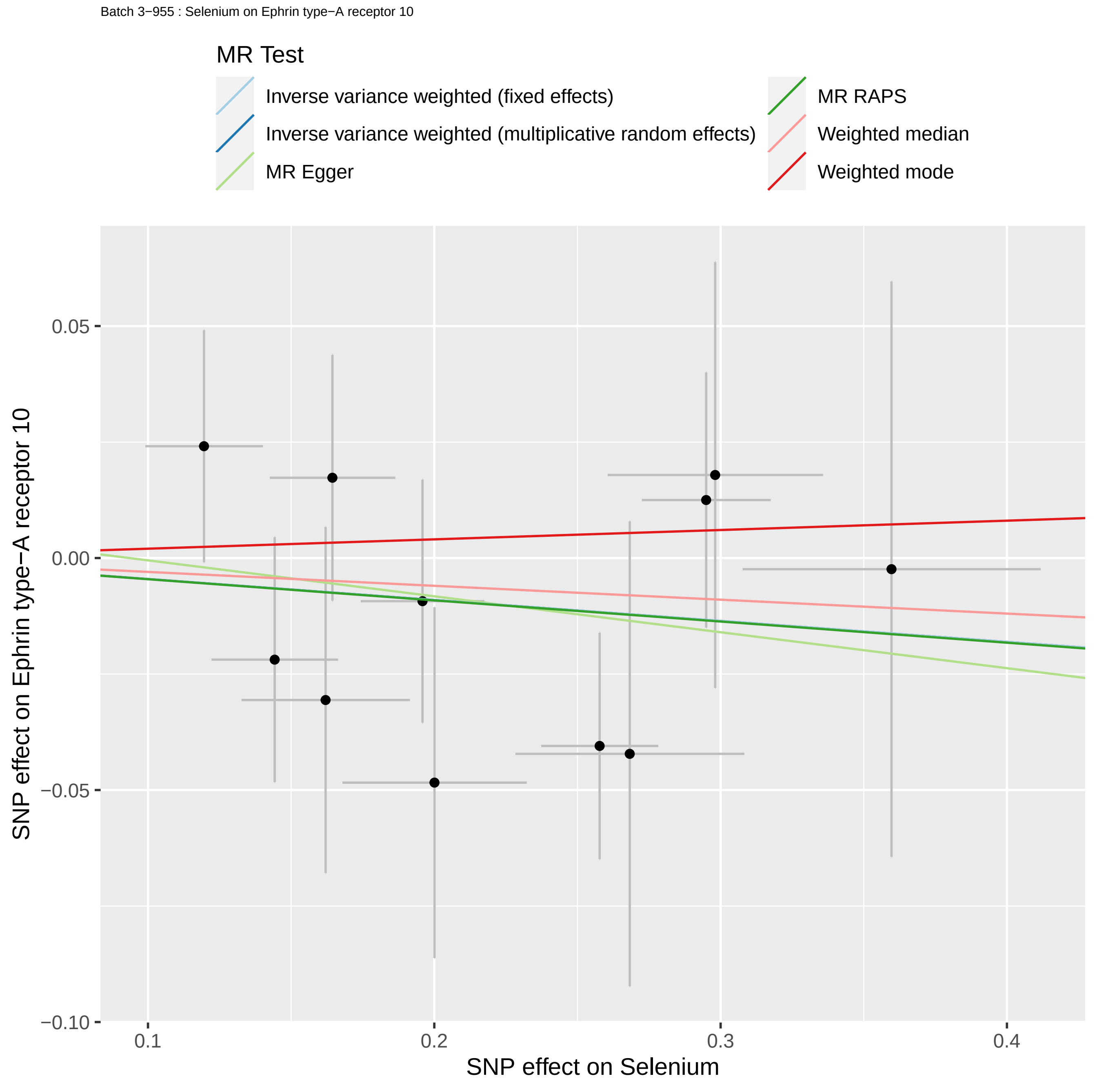
Batch 3−955 : Selenium on Ephrin type−A receptor 10
MR Test
Inverse variance weighted (fixed effects)
Inverse variance weighted (multiplicative random effects)
MR Egger
MR RAPS
Weighted median
Weighted mode
0.05
0.00
−0.05
−0.10
0.1
0.2
0.3
0.4
SNP effect on Ephrin type−A receptor 10
SNP effect on Selenium

Batch 3−956 : Selenium on Ephrin type−A receptor 1

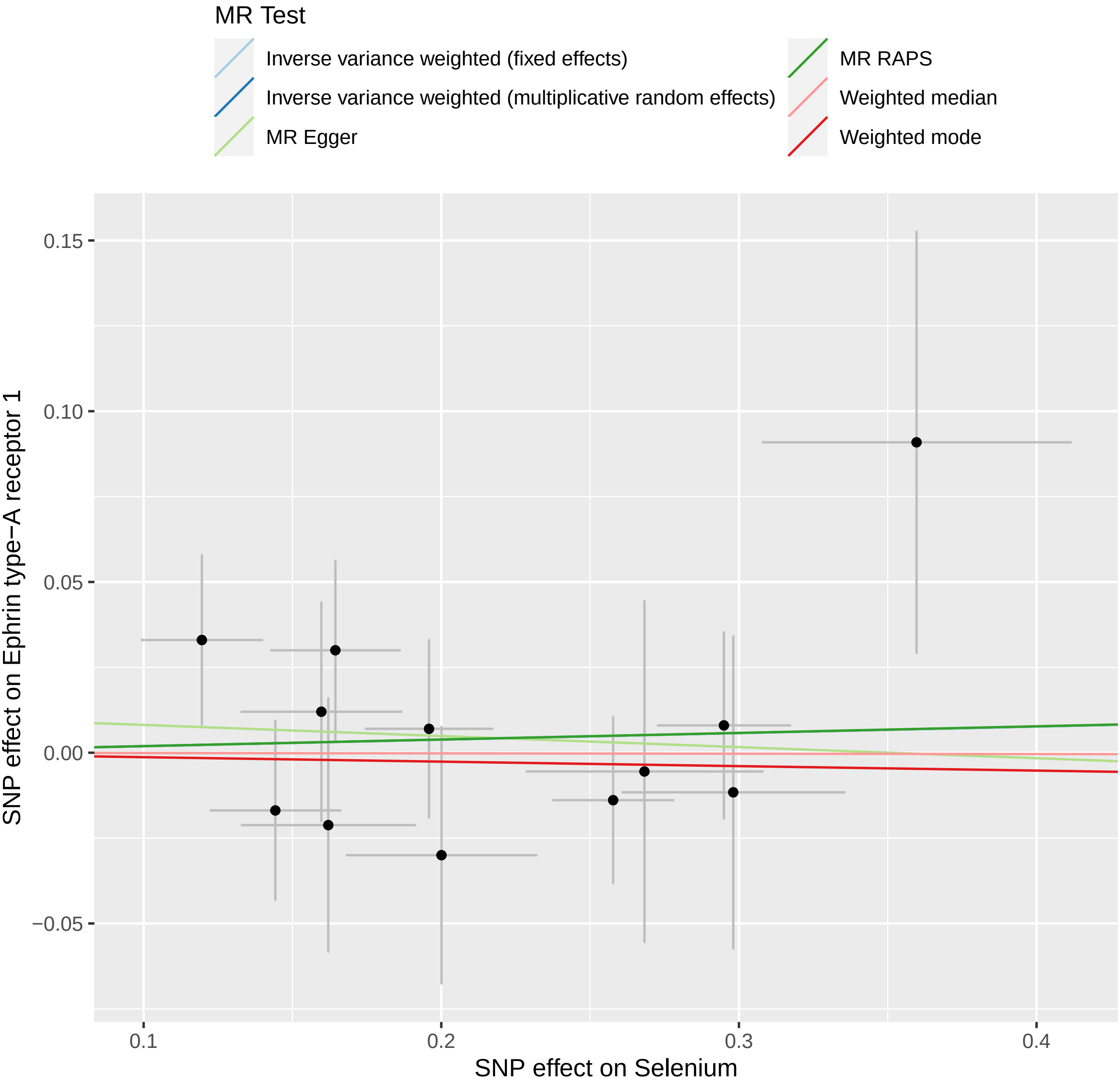

Batch 3−957 : Selenium on Ephrin type−A receptor 2

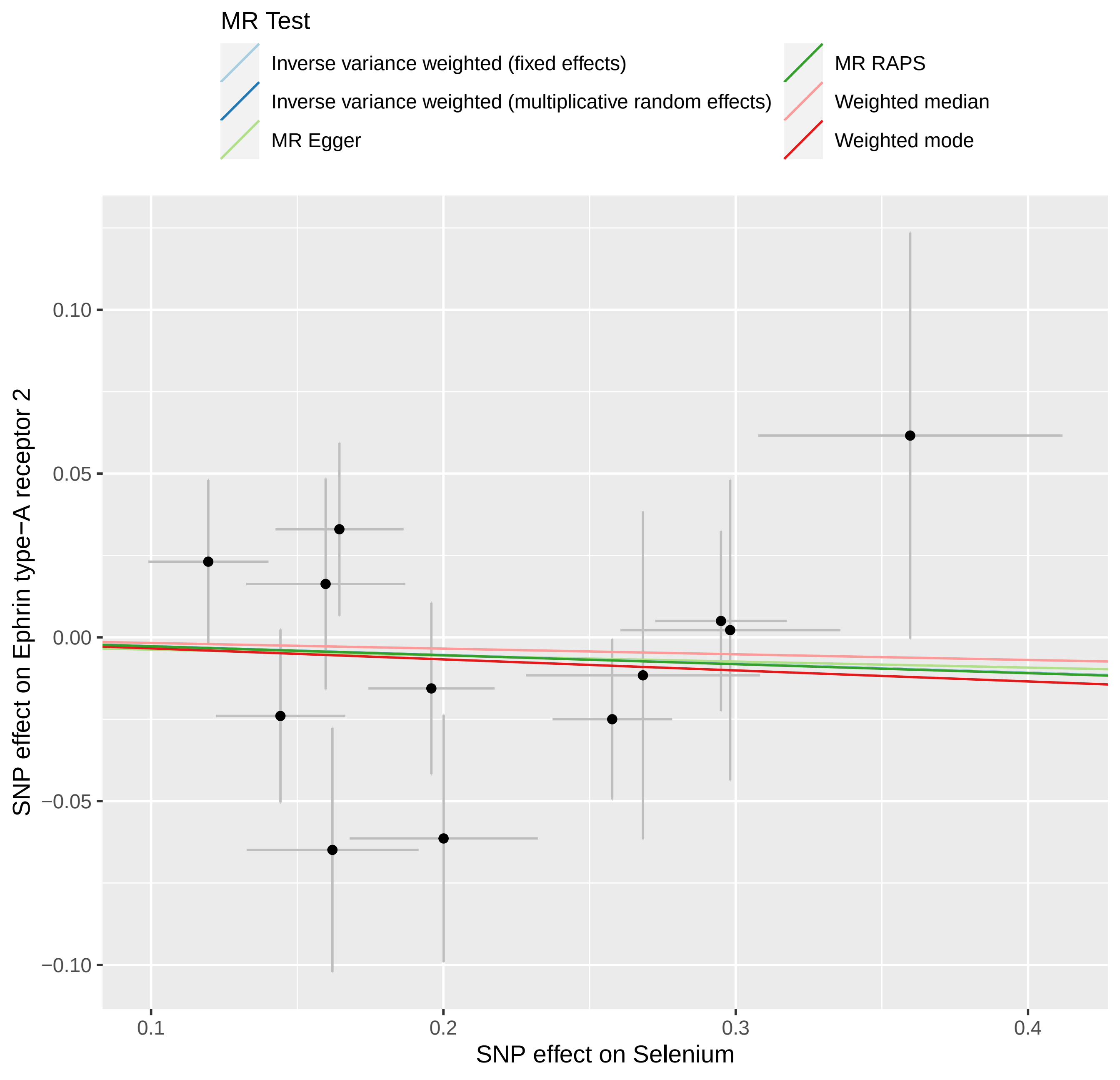

Batch 3−958 : Selenium on Ephrin type−A receptor 5

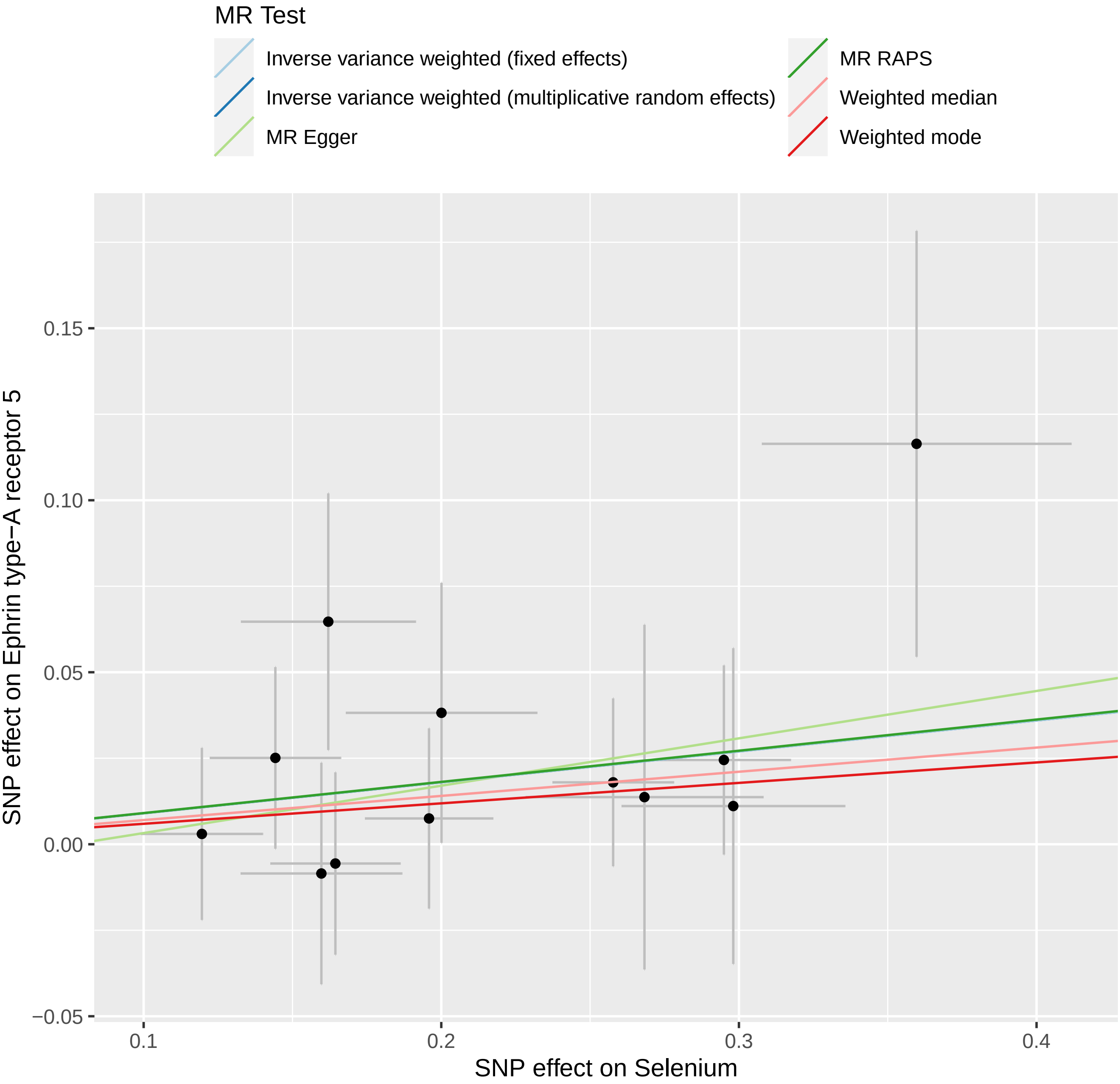

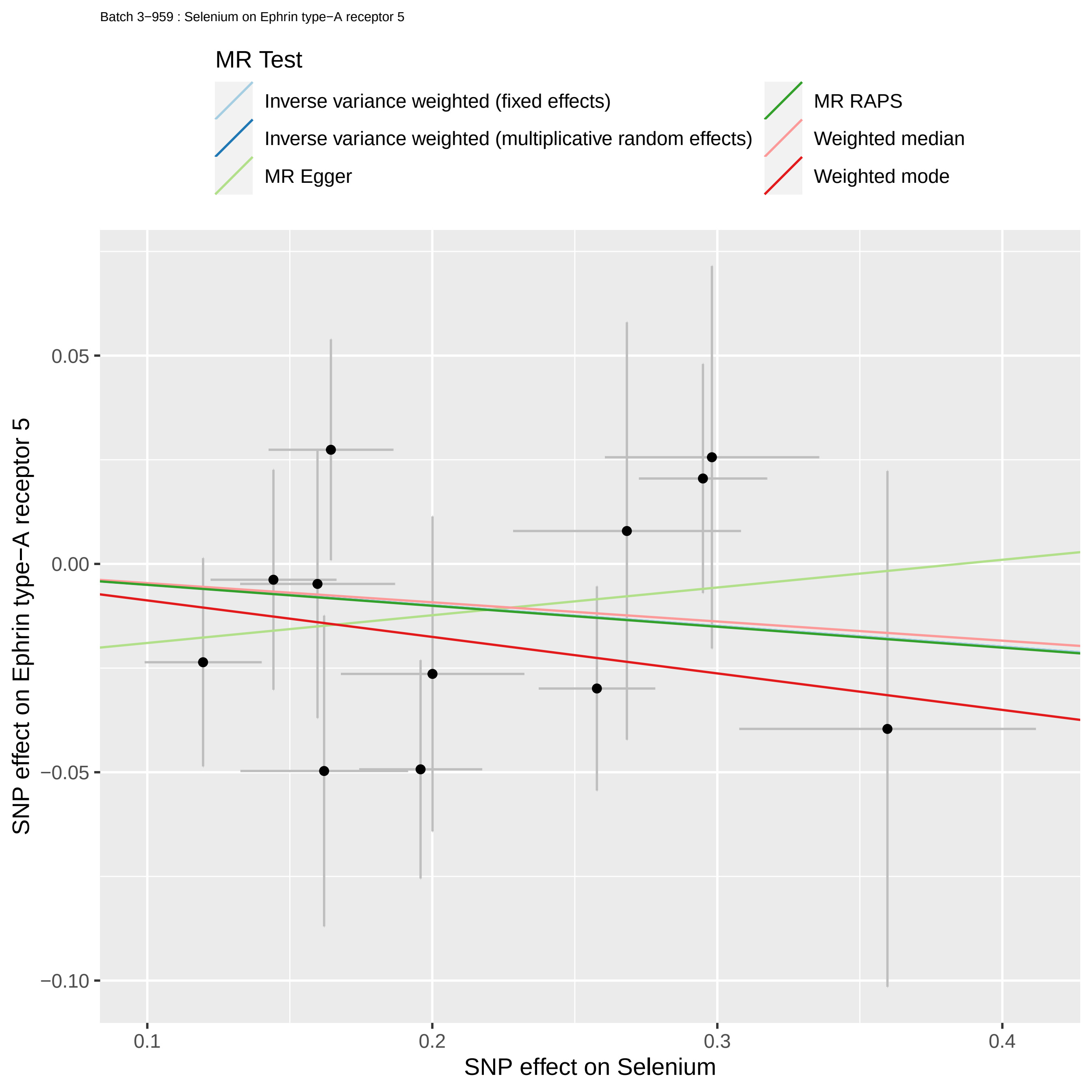
Batch 3−959 : Selenium on Ephrin type−A receptor 5
MR Test
Inverse variance weighted (fixed effects)
Inverse variance weighted (multiplicative random effects)
MR Egger
MR RAPS
Weighted median
Weighted mode
0.05
0.00
−0.05
−0.10
0.1
0.2
0.3
0.4
SNP effect on Ephrin type−A receptor 5
SNP effect on Selenium

Batch 3−960 : Selenium on Ephrin type−B receptor 2

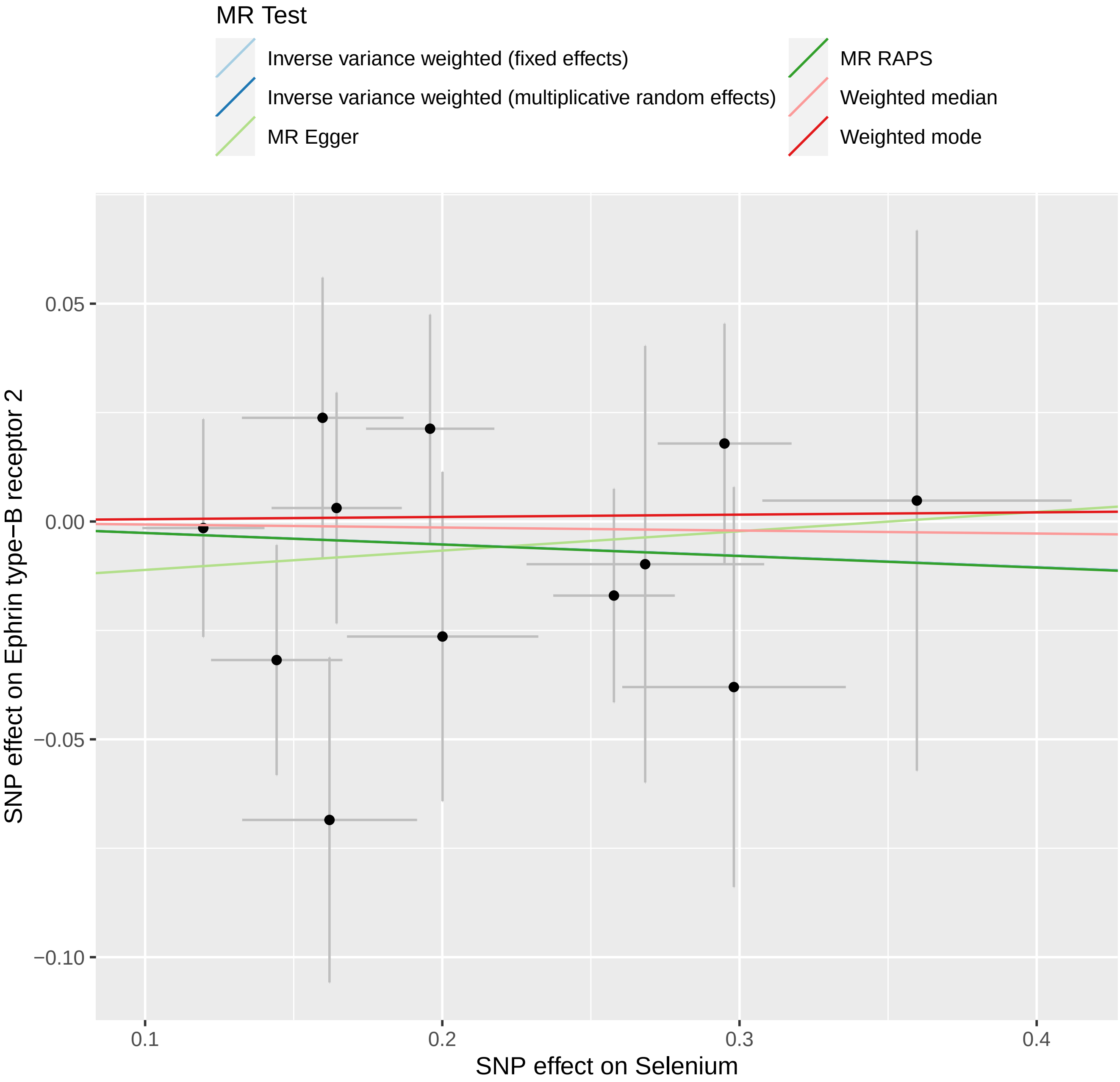

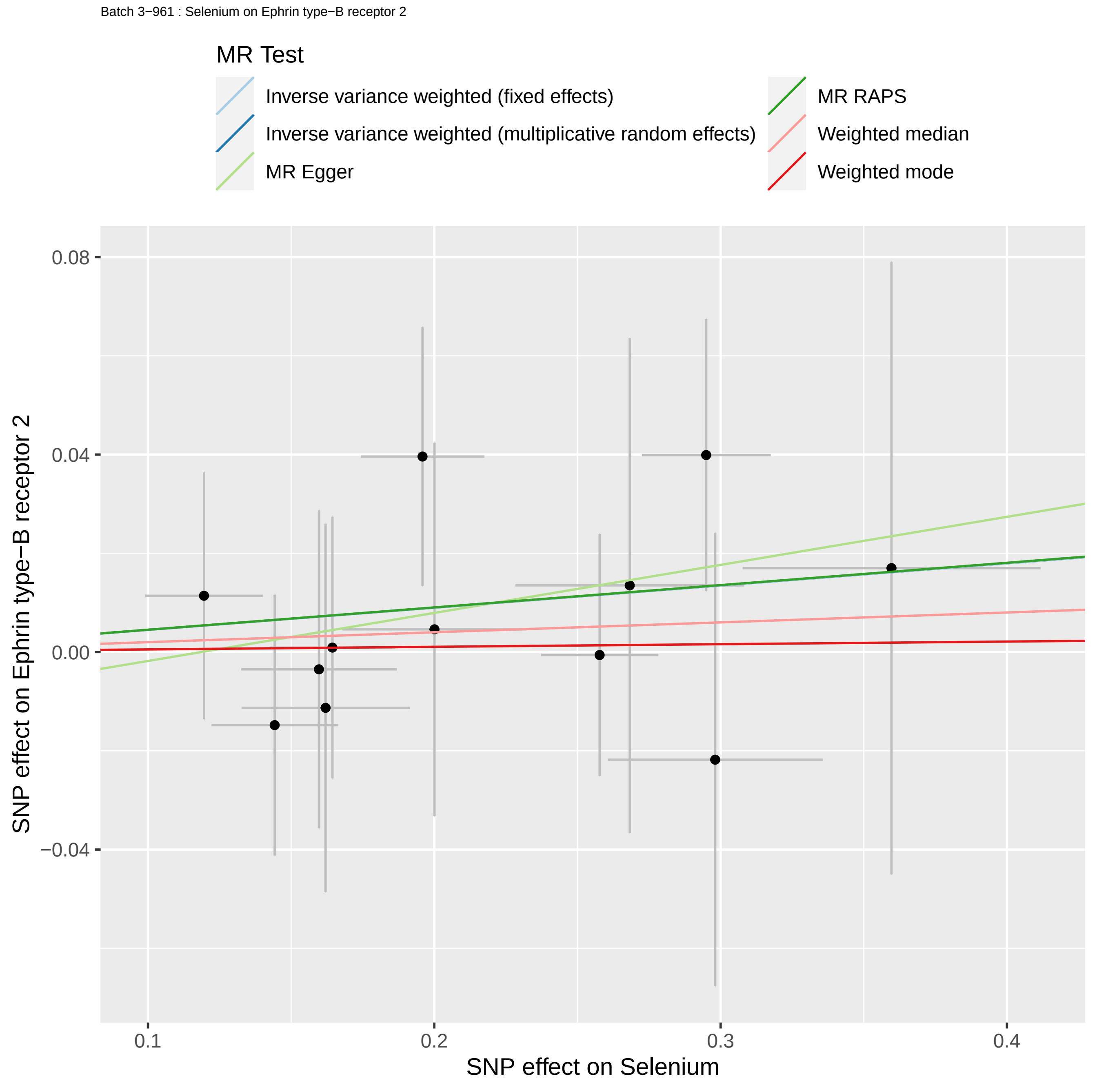

Batch 3−961 : Selenium on Ephrin type−B receptor 2
MR Test
Inverse variance weighted (fixed effects)
Inverse variance weighted (multiplicative random effects)
MR Egger
MR RAPS
Weighted median
Weighted mode
0.08
0.04
0.00
−0.04
0.1
0.2
0.3
0.4
SNP effect on Ephrin type−B receptor 2
SNP effect on Selenium

Batch 3−962 : Selenium on Ephrin type−B receptor 2

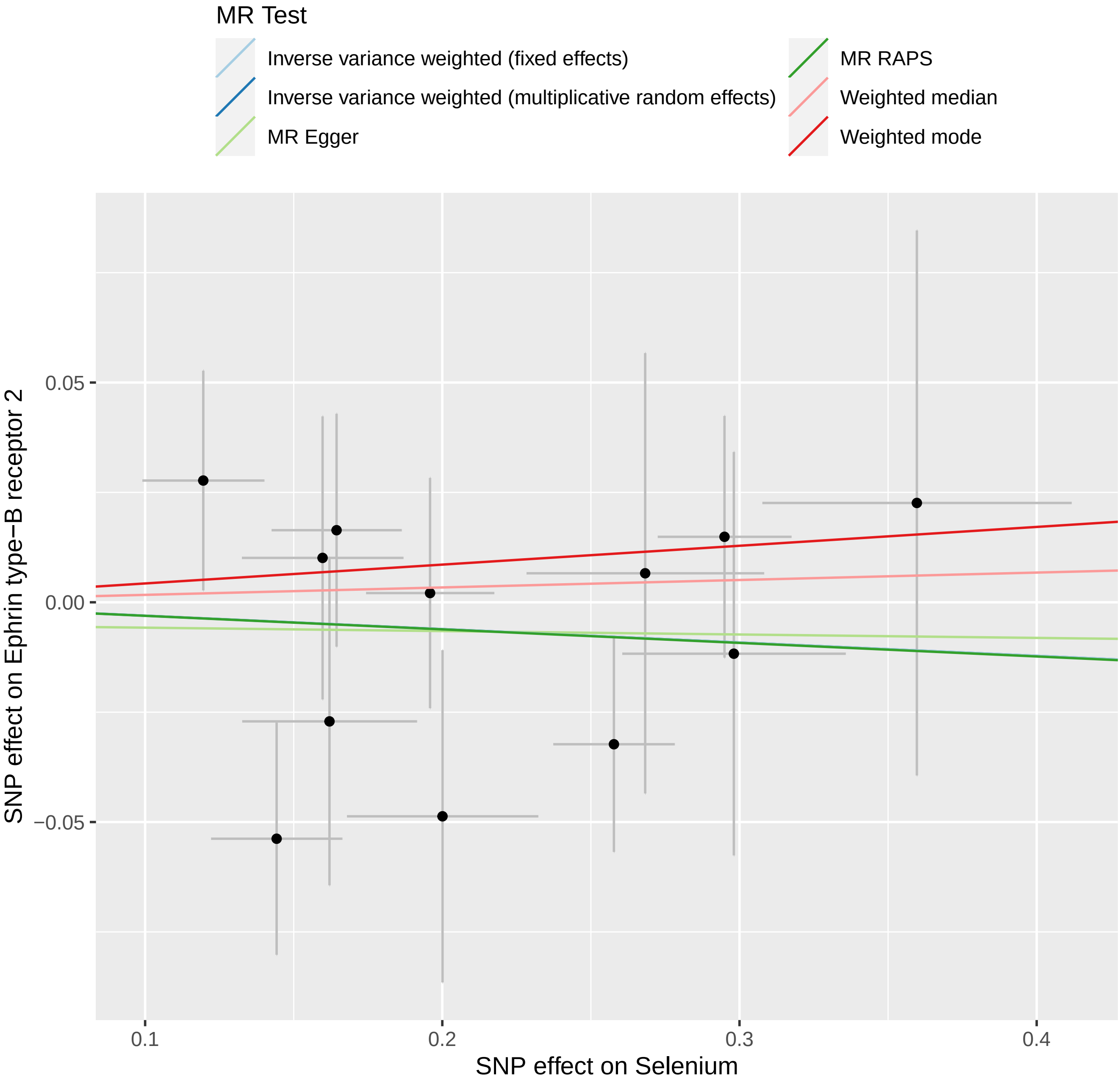

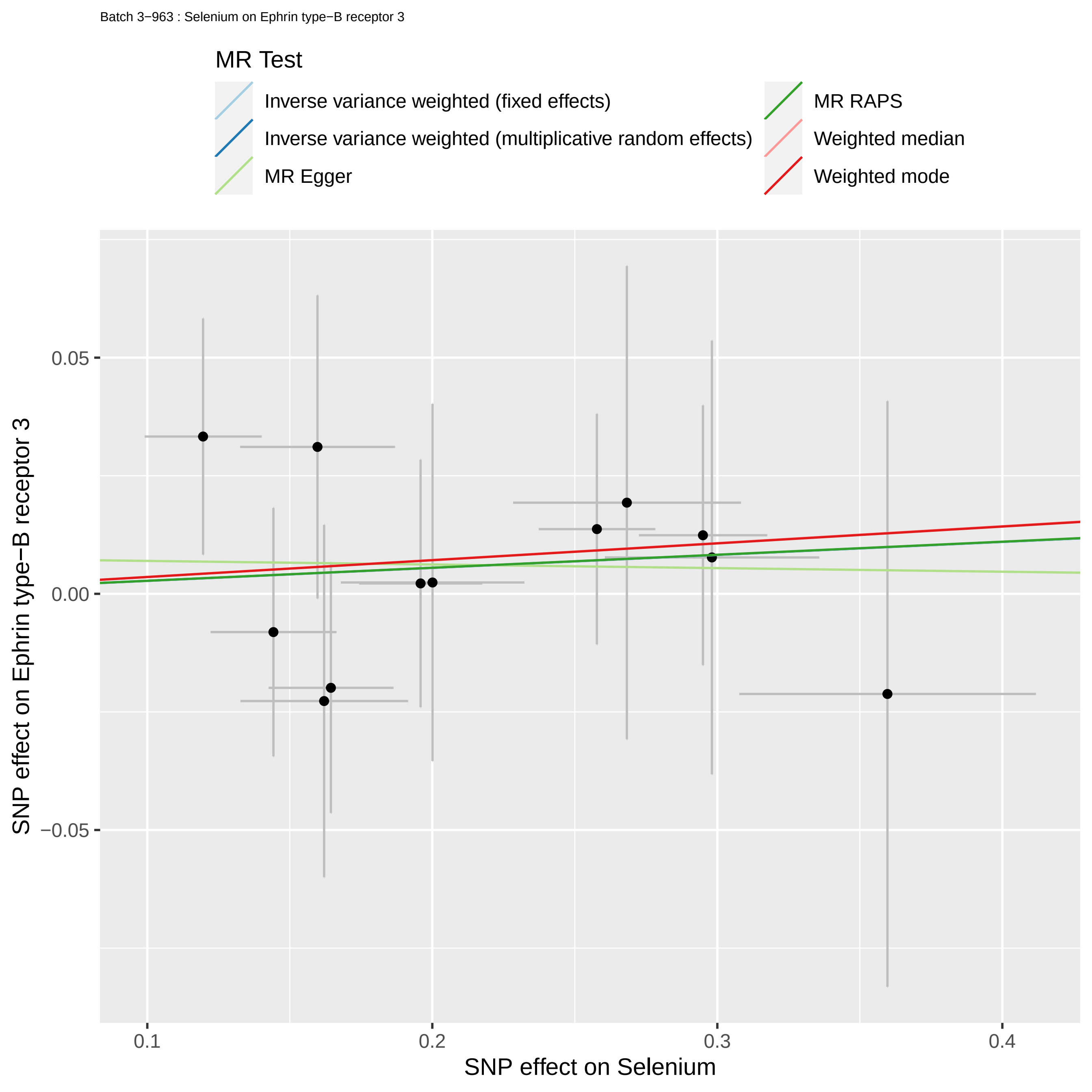

Batch 3−963 : Selenium on Ephrin type−B receptor 3
MR Test
Inverse variance weighted (fixed effects)
Inverse variance weighted (multiplicative random effects)
MR Egger
MR RAPS
Weighted median
Weighted mode
0.05
0.00
−0.05
0.1
0.2
0.3
0.4
SNP effect on Ephrin type−B receptor 3
SNP effect on Selenium

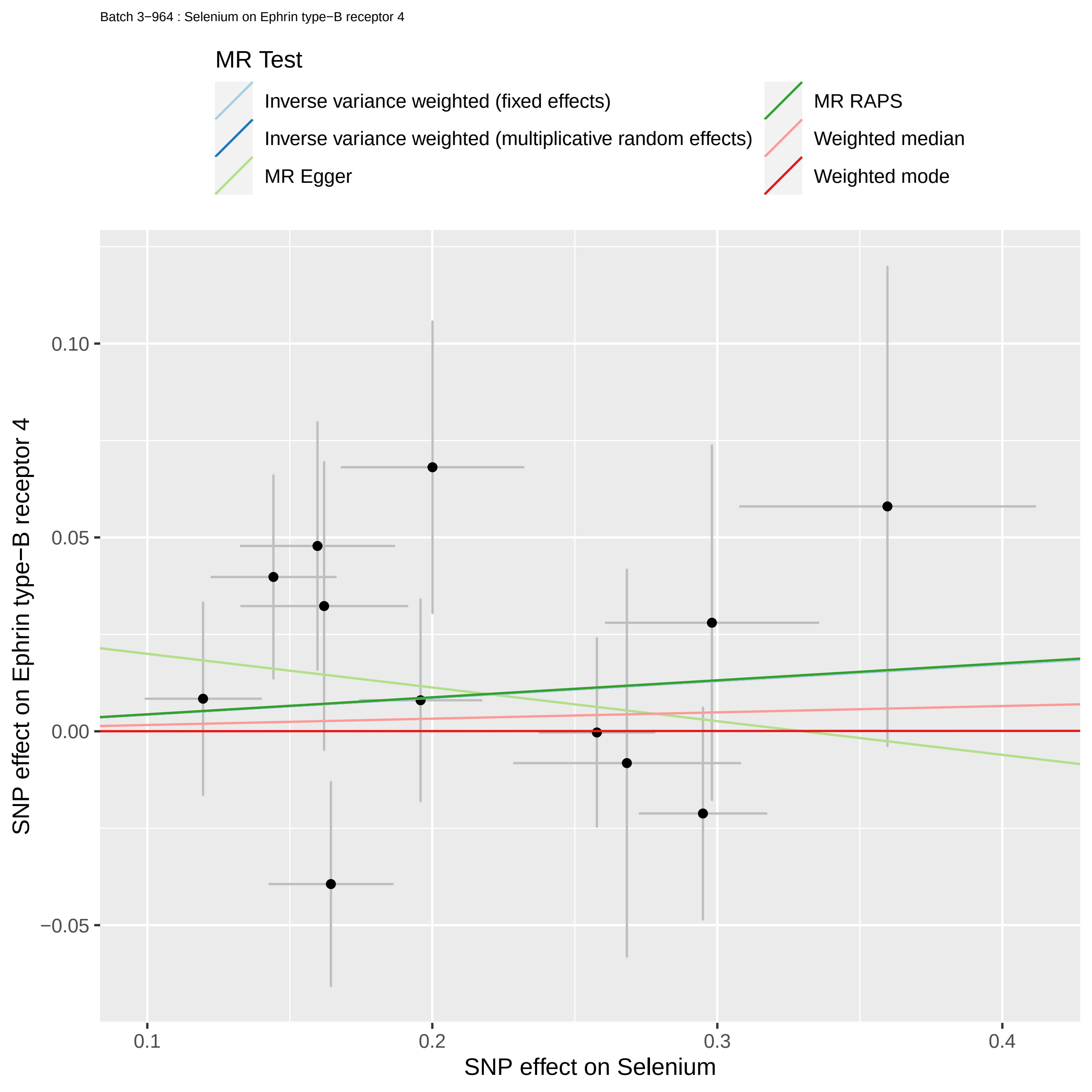
Batch 3−964 : Selenium on Ephrin type−B receptor 4
MR Test
Inverse variance weighted (fixed effects)
Inverse variance weighted (multiplicative random effects)
MR Egger
MR RAPS
Weighted median
Weighted mode
0.10
0.05
0.00
−0.05
0.1
0.2
0.3
0.4
SNP effect on Ephrin type−B receptor 4
SNP effect on Selenium

Batch 3−965 : Selenium on Ephrin type−B receptor 6

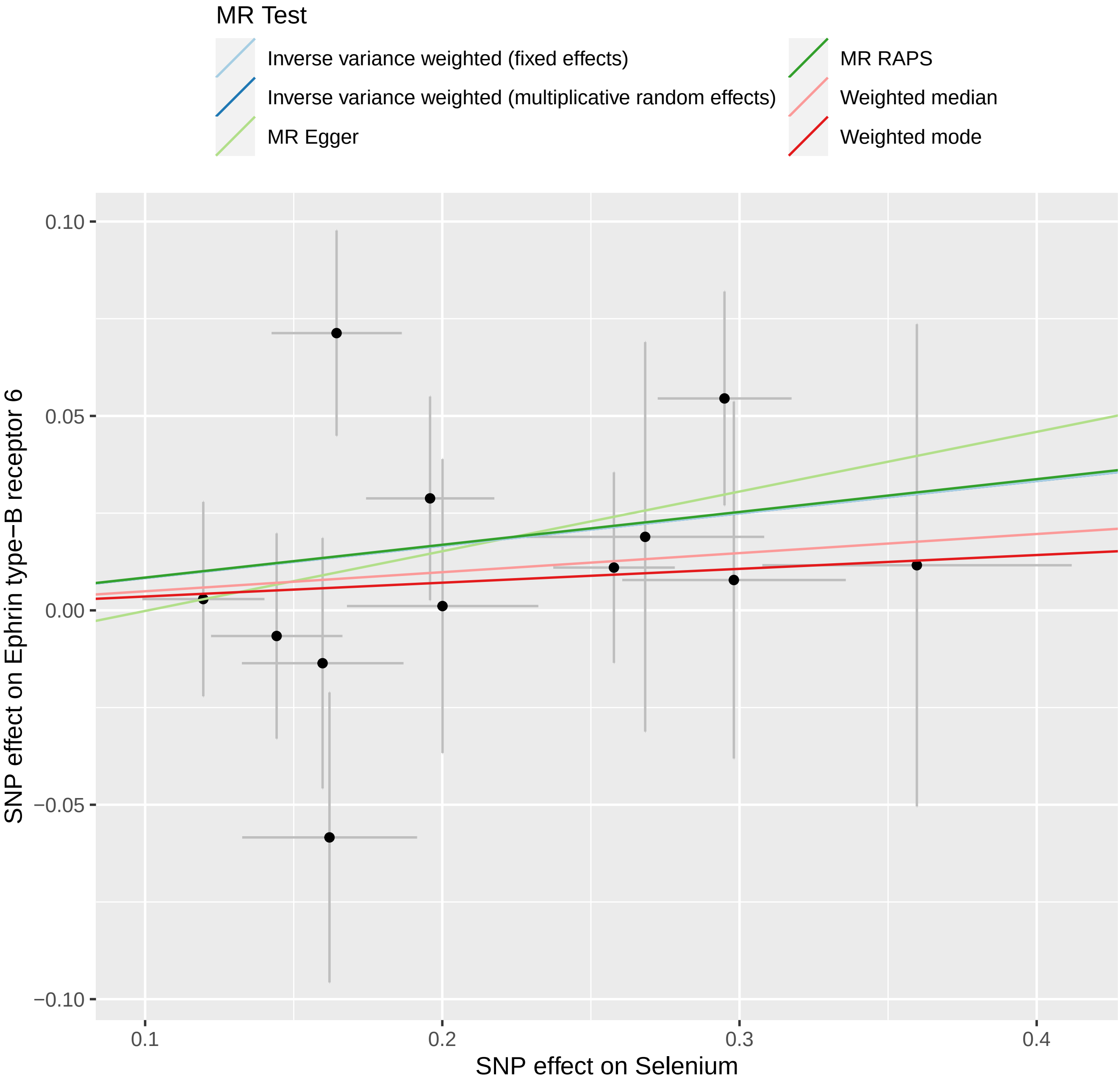

Batch 3−966 : Selenium on Ephrin type−B receptor 6

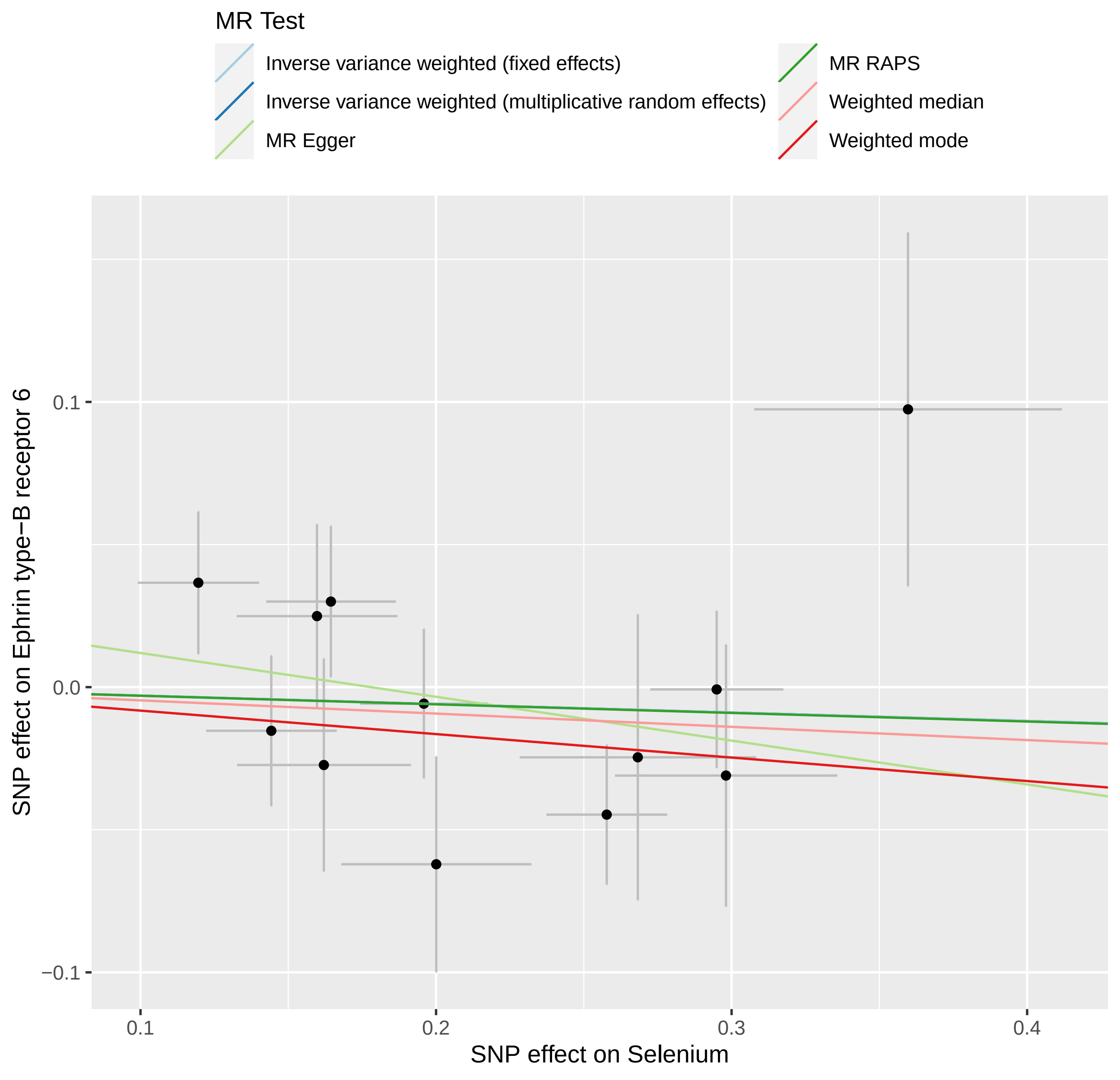

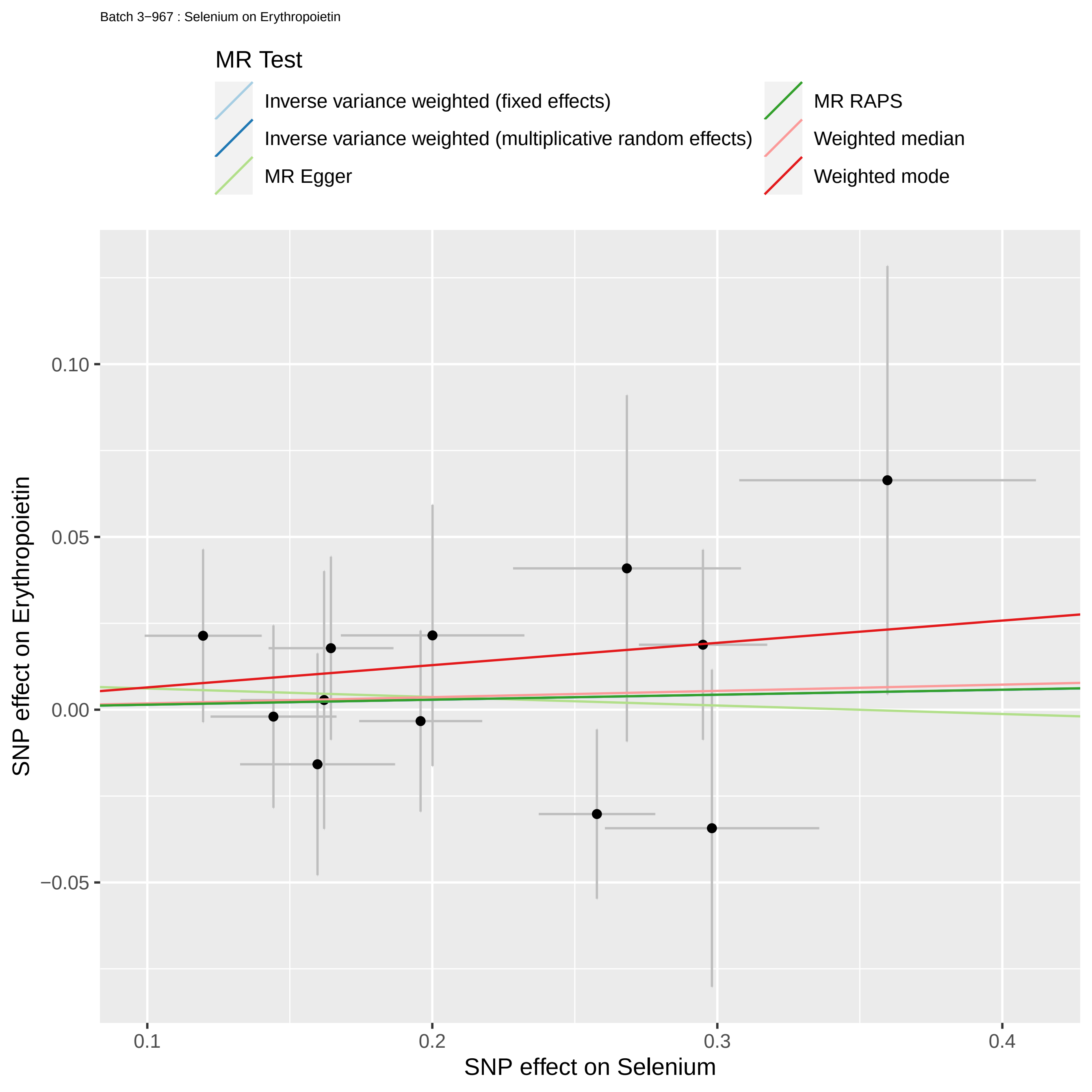
Batch 3−967 : Selenium on Erythropoietin
MR Test
Inverse variance weighted (fixed effects)
Inverse variance weighted (multiplicative random effects)
MR Egger
MR RAPS
Weighted median
Weighted mode
0.10
0.05
0.00
−0.05
0.1
0.2
0.3
0.4
SNP effect on Selenium
SNP effect on Erythropoietin

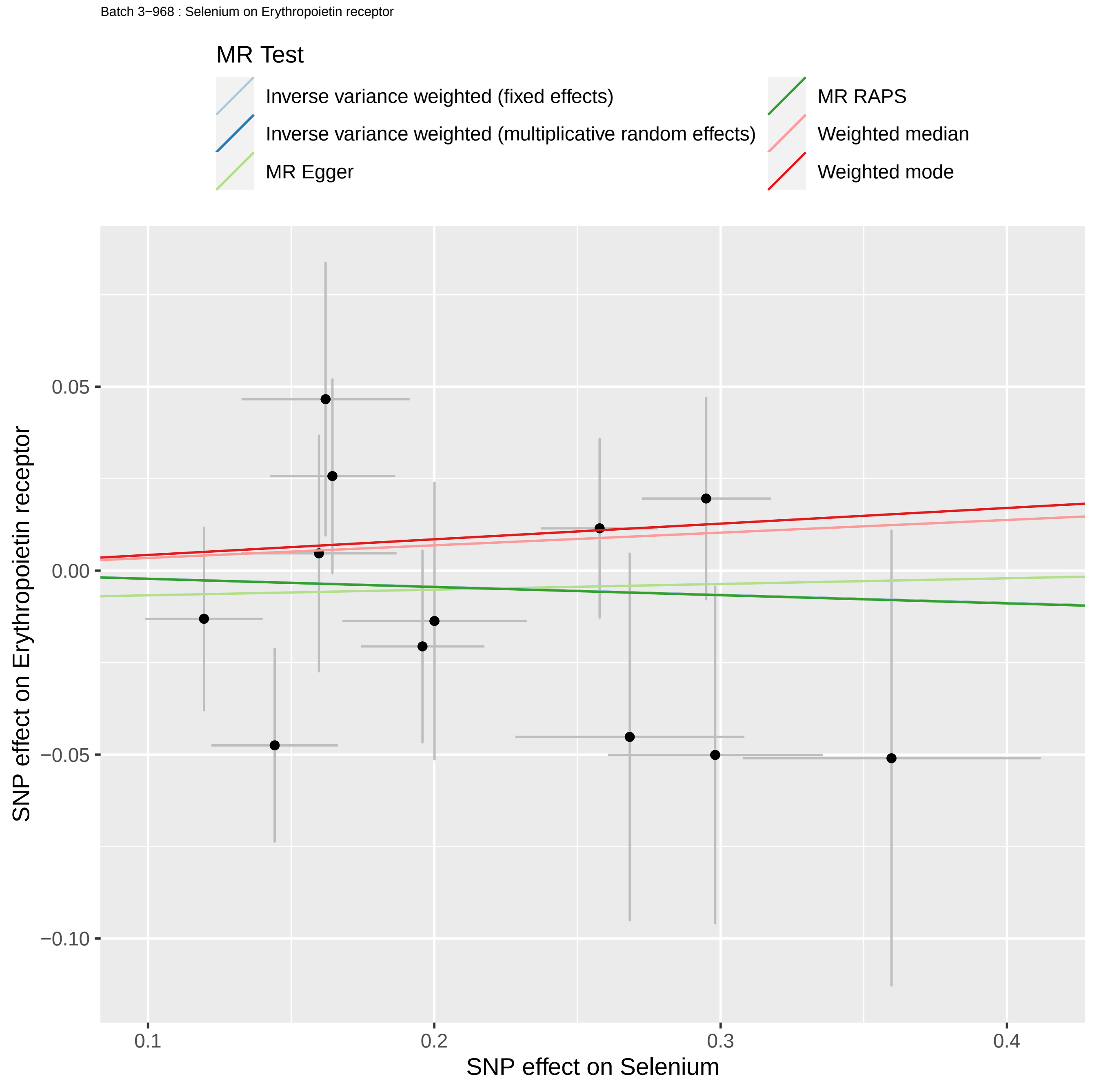
Batch 3−968 : Selenium on Erythropoietin receptor
MR Test
Inverse variance weighted (fixed effects)
Inverse variance weighted (multiplicative random effects)
MR Egger
MR RAPS
Weighted median
Weighted mode
SNP effect on Erythropoietin receptor
0.05
0.00
−0.05
−0.10
0.1
0.2
0.3
0.4
SNP effect on Selenium

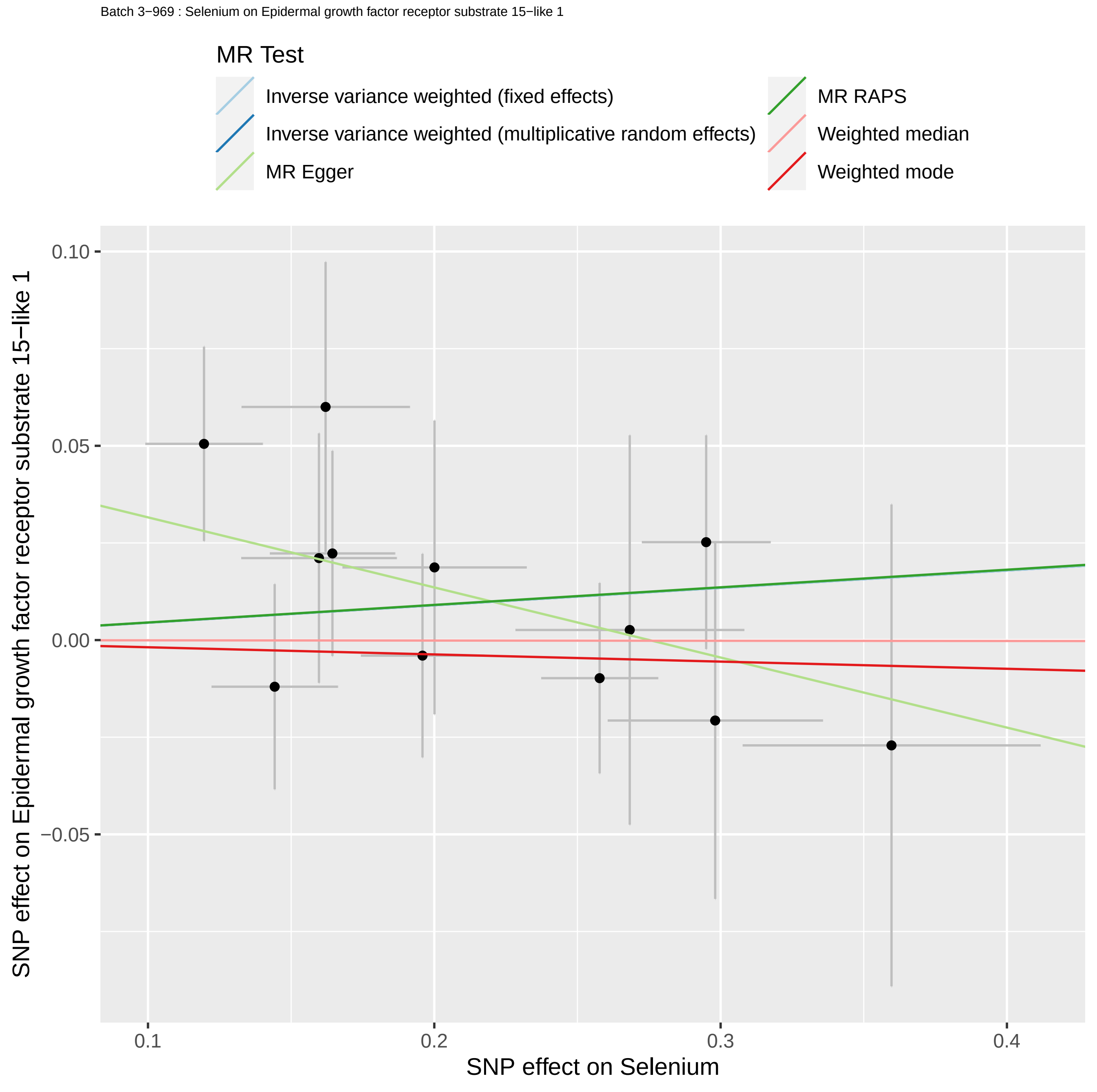

Batch 3−969 : Selenium on Epidermal growth factor receptor substrate 15−like 1
MR Test
Inverse variance weighted (fixed effects)
Inverse variance weighted (multiplicative random effects)
MR Egger
MR RAPS
Weighted median
Weighted mode
0.10
0.05
0.00
−0.05
0.1
0.2
0.3
0.4
SNP effect on Epidermal growth factor receptor substrate 15−like 1
SNP effect on Selenium

Batch 3−970 : Selenium on Epiphycan

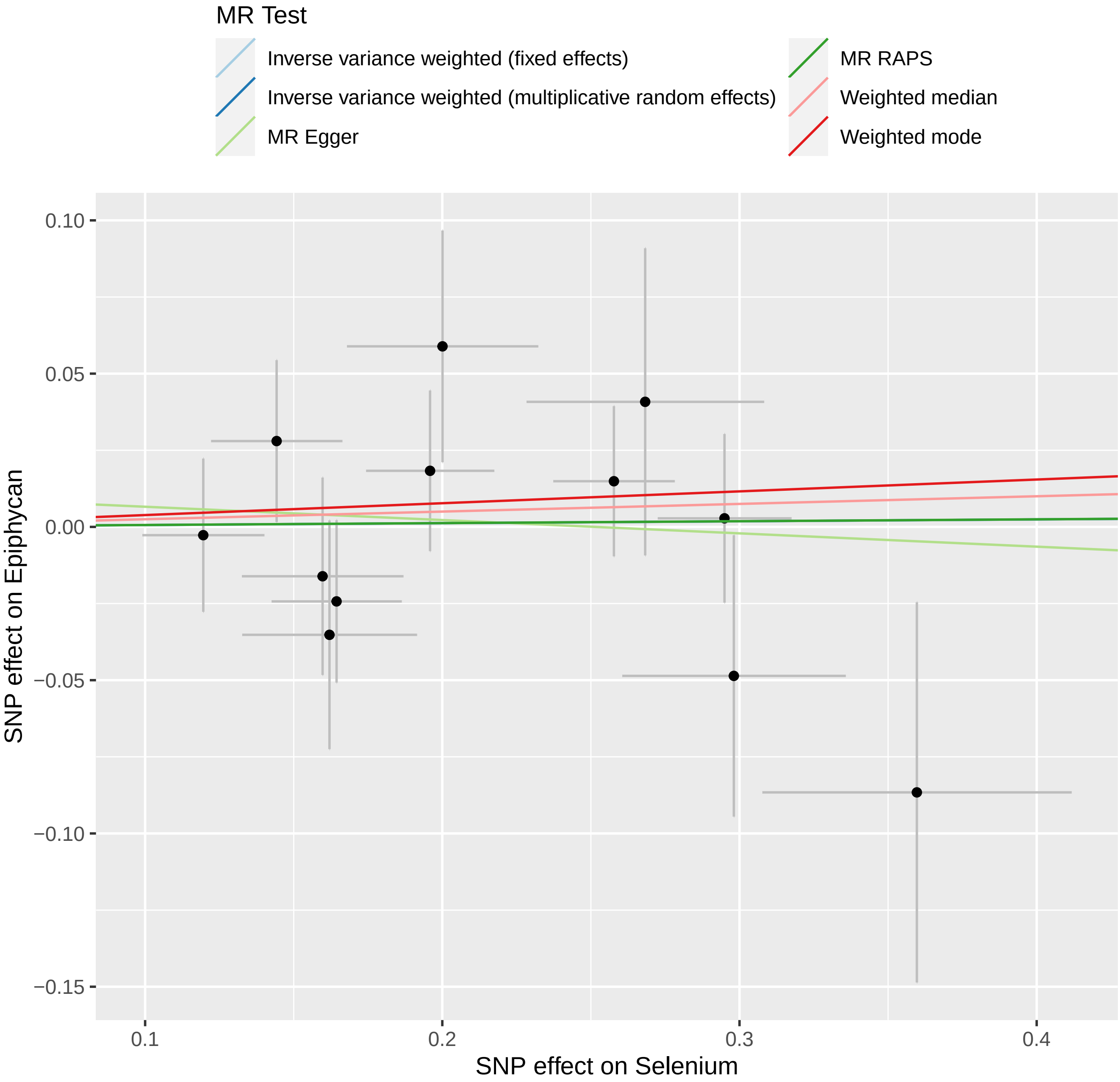

Batch 3−971 : Selenium on Endoplasmic reticulum aminopeptidase 1

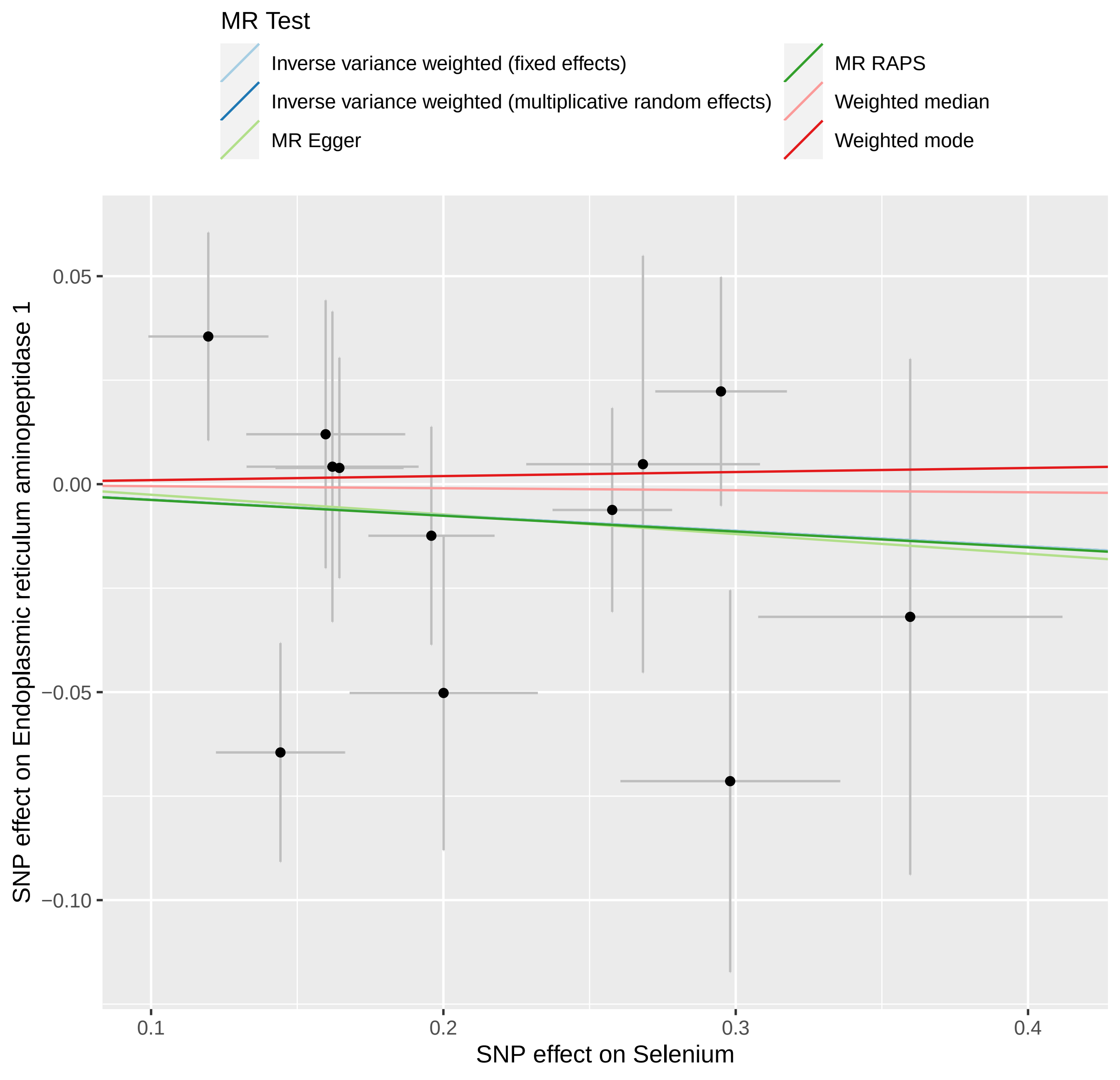

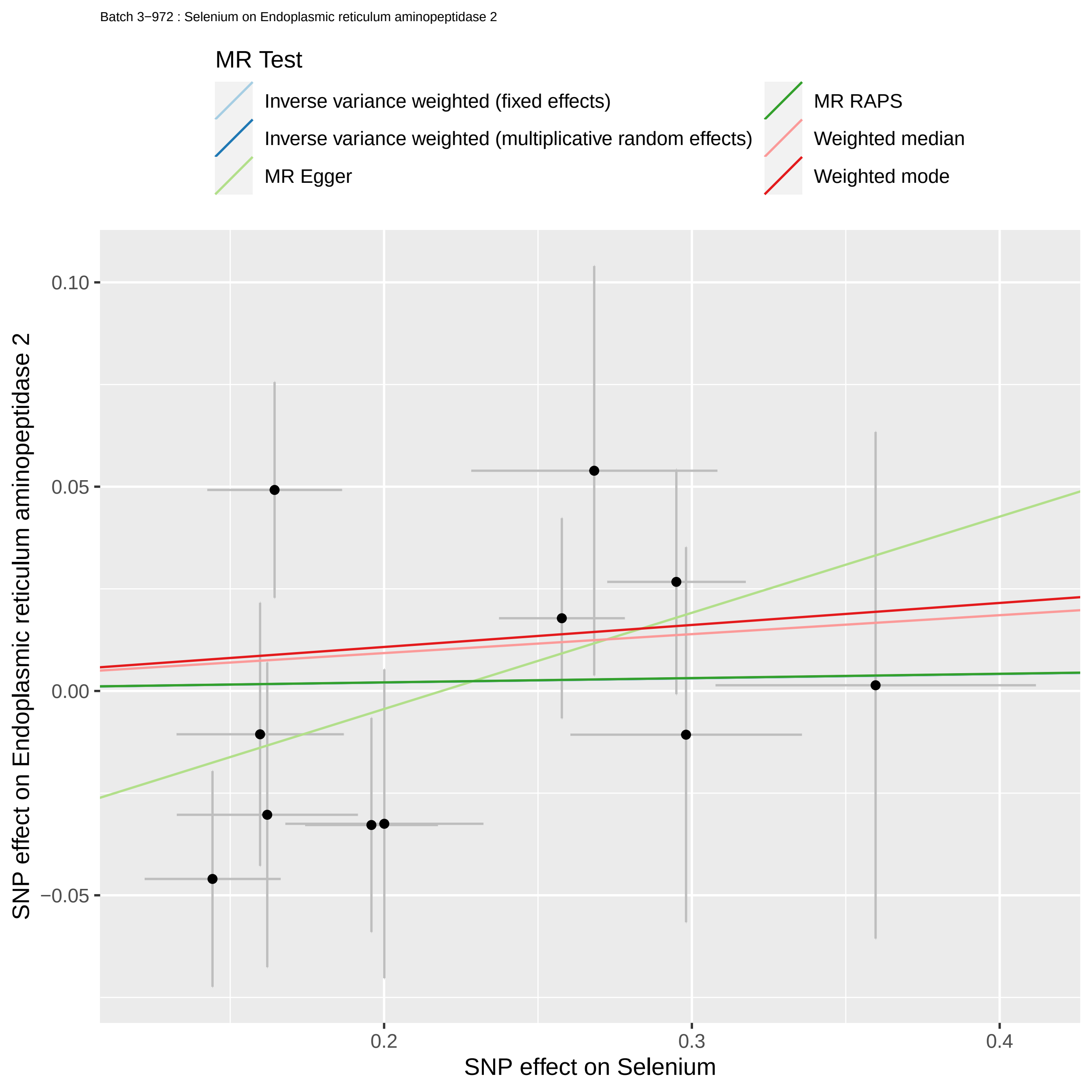
Batch 3−972 : Selenium on Endoplasmic reticulum aminopeptidase 2
MR Test
Inverse variance weighted (fixed effects)
Inverse variance weighted (multiplicative random effects)
MR Egger
MR RAPS
Weighted median
Weighted mode
0.10
0.05
0.00
−0.05
0.2
0.3
0.4
SNP effect on Endoplasmic reticulum aminopeptidase 2
SNP effect on Selenium

Batch 3−973 : Selenium on Receptor tyrosine−protein kinase erbB−2

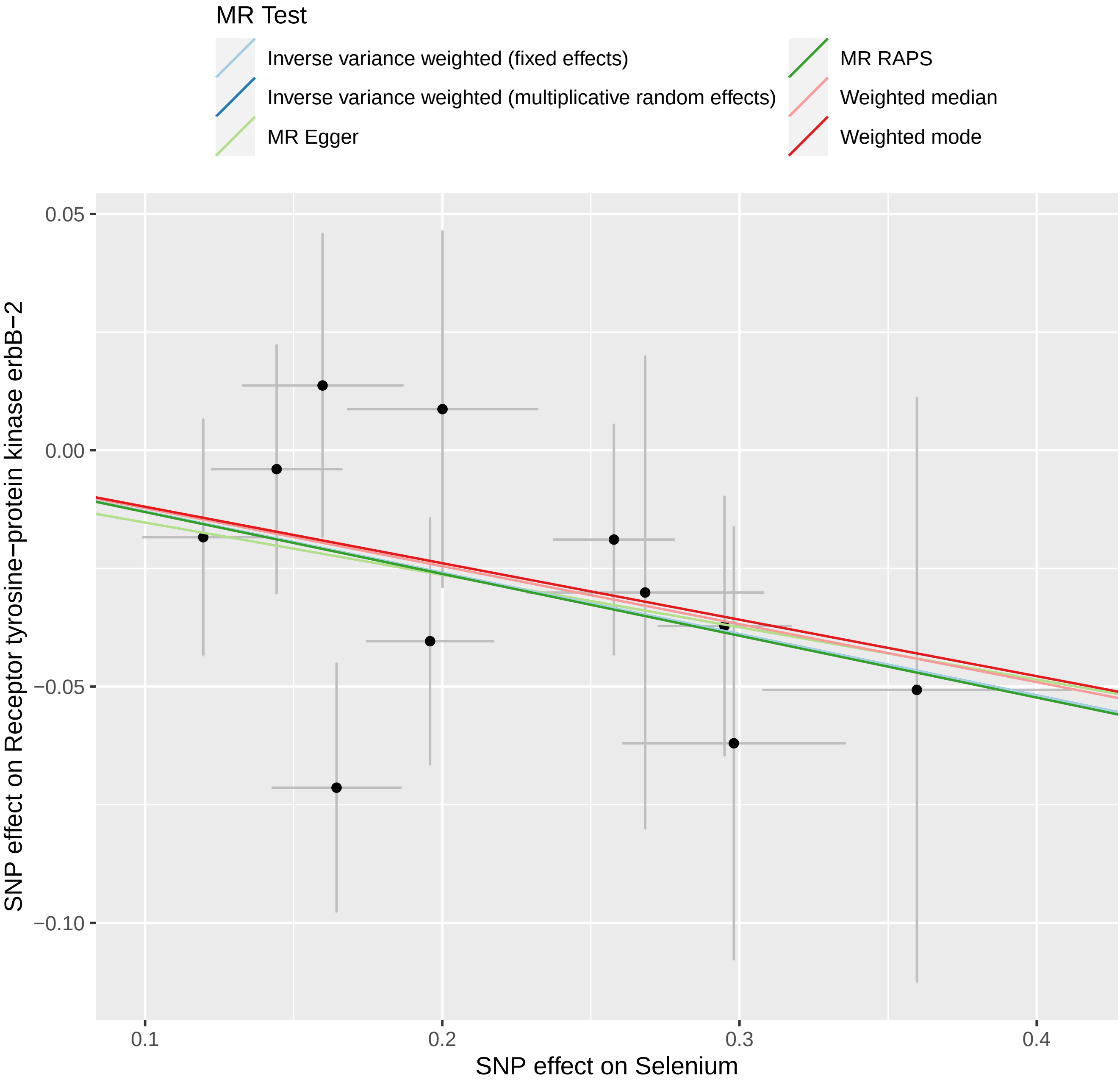

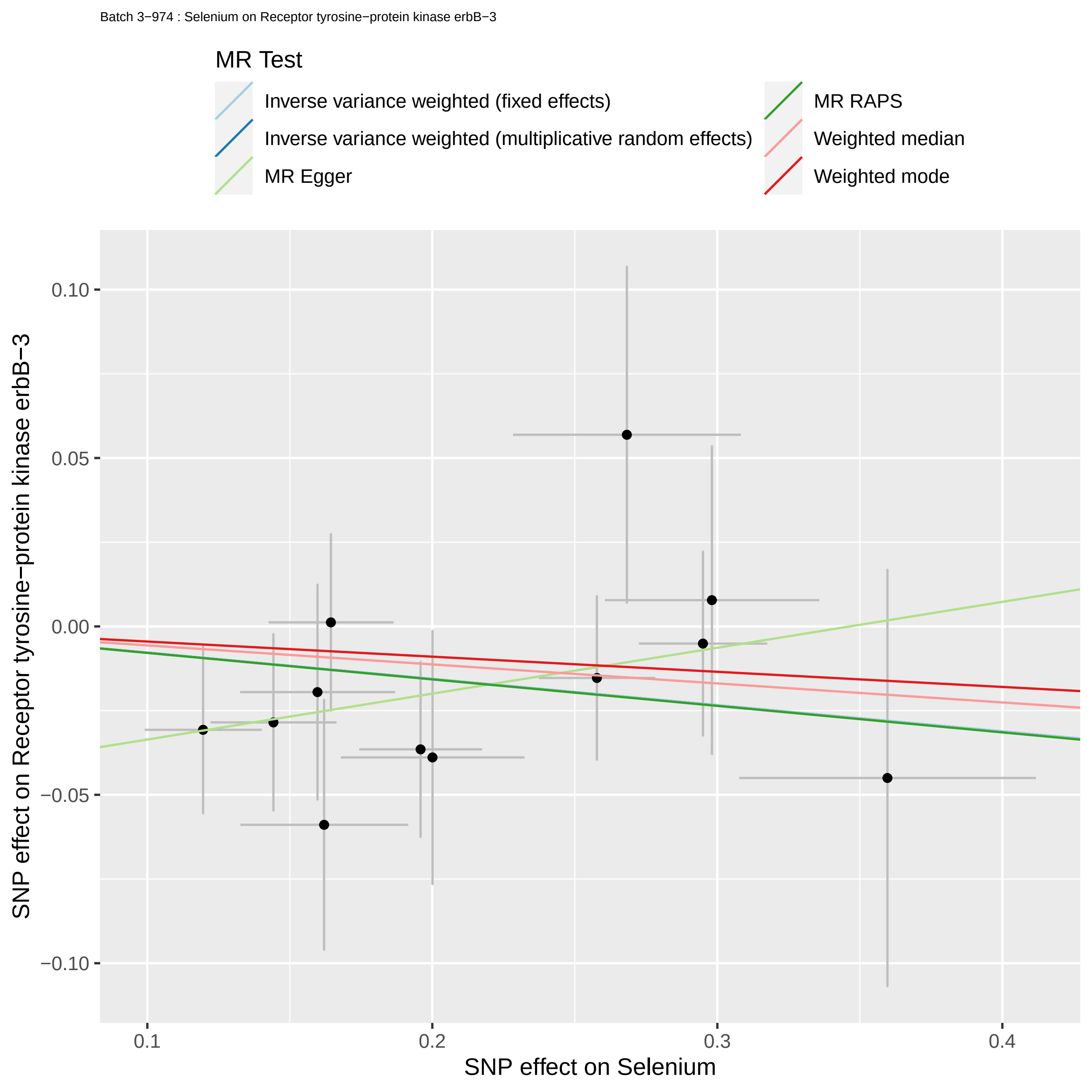

Batch 3−974 : Selenium on Receptor tyrosine−protein kinase erbB−3
MR Test
Inverse variance weighted (fixed effects)
Inverse variance weighted (multiplicative random effects)
MR Egger
MR RAPS
Weighted median
Weighted mode
0.10
0.05
0.00
−0.05
−0.10
0.1
0.2
0.3
0.4
SNP effect on Receptor tyrosine−protein kinase erbB−3
SNP effect on Selenium

Batch 3−975 : Selenium on DNA excision repair protein ERCC−1

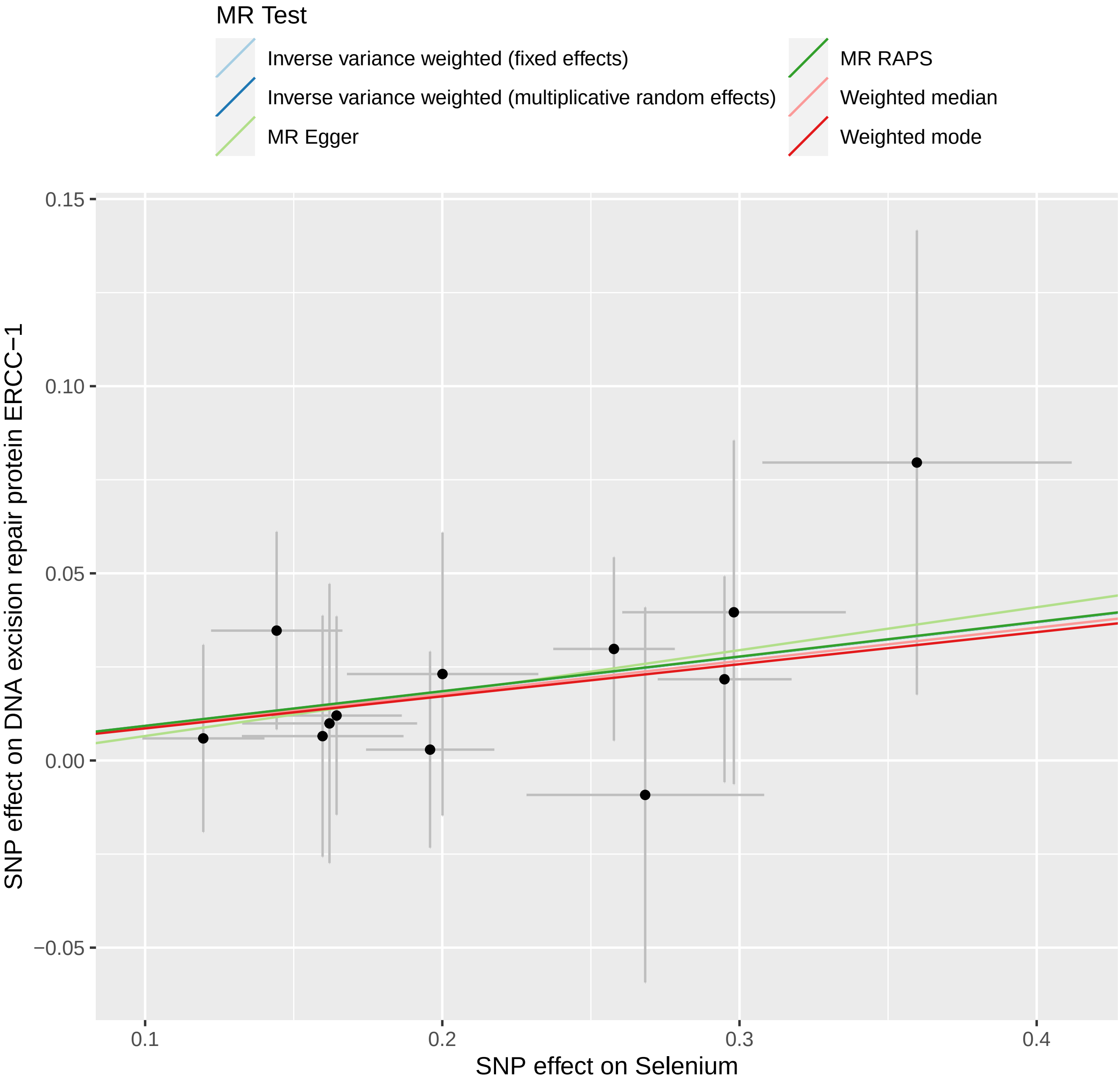

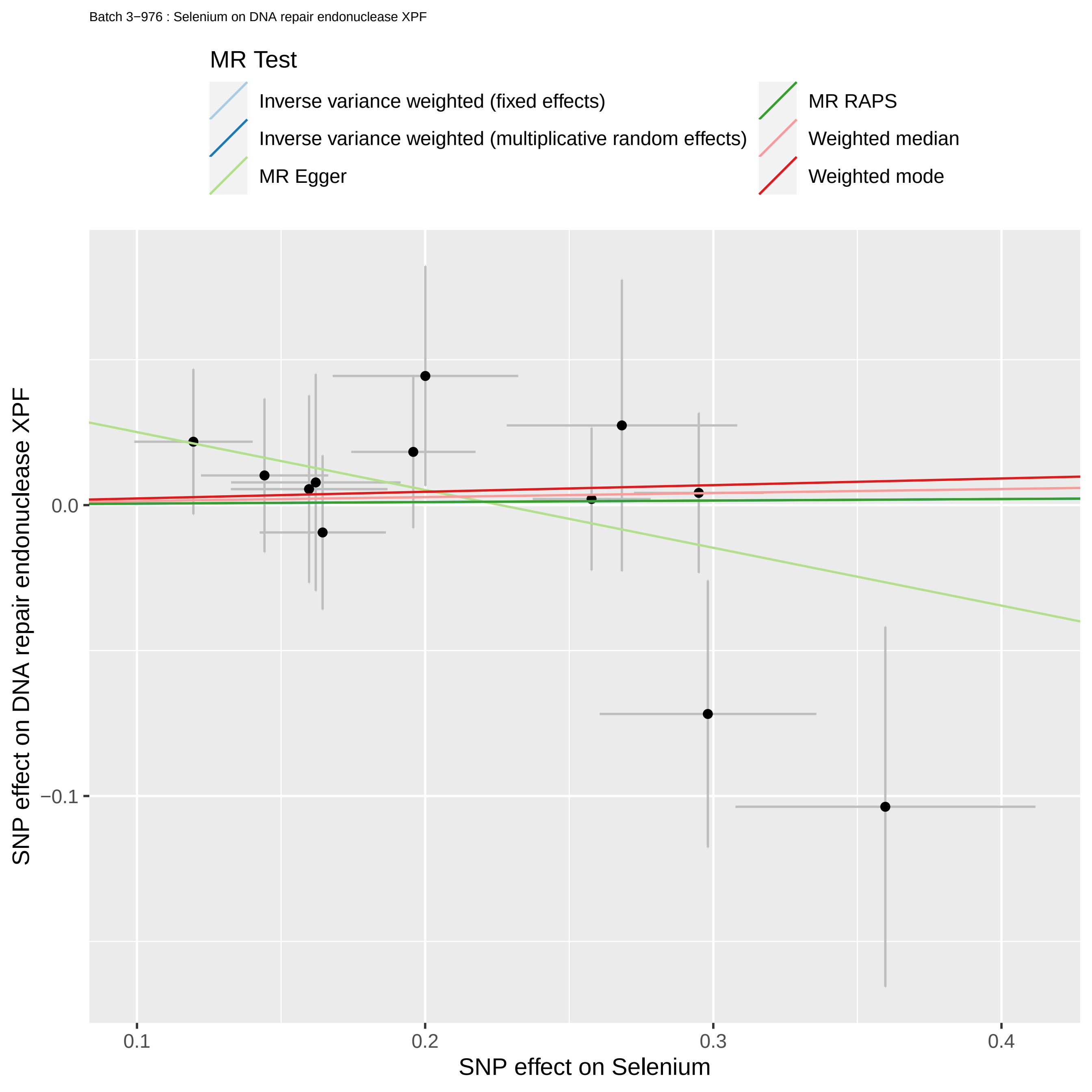

Batch 3−976 : Selenium on DNA repair endonuclease XPF
MR Test
Inverse variance weighted (fixed effects)
Inverse variance weighted (multiplicative random effects)
MR Egger
MR RAPS
Weighted median
Weighted mode
SNP effect on DNA repair endonuclease XPF
0.0
−0.1
0.1
0.2
0.3
0.4
SNP effect on Selenium

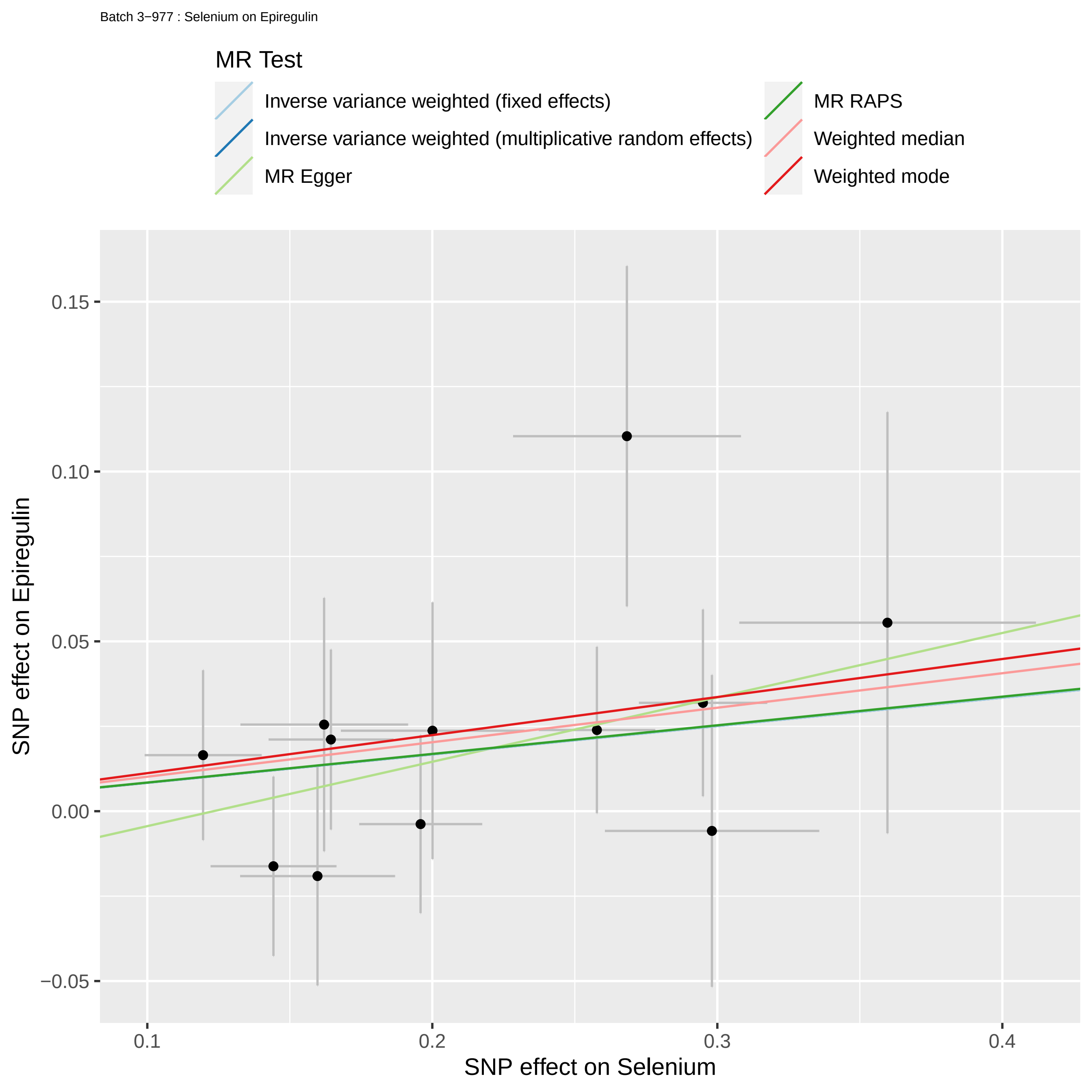

Batch 3−977 : Selenium on Epiregulin
MR Test
Inverse variance weighted (fixed effects)
Inverse variance weighted (multiplicative random effects)
MR Egger
MR RAPS
Weighted median
Weighted mode
0.15
0.10
0.05
0.00
−0.05
0.1
0.2
0.3
0.4
SNP effect on Epiregulin
SNP effect on Selenium

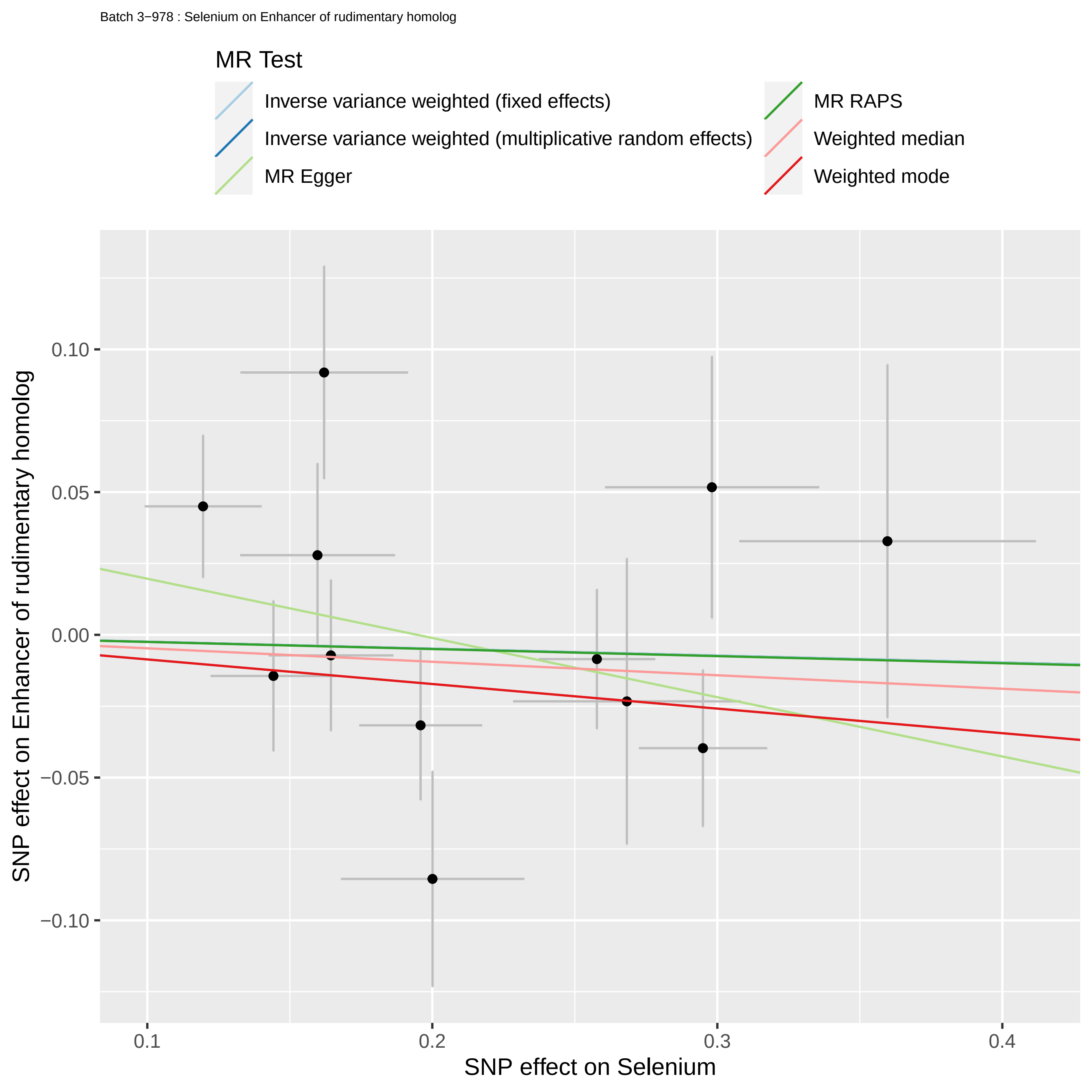
Batch 3−978 : Selenium on Enhancer of rudimentary homolog
MR Test
Inverse variance weighted (fixed effects)
Inverse variance weighted (multiplicative random effects)
MR Egger
MR RAPS
Weighted median
Weighted mode
SNP effect on Enhancer of rudimentary homolog
0.10
0.05
0.00
−0.05
−0.10
0.1
0.2
0.3
0.4
SNP effect on Selenium

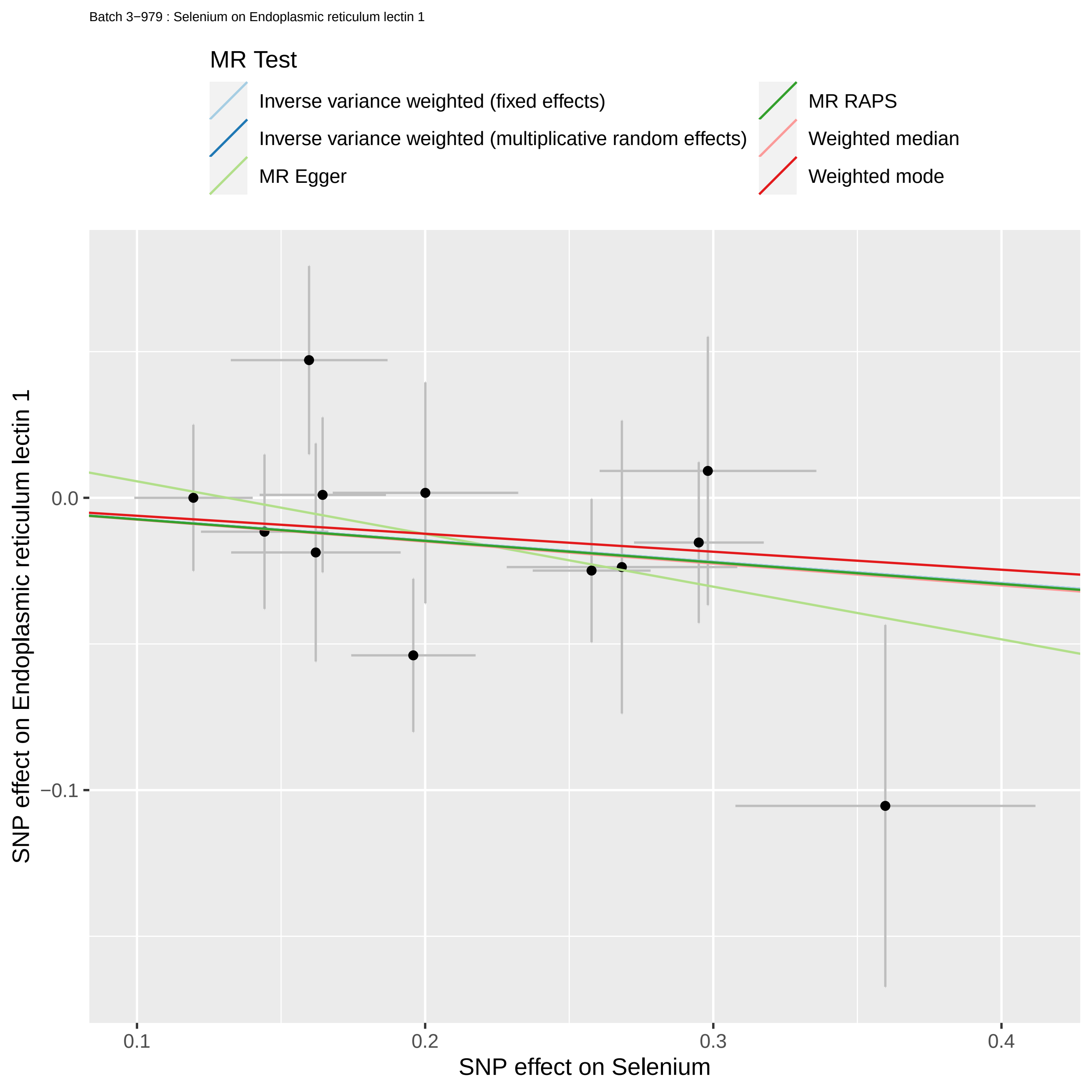

Batch 3−979 : Selenium on Endoplasmic reticulum lectin 1
MR Test
Inverse variance weighted (fixed effects)
Inverse variance weighted (multiplicative random effects)
MR Egger
MR RAPS
Weighted median
Weighted mode
SNP effect on Endoplasmic reticulum lectin 1
0.0
−0.1
0.1
0.2
0.3
0.4
SNP effect on Selenium

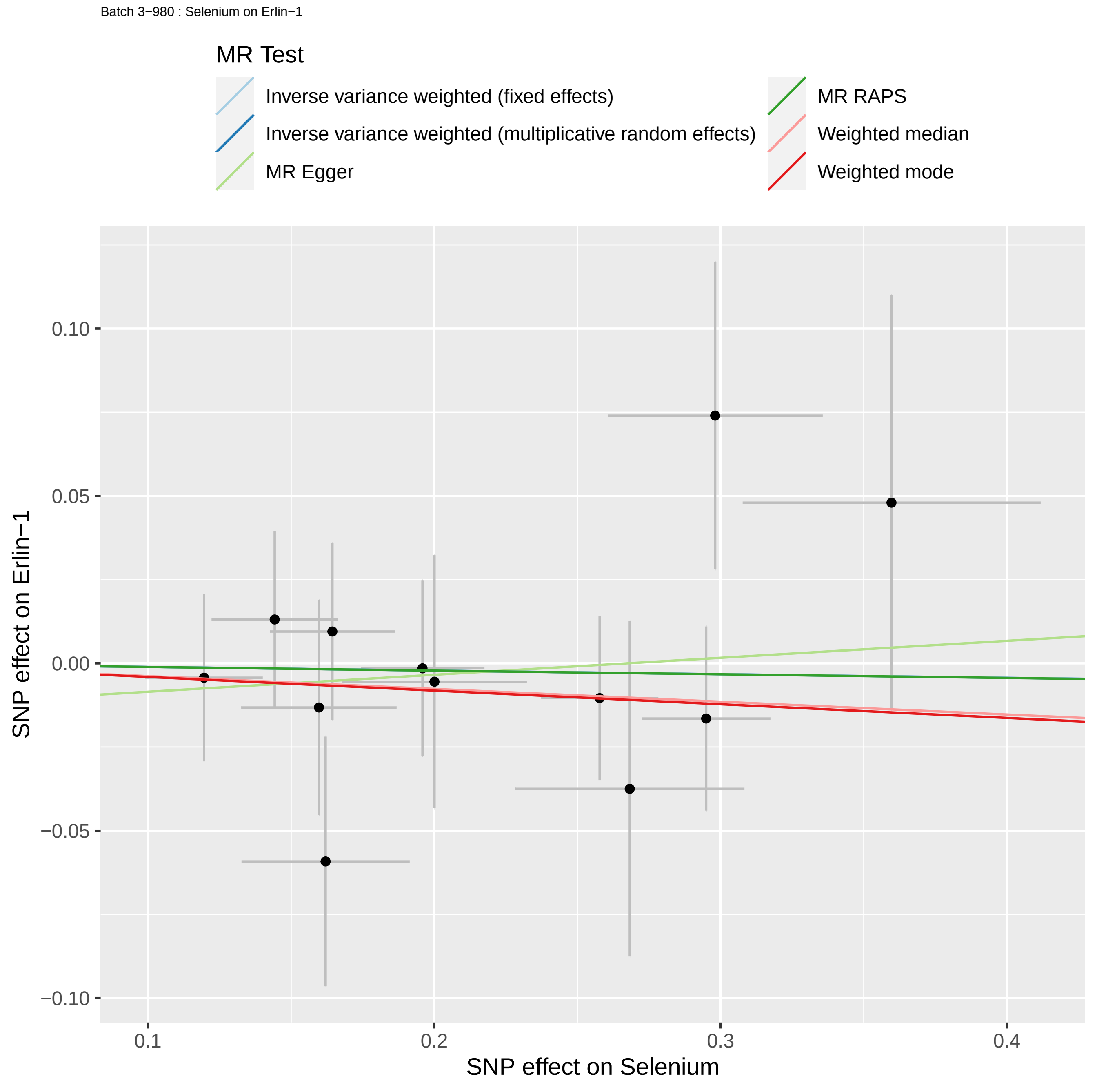
Batch 3−980 : Selenium on Erlin−1
MR Test
Inverse variance weighted (fixed effects)
Inverse variance weighted (multiplicative random effects)
MR Egger
MR RAPS
Weighted median
Weighted mode
0.10
0.05
0.00
−0.05
−0.10
0.1
0.2
0.3
0.4
SNP effect on Selenium
SNP effect on Erlin−1

Batch 3−981 : Selenium on Erythroid membrane−associated protein

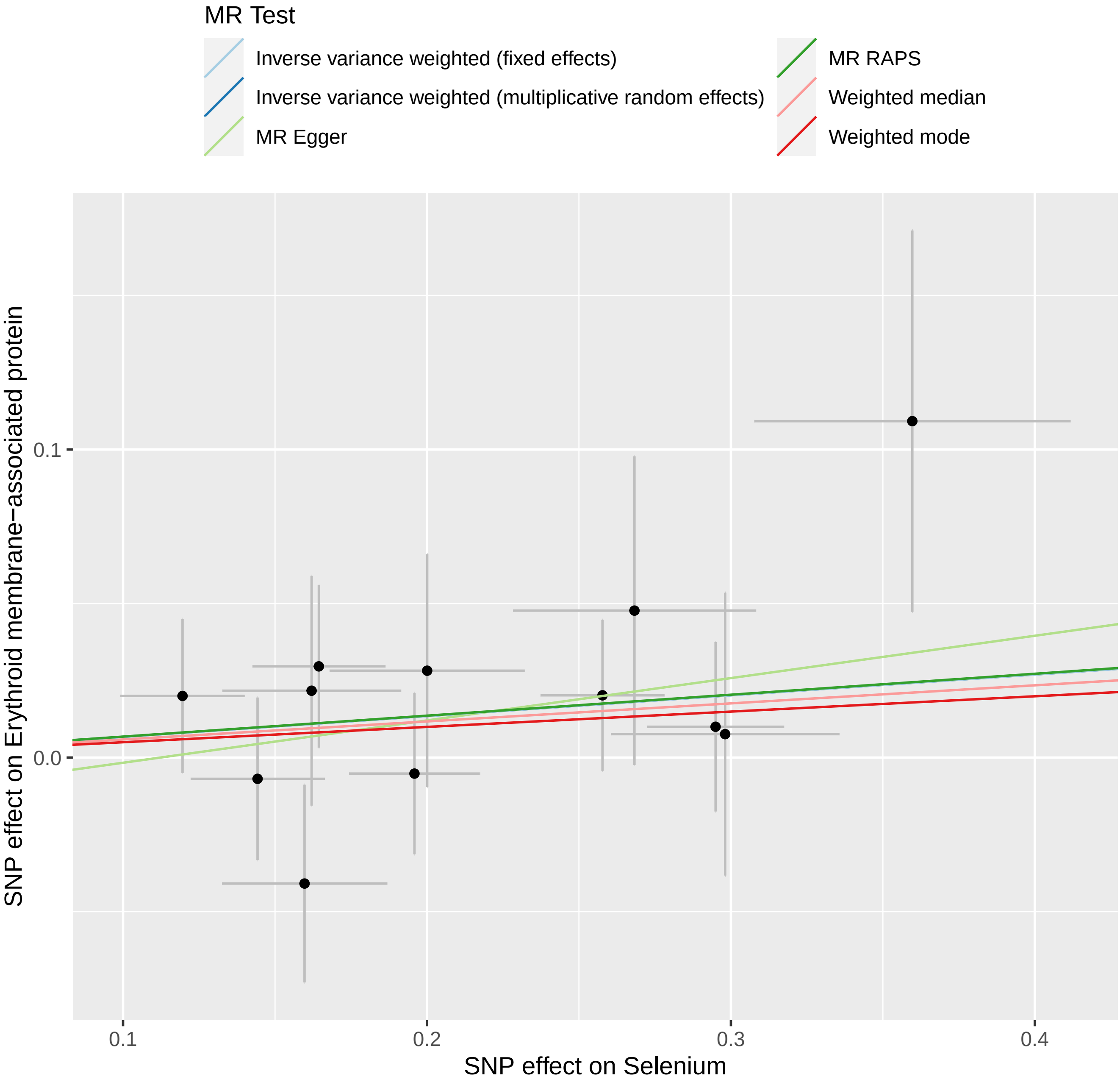

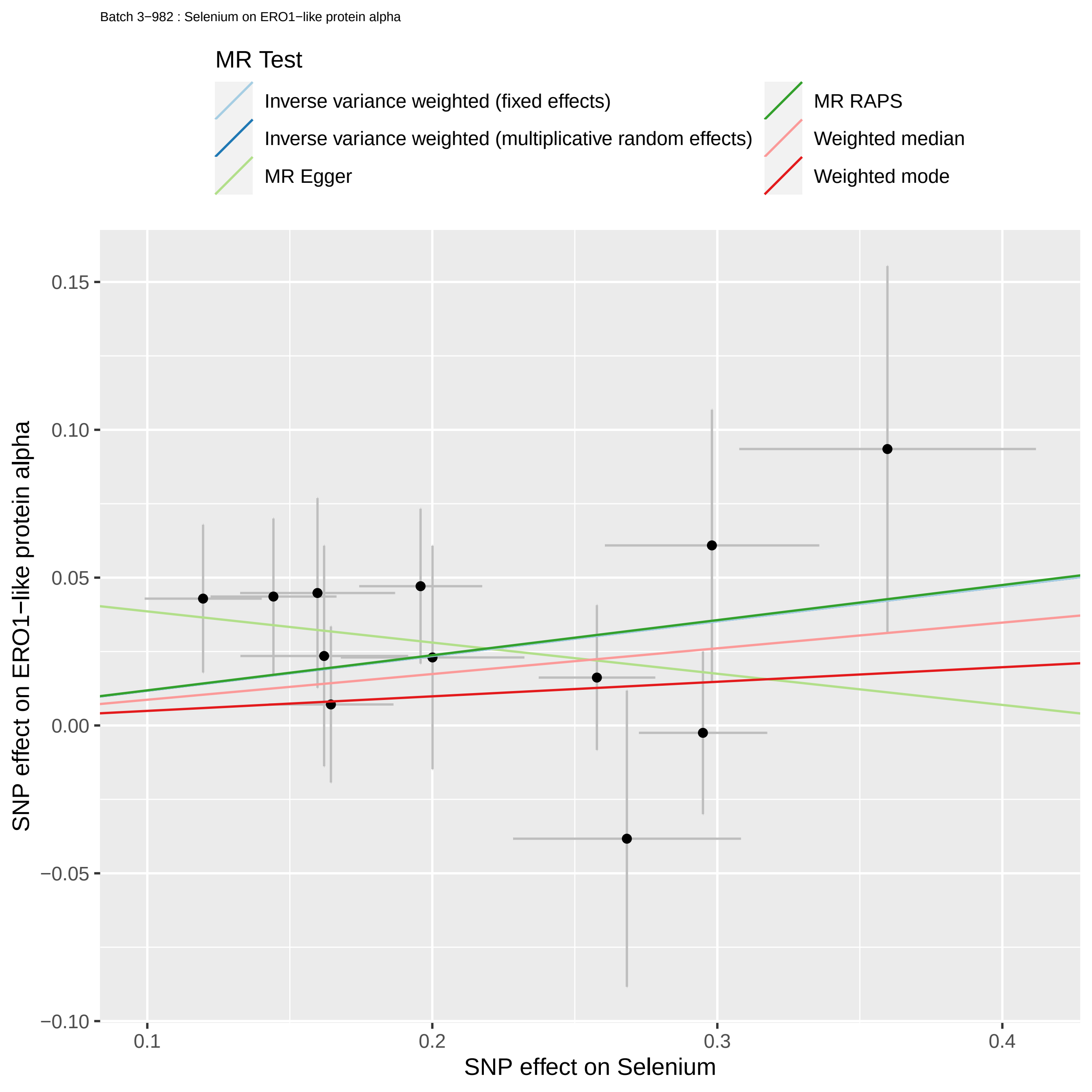
Batch 3−982 : Selenium on ERO1−like protein alpha
MR Test
Inverse variance weighted (fixed effects)
Inverse variance weighted (multiplicative random effects)
MR Egger
MR RAPS
Weighted median
Weighted mode
SNP effect on ERO1−like protein alpha
SNP effect on Selenium
0.15
0.10
0.05
0.00
−0.05
−0.10
0.1
0.2
0.3
0.4

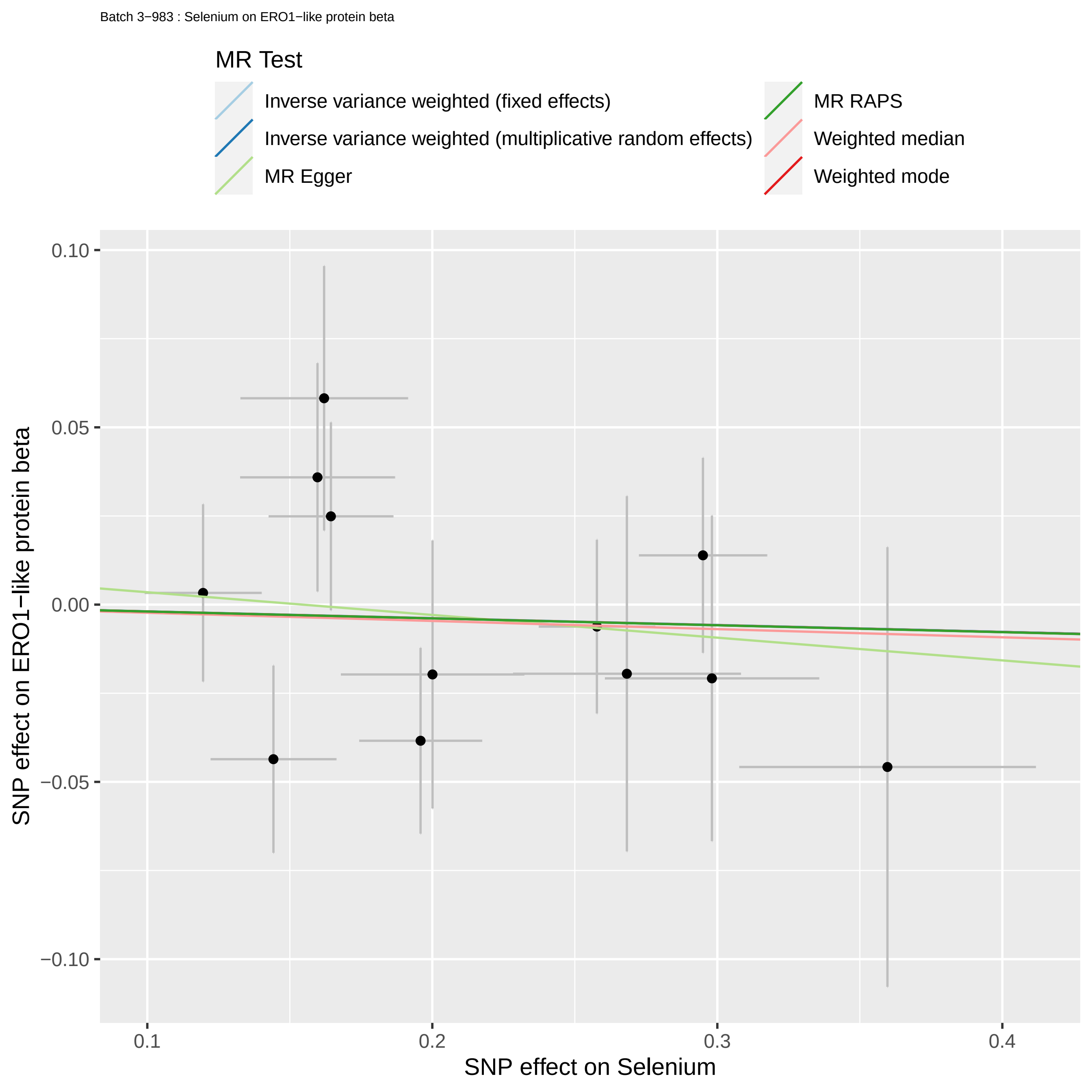

Batch 3−983 : Selenium on ERO1−like protein beta
MR Test
Inverse variance weighted (fixed effects)
Inverse variance weighted (multiplicative random effects)
MR Egger
MR RAPS
Weighted median
Weighted mode
0.10
0.05
0.00
−0.05
−0.10
0.1
0.2
0.3
0.4
SNP effect on ERO1−like protein beta
SNP effect on Selenium

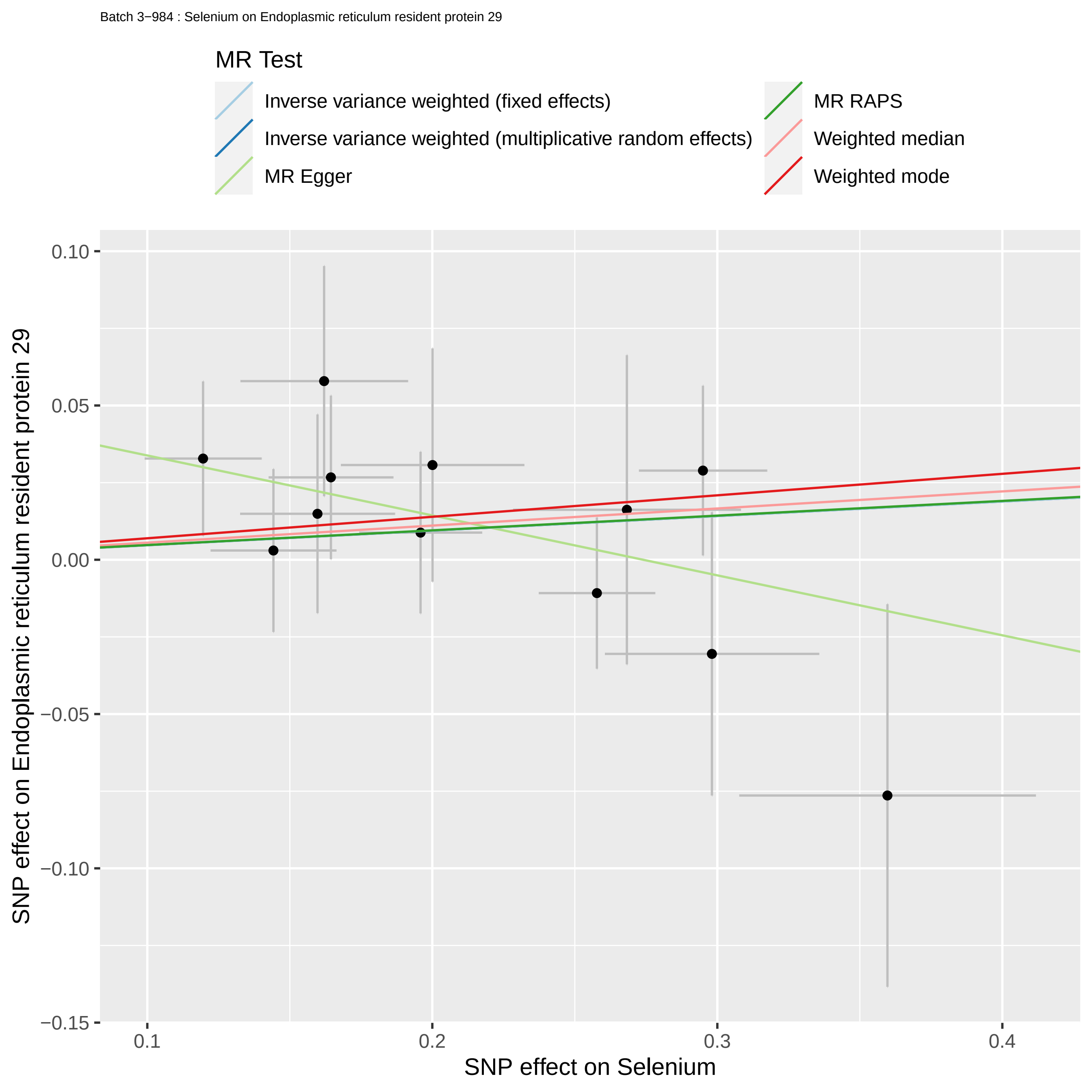

Batch 3−984 : Selenium on Endoplasmic reticulum resident protein 29
MR Test
Inverse variance weighted (fixed effects)
Inverse variance weighted (multiplicative random effects)
MR Egger
MR RAPS
Weighted median
Weighted mode
0.10
0.05
0.00
−0.05
−0.10
−0.15
0.1
0.2
0.3
0.4
SNP effect on Endoplasmic reticulum resident protein 29
SNP effect on Selenium

Batch 3−985 : Selenium on Endoplasmic reticulum resident protein 44

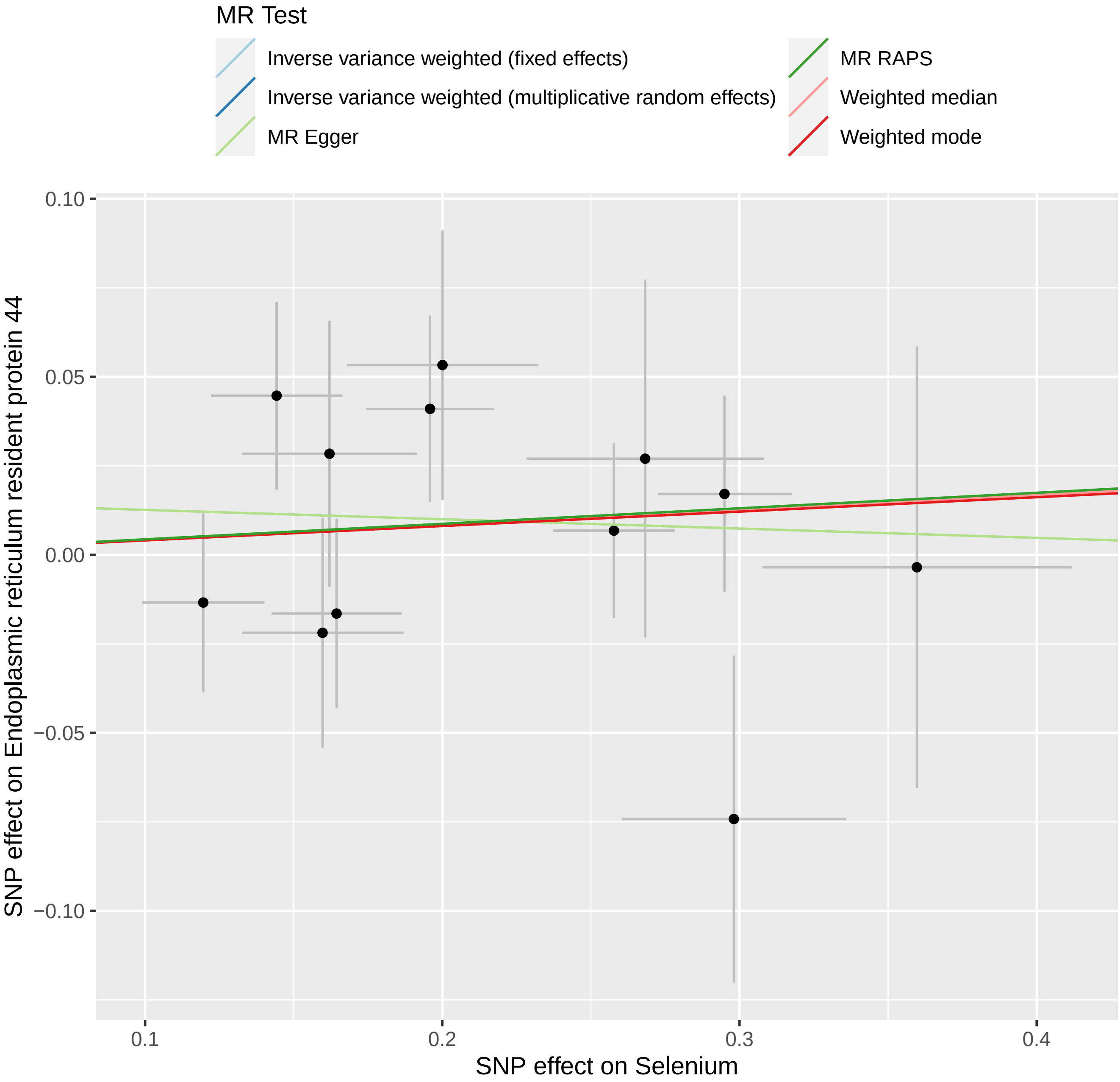

Batch 3−986 : Selenium on Endogenous retrovirus group V member 1 Env polyprotein

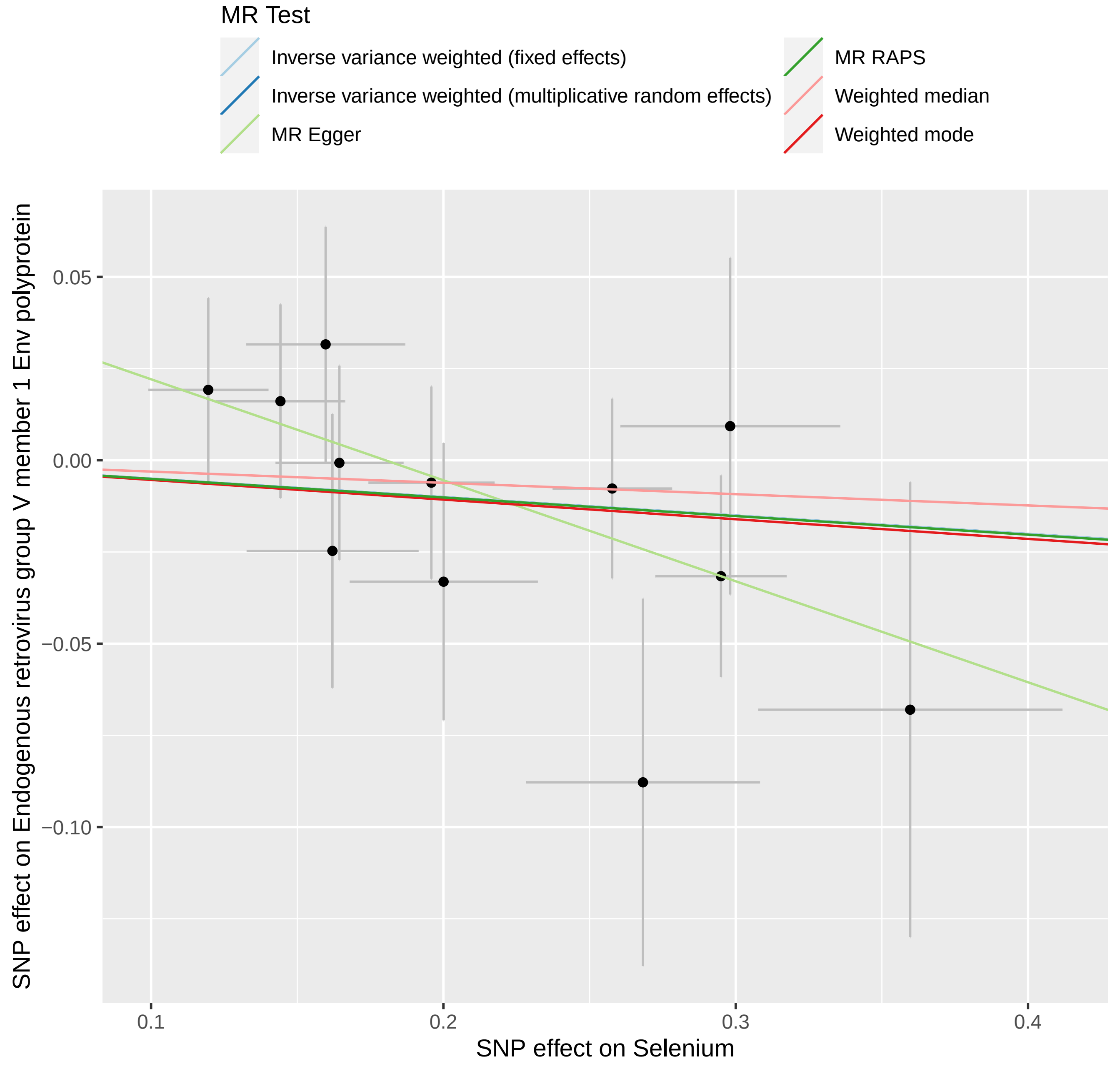

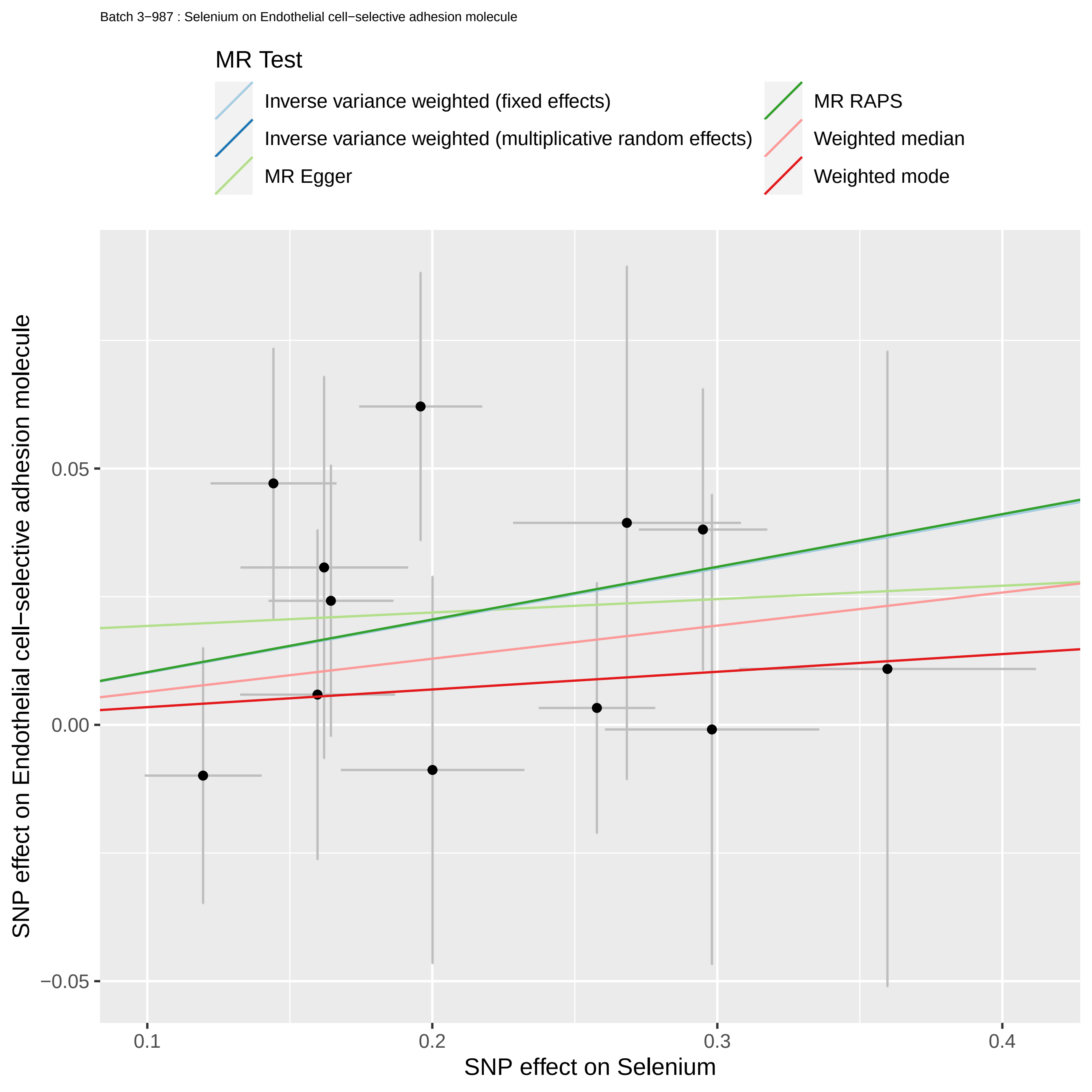

Batch 3−987 : Selenium on Endothelial cell−selective adhesion molecule
MR Test
Inverse variance weighted (fixed effects)
Inverse variance weighted (multiplicative random effects)
MR Egger
MR RAPS
Weighted median
Weighted mode
0.05
0.00
−0.05
0.1
0.2
0.3
0.4
SNP effect on Endothelial cell−selective adhesion molecule
SNP effect on Selenium

Batch 3−988 : Selenium on Endothelial cell−selective adhesion molecule

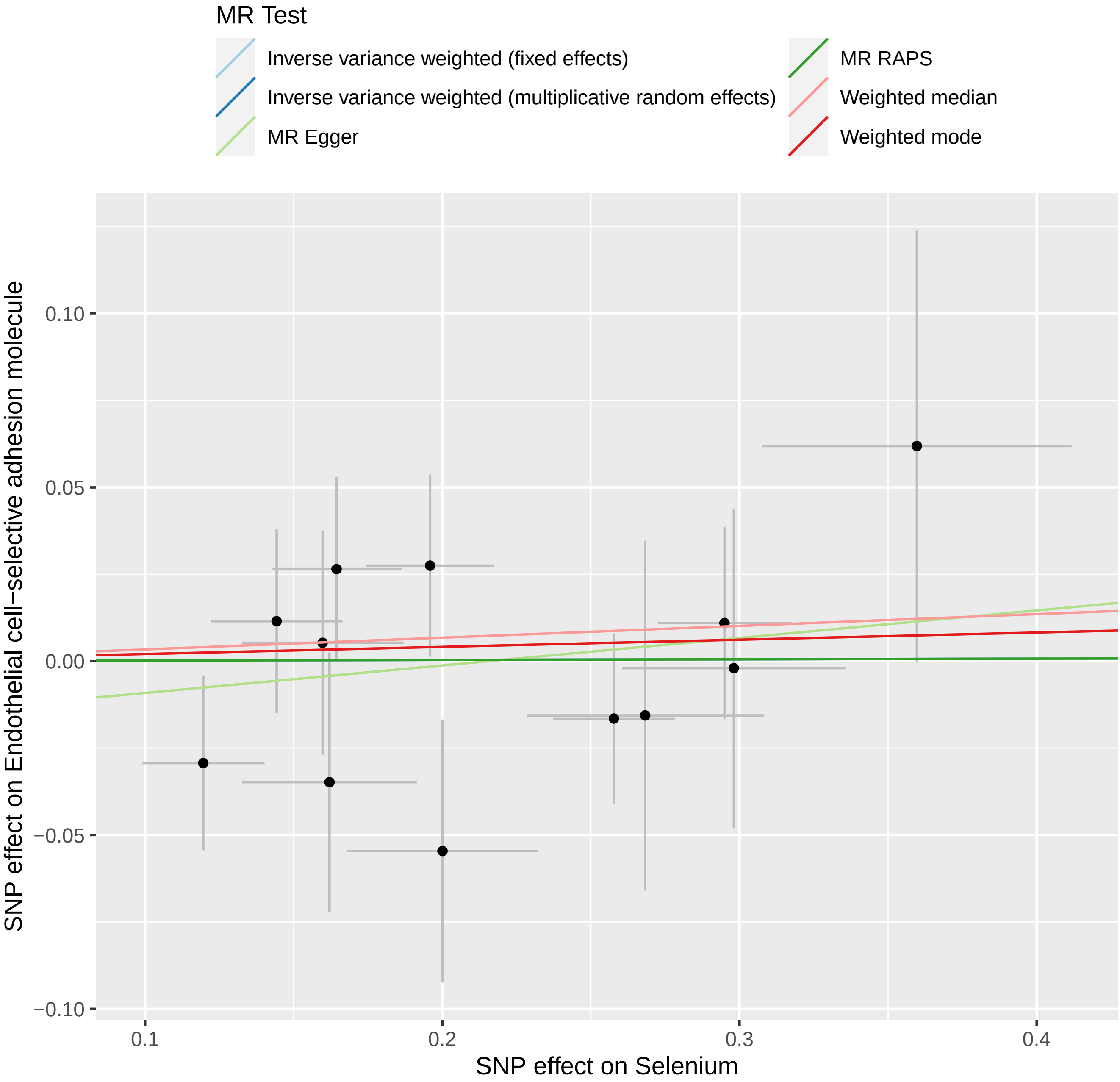

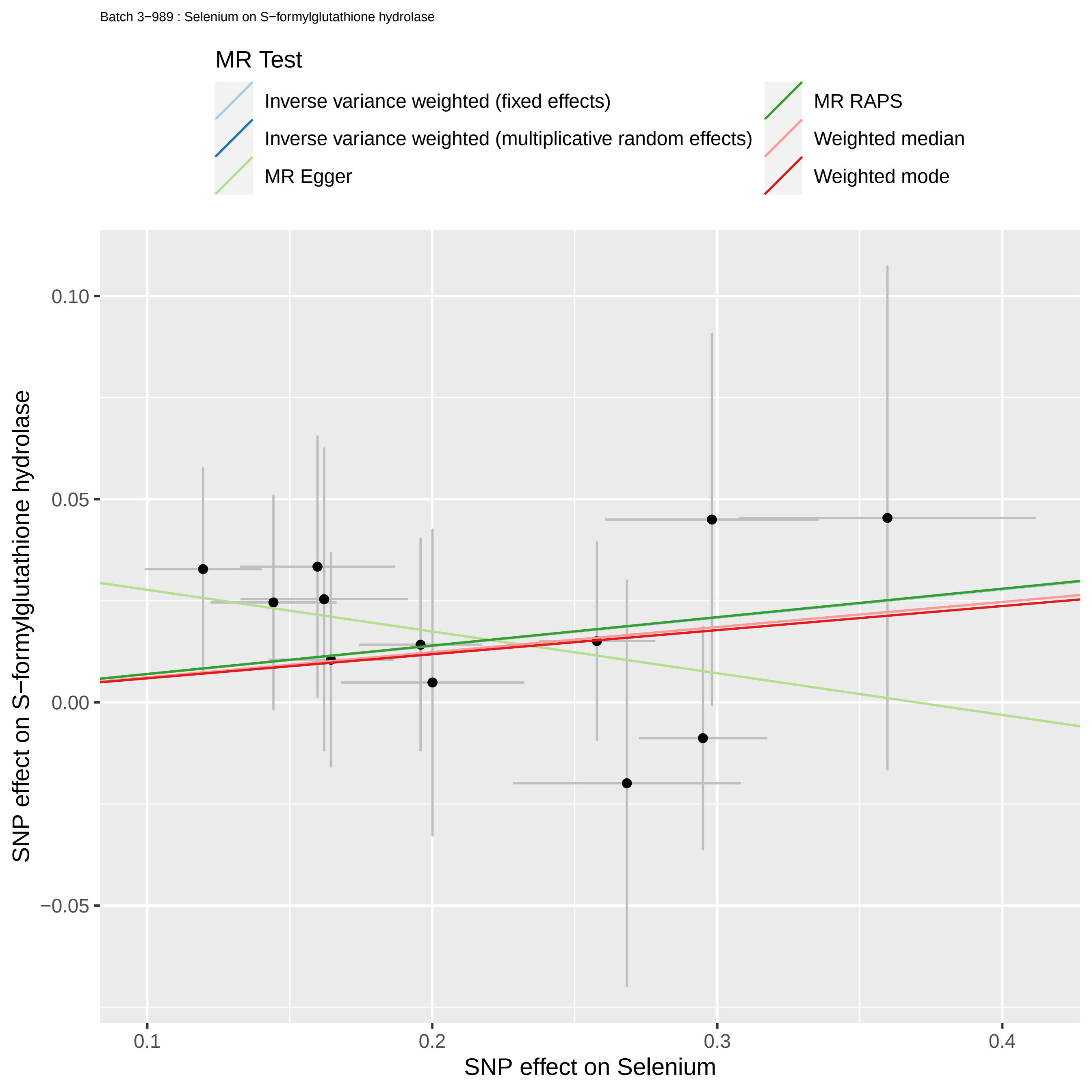
Batch 3−989 : Selenium on S−formylglutathione hydrolase
MR Test
Inverse variance weighted (fixed effects)
Inverse variance weighted (multiplicative random effects)
MR Egger
MR RAPS
Weighted median
Weighted mode
SNP effect on S−formylglutathione hydrolase
0.10
0.05
0.00
−0.05
0.1
0.2
0.3
0.4
SNP effect on Selenium

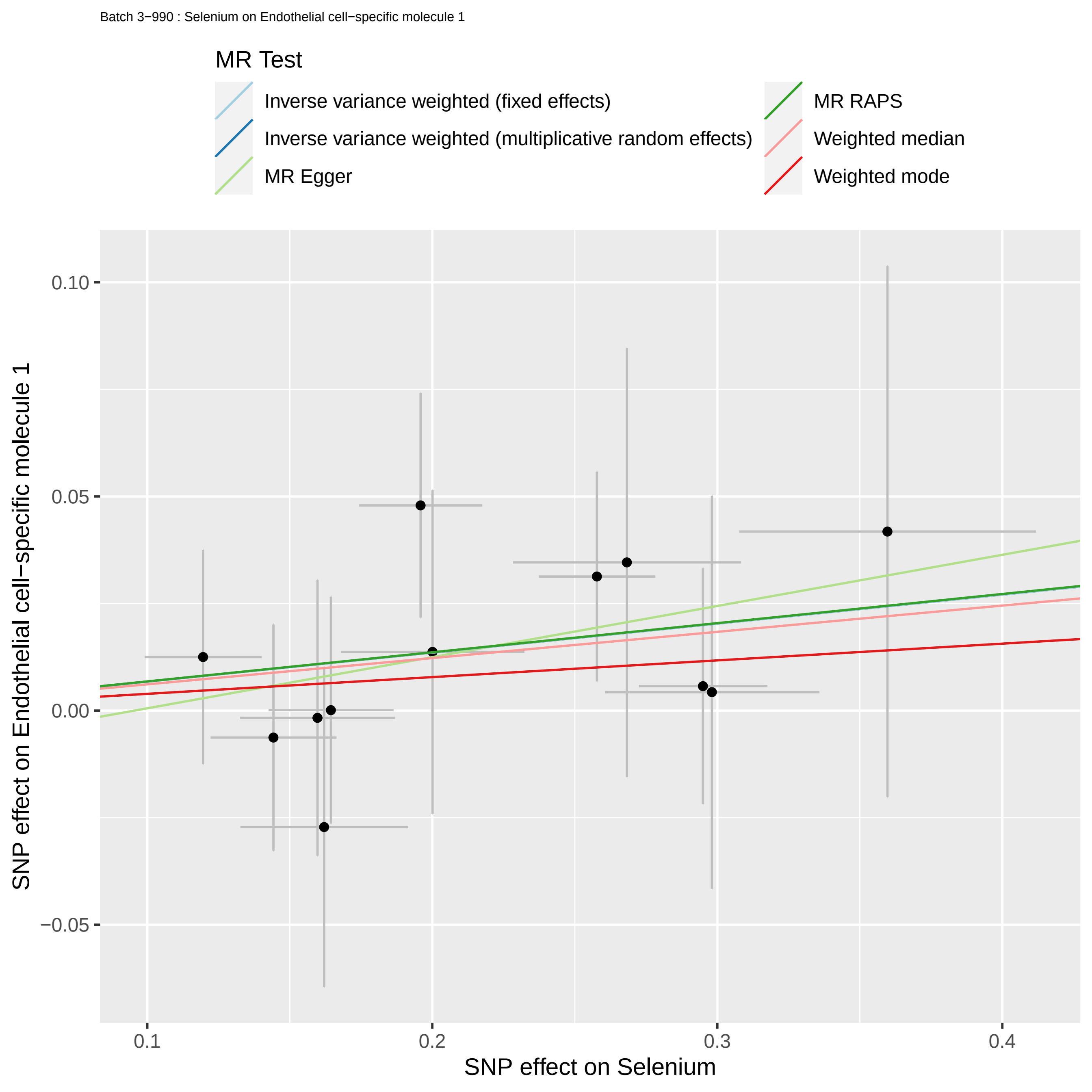
Batch 3−990 : Selenium on Endothelial cell−specific molecule 1
MR Test
Inverse variance weighted (fixed effects)
Inverse variance weighted (multiplicative random effects)
MR Egger
MR RAPS
Weighted median
Weighted mode
0.10
0.05
0.00
−0.05
0.1
0.2
0.3
0.4
SNP effect on Selenium
SNP effect on Endothelial cell−specific molecule 1

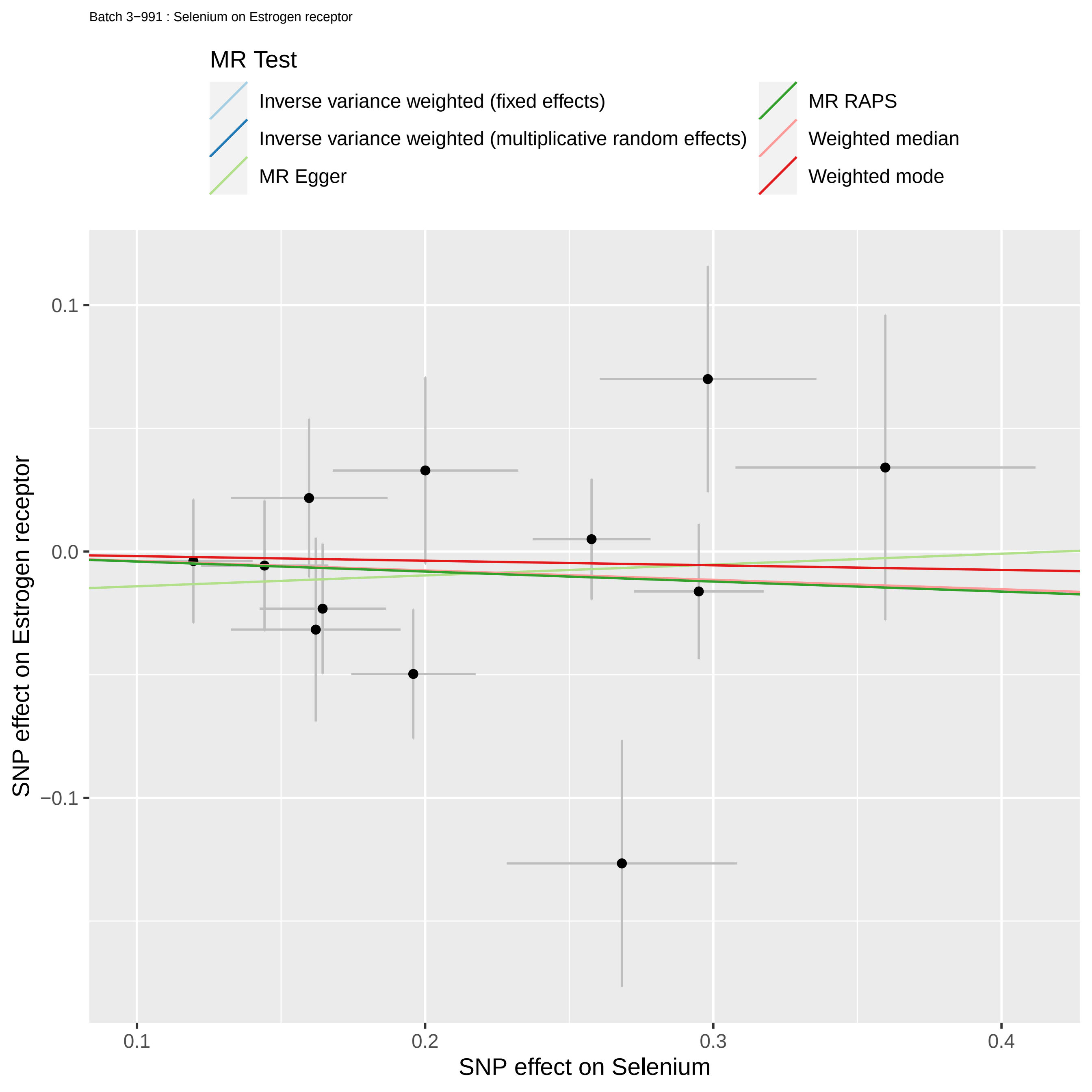
Batch 3−991 : Selenium on Estrogen receptor
MR Test
Inverse variance weighted (fixed effects)
Inverse variance weighted (multiplicative random effects)
MR Egger
MR RAPS
Weighted median
Weighted mode
0.1
0.0
−0.1
0.1
0.2
0.3
0.4
SNP effect on Estrogen receptor
SNP effect on Selenium

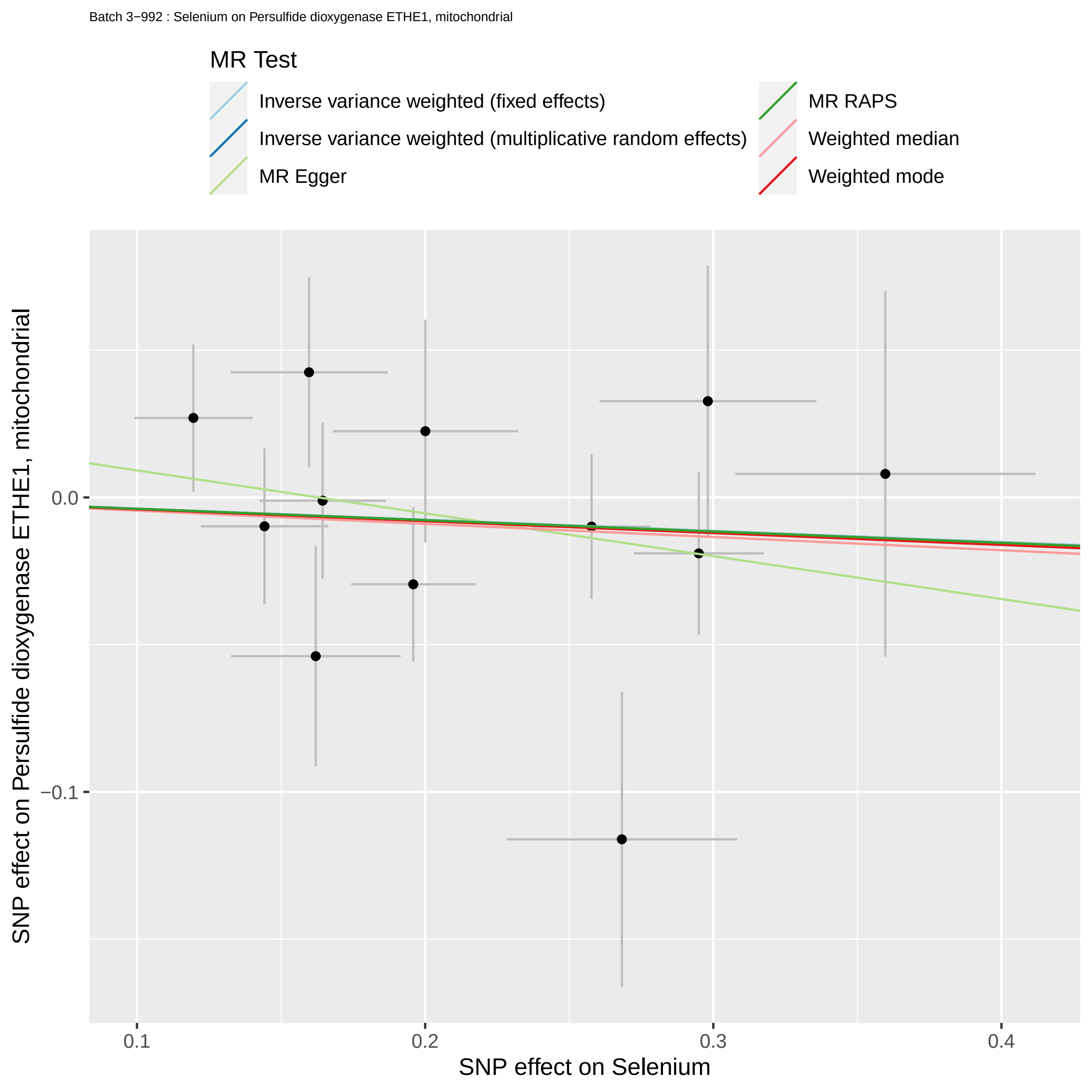
Batch 3−992 : Selenium on Persulfide dioxygenase ETHE1, mitochondrial
MR Test
Inverse variance weighted (fixed effects)
Inverse variance weighted (multiplicative random effects)
MR Egger
MR RAPS
Weighted median
Weighted mode
SNP effect on Persulfide dioxygenase ETHE1, mitochondrial
0.0
−0.1
0.1
0.2
0.3
0.4
SNP effect on Selenium

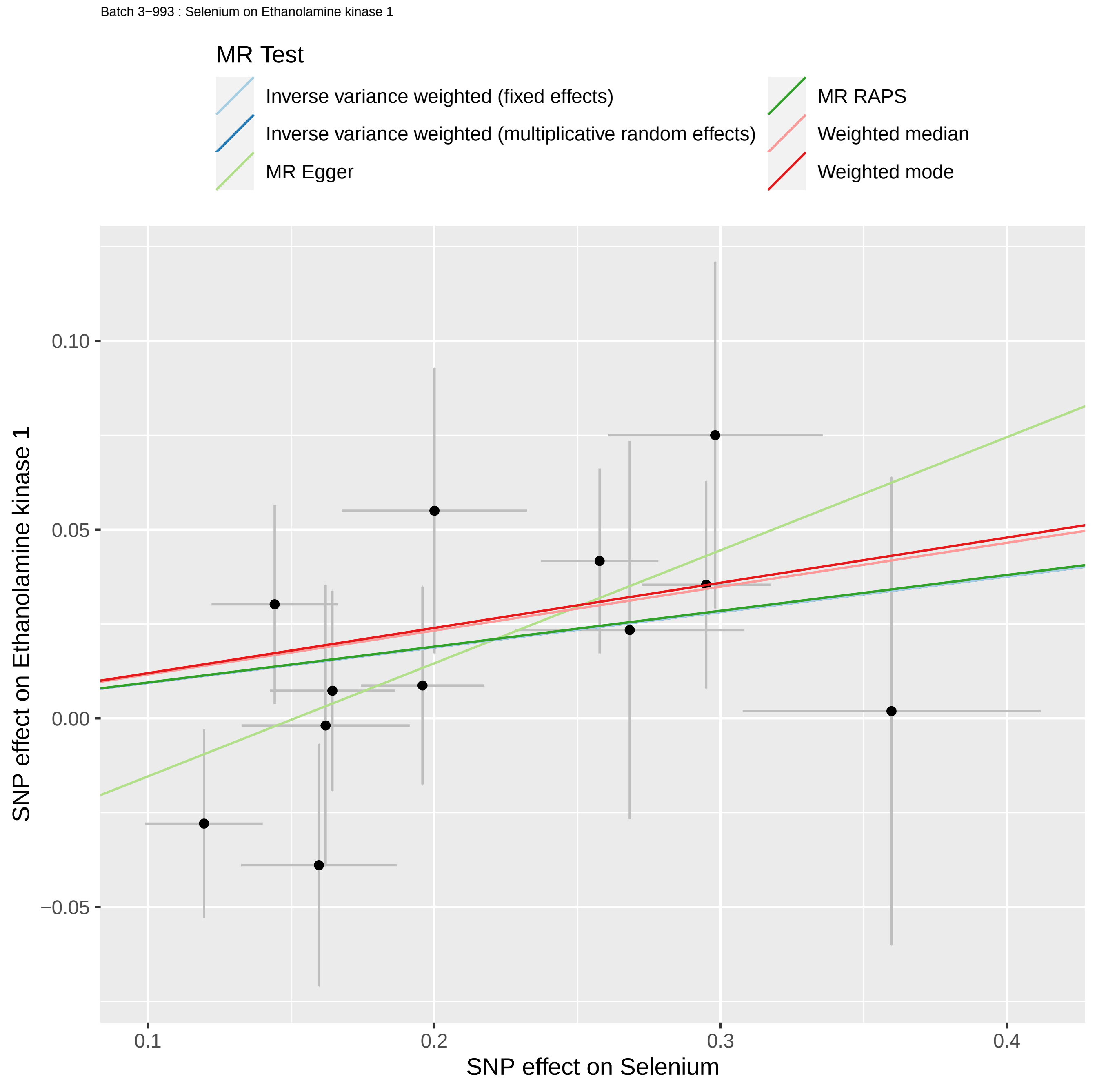
Batch 3−993 : Selenium on Ethanolamine kinase 1
MR Test
Inverse variance weighted (fixed effects)
Inverse variance weighted (multiplicative random effects)
MR Egger
MR RAPS
Weighted median
Weighted mode
SNP effect on Ethanolamine kinase 1
0.10
0.05
0.00
−0.05
0.1
0.2
0.3
0.4
SNP effect on Selenium

Batch 3−994 : Selenium on Protein C−ets−2

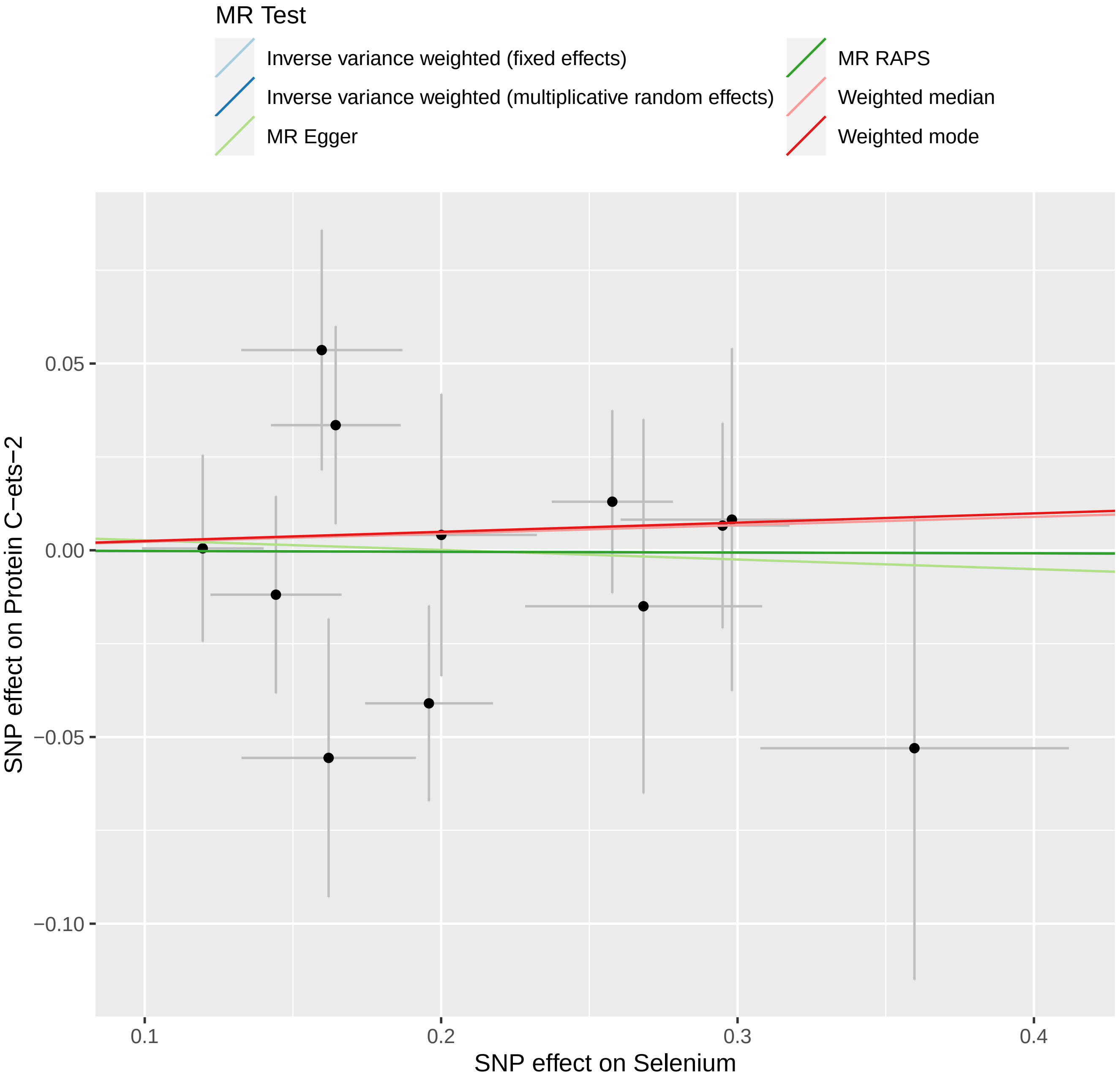

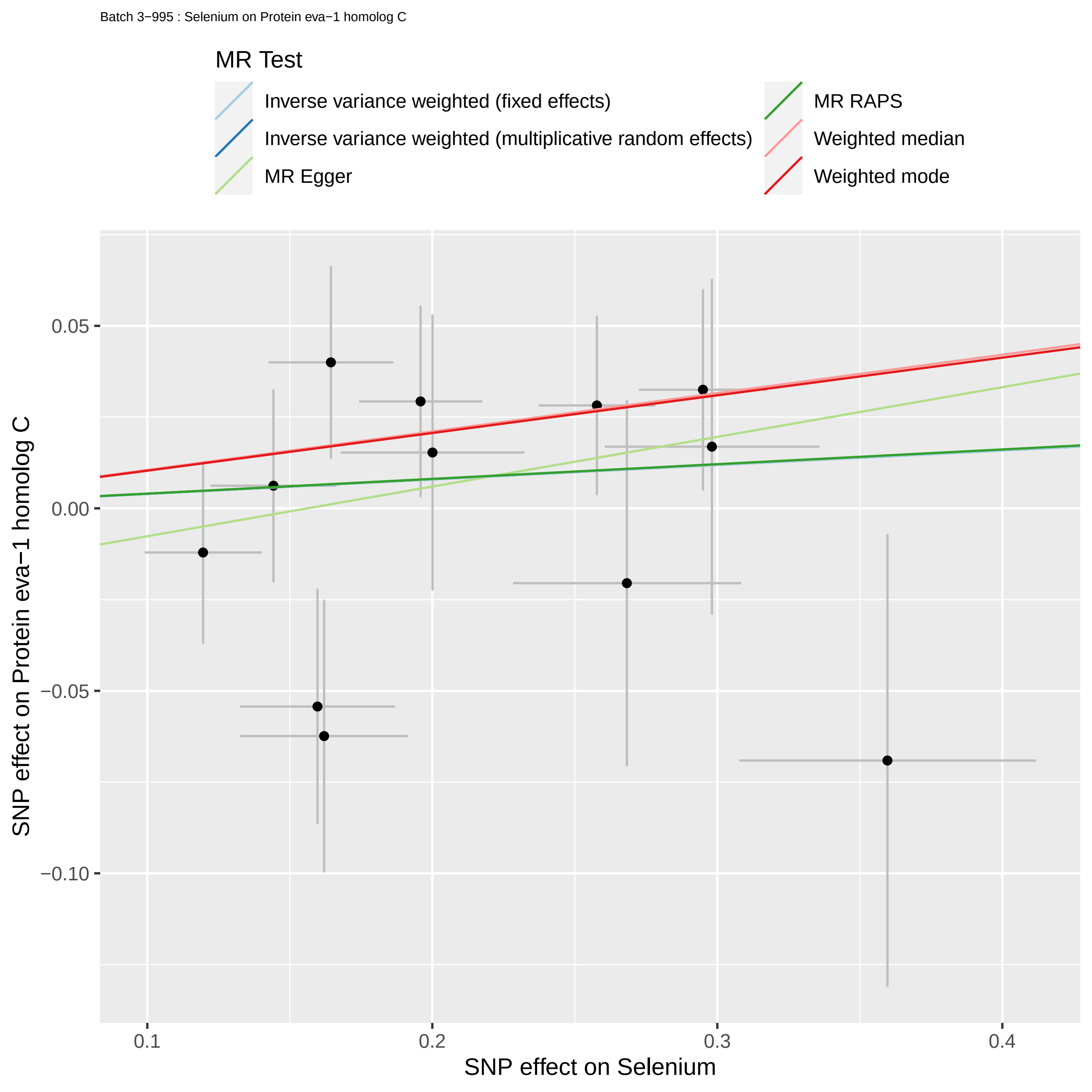
Batch 3−995 : Selenium on Protein eva−1 homolog C
MR Test
Inverse variance weighted (fixed effects)
Inverse variance weighted (multiplicative random effects)
MR Egger
MR RAPS
Weighted median
Weighted mode
0.05
0.00
−0.05
−0.10
0.1
0.2
0.3
0.4
SNP effect on Protein eva−1 homolog C
SNP effect on Selenium

Batch 3−996 : Selenium on Protein eva−1 homolog C

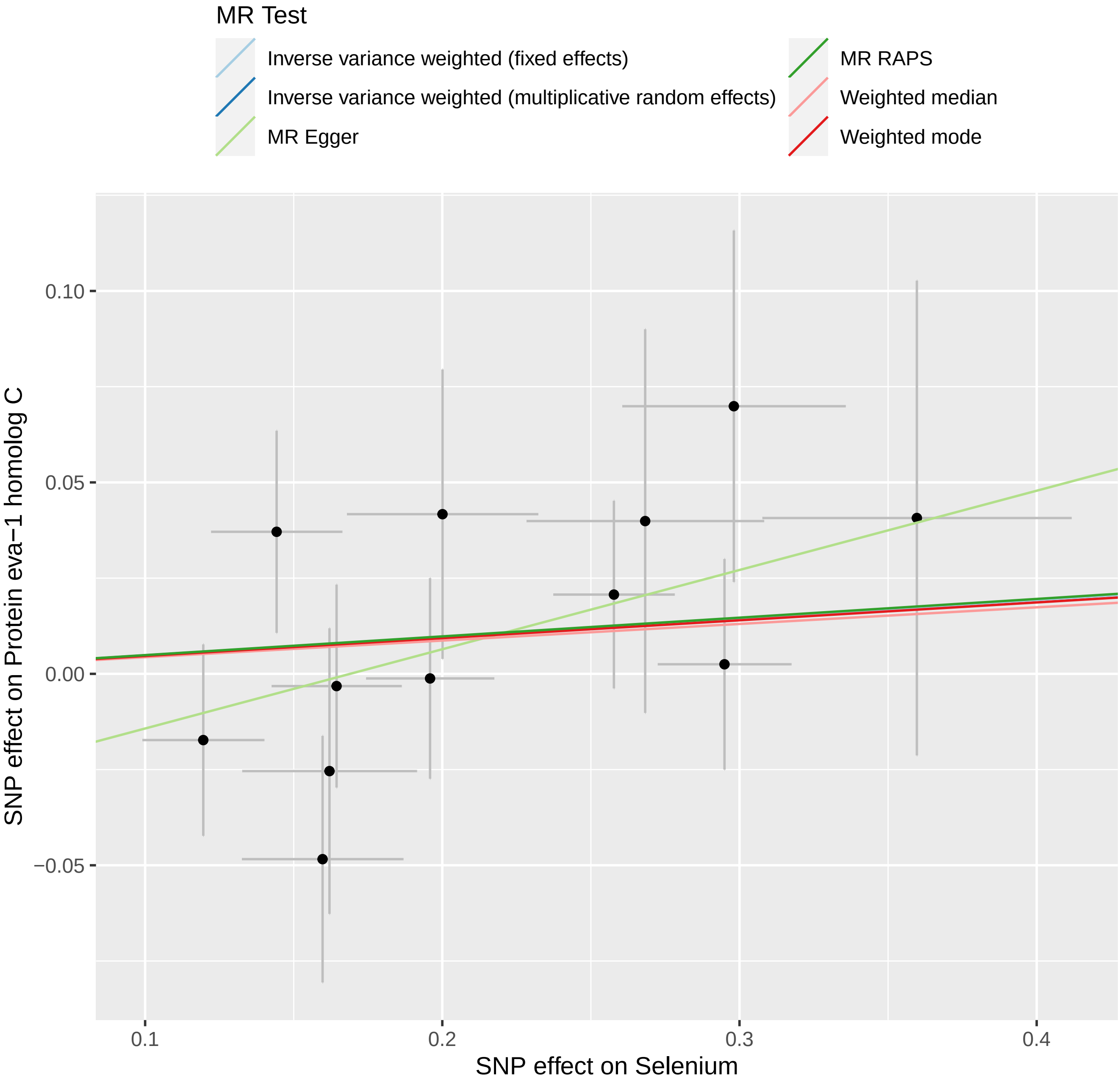

Batch 3−997 : Selenium on Protein EVI2B

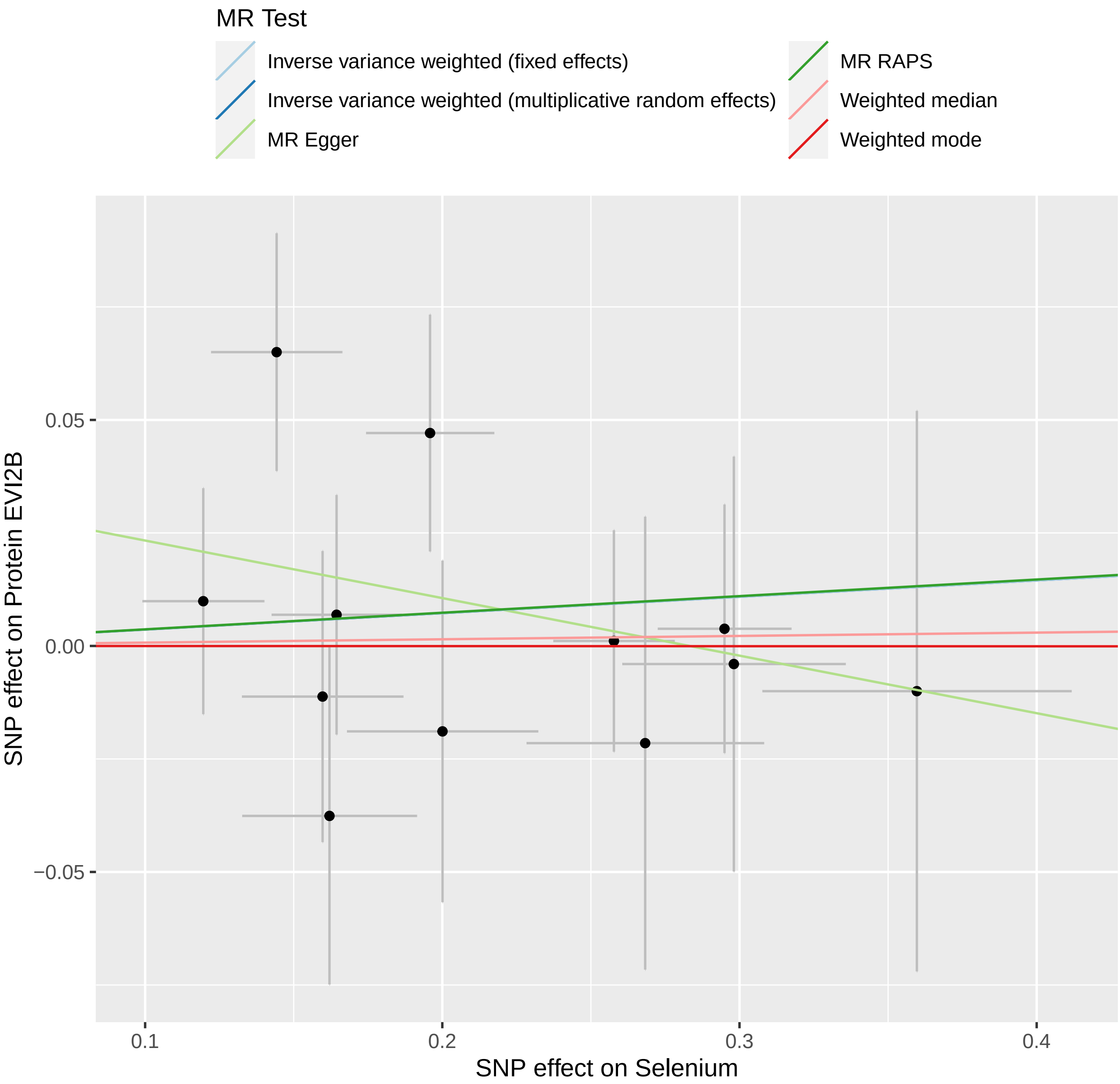

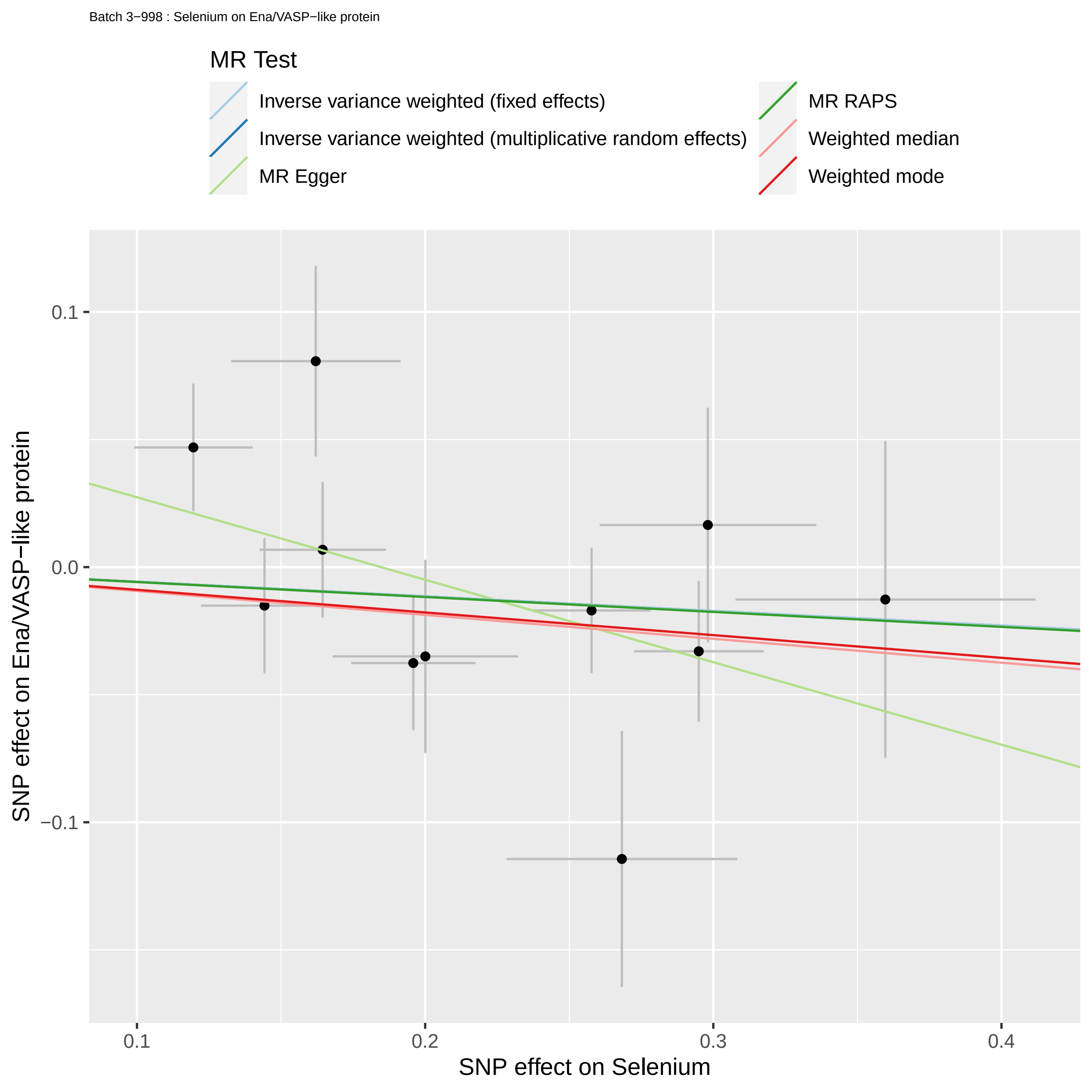

Batch 3−998 : Selenium on Ena/VASP−like protein
MR Test
Inverse variance weighted (fixed effects)
Inverse variance weighted (multiplicative random effects)
MR Egger
MR RAPS
Weighted median
Weighted mode
0.1
0.0
−0.1
0.1
0.2
0.3
0.4
SNP effect on Ena/VASP−like protein
SNP effect on Selenium

Batch 3−999 : Selenium on Envoplakin

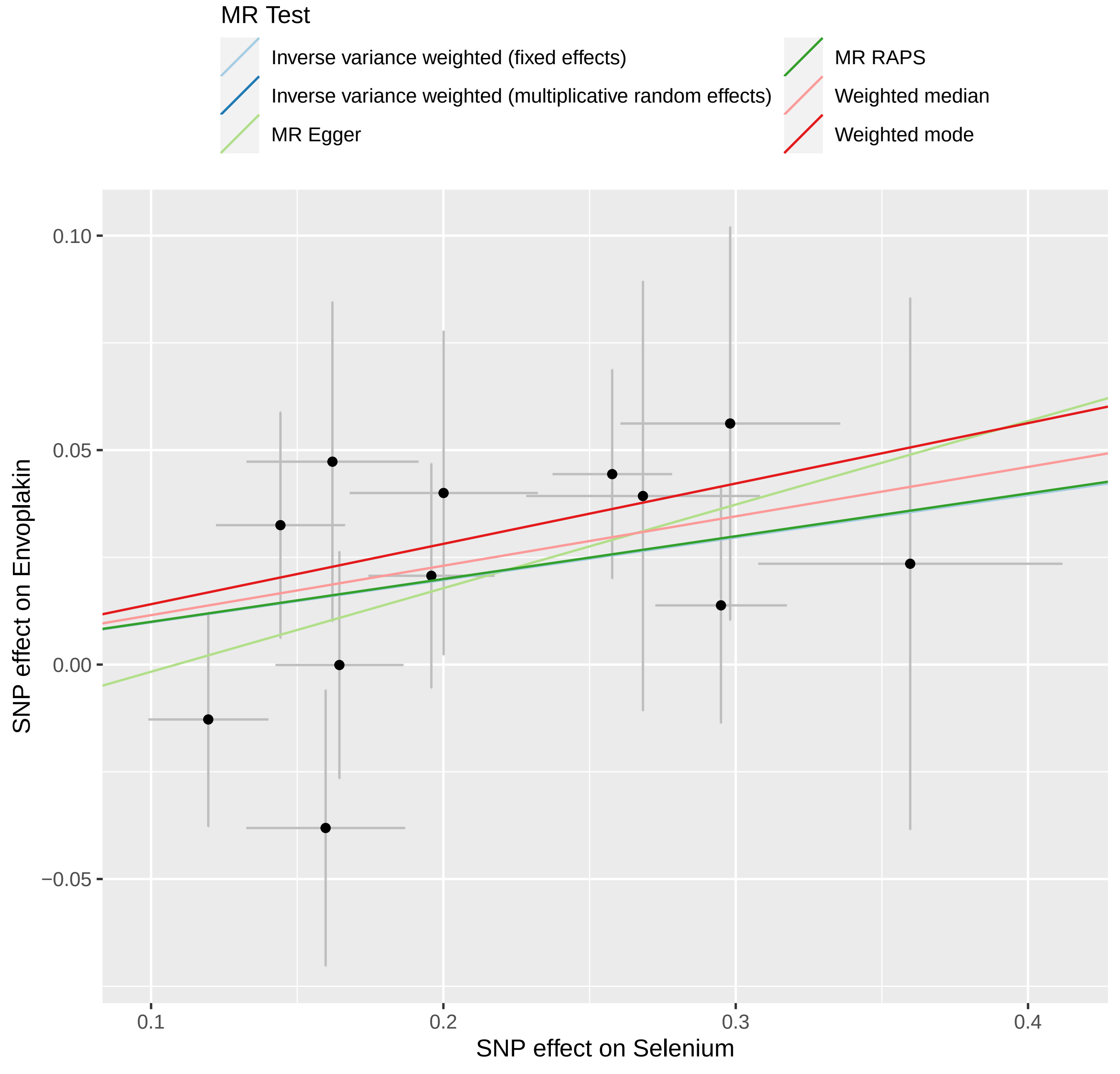

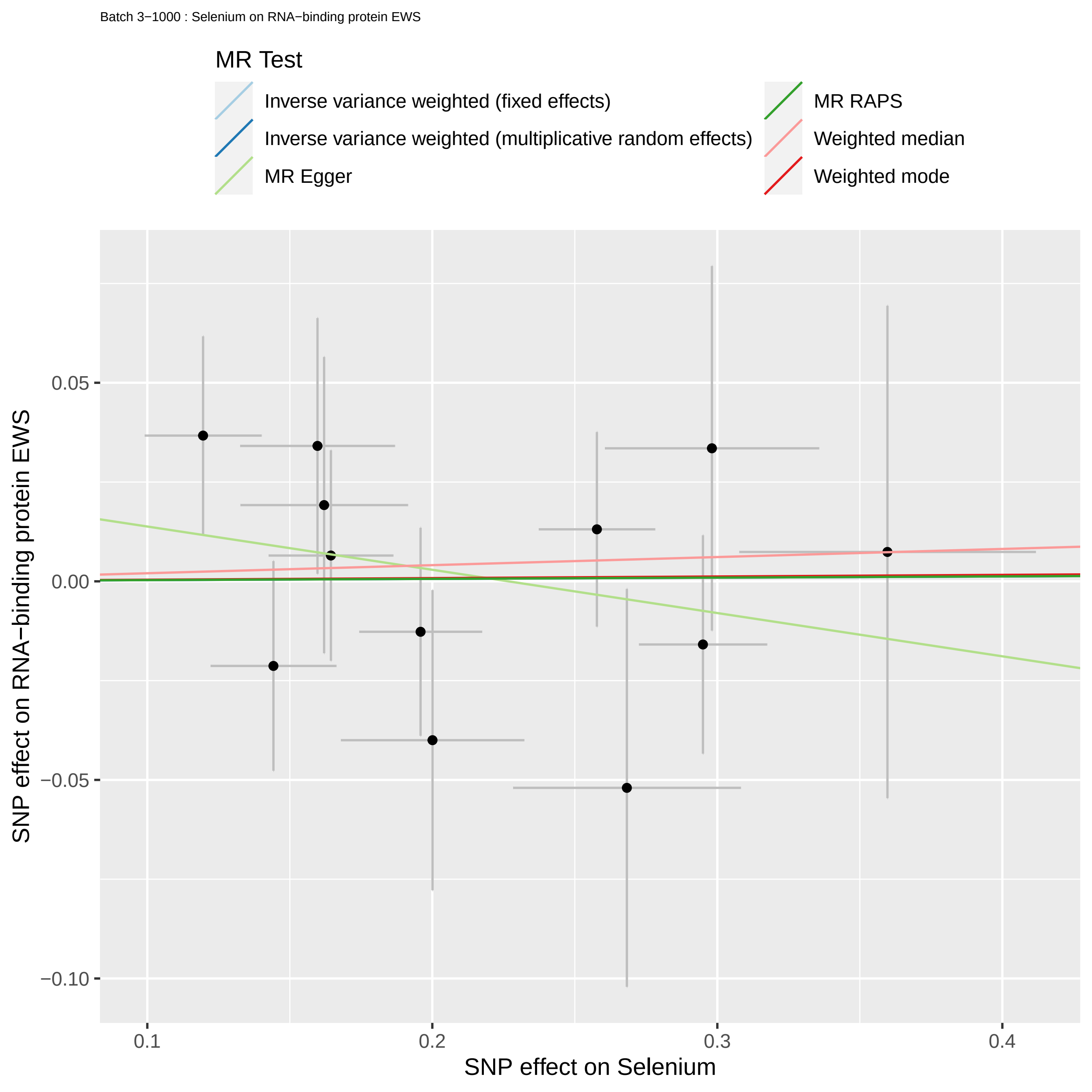

Batch 3−1000 : Selenium on RNA−binding protein EWS
MR Test
Inverse variance weighted (fixed effects)
Inverse variance weighted (multiplicative random effects)
MR Egger
MR RAPS
Weighted median
Weighted mode
0.05
0.00
−0.05
−0.10
0.1
0.2
0.3
0.4
SNP effect on Selenium
SNP effect on RNA−binding protein EWS

Batch 3−1001 : Selenium on Exosome complex component CSL4

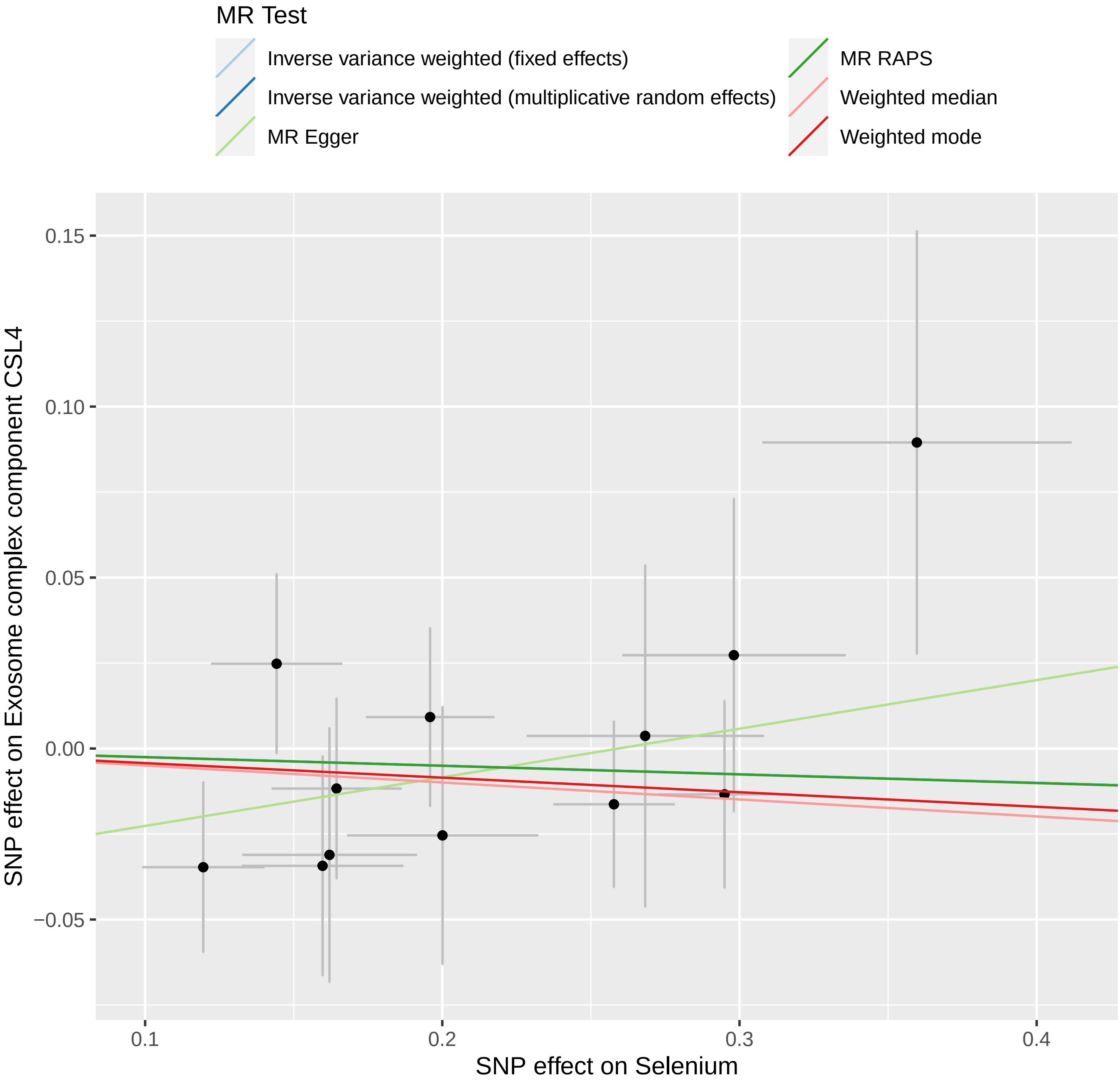

Batch 3−1002 : Selenium on Exosome complex component RRP40

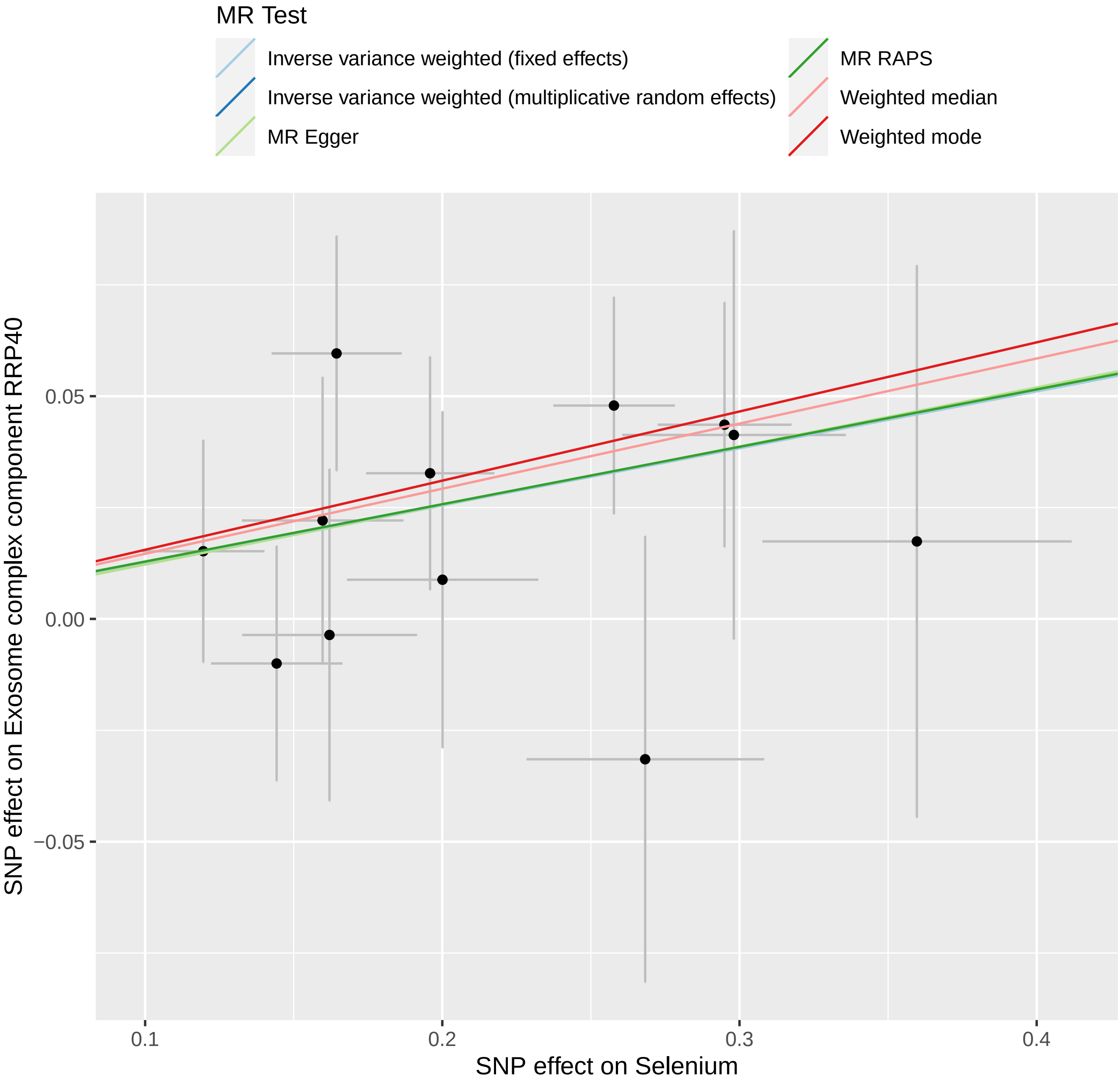

Batch 3−1003 : Selenium on Exostosin−like 2

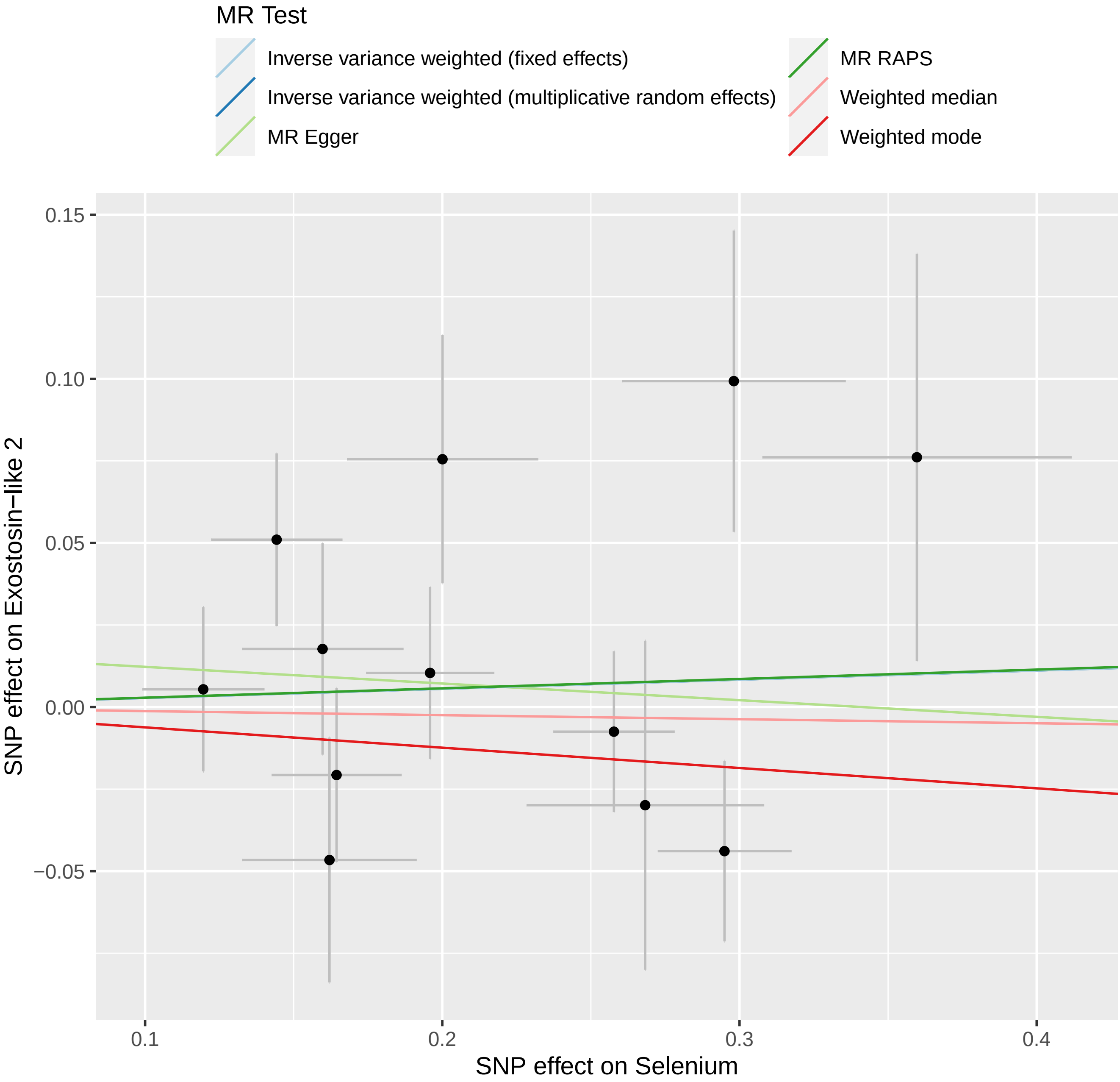

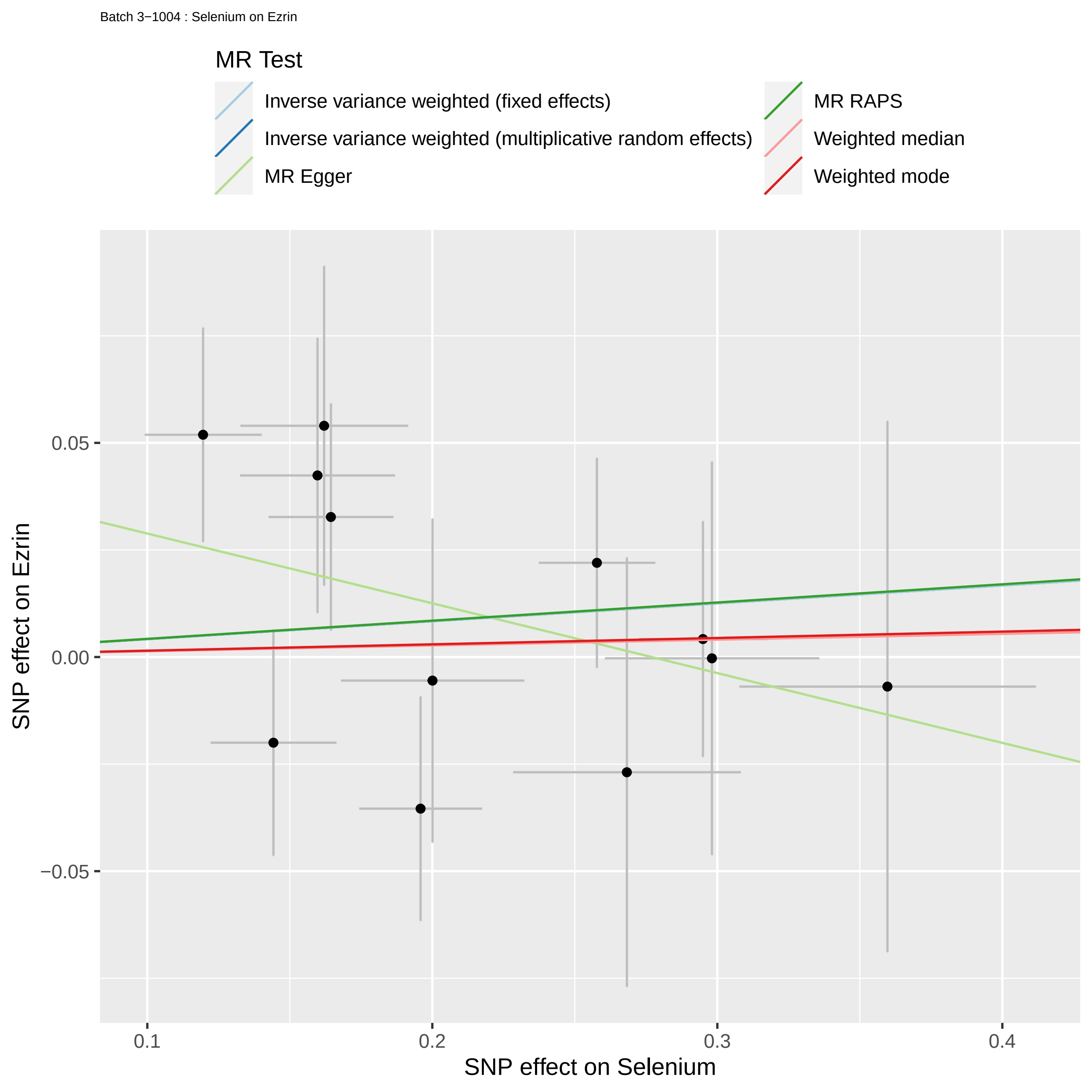
Batch 3−1004 : Selenium on Ezrin
MR Test
Inverse variance weighted (fixed effects)
Inverse variance weighted (multiplicative random effects)
MR Egger
MR RAPS
Weighted median
Weighted mode
SNP effect on Ezrin
0.05
0.00
−0.05
0.1
0.2
0.3
0.4
SNP effect on Selenium

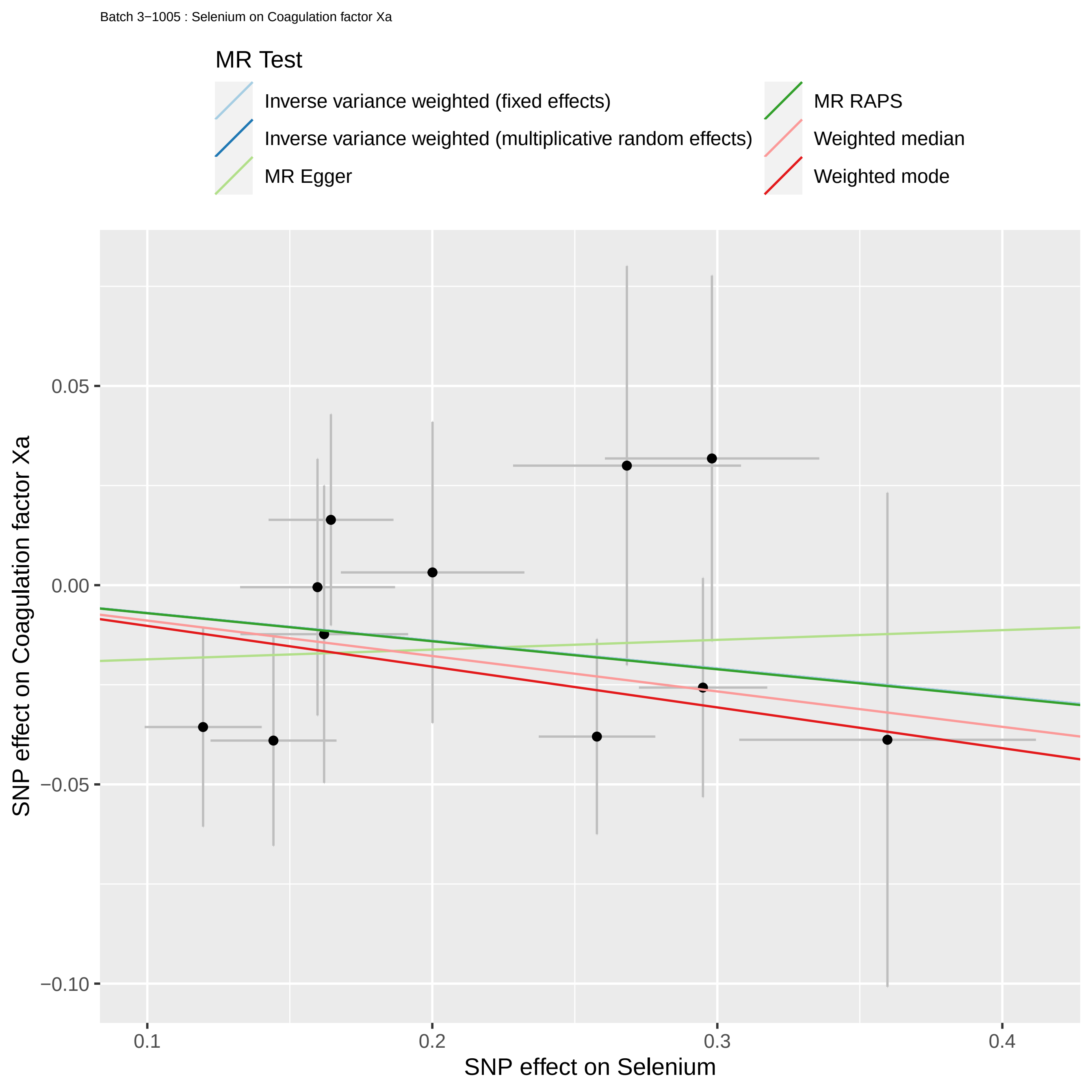
Batch 3−1005 : Selenium on Coagulation factor Xa
MR Test
Inverse variance weighted (fixed effects)
Inverse variance weighted (multiplicative random effects)
MR Egger
MR RAPS
Weighted median
Weighted mode
0.05
0.00
−0.05
−0.10
0.1
0.2
0.3
0.4
SNP effect on Coagulation factor Xa
SNP effect on Selenium

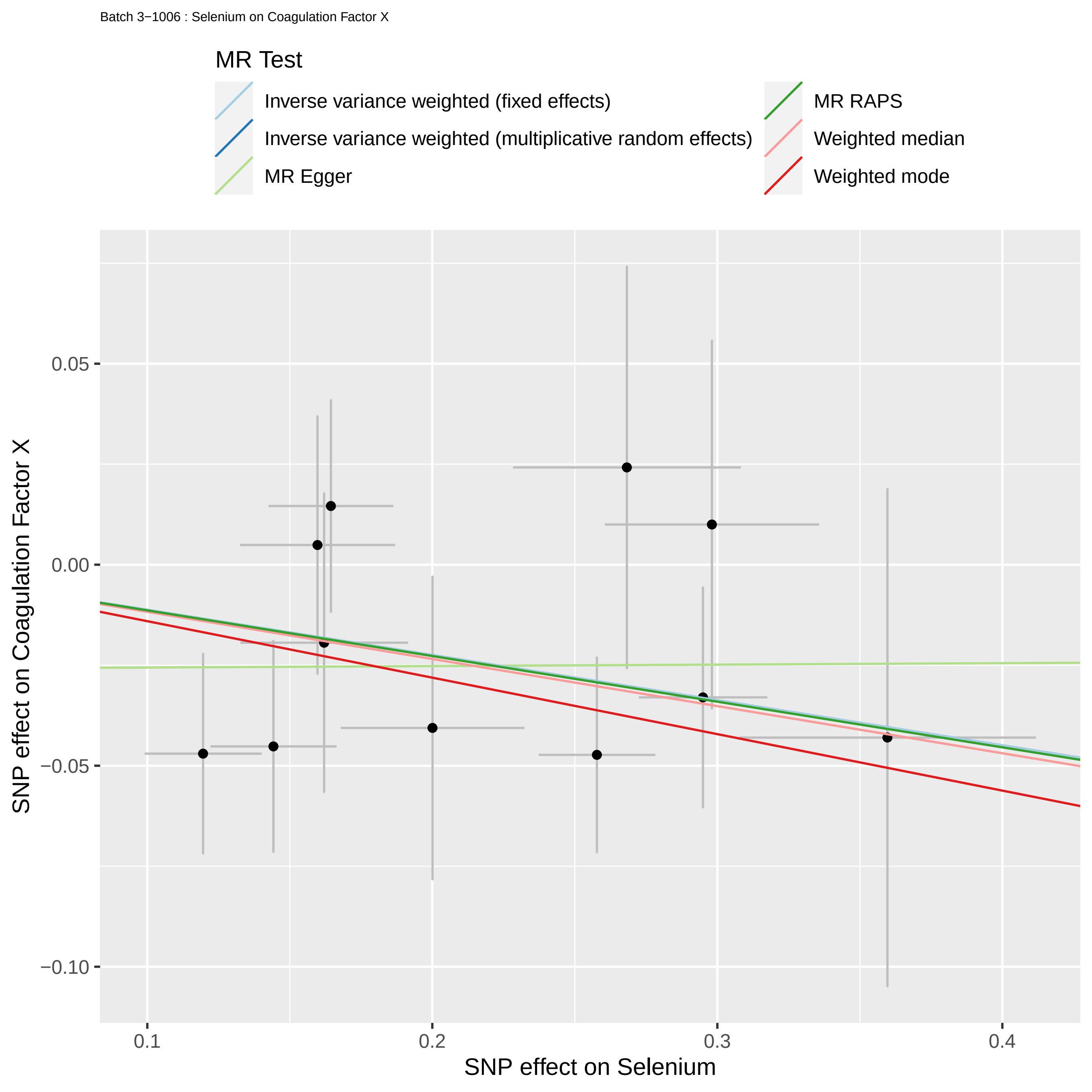

Batch 3−1006 : Selenium on Coagulation Factor X
MR Test
Inverse variance weighted (fixed effects)
Inverse variance weighted (multiplicative random effects)
MR Egger
MR RAPS
Weighted median
Weighted mode
0.05
0.00
−0.05
−0.10
0.1
0.2
0.3
0.4
SNP effect on Coagulation Factor X
SNP effect on Selenium

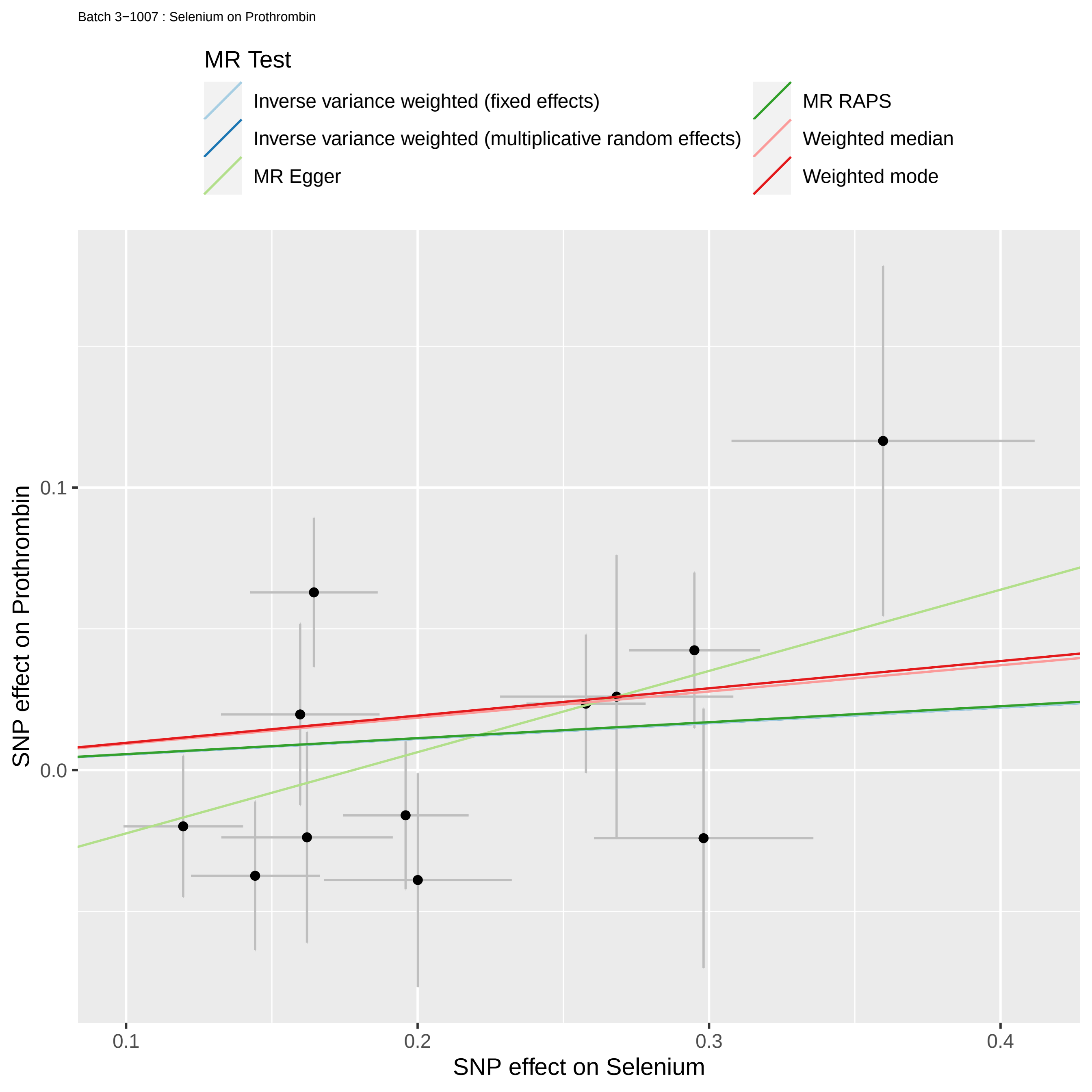

Batch 3−1007 : Selenium on Prothrombin
MR Test
Inverse variance weighted (fixed effects)
Inverse variance weighted (multiplicative random effects)
MR Egger
MR RAPS
Weighted median
Weighted mode
SNP effect on Prothrombin
0.1
0.0
0.1
0.2
0.3
0.4
SNP effect on Selenium

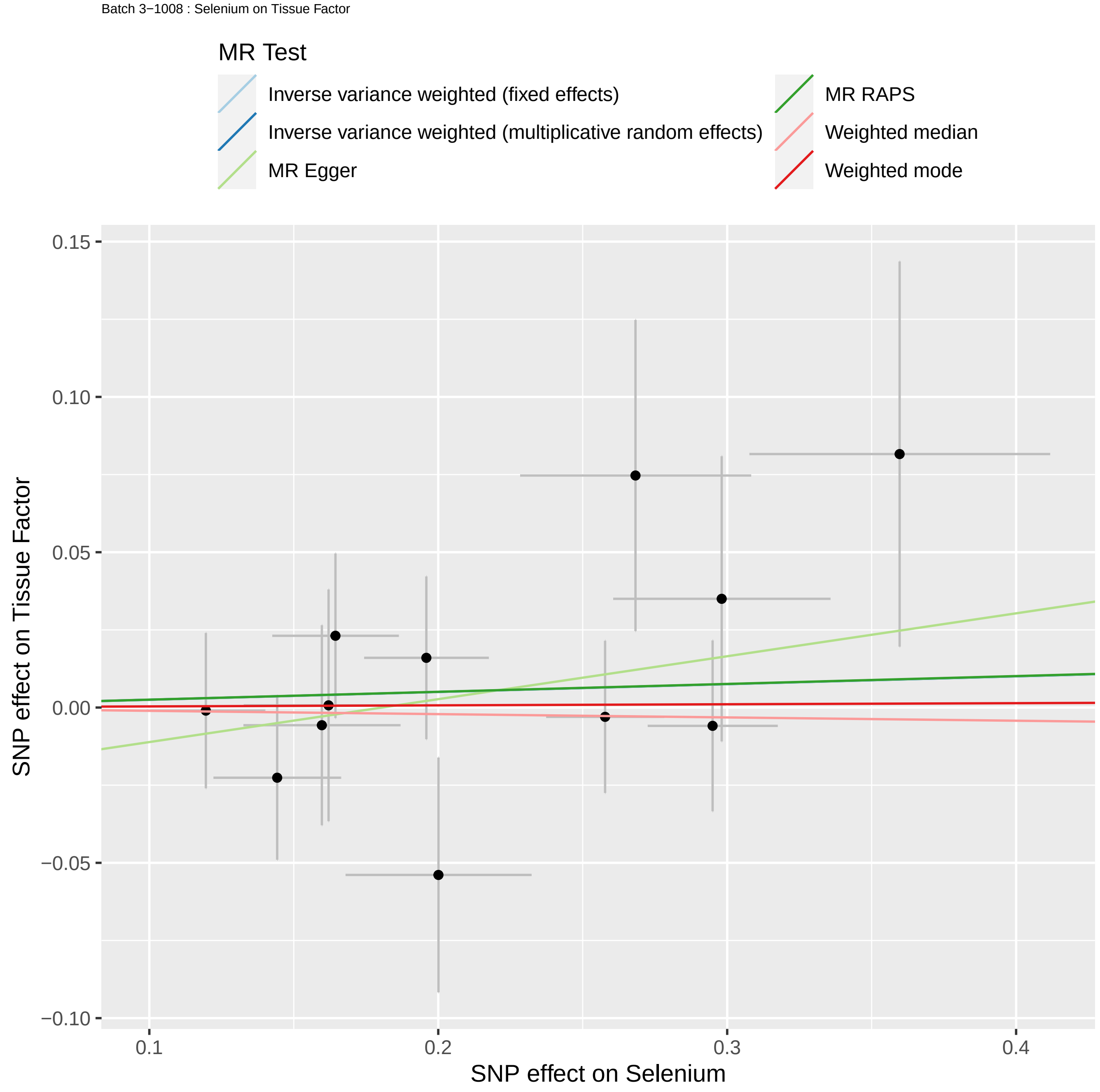
Batch 3−1008 : Selenium on Tissue Factor
MR Test
Inverse variance weighted (fixed effects)
Inverse variance weighted (multiplicative random effects)
MR Egger
MR RAPS
Weighted median
Weighted mode
0.15
0.10
0.05
0.00
−0.05
−0.10
0.1
0.2
0.3
0.4
SNP effect on Tissue Factor
SNP effect on Selenium

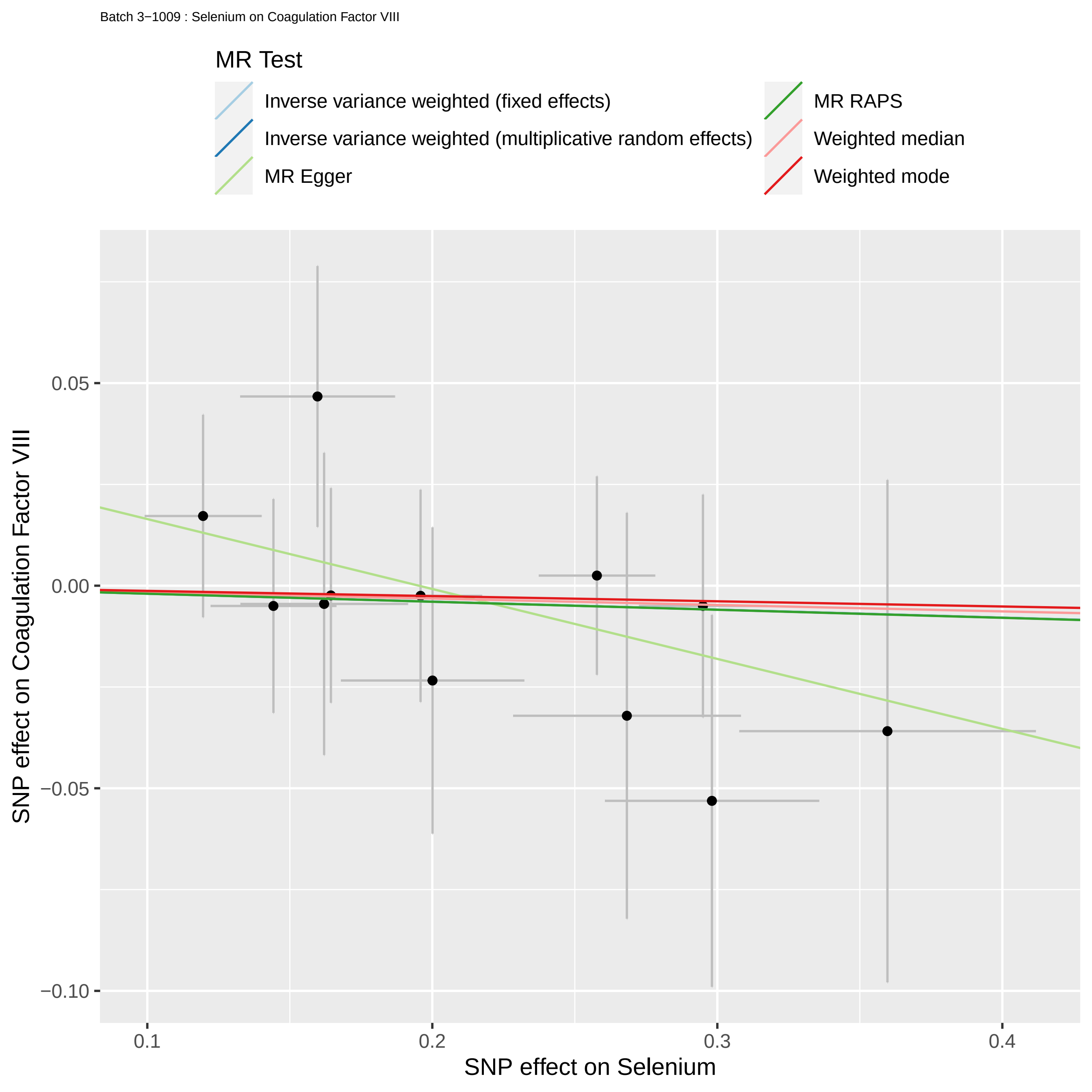

Batch 3−1009 : Selenium on Coagulation Factor VIII
MR Test
Inverse variance weighted (fixed effects)
Inverse variance weighted (multiplicative random effects)
MR Egger
MR RAPS
Weighted median
Weighted mode
SNP effect on Coagulation Factor VIII
0.05
0.00
−0.05
−0.10
0.1
0.2
0.3
0.4
SNP effect on Selenium

Batch 3−1010 : Selenium on Fatty−acid amide hydrolase 2

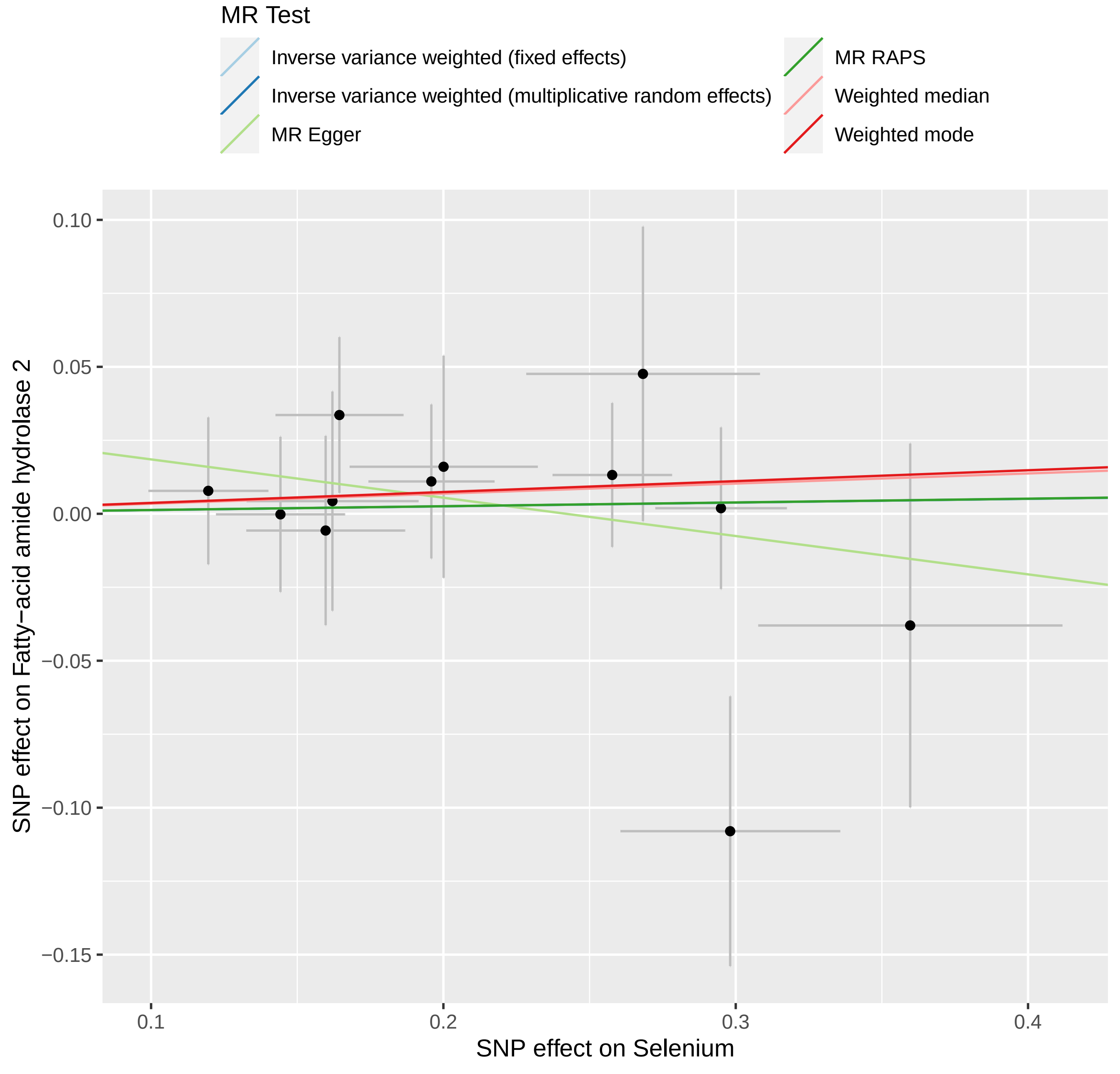

Batch 3−1011 : Selenium on Fatty acid−binding protein, liver

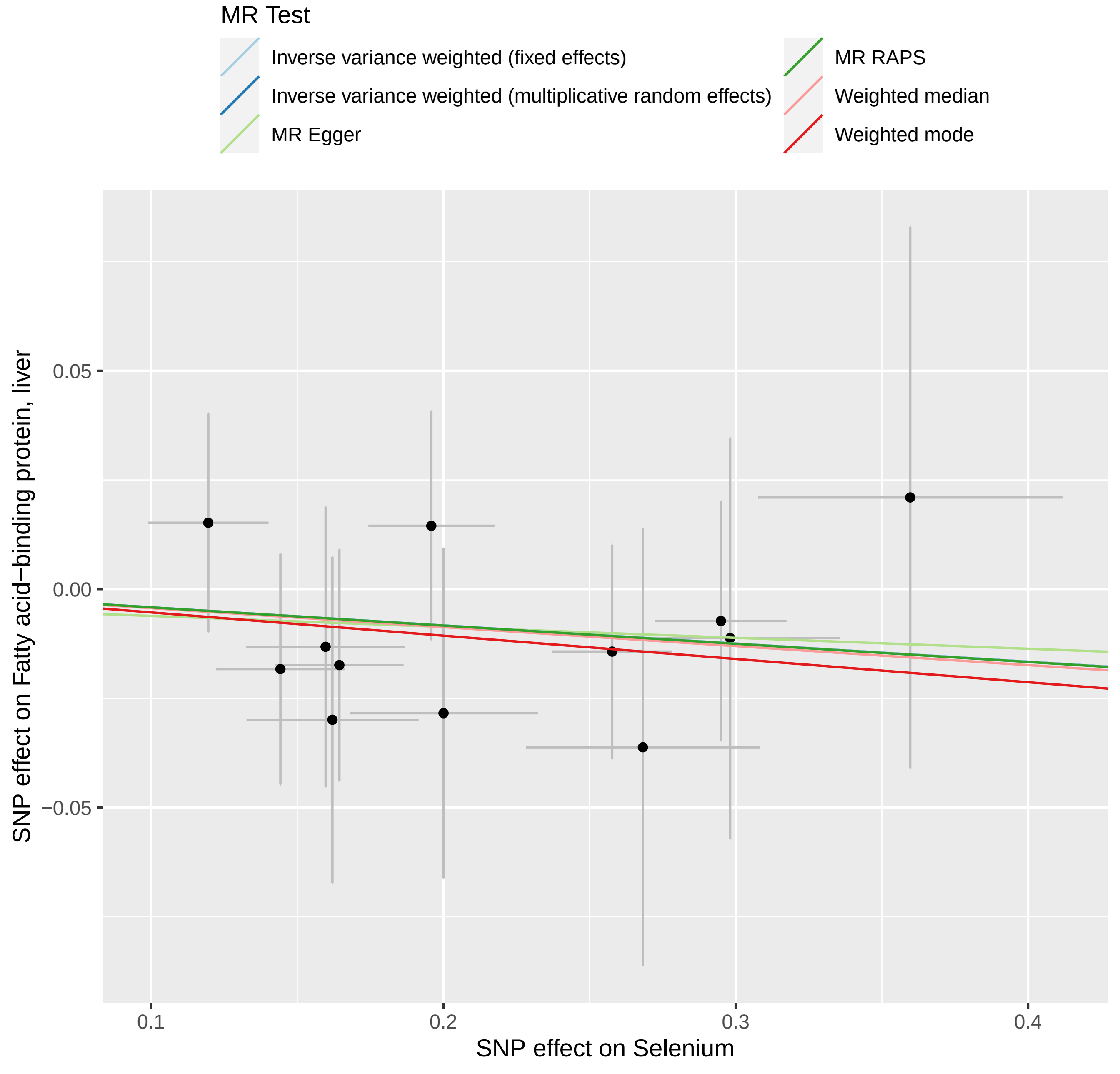

Batch 3−1012 : Selenium on Fatty acid−binding protein, heart

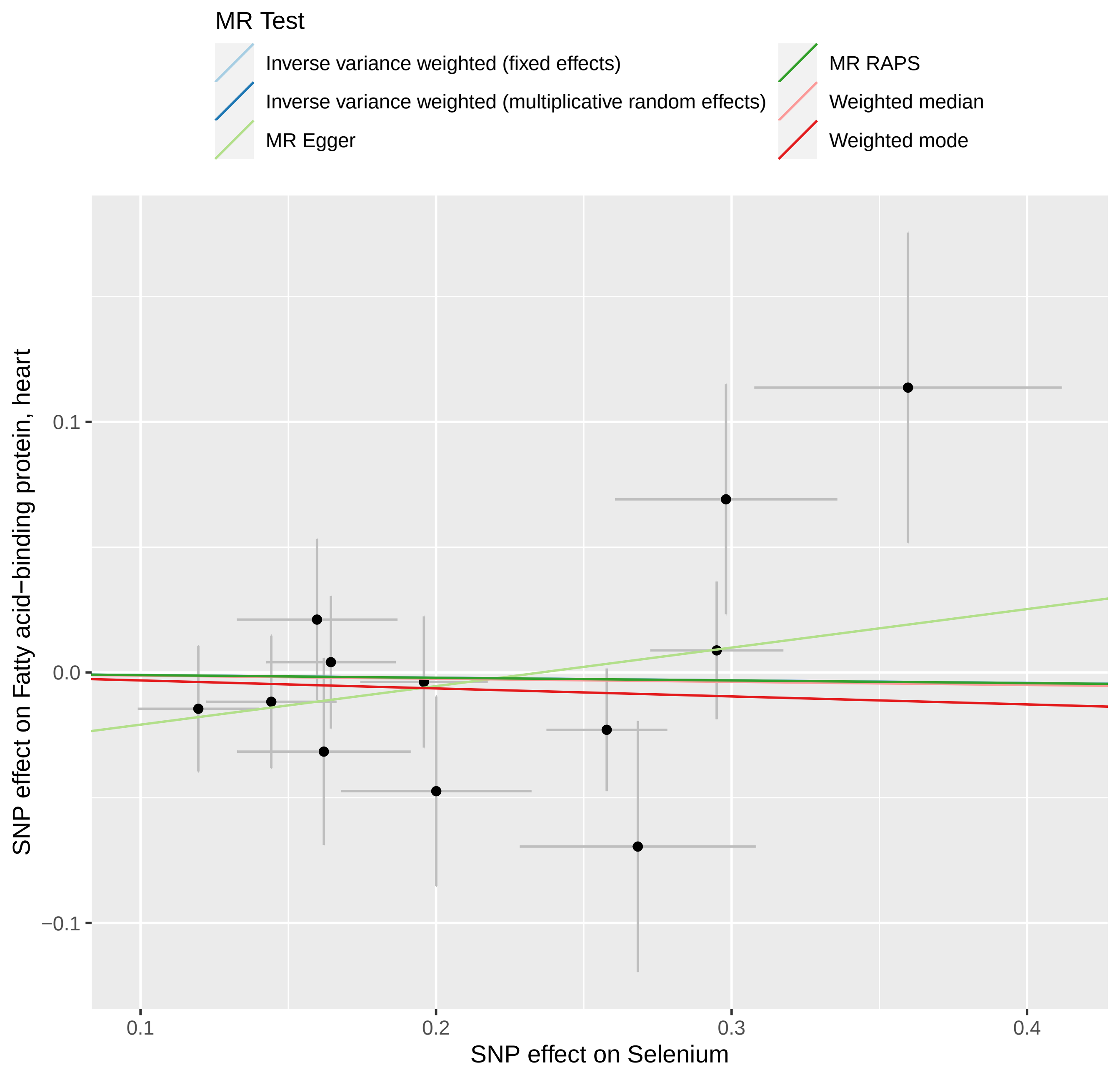

Batch 3−1013 : Selenium on Fatty acid−binding protein, adipocyte

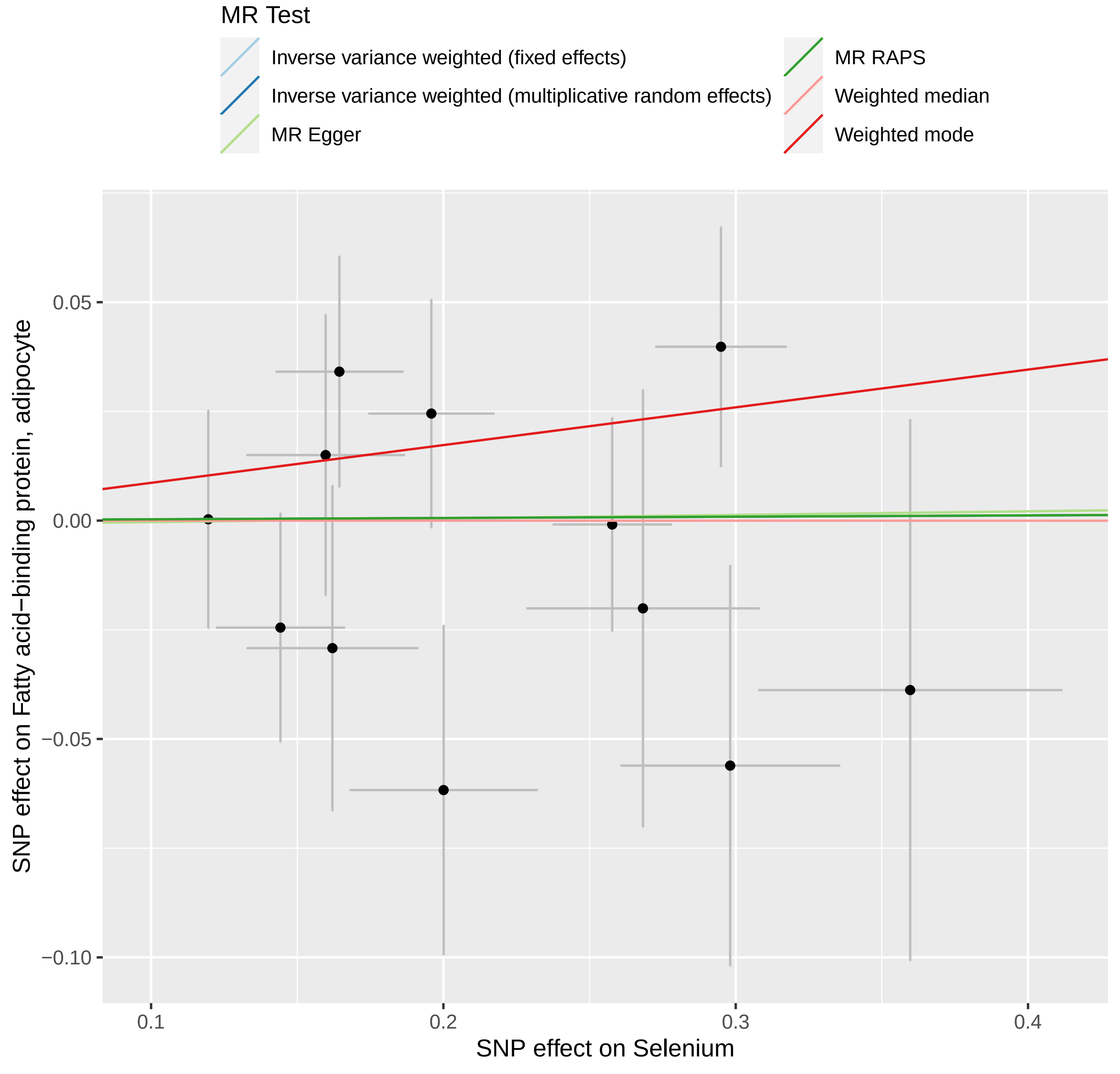

Batch 3−1014 : Selenium on Fatty acid−binding protein, epidermal

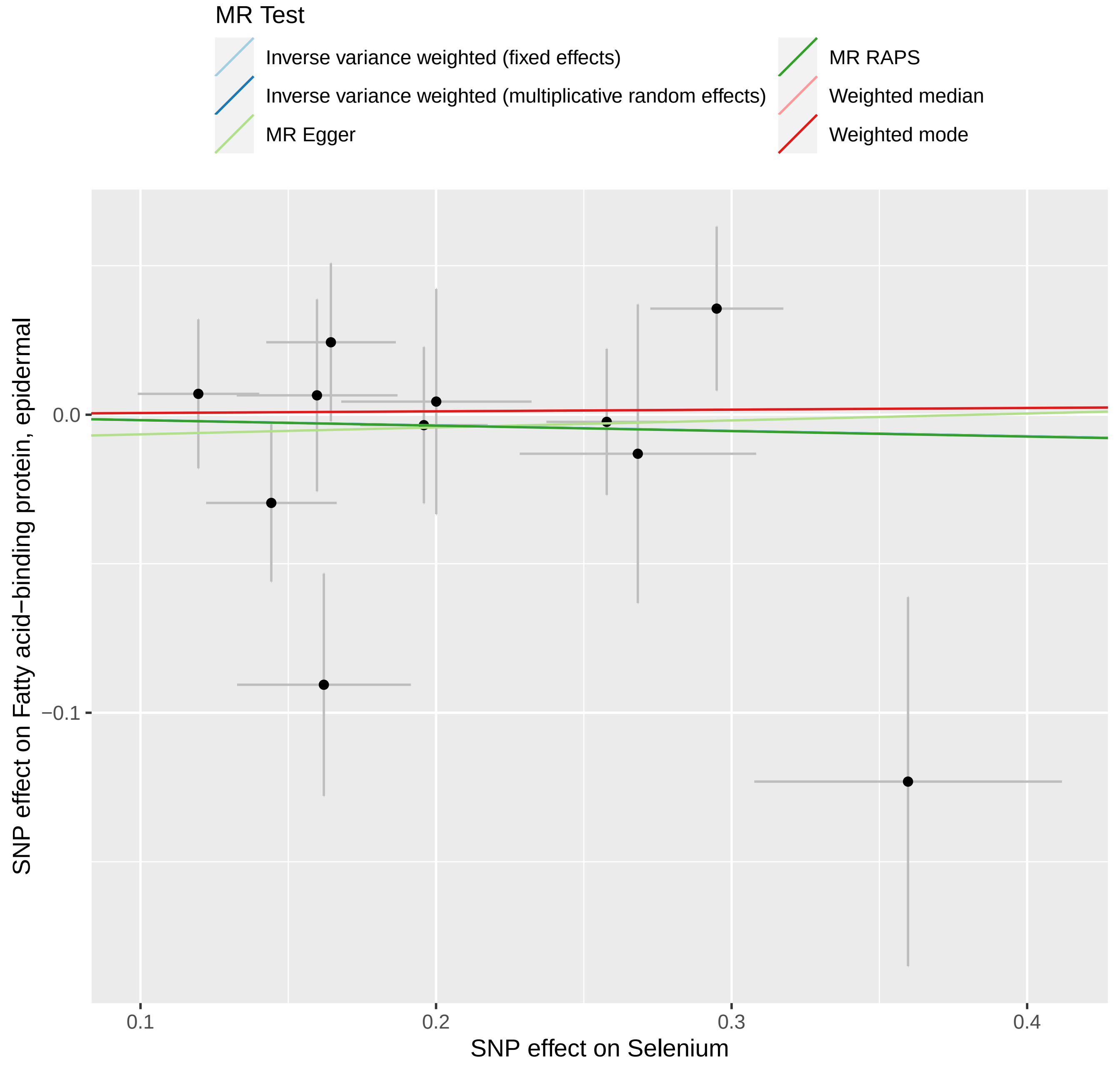

Batch 3−1015 : Selenium on FAS−associated factor 2

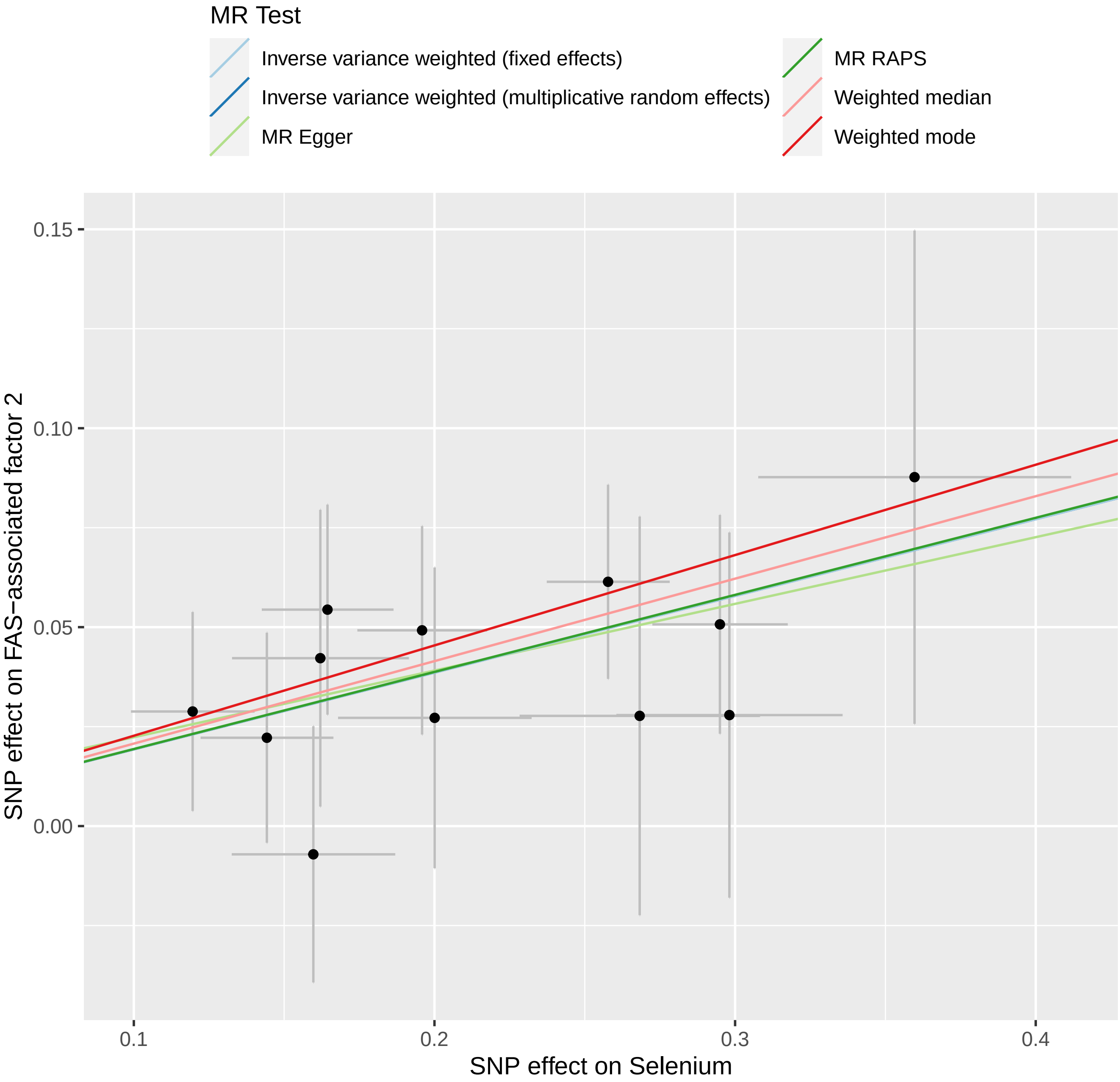

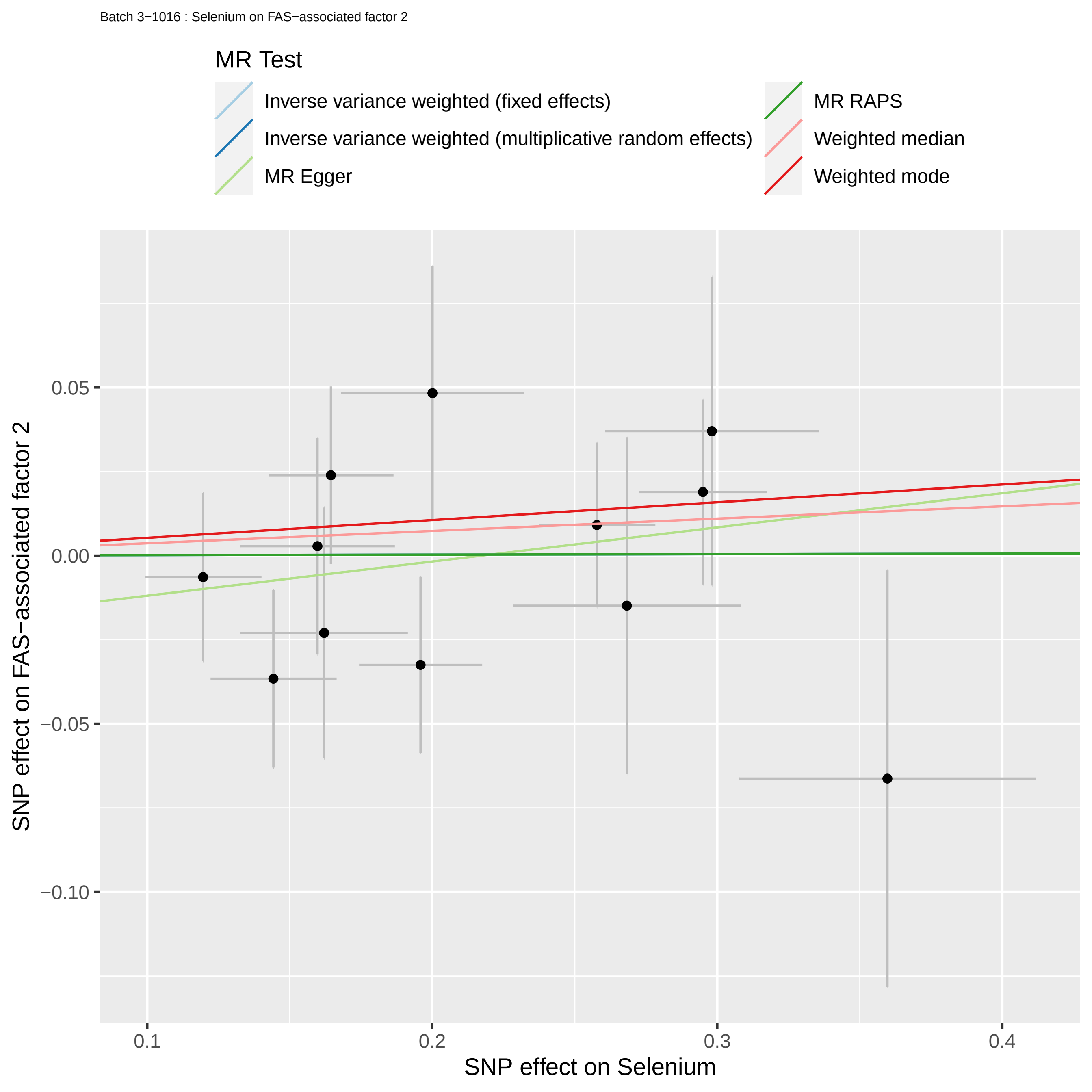

Batch 3−1016 : Selenium on FAS−associated factor 2
MR Test
Inverse variance weighted (fixed effects)
Inverse variance weighted (multiplicative random effects)
MR Egger
MR RAPS
Weighted median
Weighted mode
0.05
0.00
−0.05
−0.10
0.1
0.2
0.3
0.4
SNP effect on FAS−associated factor 2
SNP effect on Selenium

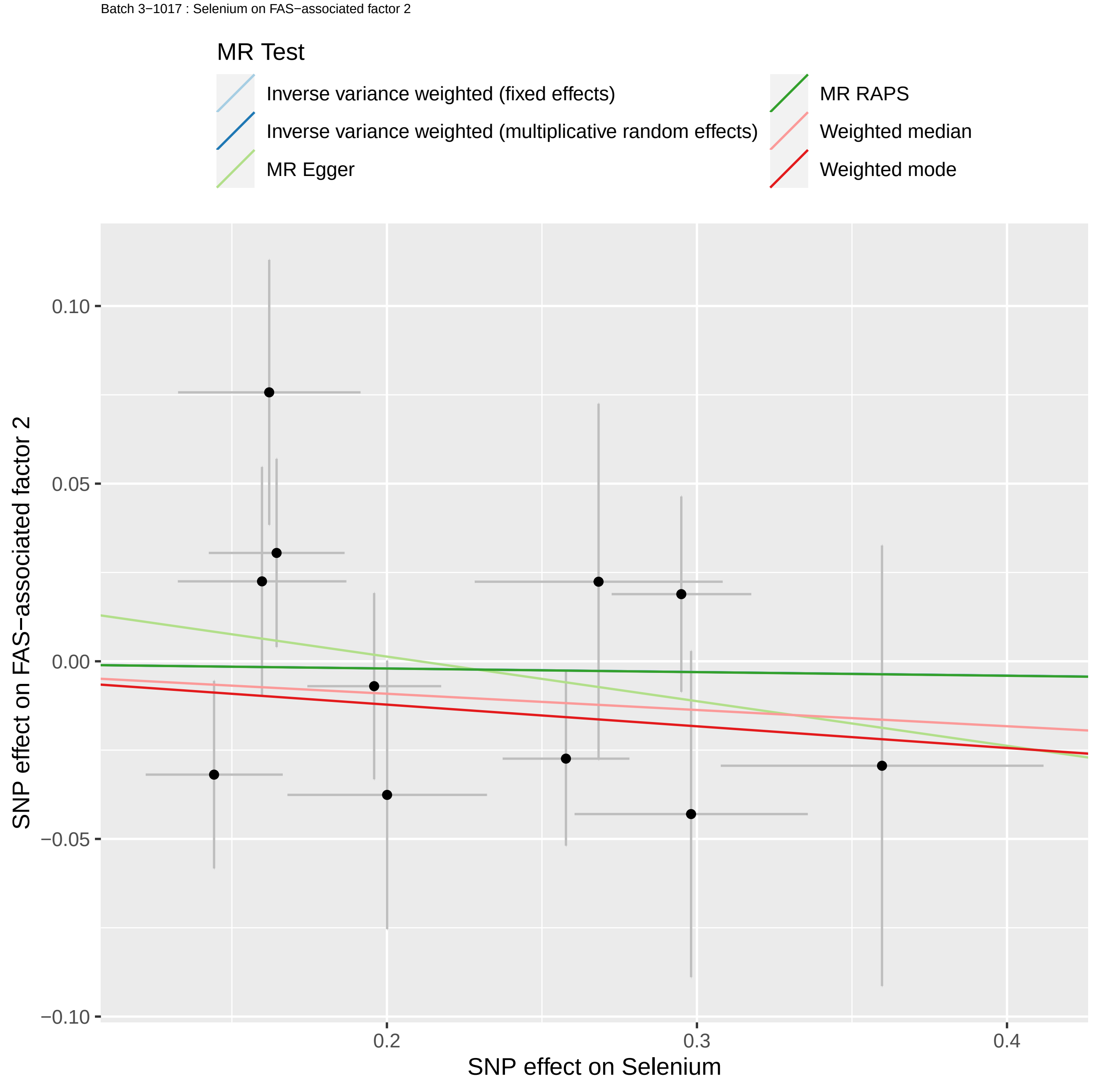

Batch 3−1017 : Selenium on FAS−associated factor 2
MR Test
Inverse variance weighted (fixed effects)
Inverse variance weighted (multiplicative random effects)
MR Egger
MR RAPS
Weighted median
Weighted mode
0.10
0.05
0.00
−0.05
−0.10
0.2
0.3
0.4
SNP effect on FAS−associated factor 2
SNP effect on Selenium

Batch 3−1018 : Selenium on Fumarylacetoacetase

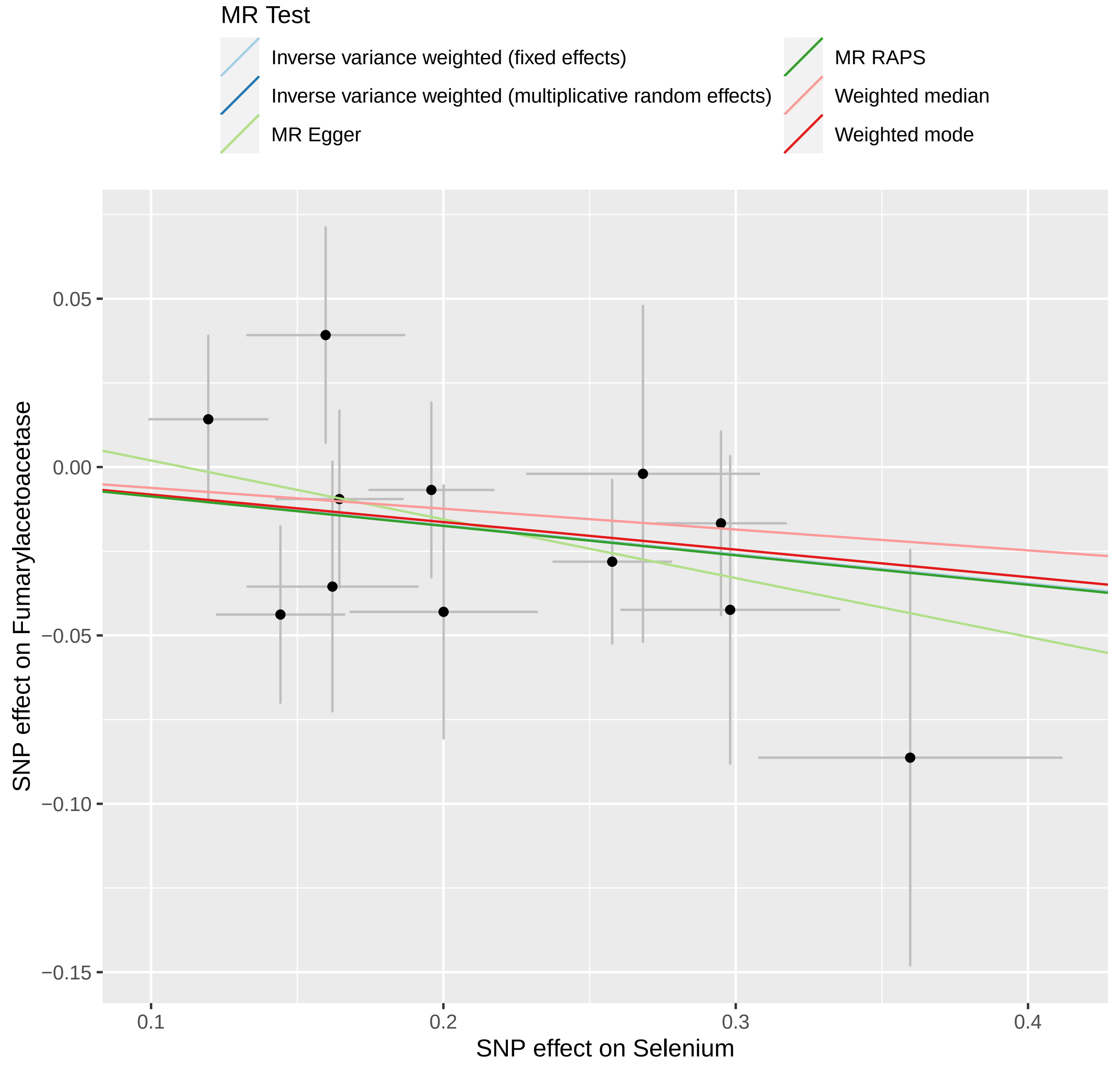

Batch 3−1019 : Selenium on Fas apoptotic inhibitory molecule 3

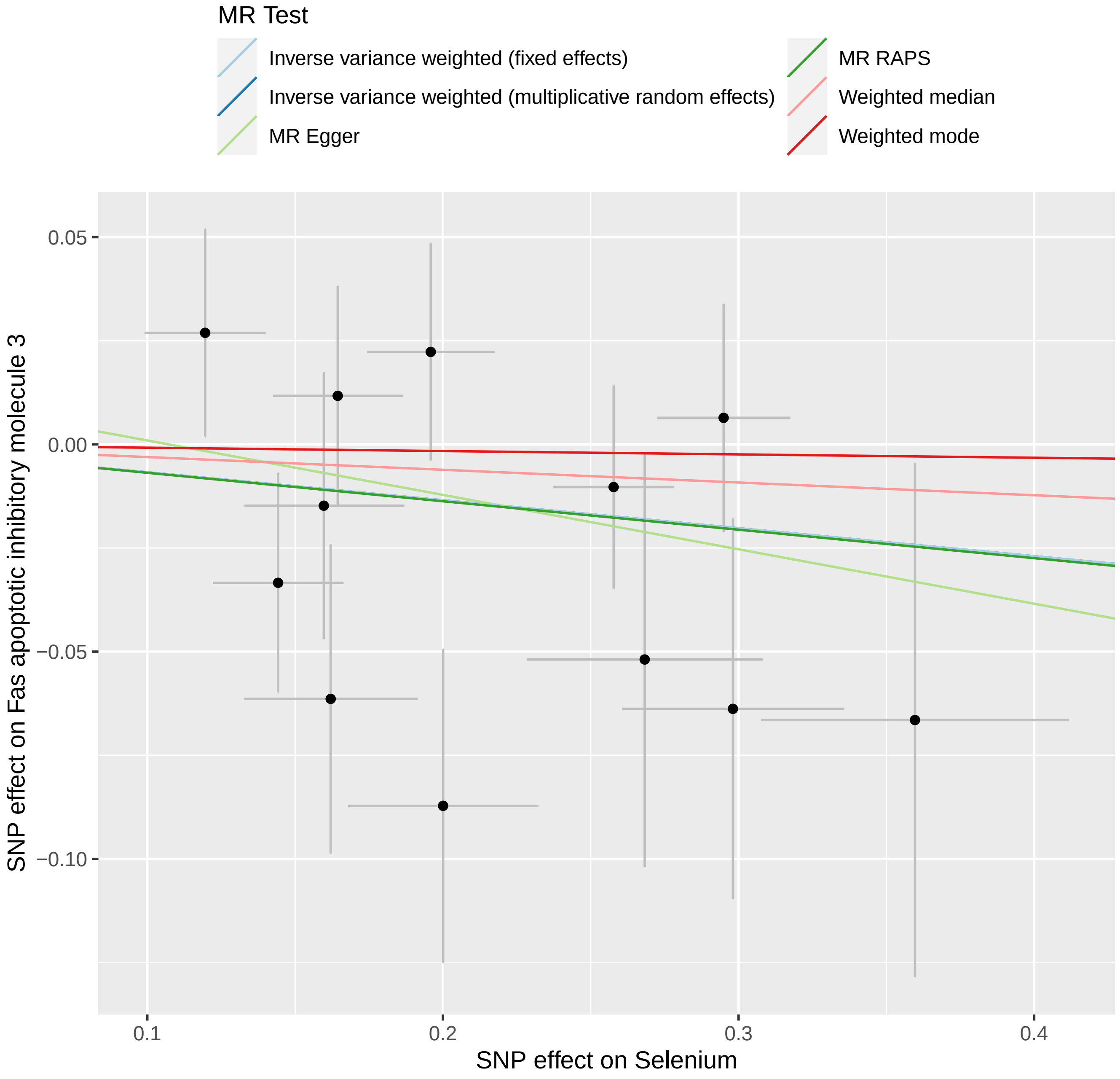

Batch 3−1020 : Selenium on Protein FAM107A

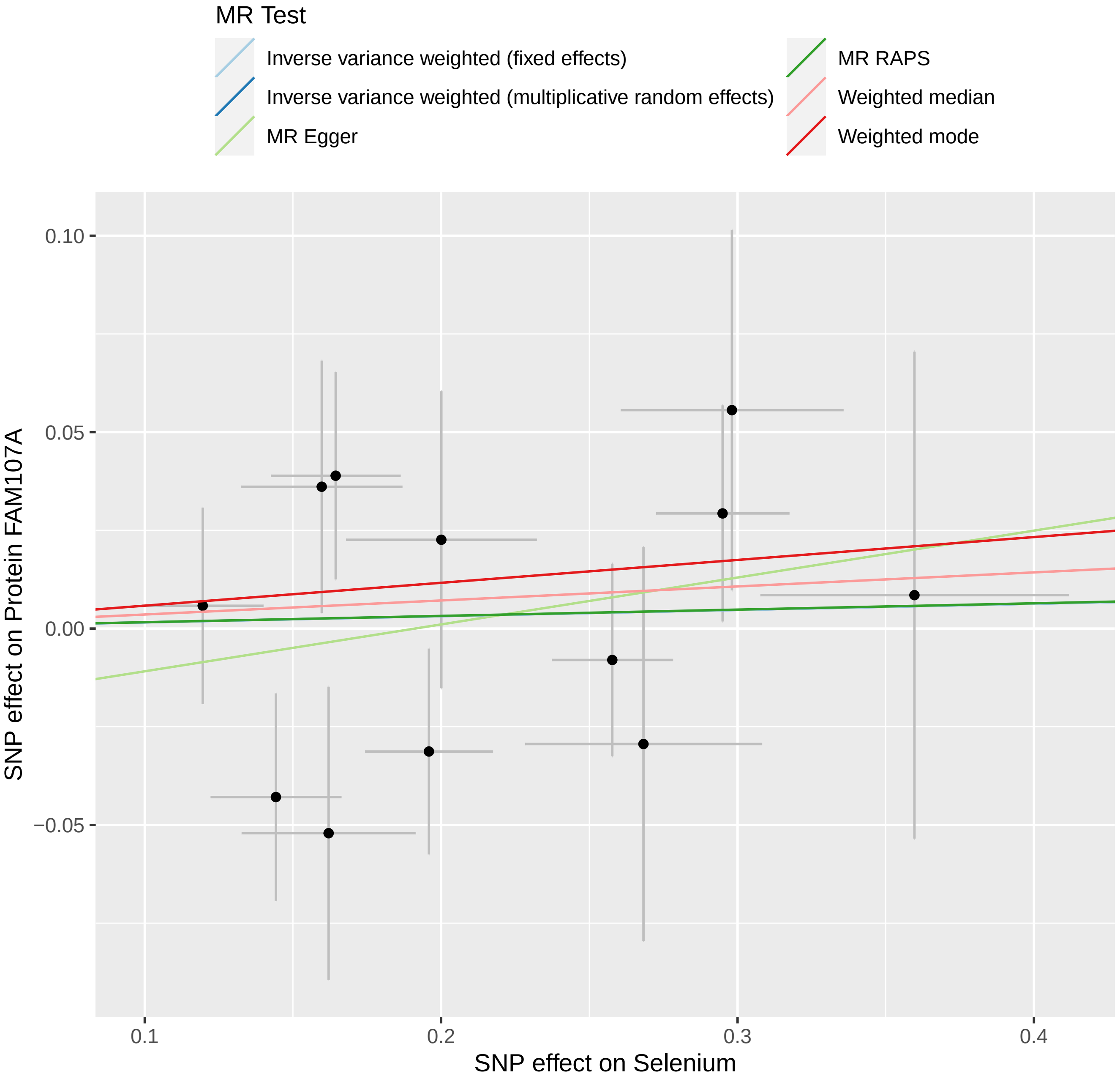

Batch 3−1021 : Selenium on Protein FAM107B

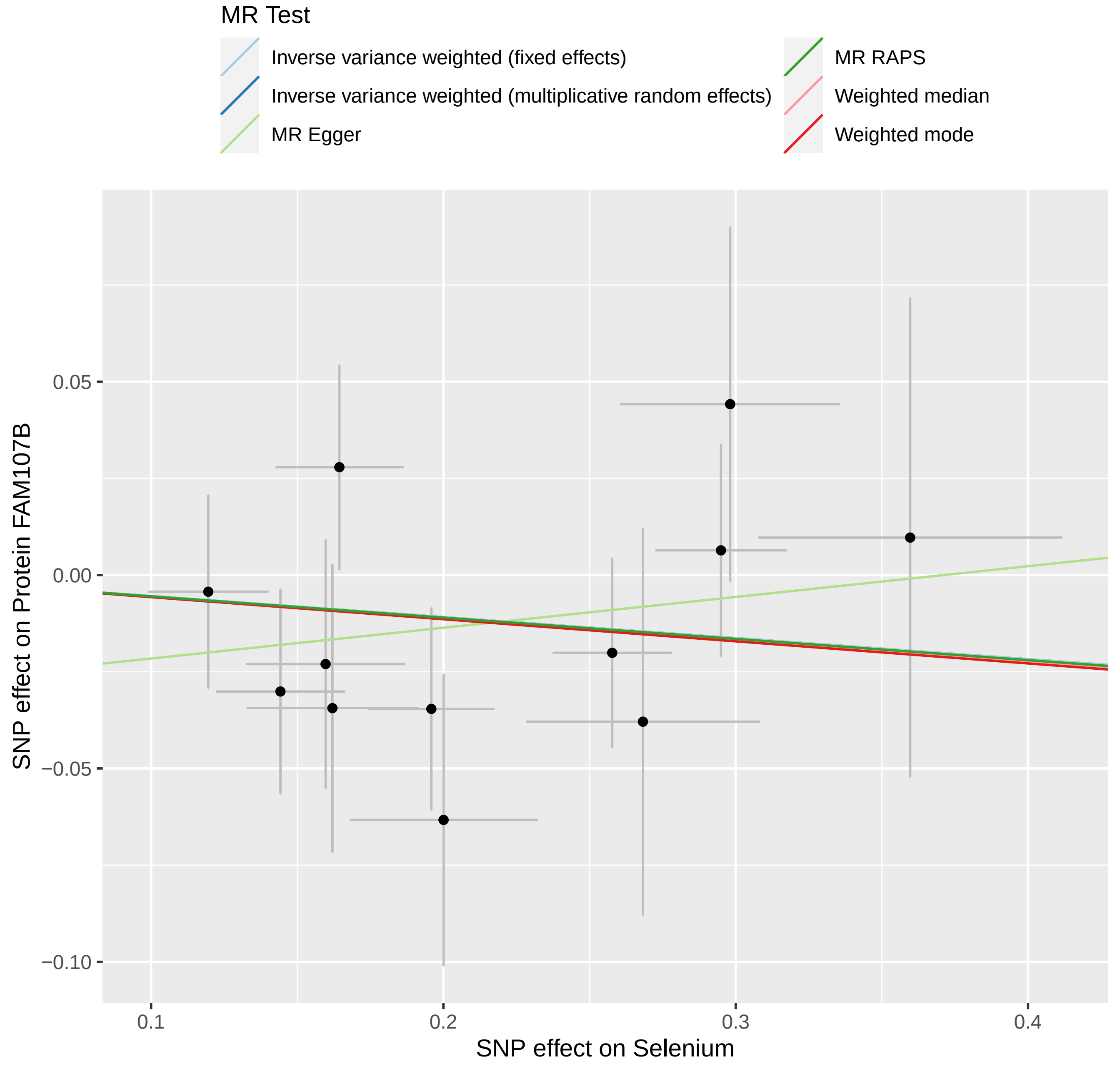

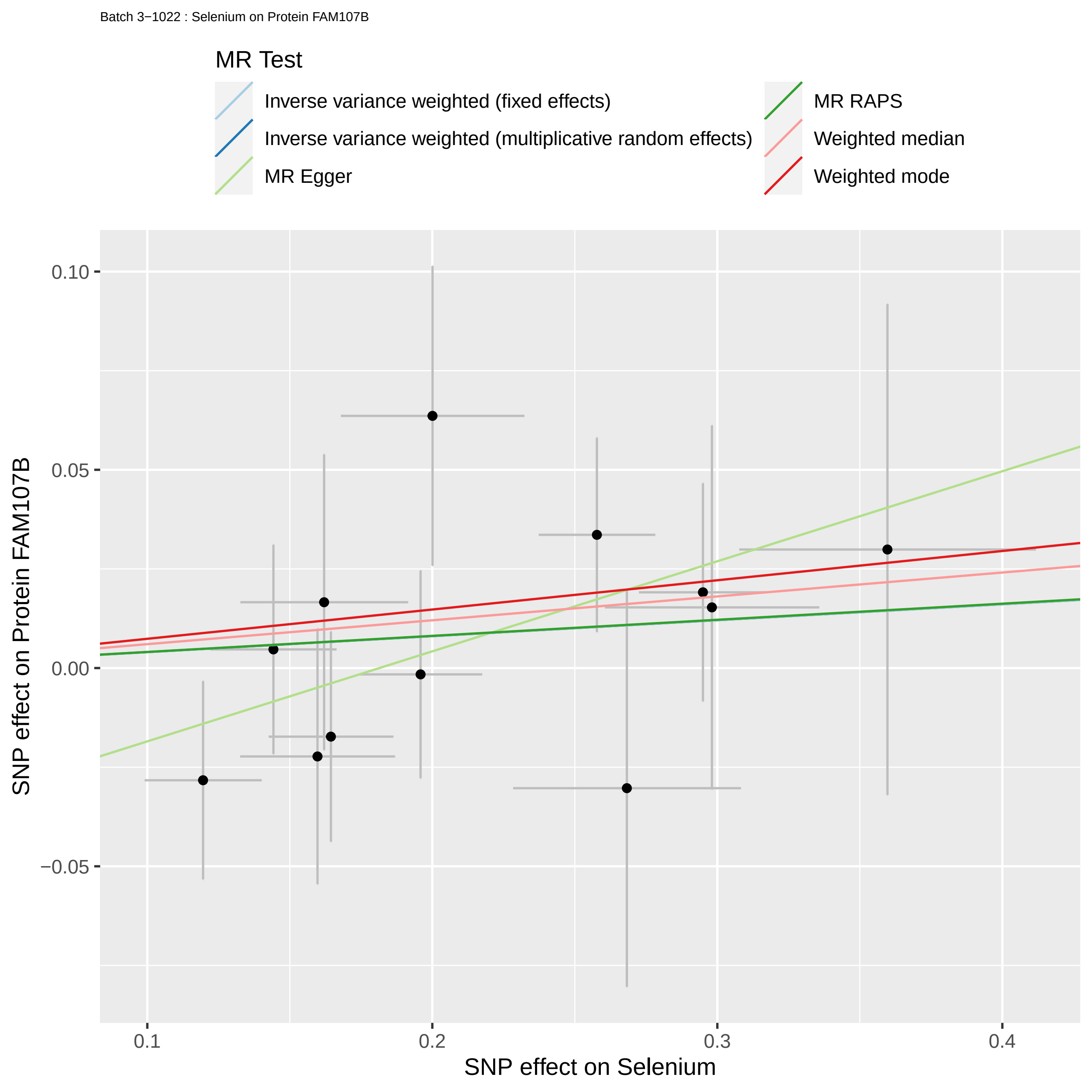
Batch 3−1022 : Selenium on Protein FAM107B
MR Test
Inverse variance weighted (fixed effects)
Inverse variance weighted (multiplicative random effects)
MR Egger
MR RAPS
Weighted median
Weighted mode
0.10
0.05
0.00
−0.05
0.1
0.2
0.3
0.4
SNP effect on Protein FAM107B
SNP effect on Selenium

Batch 3−1023 : Selenium on Protein FAM134B

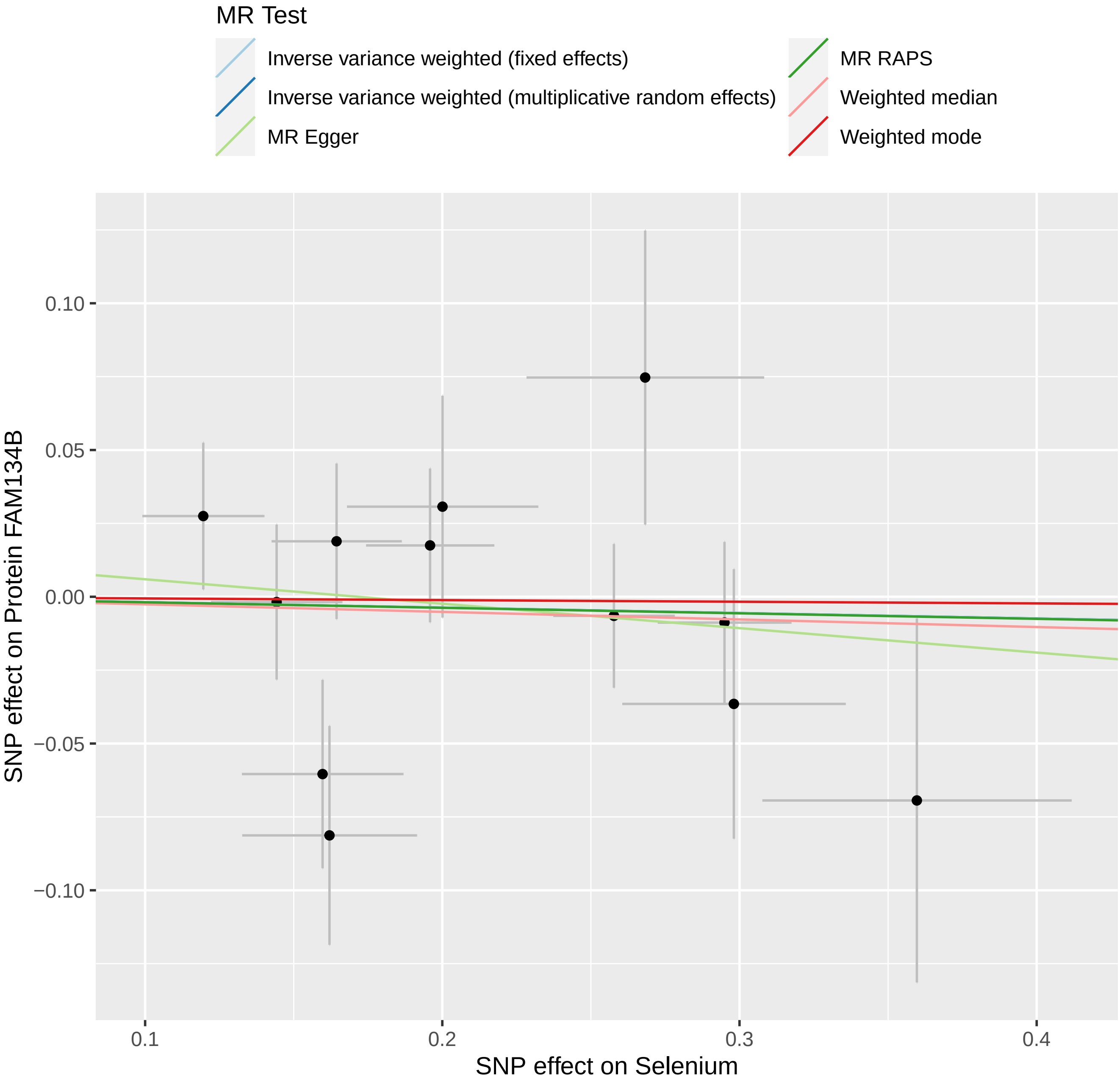

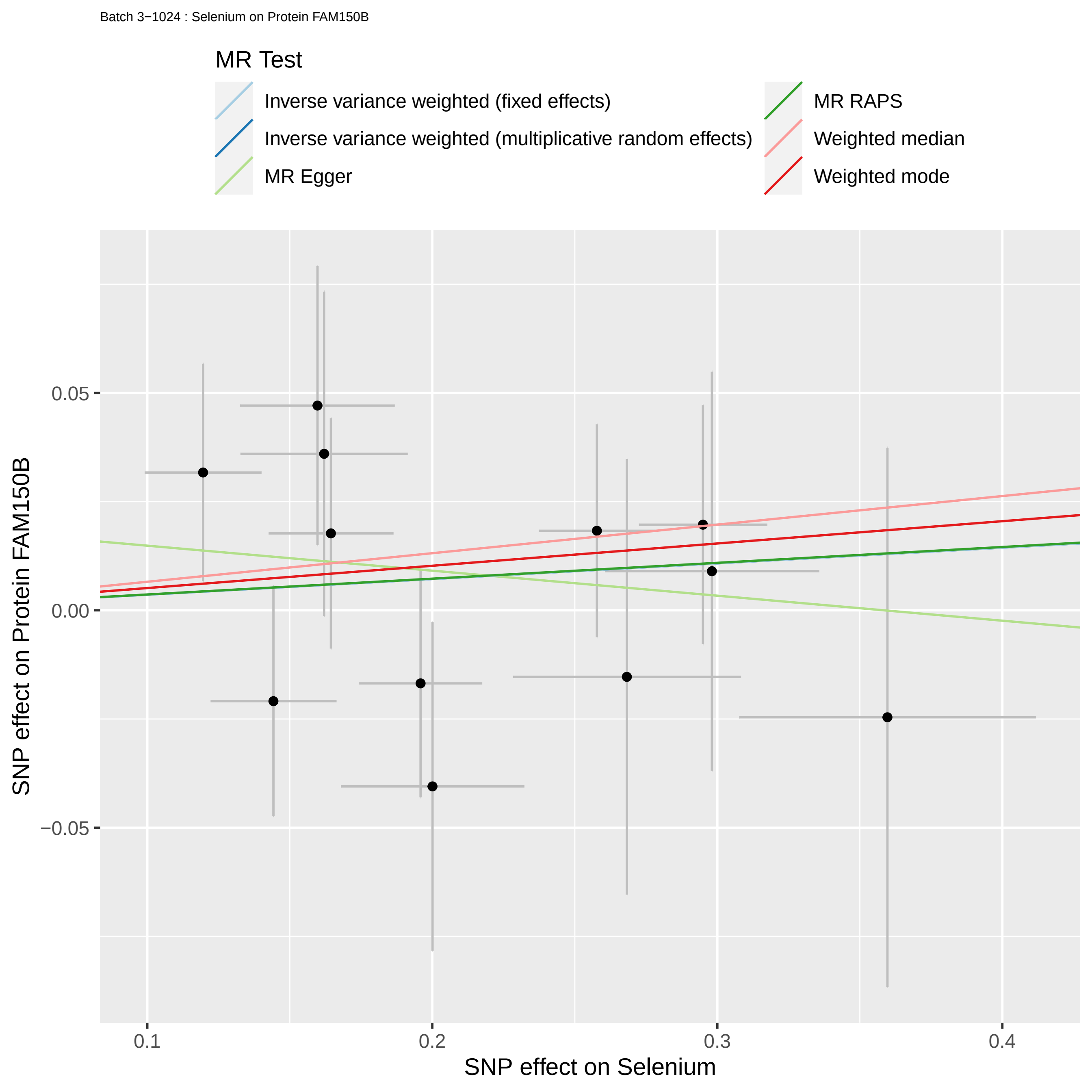
Batch 3−1024 : Selenium on Protein FAM150B
MR Test
Inverse variance weighted (fixed effects)
Inverse variance weighted (multiplicative random effects)
MR Egger
MR RAPS
Weighted median
Weighted mode
SNP effect on Protein FAM150B
SNP effect on Selenium
0.05
0.00
−0.05
0.1
0.2
0.3
0.4

Batch 3−1025 : Selenium on Protein FAM151A

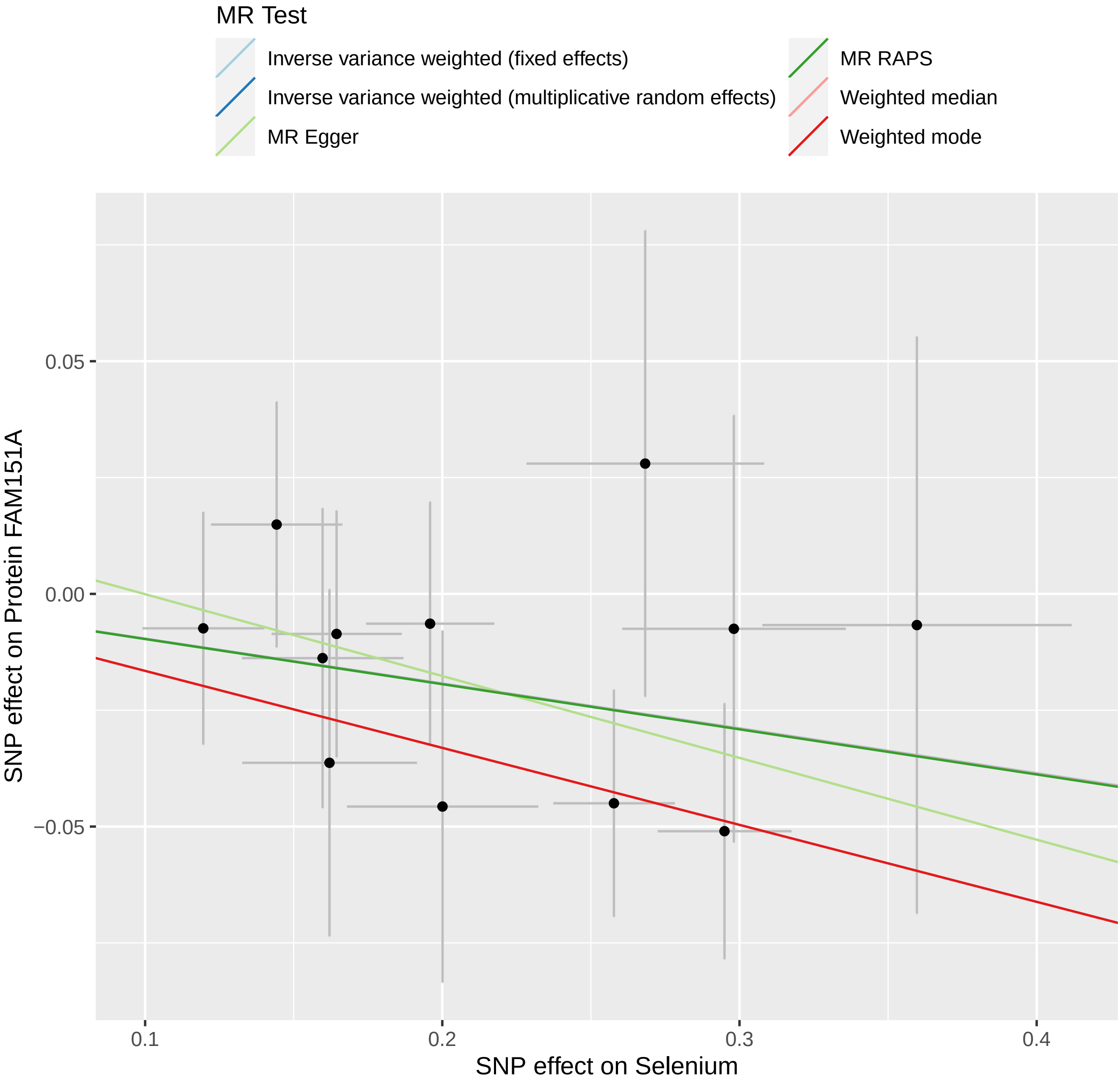

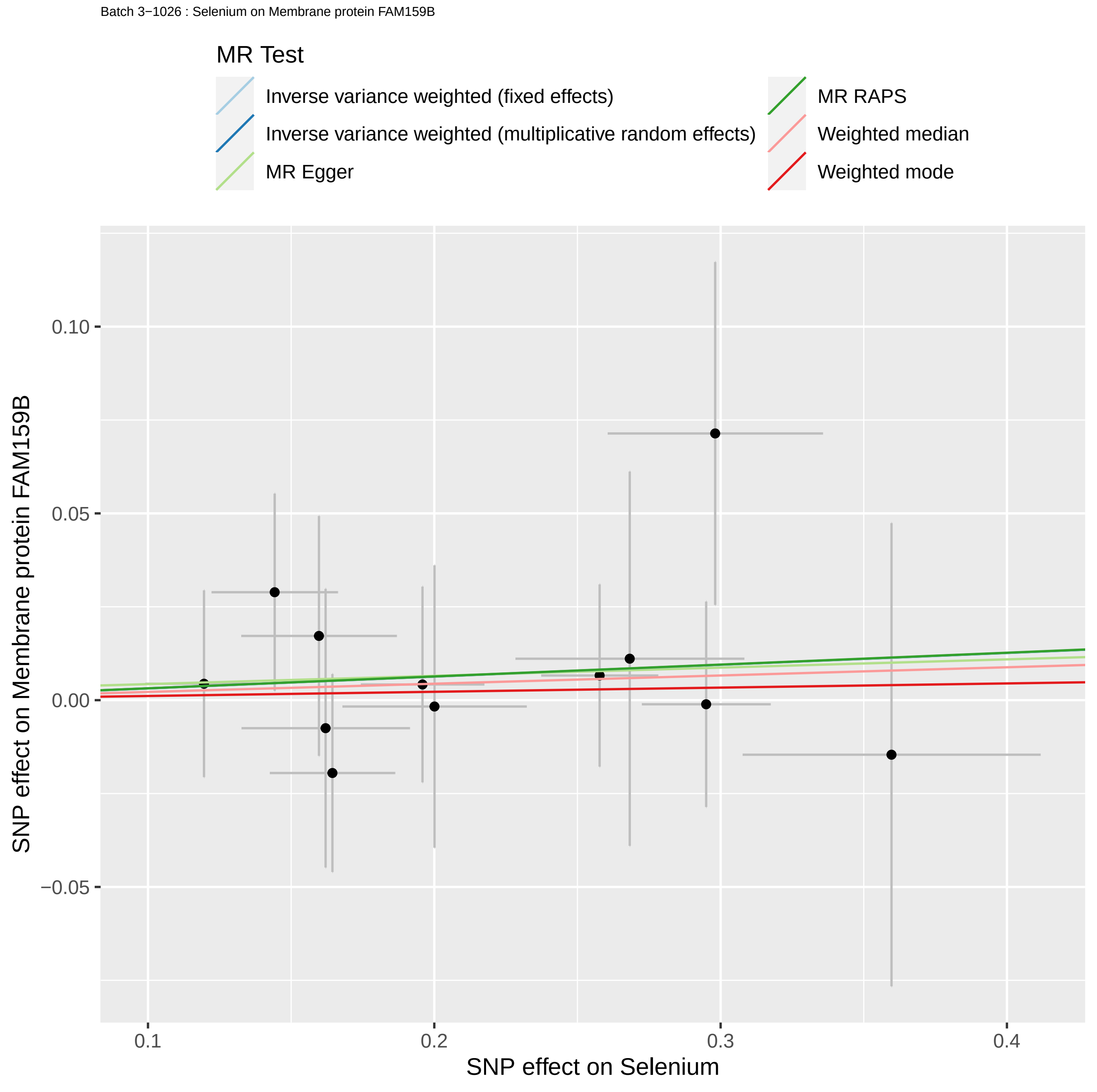

Batch 3−1026 : Selenium on Membrane protein FAM159B
MR Test
Inverse variance weighted (fixed effects)
Inverse variance weighted (multiplicative random effects)
MR Egger
MR RAPS
Weighted median
Weighted mode
0.10
0.05
0.00
−0.05
0.1
0.2
0.3
0.4
SNP effect on Membrane protein FAM159B
SNP effect on Selenium

Batch 3−1027 : Selenium on Protein FAM163A

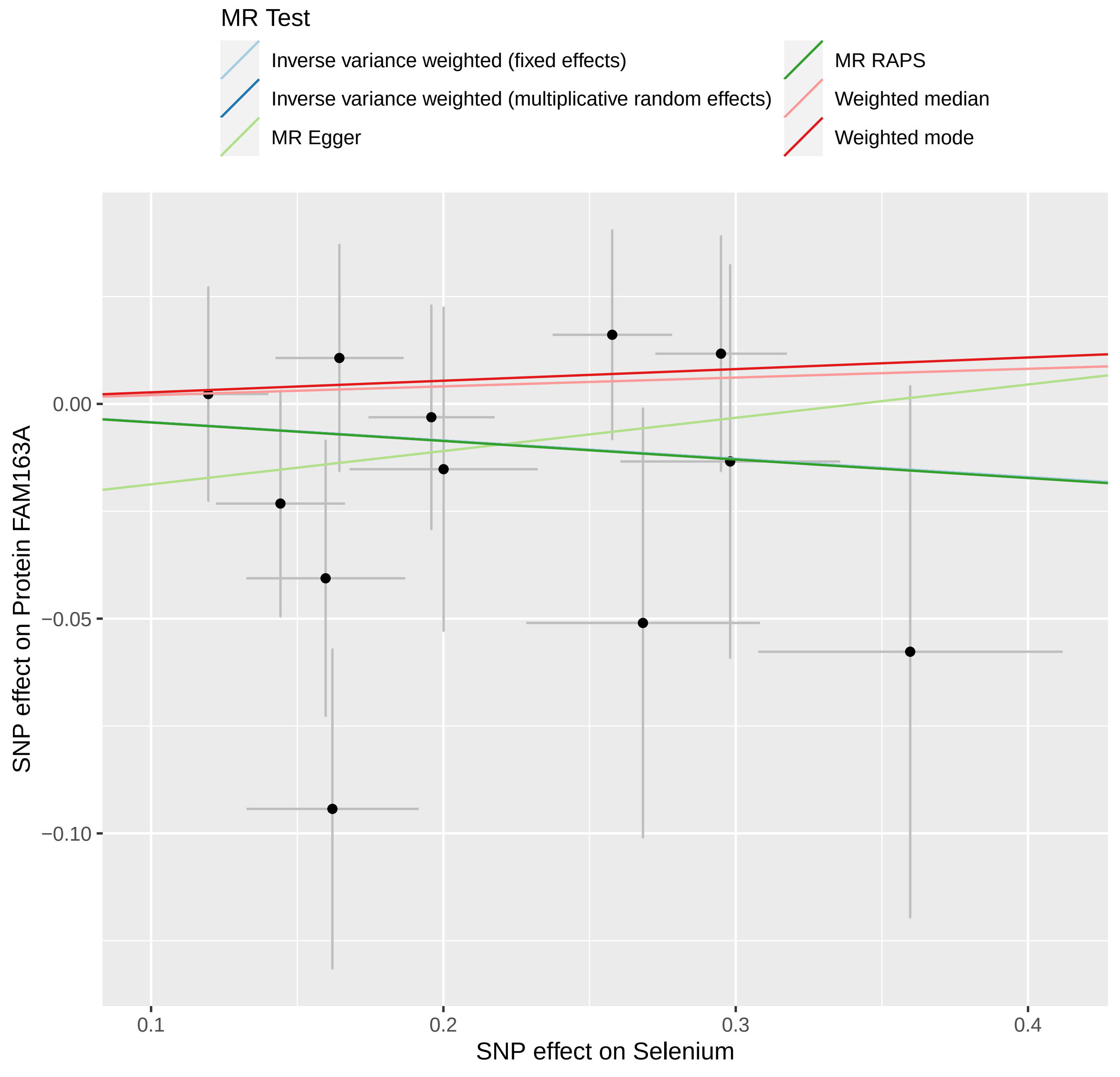

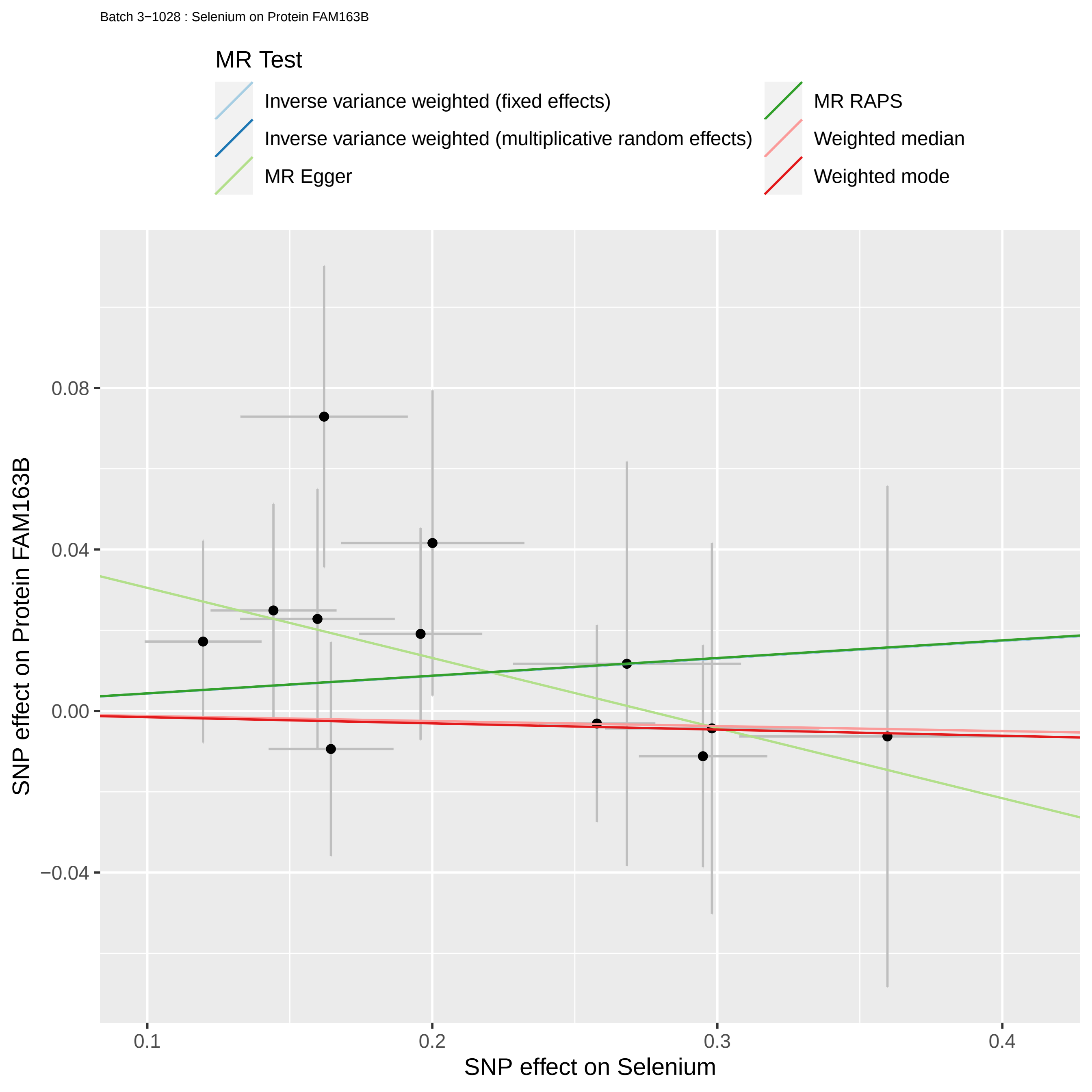

Batch 3−1028 : Selenium on Protein FAM163B
MR Test
Inverse variance weighted (fixed effects)
Inverse variance weighted (multiplicative random effects)
MR Egger
MR RAPS
Weighted median
Weighted mode
0.08
0.04
0.00
−0.04
0.1
0.2
0.3
0.4
SNP effect on Selenium
SNP effect on Protein FAM163B

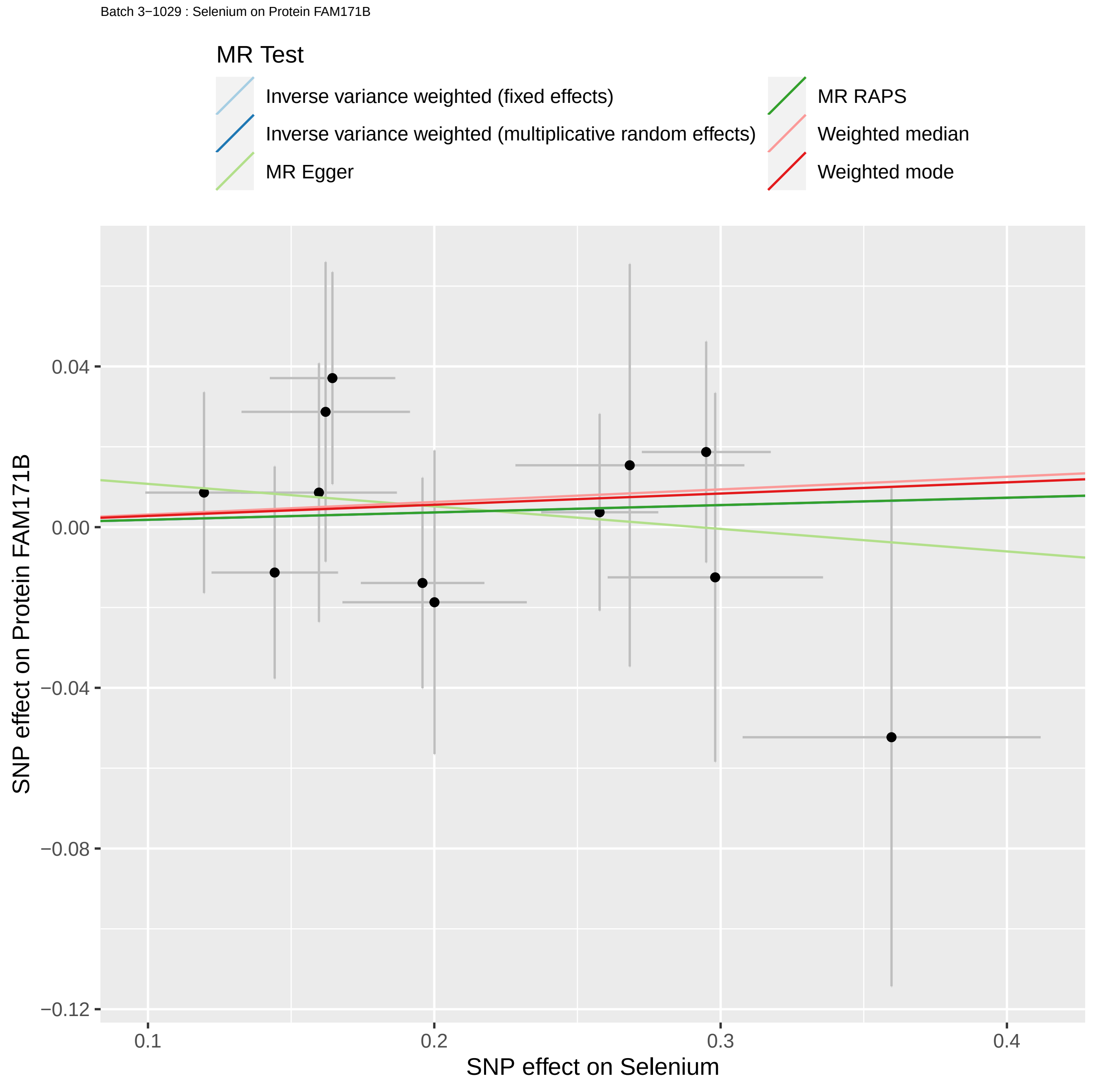

Batch 3−1029 : Selenium on Protein FAM171B
MR Test
Inverse variance weighted (fixed effects)
Inverse variance weighted (multiplicative random effects)
MR Egger
MR RAPS
Weighted median
Weighted mode
SNP effect on Protein FAM171B
0.04
0.00
−0.04
−0.08
−0.12
0.1
0.2
0.3
0.4
SNP effect on Selenium

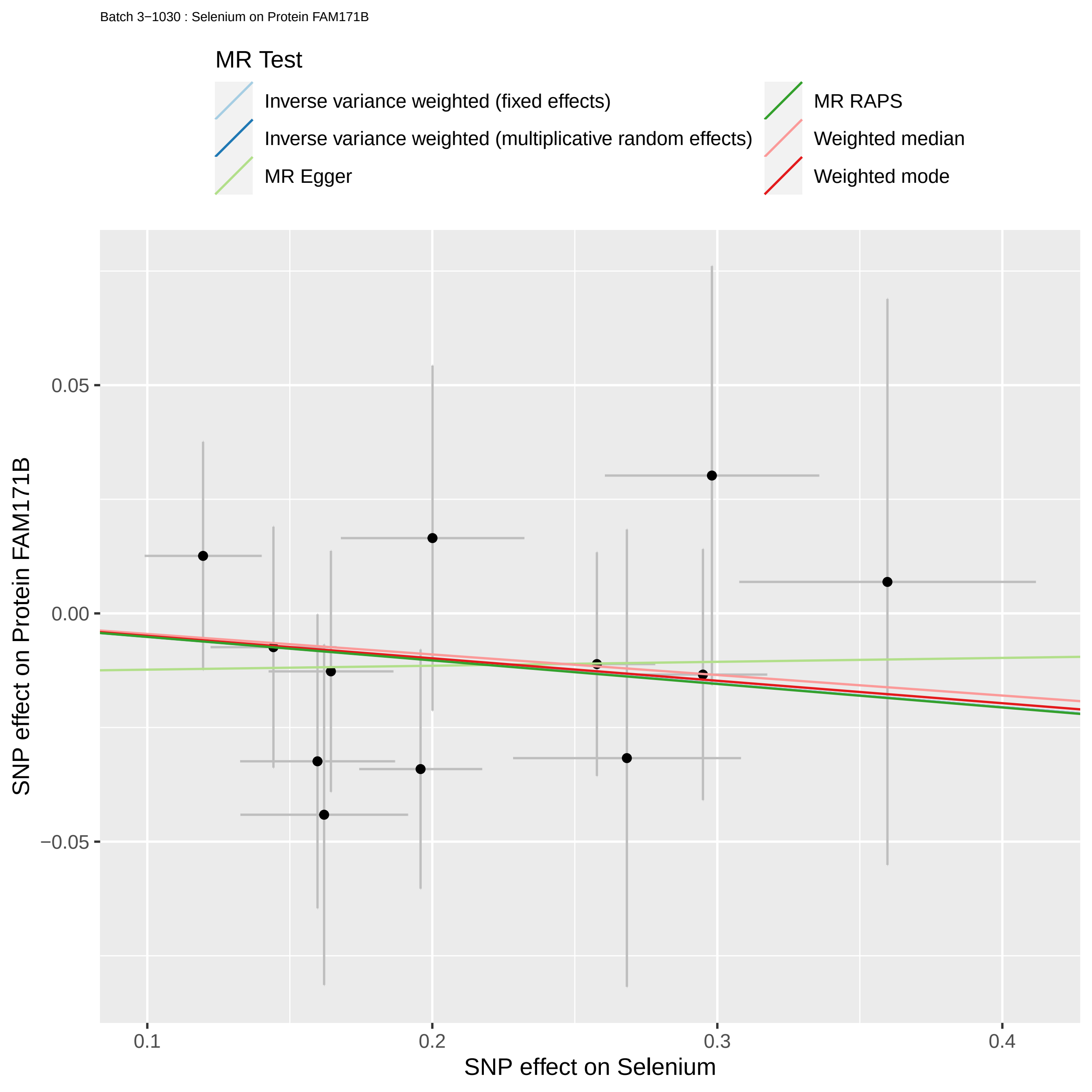

Batch 3−1030 : Selenium on Protein FAM171B
MR Test
Inverse variance weighted (fixed effects)
Inverse variance weighted (multiplicative random effects)
MR Egger
MR RAPS
Weighted median
Weighted mode
0.05
0.00
−0.05
0.1
0.2
0.3
0.4
SNP effect on Protein FAM171B
SNP effect on Selenium

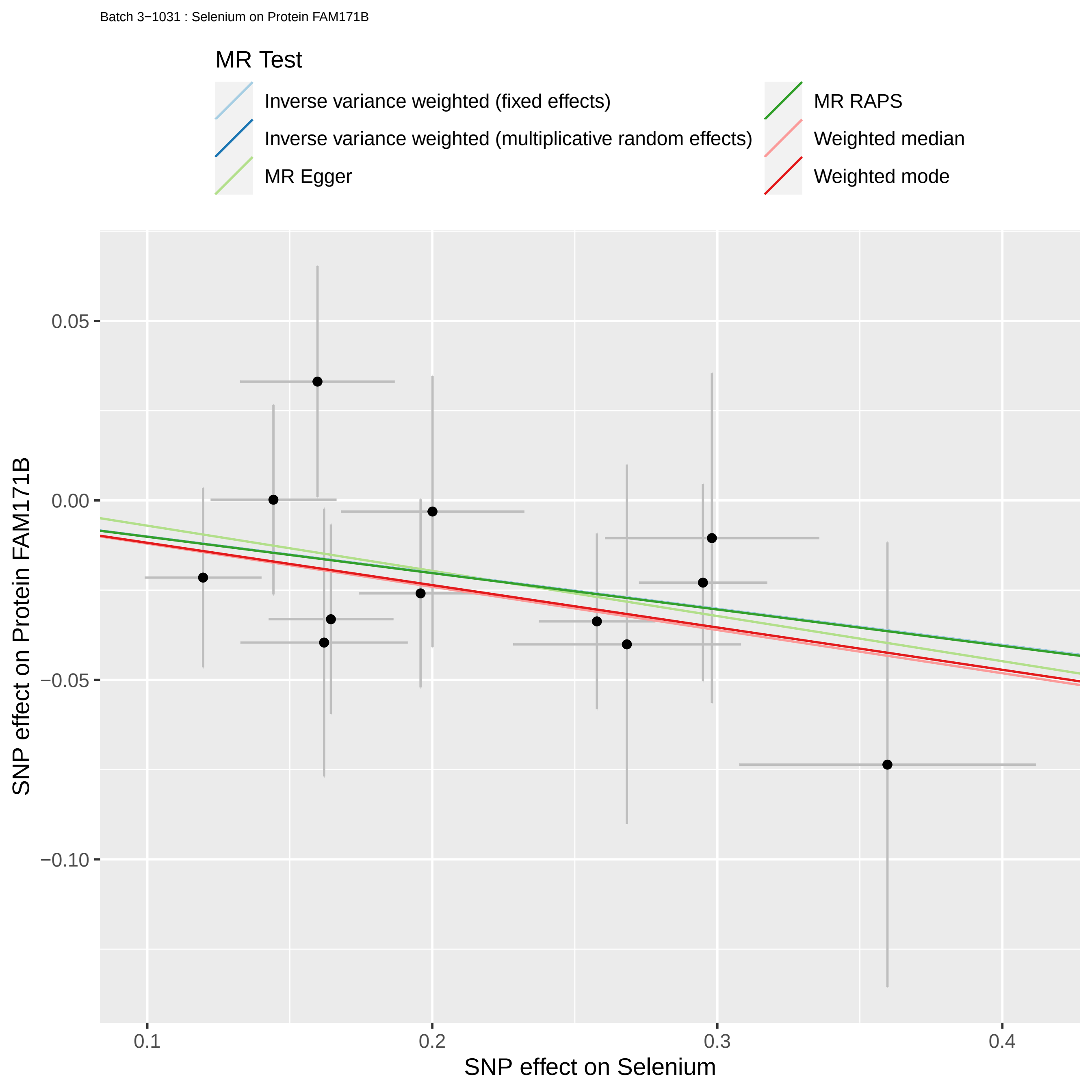

Batch 3−1031 : Selenium on Protein FAM171B
MR Test
Inverse variance weighted (fixed effects)
Inverse variance weighted (multiplicative random effects)
MR Egger
MR RAPS
Weighted median
Weighted mode
0.05
0.00
−0.05
−0.10
0.1
0.2
0.3
0.4
SNP effect on Protein FAM171B
SNP effect on Selenium

Batch 3−1032 : Selenium on Protein FAM172A

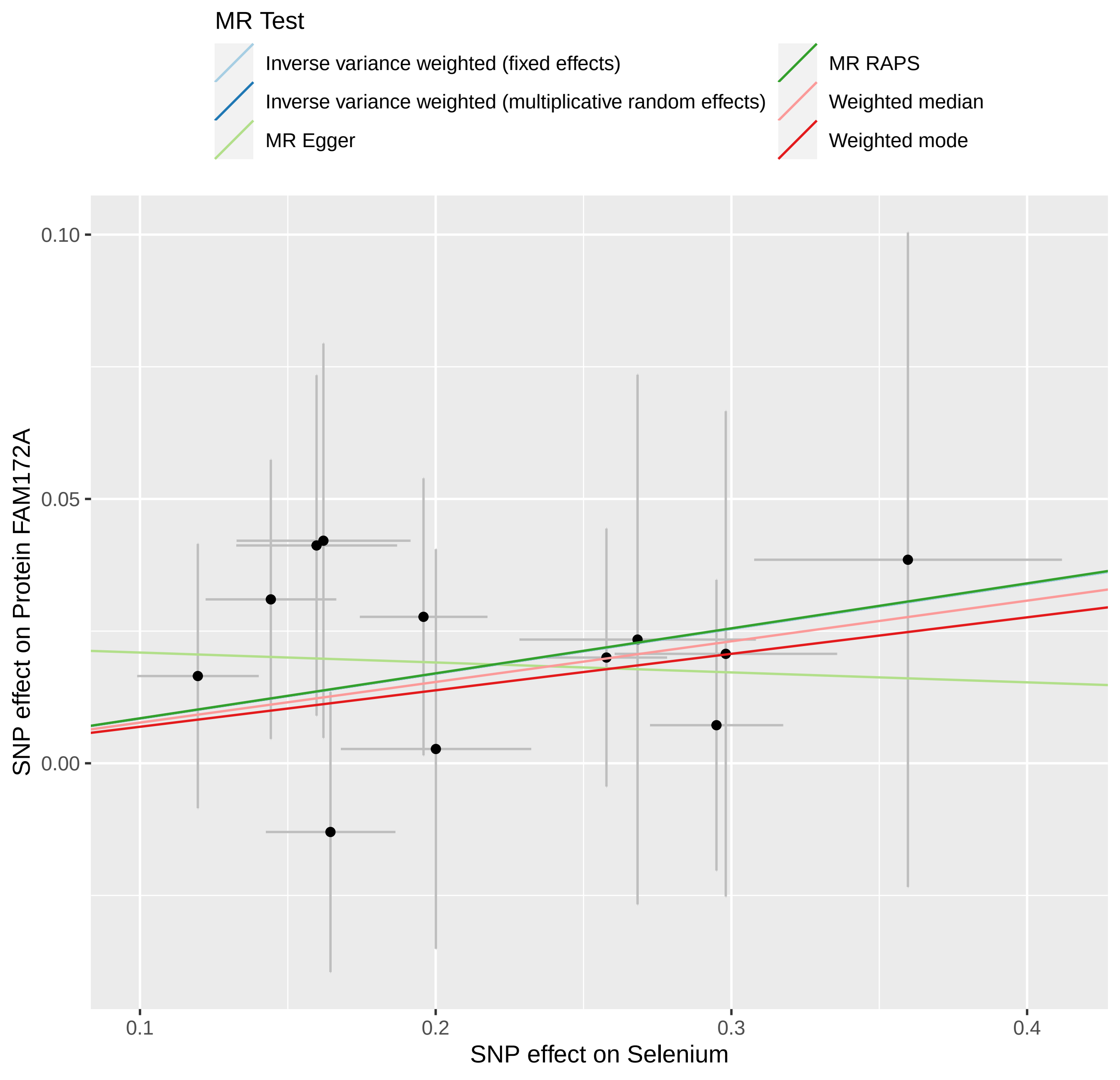

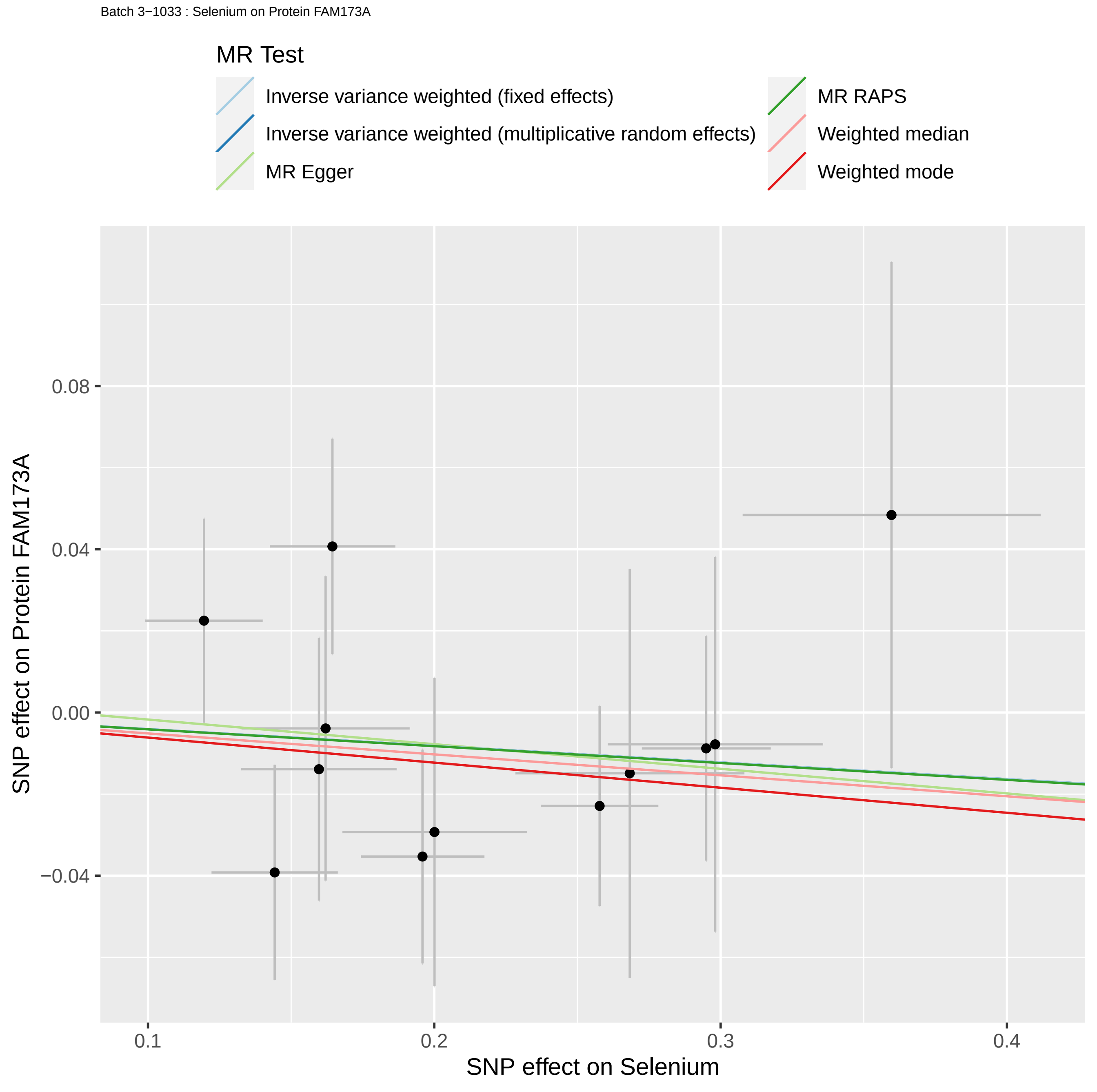

Batch 3−1033 : Selenium on Protein FAM173A
MR Test
Inverse variance weighted (fixed effects)
Inverse variance weighted (multiplicative random effects)
MR Egger
MR RAPS
Weighted median
Weighted mode
0.08
0.04
0.00
−0.04
0.1
0.2
0.3
0.4
SNP effect on Selenium
SNP effect on Protein FAM173A

Batch 3−1034 : Selenium on Membrane protein FAM174A

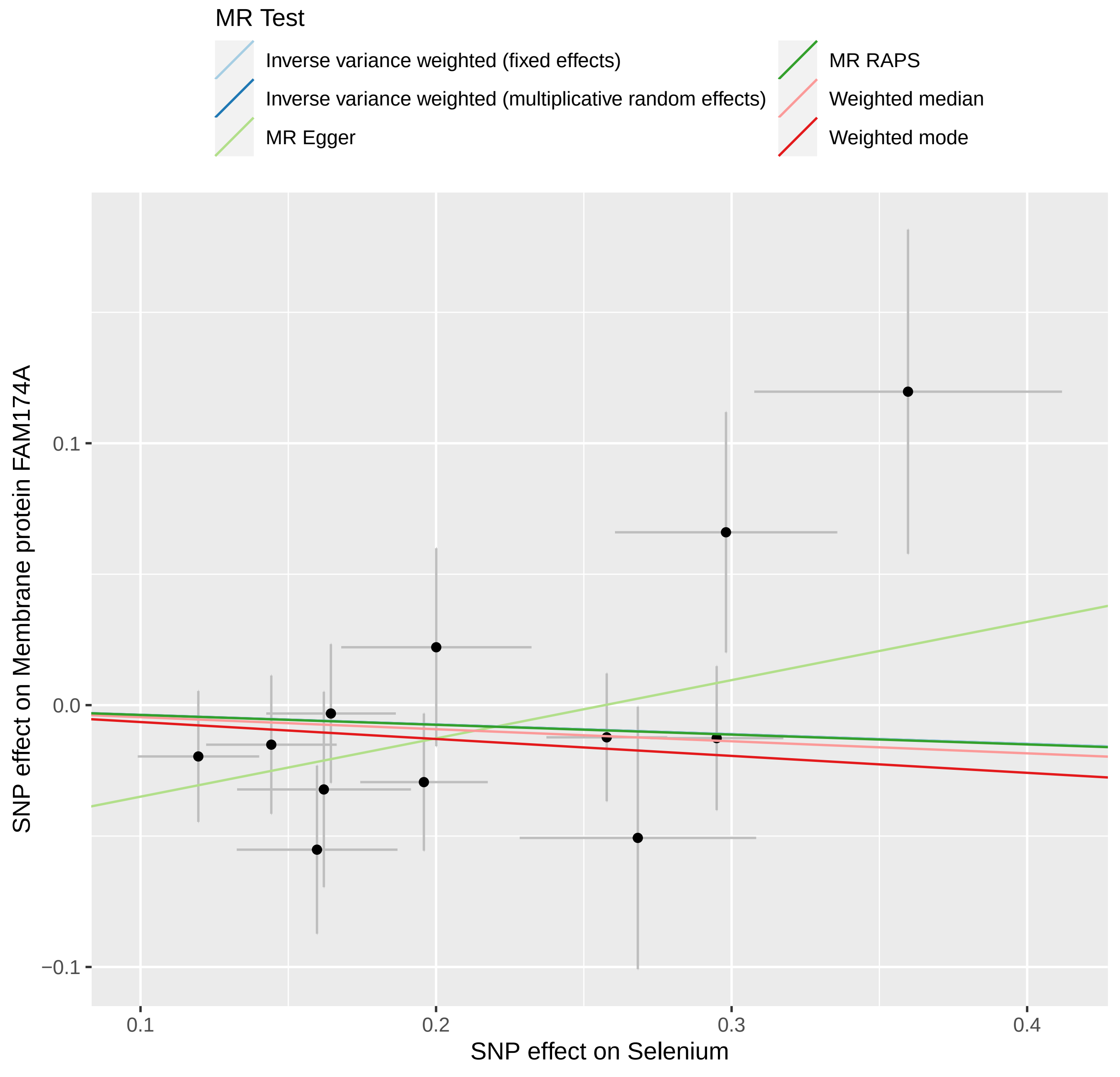

Batch 3−1035 : Selenium on Membrane protein FAM174B

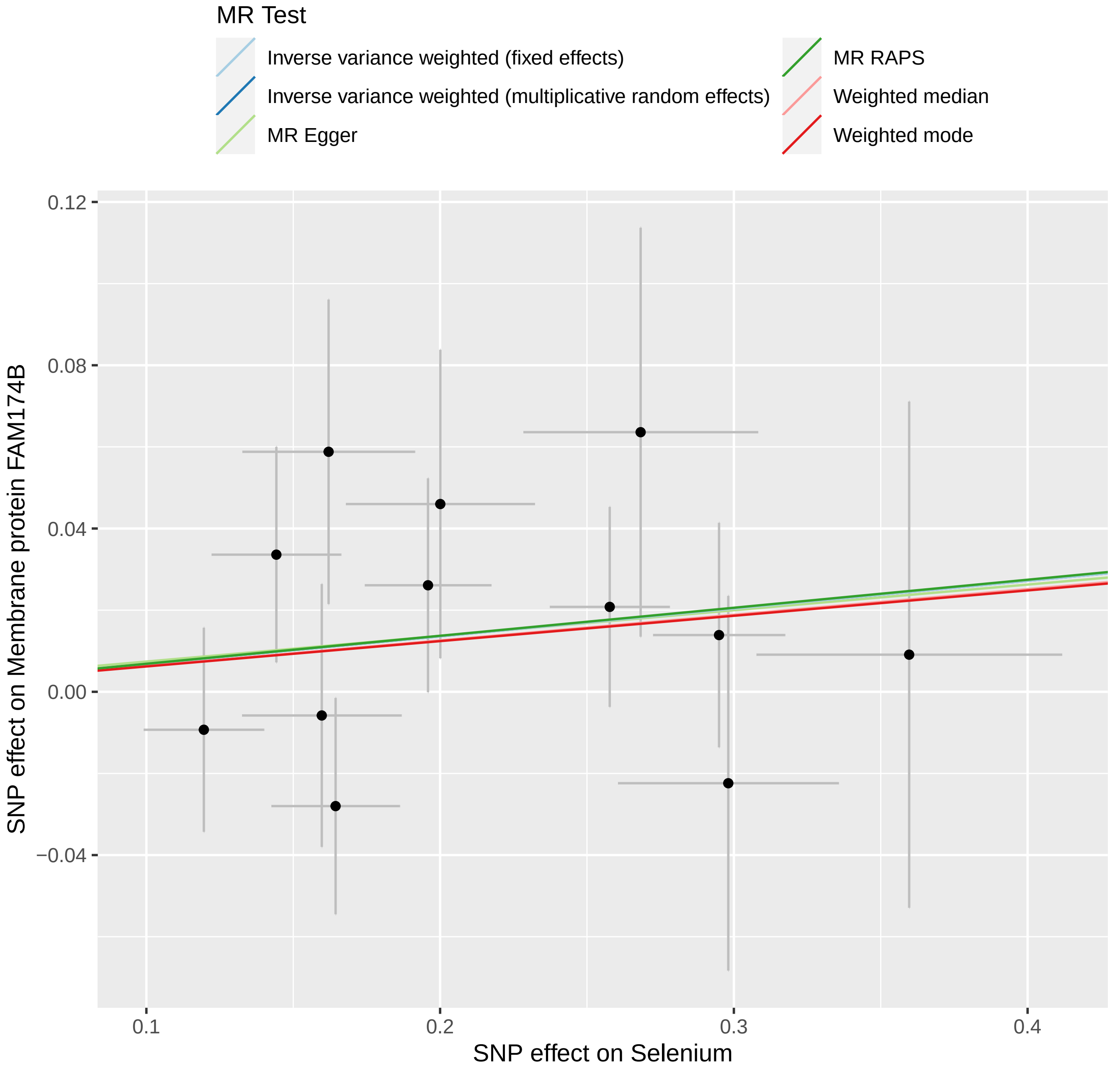

Batch 3−1036 : Selenium on BRISC complex subunit Abro1

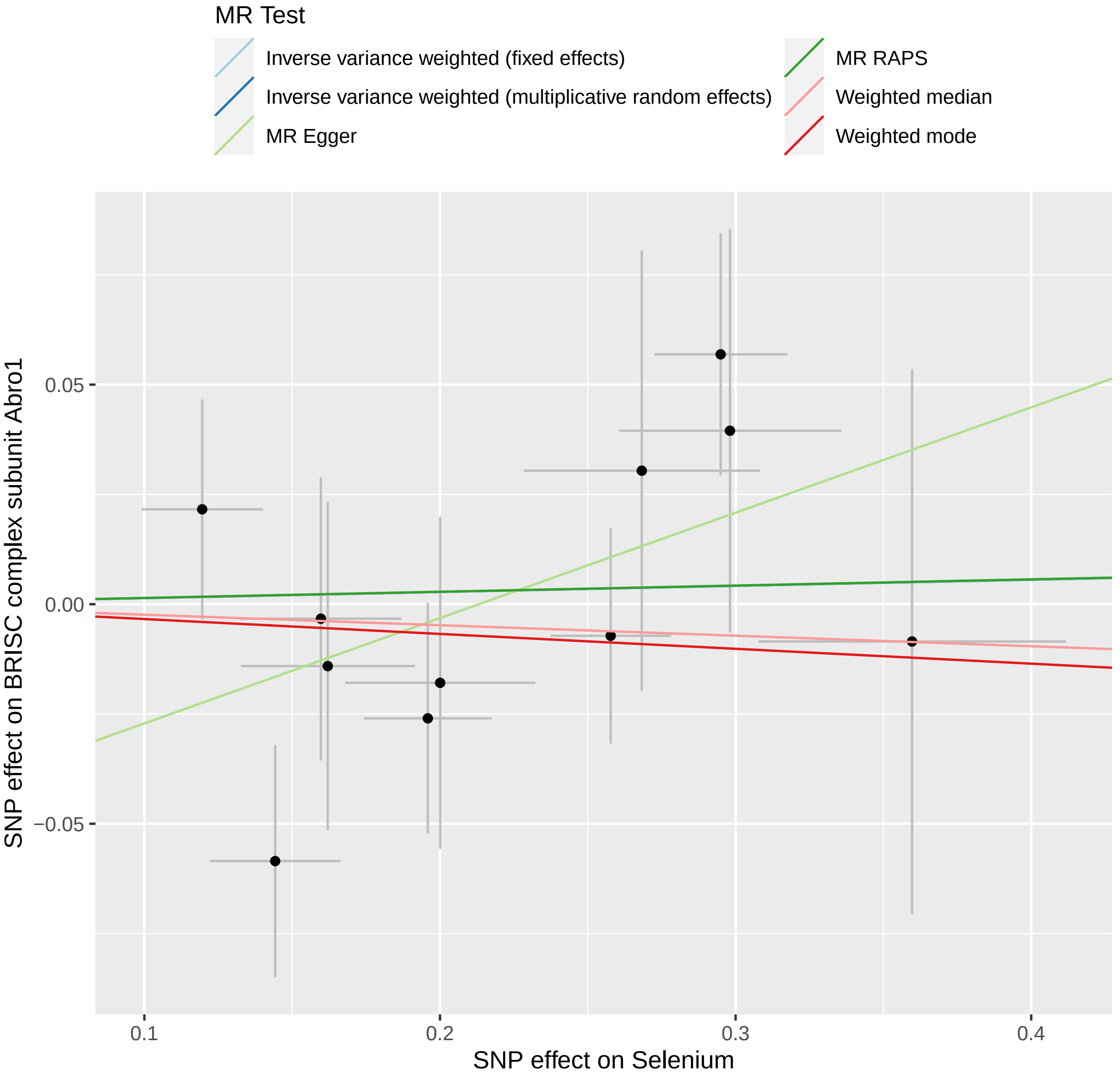

Batch 3−1037 : Selenium on Protein FAM177A1

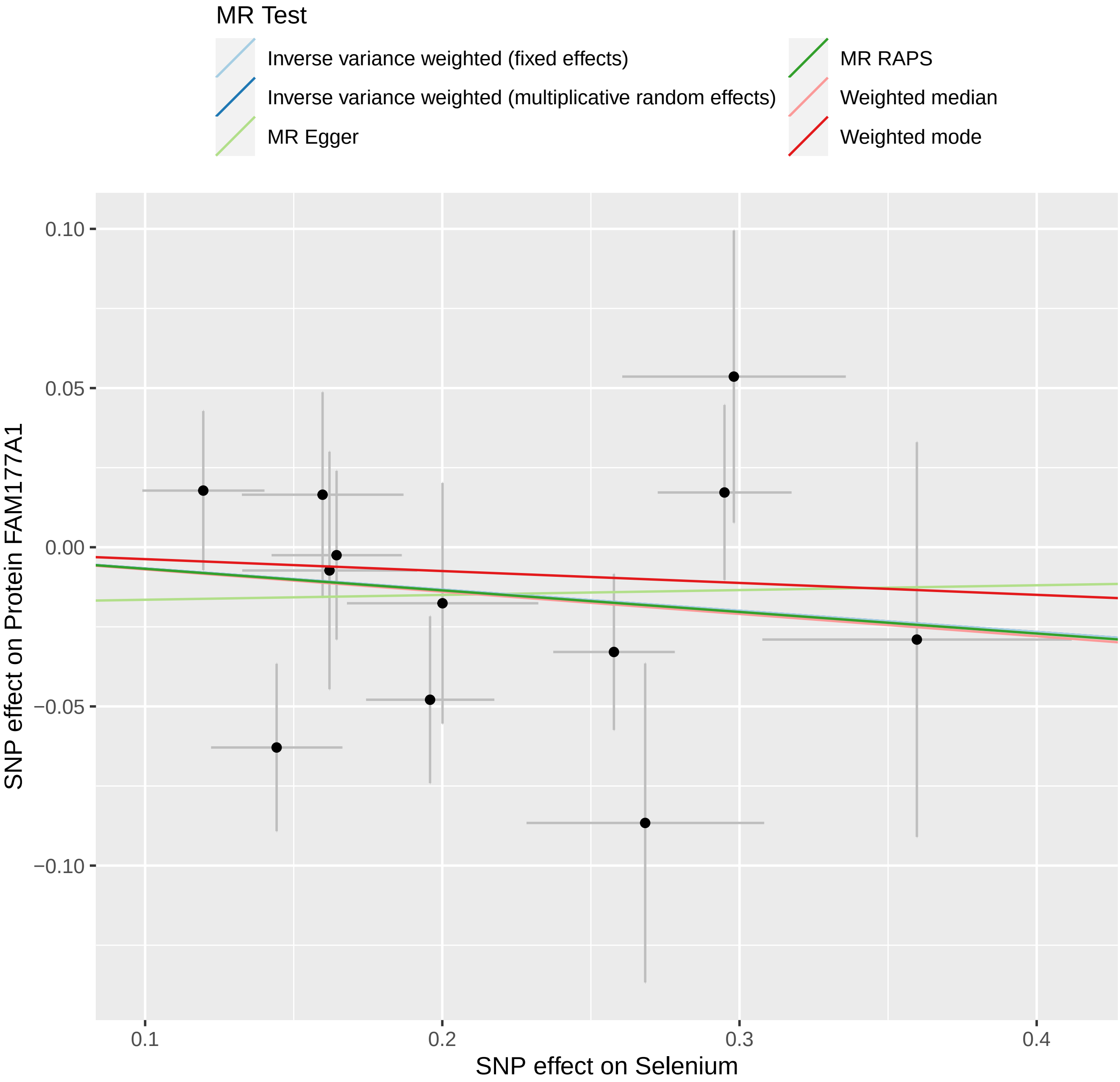

Batch 3−1038 : Selenium on Protein FAM189A2

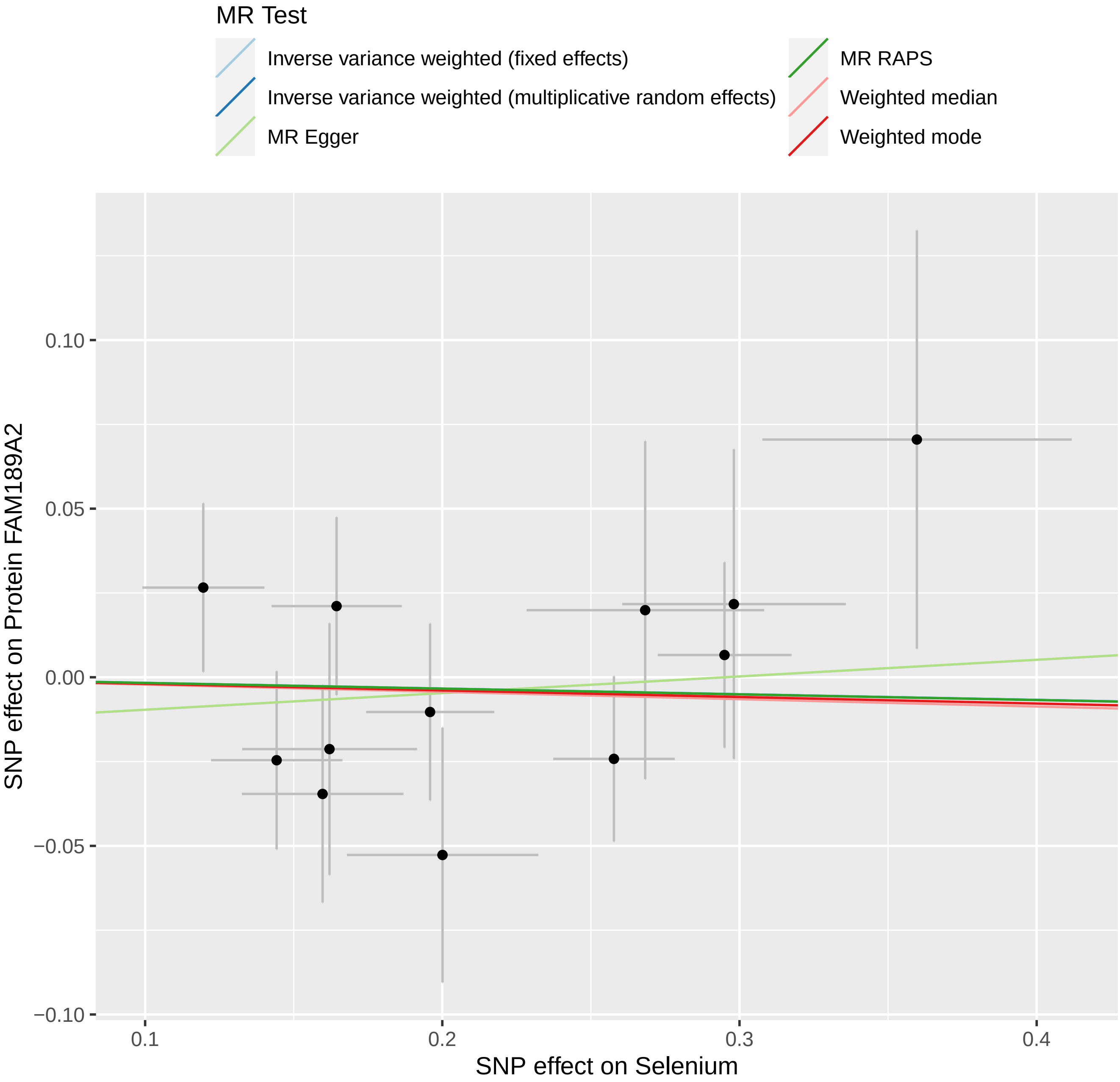

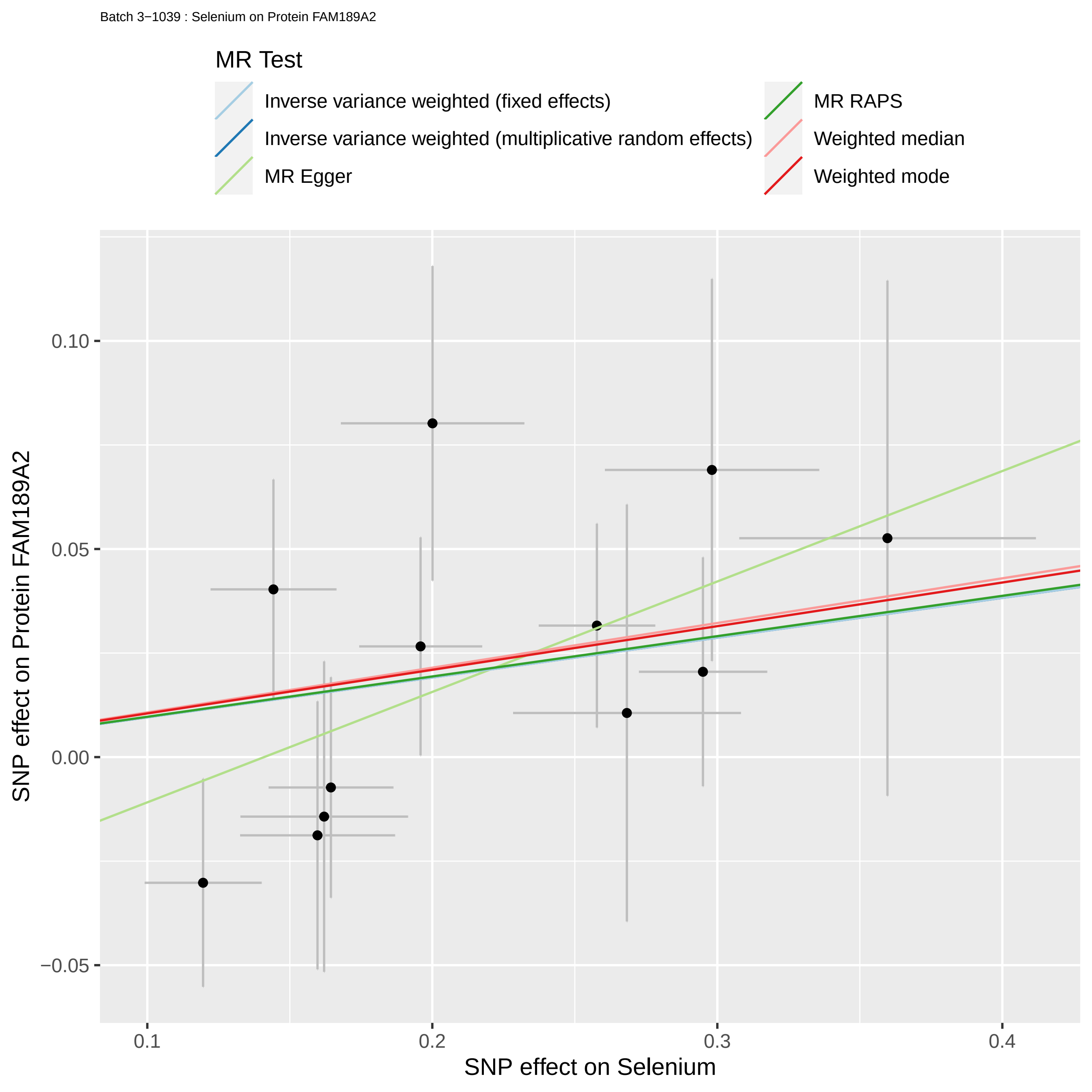

Batch 3−1039 : Selenium on Protein FAM189A2
MR Test
Inverse variance weighted (fixed effects)
Inverse variance weighted (multiplicative random effects)
MR Egger
MR RAPS
Weighted median
Weighted mode
SNP effect on Protein FAM189A2
0.10
0.05
0.00
−0.05
0.1
0.2
0.3
0.4
SNP effect on Selenium

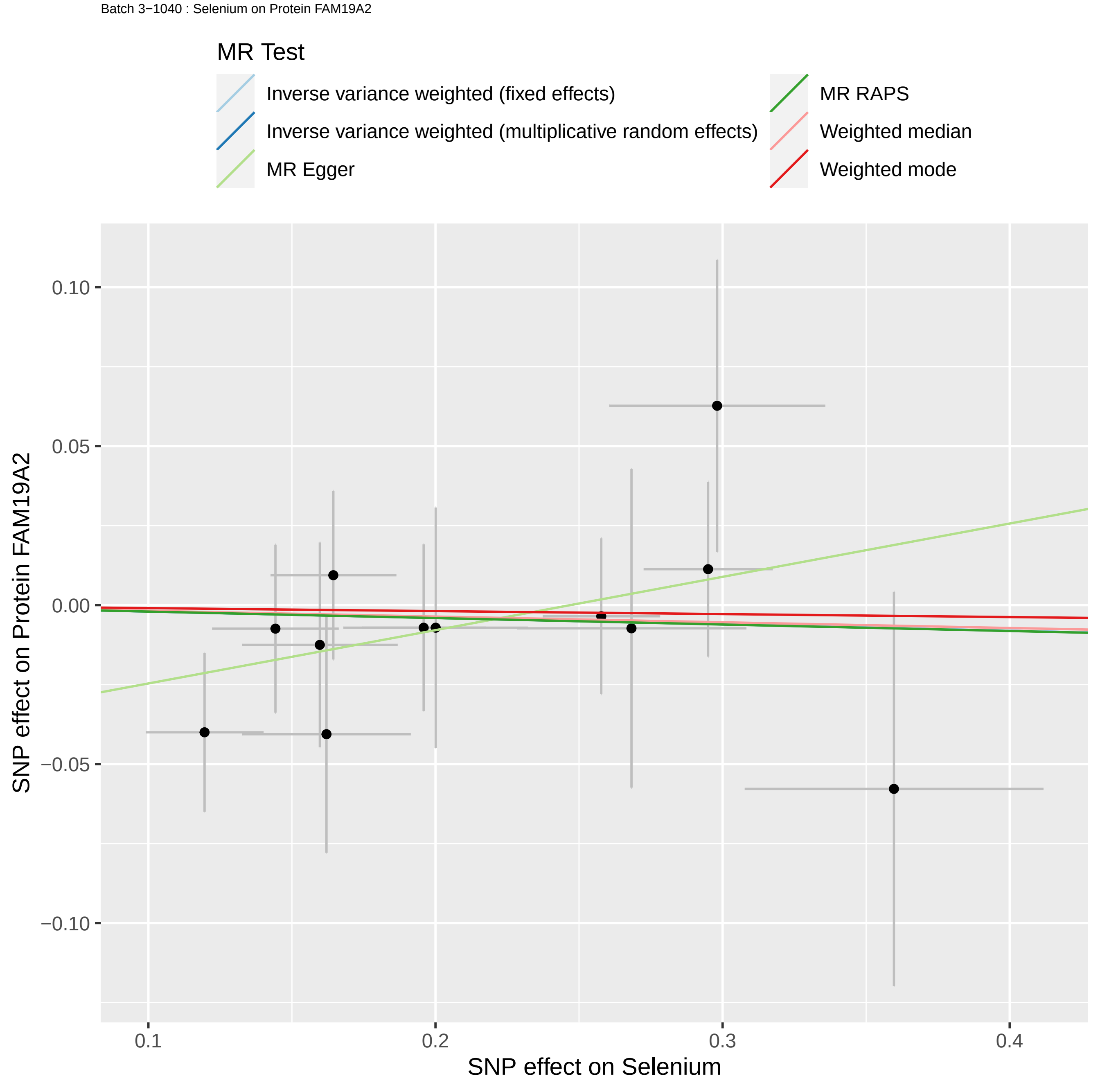

Batch 3−1040 : Selenium on Protein FAM19A2
MR Test
Inverse variance weighted (fixed effects)
Inverse variance weighted (multiplicative random effects)
MR Egger
MR RAPS
Weighted median
Weighted mode
0.10
0.05
0.00
−0.05
−0.10
0.1
0.2
0.3
0.4
SNP effect on Protein FAM19A2
SNP effect on Selenium

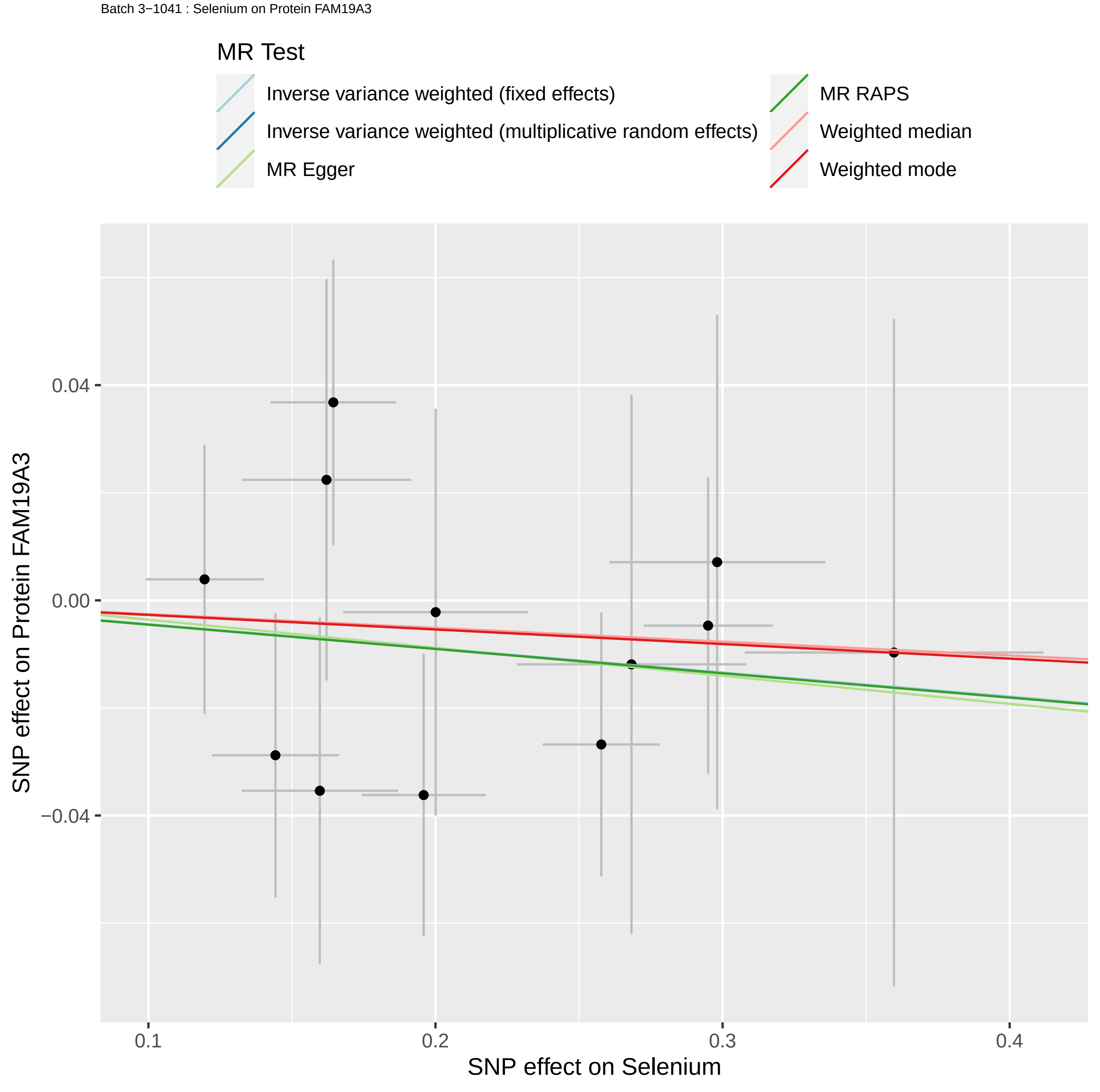
Batch 3−1041 : Selenium on Protein FAM19A3
MR Test
Inverse variance weighted (fixed effects)
Inverse variance weighted (multiplicative random effects)
MR Egger
MR RAPS
Weighted median
Weighted mode
0.04
0.00
−0.04
0.1
0.2
0.3
0.4
SNP effect on Selenium
SNP effect on Protein FAM19A3

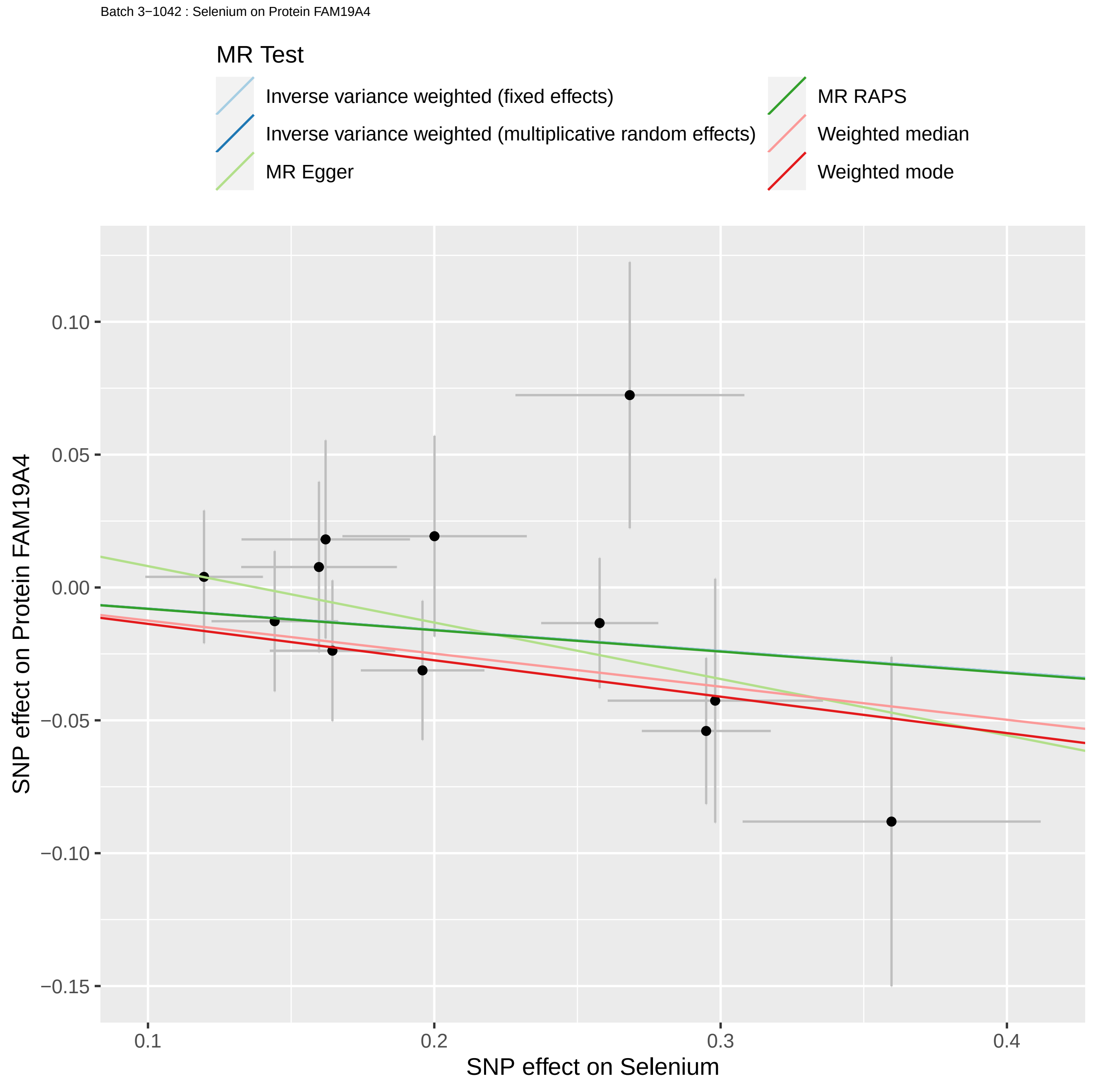

Batch 3−1042 : Selenium on Protein FAM19A4
MR Test
Inverse variance weighted (fixed effects)
Inverse variance weighted (multiplicative random effects)
MR Egger
MR RAPS
Weighted median
Weighted mode
0.10
0.05
0.00
−0.05
−0.10
−0.15
0.1
0.2
0.3
0.4
SNP effect on Protein FAM19A4
SNP effect on Selenium

Batch 3−1043 : Selenium on Protein FAM19A5

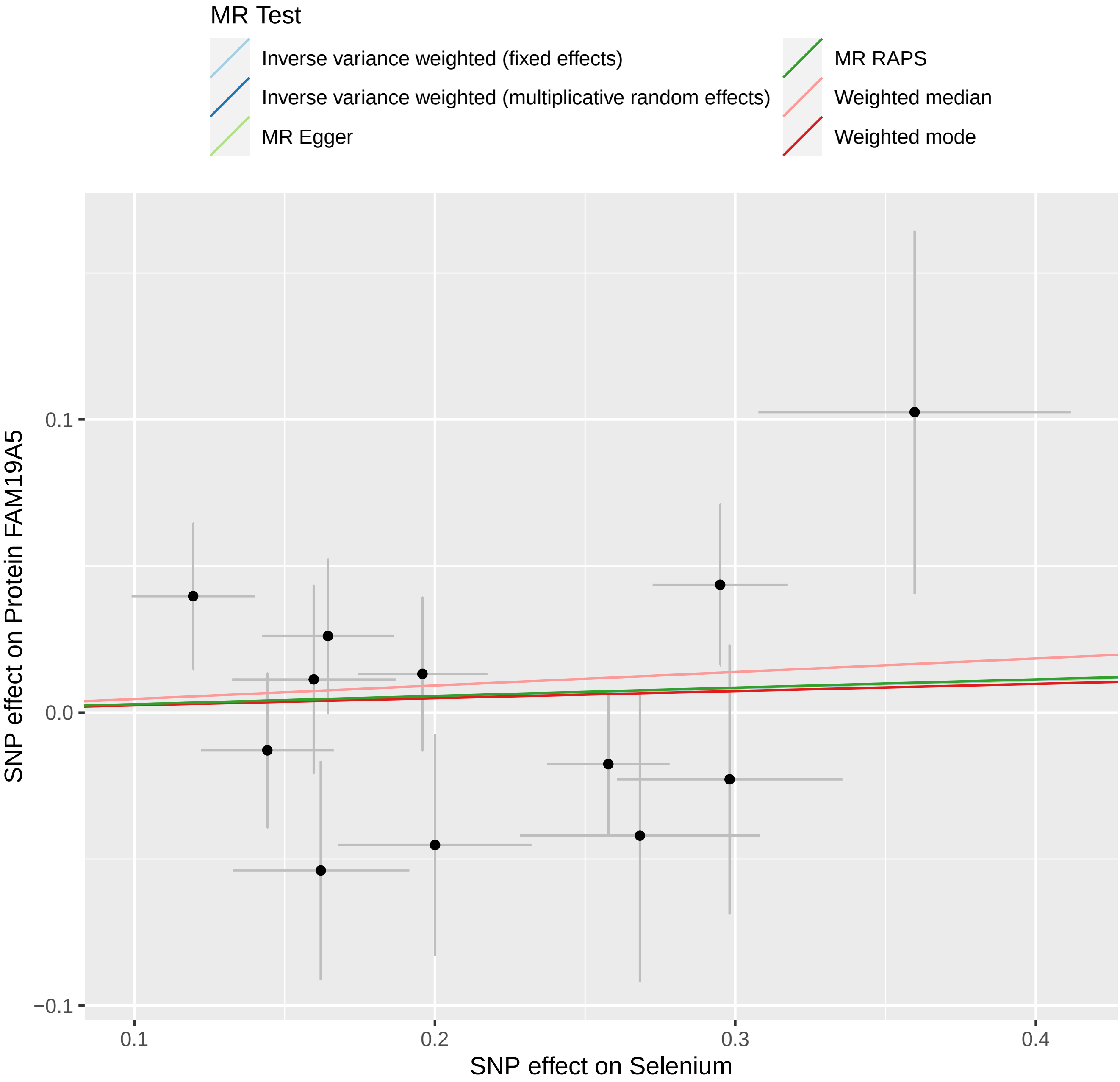

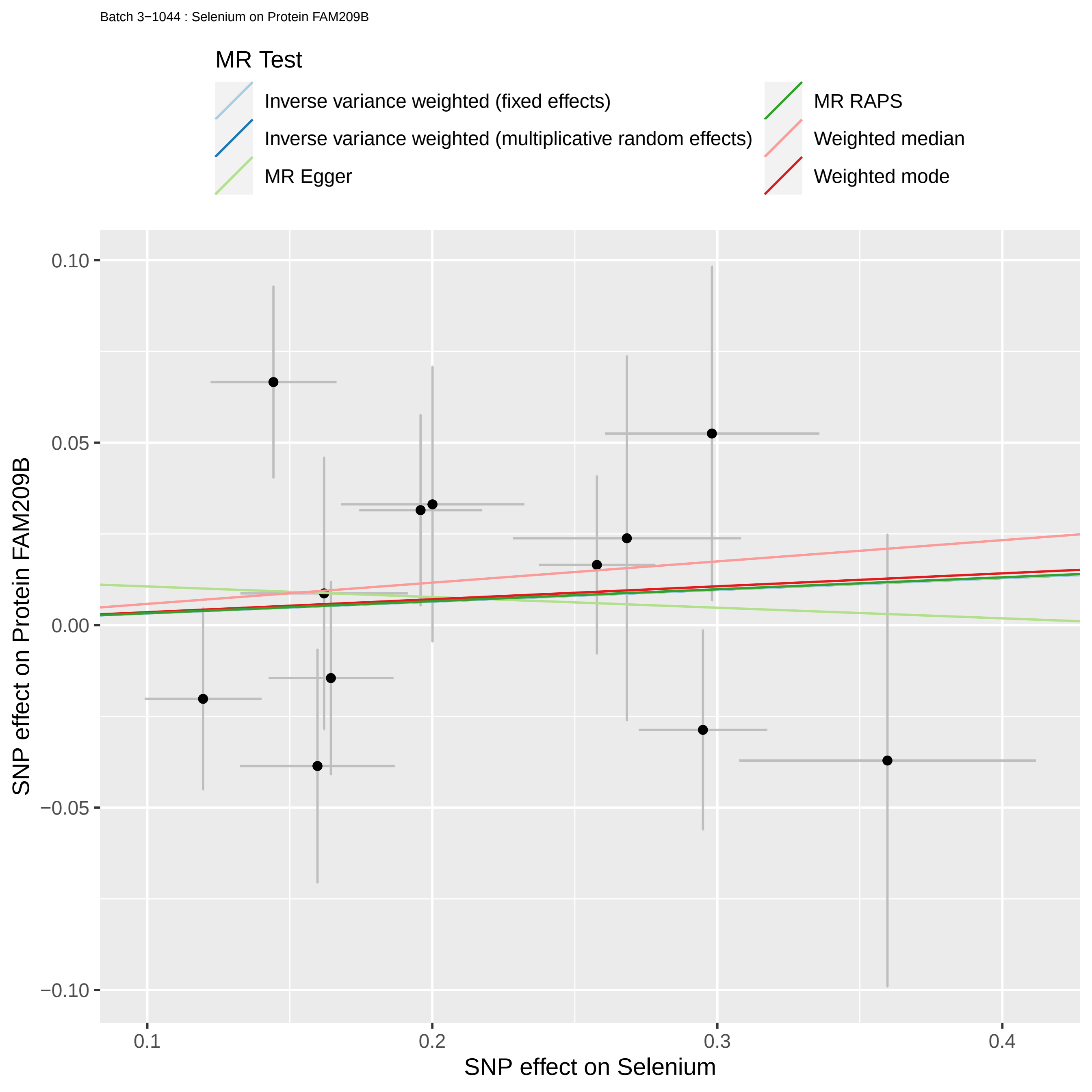

Batch 3−1044 : Selenium on Protein FAM209B
MR Test
Inverse variance weighted (fixed effects)
Inverse variance weighted (multiplicative random effects)
MR Egger
MR RAPS
Weighted median
Weighted mode
0.10
0.05
0.00
−0.05
−0.10
0.1
0.2
0.3
0.4
SNP effect on Protein FAM209B
SNP effect on Selenium

Batch 3−1045 : Selenium on Pseudokinase FAM20A

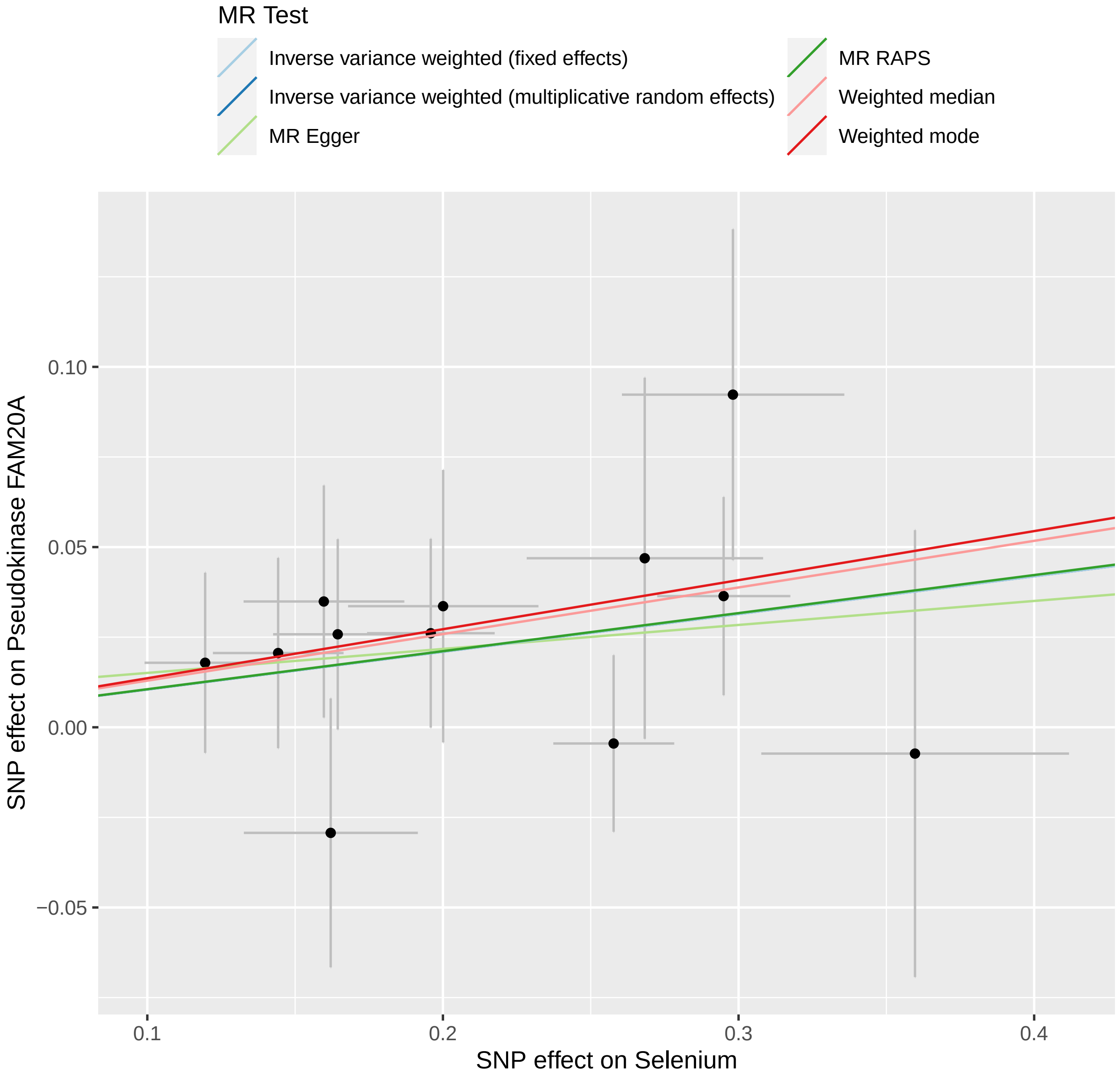

Batch 3−1046 : Selenium on Glycosaminoglycan xylosylkinase

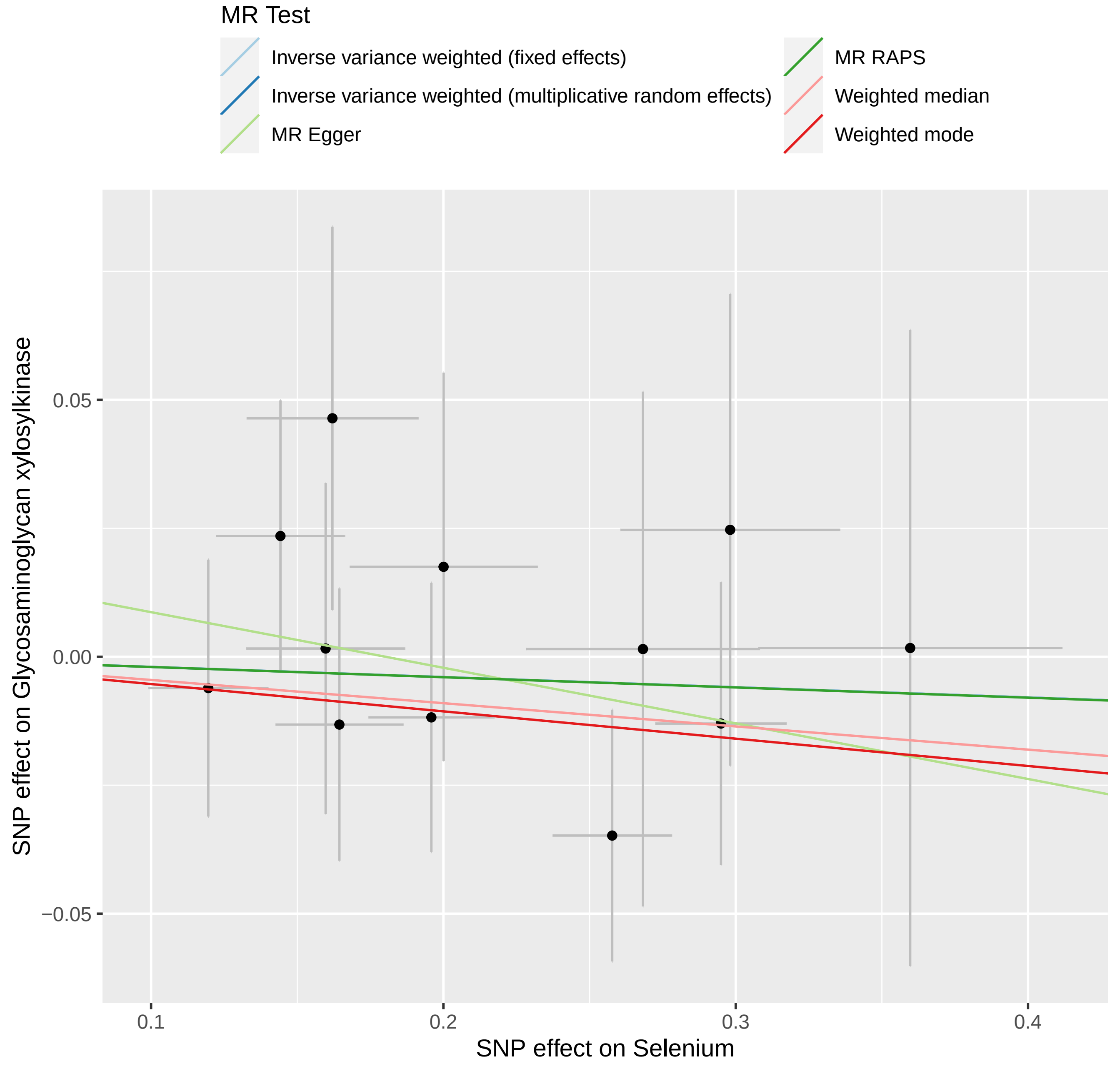

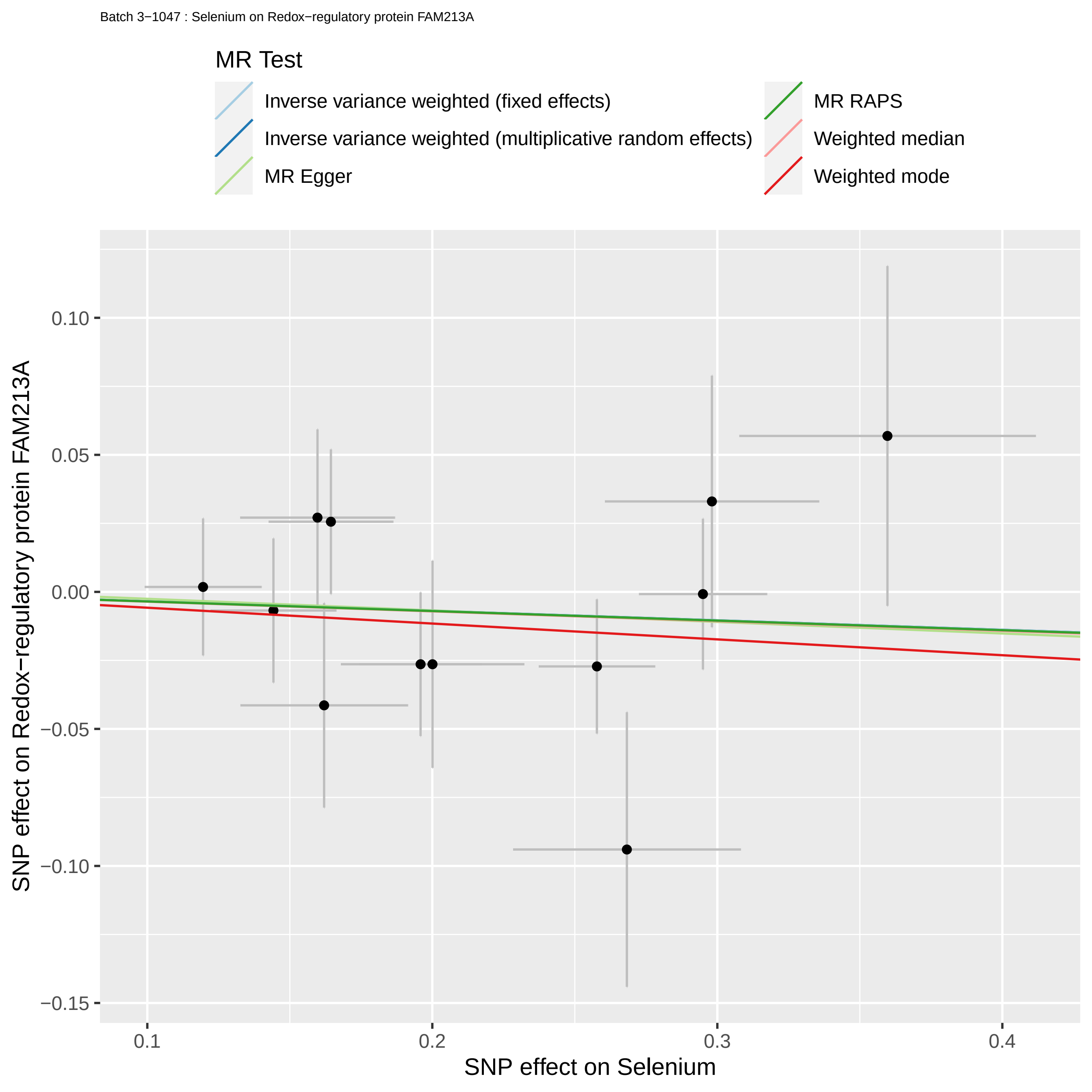
Batch 3−1047 : Selenium on Redox−regulatory protein FAM213A
MR Test
Inverse variance weighted (fixed effects)
Inverse variance weighted (multiplicative random effects)
MR Egger
MR RAPS
Weighted median
Weighted mode
SNP effect on Redox−regulatory protein FAM213A
0.10
0.05
0.00
−0.05
−0.10
−0.15
0.1
0.2
0.3
0.4
SNP effect on Selenium

Batch 3−1048 : Selenium on Protein FAM24B

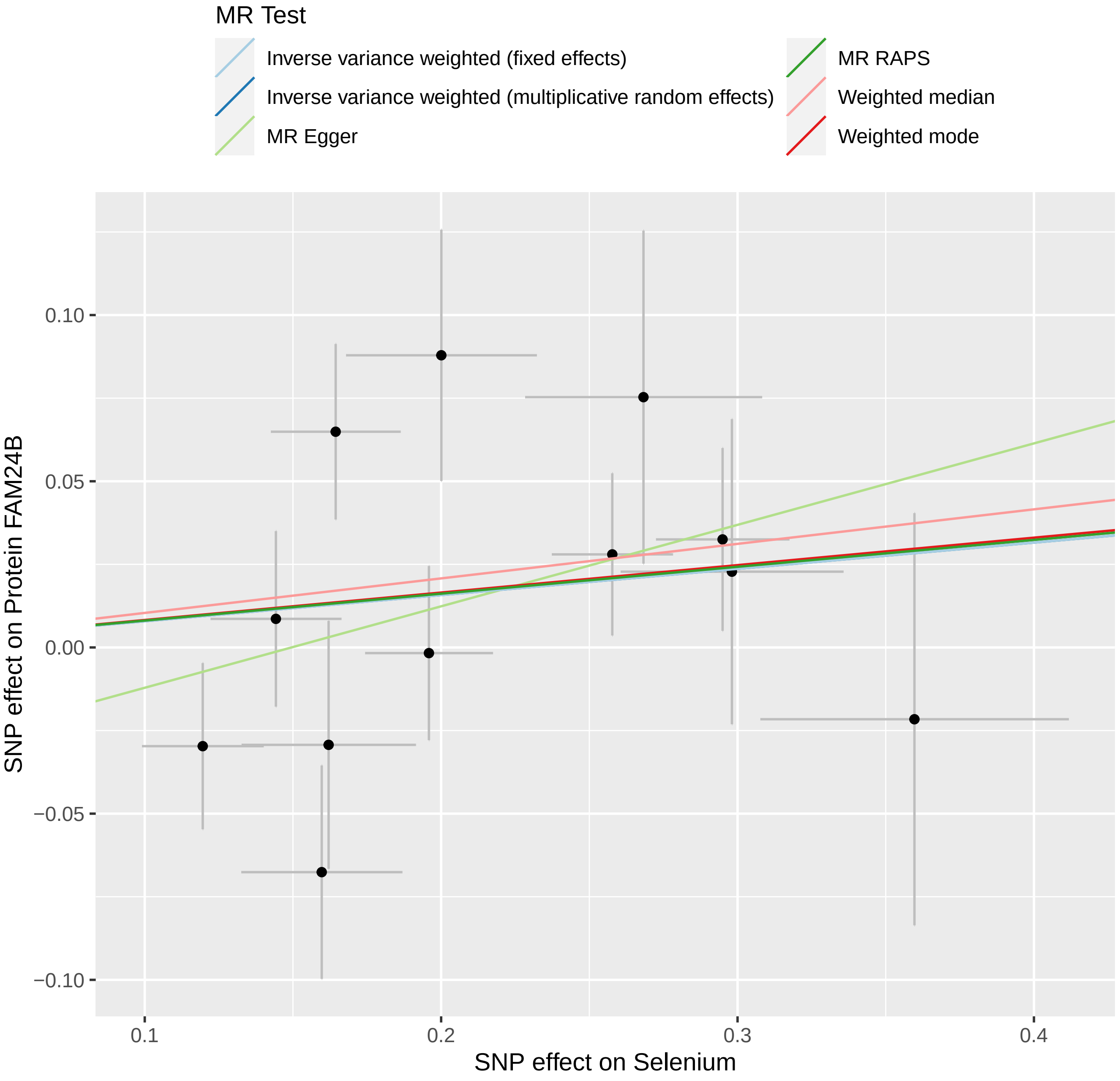

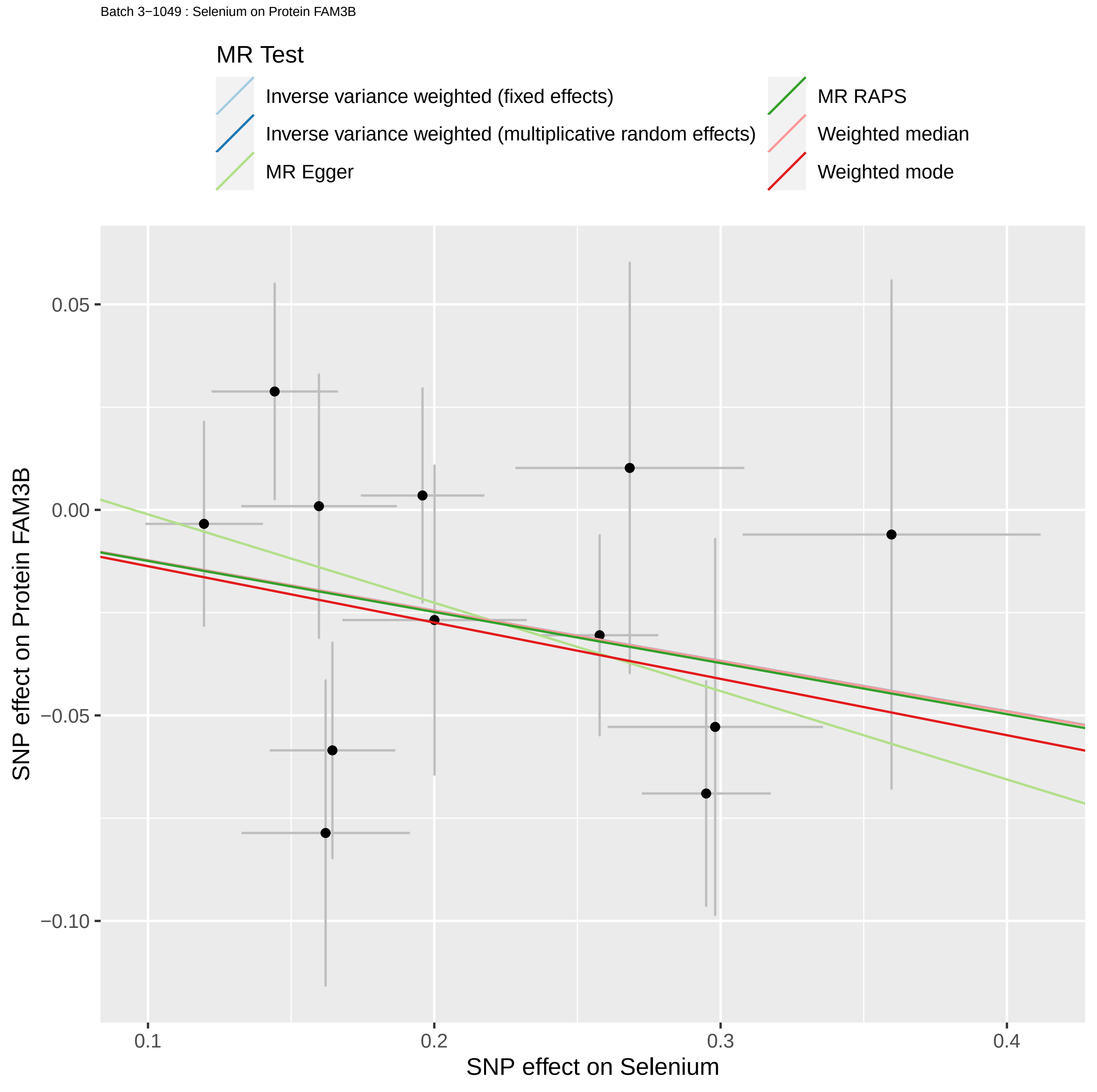

Batch 3−1049 : Selenium on Protein FAM3B
MR Test
Inverse variance weighted (fixed effects)
Inverse variance weighted (multiplicative random effects)
MR Egger
MR RAPS
Weighted median
Weighted mode
0.05
0.00
−0.05
−0.10
0.1
0.2
0.3
0.4
SNP effect on Protein FAM3B
SNP effect on Selenium

Batch 3−1050 : Selenium on Protein FAM3B

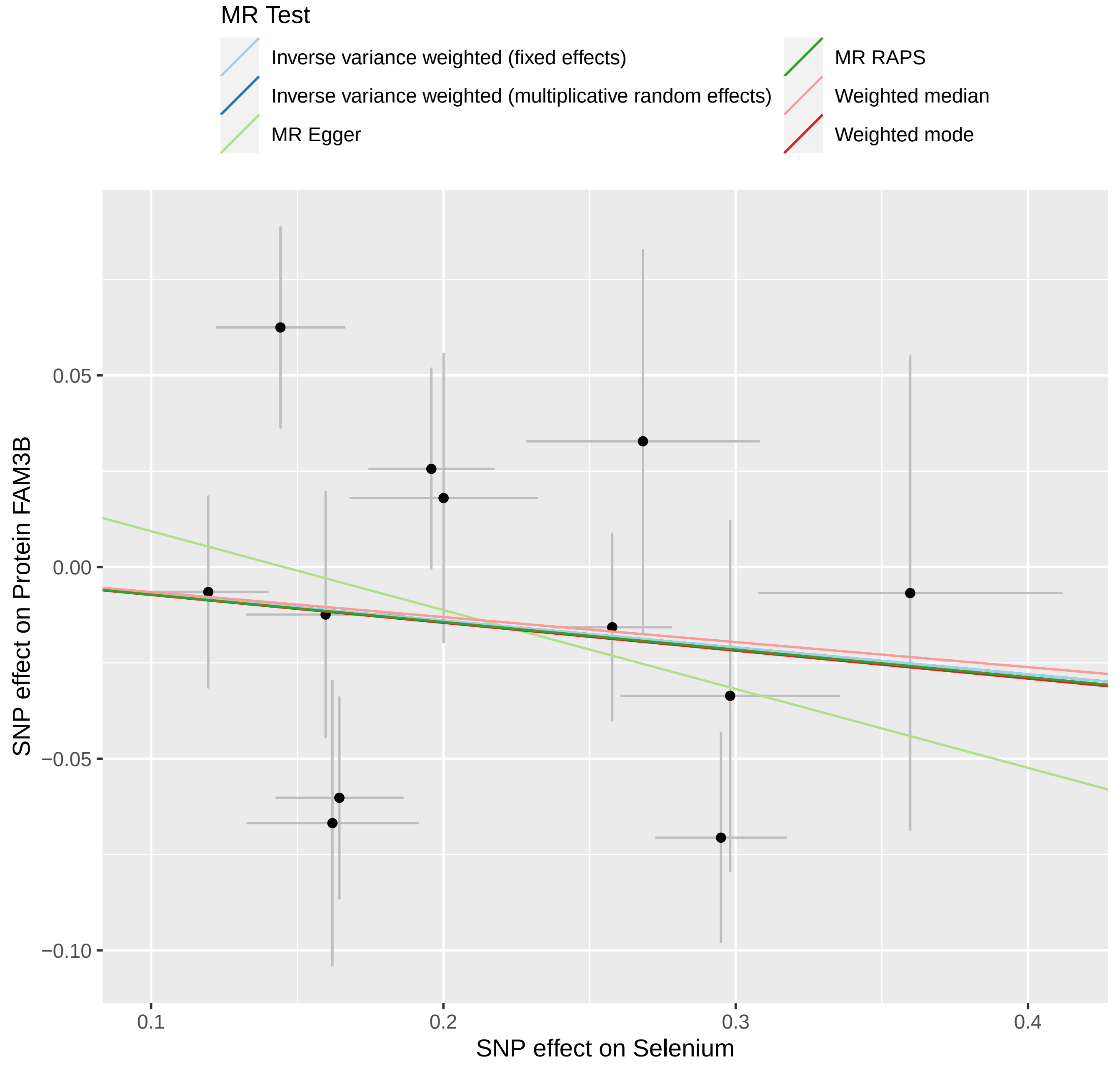

Batch 3−1051 : Selenium on Protein FAM3D

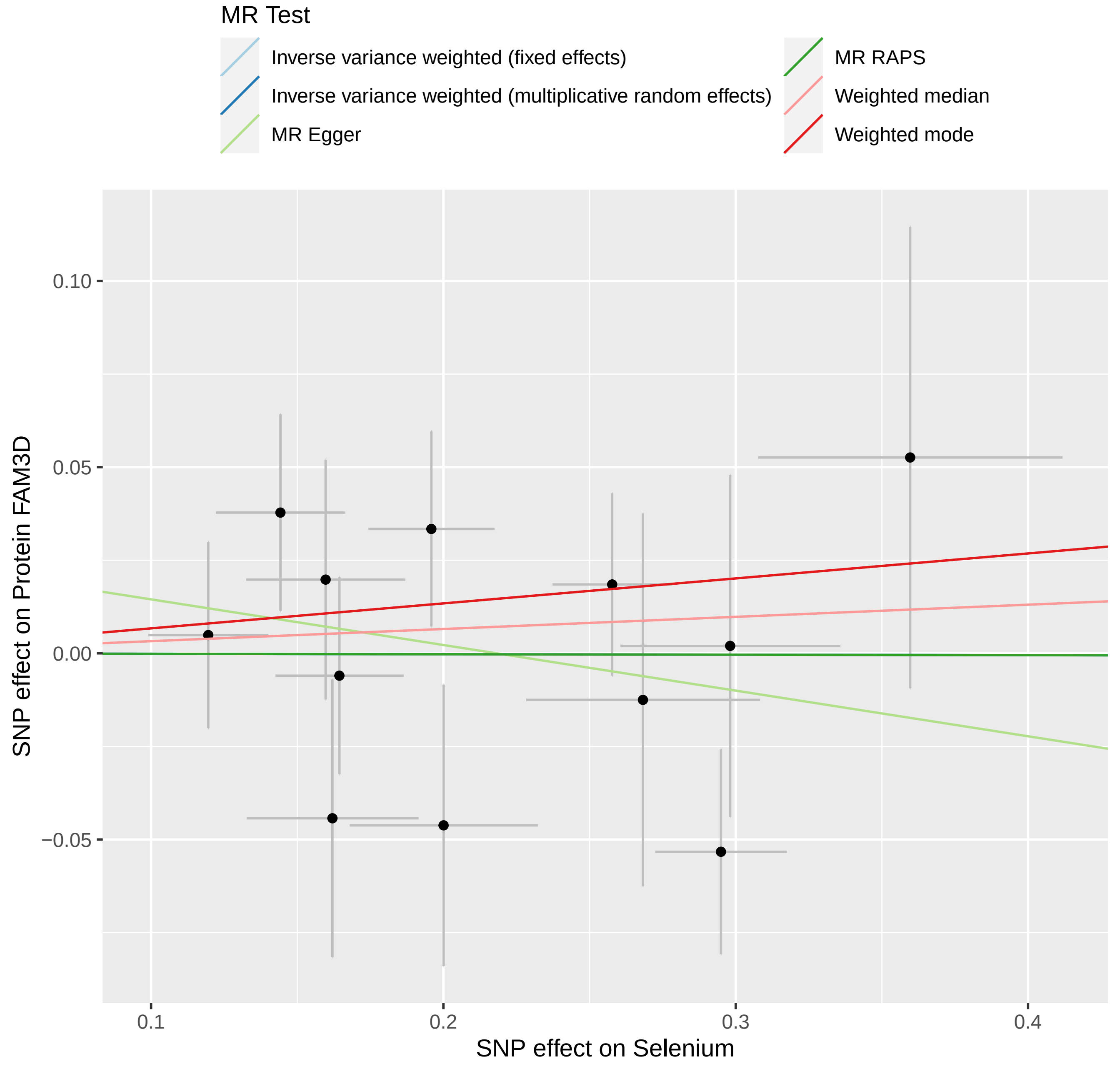

Batch 3−1052 : Selenium on MIP18 family protein FAM96A

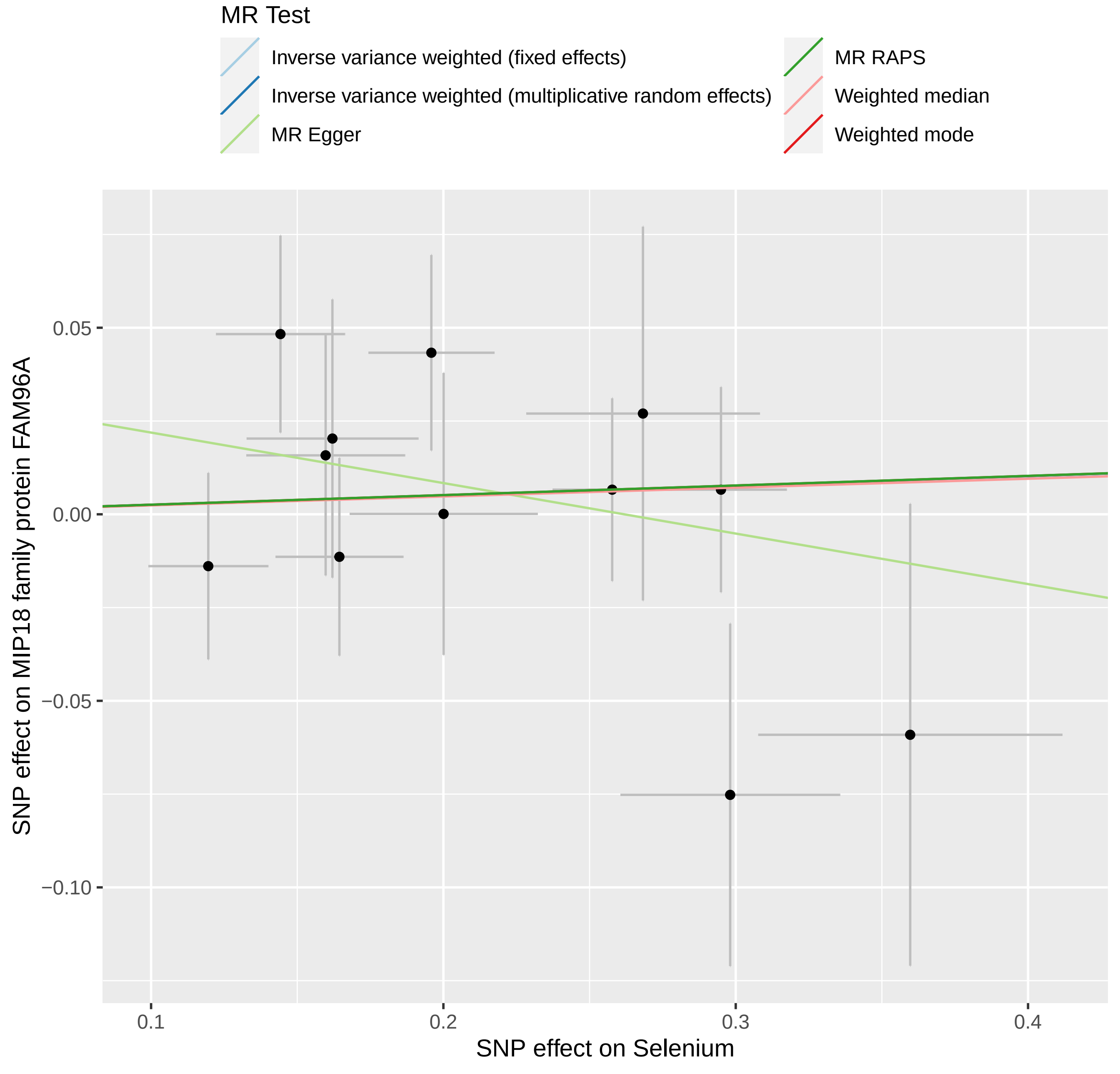

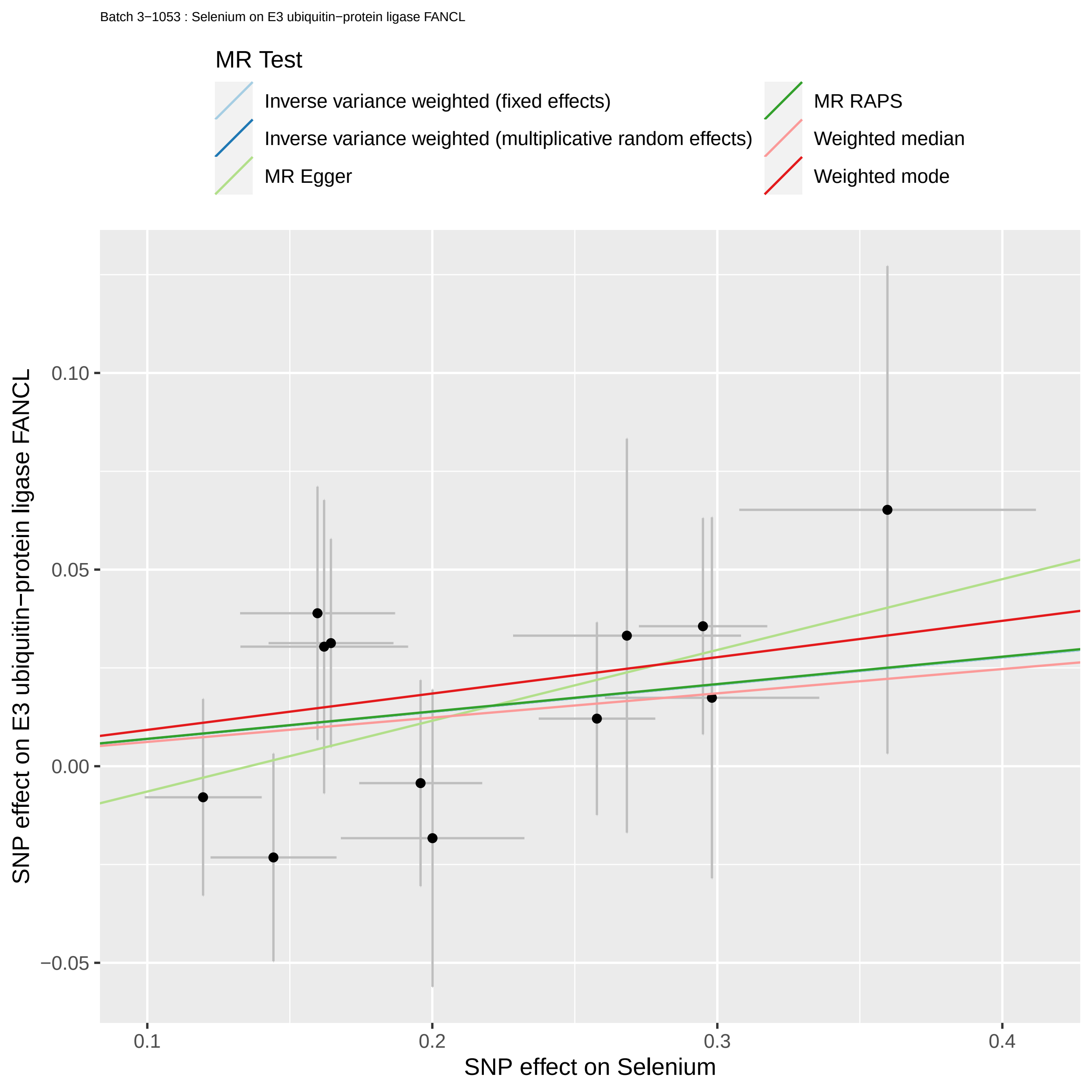

Batch 3−1053 : Selenium on E3 ubiquitin−protein ligase FANCL
MR Test
Inverse variance weighted (fixed effects)
Inverse variance weighted (multiplicative random effects)
MR Egger
MR RAPS
Weighted median
Weighted mode
SNP effect on E3 ubiquitin−protein ligase FANCL
0.10
0.05
0.00
−0.05
0.1
0.2
0.3
0.4
SNP effect on Selenium

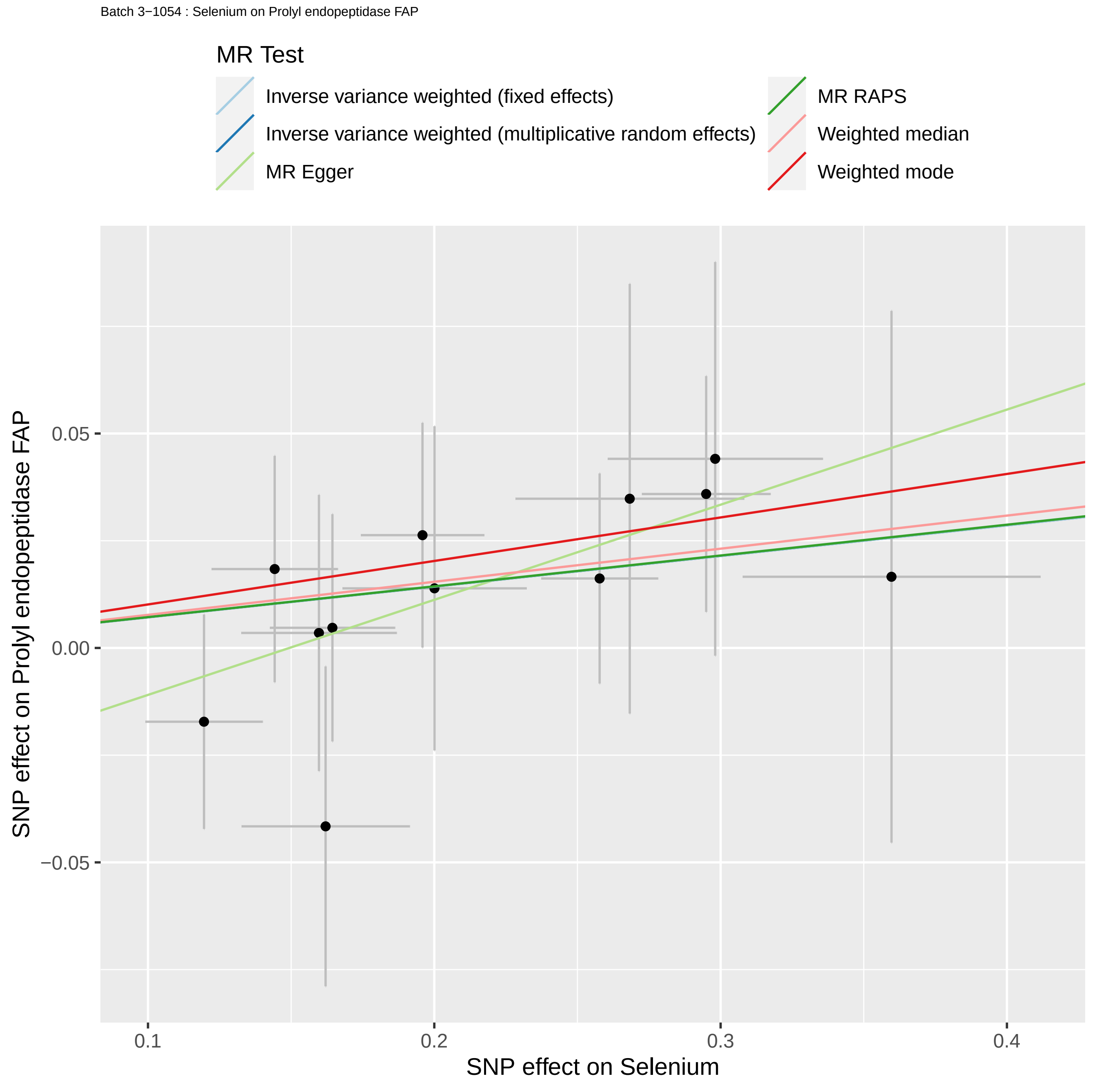
Batch 3−1054 : Selenium on Prolyl endopeptidase FAP
MR Test
Inverse variance weighted (fixed effects)
Inverse variance weighted (multiplicative random effects)
MR Egger
MR RAPS
Weighted median
Weighted mode
SNP effect on Prolyl endopeptidase FAP
0.05
0.00
−0.05
0.1
0.2
0.3
0.4
SNP effect on Selenium

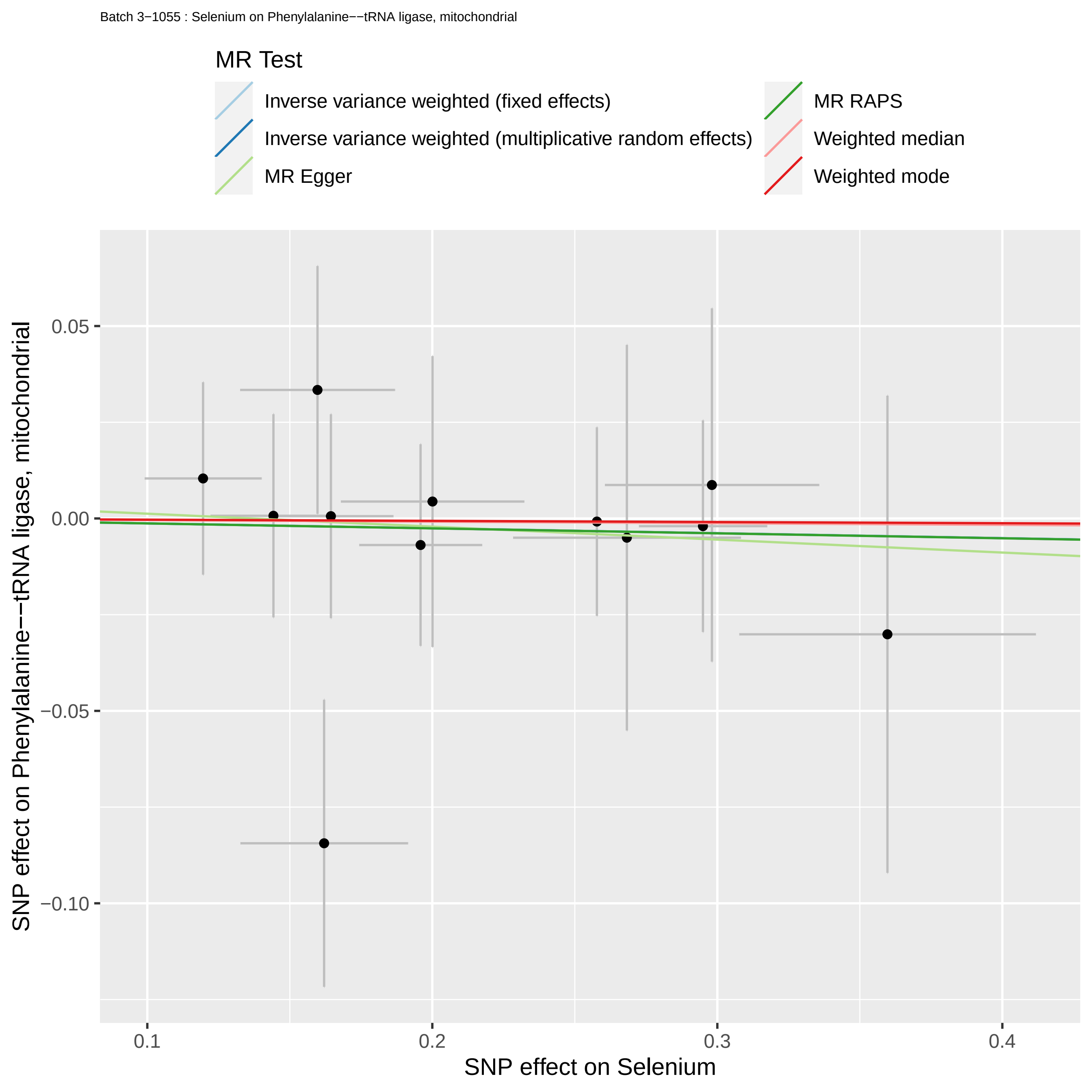

Batch 3−1055 : Selenium on Phenylalanine−−tRNA ligase, mitochondrial
MR Test
Inverse variance weighted (fixed effects)
Inverse variance weighted (multiplicative random effects)
MR Egger
MR RAPS
Weighted median
Weighted mode
0.05
0.00
−0.05
−0.10
0.1
0.2
0.3
0.4
SNP effect on Selenium
SNP effect on Phenylalanine−−tRNA ligase, mitochondrial

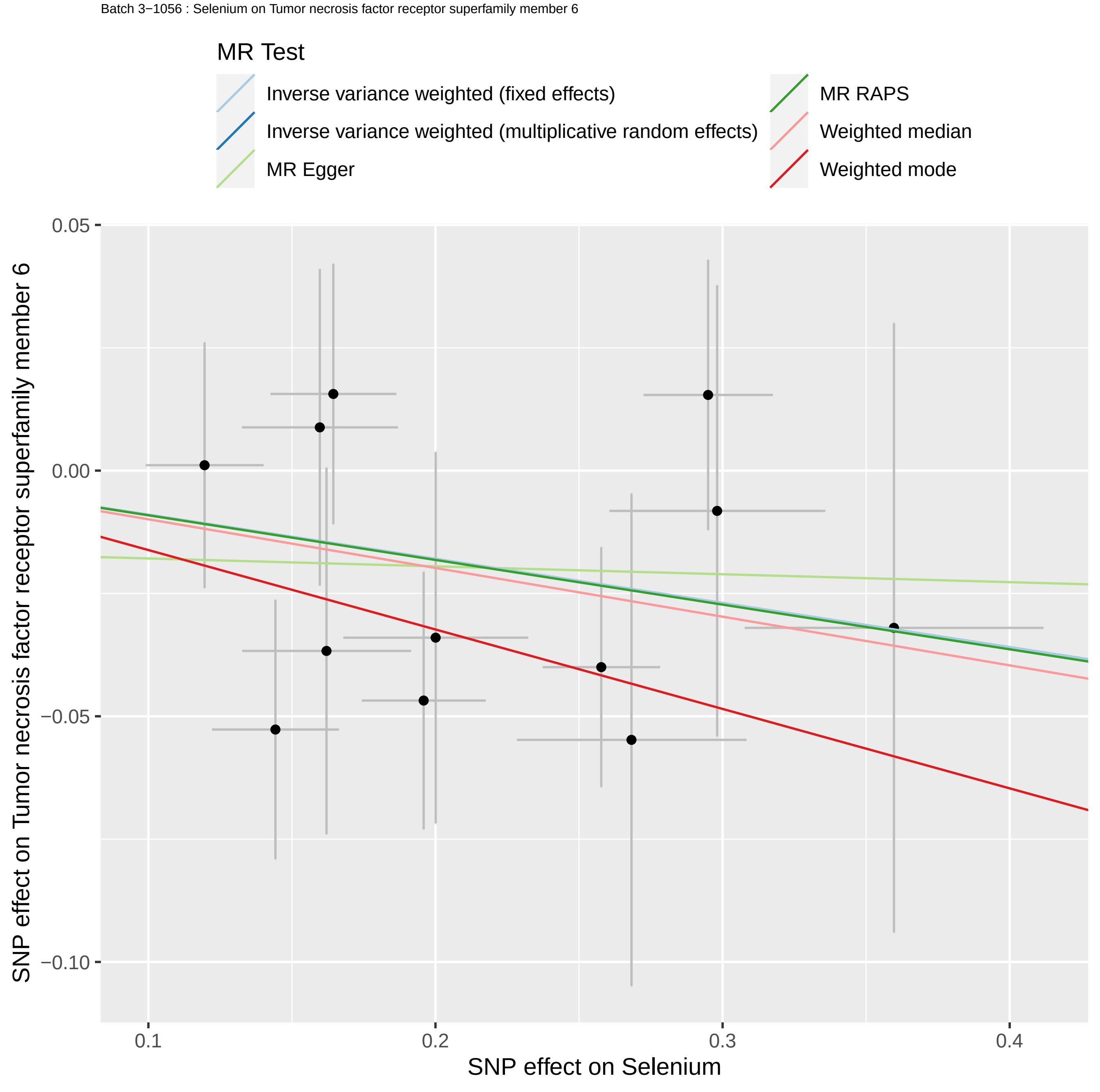

Batch 3−1056 : Selenium on Tumor necrosis factor receptor superfamily member 6
MR Test
Inverse variance weighted (fixed effects)
Inverse variance weighted (multiplicative random effects)
MR Egger
MR RAPS
Weighted median
Weighted mode
0.05
0.00
−0.05
−0.10
0.1
0.2
0.3
0.4
SNP effect on Selenium
SNP effect on Tumor necrosis factor receptor superfamily member 6

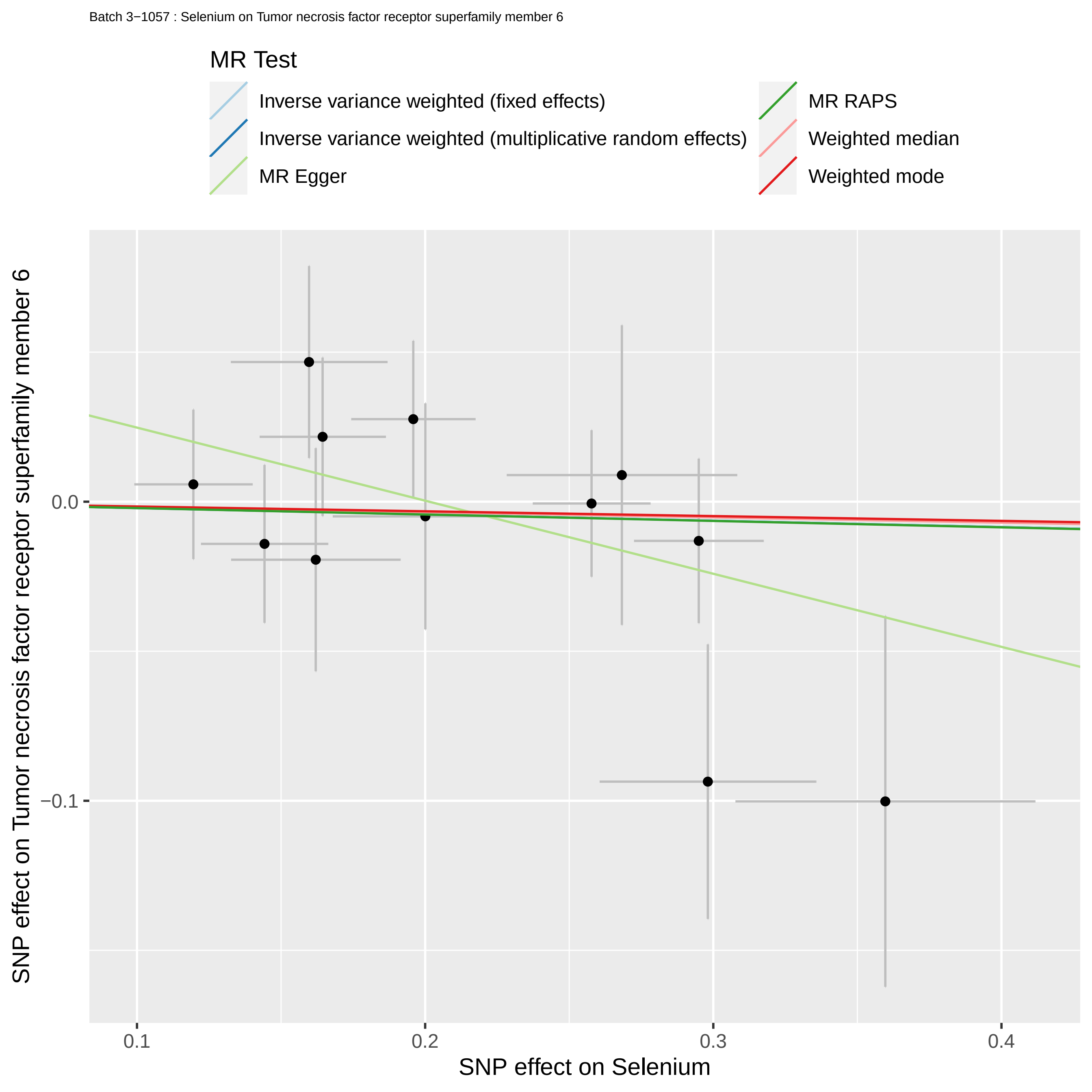
Batch 3−1057 : Selenium on Tumor necrosis factor receptor superfamily member 6
MR Test
Inverse variance weighted (fixed effects)
Inverse variance weighted (multiplicative random effects)
MR Egger
MR RAPS
Weighted median
Weighted mode
SNP effect on Tumor necrosis factor receptor superfamily member 6
0.0
−0.1
0.1
0.2
0.3
0.4
SNP effect on Selenium

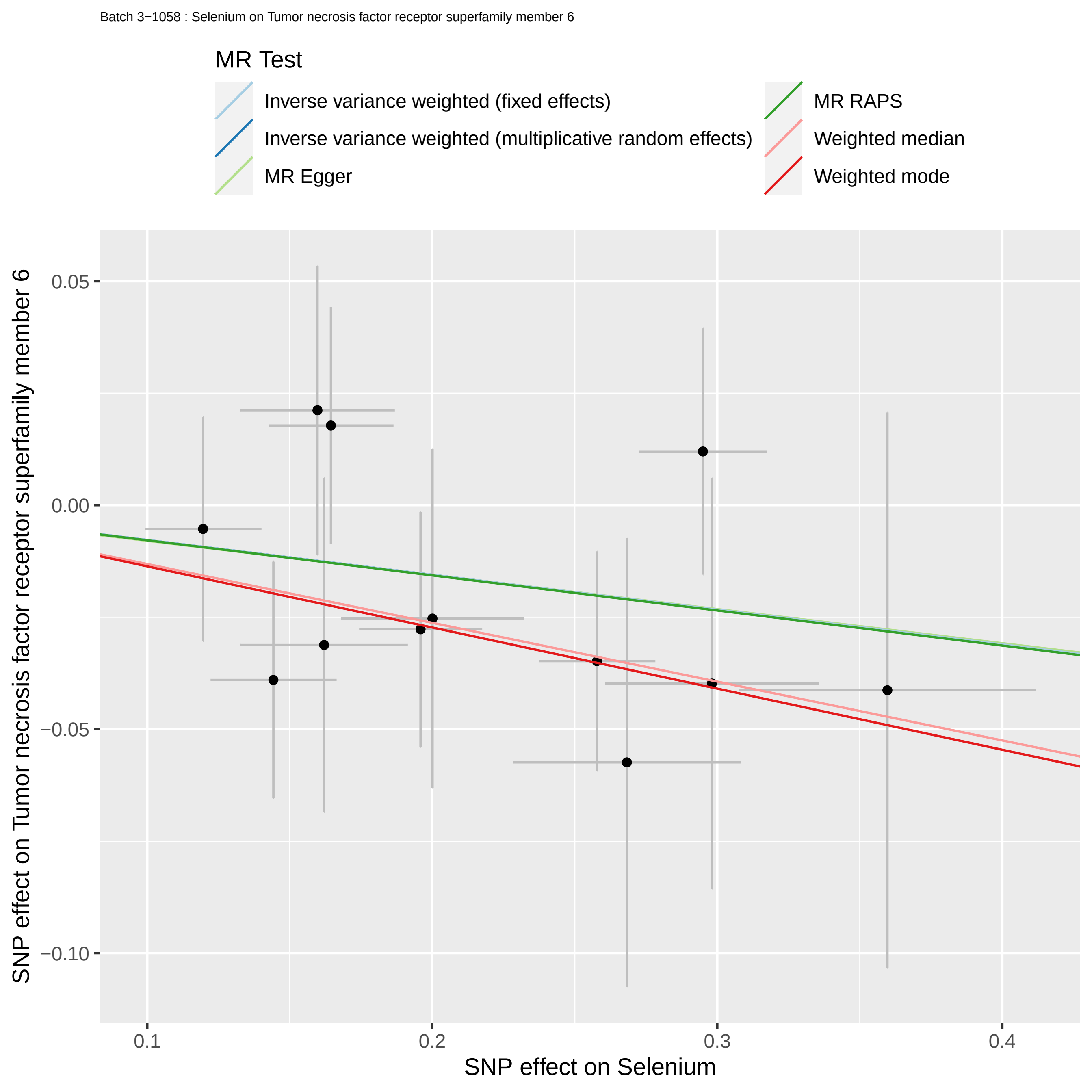
Batch 3−1058 : Selenium on Tumor necrosis factor receptor superfamily member 6
MR Test
Inverse variance weighted (fixed effects)
Inverse variance weighted (multiplicative random effects)
MR Egger
MR RAPS
Weighted median
Weighted mode
SNP effect on Tumor necrosis factor receptor superfamily member 6
0.05
0.00
−0.05
−0.10
0.1
0.2
0.3
0.4
SNP effect on Selenium

Batch 3−1059 : Selenium on Tumor necrosis factor ligand superfamily member 6, soluble form

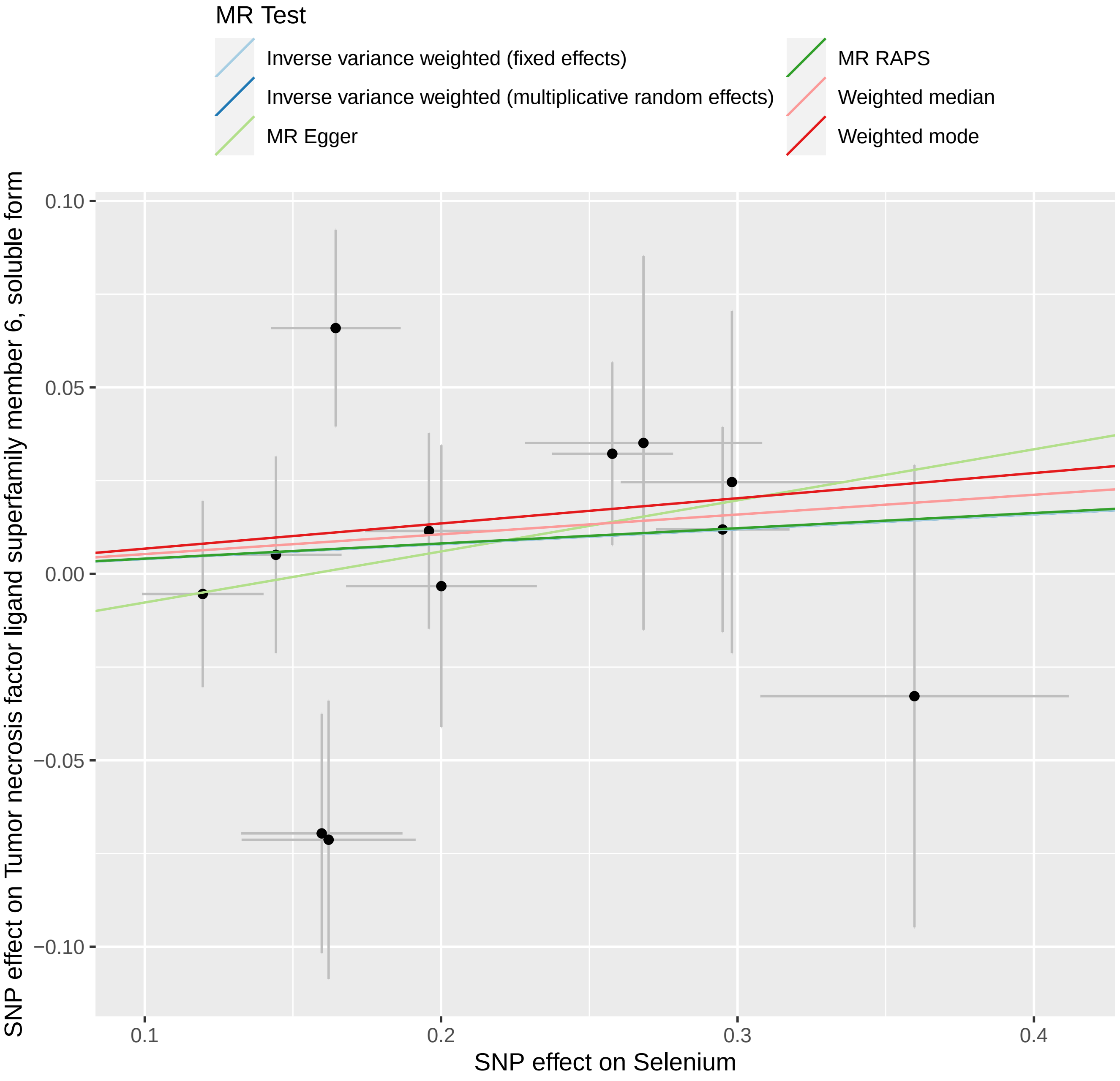

Batch 3−1060 : Selenium on Fetal and adult testis−expressed transcript protein

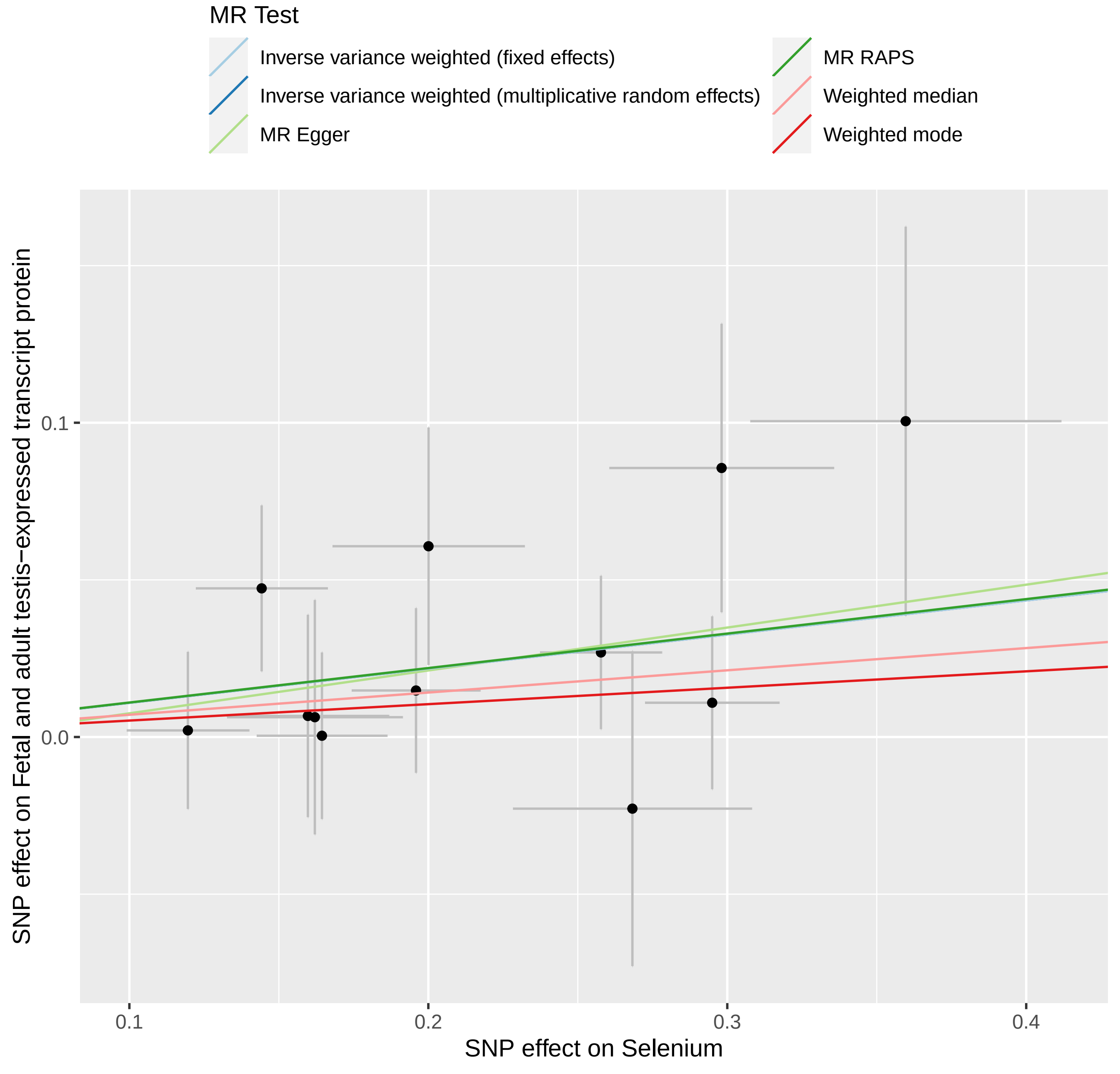

Batch 3−1061 : Selenium on Fatty acid hydroxylase domain−containing protein 2

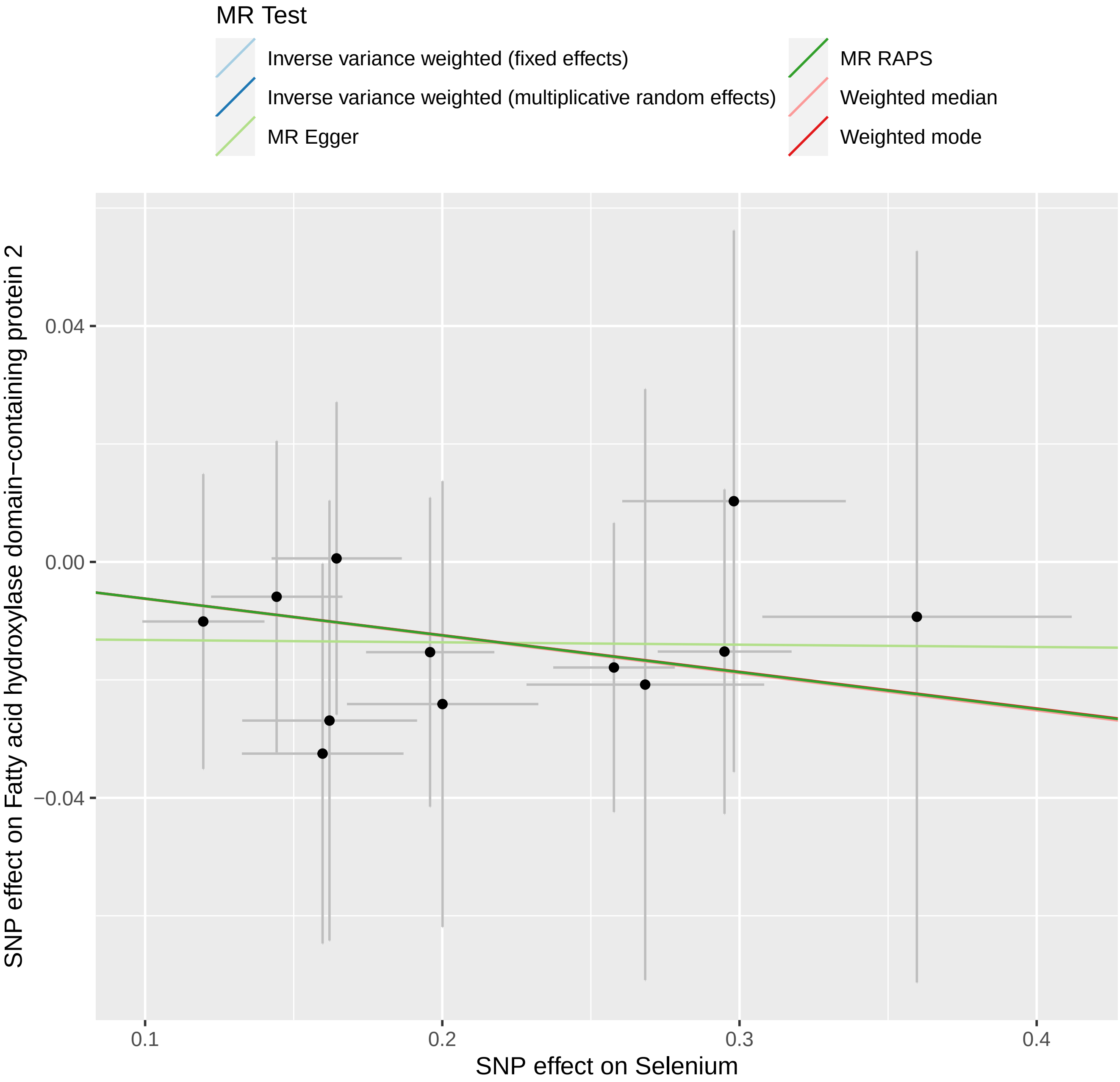

Batch 3−1062 : Selenium on Fibulin−1

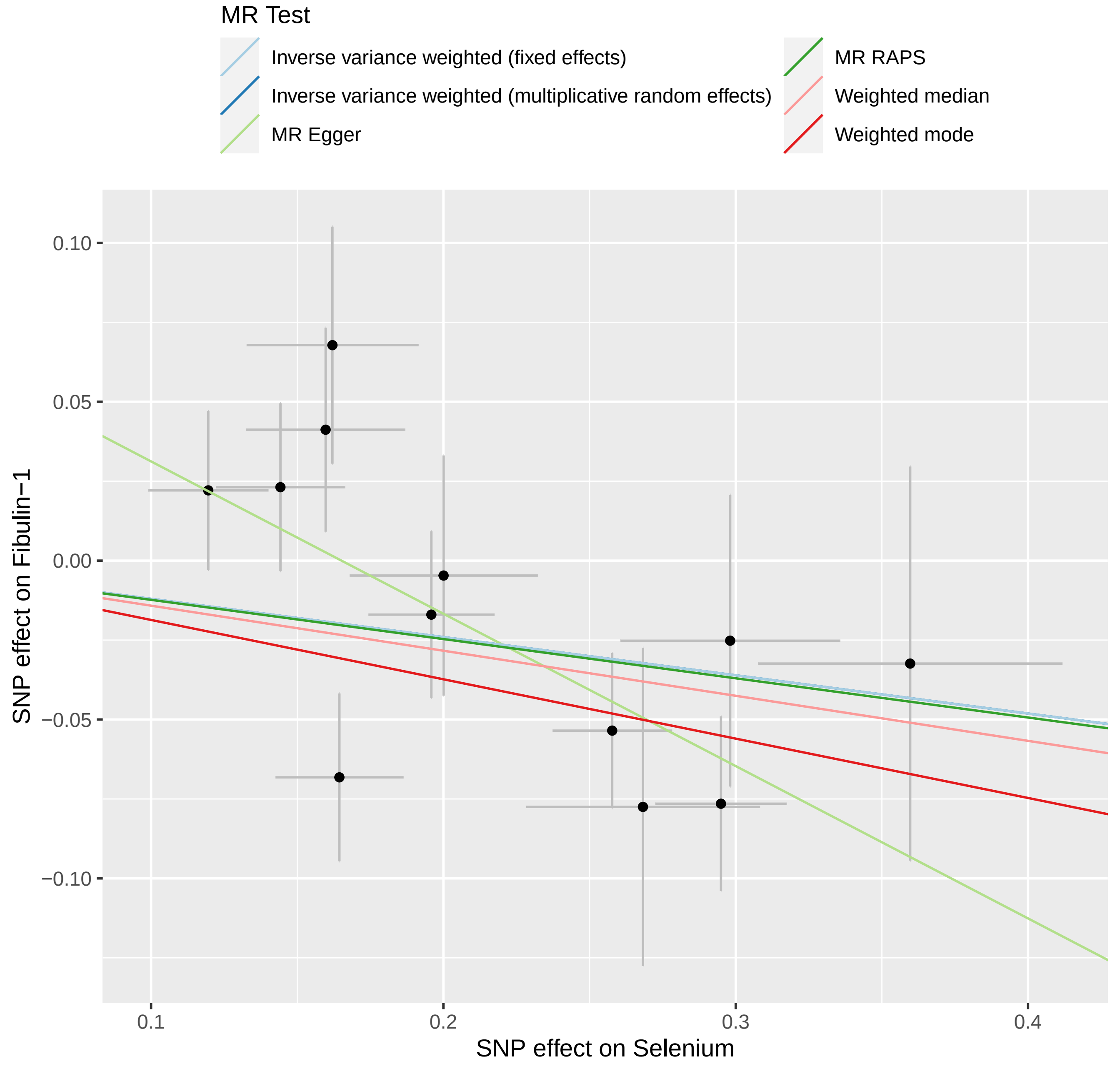

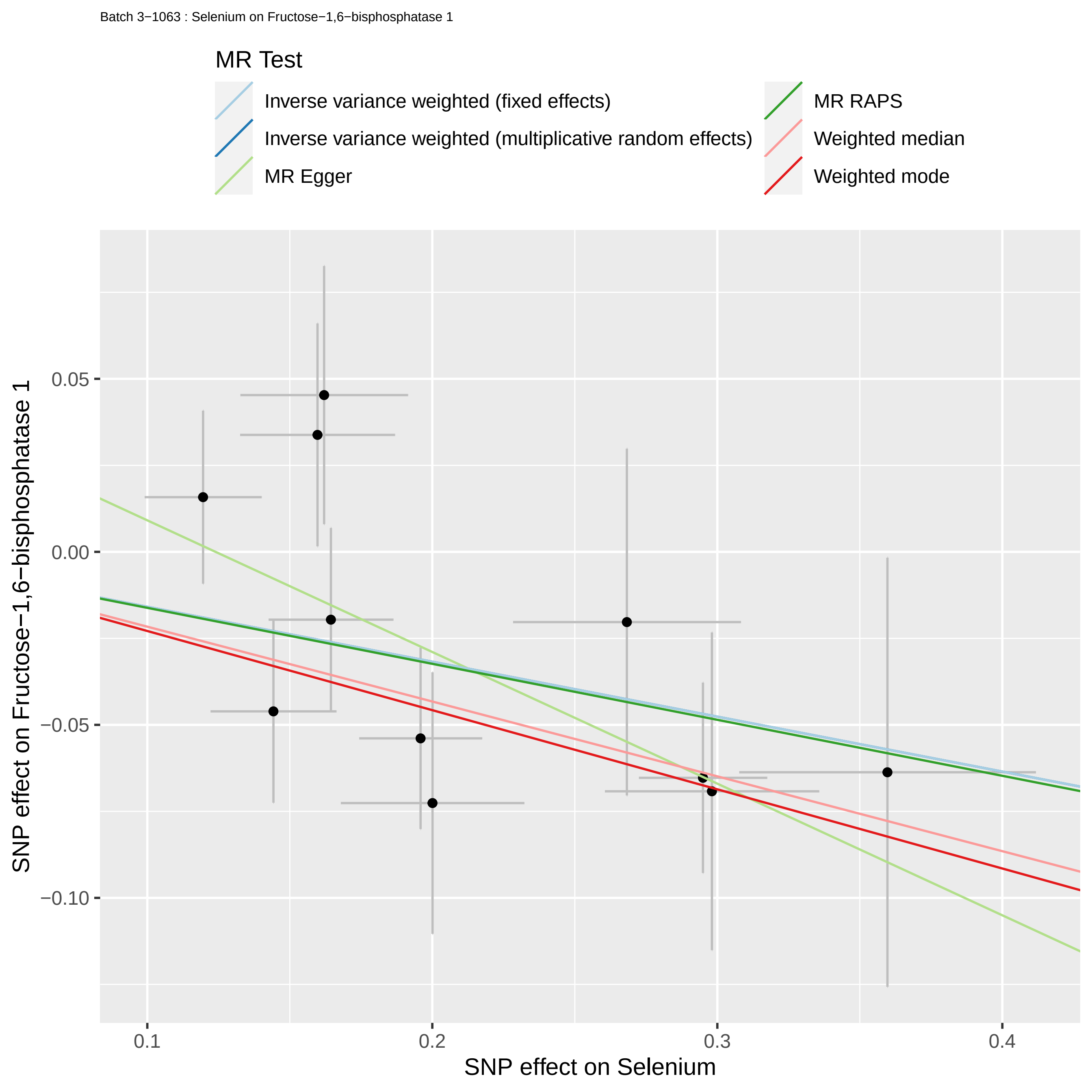

Batch 3−1063 : Selenium on Fructose−1,6−bisphosphatase 1
MR Test
Inverse variance weighted (fixed effects)
Inverse variance weighted (multiplicative random effects)
MR Egger
MR RAPS
Weighted median
Weighted mode
0.05
0.00
−0.05
−0.10
0.1
0.2
0.3
0.4
SNP effect on Fructose−1,6−bisphosphatase 1
SNP effect on Selenium

Batch 3−1064 : Selenium on Fructose−1,6−bisphosphatase isozyme 2

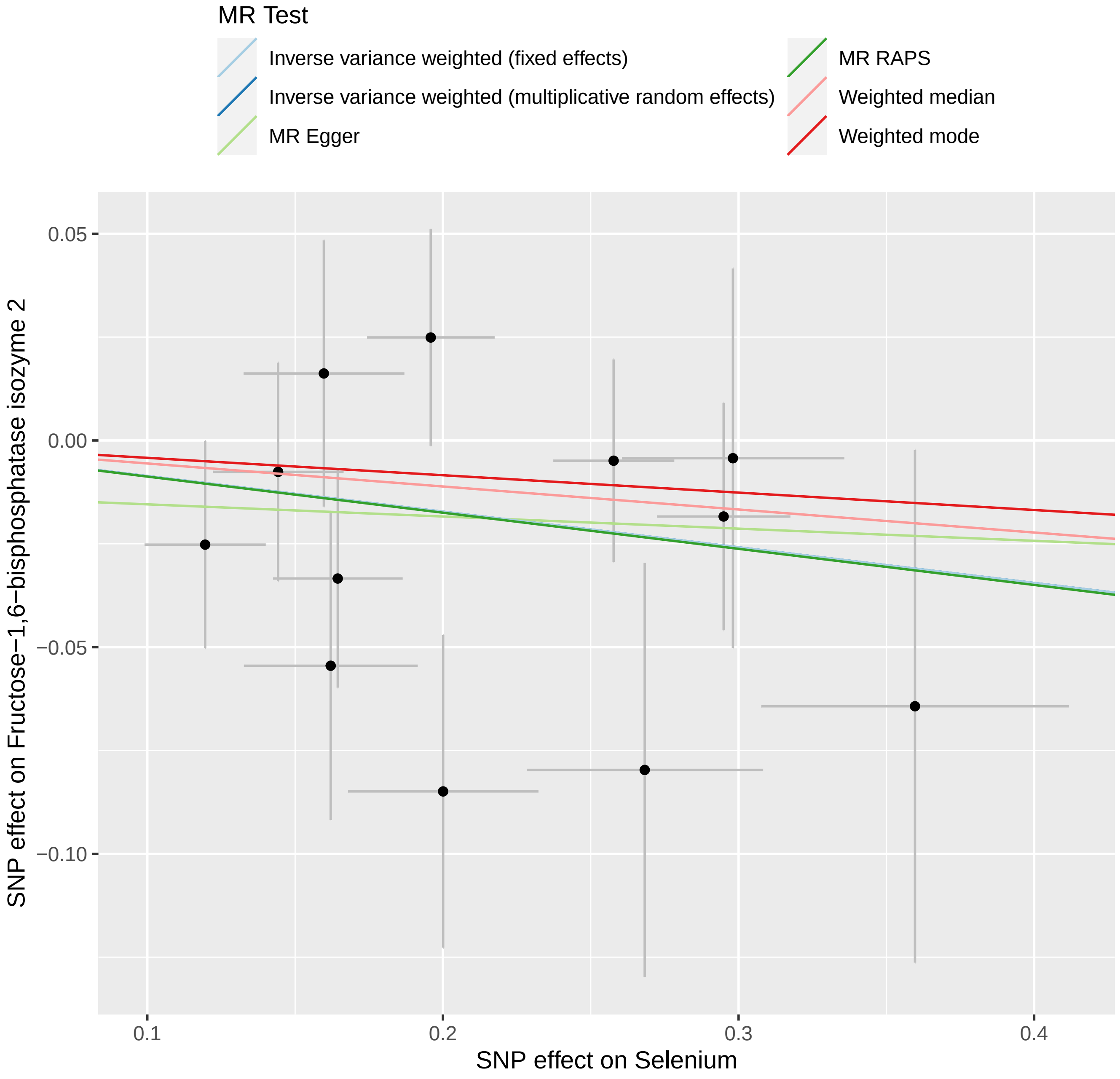

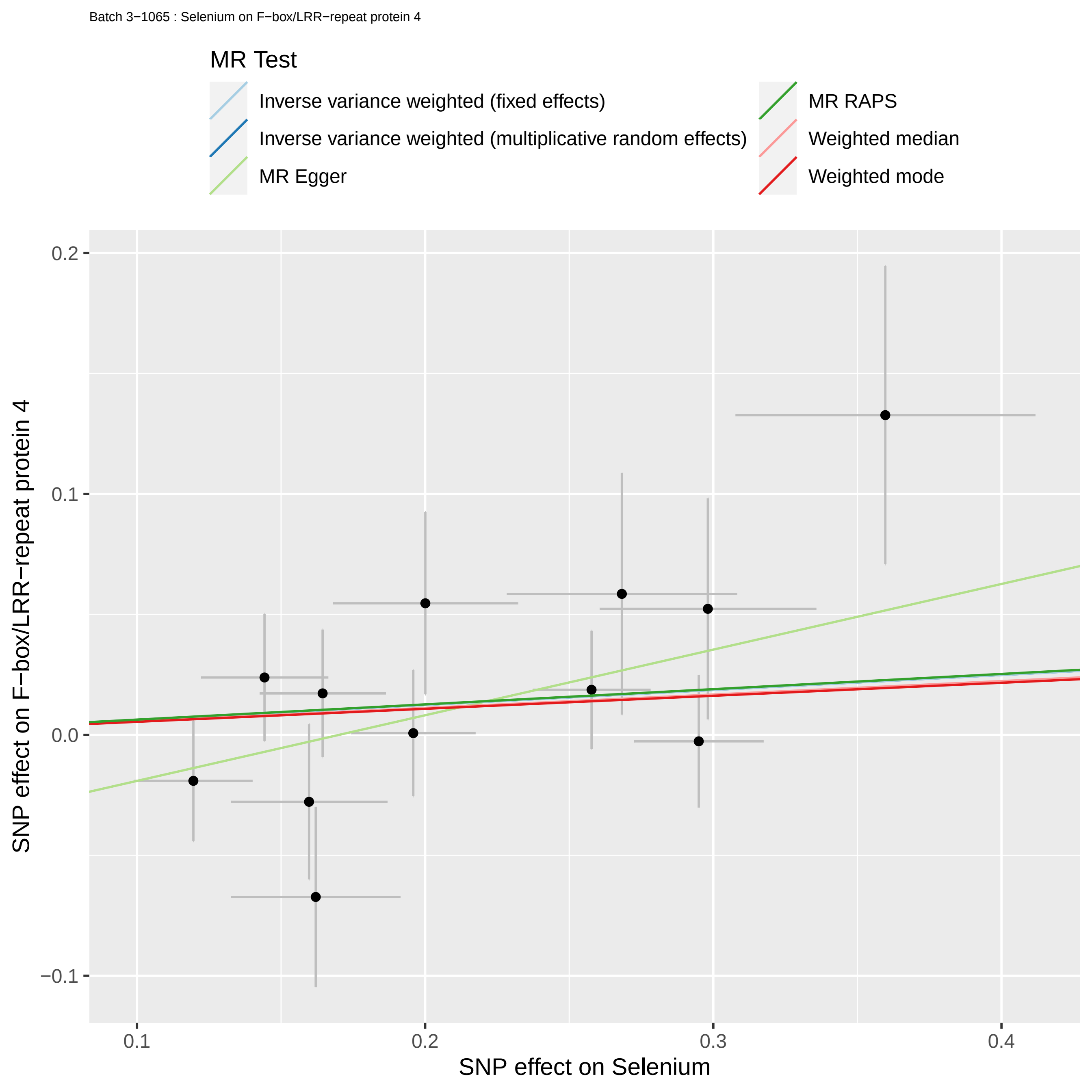

Batch 3−1065 : Selenium on F−box/LRR−repeat protein 4
MR Test
Inverse variance weighted (fixed effects)
Inverse variance weighted (multiplicative random effects)
MR Egger
MR RAPS
Weighted median
Weighted mode
0.2
0.1
0.0
−0.1
0.1
0.2
0.3
0.4
SNP effect on Selenium
SNP effect on F−box/LRR−repeat protein 4

Batch 3−1066 : Selenium on F−box/LRR−repeat protein 4

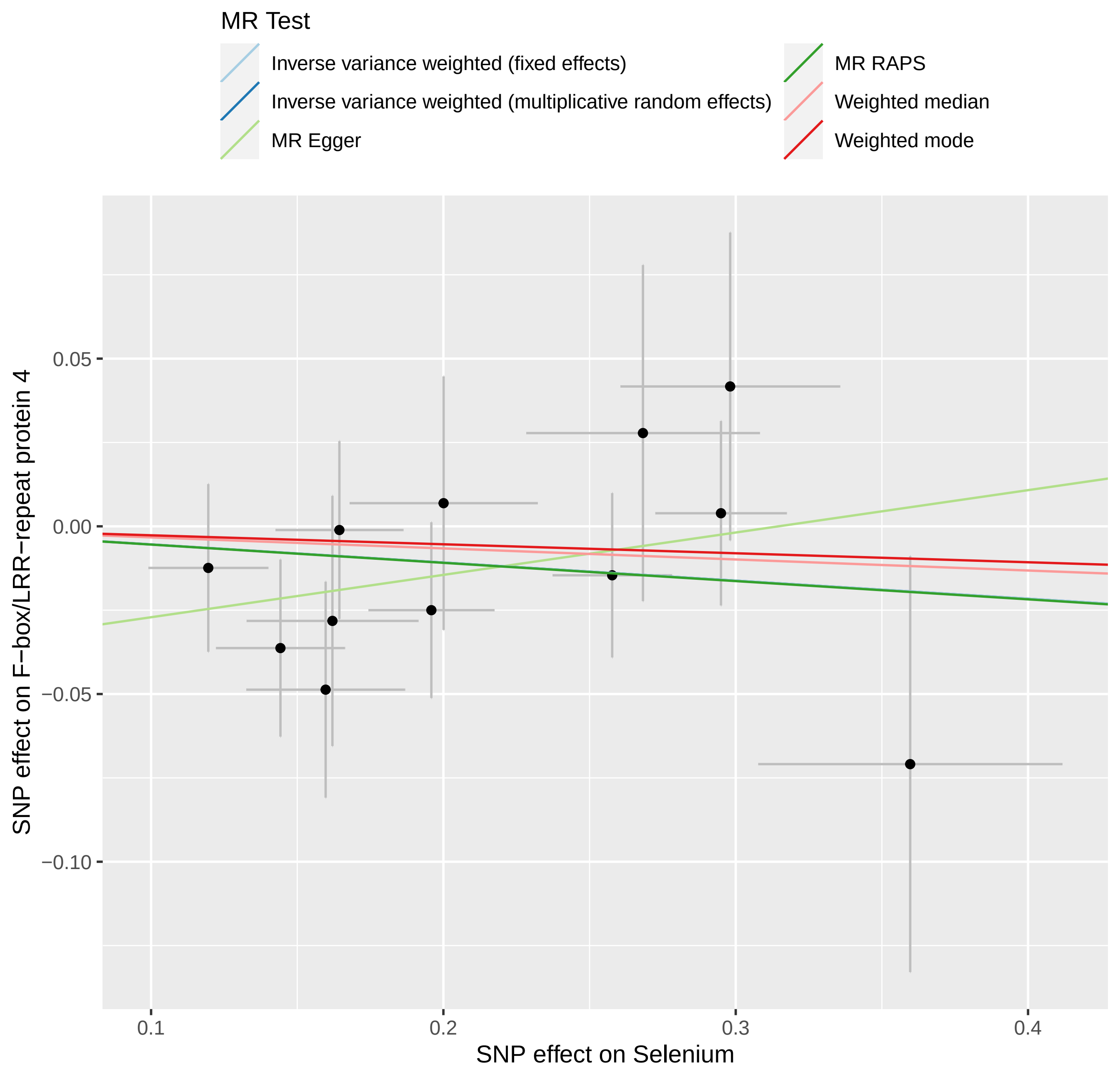

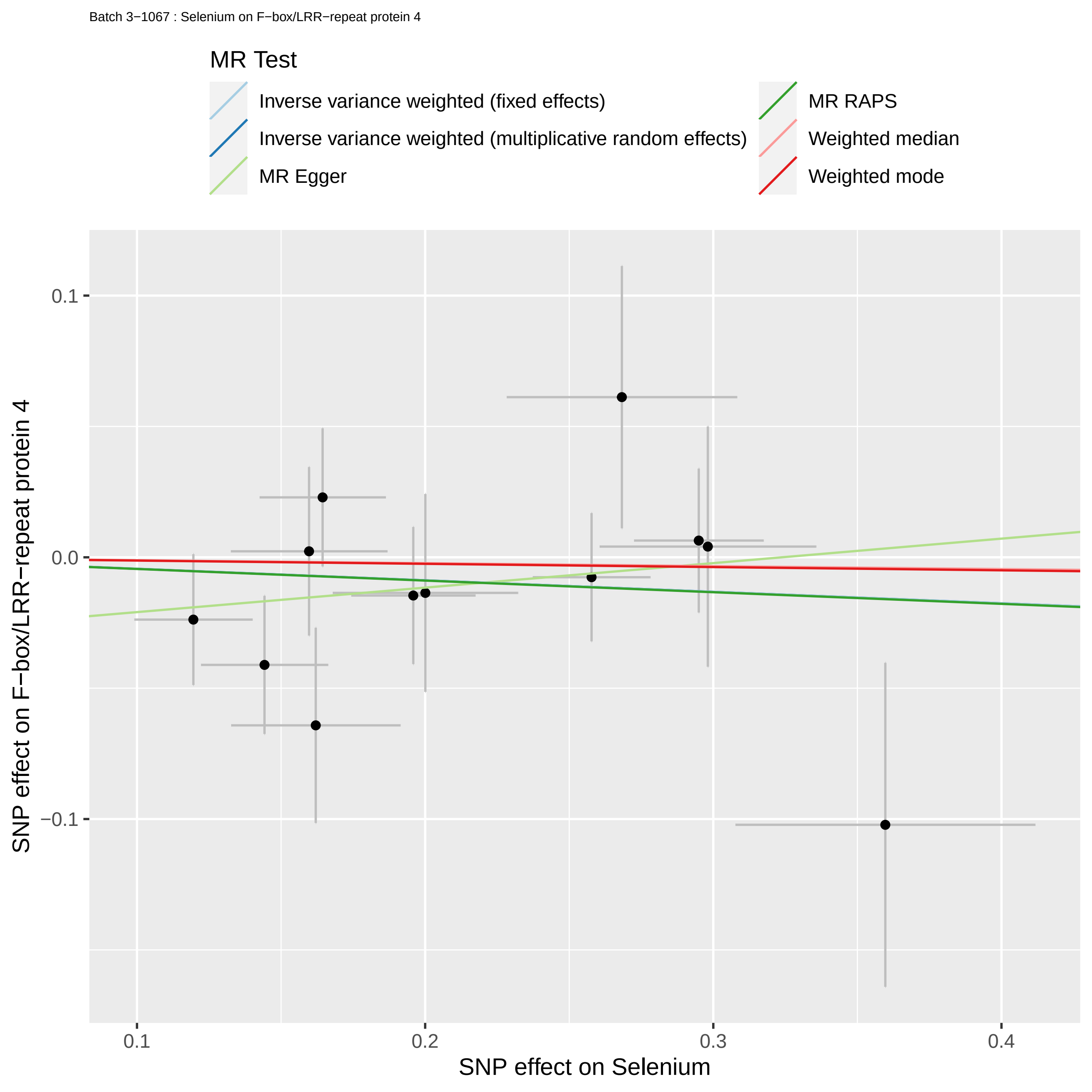

Batch 3−1067 : Selenium on F−box/LRR−repeat protein 4
MR Test
Inverse variance weighted (fixed effects)
Inverse variance weighted (multiplicative random effects)
MR Egger
MR RAPS
Weighted median
Weighted mode
0.1
0.0
−0.1
0.1
0.2
0.3
0.4
SNP effect on F−box/LRR−repeat protein 4
SNP effect on Selenium

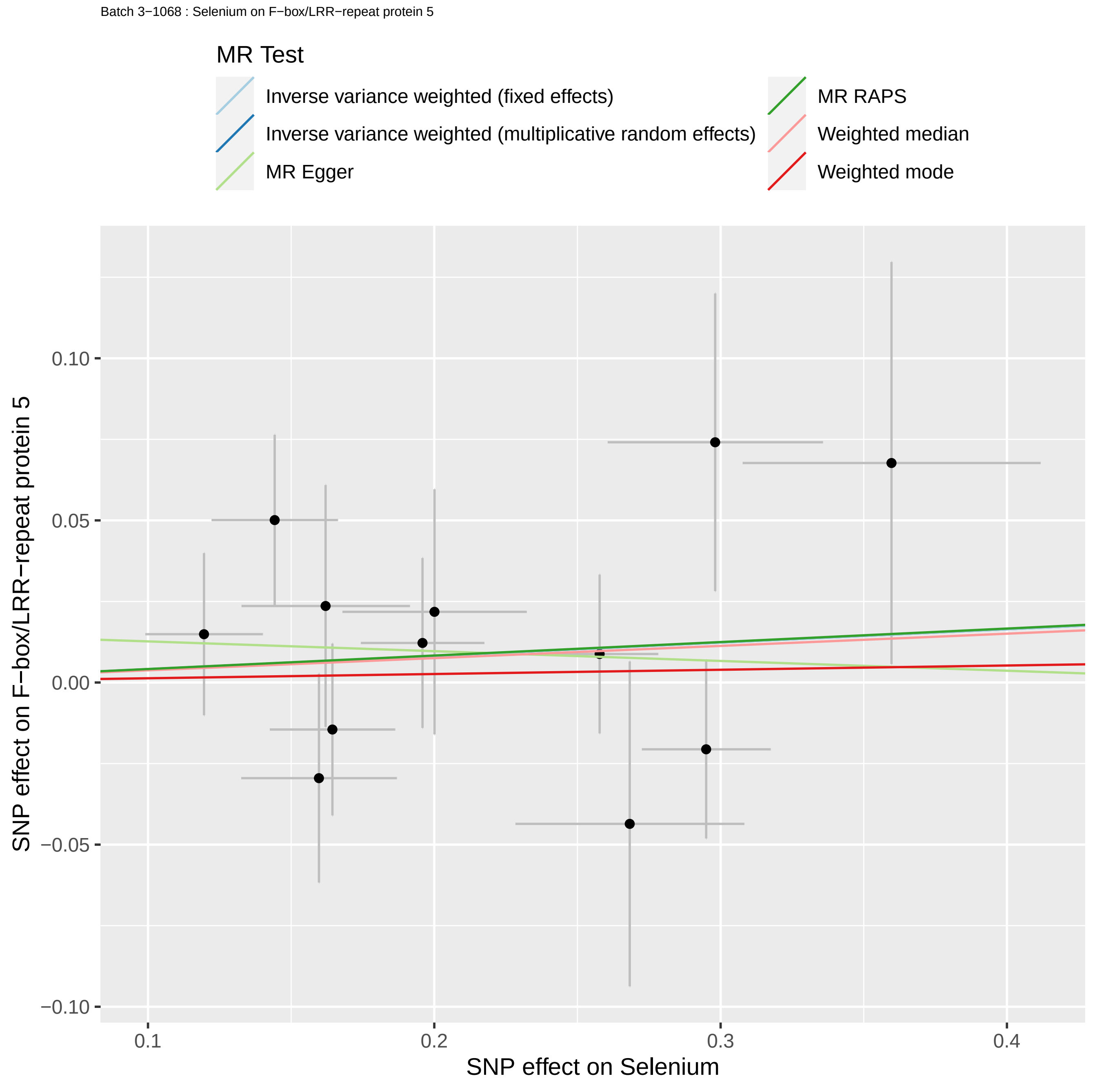

Batch 3−1068 : Selenium on F−box/LRR−repeat protein 5
MR Test
Inverse variance weighted (fixed effects)
Inverse variance weighted (multiplicative random effects)
MR Egger
MR RAPS
Weighted median
Weighted mode
0.10
0.05
0.00
−0.05
−0.10
0.1
0.2
0.3
0.4
SNP effect on F−box/LRR−repeat protein 5
SNP effect on Selenium

Batch 3−1069 : Selenium on F−box only protein 3

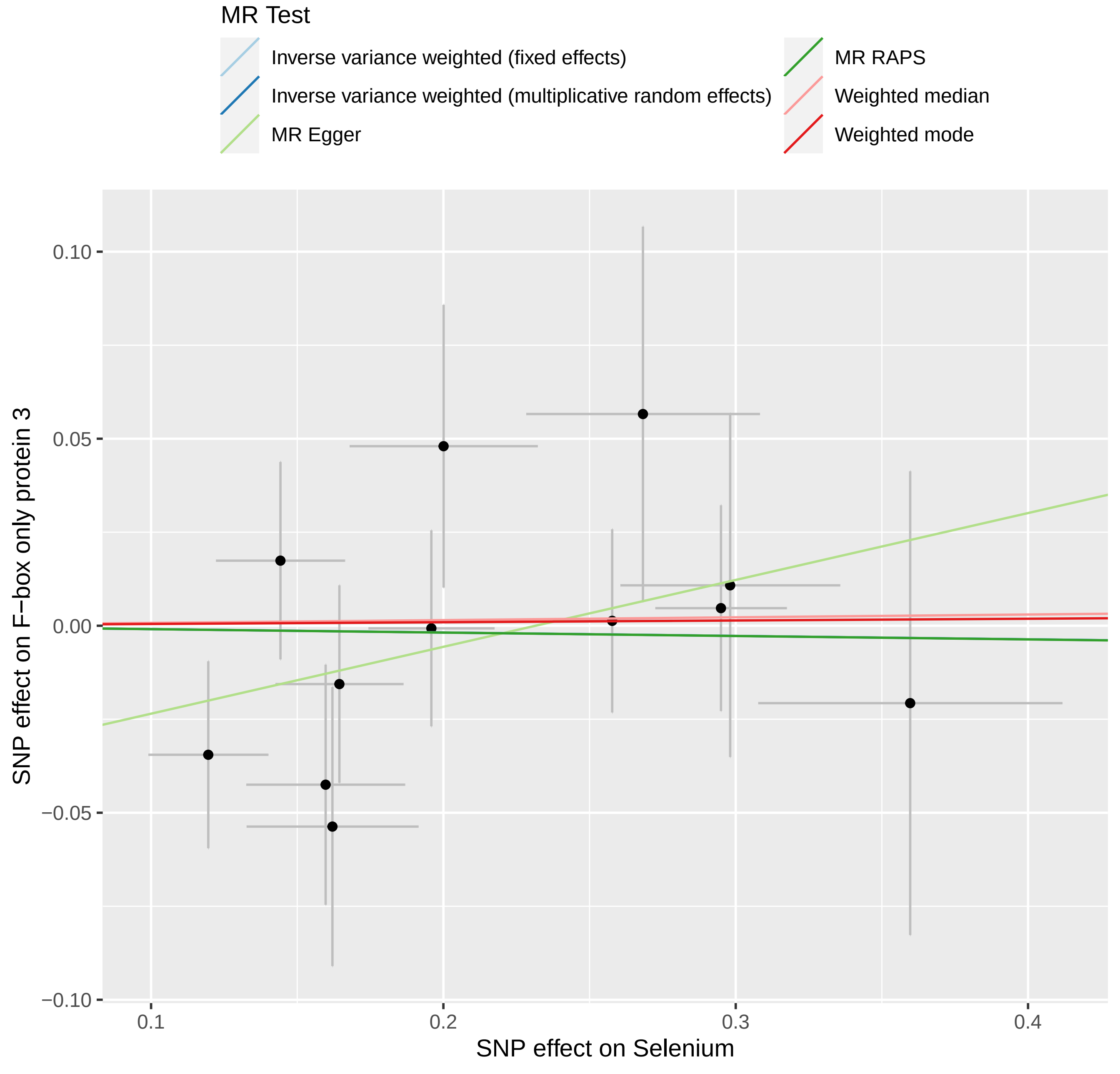

Batch 3−1070 : Selenium on High affinity immunoglobulin alpha and immunoglobulin mu Fc receptor

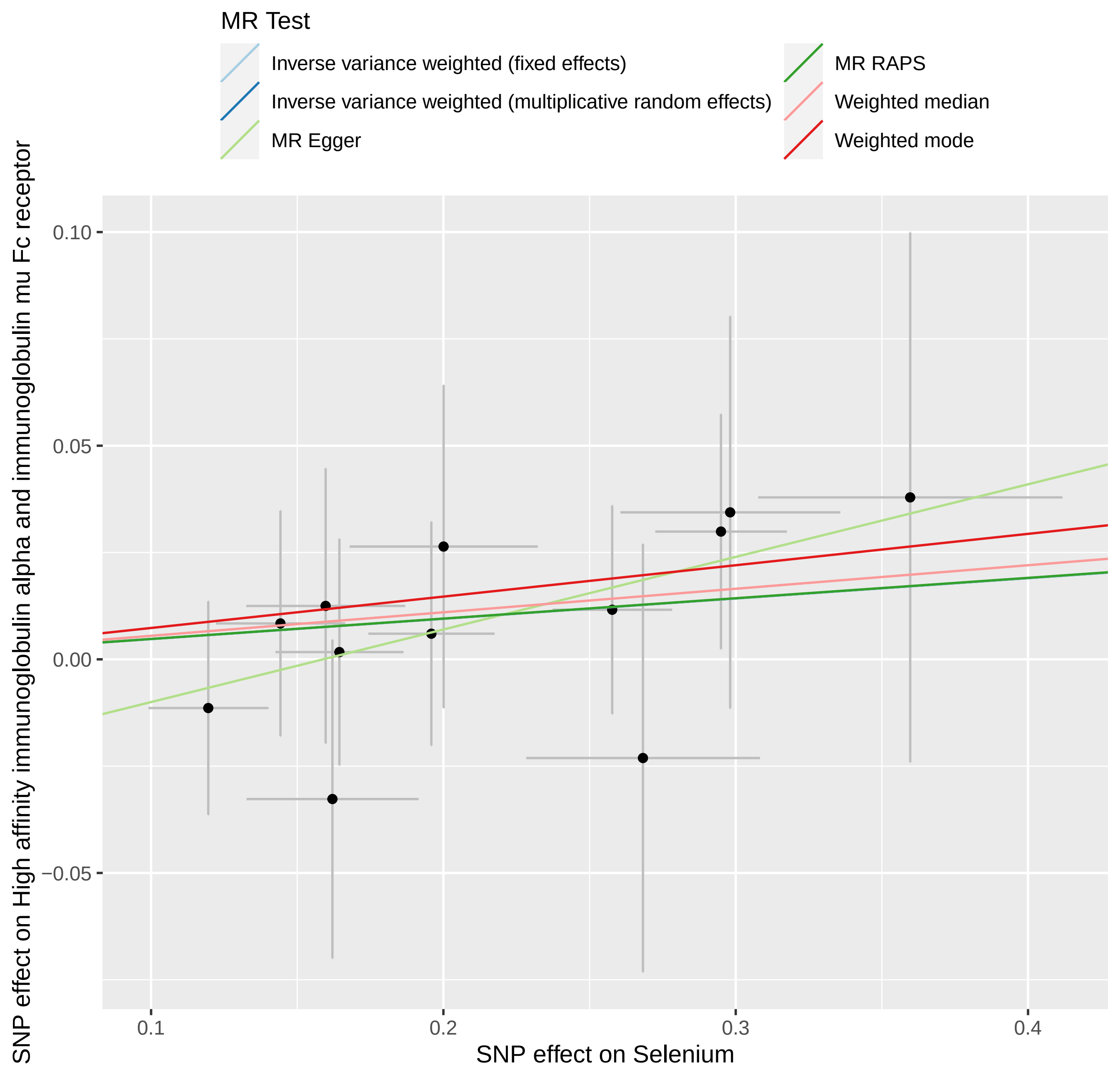

Batch 3−1071 : Selenium on Immunoglobulin alpha Fc receptor

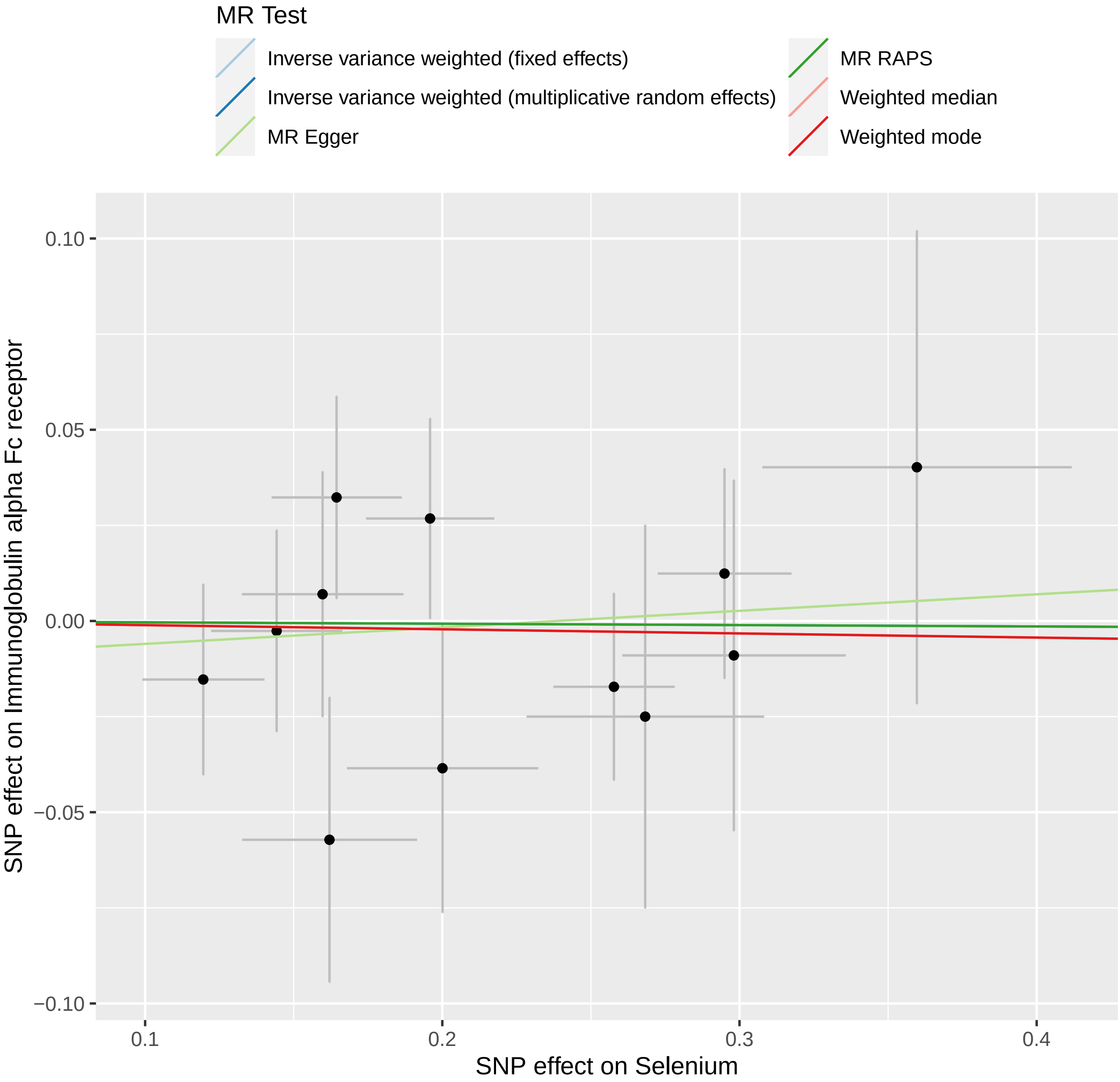

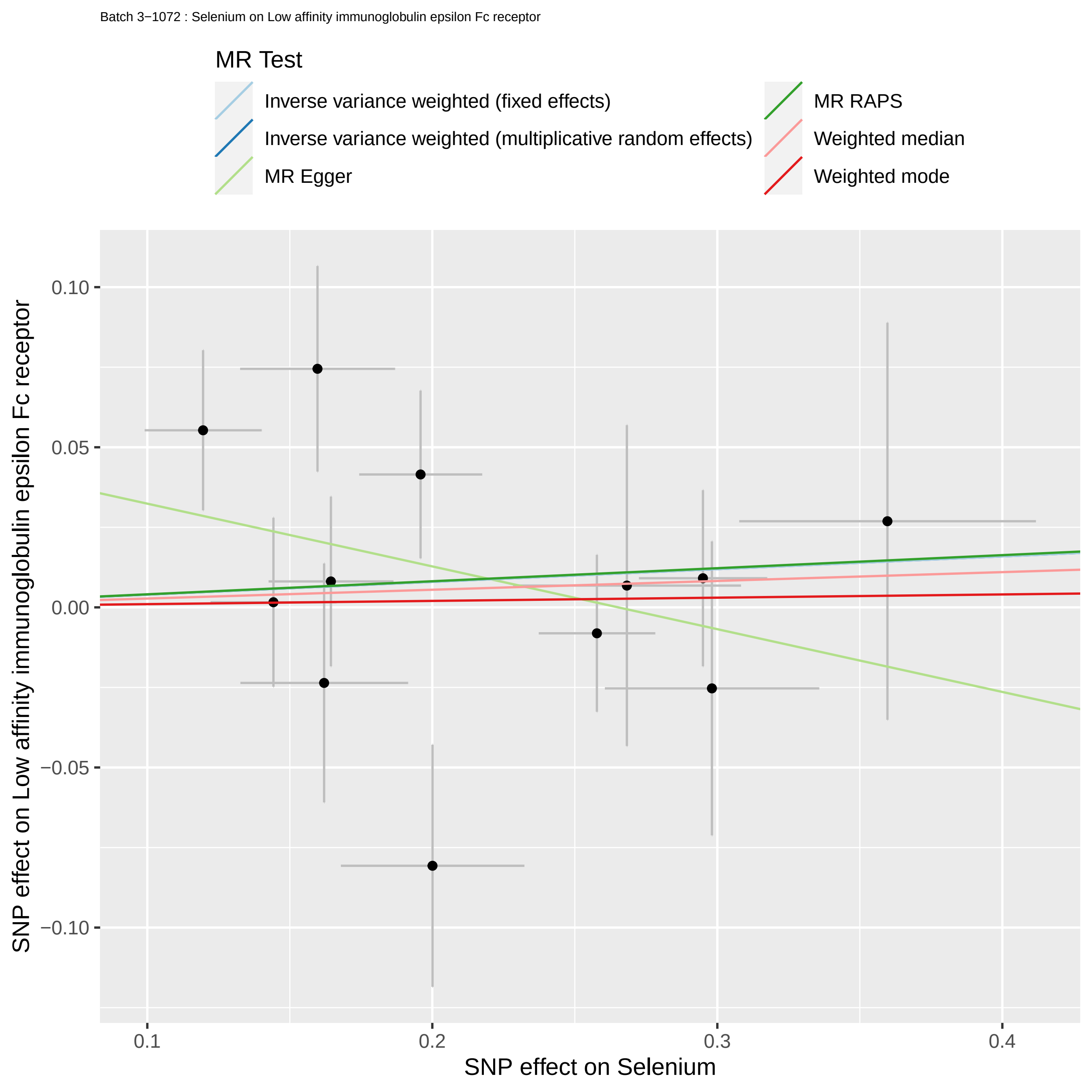
Batch 3−1072 : Selenium on Low affinity immunoglobulin epsilon Fc receptor
MR Test
Inverse variance weighted (fixed effects)
Inverse variance weighted (multiplicative random effects)
MR Egger
MR RAPS
Weighted median
Weighted mode
SNP effect on Low affinity immunoglobulin epsilon Fc receptor
0.10
0.05
0.00
−0.05
−0.10
0.1
0.2
0.3
0.4
SNP effect on Selenium

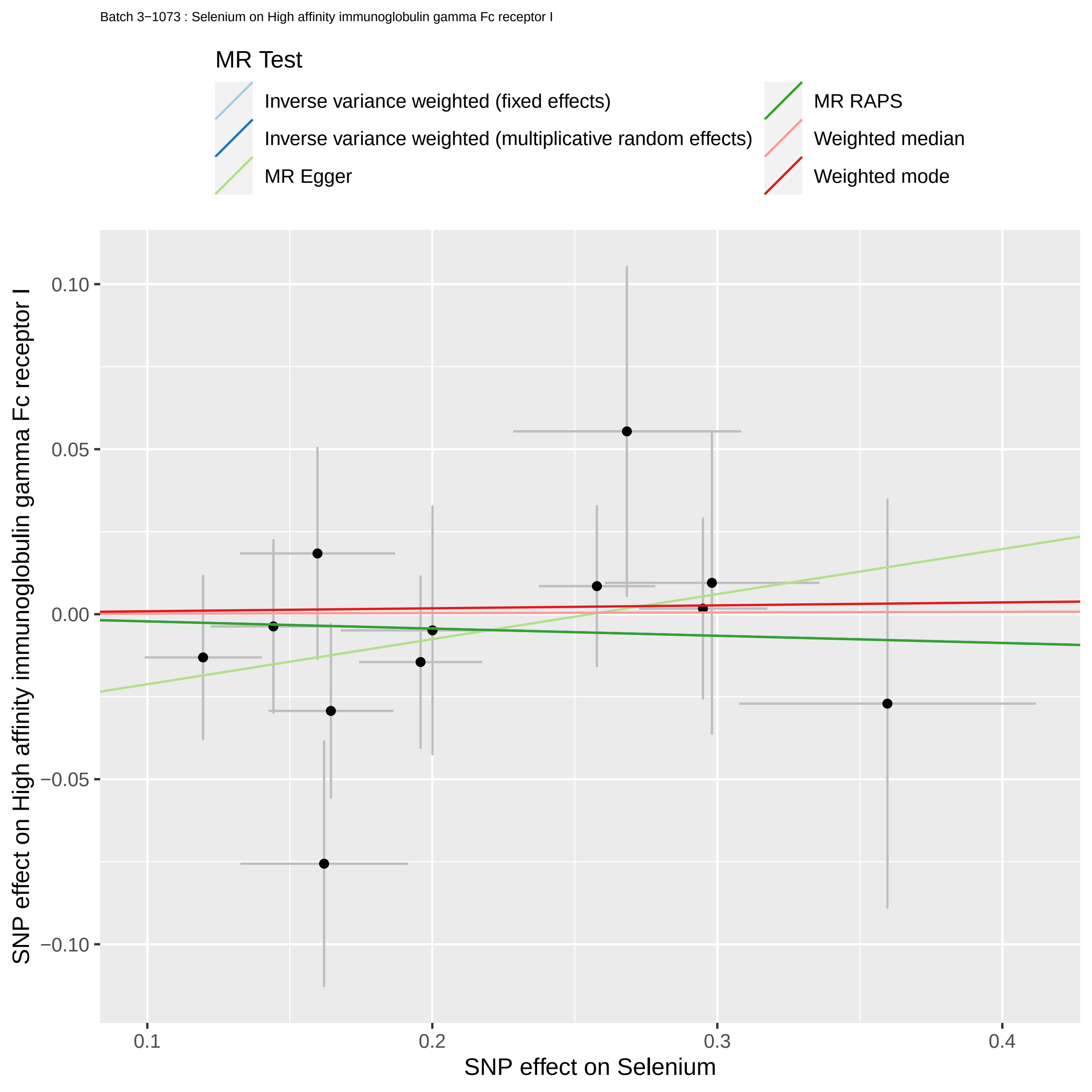

Batch 3−1073 : Selenium on High affinity immunoglobulin gamma Fc receptor I
MR Test
Inverse variance weighted (fixed effects)
Inverse variance weighted (multiplicative random effects)
MR Egger
MR RAPS
Weighted median
Weighted mode
0.10
0.05
0.00
−0.05
−0.10
0.1
0.2
0.3
0.4
SNP effect on Selenium
SNP effect on High affinity immunoglobulin gamma Fc receptor I

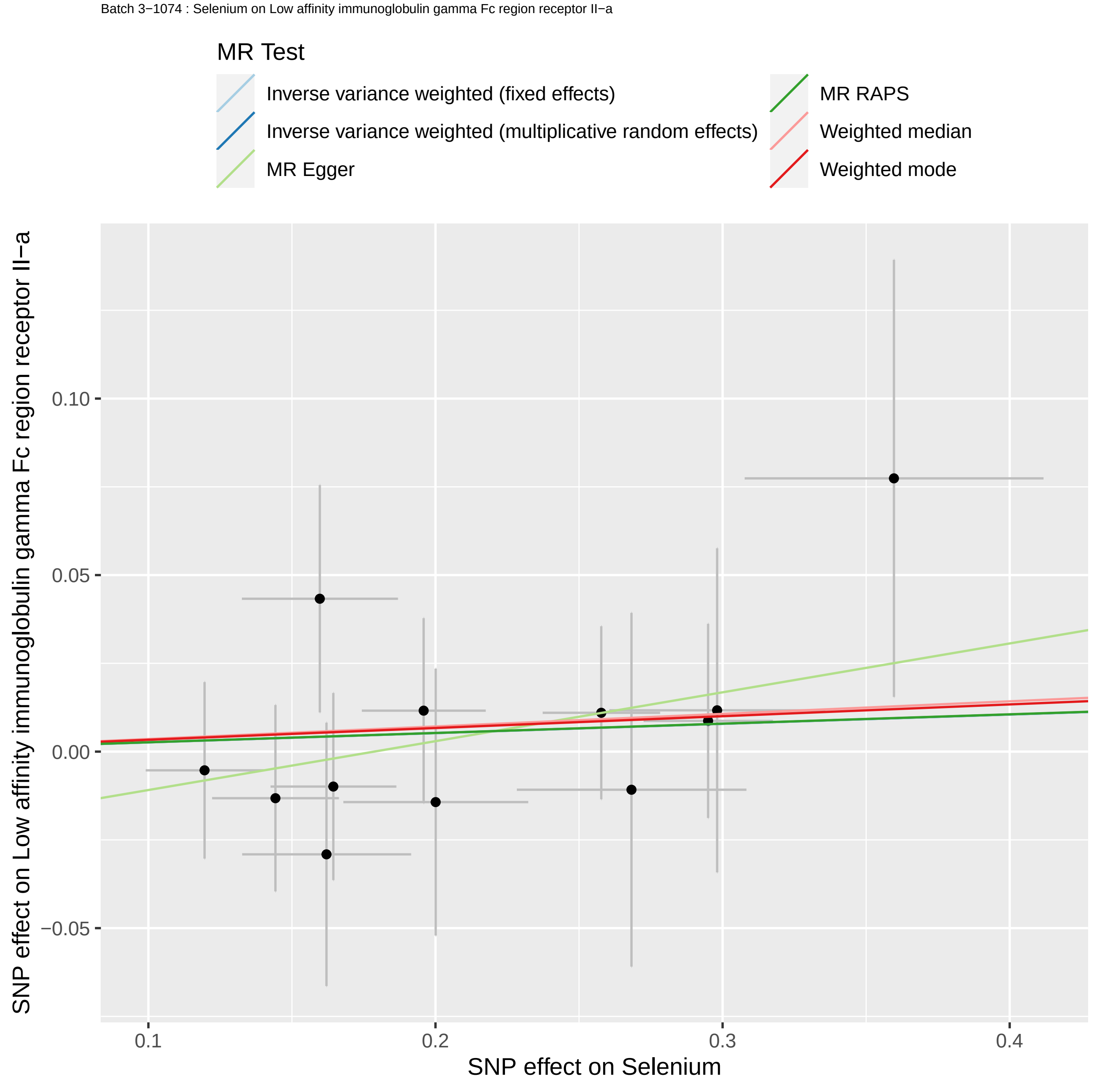

Batch 3−1074 : Selenium on Low affinity immunoglobulin gamma Fc region receptor II−a
MR Test
Inverse variance weighted (fixed effects)
Inverse variance weighted (multiplicative random effects)
MR Egger
MR RAPS
Weighted median
Weighted mode
SNP effect on Low affinity immunoglobulin gamma Fc region receptor II−a
0.10
0.05
0.00
−0.05
0.1
0.2
0.3
0.4
SNP effect on Selenium

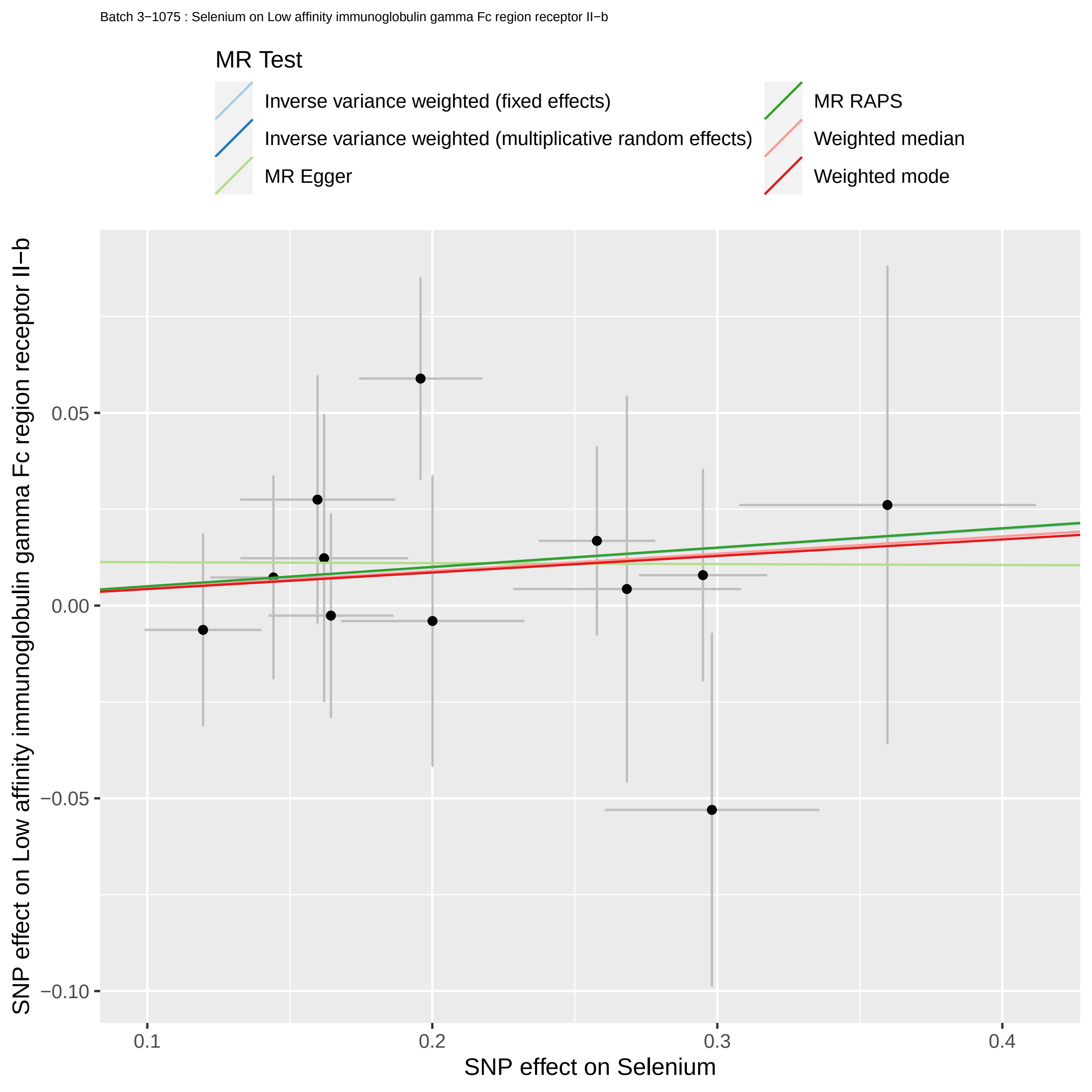
Batch 3−1075 : Selenium on Low affinity immunoglobulin gamma Fc region receptor II−b
MR Test
Inverse variance weighted (fixed effects)
Inverse variance weighted (multiplicative random effects)
MR Egger
MR RAPS
Weighted median
Weighted mode
SNP effect on Low affinity immunoglobulin gamma Fc region receptor II−b
0.05
0.00
−0.05
−0.10
0.1
0.2
0.3
0.4
SNP effect on Selenium

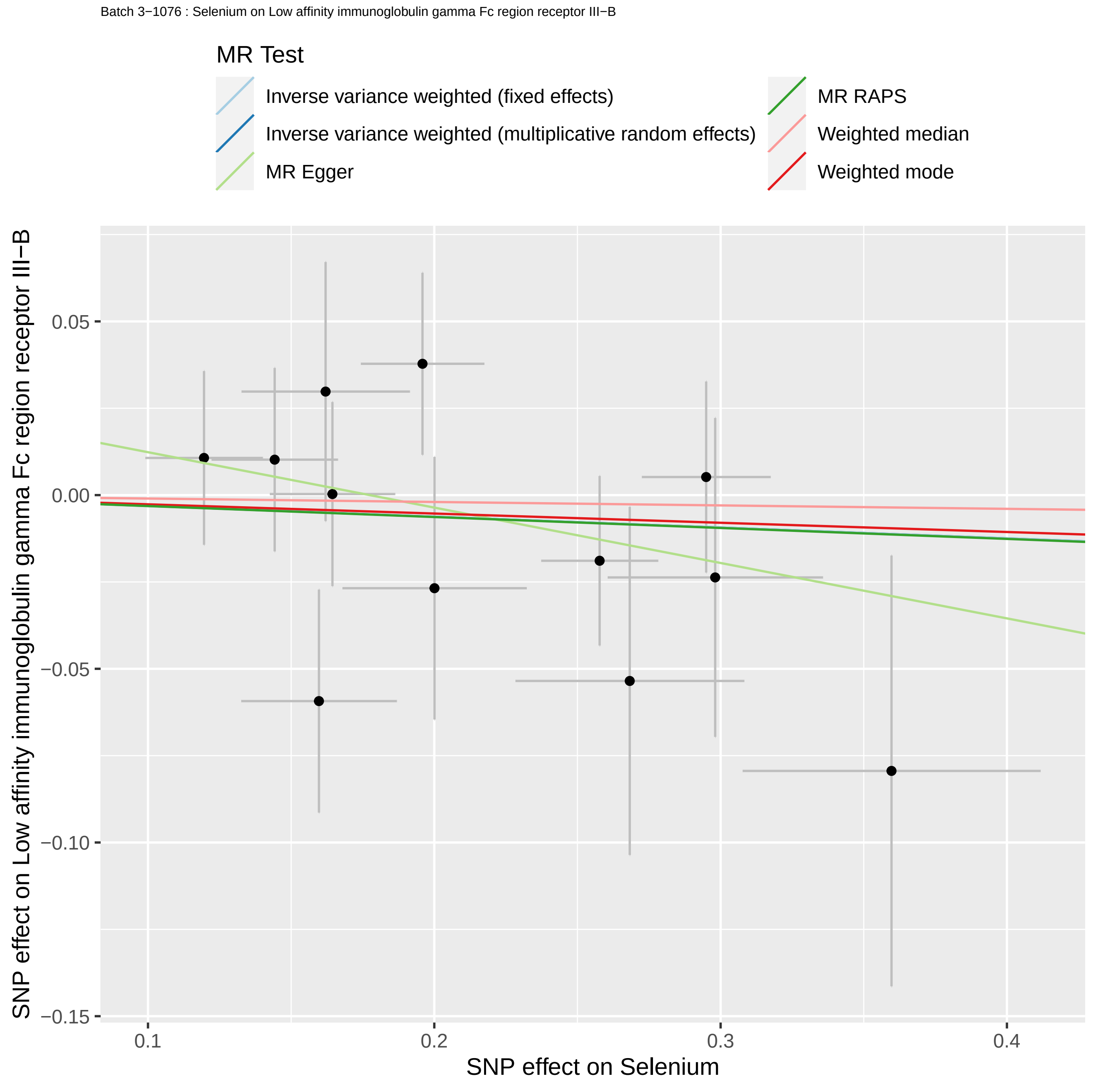

Batch 3−1076 : Selenium on Low affinity immunoglobulin gamma Fc region receptor III−B
MR Test
Inverse variance weighted (fixed effects)
Inverse variance weighted (multiplicative random effects)
MR Egger
MR RAPS
Weighted median
Weighted mode
SNP effect on Low affinity immunoglobulin gamma Fc region receptor III−B
0.05
0.00
−0.05
−0.10
−0.15
0.1
0.2
0.3
0.4
SNP effect on Selenium

Batch 3−1077 : Selenium on Ficolin−1

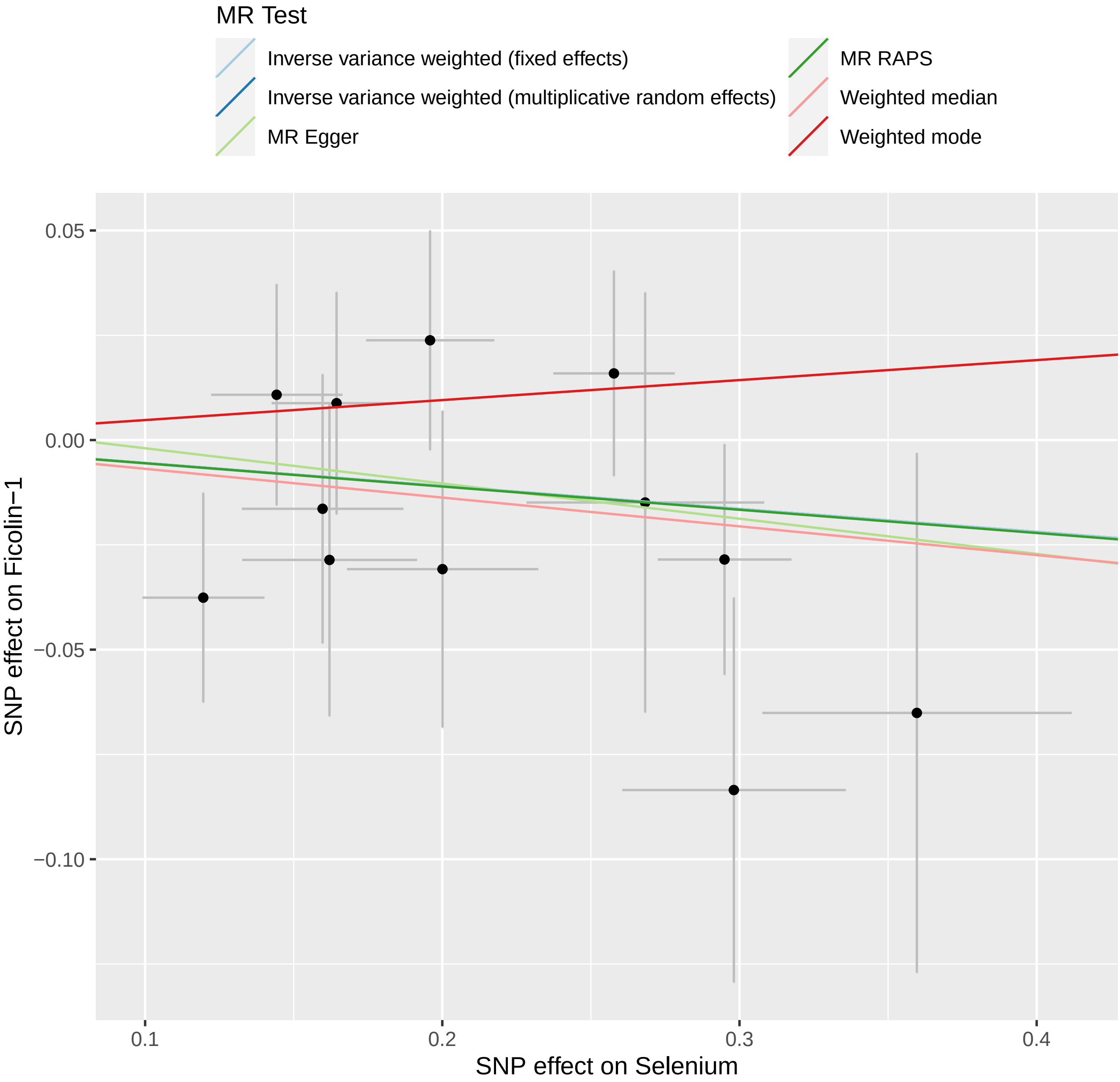

Batch 3−1078 : Selenium on Ficolin−2

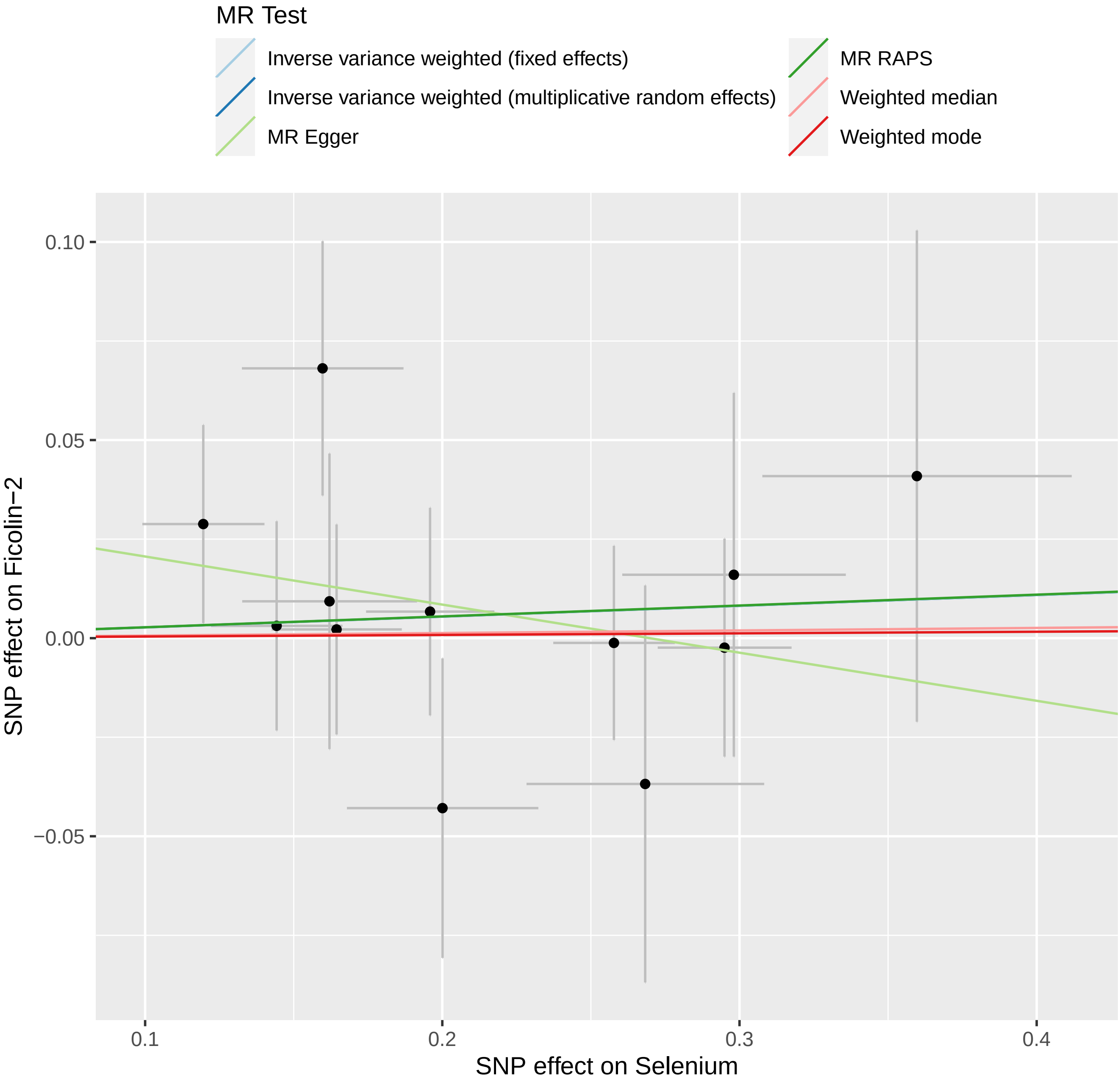

Batch 3−1079 : Selenium on Fc receptor−like protein 1

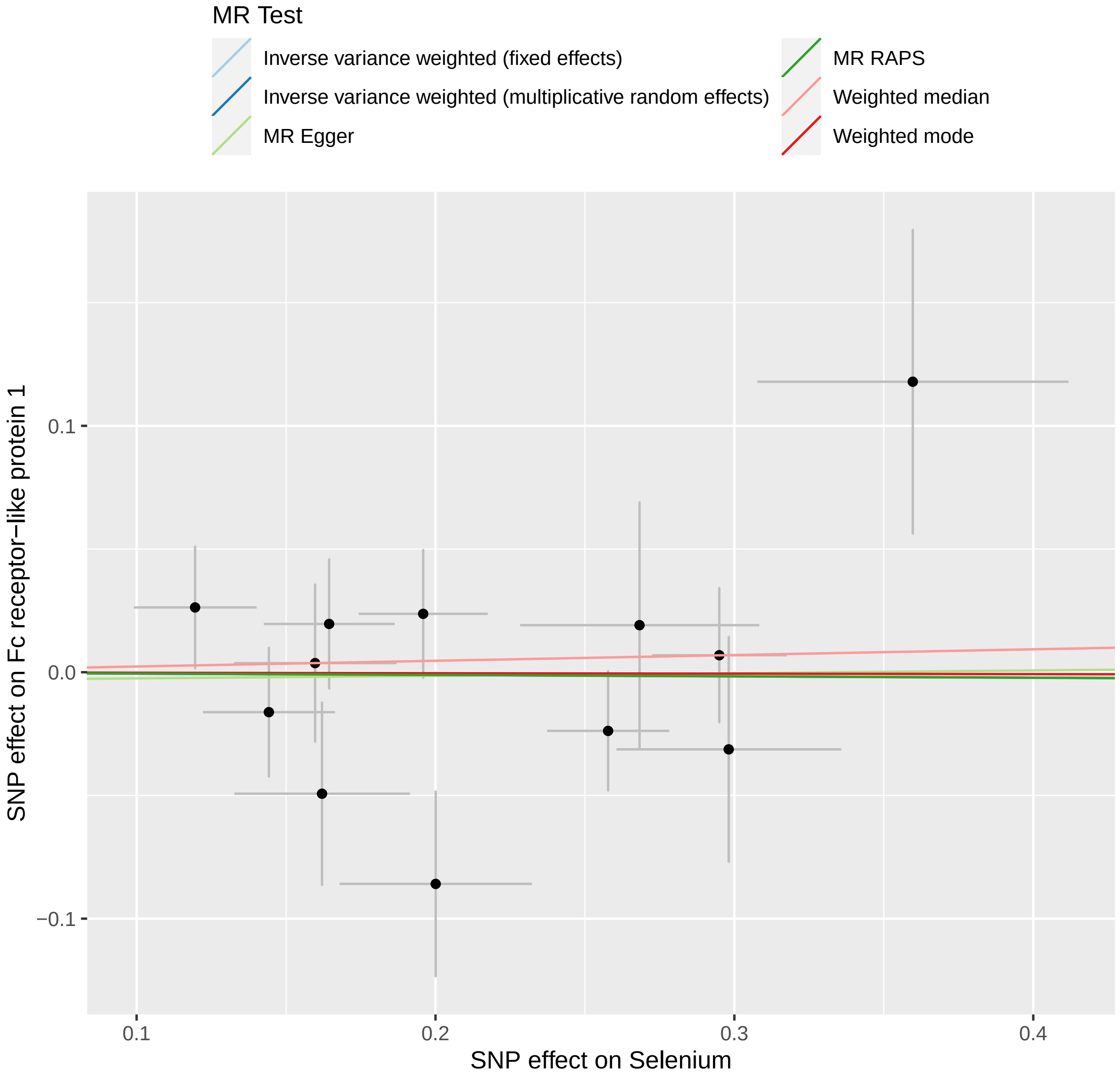

Batch 3−1080 : Selenium on Fc receptor−like protein 2

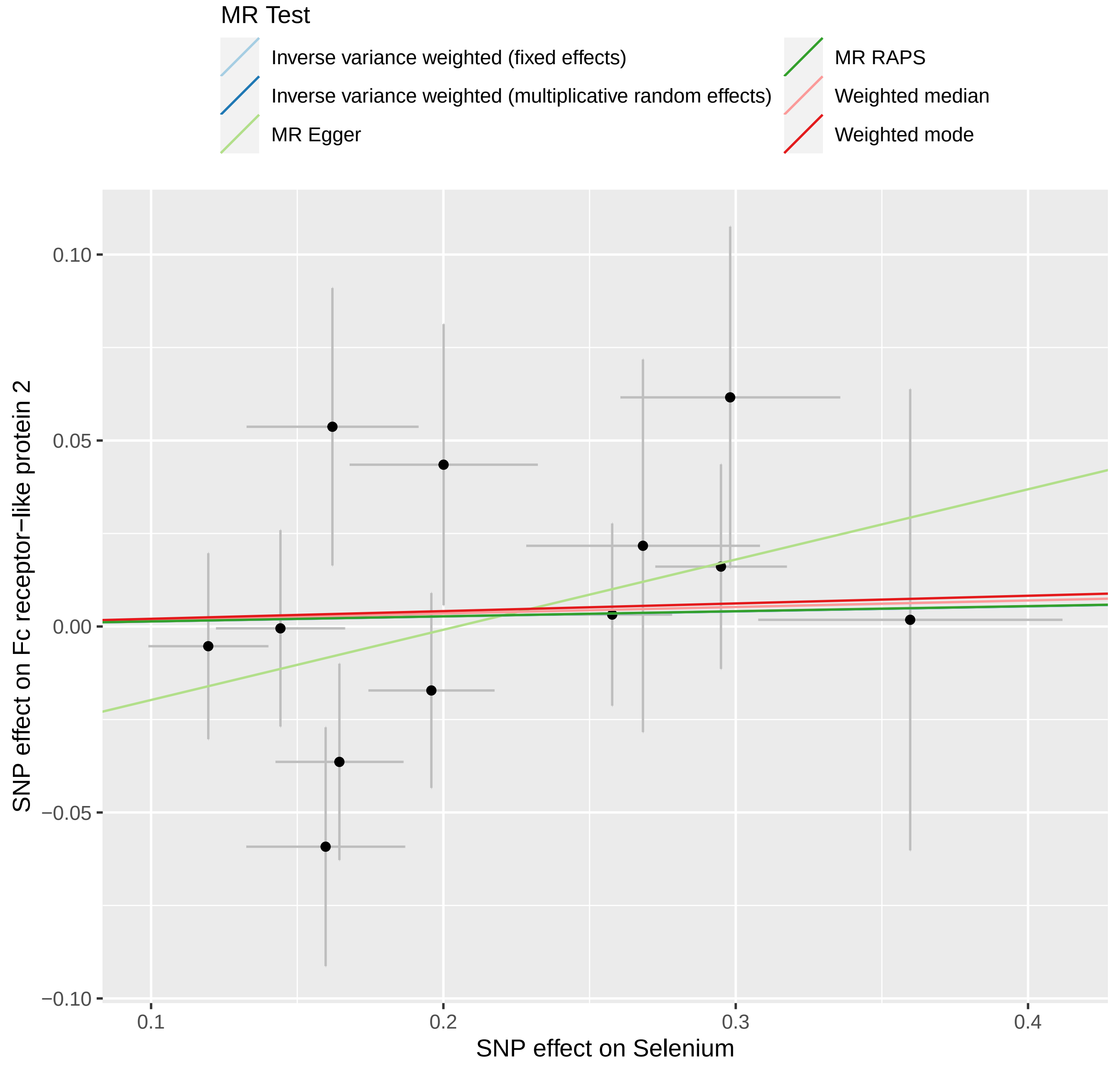

Batch 3−1081 : Selenium on Fc receptor−like protein 3

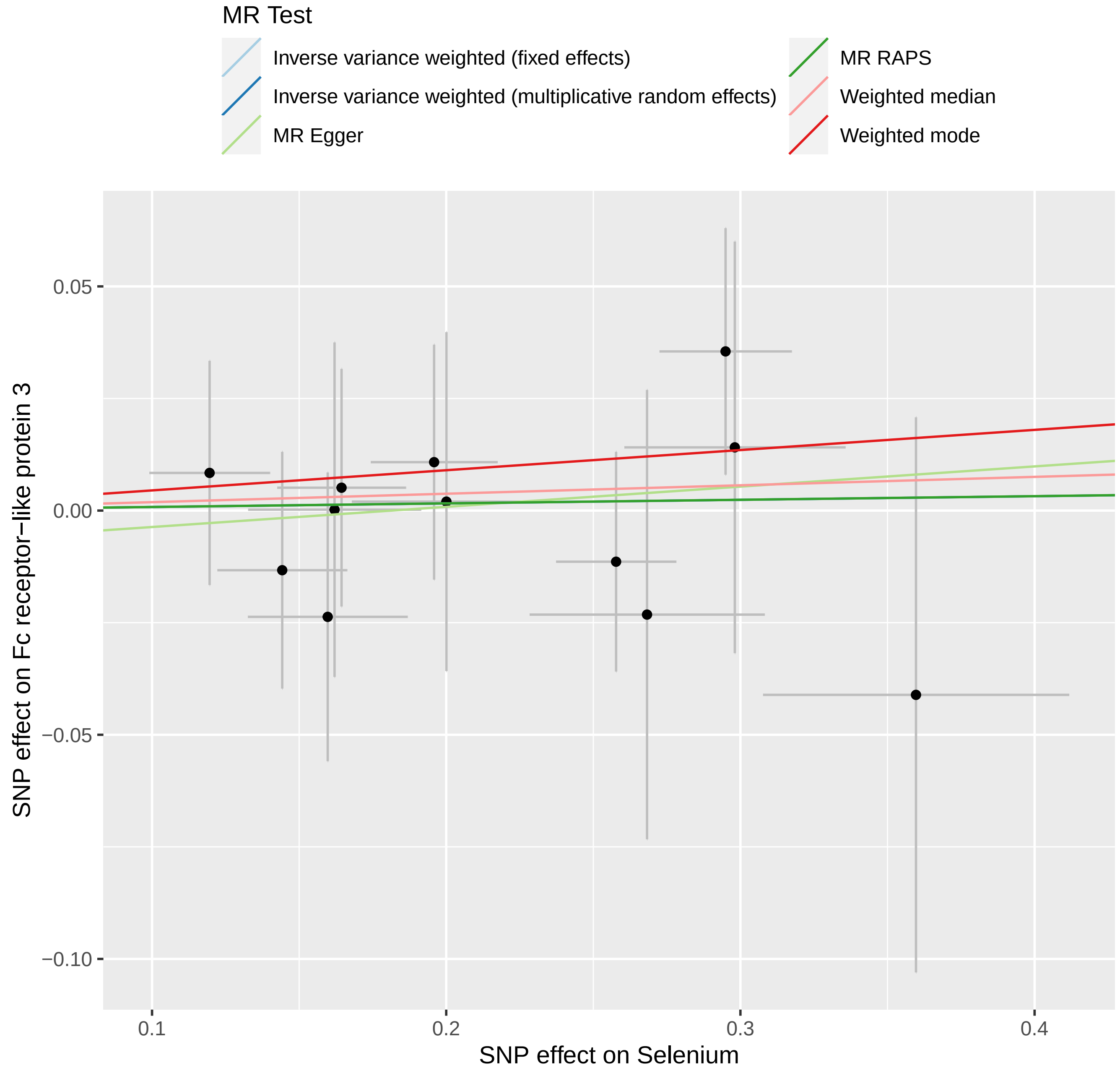

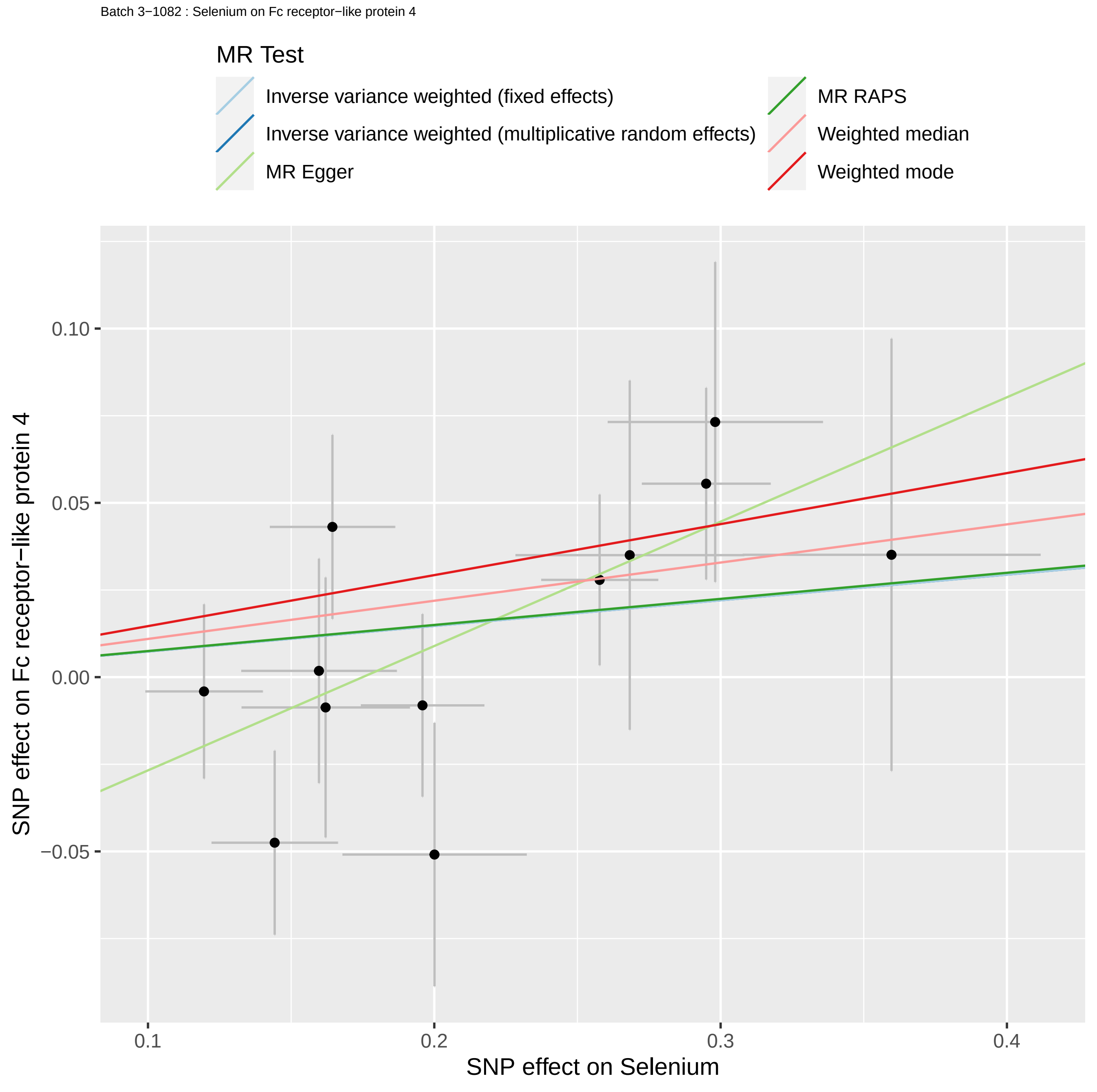

Batch 3−1082 : Selenium on Fc receptor−like protein 4
MR Test
Inverse variance weighted (fixed effects)
Inverse variance weighted (multiplicative random effects)
MR Egger
MR RAPS
Weighted median
Weighted mode
0.10
0.05
0.00
−0.05
0.1
0.2
0.3
0.4
SNP effect on Fc receptor−like protein 4
SNP effect on Selenium

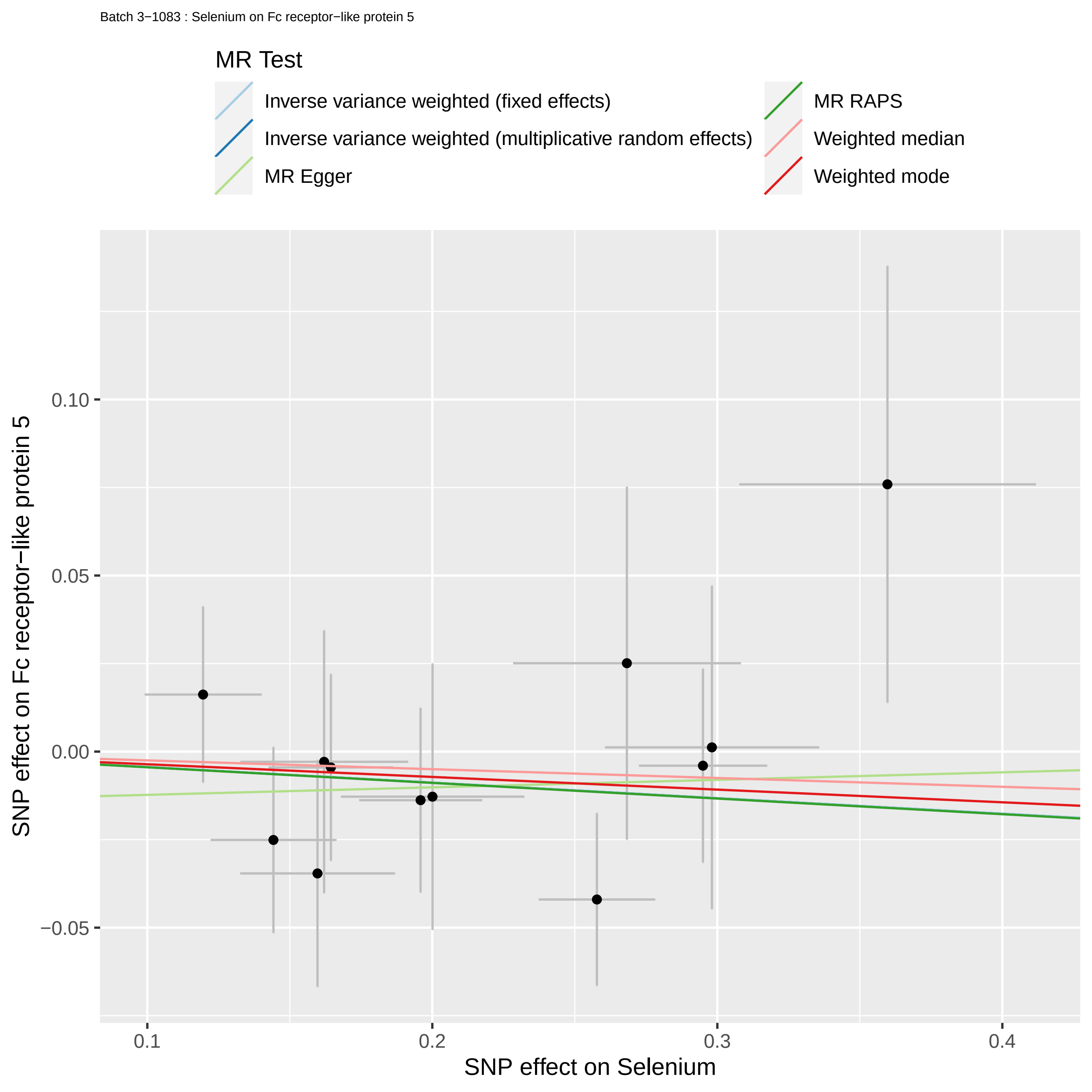
Batch 3−1083 : Selenium on Fc receptor−like protein 5
MR Test
Inverse variance weighted (fixed effects)
Inverse variance weighted (multiplicative random effects)
MR Egger
MR RAPS
Weighted median
Weighted mode
0.10
0.05
0.00
−0.05
0.1
0.2
0.3
0.4
SNP effect on Fc receptor−like protein 5
SNP effect on Selenium

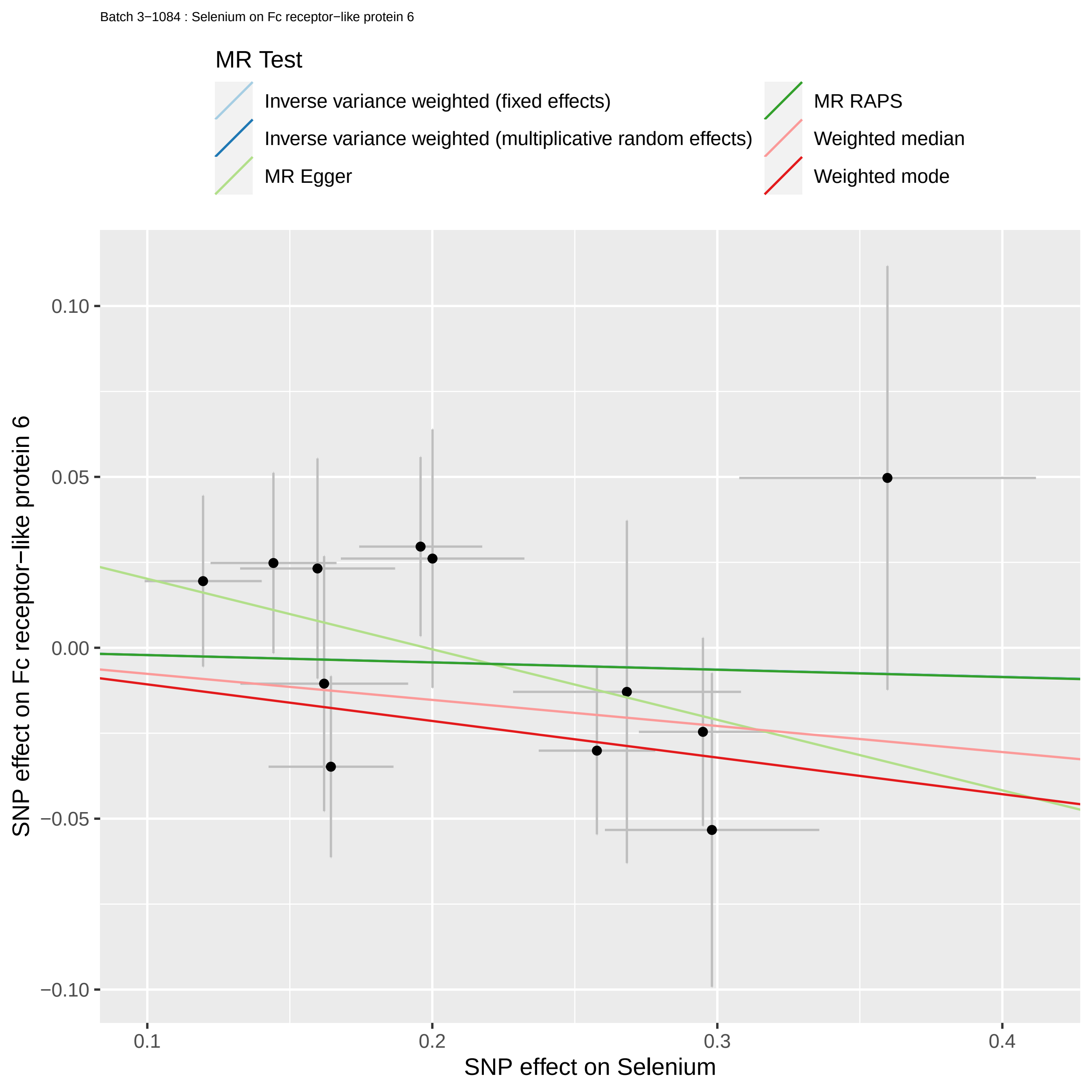
Batch 3−1084 : Selenium on Fc receptor−like protein 6
MR Test
Inverse variance weighted (fixed effects)
Inverse variance weighted (multiplicative random effects)
MR Egger
MR RAPS
Weighted median
Weighted mode
0.10
0.05
0.00
−0.05
−0.10
0.1
0.2
0.3
0.4
SNP effect on Selenium
SNP effect on Fc receptor−like protein 6

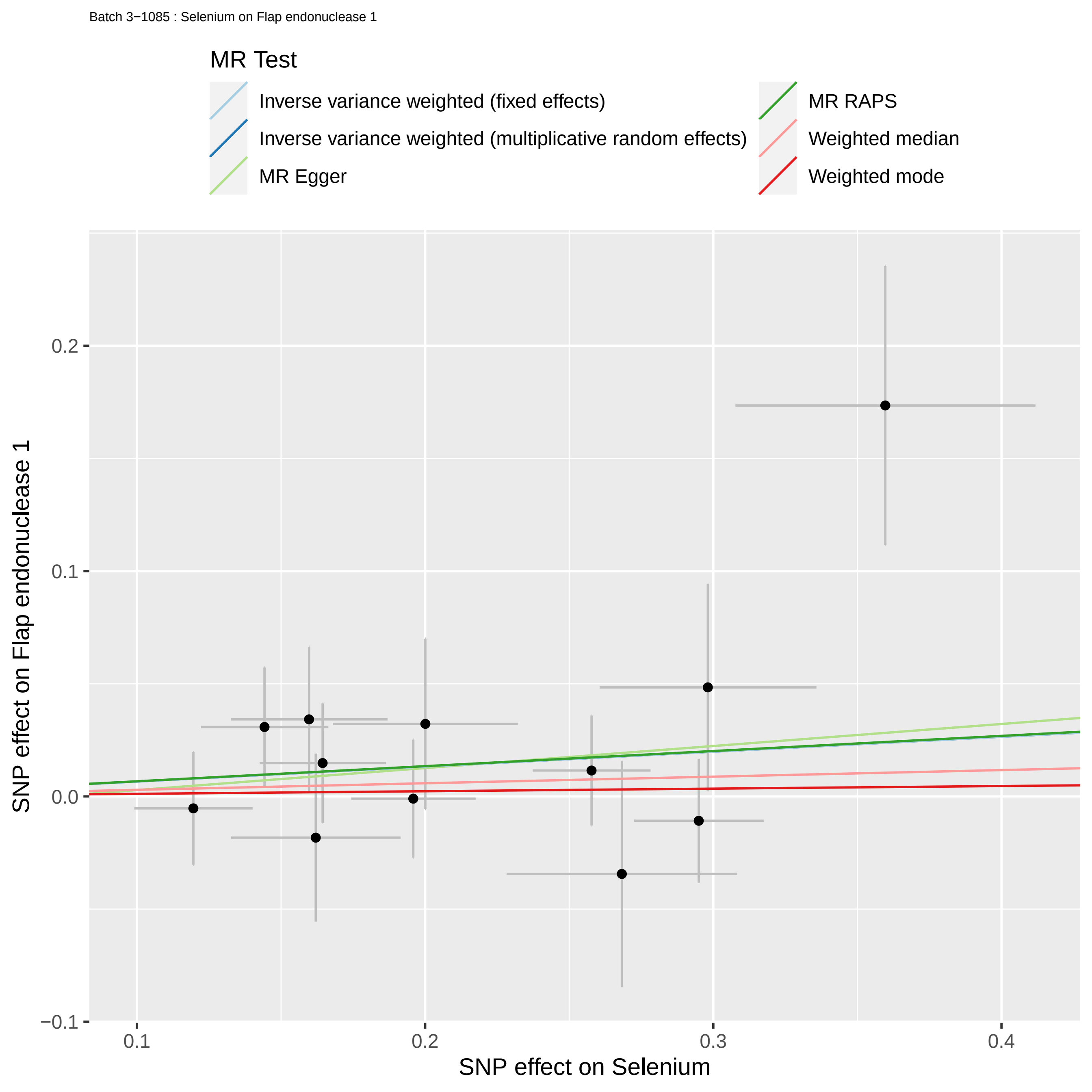

Batch 3−1085 : Selenium on Flap endonuclease 1
MR Test
Inverse variance weighted (fixed effects)
Inverse variance weighted (multiplicative random effects)
MR Egger
MR RAPS
Weighted median
Weighted mode
SNP effect on Flap endonuclease 1
0.2
0.1
0.0
−0.1
0.1
0.2
0.3
0.4
SNP effect on Selenium

Batch 3−1086 : Selenium on D−dimer

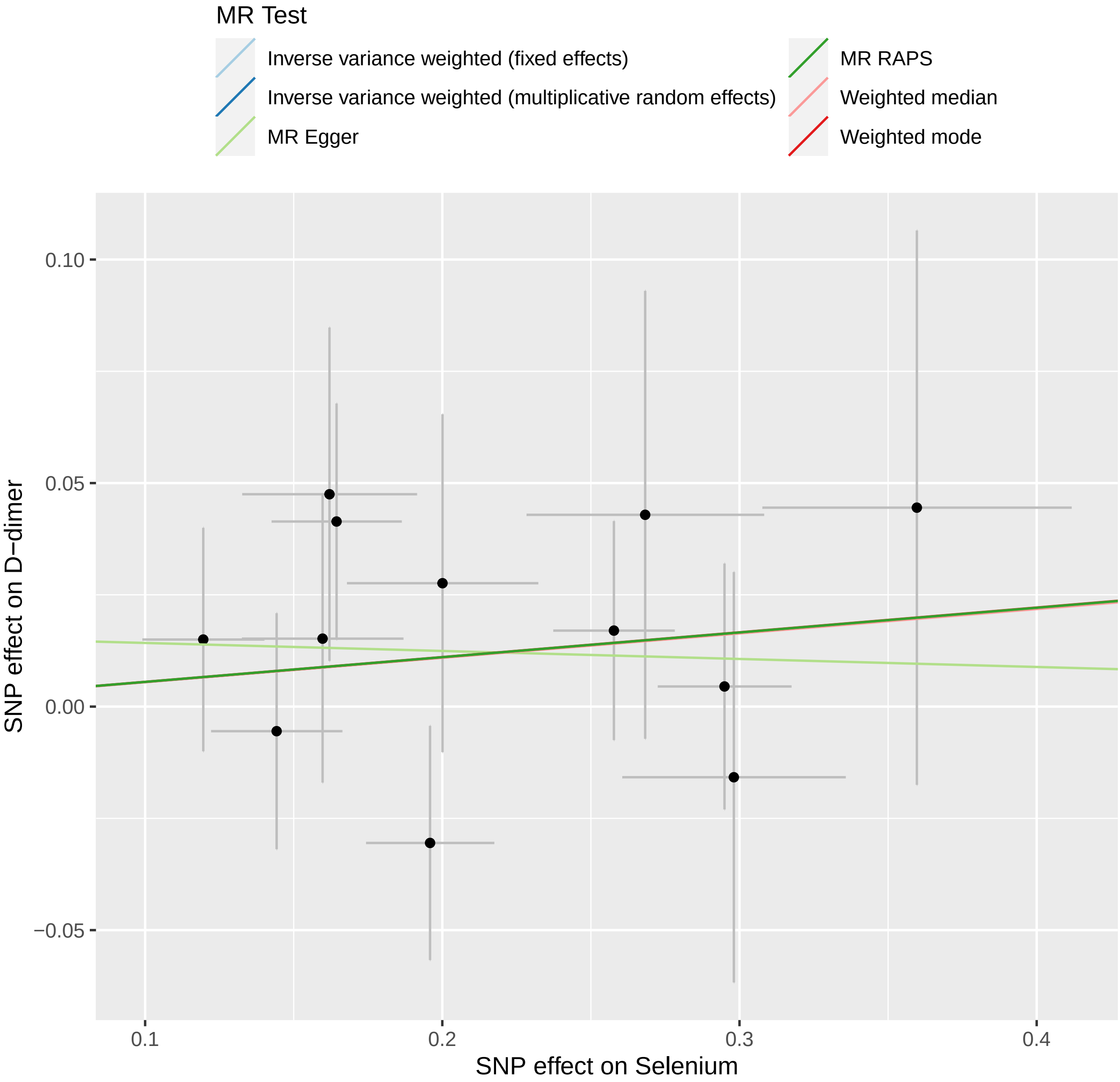

Batch 3−1087 : Selenium on Fibroblast growth factor 10

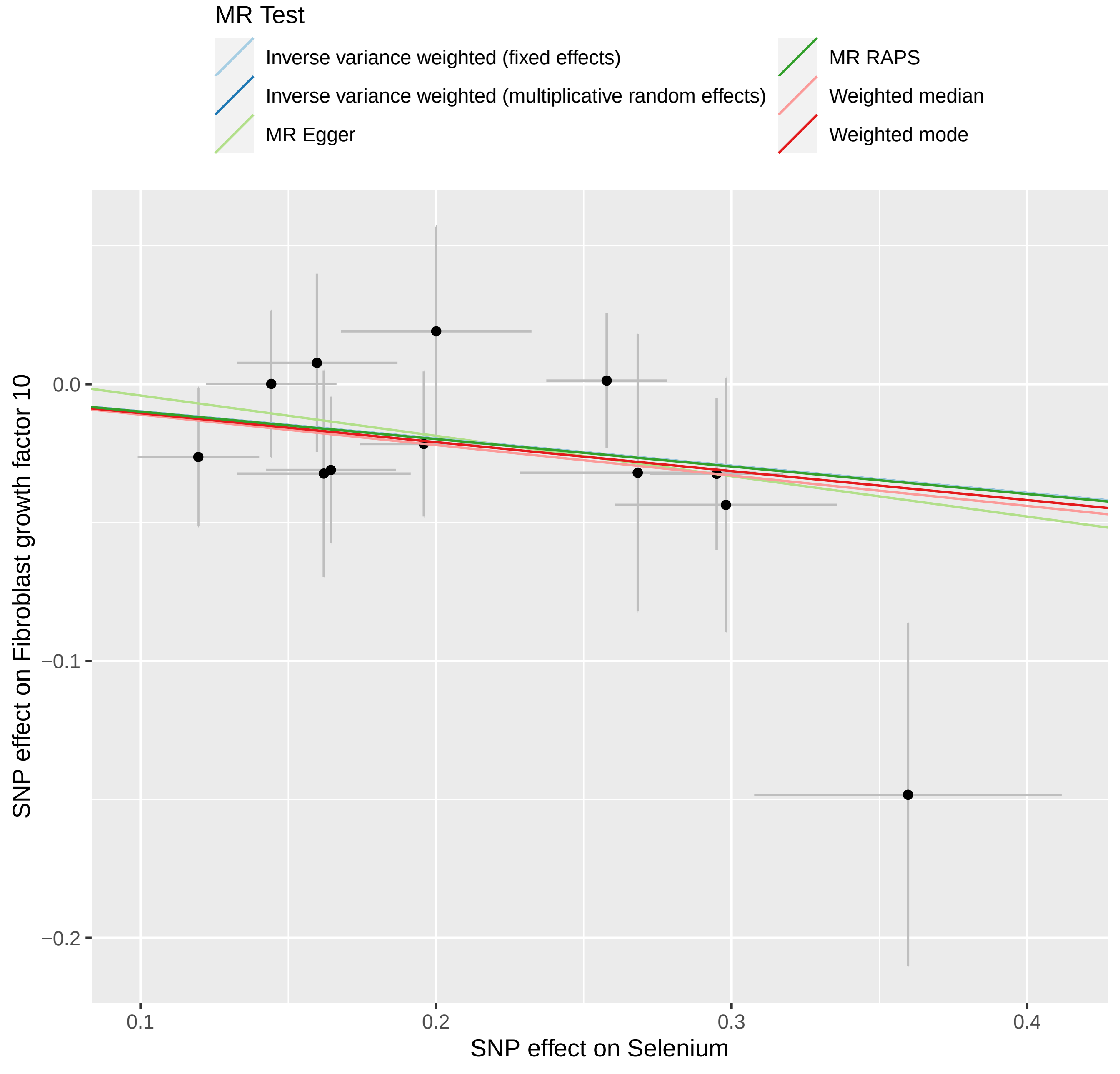

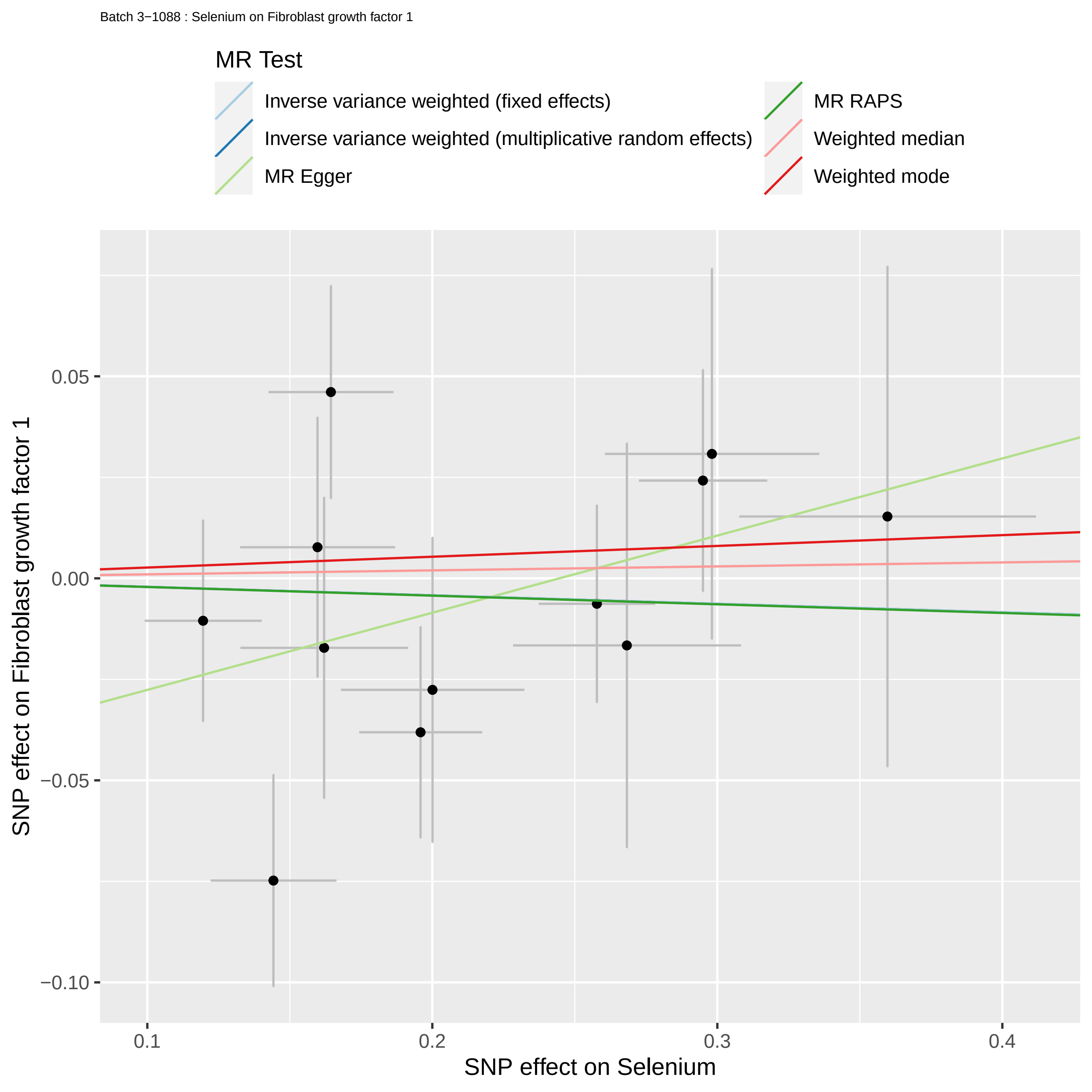
Batch 3−1088 : Selenium on Fibroblast growth factor 1
MR Test
Inverse variance weighted (fixed effects)
Inverse variance weighted (multiplicative random effects)
MR Egger
MR RAPS
Weighted median
Weighted mode
0.05
0.00
−0.05
−0.10
0.1
0.2
0.3
0.4
SNP effect on Selenium
SNP effect on Fibroblast growth factor 1

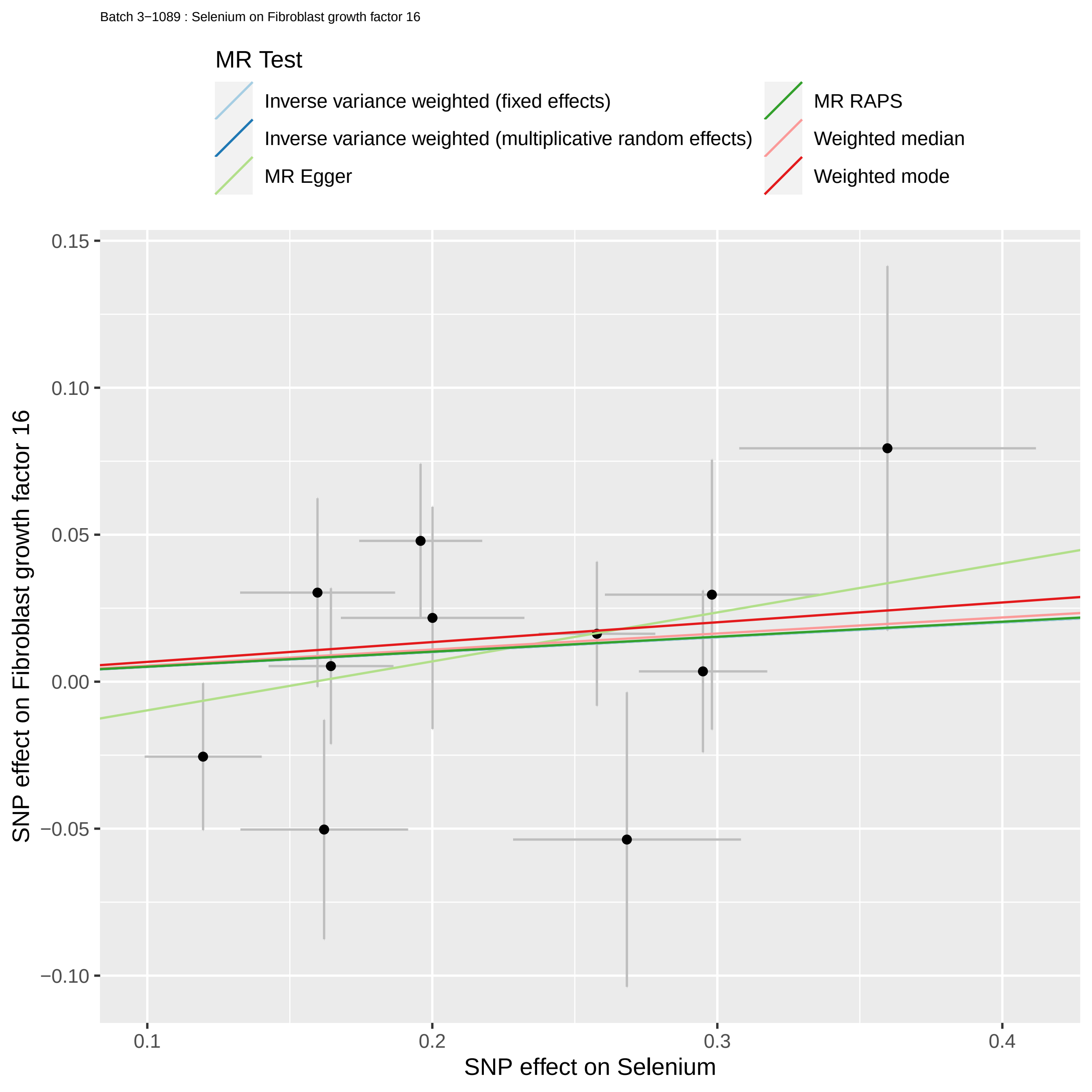
Batch 3−1089 : Selenium on Fibroblast growth factor 16
MR Test
Inverse variance weighted (fixed effects)
Inverse variance weighted (multiplicative random effects)
MR Egger
MR RAPS
Weighted median
Weighted mode
0.15
0.10
0.05
0.00
−0.05
−0.10
0.1
0.2
0.3
0.4
SNP effect on Fibroblast growth factor 16
SNP effect on Selenium

Batch 3−1090 : Selenium on Fibroblast growth factor 16

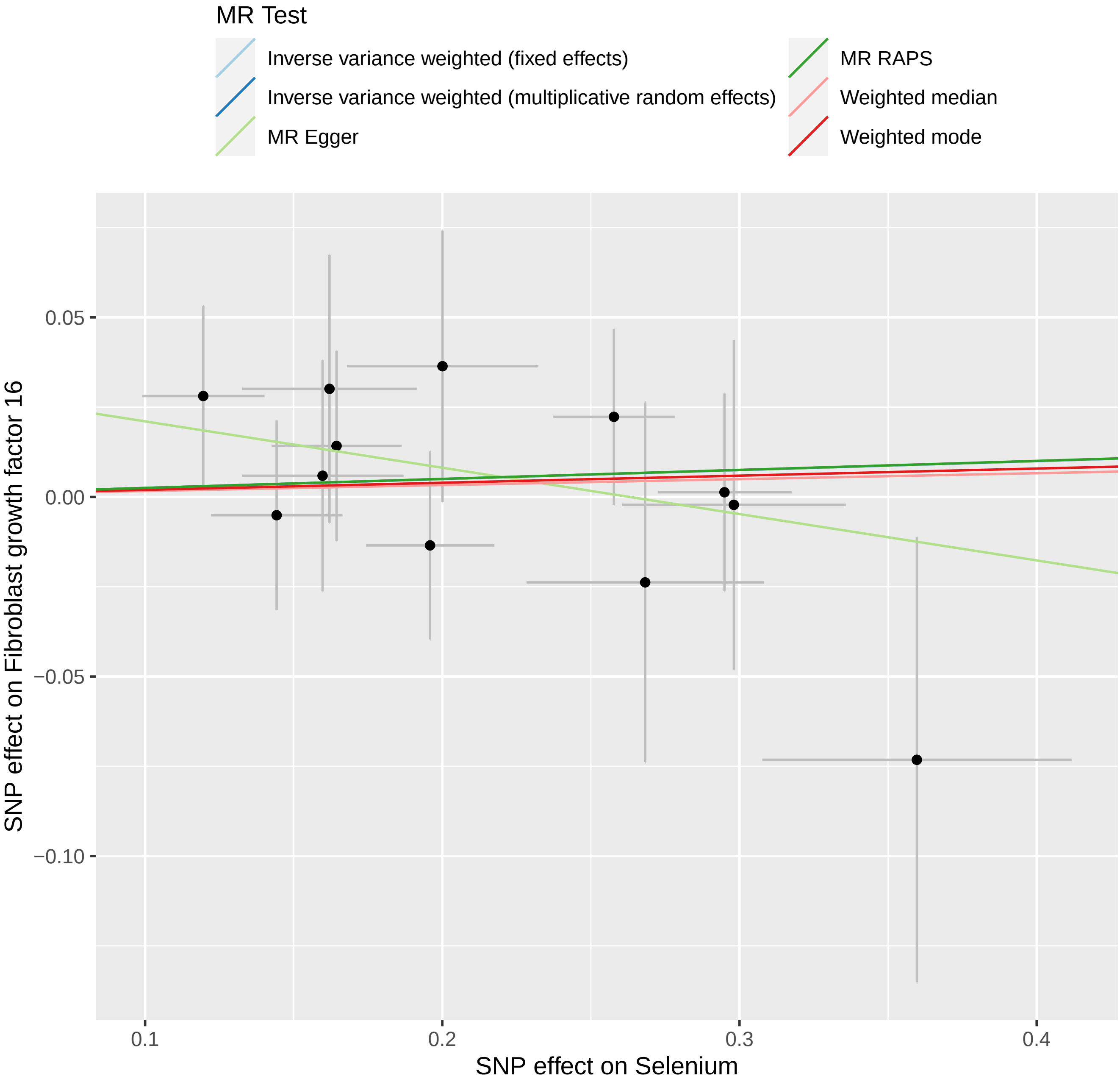

Batch 3−1091 : Selenium on Fibroblast growth factor 19

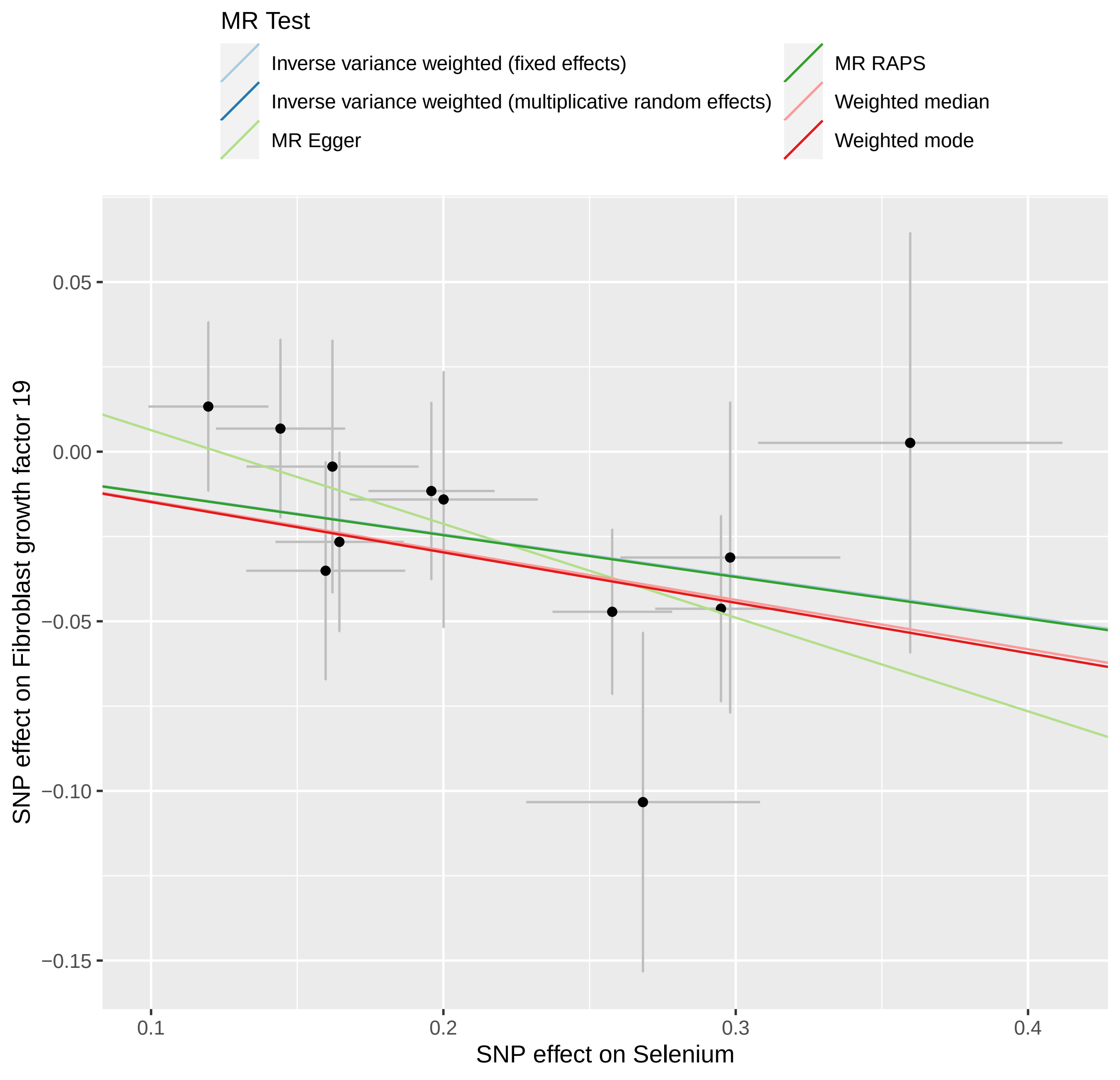

Batch 3−1092 : Selenium on Fibroblast growth factor 20

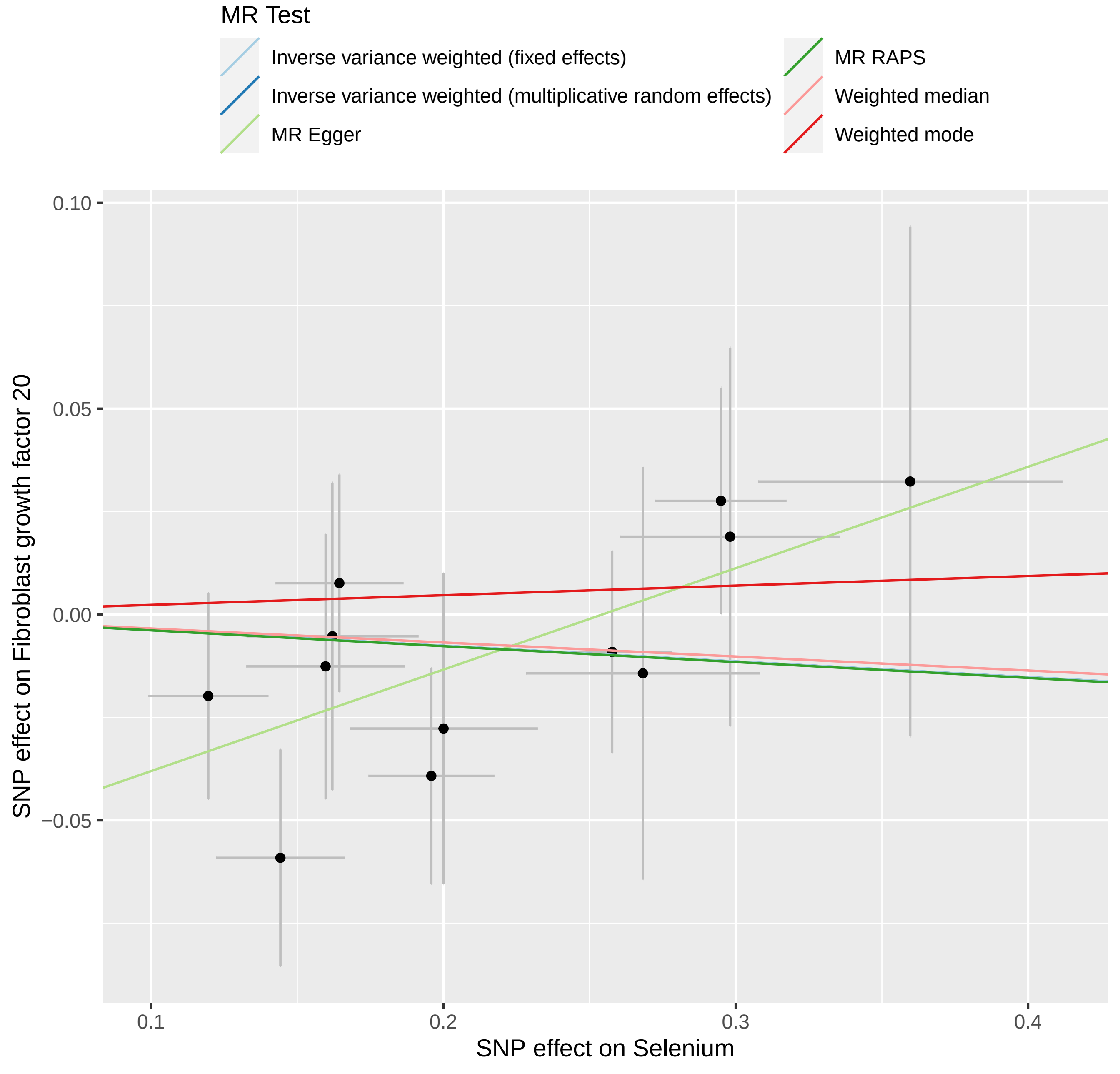

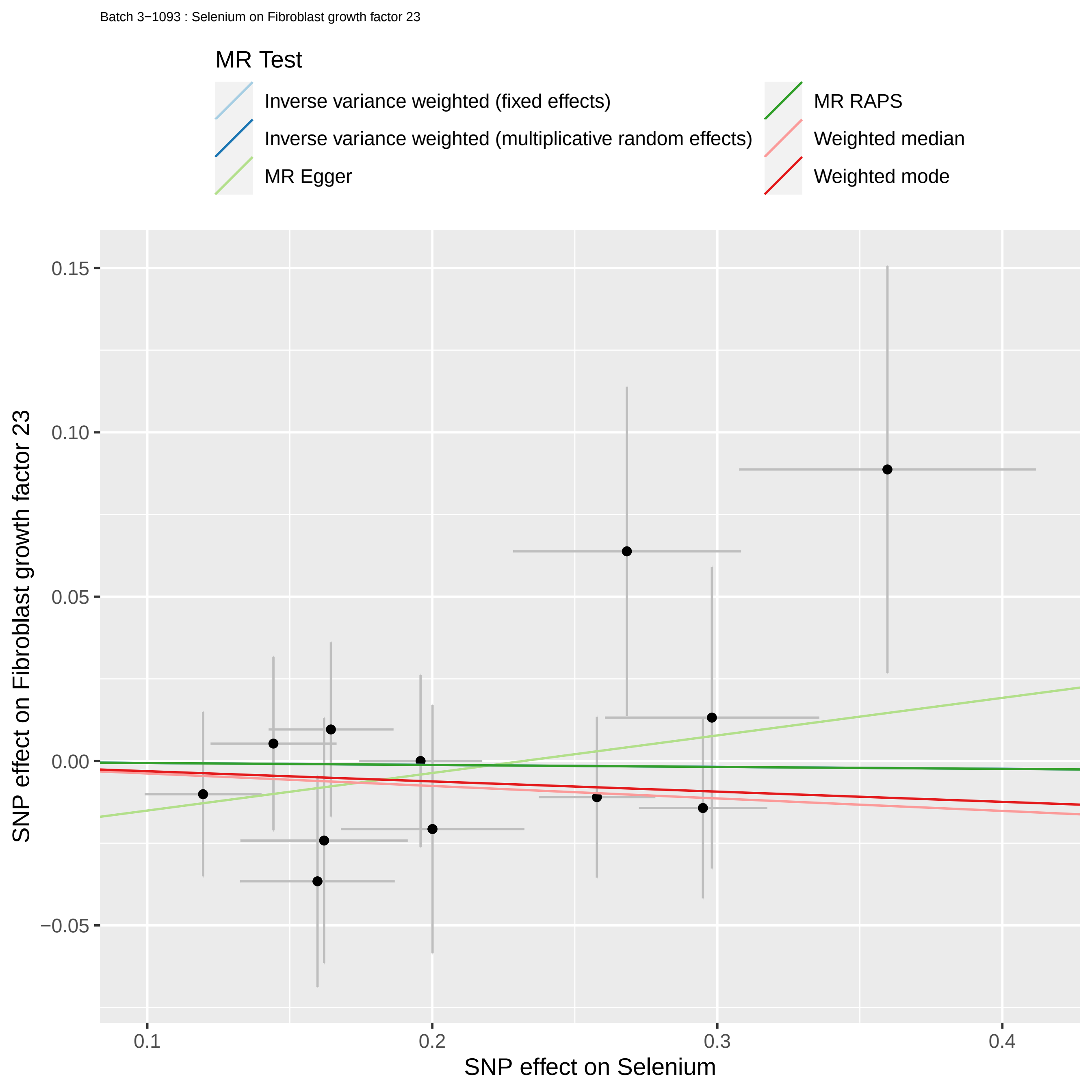
Batch 3−1093 : Selenium on Fibroblast growth factor 23
MR Test
Inverse variance weighted (fixed effects)
Inverse variance weighted (multiplicative random effects)
MR Egger
MR RAPS
Weighted median
Weighted mode
SNP effect on Fibroblast growth factor 23
0.15
0.10
0.05
0.00
−0.05
0.1
0.2
0.3
0.4
SNP effect on Selenium

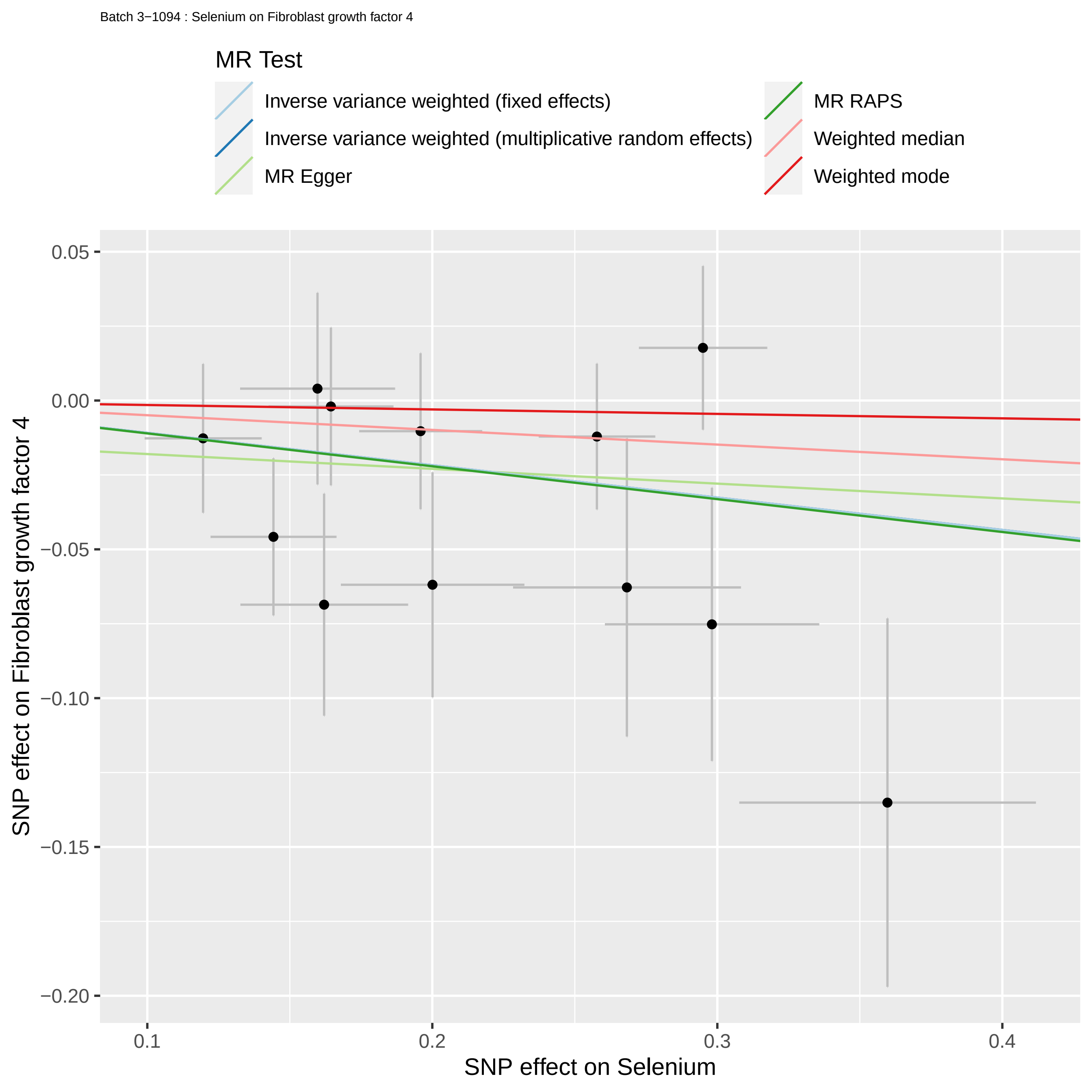

Batch 3−1094 : Selenium on Fibroblast growth factor 4
MR Test
Inverse variance weighted (fixed effects)
Inverse variance weighted (multiplicative random effects)
MR Egger
MR RAPS
Weighted median
Weighted mode
0.05
0.00
−0.05
−0.10
−0.15
−0.20
0.1
0.2
0.3
0.4
SNP effect on Fibroblast growth factor 4
SNP effect on Selenium

Batch 3−1095 : Selenium on Fibroblast growth factor 5

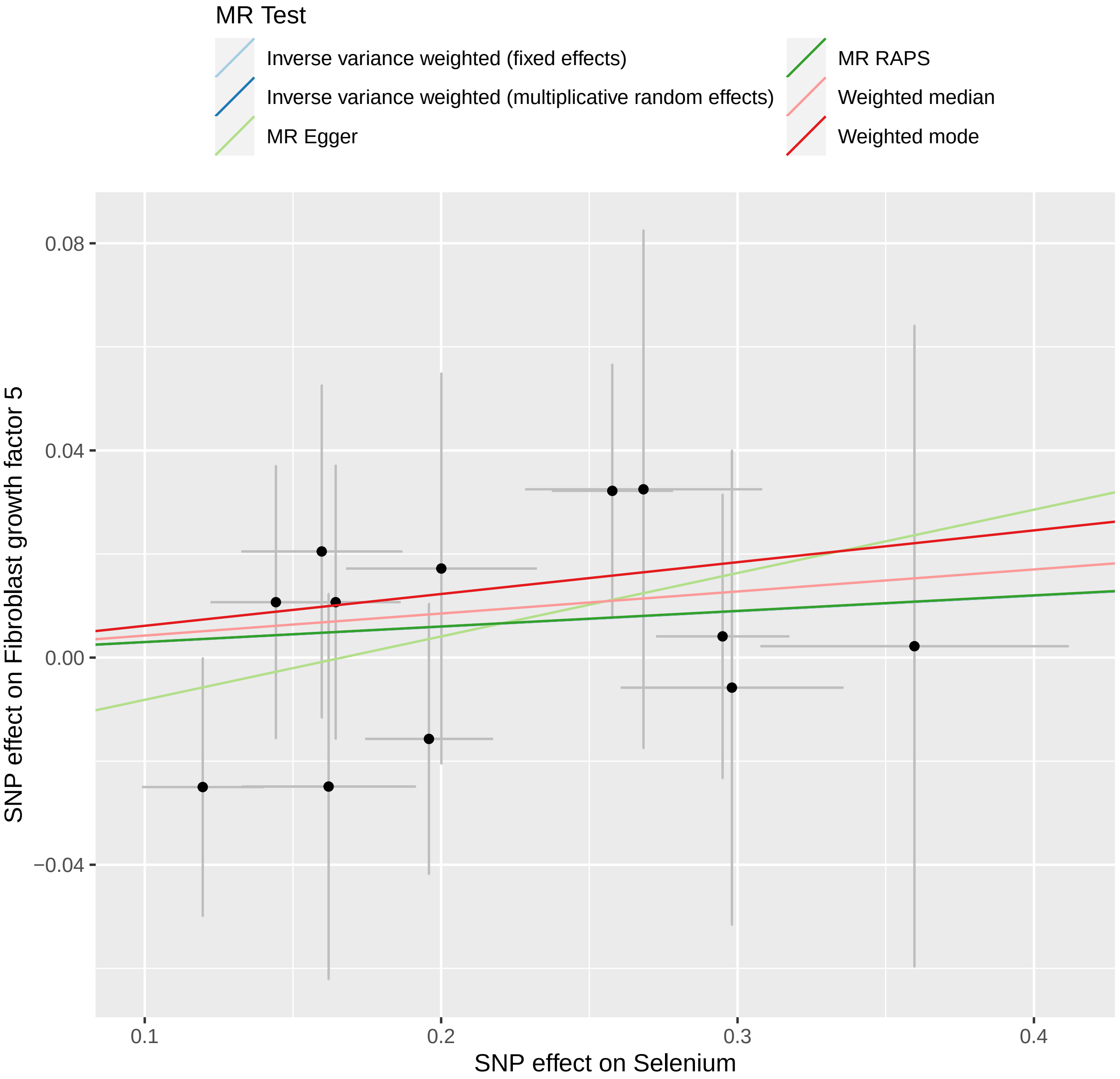

Batch 3−1096 : Selenium on Fibroblast growth factor 6

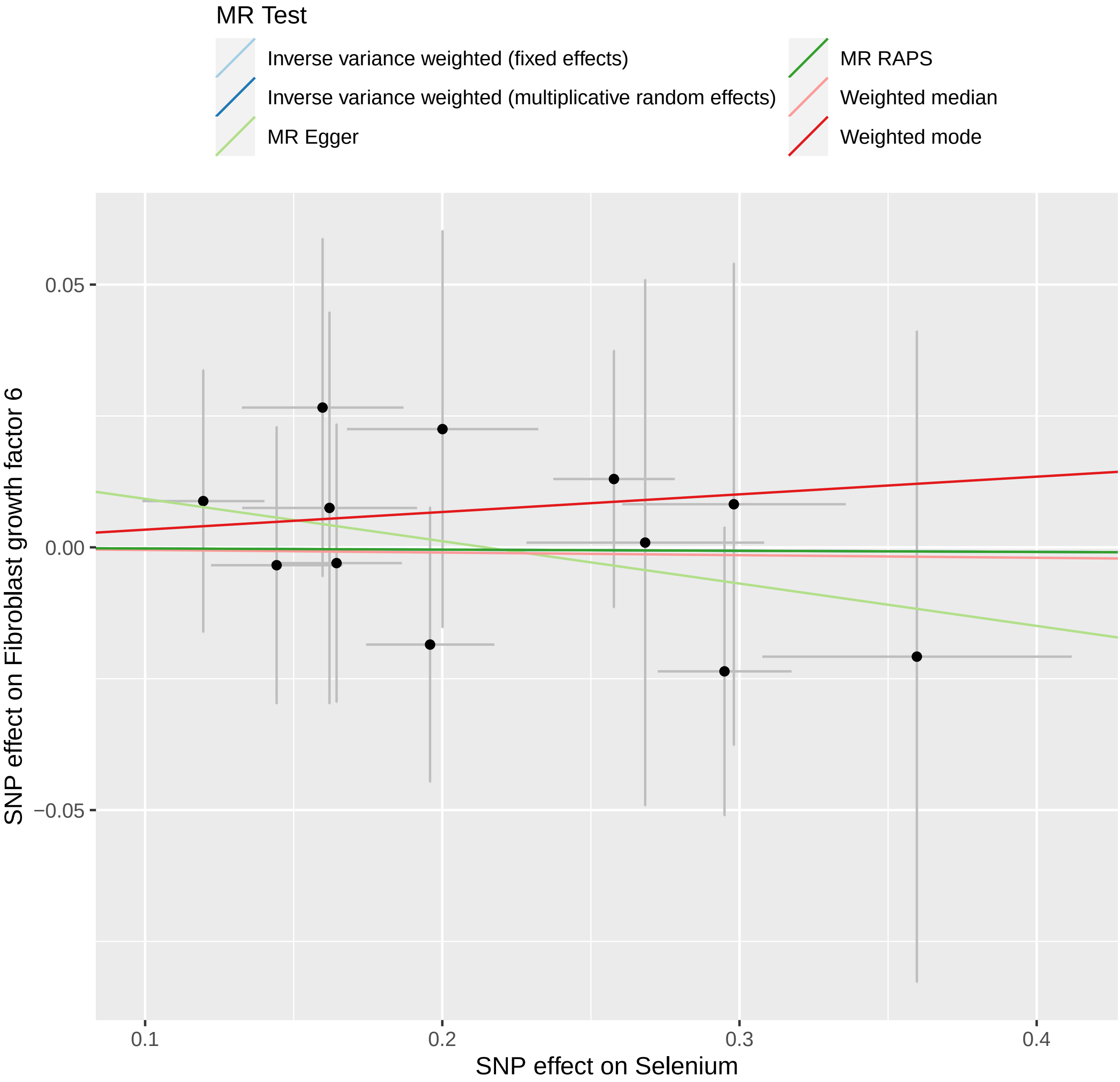

Batch 3−1097 : Selenium on Fibroblast growth factor 7

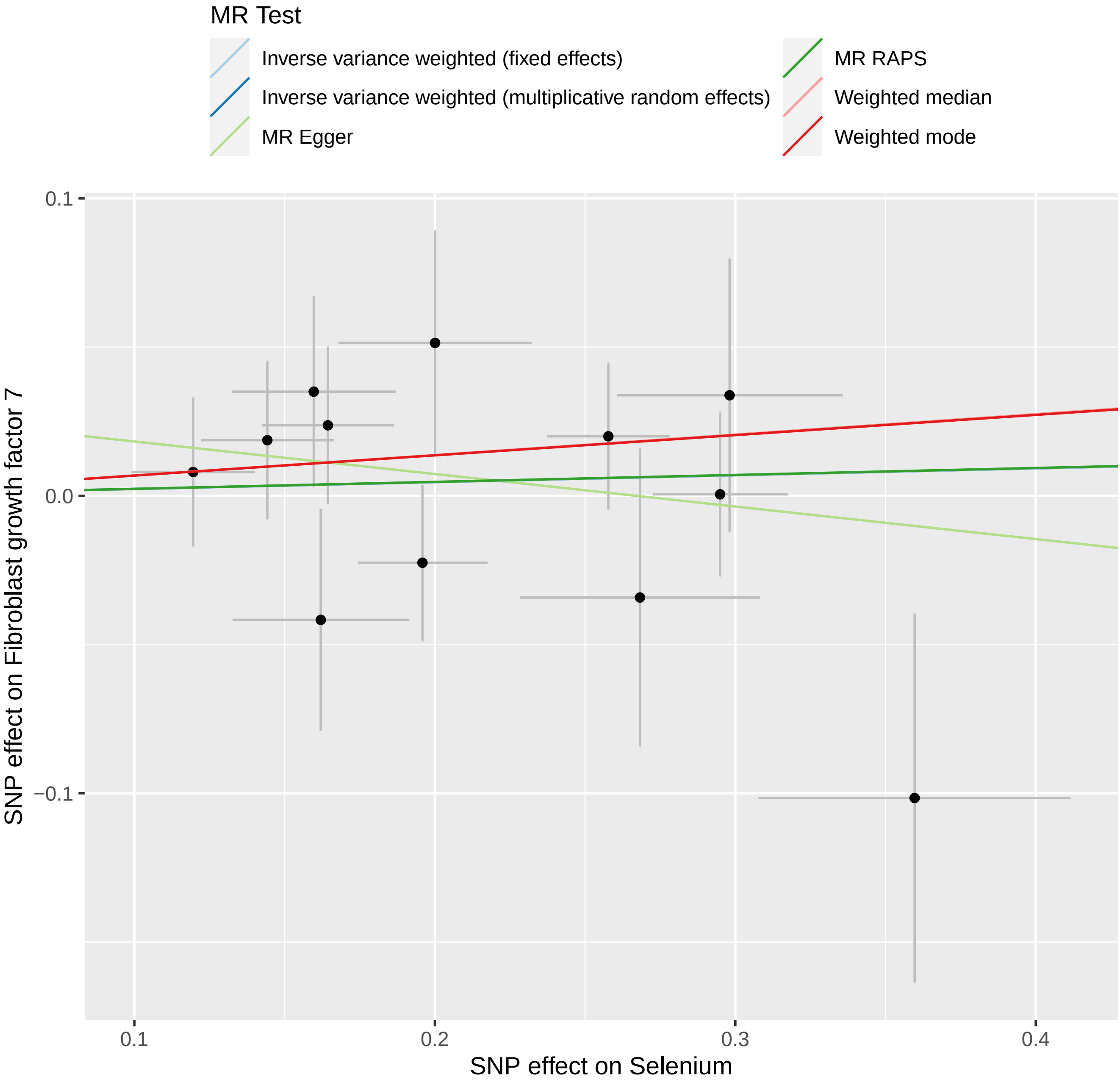

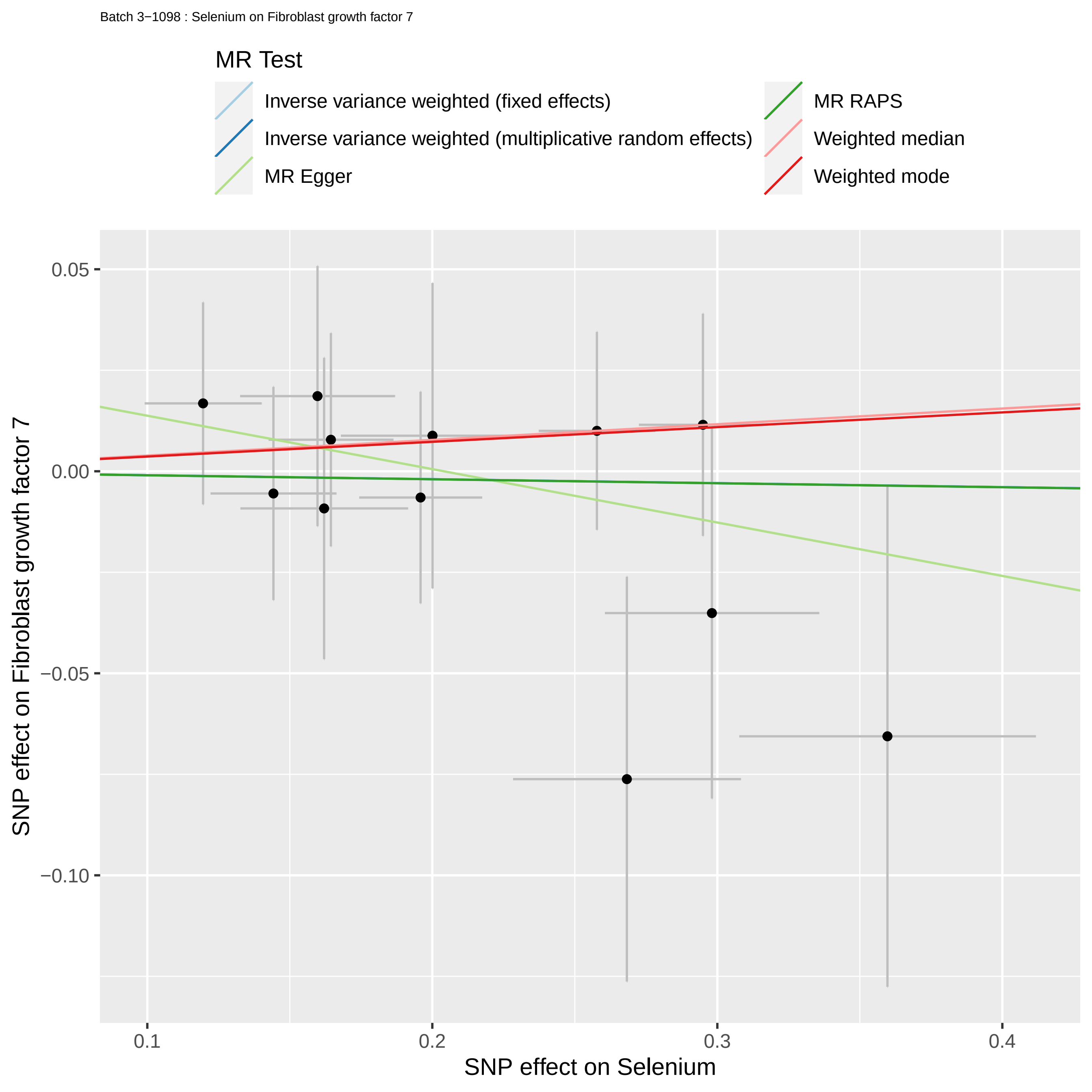

Batch 3−1098 : Selenium on Fibroblast growth factor 7
MR Test
Inverse variance weighted (fixed effects)
Inverse variance weighted (multiplicative random effects)
MR Egger
MR RAPS
Weighted median
Weighted mode
0.05
0.00
−0.05
−0.10
0.1
0.2
0.3
0.4
SNP effect on Fibroblast growth factor 7
SNP effect on Selenium

Batch 3−1099 : Selenium on Fibroblast growth factor 8 isoform B

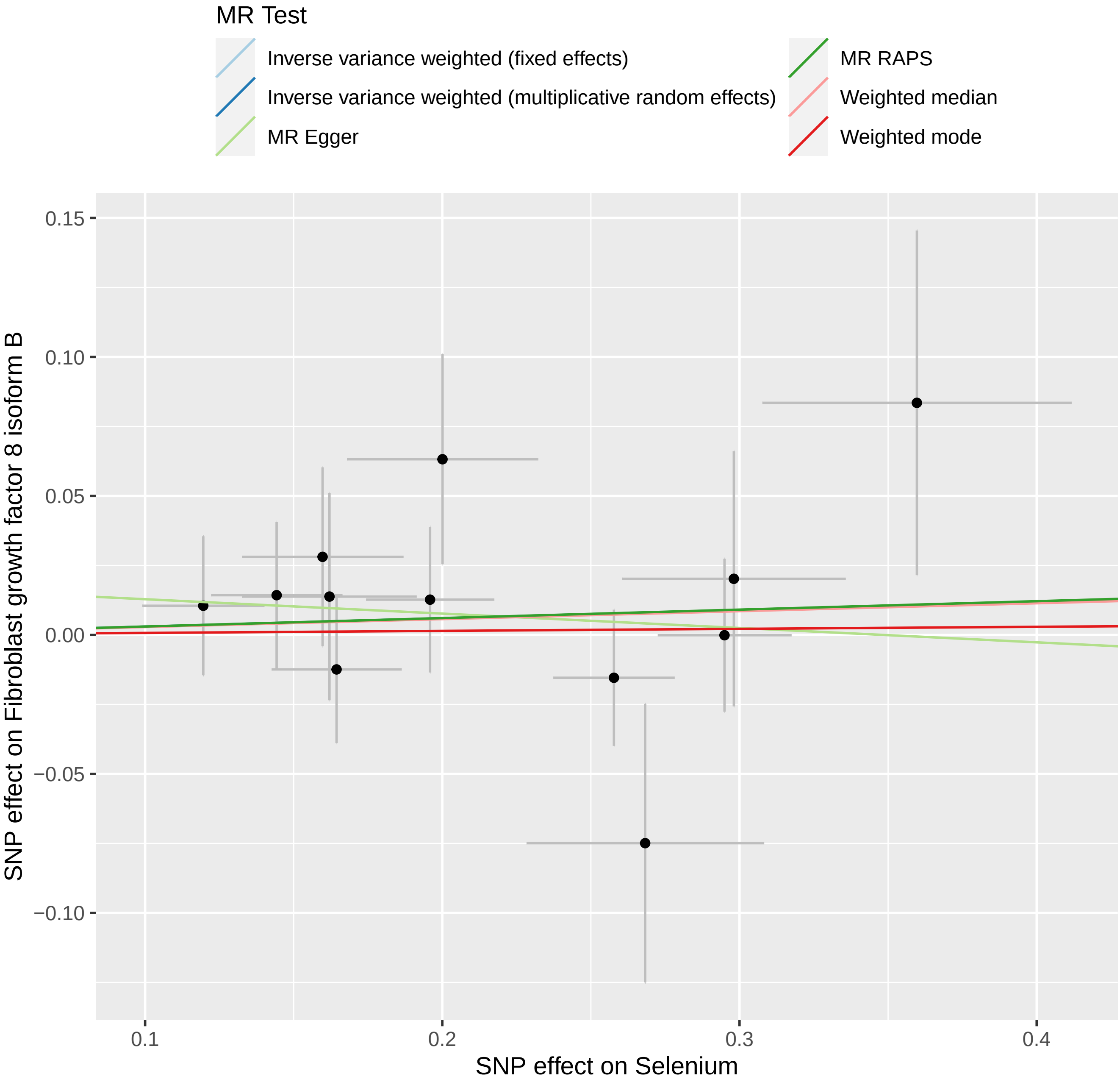

Batch 3−1100 : Selenium on Fibroblast growth factor 8 isoform A

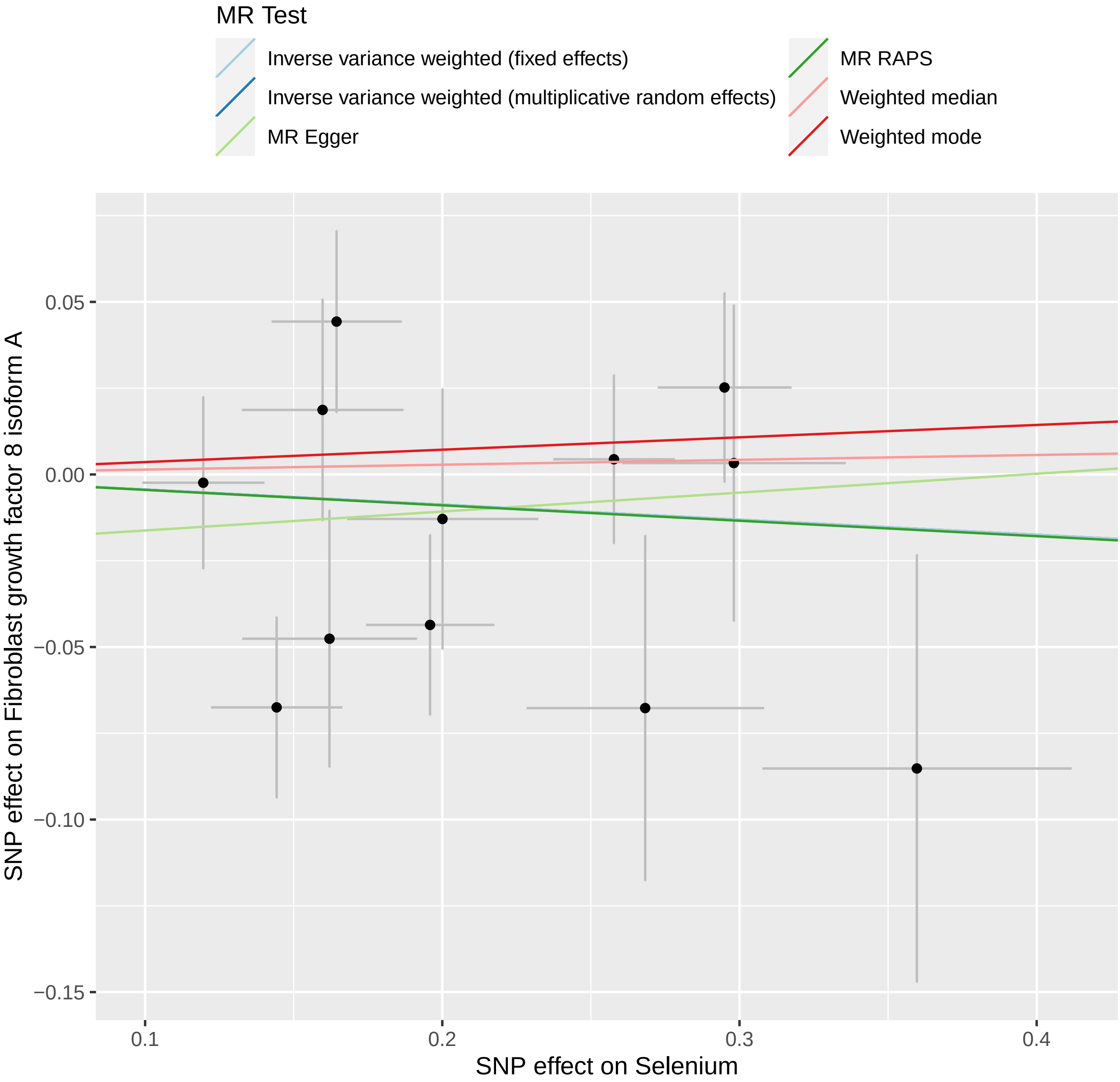

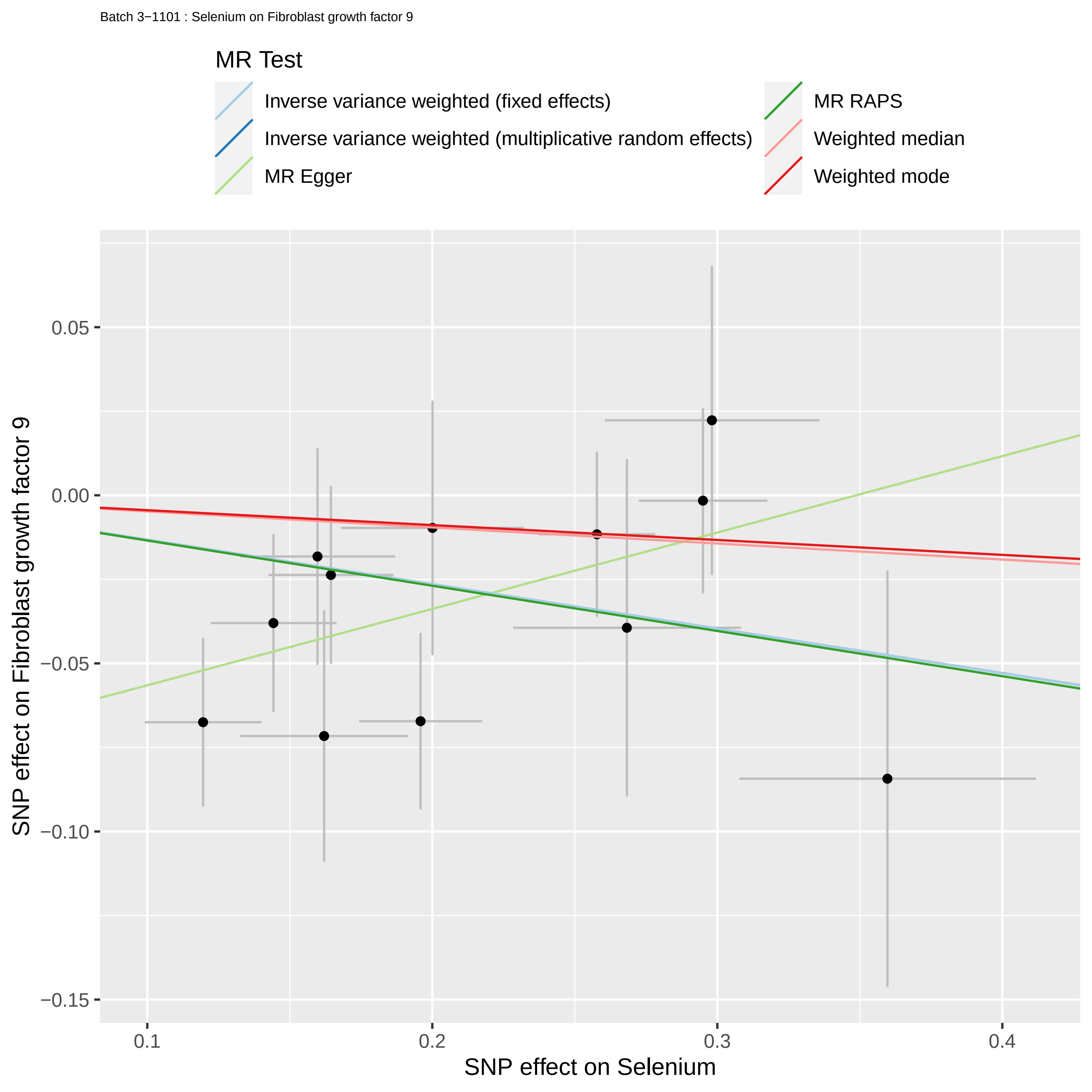
Batch 3−1101 : Selenium on Fibroblast growth factor 9
MR Test
Inverse variance weighted (fixed effects)
Inverse variance weighted (multiplicative random effects)
MR Egger
MR RAPS
Weighted median
Weighted mode
0.05
0.00
−0.05
−0.10
−0.15
0.1
0.2
0.3
0.4
SNP effect on Fibroblast growth factor 9
SNP effect on Selenium

Batch 3−1102 : Selenium on Fibroblast growth factor receptor 2

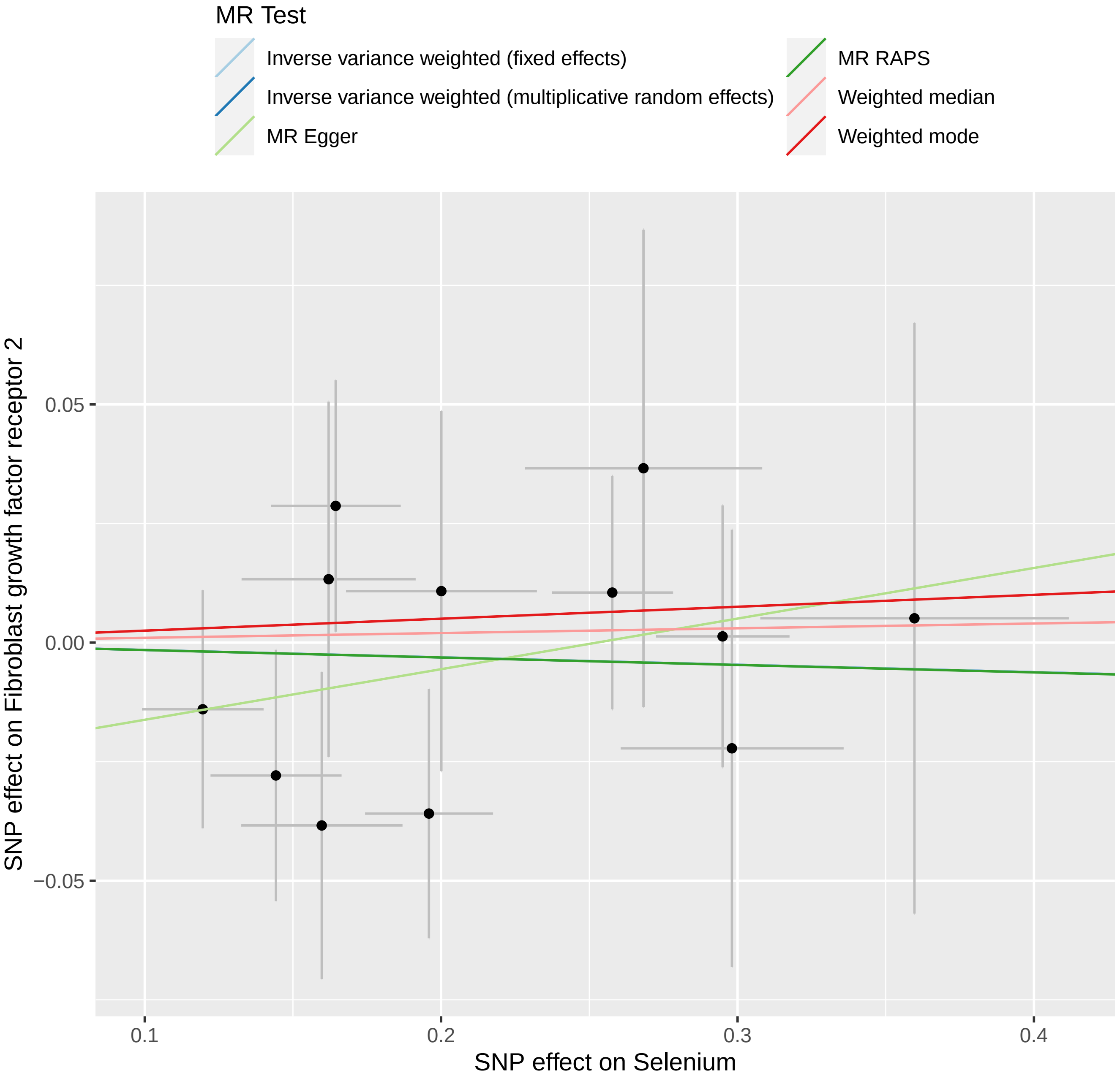

Batch 3−1103 : Selenium on Fibroblast growth factor receptor 3

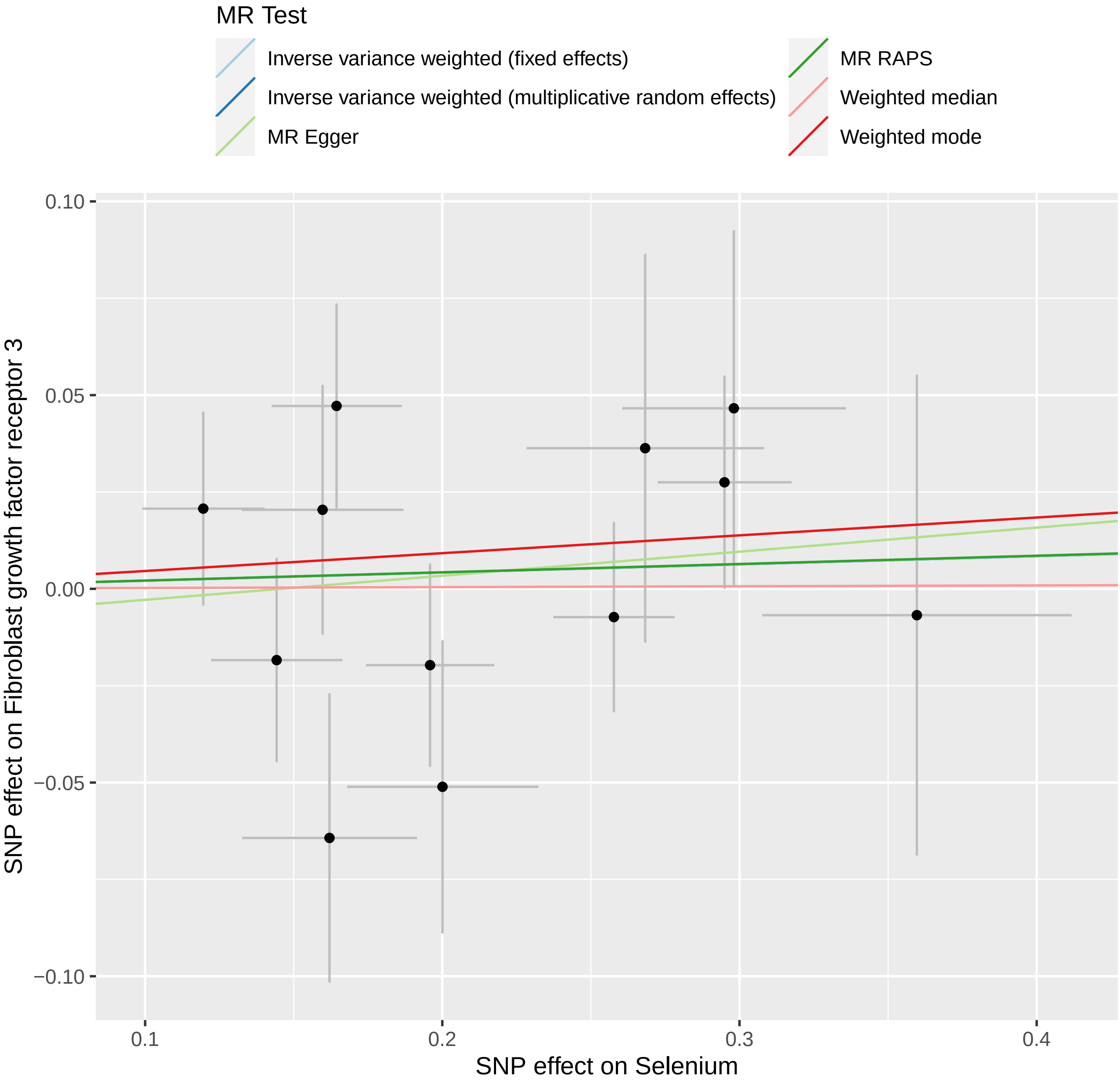

Batch 3−1104 : Selenium on Fibroblast growth factor receptor 3

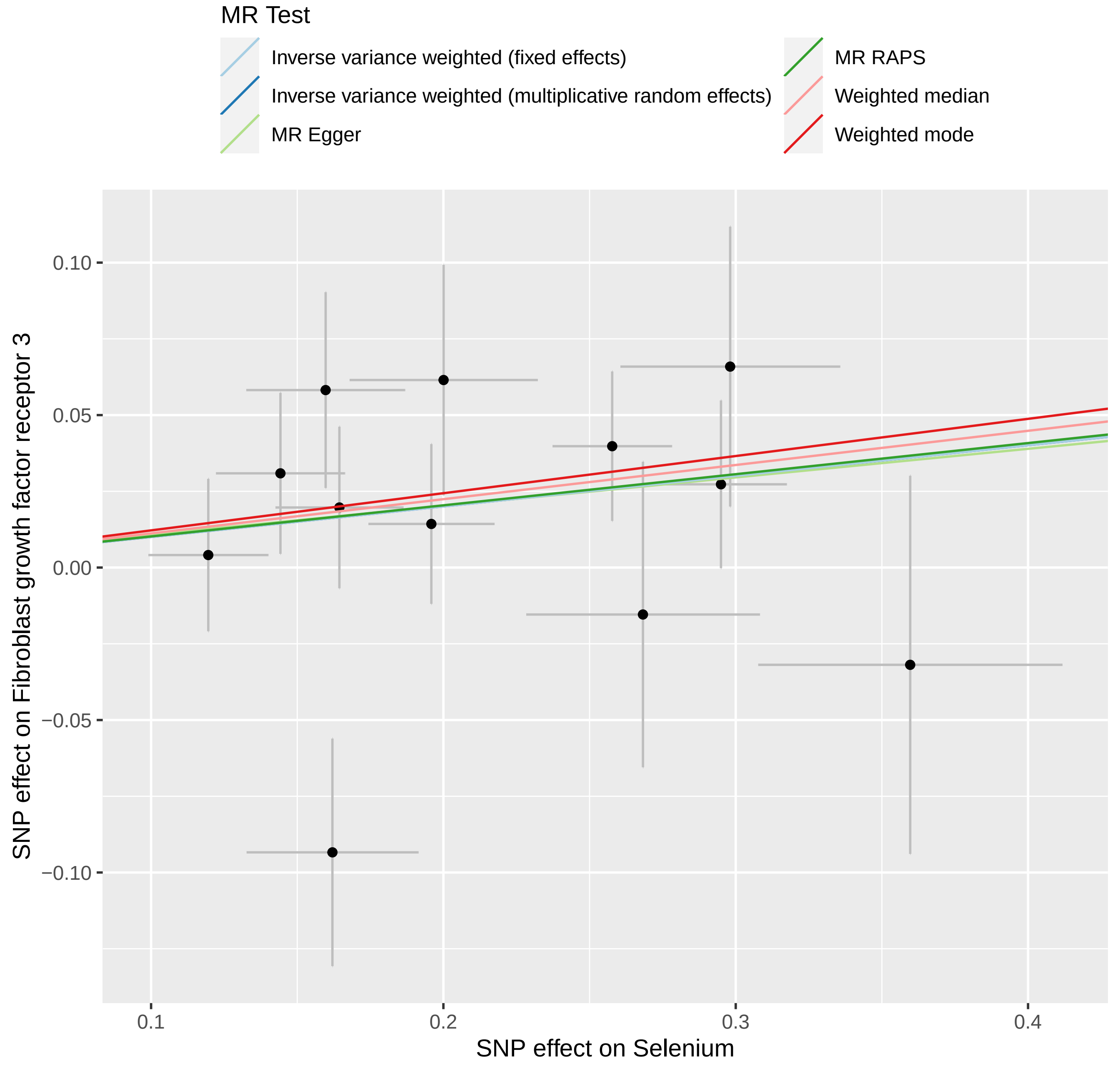

Batch 3−1105 : Selenium on Fibroblast growth factor receptor 4

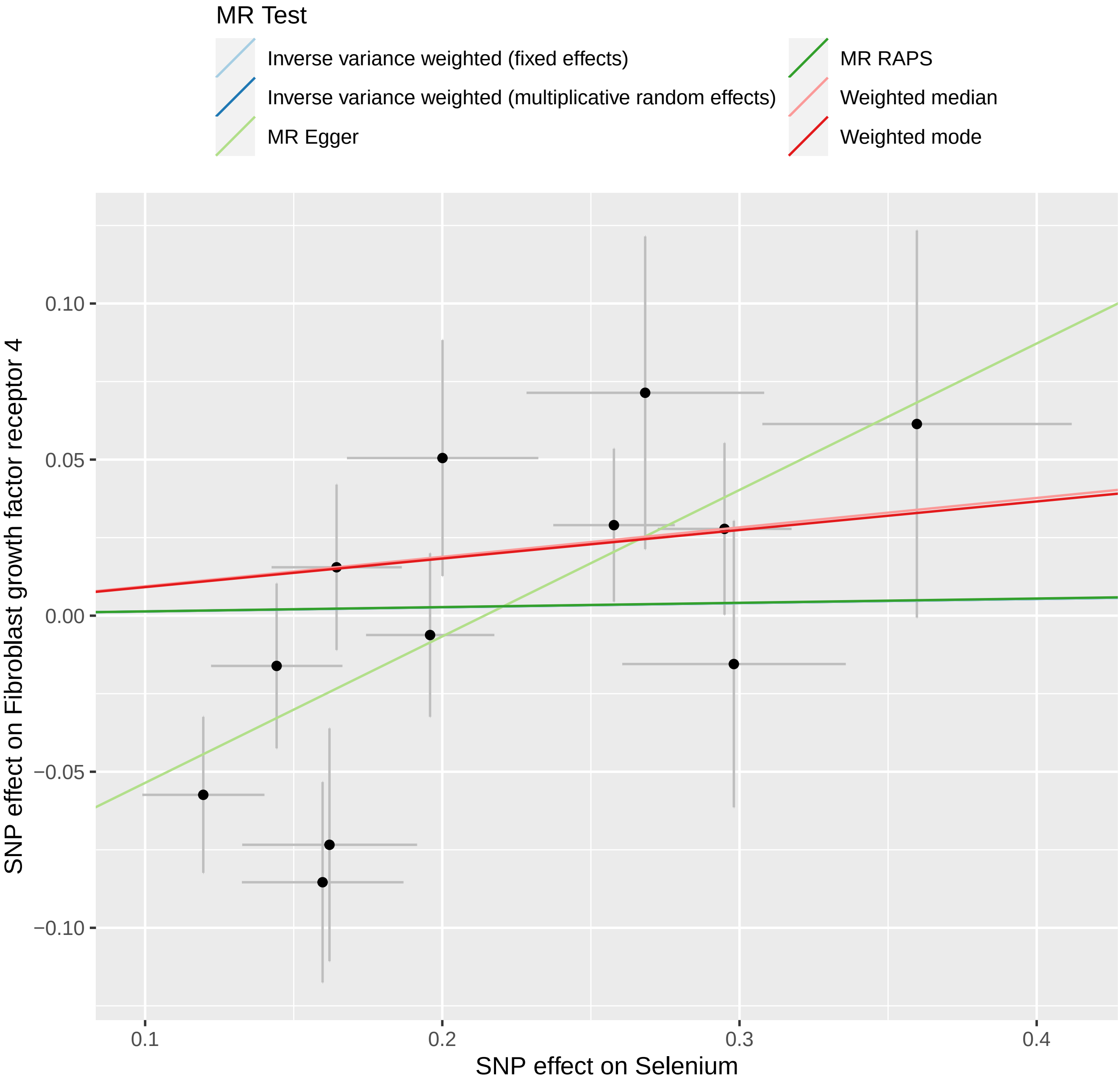

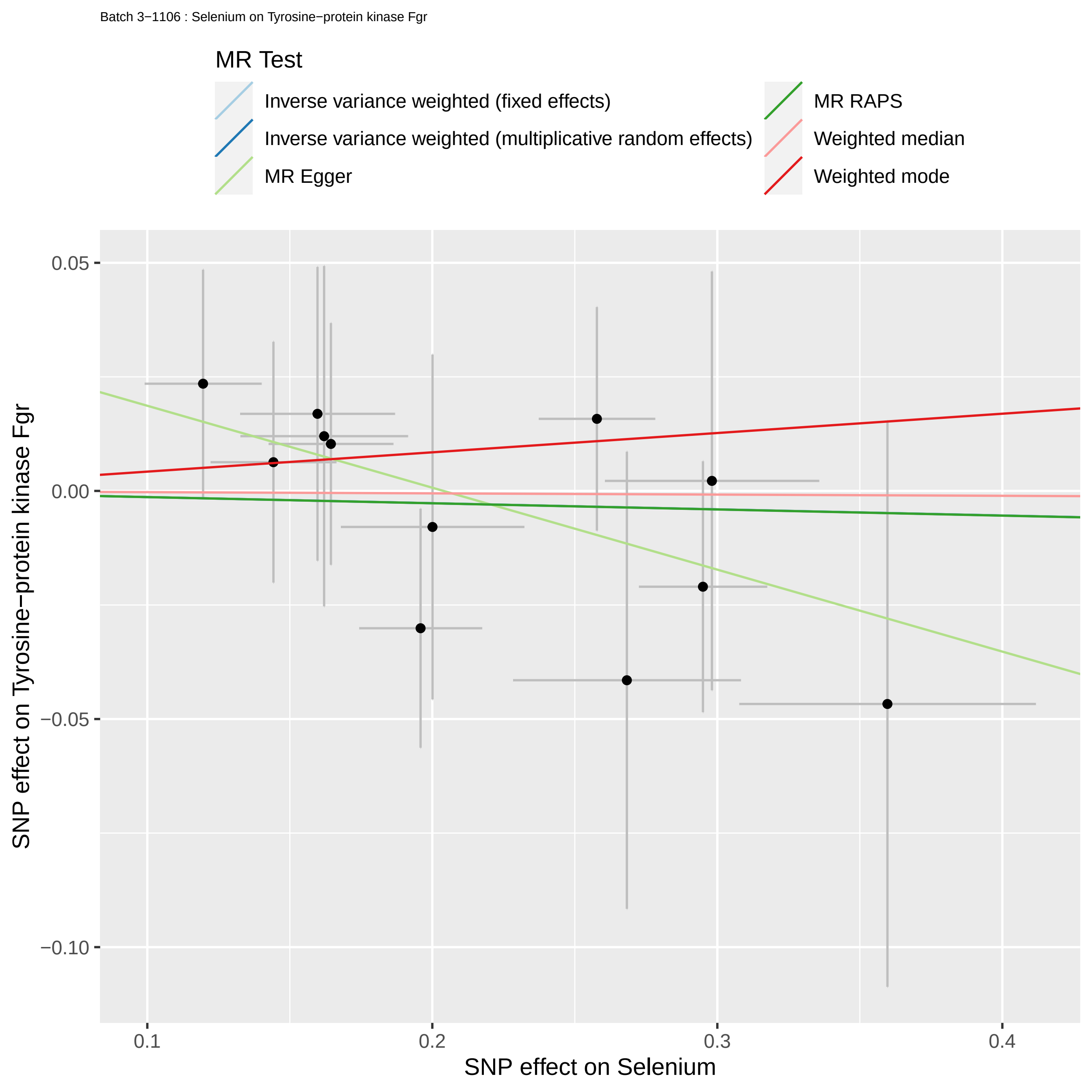

Batch 3−1106 : Selenium on Tyrosine−protein kinase Fgr
MR Test
Inverse variance weighted (fixed effects)
Inverse variance weighted (multiplicative random effects)
MR Egger
MR RAPS
Weighted median
Weighted mode
0.05
0.00
−0.05
−0.10
SNP effect on Tyrosine−protein kinase Fgr
0.1
0.2
0.3
0.4
SNP effect on Selenium

Batch 3−1107 : Selenium on Bis(5'−adenosyl)−triphosphatase

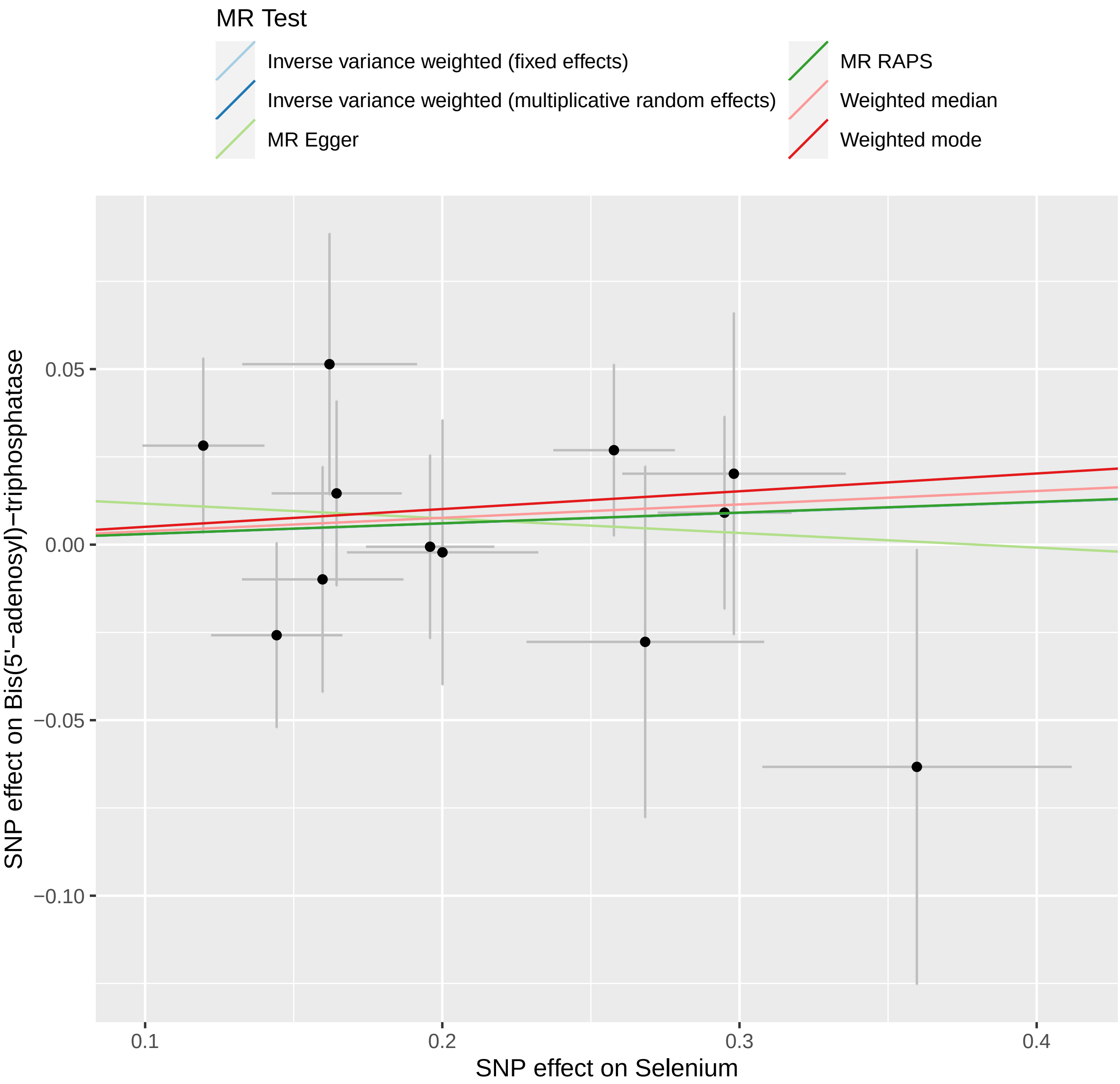

Batch 3−1108 : Selenium on Fibrinogen C domain−containing protein 1

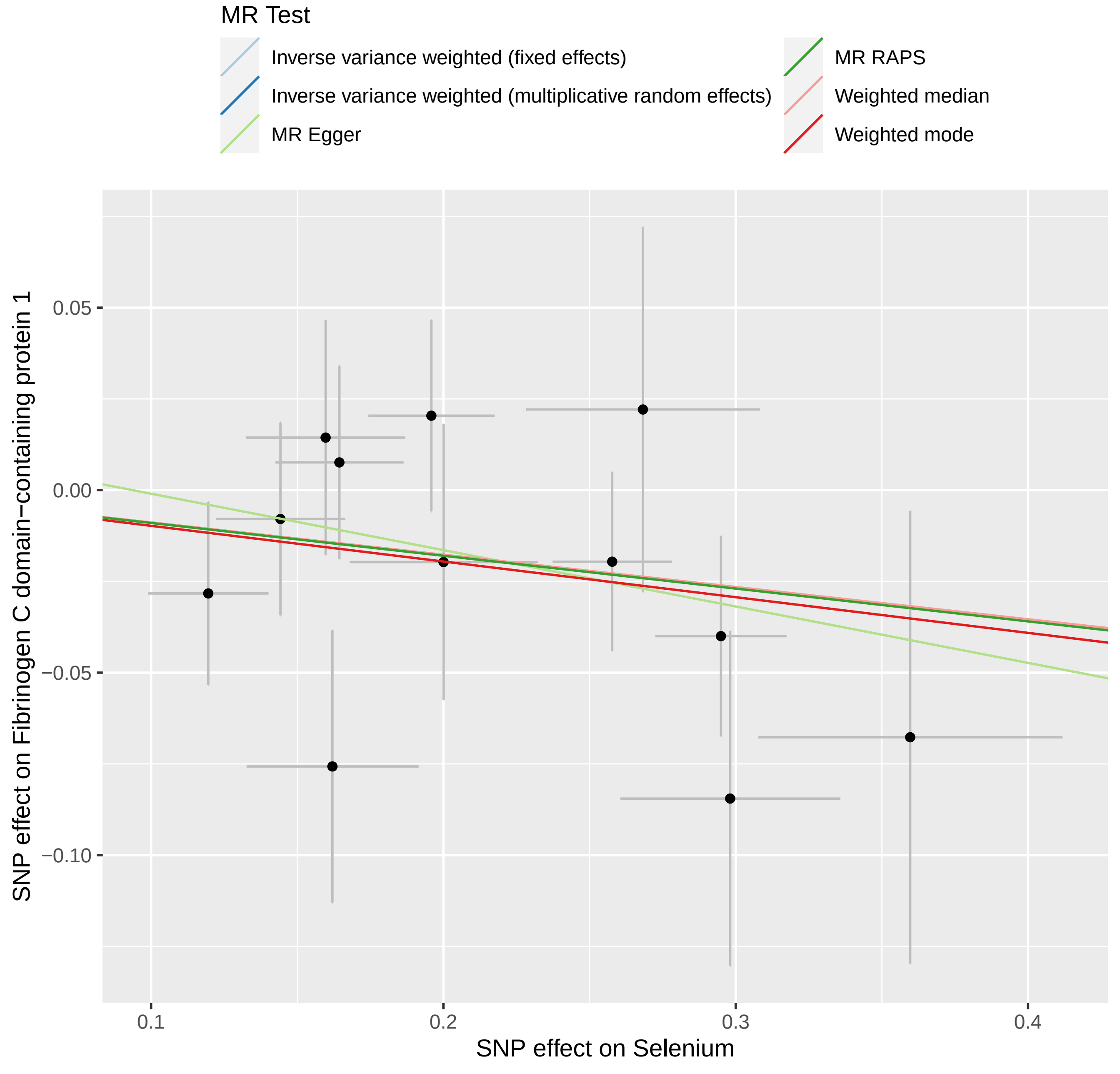

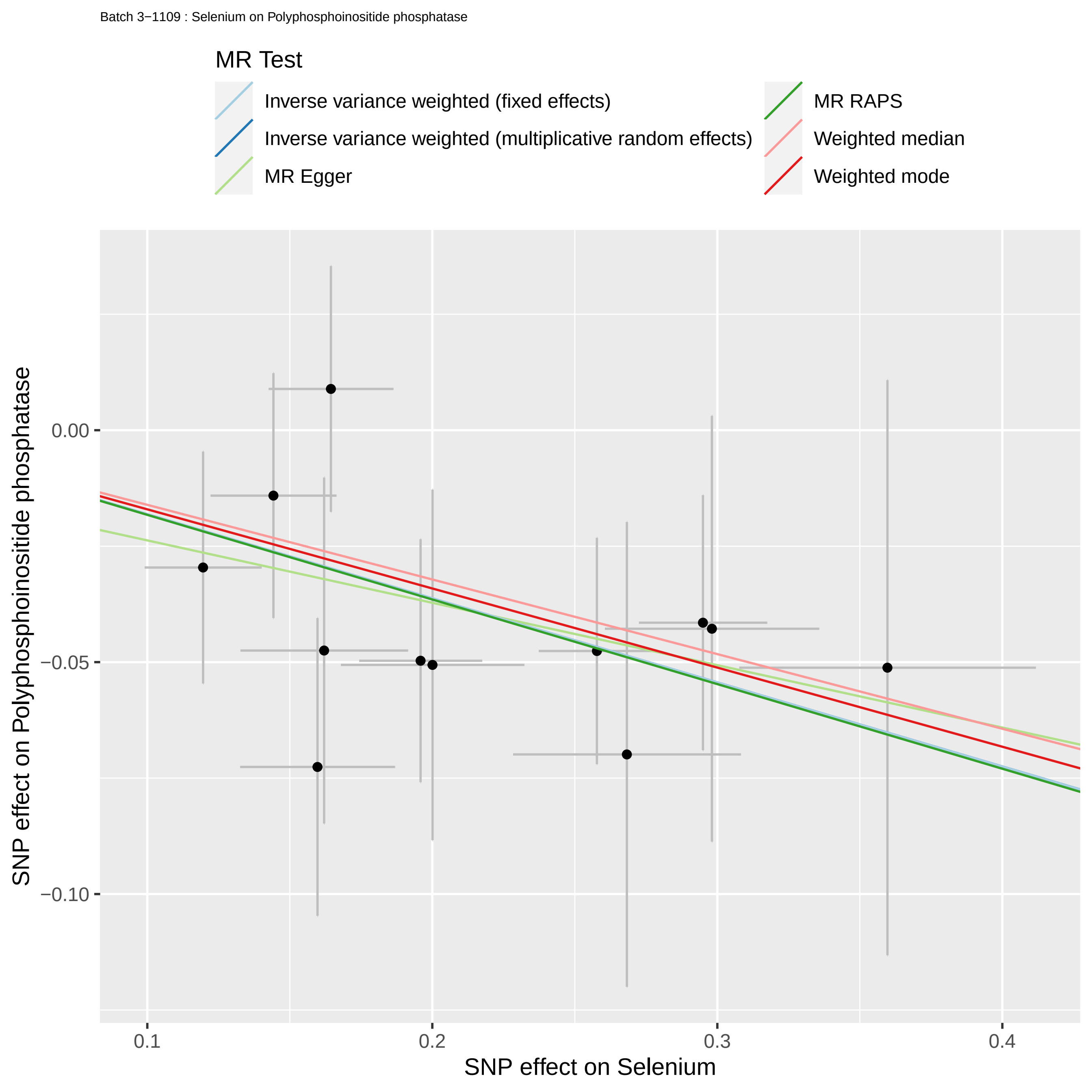
Batch 3−1109 : Selenium on Polyphosphoinositide phosphatase
MR Test
Inverse variance weighted (fixed effects)
Inverse variance weighted (multiplicative random effects)
MR Egger
MR RAPS
Weighted median
Weighted mode
SNP effect on Polyphosphoinositide phosphatase
0.00
−0.05
−0.10
0.1
0.2
0.3
0.4
SNP effect on Selenium

Batch 3−1110 : Selenium on Vascular endothelial growth factor D

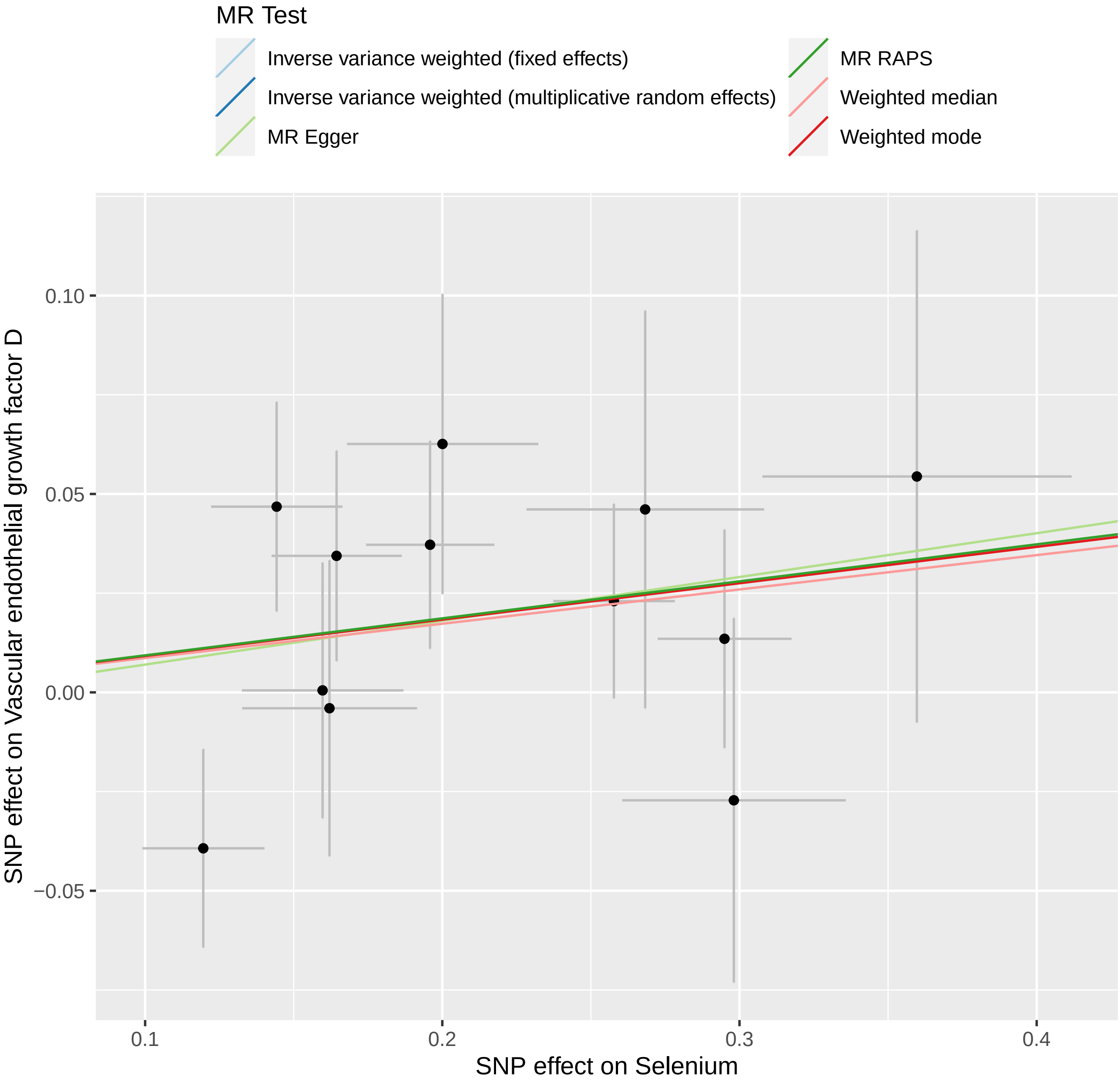

Batch 3−1111 : Selenium on Vascular endothelial growth factor D

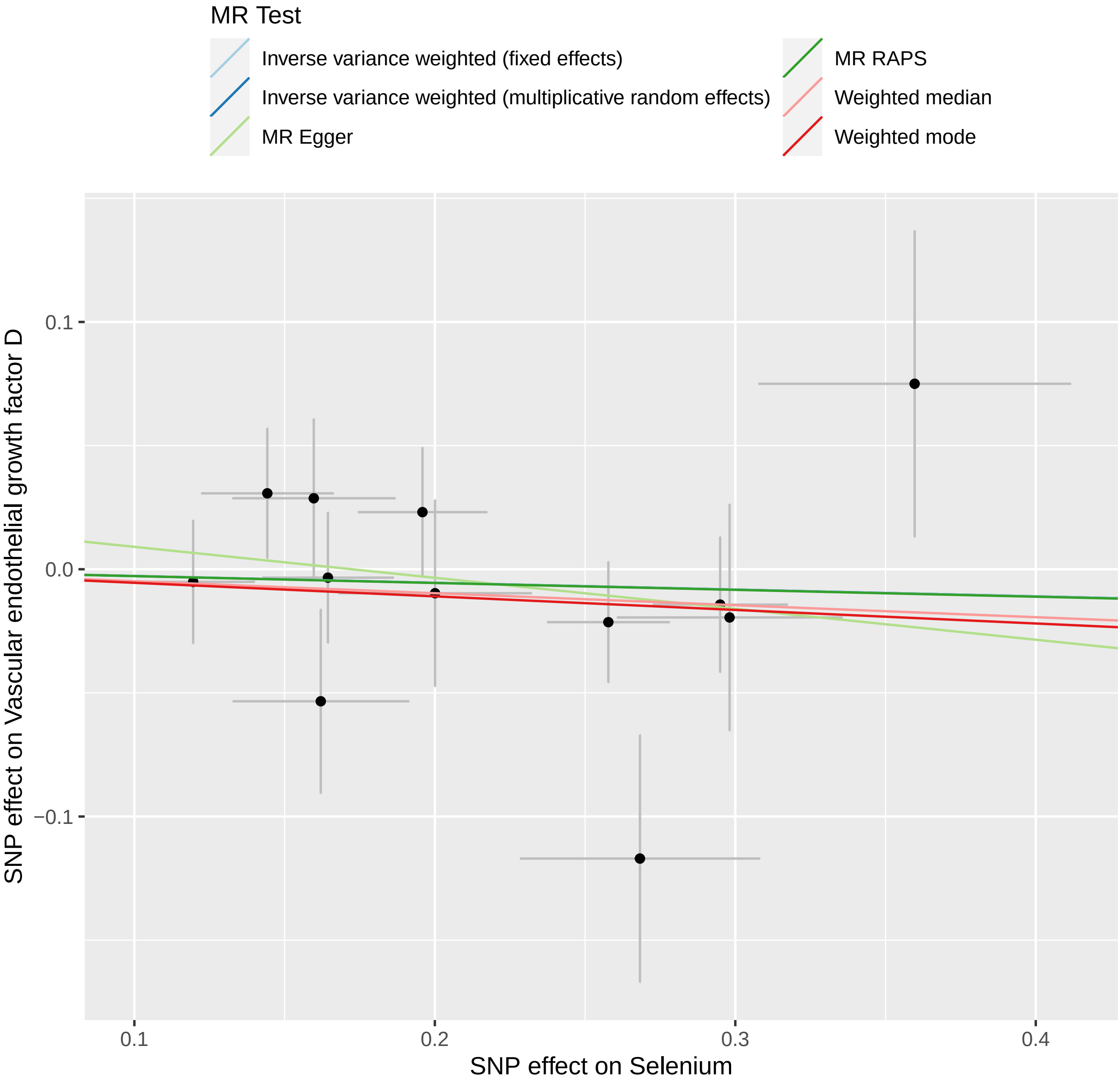

Batch 3−1112 : Selenium on Four−jointed box protein 1

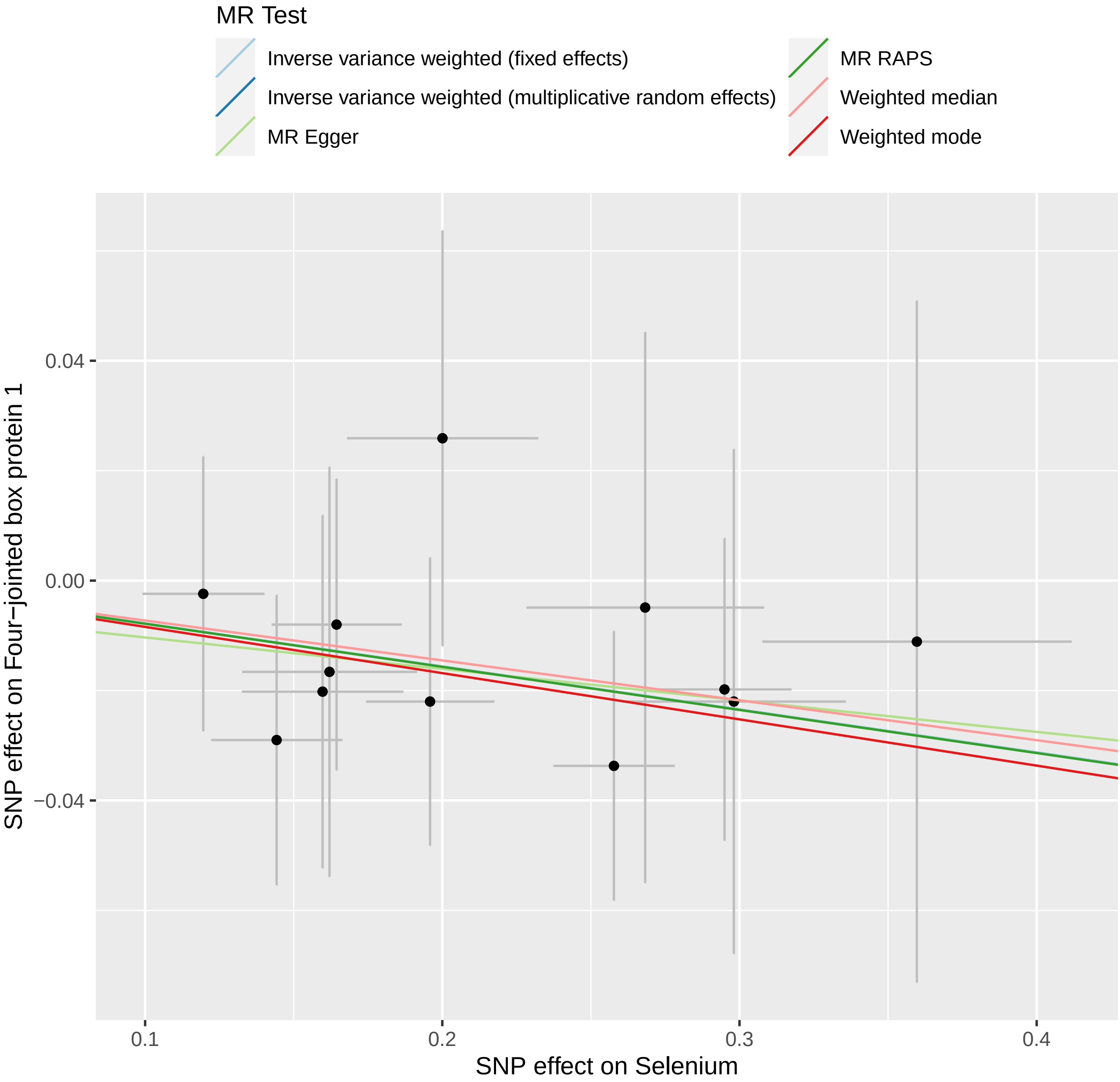

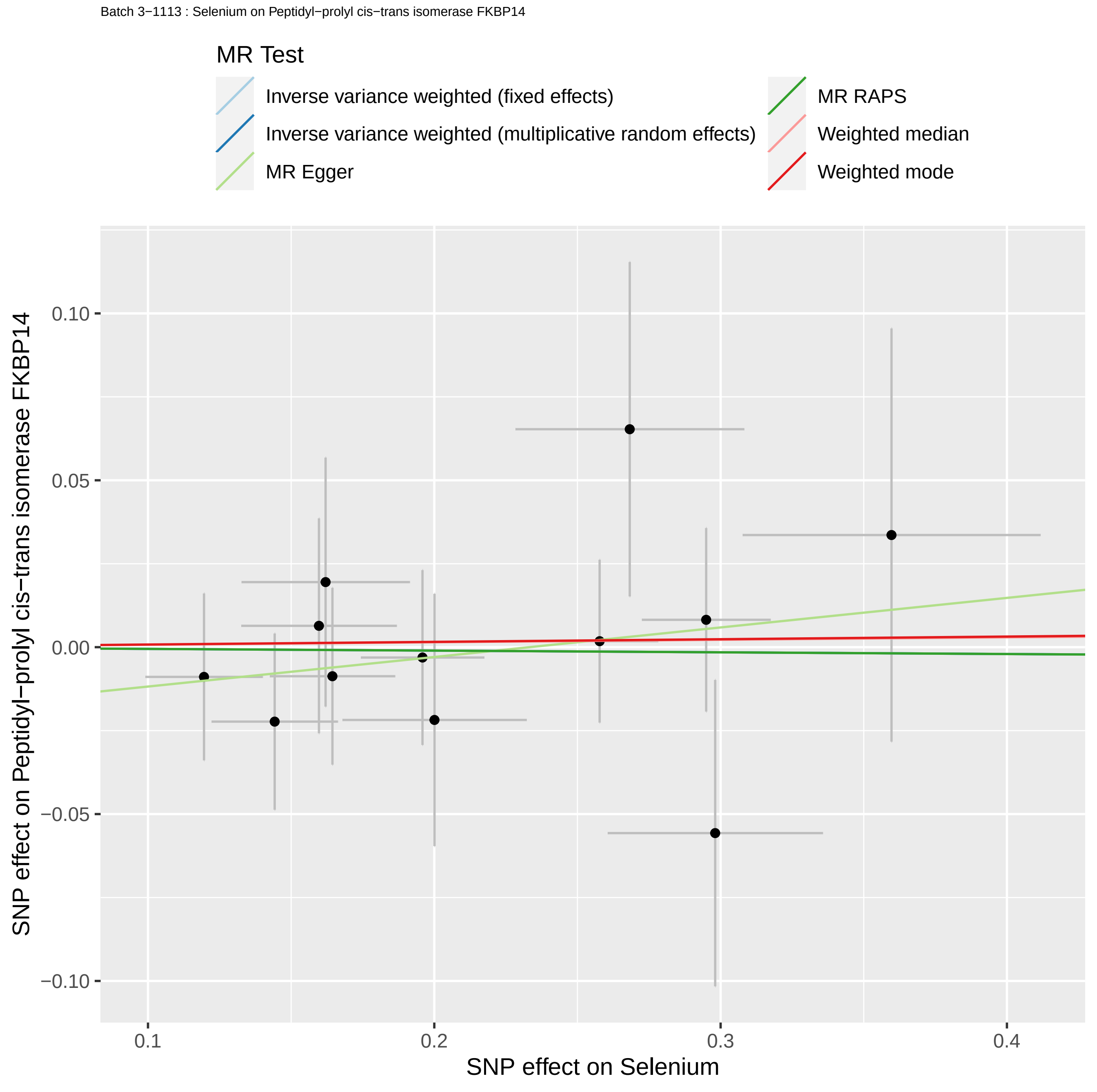
Batch 3−1113 : Selenium on Peptidyl−prolyl cis−trans isomerase FKBP14
MR Test
Inverse variance weighted (fixed effects)
Inverse variance weighted (multiplicative random effects)
MR Egger
MR RAPS
Weighted median
Weighted mode
0.10
0.05
0.00
−0.05
−0.10
0.1
0.2
0.3
0.4
SNP effect on Peptidyl−prolyl cis−trans isomerase FKBP14
SNP effect on Selenium

Batch 3−1114 : Selenium on Peptidyl−prolyl cis−trans isomerase FKBP2

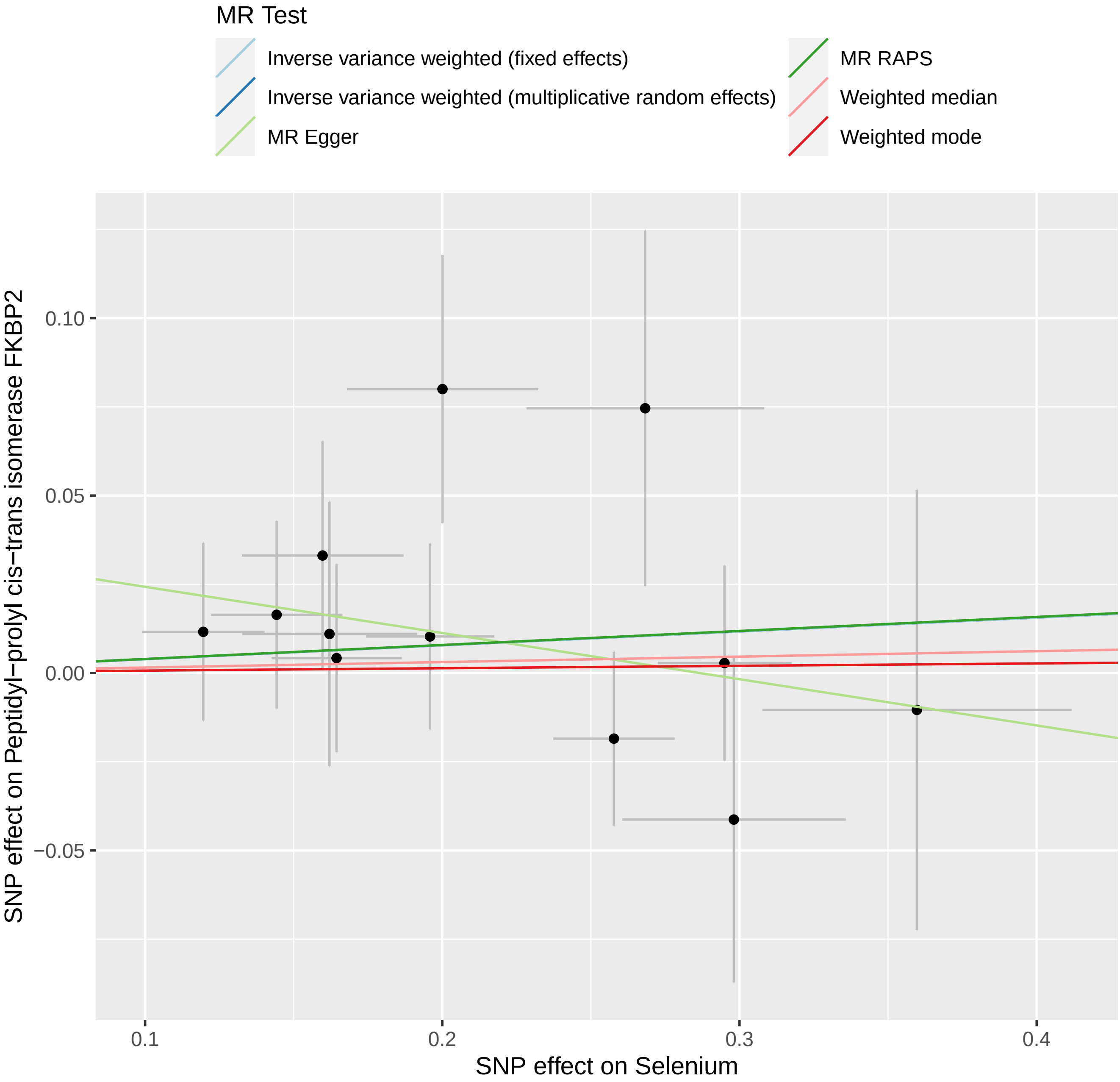

Batch 3−1115 : Selenium on Inactive peptidyl−prolyl cis−trans isomerase FKBP6

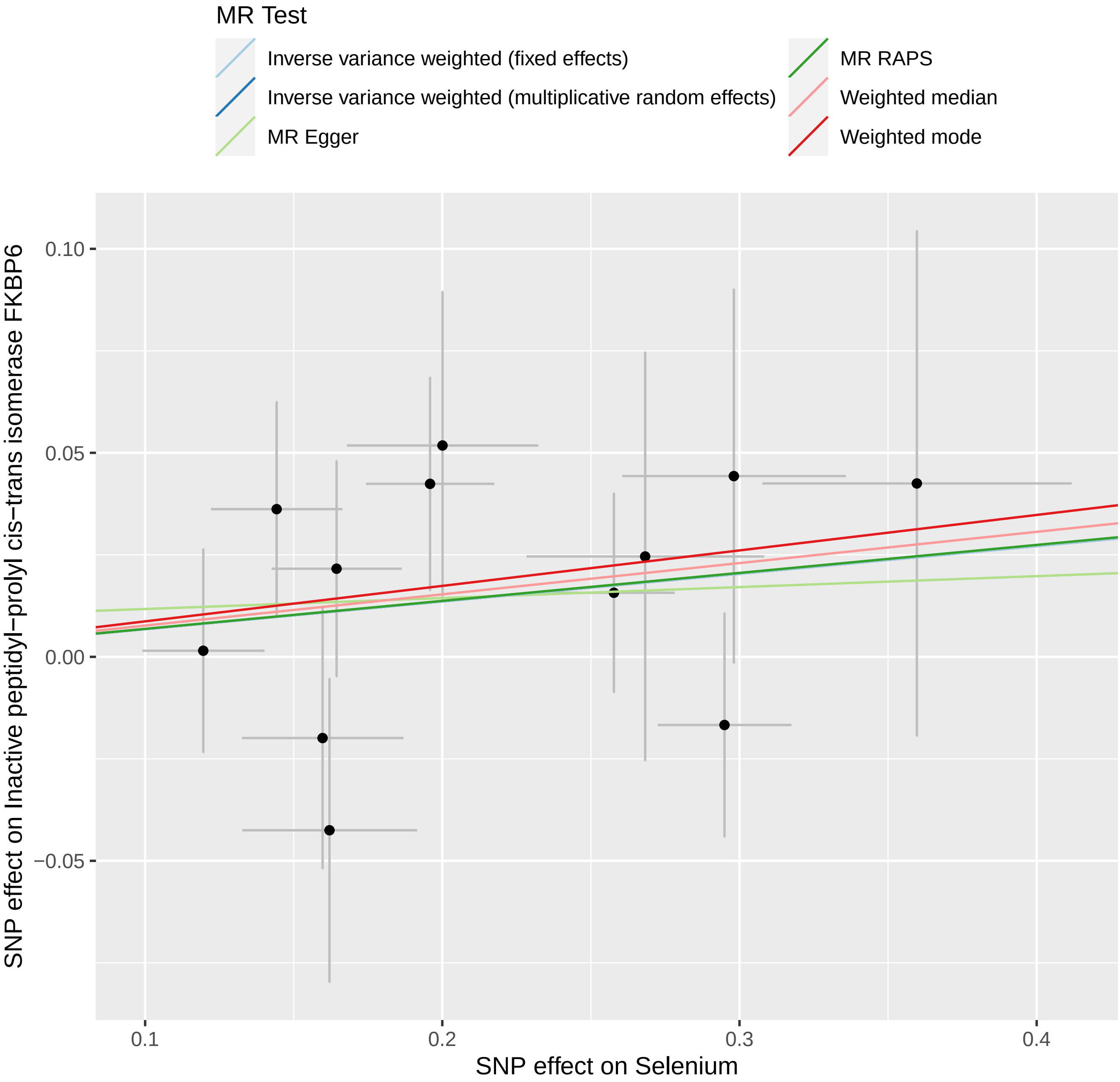

Batch 3−1116 : Selenium on Peptidyl−prolyl cis−trans isomerase FKBP7

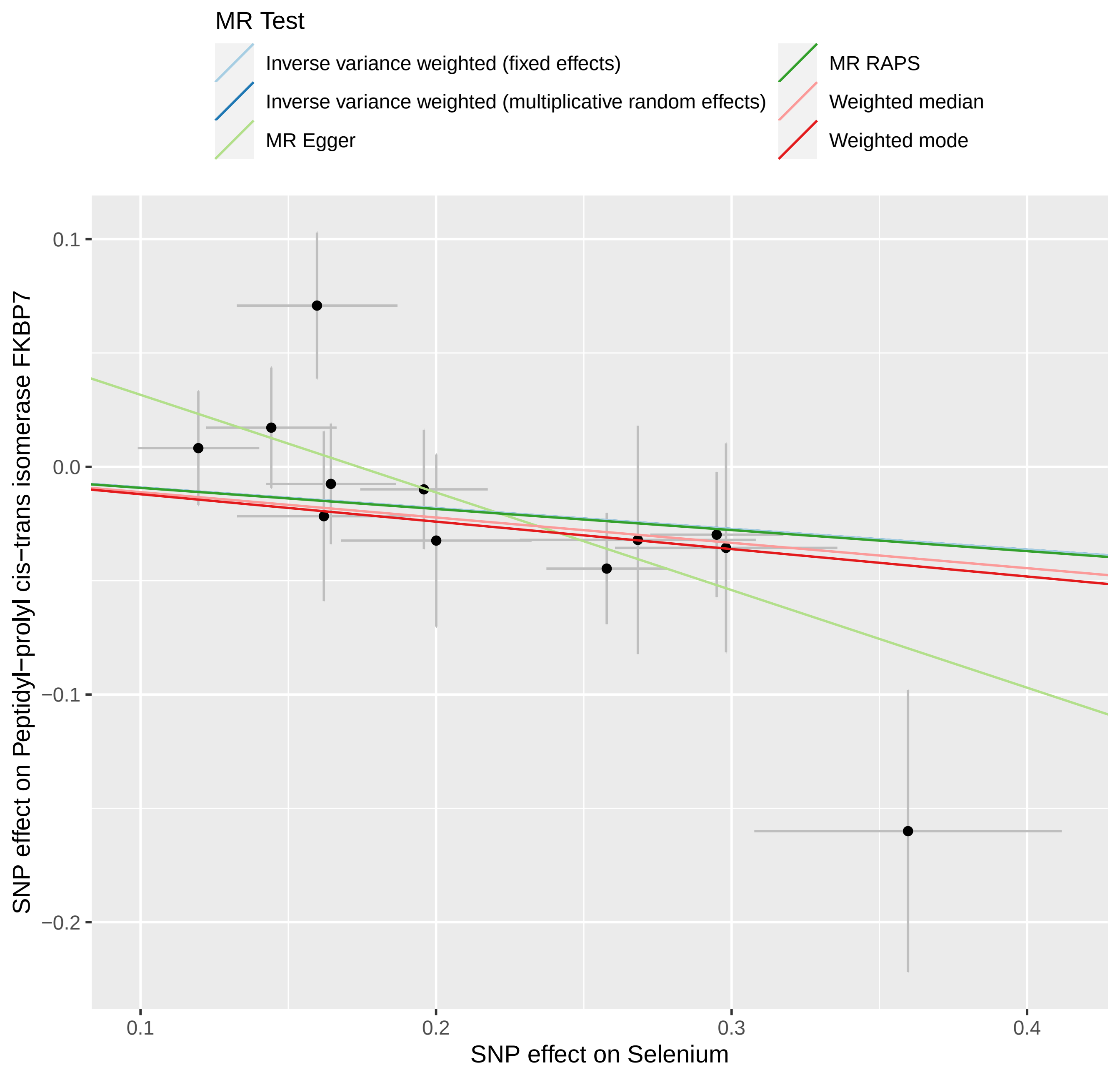

Batch 3−1117 : Selenium on Filamin−A

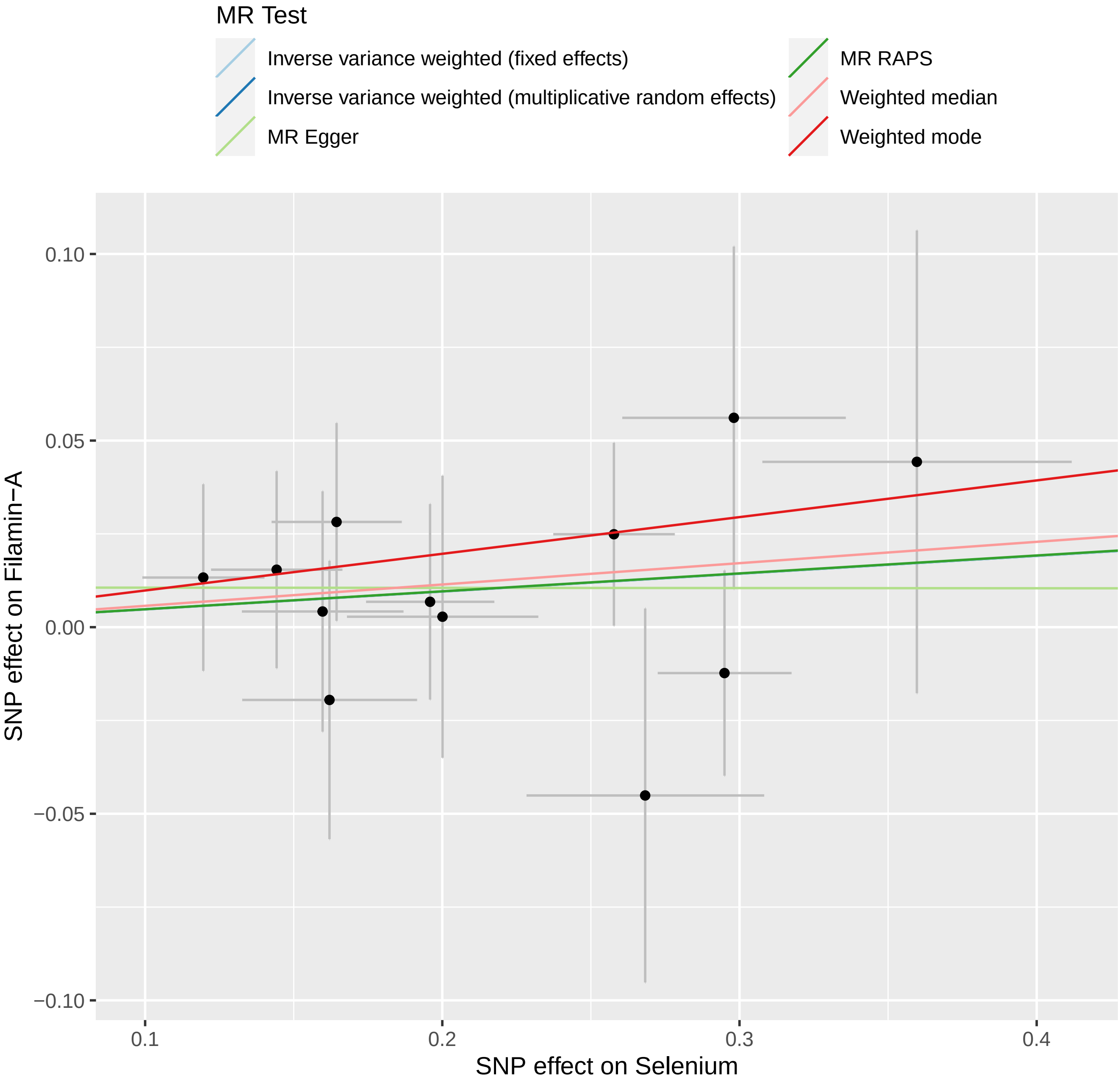

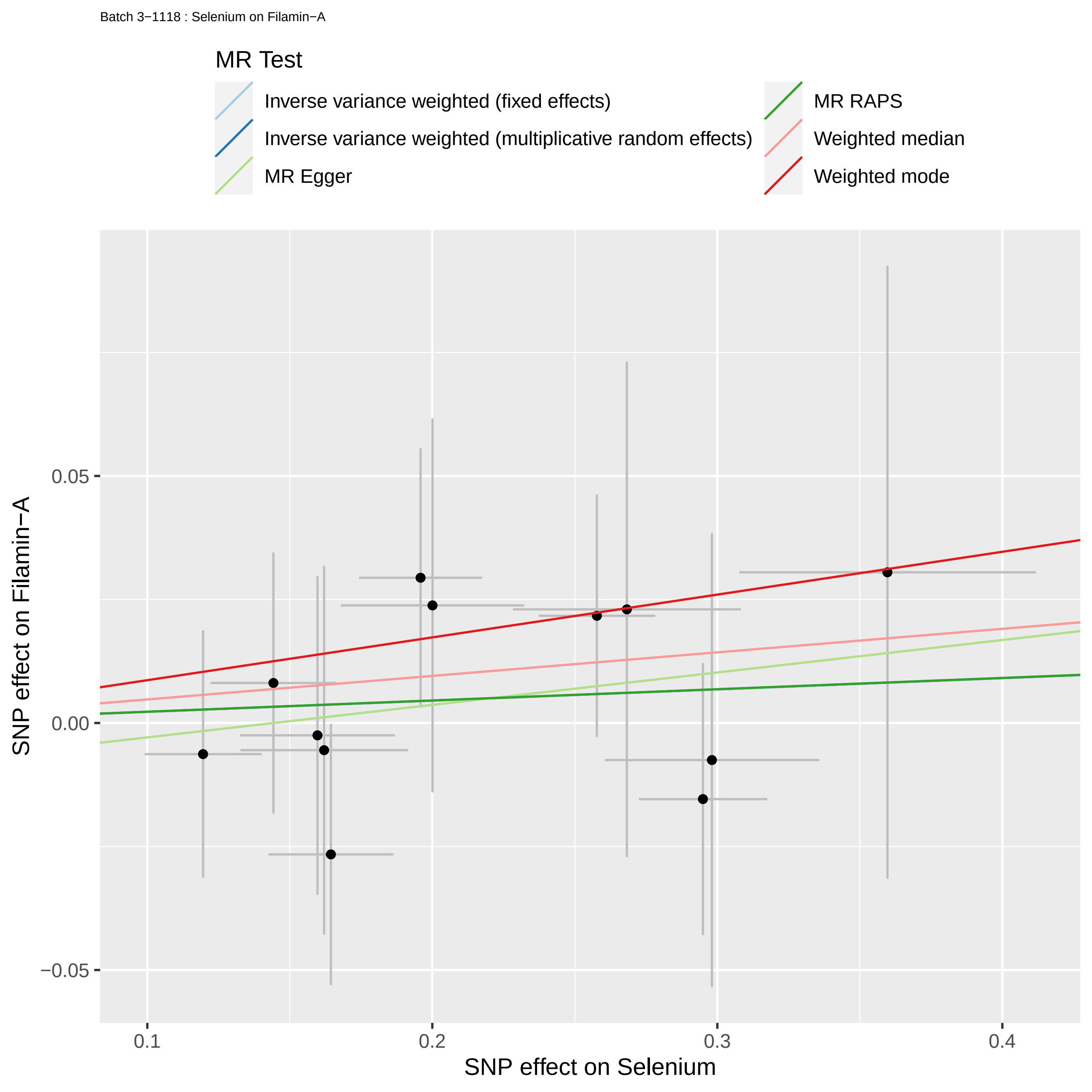
Batch 3−1118 : Selenium on Filamin−A
MR Test
Inverse variance weighted (fixed effects)
Inverse variance weighted (multiplicative random effects)
MR Egger
MR RAPS
Weighted median
Weighted mode
0.05
0.00
−0.05
0.1
0.2
0.3
0.4
SNP effect on Filamin−A
SNP effect on Selenium

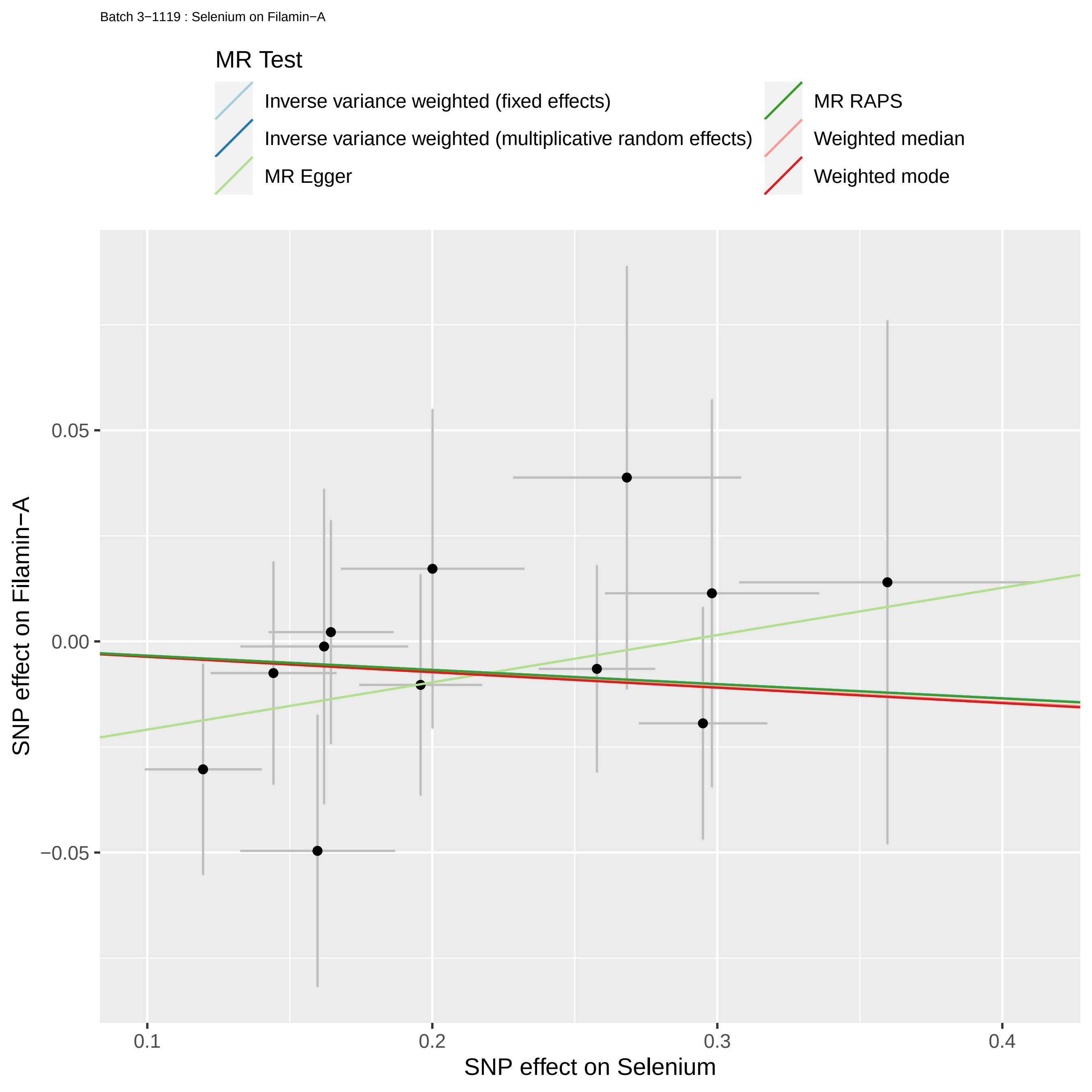

Batch 3−1119 : Selenium on Filamin−A
MR Test
Inverse variance weighted (fixed effects)
Inverse variance weighted (multiplicative random effects)
MR Egger
MR RAPS
Weighted median
Weighted mode
0.05
0.00
−0.05
0.1
0.2
0.3
0.4
SNP effect on Filamin−A
SNP effect on Selenium

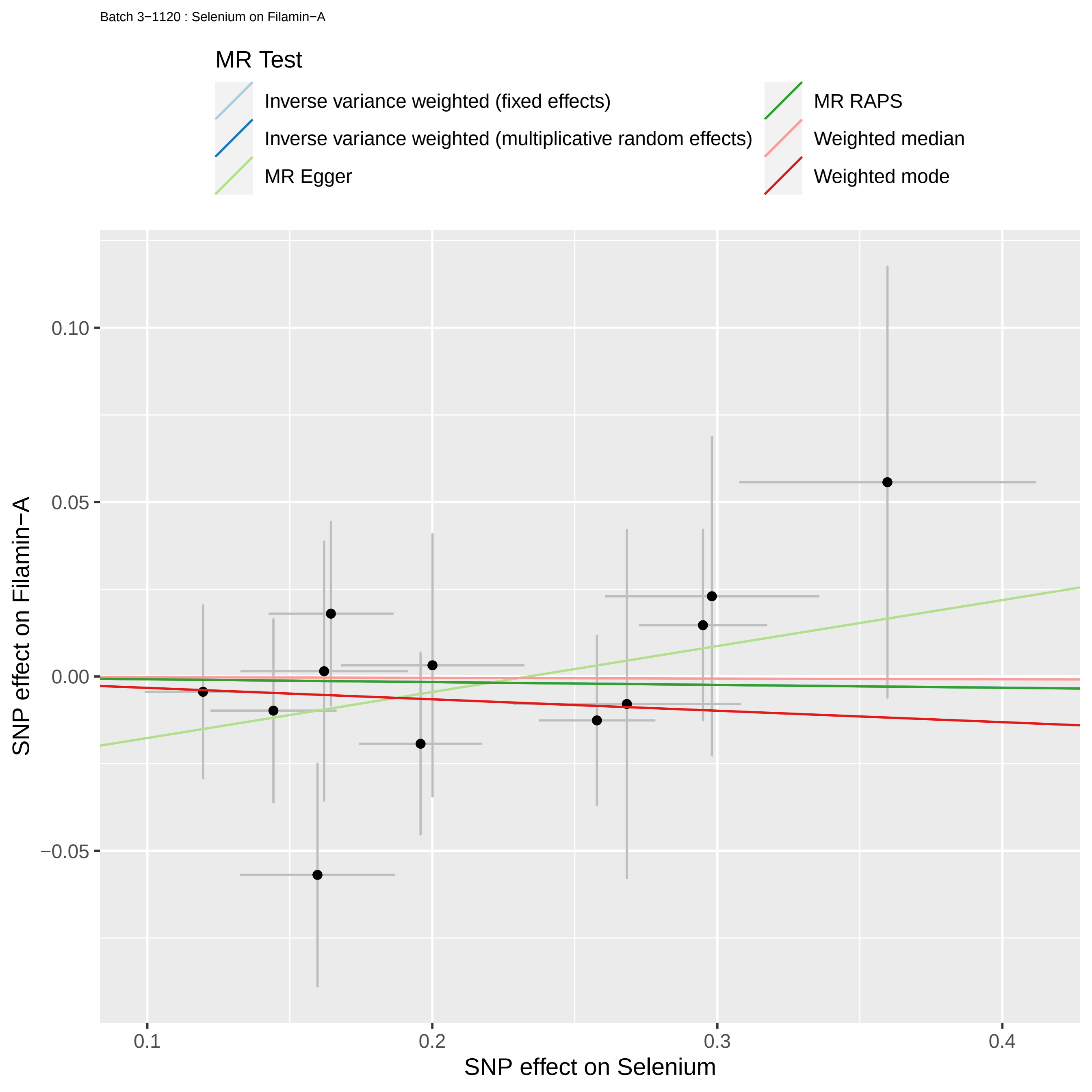

Batch 3−1120 : Selenium on Filamin−A
MR Test
Inverse variance weighted (fixed effects)
Inverse variance weighted (multiplicative random effects)
MR Egger
MR RAPS
Weighted median
Weighted mode
0.10
0.05
0.00
−0.05
0.1
0.2
0.3
0.4
SNP effect on Filamin−A
SNP effect on Selenium

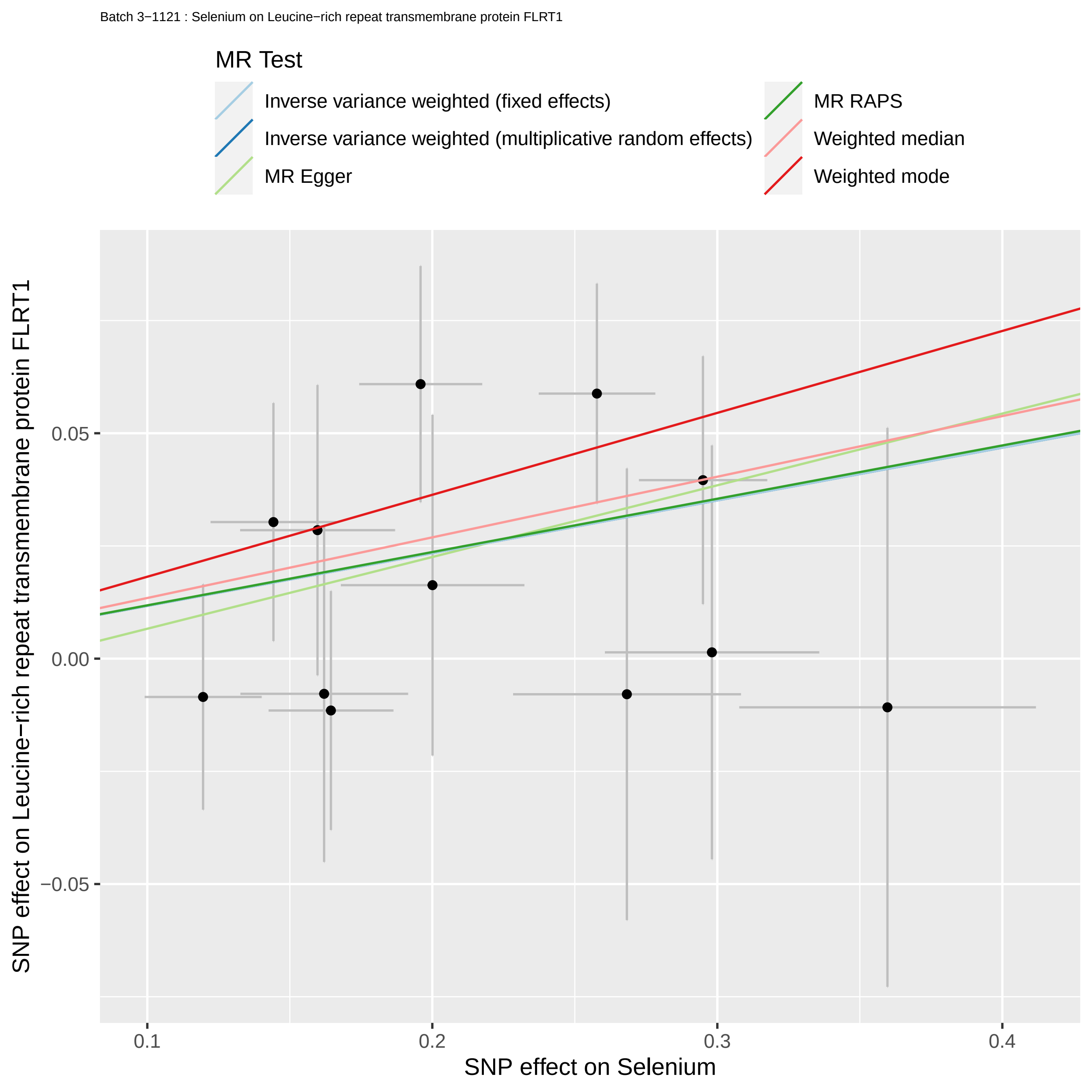

Batch 3−1121 : Selenium on Leucine−rich repeat transmembrane protein FLRT1
MR Test
Inverse variance weighted (fixed effects)
Inverse variance weighted (multiplicative random effects)
MR Egger
MR RAPS
Weighted median
Weighted mode
0.05
0.00
−0.05
0.1
0.2
0.3
0.4
SNP effect on Leucine−rich repeat transmembrane protein FLRT1
SNP effect on Selenium

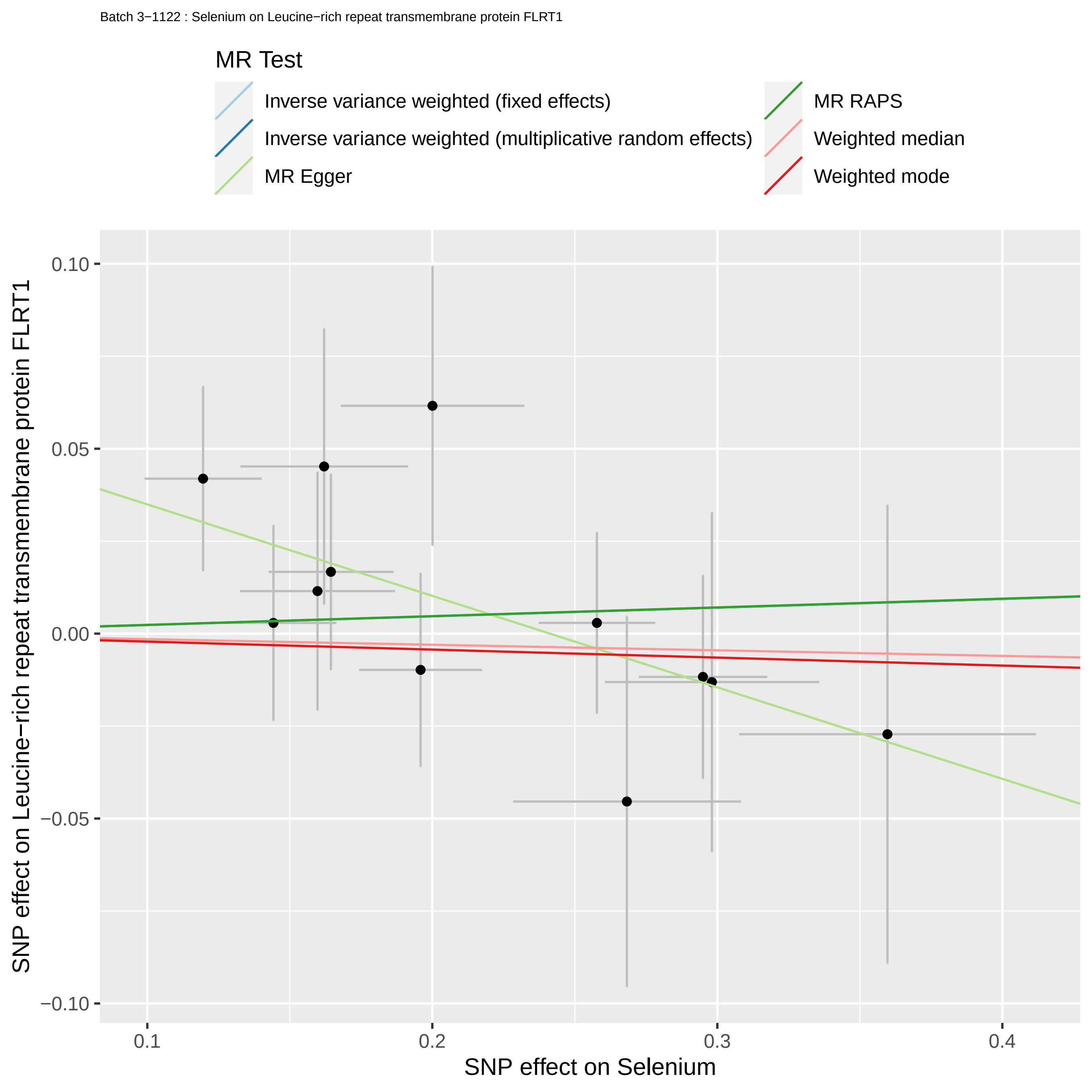
Batch 3−1122 : Selenium on Leucine−rich repeat transmembrane protein FLRT1
MR Test
Inverse variance weighted (fixed effects)
Inverse variance weighted (multiplicative random effects)
MR Egger
MR RAPS
Weighted median
Weighted mode
SNP effect on Leucine−rich repeat transmembrane protein FLRT1
0.10
0.05
0.00
−0.05
−0.10
0.1
0.2
0.3
0.4
SNP effect on Selenium

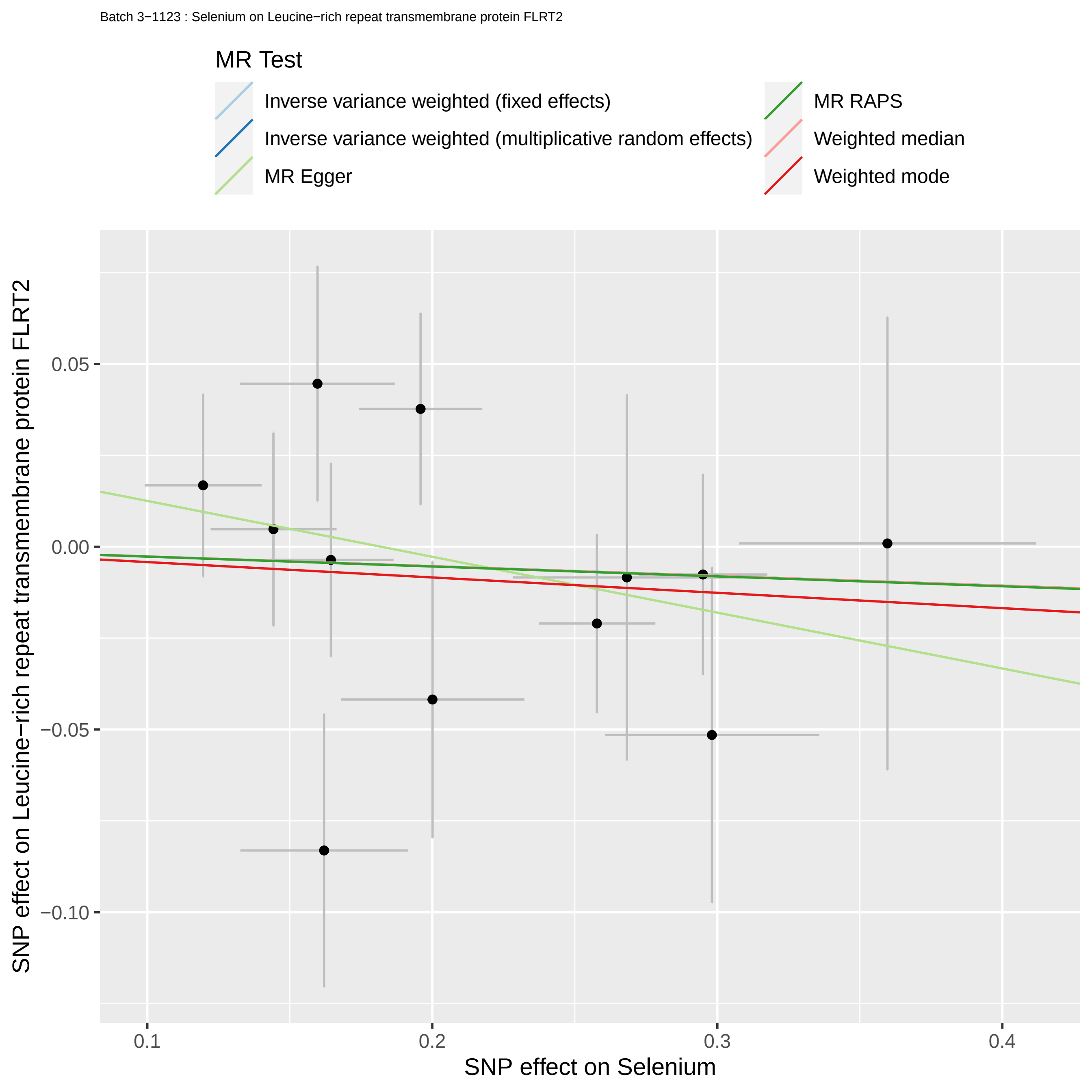

Batch 3−1123 : Selenium on Leucine−rich repeat transmembrane protein FLRT2
MR Test
Inverse variance weighted (fixed effects)
Inverse variance weighted (multiplicative random effects)
MR Egger
MR RAPS
Weighted median
Weighted mode
SNP effect on Leucine−rich repeat transmembrane protein FLRT2
0.05
0.00
−0.05
−0.10
0.1
0.2
0.3
0.4
SNP effect on Selenium

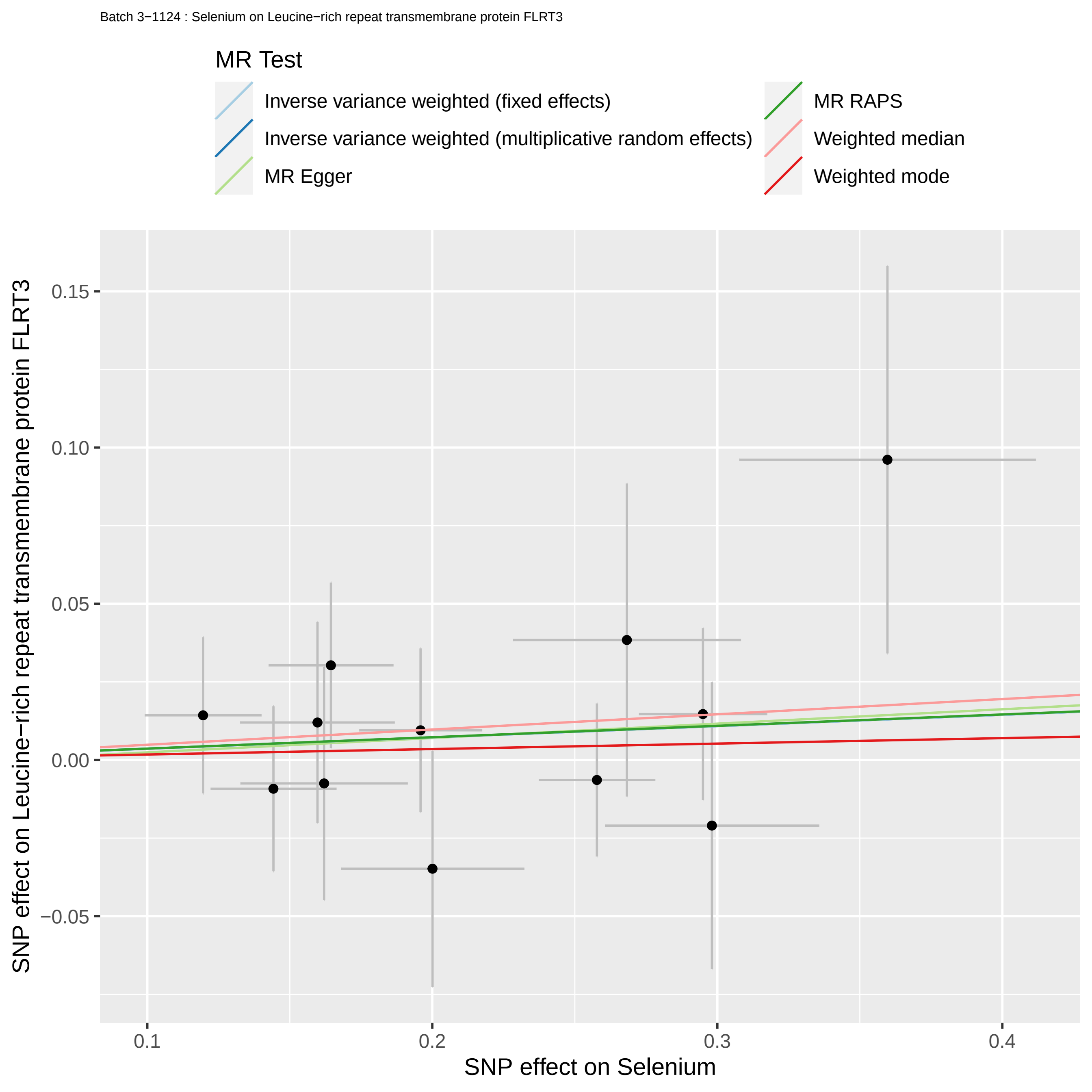

Batch 3−1124 : Selenium on Leucine−rich repeat transmembrane protein FLRT3
MR Test
Inverse variance weighted (fixed effects)
Inverse variance weighted (multiplicative random effects)
MR Egger
MR RAPS
Weighted median
Weighted mode
0.15
0.10
0.05
0.00
−0.05
0.1
0.2
0.3
0.4
SNP effect on Selenium
SNP effect on Leucine−rich repeat transmembrane protein FLRT3

Batch 3−1125 : Selenium on Leucine−rich repeat transmembrane protein FLRT3

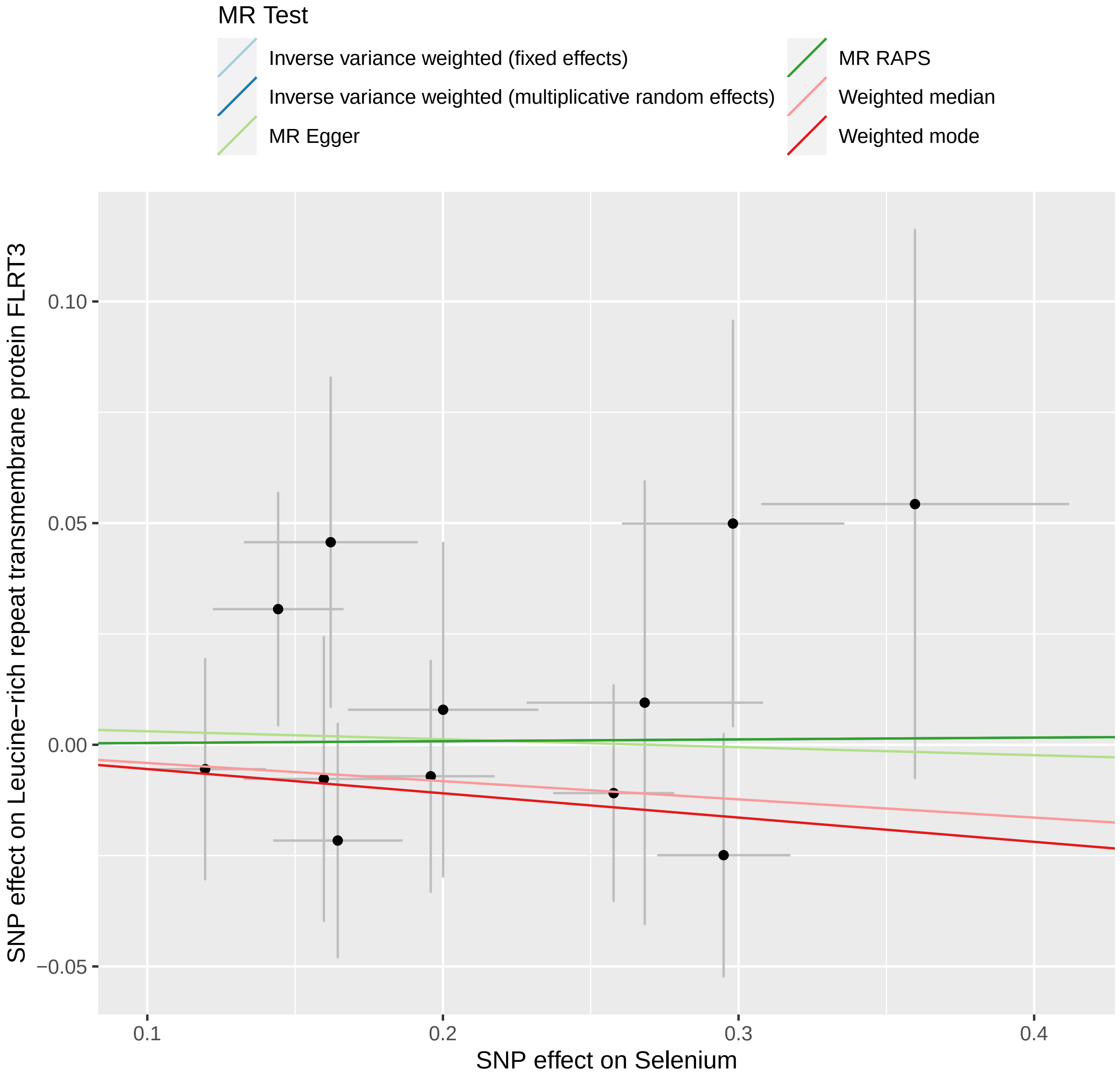

Batch 3−1126 : Selenium on Receptor−type tyrosine−protein kinase FLT3

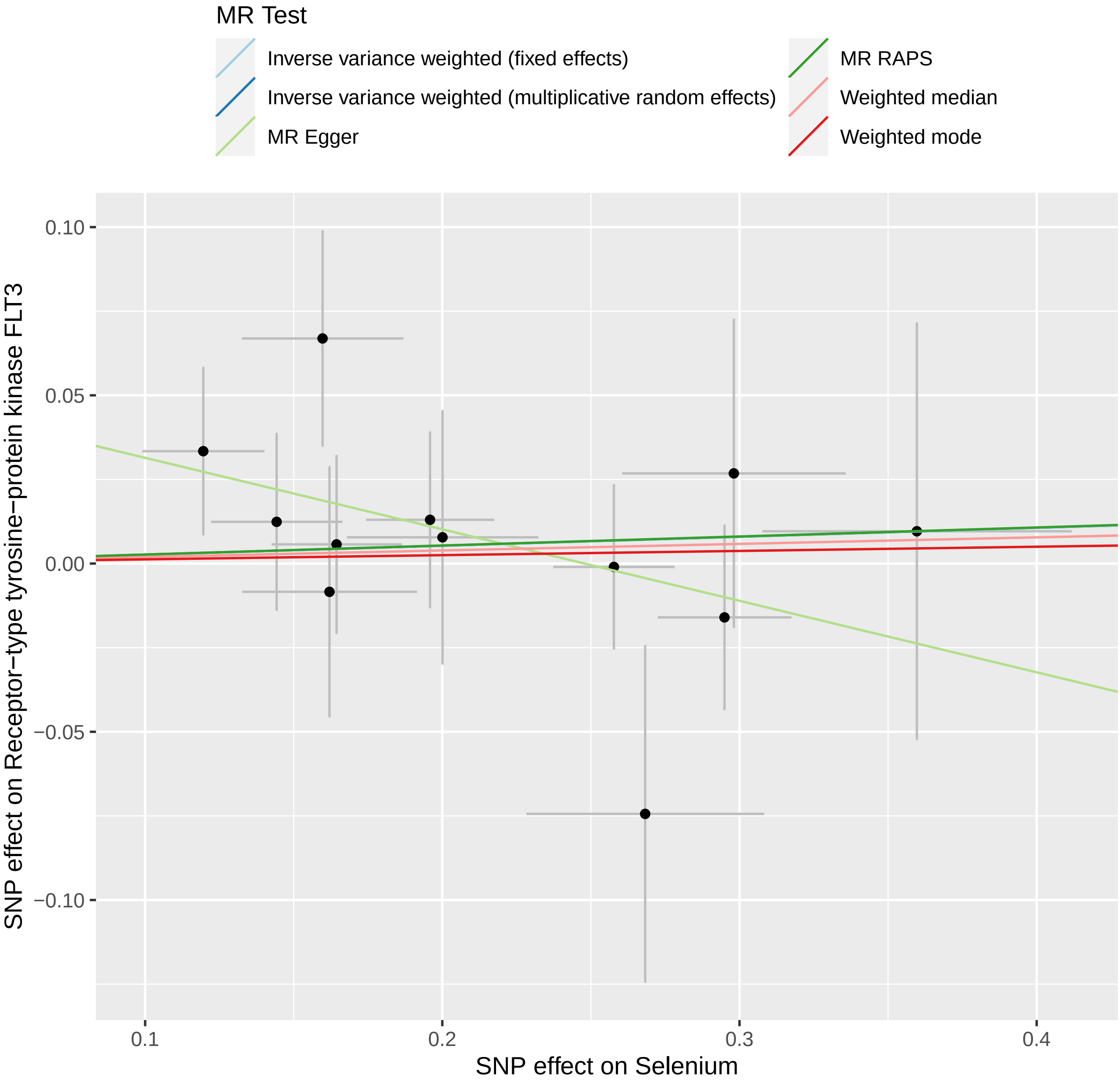

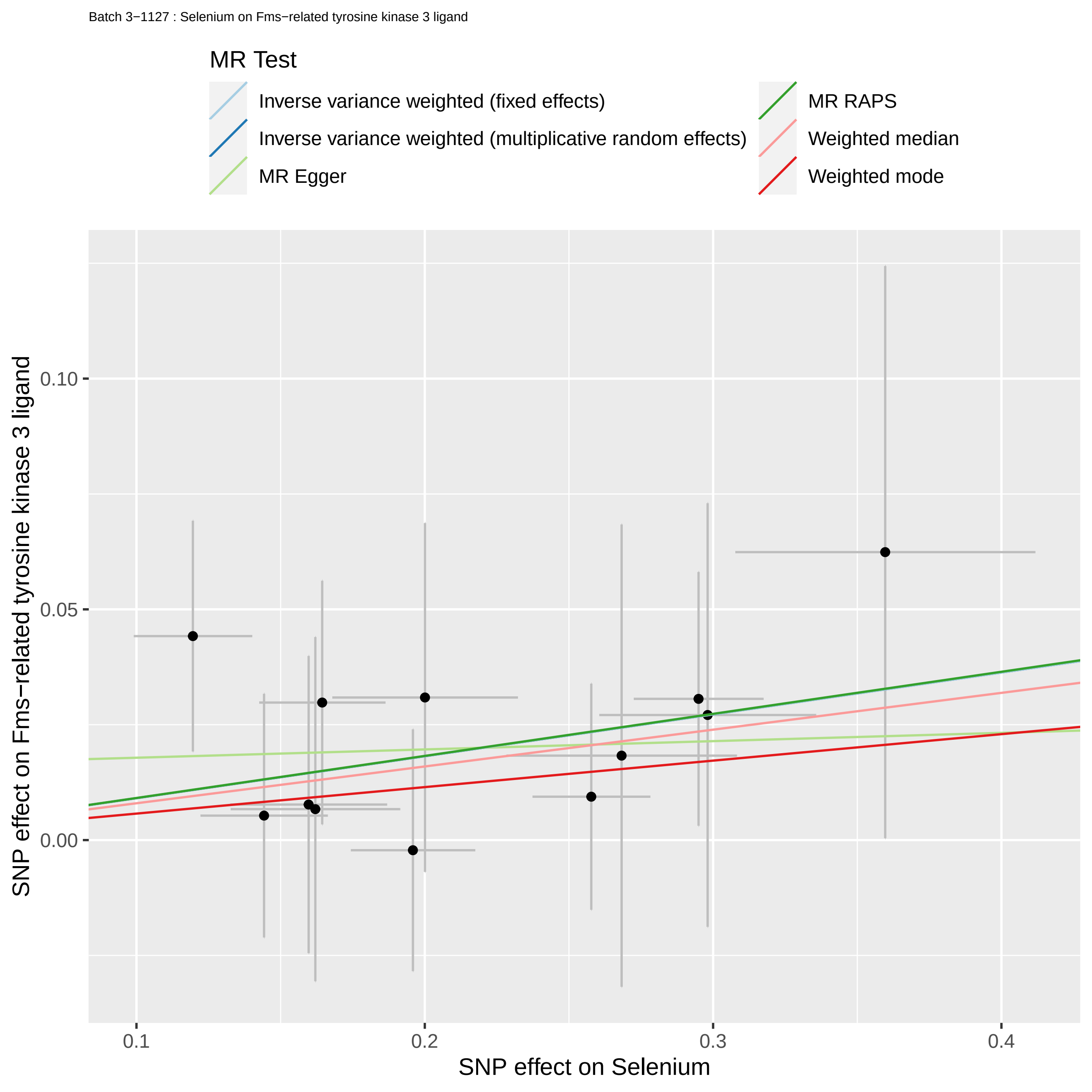

Batch 3−1127 : Selenium on Fms−related tyrosine kinase 3 ligand
MR Test
Inverse variance weighted (fixed effects)
Inverse variance weighted (multiplicative random effects)
MR Egger
MR RAPS
Weighted median
Weighted mode
0.10
0.05
0.00
0.1
0.2
0.3
0.4
SNP effect on Fms−related tyrosine kinase 3 ligand
SNP effect on Selenium

Batch 3−1128 : Selenium on Fms−related tyrosine kinase 3 ligand

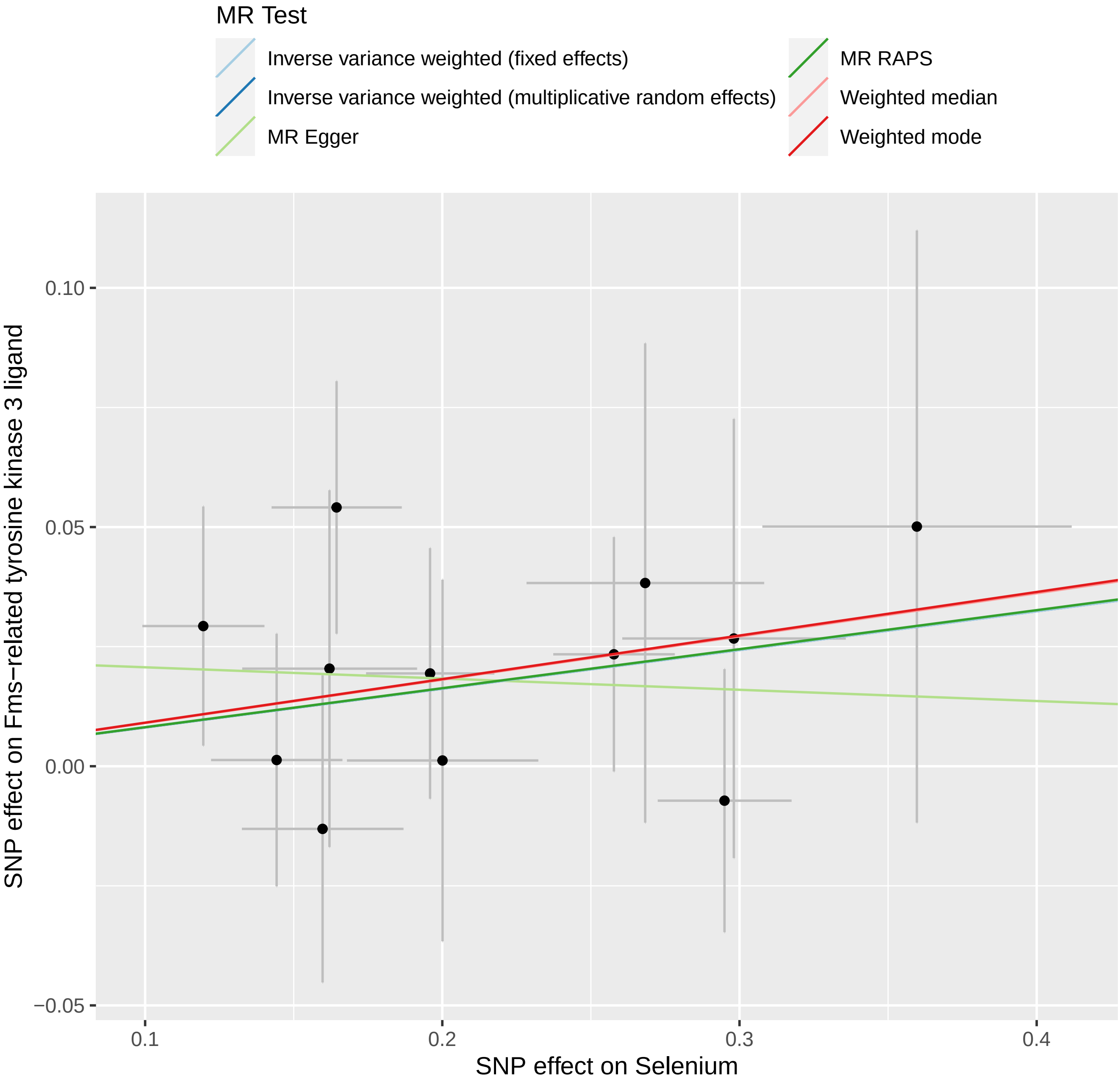

Batch 3−1129 : Selenium on Vascular endothelial growth factor receptor 3

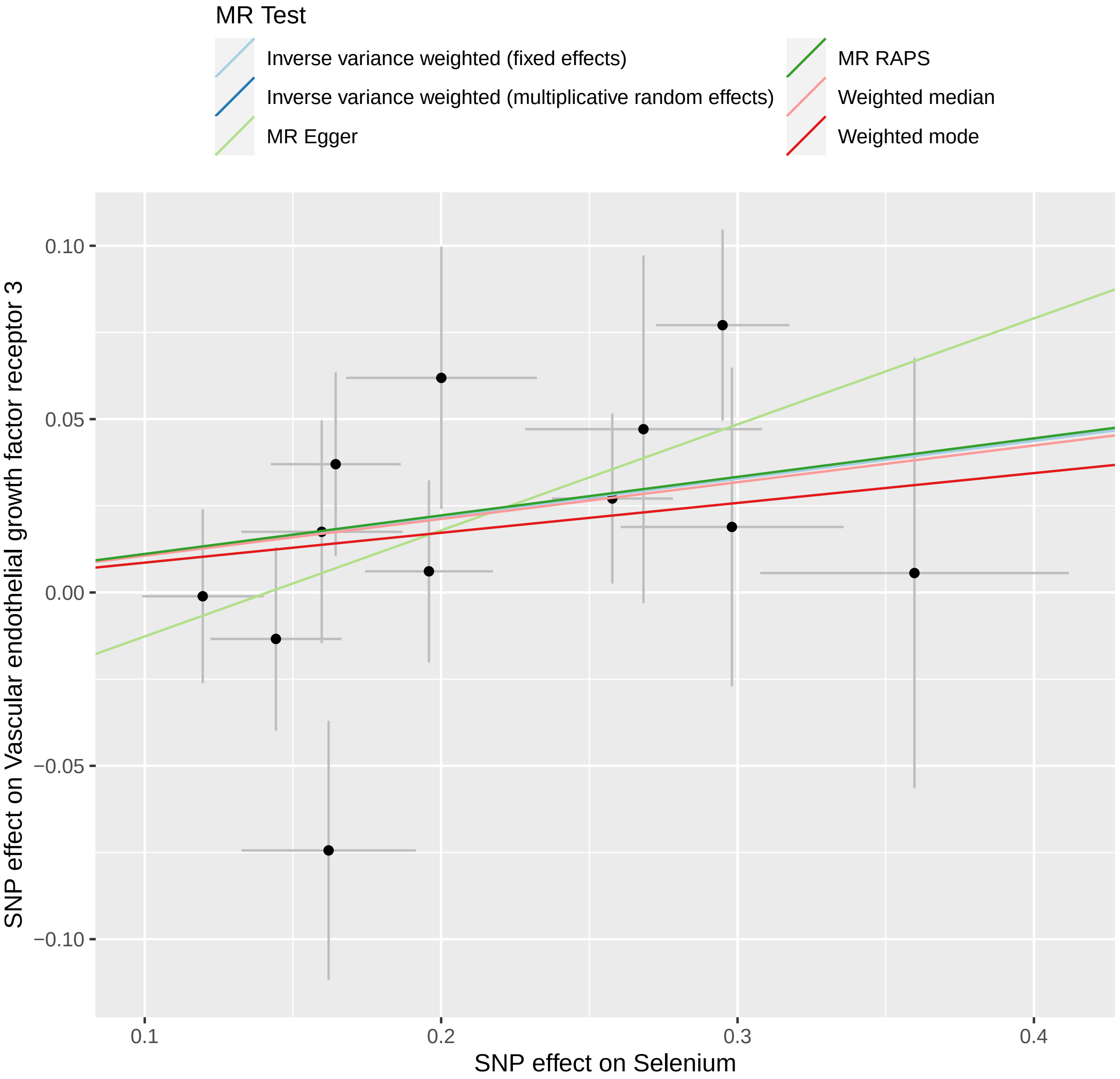

Batch 3−1130 : Selenium on Feline leukemia virus subgroup C receptor−related protein 1

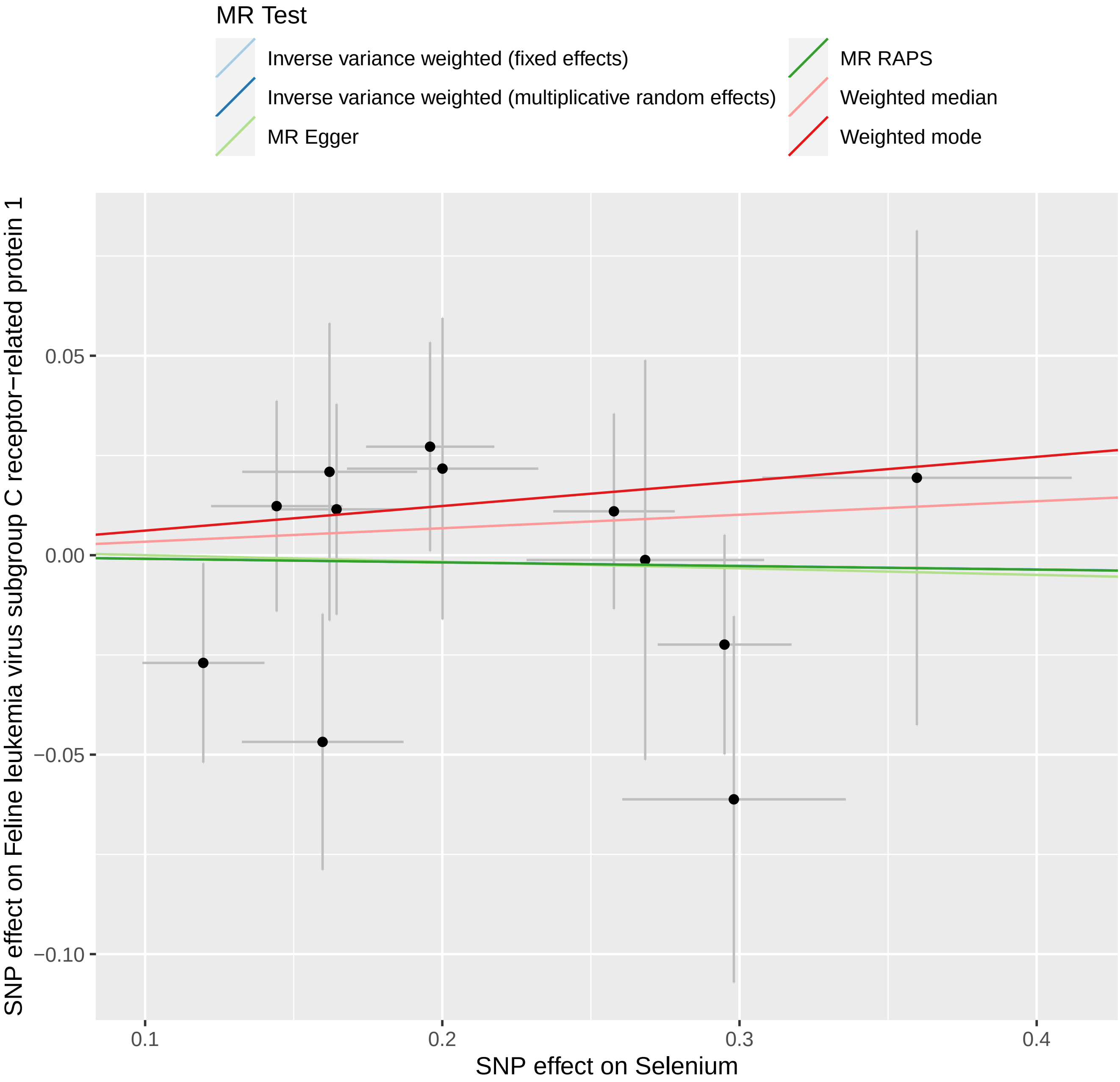

Batch 3−1131 : Selenium on Fibronectin Fragment 4

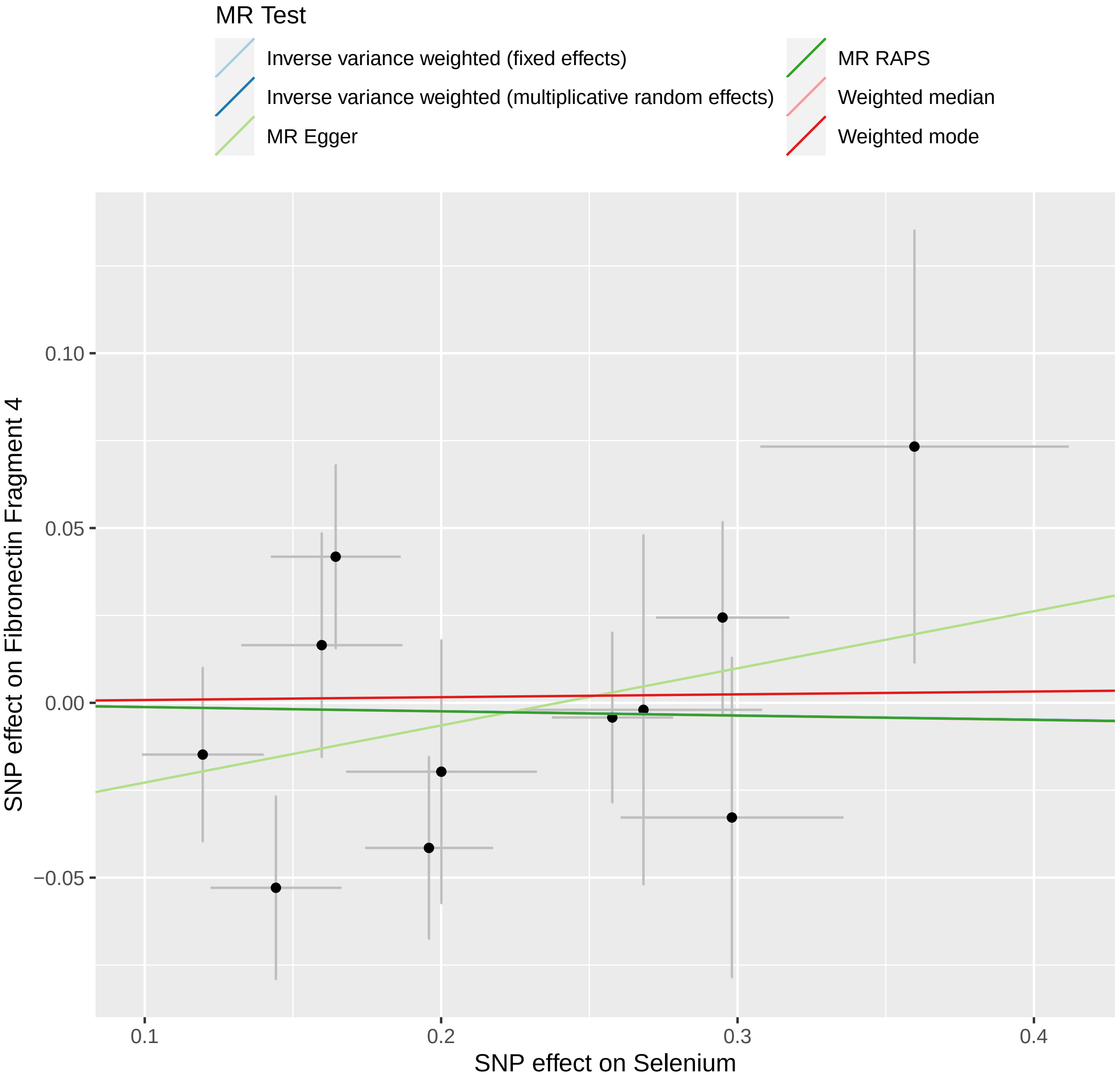

Batch 3−1132 : Selenium on Fibronectin type III domain−containing protein 4

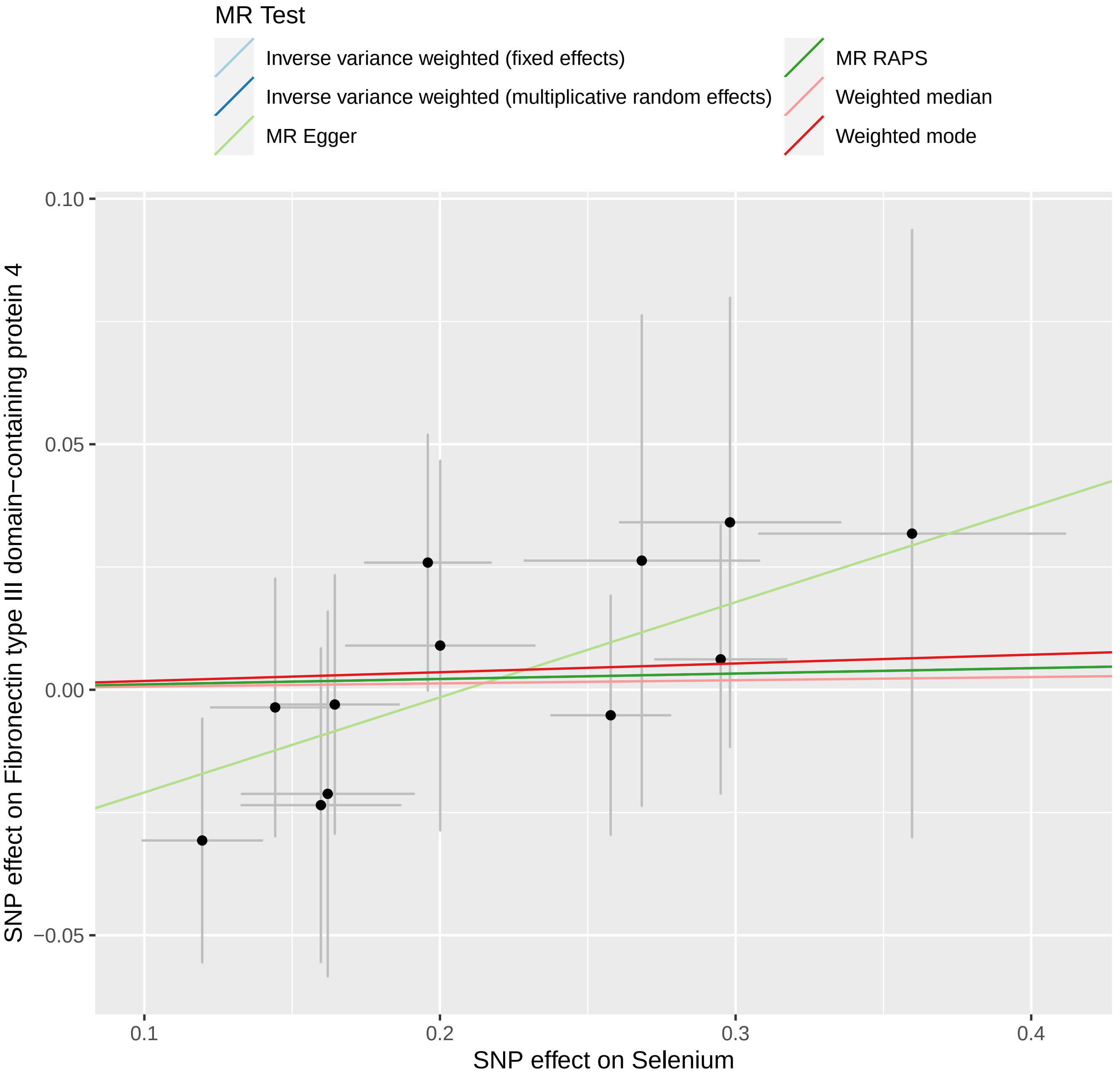

Batch 3−1133 : Selenium on Fibronectin type III domain−containing protein 5

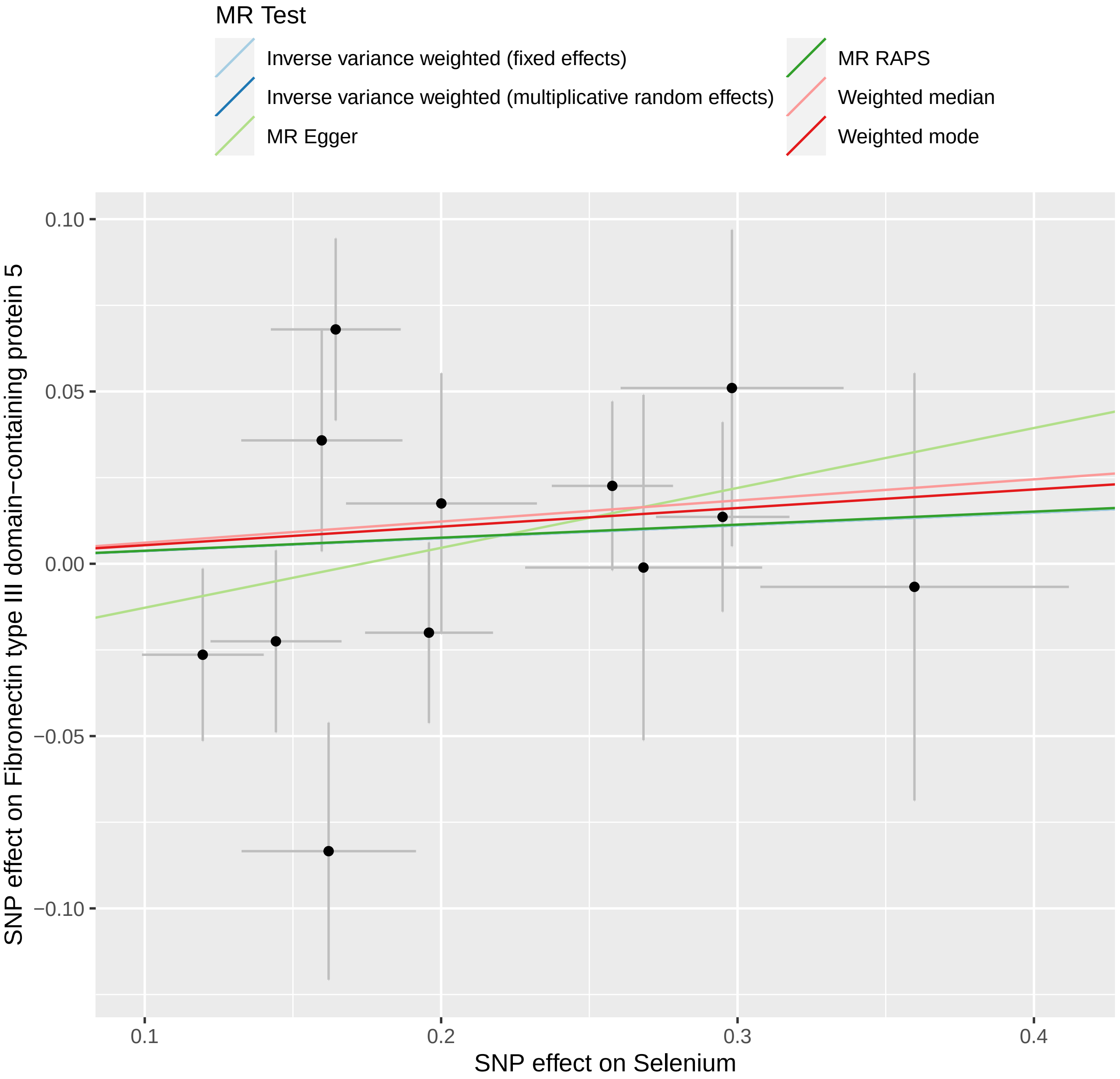

Batch 3−1134 : Selenium on Glutamate carboxypeptidase 2

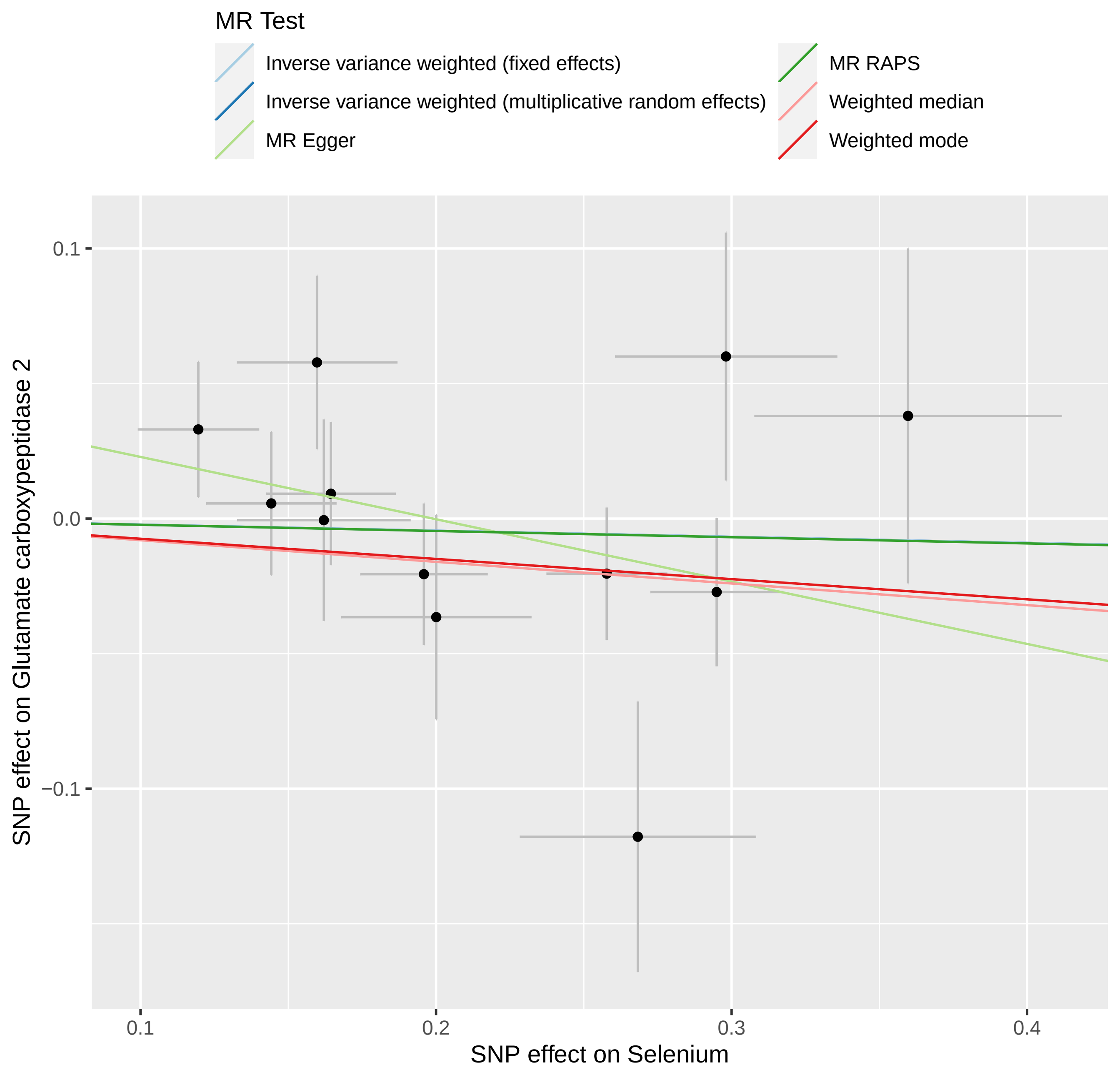

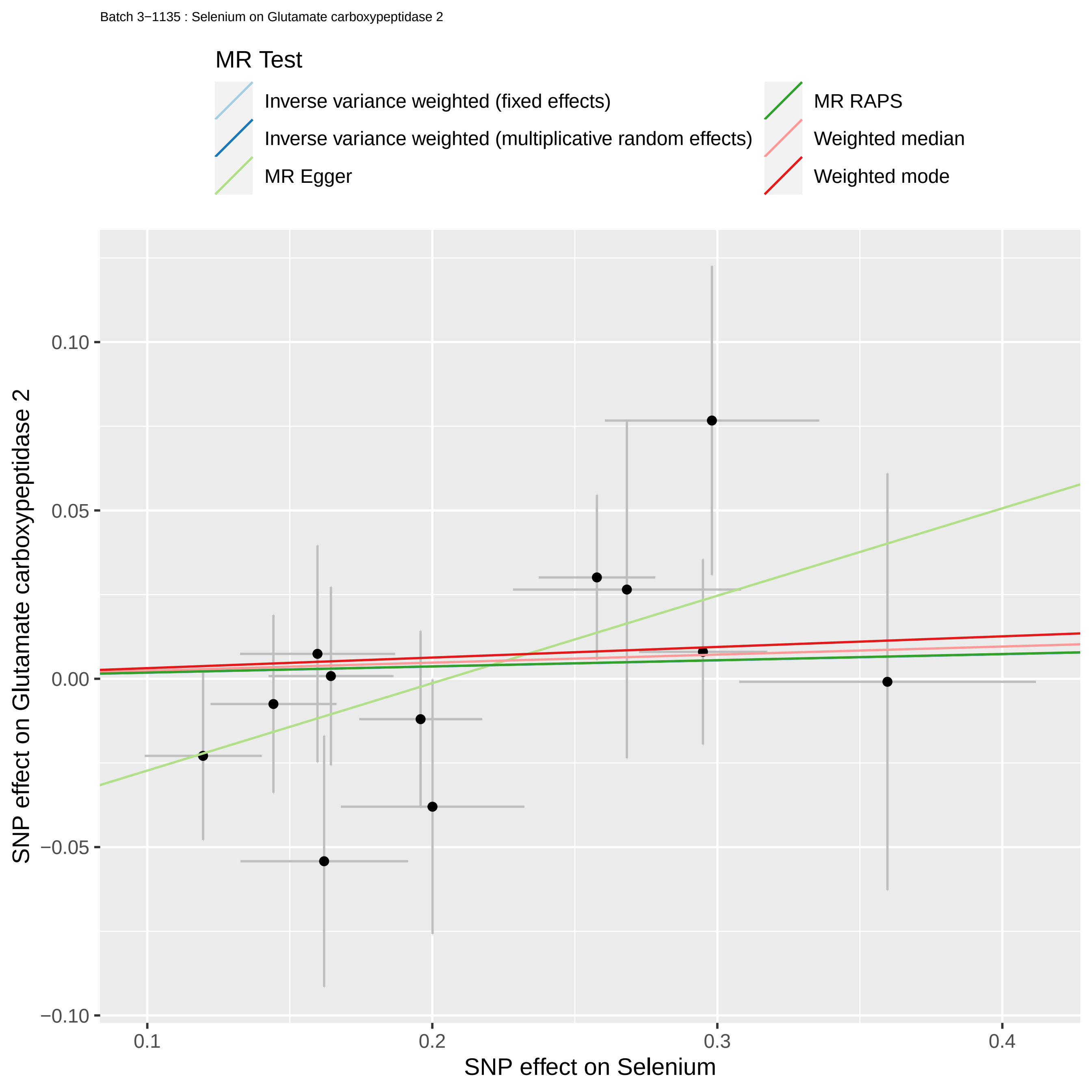
Batch 3−1135 : Selenium on Glutamate carboxypeptidase 2
MR Test
Inverse variance weighted (fixed effects)
Inverse variance weighted (multiplicative random effects)
MR Egger
MR RAPS
Weighted median
Weighted mode
SNP effect on Glutamate carboxypeptidase 2
0.10
0.05
0.00
−0.05
−0.10
0.1
0.2
0.3
0.4
SNP effect on Selenium

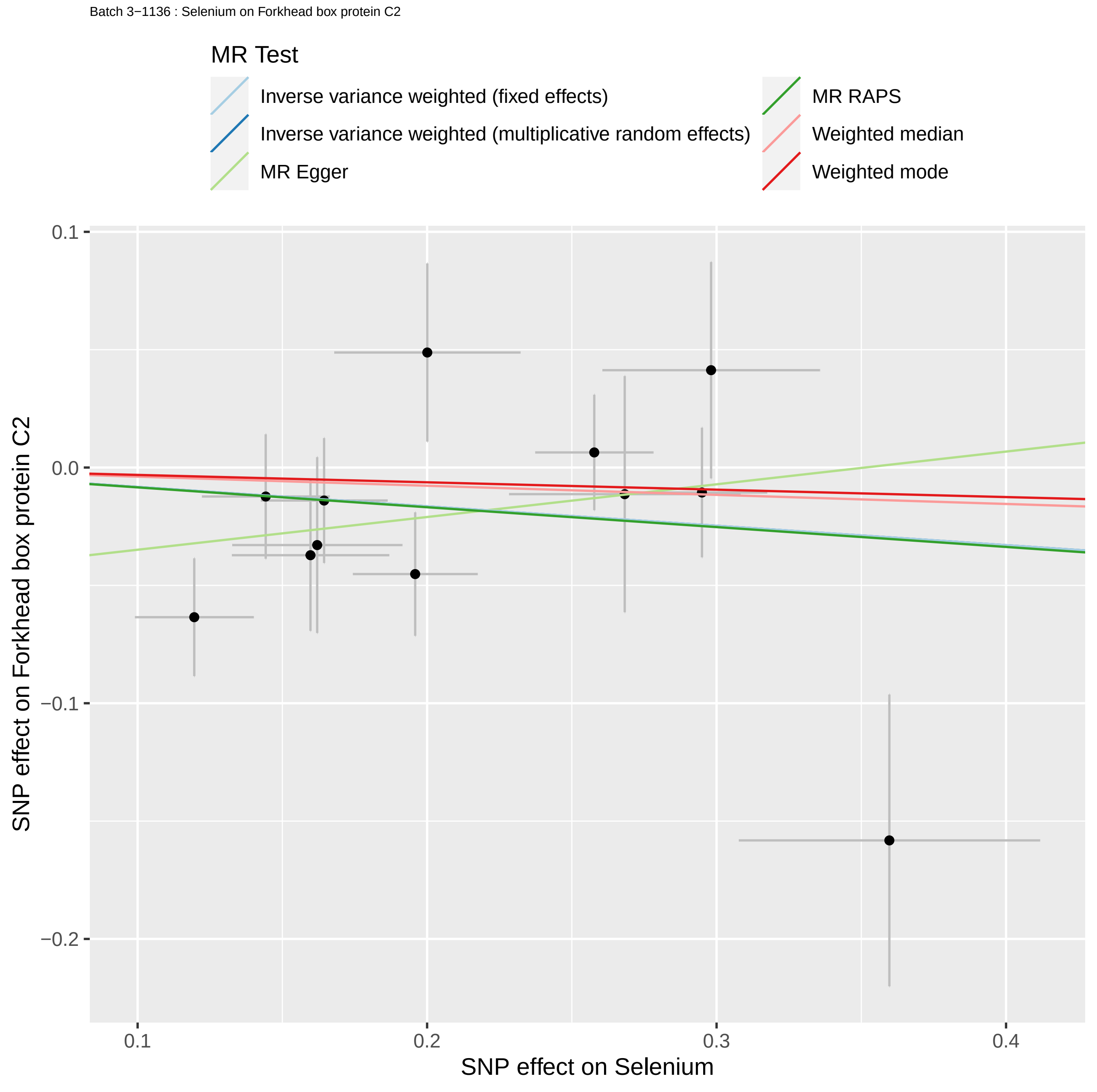

Batch 3−1136 : Selenium on Forkhead box protein C2
MR Test
Inverse variance weighted (fixed effects)
Inverse variance weighted (multiplicative random effects)
MR Egger
MR RAPS
Weighted median
Weighted mode
0.1
0.0
−0.1
−0.2
SNP effect on Forkhead box protein C2
0.1
0.2
0.3
0.4
SNP effect on Selenium

Batch 3−1137 : Selenium on Forkhead box protein J2

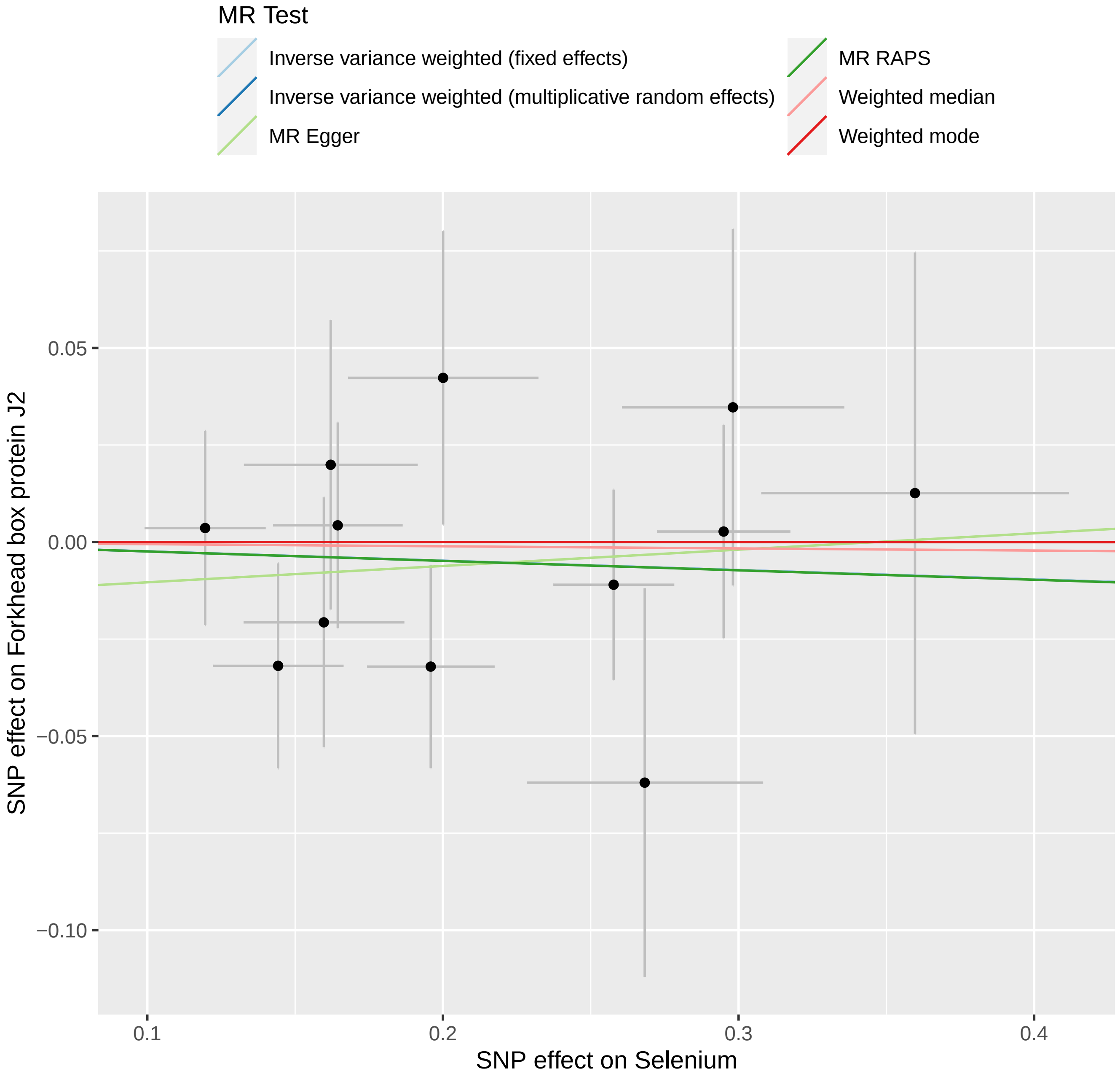

Batch 3−1138 : Selenium on Forkhead box protein L2

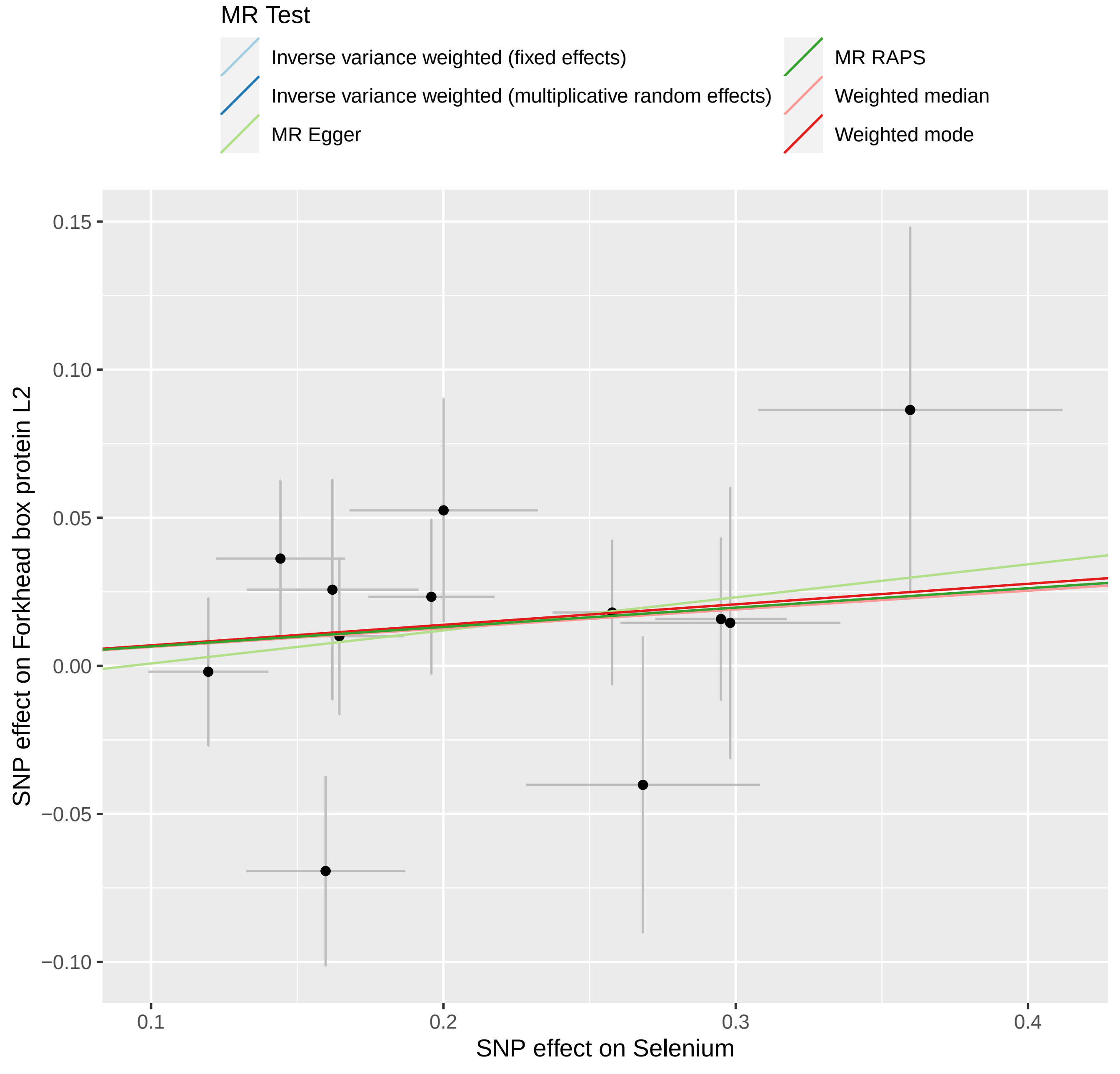

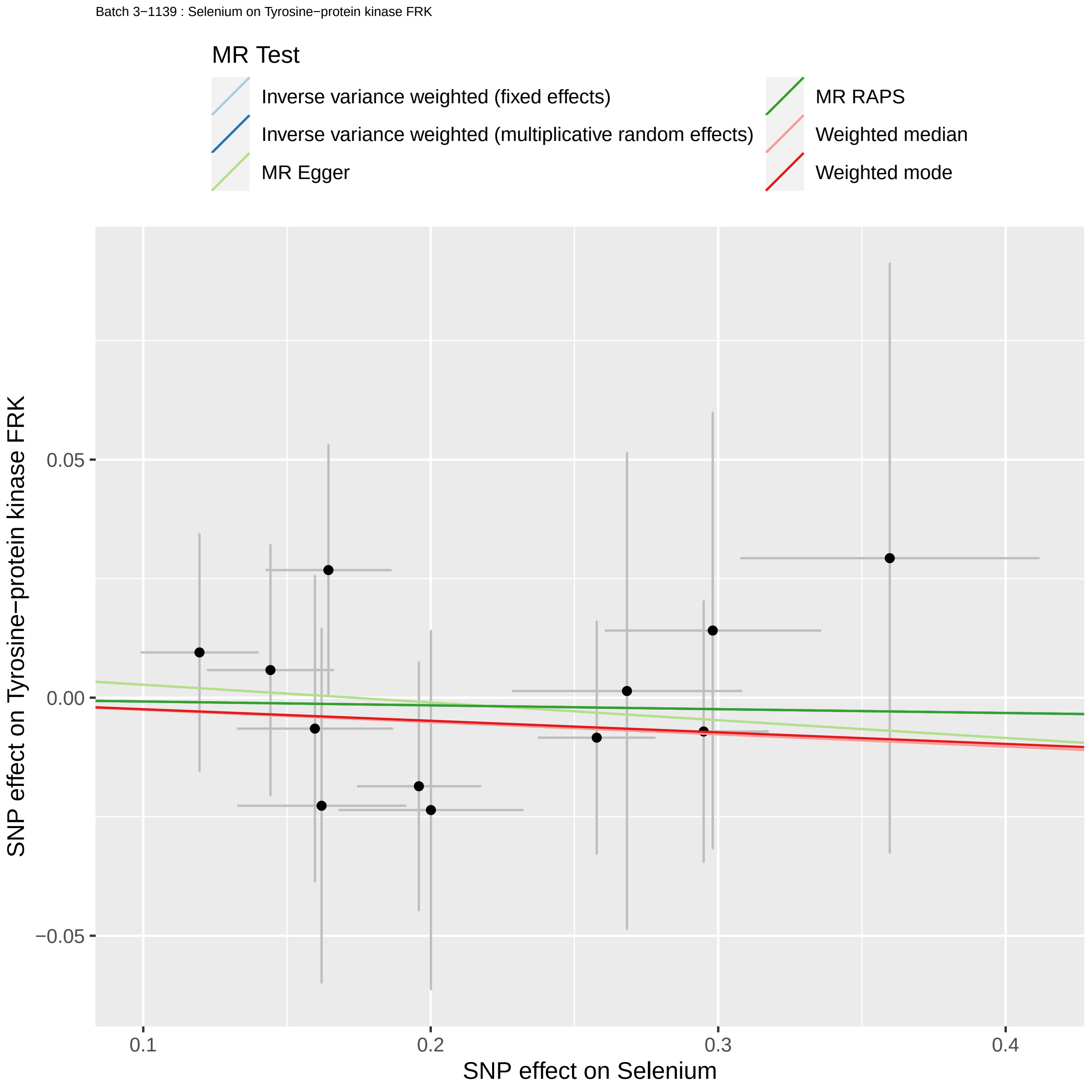

Batch 3−1139 : Selenium on Tyrosine−protein kinase FRK
MR Test
Inverse variance weighted (fixed effects)
Inverse variance weighted (multiplicative random effects)
MR Egger
MR RAPS
Weighted median
Weighted mode
SNP effect on Tyrosine−protein kinase FRK
0.05
0.00
−0.05
0.1
0.2
0.3
0.4
SNP effect on Selenium

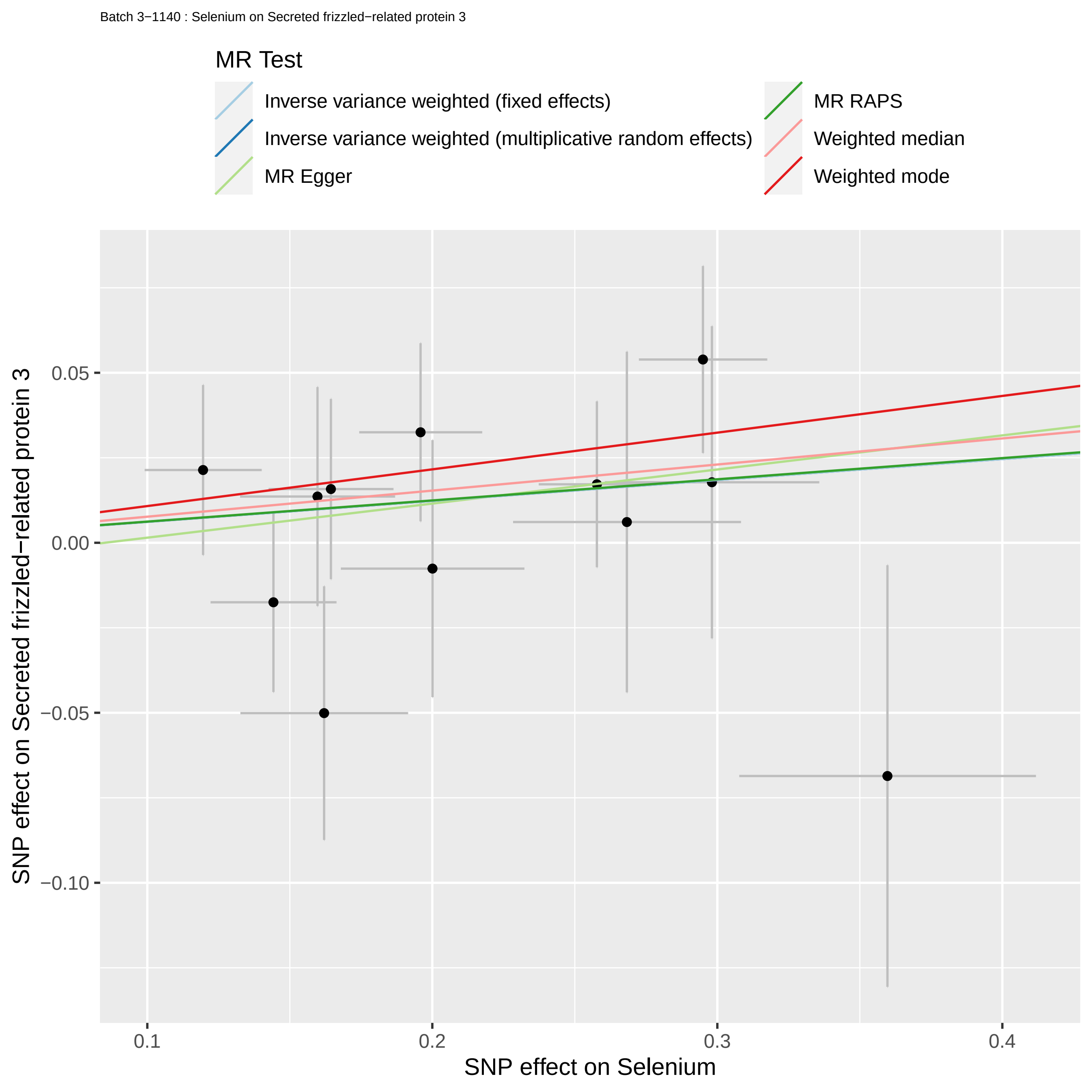

Batch 3−1140 : Selenium on Secreted frizzled−related protein 3
MR Test
Inverse variance weighted (fixed effects)
Inverse variance weighted (multiplicative random effects)
MR Egger
MR RAPS
Weighted median
Weighted mode
0.05
0.00
−0.05
−0.10
0.1
0.2
0.3
0.4
SNP effect on Secreted frizzled−related protein 3
SNP effect on Selenium

Batch 3−1141 : Selenium on Secreted frizzled−related protein 3

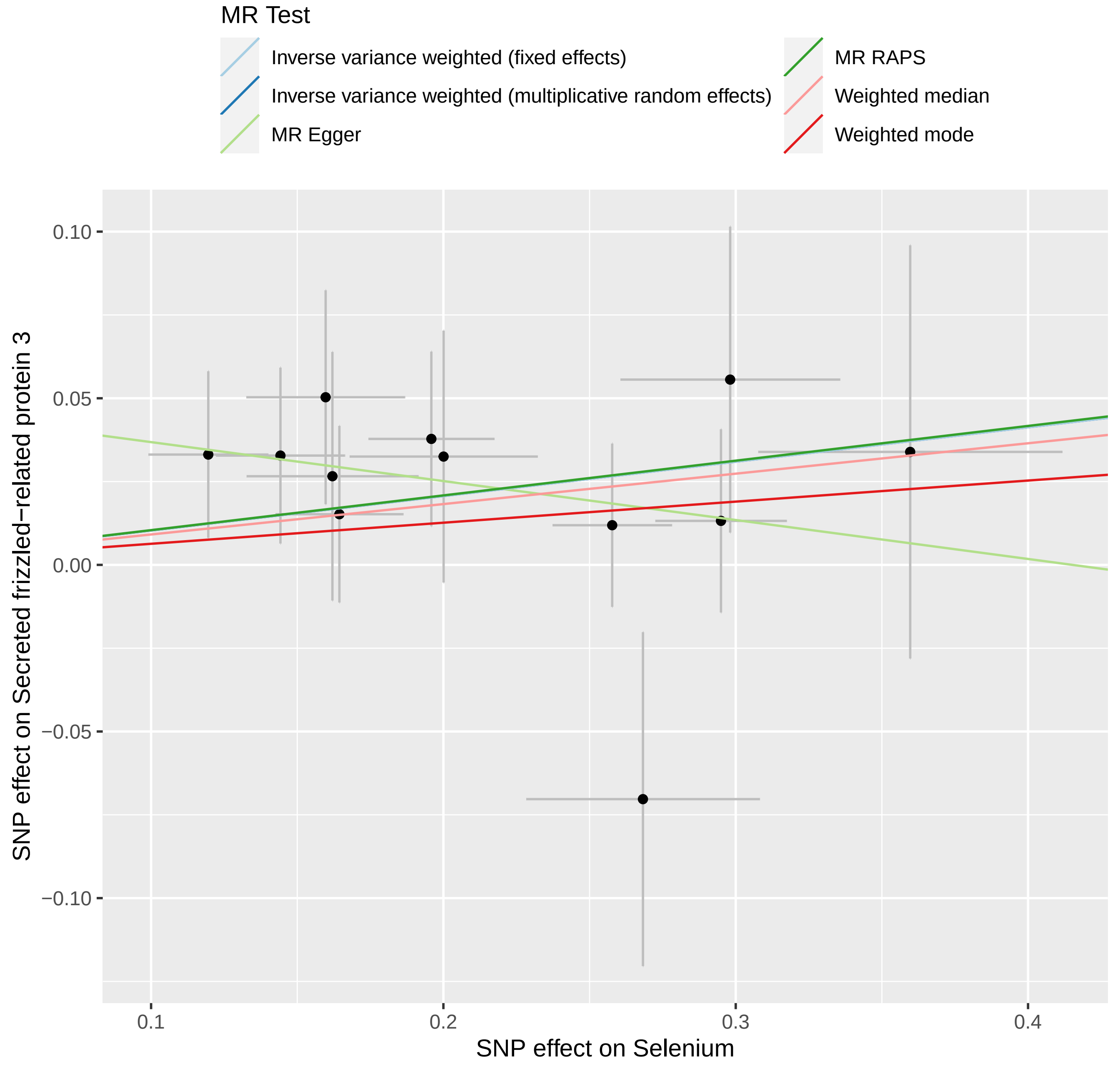

Batch 3−1142 : Selenium on Follistatin

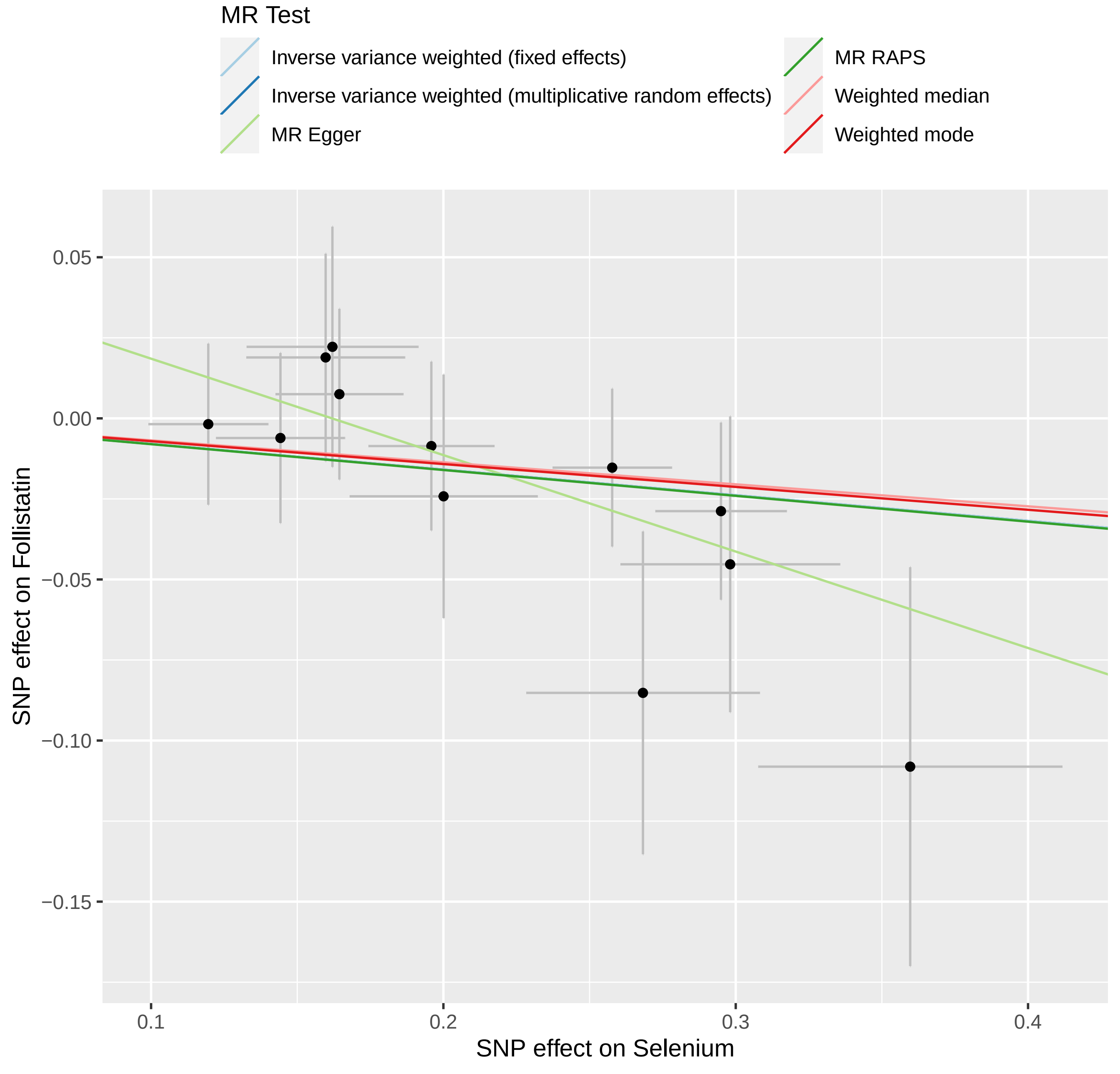

Batch 3−1143 : Selenium on Follistatin−related protein 1

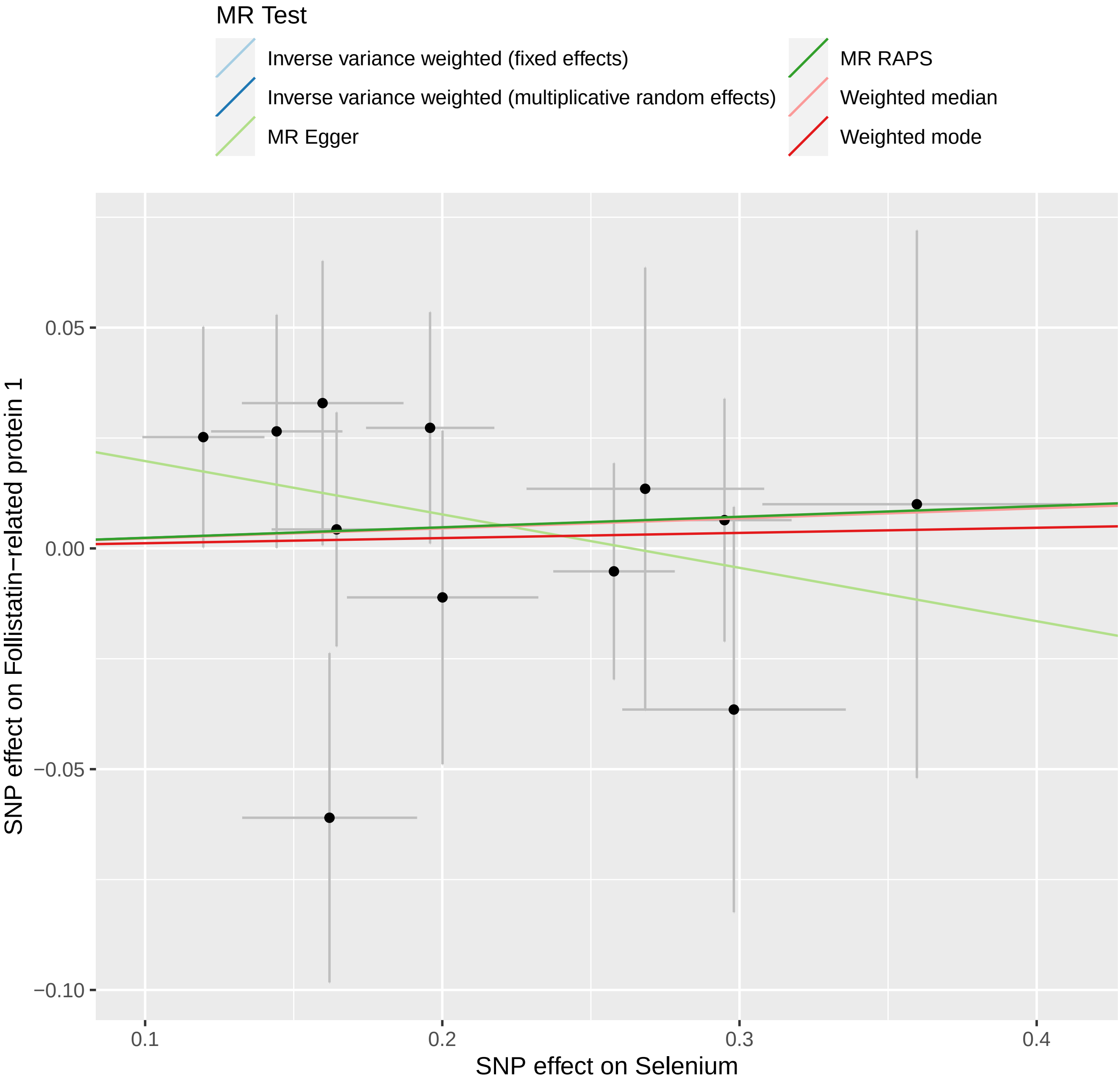

Batch 3−1144 : Selenium on Follistatin−related protein 1

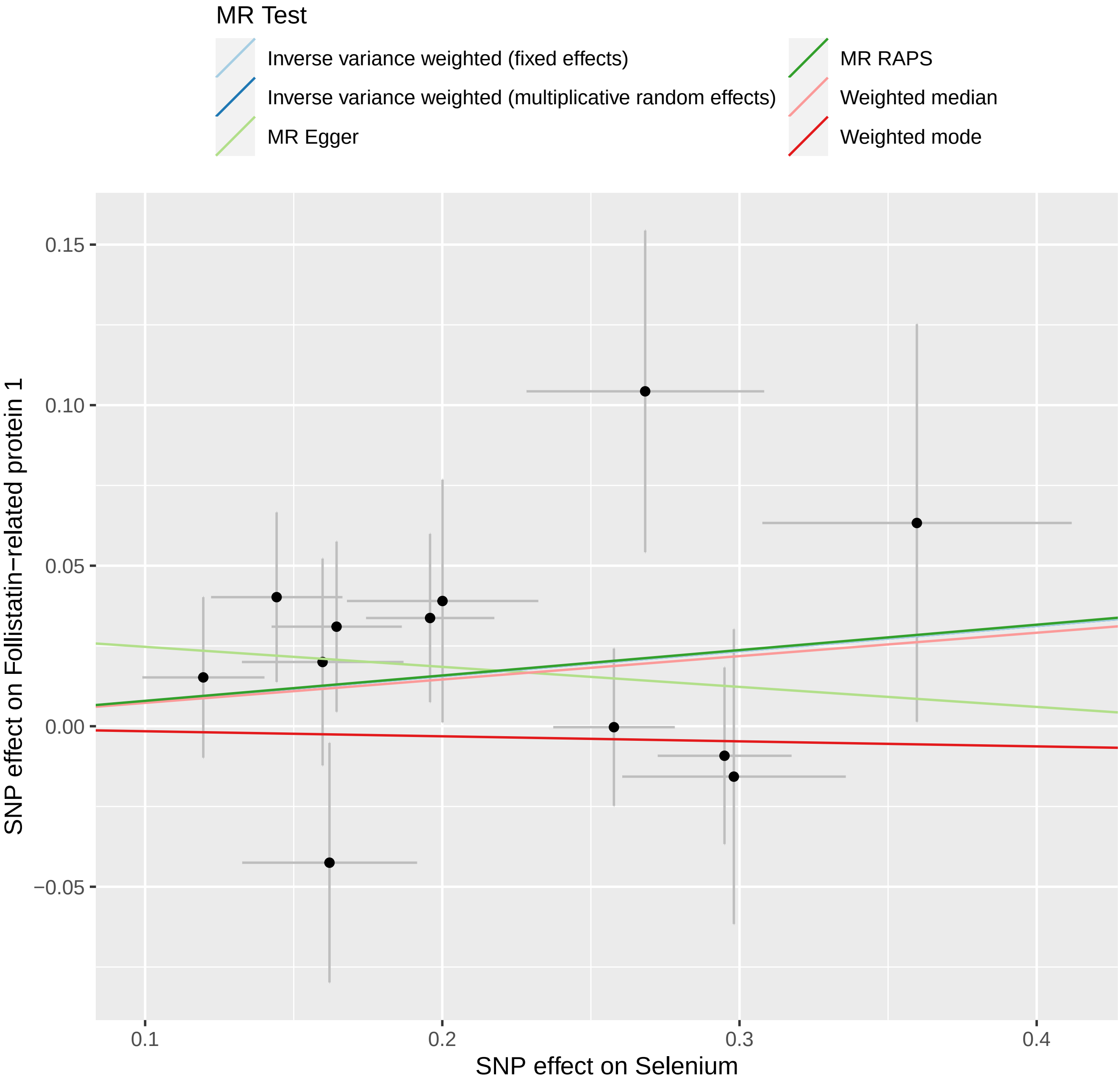

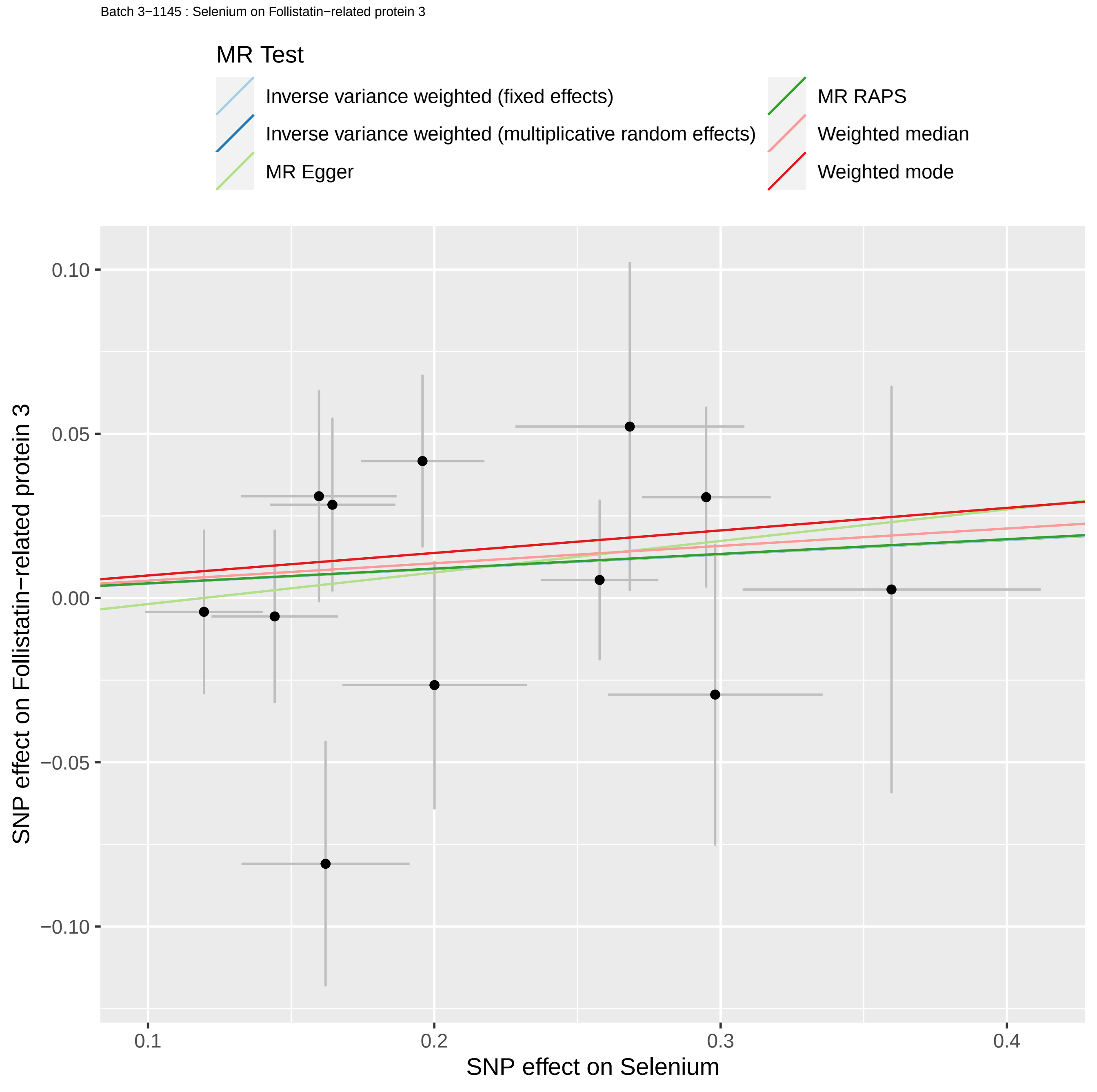

Batch 3−1145 : Selenium on Follistatin−related protein 3
MR Test
Inverse variance weighted (fixed effects)
Inverse variance weighted (multiplicative random effects)
MR Egger
MR RAPS
Weighted median
Weighted mode
0.10
0.05
0.00
−0.05
−0.10
0.1
0.2
0.3
0.4
SNP effect on Selenium
SNP effect on Follistatin−related protein 3

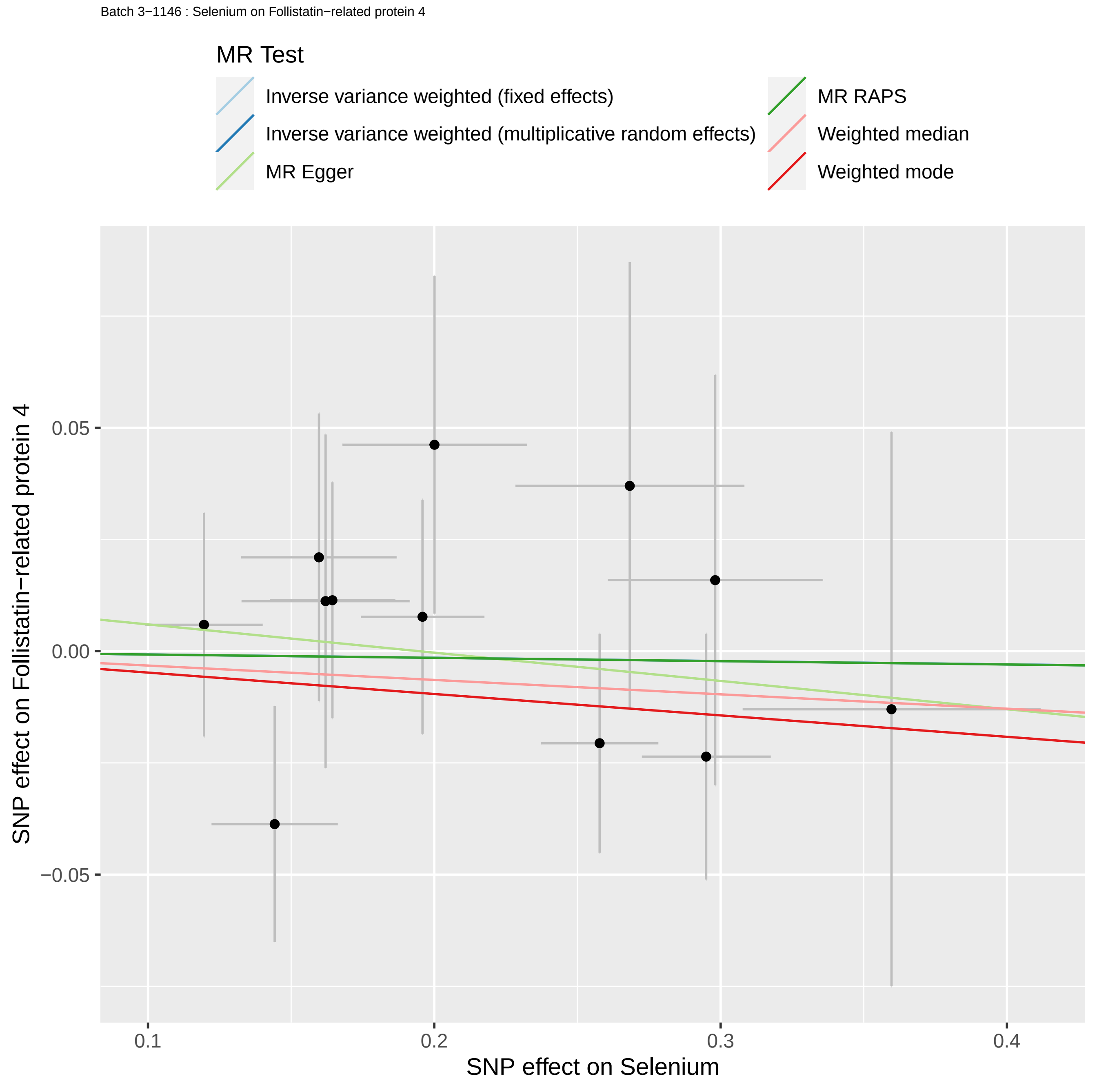
Batch 3−1146 : Selenium on Follistatin−related protein 4
MR Test
Inverse variance weighted (fixed effects)
Inverse variance weighted (multiplicative random effects)
MR Egger
MR RAPS
Weighted median
Weighted mode
0.05
0.00
−0.05
0.1
0.2
0.3
0.4
SNP effect on Selenium
SNP effect on Follistatin−related protein 4

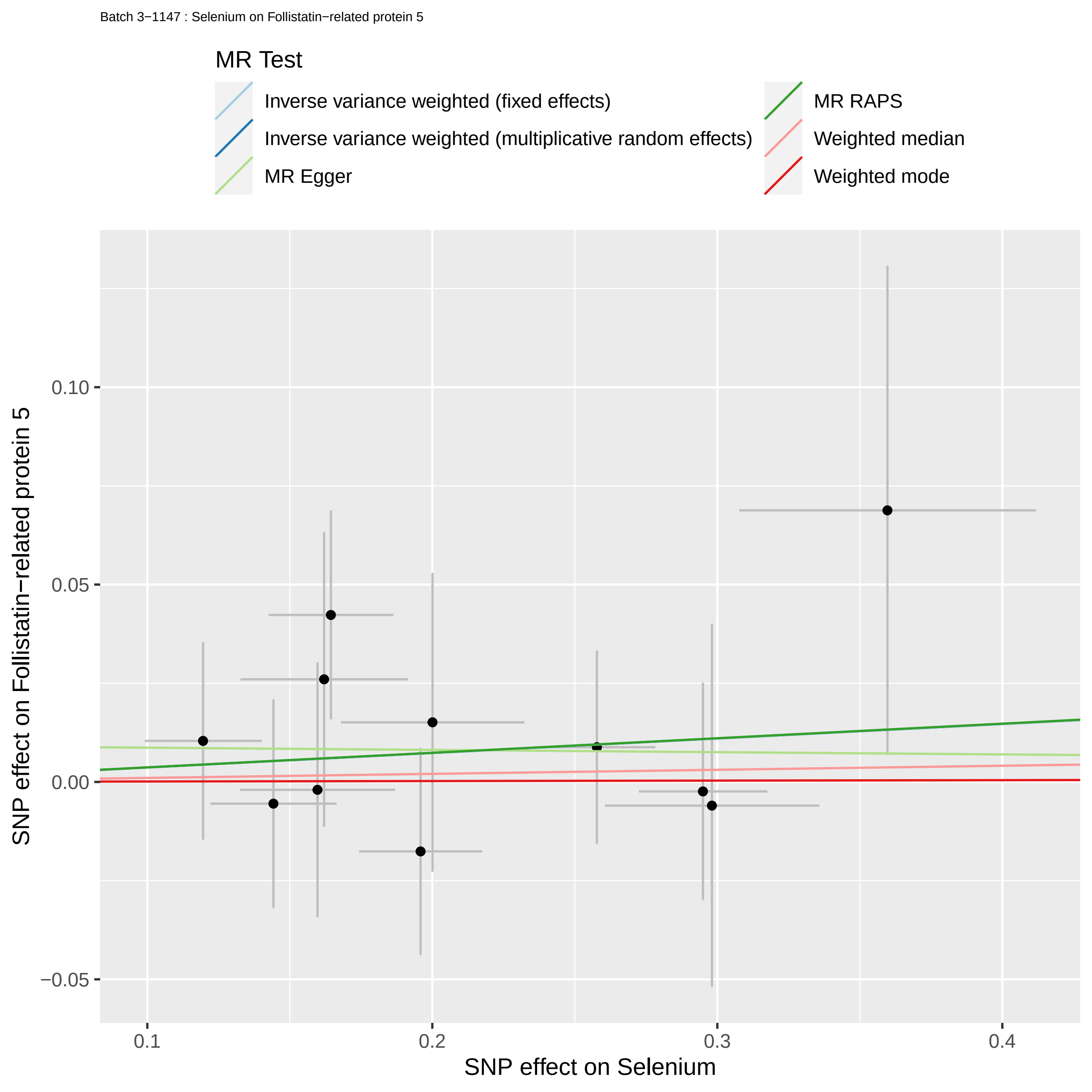

Batch 3−1147 : Selenium on Follistatin−related protein 5
MR Test
Inverse variance weighted (fixed effects)
Inverse variance weighted (multiplicative random effects)
MR Egger
MR RAPS
Weighted median
Weighted mode
0.10
0.05
0.00
−0.05
0.1
0.2
0.3
0.4
SNP effect on Follistatin−related protein 5
SNP effect on Selenium

Batch 3−1148 : Selenium on Ferritin

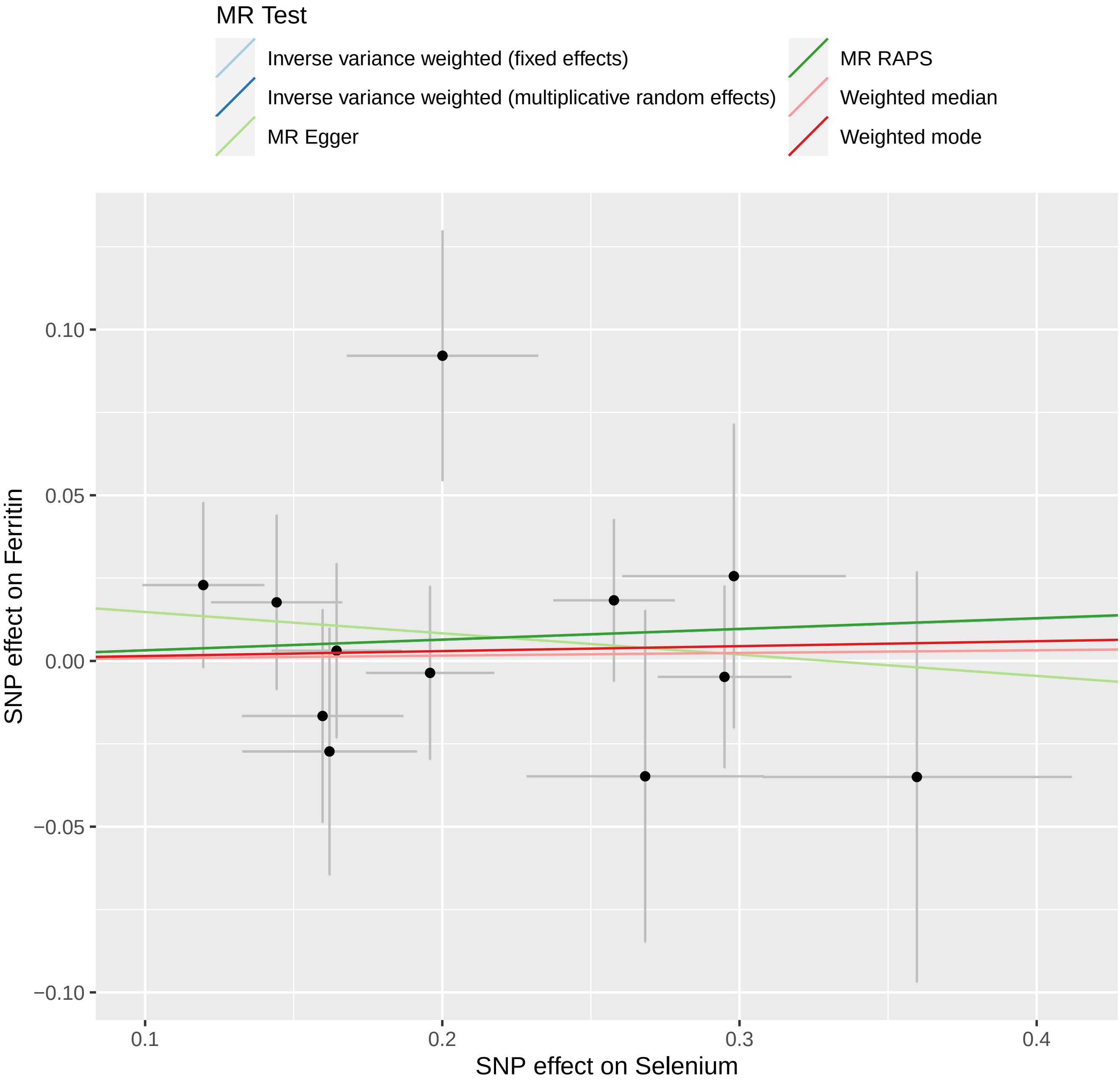

Batch 3−1149 : Selenium on Ferritin

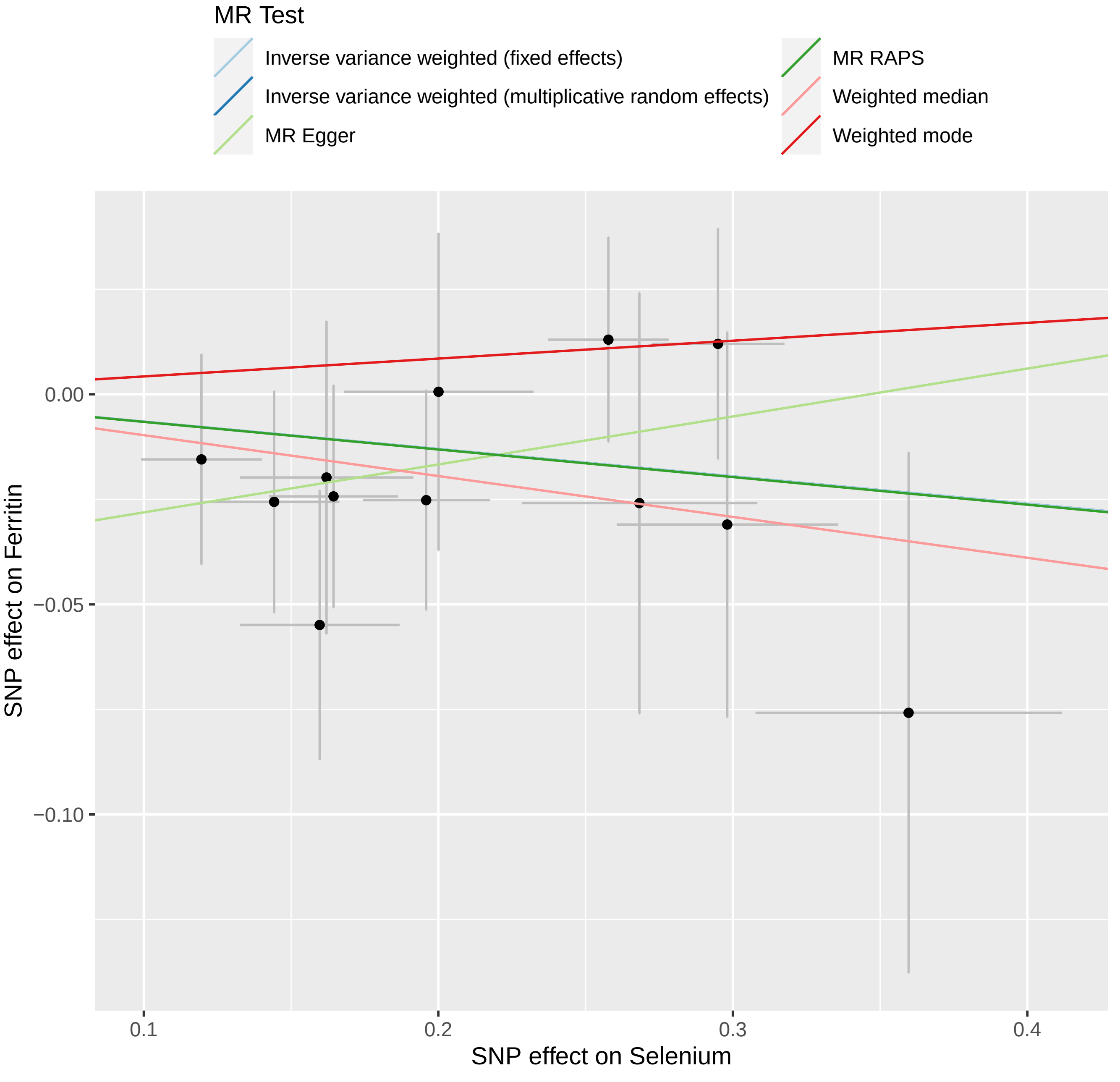

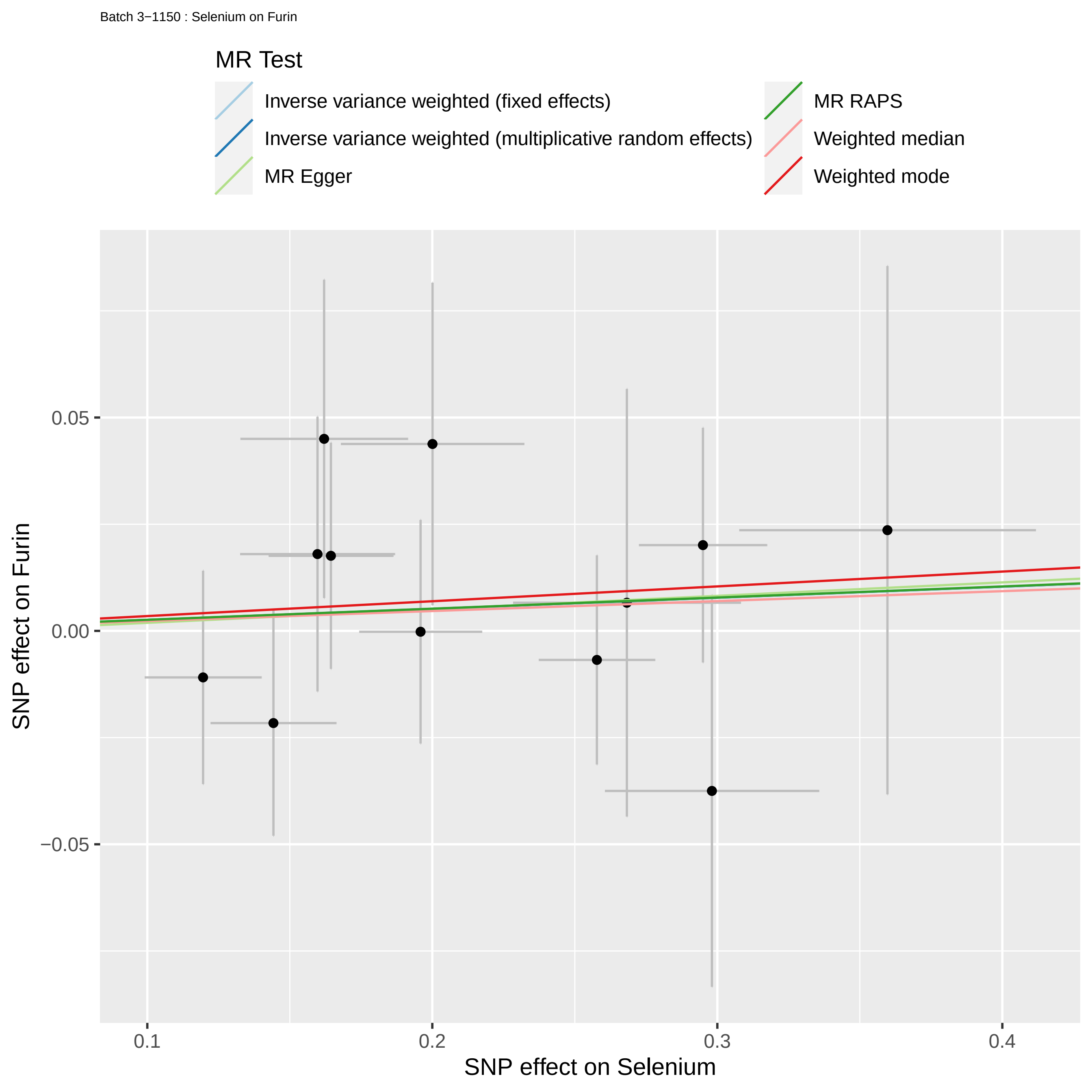
Batch 3−1150 : Selenium on Furin
MR Test
Inverse variance weighted (fixed effects)
Inverse variance weighted (multiplicative random effects)
MR Egger
MR RAPS
Weighted median
Weighted mode
SNP effect on Furin
0.05
0.00
−0.05
0.1
0.2
0.3
0.4
SNP effect on Selenium

Batch 3−1151 : Selenium on Alpha−(1,3)−fucosyltransferase 10

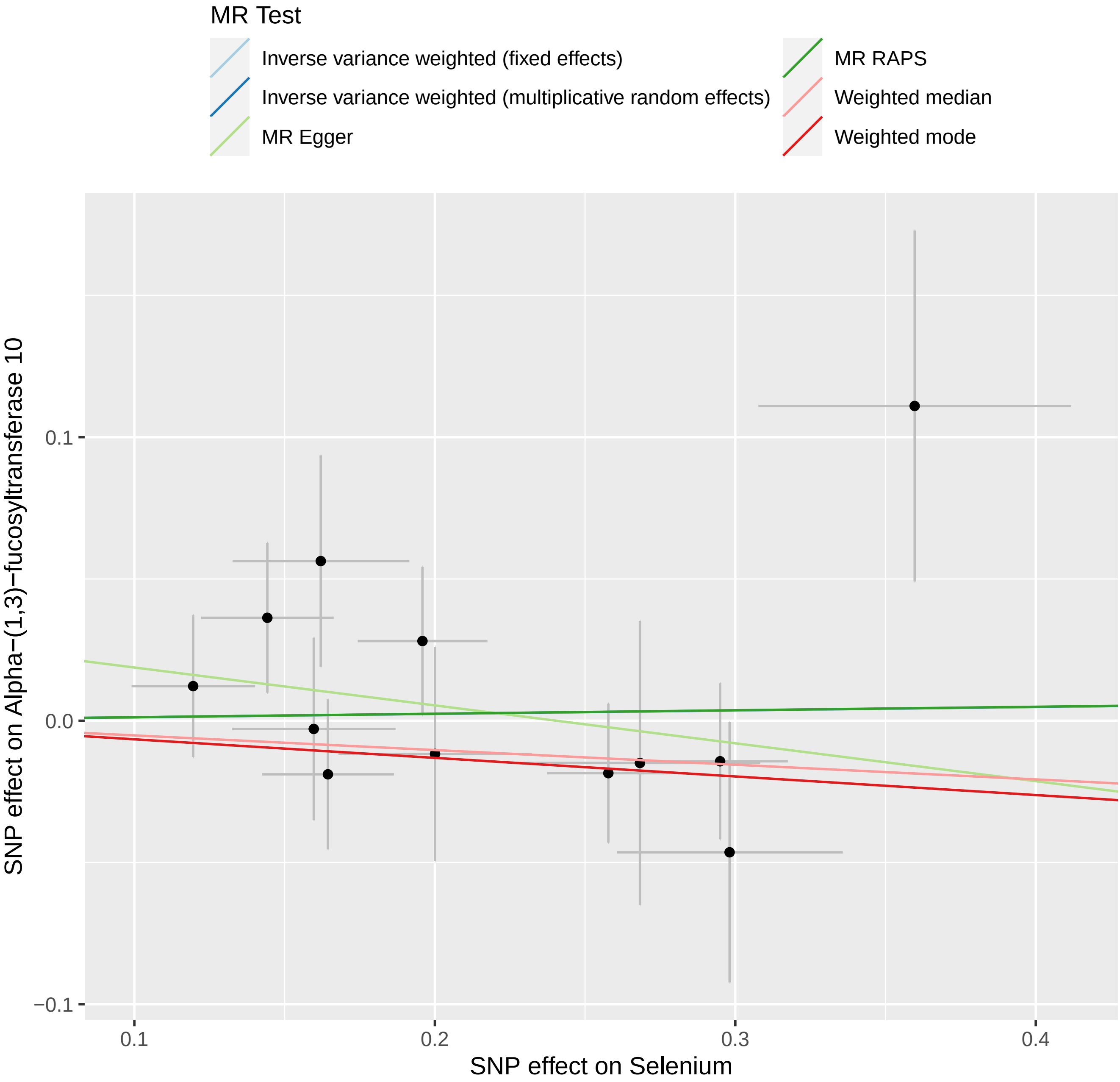

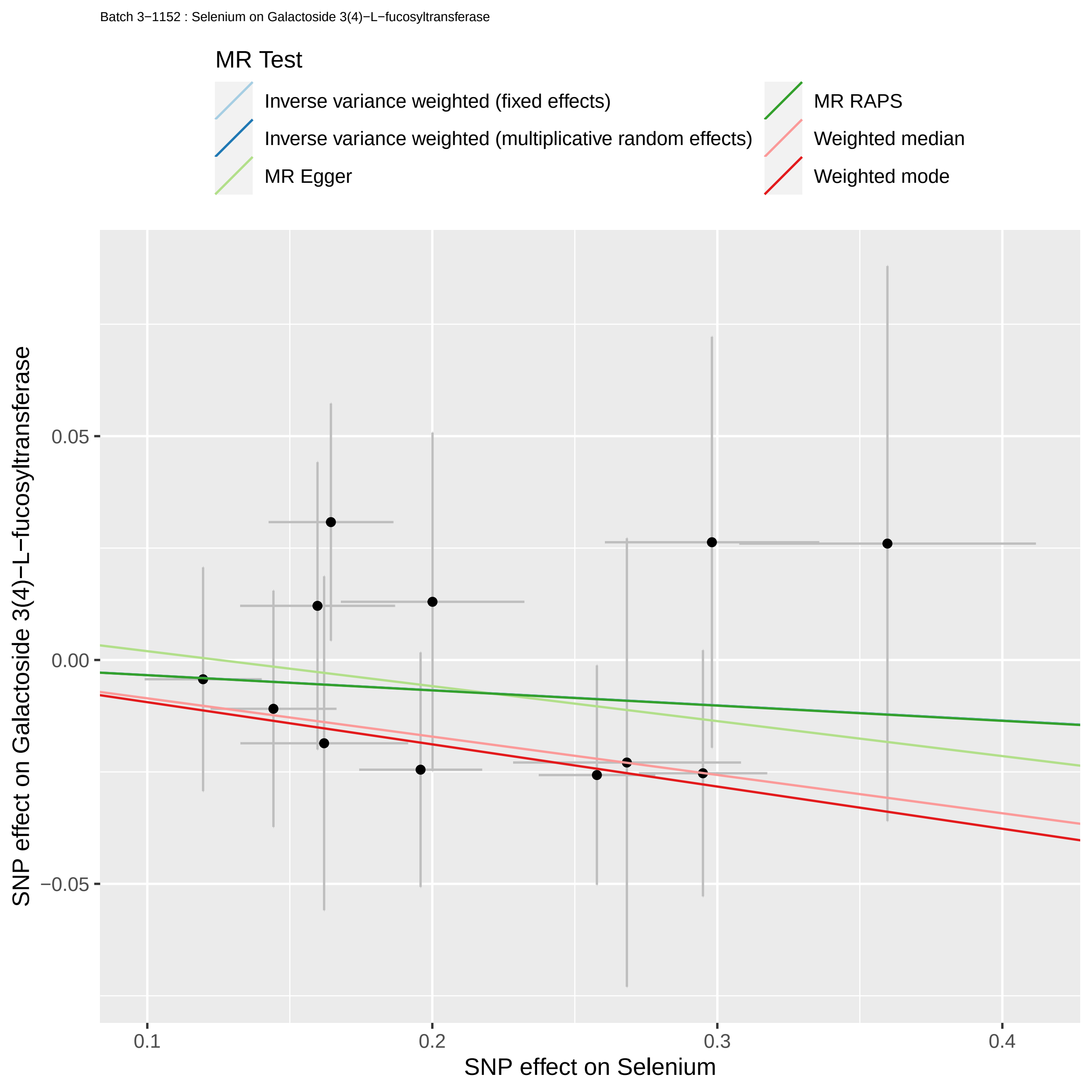
Batch 3−1152 : Selenium on Galactoside 3(4)−L−fucosyltransferase
MR Test
Inverse variance weighted (fixed effects)
Inverse variance weighted (multiplicative random effects)
MR Egger
MR RAPS
Weighted median
Weighted mode
SNP effect on Galactoside 3(4)−L−fucosyltransferase
0.05
0.00
−0.05
0.1
0.2
0.3
0.4
SNP effect on Selenium

Batch 3−1153 : Selenium on Alpha−(1,3)−fucosyltransferase 5

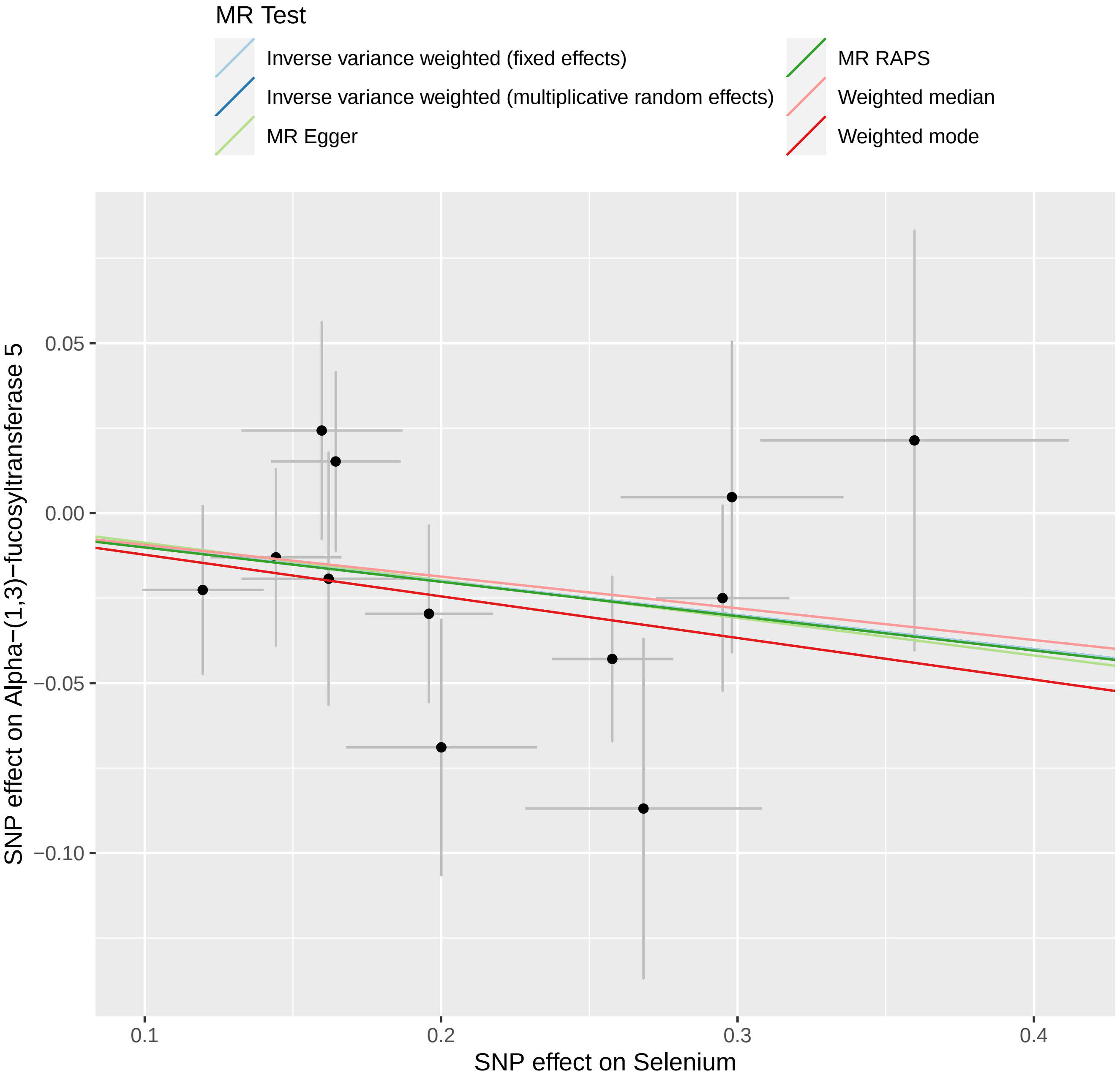

Batch 3−1154 : Selenium on Alpha−(1,6)−fucosyltransferase

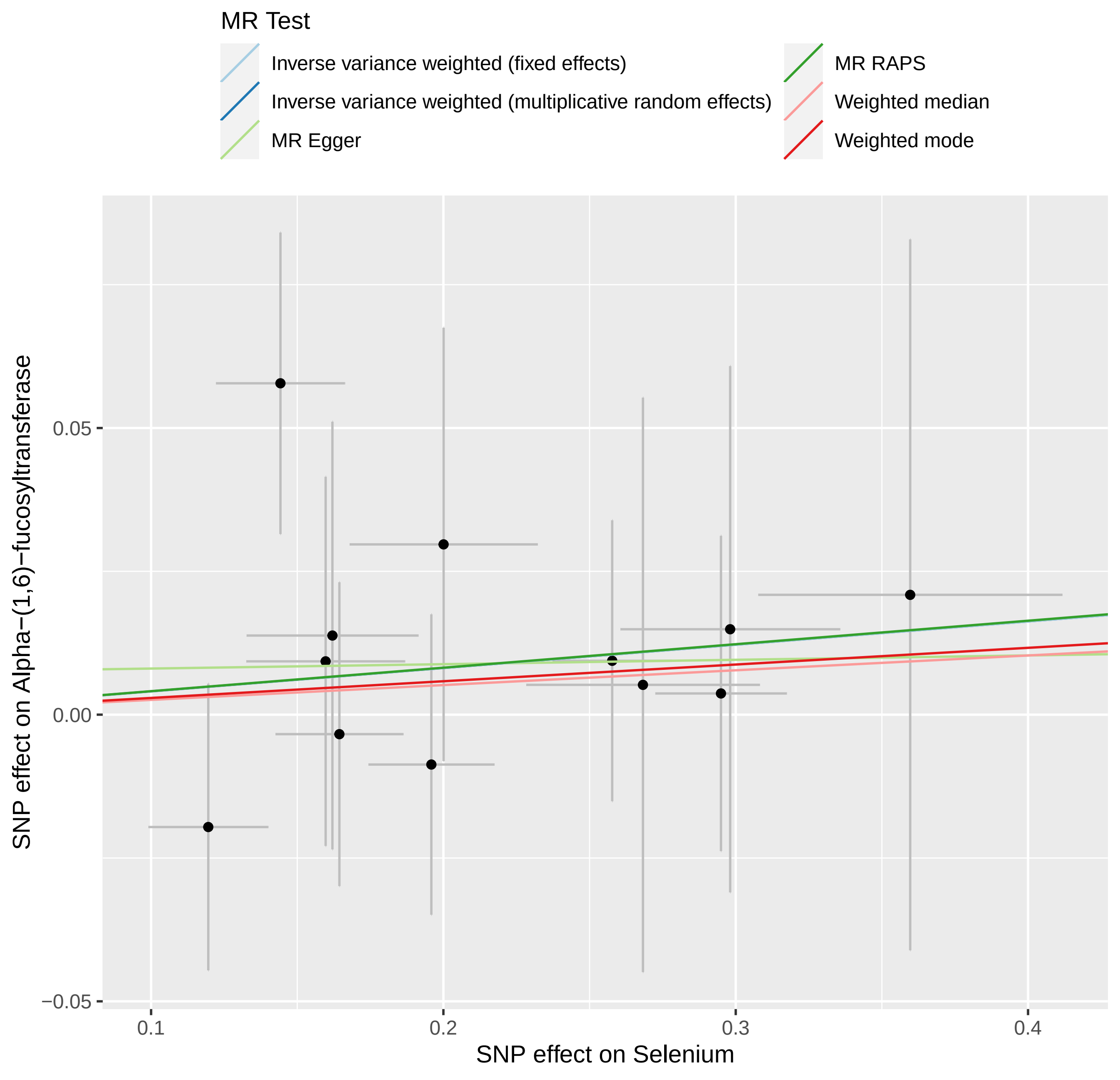

Batch 3−1155 : Selenium on Alpha−(1,3)−fucosyltransferase 9

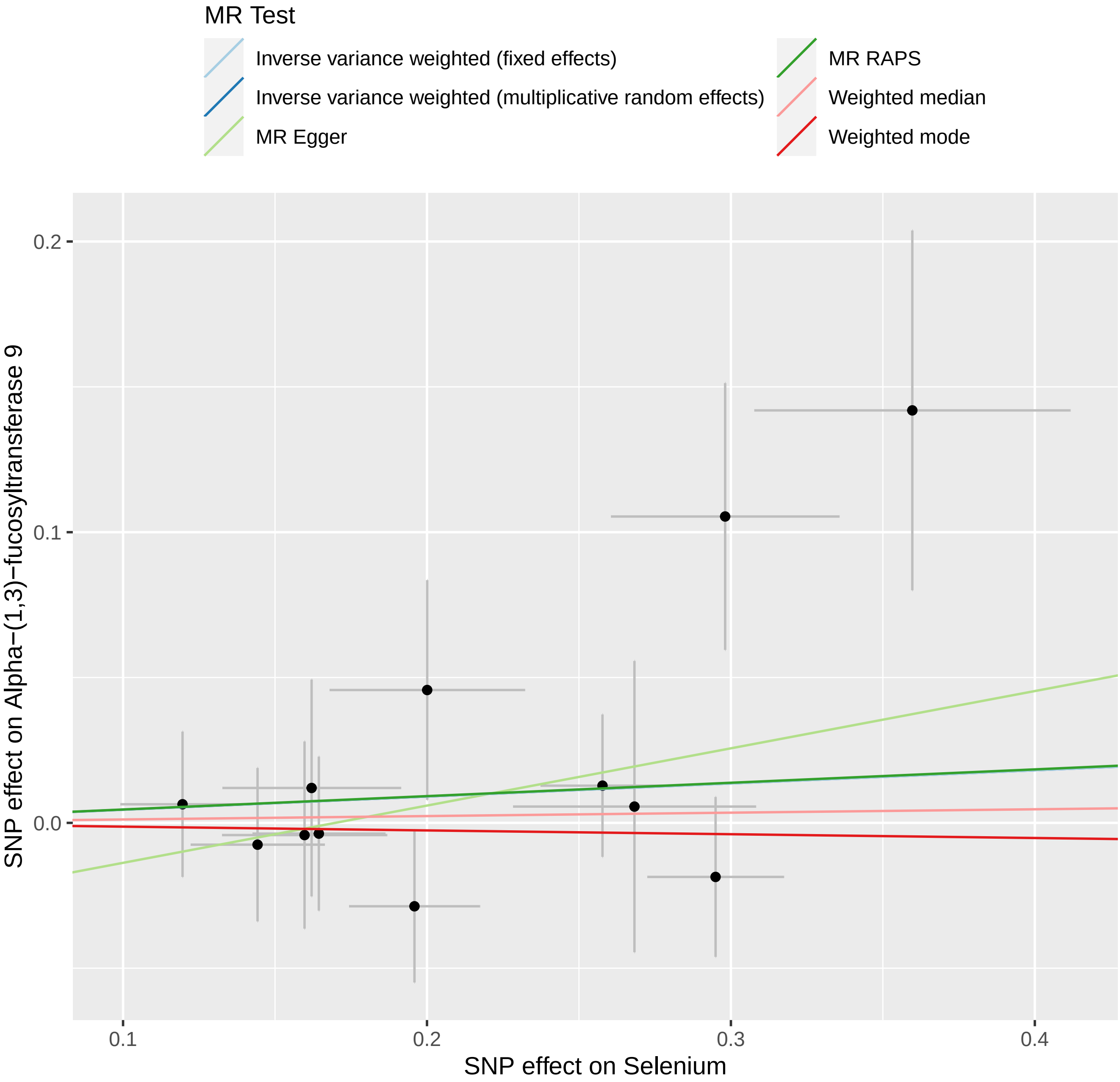

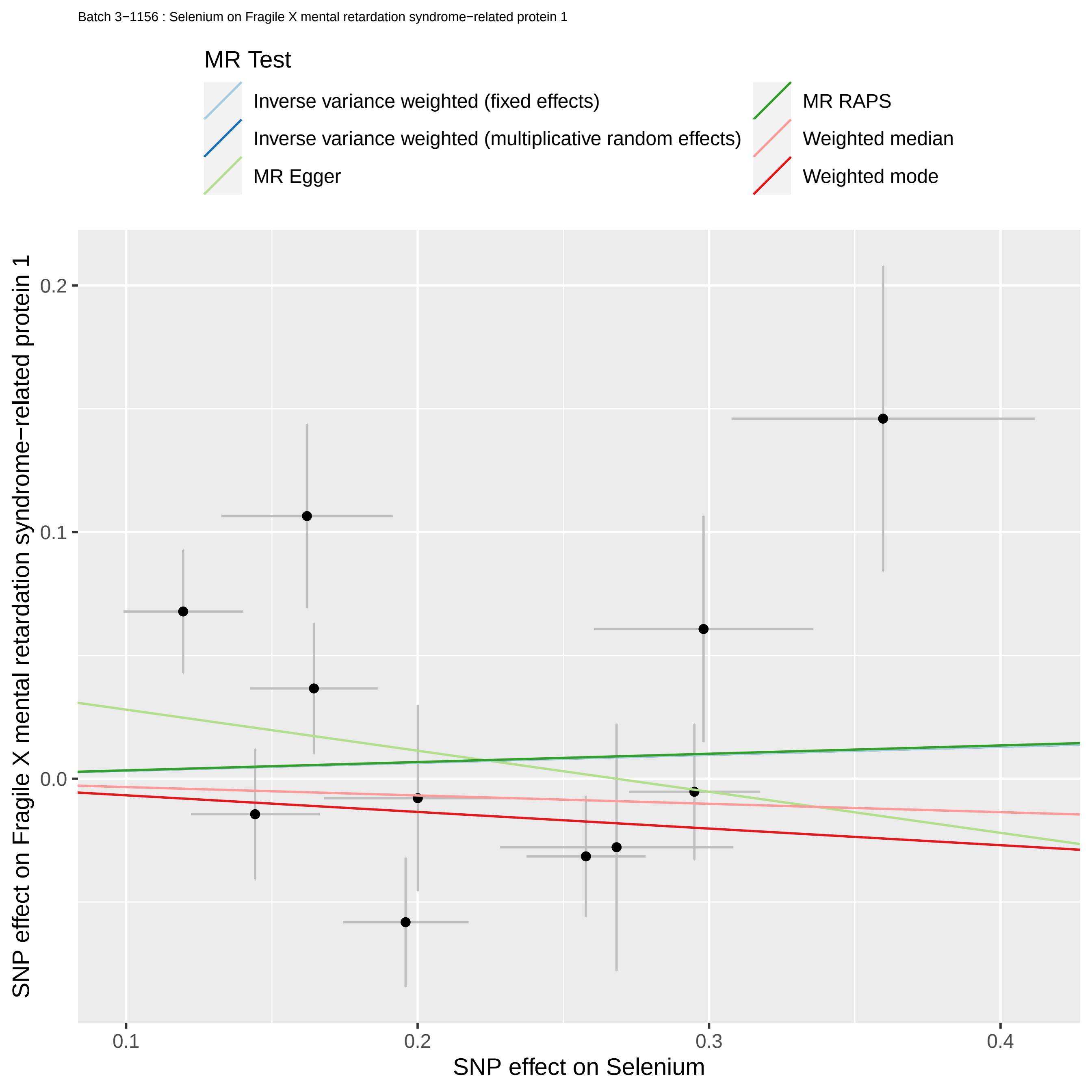

Batch 3−1156 : Selenium on Fragile X mental retardation syndrome−related protein 1
MR Test
Inverse variance weighted (fixed effects)
Inverse variance weighted (multiplicative random effects)
MR Egger
MR RAPS
Weighted median
Weighted mode
SNP effect on Fragile X mental retardation syndrome−related protein 1
0.2
0.1
0.0
0.1
0.2
0.3
0.4
SNP effect on Selenium

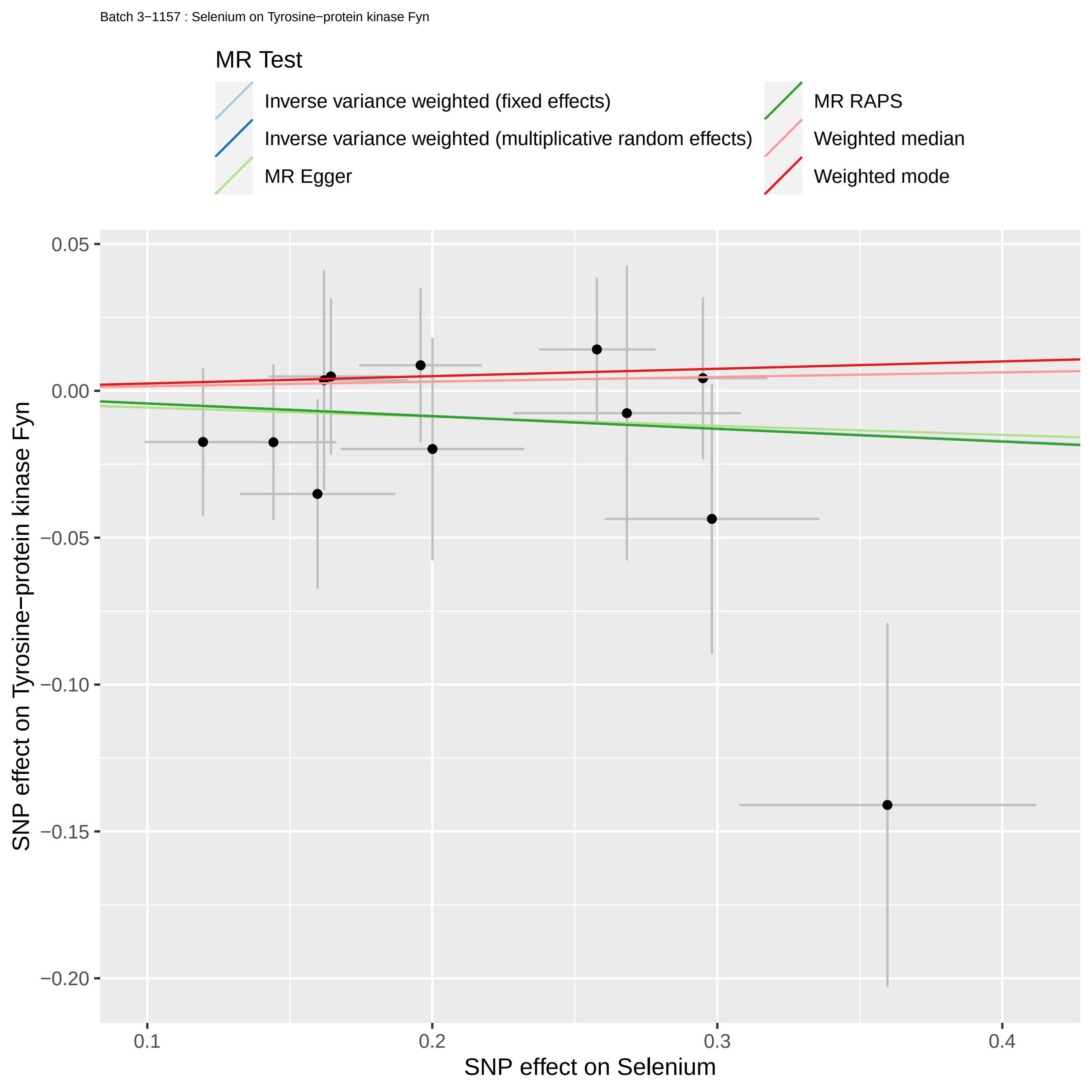

Batch 3−1157 : Selenium on Tyrosine−protein kinase Fyn
MR Test
Inverse variance weighted (fixed effects)
Inverse variance weighted (multiplicative random effects)
MR Egger
MR RAPS
Weighted median
Weighted mode
0.05
0.00
−0.05
−0.10
−0.15
−0.20
0.1
0.2
0.3
0.4
SNP effect on Selenium
SNP effect on Tyrosine−protein kinase Fyn

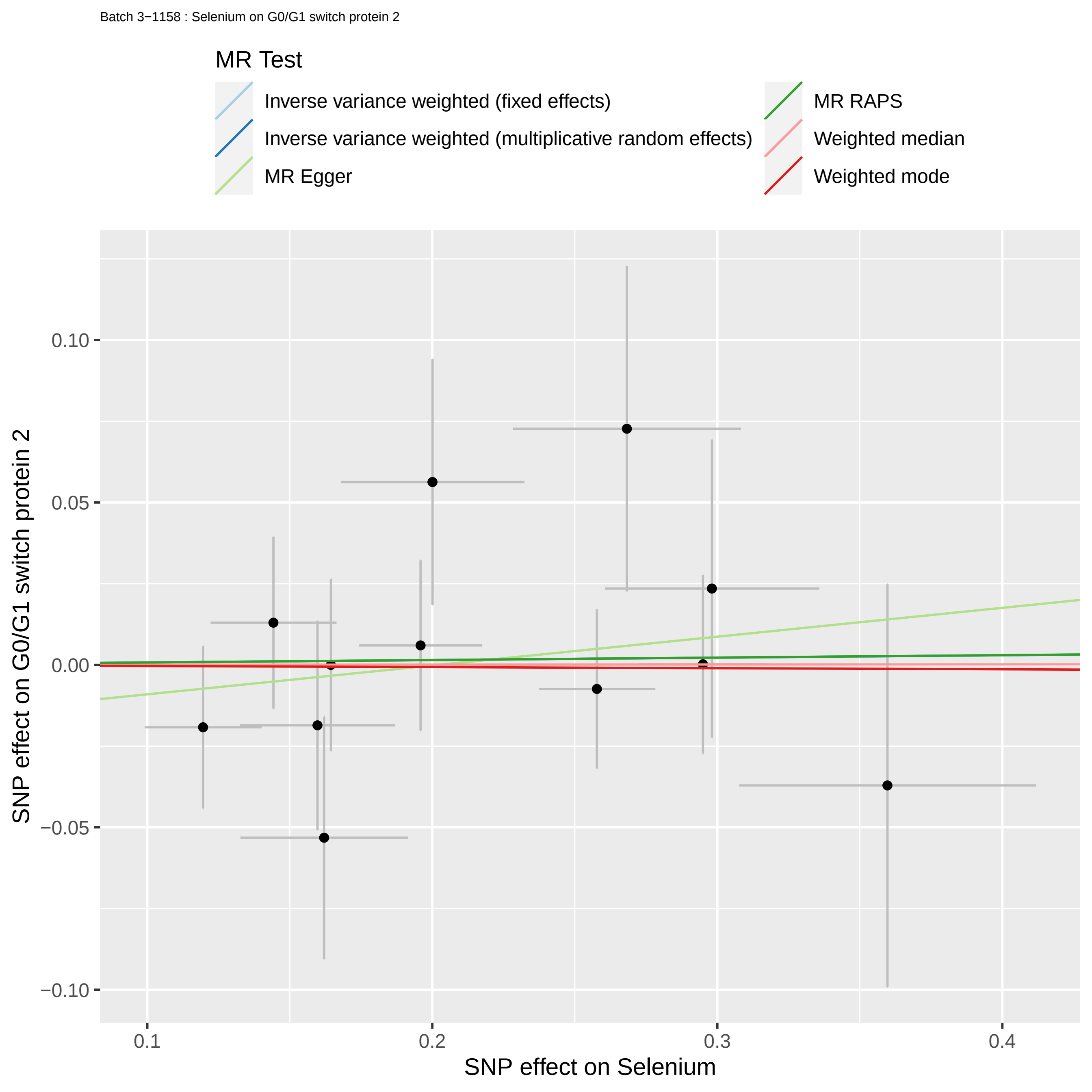
Batch 3−1158 : Selenium on G0/G1 switch protein 2
MR Test
Inverse variance weighted (fixed effects)
Inverse variance weighted (multiplicative random effects)
MR Egger
MR RAPS
Weighted median
Weighted mode
SNP effect on G0/G1 switch protein 2
0.10
0.05
0.00
−0.05
−0.10
0.1
0.2
0.3
0.4
SNP effect on Selenium

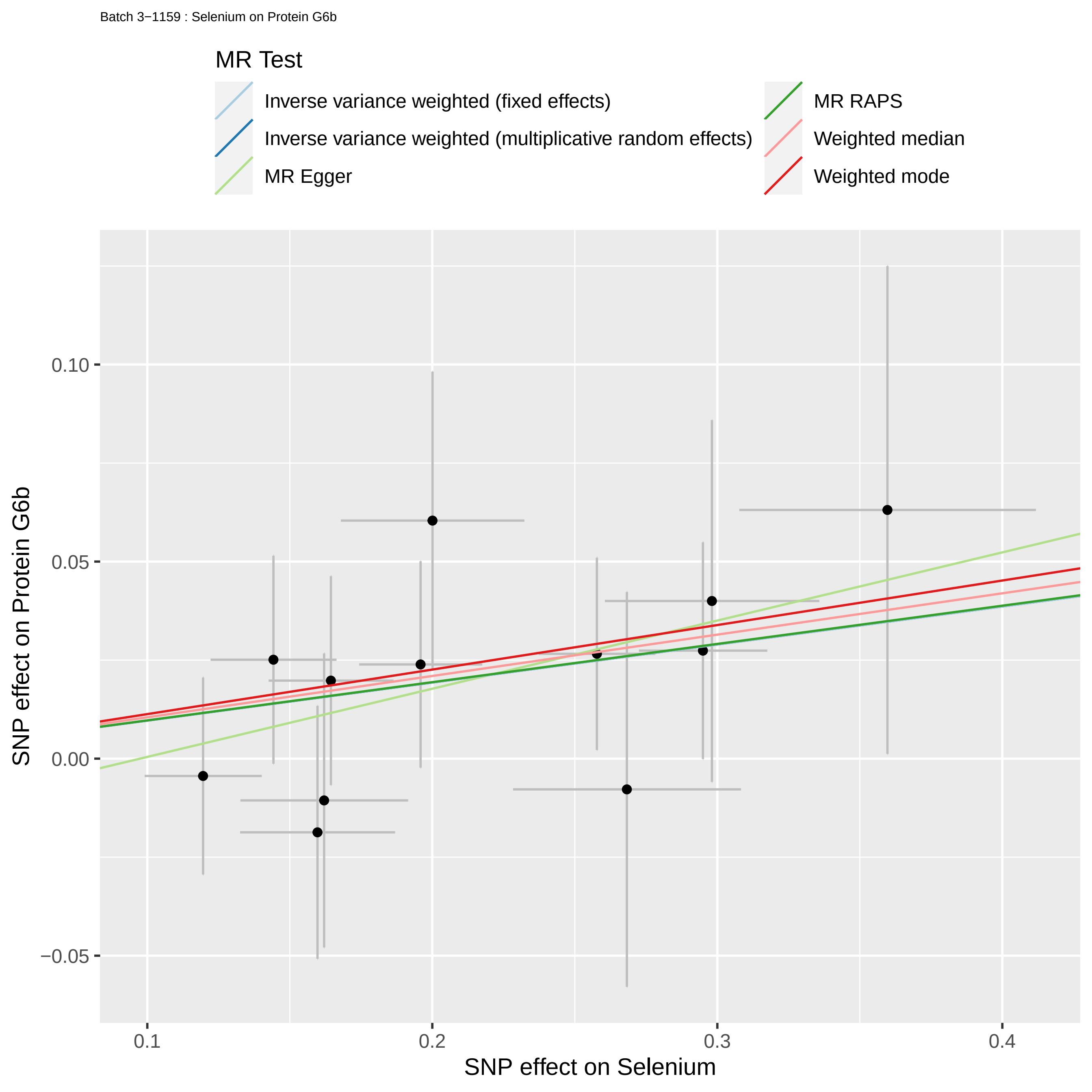

Batch 3−1159 : Selenium on Protein G6b
MR Test
Inverse variance weighted (fixed effects)
Inverse variance weighted (multiplicative random effects)
MR Egger
MR RAPS
Weighted median
Weighted mode
0.10
0.05
0.00
−0.05
0.1
0.2
0.3
0.4
SNP effect on Selenium
SNP effect on Protein G6b

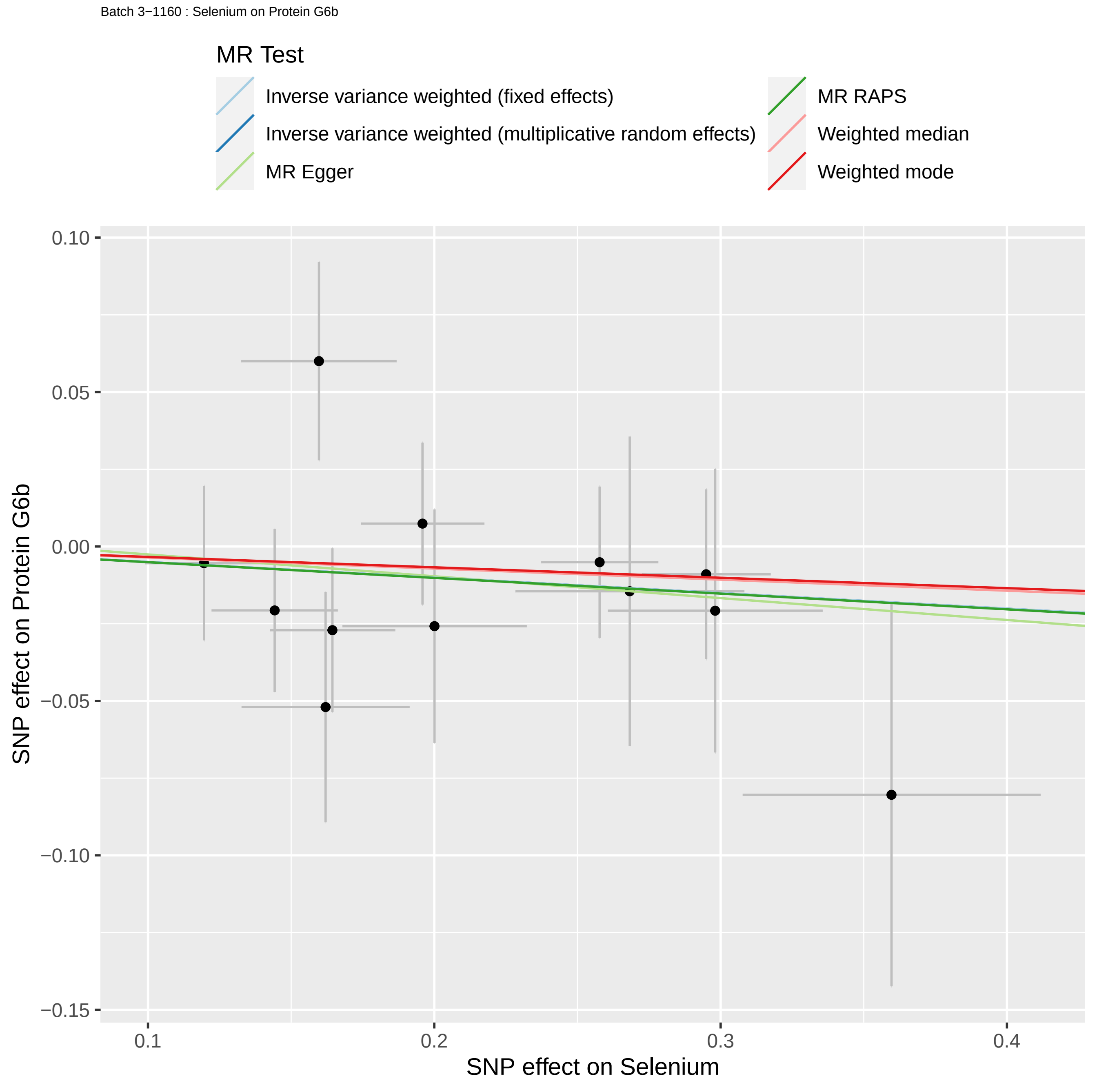

Batch 3−1160 : Selenium on Protein G6b
MR Test
Inverse variance weighted (fixed effects)
Inverse variance weighted (multiplicative random effects)
MR Egger
MR RAPS
Weighted median
Weighted mode
0.10
0.05
0.00
−0.05
−0.10
−0.15
0.1
0.2
0.3
0.4
SNP effect on Protein G6b
SNP effect on Selenium

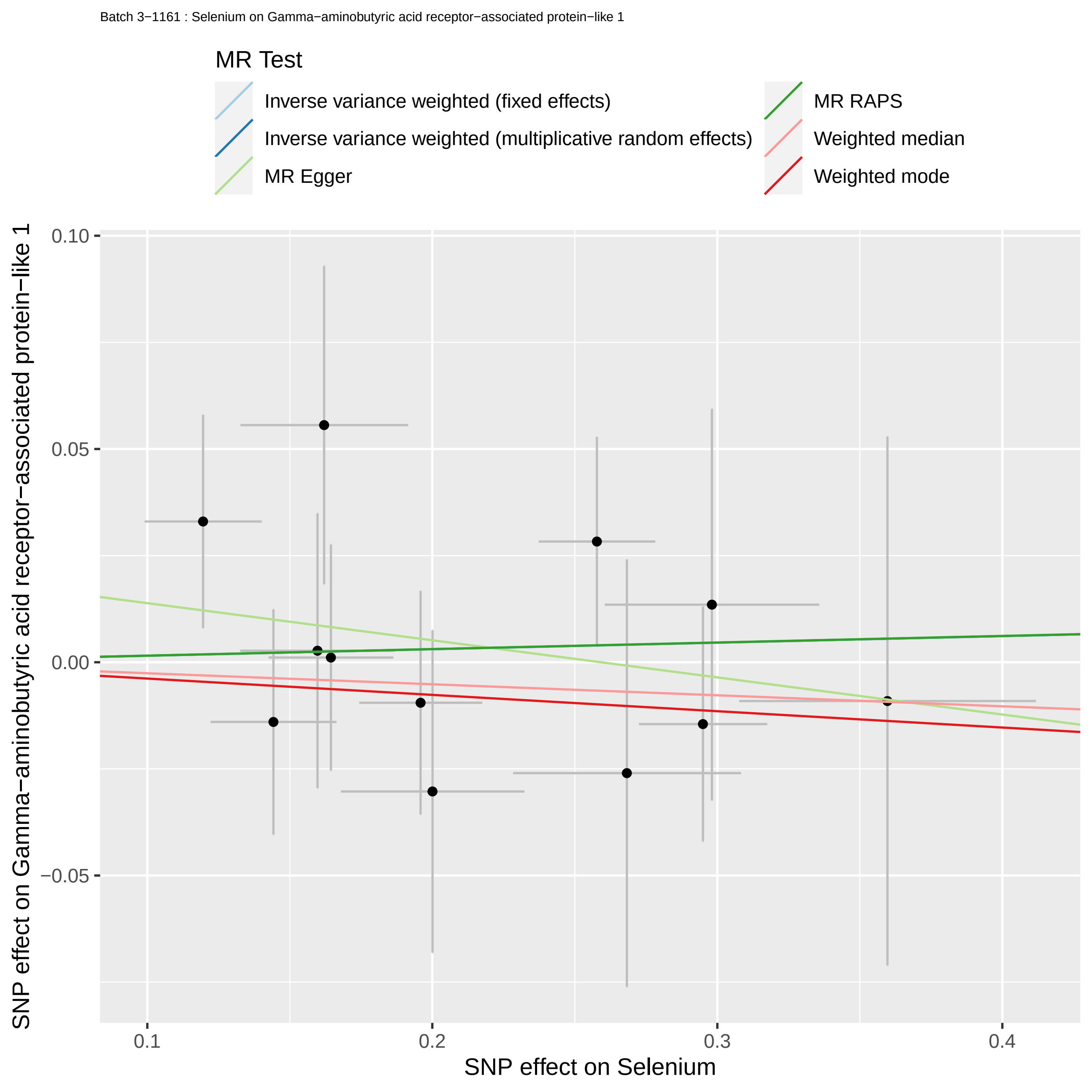
Batch 3−1161 : Selenium on Gamma−aminobutyric acid receptor−associated protein−like 1
MR Test
Inverse variance weighted (fixed effects)
Inverse variance weighted (multiplicative random effects)
MR Egger
MR RAPS
Weighted median
Weighted mode
0.10
0.05
0.00
−0.05
0.1
0.2
0.3
0.4
SNP effect on Gamma−aminobutyric acid receptor−associated protein−like 1
SNP effect on Selenium

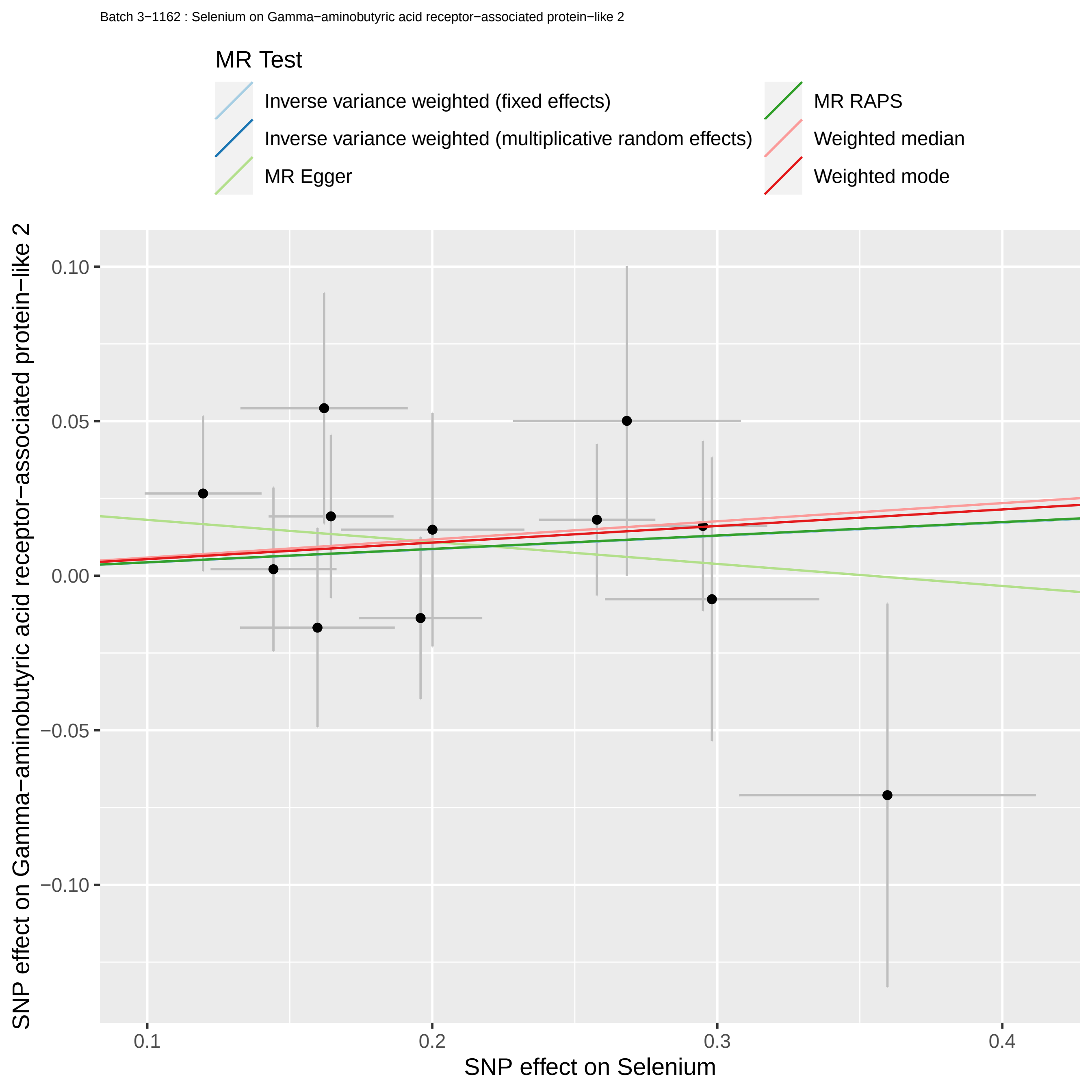

Batch 3−1162 : Selenium on Gamma−aminobutyric acid receptor−associated protein−like 2
MR Test
Inverse variance weighted (fixed effects)
Inverse variance weighted (multiplicative random effects)
MR Egger
MR RAPS
Weighted median
Weighted mode
0.10
0.05
0.00
−0.05
−0.10
0.1
0.2
0.3
0.4
SNP effect on Selenium
SNP effect on Gamma−aminobutyric acid receptor−associated protein−like 2

Batch 3−1163 : Selenium on Gamma−aminobutyric acid type B receptor subunit 2

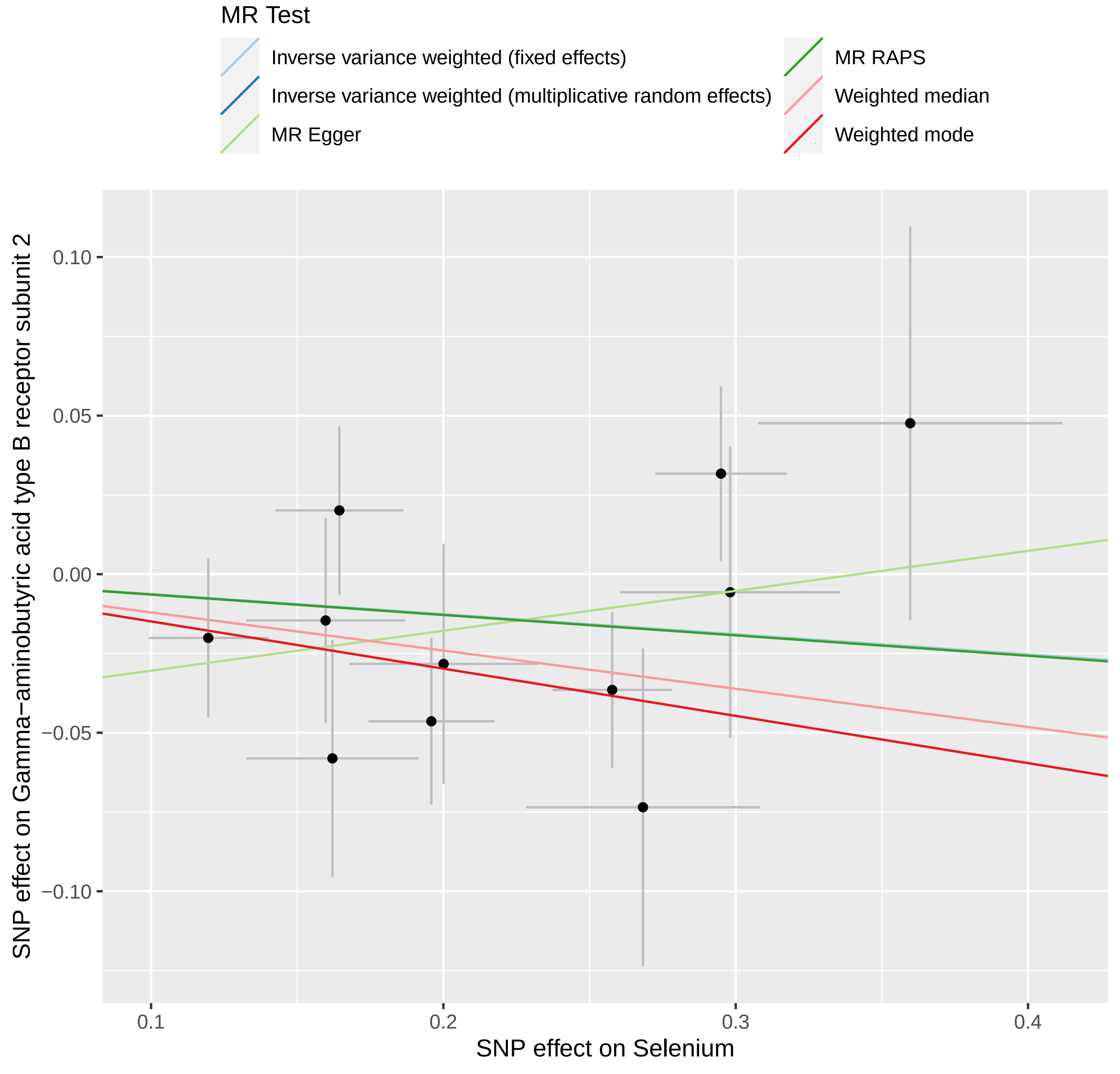

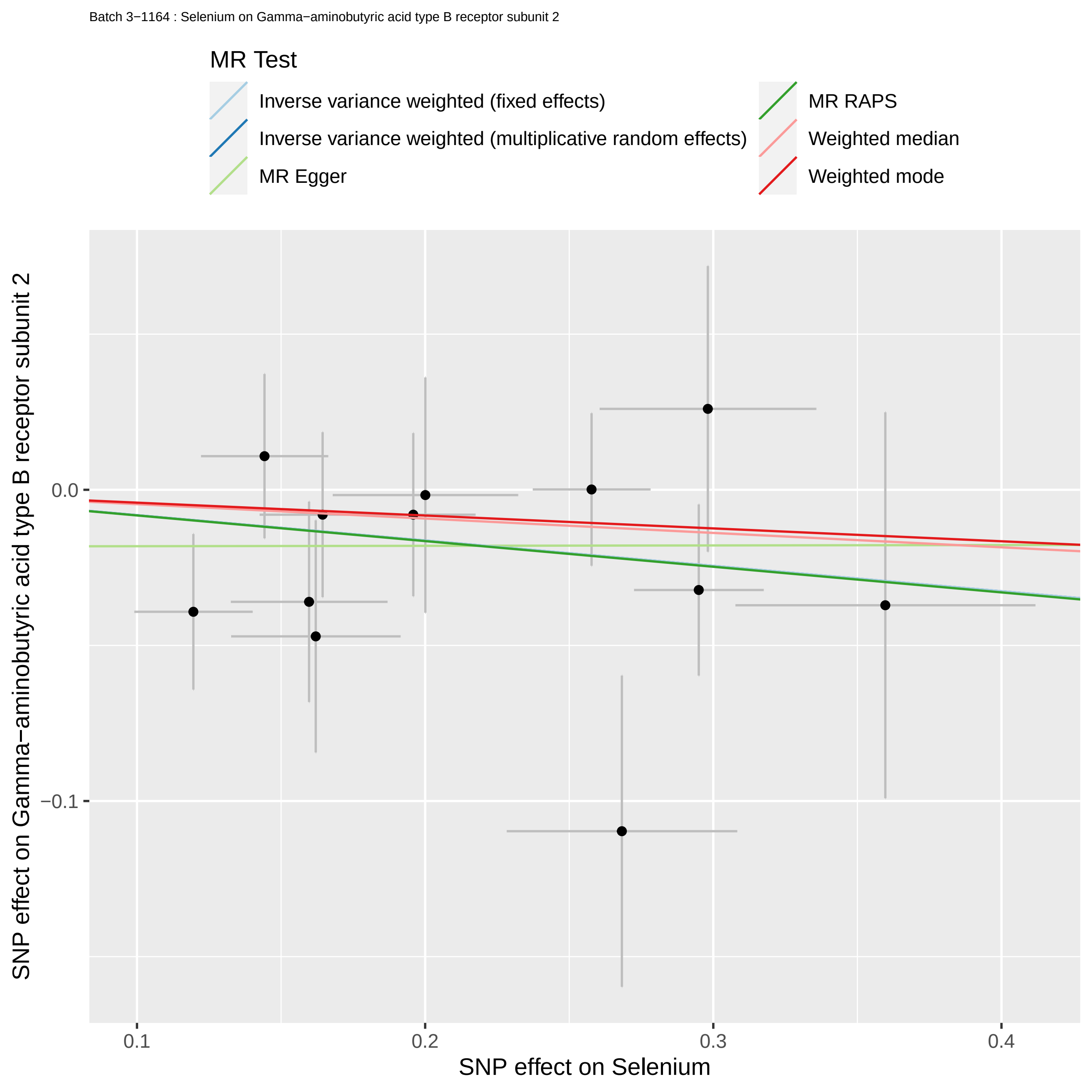
Batch 3−1164 : Selenium on Gamma−aminobutyric acid type B receptor subunit 2
MR Test
Inverse variance weighted (fixed effects)
Inverse variance weighted (multiplicative random effects)
MR Egger
MR RAPS
Weighted median
Weighted mode
SNP effect on Gamma−aminobutyric acid type B receptor subunit 2
0.0
−0.1
0.1
0.2
0.3
0.4
SNP effect on Selenium

Batch 3−1165 : Selenium on Growth arrest and DNA damage−inducible proteins−interacting protein 1

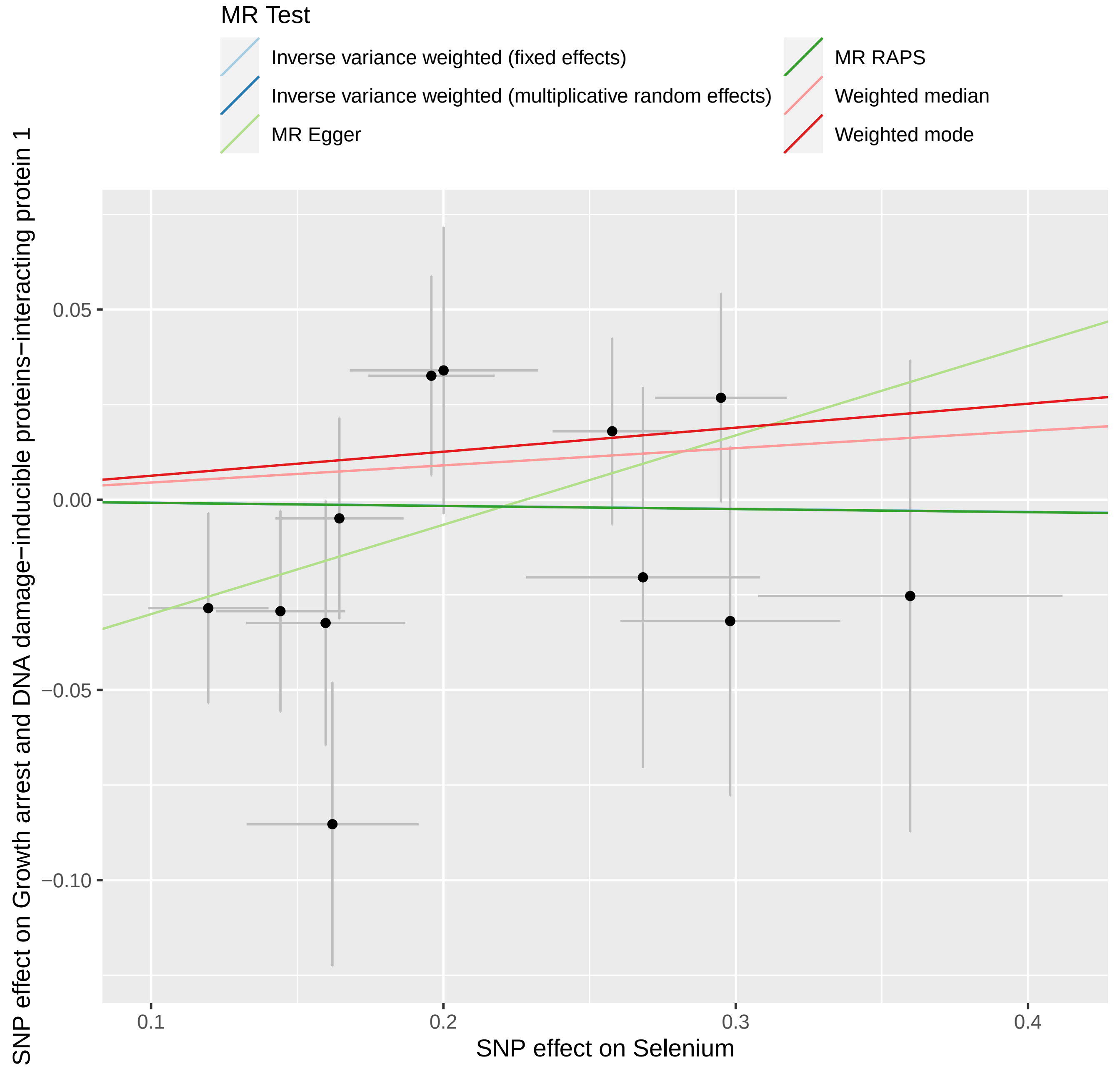

Batch 3−1166 : Selenium on Galanin peptides

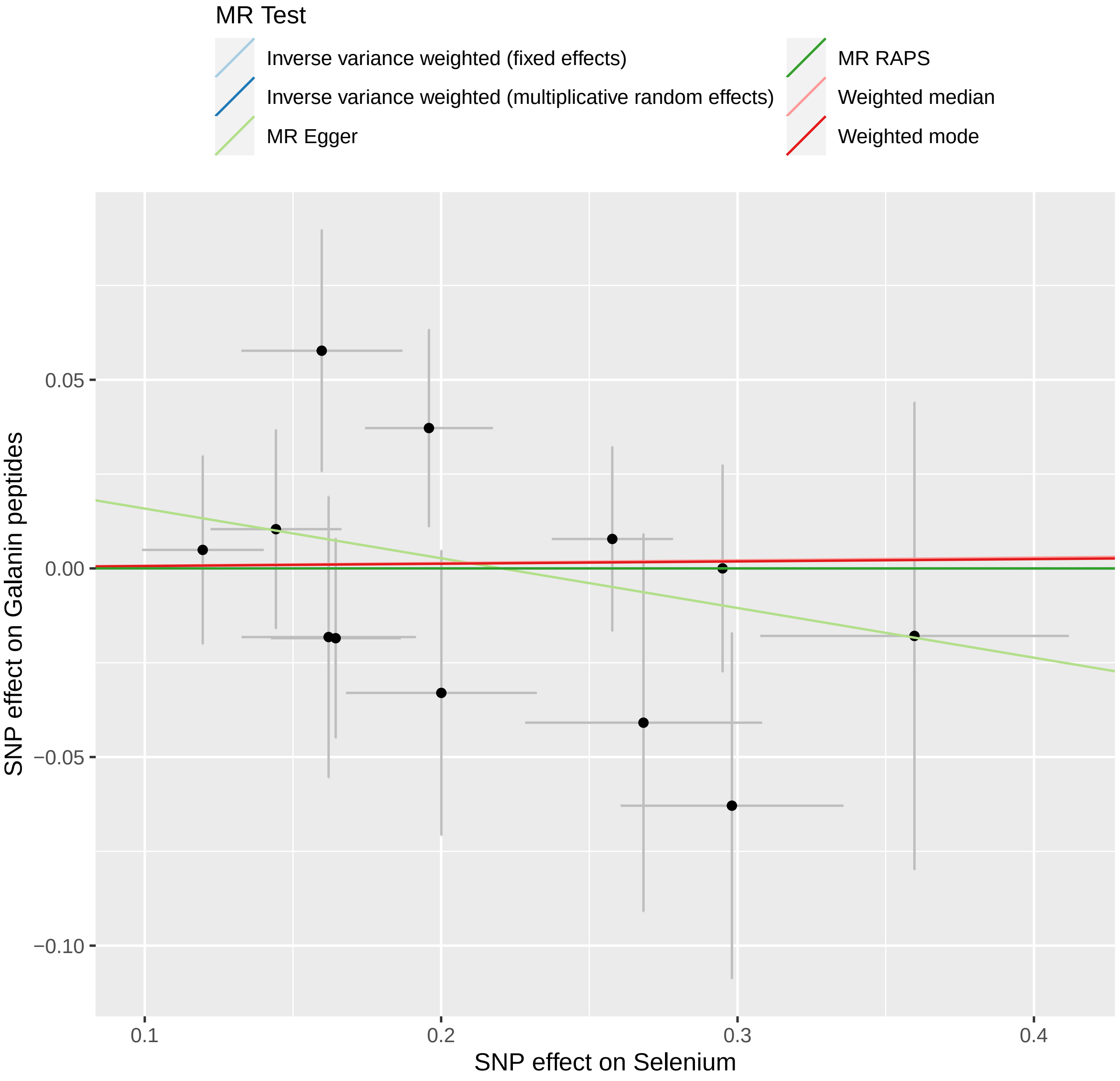

Batch 3−1167 : Selenium on Polypeptide N−acetylgalactosaminyltransferase 10

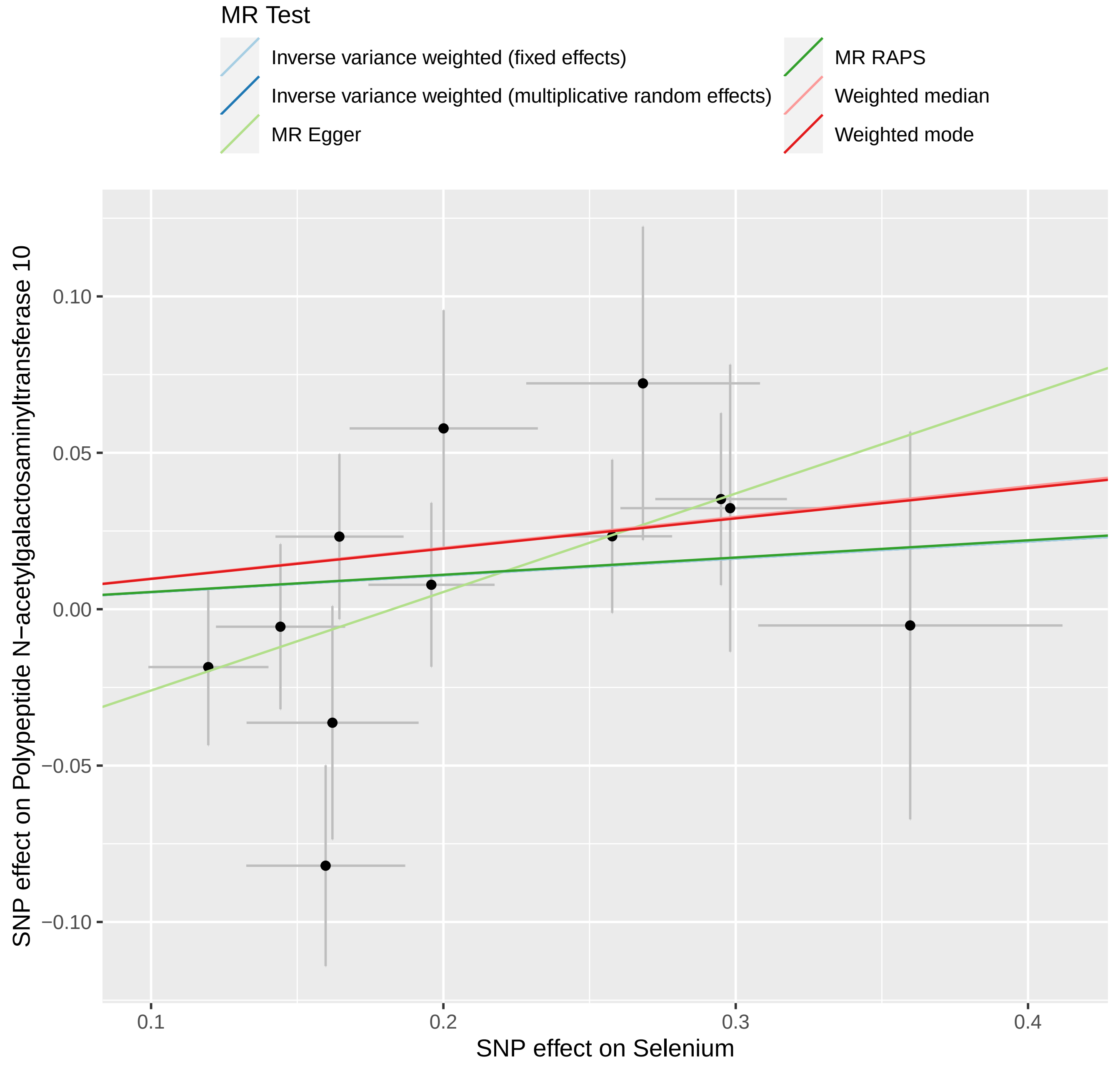

Batch 3−1168 : Selenium on Polypeptide N−acetylgalactosaminyltransferase 11

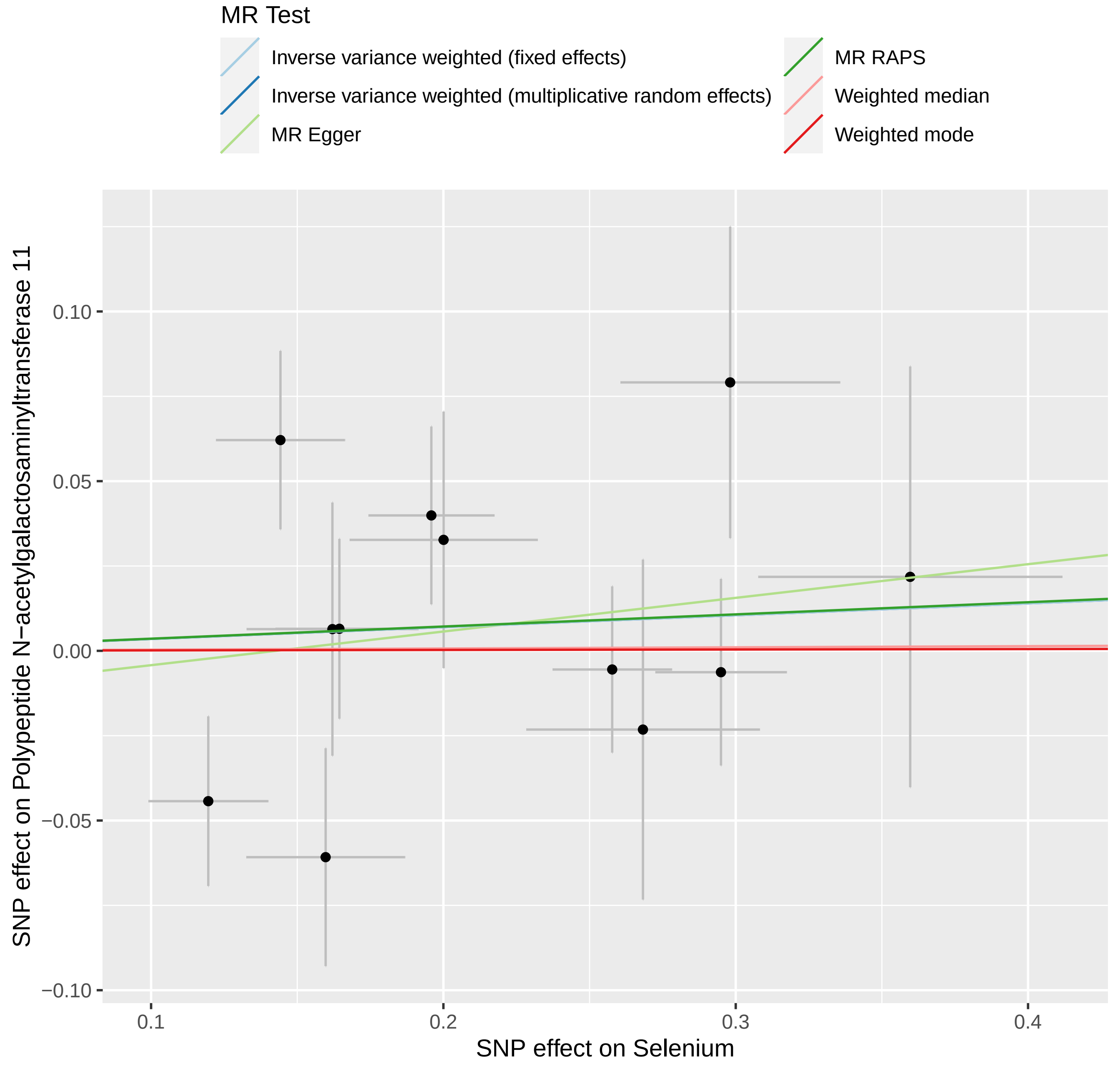

Batch 3−1169 : Selenium on Polypeptide N−acetylgalactosaminyltransferase 16

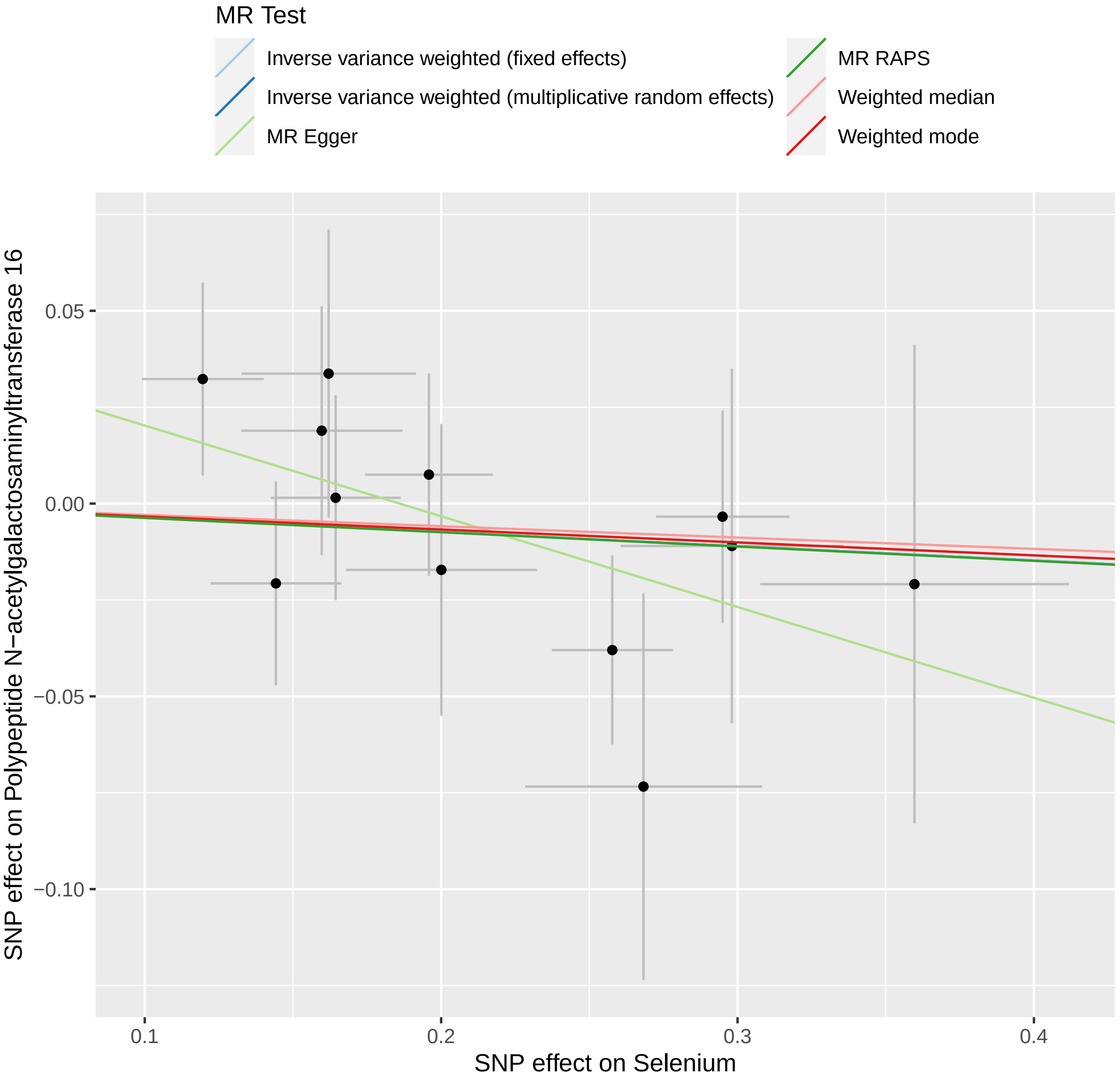

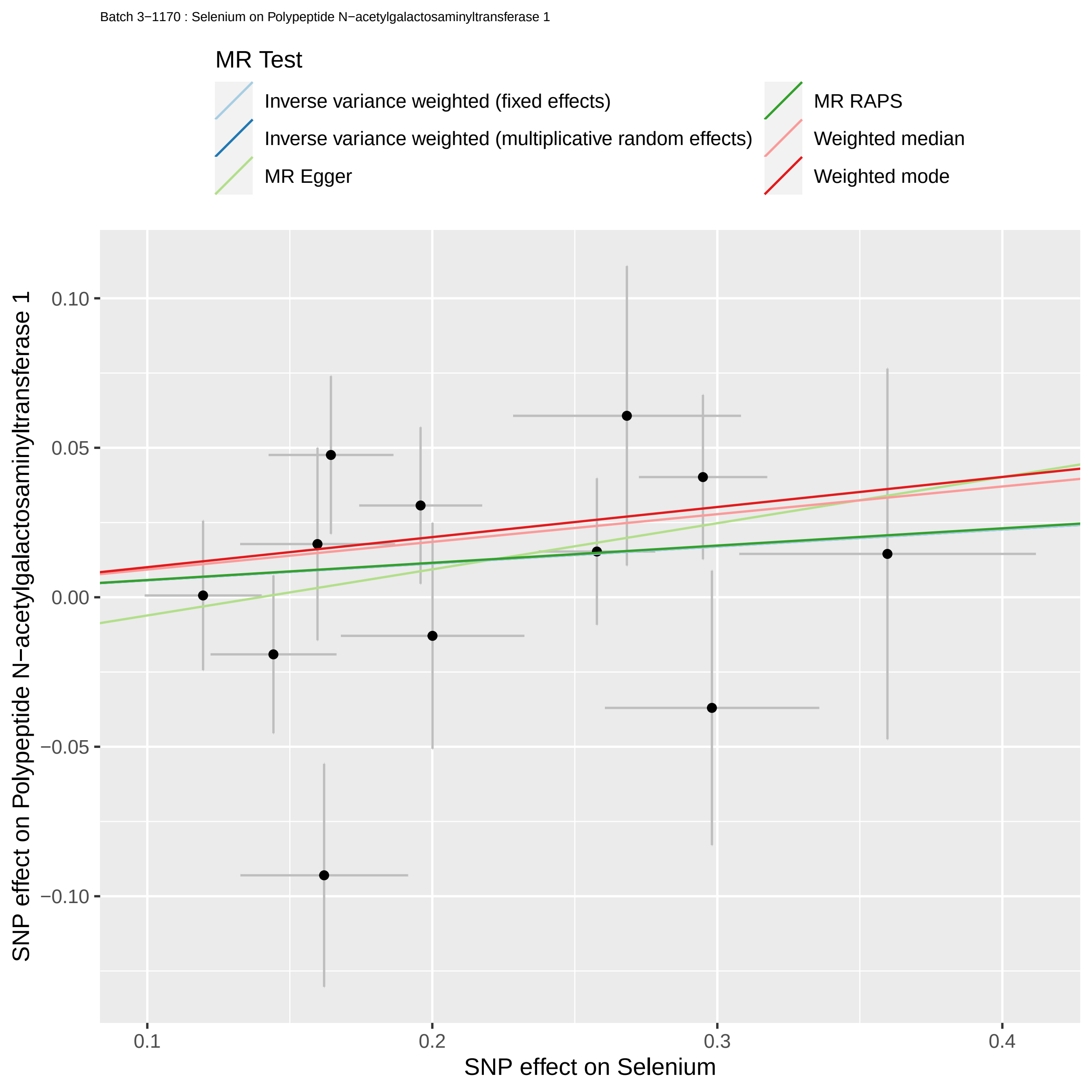

Batch 3−1170 : Selenium on Polypeptide N−acetylgalactosaminyltransferase 1
MR Test
Inverse variance weighted (fixed effects)
Inverse variance weighted (multiplicative random effects)
MR Egger
MR RAPS
Weighted median
Weighted mode
0.10
0.05
0.00
−0.05
−0.10
0.1
0.2
0.3
0.4
SNP effect on Polypeptide N−acetylgalactosaminyltransferase 1
SNP effect on Selenium

Batch 3−1171 : Selenium on Polypeptide N−acetylgalactosaminyltransferase 2

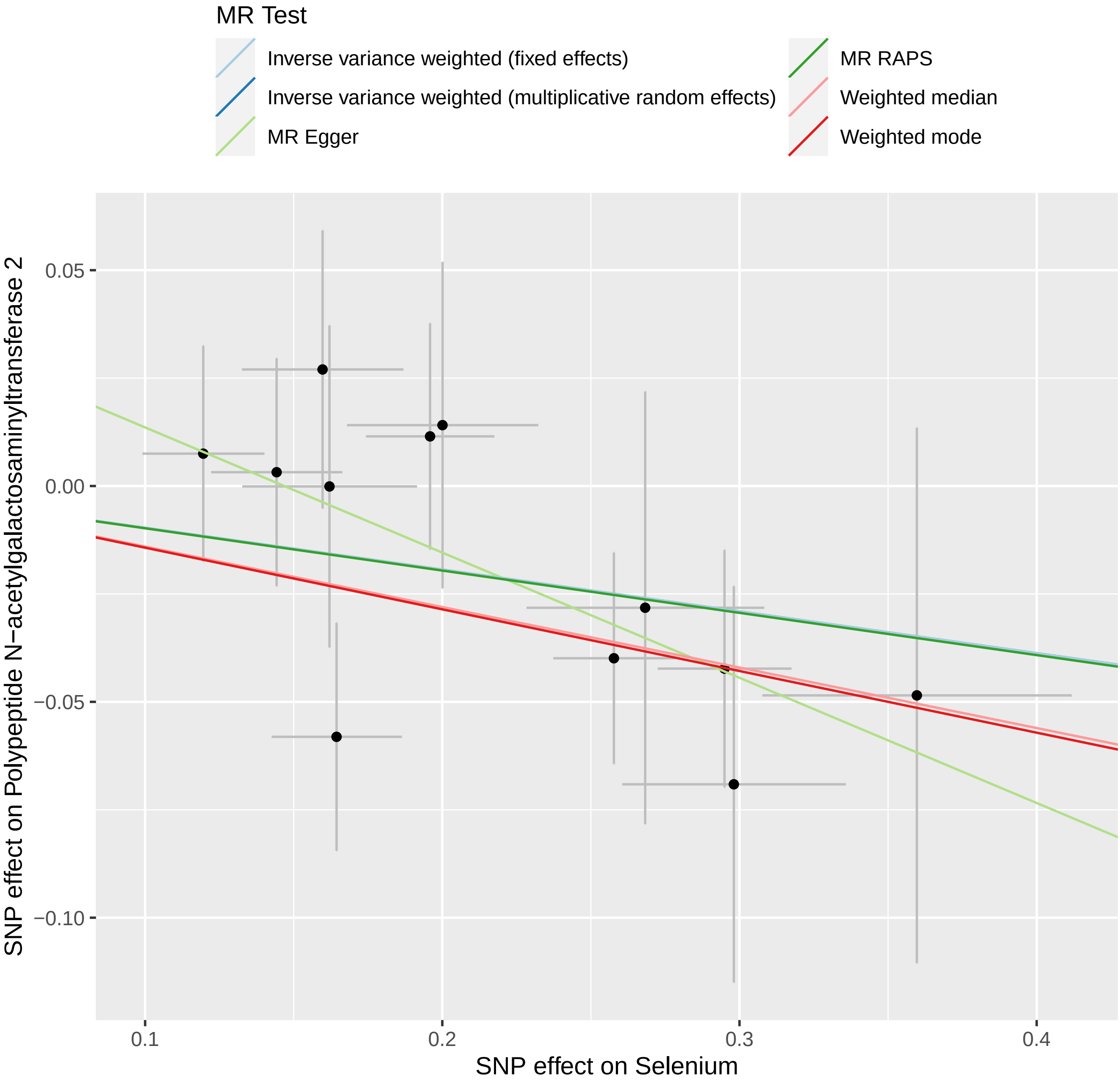

Batch 3−1172 : Selenium on Polypeptide N−acetylgalactosaminyltransferase 3

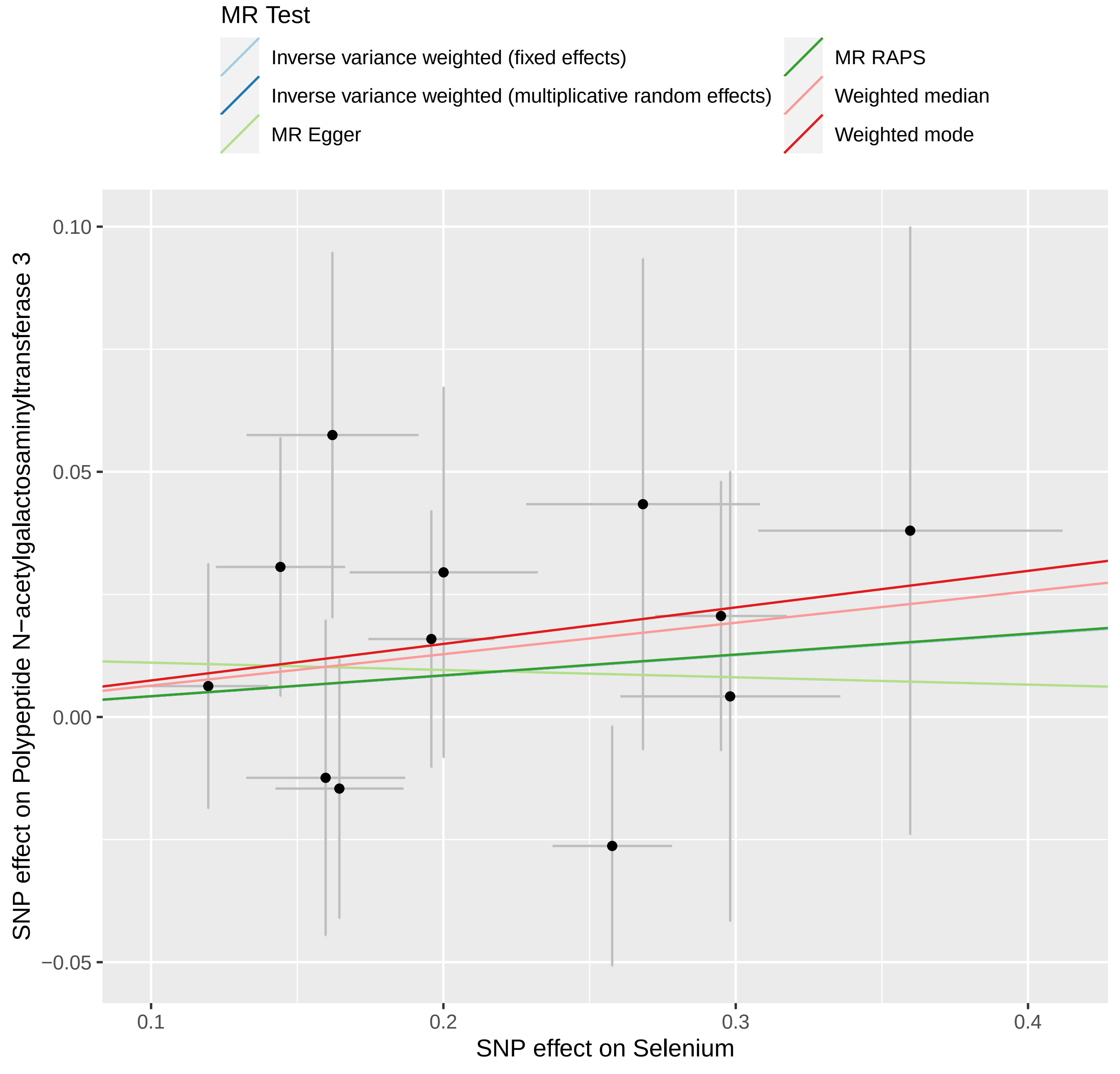

Batch 3−1173 : Selenium on N−acetylgalactosaminyltransferase 7

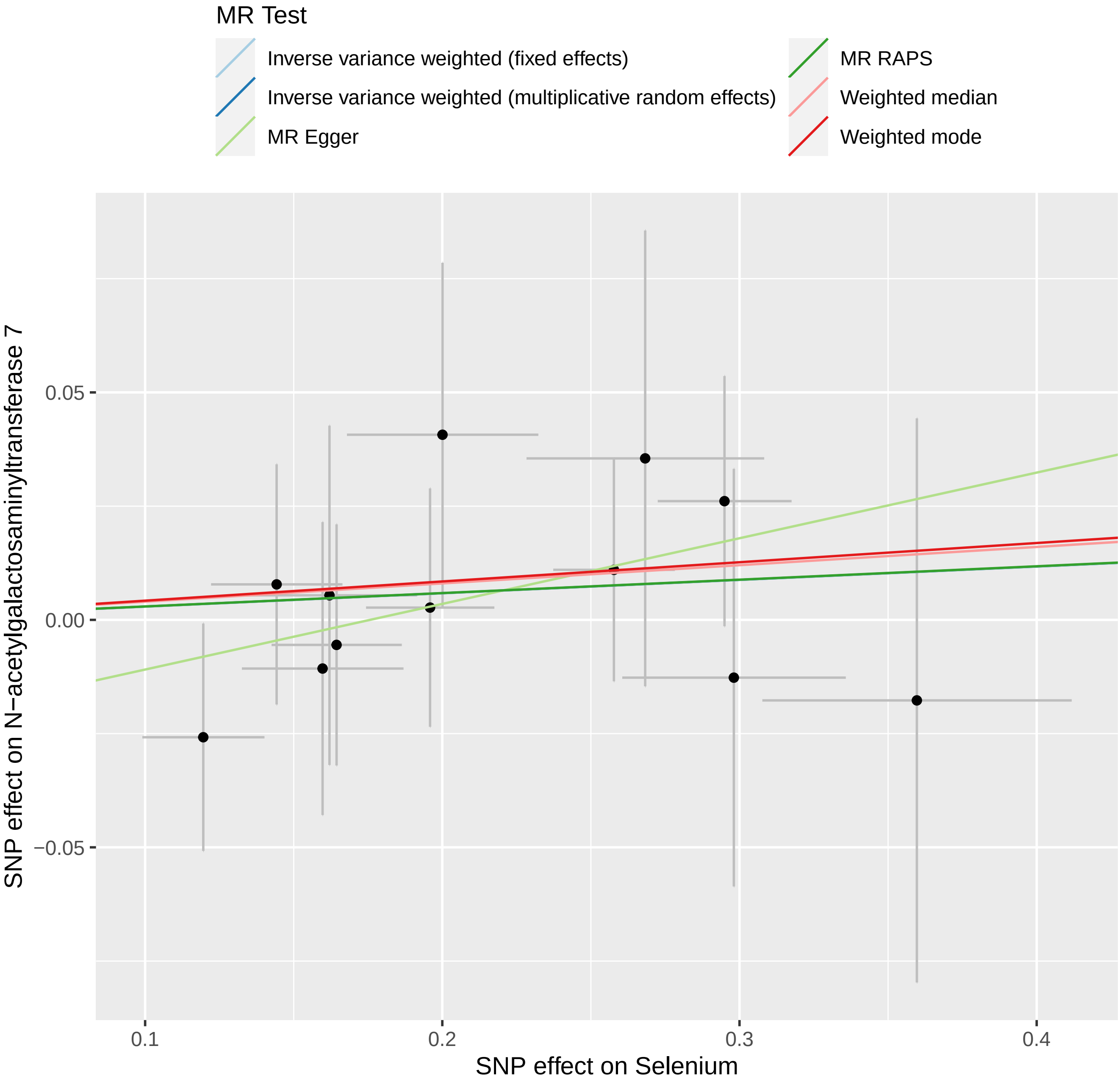

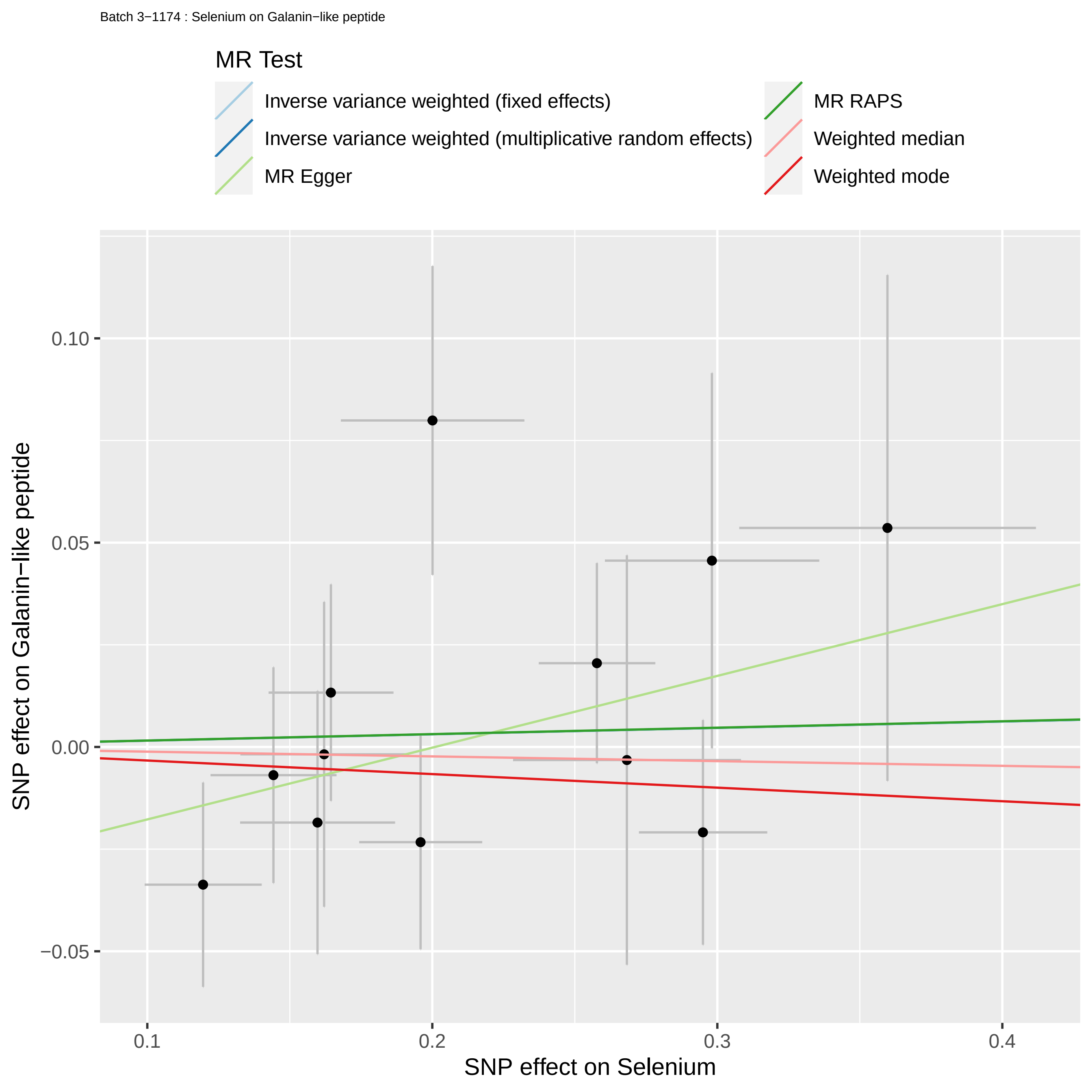
Batch 3−1174 : Selenium on Galanin−like peptide
MR Test
Inverse variance weighted (fixed effects)
Inverse variance weighted (multiplicative random effects)
MR Egger
MR RAPS
Weighted median
Weighted mode
SNP effect on Galanin−like peptide
0.10
0.05
0.00
−0.05
0.1
0.2
0.3
0.4
SNP effect on Selenium

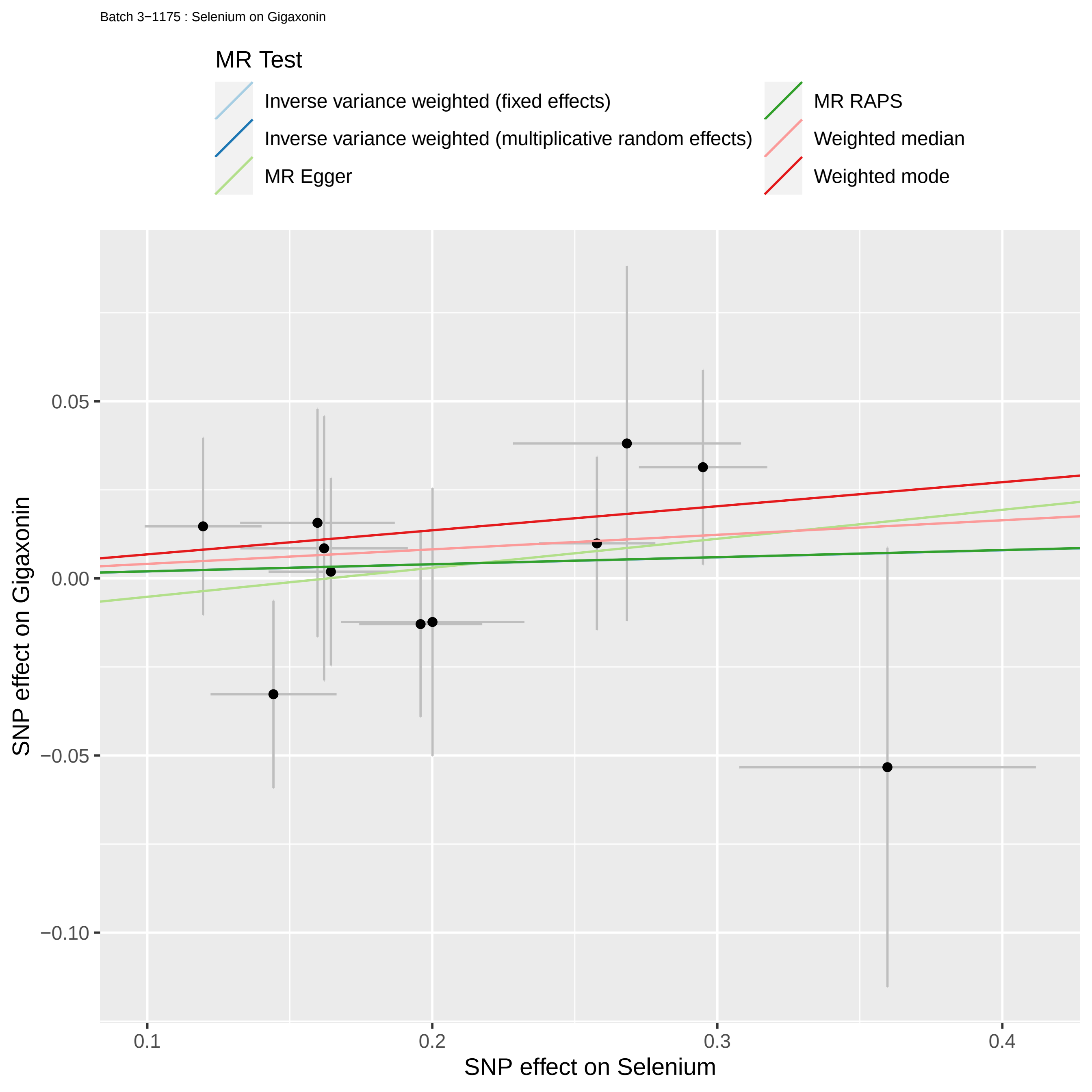
Batch 3−1175 : Selenium on Gigaxonin
MR Test
Inverse variance weighted (fixed effects)
Inverse variance weighted (multiplicative random effects)
MR Egger
MR RAPS
Weighted median
Weighted mode
0.05
0.00
−0.05
−0.10
0.1
0.2
0.3
0.4
SNP effect on Gigaxonin
SNP effect on Selenium

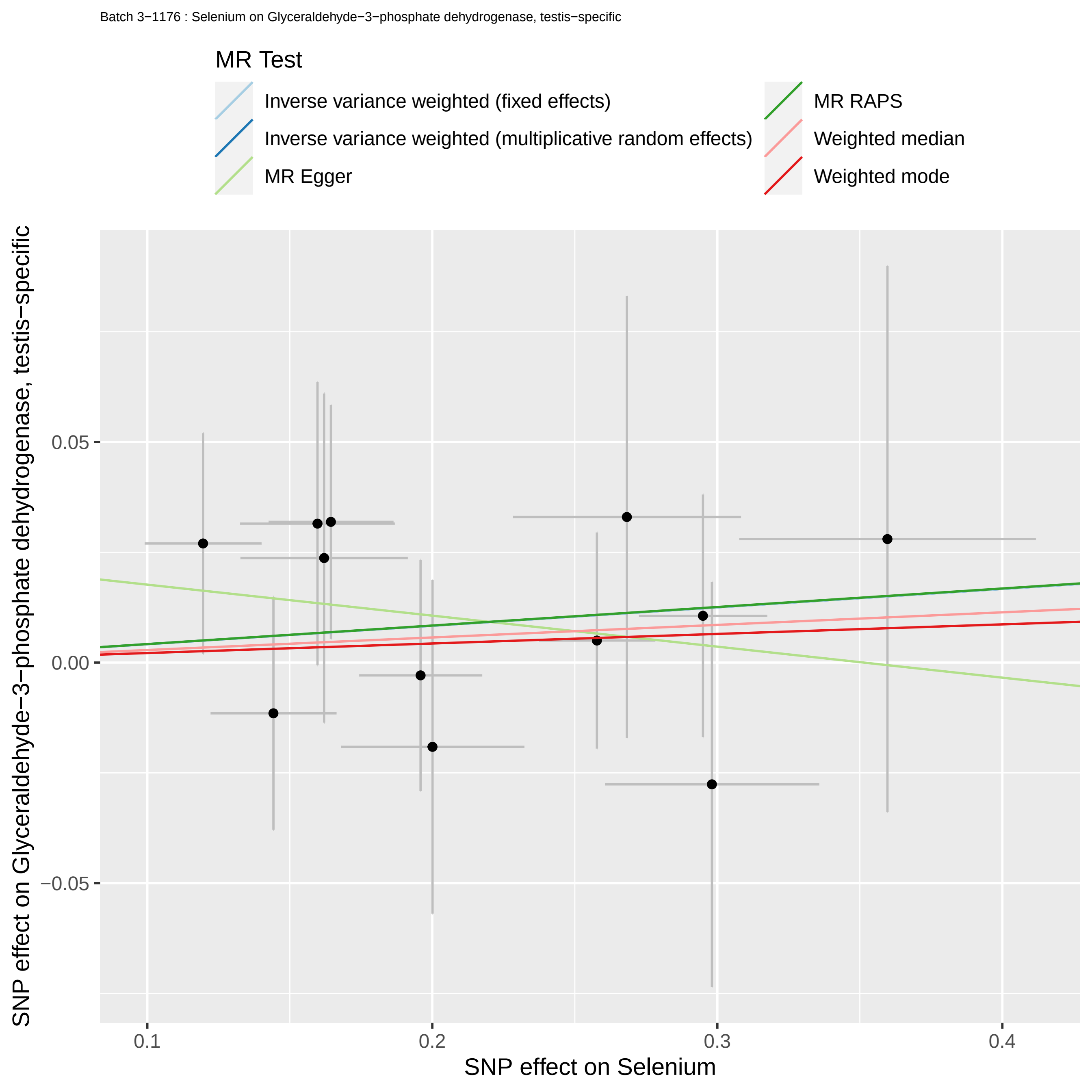
Batch 3−1176 : Selenium on Glyceraldehyde−3−phosphate dehydrogenase, testis−specific
MR Test
Inverse variance weighted (fixed effects)
Inverse variance weighted (multiplicative random effects)
MR Egger
MR RAPS
Weighted median
Weighted mode
0.05
0.00
−0.05
0.1
0.2
0.3
0.4
SNP effect on Selenium
SNP effect on Glyceraldehyde−3−phosphate dehydrogenase, testis−specific

Batch 3−1177 : Selenium on Growth arrest−specific protein 1

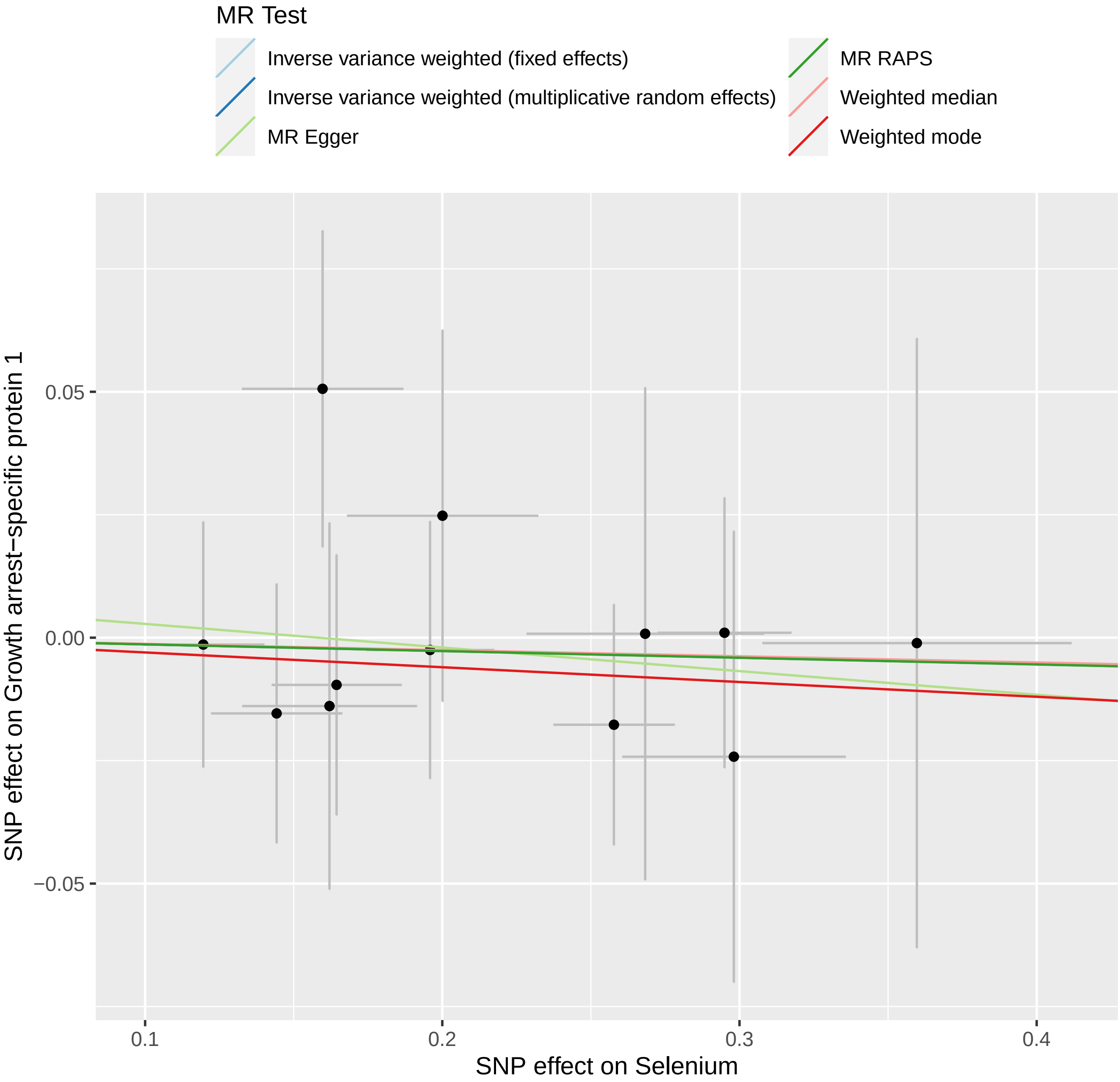

Batch 3−1178 : Selenium on Guanylate−binding protein 6

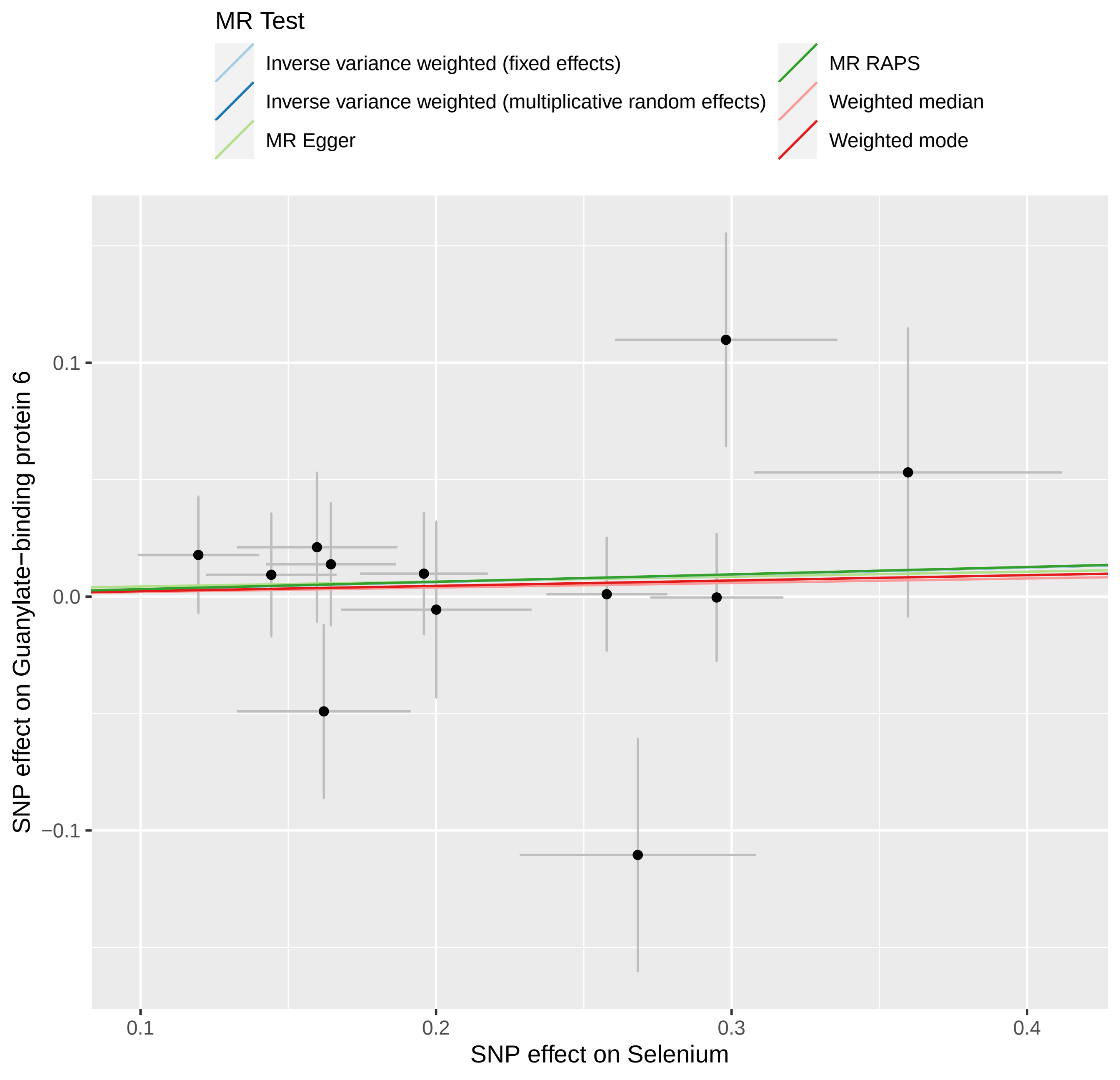

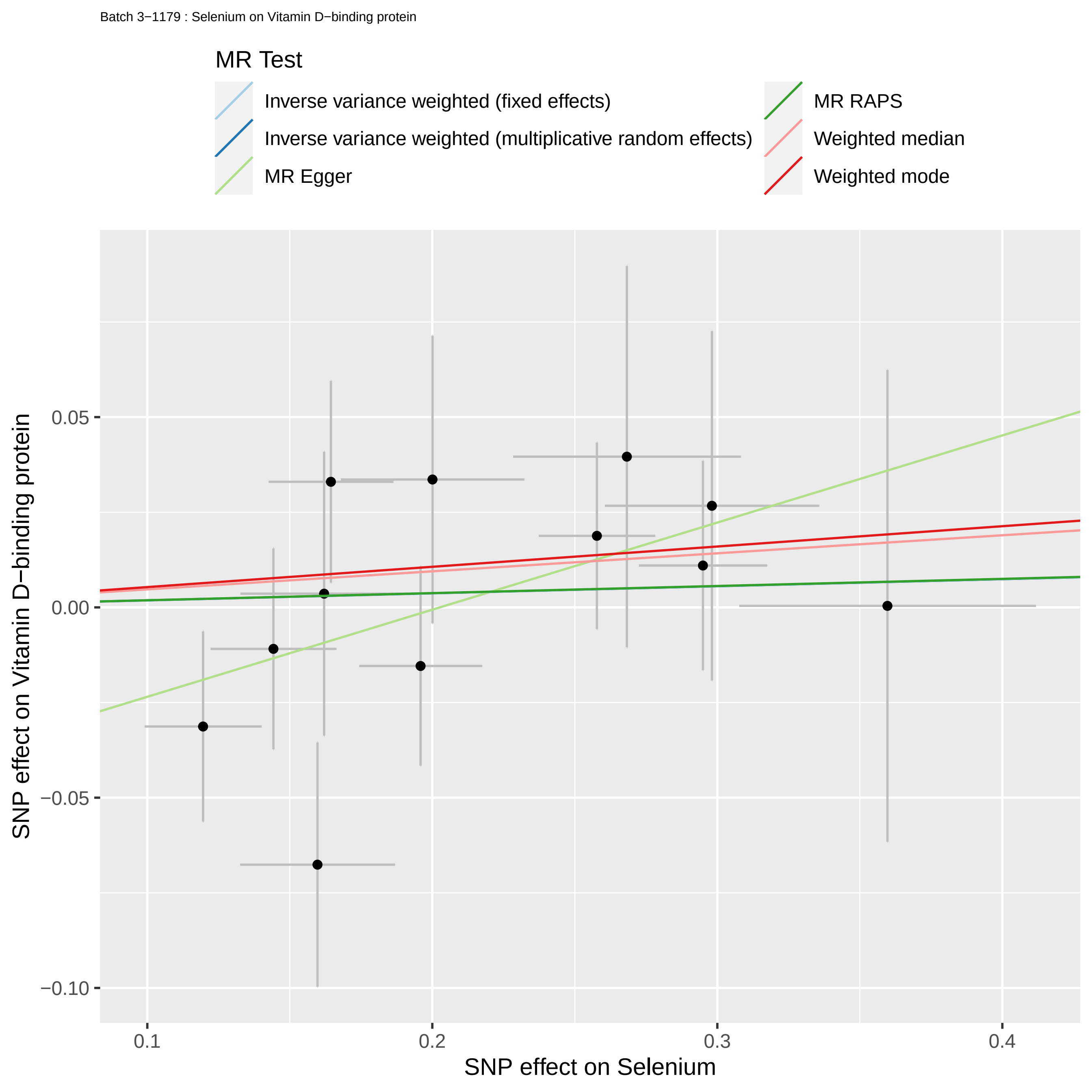

Batch 3−1179 : Selenium on Vitamin D−binding protein
MR Test
Inverse variance weighted (fixed effects)
Inverse variance weighted (multiplicative random effects)
MR Egger
MR RAPS
Weighted median
Weighted mode
0.05
0.00
−0.05
−0.10
0.1
0.2
0.3
0.4
SNP effect on Vitamin D−binding protein
SNP effect on Selenium

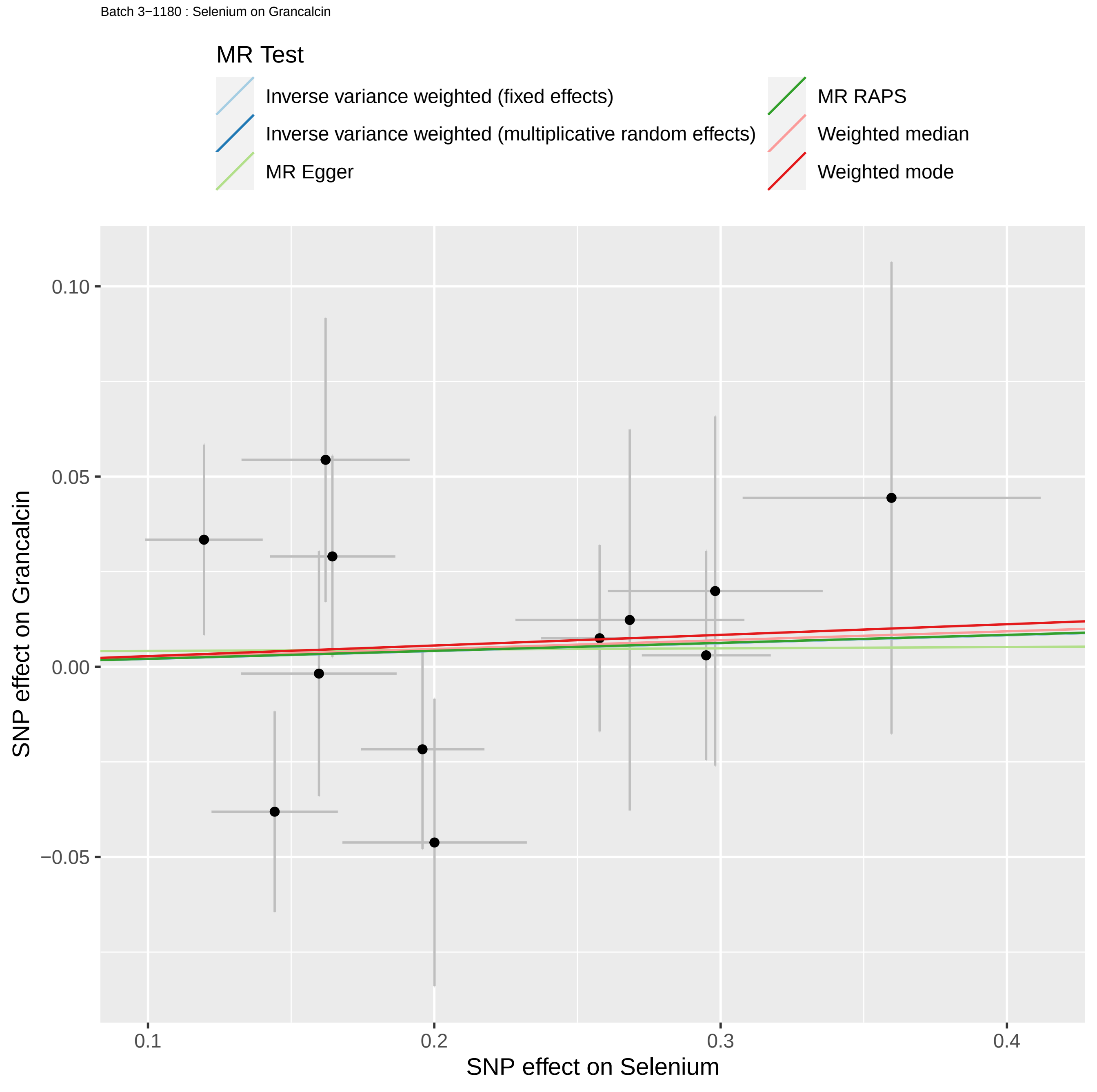
Batch 3−1180 : Selenium on Grancalcin
MR Test
Inverse variance weighted (fixed effects)
Inverse variance weighted (multiplicative random effects)
MR Egger
MR RAPS
Weighted median
Weighted mode
0.10
0.05
0.00
−0.05
0.1
0.2
0.3
0.4
SNP effect on Grancalcin
SNP effect on Selenium

Batch 3−1181 : Selenium on Glucagon

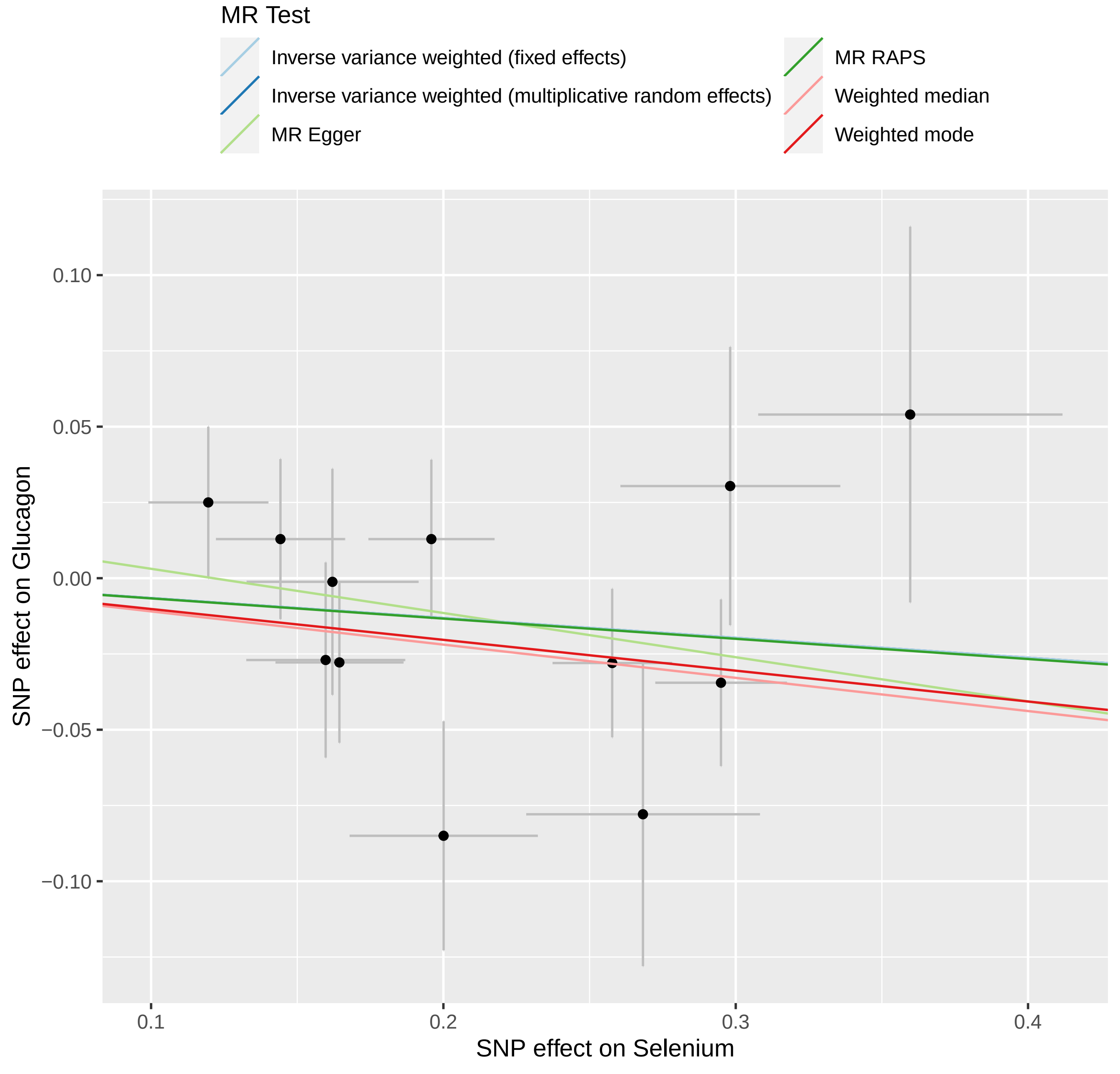

Batch 3−1182 : Selenium on GTP cyclohydrolase 1

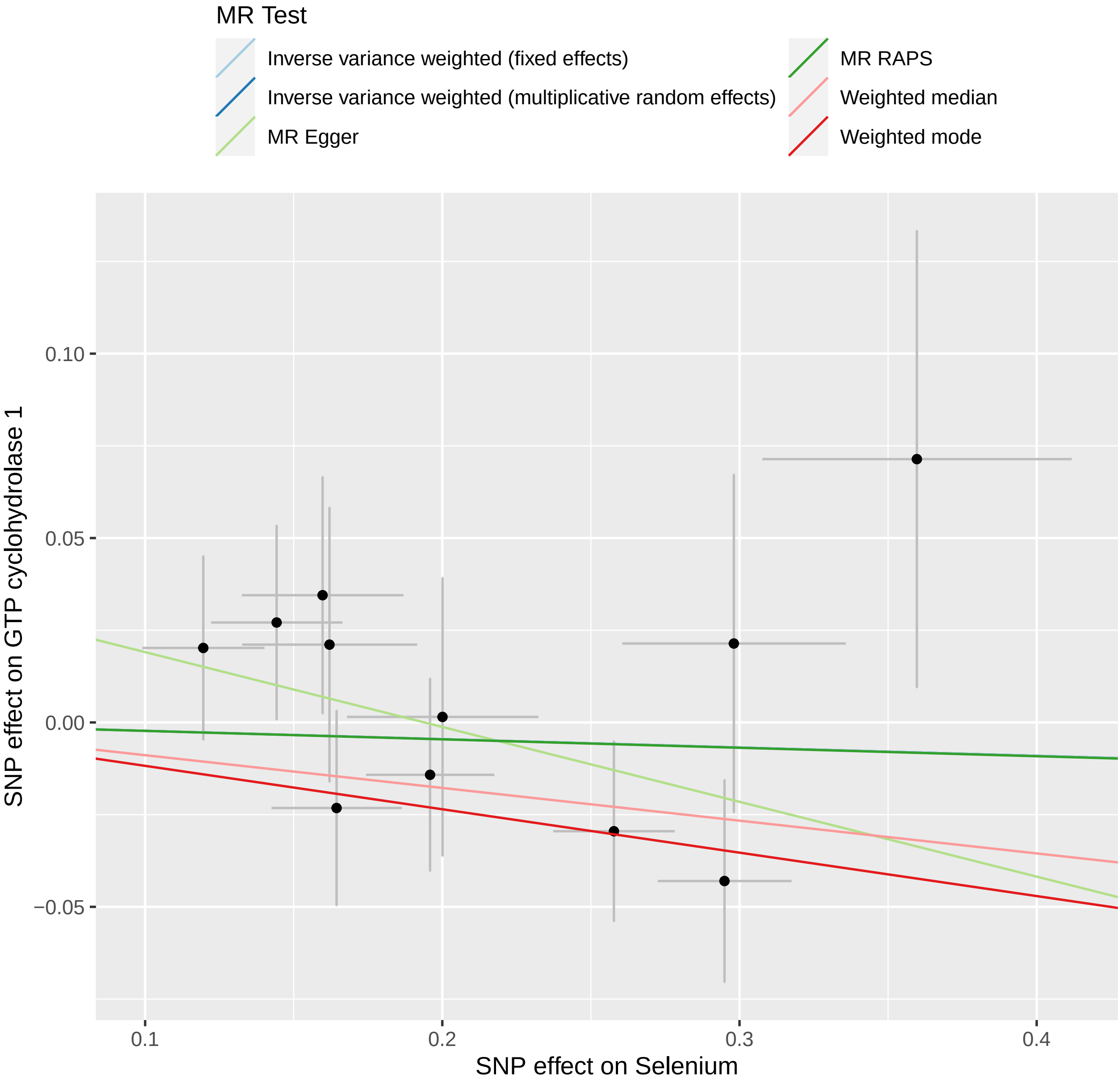

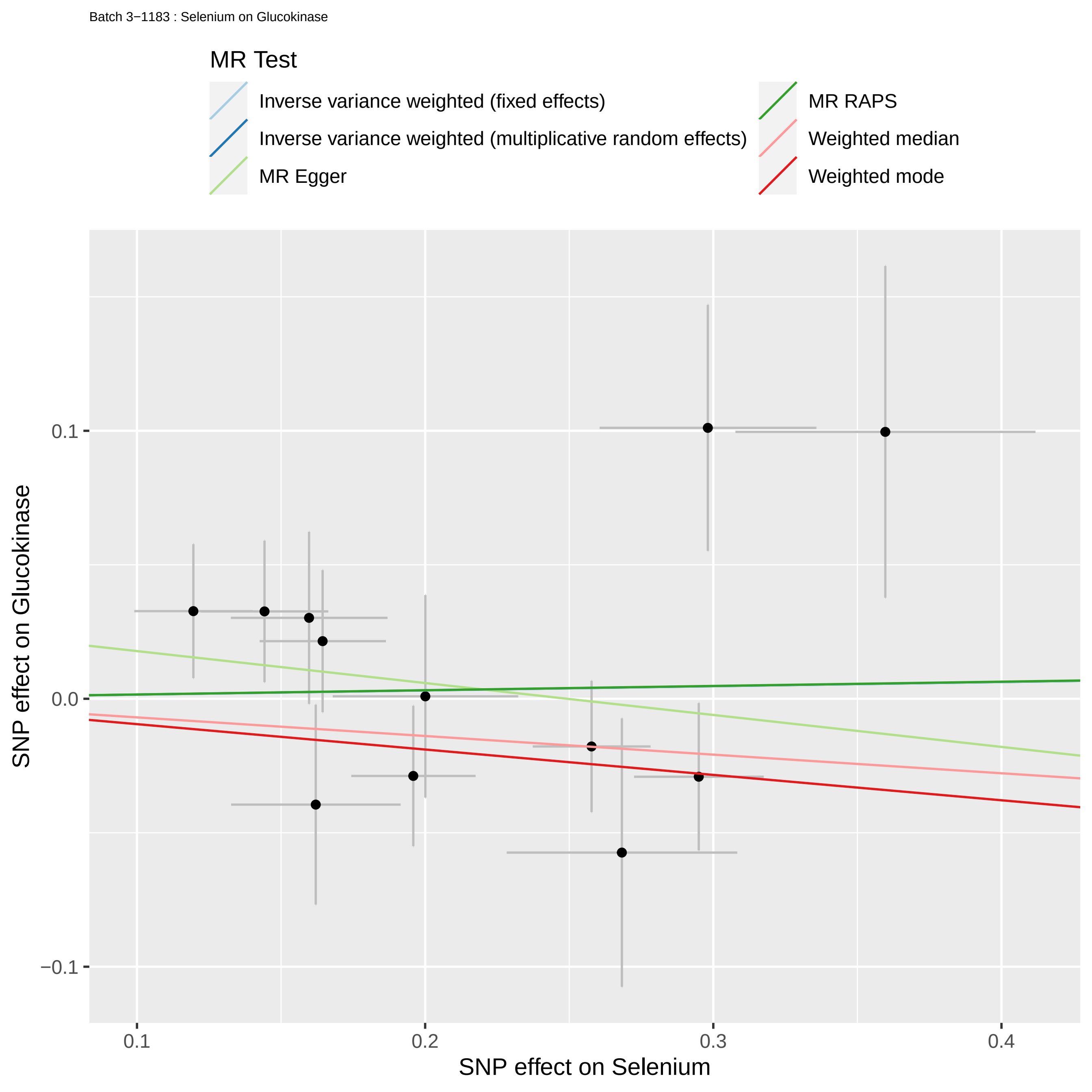

Batch 3−1183 : Selenium on Glucokinase
MR Test
Inverse variance weighted (fixed effects)
Inverse variance weighted (multiplicative random effects)
MR Egger
MR RAPS
Weighted median
Weighted mode
SNP effect on Glucokinase
0.1
0.0
−0.1
0.1
0.2
0.3
0.4
SNP effect on Selenium

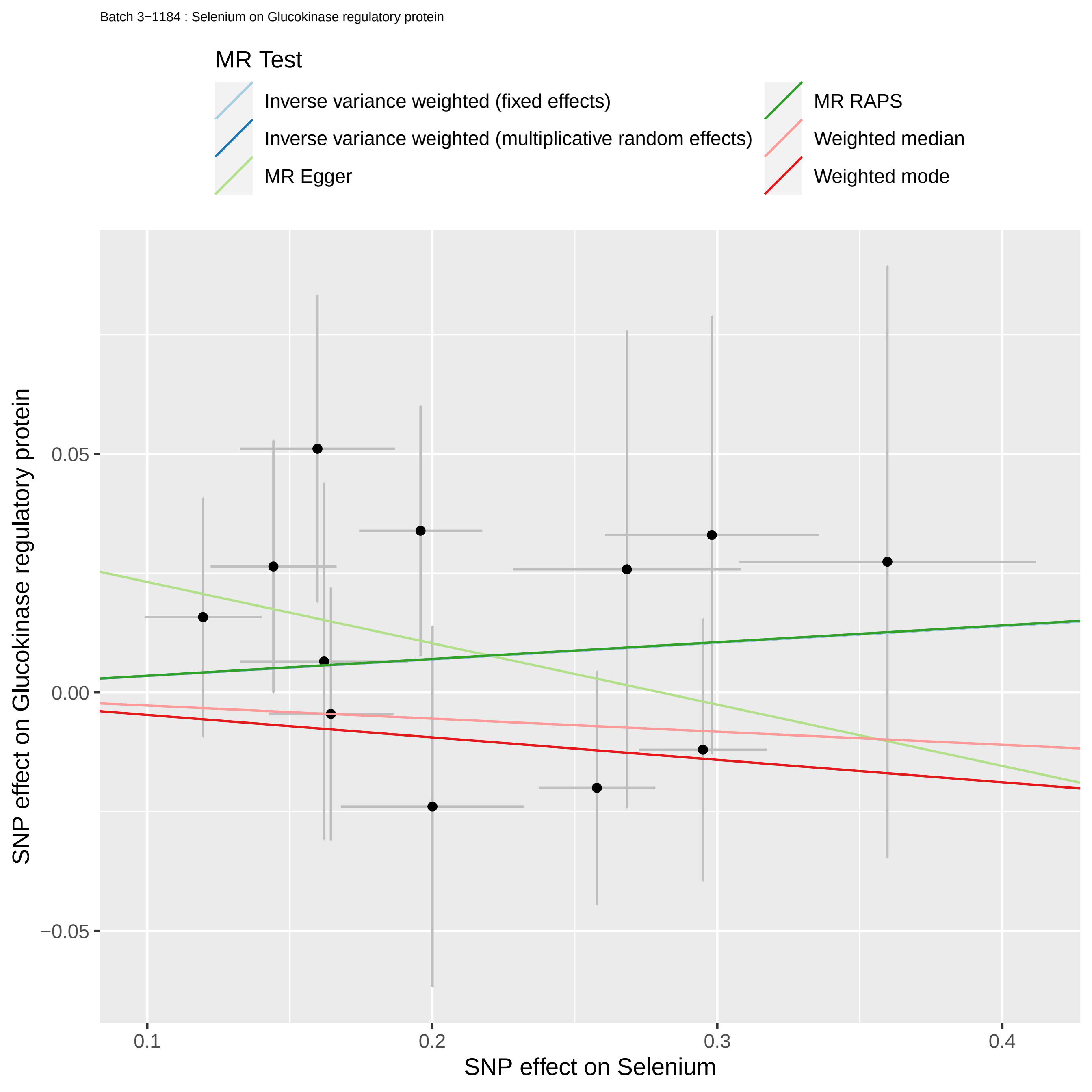
Batch 3−1184 : Selenium on Glucokinase regulatory protein
MR Test
Inverse variance weighted (fixed effects)
Inverse variance weighted (multiplicative random effects)
MR Egger
MR RAPS
Weighted median
Weighted mode
SNP effect on Glucokinase regulatory protein
0.05
0.00
−0.05
0.1
0.2
0.3
0.4
SNP effect on Selenium

Batch 3−1185 : Selenium on Beta−1,3−galactosyl−O−glycosyl−glycoprotein beta−1,6−N−acetylglucosaminyltransferase

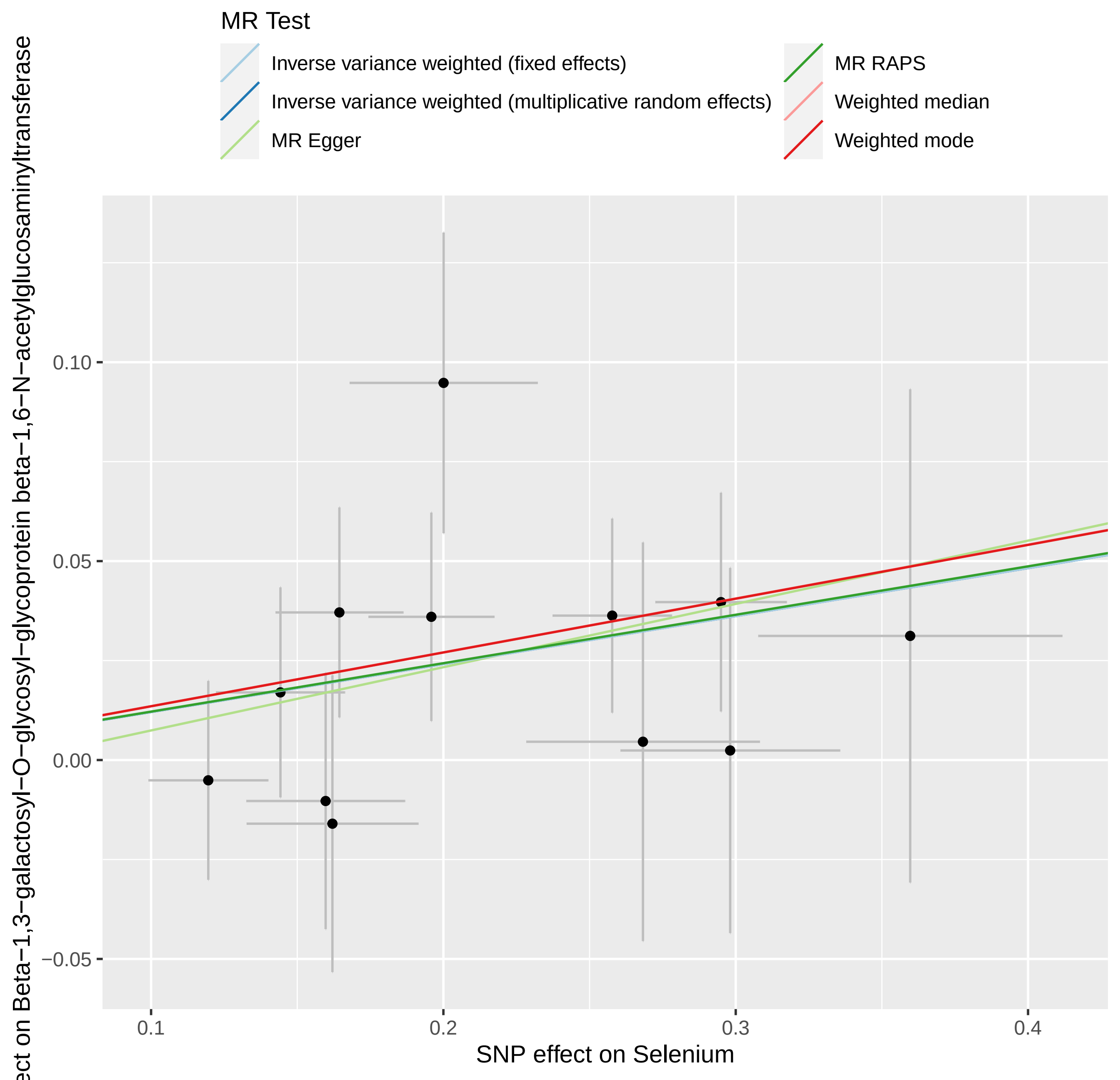

Batch 3−1186 : Selenium on N−acetyllactosaminide beta−1,6−N−acetylglucosaminyl−transferase, isoform C

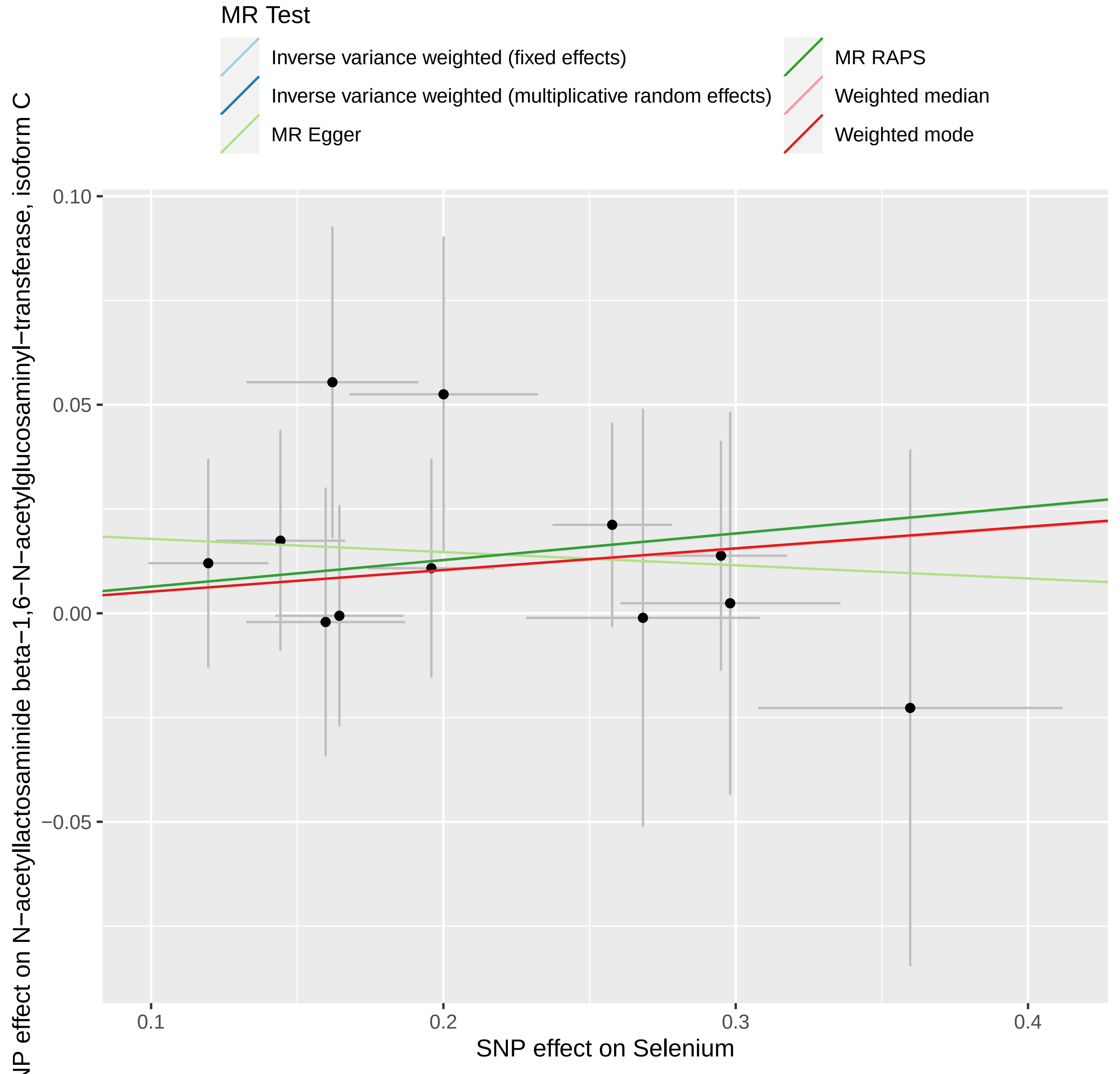

Batch 3−1187 : Selenium on Beta−1,3−galactosyl−O−glycosyl−glycoprotein beta−1,6−N−acetylglucosaminyltransferase 4

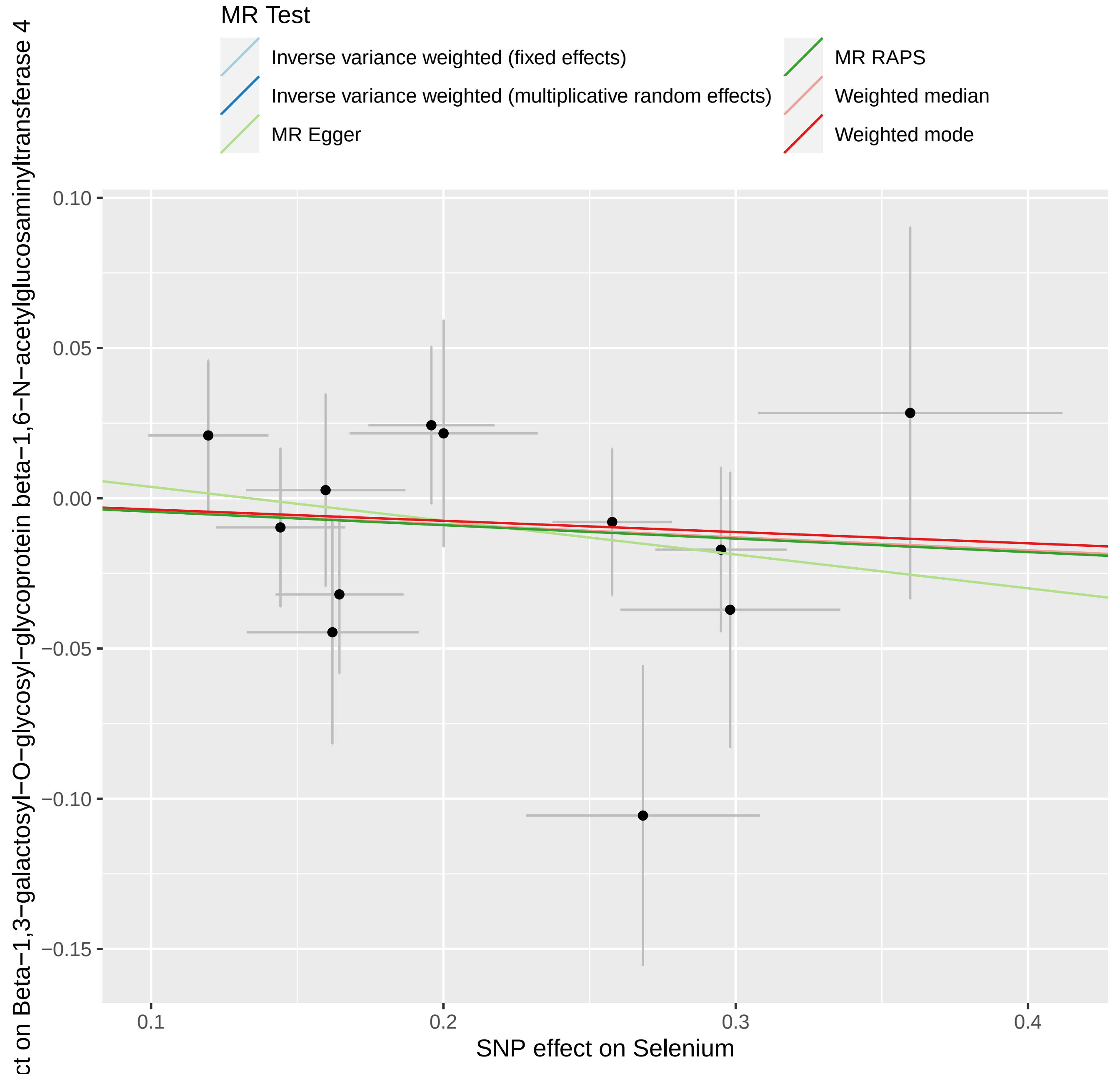

Batch 3−1188 : Selenium on Beta−1,3−galactosyl−O−glycosyl−glycoprotein beta−1,6−N−acetylglucosaminyltransferase 4

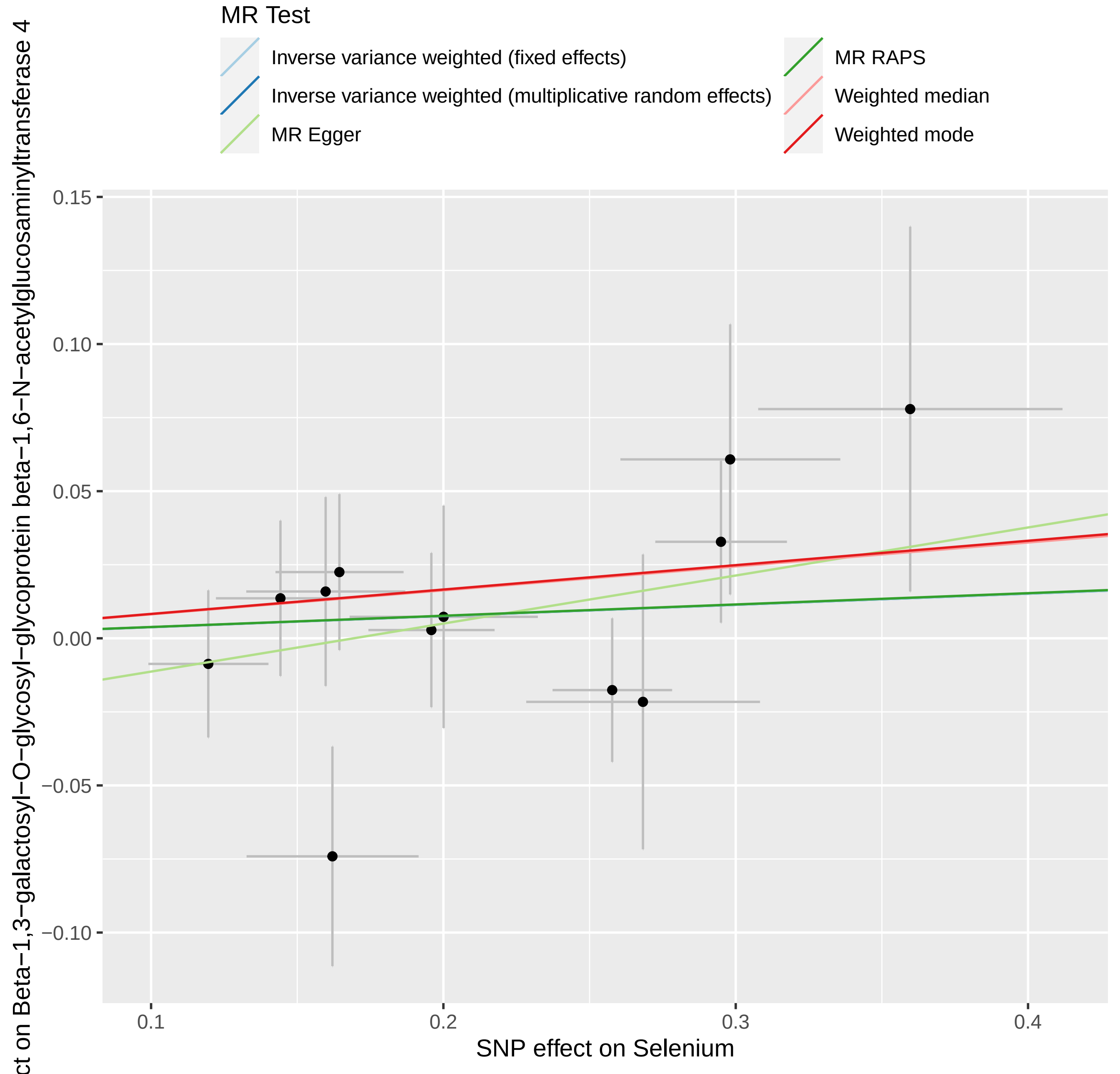

Batch 3−1189 : Selenium on Guanine deaminase

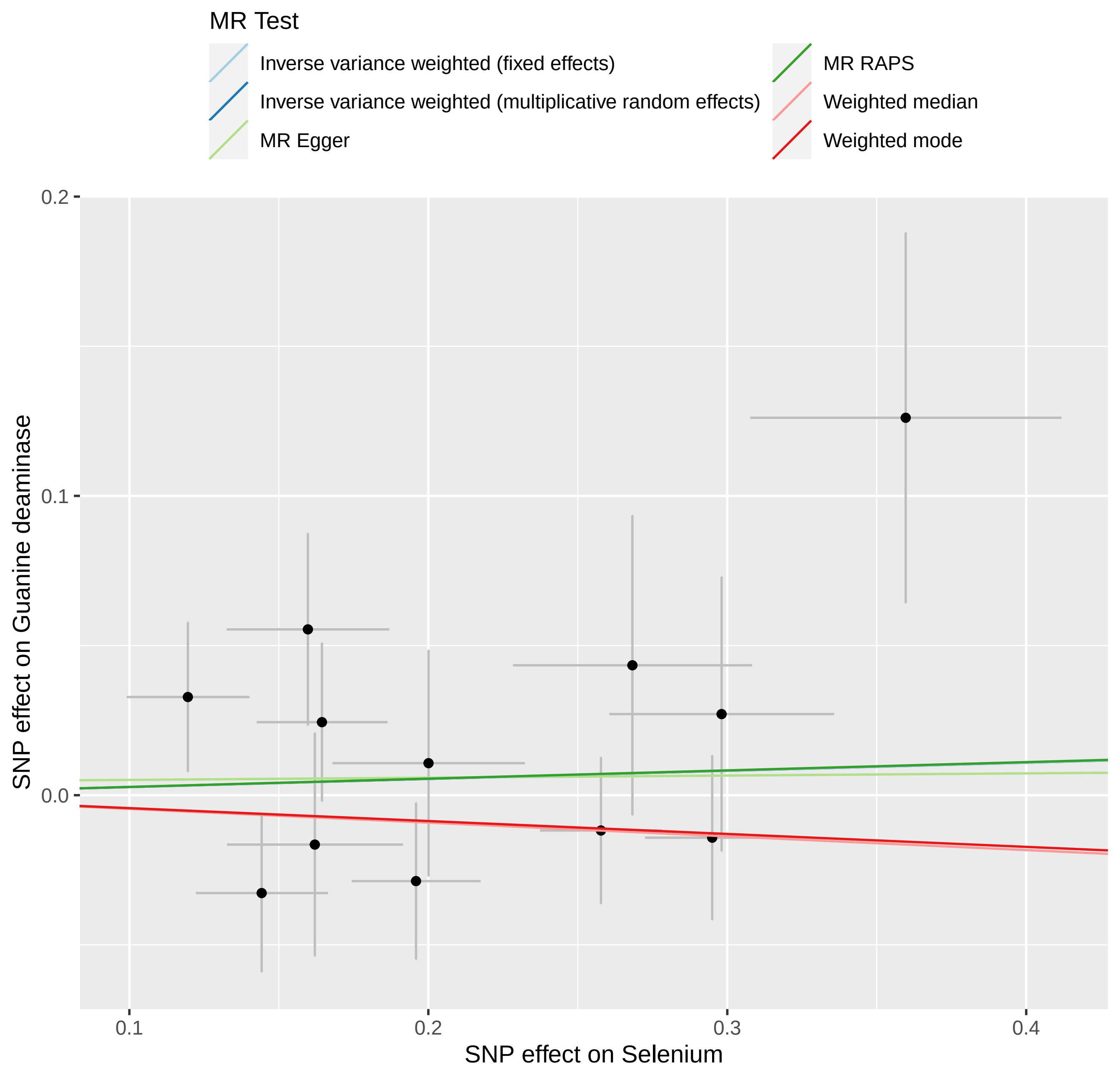

Batch 3−1190 : Selenium on Growth/differentiation factor 11

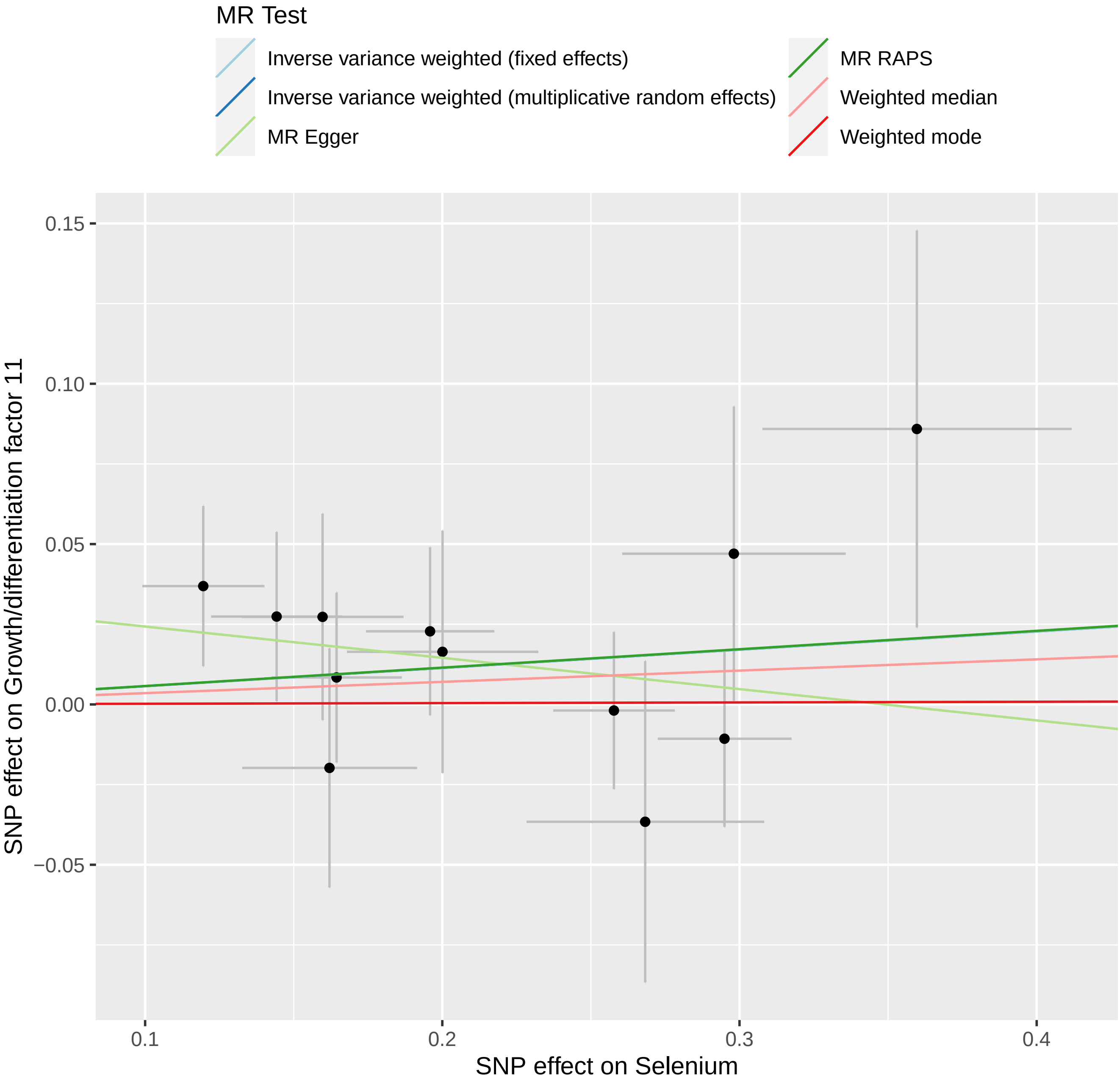

Batch 3−1191 : Selenium on Growth/differentiation factor 11

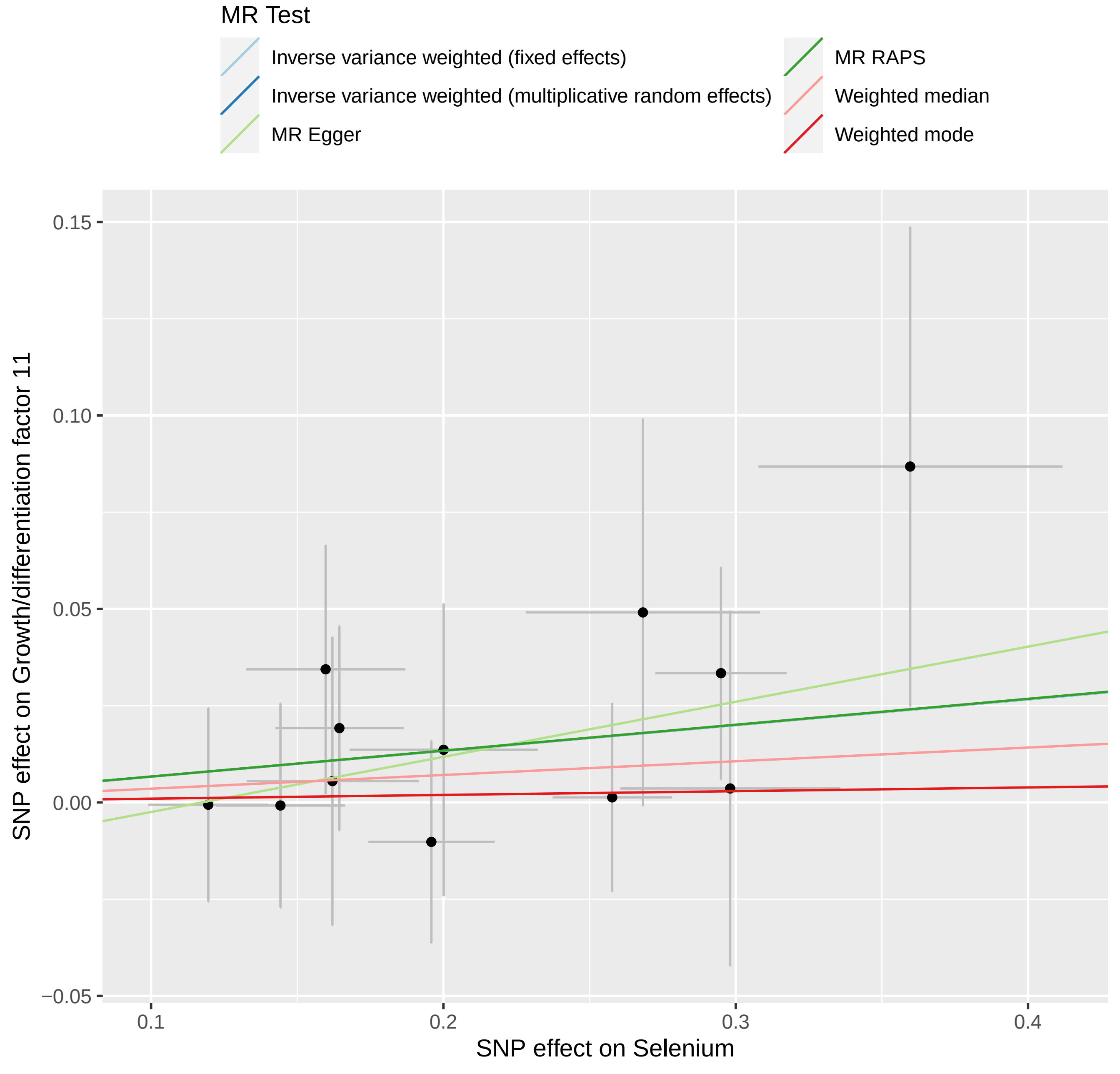

Batch 3−1192 : Selenium on Growth/differentiation factor 11

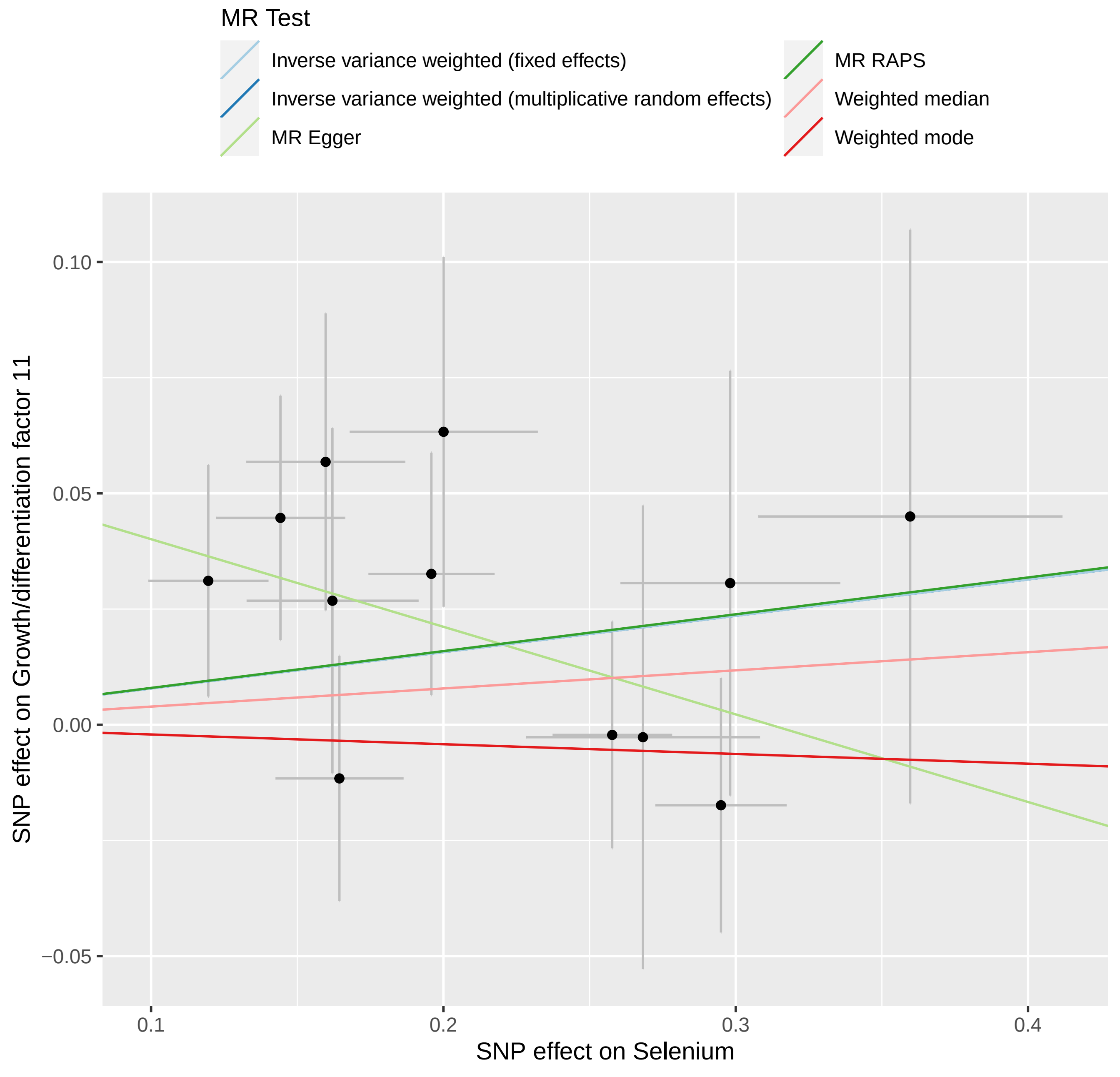

Batch 3−1193 : Selenium on Growth/differentiation factor 11

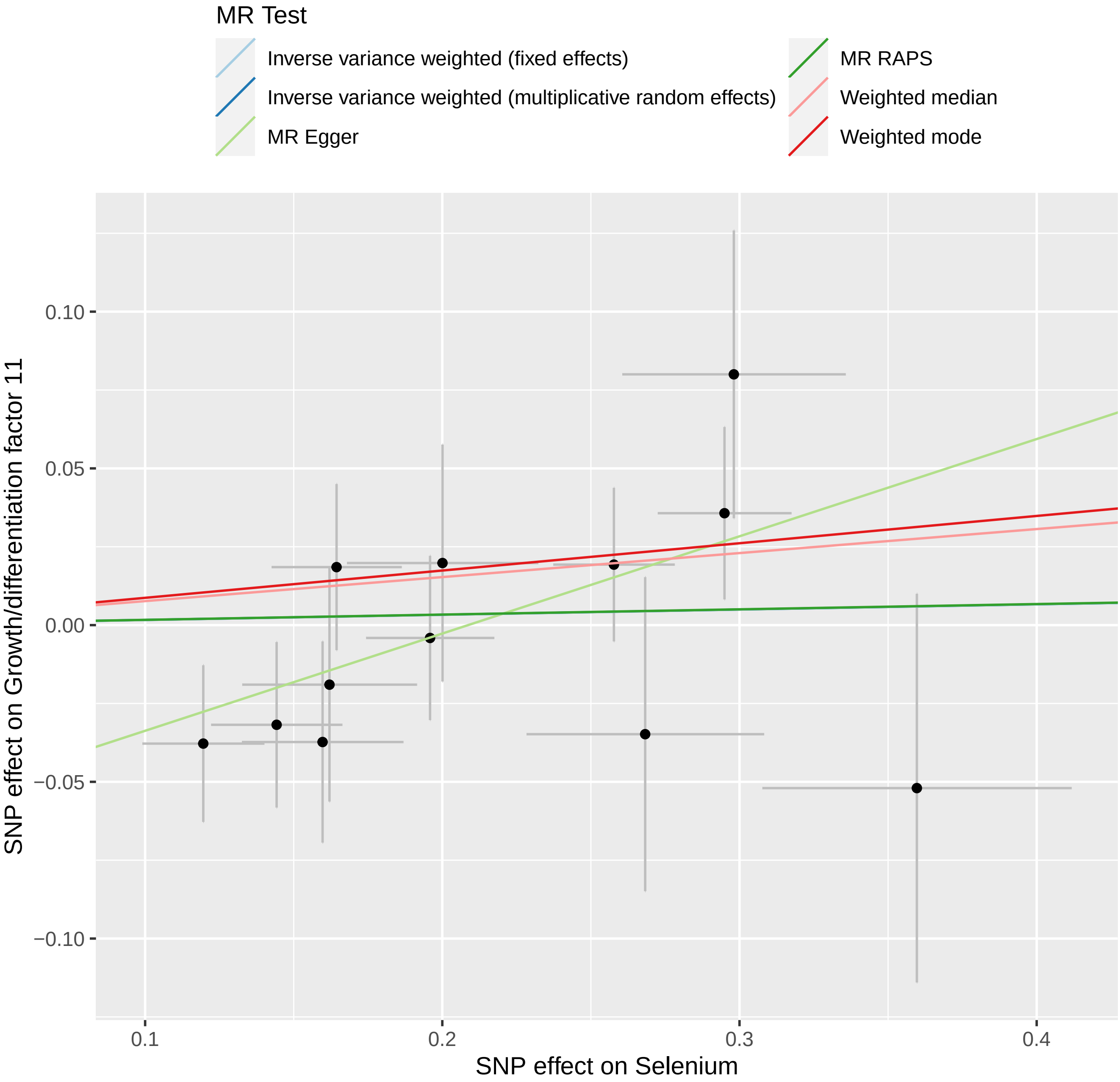

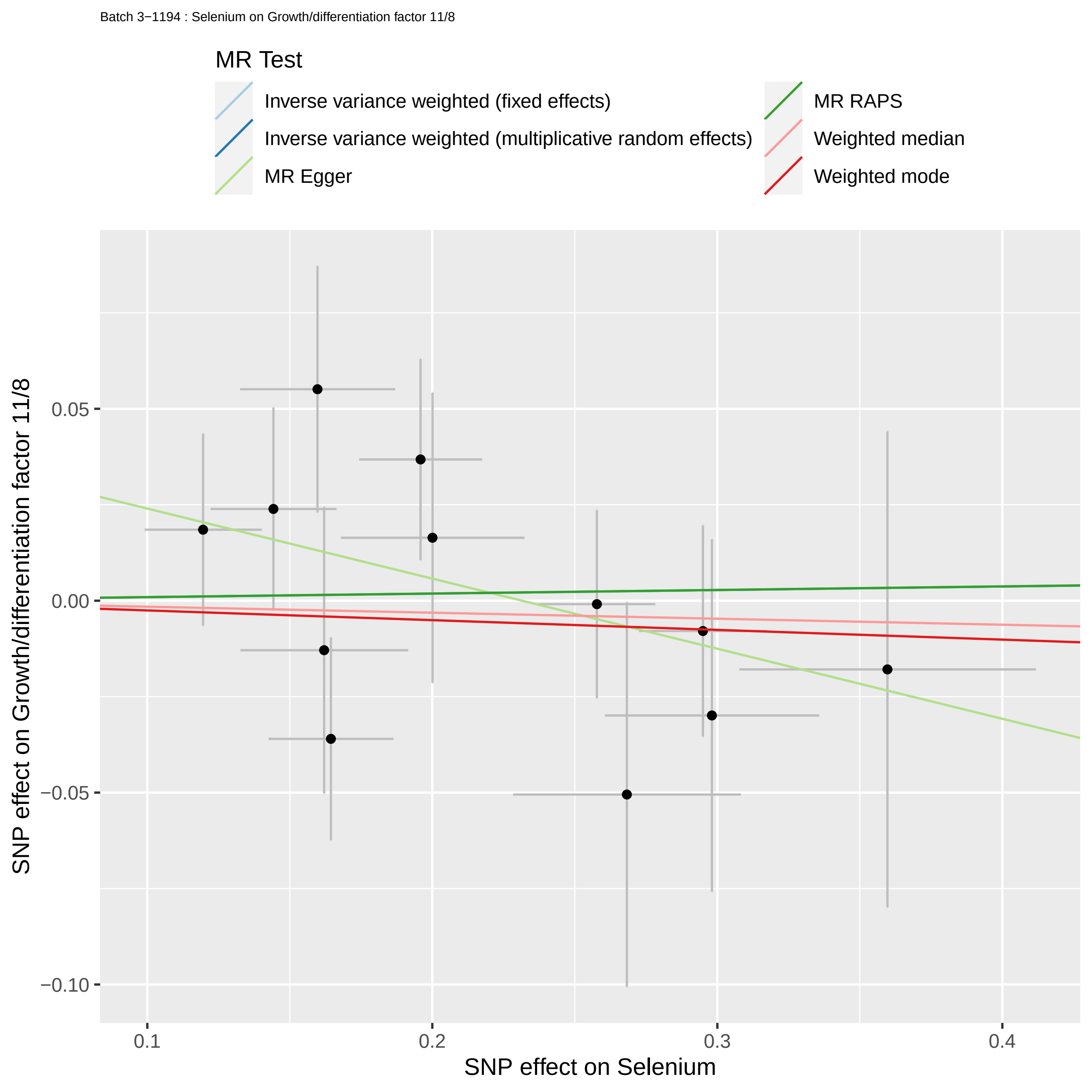

Batch 3−1194 : Selenium on Growth/differentiation factor 11/8
MR Test
Inverse variance weighted (fixed effects)
Inverse variance weighted (multiplicative random effects)
MR Egger
MR RAPS
Weighted median
Weighted mode
0.05
0.00
−0.05
−0.10
0.1
0.2
0.3
0.4
SNP effect on Growth/differentiation factor 11/8
SNP effect on Selenium

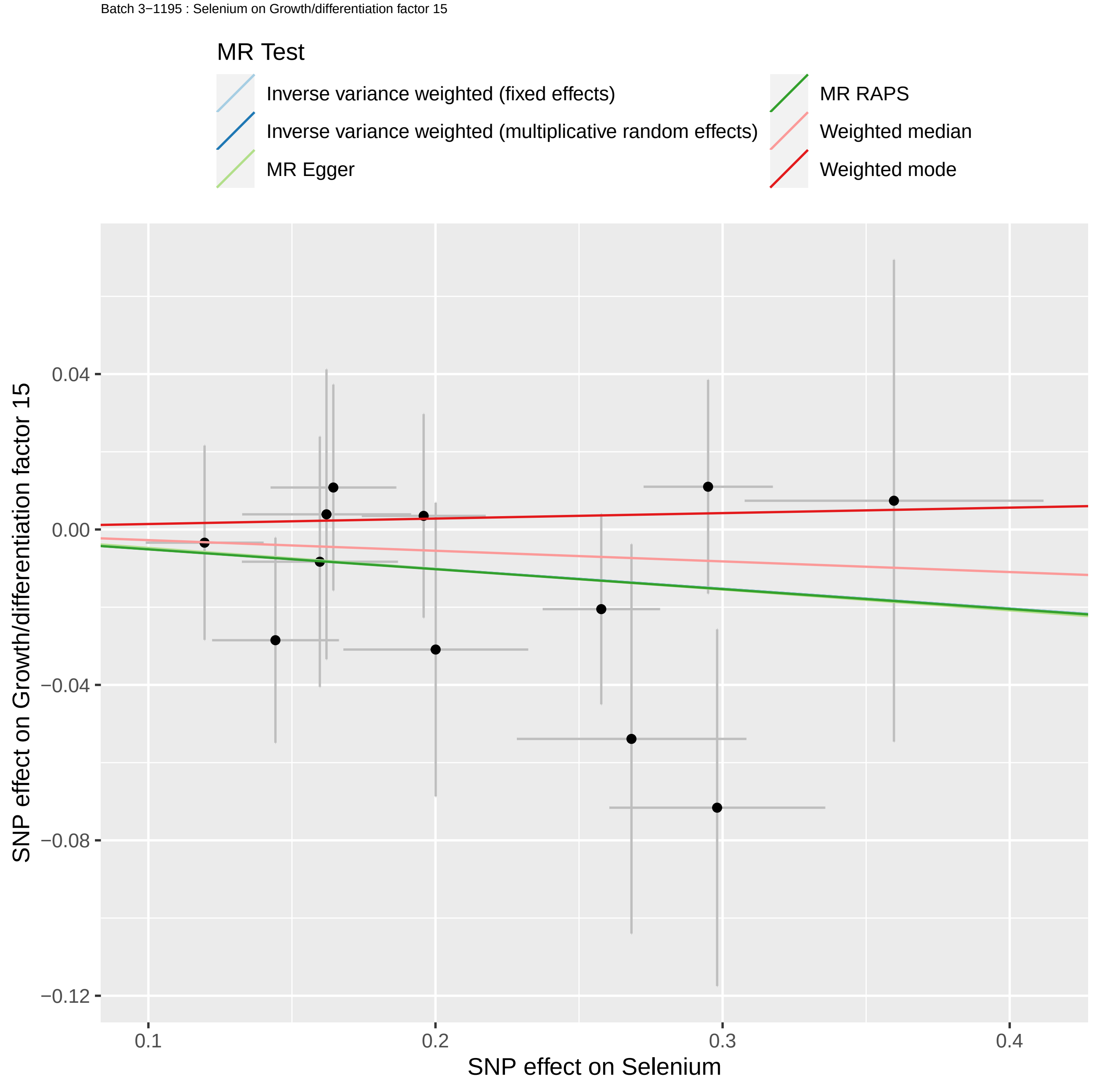
Batch 3−1195 : Selenium on Growth/differentiation factor 15
MR Test
Inverse variance weighted (fixed effects)
Inverse variance weighted (multiplicative random effects)
MR Egger
MR RAPS
Weighted median
Weighted mode
SNP effect on Growth/differentiation factor 15
0.04
0.00
−0.04
−0.08
−0.12
0.1
0.2
0.3
0.4
SNP effect on Selenium

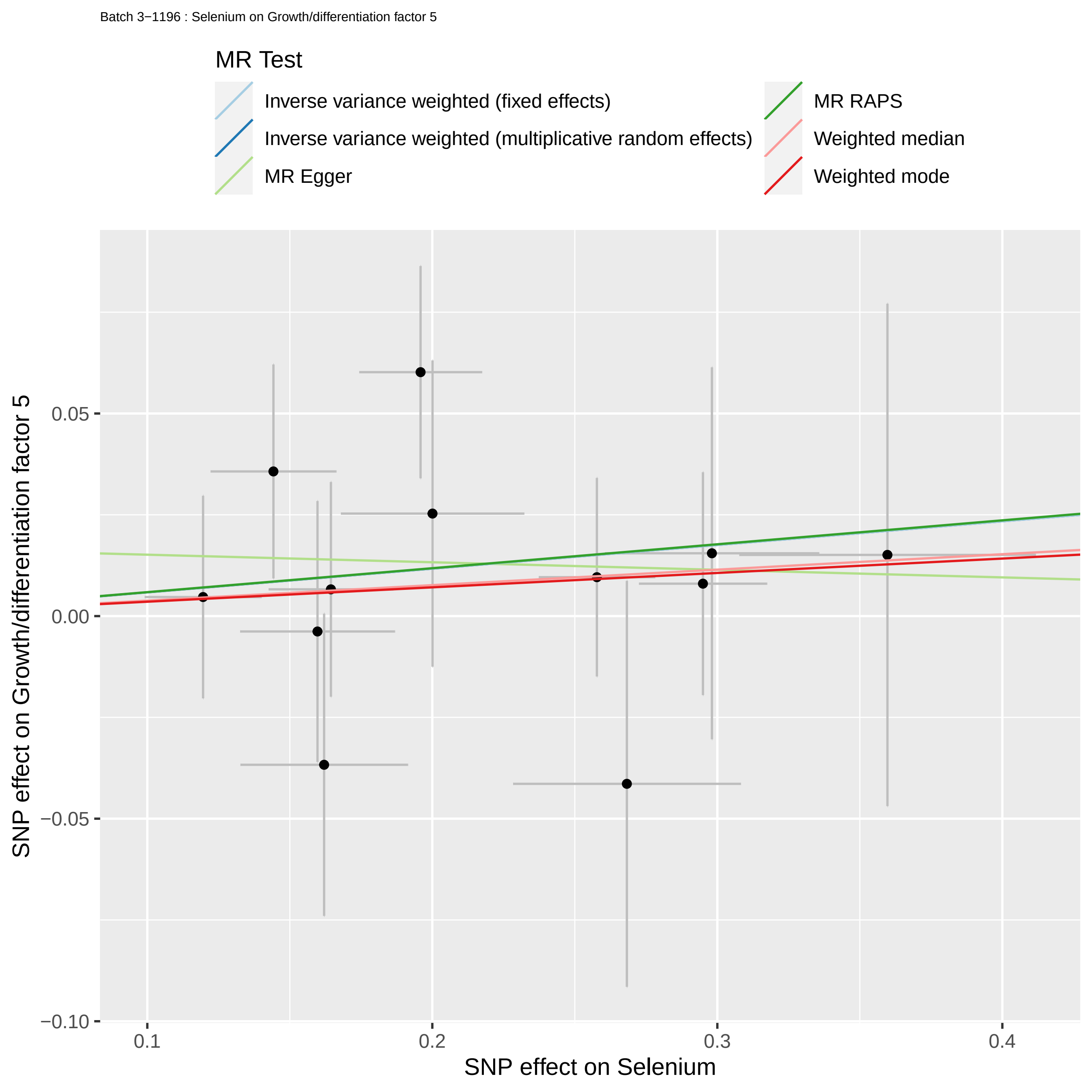

Batch 3−1196 : Selenium on Growth/differentiation factor 5
MR Test
Inverse variance weighted (fixed effects)
Inverse variance weighted (multiplicative random effects)
MR Egger
MR RAPS
Weighted median
Weighted mode
0.05
0.00
−0.05
−0.10
0.1
0.2
0.3
0.4
SNP effect on Growth/differentiation factor 5
SNP effect on Selenium

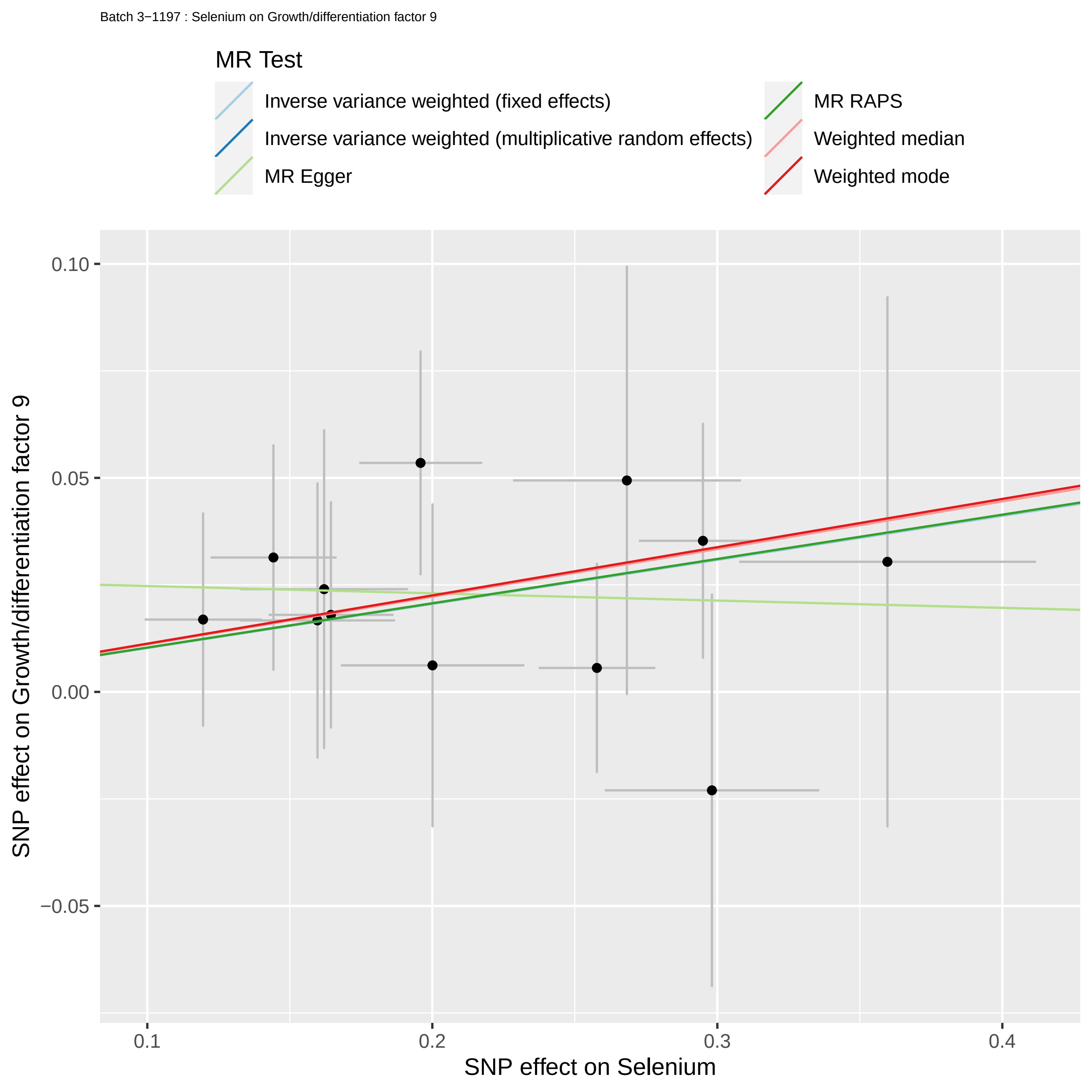

Batch 3−1197 : Selenium on Growth/differentiation factor 9
MR Test
Inverse variance weighted (fixed effects)
Inverse variance weighted (multiplicative random effects)
MR Egger
MR RAPS
Weighted median
Weighted mode
0.10
0.05
0.00
−0.05
0.1
0.2
0.3
0.4
SNP effect on Growth/differentiation factor 9
SNP effect on Selenium

Batch 3−1198 : Selenium on Glial cell line−derived neurotrophic factor

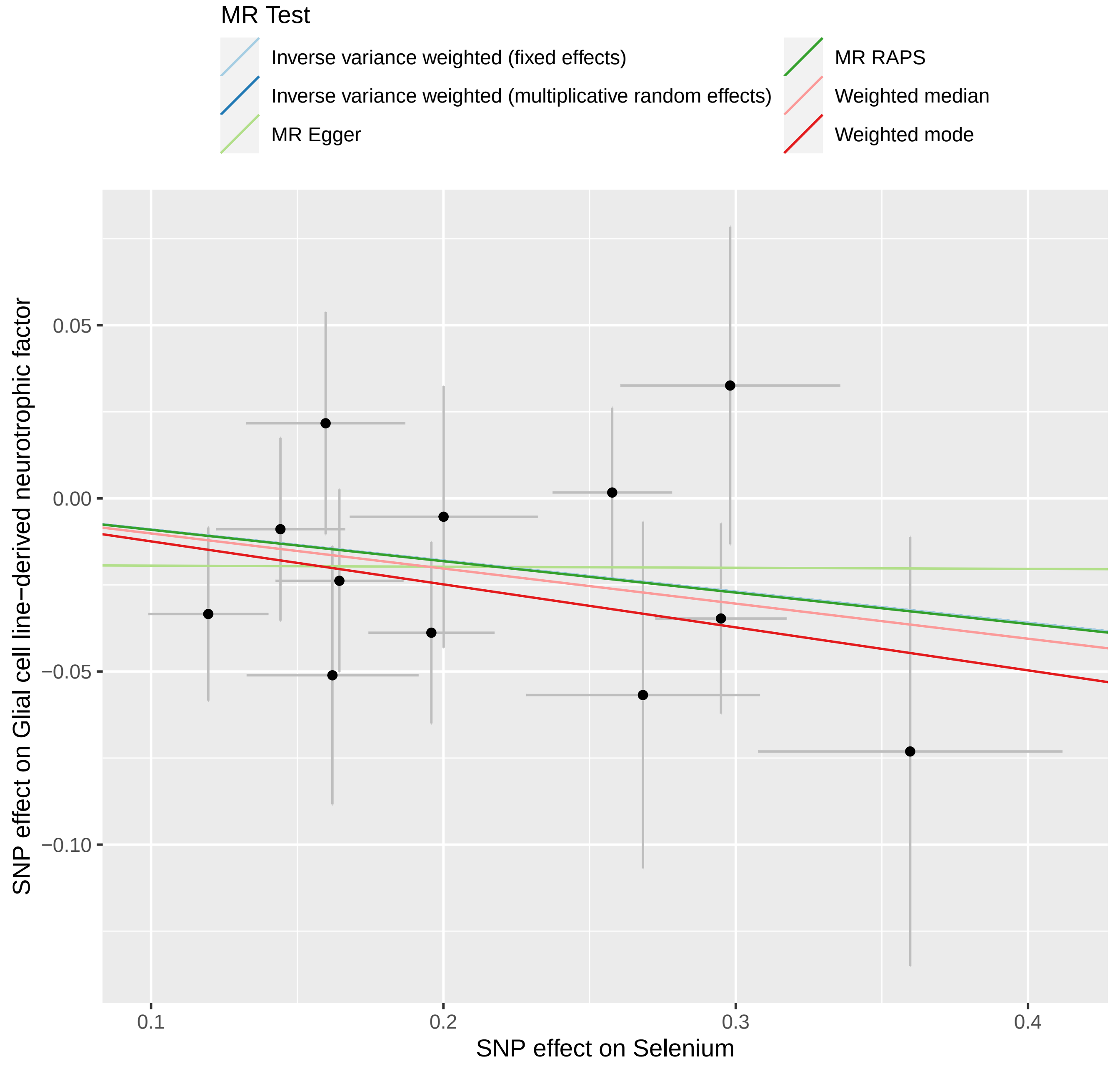

Batch 3−1199 : Selenium on GTP−binding protein GEM

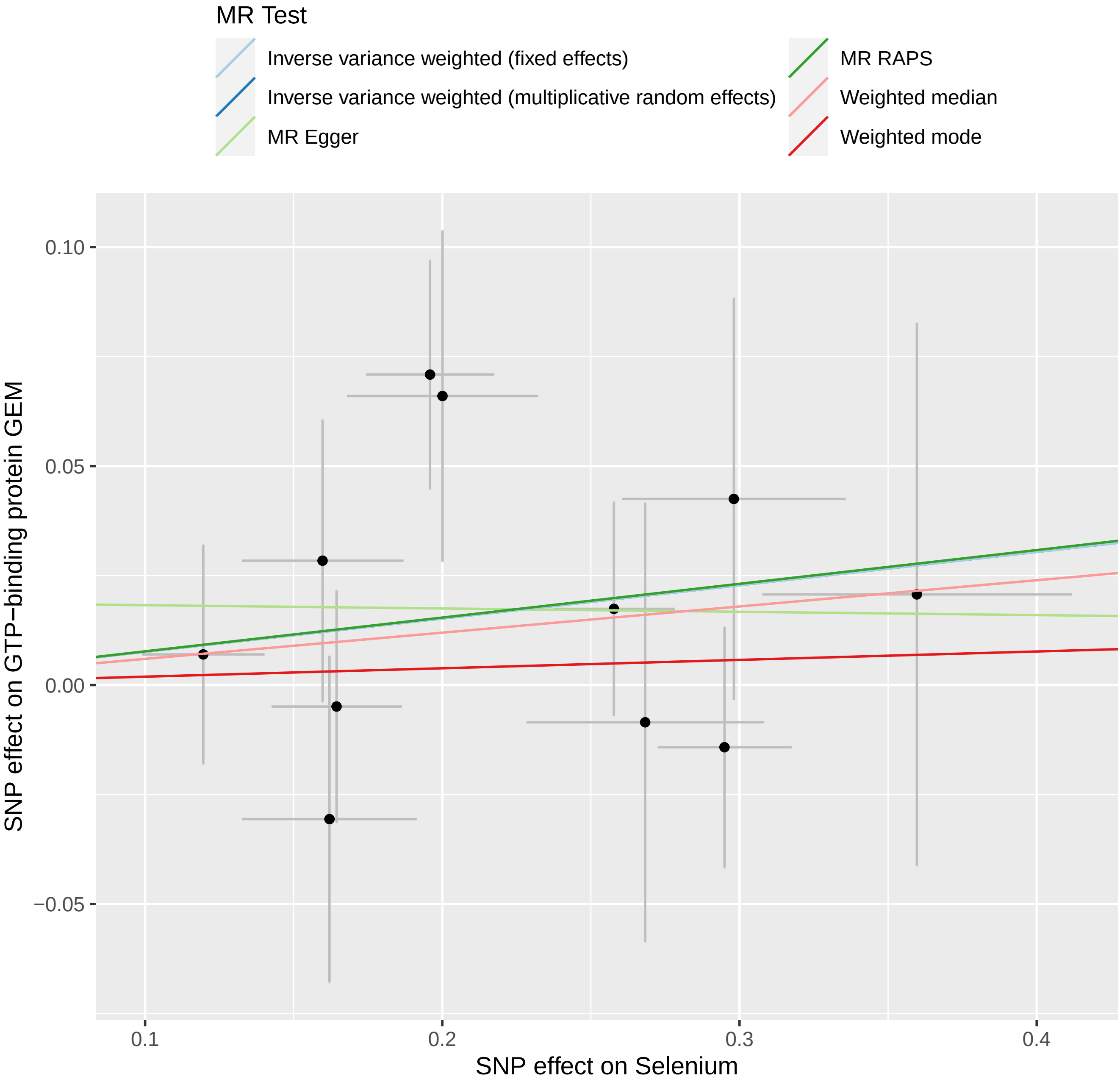

Batch 3−1200 : Selenium on Glial fibrillary acidic protein

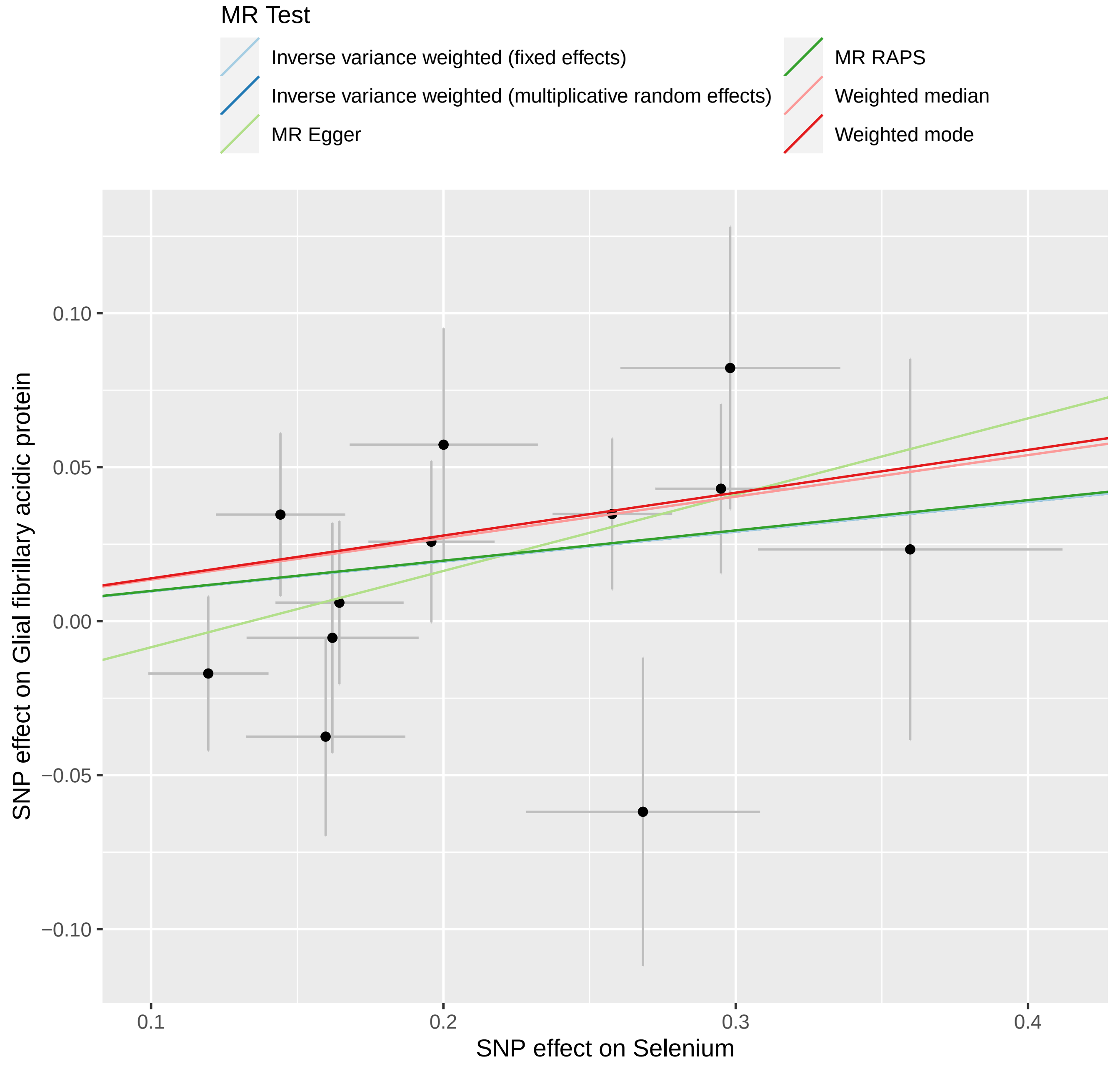

Batch 3−1201 : Selenium on GDNF family receptor alpha−1

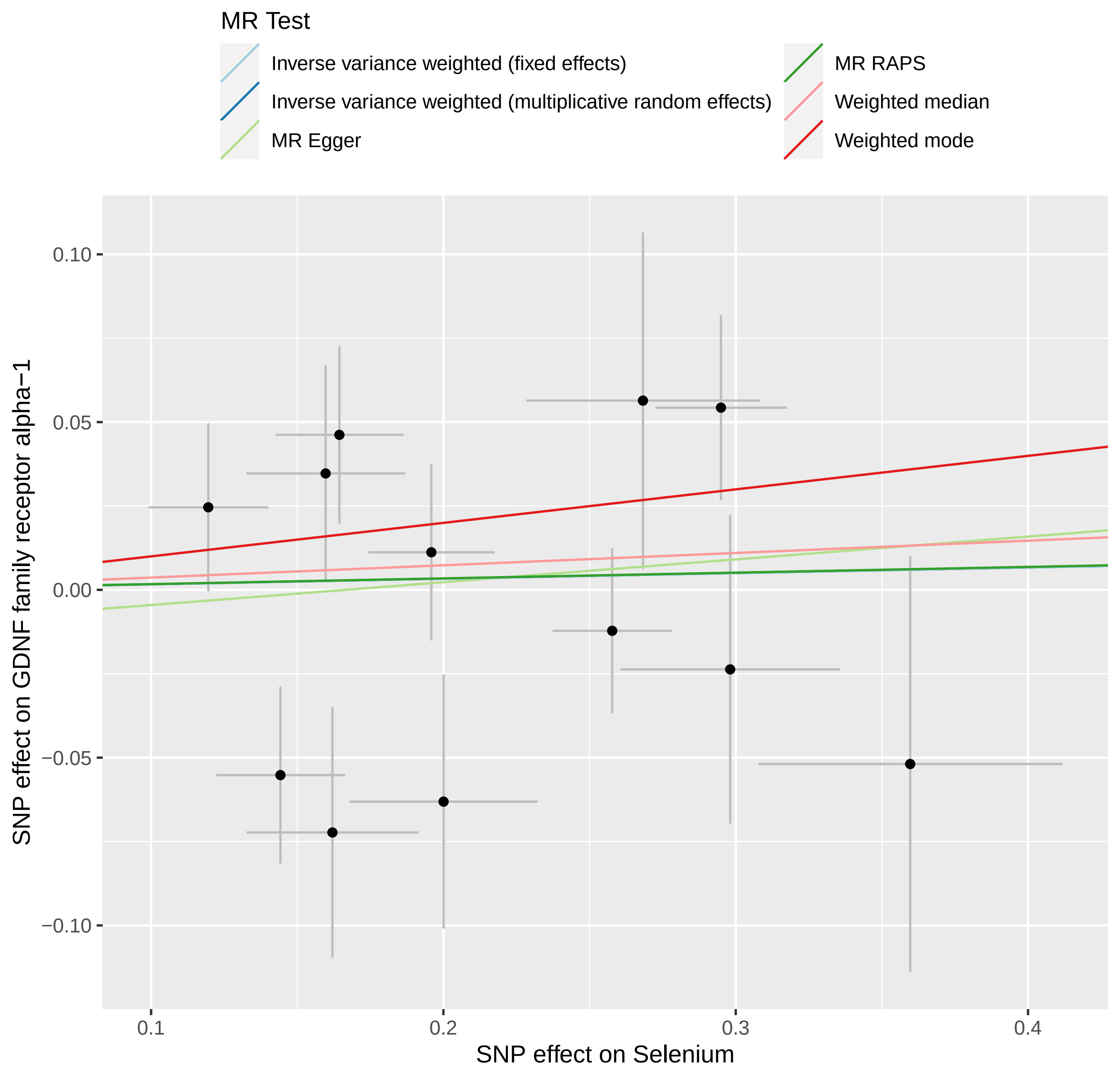

Batch 3−1202 : Selenium on GDNF family receptor alpha−2

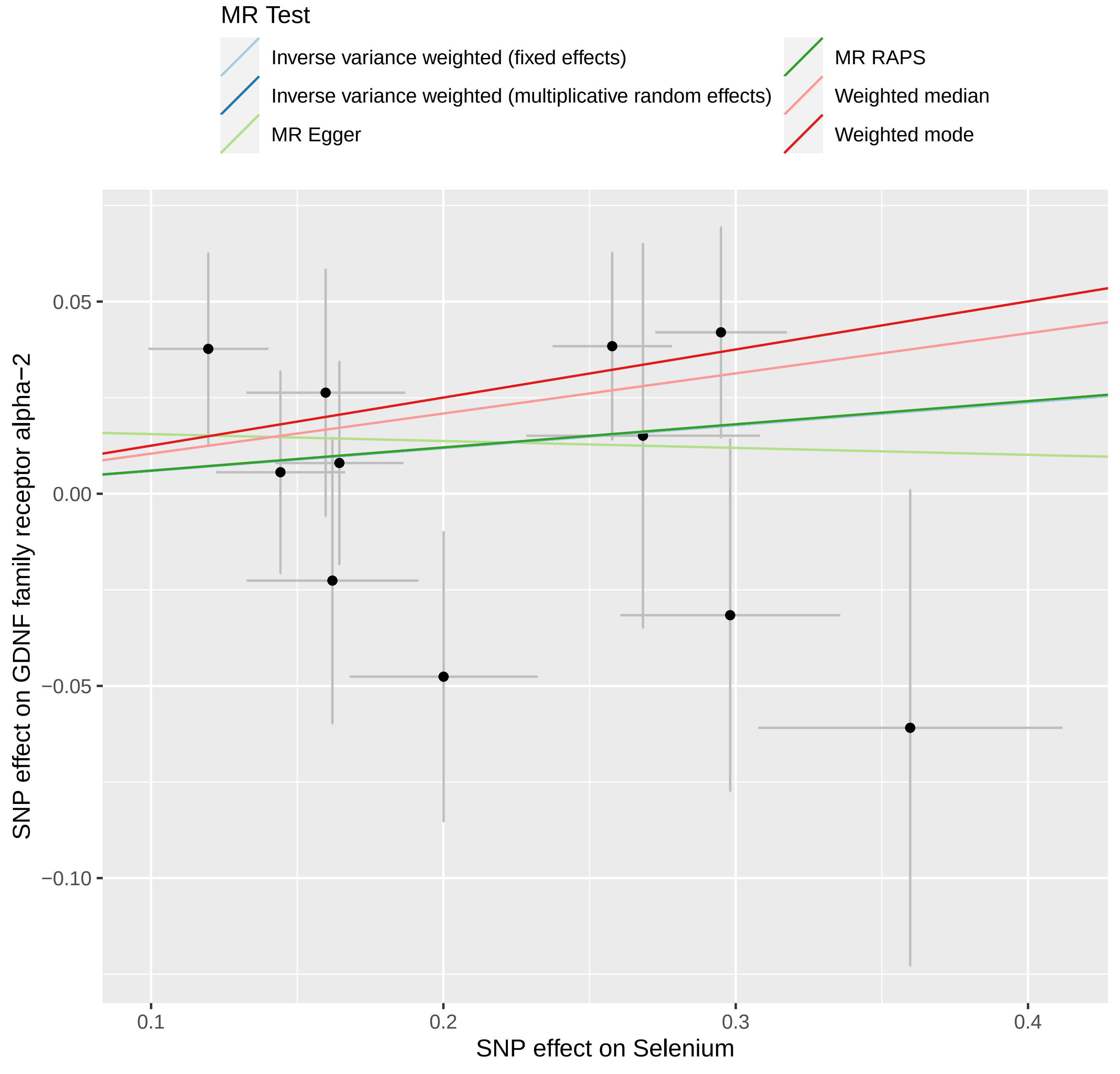

Batch 3−1203 : Selenium on GDNF family receptor alpha−3

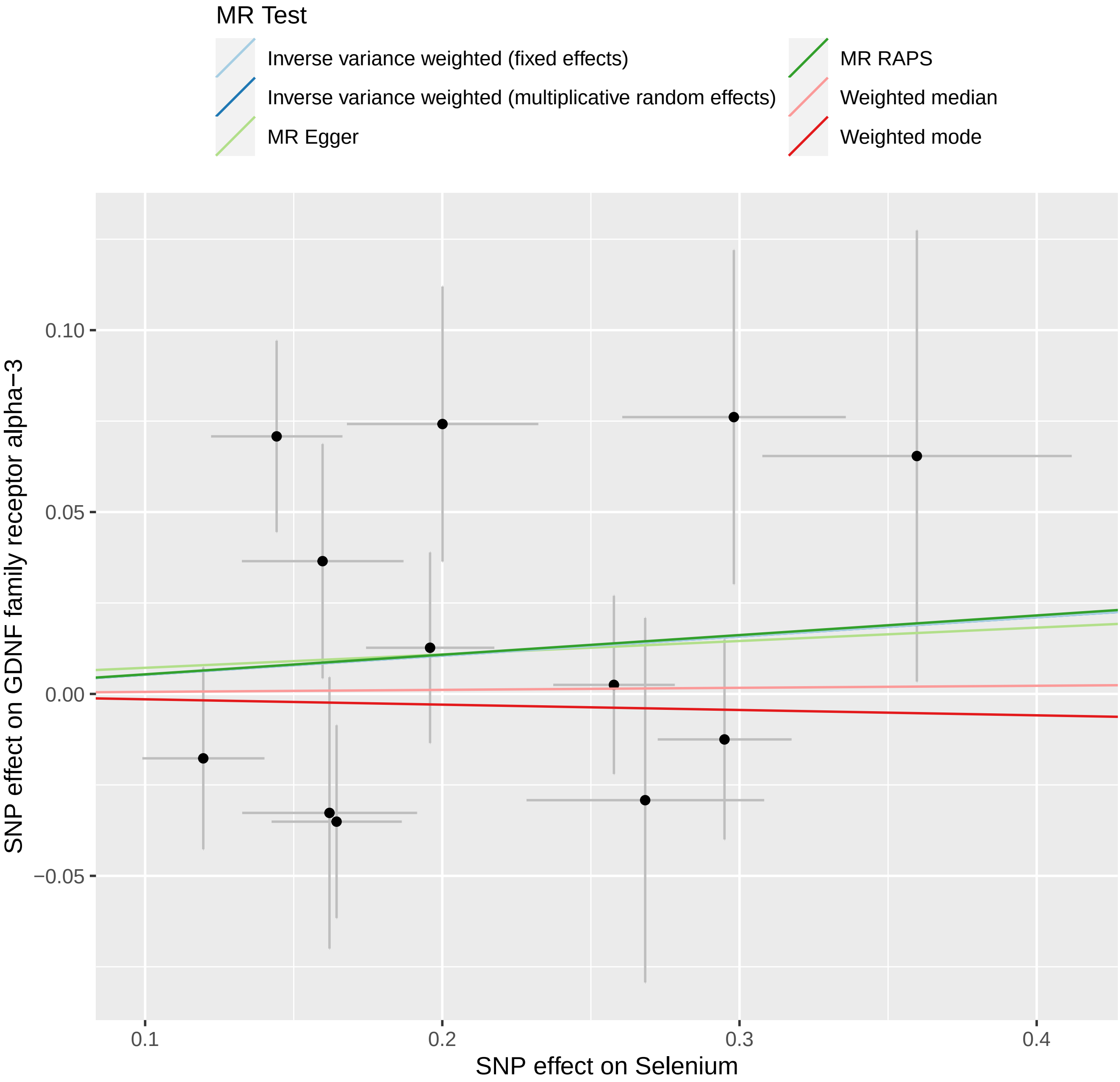

Batch 3−1204 : Selenium on GDNF family receptor alpha−like

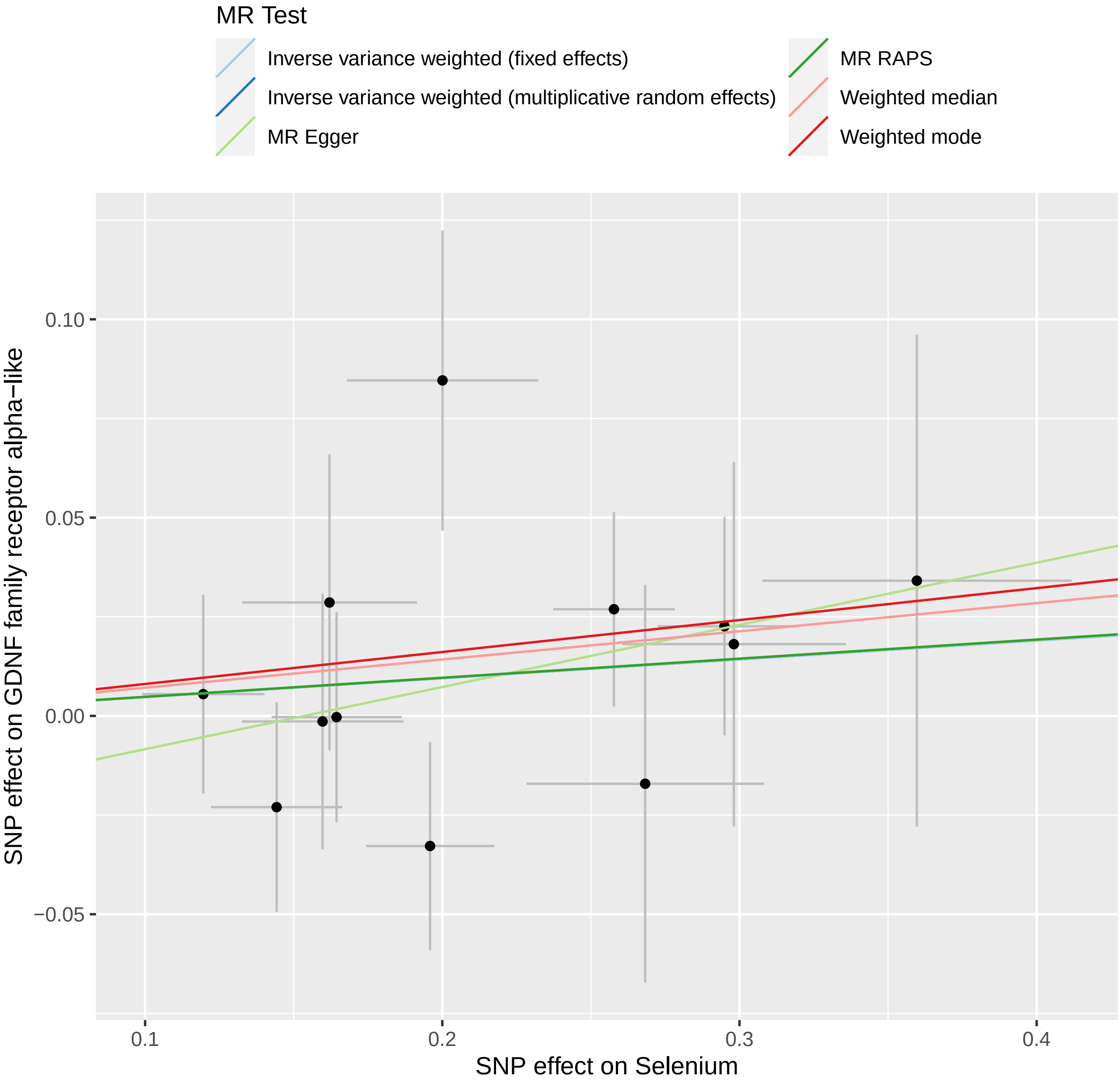

Batch 3−1205 : Selenium on ADP−ribosylation factor−binding protein GGA1

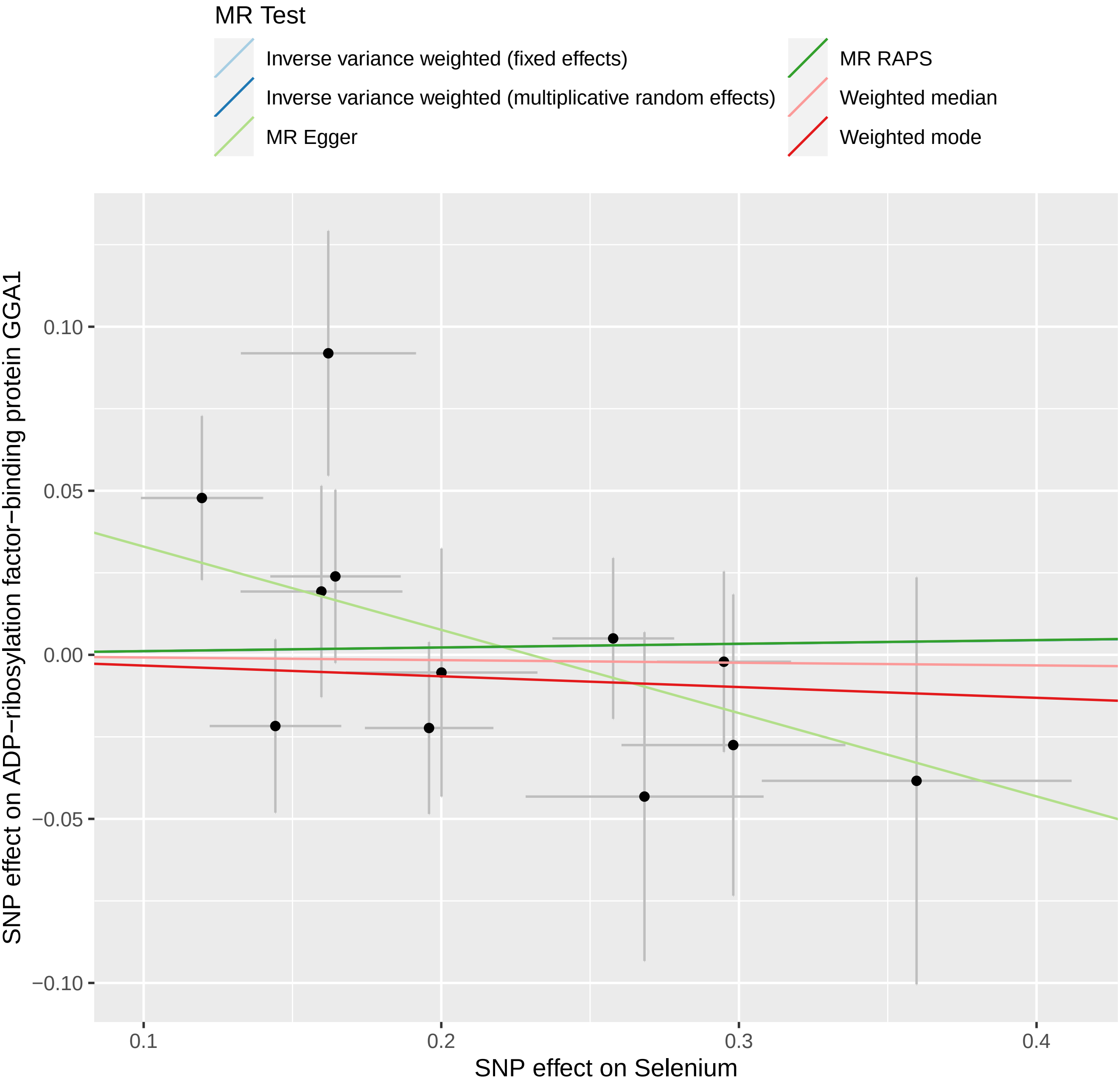

Batch 3−1206 : Selenium on ADP−ribosylation factor−binding protein GGA3

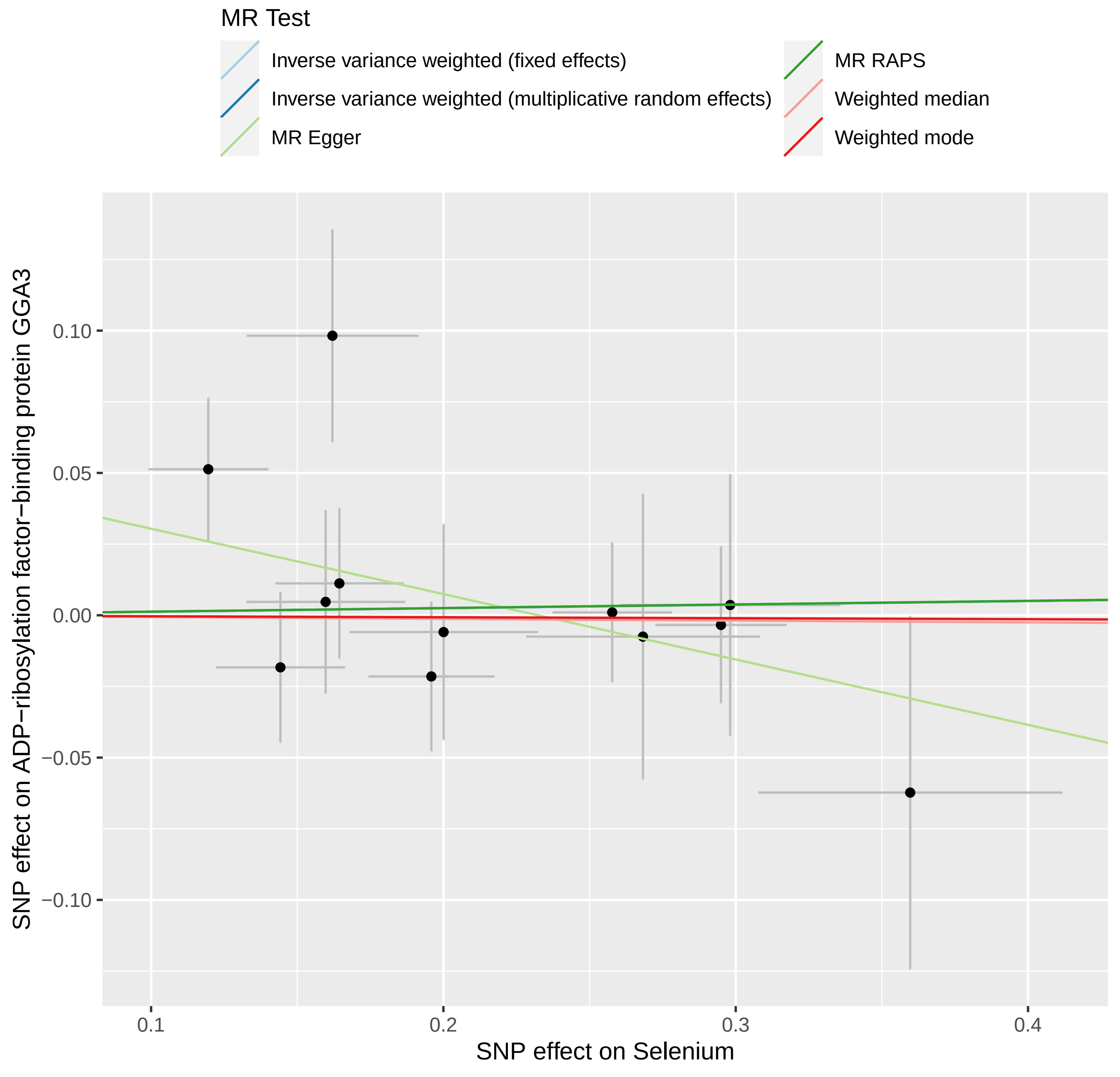

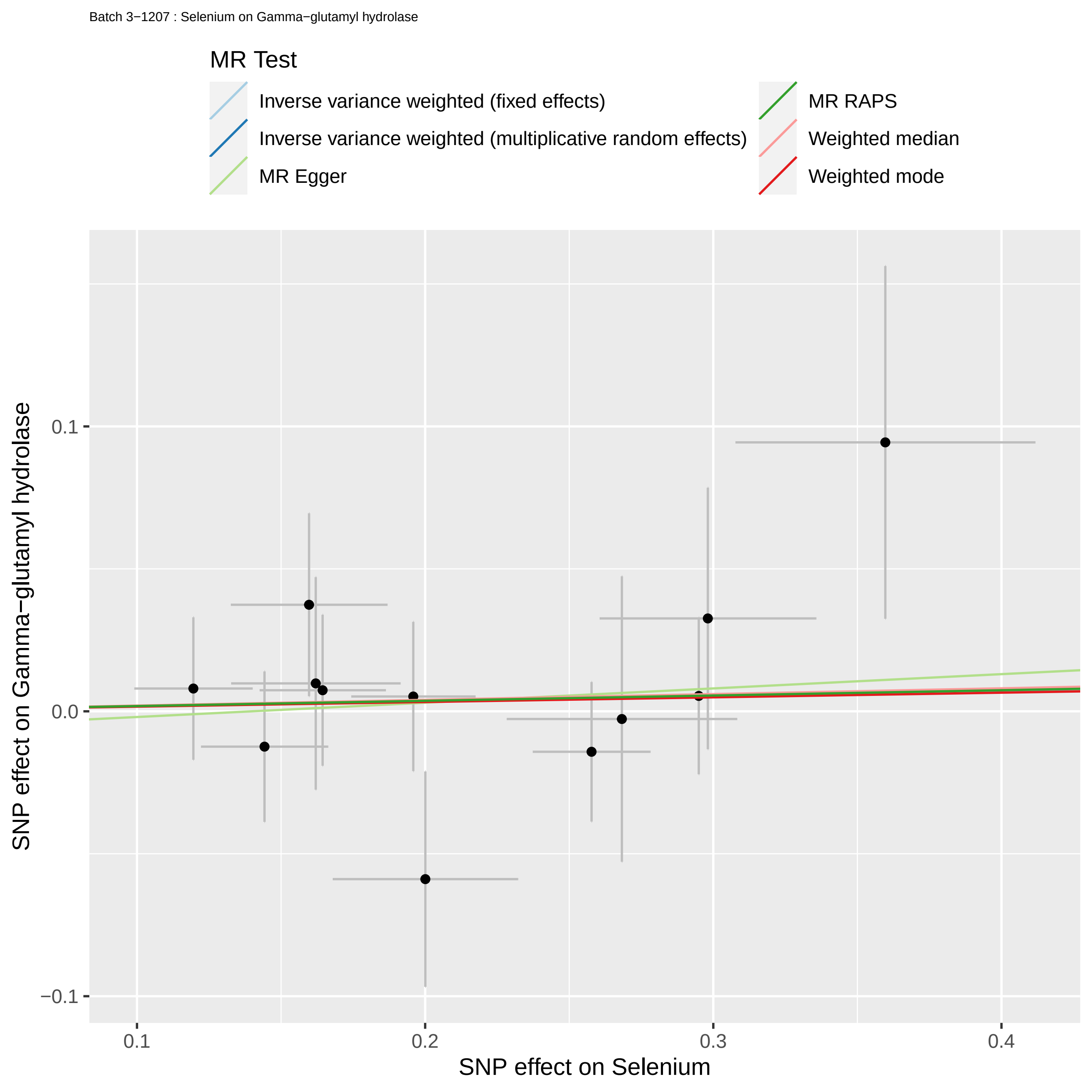

Batch 3−1207 : Selenium on Gamma−glutamyl hydrolase
MR Test
Inverse variance weighted (fixed effects)
Inverse variance weighted (multiplicative random effects)
MR Egger
MR RAPS
Weighted median
Weighted mode
SNP effect on Gamma−glutamyl hydrolase
0.1
0.0
−0.1
0.1
0.2
0.3
0.4
SNP effect on Selenium

Batch 3−1208 : Selenium on Inactive gamma−glutamyltranspeptidase 2

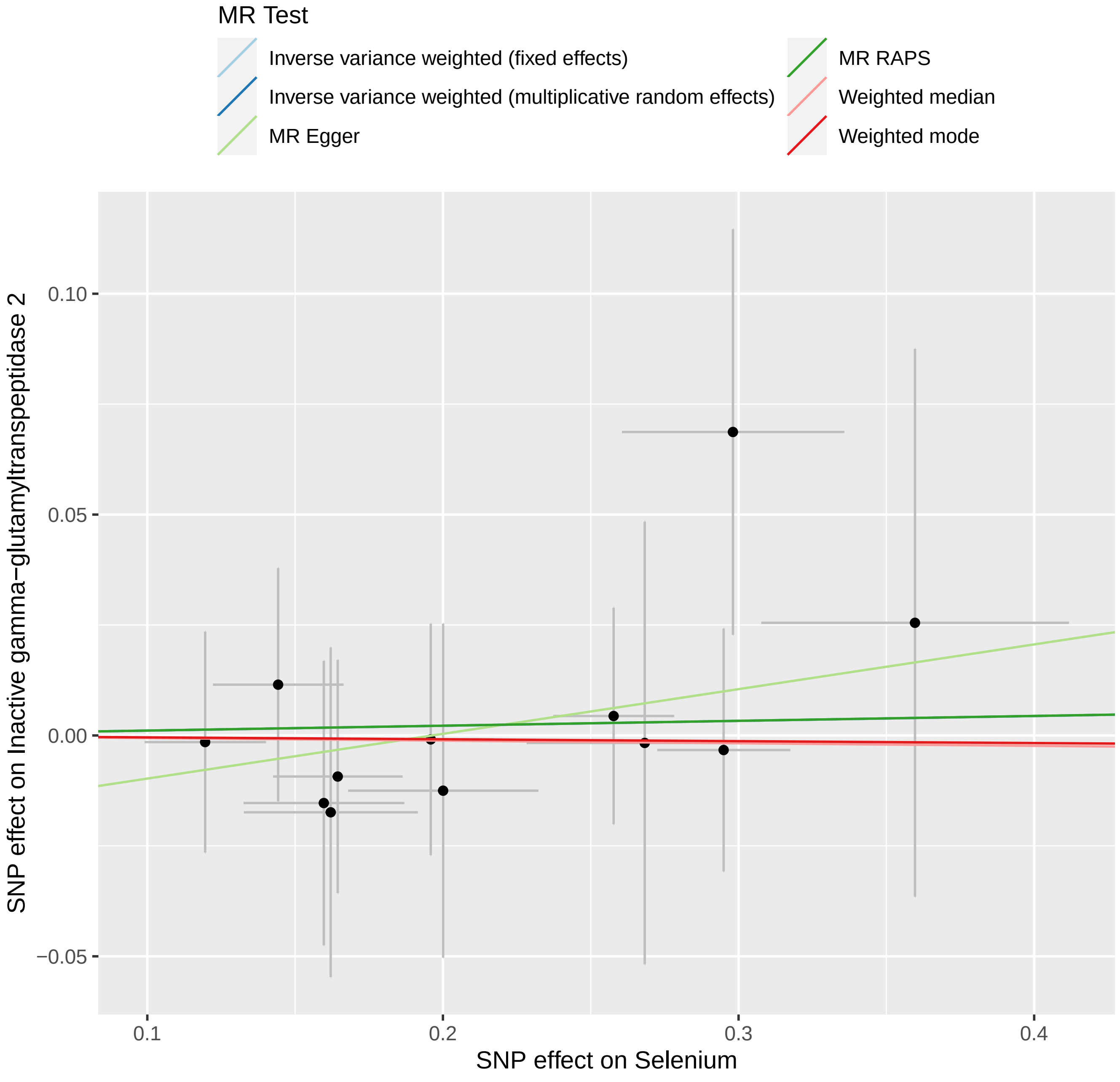

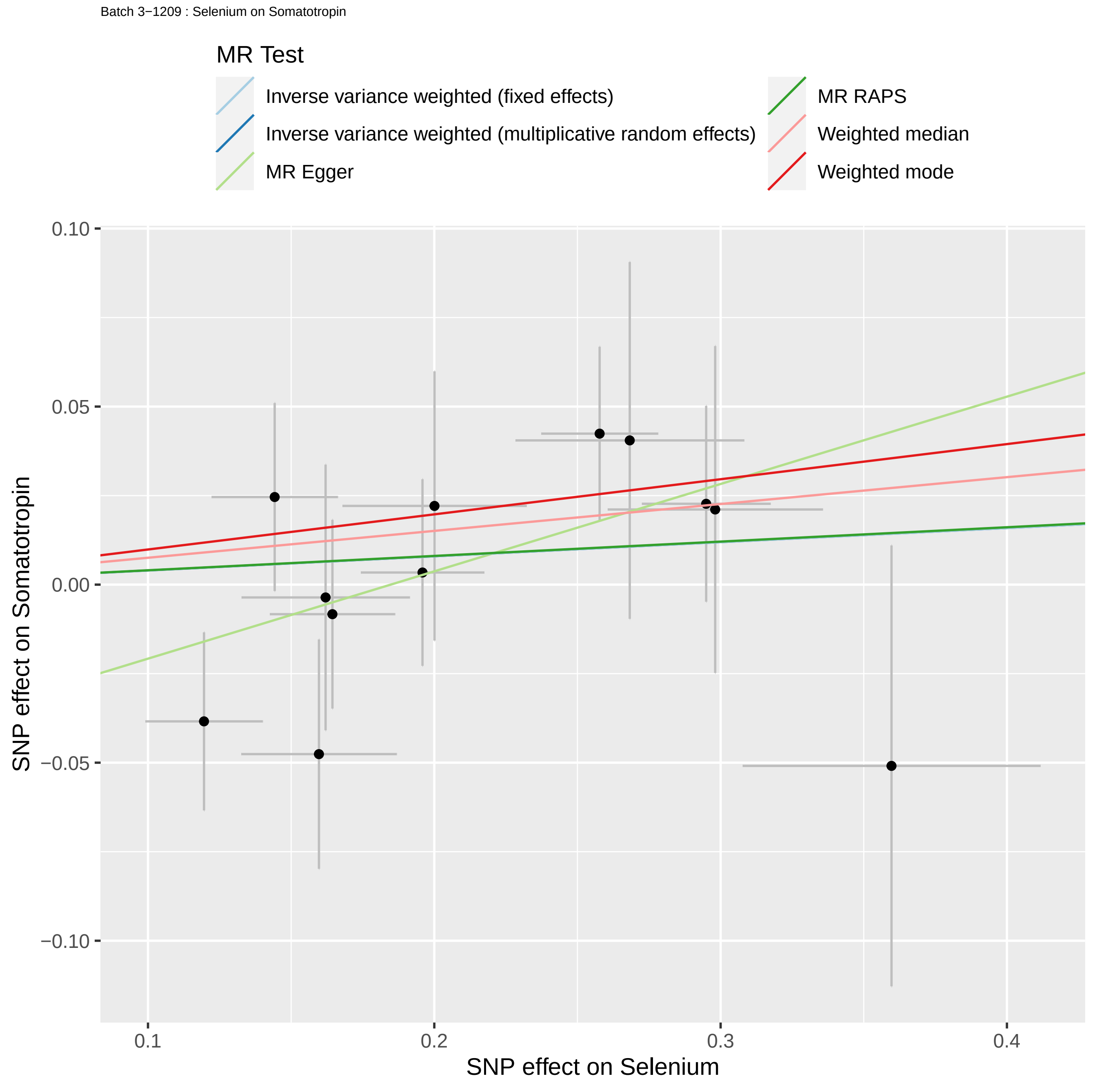

Batch 3−1209 : Selenium on Somatotropin
MR Test
Inverse variance weighted (fixed effects)
Inverse variance weighted (multiplicative random effects)
MR Egger
MR RAPS
Weighted median
Weighted mode
0.10
0.05
0.00
−0.05
−0.10
0.1
0.2
0.3
0.4
SNP effect on Somatotropin
SNP effect on Selenium

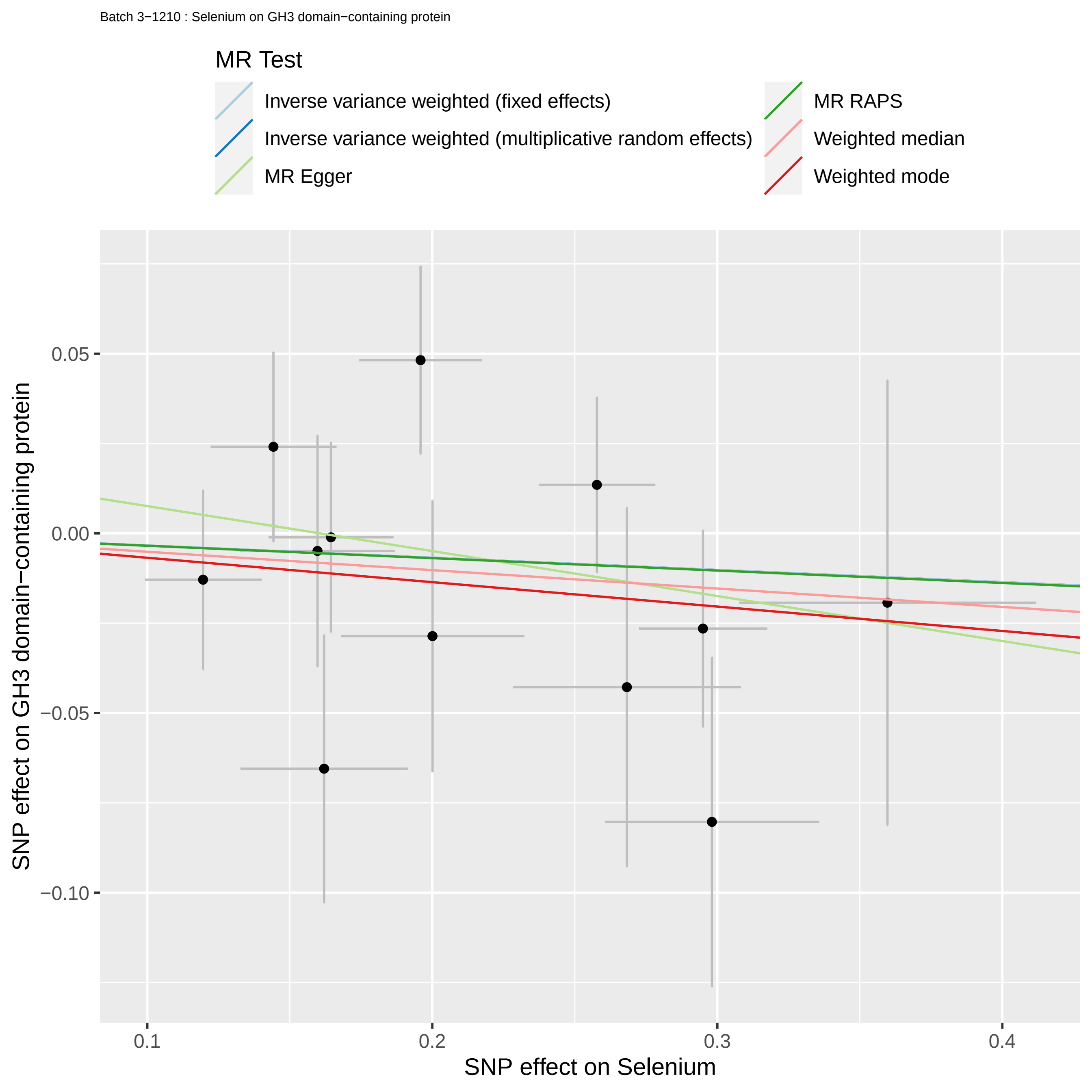
Batch 3−1210 : Selenium on GH3 domain−containing protein
MR Test
Inverse variance weighted (fixed effects)
Inverse variance weighted (multiplicative random effects)
MR Egger
MR RAPS
Weighted median
Weighted mode
0.05
0.00
−0.05
−0.10
0.1
0.2
0.3
0.4
SNP effect on GH3 domain−containing protein
SNP effect on Selenium

Batch 3−1211 : Selenium on Growth hormone receptor

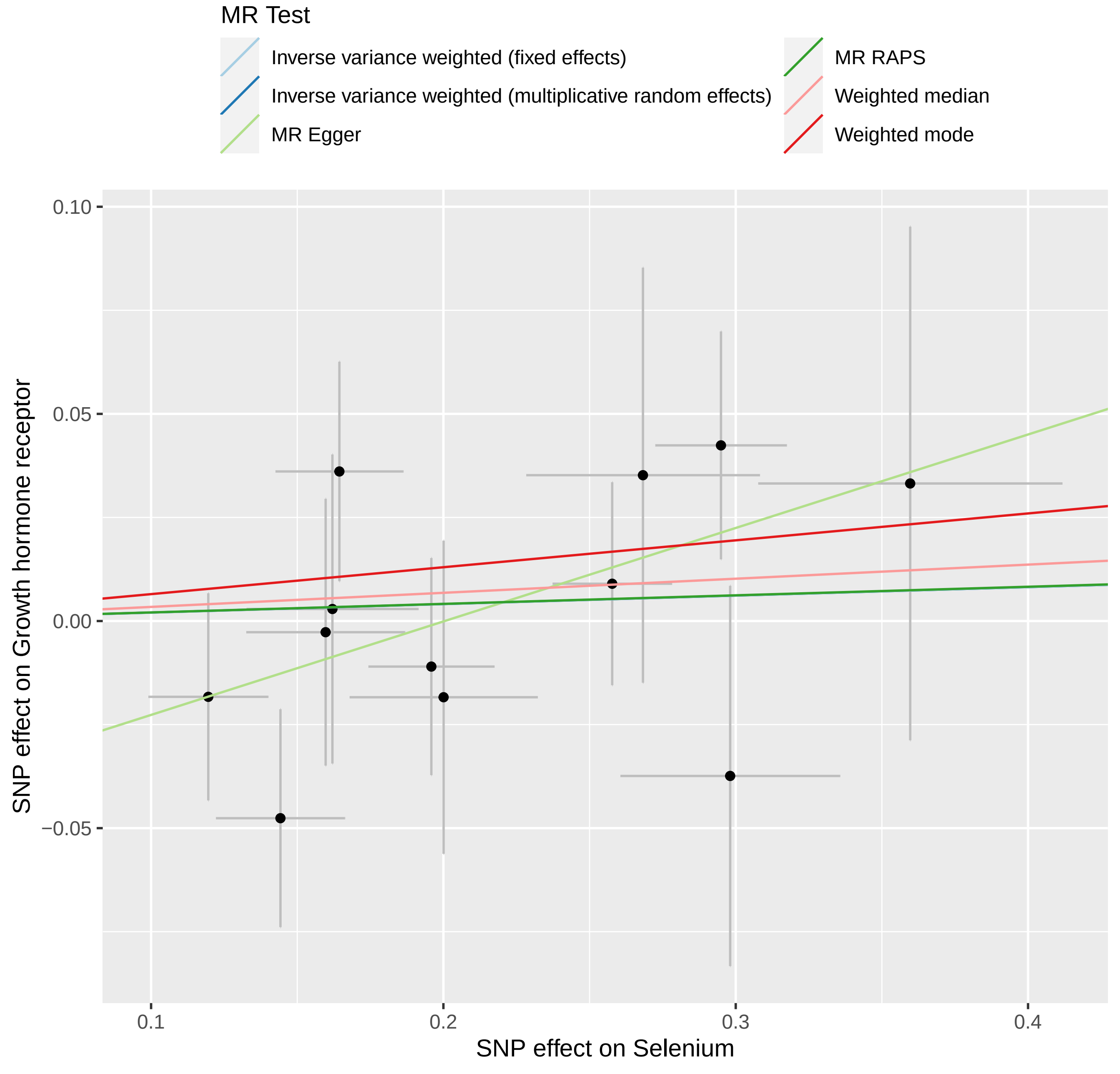

Batch 3−1212 : Selenium on Appetite−regulating hormone

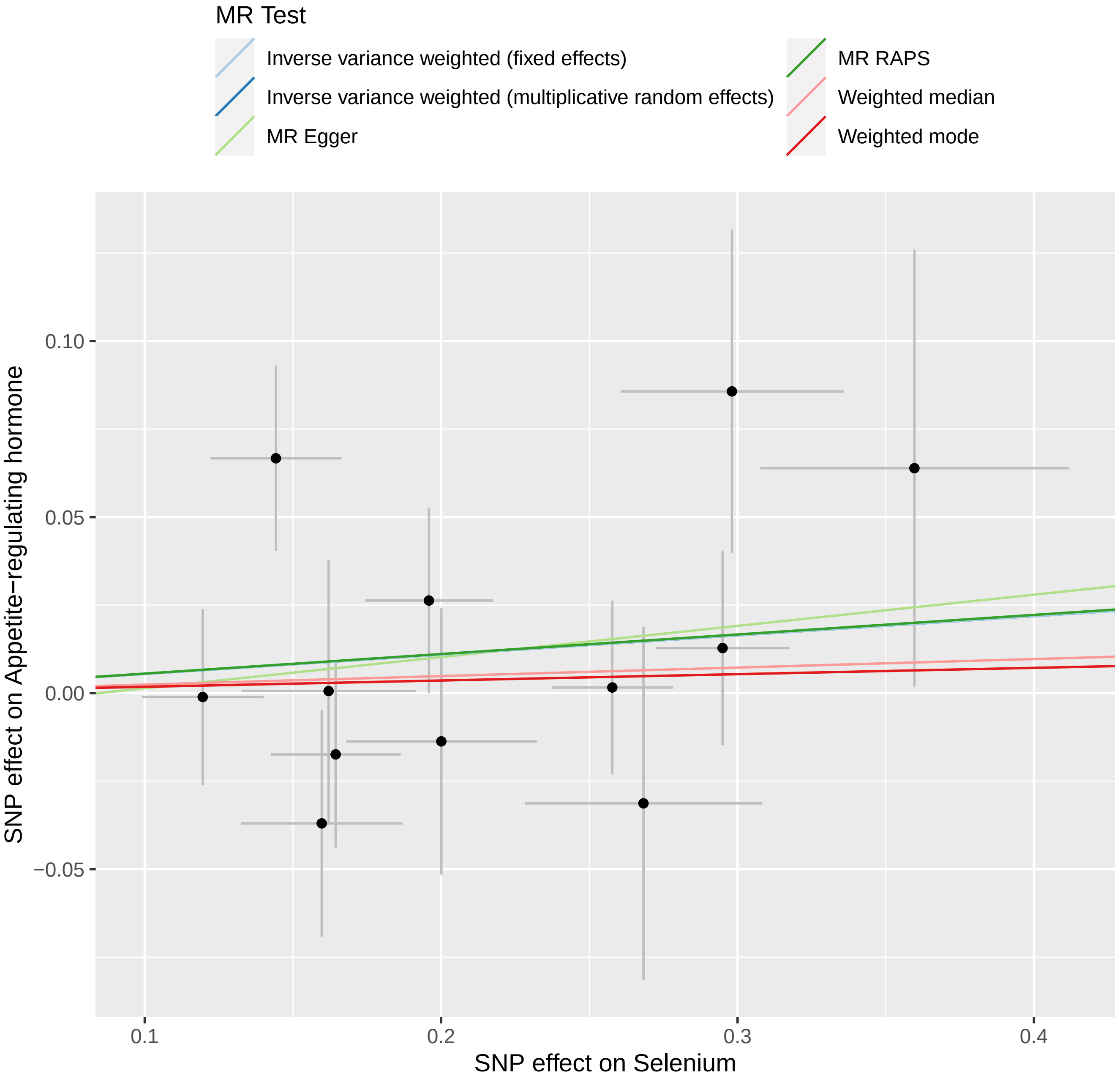

Batch 3−1213 : Selenium on Gap junction alpha−1 protein

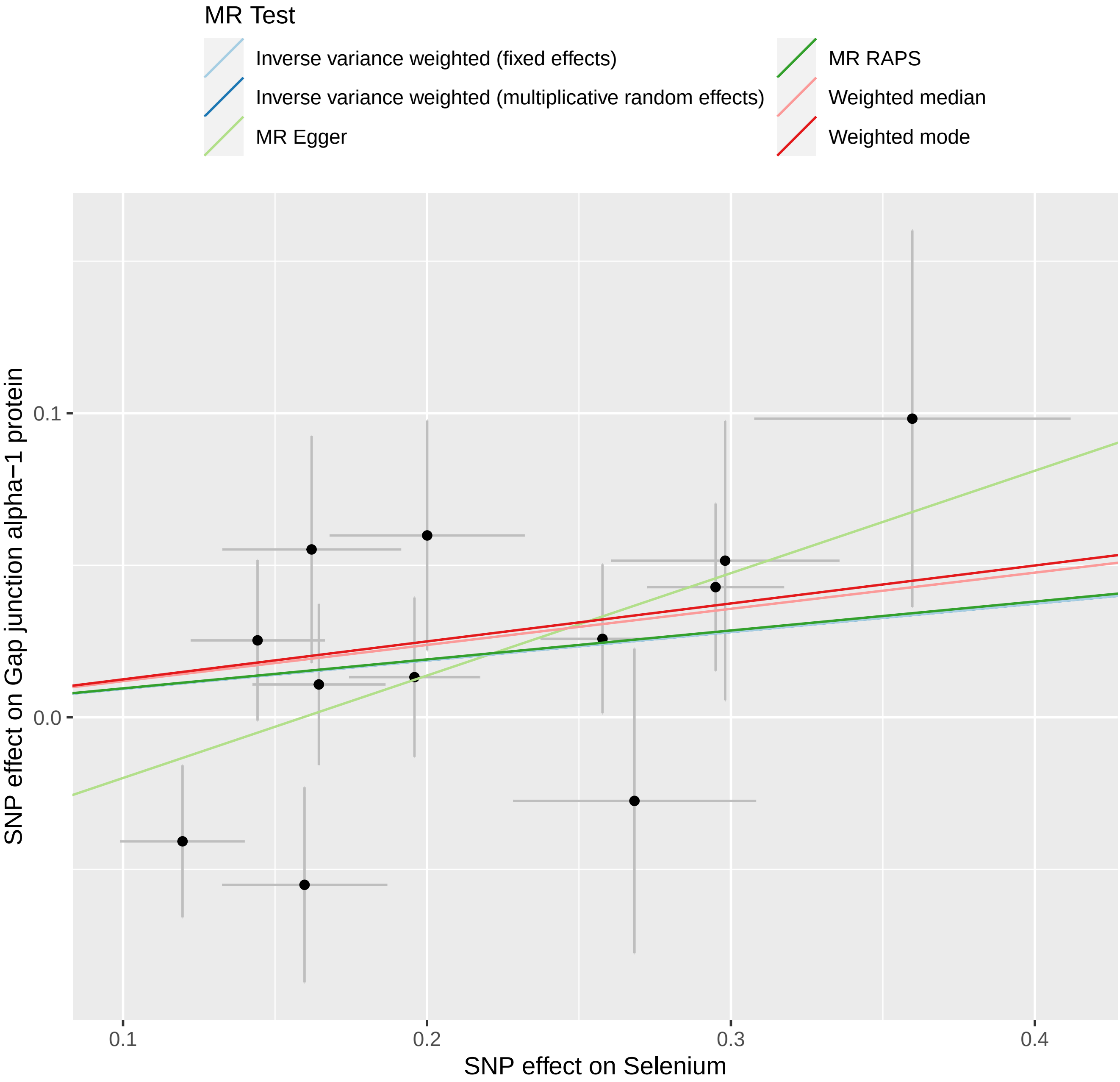

Batch 3−1214 : Selenium on Gap junction alpha−8 protein

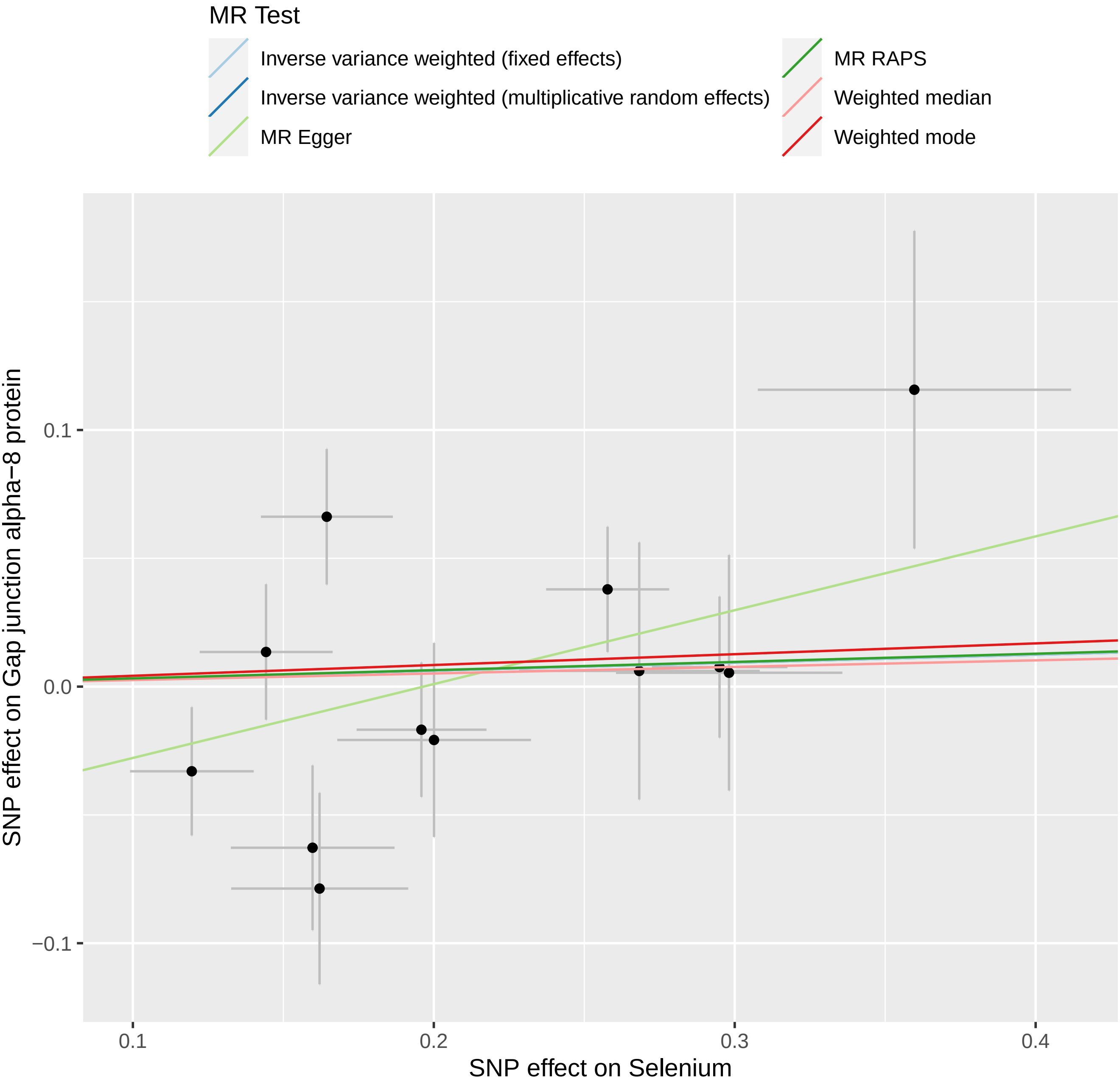

Batch 3−1215 : Selenium on Gap junction delta−2 protein

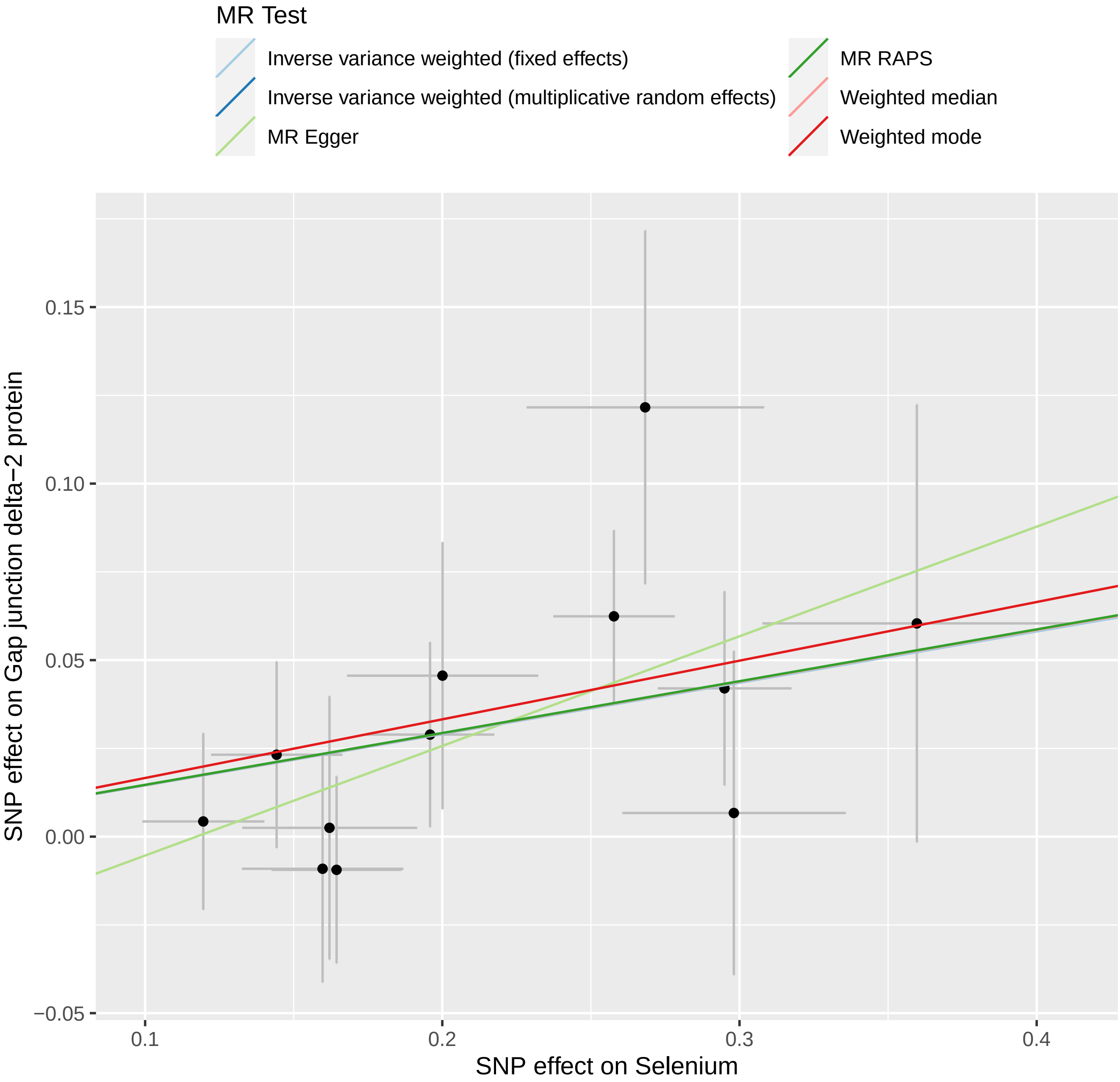

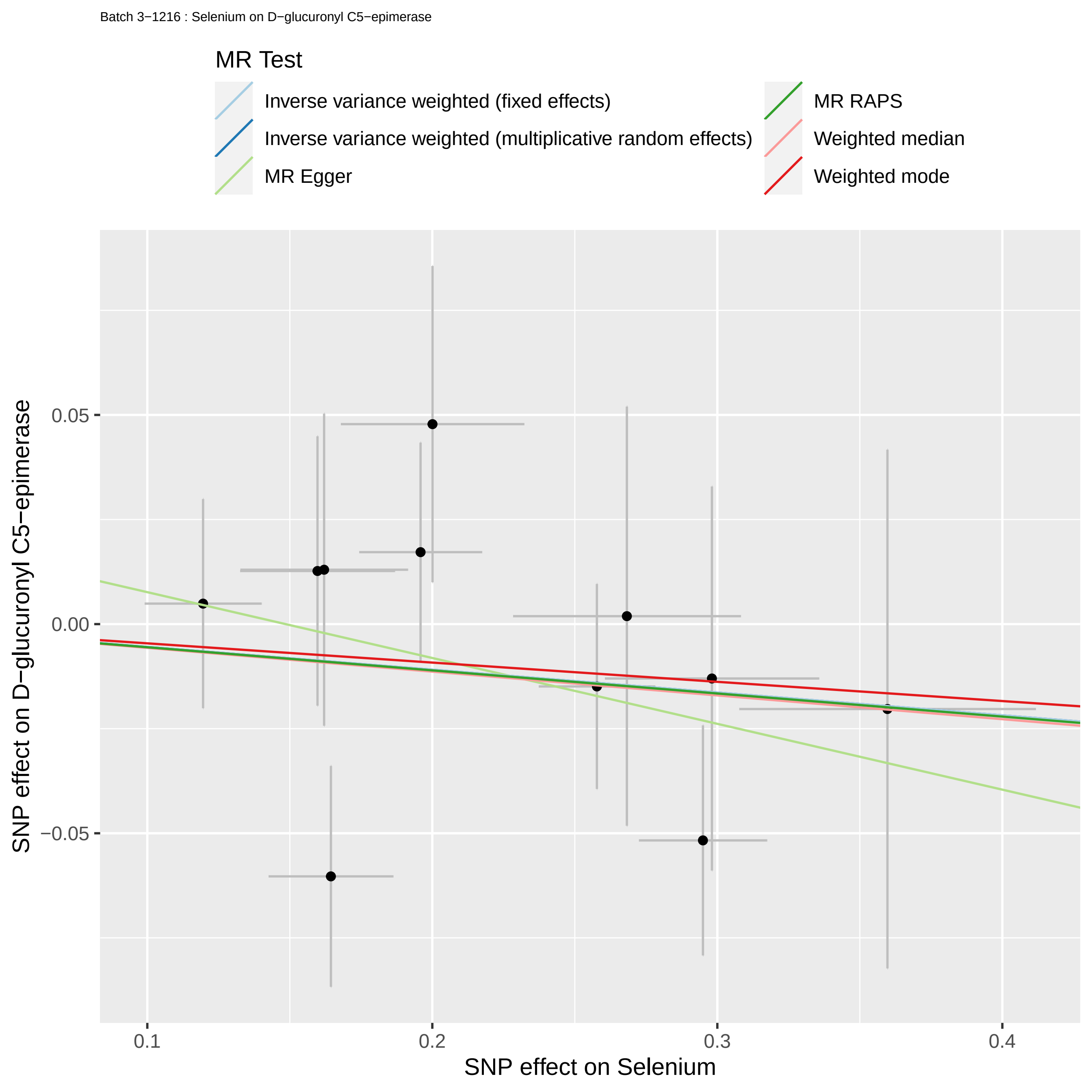

Batch 3−1216 : Selenium on D−glucuronyl C5−epimerase
MR Test
Inverse variance weighted (fixed effects)
Inverse variance weighted (multiplicative random effects)
MR Egger
MR RAPS
Weighted median
Weighted mode
SNP effect on D−glucuronyl C5−epimerase
0.05
0.00
−0.05
0.1
0.2
0.3
0.4
SNP effect on Selenium

Batch 3−1217 : Selenium on Glioma pathogenesis−related protein 1

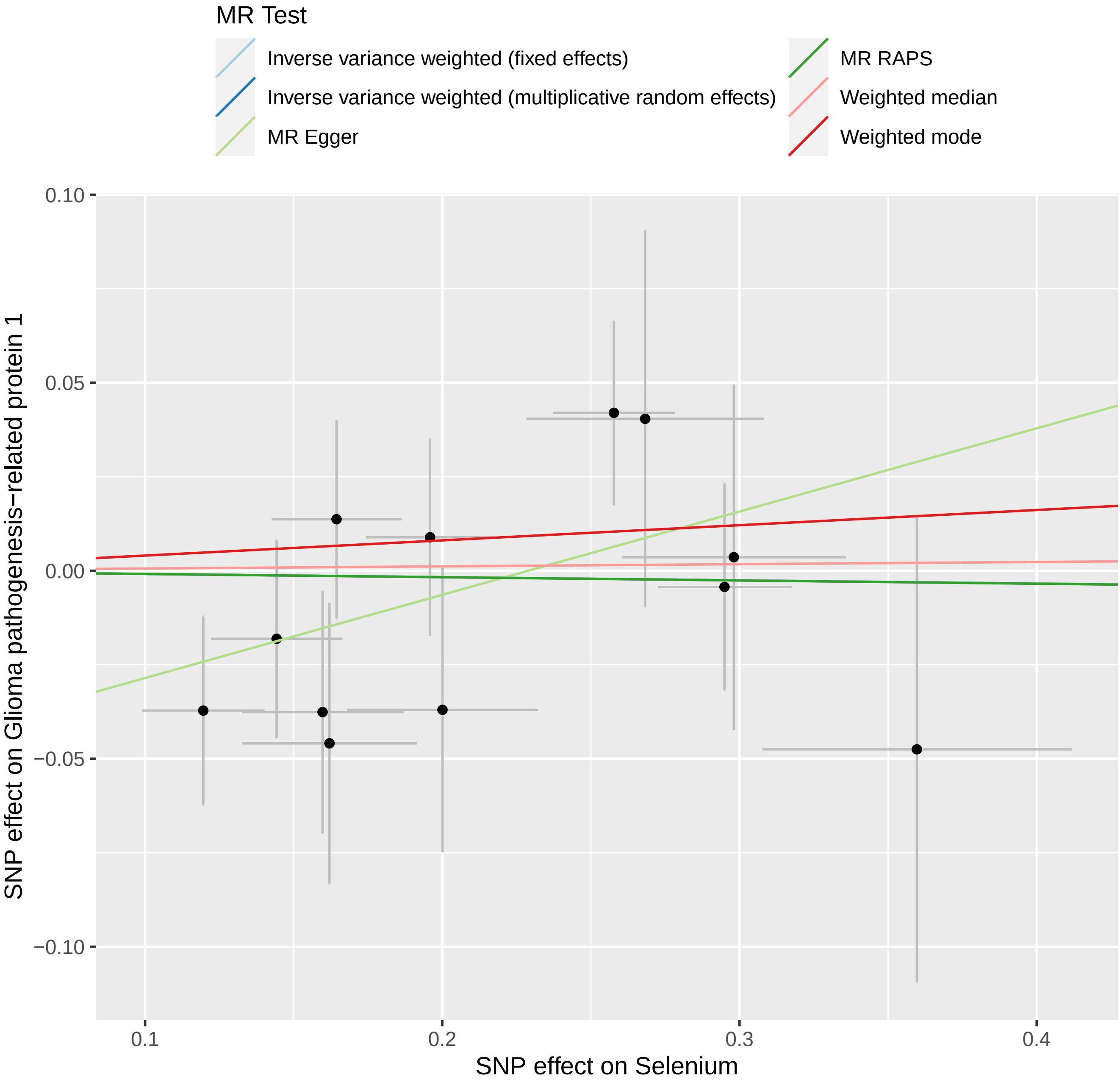

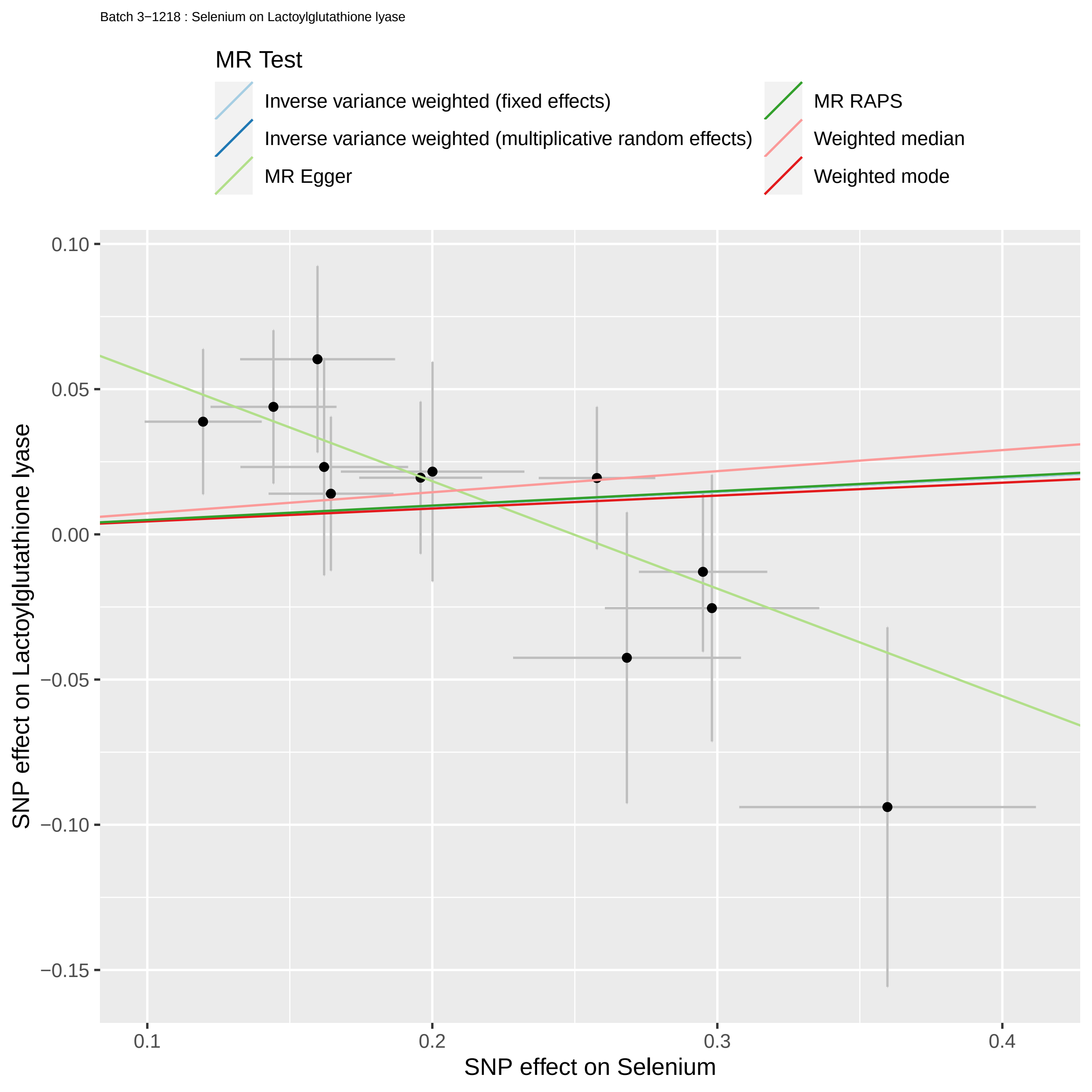
Batch 3−1218 : Selenium on Lactoylglutathione lyase
MR Test
Inverse variance weighted (fixed effects)
Inverse variance weighted (multiplicative random effects)
MR Egger
MR RAPS
Weighted median
Weighted mode
0.10
0.05
0.00
−0.05
−0.10
−0.15
0.1
0.2
0.3
0.4
SNP effect on Lactoylglutathione lyase
SNP effect on Selenium

Batch 3−1219 : Selenium on Glucagon−like peptide 1 receptor

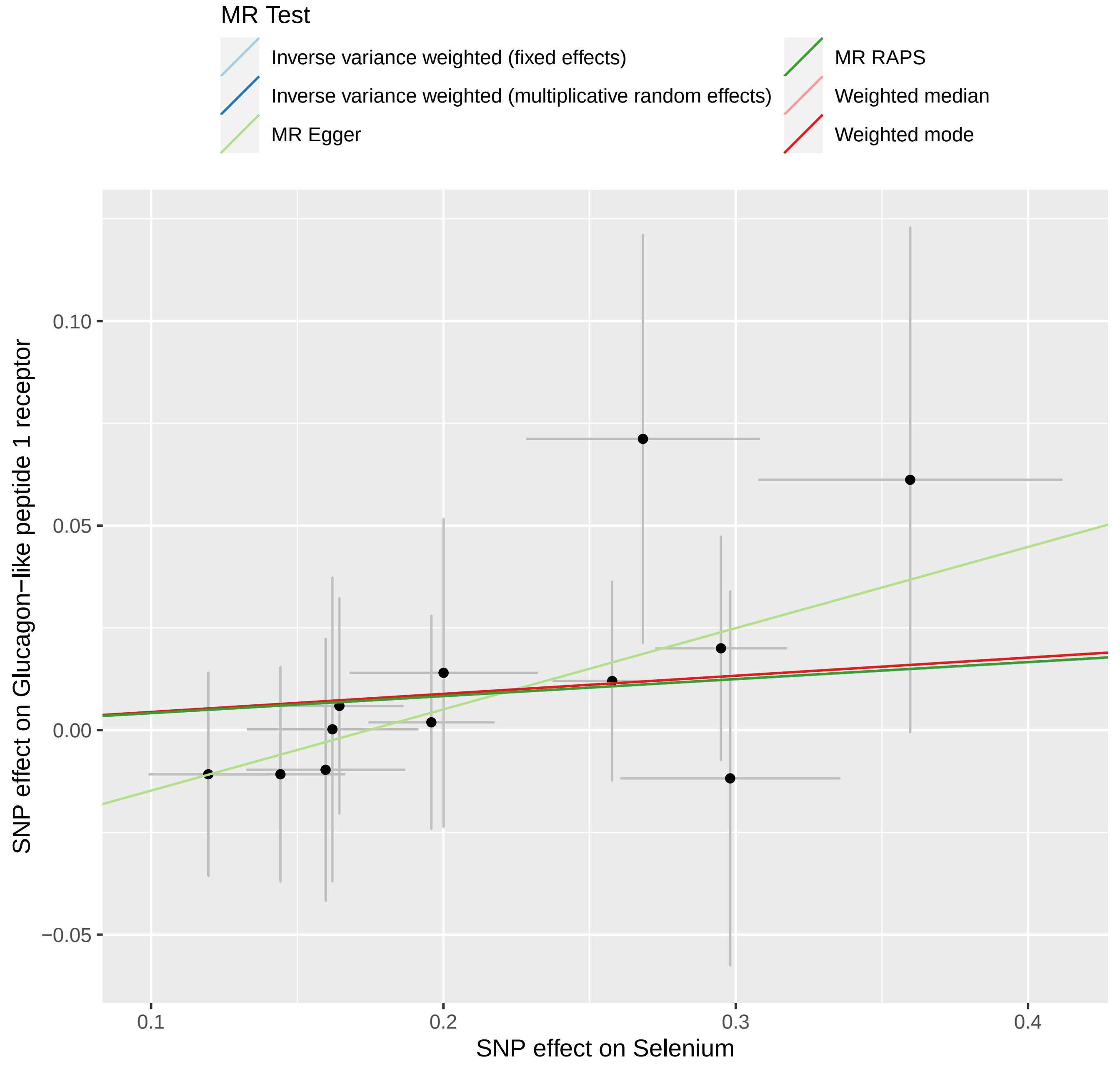

Batch 3−1220 : Selenium on Glutaredoxin−2, mitochondrial

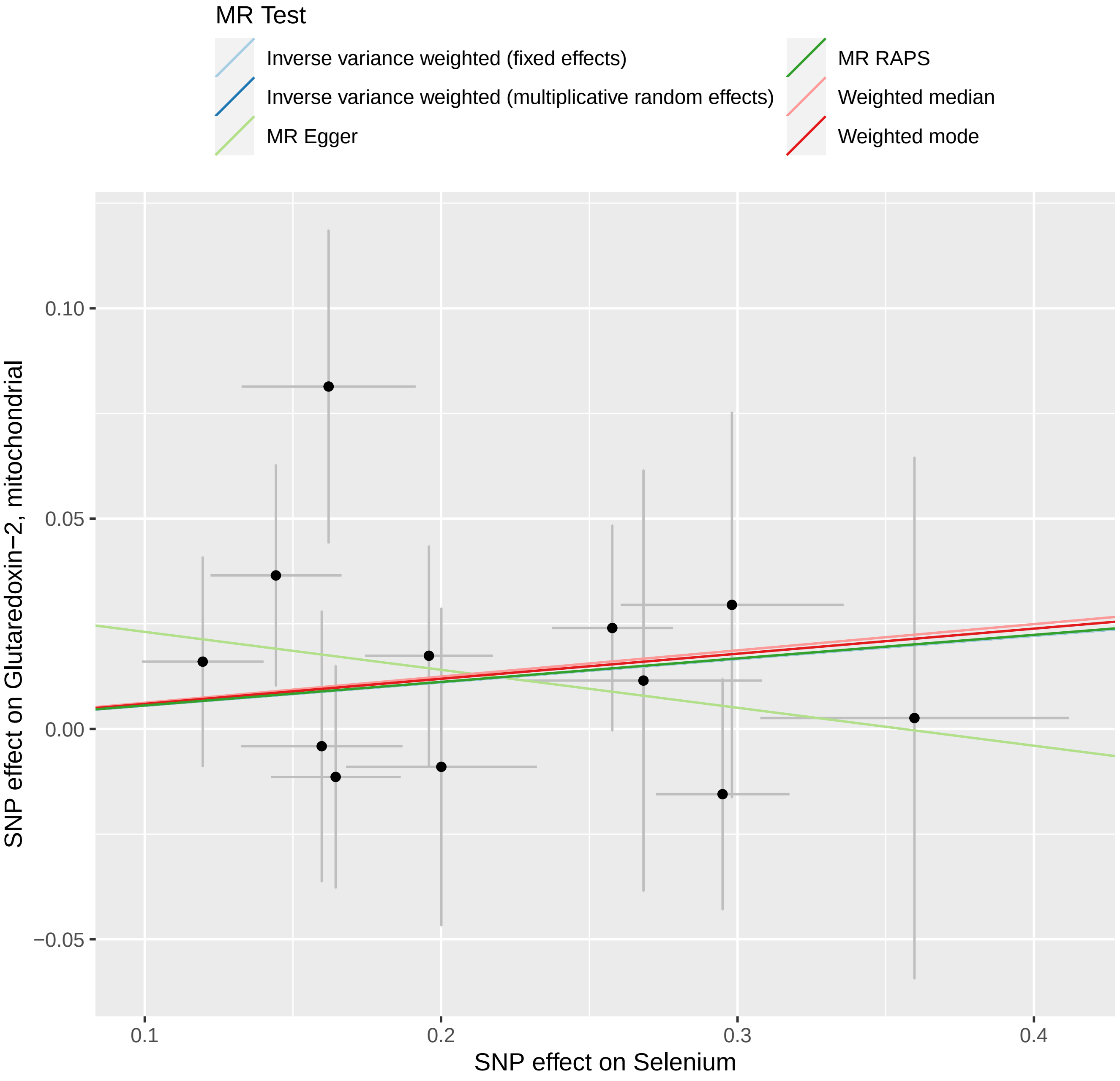

Batch 3−1221 : Selenium on Glycosyltransferase 8 domain−containing protein 1

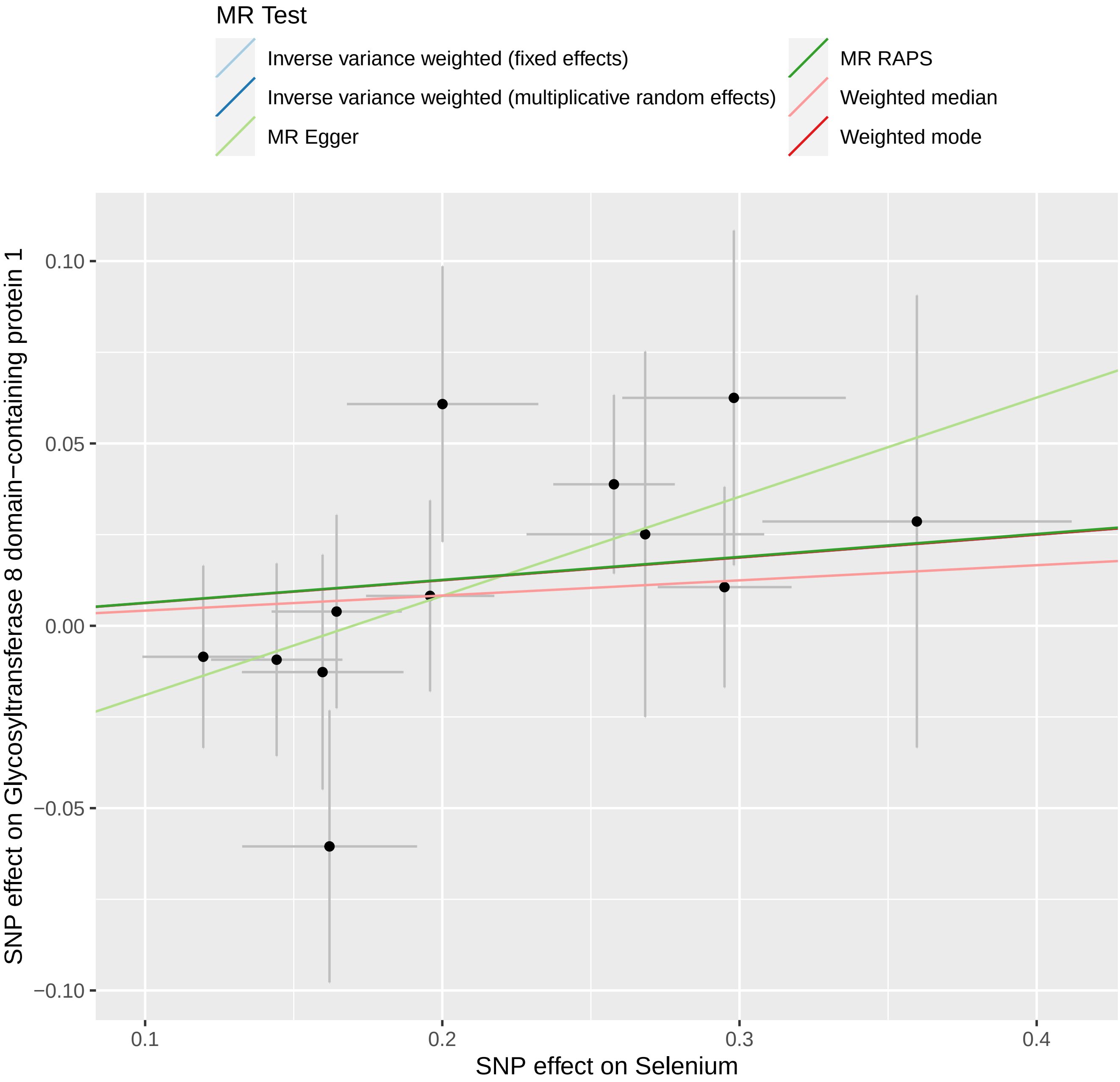

Batch 3−1222 : Selenium on Glycolipid transfer protein

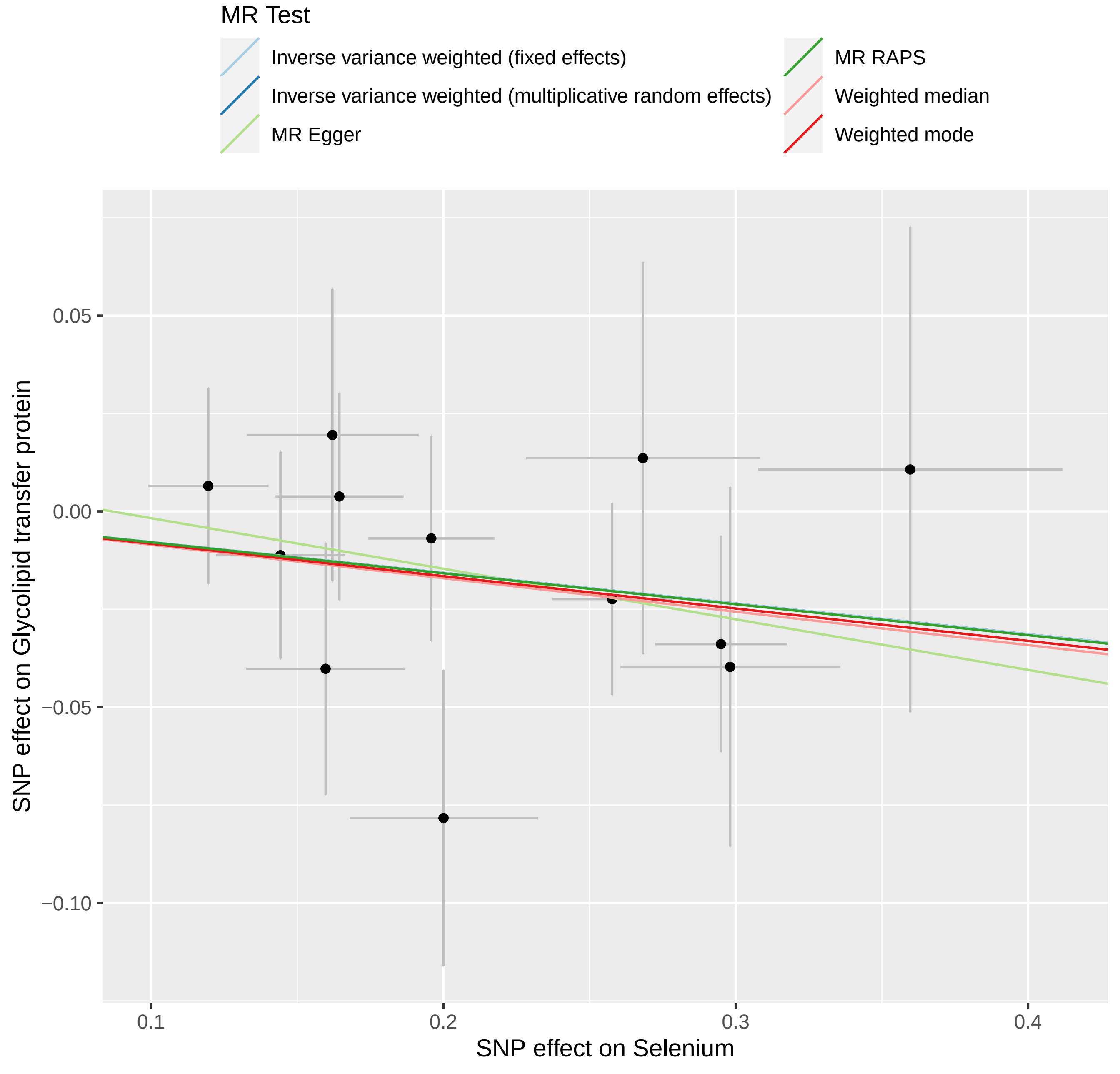

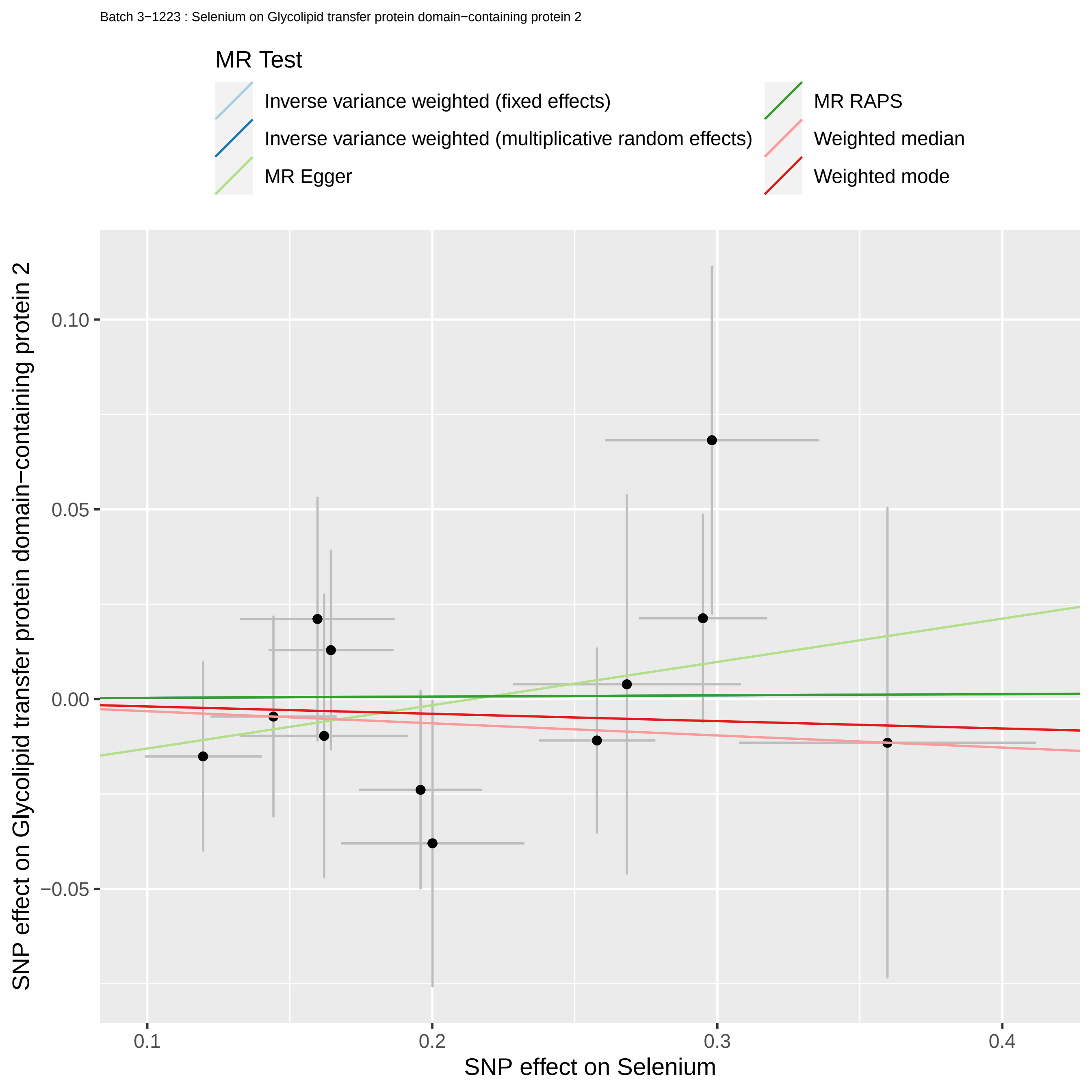

Batch 3−1223 : Selenium on Glycolipid transfer protein domain−containing protein 2
MR Test
Inverse variance weighted (fixed effects)
Inverse variance weighted (multiplicative random effects)
MR Egger
MR RAPS
Weighted median
Weighted mode
SNP effect on Glycolipid transfer protein domain−containing protein 2
0.10
0.05
0.00
−0.05
0.1
0.2
0.3
0.4
SNP effect on Selenium

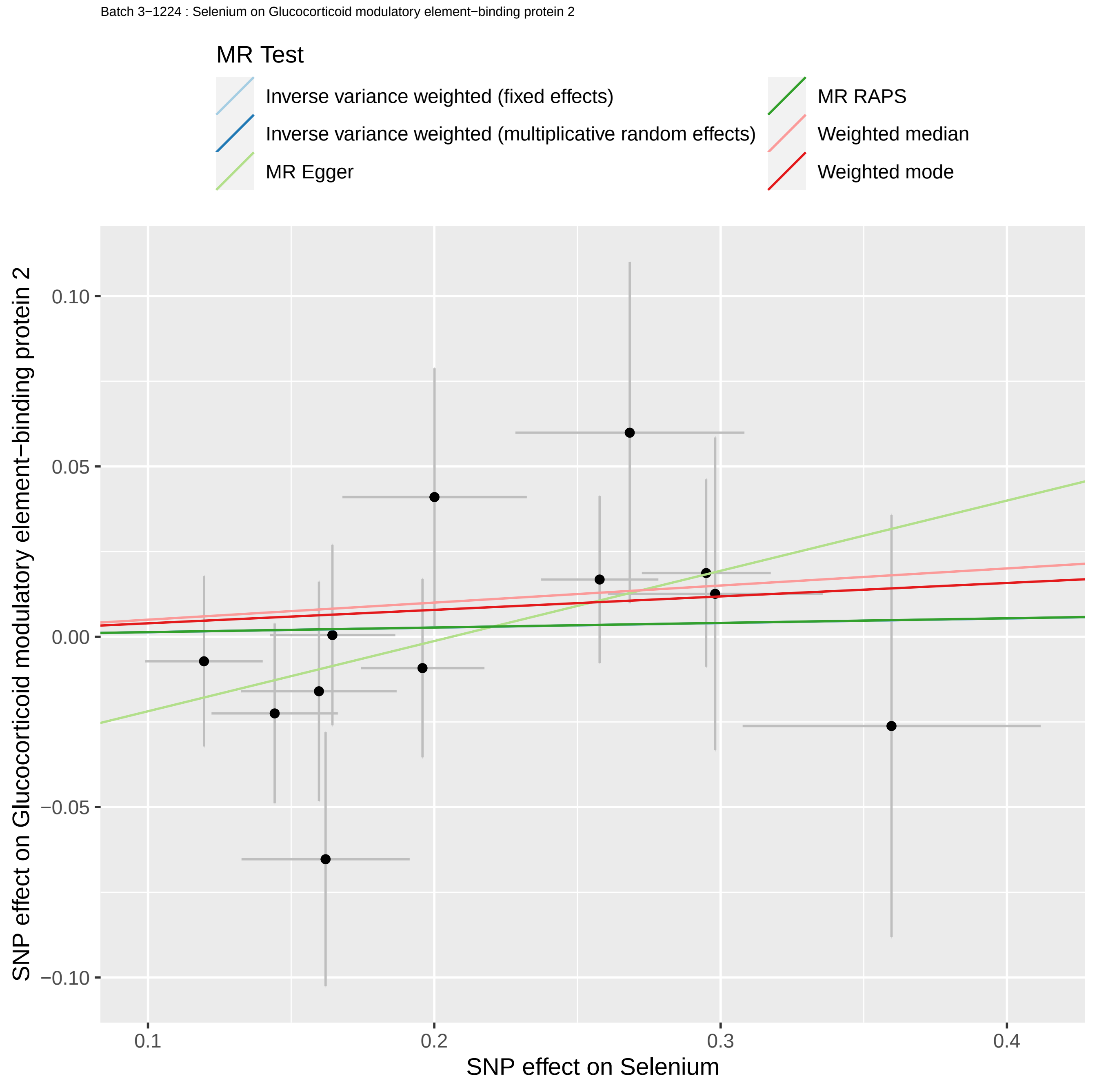

Batch 3−1224 : Selenium on Glucocorticoid modulatory element−binding protein 2
MR Test
Inverse variance weighted (fixed effects)
Inverse variance weighted (multiplicative random effects)
MR Egger
MR RAPS
Weighted median
Weighted mode
SNP effect on Glucocorticoid modulatory element−binding protein 2
0.10
0.05
0.00
−0.05
−0.10
0.1
0.2
0.3
0.4
SNP effect on Selenium

Batch 3−1225 : Selenium on Glia maturation factor gamma

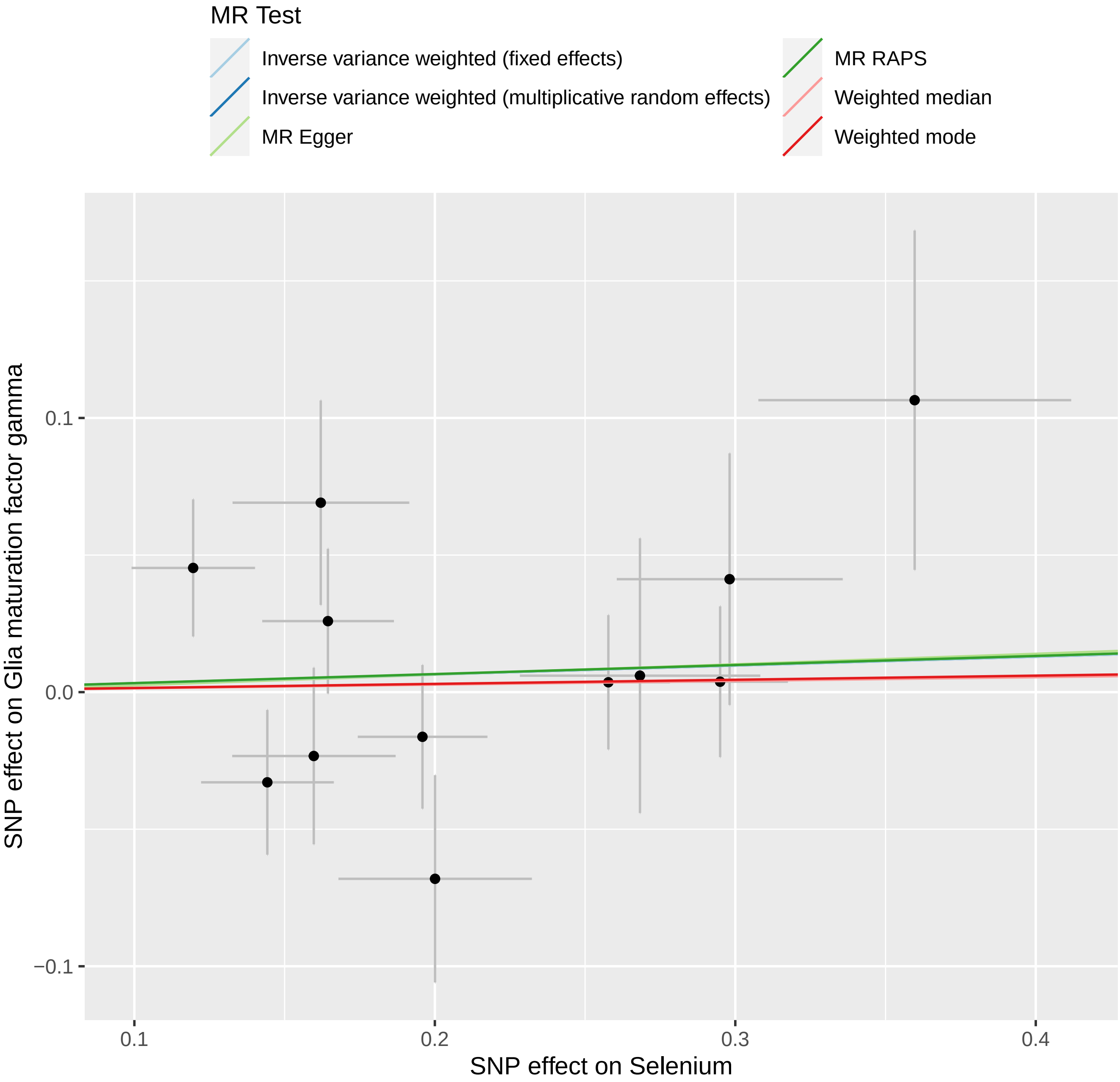

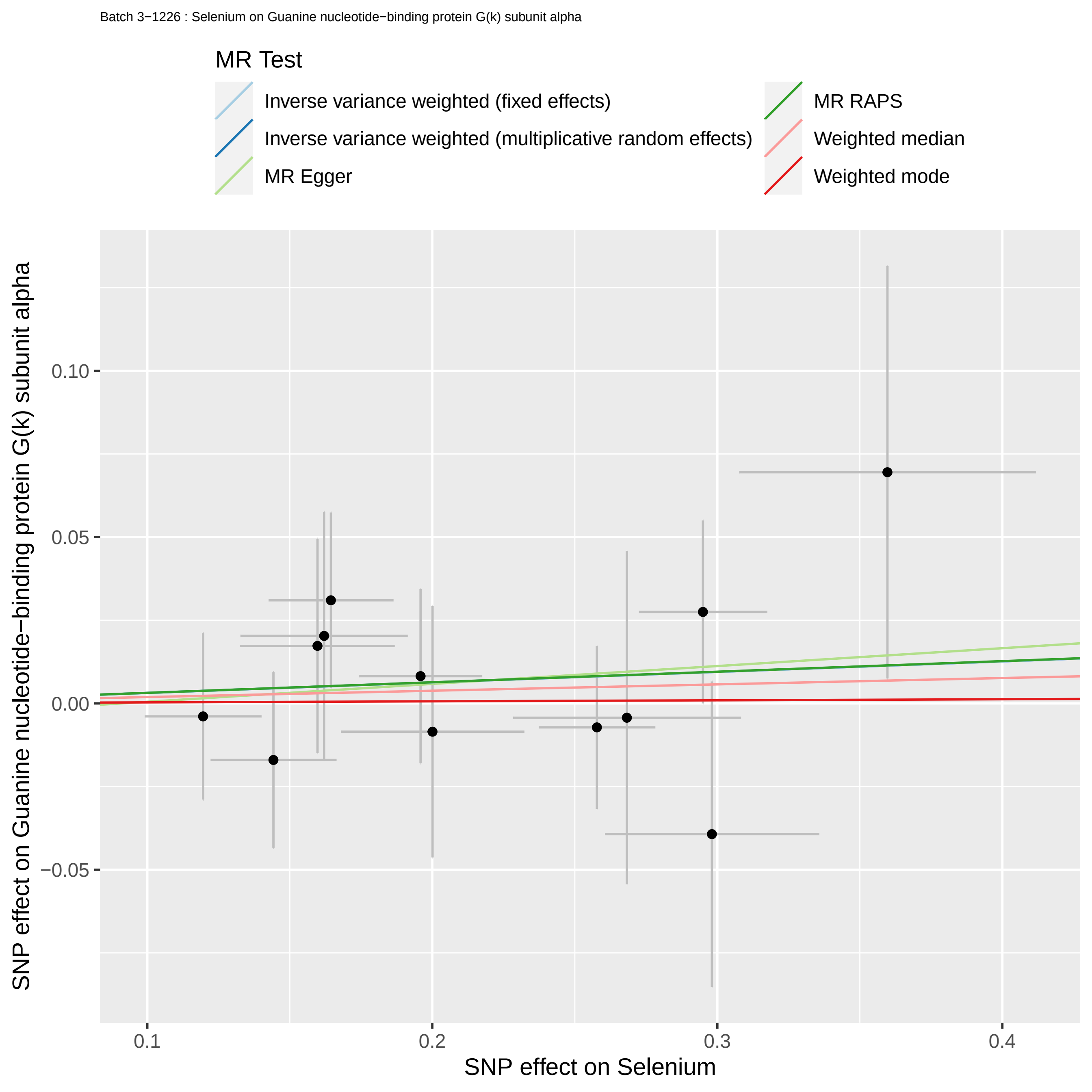

Batch 3−1226 : Selenium on Guanine nucleotide−binding protein G(k) subunit alpha
MR Test
Inverse variance weighted (fixed effects)
Inverse variance weighted (multiplicative random effects)
MR Egger
MR RAPS
Weighted median
Weighted mode
SNP effect on Guanine nucleotide−binding protein G(k) subunit alpha
0.10
0.05
0.00
−0.05
0.1
0.2
0.3
0.4
SNP effect on Selenium

Batch 3−1227 : Selenium on Guanine nucleotide−binding protein G(I)/G(S)/G(O) subunit gamma−T2

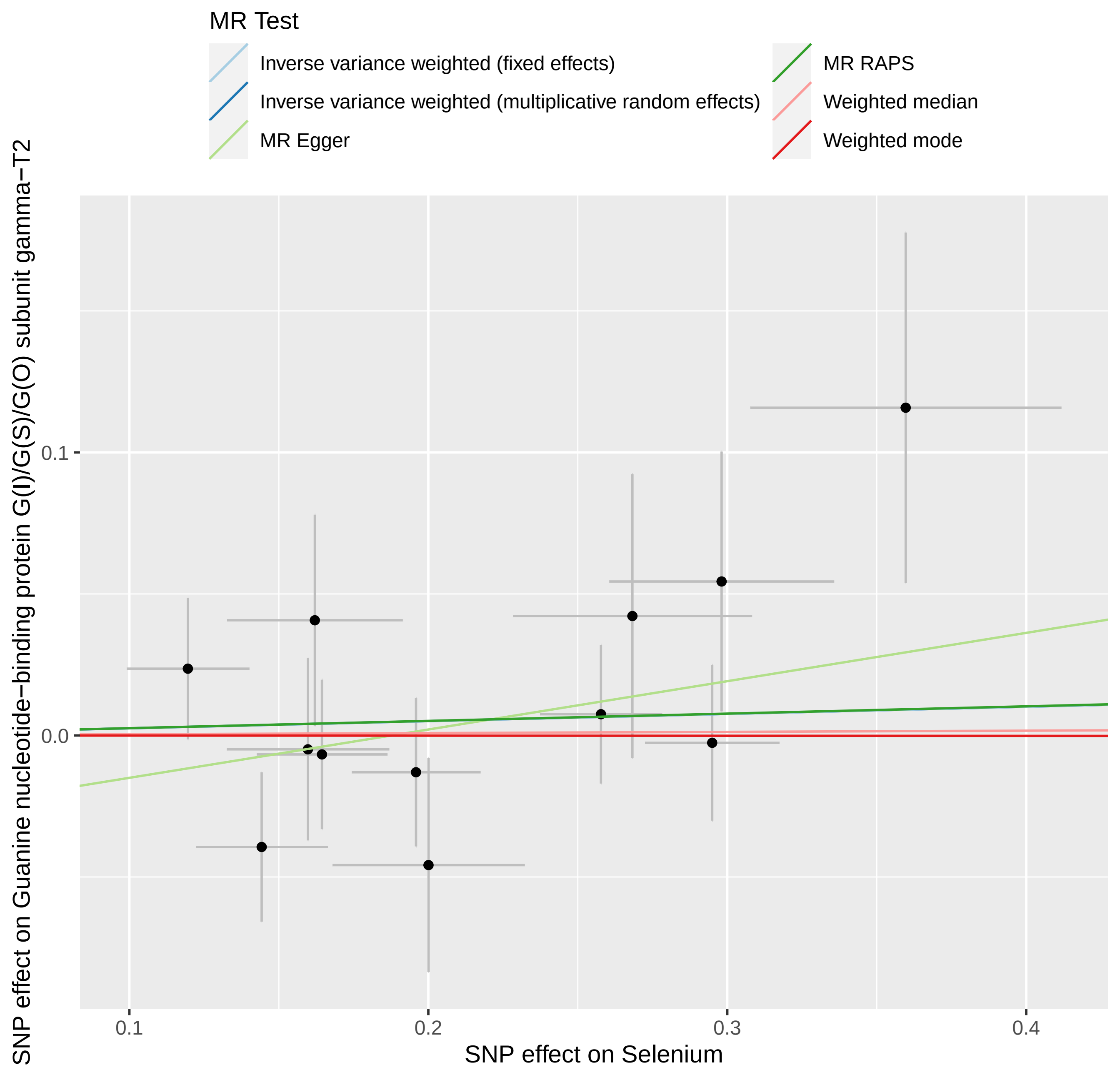

Batch 3−1228 : Selenium on Granulysin

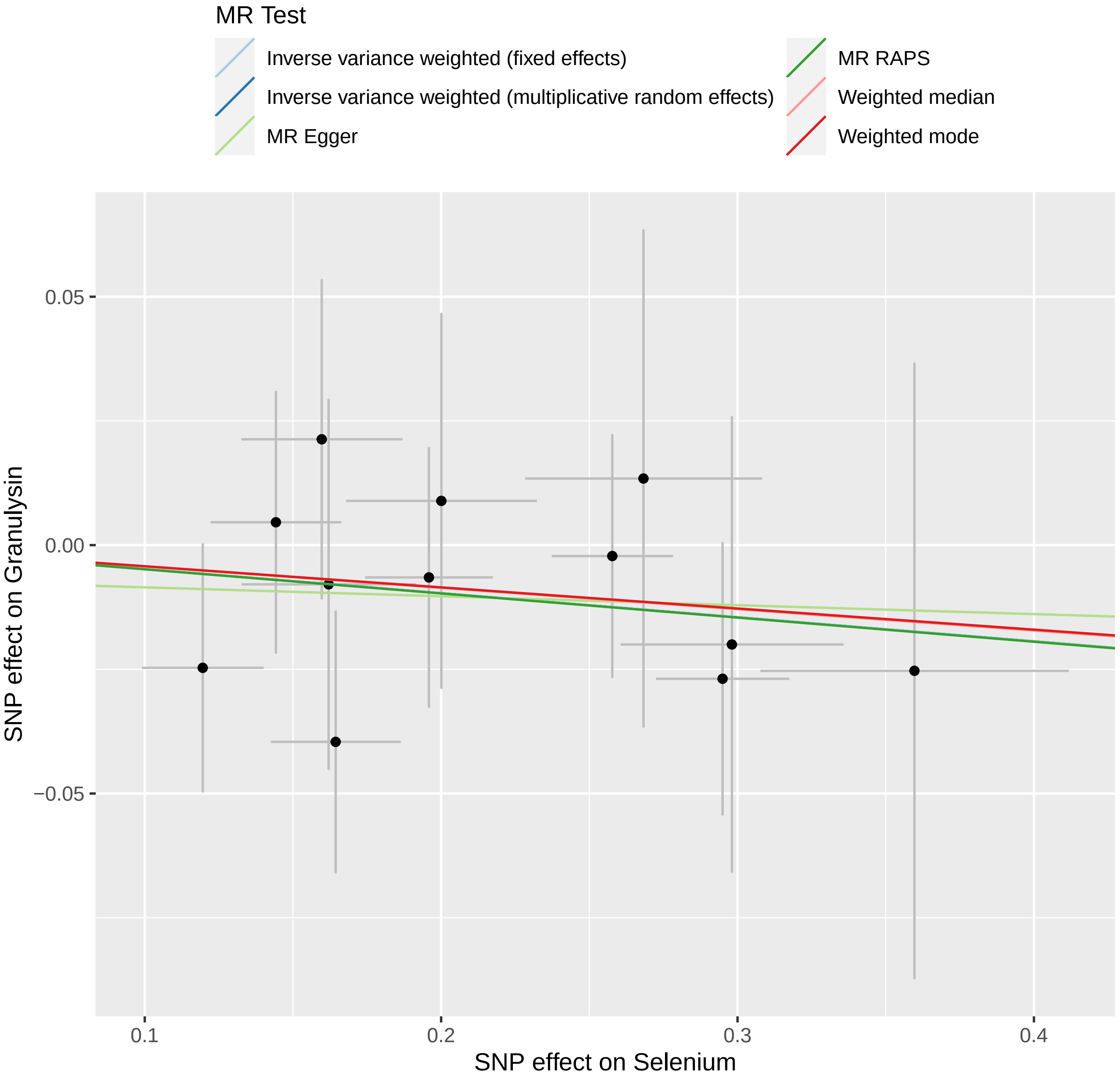

Batch 3−1229 : Selenium on Glycine N−methyltransferase

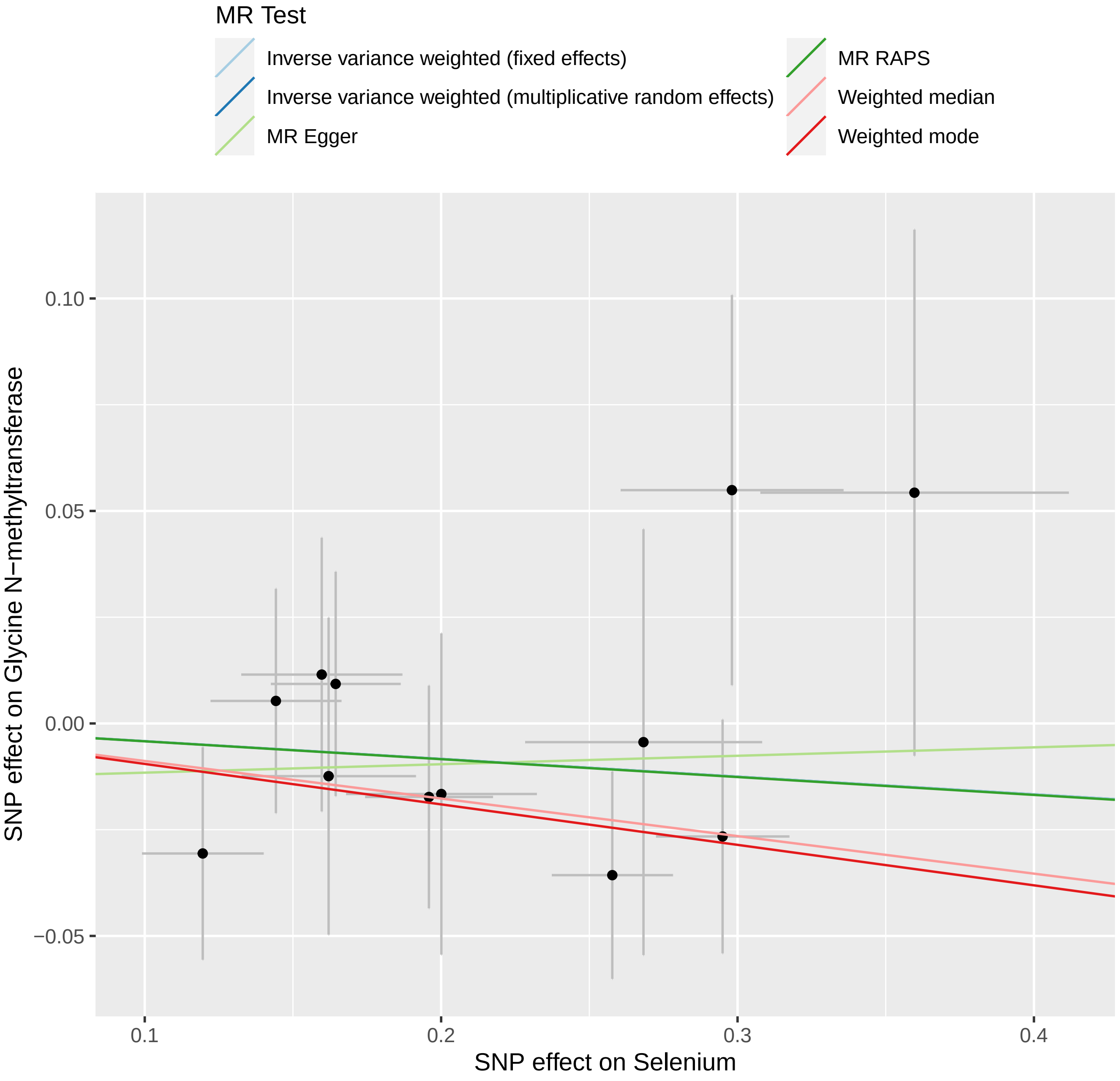

Batch 3−1230 : Selenium on Glucosamine−6−phosphate isomerase 1

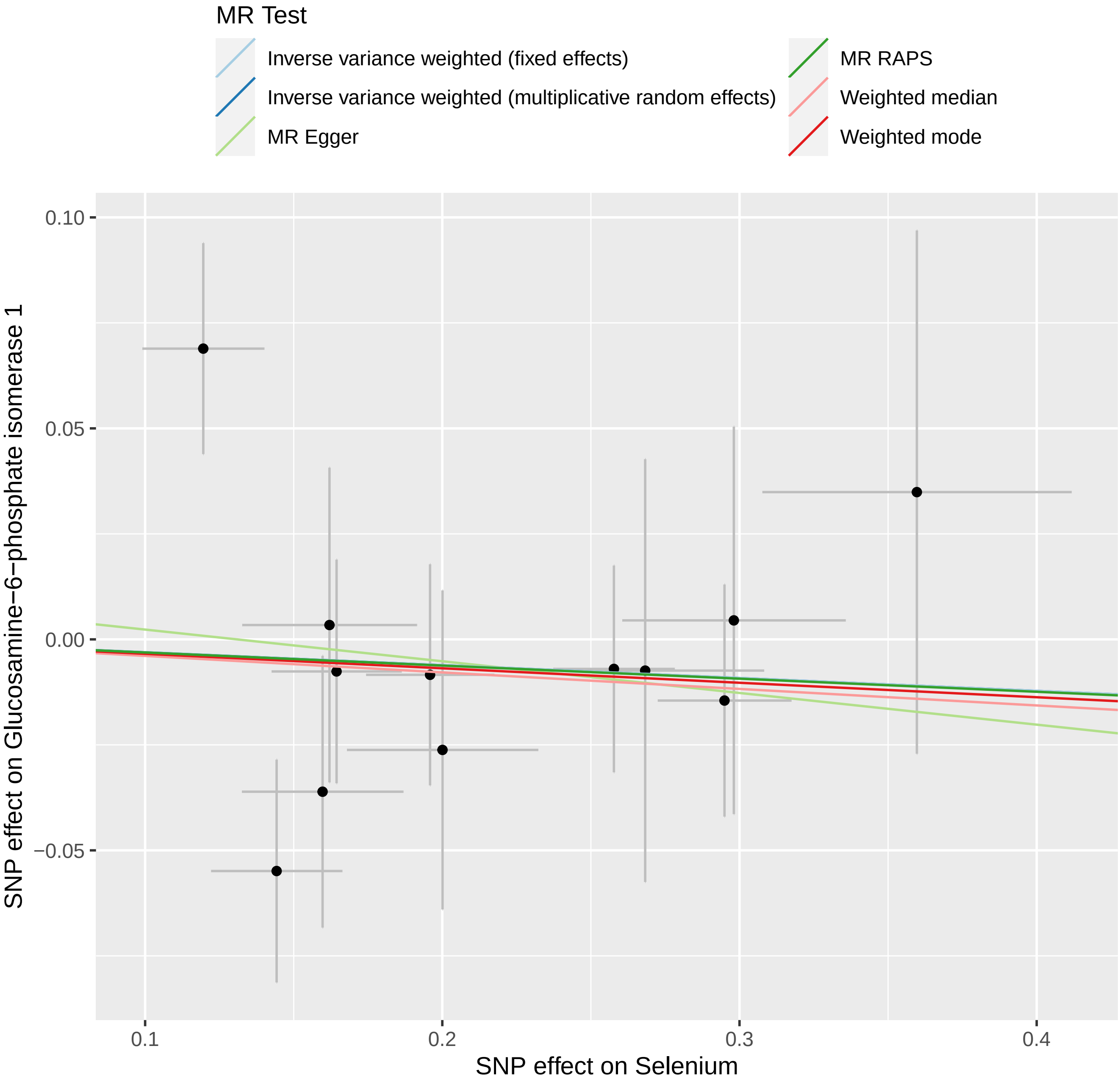

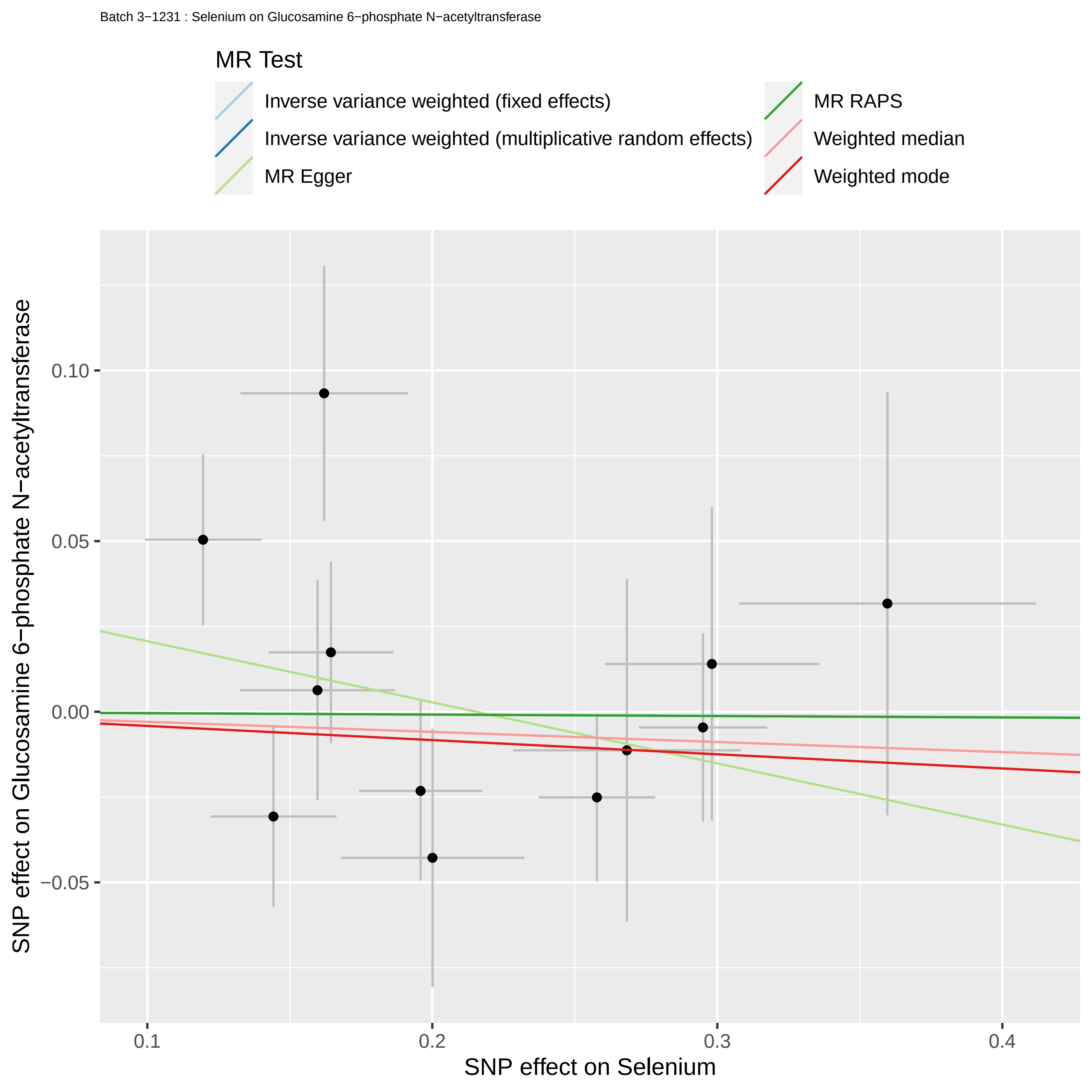
Batch 3−1231 : Selenium on Glucosamine 6−phosphate N−acetyltransferase
MR Test
Inverse variance weighted (fixed effects)
Inverse variance weighted (multiplicative random effects)
MR Egger
MR RAPS
Weighted median
Weighted mode
0.10
0.05
0.00
−0.05
0.1
0.2
0.3
0.4
SNP effect on Selenium
SNP effect on Glucosamine 6−phosphate N−acetyltransferase

Batch 3−1232 : Selenium on N−acetylglucosamine−1−phosphotransferase subunit gamma

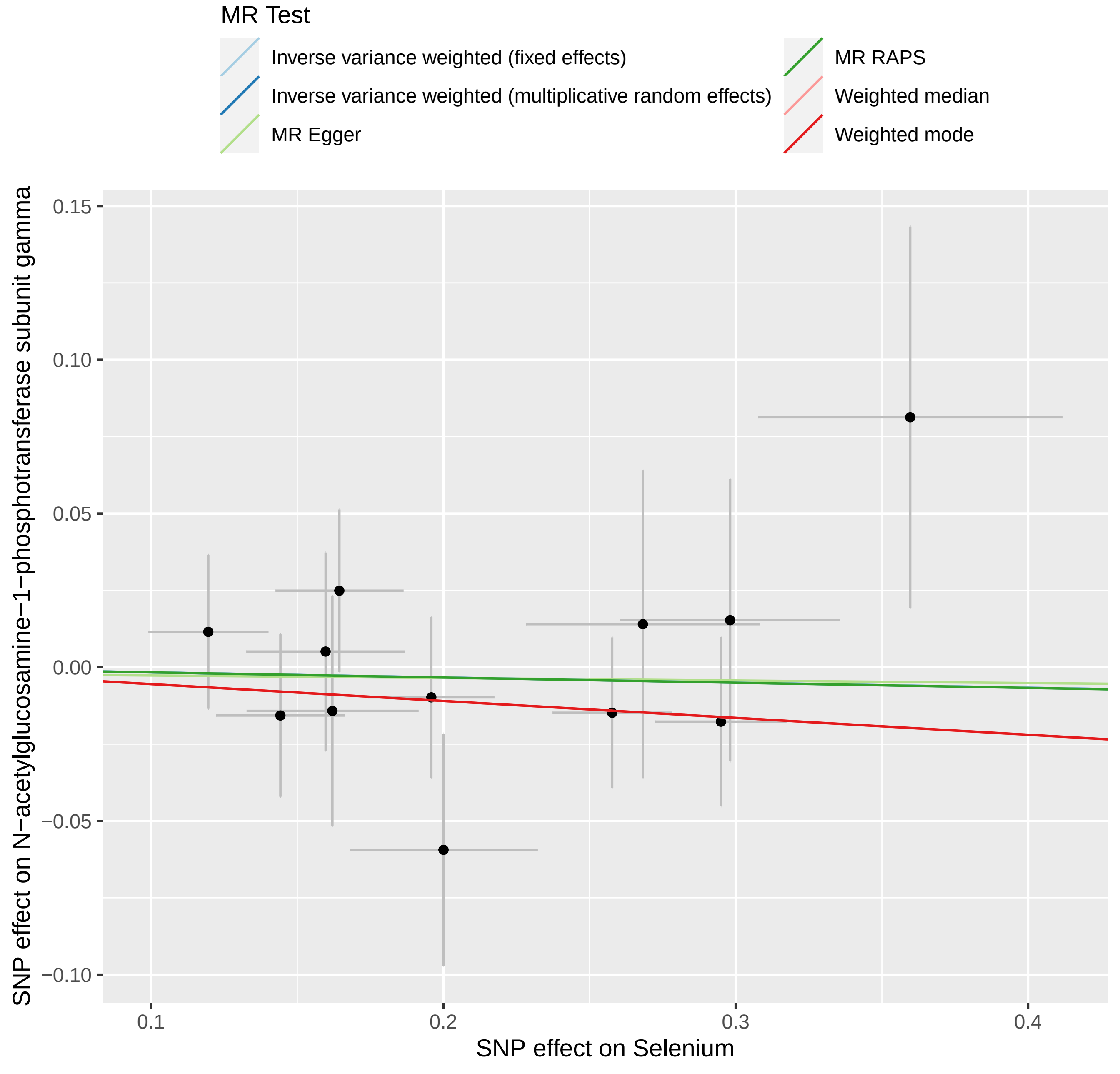

Batch 3−1233 : Selenium on Progonadoliberin−1

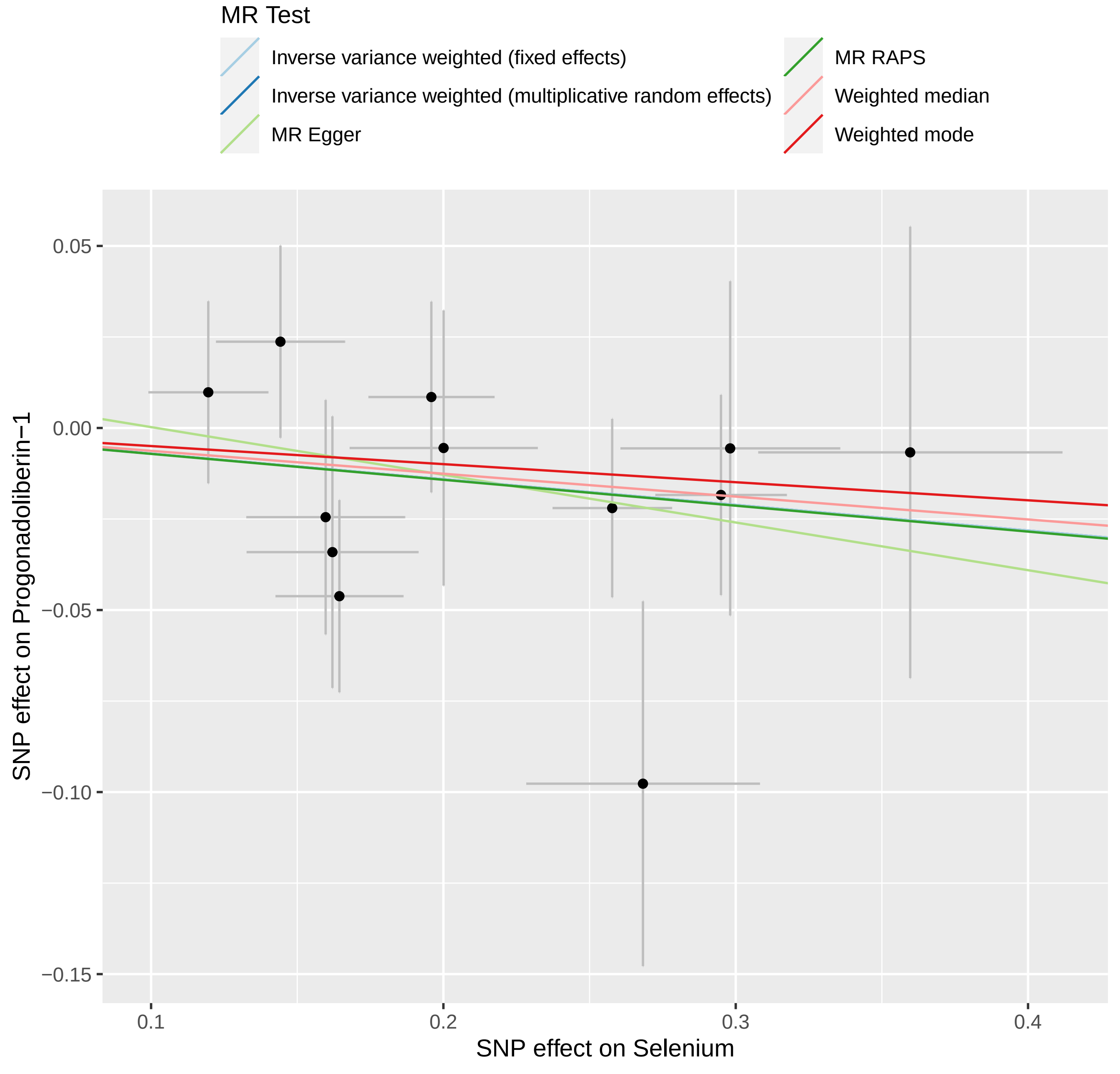

Batch 3−1234 : Selenium on Progonadoliberin−2

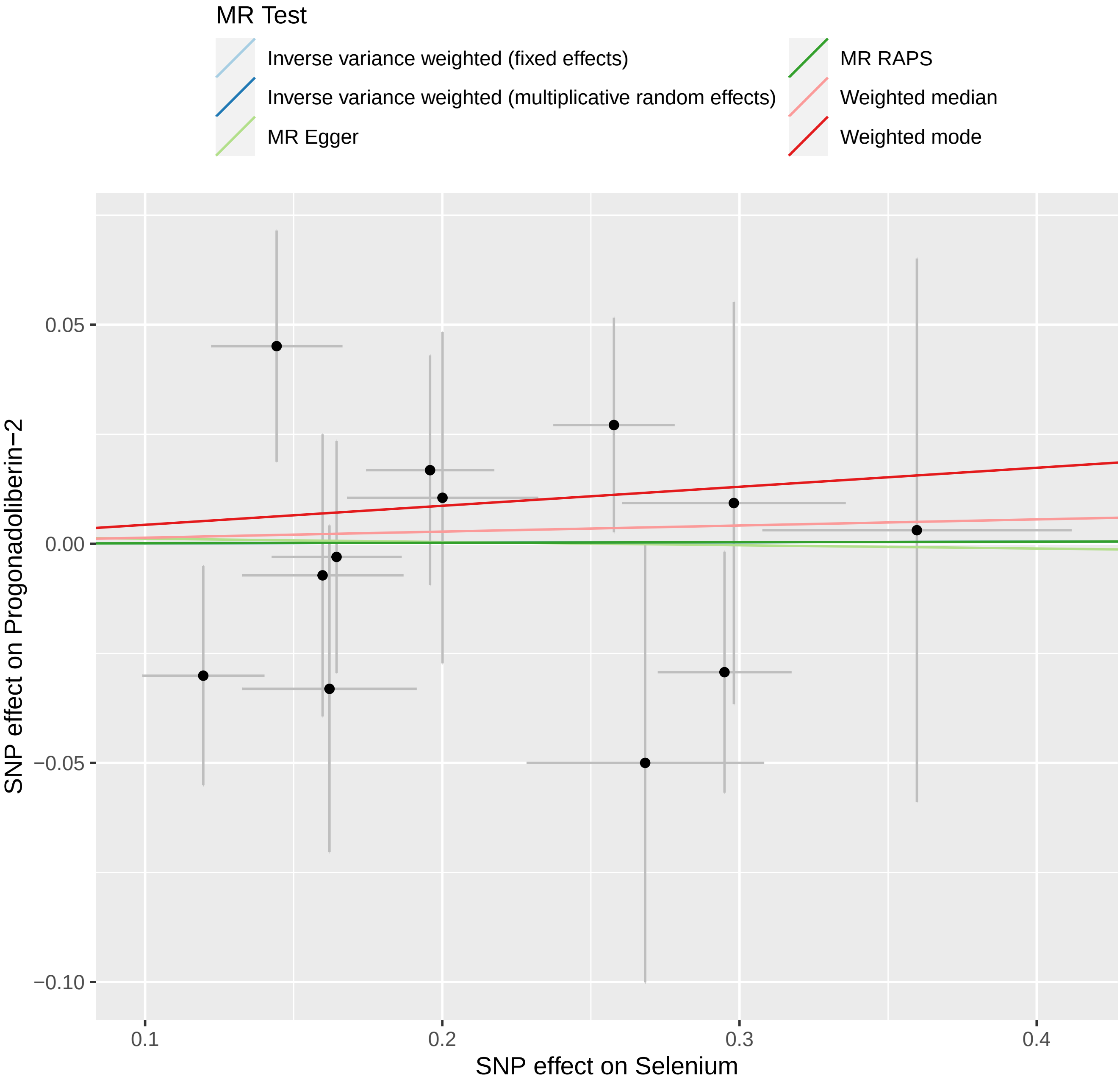

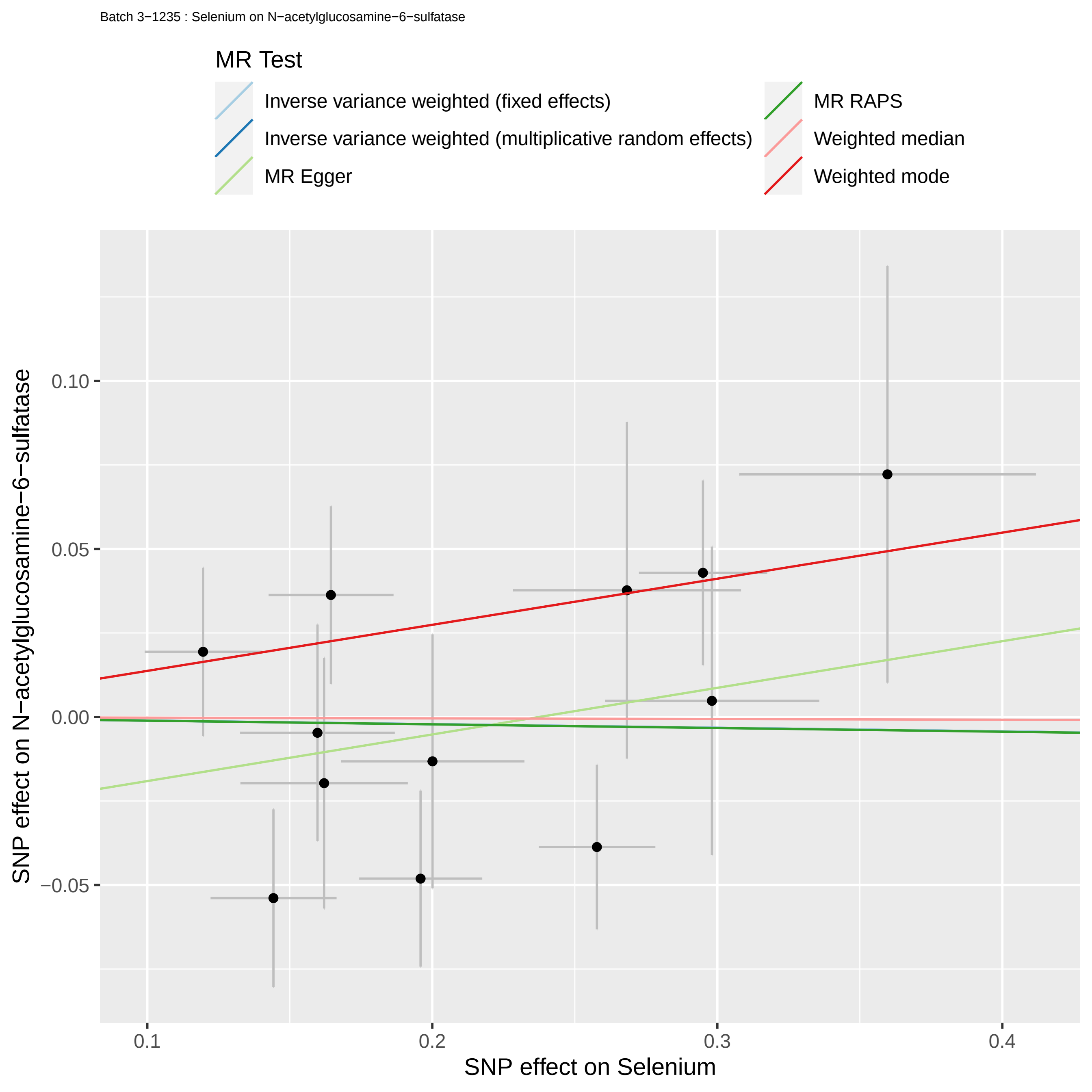
Batch 3−1235 : Selenium on N−acetylglucosamine−6−sulfatase
MR Test
Inverse variance weighted (fixed effects)
Inverse variance weighted (multiplicative random effects)
MR Egger
MR RAPS
Weighted median
Weighted mode
SNP effect on N−acetylglucosamine−6−sulfatase
0.10
0.05
0.00
−0.05
0.1
0.2
0.3
0.4
SNP effect on Selenium

Batch 3−1236 : Selenium on N−acetylglucosamine−6−sulfatase

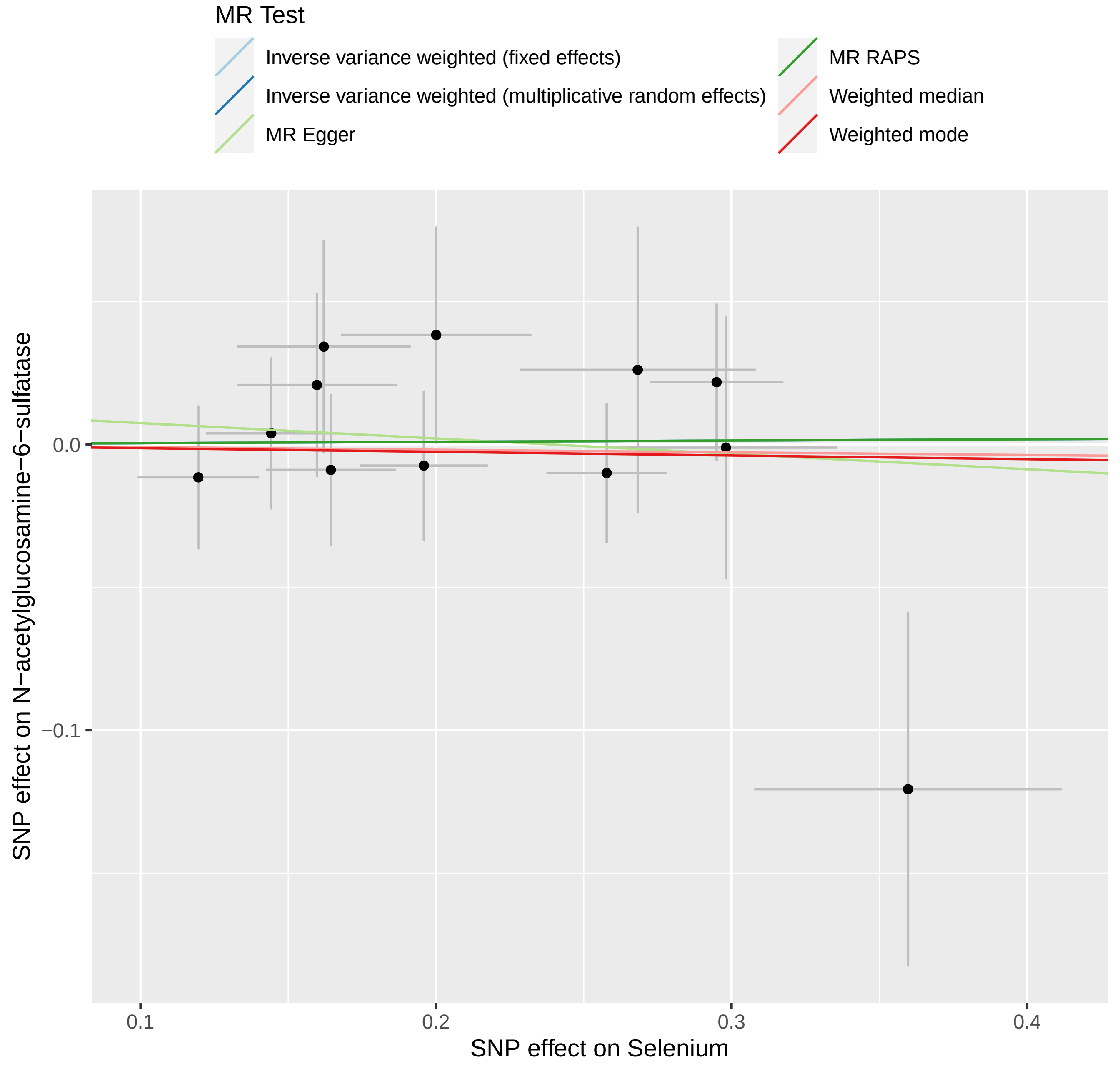

Batch 3−1237 : Selenium on Golgi membrane protein 1

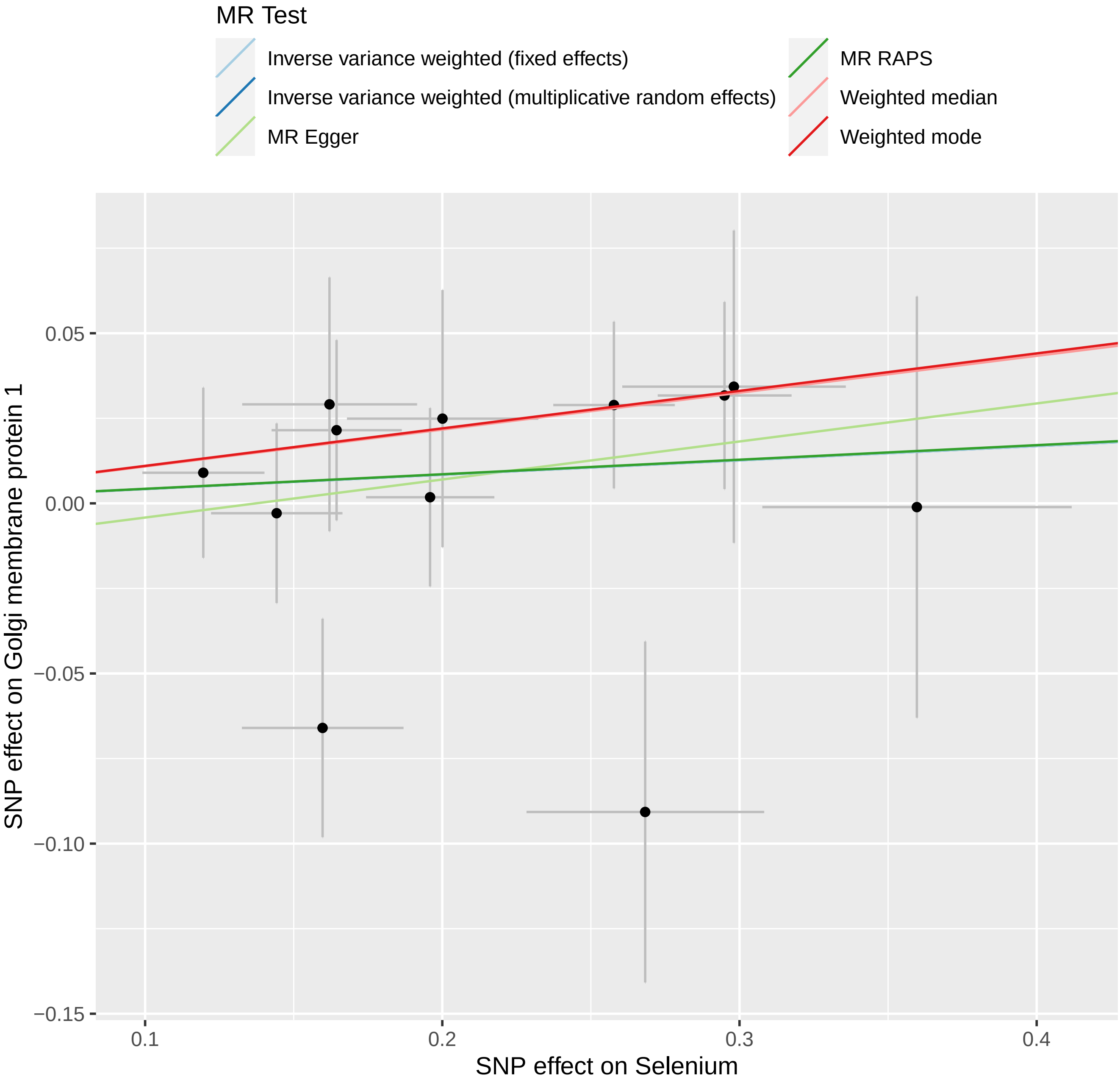

Batch 3−1238 : Selenium on Golgi membrane protein 1

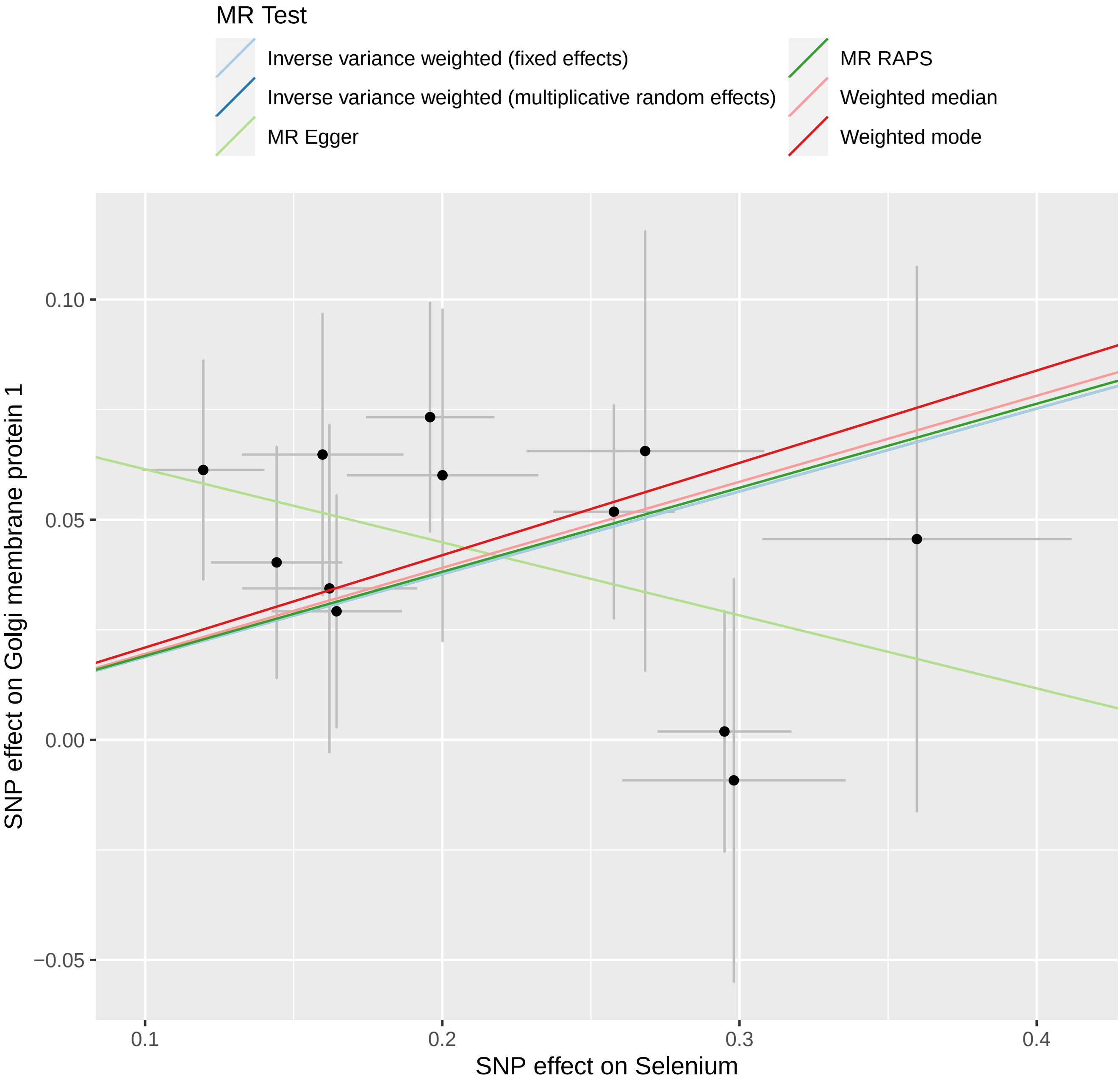

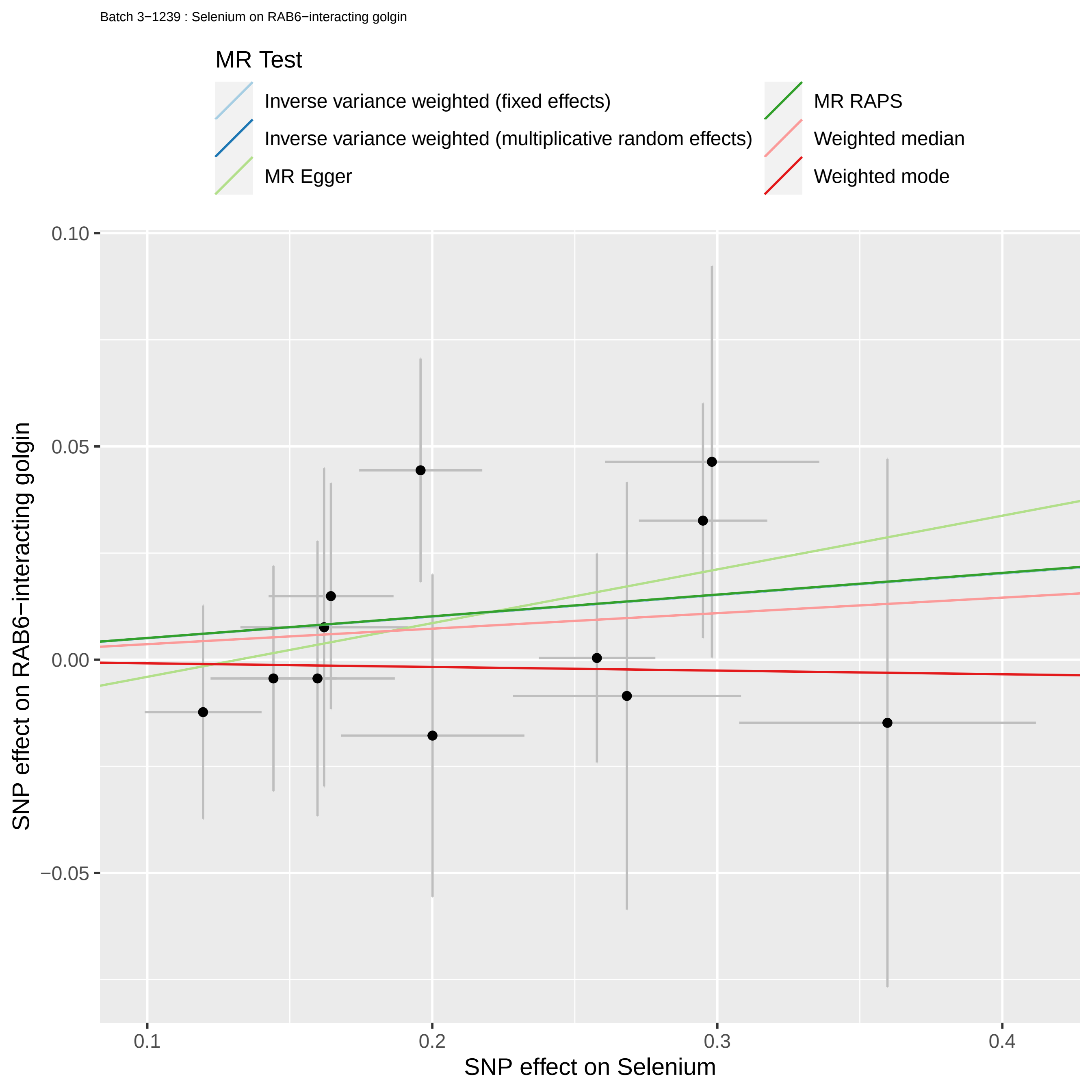

Batch 3−1239 : Selenium on RAB6−interacting golgin
MR Test
Inverse variance weighted (fixed effects)
Inverse variance weighted (multiplicative random effects)
MR Egger
MR RAPS
Weighted median
Weighted mode
0.10
0.05
0.00
−0.05
0.1
0.2
0.3
0.4
SNP effect on RAB6−interacting golgin
SNP effect on Selenium

Batch 3−1240 : Selenium on Golgi SNAP receptor complex member 1

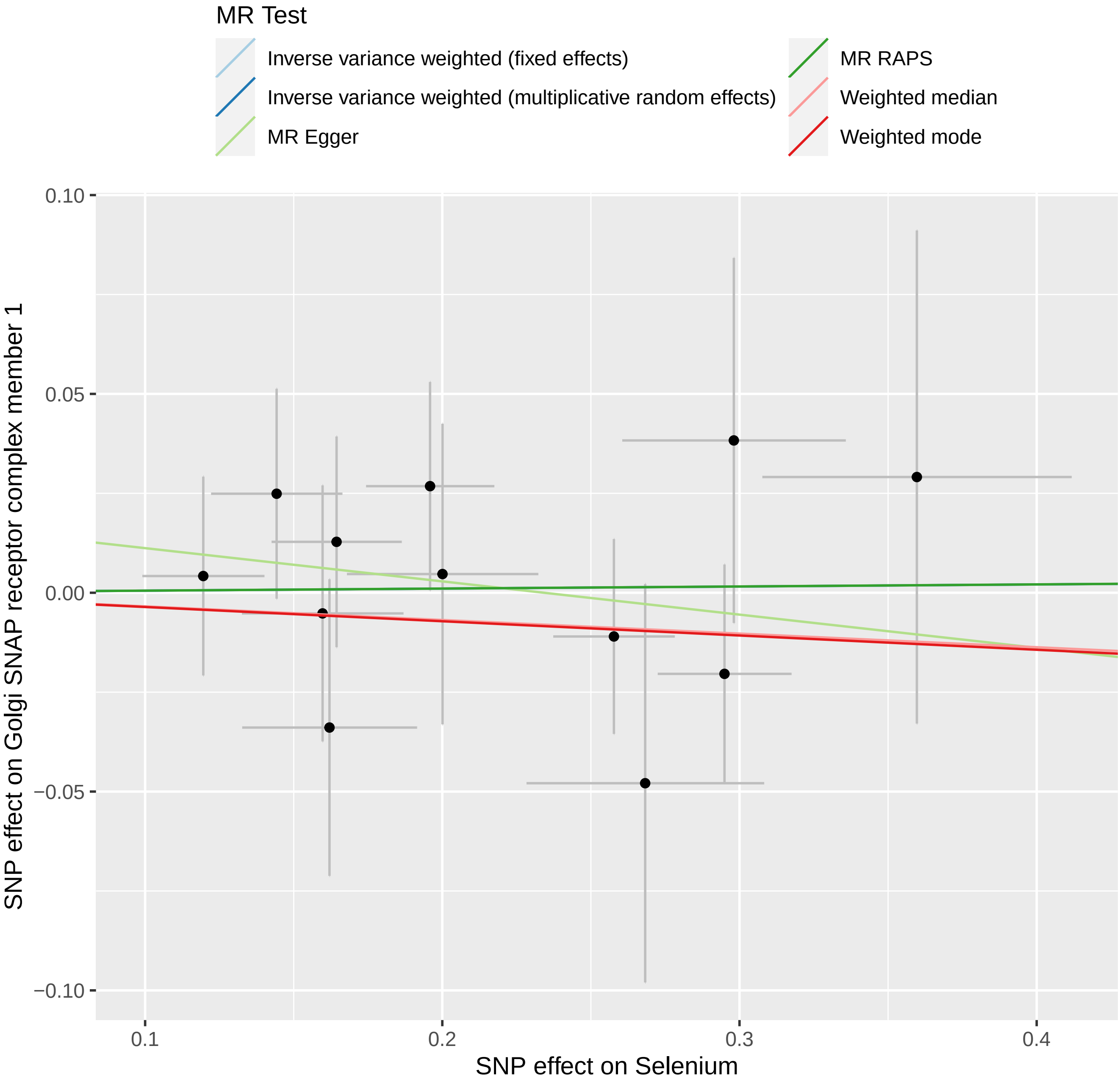

Batch 3−1241 : Selenium on Aspartate aminotransferase, cytoplasmic

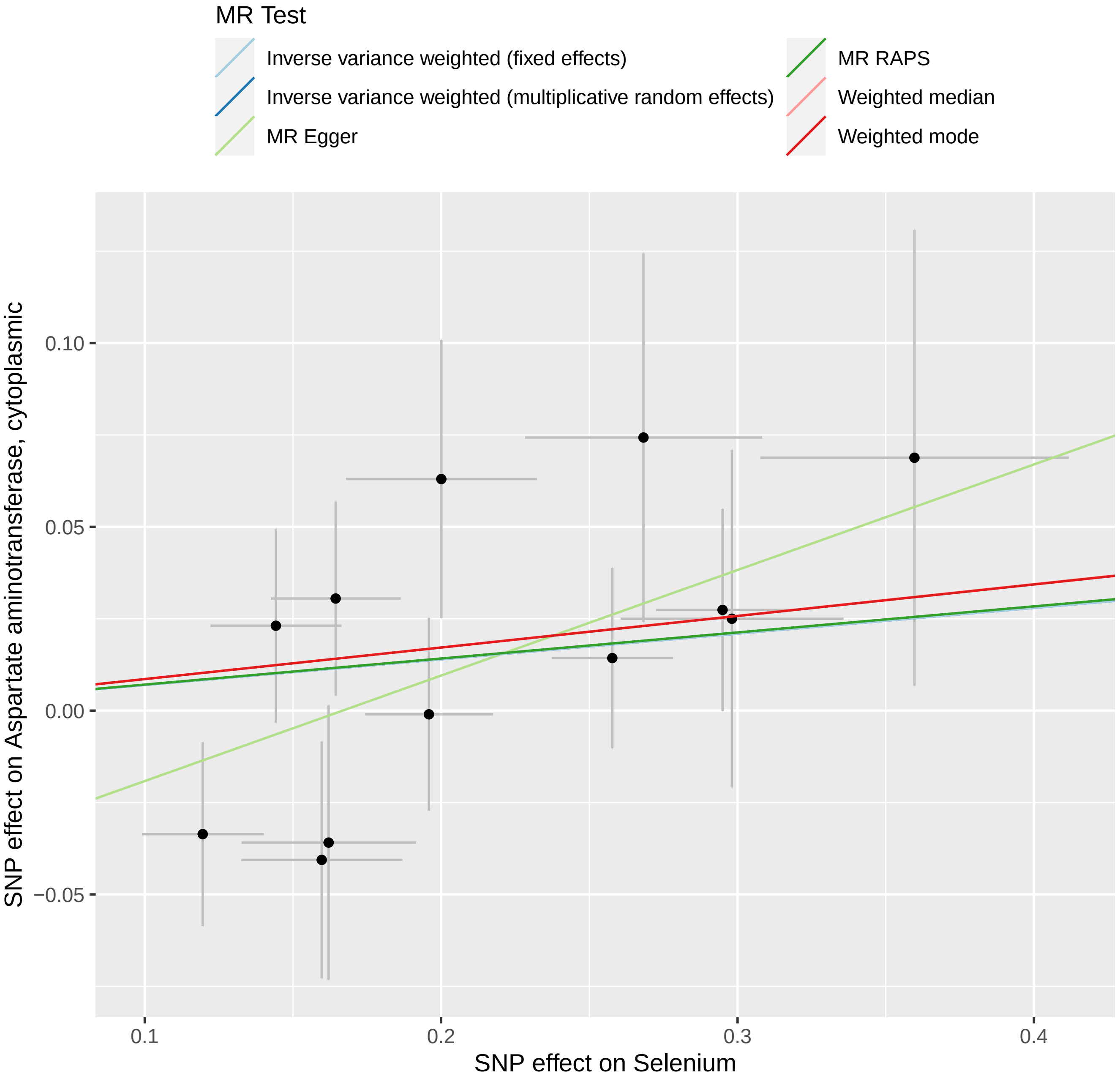

Batch 3−1242 : Selenium on Platelet glycoprotein Ib alpha chain

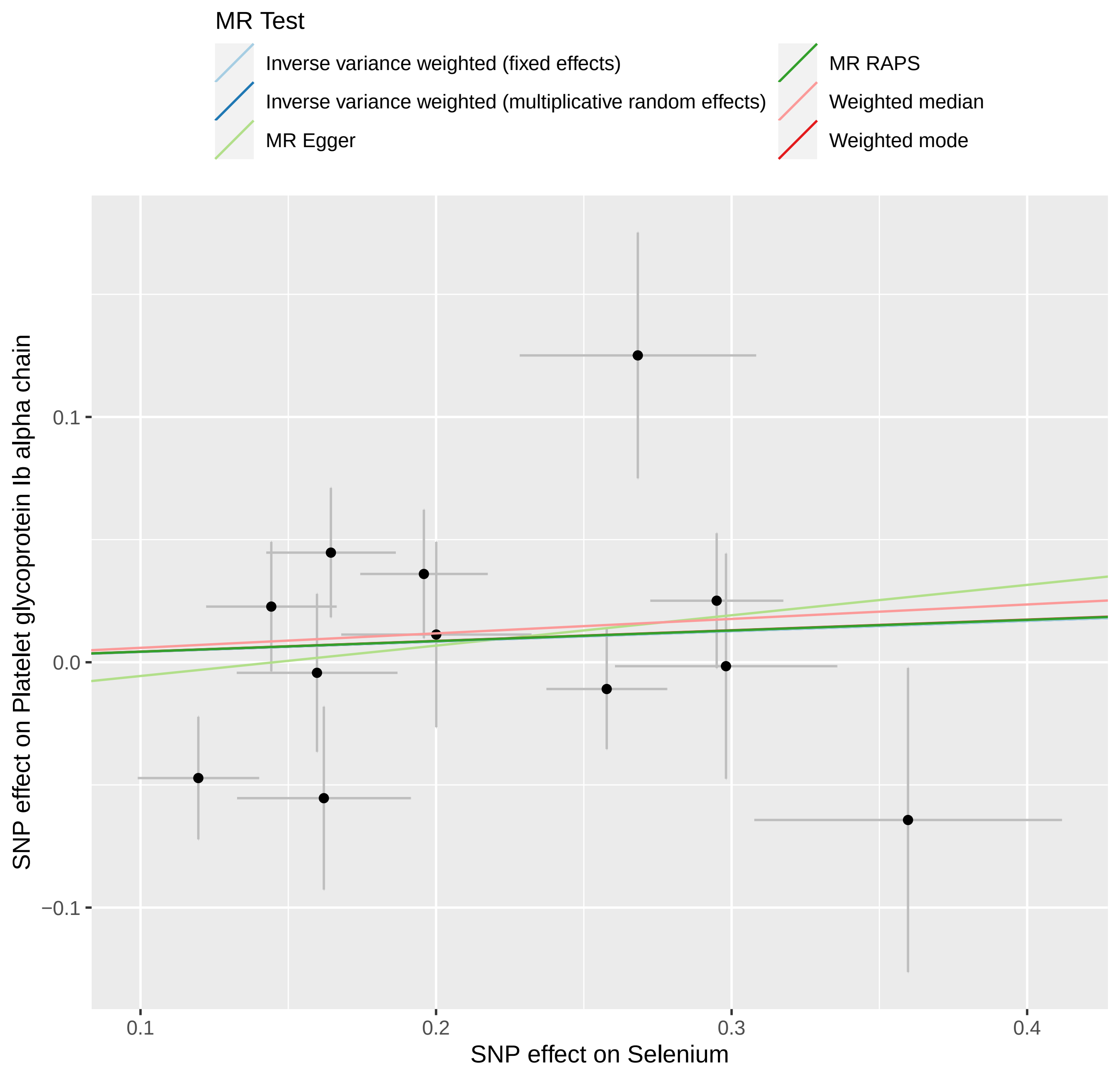

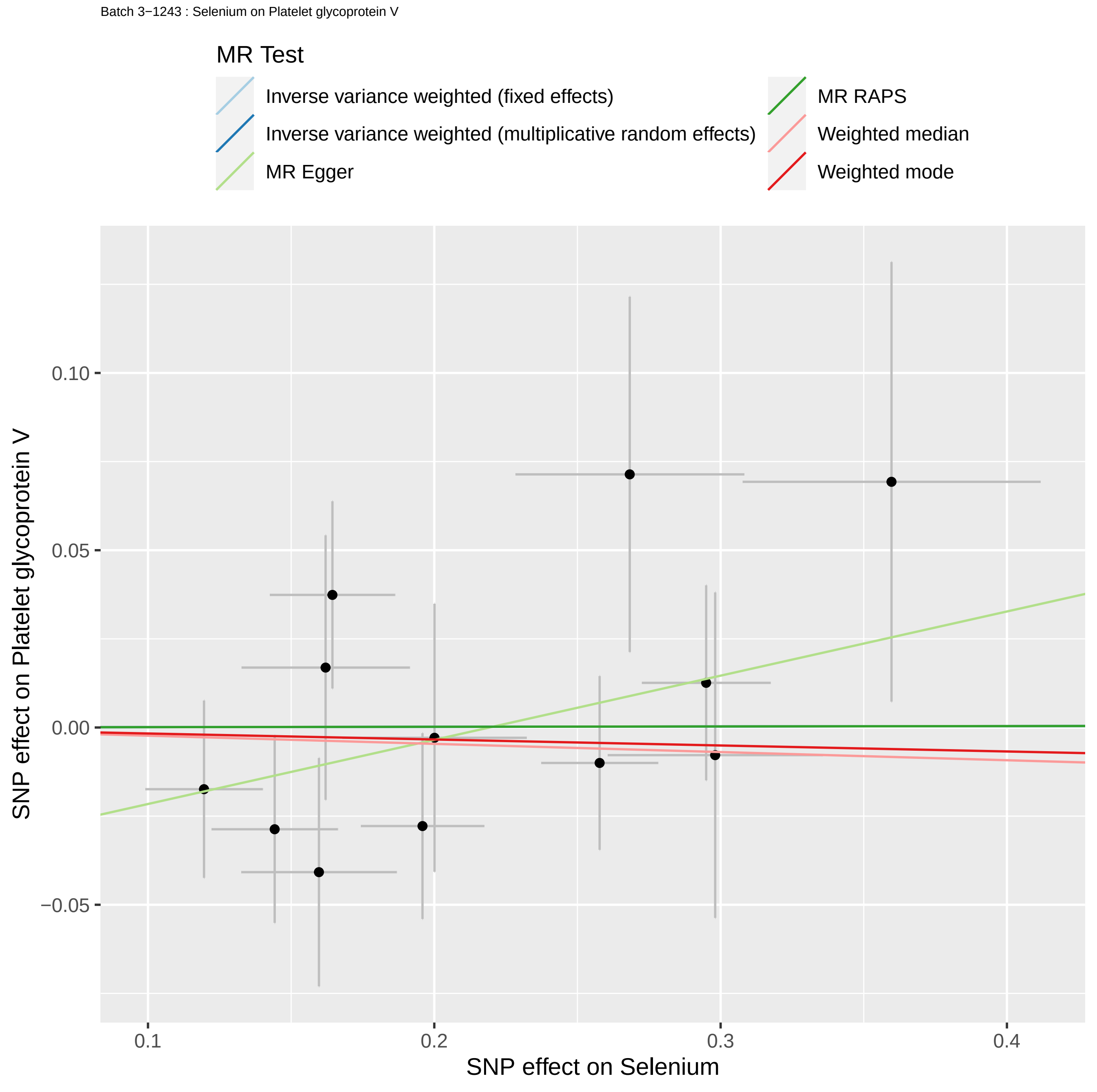
Batch 3−1243 : Selenium on Platelet glycoprotein V
MR Test
Inverse variance weighted (fixed effects)
Inverse variance weighted (multiplicative random effects)
MR Egger
MR RAPS
Weighted median
Weighted mode
SNP effect on Platelet glycoprotein V
0.10
0.05
0.00
−0.05
0.1
0.2
0.3
0.4
SNP effect on Selenium

Batch 3−1244 : Selenium on Cell surface A33 antigen

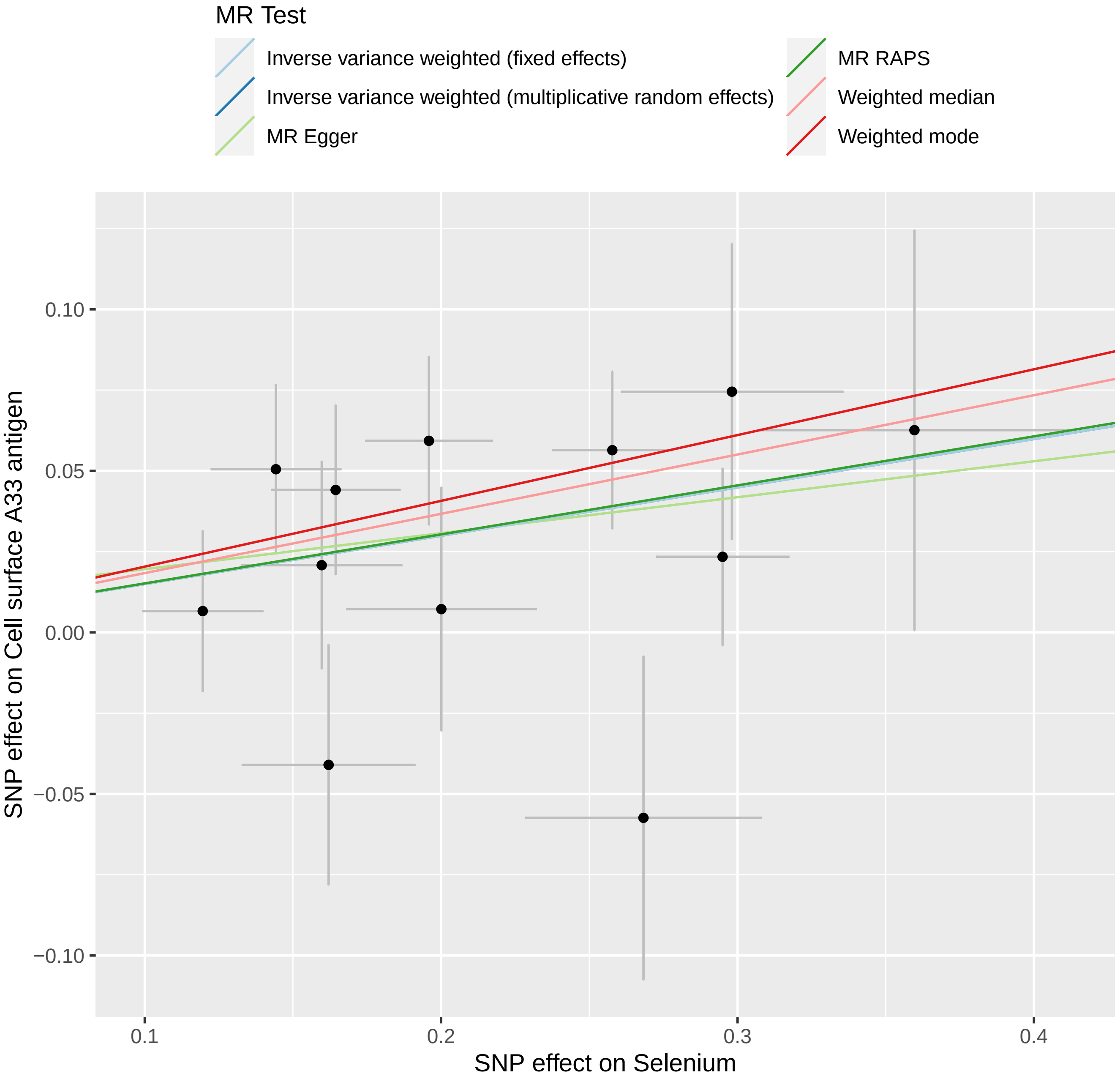

Batch 3−1245 : Selenium on Glypican−1

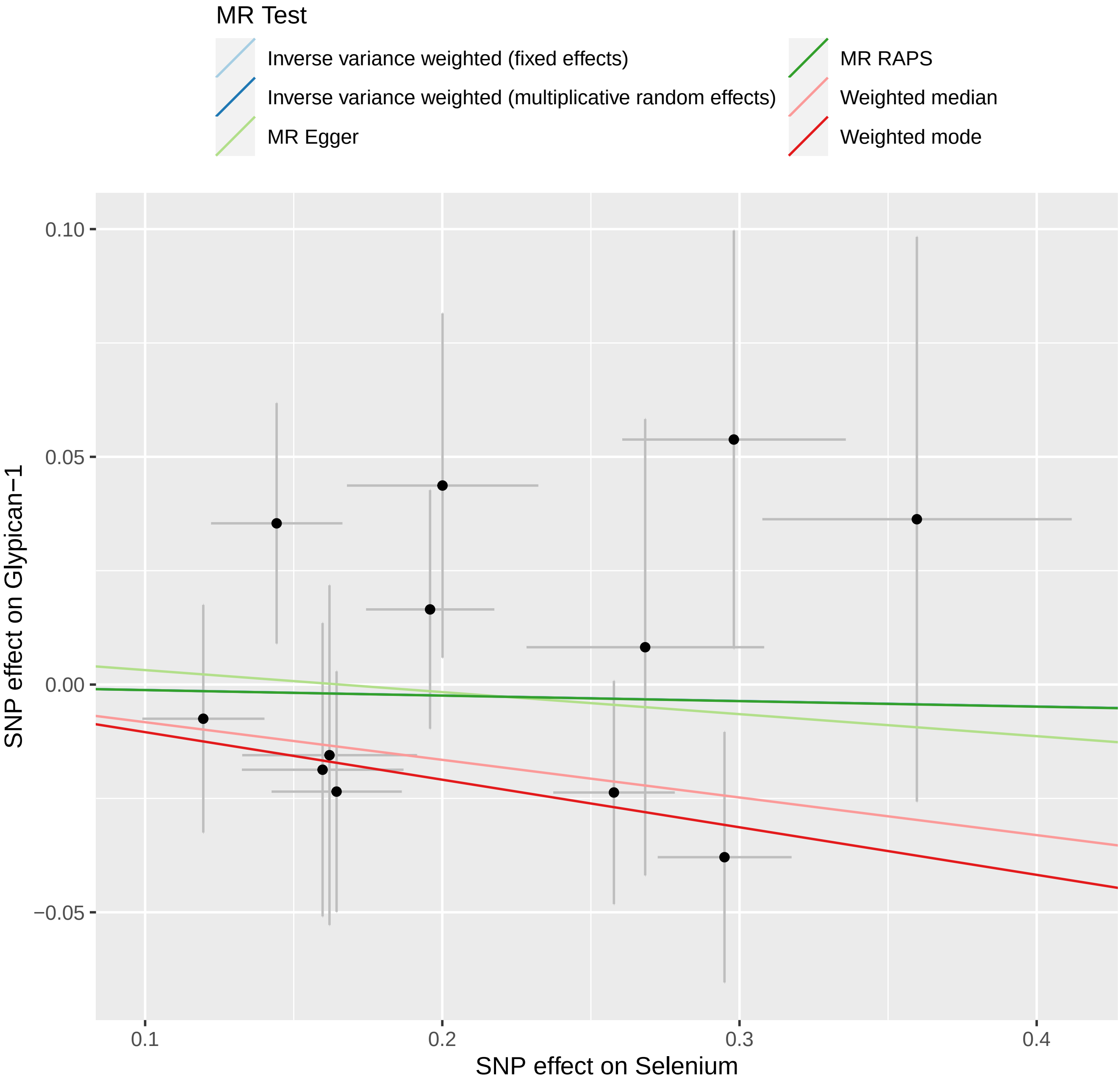

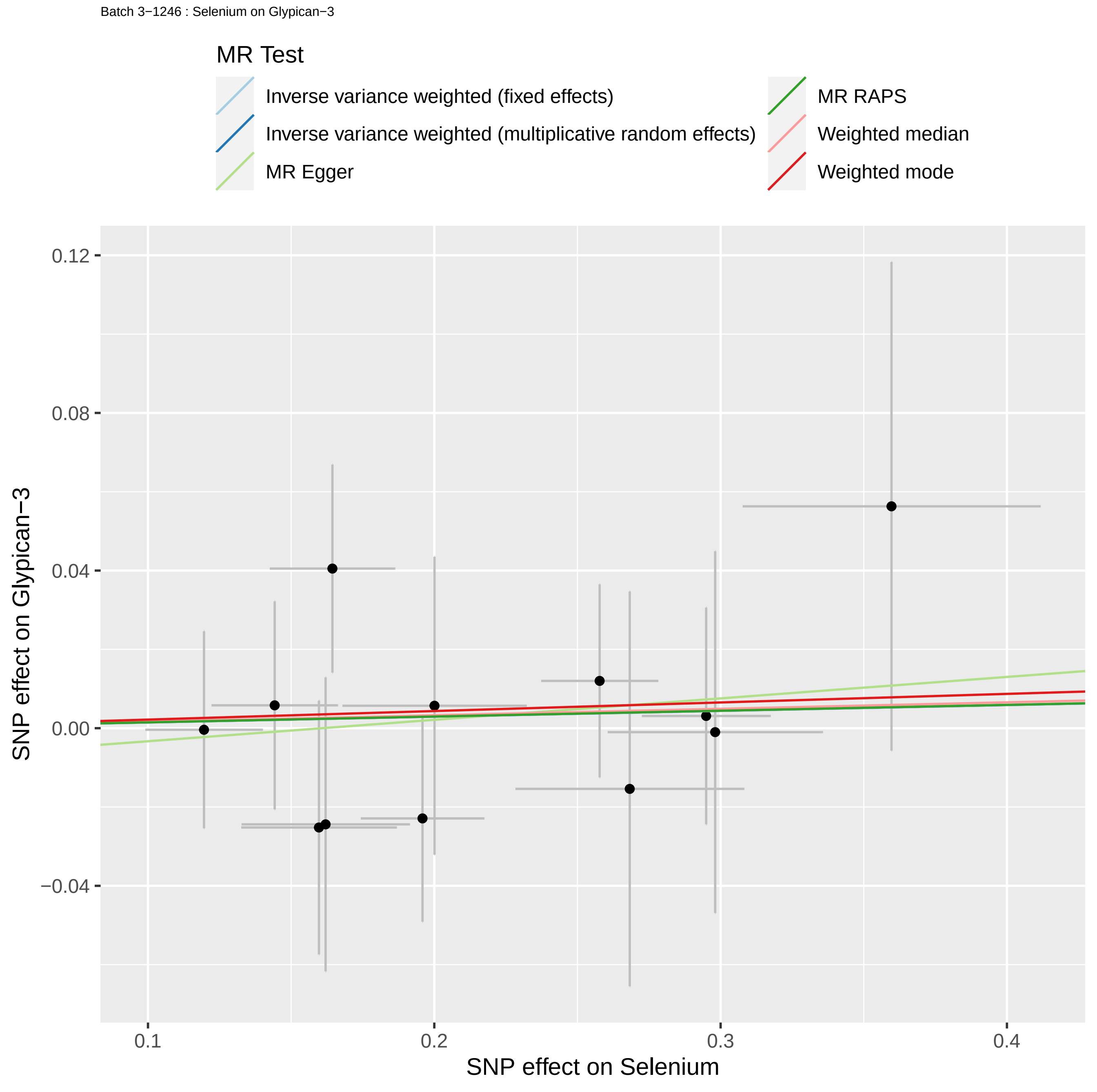
Batch 3−1246 : Selenium on Glypican−3
MR Test
Inverse variance weighted (fixed effects)
Inverse variance weighted (multiplicative random effects)
MR Egger
MR RAPS
Weighted median
Weighted mode
0.12
0.08
0.04
0.00
−0.04
0.1
0.2
0.3
0.4
SNP effect on Glypican−3
SNP effect on Selenium

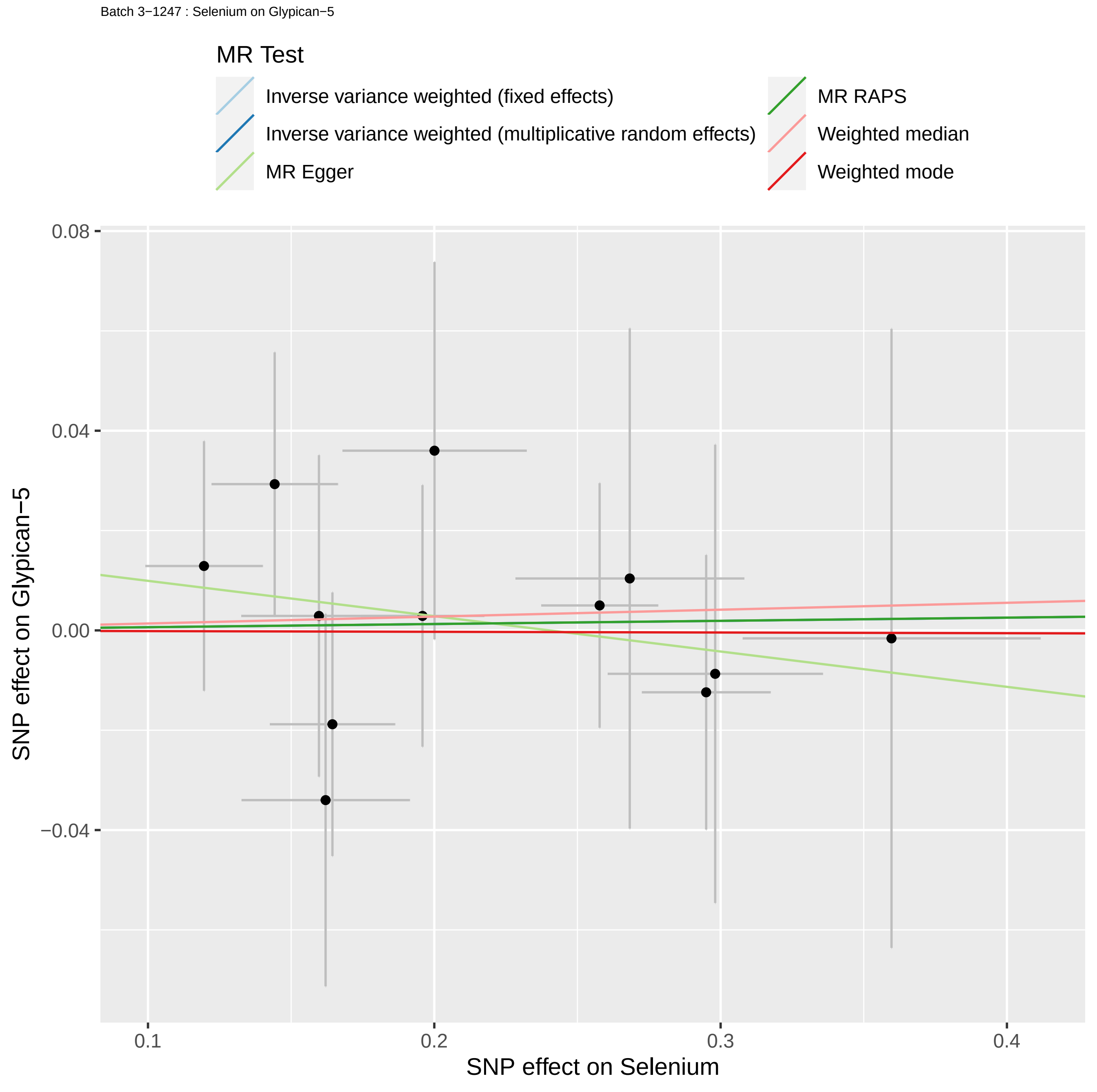
Batch 3−1247 : Selenium on Glypican−5
MR Test
Inverse variance weighted (fixed effects)
Inverse variance weighted (multiplicative random effects)
MR Egger
MR RAPS
Weighted median
Weighted mode
0.08
0.04
0.00
−0.04
0.1
0.2
0.3
0.4
SNP effect on Glypican−5
SNP effect on Selenium

Batch 3−1248 : Selenium on Glypican−6

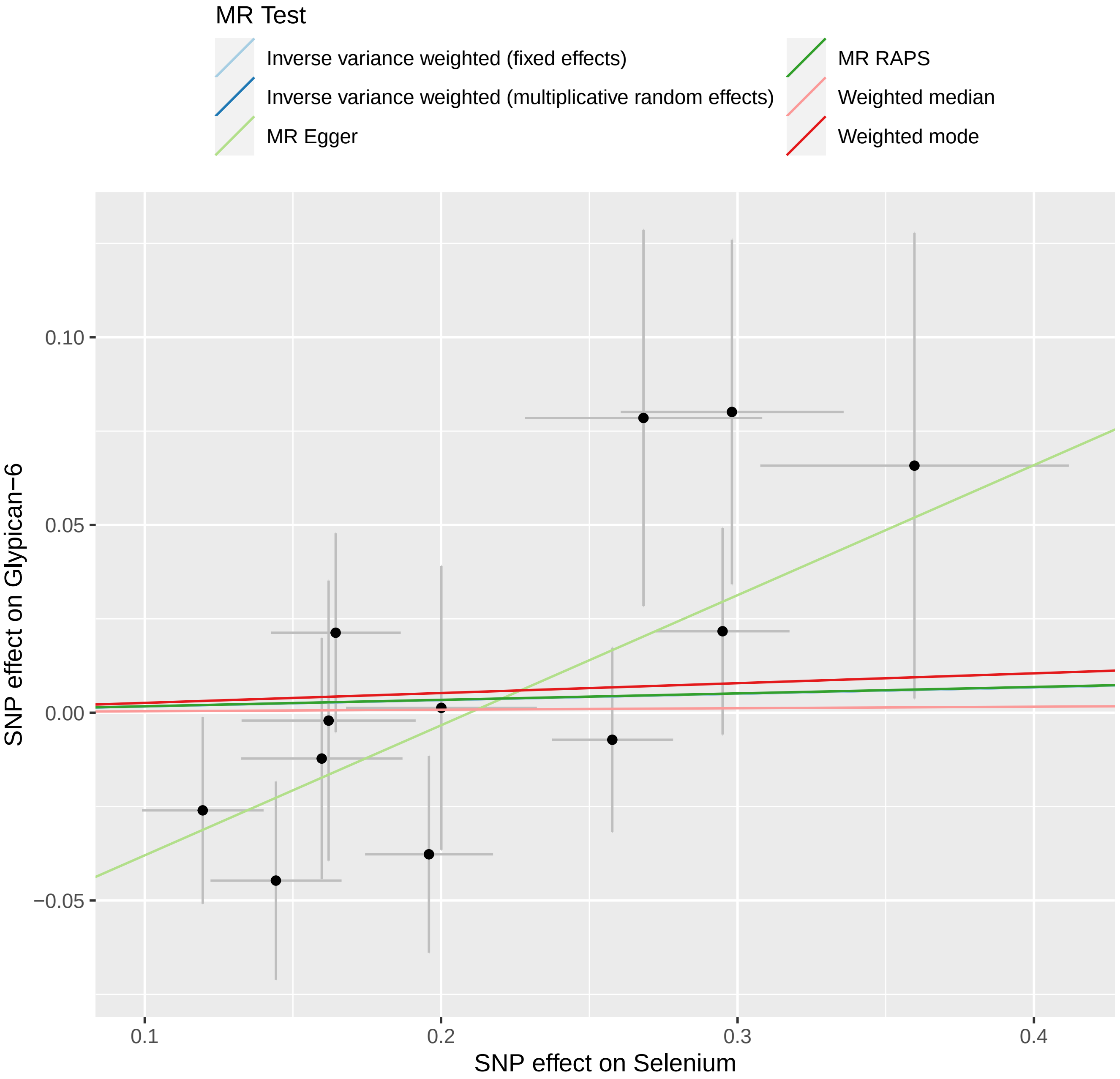

Batch 3−1249 : Selenium on Glycerophosphocholine phosphodiesterase GPCPD1

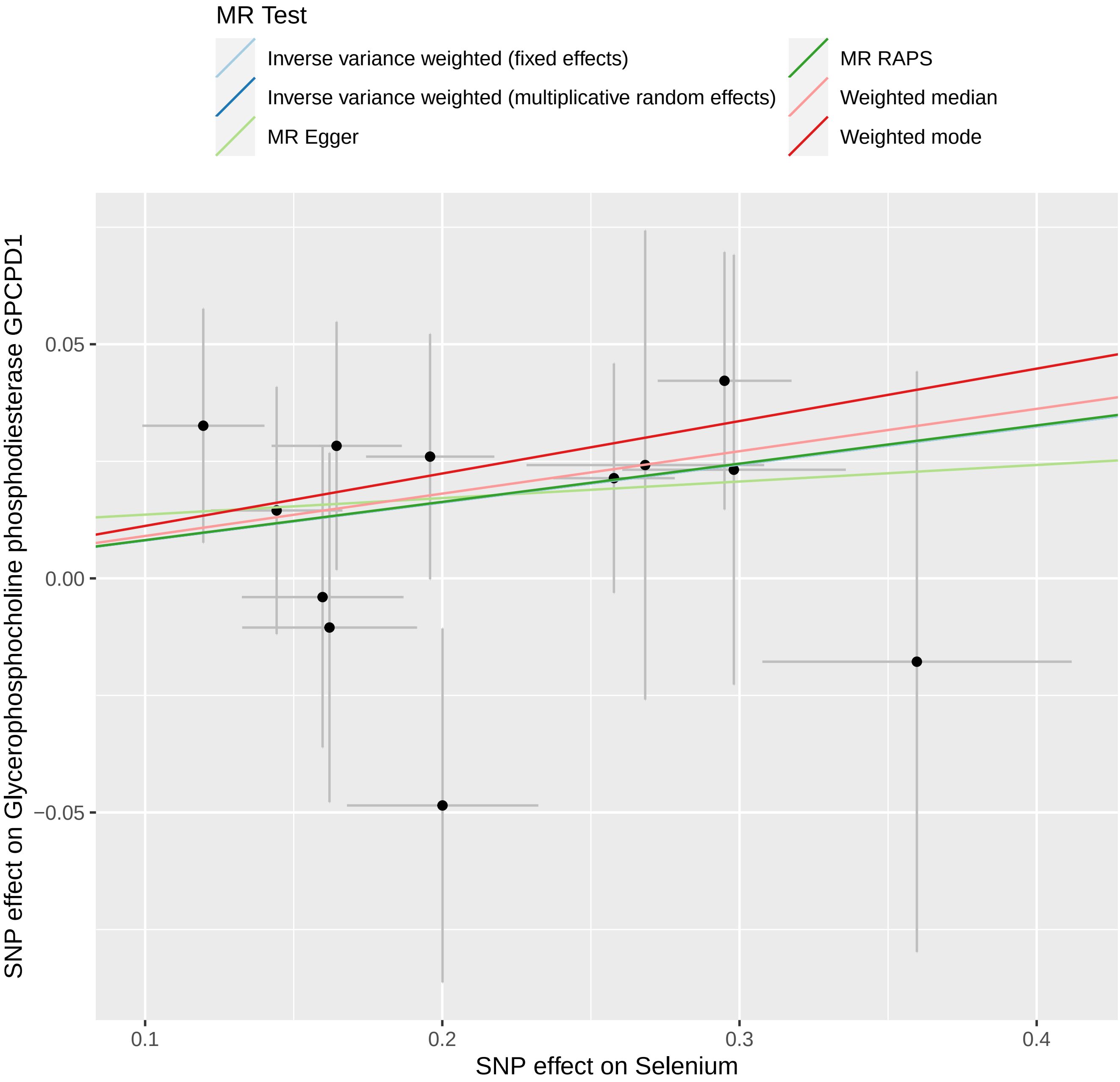

Batch 3−1250 : Selenium on Glycerol−3−phosphate dehydrogenase 1−like protein

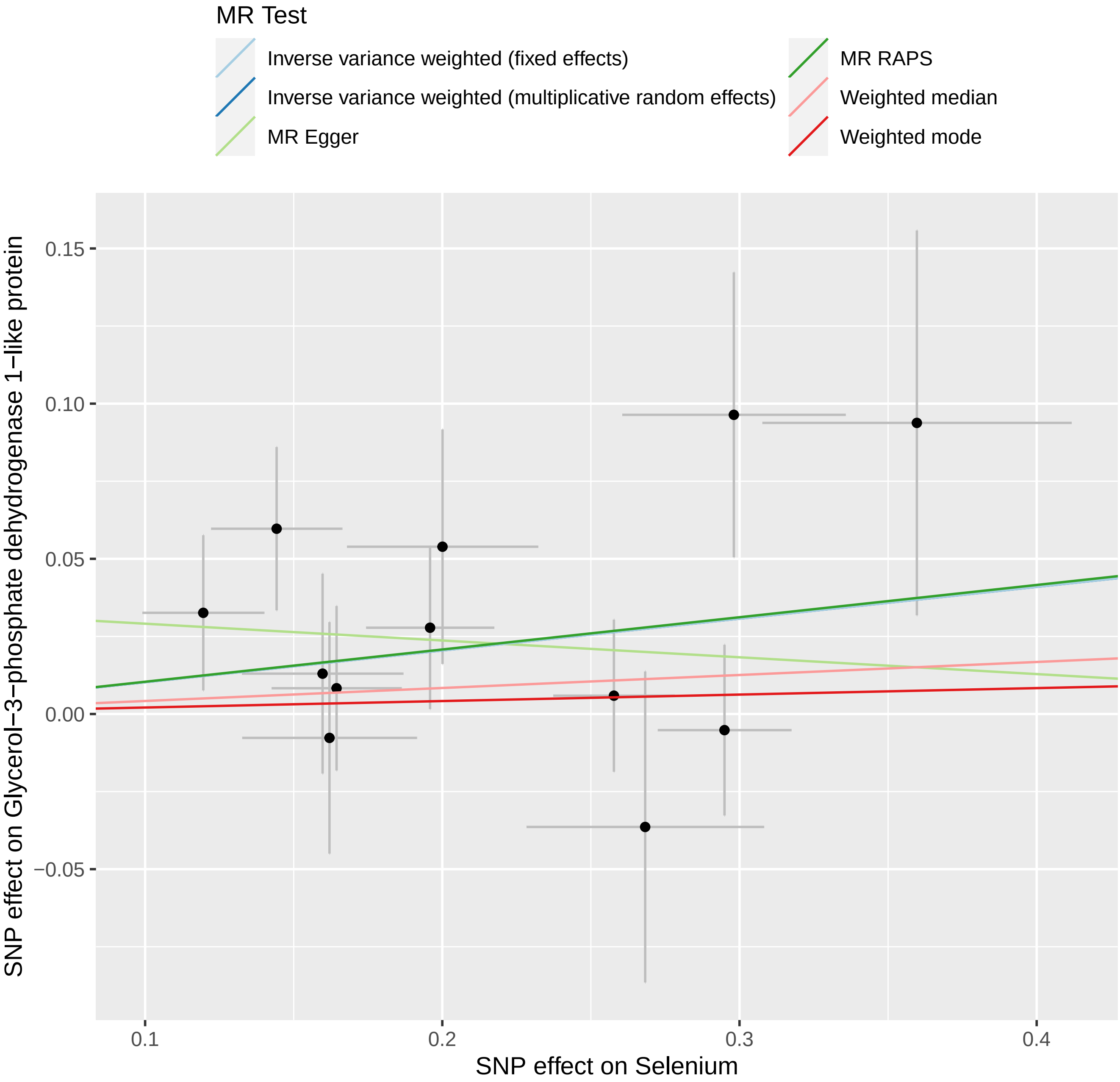

Batch 3−1251 : Selenium on Glycoprotein hormone alpha−2

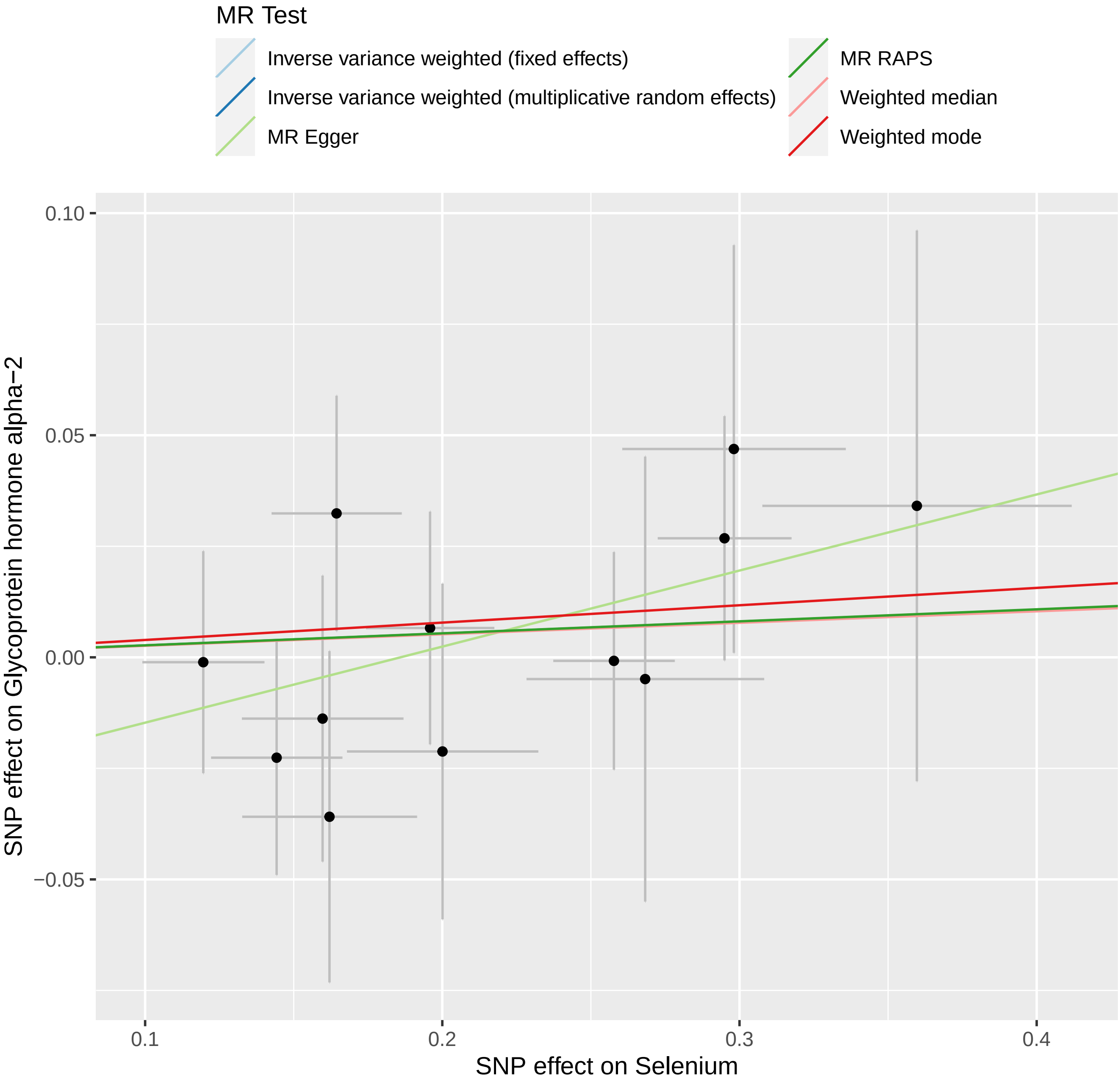

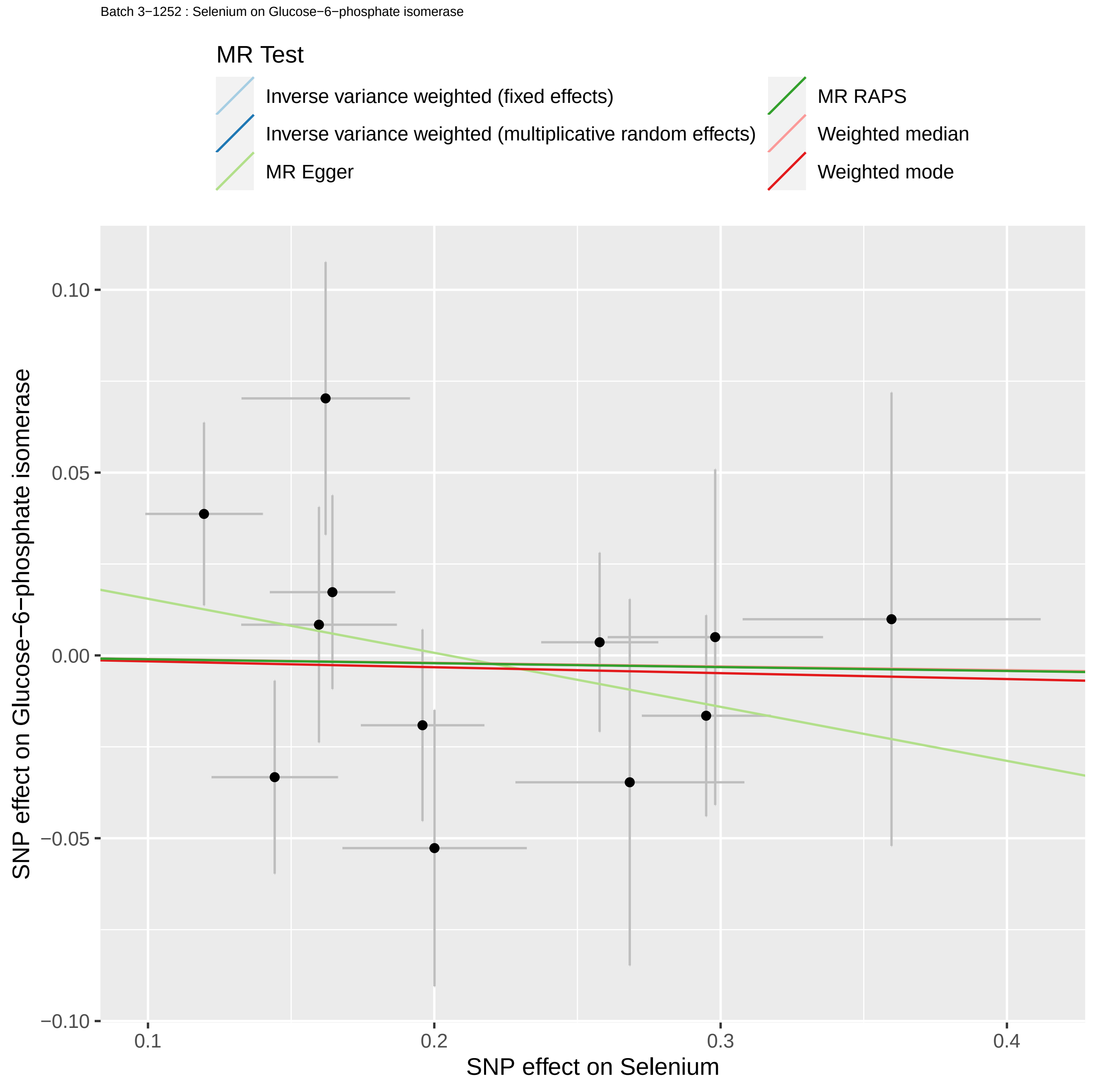

Batch 3−1252 : Selenium on Glucose−6−phosphate isomerase
MR Test
Inverse variance weighted (fixed effects)
Inverse variance weighted (multiplicative random effects)
MR Egger
MR RAPS
Weighted median
Weighted mode
0.10
0.05
0.00
−0.05
−0.10
0.1
0.2
0.3
0.4
SNP effect on Selenium
SNP effect on Glucose−6−phosphate isomerase

Batch 3−1253 : Selenium on Phosphatidylinositol−glycan−specific phospholipase D

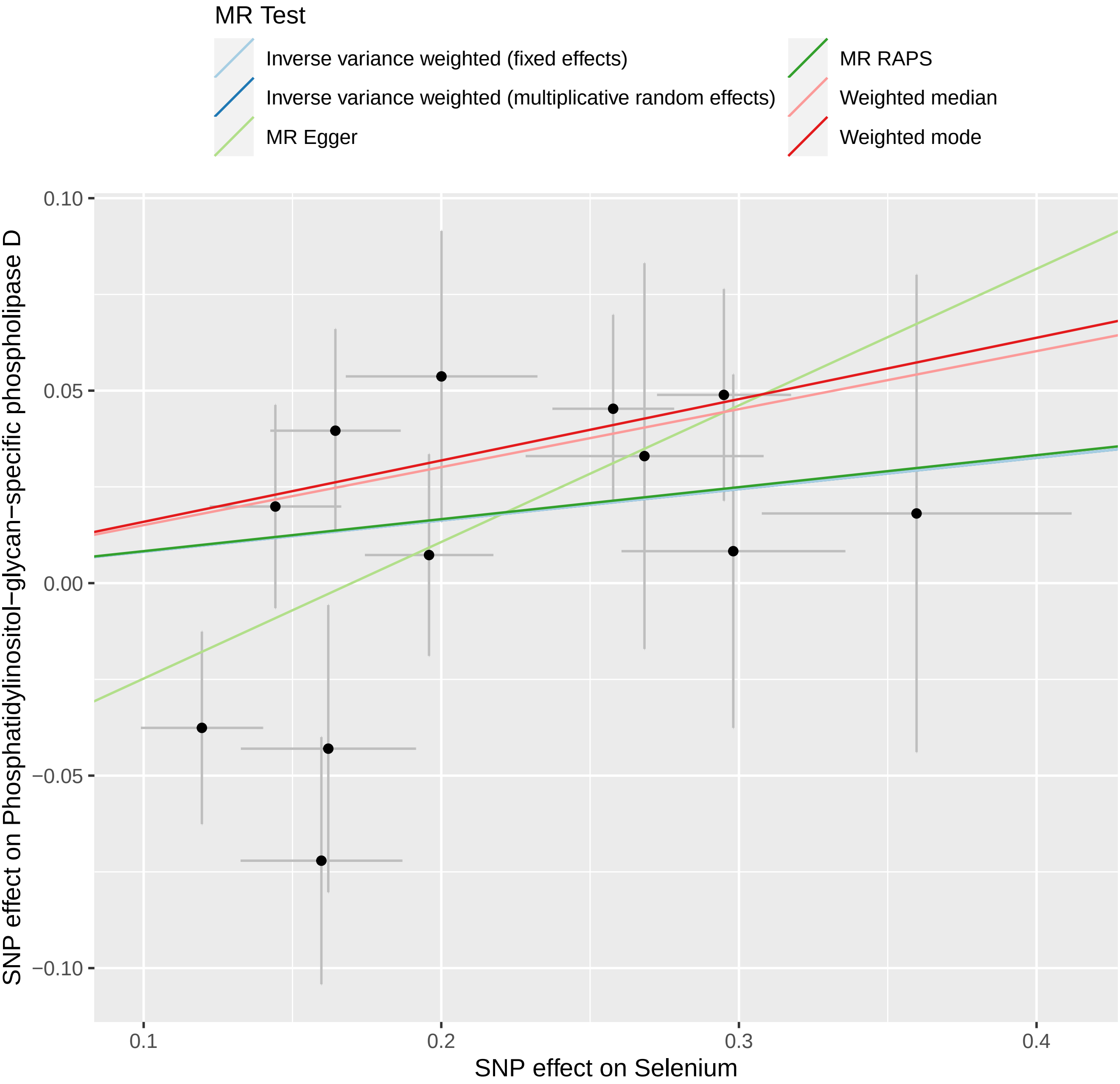

Batch 3−1254 : Selenium on Phosphatidylinositol−glycan−specific phospholipase D

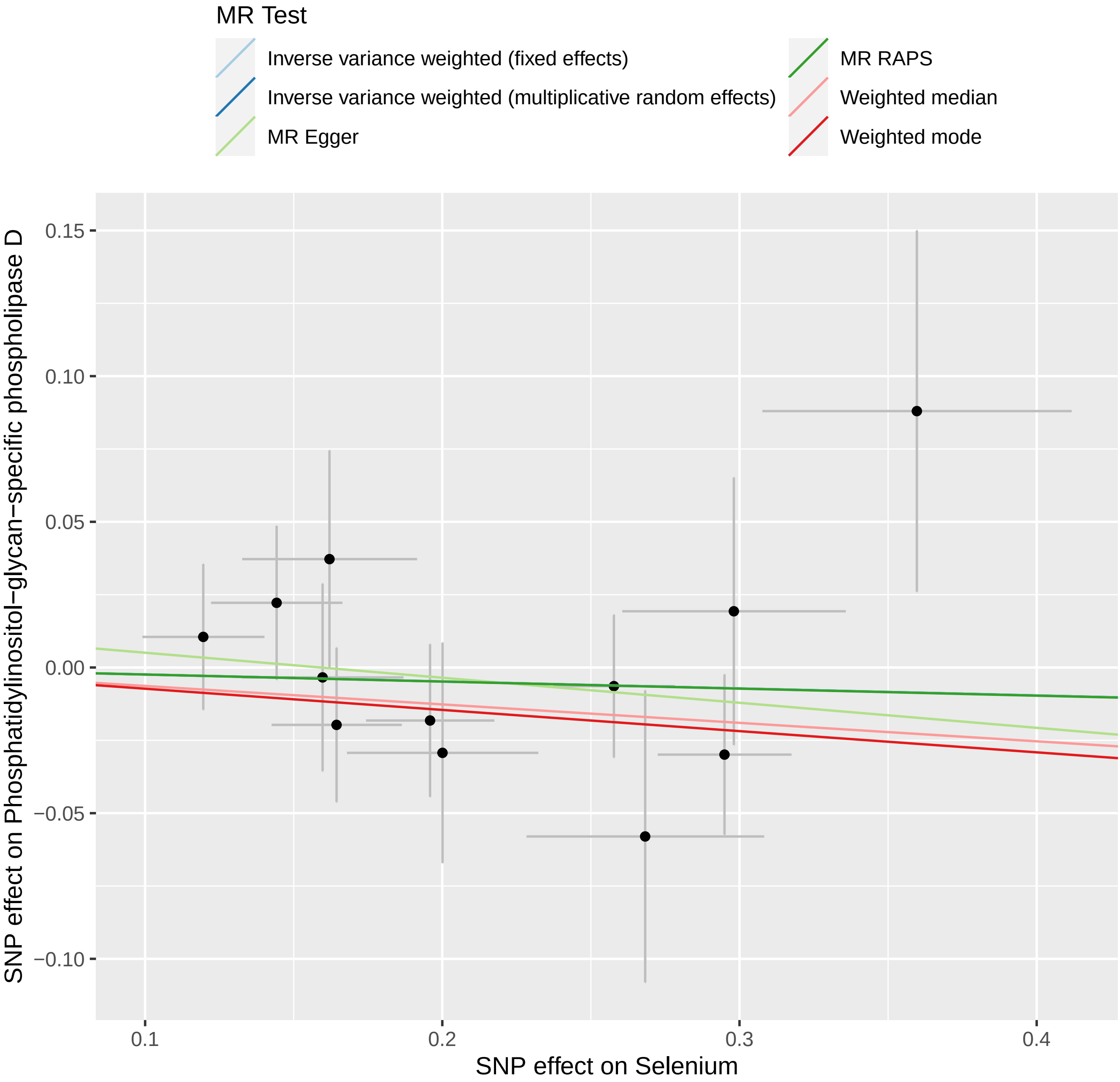

Batch 3−1255 : Selenium on Transmembrane glycoprotein NMB

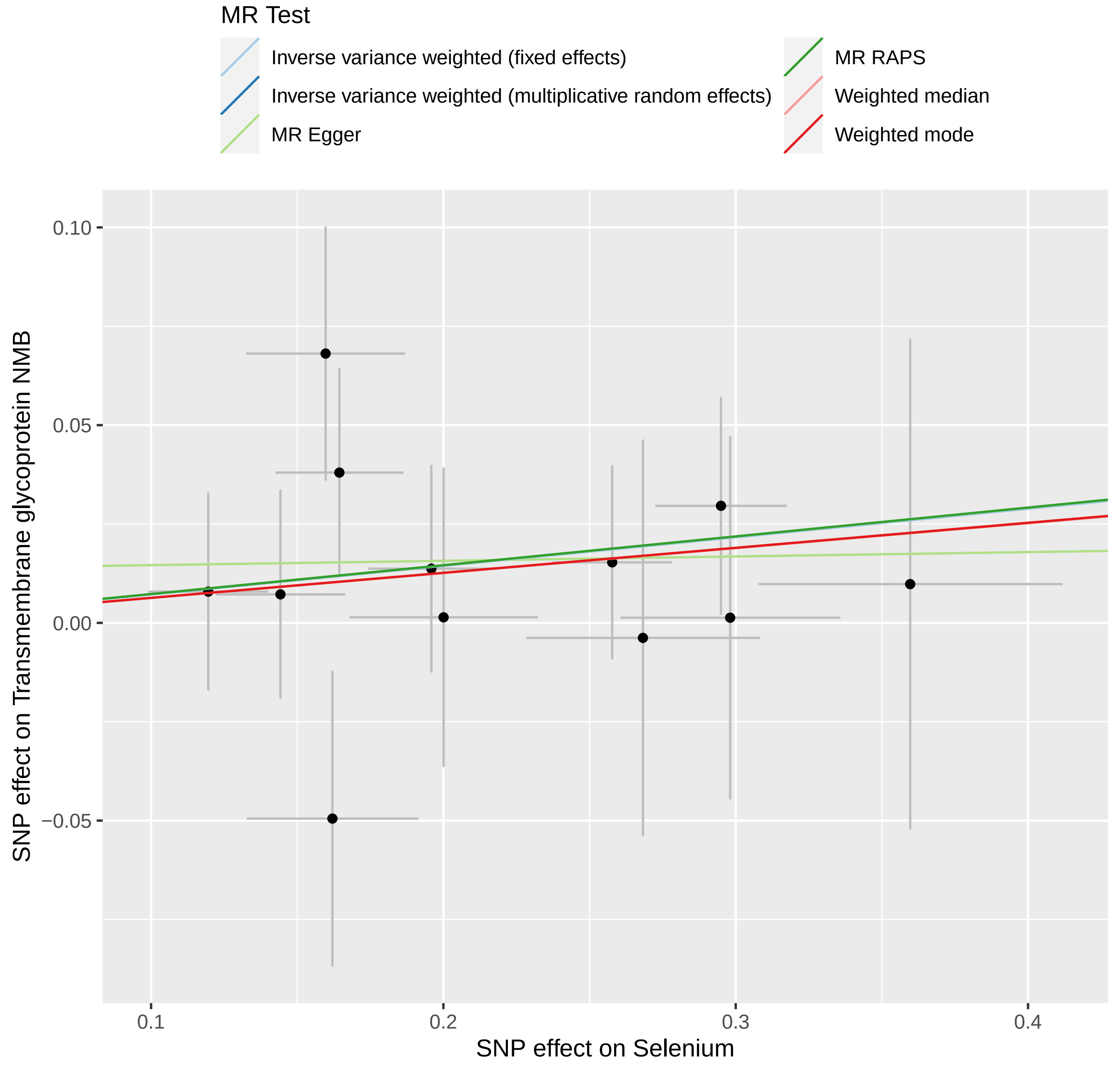

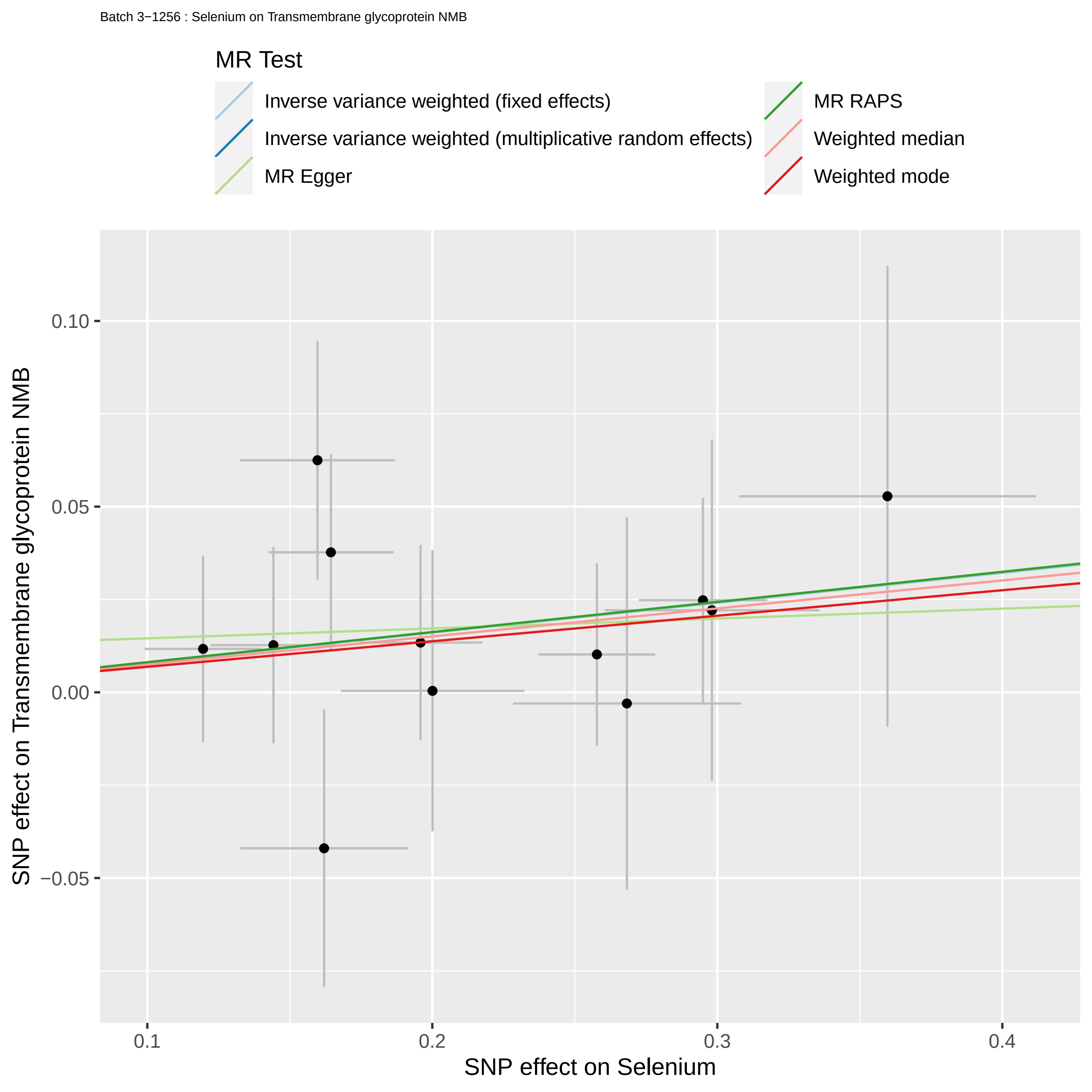
Batch 3−1256 : Selenium on Transmembrane glycoprotein NMB
MR Test
Inverse variance weighted (fixed effects)
Inverse variance weighted (multiplicative random effects)
MR Egger
MR RAPS
Weighted median
Weighted mode
0.10
0.05
0.00
−0.05
0.1
0.2
0.3
0.4
SNP effect on Transmembrane glycoprotein NMB
SNP effect on Selenium

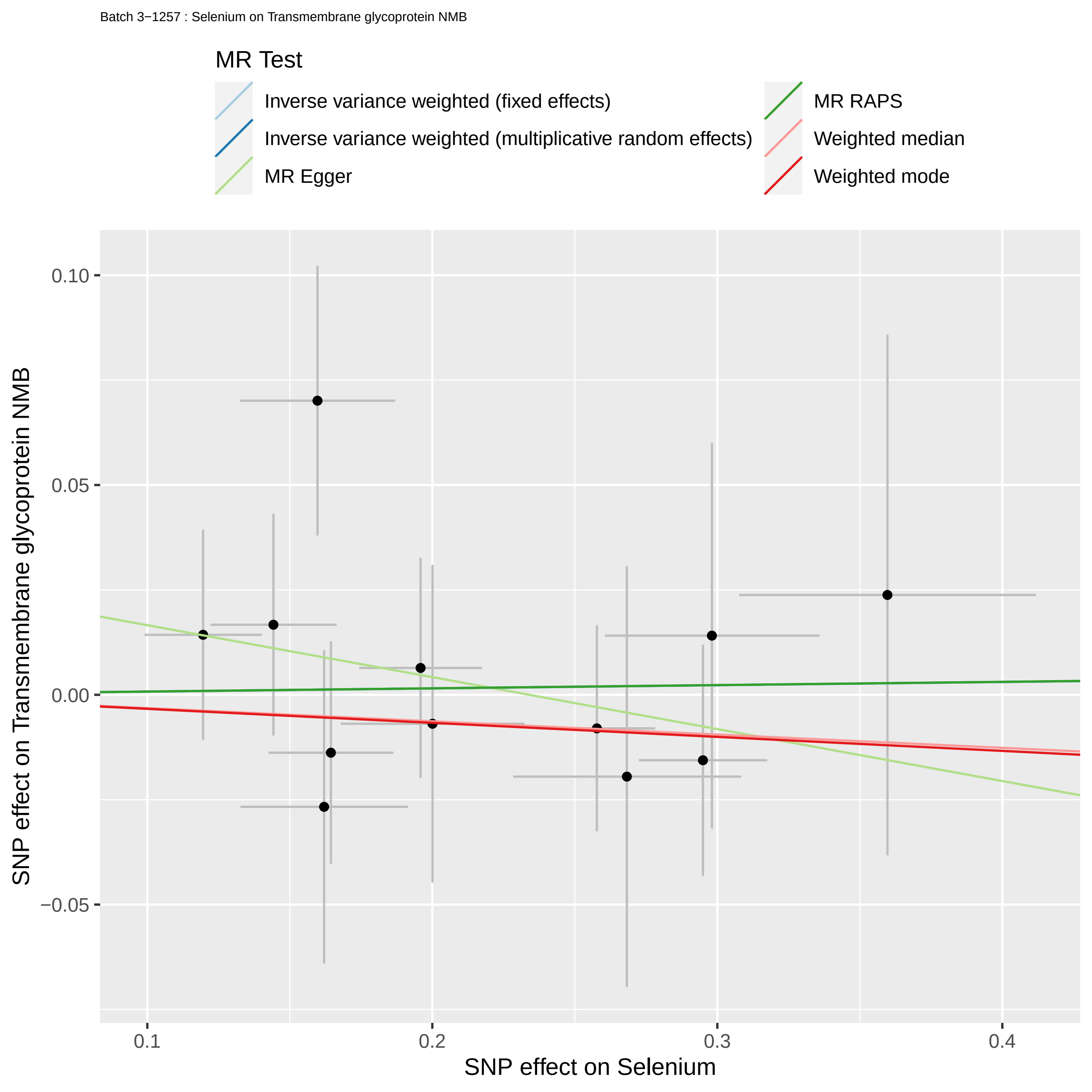
Batch 3−1257 : Selenium on Transmembrane glycoprotein NMB
MR Test
Inverse variance weighted (fixed effects)
Inverse variance weighted (multiplicative random effects)
MR Egger
MR RAPS
Weighted median
Weighted mode
0.10
0.05
0.00
−0.05
0.1
0.2
0.3
0.4
SNP effect on Transmembrane glycoprotein NMB
SNP effect on Selenium

Batch 3−1258 : Selenium on Transmembrane glycoprotein NMB

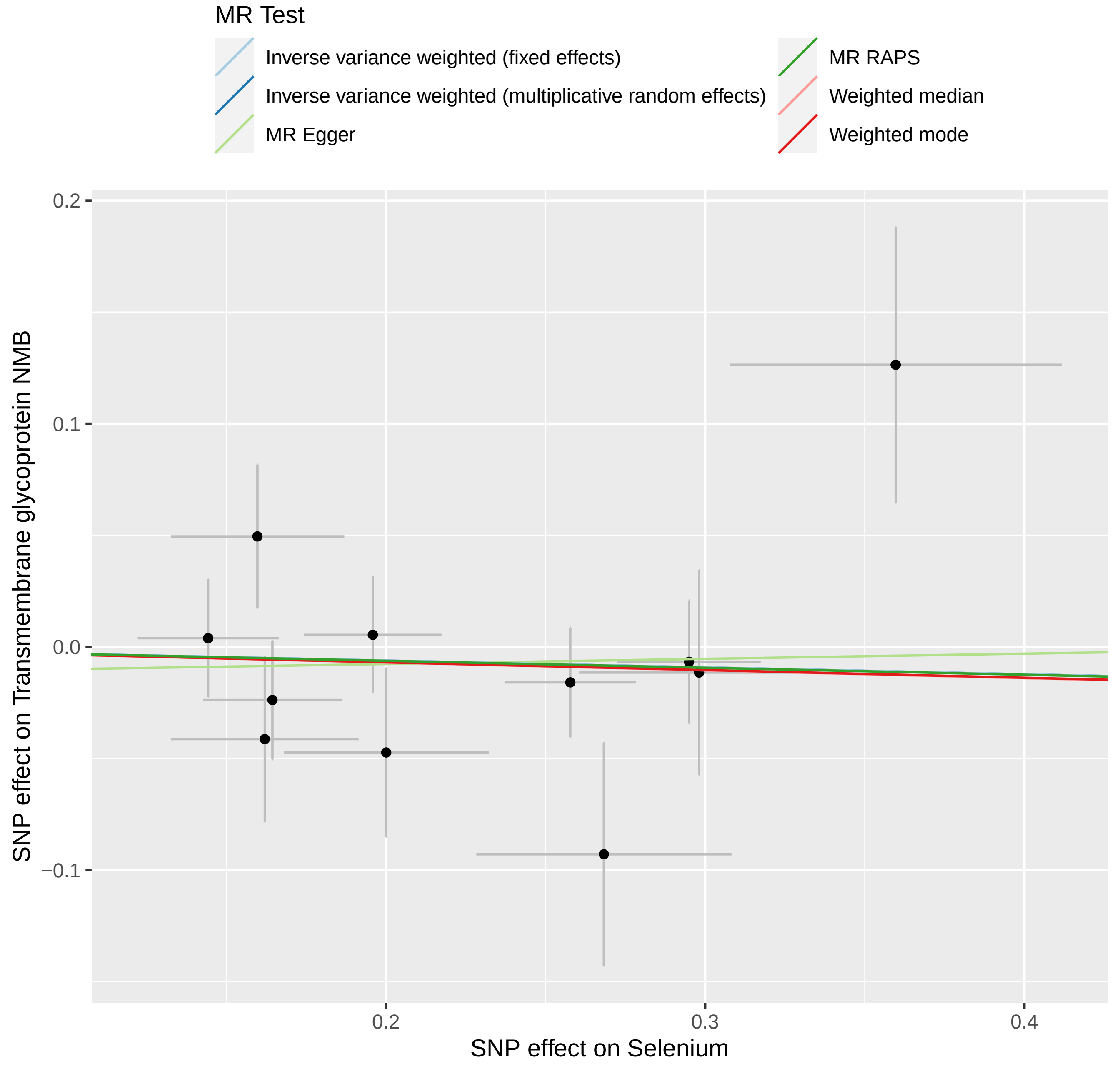

Batch 3−1259 : Selenium on Probable G−protein coupled receptor 101

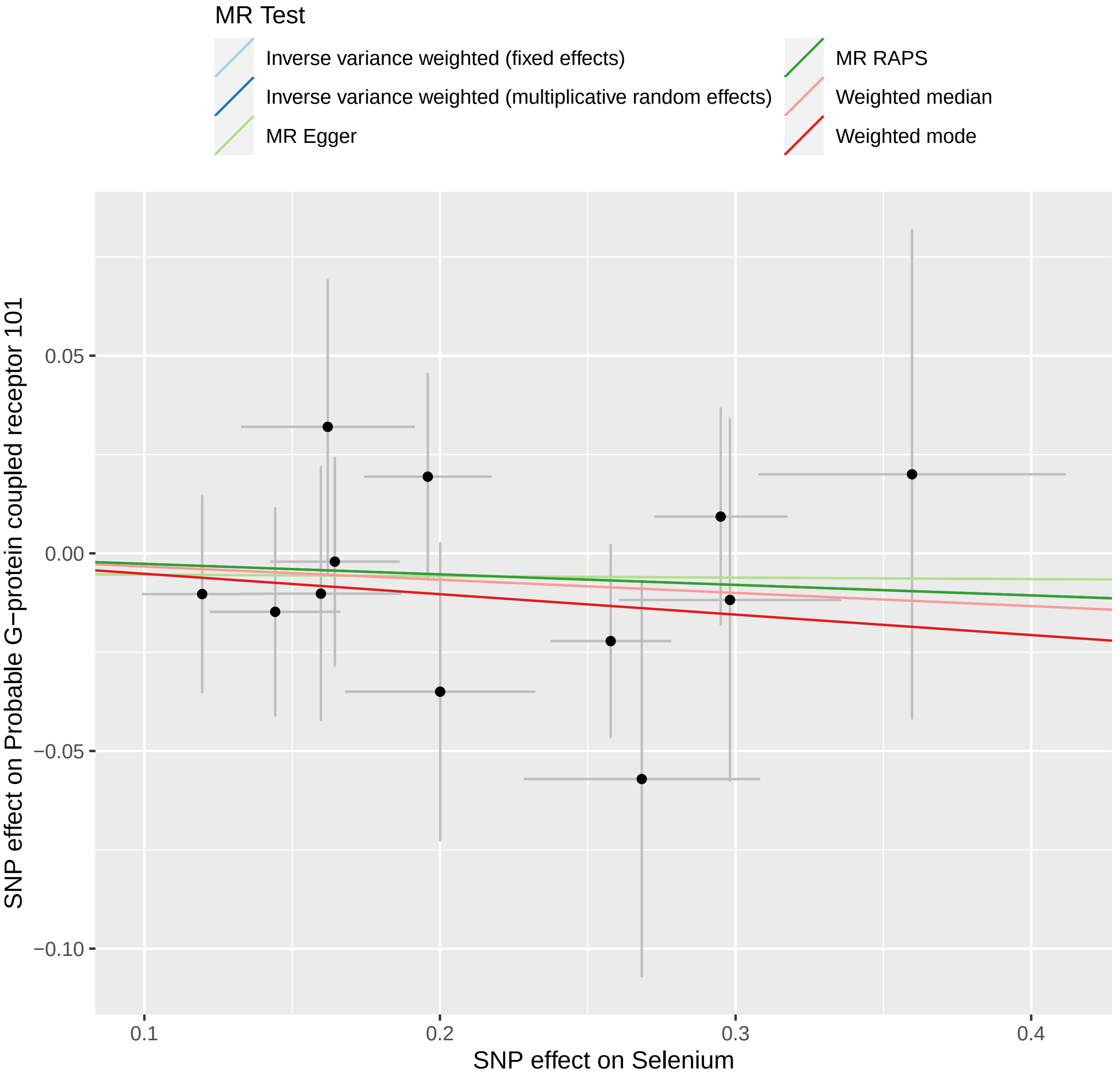

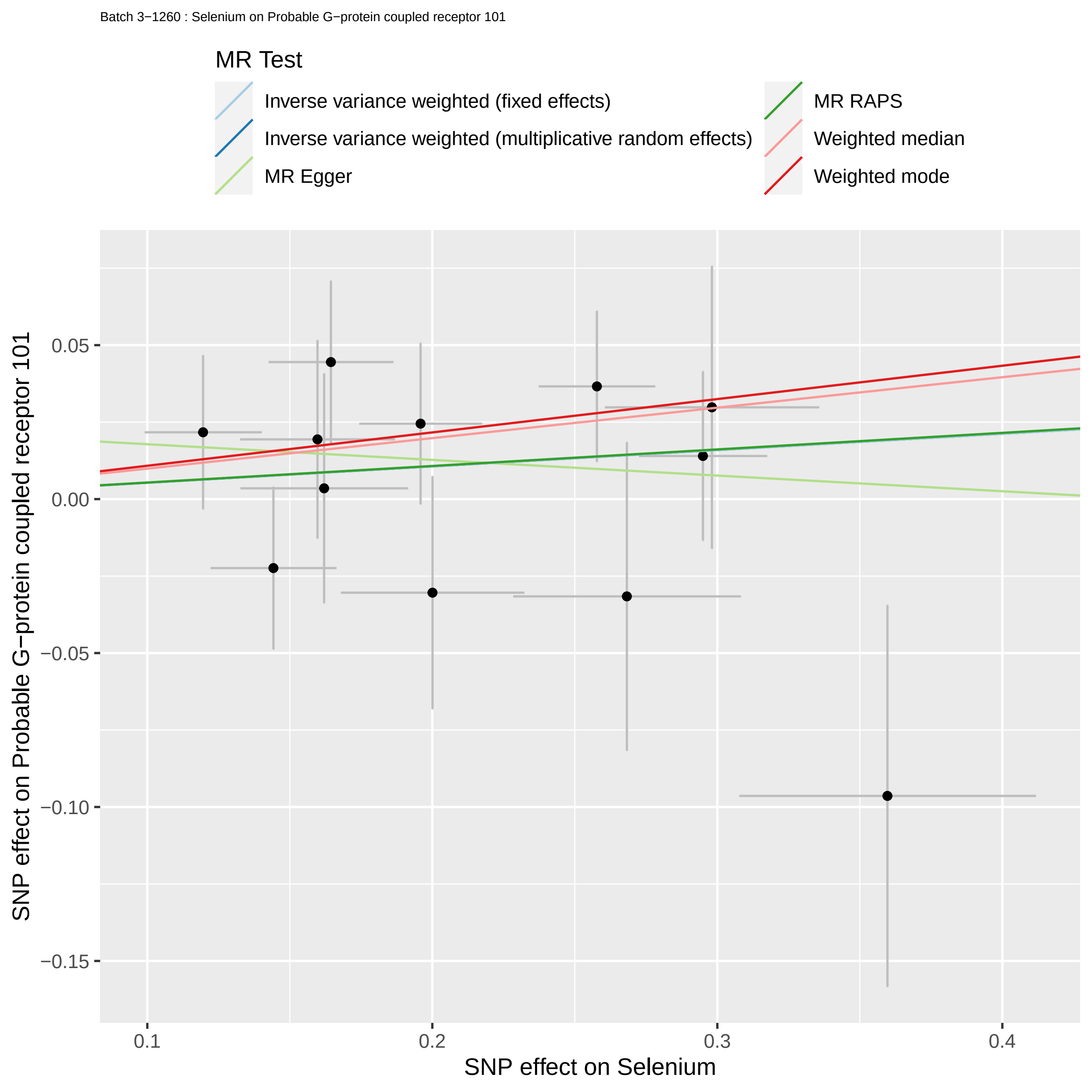
Batch 3−1260 : Selenium on Probable G−protein coupled receptor 101
MR Test
Inverse variance weighted (fixed effects)
Inverse variance weighted (multiplicative random effects)
MR Egger
MR RAPS
Weighted median
Weighted mode
0.05
0.00
−0.05
−0.10
−0.15
0.1
0.2
0.3
0.4
SNP effect on Probable G−protein coupled receptor 101
SNP effect on Selenium

Batch 3−1261 : Selenium on Protein GPR107

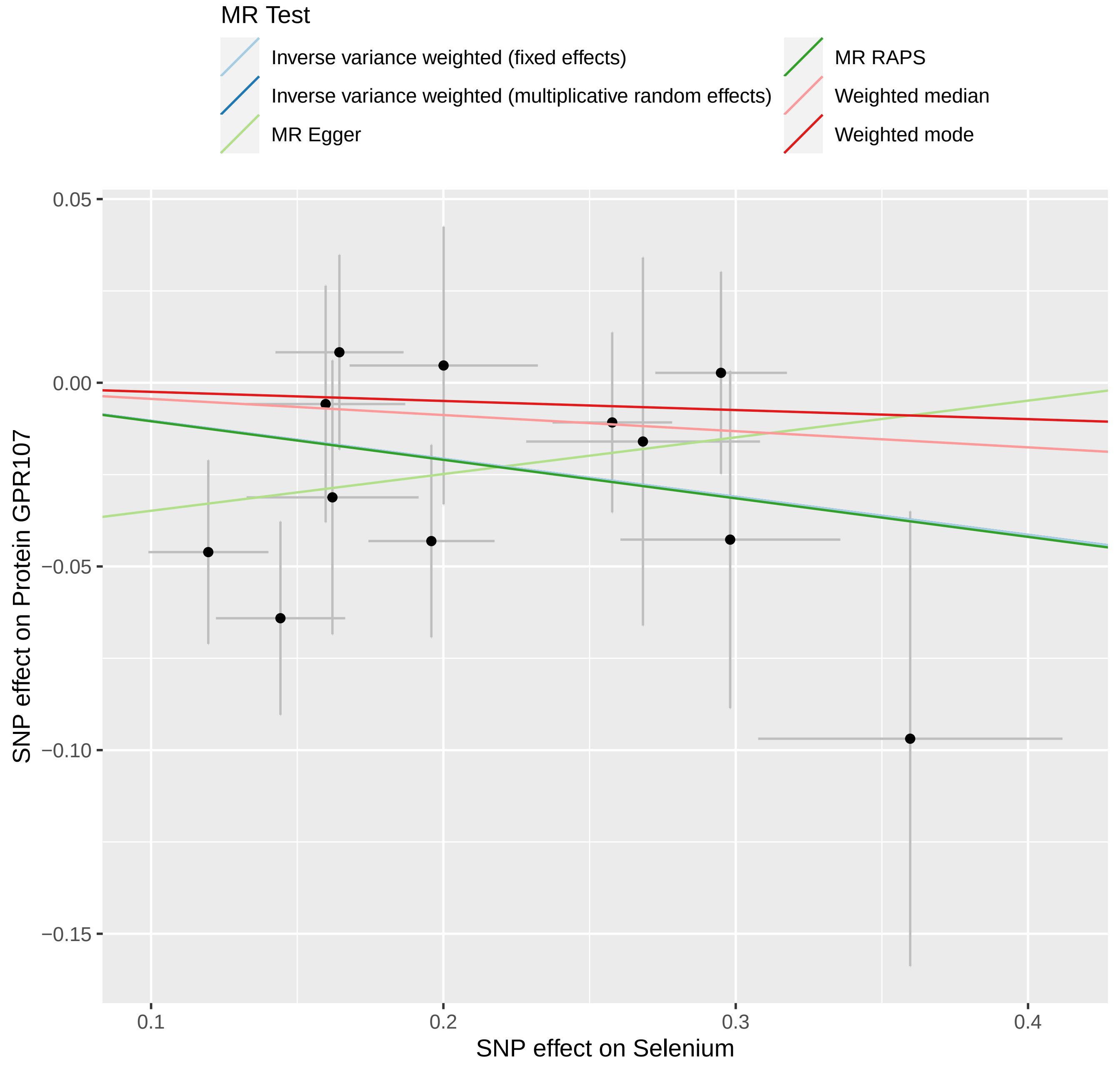

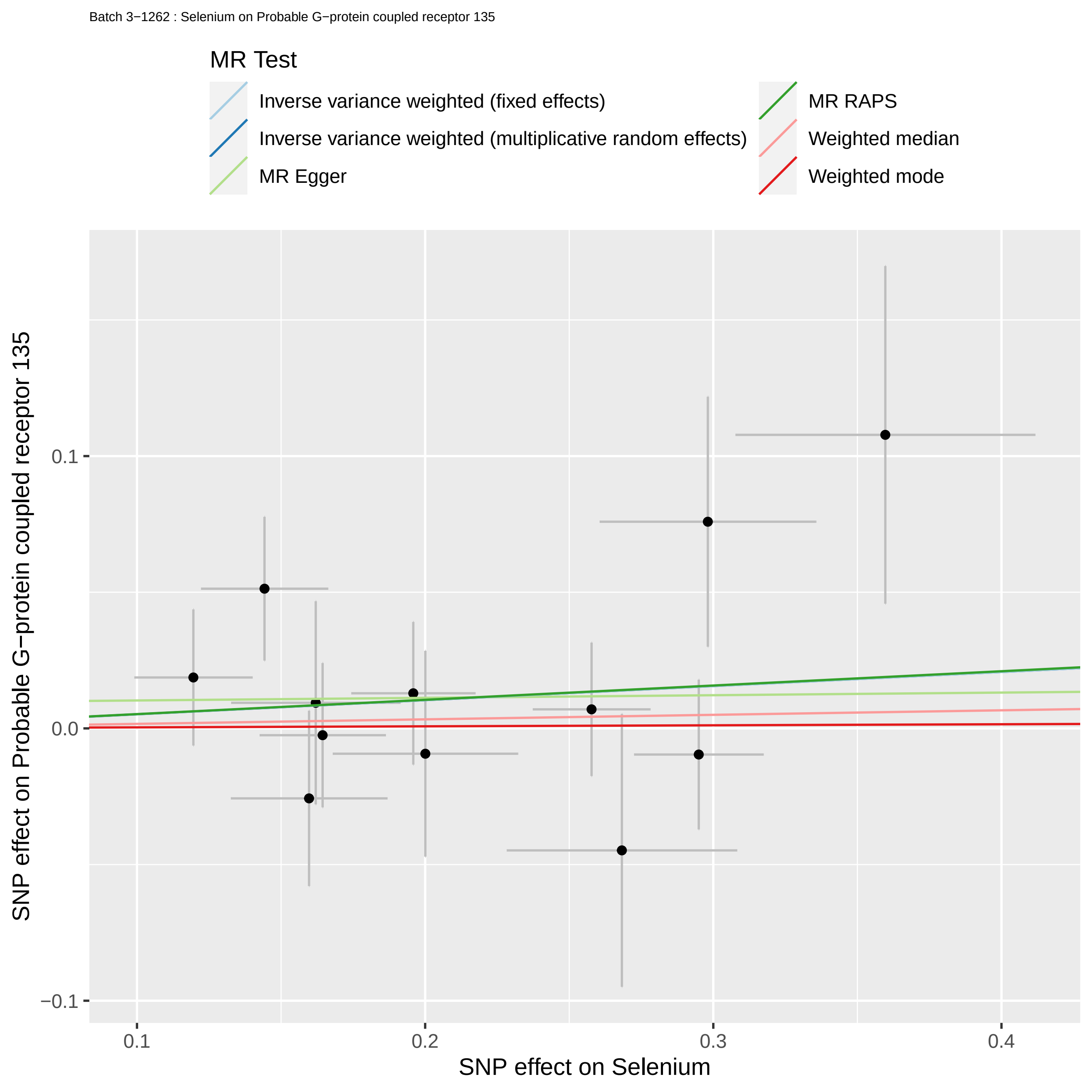

Batch 3−1262 : Selenium on Probable G−protein coupled receptor 135
MR Test
Inverse variance weighted (fixed effects)
Inverse variance weighted (multiplicative random effects)
MR Egger
MR RAPS
Weighted median
Weighted mode
SNP effect on Probable G−protein coupled receptor 135
0.1
0.0
−0.1
0.1
0.2
0.3
0.4
SNP effect on Selenium

Batch 3−1263 : Selenium on G−protein coupled receptor 26

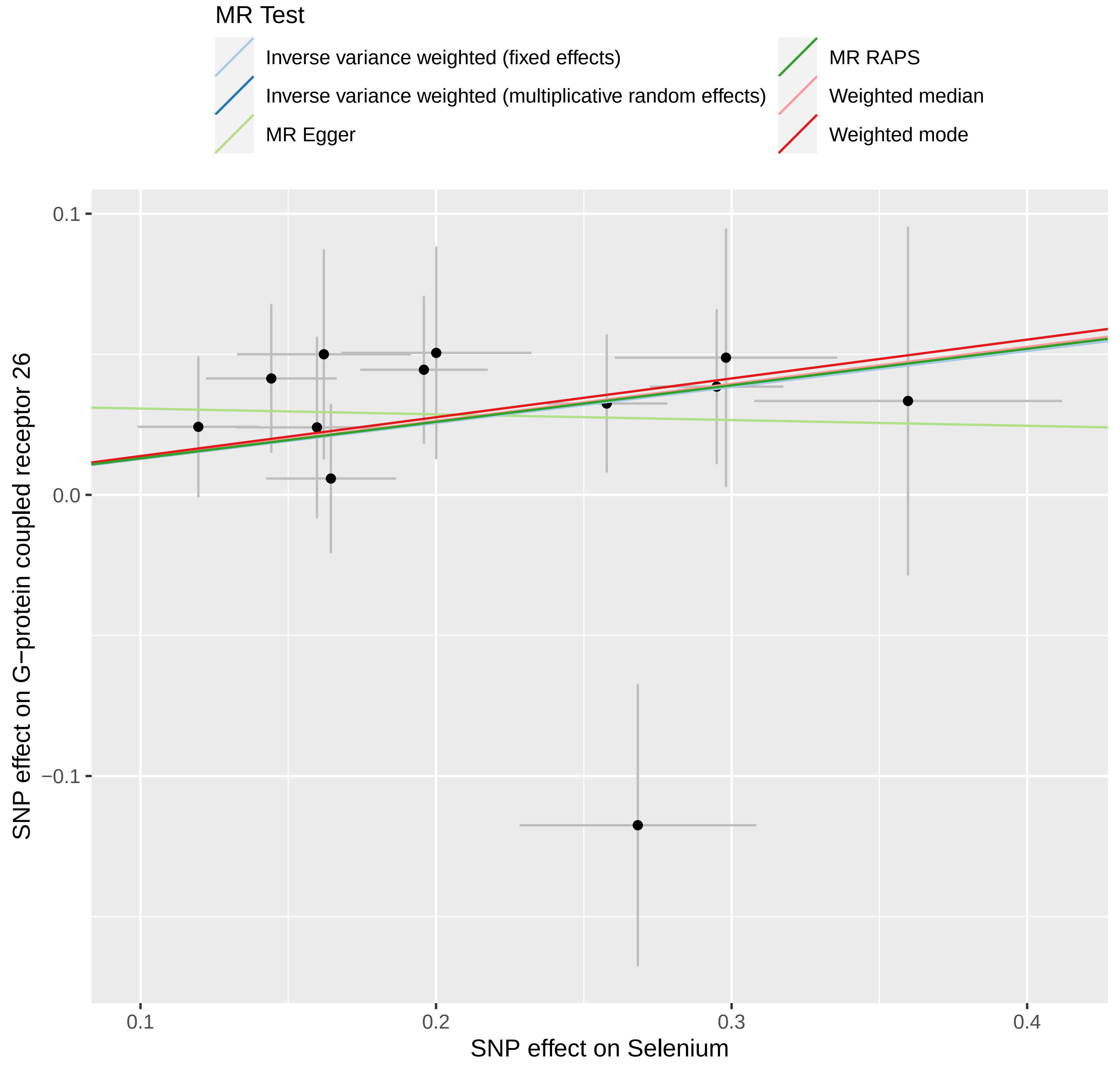

Batch 3−1264 : Selenium on Alanine aminotransferase 1

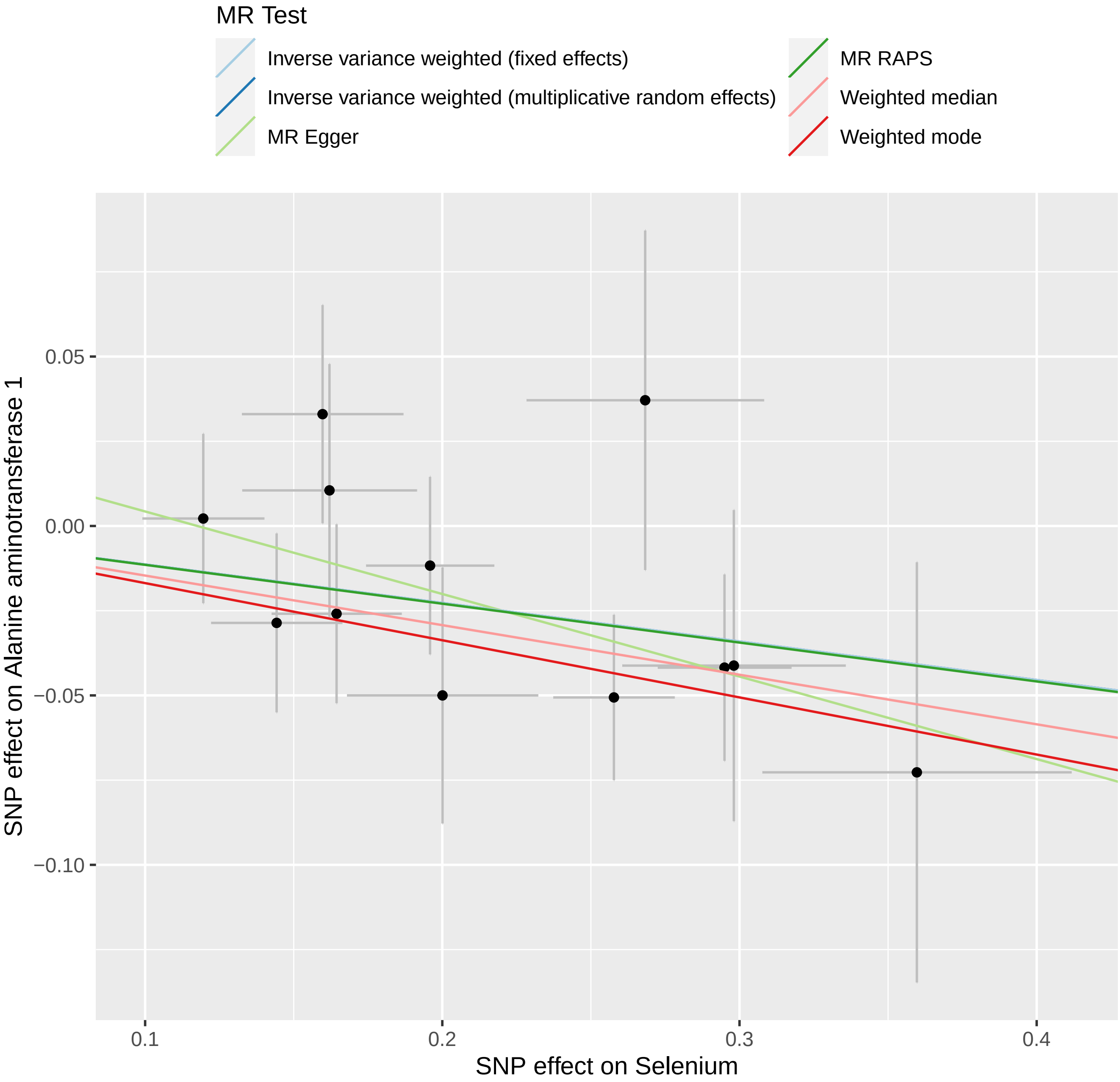

Batch 3−1265 : Selenium on Glutathione peroxidase 7

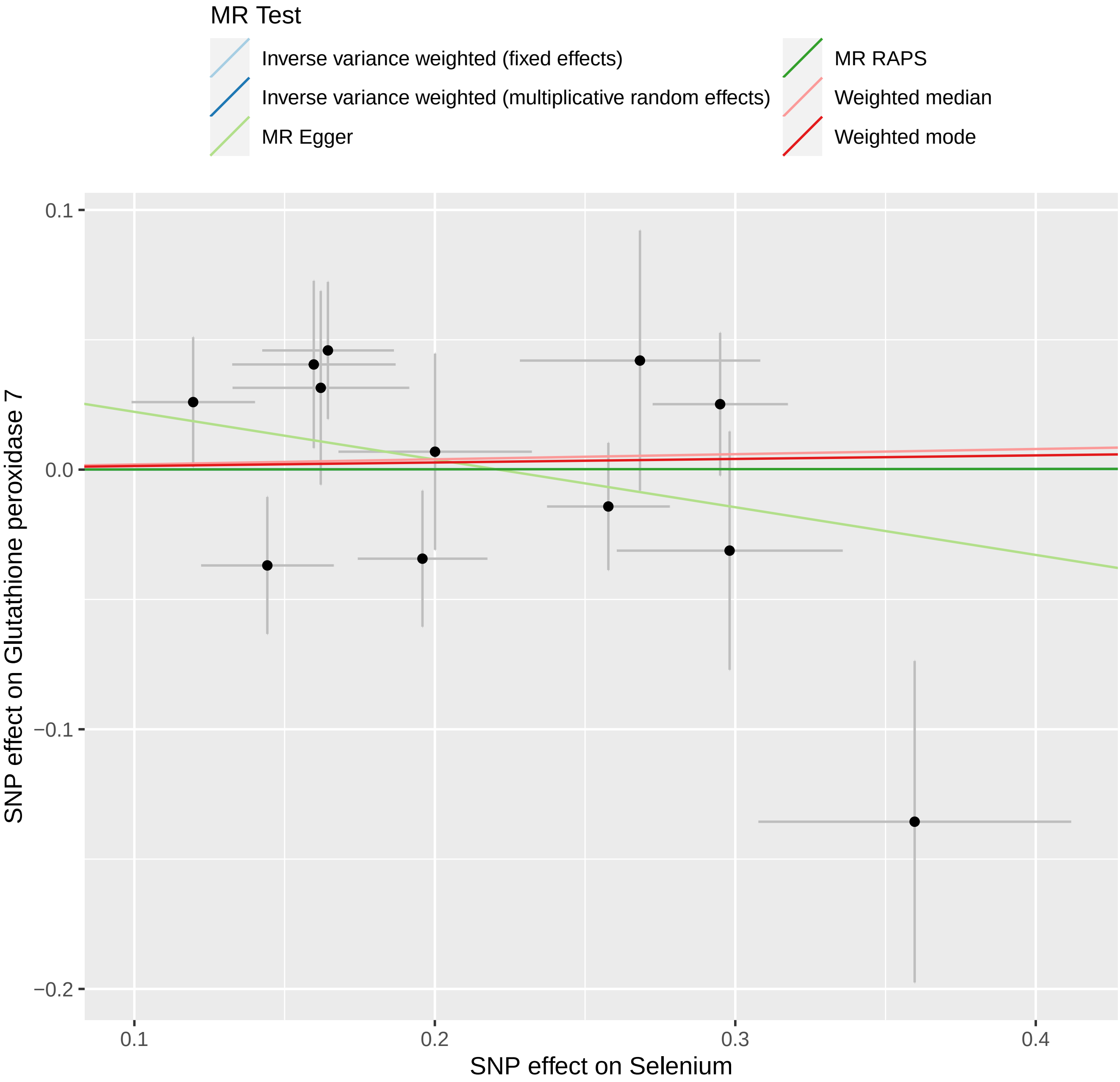

Batch 3−1266 : Selenium on GRAM domain−containing protein 1C

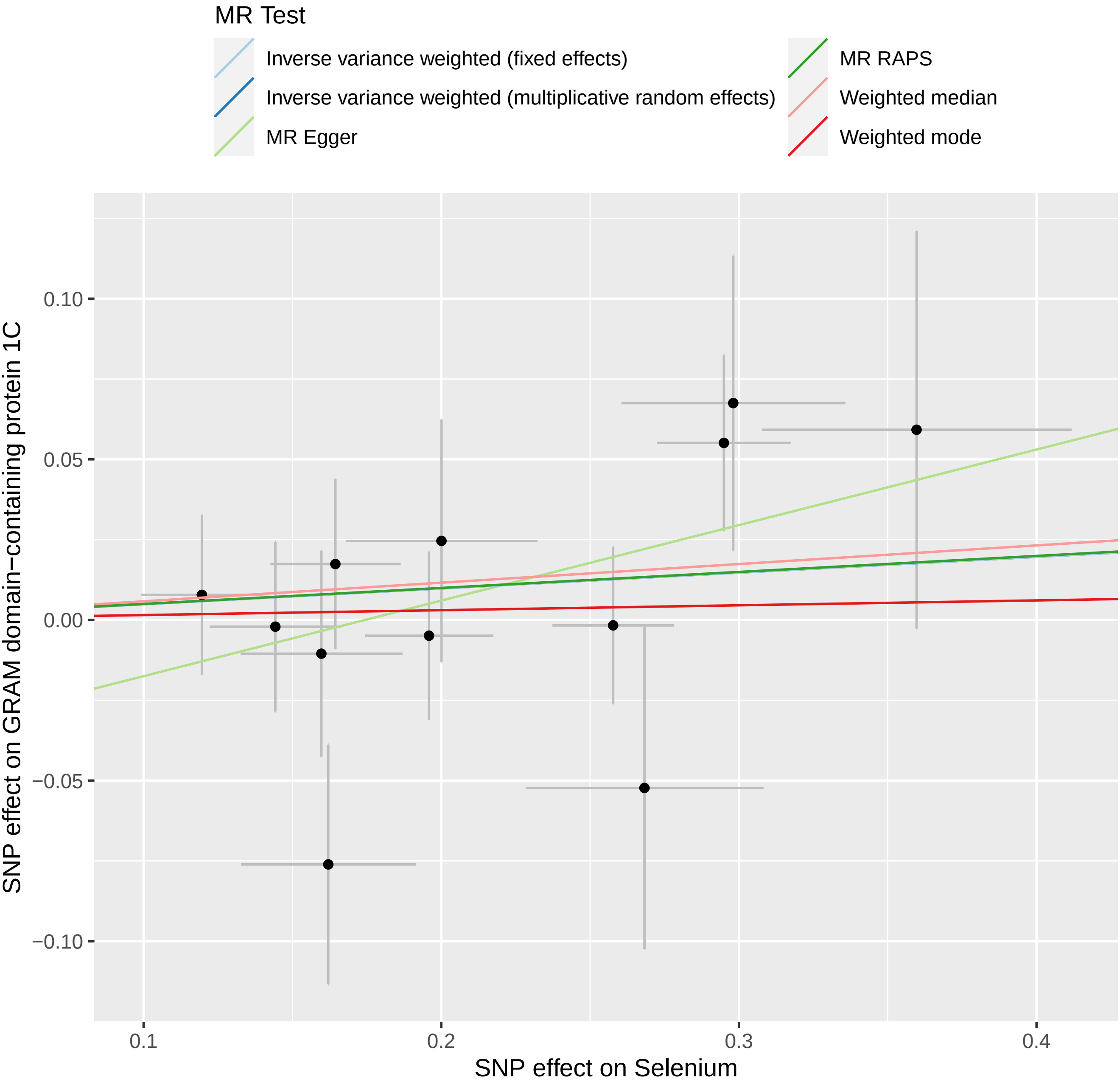

Batch 3−1267 : Selenium on GRAM domain−containing protein 1C

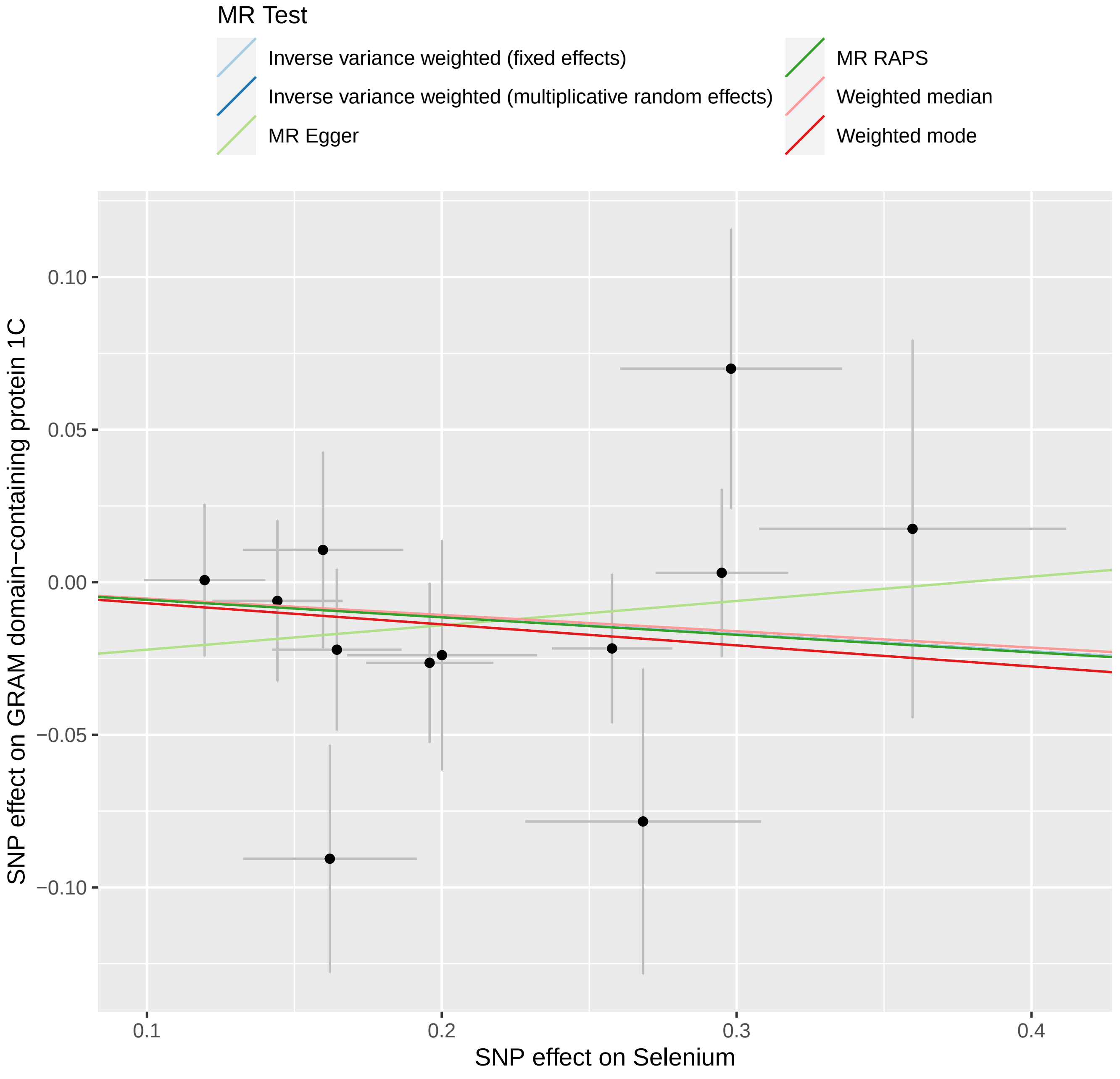

Batch 3−1268 : Selenium on GRB2−related adapter protein

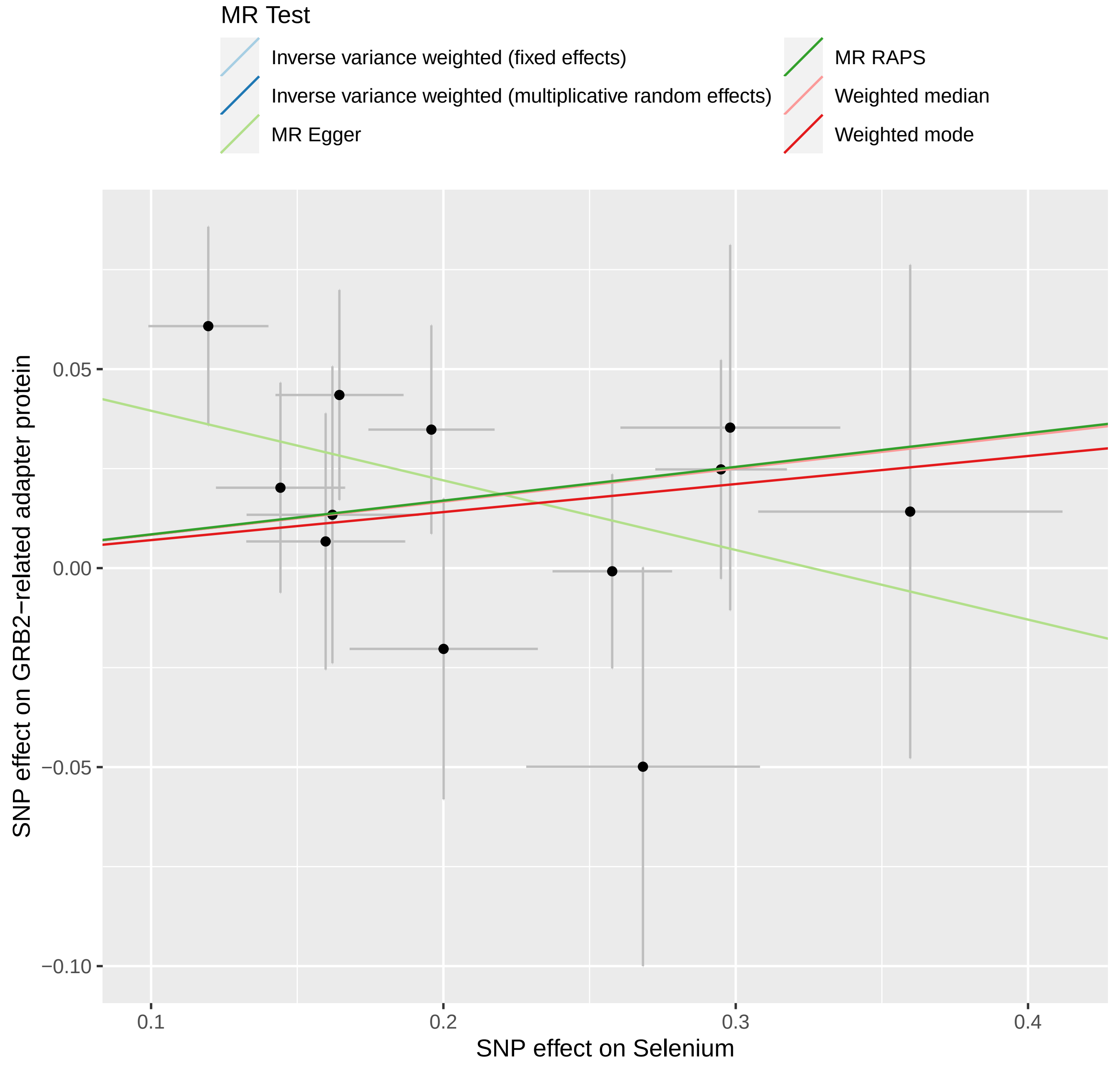

Batch 3−1269 : Selenium on GRB2−related adapter protein 2

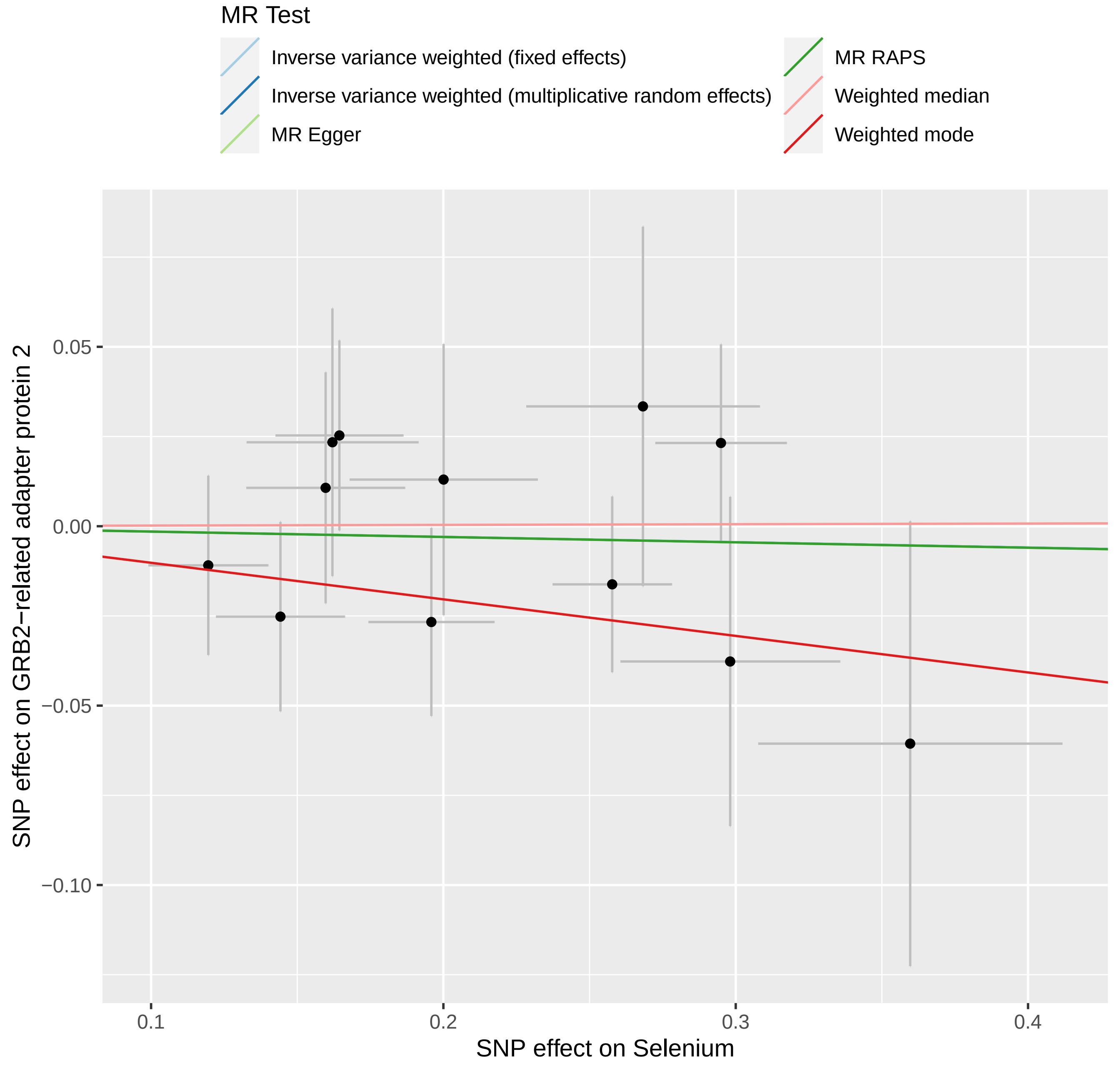

Batch 3−1270 : Selenium on Growth factor receptor−bound protein 14

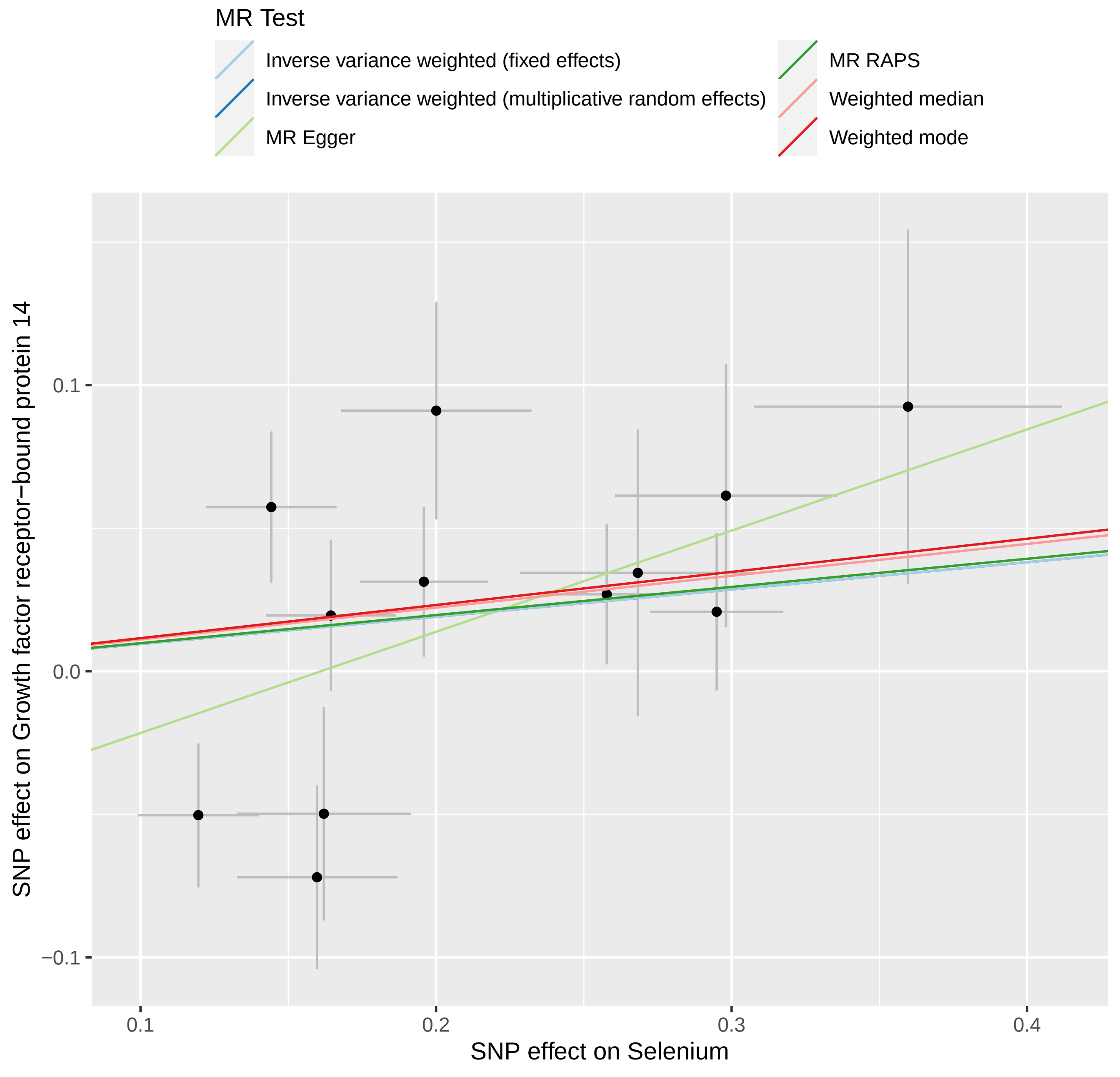

Batch 3−1271 : Selenium on Growth factor receptor−bound protein 2

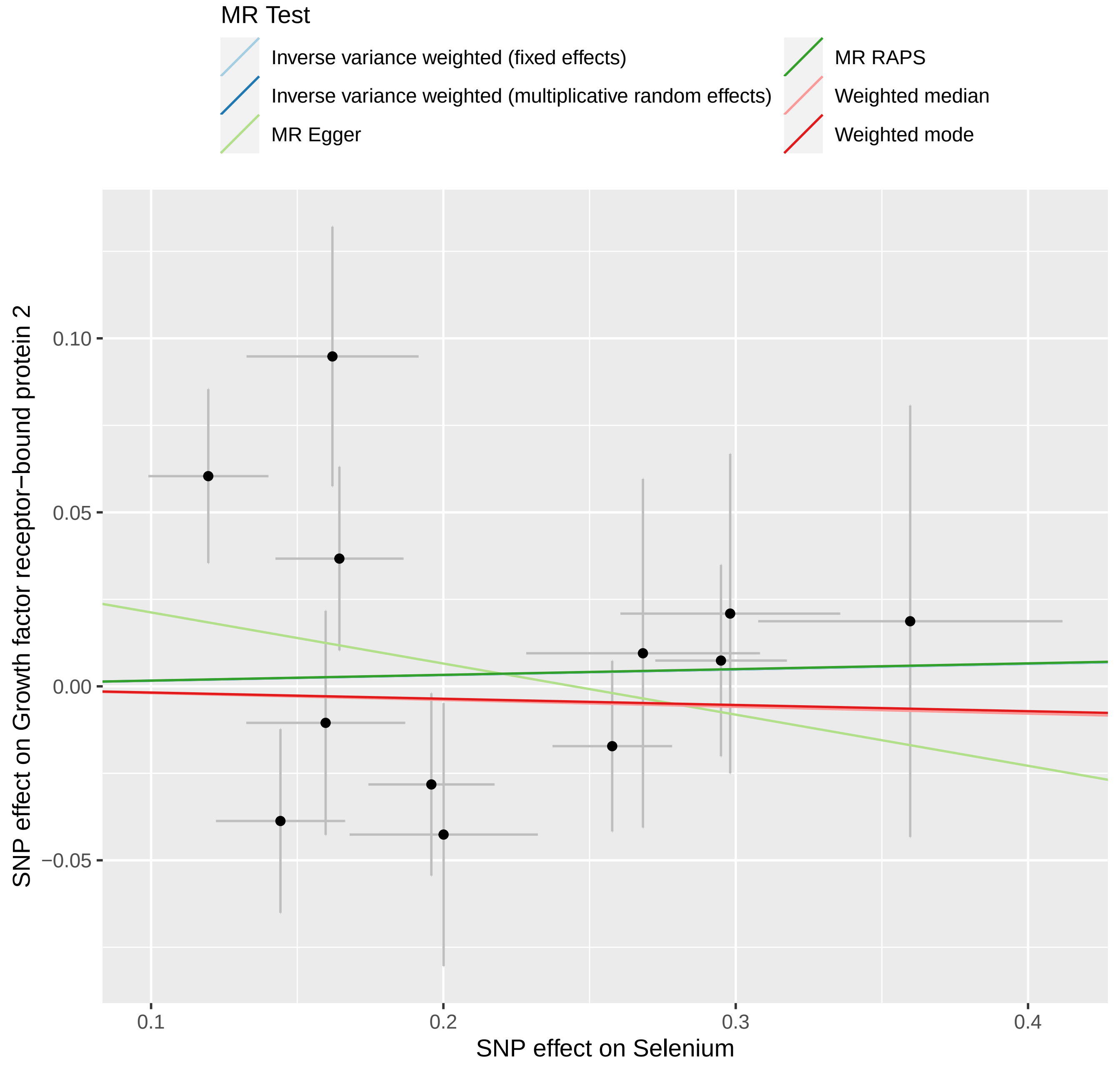

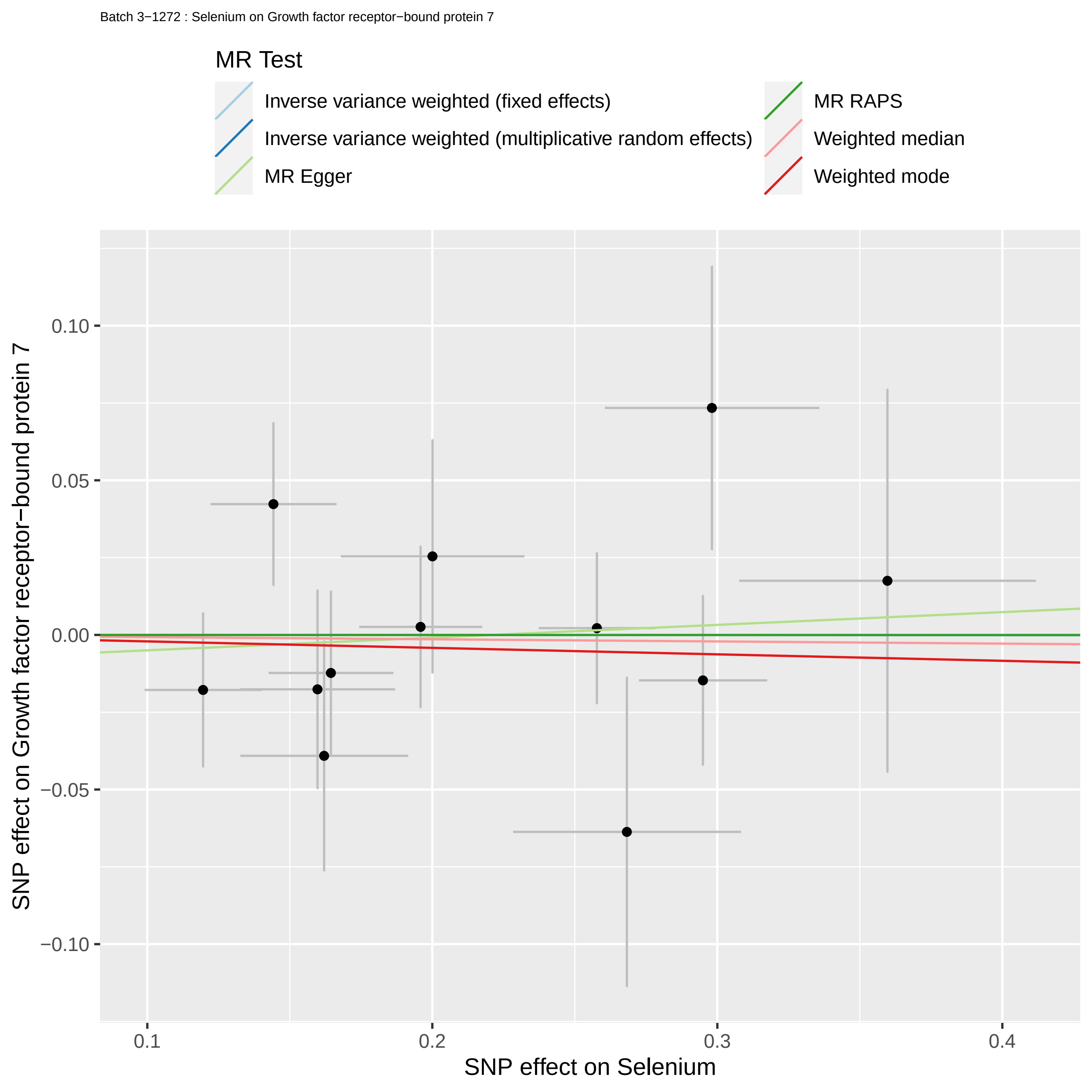

Batch 3−1272 : Selenium on Growth factor receptor−bound protein 7
MR Test
Inverse variance weighted (fixed effects)
Inverse variance weighted (multiplicative random effects)
MR Egger
MR RAPS
Weighted median
Weighted mode
0.10
0.05
0.00
−0.05
−0.10
0.1
0.2
0.3
0.4
SNP effect on Growth factor receptor−bound protein 7
SNP effect on Selenium

Batch 3−1273 : Selenium on Gremlin−1

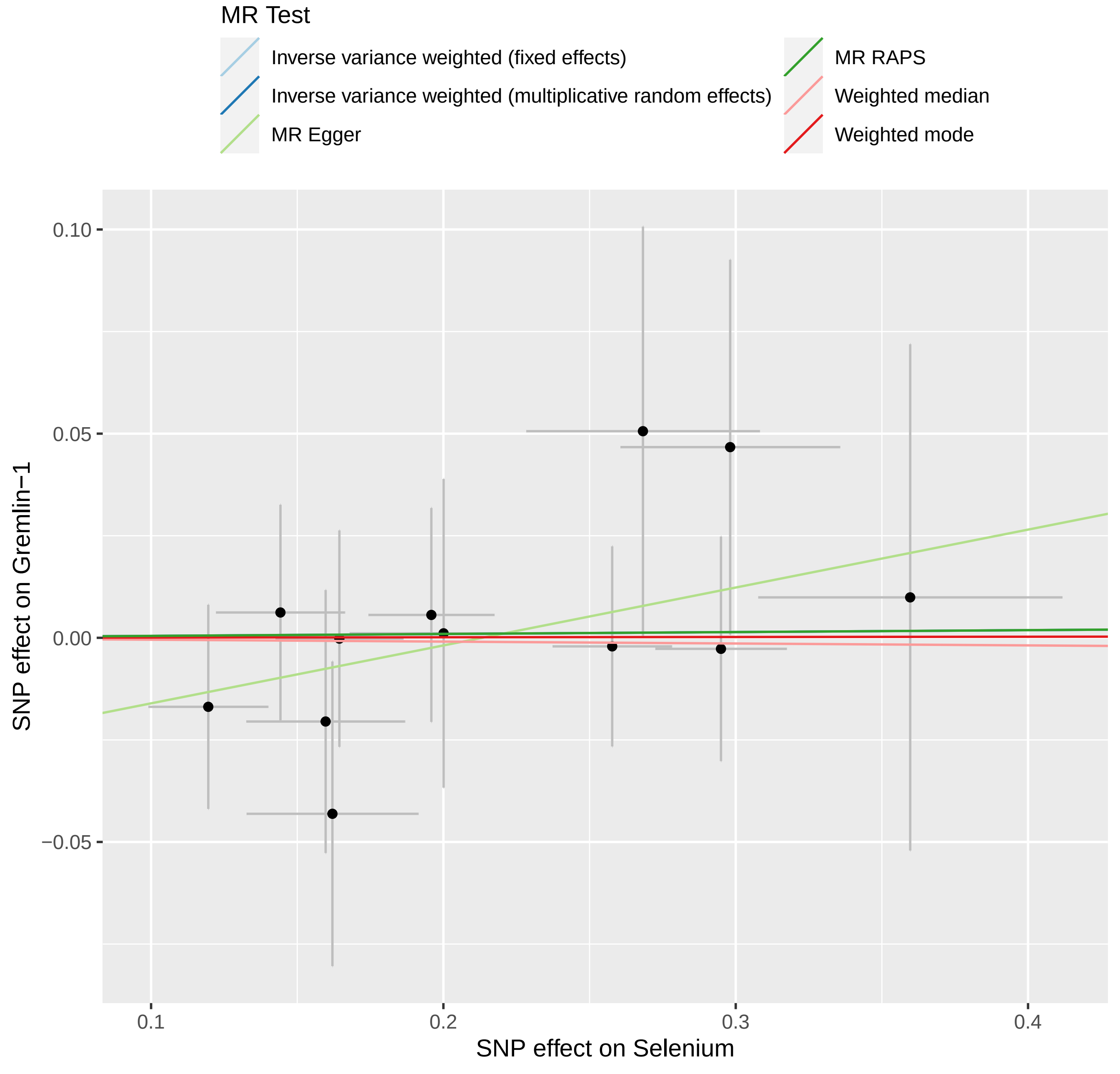

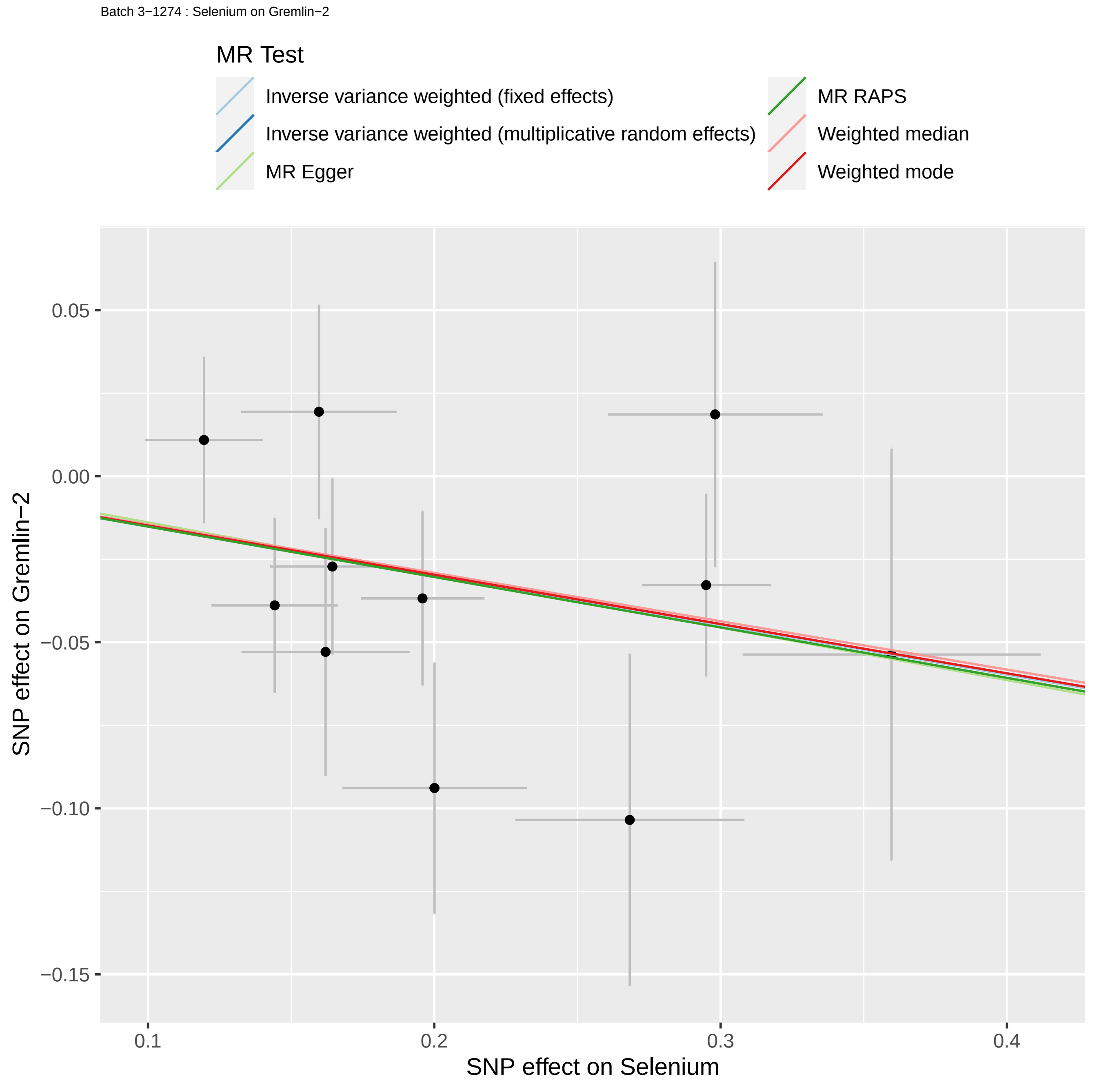
Batch 3−1274 : Selenium on Gremlin−2
MR Test
Inverse variance weighted (fixed effects)
Inverse variance weighted (multiplicative random effects)
MR Egger
MR RAPS
Weighted median
Weighted mode
0.05
0.00
−0.05
−0.10
−0.15
0.1
0.2
0.3
0.4
SNP effect on Gremlin−2
SNP effect on Selenium

Batch 3−1275 : Selenium on Glutamate receptor 4

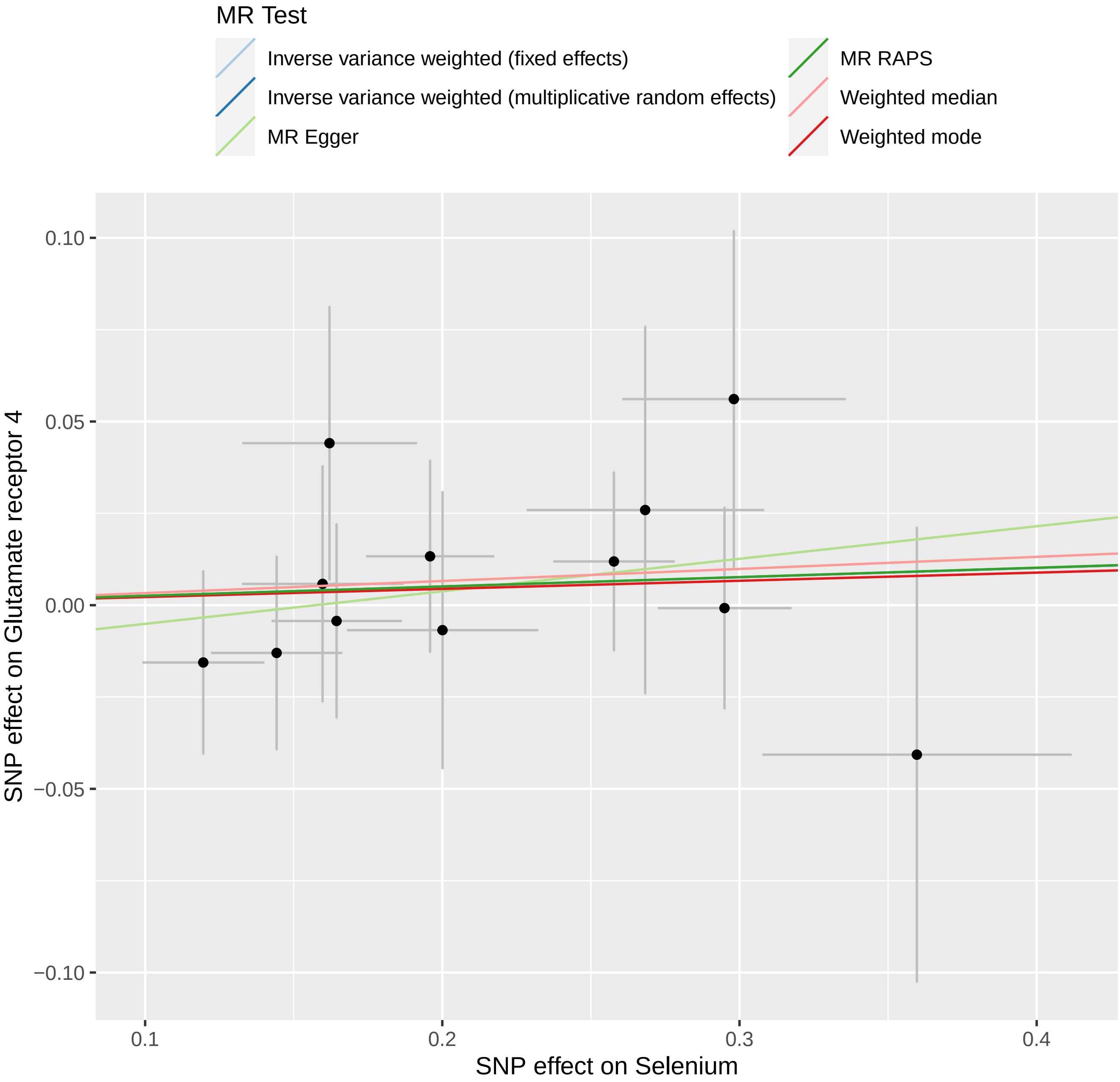

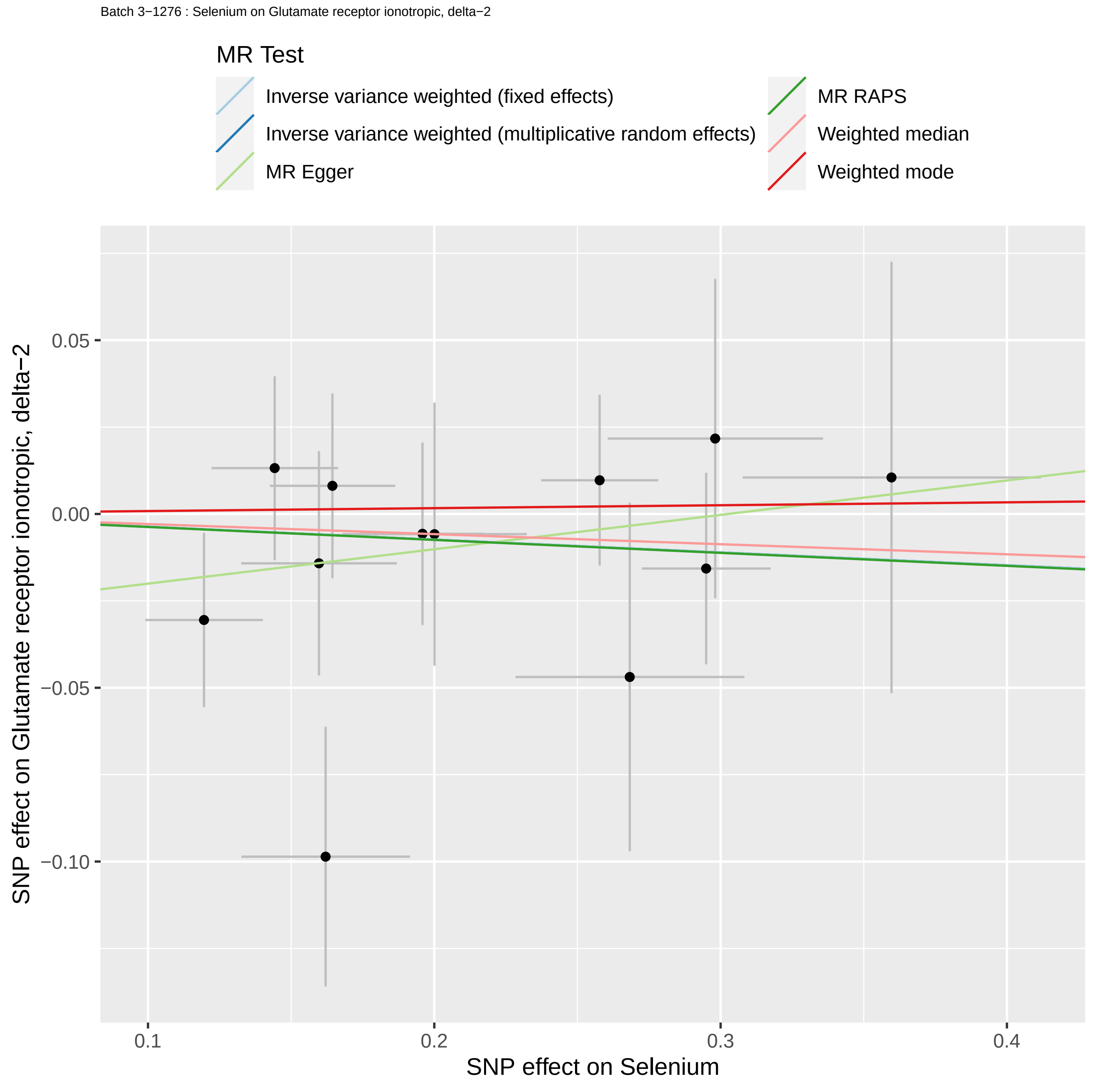

Batch 3−1276 : Selenium on Glutamate receptor ionotropic, delta−2
MR Test
Inverse variance weighted (fixed effects)
Inverse variance weighted (multiplicative random effects)
MR Egger
MR RAPS
Weighted median
Weighted mode
0.05
0.00
−0.05
−0.10
0.1
0.2
0.3
0.4
SNP effect on Glutamate receptor ionotropic, delta−2
SNP effect on Selenium

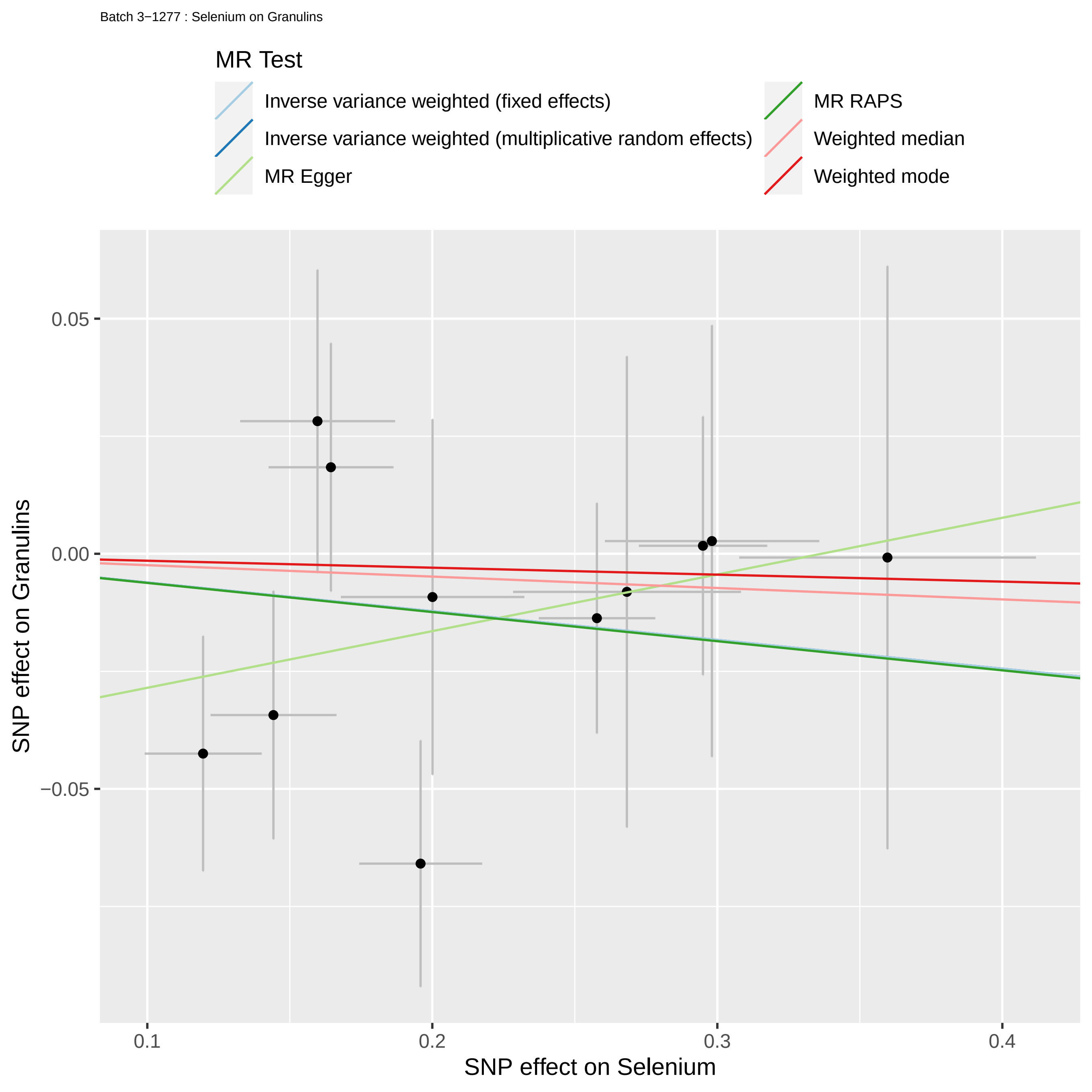
Batch 3−1277 : Selenium on Granulins
MR Test
Inverse variance weighted (fixed effects)
Inverse variance weighted (multiplicative random effects)
MR Egger
MR RAPS
Weighted median
Weighted mode
0.05
0.00
−0.05
0.1
0.2
0.3
0.4
SNP effect on Granulins
SNP effect on Selenium

Batch 3−1278 : Selenium on Gastrin−releasing peptide

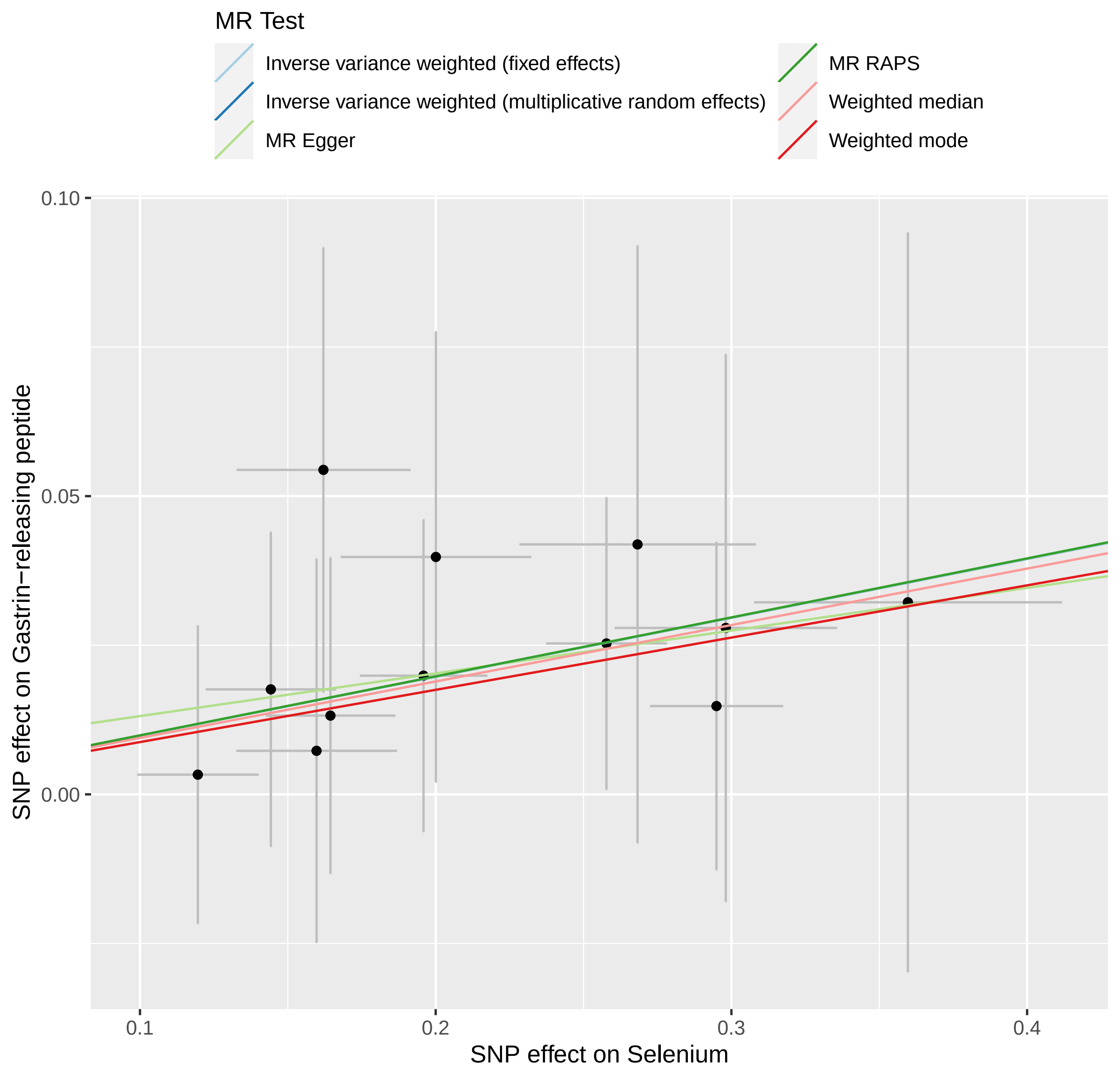

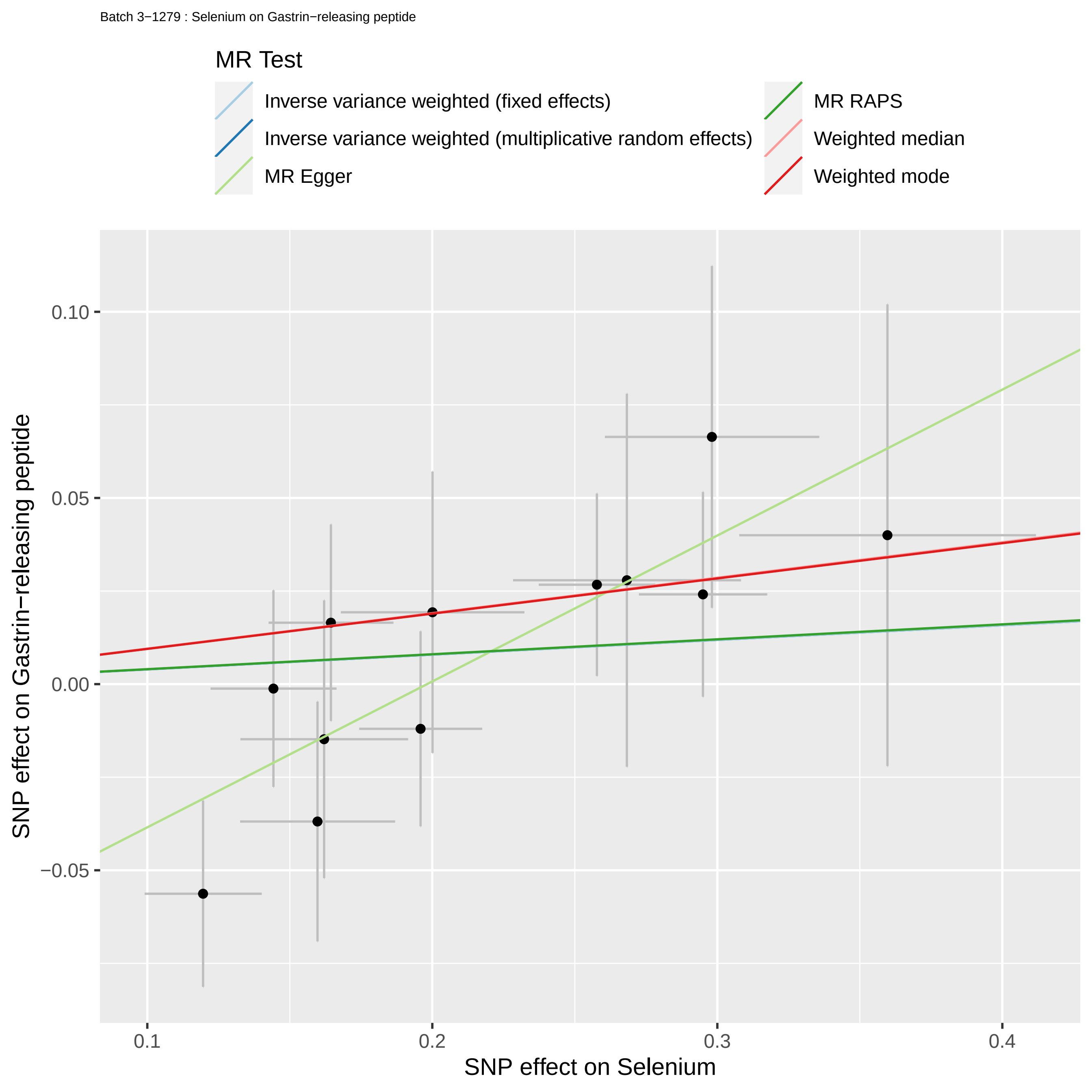

Batch 3−1279 : Selenium on Gastrin−releasing peptide
MR Test
Inverse variance weighted (fixed effects)
Inverse variance weighted (multiplicative random effects)
MR Egger
MR RAPS
Weighted median
Weighted mode
0.10
0.05
0.00
−0.05
0.1
0.2
0.3
0.4
SNP effect on Gastrin−releasing peptide
SNP effect on Selenium

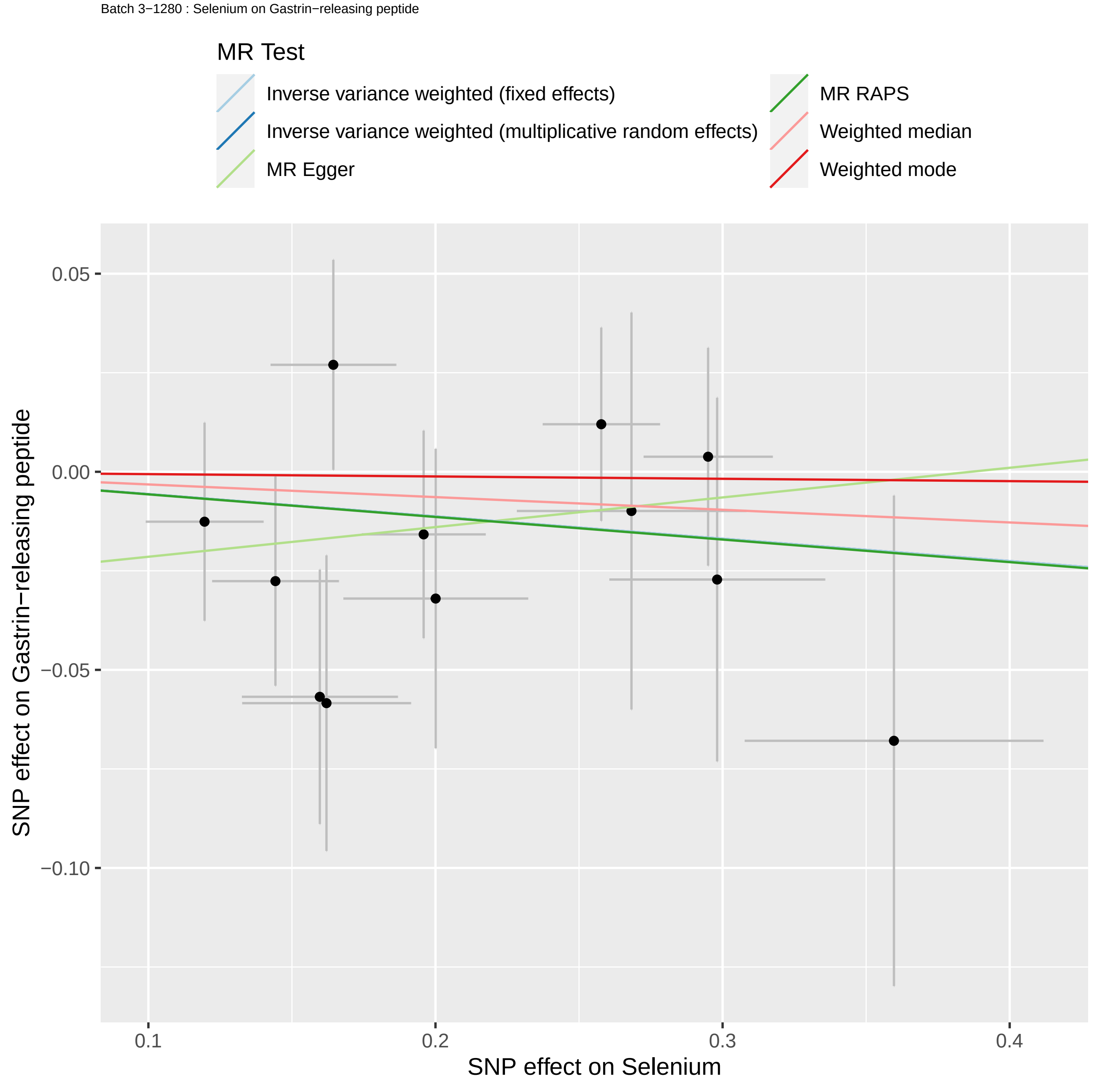

Batch 3−1280 : Selenium on Gastrin−releasing peptide
MR Test
Inverse variance weighted (fixed effects)
Inverse variance weighted (multiplicative random effects)
MR Egger
MR RAPS
Weighted median
Weighted mode
0.05
0.00
−0.05
−0.10
0.1
0.2
0.3
0.4
SNP effect on Gastrin−releasing peptide
SNP effect on Selenium

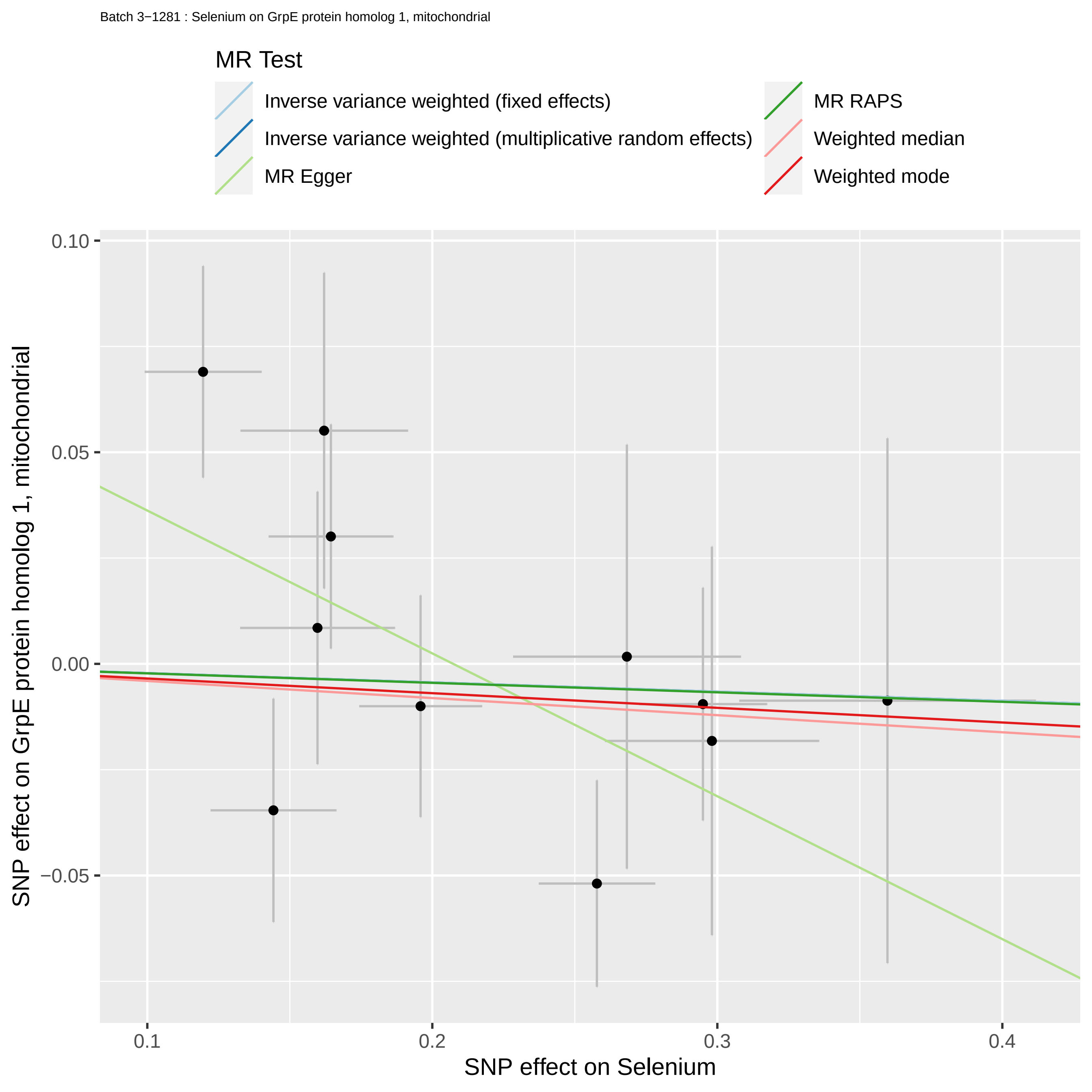

Batch 3−1281 : Selenium on GrpE protein homolog 1, mitochondrial
MR Test
Inverse variance weighted (fixed effects)
Inverse variance weighted (multiplicative random effects)
MR Egger
MR RAPS
Weighted median
Weighted mode
0.10
0.05
0.00
−0.05
0.1
0.2
0.3
0.4
SNP effect on GrpE protein homolog 1, mitochondrial
SNP effect on Selenium

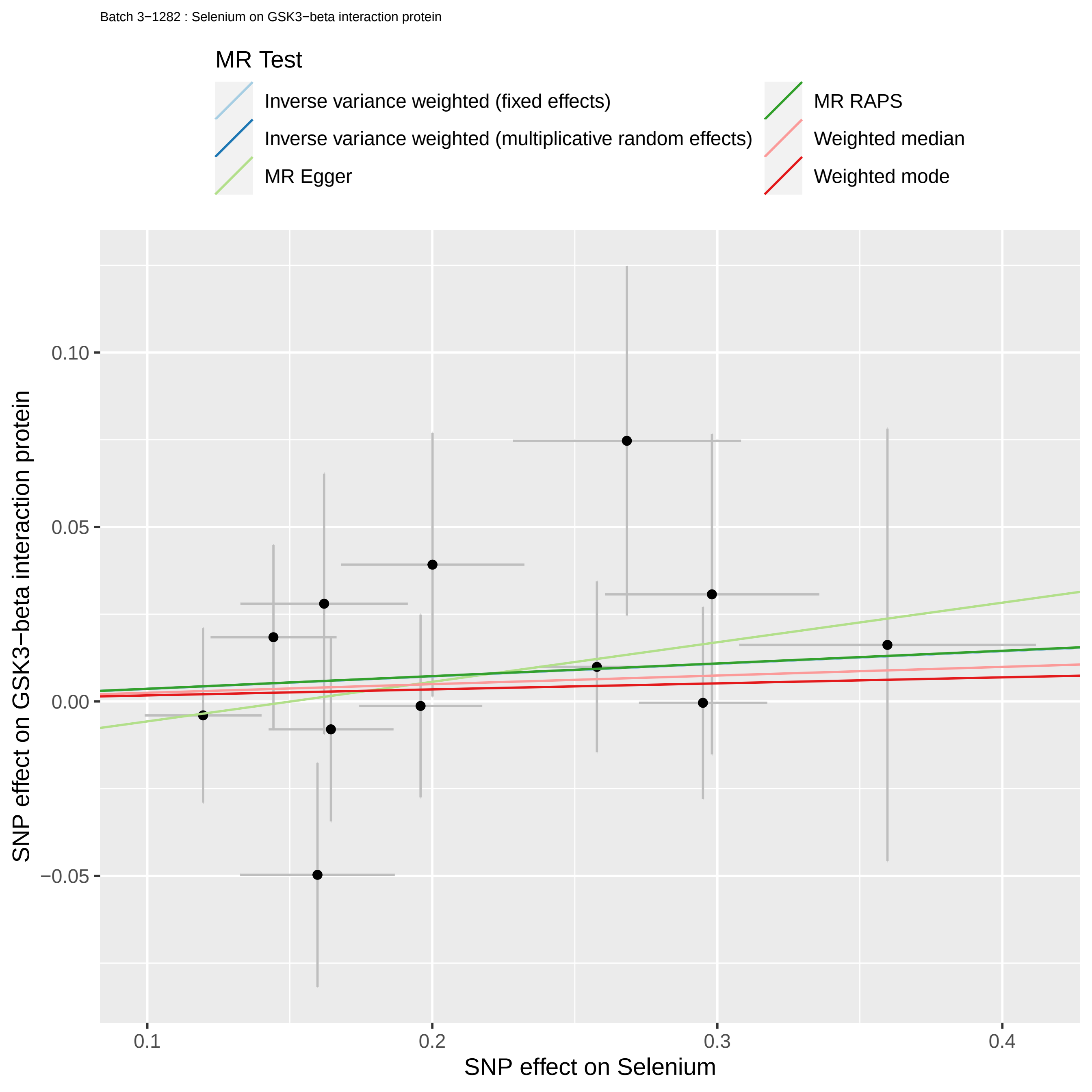
Batch 3−1282 : Selenium on GSK3−beta interaction protein
MR Test
Inverse variance weighted (fixed effects)
Inverse variance weighted (multiplicative random effects)
MR Egger
MR RAPS
Weighted median
Weighted mode
0.10
0.05
0.00
−0.05
0.1
0.2
0.3
0.4
SNP effect on GSK3−beta interaction protein
SNP effect on Selenium

Batch 3−1283 : Selenium on Glutathione S−transferase A1

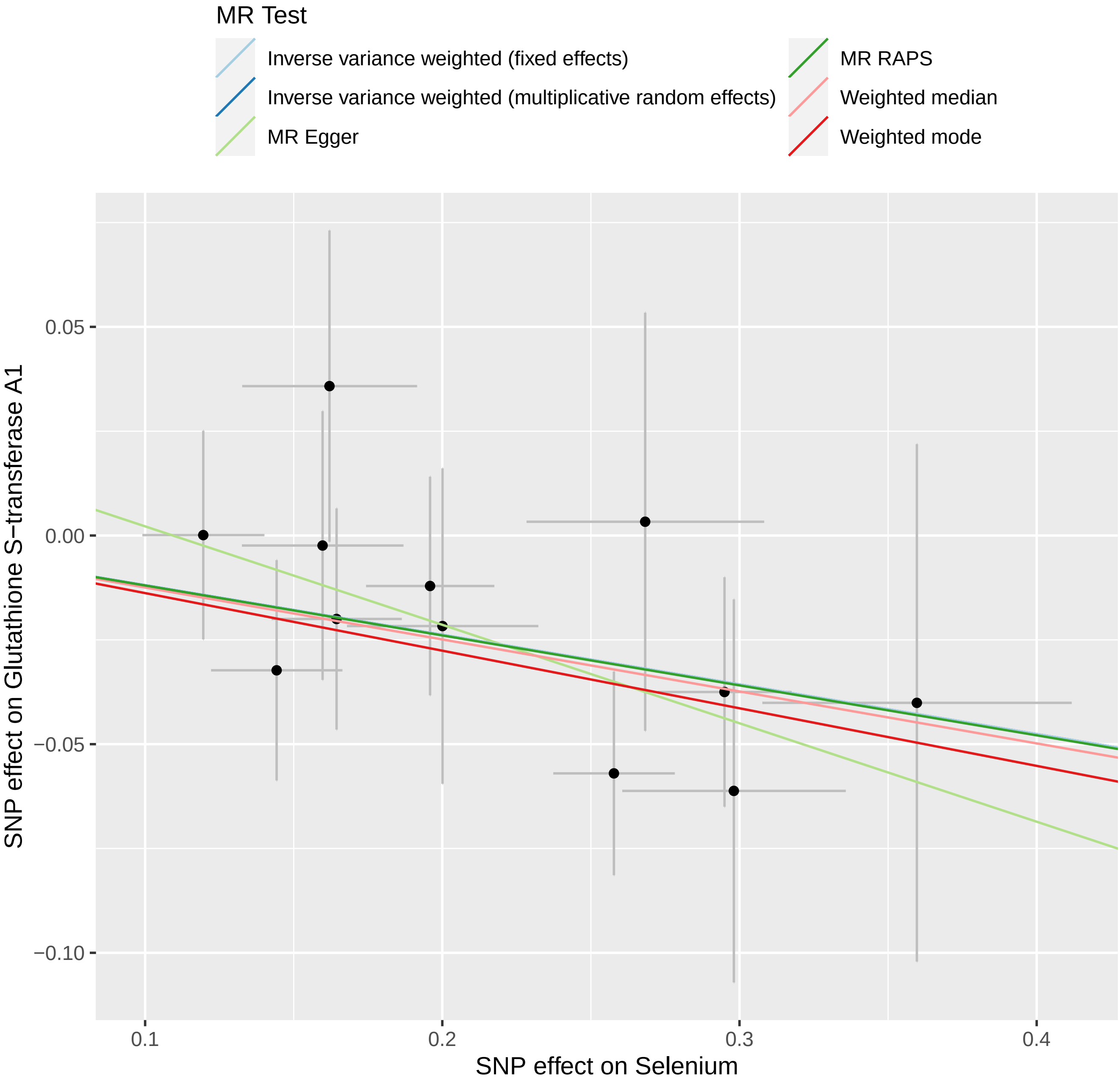

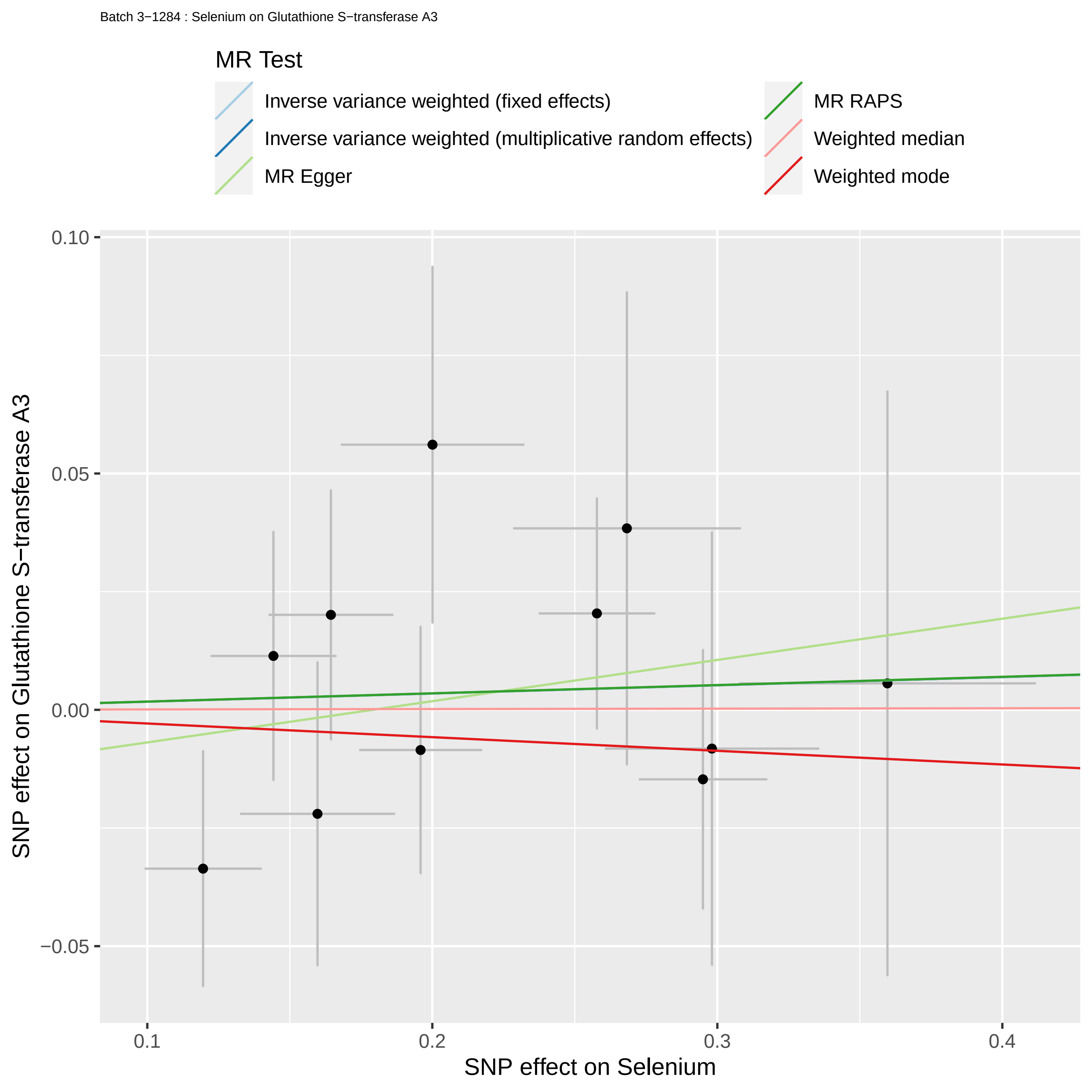
Batch 3−1284 : Selenium on Glutathione S−transferase A3
MR Test
Inverse variance weighted (fixed effects)
Inverse variance weighted (multiplicative random effects)
MR Egger
MR RAPS
Weighted median
Weighted mode
0.10
0.05
0.00
−0.05
0.1
0.2
0.3
0.4
SNP effect on Glutathione S−transferase A3
SNP effect on Selenium

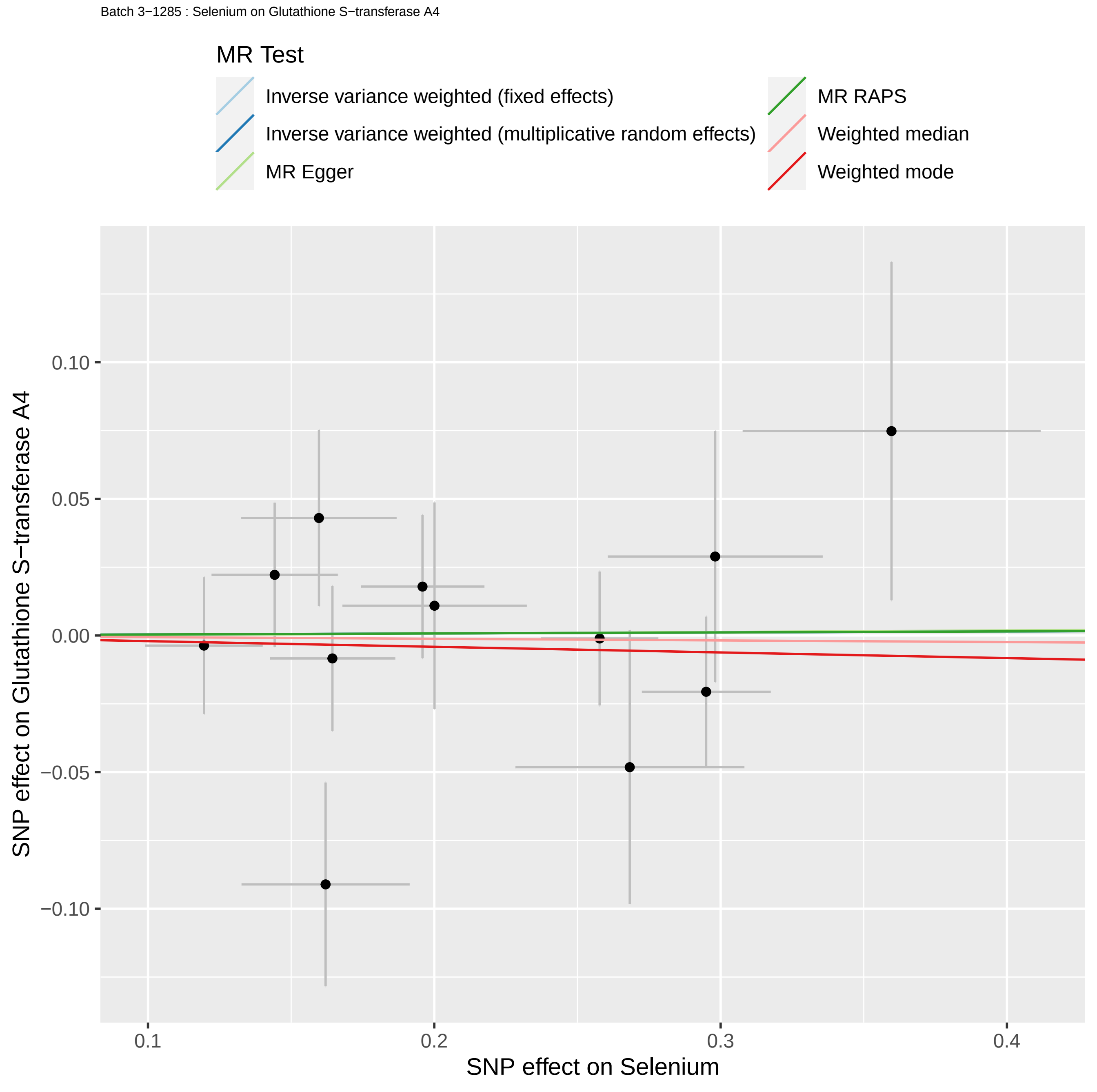
Batch 3−1285 : Selenium on Glutathione S−transferase A4
MR Test
Inverse variance weighted (fixed effects)
Inverse variance weighted (multiplicative random effects)
MR Egger
MR RAPS
Weighted median
Weighted mode
0.10
0.05
0.00
−0.05
−0.10
0.1
0.2
0.3
0.4
SNP effect on Glutathione S−transferase A4
SNP effect on Selenium

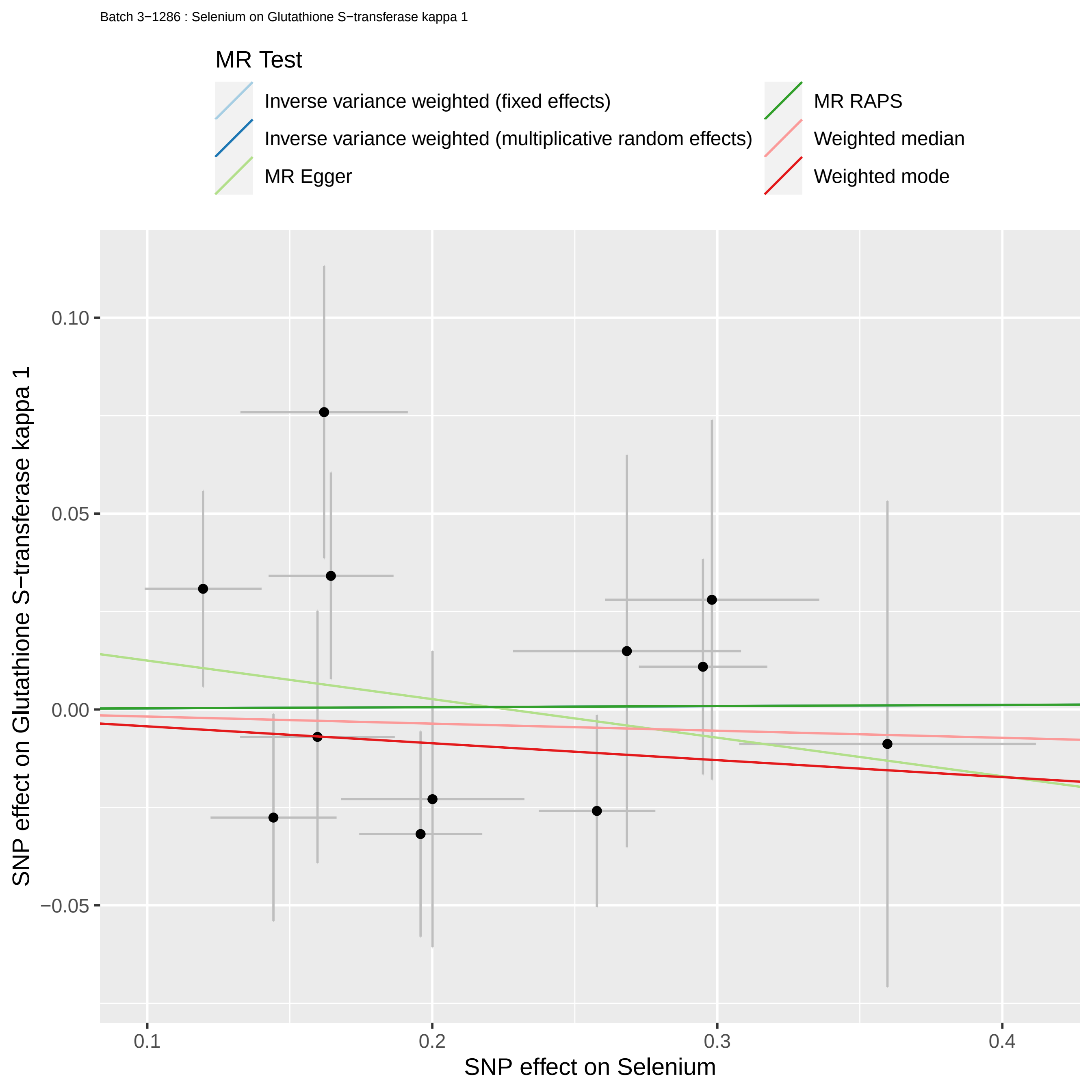

Batch 3−1286 : Selenium on Glutathione S−transferase kappa 1
MR Test
Inverse variance weighted (fixed effects)
Inverse variance weighted (multiplicative random effects)
MR Egger
MR RAPS
Weighted median
Weighted mode
0.10
0.05
0.00
−0.05
0.1
0.2
0.3
0.4
SNP effect on Glutathione S−transferase kappa 1
SNP effect on Selenium

Batch 3−1287 : Selenium on Glutathione S−transferase Mu 1

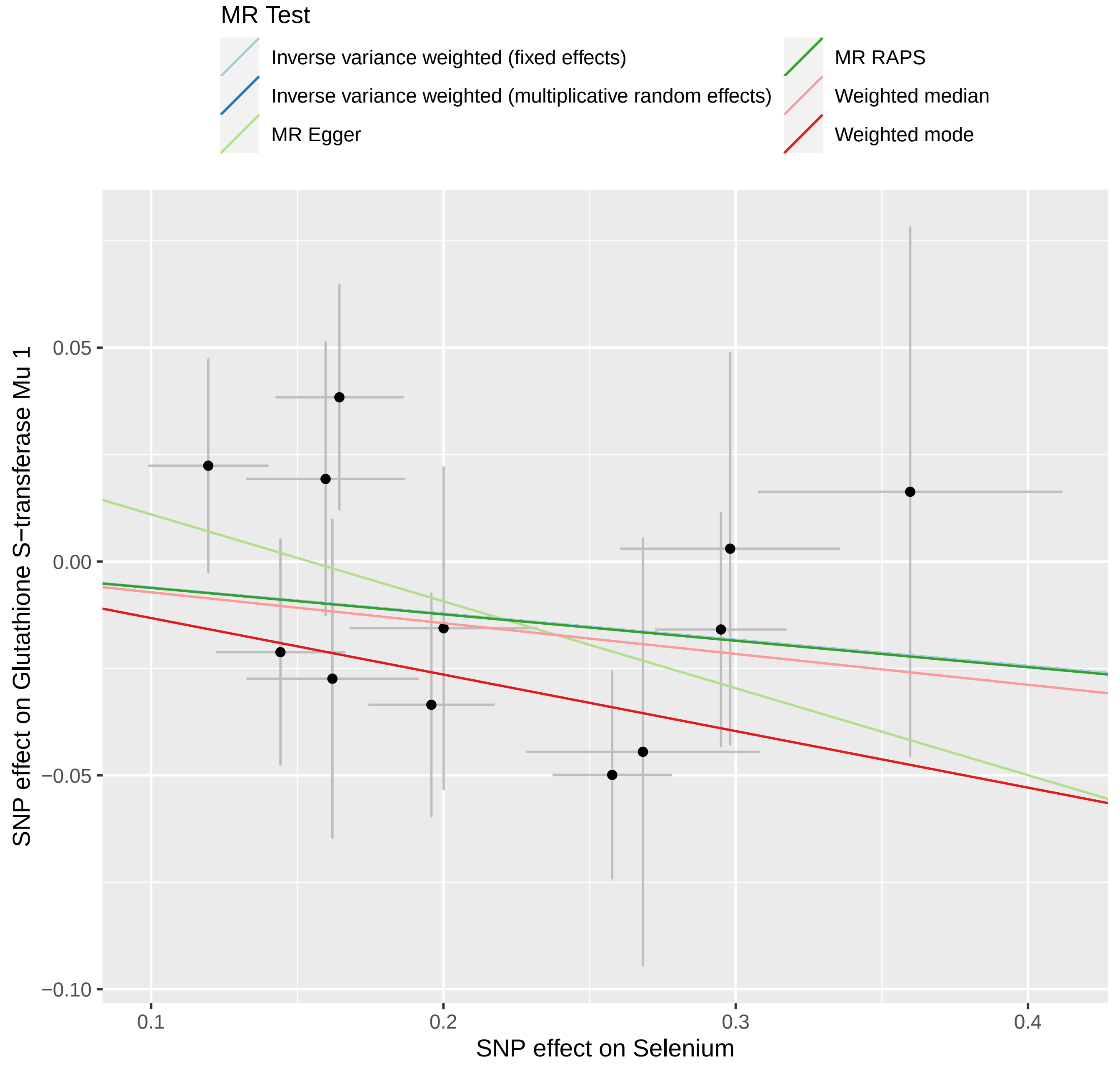

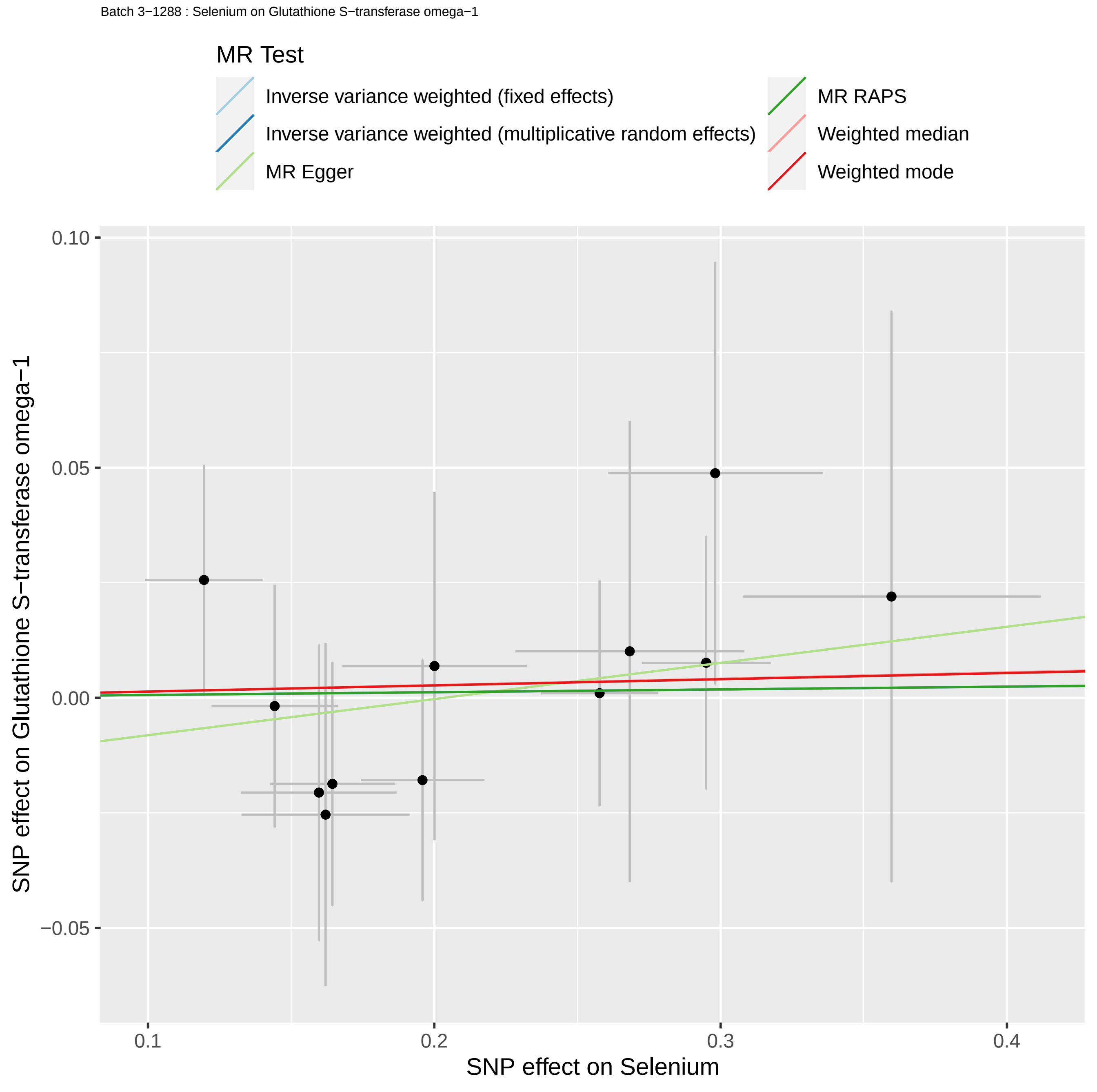

Batch 3−1288 : Selenium on Glutathione S−transferase omega−1
MR Test
Inverse variance weighted (fixed effects)
Inverse variance weighted (multiplicative random effects)
MR Egger
MR RAPS
Weighted median
Weighted mode
0.10
0.05
0.00
−0.05
0.1
0.2
0.3
0.4
SNP effect on Selenium
SNP effect on Glutathione S−transferase omega−1

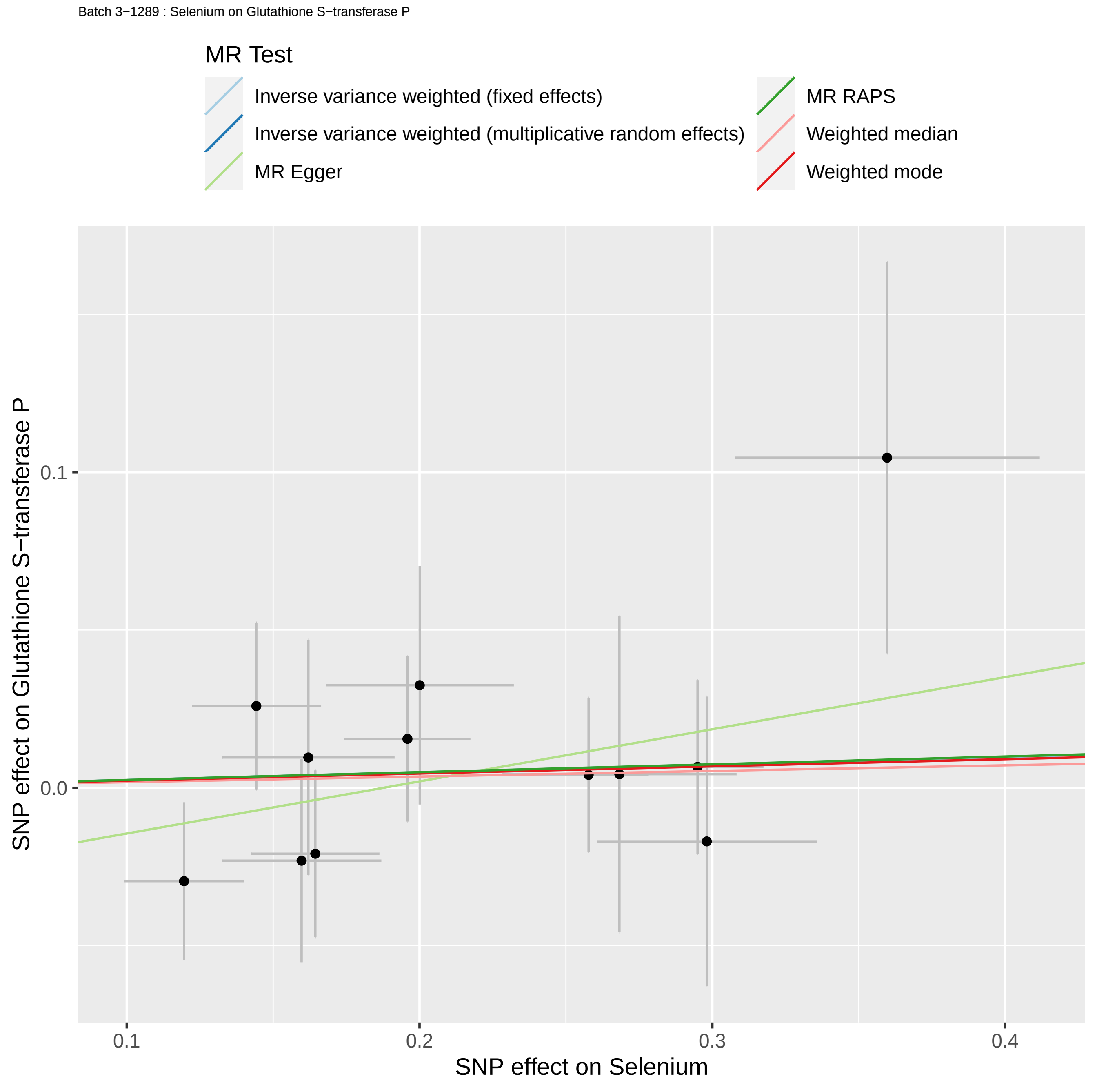

Batch 3−1289 : Selenium on Glutathione S−transferase P
MR Test
Inverse variance weighted (fixed effects)
Inverse variance weighted (multiplicative random effects)
MR Egger
MR RAPS
Weighted median
Weighted mode
SNP effect on Glutathione S−transferase P
0.1
0.0
0.1
0.2
0.3
0.4
SNP effect on Selenium

Batch 3−1290 : Selenium on Glutathione S−transferase P

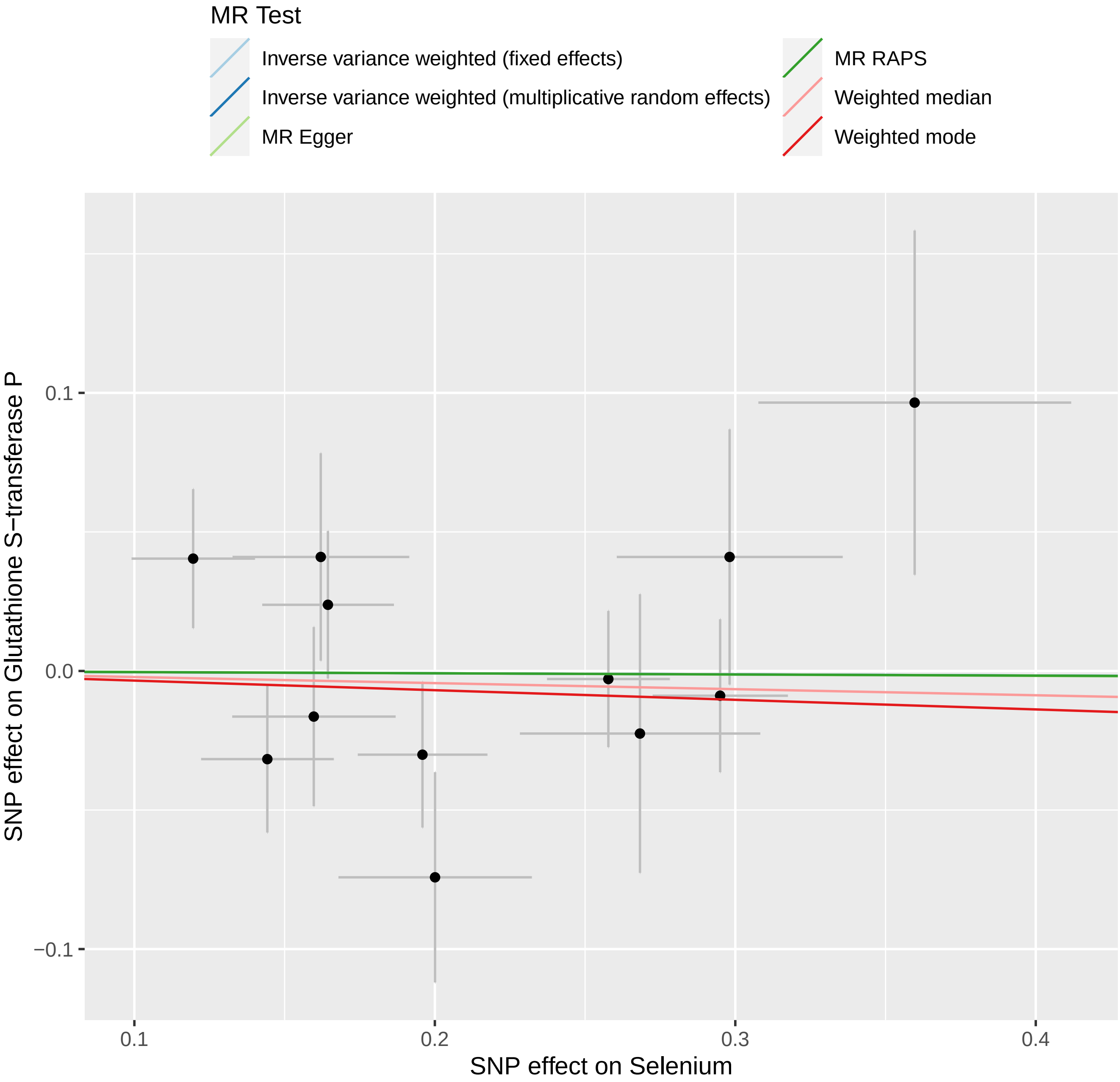

Batch 3−1291 : Selenium on Glutathione S−transferase theta−2B

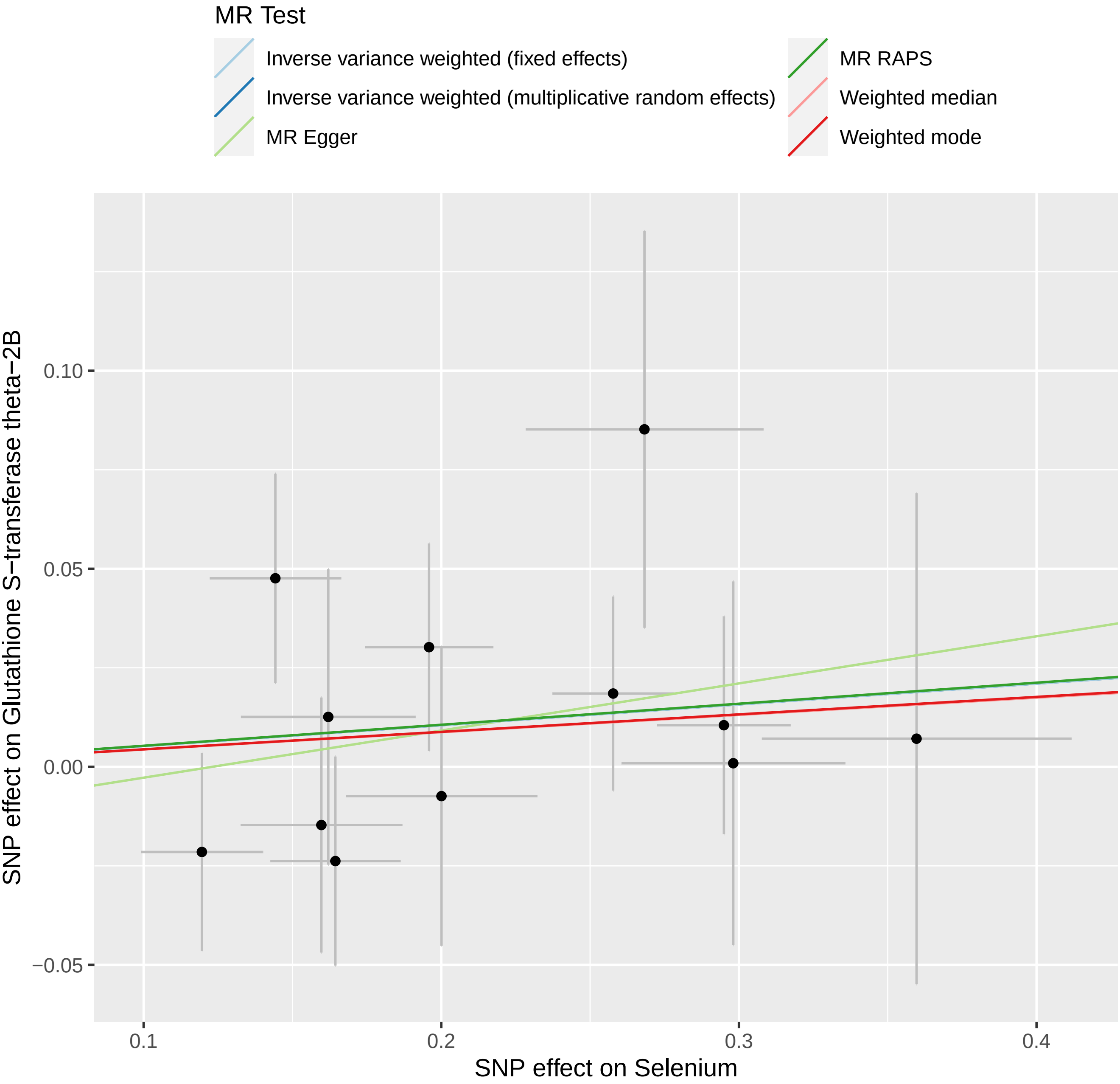

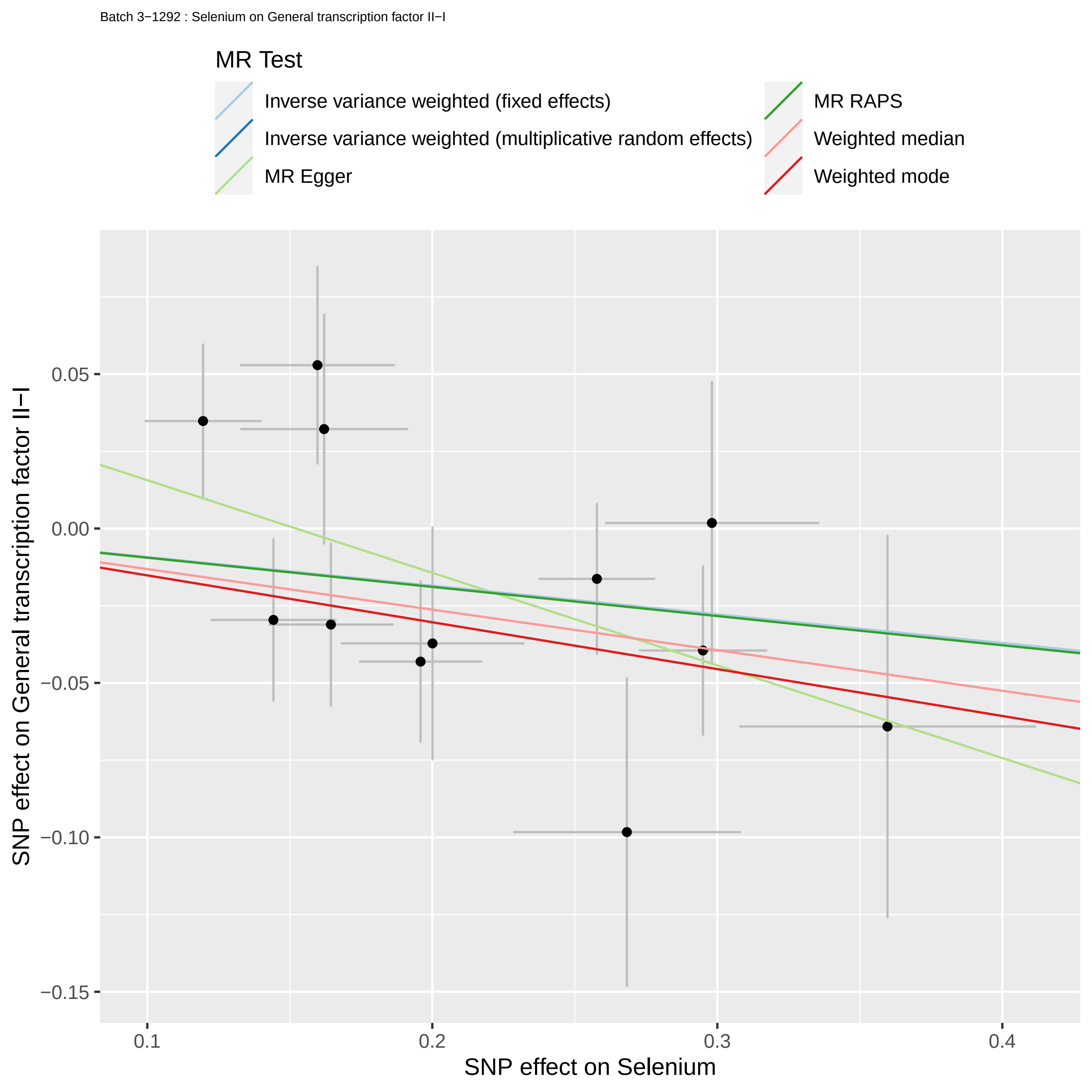
Batch 3−1292 : Selenium on General transcription factor II−I
MR Test
Inverse variance weighted (fixed effects)
Inverse variance weighted (multiplicative random effects)
MR Egger
MR RAPS
Weighted median
Weighted mode
0.05
0.00
−0.05
−0.10
−0.15
0.1
0.2
0.3
0.4
SNP effect on General transcription factor II−I
SNP effect on Selenium

Batch 3−1293 : Selenium on Guanylyl cyclase−activating protein 1

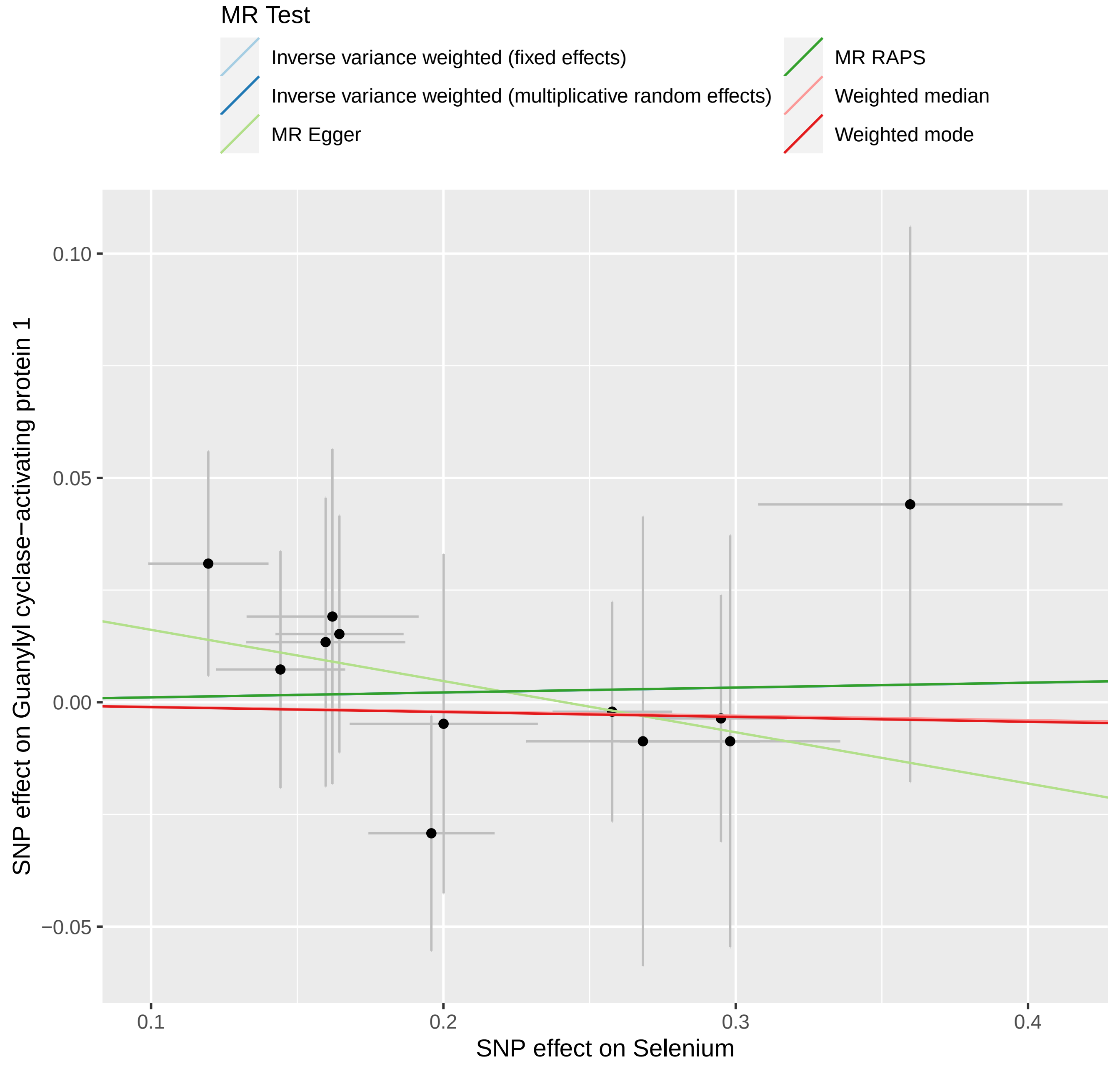

Batch 3−1294 : Selenium on Guanylate cyclase activator 2B

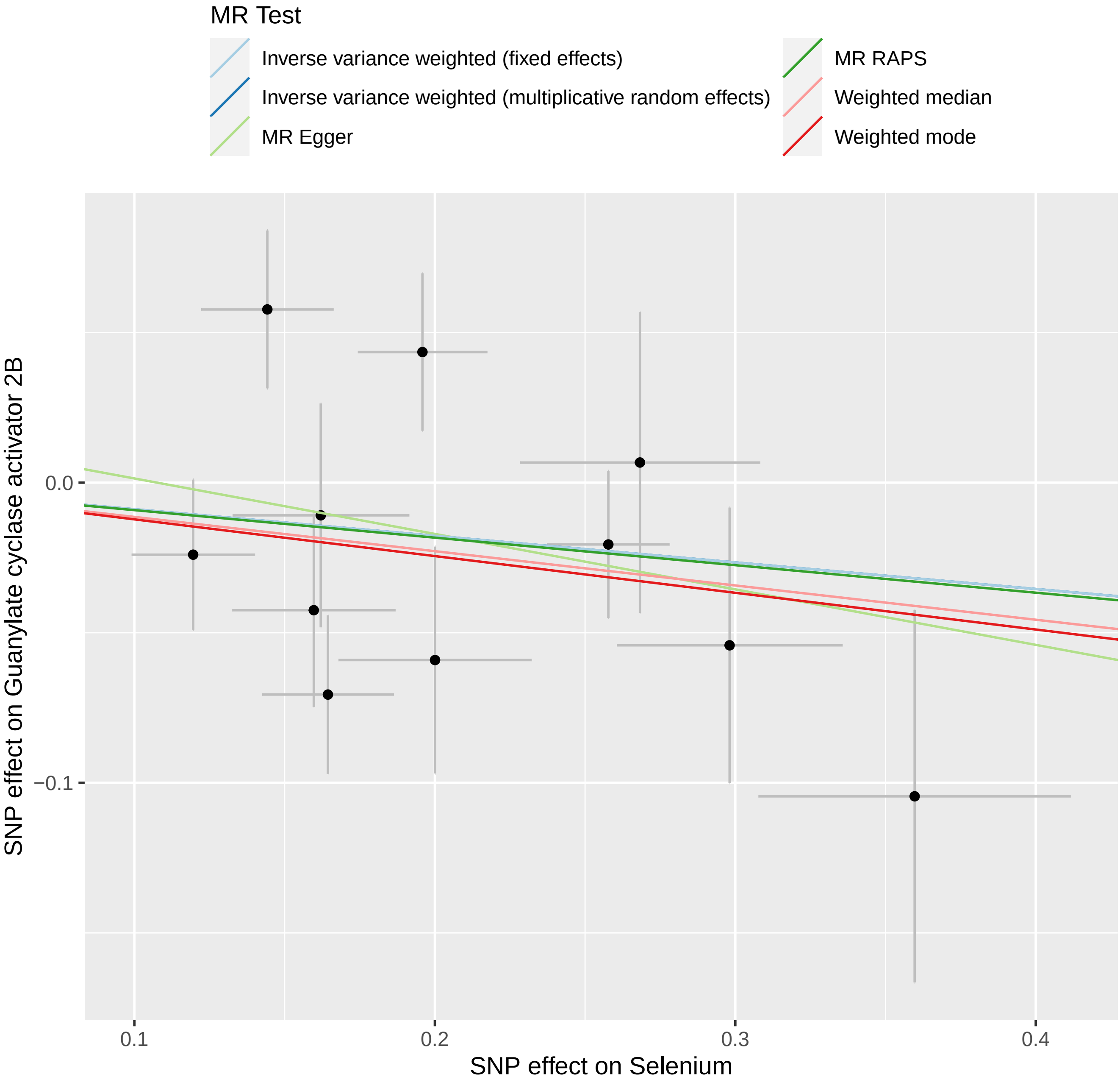

Batch 3−1295 : Selenium on Glucoside xylosyltransferase 1

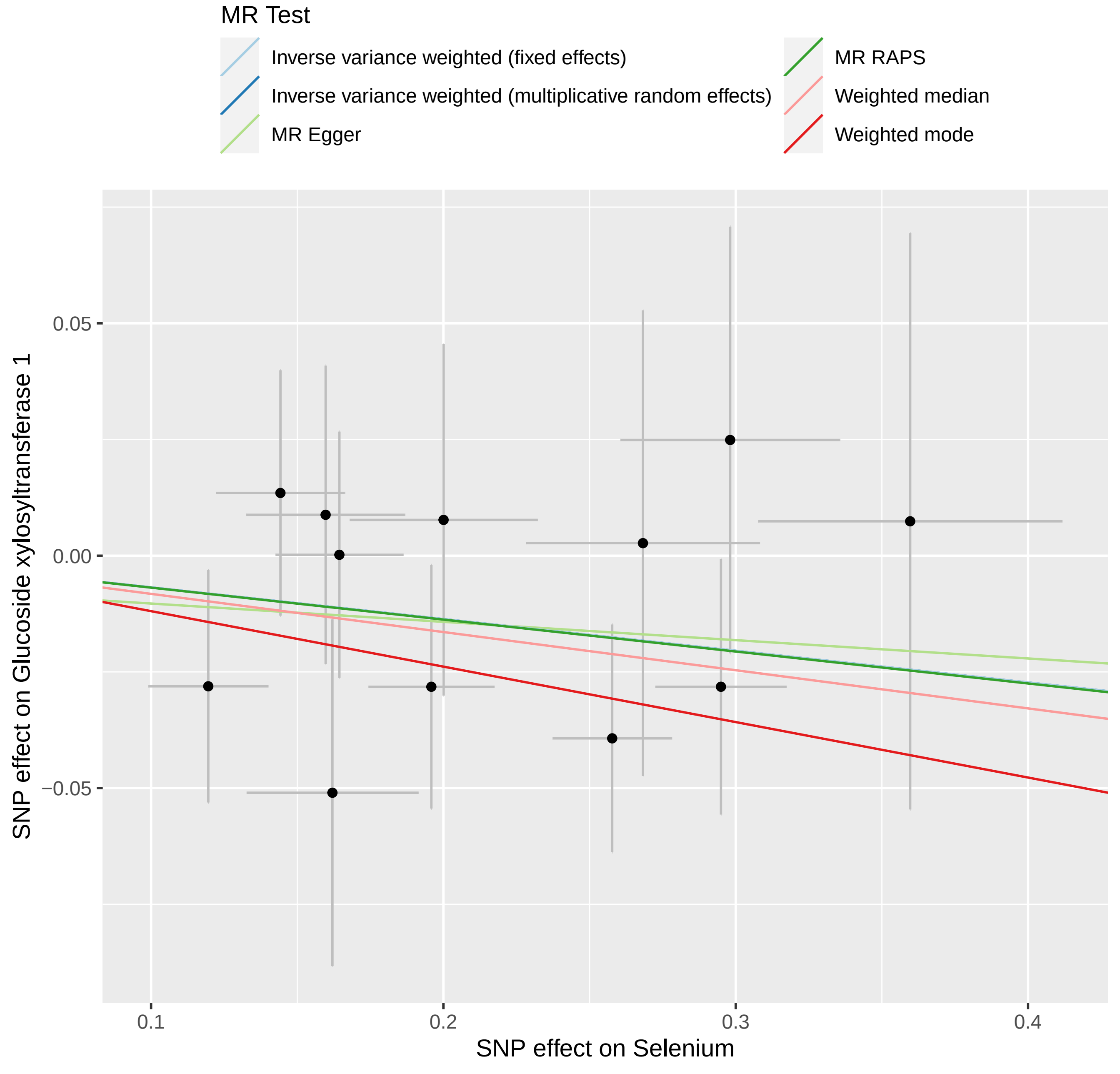

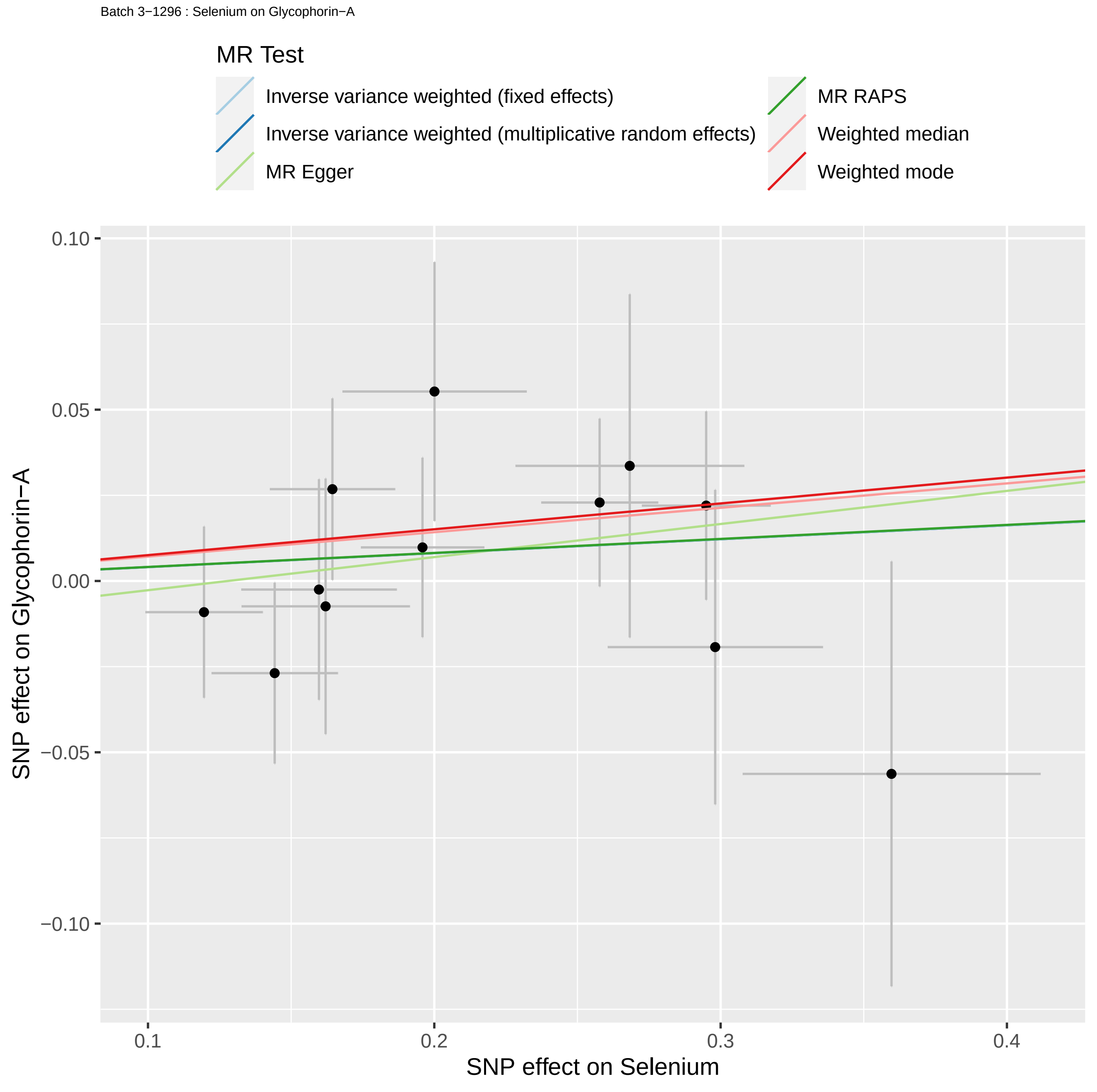

Batch 3−1296 : Selenium on Glycophorin−A
MR Test
Inverse variance weighted (fixed effects)
Inverse variance weighted (multiplicative random effects)
MR Egger
MR RAPS
Weighted median
Weighted mode
0.10
0.05
0.00
−0.05
−0.10
SNP effect on Glycophorin−A
0.1
0.2
0.3
0.4
SNP effect on Selenium

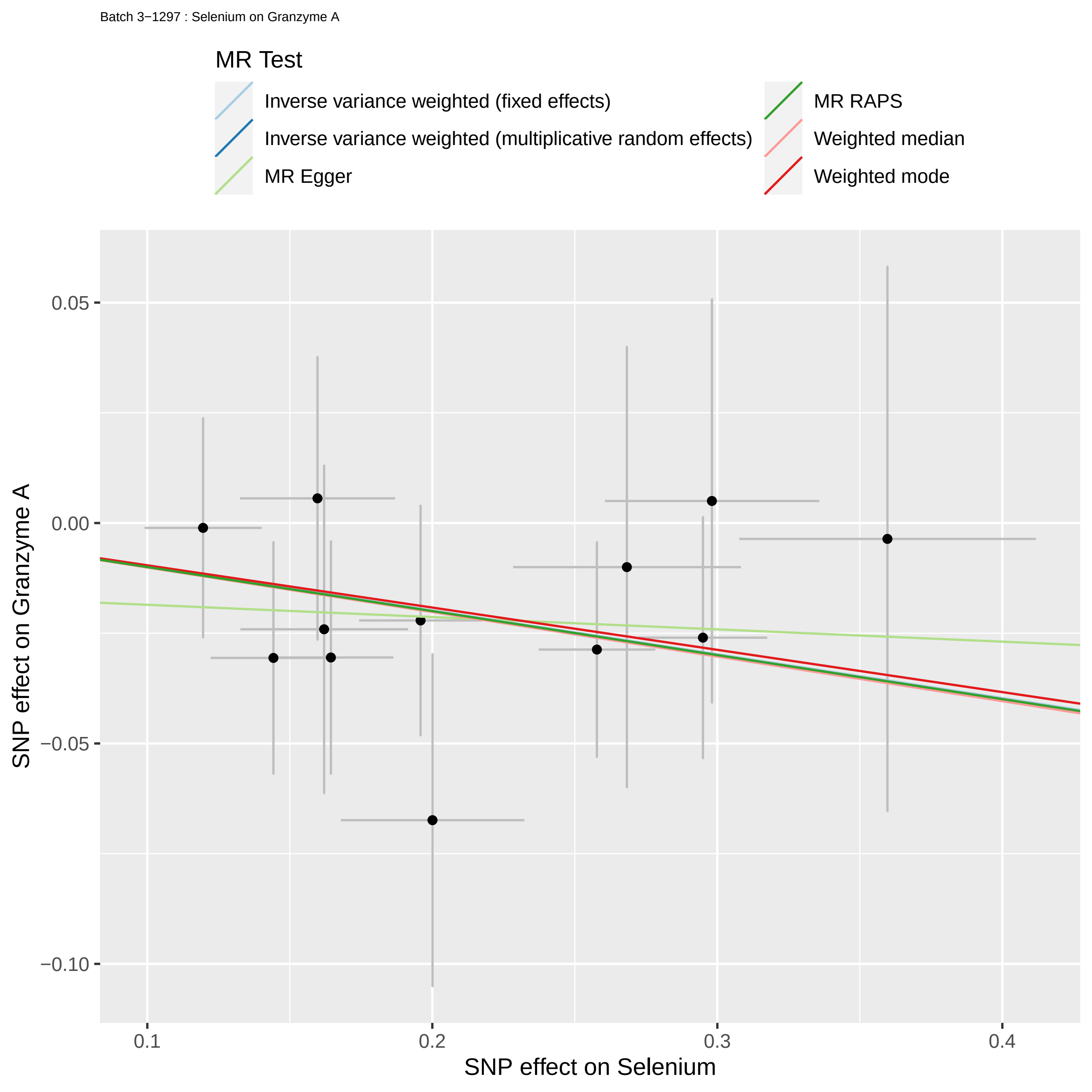
Batch 3−1297 : Selenium on Granzyme A
MR Test
Inverse variance weighted (fixed effects)
Inverse variance weighted (multiplicative random effects)
MR Egger
MR RAPS
Weighted median
Weighted mode
0.05
0.00
−0.05
−0.10
0.1
0.2
0.3
0.4
SNP effect on Granzyme A
SNP effect on Selenium

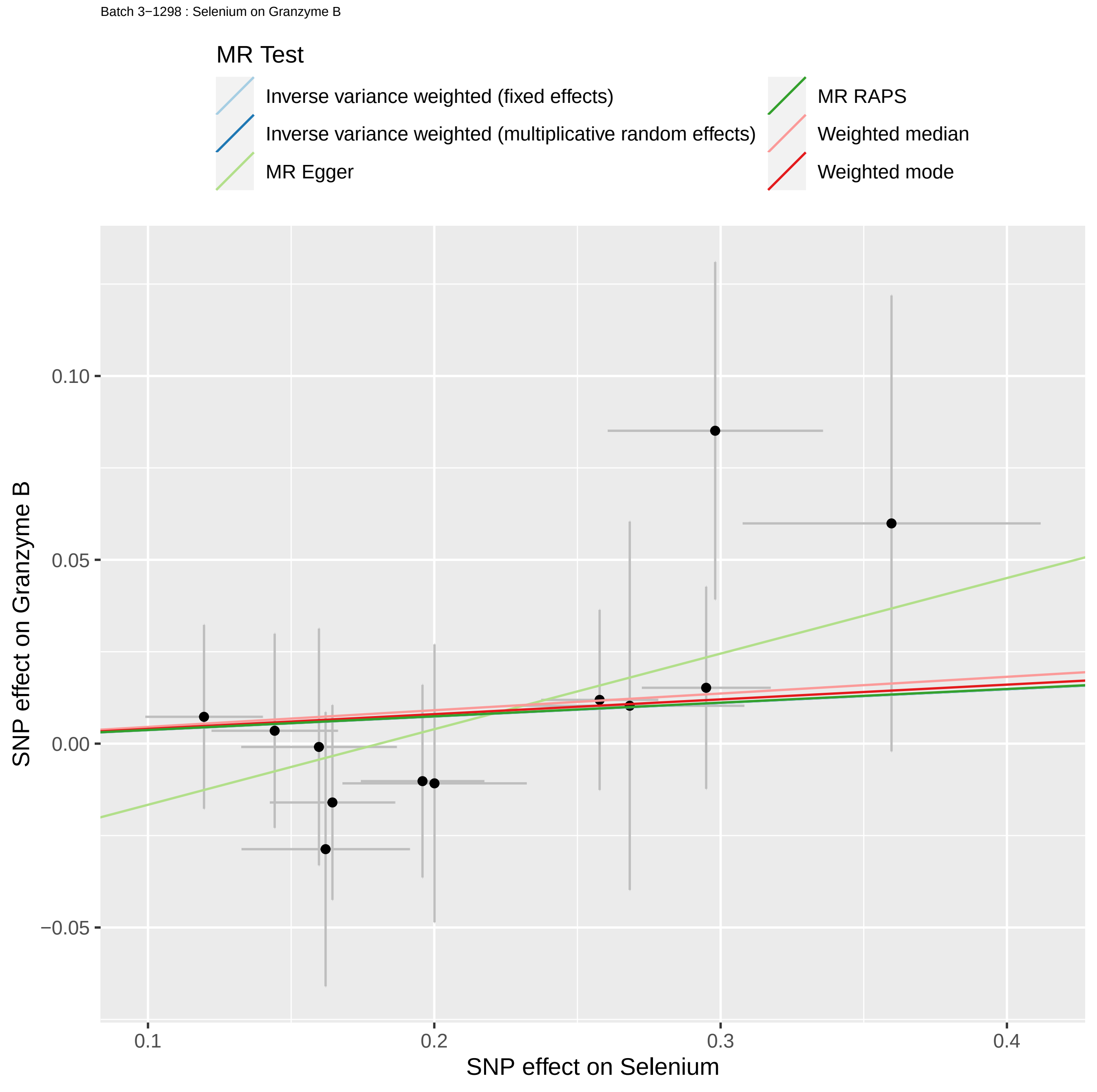
Batch 3−1298 : Selenium on Granzyme B
MR Test
Inverse variance weighted (fixed effects)
Inverse variance weighted (multiplicative random effects)
MR Egger
MR RAPS
Weighted median
Weighted mode
SNP effect on Granzyme B
0.10
0.05
0.00
−0.05
0.1
0.2
0.3
0.4
SNP effect on Selenium

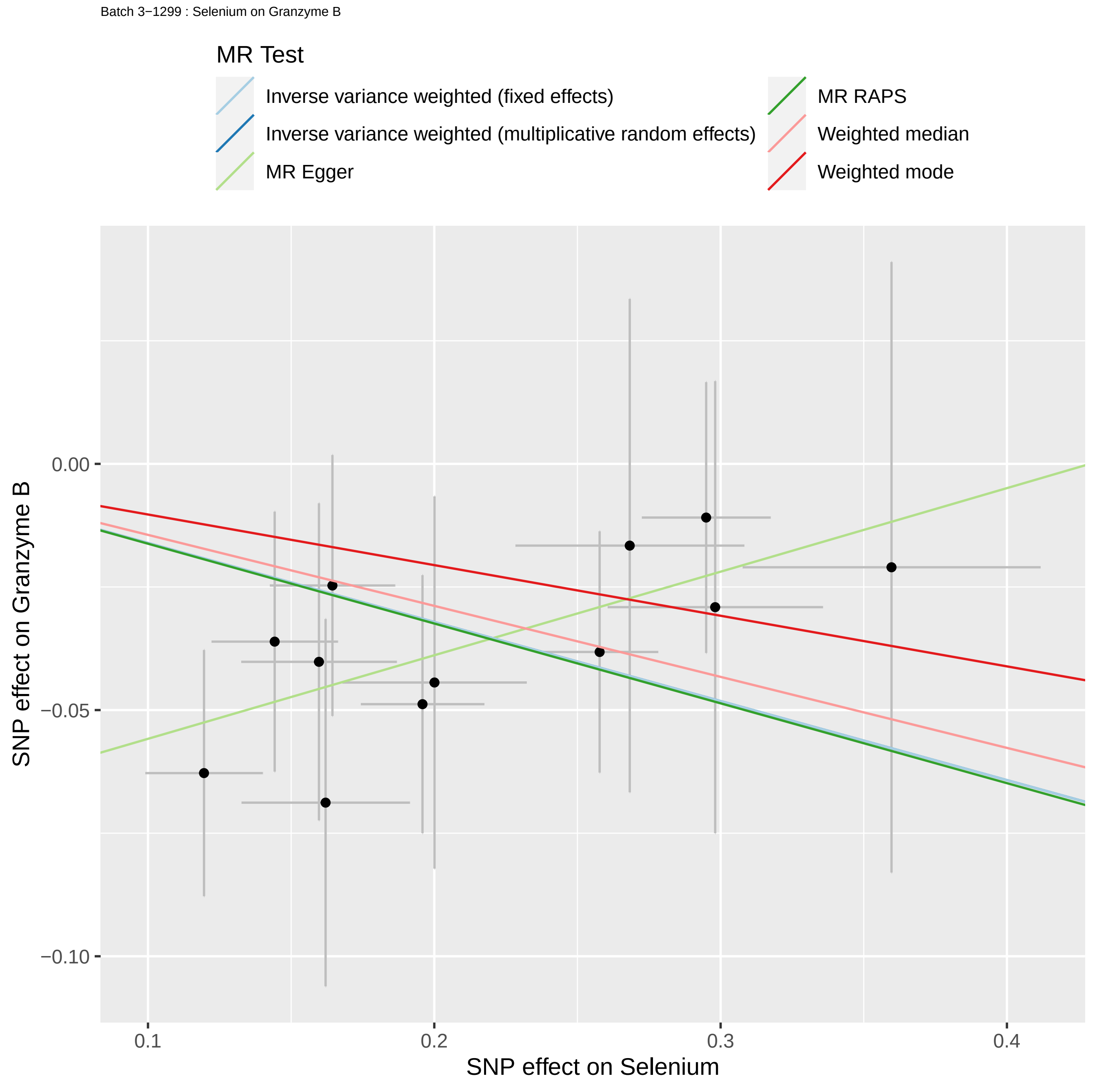

Batch 3−1299 : Selenium on Granzyme B
MR Test
Inverse variance weighted (fixed effects)
Inverse variance weighted (multiplicative random effects)
MR Egger
MR RAPS
Weighted median
Weighted mode
0.00
−0.05
−0.10
0.1
0.2
0.3
0.4
SNP effect on Granzyme B
SNP effect on Selenium

Batch 3−1300 : Selenium on Granzyme H

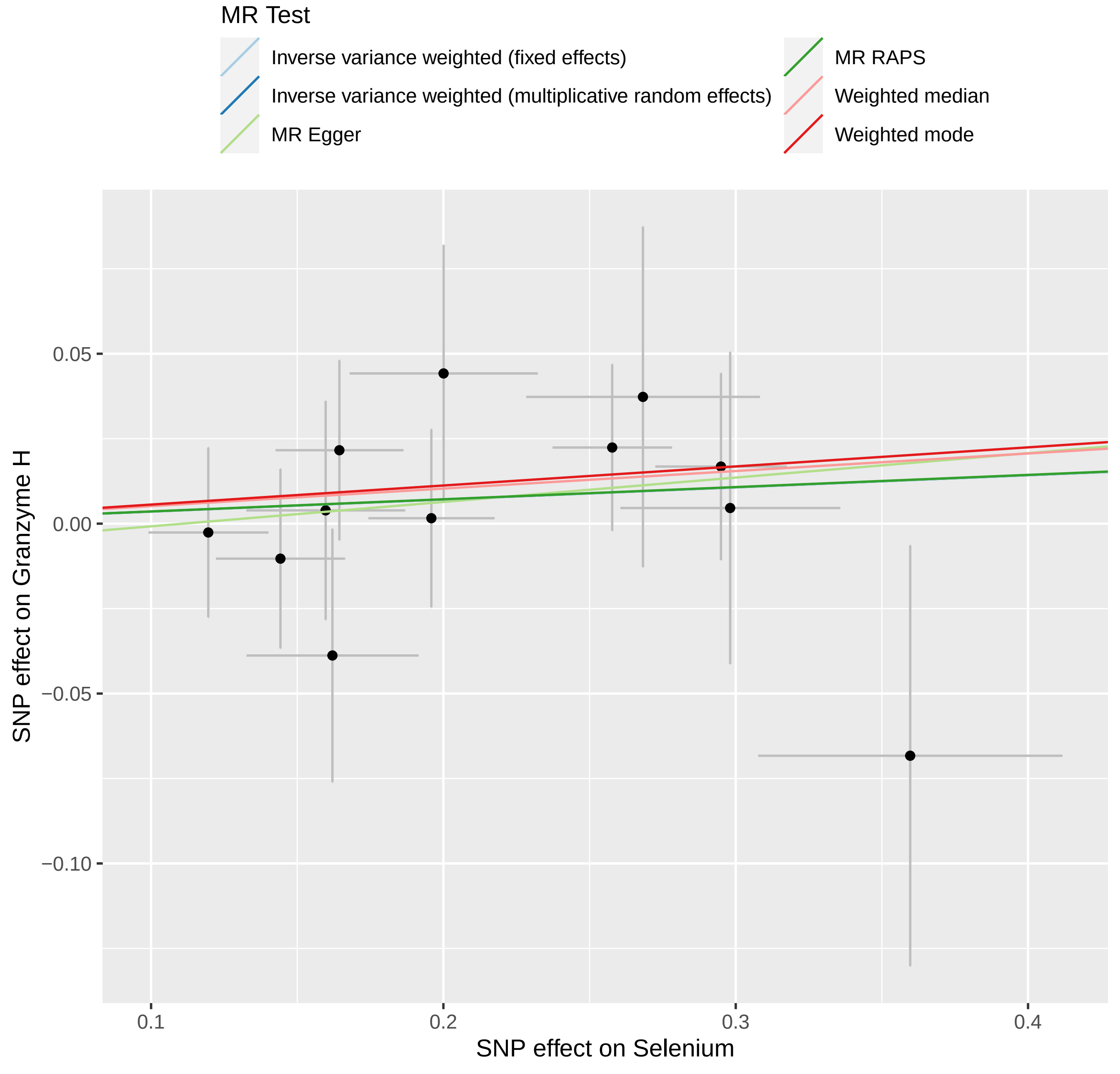

Batch 3−1301 : Selenium on Granzyme K

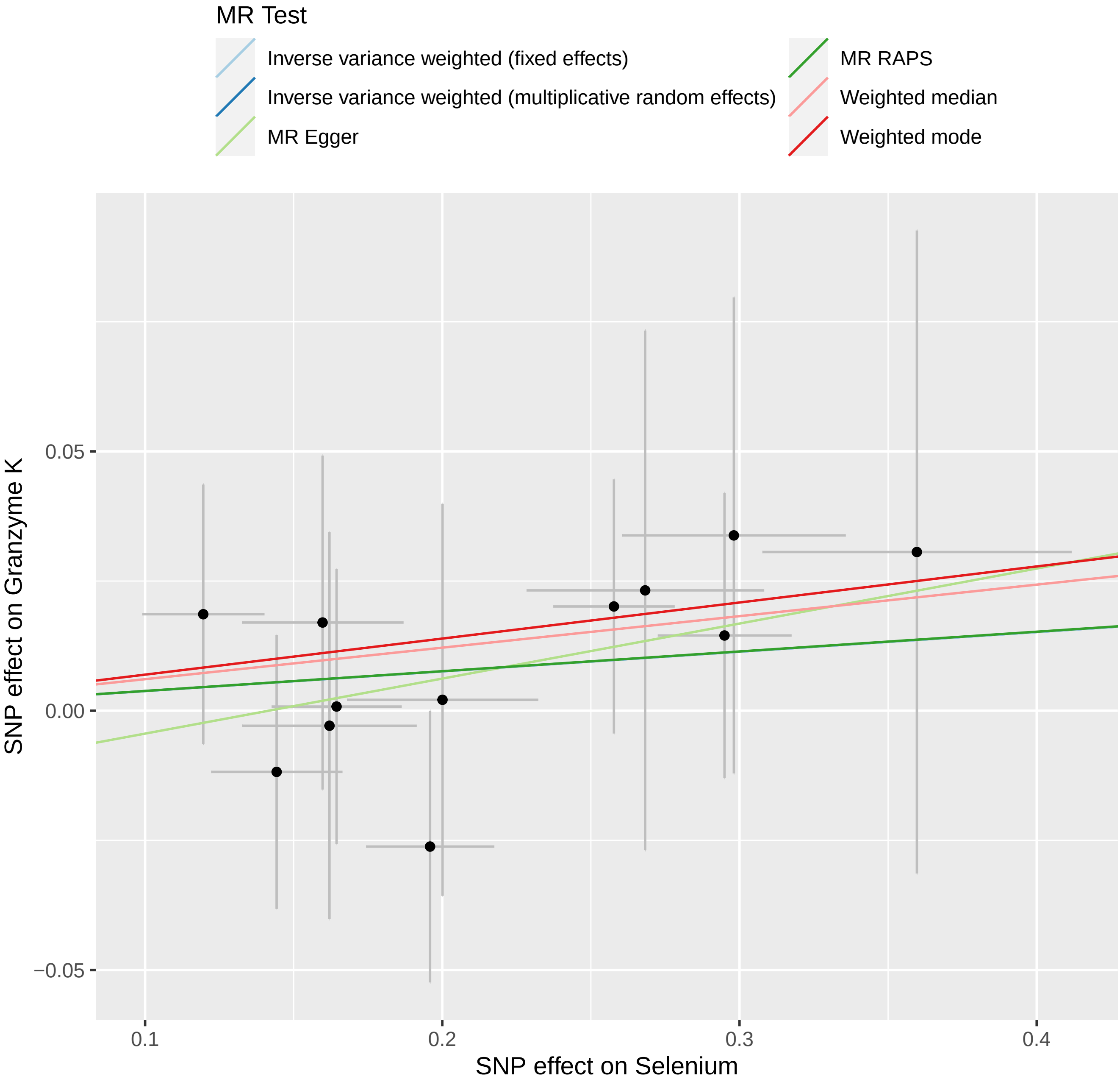

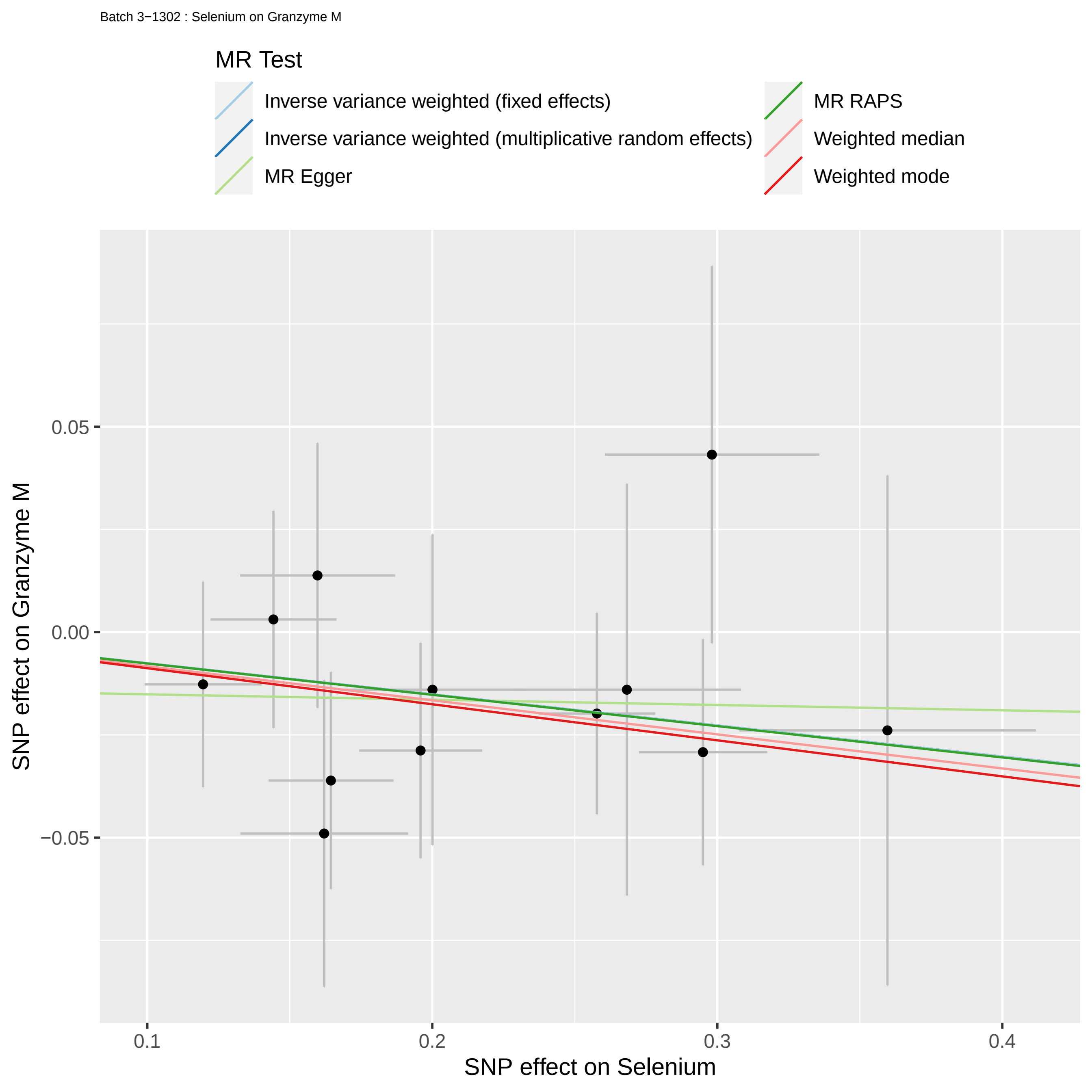
Batch 3−1302 : Selenium on Granzyme M
MR Test
Inverse variance weighted (fixed effects)
Inverse variance weighted (multiplicative random effects)
MR Egger
MR RAPS
Weighted median
Weighted mode
0.05
0.00
−0.05
0.1
0.2
0.3
0.4
SNP effect on Granzyme M
SNP effect on Selenium

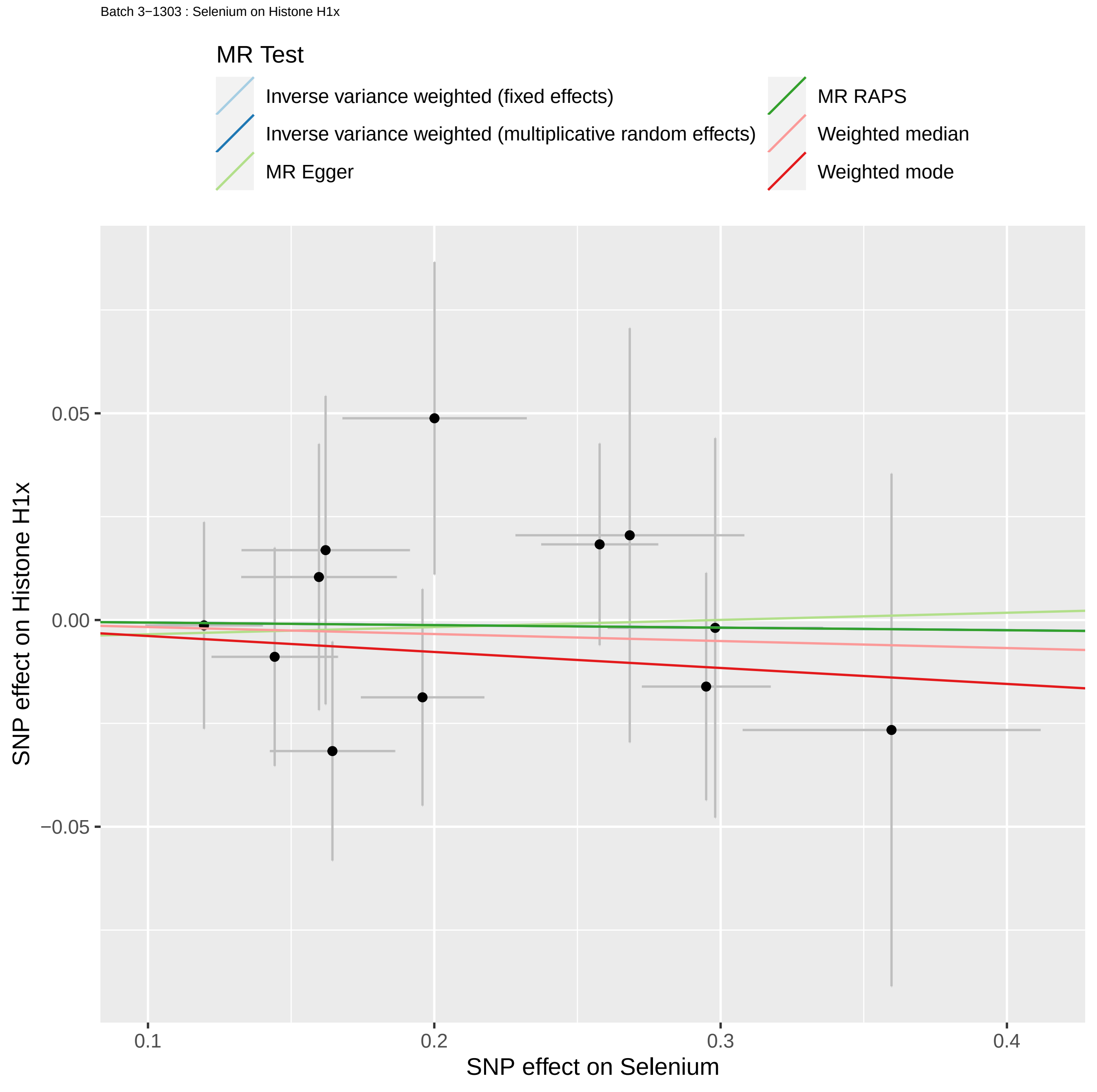

Batch 3−1303 : Selenium on Histone H1x
MR Test
Inverse variance weighted (fixed effects)
Inverse variance weighted (multiplicative random effects)
MR Egger
MR RAPS
Weighted median
Weighted mode
0.05
0.00
−0.05
0.1
0.2
0.3
0.4
SNP effect on Histone H1x
SNP effect on Selenium

Batch 3−1304 : Selenium on Histone H2A.z

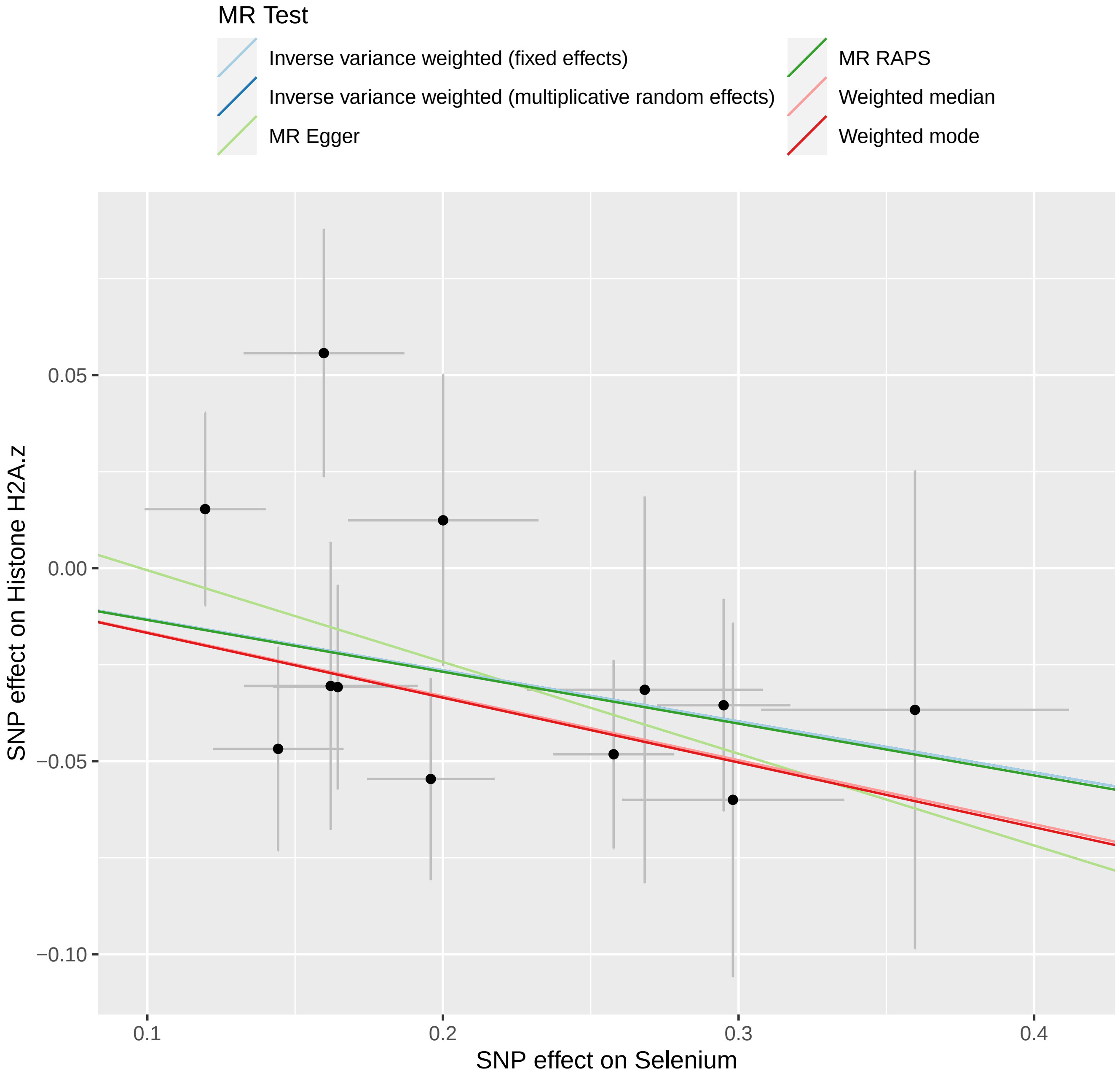

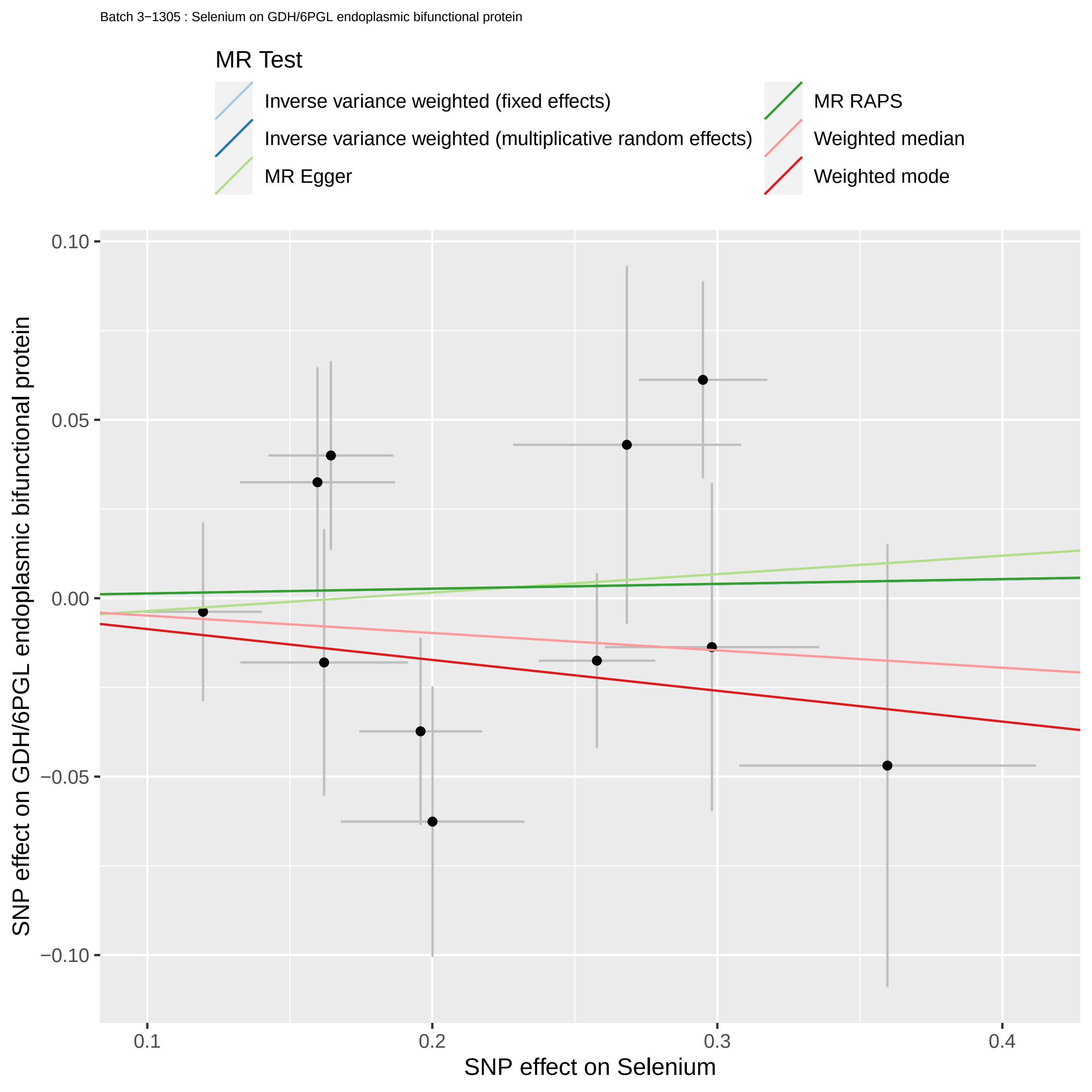
Batch 3−1305 : Selenium on GDH/6PGL endoplasmic bifunctional protein
MR Test
Inverse variance weighted (fixed effects)
Inverse variance weighted (multiplicative random effects)
MR Egger
MR RAPS
Weighted median
Weighted mode
0.10
0.05
0.00
−0.05
−0.10
SNP effect on GDH/6PGL endoplasmic bifunctional protein
0.1
0.2
0.3
0.4
SNP effect on Selenium

Batch 3−1306 : Selenium on 3−hydroxyanthranilate 3,4−dioxygenase

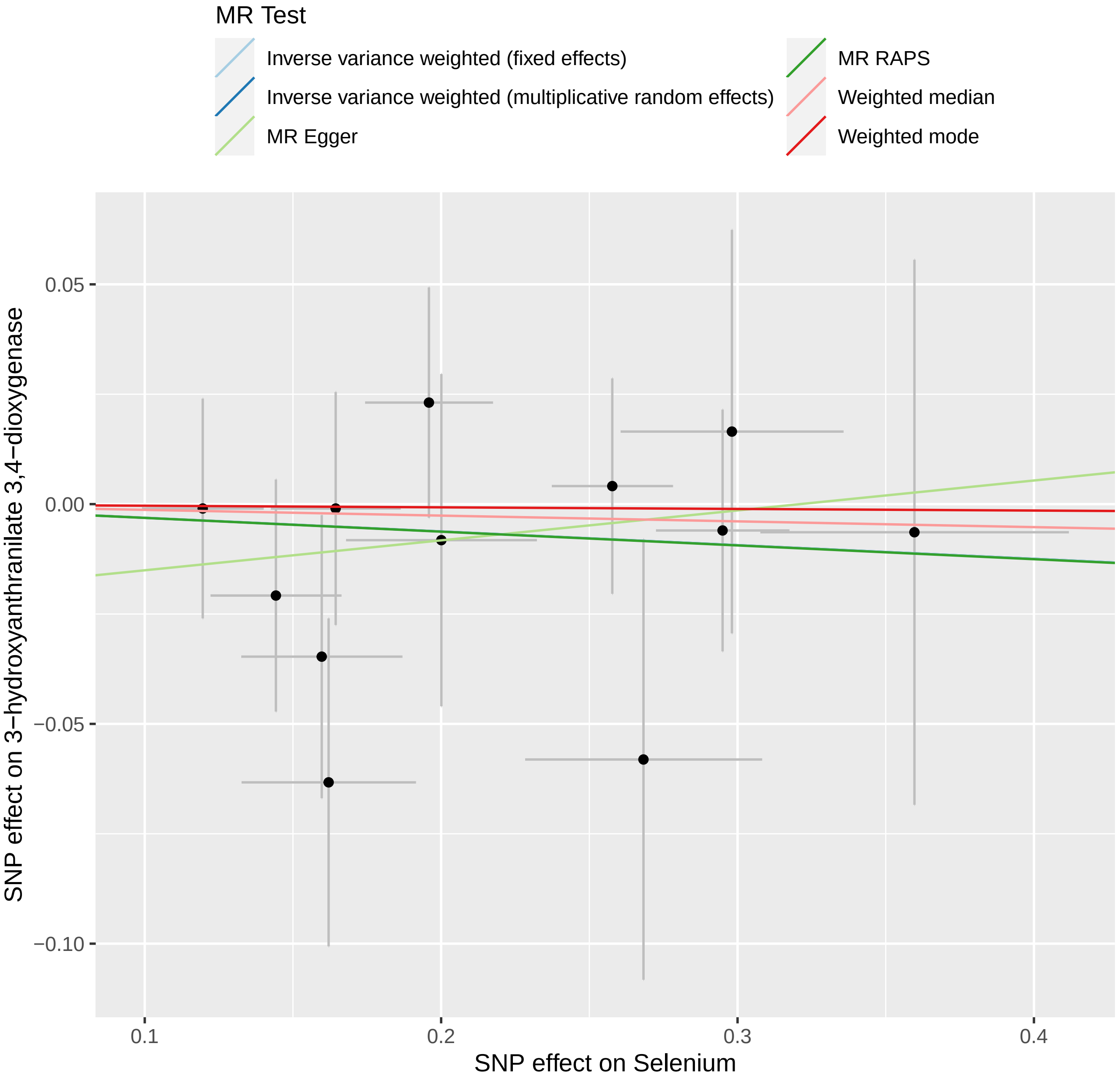

Batch 3−1307 : Selenium on Hepcidin

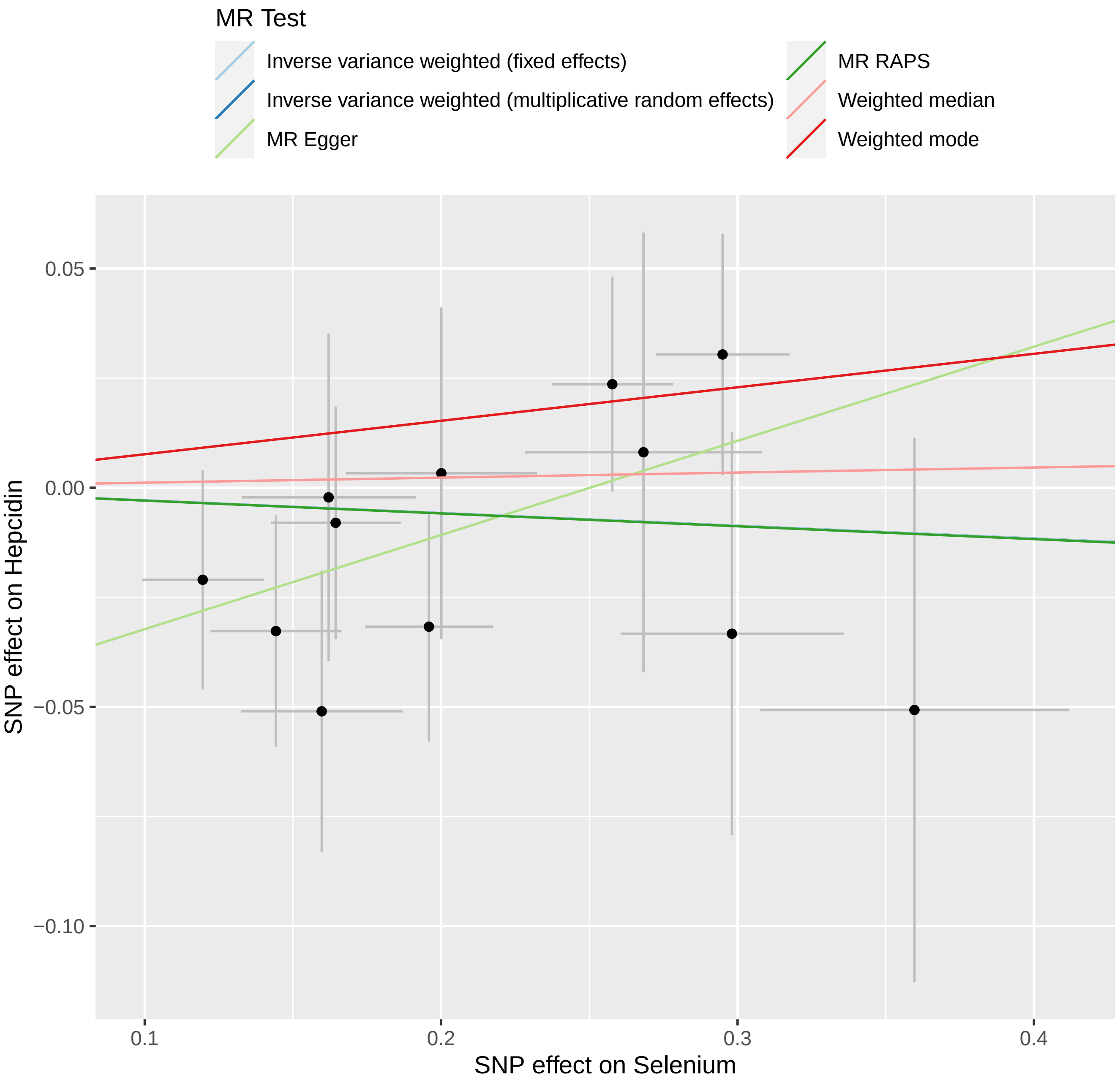

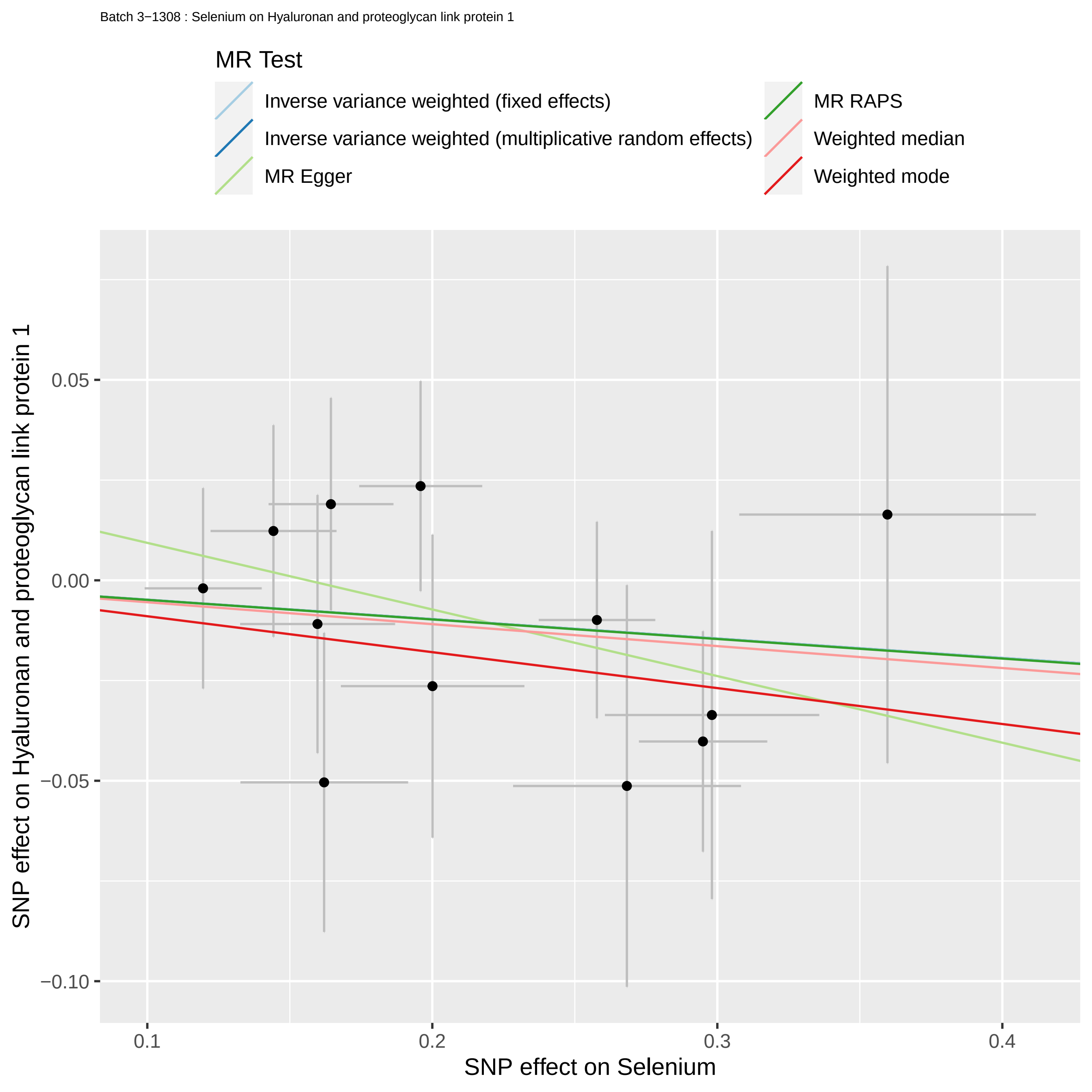

Batch 3−1308 : Selenium on Hyaluronan and proteoglycan link protein 1
MR Test
Inverse variance weighted (fixed effects)
Inverse variance weighted (multiplicative random effects)
MR Egger
MR RAPS
Weighted median
Weighted mode
0.05
0.00
−0.05
−0.10
0.1
0.2
0.3
0.4
SNP effect on Hyaluronan and proteoglycan link protein 1
SNP effect on Selenium

Batch 3−1309 : Selenium on Histone acetyltransferase type B catalytic subunit

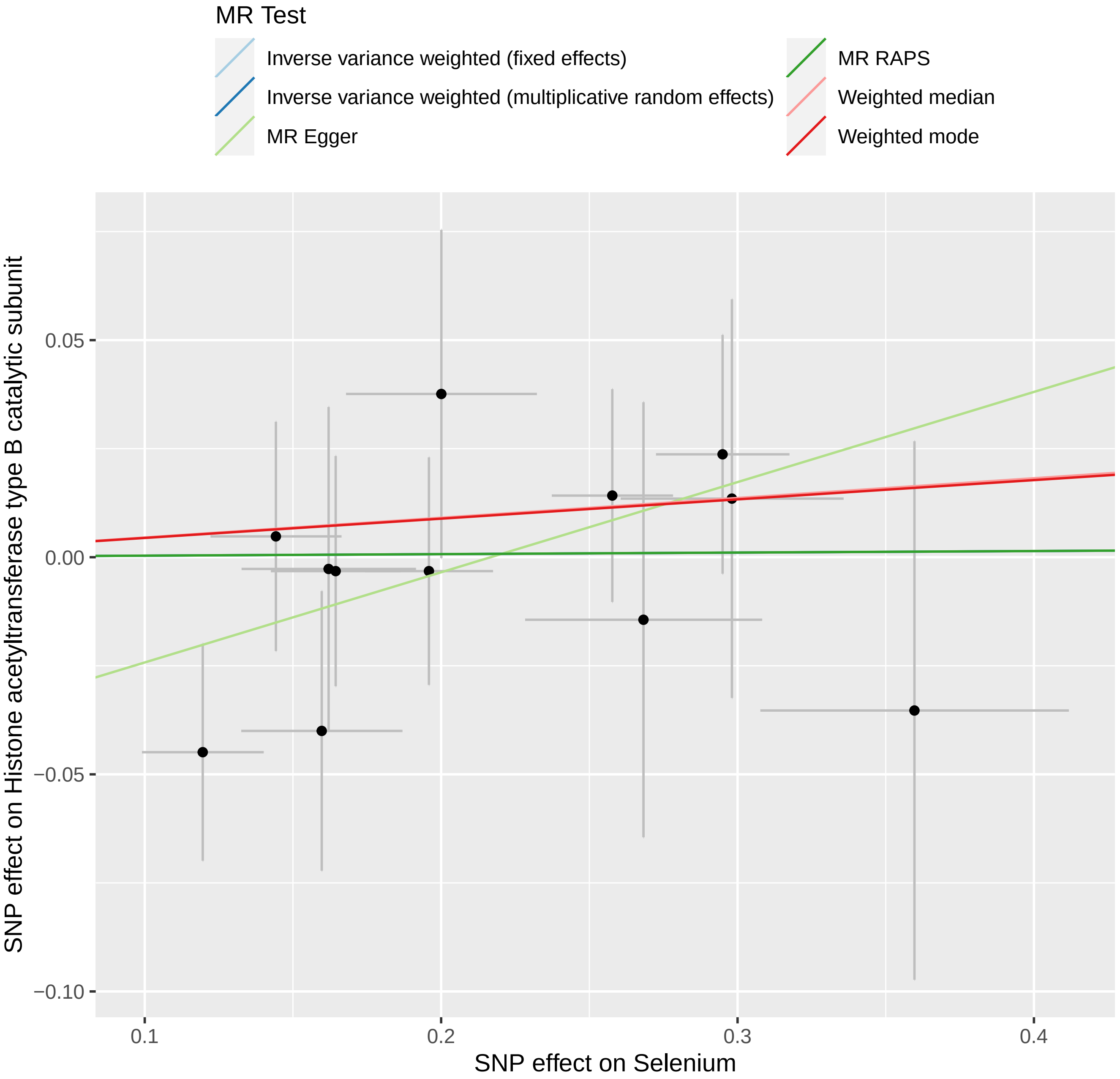

Batch 3−1310 : Selenium on Hepatitis A virus cellular receptor 1

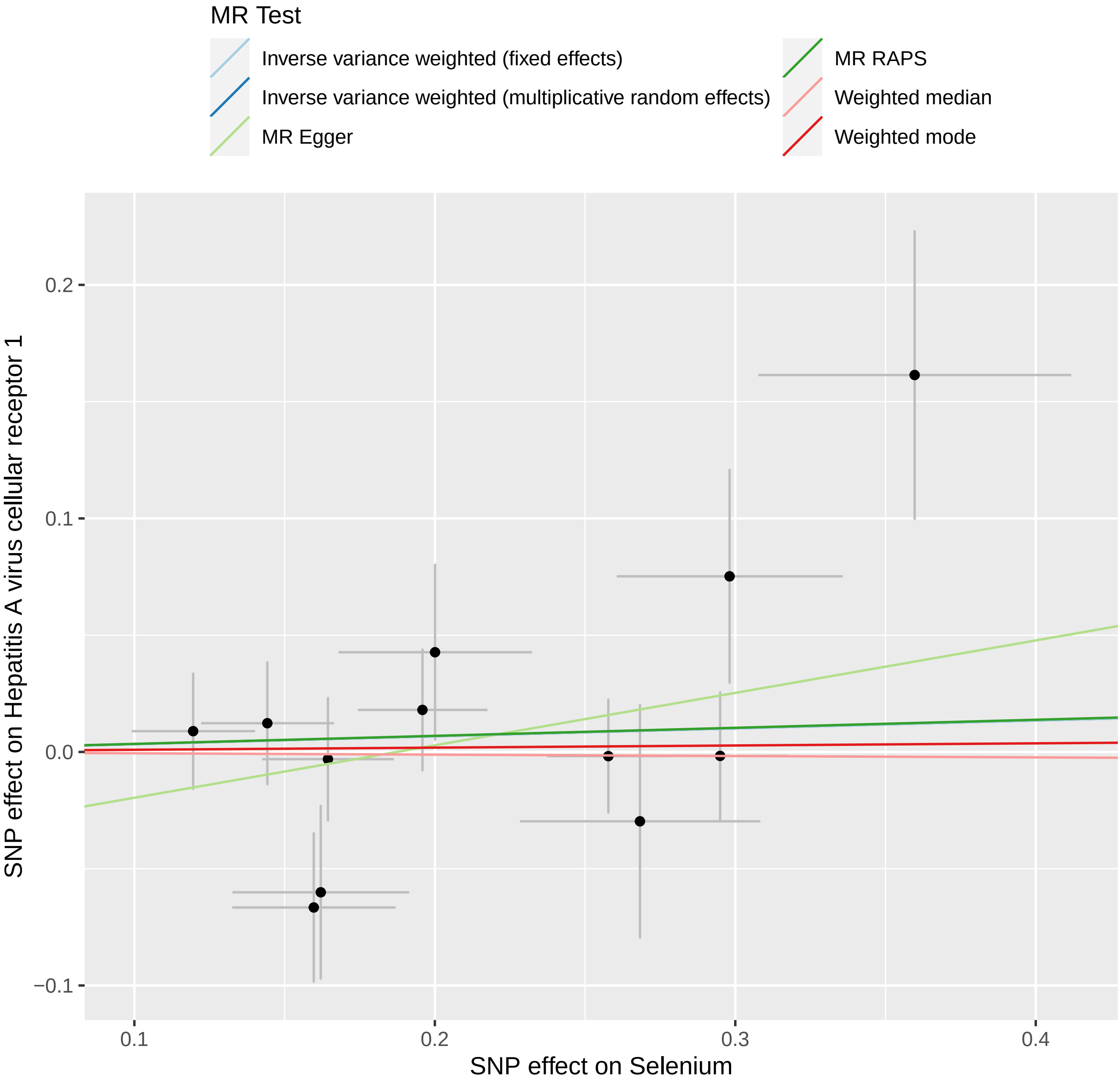

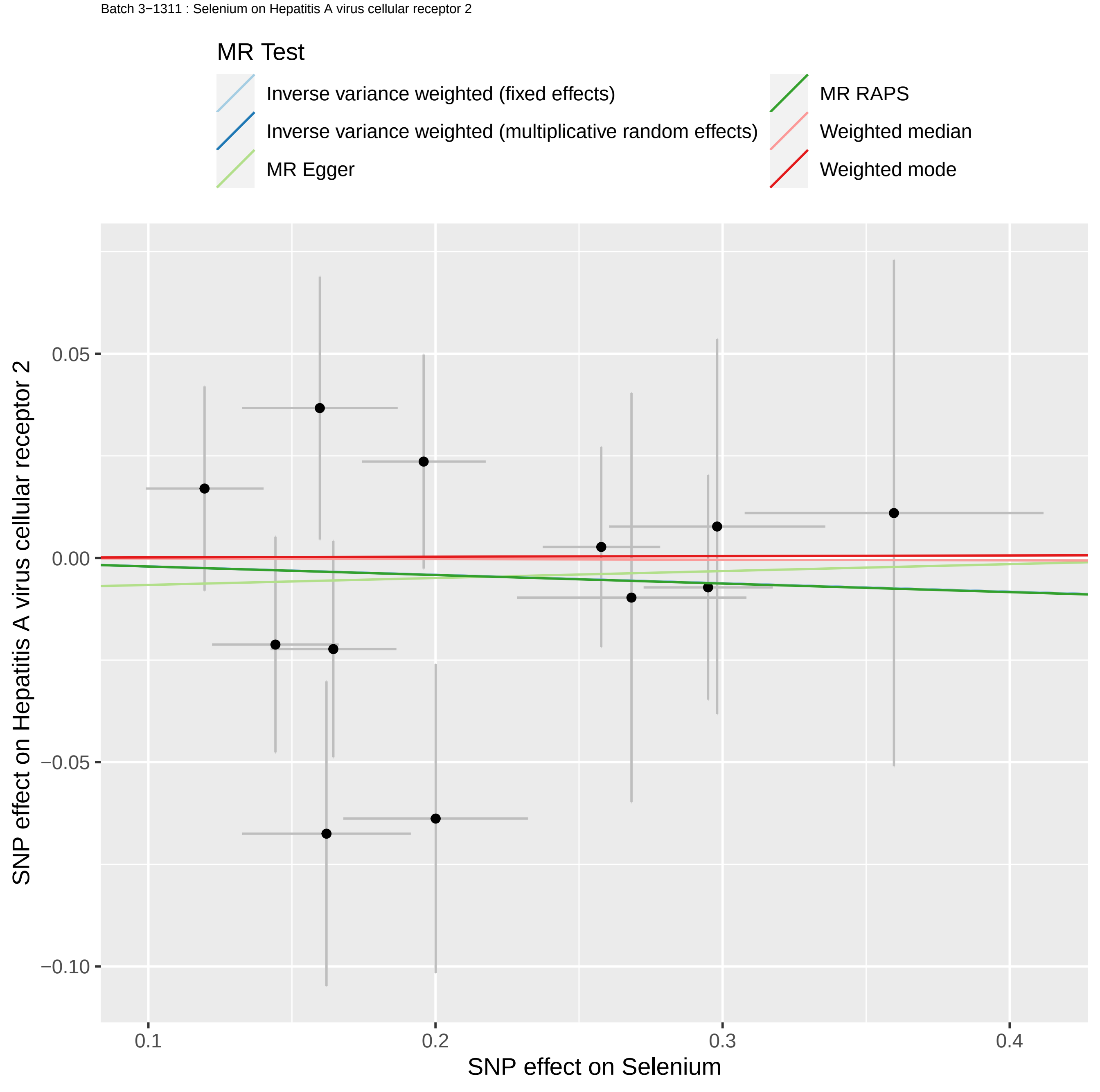
Batch 3−1311 : Selenium on Hepatitis A virus cellular receptor 2
MR Test
Inverse variance weighted (fixed effects)
Inverse variance weighted (multiplicative random effects)
MR Egger
MR RAPS
Weighted median
Weighted mode
SNP effect on Hepatitis A virus cellular receptor 2
0.05
0.00
−0.05
−0.10
0.1
0.2
0.3
0.4
SNP effect on Selenium

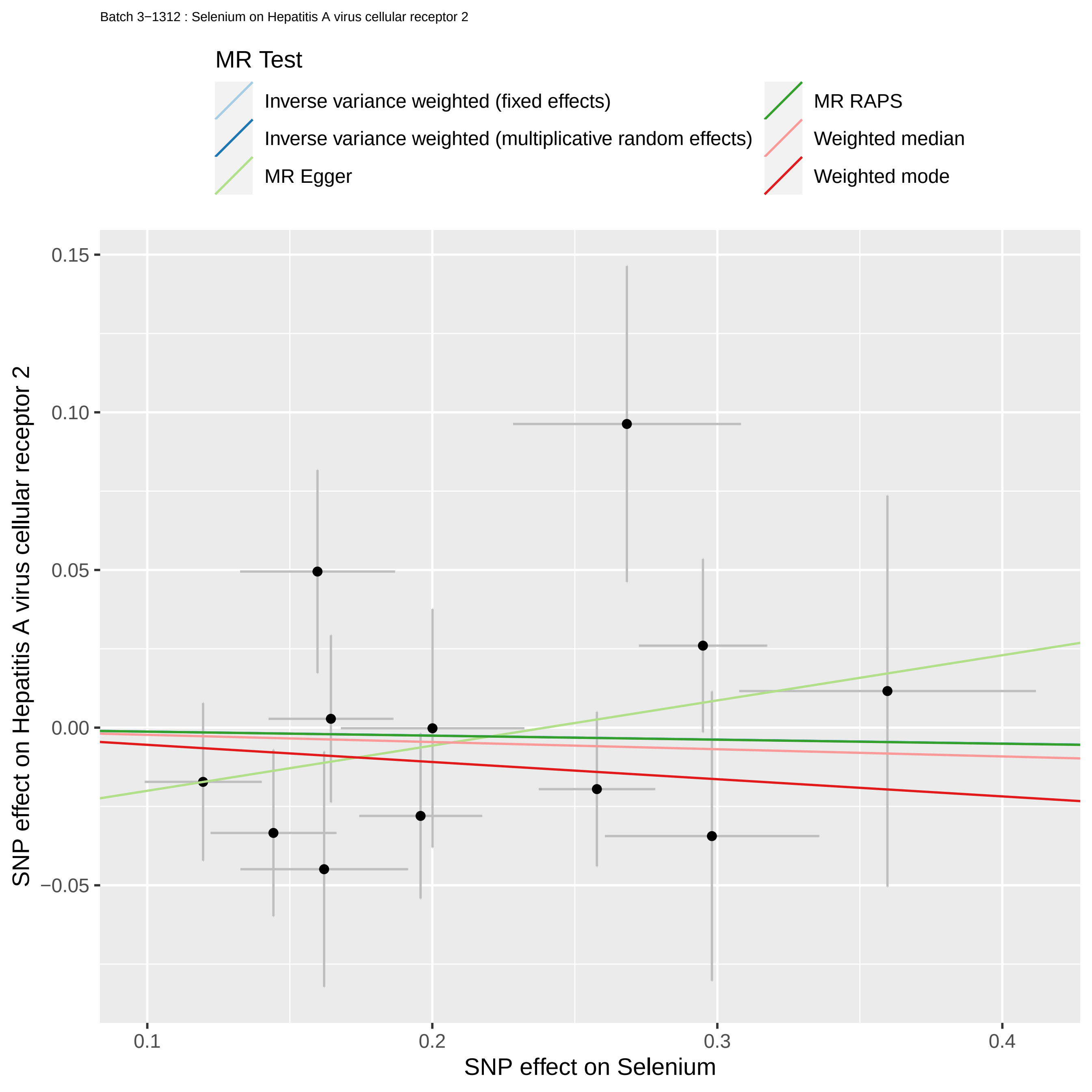

Batch 3−1312 : Selenium on Hepatitis A virus cellular receptor 2
MR Test
Inverse variance weighted (fixed effects)
Inverse variance weighted (multiplicative random effects)
MR Egger
MR RAPS
Weighted median
Weighted mode
0.15
0.10
0.05
0.00
−0.05
0.1
0.2
0.3
0.4
SNP effect on Selenium
SNP effect on Hepatitis A virus cellular receptor 2

Batch 3−1313 : Selenium on Heparin−binding EGF−like growth factor

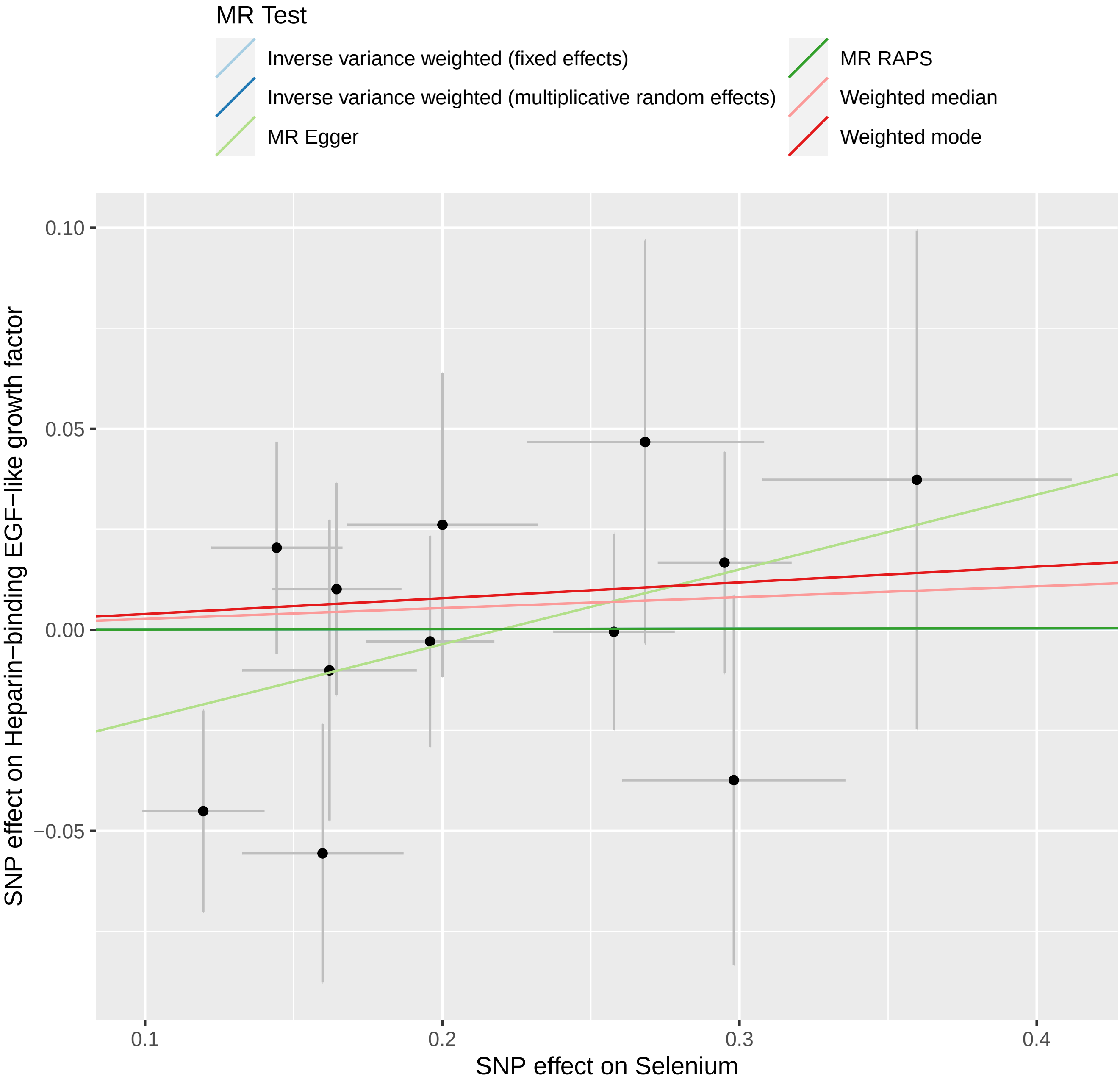

Batch 3−1314 : Selenium on Heparin−binding EGF−like growth factor

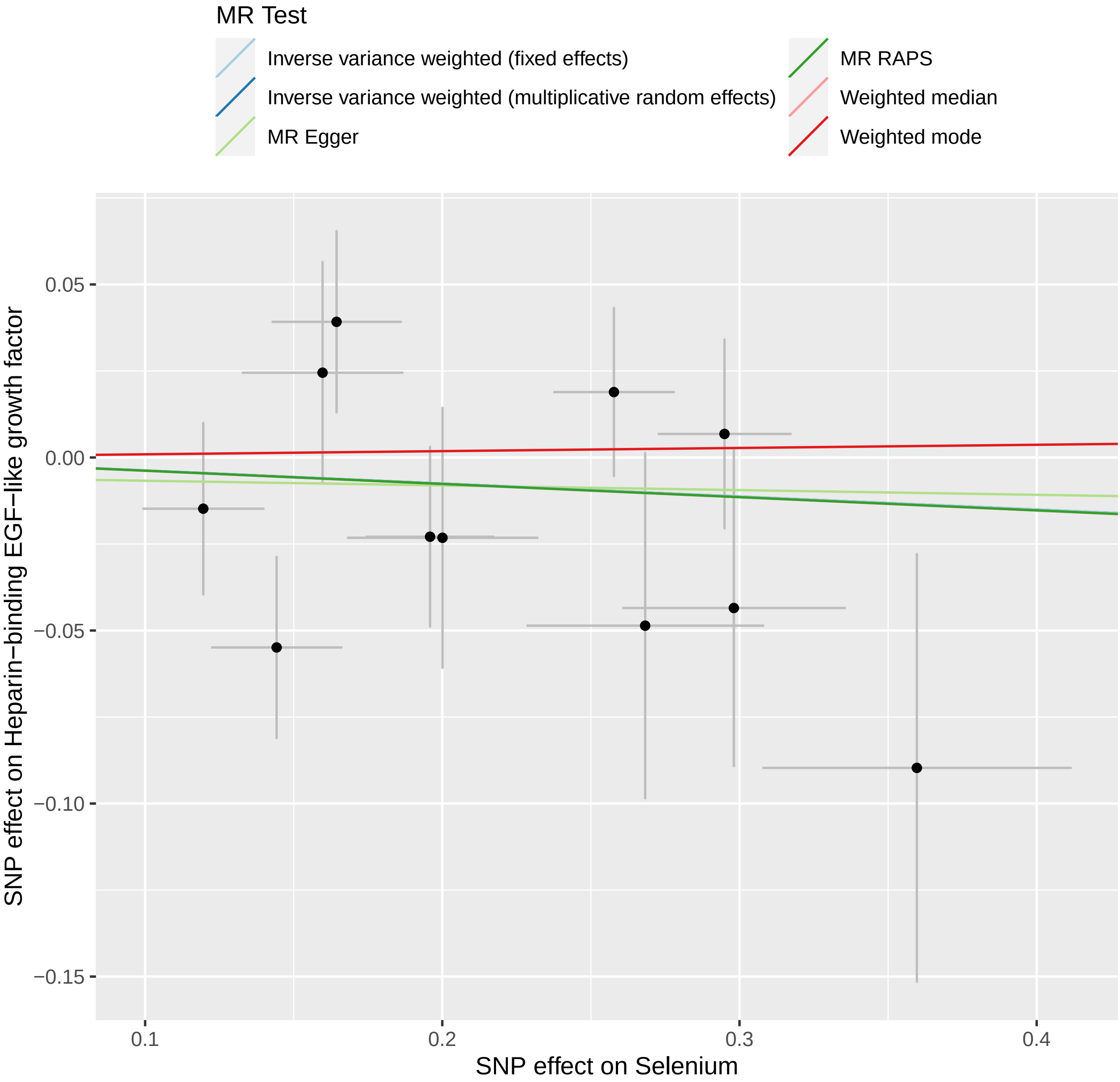

Batch 3−1315 : Selenium on Hemoglobin subunit gamma−1

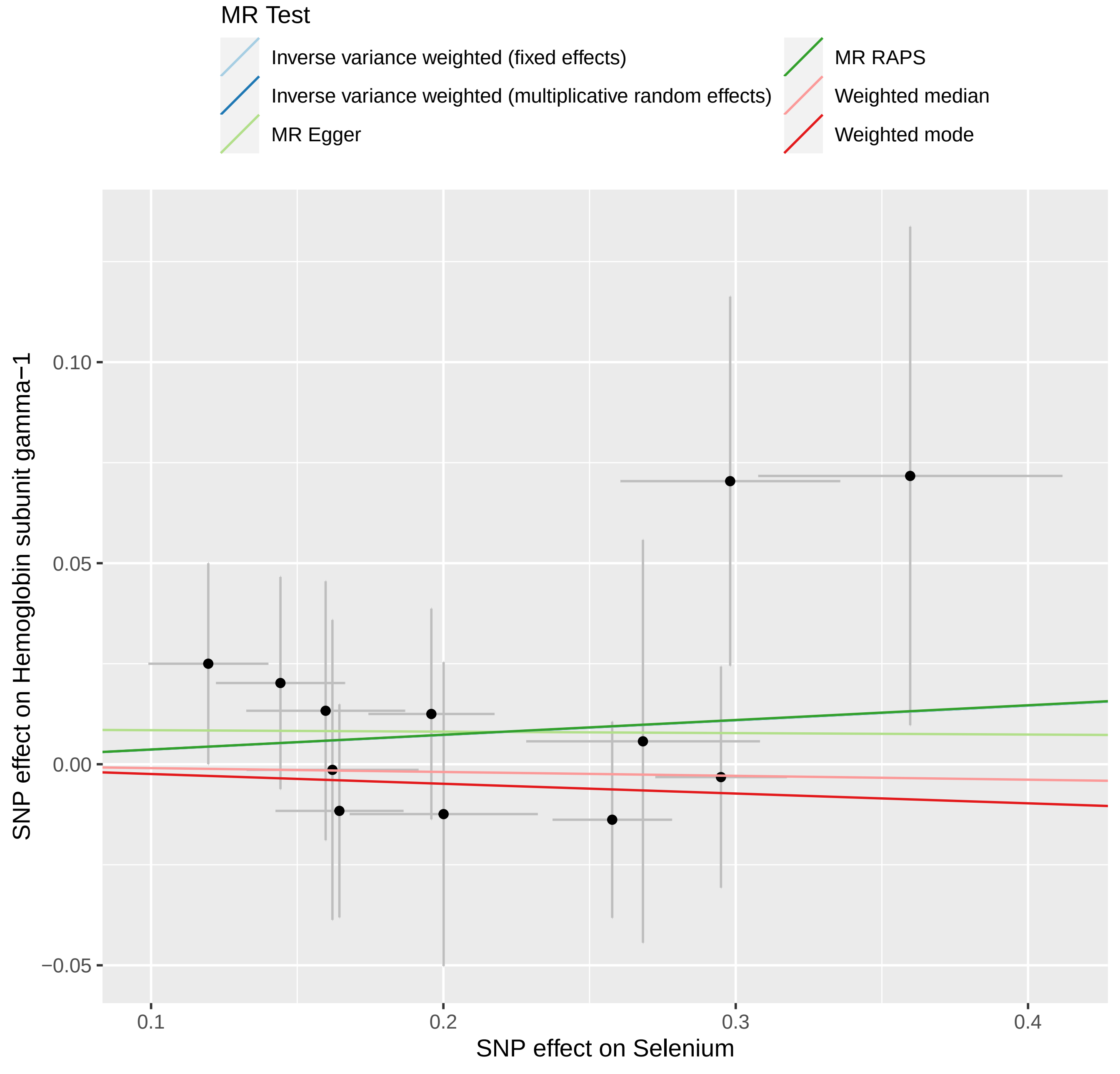

Batch 3−1316 : Selenium on Hemoglobin subunit theta−1

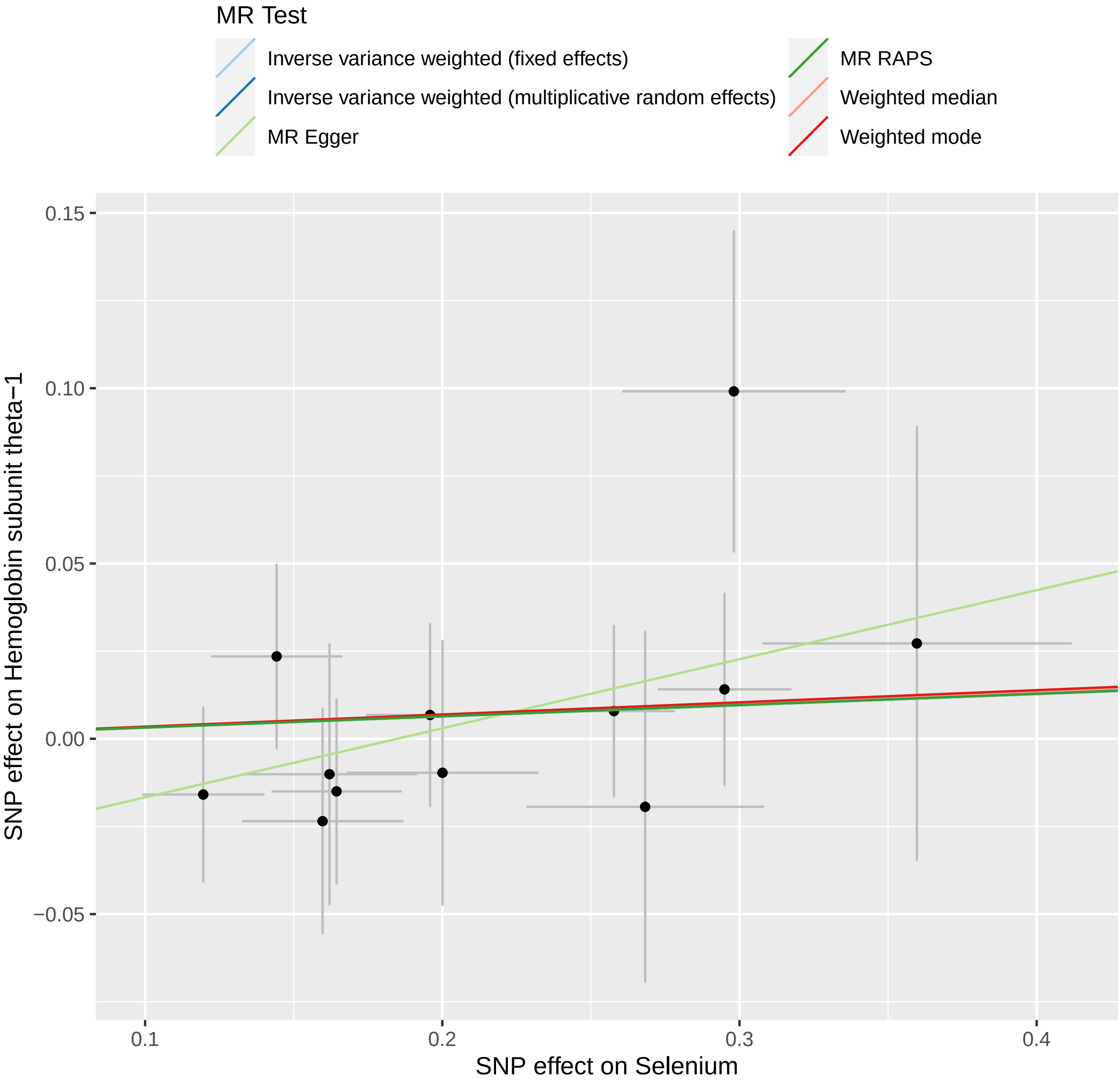

Batch 3−1317 : Selenium on Hemoglobin subunit zeta

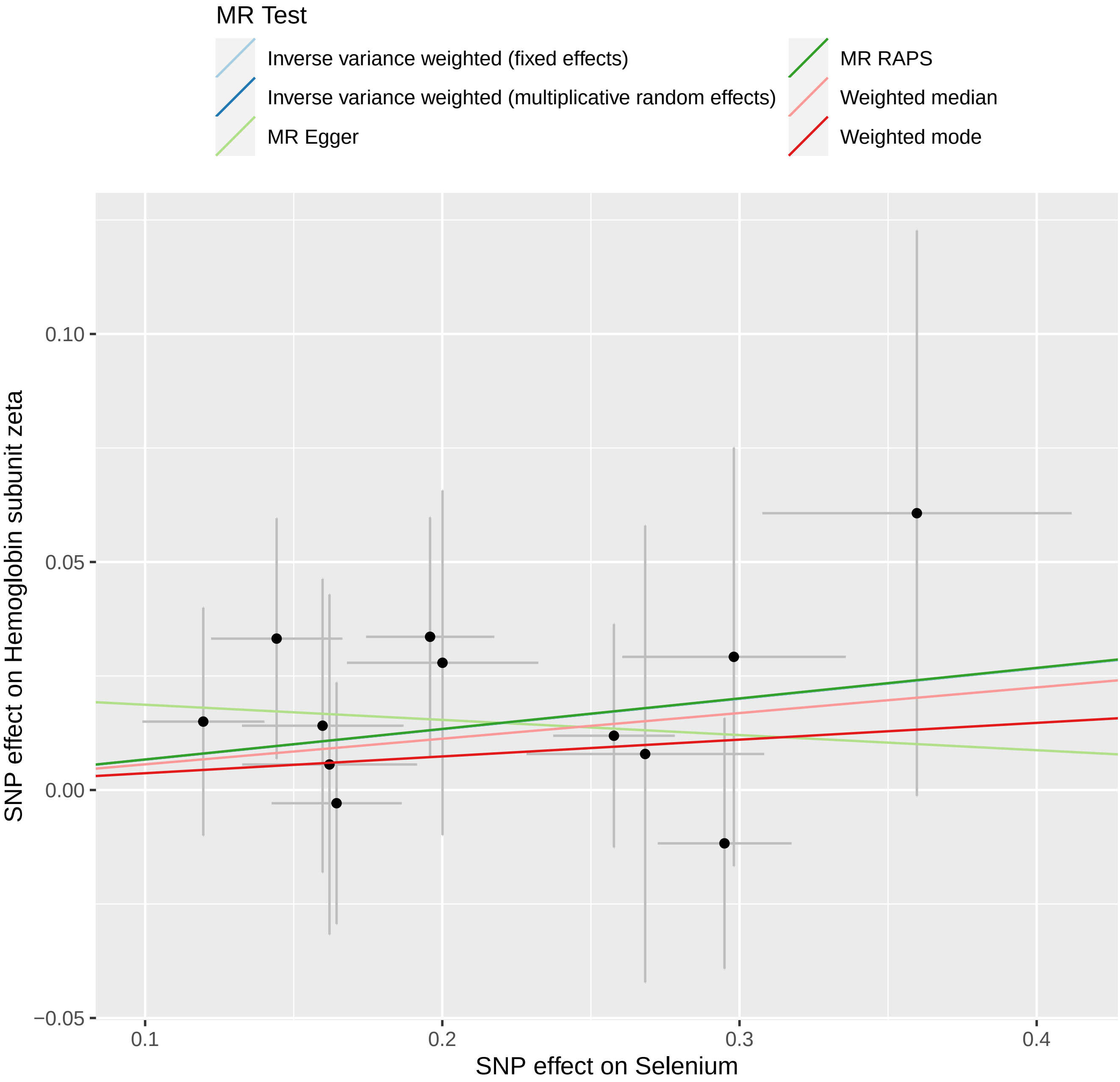

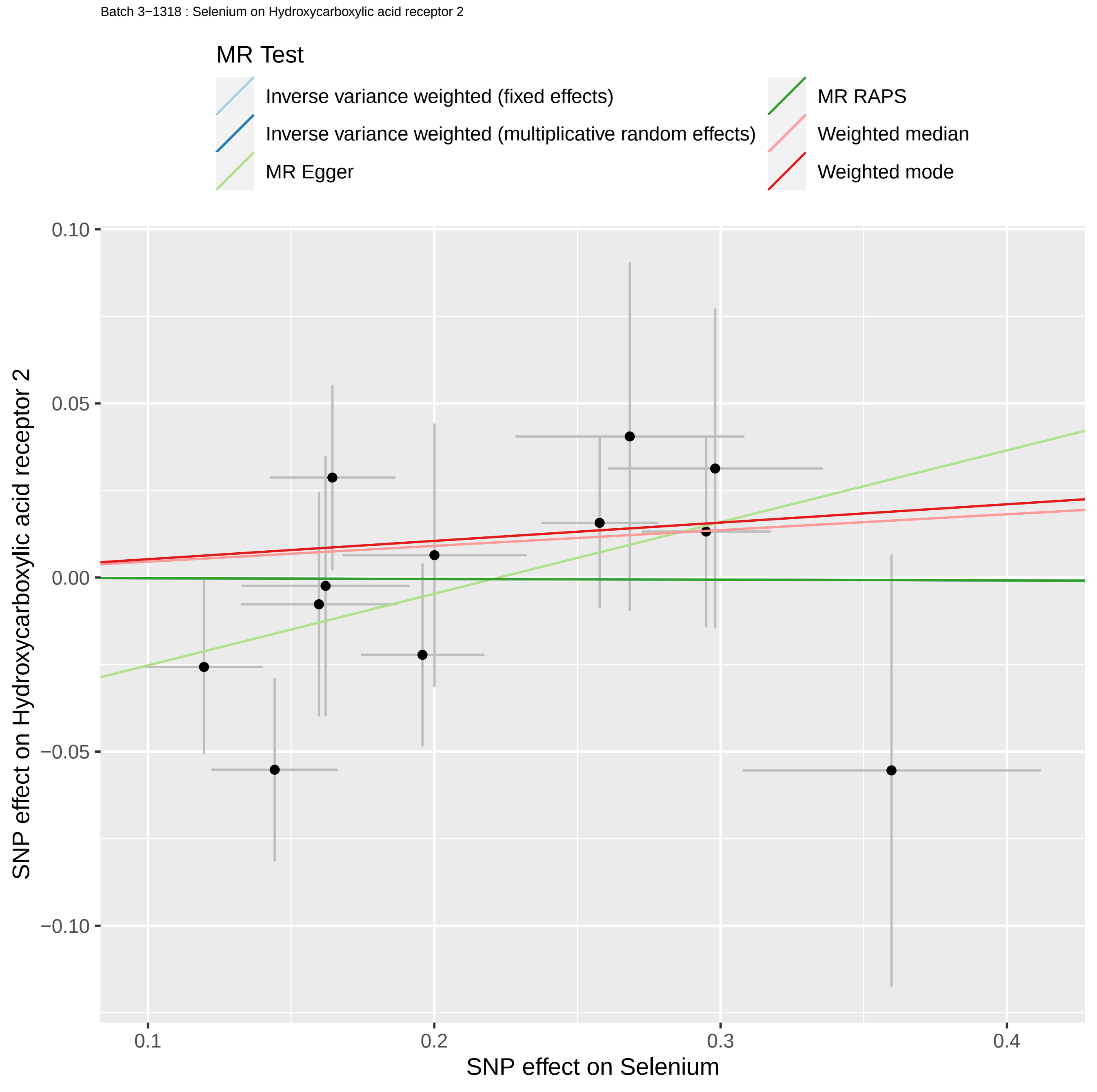

Batch 3−1318 : Selenium on Hydroxycarboxylic acid receptor 2
MR Test
Inverse variance weighted (fixed effects)
Inverse variance weighted (multiplicative random effects)
MR Egger
MR RAPS
Weighted median
Weighted mode
0.10
0.05
0.00
−0.05
−0.10
0.1
0.2
0.3
0.4
SNP effect on Hydroxycarboxylic acid receptor 2
SNP effect on Selenium

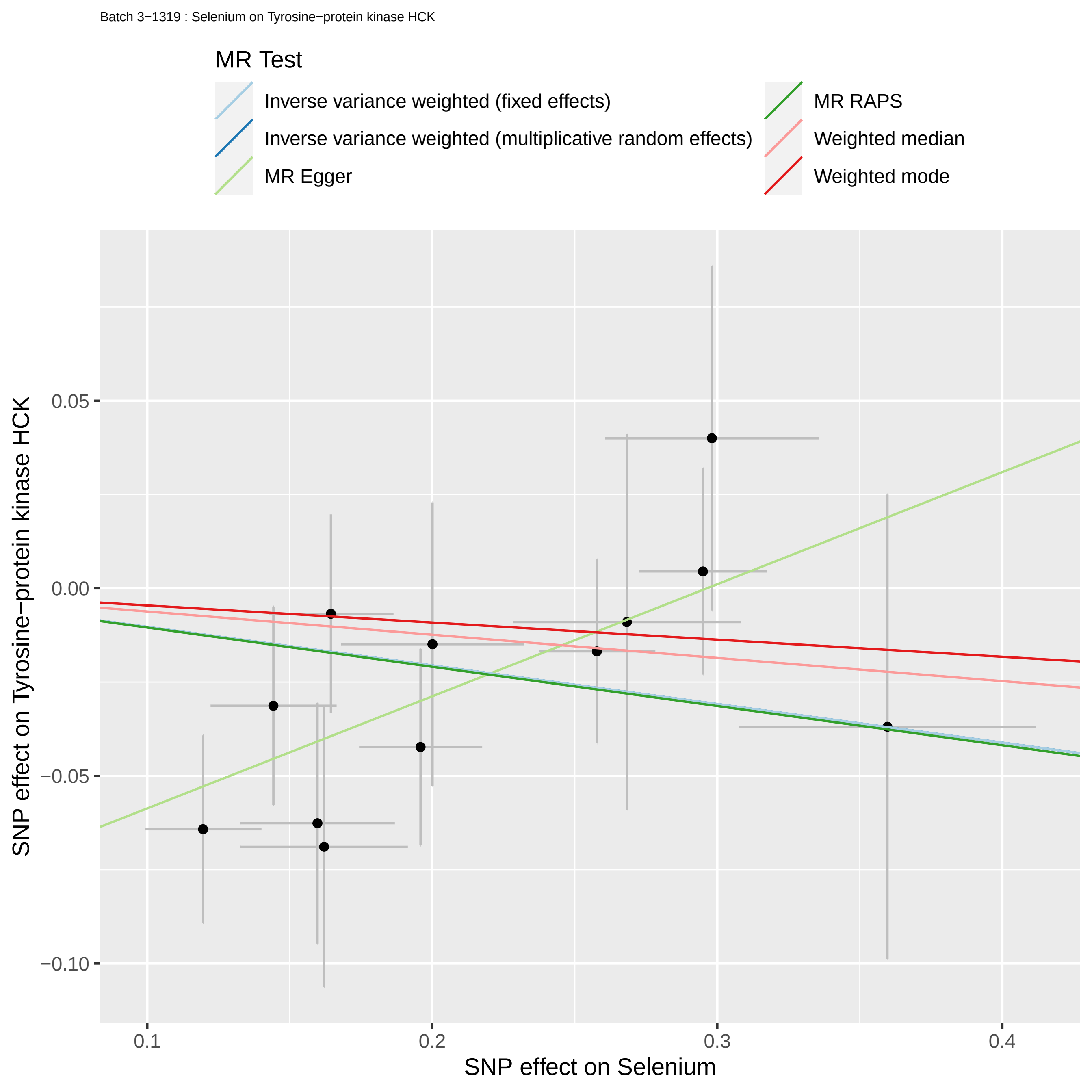

Batch 3−1319 : Selenium on Tyrosine−protein kinase HCK
MR Test
Inverse variance weighted (fixed effects)
Inverse variance weighted (multiplicative random effects)
MR Egger
MR RAPS
Weighted median
Weighted mode
0.05
0.00
−0.05
−0.10
0.1
0.2
0.3
0.4
SNP effect on Tyrosine−protein kinase HCK
SNP effect on Selenium

Batch 3−1320 : Selenium on Histone deacetylase 8

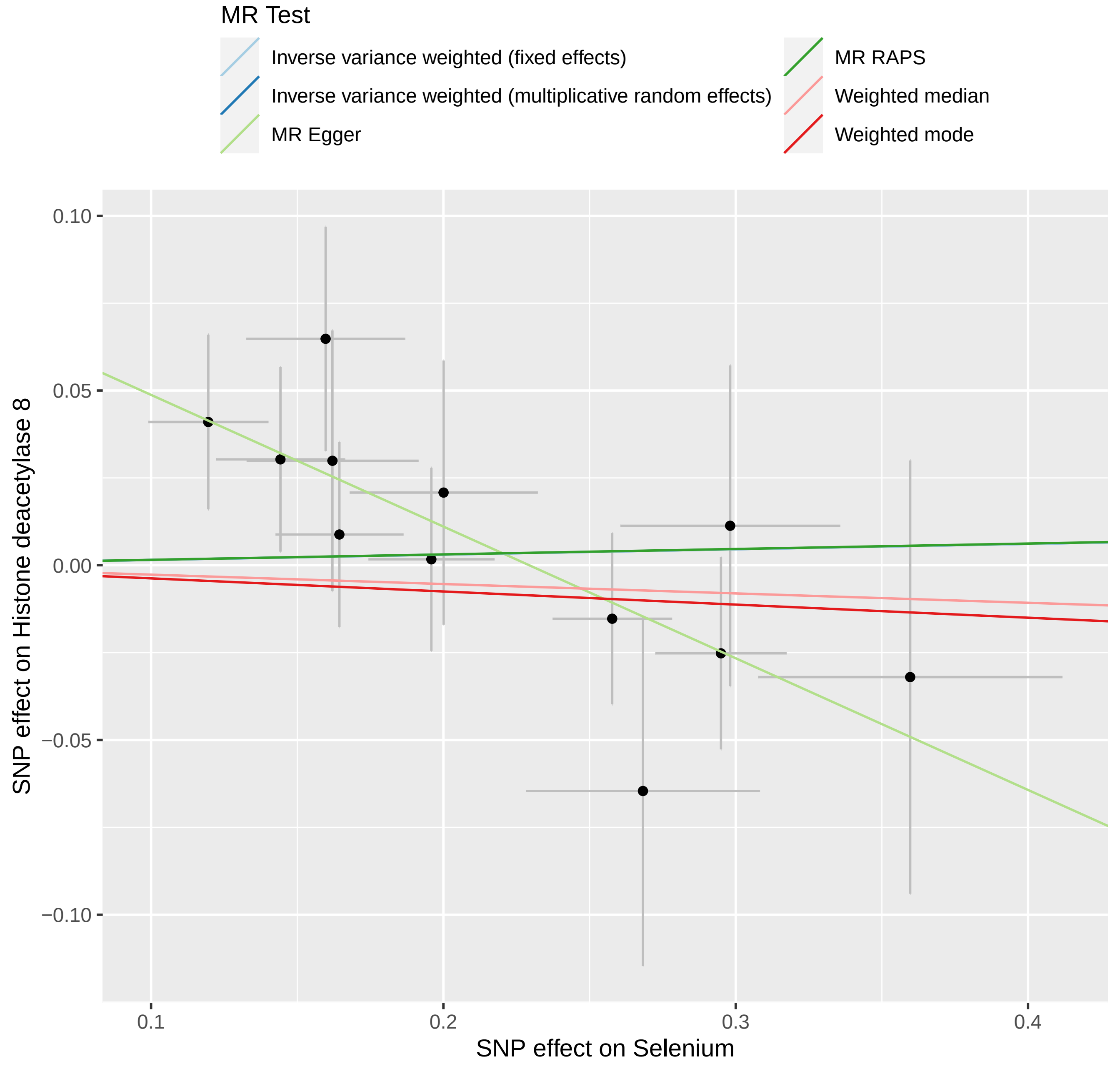

Batch 3−1321 : Selenium on Hepatoma−derived growth factor−related protein 2

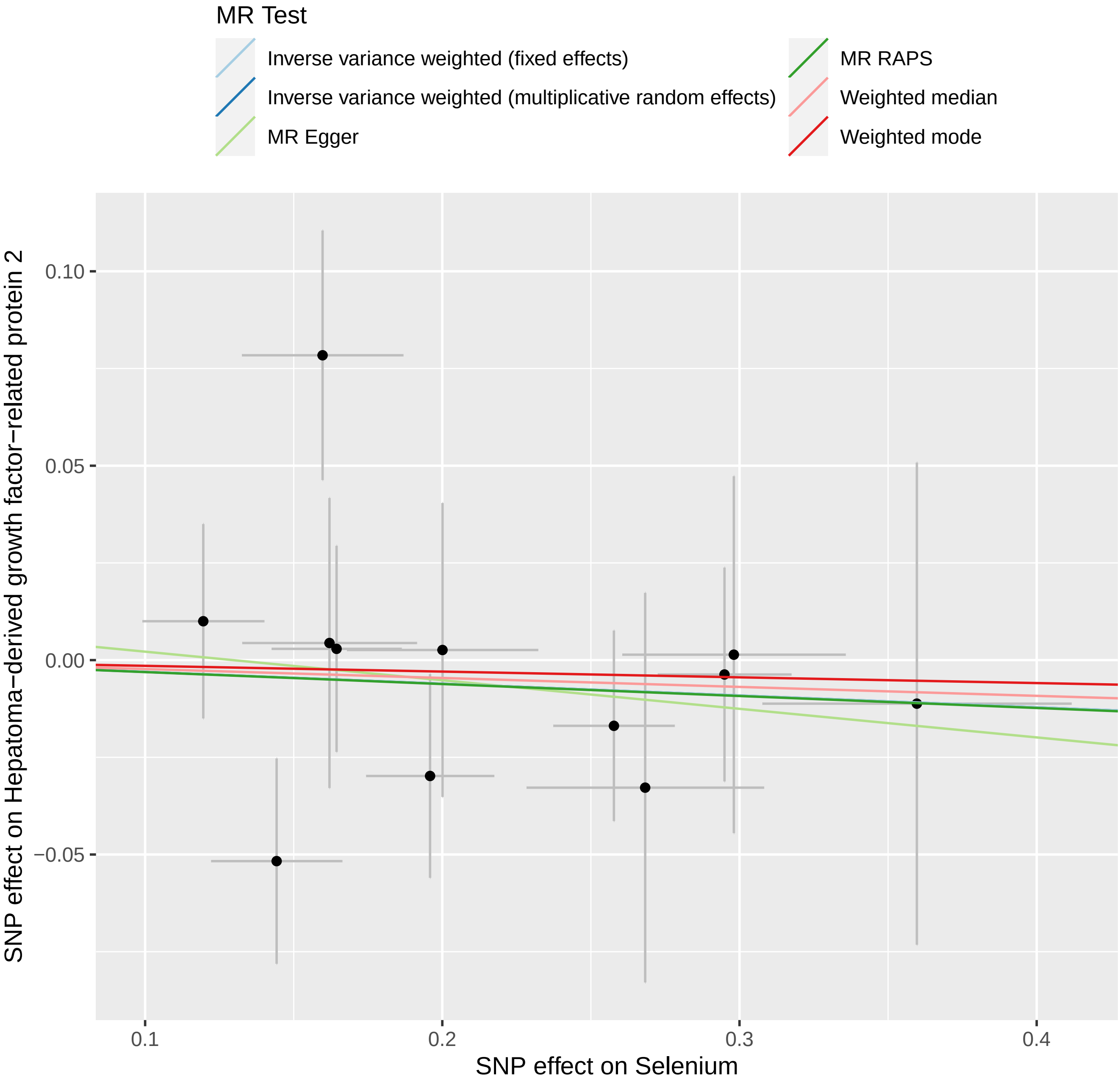

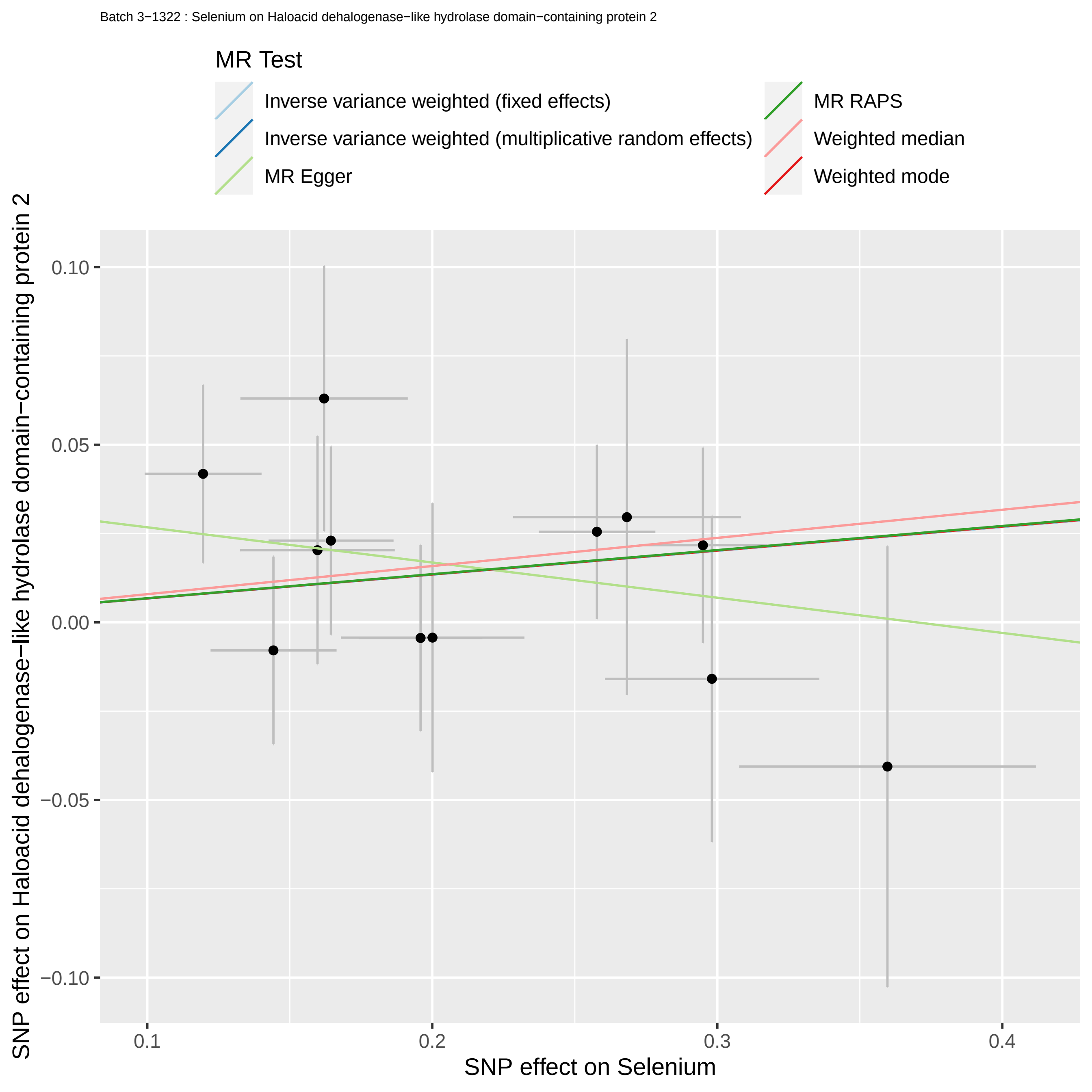
Batch 3−1322 : Selenium on Haloacid dehalogenase−like hydrolase domain−containing protein 2
MR Test
Inverse variance weighted (fixed effects)
Inverse variance weighted (multiplicative random effects)
MR Egger
MR RAPS
Weighted median
Weighted mode
SNP effect on Haloacid dehalogenase−like hydrolase domain−containing protein 2
0.10
0.05
0.00
−0.05
−0.10
0.1
0.2
0.3
0.4
SNP effect on Selenium

Batch 3−1323 : Selenium on Vigilin

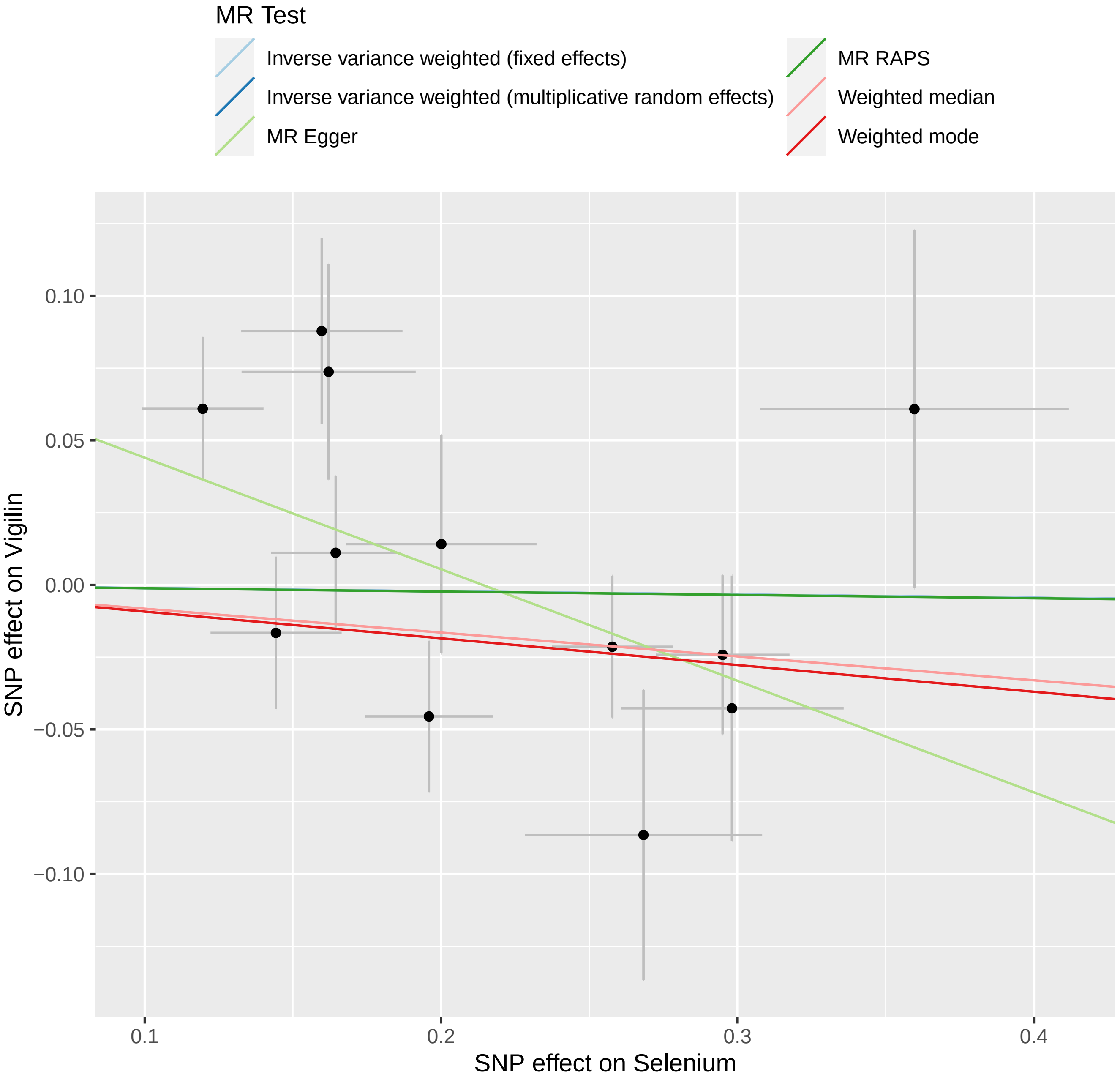

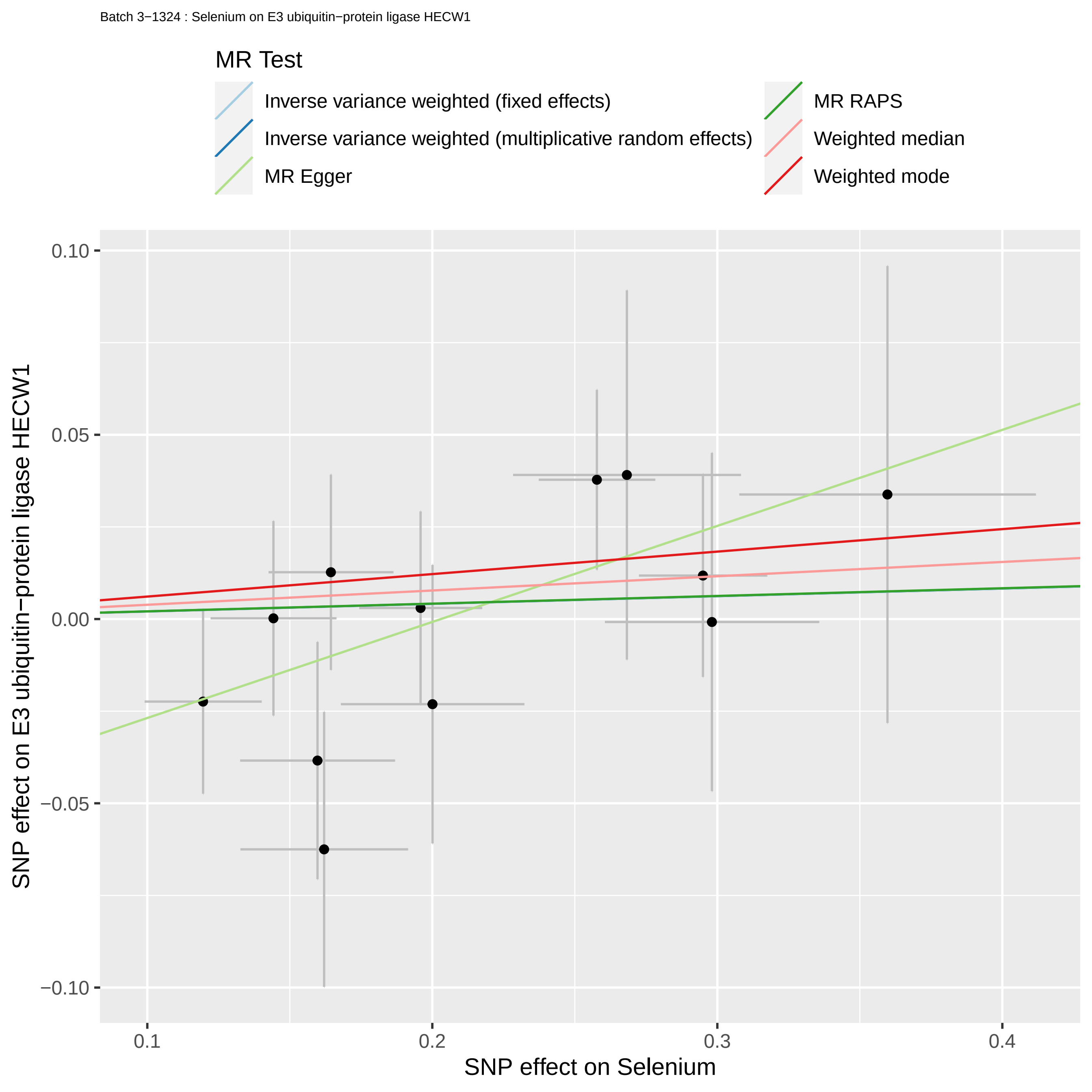
Batch 3−1324 : Selenium on E3 ubiquitin−protein ligase HECW1
MR Test
Inverse variance weighted (fixed effects)
Inverse variance weighted (multiplicative random effects)
MR Egger
MR RAPS
Weighted median
Weighted mode
0.10
0.05
0.00
−0.05
−0.10
0.1
0.2
0.3
0.4
SNP effect on Selenium
SNP effect on E3 ubiquitin−protein ligase HECW1

Batch 3−1325 : Selenium on Protein HEG homolog 1

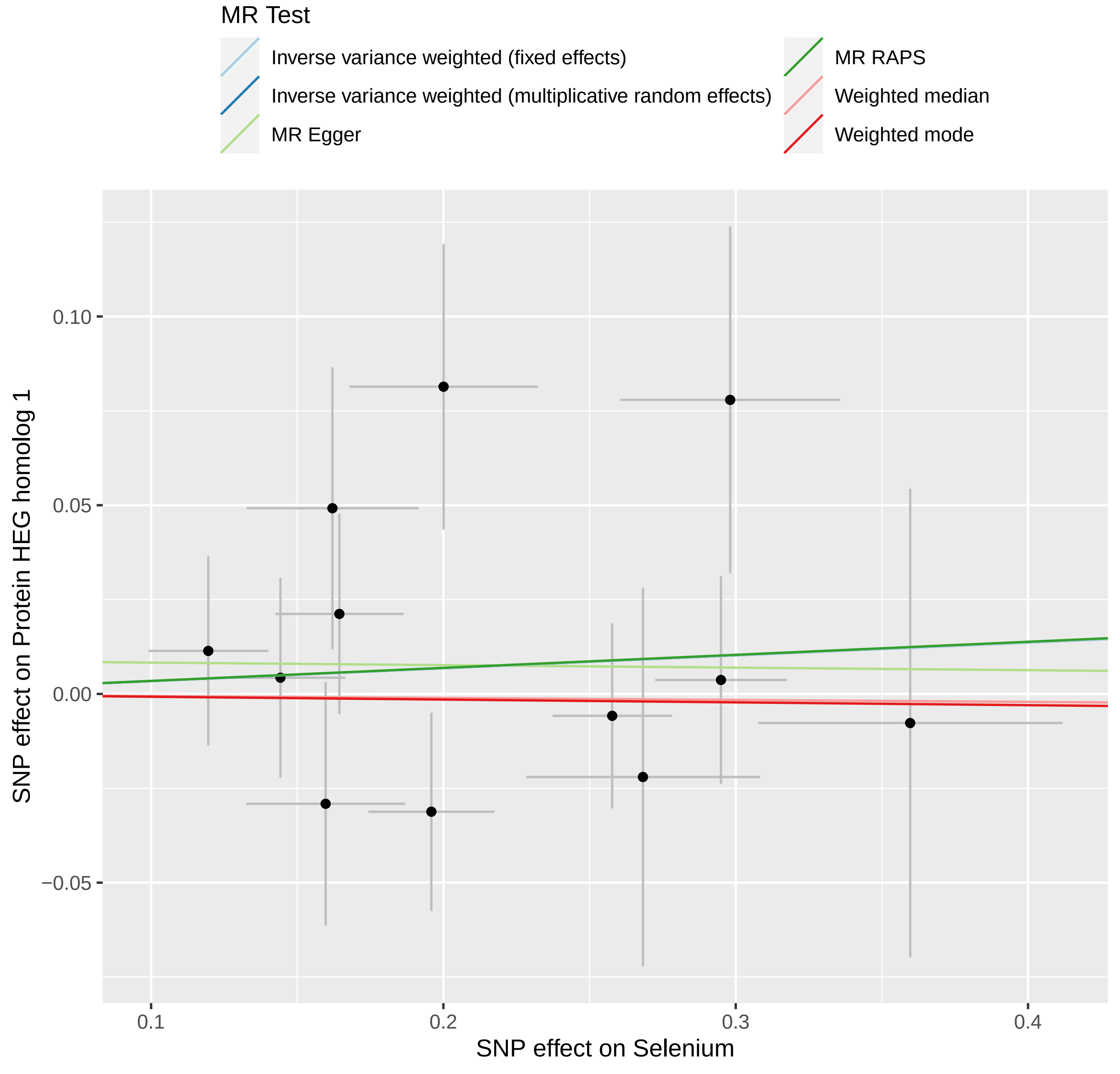

Batch 3−1326 : Selenium on HEPACAM family member 2

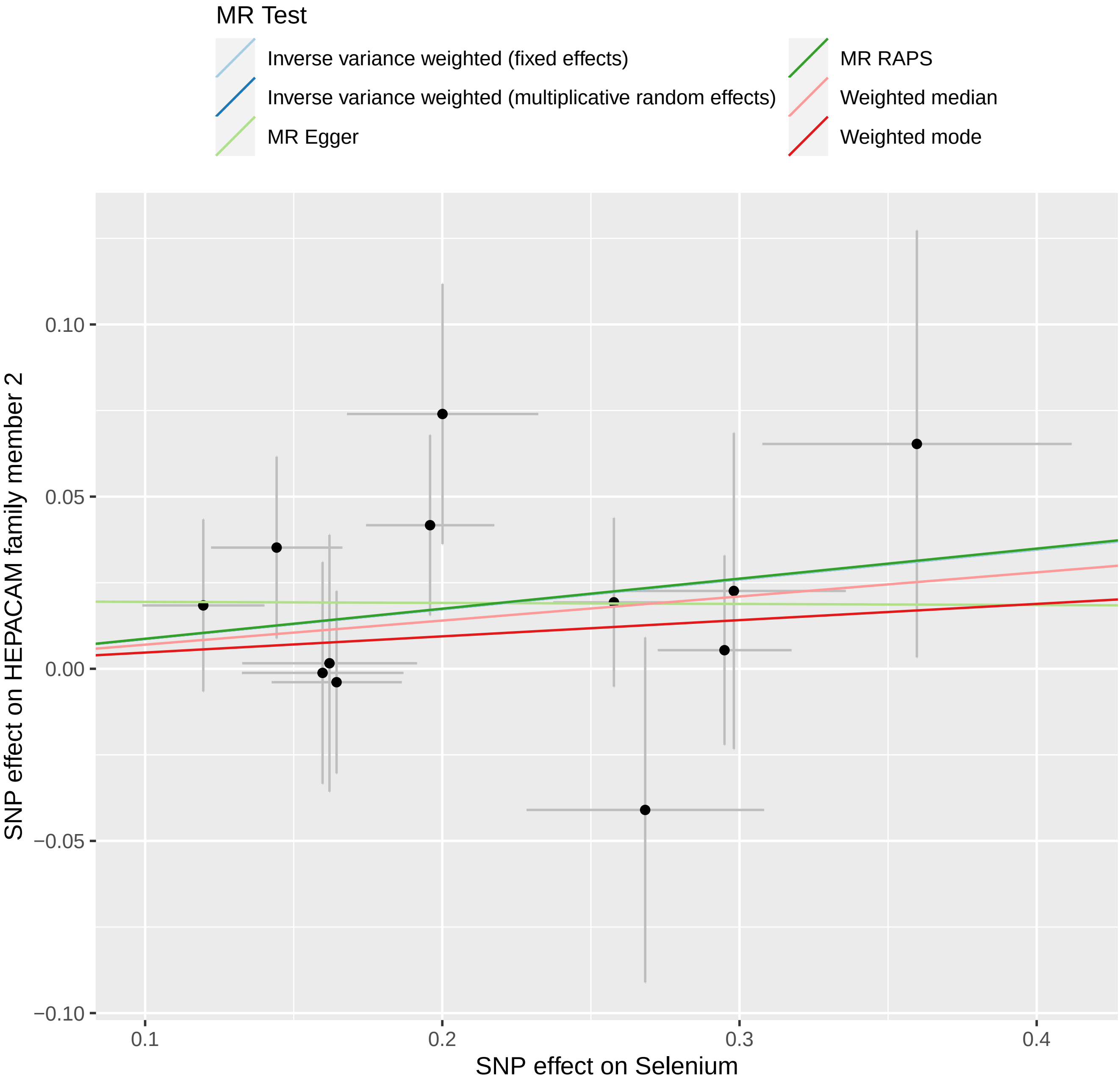

Batch 3−1327 : Selenium on HEPACAM family member 2

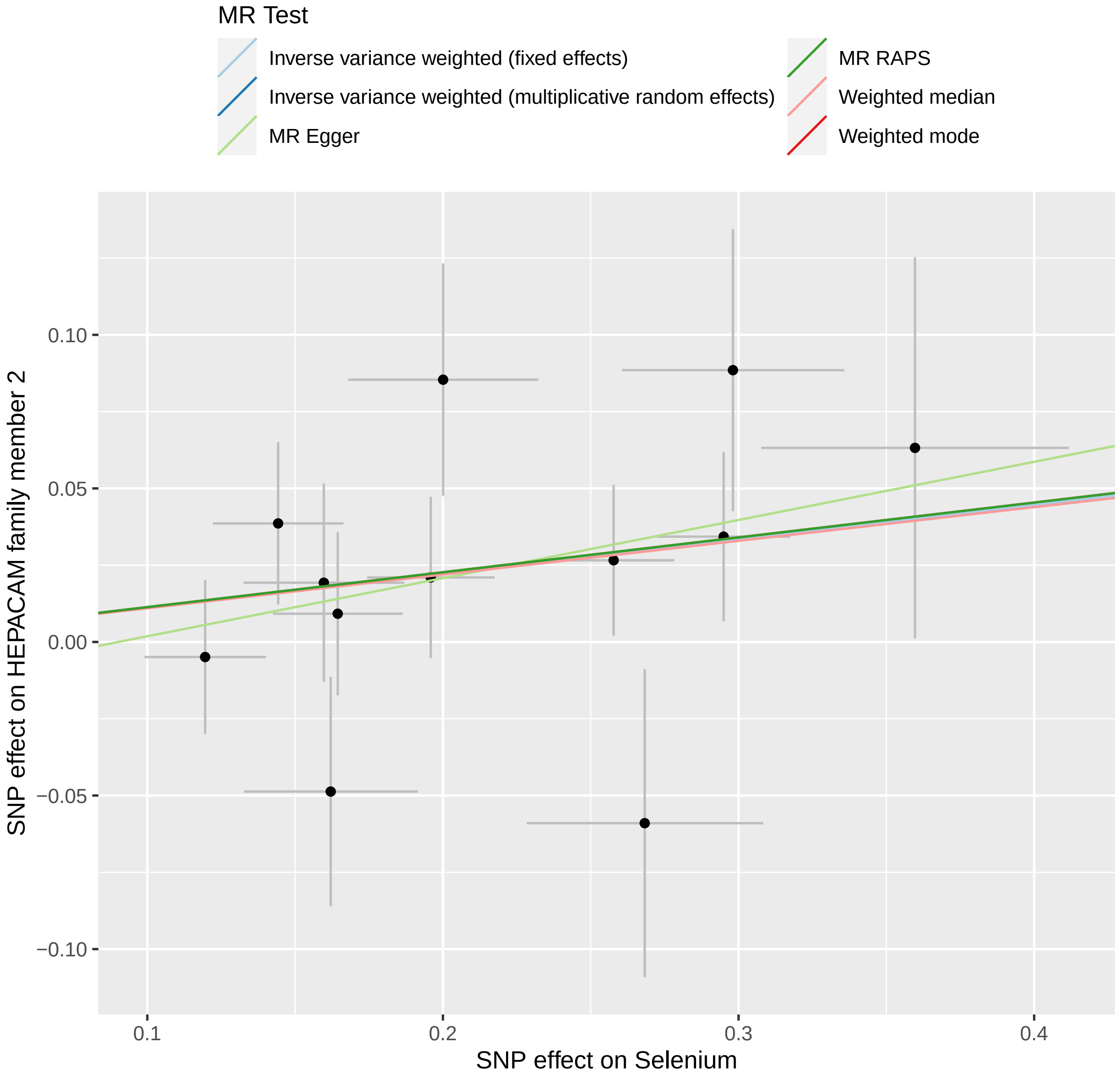

Batch 3−1328 : Selenium on Hephaestin−like protein 1

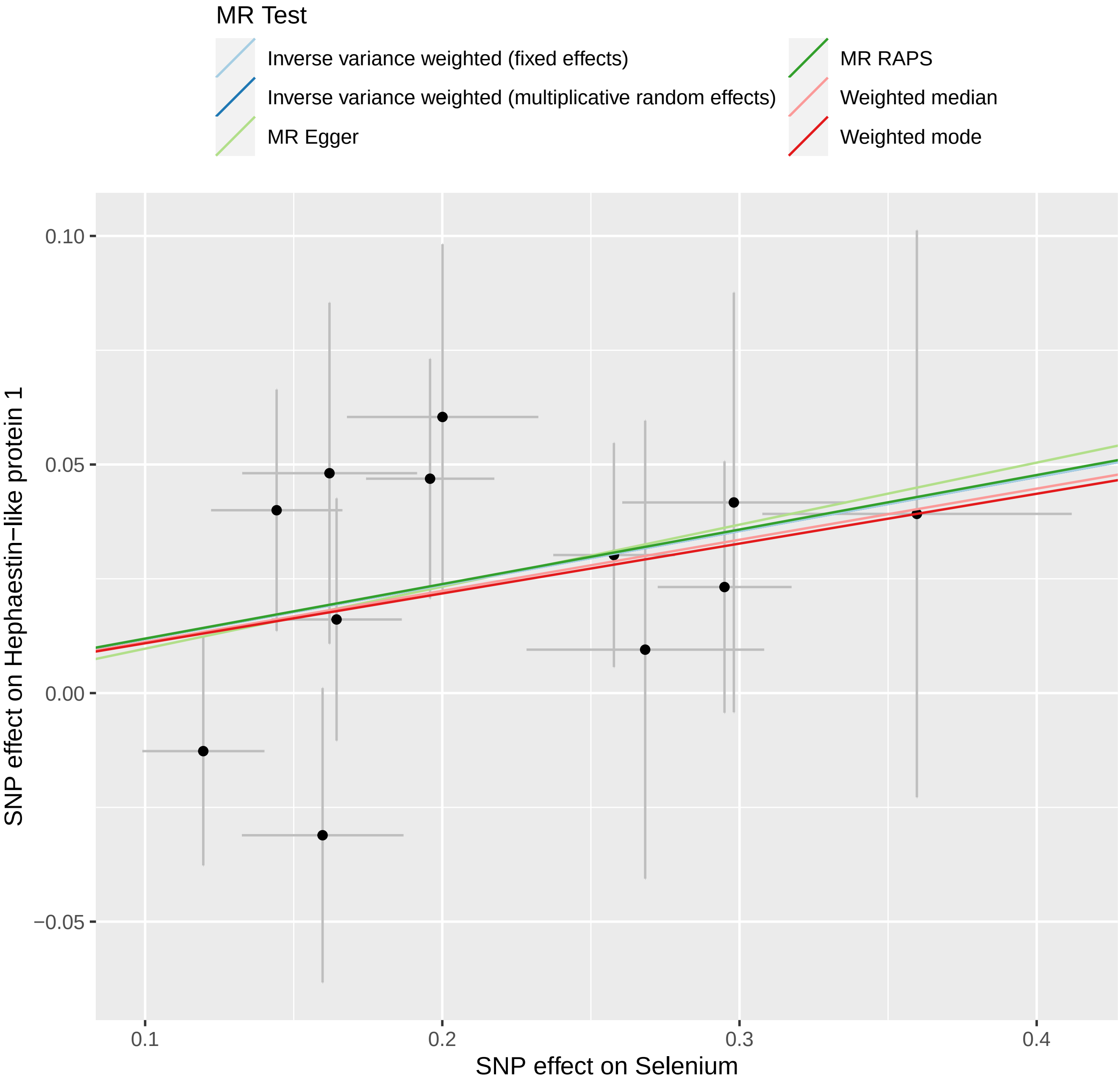

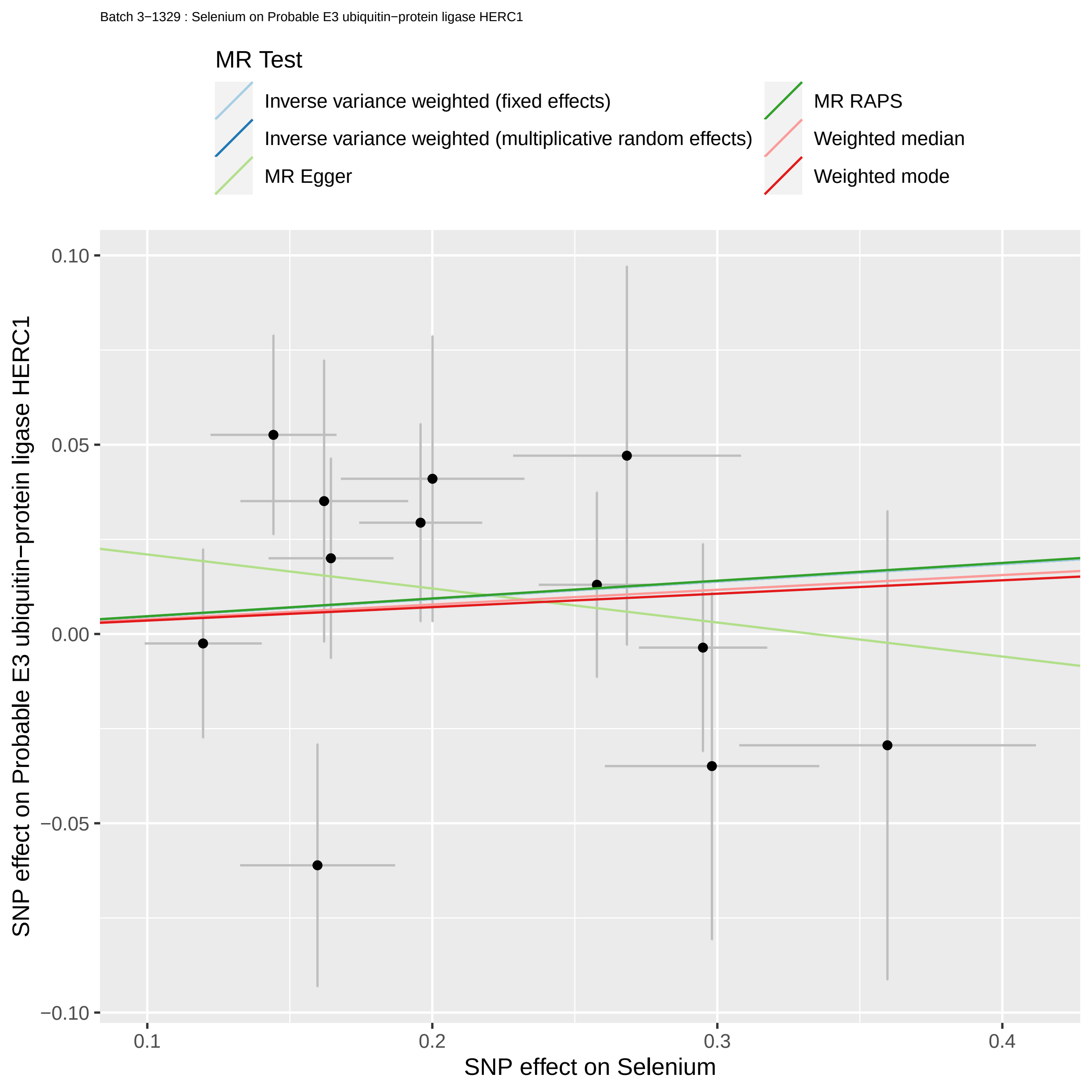
Batch 3−1329 : Selenium on Probable E3 ubiquitin−protein ligase HERC1
MR Test
Inverse variance weighted (fixed effects)
Inverse variance weighted (multiplicative random effects)
MR Egger
MR RAPS
Weighted median
Weighted mode
0.10
0.05
0.00
−0.05
−0.10
0.1
0.2
0.3
0.4
SNP effect on Selenium
SNP effect on Probable E3 ubiquitin−protein ligase HERC1

Batch 3−1330 : Selenium on Probable E3 ubiquitin−protein ligase HERC4

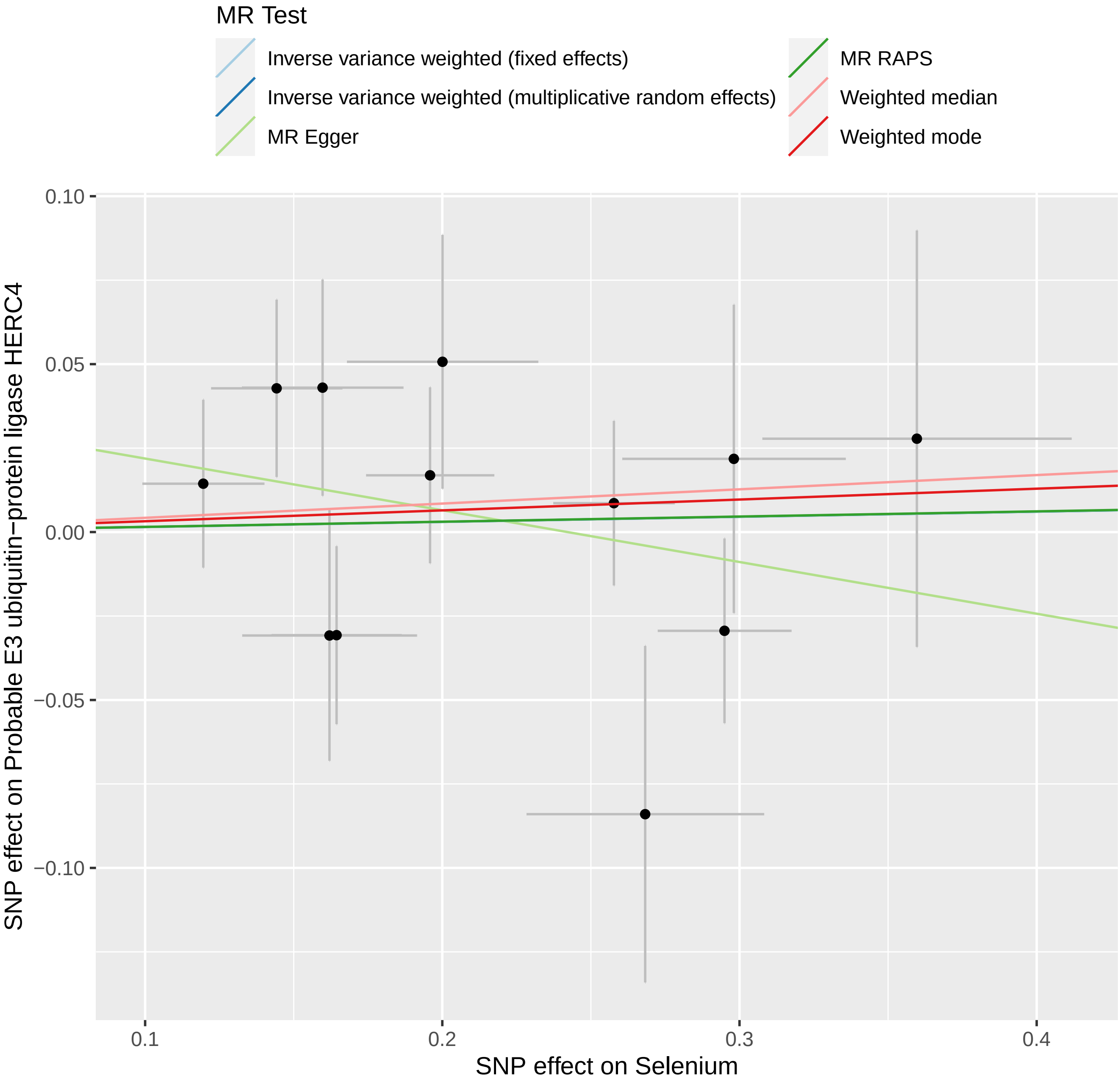

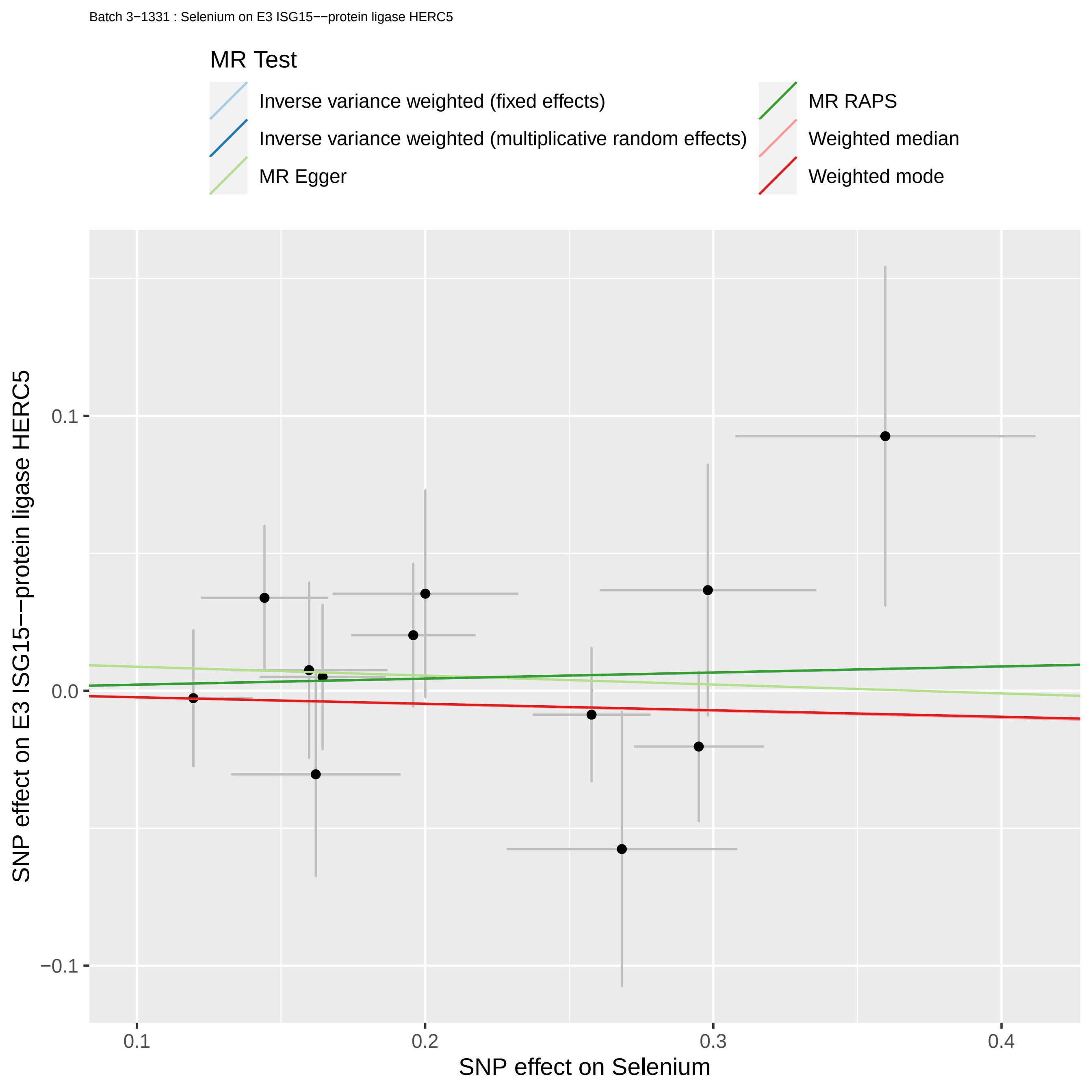
Batch 3−1331 : Selenium on E3 ISG15−−protein ligase HERC5
MR Test
Inverse variance weighted (fixed effects)
Inverse variance weighted (multiplicative random effects)
MR Egger
MR RAPS
Weighted median
Weighted mode
SNP effect on E3 ISG15−−protein ligase HERC5
0.1
0.0
−0.1
0.1
0.2
0.3
0.4
SNP effect on Selenium

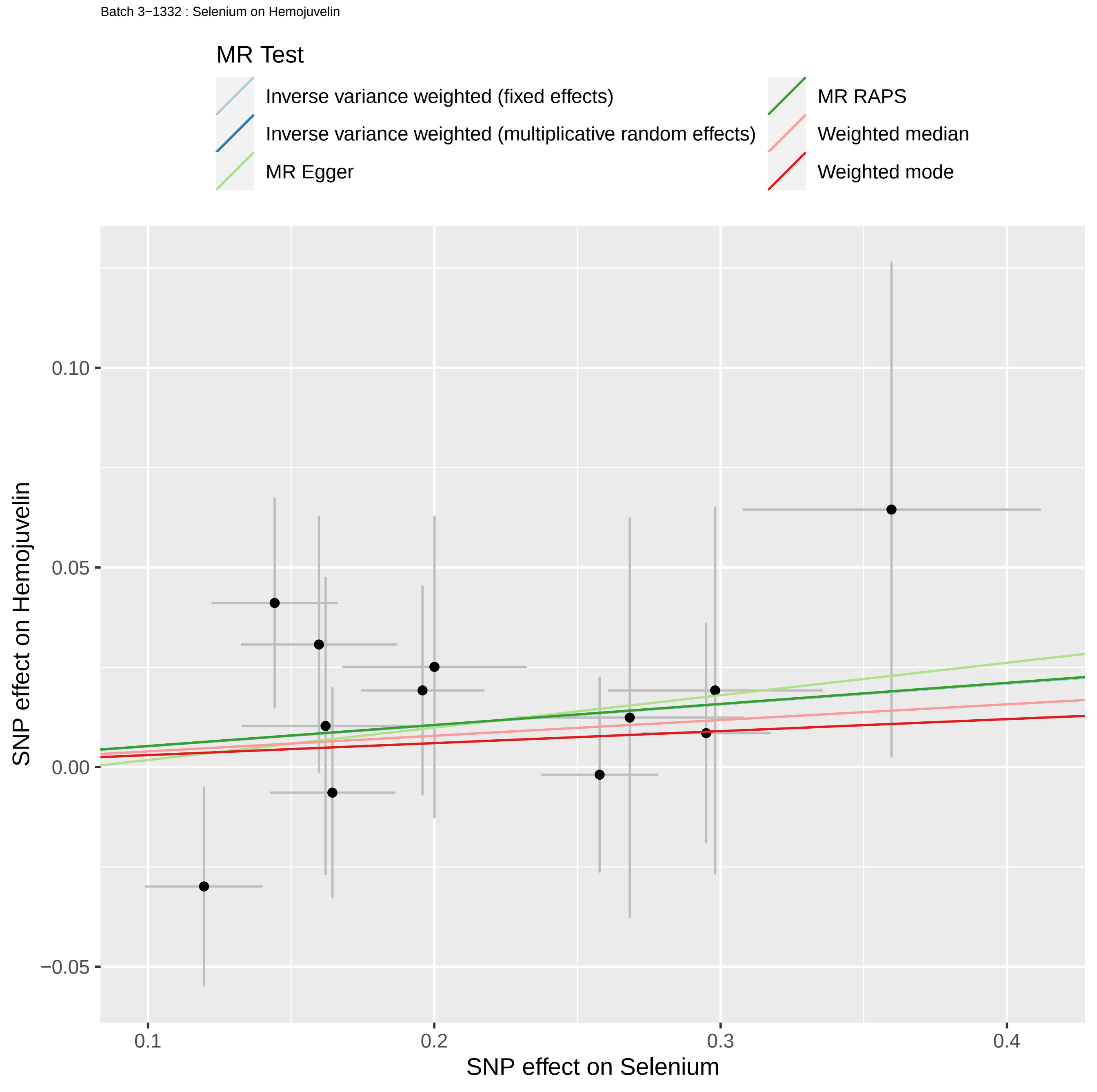

Batch 3−1332 : Selenium on Hemojuvelin
MR Test
Inverse variance weighted (fixed effects)
Inverse variance weighted (multiplicative random effects)
MR Egger
MR RAPS
Weighted median
Weighted mode
SNP effect on Hemojuvelin
SNP effect on Selenium
0.10
0.05
0.00
−0.05
0.1
0.2
0.3
0.4

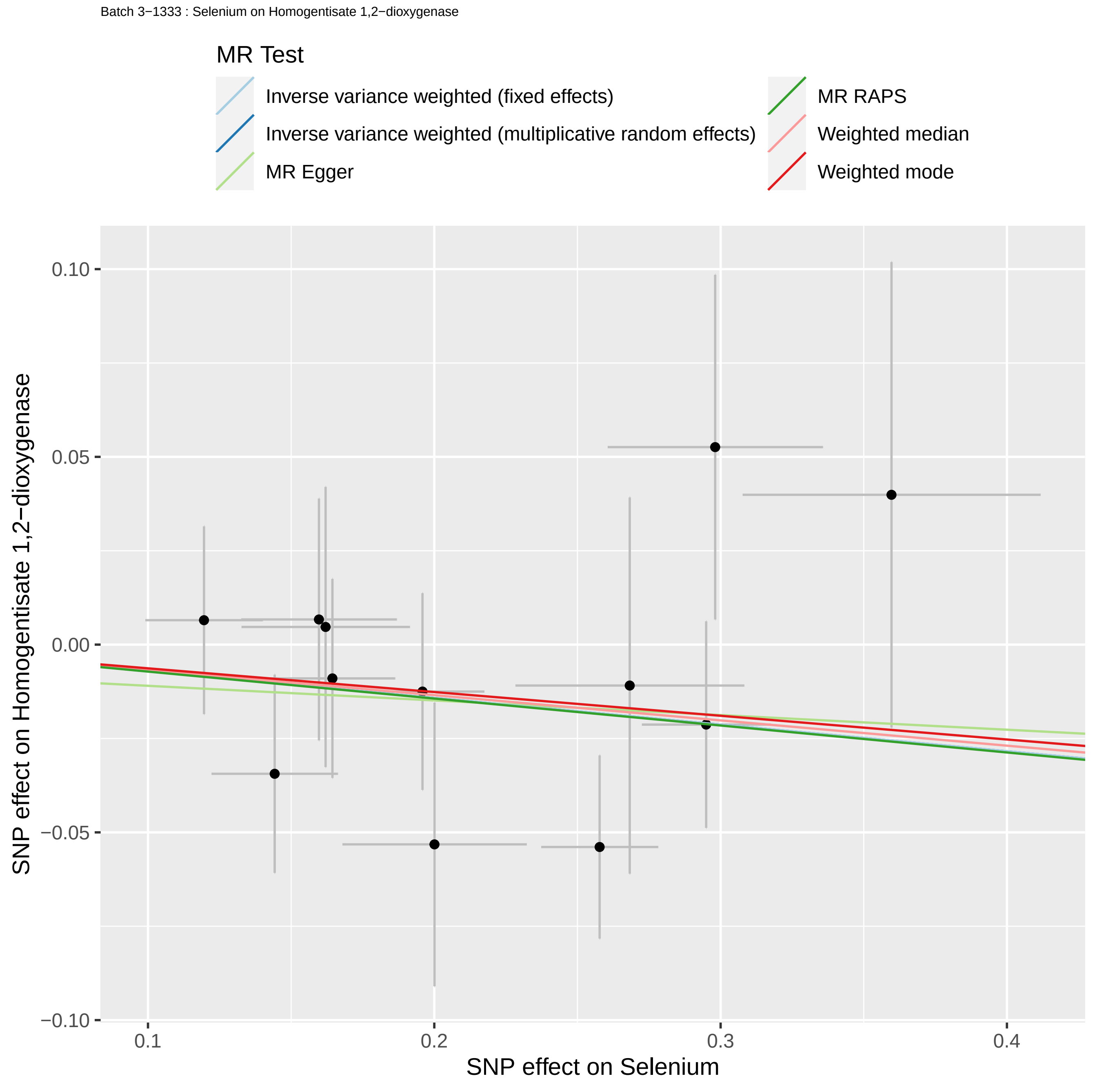
Batch 3−1333 : Selenium on Homogentisate 1,2−dioxygenase
MR Test
Inverse variance weighted (fixed effects)
Inverse variance weighted (multiplicative random effects)
MR Egger
MR RAPS
Weighted median
Weighted mode
0.10
0.05
0.00
−0.05
−0.10
0.1
0.2
0.3
0.4
SNP effect on Homogentisate 1,2−dioxygenase
SNP effect on Selenium

Batch 3−1334 : Selenium on Hepatocyte growth factor

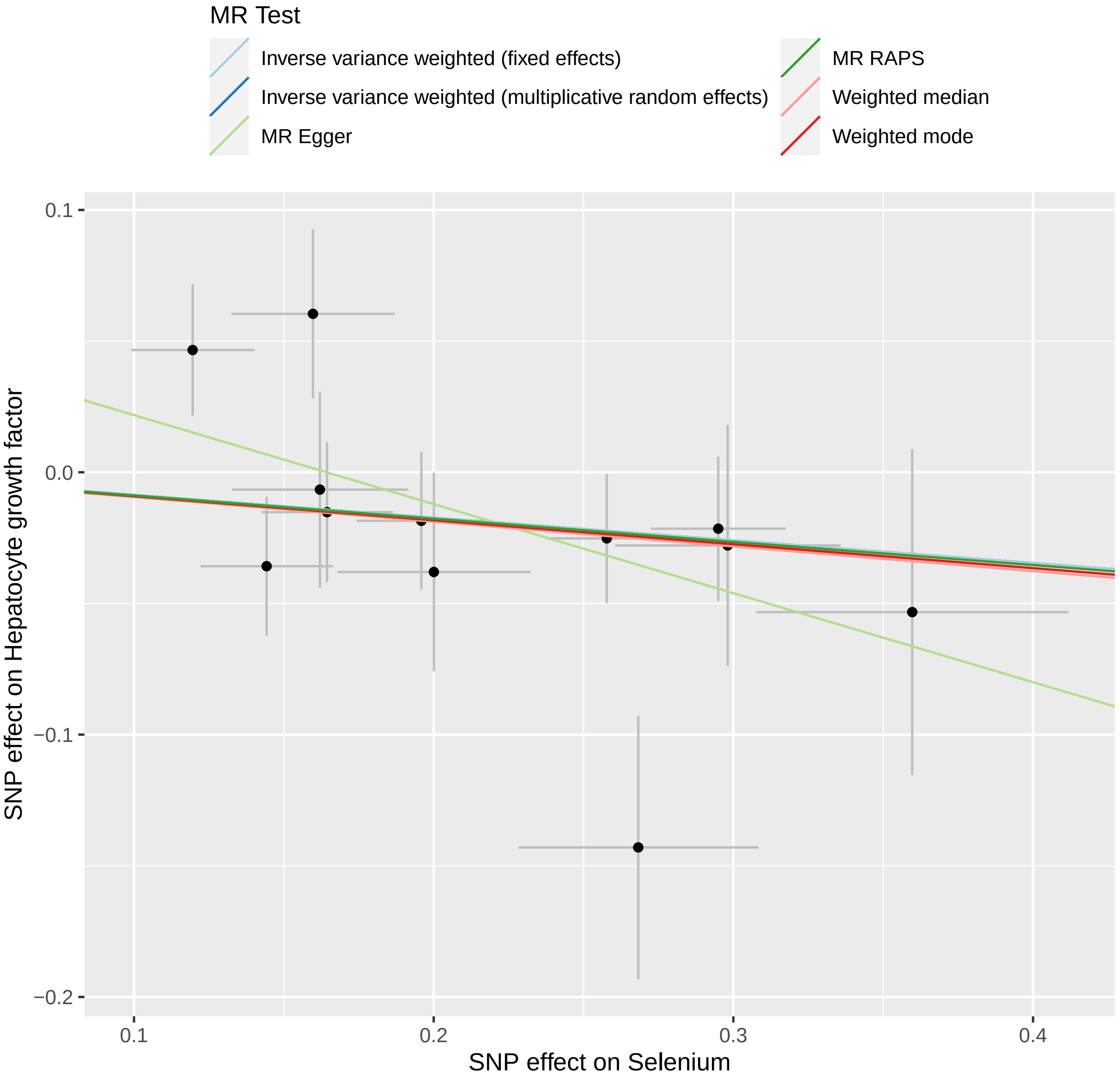

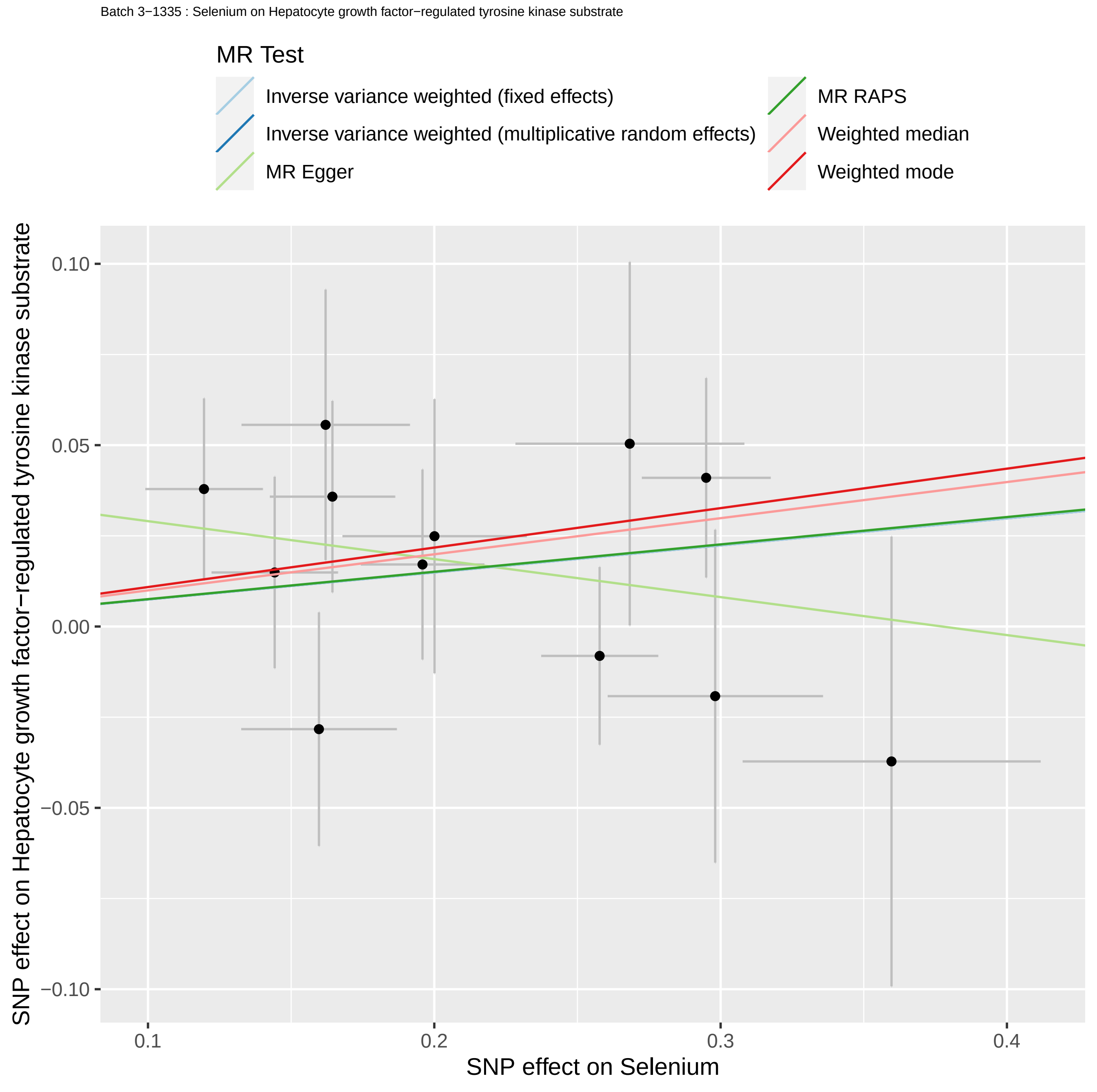

Batch 3−1335 : Selenium on Hepatocyte growth factor−regulated tyrosine kinase substrate
MR Test
Inverse variance weighted (fixed effects)
Inverse variance weighted (multiplicative random effects)
MR Egger
MR RAPS
Weighted median
Weighted mode
SNP effect on Hepatocyte growth factor−regulated tyrosine kinase substrate
0.10
0.05
0.00
−0.05
−0.10
0.1
0.2
0.3
0.4
SNP effect on Selenium

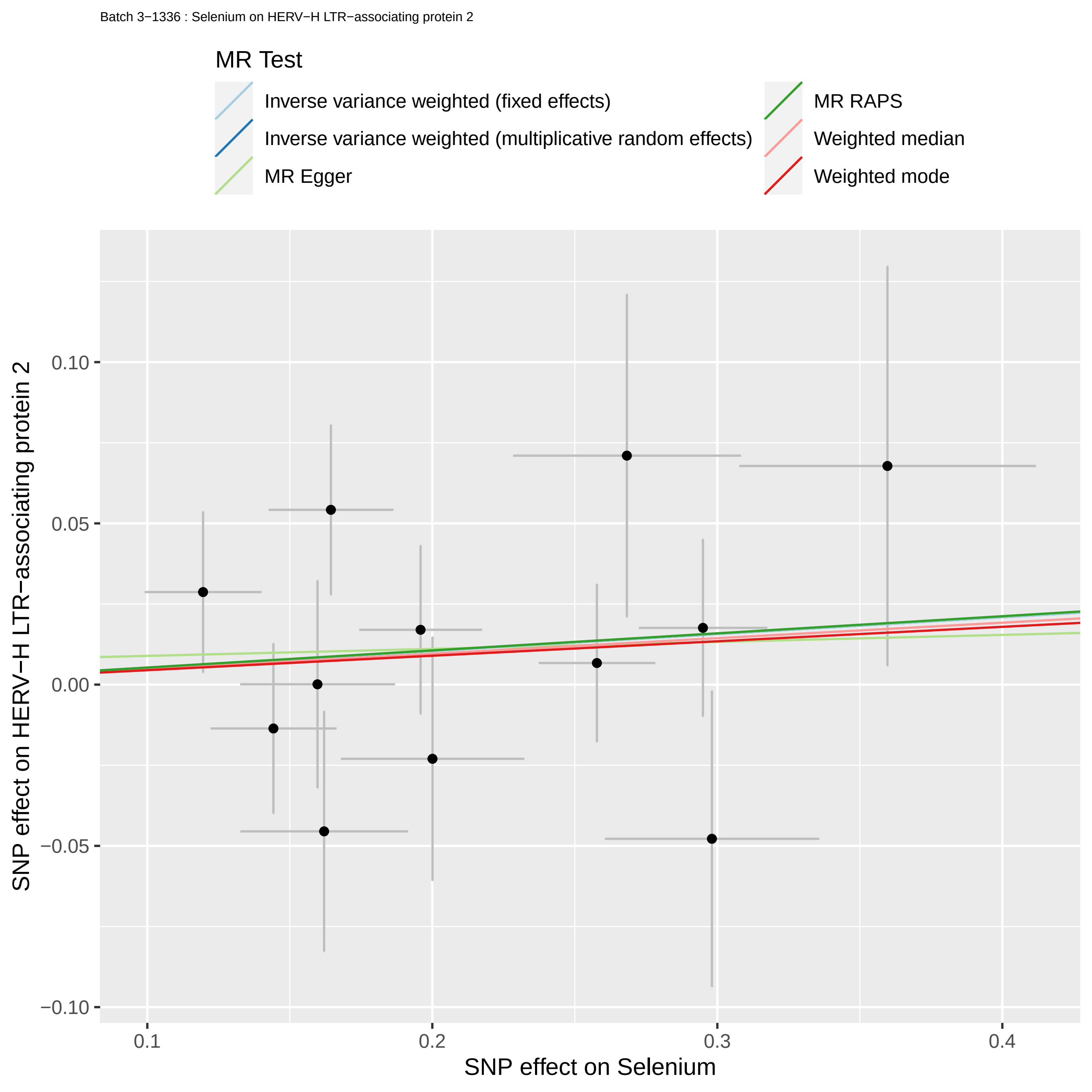
Batch 3−1336 : Selenium on HERV−H LTR−associating protein 2
MR Test
Inverse variance weighted (fixed effects)
Inverse variance weighted (multiplicative random effects)
MR Egger
MR RAPS
Weighted median
Weighted mode
0.10
0.05
0.00
−0.05
−0.10
0.1
0.2
0.3
0.4
SNP effect on HERV−H LTR−associating protein 2
SNP effect on Selenium

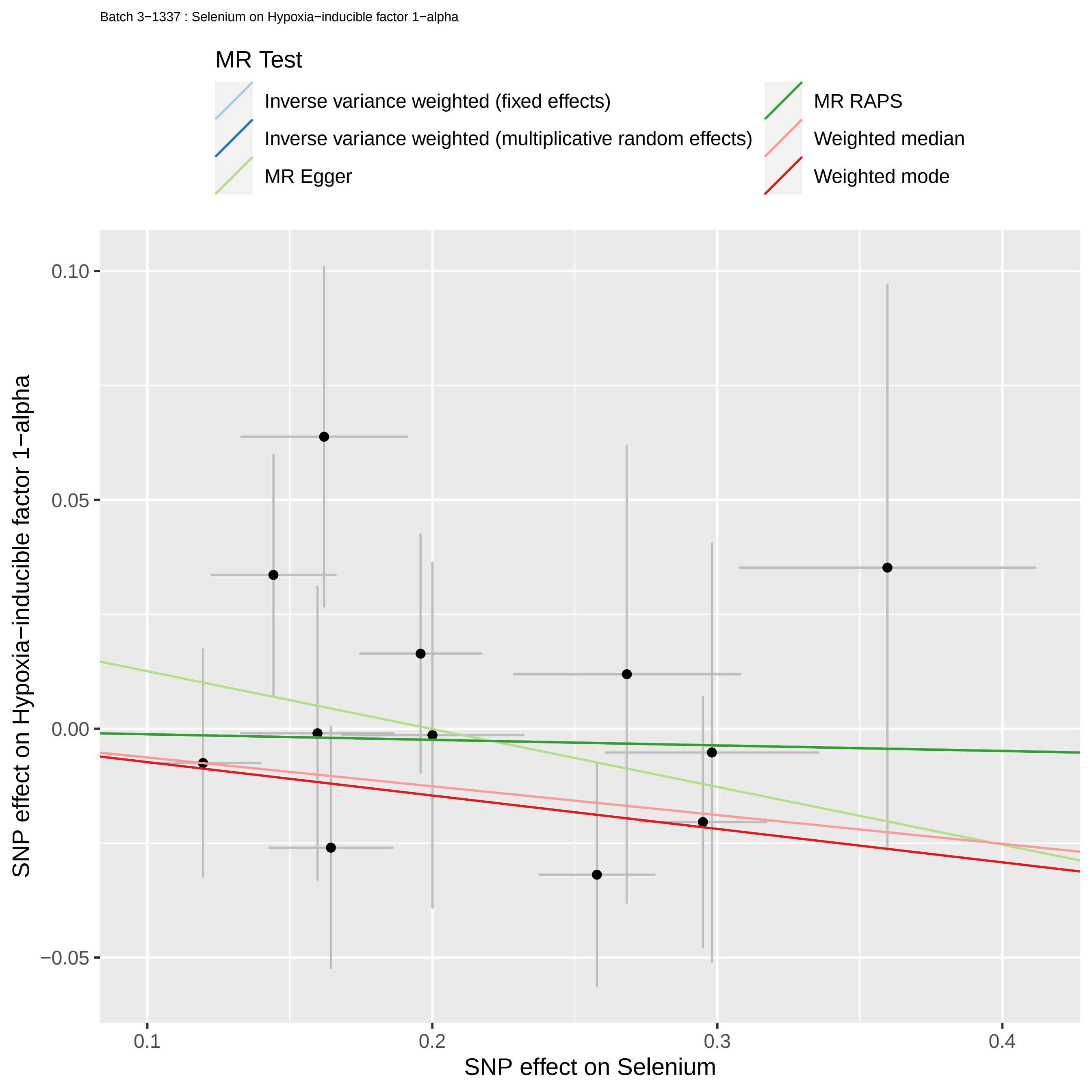

Batch 3−1337 : Selenium on Hypoxia−inducible factor 1−alpha
MR Test
Inverse variance weighted (fixed effects)
Inverse variance weighted (multiplicative random effects)
MR Egger
MR RAPS
Weighted median
Weighted mode
0.10
0.05
0.00
−0.05
0.1
0.2
0.3
0.4
SNP effect on Hypoxia−inducible factor 1−alpha
SNP effect on Selenium

Batch 3−1338 : Selenium on Histidine triad nucleotide−binding protein 1

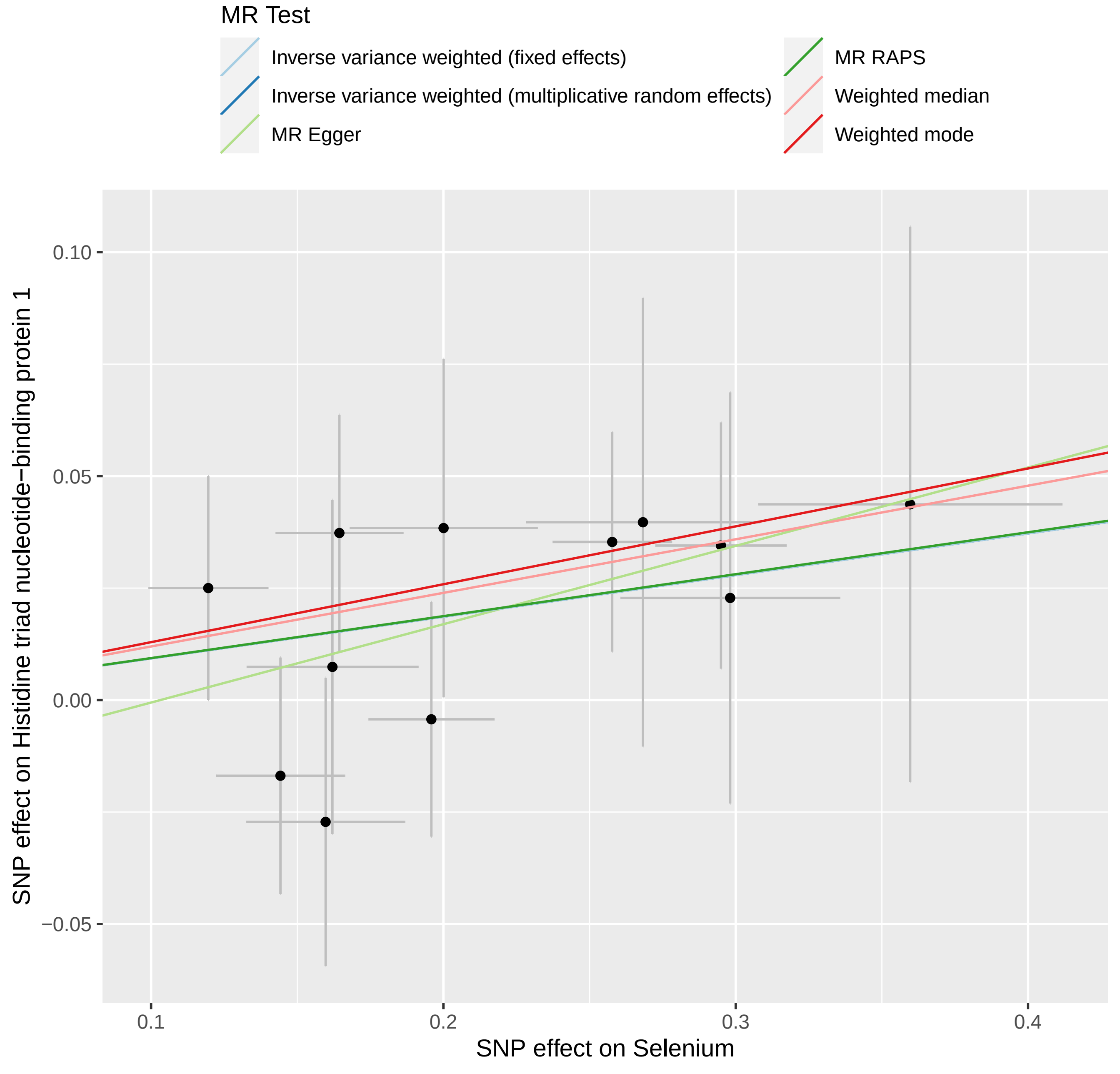

Batch 3−1339 : Selenium on Histidine triad nucleotide−binding protein 2, mitochondrial

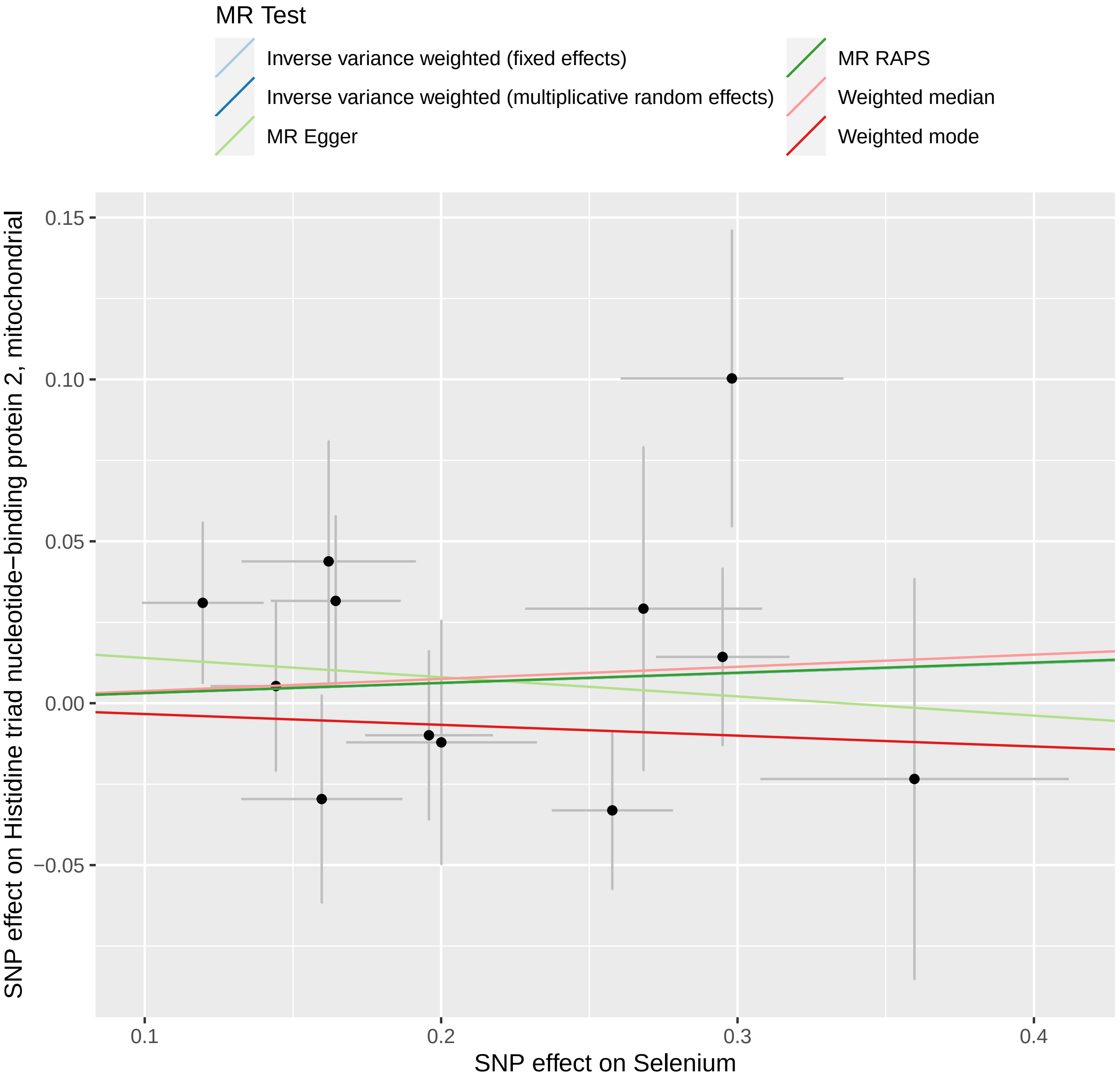

Batch 3−1340 : Selenium on Homeodomain−interacting protein kinase 3

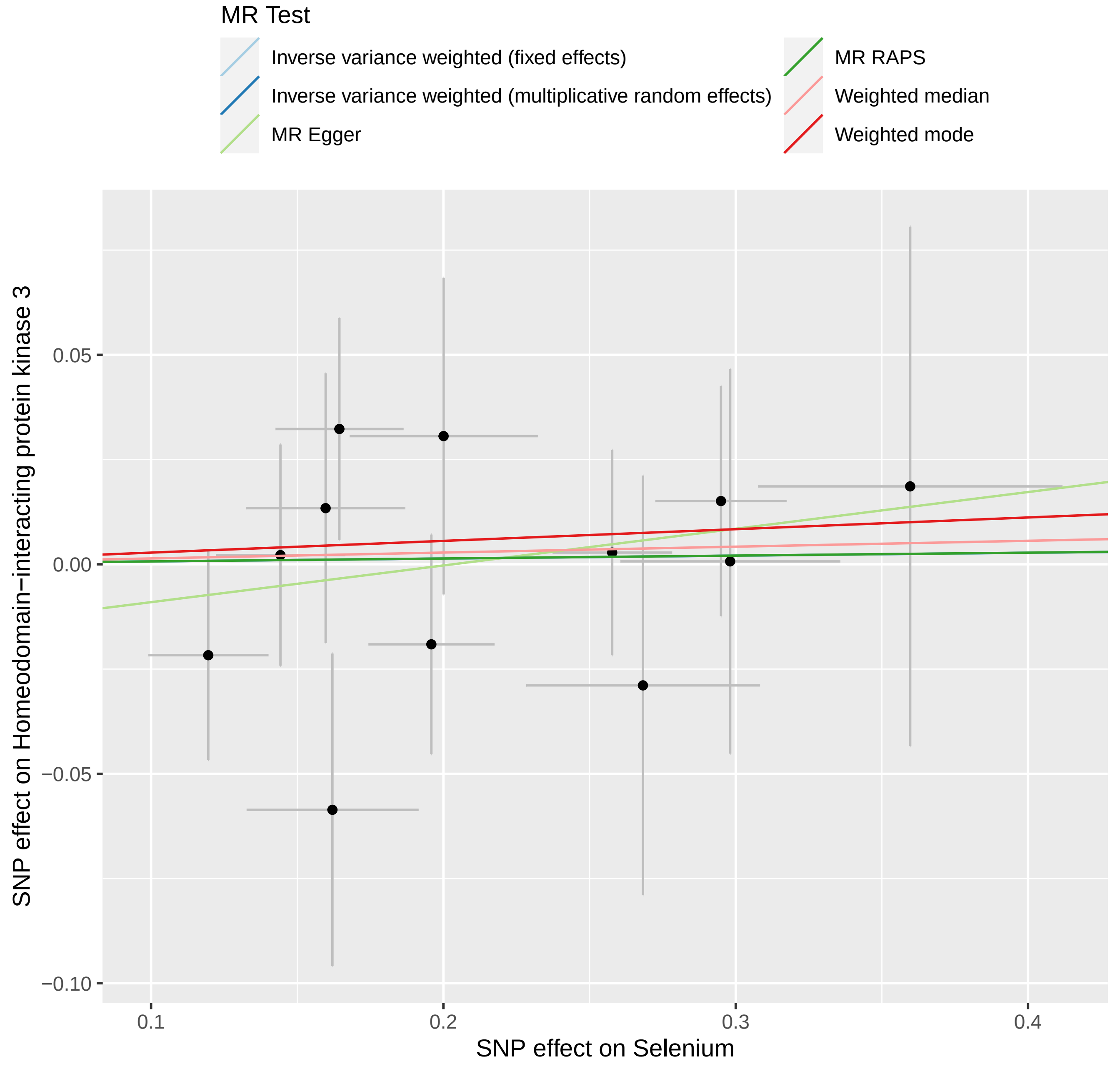

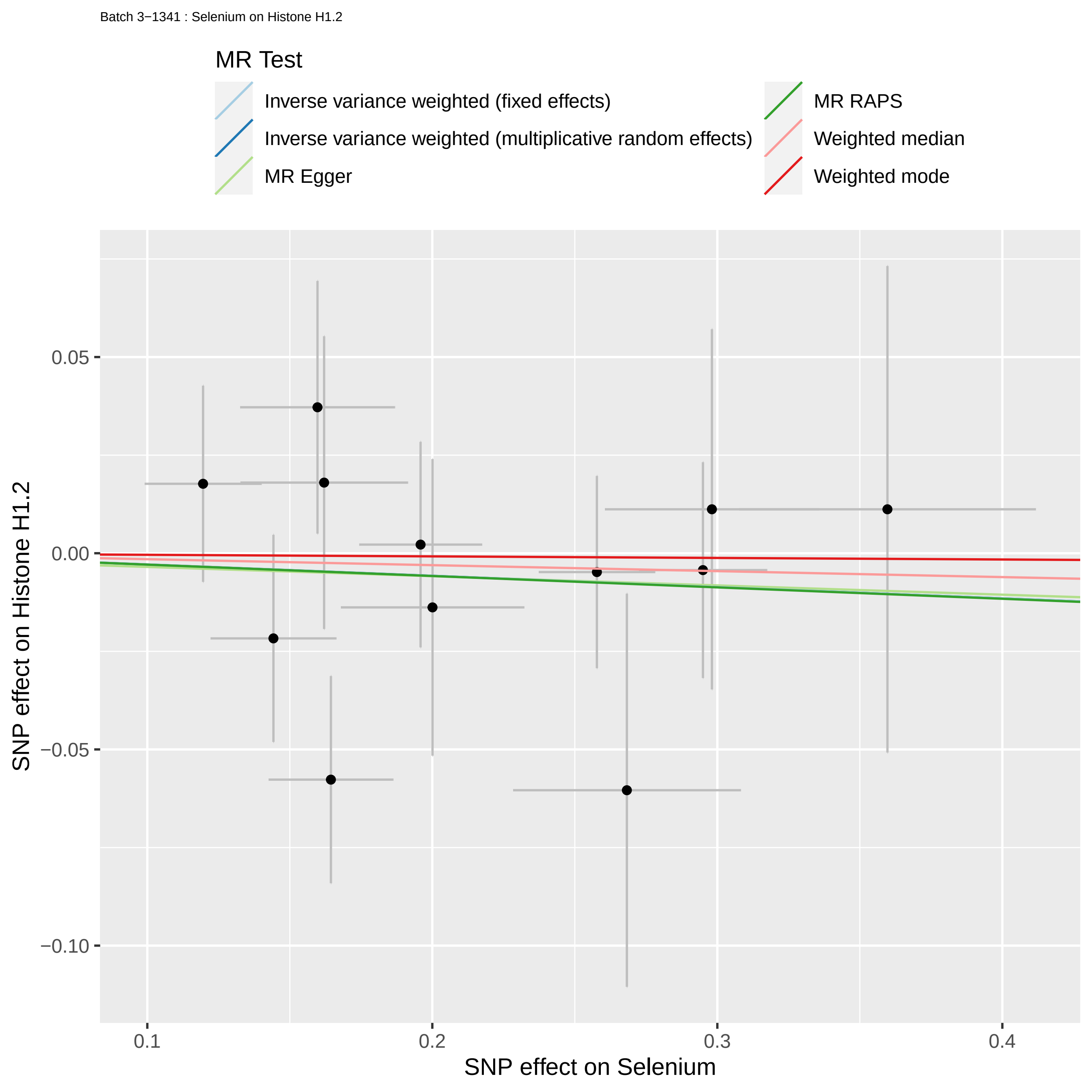

Batch 3−1341 : Selenium on Histone H1.2
MR Test
Inverse variance weighted (fixed effects)
Inverse variance weighted (multiplicative random effects)
MR Egger
MR RAPS
Weighted median
Weighted mode
0.05
0.00
−0.05
−0.10
0.1
0.2
0.3
0.4
SNP effect on Histone H1.2
SNP effect on Selenium

Batch 3−1342 : Selenium on Histone H2A type 3

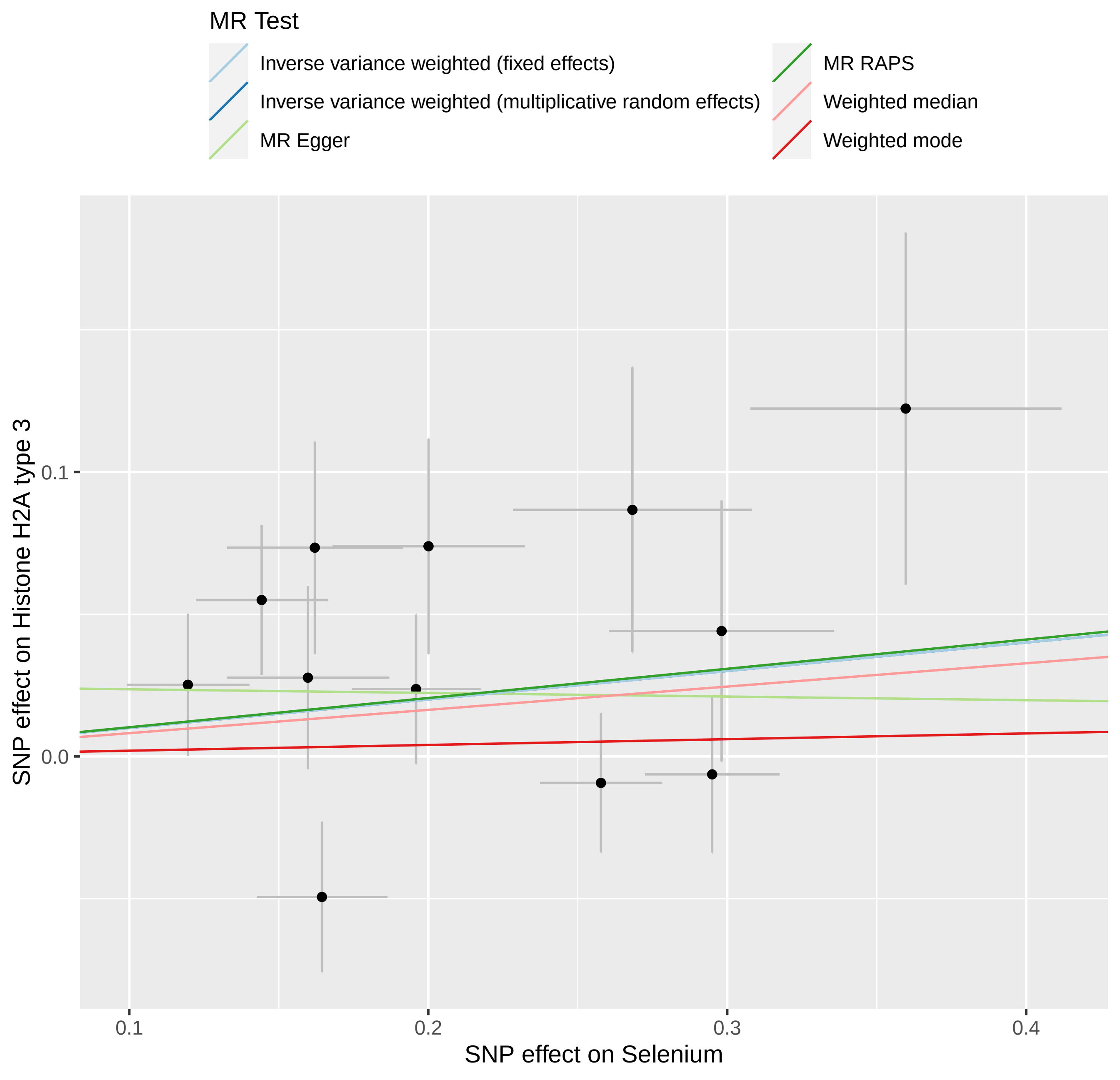

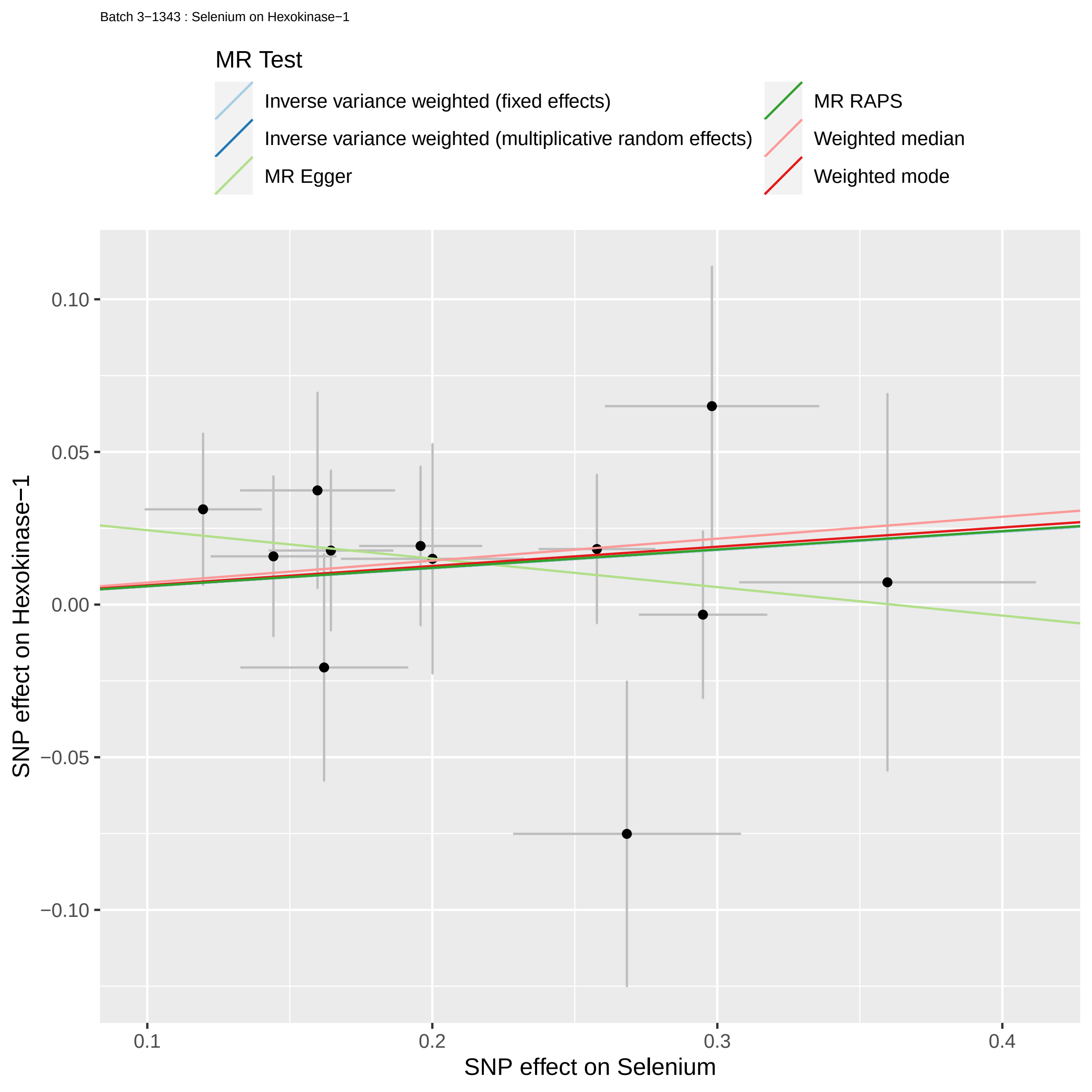

Batch 3−1343 : Selenium on Hexokinase−1
MR Test
Inverse variance weighted (fixed effects)
Inverse variance weighted (multiplicative random effects)
MR Egger
MR RAPS
Weighted median
Weighted mode
0.10
0.05
0.00
−0.05
−0.10
SNP effect on Hexokinase−1
0.1
0.2
0.3
0.4
SNP effect on Selenium

Batch 3−1344 : Selenium on Hexokinase−2

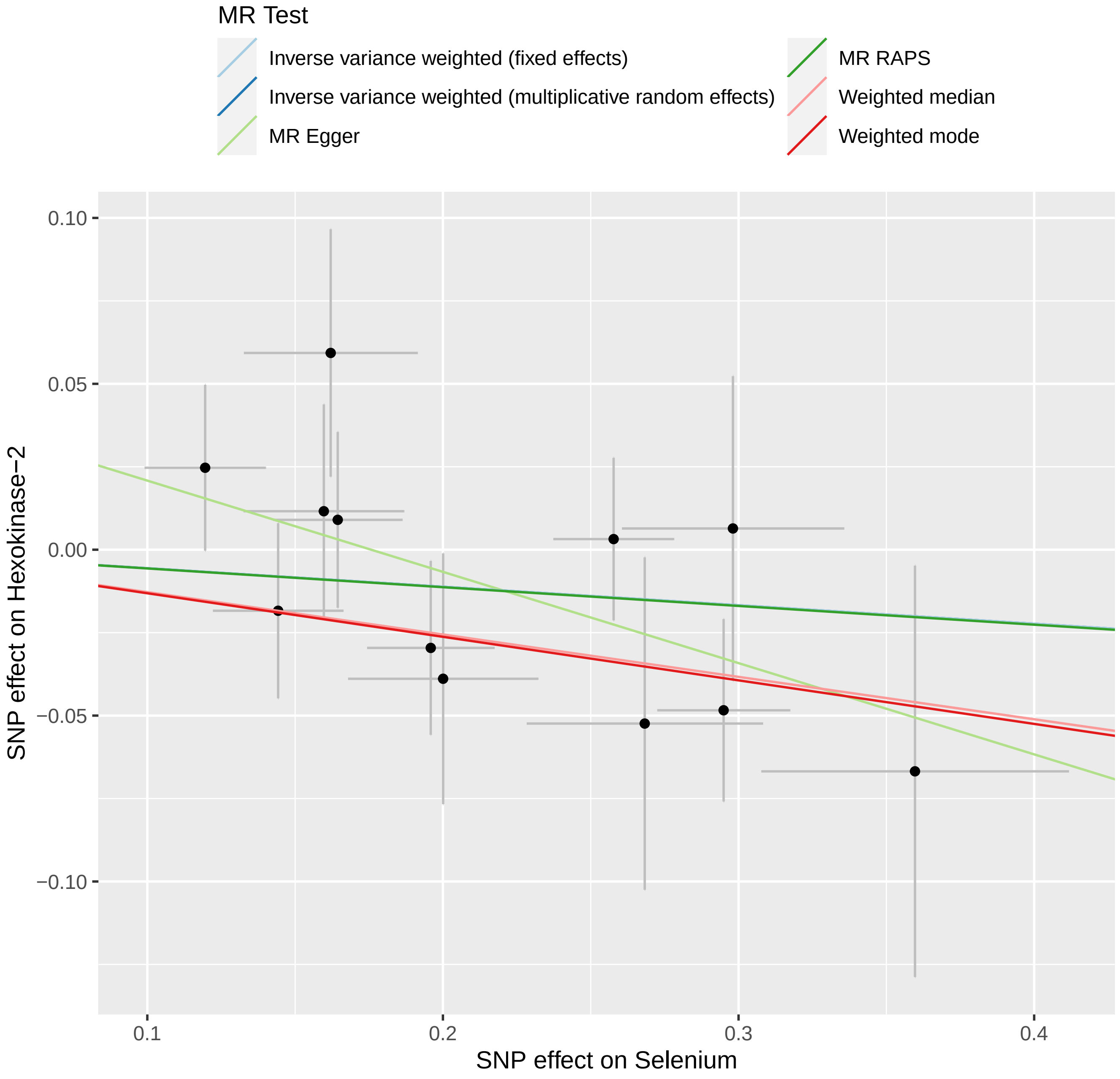

Batch 3−1345 : Selenium on HLA class II histocompatibility antigen, DM alpha chain

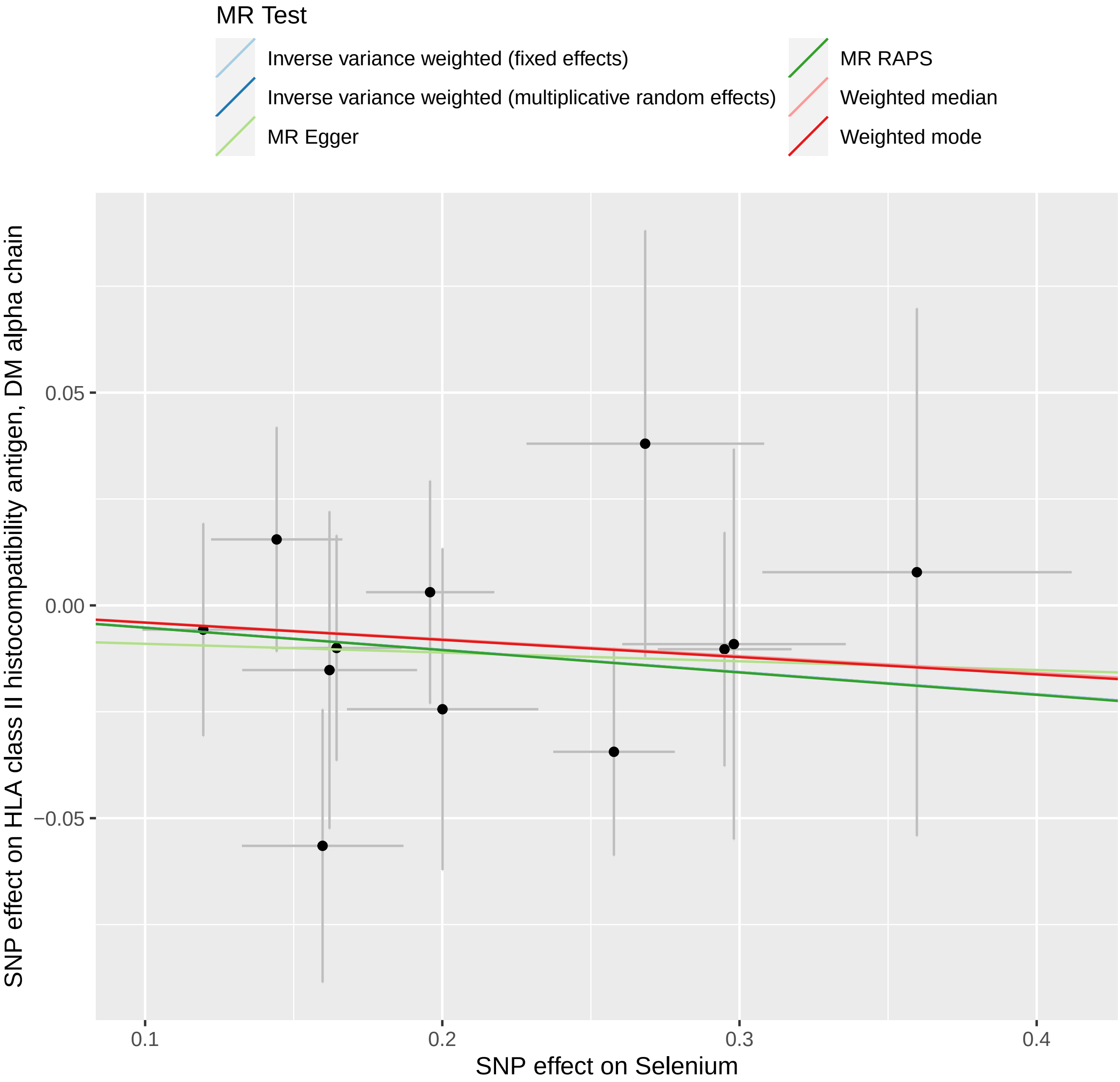

Batch 3−1346 : Selenium on HLA class II histocompatibility antigen, DP beta 1 chain

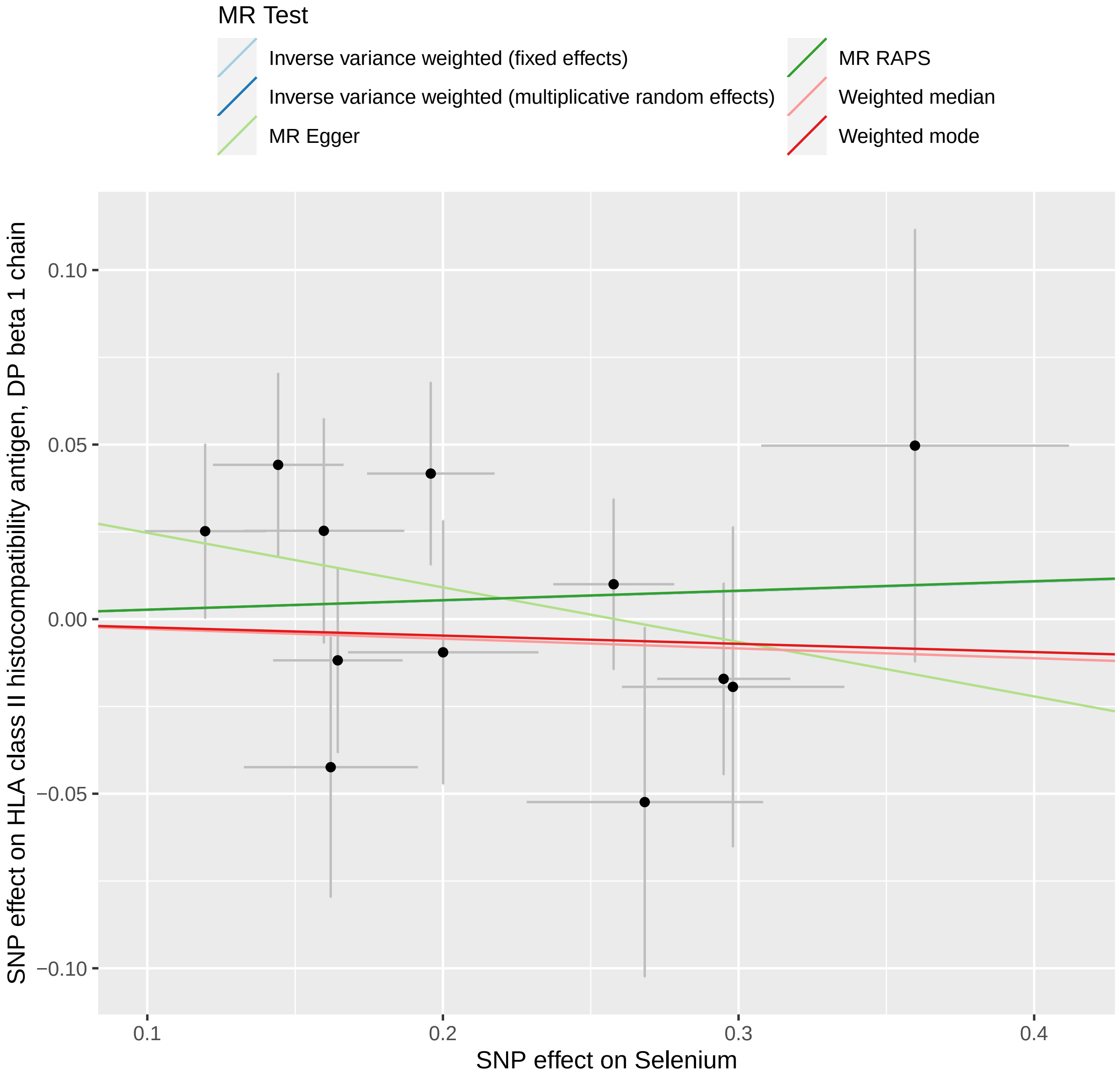

Batch 3−1347 : Selenium on HLA class II histocompatibility antigen, DQ alpha 2 chain

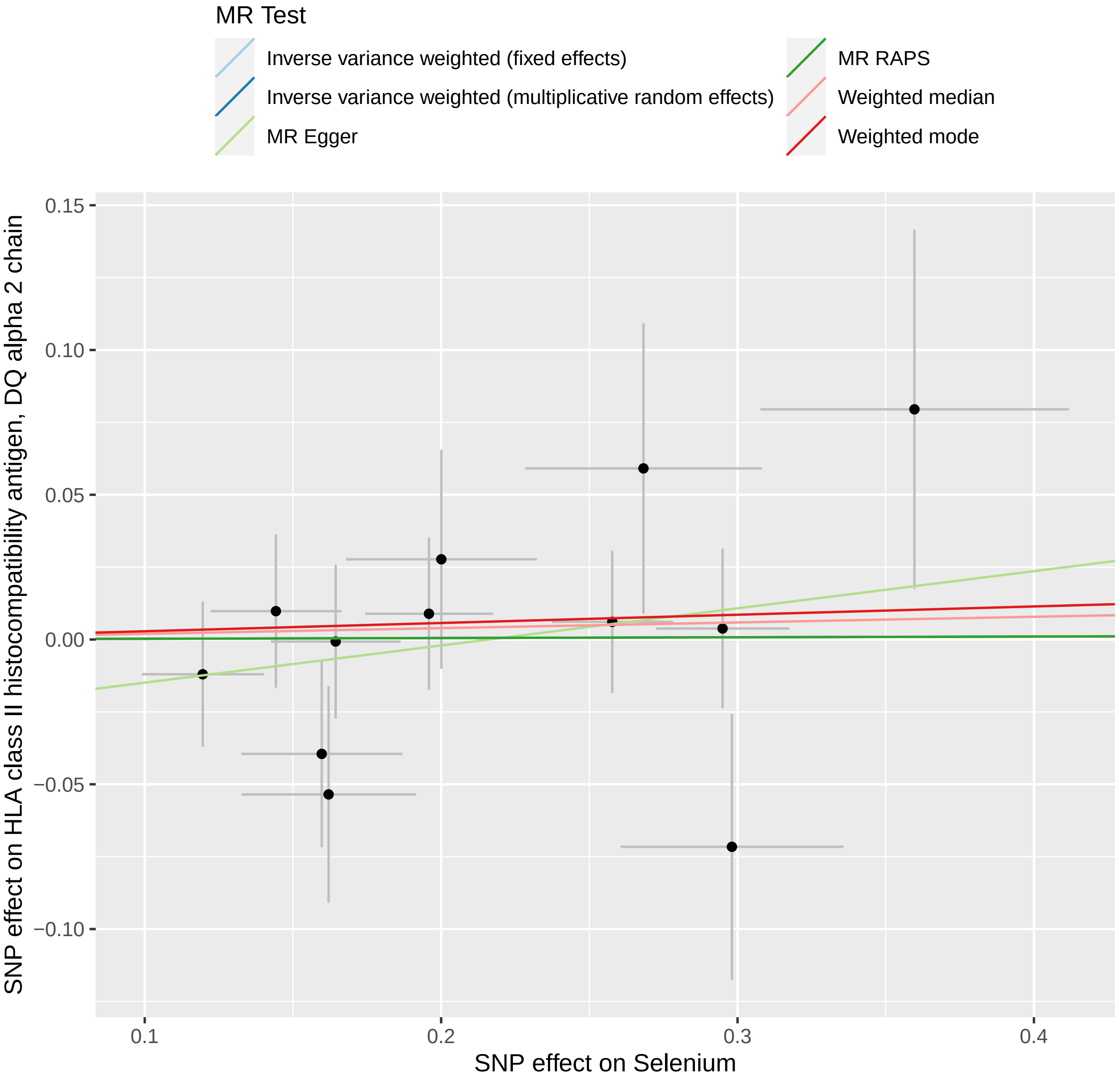

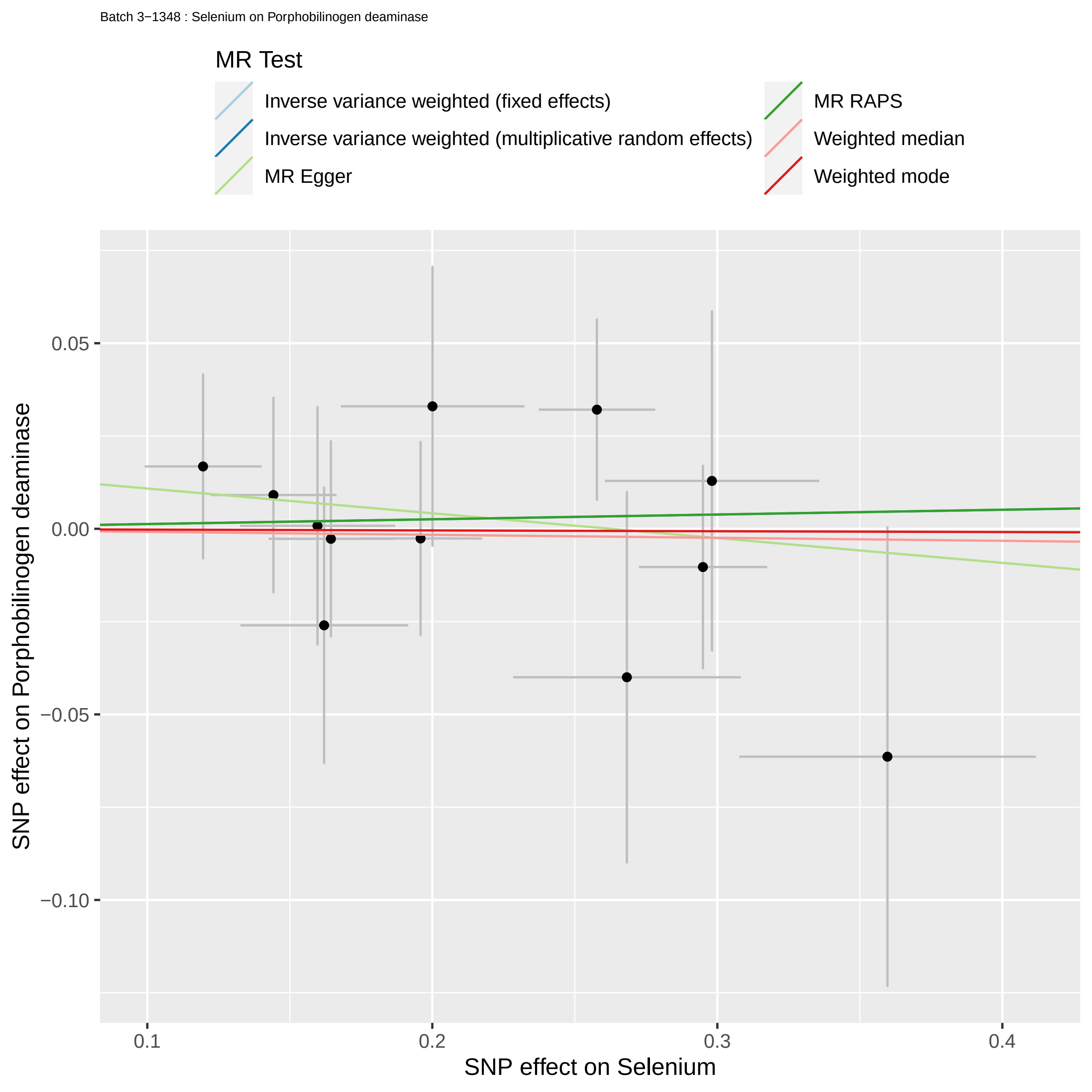
Batch 3−1348 : Selenium on Porphobilinogen deaminase
MR Test
Inverse variance weighted (fixed effects)
Inverse variance weighted (multiplicative random effects)
MR Egger
MR RAPS
Weighted median
Weighted mode
0.05
0.00
−0.05
−0.10
0.1
0.2
0.3
0.4
SNP effect on Porphobilinogen deaminase
SNP effect on Selenium

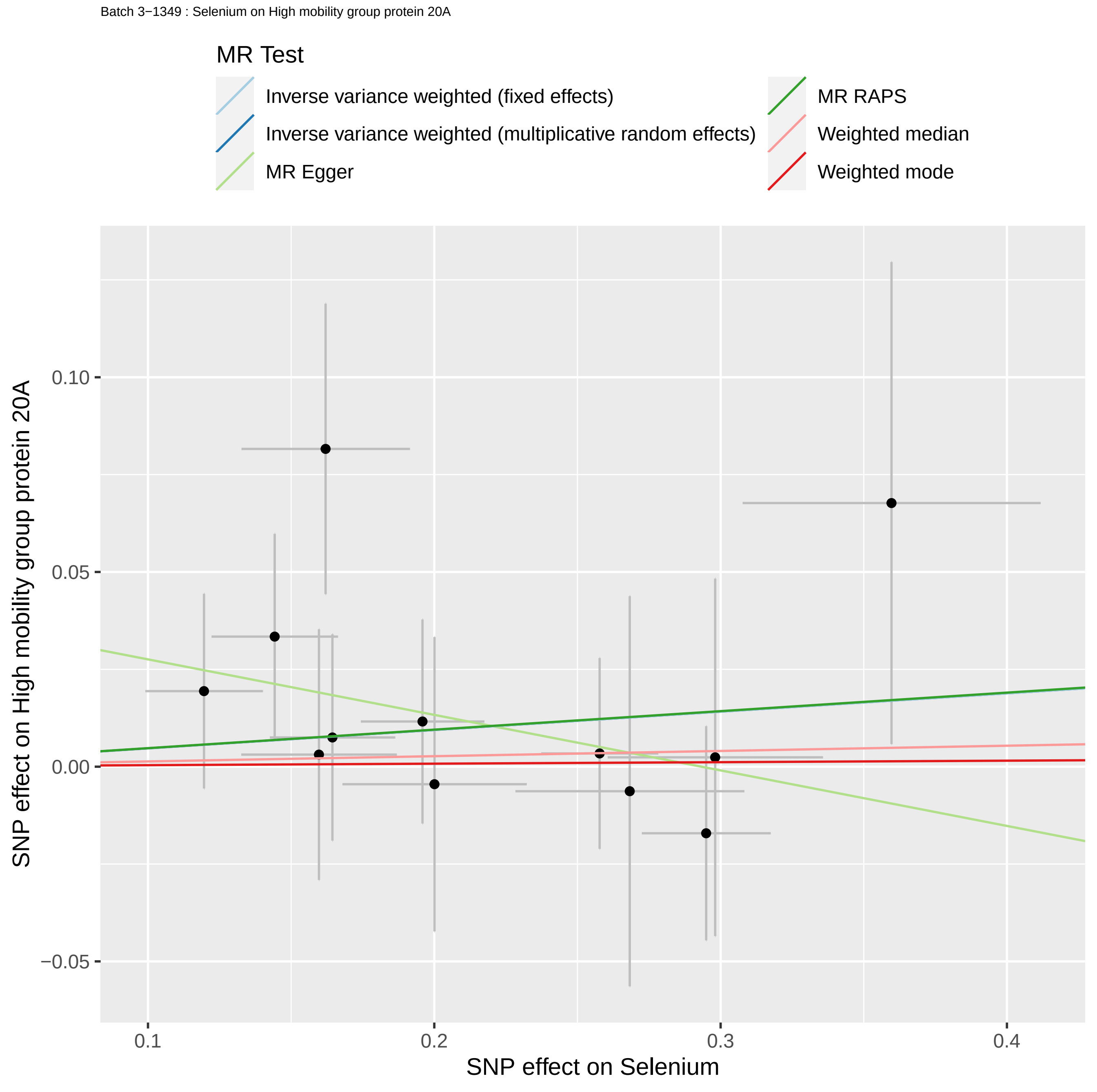

Batch 3−1349 : Selenium on High mobility group protein 20A
MR Test
Inverse variance weighted (fixed effects)
Inverse variance weighted (multiplicative random effects)
MR Egger
MR RAPS
Weighted median
Weighted mode
SNP effect on High mobility group protein 20A
0.10
0.05
0.00
−0.05
0.1
0.2
0.3
0.4
SNP effect on Selenium

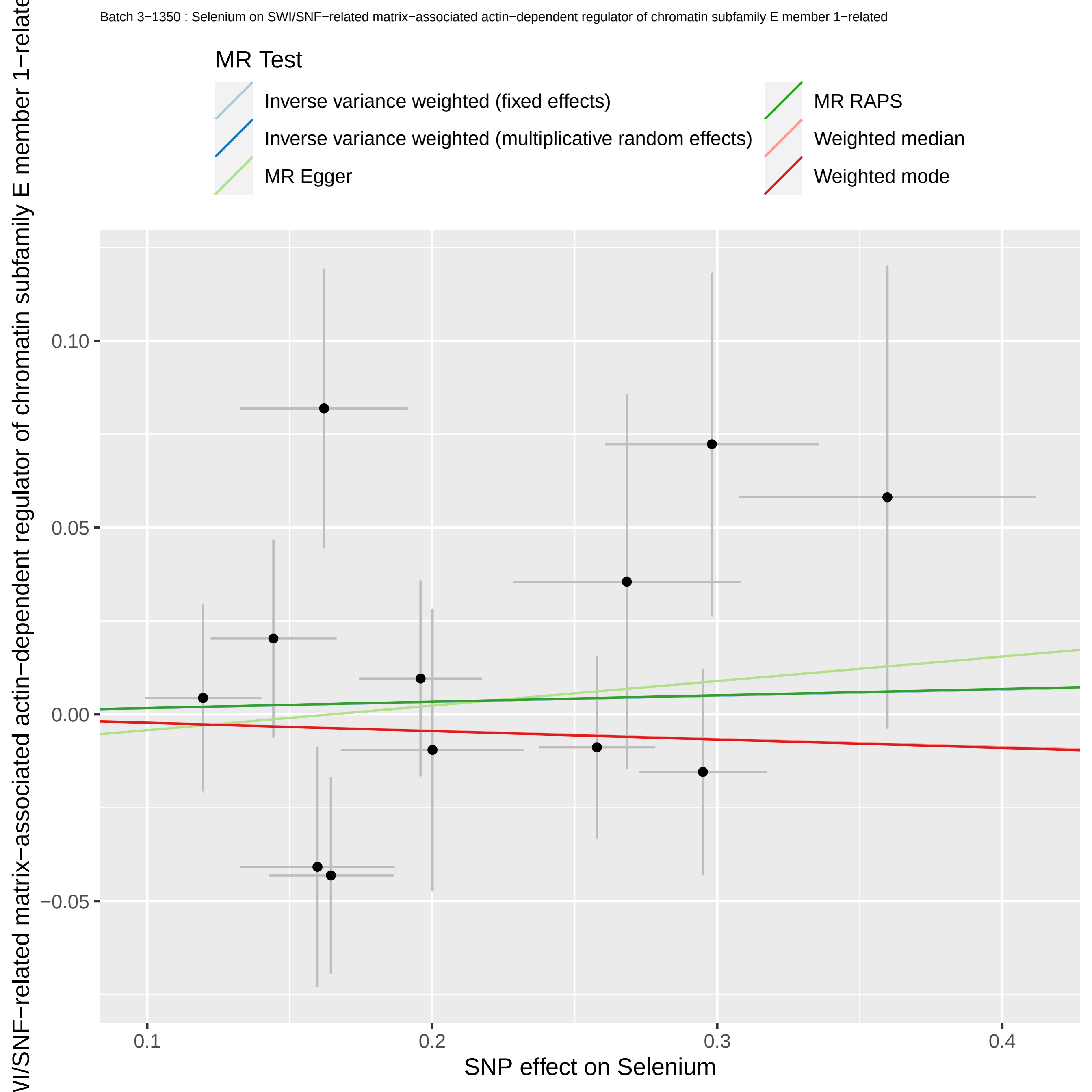

Batch 3−1350 : Selenium on SWI/SNF−related matrix−associated actin−dependent regulator of chromatin subfamily E member 1−related
MR Test
Inverse variance weighted (fixed effects)
Inverse variance weighted (multiplicative random effects)
MR Egger
MR RAPS
Weighted median
Weighted mode
0.10
0.05
0.00
−0.05
0.1
0.2
0.3
0.4
SNP effect on Selenium

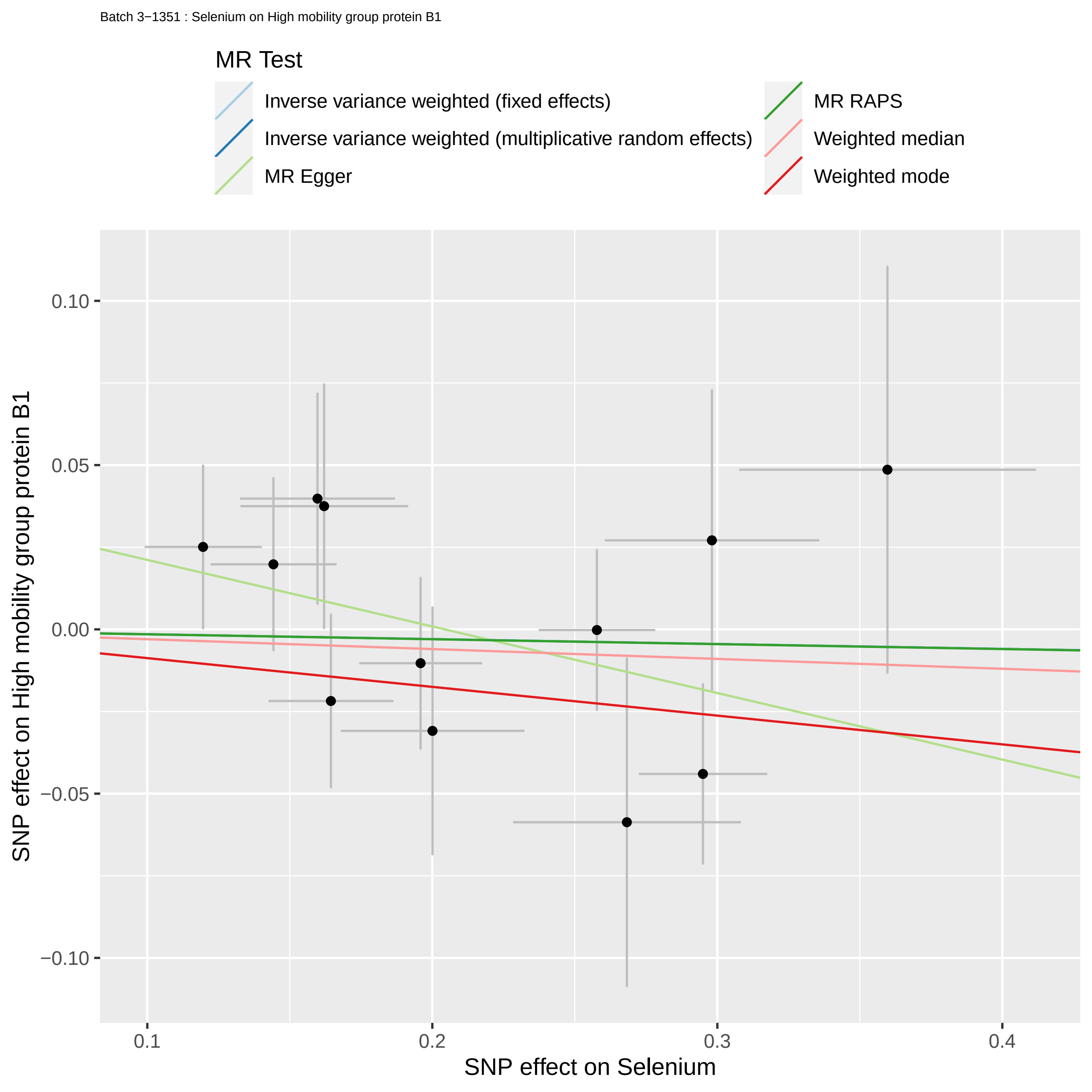
Batch 3−1351 : Selenium on High mobility group protein B1
MR Test
Inverse variance weighted (fixed effects)
Inverse variance weighted (multiplicative random effects)
MR Egger
MR RAPS
Weighted median
Weighted mode
0.10
0.05
0.00
−0.05
−0.10
0.1
0.2
0.3
0.4
SNP effect on High mobility group protein B1
SNP effect on Selenium

Batch 3−1352 : Selenium on High mobility group protein B2

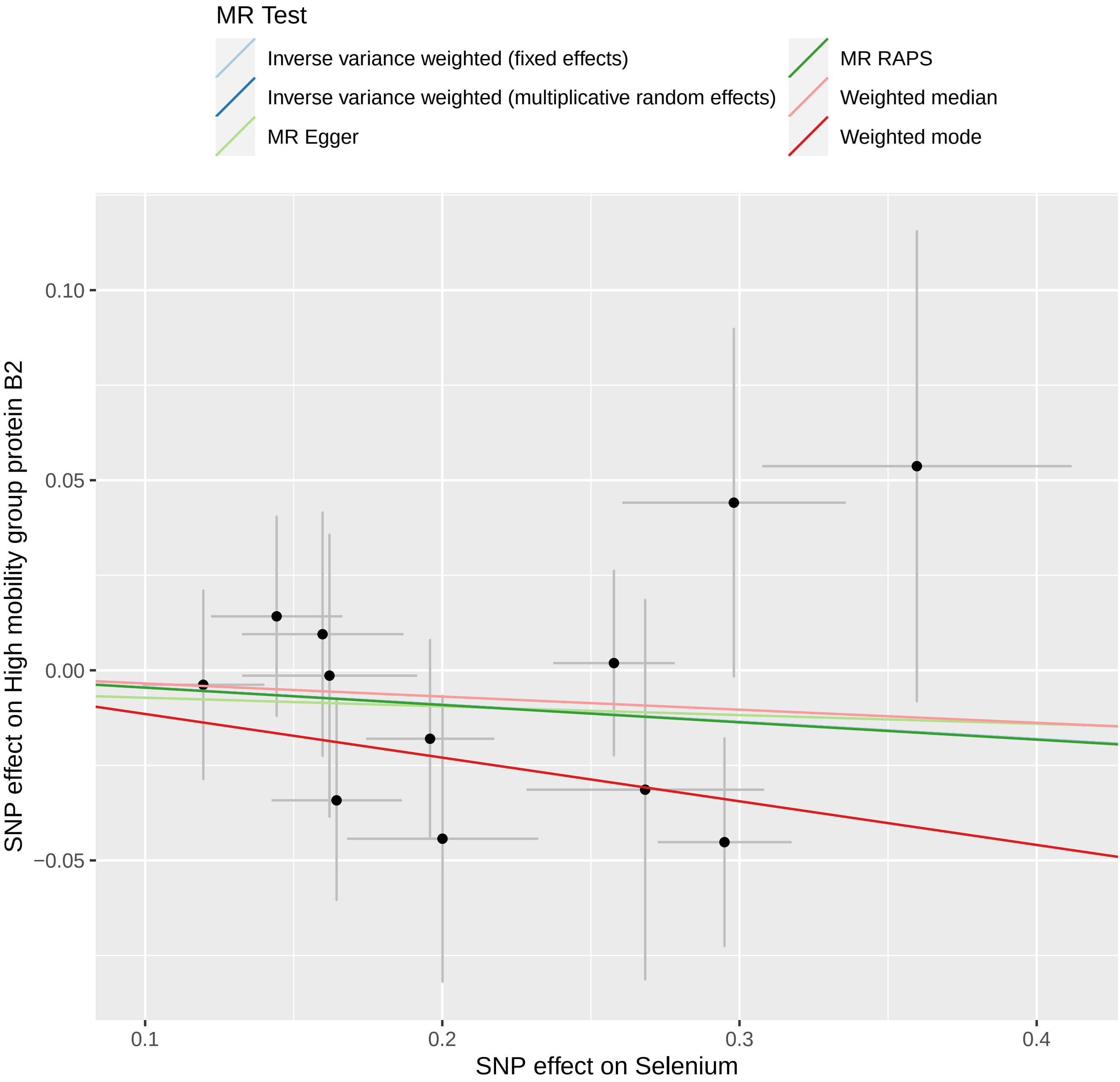

Batch 3−1353 : Selenium on High mobility group protein B3

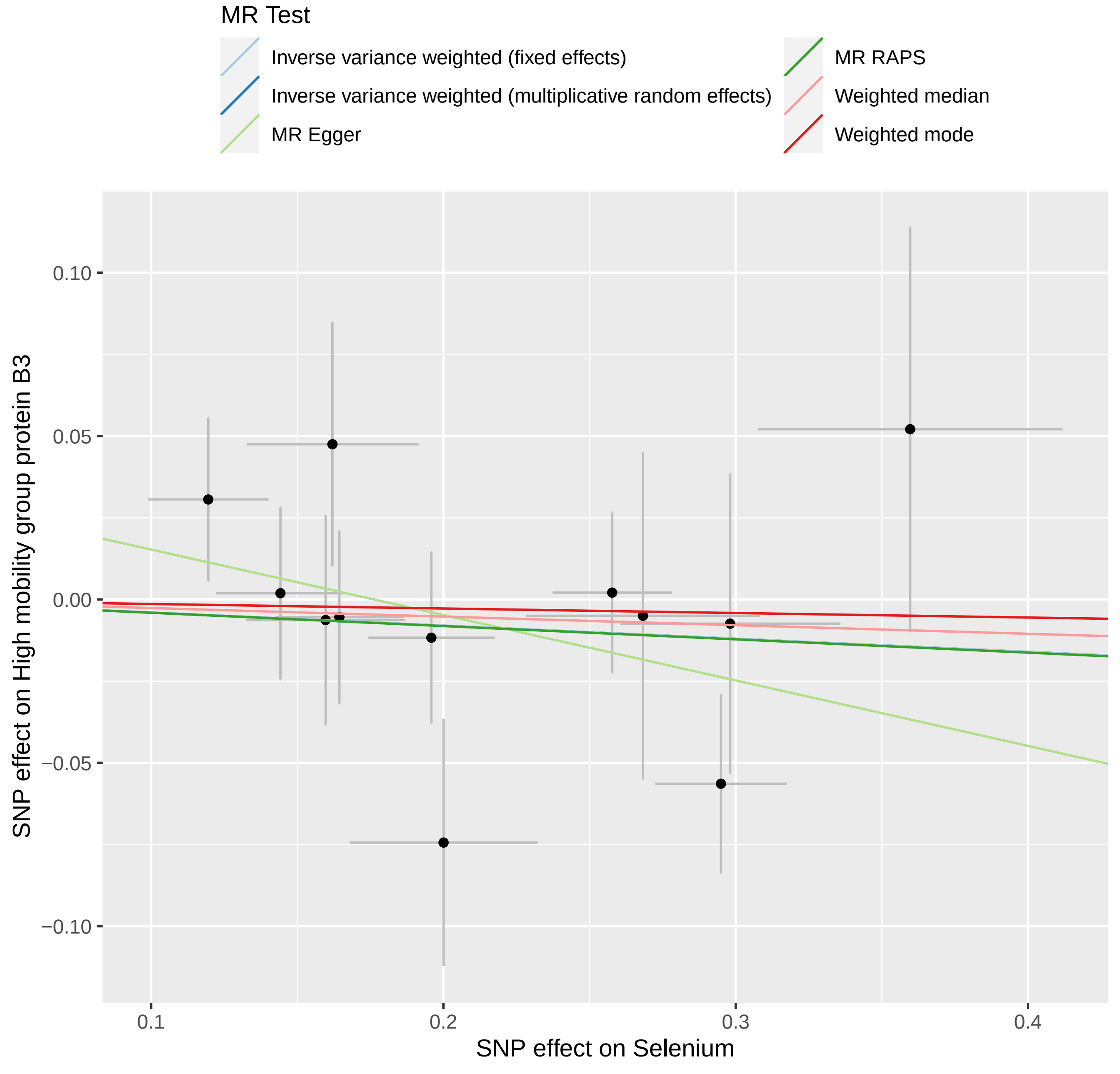

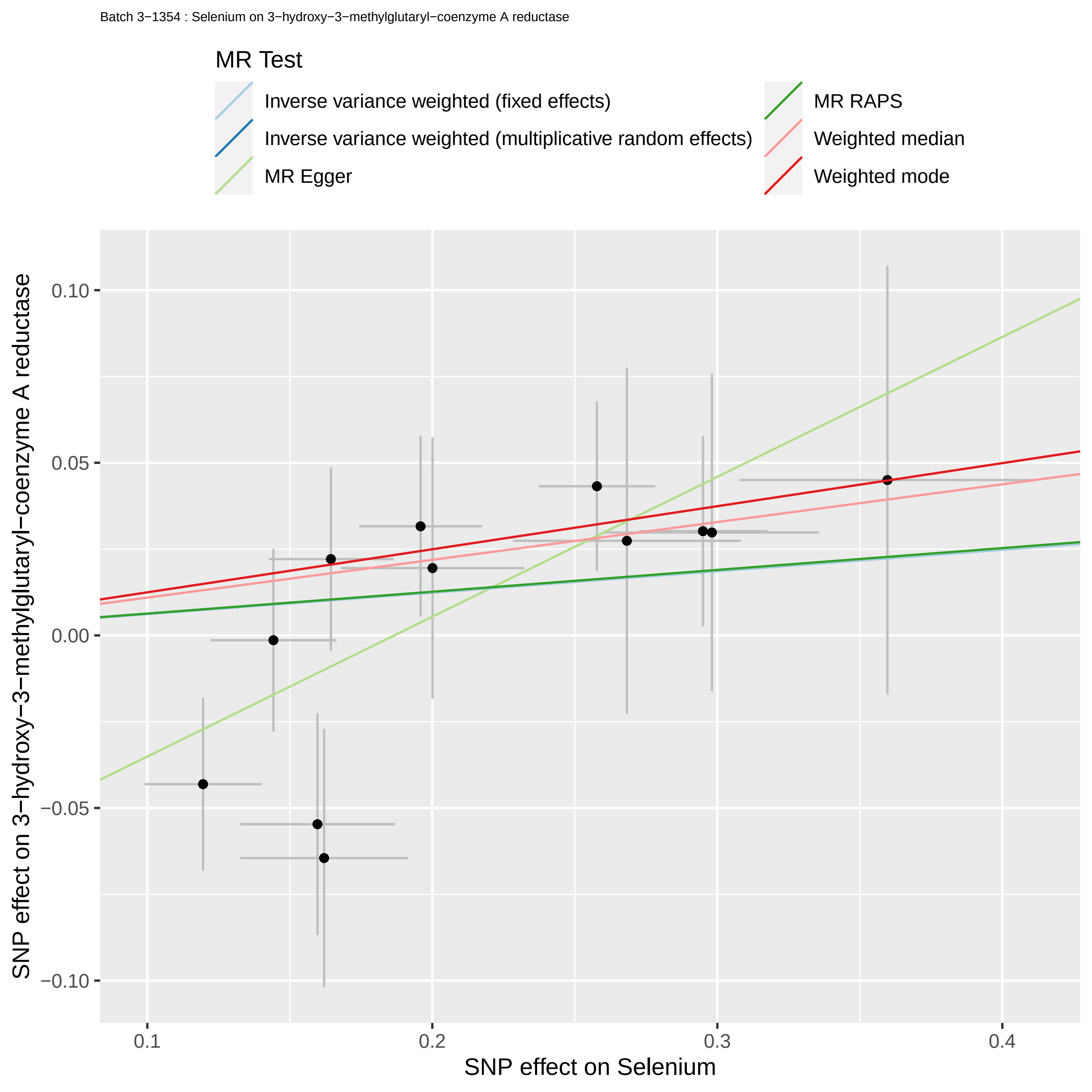

Batch 3−1354 : Selenium on 3−hydroxy−3−methylglutaryl−coenzyme A reductase
MR Test
Inverse variance weighted (fixed effects)
Inverse variance weighted (multiplicative random effects)
MR Egger
MR RAPS
Weighted median
Weighted mode
0.10
0.05
0.00
−0.05
−0.10
0.1
0.2
0.3
0.4
SNP effect on 3−hydroxy−3−methylglutaryl−coenzyme A reductase
SNP effect on Selenium

Batch 3−1355 : Selenium on Hydroxymethylglutaryl−CoA synthase, cytoplasmic

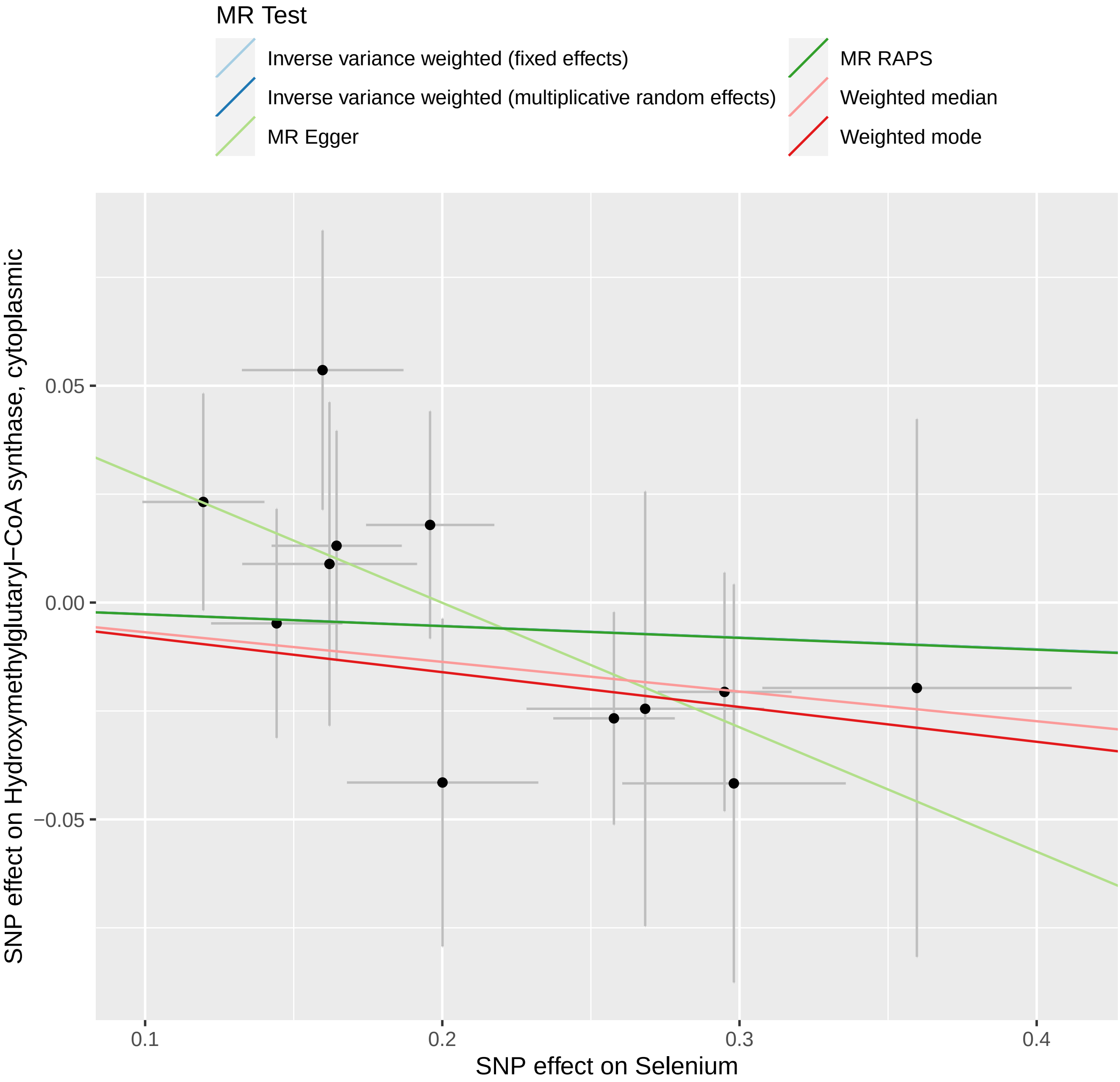

Batch 3−1356 : Selenium on Hydroxymethylglutaryl−CoA synthase, mitochondrial

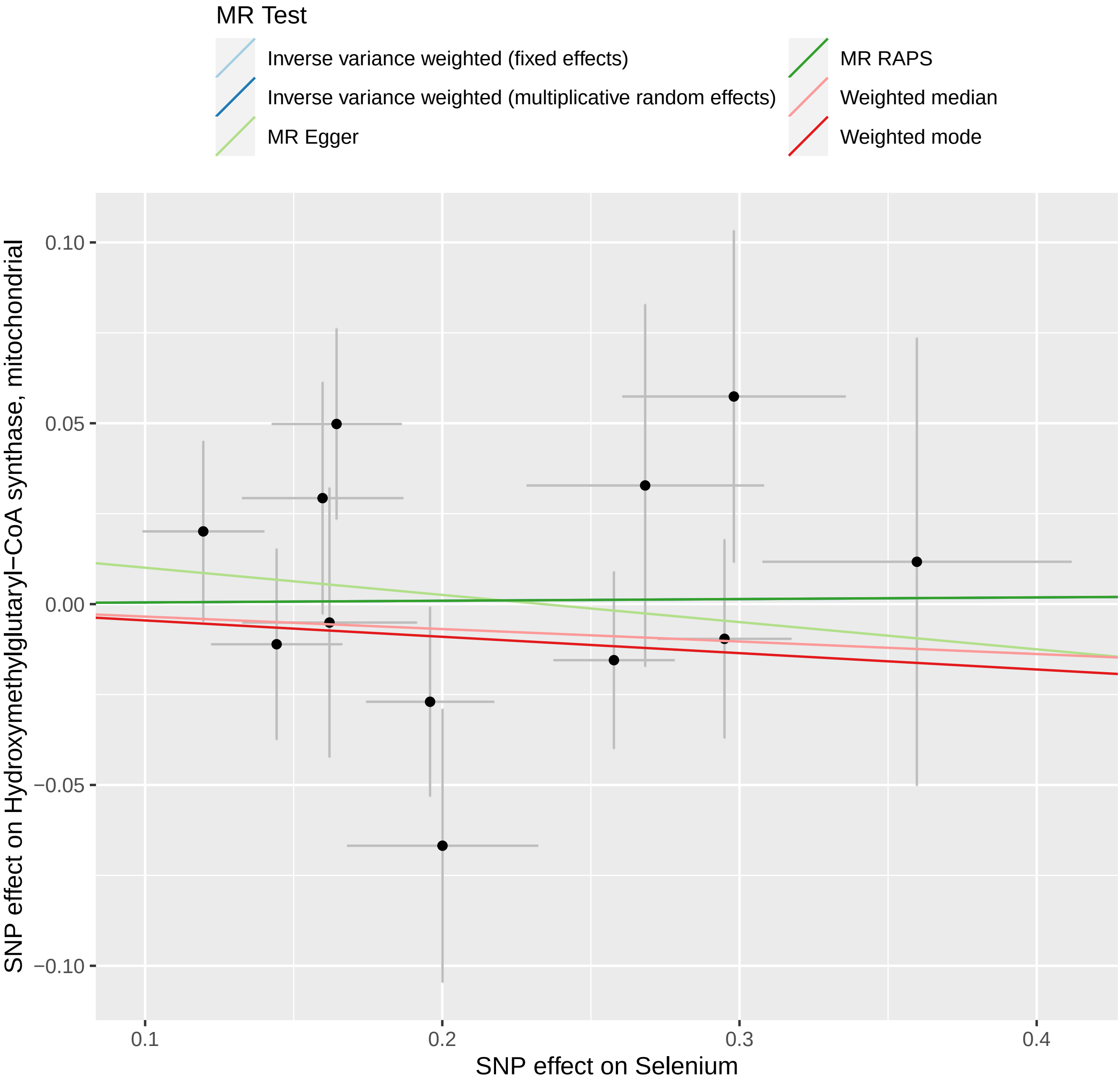

Batch 3−1357 : Selenium on Non−histone chromosomal protein HMG−14

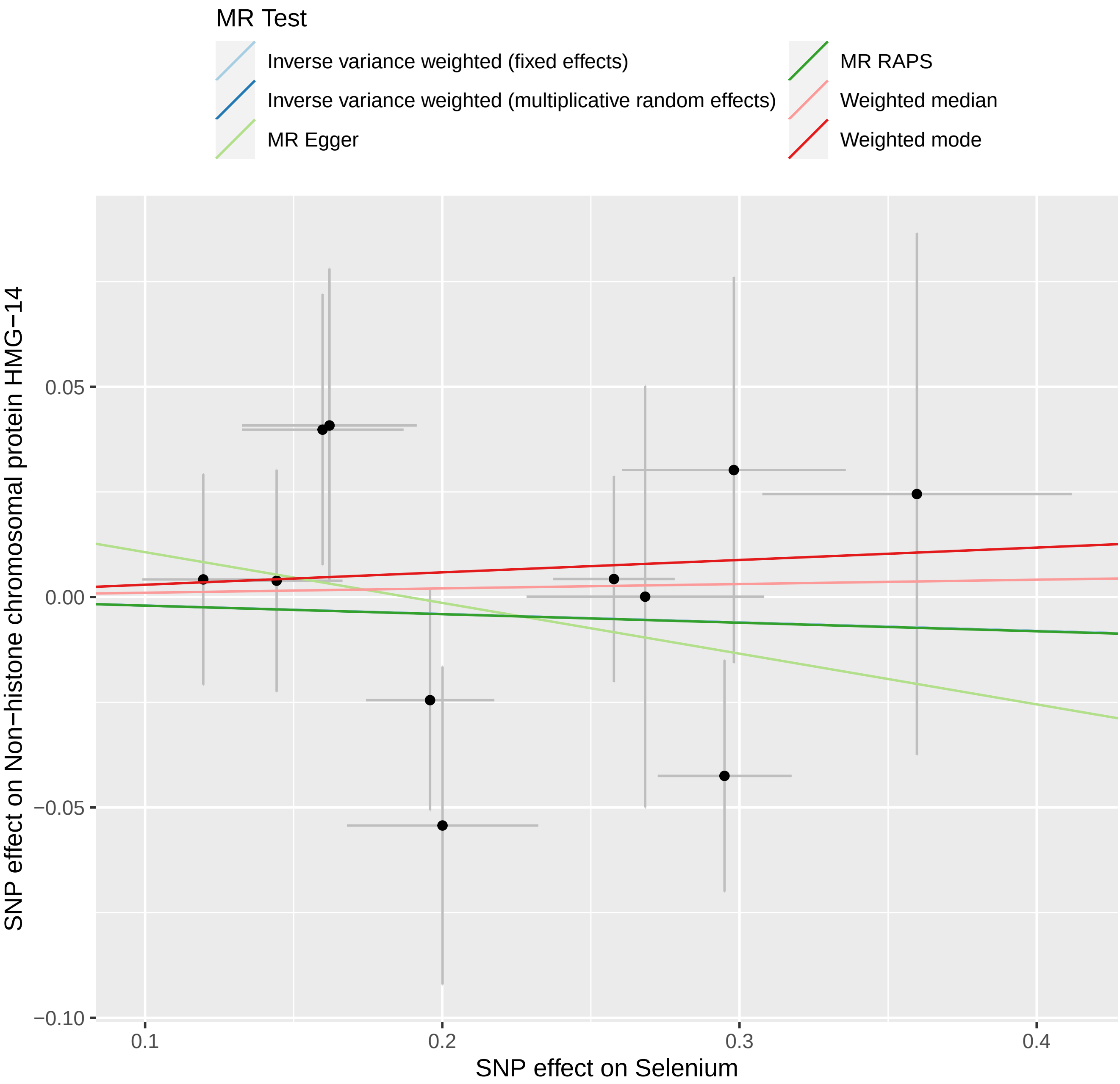

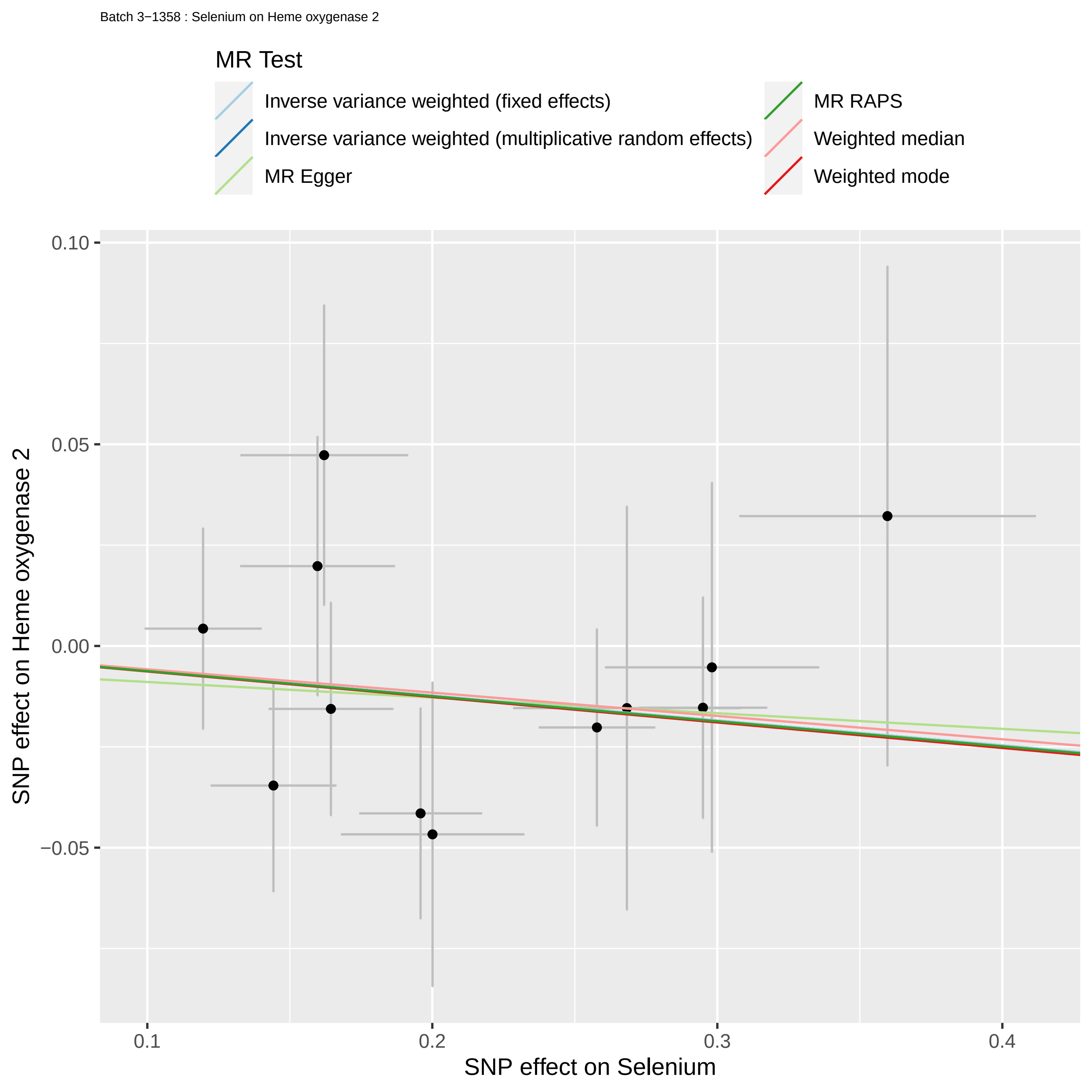

Batch 3−1358 : Selenium on Heme oxygenase 2
MR Test
Inverse variance weighted (fixed effects)
Inverse variance weighted (multiplicative random effects)
MR Egger
MR RAPS
Weighted median
Weighted mode
0.10
0.05
0.00
−0.05
0.1
0.2
0.3
0.4
SNP effect on Heme oxygenase 2
SNP effect on Selenium

Batch 3−1359 : Selenium on Hepatocyte nuclear factor 1−alpha

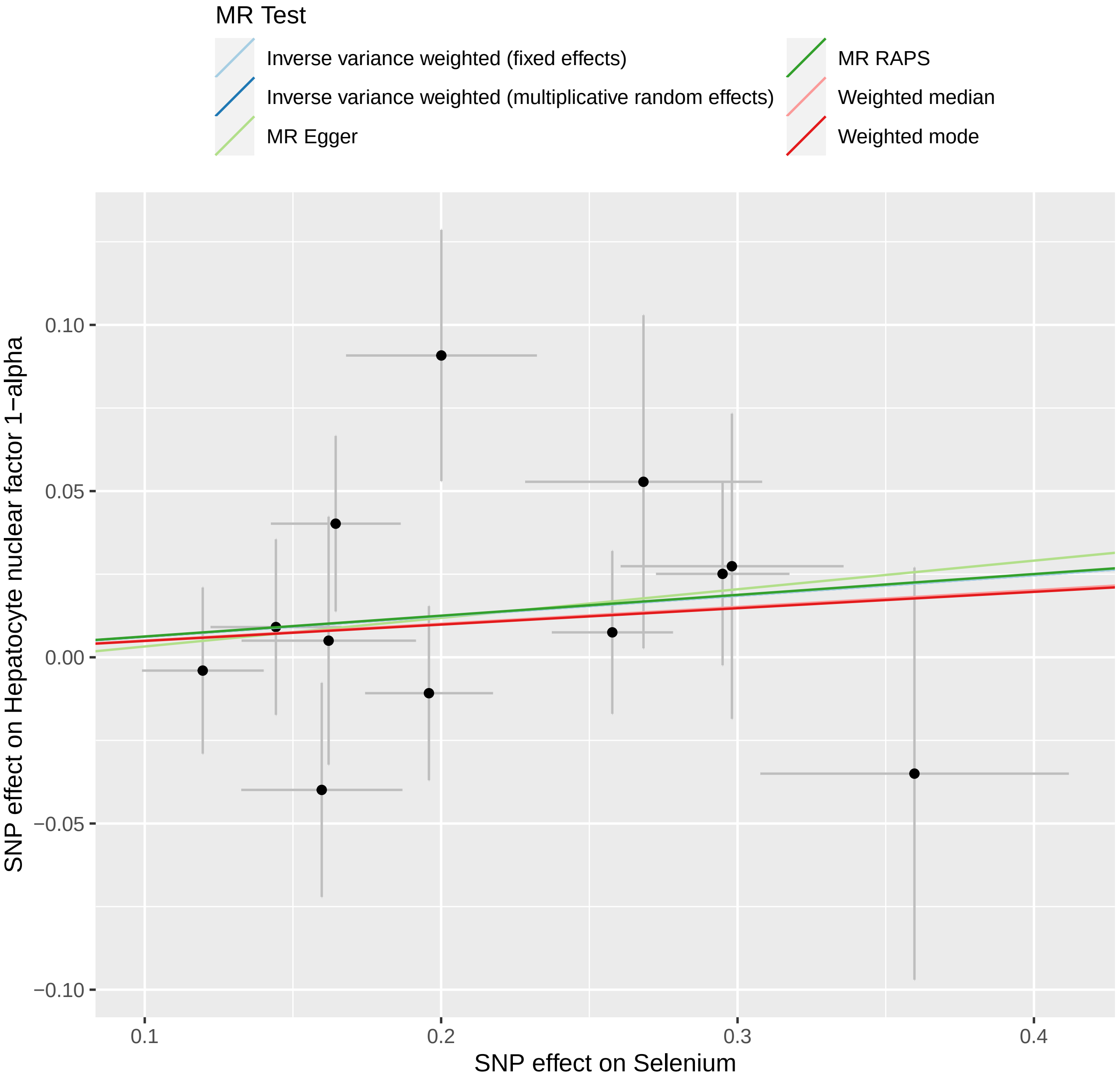

Batch 3−1360 : Selenium on Hepatocyte nuclear factor 4−alpha

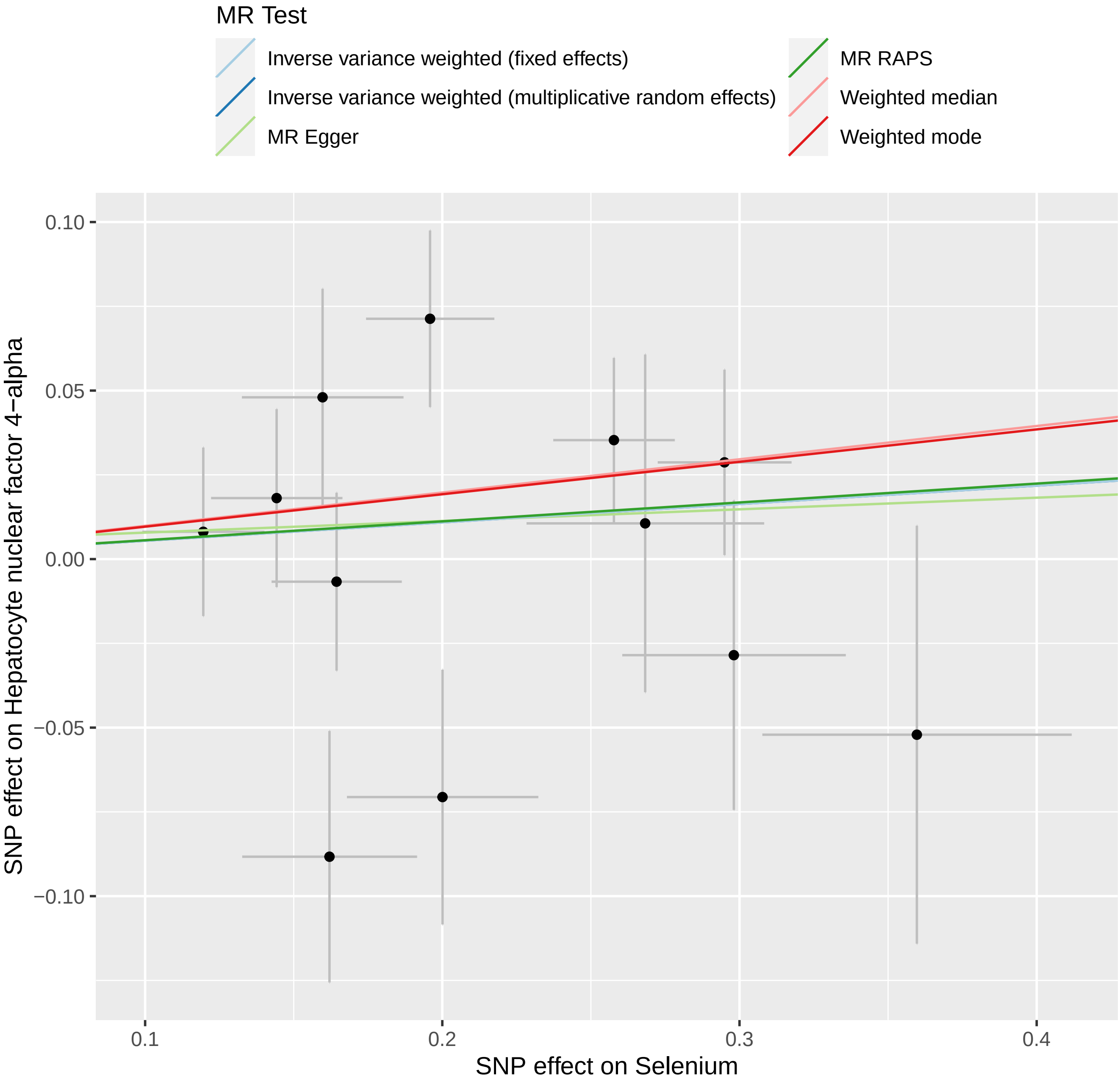

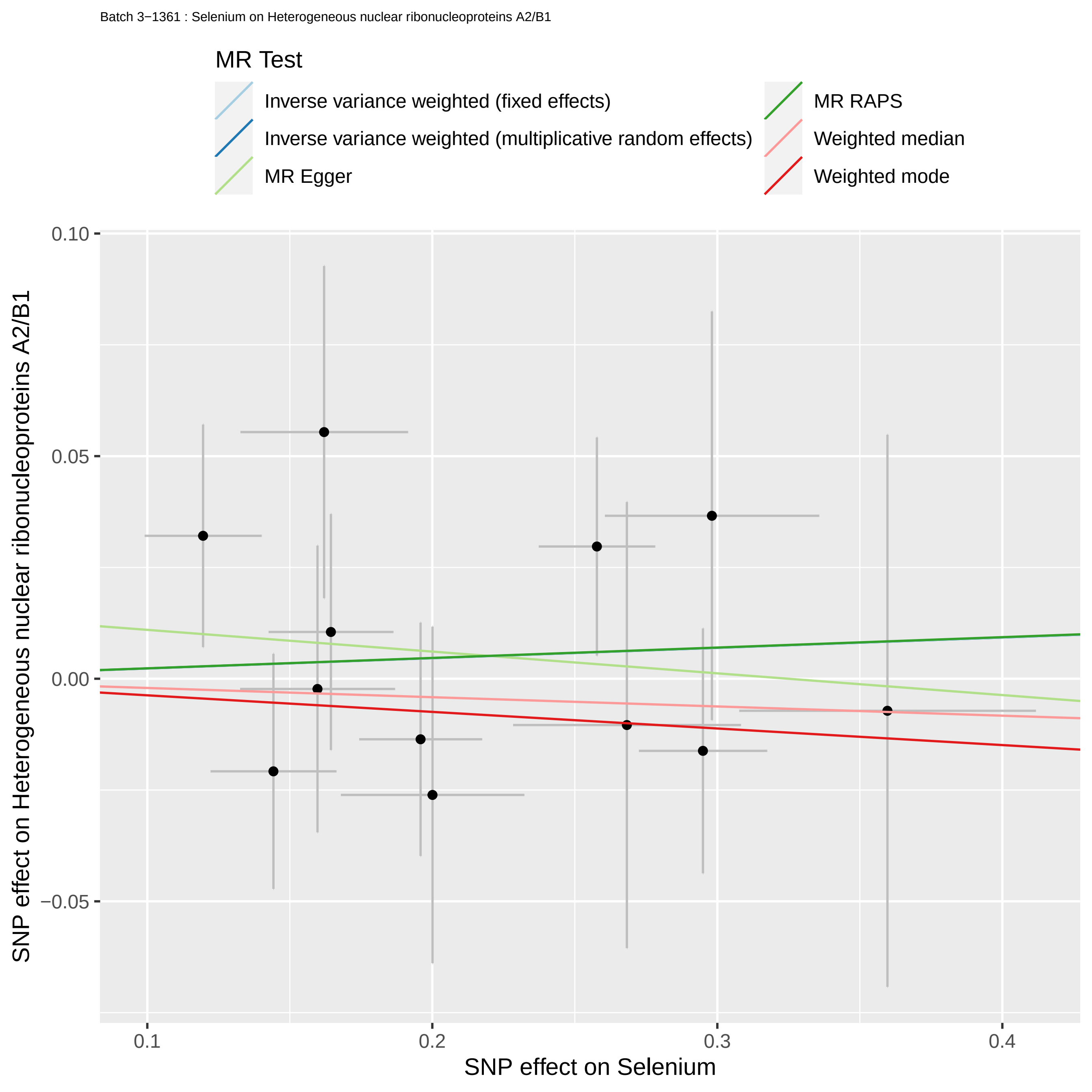
Batch 3−1361 : Selenium on Heterogeneous nuclear ribonucleoproteins A2/B1
MR Test
Inverse variance weighted (fixed effects)
Inverse variance weighted (multiplicative random effects)
MR Egger
MR RAPS
Weighted median
Weighted mode
0.10
0.05
0.00
−0.05
0.1
0.2
0.3
0.4
SNP effect on Selenium
SNP effect on Heterogeneous nuclear ribonucleoproteins A2/B1

Batch 3−1362 : Selenium on Heterogeneous nuclear ribonucleoprotein A/B

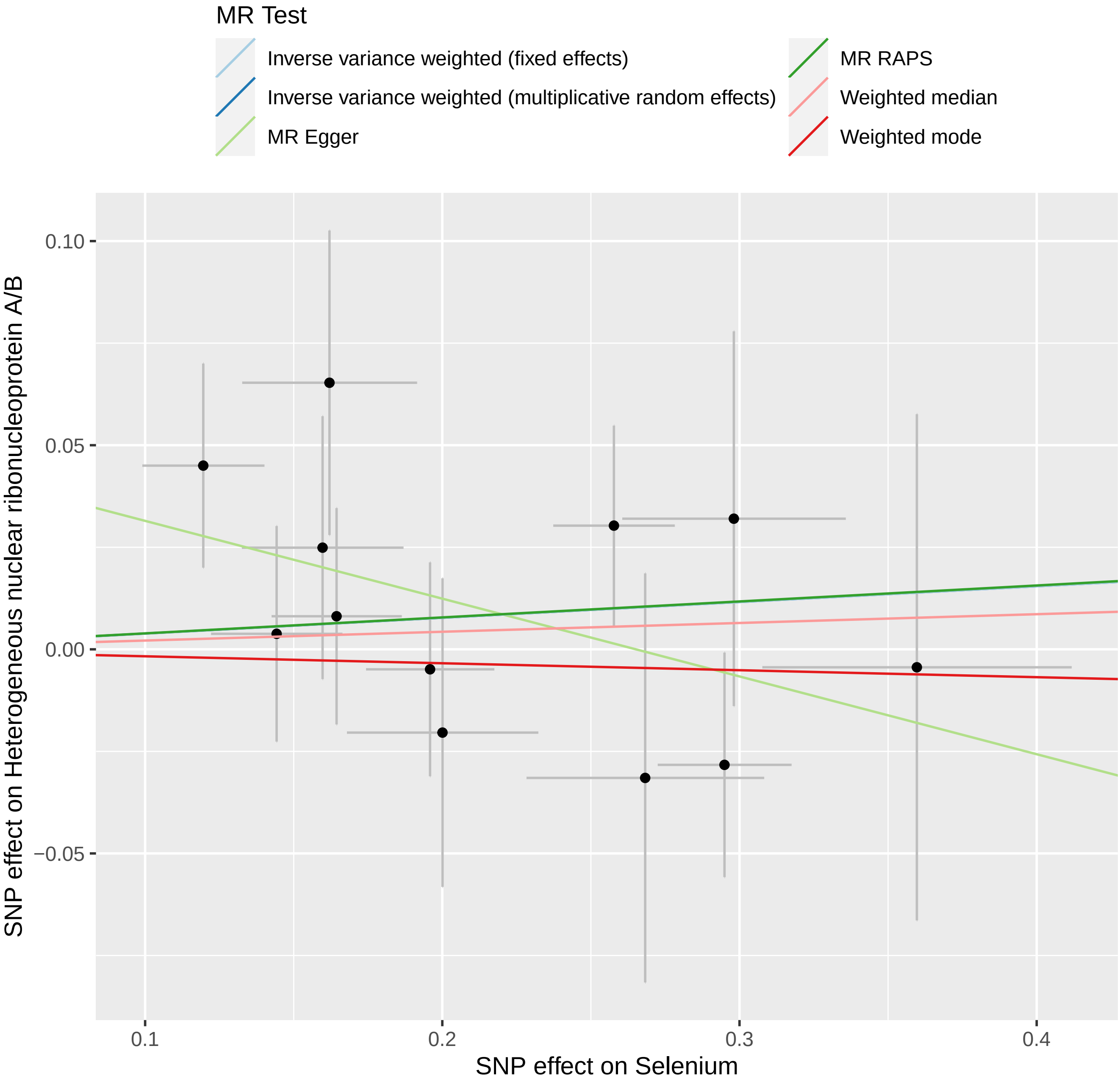

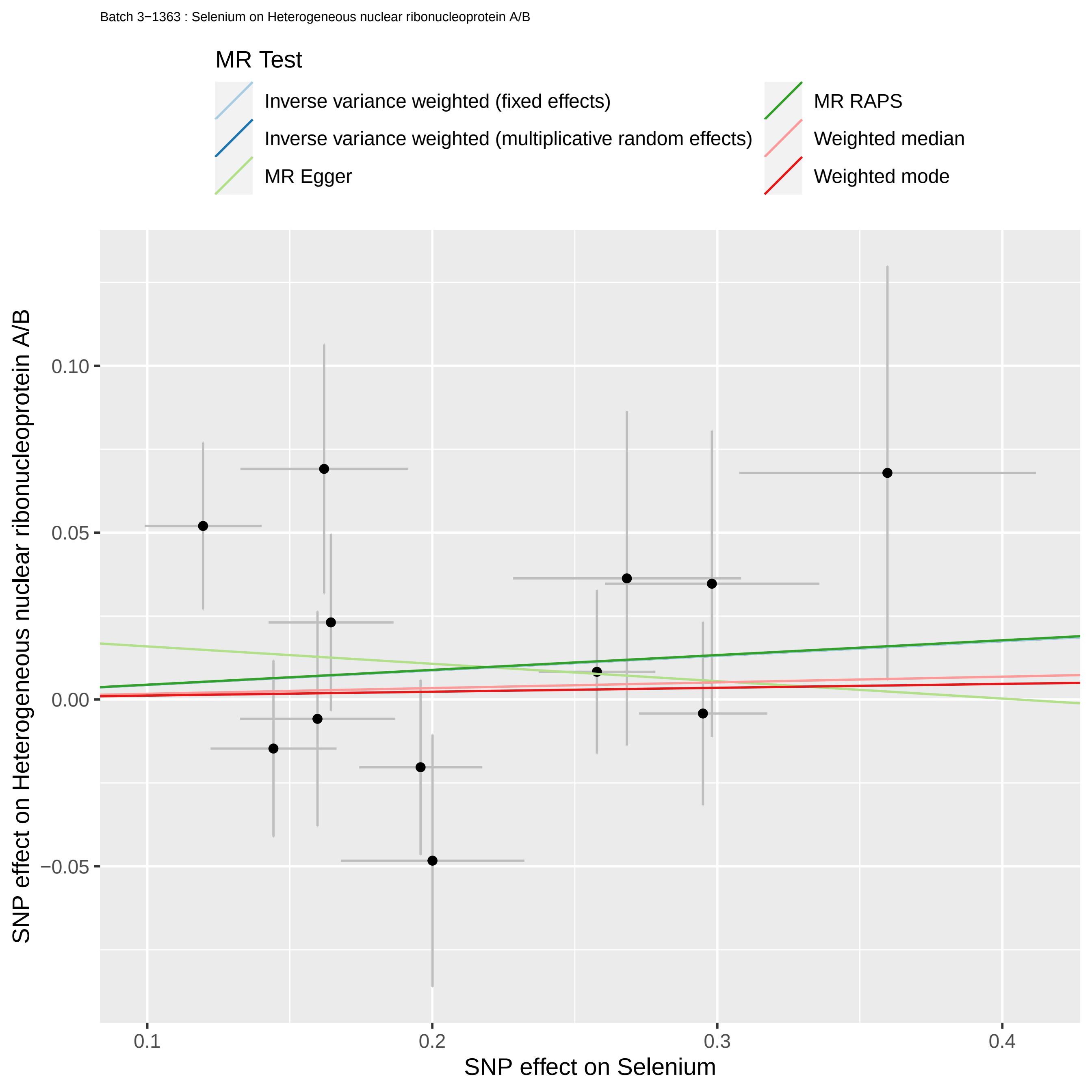

Batch 3−1363 : Selenium on Heterogeneous nuclear ribonucleoprotein A/B
MR Test
Inverse variance weighted (fixed effects)
Inverse variance weighted (multiplicative random effects)
MR Egger
MR RAPS
Weighted median
Weighted mode
SNP effect on Heterogeneous nuclear ribonucleoprotein A/B
0.10
0.05
0.00
−0.05
0.1
0.2
0.3
0.4
SNP effect on Selenium

Batch 3−1364 : Selenium on Heterogeneous nuclear ribonucleoproteins C1/C2

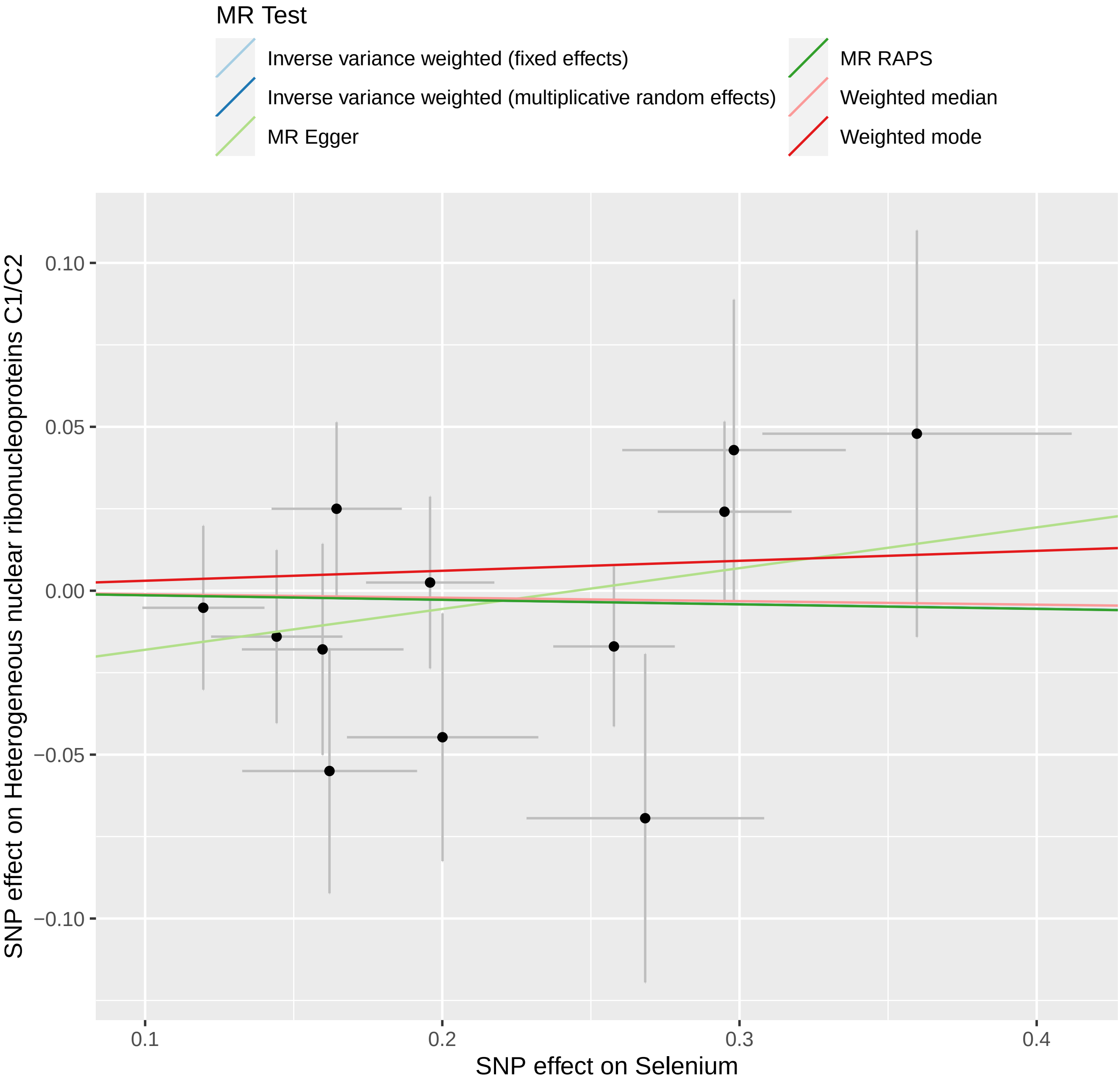

Batch 3−1365 : Selenium on Heterogeneous nuclear ribonucleoprotein D−like

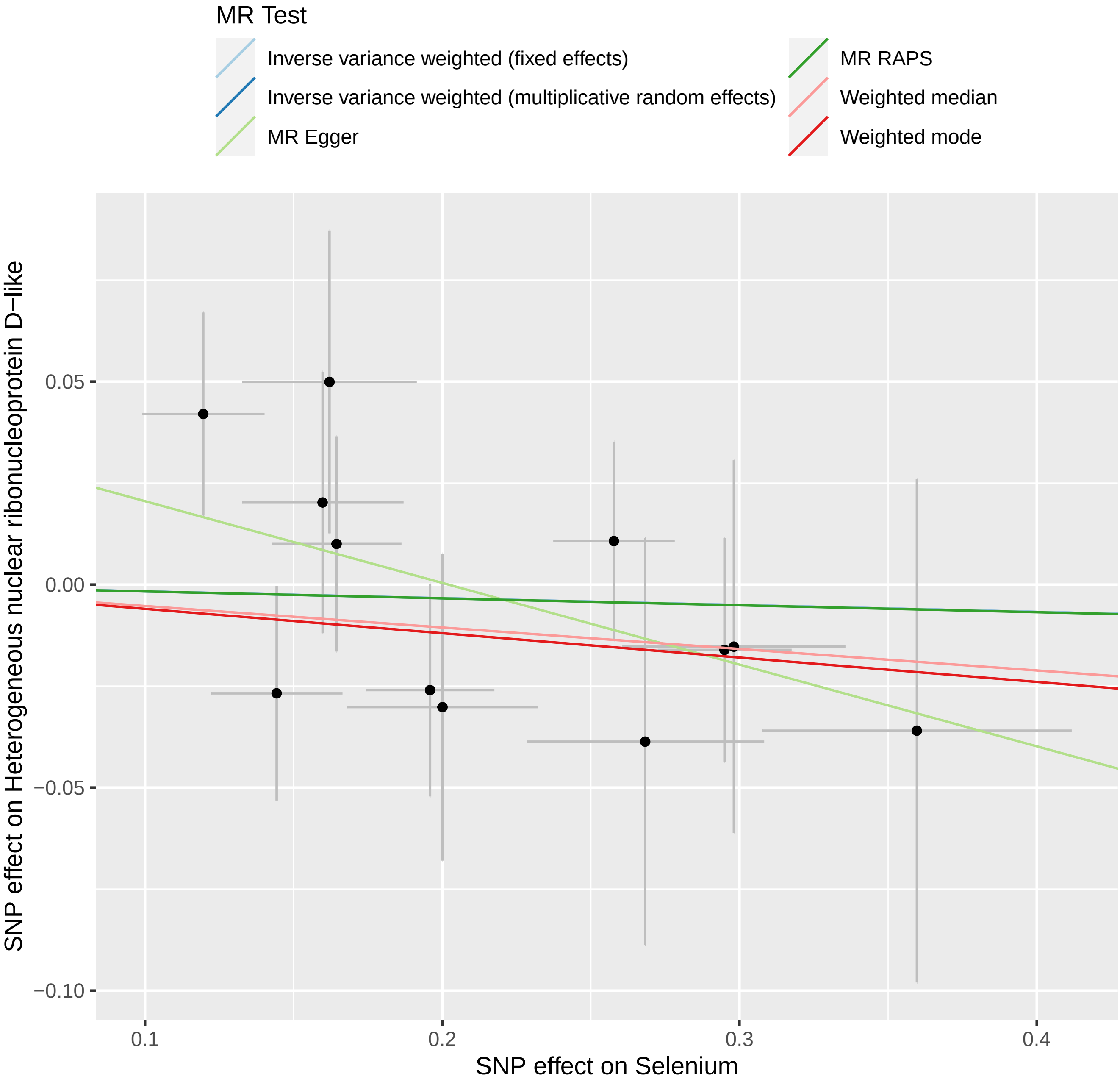

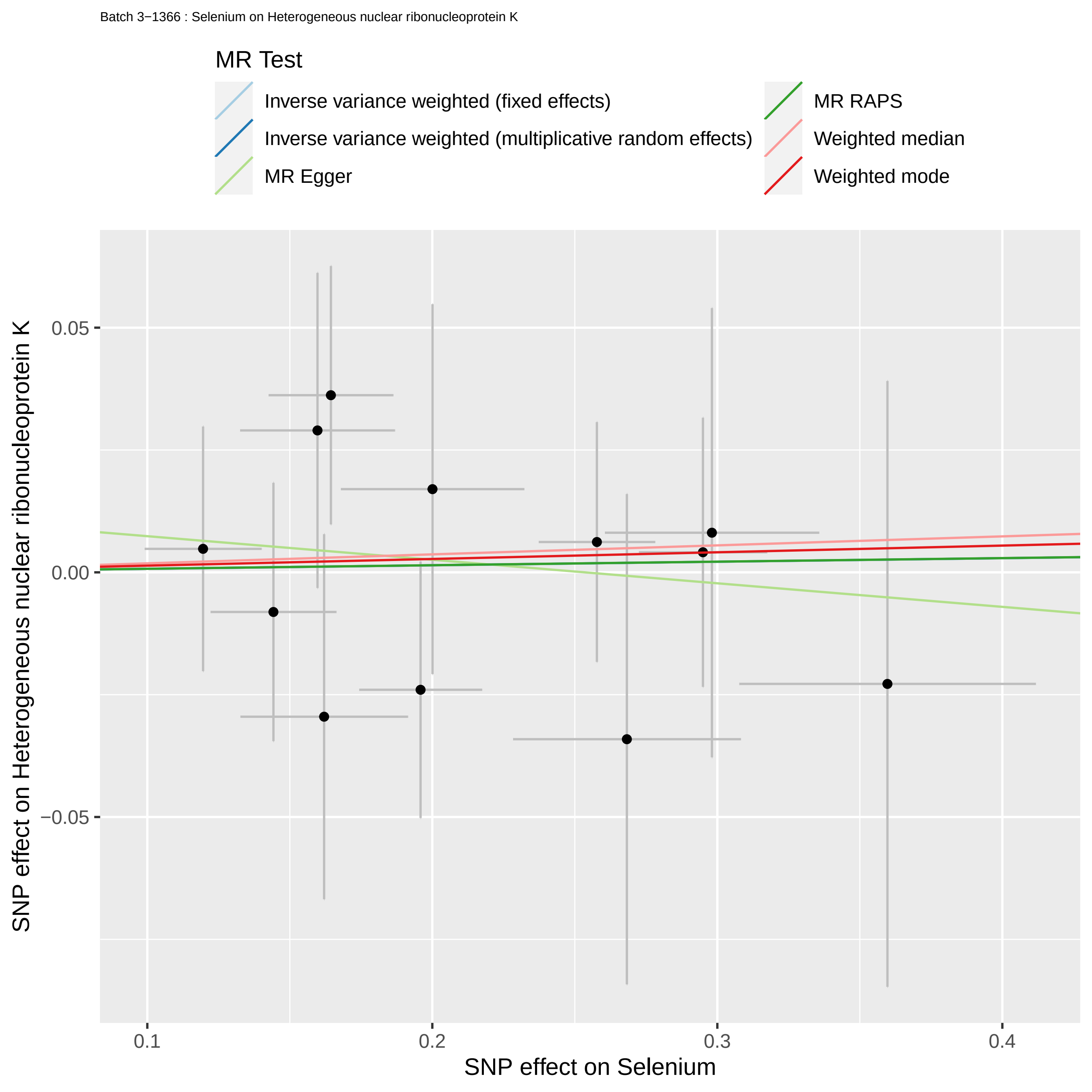
Batch 3−1366 : Selenium on Heterogeneous nuclear ribonucleoprotein K
MR Test
Inverse variance weighted (fixed effects)
Inverse variance weighted (multiplicative random effects)
MR Egger
MR RAPS
Weighted median
Weighted mode
0.05
0.00
−0.05
0.1
0.2
0.3
0.4
SNP effect on Heterogeneous nuclear ribonucleoprotein K
SNP effect on Selenium

Batch 3−1367 : Selenium on Heterogeneous nuclear ribonucleoprotein M

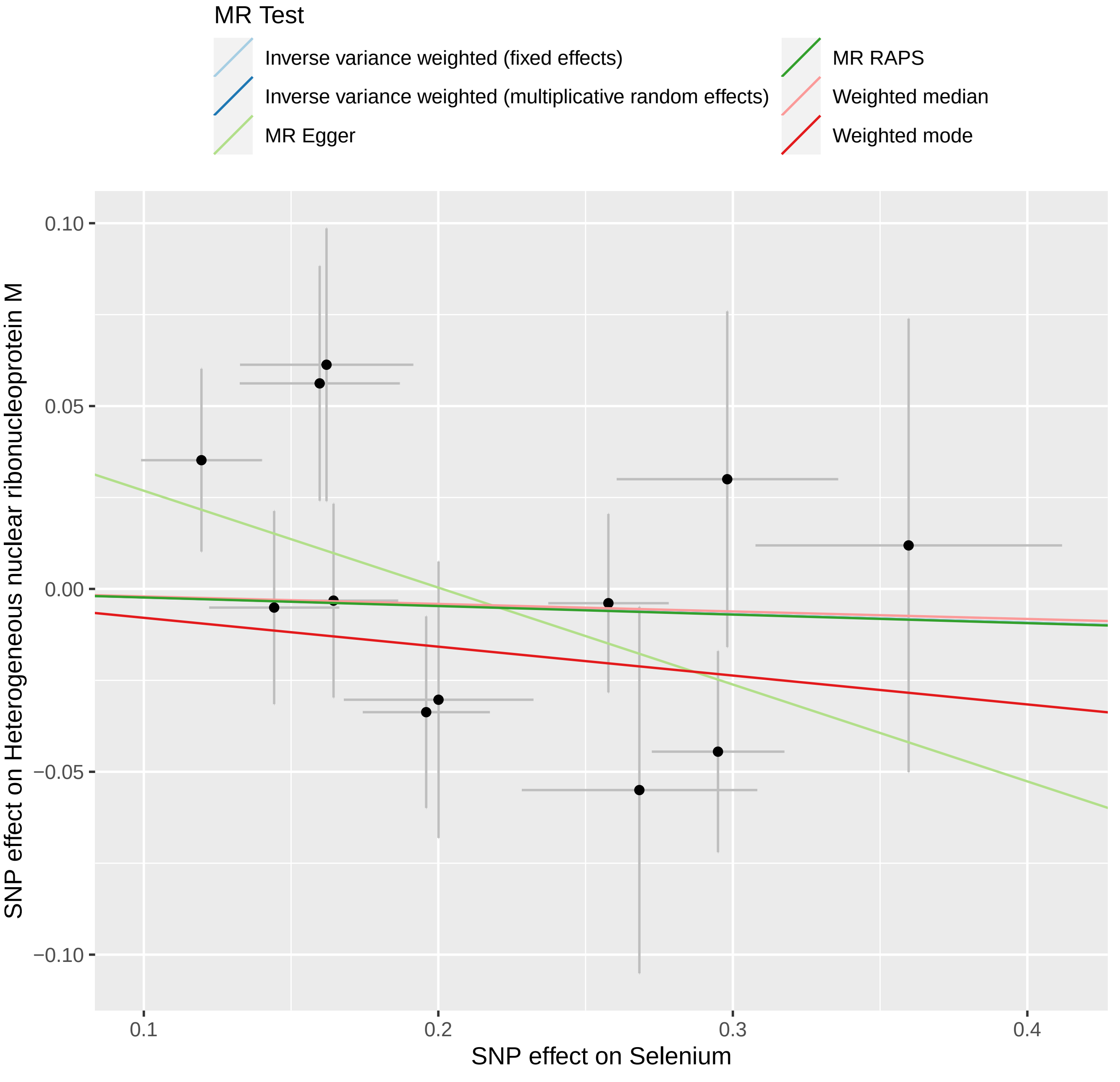

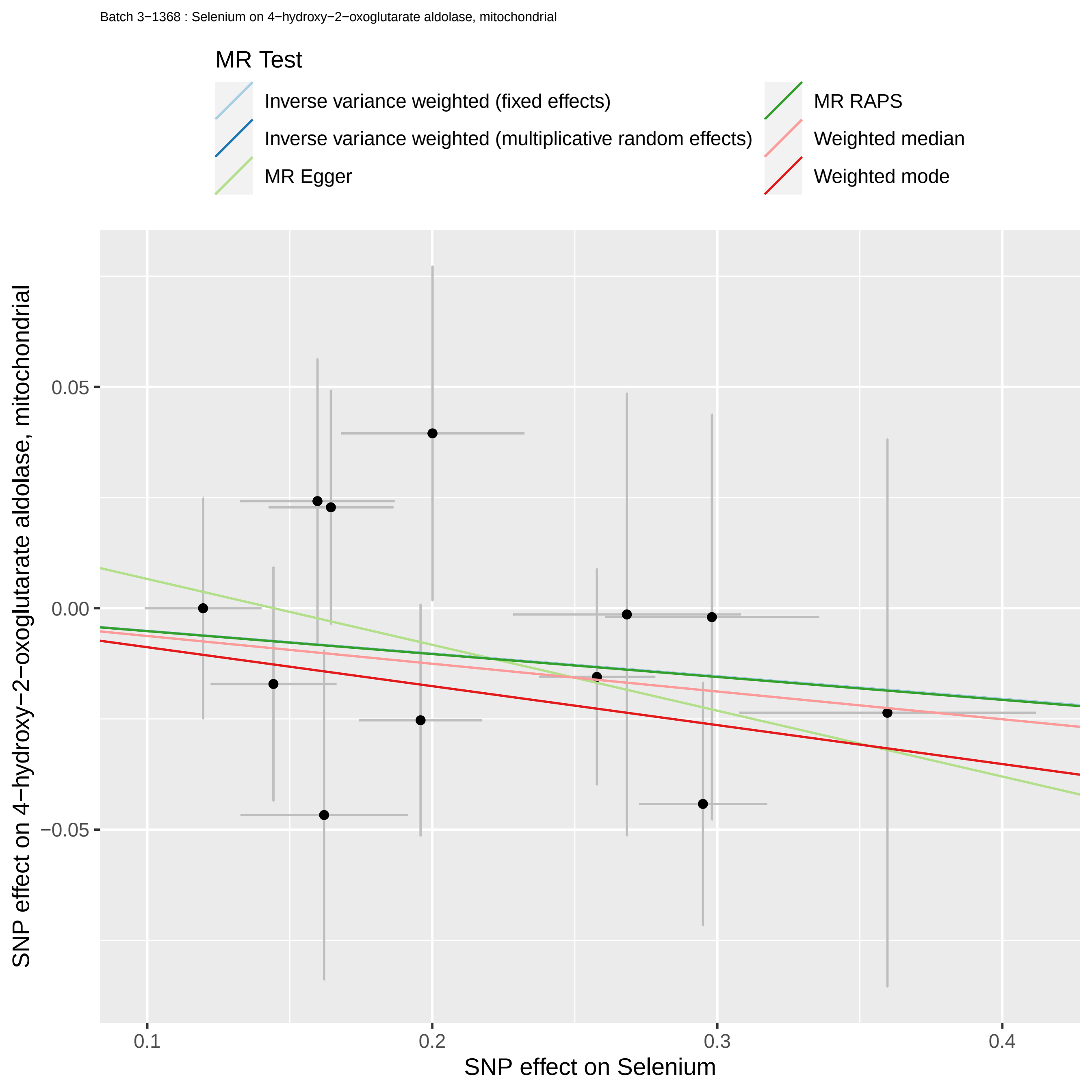

Batch 3−1368 : Selenium on 4−hydroxy−2−oxoglutarate aldolase, mitochondrial
MR Test
Inverse variance weighted (fixed effects)
Inverse variance weighted (multiplicative random effects)
MR Egger
MR RAPS
Weighted median
Weighted mode
SNP effect on 4−hydroxy−2−oxoglutarate aldolase, mitochondrial
0.05
0.00
−0.05
0.1
0.2
0.3
0.4
SNP effect on Selenium

Batch 3−1369 : Selenium on Haptoglobin

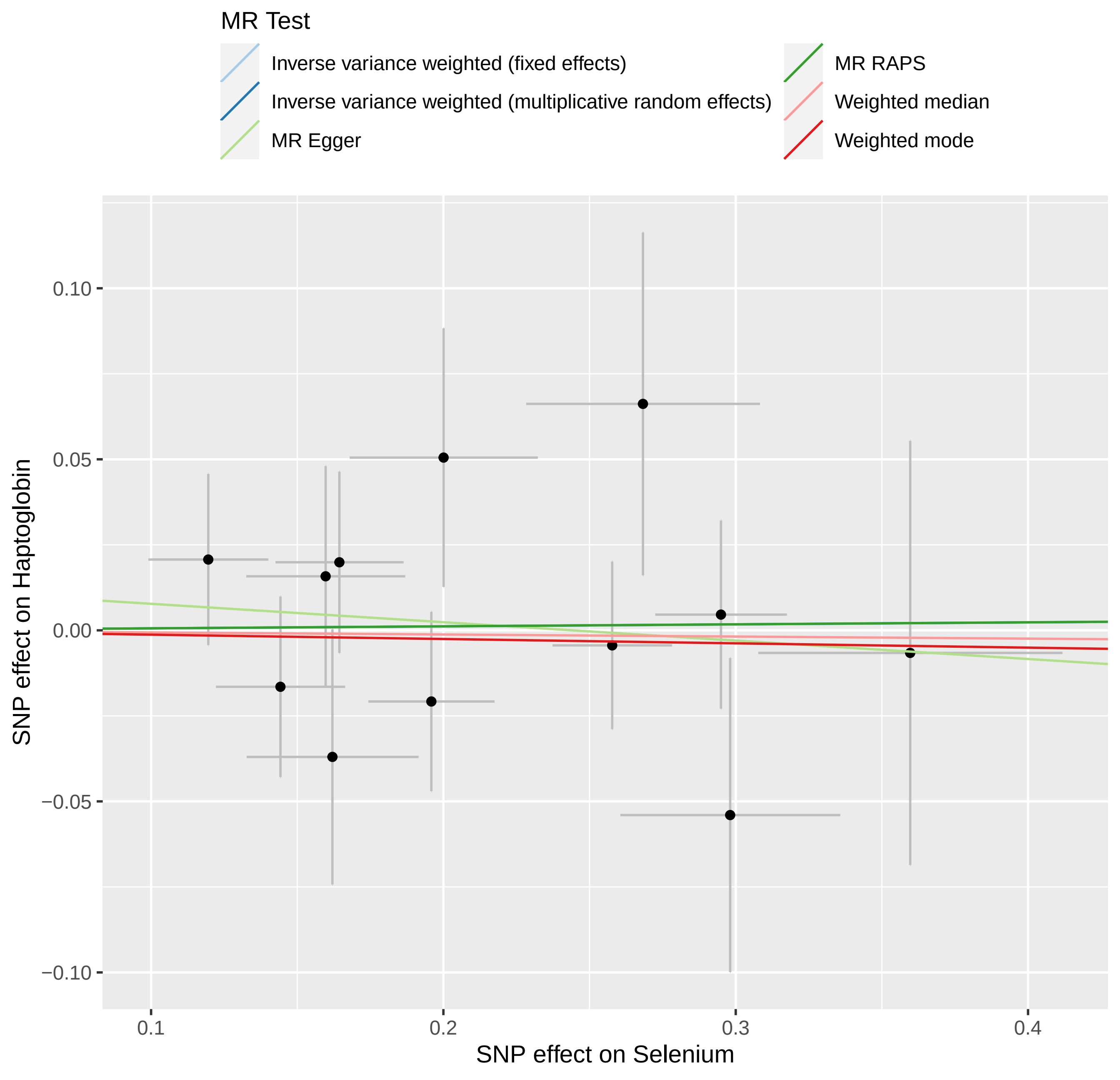

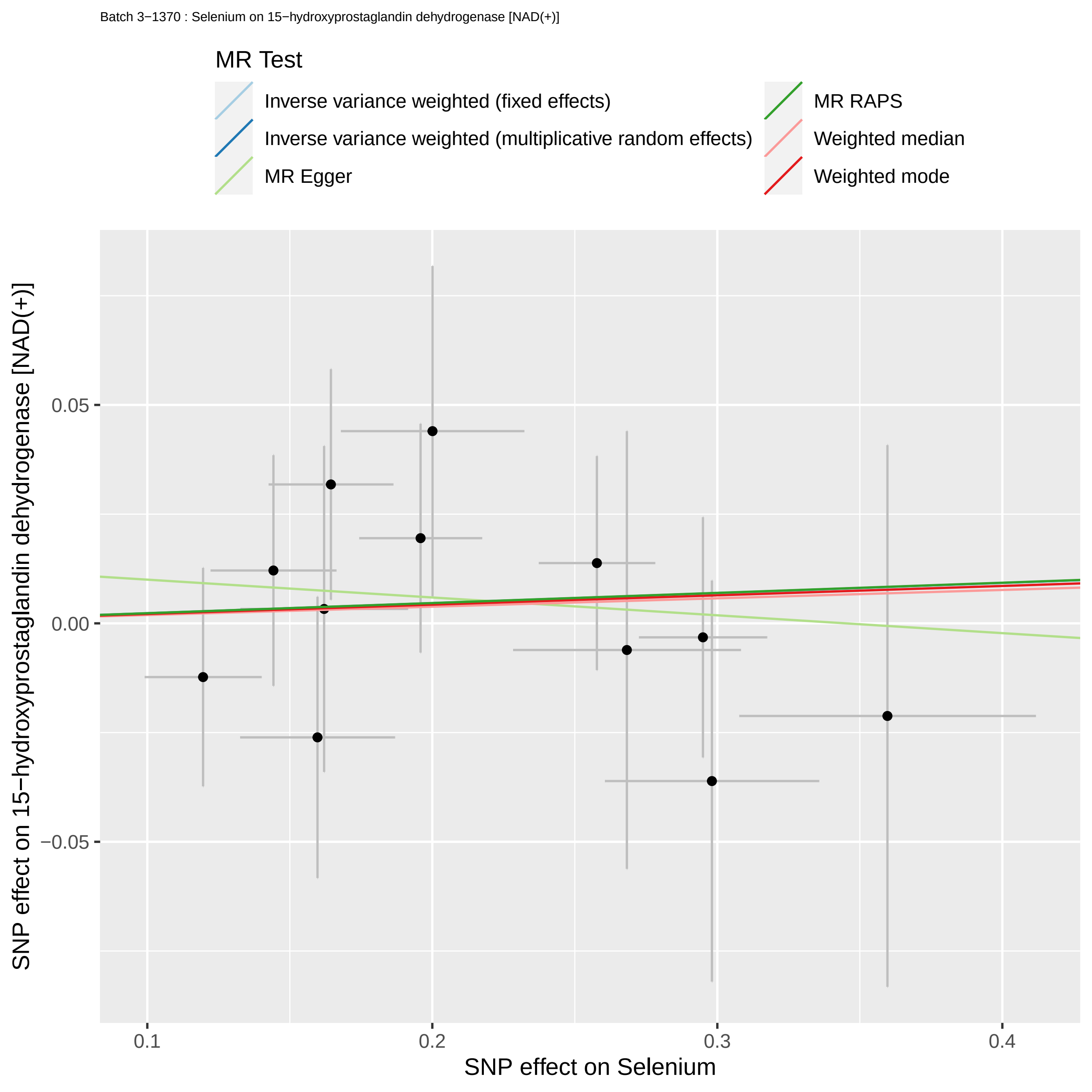

Batch 3−1370 : Selenium on 15−hydroxyprostaglandin dehydrogenase [NAD(+)]
MR Test
Inverse variance weighted (fixed effects)
Inverse variance weighted (multiplicative random effects)
MR Egger
MR RAPS
Weighted median
Weighted mode
0.05
0.00
−0.05
0.1
0.2
0.3
0.4
SNP effect on Selenium
SNP effect on 15−hydroxyprostaglandin dehydrogenase [NAD(+)]

Batch 3−1371 : Selenium on Hematopoietic prostaglandin D synthase

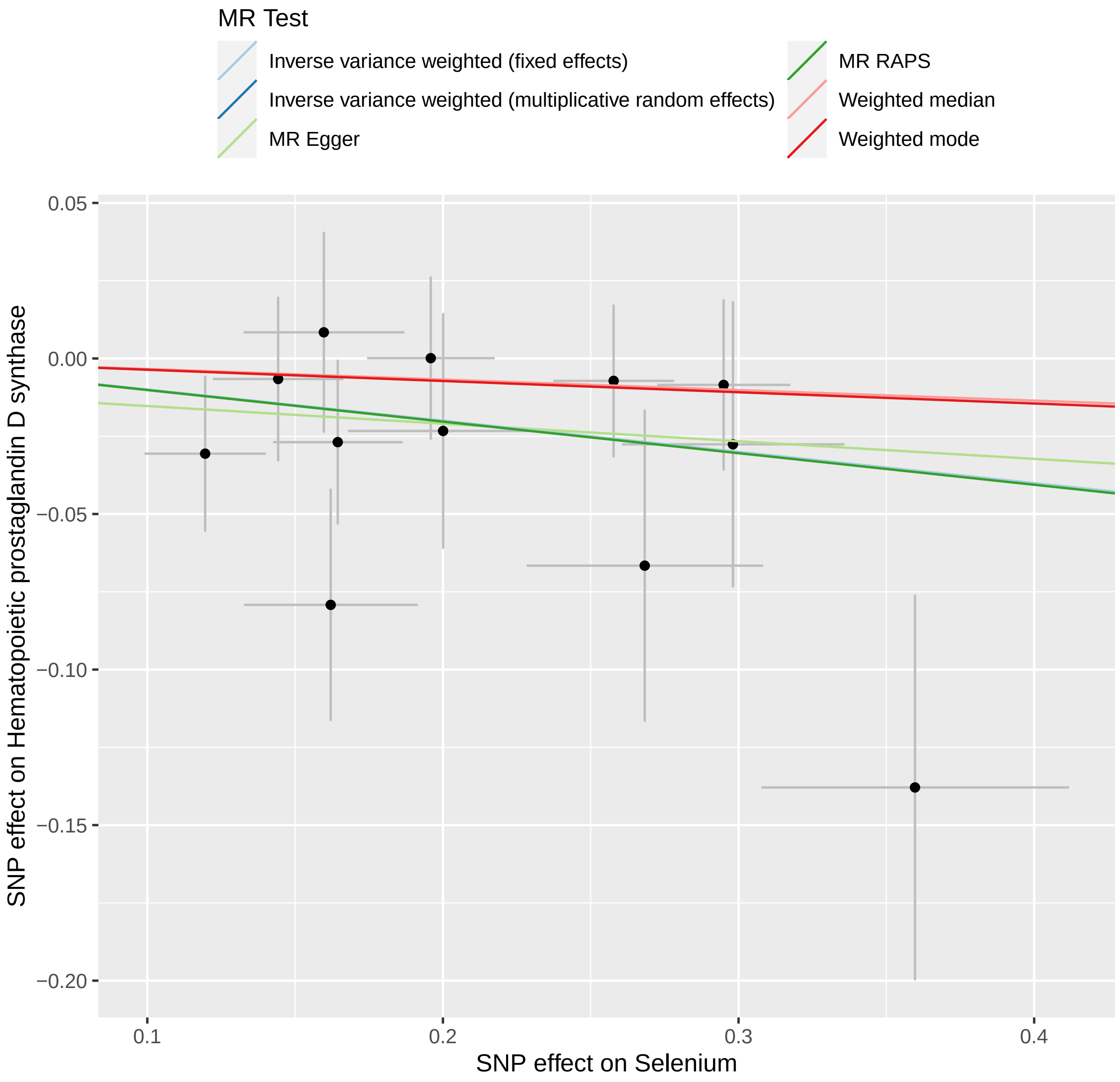

Batch 3−1372 : Selenium on HRAS−like suppressor 2

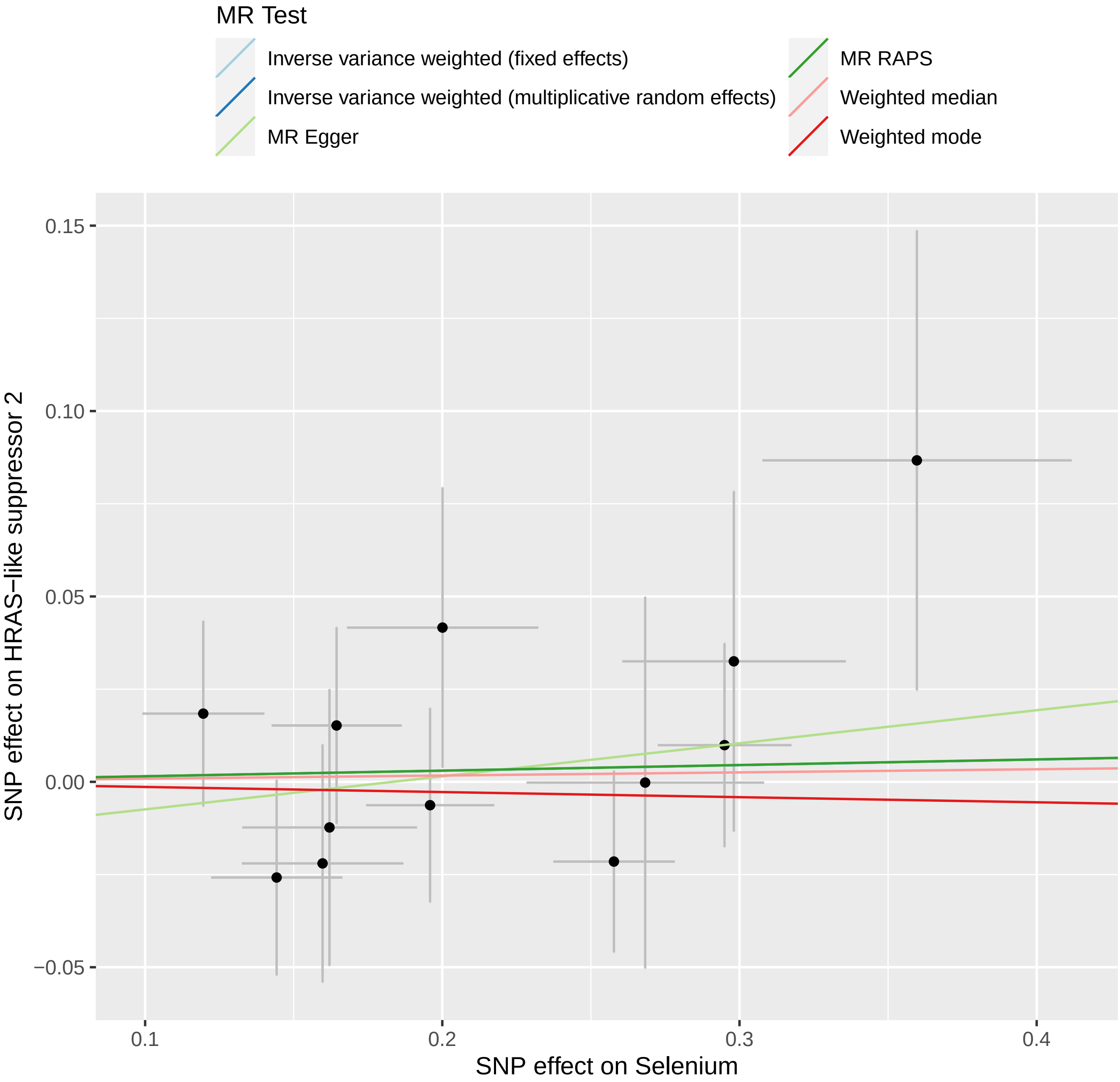

Batch 3−1373 : Selenium on Activator of apoptosis harakiri

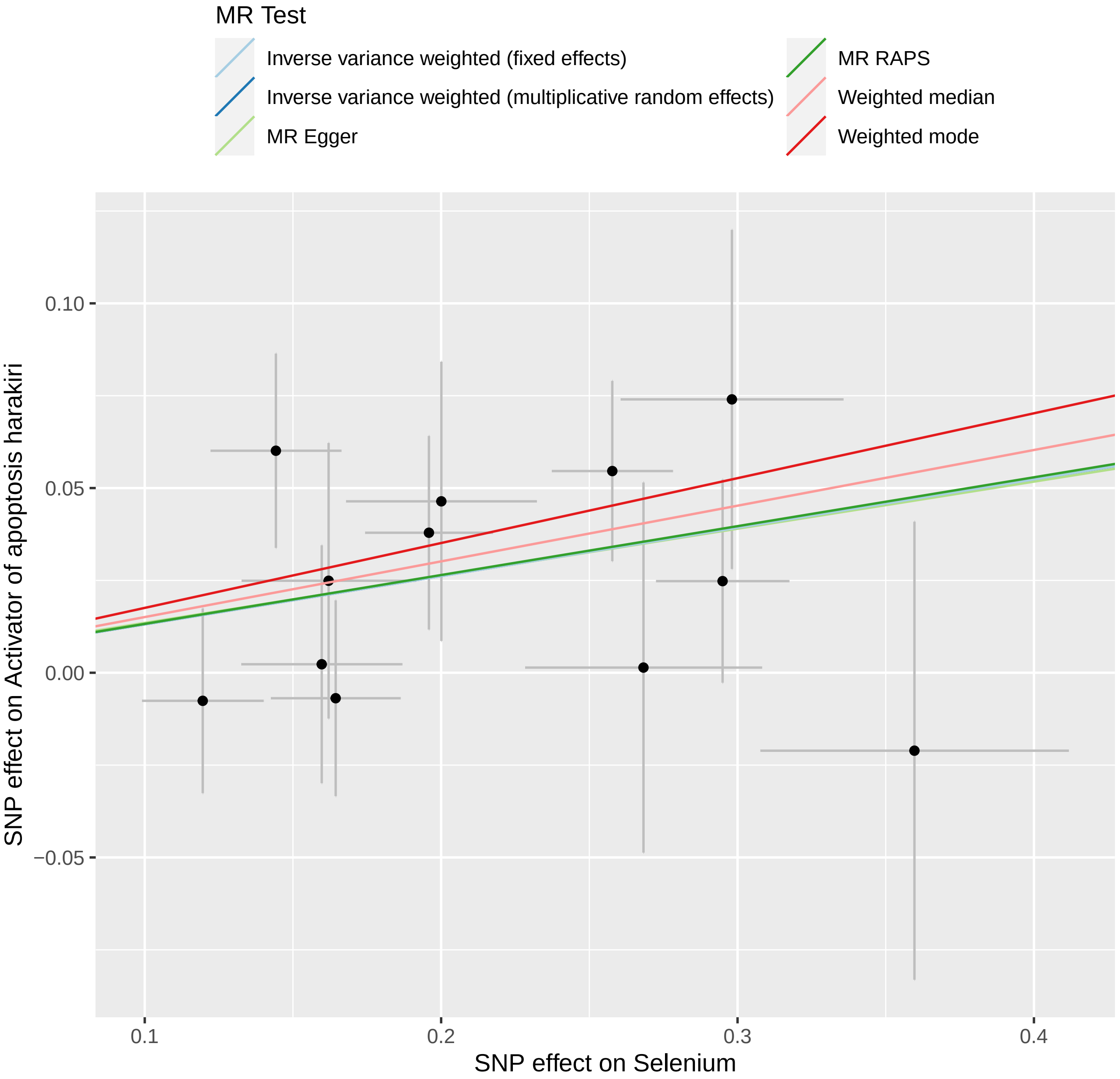

Batch 3−1374 : Selenium on Ribonuclease UK114

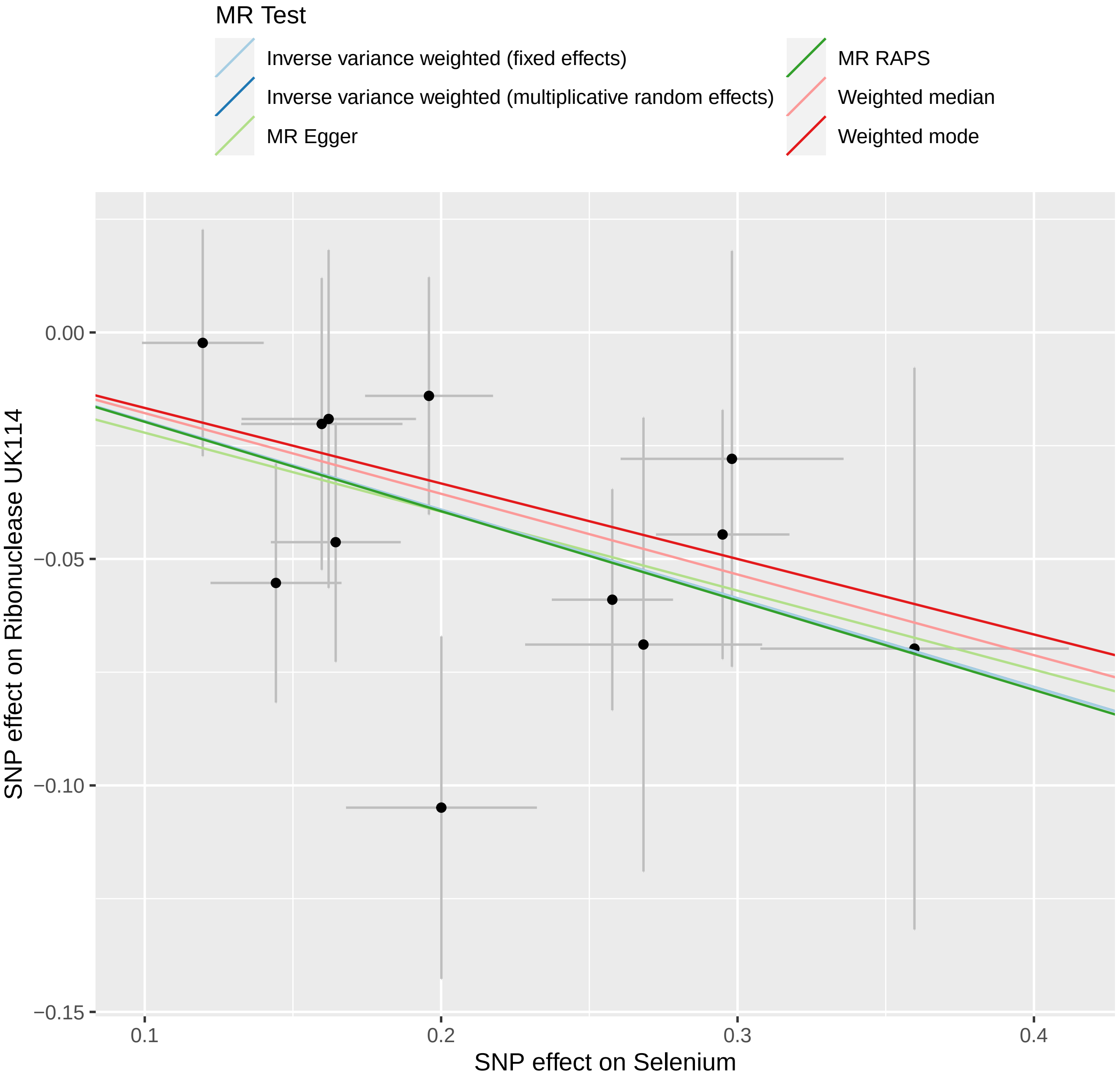

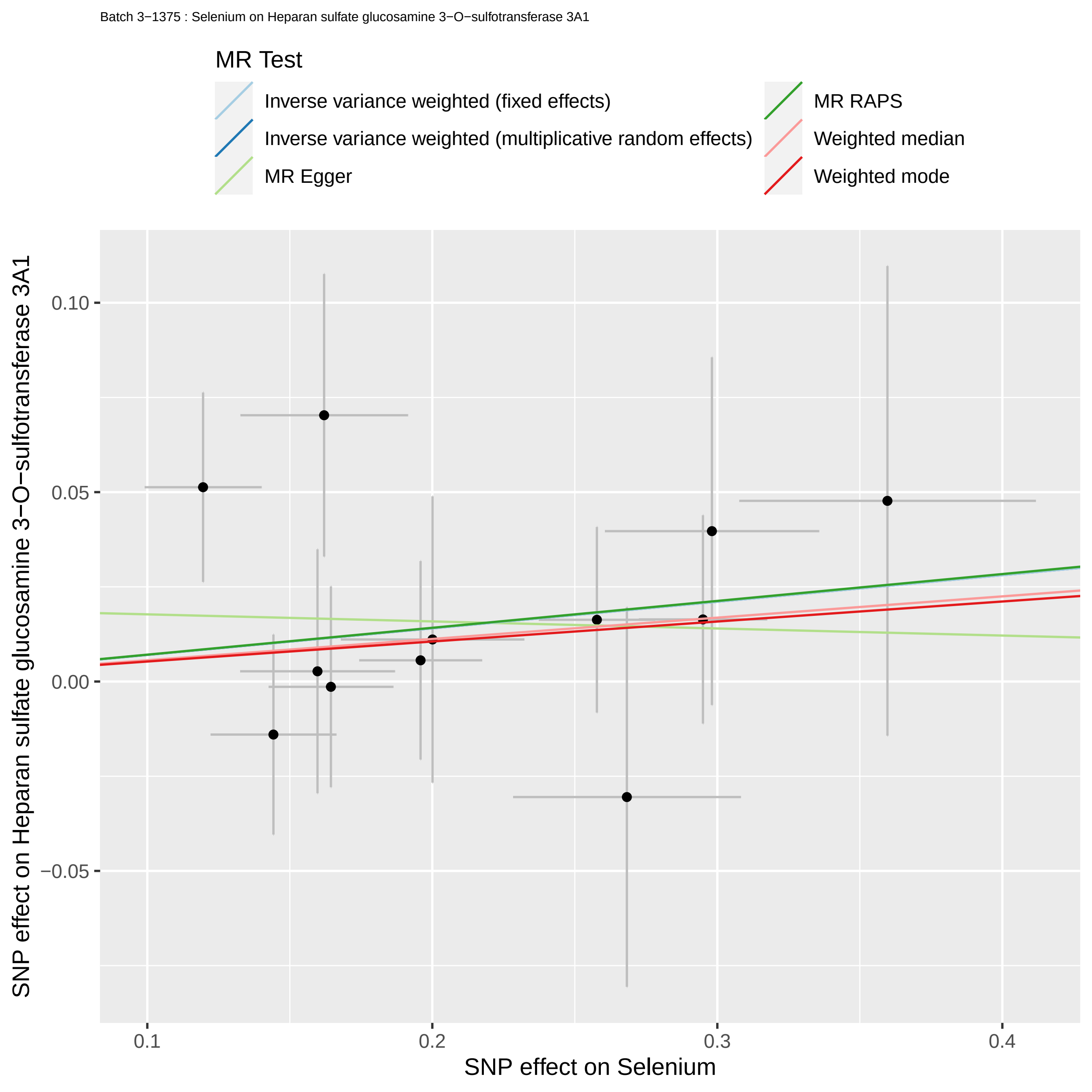
Batch 3−1375 : Selenium on Heparan sulfate glucosamine 3−O−sulfotransferase 3A1
MR Test
Inverse variance weighted (fixed effects)
Inverse variance weighted (multiplicative random effects)
MR Egger
MR RAPS
Weighted median
Weighted mode
SNP effect on Heparan sulfate glucosamine 3−O−sulfotransferase 3A1
0.10
0.05
0.00
−0.05
0.1
0.2
0.3
0.4
SNP effect on Selenium

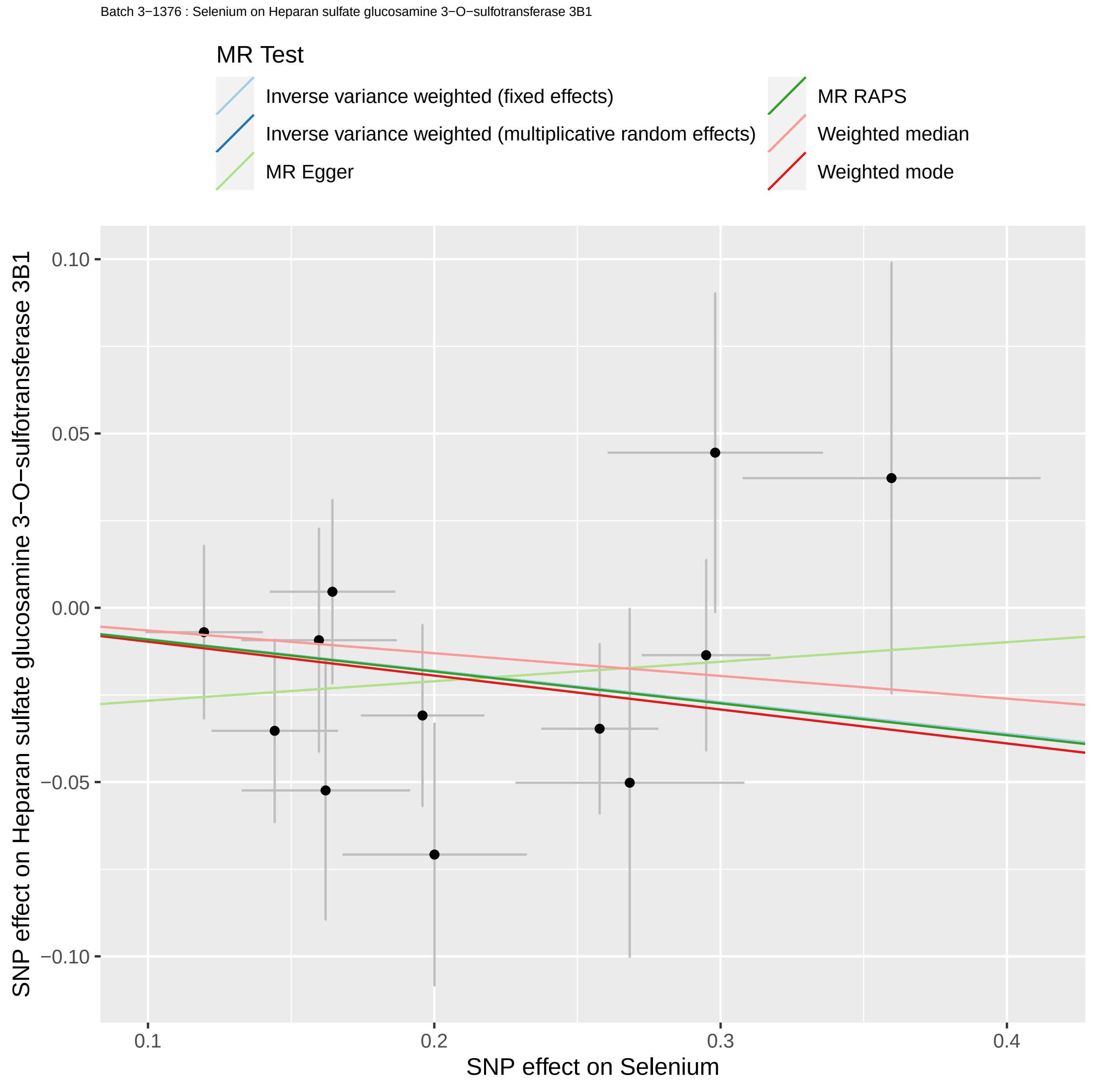

Batch 3−1376 : Selenium on Heparan sulfate glucosamine 3−O−sulfotransferase 3B1
MR Test
Inverse variance weighted (fixed effects)
Inverse variance weighted (multiplicative random effects)
MR Egger
MR RAPS
Weighted median
Weighted mode
0.10
0.05
0.00
−0.05
−0.10
0.1
0.2
0.3
0.4
SNP effect on Selenium
SNP effect on Heparan sulfate glucosamine 3−O−sulfotransferase 3B1

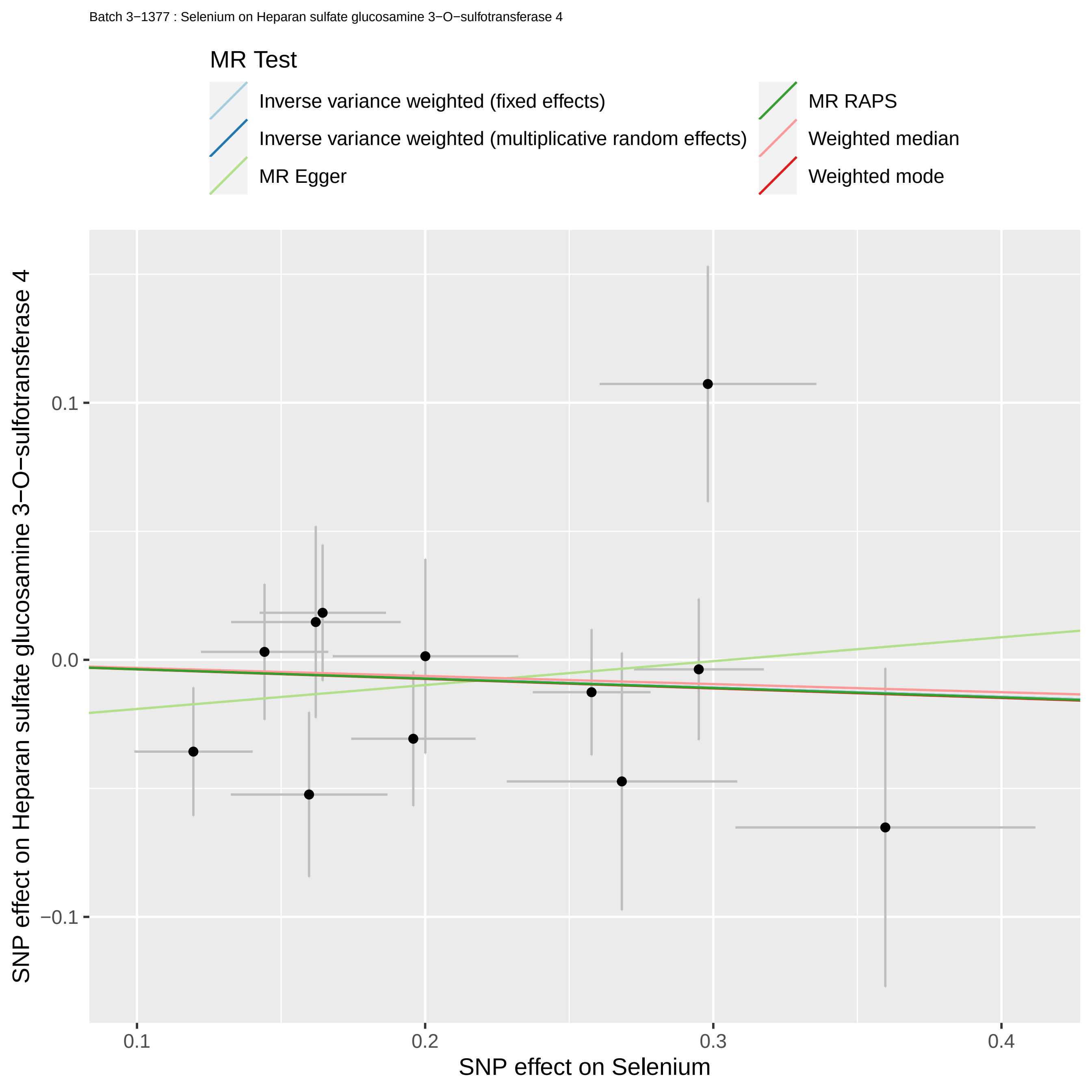
Batch 3−1377 : Selenium on Heparan sulfate glucosamine 3−O−sulfotransferase 4
MR Test
Inverse variance weighted (fixed effects)
Inverse variance weighted (multiplicative random effects)
MR Egger
MR RAPS
Weighted median
Weighted mode
SNP effect on Heparan sulfate glucosamine 3−O−sulfotransferase 4
0.1
0.0
−0.1
0.1
0.2
0.3
0.4
SNP effect on Selenium

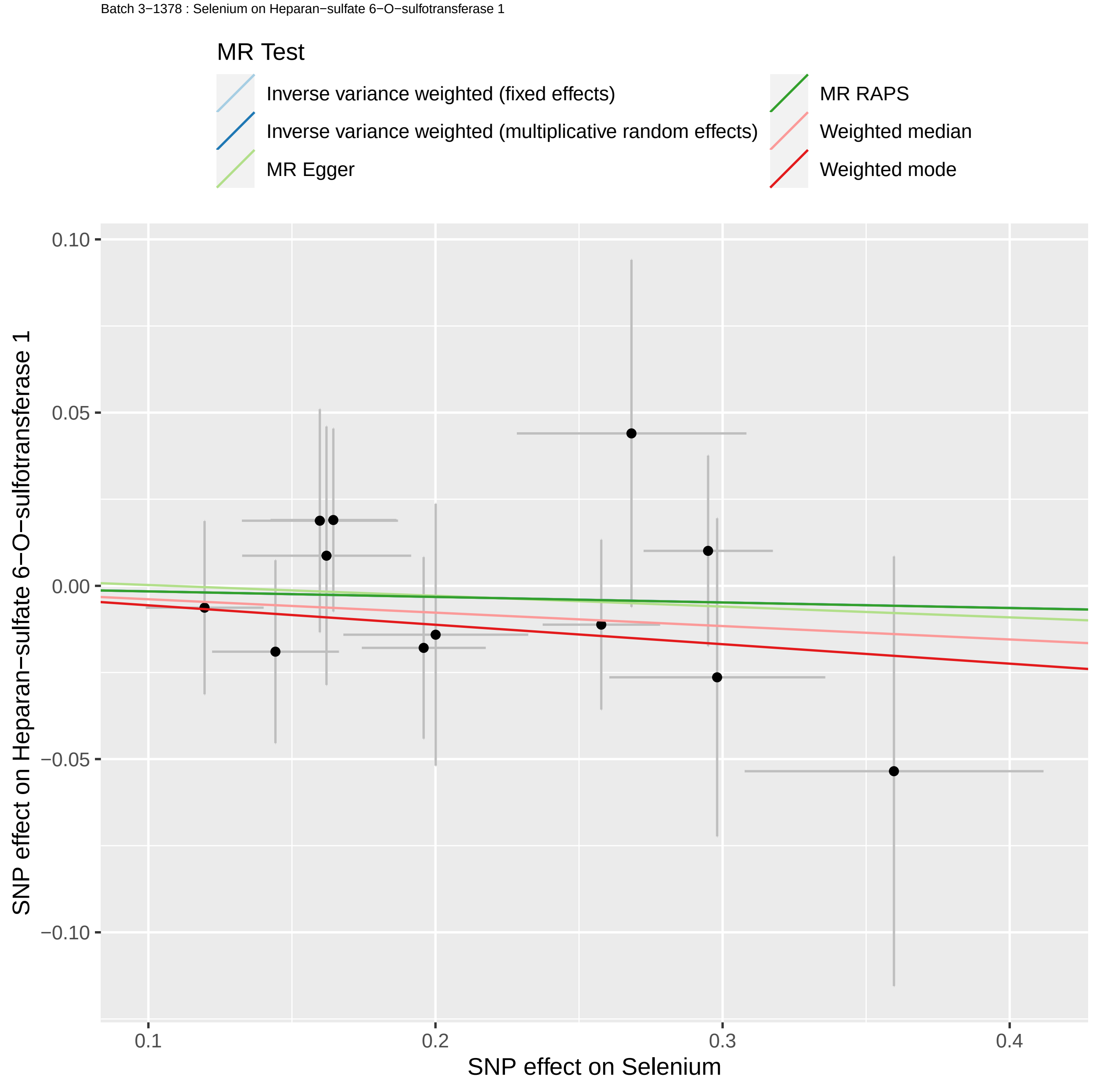

Batch 3−1378 : Selenium on Heparan−sulfate 6−O−sulfotransferase 1
MR Test
Inverse variance weighted (fixed effects)
Inverse variance weighted (multiplicative random effects)
MR Egger
MR RAPS
Weighted median
Weighted mode
0.10
0.05
0.00
−0.05
−0.10
0.1
0.2
0.3
0.4
SNP effect on Heparan−sulfate 6−O−sulfotransferase 1
SNP effect on Selenium

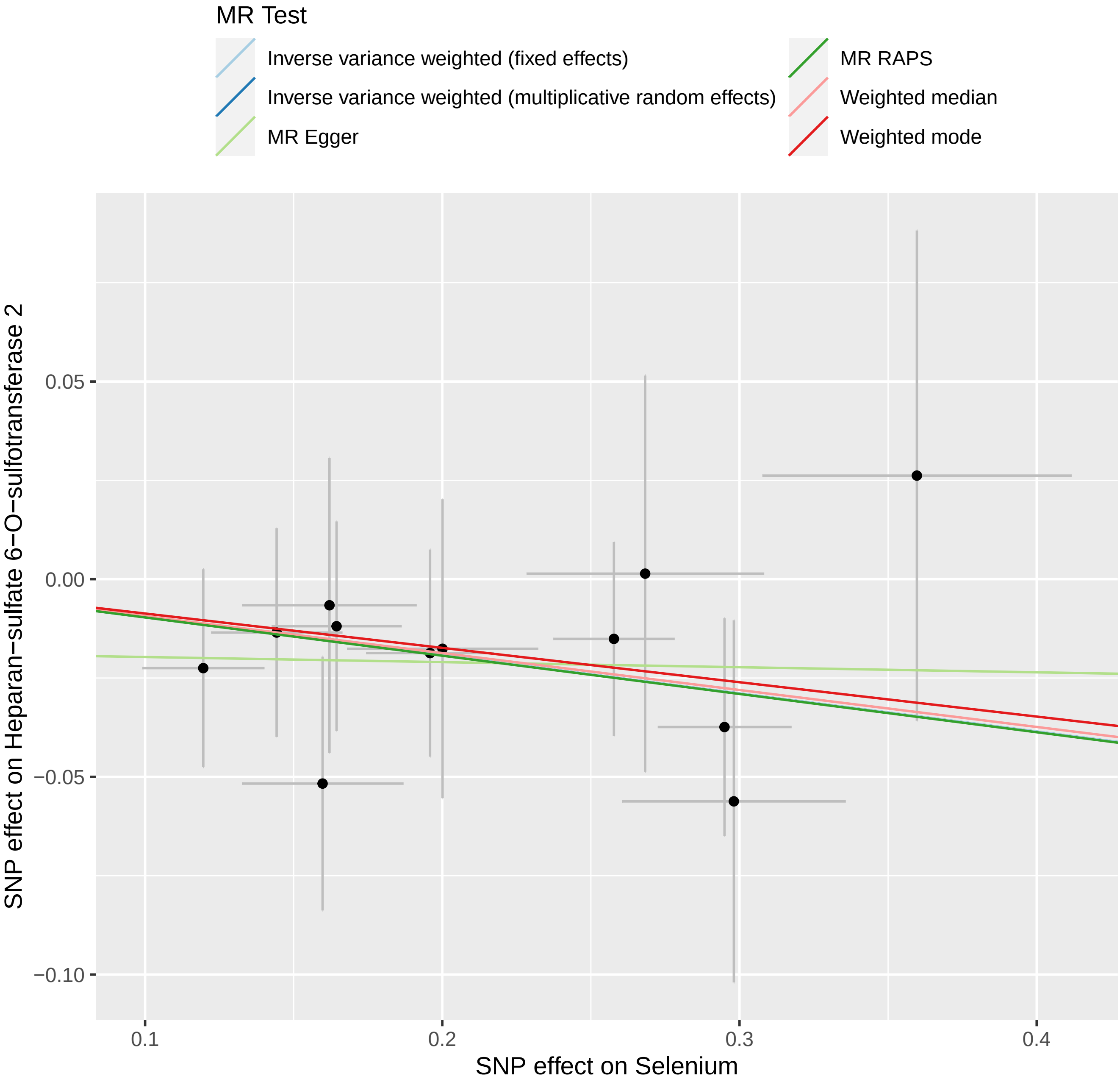
MR Test
Inverse variance weighted (fixed effects)
Inverse variance weighted (multiplicative random effects)
MR Egger
MR RAPS
Weighted median
Weighted mode
0.05
0.00
−0.05
−0.10
0.1
0.2
0.3
0.4
SNP effect on Heparan−sulfate 6−O−sulfotransferase 2
SNP effect on Selenium

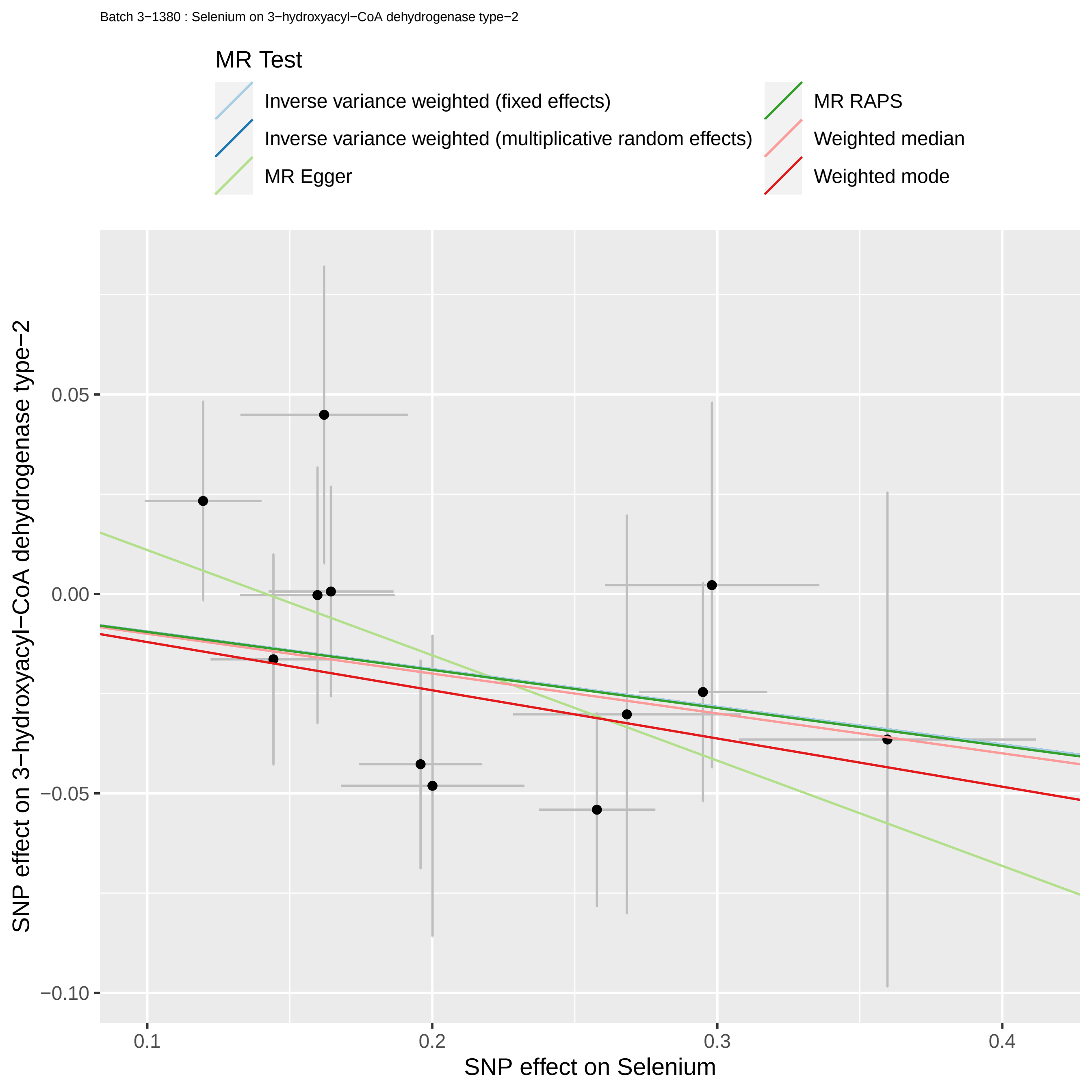
Batch 3−1380 : Selenium on 3−hydroxyacyl−CoA dehydrogenase type−2
MR Test
Inverse variance weighted (fixed effects)
Inverse variance weighted (multiplicative random effects)
MR Egger
MR RAPS
Weighted median
Weighted mode
SNP effect on 3−hydroxyacyl−CoA dehydrogenase type−2
0.05
0.00
−0.05
−0.10
0.1
0.2
0.3
0.4
SNP effect on Selenium

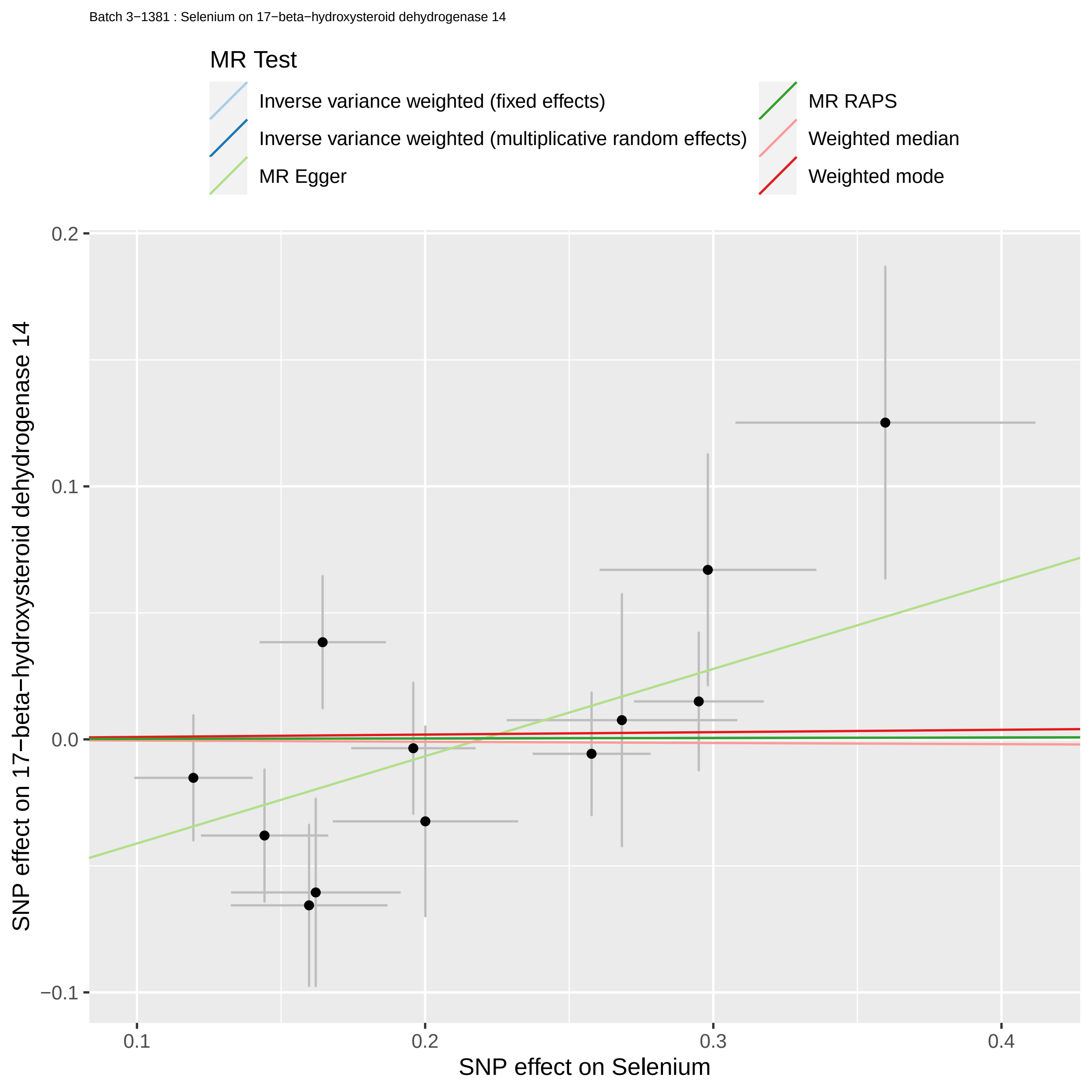
Batch 3−1381 : Selenium on 17−beta−hydroxysteroid dehydrogenase 14
MR Test
Inverse variance weighted (fixed effects)
Inverse variance weighted (multiplicative random effects)
MR Egger
MR RAPS
Weighted median
Weighted mode
0.2
0.1
0.0
−0.1
0.1
0.2
0.3
0.4
SNP effect on 17−beta−hydroxysteroid dehydrogenase 14
SNP effect on Selenium

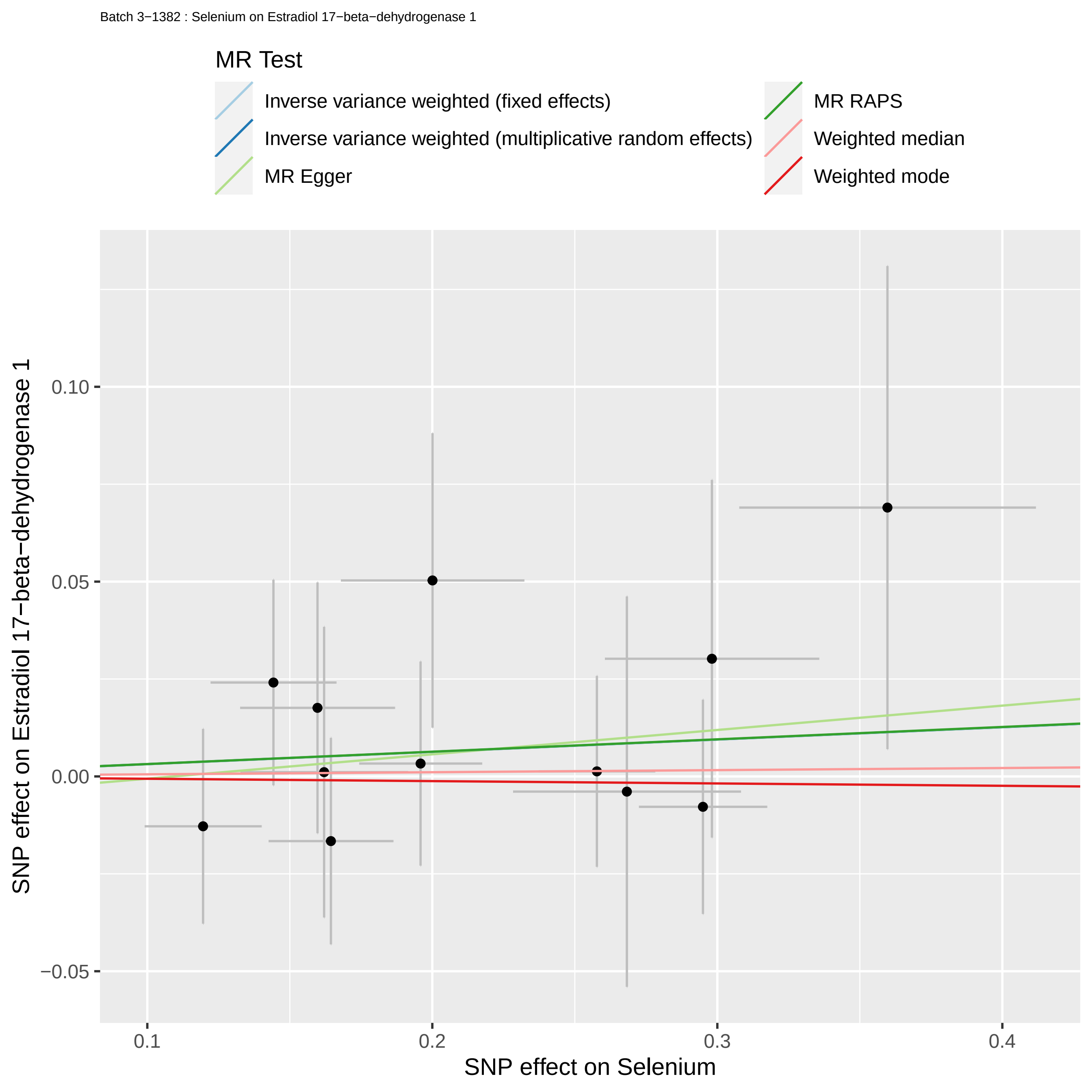

Batch 3−1382 : Selenium on Estradiol 17−beta−dehydrogenase 1
MR Test
Inverse variance weighted (fixed effects)
Inverse variance weighted (multiplicative random effects)
MR Egger
MR RAPS
Weighted median
Weighted mode
SNP effect on Estradiol 17−beta−dehydrogenase 1
0.10
0.05
0.00
−0.05
0.1
0.2
0.3
0.4
SNP effect on Selenium

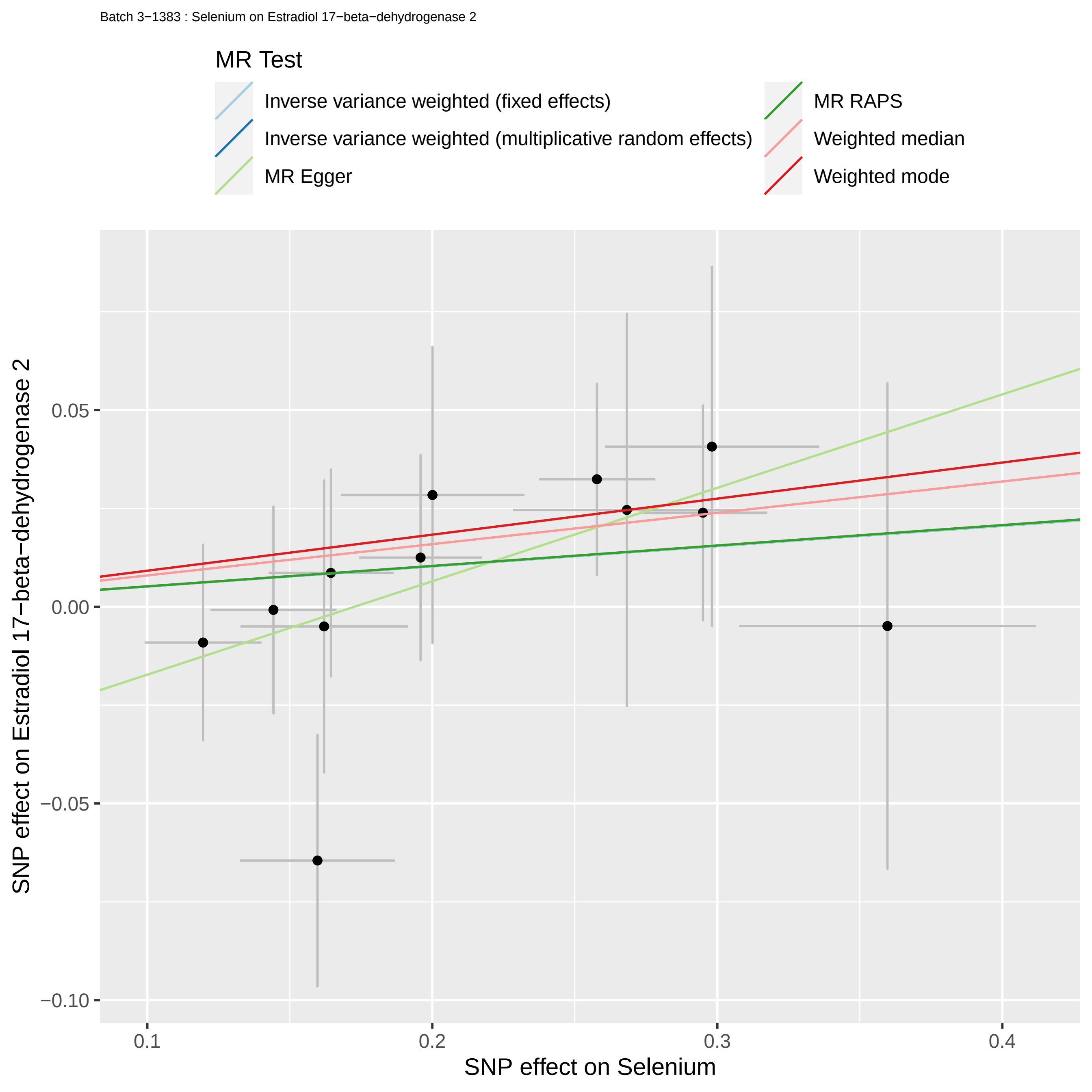

Batch 3−1383 : Selenium on Estradiol 17−beta−dehydrogenase 2
MR Test
Inverse variance weighted (fixed effects)
Inverse variance weighted (multiplicative random effects)
MR Egger
MR RAPS
Weighted median
Weighted mode
SNP effect on Estradiol 17−beta−dehydrogenase 2
0.05
0.00
−0.05
−0.10
0.1
0.2
0.3
0.4
SNP effect on Selenium

Batch 3−1384 : Selenium on Heat shock protein HSP 90−alpha/beta

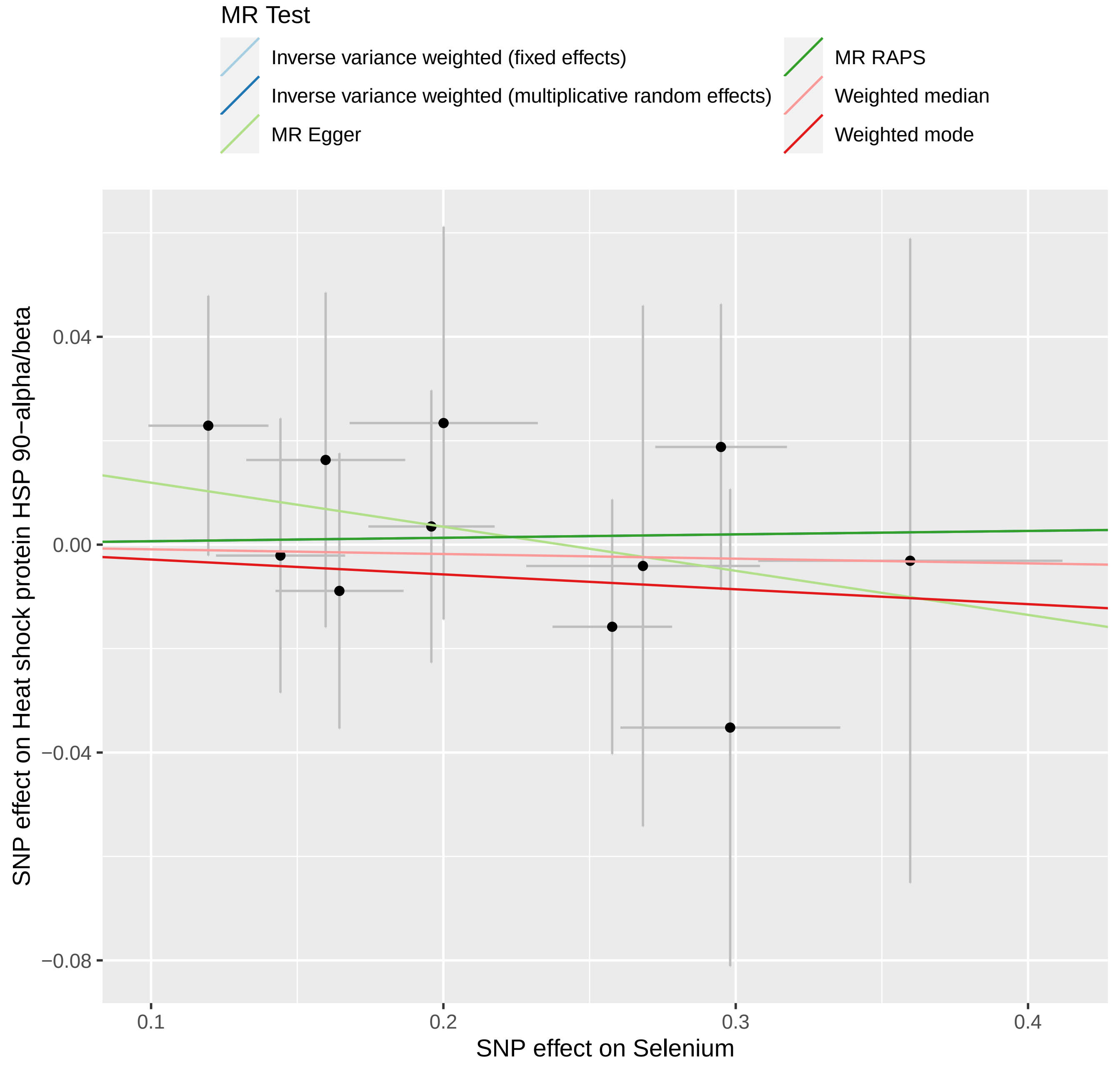

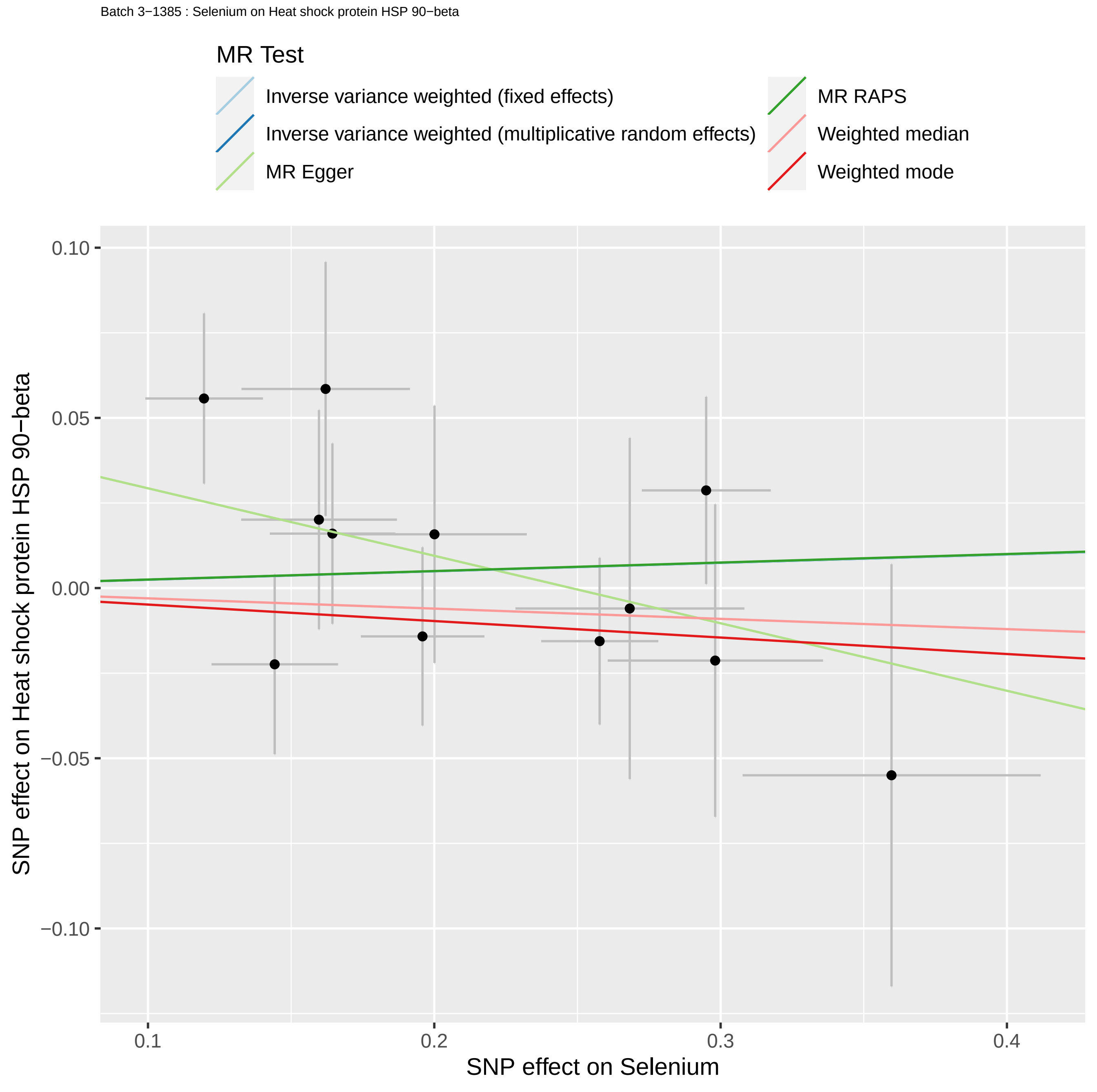

Batch 3−1385 : Selenium on Heat shock protein HSP 90−beta
MR Test
Inverse variance weighted (fixed effects)
Inverse variance weighted (multiplicative random effects)
MR Egger
MR RAPS
Weighted median
Weighted mode
SNP effect on Heat shock protein HSP 90−beta
0.10
0.05
0.00
−0.05
−0.10
0.1
0.2
0.3
0.4
SNP effect on Selenium

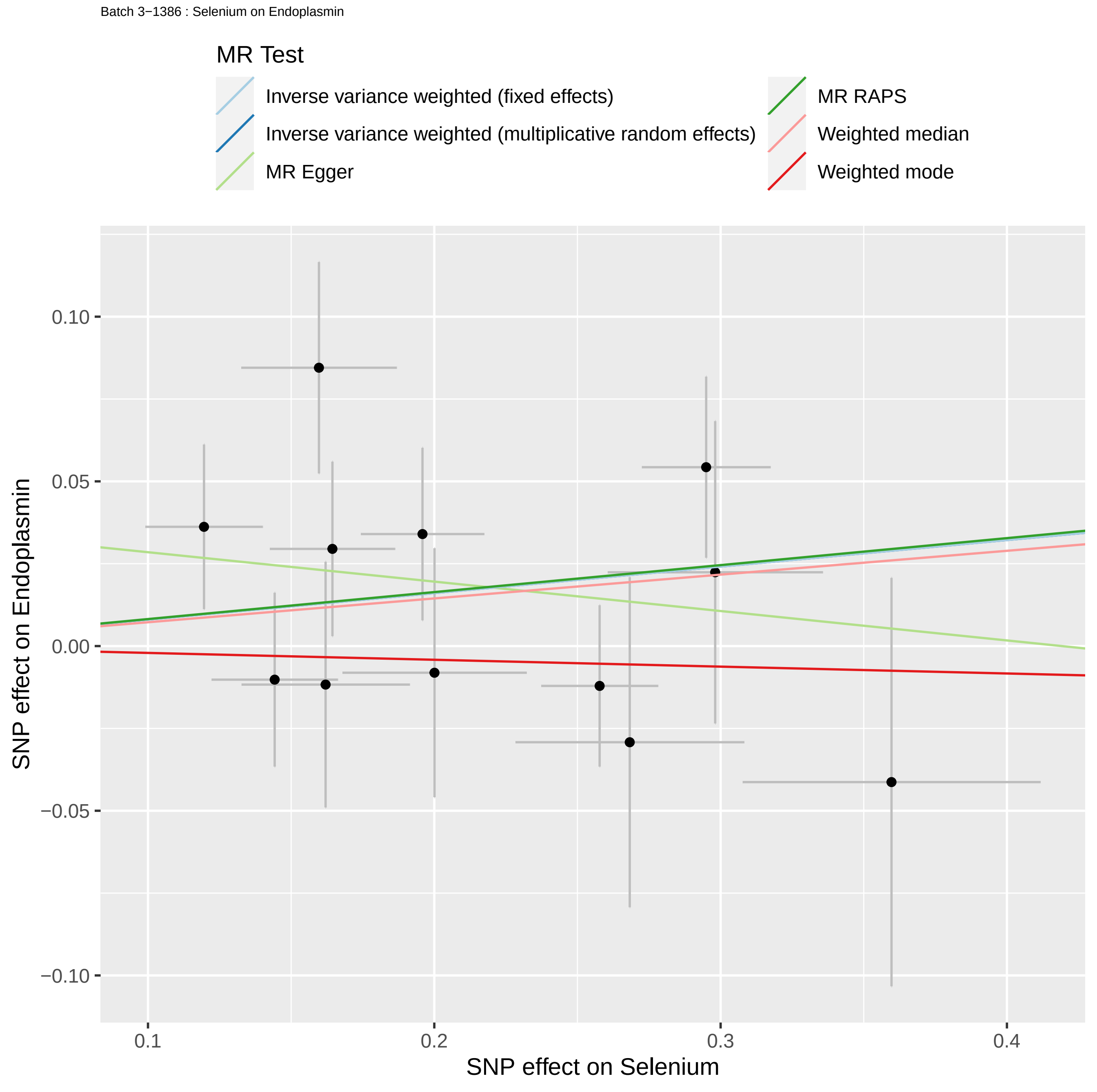
Batch 3−1386 : Selenium on Endoplasmin
MR Test
Inverse variance weighted (fixed effects)
Inverse variance weighted (multiplicative random effects)
MR Egger
MR RAPS
Weighted median
Weighted mode
SNP effect on Endoplasmin
0.10
0.05
0.00
−0.05
−0.10
0.1
0.2
0.3
0.4
SNP effect on Selenium

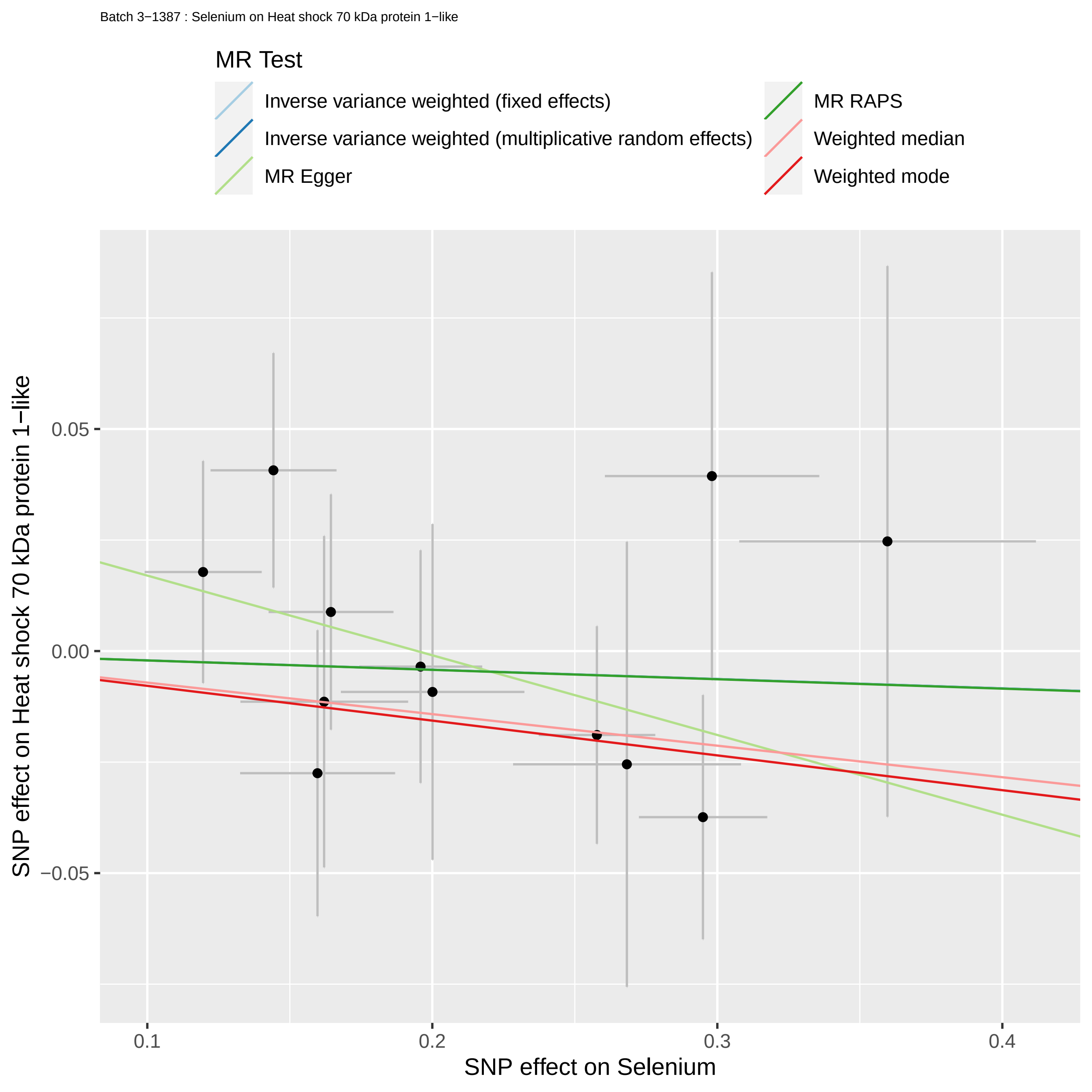

Batch 3−1387 : Selenium on Heat shock 70 kDa protein 1−like
MR Test
Inverse variance weighted (fixed effects)
Inverse variance weighted (multiplicative random effects)
MR Egger
MR RAPS
Weighted median
Weighted mode
SNP effect on Heat shock 70 kDa protein 1−like
0.05
0.00
−0.05
0.1
0.2
0.3
0.4
SNP effect on Selenium

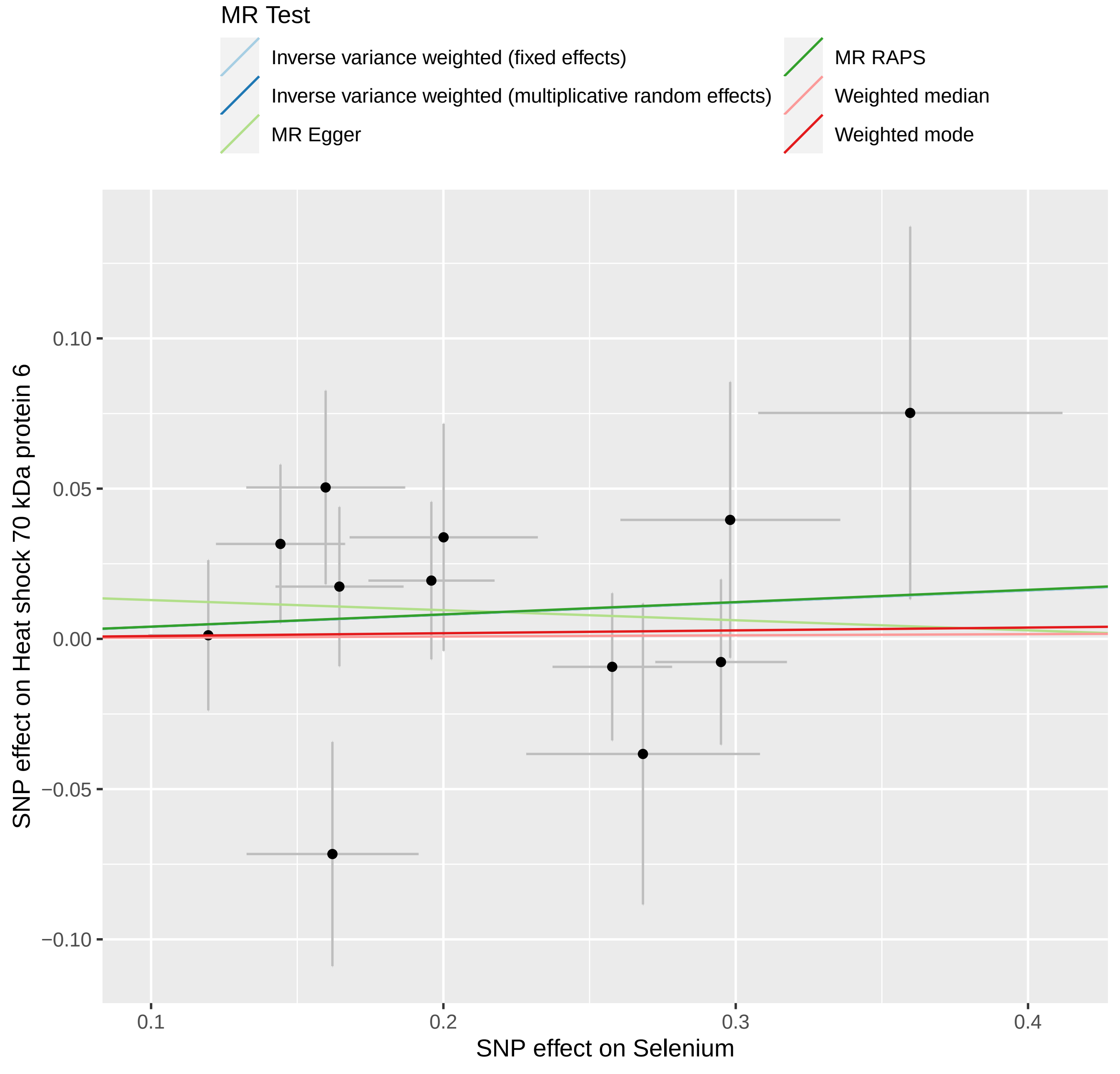
MR Test
Inverse variance weighted (fixed effects)
Inverse variance weighted (multiplicative random effects)
MR Egger
MR RAPS
Weighted median
Weighted mode
0.10
0.05
0.00
−0.05
−0.10
0.1
0.2
0.3
0.4
SNP effect on Heat shock 70 kDa protein 6
SNP effect on Selenium

Batch 3−1389 : Selenium on Oxidoreductase HTATIP2

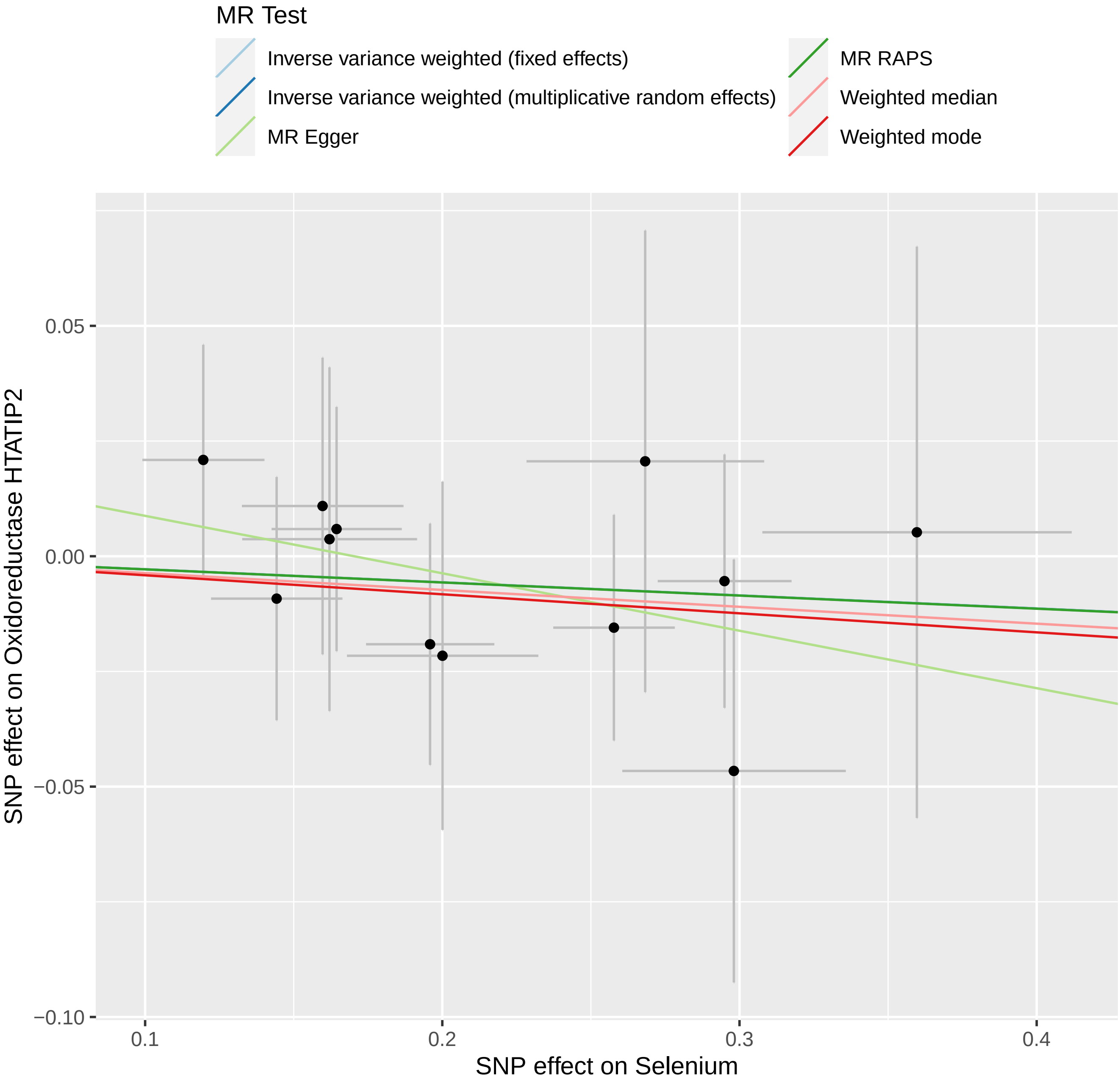

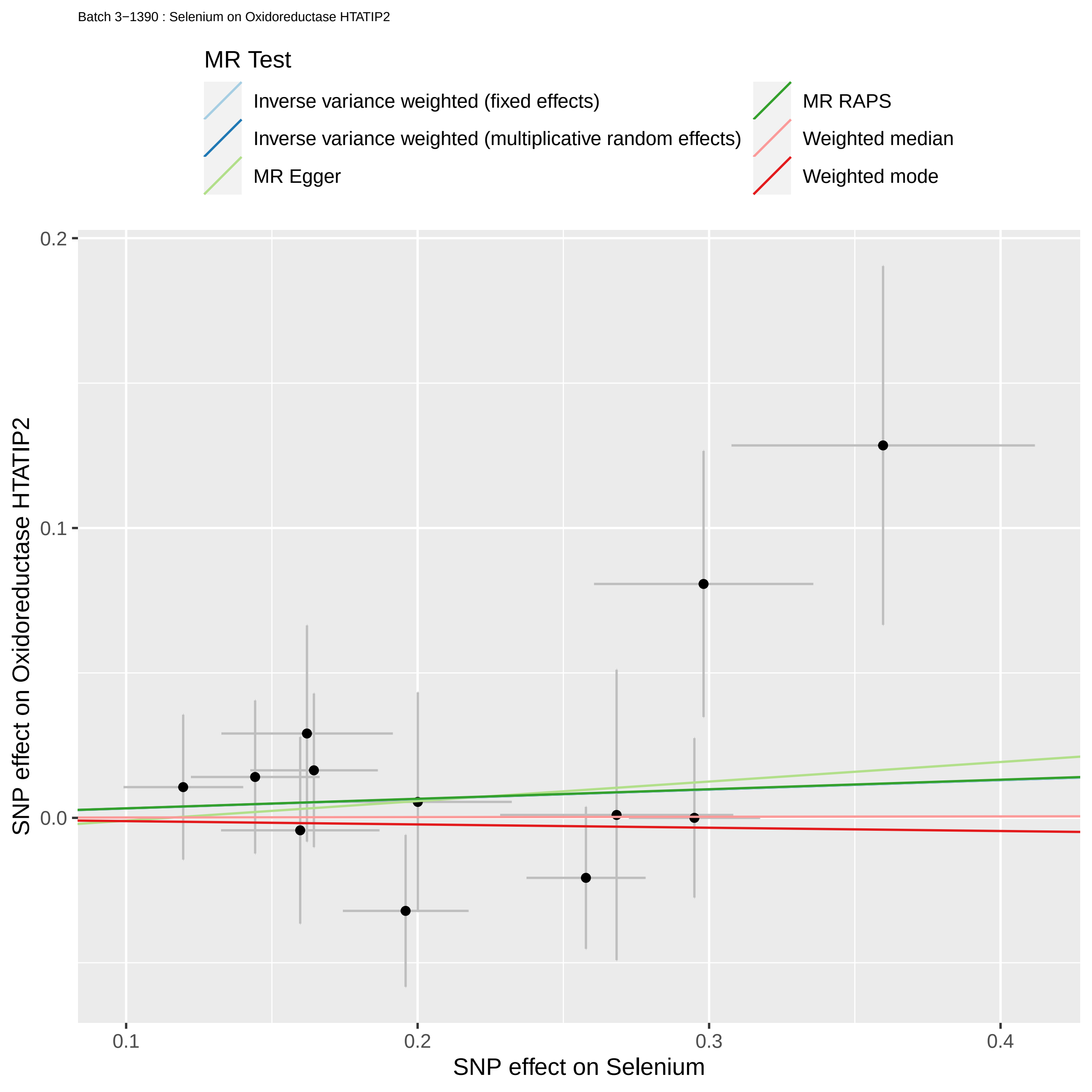

Batch 3−1390 : Selenium on Oxidoreductase HTATIP2
MR Test
Inverse variance weighted (fixed effects)
Inverse variance weighted (multiplicative random effects)
MR Egger
MR RAPS
Weighted median
Weighted mode
0.2
0.1
0.0
0.1
0.2
0.3
0.4
SNP effect on Oxidoreductase HTATIP2
SNP effect on Selenium

Batch 3−1391 : Selenium on Histatin−1

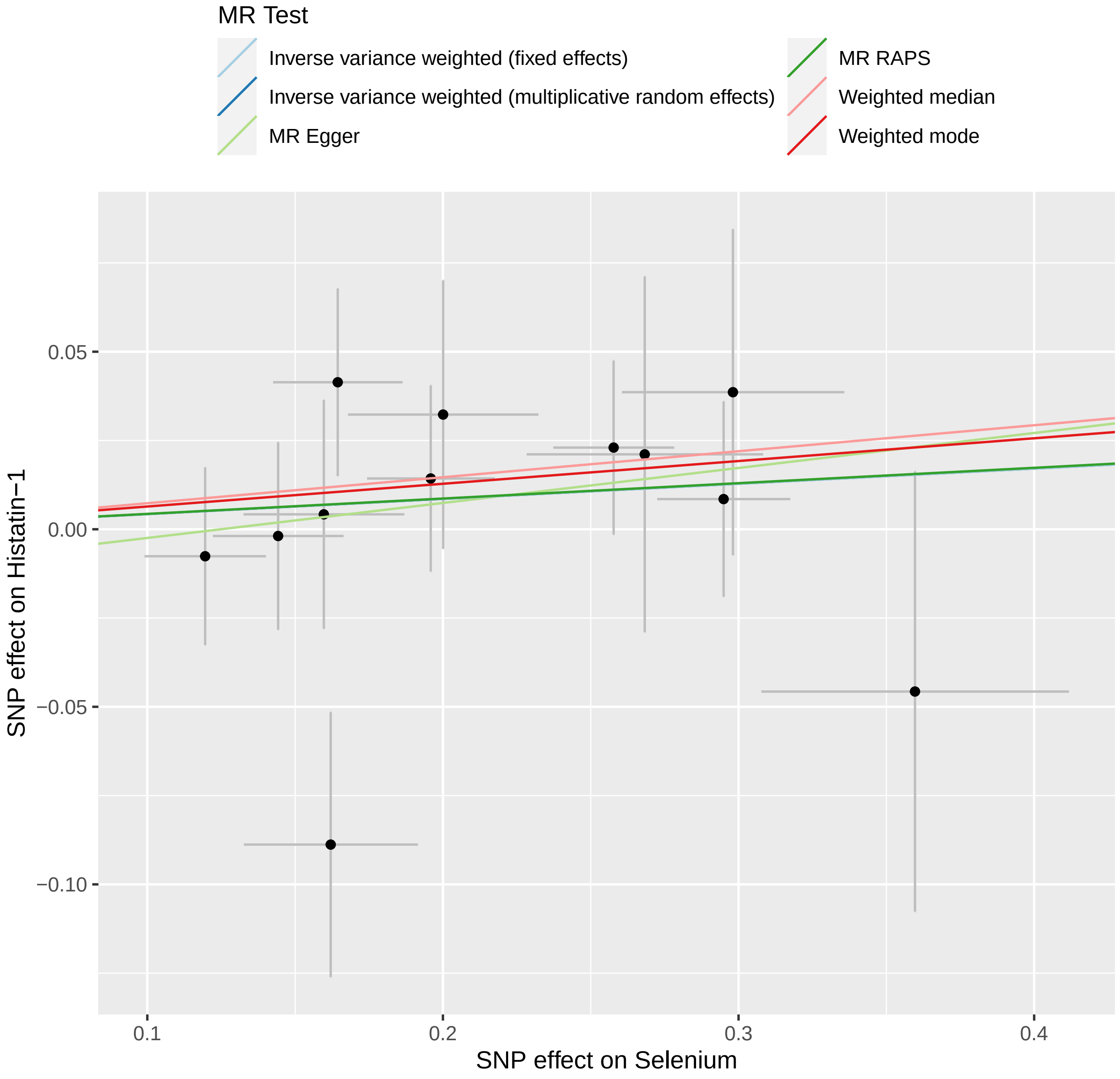

Batch 3−1392 : Selenium on Serine protease HTRA2, mitochondrial

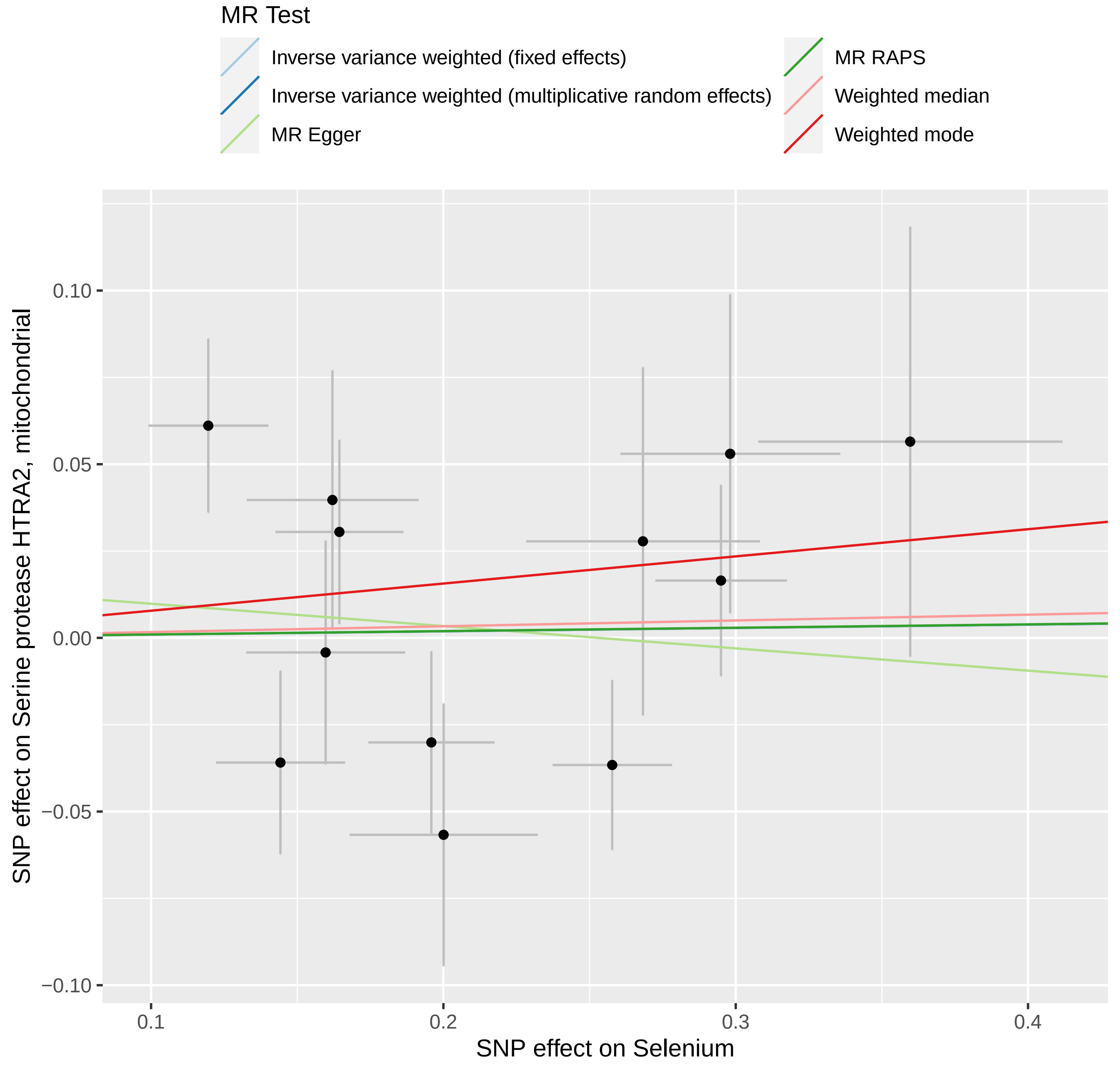

Batch 3−1393 : Selenium on Hyaluronidase−1

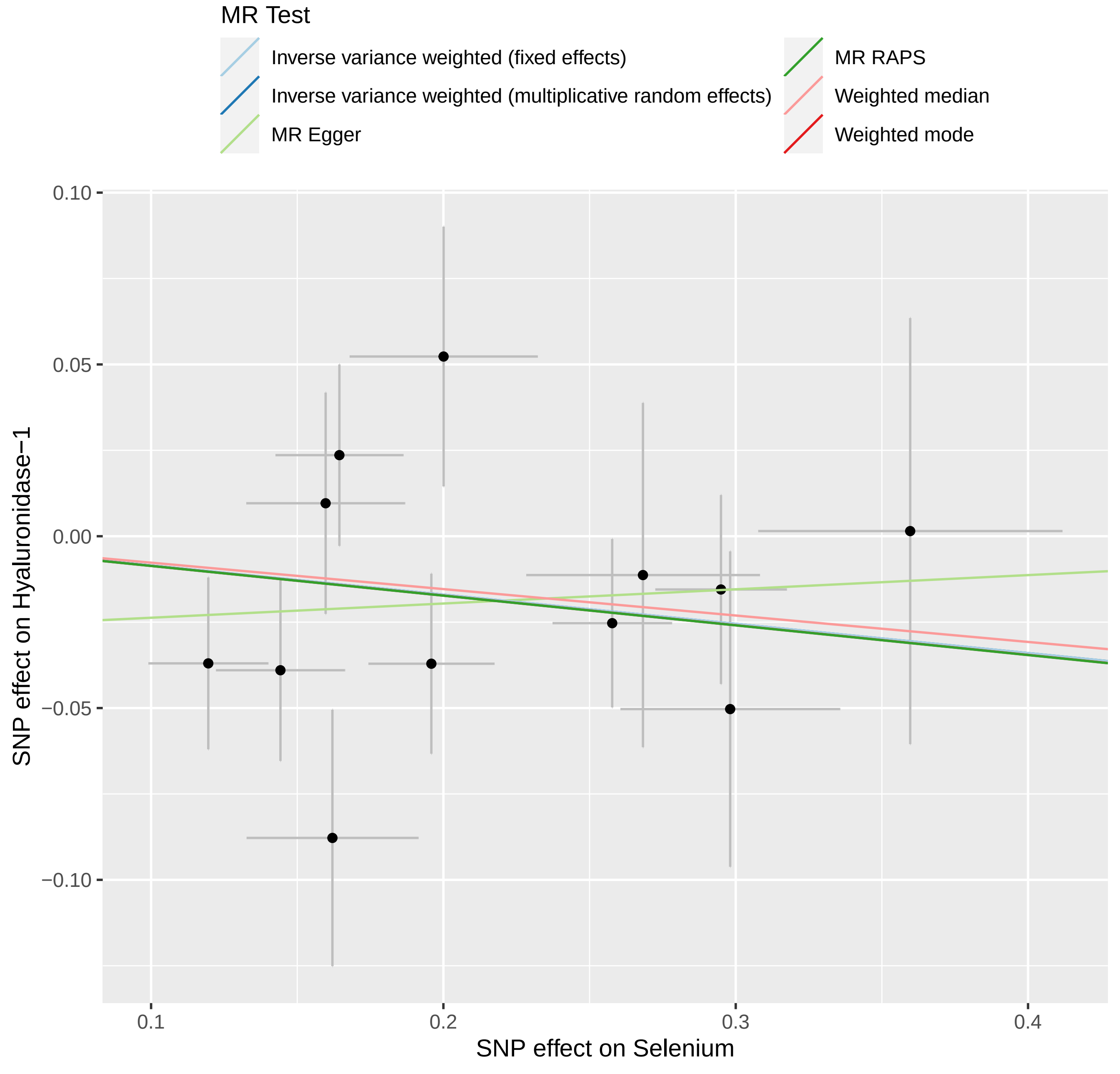

Batch 3−1394 : Selenium on Isoleucine−−tRNA ligase, cytoplasmic

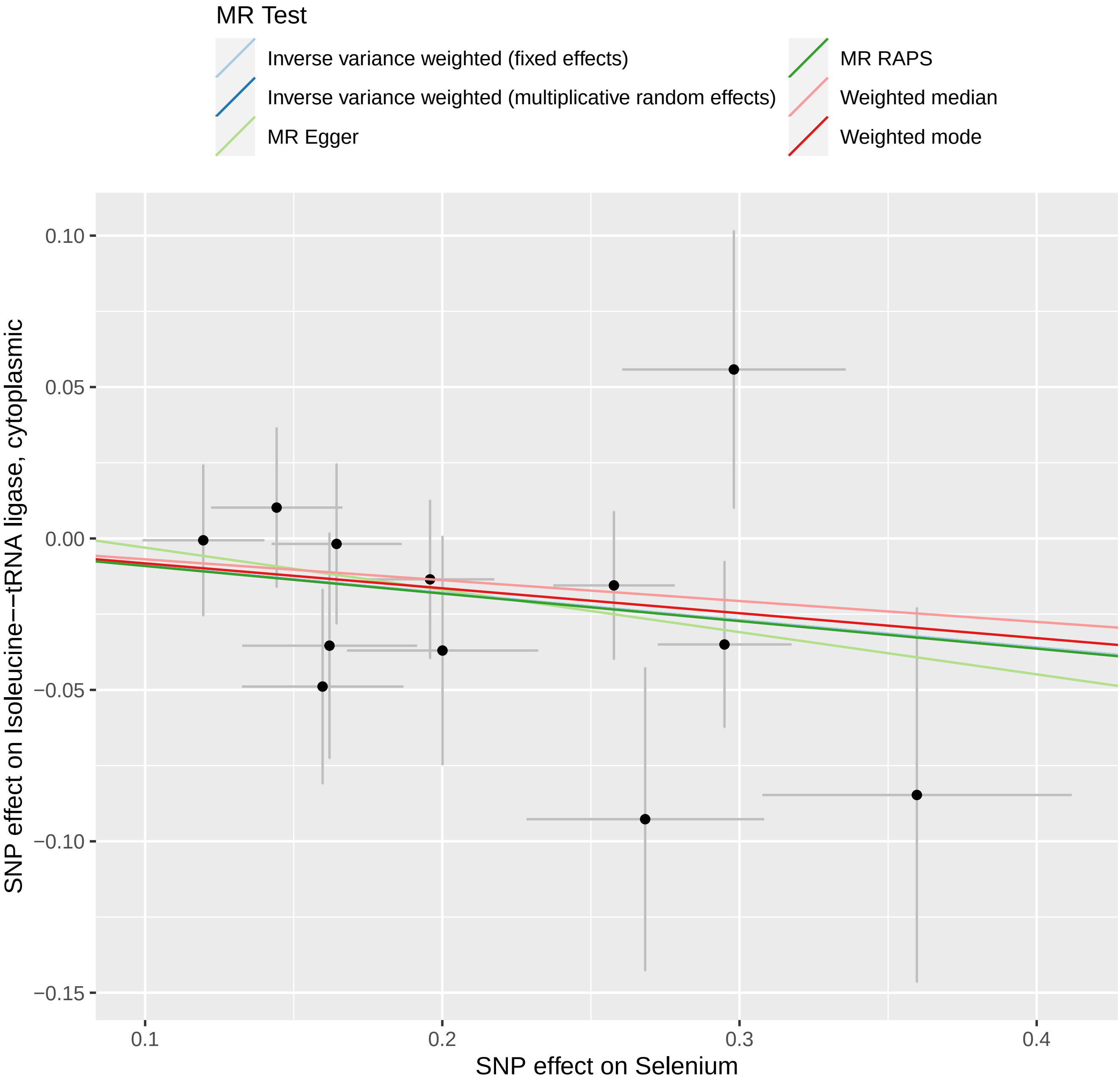

Batch 3−1395 : Selenium on Bone sialoprotein 2

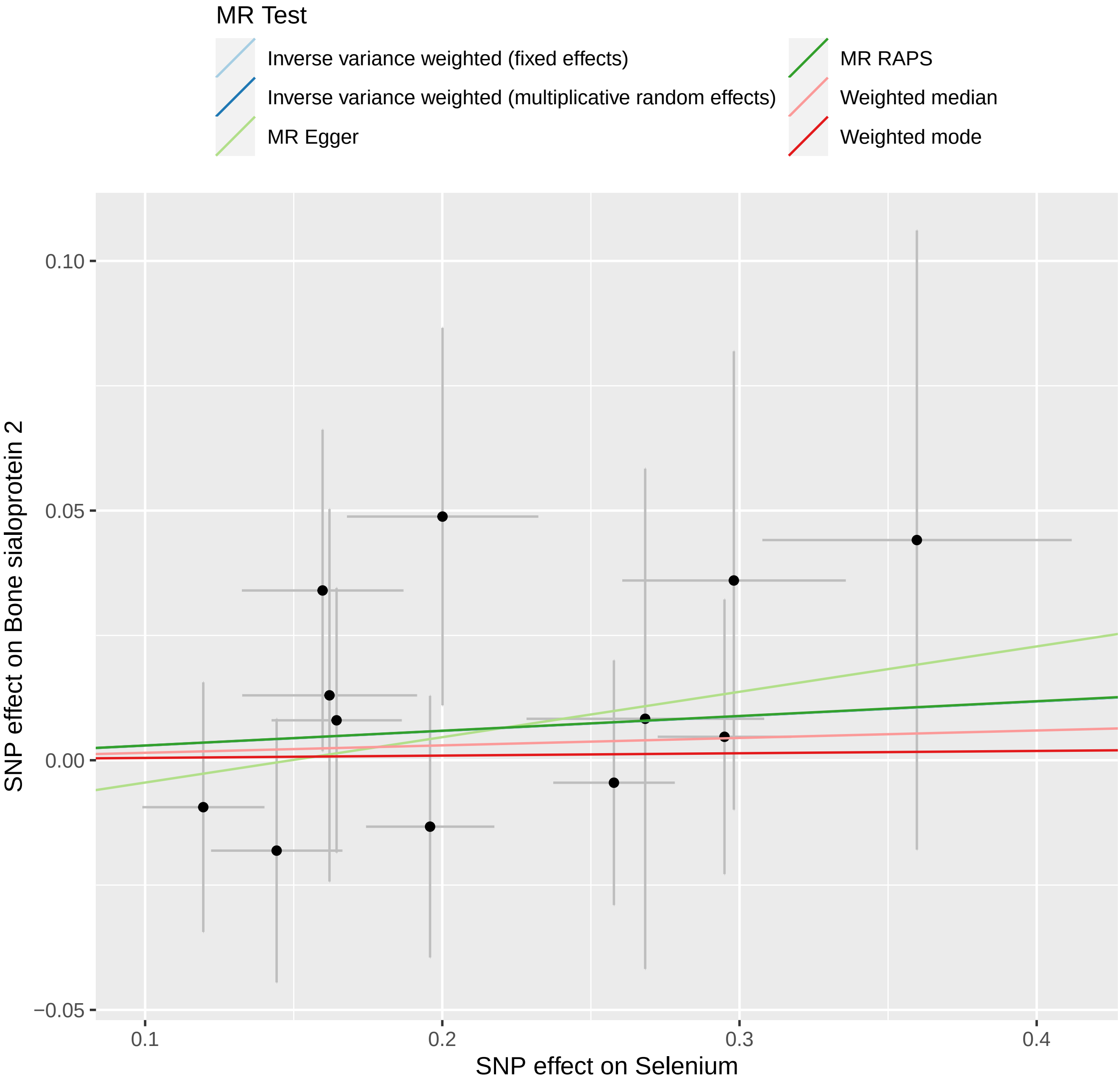

Batch 3−1396 : Selenium on Bone sialoprotein 2

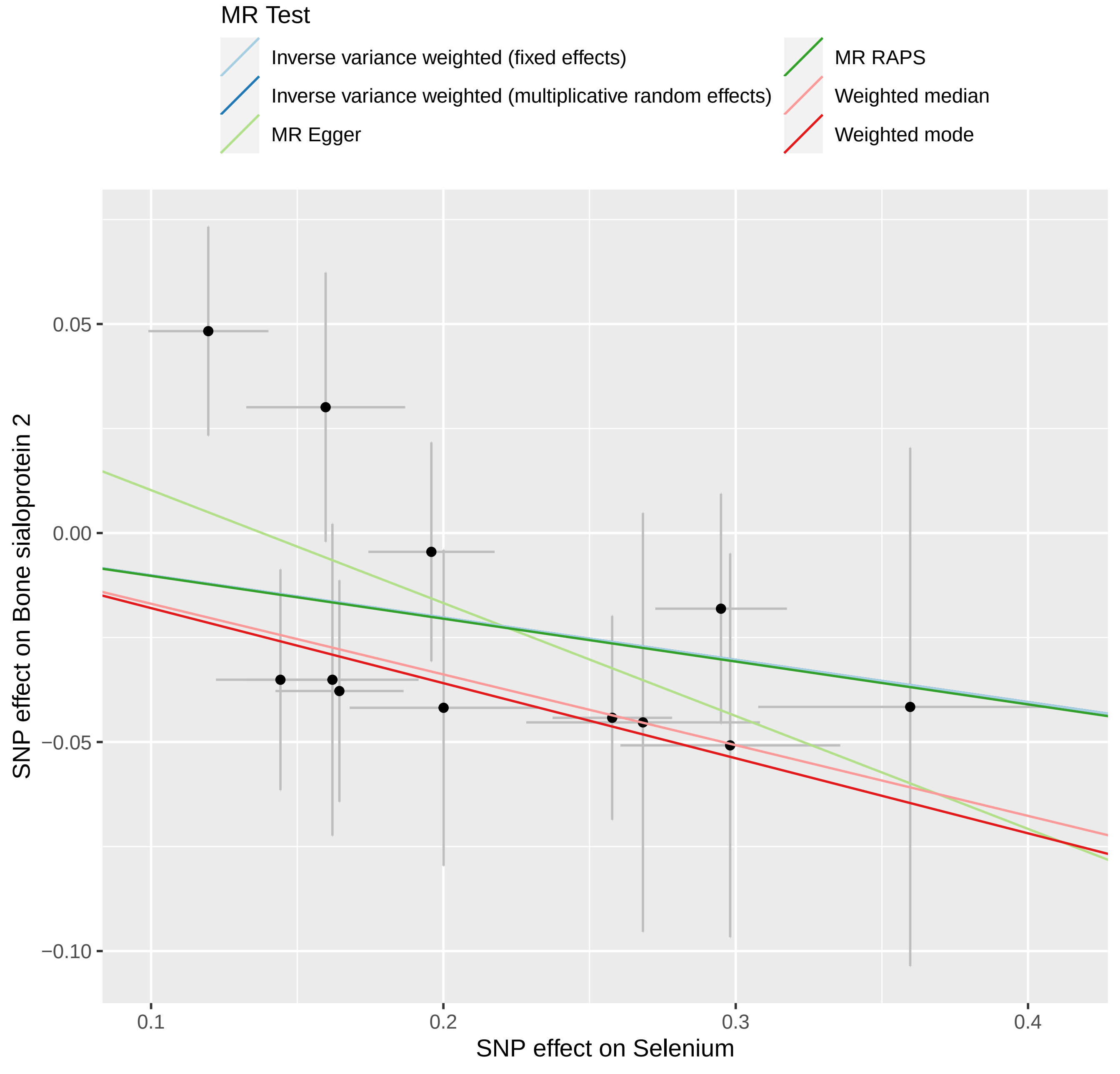

Batch 3−1397 : Selenium on Intercellular adhesion molecule 1

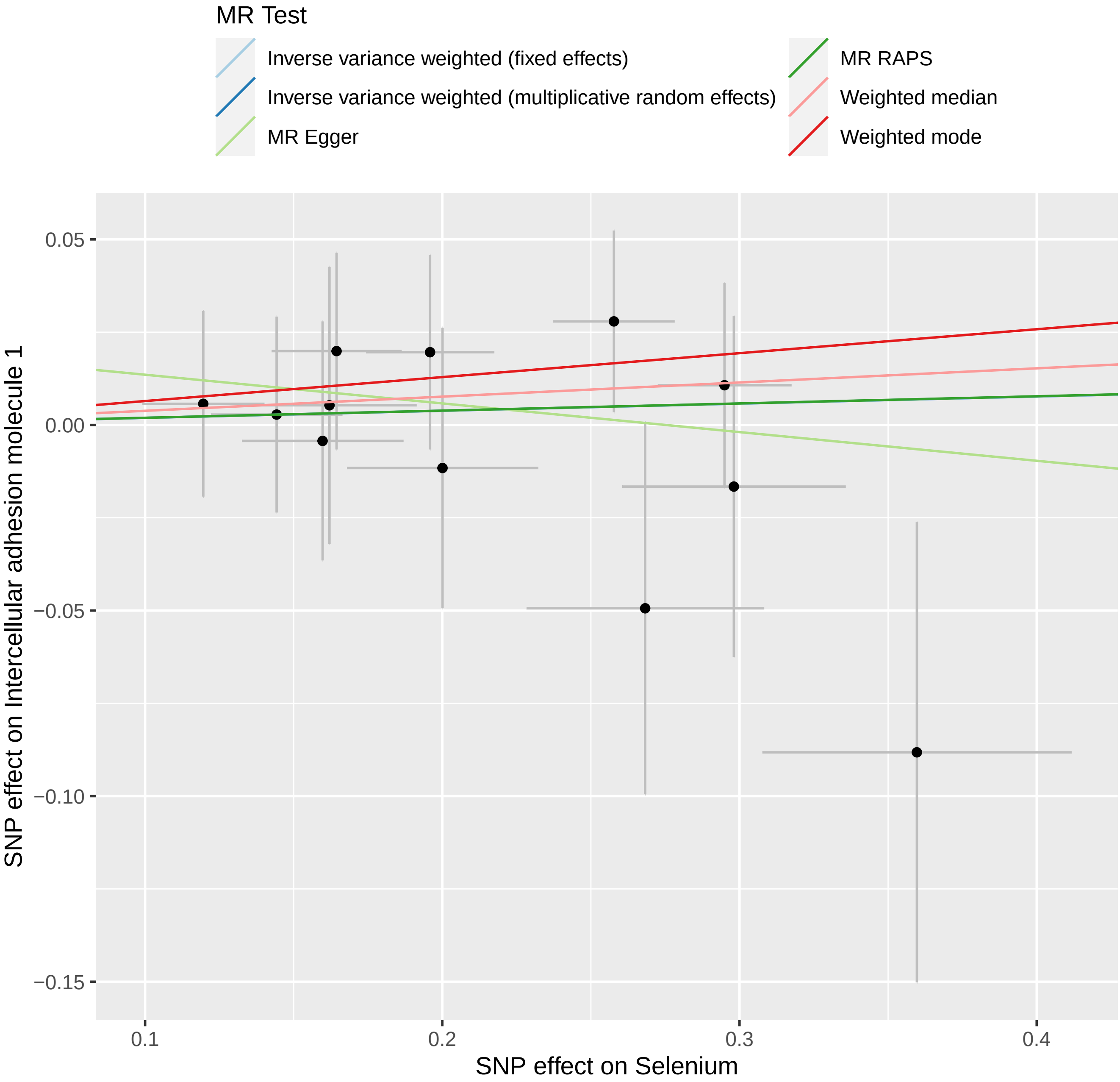

Batch 3−1398 : Selenium on Intercellular adhesion molecule 2

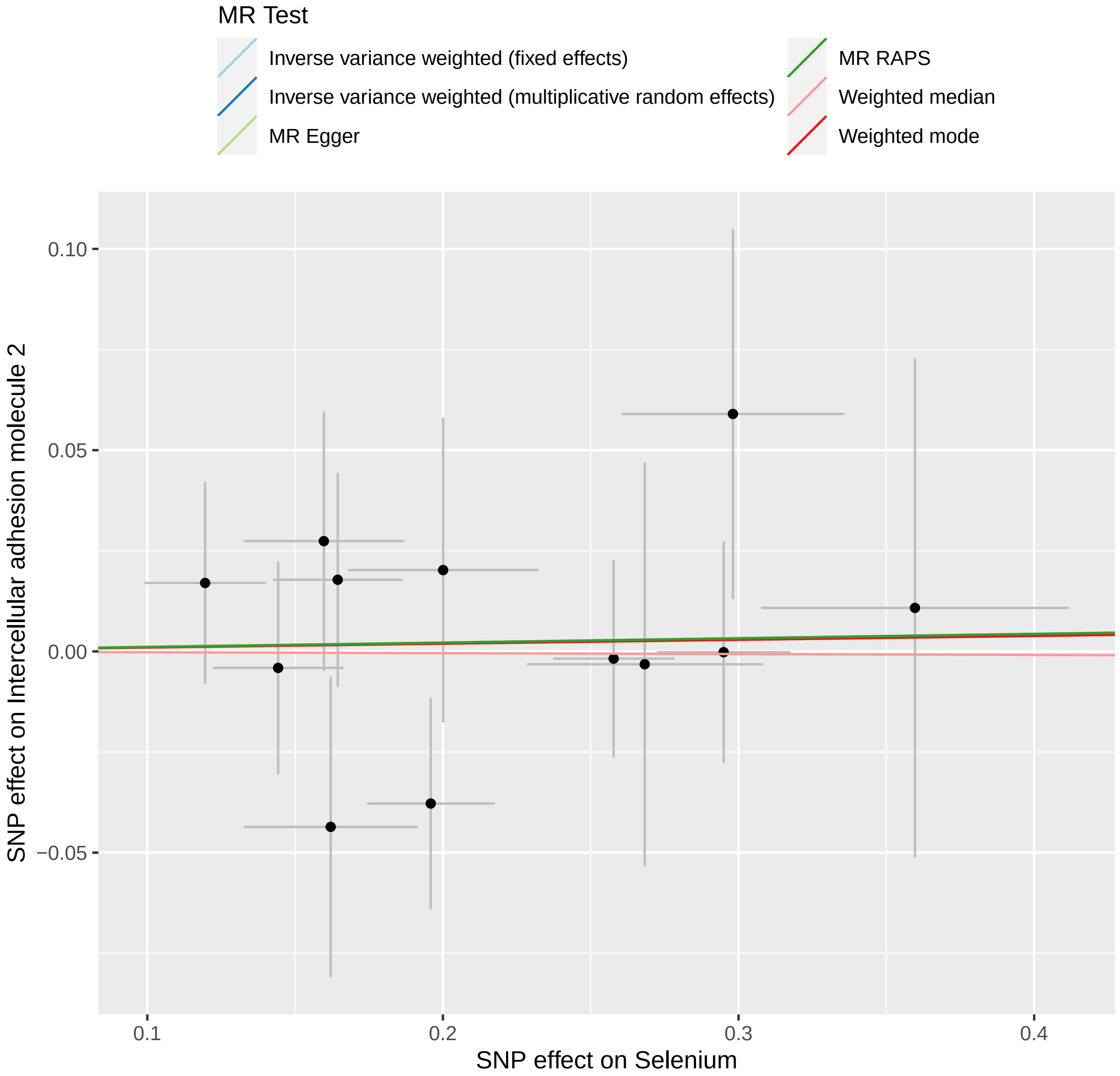

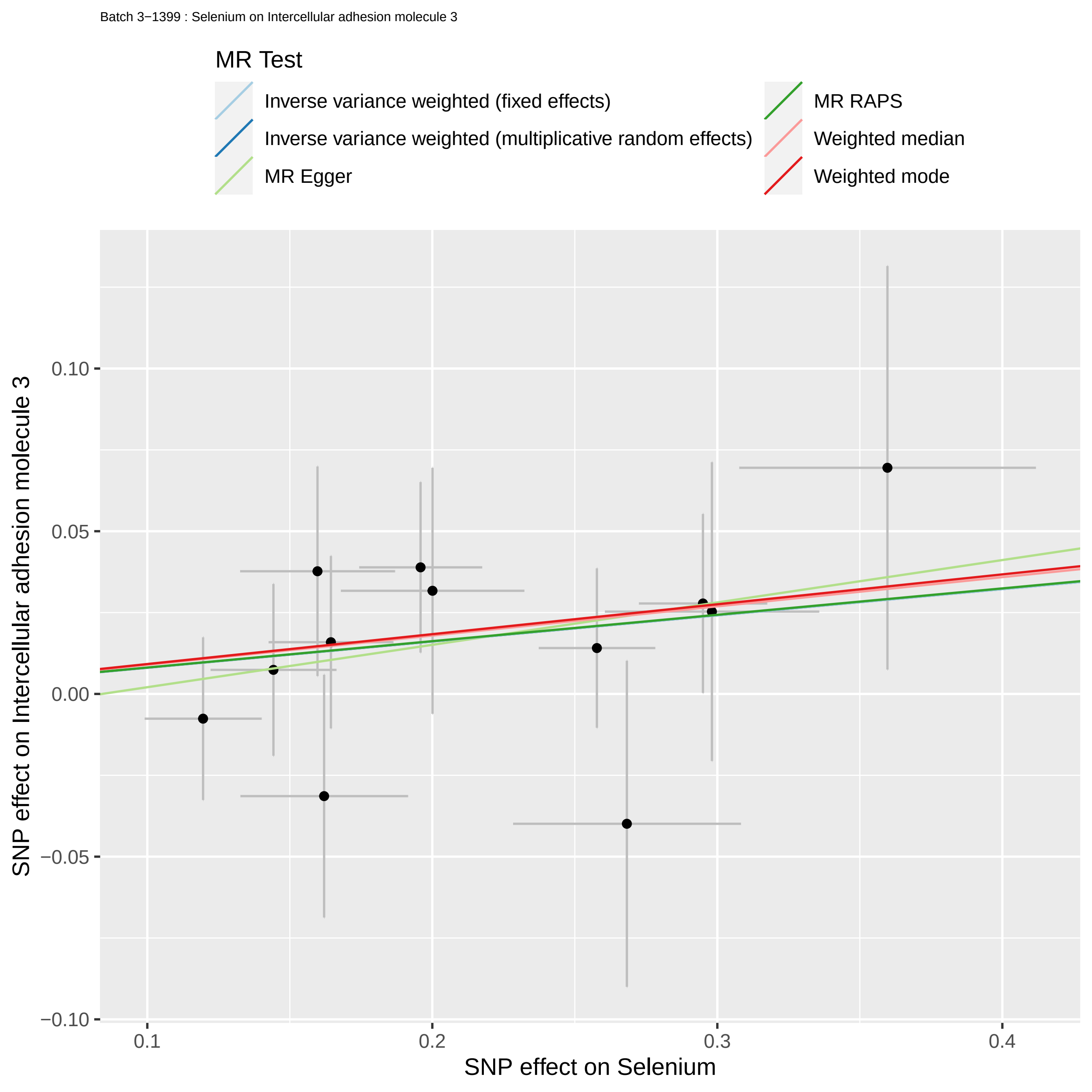
Batch 3−1399 : Selenium on Intercellular adhesion molecule 3
MR Test
Inverse variance weighted (fixed effects)
Inverse variance weighted (multiplicative random effects)
MR Egger
MR RAPS
Weighted median
Weighted mode
0.10
0.05
0.00
−0.05
−0.10
0.1
0.2
0.3
0.4
SNP effect on Intercellular adhesion molecule 3
SNP effect on Selenium

Batch 3−1400 : Selenium on Intercellular adhesion molecule 4

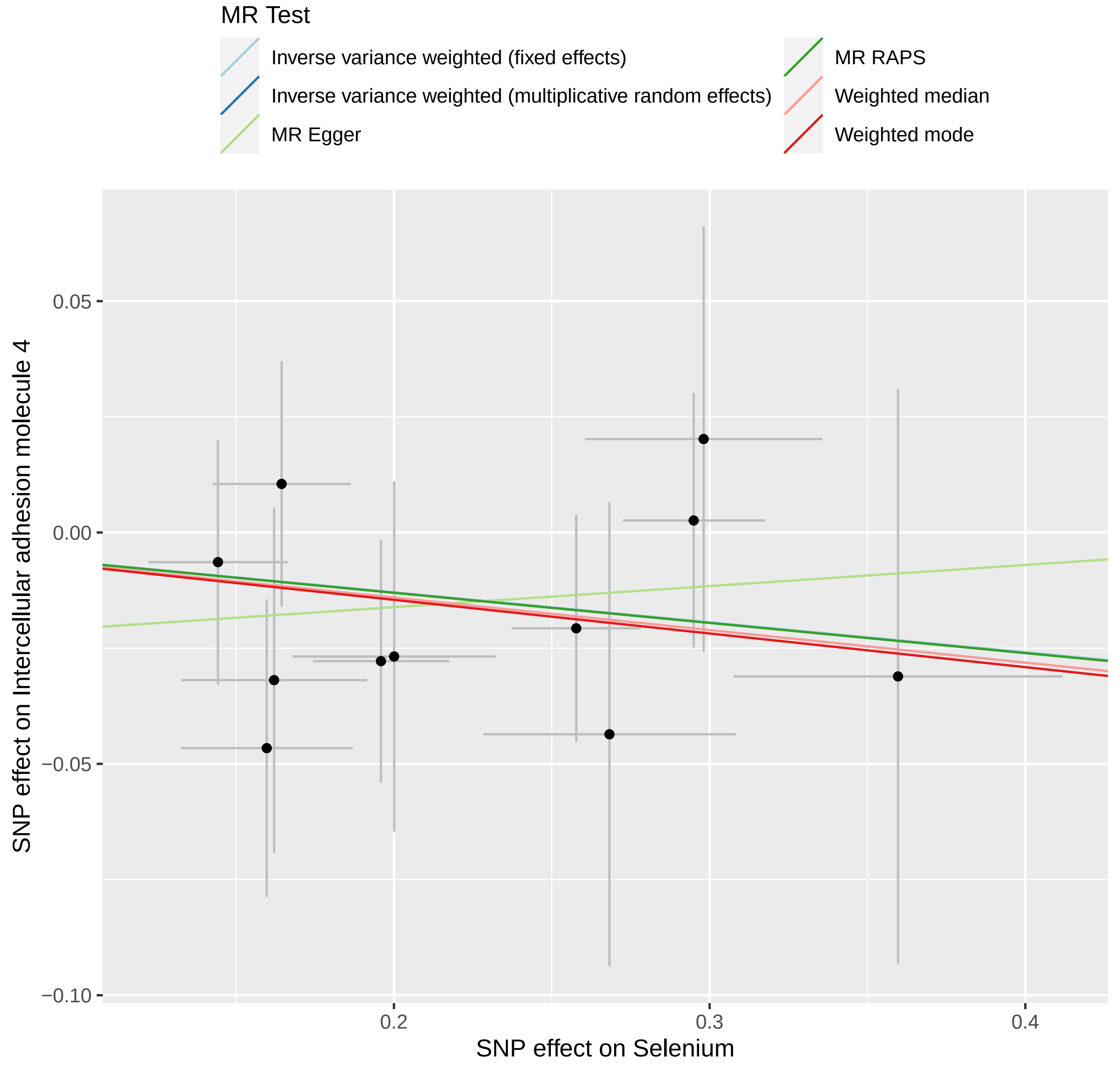

Batch 3−1401 : Selenium on Intercellular adhesion molecule 5

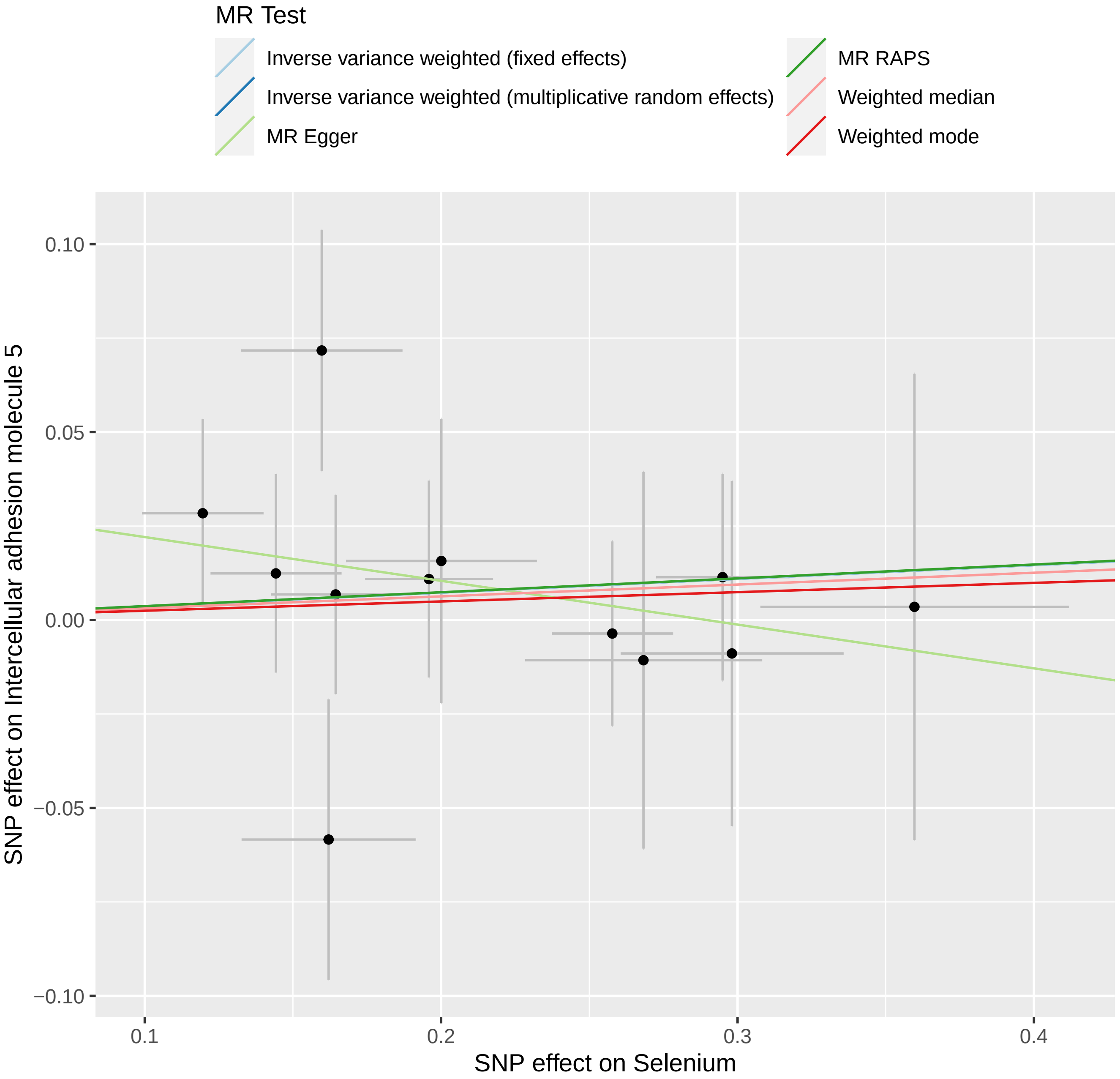

Batch 3−1402 : Selenium on Intercellular adhesion molecule 5

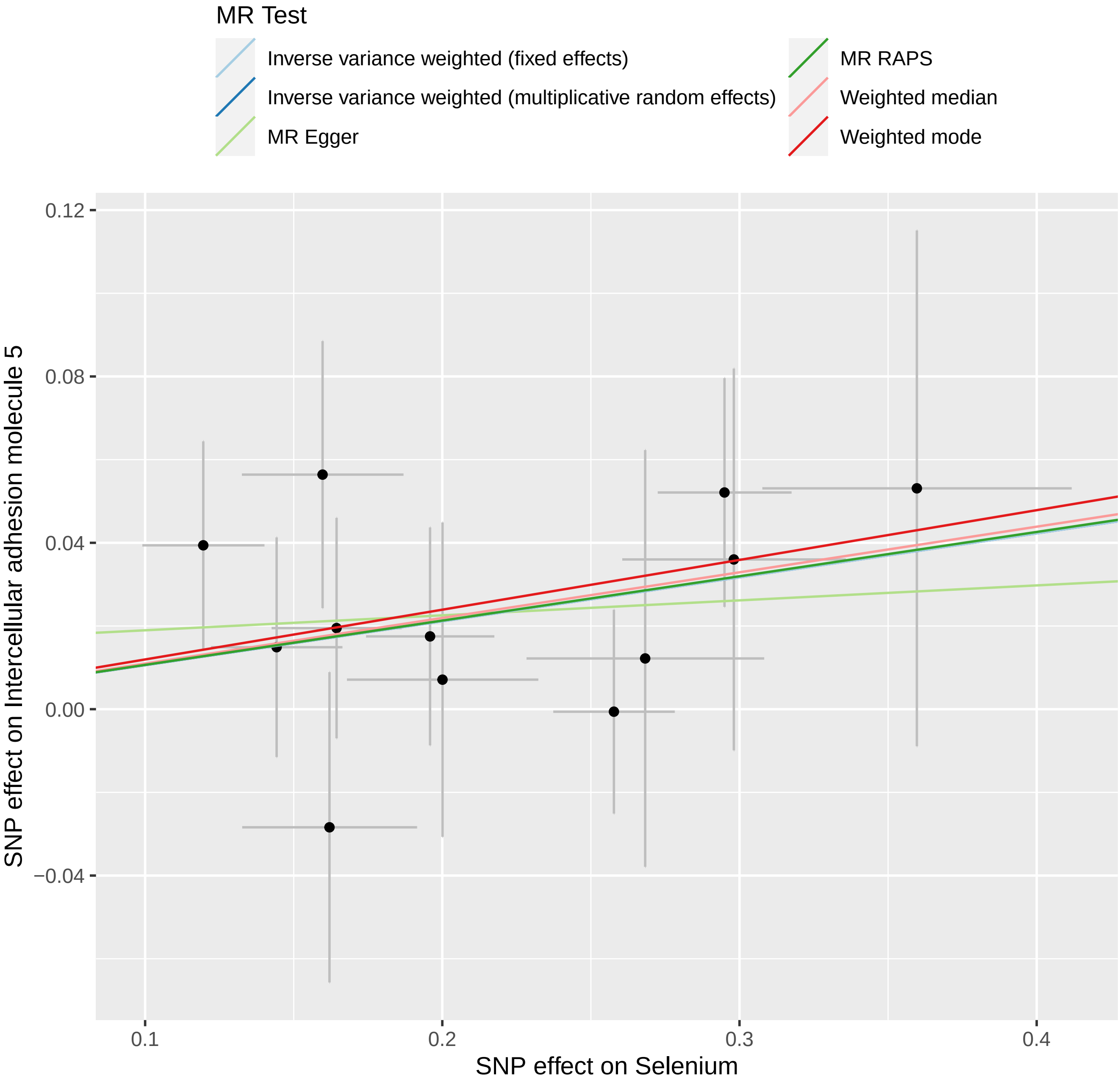

Batch 3−1403 : Selenium on Inducible T−cell costimulator

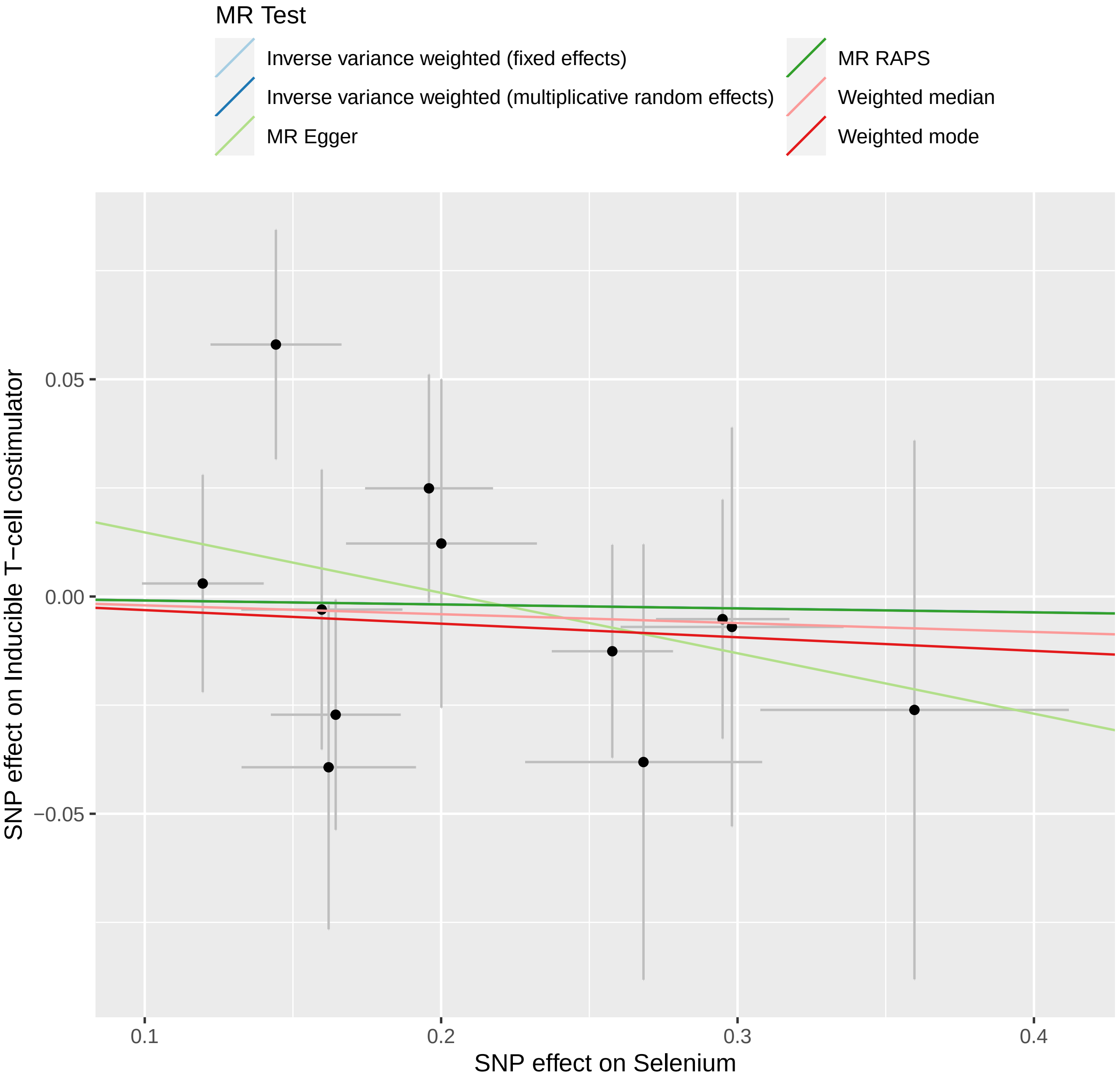

Batch 3−1404 : Selenium on Inducible T−cell costimulator

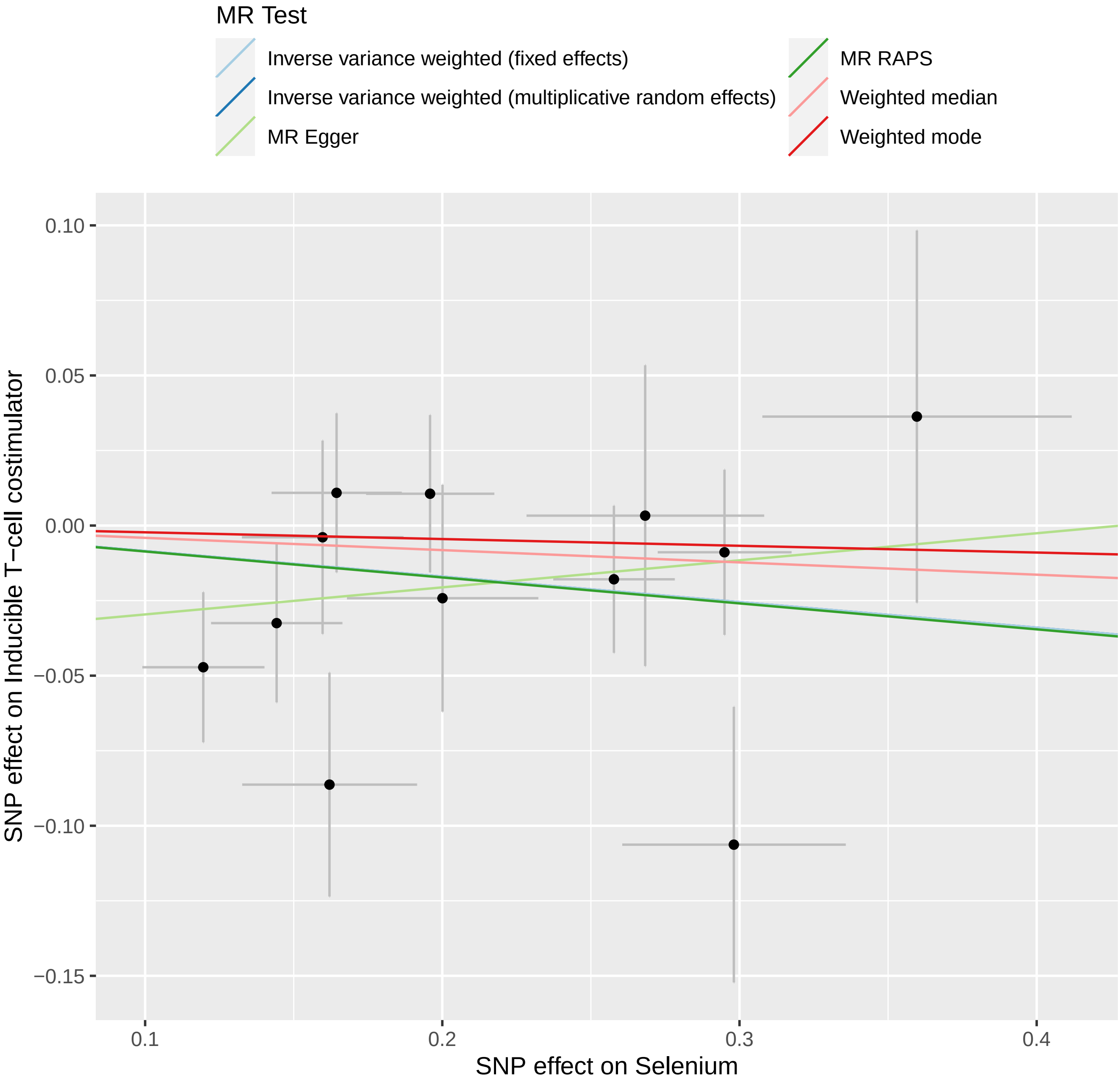

Batch 3−1405 : Selenium on ICOS ligand

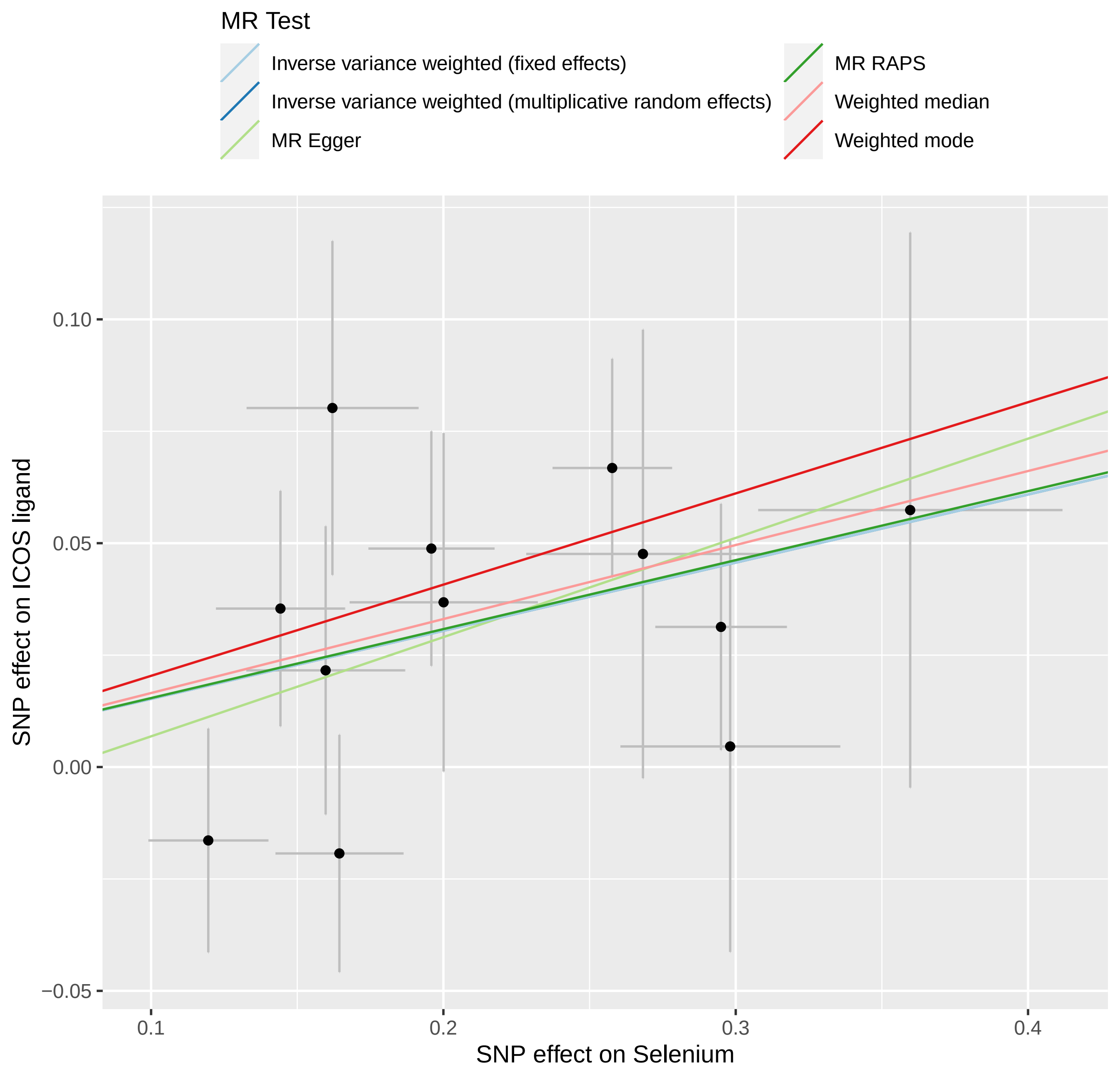

Batch 3−1406 : Selenium on ICOS ligand

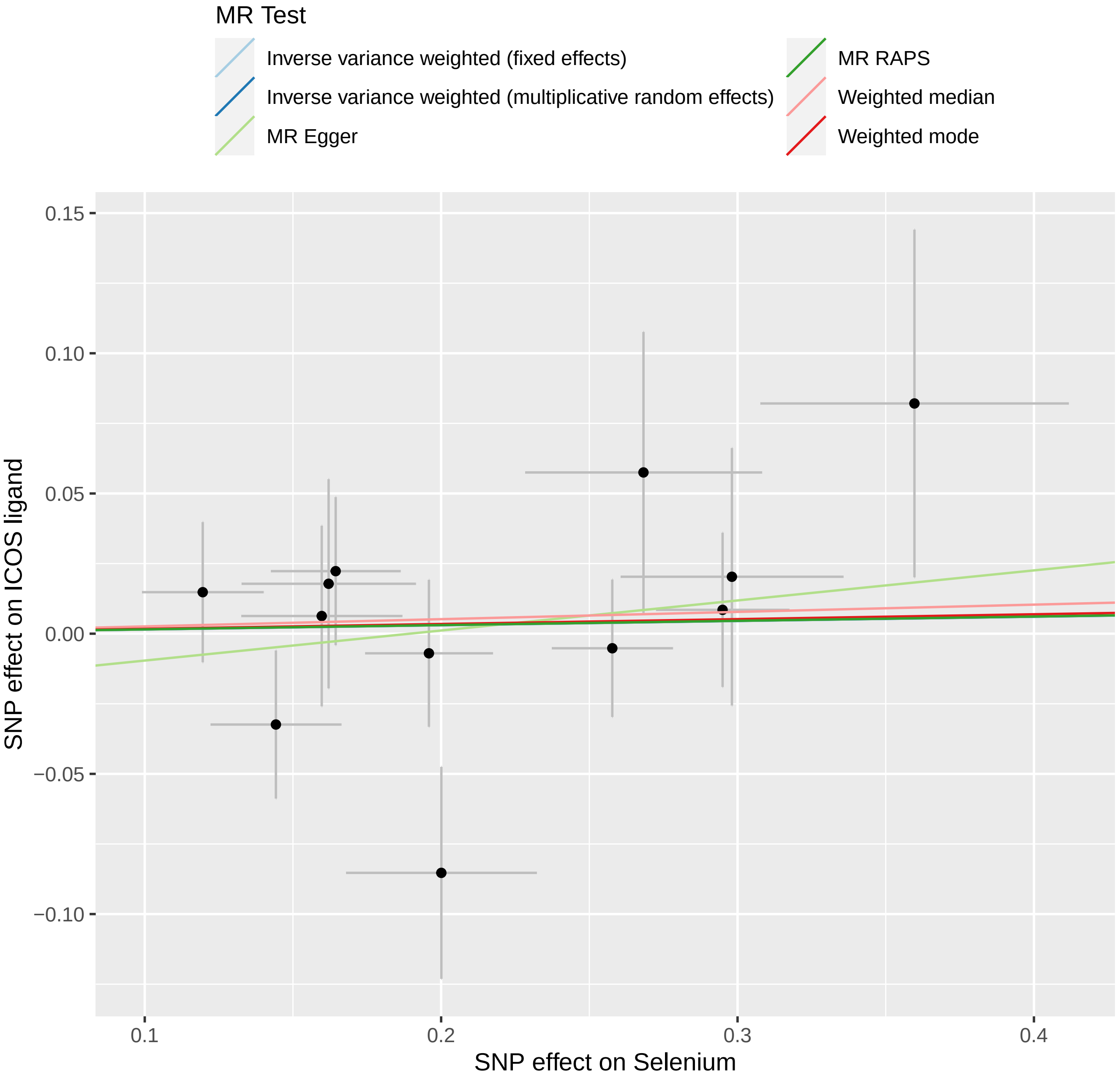

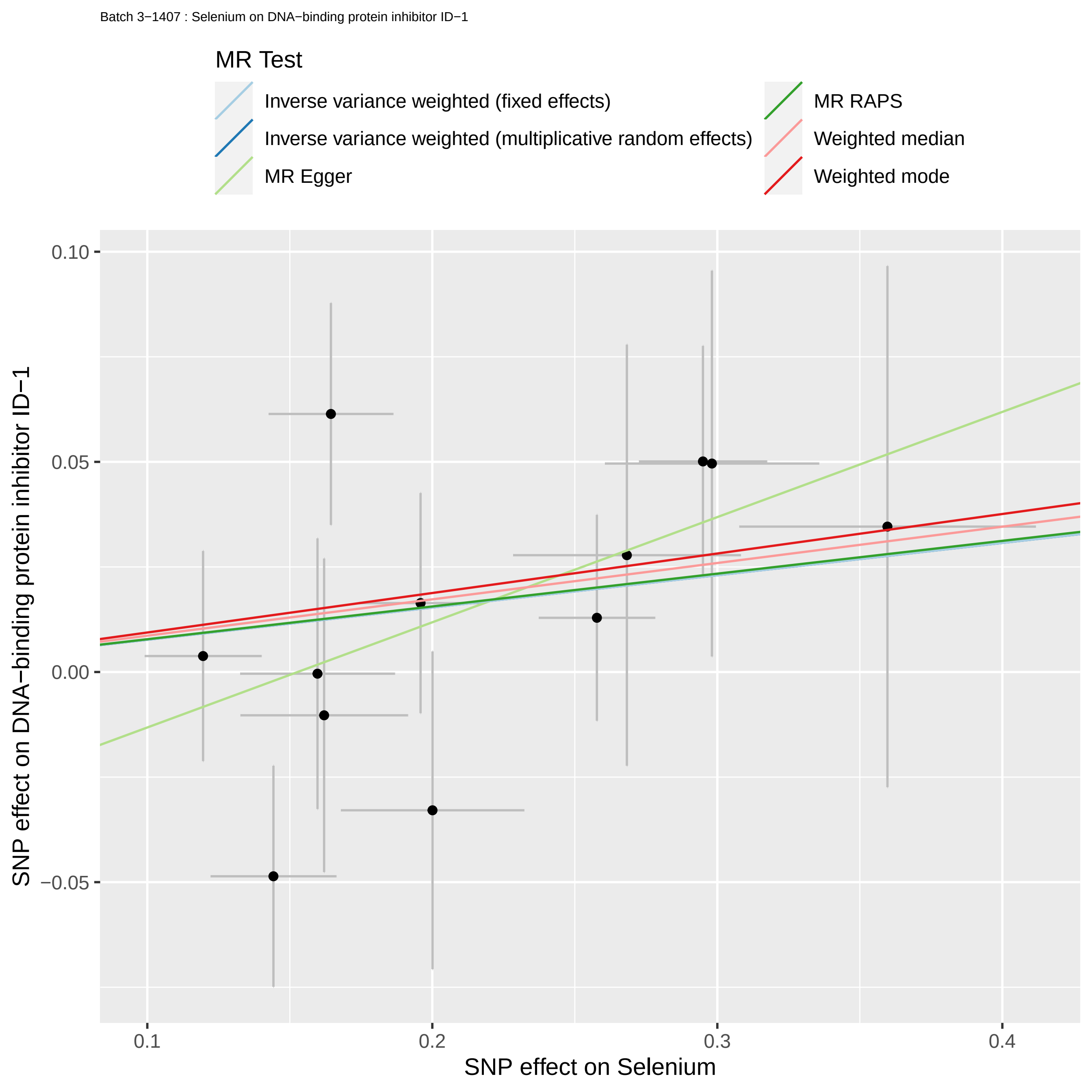

Batch 3−1407 : Selenium on DNA−binding protein inhibitor ID−1
MR Test
Inverse variance weighted (fixed effects)
Inverse variance weighted (multiplicative random effects)
MR Egger
MR RAPS
Weighted median
Weighted mode
SNP effect on DNA−binding protein inhibitor ID−1
0.10
0.05
0.00
−0.05
0.1
0.2
0.3
0.4
SNP effect on Selenium

Batch 3−1408 : Selenium on DNA−binding protein inhibitor ID−2

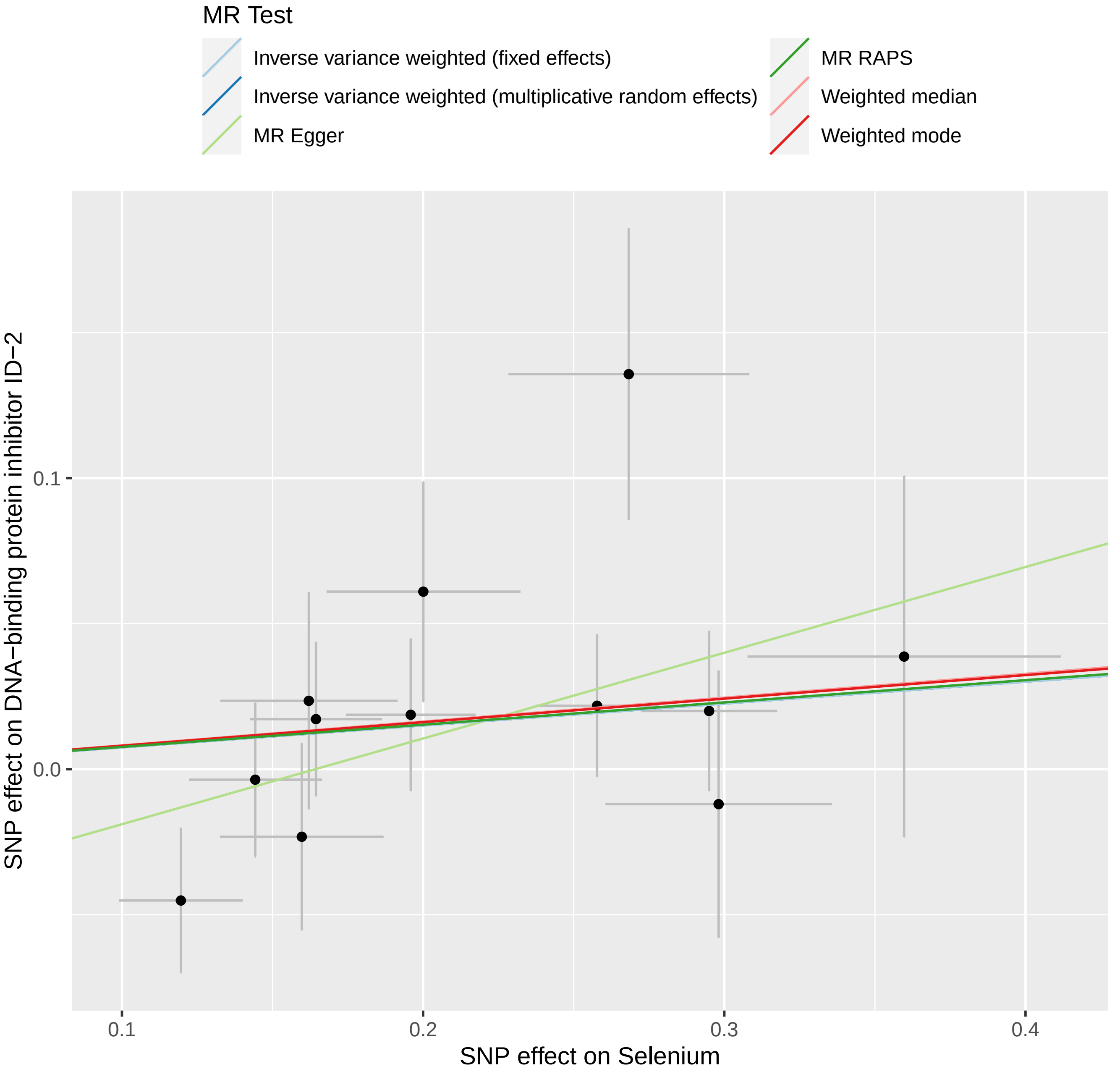

Batch 3−1409 : Selenium on Insulin−degrading enzyme

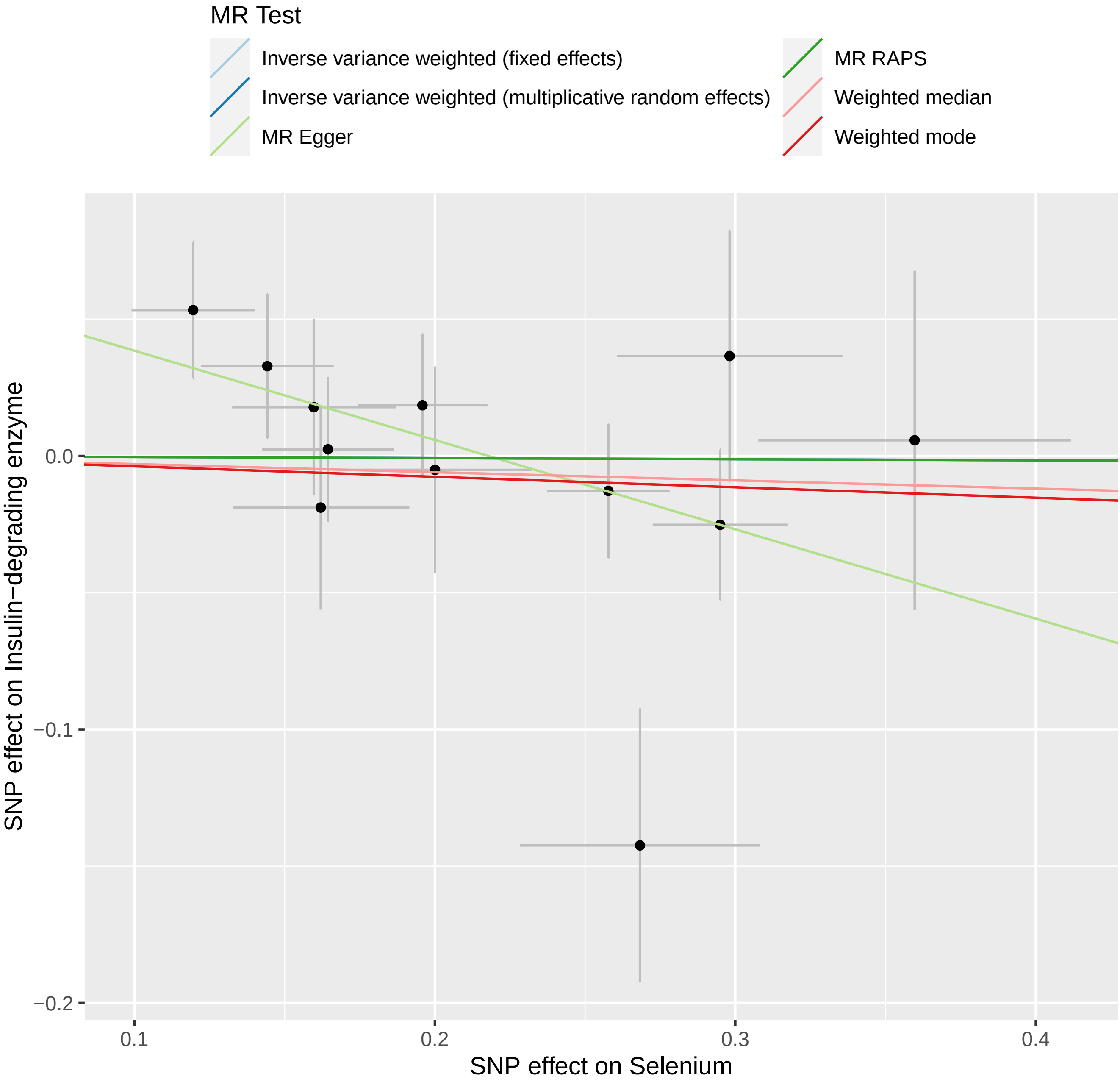

Batch 3−1410 : Selenium on Indoleamine 2,3−dioxygenase 1

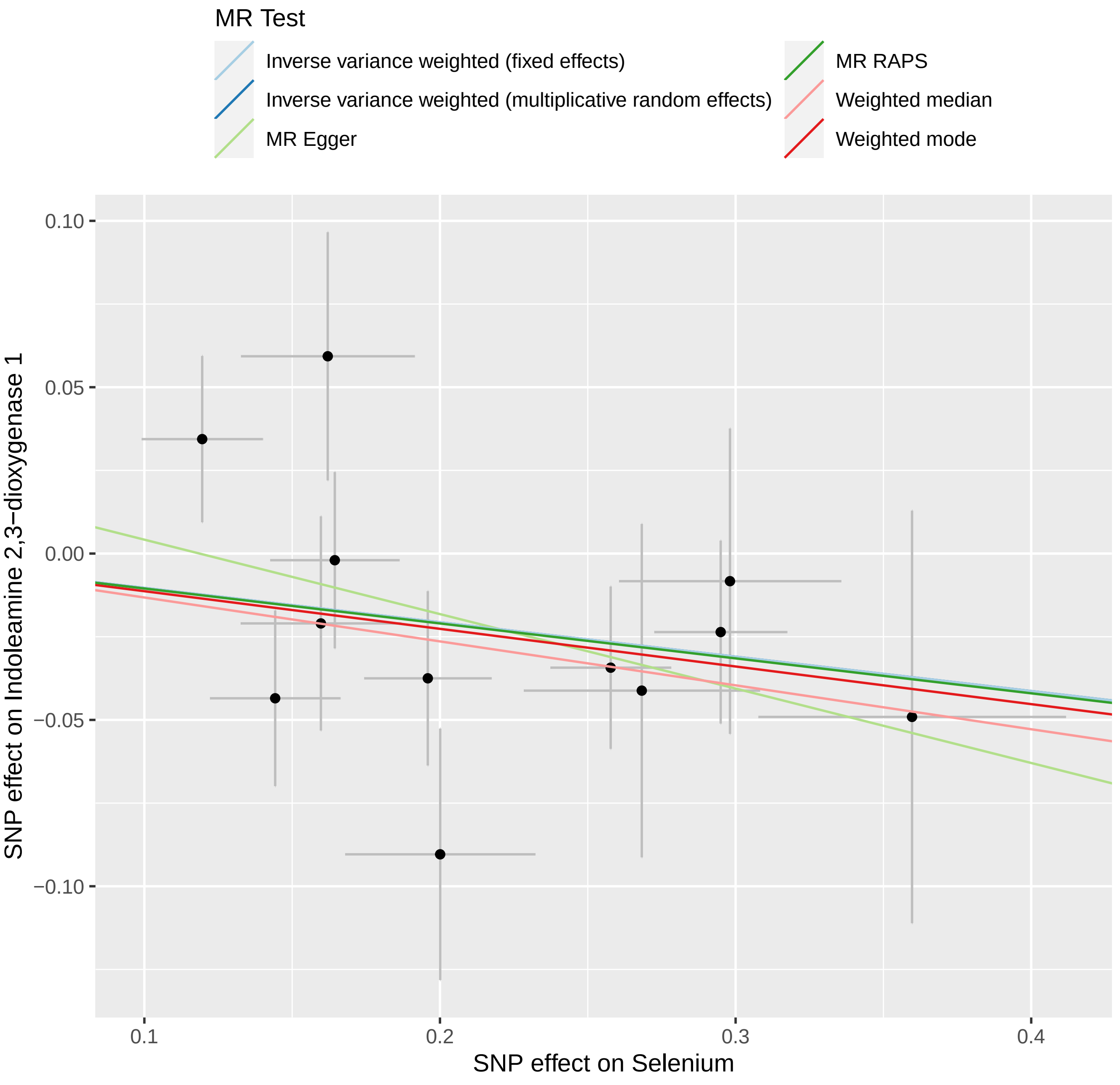

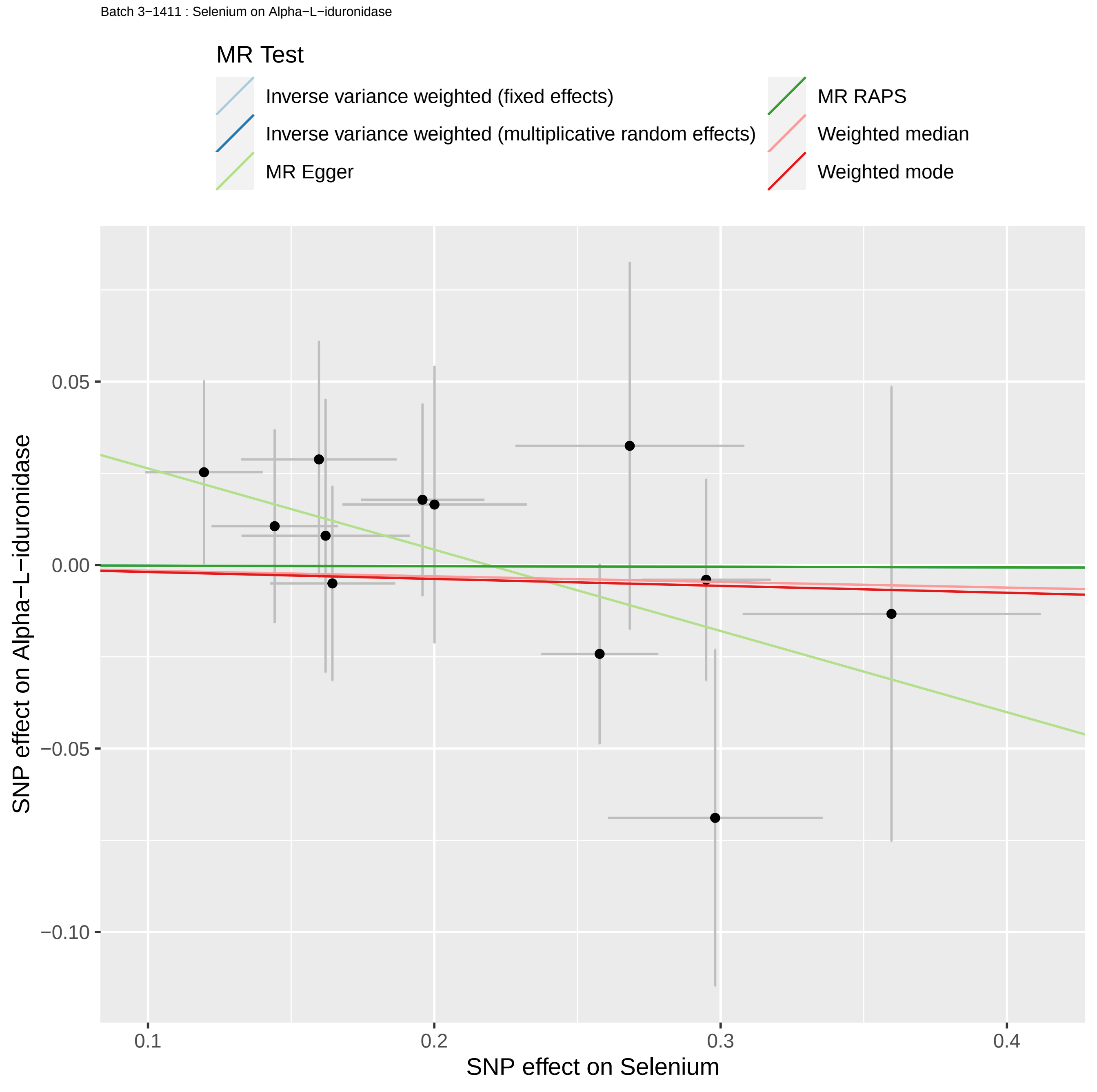
Batch 3−1411 : Selenium on Alpha−L−iduronidase
MR Test
Inverse variance weighted (fixed effects)
Inverse variance weighted (multiplicative random effects)
MR Egger
MR RAPS
Weighted median
Weighted mode
0.05
0.00
−0.05
−0.10
0.1
0.2
0.3
0.4
SNP effect on Alpha−L−iduronidase
SNP effect on Selenium

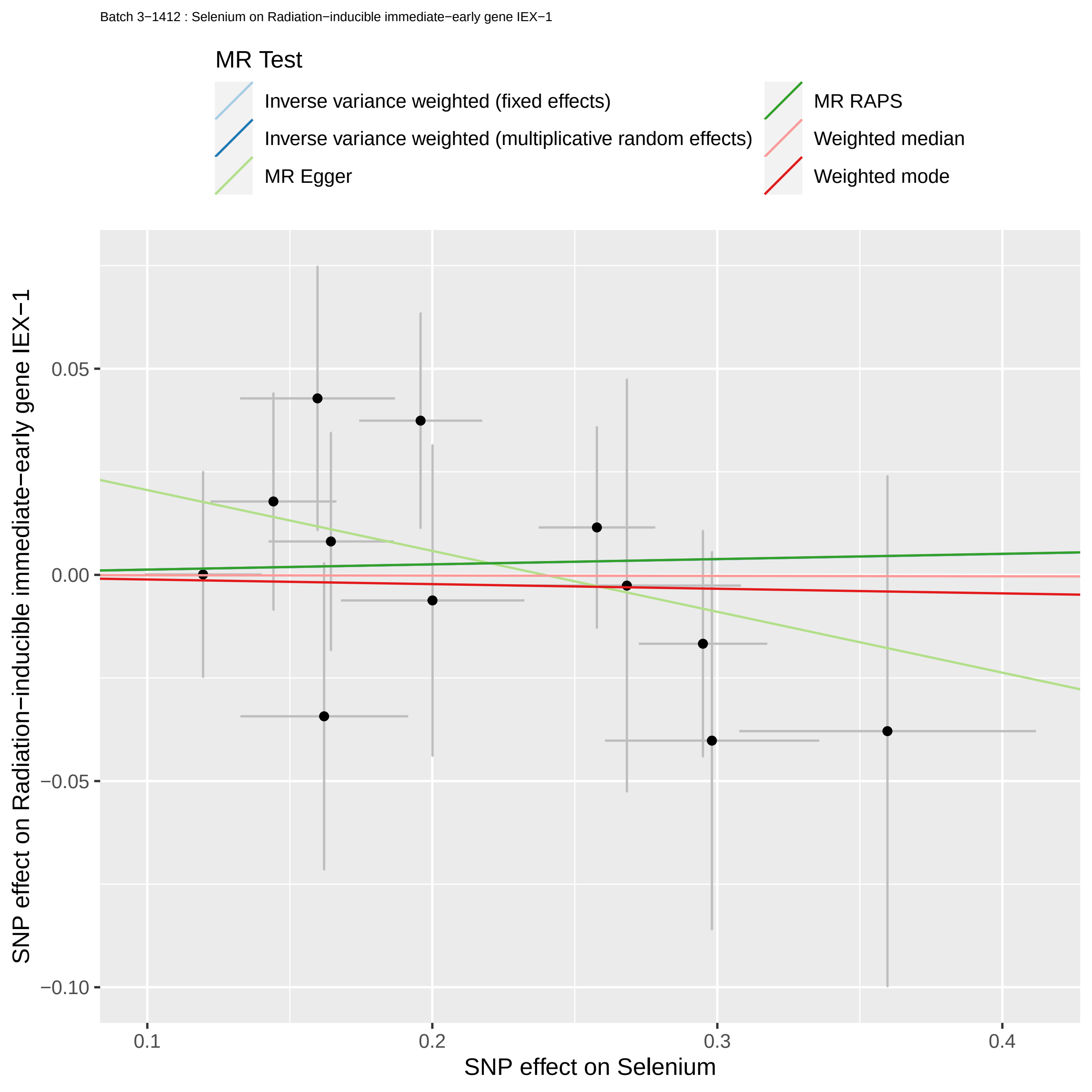

Batch 3−1412 : Selenium on Radiation−inducible immediate−early gene IEX−1
MR Test
Inverse variance weighted (fixed effects)
Inverse variance weighted (multiplicative random effects)
MR Egger
MR RAPS
Weighted median
Weighted mode
SNP effect on Radiation−inducible immediate−early gene IEX−1
0.05
0.00
−0.05
−0.10
0.1
0.2
0.3
0.4
SNP effect on Selenium

Batch 3−1413 : Selenium on Immediate early response 3−interacting protein 1

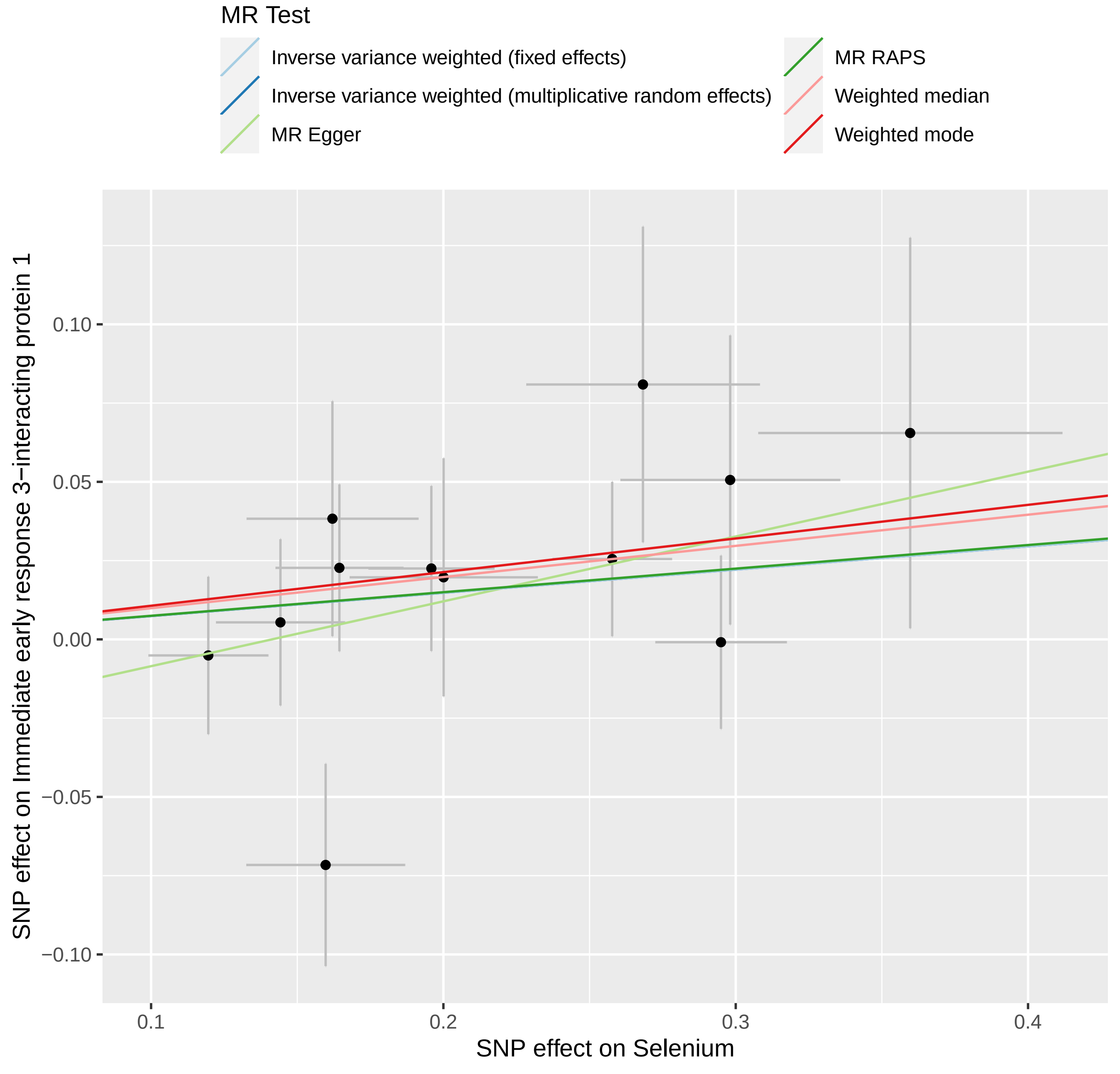

Batch 3−1414 : Selenium on Gamma−interferon−inducible protein 16

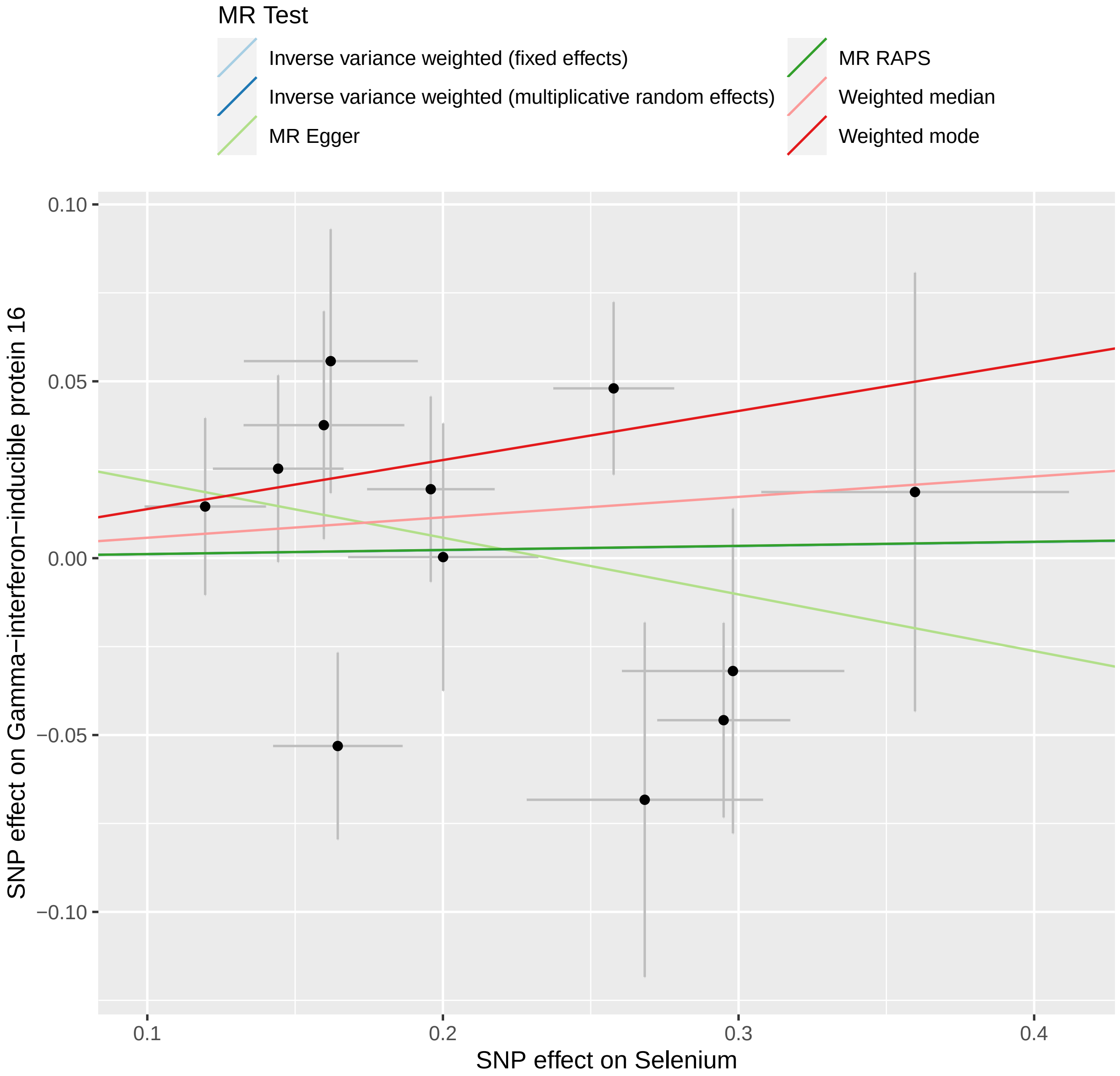

Batch 3−1415 : Selenium on Gamma−interferon−inducible protein 16

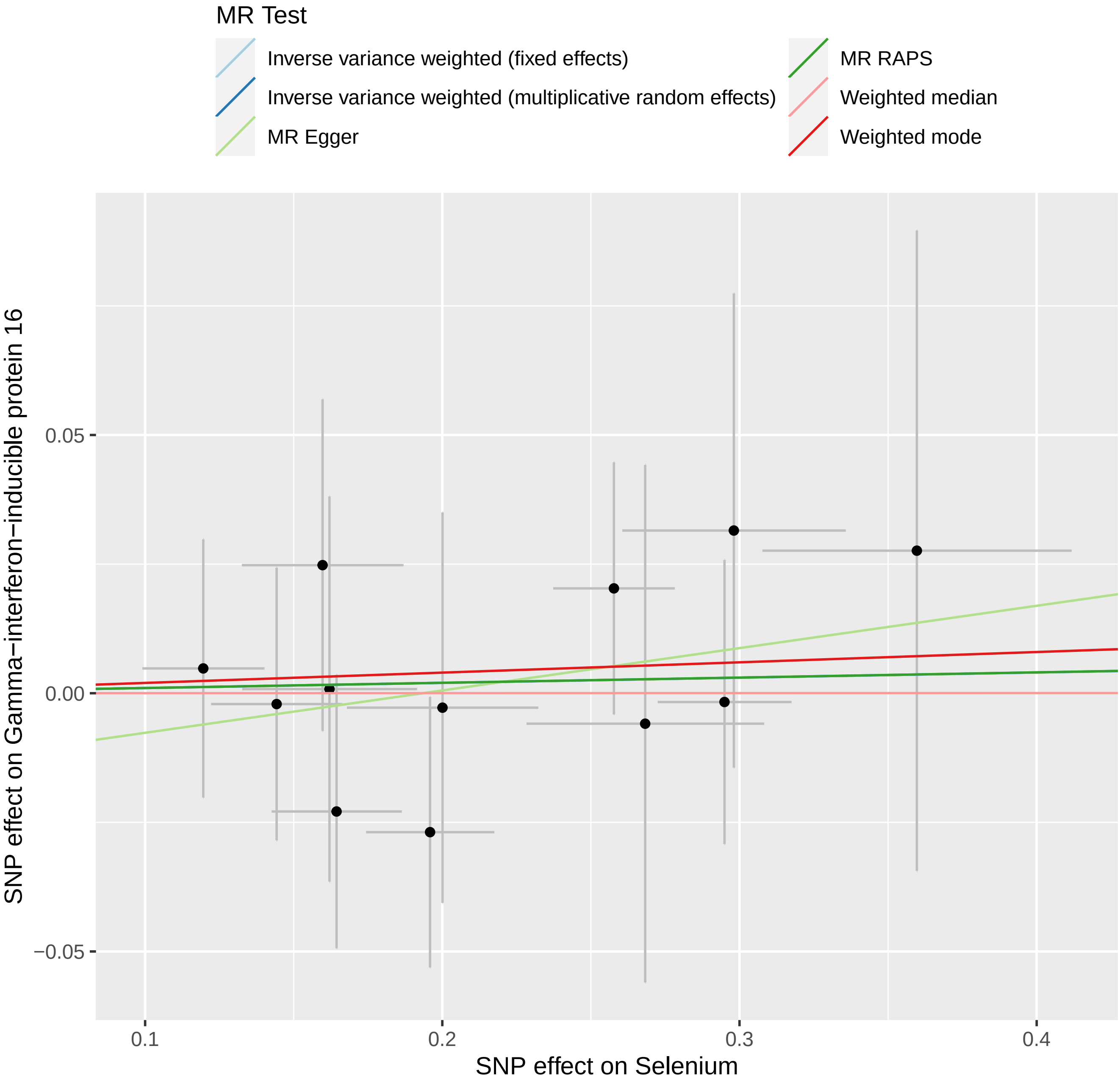

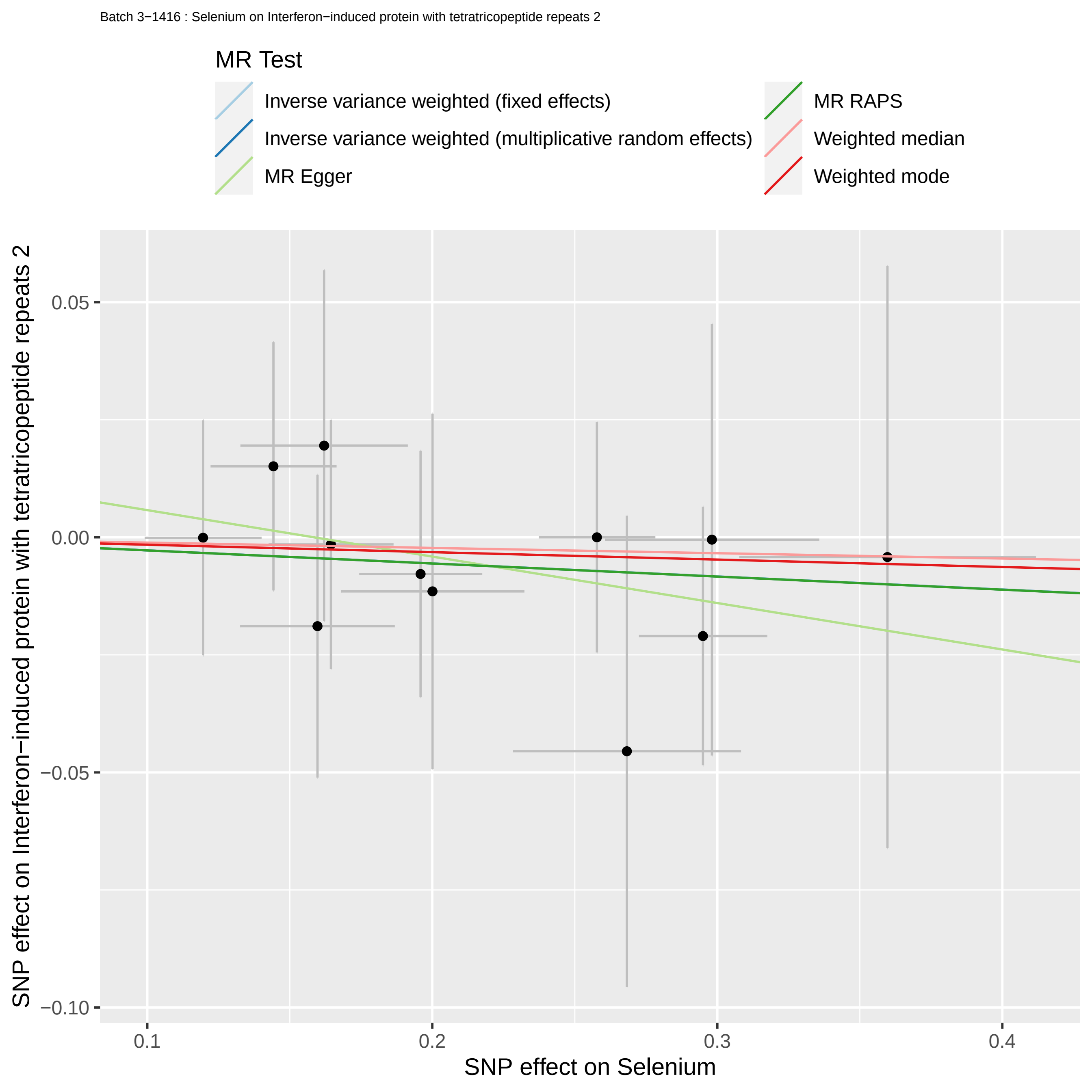
Batch 3−1416 : Selenium on Interferon−induced protein with tetratricopeptide repeats 2
MR Test
Inverse variance weighted (fixed effects)
Inverse variance weighted (multiplicative random effects)
MR Egger
MR RAPS
Weighted median
Weighted mode
SNP effect on Interferon−induced protein with tetratricopeptide repeats 2
0.05
0.00
−0.05
−0.10
0.1
0.2
0.3
0.4
SNP effect on Selenium

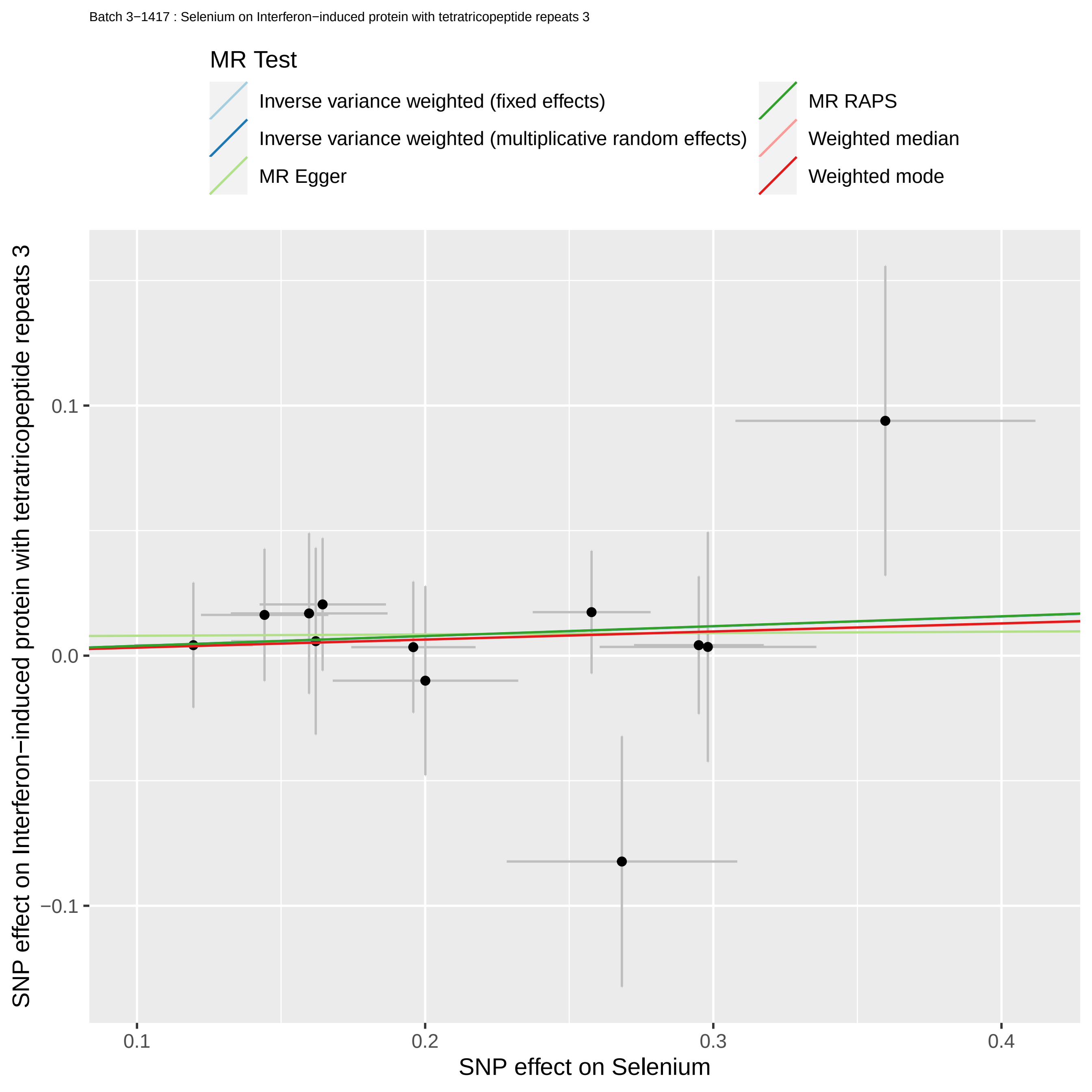

Batch 3−1417 : Selenium on Interferon−induced protein with tetratricopeptide repeats 3
MR Test
Inverse variance weighted (fixed effects)
Inverse variance weighted (multiplicative random effects)
MR Egger
MR RAPS
Weighted median
Weighted mode
SNP effect on Interferon−induced protein with tetratricopeptide repeats 3
0.1
0.0
−0.1
0.1
0.2
0.3
0.4
SNP effect on Selenium

Batch 3−1418 : Selenium on Interferon alpha−10

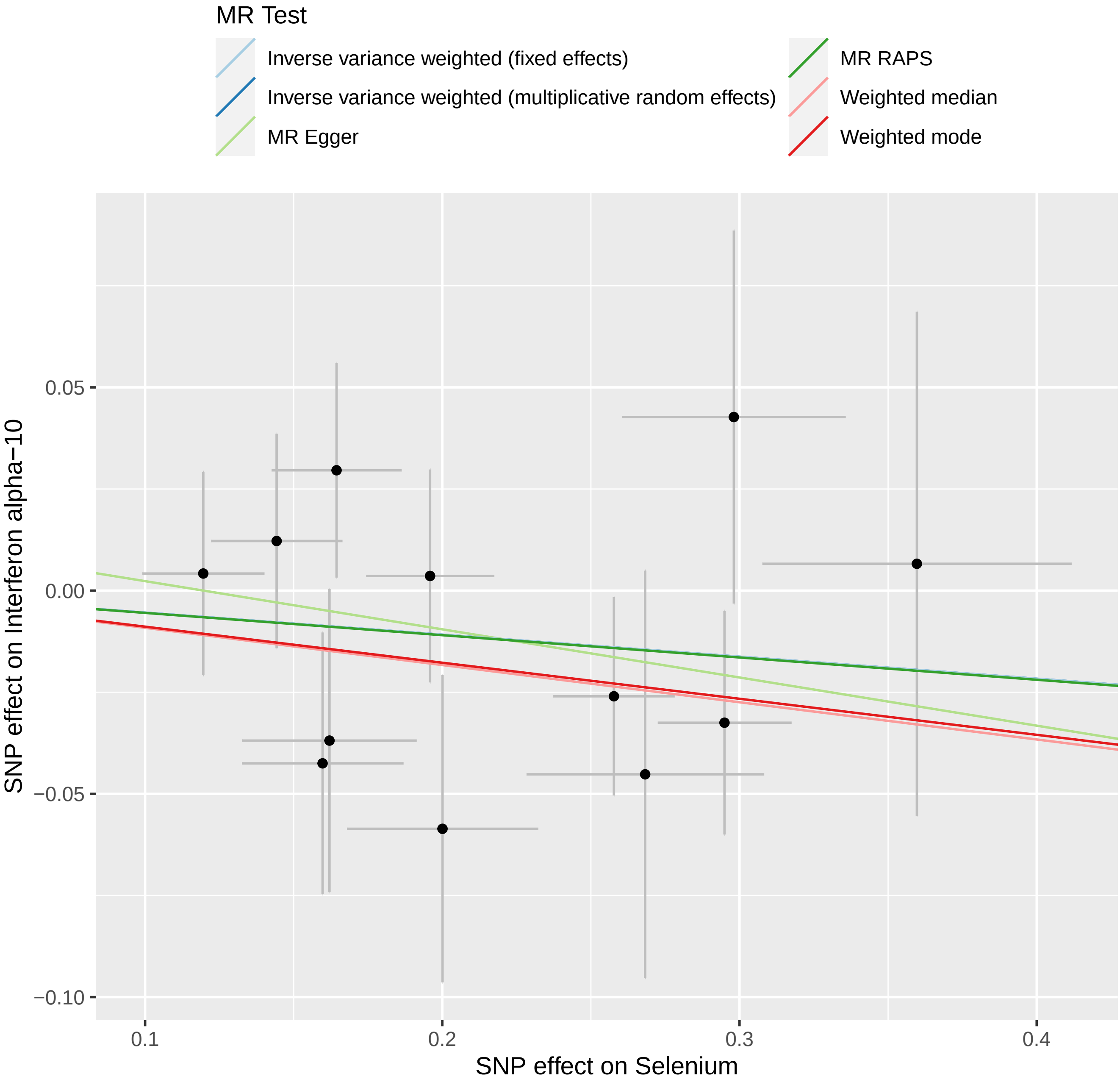

Batch 3−1419 : Selenium on Interferon alpha−10

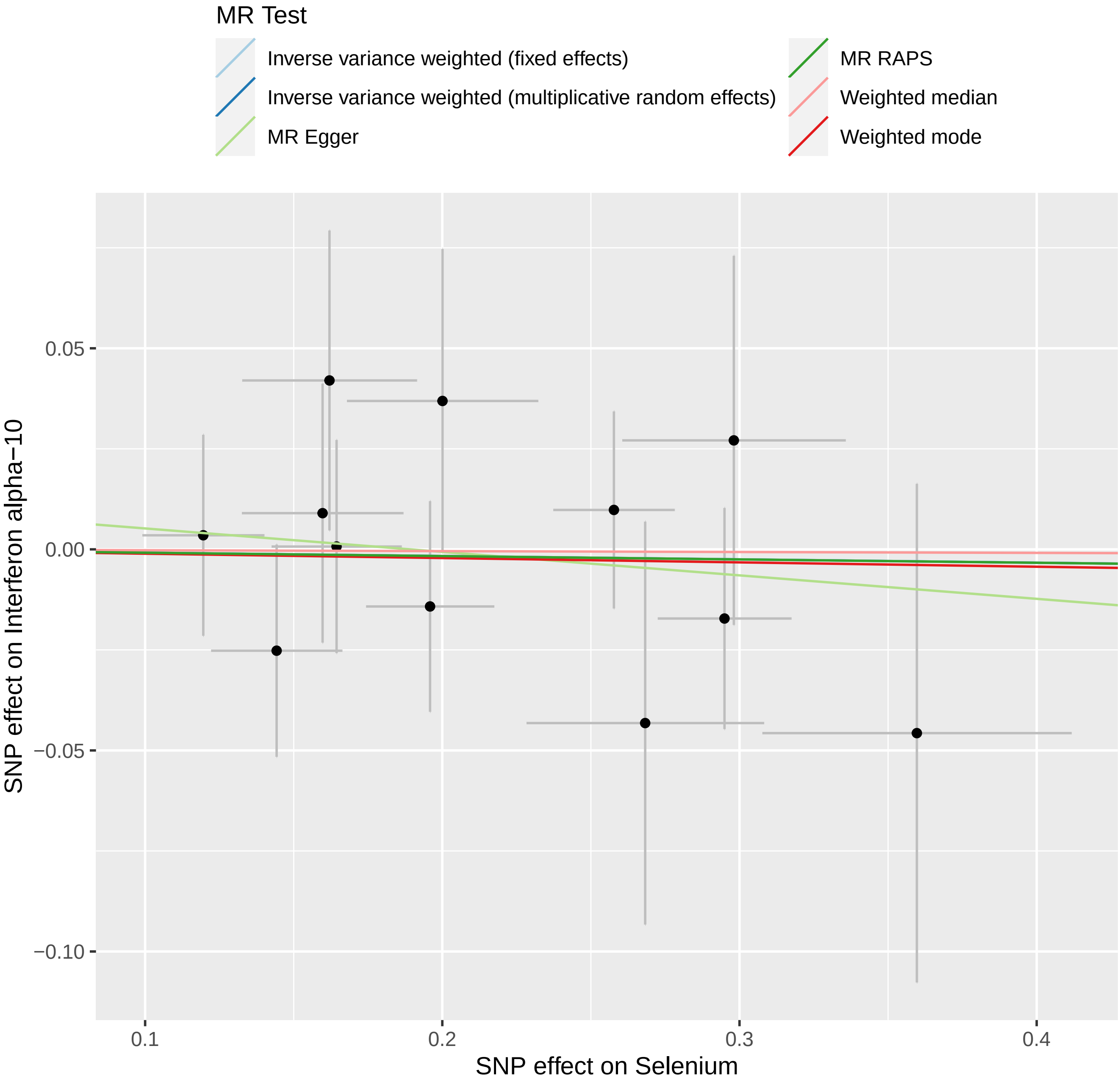

Batch 3−1420 : Selenium on Interferon alpha−14

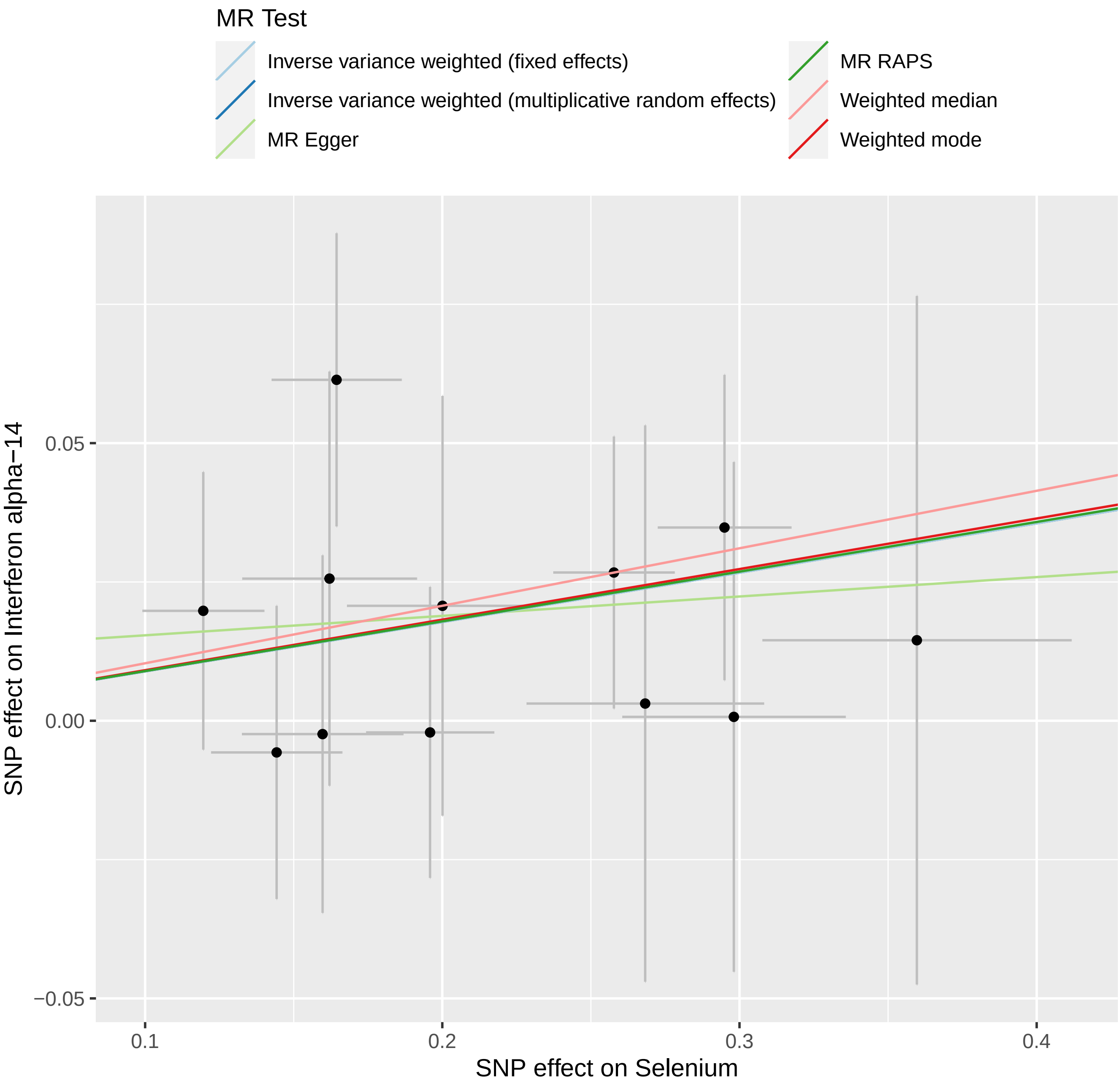

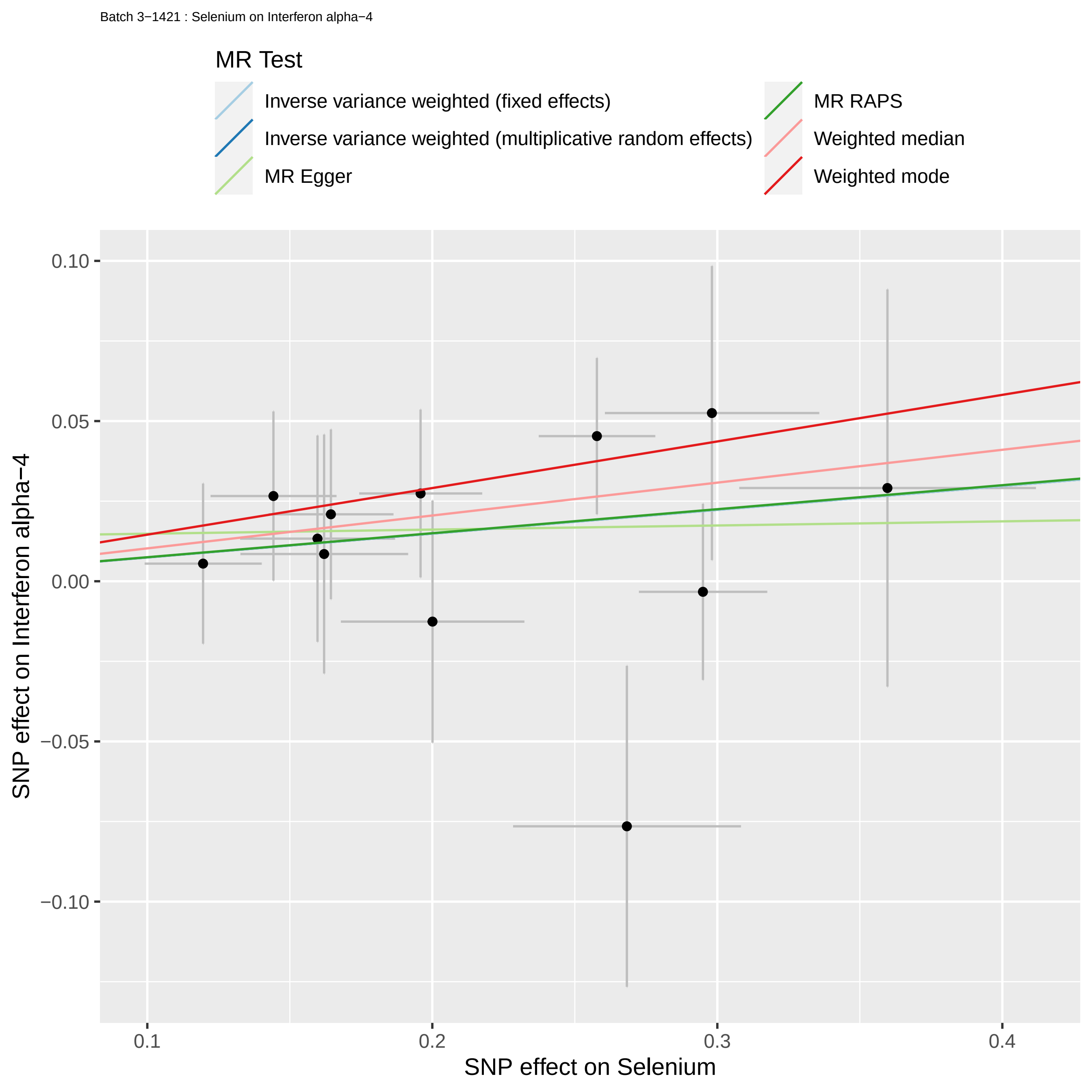

Batch 3−1421 : Selenium on Interferon alpha−4
MR Test
Inverse variance weighted (fixed effects)
Inverse variance weighted (multiplicative random effects)
MR Egger
MR RAPS
Weighted median
Weighted mode
0.10
0.05
0.00
−0.05
−0.10
0.1
0.2
0.3
0.4
SNP effect on Interferon alpha−4
SNP effect on Selenium

Batch 3−1422 : Selenium on Interferon alpha−5

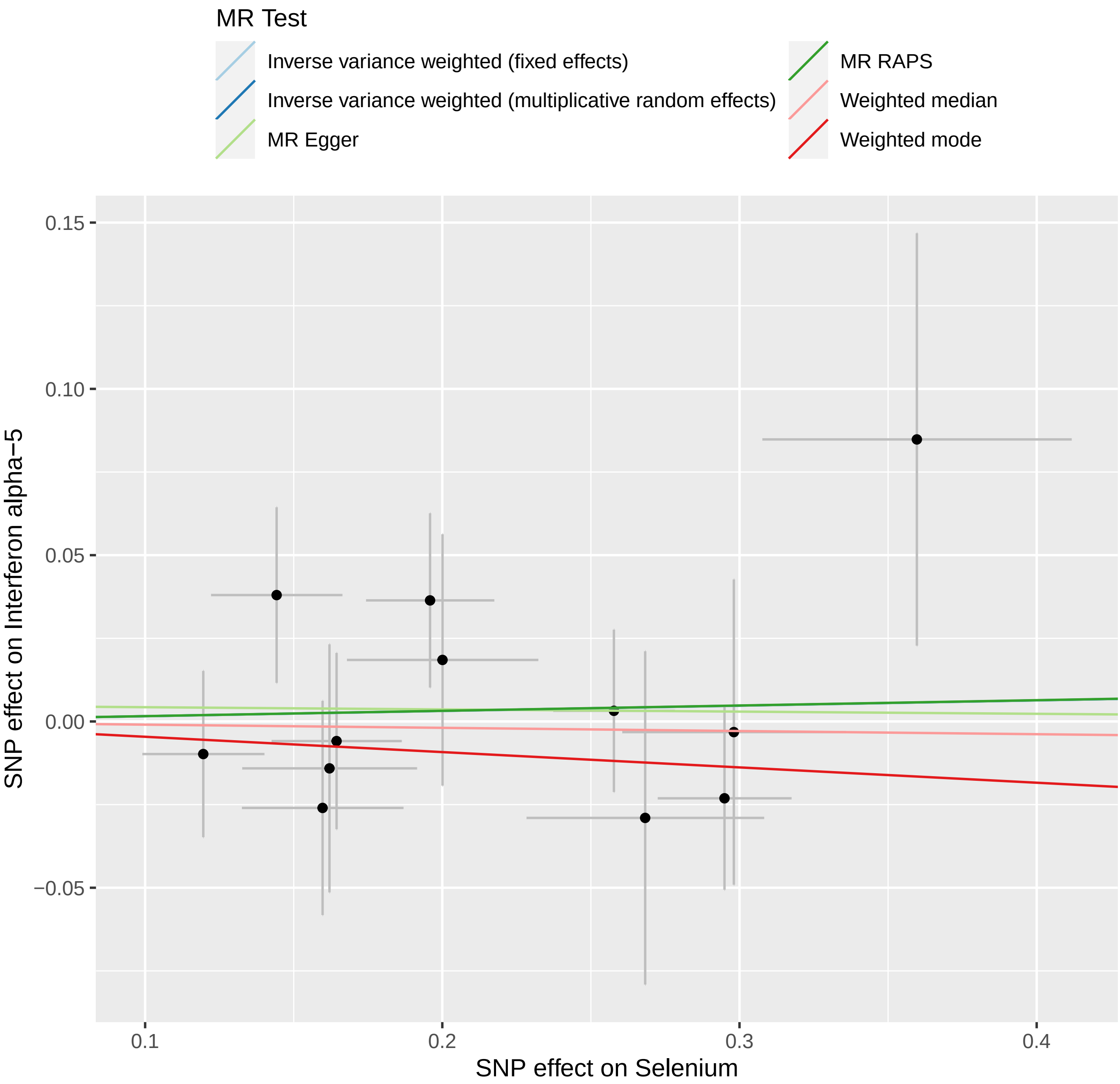

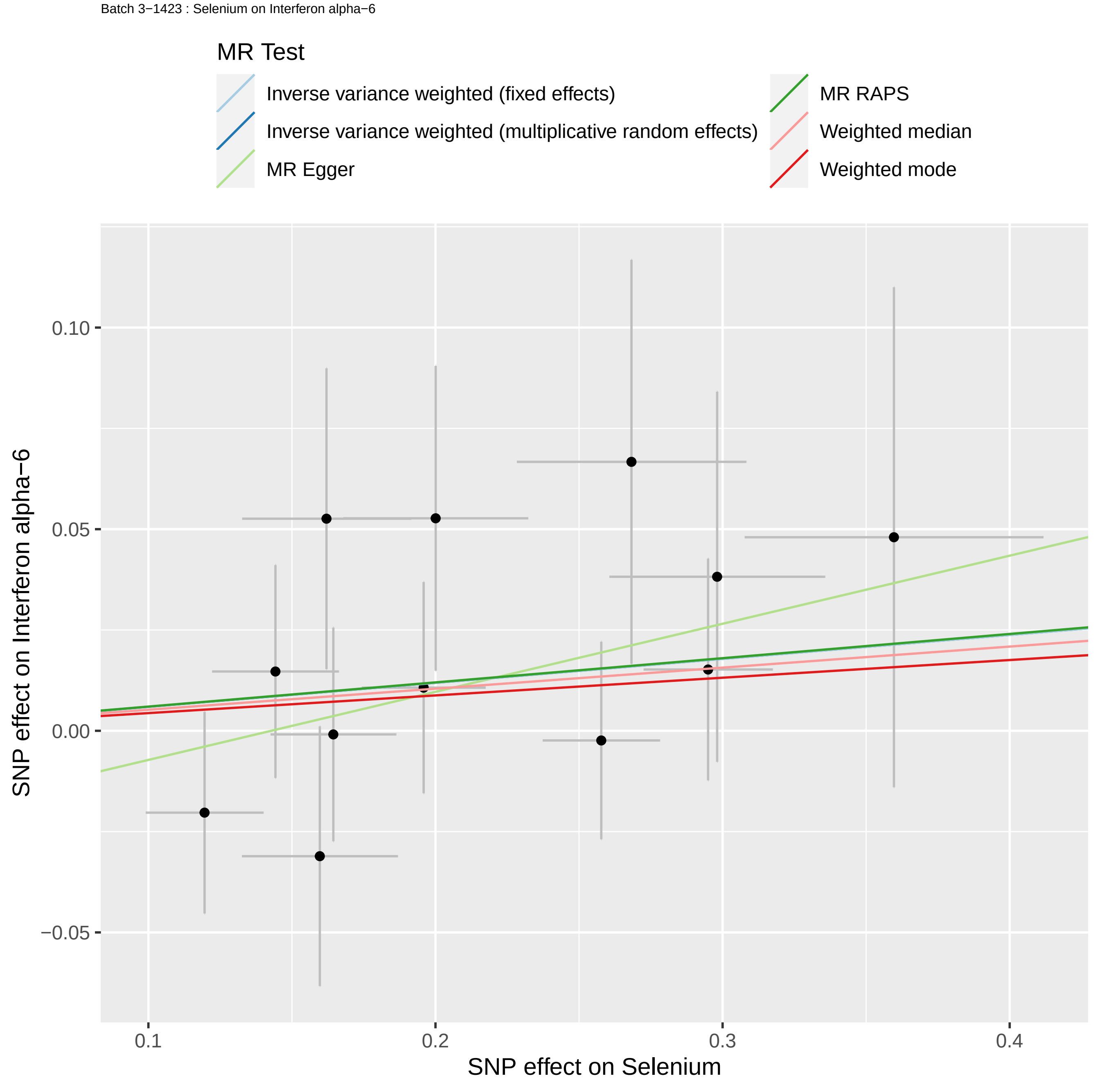
Batch 3−1423 : Selenium on Interferon alpha−6
MR Test
Inverse variance weighted (fixed effects)
Inverse variance weighted (multiplicative random effects)
MR Egger
MR RAPS
Weighted median
Weighted mode
SNP effect on Interferon alpha−6
0.10
0.05
0.00
−0.05
0.1
0.2
0.3
0.4
SNP effect on Selenium

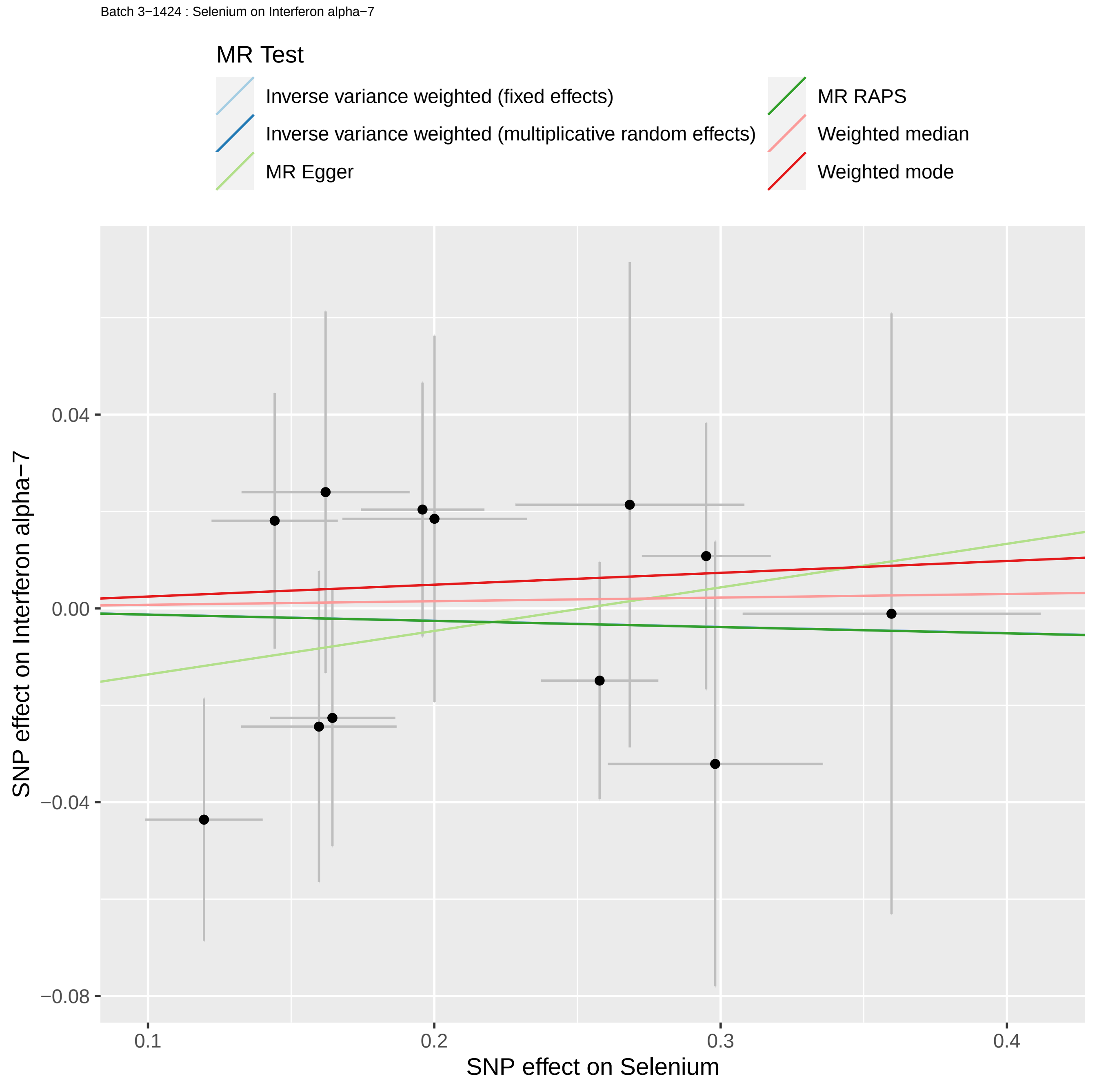

Batch 3−1424 : Selenium on Interferon alpha−7
MR Test
Inverse variance weighted (fixed effects)
Inverse variance weighted (multiplicative random effects)
MR Egger
MR RAPS
Weighted median
Weighted mode
0.04
0.00
−0.04
−0.08
0.1
0.2
0.3
0.4
SNP effect on Interferon alpha−7
SNP effect on Selenium

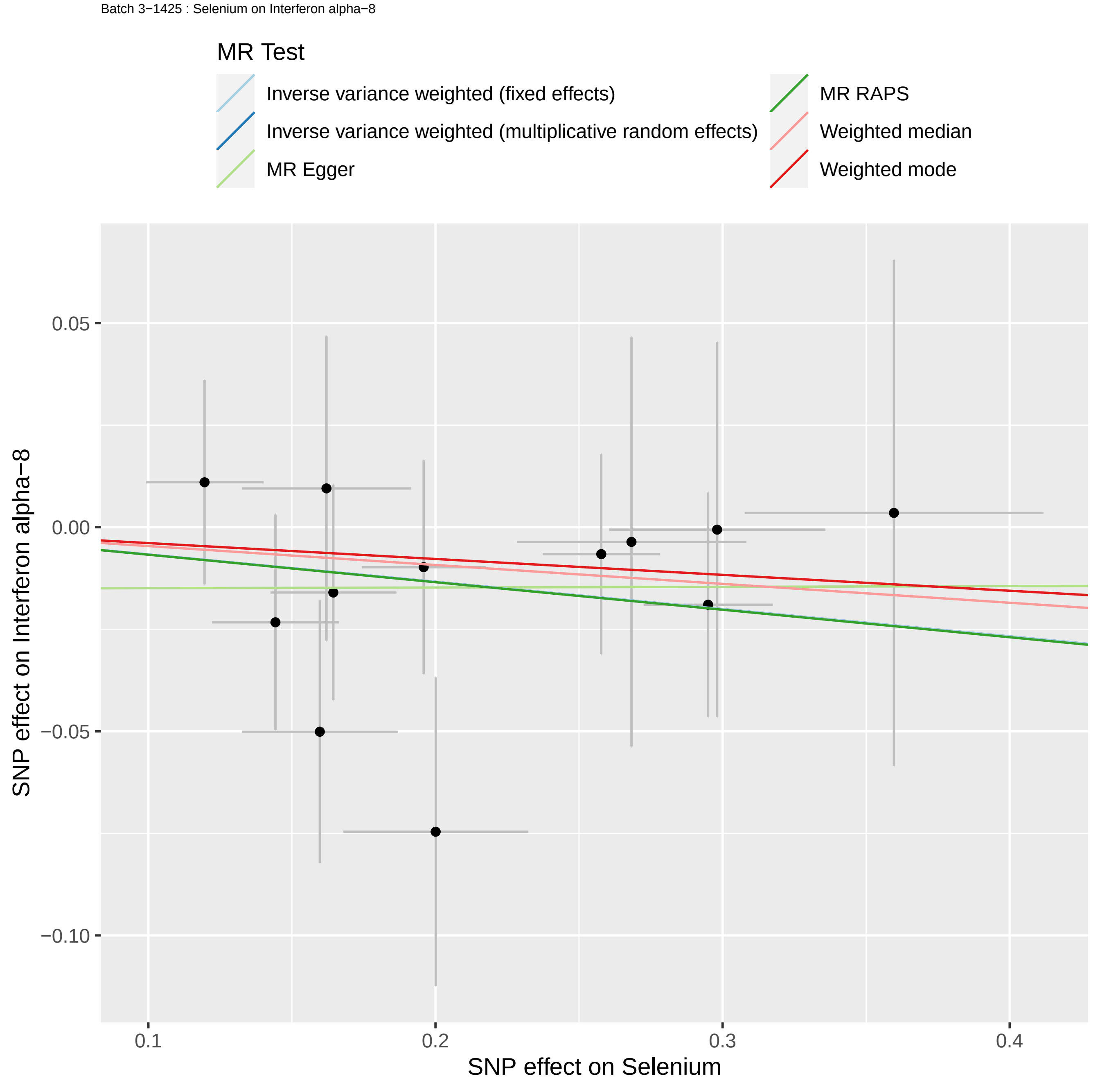
Batch 3−1425 : Selenium on Interferon alpha−8
MR Test
Inverse variance weighted (fixed effects)
Inverse variance weighted (multiplicative random effects)
MR Egger
MR RAPS
Weighted median
Weighted mode
0.05
0.00
−0.05
−0.10
0.1
0.2
0.3
0.4
SNP effect on Interferon alpha−8
SNP effect on Selenium

Batch 3−1426 : Selenium on Interferon alpha/beta receptor 1

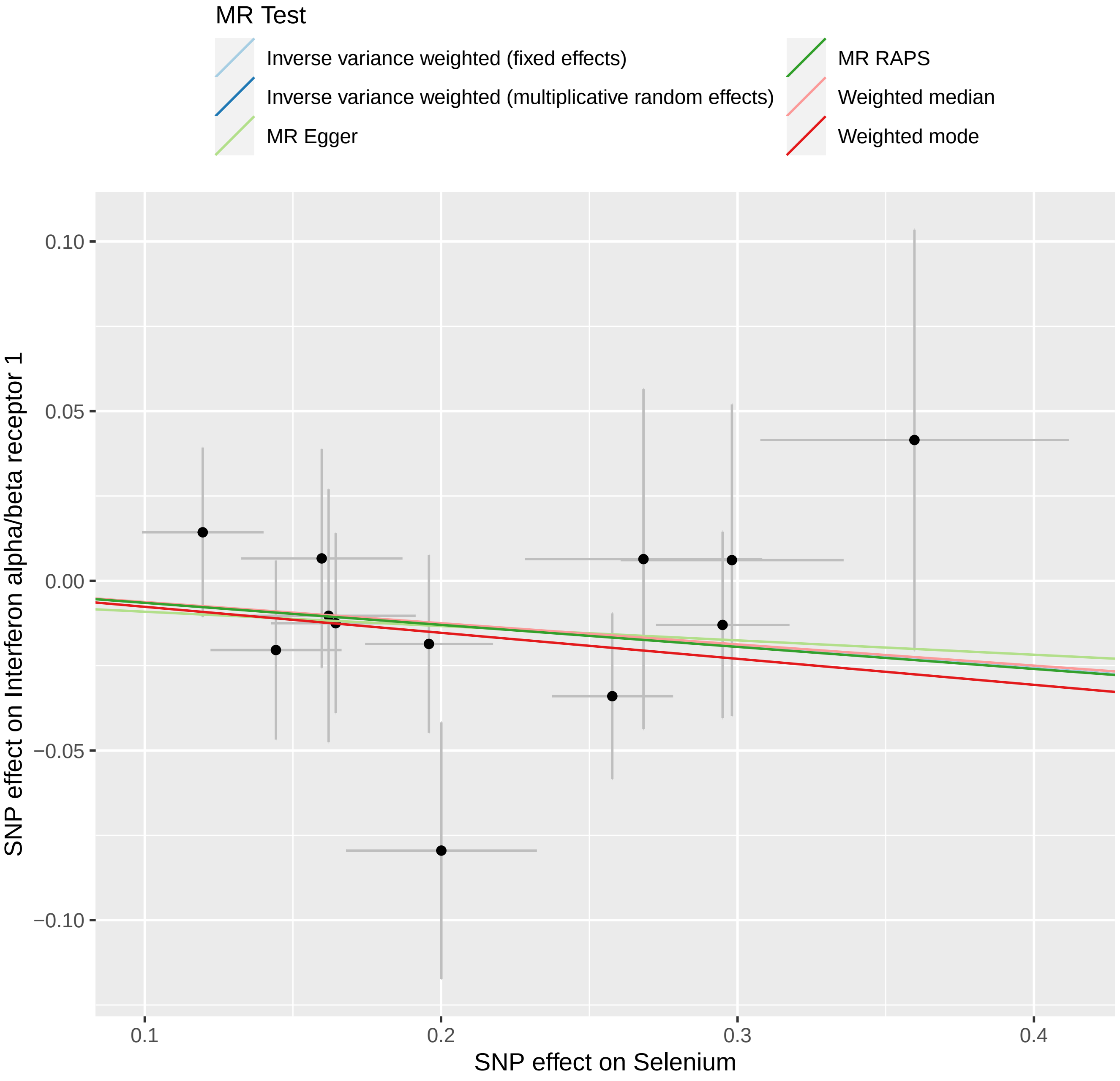

Batch 3−1427 : Selenium on Interferon beta

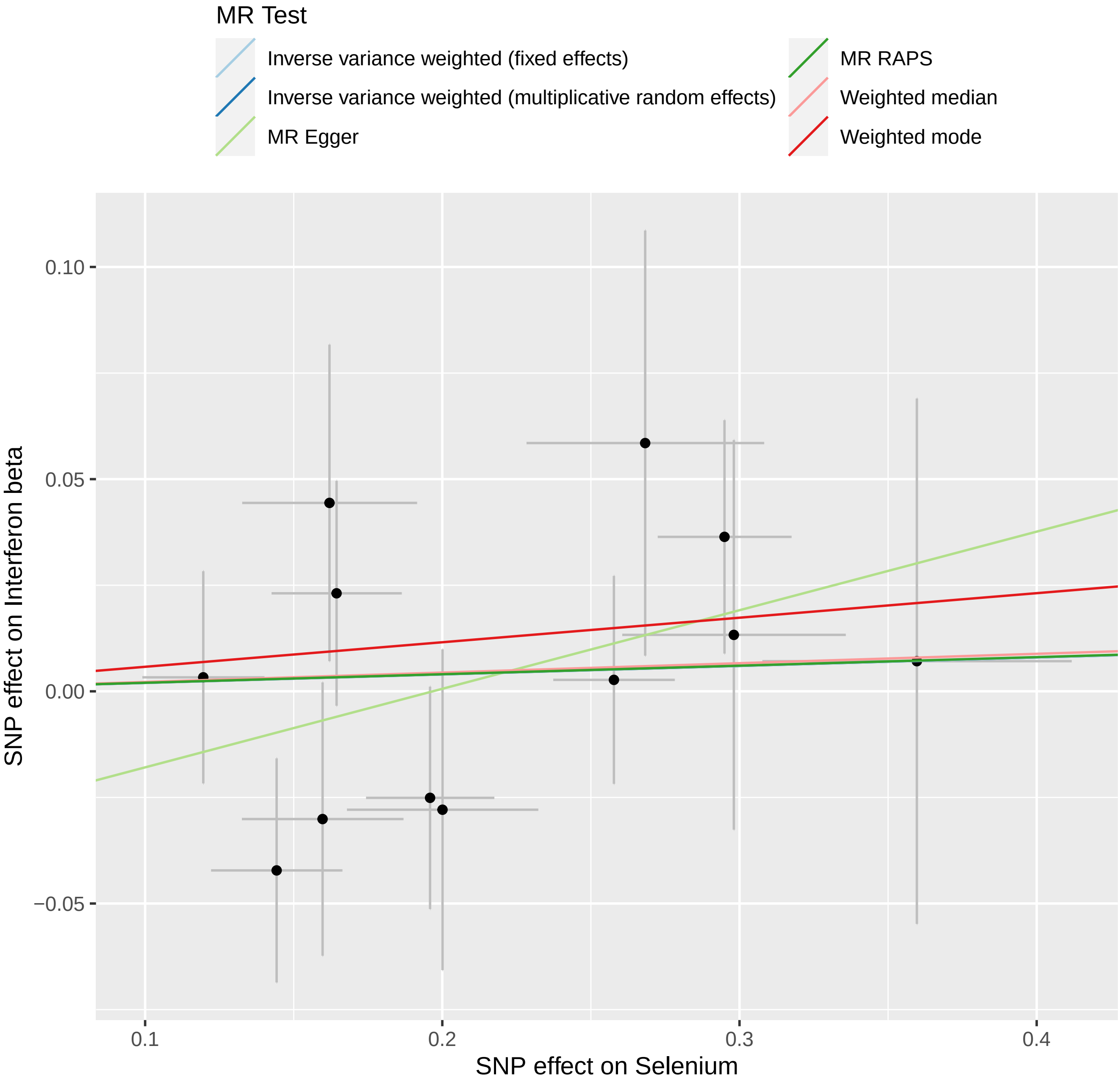

Batch 3−1428 : Selenium on Interferon gamma

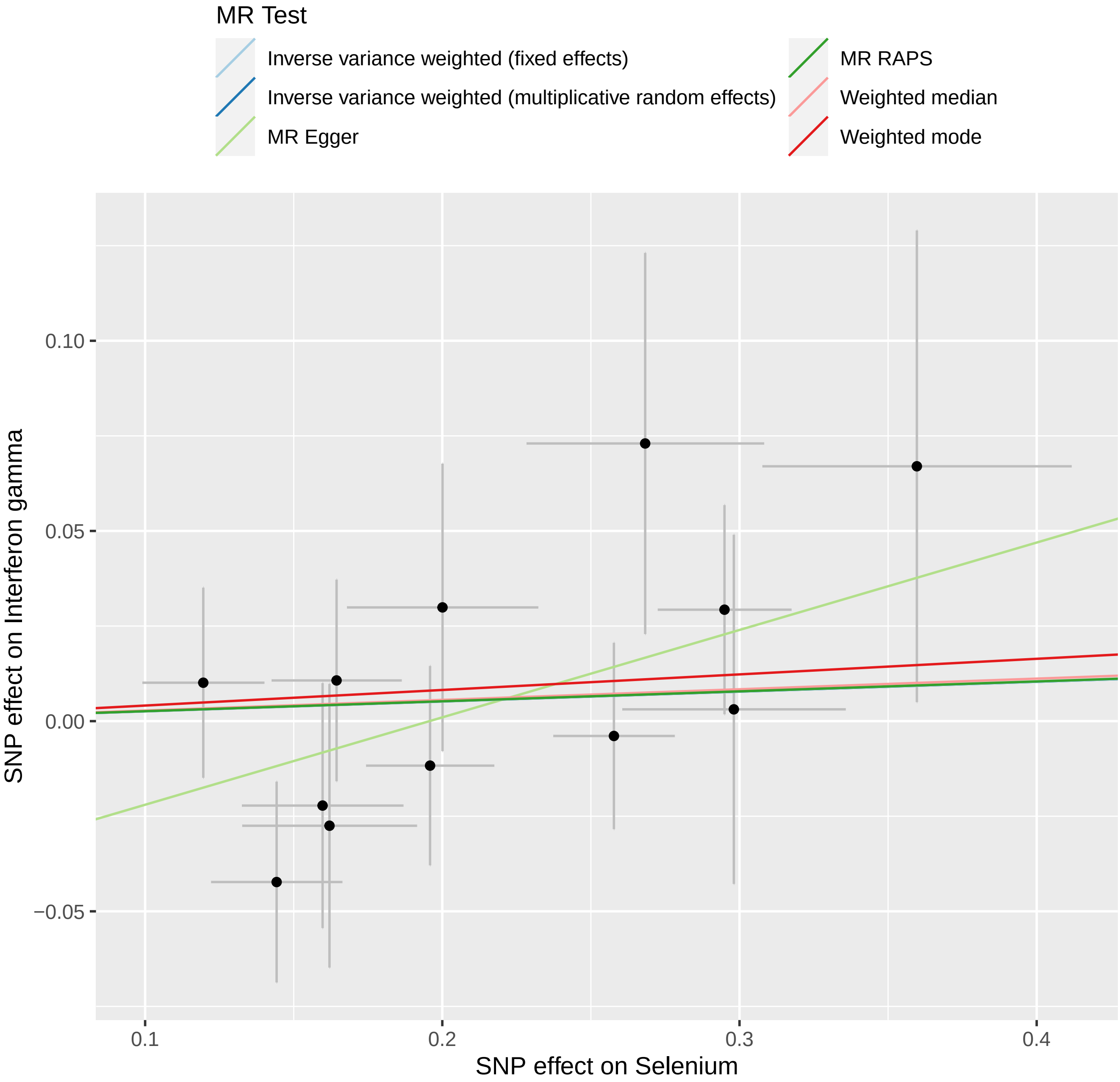

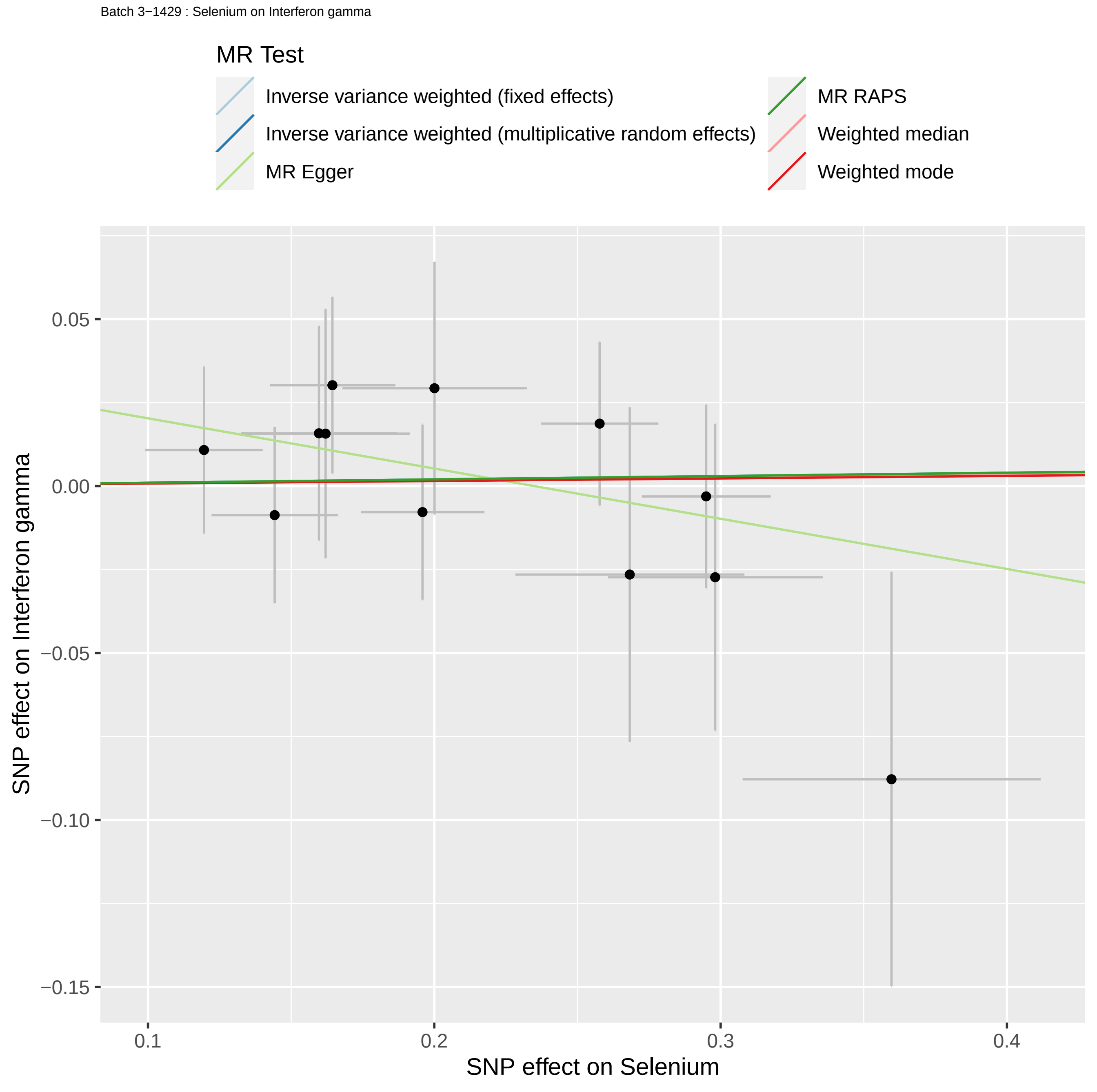

Batch 3−1429 : Selenium on Interferon gamma
MR Test
Inverse variance weighted (fixed effects)
Inverse variance weighted (multiplicative random effects)
MR Egger
MR RAPS
Weighted median
Weighted mode
0.05
0.00
−0.05
−0.10
−0.15
0.1
0.2
0.3
0.4
SNP effect on Interferon gamma
SNP effect on Selenium

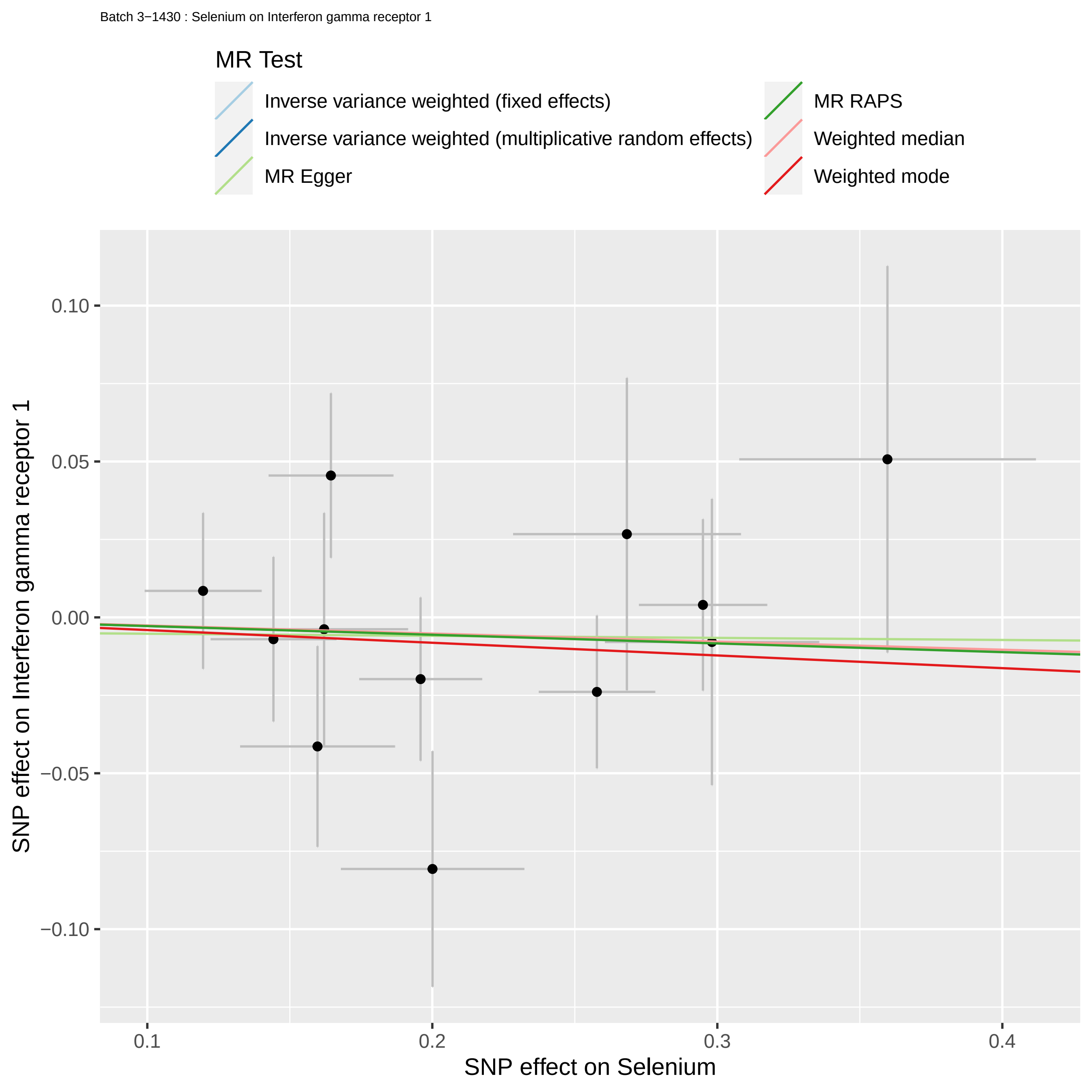
Batch 3−1430 : Selenium on Interferon gamma receptor 1
MR Test
Inverse variance weighted (fixed effects)
Inverse variance weighted (multiplicative random effects)
MR Egger
MR RAPS
Weighted median
Weighted mode
0.10
0.05
0.00
−0.05
−0.10
0.1
0.2
0.3
0.4
SNP effect on Interferon gamma receptor 1
SNP effect on Selenium

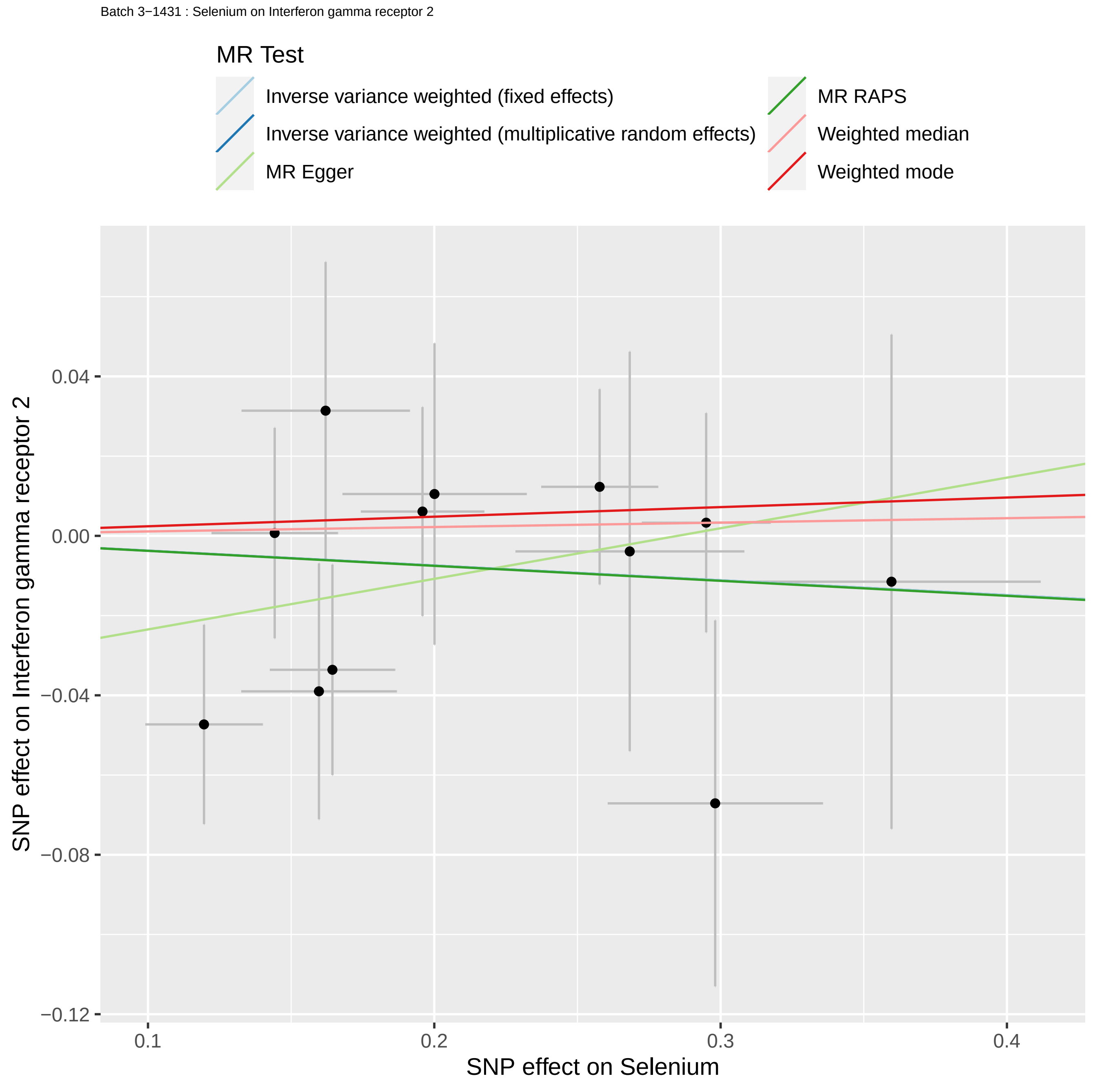
Batch 3−1431 : Selenium on Interferon gamma receptor 2
MR Test
Inverse variance weighted (fixed effects)
Inverse variance weighted (multiplicative random effects)
MR Egger
MR RAPS
Weighted median
Weighted mode
0.04
0.00
−0.04
−0.08
−0.12
0.1
0.2
0.3
0.4
SNP effect on Interferon gamma receptor 2
SNP effect on Selenium

Batch 3−1432 : Selenium on Interferon gamma receptor 2

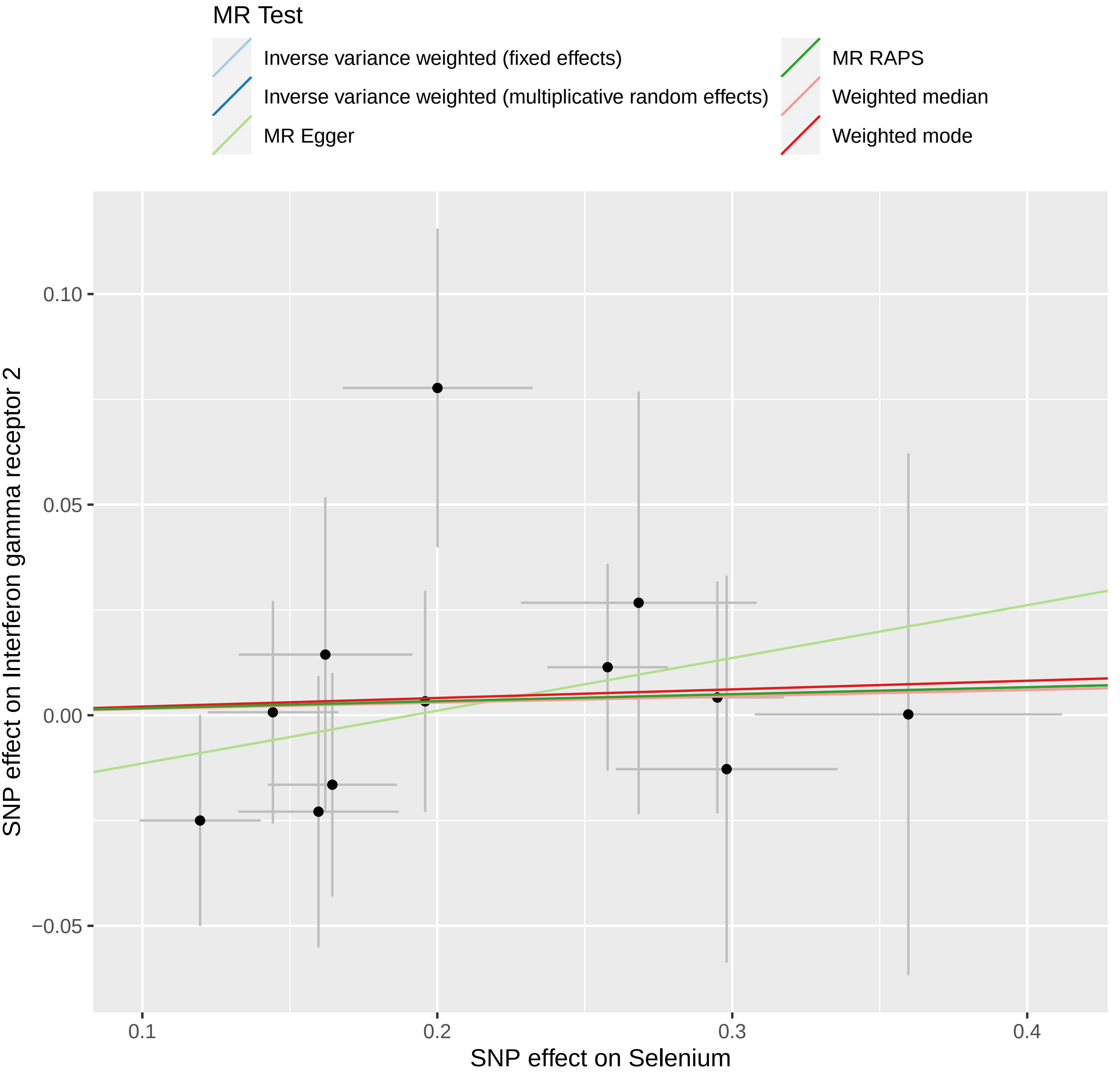

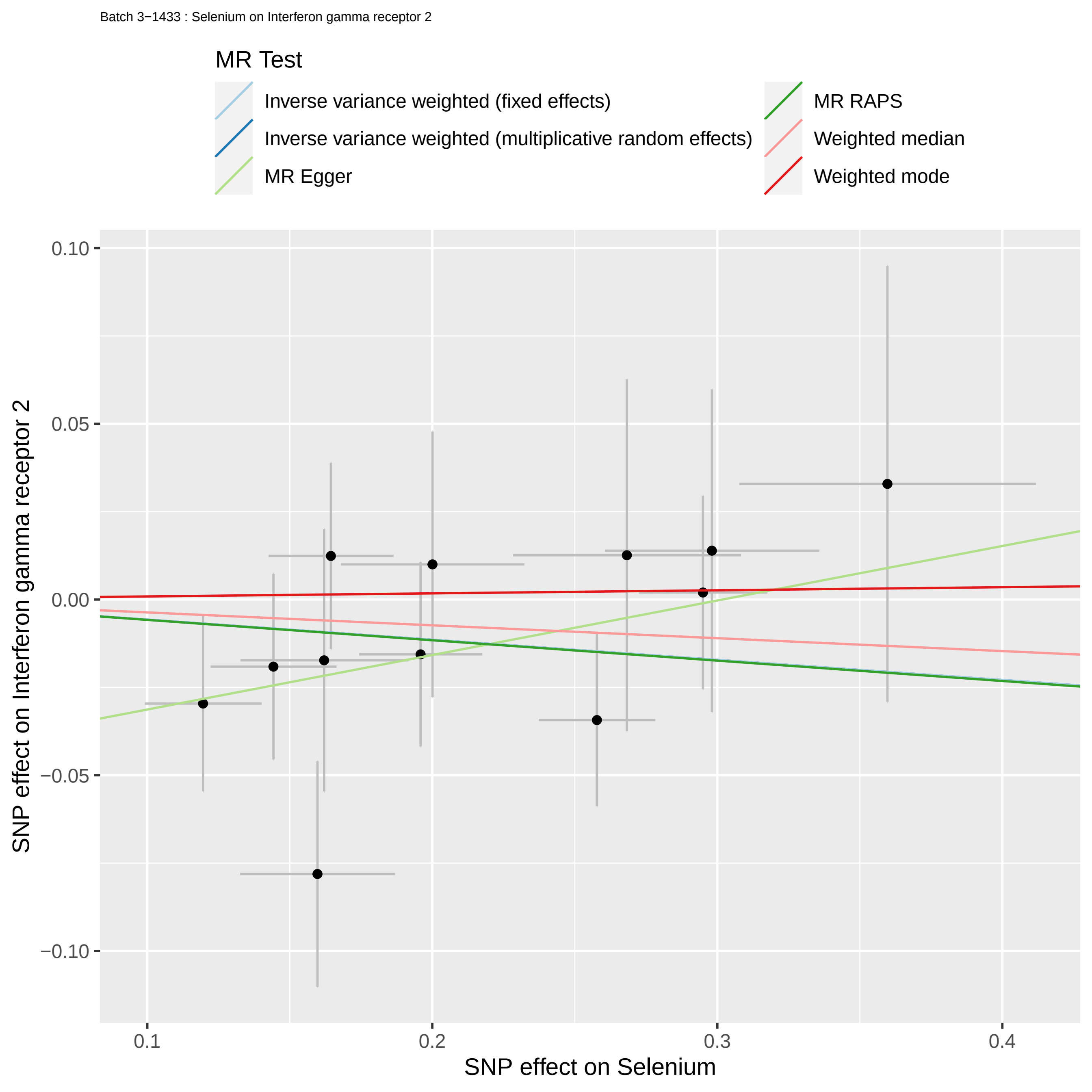
Batch 3−1433 : Selenium on Interferon gamma receptor 2
MR Test
Inverse variance weighted (fixed effects)
Inverse variance weighted (multiplicative random effects)
MR Egger
MR RAPS
Weighted median
Weighted mode
0.10
0.05
0.00
−0.05
−0.10
0.1
0.2
0.3
0.4
SNP effect on Selenium
SNP effect on Interferon gamma receptor 2

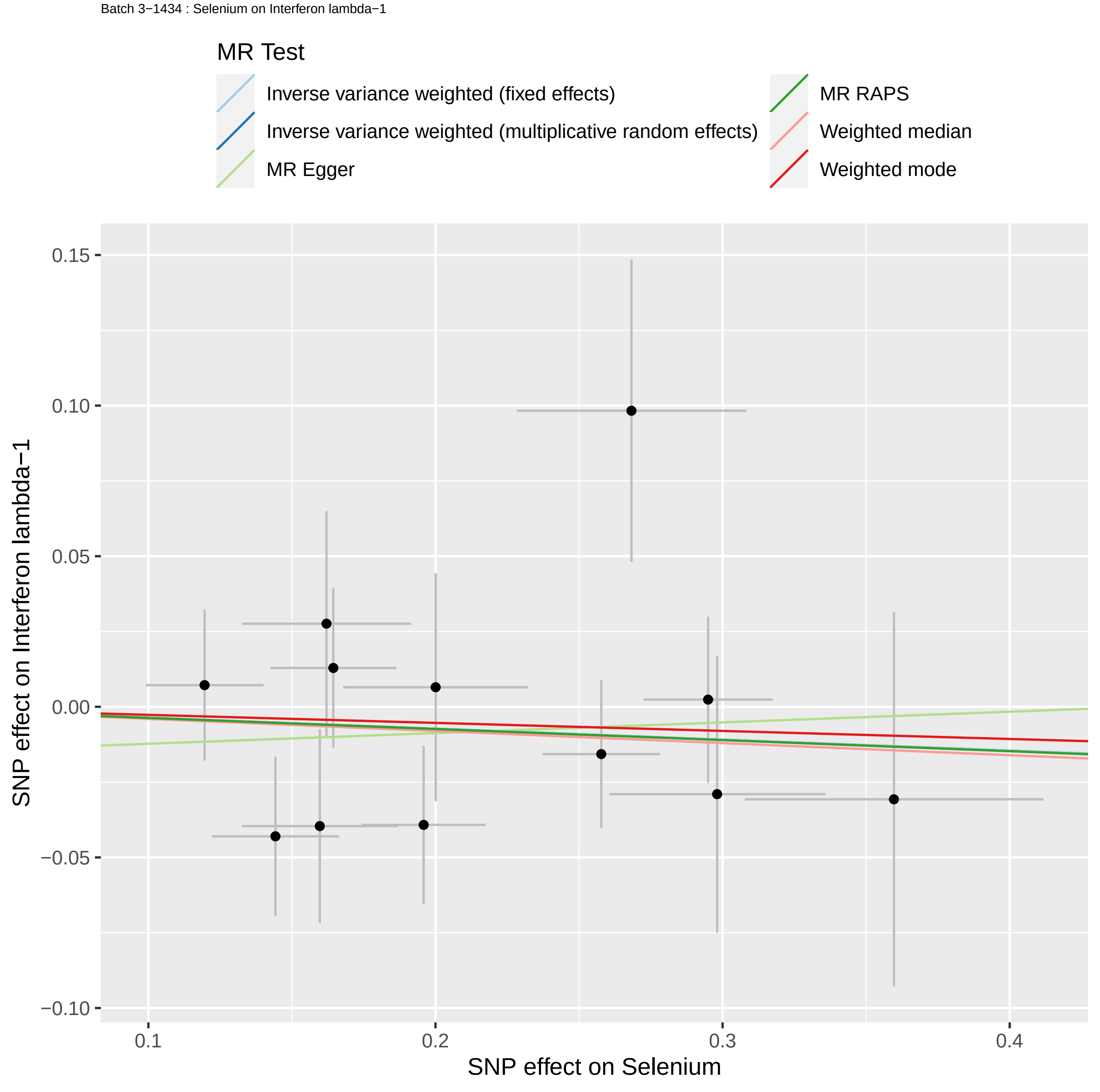

Batch 3−1434 : Selenium on Interferon lambda−1
MR Test
Inverse variance weighted (fixed effects)
Inverse variance weighted (multiplicative random effects)
MR Egger
MR RAPS
Weighted median
Weighted mode
0.15
0.10
0.05
0.00
−0.05
−0.10
0.1
0.2
0.3
0.4
SNP effect on Interferon lambda−1
SNP effect on Selenium

Batch 3−1435 : Selenium on Interferon lambda−1

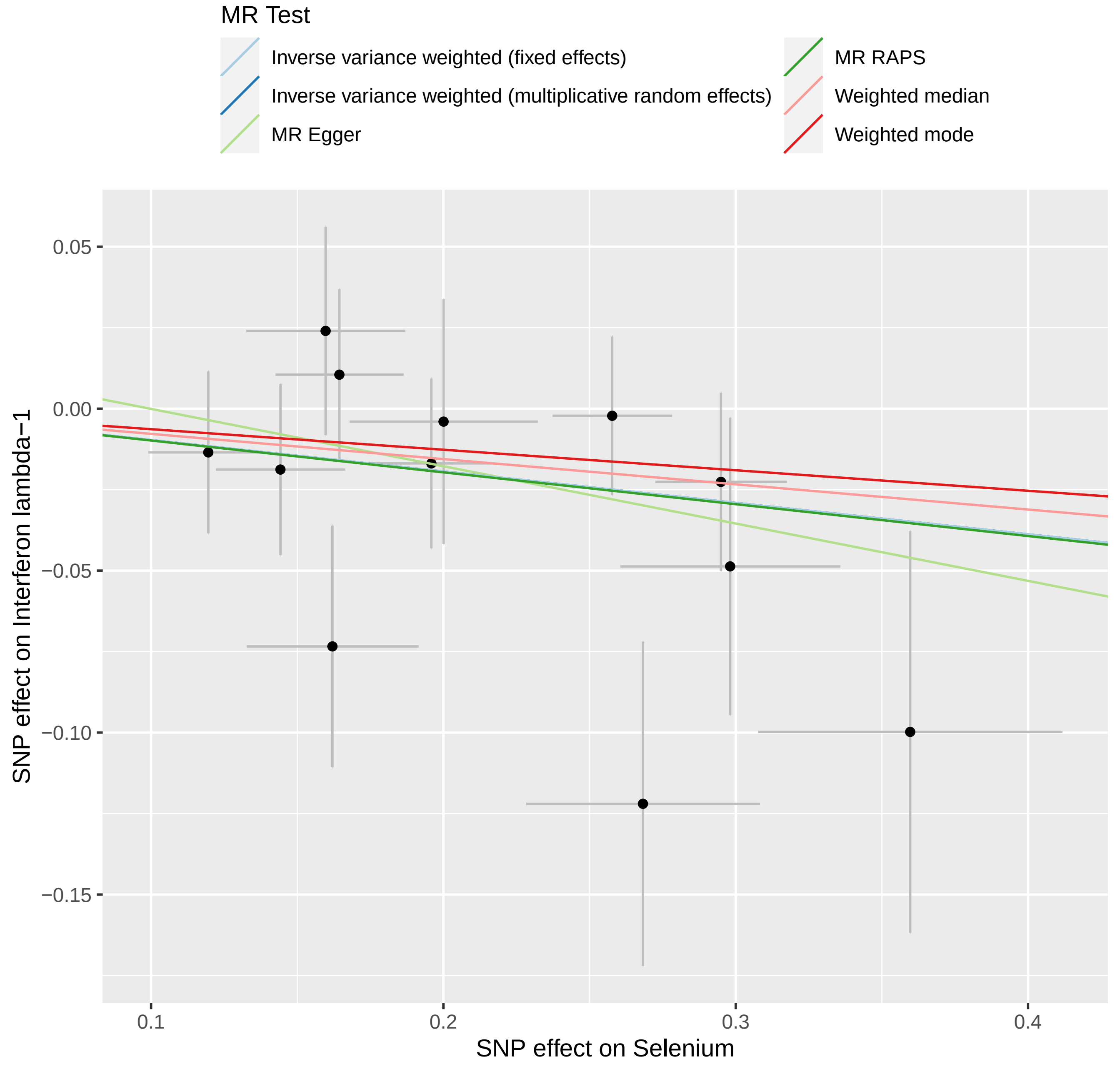

Batch 3−1436 : Selenium on Interferon lambda−2

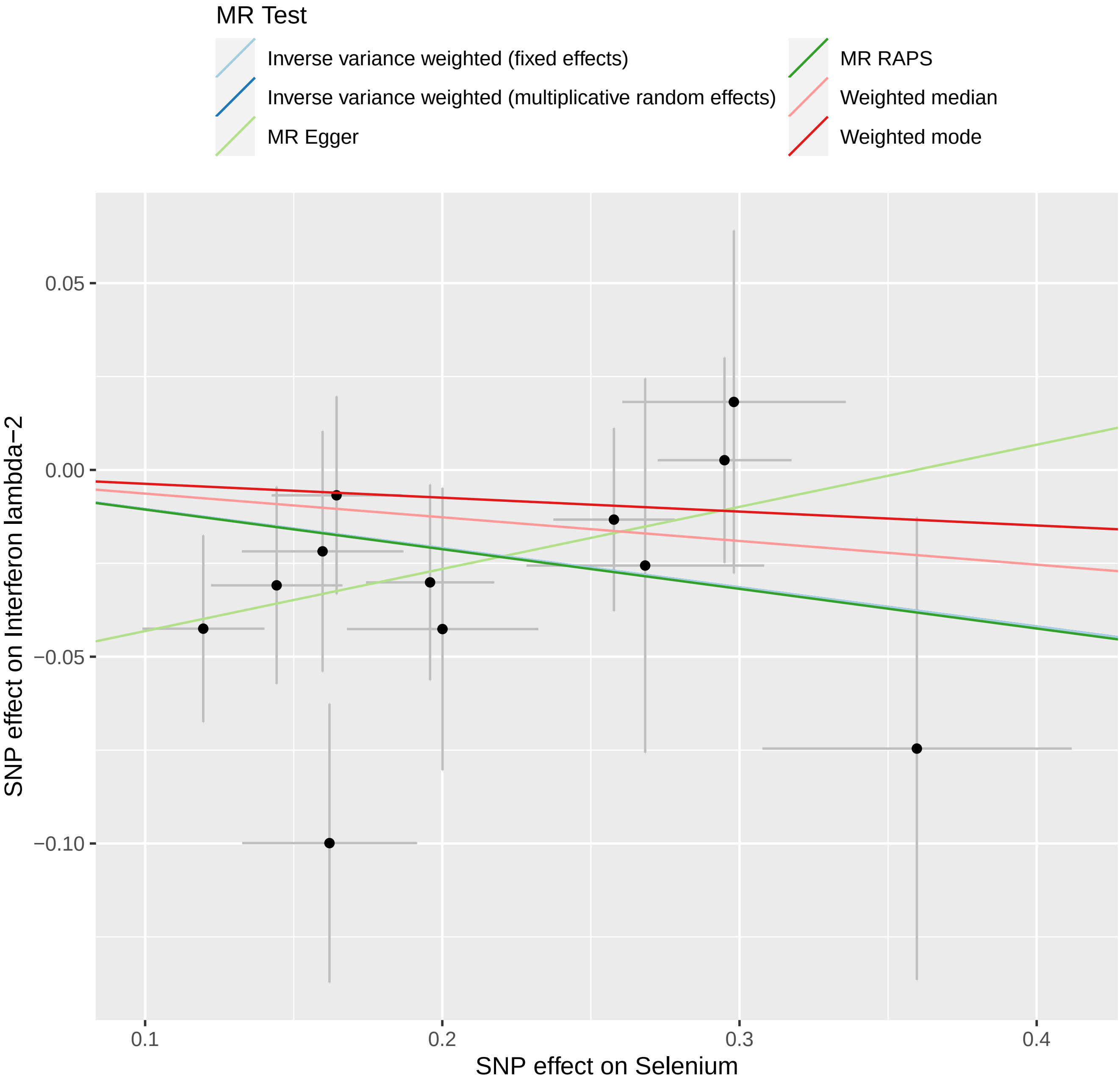

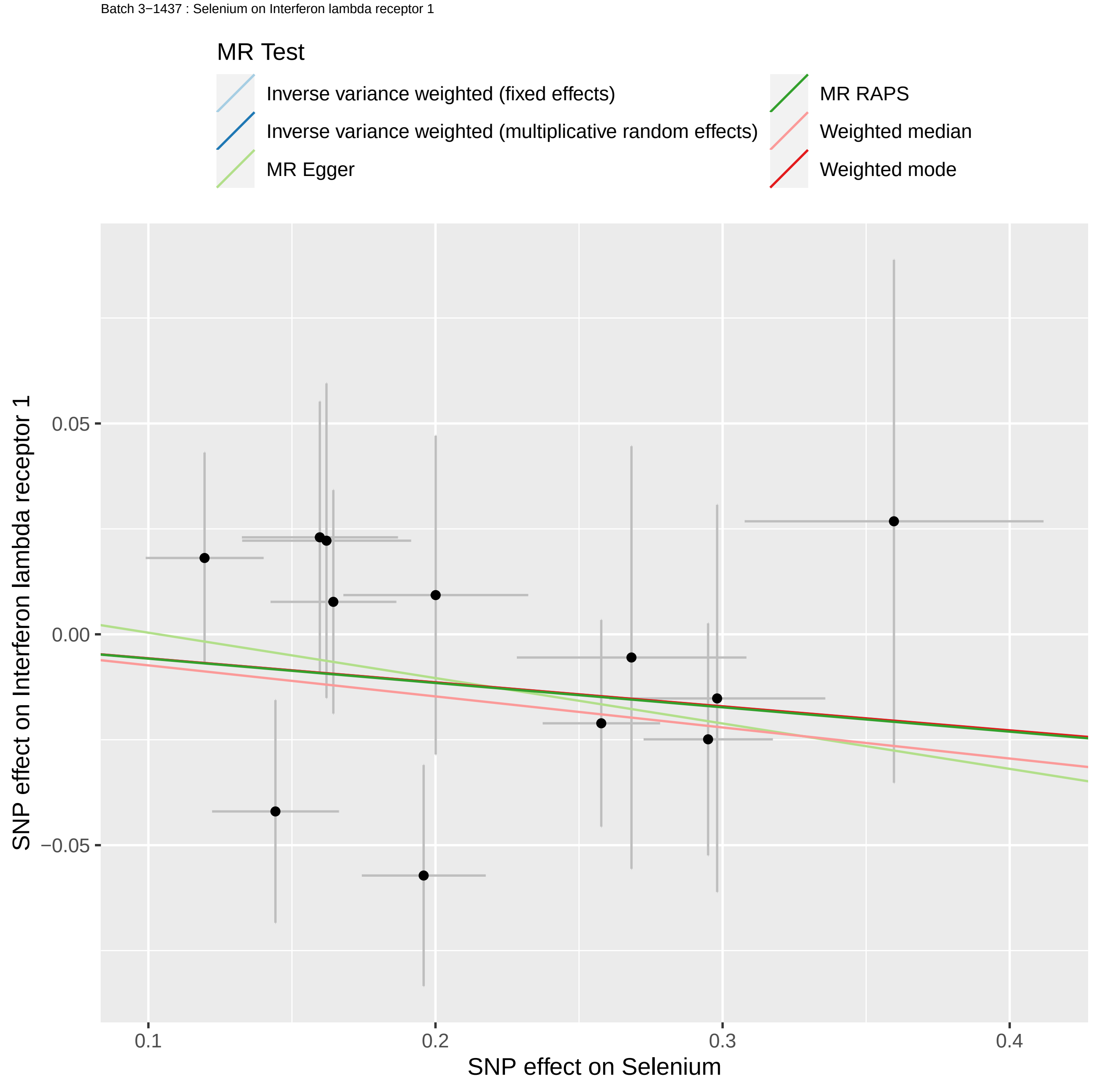

Batch 3−1437 : Selenium on Interferon lambda receptor 1
MR Test
Inverse variance weighted (fixed effects)
Inverse variance weighted (multiplicative random effects)
MR Egger
MR RAPS
Weighted median
Weighted mode
0.05
0.00
−0.05
0.1
0.2
0.3
0.4
SNP effect on Interferon lambda receptor 1
SNP effect on Selenium

Batch 3−1438 : Selenium on Interferon omega−1

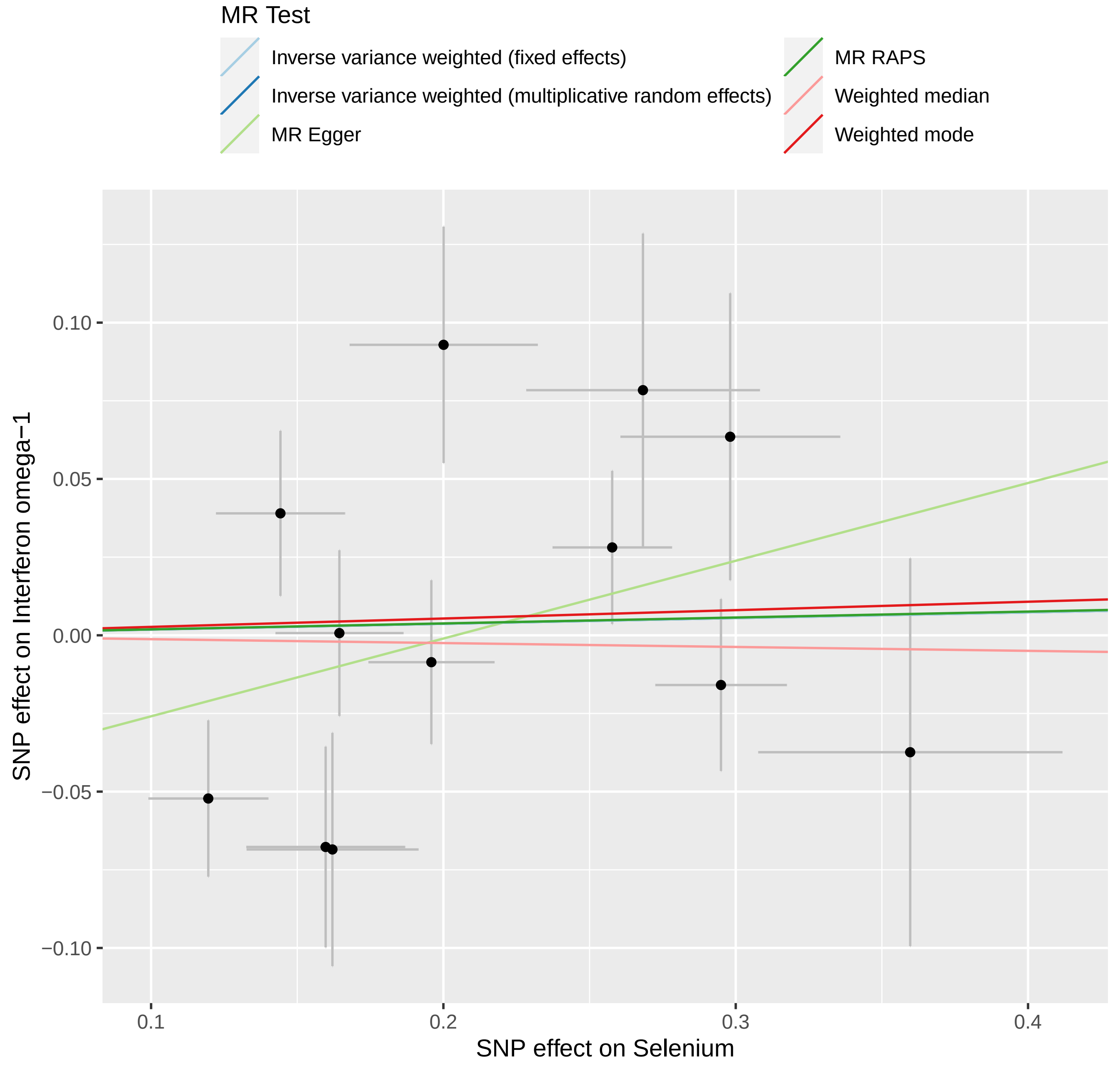

Batch 3−1439 : Selenium on Immunoglobulin−binding protein 1

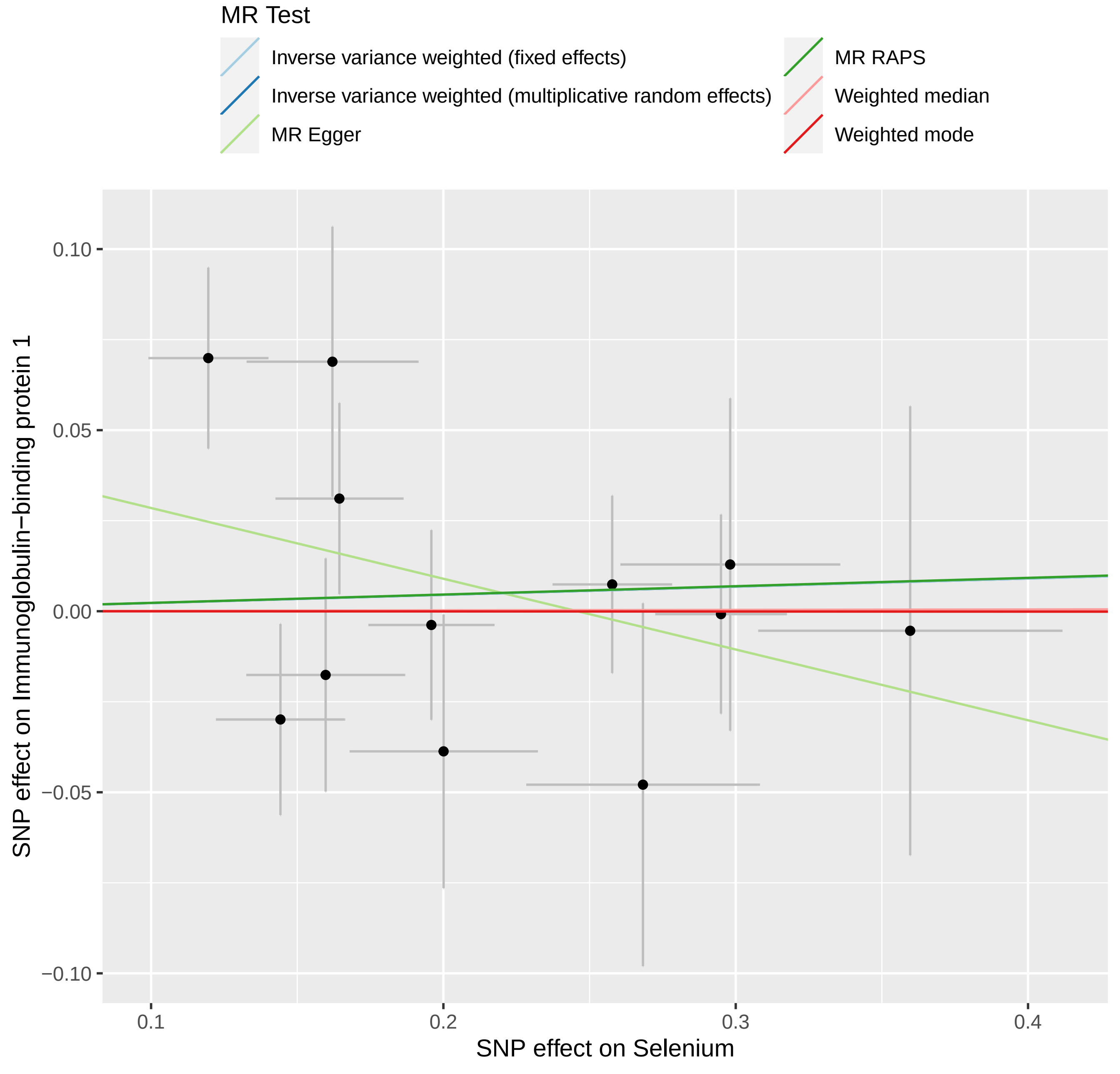

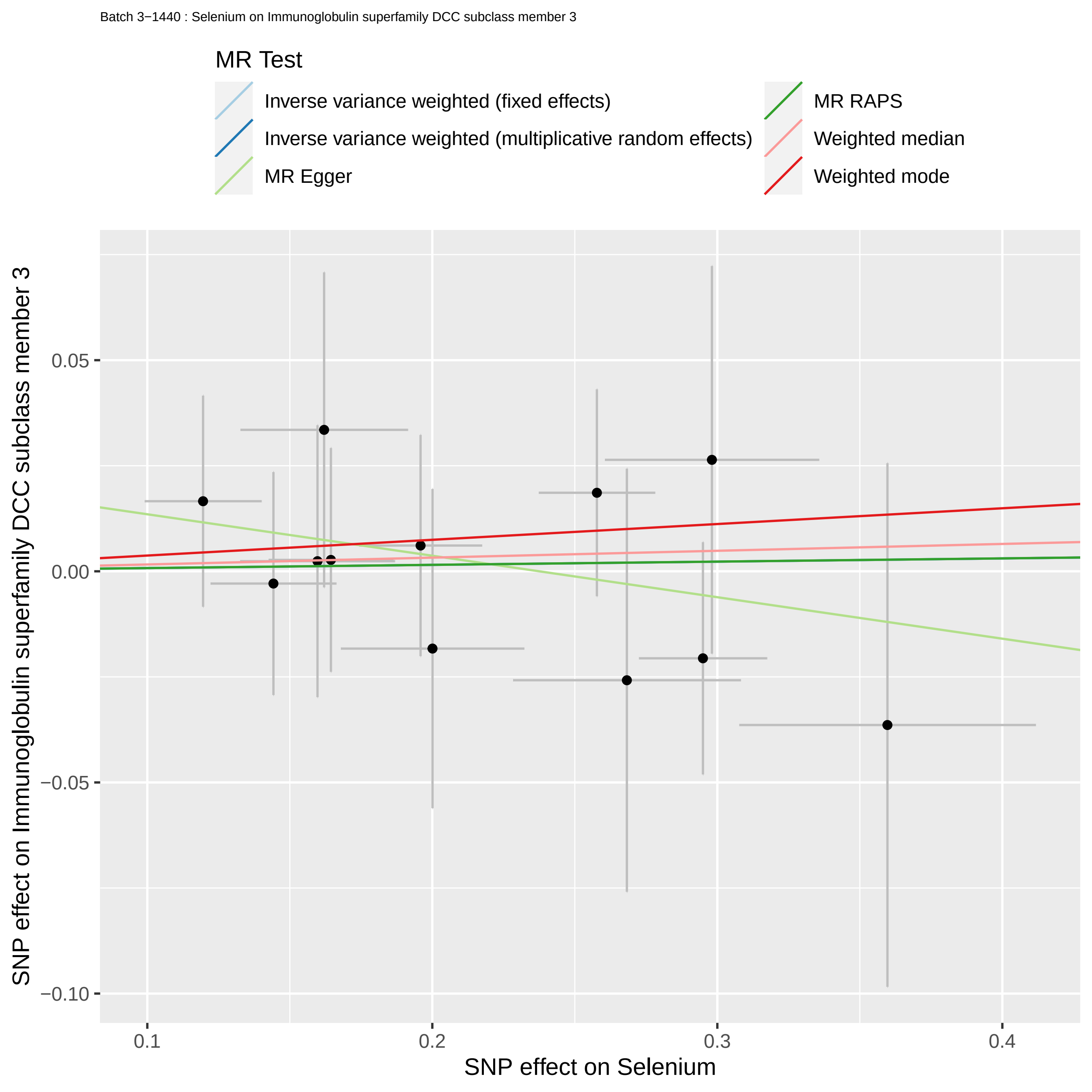

Batch 3−1440 : Selenium on Immunoglobulin superfamily DCC subclass member 3
MR Test
Inverse variance weighted (fixed effects)
Inverse variance weighted (multiplicative random effects)
MR Egger
MR RAPS
Weighted median
Weighted mode
0.05
0.00
−0.05
−0.10
0.1
0.2
0.3
0.4
SNP effect on Immunoglobulin superfamily DCC subclass member 3
SNP effect on Selenium

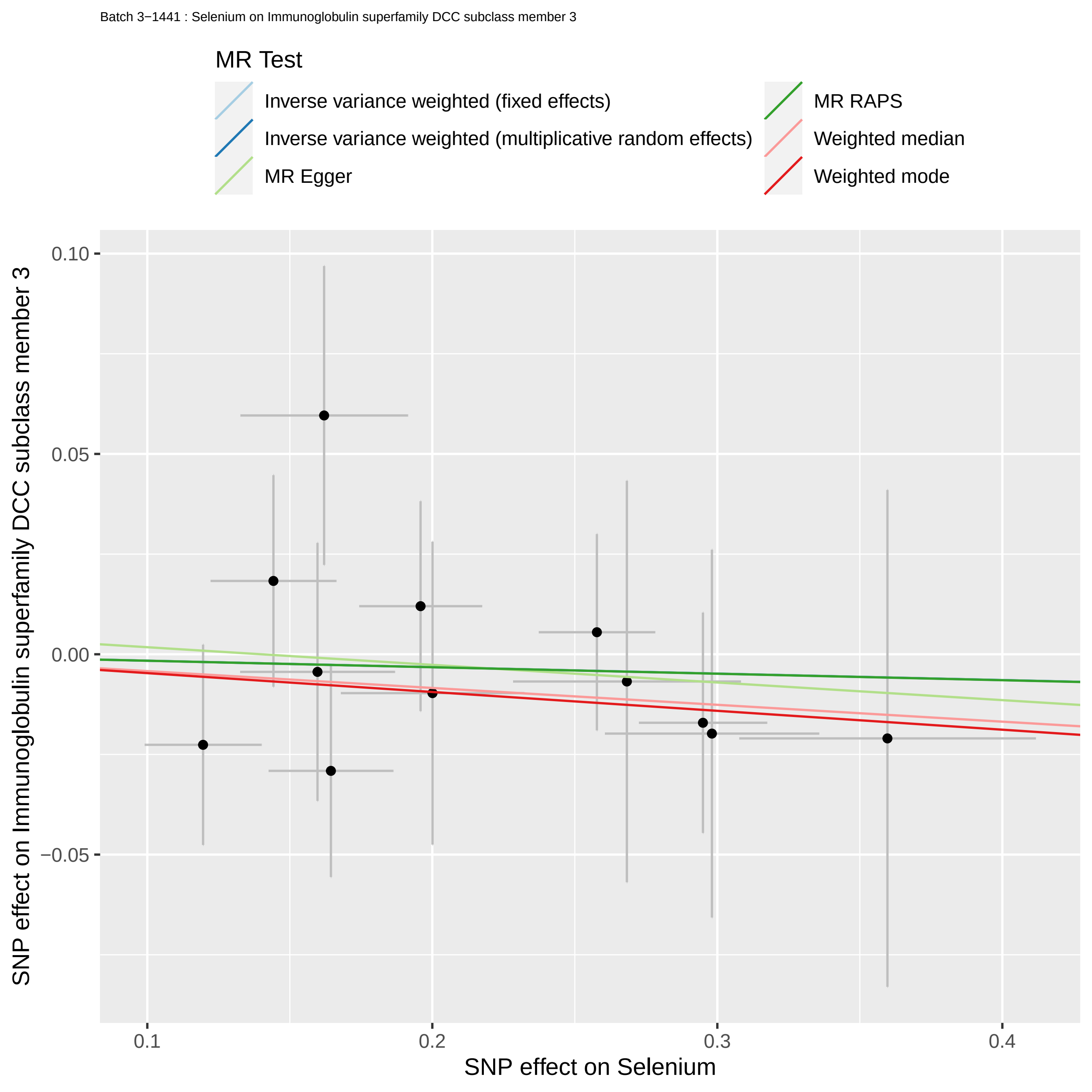

Batch 3−1441 : Selenium on Immunoglobulin superfamily DCC subclass member 3
MR Test
Inverse variance weighted (fixed effects)
Inverse variance weighted (multiplicative random effects)
MR Egger
MR RAPS
Weighted median
Weighted mode
0.10
0.05
0.00
−0.05
0.1
0.2
0.3
0.4
SNP effect on Selenium
SNP effect on Immunoglobulin superfamily DCC subclass member 3

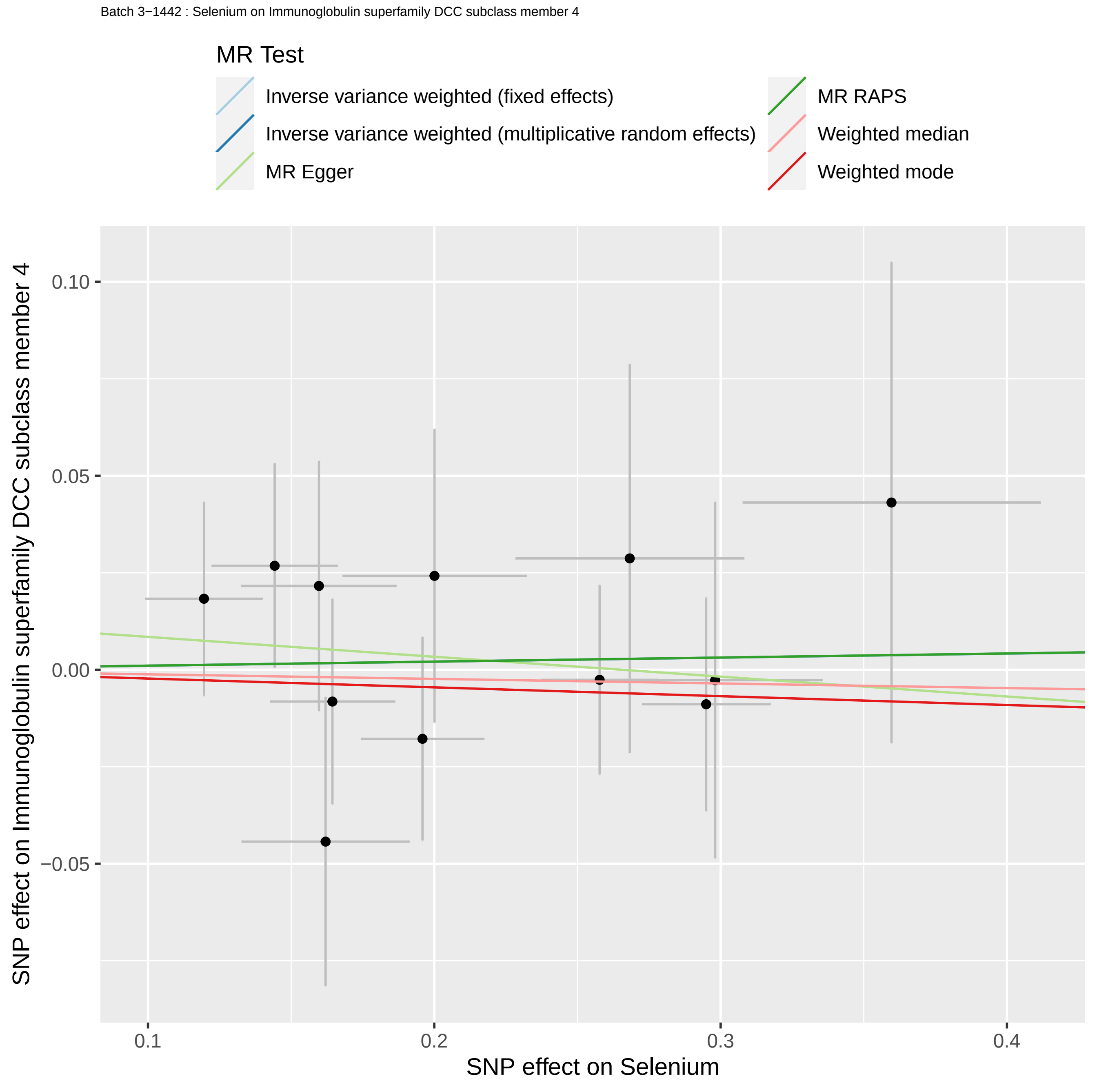

Batch 3−1442 : Selenium on Immunoglobulin superfamily DCC subclass member 4
MR Test
Inverse variance weighted (fixed effects)
Inverse variance weighted (multiplicative random effects)
MR Egger
MR RAPS
Weighted median
Weighted mode
SNP effect on Immunoglobulin superfamily DCC subclass member 4
0.10
0.05
0.00
−0.05
0.1
0.2
0.3
0.4
SNP effect on Selenium

Batch 3−1443 : Selenium on Insulin−like growth factor I

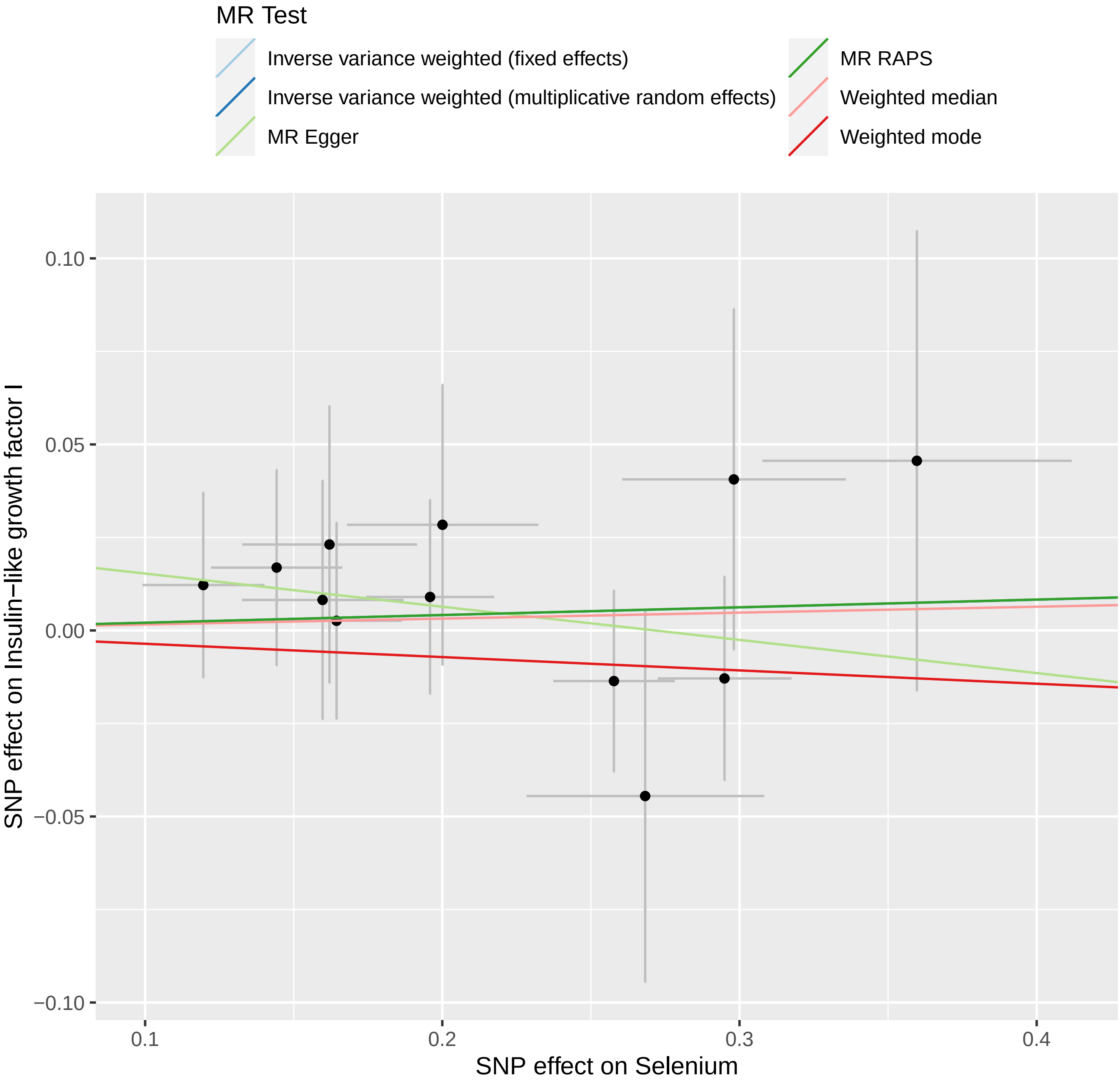

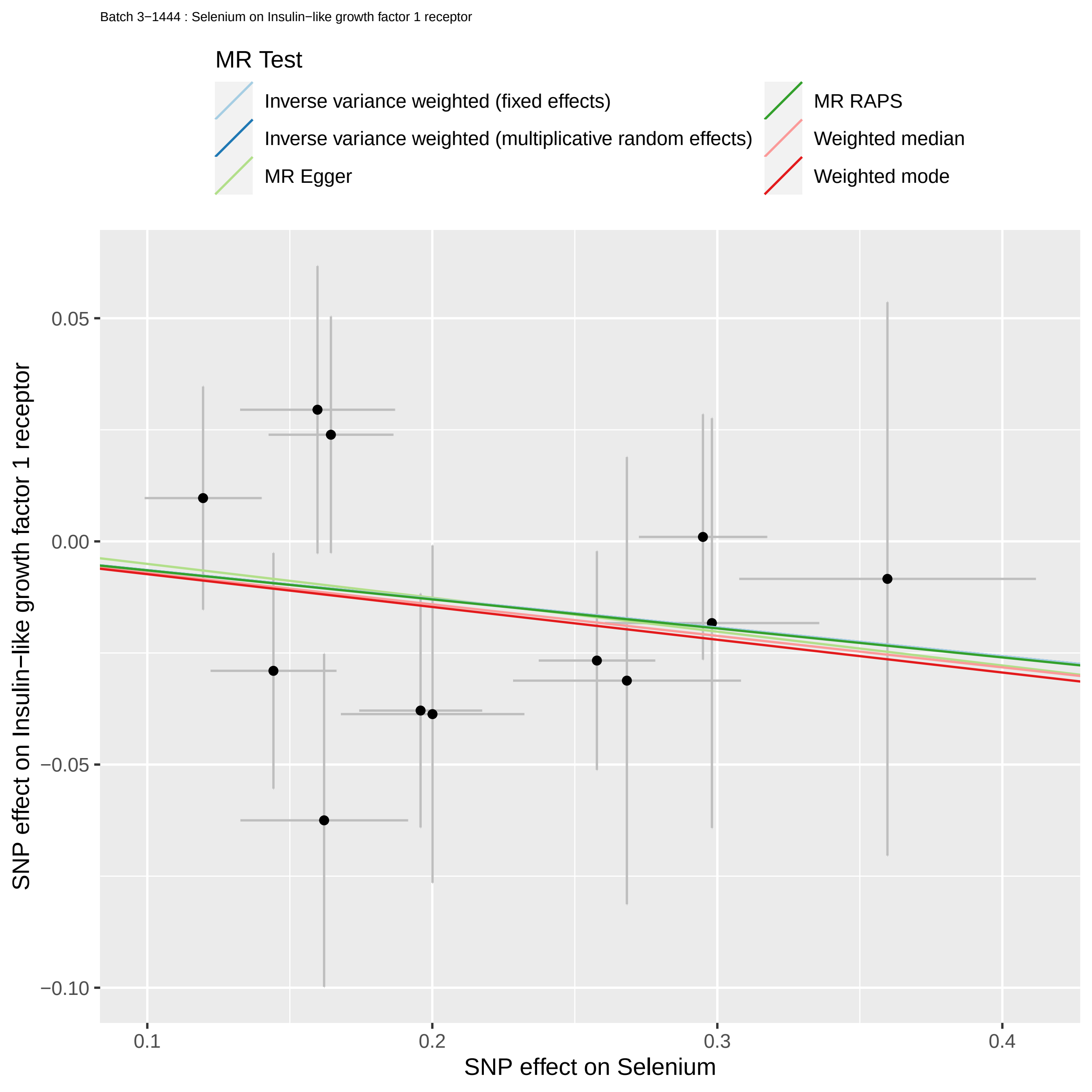

Batch 3−1444 : Selenium on Insulin−like growth factor 1 receptor
MR Test
Inverse variance weighted (fixed effects)
Inverse variance weighted (multiplicative random effects)
MR Egger
MR RAPS
Weighted median
Weighted mode
0.05
0.00
−0.05
−0.10
0.1
0.2
0.3
0.4
SNP effect on Insulin−like growth factor 1 receptor
SNP effect on Selenium

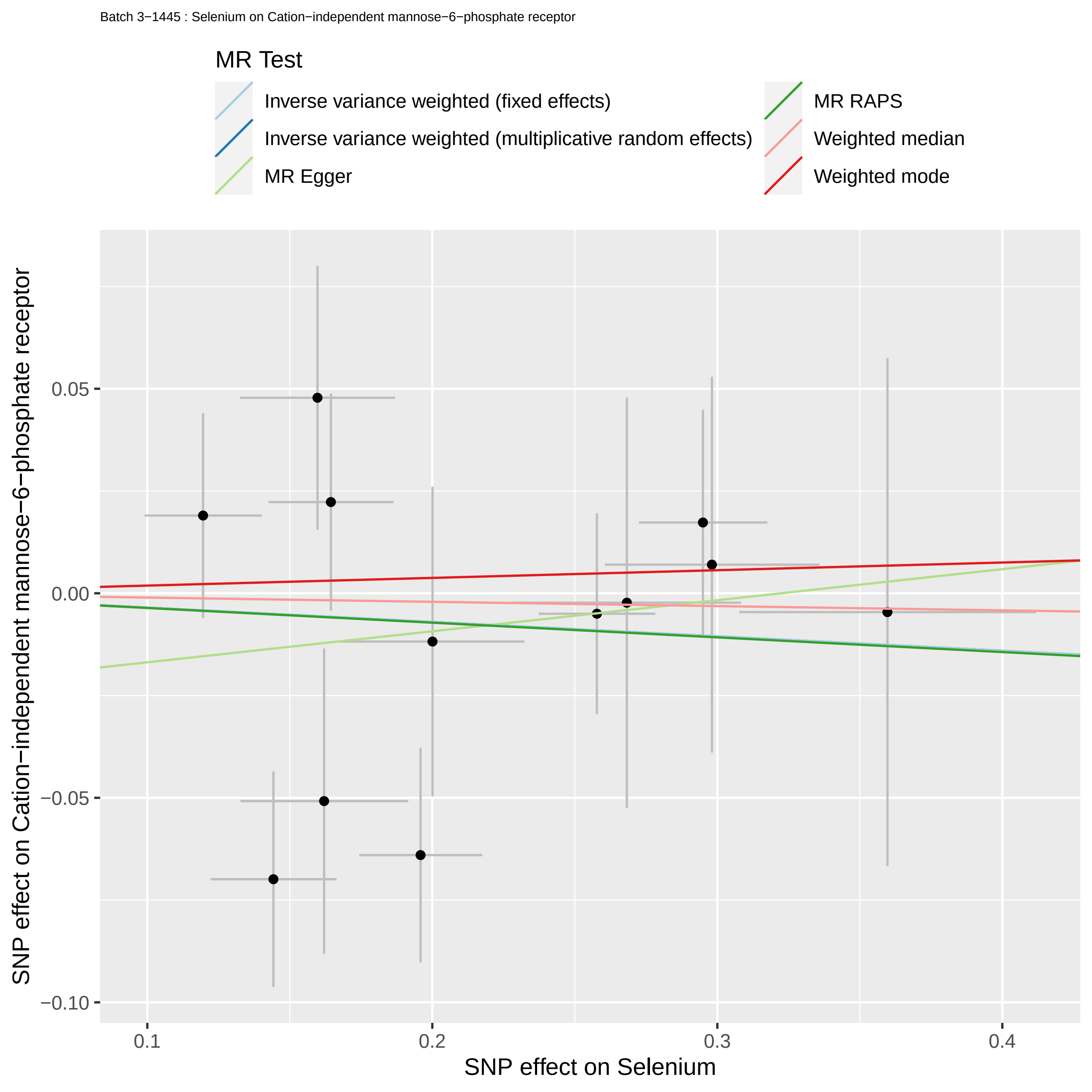
Batch 3−1445 : Selenium on Cation−independent mannose−6−phosphate receptor
MR Test
Inverse variance weighted (fixed effects)
Inverse variance weighted (multiplicative random effects)
MR Egger
MR RAPS
Weighted median
Weighted mode
SNP effect on Cation−independent mannose−6−phosphate receptor
0.05
0.00
−0.05
−0.10
0.1
0.2
0.3
0.4
SNP effect on Selenium

Batch 3−1446 : Selenium on Insulin−like growth factor−binding protein 1

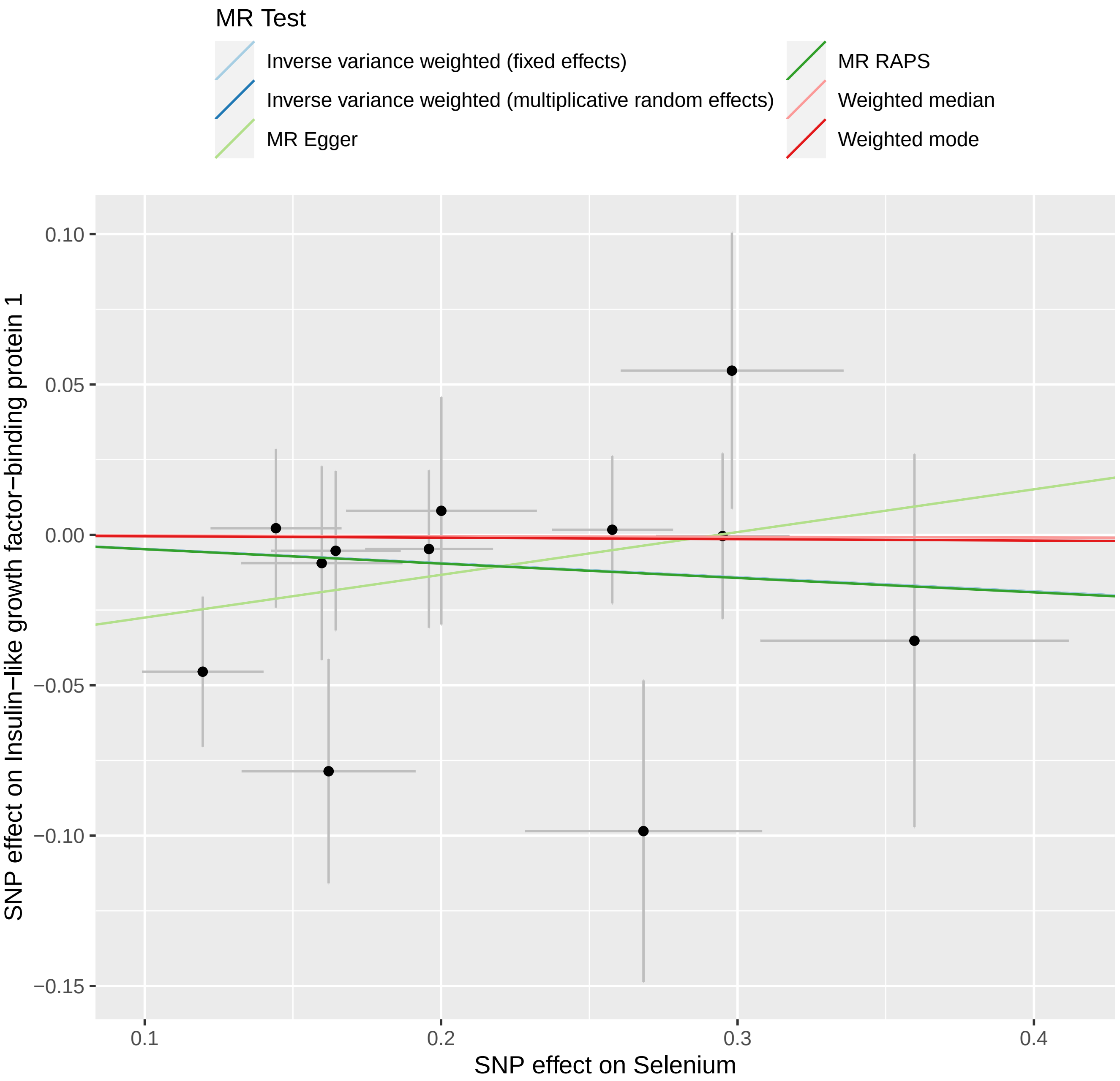

Batch 3−1447 : Selenium on Insulin−like growth factor−binding protein 1

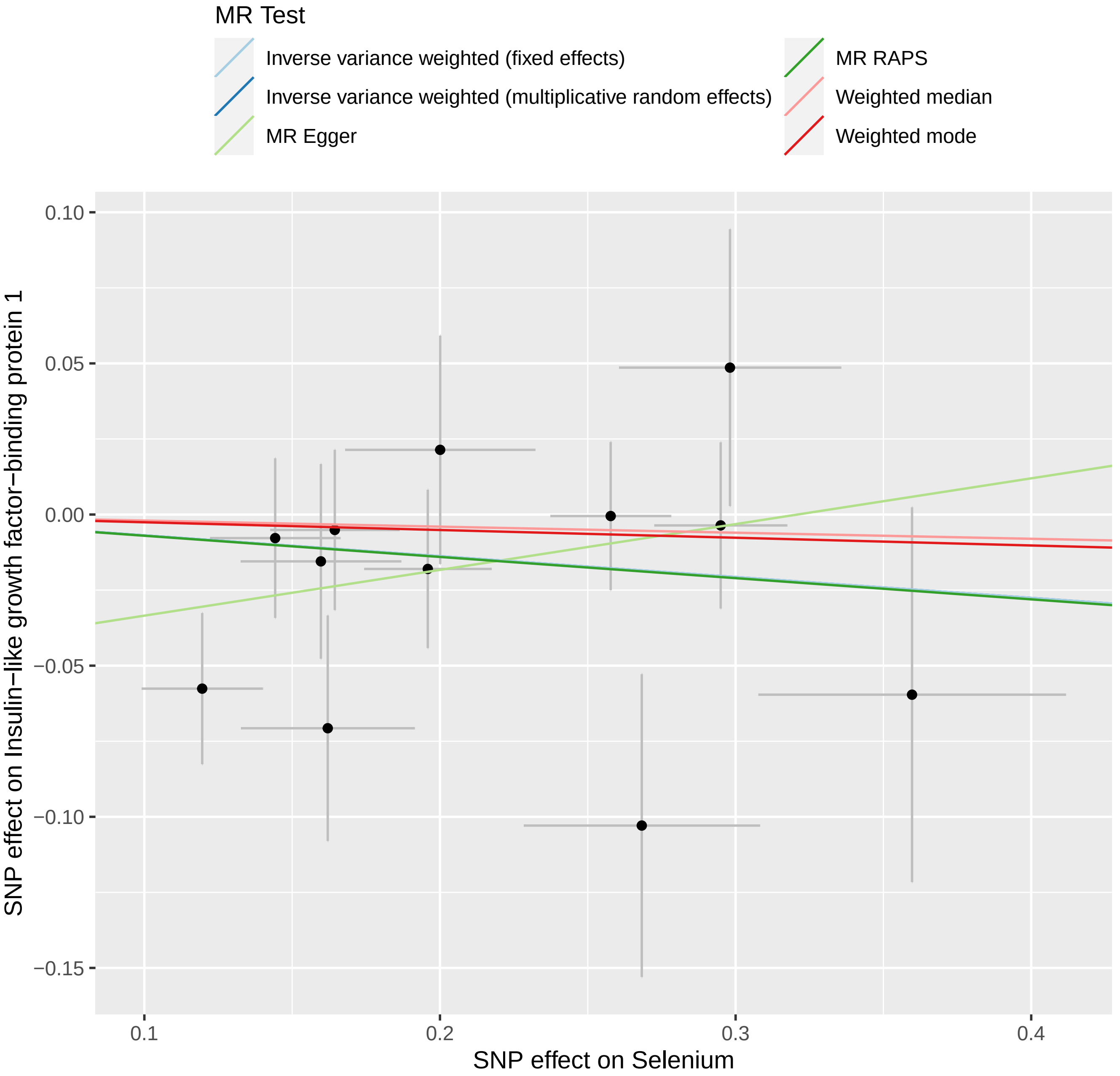

Batch 3−1448 : Selenium on Insulin−like growth factor−binding protein 2

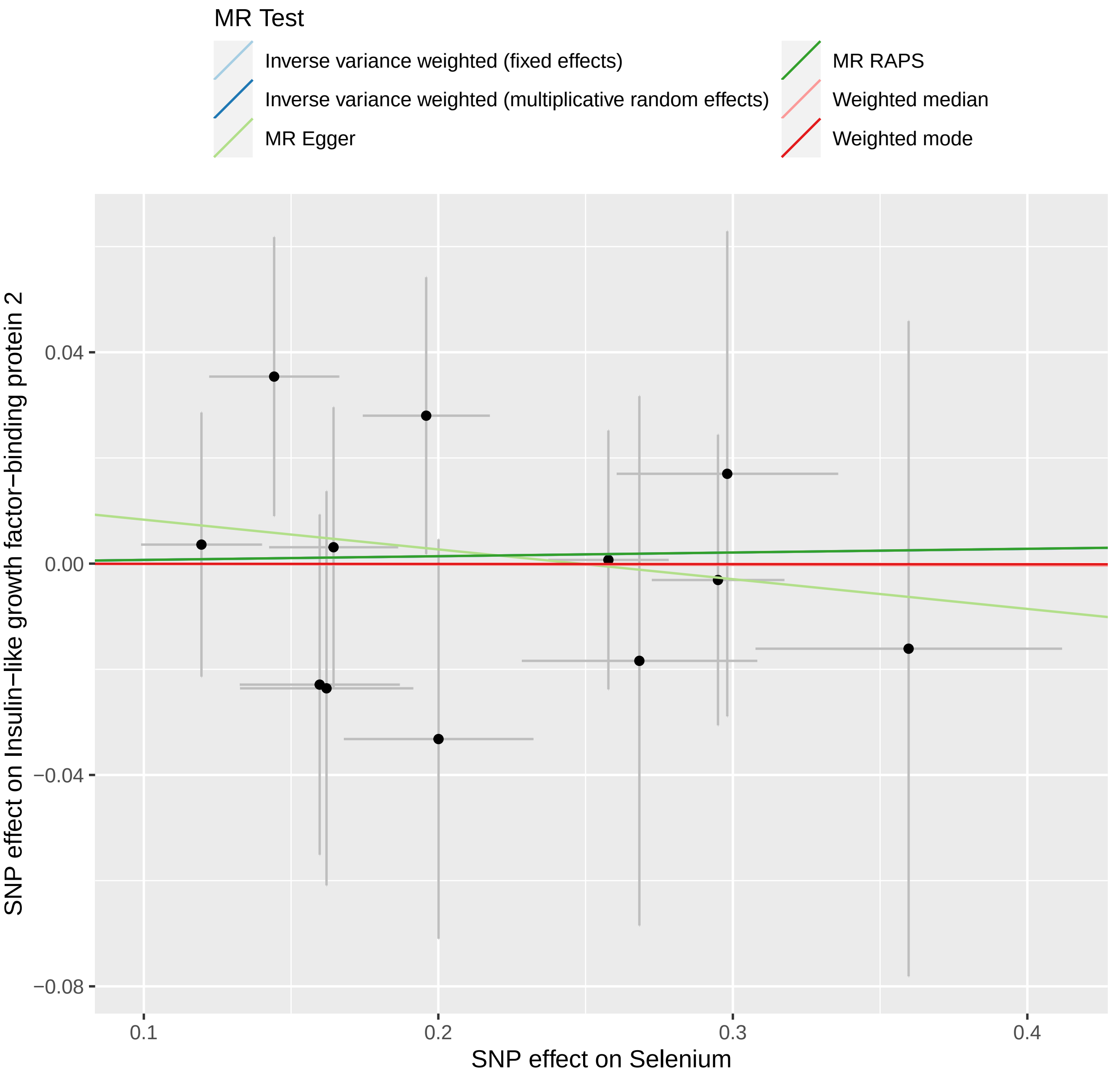

Batch 3−1449 : Selenium on Insulin−like growth factor−binding protein 3

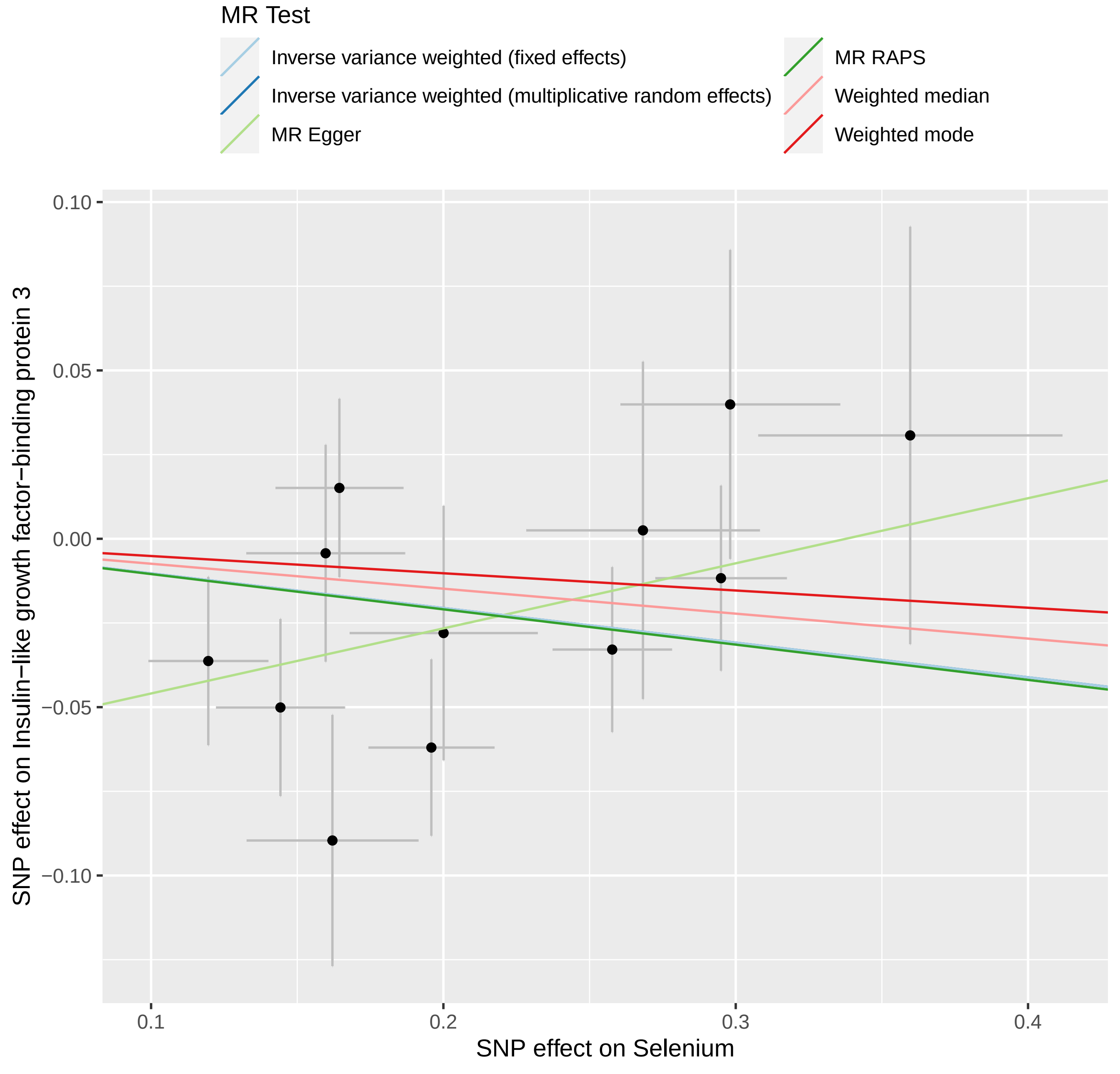

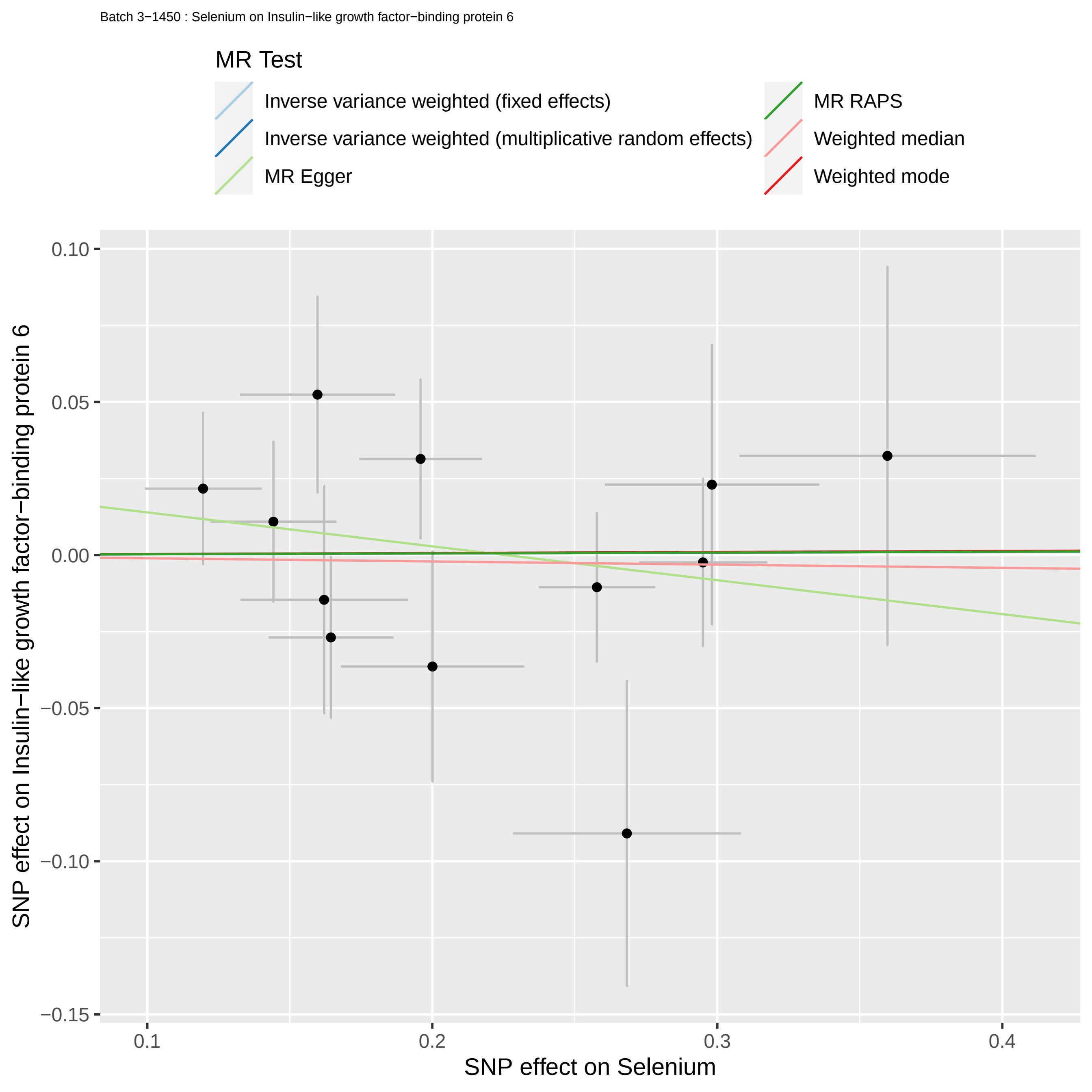

Batch 3−1450 : Selenium on Insulin−like growth factor−binding protein 6
MR Test
Inverse variance weighted (fixed effects)
Inverse variance weighted (multiplicative random effects)
MR Egger
MR RAPS
Weighted median
Weighted mode
0.10
0.05
0.00
−0.05
−0.10
−0.15
0.1
0.2
0.3
0.4
SNP effect on Insulin−like growth factor−binding protein 6
SNP effect on Selenium

Batch 3−1451 : Selenium on Insulin−like growth factor−binding protein 7

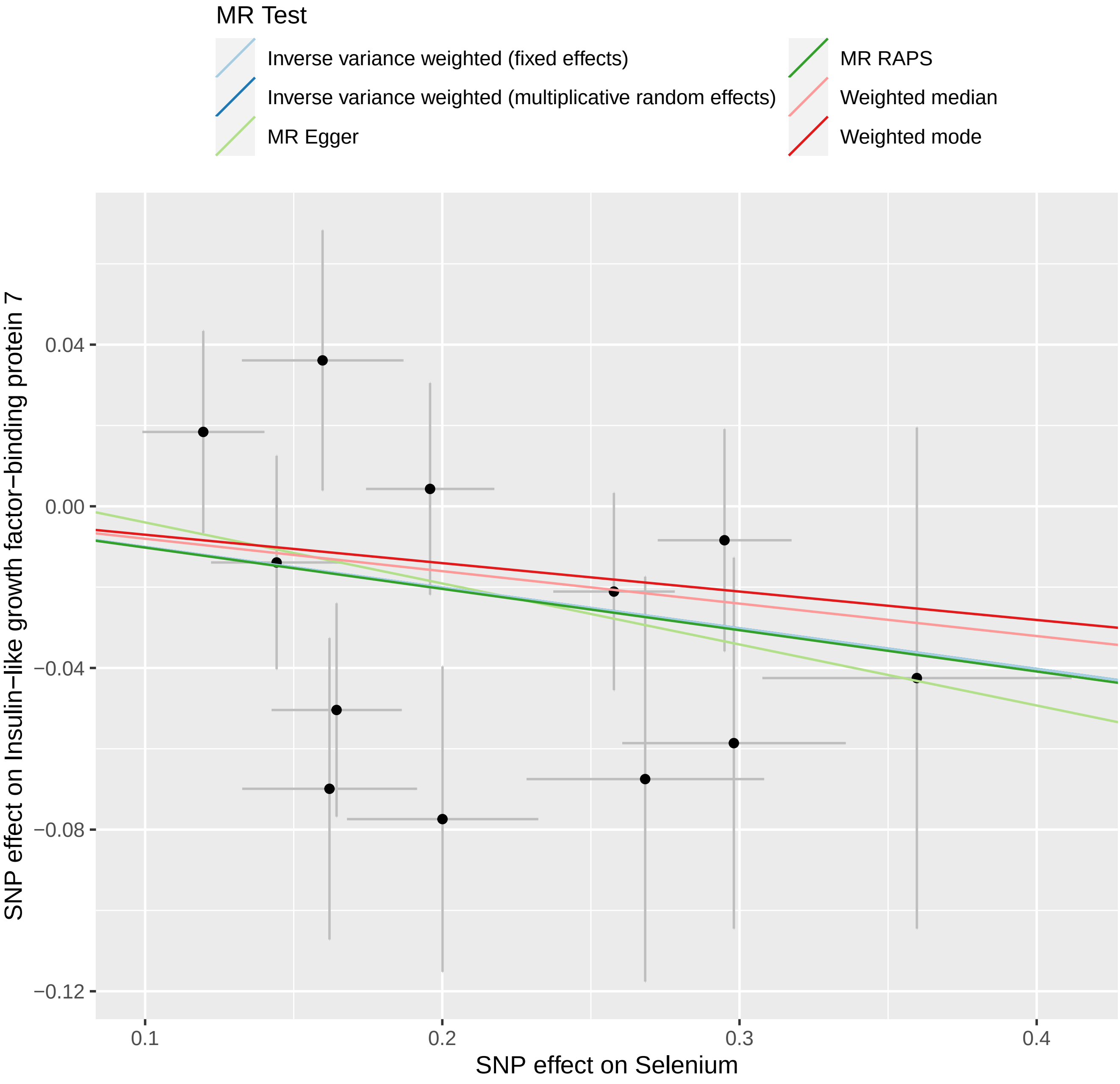

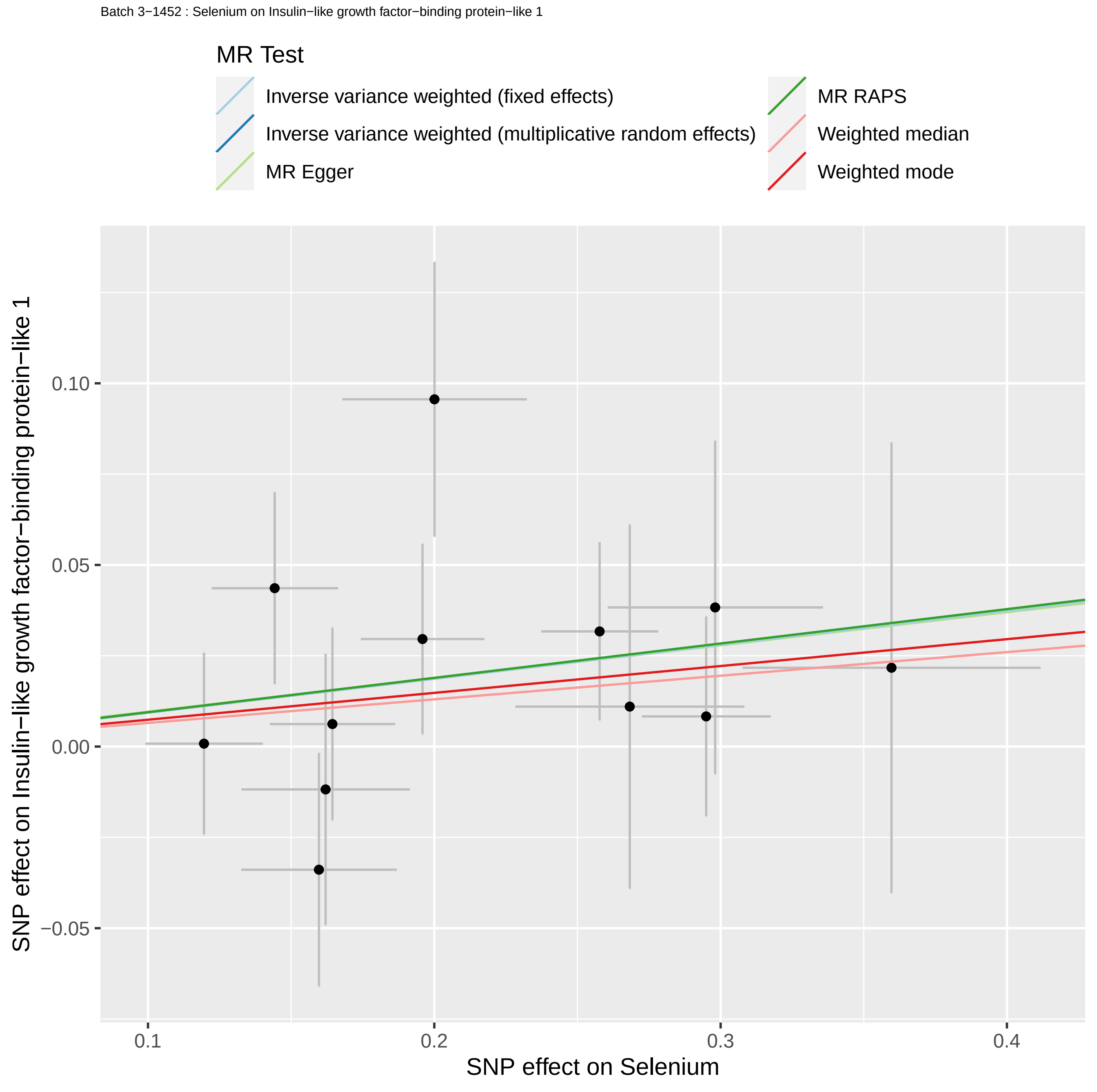
Batch 3−1452 : Selenium on Insulin−like growth factor−binding protein−like 1
MR Test
Inverse variance weighted (fixed effects)
Inverse variance weighted (multiplicative random effects)
MR Egger
MR RAPS
Weighted median
Weighted mode
SNP effect on Insulin−like growth factor−binding protein−like 1
0.10
0.05
0.00
−0.05
0.1
0.2
0.3
0.4
SNP effect on Selenium

Batch 3−1453 : Selenium on Insulin growth factor−like family member 3

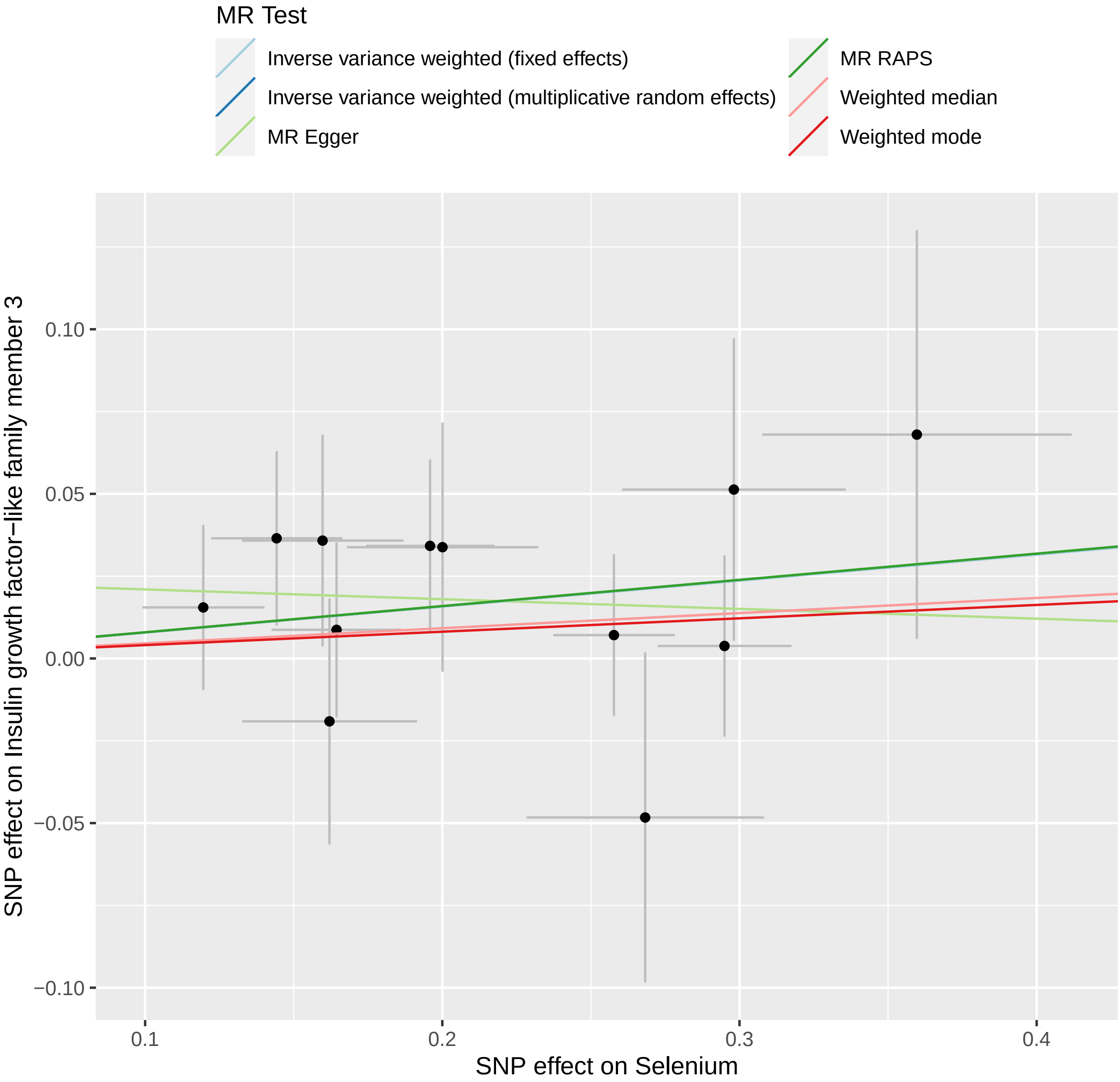

Batch 3−1454 : Selenium on Insulin growth factor−like family member 4

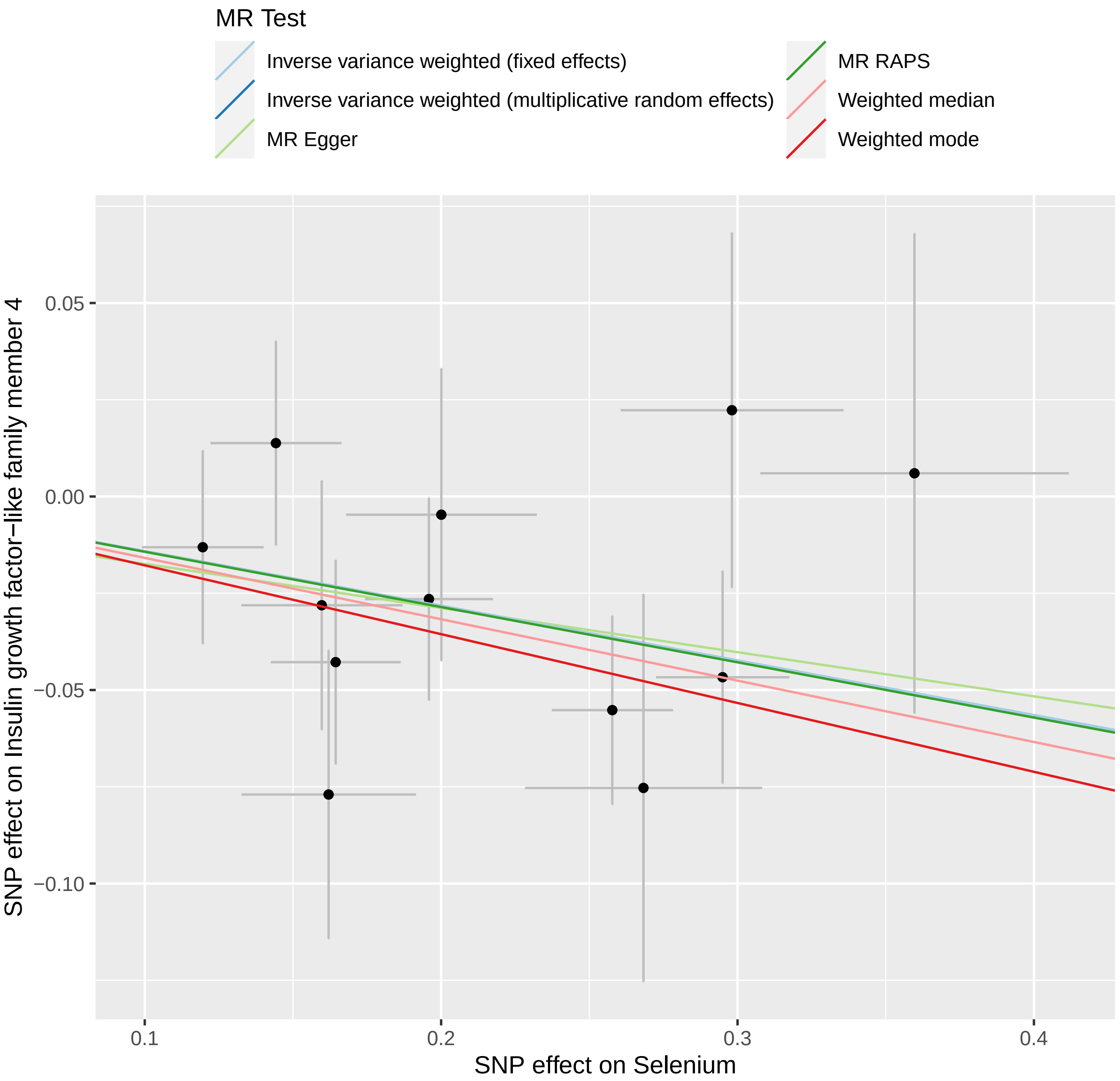

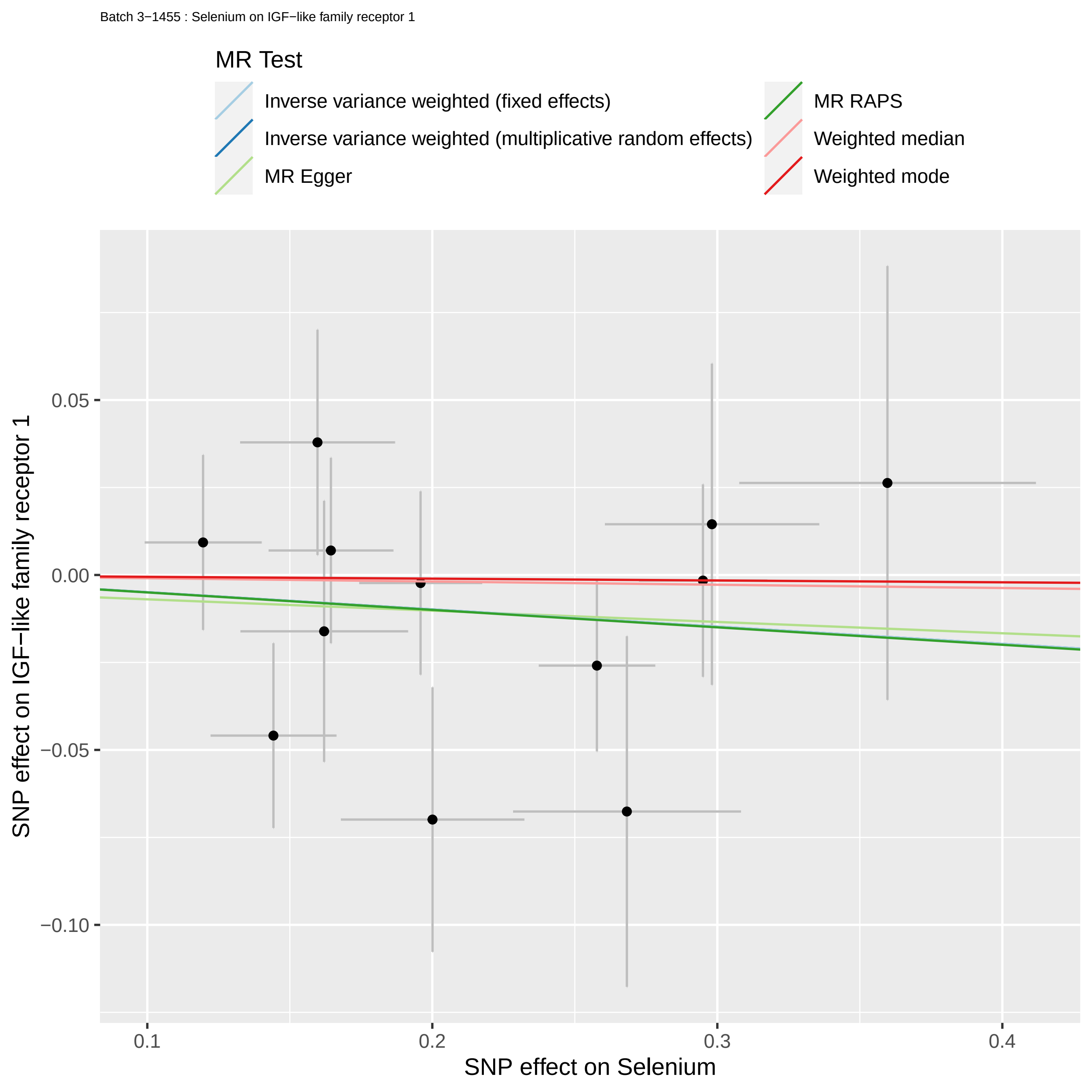

Batch 3−1455 : Selenium on IGF−like family receptor 1
MR Test
Inverse variance weighted (fixed effects)
Inverse variance weighted (multiplicative random effects)
MR Egger
MR RAPS
Weighted median
Weighted mode
0.05
0.00
−0.05
−0.10
0.1
0.2
0.3
0.4
SNP effect on IGF−like family receptor 1
SNP effect on Selenium

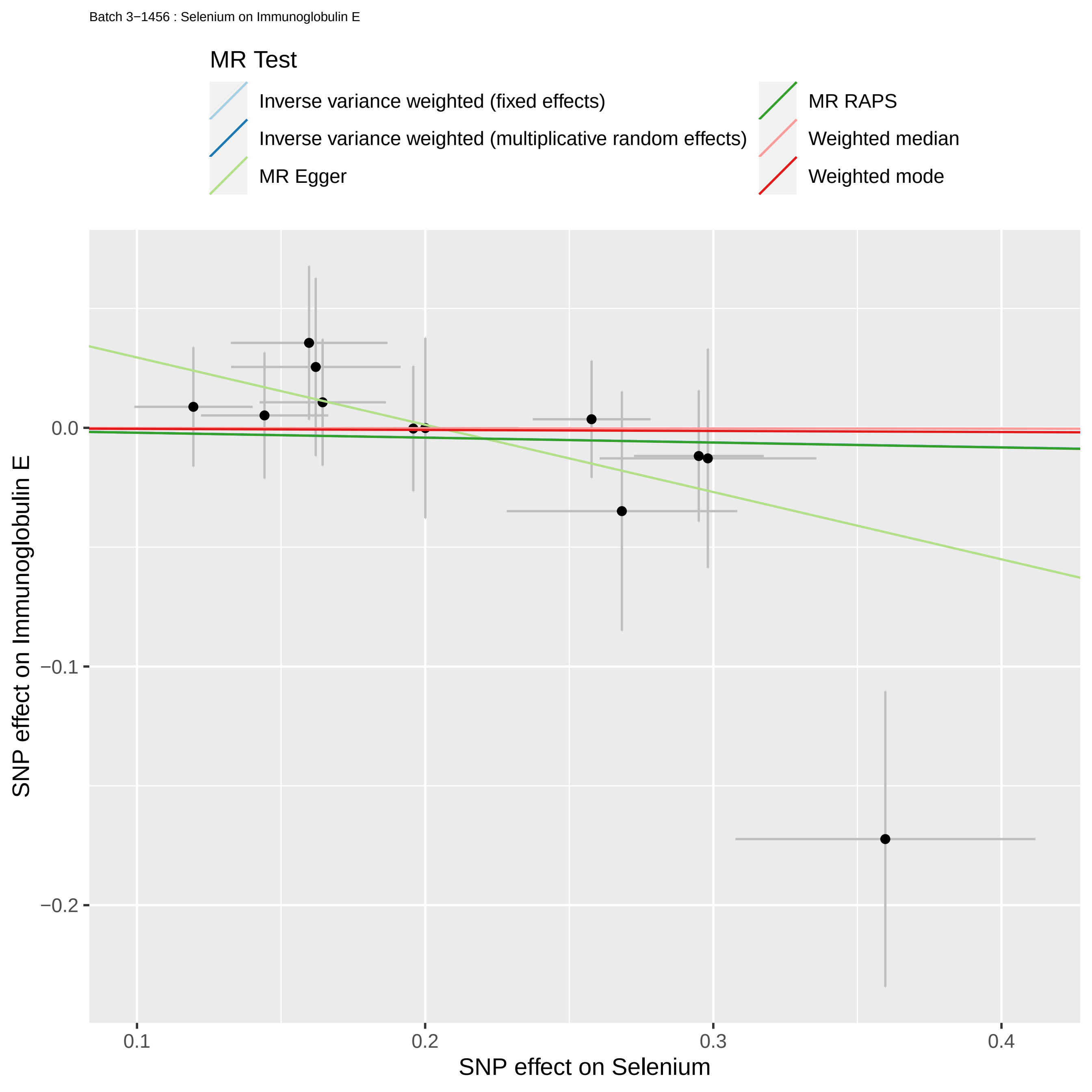

Batch 3−1456 : Selenium on Immunoglobulin E
MR Test
Inverse variance weighted (fixed effects)
Inverse variance weighted (multiplicative random effects)
MR Egger
MR RAPS
Weighted median
Weighted mode
0.0
−0.1
−0.2
SNP effect on Immunoglobulin E
0.1
0.2
0.3
0.4
SNP effect on Selenium

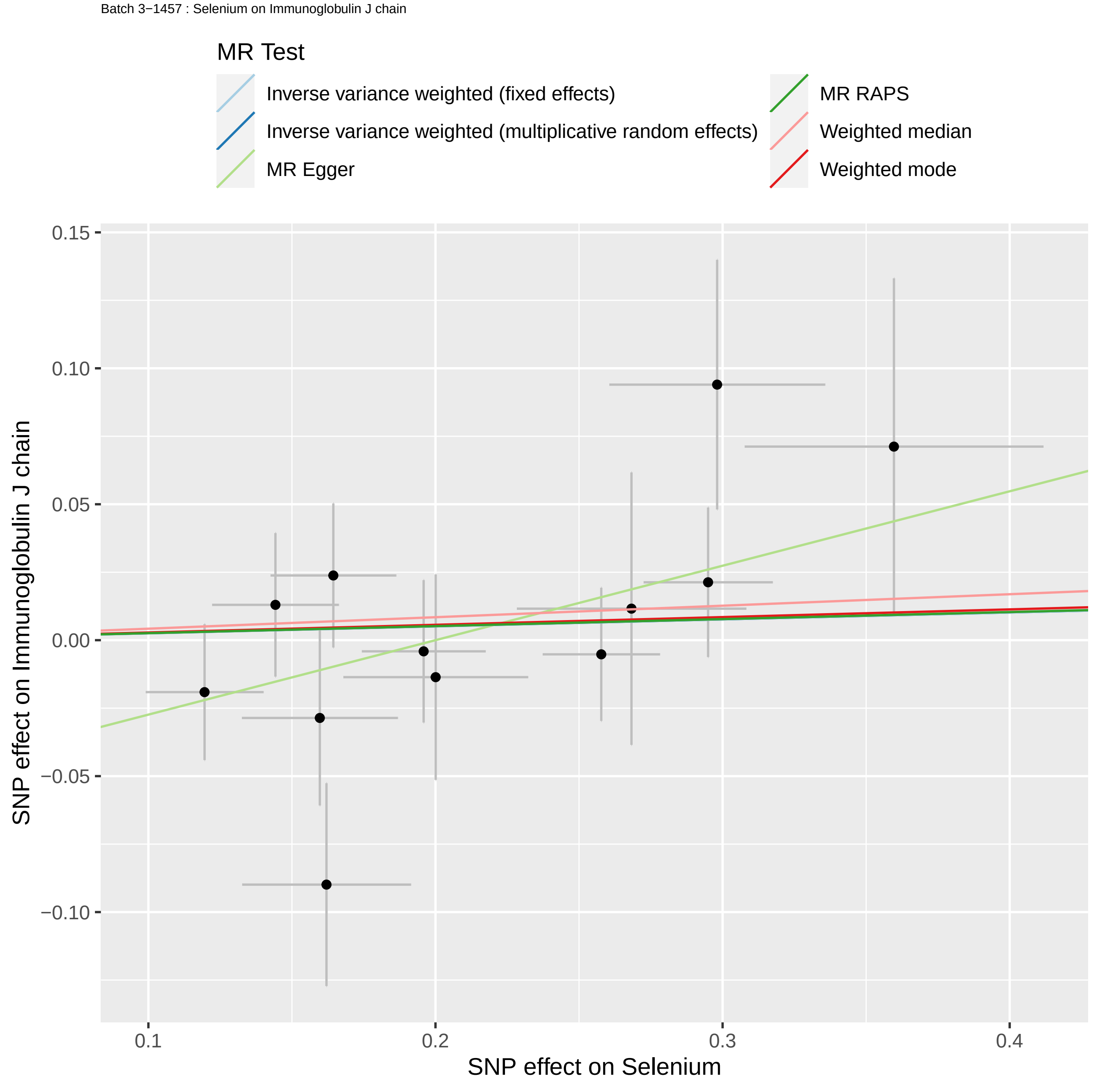
Batch 3−1457 : Selenium on Immunoglobulin J chain
MR Test
Inverse variance weighted (fixed effects)
Inverse variance weighted (multiplicative random effects)
MR Egger
MR RAPS
Weighted median
Weighted mode
0.15
0.10
0.05
0.00
−0.05
−0.10
0.1
0.2
0.3
0.4
SNP effect on Immunoglobulin J chain
SNP effect on Selenium

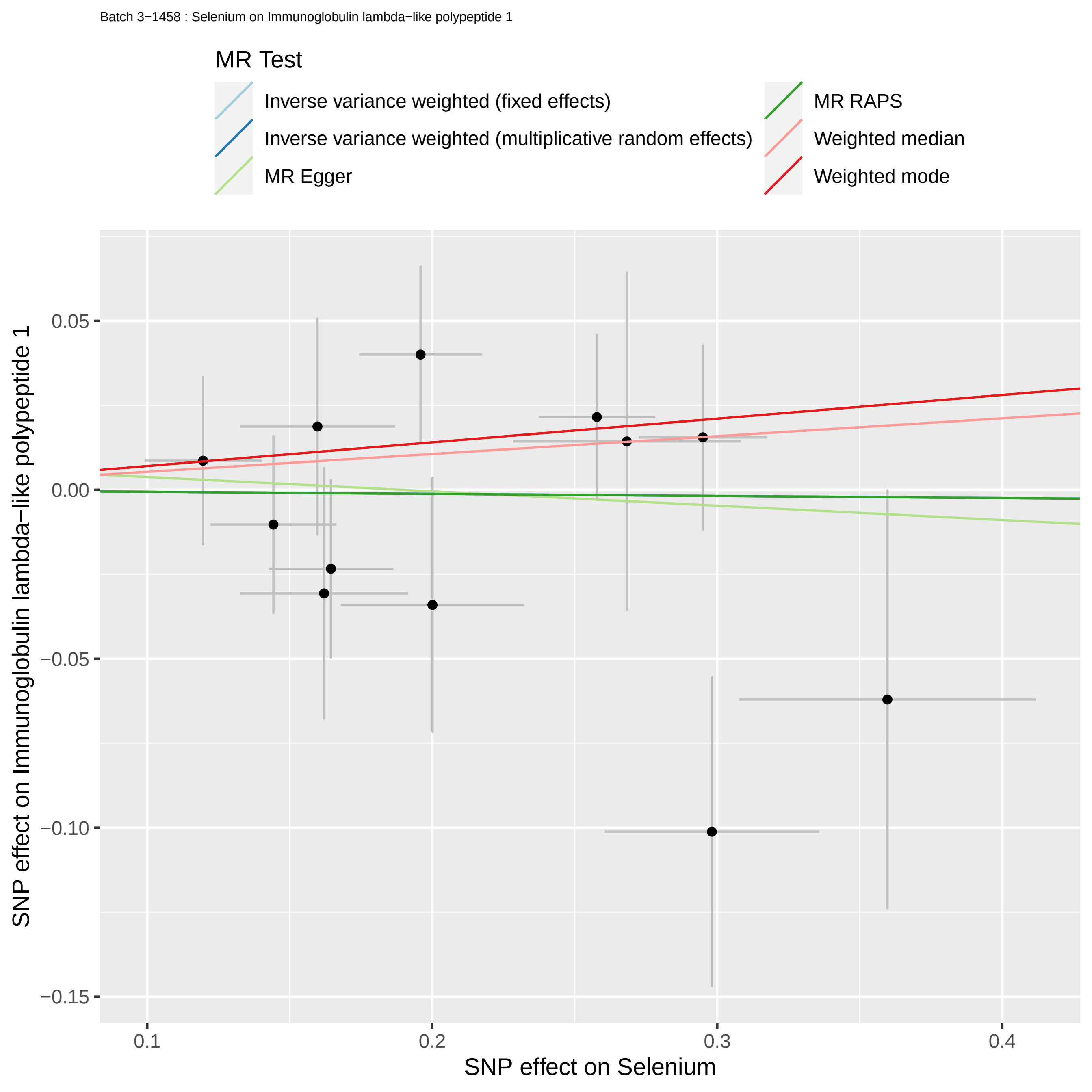
Batch 3−1458 : Selenium on Immunoglobulin lambda−like polypeptide 1
MR Test
Inverse variance weighted (fixed effects)
Inverse variance weighted (multiplicative random effects)
MR Egger
MR RAPS
Weighted median
Weighted mode
SNP effect on Immunoglobulin lambda−like polypeptide 1
0.05
0.00
−0.05
−0.10
−0.15
0.1
0.2
0.3
0.4
SNP effect on Selenium

Batch 3−1459 : Selenium on IgLON family member 5

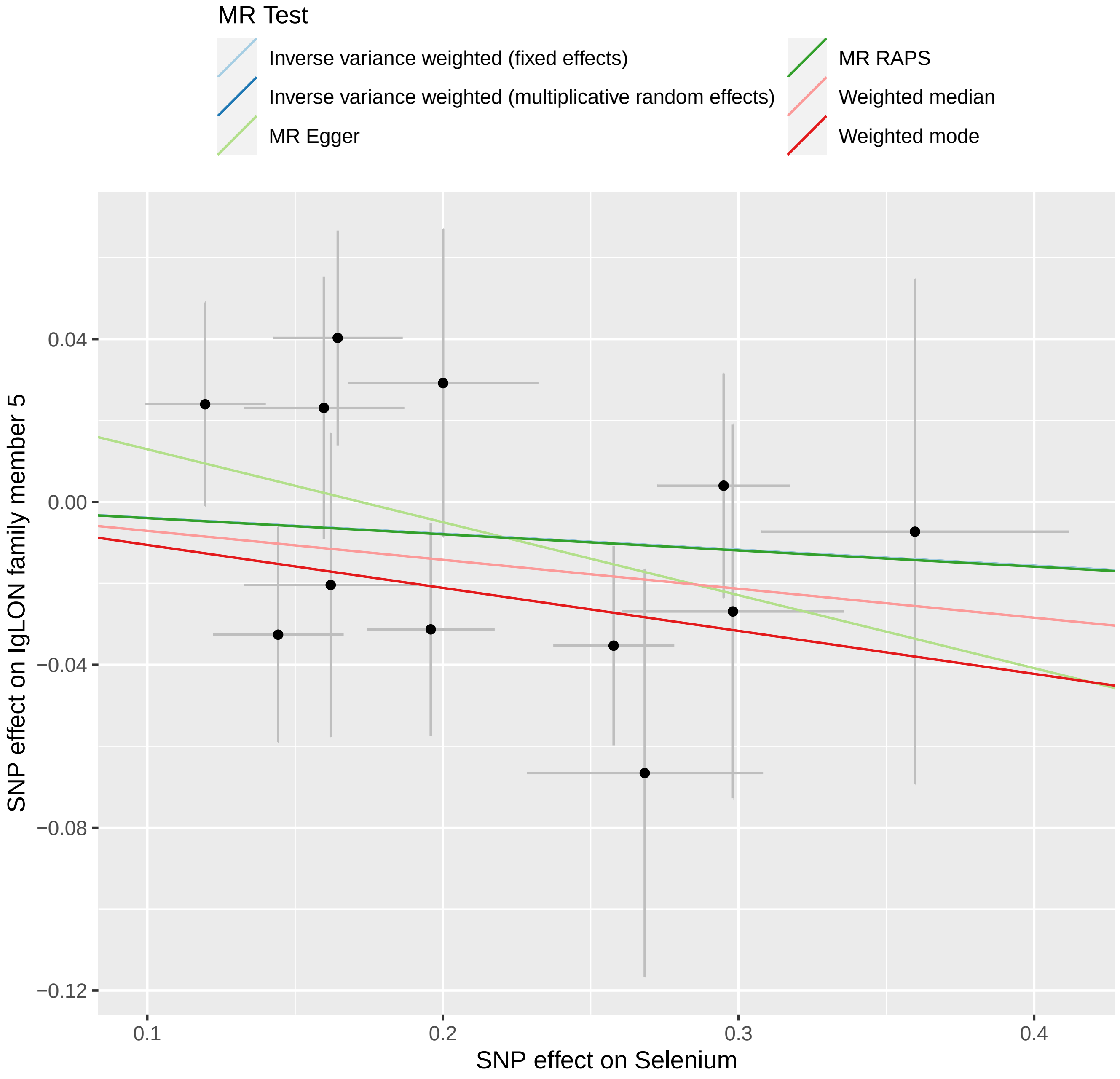

Batch 3−1460 : Selenium on Immunoglobulin superfamily member 11

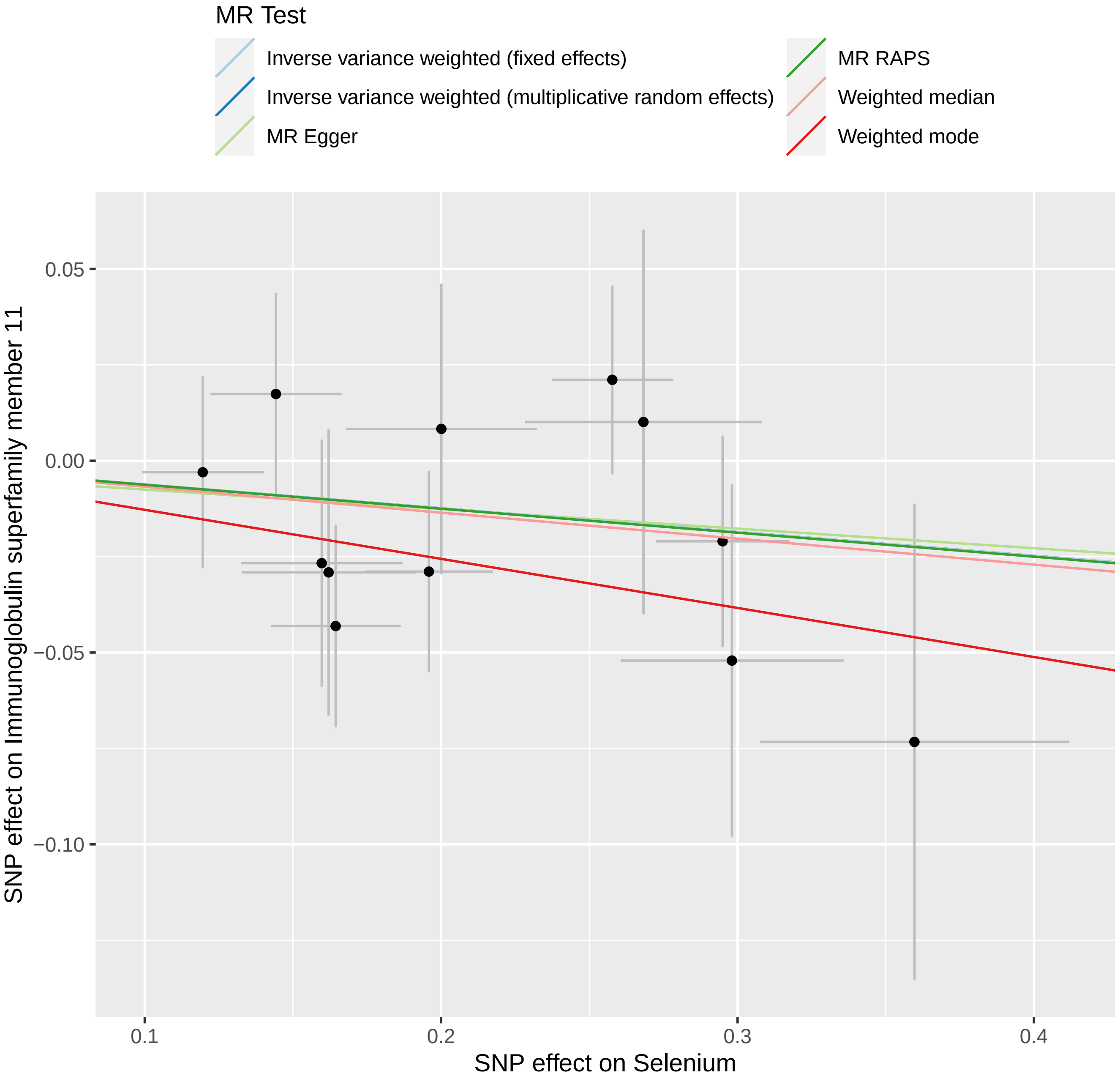

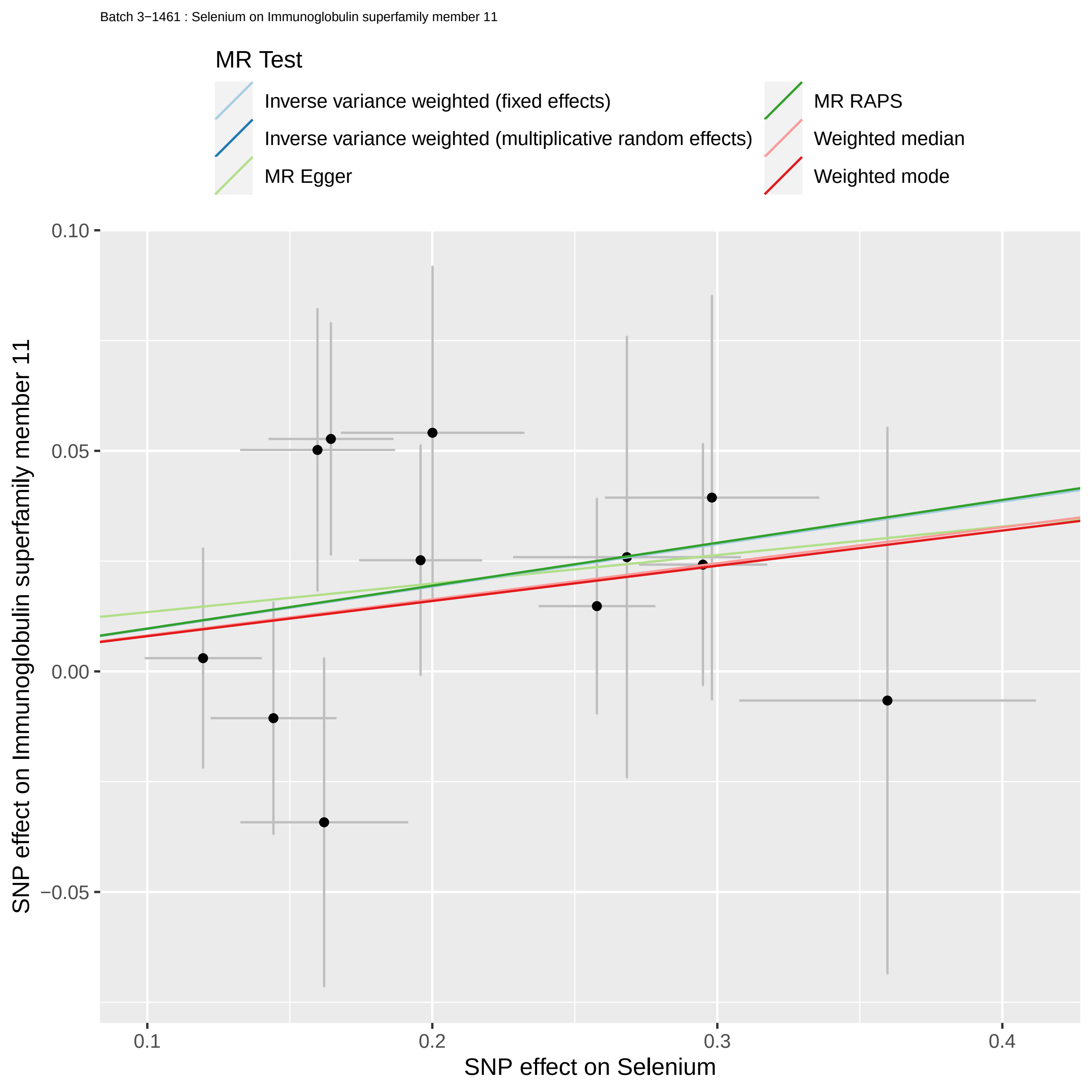

Batch 3−1461 : Selenium on Immunoglobulin superfamily member 11
MR Test
Inverse variance weighted (fixed effects)
Inverse variance weighted (multiplicative random effects)
MR Egger
MR RAPS
Weighted median
Weighted mode
0.10
0.05
0.00
−0.05
0.1
0.2
0.3
0.4
SNP effect on Immunoglobulin superfamily member 11
SNP effect on Selenium

Batch 3−1462 : Selenium on Immunoglobulin superfamily member 8

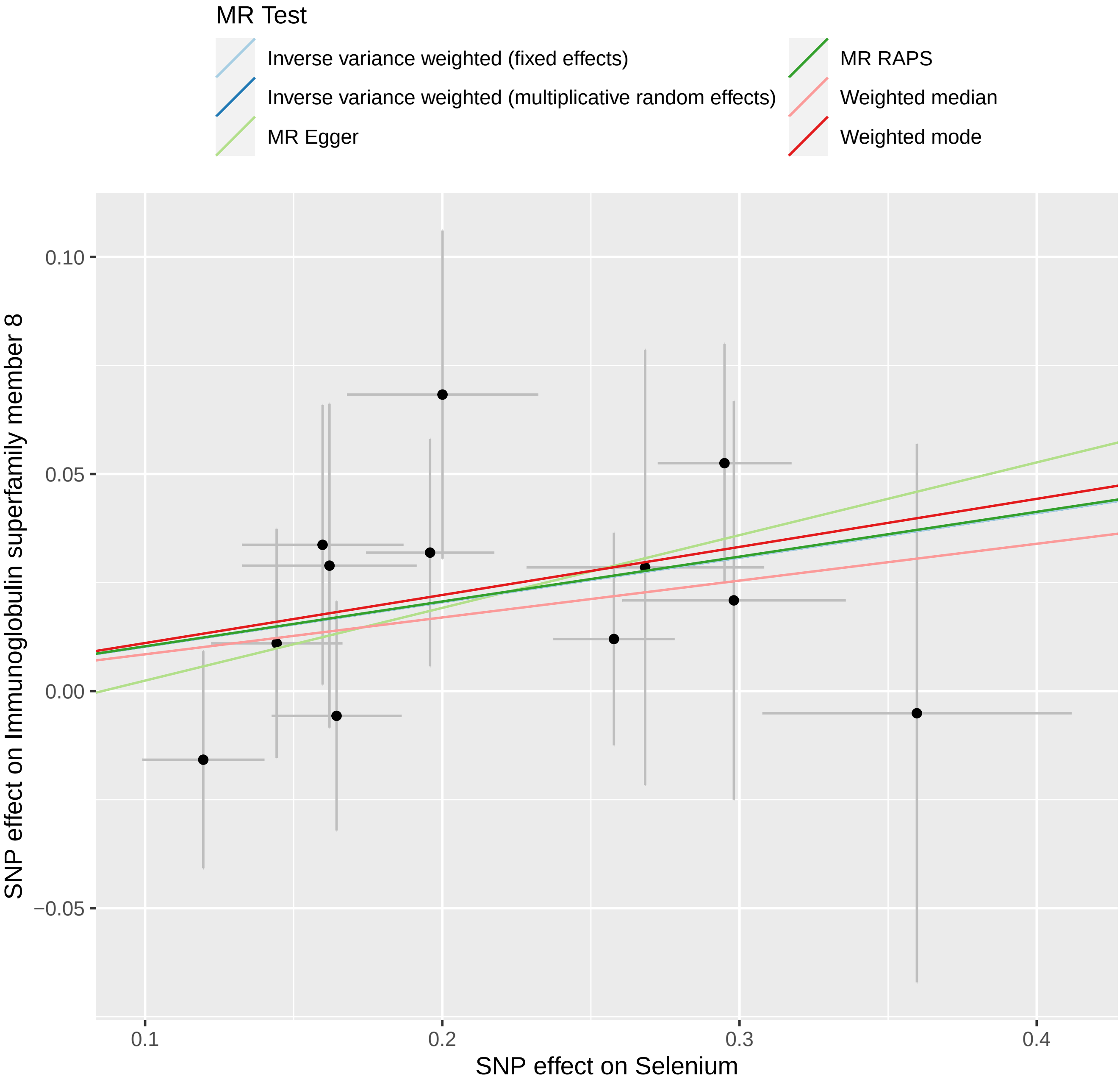

Batch 3−1463 : Selenium on Protein turtle homolog A

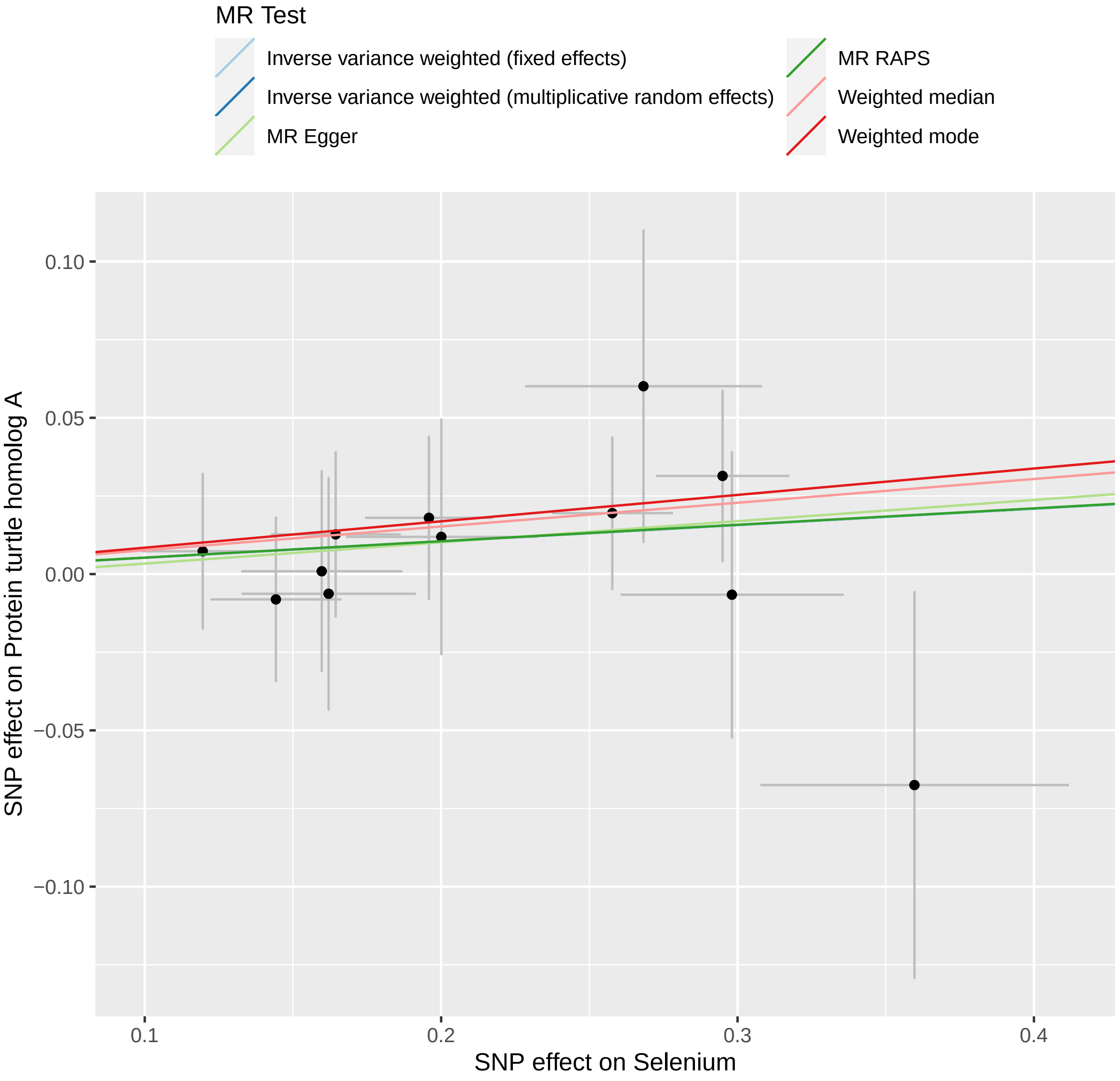

Batch 3−1464 : Selenium on Interleukin−10

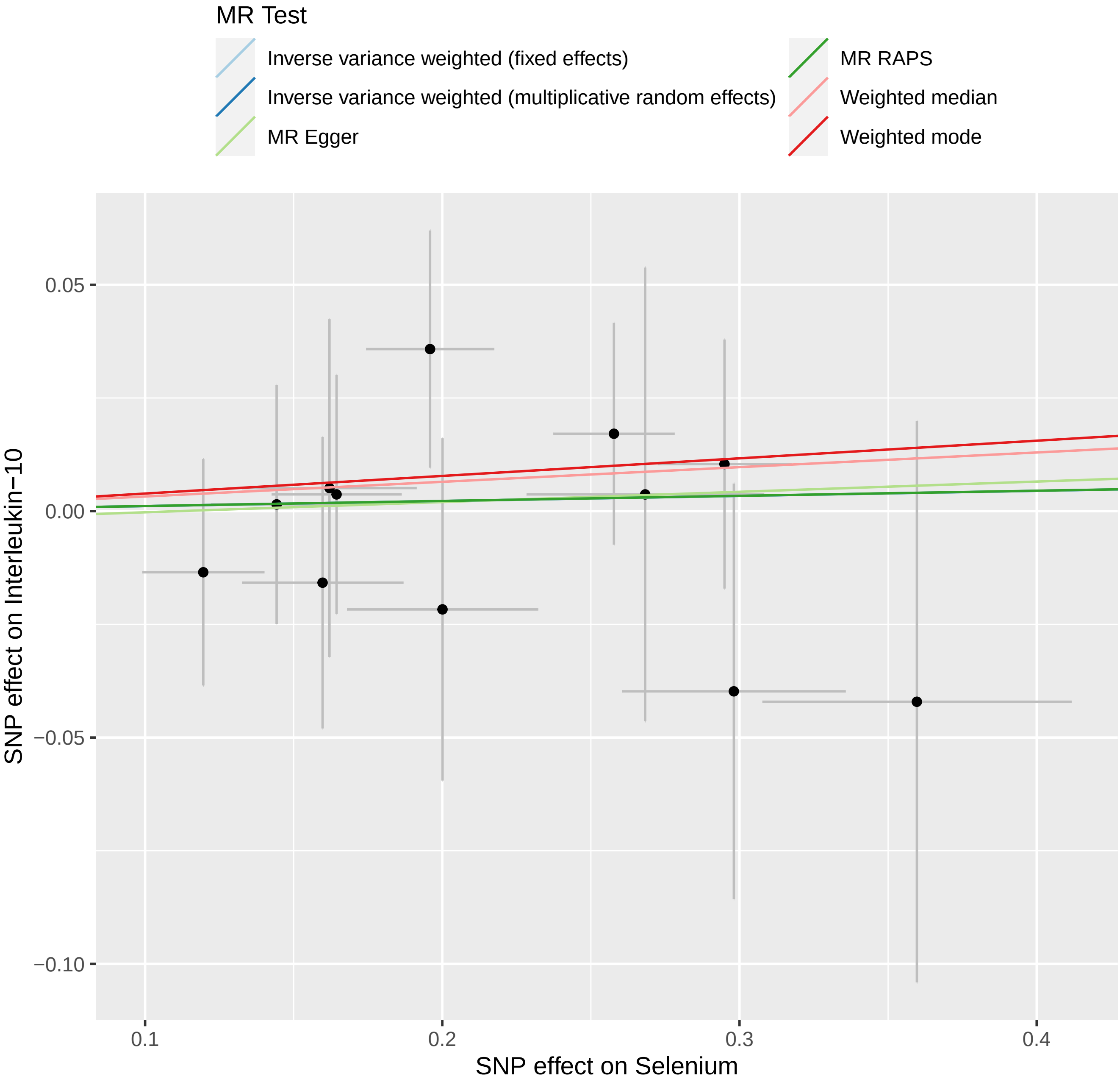

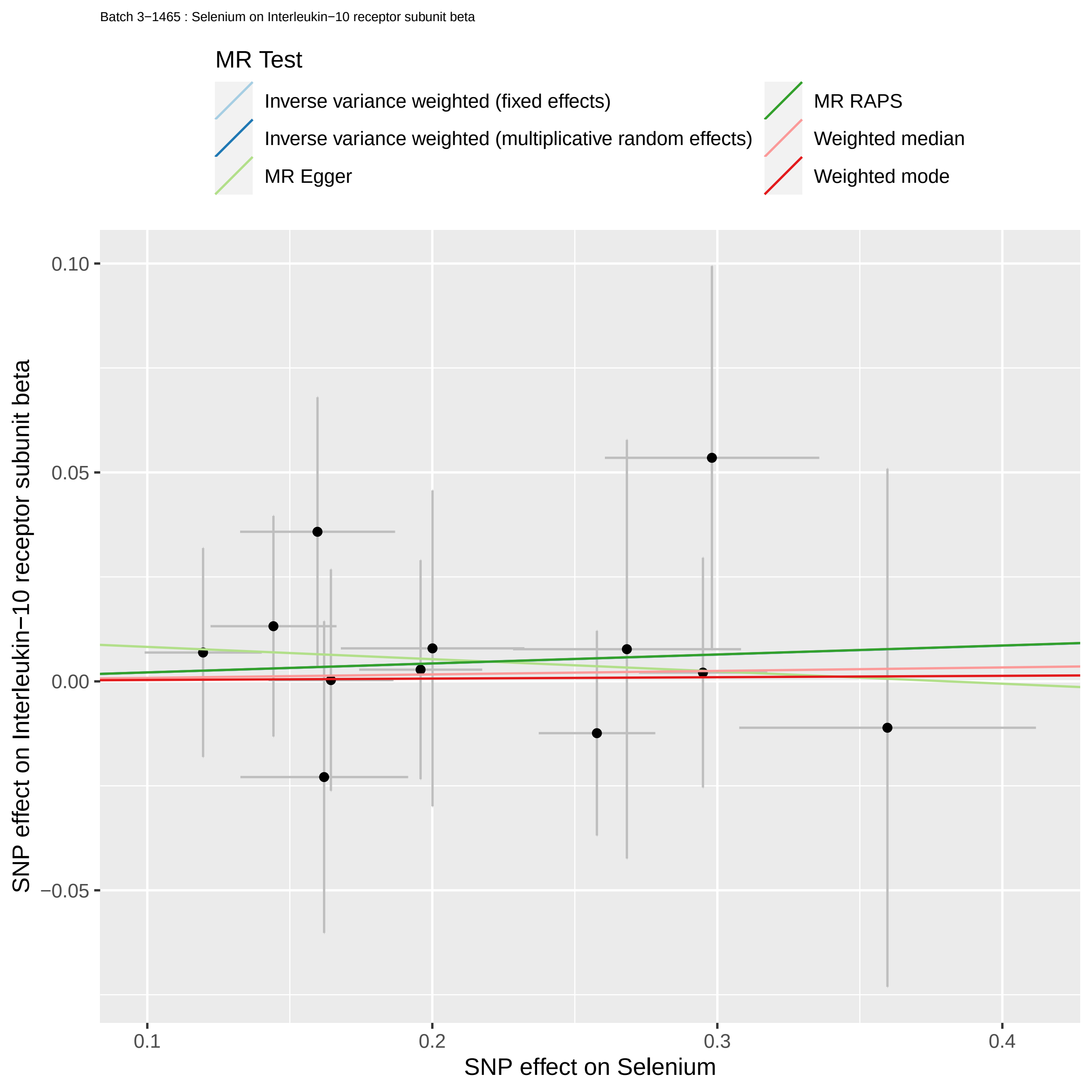

Batch 3−1465 : Selenium on Interleukin−10 receptor subunit beta
MR Test
Inverse variance weighted (fixed effects)
Inverse variance weighted (multiplicative random effects)
MR Egger
MR RAPS
Weighted median
Weighted mode
0.10
0.05
0.00
−0.05
0.1
0.2
0.3
0.4
SNP effect on Interleukin−10 receptor subunit beta
SNP effect on Selenium

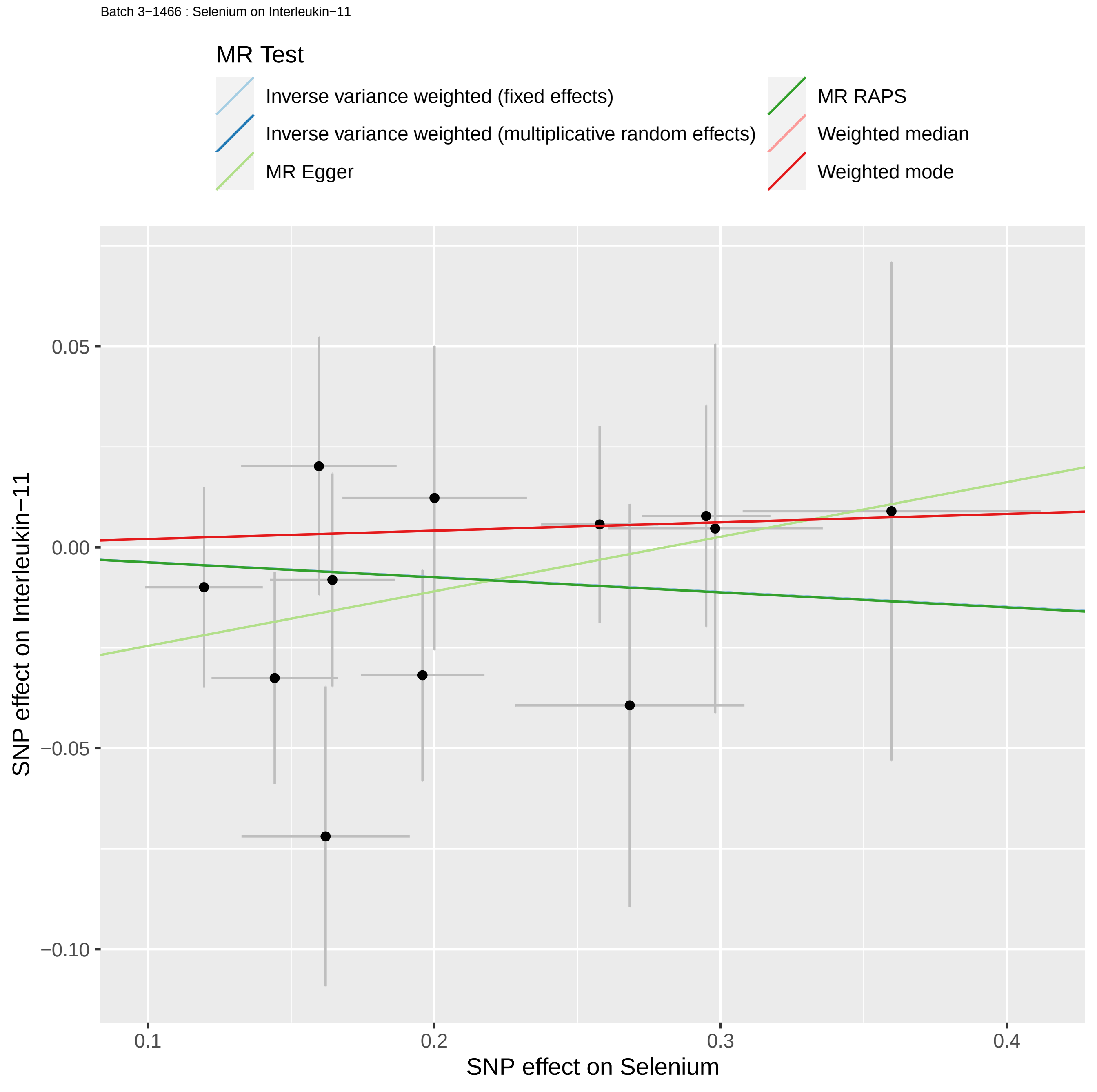

Batch 3−1466 : Selenium on Interleukin−11
MR Test
Inverse variance weighted (fixed effects)
Inverse variance weighted (multiplicative random effects)
MR Egger
MR RAPS
Weighted median
Weighted mode
0.05
0.00
−0.05
−0.10
0.1
0.2
0.3
0.4
SNP effect on Interleukin−11
SNP effect on Selenium

Batch 3−1467 : Selenium on Interleukin−11 receptor subunit alpha

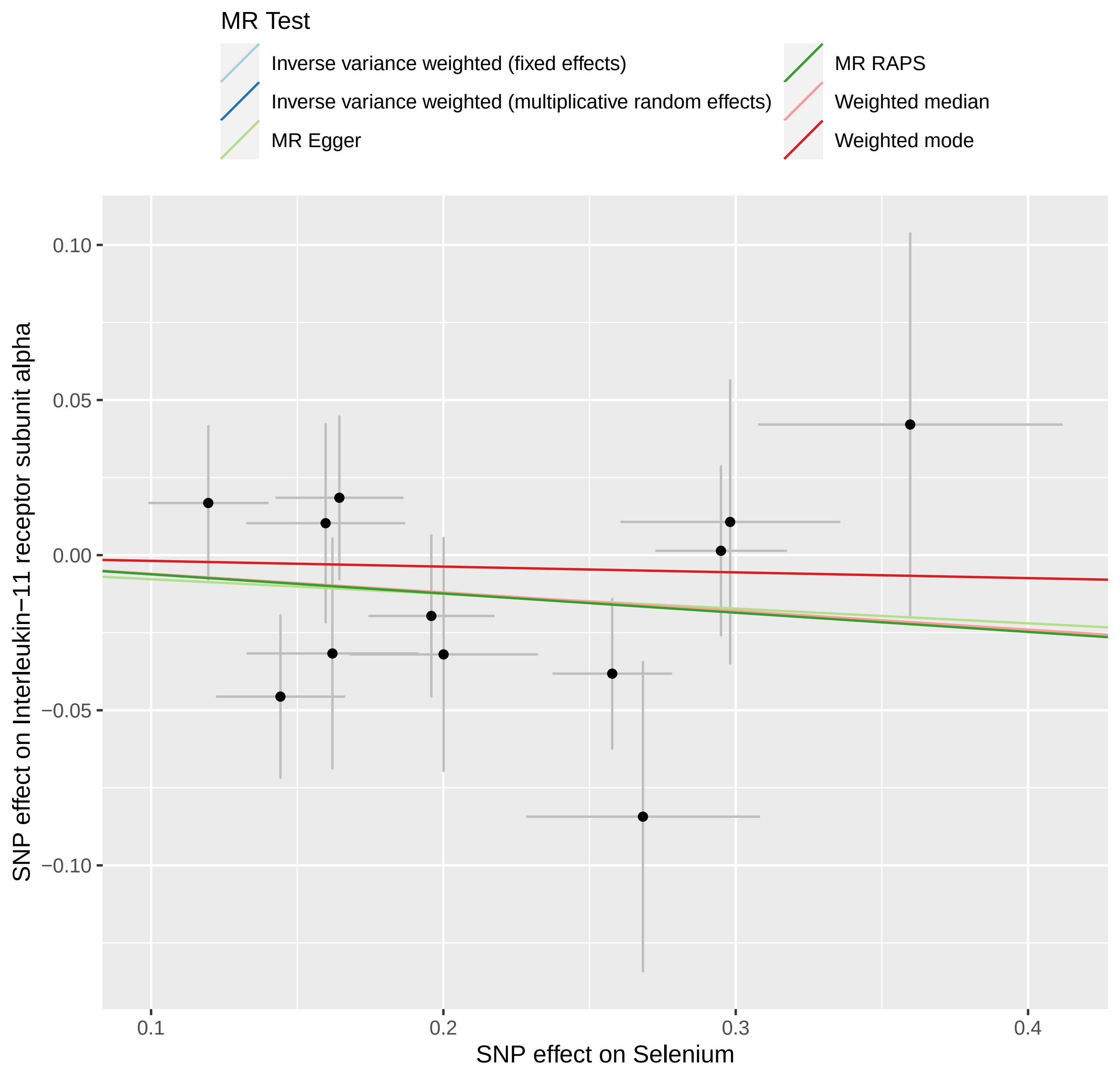

Batch 3−1468 : Selenium on Interleukin−12

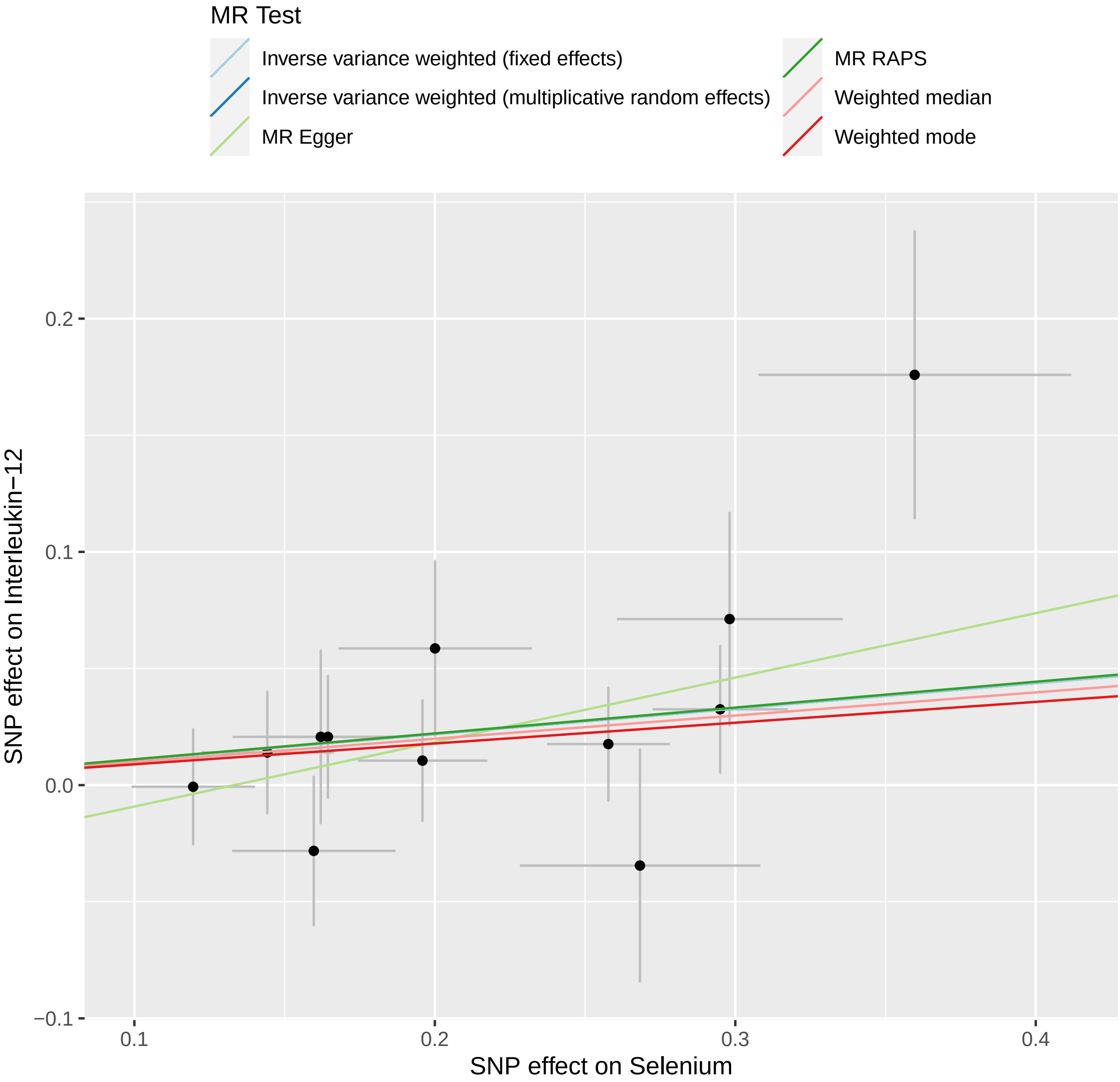

Batch 3−1469 : Selenium on Interleukin−12

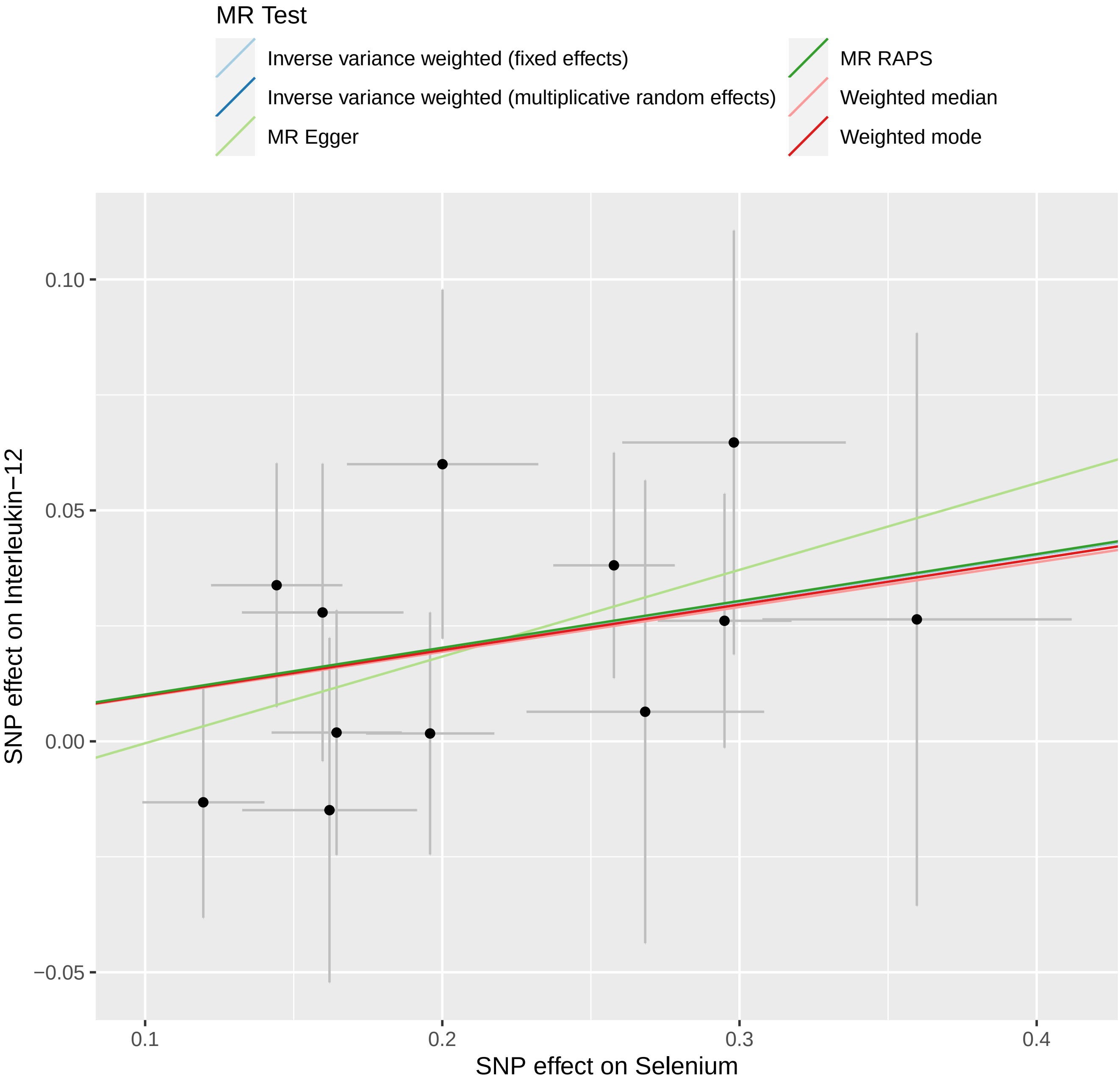

Batch 3−1470 : Selenium on Interleukin−12

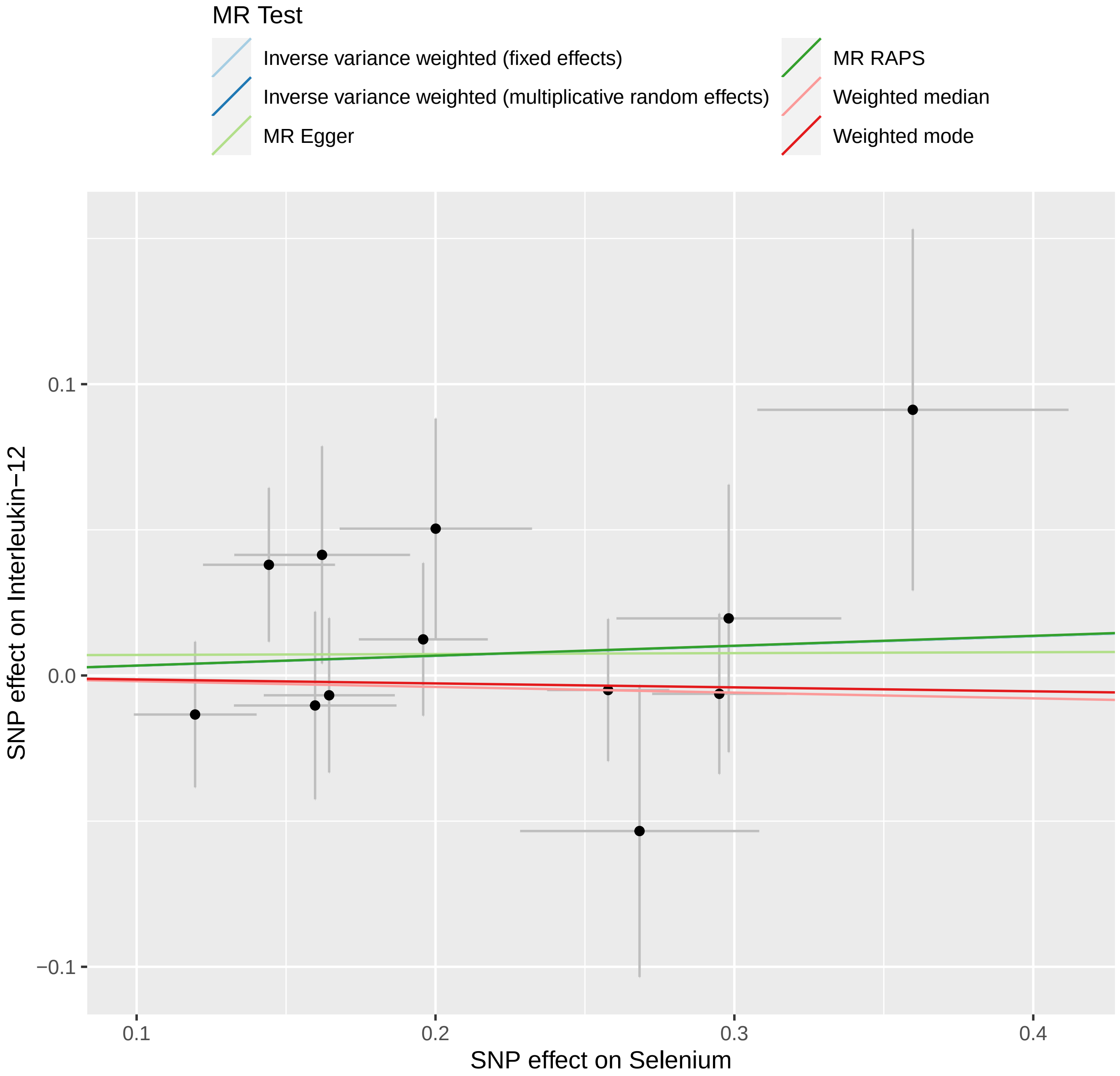

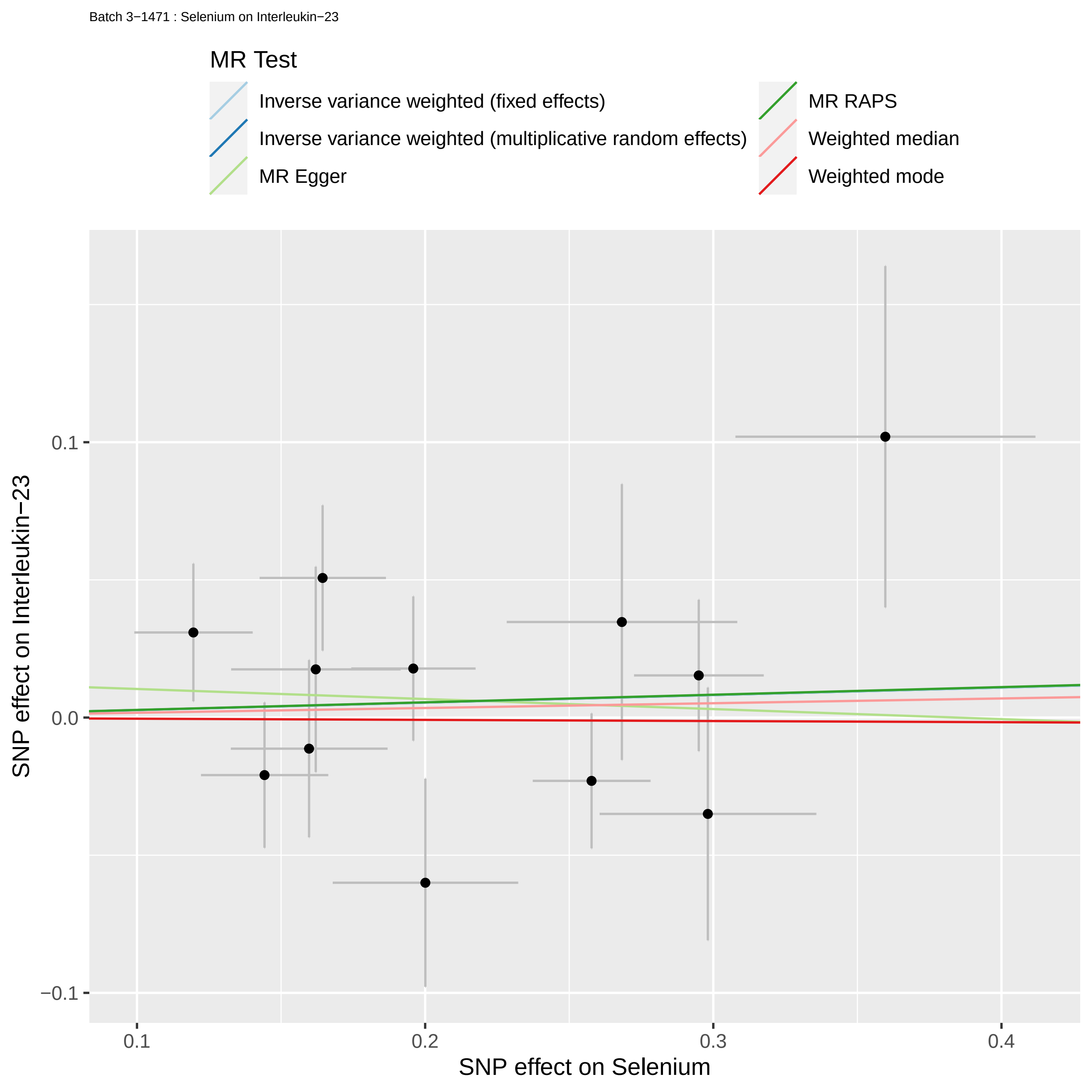

Batch 3−1471 : Selenium on Interleukin−23
MR Test
Inverse variance weighted (fixed effects)
Inverse variance weighted (multiplicative random effects)
MR Egger
MR RAPS
Weighted median
Weighted mode
0.1
0.0
−0.1
0.1
0.2
0.3
0.4
SNP effect on Interleukin−23
SNP effect on Selenium

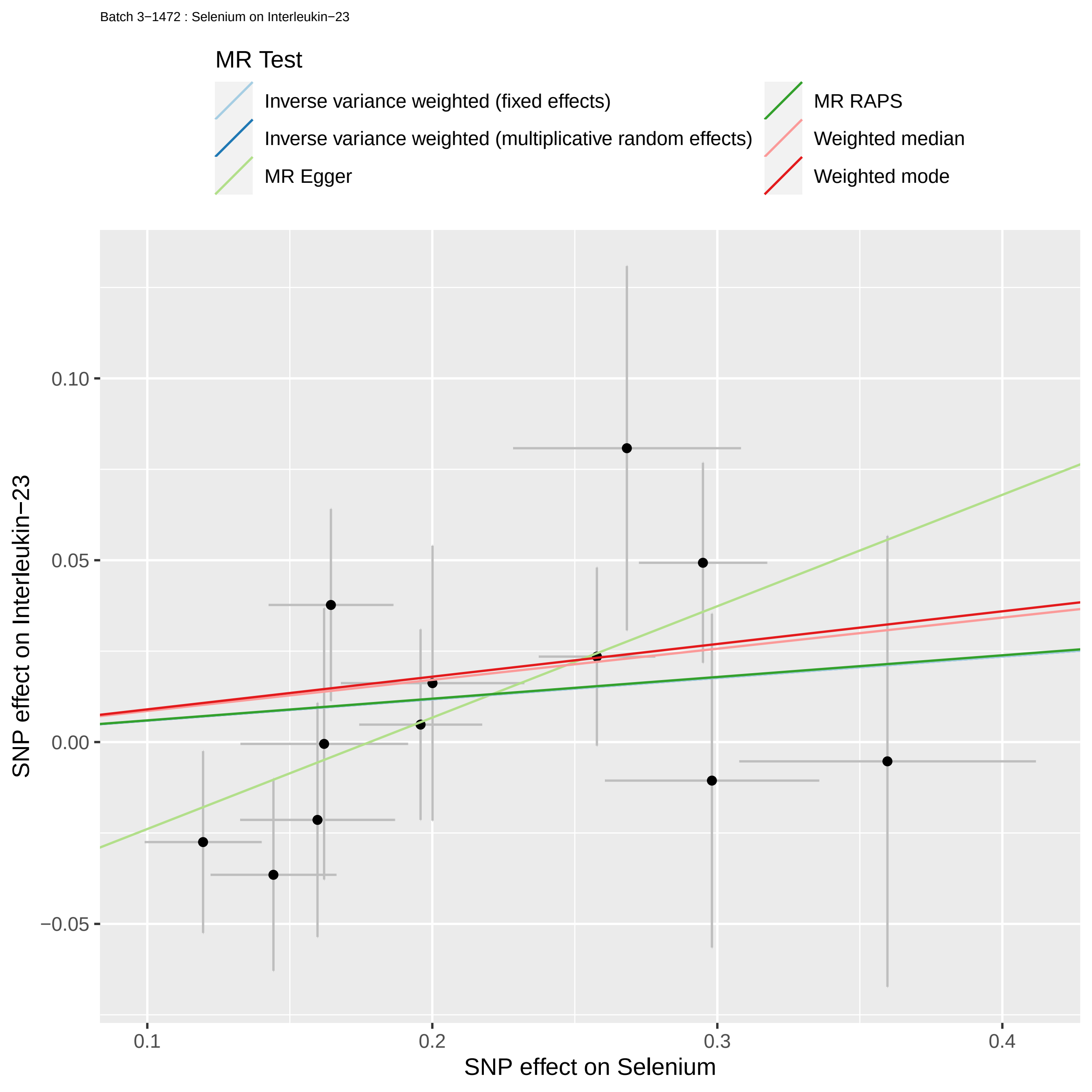

Batch 3−1472 : Selenium on Interleukin−23
MR Test
Inverse variance weighted (fixed effects)
Inverse variance weighted (multiplicative random effects)
MR Egger
MR RAPS
Weighted median
Weighted mode
0.10
0.05
0.00
−0.05
0.1
0.2
0.3
0.4
SNP effect on Interleukin−23
SNP effect on Selenium

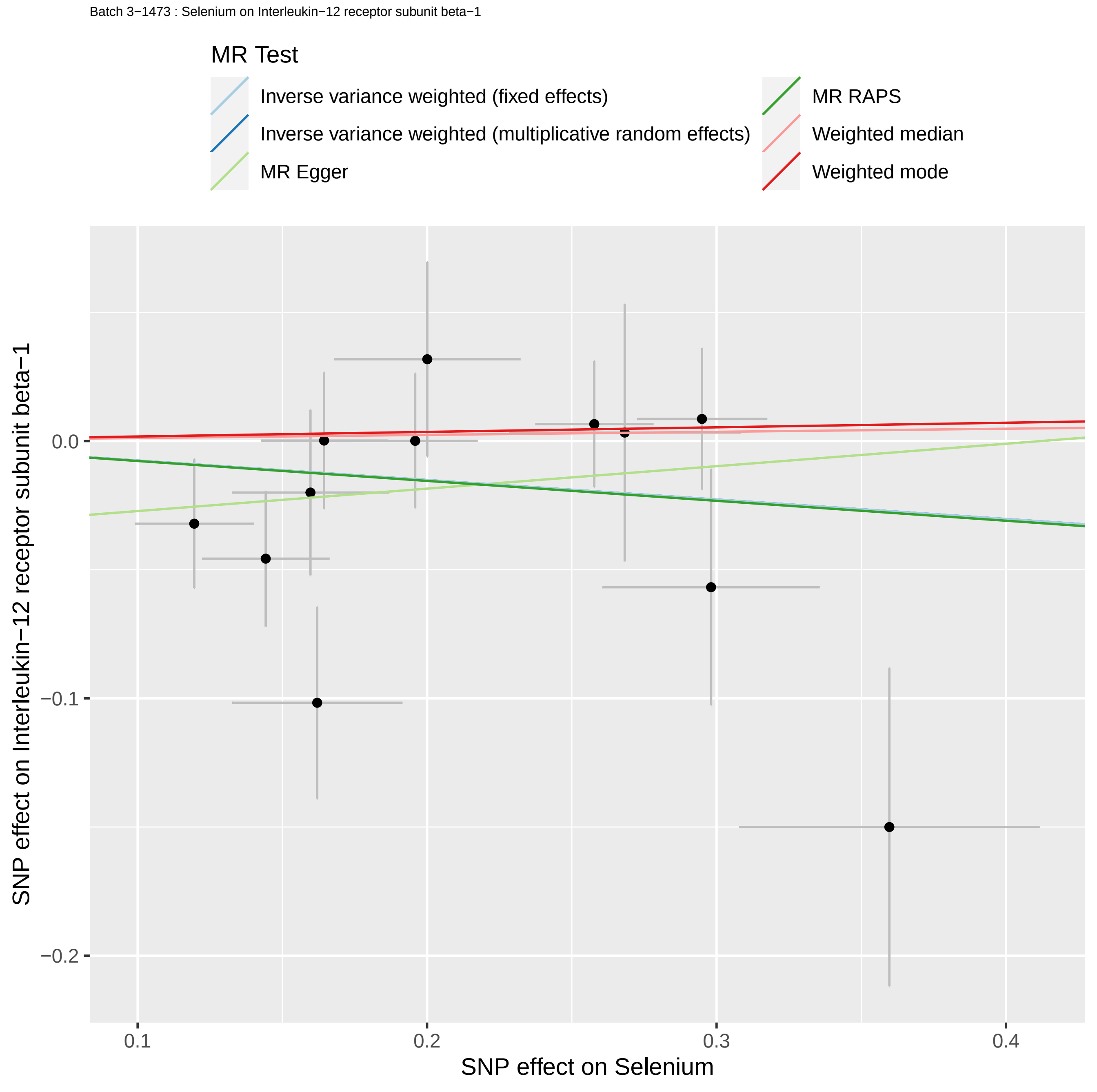
Batch 3−1473 : Selenium on Interleukin−12 receptor subunit beta−1
MR Test
Inverse variance weighted (fixed effects)
Inverse variance weighted (multiplicative random effects)
MR Egger
MR RAPS
Weighted median
Weighted mode
0.0
−0.1
−0.2
0.1
0.2
0.3
0.4
SNP effect on Interleukin−12 receptor subunit beta−1
SNP effect on Selenium

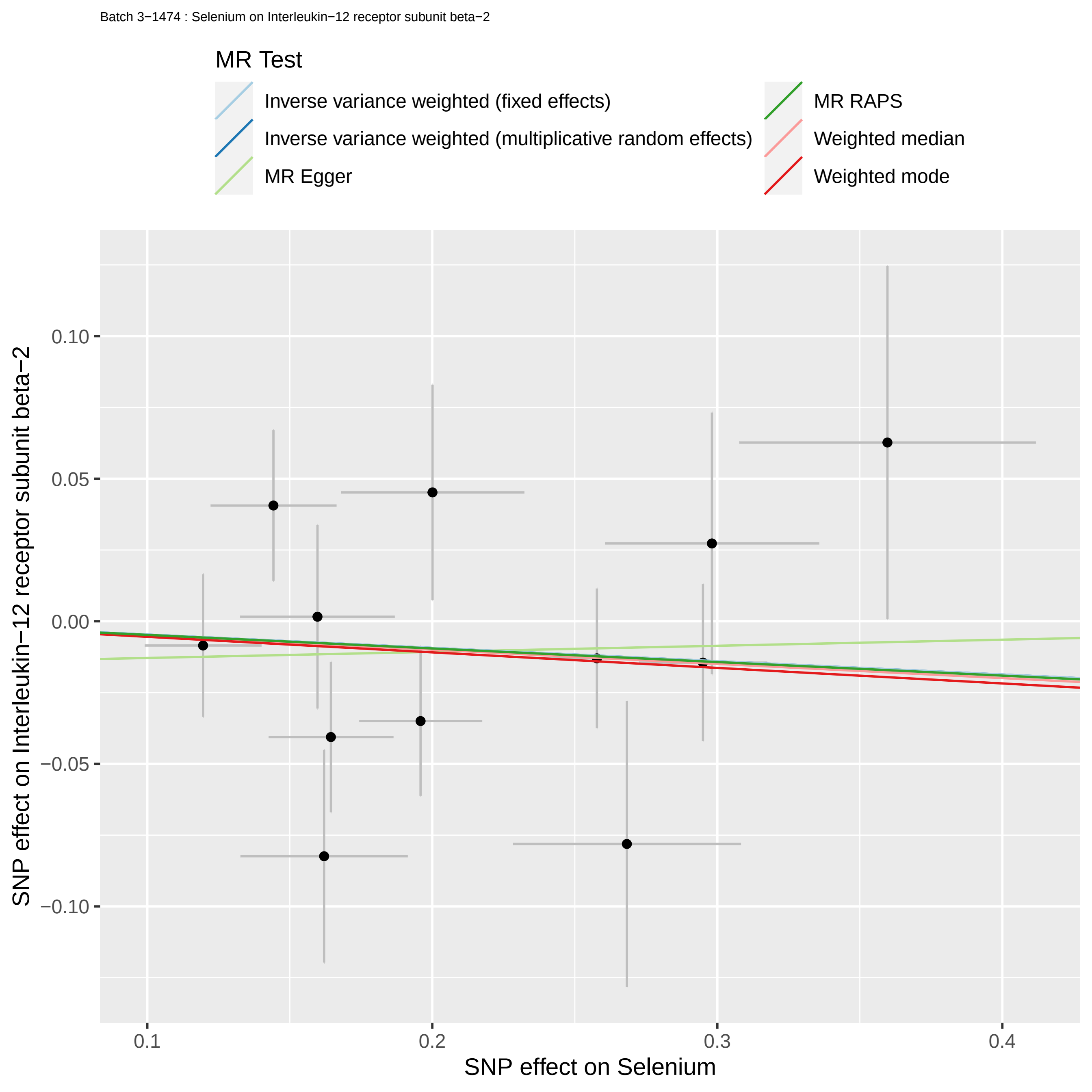

Batch 3−1474 : Selenium on Interleukin−12 receptor subunit beta−2
MR Test
Inverse variance weighted (fixed effects)
Inverse variance weighted (multiplicative random effects)
MR Egger
MR RAPS
Weighted median
Weighted mode
SNP effect on Interleukin−12 receptor subunit beta−2
0.10
0.05
0.00
−0.05
−0.10
0.1
0.2
0.3
0.4
SNP effect on Selenium

Batch 3−1475 : Selenium on Interleukin−13

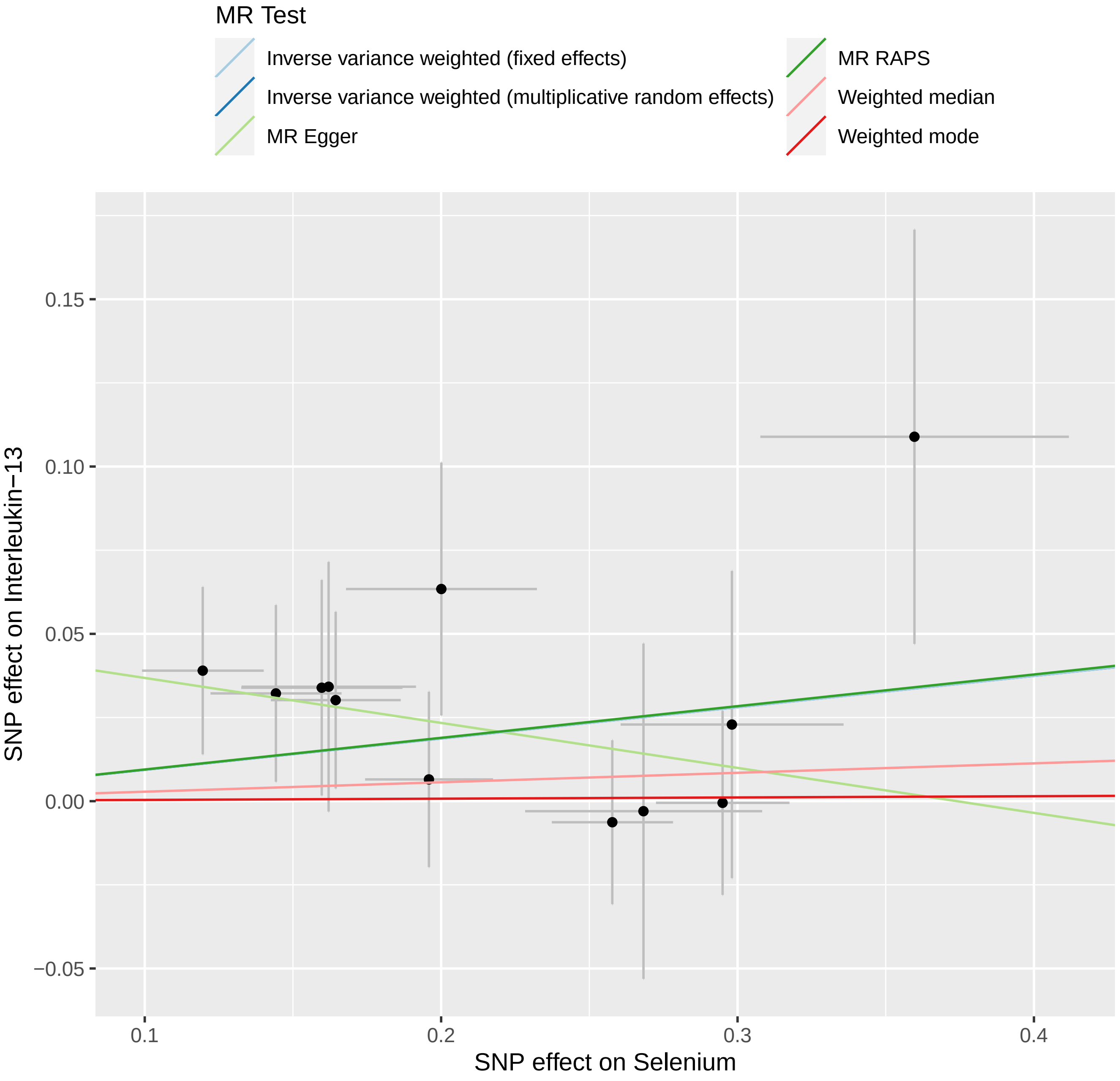

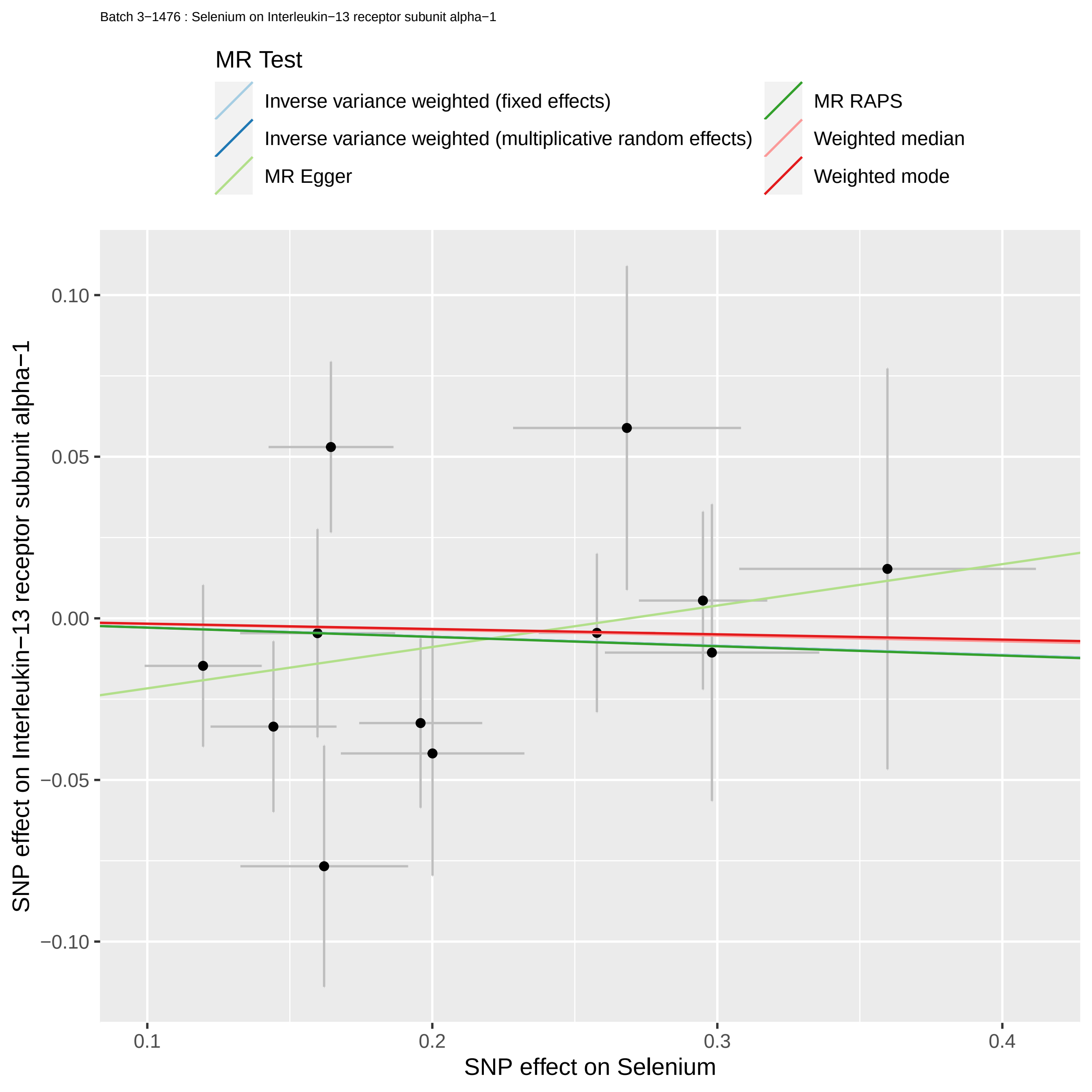
Batch 3−1476 : Selenium on Interleukin−13 receptor subunit alpha−1
MR Test
Inverse variance weighted (fixed effects)
Inverse variance weighted (multiplicative random effects)
MR Egger
MR RAPS
Weighted median
Weighted mode
SNP effect on Interleukin−13 receptor subunit alpha−1
0.10
0.05
0.00
−0.05
−0.10
0.1
0.2
0.3
0.4
SNP effect on Selenium

Batch 3−1477 : Selenium on Interleukin−15 receptor subunit alpha

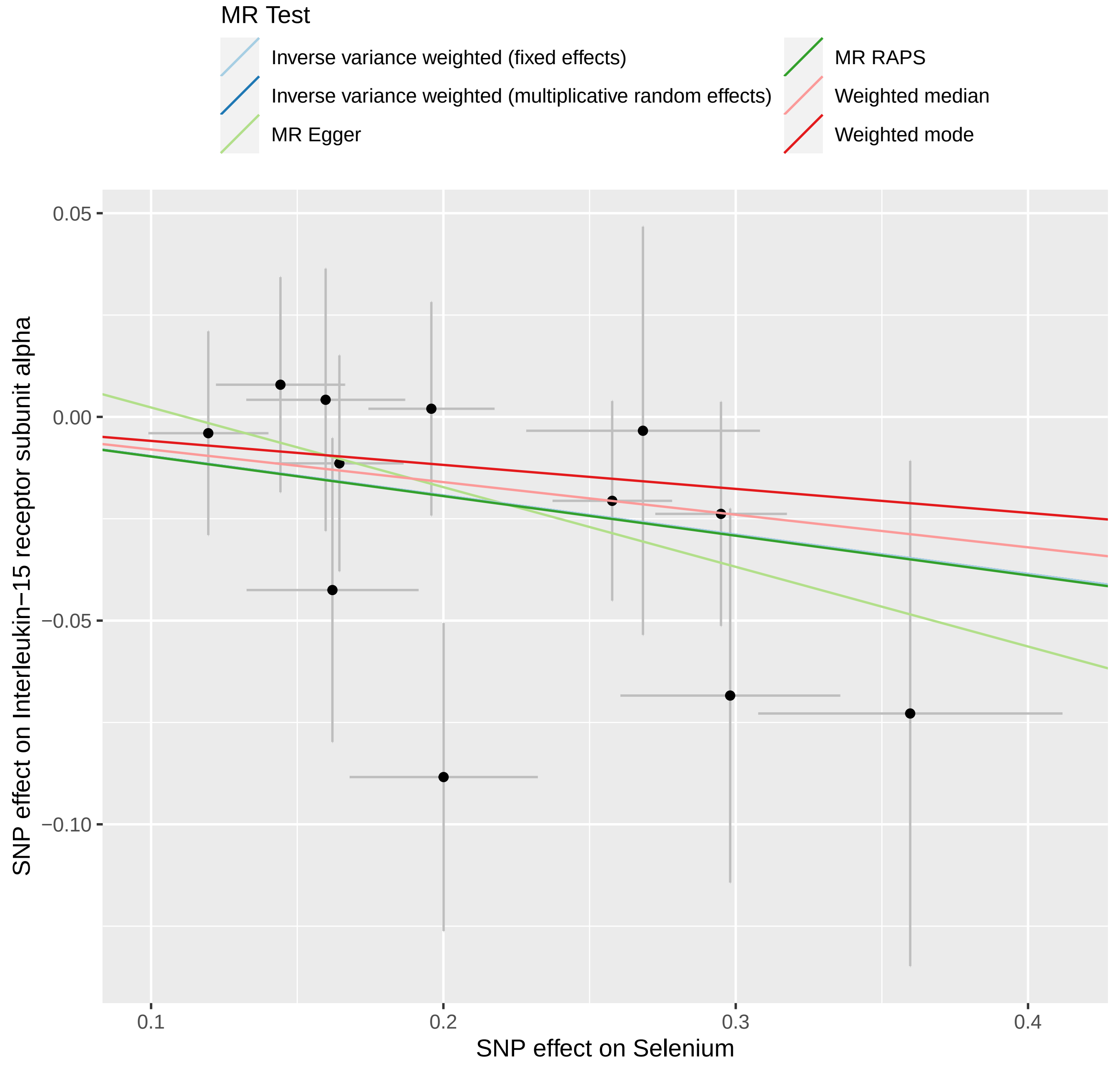

Batch 3−1478 : Selenium on Interleukin−15 receptor subunit alpha

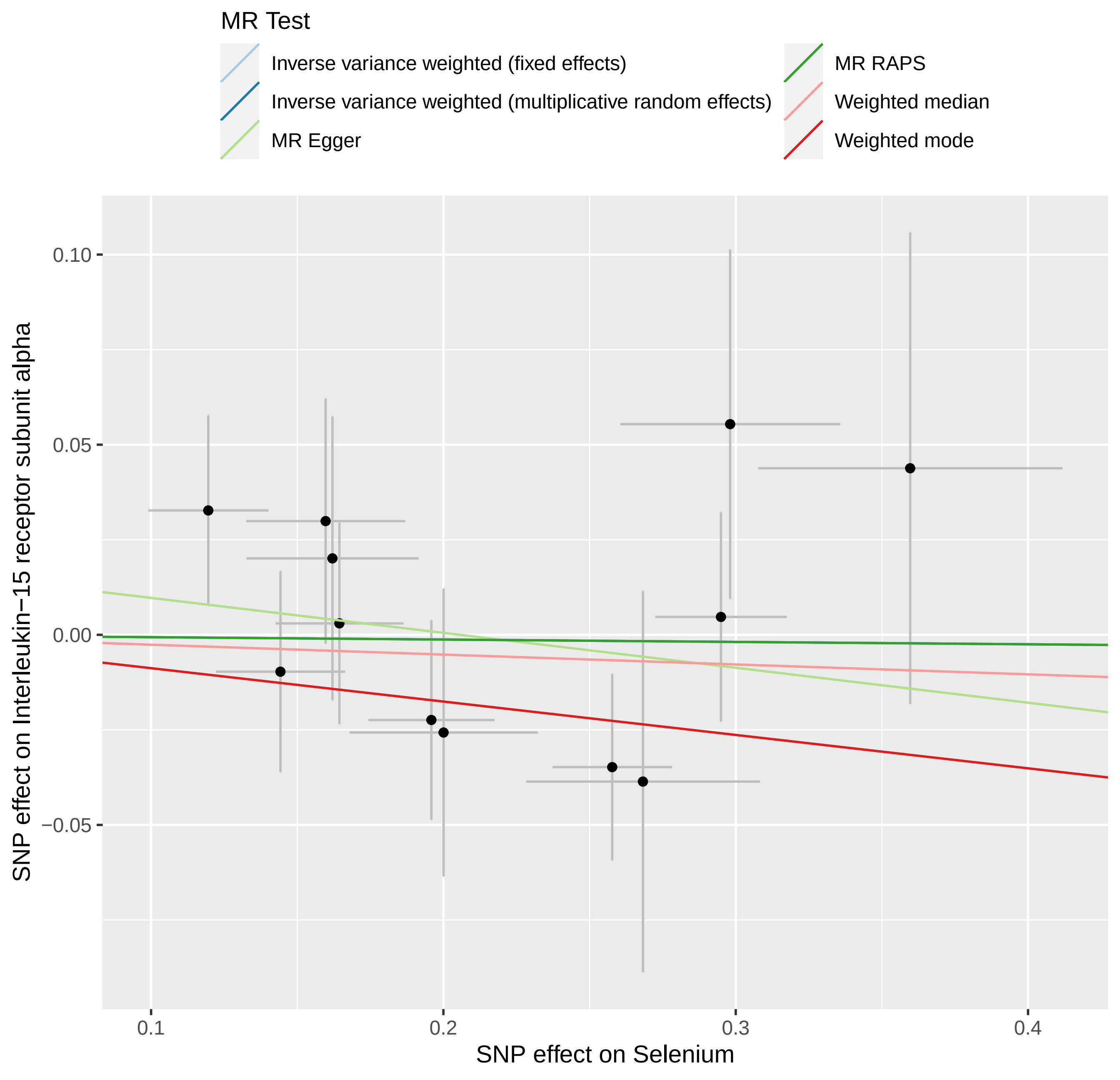

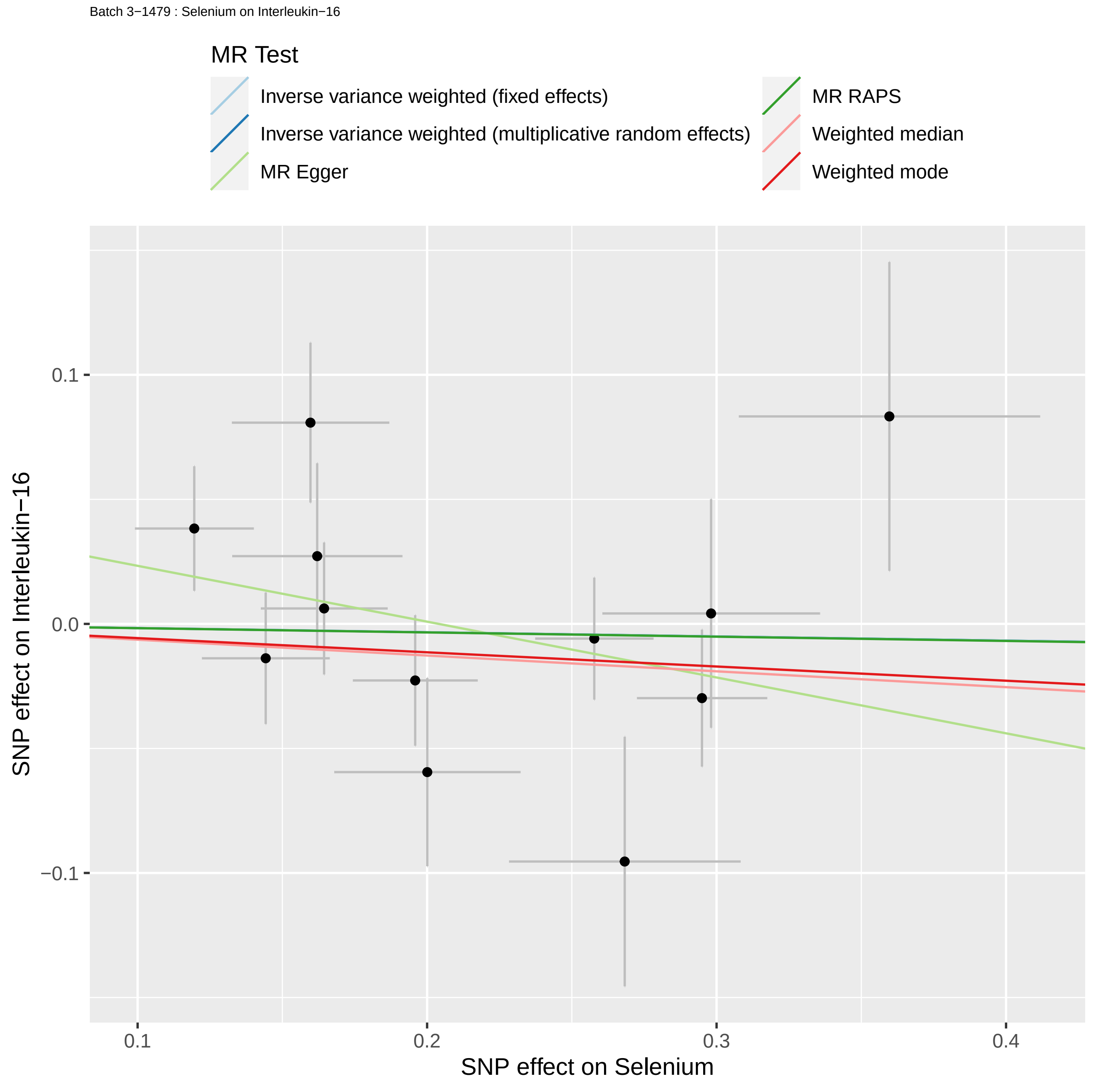

Batch 3−1479 : Selenium on Interleukin−16
MR Test
Inverse variance weighted (fixed effects)
Inverse variance weighted (multiplicative random effects)
MR Egger
MR RAPS
Weighted median
Weighted mode
0.1
0.0
−0.1
0.1
0.2
0.3
0.4
SNP effect on Interleukin−16
SNP effect on Selenium

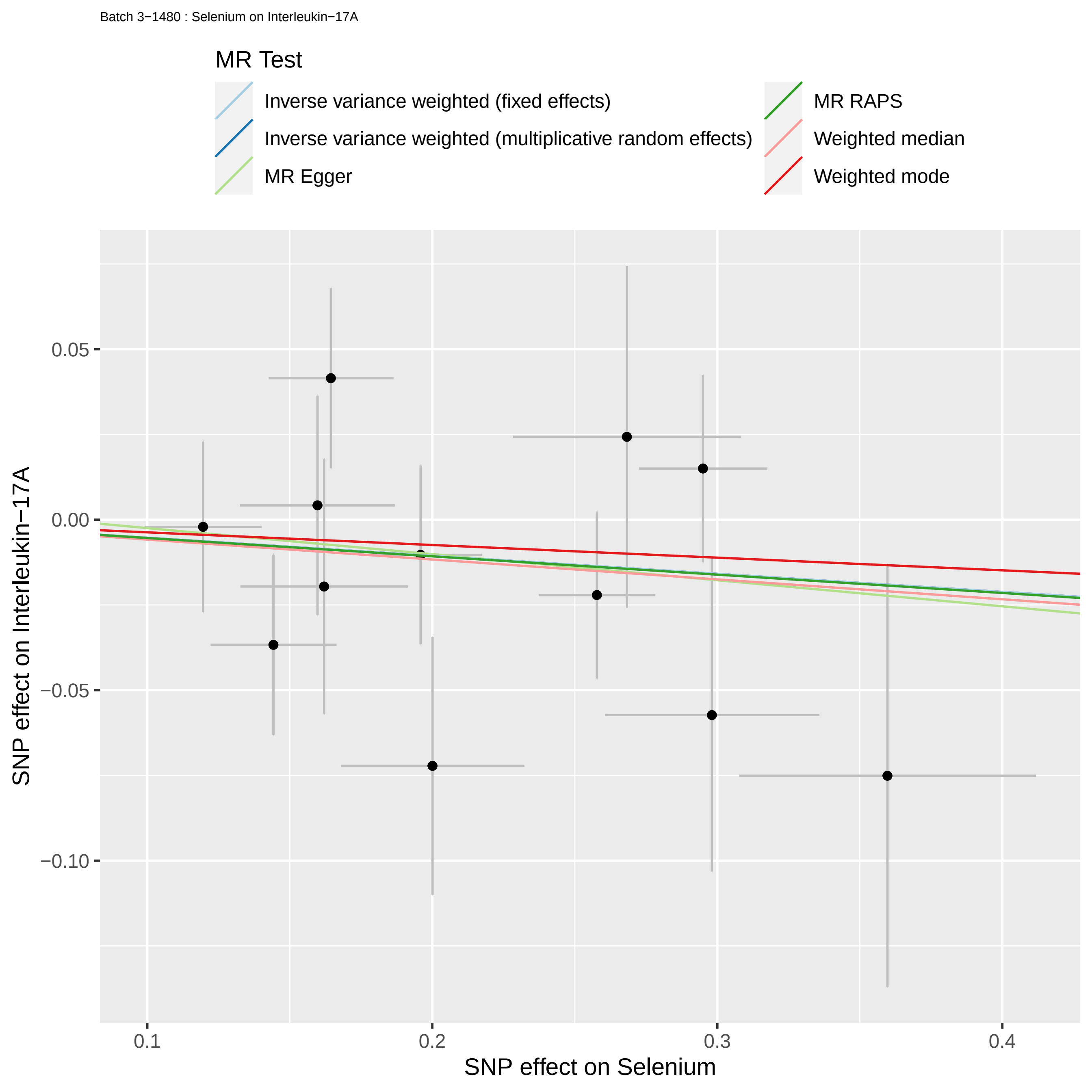
Batch 3−1480 : Selenium on Interleukin−17A
MR Test
Inverse variance weighted (fixed effects)
Inverse variance weighted (multiplicative random effects)
MR Egger
MR RAPS
Weighted median
Weighted mode
0.05
0.00
−0.05
−0.10
0.1
0.2
0.3
0.4
SNP effect on Interleukin−17A
SNP effect on Selenium

Batch 3−1481 : Selenium on Interleukin−17A

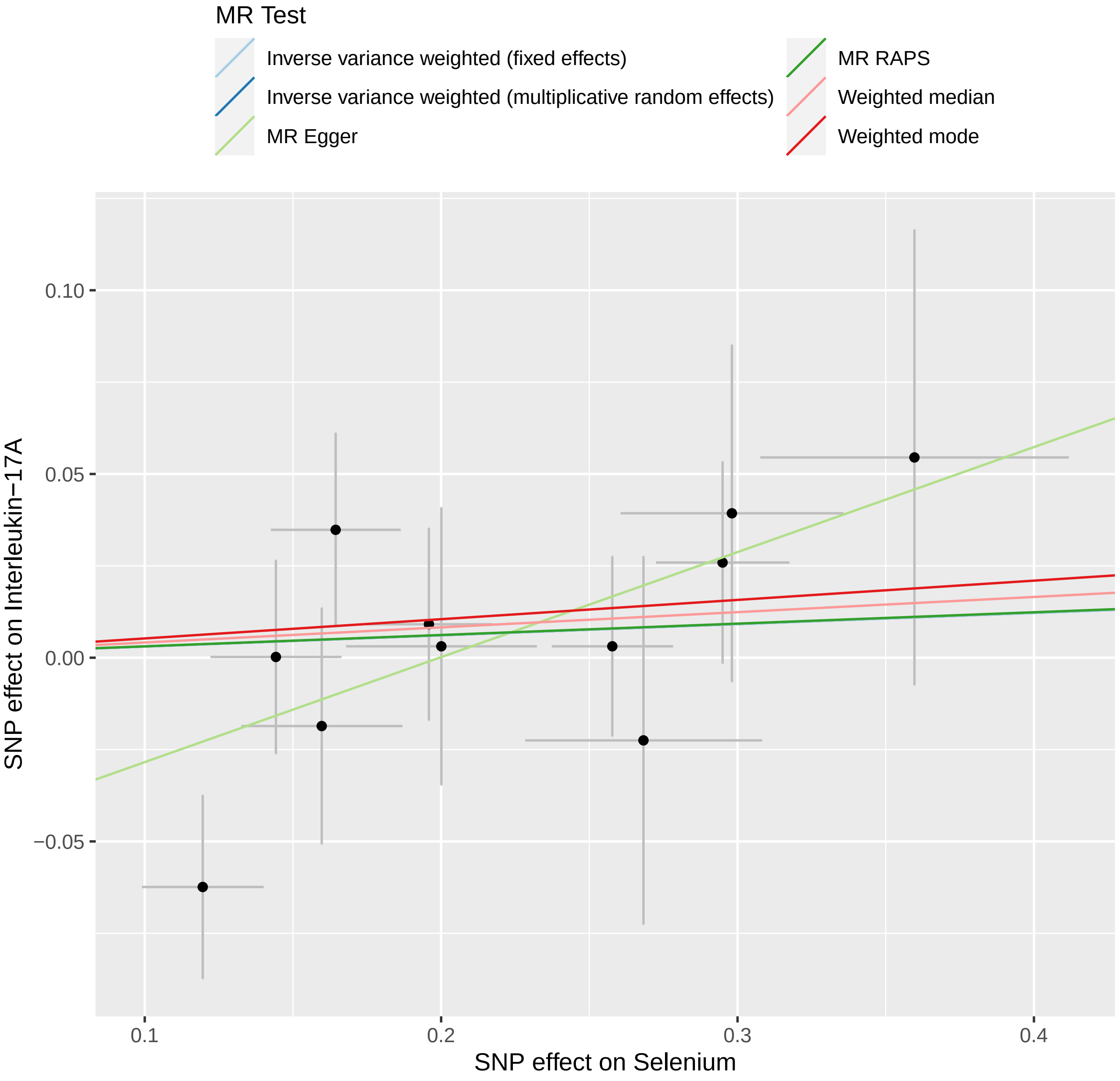

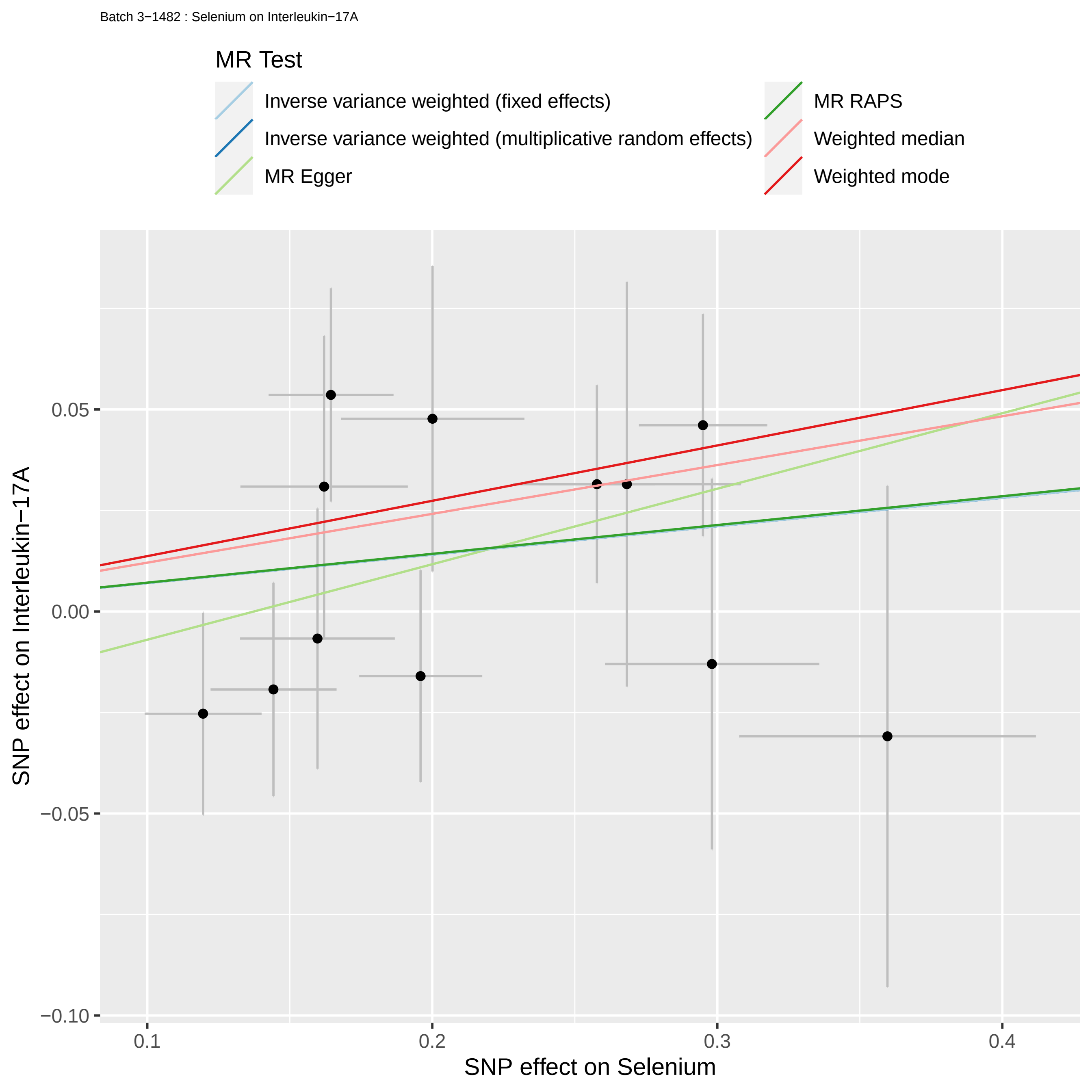

Batch 3−1482 : Selenium on Interleukin−17A
MR Test
Inverse variance weighted (fixed effects)
Inverse variance weighted (multiplicative random effects)
MR Egger
MR RAPS
Weighted median
Weighted mode
0.05
0.00
−0.05
−0.10
0.1
0.2
0.3
0.4
SNP effect on Interleukin−17A
SNP effect on Selenium

Batch 3−1483 : Selenium on Interleukin−17C

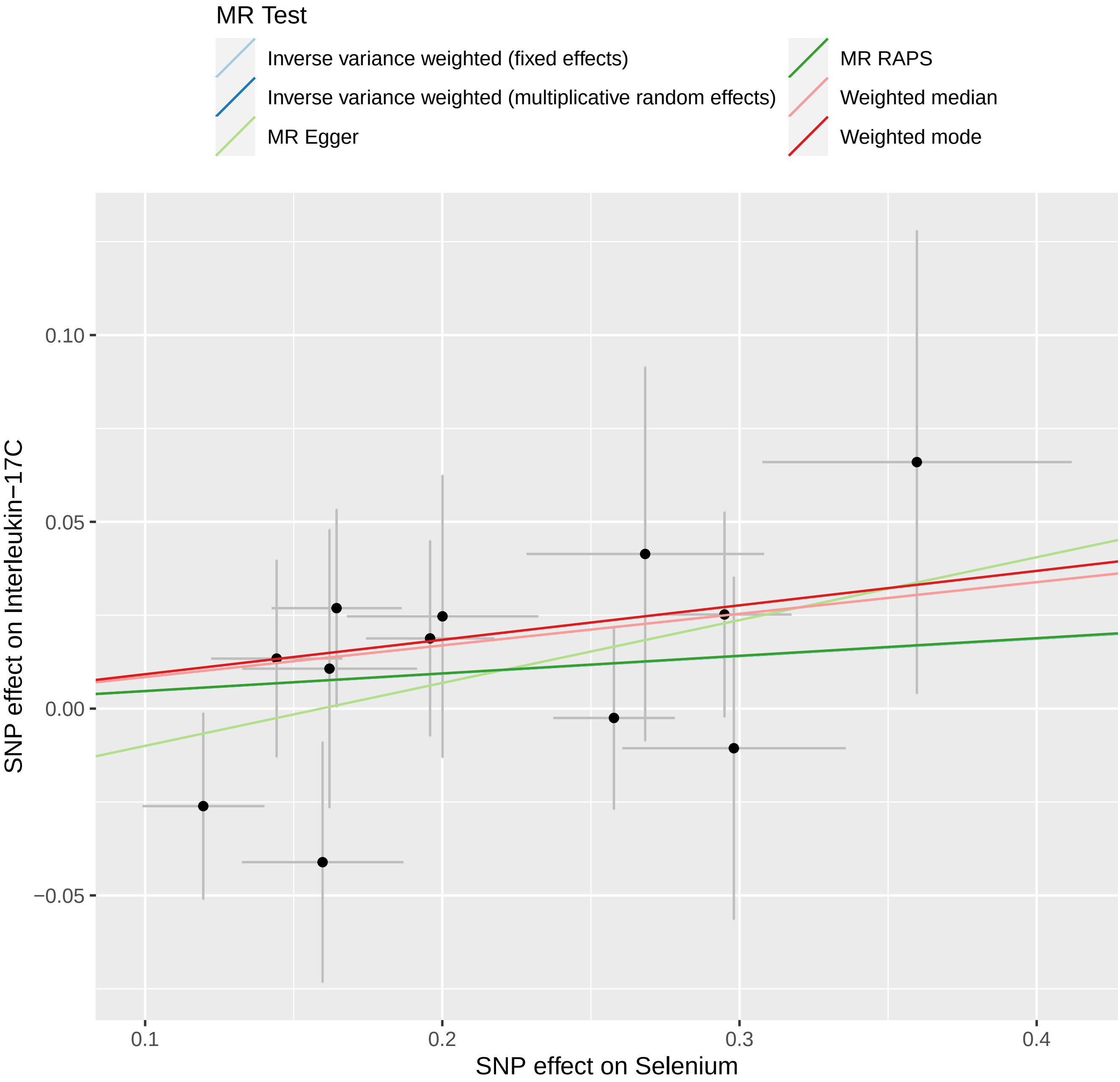

Batch 3−1484 : Selenium on Interleukin−17D

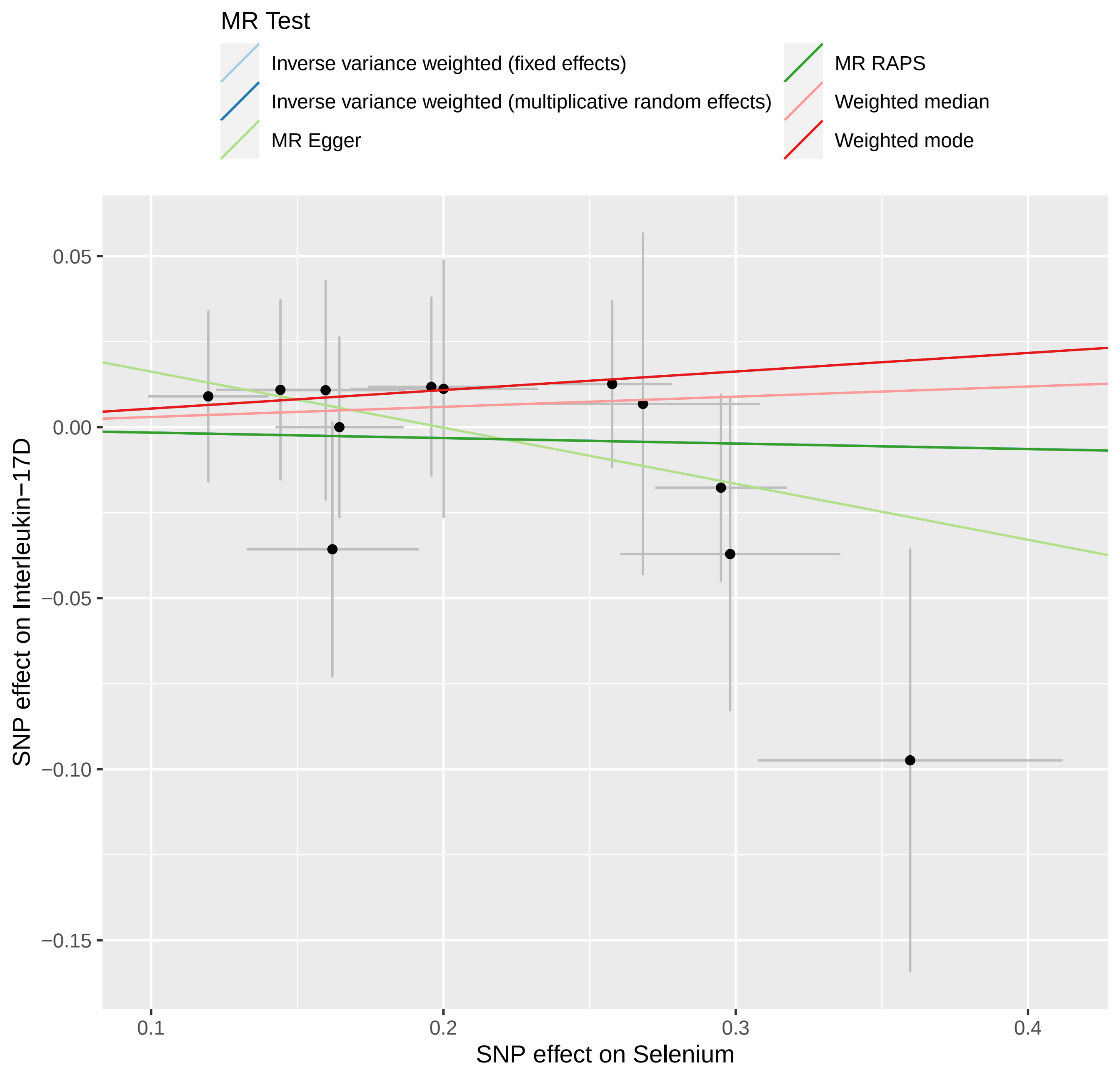

Batch 3−1485 : Selenium on Interleukin−17F

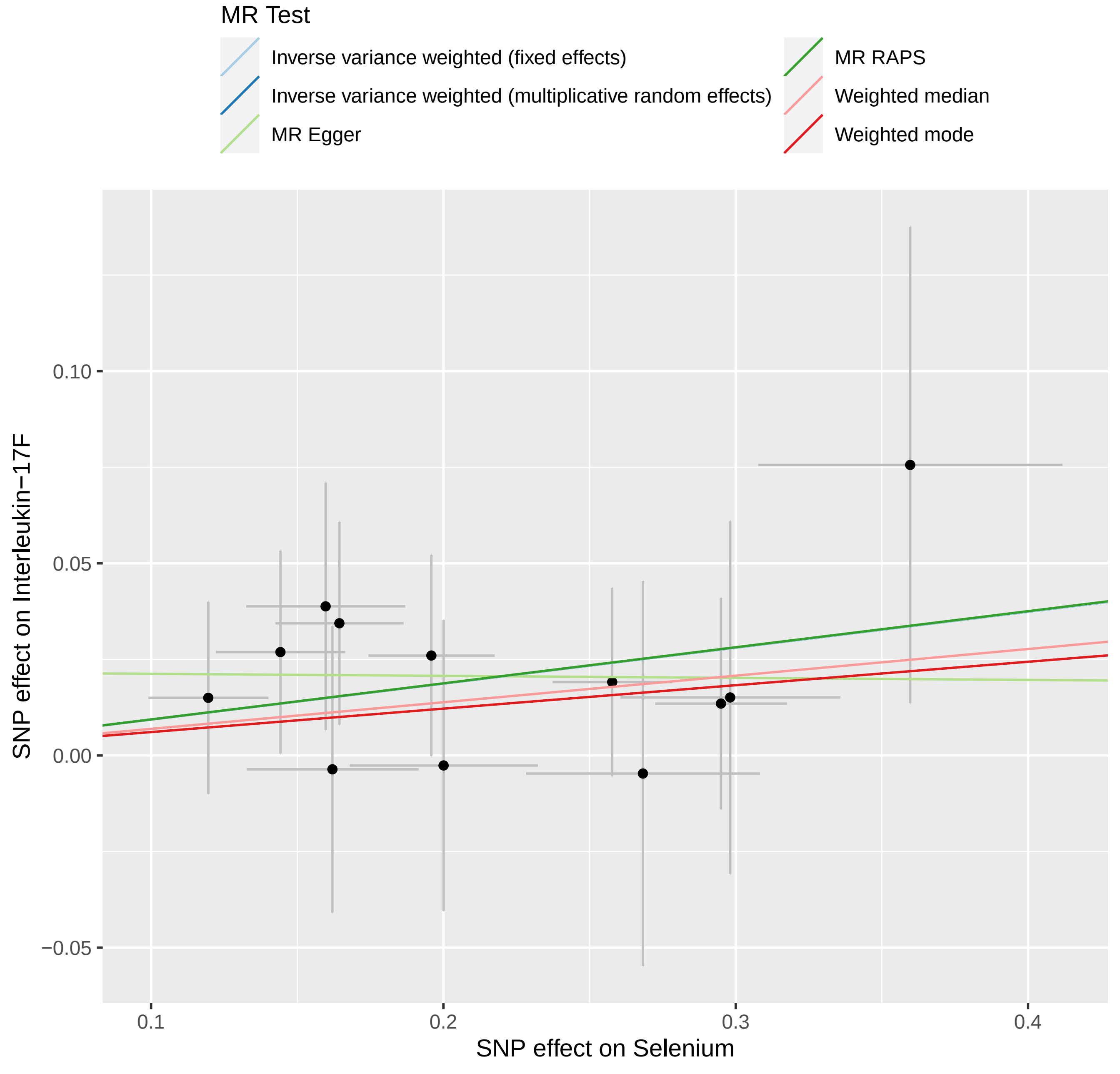

Batch 3−1486 : Selenium on Interleukin−17 receptor A

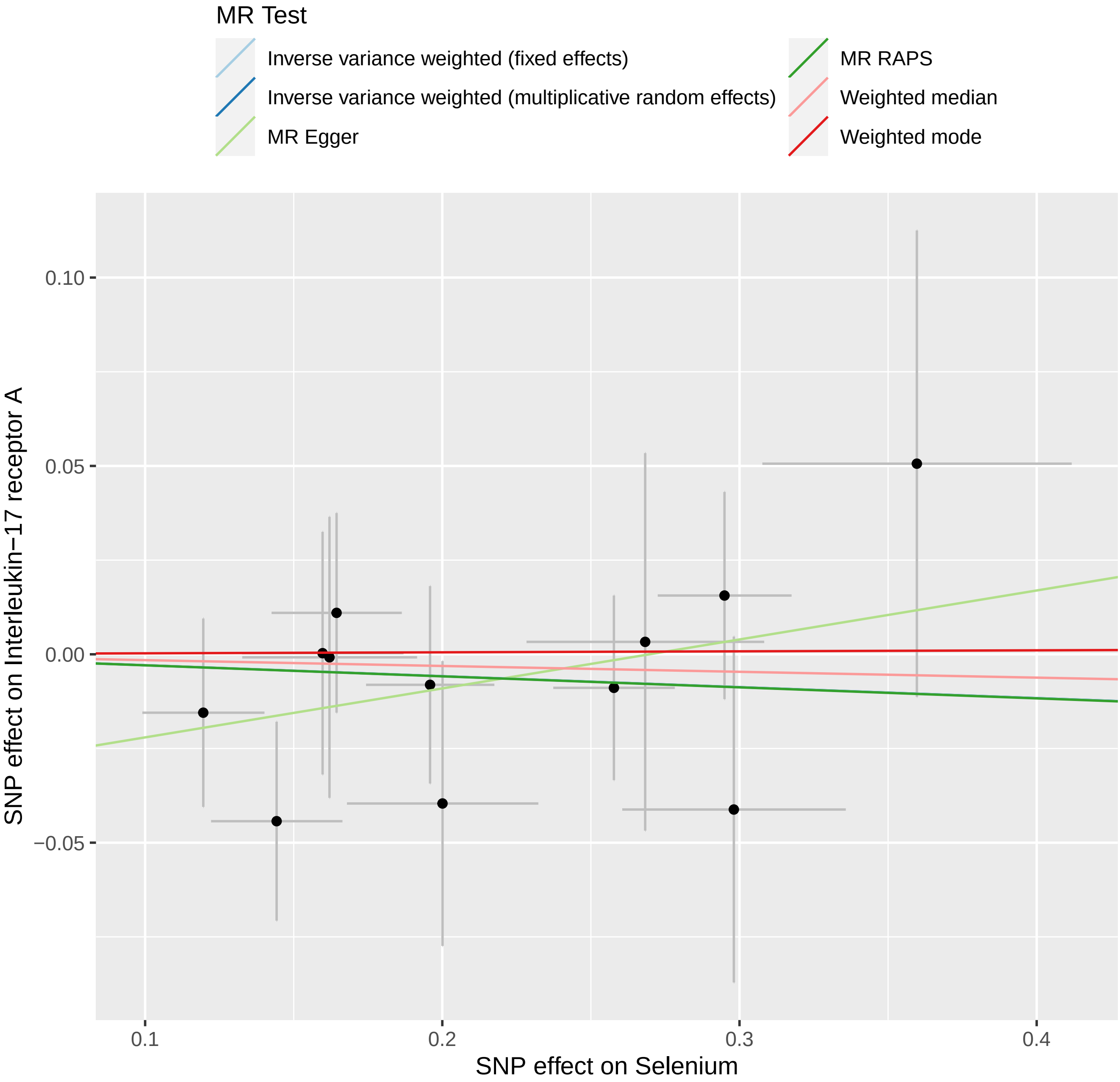

Batch 3−1487 : Selenium on Interleukin−17 receptor B

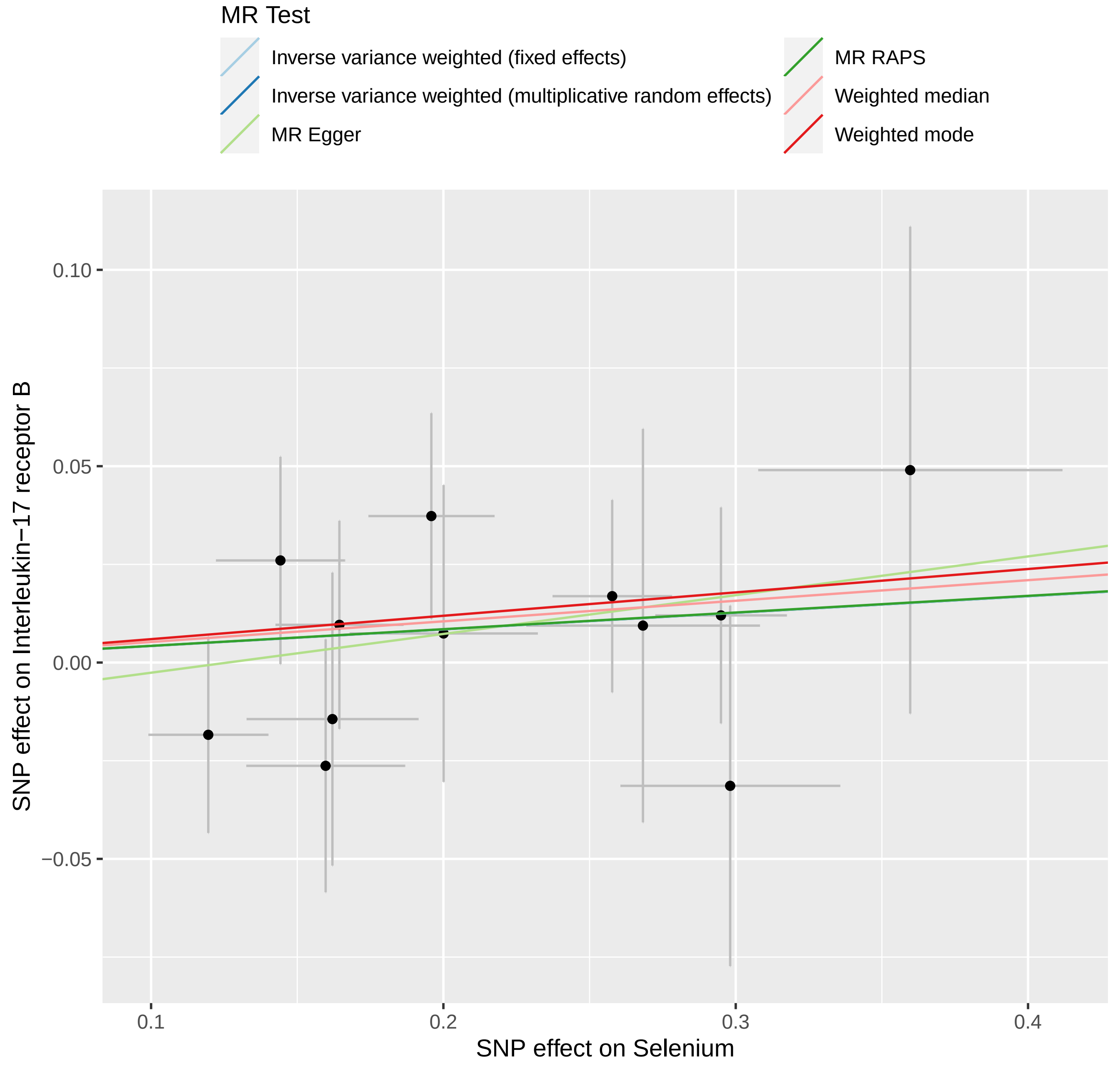

Batch 3−1488 : Selenium on Interleukin−17 receptor C

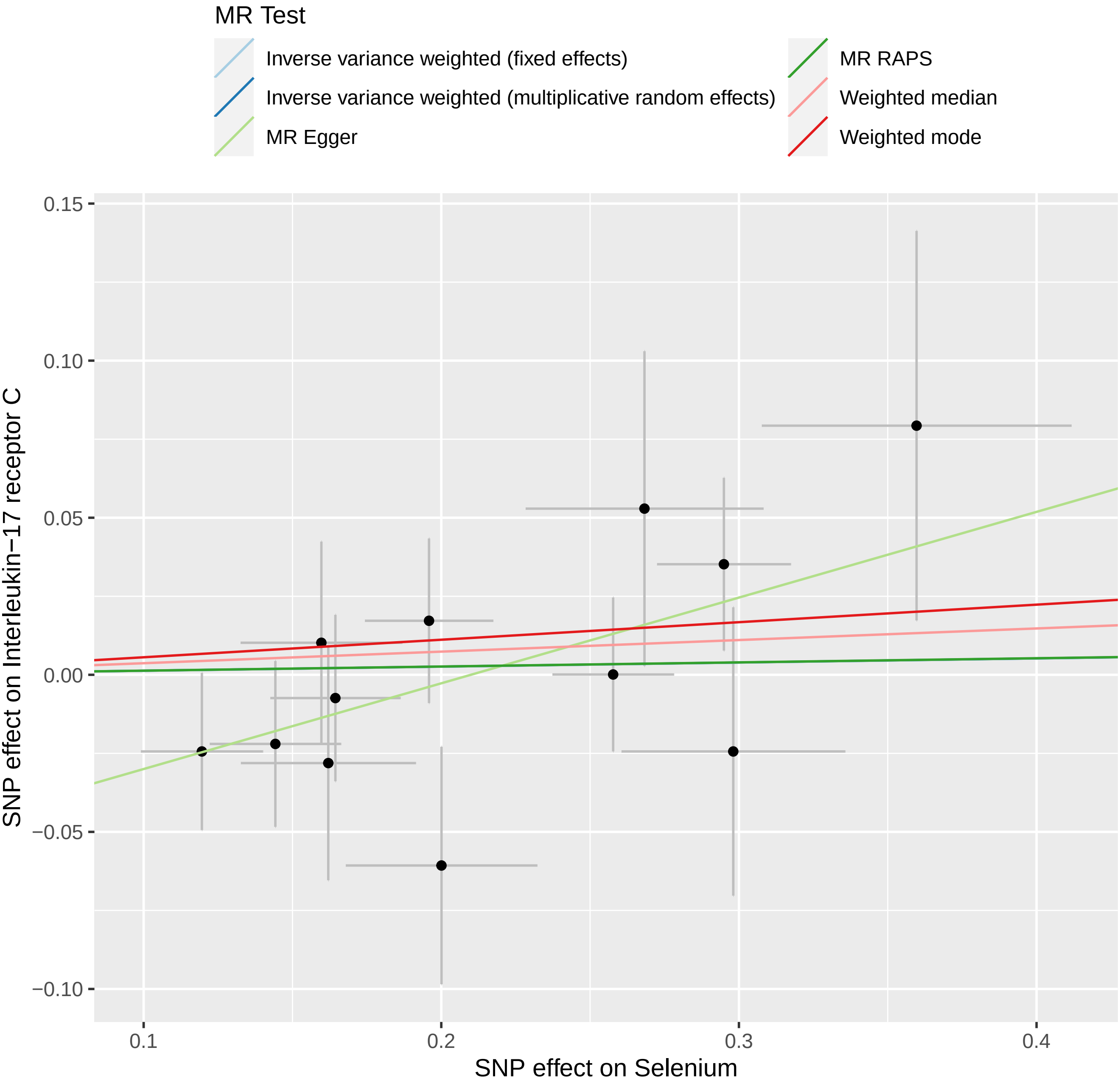

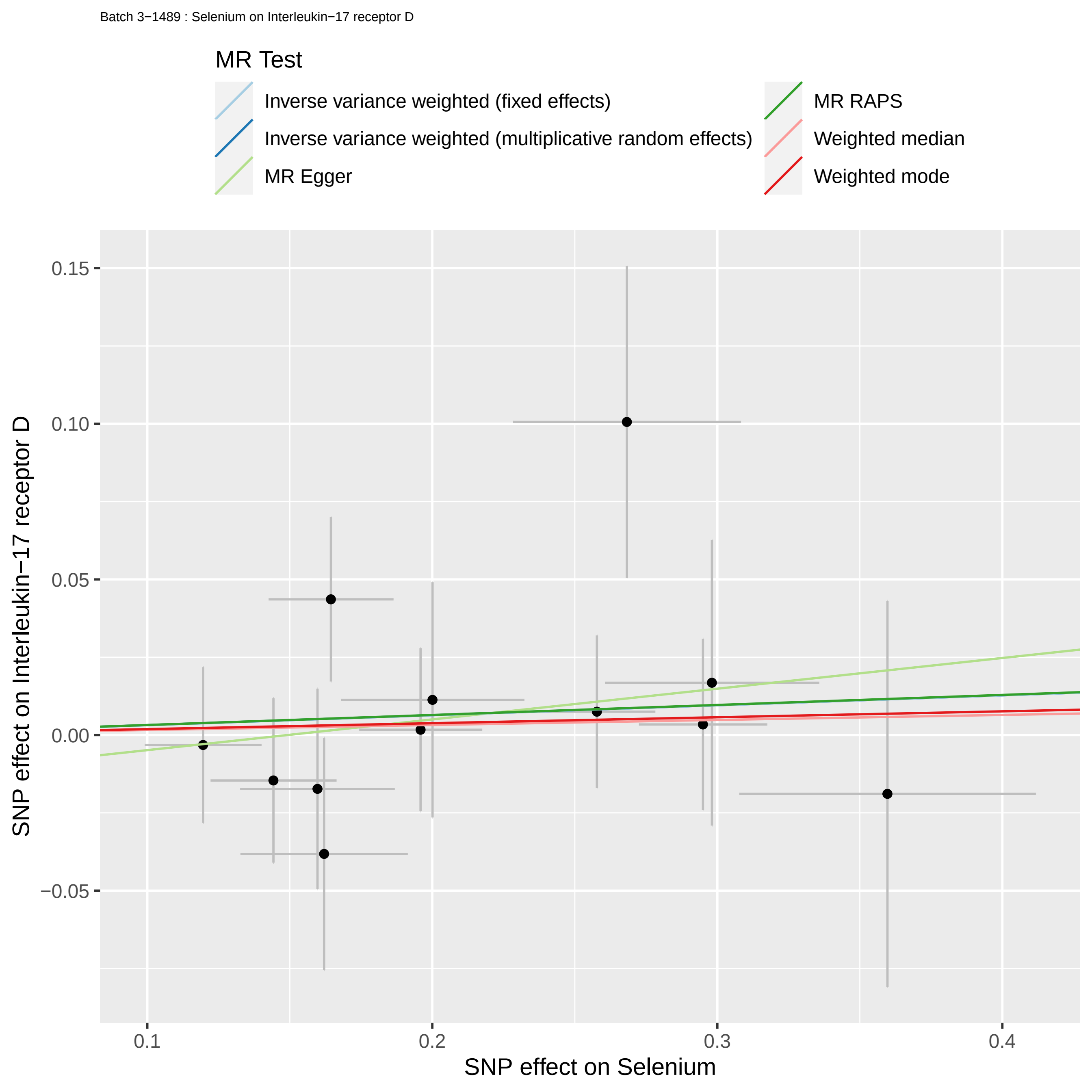

Batch 3−1489 : Selenium on Interleukin−17 receptor D
MR Test
Inverse variance weighted (fixed effects)
Inverse variance weighted (multiplicative random effects)
MR Egger
MR RAPS
Weighted median
Weighted mode
0.15
0.10
0.05
0.00
−0.05
0.1
0.2
0.3
0.4
SNP effect on Interleukin−17 receptor D
SNP effect on Selenium

Batch 3−1490 : Selenium on Interleukin−18−binding protein

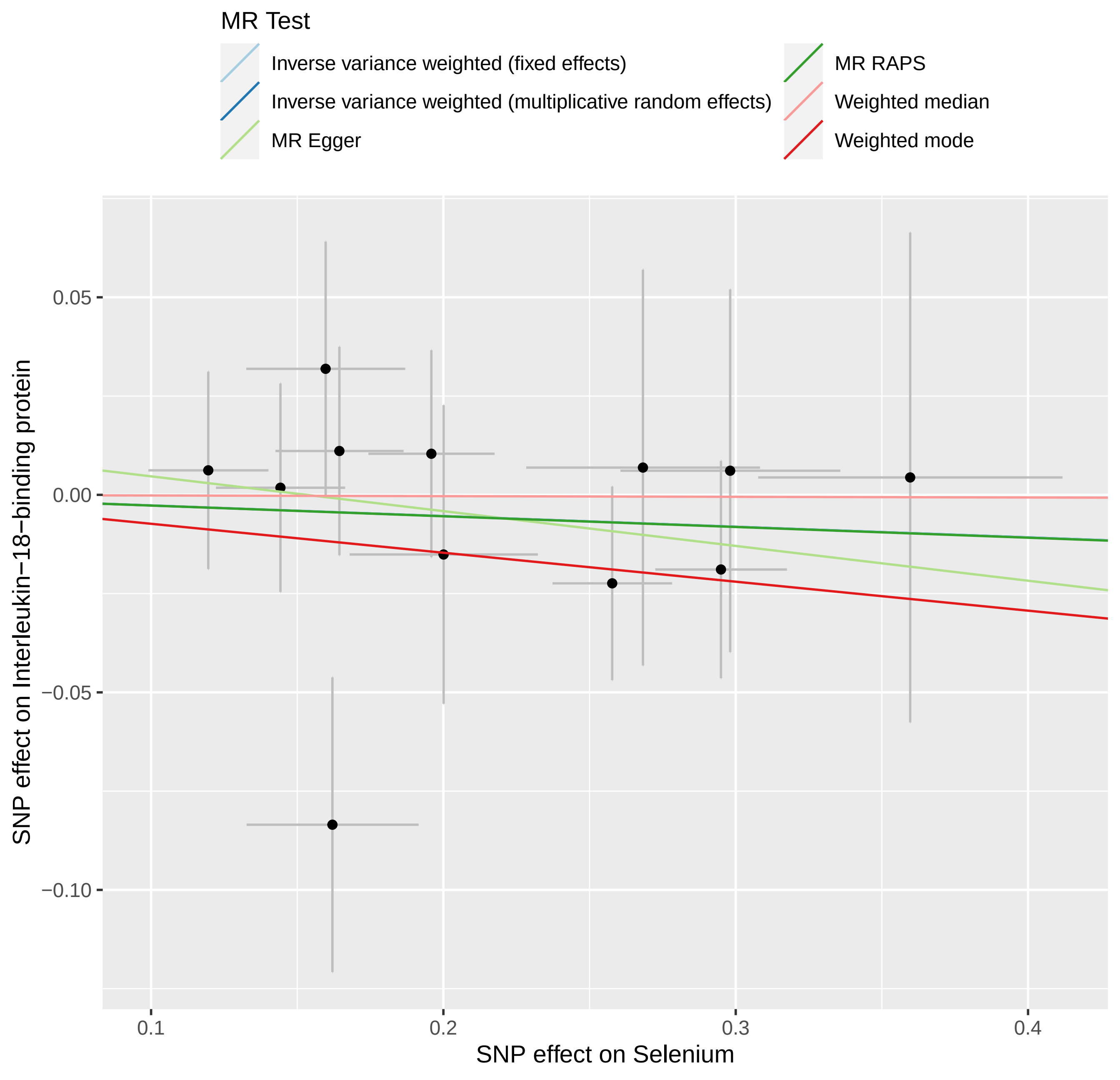

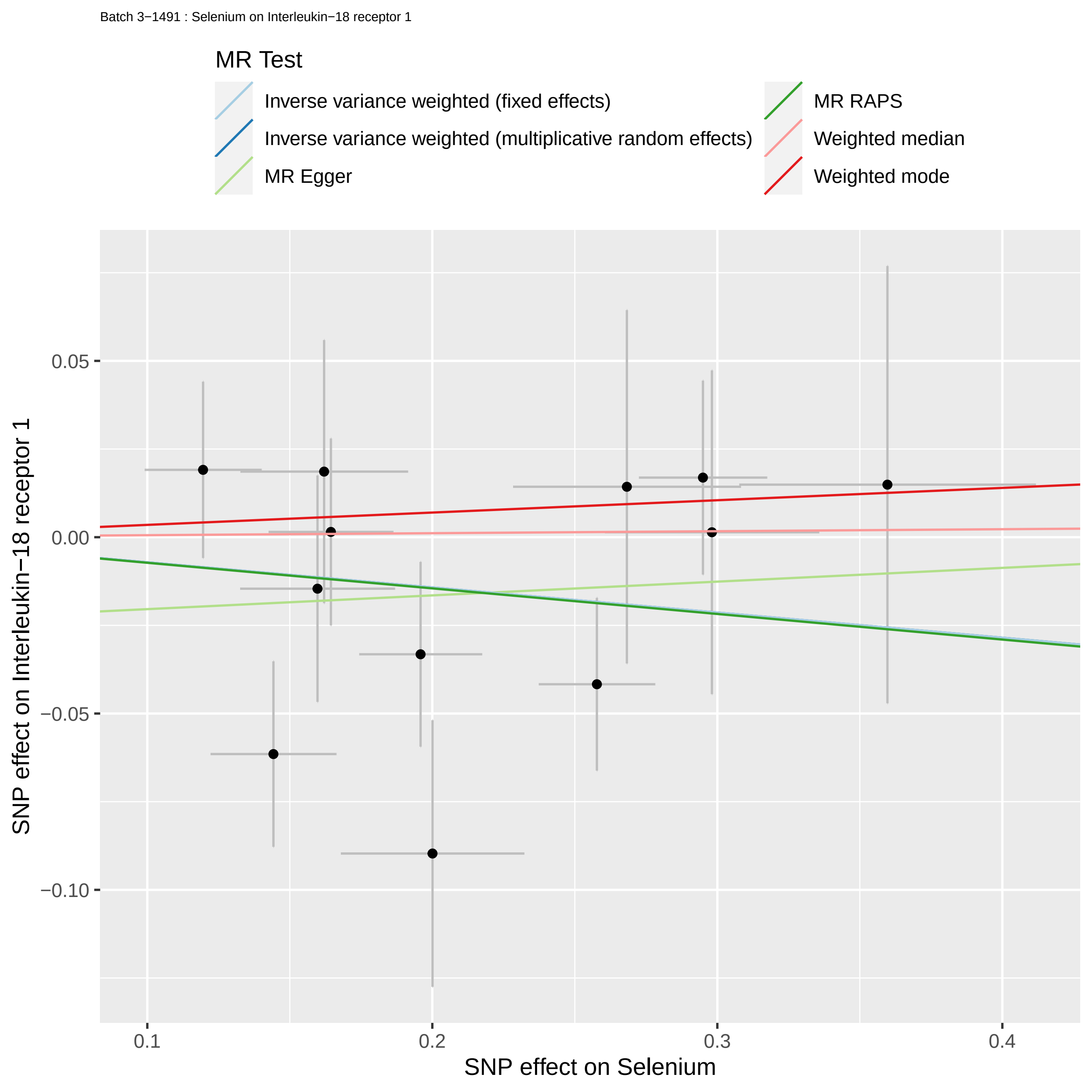

Batch 3−1491 : Selenium on Interleukin−18 receptor 1
MR Test
Inverse variance weighted (fixed effects)
Inverse variance weighted (multiplicative random effects)
MR Egger
MR RAPS
Weighted median
Weighted mode
0.05
0.00
−0.05
−0.10
0.1
0.2
0.3
0.4
SNP effect on Interleukin−18 receptor 1
SNP effect on Selenium

Batch 3−1492 : Selenium on Interleukin−18 receptor 1

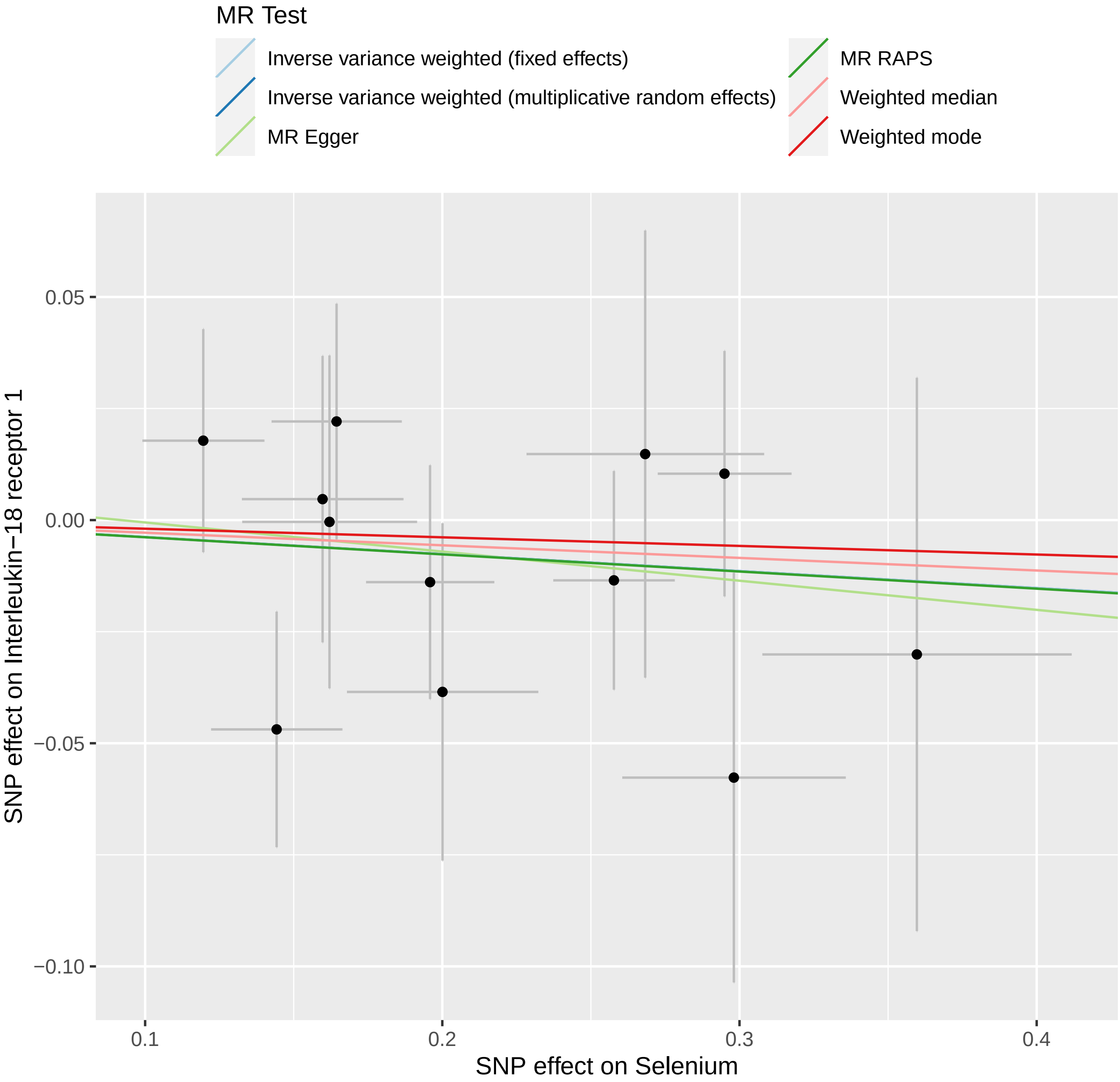

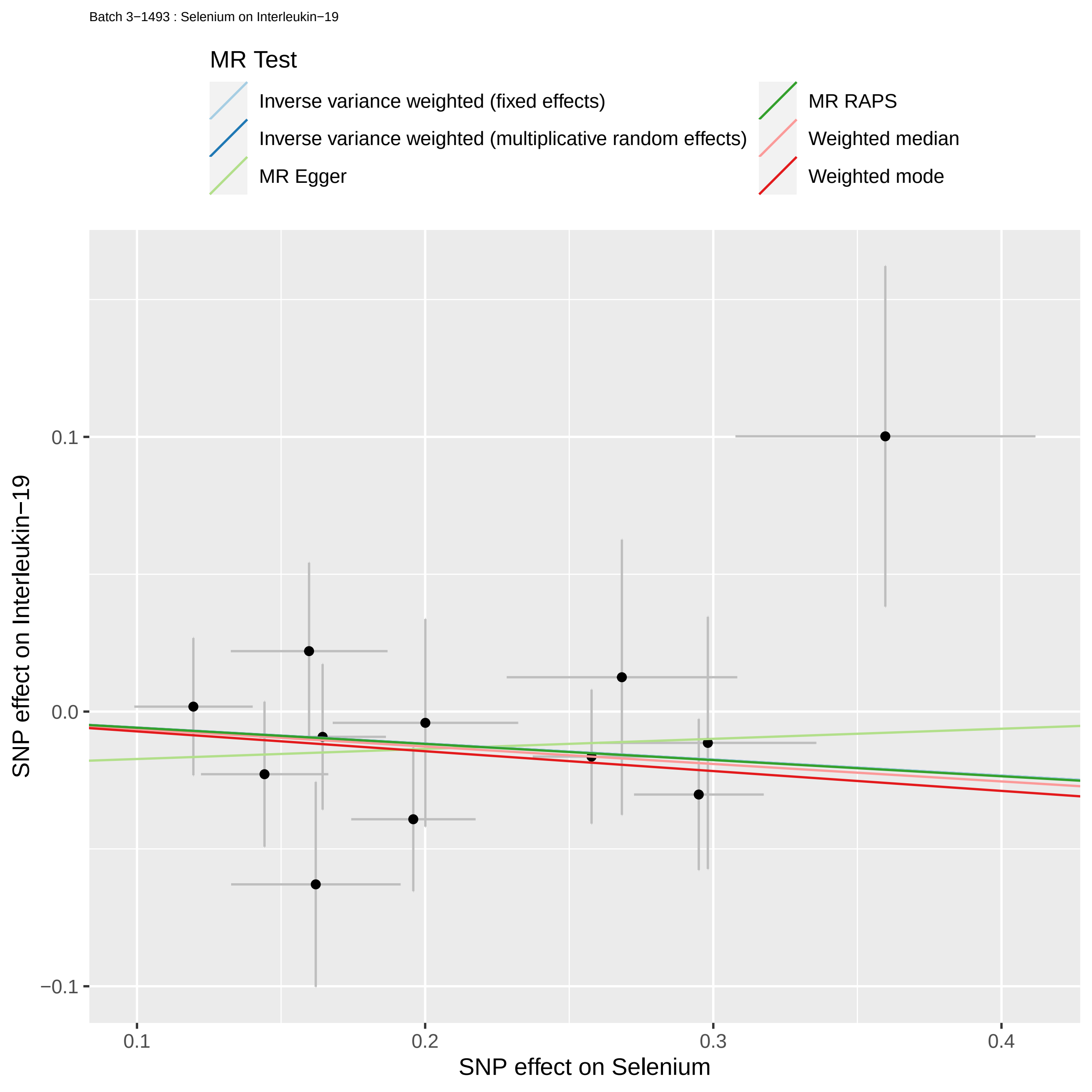

Batch 3−1493 : Selenium on Interleukin−19
MR Test
Inverse variance weighted (fixed effects)
Inverse variance weighted (multiplicative random effects)
MR Egger
MR RAPS
Weighted median
Weighted mode
0.1
0.0
−0.1
0.1
0.2
0.3
0.4
SNP effect on Interleukin−19
SNP effect on Selenium

Batch 3−1494 : Selenium on Interleukin−1 alpha

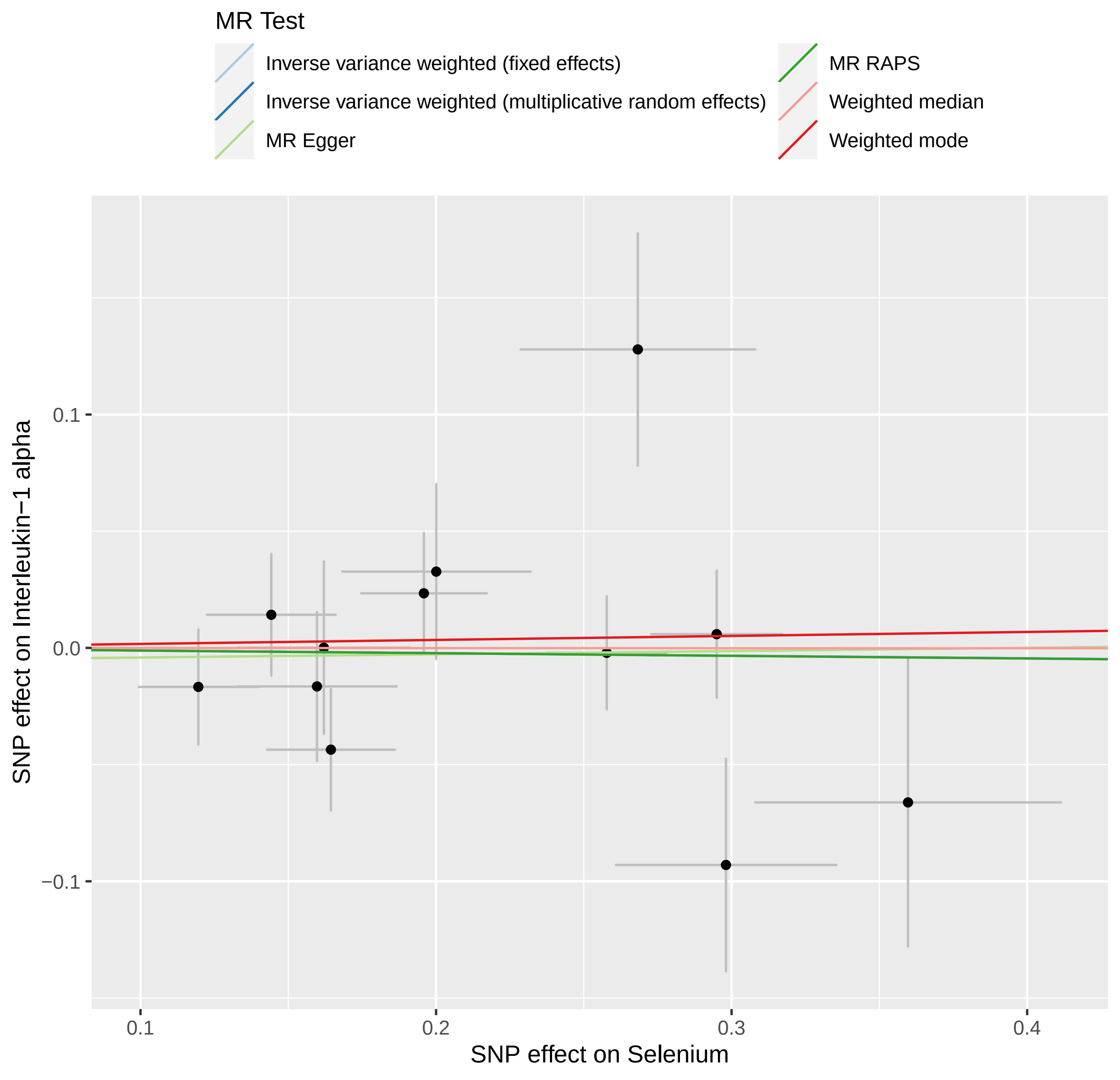

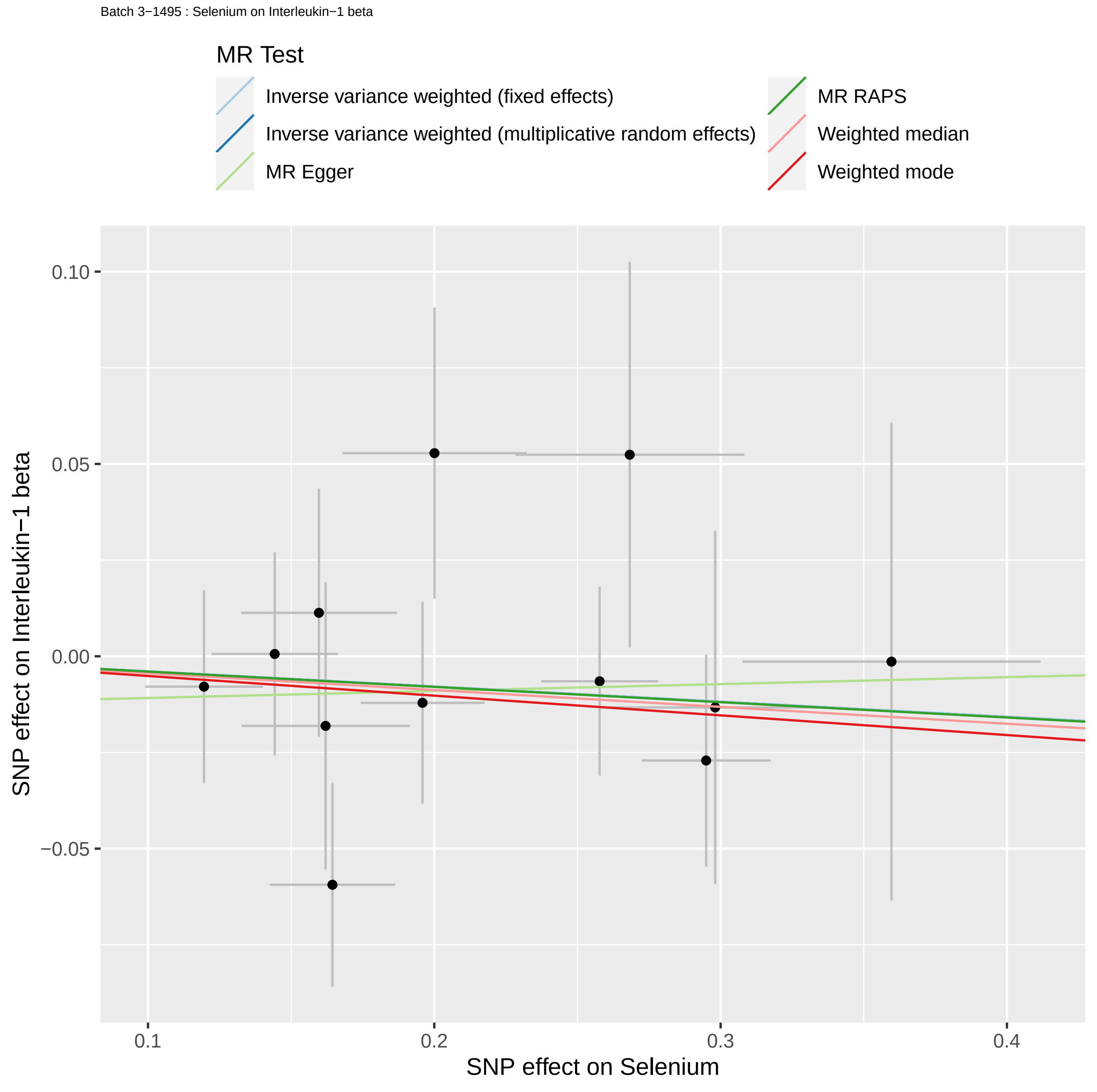

Batch 3−1495 : Selenium on Interleukin−1 beta
MR Test
Inverse variance weighted (fixed effects)
Inverse variance weighted (multiplicative random effects)
MR Egger
MR RAPS
Weighted median
Weighted mode
0.10
0.05
0.00
−0.05
0.1
0.2
0.3
0.4
SNP effect on Interleukin−1 beta
SNP effect on Selenium

Batch 3−1496 : Selenium on Interleukin−1 receptor type 1

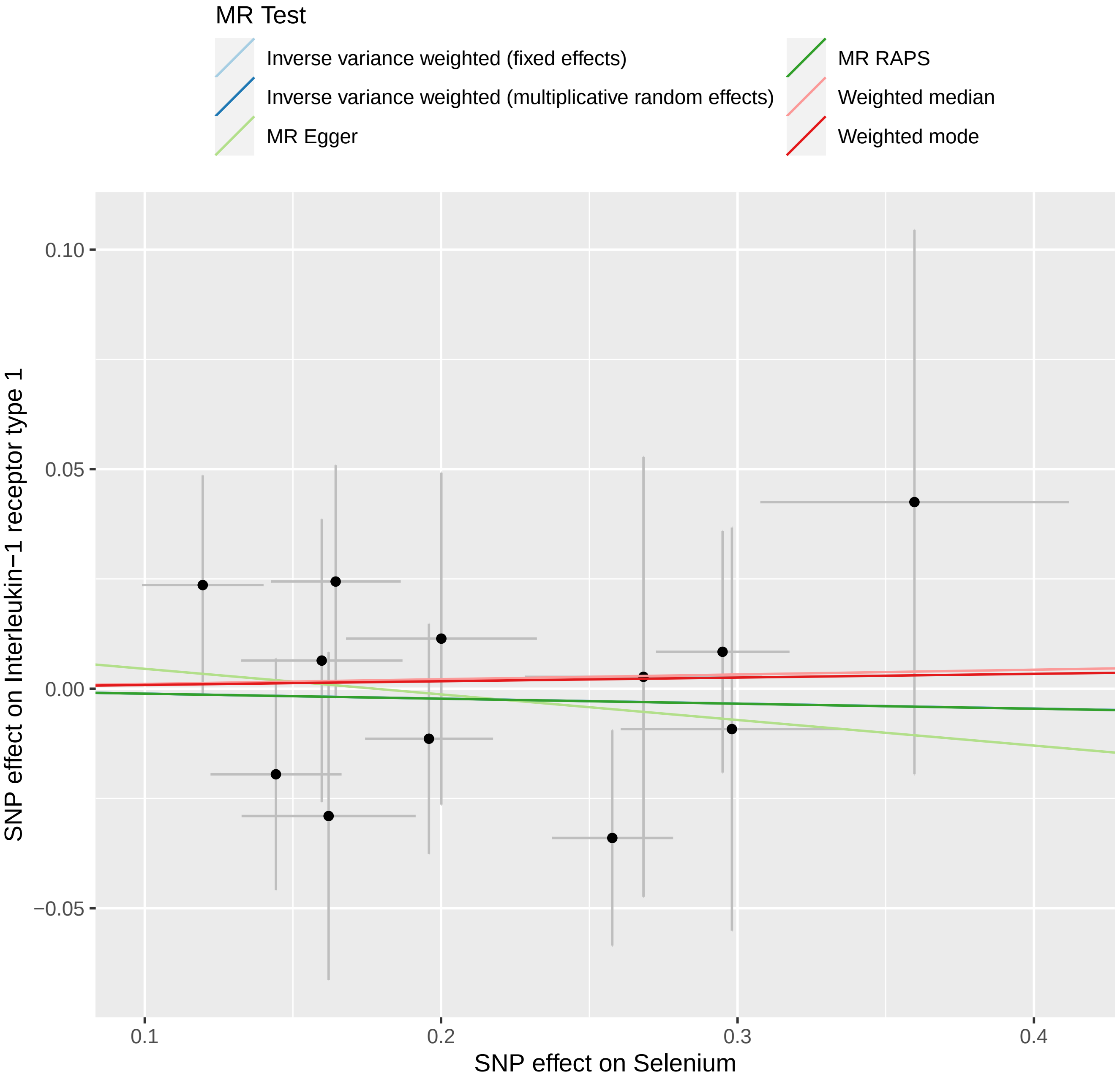

Batch 3−1497 : Selenium on Interleukin−1 receptor type 2

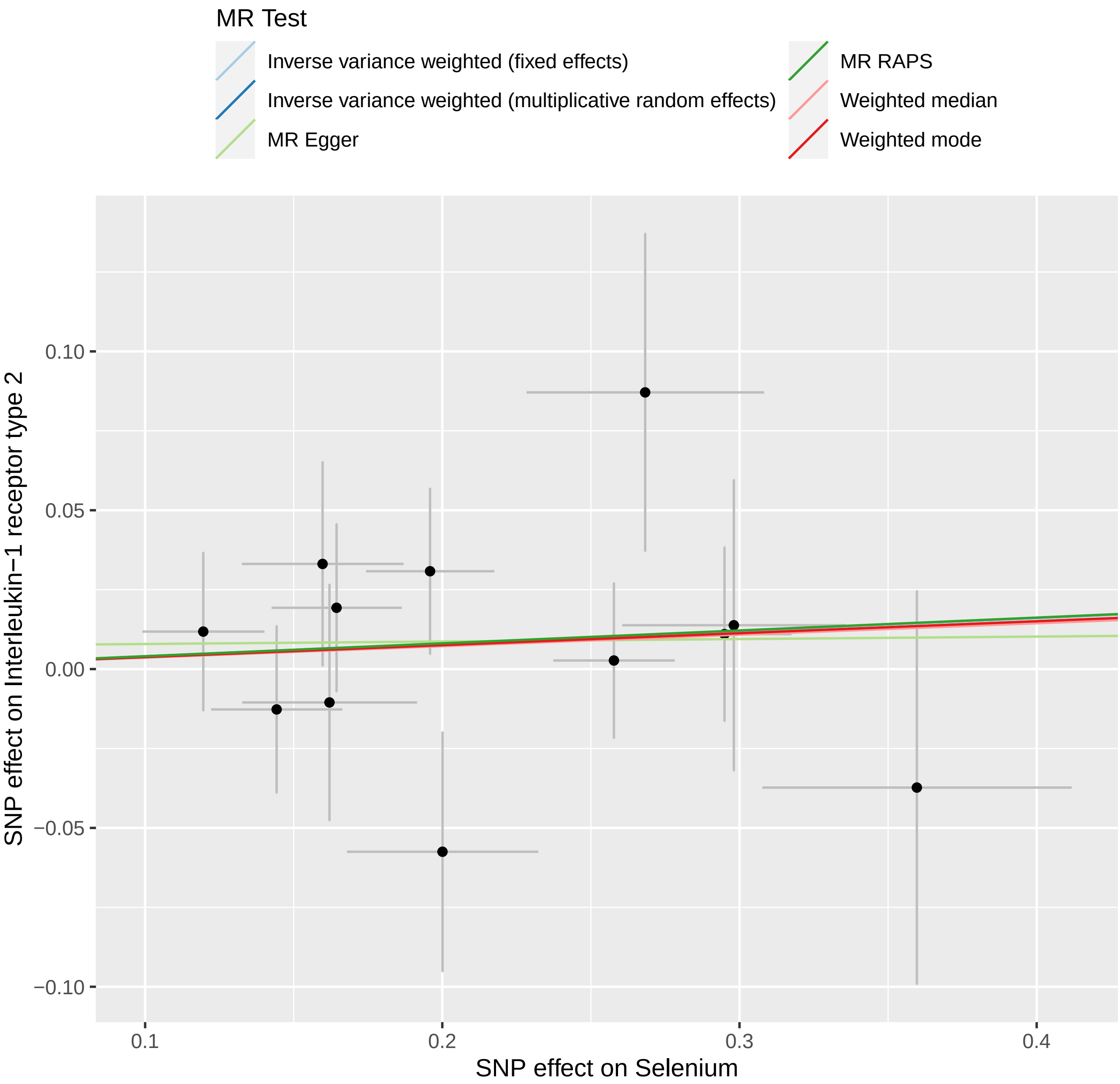

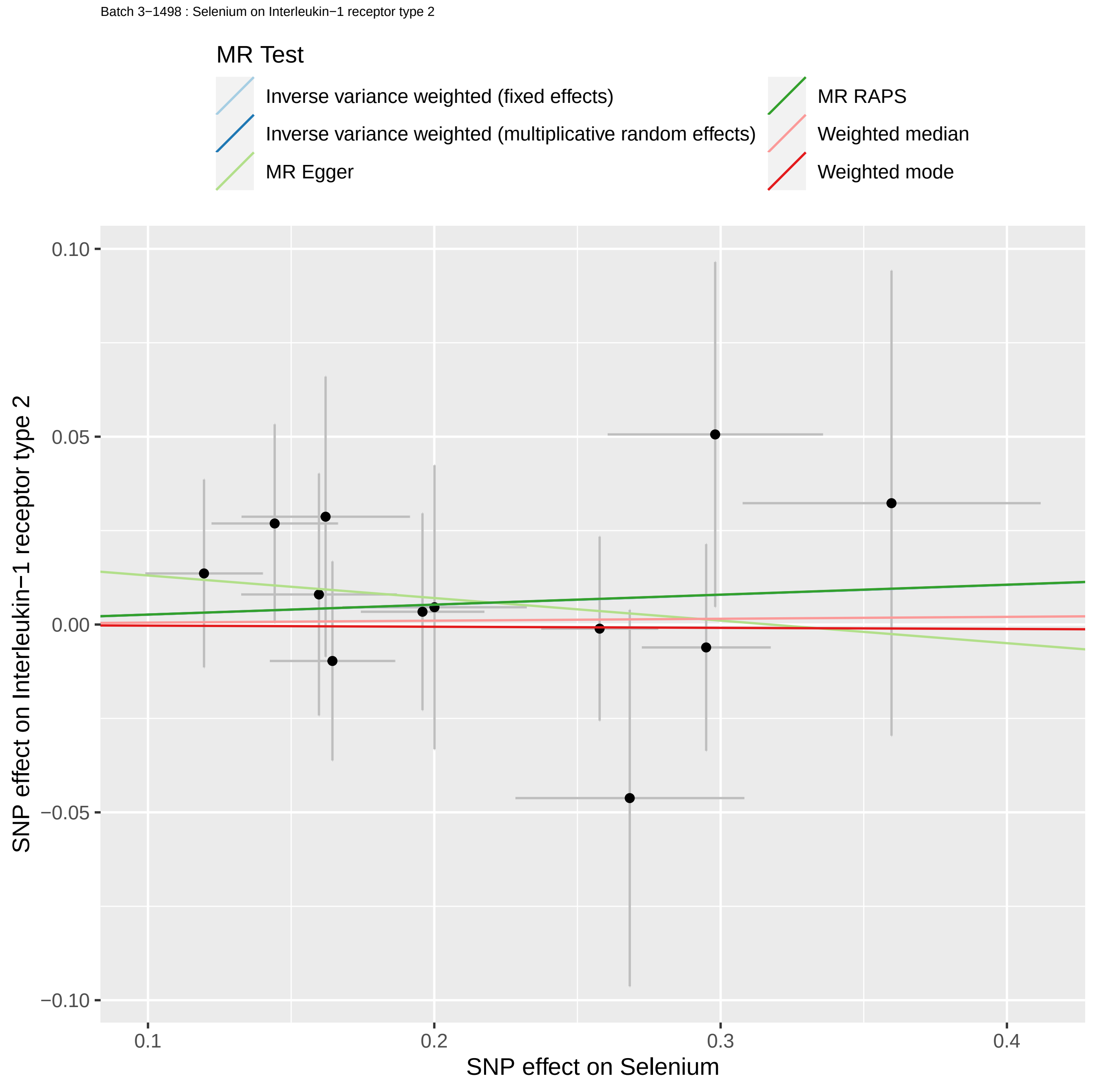
Batch 3−1498 : Selenium on Interleukin−1 receptor type 2
MR Test
Inverse variance weighted (fixed effects)
Inverse variance weighted (multiplicative random effects)
MR Egger
MR RAPS
Weighted median
Weighted mode
0.10
0.05
0.00
−0.05
−0.10
0.1
0.2
0.3
0.4
SNP effect on Interleukin−1 receptor type 2
SNP effect on Selenium

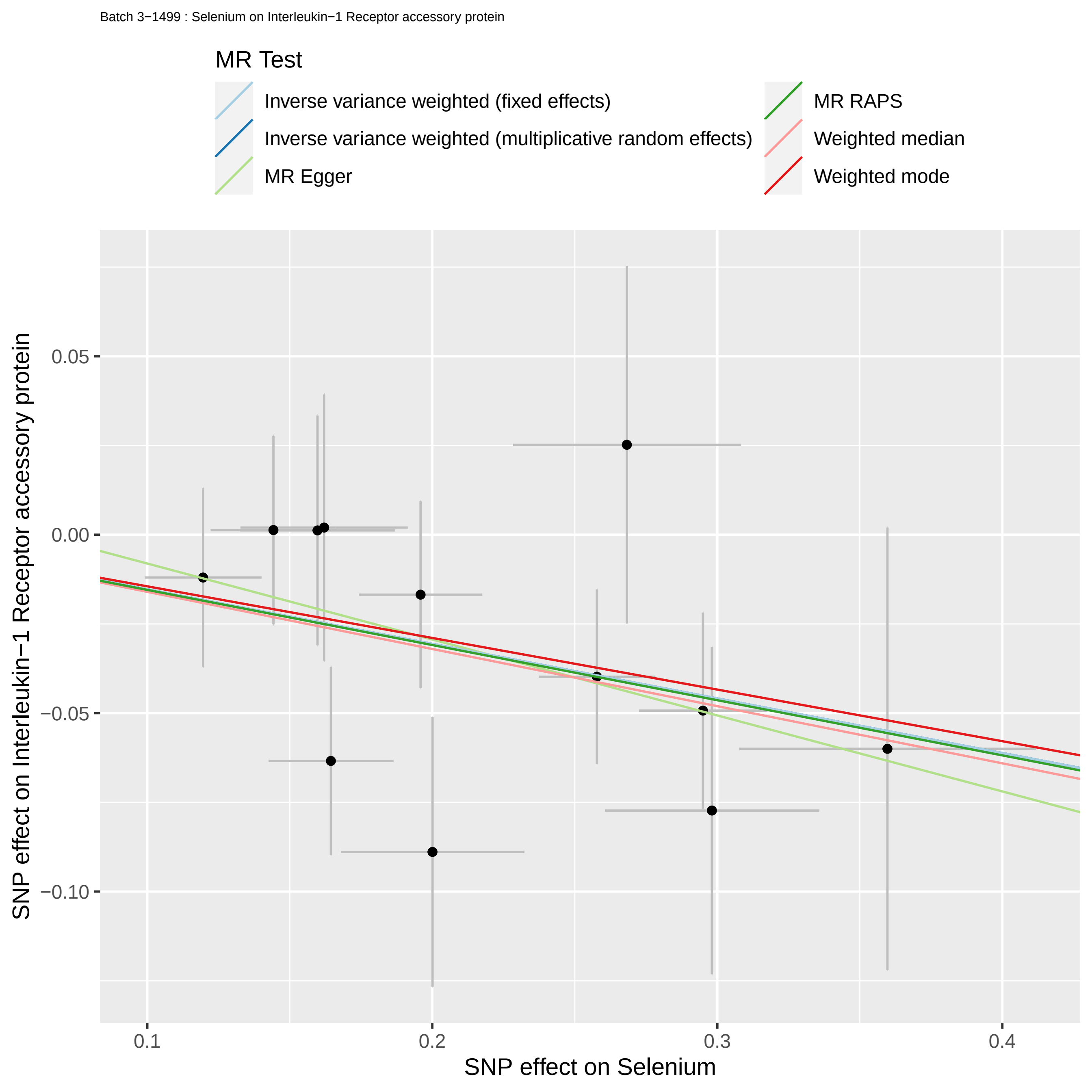

Batch 3−1499 : Selenium on Interleukin−1 Receptor accessory protein
MR Test
Inverse variance weighted (fixed effects)
Inverse variance weighted (multiplicative random effects)
MR Egger
MR RAPS
Weighted median
Weighted mode
0.05
0.00
−0.05
−0.10
0.1
0.2
0.3
0.4
SNP effect on Interleukin−1 Receptor accessory protein
SNP effect on Selenium

Batch 3−1500 : Selenium on Interleukin−1 Receptor accessory protein

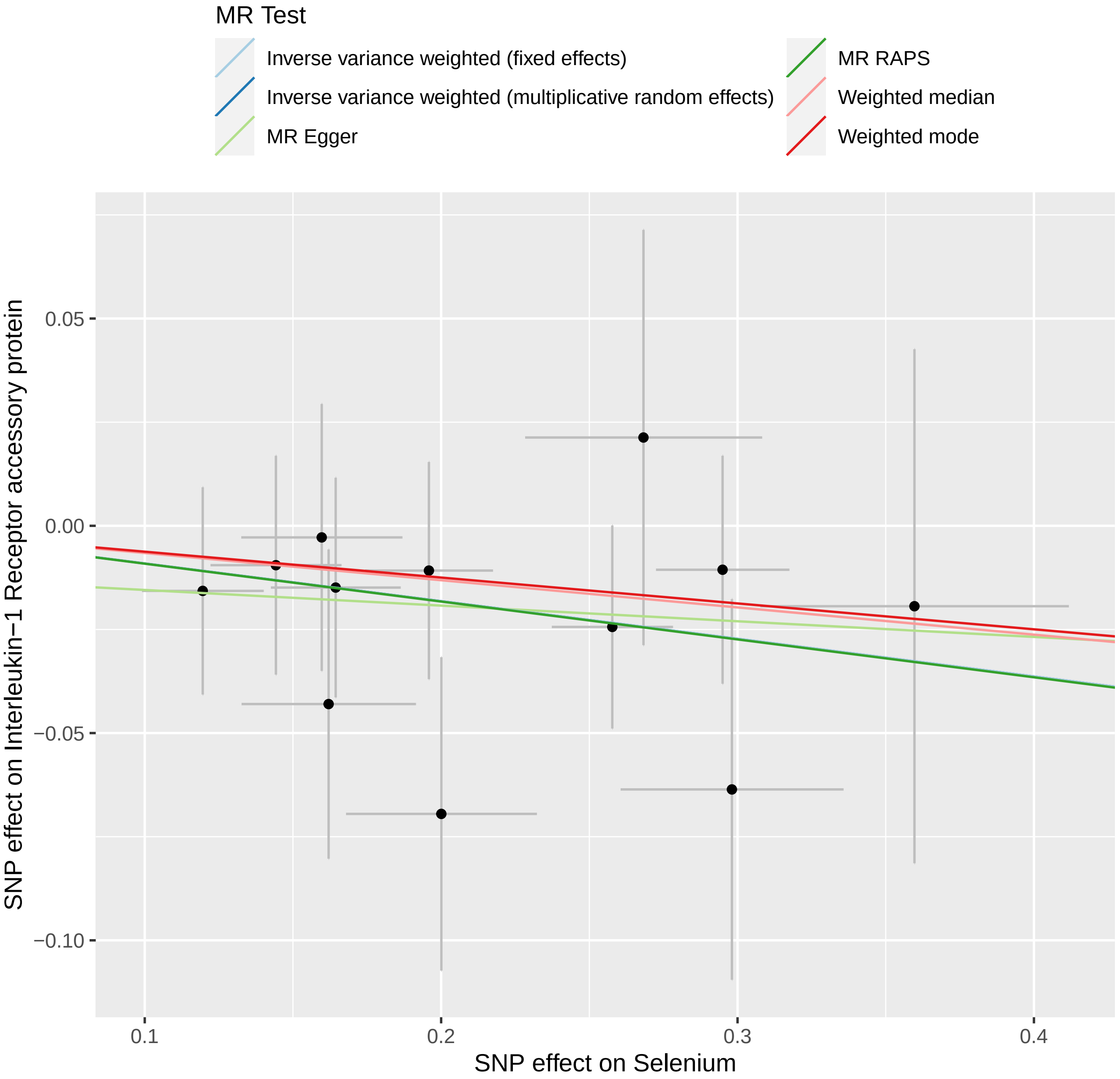

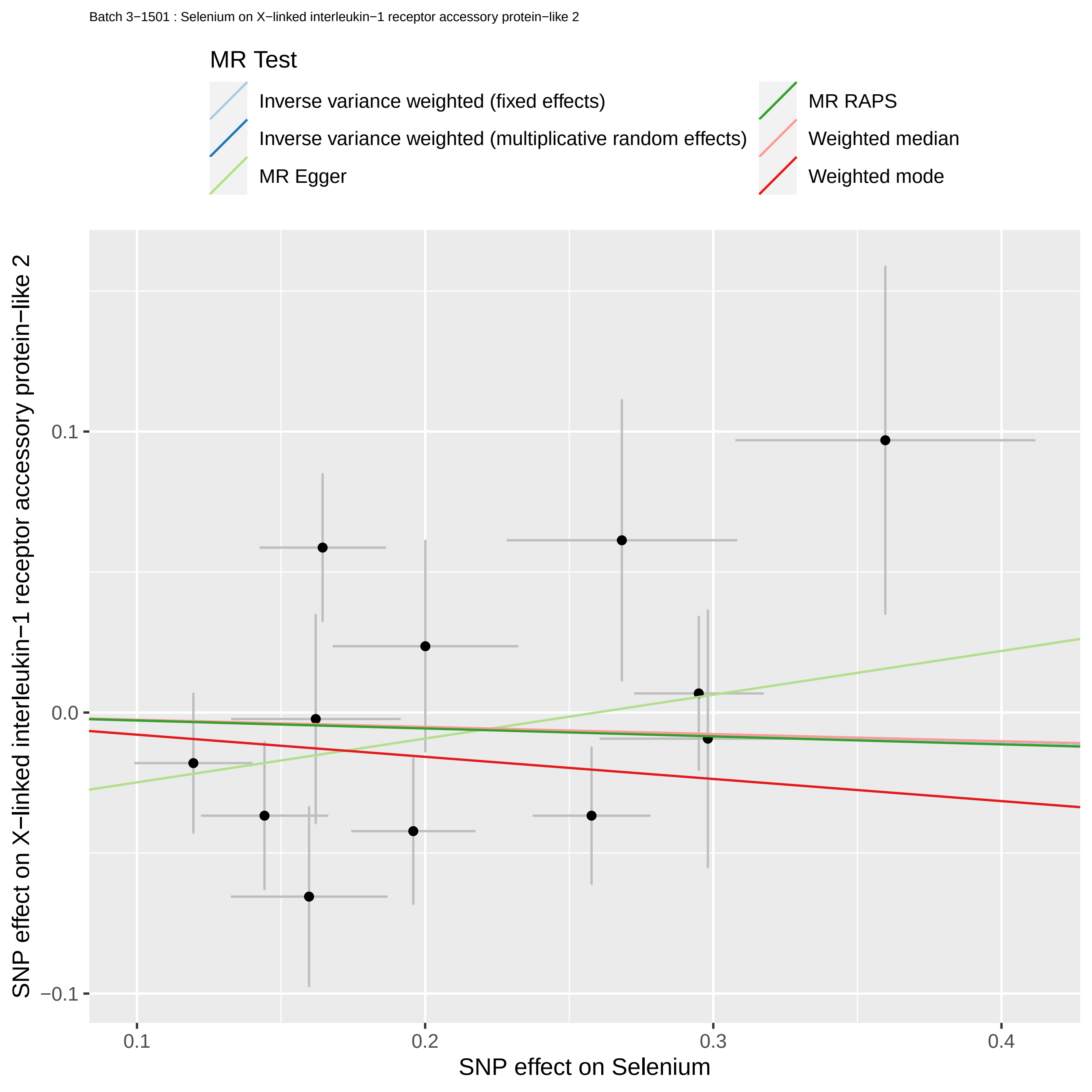

Batch 3−1501 : Selenium on X−linked interleukin−1 receptor accessory protein−like 2
MR Test
Inverse variance weighted (fixed effects)
Inverse variance weighted (multiplicative random effects)
MR Egger
MR RAPS
Weighted median
Weighted mode
0.1
0.0
−0.1
0.1
0.2
0.3
0.4
SNP effect on X−linked interleukin−1 receptor accessory protein−like 2
SNP effect on Selenium

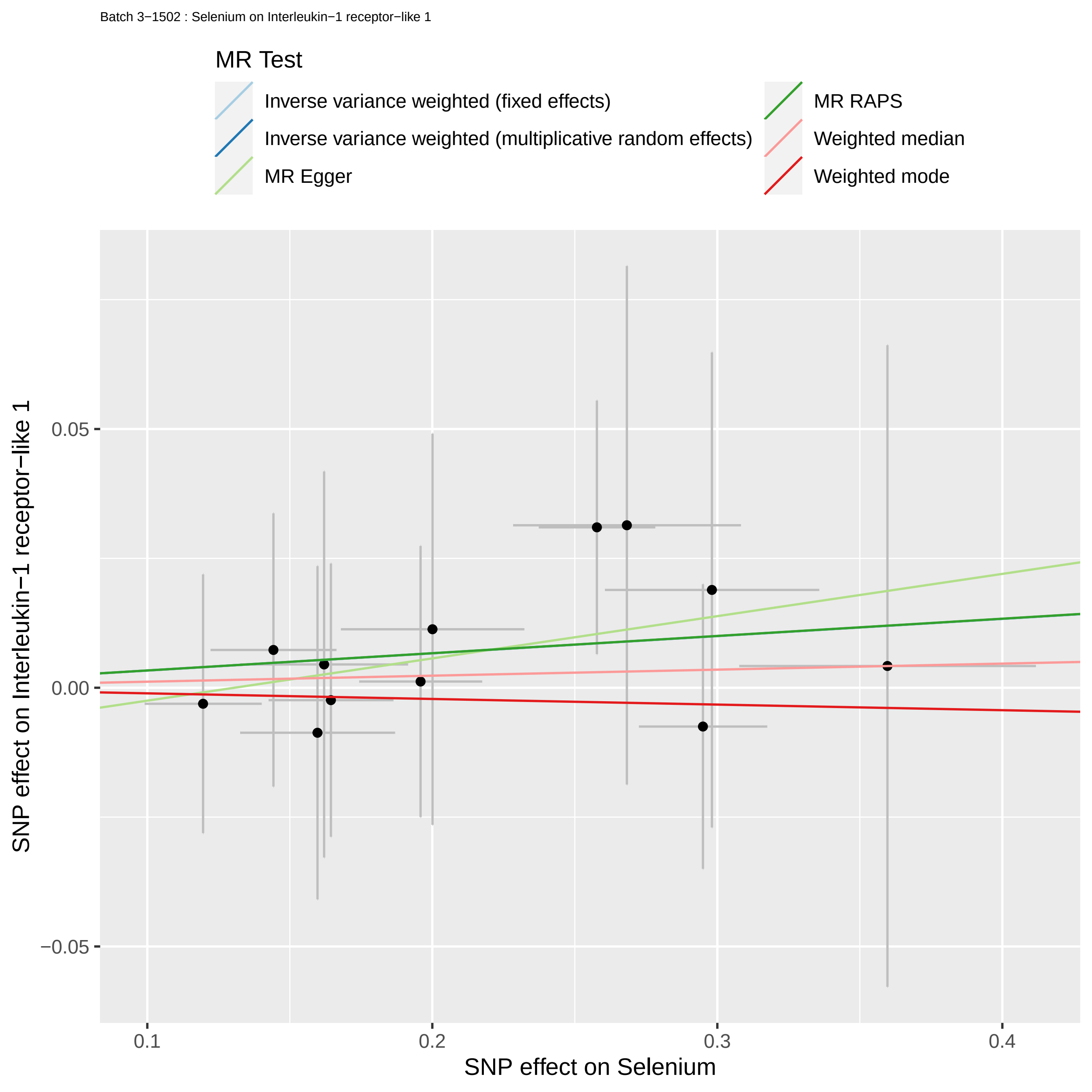
Batch 3−1502 : Selenium on Interleukin−1 receptor−like 1
MR Test
Inverse variance weighted (fixed effects)
Inverse variance weighted (multiplicative random effects)
MR Egger
MR RAPS
Weighted median
Weighted mode
0.05
0.00
−0.05
0.1
0.2
0.3
0.4
SNP effect on Interleukin−1 receptor−like 1
SNP effect on Selenium

Batch 3−1503 : Selenium on Interleukin−1 receptor−like 2

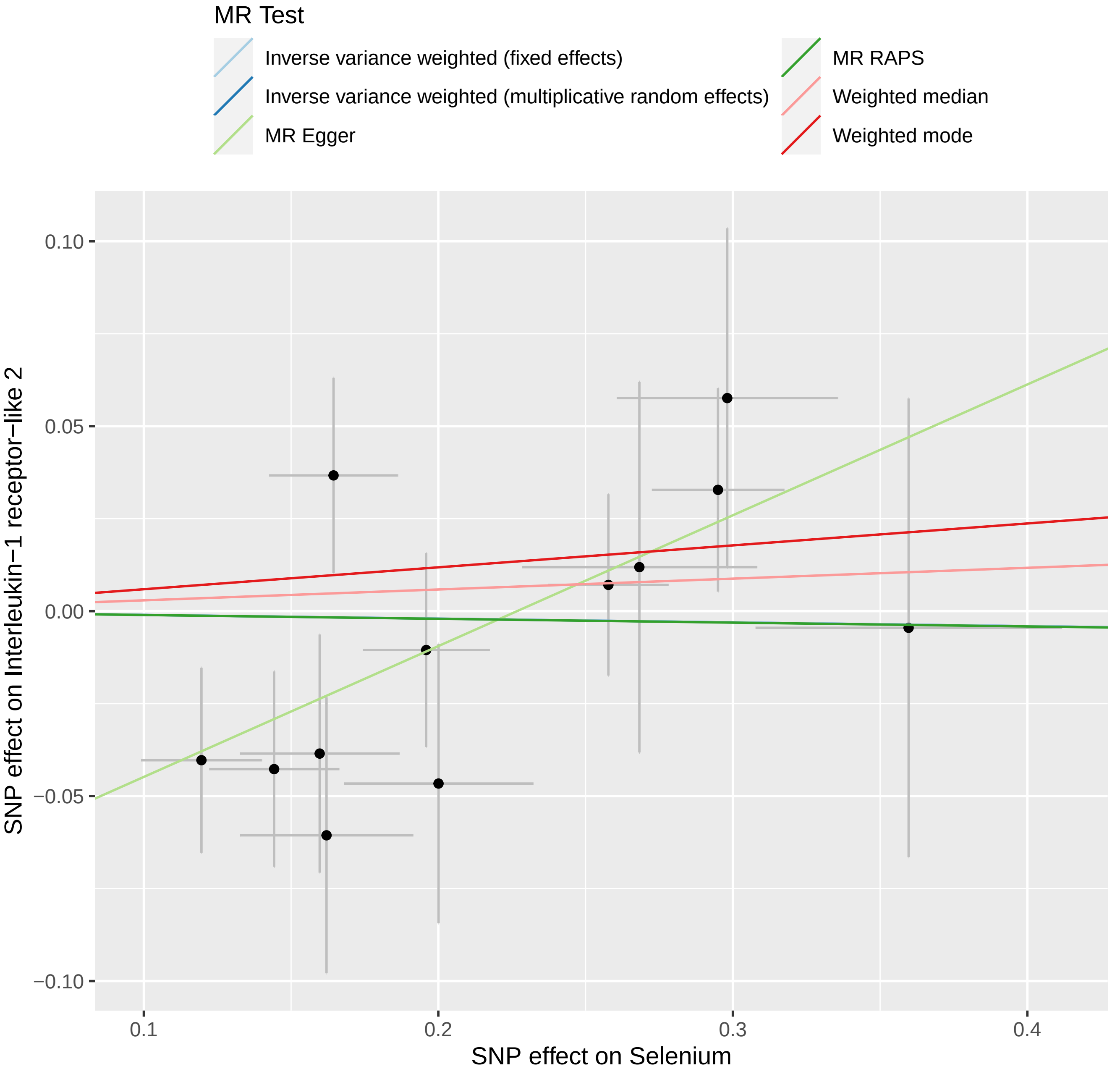

Batch 3−1504 : Selenium on Interleukin−1 receptor antagonist protein

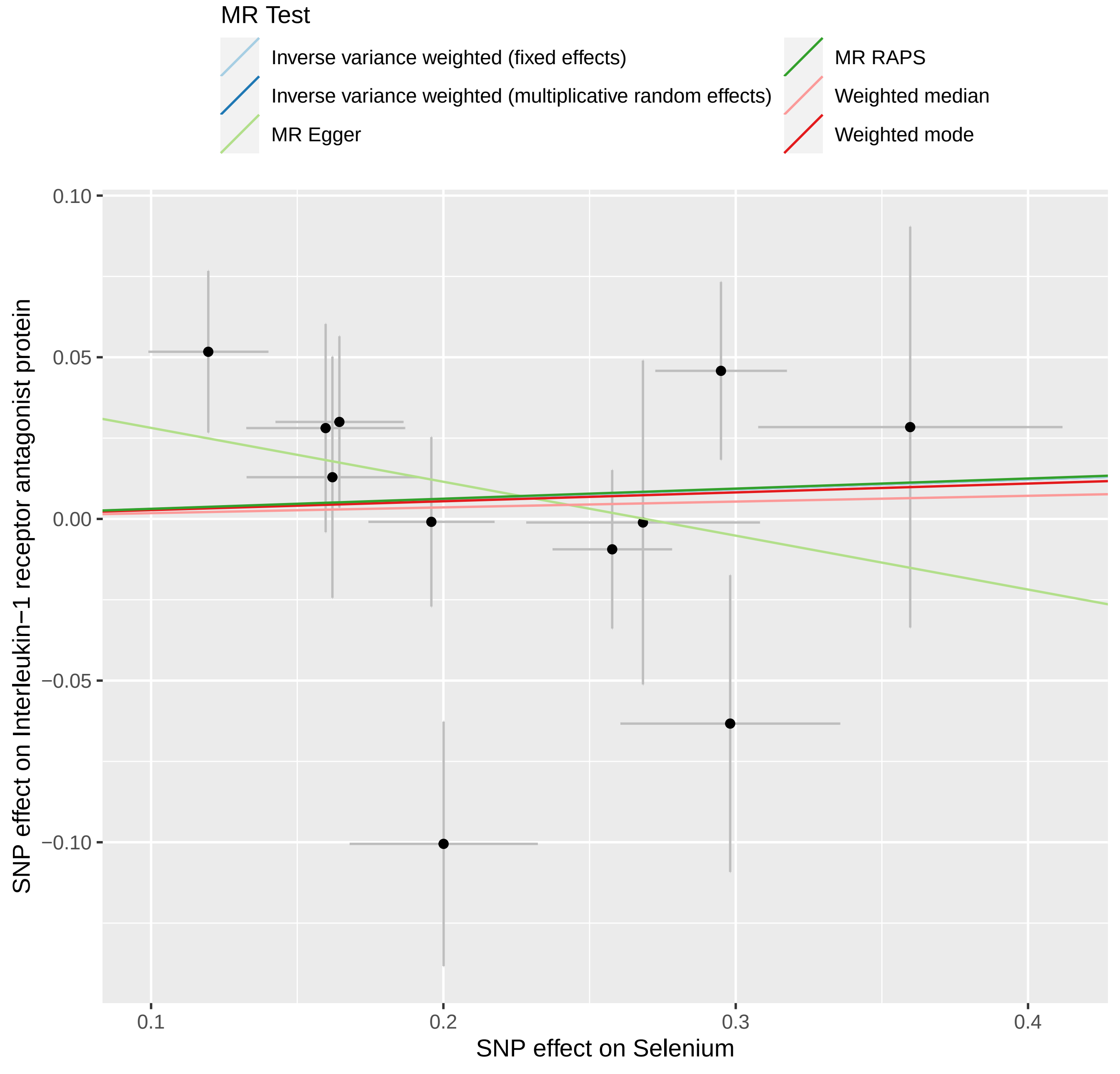

Batch 3−1505 : Selenium on Interleukin−20

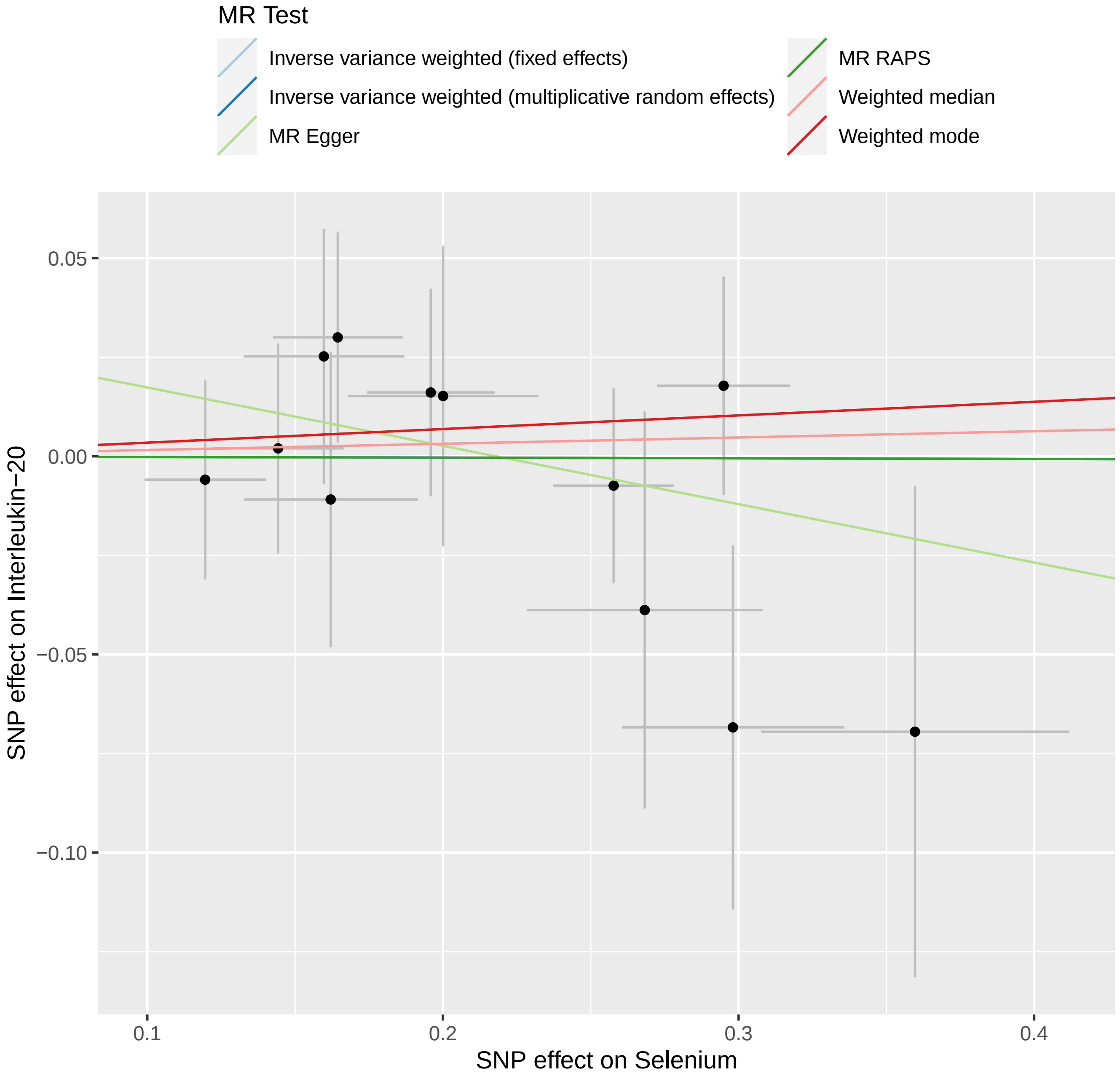

Batch 3−1506 : Selenium on Interleukin−21

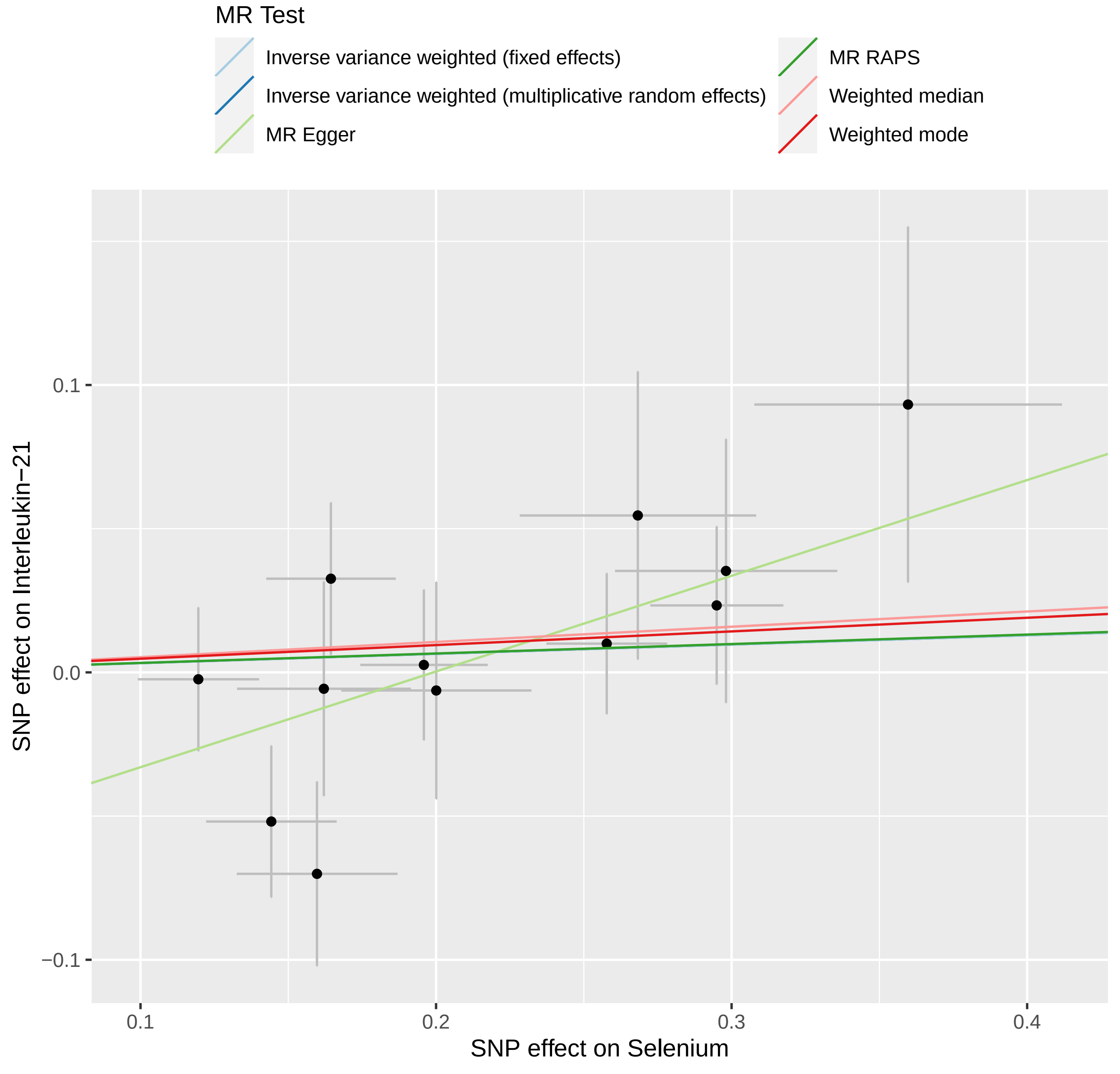

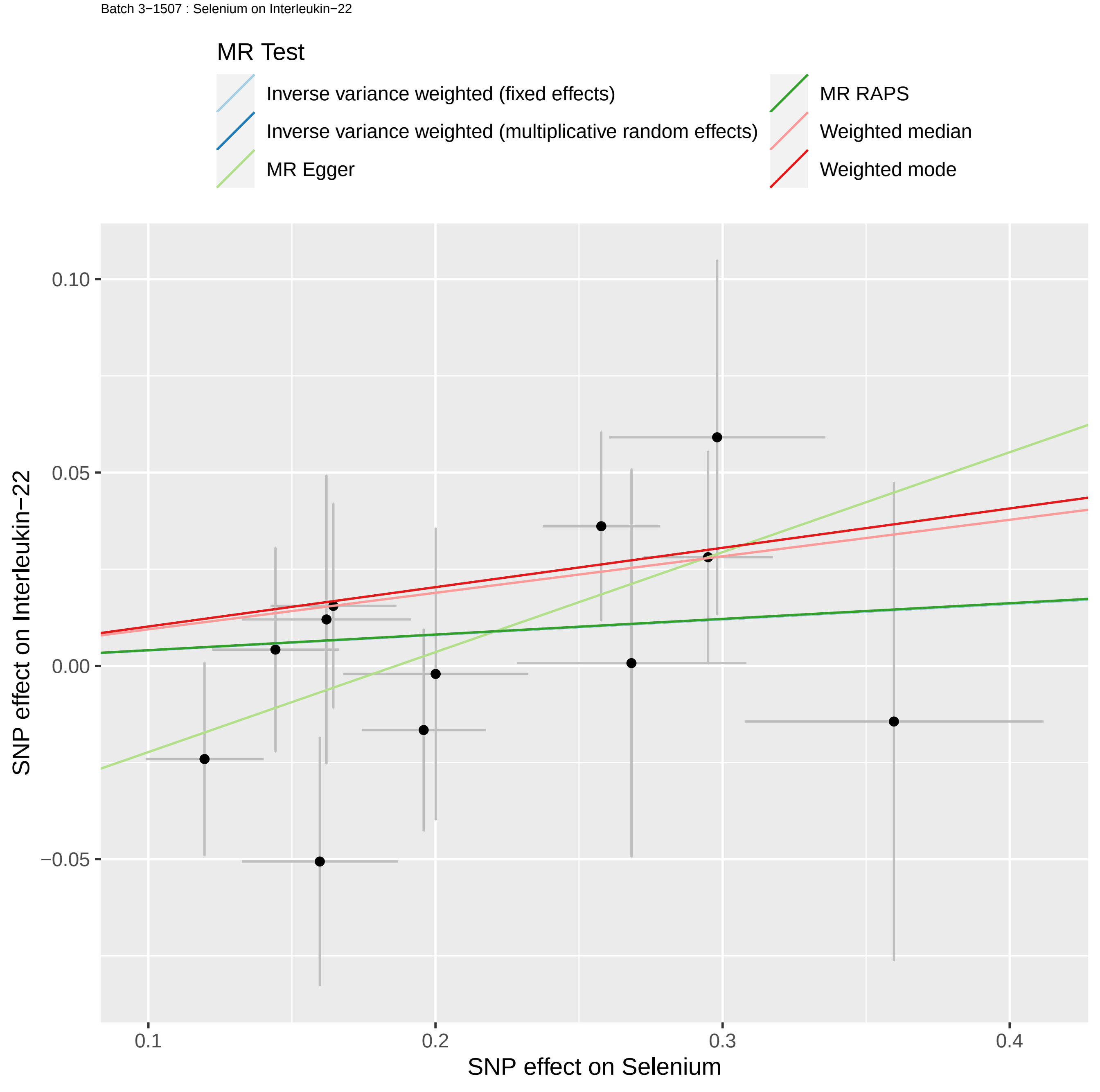

Batch 3−1507 : Selenium on Interleukin−22
MR Test
Inverse variance weighted (fixed effects)
Inverse variance weighted (multiplicative random effects)
MR Egger
MR RAPS
Weighted median
Weighted mode
0.10
0.05
0.00
−0.05
0.1
0.2
0.3
0.4
SNP effect on Selenium
SNP effect on Interleukin−22

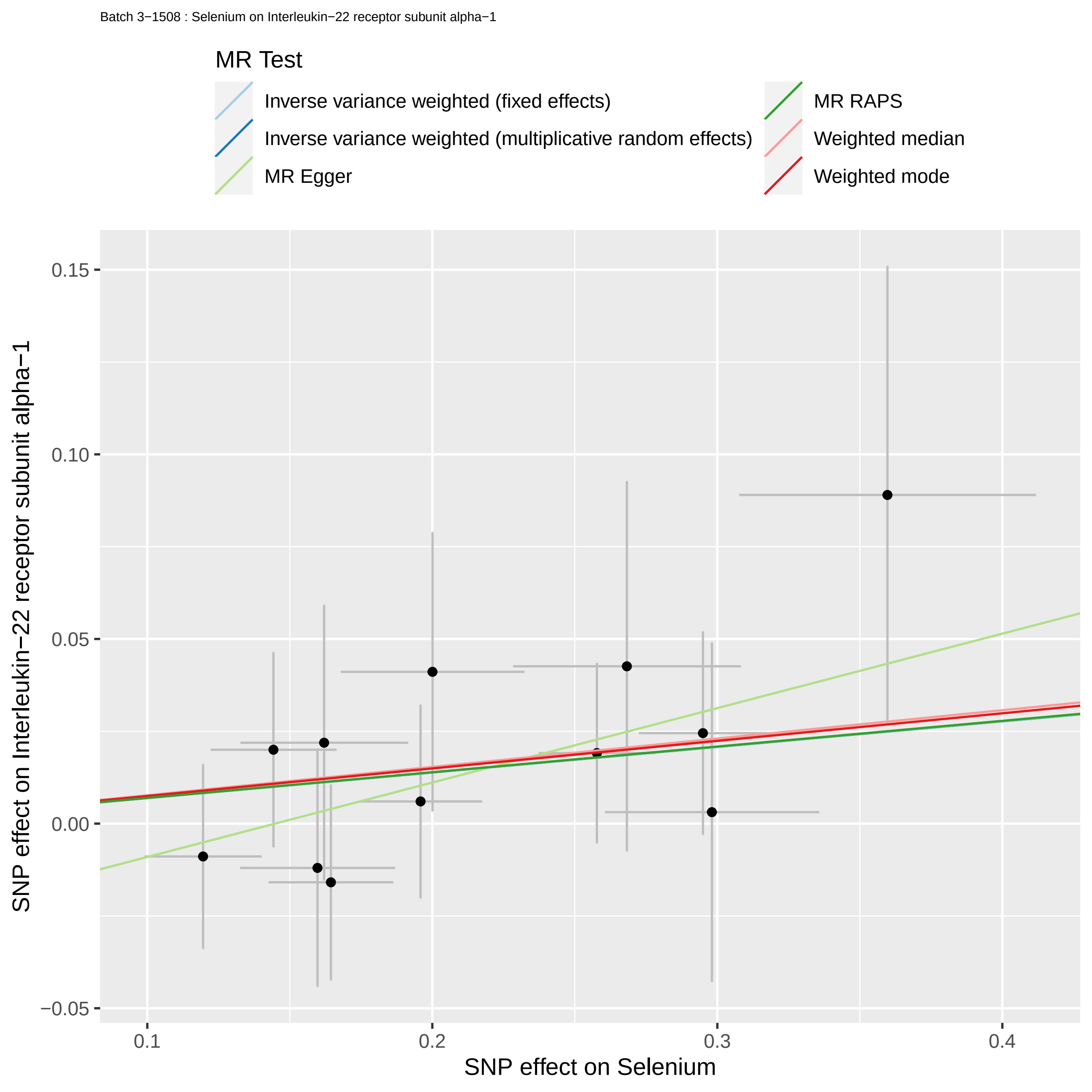

Batch 3−1508 : Selenium on Interleukin−22 receptor subunit alpha−1
MR Test
Inverse variance weighted (fixed effects)
Inverse variance weighted (multiplicative random effects)
MR Egger
MR RAPS
Weighted median
Weighted mode
0.15
0.10
0.05
0.00
−0.05
0.1
0.2
0.3
0.4
SNP effect on Interleukin−22 receptor subunit alpha−1
SNP effect on Selenium

Batch 3−1509 : Selenium on Interleukin−22 receptor subunit alpha−1

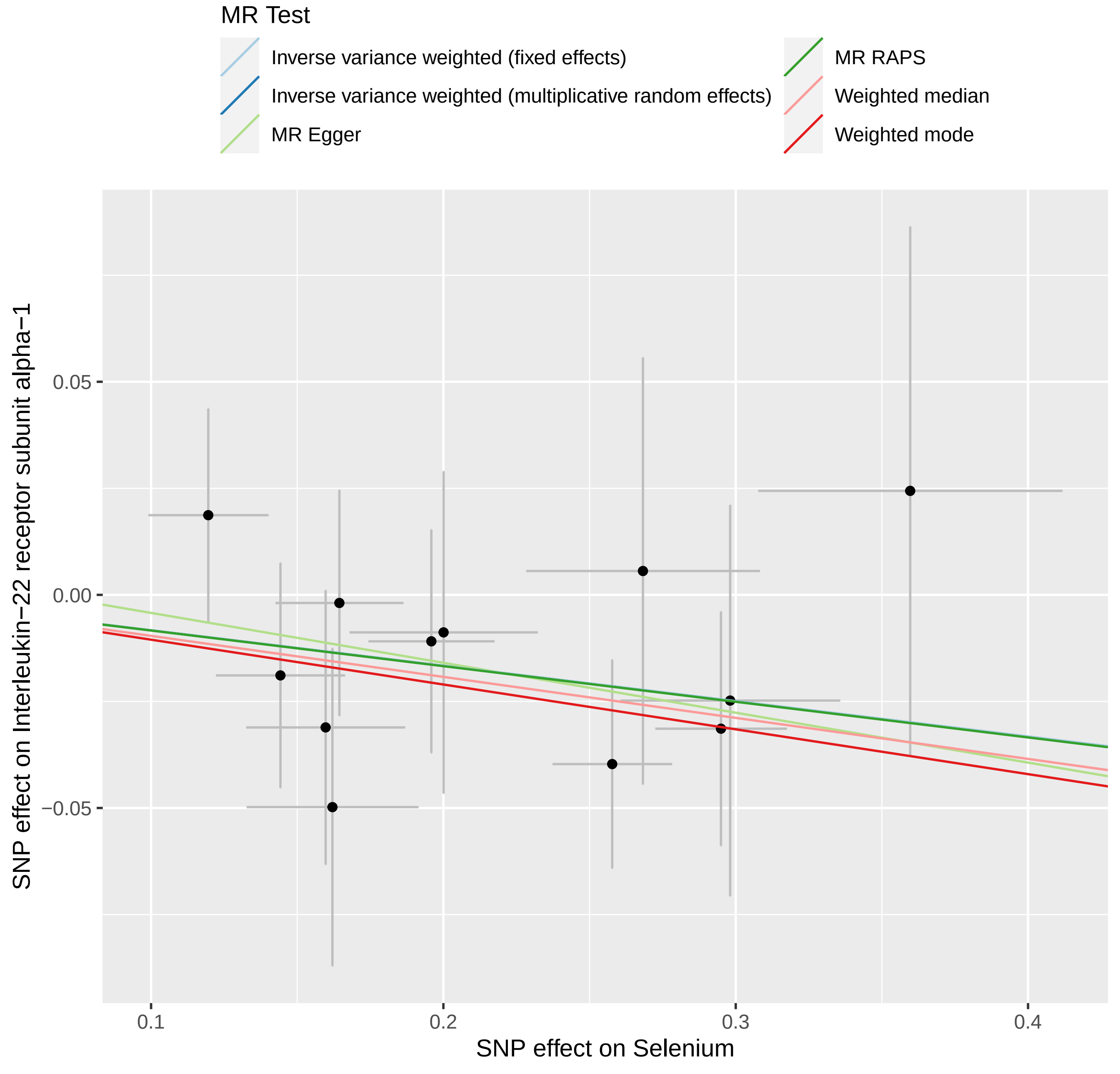

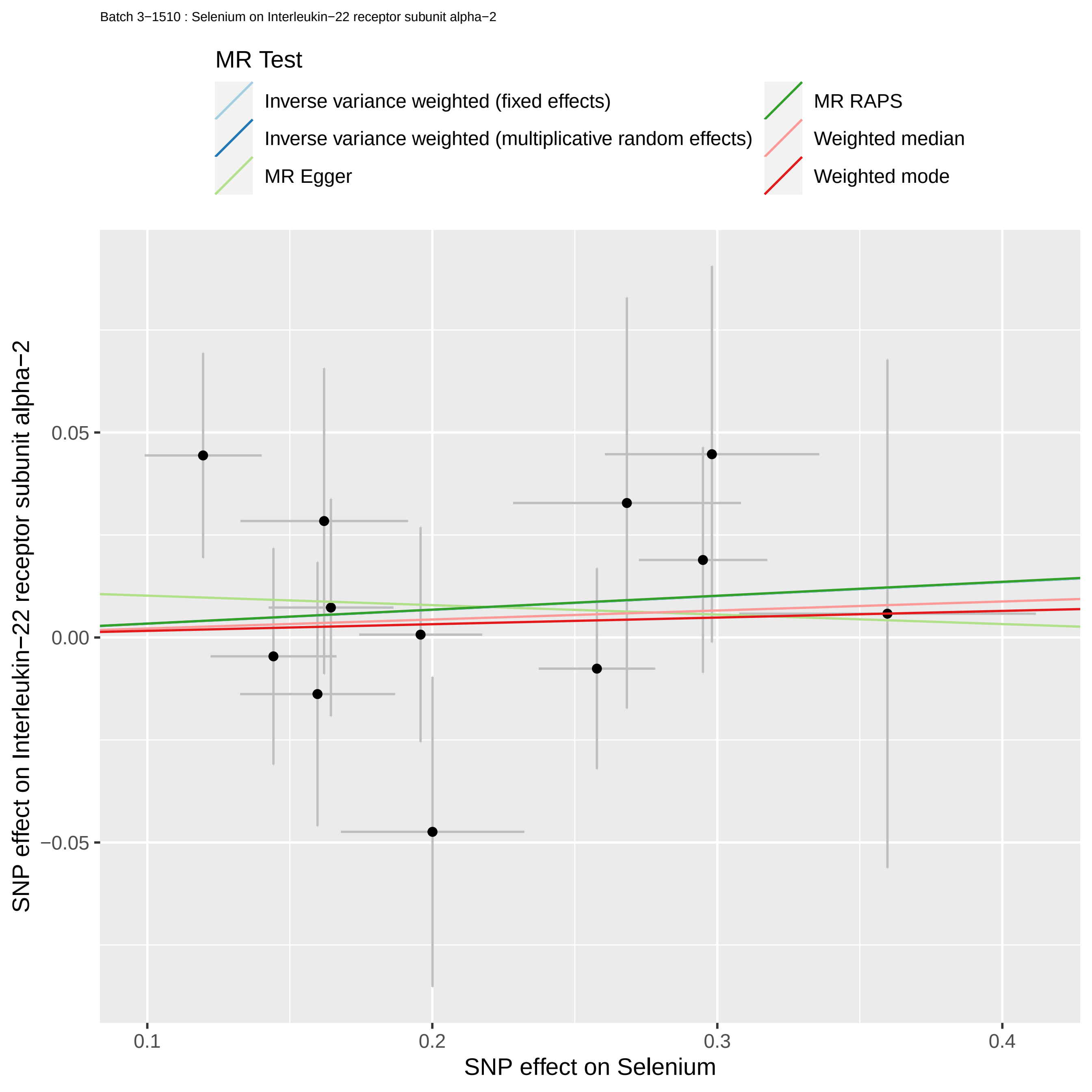

Batch 3−1510 : Selenium on Interleukin−22 receptor subunit alpha−2
MR Test
Inverse variance weighted (fixed effects)
Inverse variance weighted (multiplicative random effects)
MR Egger
MR RAPS
Weighted median
Weighted mode
0.05
0.00
−0.05
0.1
0.2
0.3
0.4
SNP effect on Interleukin−22 receptor subunit alpha−2
SNP effect on Selenium

Batch 3−1511 : Selenium on Interleukin−22 receptor subunit alpha−2

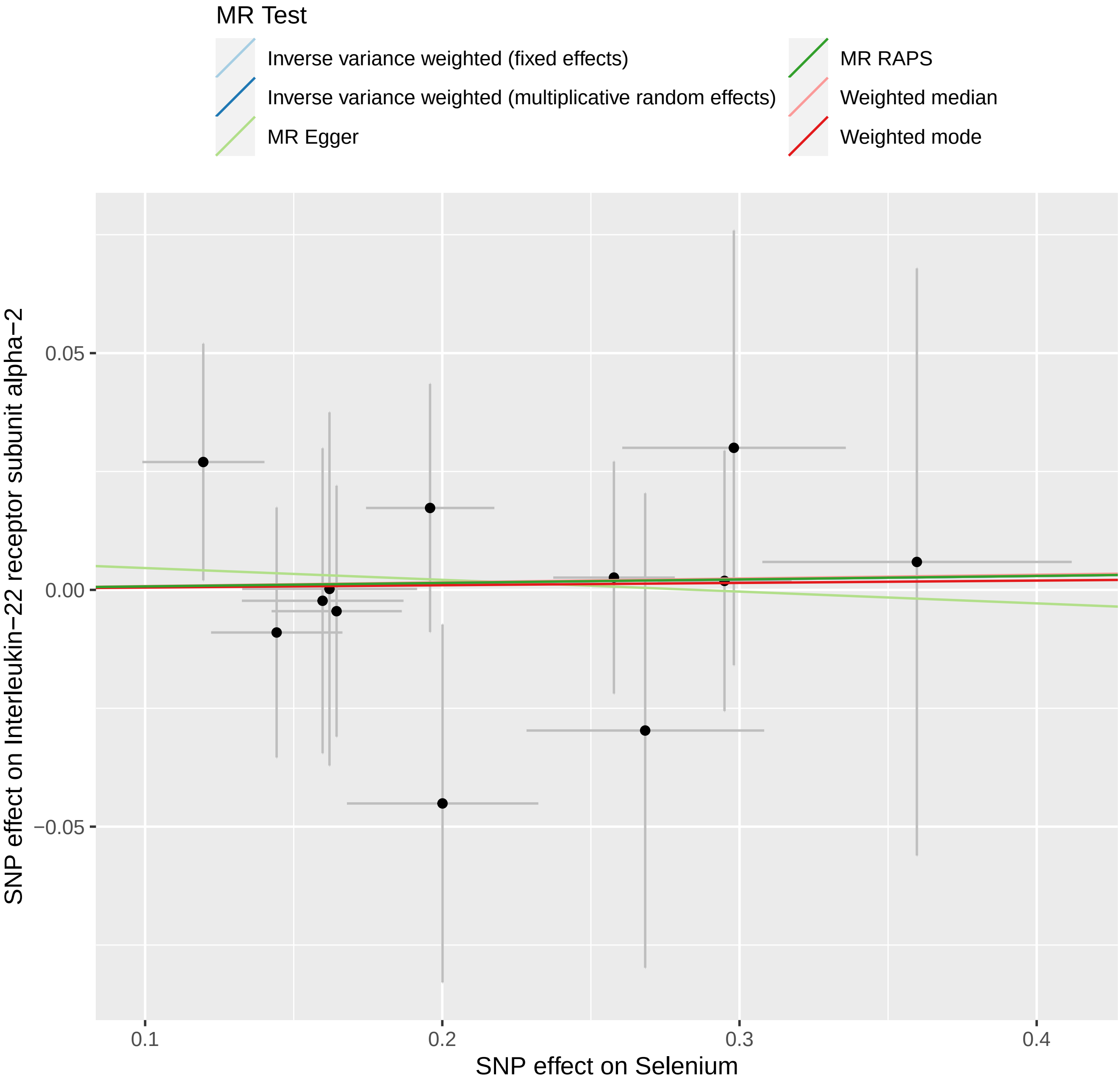

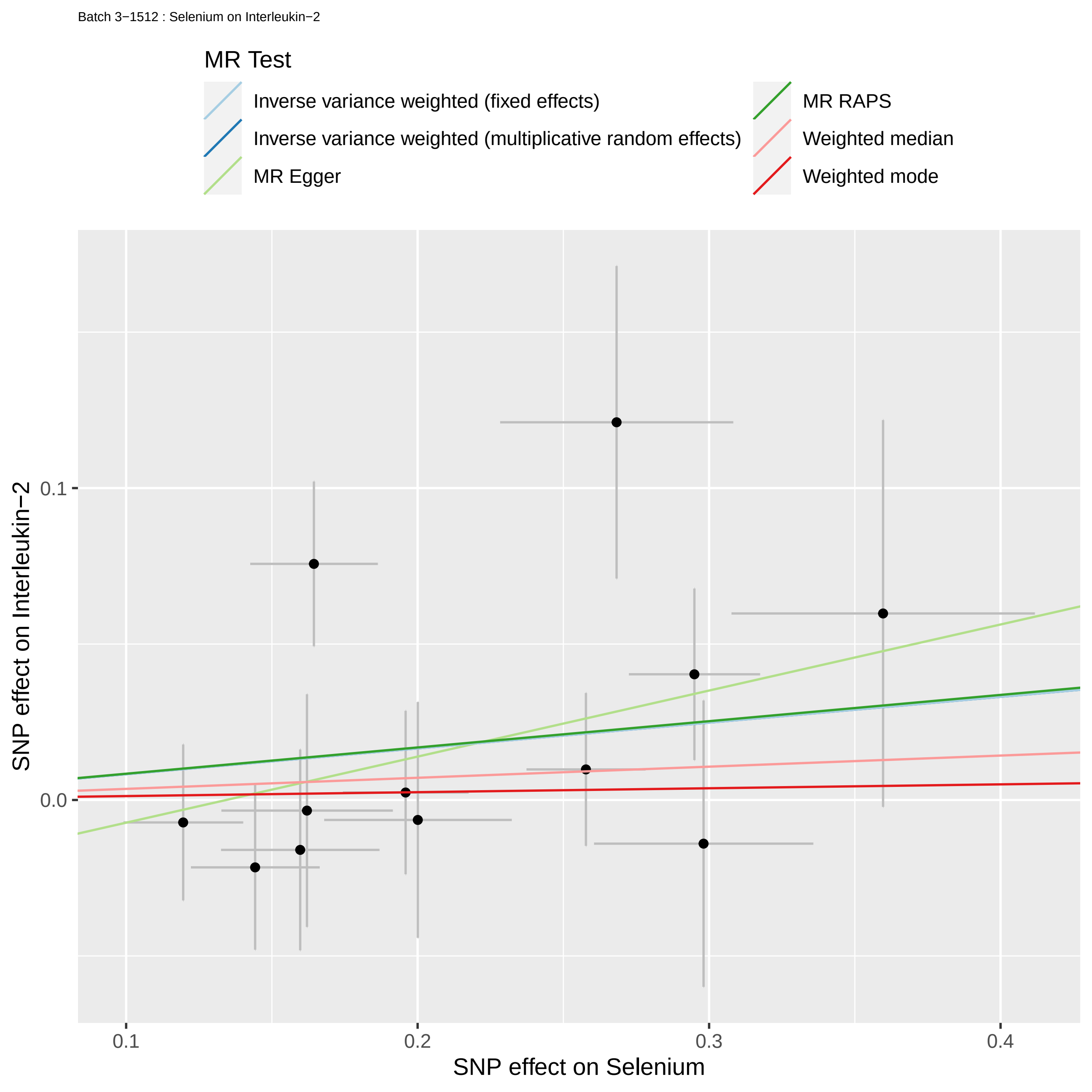
Batch 3−1512 : Selenium on Interleukin−2
MR Test
Inverse variance weighted (fixed effects)
Inverse variance weighted (multiplicative random effects)
MR Egger
MR RAPS
Weighted median
Weighted mode
SNP effect on Interleukin−2
0.1
0.0
0.1
0.2
0.3
0.4
SNP effect on Selenium

Batch 3−1513 : Selenium on Interleukin−23 receptor

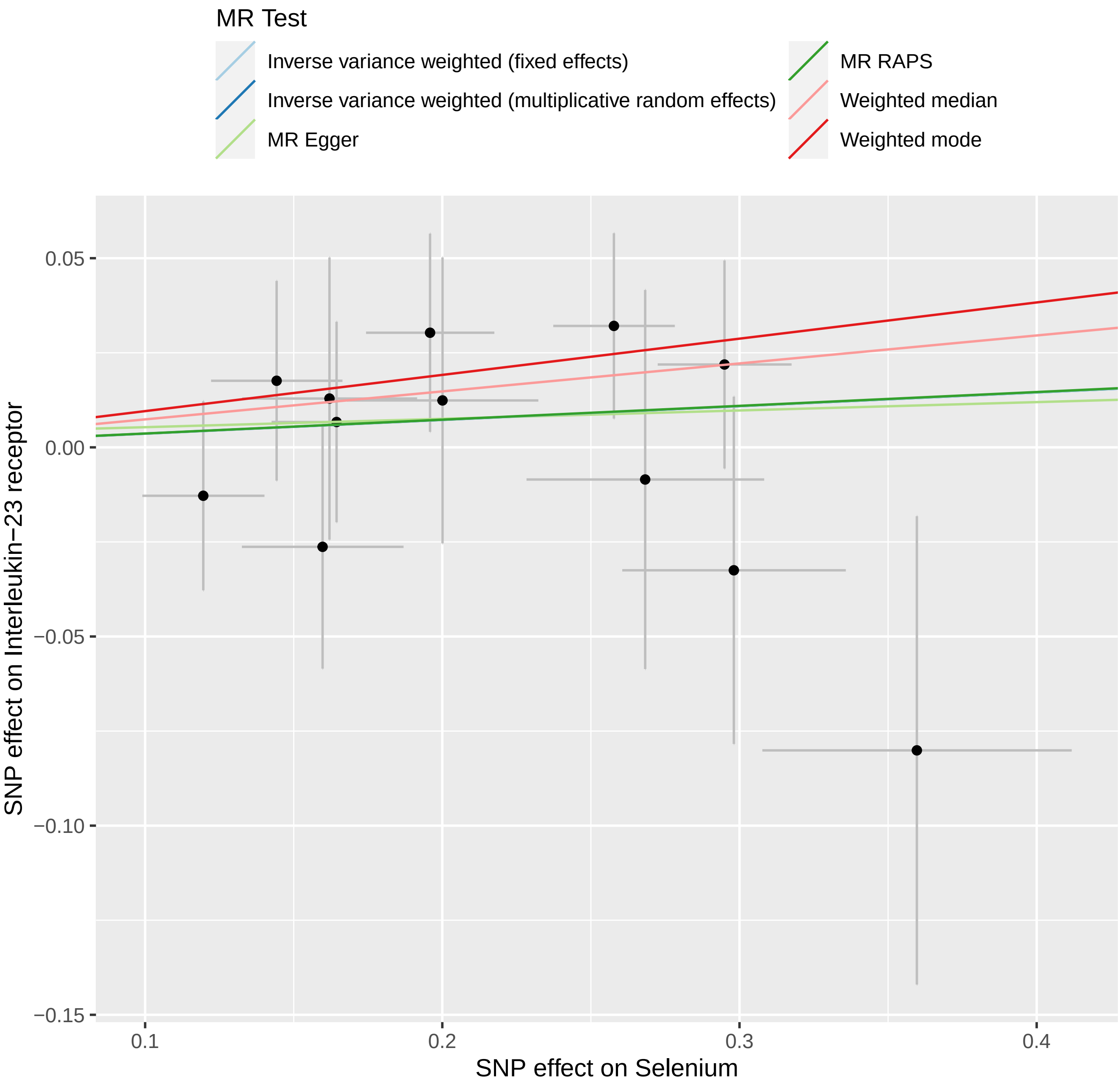

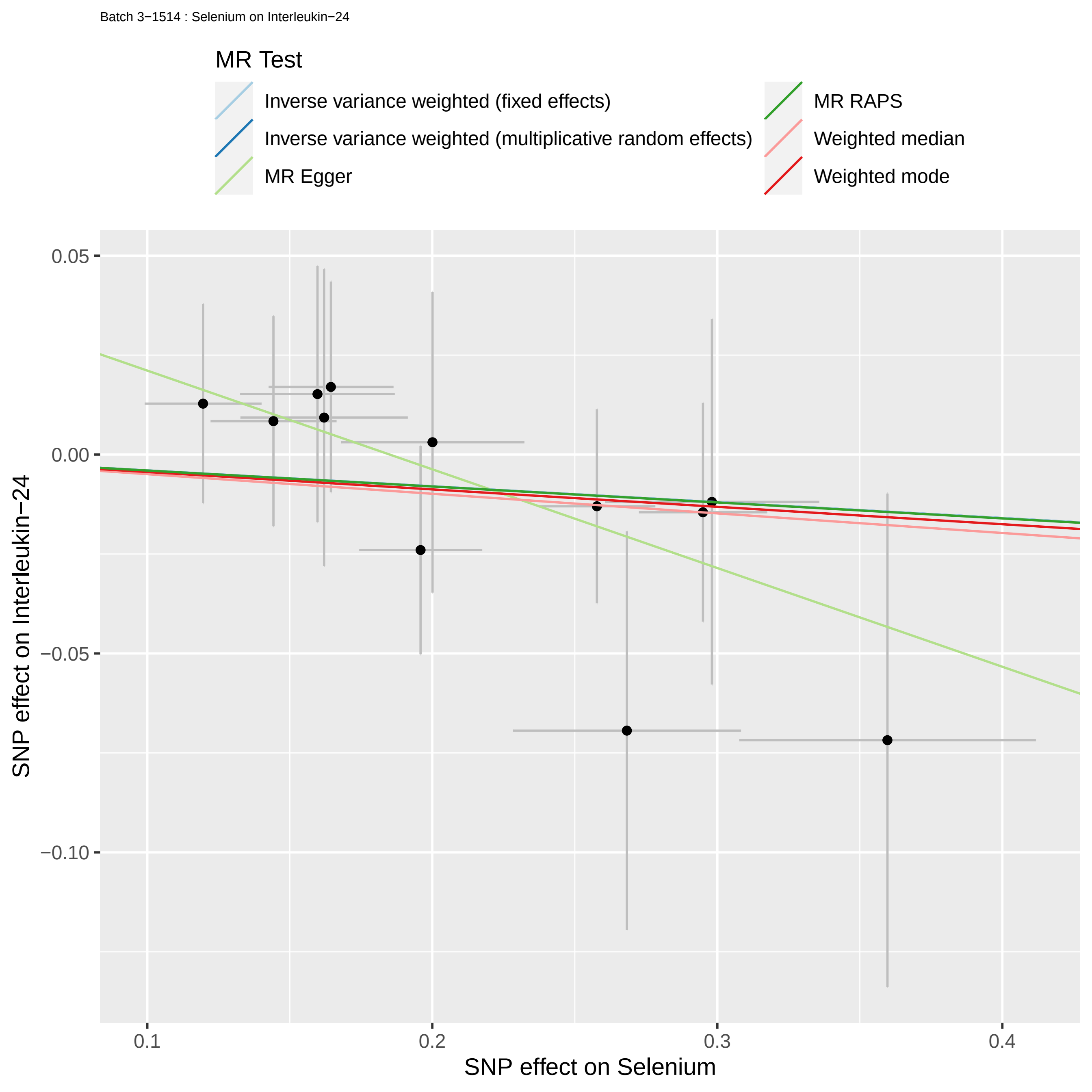
Batch 3−1514 : Selenium on Interleukin−24
MR Test
Inverse variance weighted (fixed effects)
Inverse variance weighted (multiplicative random effects)
MR Egger
MR RAPS
Weighted median
Weighted mode
0.05
0.00
−0.05
−0.10
0.1
0.2
0.3
0.4
SNP effect on Interleukin−24
SNP effect on Selenium

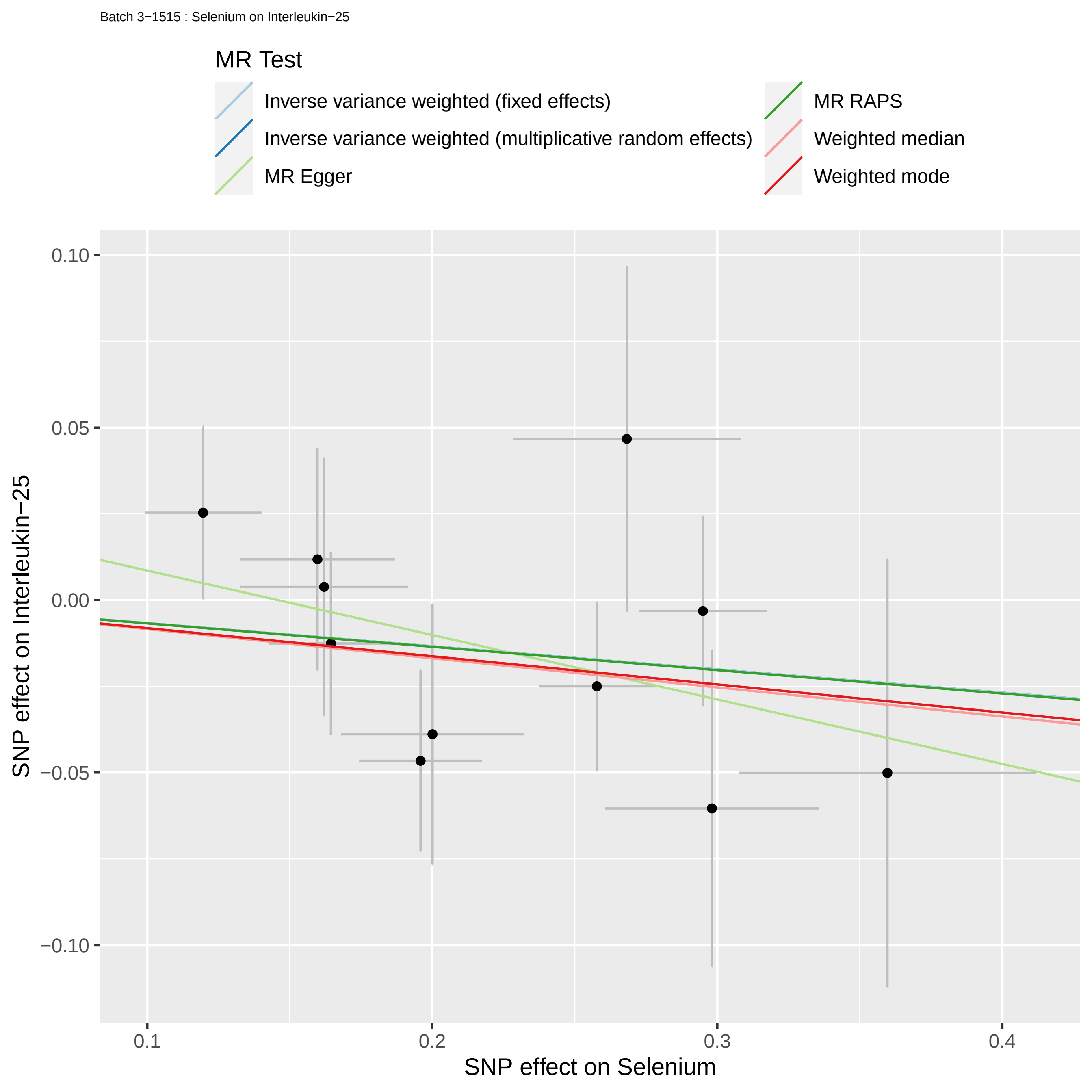

Batch 3−1515 : Selenium on Interleukin−25
MR Test
Inverse variance weighted (fixed effects)
Inverse variance weighted (multiplicative random effects)
MR Egger
MR RAPS
Weighted median
Weighted mode
0.10
0.05
0.00
−0.05
−0.10
0.1
0.2
0.3
0.4
SNP effect on Interleukin−25
SNP effect on Selenium

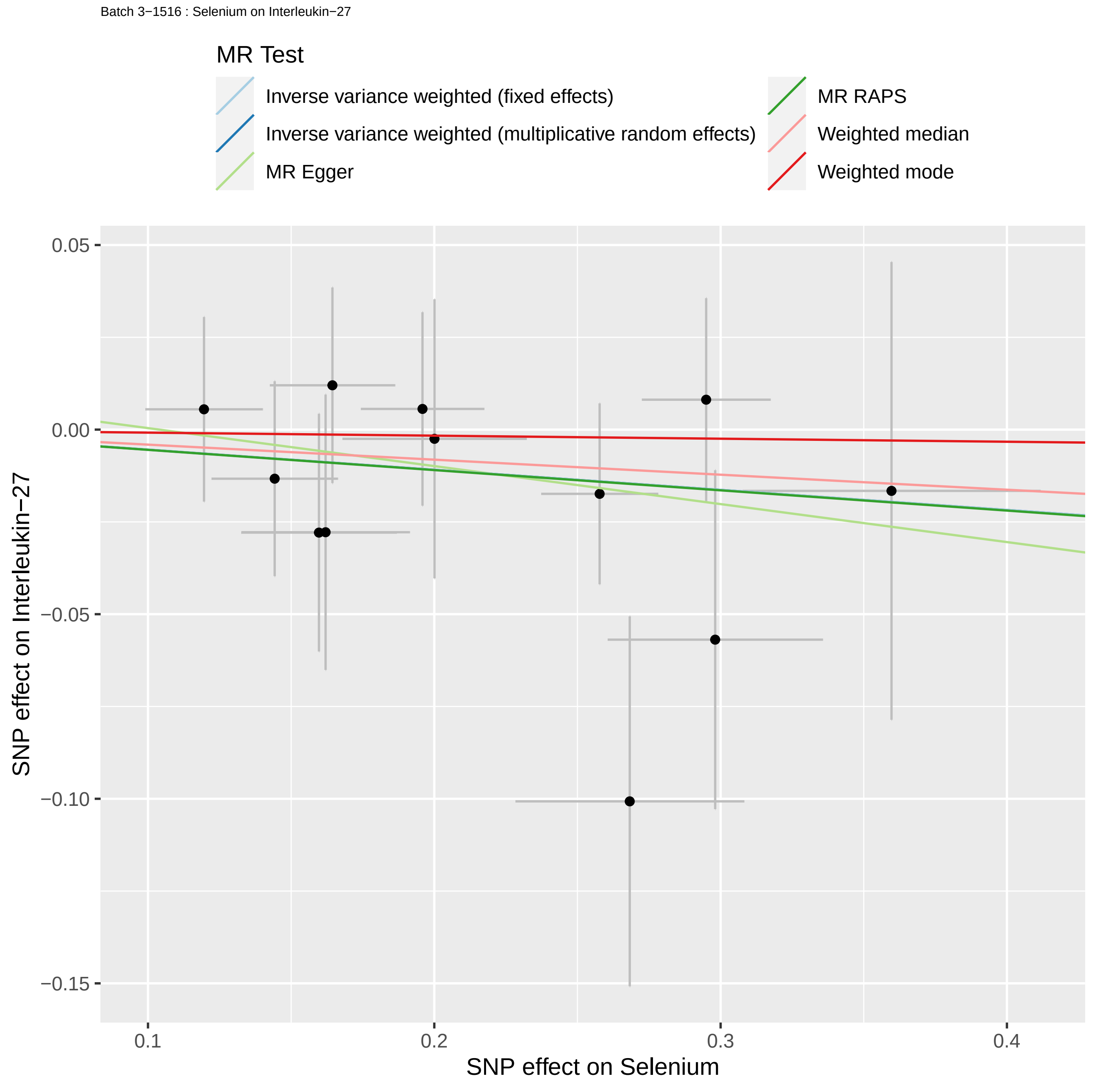

Batch 3−1516 : Selenium on Interleukin−27
MR Test
Inverse variance weighted (fixed effects)
Inverse variance weighted (multiplicative random effects)
MR Egger
MR RAPS
Weighted median
Weighted mode
0.05
0.00
−0.05
−0.10
−0.15
0.1
0.2
0.3
0.4
SNP effect on Interleukin−27
SNP effect on Selenium

Batch 3−1517 : Selenium on Interleukin−27 receptor subunit alpha

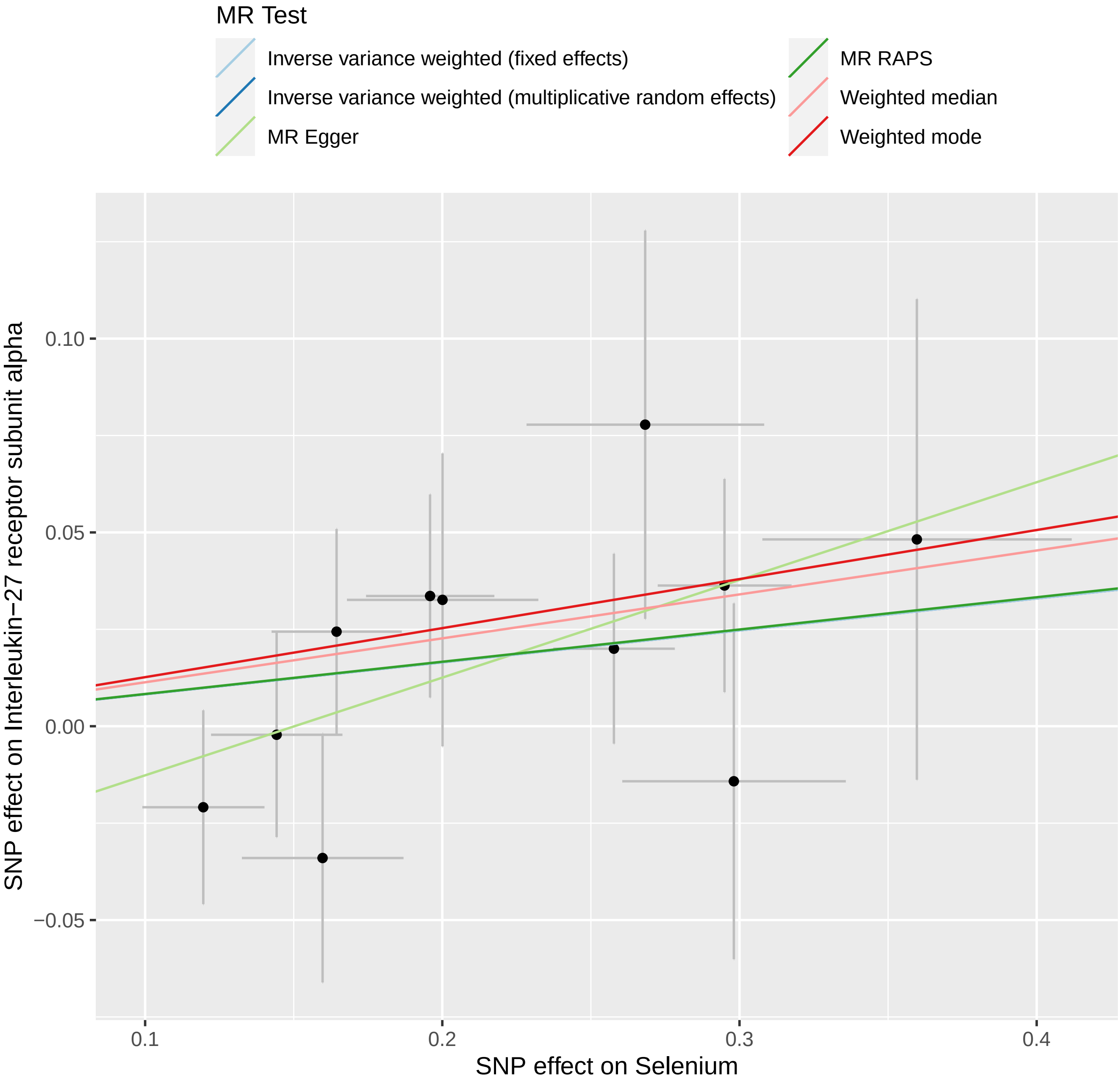

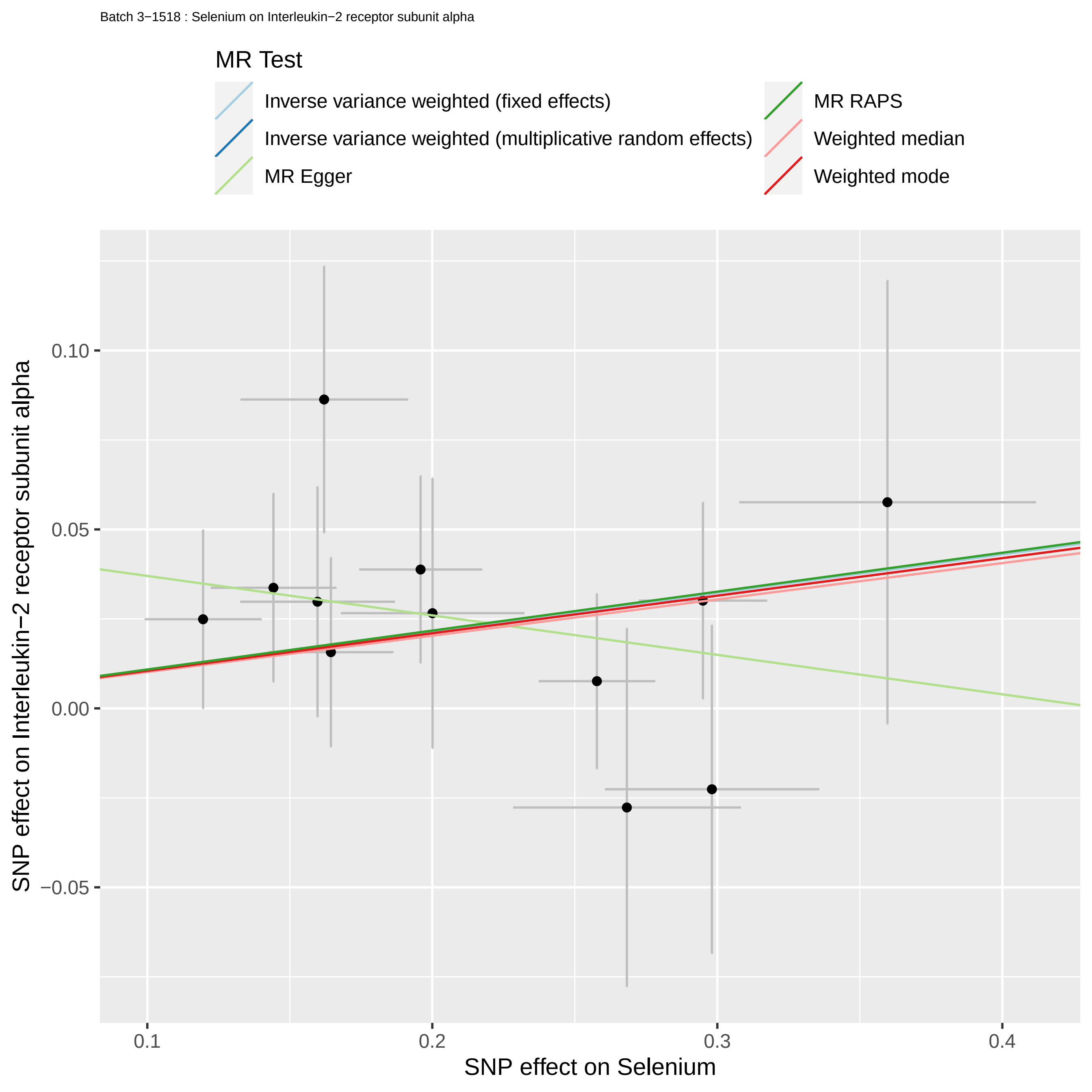

Batch 3−1518 : Selenium on Interleukin−2 receptor subunit alpha
MR Test
Inverse variance weighted (fixed effects)
Inverse variance weighted (multiplicative random effects)
MR Egger
MR RAPS
Weighted median
Weighted mode
0.10
0.05
0.00
−0.05
0.1
0.2
0.3
0.4
SNP effect on Interleukin−2 receptor subunit alpha
SNP effect on Selenium

Batch 3−1519 : Selenium on Interleukin−2 receptor subunit beta

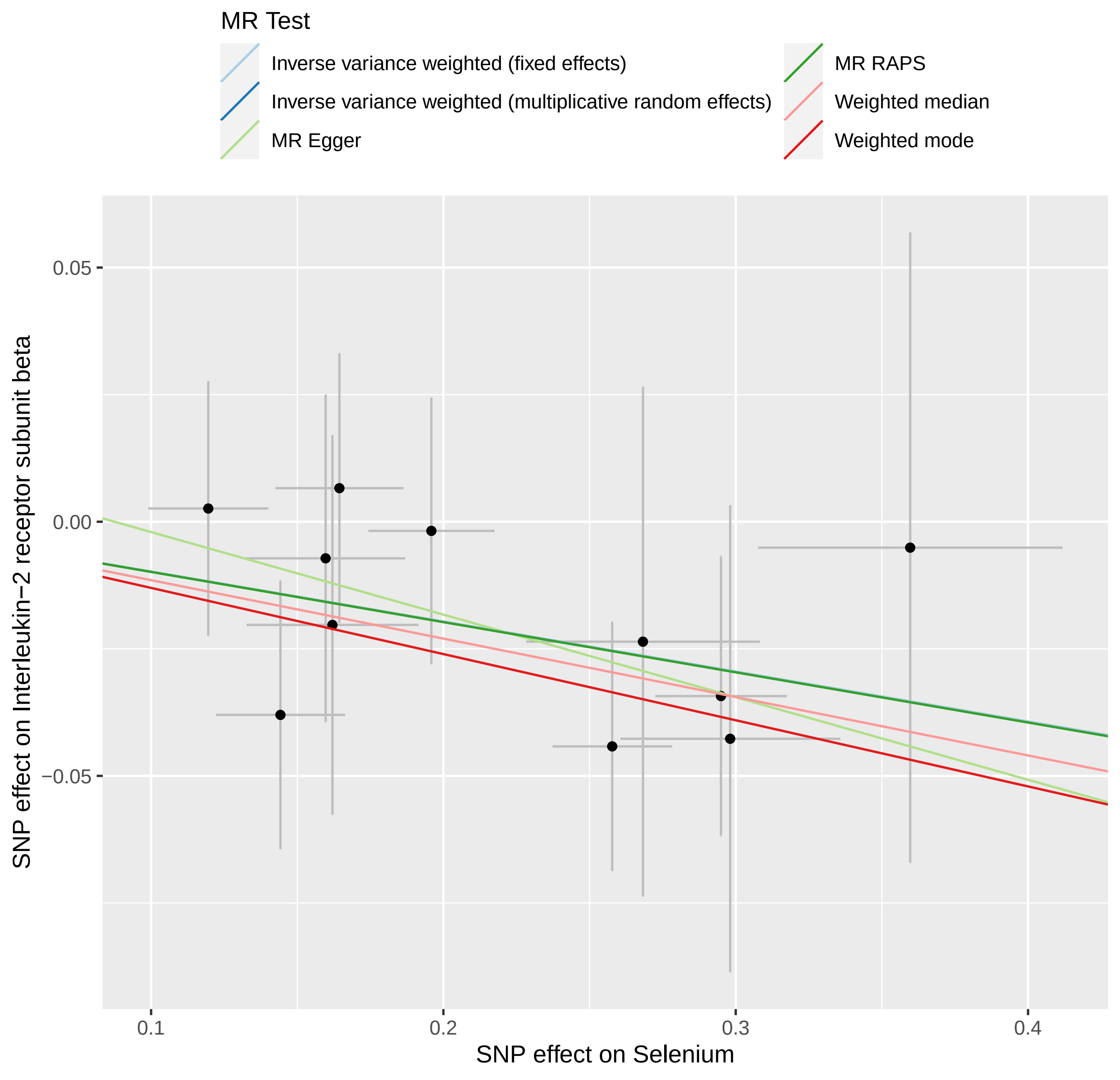

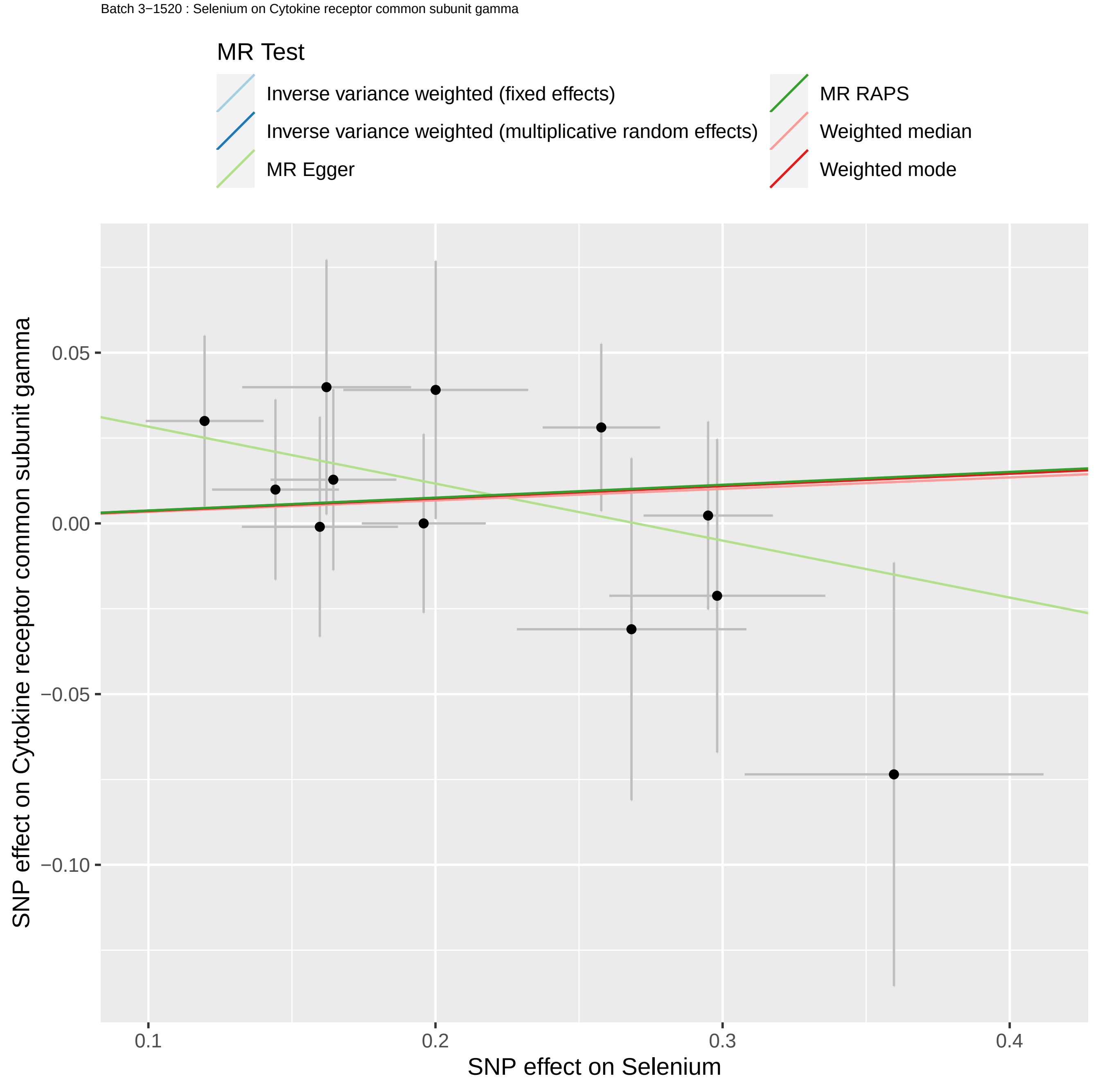
Batch 3−1520 : Selenium on Cytokine receptor common subunit gamma
MR Test
Inverse variance weighted (fixed effects)
Inverse variance weighted (multiplicative random effects)
MR Egger
MR RAPS
Weighted median
Weighted mode
0.05
0.00
−0.05
−0.10
0.1
0.2
0.3
0.4
SNP effect on Selenium
SNP effect on Cytokine receptor common subunit gamma

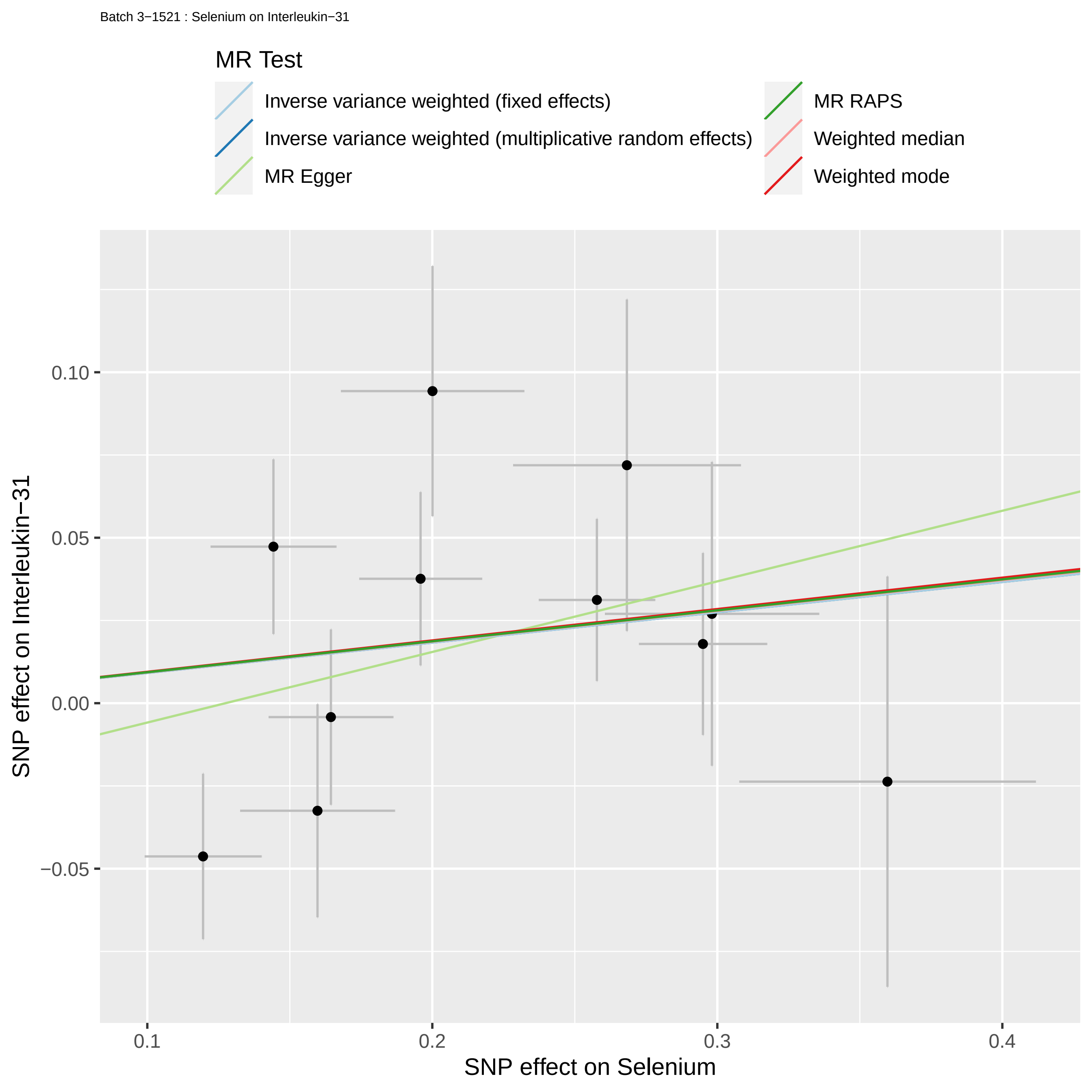
Batch 3−1521 : Selenium on Interleukin−31
MR Test
Inverse variance weighted (fixed effects)
Inverse variance weighted (multiplicative random effects)
MR Egger
MR RAPS
Weighted median
Weighted mode
SNP effect on Interleukin−31
0.10
0.05
0.00
−0.05
0.1
0.2
0.3
0.4
SNP effect on Selenium

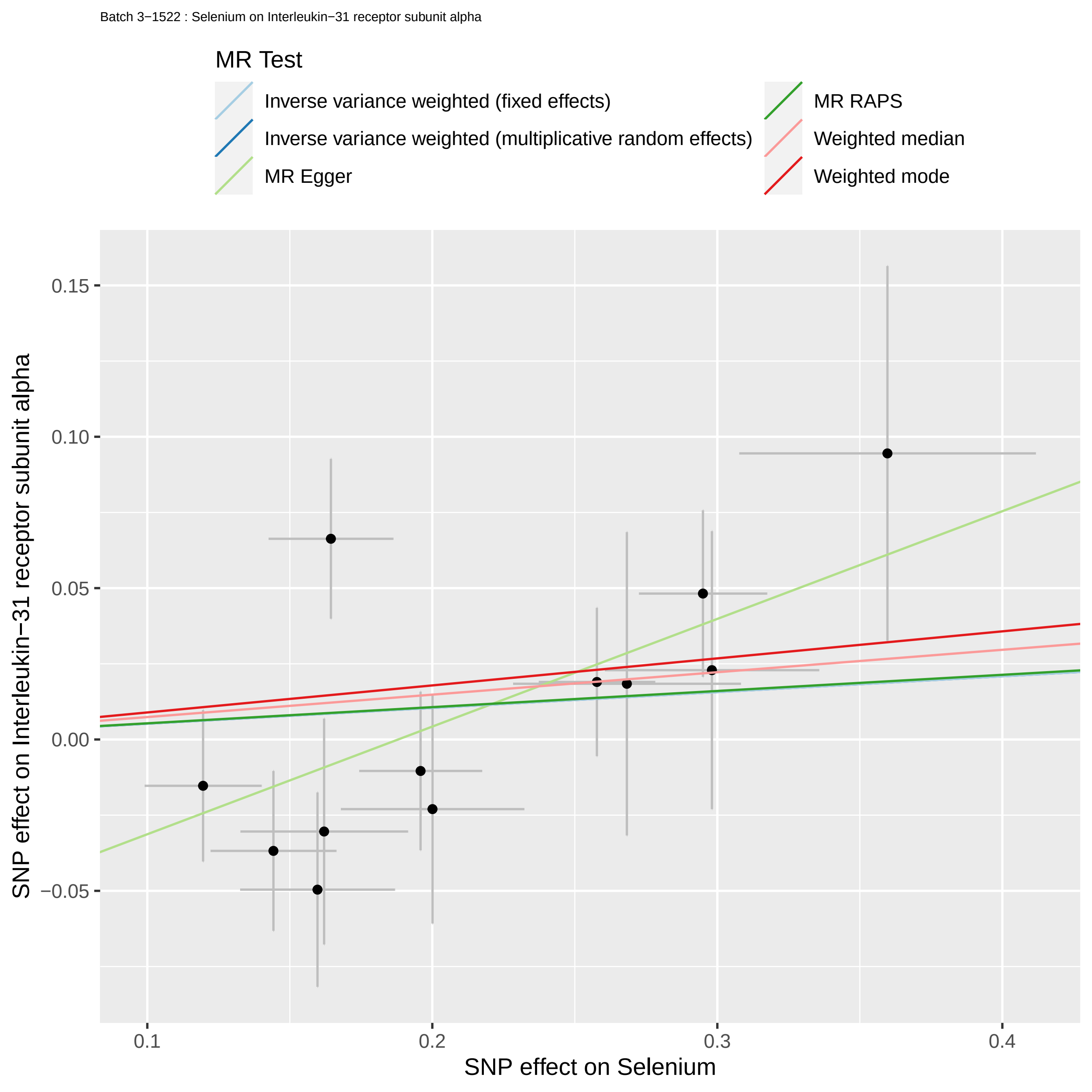
Batch 3−1522 : Selenium on Interleukin−31 receptor subunit alpha
MR Test
Inverse variance weighted (fixed effects)
Inverse variance weighted (multiplicative random effects)
MR Egger
MR RAPS
Weighted median
Weighted mode
SNP effect on Interleukin−31 receptor subunit alpha
0.15
0.10
0.05
0.00
−0.05
0.1
0.2
0.3
0.4
SNP effect on Selenium

Batch 3−1523 : Selenium on Interleukin−32

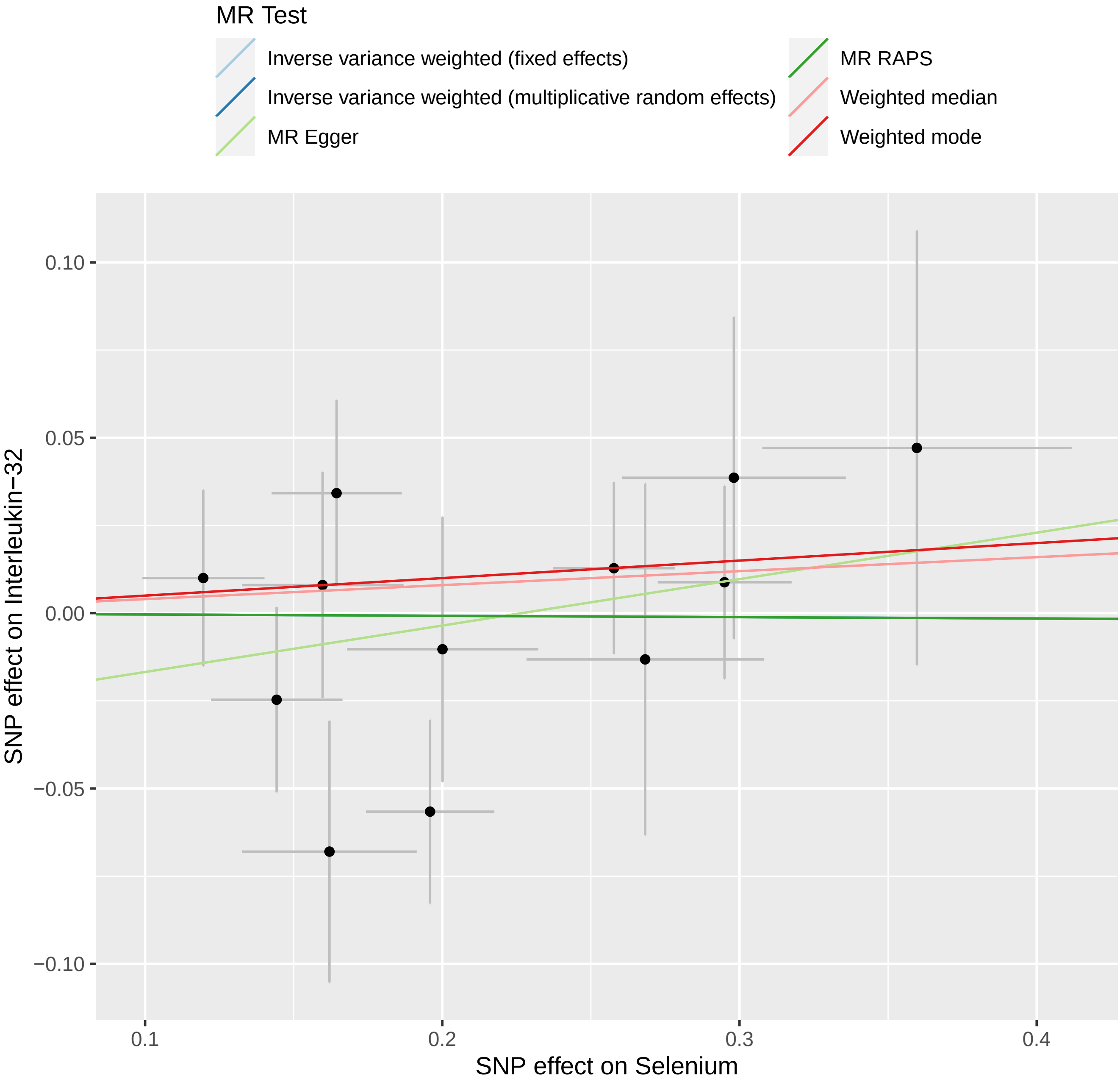

Batch 3−1524 : Selenium on Interleukin−34

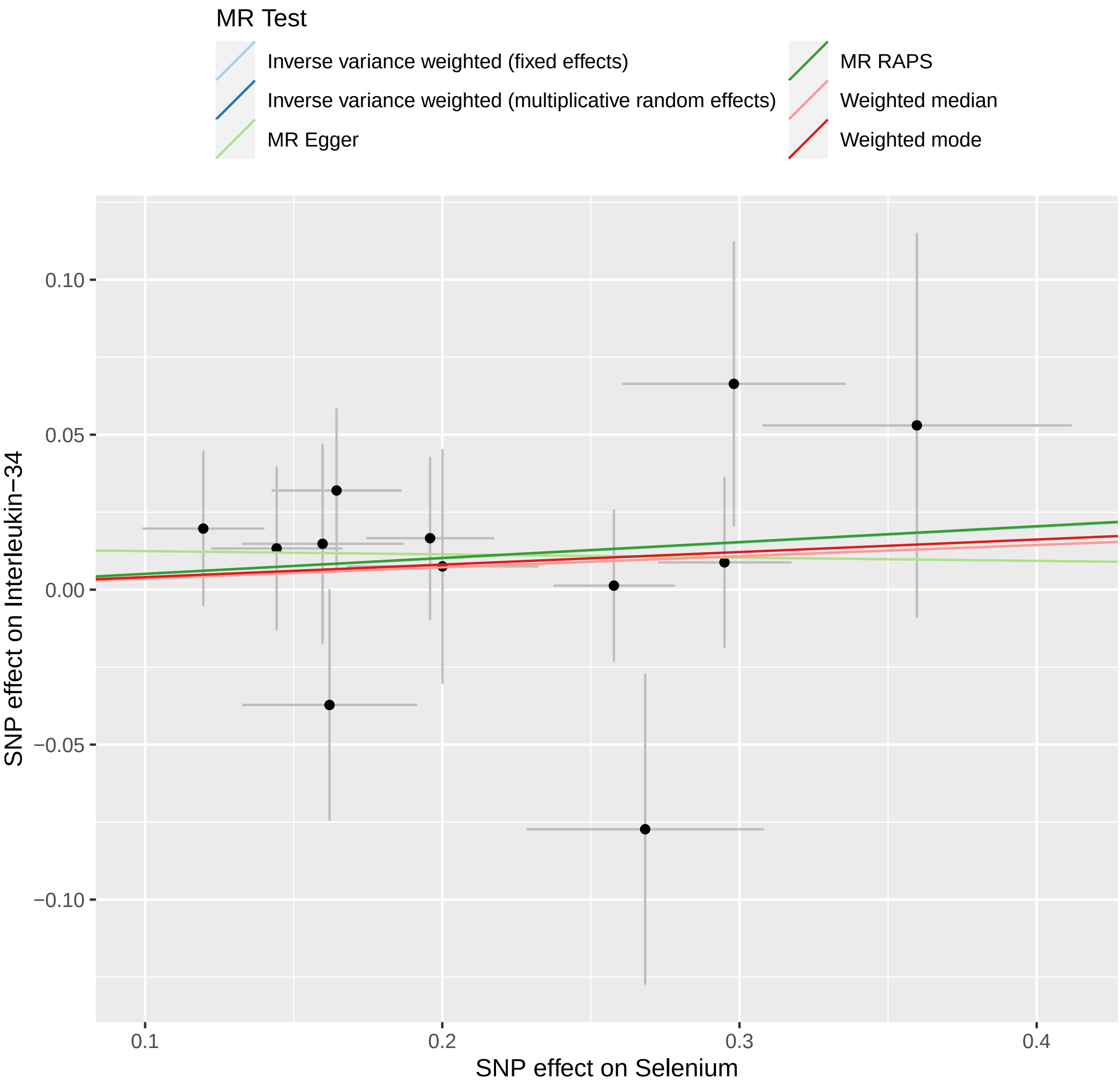

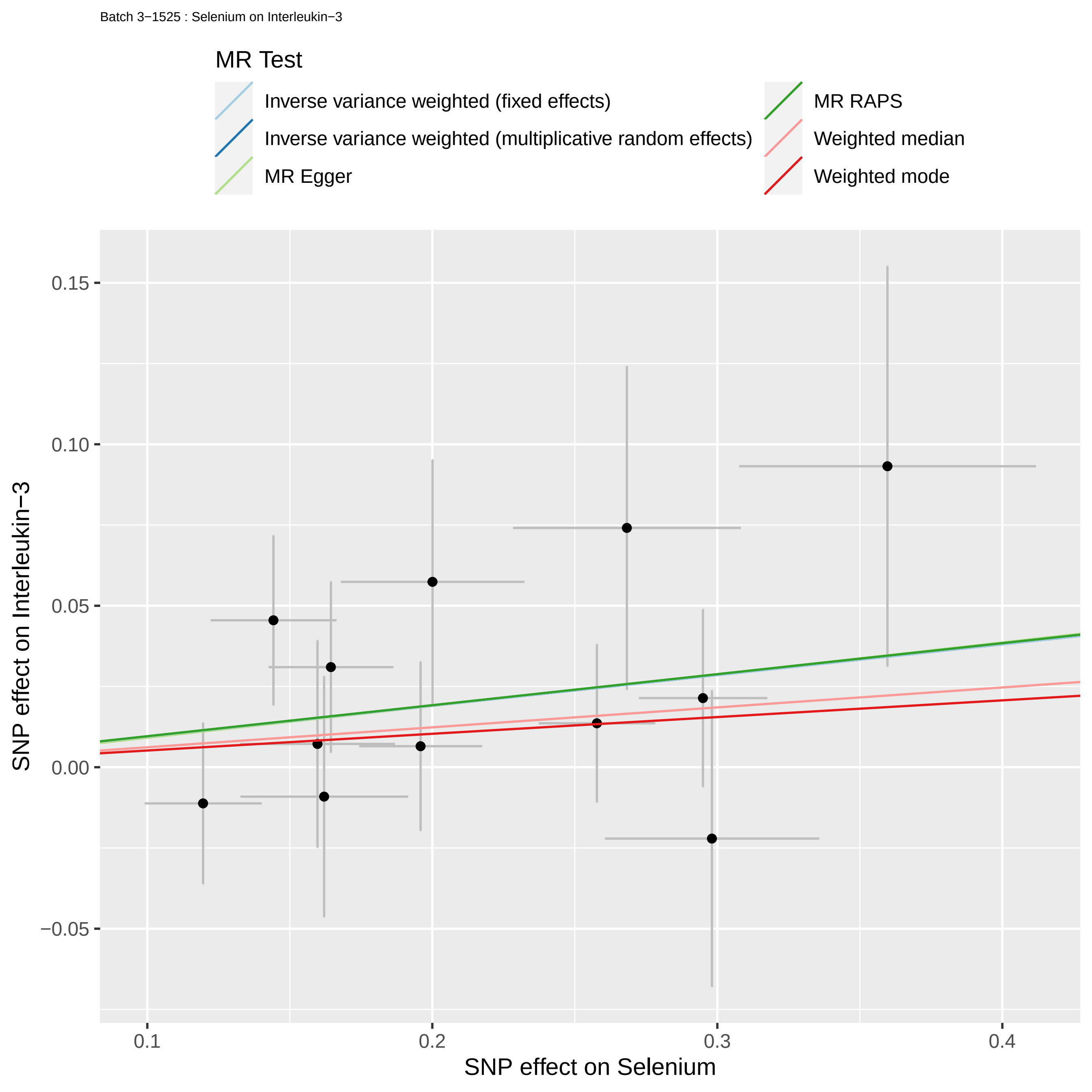
Batch 3−1525 : Selenium on Interleukin−3
MR Test
Inverse variance weighted (fixed effects)
Inverse variance weighted (multiplicative random effects)
MR Egger
MR RAPS
Weighted median
Weighted mode
0.15
0.10
0.05
0.00
−0.05
0.1
0.2
0.3
0.4
SNP effect on Interleukin−3
SNP effect on Selenium

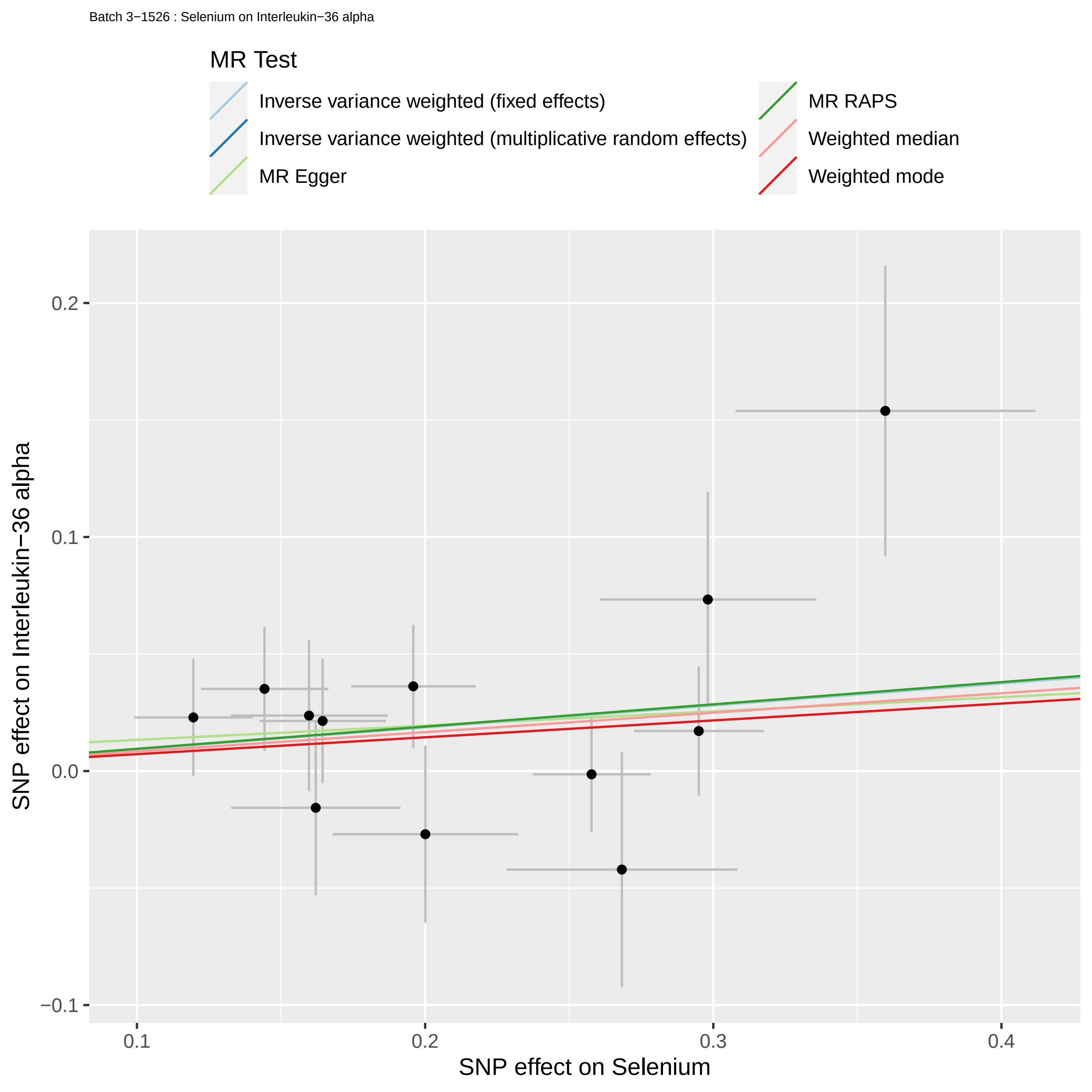
Batch 3−1526 : Selenium on Interleukin−36 alpha
MR Test
Inverse variance weighted (fixed effects)
Inverse variance weighted (multiplicative random effects)
MR Egger
MR RAPS
Weighted median
Weighted mode
0.2
0.1
0.0
−0.1
0.1
0.2
0.3
0.4
SNP effect on Selenium
SNP effect on Interleukin−36 alpha

Batch 3−1527 : Selenium on Interleukin−36 beta

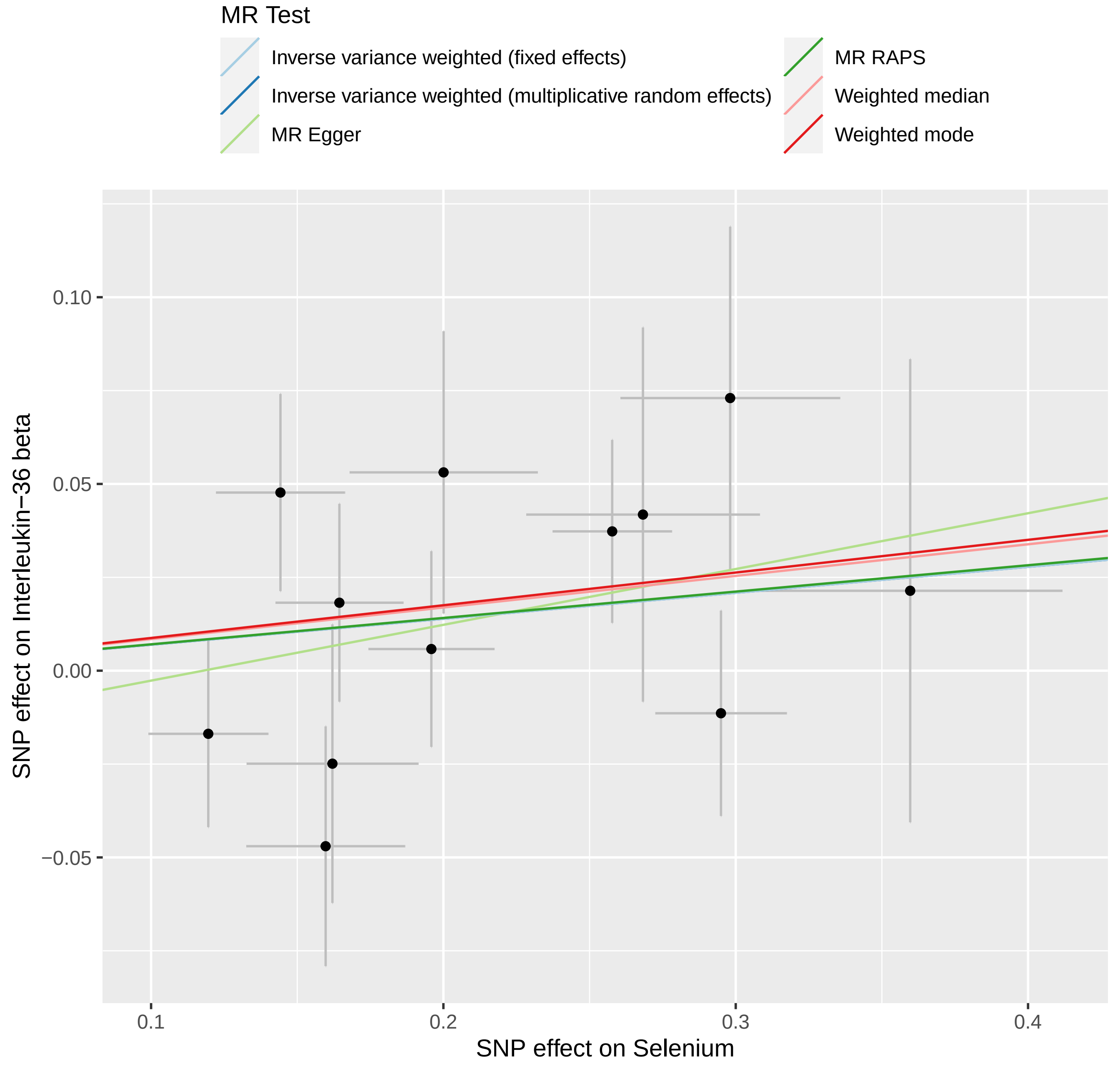

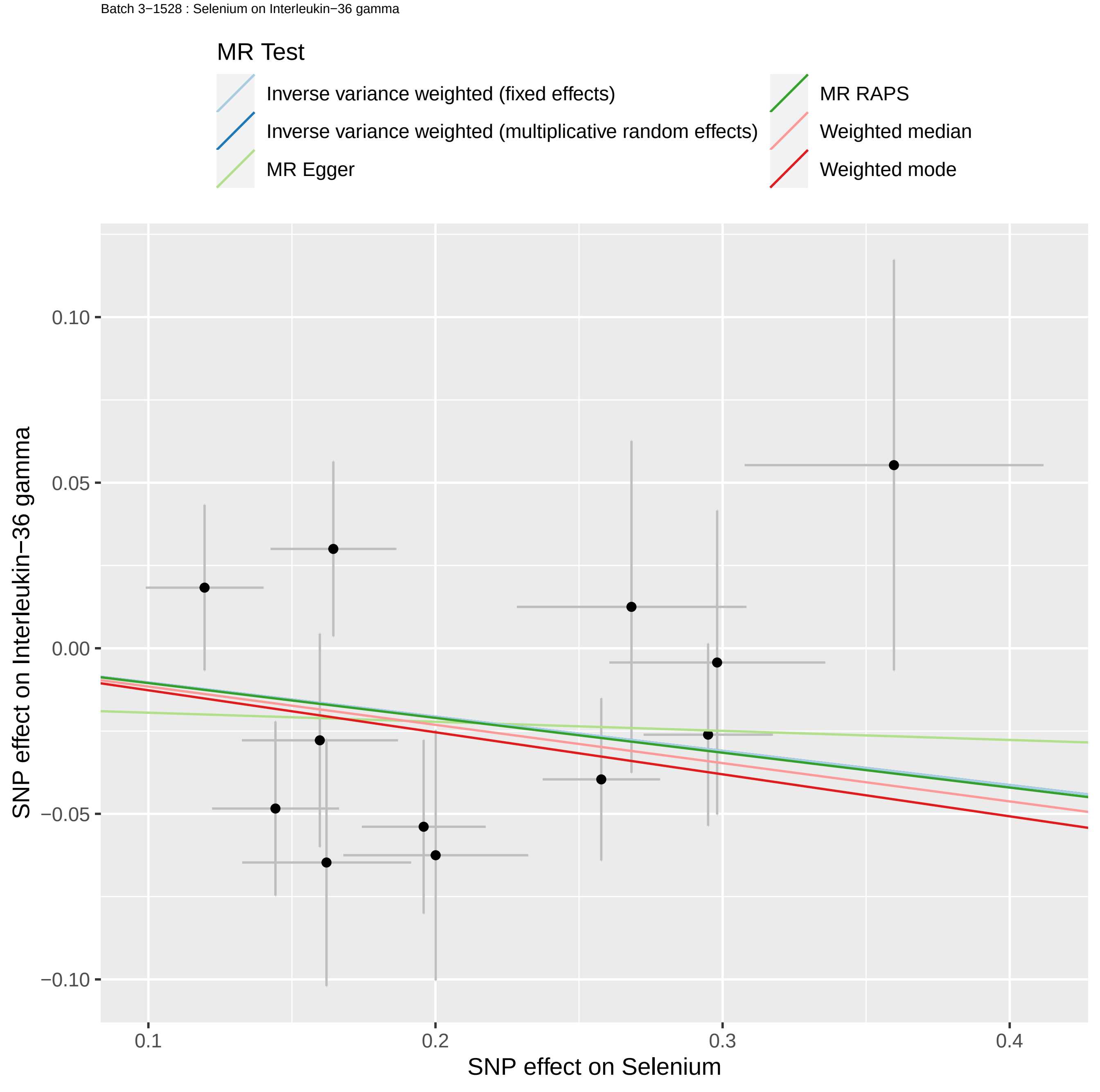

Batch 3−1528 : Selenium on Interleukin−36 gamma
MR Test
Inverse variance weighted (fixed effects)
Inverse variance weighted (multiplicative random effects)
MR Egger
MR RAPS
Weighted median
Weighted mode
SNP effect on Interleukin−36 gamma
0.10
0.05
0.00
−0.05
−0.10
SNP effect on Selenium
0.1
0.2
0.3
0.4

Batch 3−1529 : Selenium on Interleukin−37

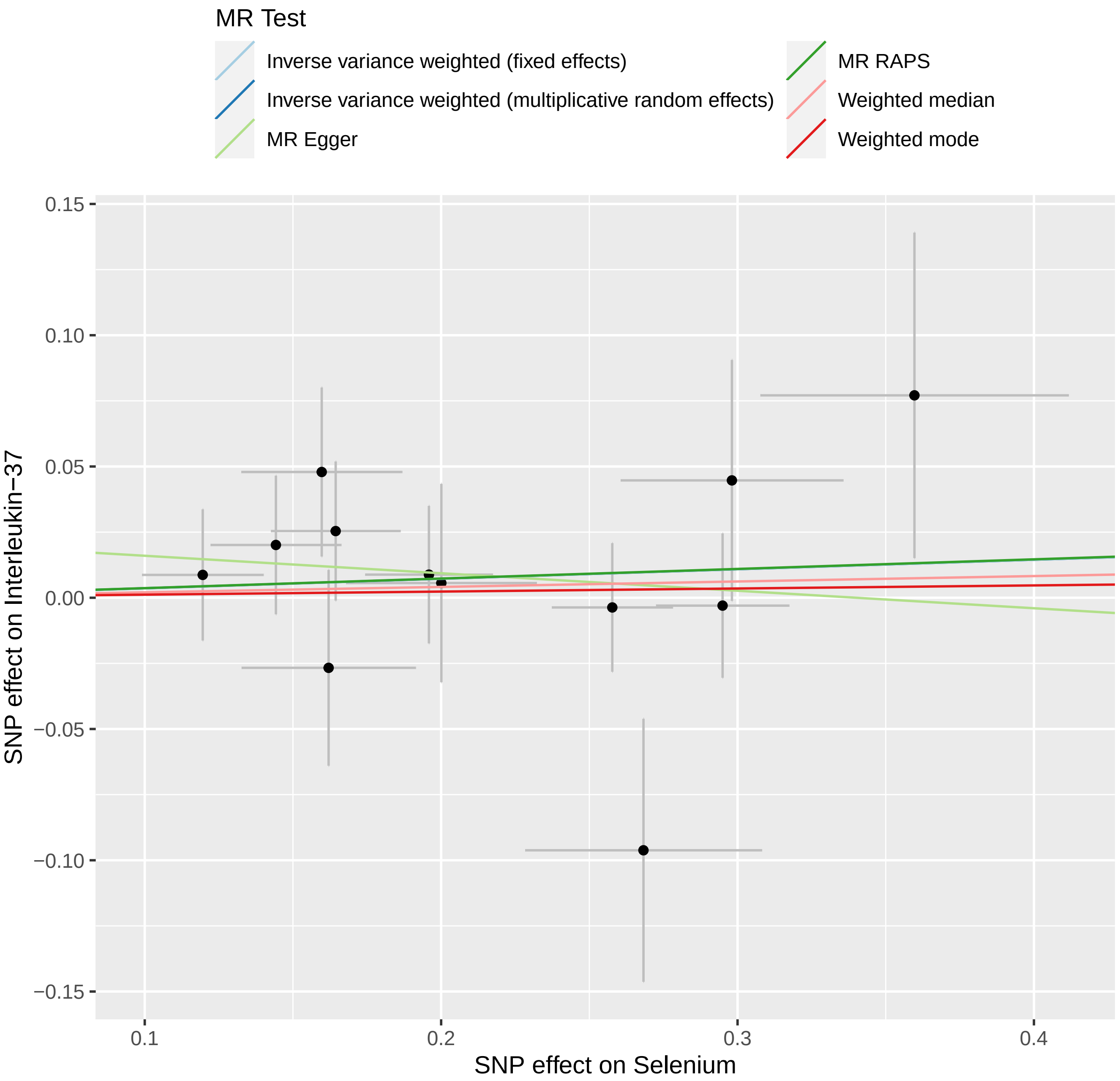

Batch 3−1530 : Selenium on Interleukin−3 receptor subunit alpha

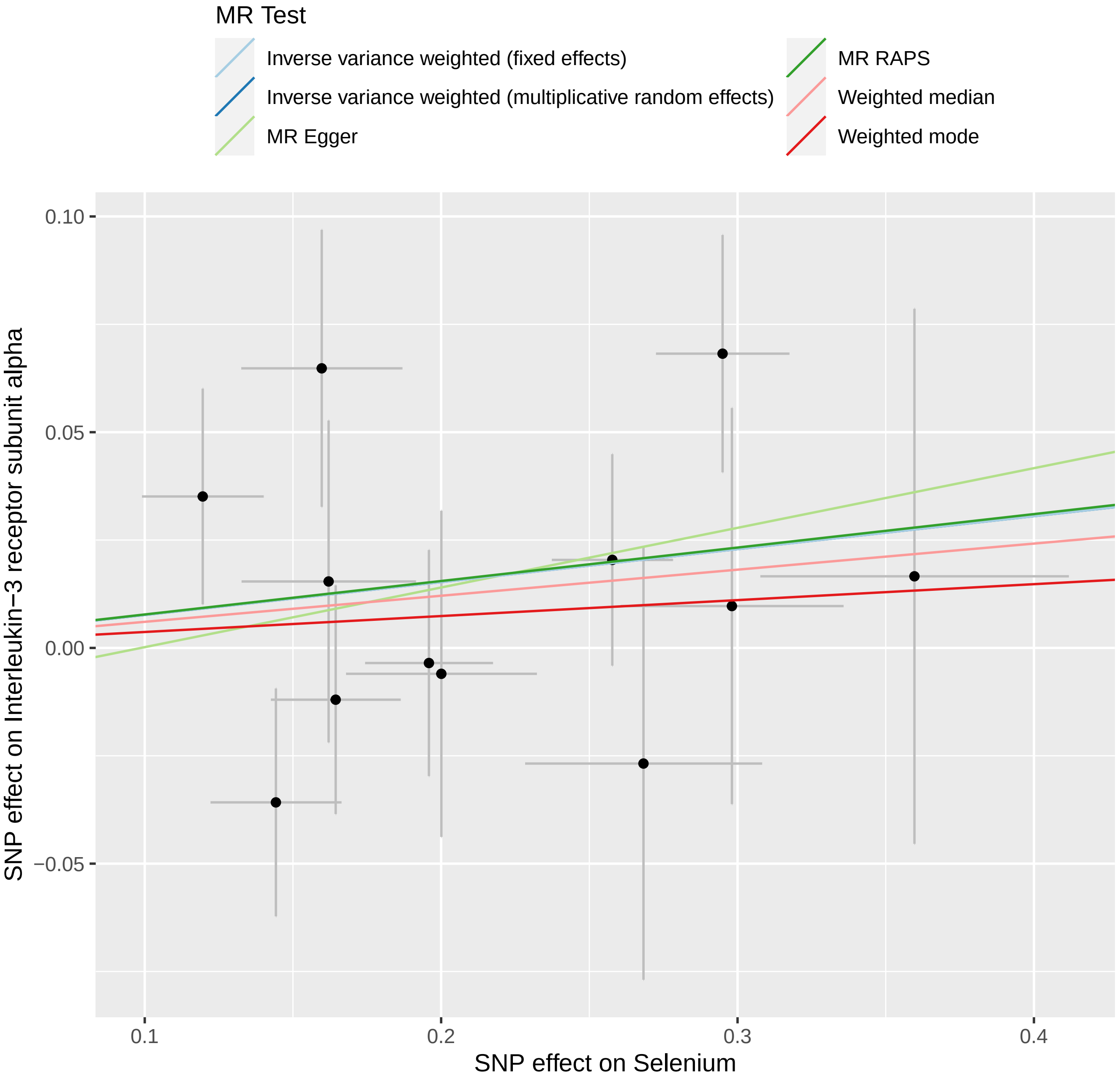

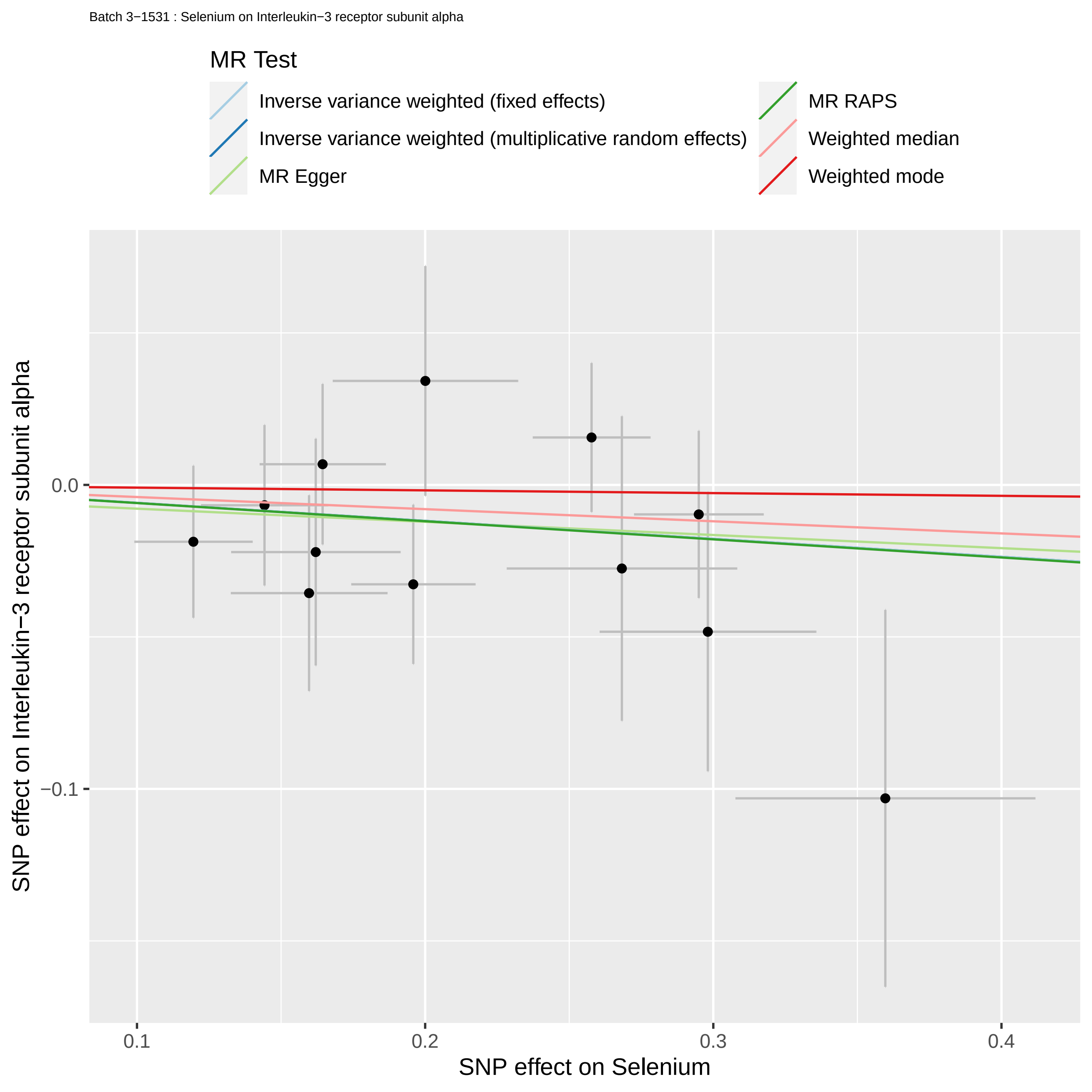

Batch 3−1531 : Selenium on Interleukin−3 receptor subunit alpha
MR Test
Inverse variance weighted (fixed effects)
Inverse variance weighted (multiplicative random effects)
MR Egger
MR RAPS
Weighted median
Weighted mode
0.0
−0.1
0.1
0.2
0.3
0.4
SNP effect on Interleukin−3 receptor subunit alpha
SNP effect on Selenium

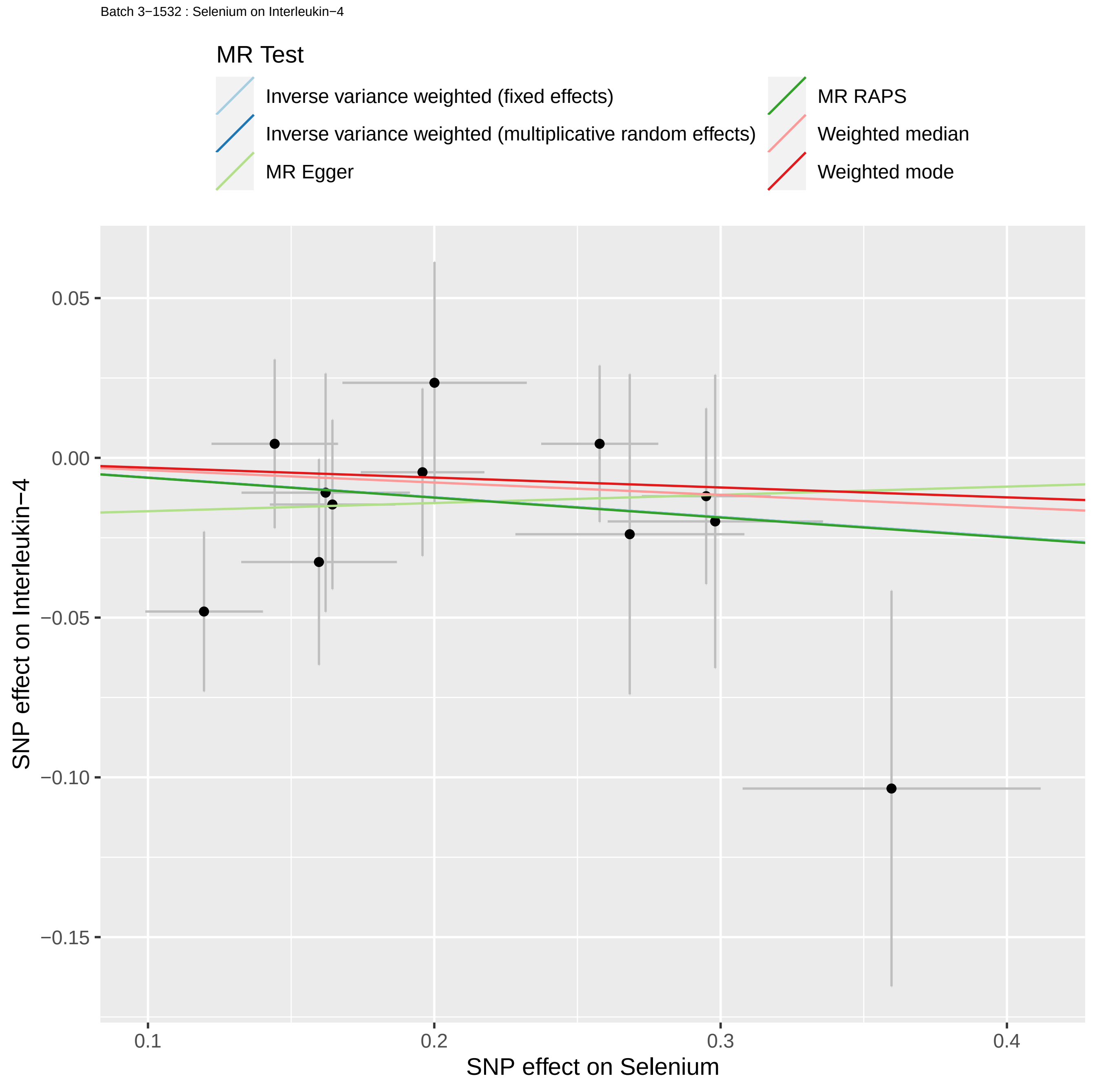
Batch 3−1532 : Selenium on Interleukin−4
MR Test
Inverse variance weighted (fixed effects)
Inverse variance weighted (multiplicative random effects)
MR Egger
MR RAPS
Weighted median
Weighted mode
0.05
0.00
−0.05
−0.10
−0.15
0.1
0.2
0.3
0.4
SNP effect on Interleukin−4
SNP effect on Selenium

Batch 3−1533 : Selenium on Interleukin−4 receptor subunit alpha

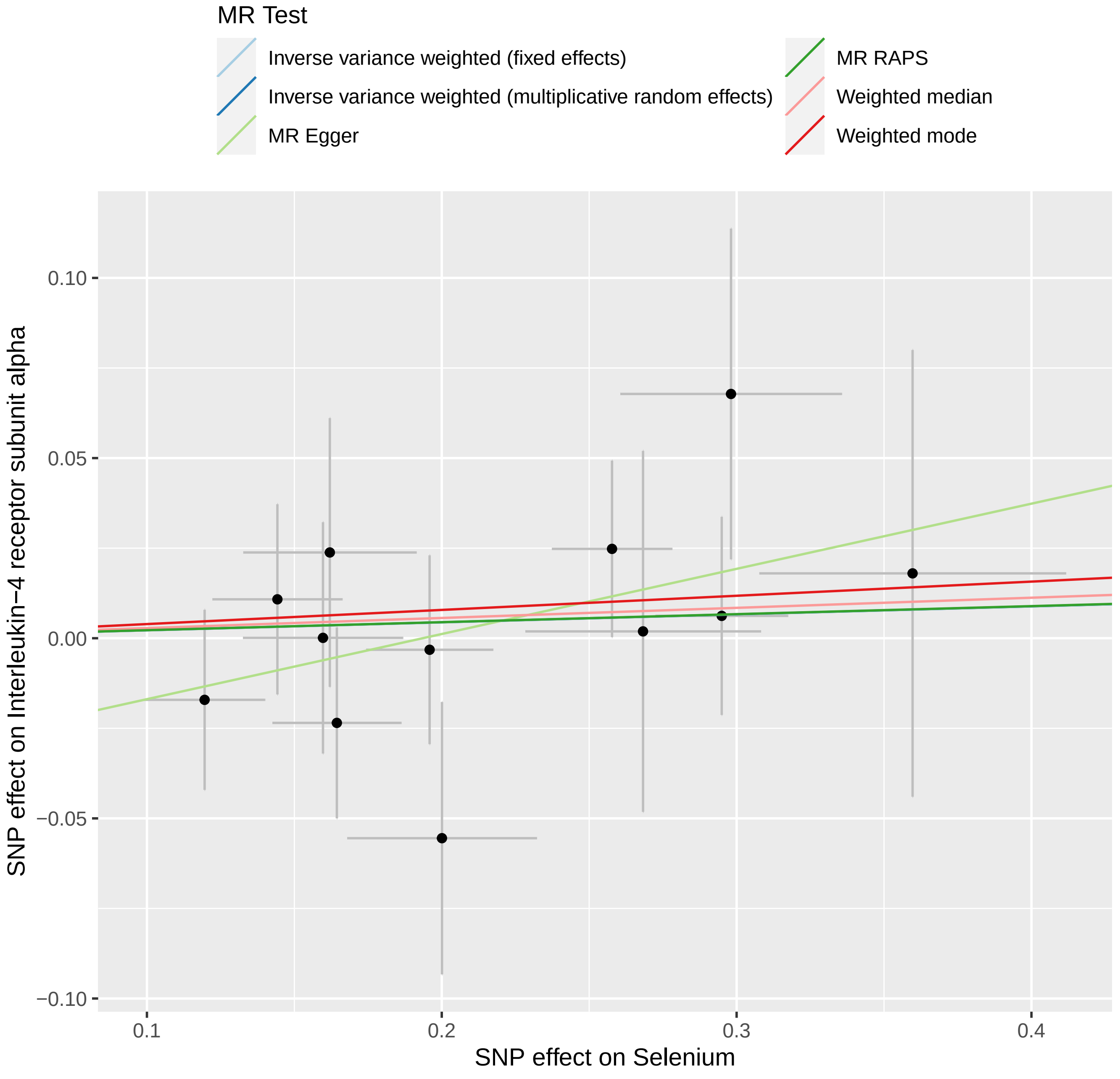

Batch 3−1534 : Selenium on Interleukin−5

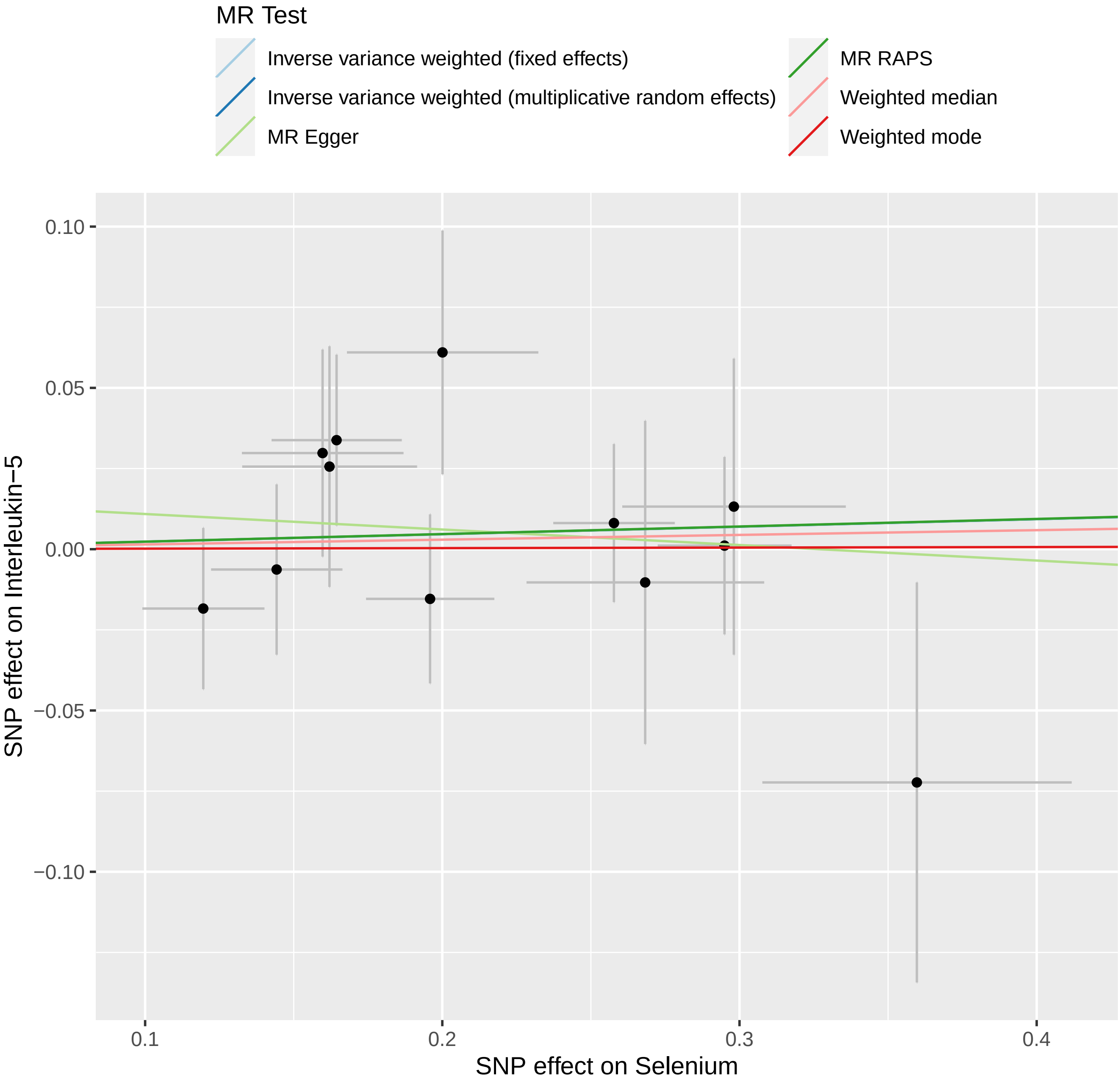

Batch 3−1535 : Selenium on Interleukin−5

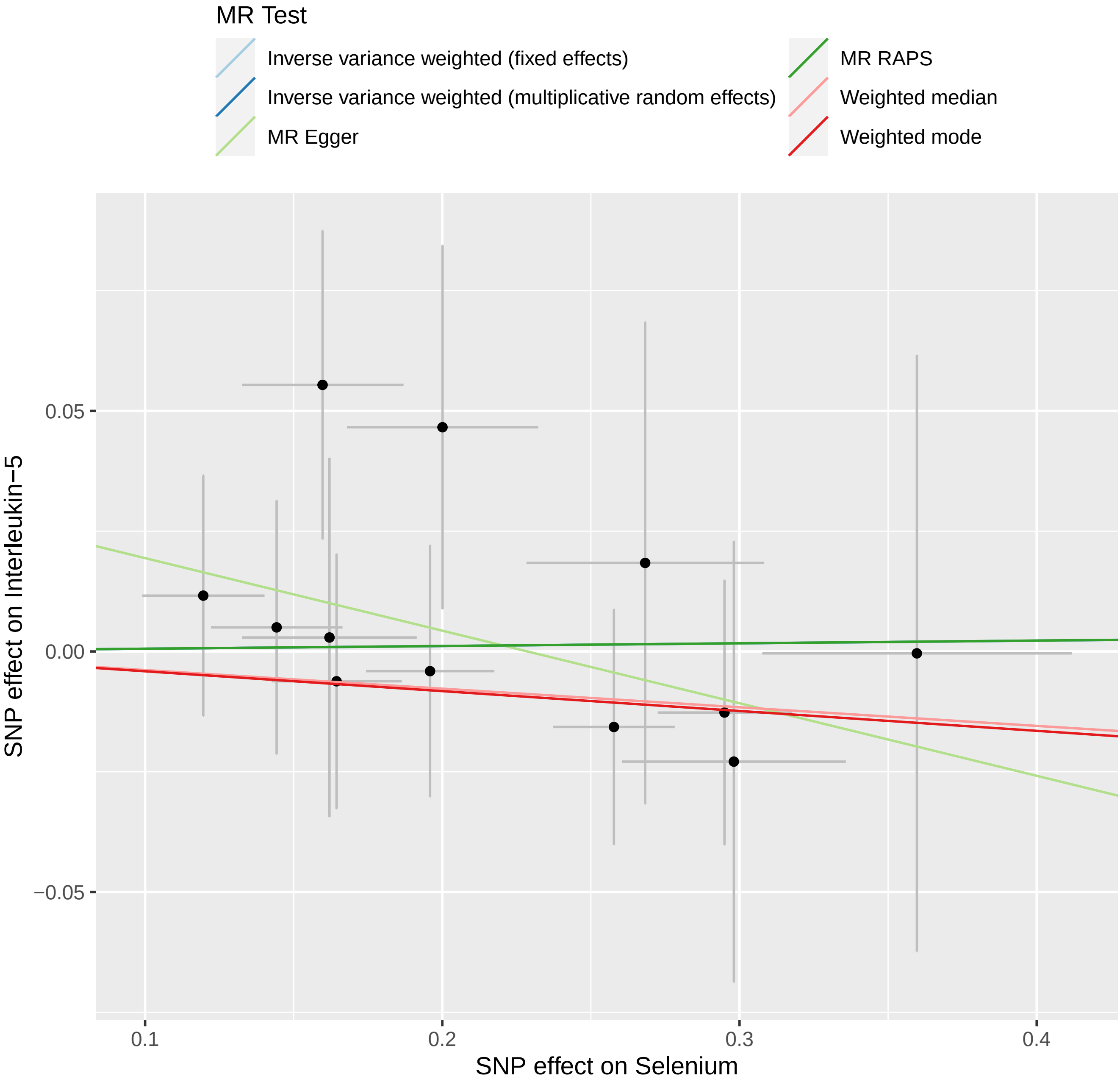

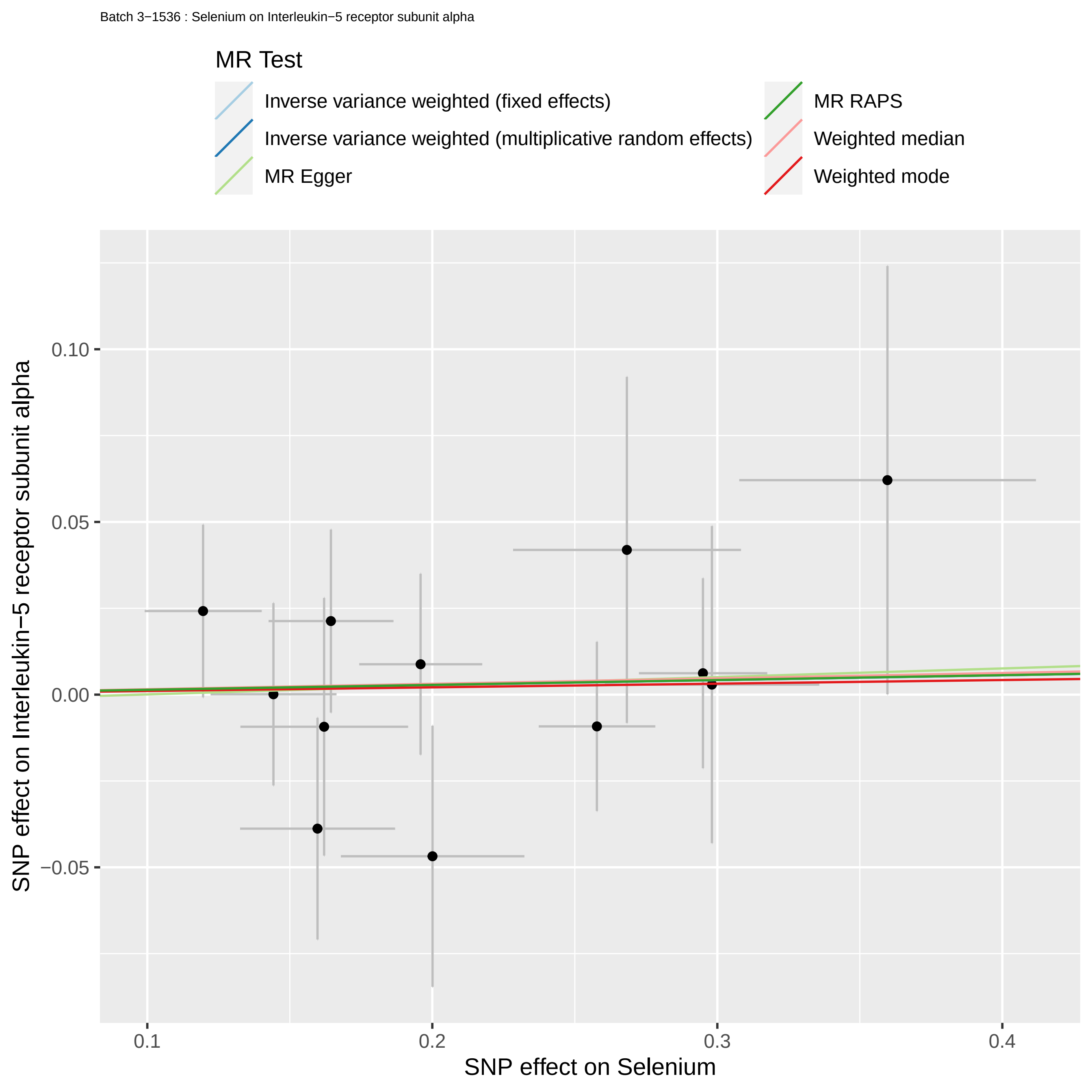

Batch 3−1536 : Selenium on Interleukin−5 receptor subunit alpha
MR Test
Inverse variance weighted (fixed effects)
Inverse variance weighted (multiplicative random effects)
MR Egger
MR RAPS
Weighted median
Weighted mode
SNP effect on Interleukin−5 receptor subunit alpha
0.10
0.05
0.00
−0.05
0.1
0.2
0.3
0.4
SNP effect on Selenium

Batch 3−1537 : Selenium on Interleukin−5 receptor subunit alpha

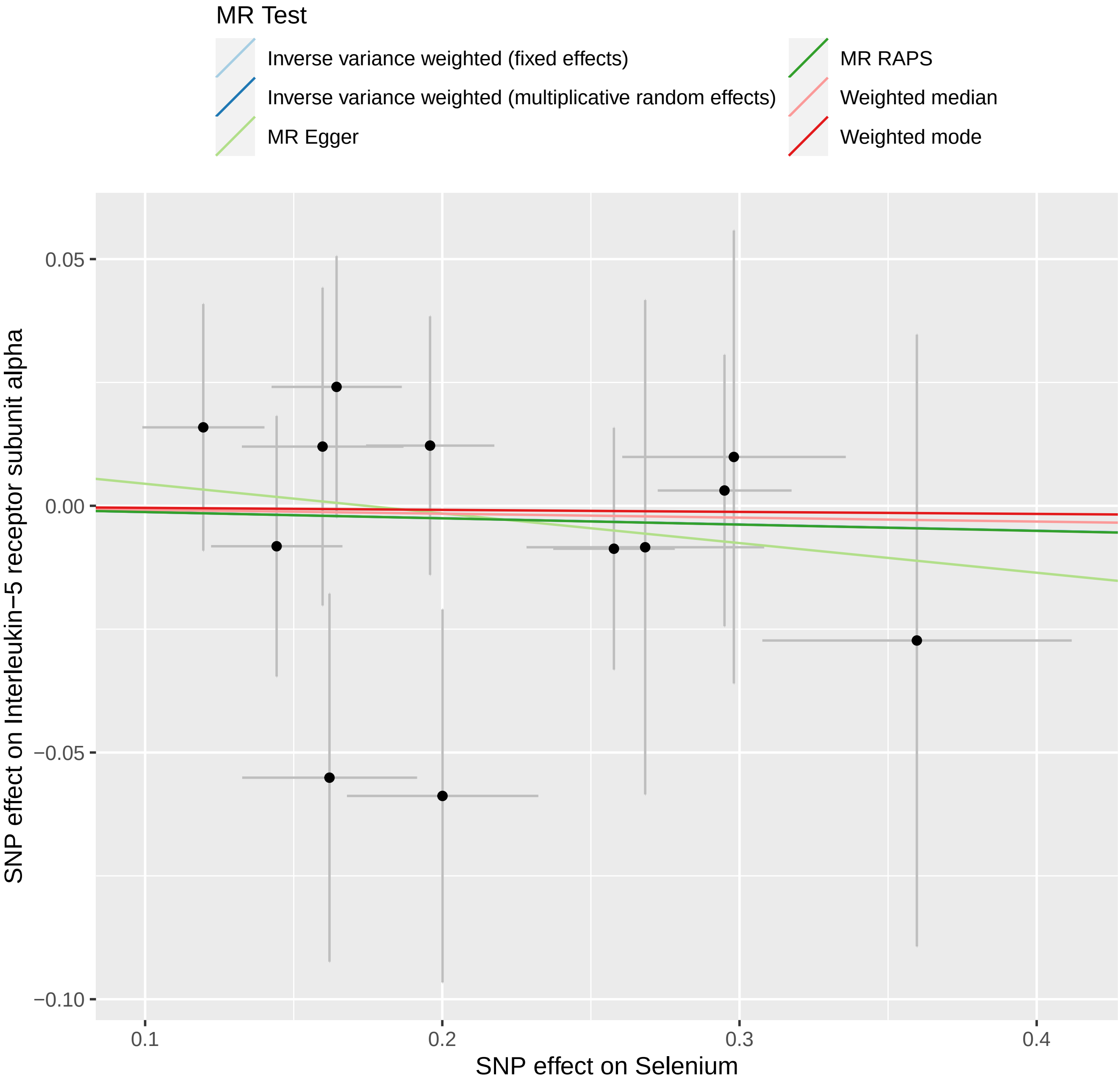

Batch 3−1538 : Selenium on Interleukin−6

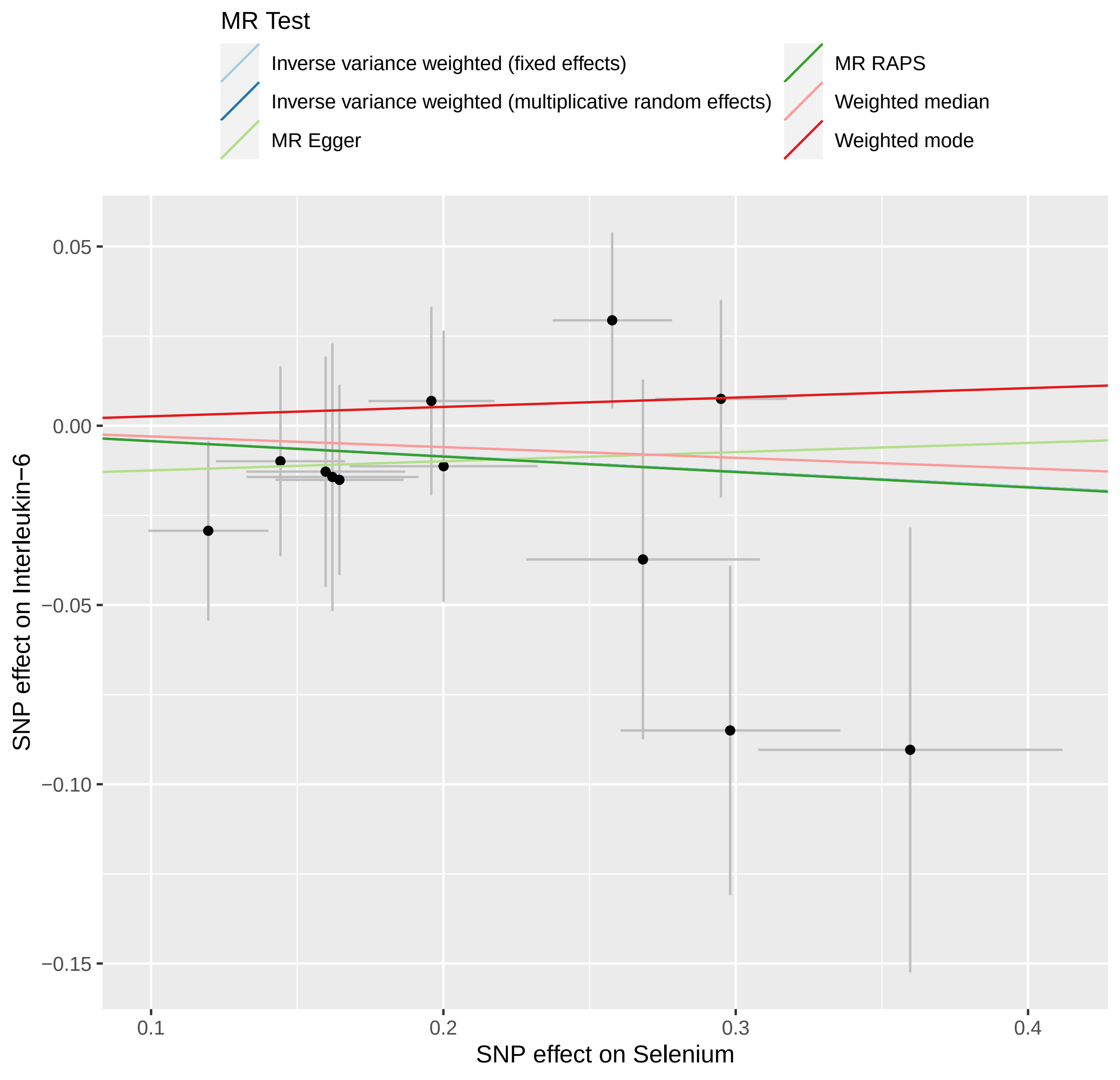

Batch 3−1539 : Selenium on Interleukin−6

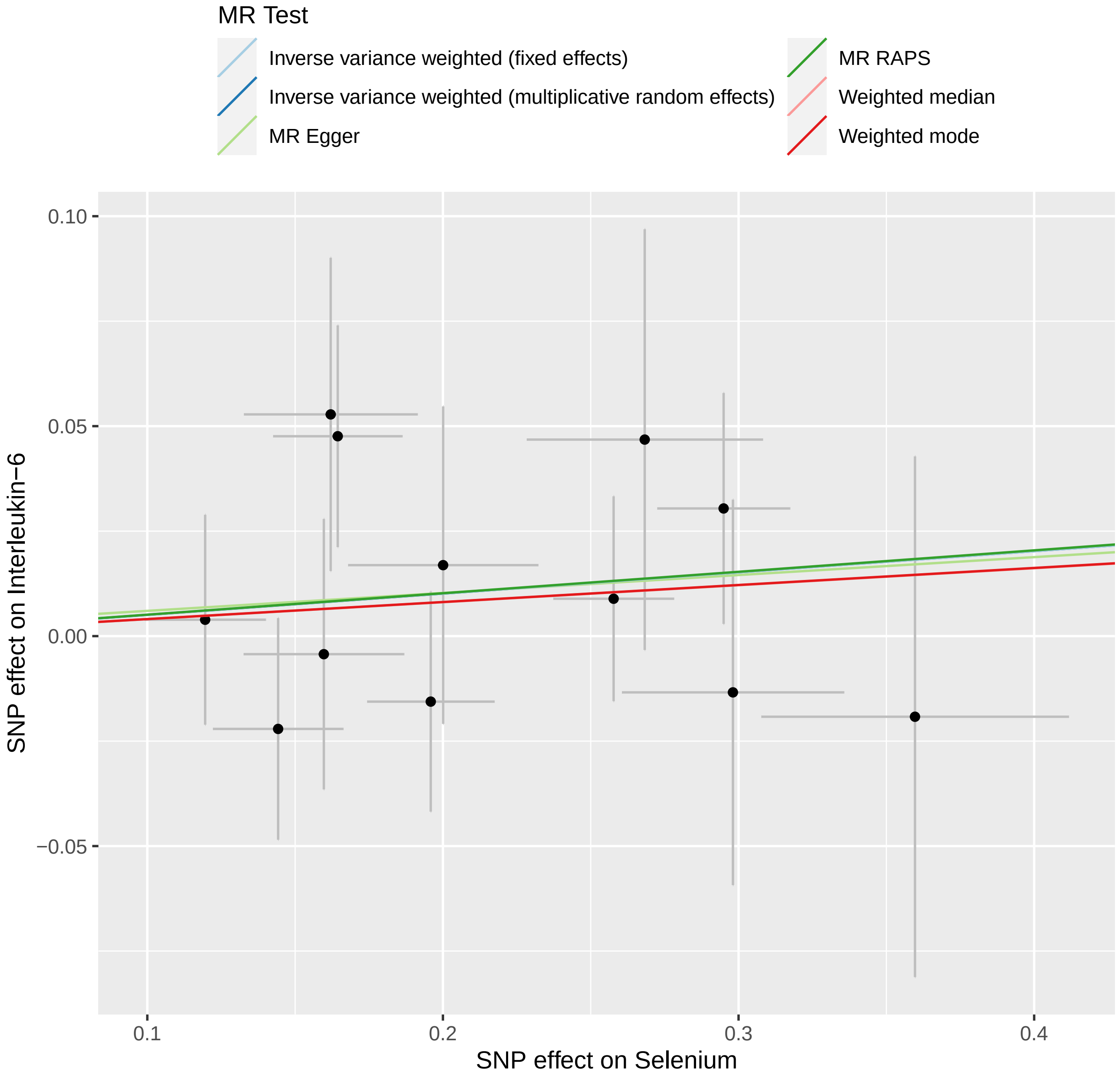

Batch 3−1540 : Selenium on Interleukin−6 receptor subunit alpha

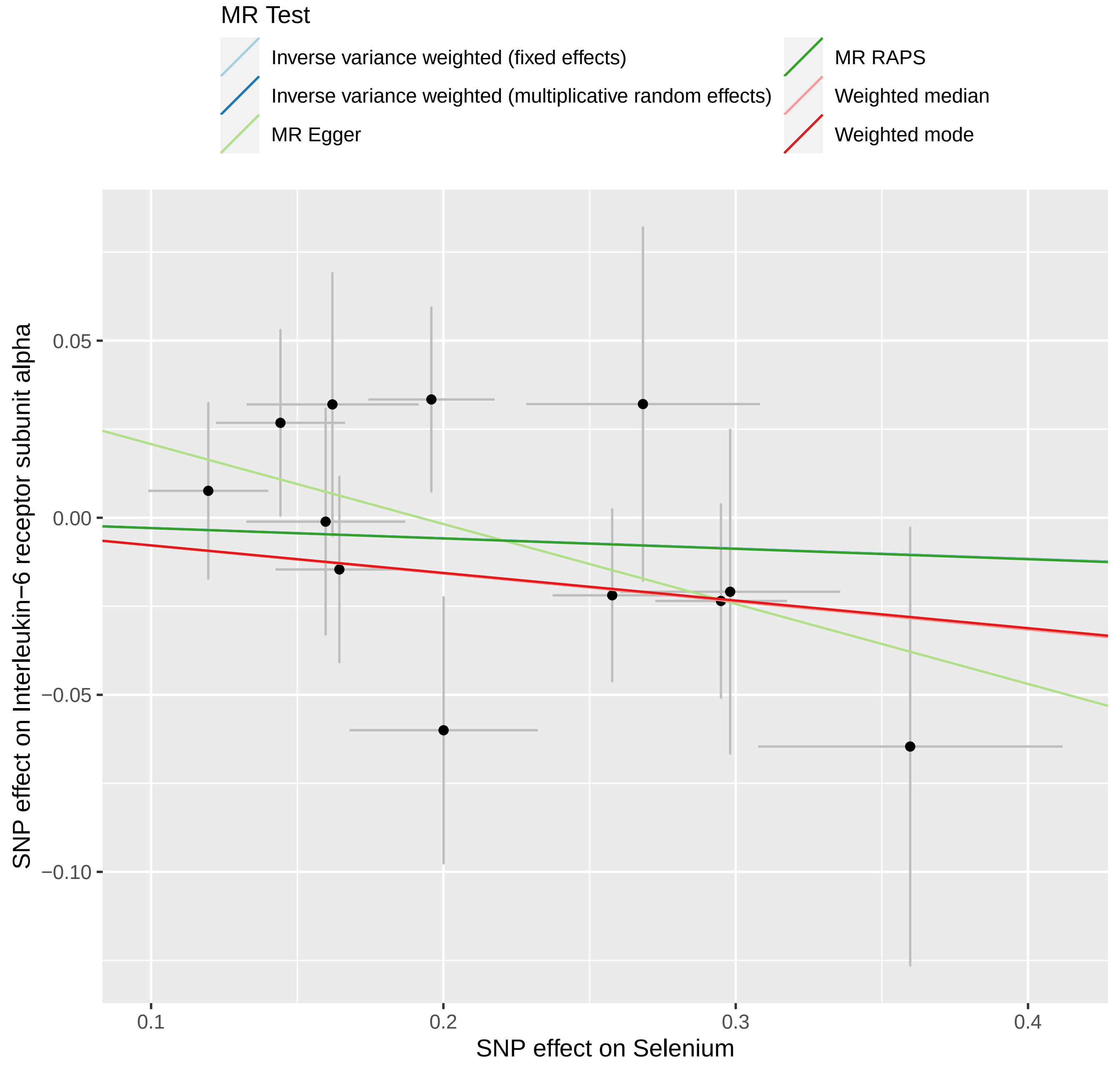

Batch 3−1541 : Selenium on Interleukin−6 receptor subunit alpha

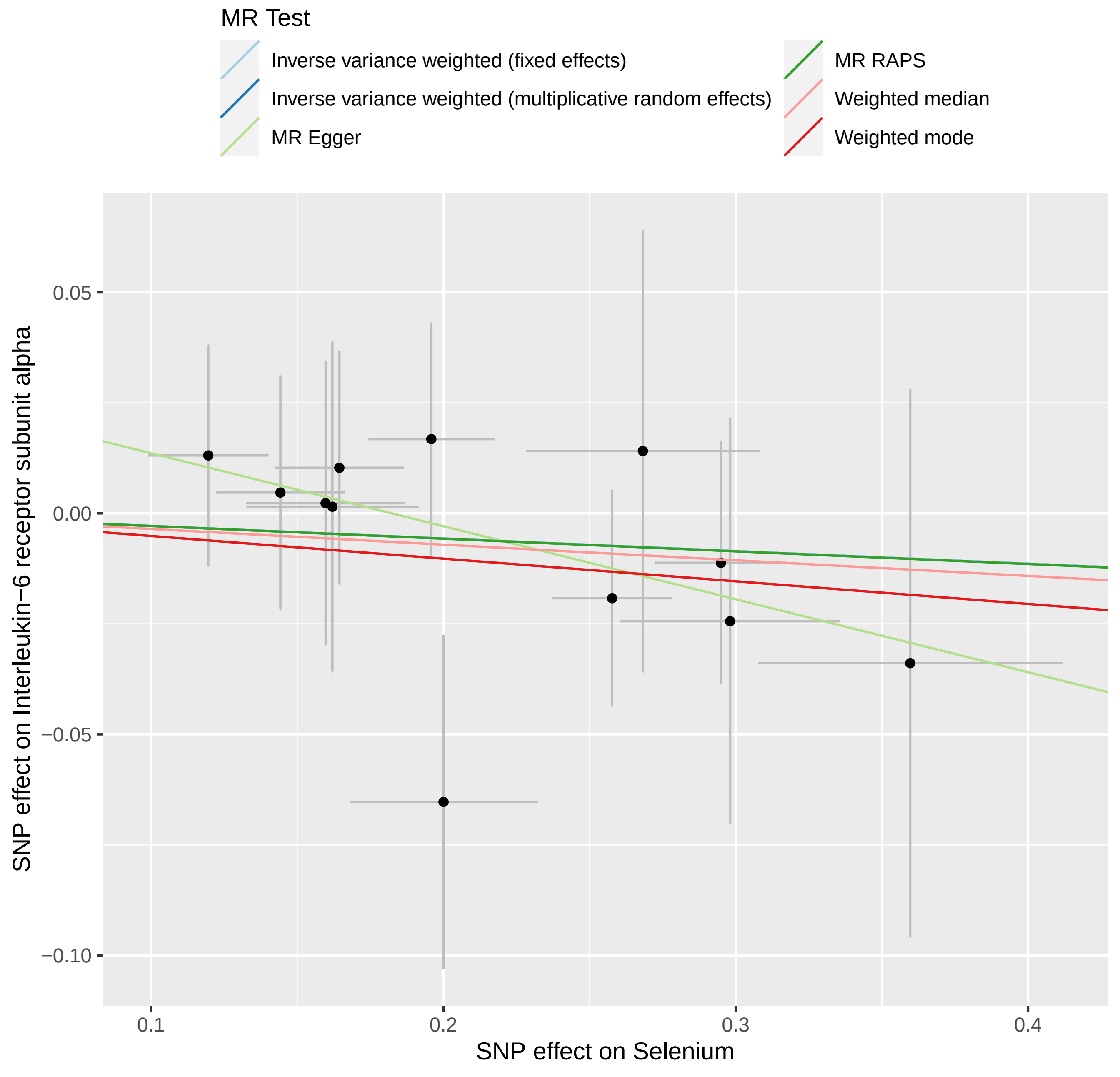

Batch 3−1542 : Selenium on Interleukin−6 receptor subunit beta

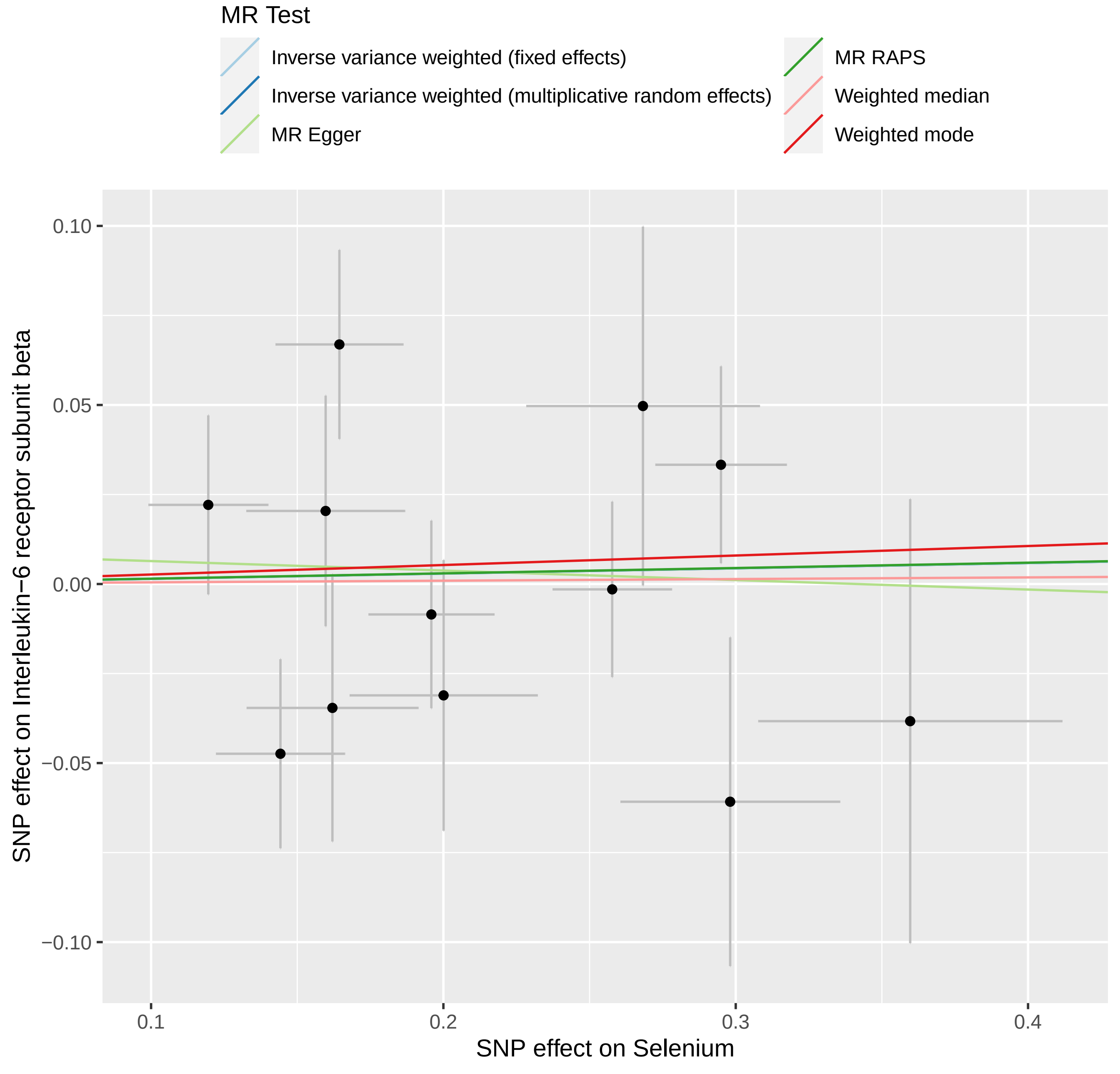

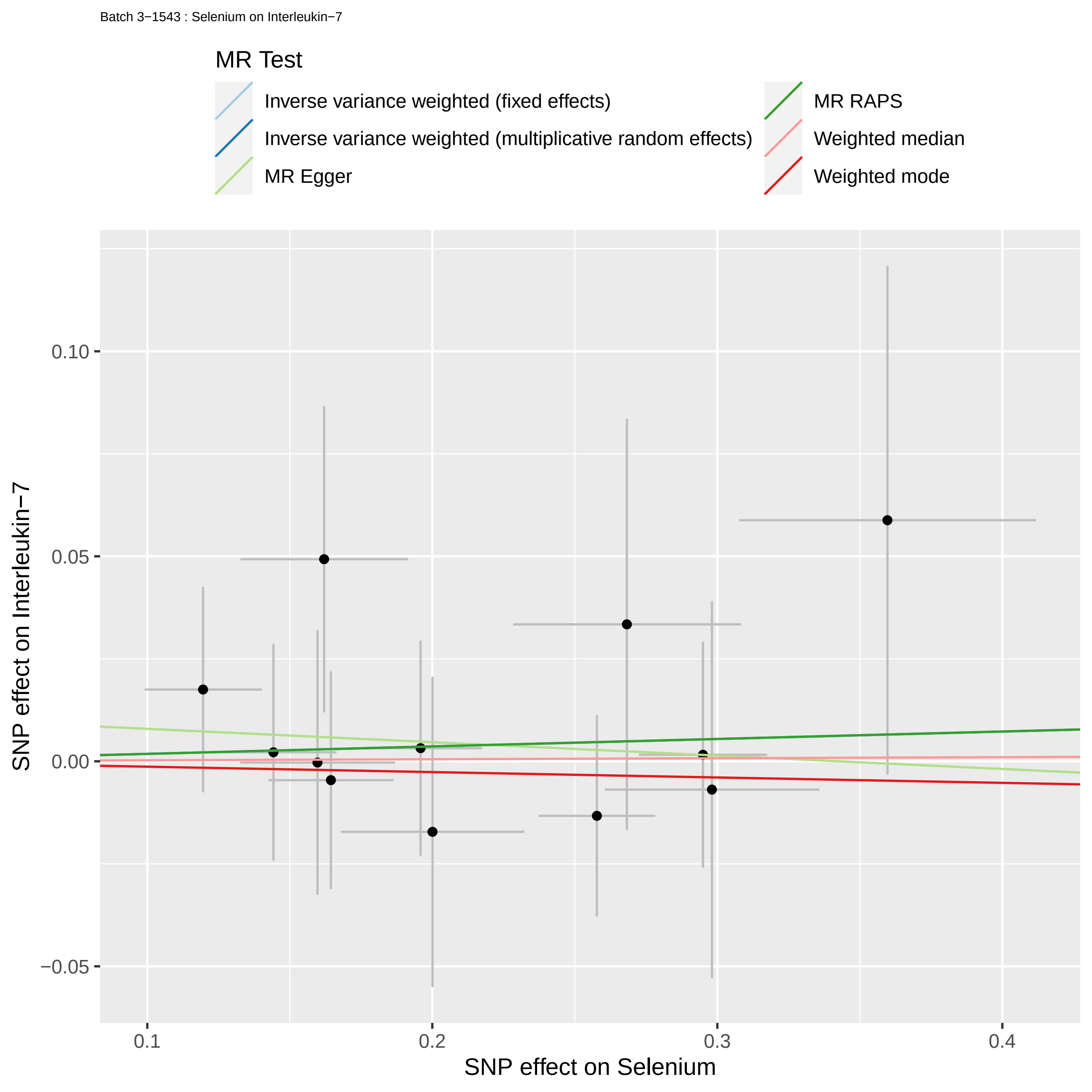

Batch 3−1543 : Selenium on Interleukin−7
MR Test
Inverse variance weighted (fixed effects)
Inverse variance weighted (multiplicative random effects)
MR Egger
MR RAPS
Weighted median
Weighted mode
SNP effect on Interleukin−7
0.10
0.05
0.00
−0.05
0.1
0.2
0.3
0.4
SNP effect on Selenium

Batch 3−1544 : Selenium on Interleukin−7 receptor subunit alpha

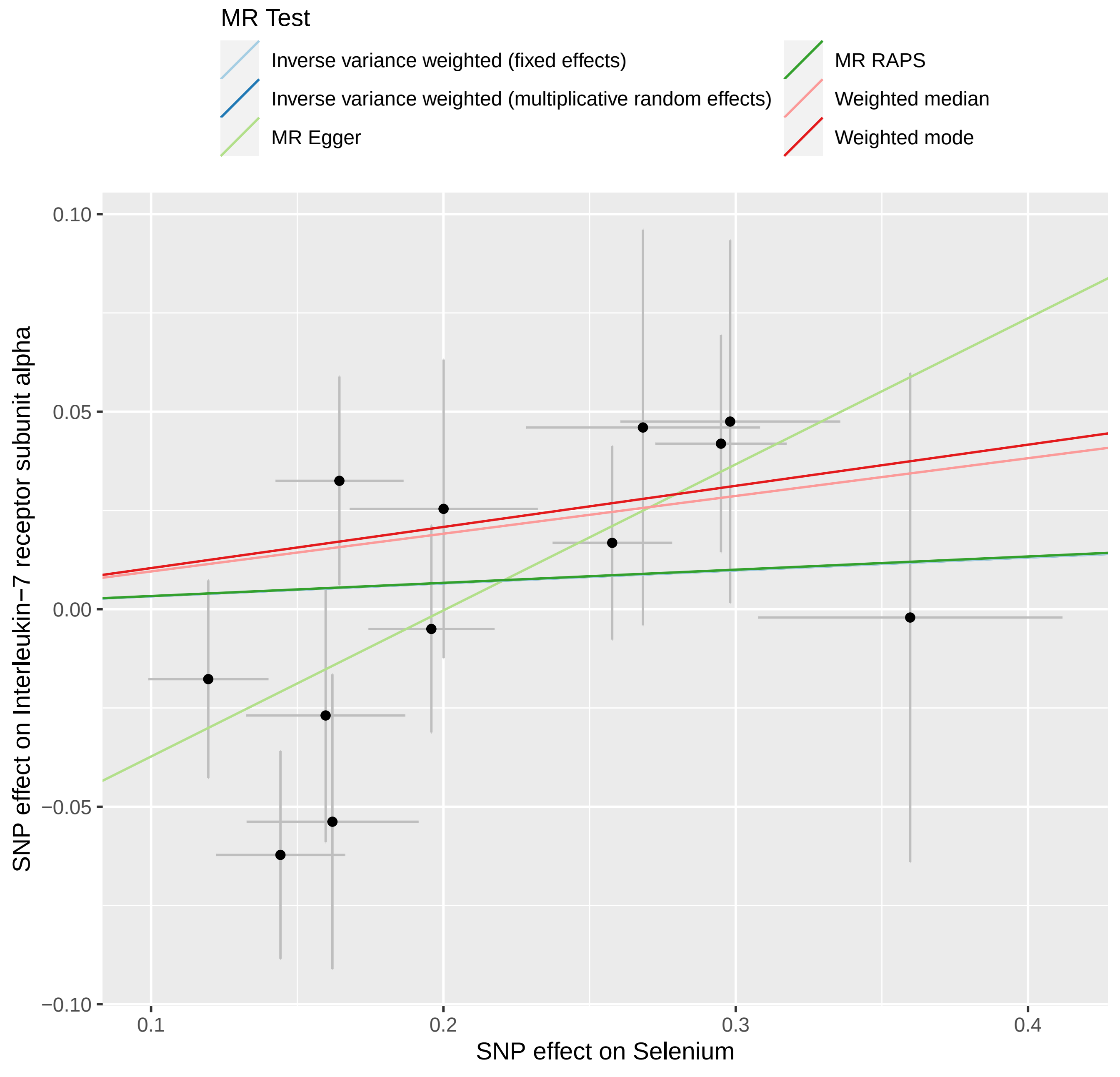

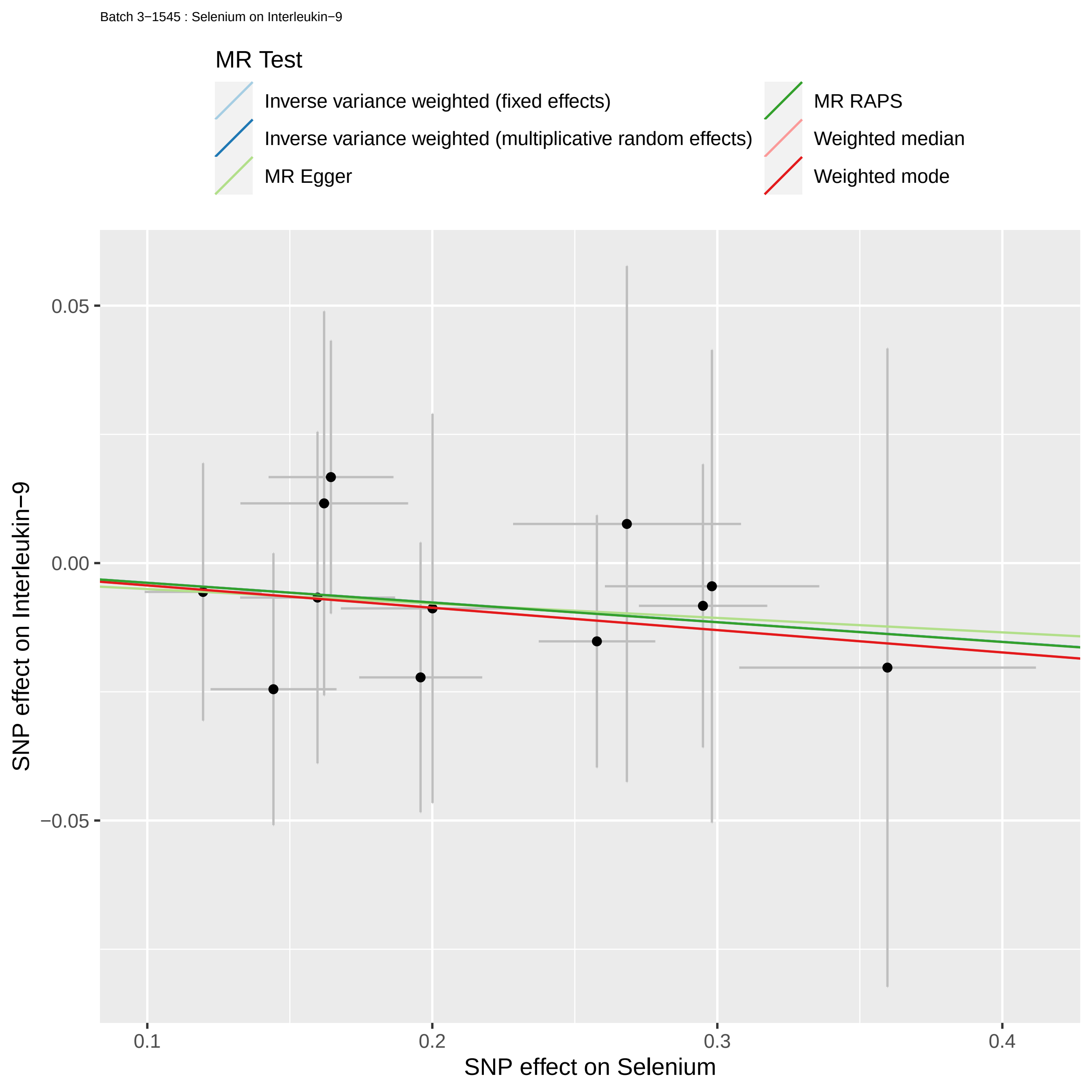
Batch 3−1545 : Selenium on Interleukin−9
MR Test
Inverse variance weighted (fixed effects)
Inverse variance weighted (multiplicative random effects)
MR Egger
MR RAPS
Weighted median
Weighted mode
0.05
0.00
−0.05
0.1
0.2
0.3
0.4
SNP effect on Interleukin−9
SNP effect on Selenium

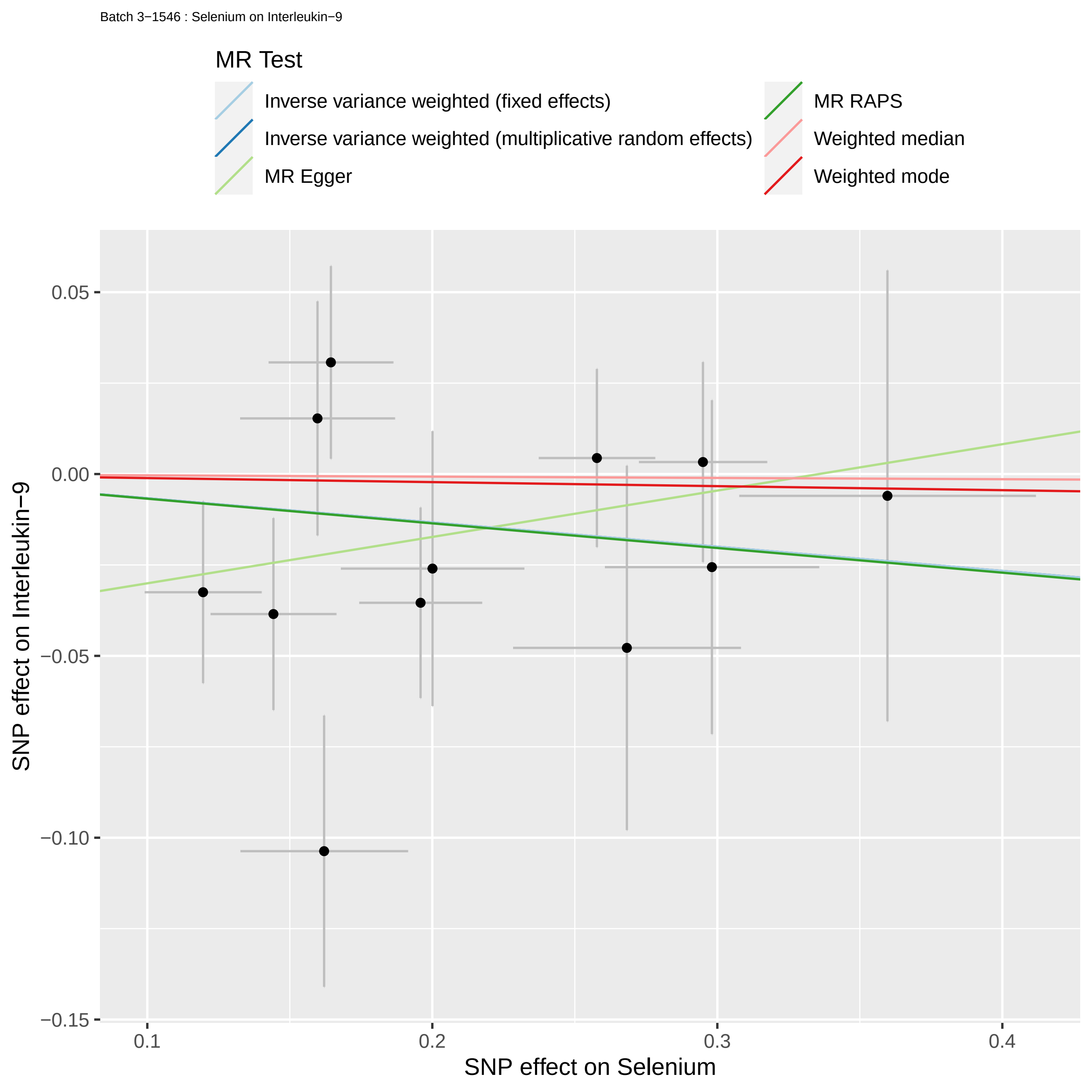
Batch 3−1546 : Selenium on Interleukin−9
MR Test
Inverse variance weighted (fixed effects)
Inverse variance weighted (multiplicative random effects)
MR Egger
MR RAPS
Weighted median
Weighted mode
0.05
0.00
−0.05
−0.10
−0.15
0.1
0.2
0.3
0.4
SNP effect on Interleukin−9
SNP effect on Selenium

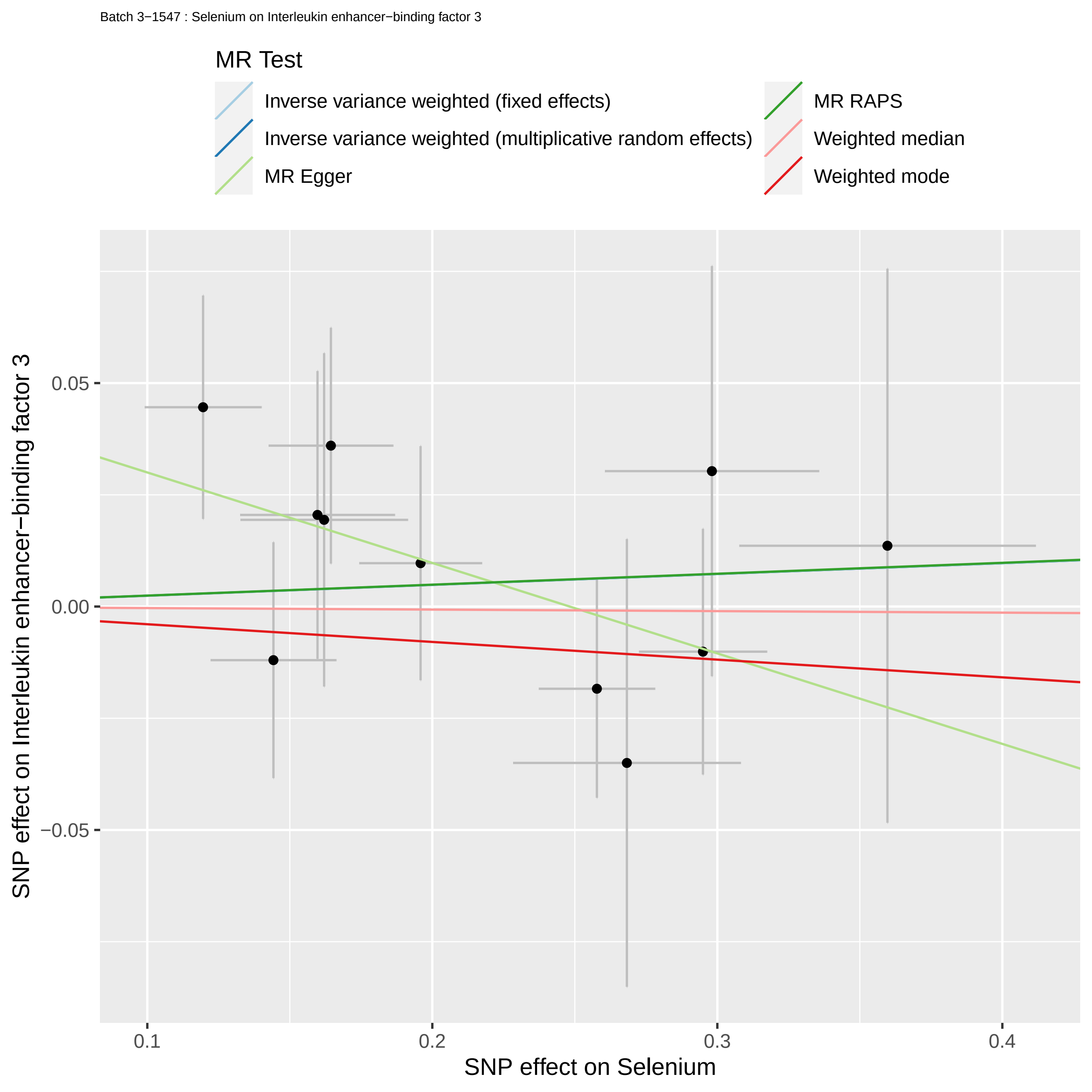

Batch 3−1547 : Selenium on Interleukin enhancer−binding factor 3
MR Test
Inverse variance weighted (fixed effects)
Inverse variance weighted (multiplicative random effects)
MR Egger
MR RAPS
Weighted median
Weighted mode
SNP effect on Interleukin enhancer−binding factor 3
0.05
0.00
−0.05
0.1
0.2
0.3
0.4
SNP effect on Selenium

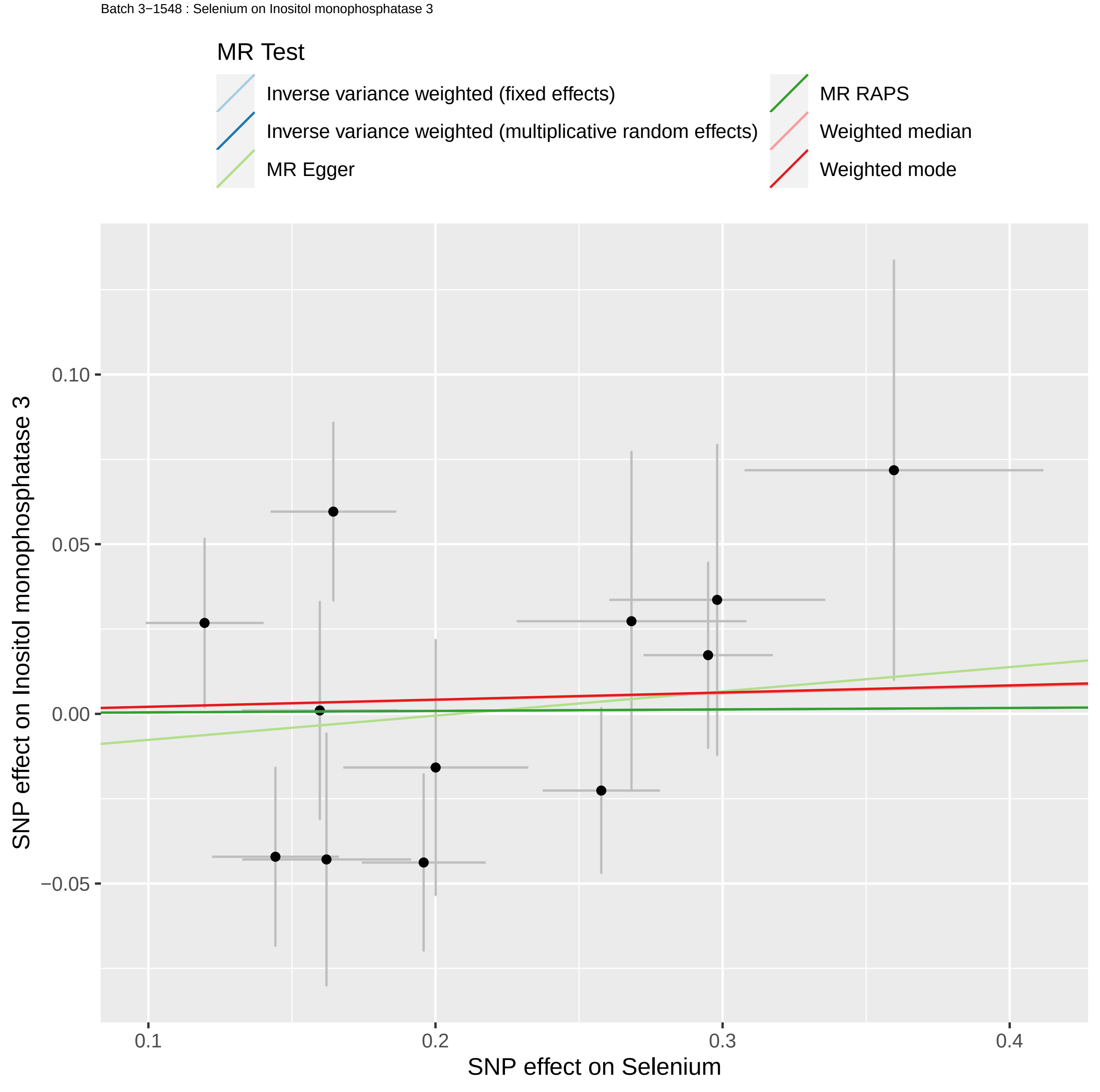

Batch 3−1548 : Selenium on Inositol monophosphatase 3
MR Test
Inverse variance weighted (fixed effects)
Inverse variance weighted (multiplicative random effects)
MR Egger
MR RAPS
Weighted median
Weighted mode
SNP effect on Inositol monophosphatase 3
0.10
0.05
0.00
−0.05
0.1
0.2
0.3
0.4
SNP effect on Selenium

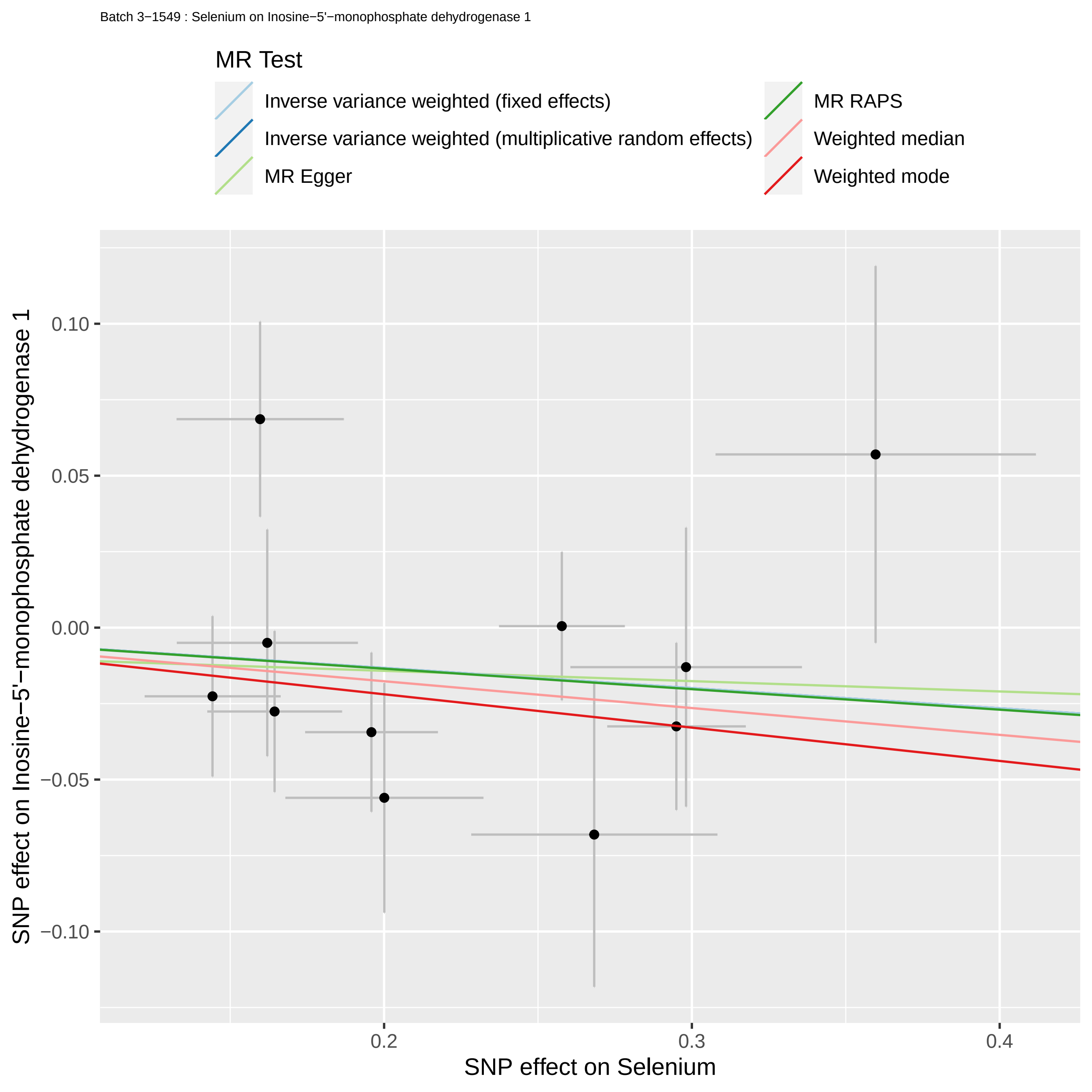
Batch 3−1549 : Selenium on Inosine−5'−monophosphate dehydrogenase 1
MR Test
Inverse variance weighted (fixed effects)
Inverse variance weighted (multiplicative random effects)
MR Egger
MR RAPS
Weighted median
Weighted mode
SNP effect on Inosine−5'−monophosphate dehydrogenase 1
0.10
0.05
0.00
−0.05
−0.10
0.2
0.3
0.4
SNP effect on Selenium

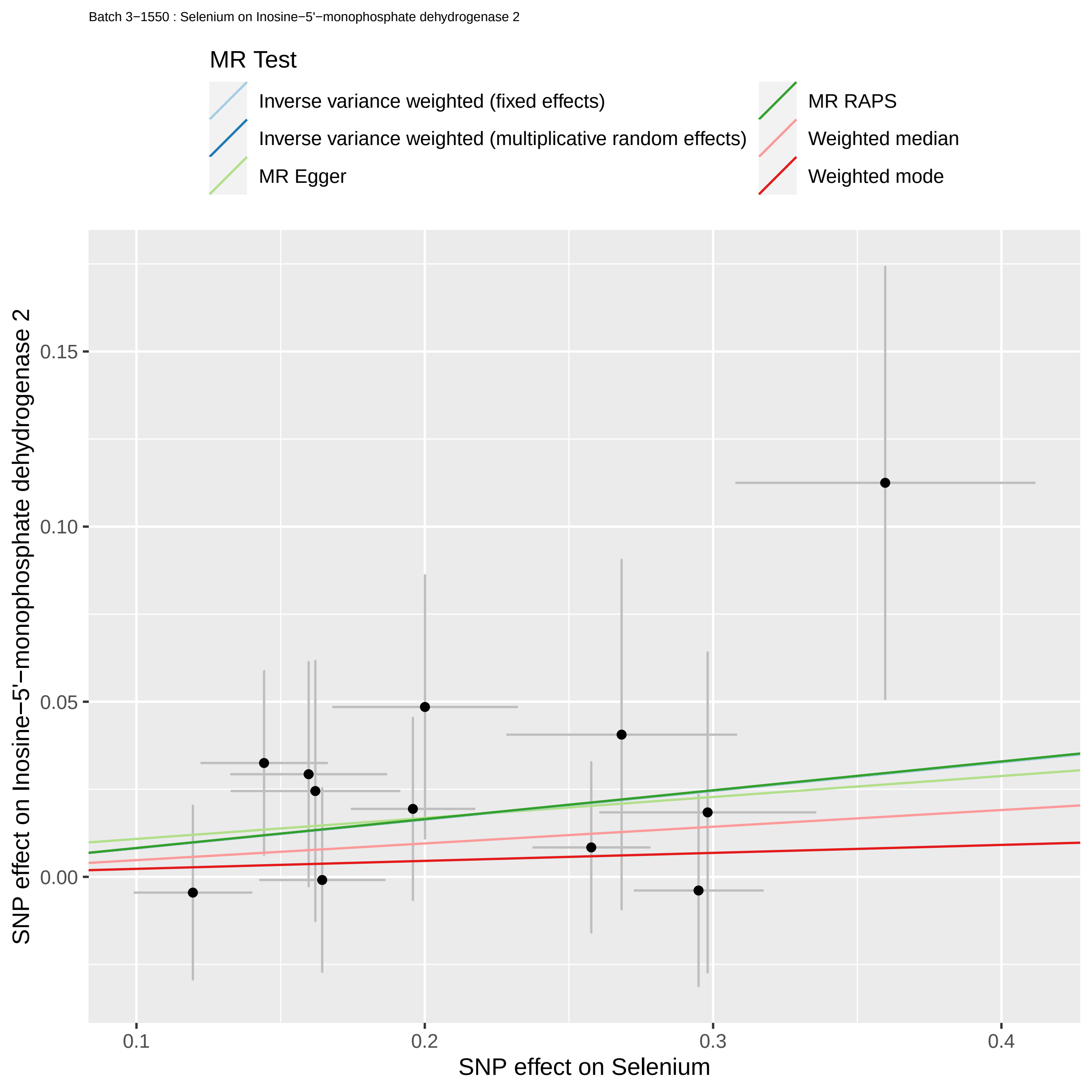

Batch 3−1550 : Selenium on Inosine−5'−monophosphate dehydrogenase 2
MR Test
Inverse variance weighted (fixed effects)
Inverse variance weighted (multiplicative random effects)
MR Egger
MR RAPS
Weighted median
Weighted mode
SNP effect on Inosine−5'−monophosphate dehydrogenase 2
0.15
0.10
0.05
0.00
0.1
0.2
0.3
0.4
SNP effect on Selenium

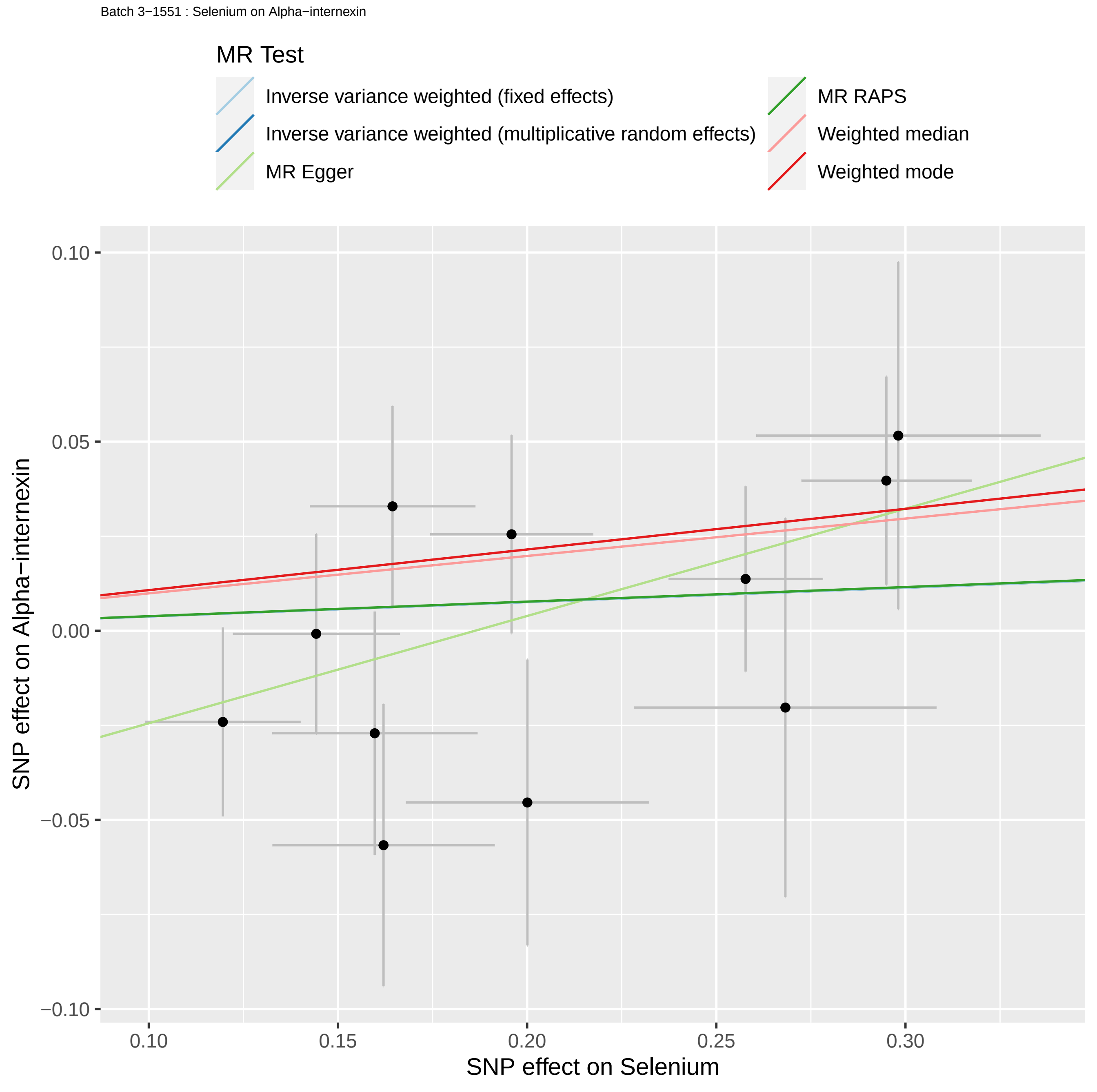

Batch 3−1551 : Selenium on Alpha−internexin
MR Test
Inverse variance weighted (fixed effects)
Inverse variance weighted (multiplicative random effects)
MR Egger
MR RAPS
Weighted median
Weighted mode
0.10
0.05
0.00
−0.05
−0.10
SNP effect on Alpha−internexin
0.10
0.15
0.20
0.25
0.30
SNP effect on Selenium

Batch 3−1552 : Selenium on Inhibitor of growth protein 1

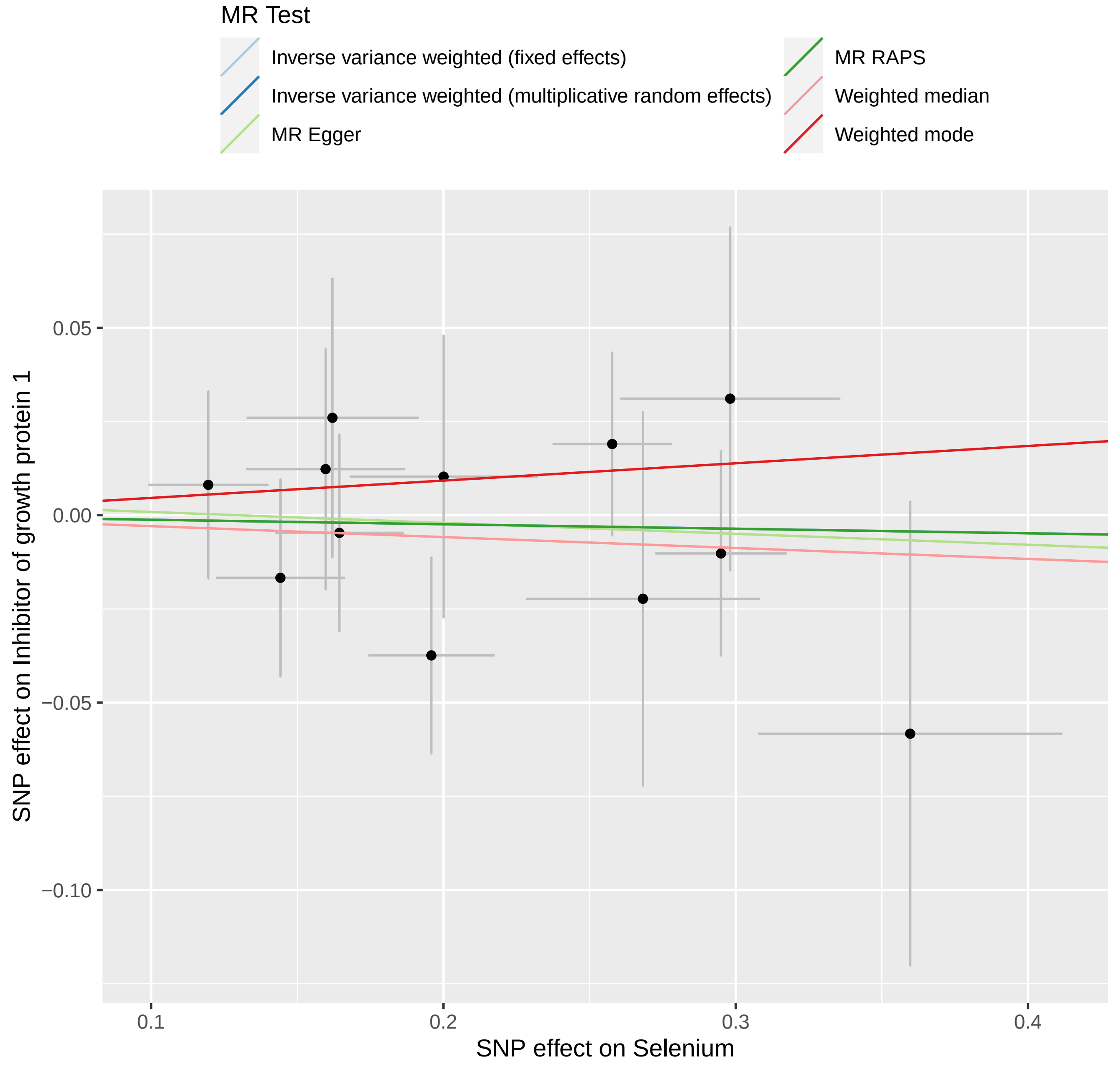

Batch 3−1553 : Selenium on Inhibin beta A chain

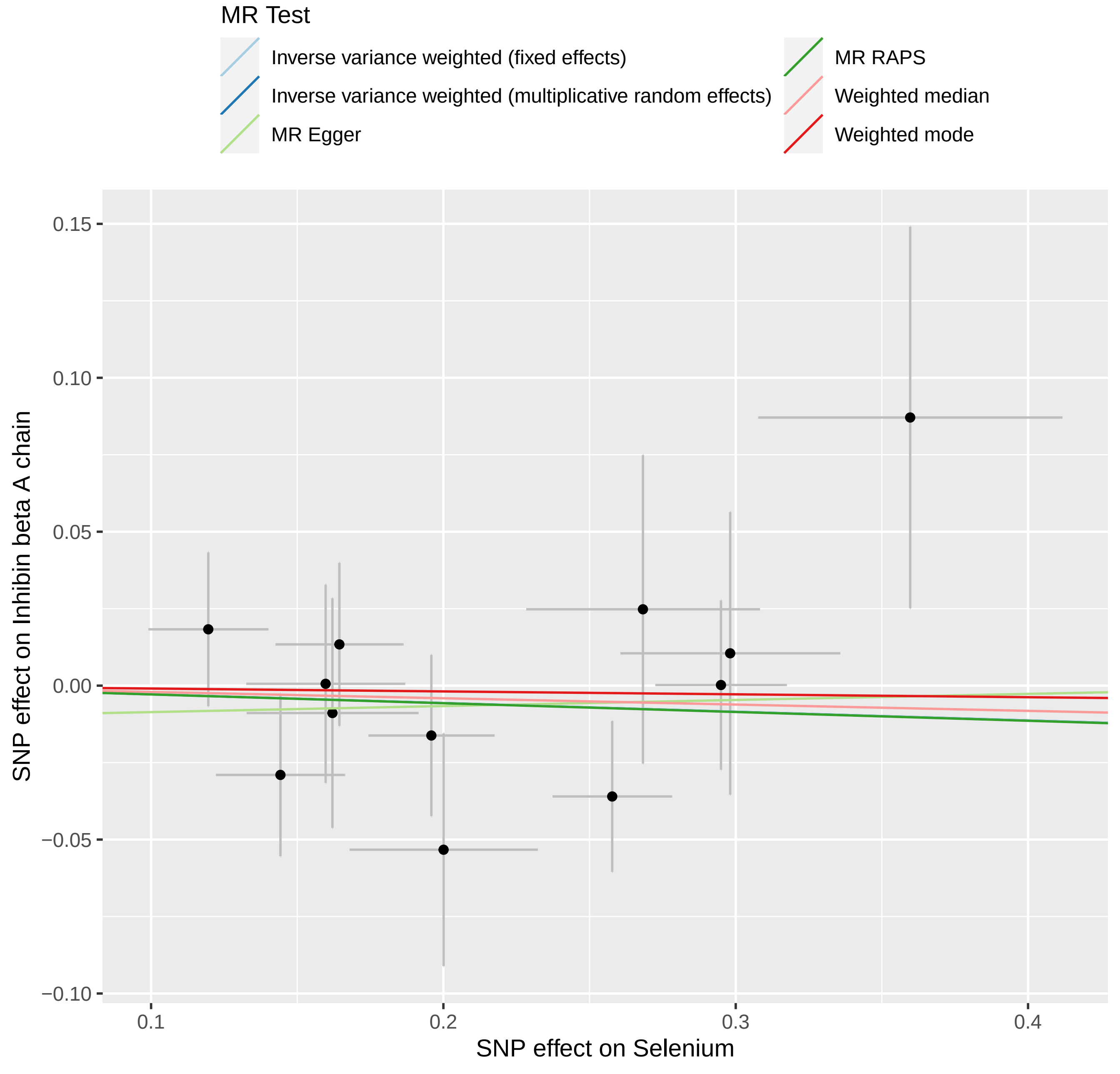

Batch 3−1554 : Selenium on Inhibin beta A chain:Inhibin beta B chain heterodimer

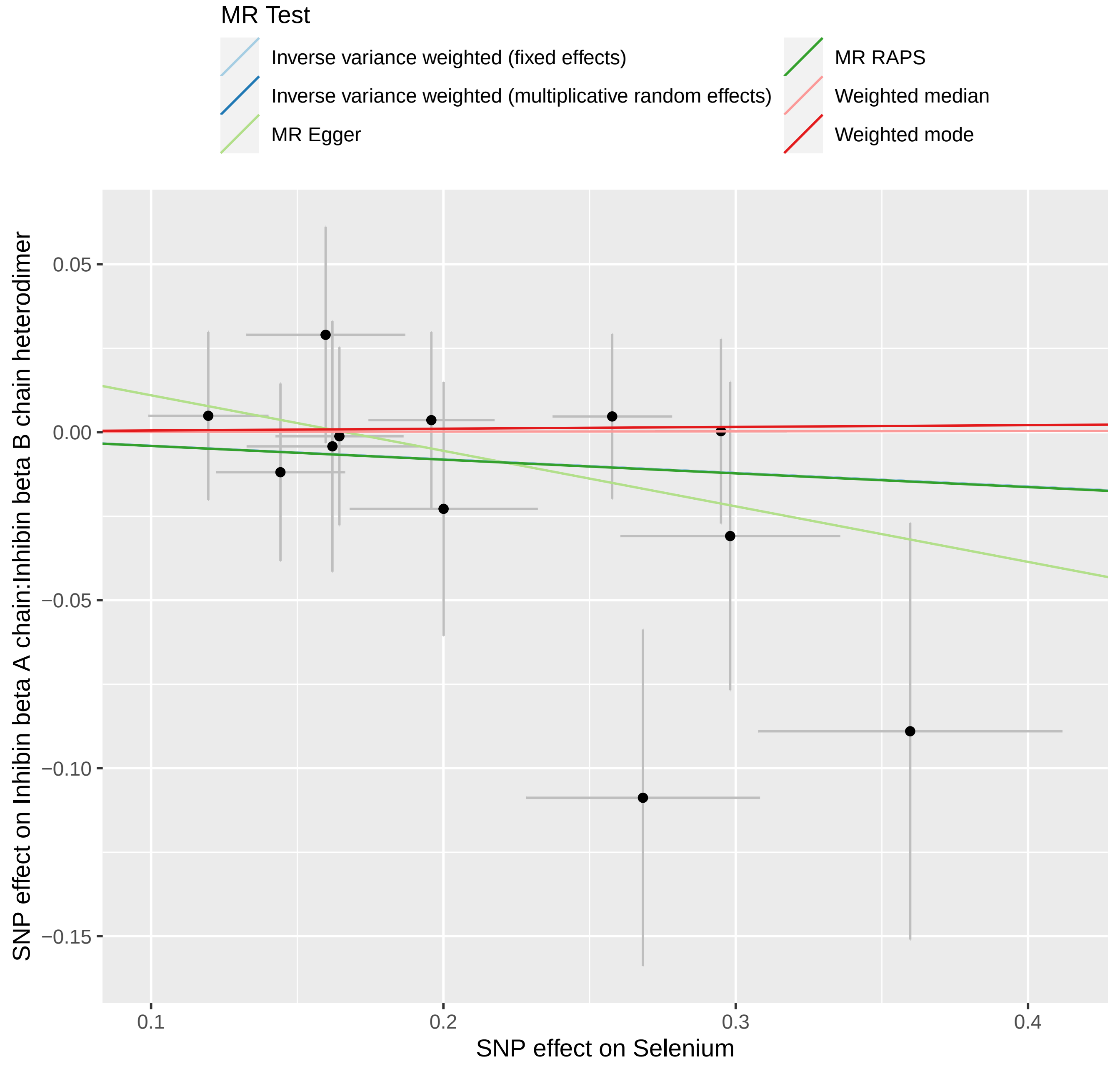

Batch 3−1555 : Selenium on Inhibin beta A chain:Inhibin beta B chain heterodimer

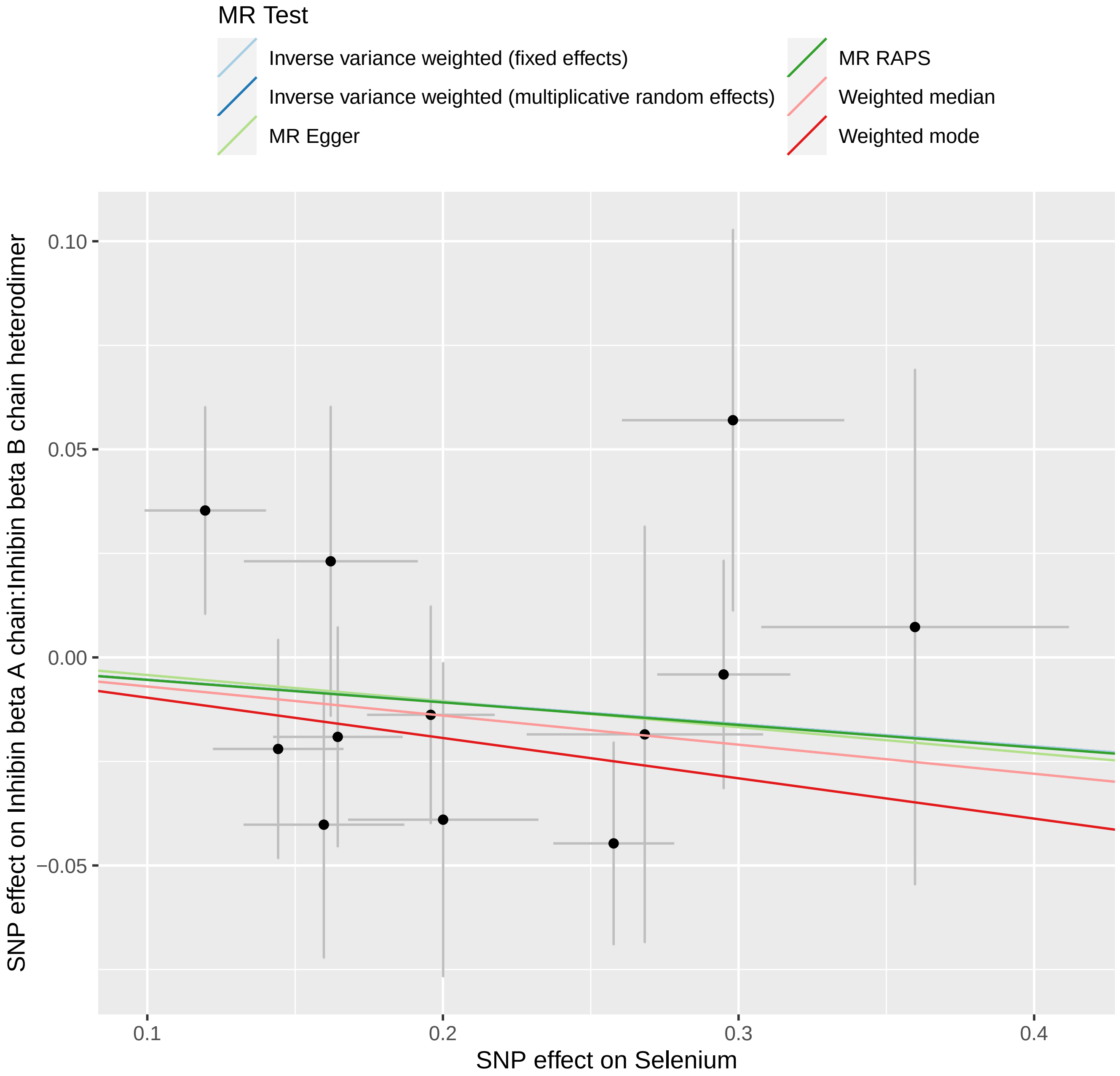

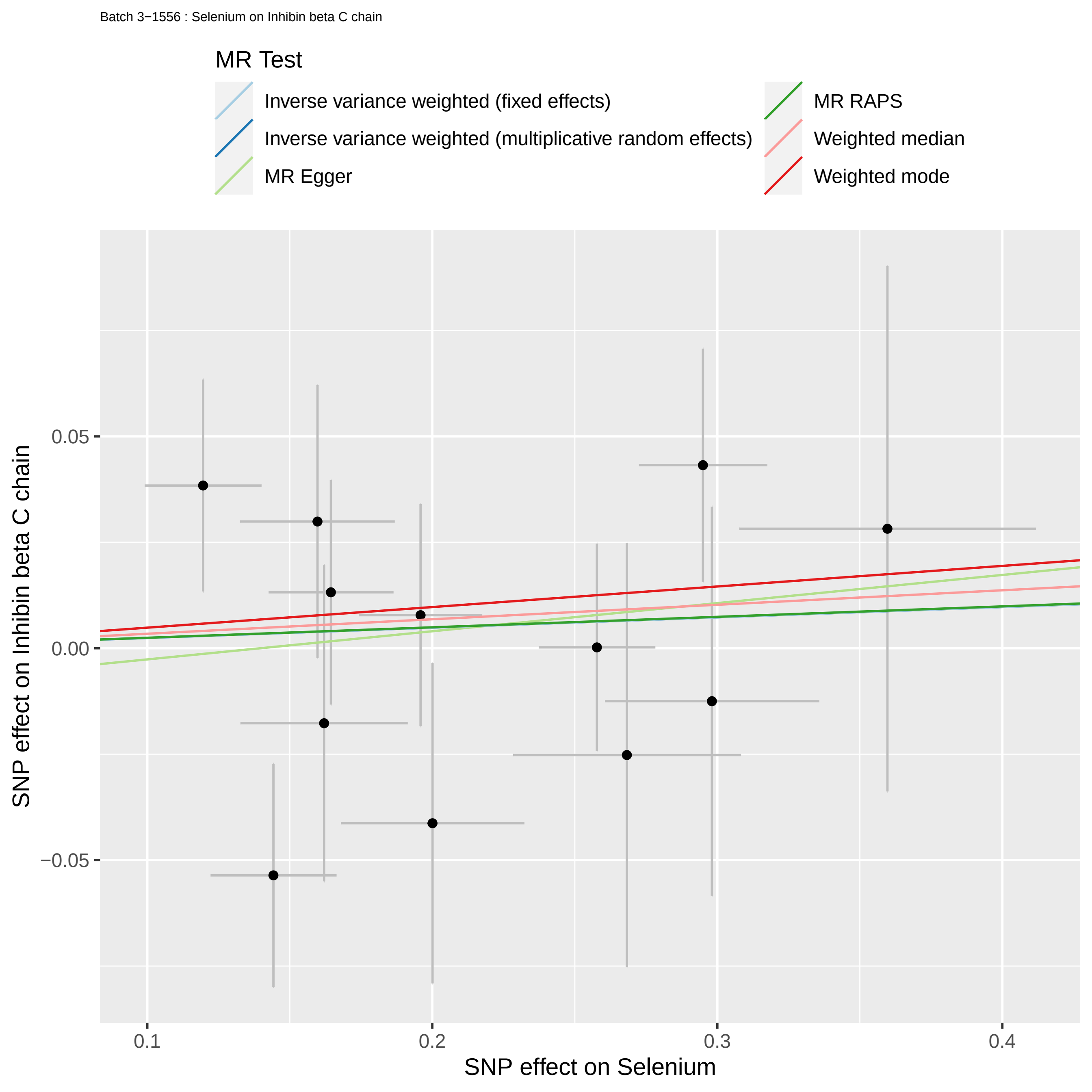

Batch 3−1556 : Selenium on Inhibin beta C chain
MR Test
Inverse variance weighted (fixed effects)
Inverse variance weighted (multiplicative random effects)
MR Egger
MR RAPS
Weighted median
Weighted mode
0.05
0.00
−0.05
0.1
0.2
0.3
0.4
SNP effect on Inhibin beta C chain
SNP effect on Selenium

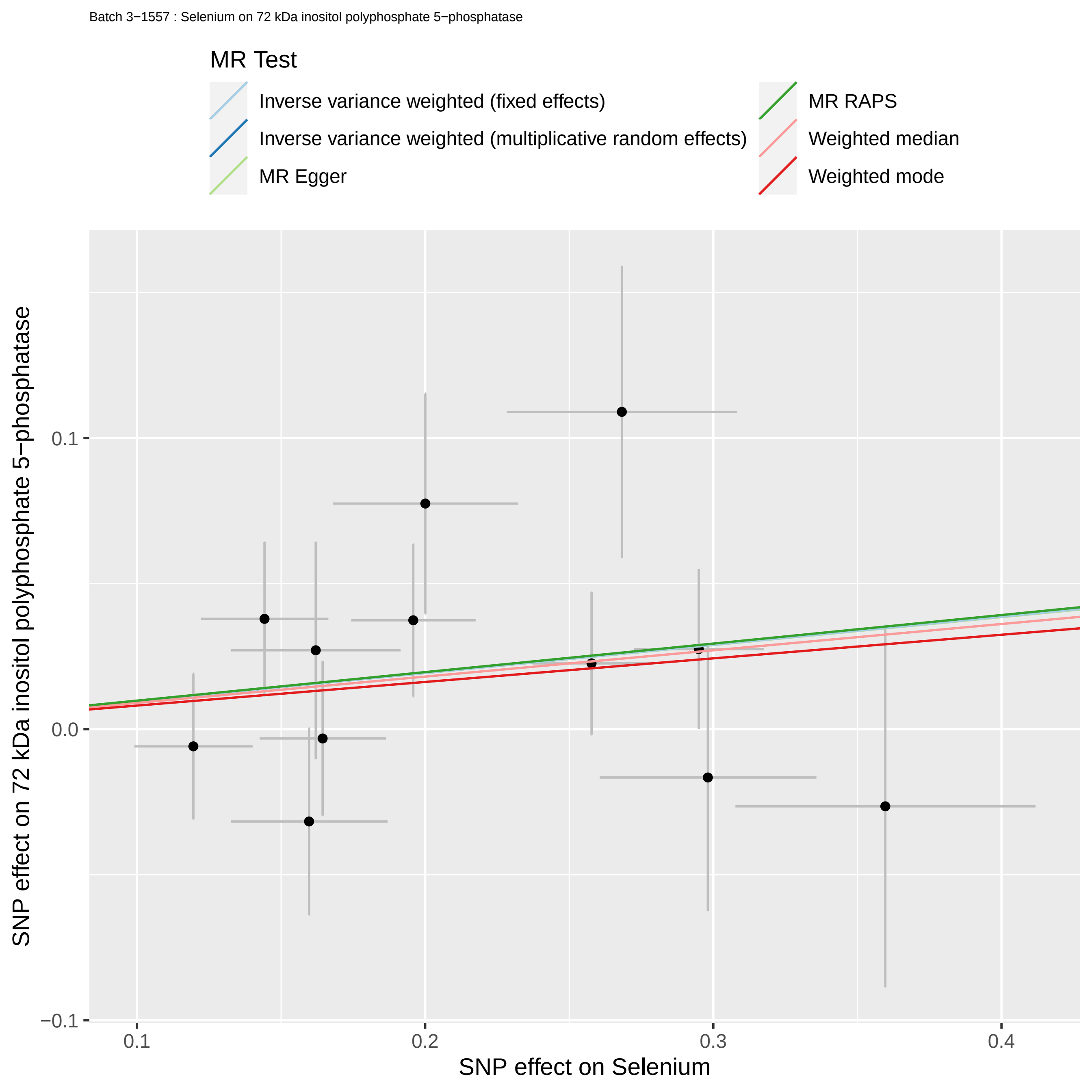

Batch 3−1557 : Selenium on 72 kDa inositol polyphosphate 5−phosphatase
MR Test
Inverse variance weighted (fixed effects)
Inverse variance weighted (multiplicative random effects)
MR Egger
MR RAPS
Weighted median
Weighted mode
SNP effect on 72 kDa inositol polyphosphate 5−phosphatase
0.1
0.0
−0.1
0.1
0.2
0.3
0.4
SNP effect on Selenium

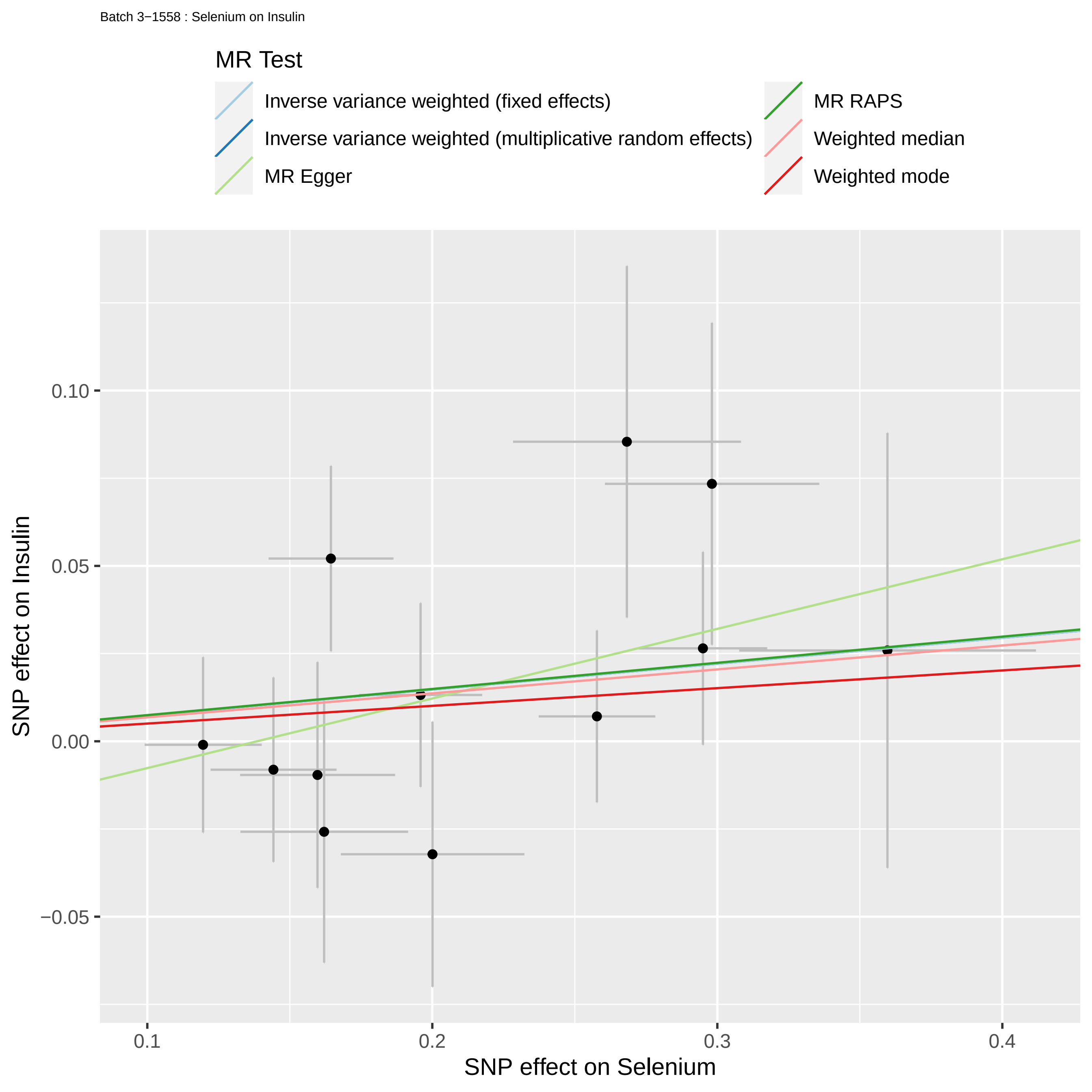
Batch 3−1558 : Selenium on Insulin
MR Test
Inverse variance weighted (fixed effects)
Inverse variance weighted (multiplicative random effects)
MR Egger
MR RAPS
Weighted median
Weighted mode
0.10
0.05
0.00
−0.05
0.1
0.2
0.3
0.4
SNP effect on Insulin
SNP effect on Selenium

Batch 3−1559 : Selenium on Insulin−induced gene 1 protein

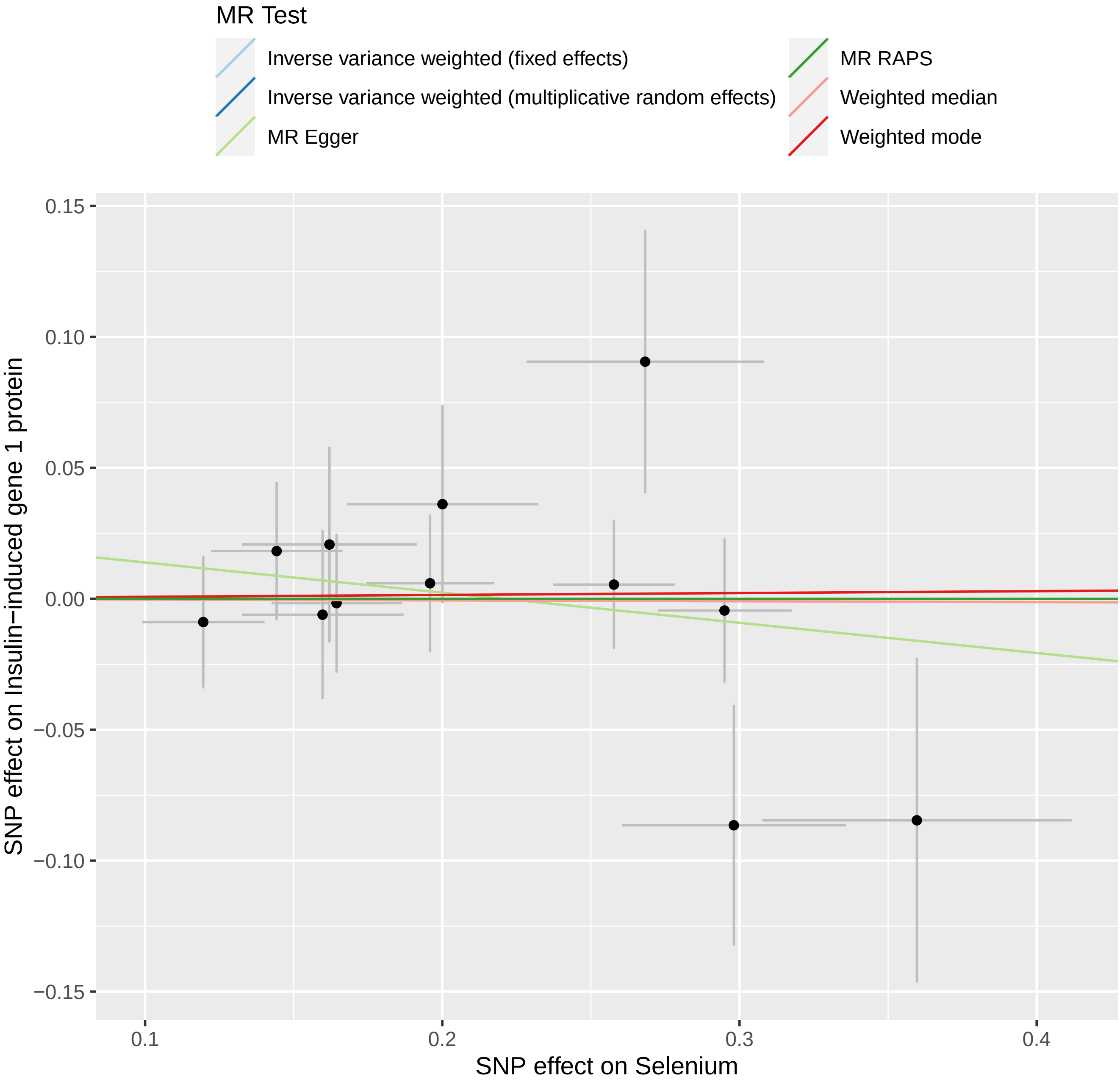

Batch 3−1560 : Selenium on Insulin−like 3

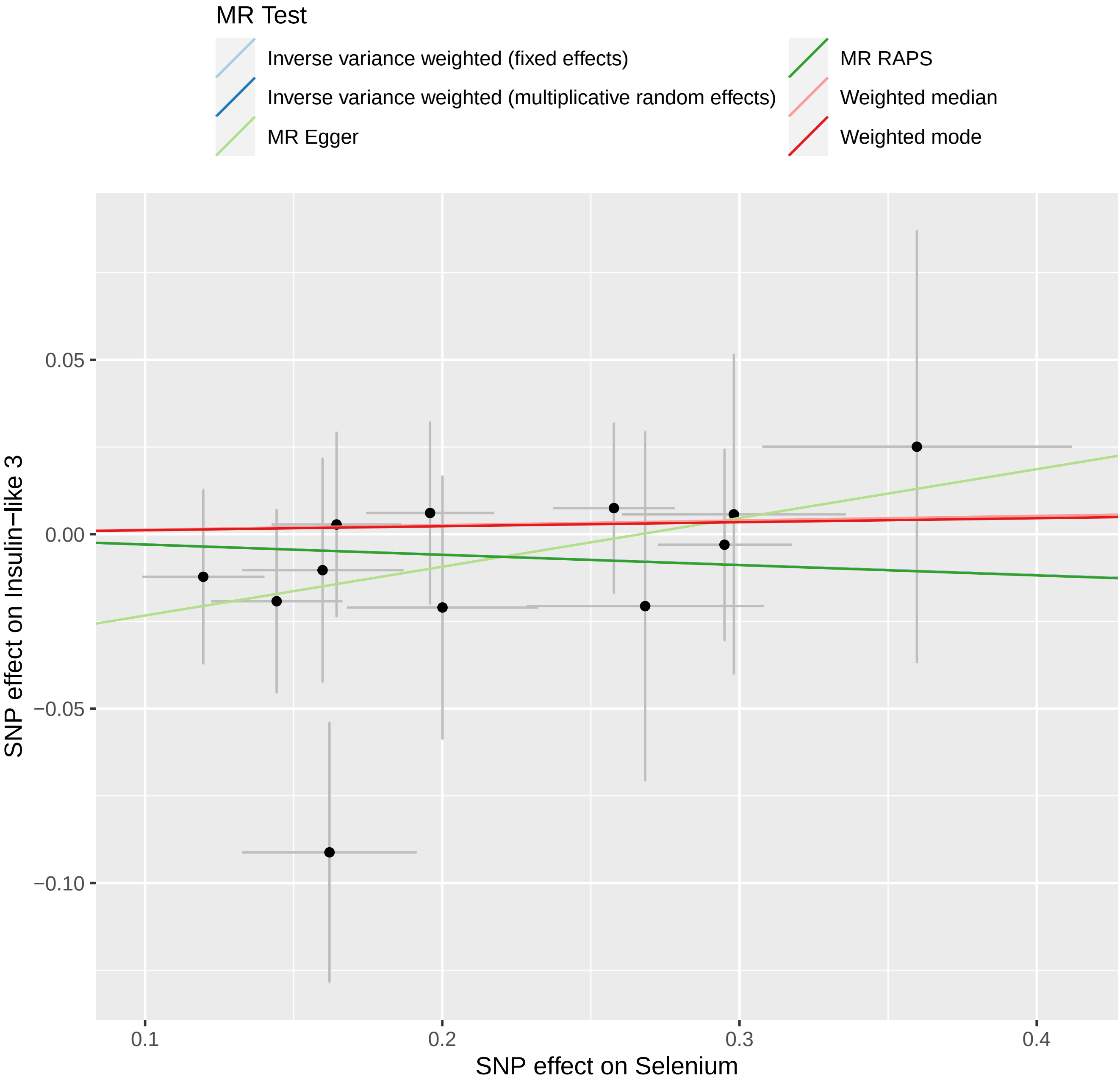

Batch 3−1561 : Selenium on Early placenta insulin−like peptide

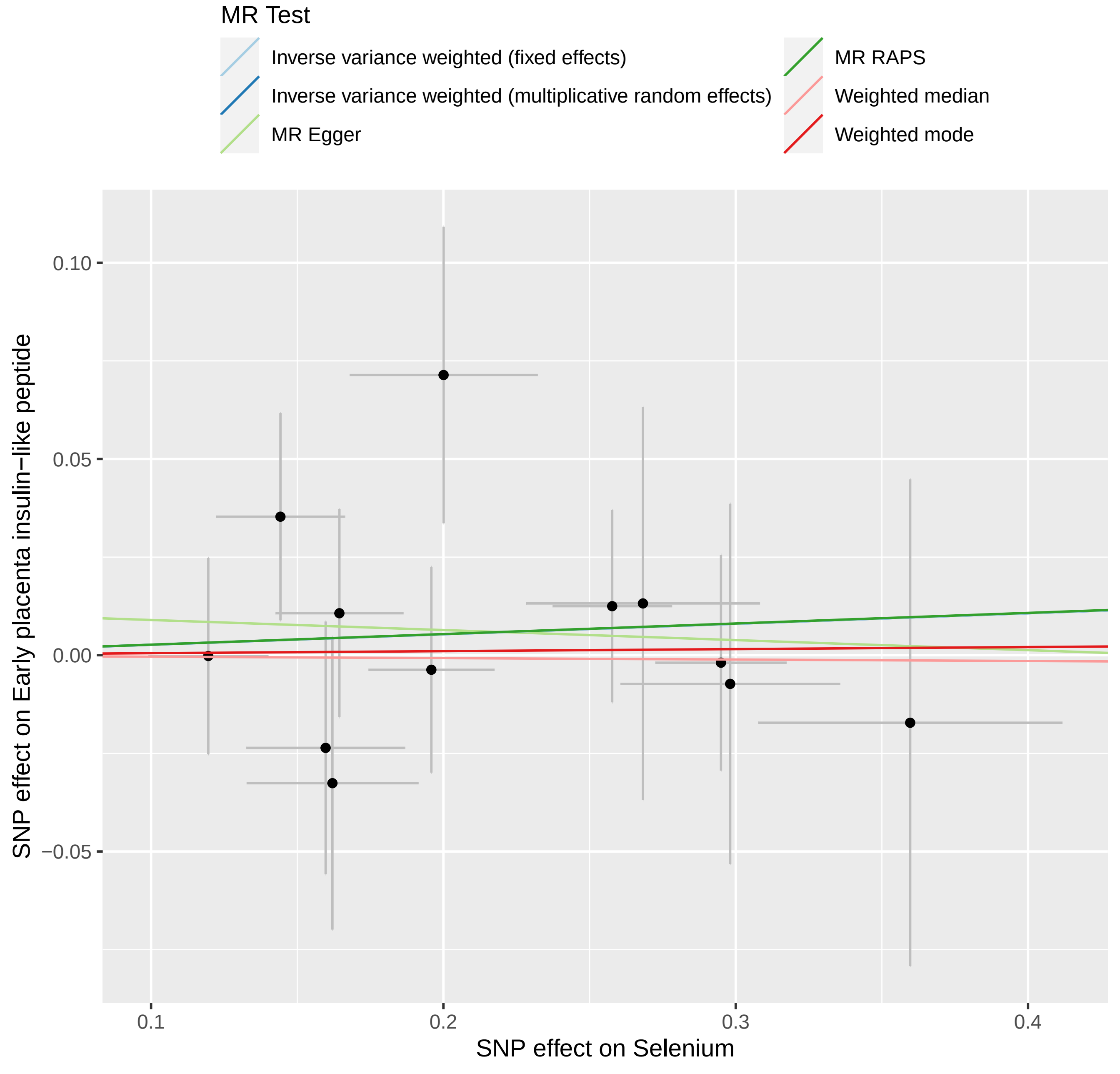

Batch 3−1562 : Selenium on Insulin−like peptide INSL5

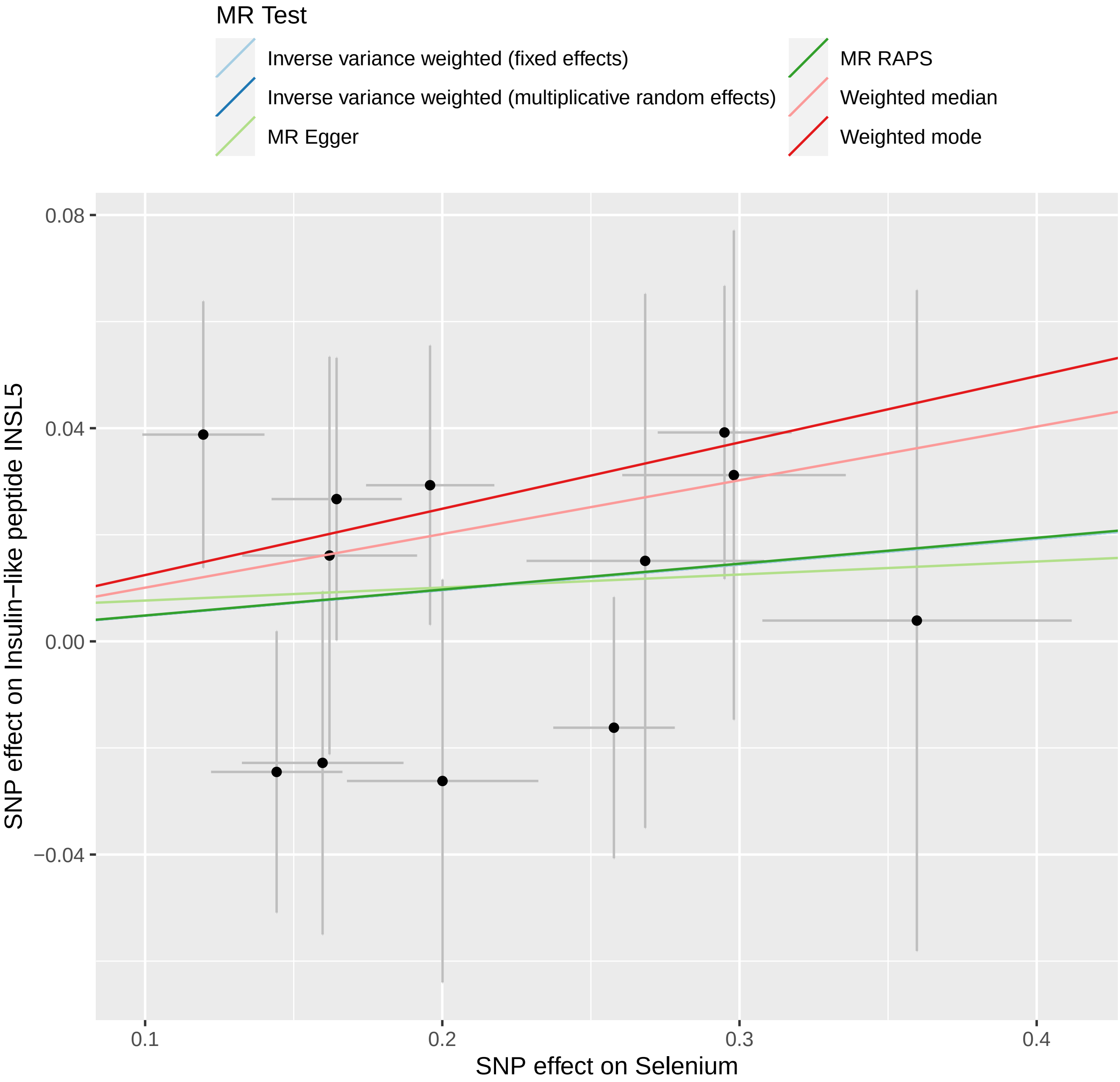

Batch 3−1563 : Selenium on Insulin−like peptide INSL6

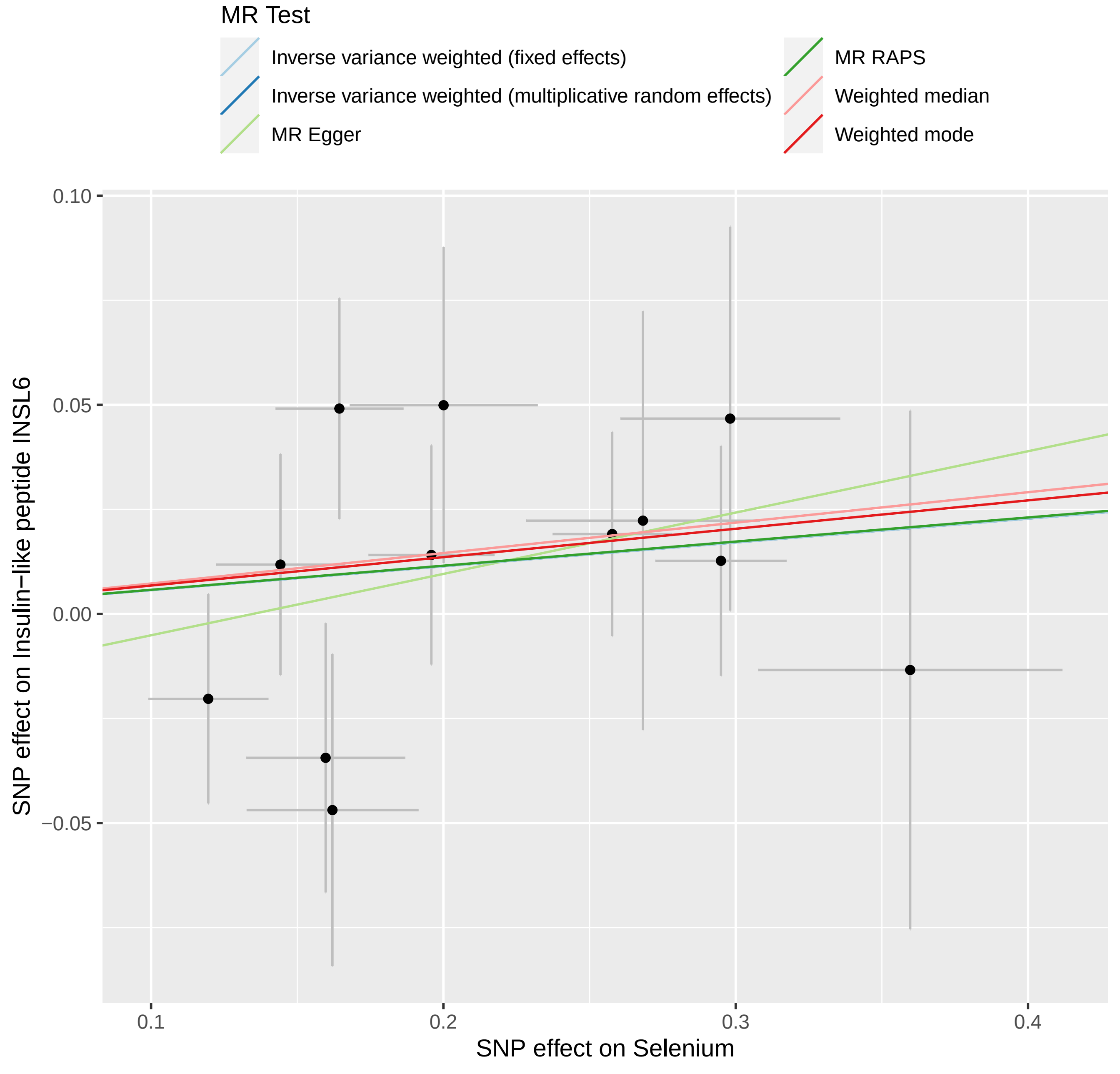

Batch 3−1564 : Selenium on Insulin receptor

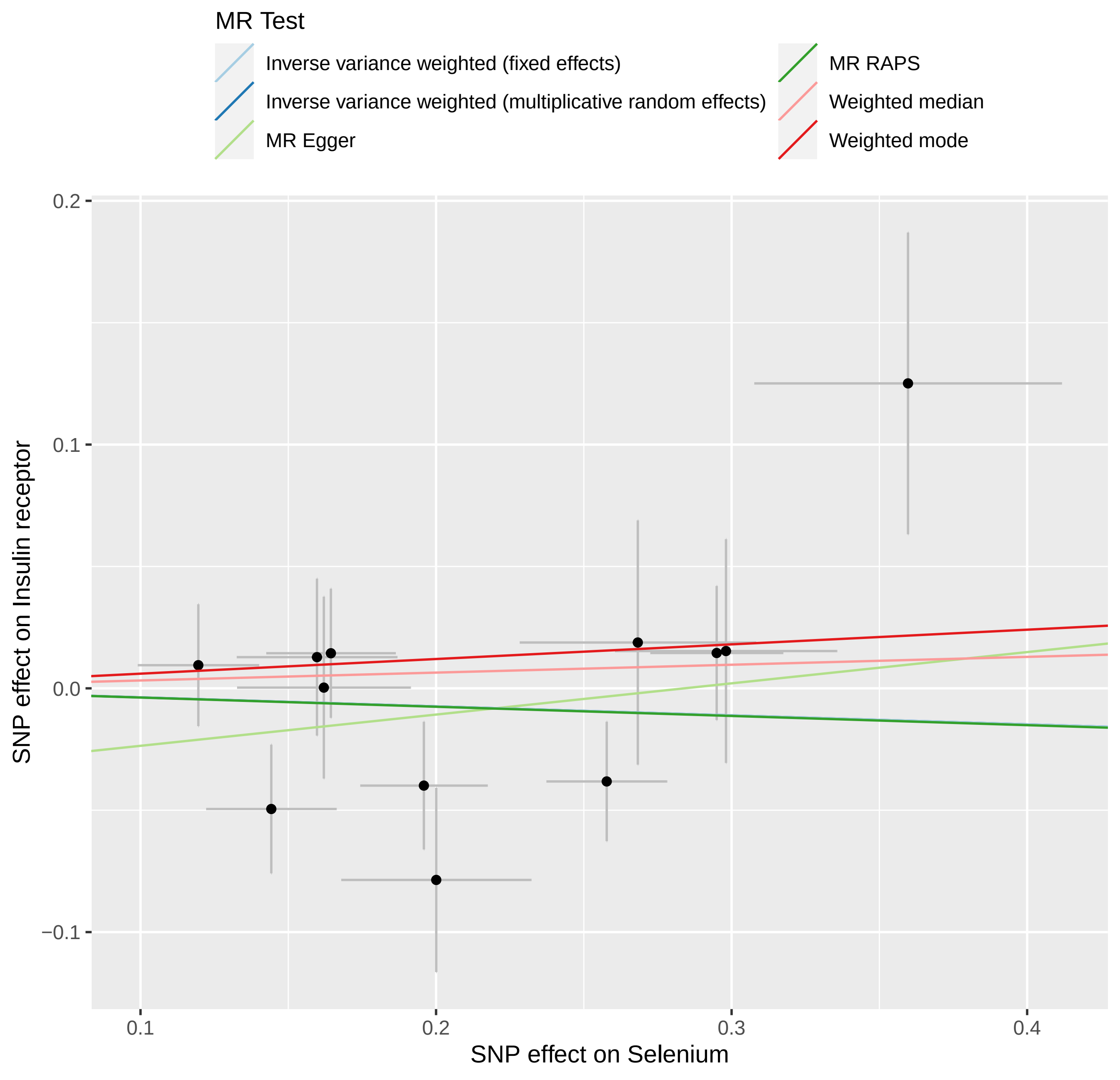

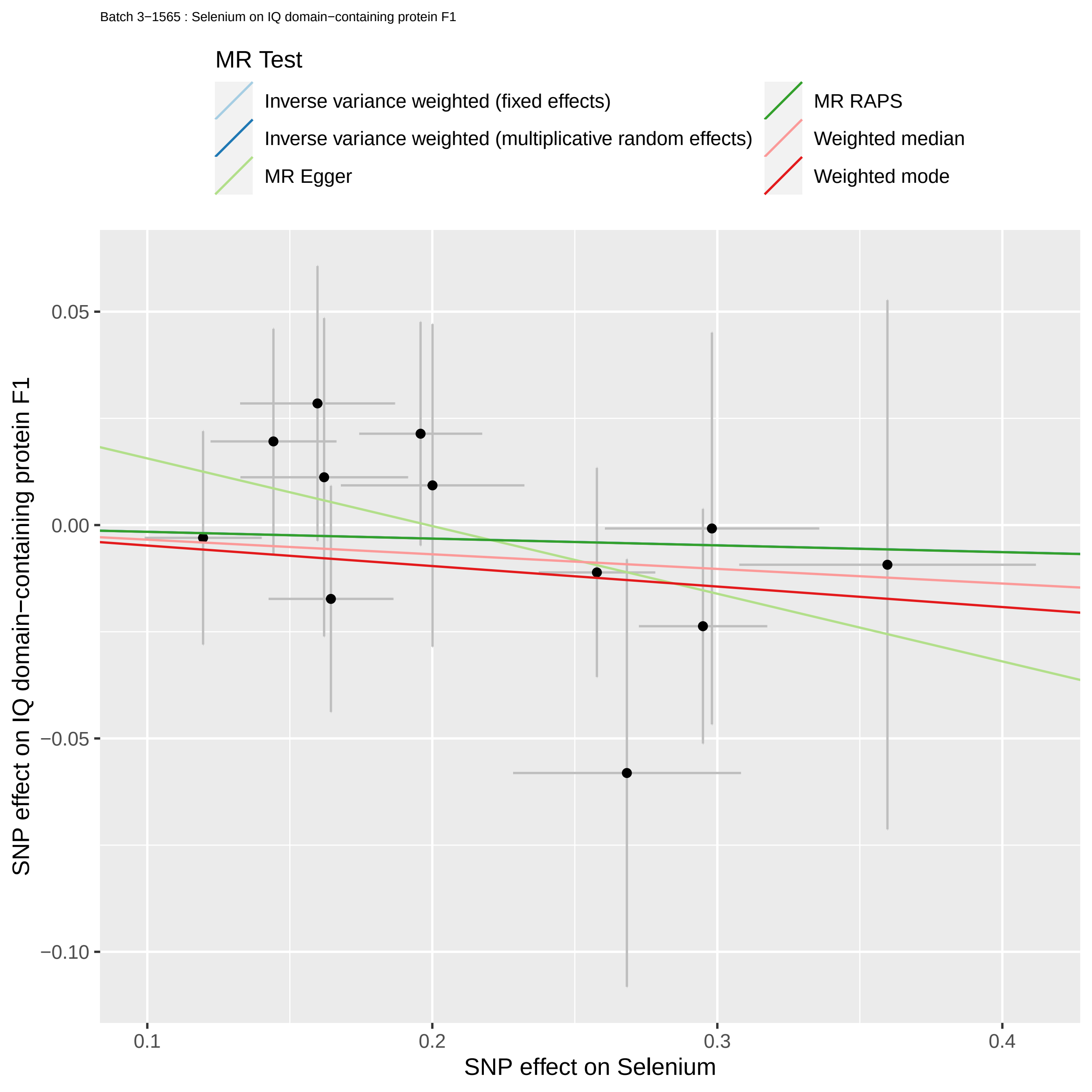

Batch 3−1565 : Selenium on IQ domain−containing protein F1
MR Test
Inverse variance weighted (fixed effects)
Inverse variance weighted (multiplicative random effects)
MR Egger
MR RAPS
Weighted median
Weighted mode
0.05
0.00
−0.05
−0.10
0.1
0.2
0.3
0.4
SNP effect on Selenium
SNP effect on IQ domain−containing protein F1

Batch 3−1566 : Selenium on Interferon regulatory factor 1

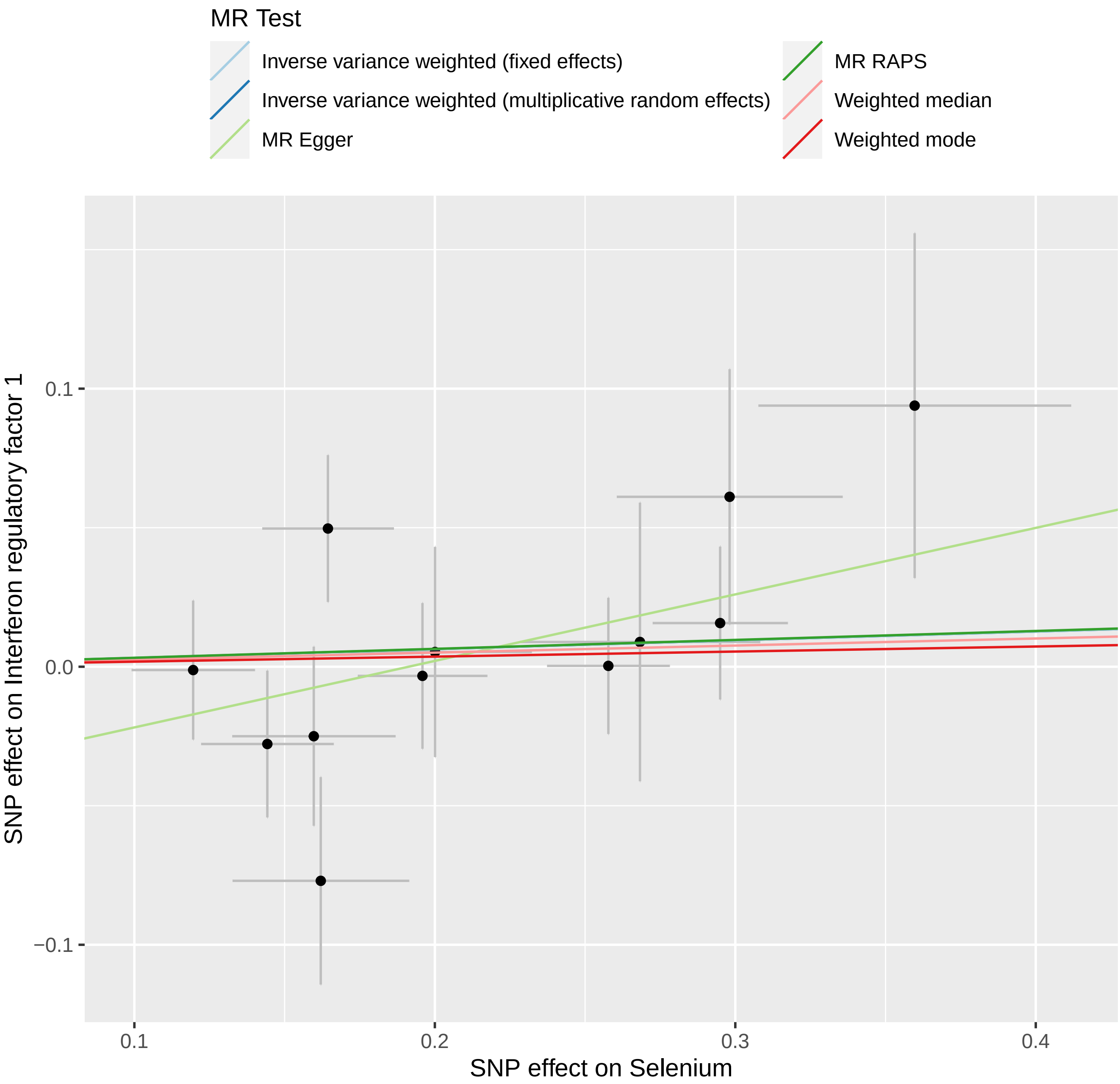

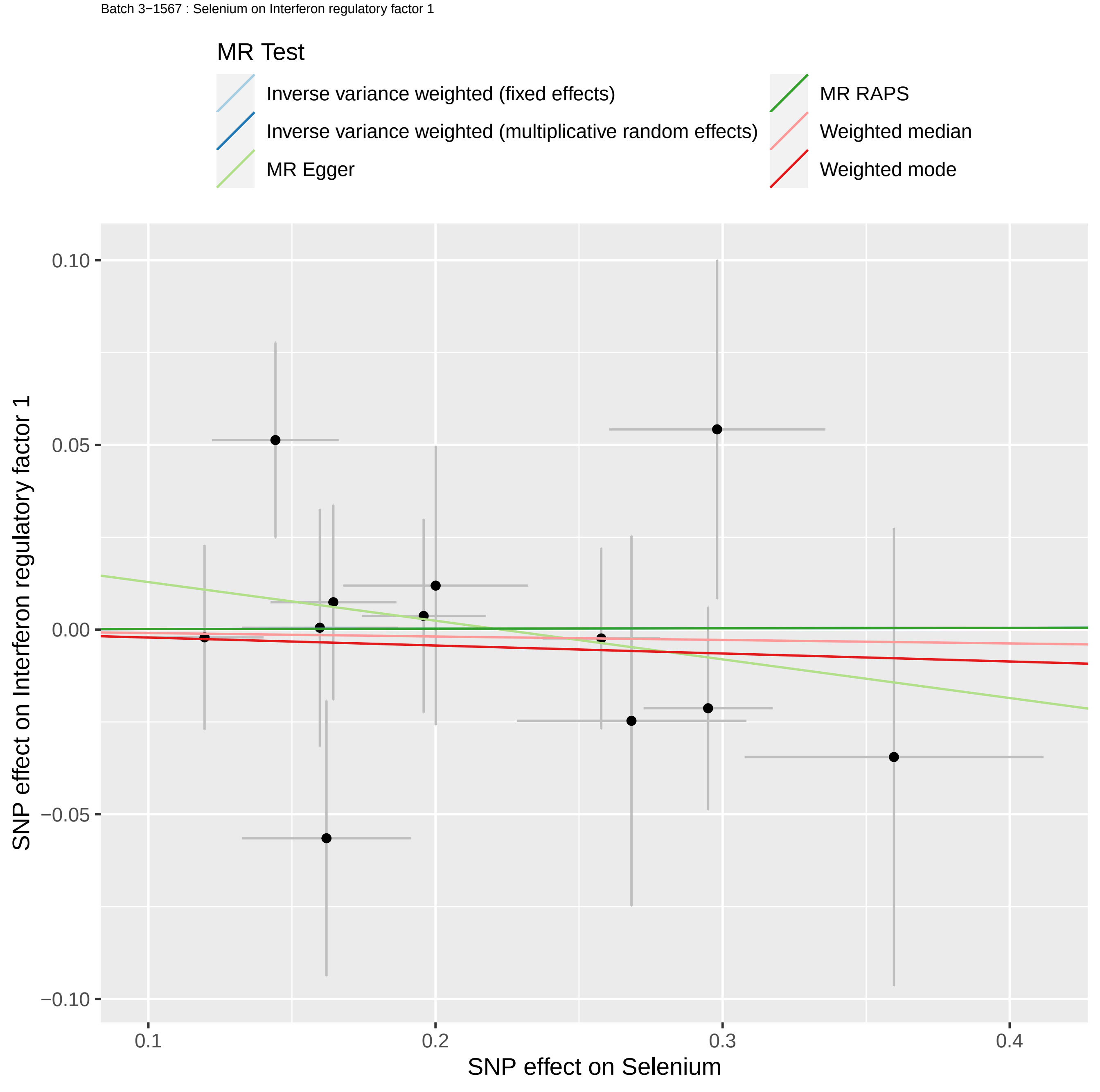
Batch 3−1567 : Selenium on Interferon regulatory factor 1
MR Test
Inverse variance weighted (fixed effects)
Inverse variance weighted (multiplicative random effects)
MR Egger
MR RAPS
Weighted median
Weighted mode
0.10
0.05
0.00
−0.05
−0.10
0.1
0.2
0.3
0.4
SNP effect on Interferon regulatory factor 1
SNP effect on Selenium

Batch 3−1568 : Selenium on Interferon regulatory factor 2

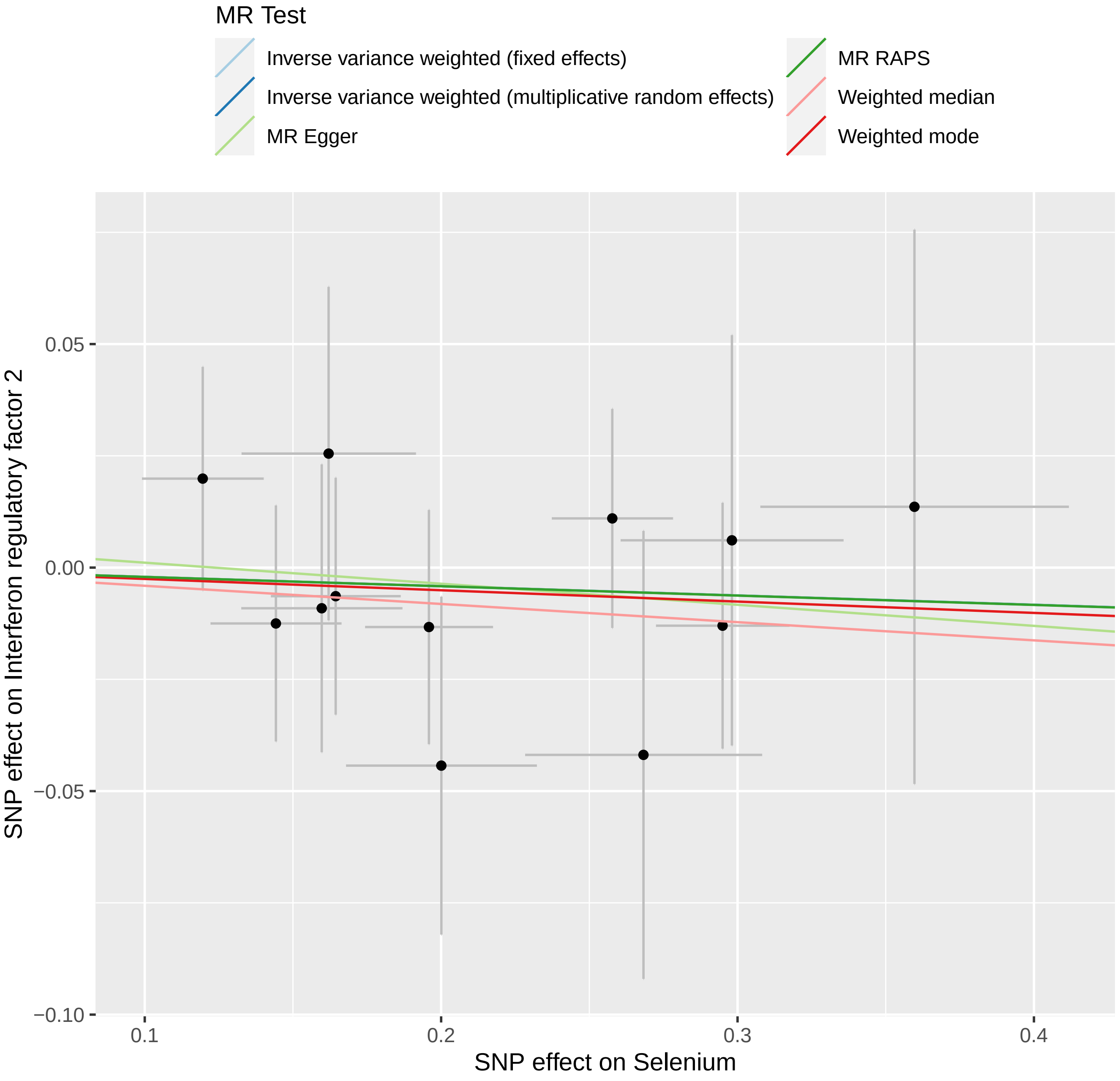

Batch 3−1569 : Selenium on Interferon regulatory factor 4

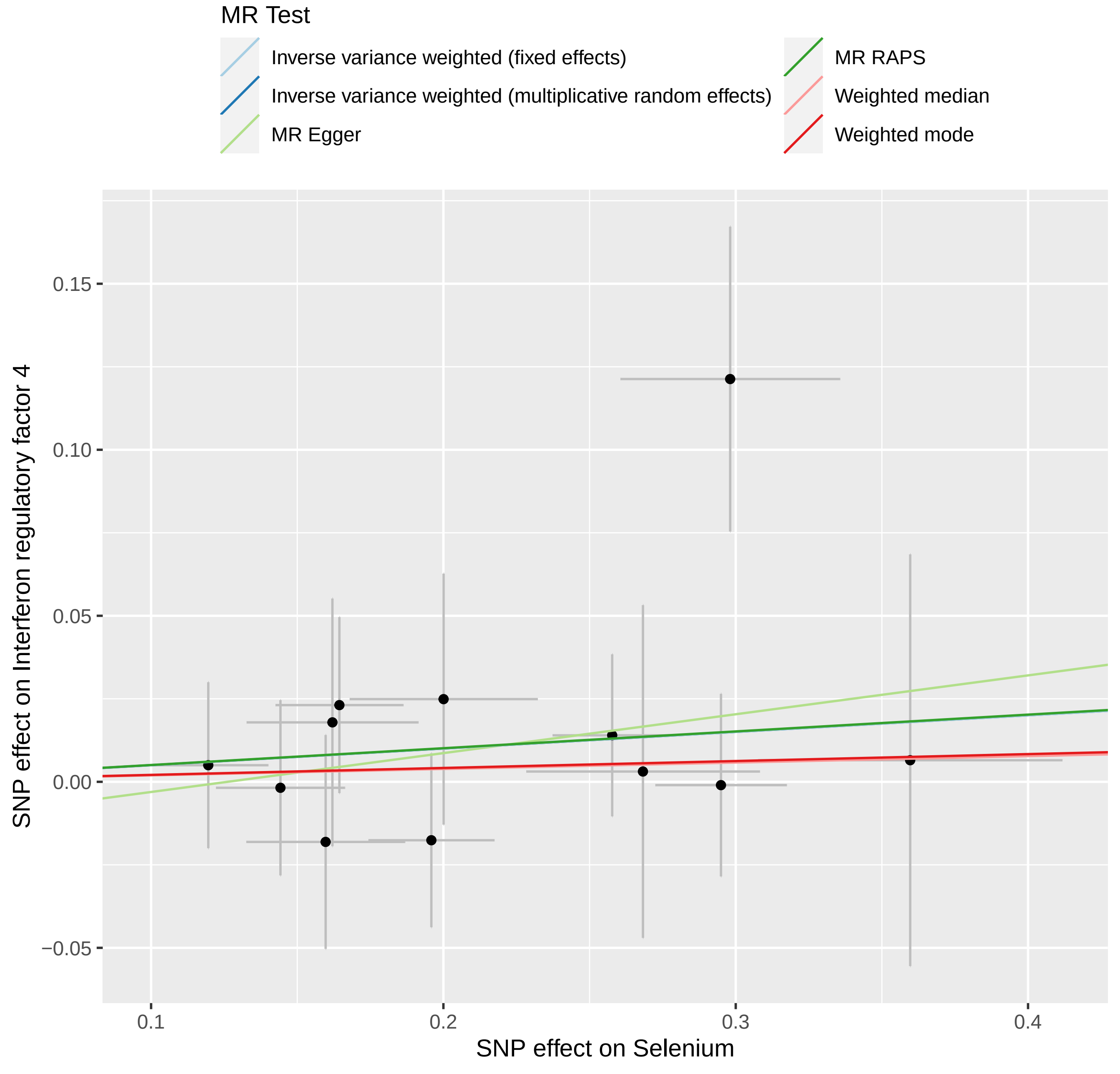

Batch 3−1570 : Selenium on Interferon regulatory factor 6

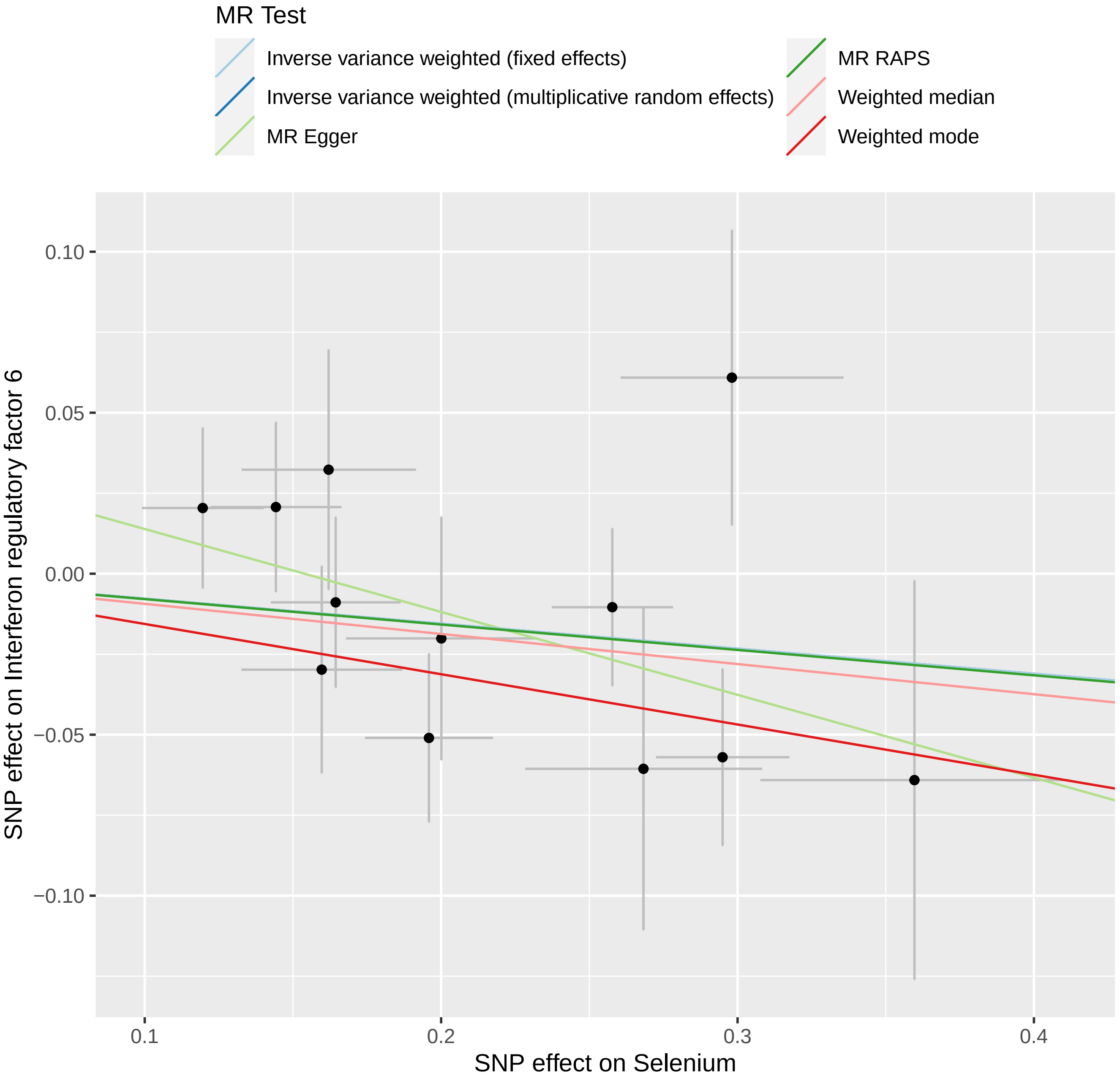

Batch 3−1571 : Selenium on Interferon regulatory factor 9

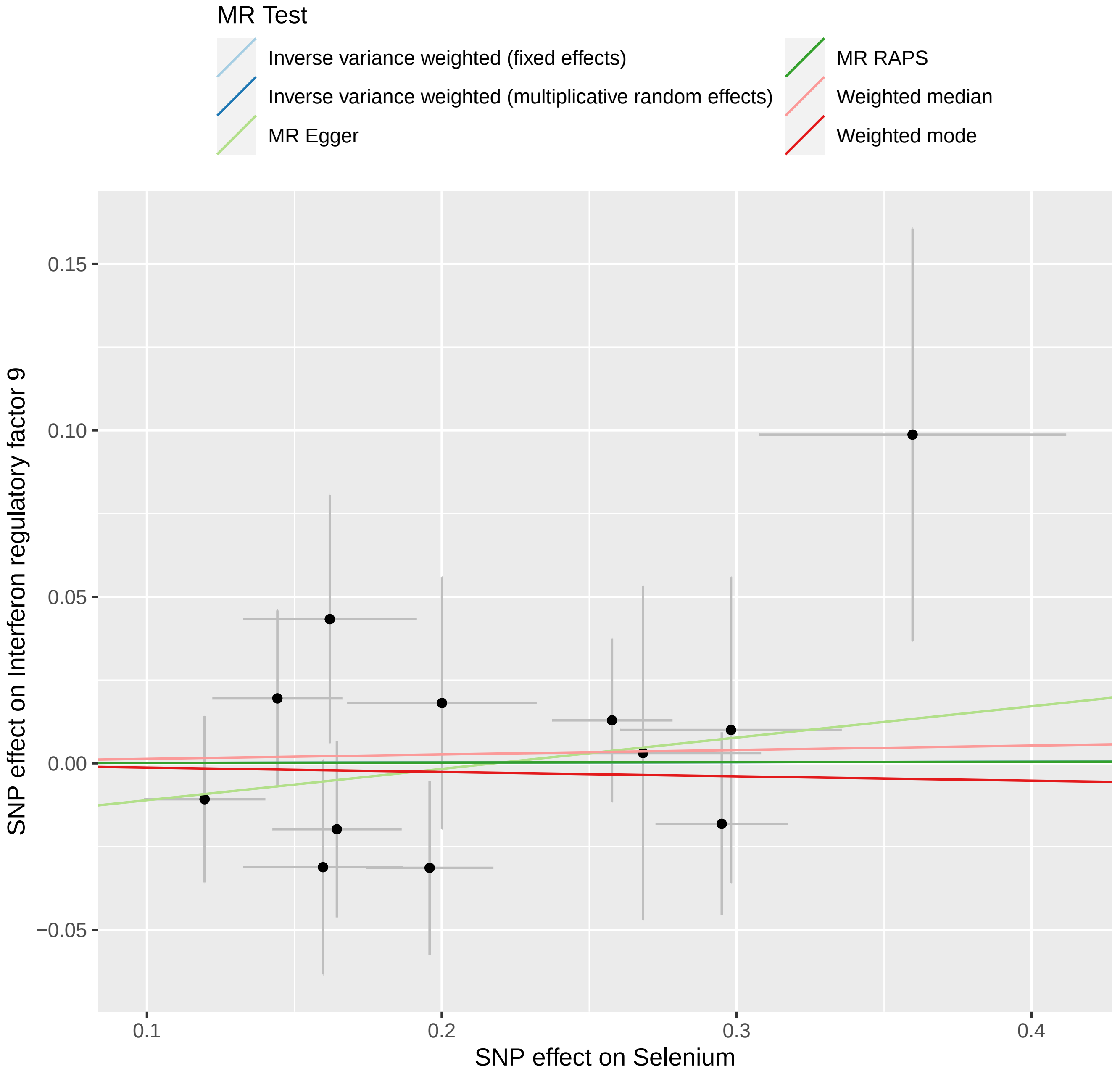

Batch 3−1572 : Selenium on Iron−sulfur cluster assembly enzyme ISCU, mitochondrial

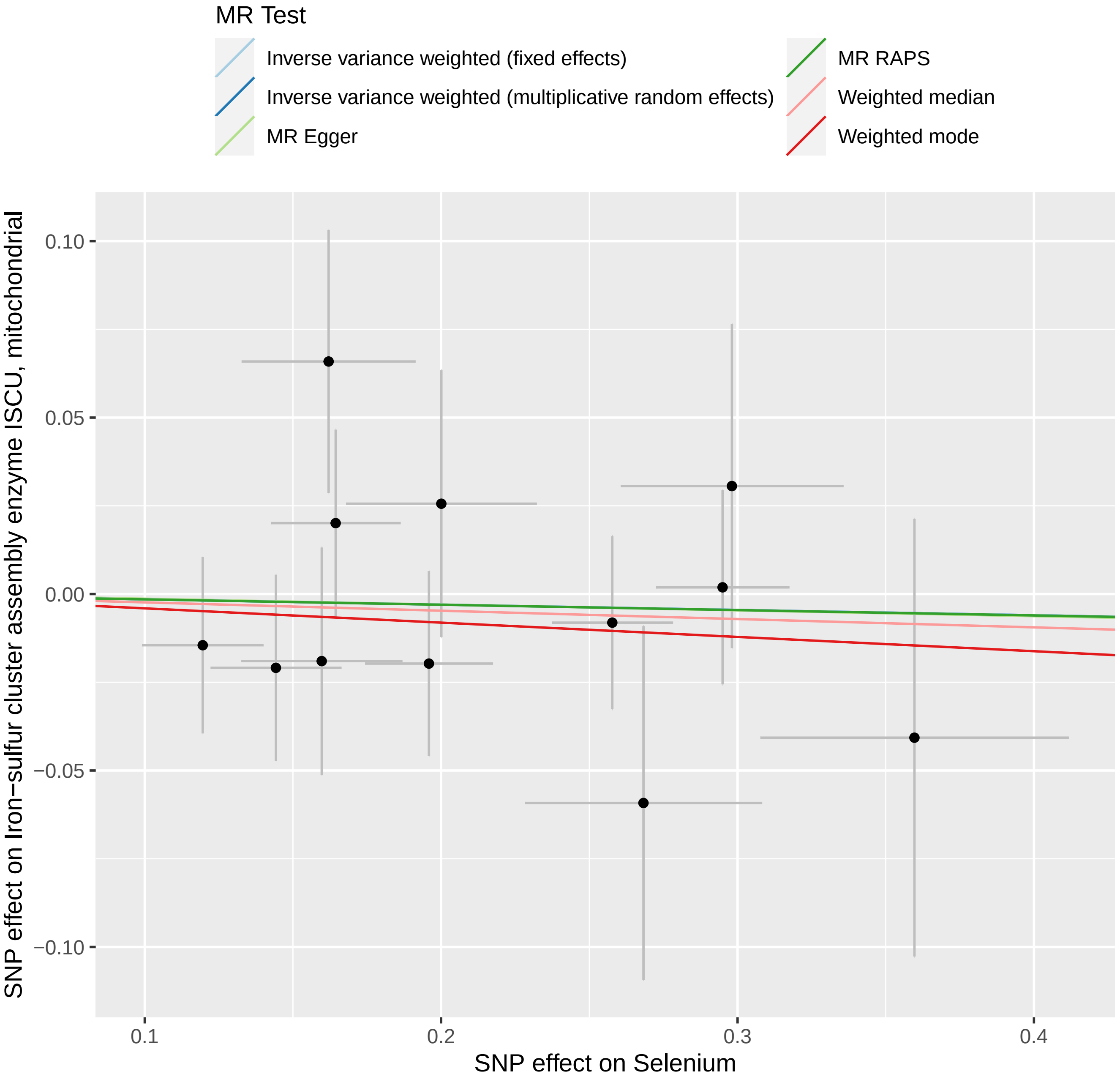

Batch 3−1573 : Selenium on Ubiquitin−like protein ISG15

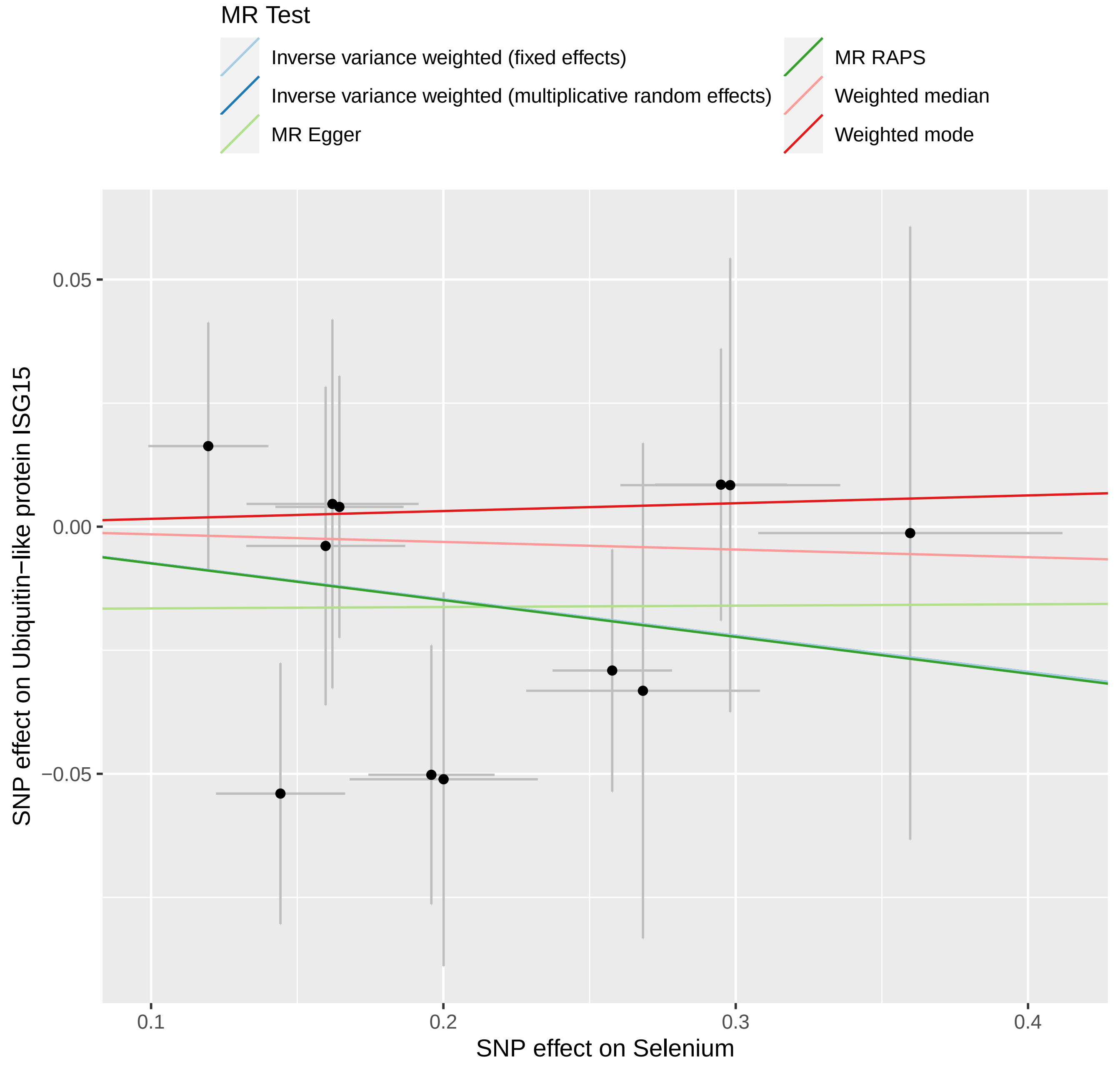

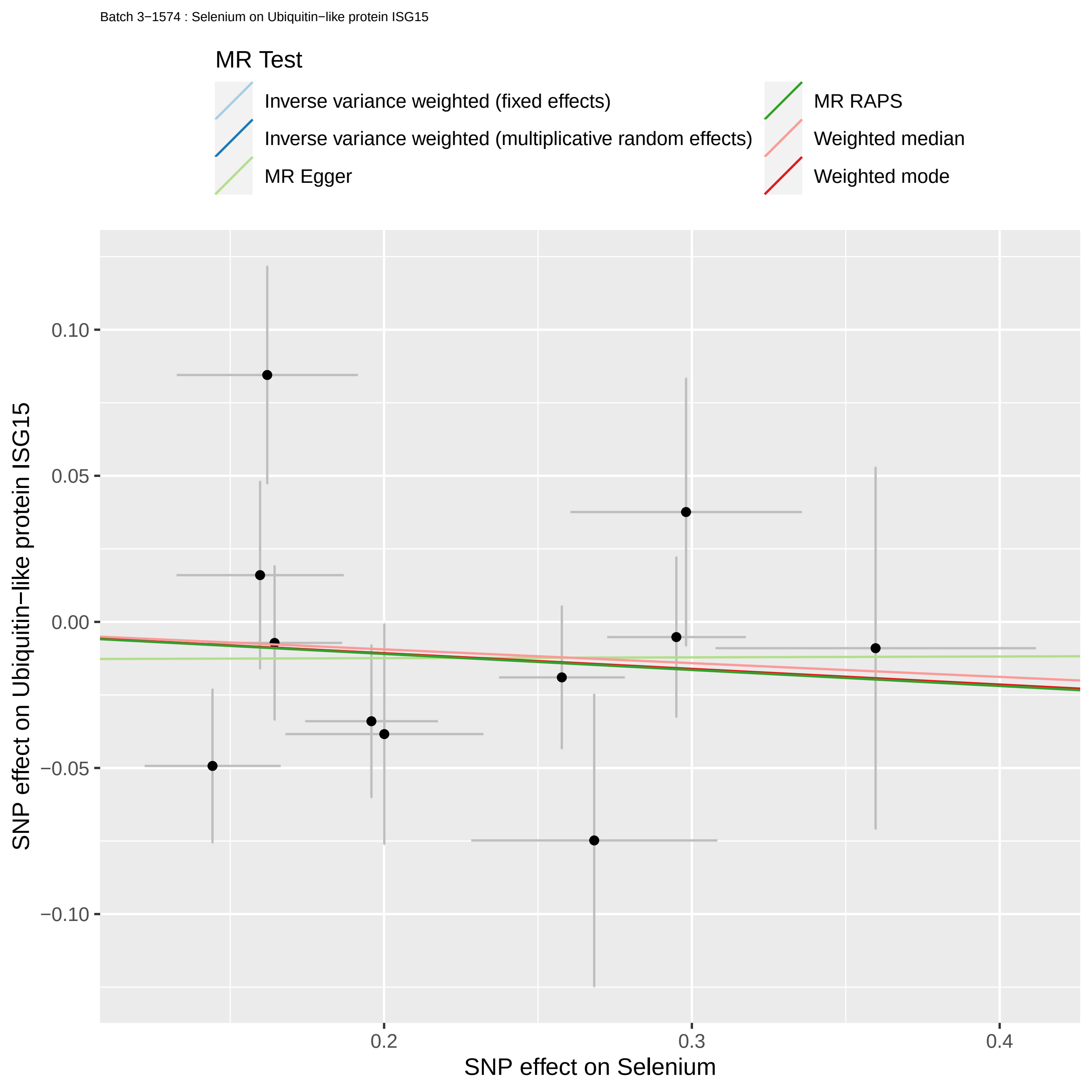
Batch 3−1574 : Selenium on Ubiquitin−like protein ISG15
MR Test
Inverse variance weighted (fixed effects)
Inverse variance weighted (multiplicative random effects)
MR Egger
MR RAPS
Weighted median
Weighted mode
0.10
0.05
0.00
−0.05
−0.10
0.2
0.3
0.4
SNP effect on Ubiquitin−like protein ISG15
SNP effect on Selenium

Batch 3−1575 : Selenium on Insulin gene enhancer protein ISL−1

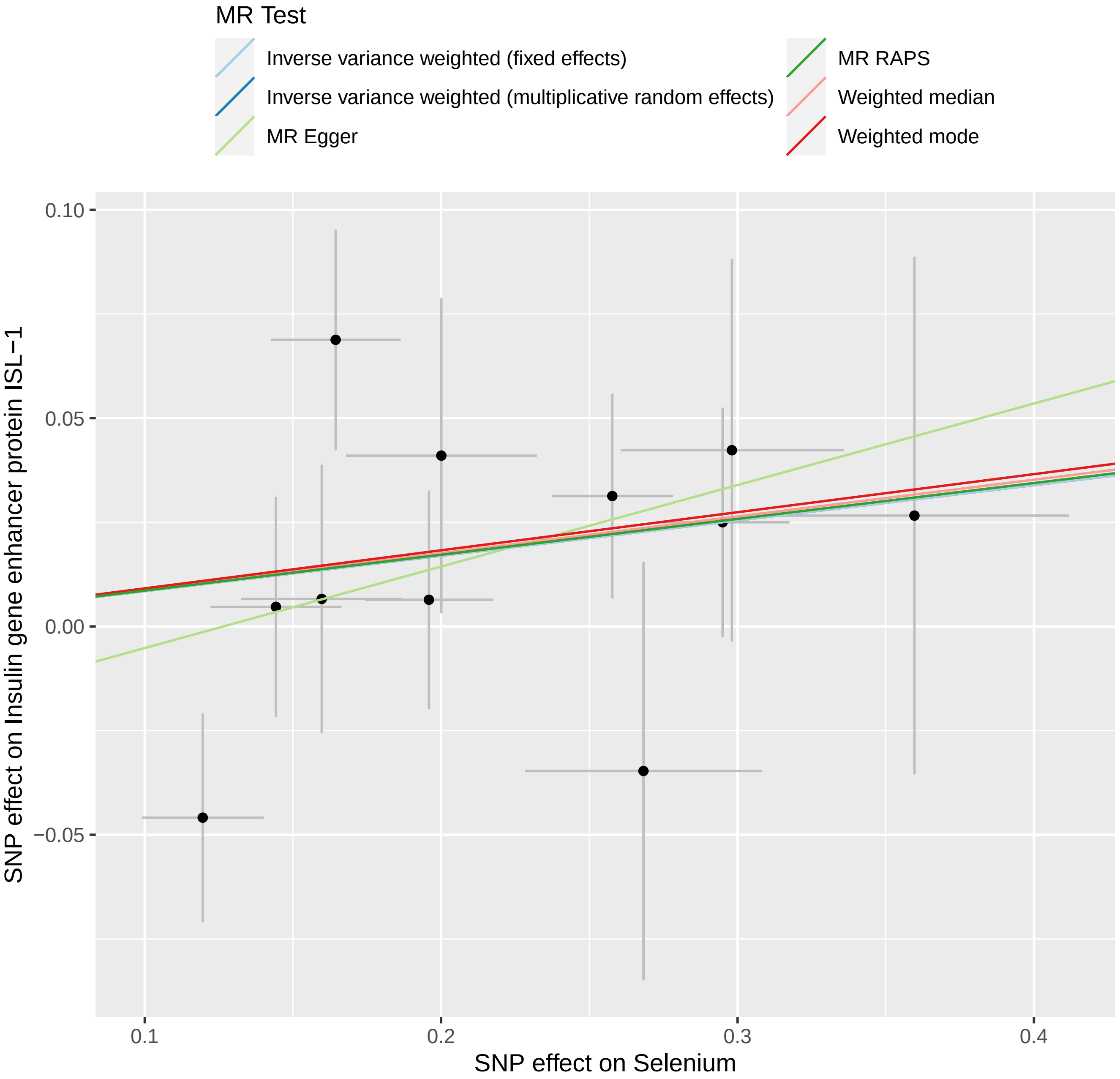

Batch 3−1576 : Selenium on Immunoglobulin superfamily containing leucine−rich repeat protein 2

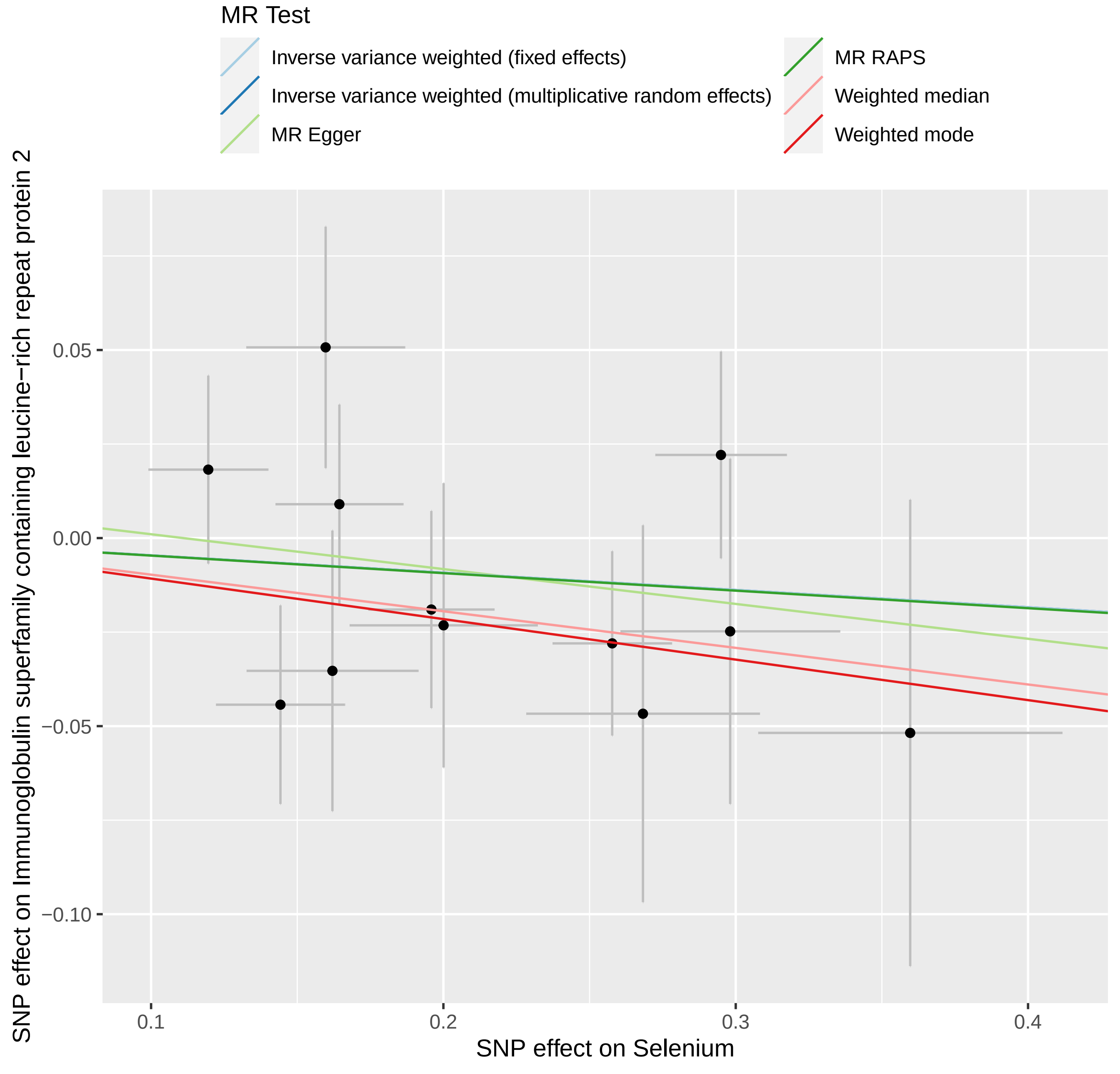

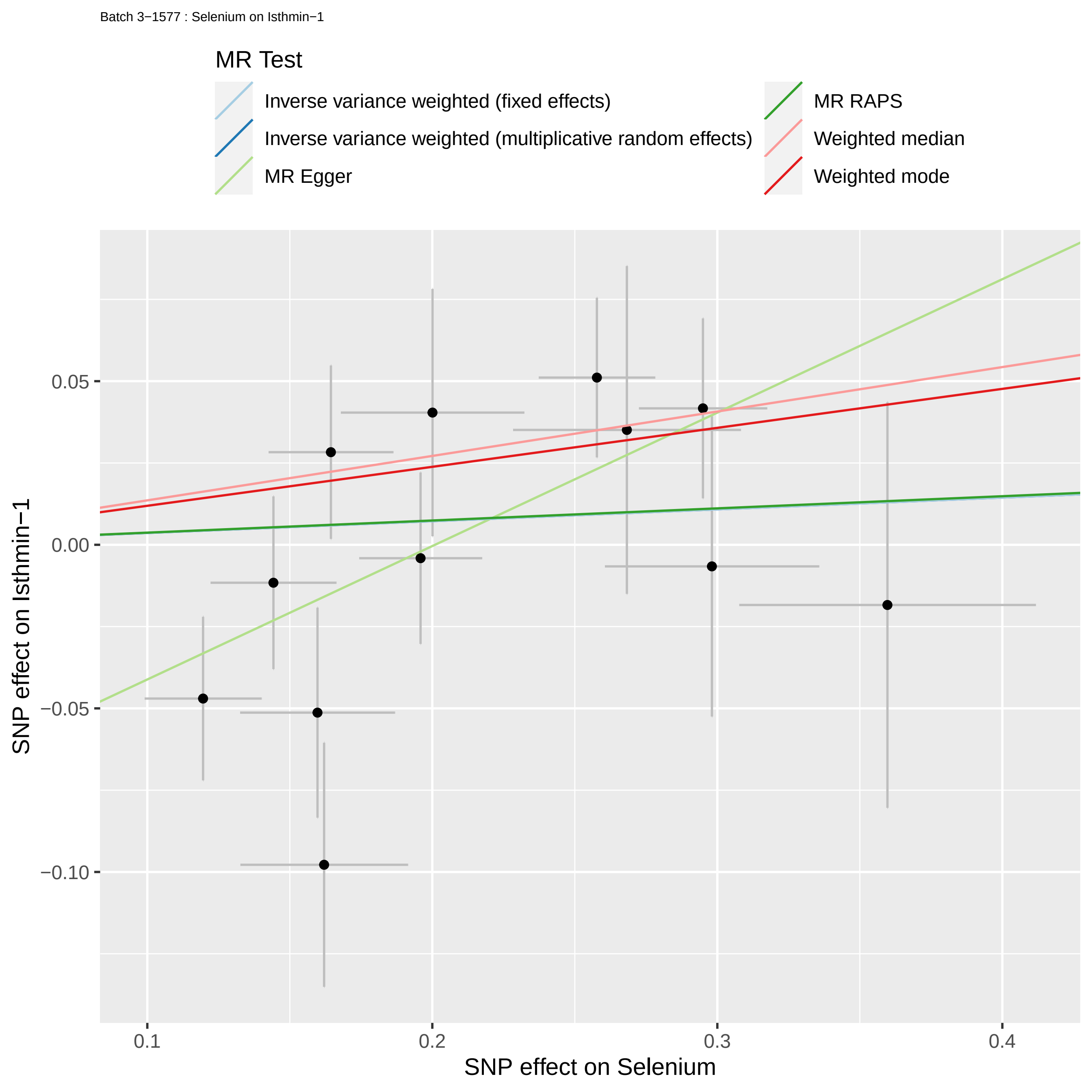

Batch 3−1577 : Selenium on Isthmin−1
MR Test
Inverse variance weighted (fixed effects)
Inverse variance weighted (multiplicative random effects)
MR Egger
MR RAPS
Weighted median
Weighted mode
0.05
0.00
−0.05
−0.10
0.1
0.2
0.3
0.4
SNP effect on Isthmin−1
SNP effect on Selenium

Batch 3−1578 : Selenium on Isochorismatase domain−containing protein 1

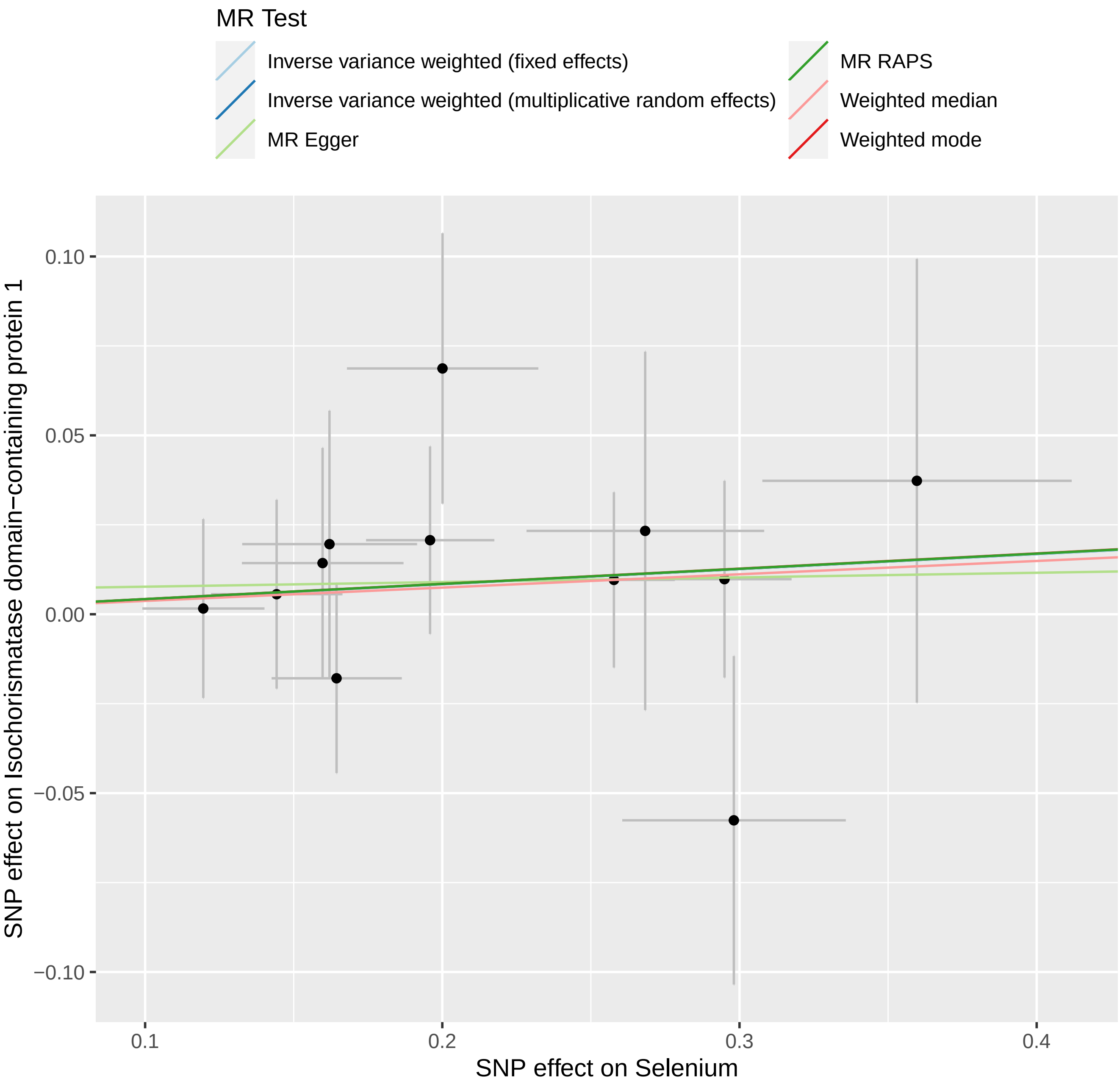

Batch 3−1579 : Selenium on IST1 homolog

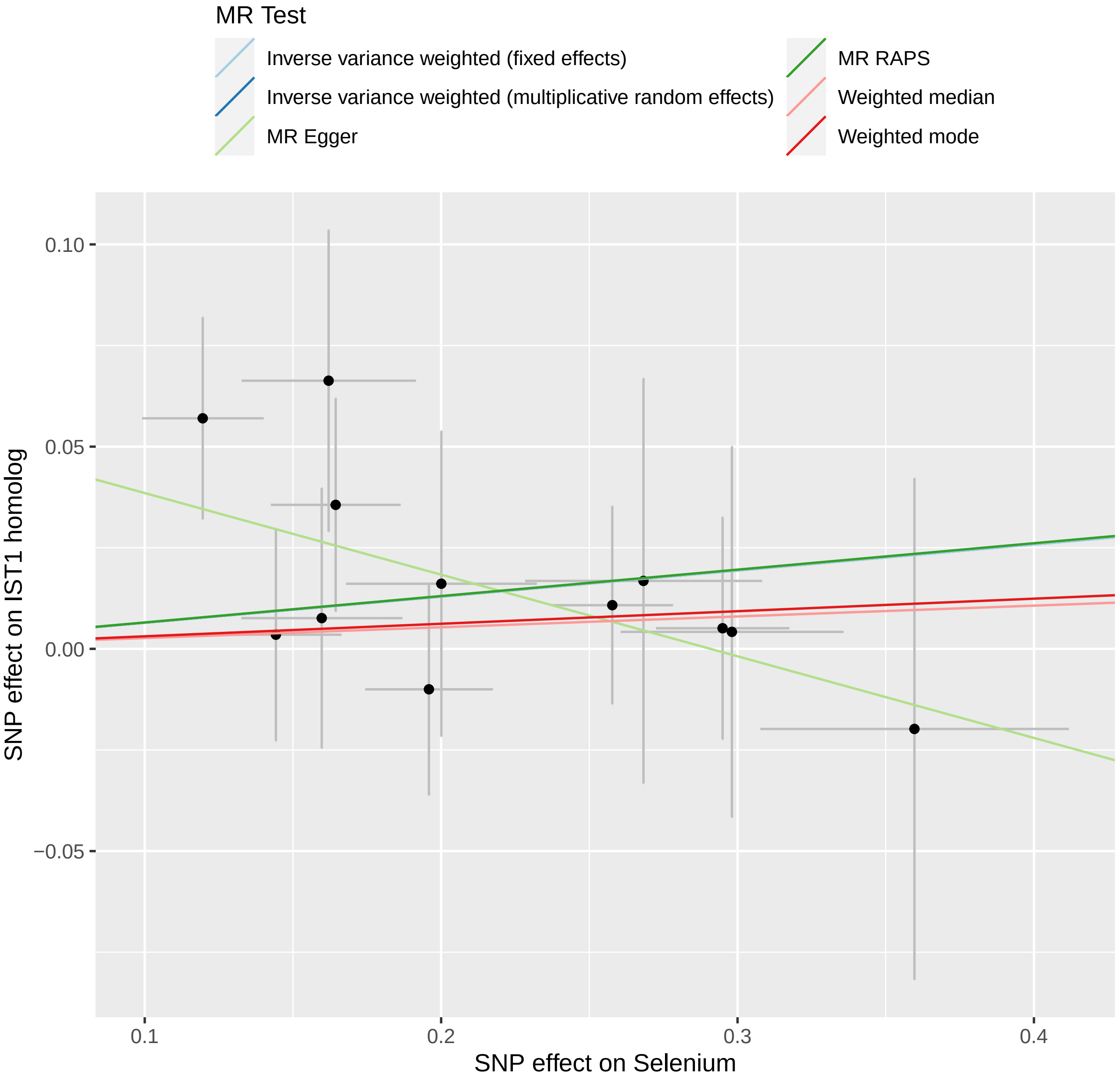

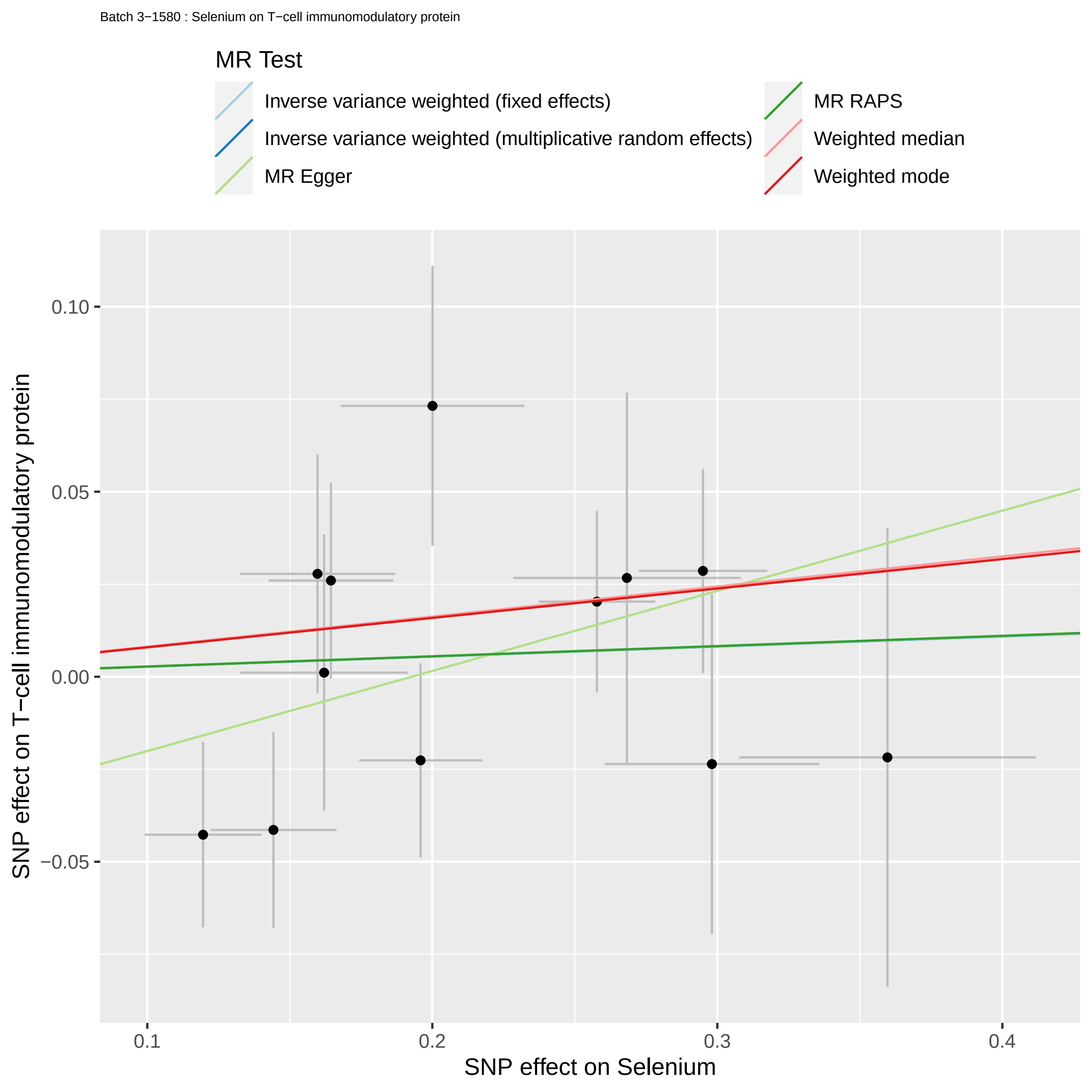
Batch 3−1580 : Selenium on T−cell immunomodulatory protein
MR Test
Inverse variance weighted (fixed effects)
Inverse variance weighted (multiplicative random effects)
MR Egger
MR RAPS
Weighted median
Weighted mode
0.10
0.05
0.00
−0.05
0.1
0.2
0.3
0.4
SNP effect on T−cell immunomodulatory protein
SNP effect on Selenium

Batch 3−1581 : Selenium on Integrin alpha−I: beta−1 complex

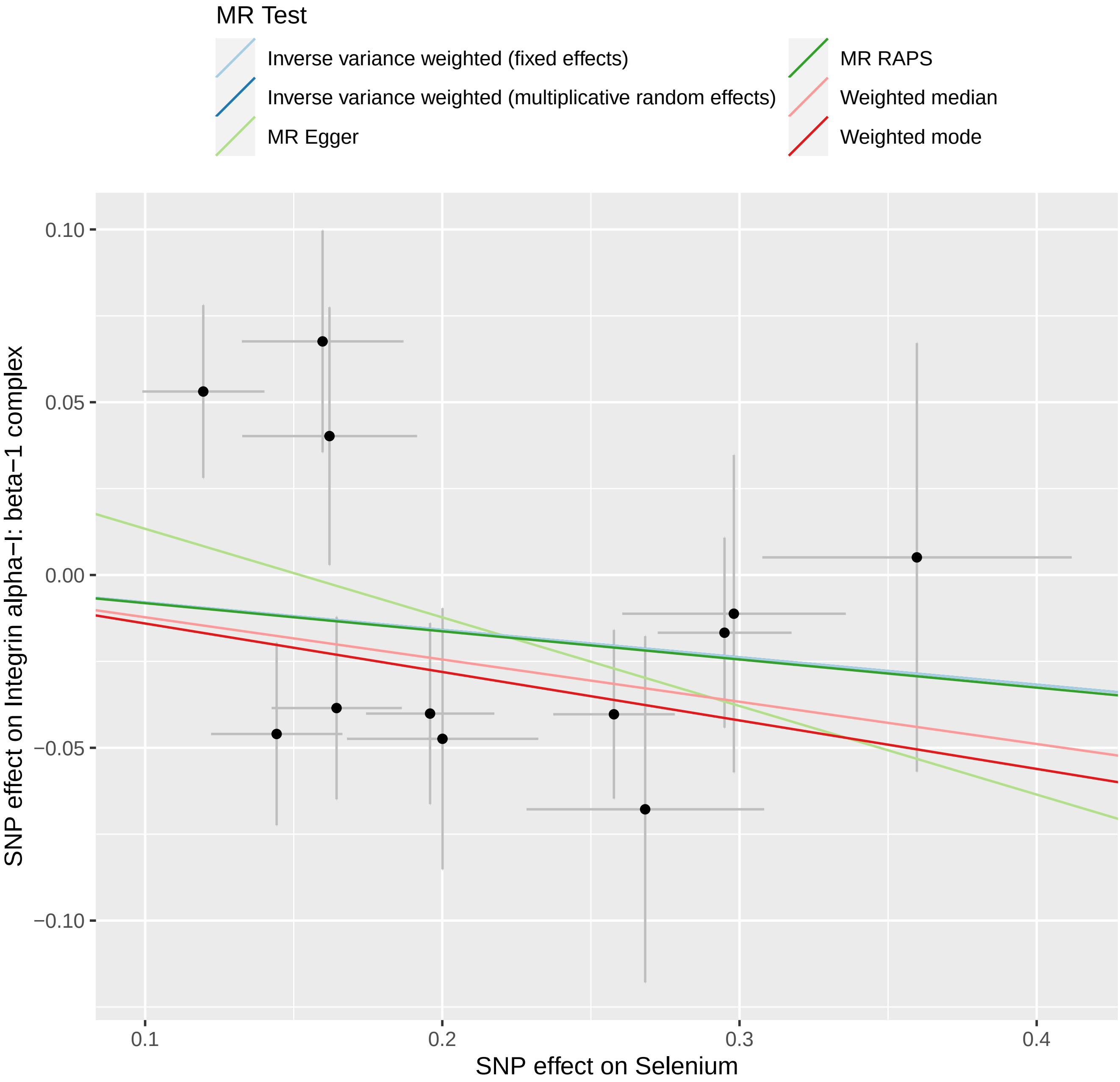

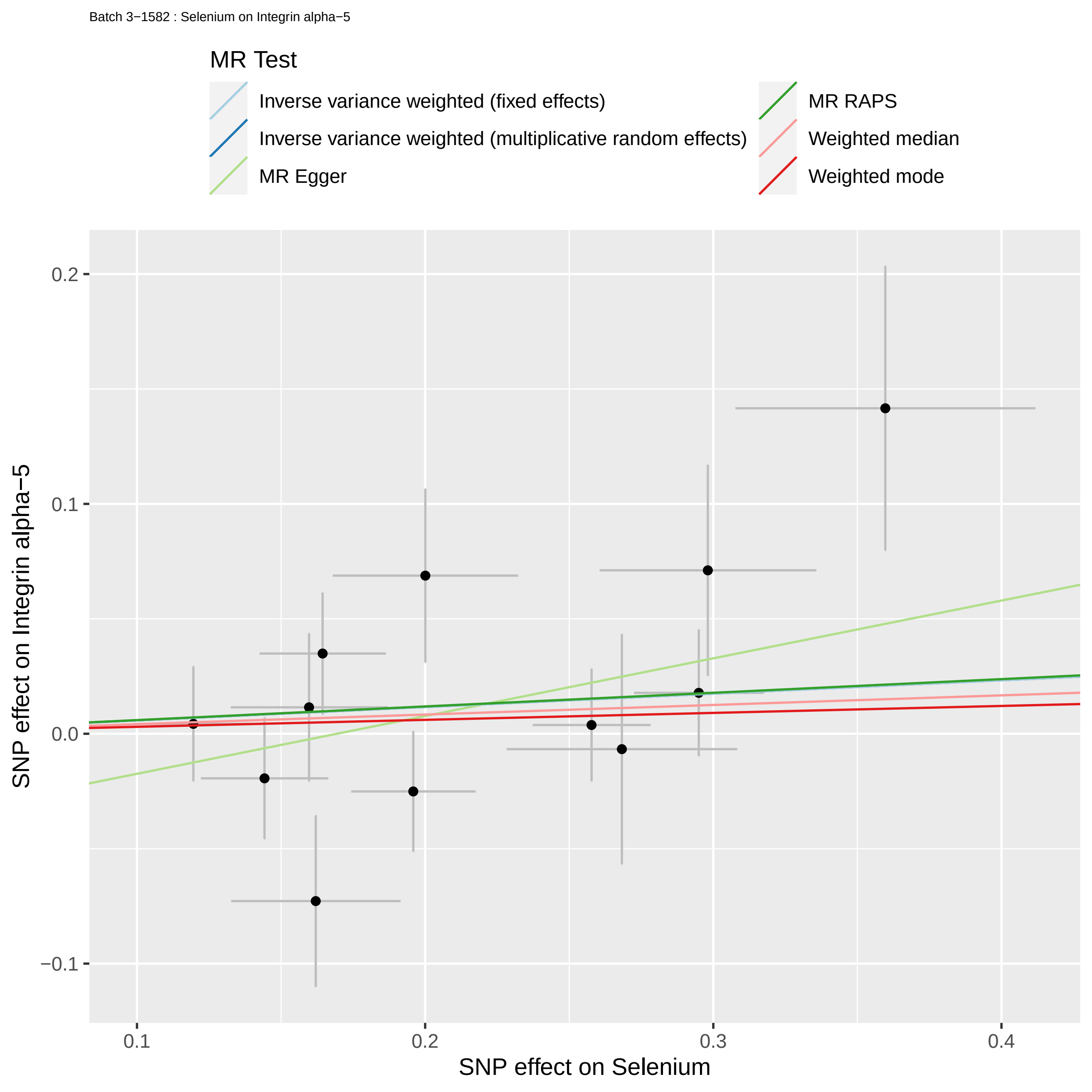

Batch 3−1582 : Selenium on Integrin alpha−5
MR Test
Inverse variance weighted (fixed effects)
Inverse variance weighted (multiplicative random effects)
MR Egger
MR RAPS
Weighted median
Weighted mode
0.2
0.1
0.0
−0.1
0.1
0.2
0.3
0.4
SNP effect on Integrin alpha−5
SNP effect on Selenium

Batch 3−1583 : Selenium on Integrin alpha−L

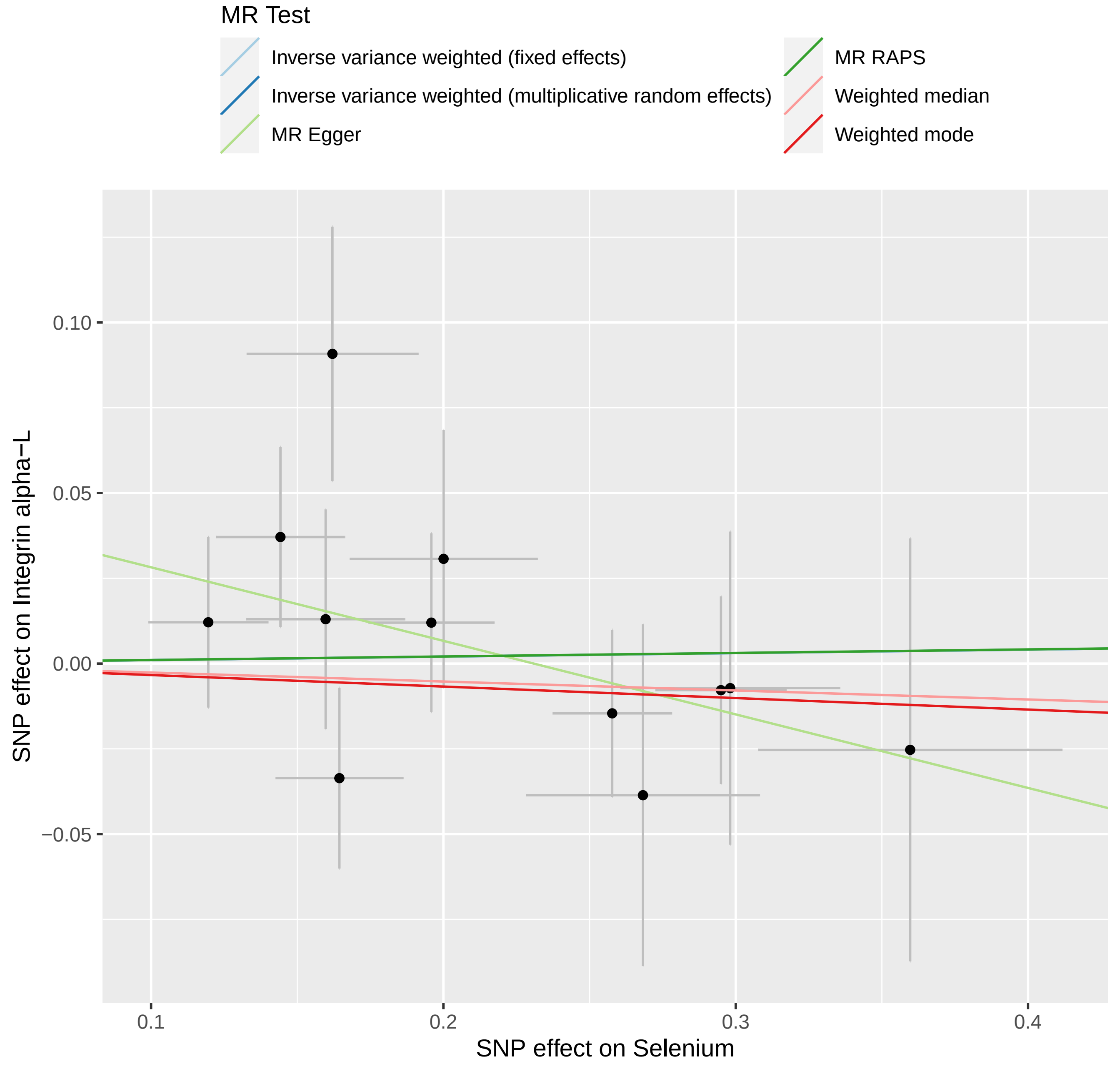

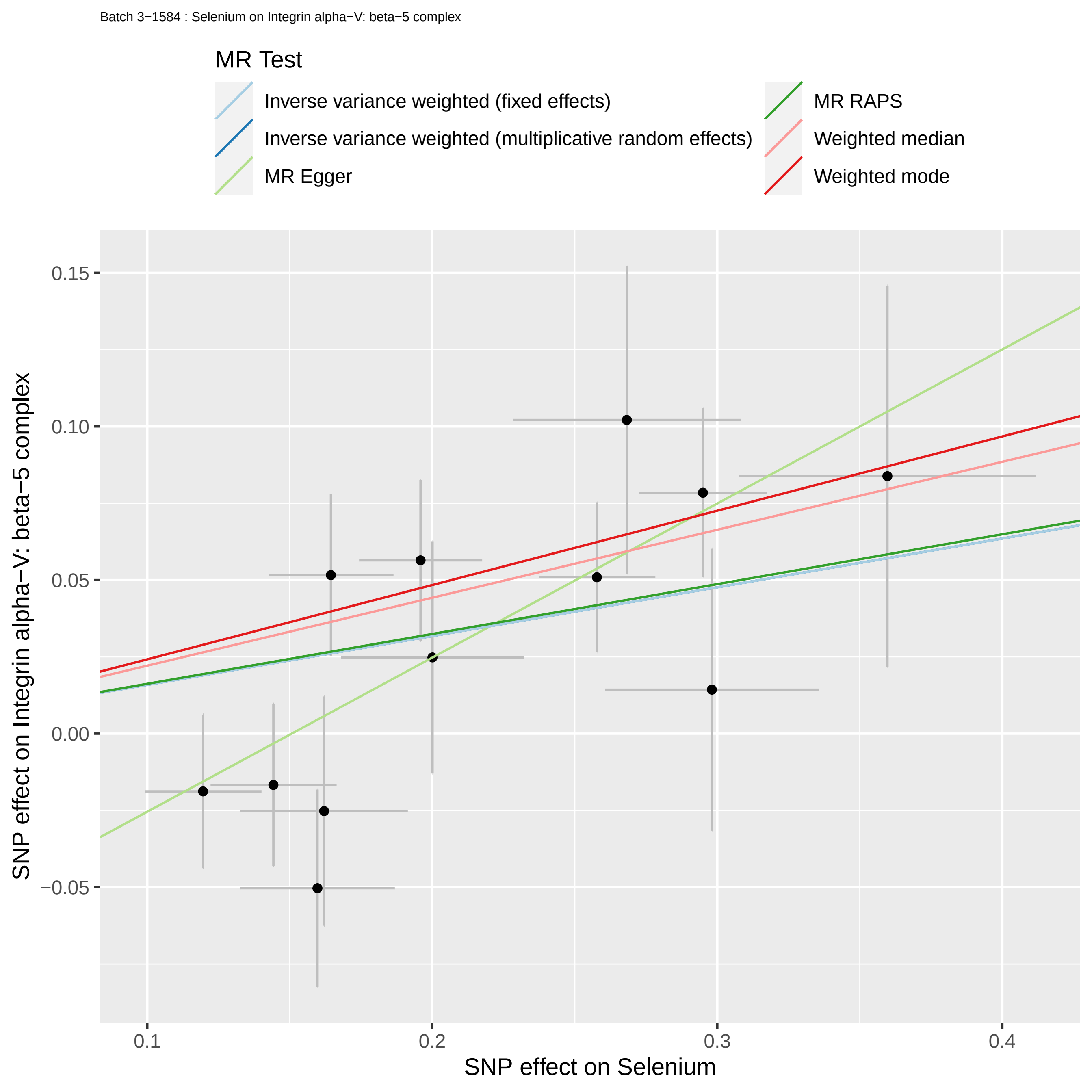
Batch 3−1584 : Selenium on Integrin alpha−V: beta−5 complex
MR Test
Inverse variance weighted (fixed effects)
Inverse variance weighted (multiplicative random effects)
MR Egger
MR RAPS
Weighted median
Weighted mode
SNP effect on Integrin alpha−V: beta−5 complex
0.15
0.10
0.05
0.00
−0.05
0.1
0.2
0.3
0.4
SNP effect on Selenium

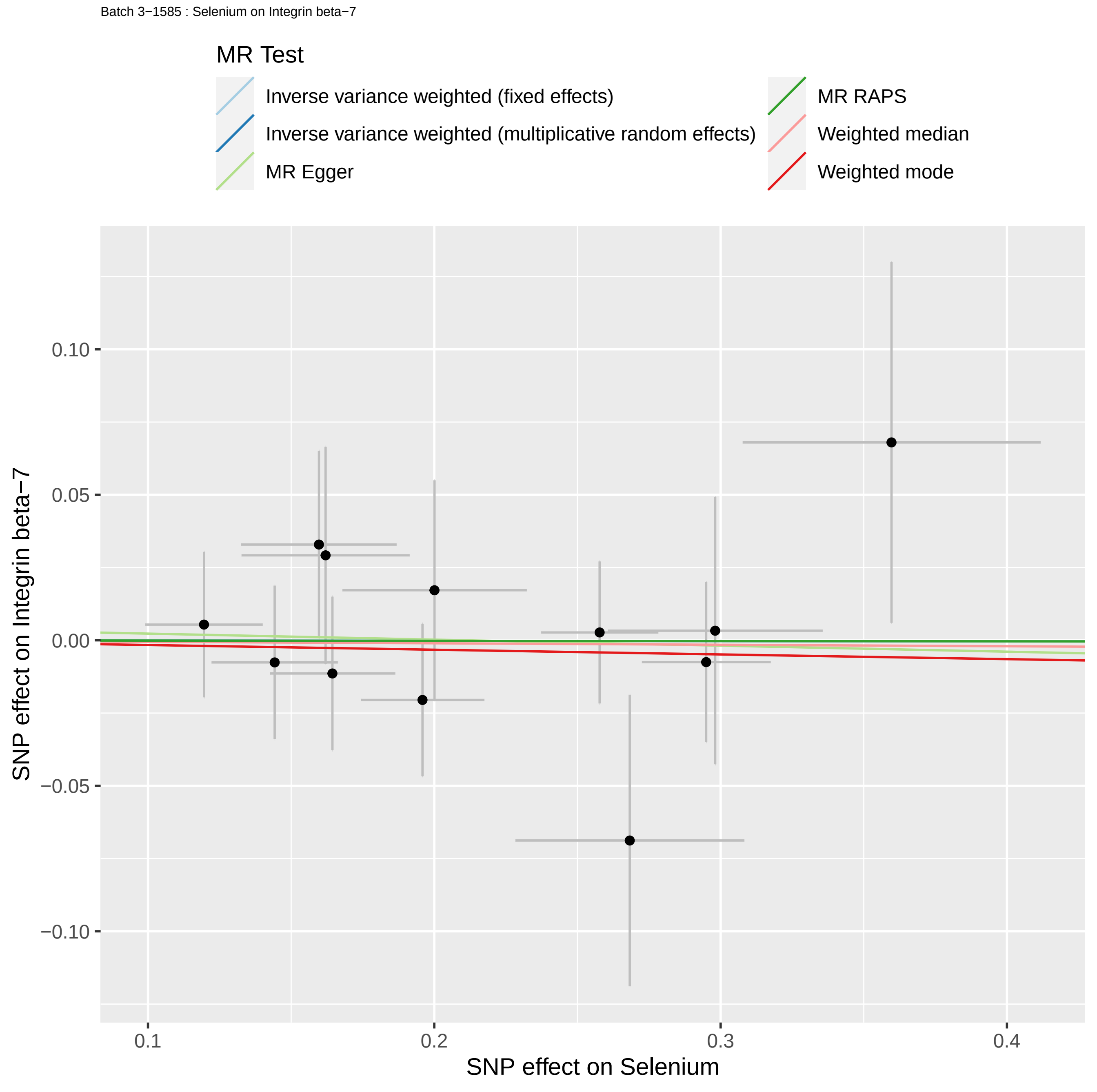
Batch 3−1585 : Selenium on Integrin beta−7
MR Test
Inverse variance weighted (fixed effects)
Inverse variance weighted (multiplicative random effects)
MR Egger
MR RAPS
Weighted median
Weighted mode
0.10
0.05
0.00
−0.05
−0.10
0.1
0.2
0.3
0.4
SNP effect on Integrin beta−7
SNP effect on Selenium

Batch 3−1586 : Selenium on Inter−alpha−trypsin inhibitor heavy chain H1

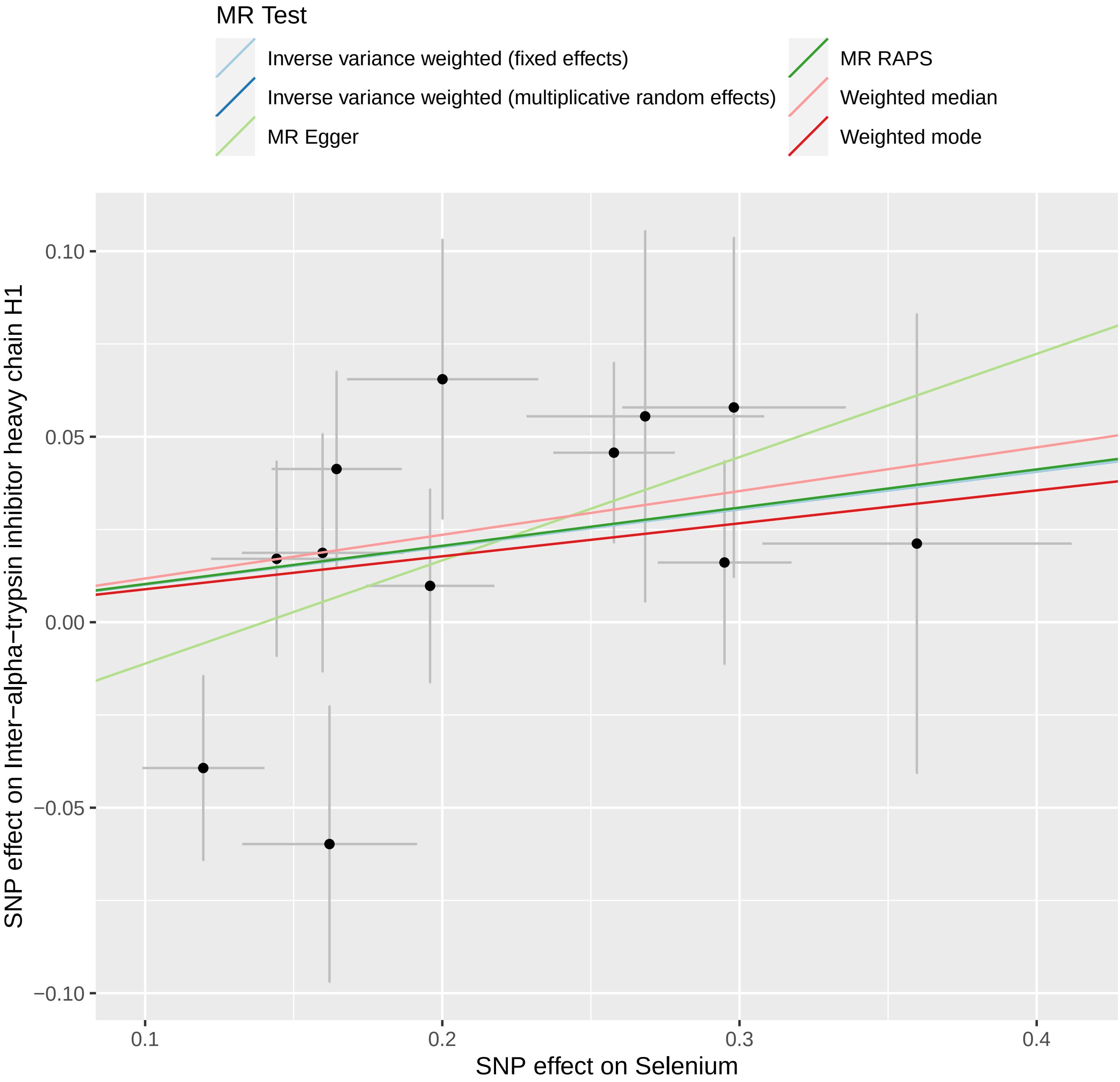

Batch 3−1587 : Selenium on Inter−alpha−trypsin inhibitor heavy chain H5

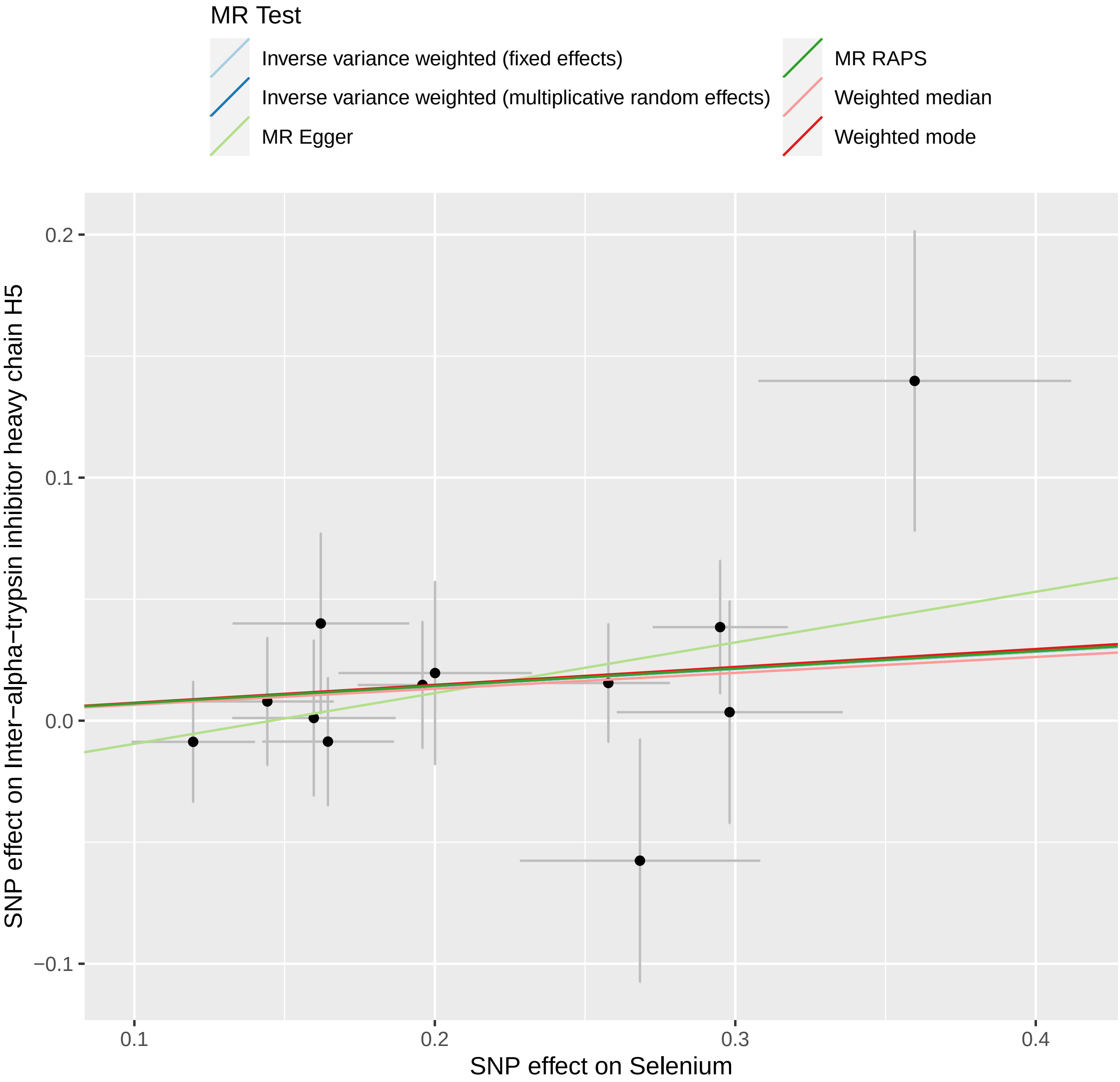

Batch 3−1588 : Selenium on Integral membrane protein 2A

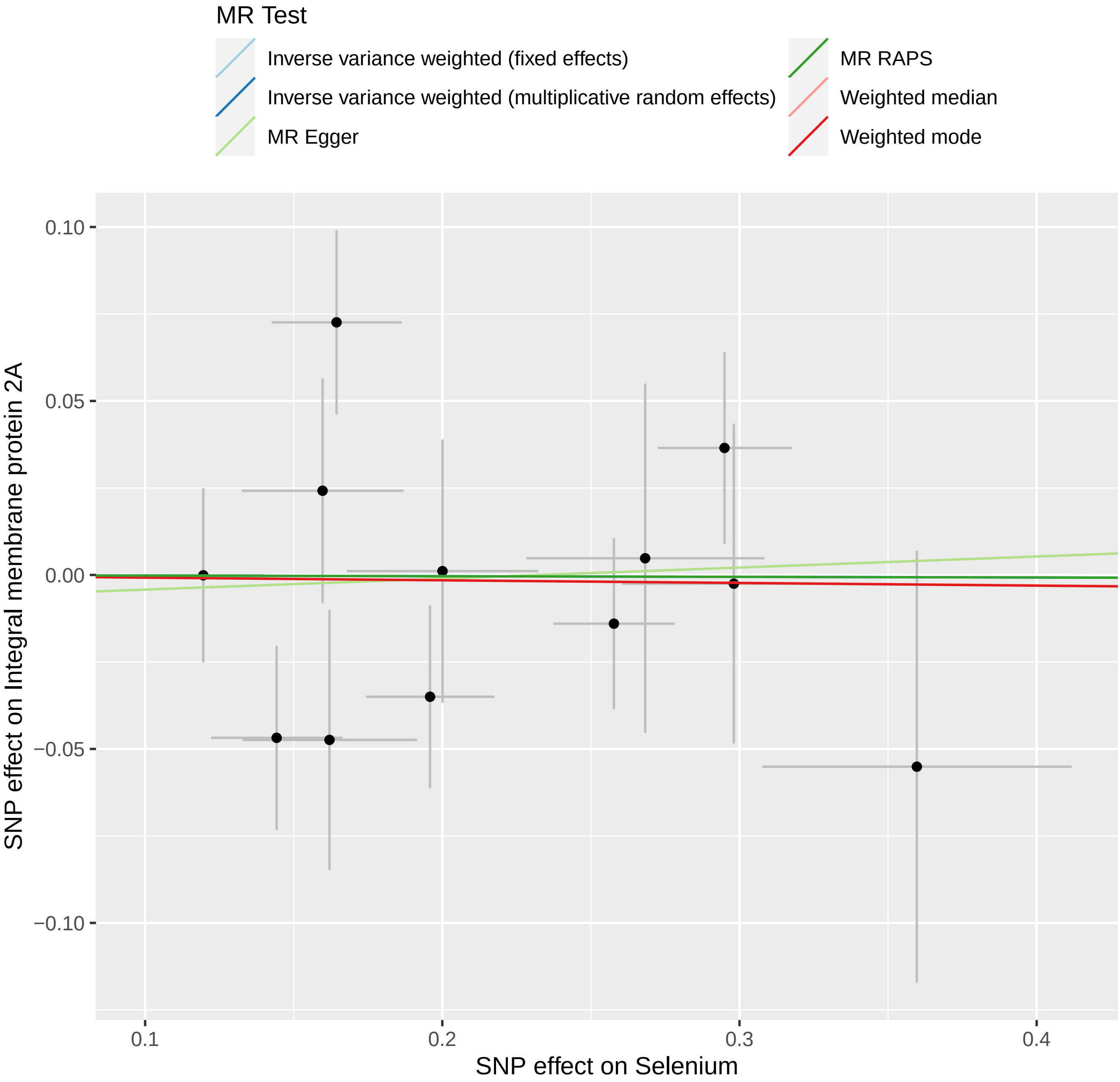

Batch 3−1589 : Selenium on Integral membrane protein 2A

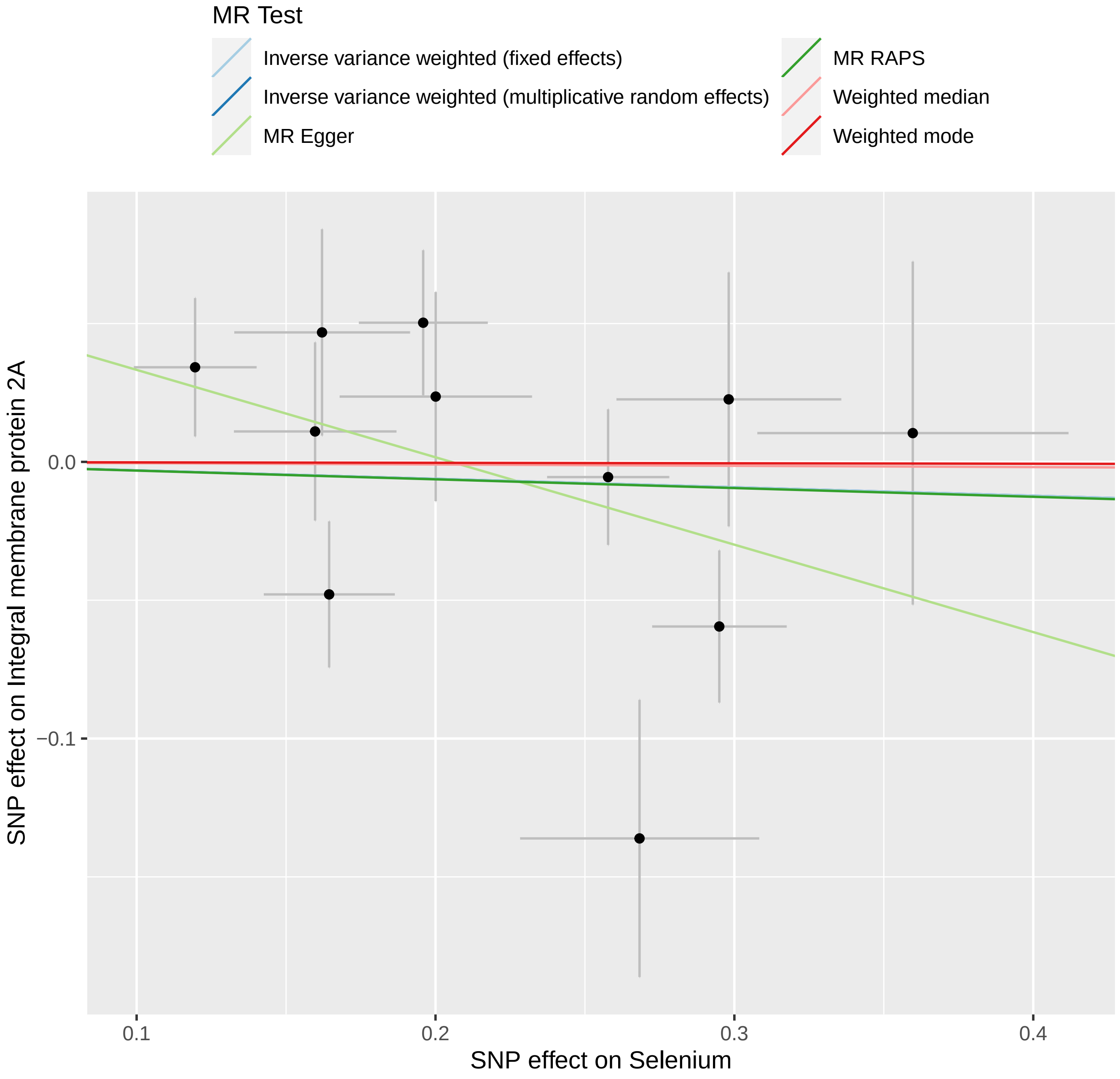

Batch 3−1590 : Selenium on Integral membrane protein 2B

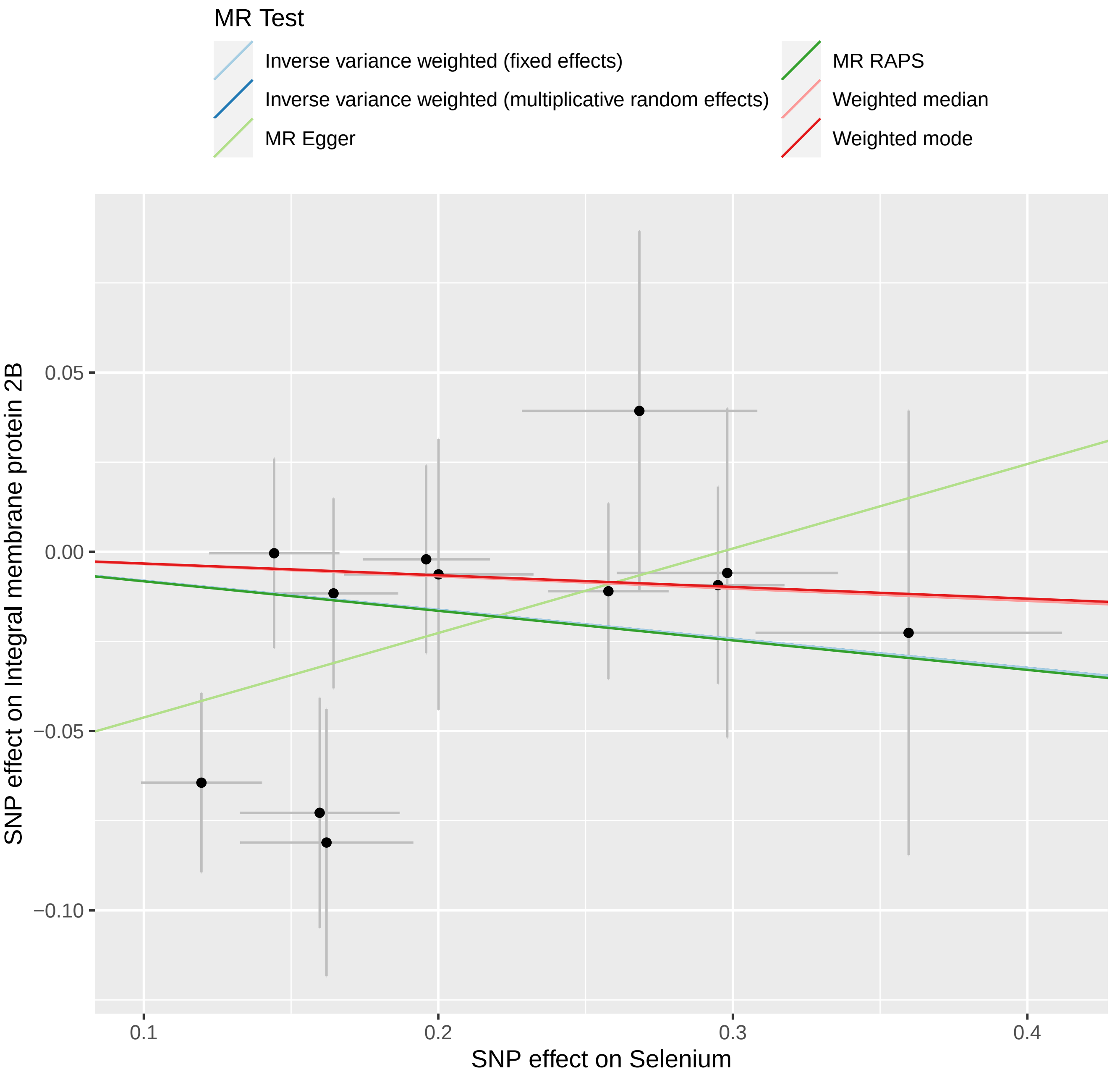

Batch 3−1591 : Selenium on Integral membrane protein 2C

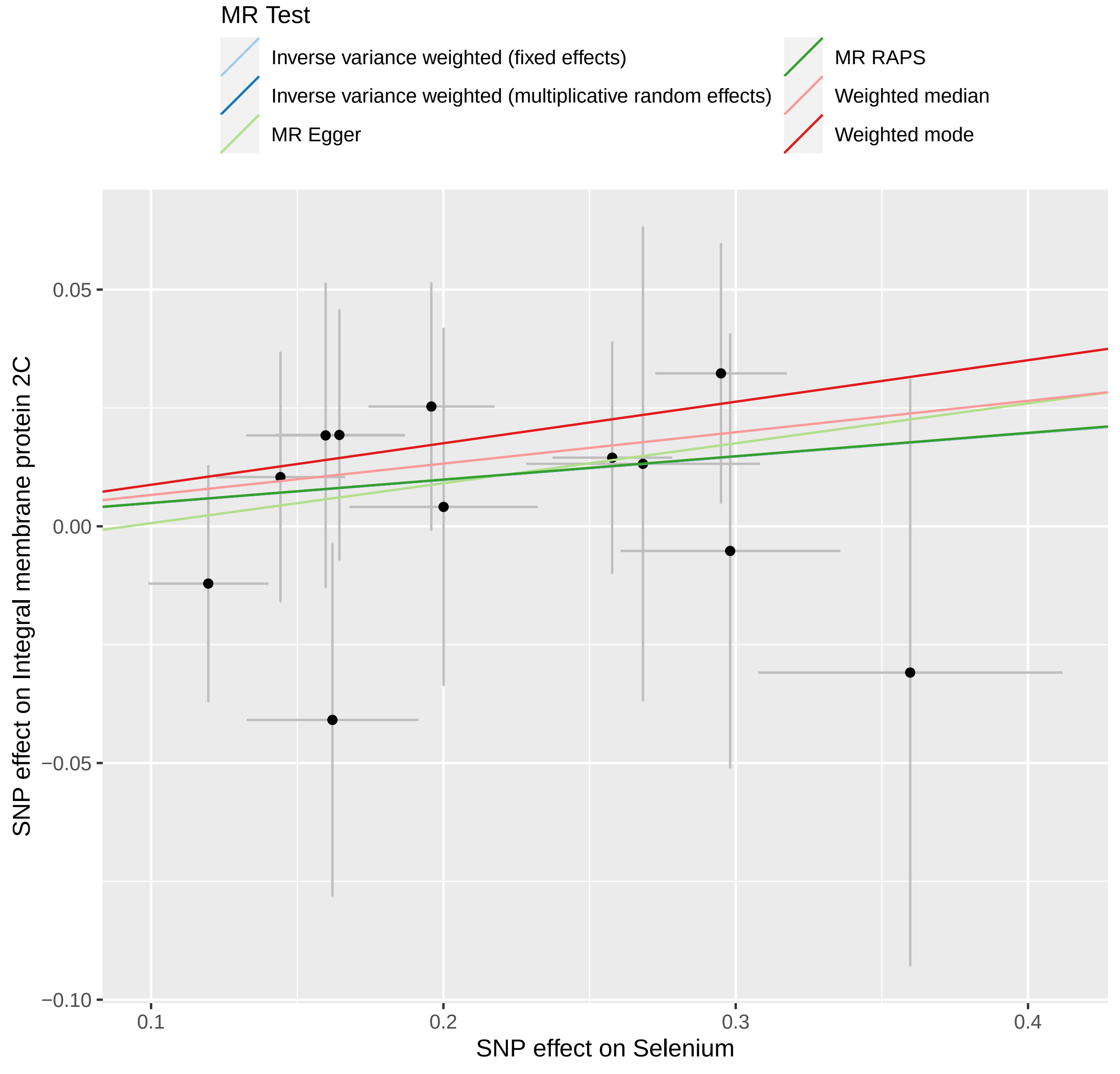

Batch 3−1592 : Selenium on Integral membrane protein 2C

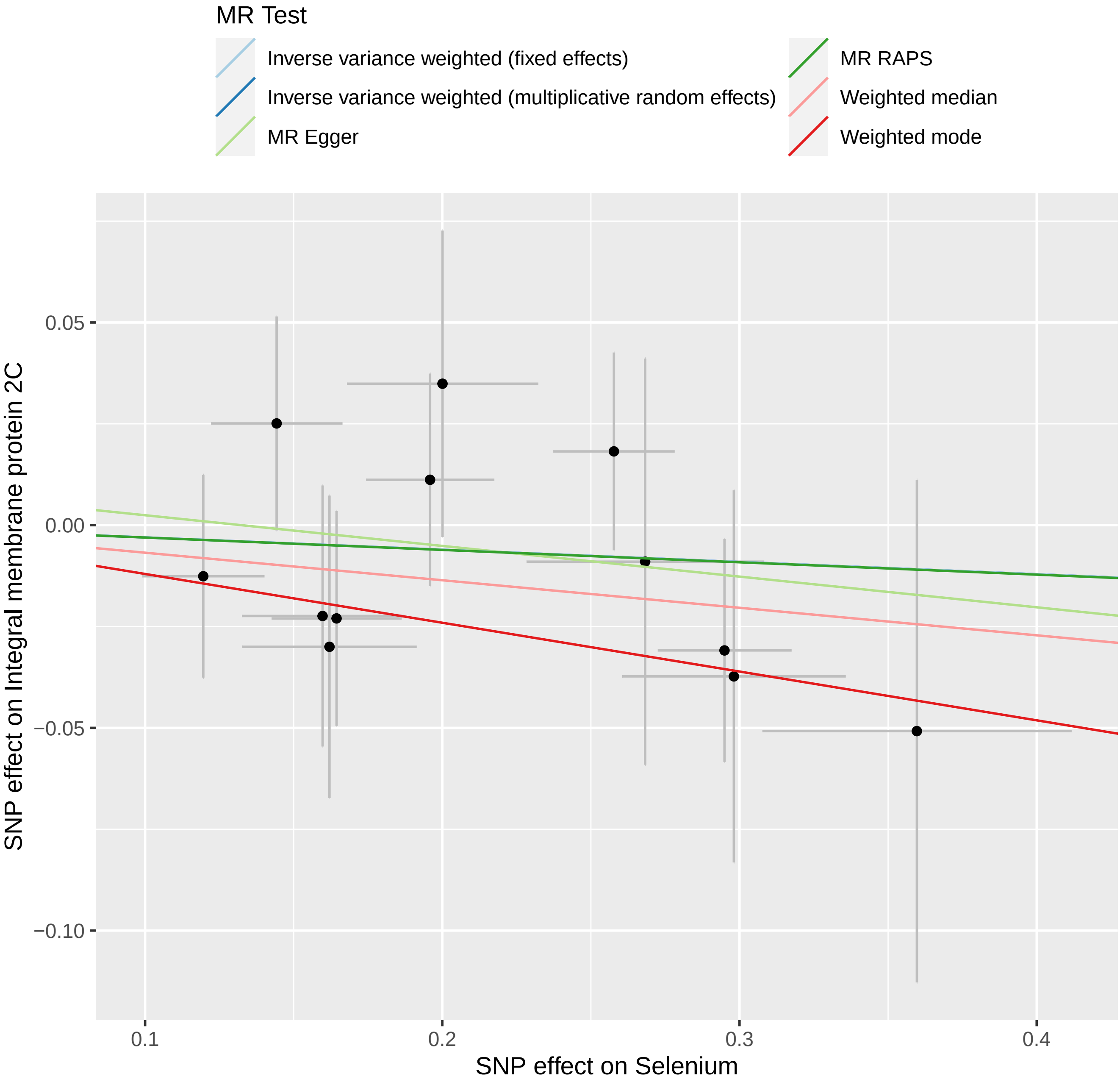

Batch 3−1593 : Selenium on Inositol−trisphosphate 3−kinase A

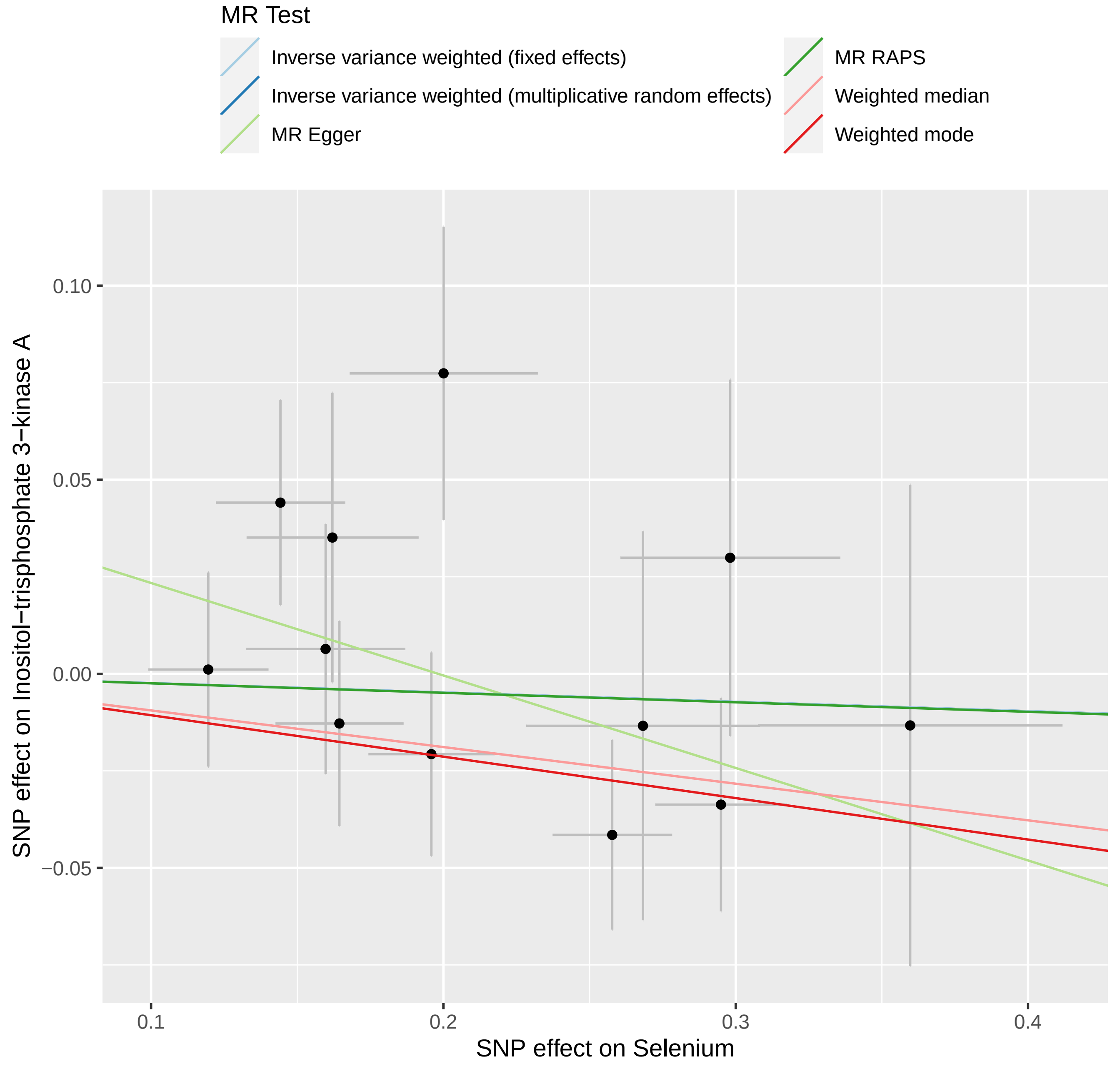

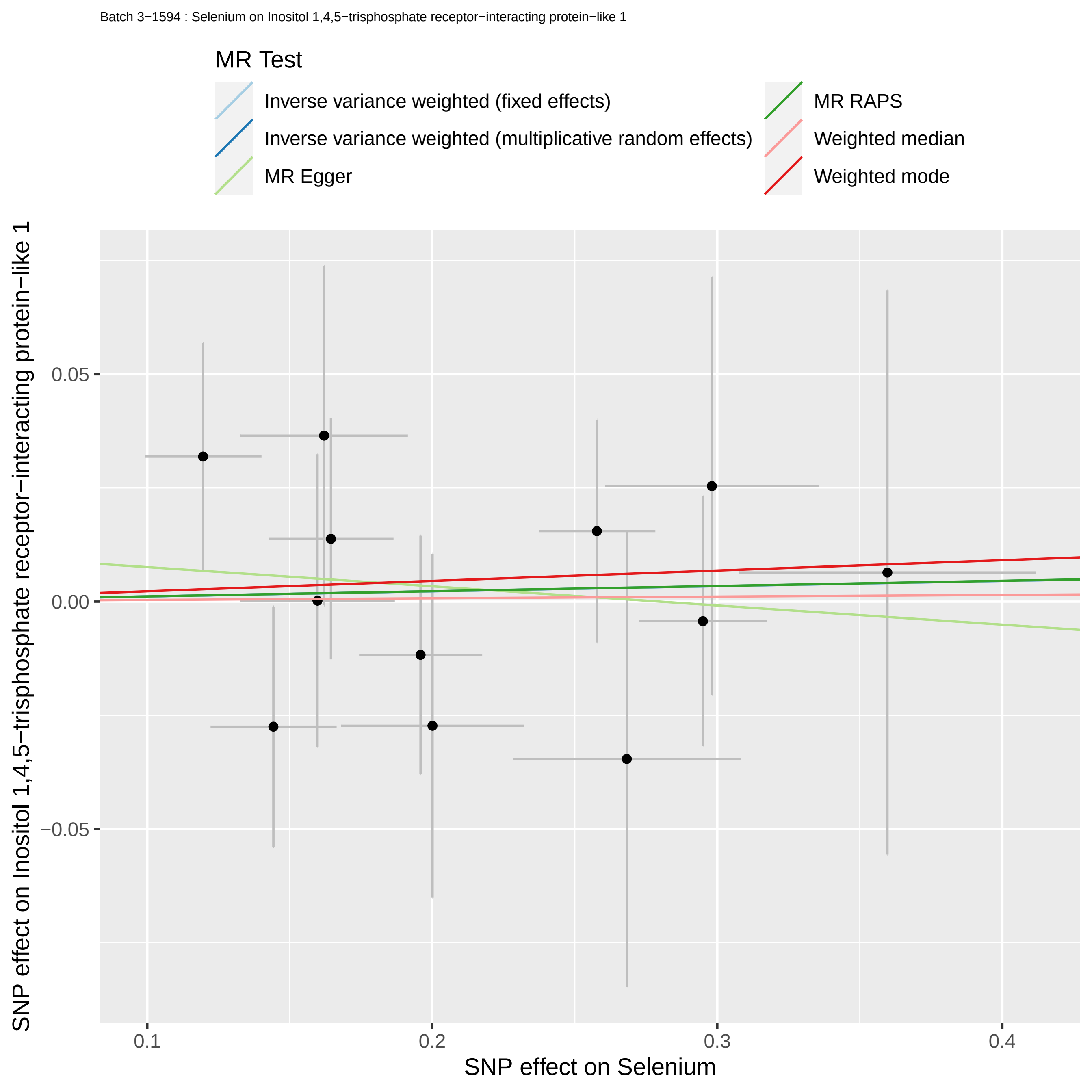

Batch 3−1594 : Selenium on Inositol 1,4,5−trisphosphate receptor−interacting protein−like 1
MR Test
Inverse variance weighted (fixed effects)
Inverse variance weighted (multiplicative random effects)
MR Egger
MR RAPS
Weighted median
Weighted mode
SNP effect on Inositol 1,4,5−trisphosphate receptor−interacting protein−like 1
0.05
0.00
−0.05
0.1
0.2
0.3
0.4
SNP effect on Selenium

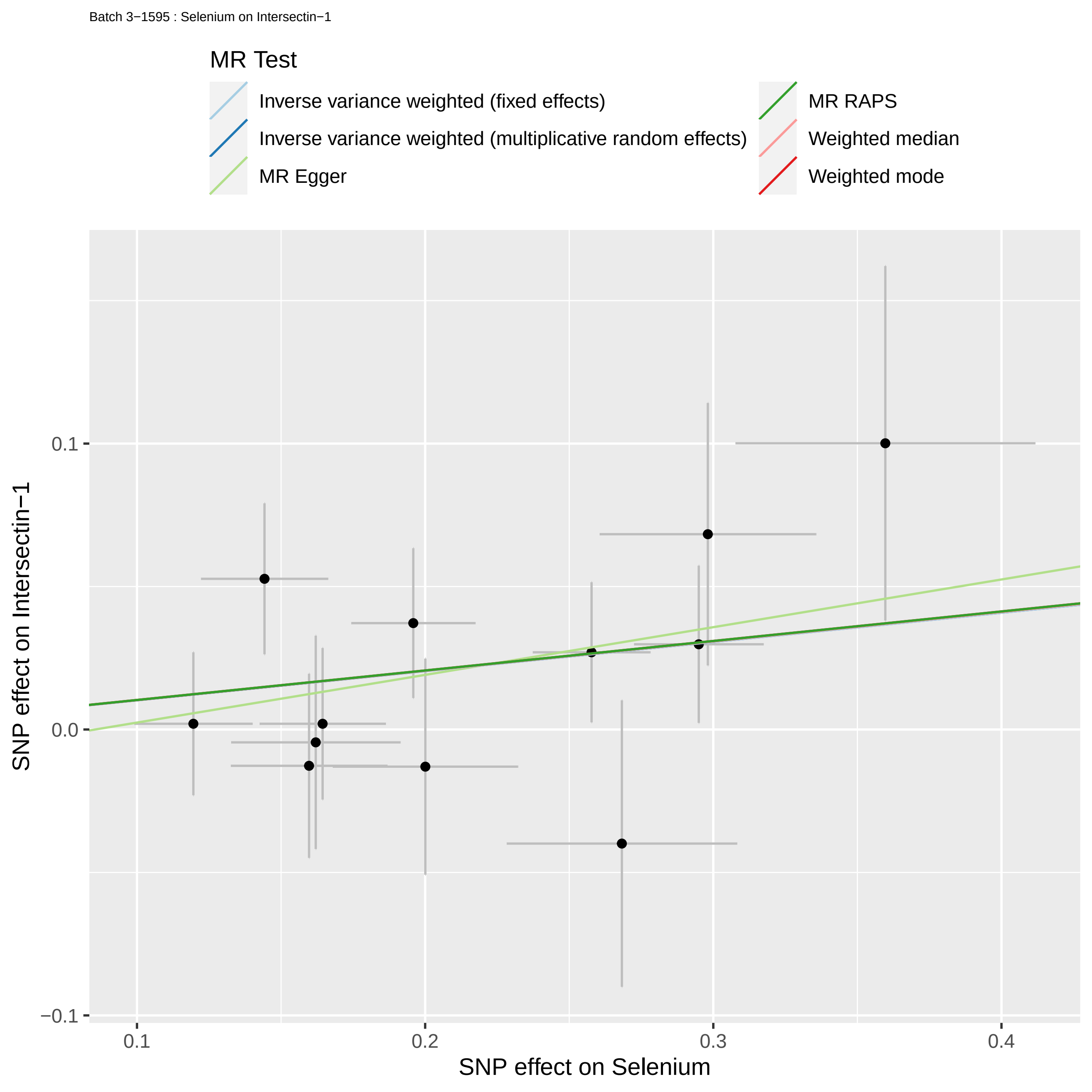

Batch 3−1595 : Selenium on Intersectin−1
MR Test
Inverse variance weighted (fixed effects)
Inverse variance weighted (multiplicative random effects)
MR Egger
MR RAPS
Weighted median
Weighted mode
SNP effect on Intersectin−1
0.1
0.0
−0.1
0.1
0.2
0.3
0.4
SNP effect on Selenium

Batch 3−1596 : Selenium on Protein jagged−1

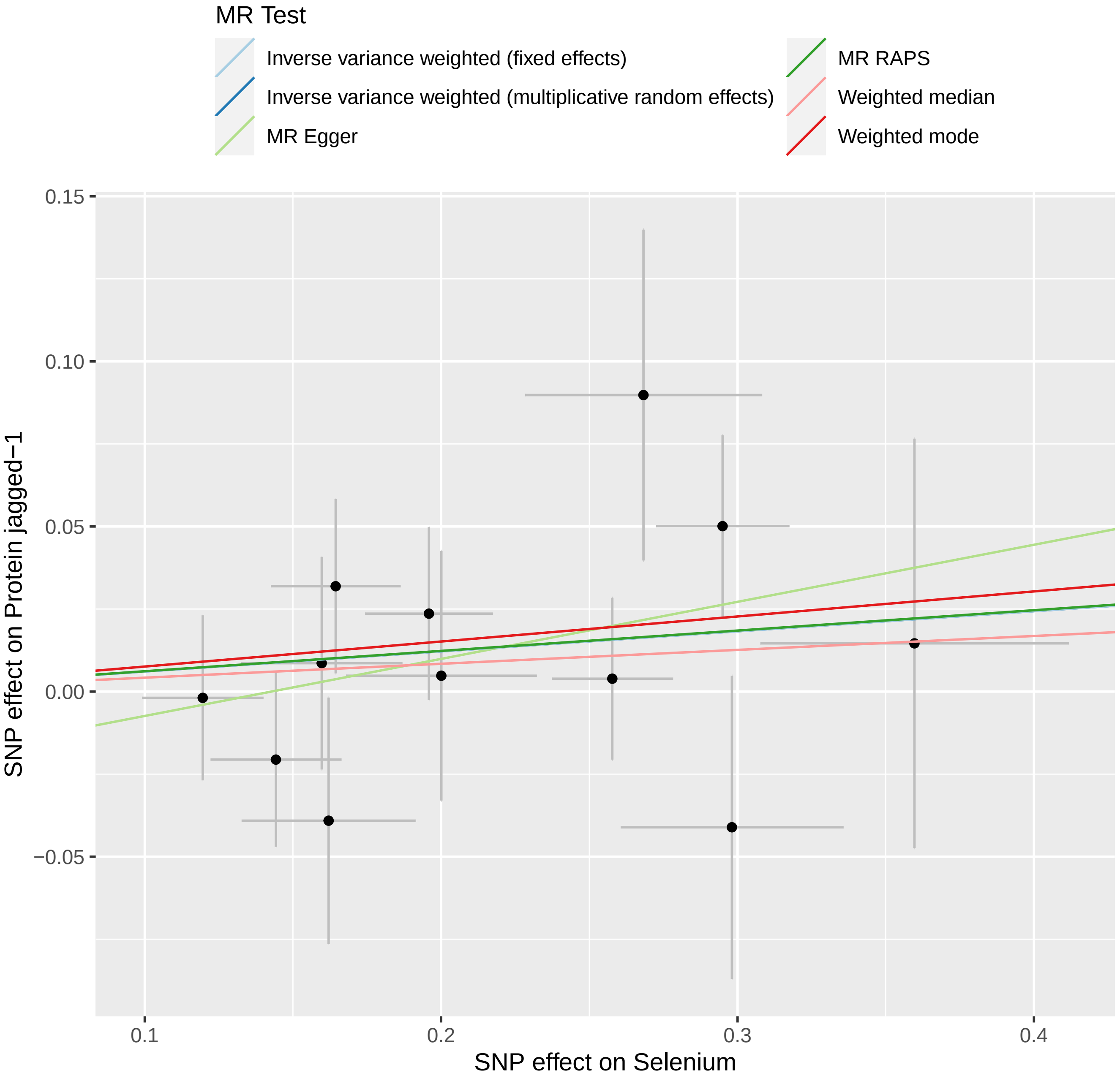

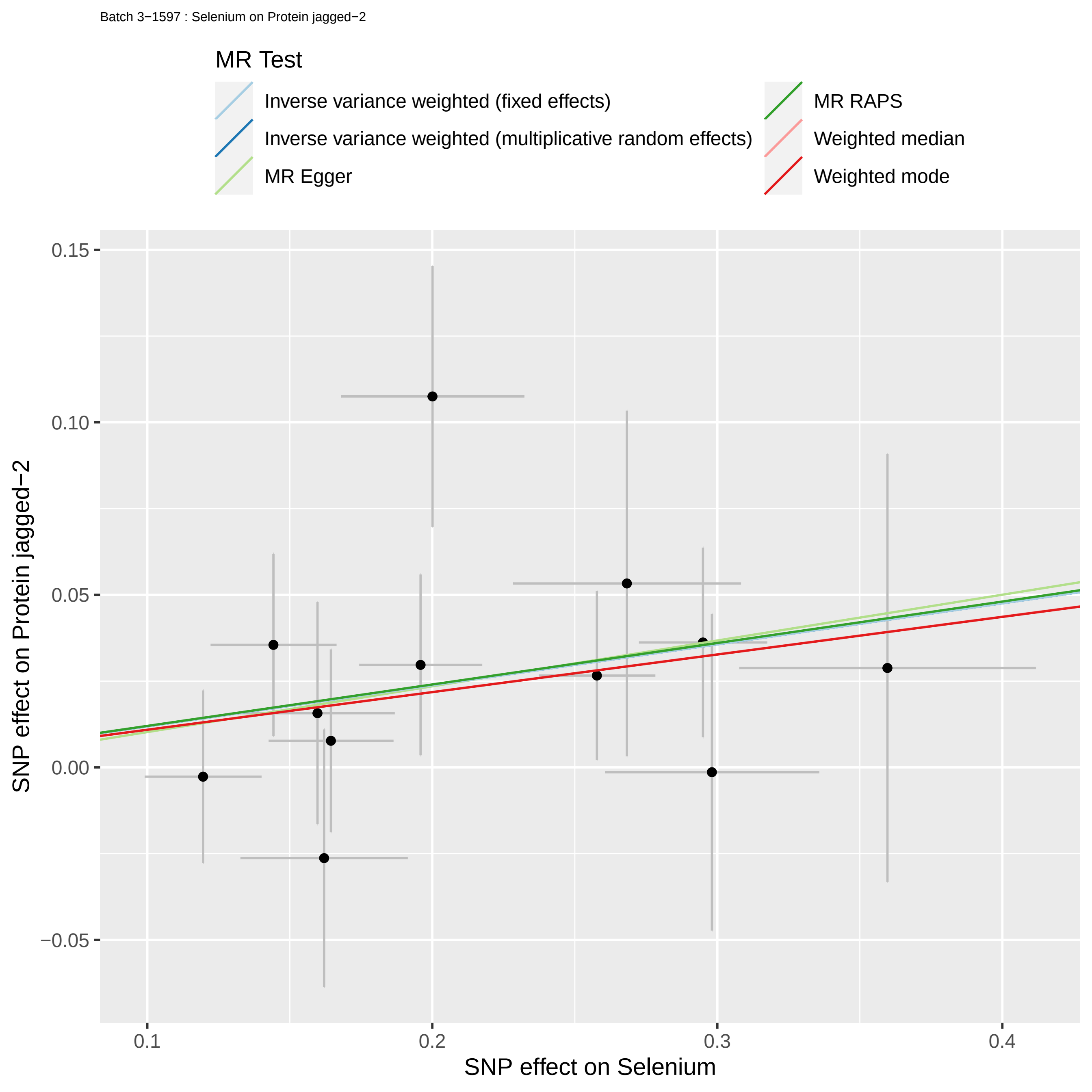
Batch 3−1597 : Selenium on Protein jagged−2
MR Test
Inverse variance weighted (fixed effects)
Inverse variance weighted (multiplicative random effects)
MR Egger
MR RAPS
Weighted median
Weighted mode
0.15
0.10
0.05
0.00
−0.05
0.1
0.2
0.3
0.4
SNP effect on Protein jagged−2
SNP effect on Selenium

Batch 3−1598 : Selenium on Tyrosine−protein kinase JAK2

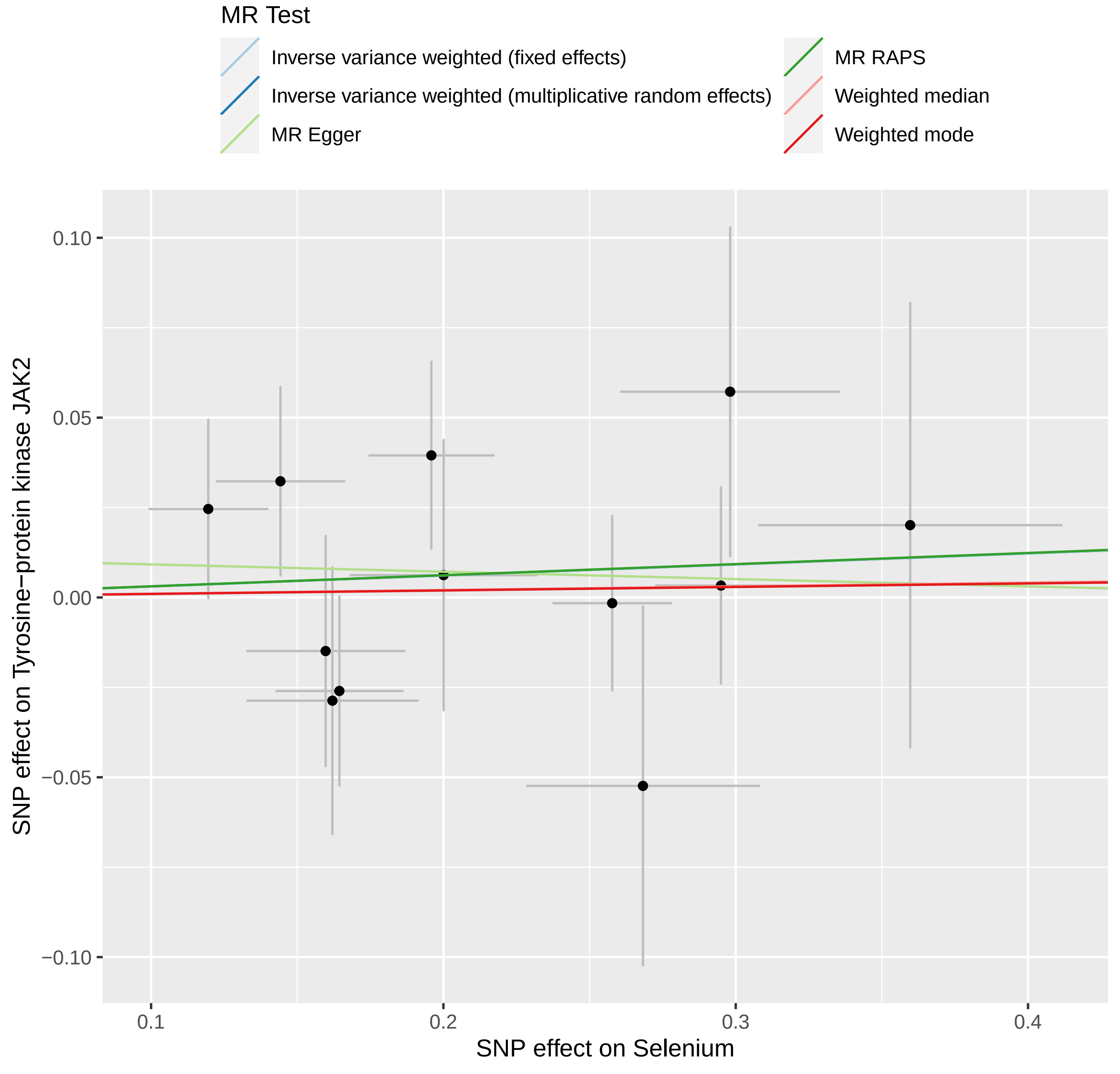

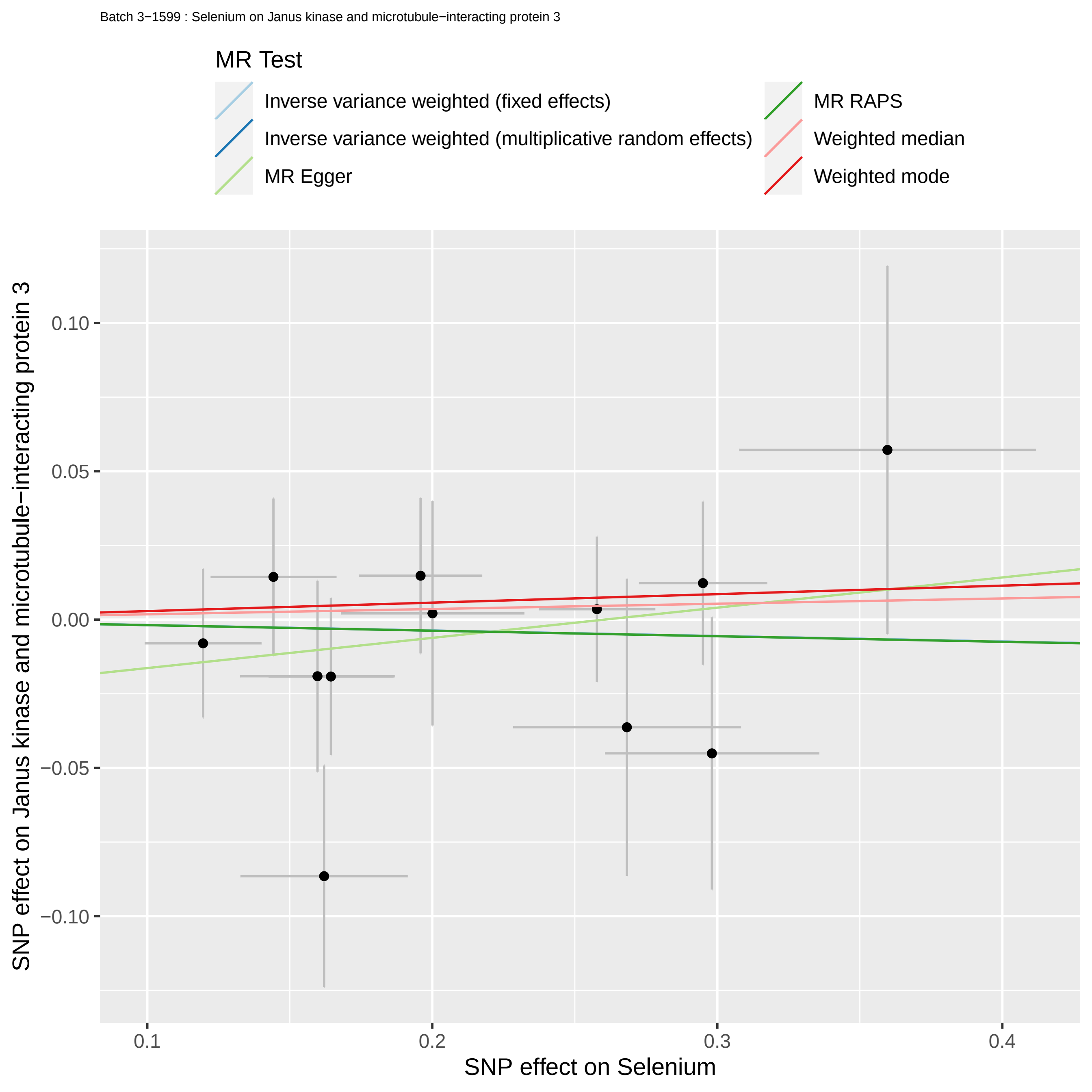

Batch 3−1599 : Selenium on Janus kinase and microtubule−interacting protein 3
MR Test
Inverse variance weighted (fixed effects)
Inverse variance weighted (multiplicative random effects)
MR Egger
MR RAPS
Weighted median
Weighted mode
0.10
0.05
0.00
−0.05
−0.10
0.1
0.2
0.3
0.4
SNP effect on Janus kinase and microtubule−interacting protein 3
SNP effect on Selenium

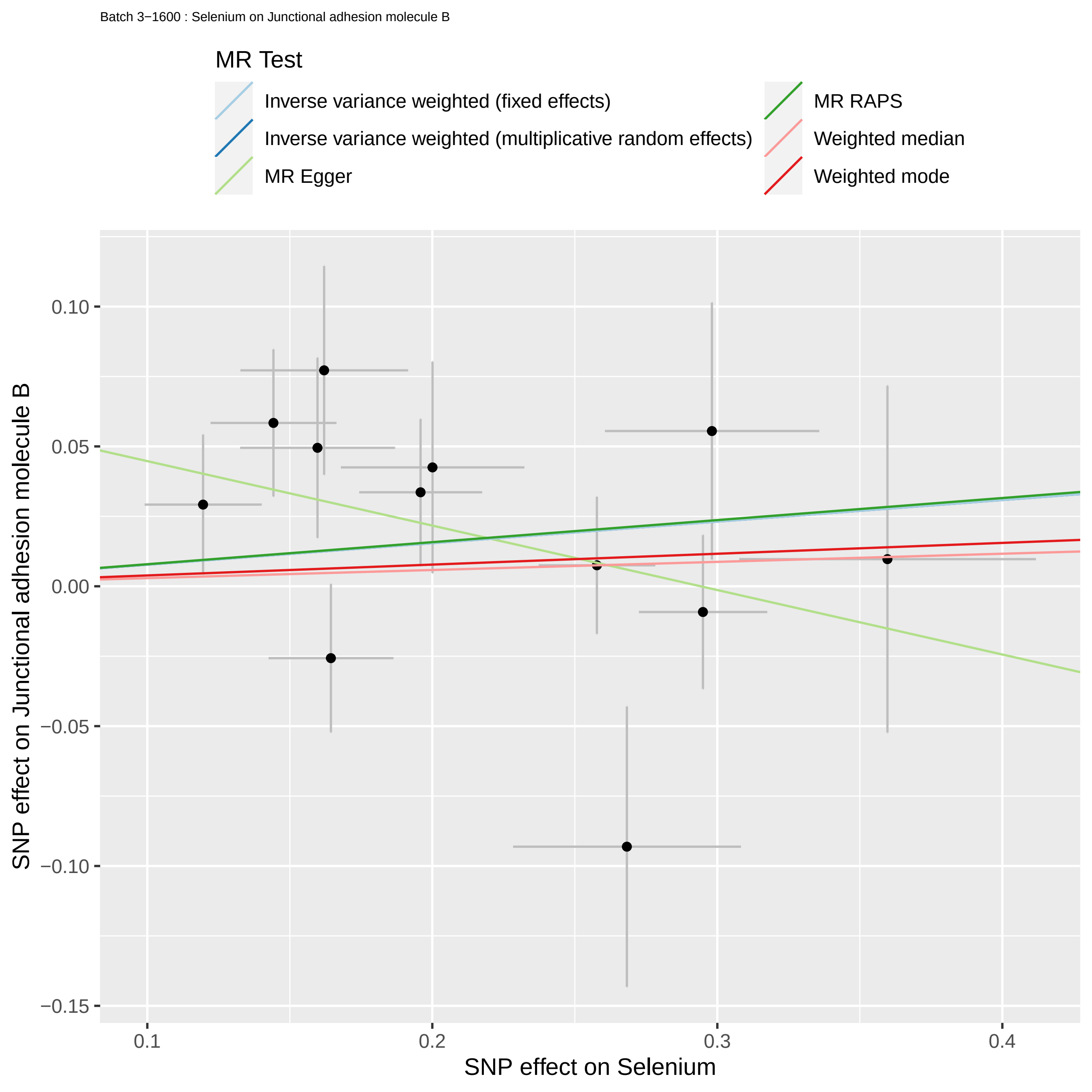
Batch 3−1600 : Selenium on Junctional adhesion molecule B
MR Test
Inverse variance weighted (fixed effects)
Inverse variance weighted (multiplicative random effects)
MR Egger
MR RAPS
Weighted median
Weighted mode
0.10
0.05
0.00
−0.05
−0.10
−0.15
0.1
0.2
0.3
0.4
SNP effect on Junctional adhesion molecule B
SNP effect on Selenium

Batch 3−1601 : Selenium on Junctional adhesion molecule C

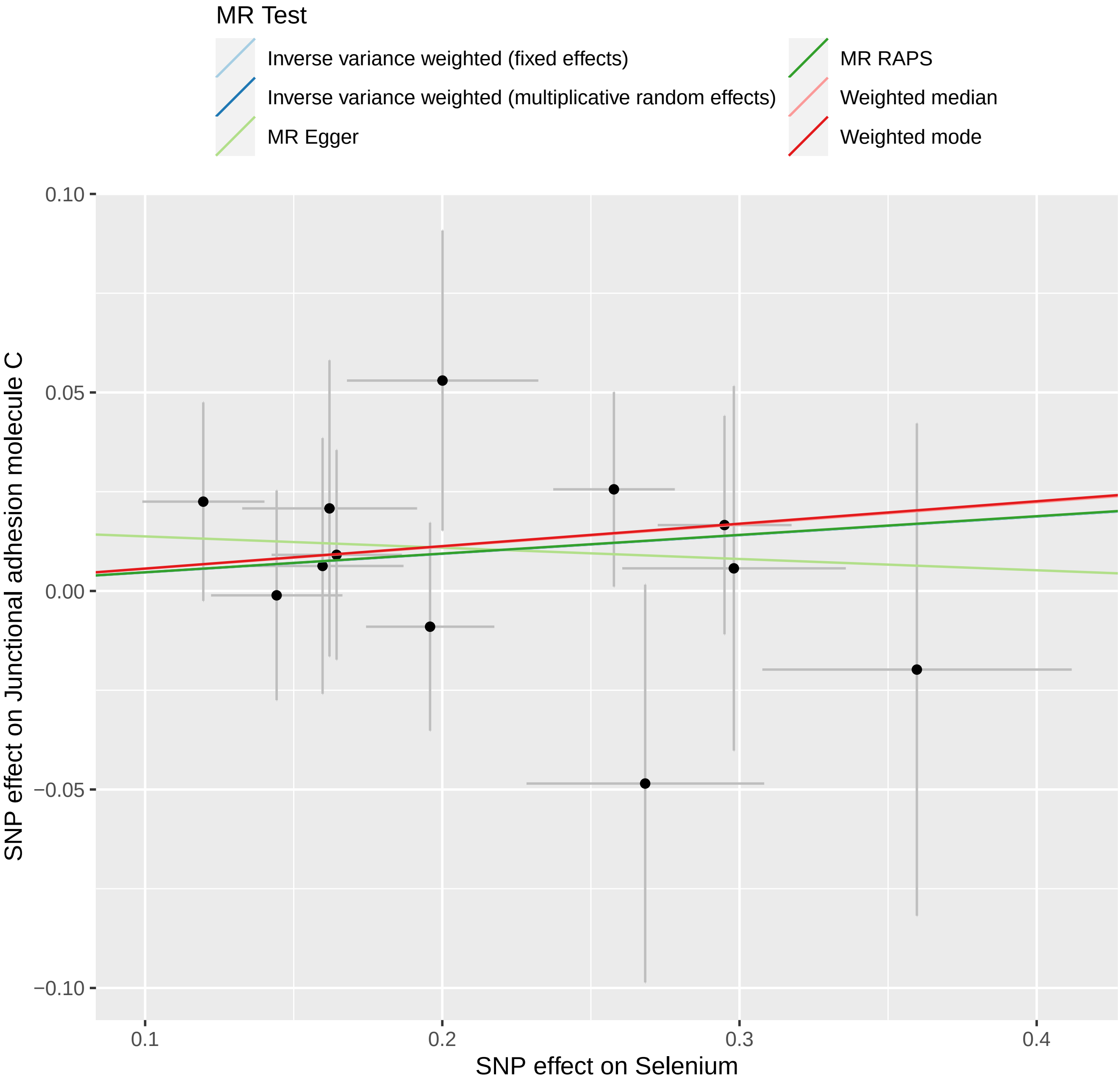

Batch 3−1602 : Selenium on Junctophilin−1

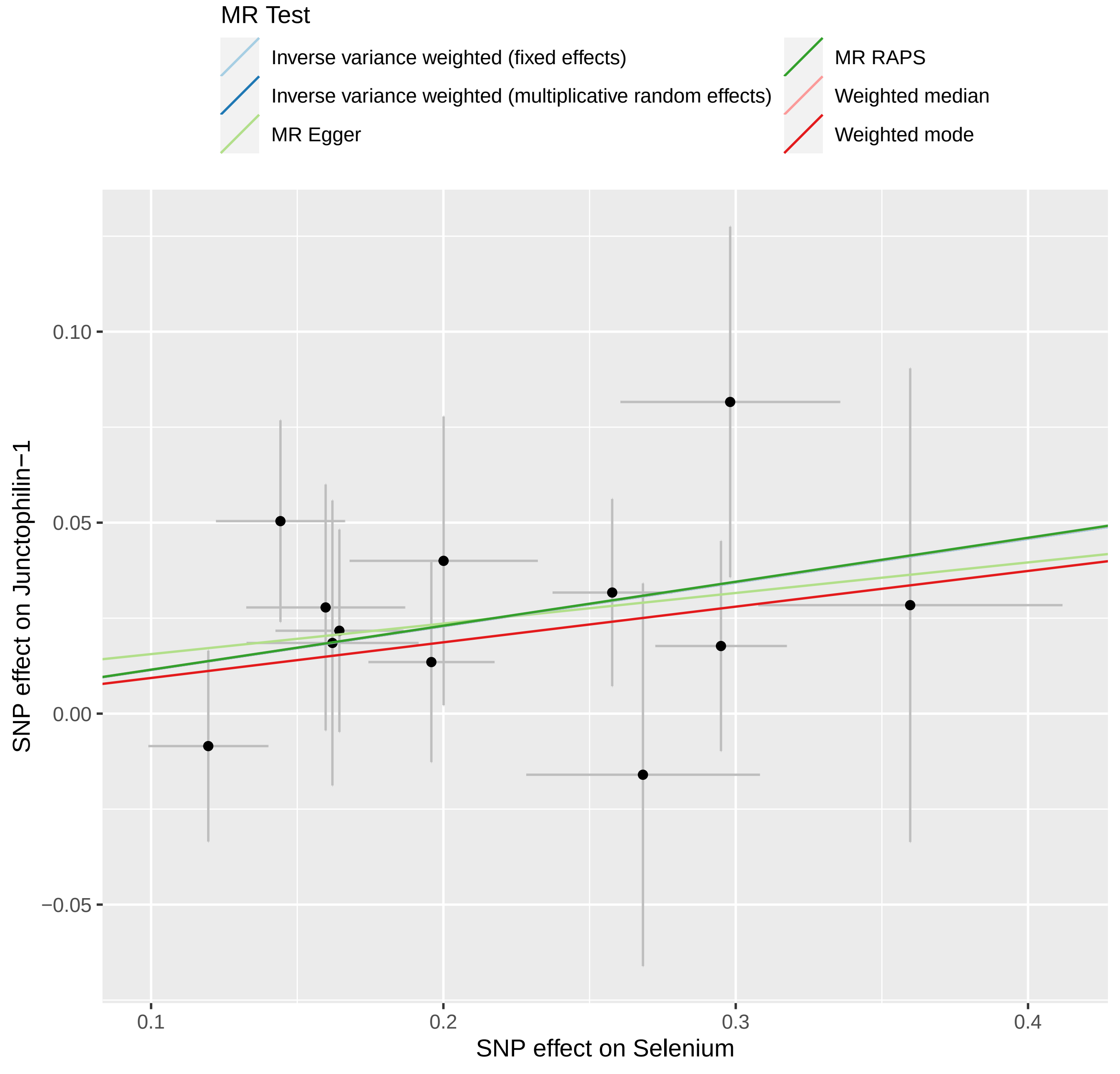

Batch 3−1603 : Selenium on Junctophilin−3

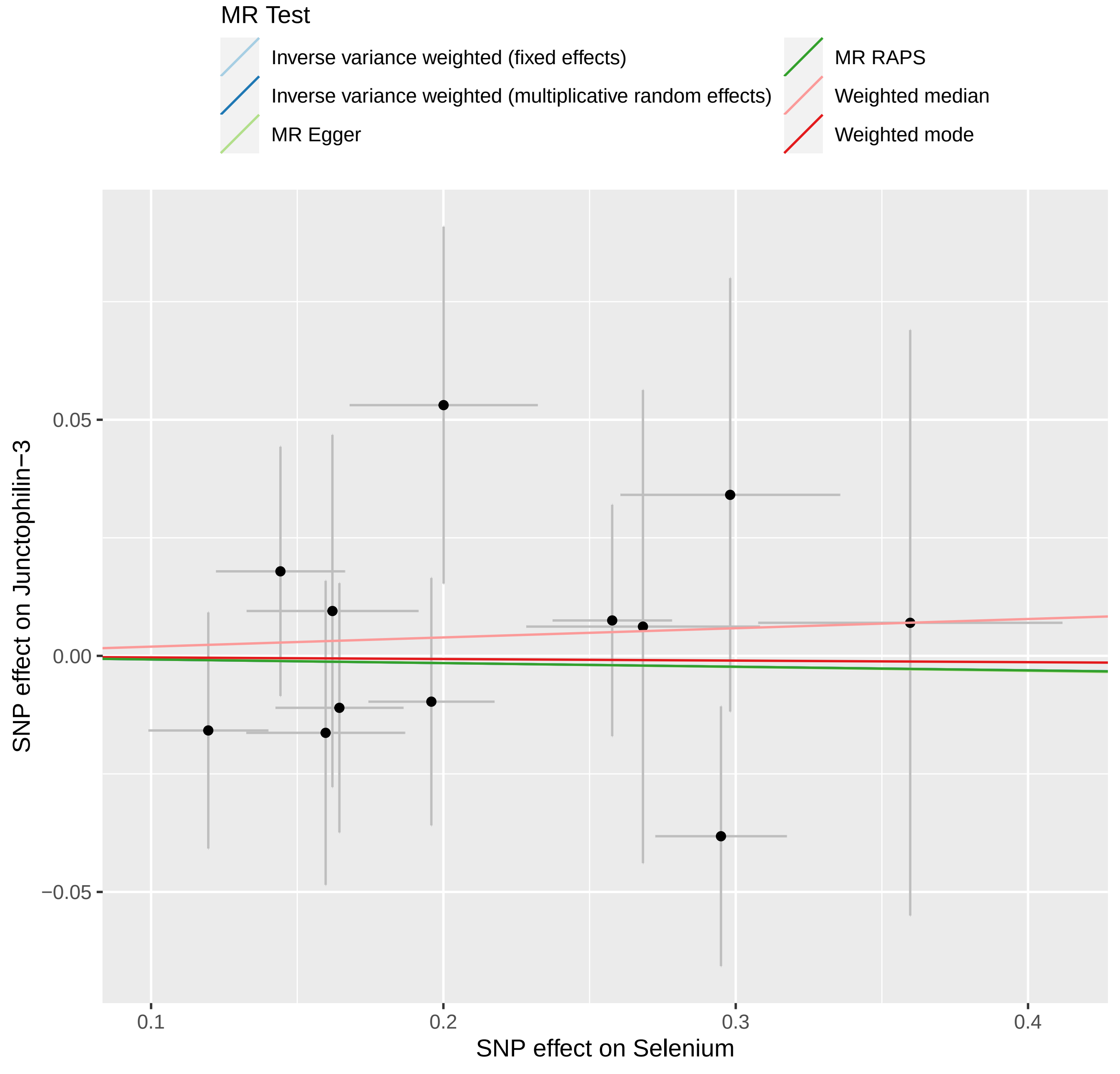

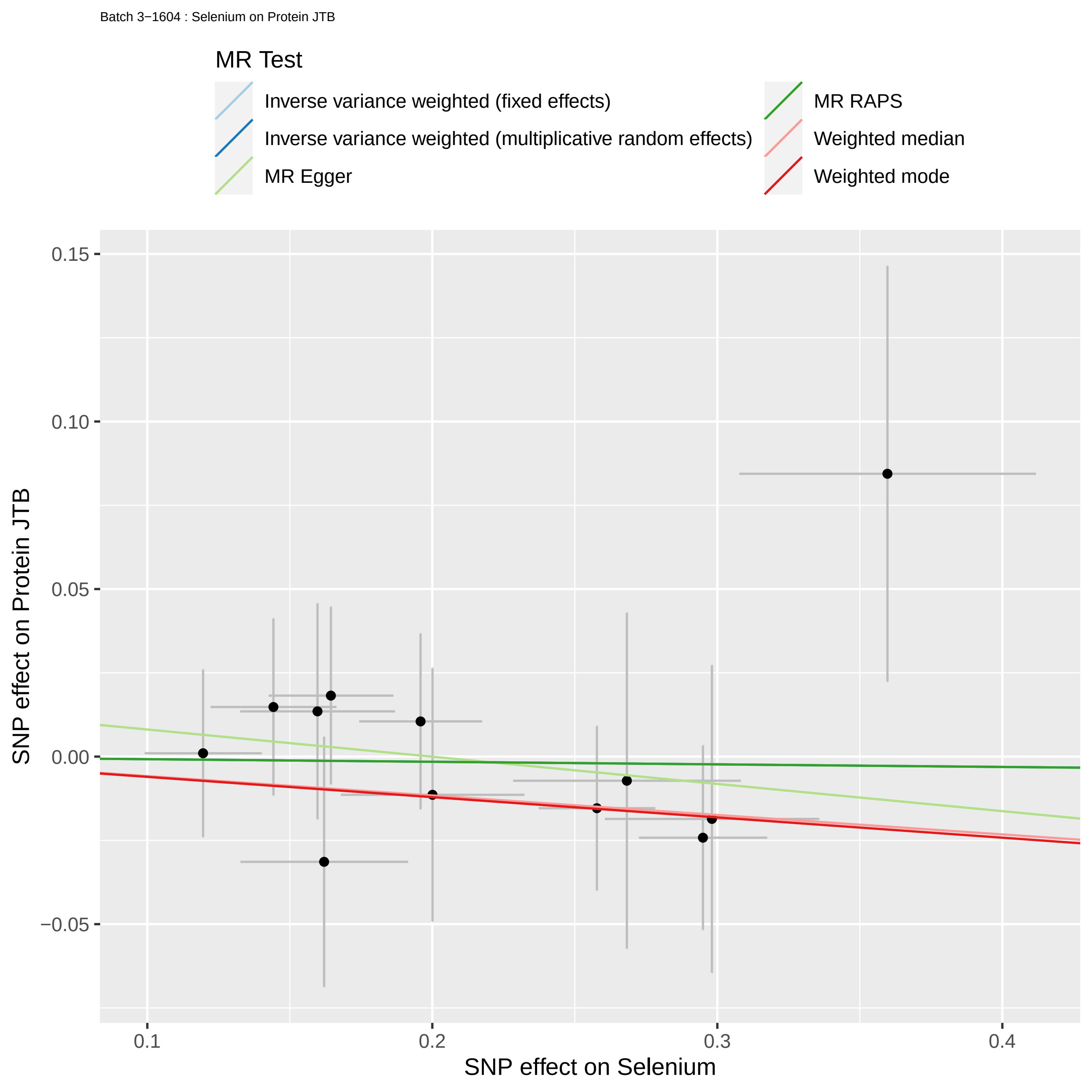

Batch 3−1604 : Selenium on Protein JTB
MR Test
Inverse variance weighted (fixed effects)
Inverse variance weighted (multiplicative random effects)
MR Egger
MR RAPS
Weighted median
Weighted mode
0.15
0.10
0.05
0.00
−0.05
0.1
0.2
0.3
0.4
SNP effect on Protein JTB
SNP effect on Selenium

Batch 3−1605 : Selenium on Transcription factor AP−1

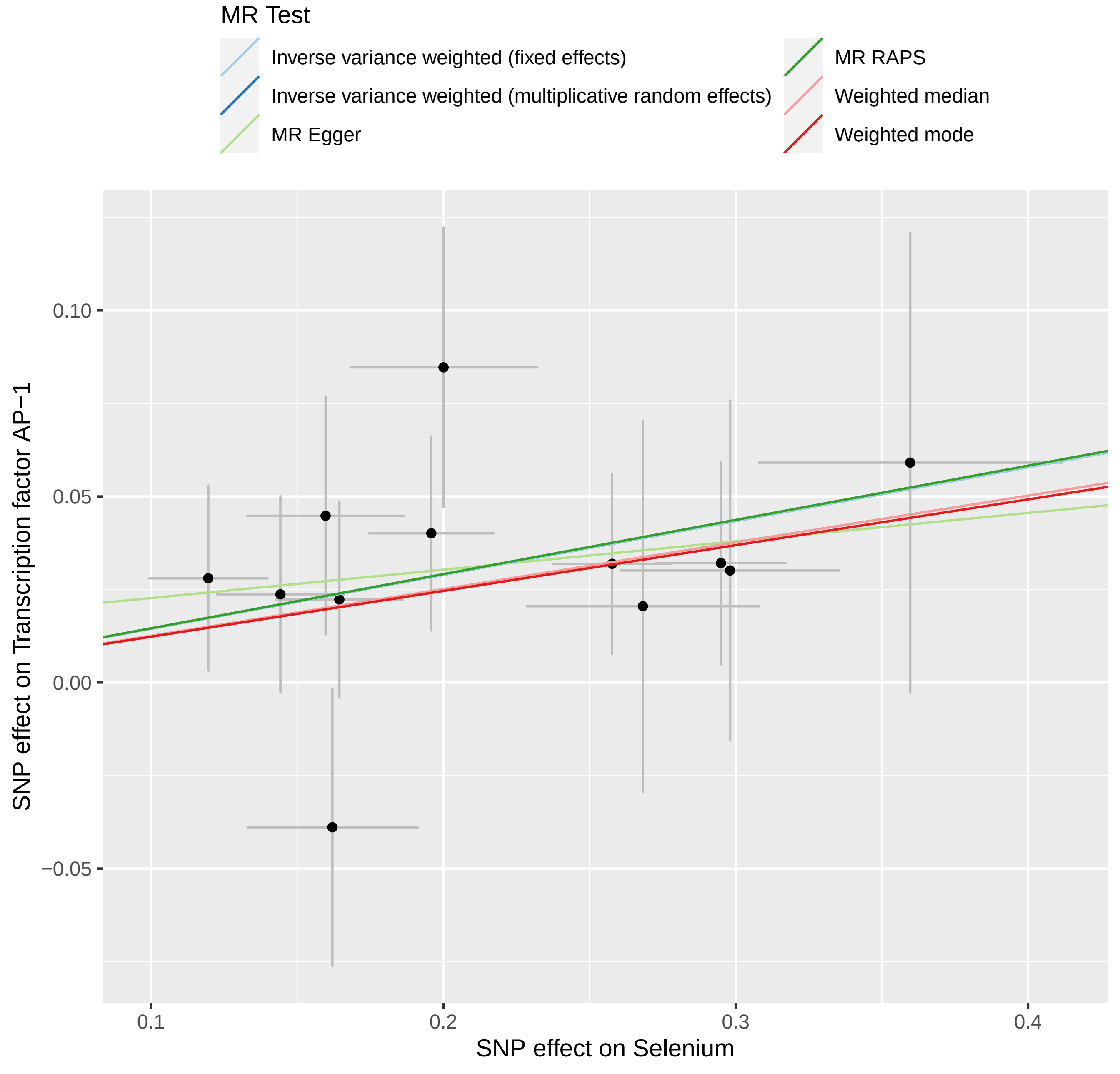

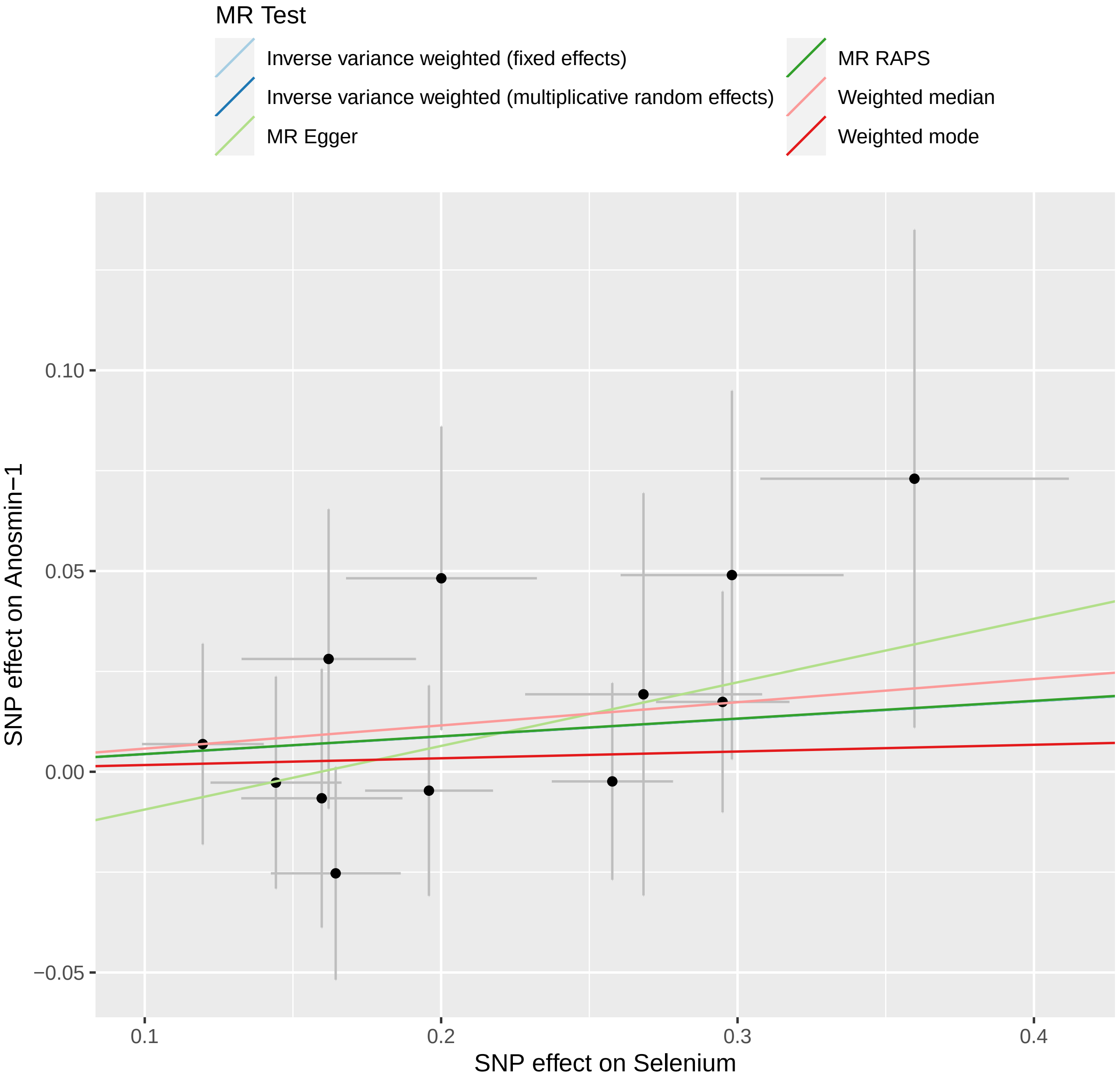
MR Test
Inverse variance weighted (fixed effects)
Inverse variance weighted (multiplicative random effects)
MR Egger
MR RAPS
Weighted median
Weighted mode
0.10
0.05
0.00
−0.05
0.1
0.2
0.3
0.4
SNP effect on Anosmin−1
SNP effect on Selenium

Batch 3−1607 : Selenium on Histone acetyltransferase KAT6A

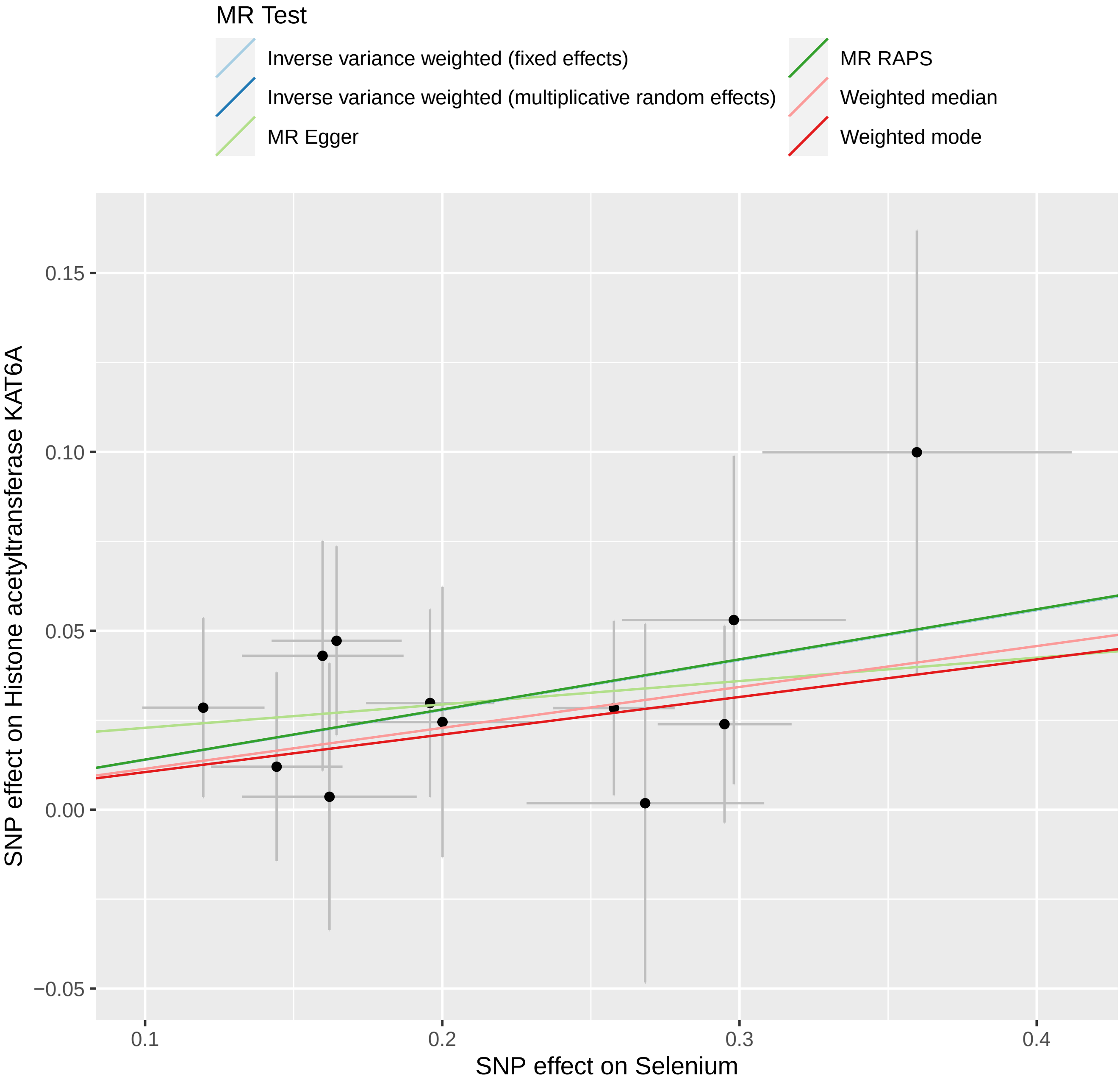

Batch 3−1608 : Selenium on Potassium voltage−gated channel subfamily A member 10

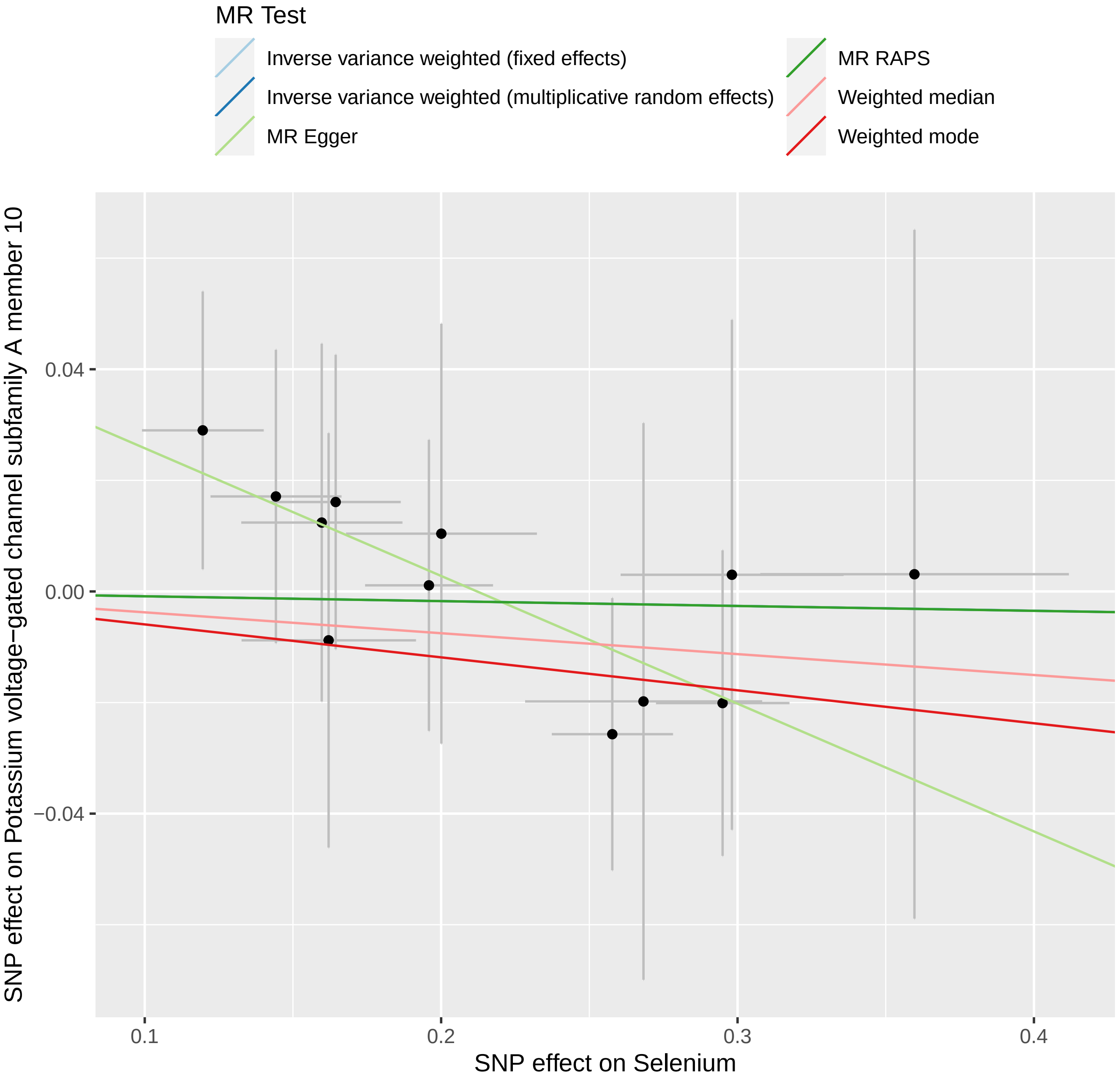

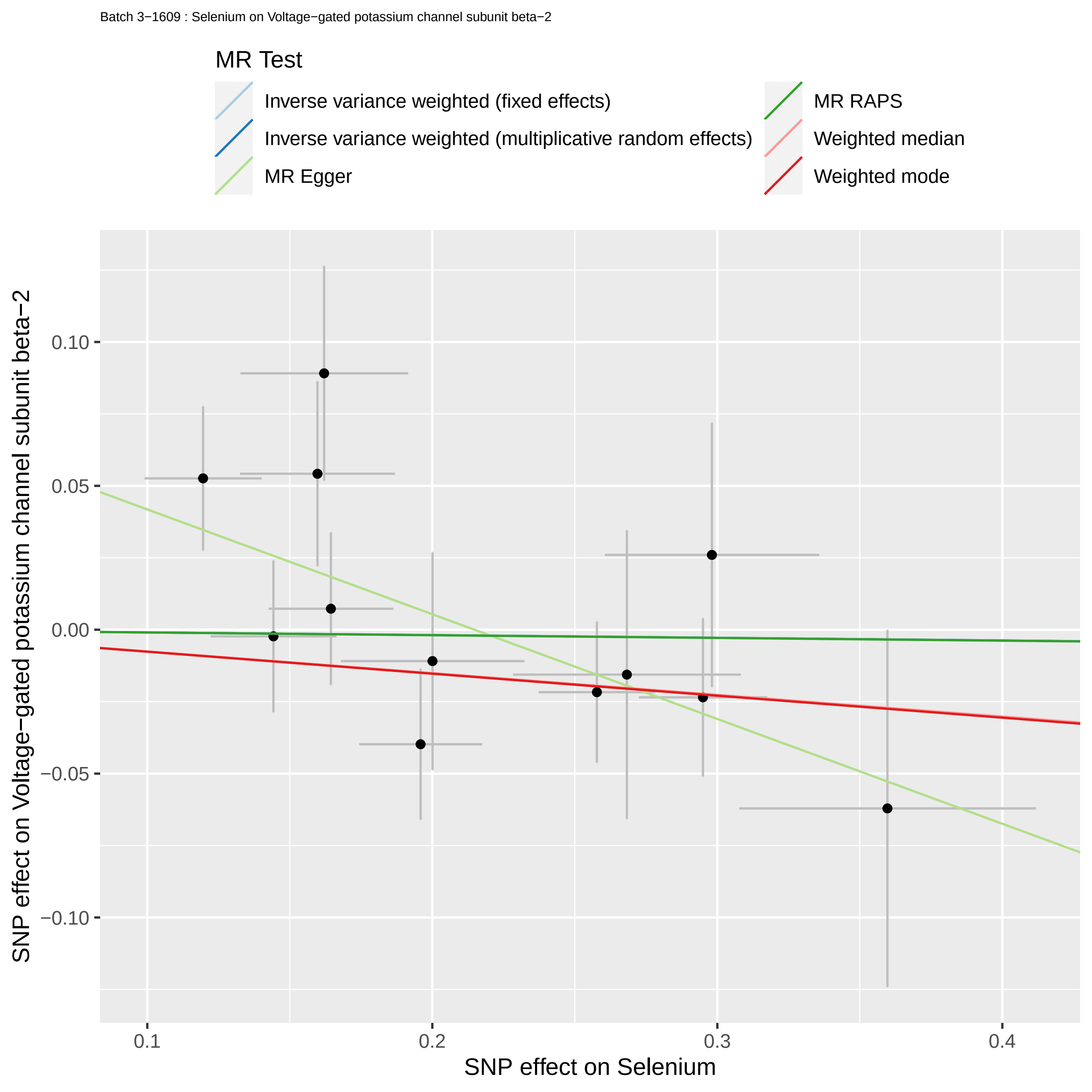
Batch 3−1609 : Selenium on Voltage−gated potassium channel subunit beta−2
MR Test
Inverse variance weighted (fixed effects)
Inverse variance weighted (multiplicative random effects)
MR Egger
MR RAPS
Weighted median
Weighted mode
SNP effect on Voltage−gated potassium channel subunit beta−2
0.10
0.05
0.00
−0.05
−0.10
0.1
0.2
0.3
0.4
SNP effect on Selenium

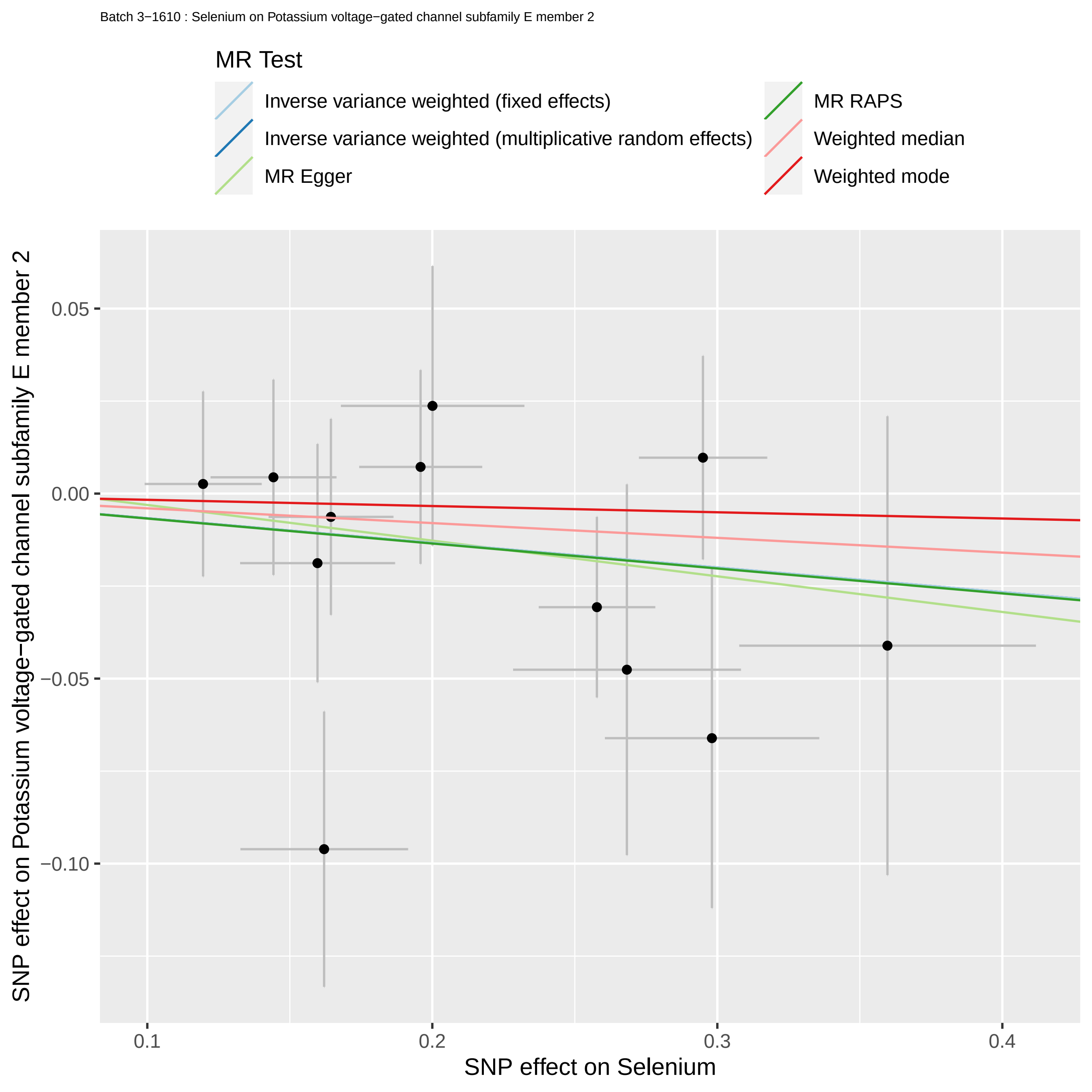

Batch 3−1610 : Selenium on Potassium voltage−gated channel subfamily E member 2
MR Test
Inverse variance weighted (fixed effects)
Inverse variance weighted (multiplicative random effects)
MR Egger
MR RAPS
Weighted median
Weighted mode
0.05
0.00
−0.05
−0.10
0.1
0.2
0.3
0.4
SNP effect on Potassium voltage−gated channel subfamily E member 2
SNP effect on Selenium

Batch 3−1611 : Selenium on Potassium voltage−gated channel subfamily E member 3

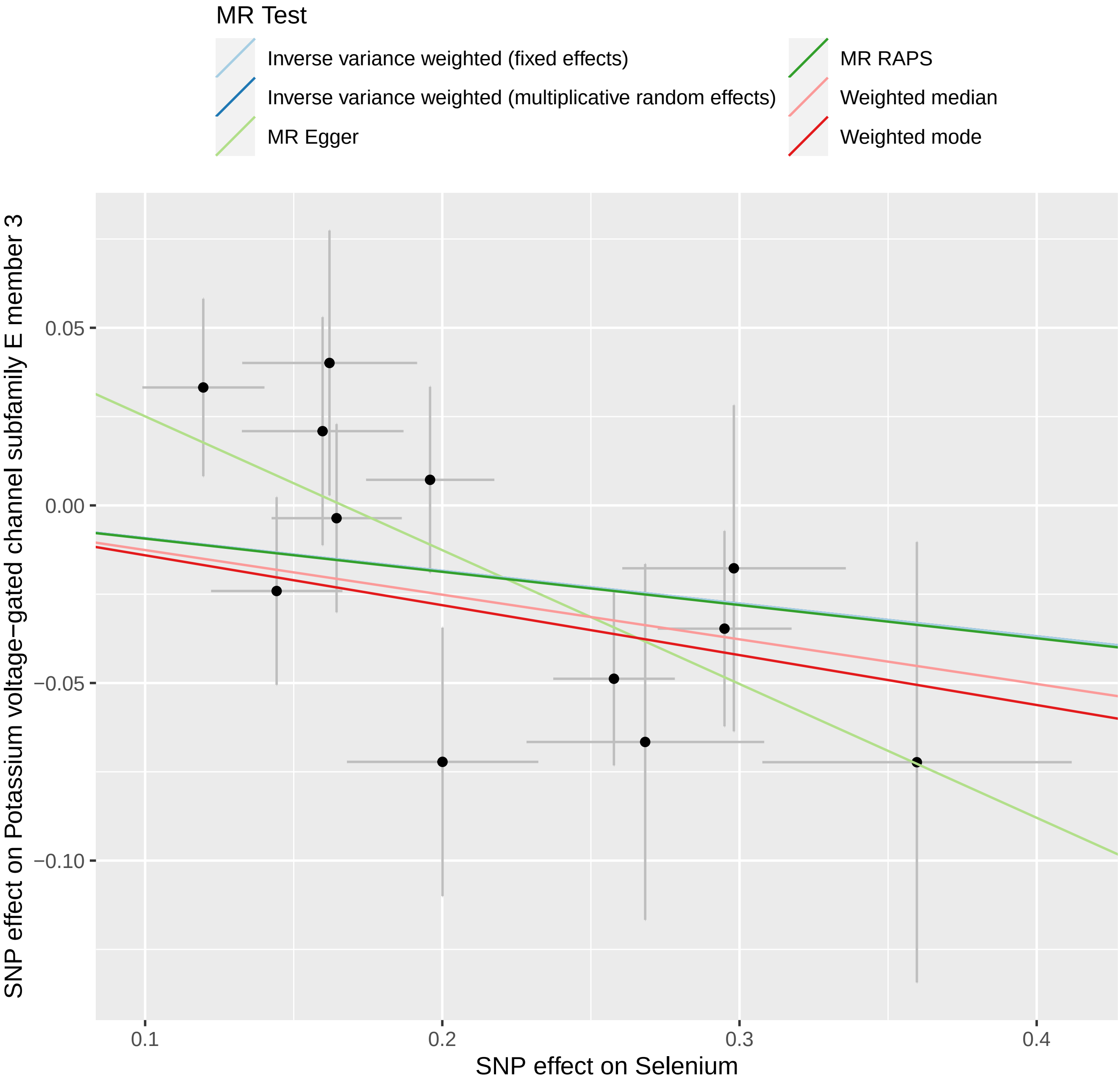

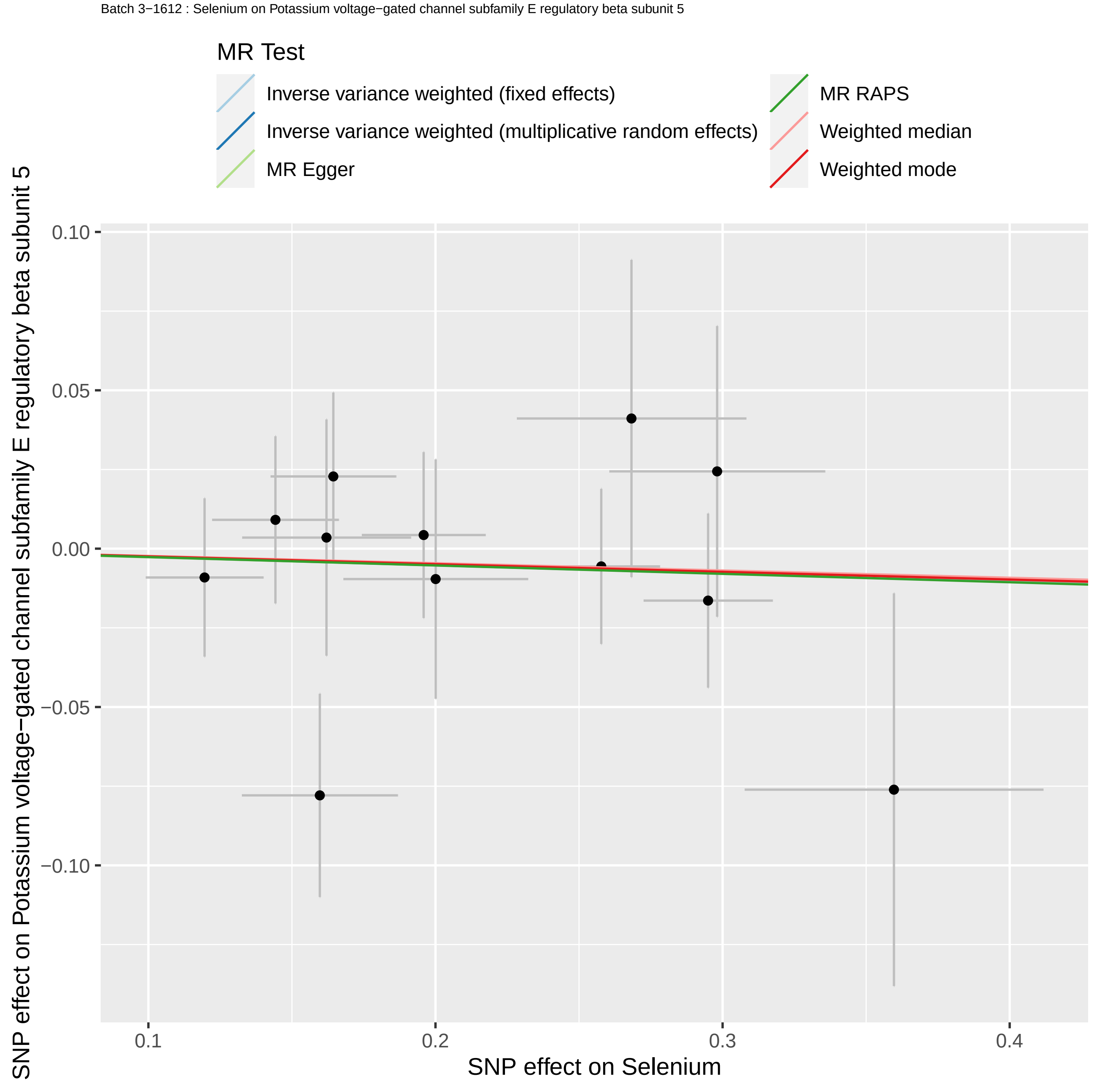

Batch 3−1612 : Selenium on Potassium voltage−gated channel subfamily E regulatory beta subunit 5
MR Test
Inverse variance weighted (fixed effects)
Inverse variance weighted (multiplicative random effects)
MR Egger
MR RAPS
Weighted median
Weighted mode
0.10
0.05
0.00
−0.05
−0.10
0.1
0.2
0.3
0.4
SNP effect on Selenium
SNP effect on Potassium voltage−gated channel subfamily E regulatory beta subunit 5

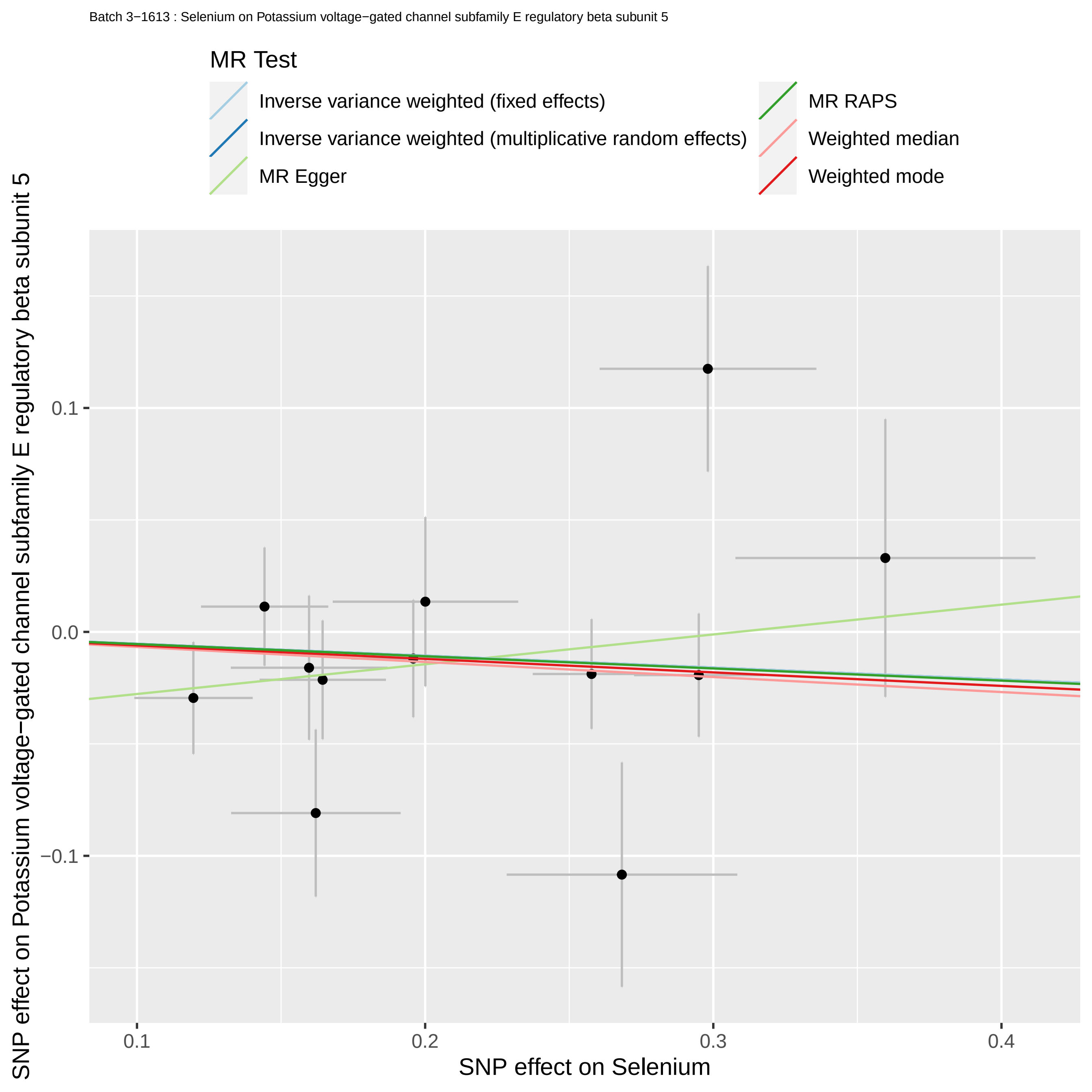

Batch 3−1613 : Selenium on Potassium voltage−gated channel subfamily E regulatory beta subunit 5
MR Test
Inverse variance weighted (fixed effects)
Inverse variance weighted (multiplicative random effects)
MR Egger
MR RAPS
Weighted median
Weighted mode
SNP effect on Potassium voltage−gated channel subfamily E regulatory beta subunit 5
0.1
0.0
−0.1
0.1
0.2
0.3
0.4
SNP effect on Selenium

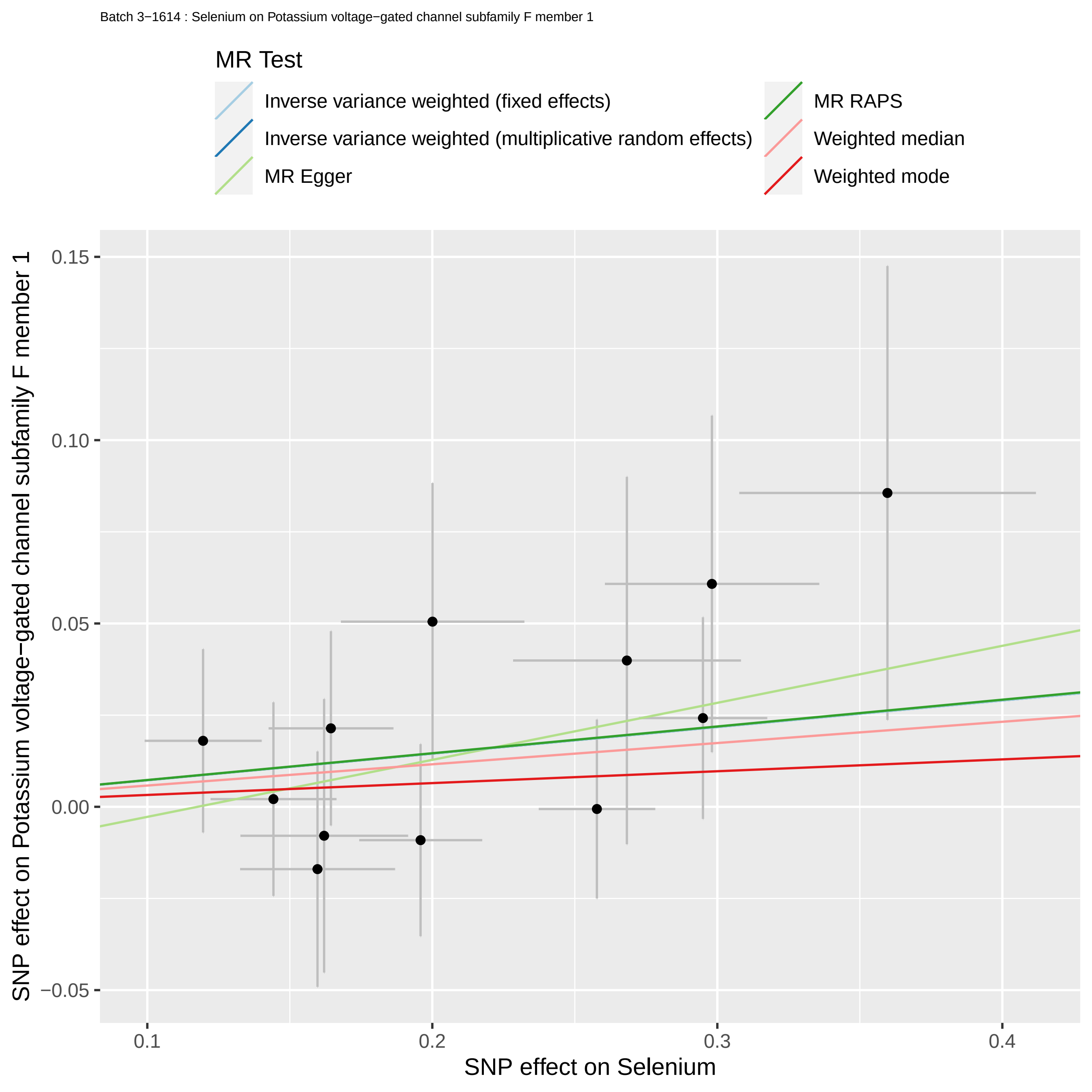

Batch 3−1614 : Selenium on Potassium voltage−gated channel subfamily F member 1
MR Test
Inverse variance weighted (fixed effects)
Inverse variance weighted (multiplicative random effects)
MR Egger
MR RAPS
Weighted median
Weighted mode
SNP effect on Potassium voltage−gated channel subfamily F member 1
0.15
0.10
0.05
0.00
−0.05
0.1
0.2
0.3
0.4
SNP effect on Selenium

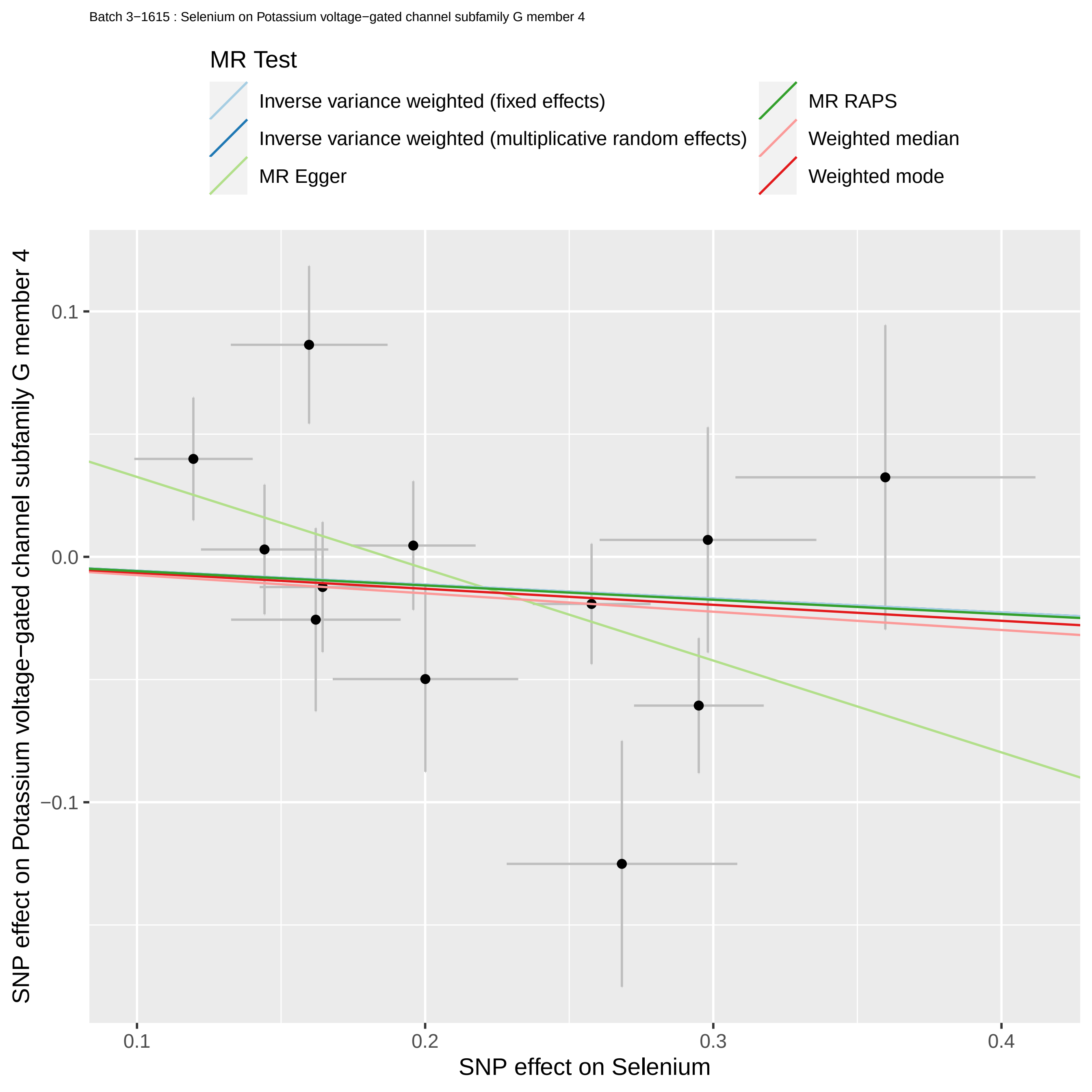

Batch 3−1615 : Selenium on Potassium voltage−gated channel subfamily G member 4
MR Test
Inverse variance weighted (fixed effects)
Inverse variance weighted (multiplicative random effects)
MR Egger
MR RAPS
Weighted median
Weighted mode
SNP effect on Potassium voltage−gated channel subfamily G member 4
0.1
0.0
−0.1
0.1
0.2
0.3
0.4
SNP effect on Selenium

Batch 3−1616 : Selenium on Kv channel−interacting protein 1

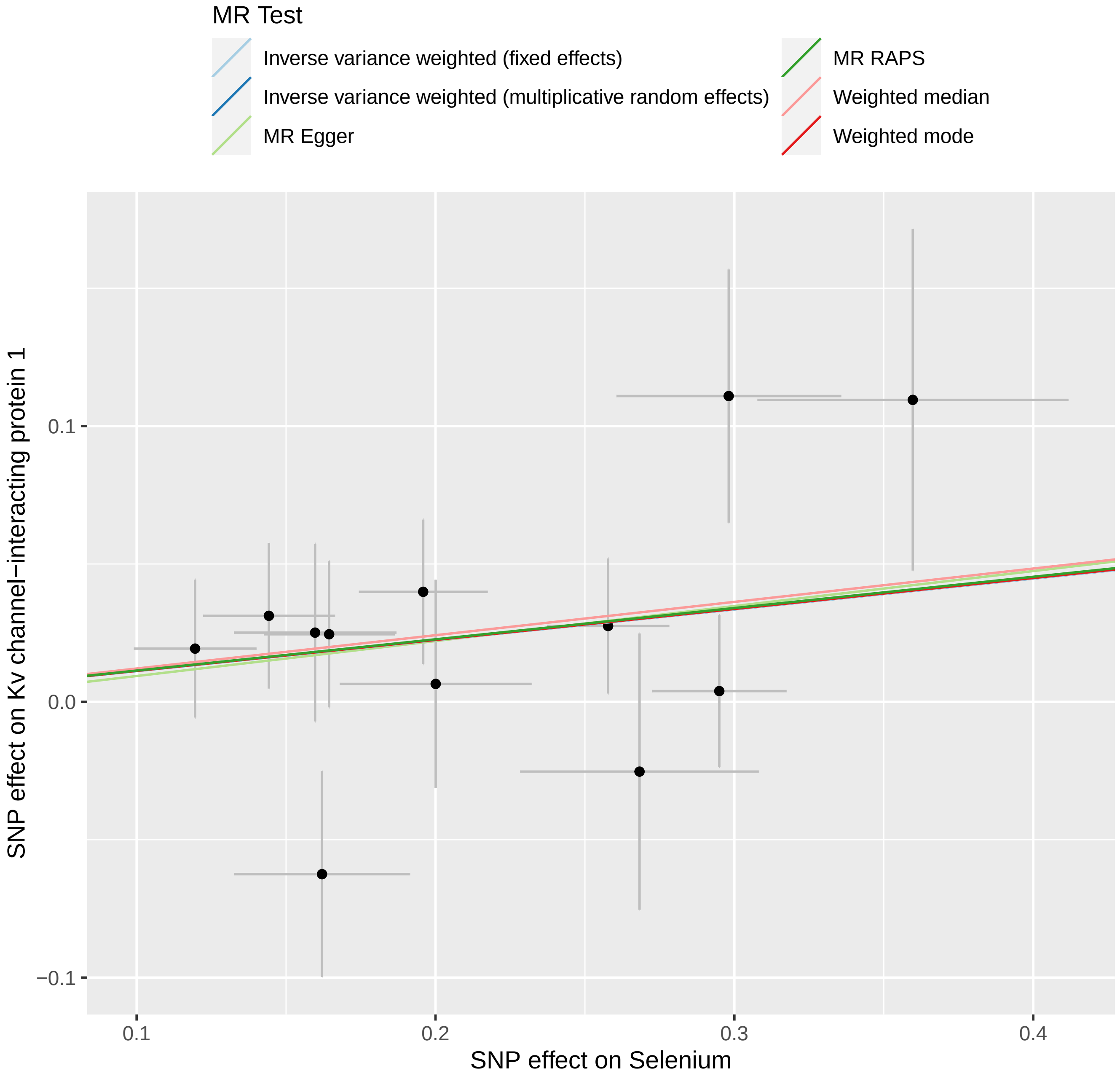

Batch 3−1617 : Selenium on Calsenilin

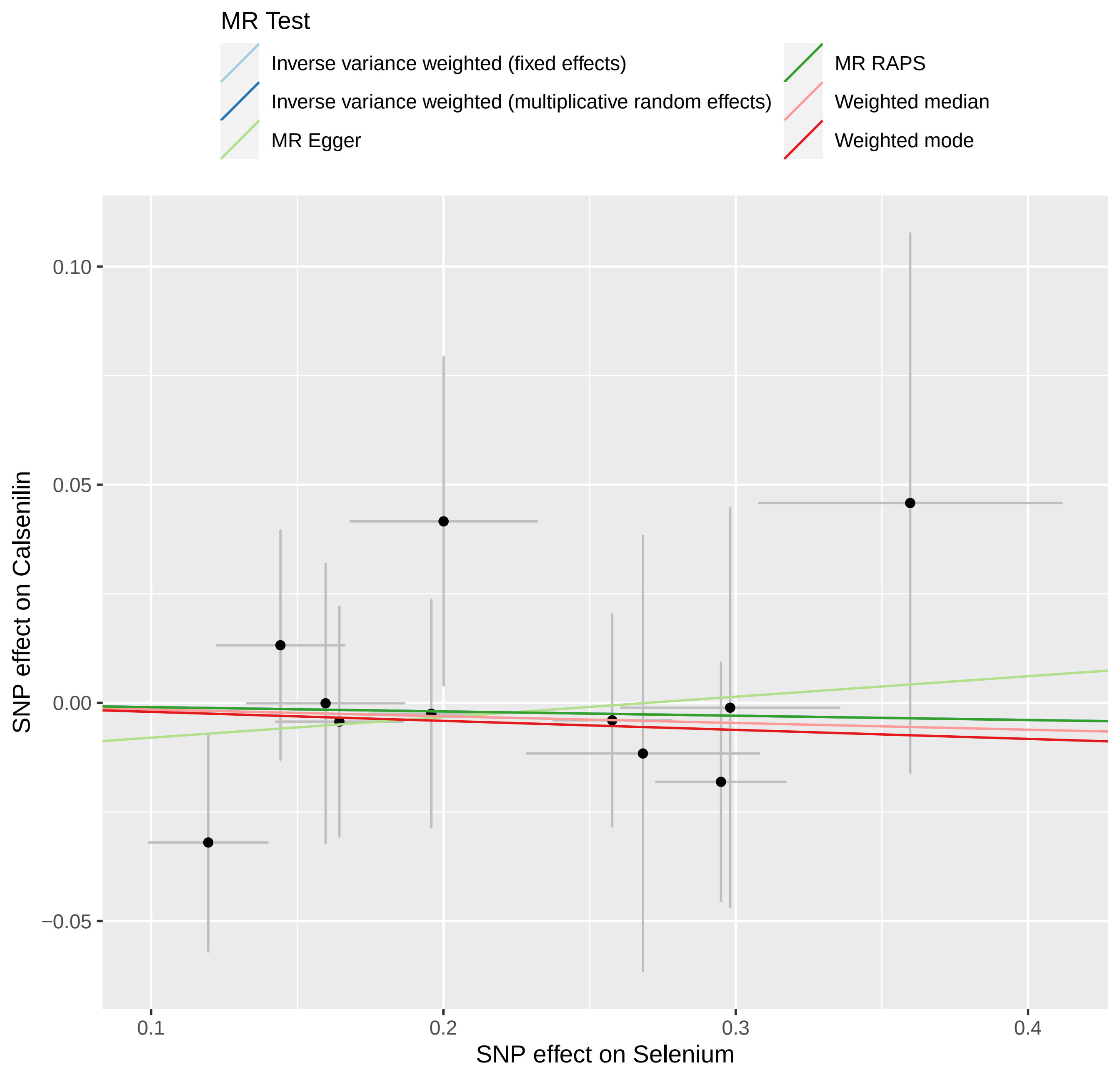

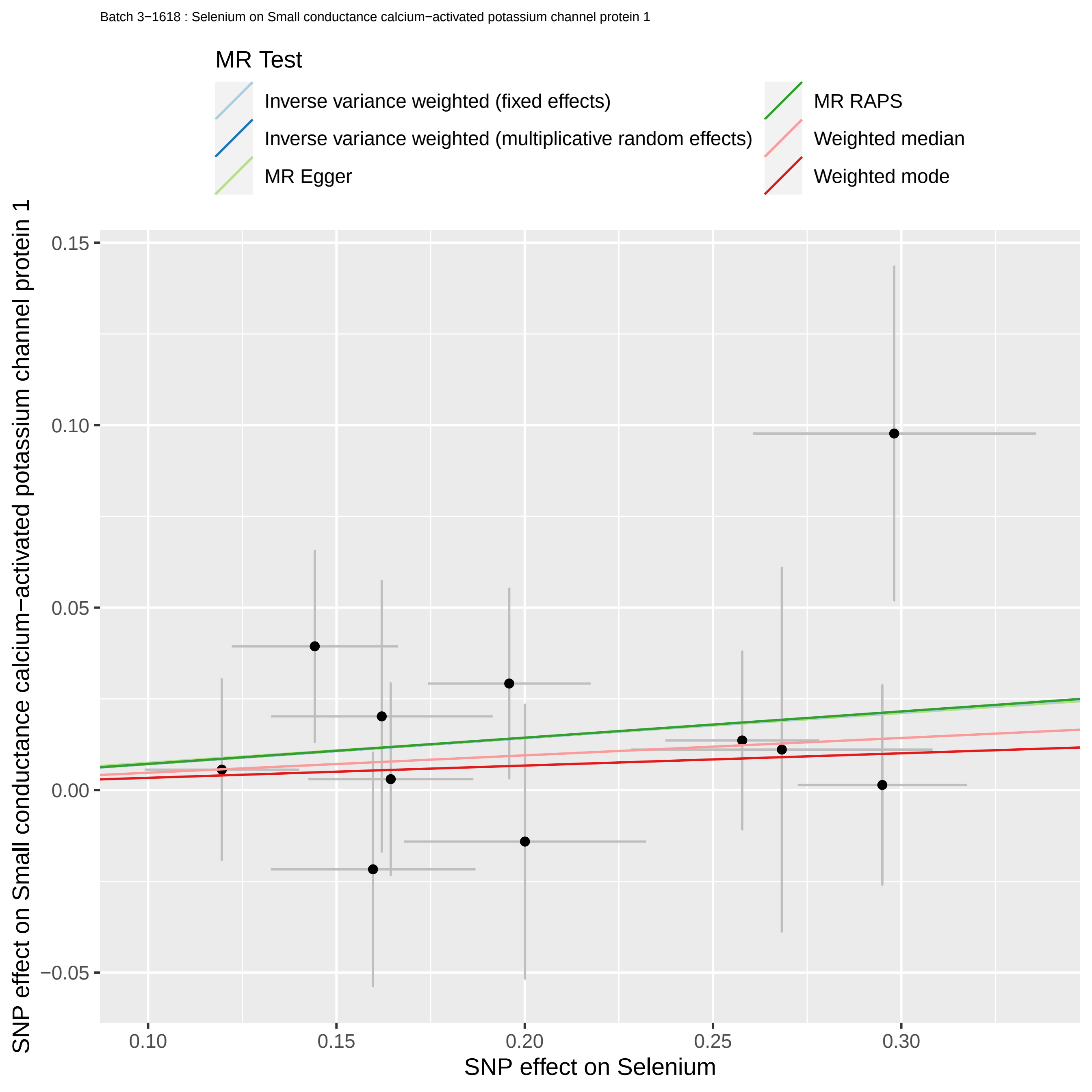

Batch 3−1618 : Selenium on Small conductance calcium−activated potassium channel protein 1
MR Test
Inverse variance weighted (fixed effects)
Inverse variance weighted (multiplicative random effects)
MR Egger
MR RAPS
Weighted median
Weighted mode
SNP effect on Small conductance calcium−activated potassium channel protein 1
0.15
0.10
0.05
0.00
−0.05
0.10
0.15
0.20
0.25
0.30
SNP effect on Selenium

Batch 3−1619 : Selenium on Keratinocyte−associated transmembrane protein 2

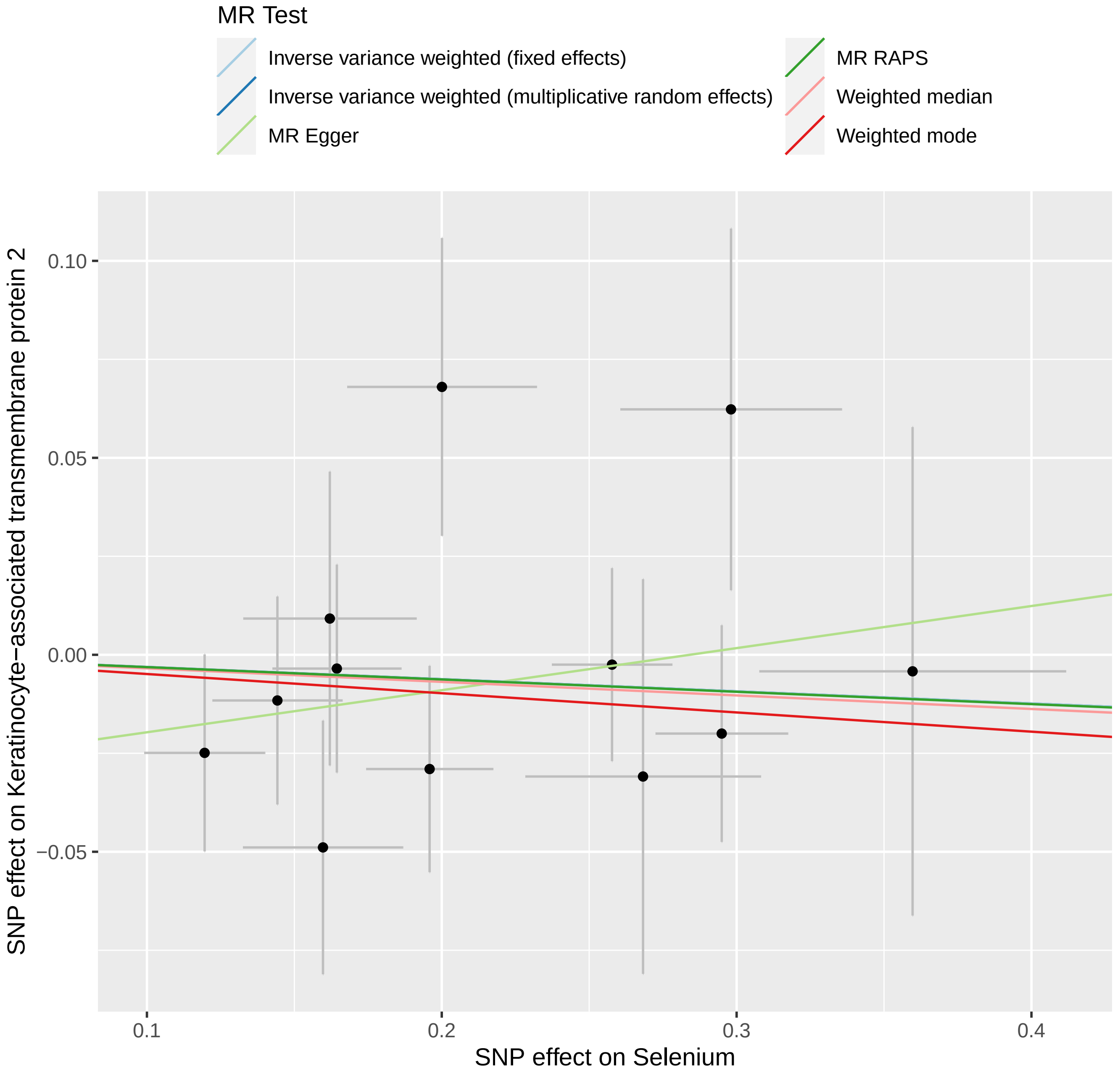

Batch 3−1620 : Selenium on BTB/POZ domain−containing protein KCTD5

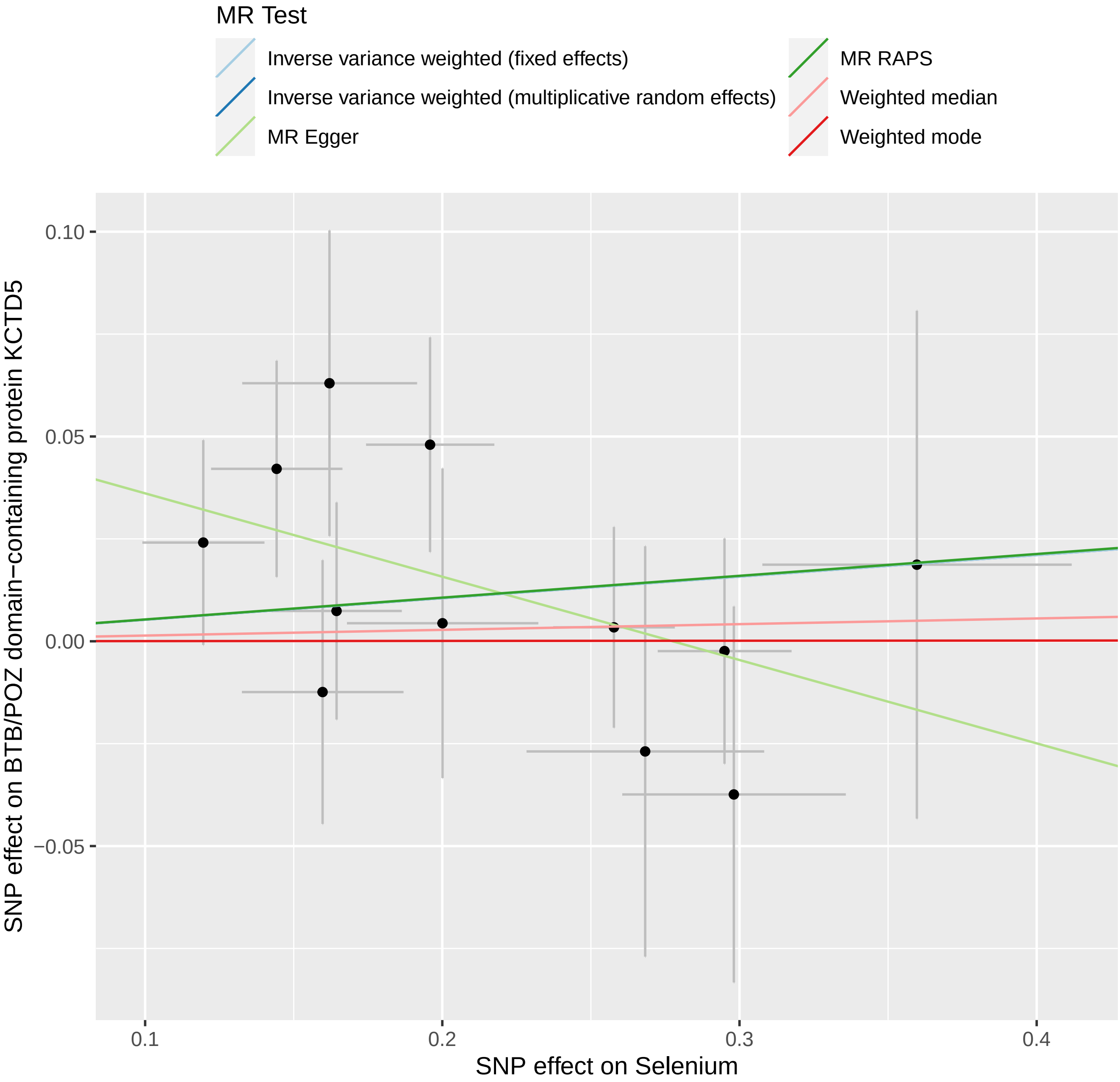

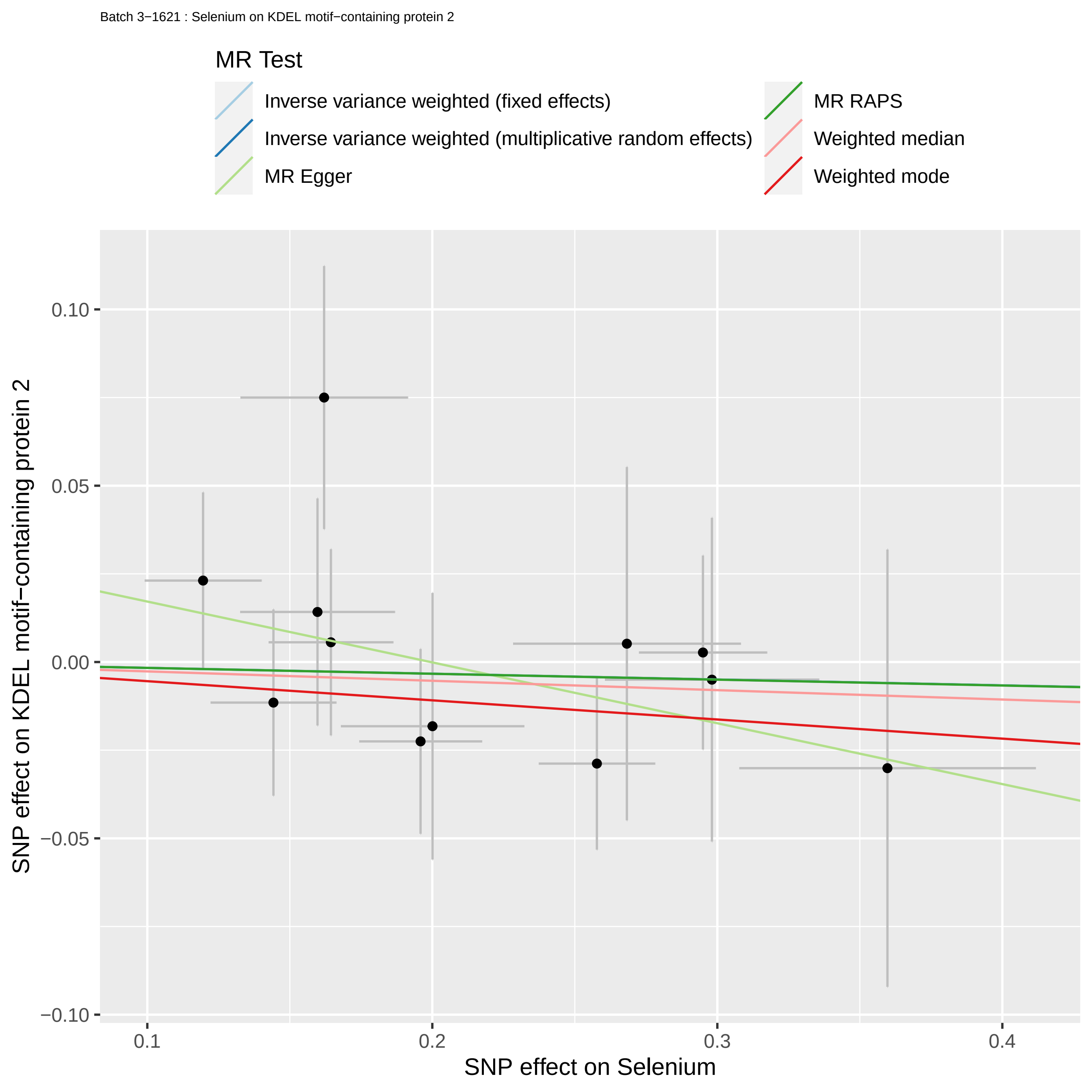
Batch 3−1621 : Selenium on KDEL motif−containing protein 2
MR Test
Inverse variance weighted (fixed effects)
Inverse variance weighted (multiplicative random effects)
MR Egger
MR RAPS
Weighted median
Weighted mode
0.10
0.05
0.00
−0.05
−0.10
0.1
0.2
0.3
0.4
SNP effect on KDEL motif−containing protein 2
SNP effect on Selenium

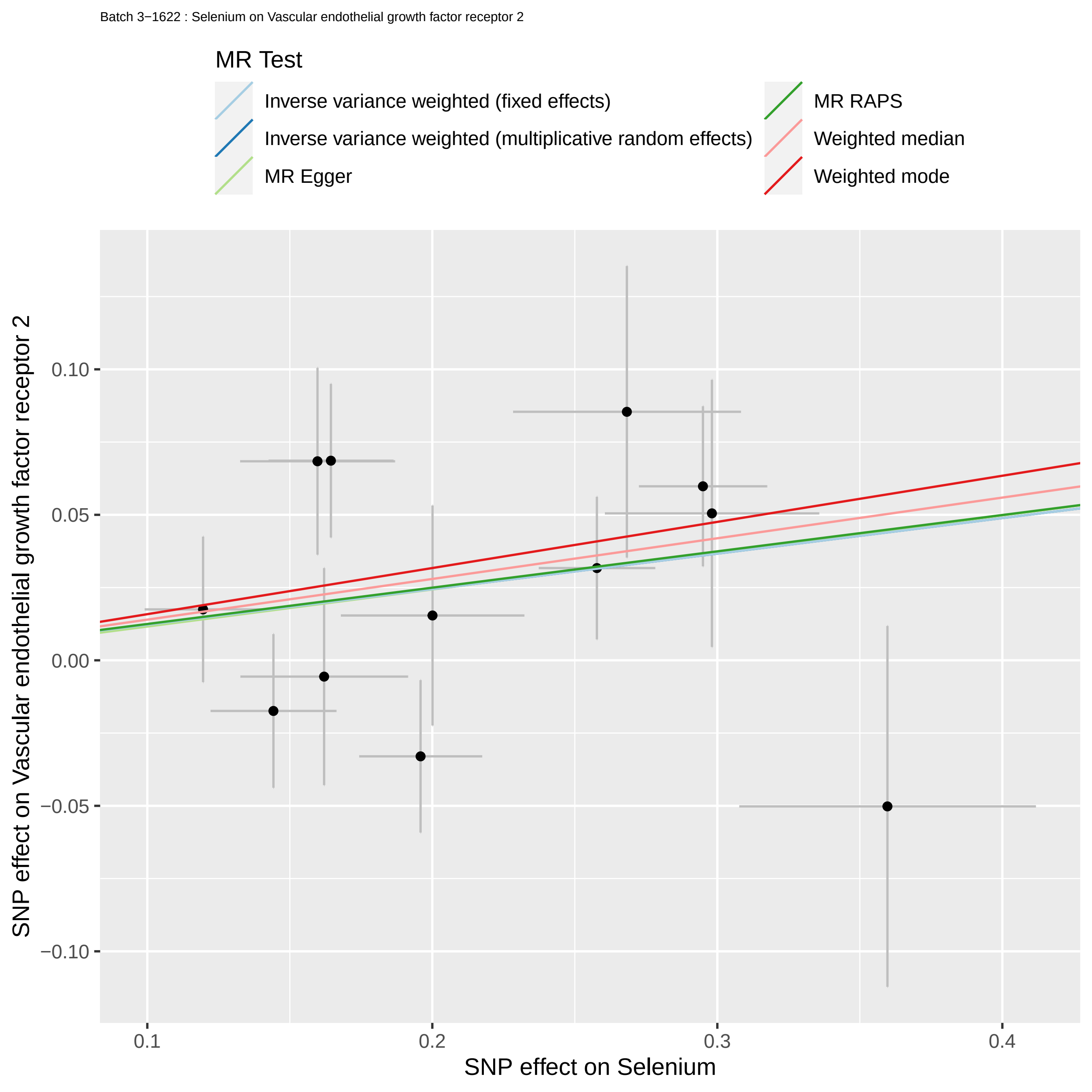
Batch 3−1622 : Selenium on Vascular endothelial growth factor receptor 2
MR Test
Inverse variance weighted (fixed effects)
Inverse variance weighted (multiplicative random effects)
MR Egger
MR RAPS
Weighted median
Weighted mode
0.10
0.05
0.00
−0.05
−0.10
0.1
0.2
0.3
0.4
SNP effect on Vascular endothelial growth factor receptor 2
SNP effect on Selenium

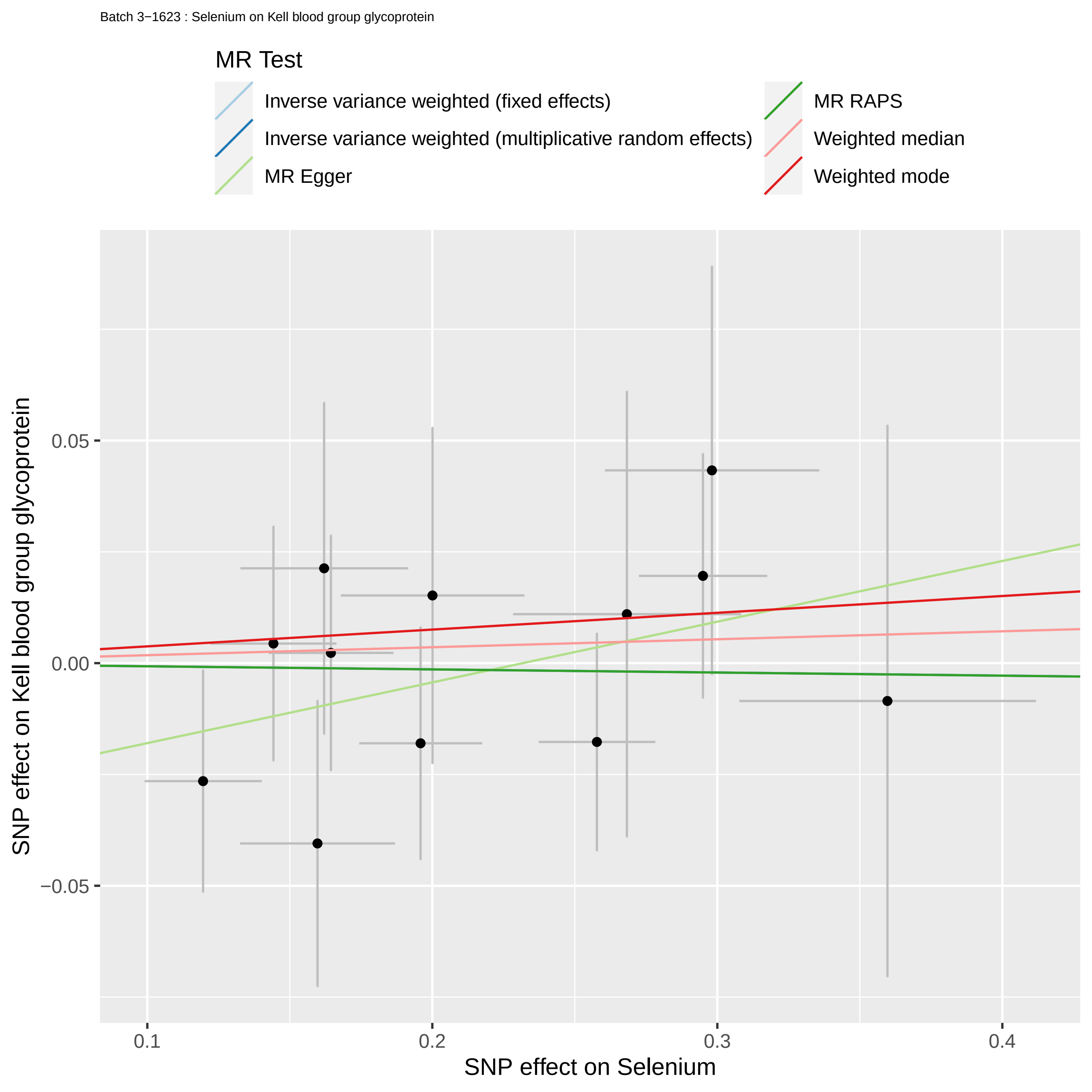
Batch 3−1623 : Selenium on Kell blood group glycoprotein
MR Test
Inverse variance weighted (fixed effects)
Inverse variance weighted (multiplicative random effects)
MR Egger
MR RAPS
Weighted median
Weighted mode
SNP effect on Kell blood group glycoprotein
0.05
0.00
−0.05
0.1
0.2
0.3
0.4
SNP effect on Selenium

Batch 3−1624 : Selenium on Uncharacterized protein KIAA0040

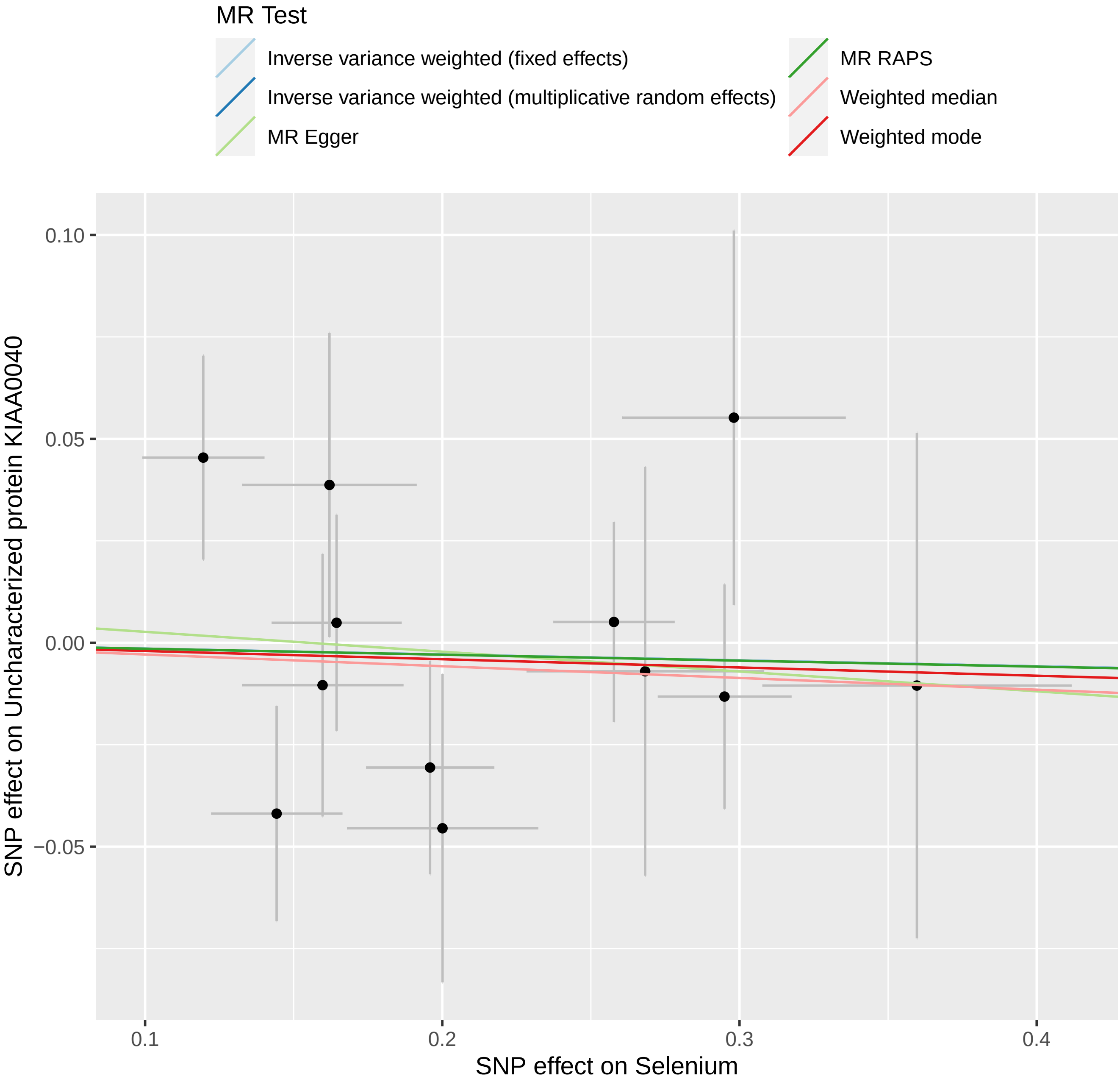

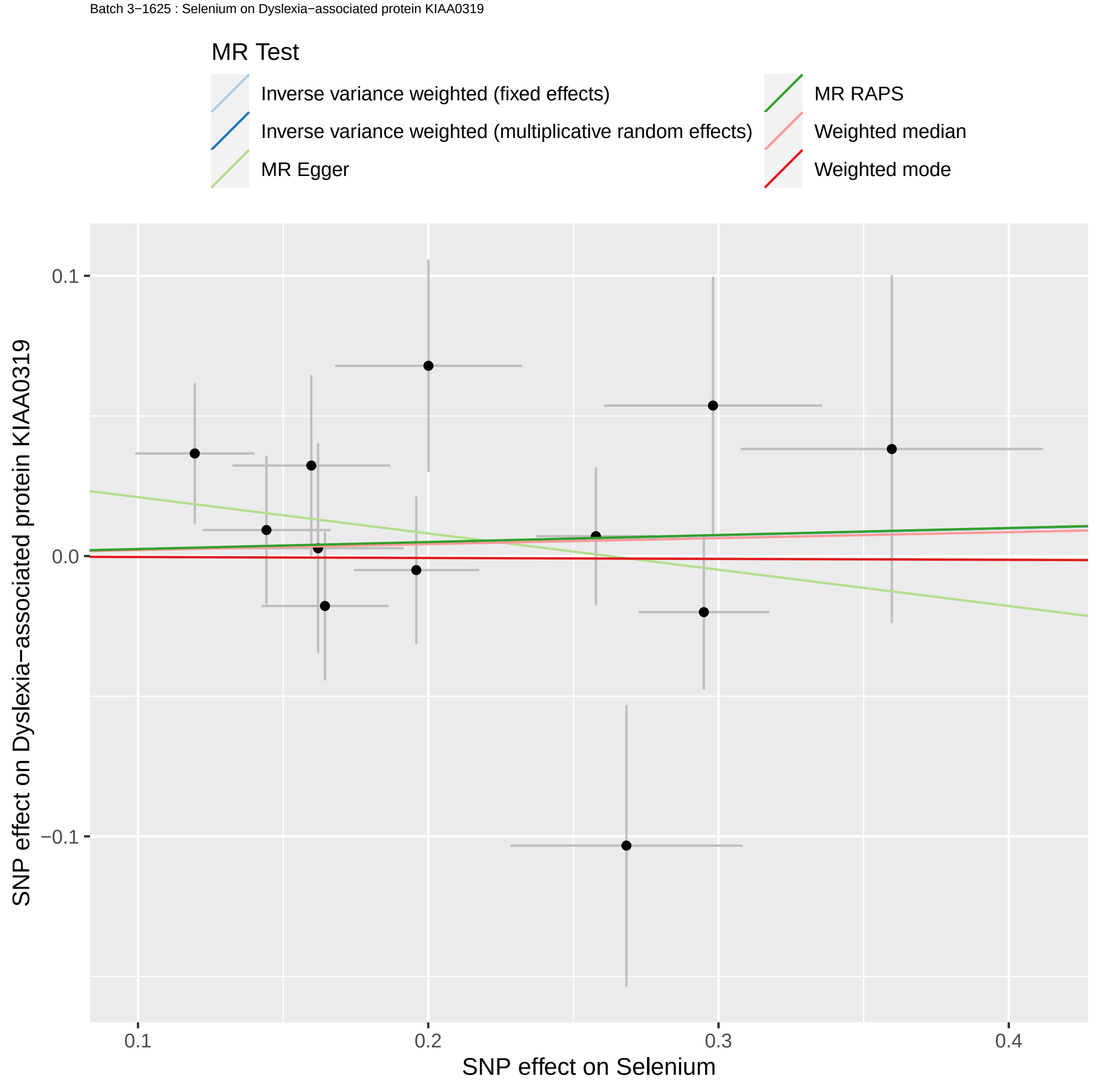

Batch 3−1625 : Selenium on Dyslexia−associated protein KIAA0319
MR Test
Inverse variance weighted (fixed effects)
Inverse variance weighted (multiplicative random effects)
MR Egger
MR RAPS
Weighted median
Weighted mode
0.1
0.0
−0.1
0.1
0.2
0.3
0.4
SNP effect on Selenium
SNP effect on Dyslexia−associated protein KIAA0319

Batch 3−1626 : Selenium on Dyslexia−associated protein KIAA0319−like protein

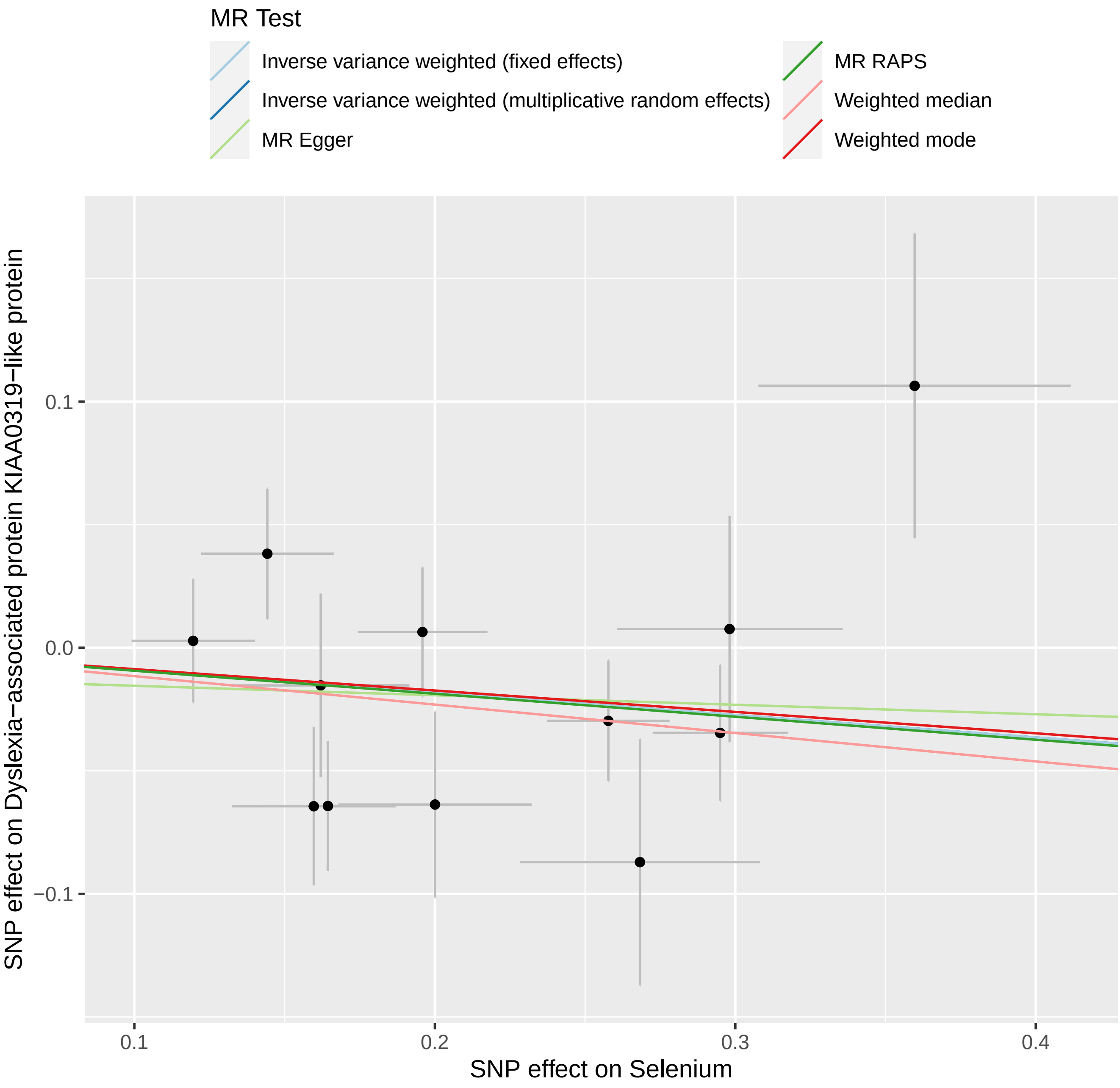

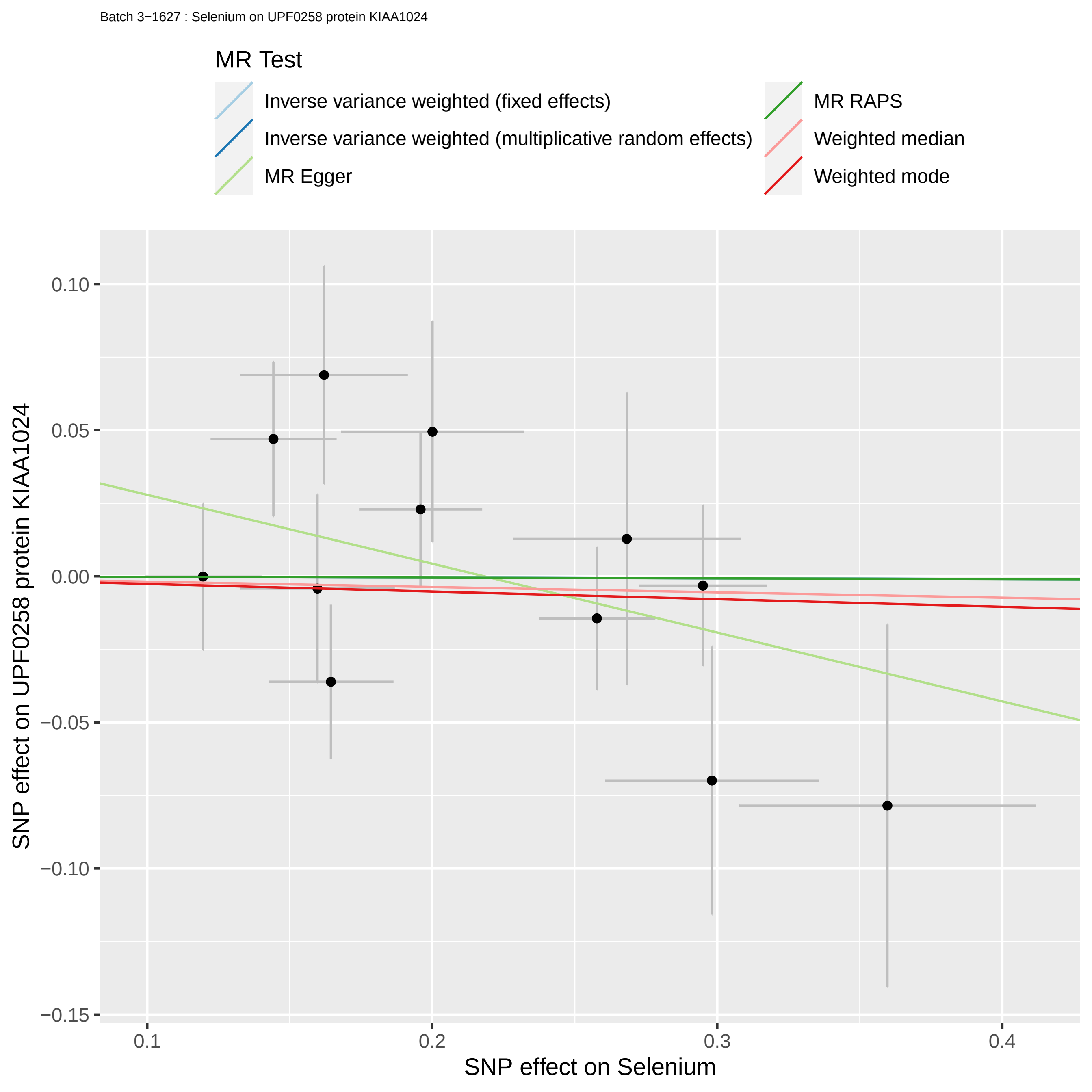

Batch 3−1627 : Selenium on UPF0258 protein KIAA1024
MR Test
Inverse variance weighted (fixed effects)
Inverse variance weighted (multiplicative random effects)
MR Egger
MR RAPS
Weighted median
Weighted mode
0.10
0.05
0.00
−0.05
−0.10
−0.15
0.1
0.2
0.3
0.4
SNP effect on UPF0258 protein KIAA1024
SNP effect on Selenium

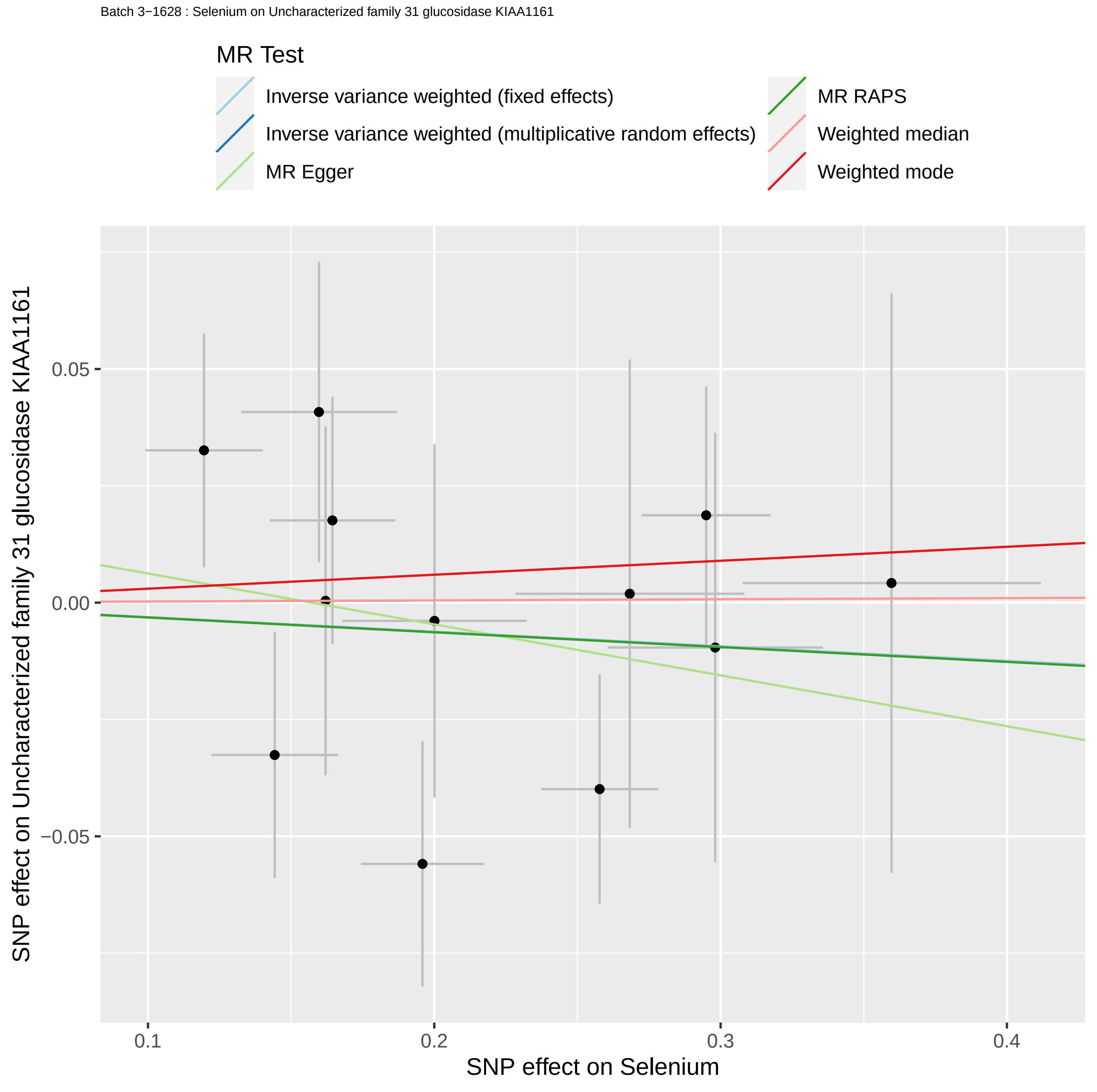
Batch 3−1628 : Selenium on Uncharacterized family 31 glucosidase KIAA1161
MR Test
Inverse variance weighted (fixed effects)
Inverse variance weighted (multiplicative random effects)
MR Egger
MR RAPS
Weighted median
Weighted mode
SNP effect on Uncharacterized family 31 glucosidase KIAA1161
0.05
0.00
−0.05
0.1
0.2
0.3
0.4
SNP effect on Selenium

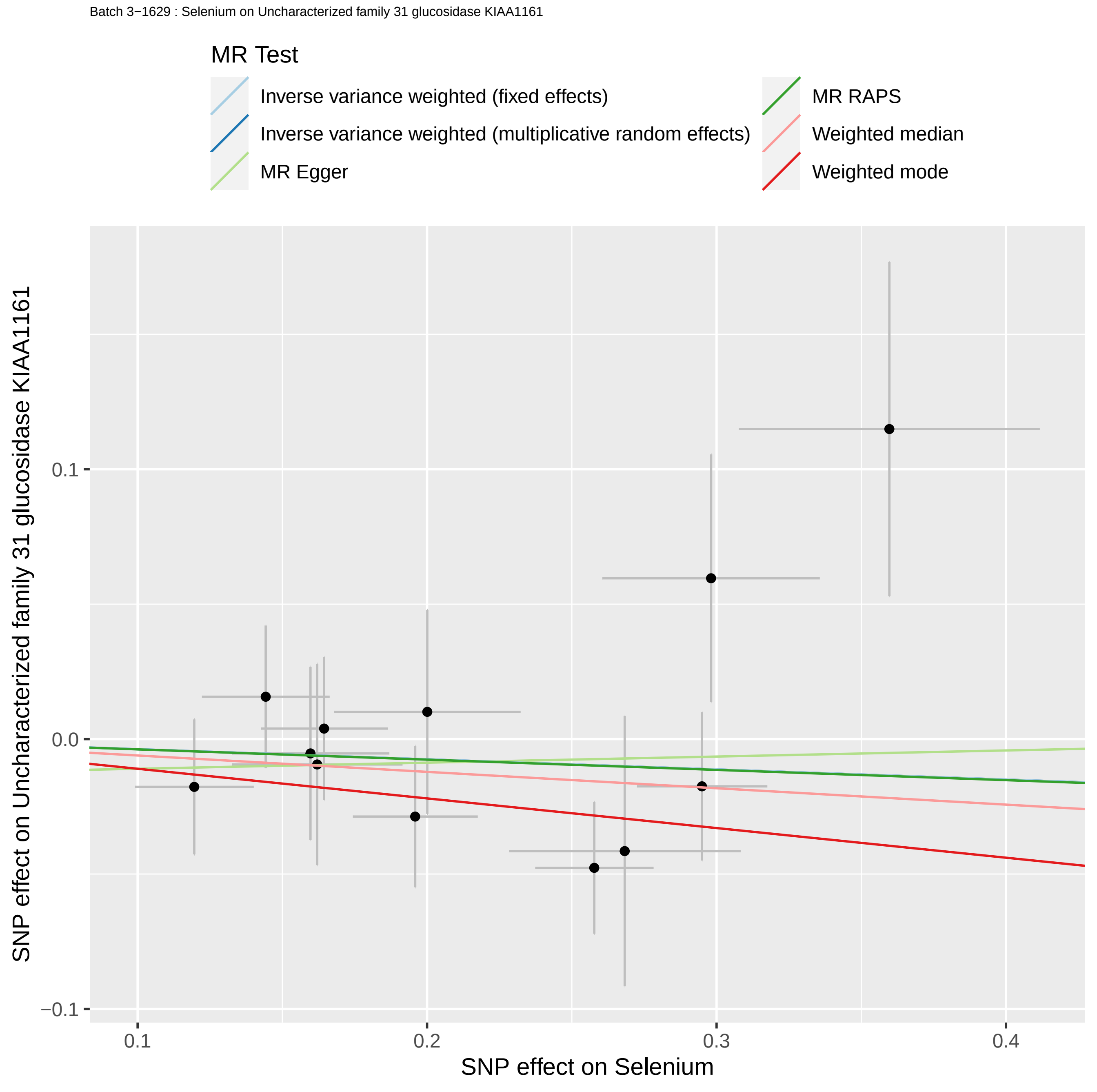
Batch 3−1629 : Selenium on Uncharacterized family 31 glucosidase KIAA1161
MR Test
Inverse variance weighted (fixed effects)
Inverse variance weighted (multiplicative random effects)
MR Egger
MR RAPS
Weighted median
Weighted mode
SNP effect on Uncharacterized family 31 glucosidase KIAA1161
0.1
0.0
−0.1
0.1
0.2
0.3
0.4
SNP effect on Selenium

Batch 3−1630 : Selenium on UPF0577 protein KIAA1324

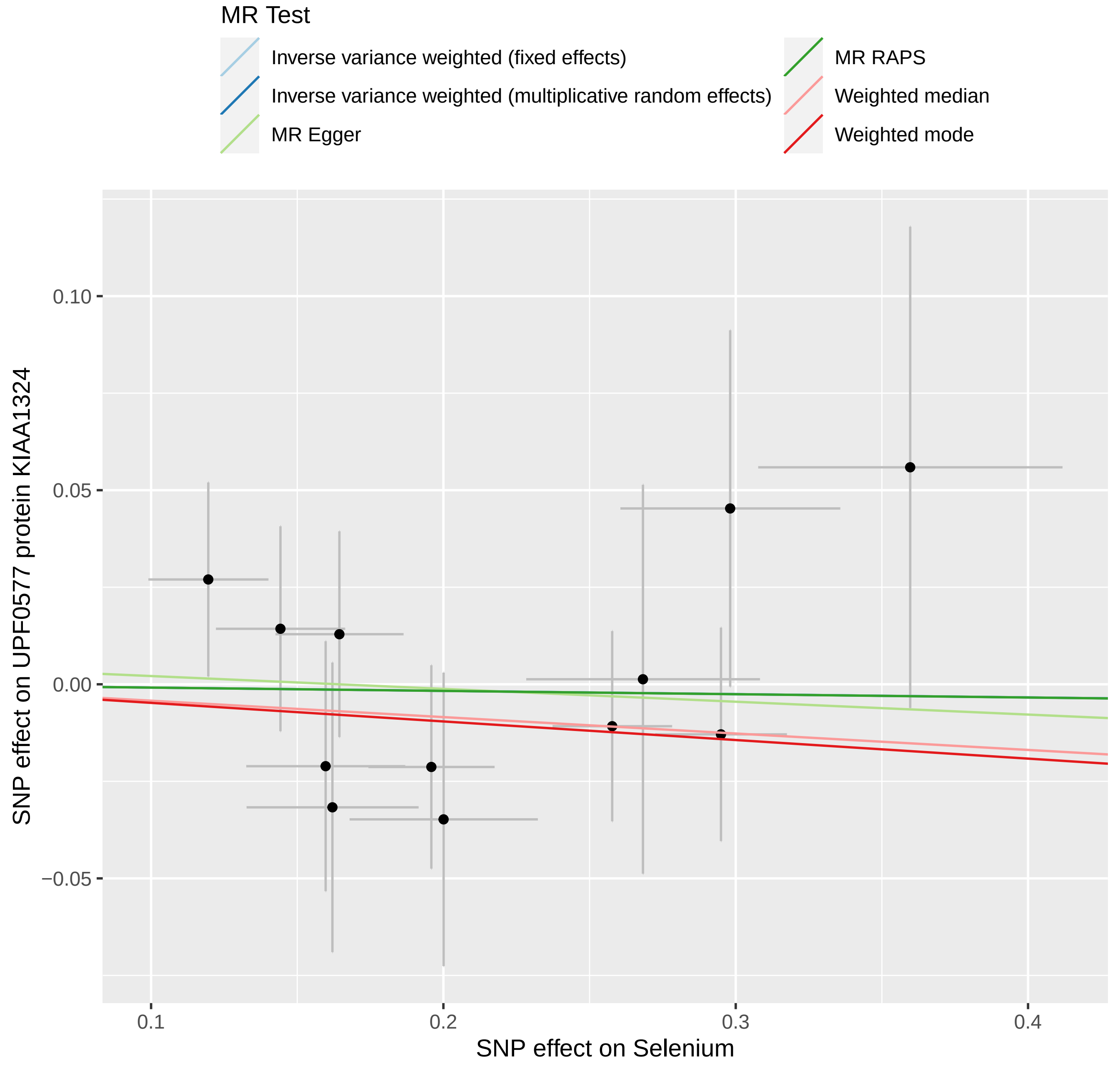

Batch 3−1631 : Selenium on UPF0577 protein KIAA1324

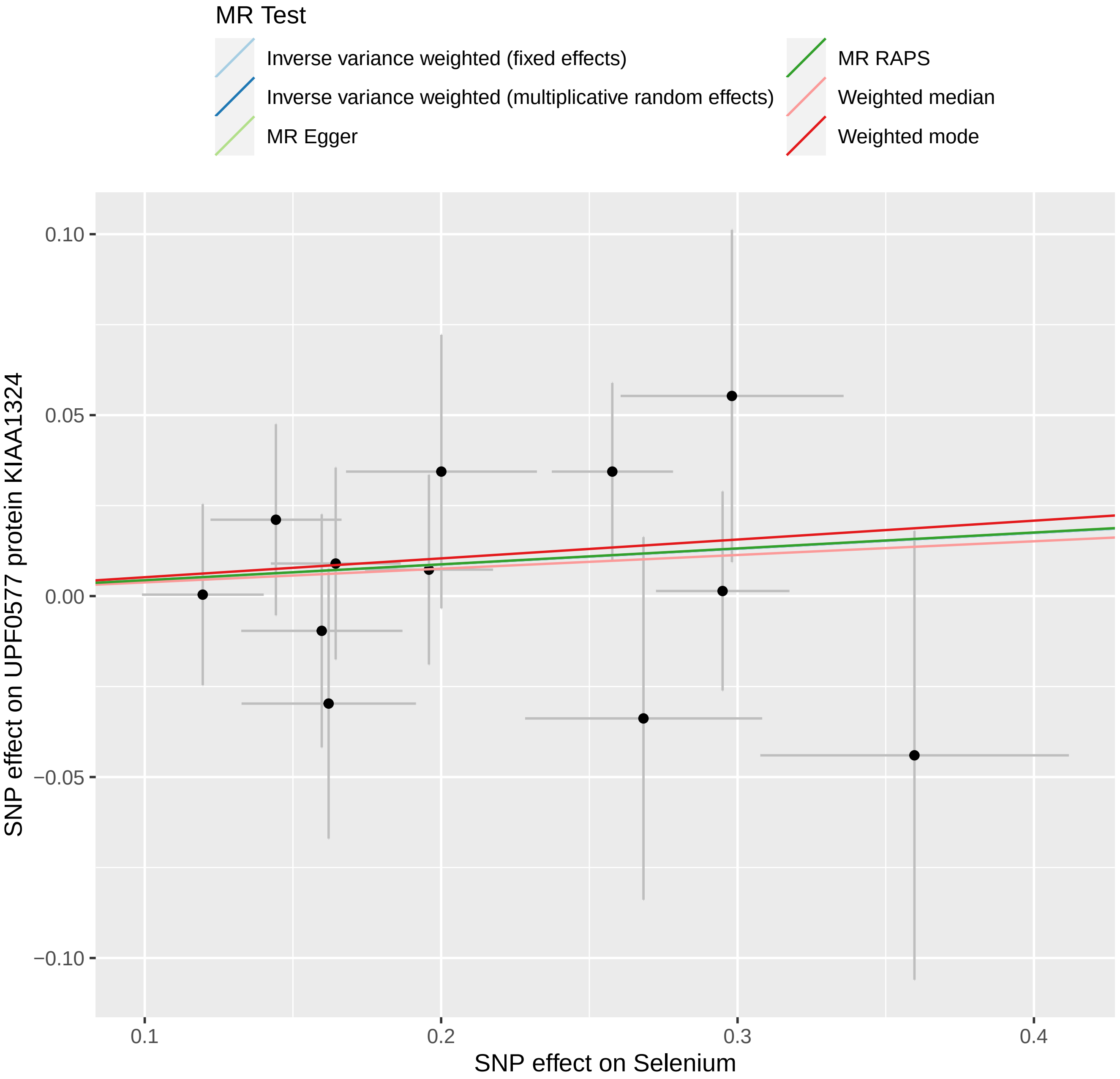

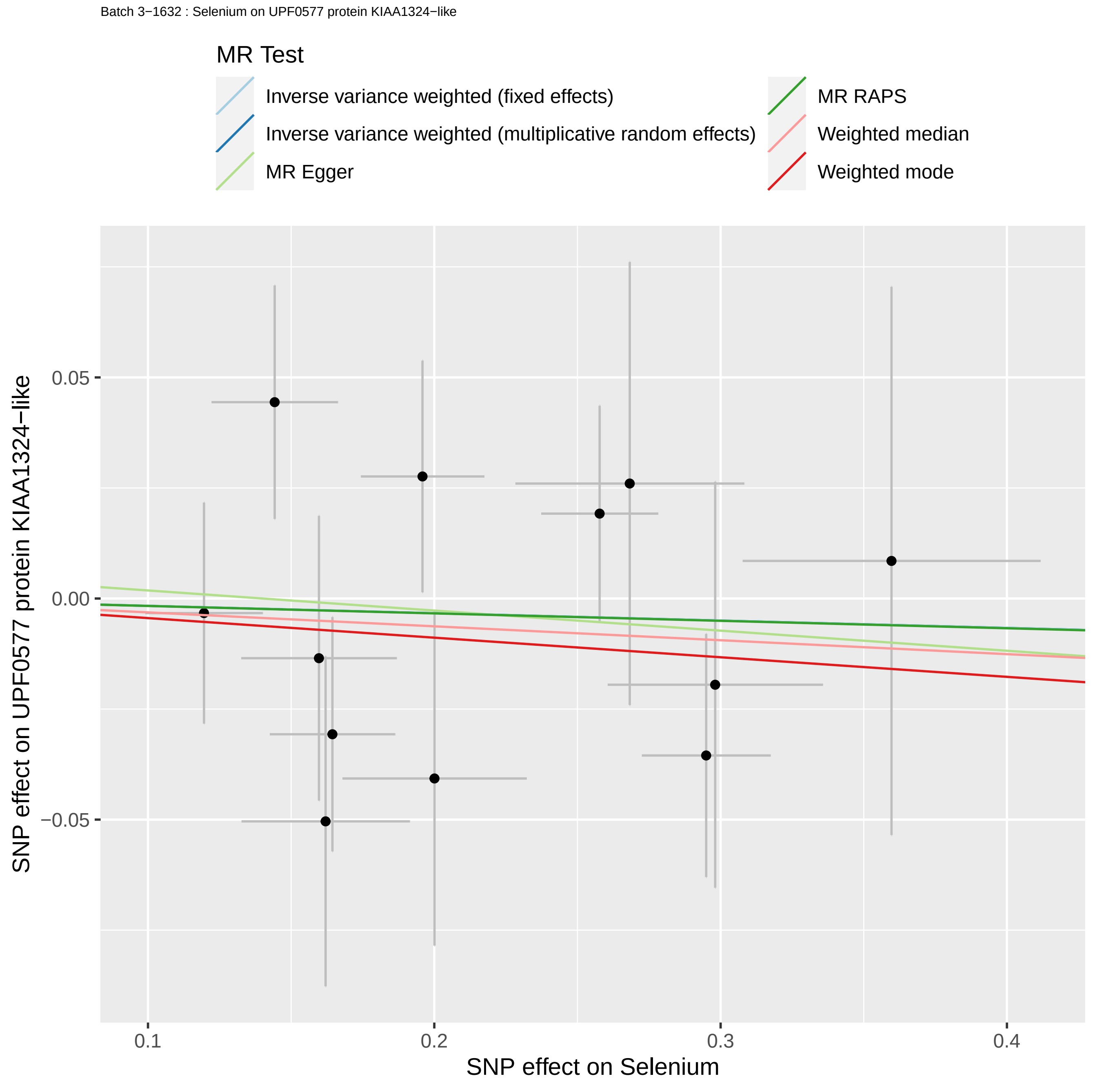
Batch 3−1632 : Selenium on UPF0577 protein KIAA1324−like
MR Test
Inverse variance weighted (fixed effects)
Inverse variance weighted (multiplicative random effects)
MR Egger
MR RAPS
Weighted median
Weighted mode
SNP effect on UPF0577 protein KIAA1324−like
0.05
0.00
−0.05
0.1
0.2
0.3
0.4
SNP effect on Selenium

Batch 3−1633 : Selenium on Uncharacterized protein KIAA1467

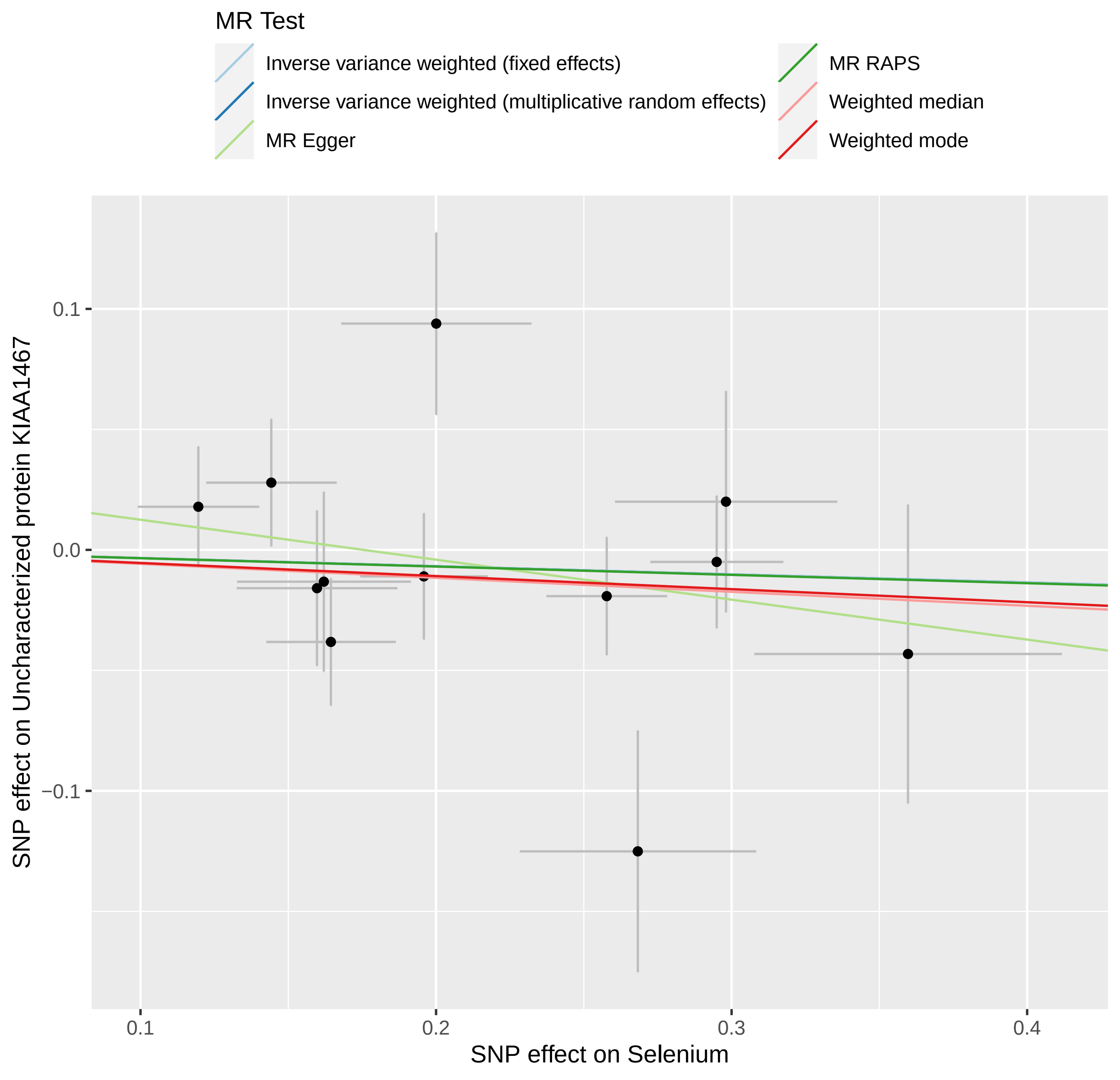

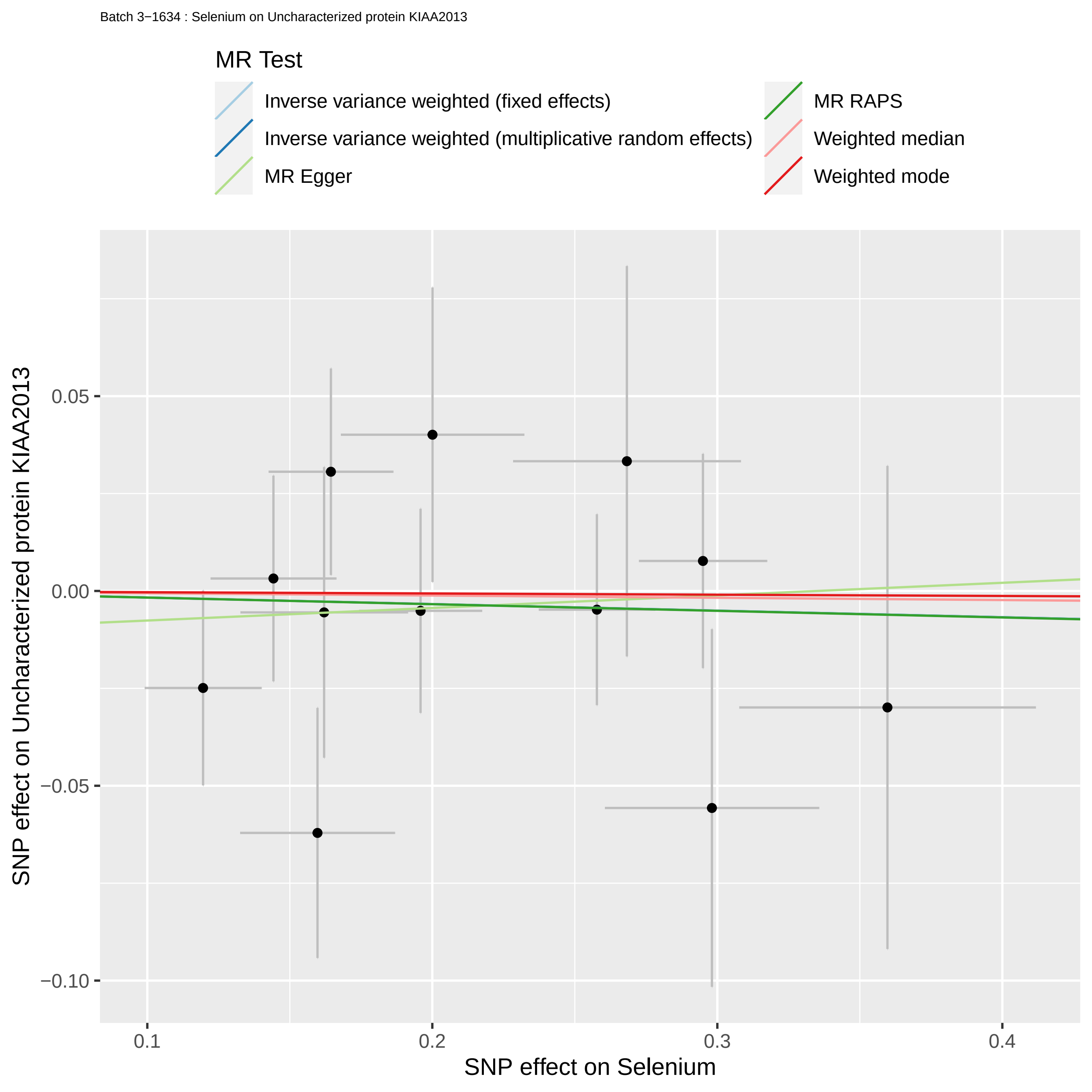
Batch 3−1634 : Selenium on Uncharacterized protein KIAA2013
MR Test
Inverse variance weighted (fixed effects)
Inverse variance weighted (multiplicative random effects)
MR Egger
MR RAPS
Weighted median
Weighted mode
SNP effect on Uncharacterized protein KIAA2013
0.05
0.00
−0.05
−0.10
0.1
0.2
0.3
0.4
SNP effect on Selenium

Batch 3−1635 : Selenium on Kinesin−like protein KIF16B

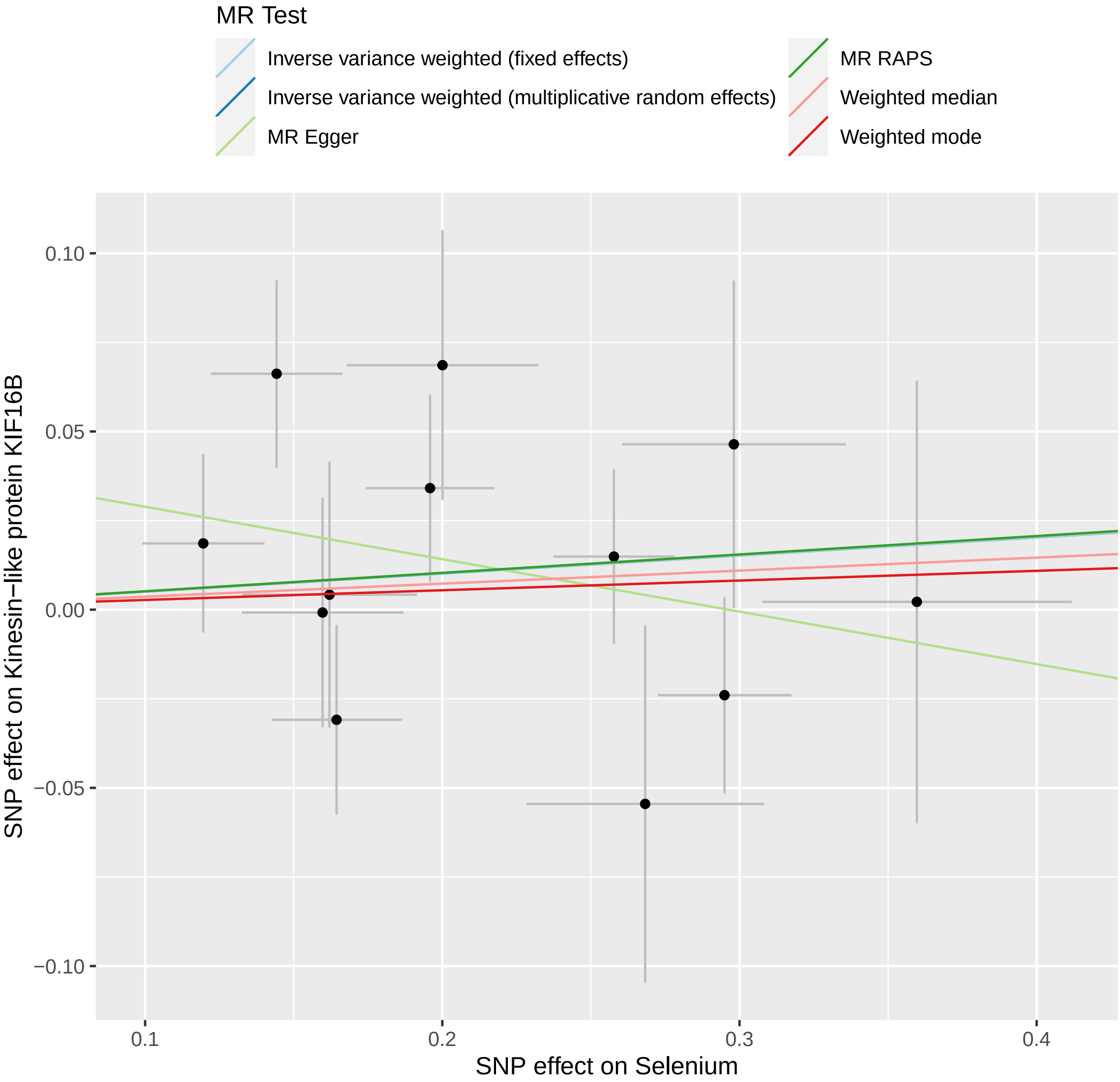

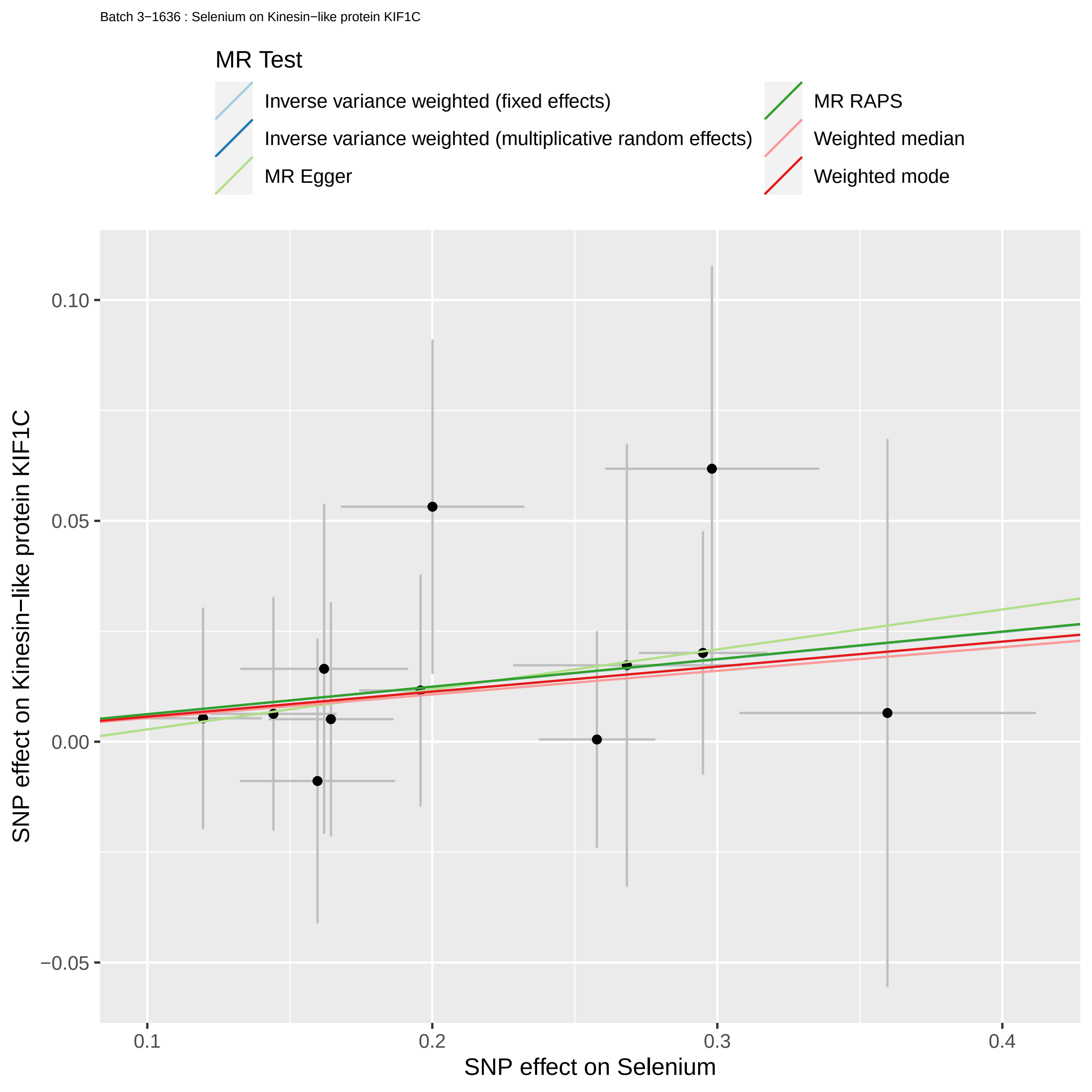

Batch 3−1636 : Selenium on Kinesin−like protein KIF1C
MR Test
Inverse variance weighted (fixed effects)
Inverse variance weighted (multiplicative random effects)
MR Egger
MR RAPS
Weighted median
Weighted mode
SNP effect on Kinesin−like protein KIF1C
0.10
0.05
0.00
−0.05
0.1
0.2
0.3
0.4
SNP effect on Selenium

Batch 3−1637 : Selenium on Kinesin−like protein KIF23

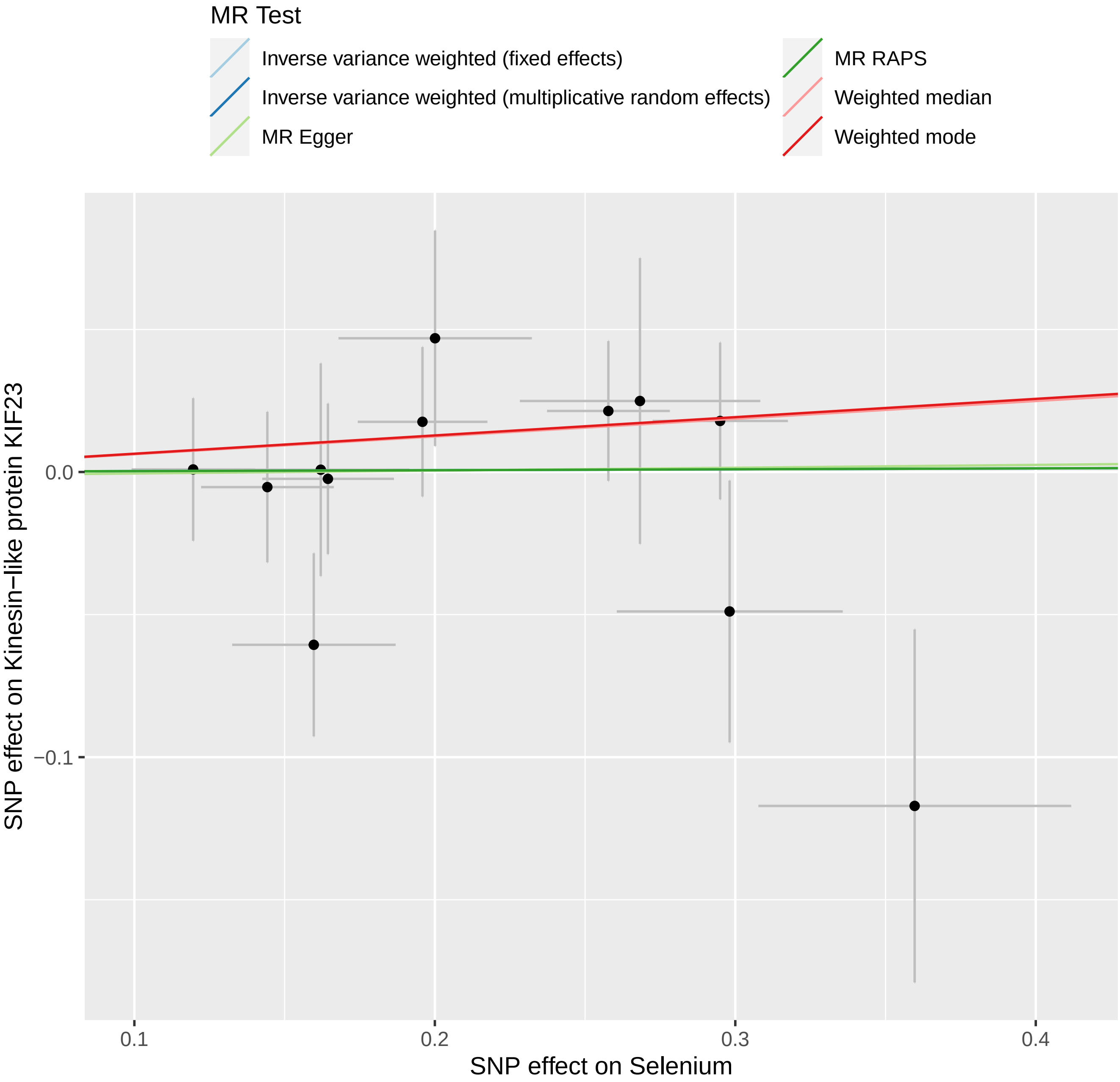

Batch 3−1638 : Selenium on DNA/RNA−binding protein KIN17

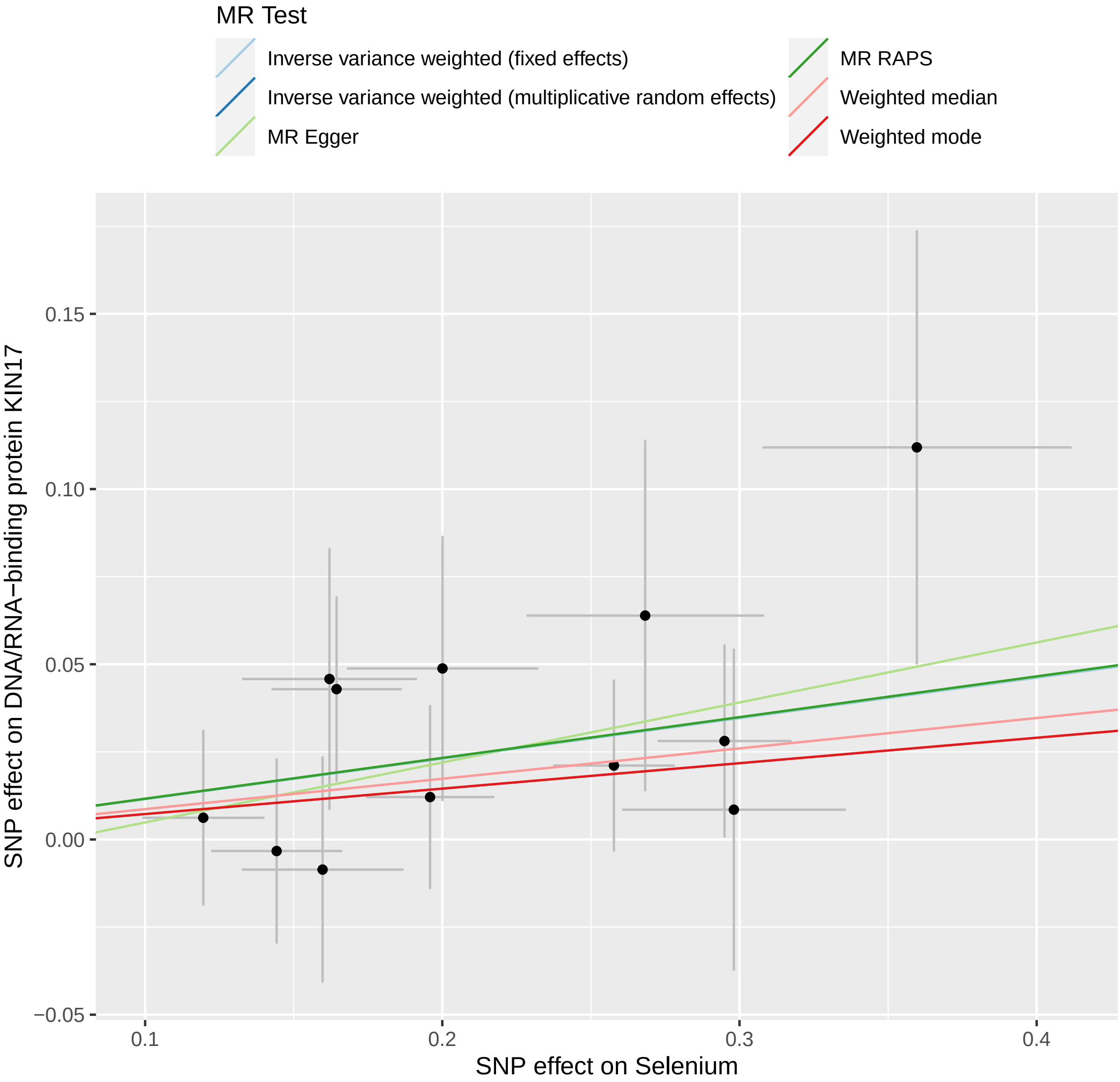

Batch 3−1639 : Selenium on DNA/RNA−binding protein KIN17

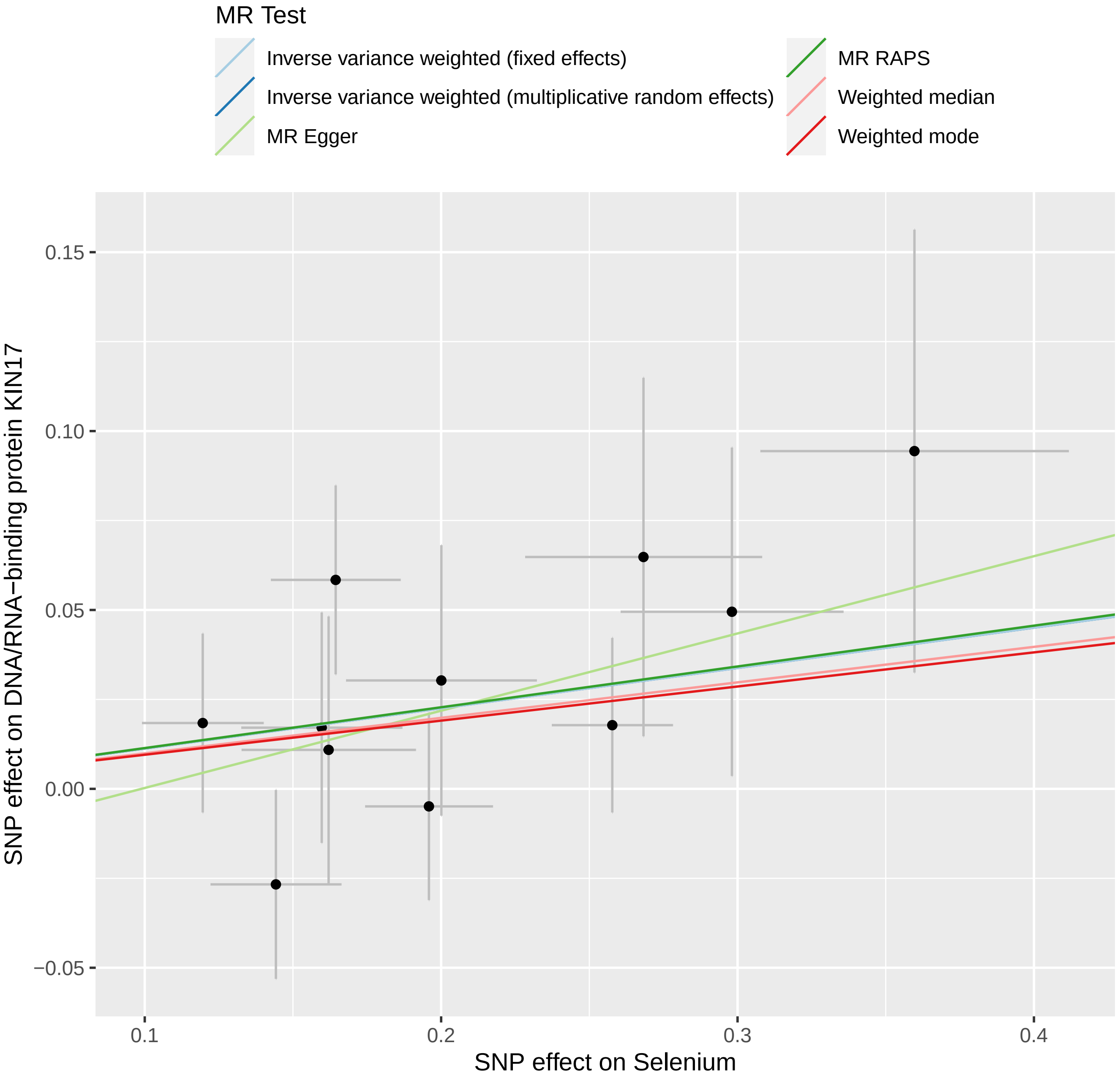

Batch 3−1640 : Selenium on Killer cell immunoglobulin−like receptor 2DL2

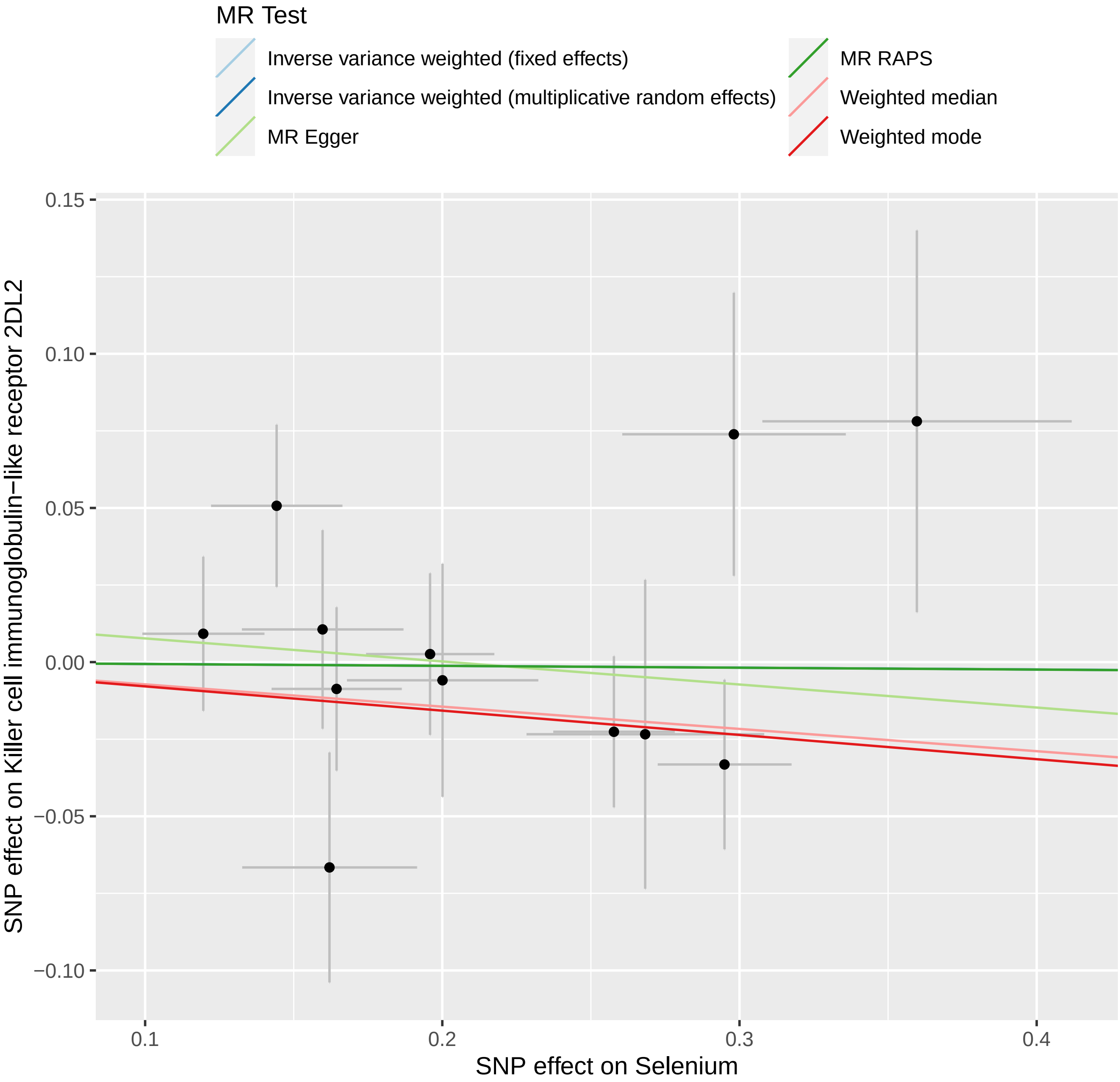

Batch 3−1641 : Selenium on Killer cell immunoglobulin−like receptor 2DL4

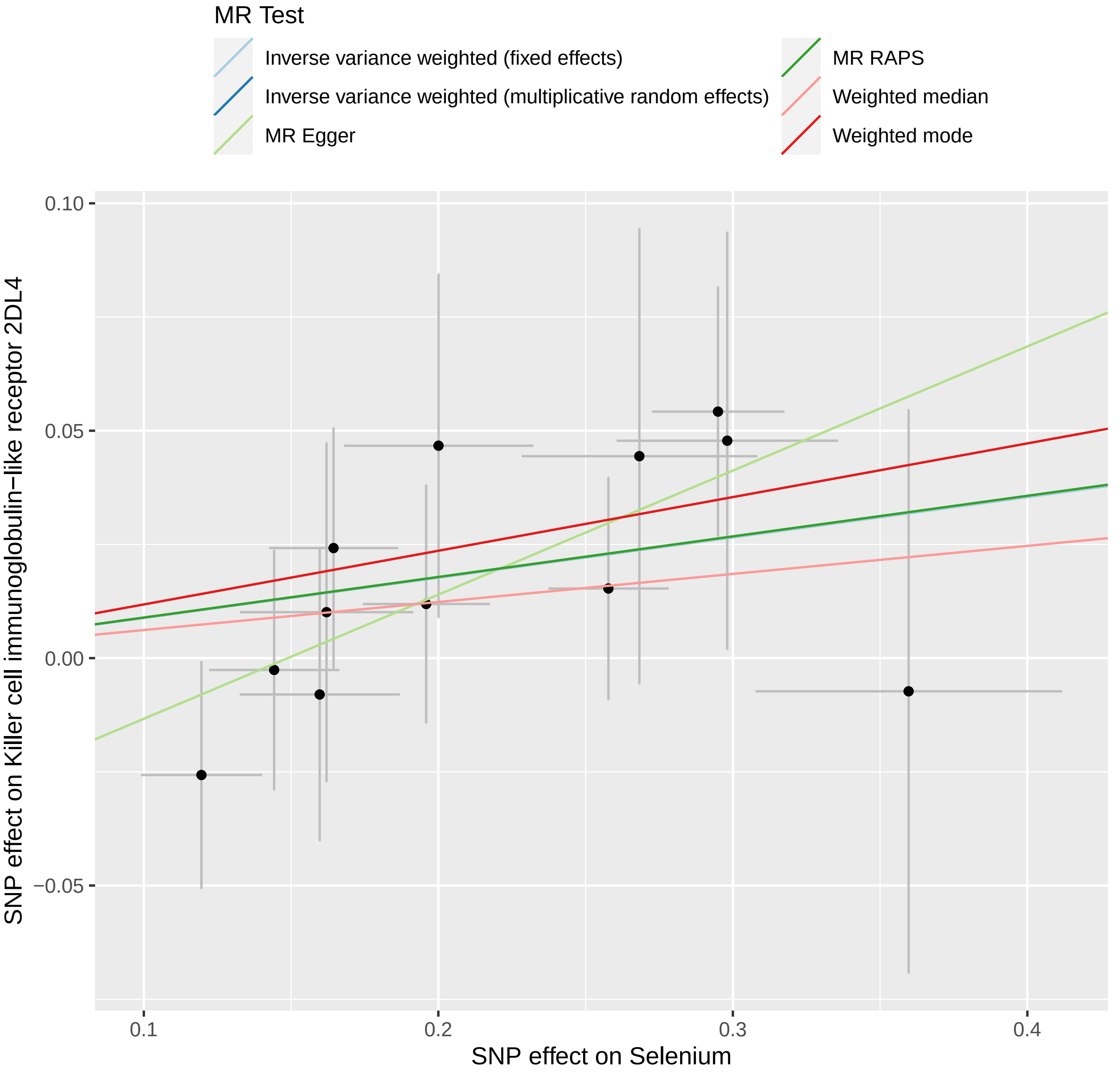

Batch 3−1642 : Selenium on Killer cell immunoglobulin−like receptor 2DL4

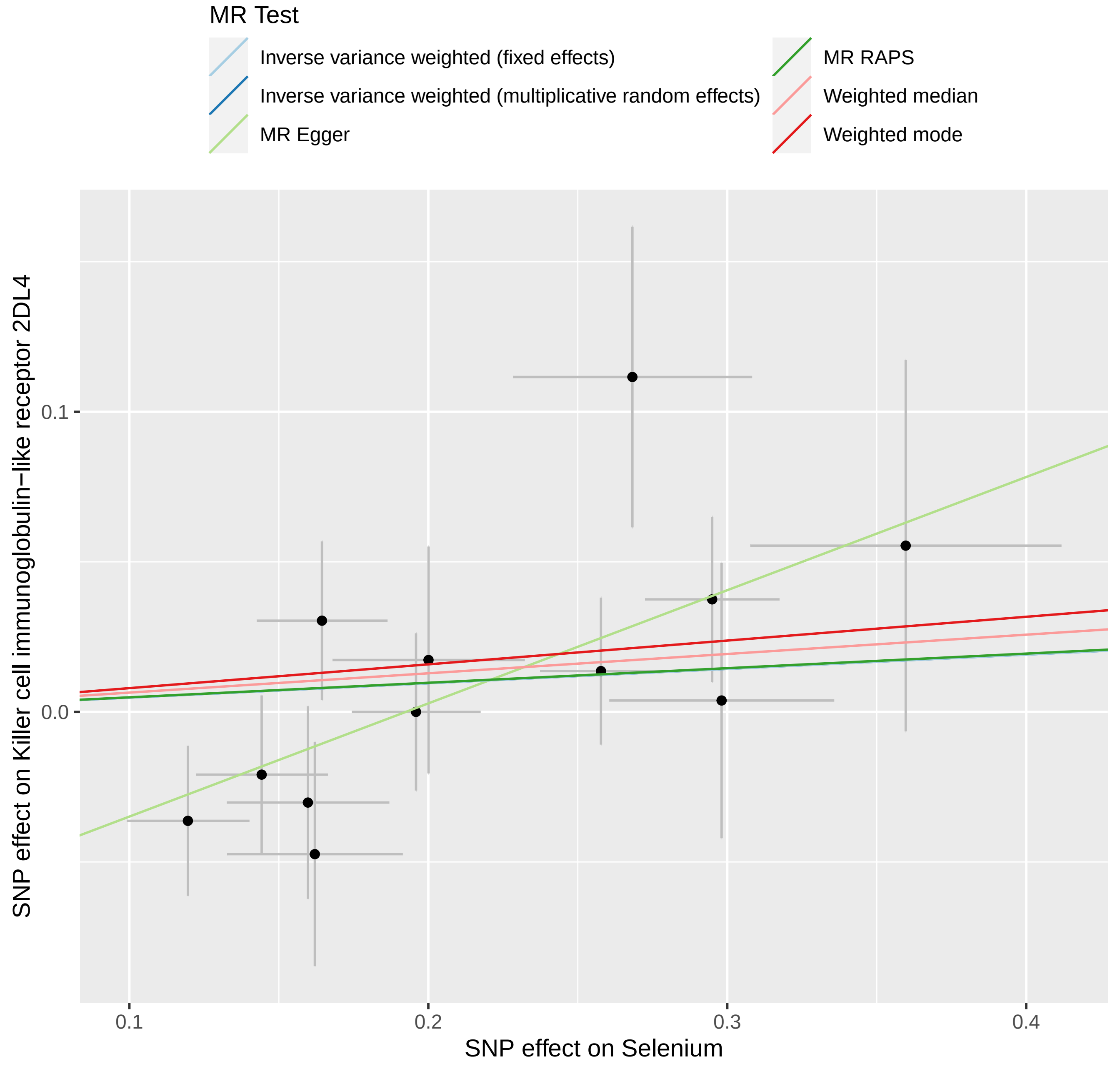

Batch 3−1643 : Selenium on Killer cell immunoglobulin−like receptor 2DL5A

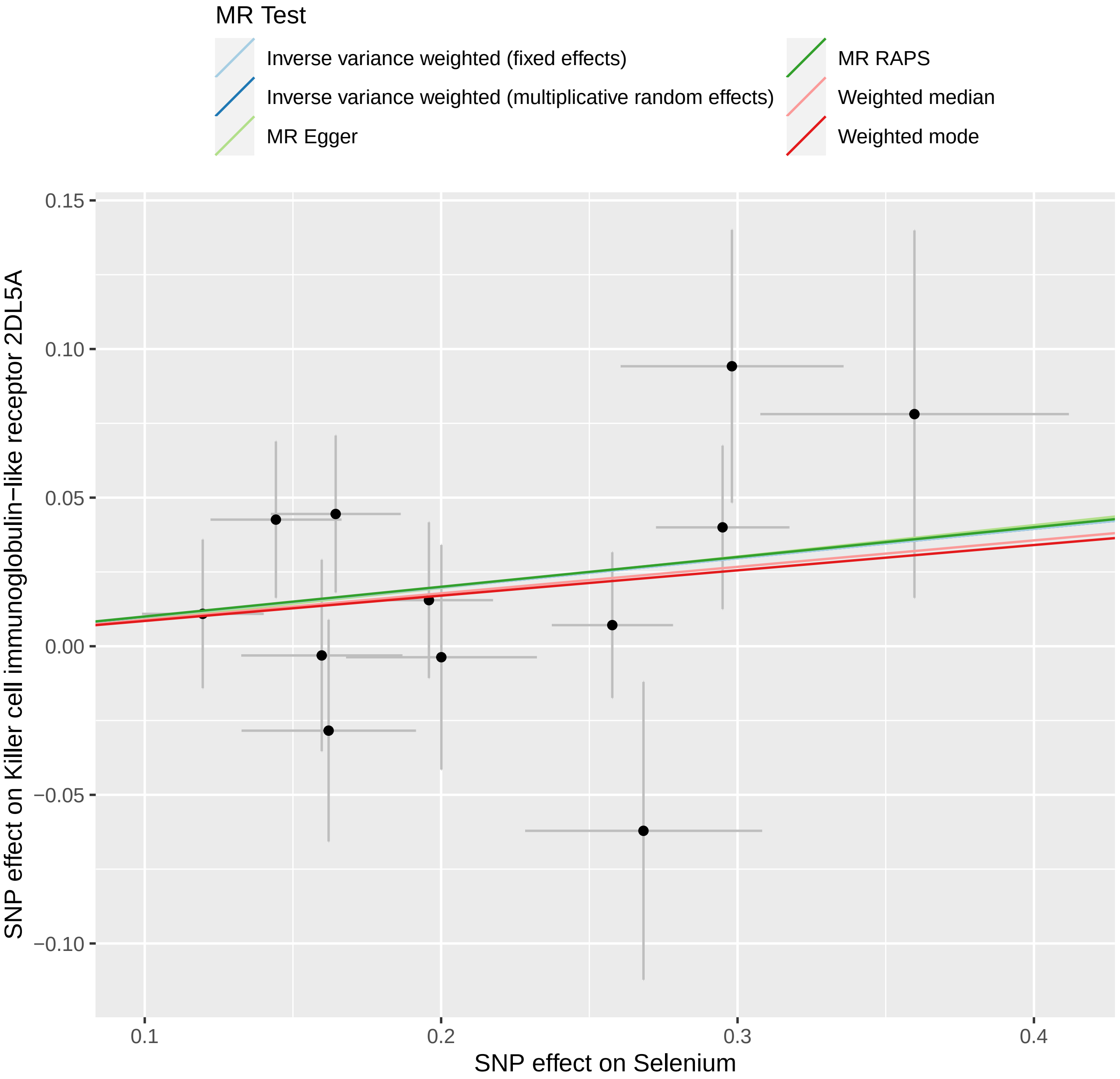

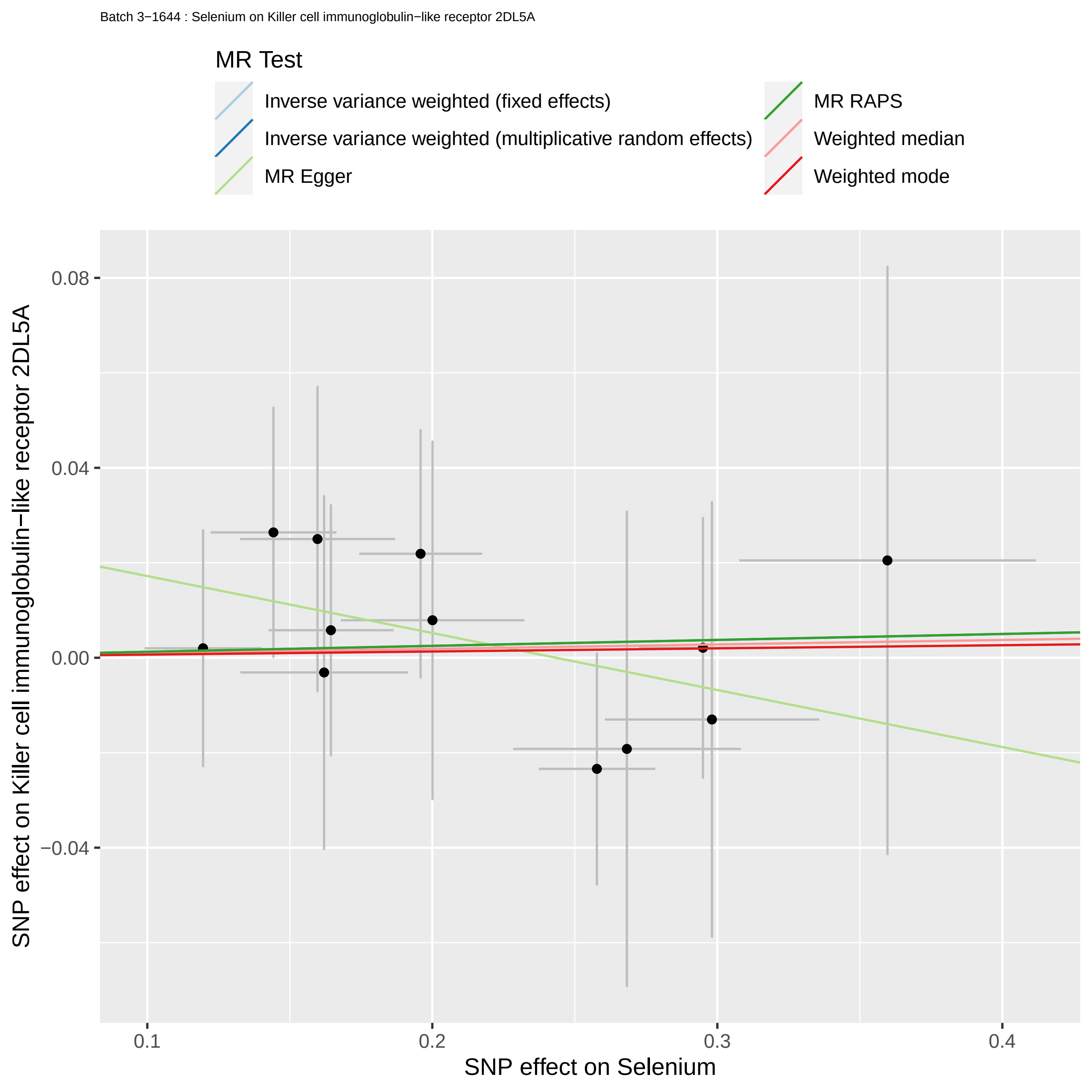

Batch 3−1644 : Selenium on Killer cell immunoglobulin−like receptor 2DL5A
MR Test
Inverse variance weighted (fixed effects)
Inverse variance weighted (multiplicative random effects)
MR Egger
MR RAPS
Weighted median
Weighted mode
0.08
0.04
0.00
−0.04
0.1
0.2
0.3
0.4
SNP effect on Selenium
SNP effect on Killer cell immunoglobulin−like receptor 2DL5A

Batch 3−1645 : Selenium on Killer cell immunoglobulin−like receptor 2DS2

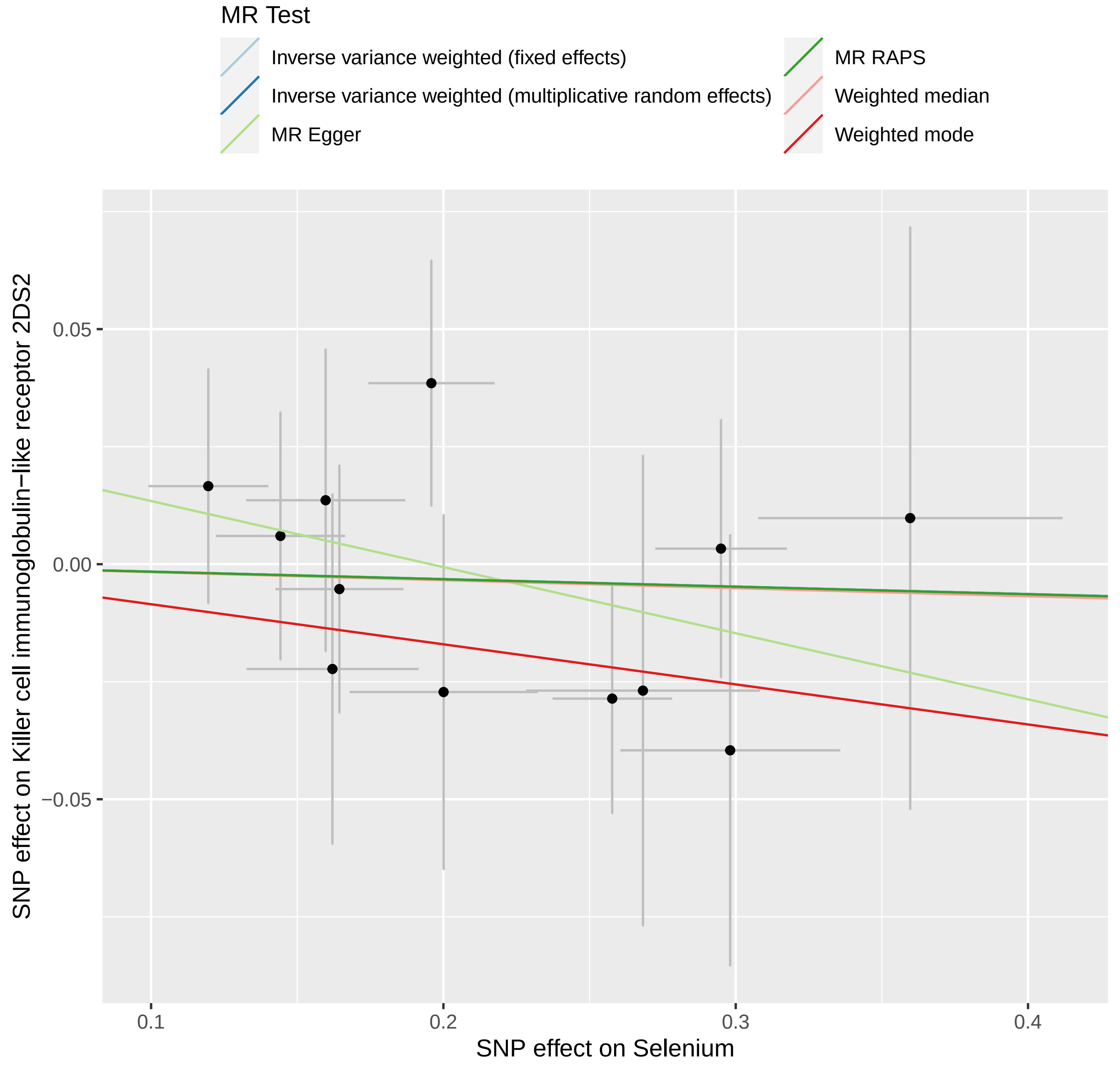

Batch 3−1646 : Selenium on Killer cell immunoglobulin−like receptor 3DL2

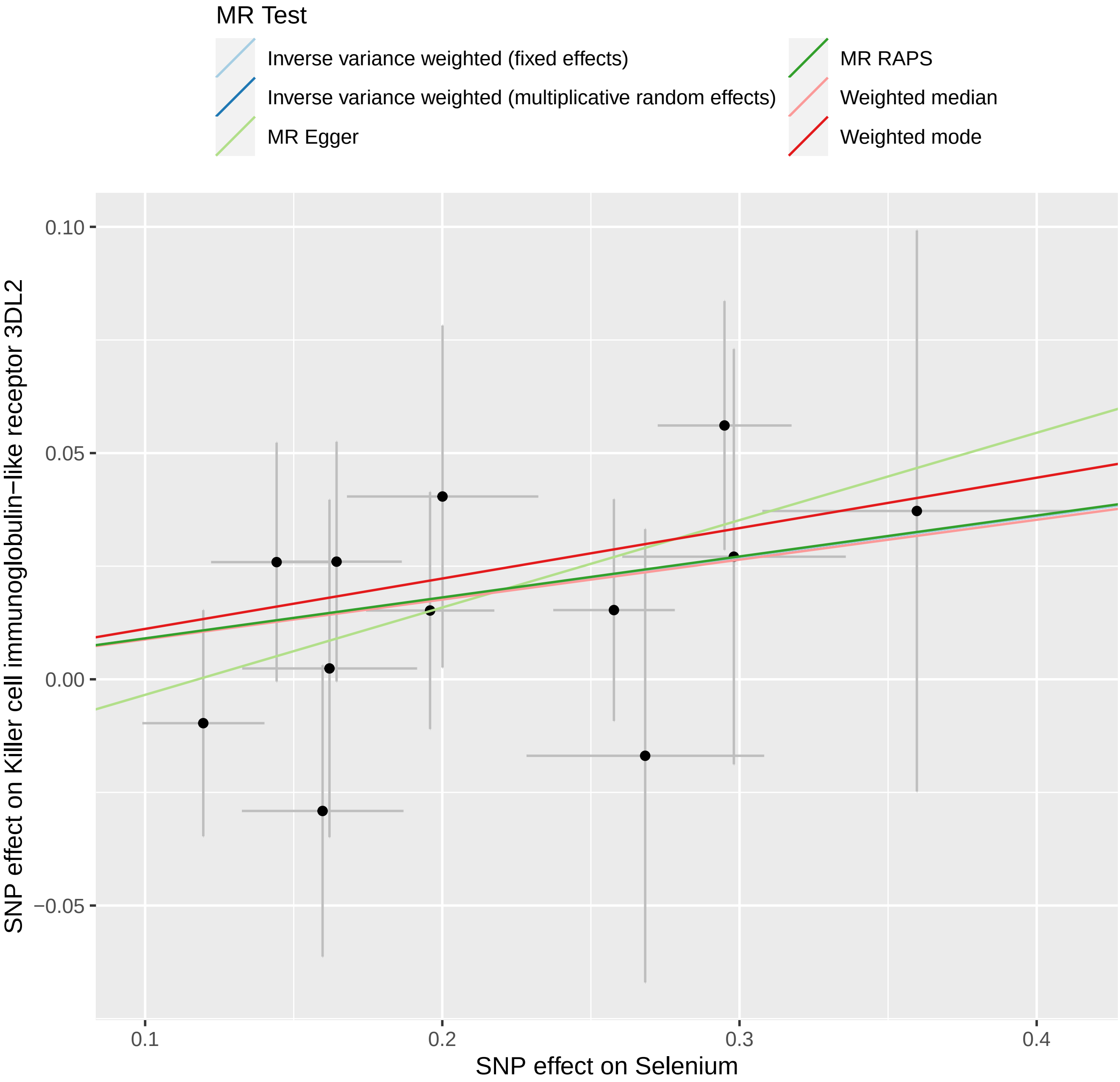

Batch 3−1647 : Selenium on Killer cell immunoglobulin−like receptor 3DL3

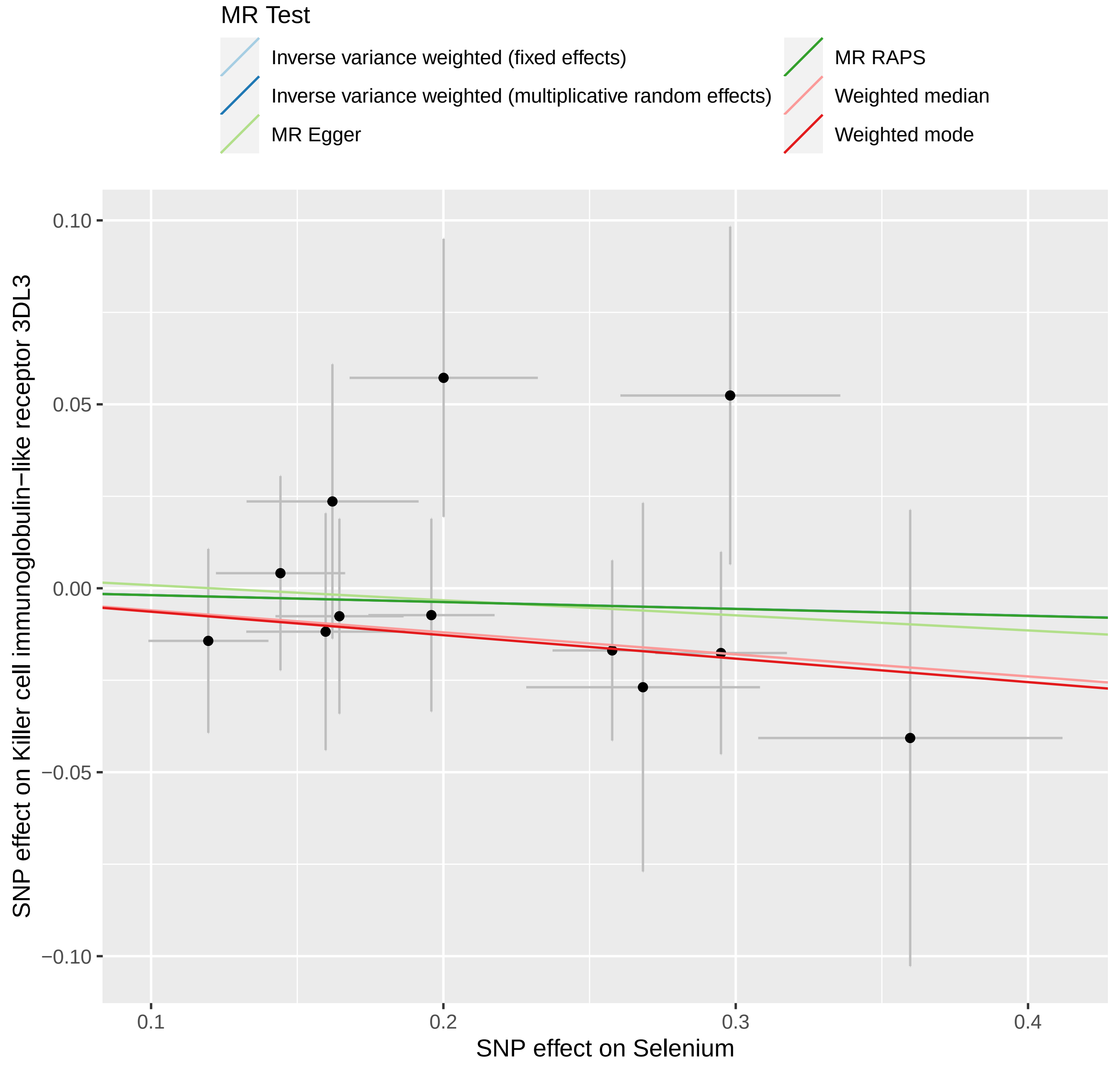

Batch 3−1648 : Selenium on Killer cell immunoglobulin−like receptor 3DS1

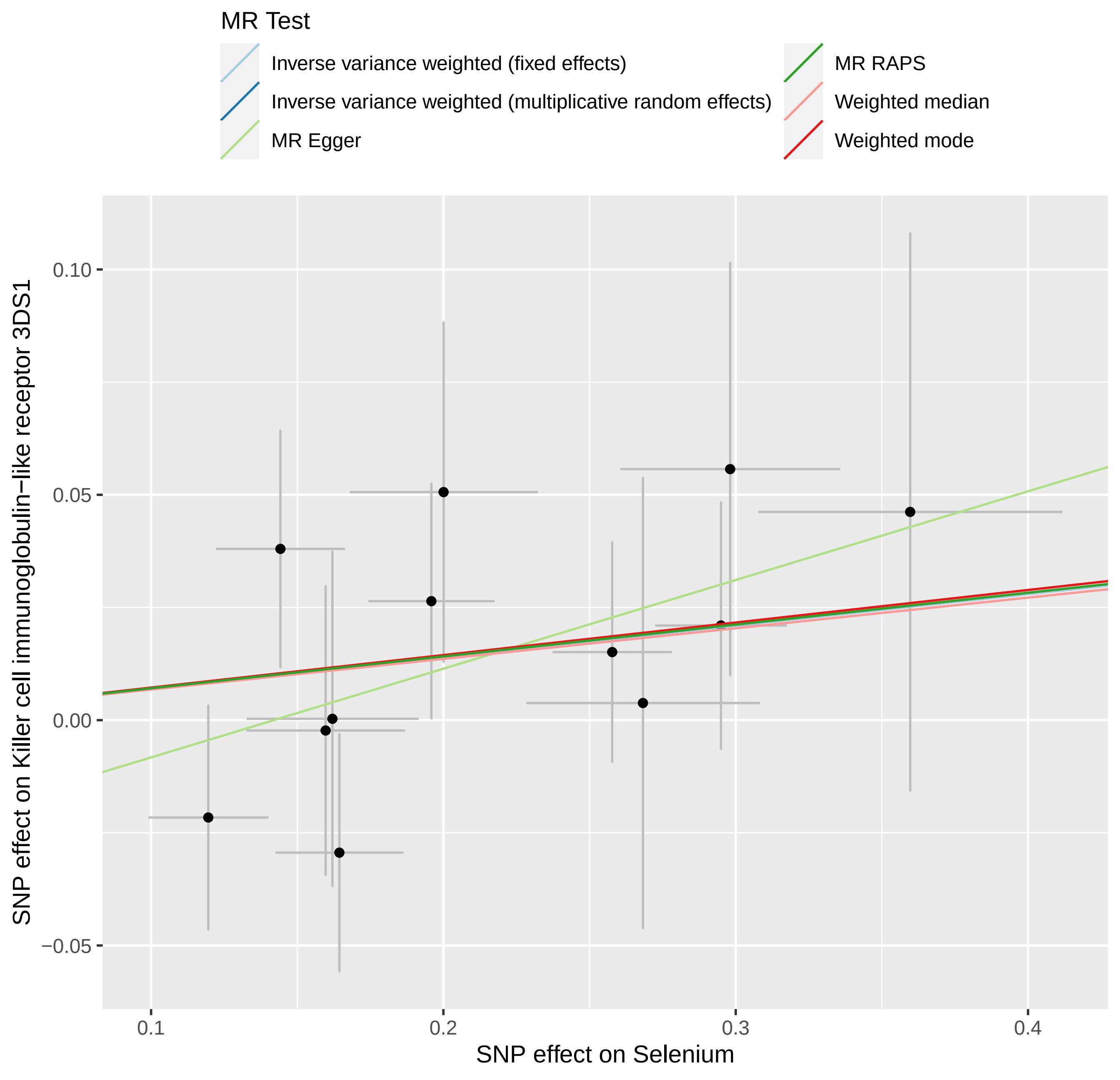

Batch 3−1649 : Selenium on Kin of IRRE−like protein 2

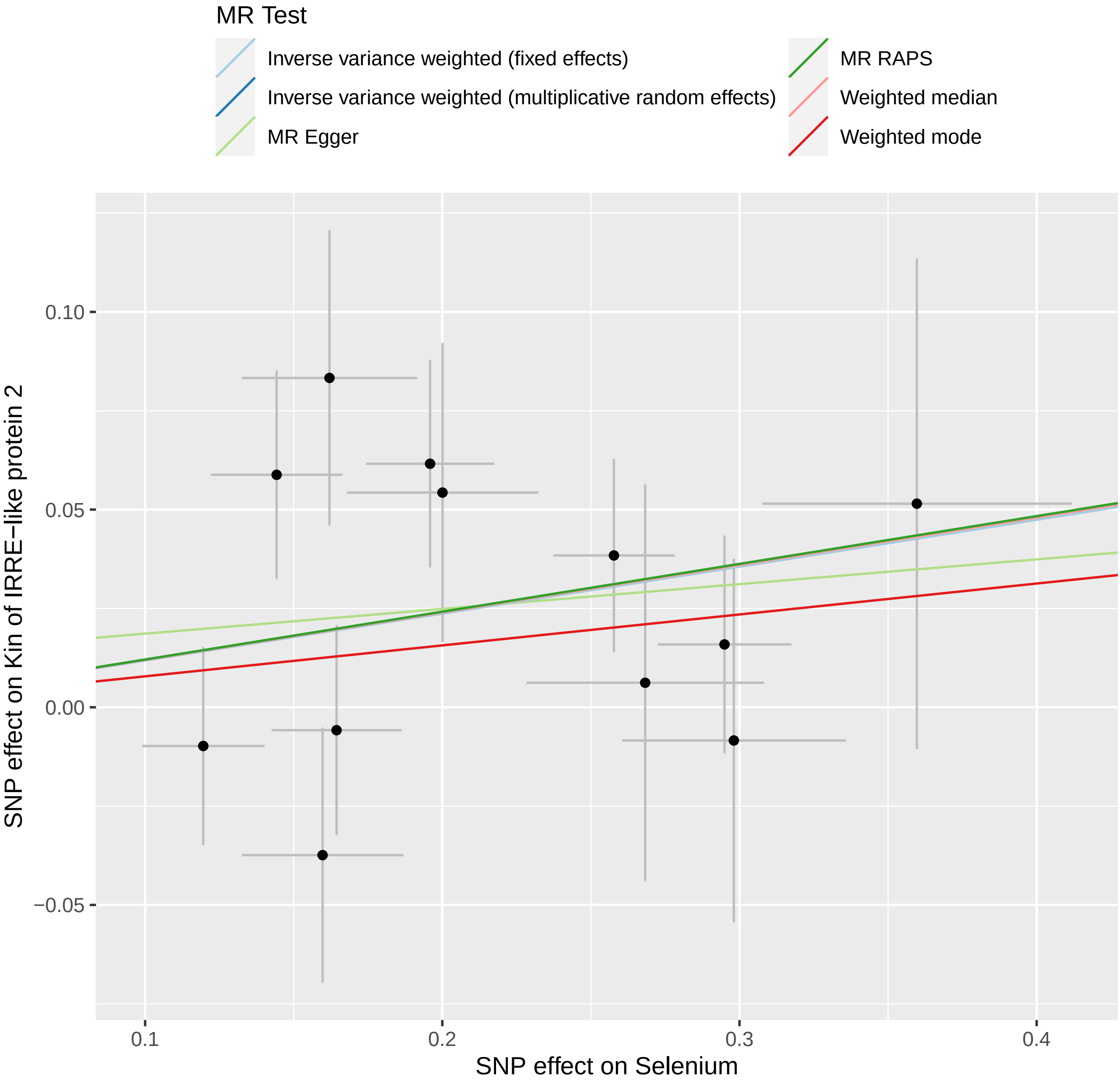

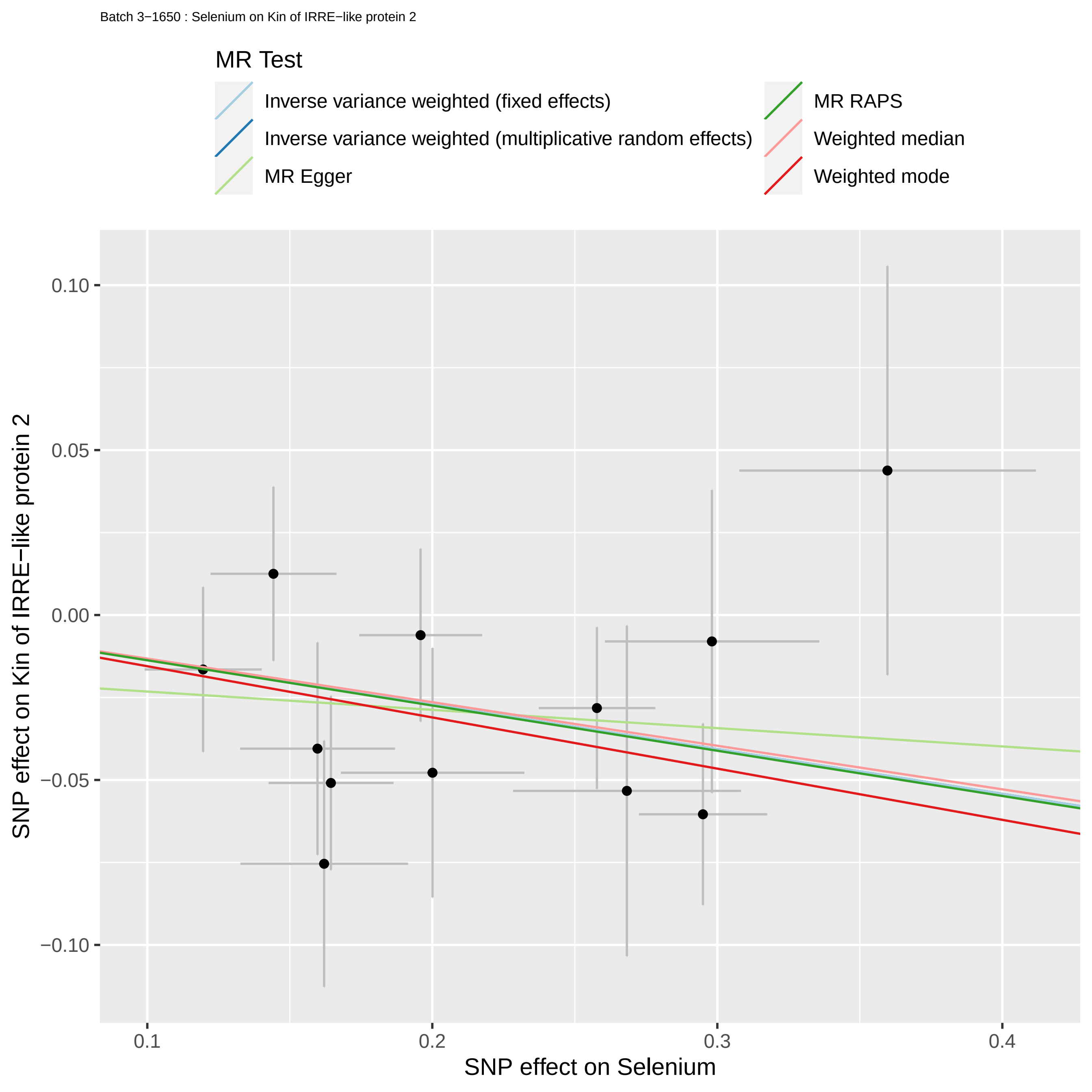

Batch 3−1650 : Selenium on Kin of IRRE−like protein 2
MR Test
Inverse variance weighted (fixed effects)
Inverse variance weighted (multiplicative random effects)
MR Egger
MR RAPS
Weighted median
Weighted mode
0.10
0.05
0.00
−0.05
−0.10
0.1
0.2
0.3
0.4
SNP effect on Kin of IRRE−like protein 2
SNP effect on Selenium

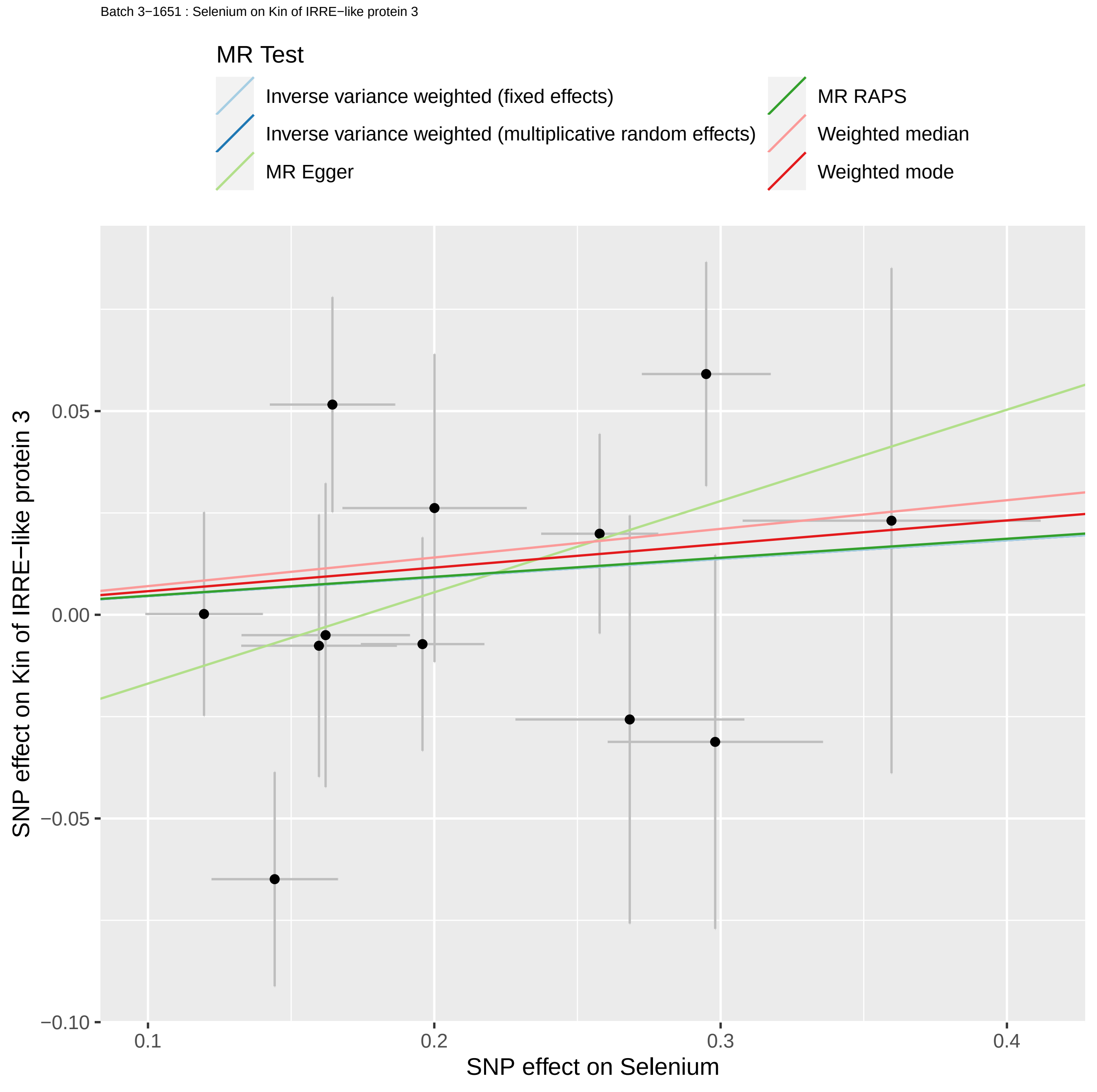

Batch 3−1651 : Selenium on Kin of IRRE−like protein 3
MR Test
Inverse variance weighted (fixed effects)
Inverse variance weighted (multiplicative random effects)
MR Egger
MR RAPS
Weighted median
Weighted mode
0.05
0.00
−0.05
−0.10
0.1
0.2
0.3
0.4
SNP effect on Kin of IRRE−like protein 3
SNP effect on Selenium

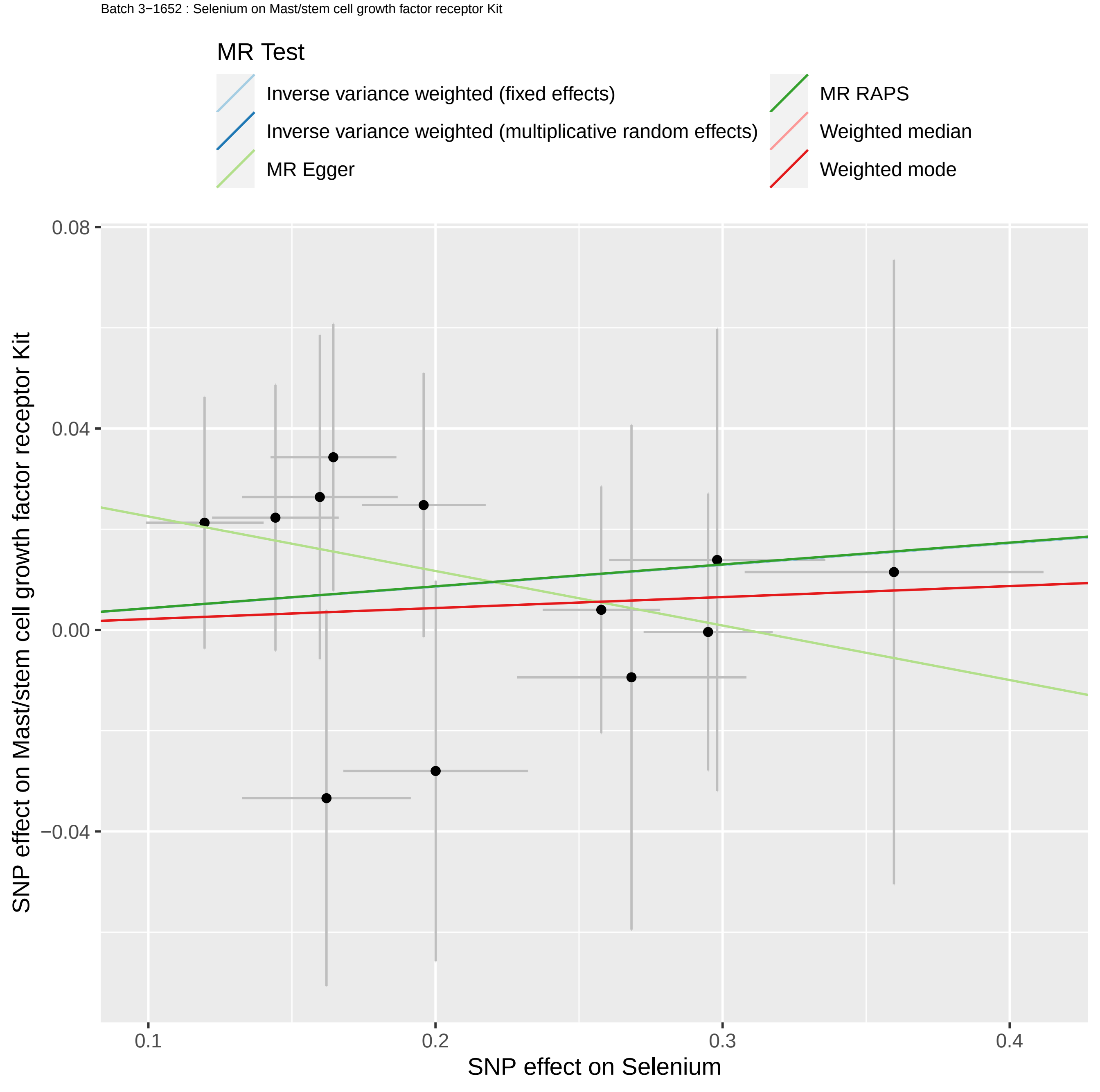

Batch 3−1652 : Selenium on Mast/stem cell growth factor receptor Kit
MR Test
Inverse variance weighted (fixed effects)
Inverse variance weighted (multiplicative random effects)
MR Egger
MR RAPS
Weighted median
Weighted mode
0.08
0.04
0.00
−0.04
0.1
0.2
0.3
0.4
SNP effect on Mast/stem cell growth factor receptor Kit
SNP effect on Selenium

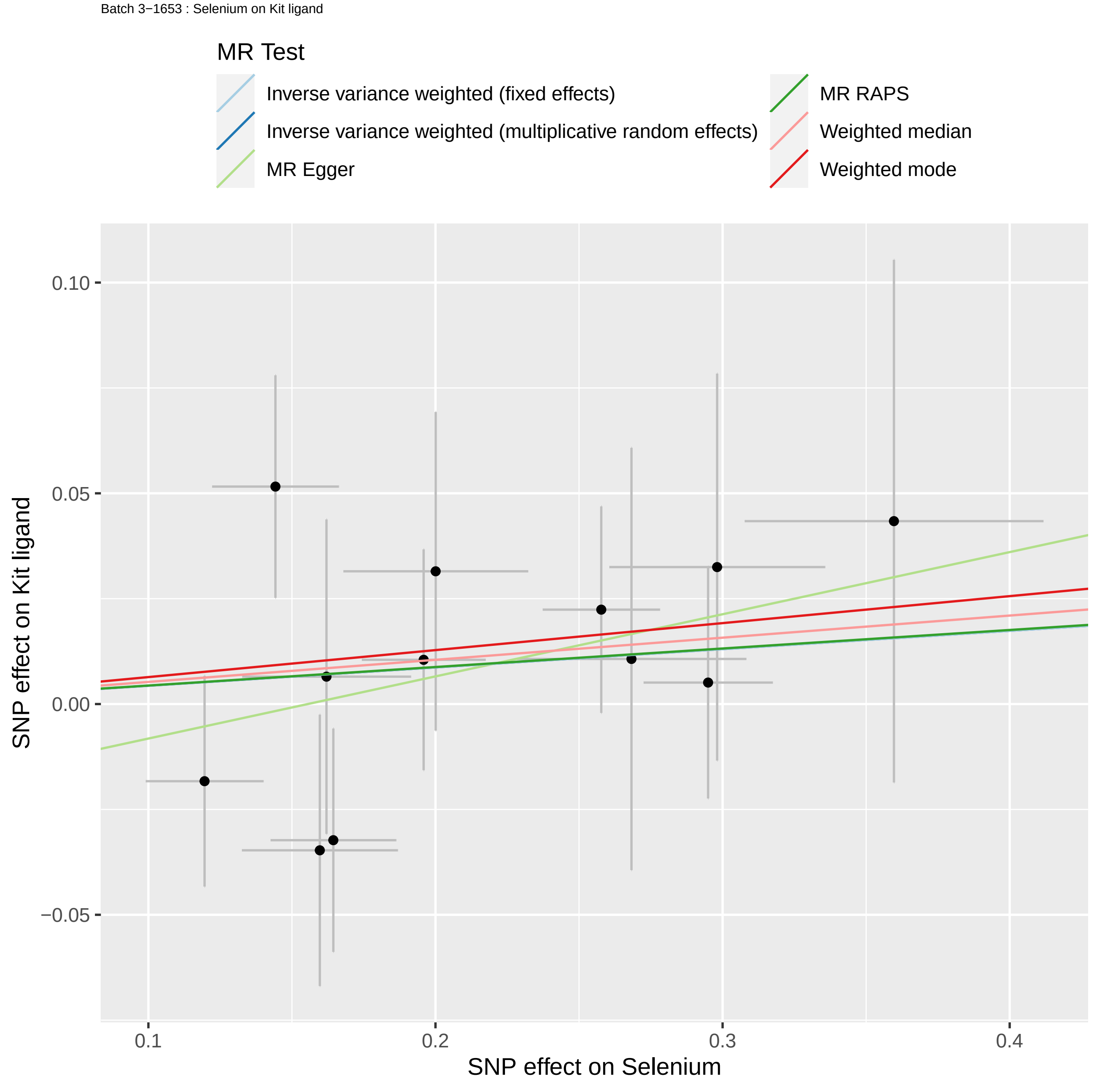

Batch 3−1653 : Selenium on Kit ligand
MR Test
Inverse variance weighted (fixed effects)
Inverse variance weighted (multiplicative random effects)
MR Egger
MR RAPS
Weighted median
Weighted mode
0.10
0.05
0.00
−0.05
0.1
0.2
0.3
0.4
SNP effect on Kit ligand
SNP effect on Selenium

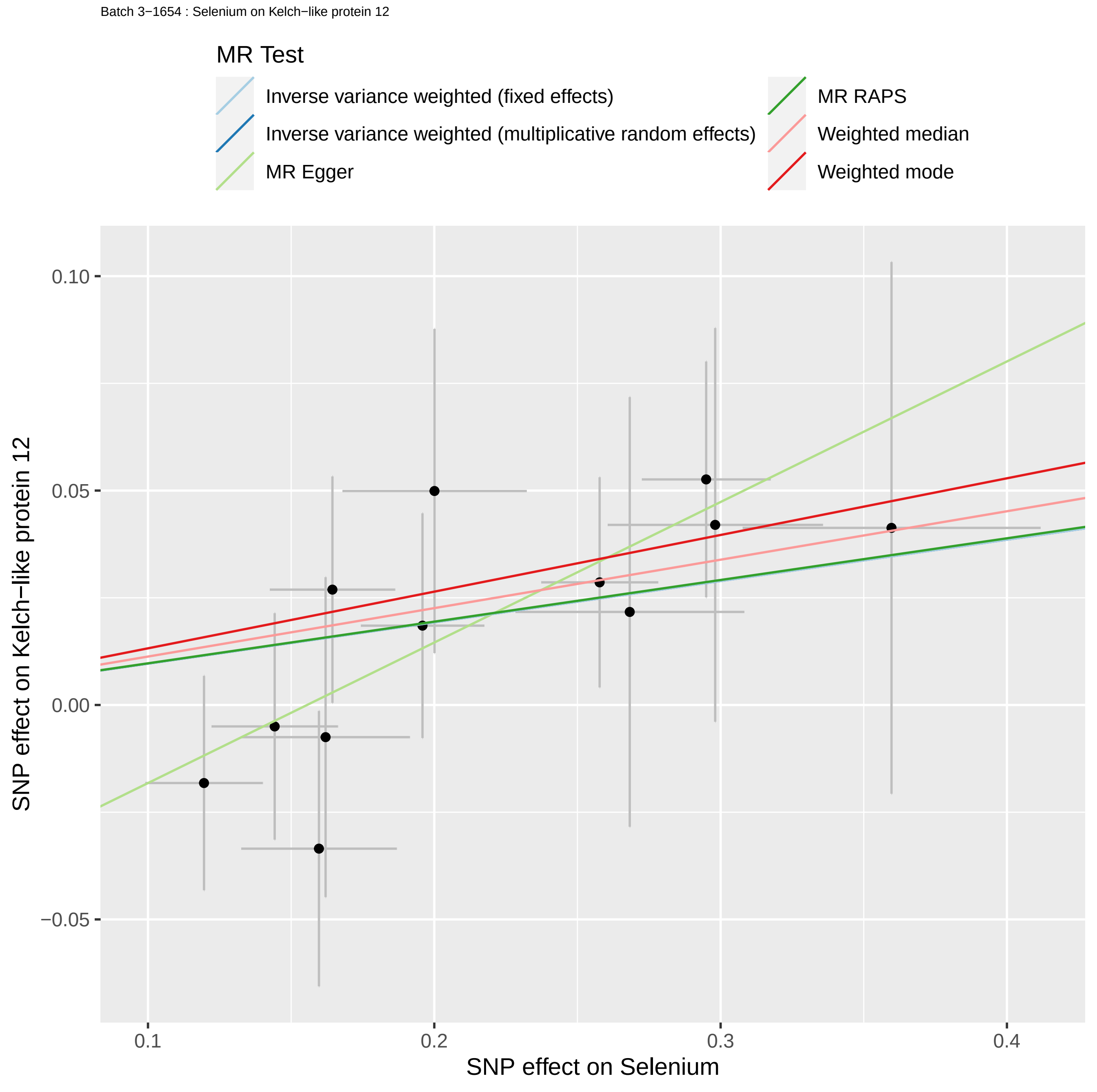
Batch 3−1654 : Selenium on Kelch−like protein 12
MR Test
Inverse variance weighted (fixed effects)
Inverse variance weighted (multiplicative random effects)
MR Egger
MR RAPS
Weighted median
Weighted mode
SNP effect on Kelch−like protein 12
0.10
0.05
0.00
−0.05
0.1
0.2
0.3
0.4
SNP effect on Selenium

Batch 3−1655 : Selenium on Kelch−like protein 13

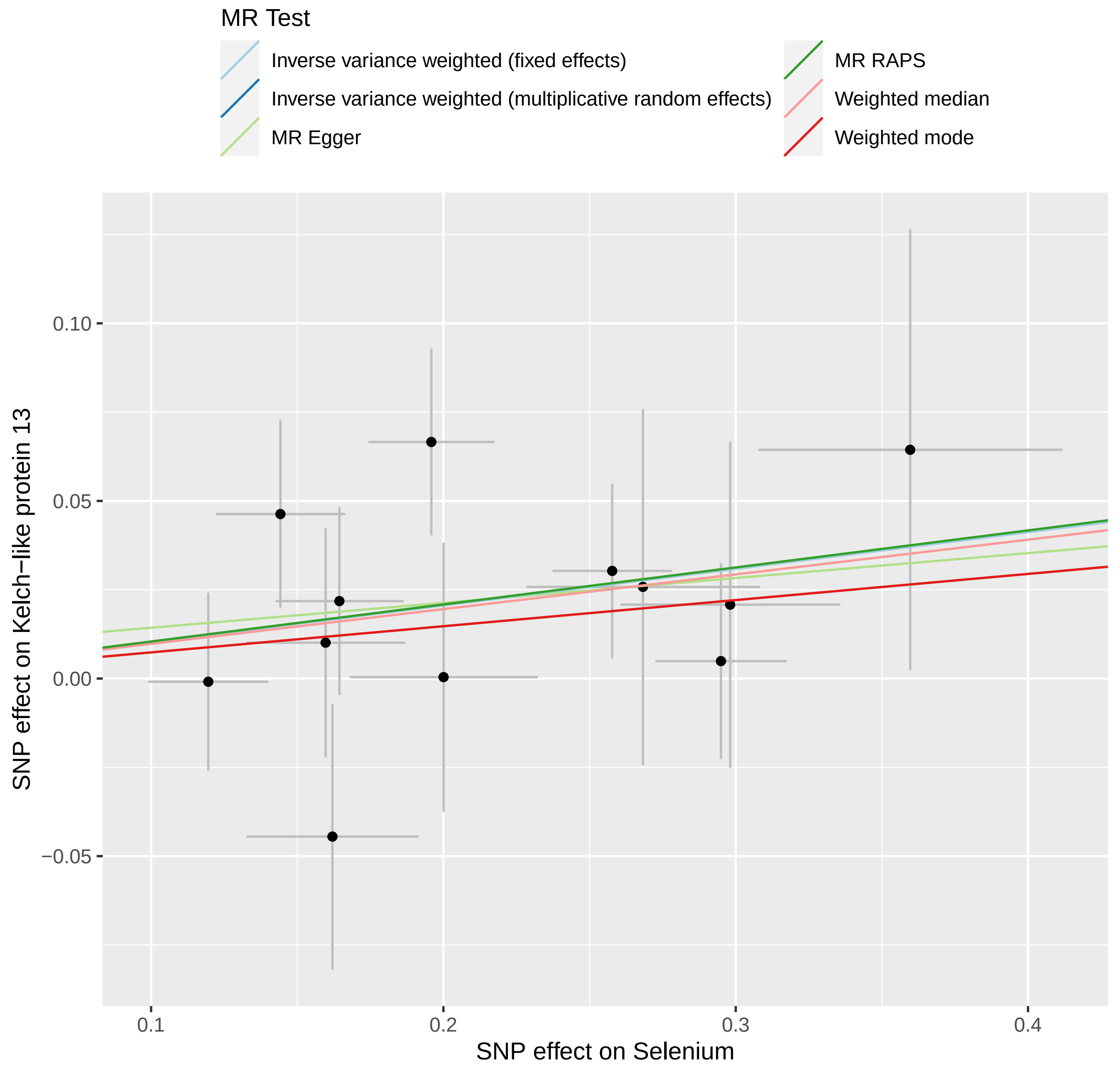

Batch 3−1656 : Selenium on Kallikrein−11

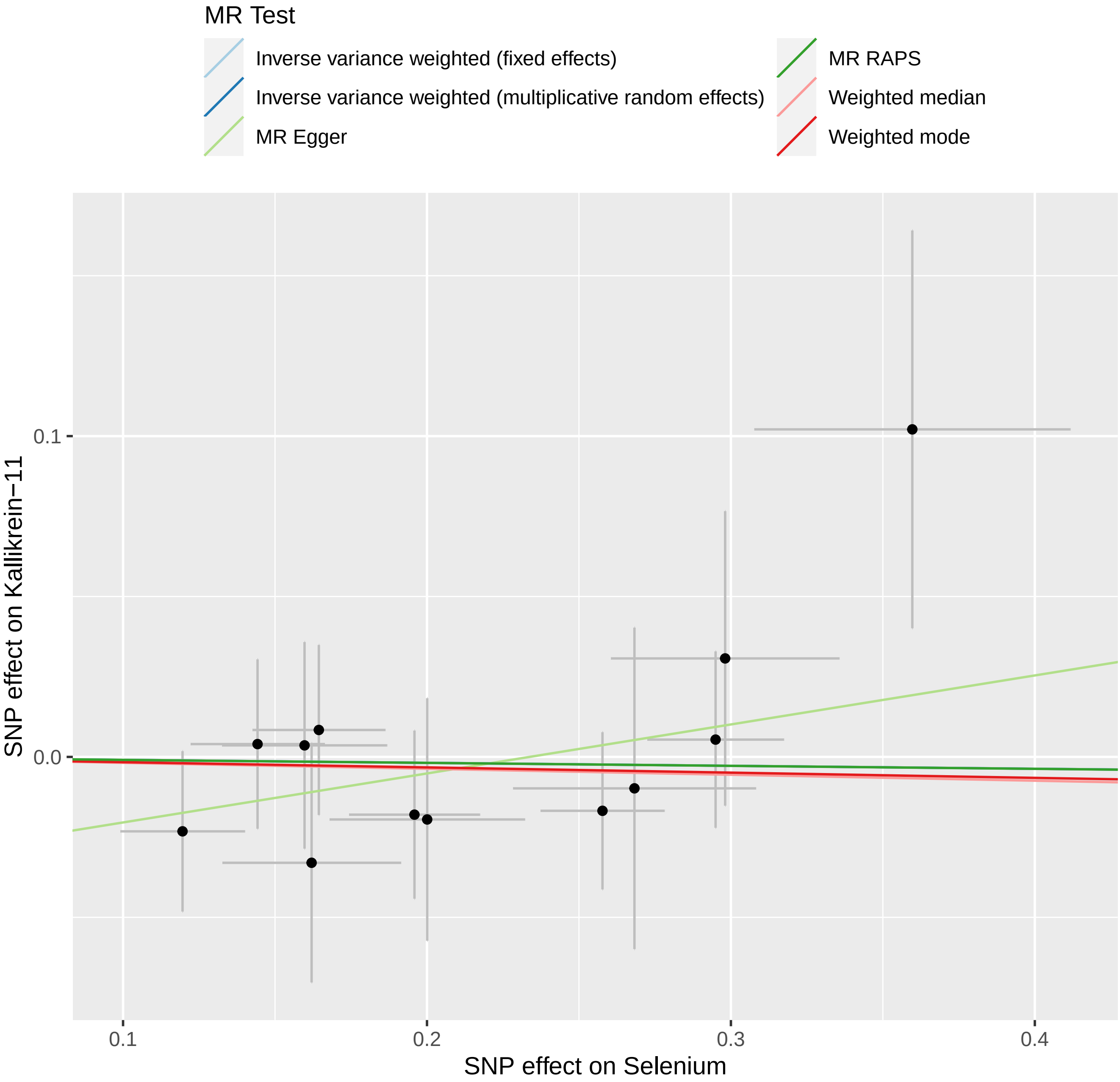

Batch 3−1657 : Selenium on Kallikrein−12

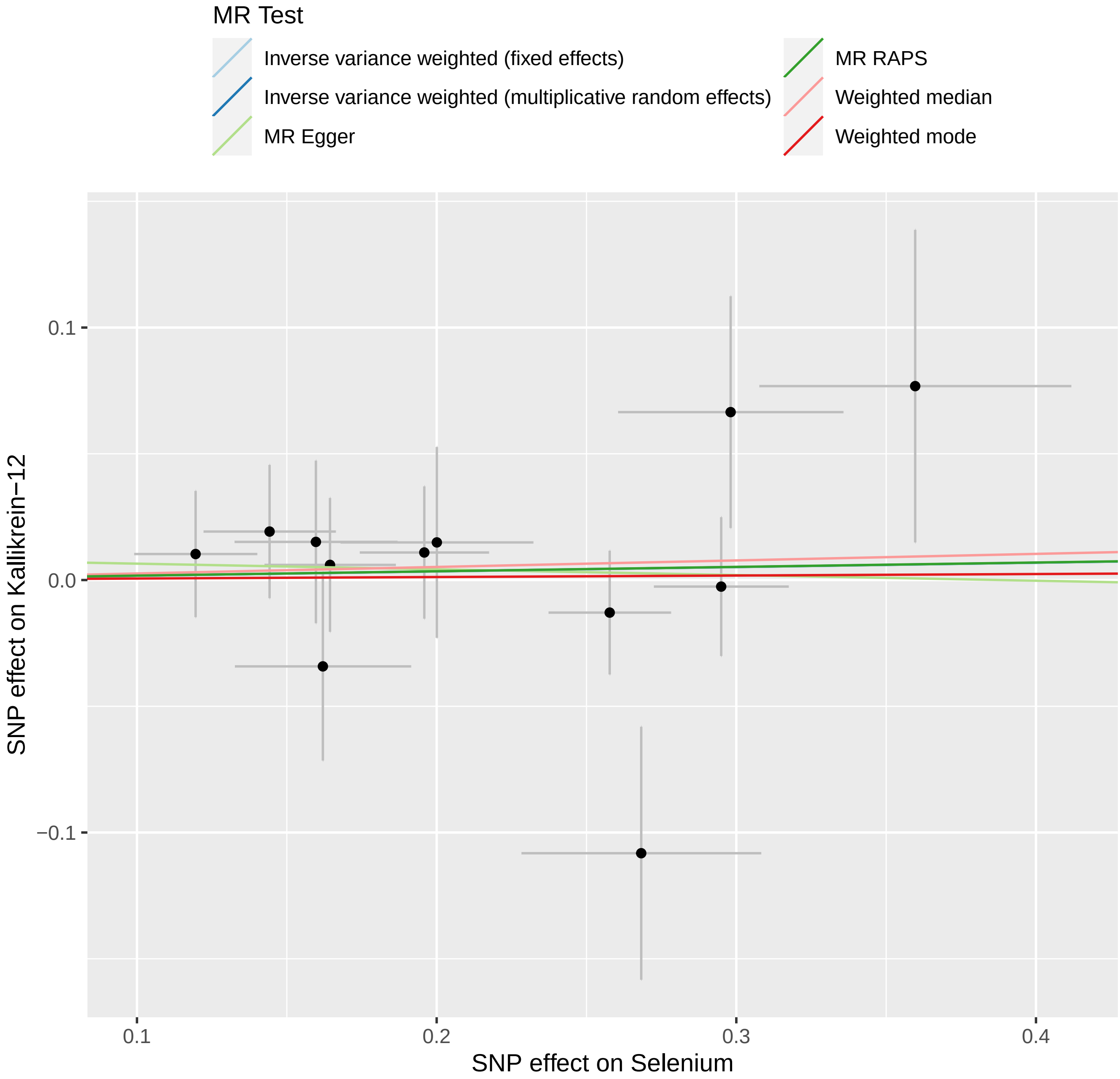

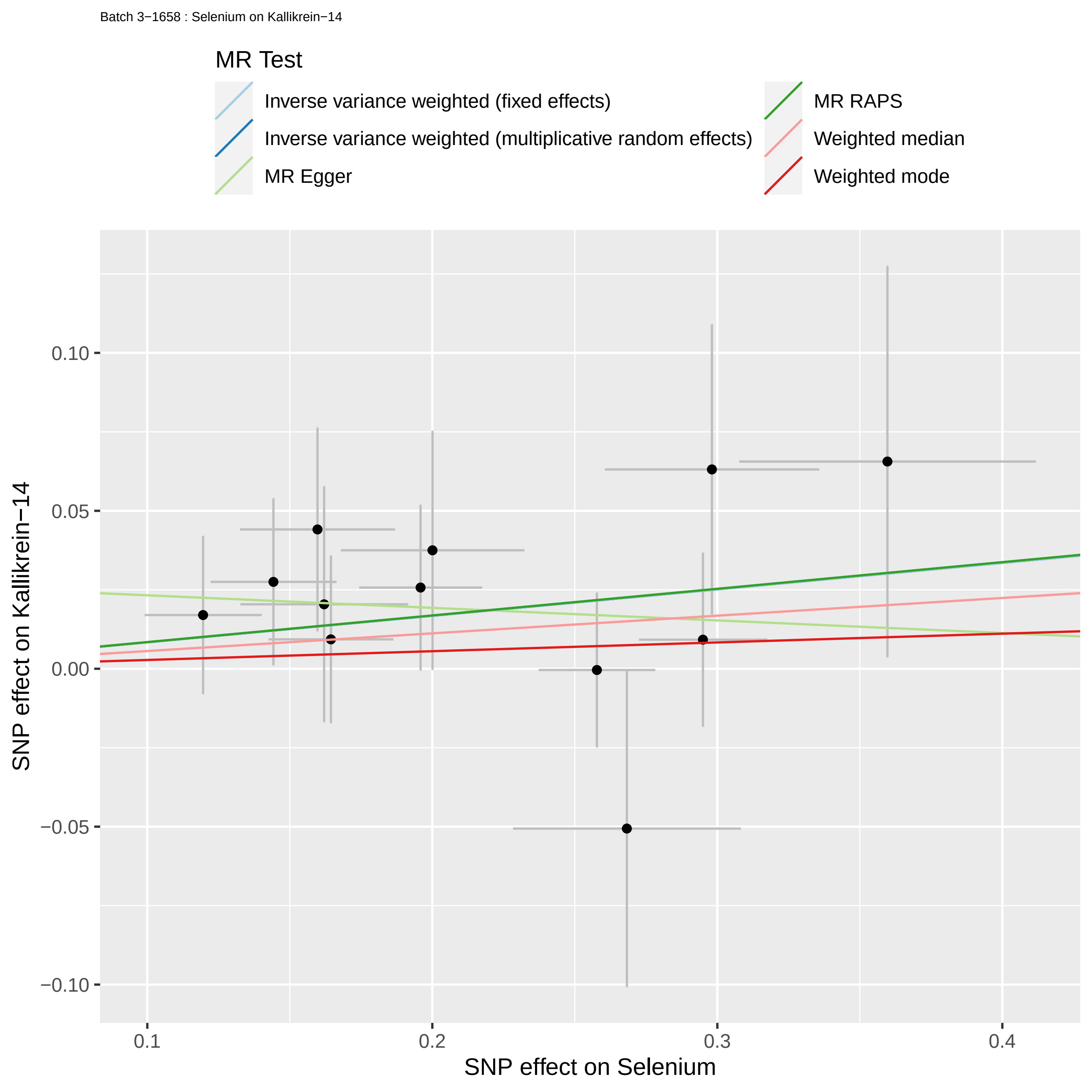

Batch 3−1658 : Selenium on Kallikrein−14
MR Test
Inverse variance weighted (fixed effects)
Inverse variance weighted (multiplicative random effects)
MR Egger
MR RAPS
Weighted median
Weighted mode
0.10
0.05
0.00
−0.05
−0.10
0.1
0.2
0.3
0.4
SNP effect on Kallikrein−14
SNP effect on Selenium

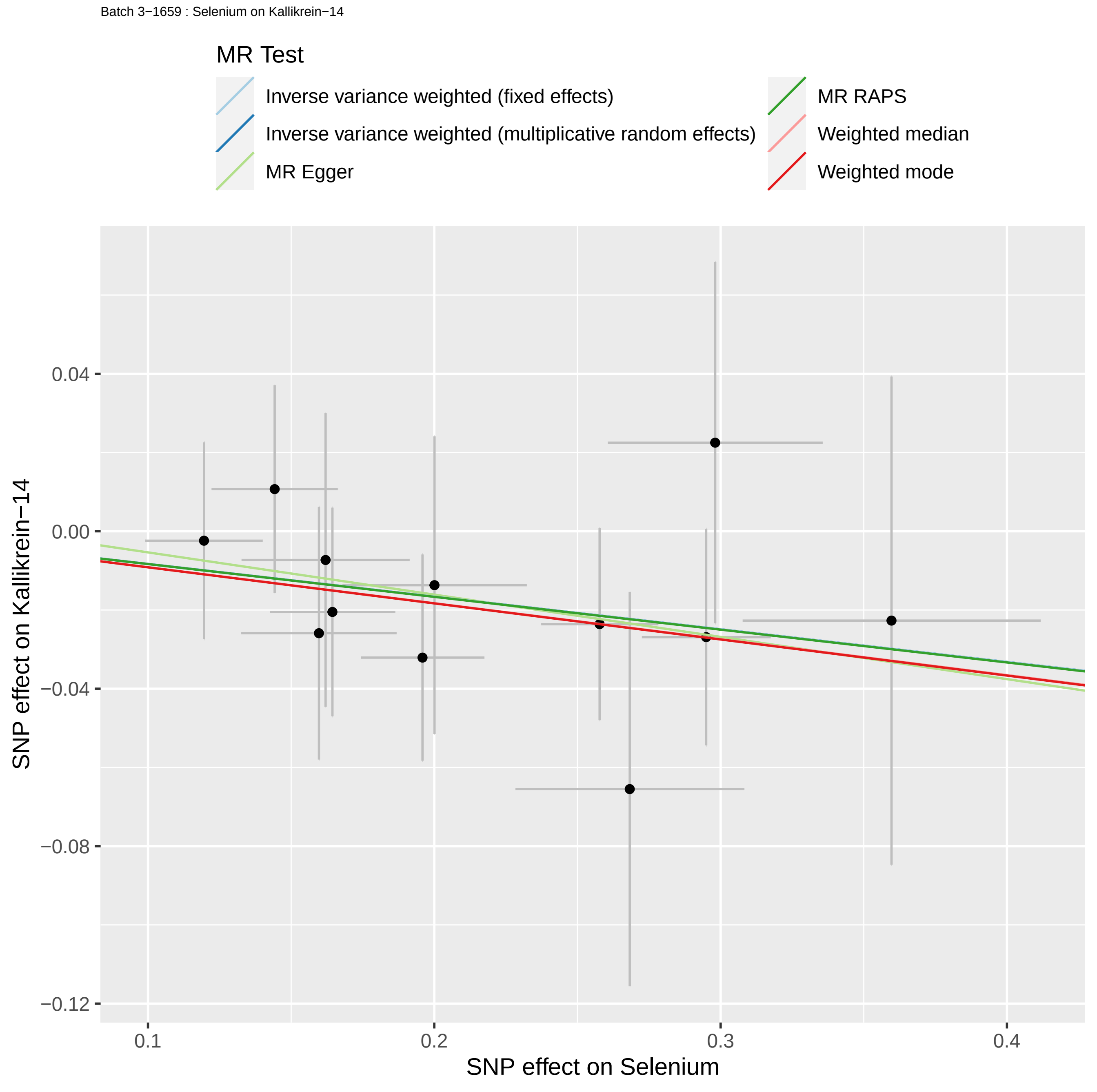

Batch 3−1659 : Selenium on Kallikrein−14
MR Test
Inverse variance weighted (fixed effects)
Inverse variance weighted (multiplicative random effects)
MR Egger
MR RAPS
Weighted median
Weighted mode
0.04
0.00
−0.04
−0.08
−0.12
0.1
0.2
0.3
0.4
SNP effect on Kallikrein−14
SNP effect on Selenium

Batch 3−1660 : Selenium on Kallikrein−15

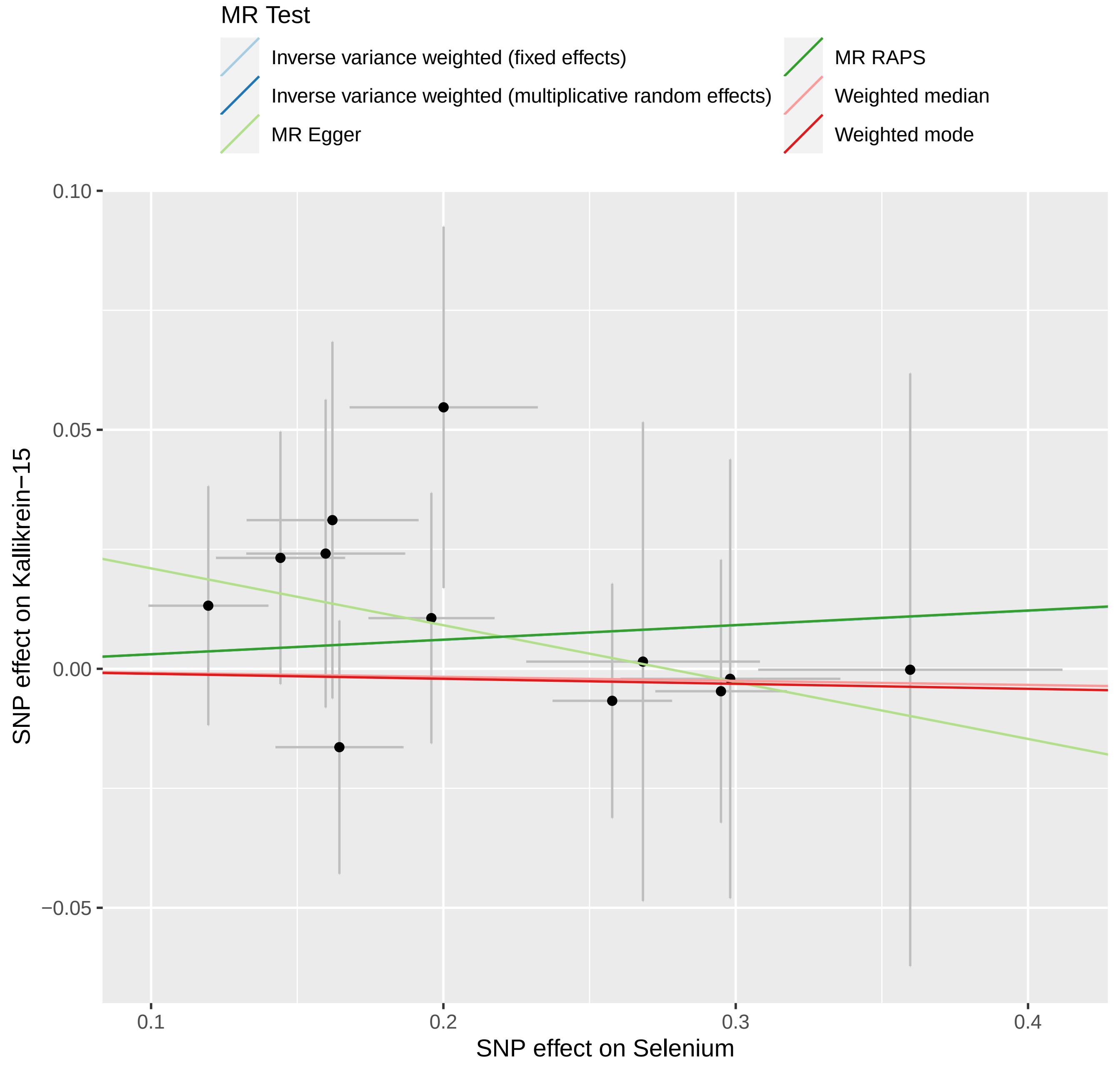

Batch 3−1661 : Selenium on Prostate−specific antigen

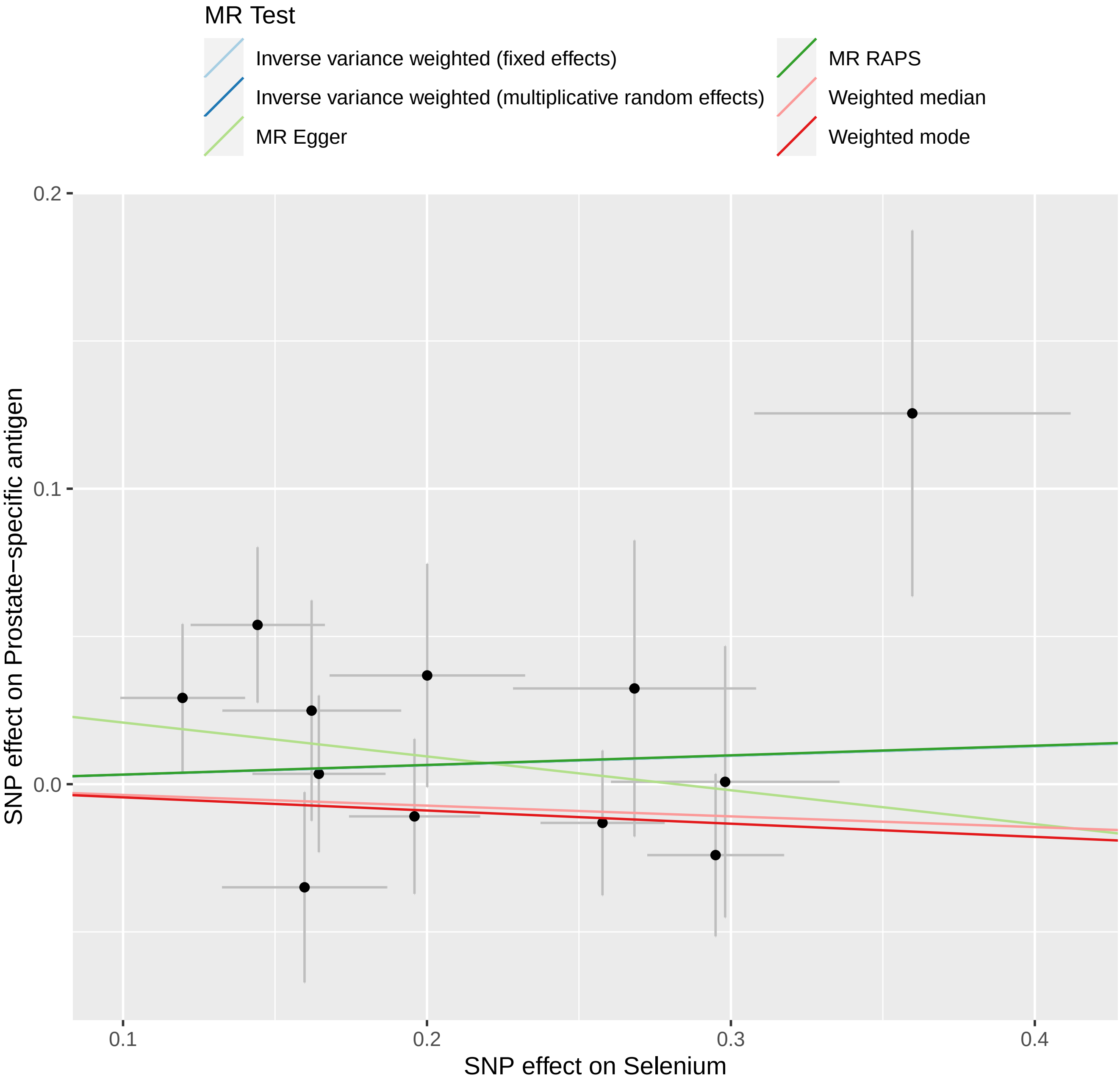

Batch 3−1662 : Selenium on Kallikrein−4

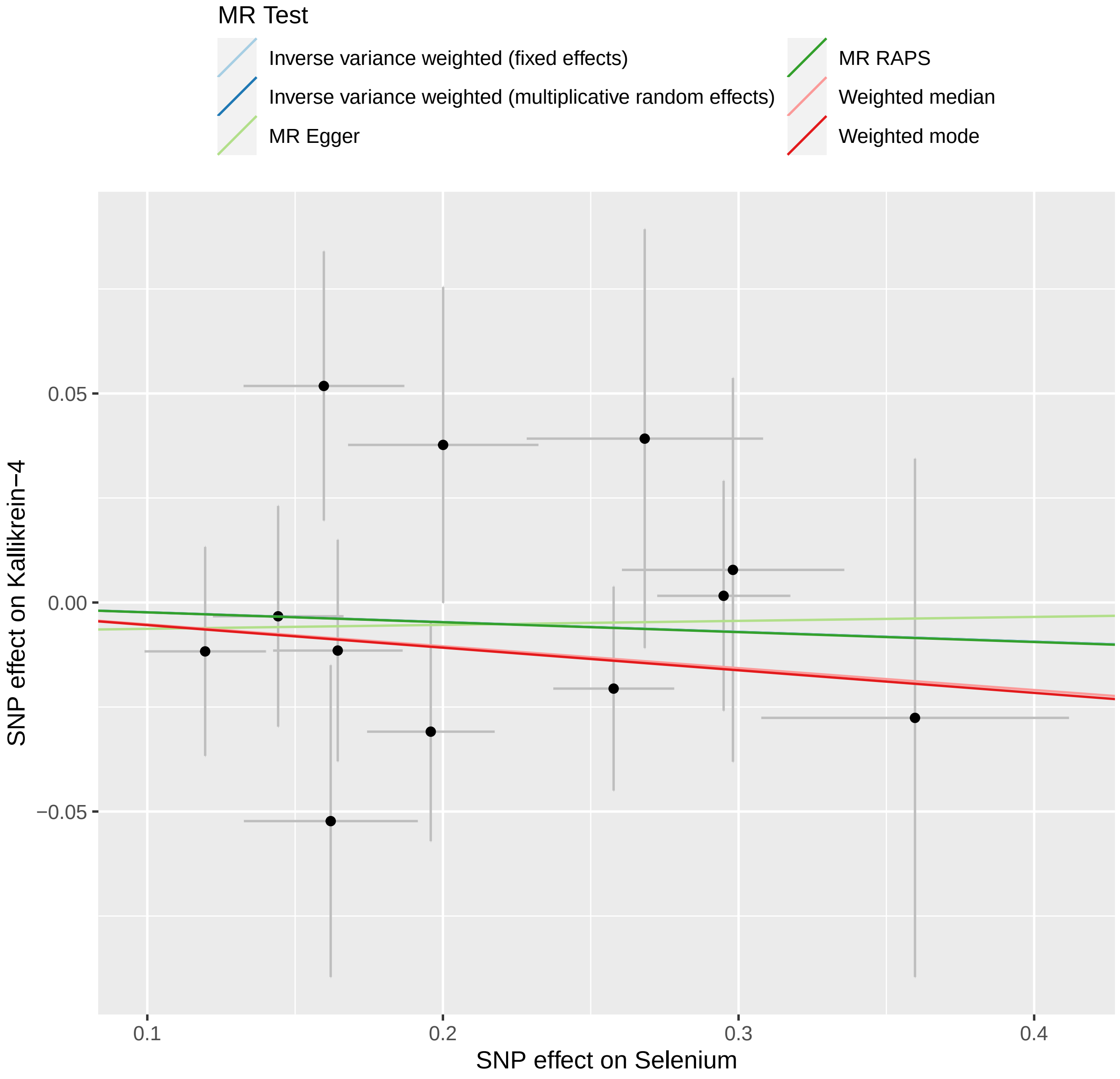

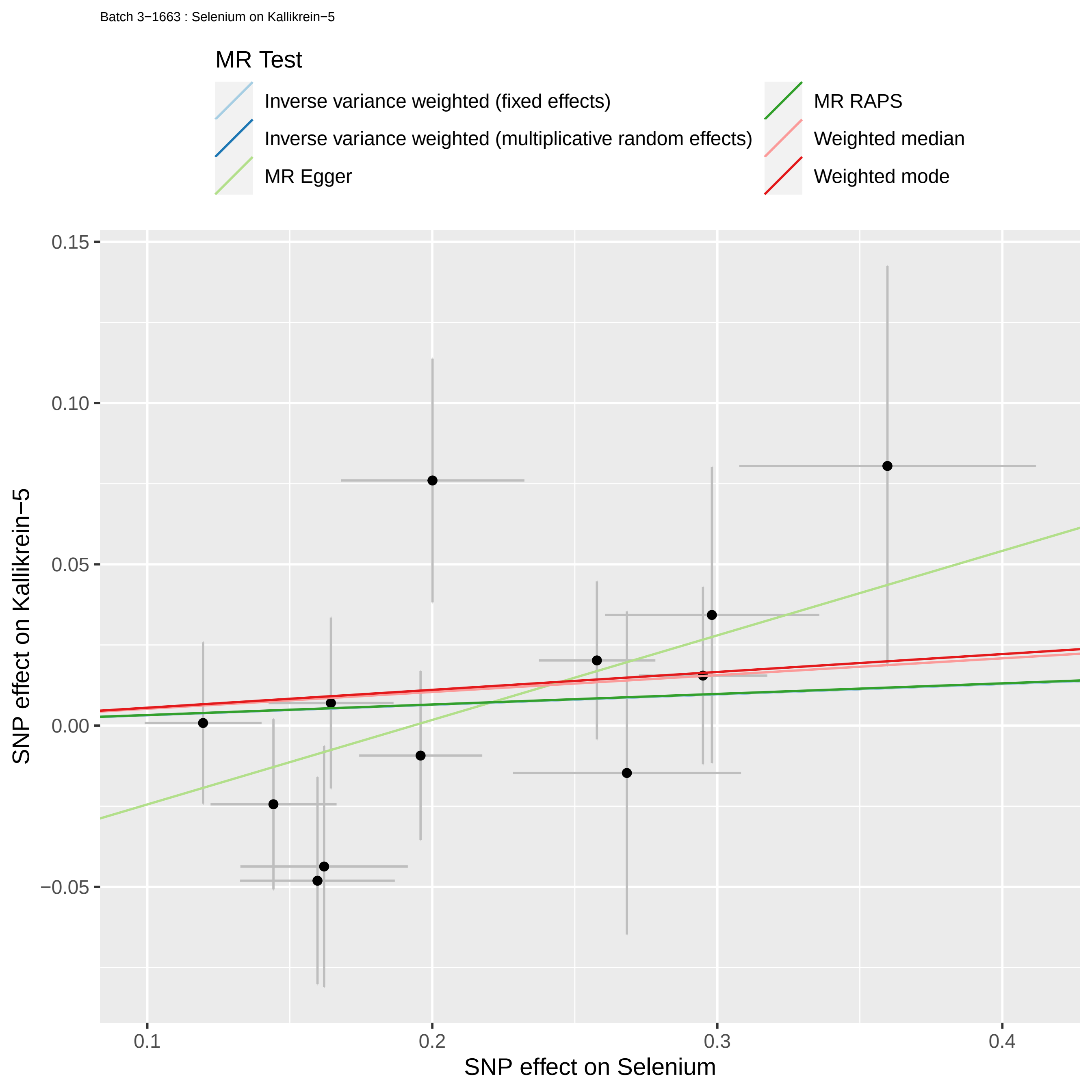
Batch 3−1663 : Selenium on Kallikrein−5
MR Test
Inverse variance weighted (fixed effects)
Inverse variance weighted (multiplicative random effects)
MR Egger
MR RAPS
Weighted median
Weighted mode
0.15
0.10
0.05
0.00
−0.05
0.1
0.2
0.3
0.4
SNP effect on Kallikrein−5
SNP effect on Selenium

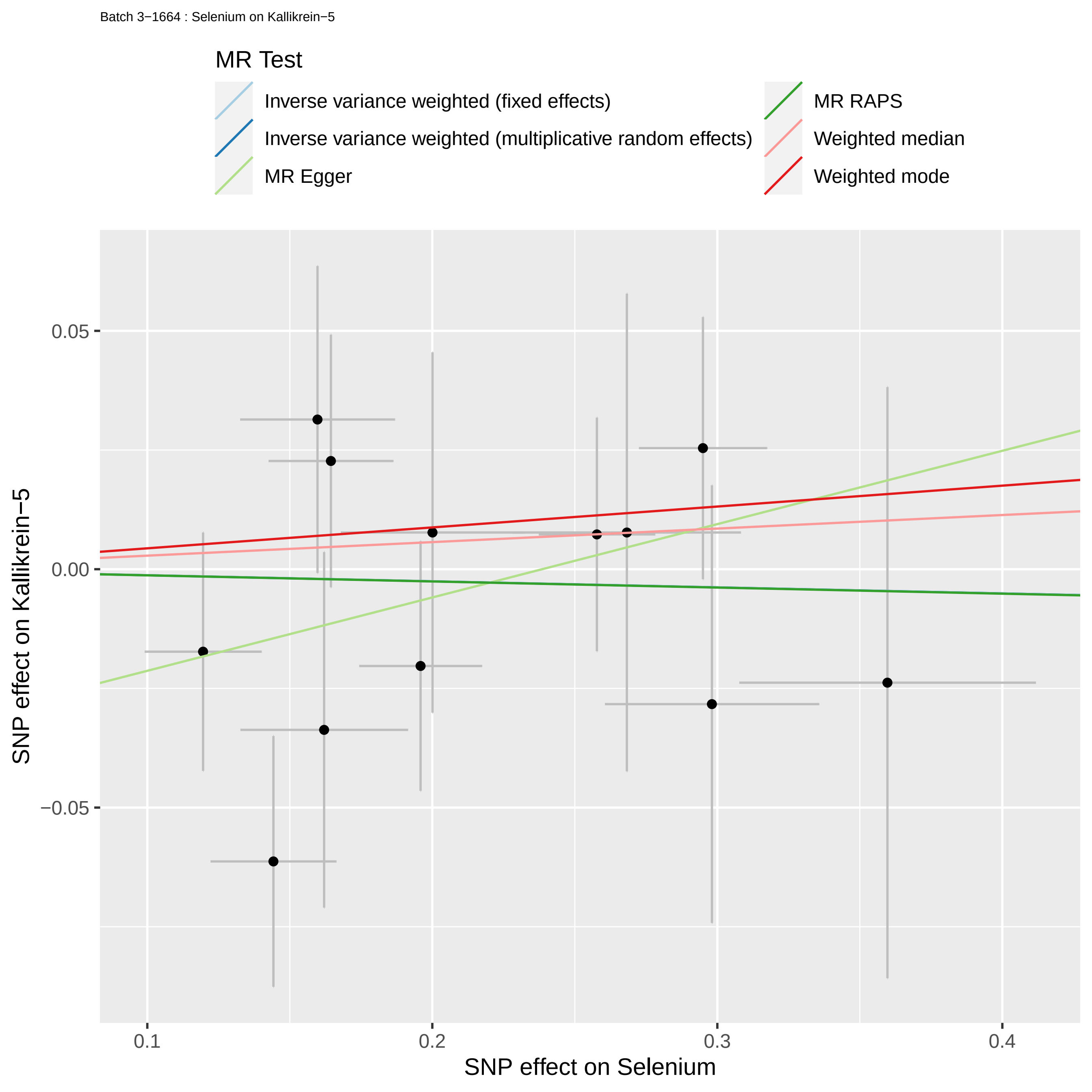
Batch 3−1664 : Selenium on Kallikrein−5
MR Test
Inverse variance weighted (fixed effects)
Inverse variance weighted (multiplicative random effects)
MR Egger
MR RAPS
Weighted median
Weighted mode
0.05
0.00
−0.05
0.1
0.2
0.3
0.4
SNP effect on Kallikrein−5
SNP effect on Selenium

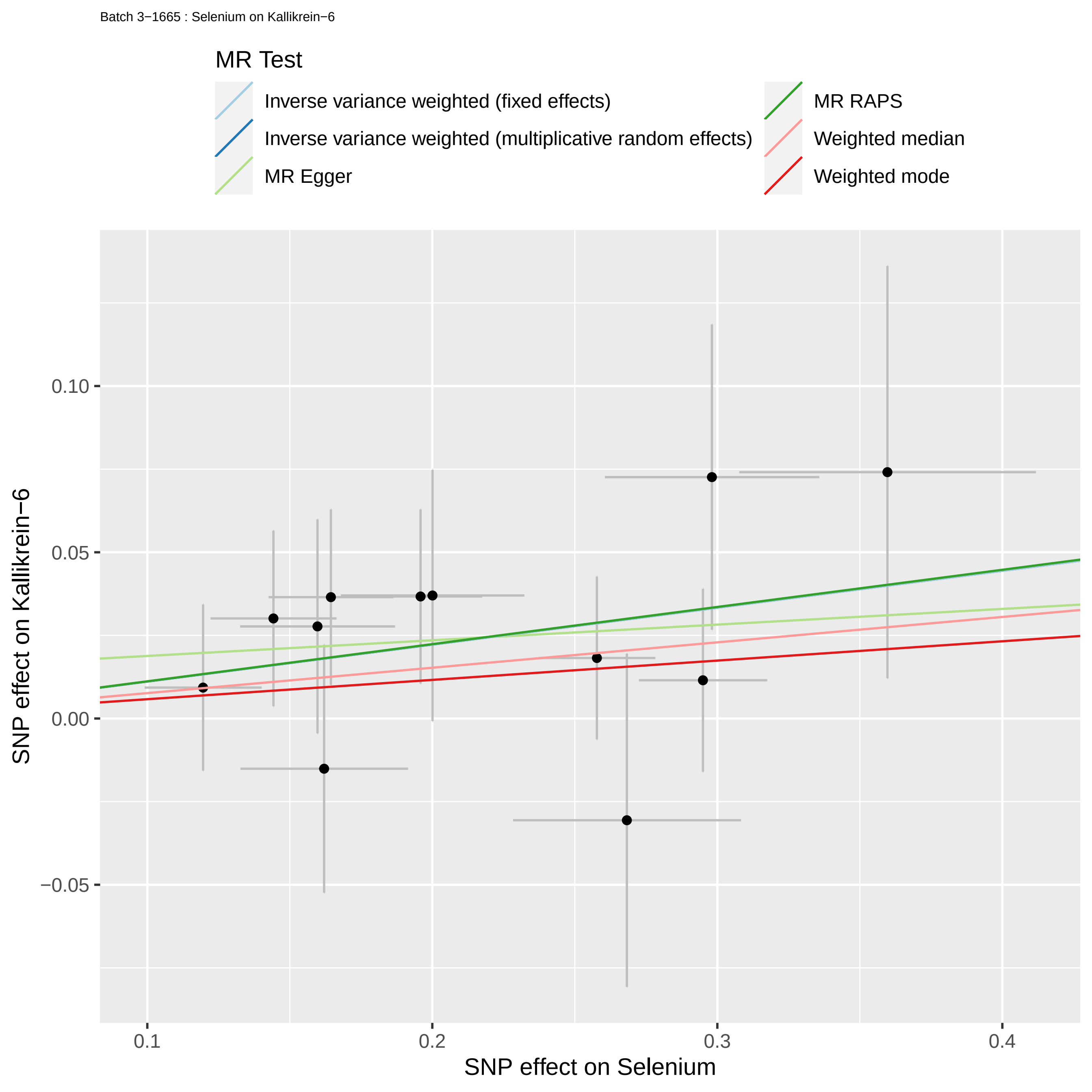
Batch 3−1665 : Selenium on Kallikrein−6
MR Test
Inverse variance weighted (fixed effects)
Inverse variance weighted (multiplicative random effects)
MR Egger
MR RAPS
Weighted median
Weighted mode
0.10
0.05
0.00
−0.05
0.1
0.2
0.3
0.4
SNP effect on Kallikrein−6
SNP effect on Selenium

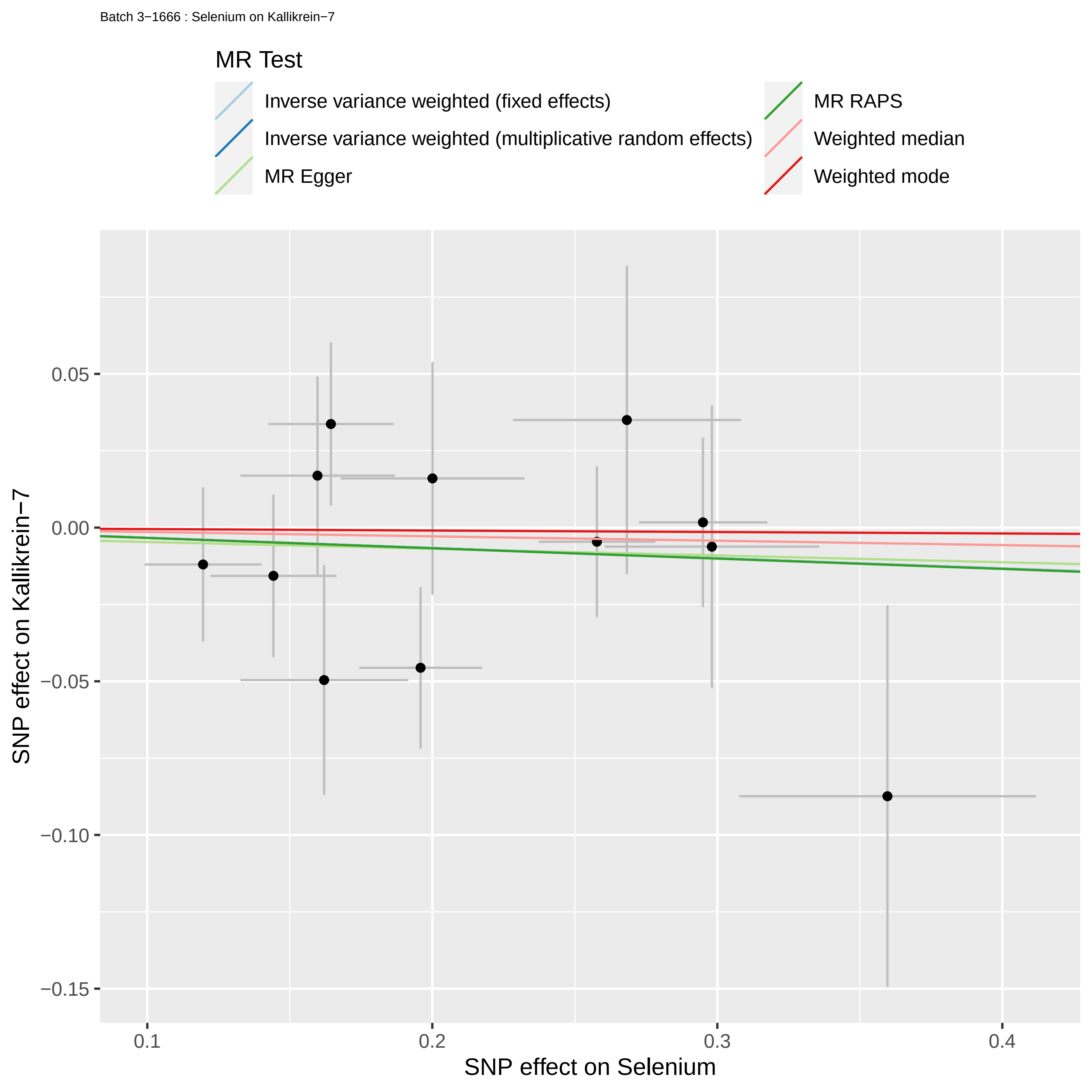

Batch 3−1666 : Selenium on Kallikrein−7
MR Test
Inverse variance weighted (fixed effects)
Inverse variance weighted (multiplicative random effects)
MR Egger
MR RAPS
Weighted median
Weighted mode
0.05
0.00
−0.05
−0.10
−0.15
0.1
0.2
0.3
0.4
SNP effect on Kallikrein−7
SNP effect on Selenium

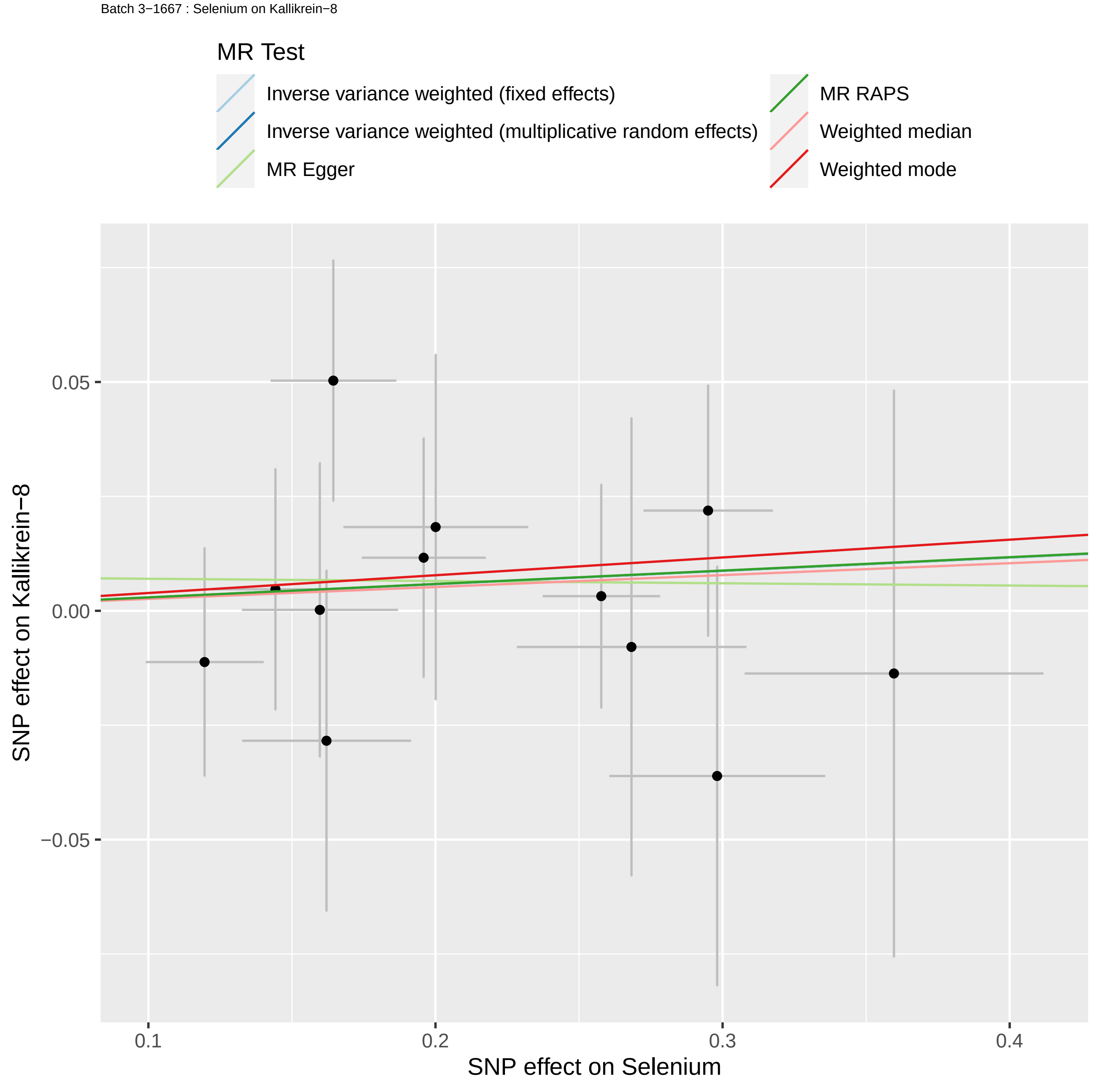
Batch 3−1667 : Selenium on Kallikrein−8
MR Test
Inverse variance weighted (fixed effects)
Inverse variance weighted (multiplicative random effects)
MR Egger
MR RAPS
Weighted median
Weighted mode
0.05
0.00
−0.05
0.1
0.2
0.3
0.4
SNP effect on Kallikrein−8
SNP effect on Selenium

Batch 3−1668 : Selenium on Kallikrein−8

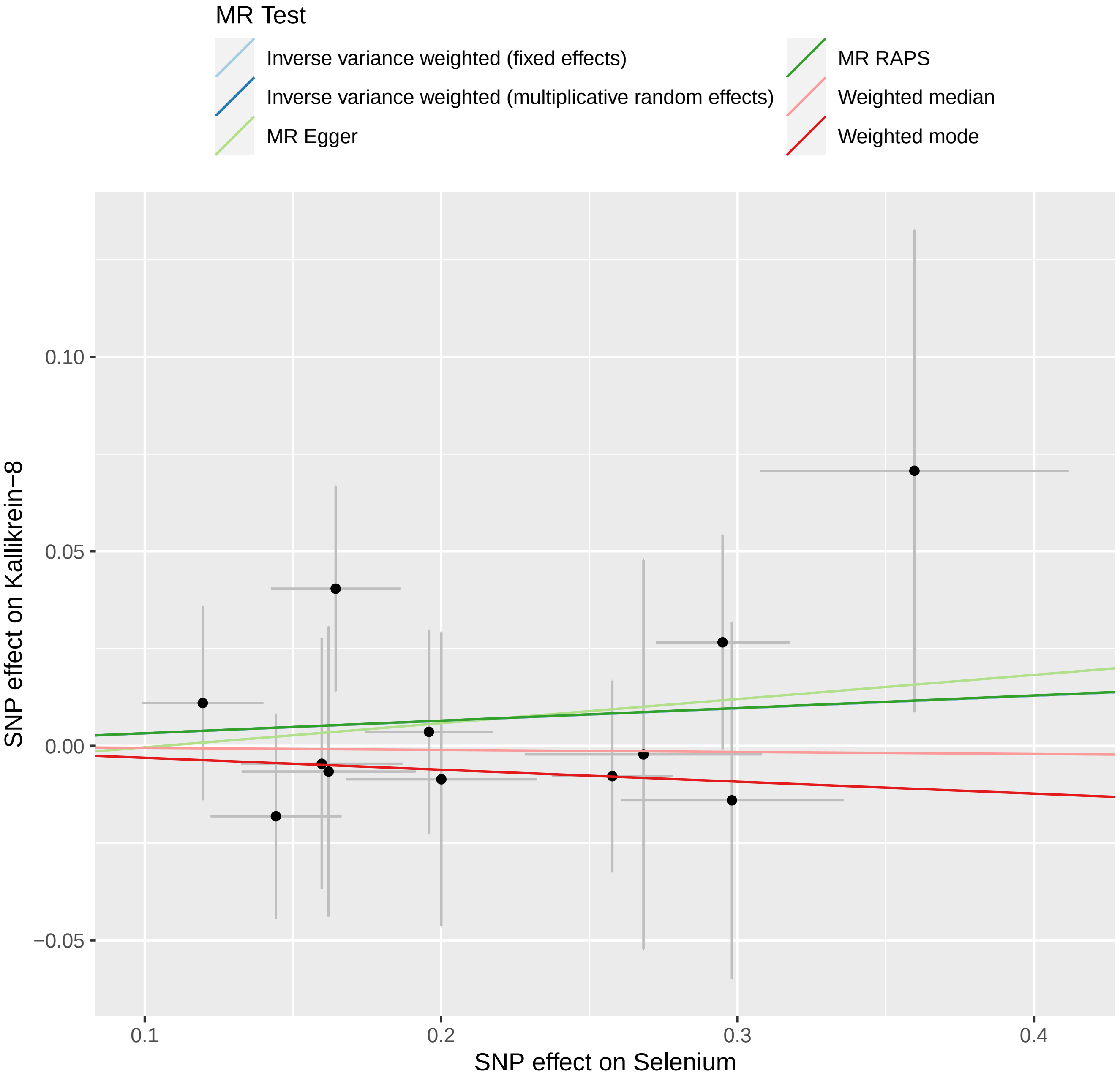

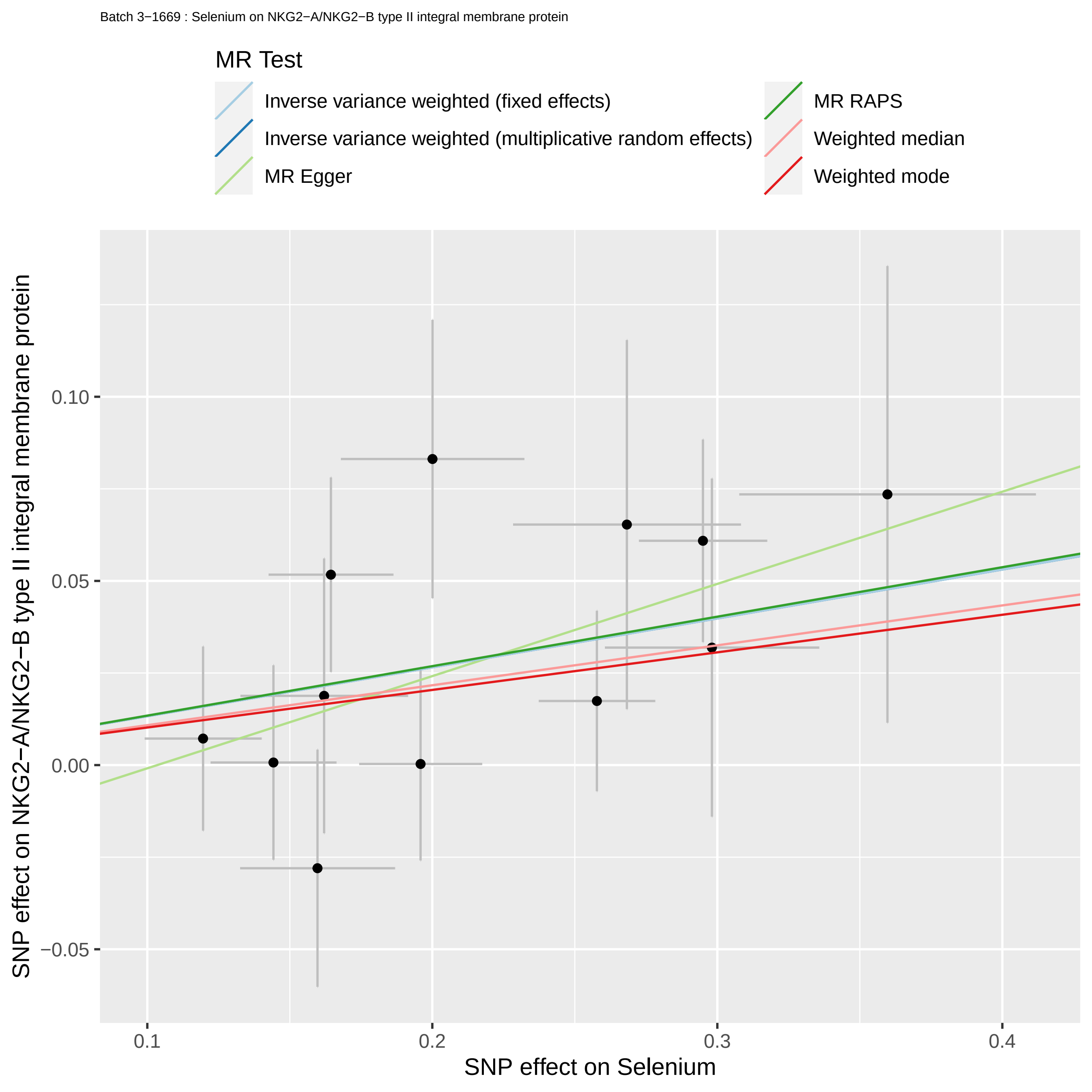

Batch 3−1669 : Selenium on NKG2−A/NKG2−B type II integral membrane protein
MR Test
Inverse variance weighted (fixed effects)
Inverse variance weighted (multiplicative random effects)
MR Egger
MR RAPS
Weighted median
Weighted mode
0.10
0.05
0.00
−0.05
0.1
0.2
0.3
0.4
SNP effect on NKG2−A/NKG2−B type II integral membrane protein
SNP effect on Selenium

Batch 3−1670 : Selenium on NKG2−E type II integral membrane protein

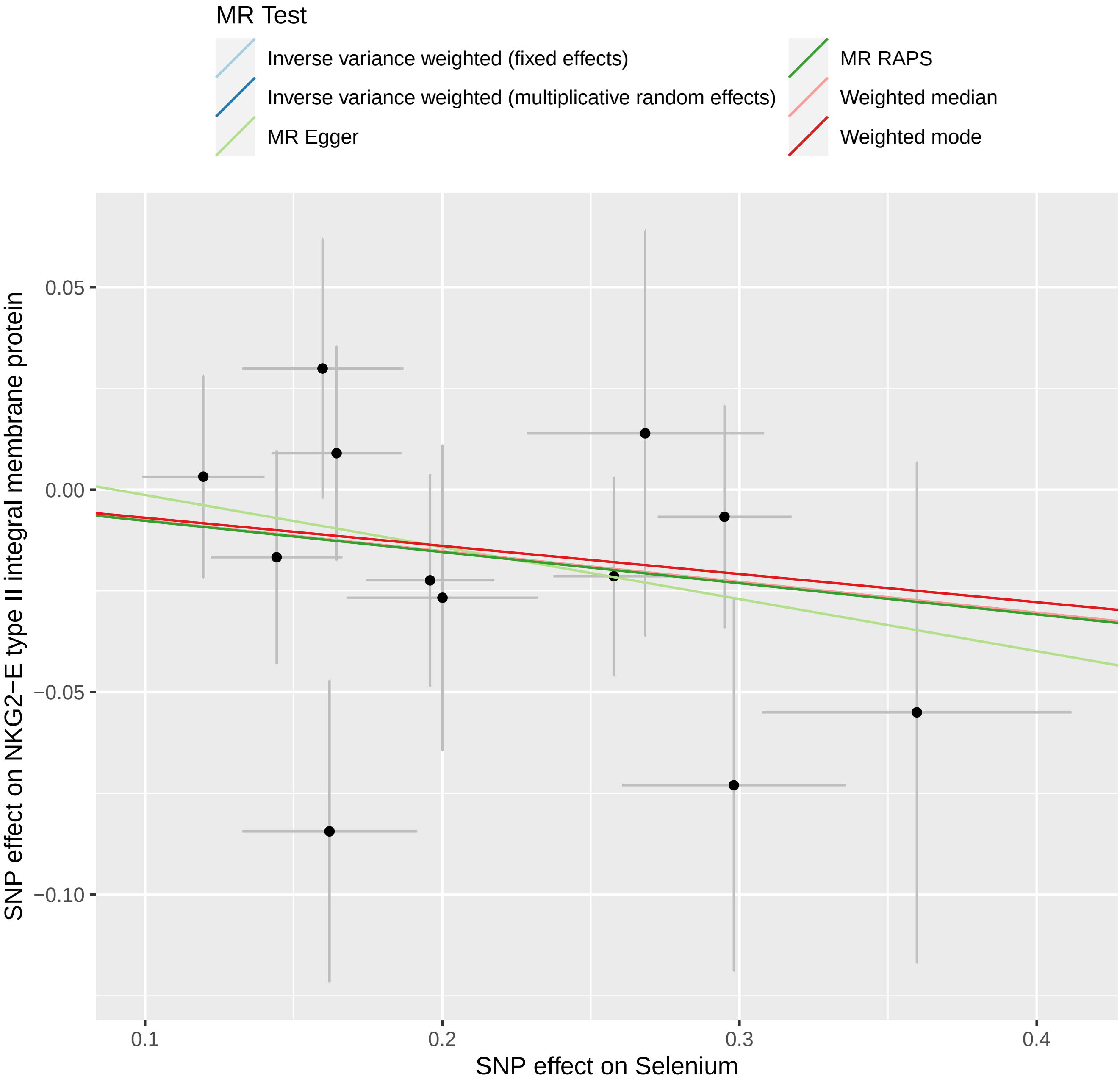

Batch 3−1671 : Selenium on NKG2−E type II integral membrane protein

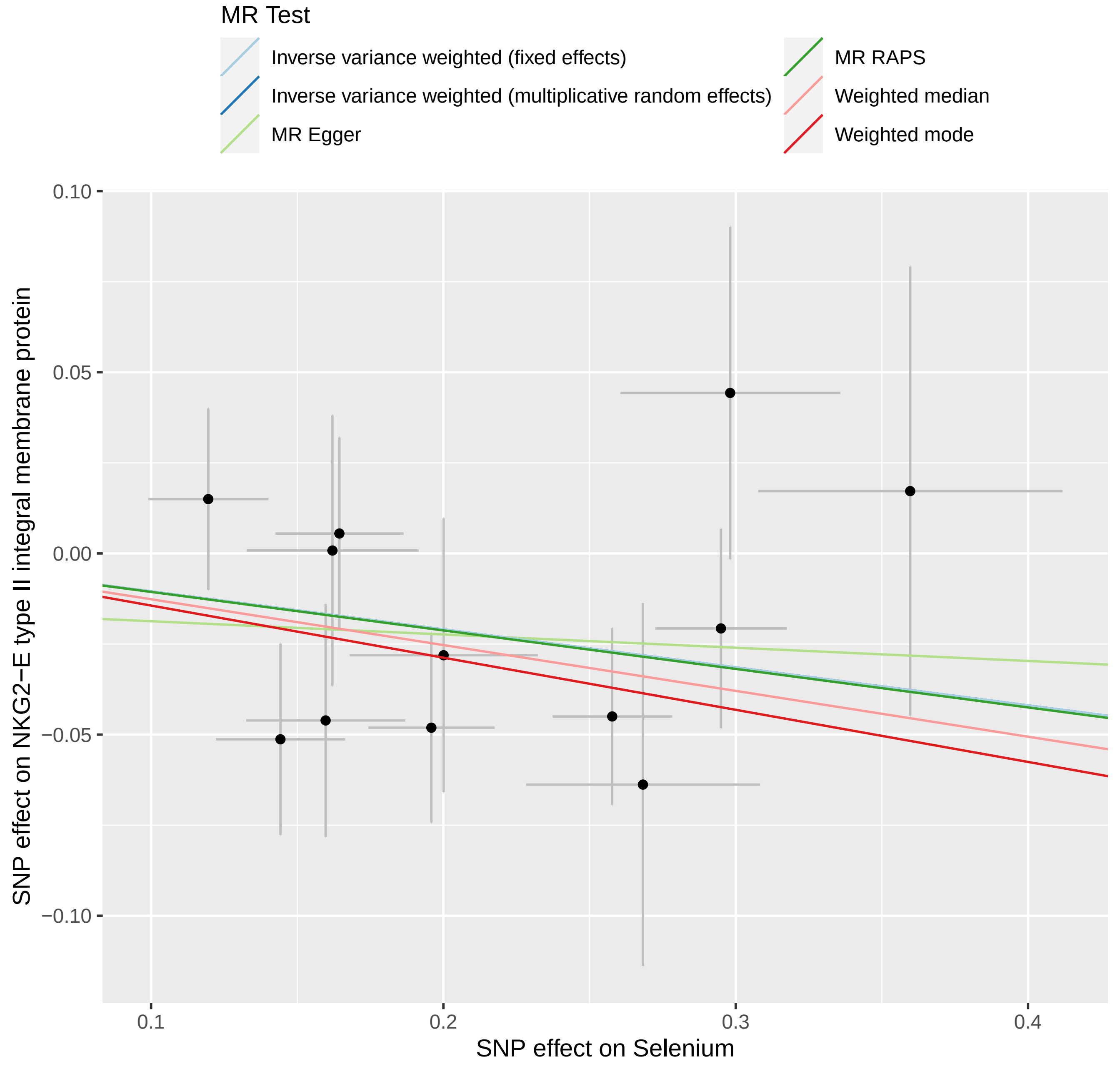

Batch 3−1672 : Selenium on Killer cell lectin−like receptor subfamily F member 1

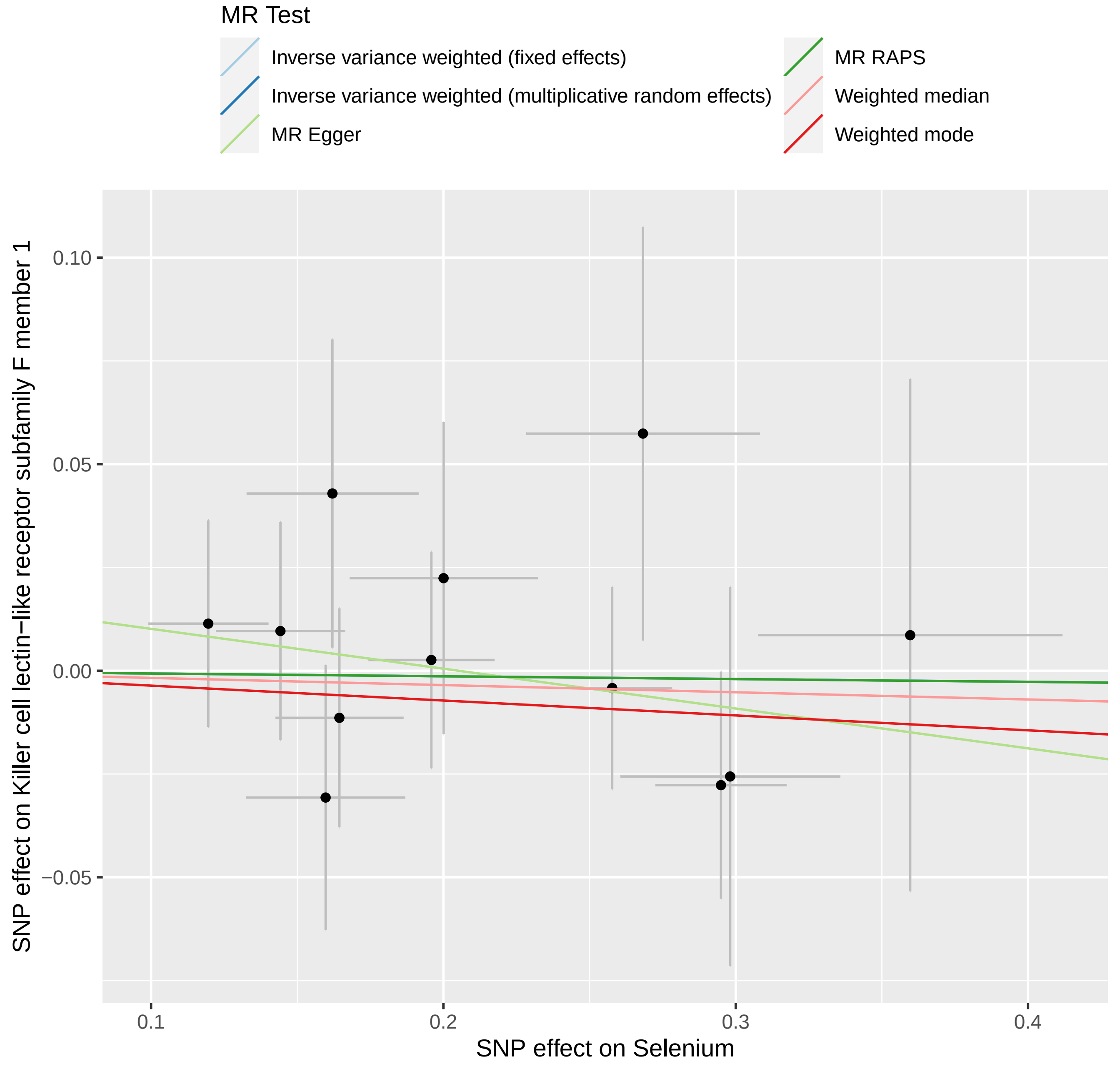

Batch 3−1673 : Selenium on Killer cell lectin−like receptor subfamily F member 1

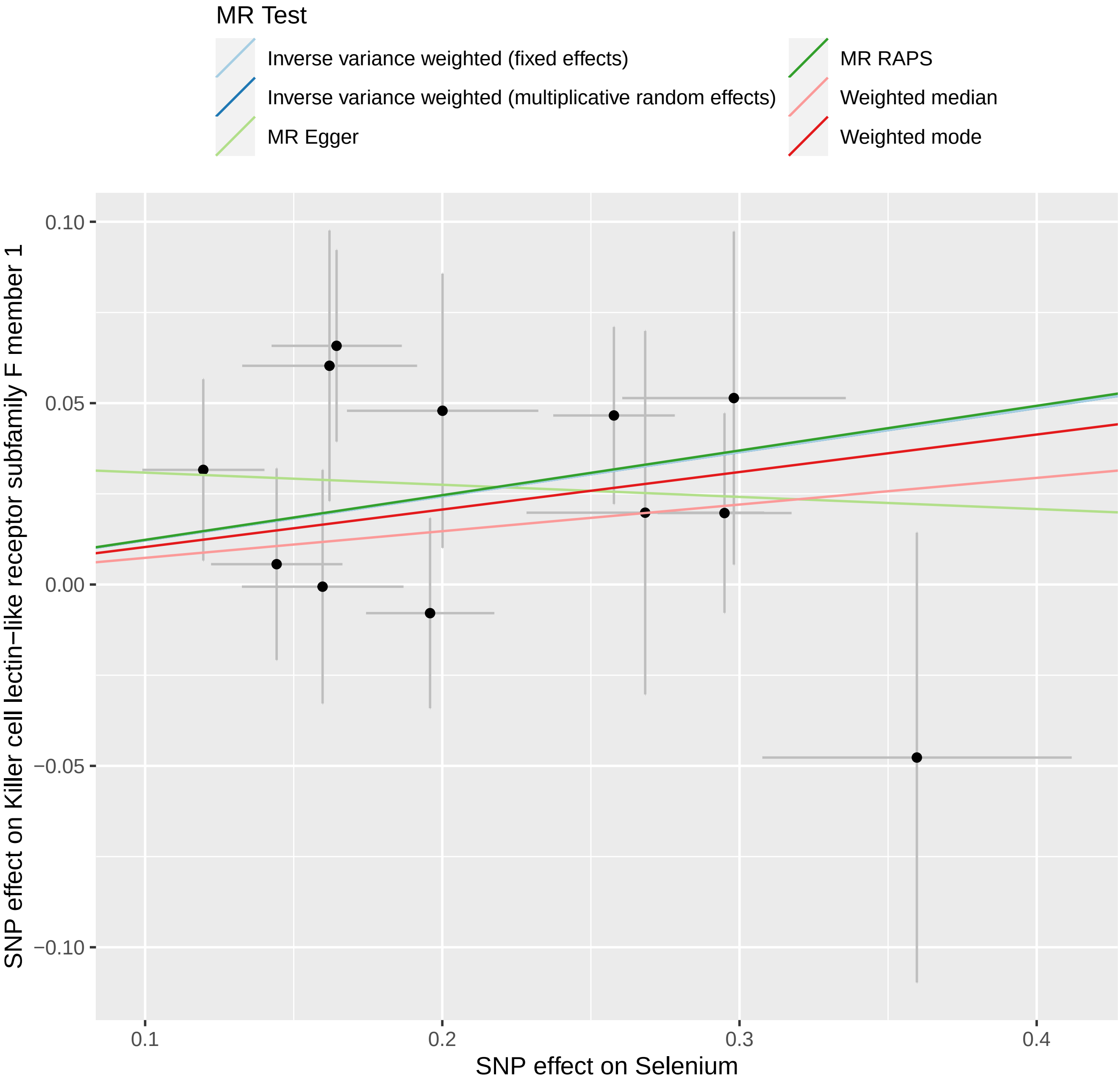

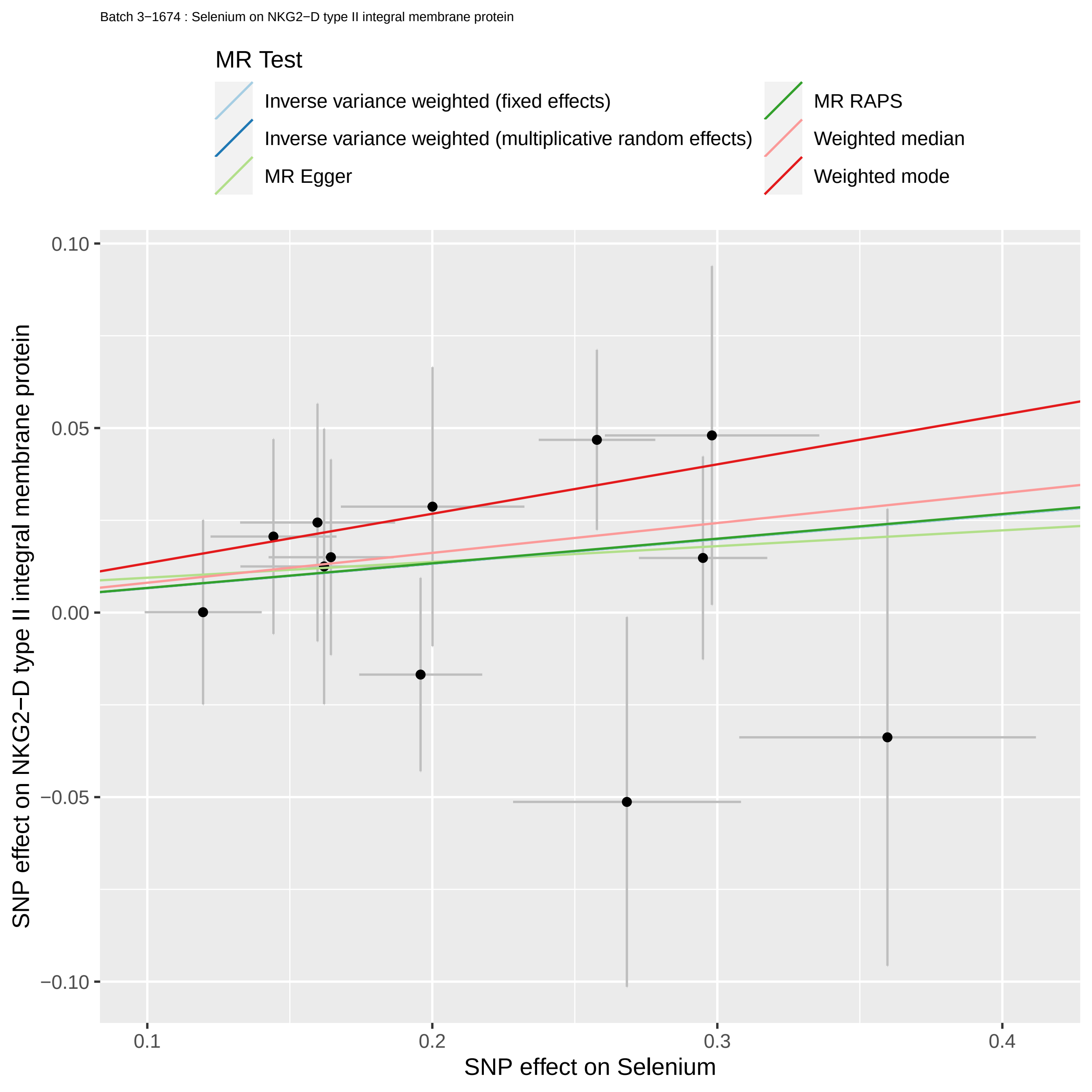

Batch 3−1674 : Selenium on NKG2−D type II integral membrane protein
MR Test
Inverse variance weighted (fixed effects)
Inverse variance weighted (multiplicative random effects)
MR Egger
MR RAPS
Weighted median
Weighted mode
0.10
0.05
0.00
−0.05
−0.10
0.1
0.2
0.3
0.4
SNP effect on Selenium
SNP effect on NKG2−D type II integral membrane protein

Batch 3−1675 : Selenium on NKG2−D type II integral membrane protein

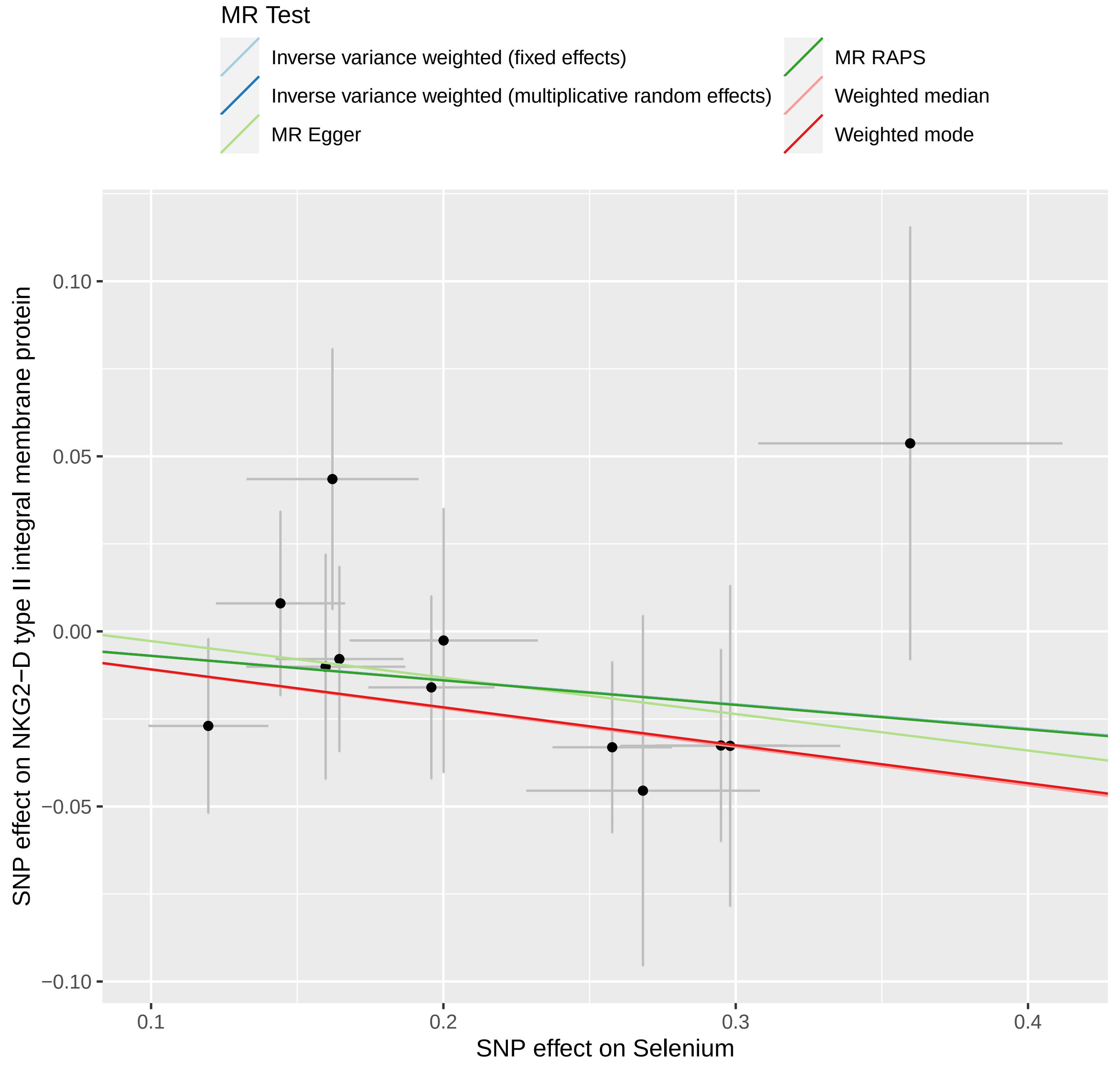

Batch 3−1676 : Selenium on Histone−lysine N−methyltransferase 2C

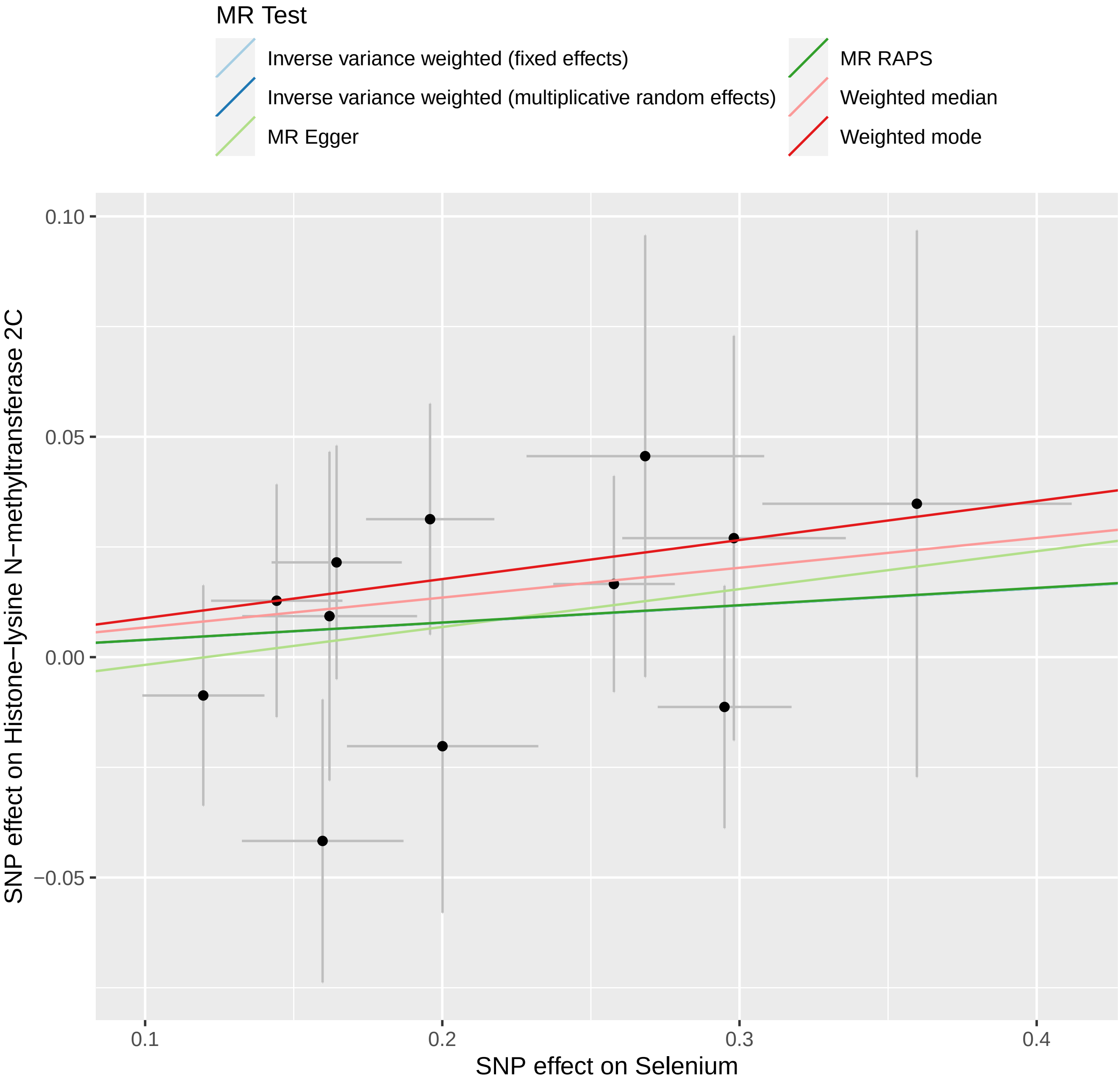

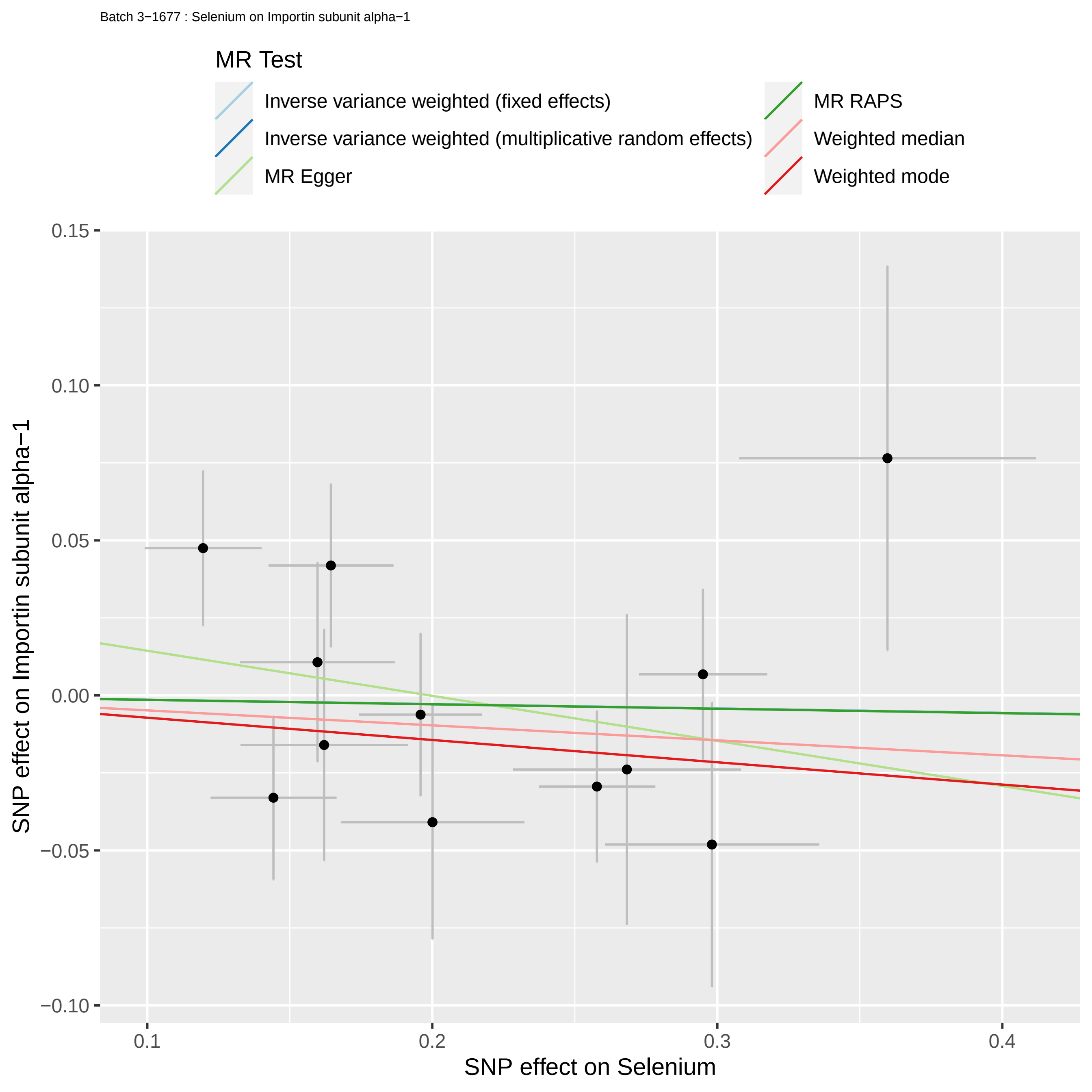

Batch 3−1677 : Selenium on Importin subunit alpha−1
MR Test
Inverse variance weighted (fixed effects)
Inverse variance weighted (multiplicative random effects)
MR Egger
MR RAPS
Weighted median
Weighted mode
0.15
0.10
0.05
0.00
−0.05
−0.10
0.1
0.2
0.3
0.4
SNP effect on Importin subunit alpha−1
SNP effect on Selenium

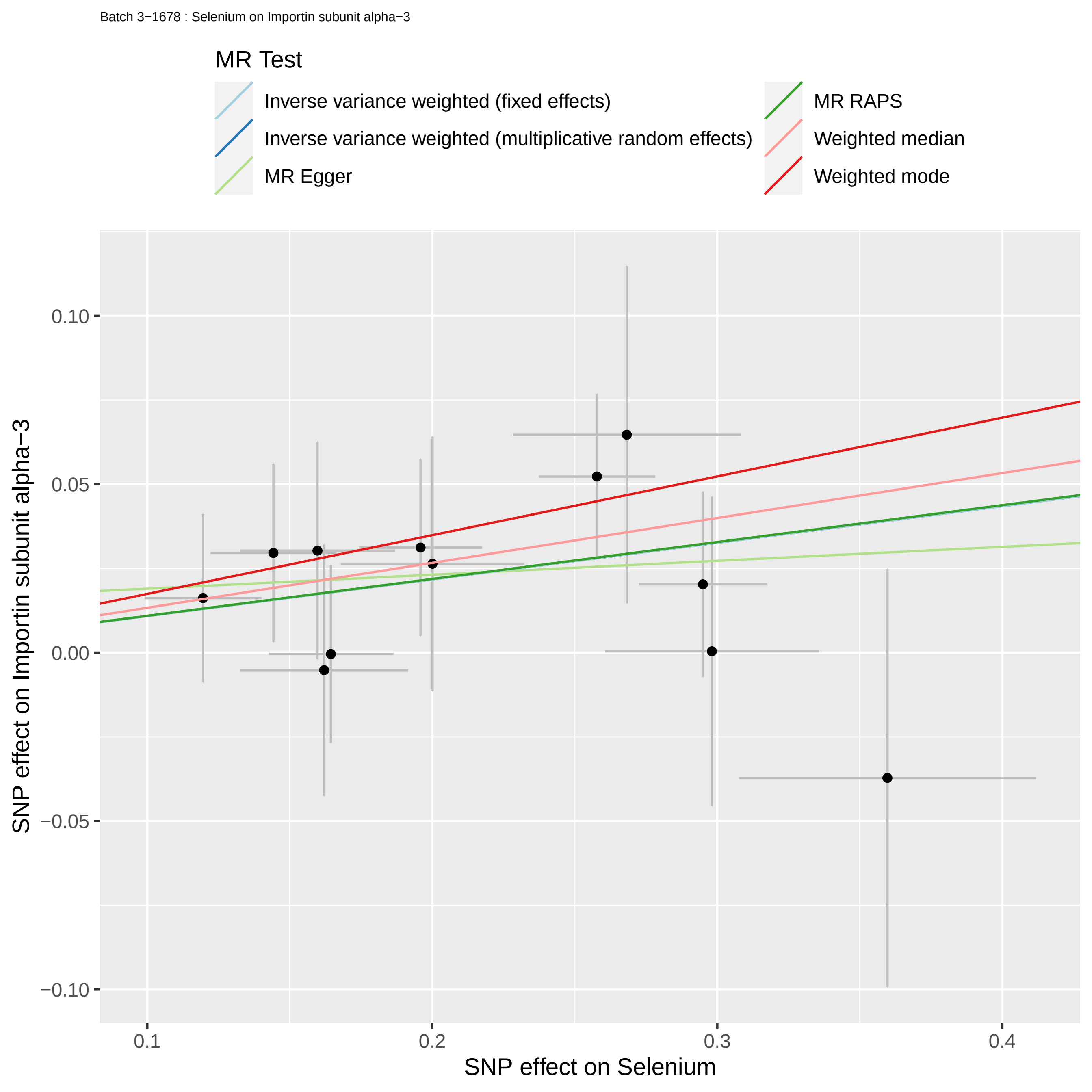

Batch 3−1678 : Selenium on Importin subunit alpha−3
MR Test
Inverse variance weighted (fixed effects)
Inverse variance weighted (multiplicative random effects)
MR Egger
MR RAPS
Weighted median
Weighted mode
0.10
0.05
0.00
−0.05
−0.10
0.1
0.2
0.3
0.4
SNP effect on Importin subunit alpha−3
SNP effect on Selenium

Batch 3−1679 : Selenium on GTPase KRas

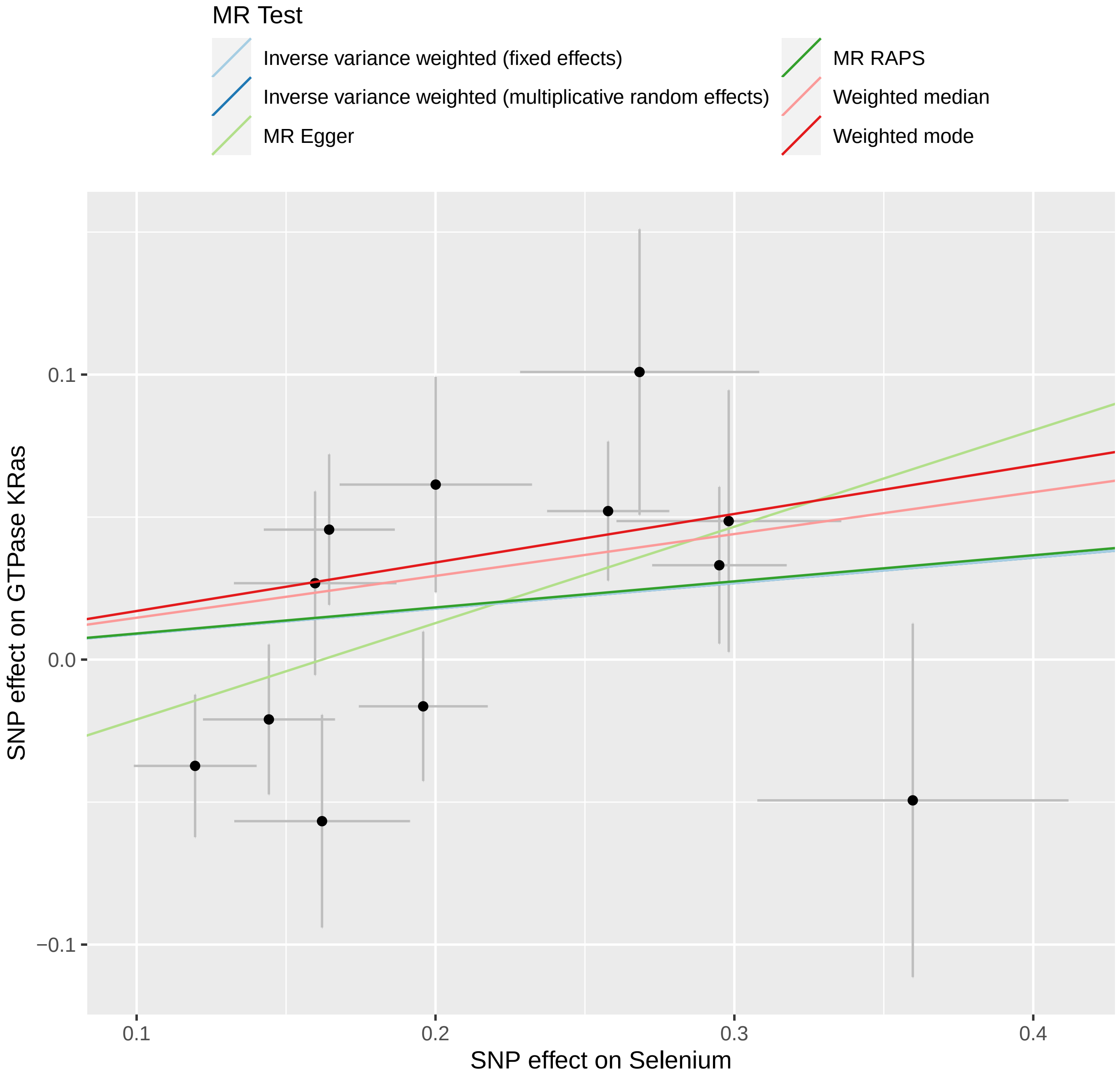

Batch 3−1680 : Selenium on Kremen protein 1

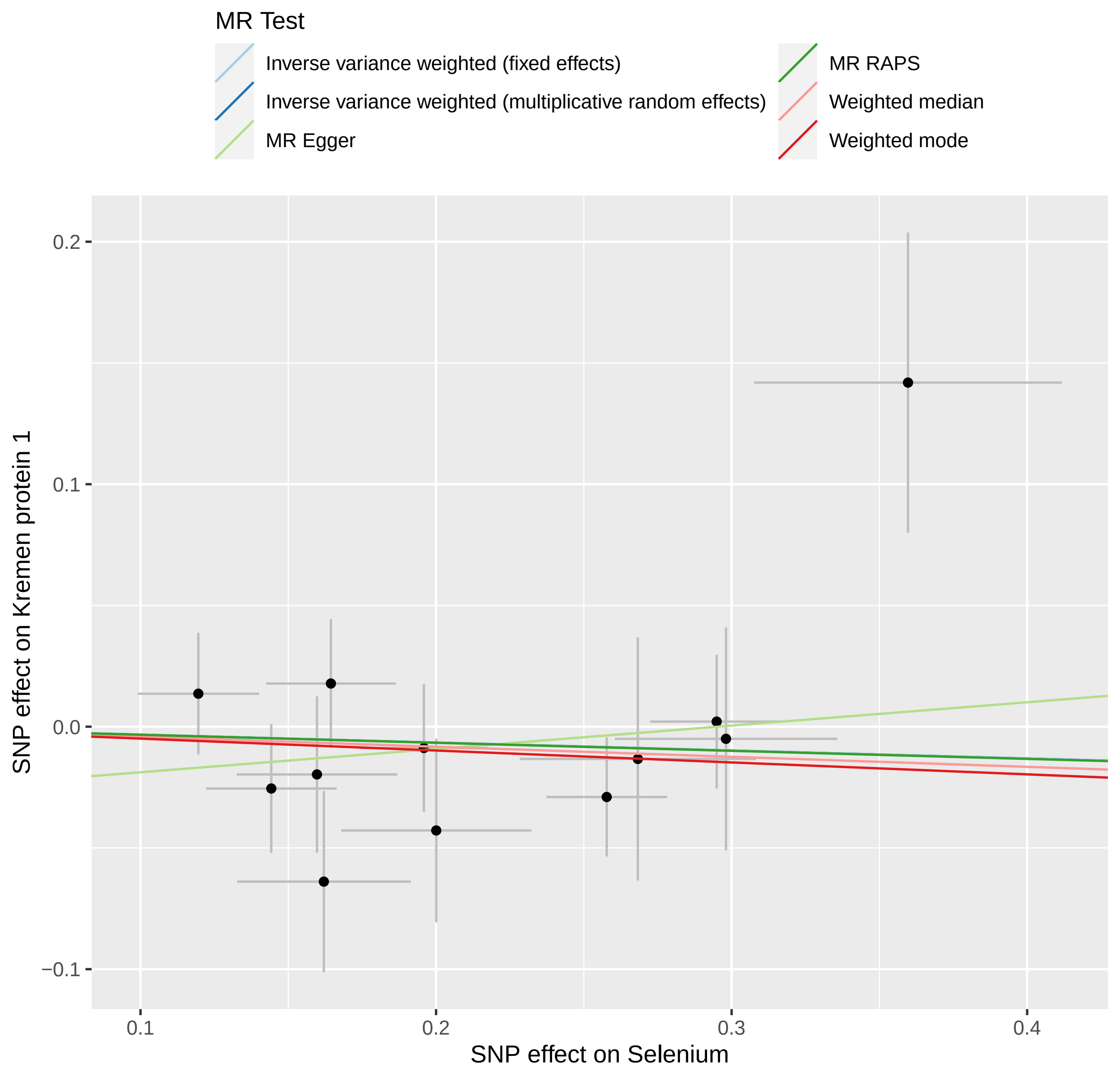

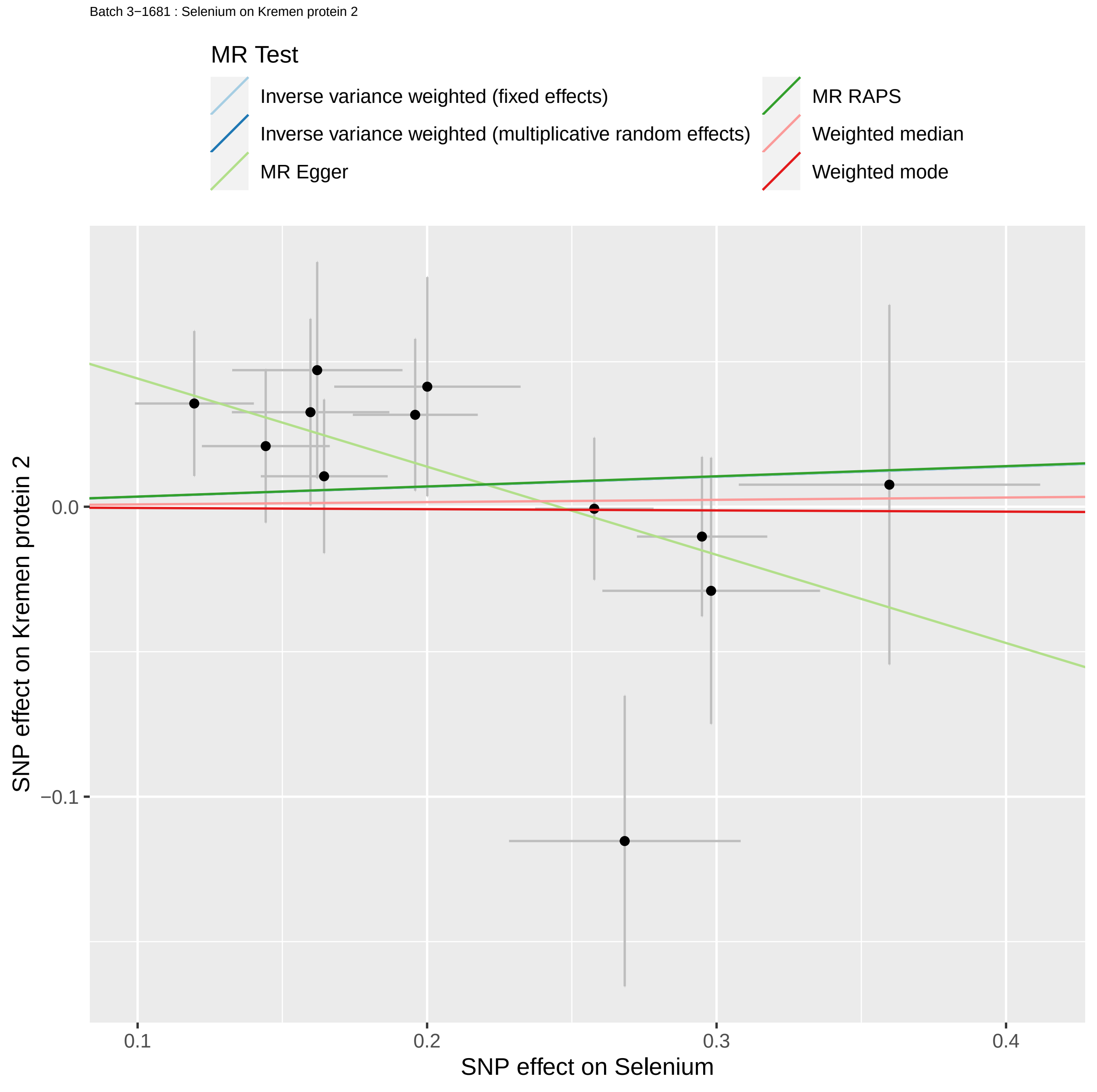

Batch 3−1681 : Selenium on Kremen protein 2
MR Test
Inverse variance weighted (fixed effects)
Inverse variance weighted (multiplicative random effects)
MR Egger
MR RAPS
Weighted median
Weighted mode
SNP effect on Kremen protein 2
0.0
−0.1
0.1
0.2
0.3
0.4
SNP effect on Selenium

Batch 3−1682 : Selenium on Keratin, type I cytoskeletal 17

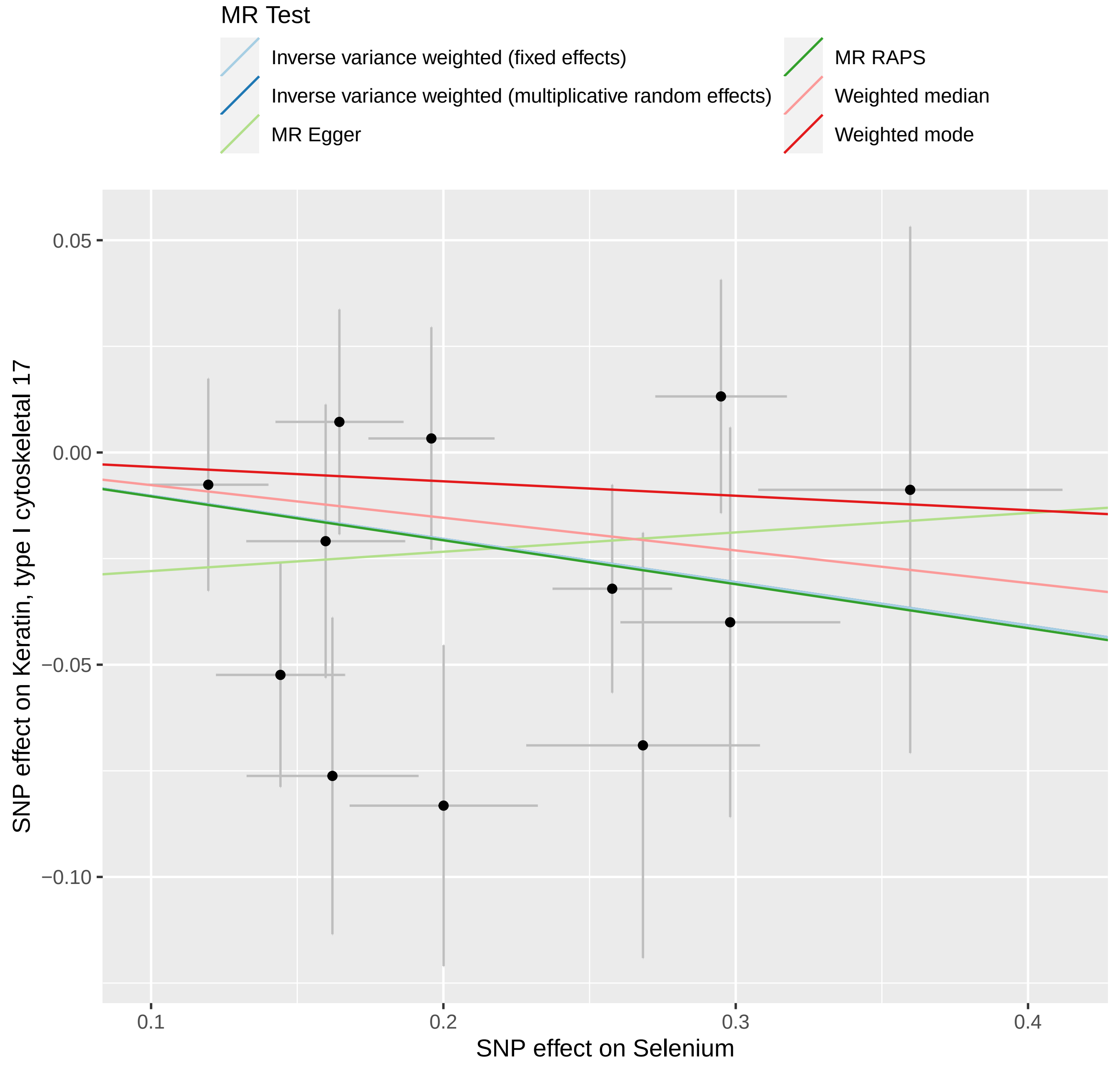

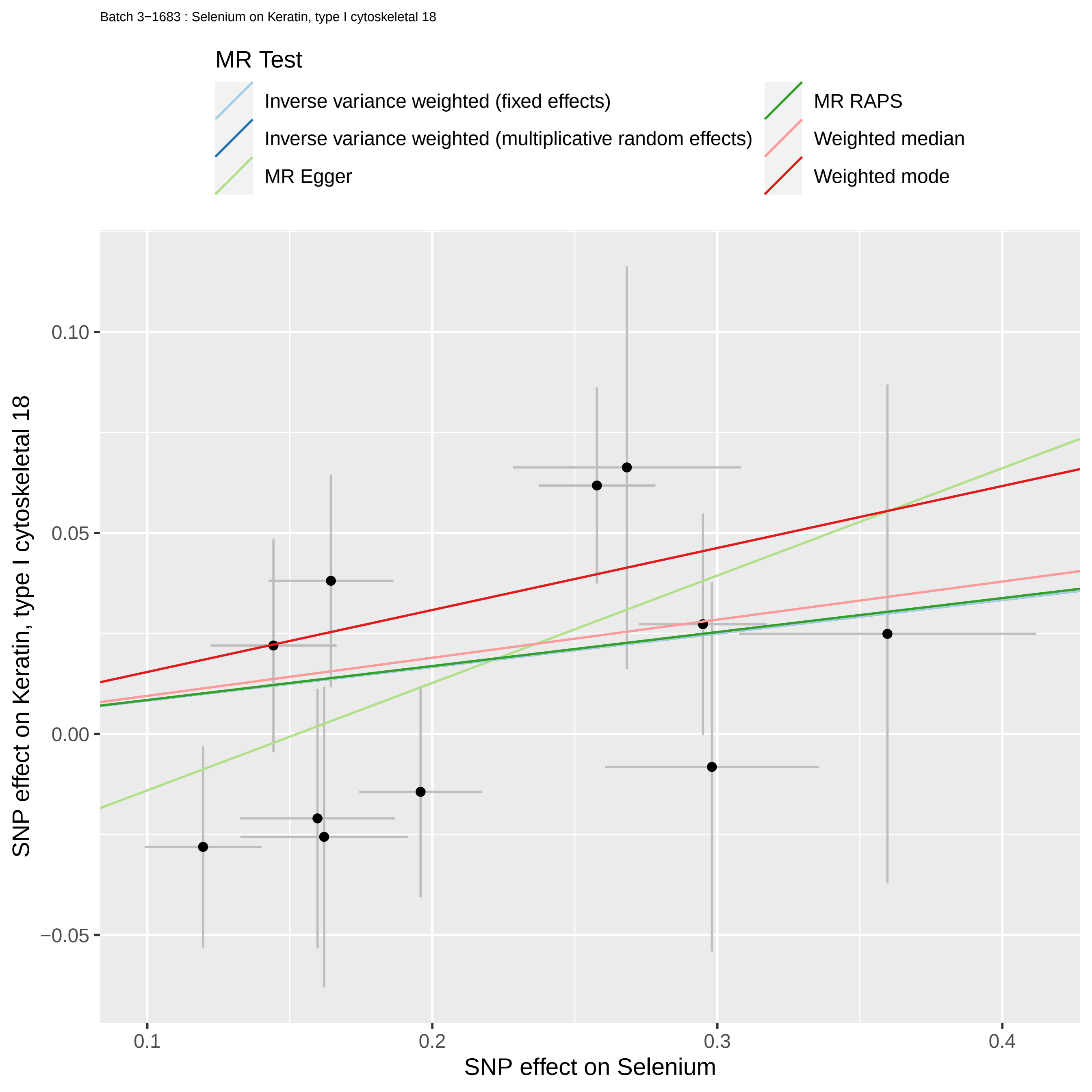

Batch 3−1683 : Selenium on Keratin, type I cytoskeletal 18
MR Test
Inverse variance weighted (fixed effects)
Inverse variance weighted (multiplicative random effects)
MR Egger
MR RAPS
Weighted median
Weighted mode
0.10
0.05
0.00
−0.05
0.1
0.2
0.3
0.4
SNP effect on Keratin, type I cytoskeletal 18
SNP effect on Selenium

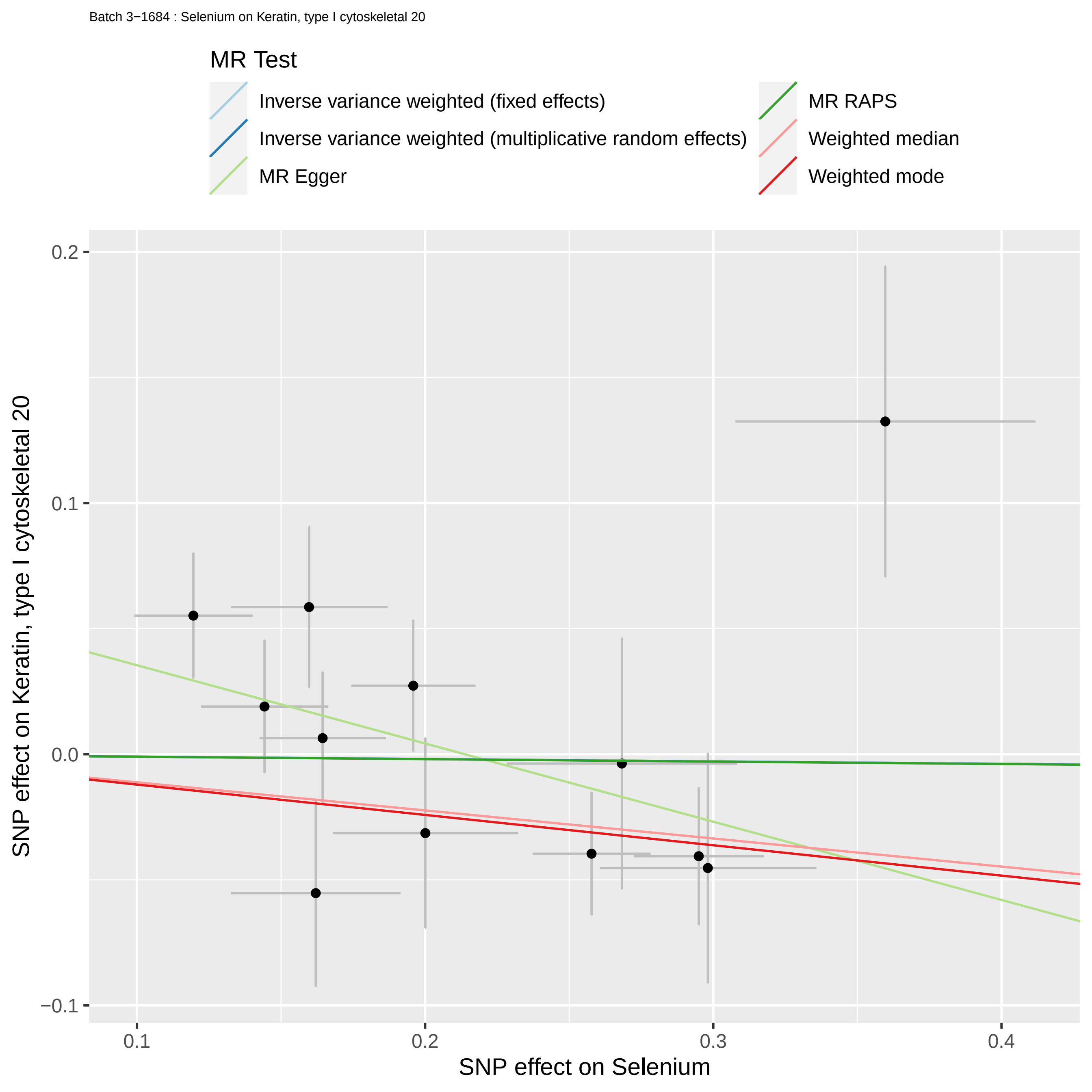

Batch 3−1684 : Selenium on Keratin, type I cytoskeletal 20
MR Test
Inverse variance weighted (fixed effects)
Inverse variance weighted (multiplicative random effects)
MR Egger
MR RAPS
Weighted median
Weighted mode
0.2
0.1
0.0
−0.1
0.1
0.2
0.3
0.4
SNP effect on Selenium
SNP effect on Keratin, type I cytoskeletal 20

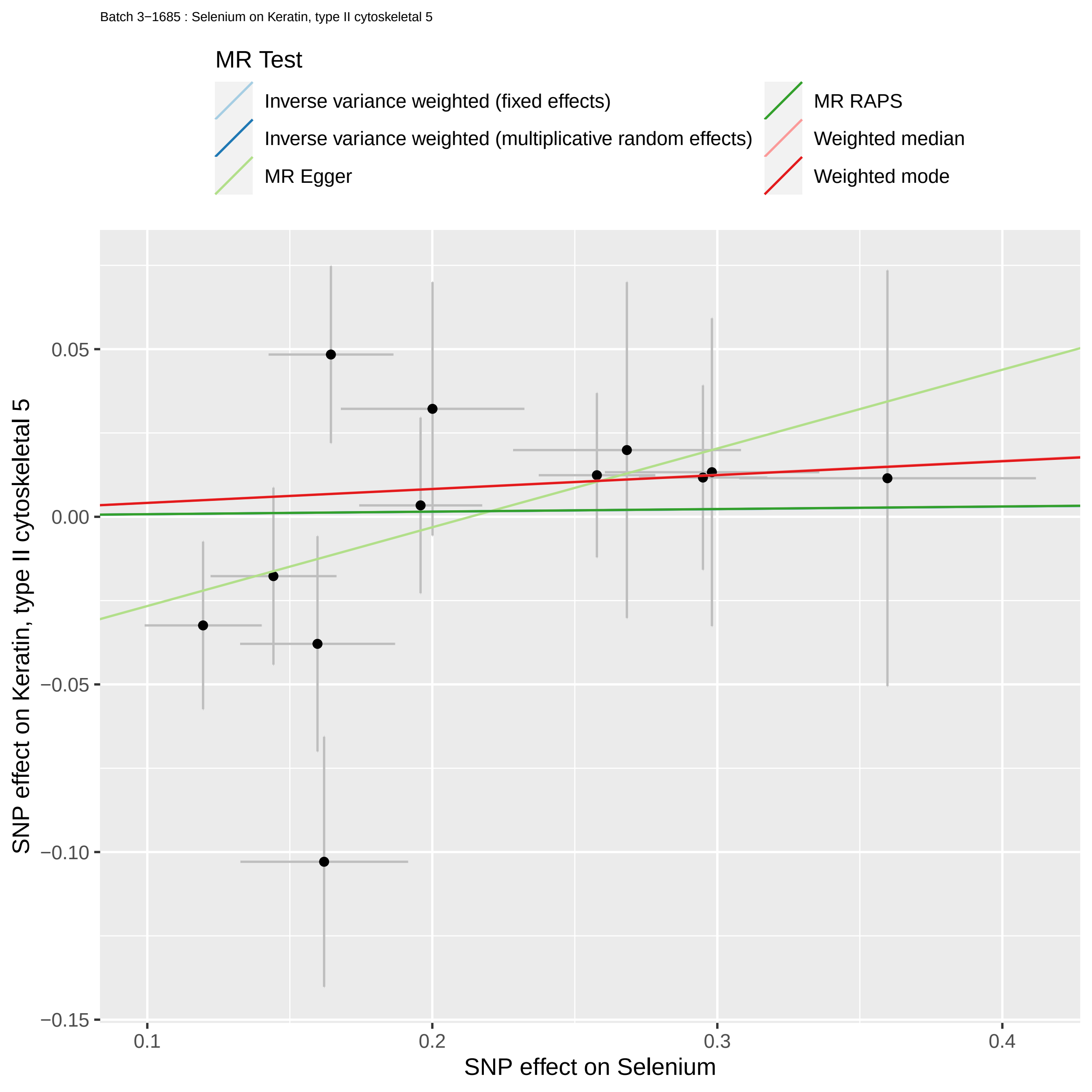

Batch 3−1685 : Selenium on Keratin, type II cytoskeletal 5
MR Test
Inverse variance weighted (fixed effects)
Inverse variance weighted (multiplicative random effects)
MR Egger
MR RAPS
Weighted median
Weighted mode
SNP effect on Keratin, type II cytoskeletal 5
0.05
0.00
−0.05
−0.10
−0.15
0.1
0.2
0.3
0.4
SNP effect on Selenium

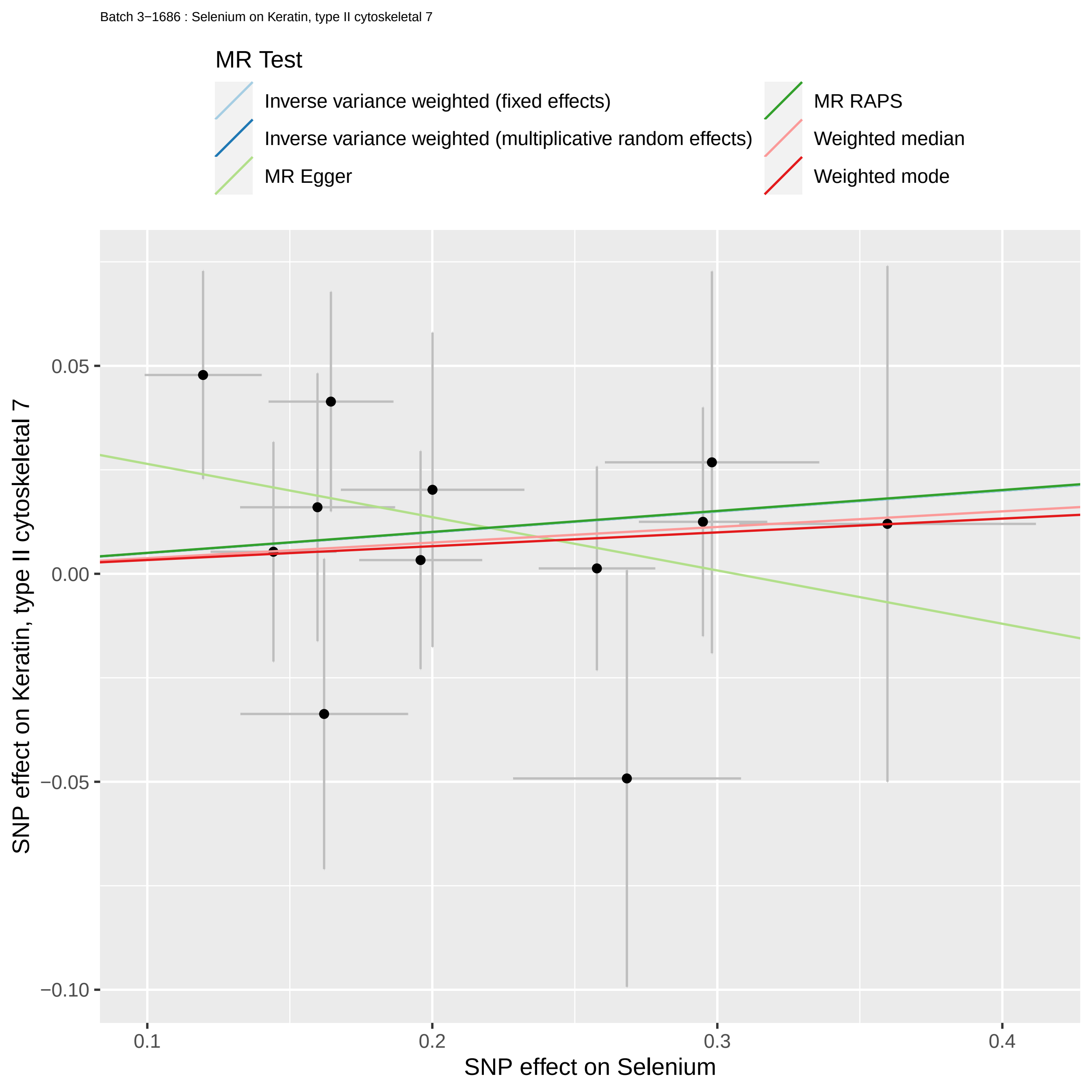

Batch 3−1686 : Selenium on Keratin, type II cytoskeletal 7
MR Test
Inverse variance weighted (fixed effects)
Inverse variance weighted (multiplicative random effects)
MR Egger
MR RAPS
Weighted median
Weighted mode
SNP effect on Keratin, type II cytoskeletal 7
0.05
0.00
−0.05
−0.10
0.1
0.2
0.3
0.4
SNP effect on Selenium

Batch 3−1687 : Selenium on Keratin−associated protein 2−4

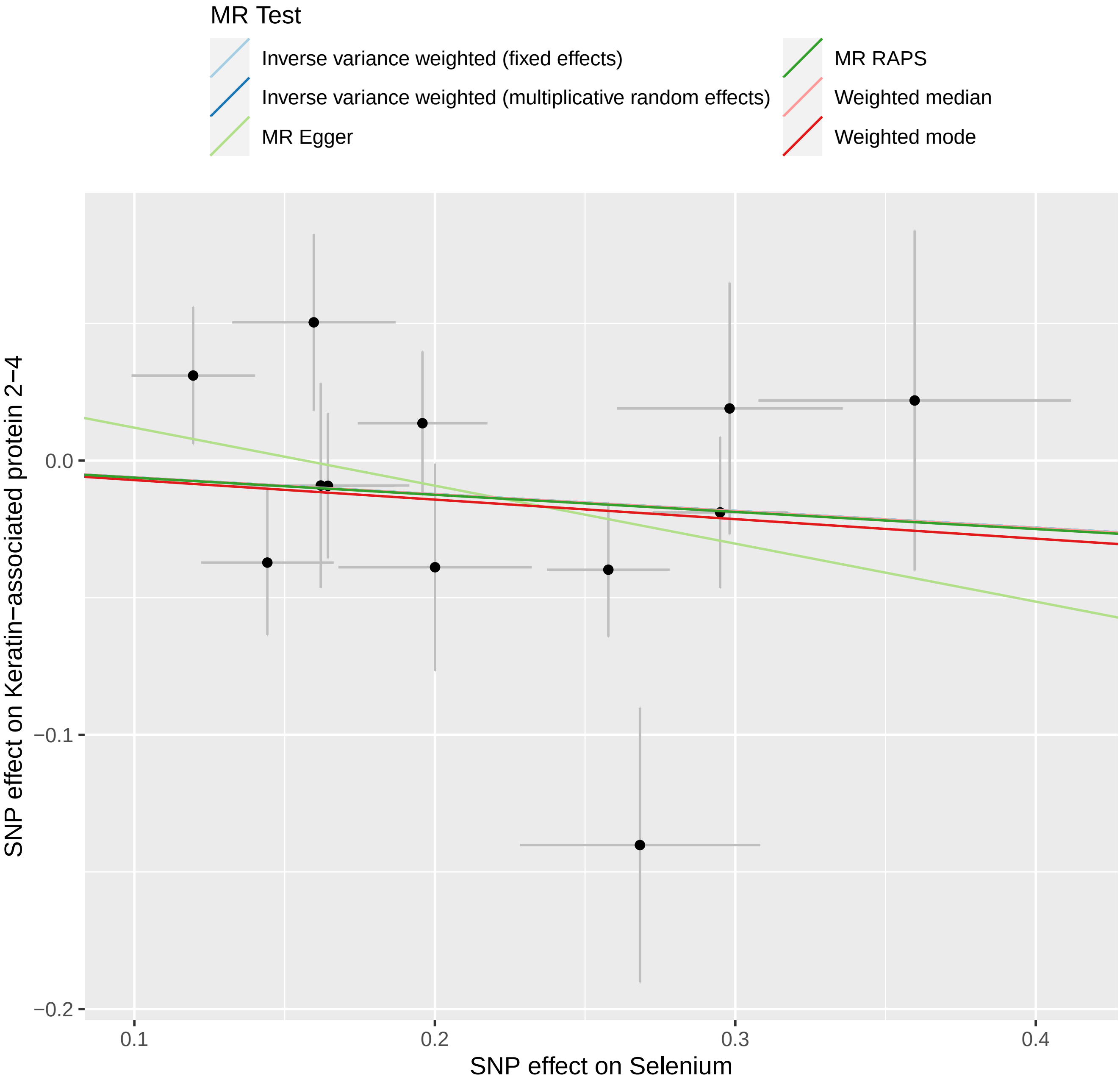

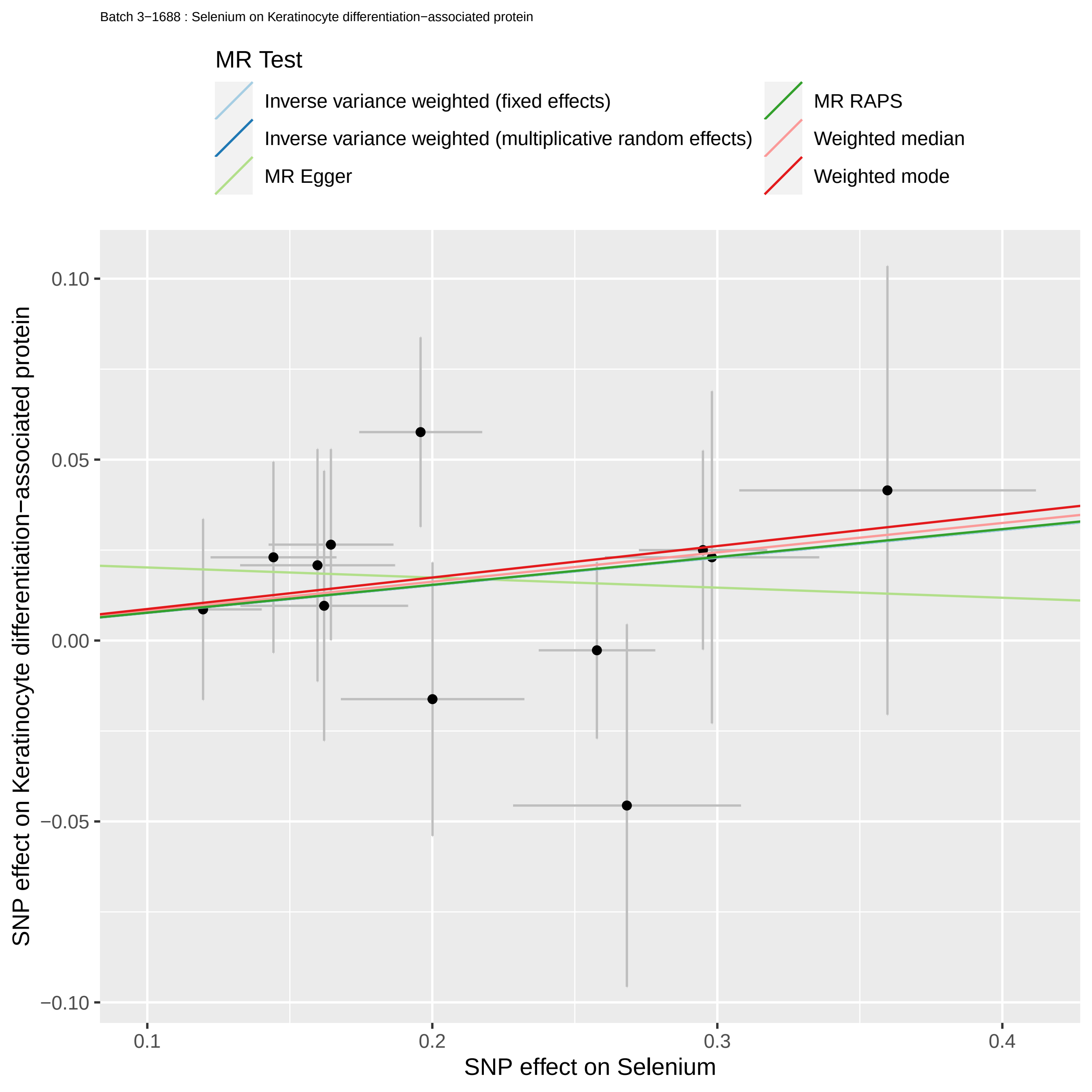
Batch 3−1688 : Selenium on Keratinocyte differentiation−associated protein
MR Test
Inverse variance weighted (fixed effects)
Inverse variance weighted (multiplicative random effects)
MR Egger
MR RAPS
Weighted median
Weighted mode
0.10
0.05
0.00
−0.05
−0.10
0.1
0.2
0.3
0.4
SNP effect on Keratinocyte differentiation−associated protein
SNP effect on Selenium

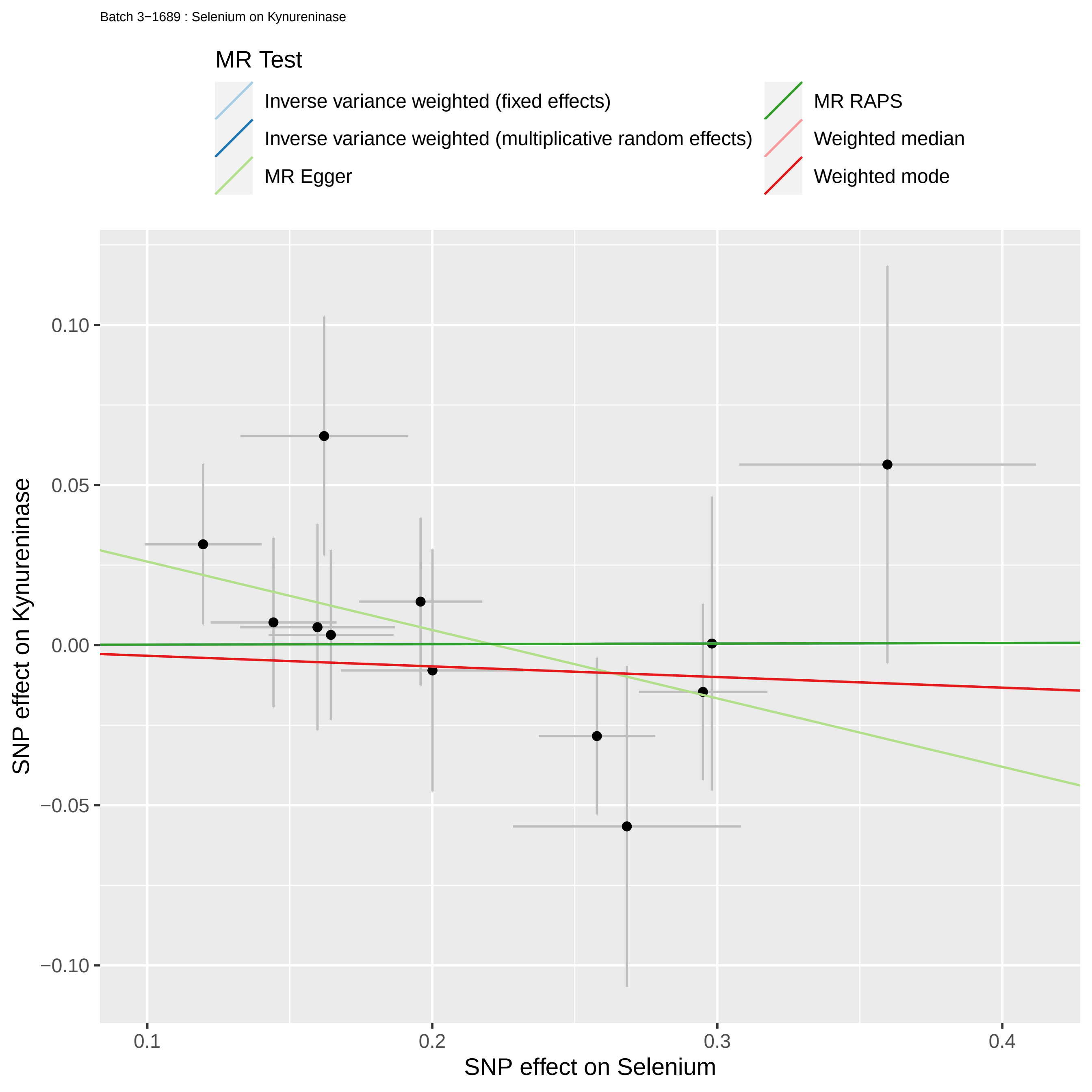
Batch 3−1689 : Selenium on Kynureninase
MR Test
Inverse variance weighted (fixed effects)
Inverse variance weighted (multiplicative random effects)
MR Egger
MR RAPS
Weighted median
Weighted mode
0.10
0.05
0.00
−0.05
−0.10
0.1
0.2
0.3
0.4
SNP effect on Kynureninase
SNP effect on Selenium

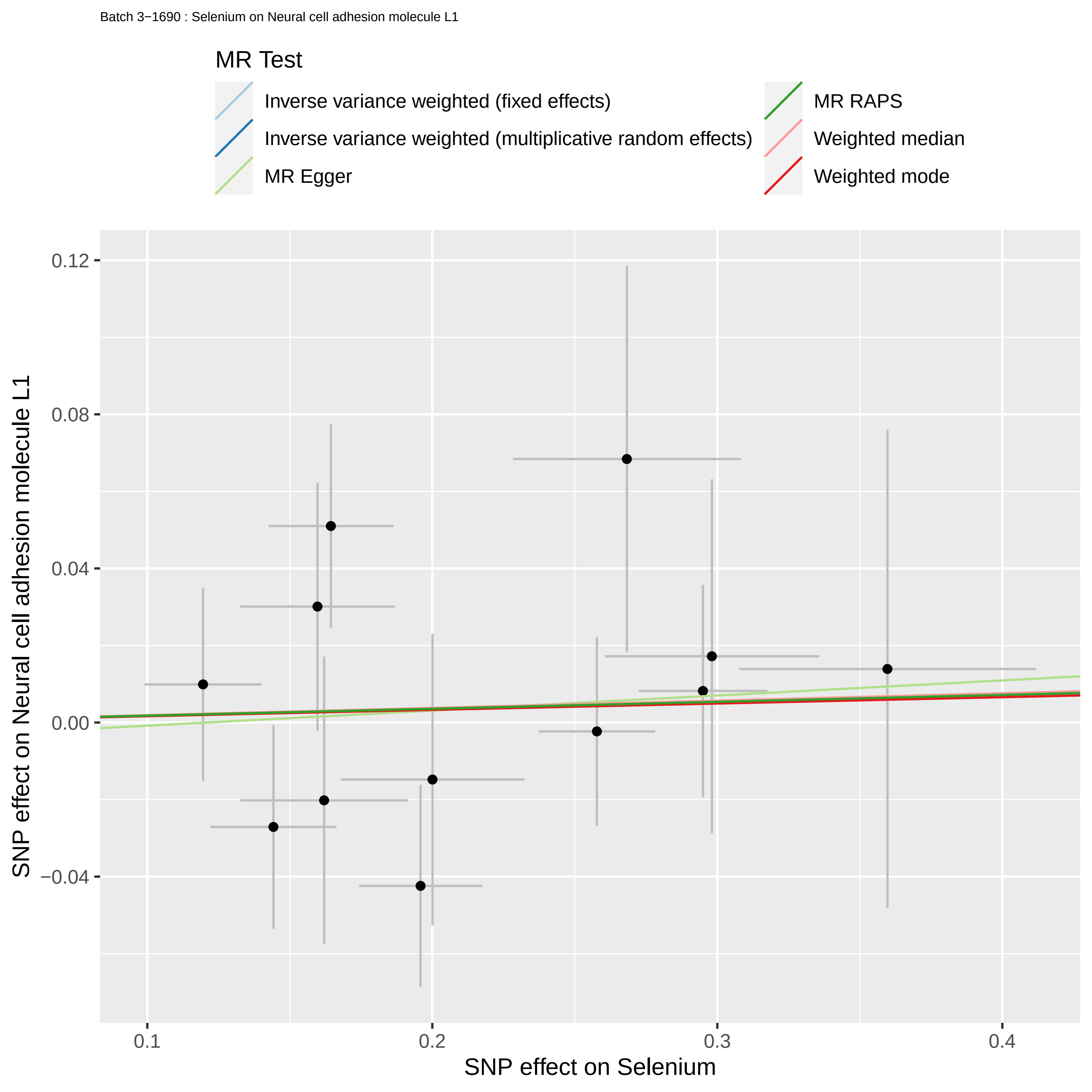

Batch 3−1690 : Selenium on Neural cell adhesion molecule L1
MR Test
Inverse variance weighted (fixed effects)
Inverse variance weighted (multiplicative random effects)
MR Egger
MR RAPS
Weighted median
Weighted mode
0.12
0.08
0.04
0.00
−0.04
0.1
0.2
0.3
0.4
SNP effect on Neural cell adhesion molecule L1
SNP effect on Selenium

Batch 3−1691 : Selenium on Ladinin−1

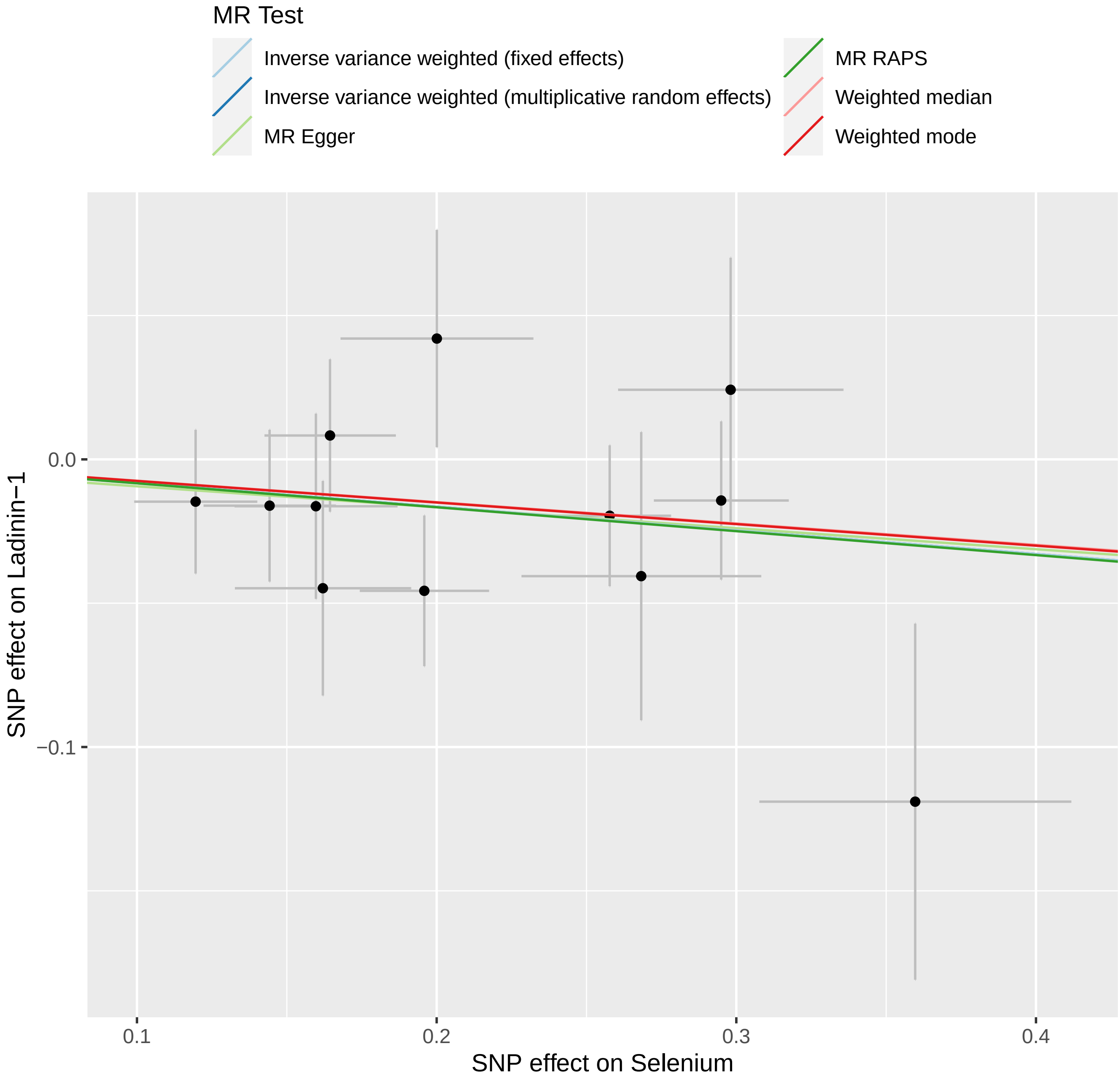

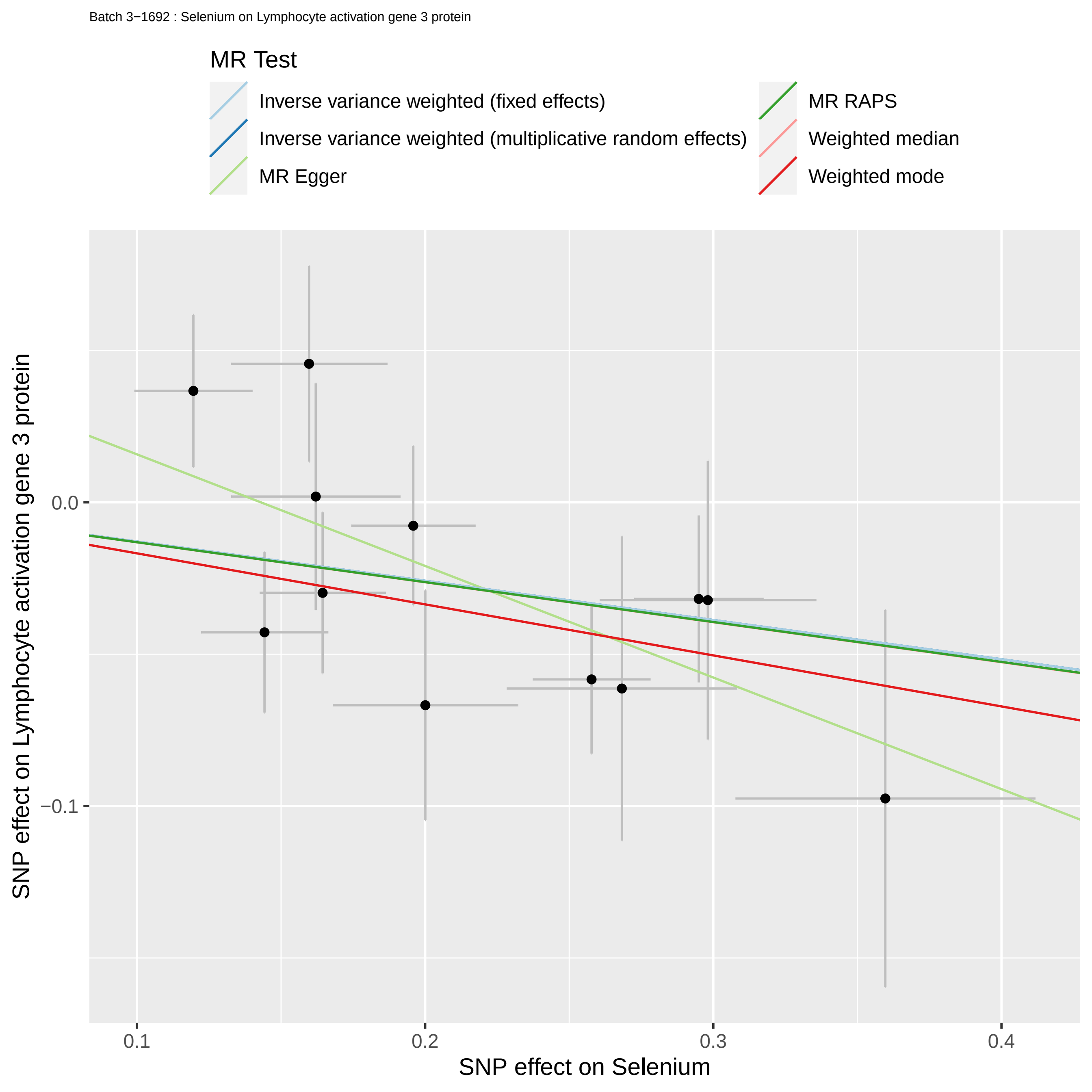

Batch 3−1692 : Selenium on Lymphocyte activation gene 3 protein
MR Test
Inverse variance weighted (fixed effects)
Inverse variance weighted (multiplicative random effects)
MR Egger
MR RAPS
Weighted median
Weighted mode
SNP effect on Lymphocyte activation gene 3 protein
0.0
−0.1
0.1
0.2
0.3
0.4
SNP effect on Selenium

Batch 3−1693 : Selenium on Lymphocyte activation gene 3 protein

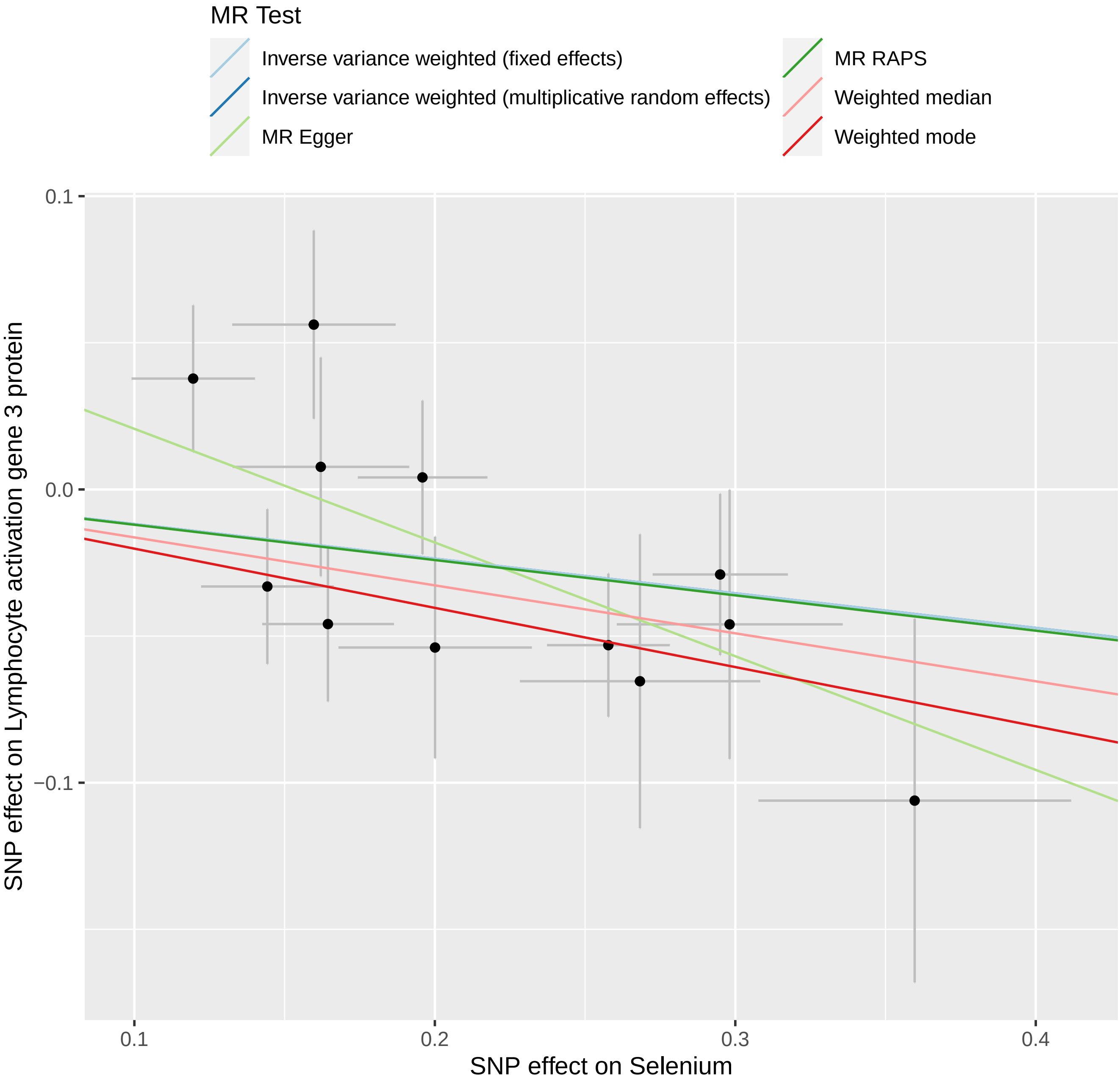

Batch 3−1694 : Selenium on Leukocyte−associated immunoglobulin−like receptor 1

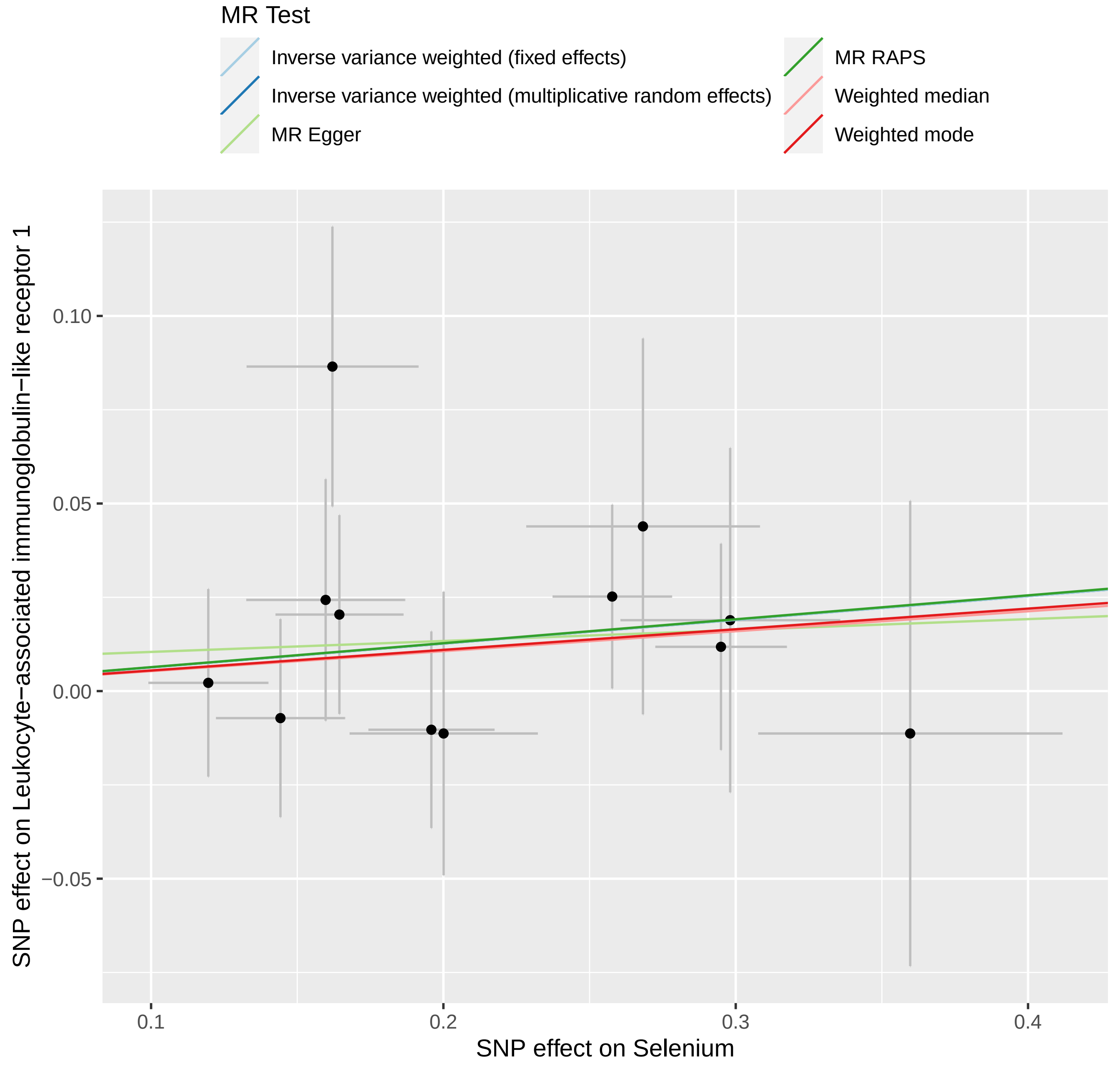

Batch 3−1695 : Selenium on Laminin

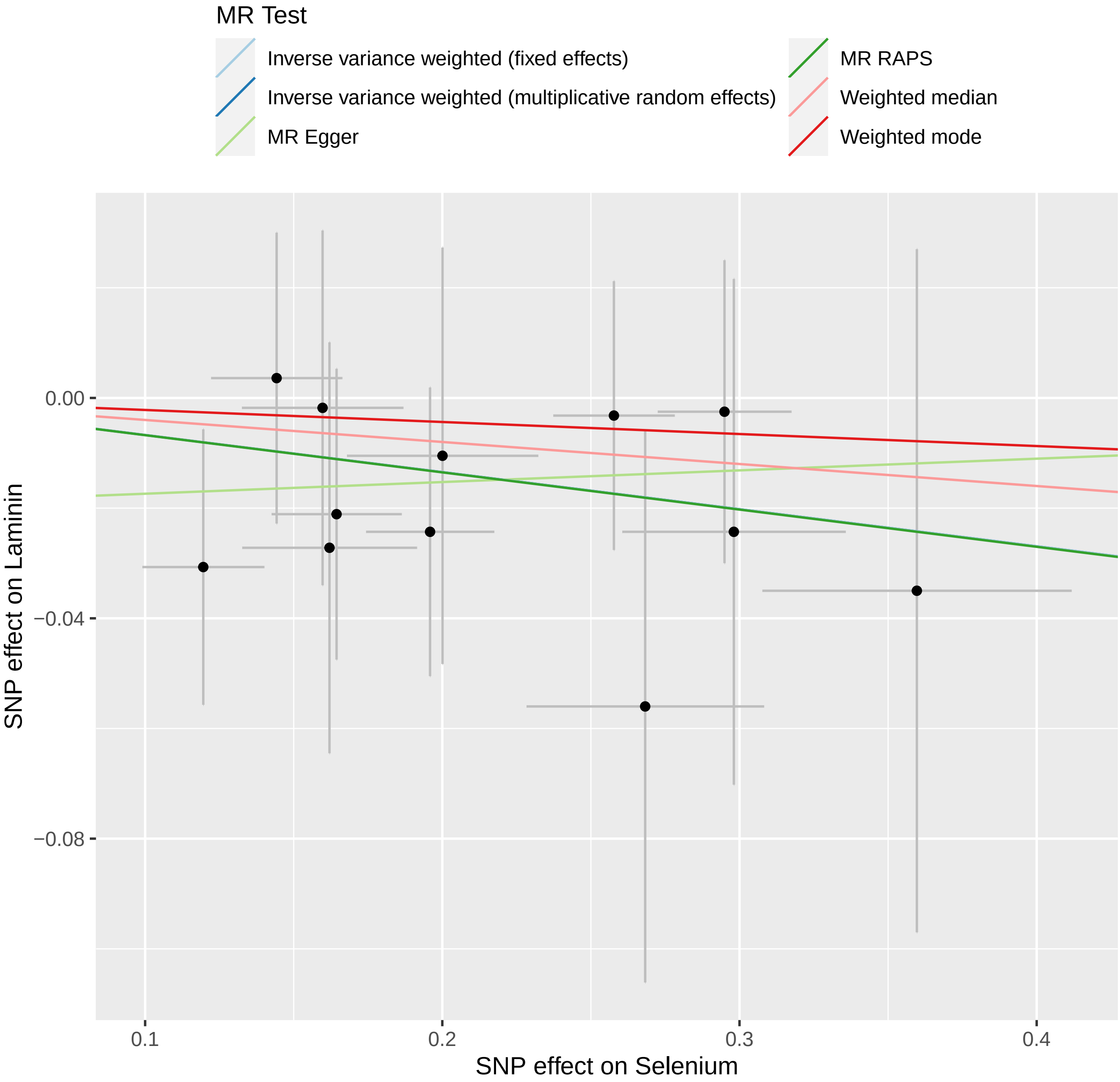

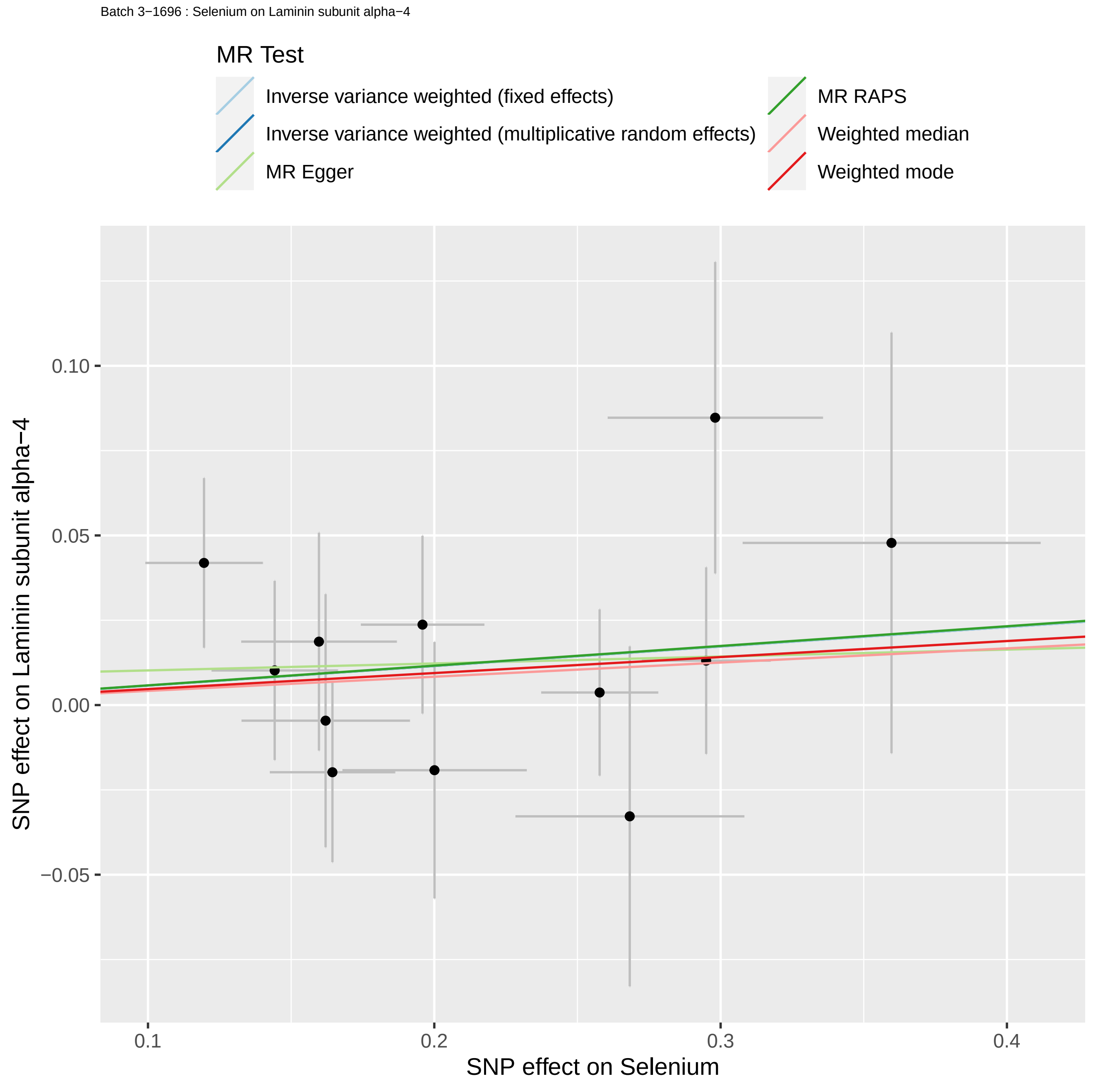
Batch 3−1696 : Selenium on Laminin subunit alpha−4
MR Test
Inverse variance weighted (fixed effects)
Inverse variance weighted (multiplicative random effects)
MR Egger
MR RAPS
Weighted median
Weighted mode
0.10
0.05
0.00
−0.05
0.1
0.2
0.3
0.4
SNP effect on Laminin subunit alpha−4
SNP effect on Selenium

Batch 3−1697 : Selenium on Laminin subunit gamma−2

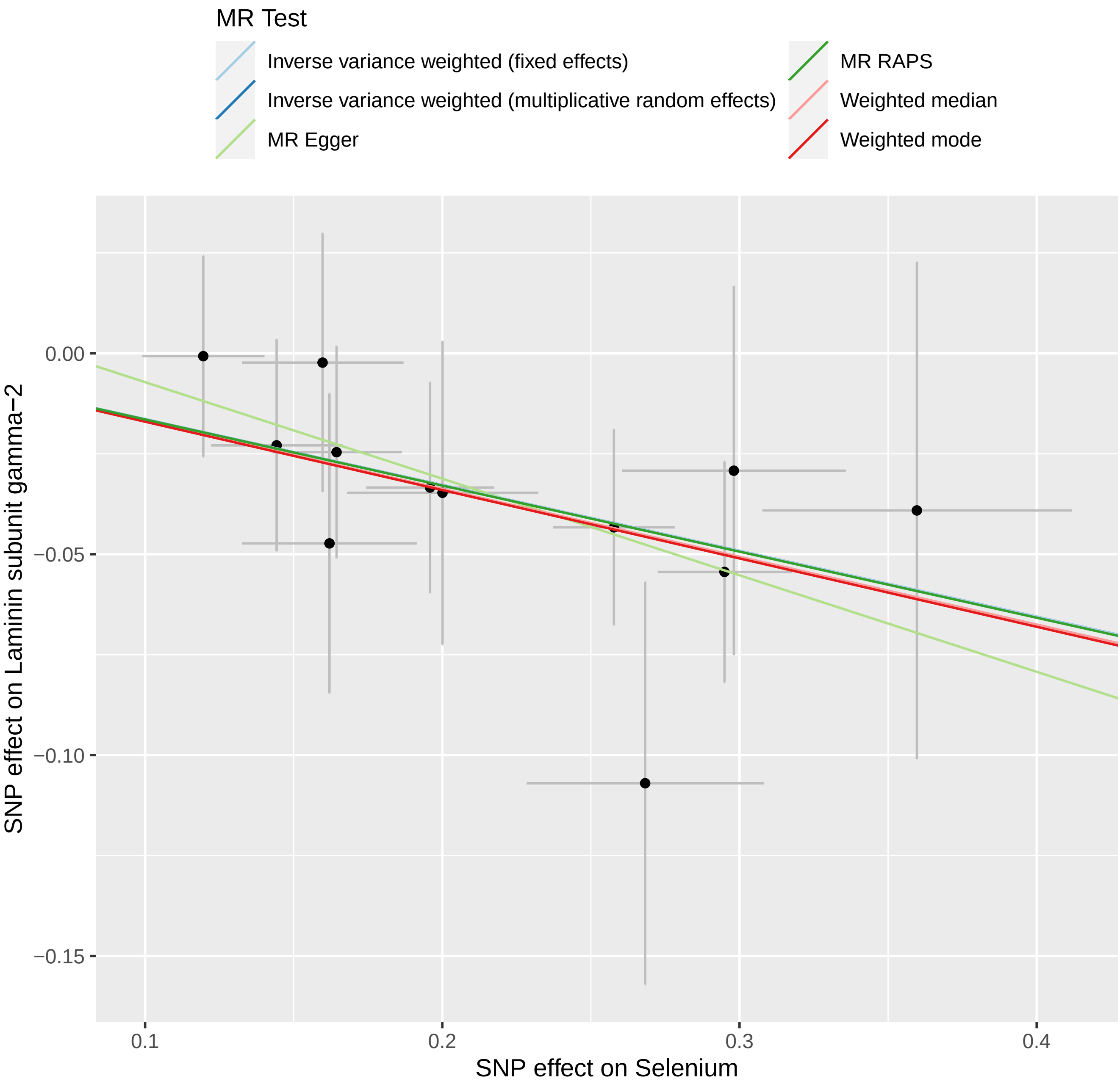

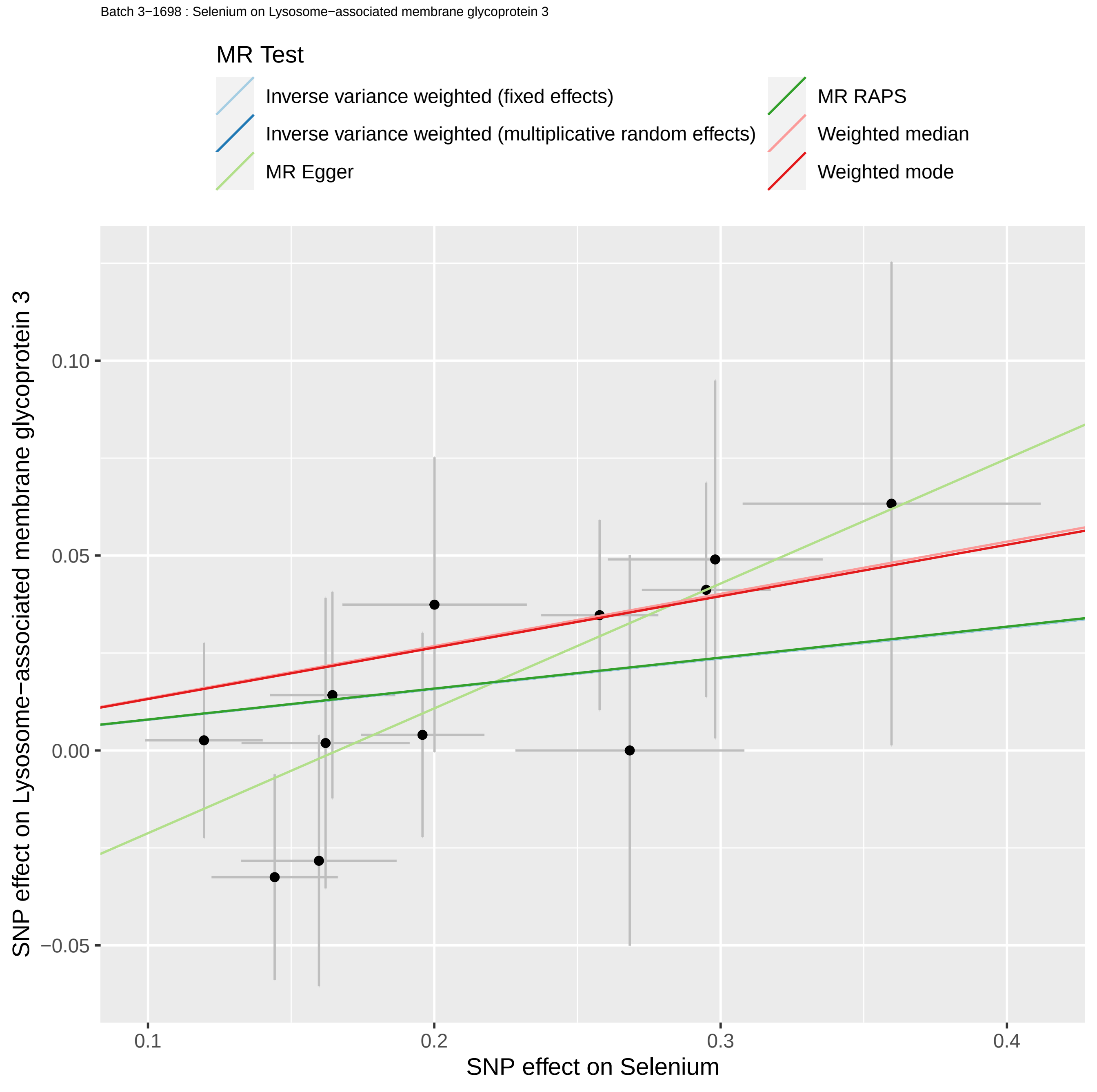

Batch 3−1698 : Selenium on Lysosome−associated membrane glycoprotein 3
MR Test
Inverse variance weighted (fixed effects)
Inverse variance weighted (multiplicative random effects)
MR Egger
MR RAPS
Weighted median
Weighted mode
SNP effect on Lysosome−associated membrane glycoprotein 3
0.10
0.05
0.00
−0.05
0.1
0.2
0.3
0.4
SNP effect on Selenium

Batch 3−1699 : Selenium on Ragulator complex protein LAMTOR3

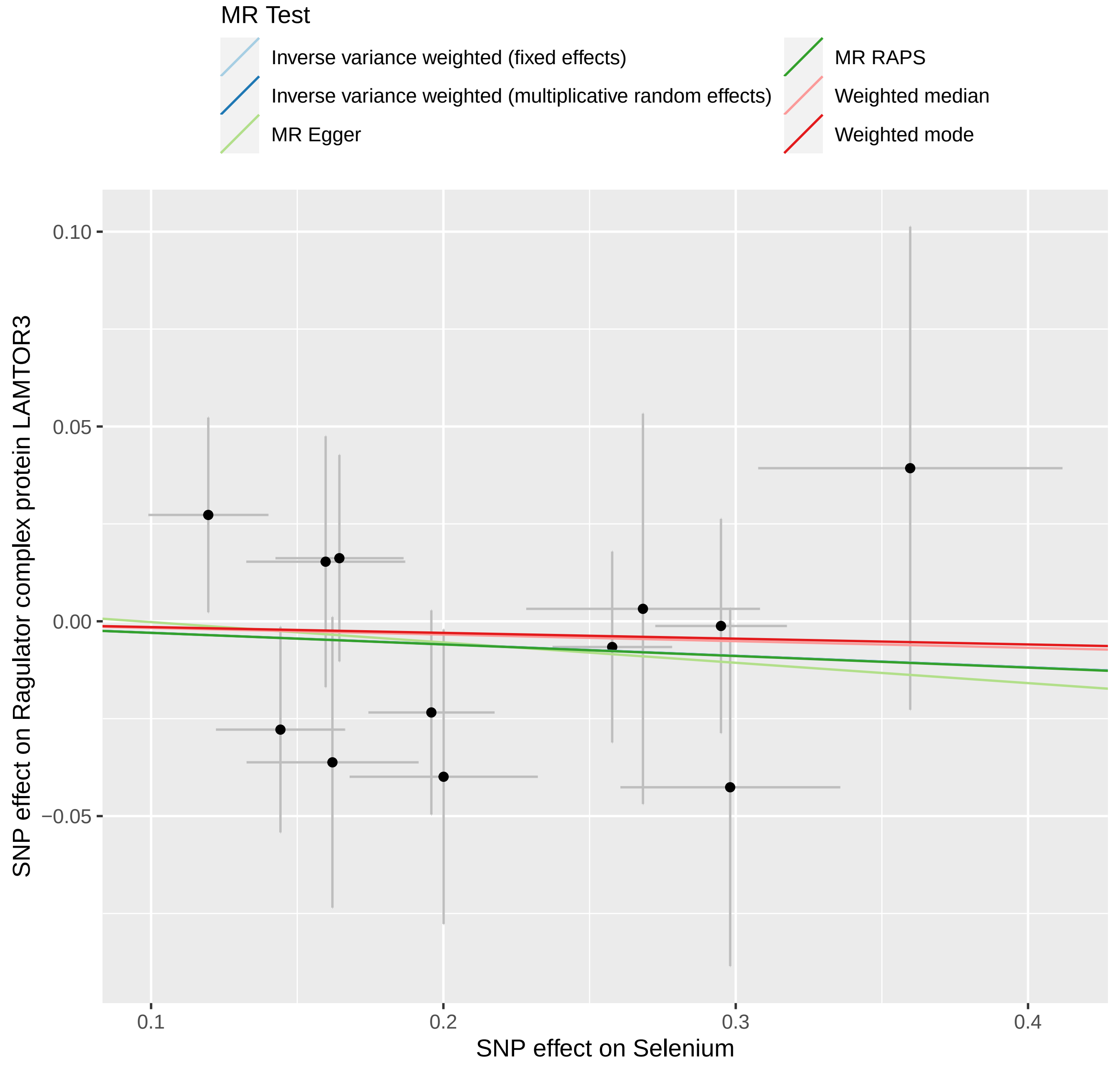

Batch 3−1700 : Selenium on Glycosyltransferase−like protein LARGE1

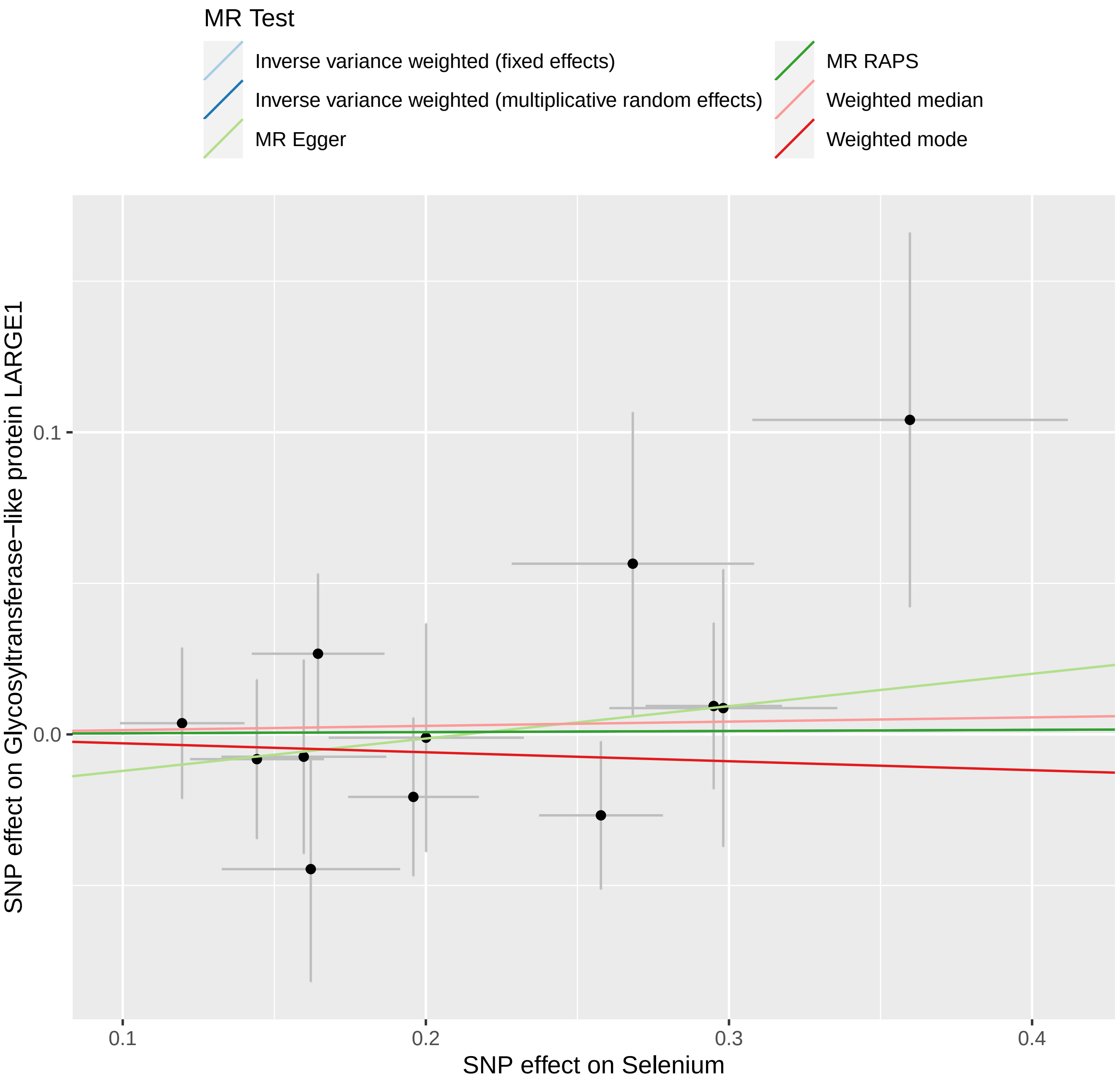

Batch 3−1701 : Selenium on Linker for activation of T−cells family member 1

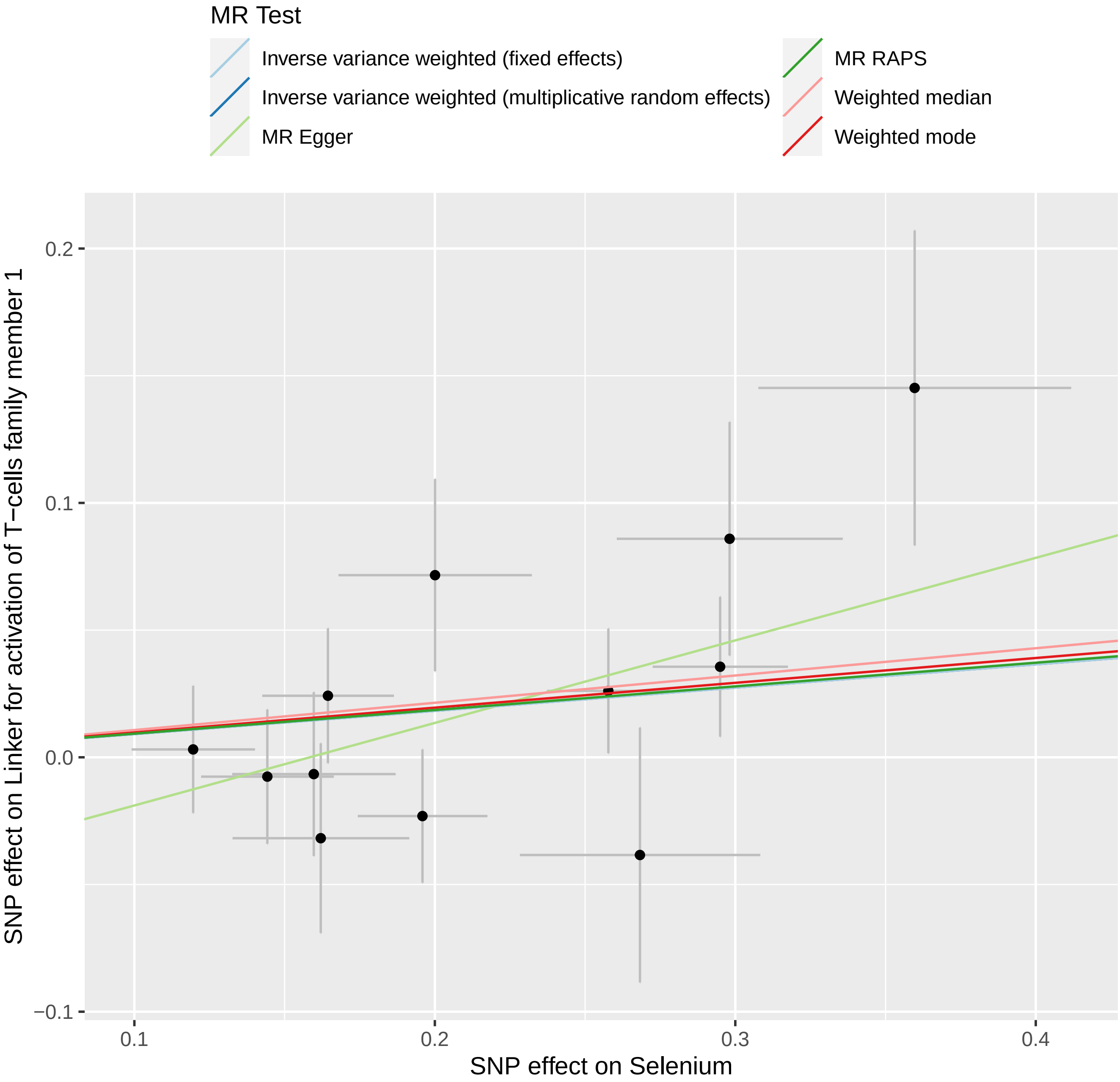

Batch 3−1702 : Selenium on Linker for activation of T−cells family member 2

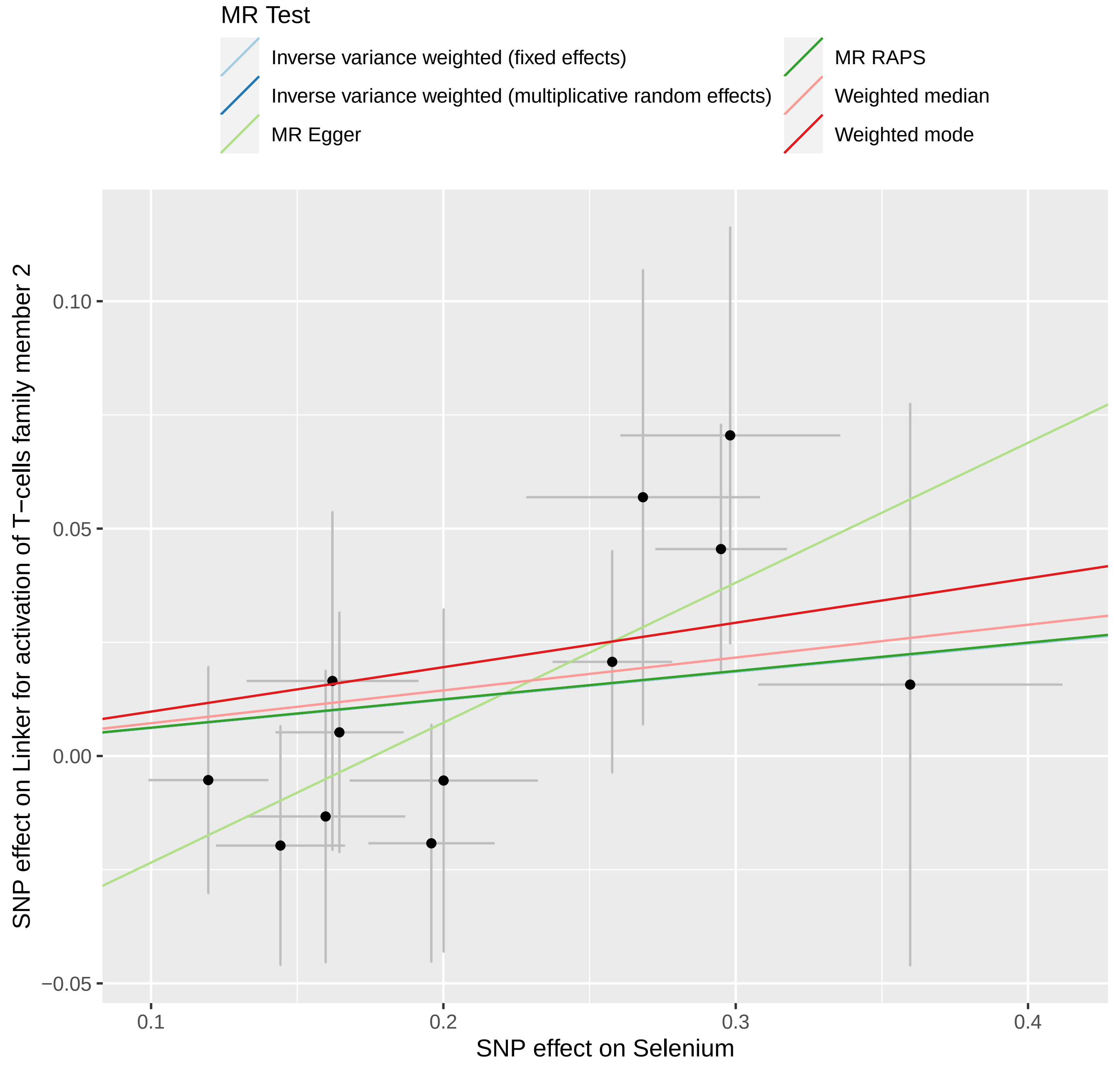

Batch 3−1703 : Selenium on Layilin

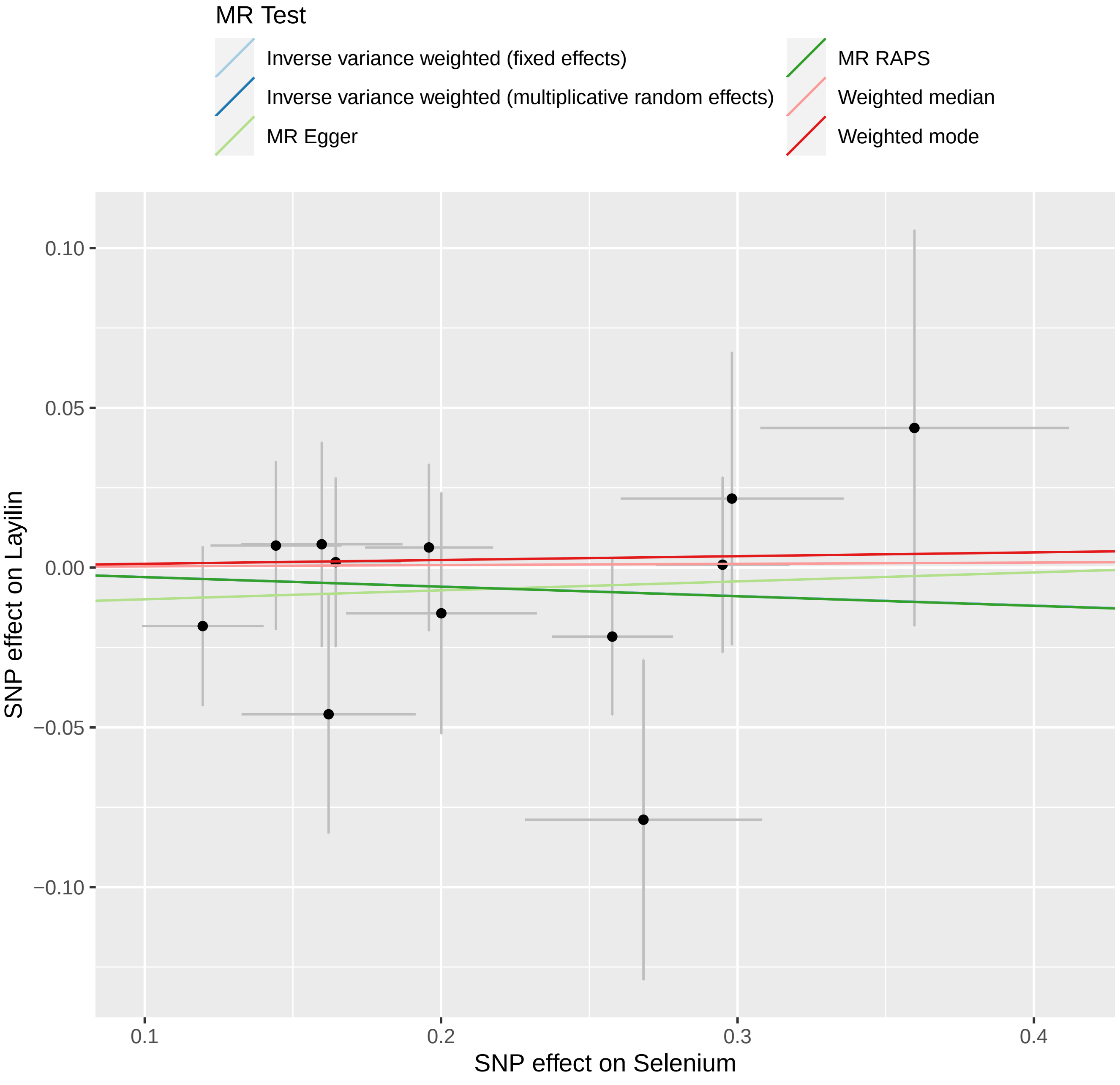

Batch 3−1704 : Selenium on Tyrosine−protein kinase Lck

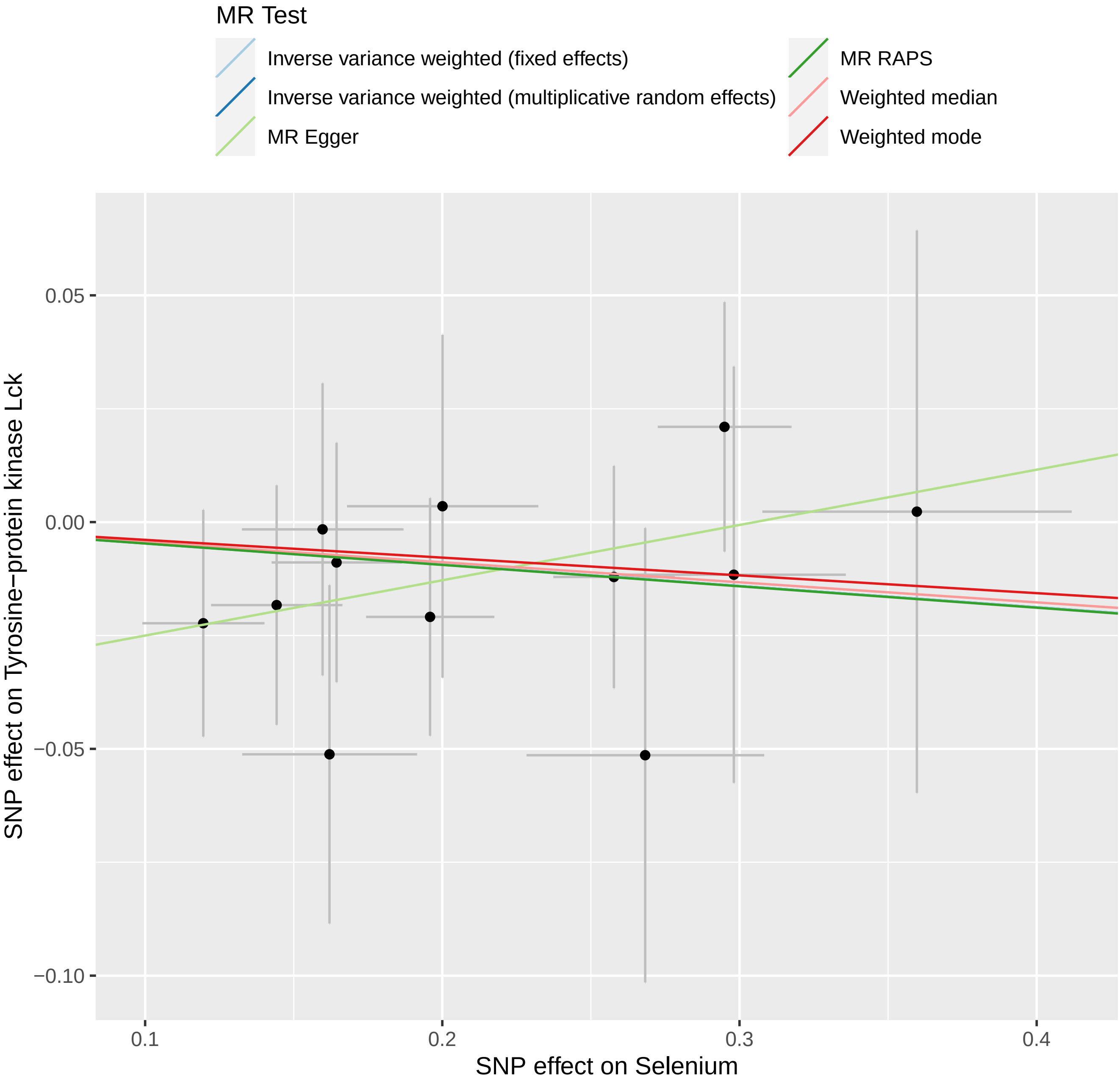

Batch 3−1705 : Selenium on Tyrosine−protein kinase Lck

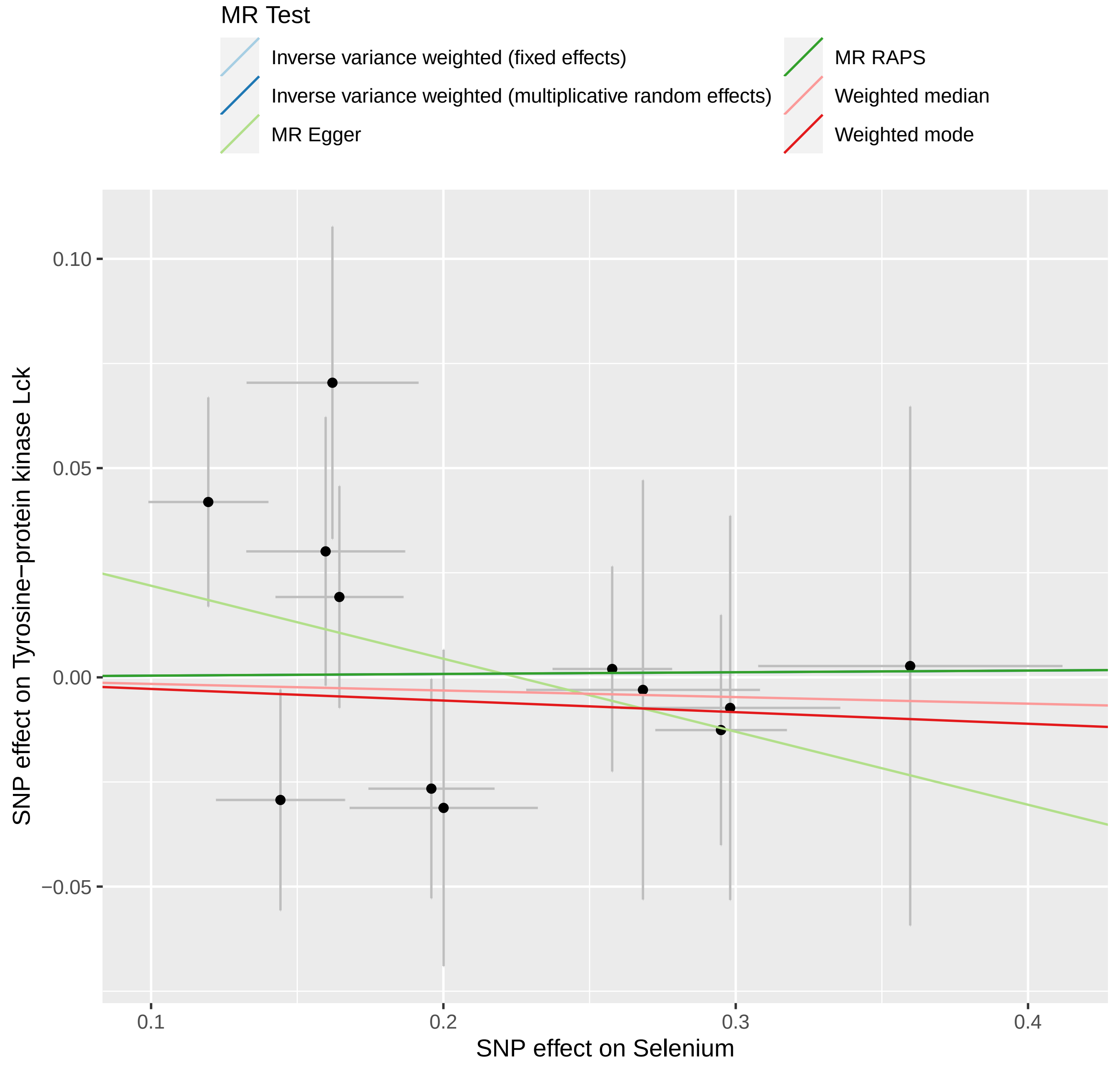

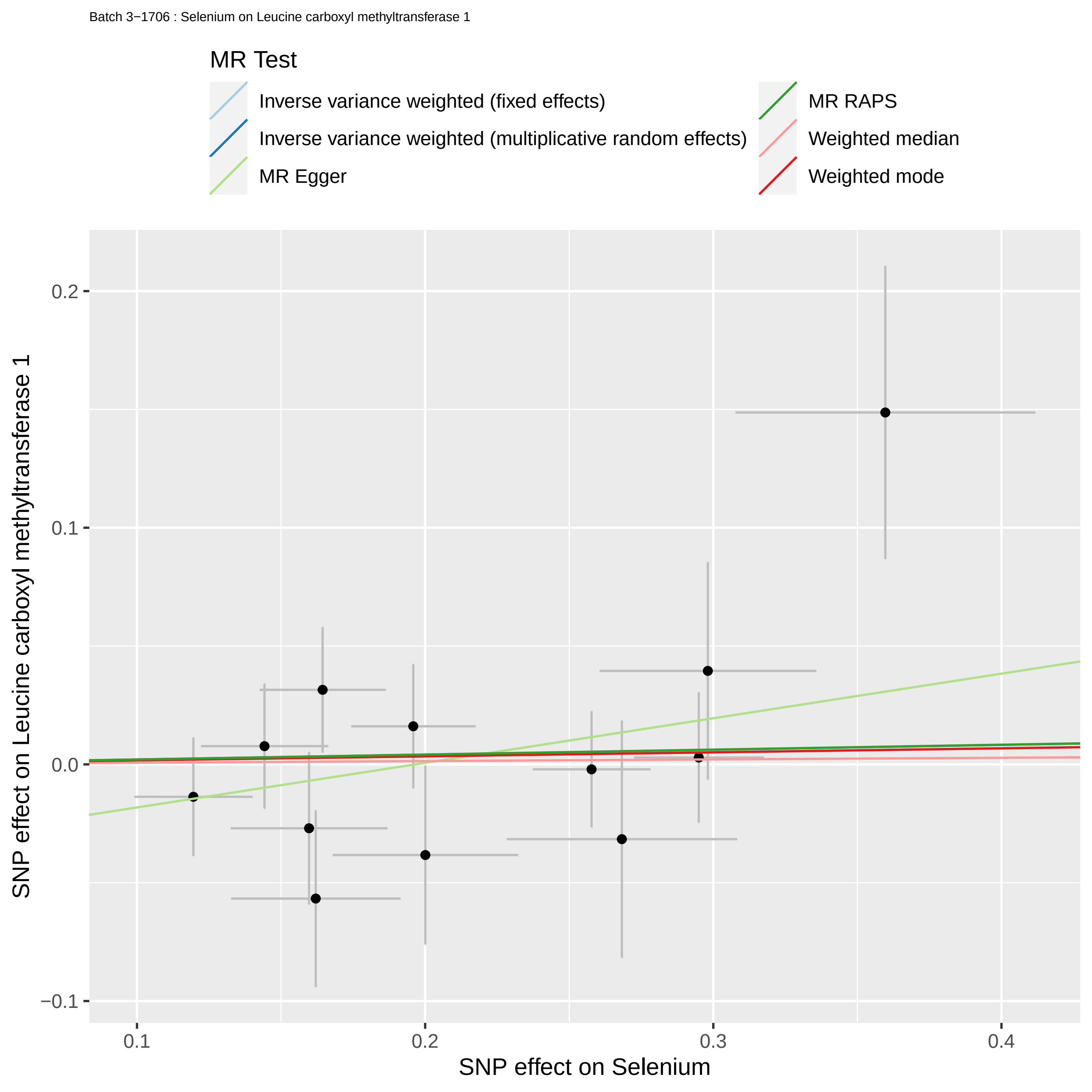

Batch 3−1706 : Selenium on Leucine carboxyl methyltransferase 1
MR Test
Inverse variance weighted (fixed effects)
Inverse variance weighted (multiplicative random effects)
MR Egger
MR RAPS
Weighted median
Weighted mode
0.2
0.1
0.0
−0.1
SNP effect on Leucine carboxyl methyltransferase 1
0.1
0.2
0.3
0.4
SNP effect on Selenium

Batch 3−1707 : Selenium on Epididymal−specific lipocalin−10

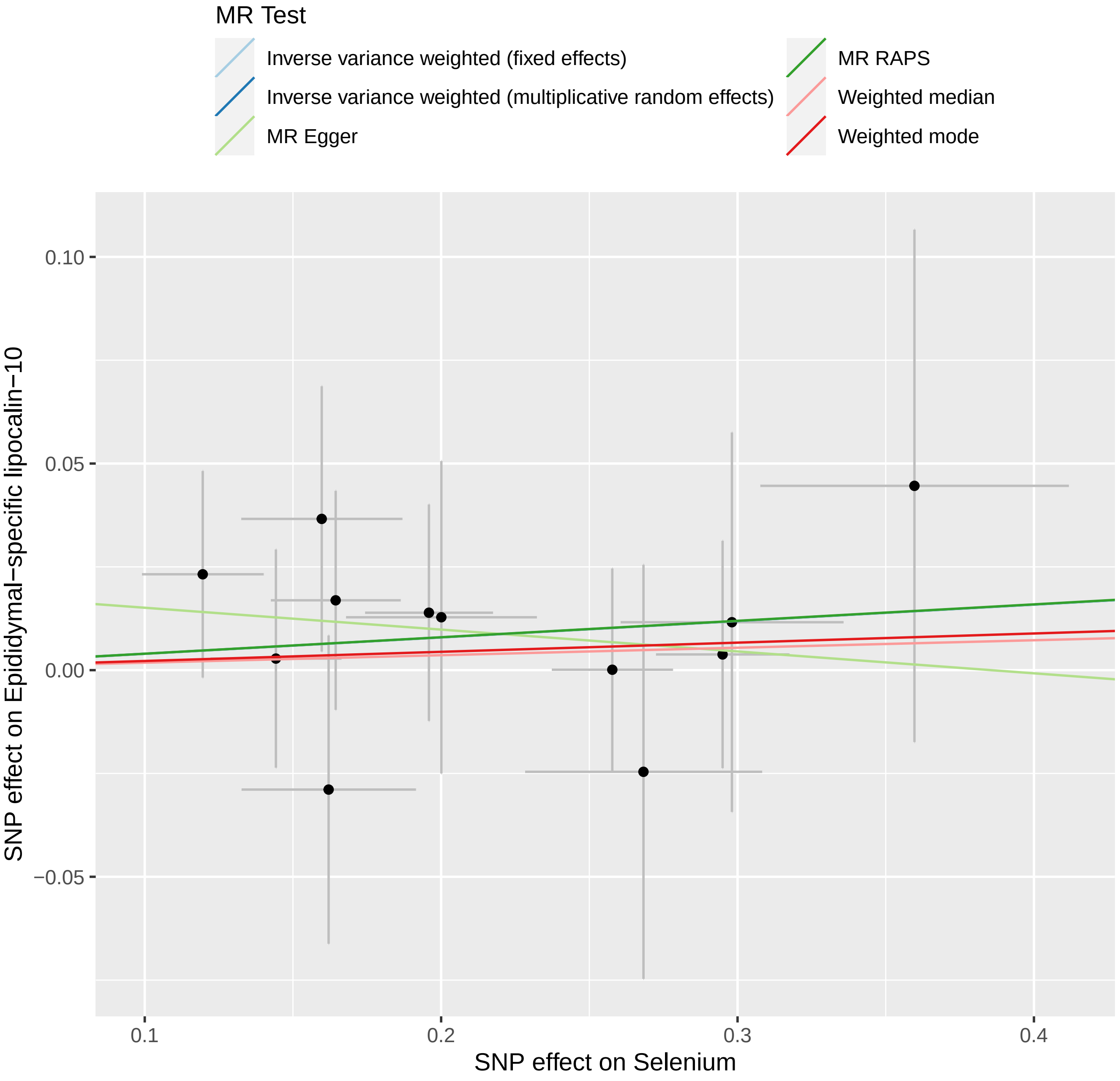

Batch 3−1708 : Selenium on Lipocalin−1

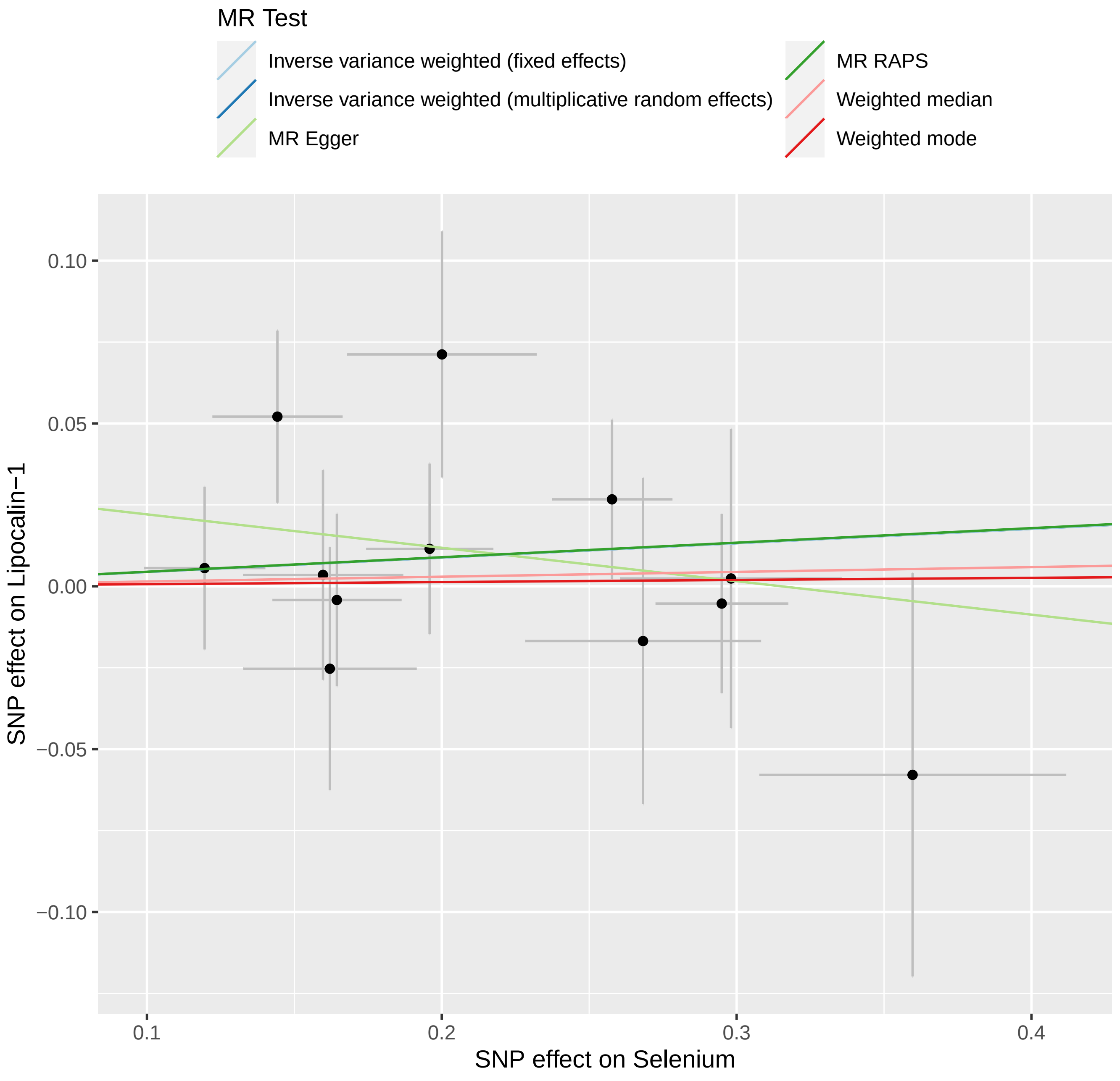

Batch 3−1709 : Selenium on Neutrophil gelatinase−associated lipocalin

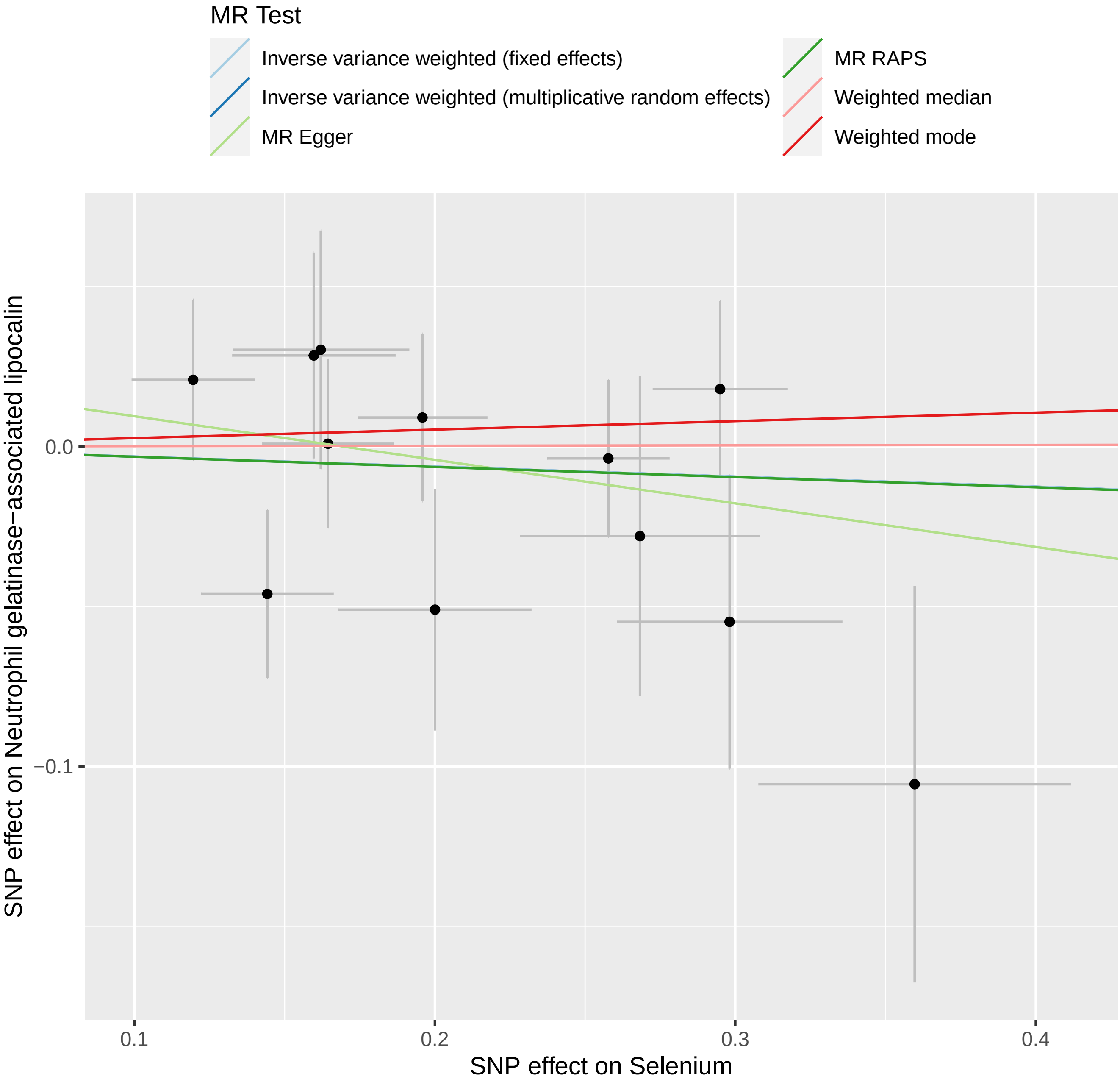

Batch 3−1710 : Selenium on Epididymal−specific lipocalin−8

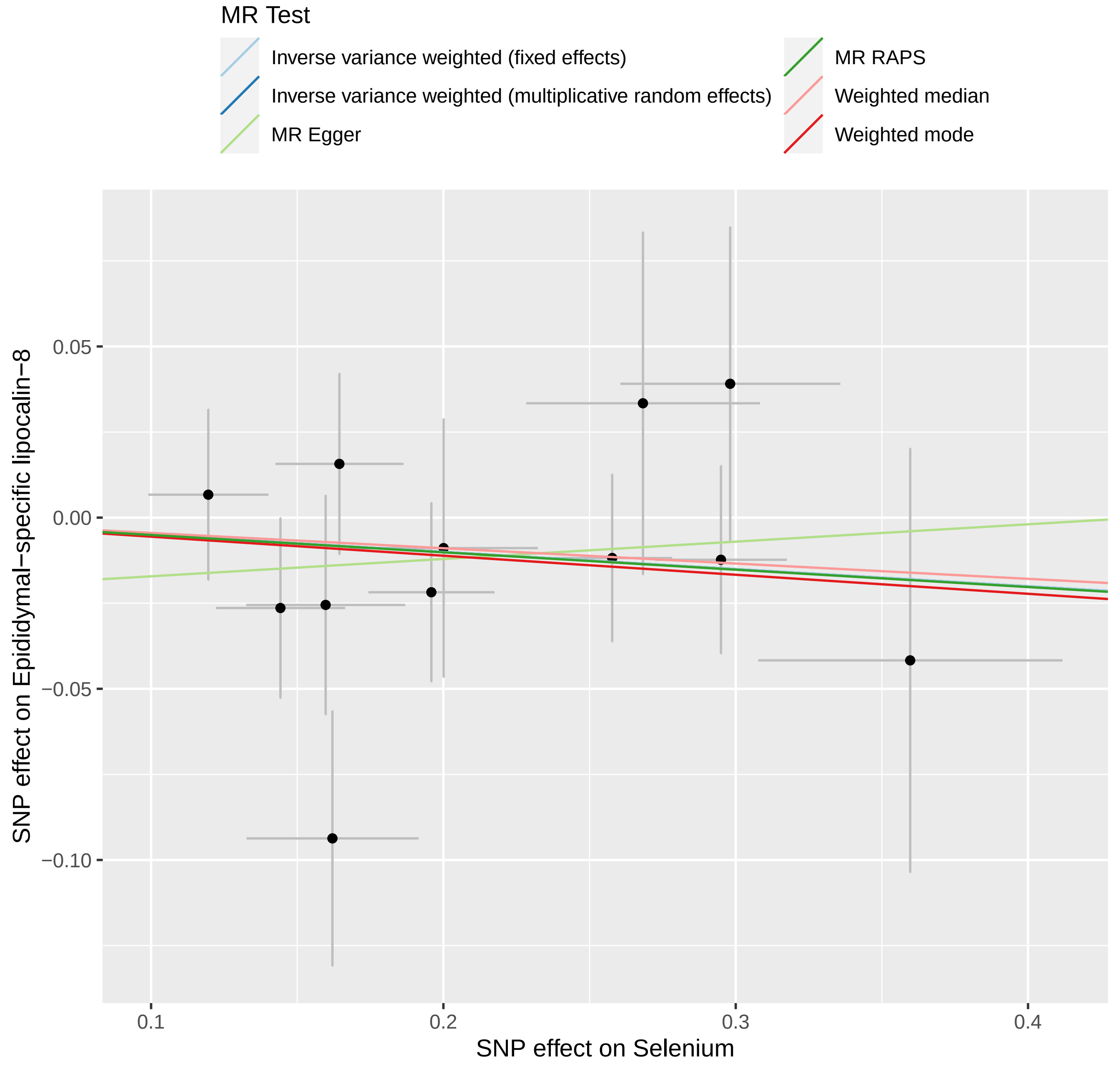

Batch 3−1711 : Selenium on Ligand−dependent nuclear receptor corepressor−like protein

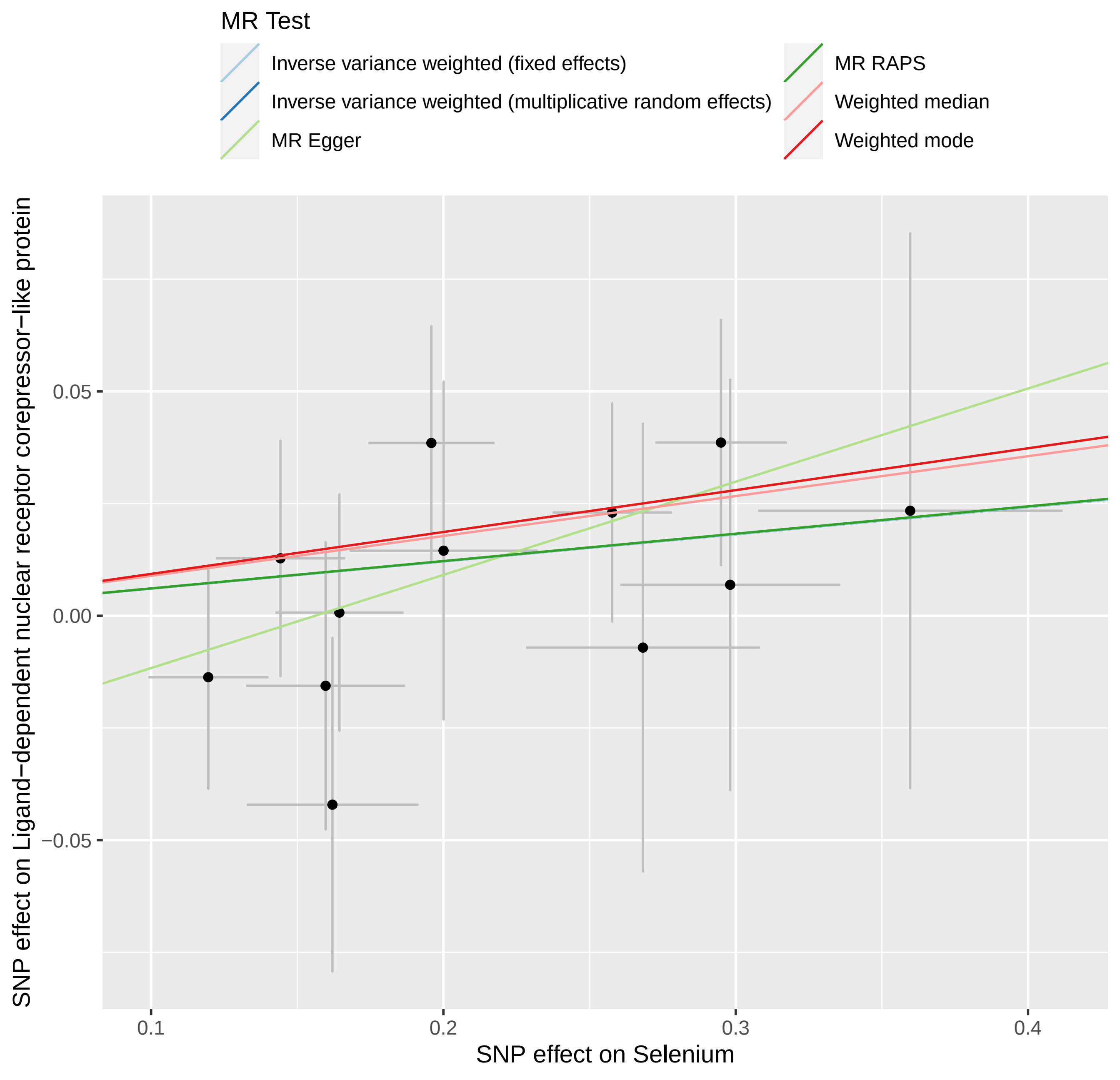

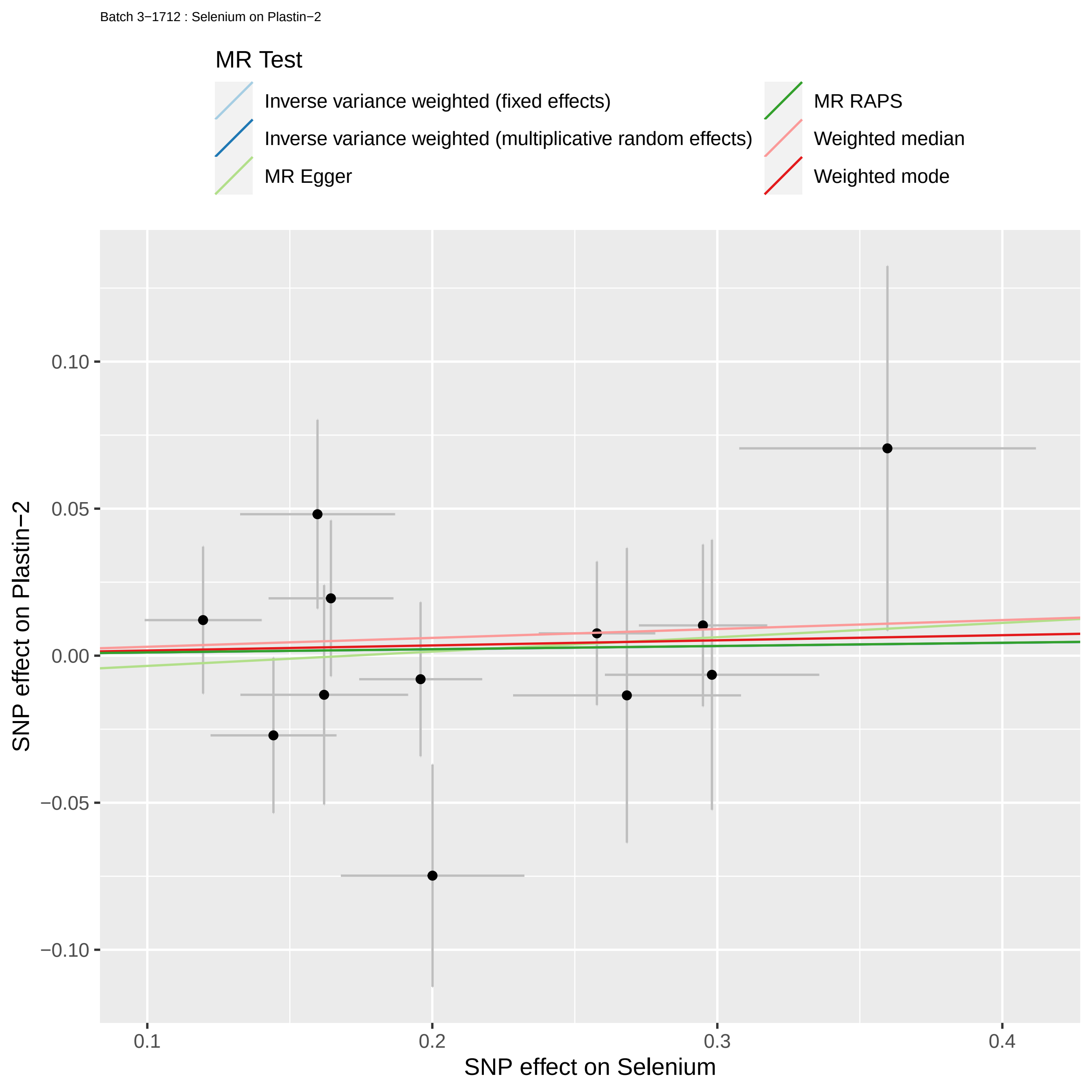

Batch 3−1712 : Selenium on Plastin−2
MR Test
Inverse variance weighted (fixed effects)
Inverse variance weighted (multiplicative random effects)
MR Egger
MR RAPS
Weighted median
Weighted mode
SNP effect on Plastin−2
0.10
0.05
0.00
−0.05
−0.10
0.1
0.2
0.3
0.4
SNP effect on Selenium

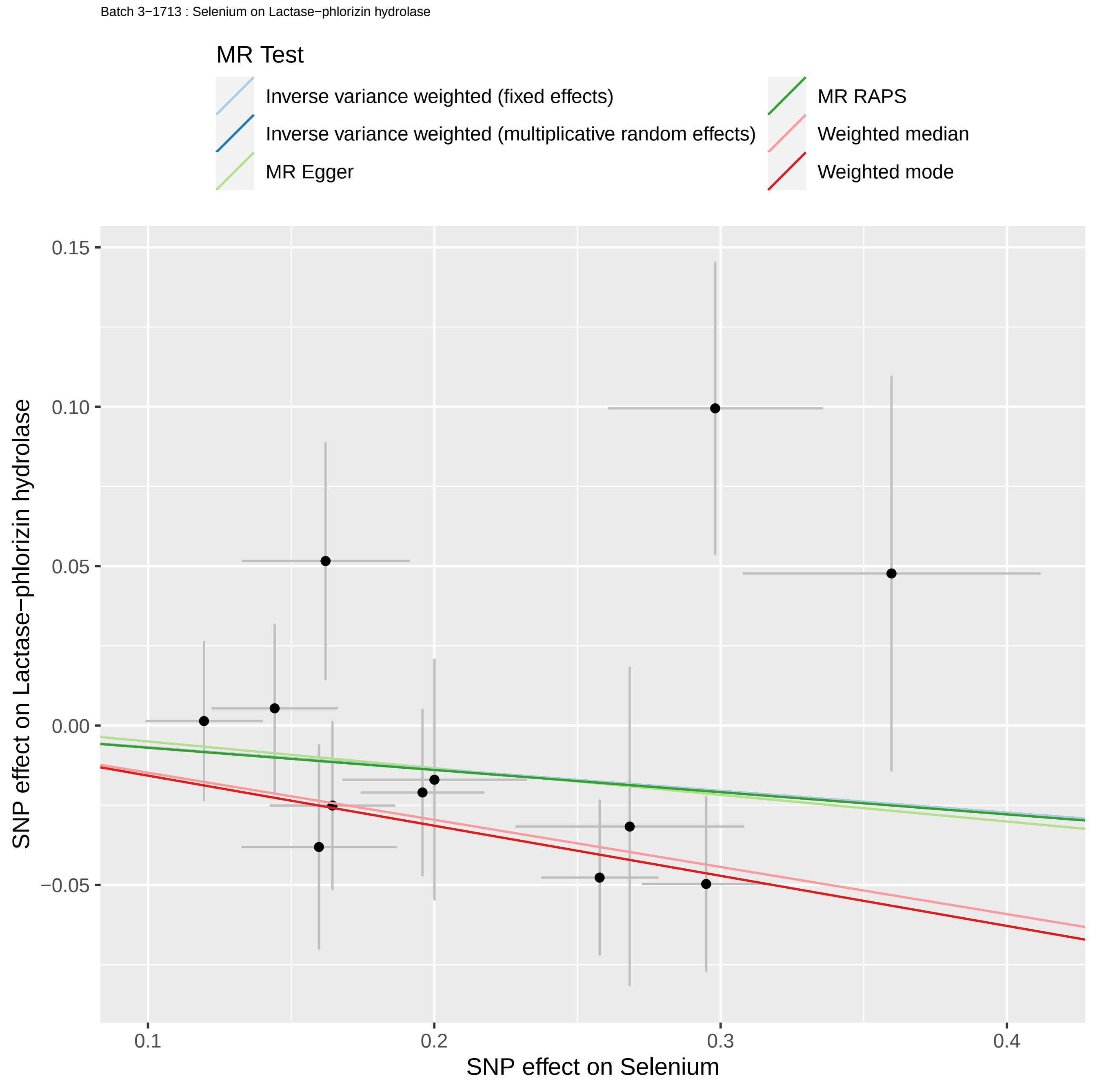
Batch 3−1713 : Selenium on Lactase−phlorizin hydrolase
MR Test
Inverse variance weighted (fixed effects)
Inverse variance weighted (multiplicative random effects)
MR Egger
MR RAPS
Weighted median
Weighted mode
SNP effect on Lactase−phlorizin hydrolase
0.15
0.10
0.05
0.00
−0.05
0.1
0.2
0.3
0.4
SNP effect on Selenium

Batch 3−1714 : Selenium on Lactase−like protein

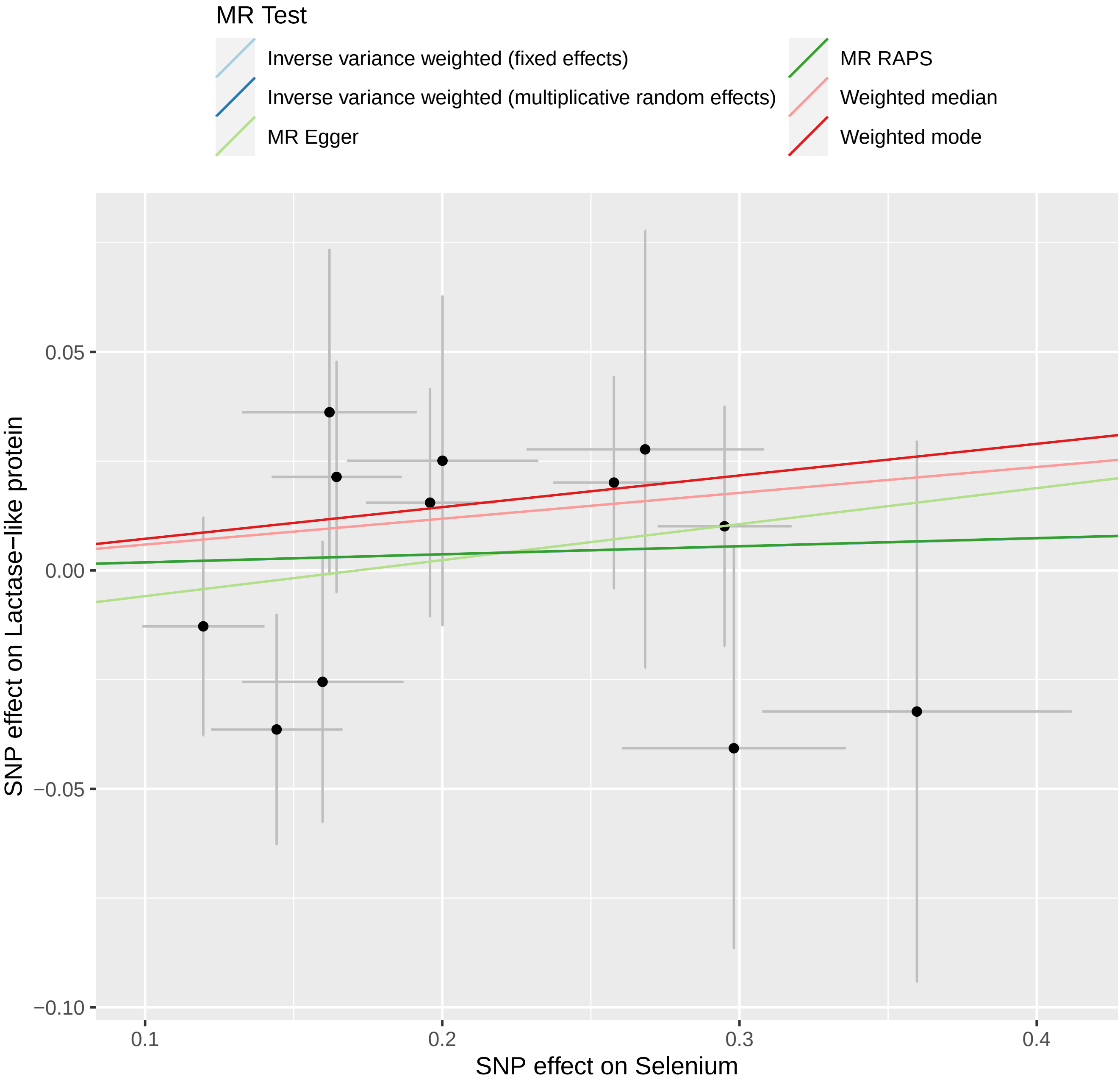

Batch 3−1715 : Selenium on L−lactate dehydrogenase B chain

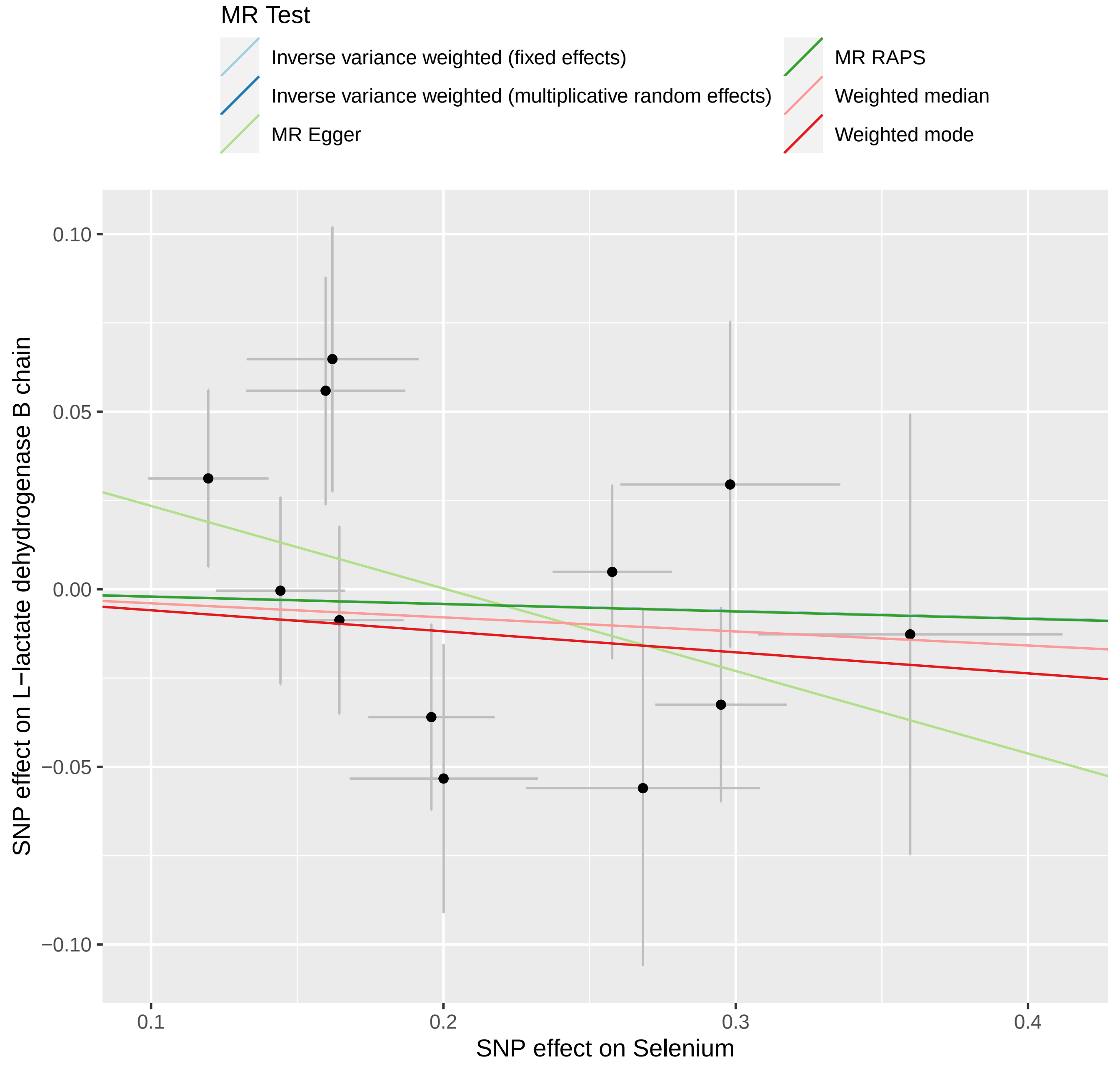

Batch 3−1716 : Selenium on L−lactate dehydrogenase C chain

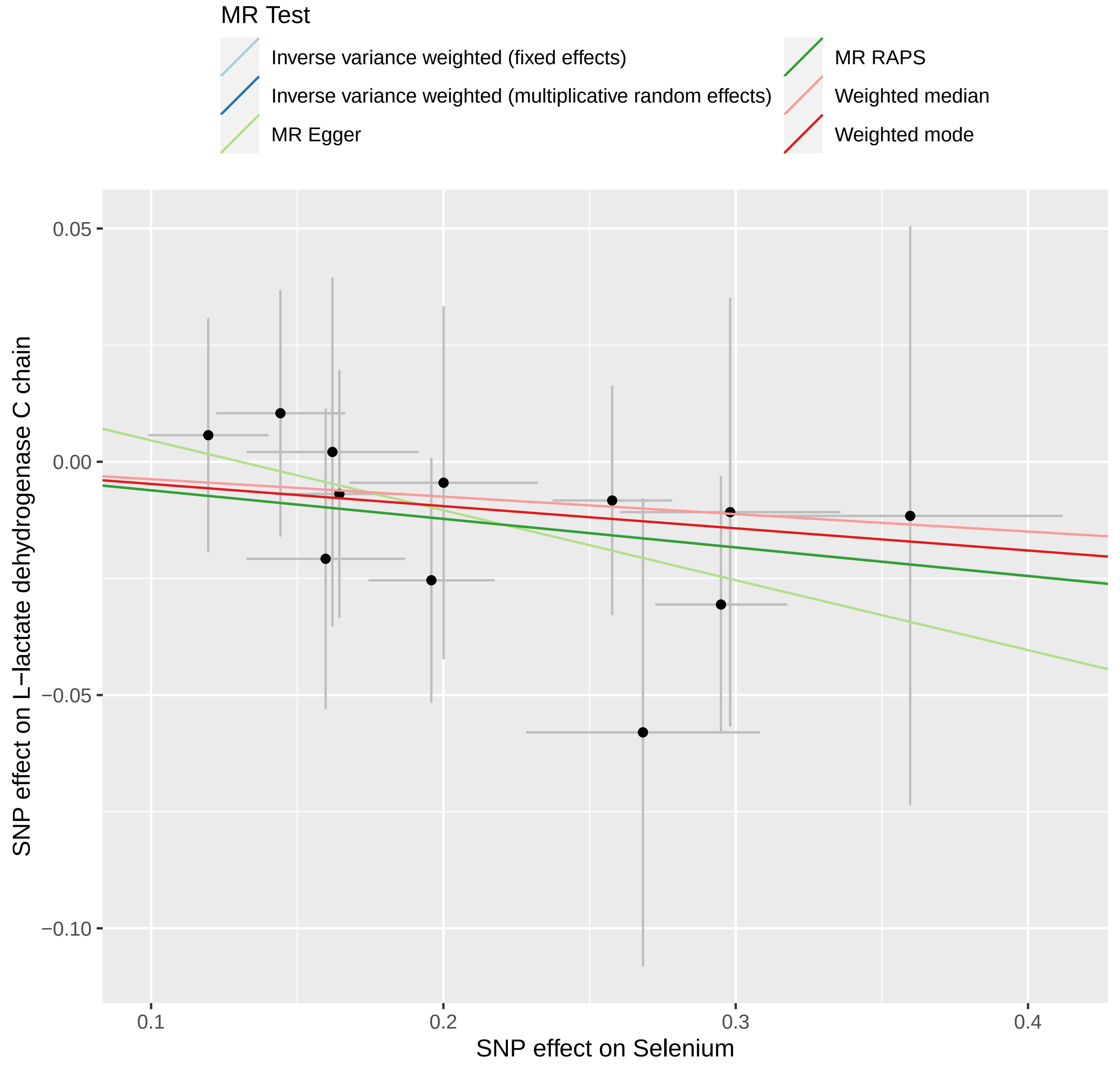

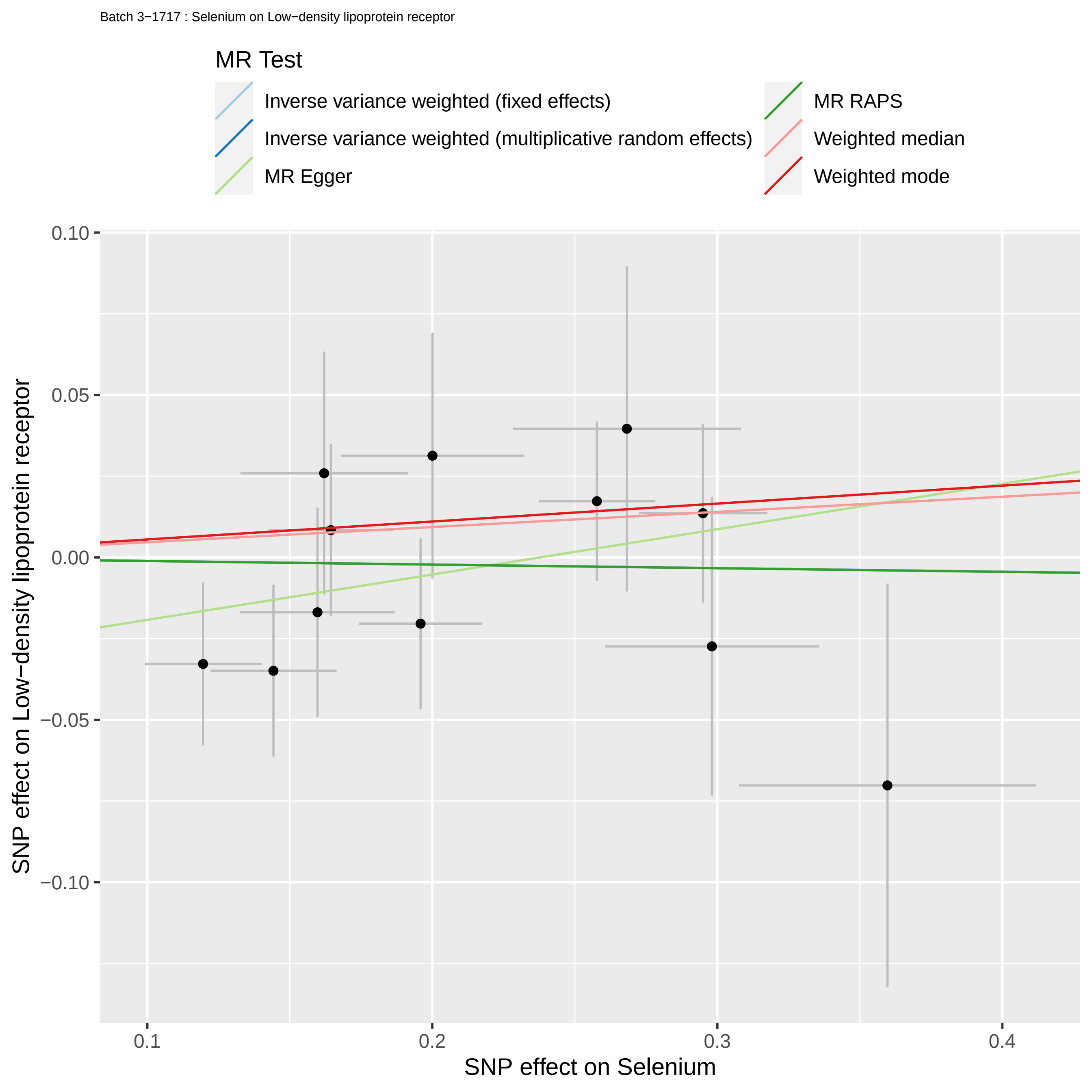

Batch 3−1717 : Selenium on Low−density lipoprotein receptor
MR Test
Inverse variance weighted (fixed effects)
Inverse variance weighted (multiplicative random effects)
MR Egger
MR RAPS
Weighted median
Weighted mode
0.10
0.05
0.00
−0.05
−0.10
0.1
0.2
0.3
0.4
SNP effect on Selenium
SNP effect on Low−density lipoprotein receptor

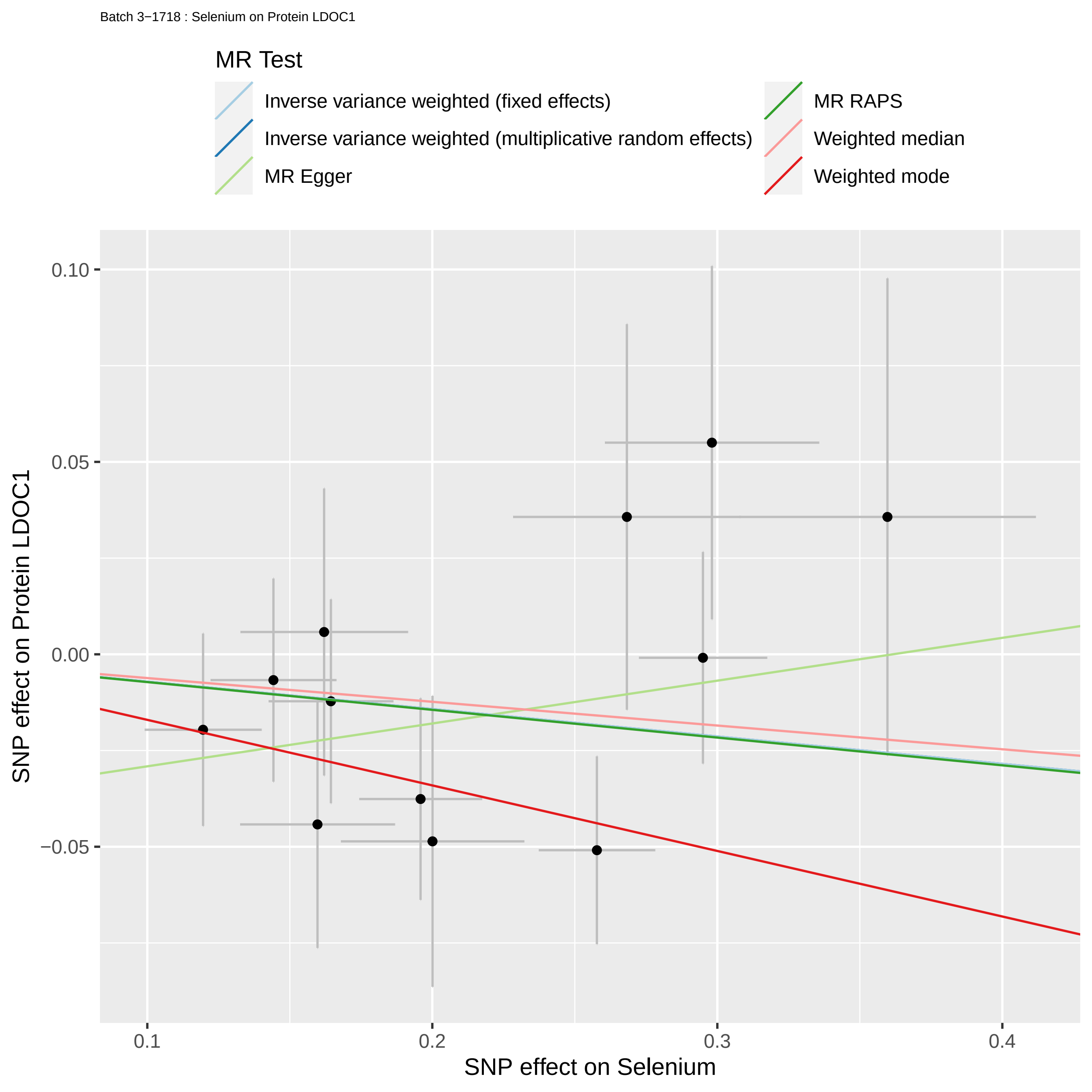
Batch 3−1718 : Selenium on Protein LDOC1
MR Test
Inverse variance weighted (fixed effects)
Inverse variance weighted (multiplicative random effects)
MR Egger
MR RAPS
Weighted median
Weighted mode
0.10
0.05
0.00
−0.05
0.1
0.2
0.3
0.4
SNP effect on Protein LDOC1
SNP effect on Selenium

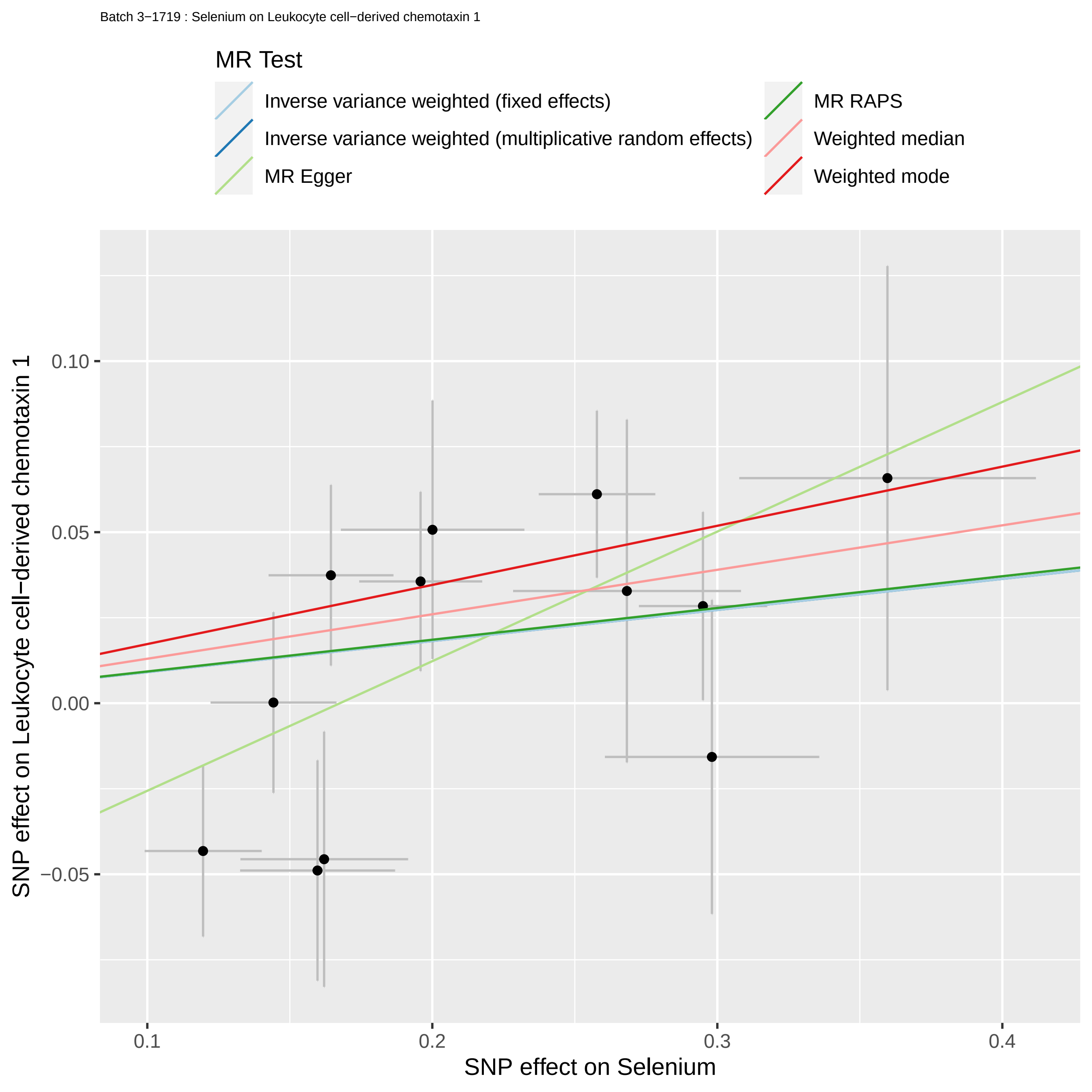

Batch 3−1719 : Selenium on Leukocyte cell−derived chemotaxin 1
MR Test
Inverse variance weighted (fixed effects)
Inverse variance weighted (multiplicative random effects)
MR Egger
MR RAPS
Weighted median
Weighted mode
0.10
0.05
0.00
−0.05
0.1
0.2
0.3
0.4
SNP effect on Selenium
SNP effect on Leukocyte cell−derived chemotaxin 1

Batch 3−1720 : Selenium on LEM domain−containing protein 1

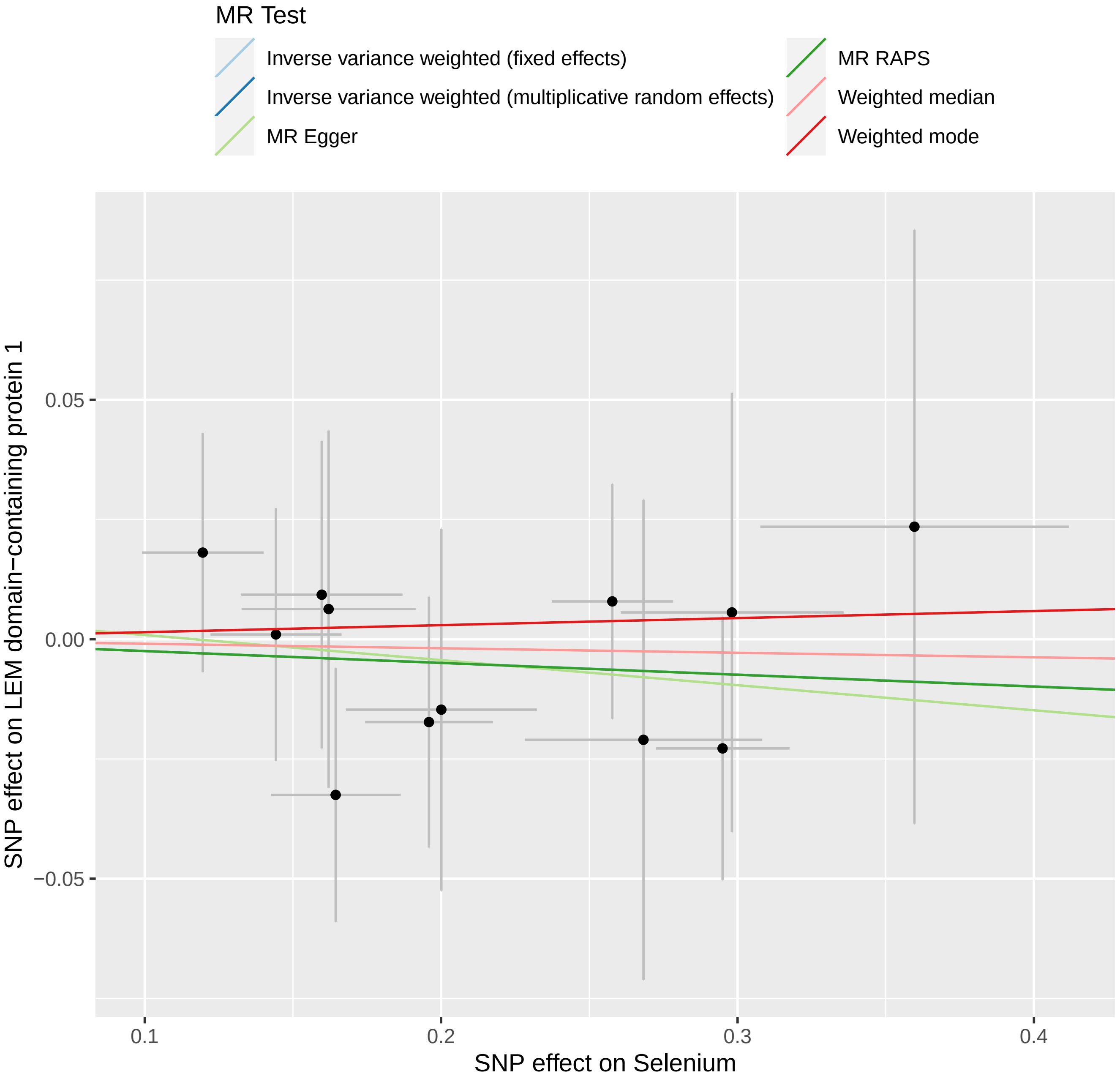

Batch 3−1721 : Selenium on Leptin

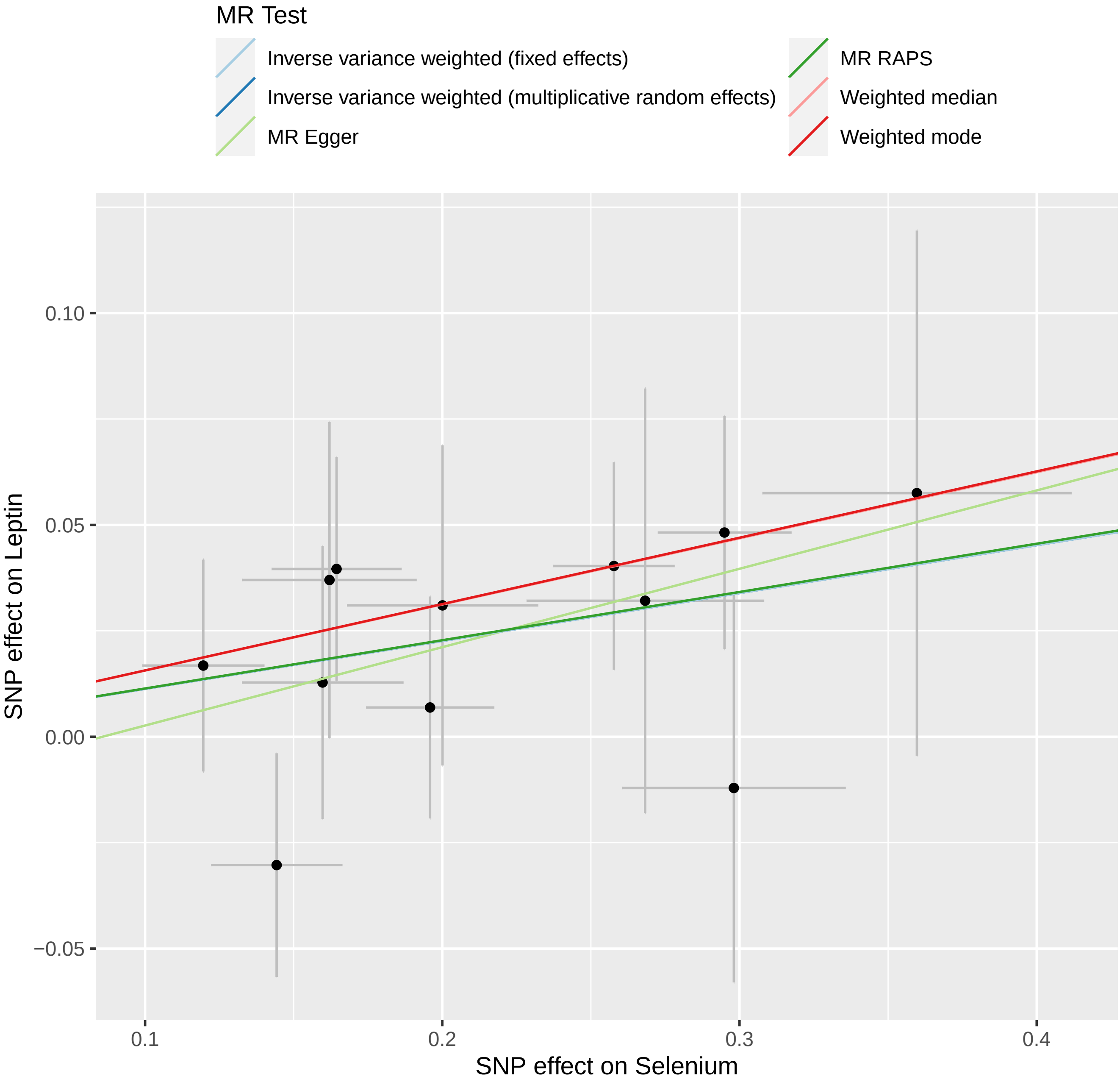

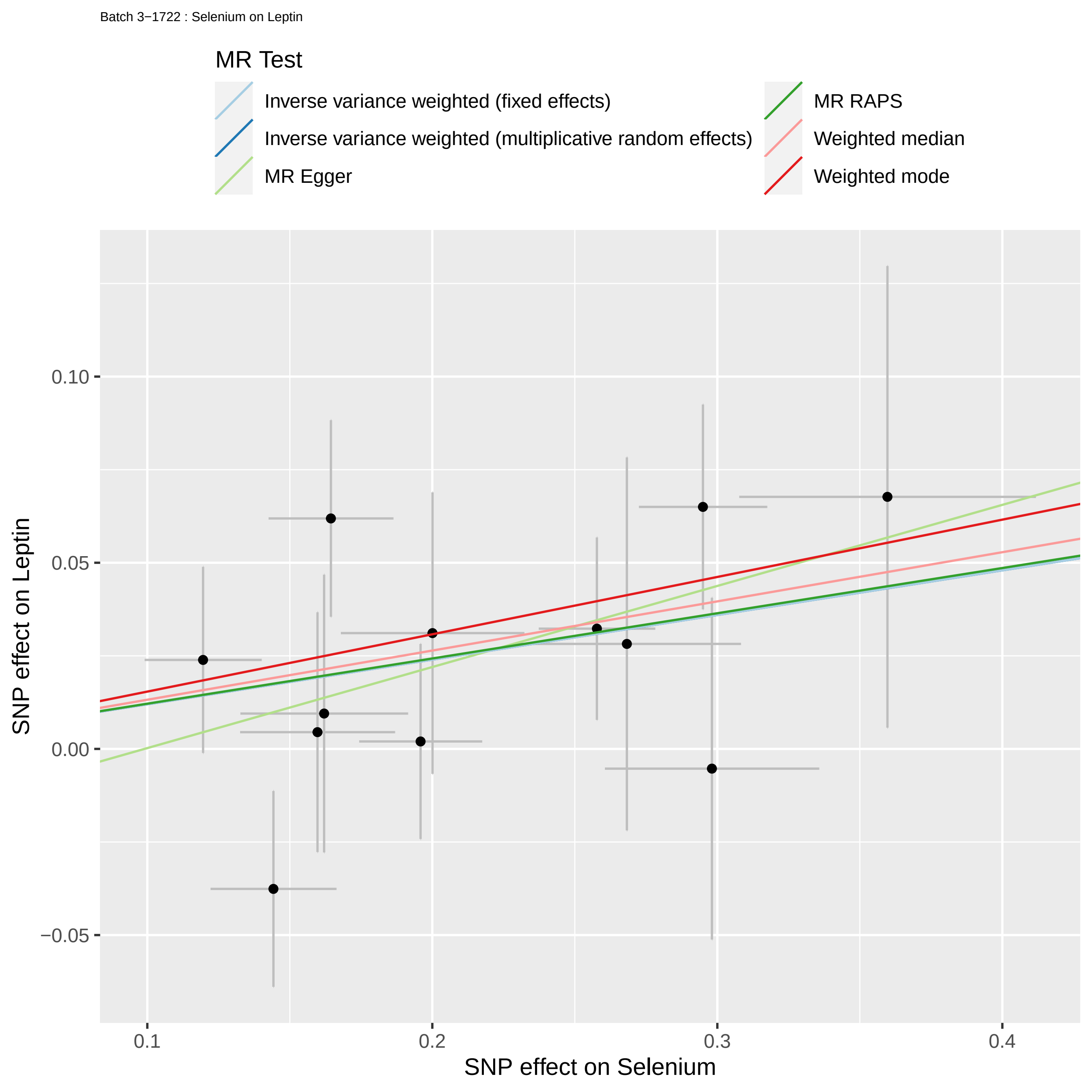
Batch 3−1722 : Selenium on Leptin
MR Test
Inverse variance weighted (fixed effects)
Inverse variance weighted (multiplicative random effects)
MR Egger
MR RAPS
Weighted median
Weighted mode
0.10
0.05
0.00
−0.05
0.1
0.2
0.3
0.4
SNP effect on Leptin
SNP effect on Selenium

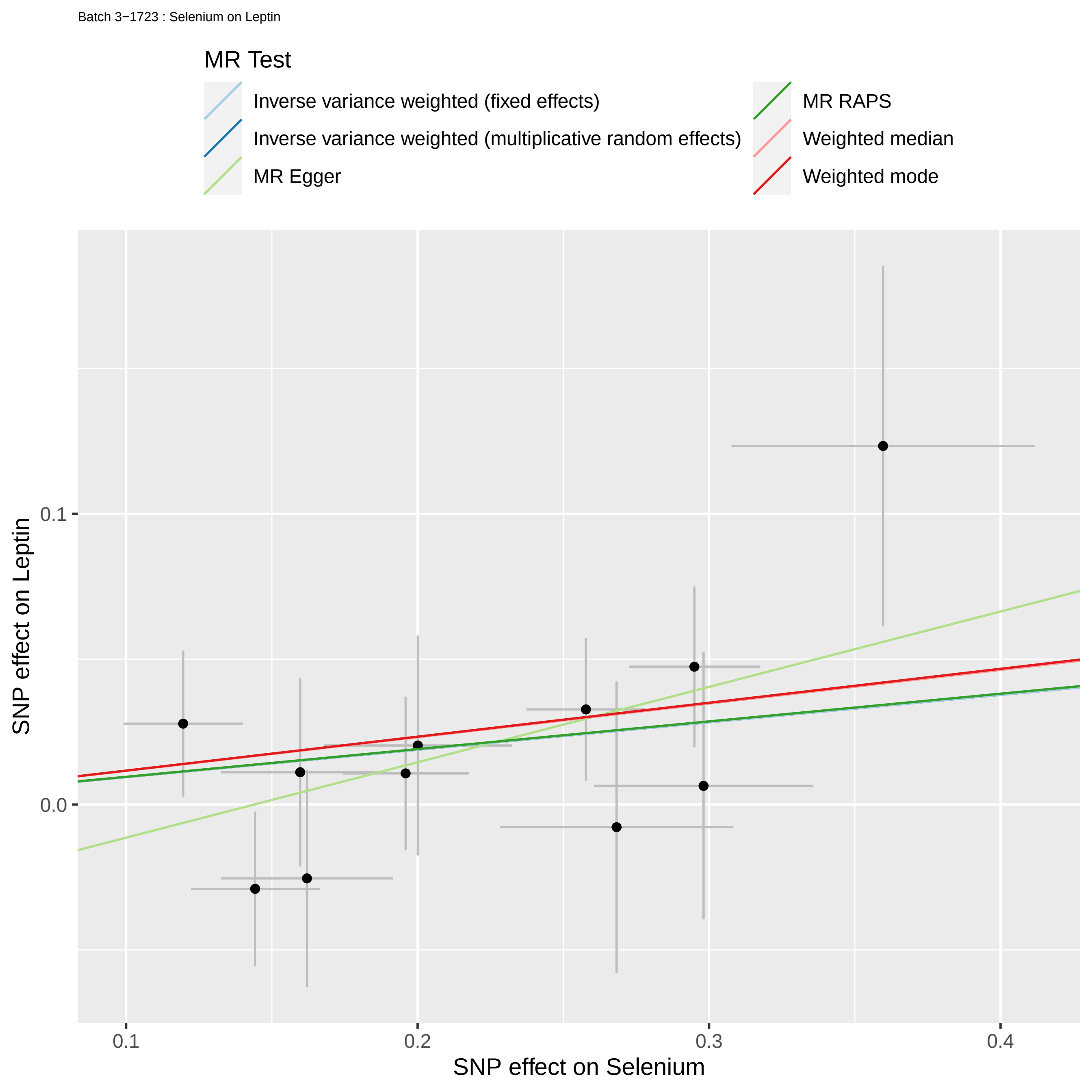

Batch 3−1723 : Selenium on Leptin
MR Test
Inverse variance weighted (fixed effects)
Inverse variance weighted (multiplicative random effects)
MR Egger
MR RAPS
Weighted median
Weighted mode
0.1
0.0
0.1
0.2
0.3
0.4
SNP effect on Leptin
SNP effect on Selenium

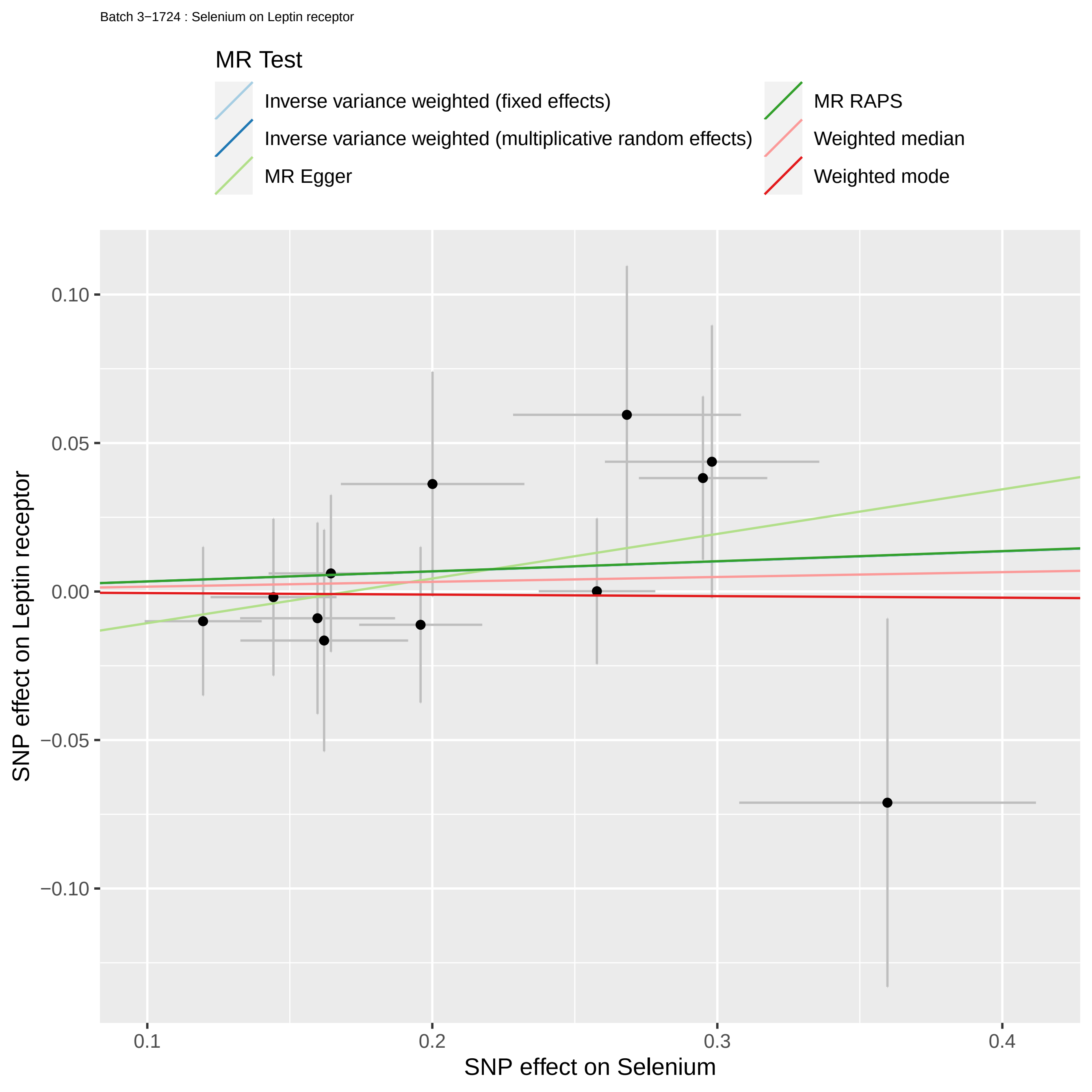
Batch 3−1724 : Selenium on Leptin receptor
MR Test
Inverse variance weighted (fixed effects)
Inverse variance weighted (multiplicative random effects)
MR Egger
MR RAPS
Weighted median
Weighted mode
0.10
0.05
0.00
−0.05
−0.10
0.1
0.2
0.3
0.4
SNP effect on Selenium
SNP effect on Leptin receptor

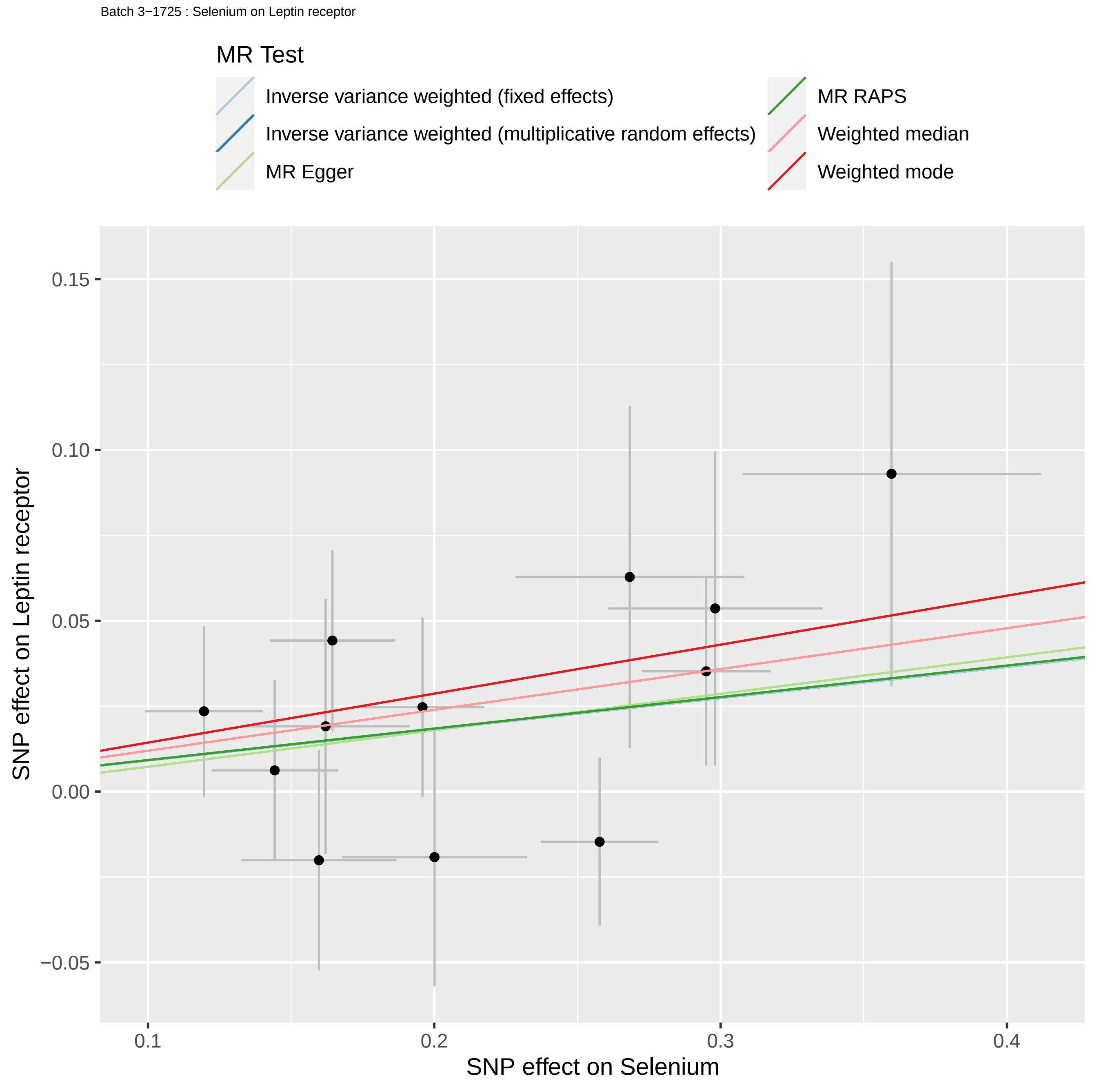
Batch 3−1725 : Selenium on Leptin receptor
MR Test
Inverse variance weighted (fixed effects)
Inverse variance weighted (multiplicative random effects)
MR Egger
MR RAPS
Weighted median
Weighted mode
SNP effect on Leptin receptor
0.15
0.10
0.05
0.00
−0.05
0.1
0.2
0.3
0.4
SNP effect on Selenium

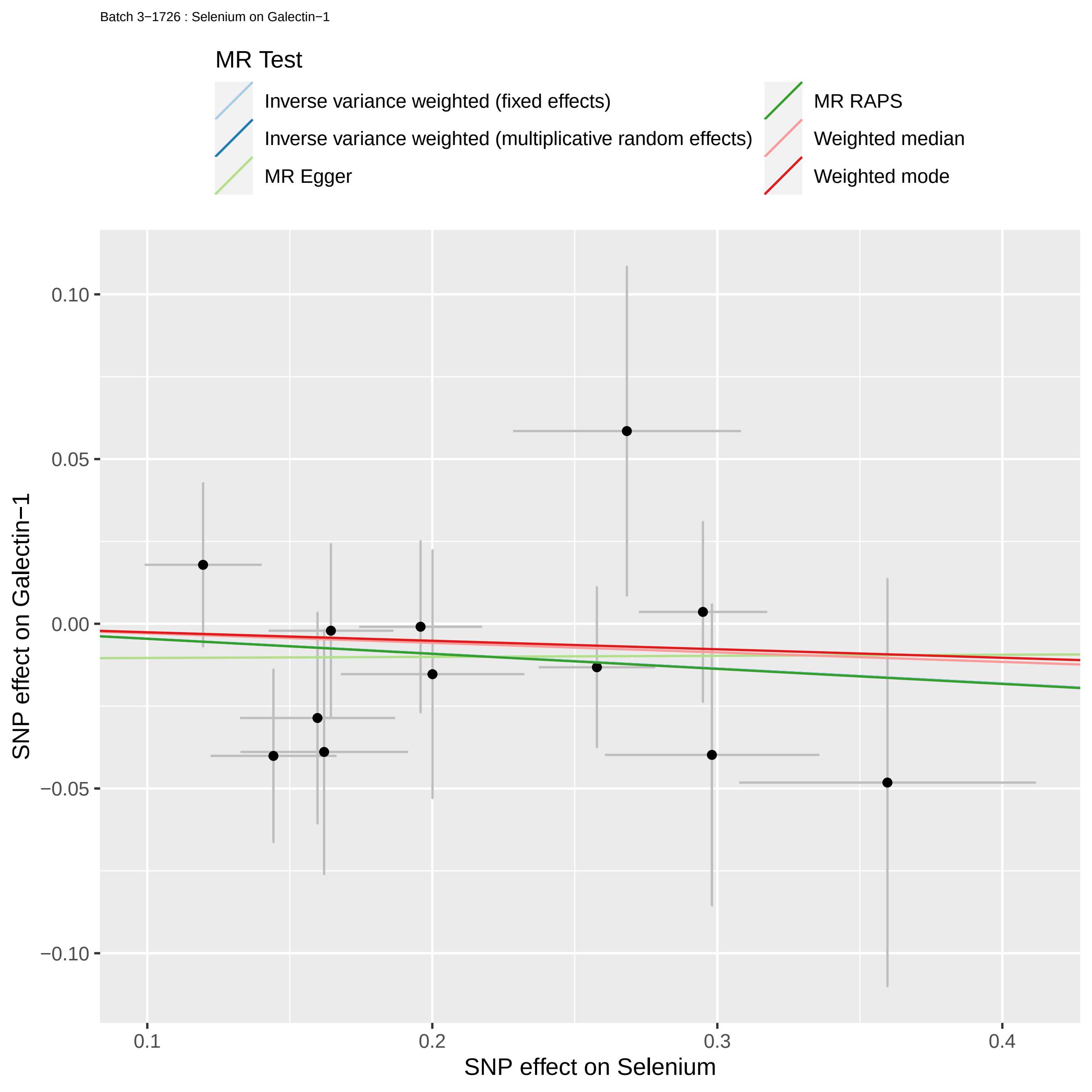

Batch 3−1726 : Selenium on Galectin−1
MR Test
Inverse variance weighted (fixed effects)
Inverse variance weighted (multiplicative random effects)
MR Egger
MR RAPS
Weighted median
Weighted mode
0.10
0.05
0.00
−0.05
−0.10
0.1
0.2
0.3
0.4
SNP effect on Galectin−1
SNP effect on Selenium

Batch 3−1727 : Selenium on Galectin−2

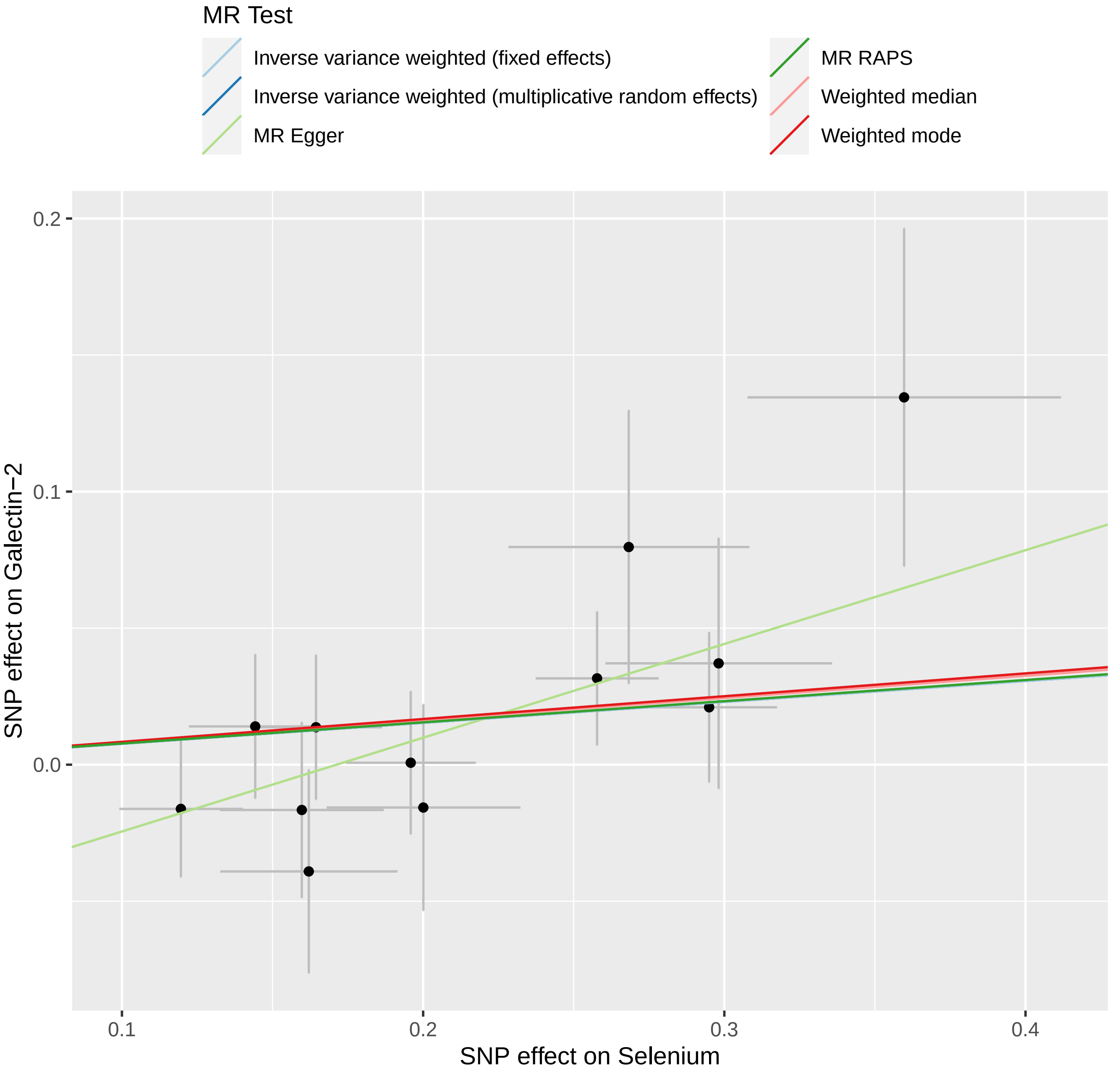

Batch 3−1728 : Selenium on Galectin−4

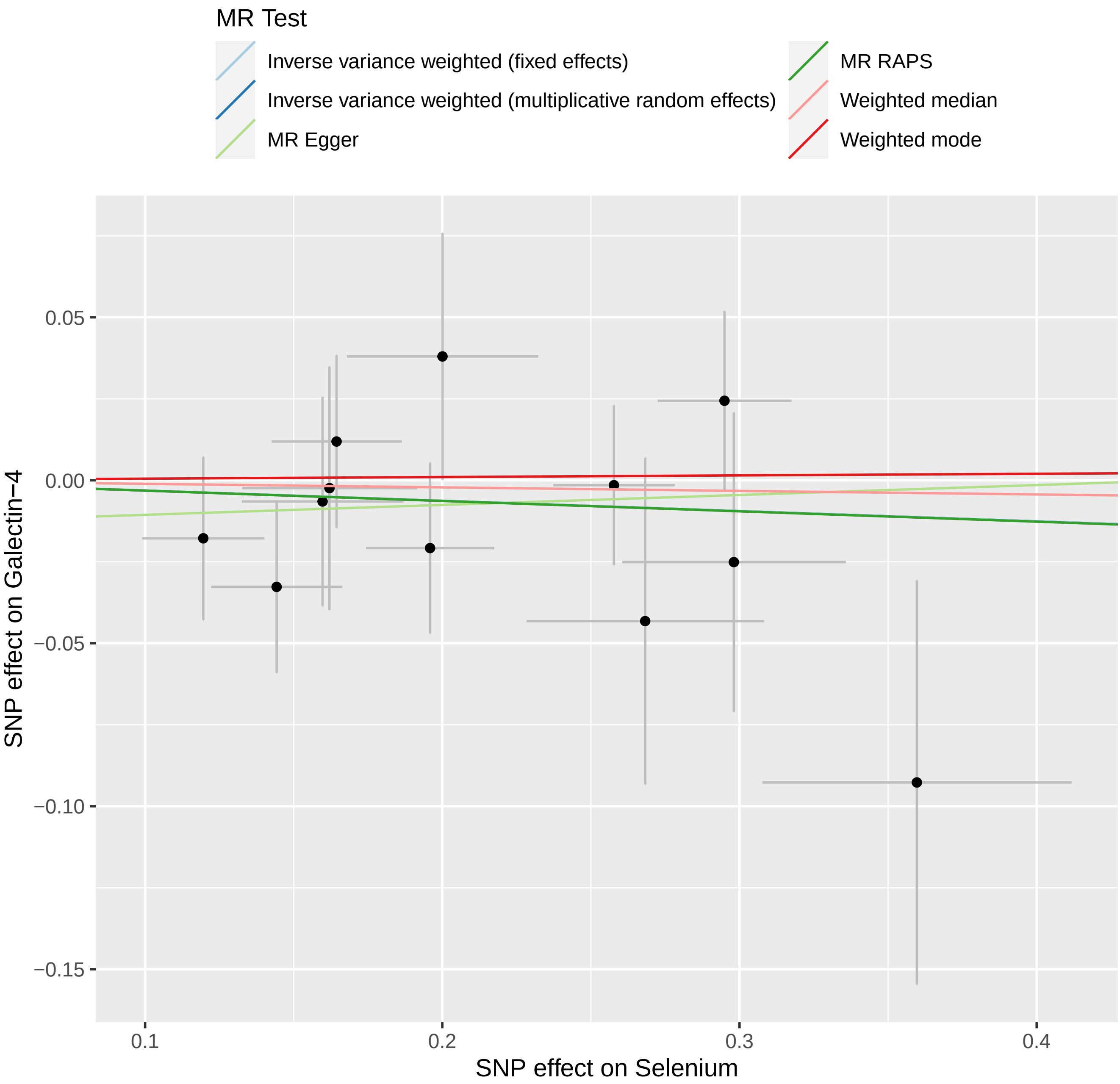

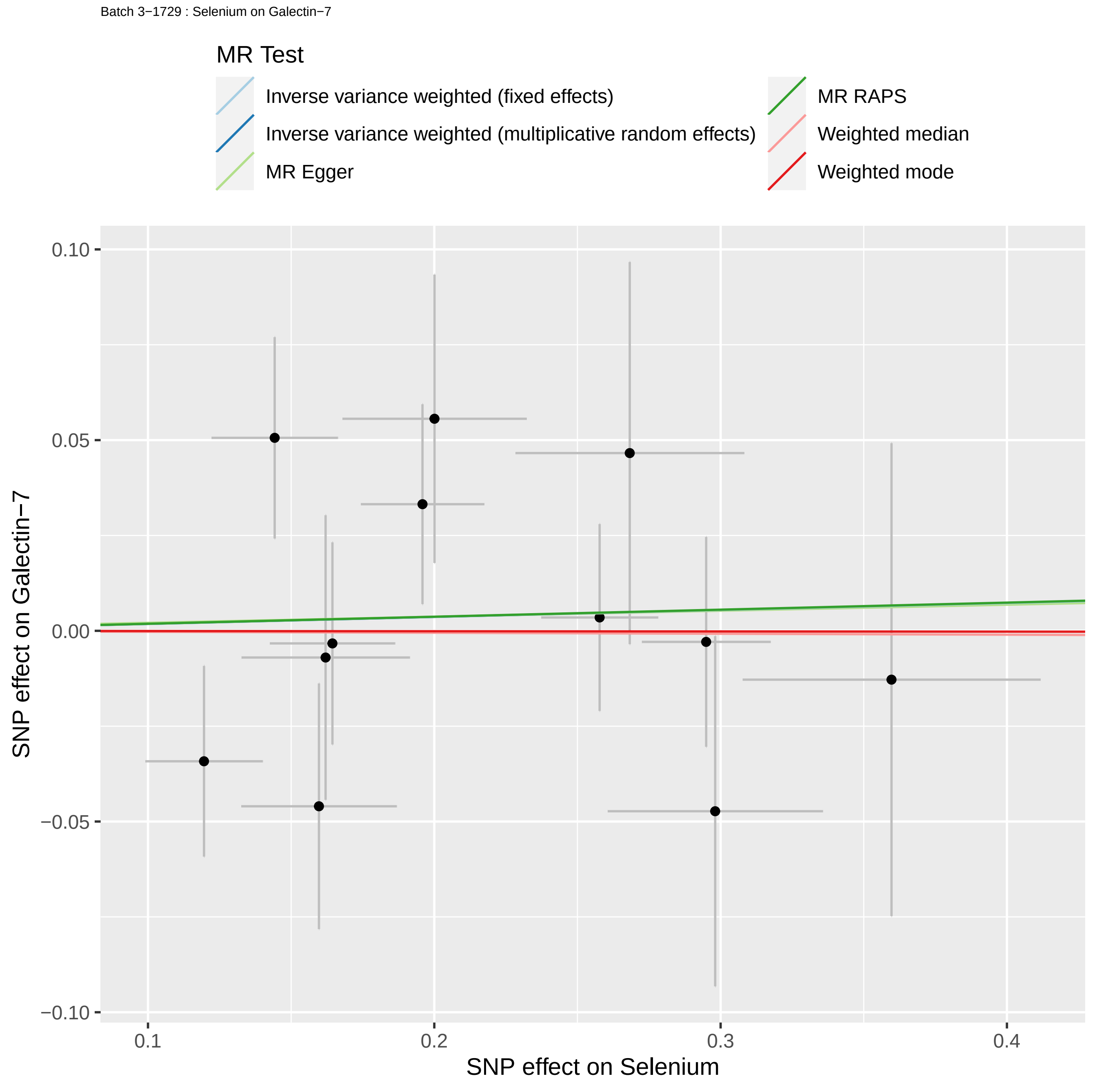

Batch 3−1729 : Selenium on Galectin−7
MR Test
Inverse variance weighted (fixed effects)
Inverse variance weighted (multiplicative random effects)
MR Egger
MR RAPS
Weighted median
Weighted mode
0.10
0.05
0.00
−0.05
−0.10
0.1
0.2
0.3
0.4
SNP effect on Galectin−7
SNP effect on Selenium

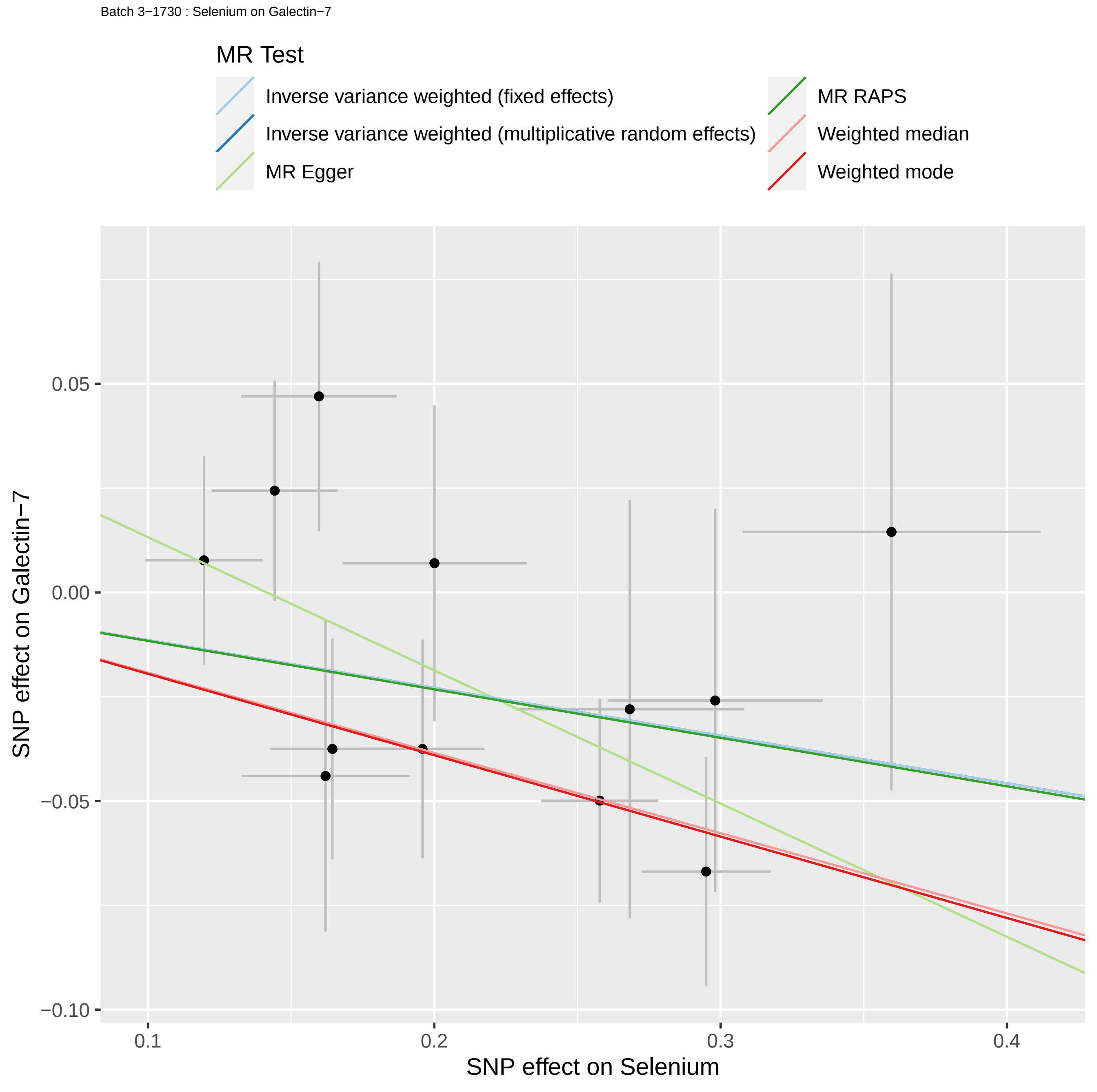
Batch 3−1730 : Selenium on Galectin−7
MR Test
Inverse variance weighted (fixed effects)
Inverse variance weighted (multiplicative random effects)
MR Egger
MR RAPS
Weighted median
Weighted mode
0.05
0.00
−0.05
−0.10
0.1
0.2
0.3
0.4
SNP effect on Selenium
SNP effect on Galectin−7

Batch 3−1731 : Selenium on Galectin−8

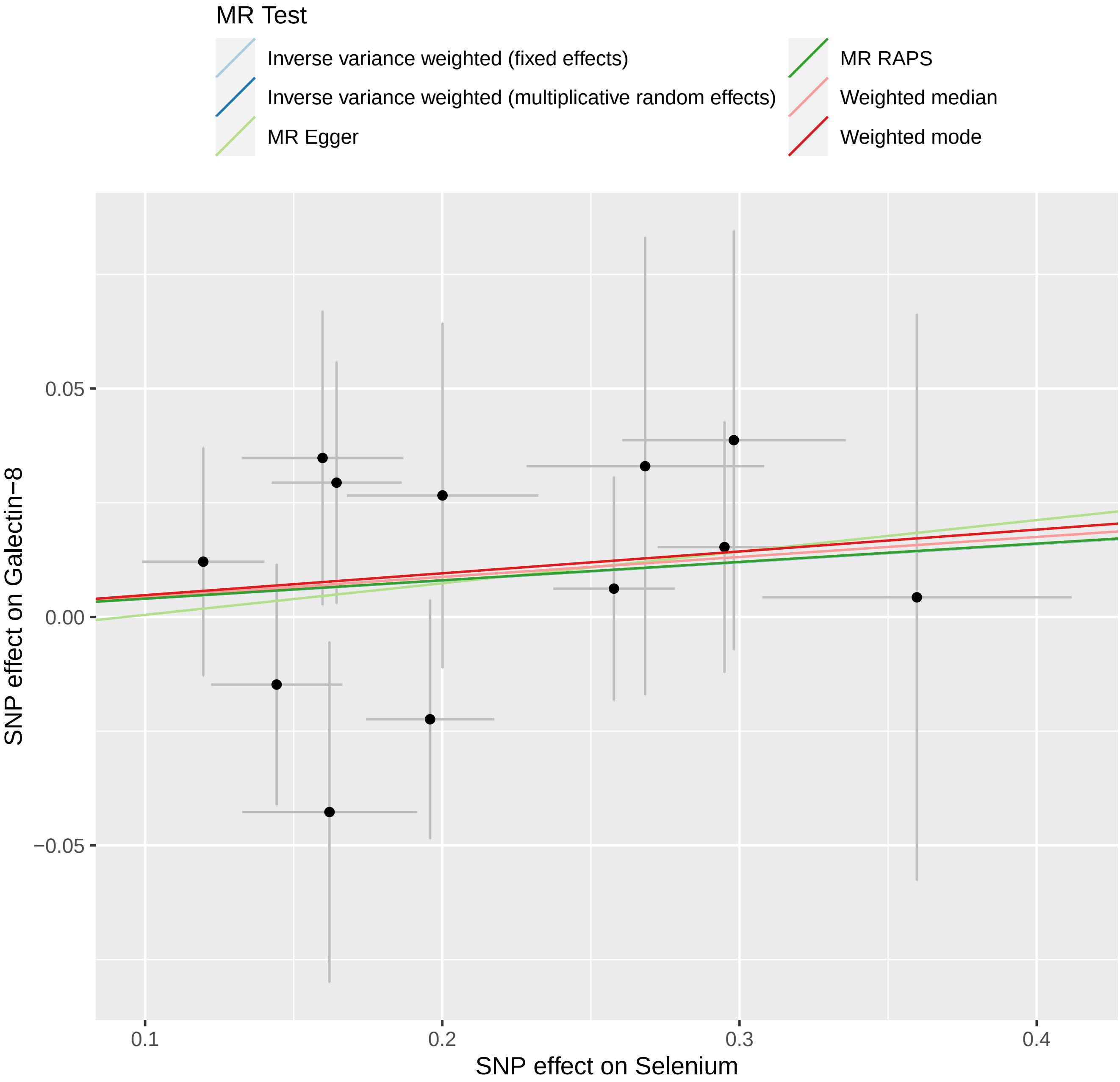

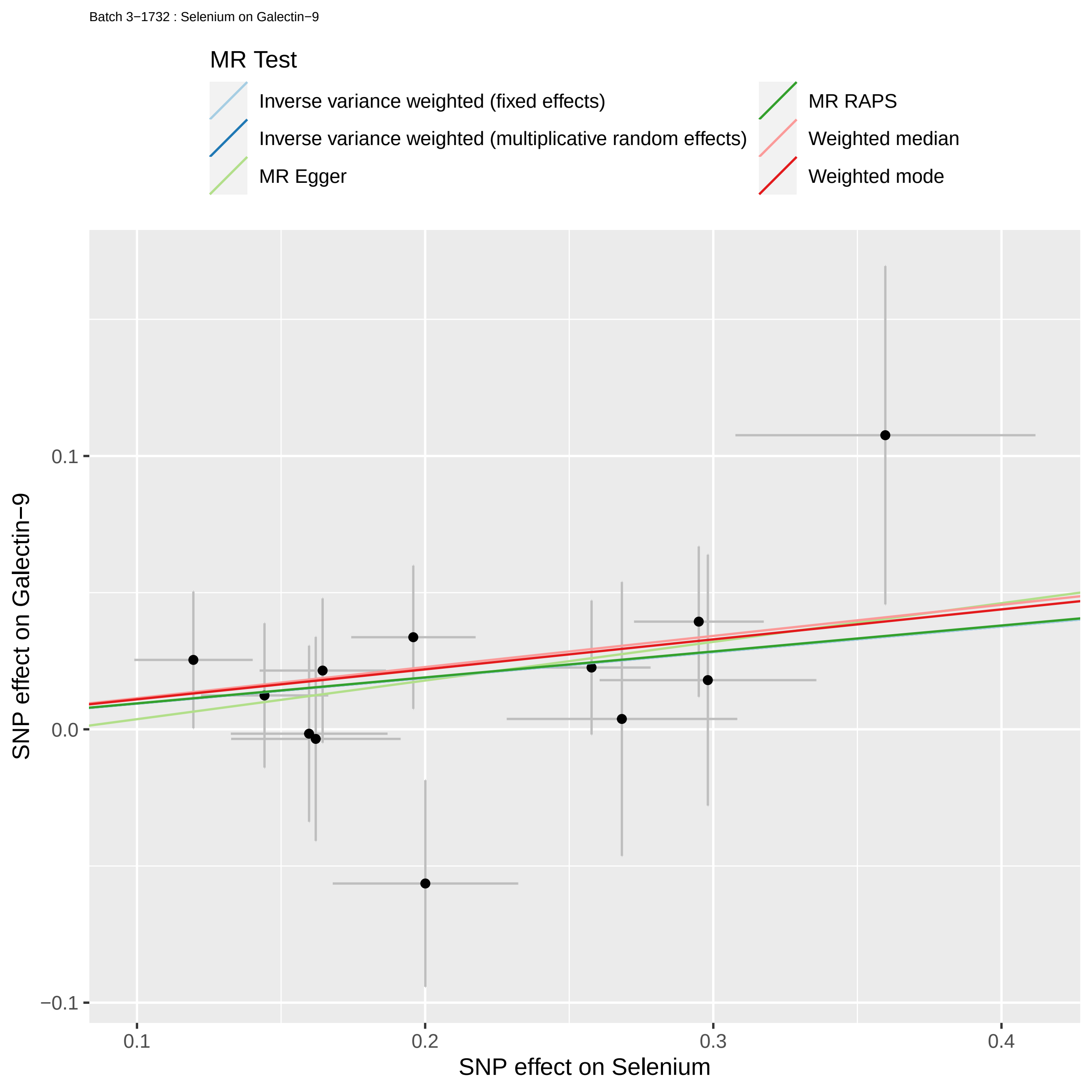
Batch 3−1732 : Selenium on Galectin−9
MR Test
Inverse variance weighted (fixed effects)
Inverse variance weighted (multiplicative random effects)
MR Egger
MR RAPS
Weighted median
Weighted mode
SNP effect on Galectin−9
0.1
0.0
−0.1
0.1
0.2
0.3
0.4
SNP effect on Selenium

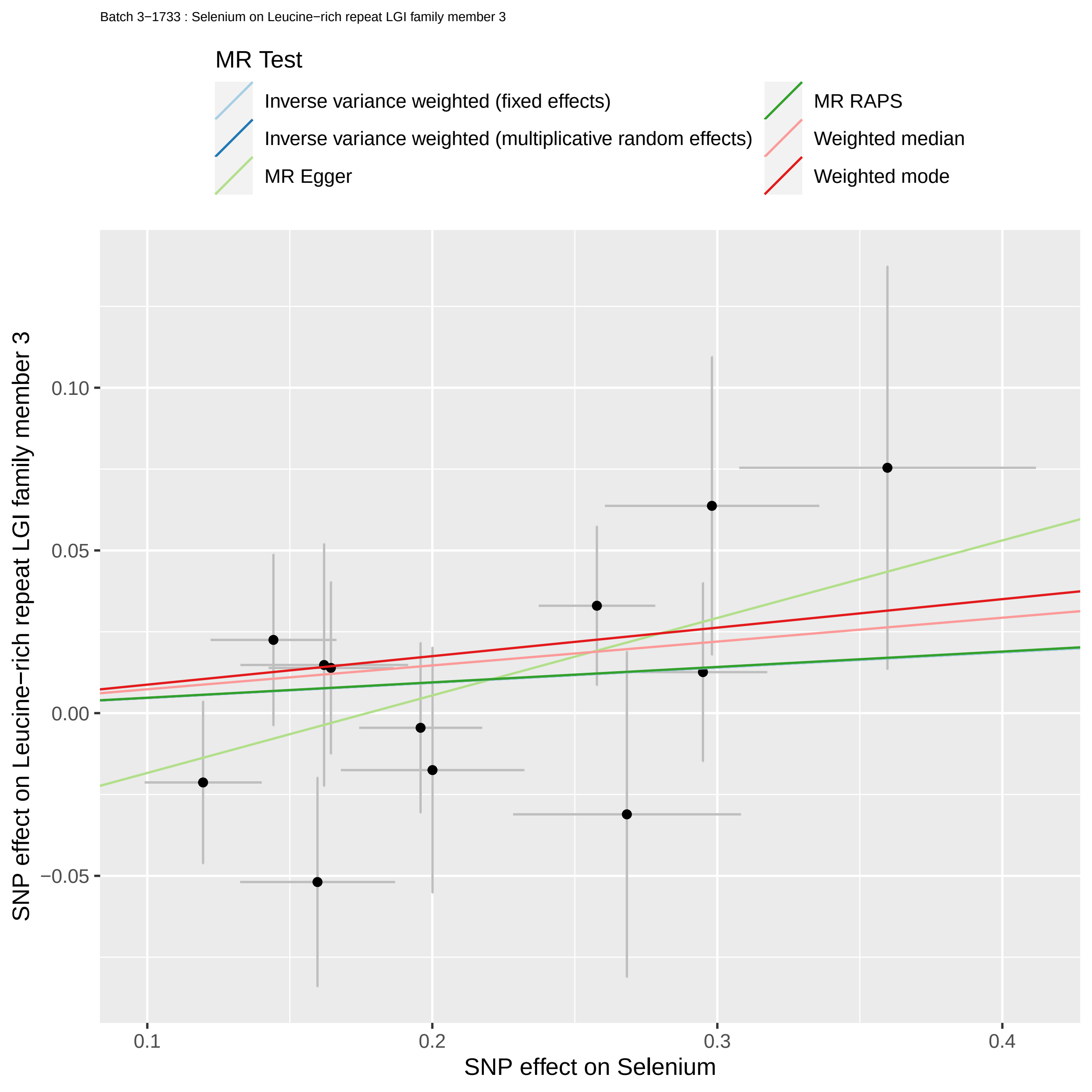
Batch 3−1733 : Selenium on Leucine−rich repeat LGI family member 3
MR Test
Inverse variance weighted (fixed effects)
Inverse variance weighted (multiplicative random effects)
MR Egger
MR RAPS
Weighted median
Weighted mode
SNP effect on Leucine−rich repeat LGI family member 3
0.10
0.05
0.00
−0.05
0.1
0.2
0.3
0.4
SNP effect on Selenium

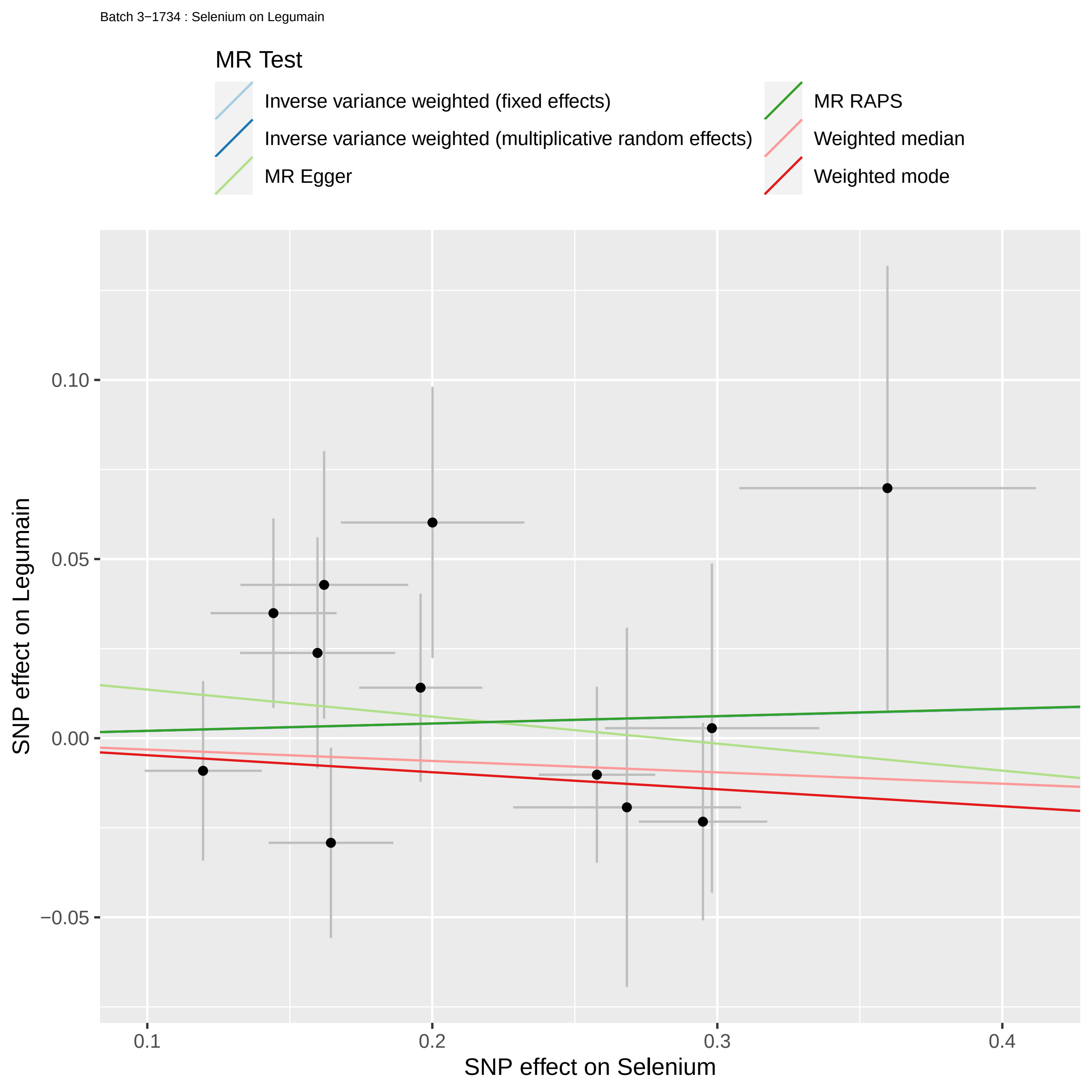
Batch 3−1734 : Selenium on Legumain
MR Test
Inverse variance weighted (fixed effects)
Inverse variance weighted (multiplicative random effects)
MR Egger
MR RAPS
Weighted median
Weighted mode
0.10
0.05
0.00
−0.05
0.1
0.2
0.3
0.4
SNP effect on Legumain
SNP effect on Selenium

Batch 3−1735 : Selenium on Lutropin subunit beta

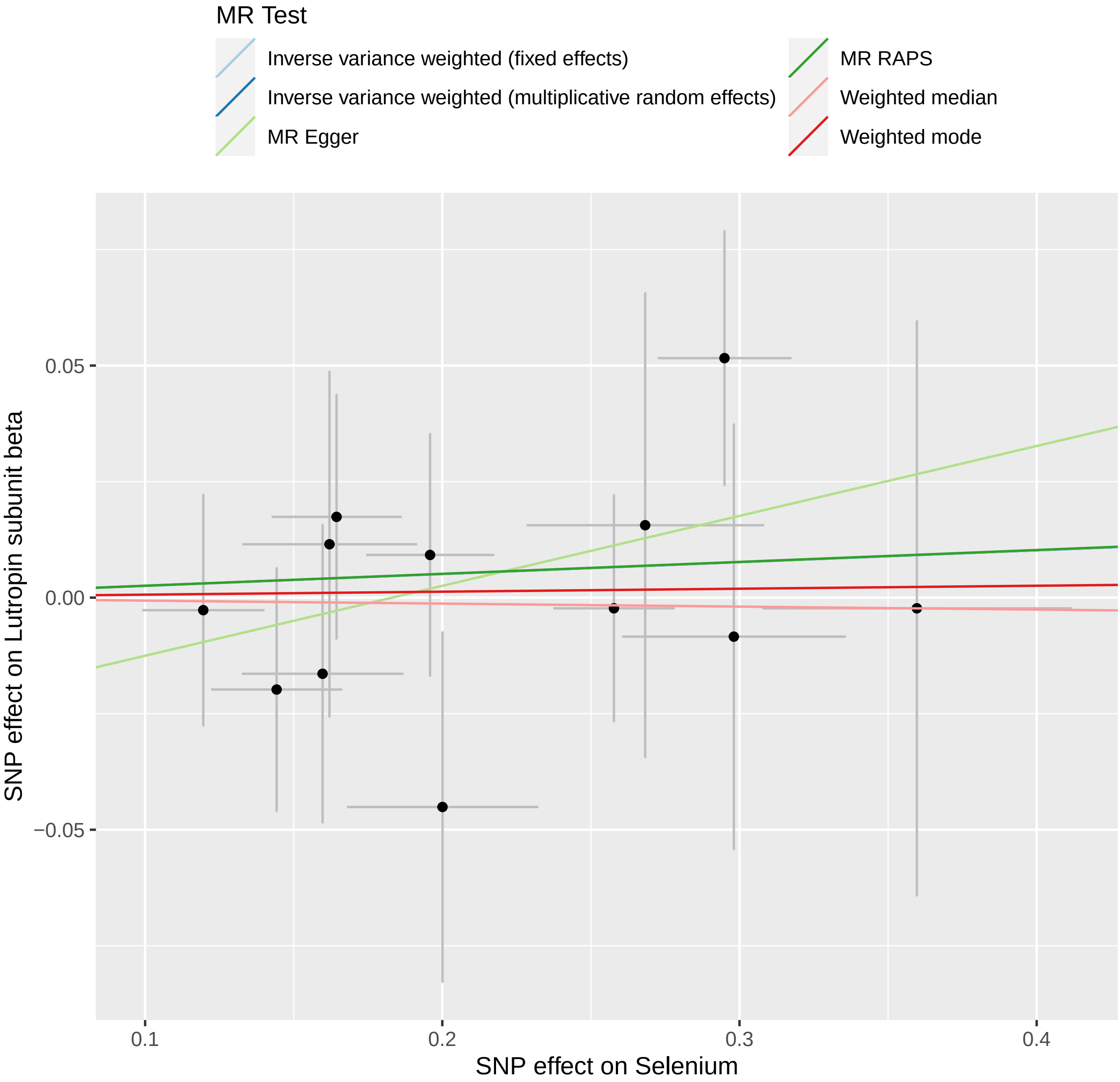

Batch 3−1736 : Selenium on Leukemia inhibitory factor

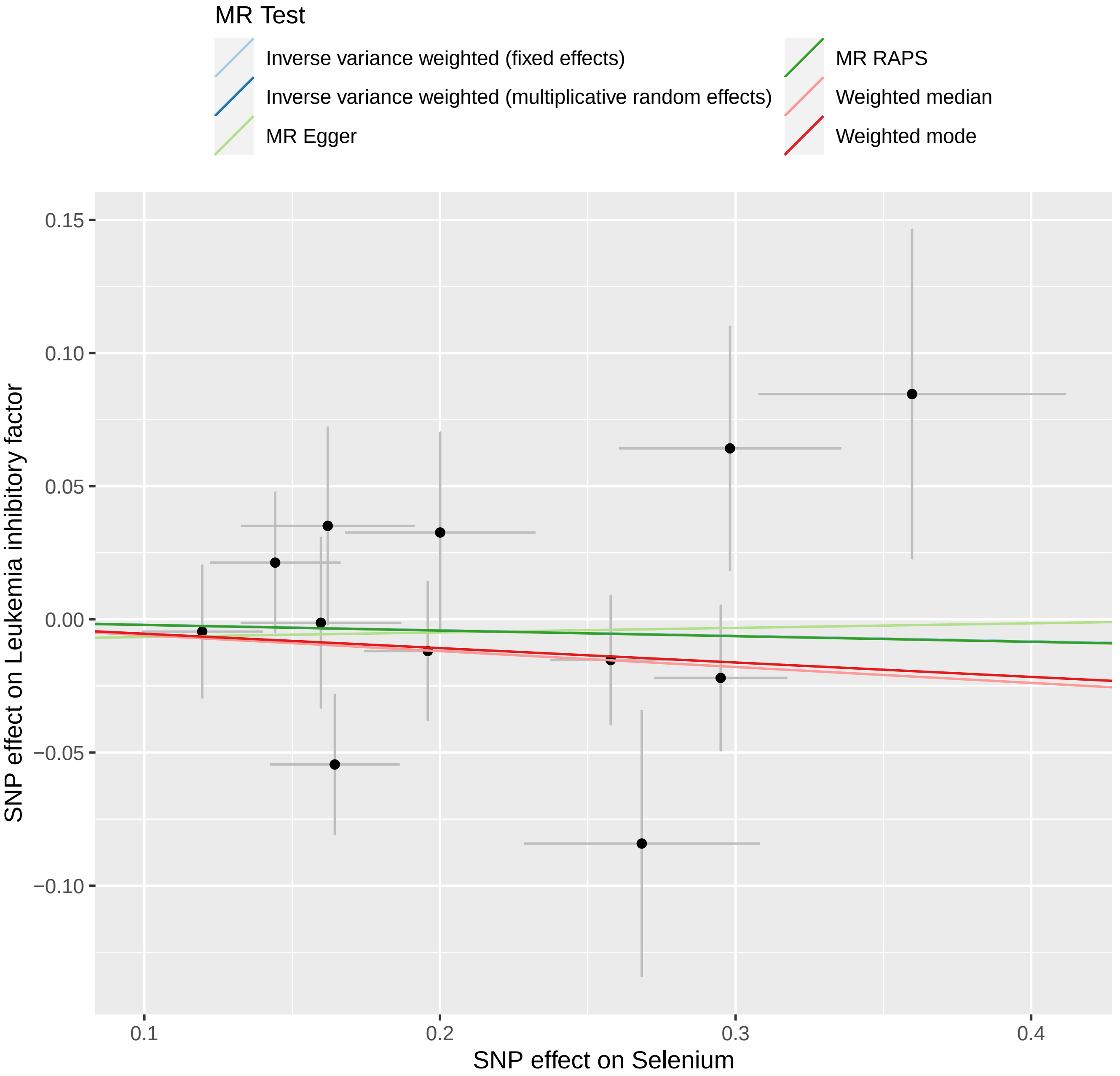

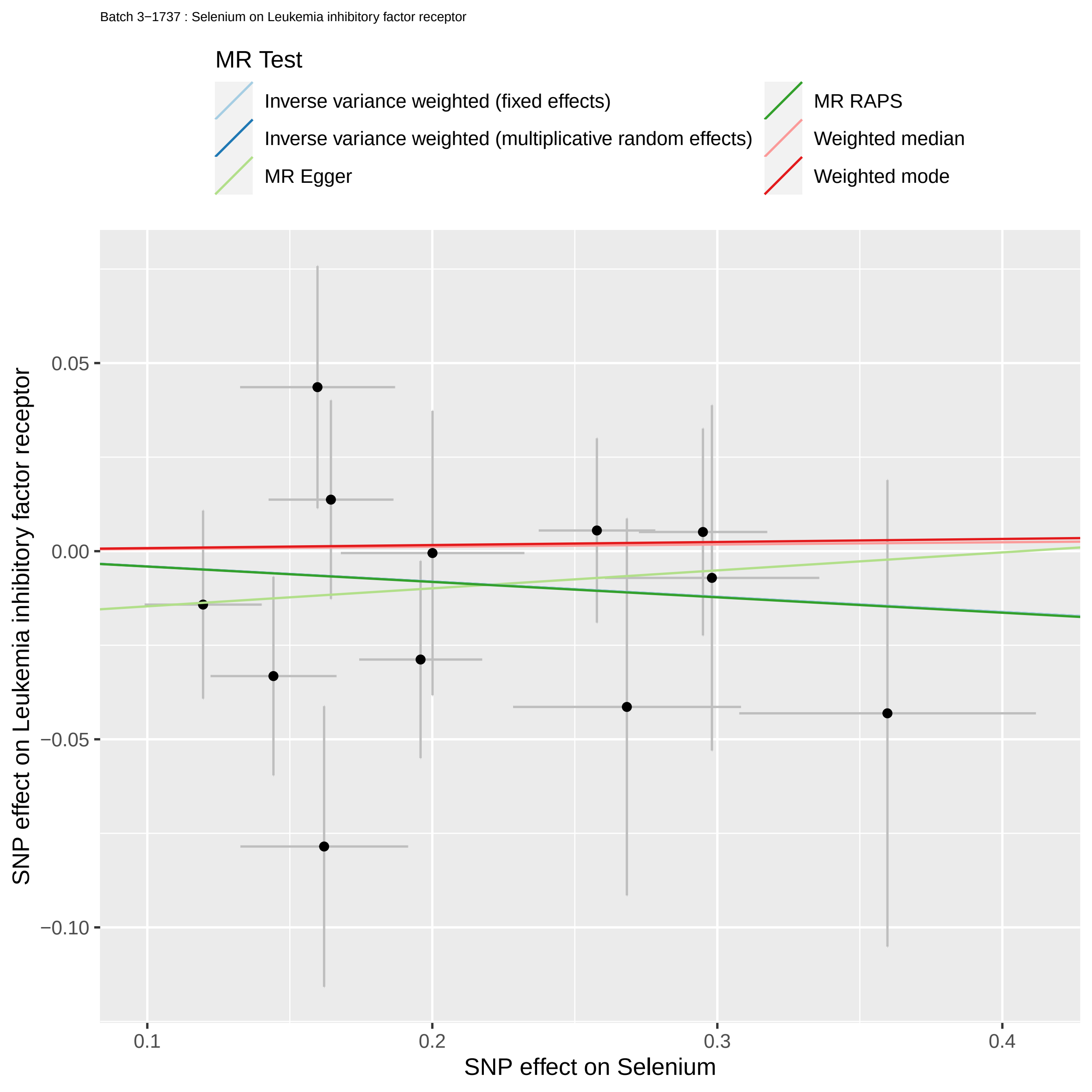
Batch 3−1737 : Selenium on Leukemia inhibitory factor receptor
MR Test
Inverse variance weighted (fixed effects)
Inverse variance weighted (multiplicative random effects)
MR Egger
MR RAPS
Weighted median
Weighted mode
SNP effect on Leukemia inhibitory factor receptor
0.05
0.00
−0.05
−0.10
0.1
0.2
0.3
0.4
SNP effect on Selenium

Batch 3−1738 : Selenium on Leukocyte immunoglobulin−like receptor subfamily A member 4

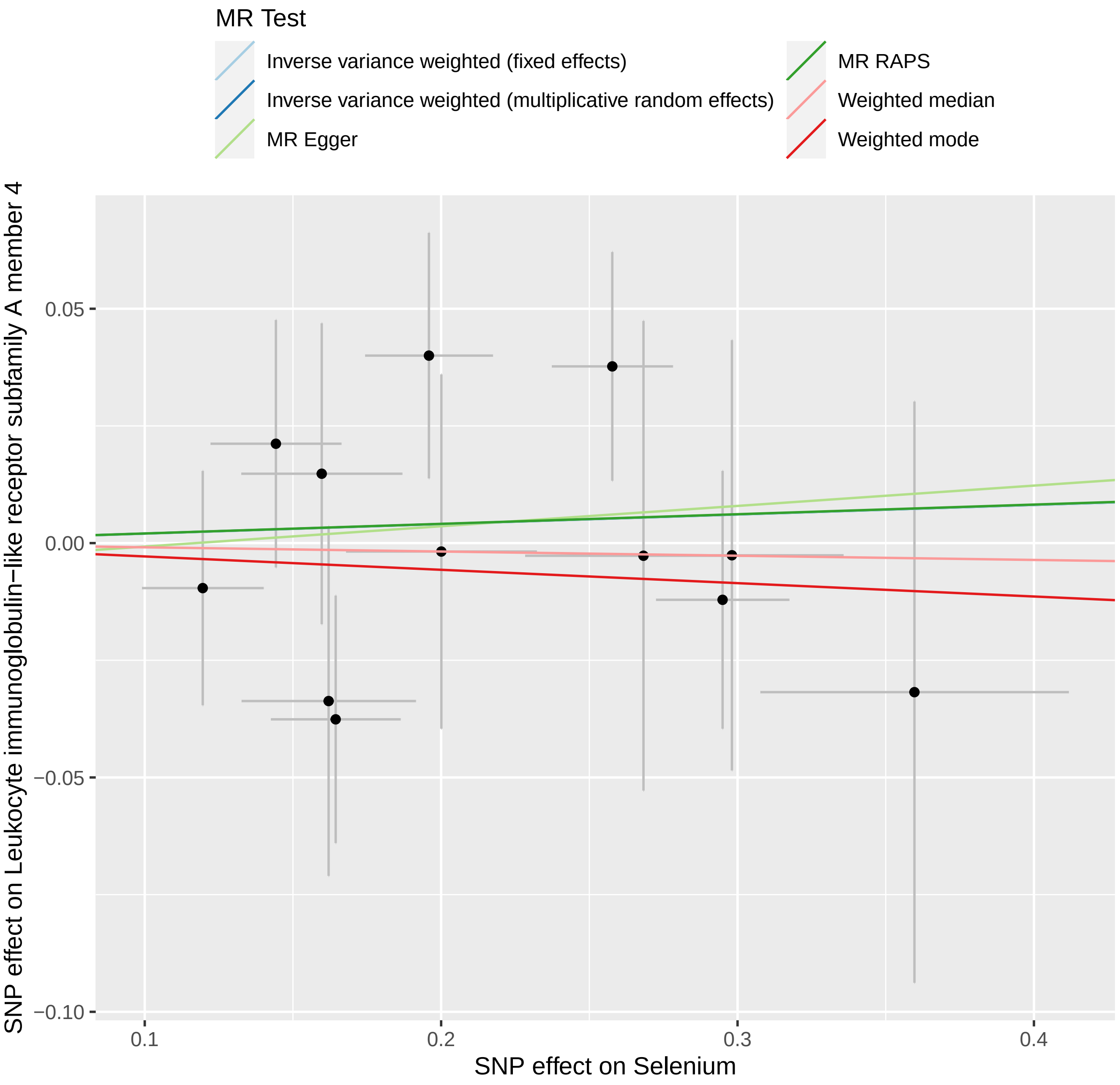

Batch 3−1739 : Selenium on Leukocyte immunoglobulin−like receptor subfamily A member 5

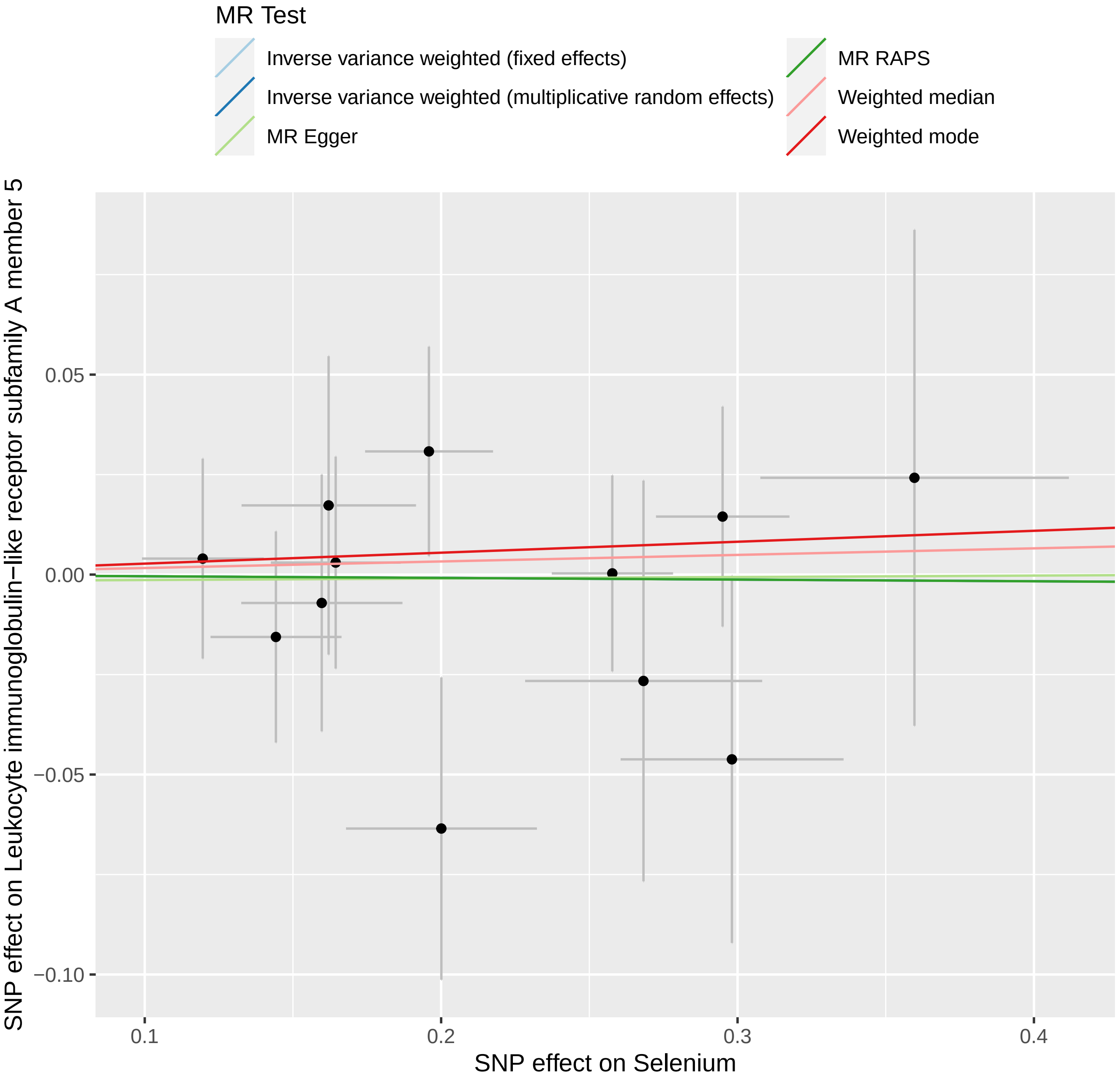

Batch 3−1740 : Selenium on Leukocyte immunoglobulin−like receptor subfamily A member 5

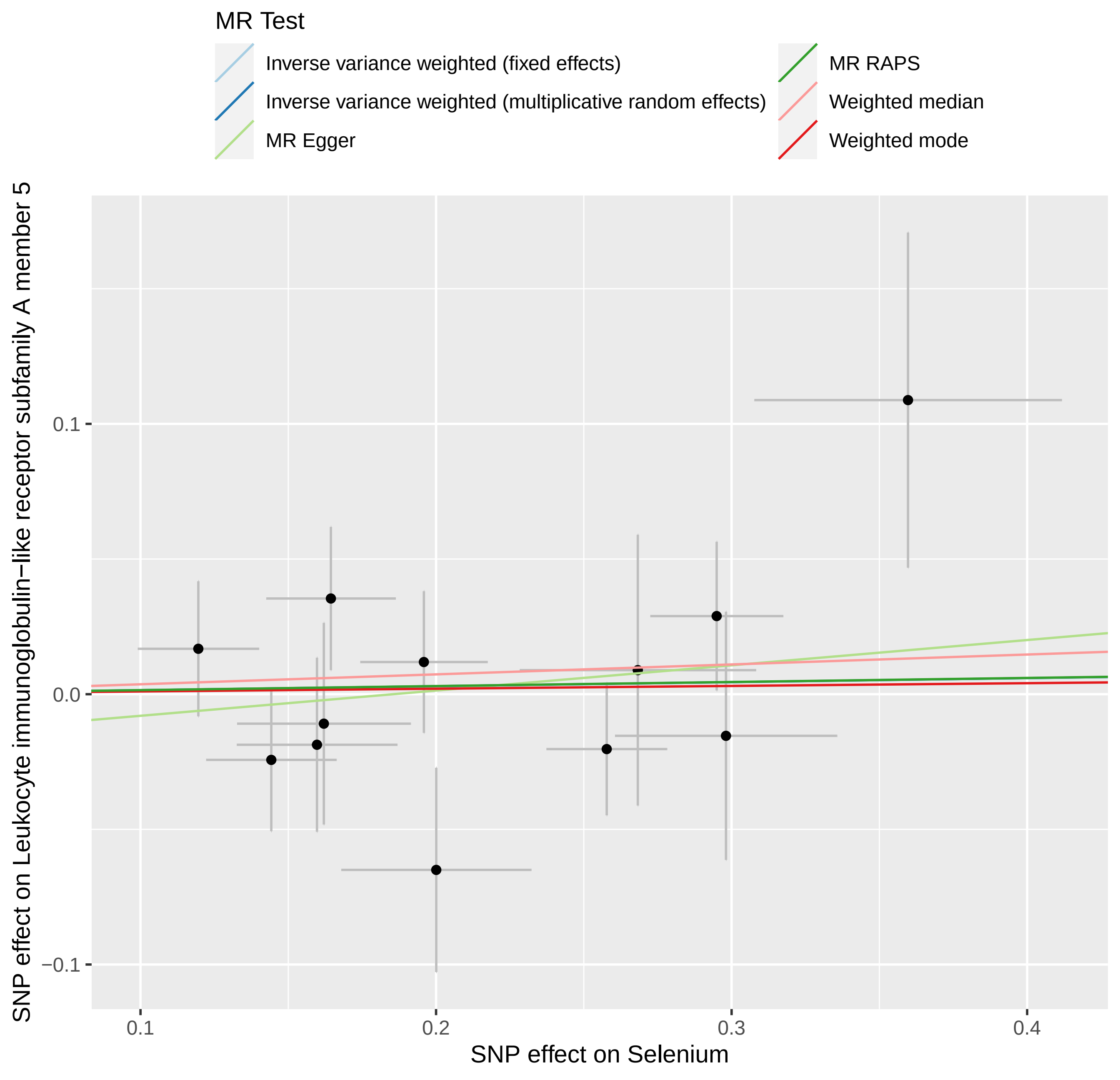

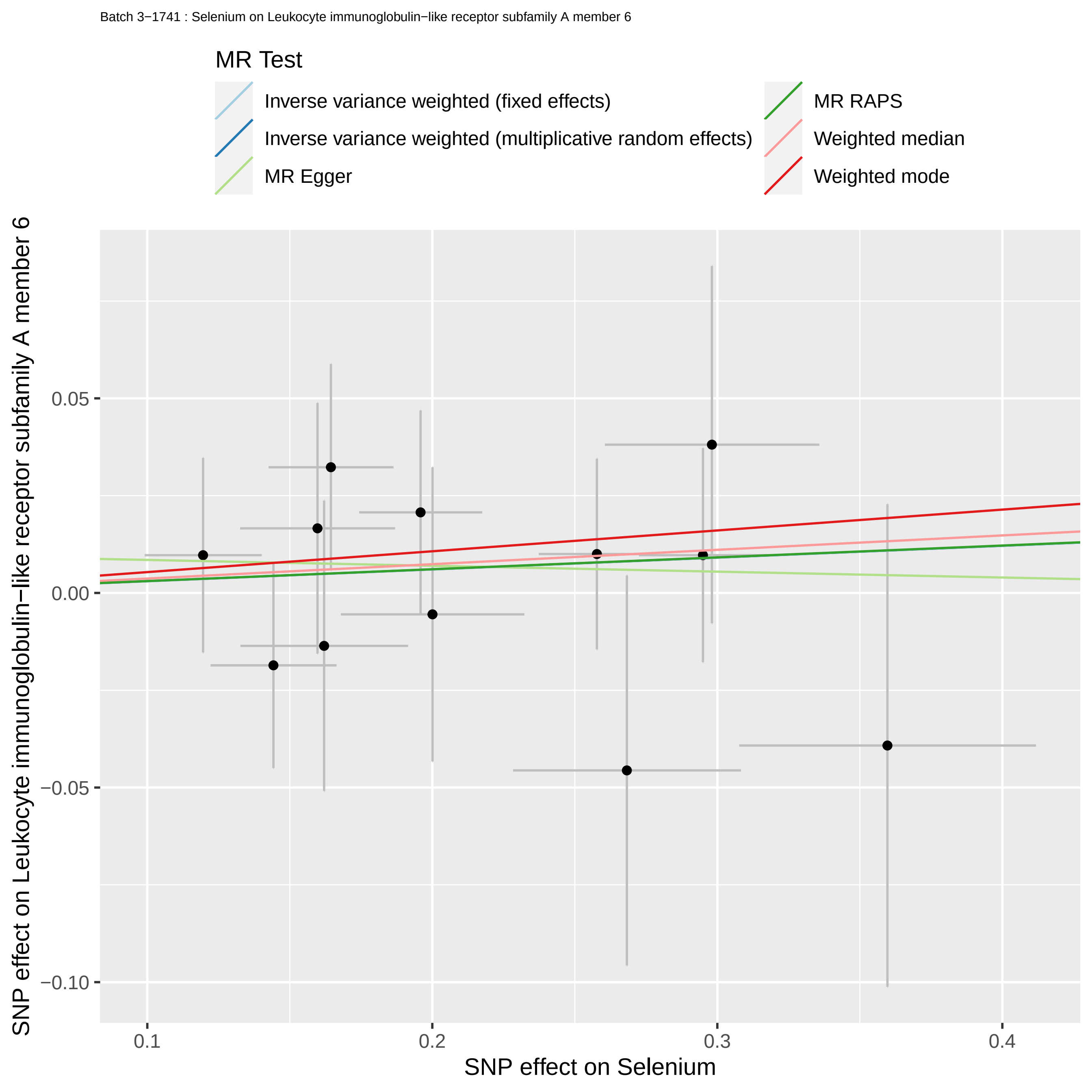
Batch 3−1741 : Selenium on Leukocyte immunoglobulin−like receptor subfamily A member 6
MR Test
Inverse variance weighted (fixed effects)
Inverse variance weighted (multiplicative random effects)
MR Egger
MR RAPS
Weighted median
Weighted mode
0.05
0.00
−0.05
−0.10
0.1
0.2
0.3
0.4
SNP effect on Leukocyte immunoglobulin−like receptor subfamily A member 6
SNP effect on Selenium

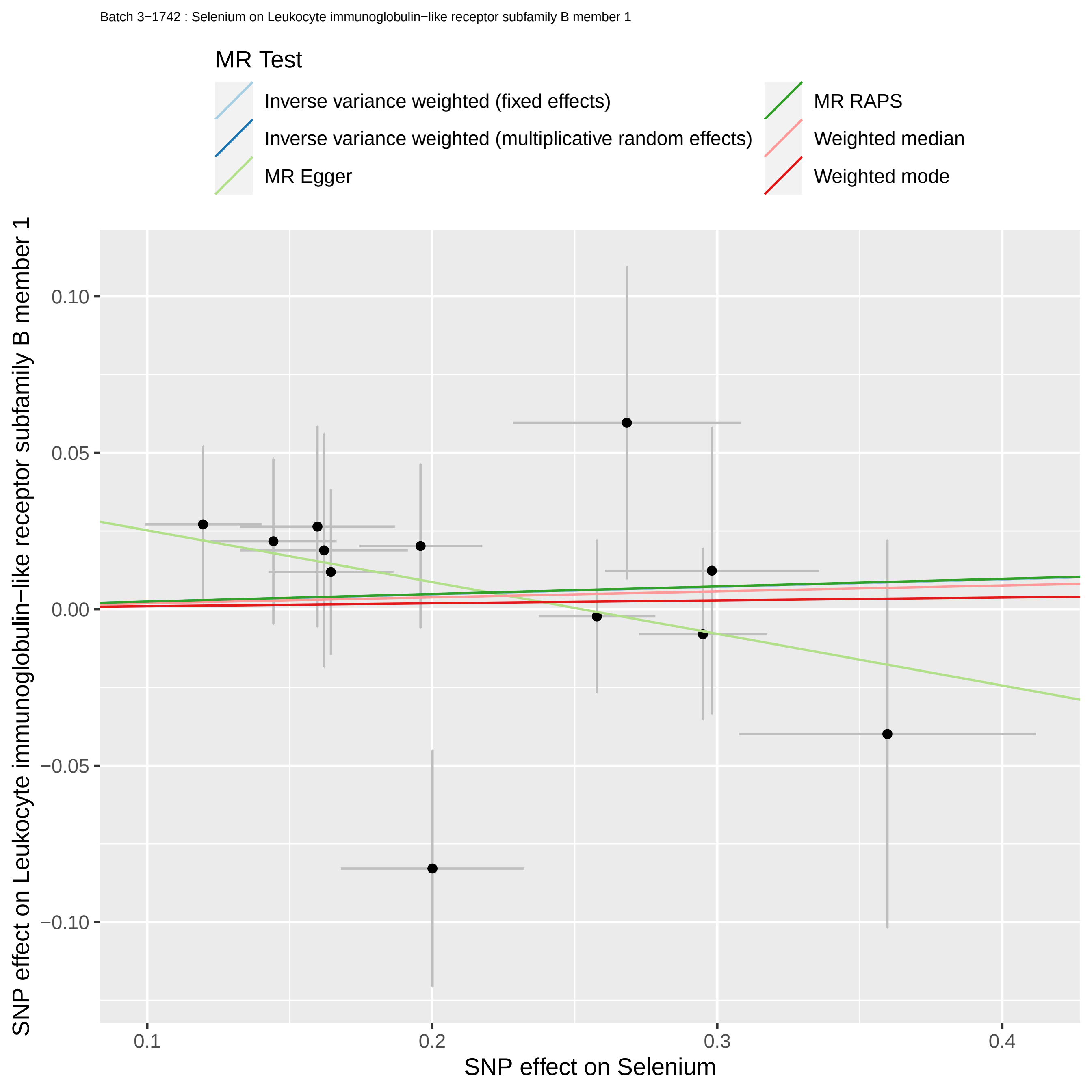

Batch 3−1742 : Selenium on Leukocyte immunoglobulin−like receptor subfamily B member 1
MR Test
Inverse variance weighted (fixed effects)
Inverse variance weighted (multiplicative random effects)
MR Egger
MR RAPS
Weighted median
Weighted mode
SNP effect on Leukocyte immunoglobulin−like receptor subfamily B member 1
0.10
0.05
0.00
−0.05
−0.10
0.1
0.2
0.3
0.4
SNP effect on Selenium

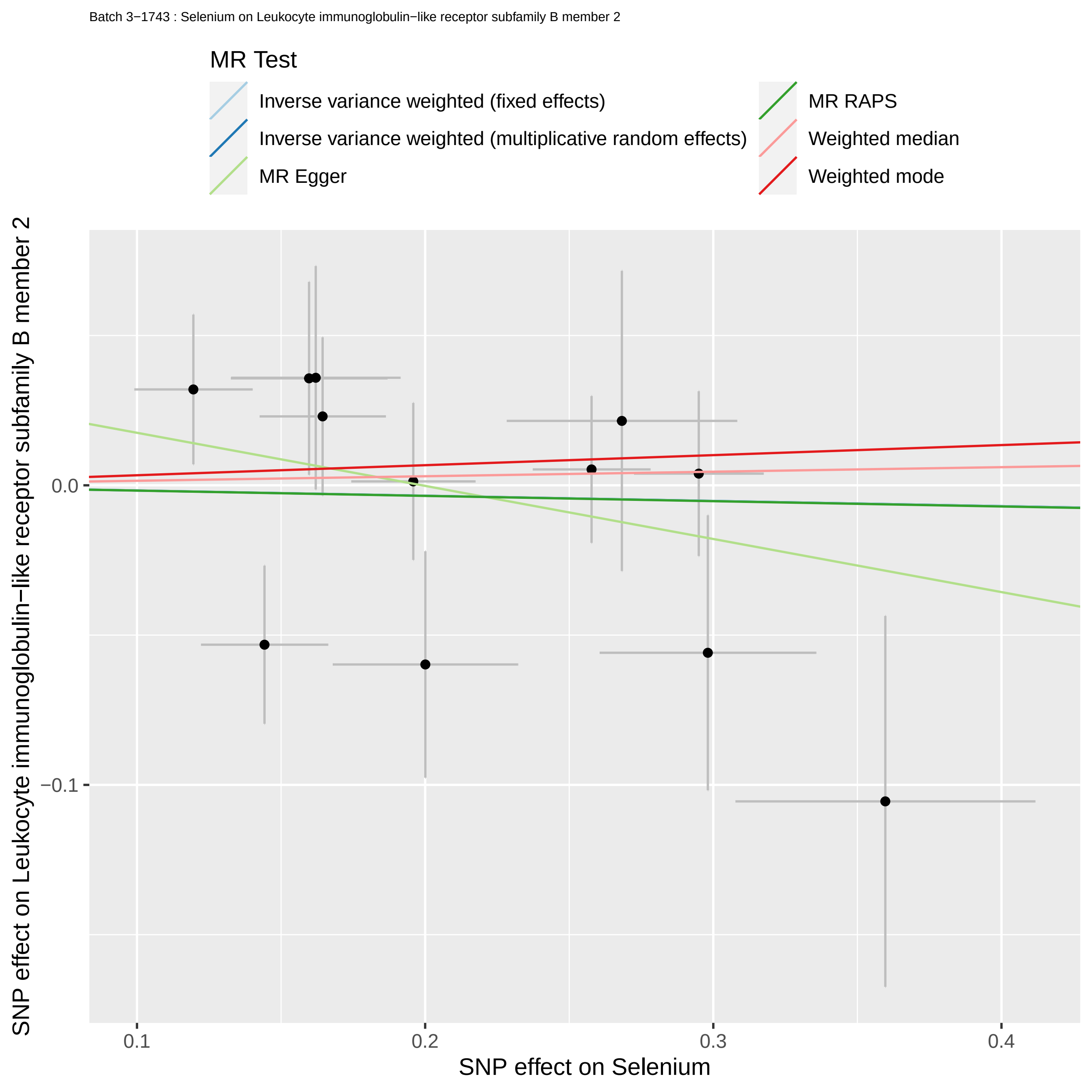

Batch 3−1743 : Selenium on Leukocyte immunoglobulin−like receptor subfamily B member 2
MR Test
Inverse variance weighted (fixed effects)
Inverse variance weighted (multiplicative random effects)
MR Egger
MR RAPS
Weighted median
Weighted mode
SNP effect on Leukocyte immunoglobulin−like receptor subfamily B member 2
0.0
−0.1
0.1
0.2
0.3
0.4
SNP effect on Selenium

Batch 3−1744 : Selenium on Leukocyte immunoglobulin−like receptor subfamily B member 2

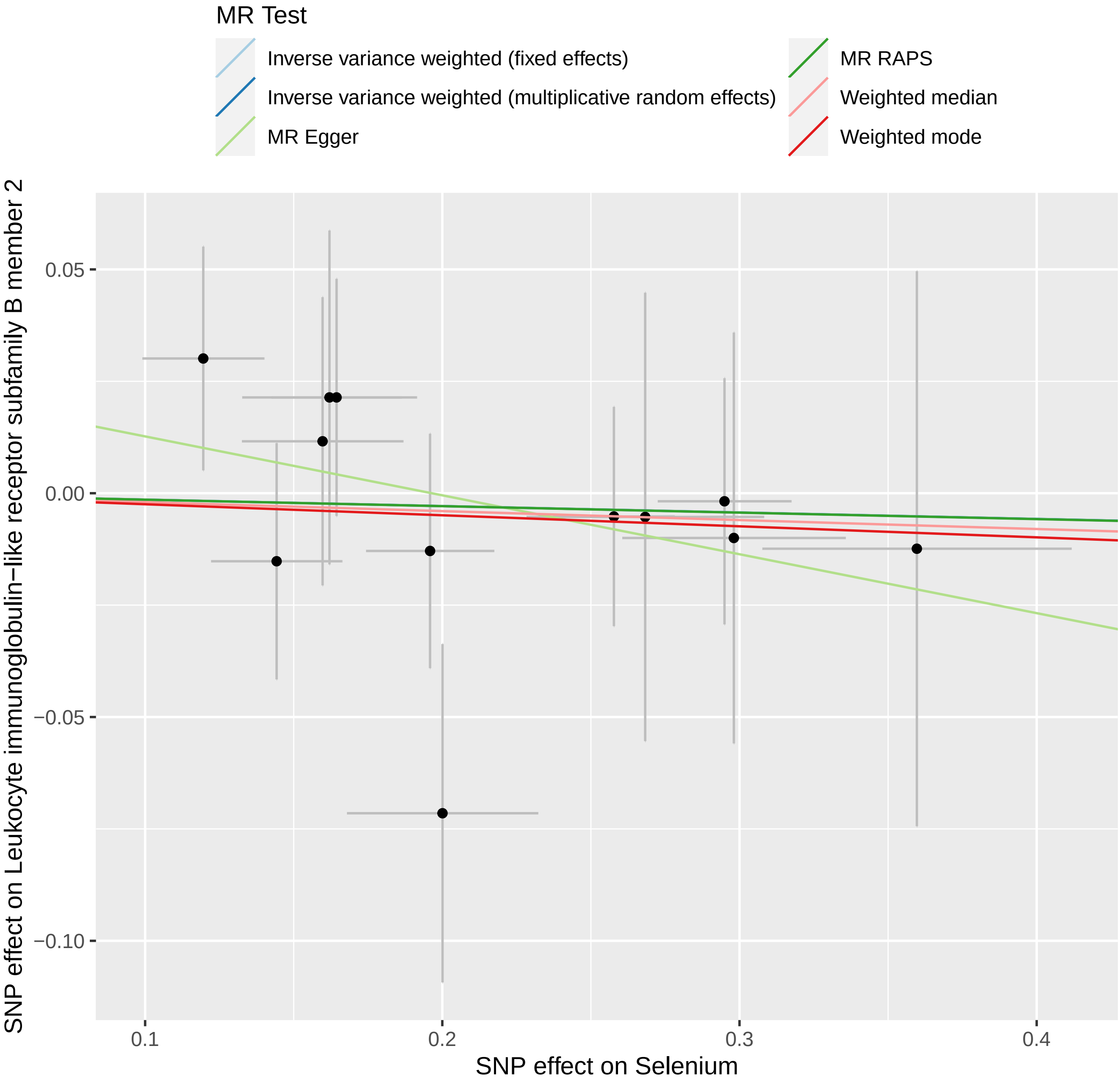

Batch 3−1745 : Selenium on Leukocyte immunoglobulin−like receptor subfamily B member 3

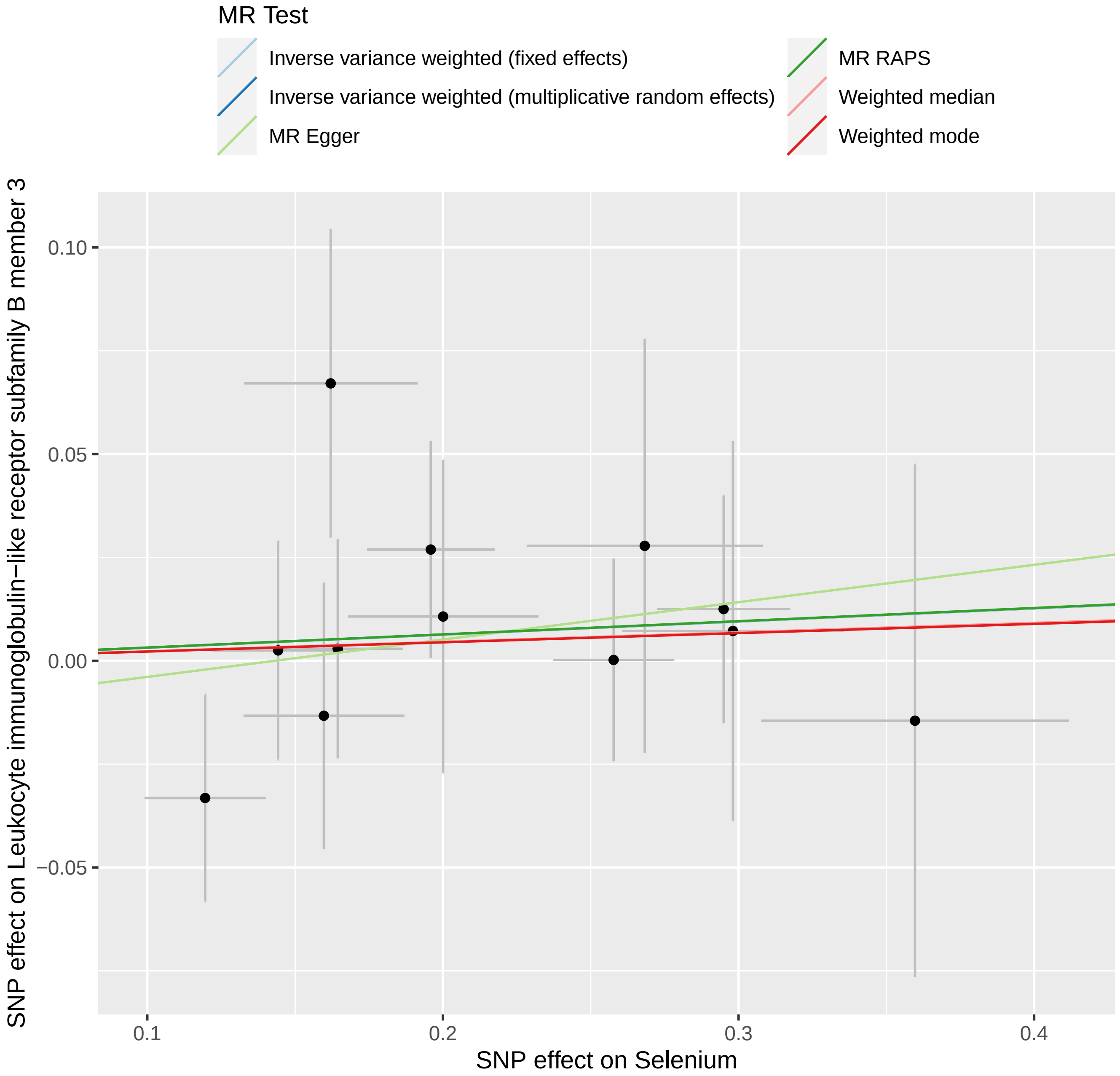

Batch 3−1746 : Selenium on Leukocyte immunoglobulin−like receptor subfamily B member 4

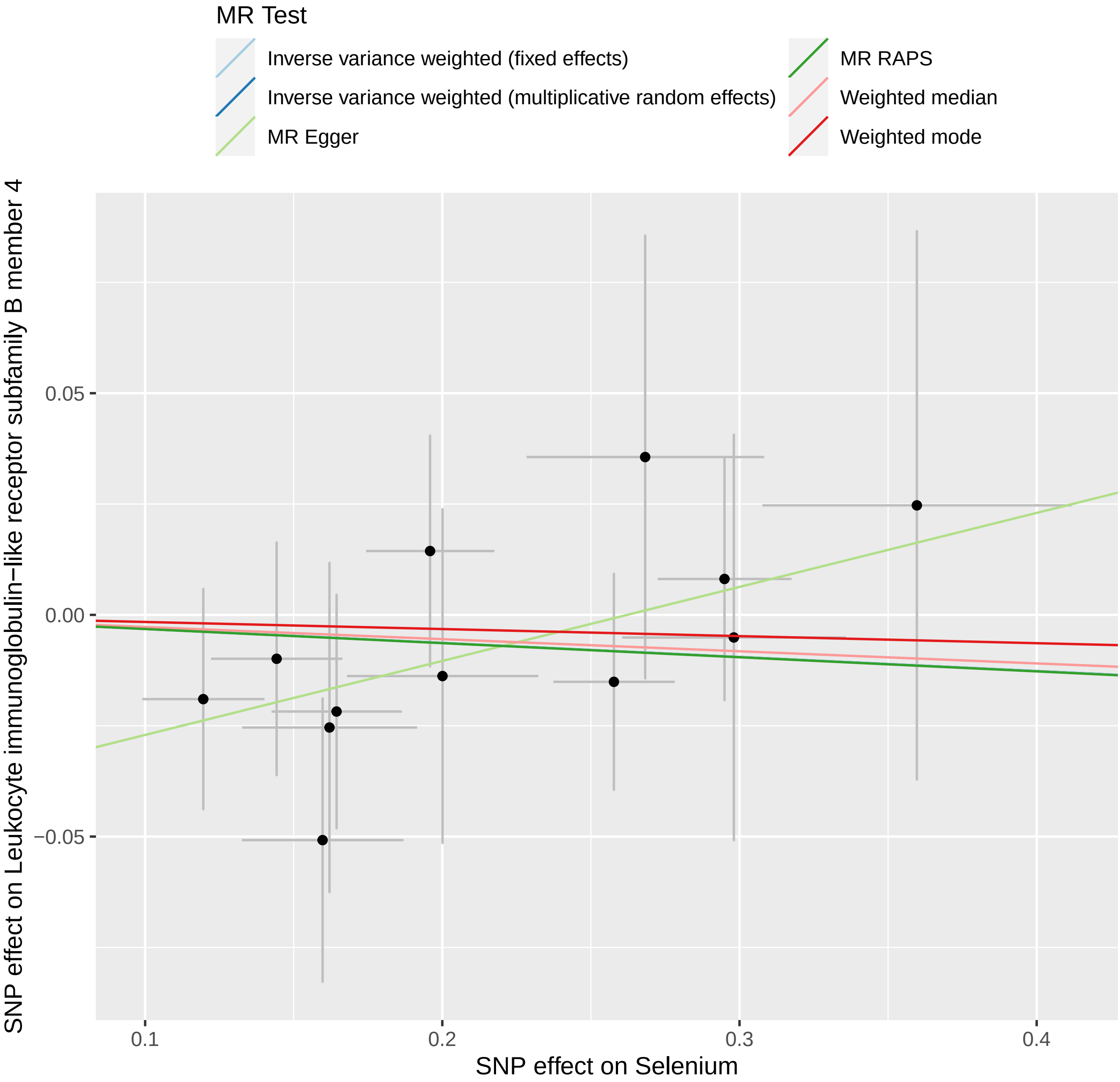

Batch 3−1747 : Selenium on Leukocyte immunoglobulin−like receptor subfamily B member 5

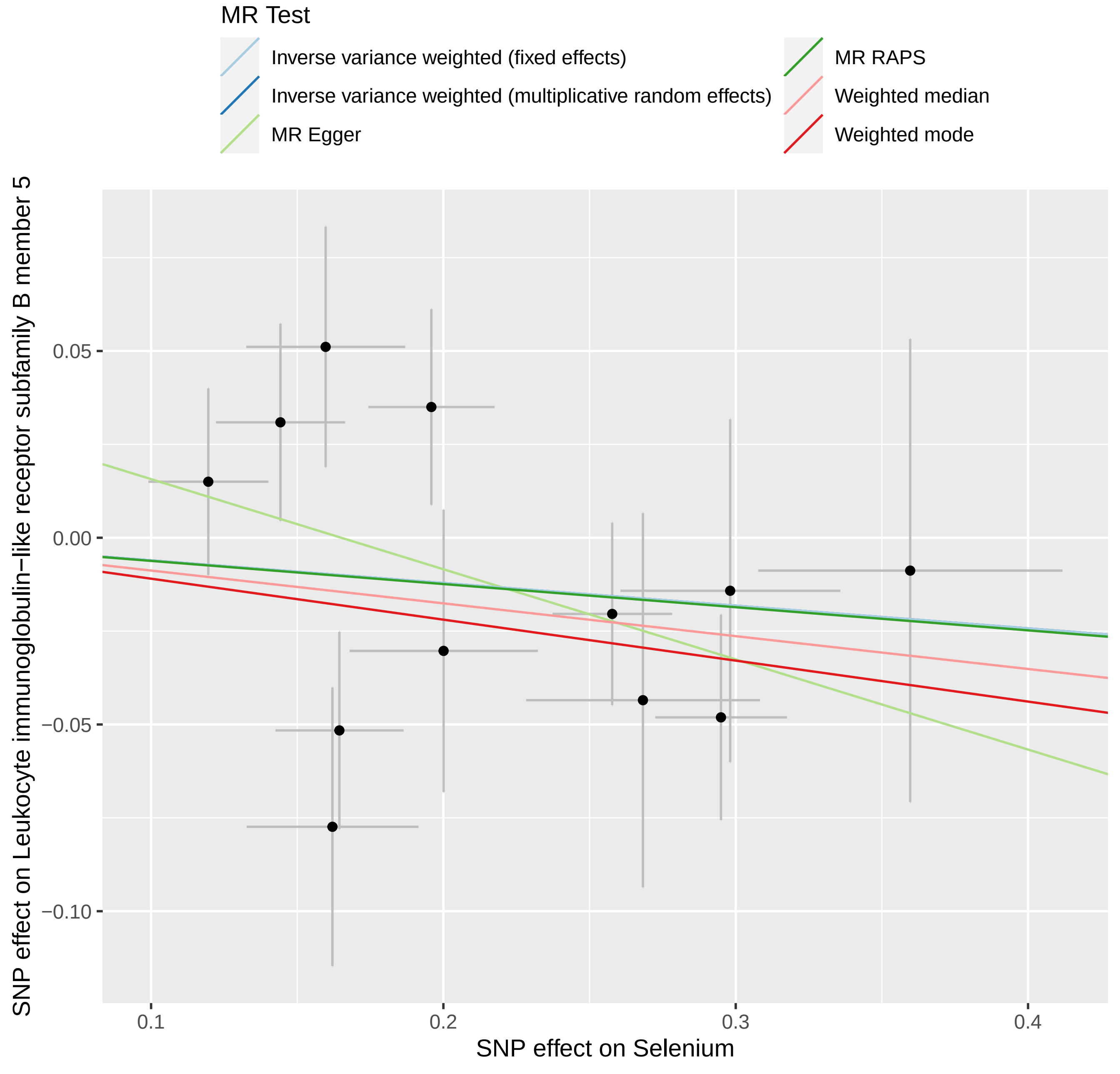

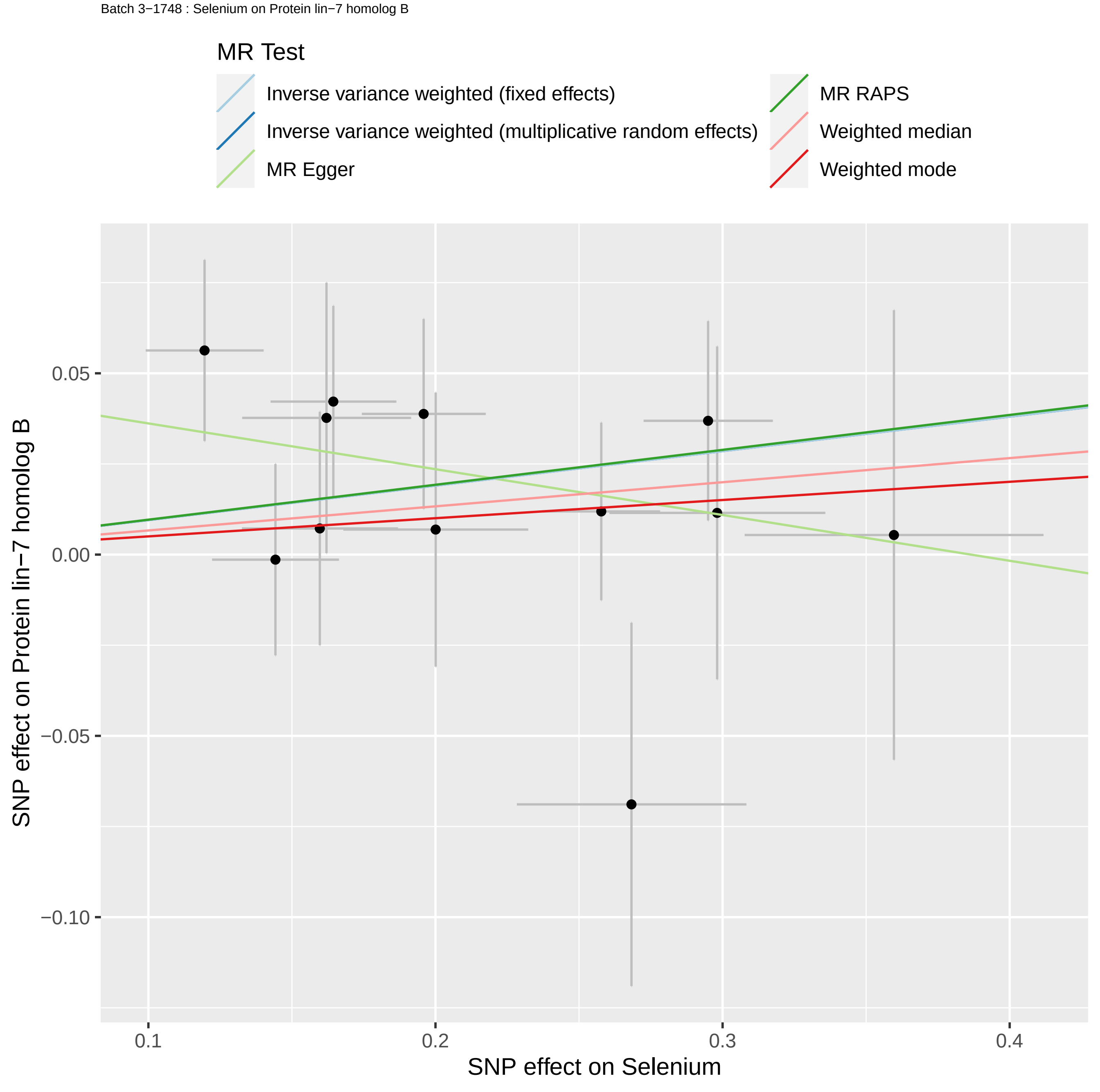

Batch 3−1748 : Selenium on Protein lin−7 homolog B
MR Test
Inverse variance weighted (fixed effects)
Inverse variance weighted (multiplicative random effects)
MR Egger
MR RAPS
Weighted median
Weighted mode
SNP effect on Protein lin−7 homolog B
0.05
0.00
−0.05
−0.10
0.1
0.2
0.3
0.4
SNP effect on Selenium

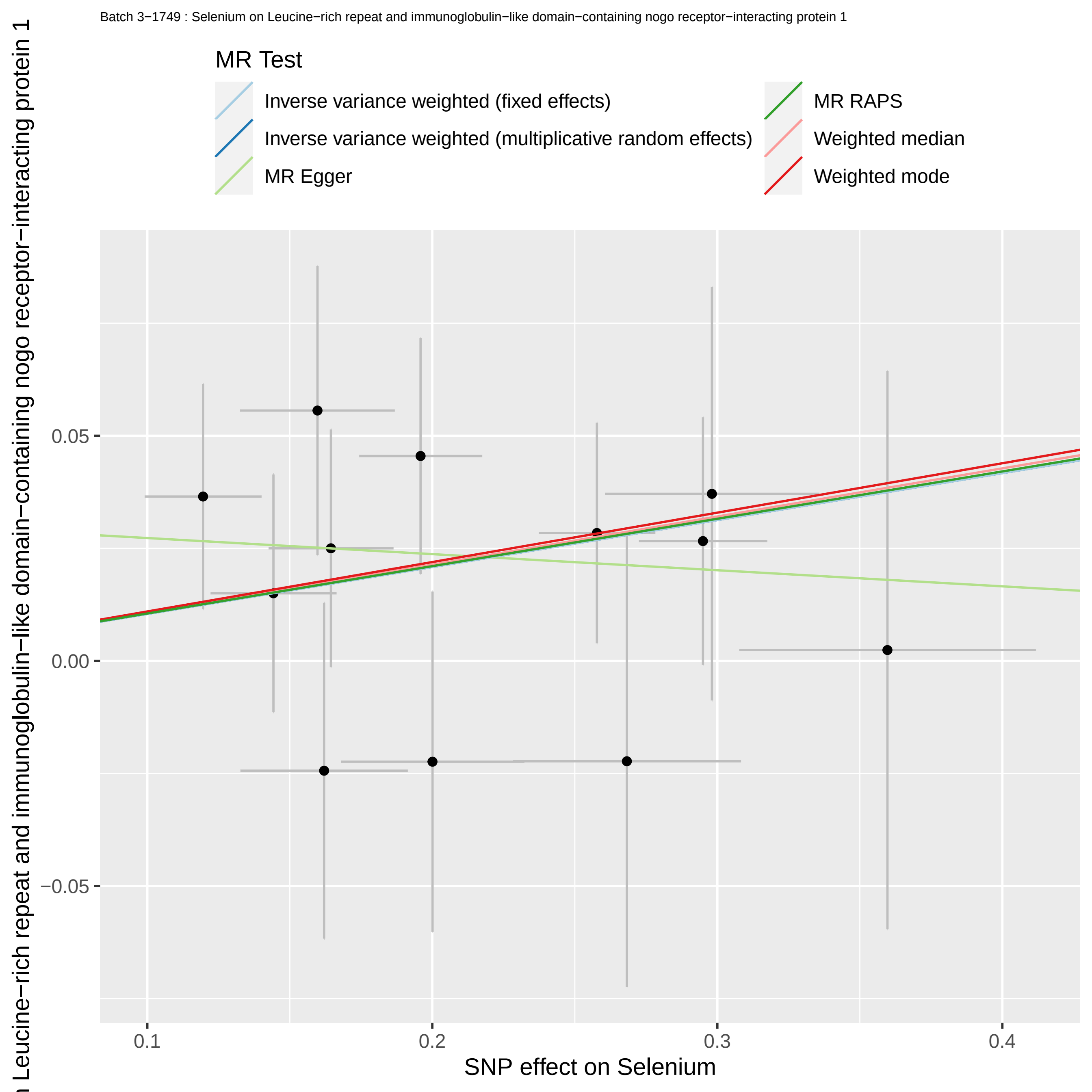

Batch 3−1749 : Selenium on Leucine−rich repeat and immunoglobulin−like domain−containing nogo receptor−interacting protein 1
MR Test
Inverse variance weighted (fixed effects)
Inverse variance weighted (multiplicative random effects)
MR Egger
MR RAPS
Weighted median
Weighted mode
0.05
0.00
−0.05
0.1
0.2
0.3
0.4
SNP effect on Selenium
n Leucine−rich repeat and immunoglobulin−like domain−containing nogo receptor−interacting protein 1

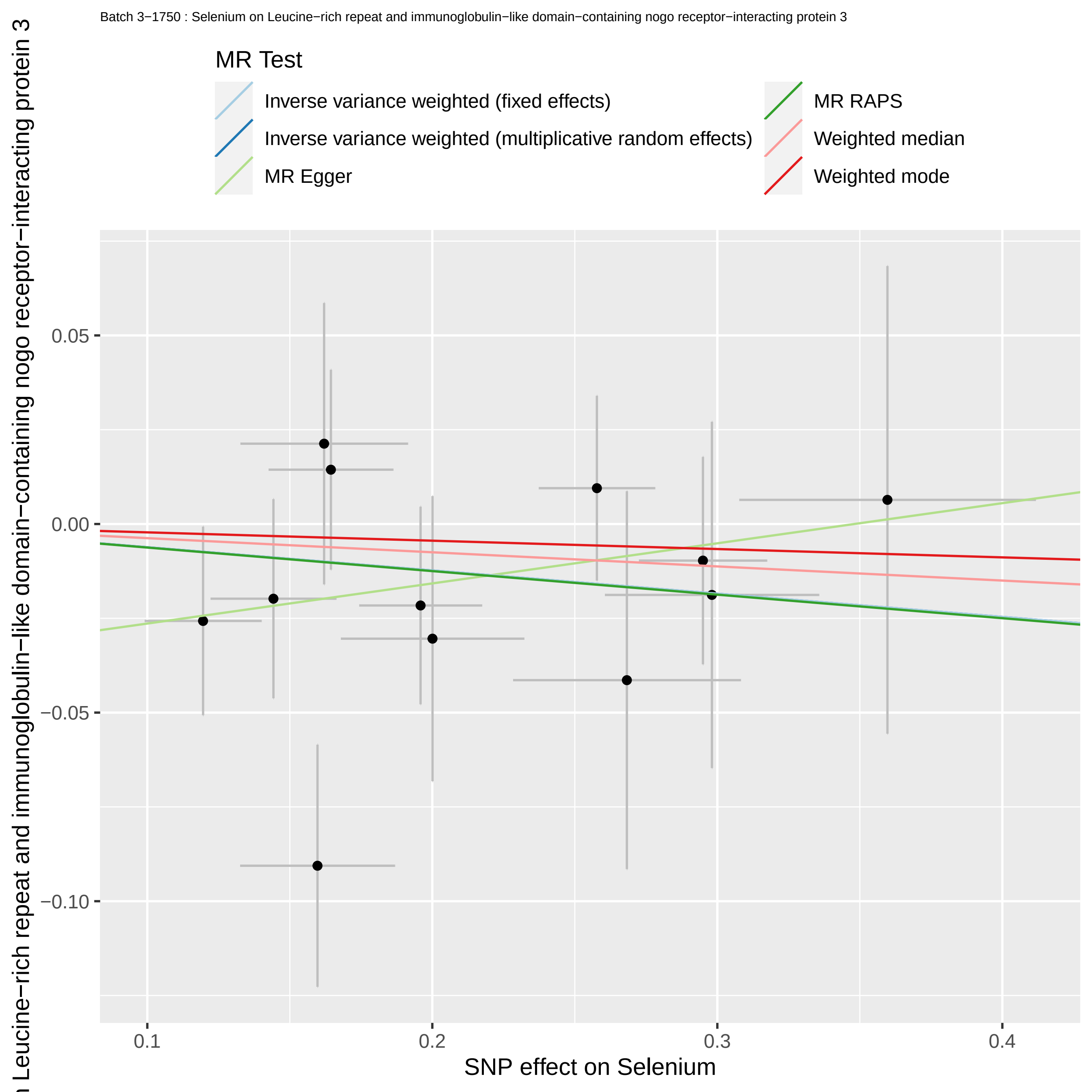

Batch 3−1750 : Selenium on Leucine−rich repeat and immunoglobulin−like domain−containing nogo receptor−interacting protein 3
MR Test
Inverse variance weighted (fixed effects)
Inverse variance weighted (multiplicative random effects)
MR Egger
MR RAPS
Weighted median
Weighted mode
0.05
0.00
−0.05
−0.10
0.1
0.2
0.3
0.4
SNP effect on Selenium
n Leucine−rich repeat and immunoglobulin−like domain−containing nogo receptor−interacting protein 3

Batch 3−1751 : Selenium on Gastric triacylglycerol lipase

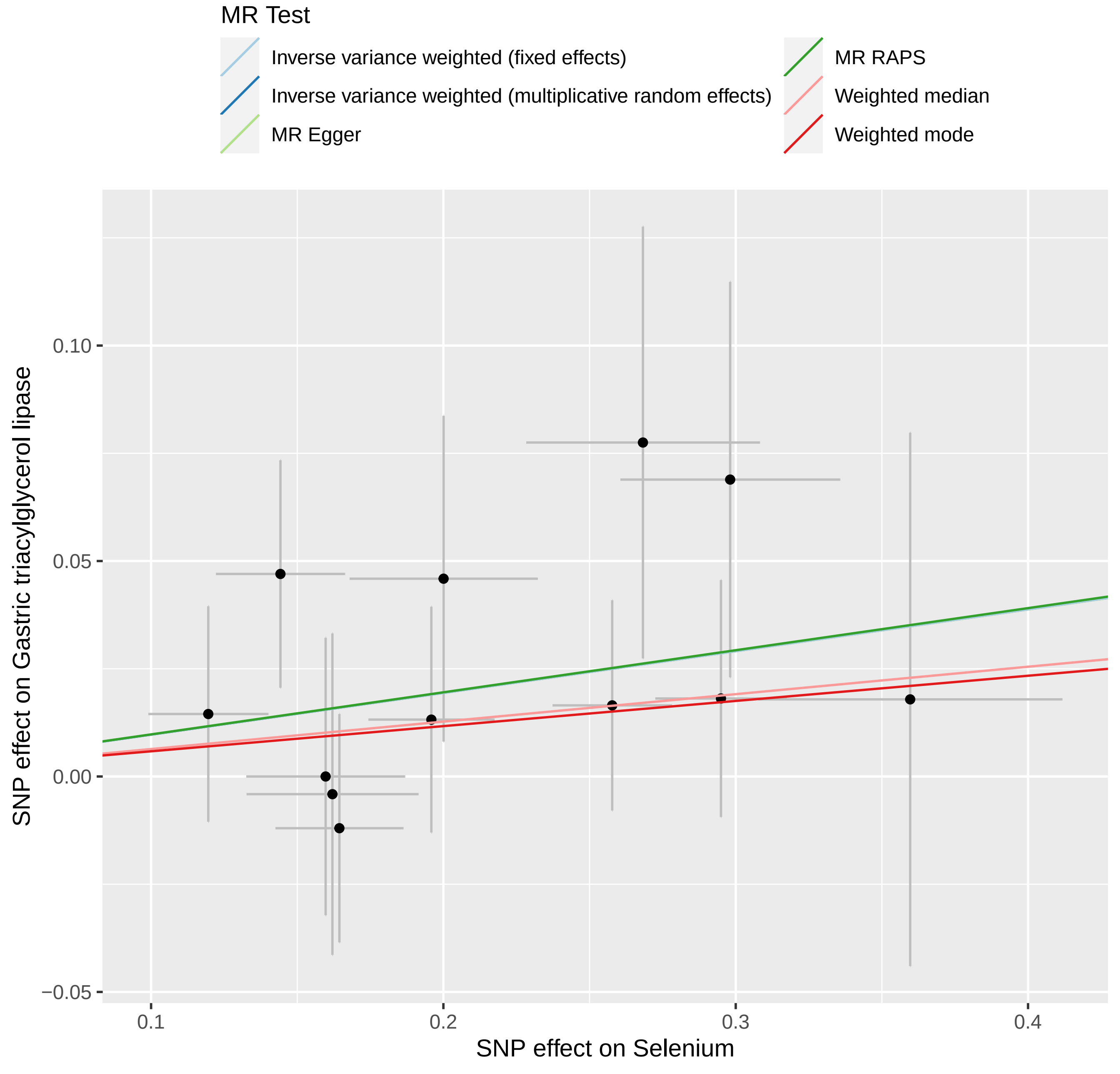

Batch 3−1752 : Selenium on Lipase member K

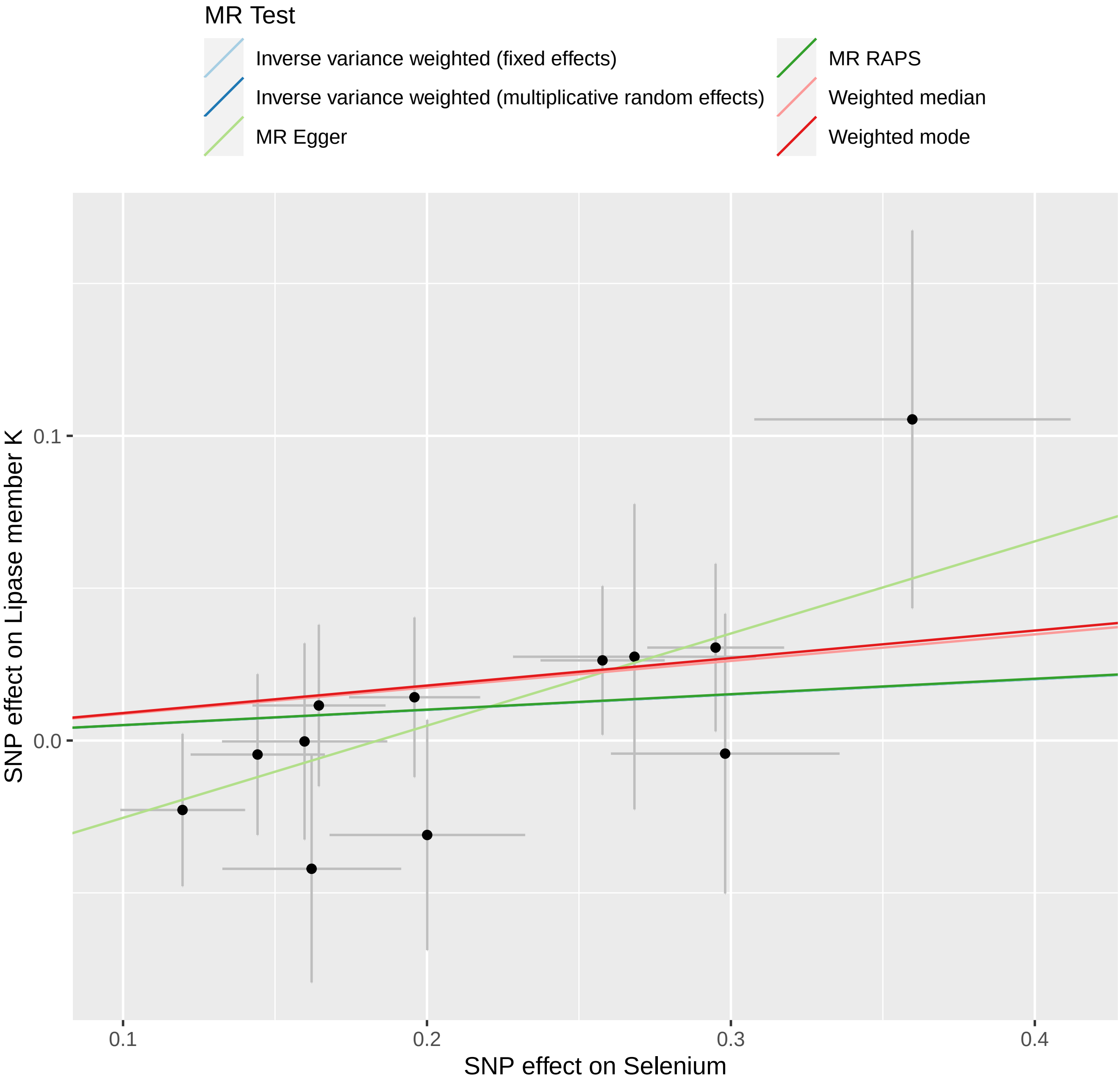

Batch 3−1753 : Selenium on Lipase member N

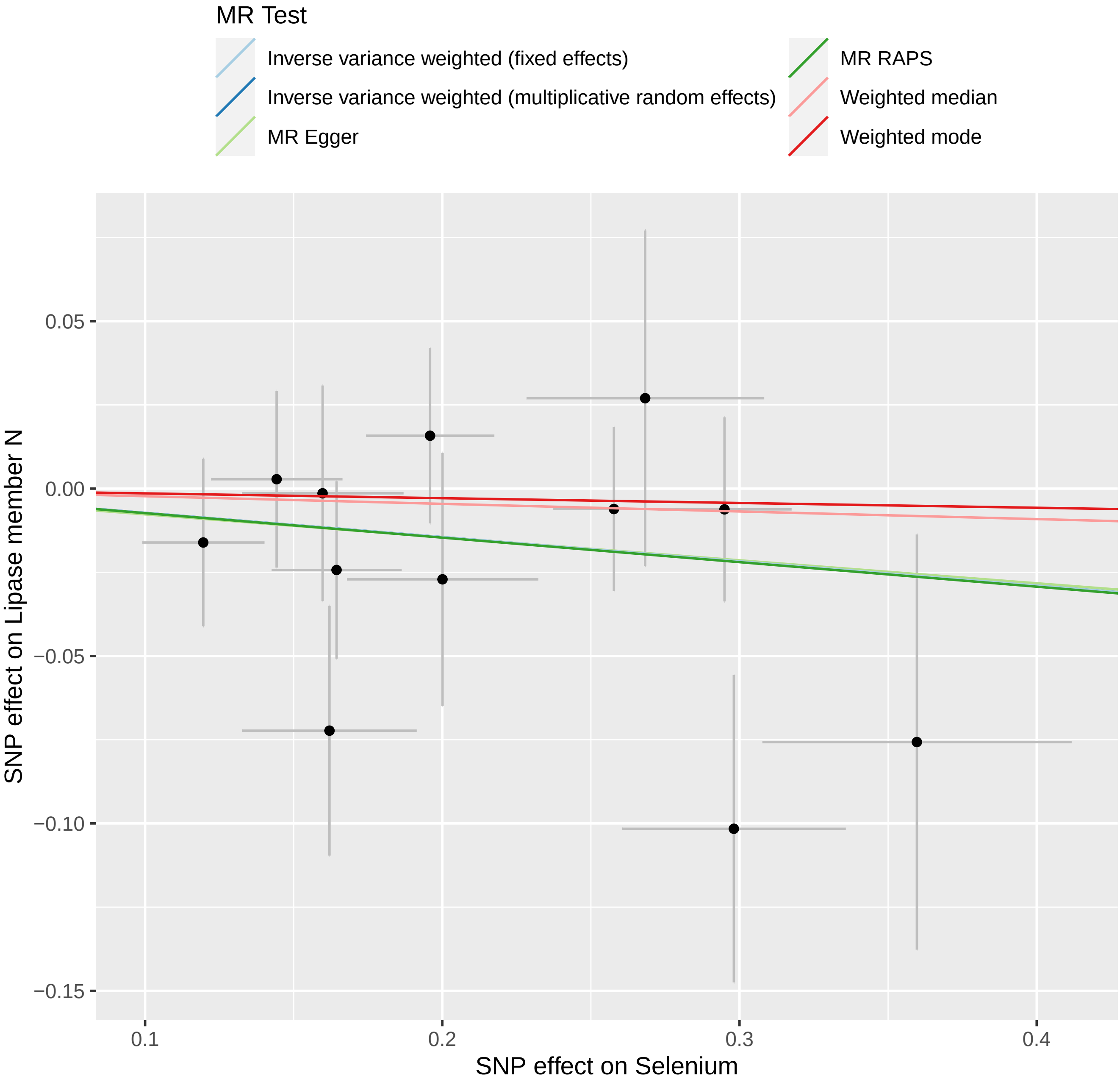

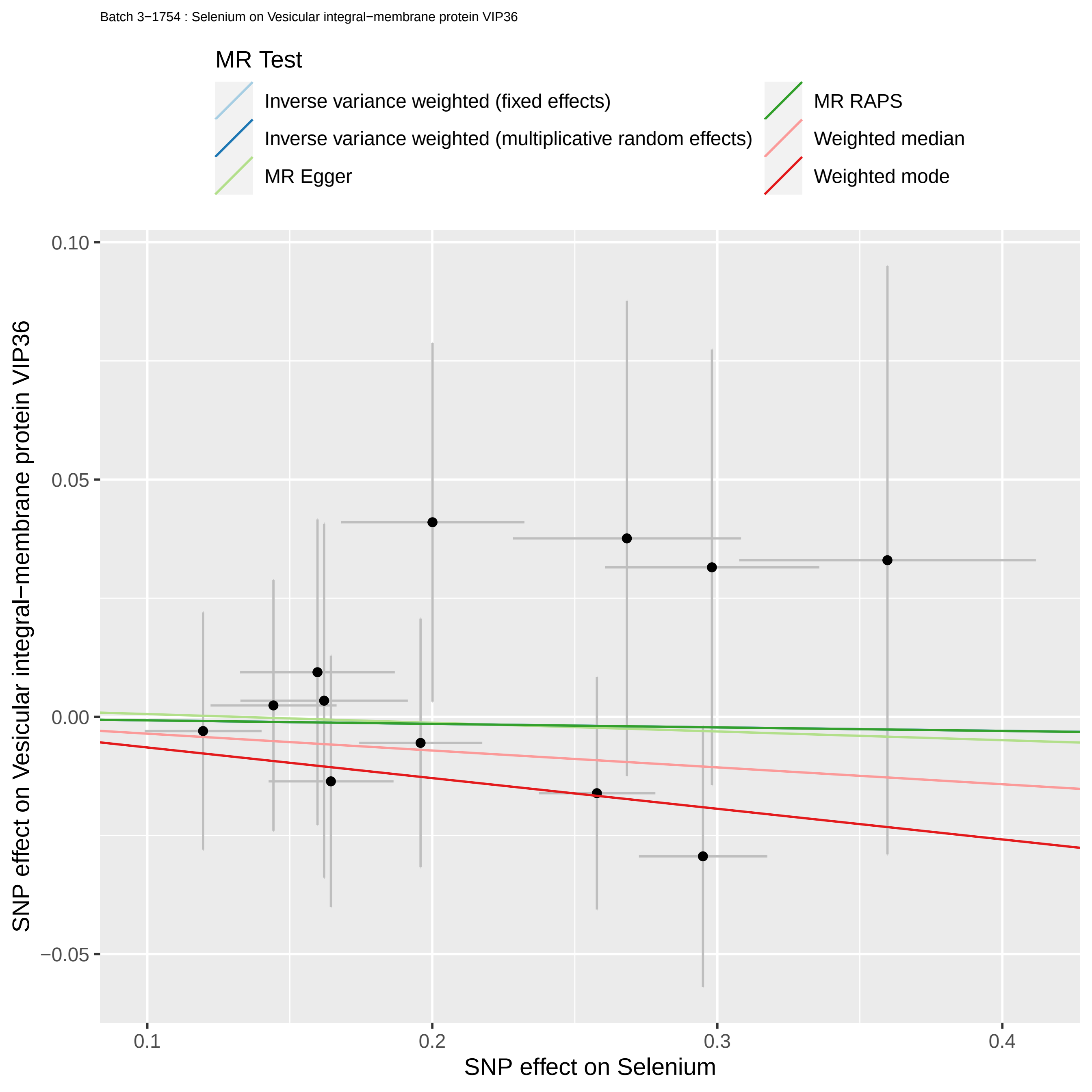
Batch 3−1754 : Selenium on Vesicular integral−membrane protein VIP36
MR Test
Inverse variance weighted (fixed effects)
Inverse variance weighted (multiplicative random effects)
MR Egger
MR RAPS
Weighted median
Weighted mode
0.10
0.05
0.00
−0.05
0.1
0.2
0.3
0.4
SNP effect on Vesicular integral−membrane protein VIP36
SNP effect on Selenium

Batch 3−1755 : Selenium on Vesicular integral−membrane protein VIP36

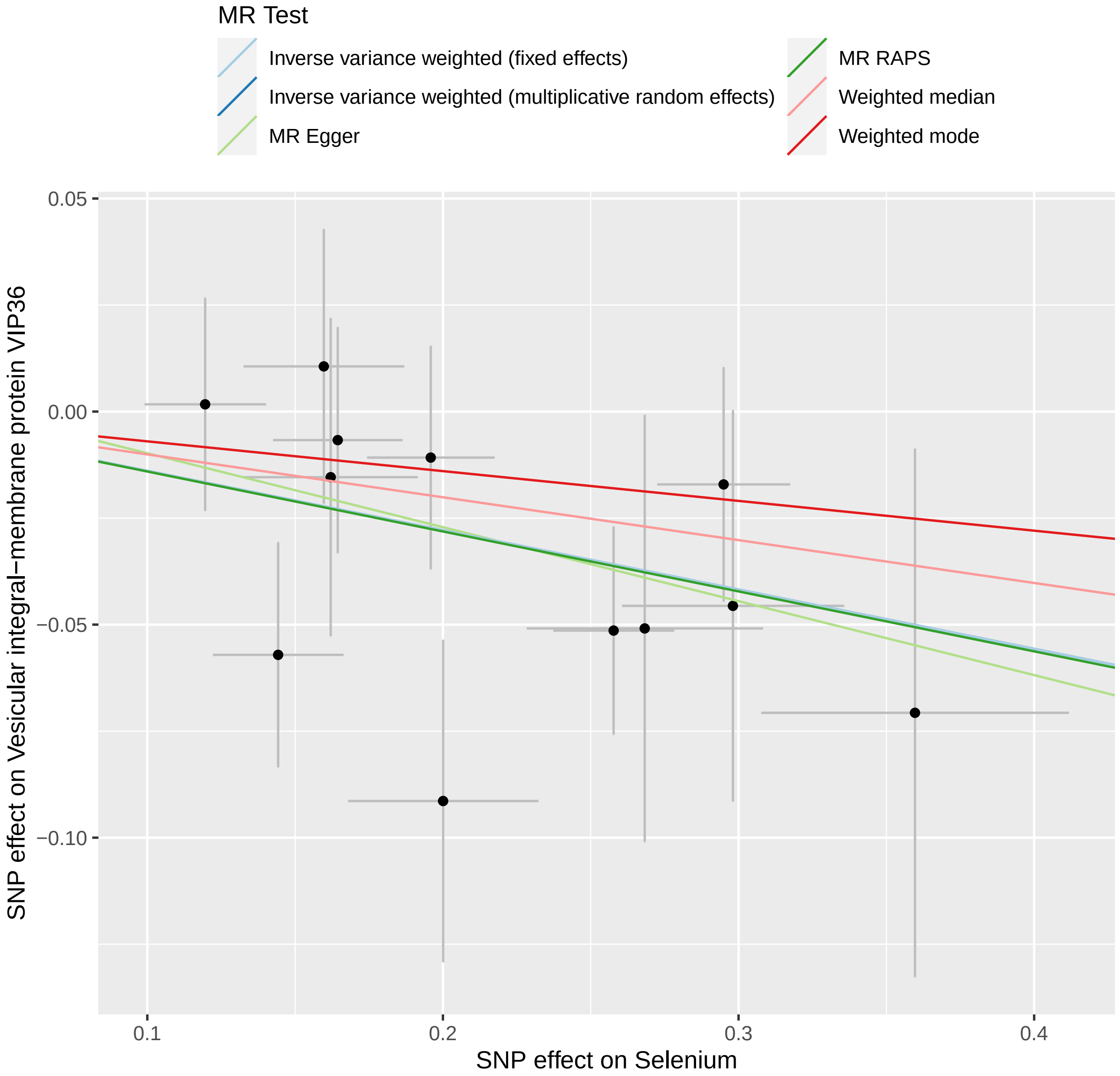

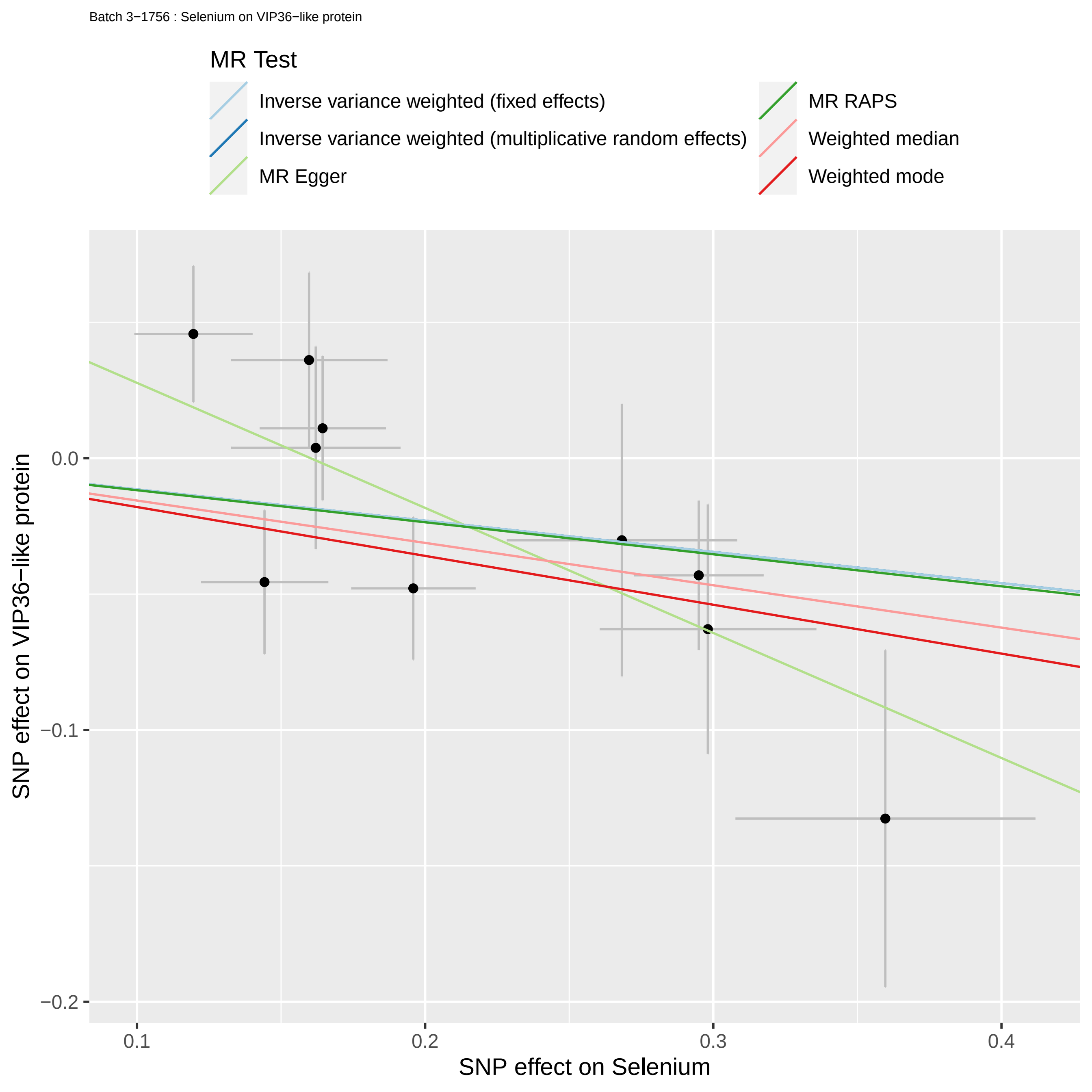
Batch 3−1756 : Selenium on VIP36−like protein
MR Test
Inverse variance weighted (fixed effects)
Inverse variance weighted (multiplicative random effects)
MR Egger
MR RAPS
Weighted median
Weighted mode
0.0
−0.1
−0.2
0.1
0.2
0.3
0.4
SNP effect on VIP36−like protein
SNP effect on Selenium

Batch 3−1757 : Selenium on LIM and cysteine−rich domains protein 1

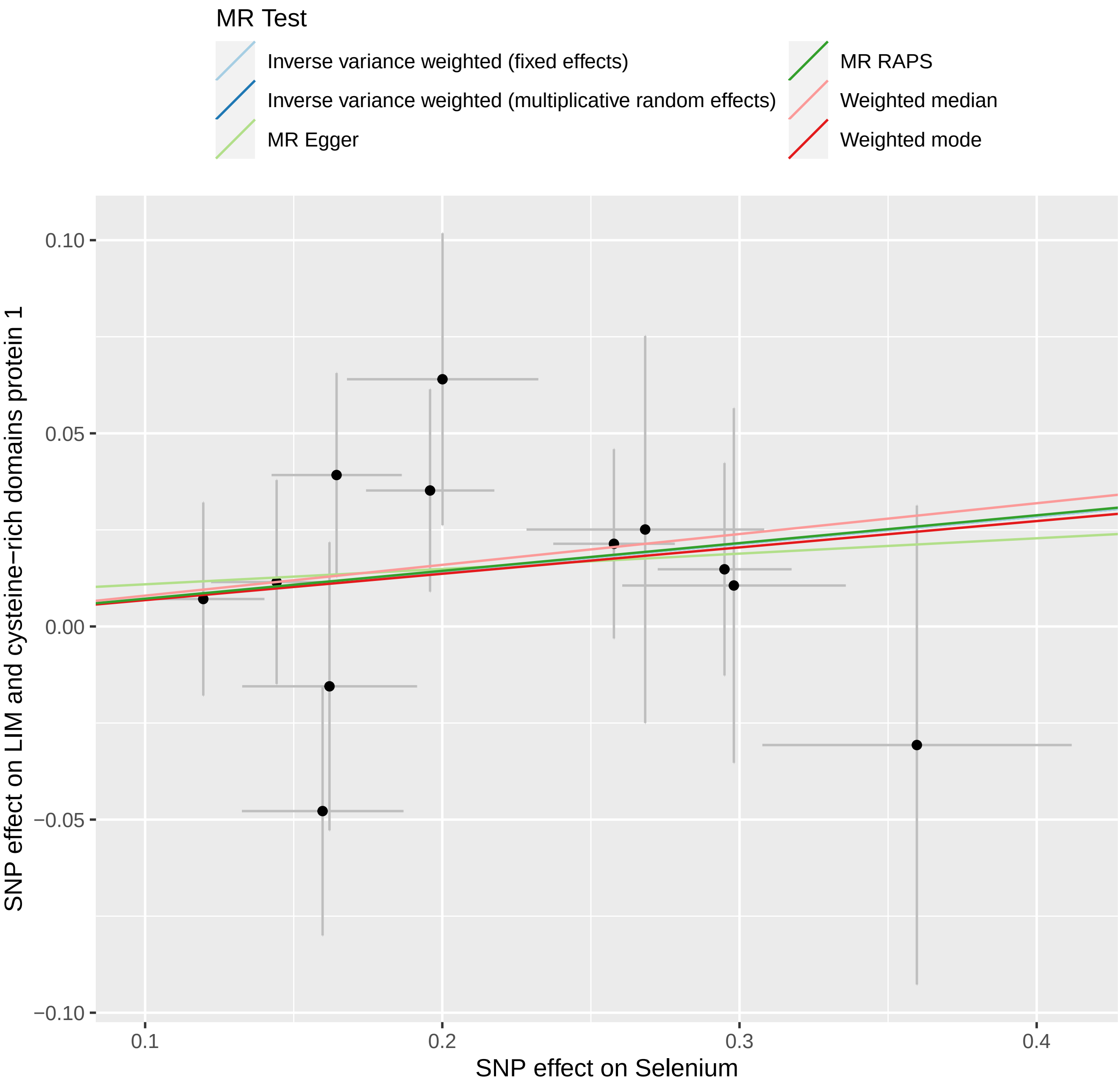

Batch 3−1758 : Selenium on Lamin−B1

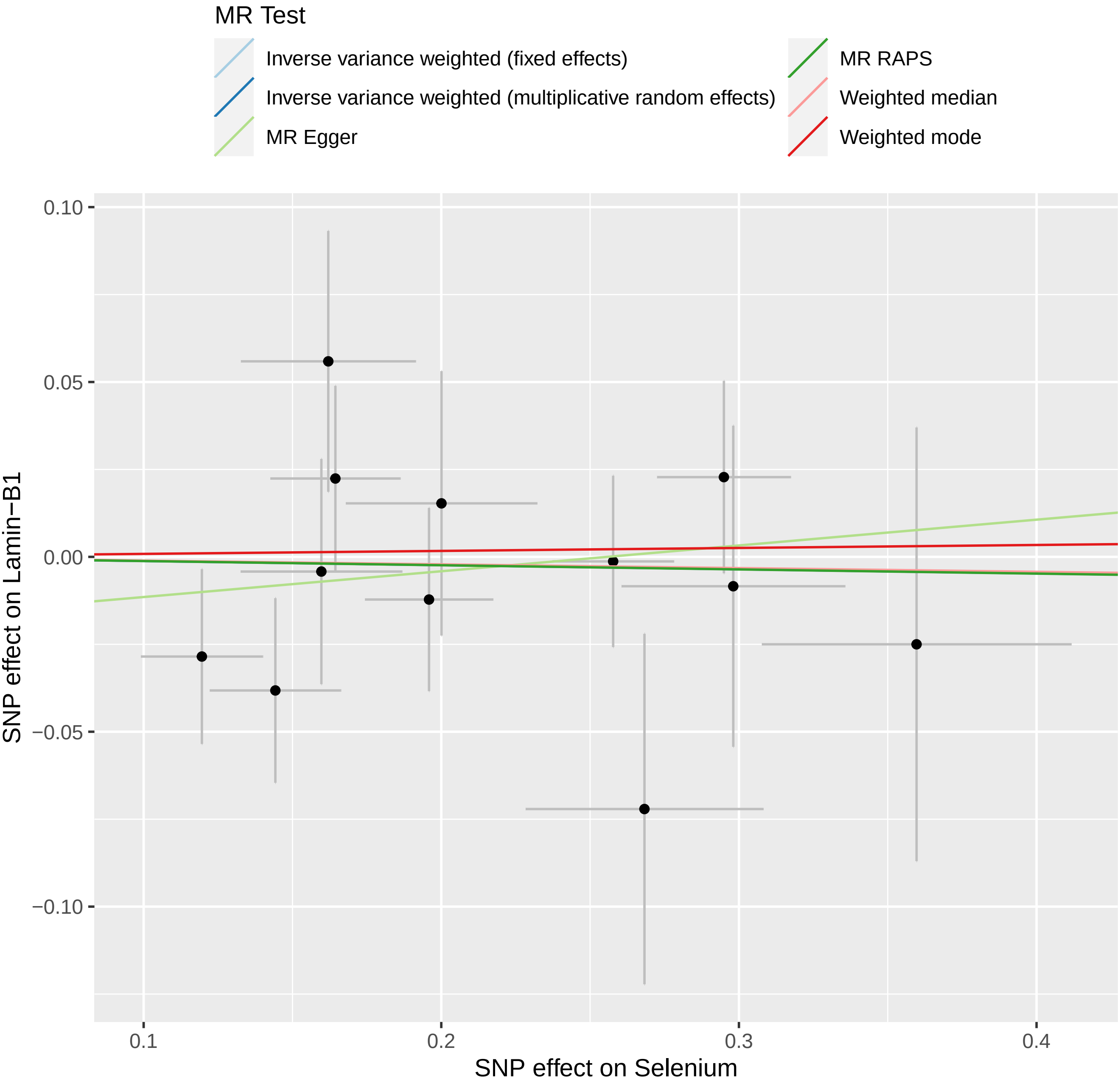

Batch 3−1759 : Selenium on Leiomodin−1

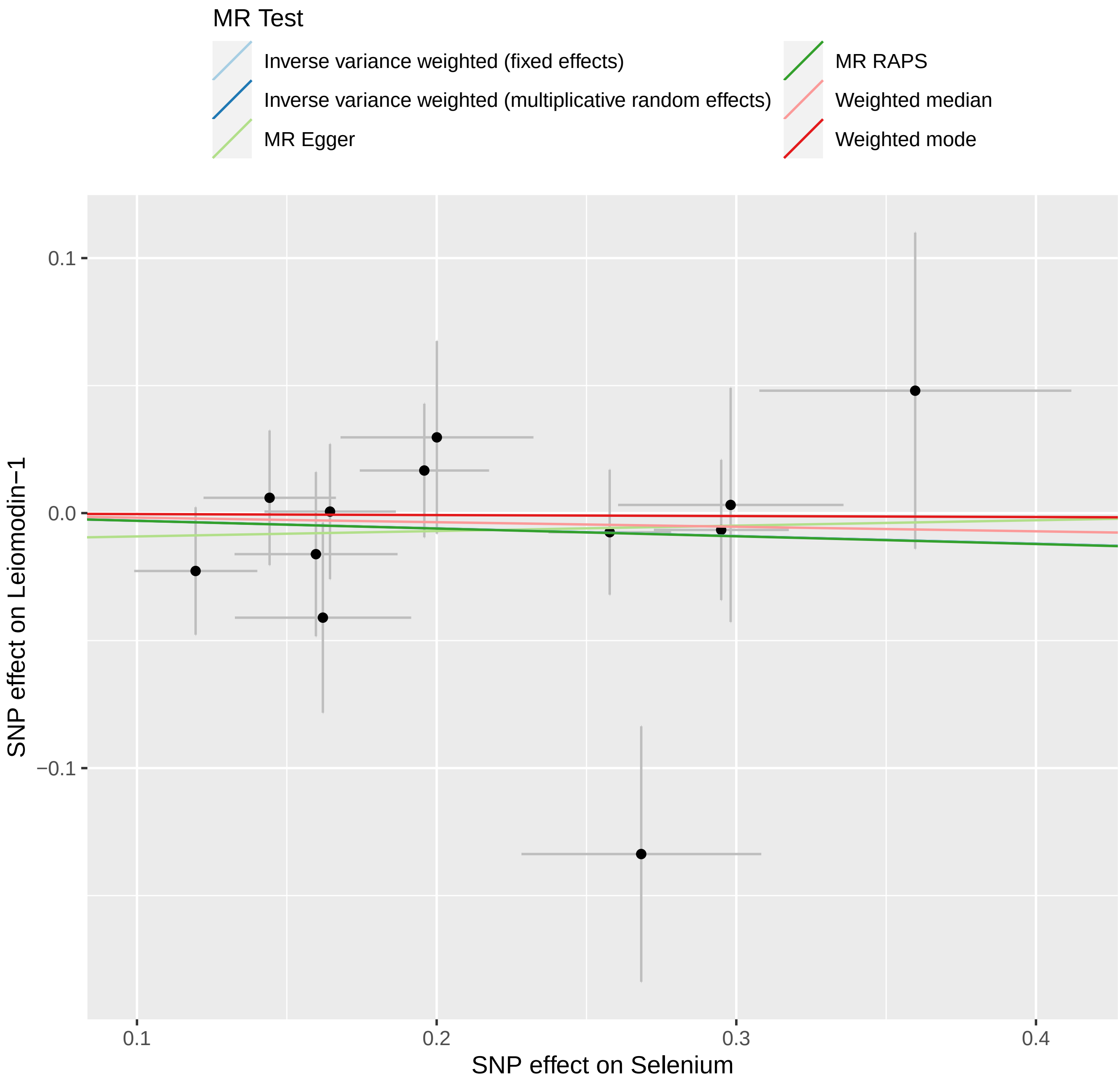

Batch 3−1760 : Selenium on Ig Kappa chain V−I region HK102− like

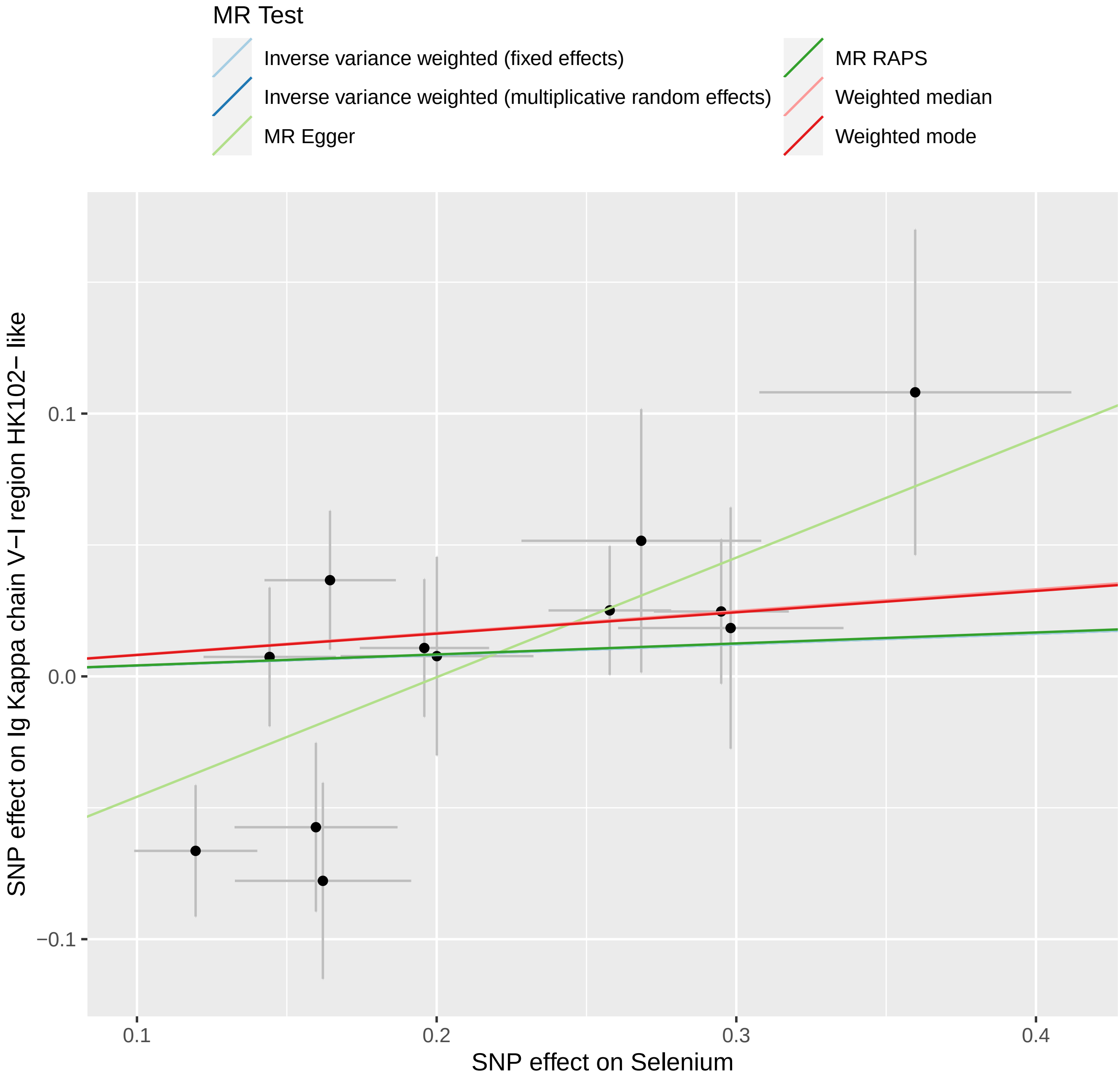

Batch 3−1761 : Selenium on Lon protease homolog, mitochondrial

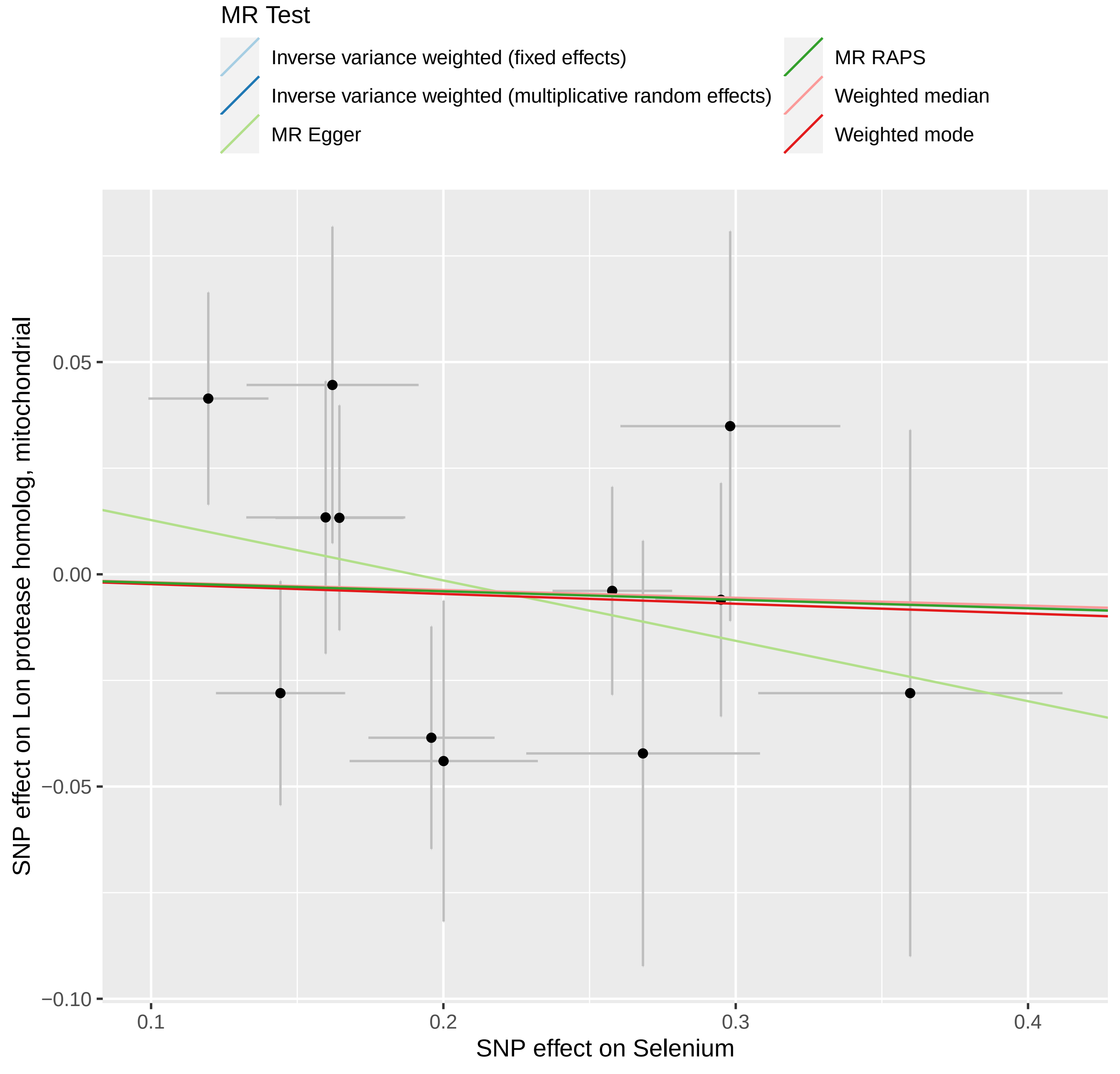

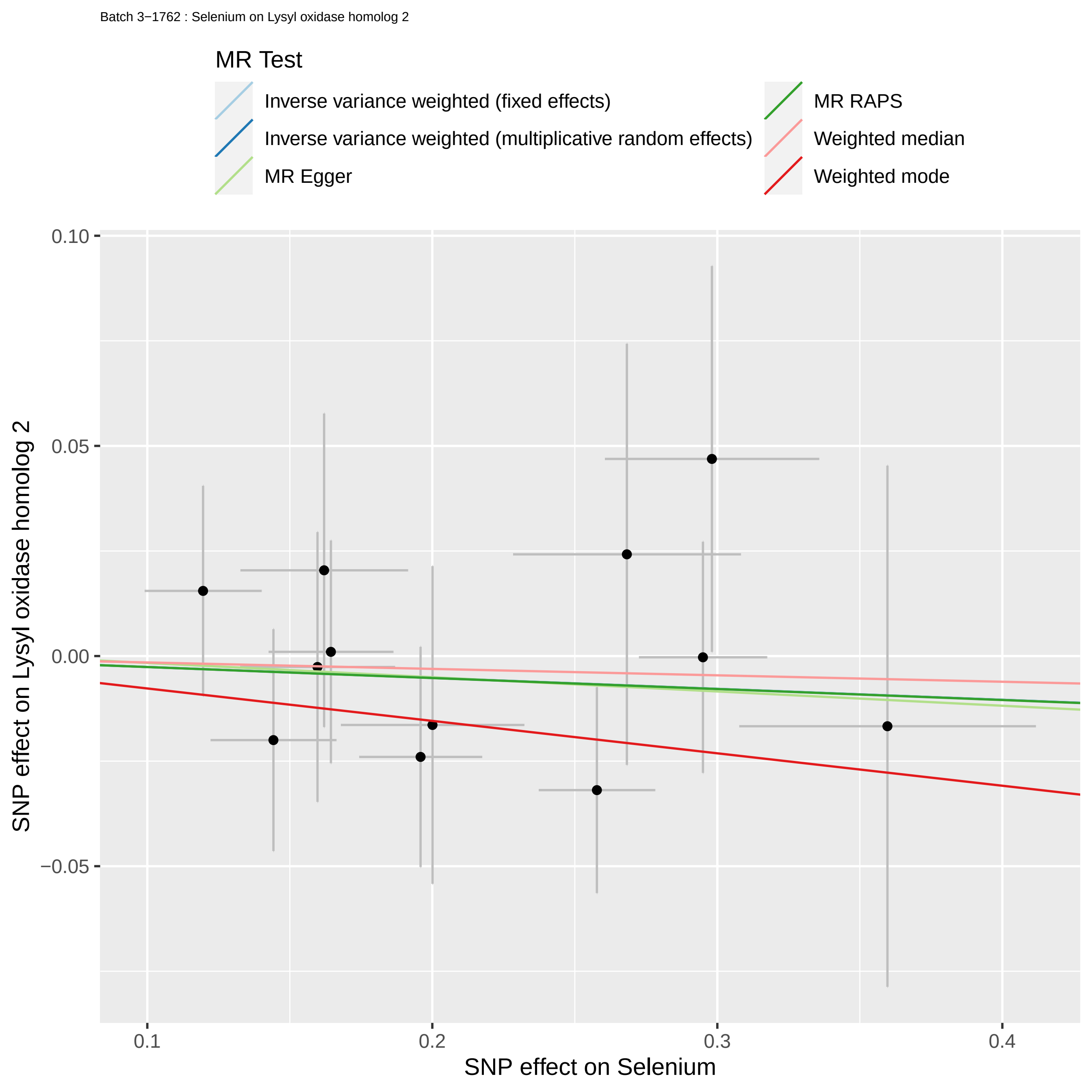

Batch 3−1762 : Selenium on Lysyl oxidase homolog 2
MR Test
Inverse variance weighted (fixed effects)
Inverse variance weighted (multiplicative random effects)
MR Egger
MR RAPS
Weighted median
Weighted mode
0.10
0.05
0.00
−0.05
0.1
0.2
0.3
0.4
SNP effect on Lysyl oxidase homolog 2
SNP effect on Selenium

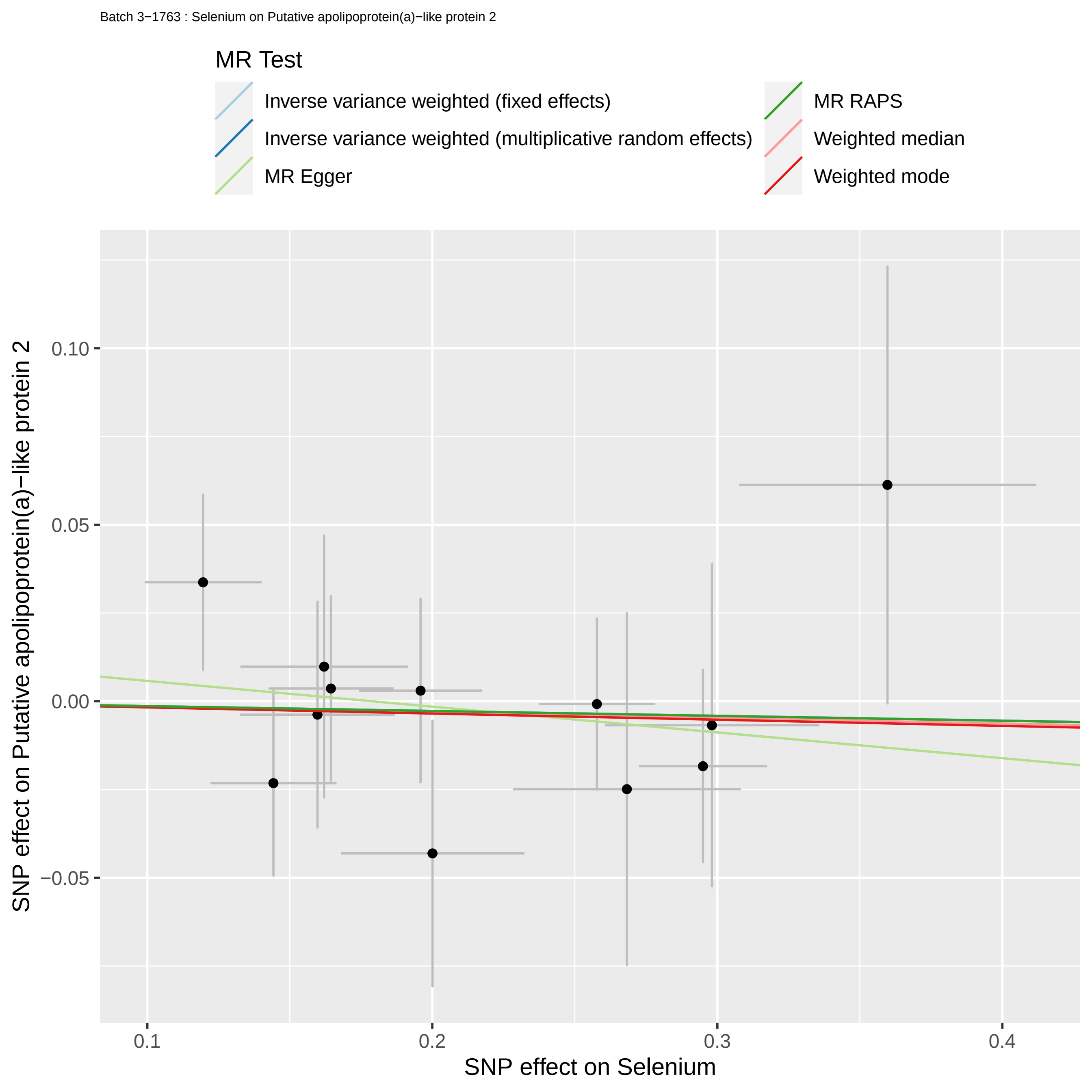

Batch 3−1763 : Selenium on Putative apolipoprotein(a)−like protein 2
MR Test
Inverse variance weighted (fixed effects)
Inverse variance weighted (multiplicative random effects)
MR Egger
MR RAPS
Weighted median
Weighted mode
SNP effect on Putative apolipoprotein(a)−like protein 2
0.10
0.05
0.00
−0.05
0.1
0.2
0.3
0.4
SNP effect on Selenium

Batch 3−1764 : Selenium on Lysophosphatidylcholine acyltransferase 2

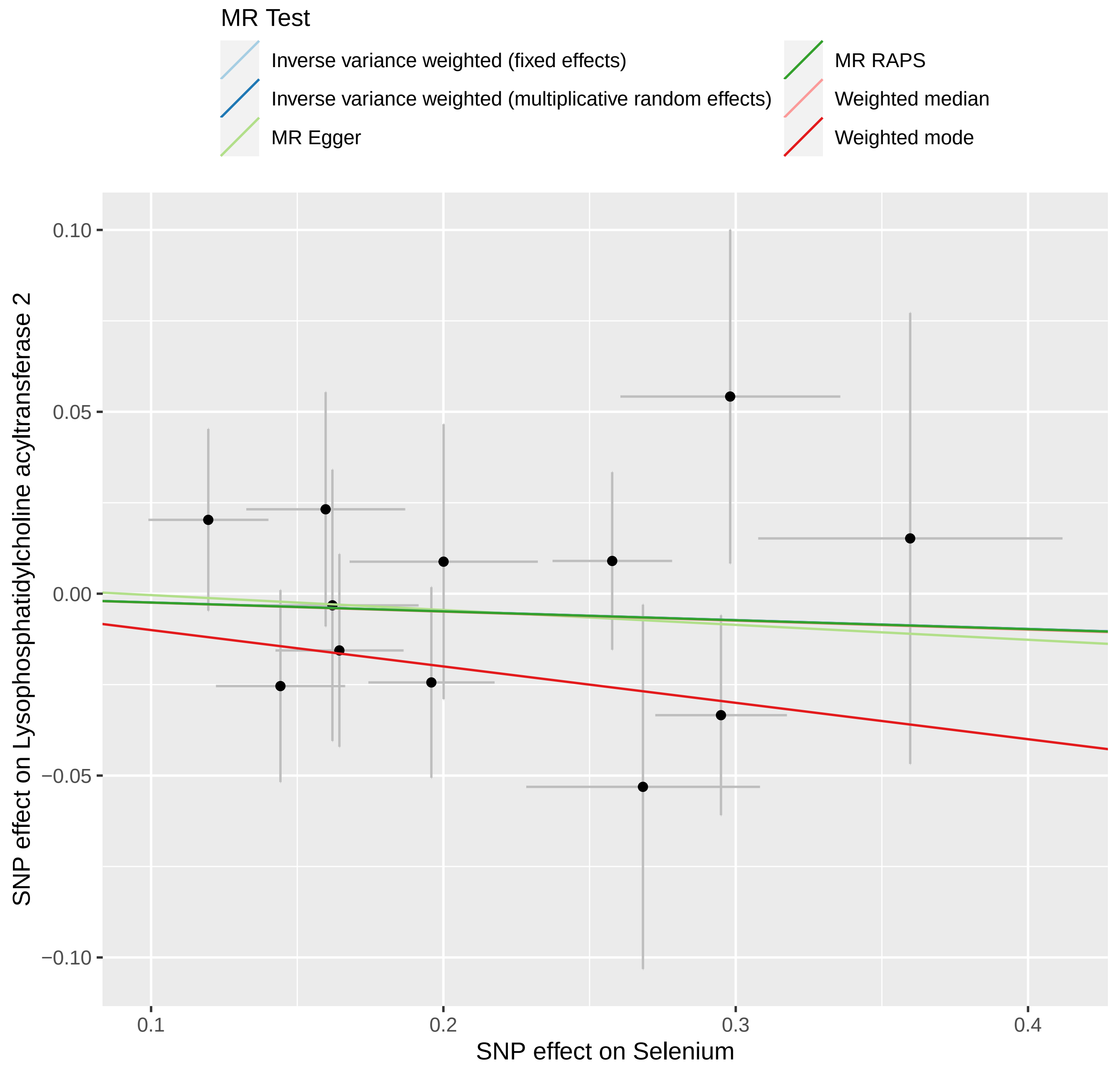

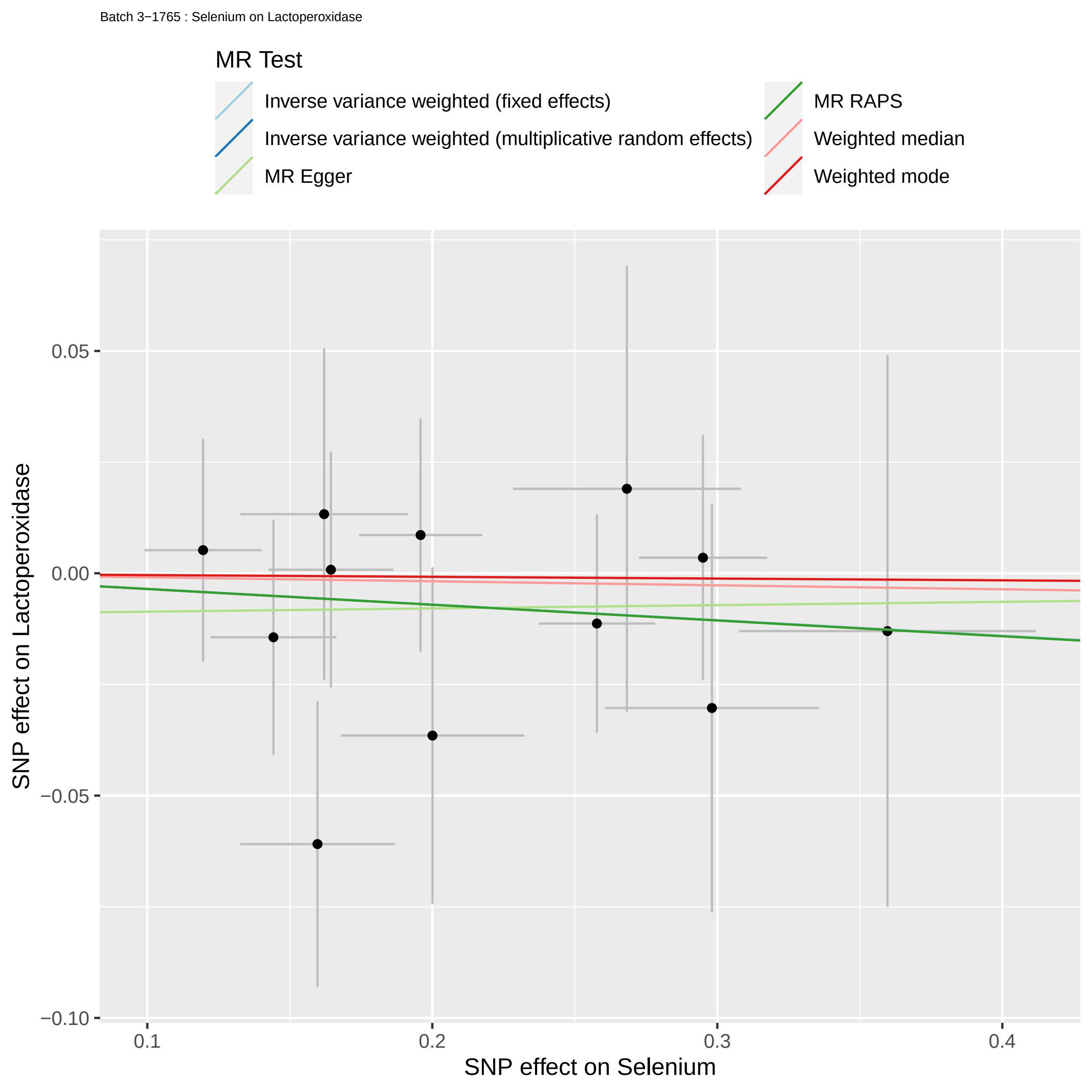
Batch 3−1765 : Selenium on Lactoperoxidase
MR Test
Inverse variance weighted (fixed effects)
Inverse variance weighted (multiplicative random effects)
MR Egger
MR RAPS
Weighted median
Weighted mode
0.05
0.00
−0.05
−0.10
0.1
0.2
0.3
0.4
SNP effect on Lactoperoxidase
SNP effect on Selenium

Batch 3−1766 : Selenium on Leucine−rich repeat and calponin homology domain−containing protein 4

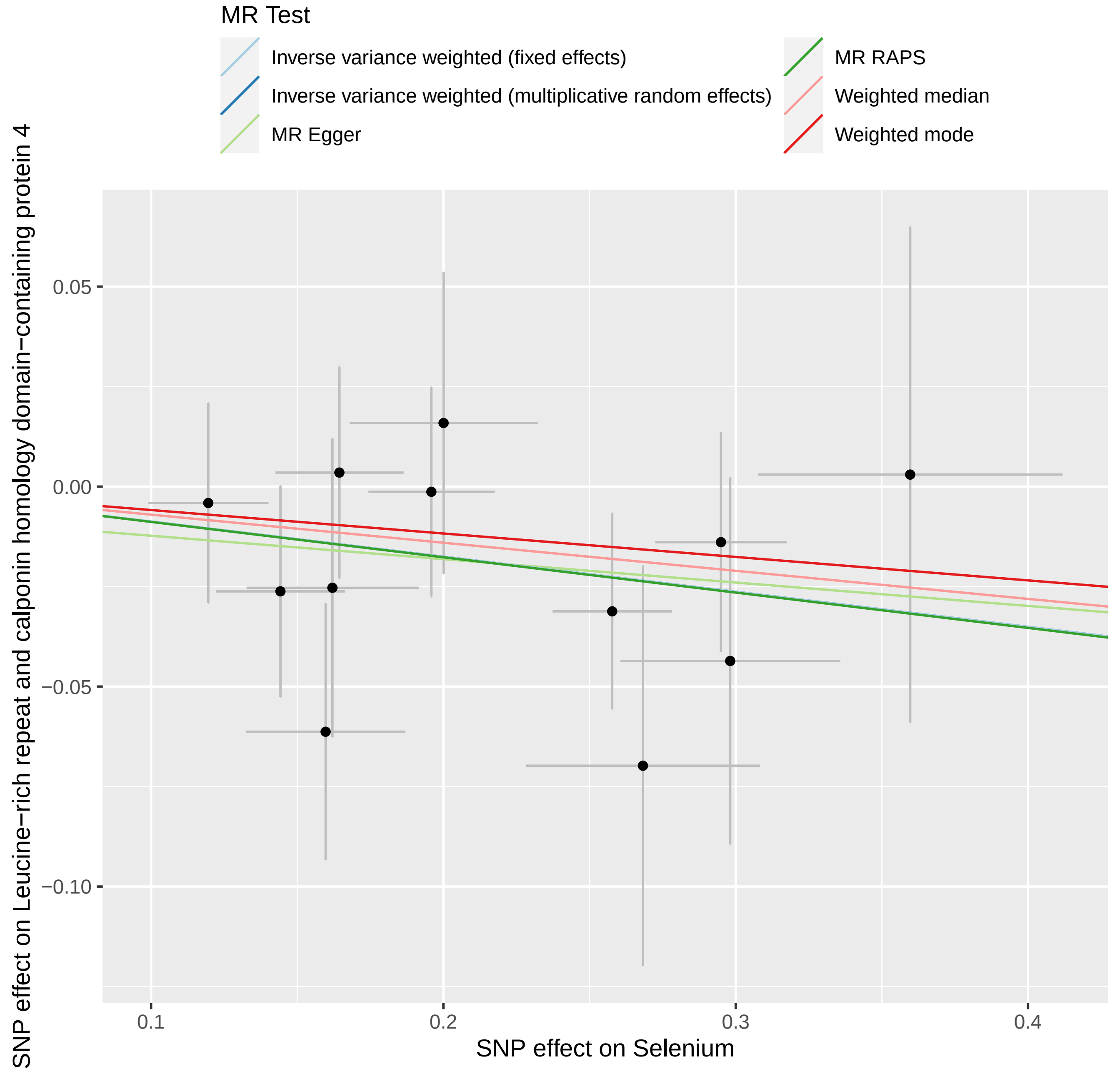

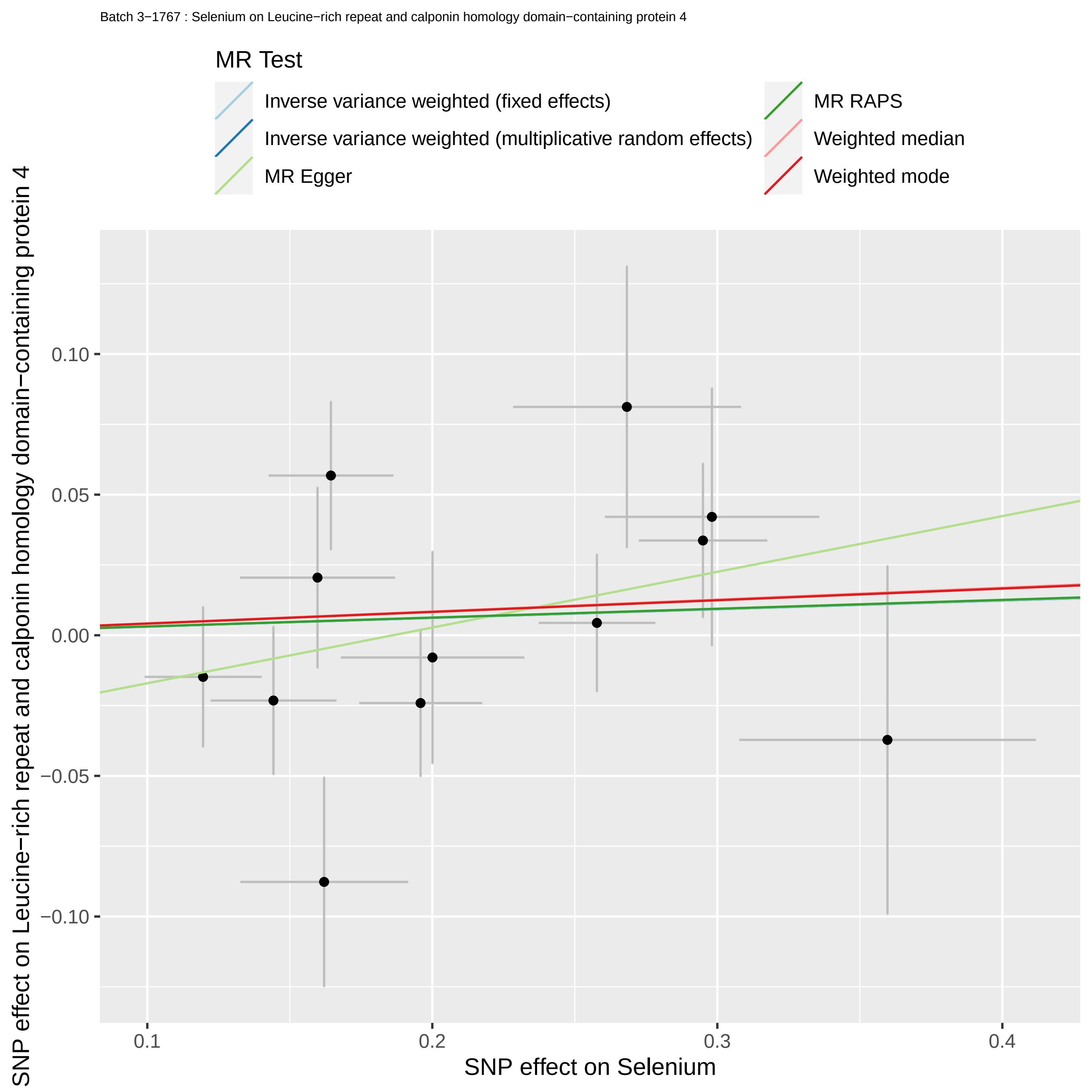

Batch 3−1767 : Selenium on Leucine−rich repeat and calponin homology domain−containing protein 4
MR Test
Inverse variance weighted (fixed effects)
Inverse variance weighted (multiplicative random effects)
MR Egger
MR RAPS
Weighted median
Weighted mode
SNP effect on Leucine−rich repeat and calponin homology domain−containing protein 4
0.10
0.05
0.00
−0.05
−0.10
0.1
0.2
0.3
0.4
SNP effect on Selenium

Batch 3−1768 : Selenium on Leucine−rich repeat and fibronectin type III domain−containing protein 1

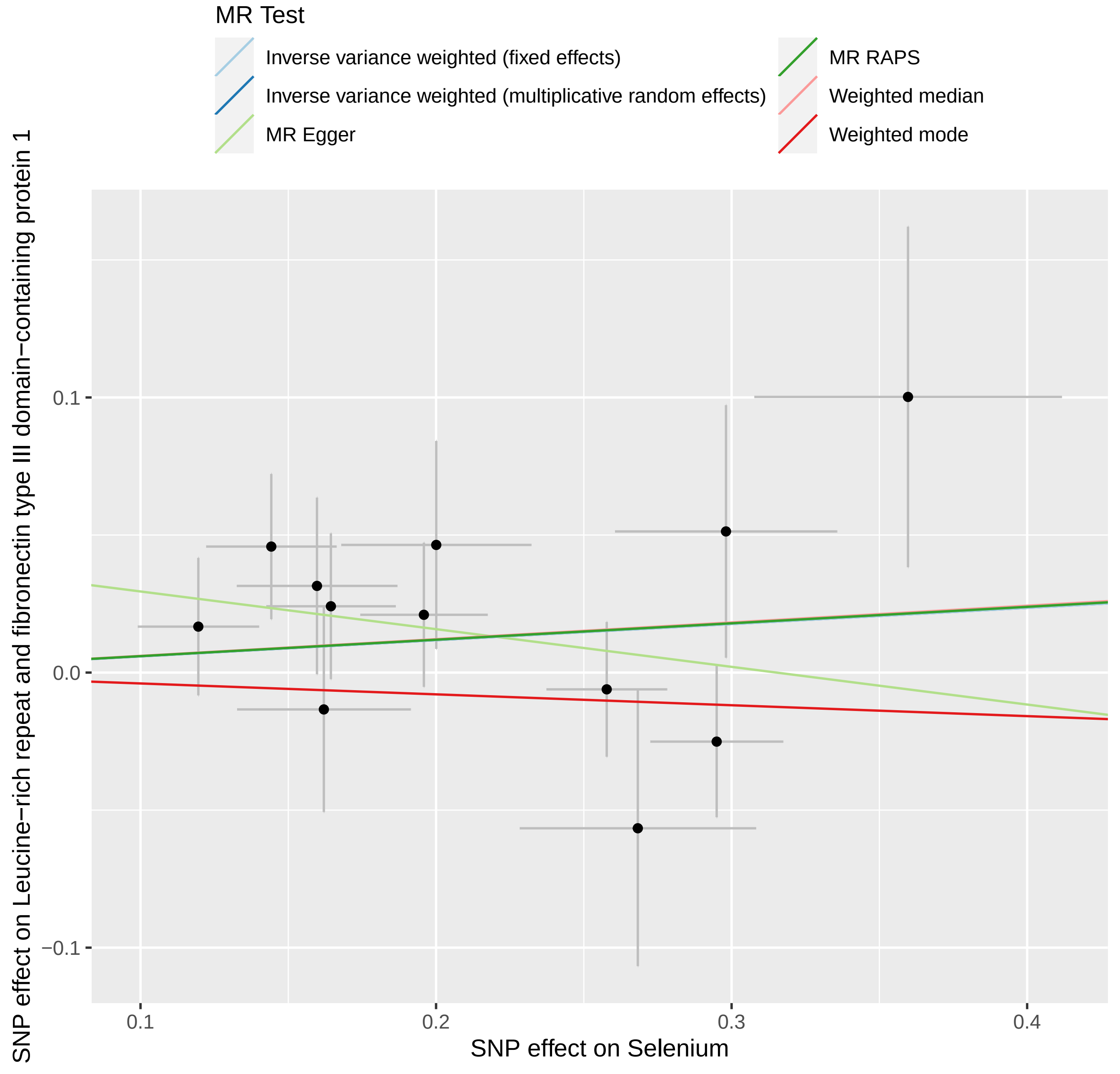

Batch 3−1769 : Selenium on Leucine−rich repeat and fibronectin type−III domain−containing protein 2

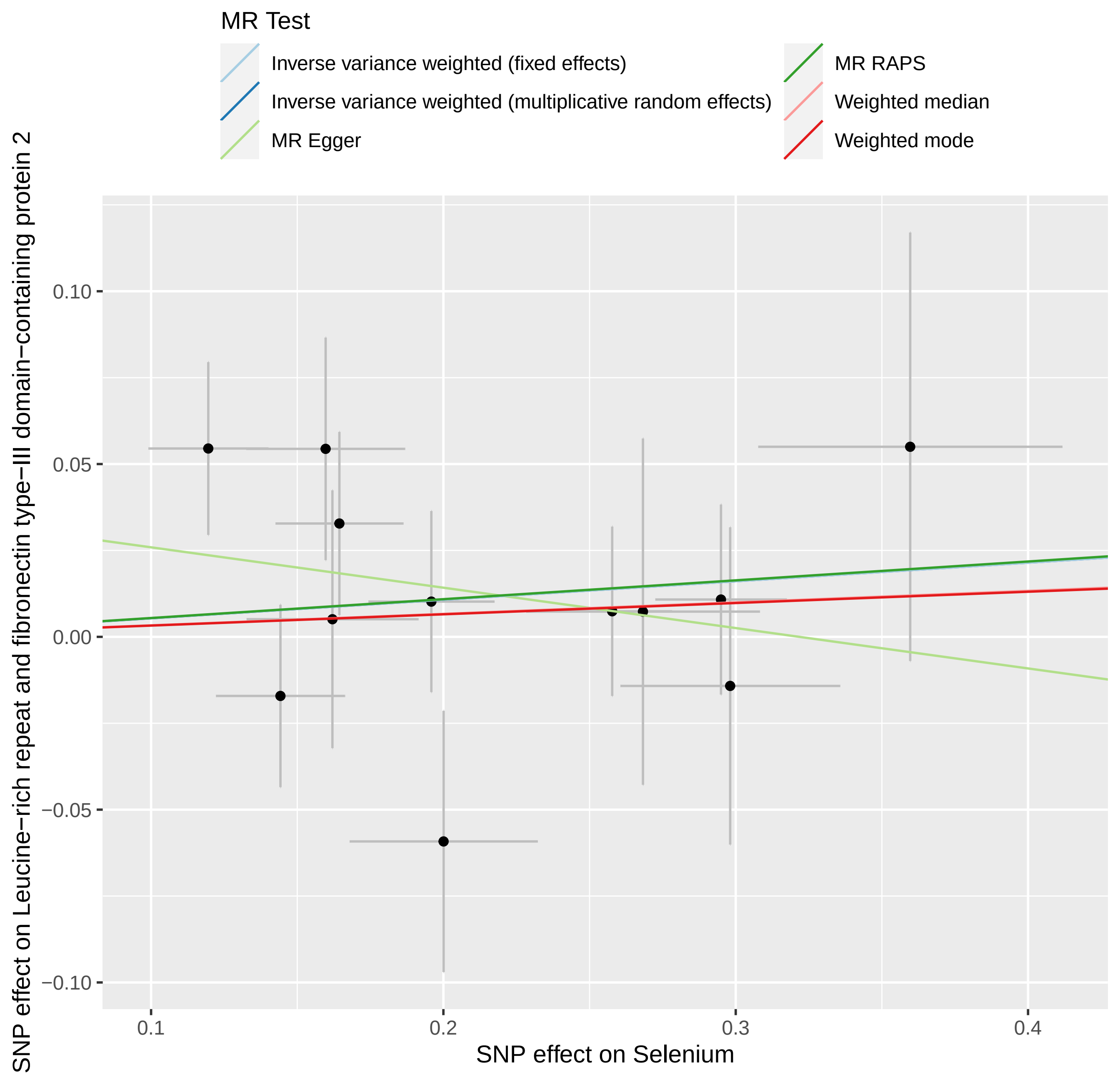

Batch 3−1770 : Selenium on Leucine−rich repeats and immunoglobulin−like domains protein 3

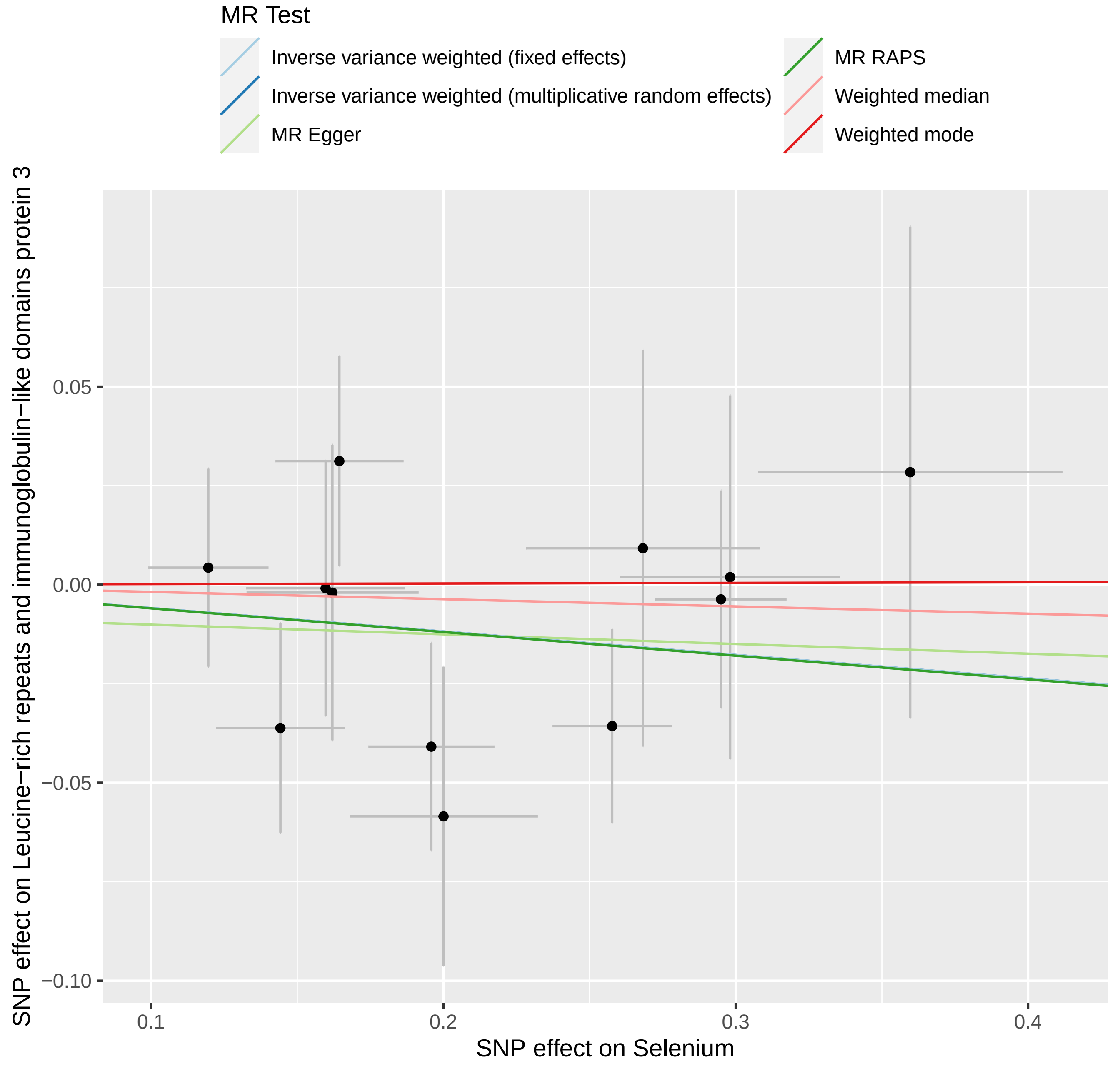

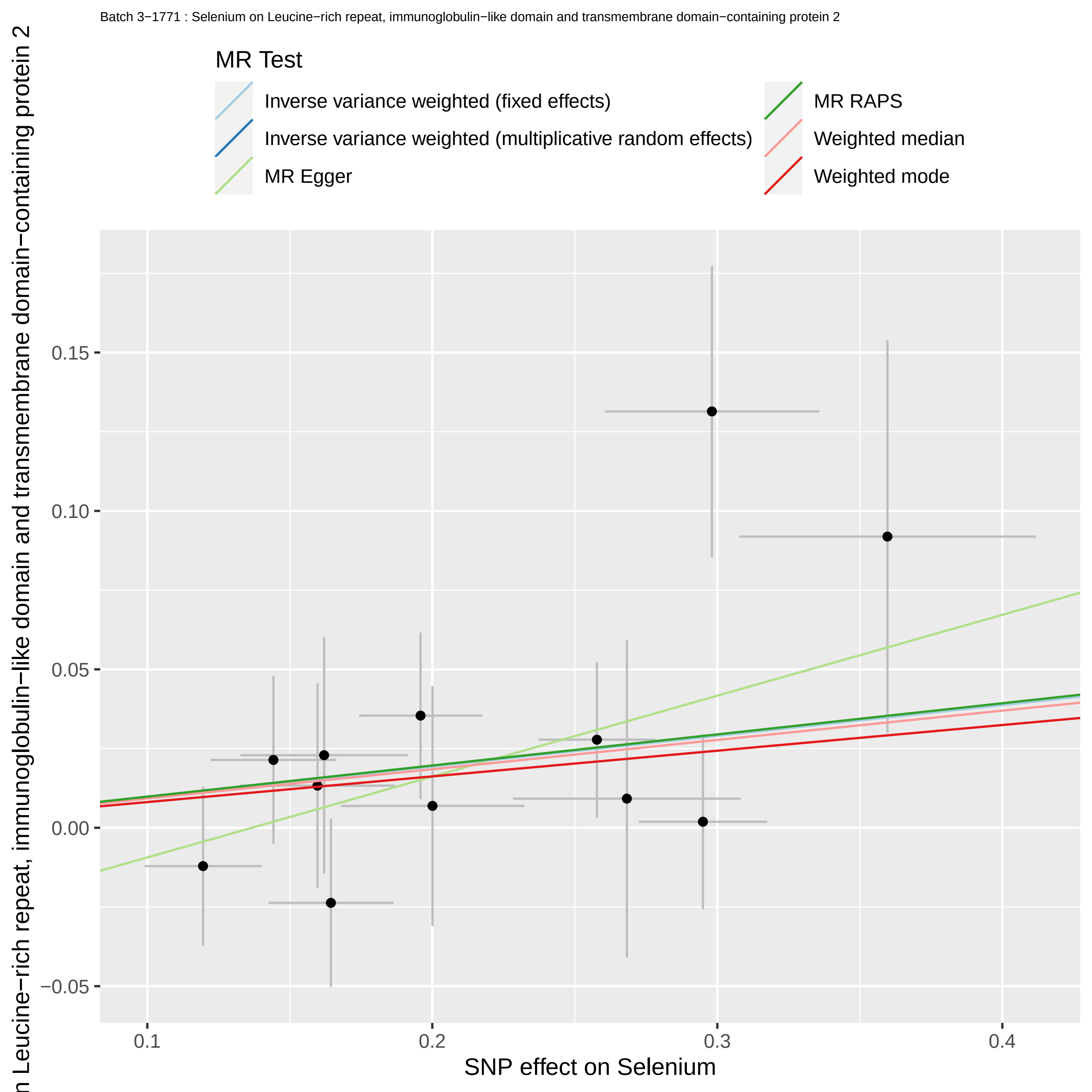

Batch 3−1771 : Selenium on Leucine−rich repeat, immunoglobulin−like domain and transmembrane domain−containing protein 2
MR Test
Inverse variance weighted (fixed effects)
Inverse variance weighted (multiplicative random effects)
MR Egger
MR RAPS
Weighted median
Weighted mode
0.15
0.10
0.05
0.00
−0.05
0.1
0.2
0.3
0.4
SNP effect on Selenium
n Leucine−rich repeat, immunoglobulin−like domain and transmembrane domain−containing protein 2

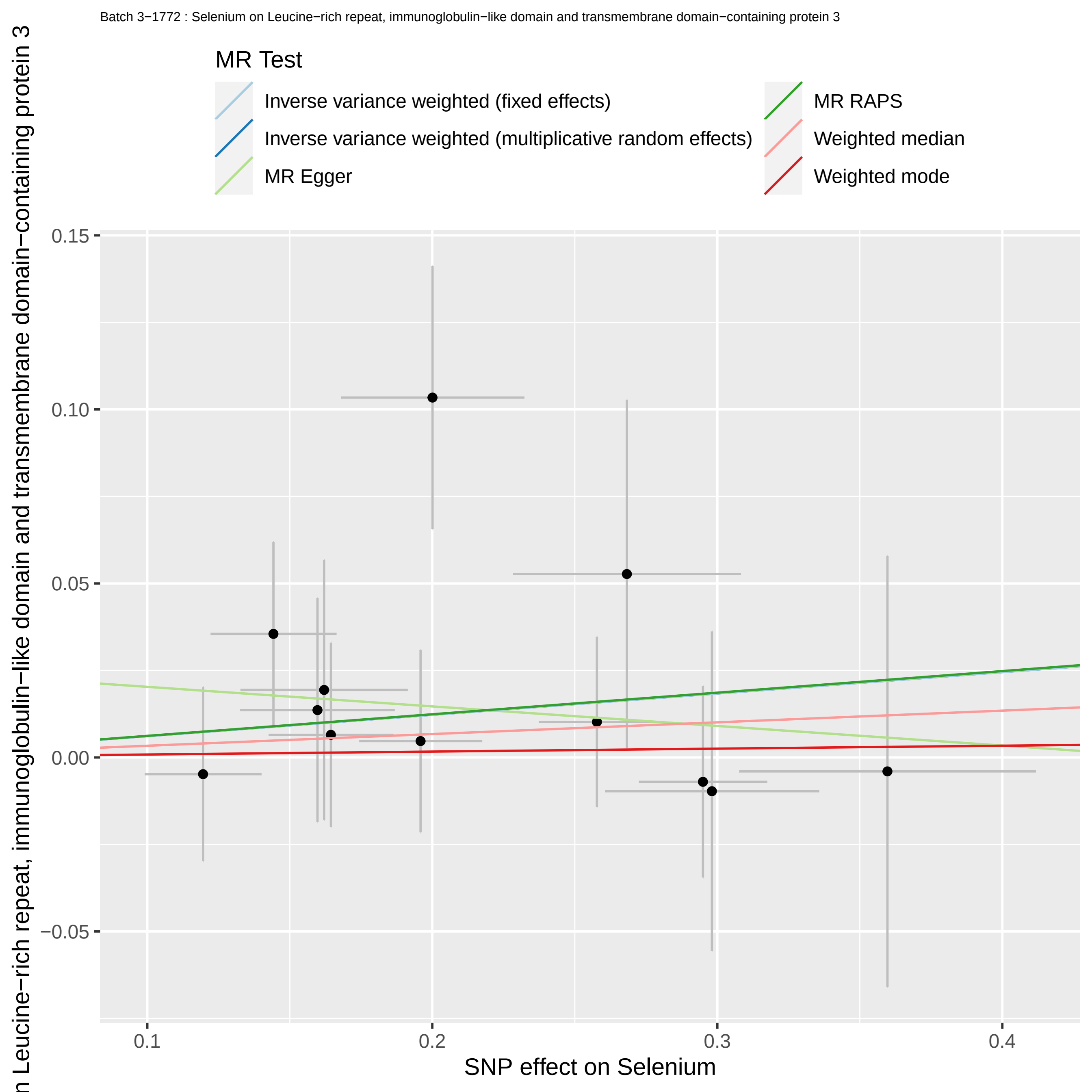

Batch 3−1772 : Selenium on Leucine−rich repeat, immunoglobulin−like domain and transmembrane domain−containing protein 3
MR Test
Inverse variance weighted (fixed effects)
Inverse variance weighted (multiplicative random effects)
MR Egger
MR RAPS
Weighted median
Weighted mode
0.15
0.10
0.05
0.00
−0.05
0.1
0.2
0.3
0.4
SNP effect on Selenium
n Leucine−rich repeat, immunoglobulin−like domain and transmembrane domain−containing protein 3

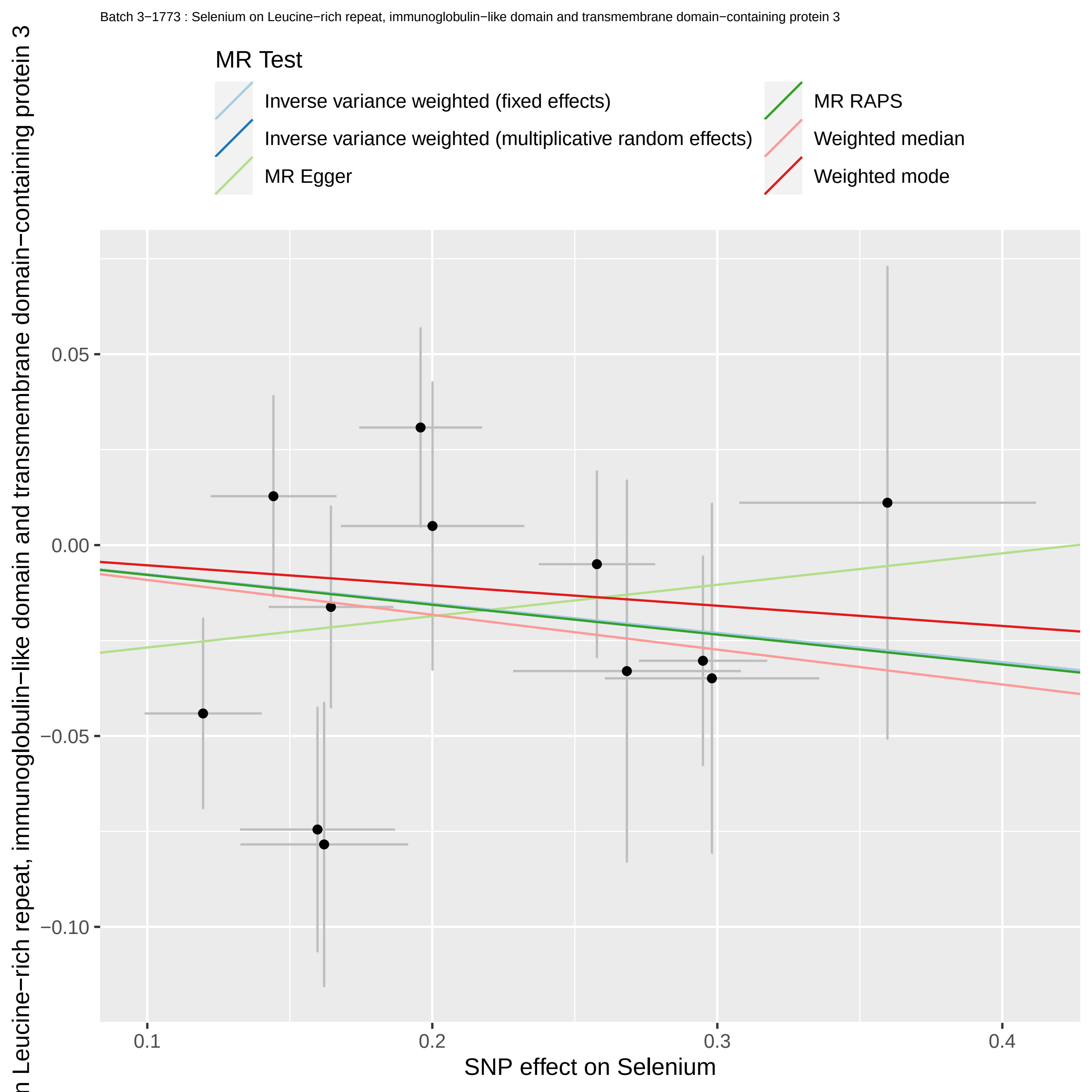
Batch 3−1773 : Selenium on Leucine−rich repeat, immunoglobulin−like domain and transmembrane domain−containing protein 3
MR Test
Inverse variance weighted (fixed effects)
Inverse variance weighted (multiplicative random effects)
MR Egger
MR RAPS
Weighted median
Weighted mode
0.05
0.00
−0.05
−0.10
0.1
0.2
0.3
0.4
SNP effect on Selenium
n Leucine−rich repeat, immunoglobulin−like domain and transmembrane domain−containing protein 3

Batch 3−1774 : Selenium on Lymphoid−restricted membrane protein

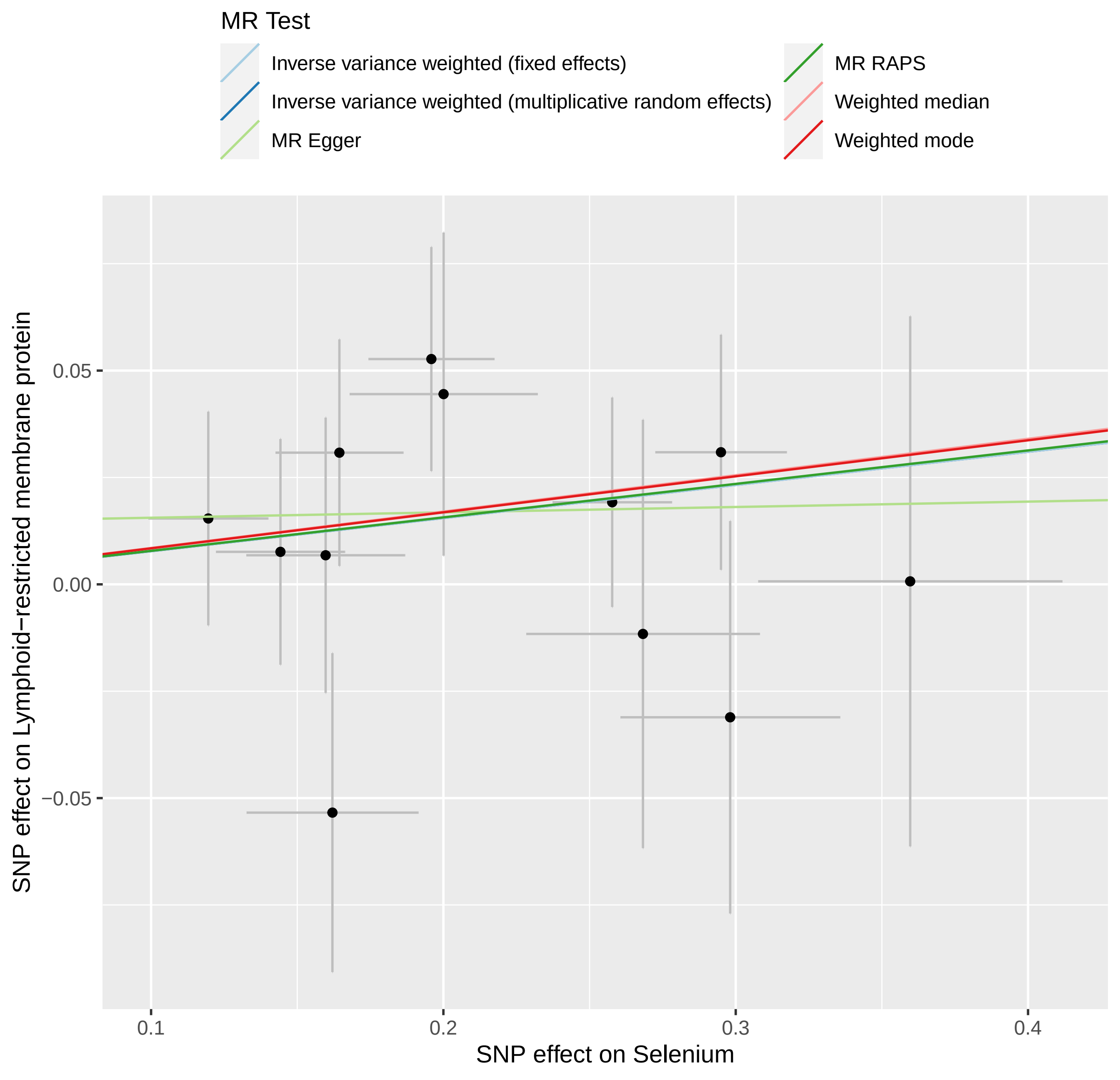

Batch 3−1775 : Selenium on Prolow−density lipoprotein receptor−related protein 1

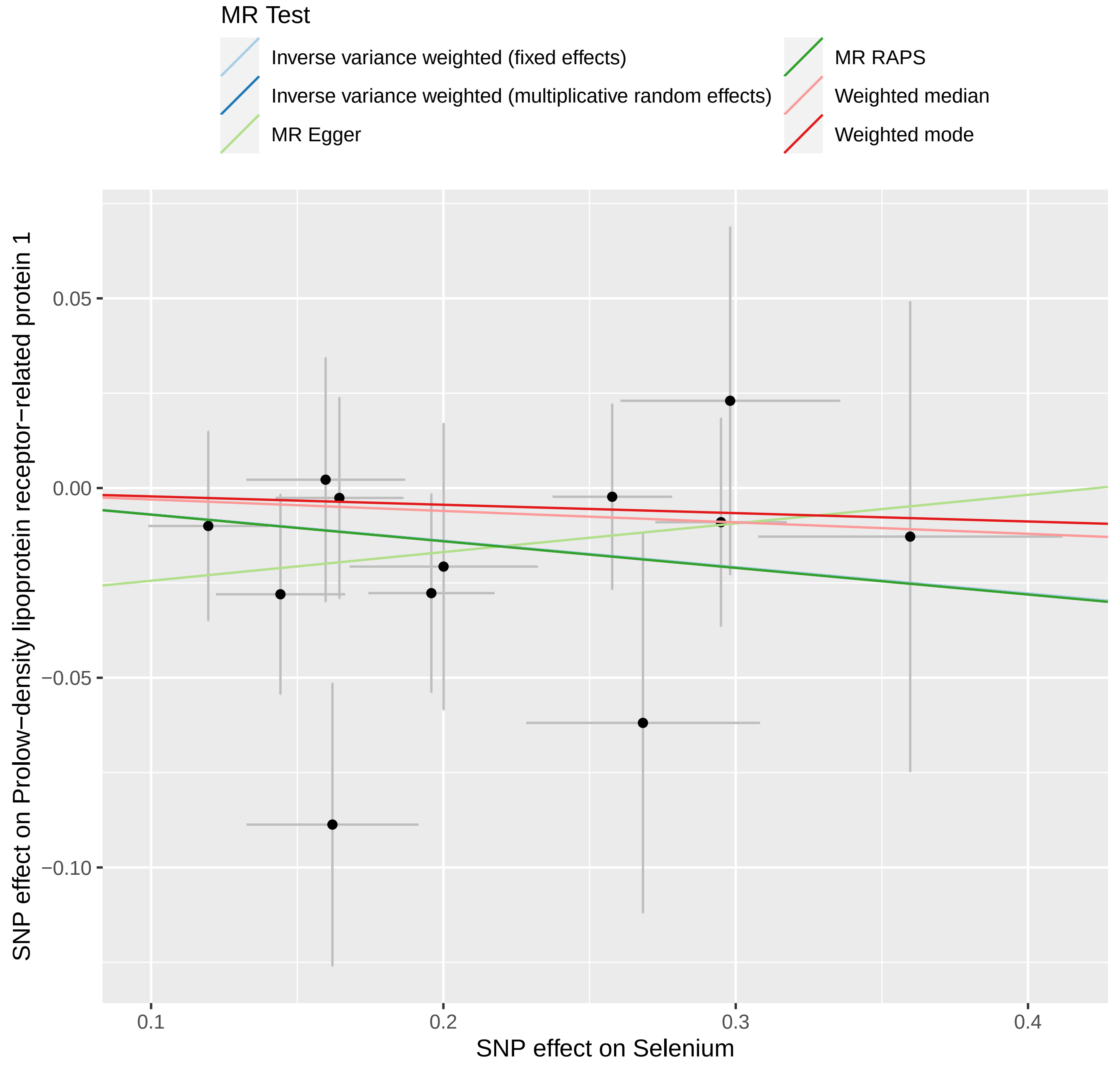

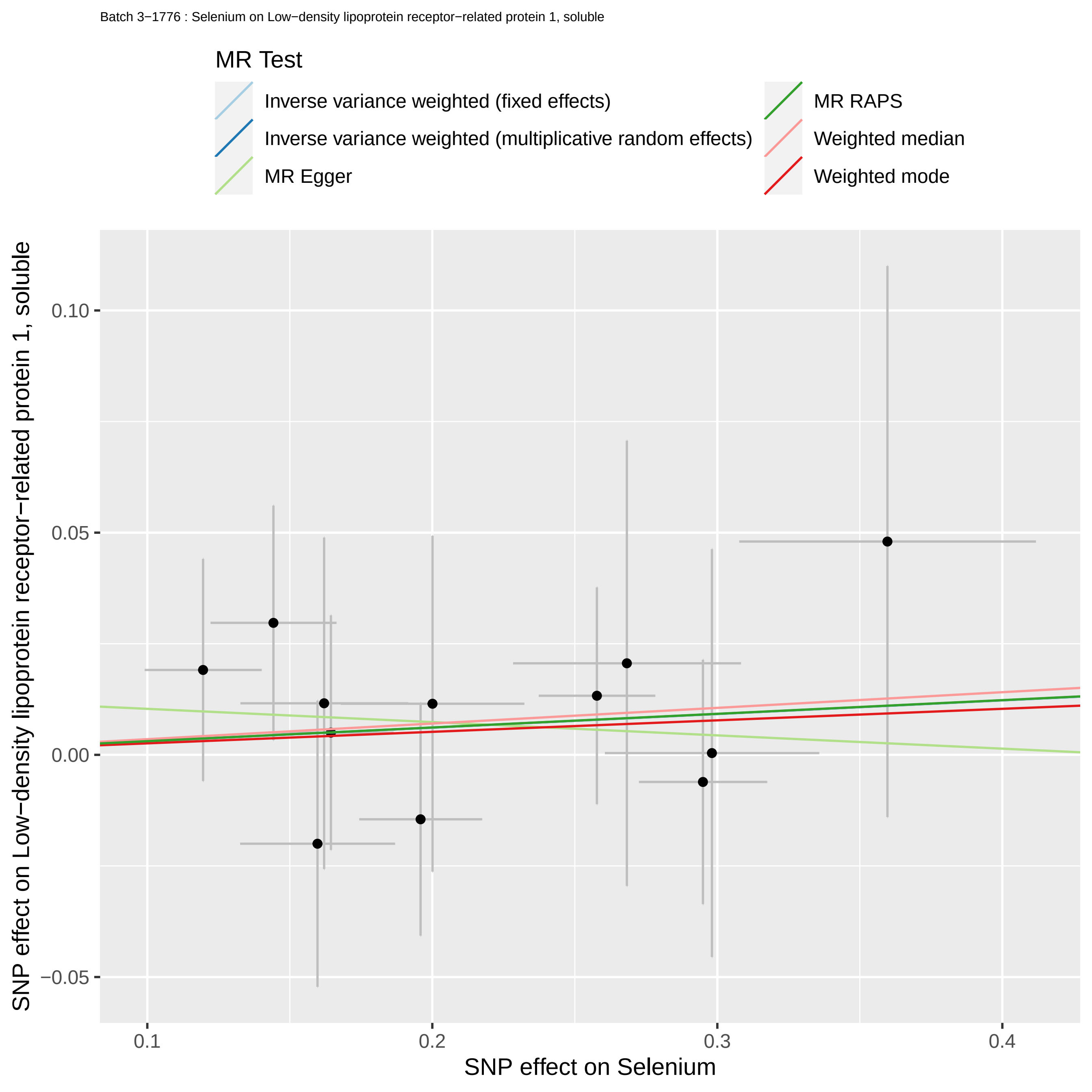
Batch 3−1776 : Selenium on Low−density lipoprotein receptor−related protein 1, soluble
MR Test
Inverse variance weighted (fixed effects)
Inverse variance weighted (multiplicative random effects)
MR Egger
MR RAPS
Weighted median
Weighted mode
SNP effect on Low−density lipoprotein receptor−related protein 1, soluble
0.10
0.05
0.00
−0.05
0.1
0.2
0.3
0.4
SNP effect on Selenium

Batch 3−1777 : Selenium on Low−density lipoprotein receptor−related protein 1, soluble

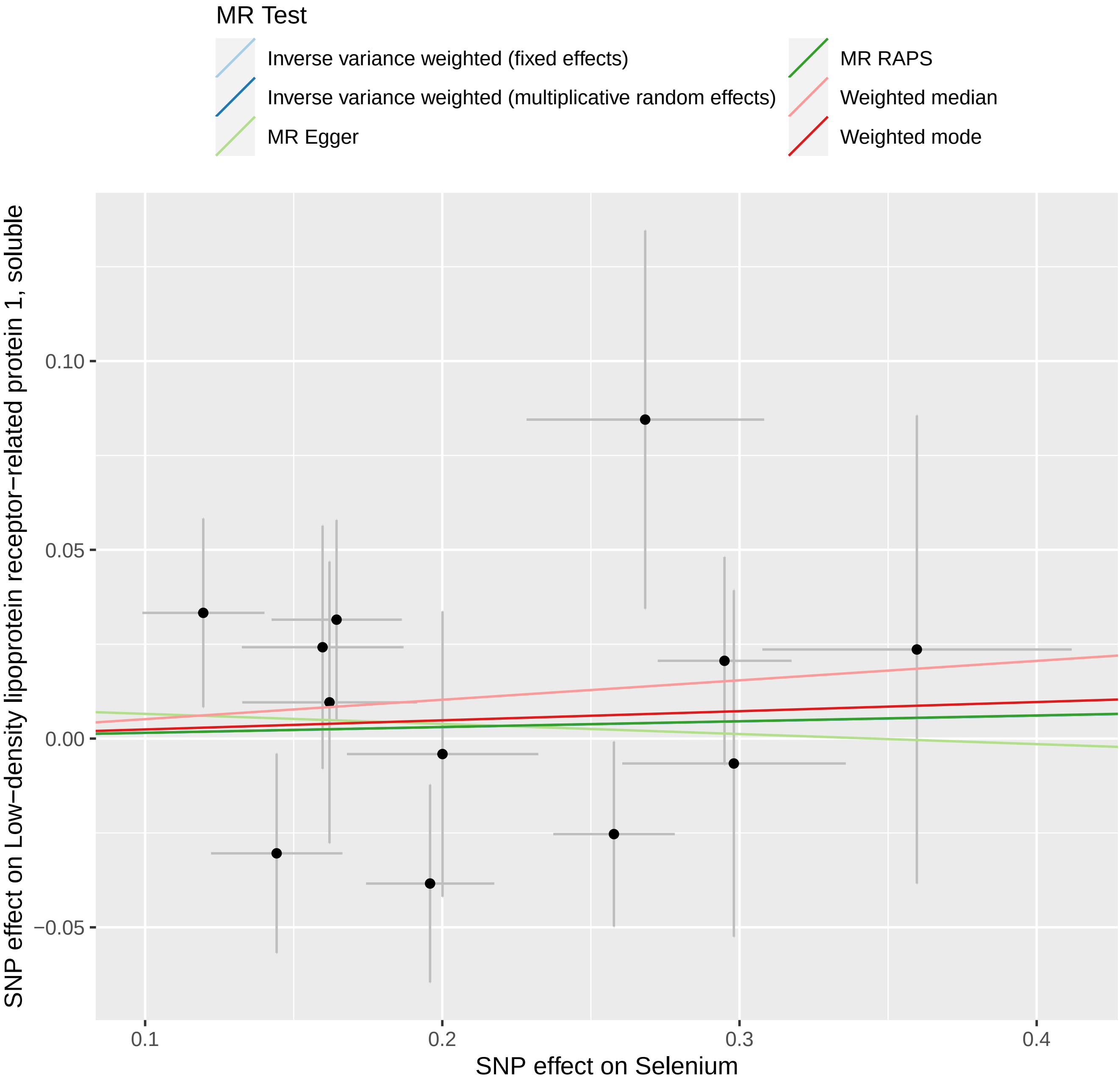

Batch 3−1778 : Selenium on Low−density lipoprotein receptor−related protein 1B

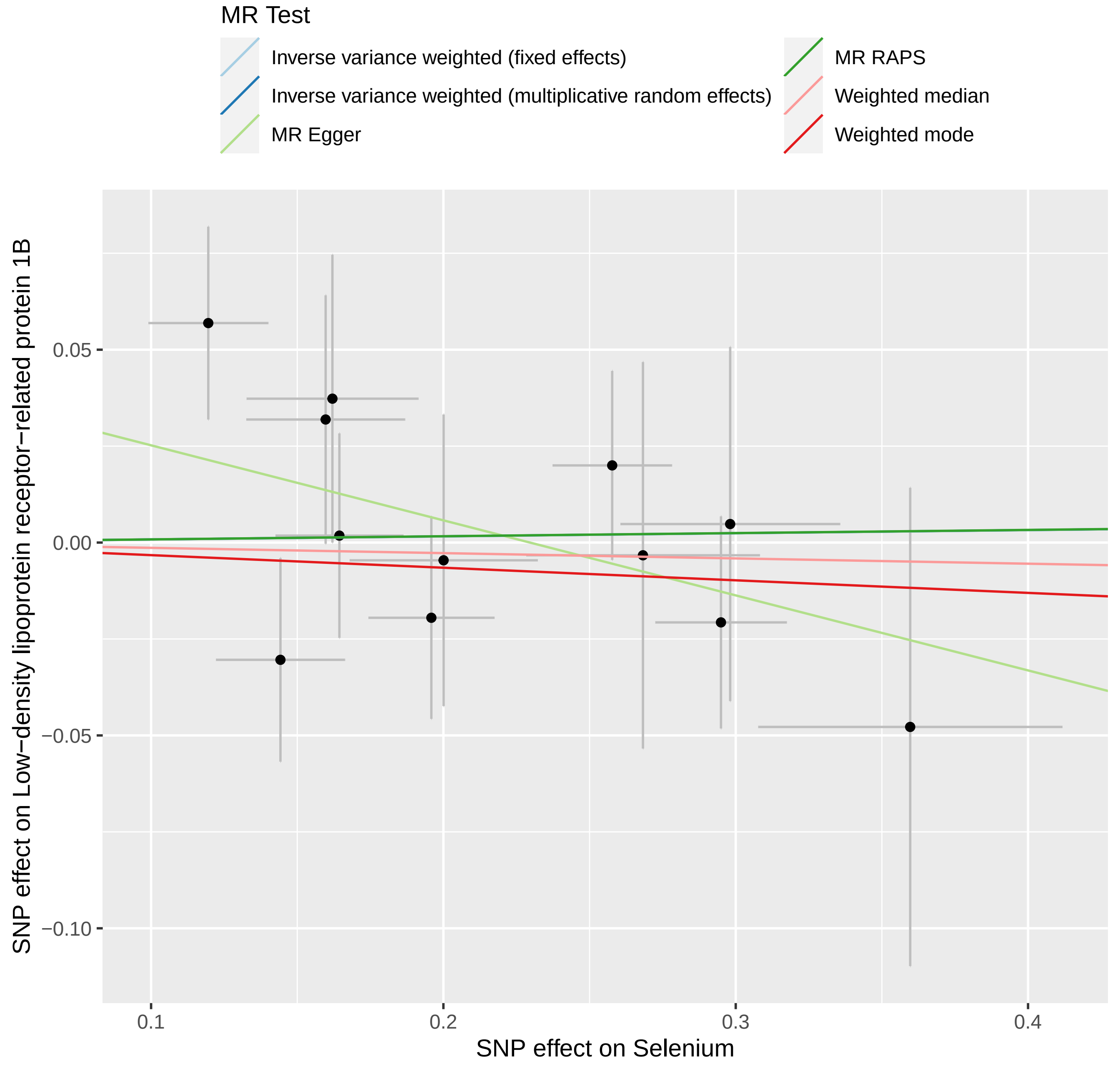

Batch 3−1779 : Selenium on Low−density lipoprotein receptor−related protein 1B

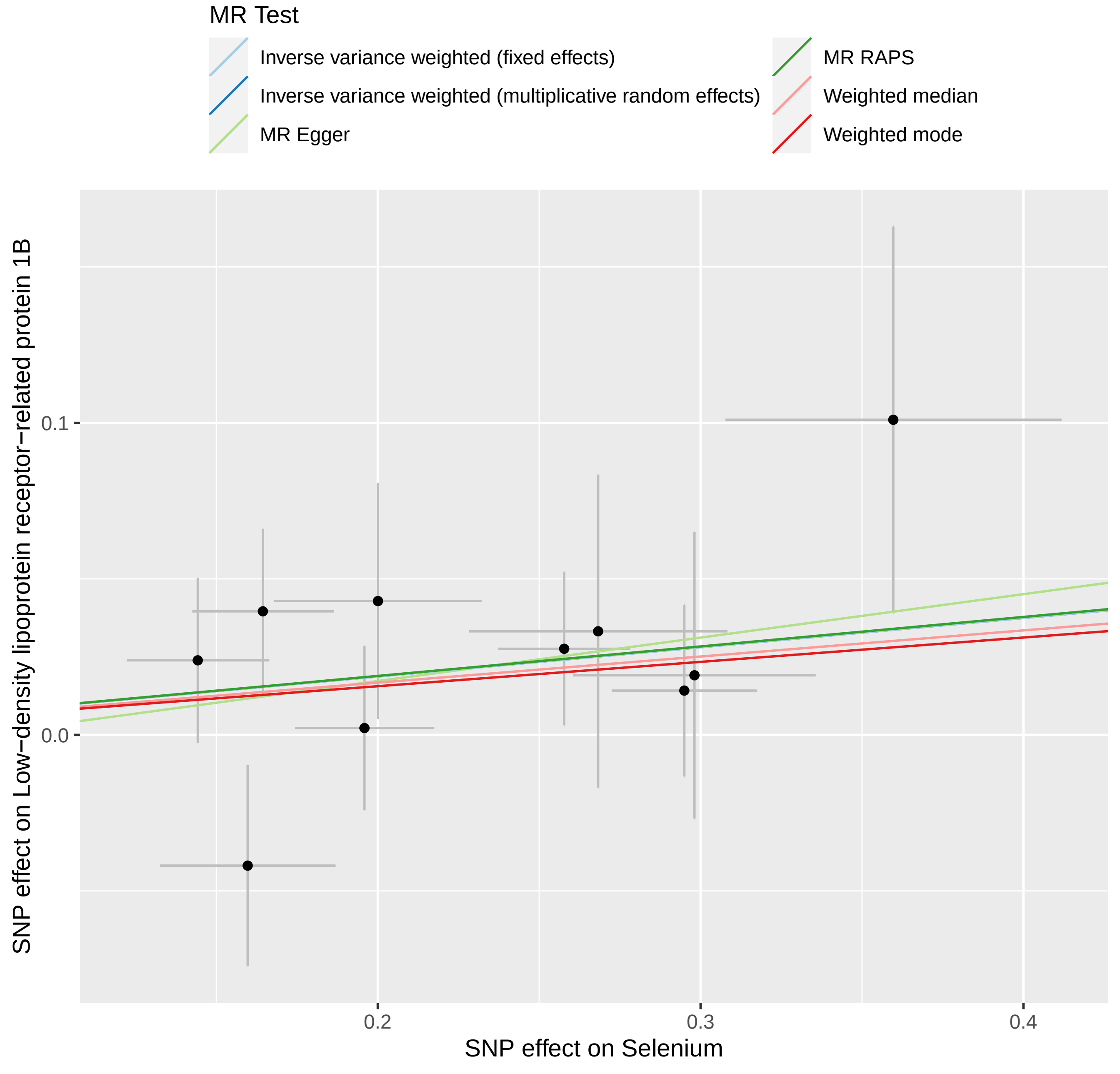

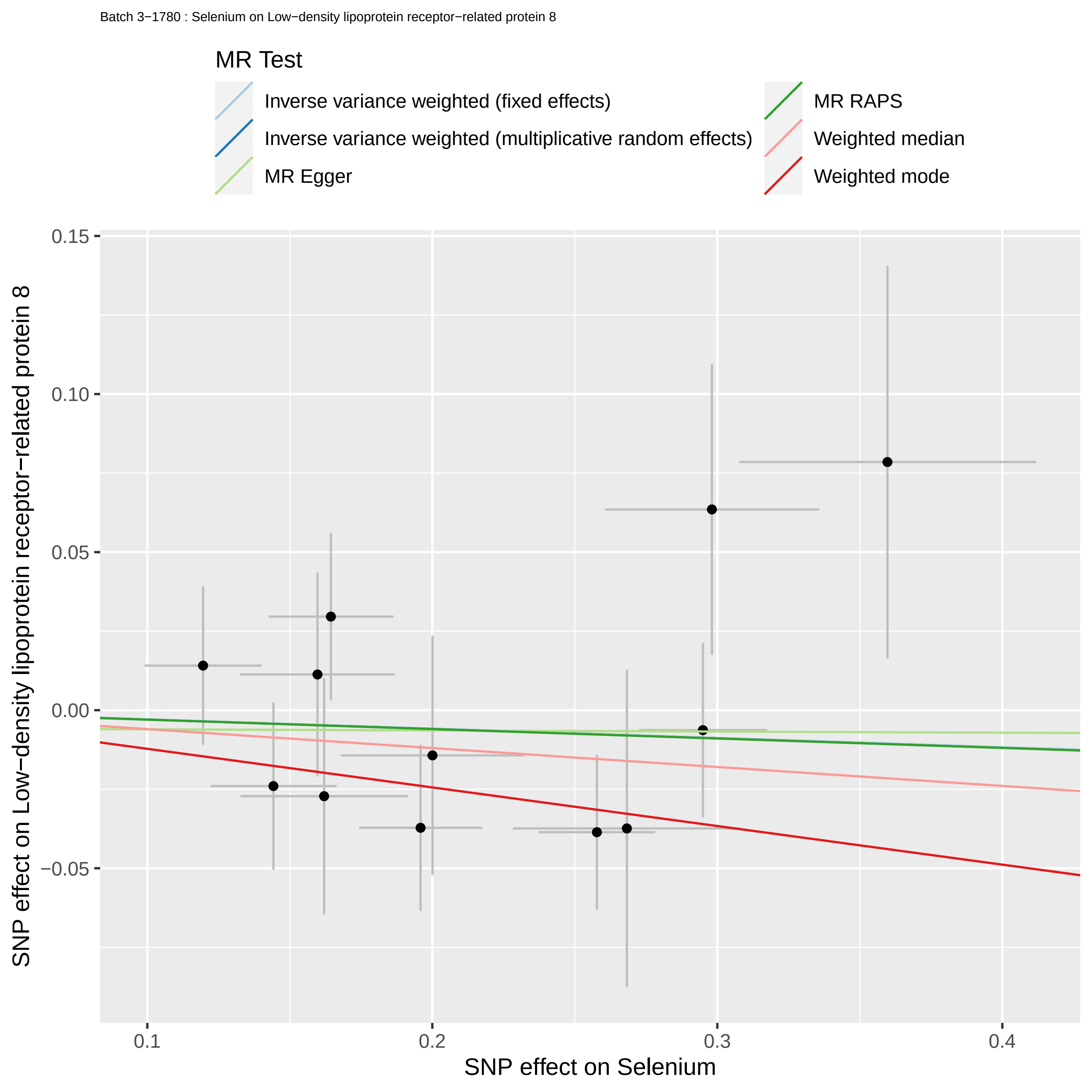

Batch 3−1780 : Selenium on Low−density lipoprotein receptor−related protein 8
MR Test
Inverse variance weighted (fixed effects)
Inverse variance weighted (multiplicative random effects)
MR Egger
MR RAPS
Weighted median
Weighted mode
0.15
0.10
0.05
0.00
−0.05
0.1
0.2
0.3
0.4
SNP effect on Low−density lipoprotein receptor−related protein 8
SNP effect on Selenium

Batch 3−1781 : Selenium on alpha−2−macroglobulin receptor−associated protein

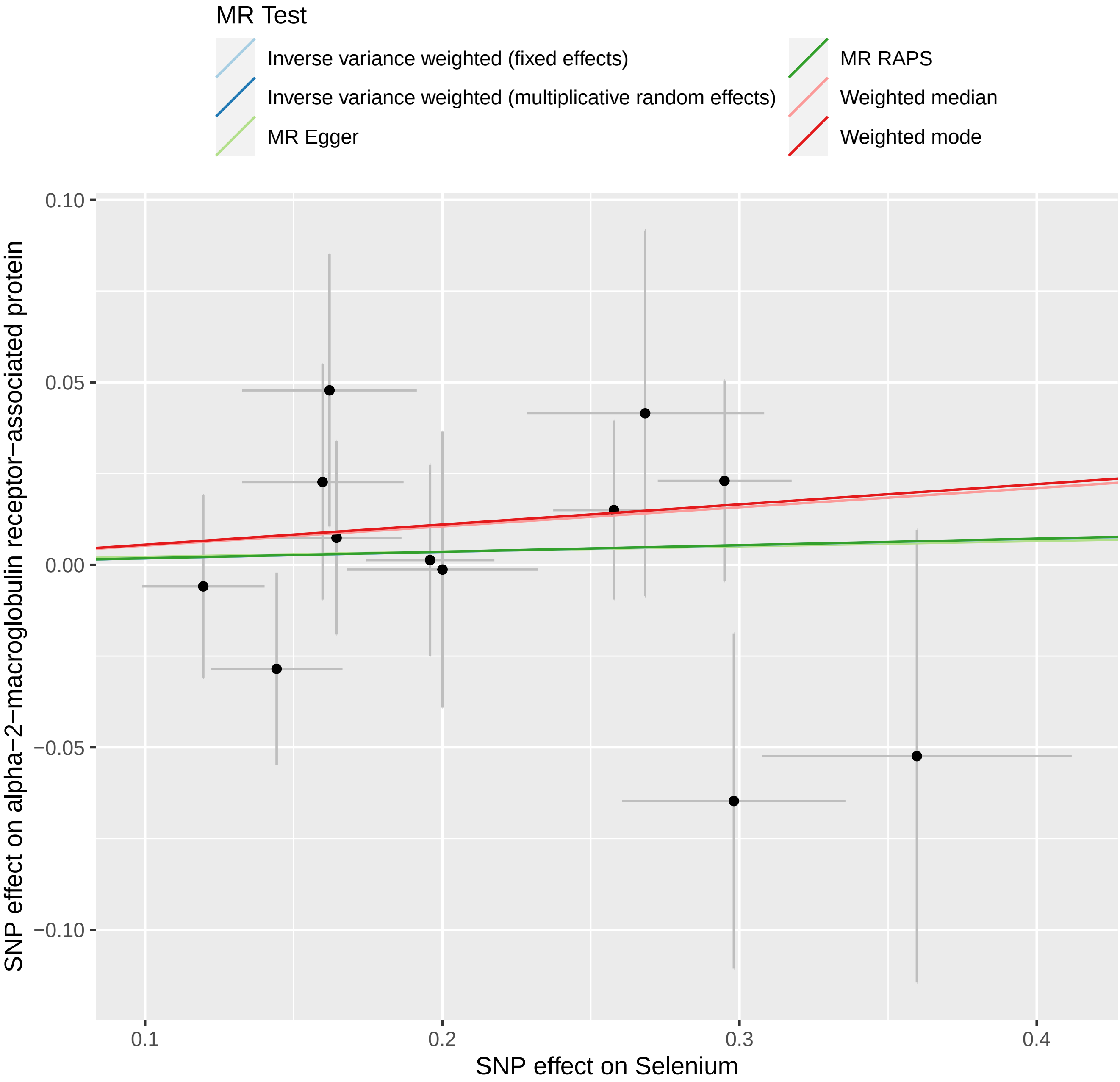

Batch 3−1782 : Selenium on alpha−2−macroglobulin receptor−associated protein

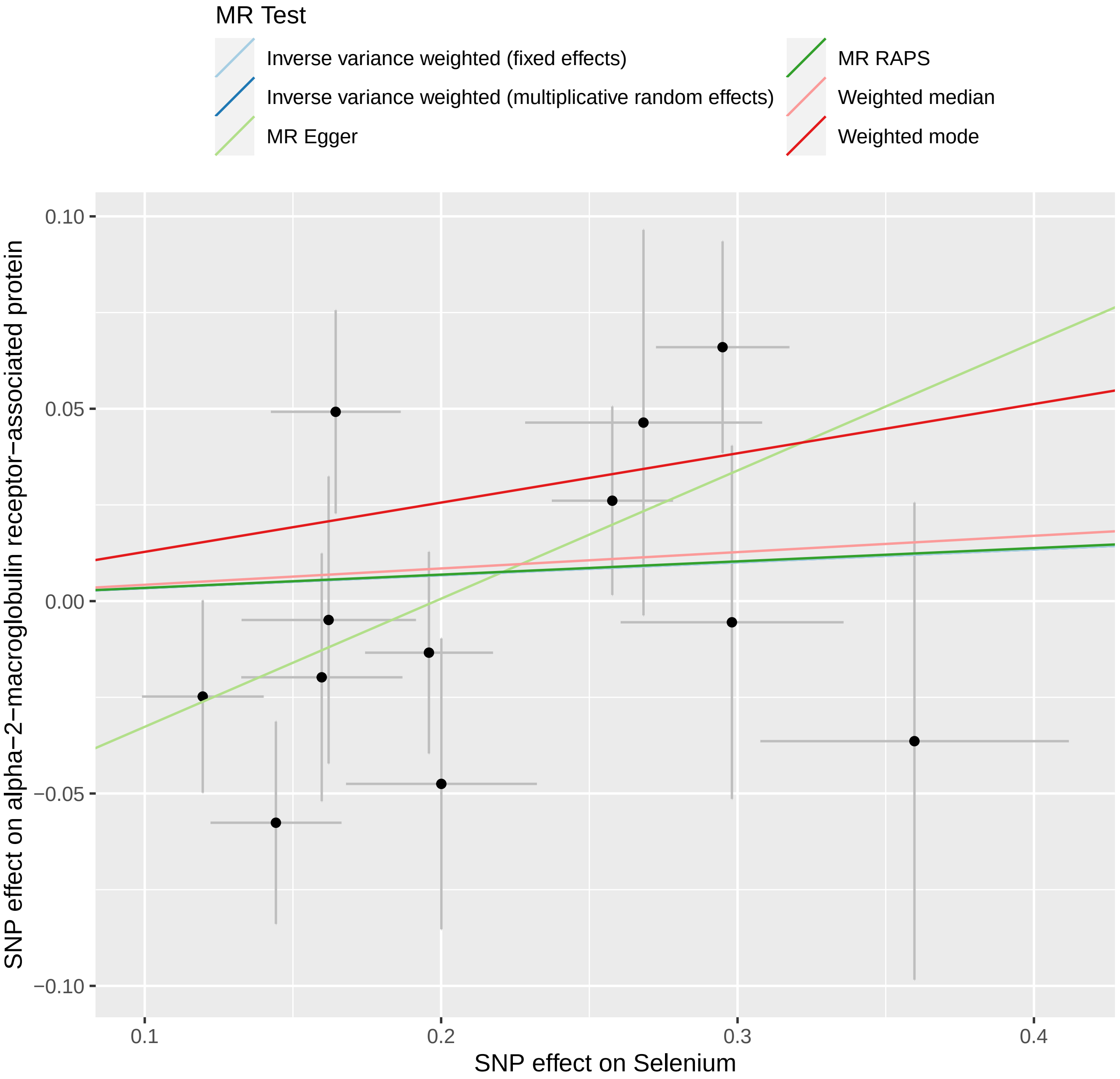

Batch 3−1783 : Selenium on Leucine−rich PPR motif−containing protein, mitochondrial

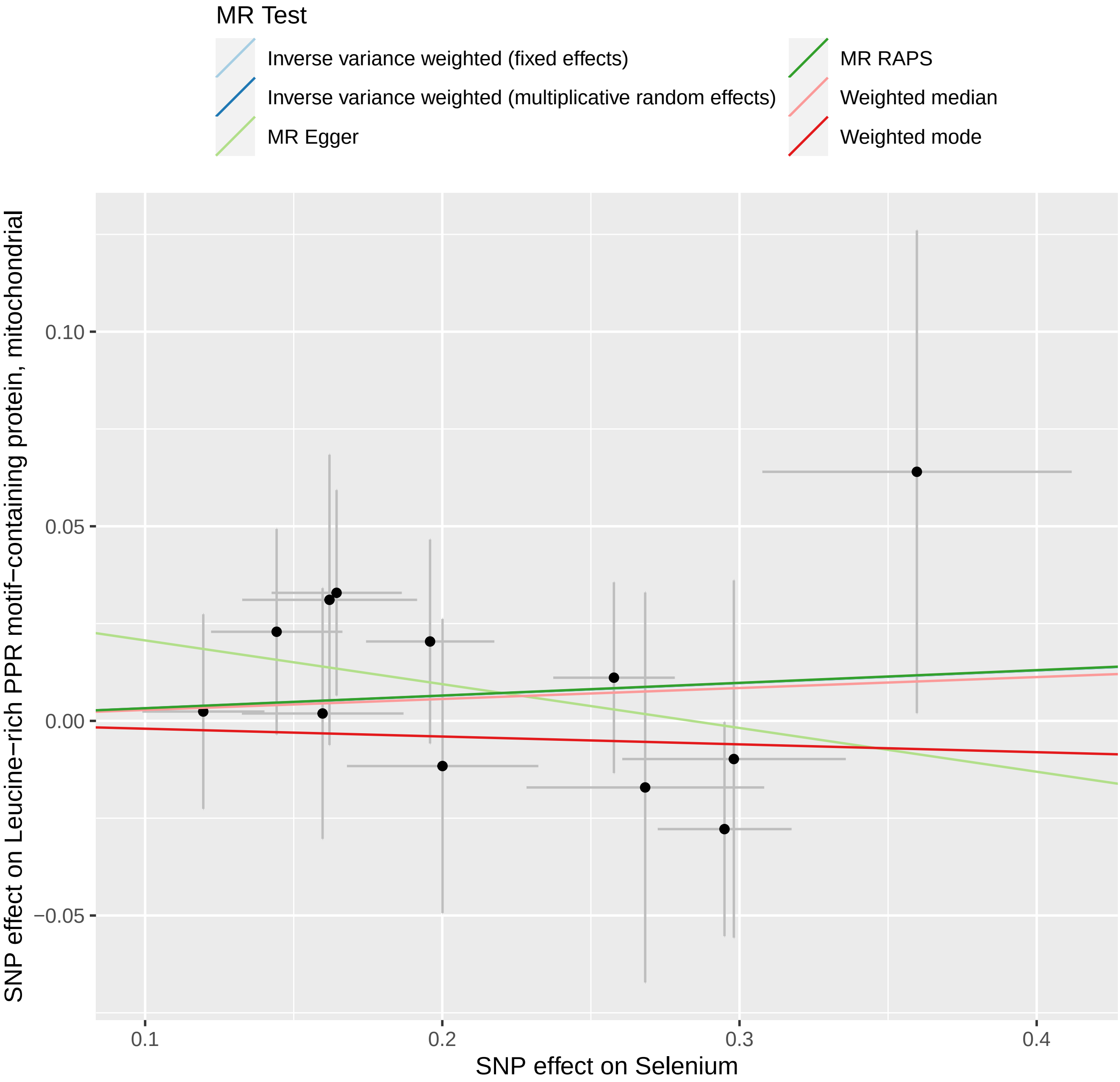

Batch 3−1784 : Selenium on Leucine−rich repeat−containing protein 15

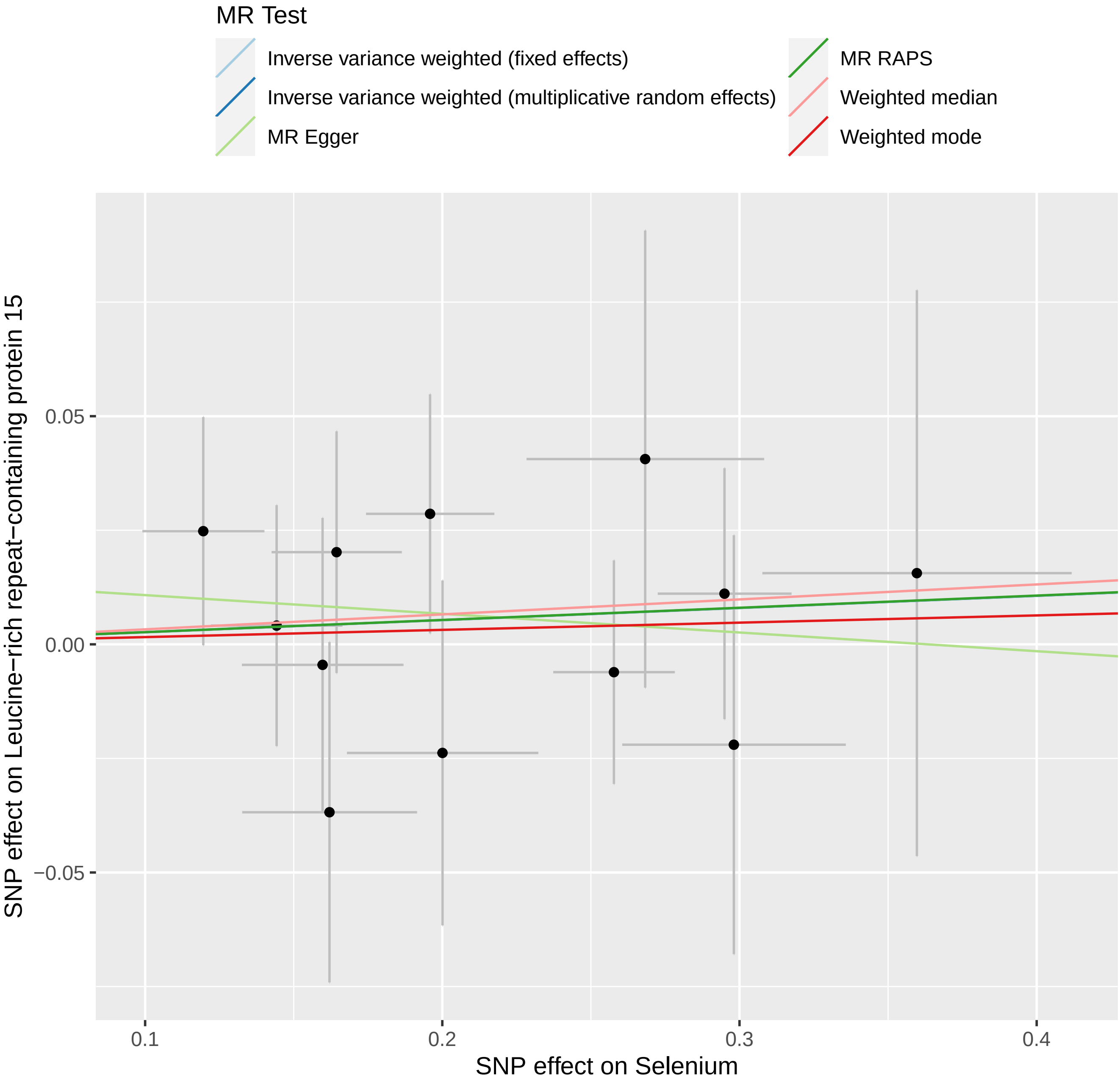

Batch 3−1785 : Selenium on Leucine−rich repeat−containing protein 19

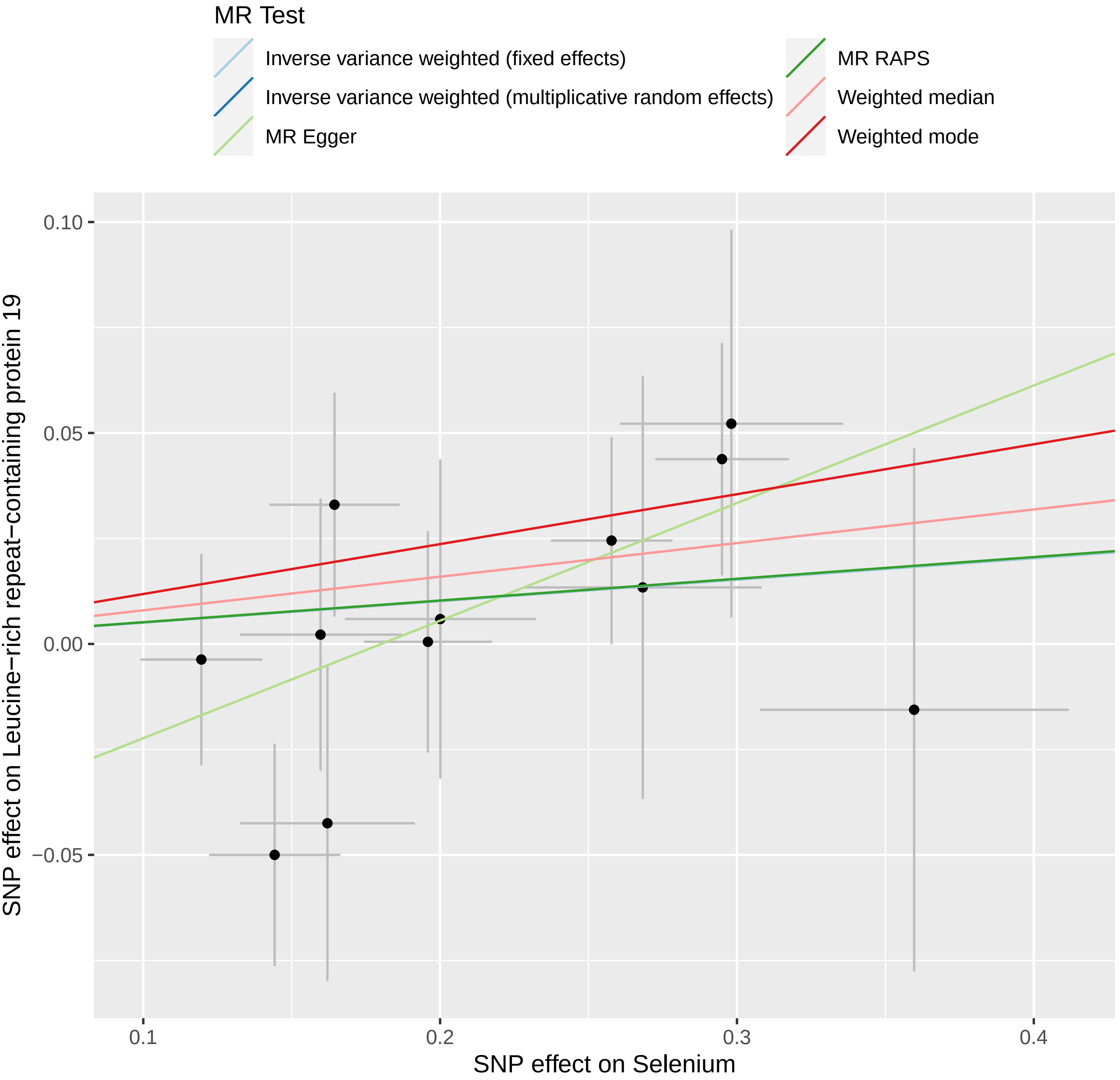

Batch 3−1786 : Selenium on Leucine−rich repeat−containing protein 3

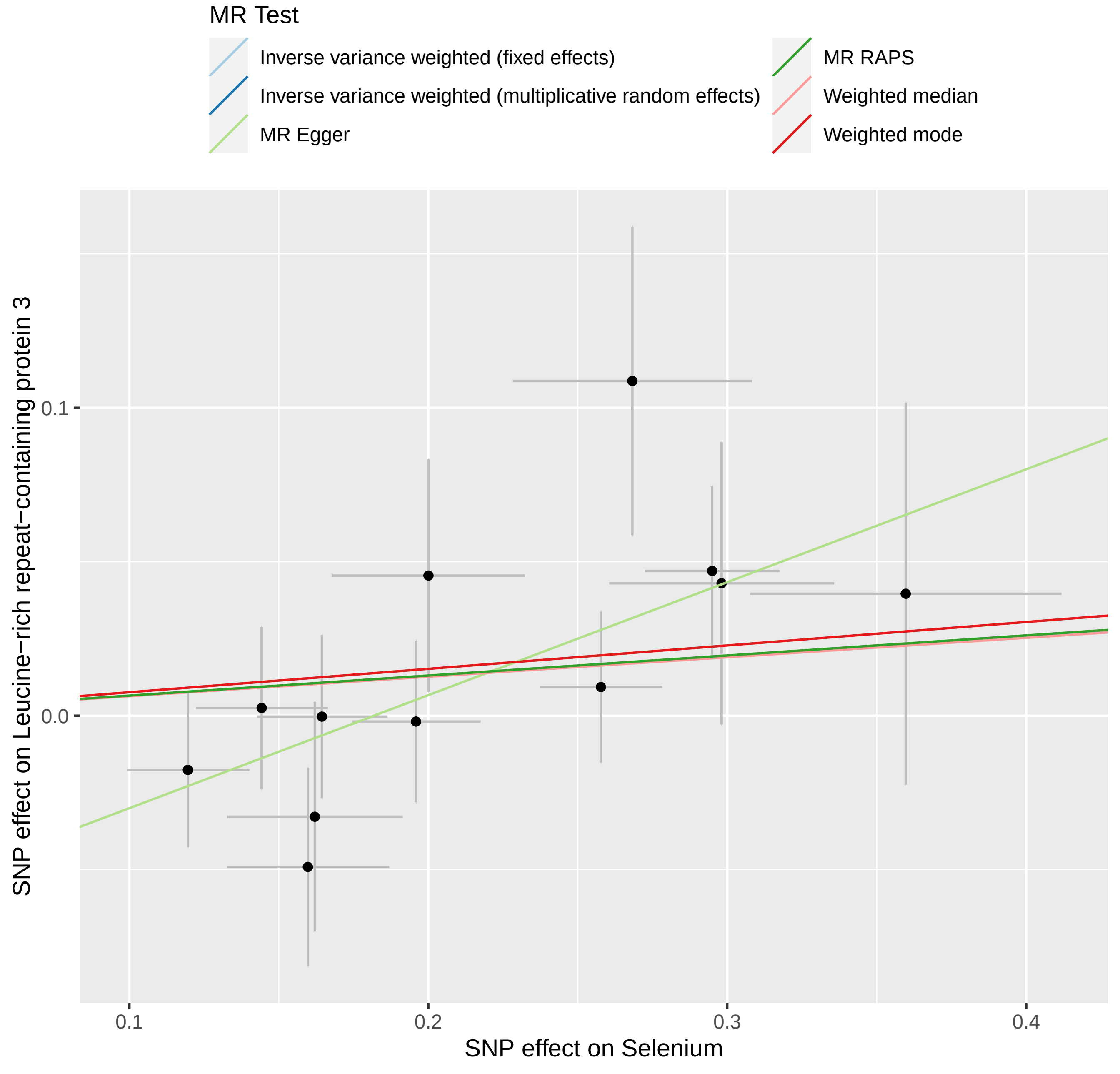

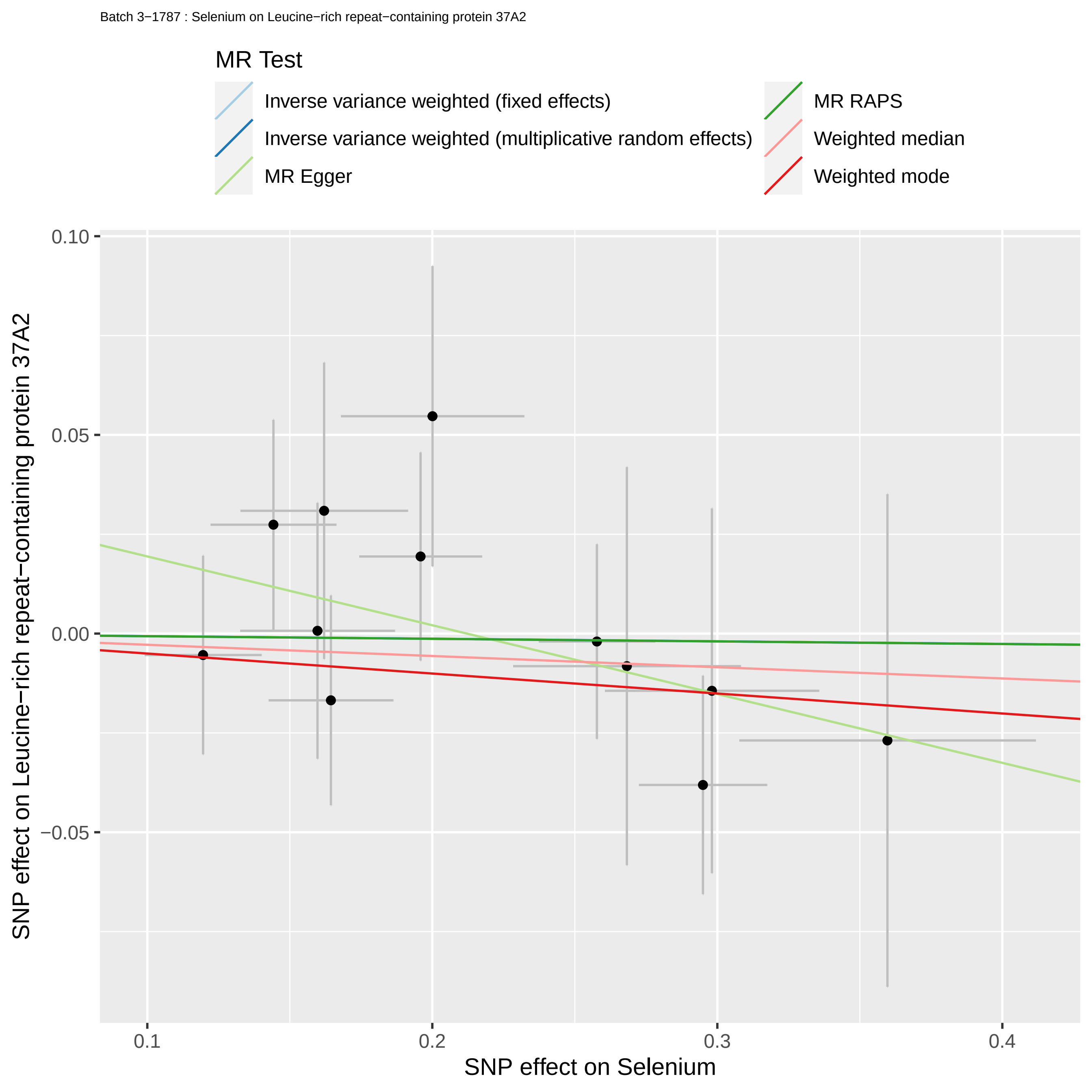
Batch 3−1787 : Selenium on Leucine−rich repeat−containing protein 37A2
MR Test
Inverse variance weighted (fixed effects)
Inverse variance weighted (multiplicative random effects)
MR Egger
MR RAPS
Weighted median
Weighted mode
0.10
0.05
0.00
−0.05
0.1
0.2
0.3
0.4
SNP effect on Leucine−rich repeat−containing protein 37A2
SNP effect on Selenium

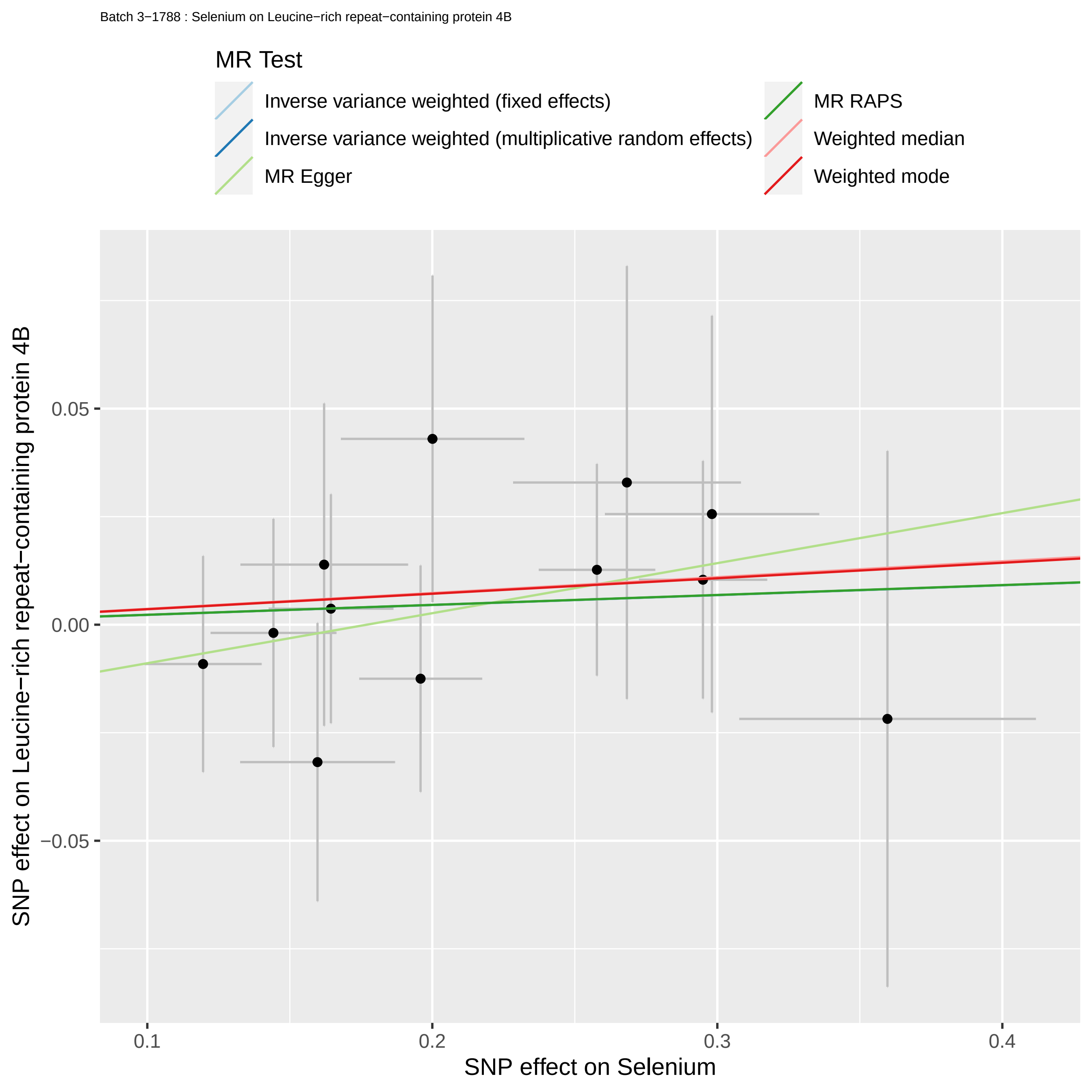
Batch 3−1788 : Selenium on Leucine−rich repeat−containing protein 4B
MR Test
Inverse variance weighted (fixed effects)
Inverse variance weighted (multiplicative random effects)
MR Egger
MR RAPS
Weighted median
Weighted mode
SNP effect on Leucine−rich repeat−containing protein 4B
0.05
0.00
−0.05
0.1
0.2
0.3
0.4
SNP effect on Selenium

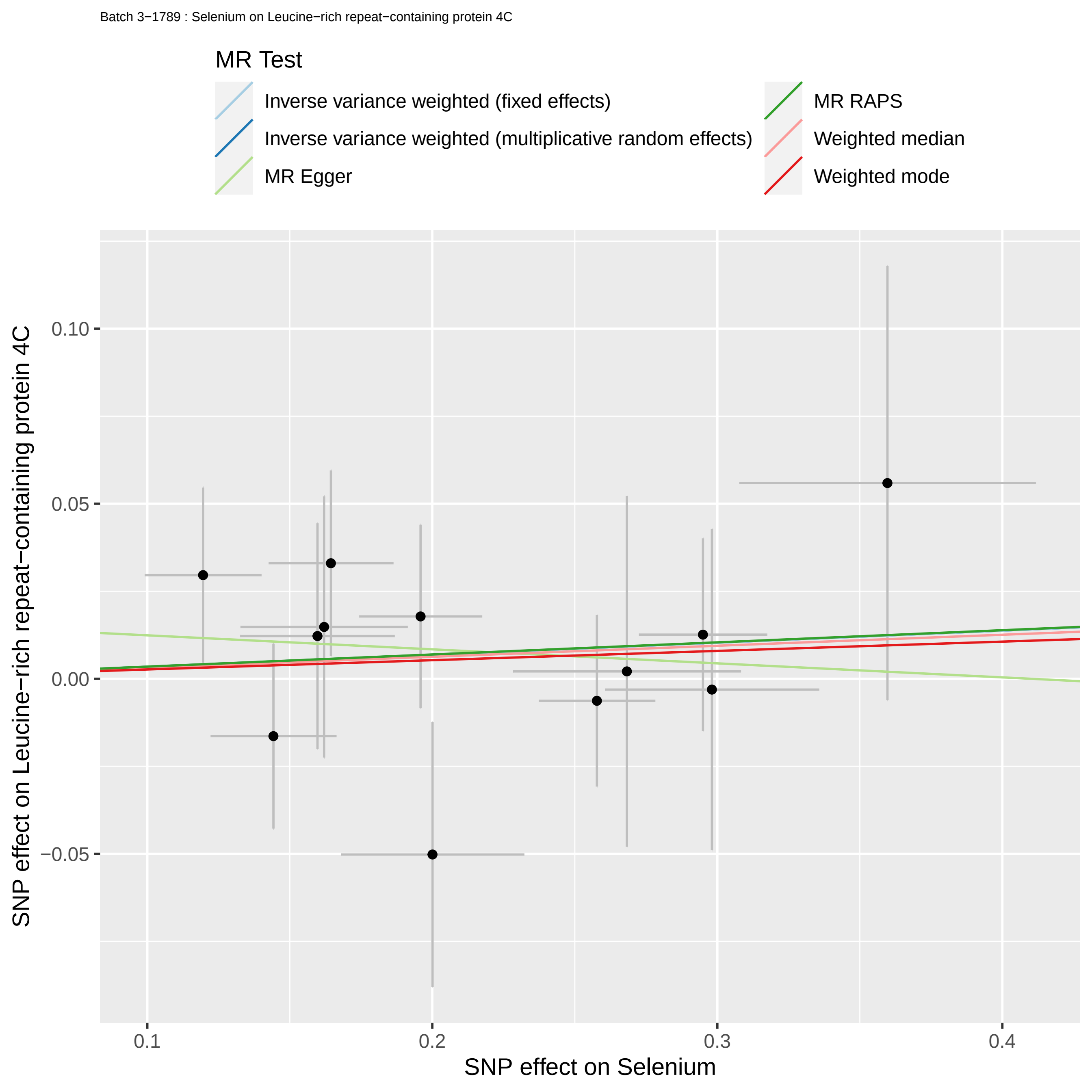
Batch 3−1789 : Selenium on Leucine−rich repeat−containing protein 4C
MR Test
Inverse variance weighted (fixed effects)
Inverse variance weighted (multiplicative random effects)
MR Egger
MR RAPS
Weighted median
Weighted mode
0.10
0.05
0.00
−0.05
0.1
0.2
0.3
0.4
SNP effect on Selenium
SNP effect on Leucine−rich repeat−containing protein 4C

Batch 3−1790 : Selenium on Leucine−rich repeat−containing protein 74A

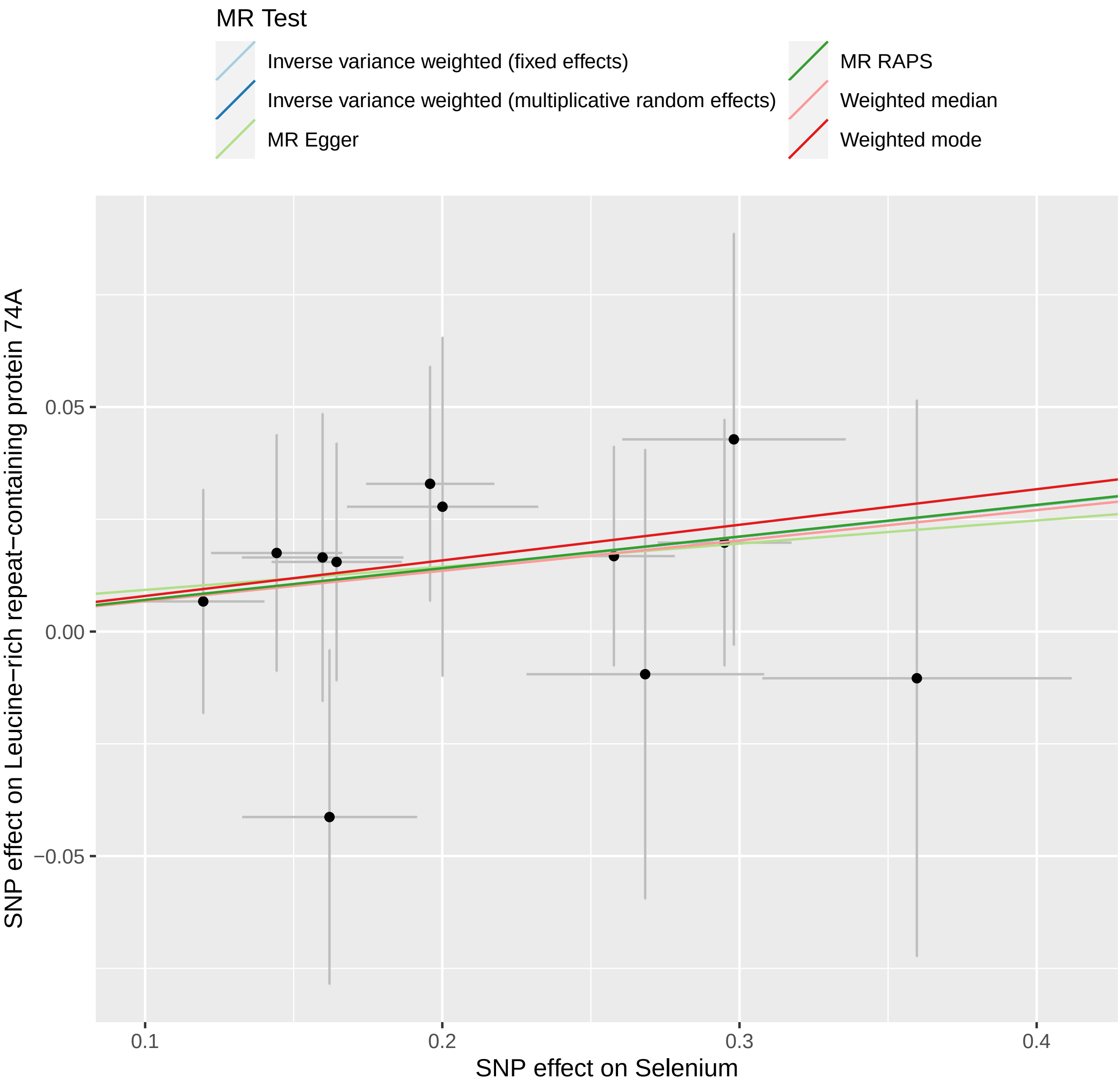

Batch 3−1791 : Selenium on Leucine−rich repeat serine/threonine−protein kinase 2

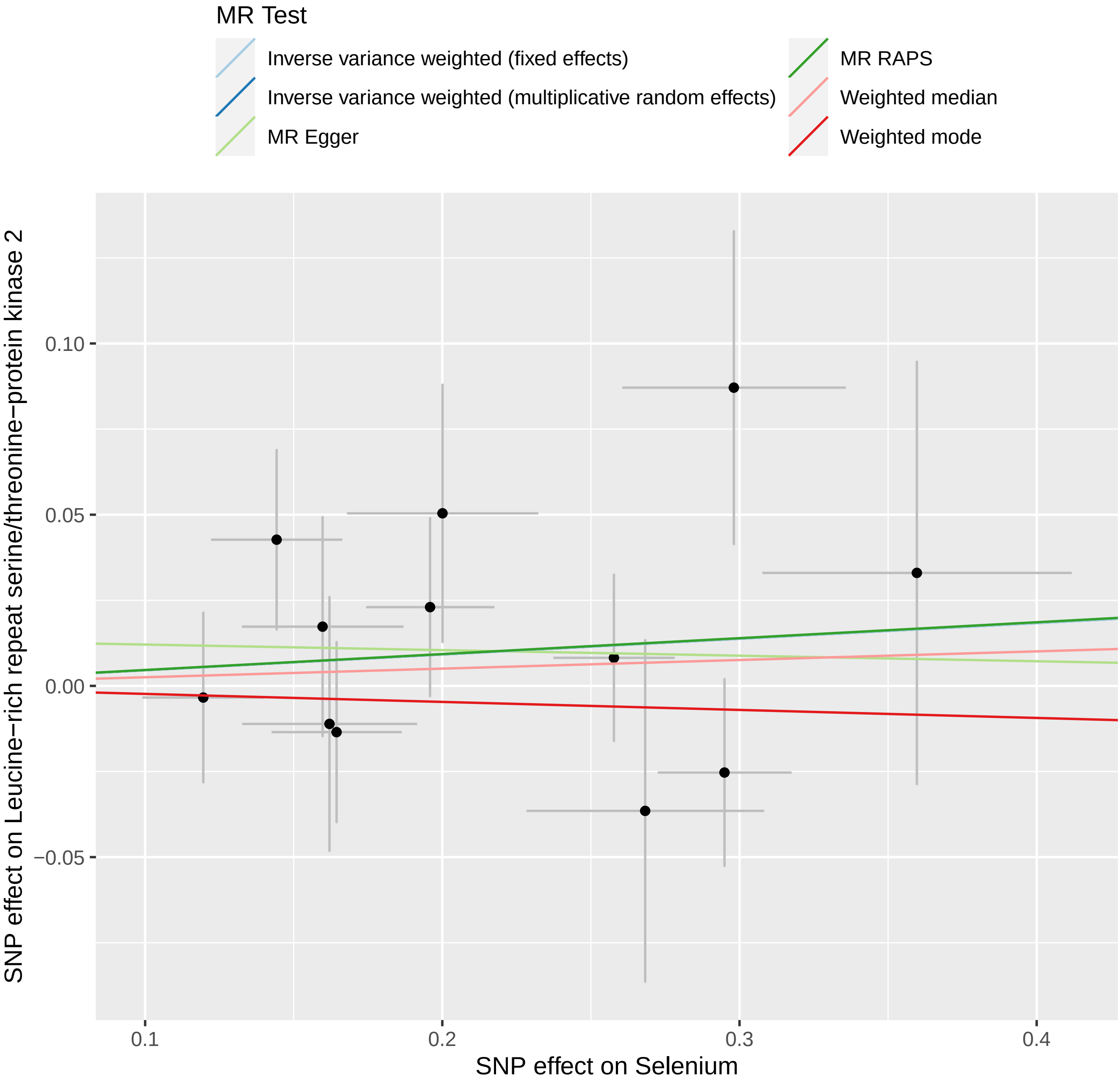

Batch 3−1792 : Selenium on Leucine−rich repeat neuronal protein 1

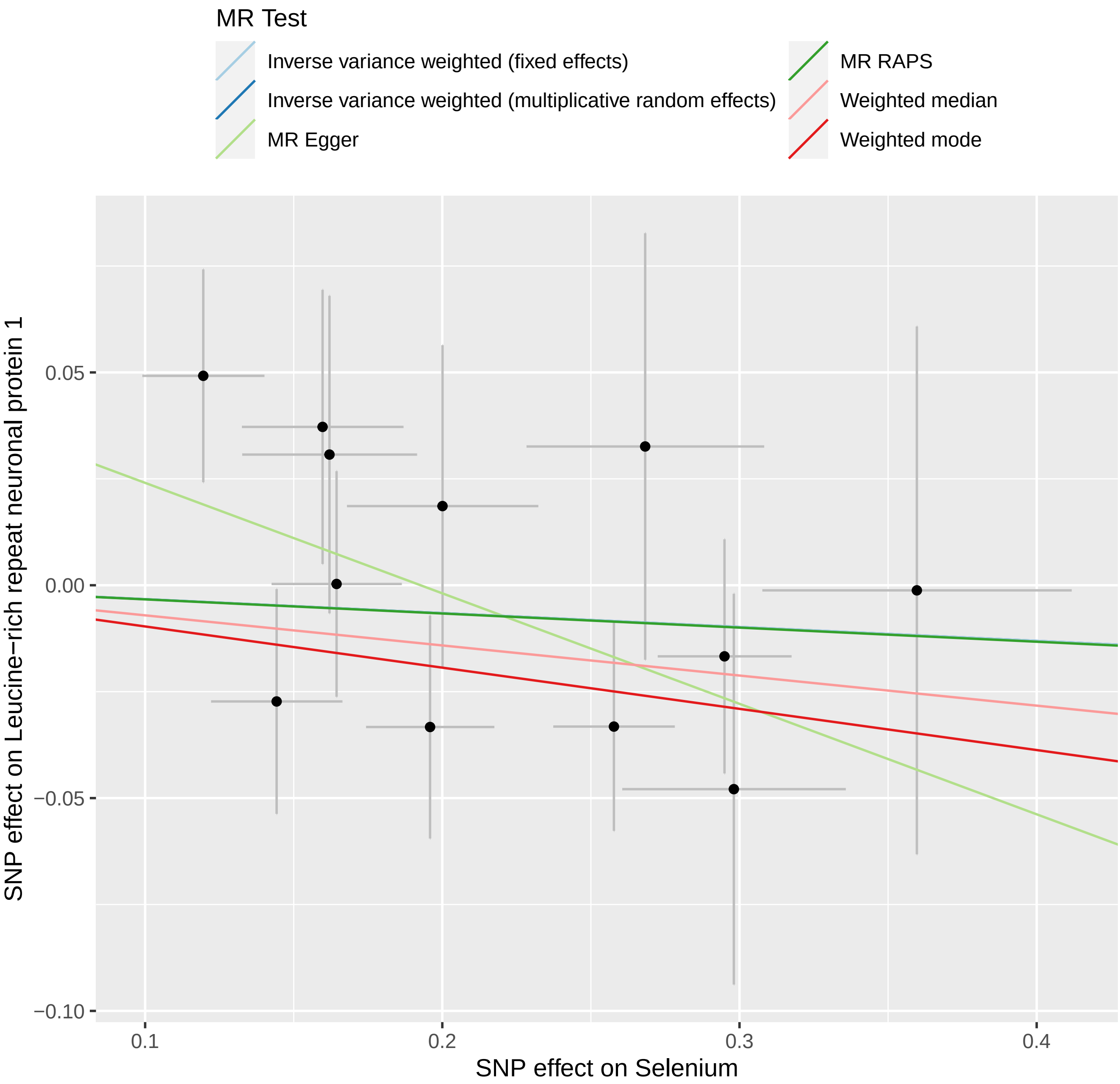

Batch 3−1793 : Selenium on Leucine−rich repeat neuronal protein 1

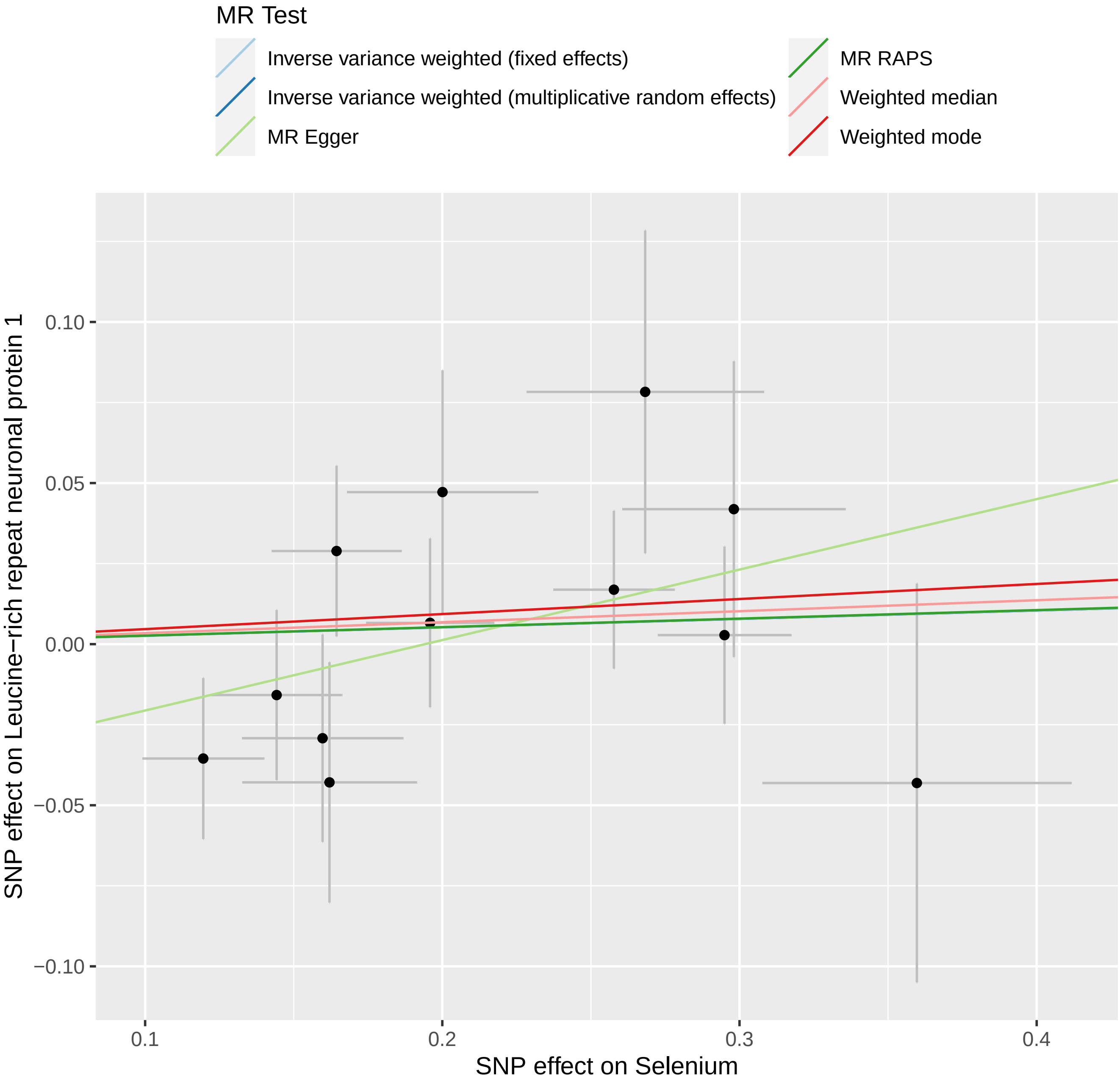

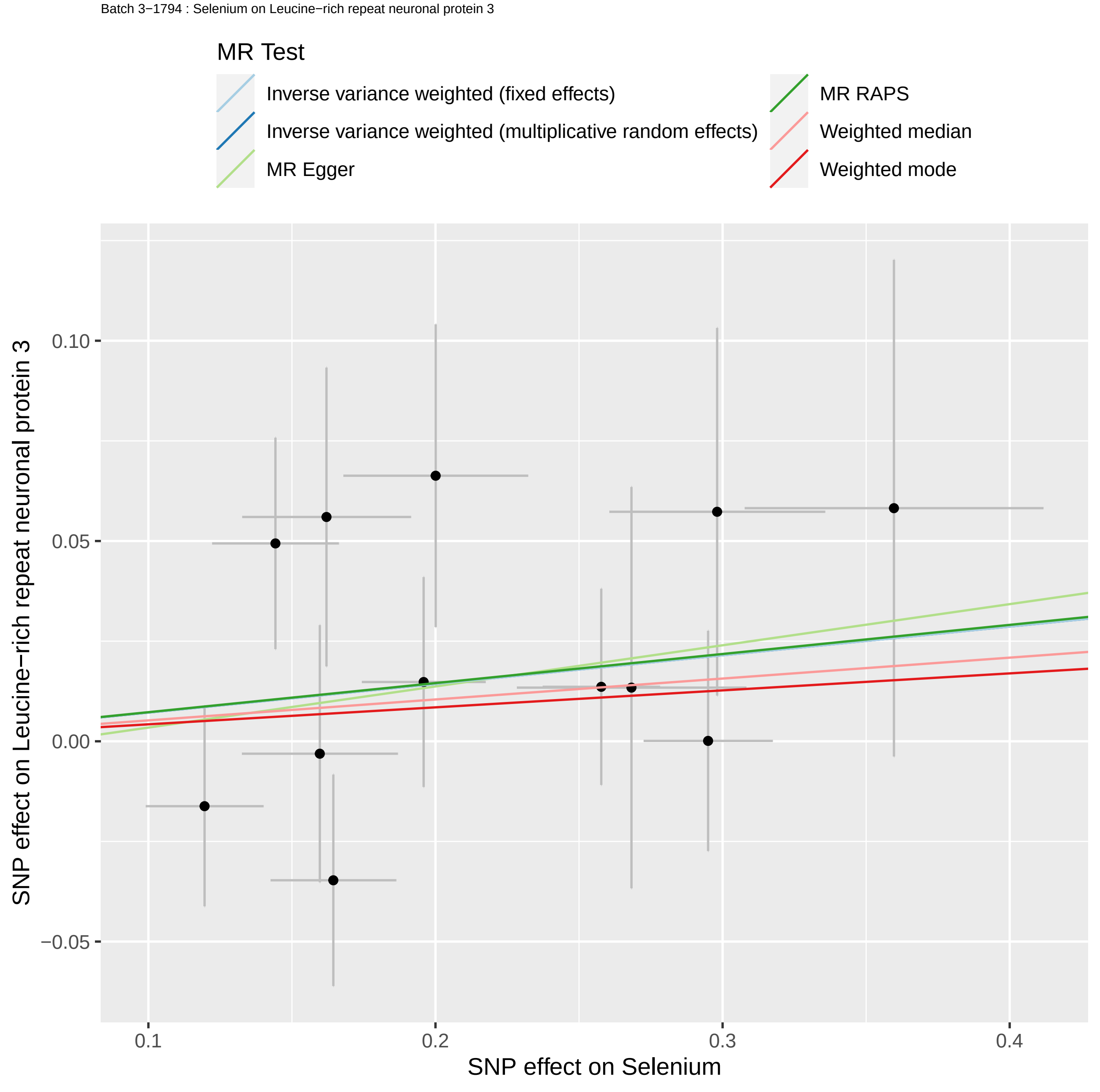

Batch 3−1794 : Selenium on Leucine−rich repeat neuronal protein 3
MR Test
Inverse variance weighted (fixed effects)
Inverse variance weighted (multiplicative random effects)
MR Egger
MR RAPS
Weighted median
Weighted mode
0.10
0.05
0.00
−0.05
0.1
0.2
0.3
0.4
SNP effect on Selenium
SNP effect on Leucine−rich repeat neuronal protein 3

Batch 3−1795 : Selenium on Leucine−rich repeat transmembrane neuronal protein 1

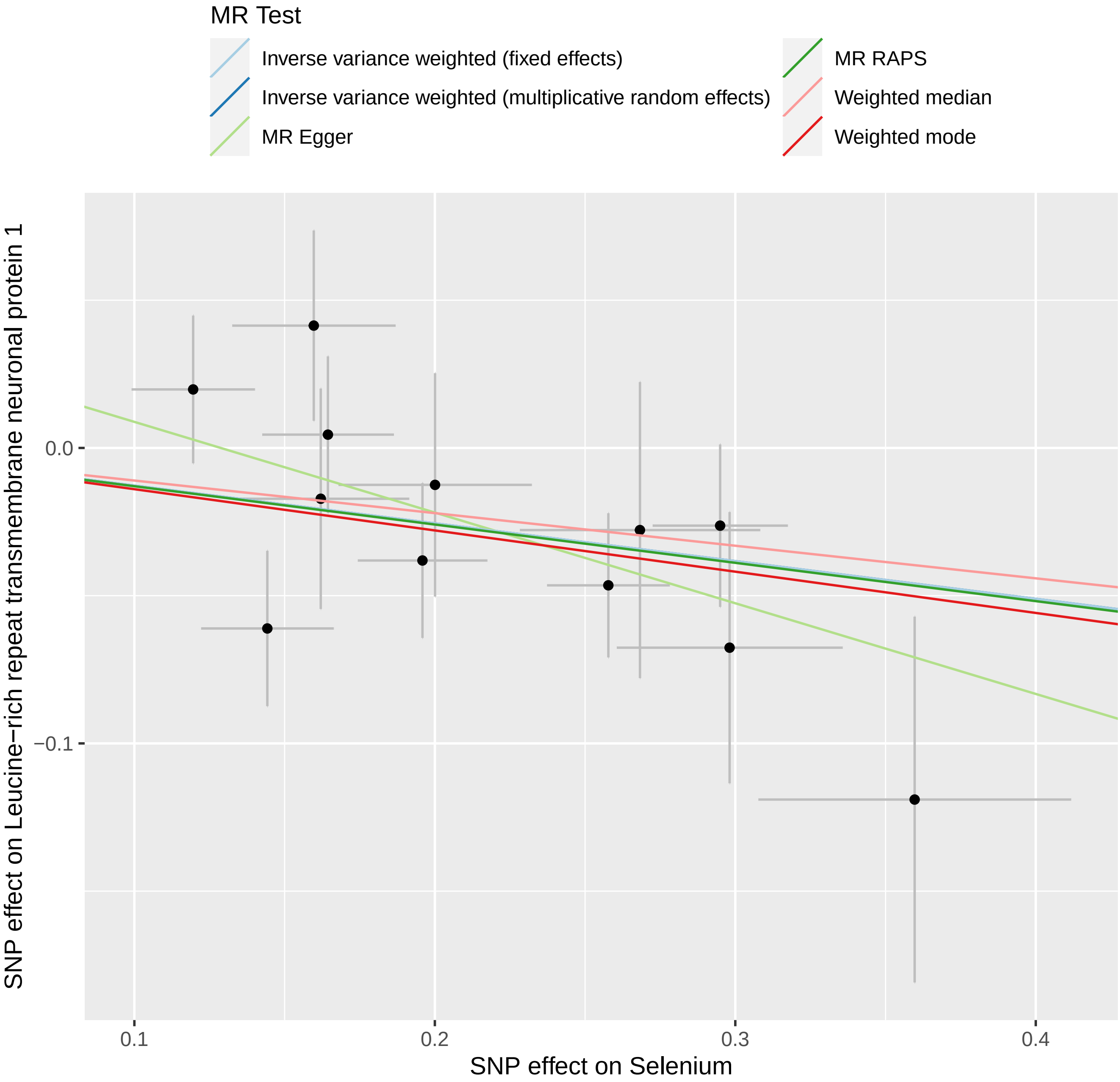

Batch 3−1796 : Selenium on Leucine−rich repeat transmembrane neuronal protein 2

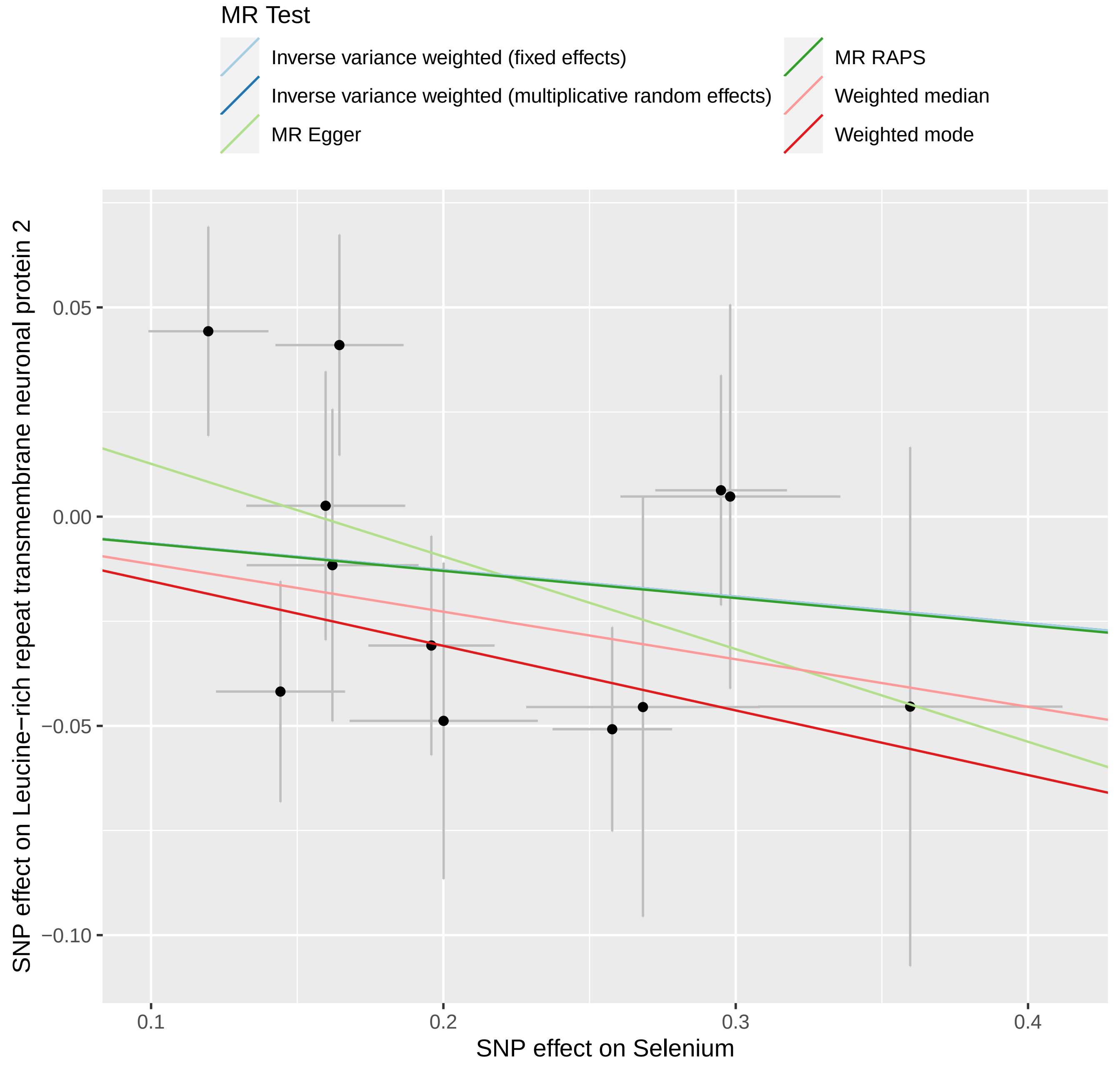

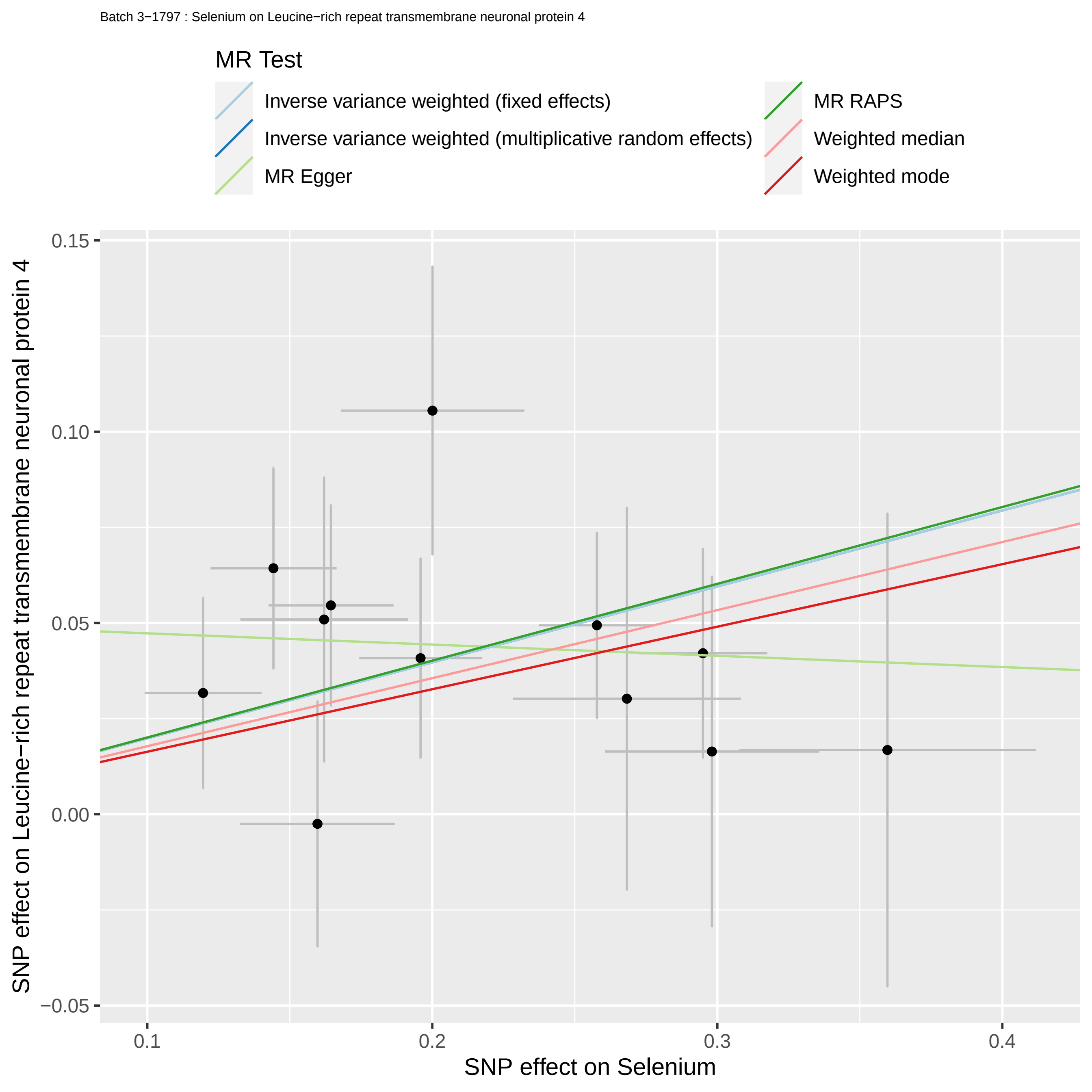

Batch 3−1797 : Selenium on Leucine−rich repeat transmembrane neuronal protein 4
MR Test
Inverse variance weighted (fixed effects)
Inverse variance weighted (multiplicative random effects)
MR Egger
MR RAPS
Weighted median
Weighted mode
0.15
0.10
0.05
0.00
−0.05
0.1
0.2
0.3
0.4
SNP effect on Selenium
SNP effect on Leucine−rich repeat transmembrane neuronal protein 4

Batch 3−1798 : Selenium on Leucine−rich repeat transmembrane neuronal protein 4

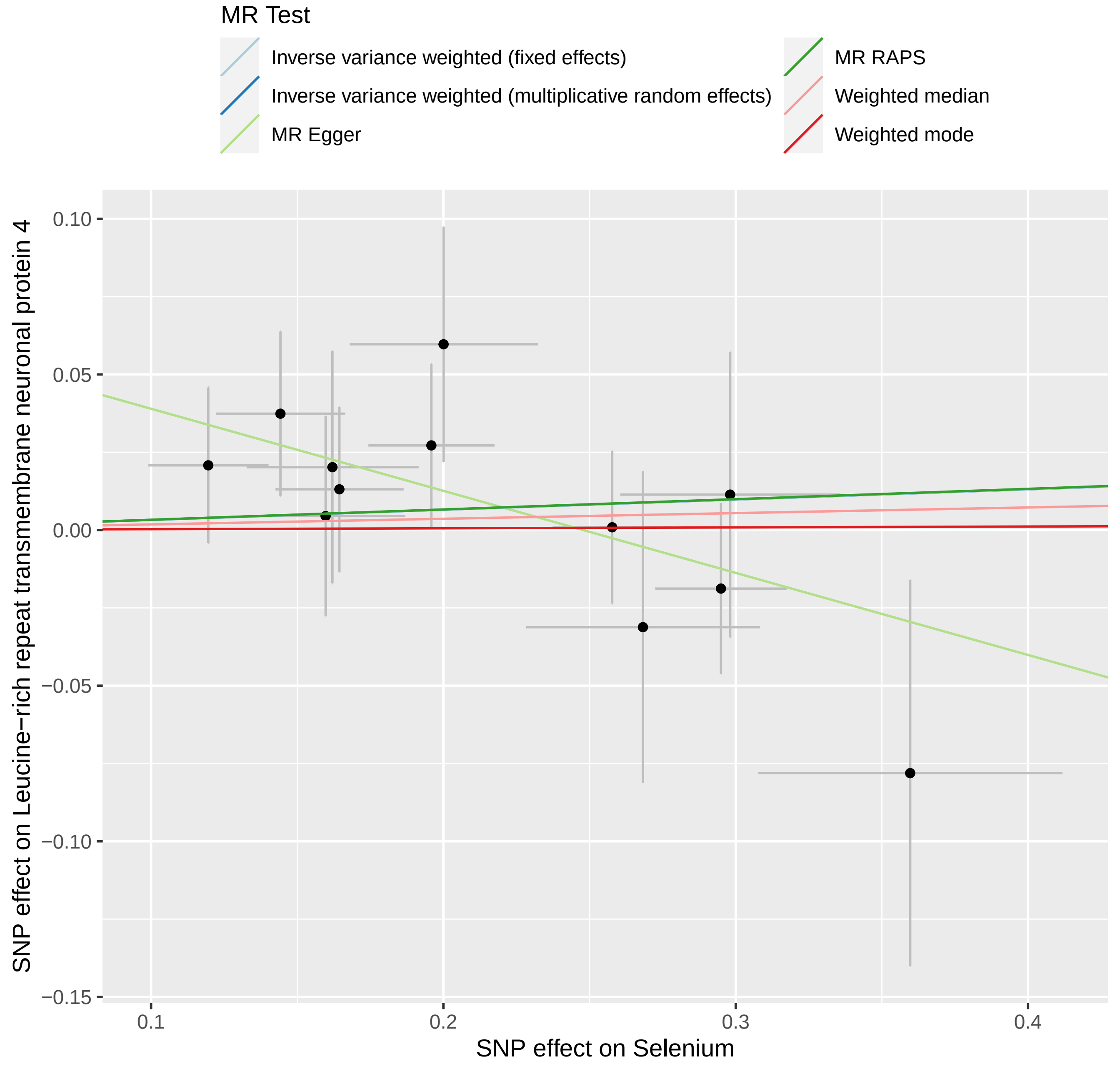

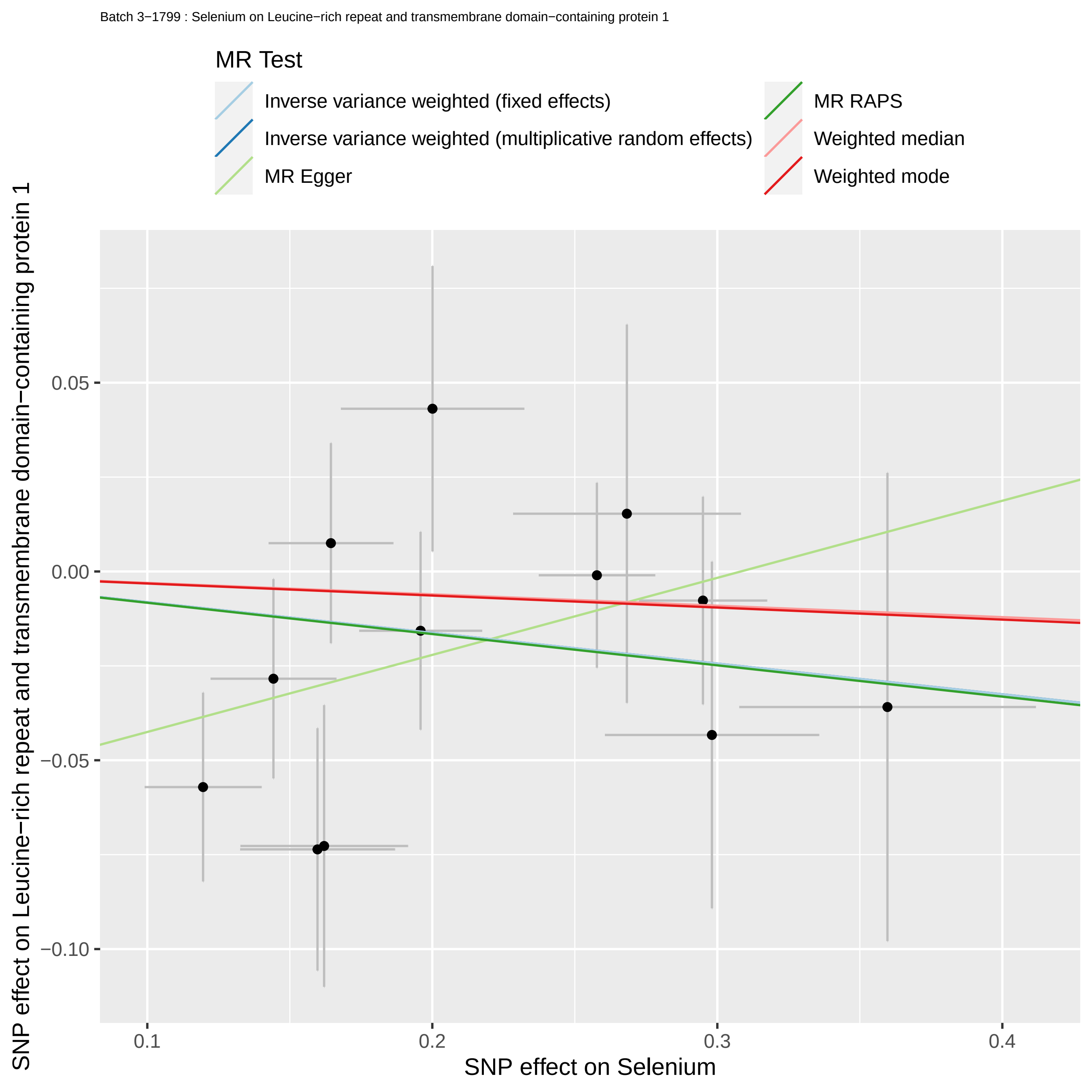

Batch 3−1799 : Selenium on Leucine−rich repeat and transmembrane domain−containing protein 1
MR Test
Inverse variance weighted (fixed effects)
Inverse variance weighted (multiplicative random effects)
MR Egger
MR RAPS
Weighted median
Weighted mode
SNP effect on Leucine−rich repeat and transmembrane domain−containing protein 1
0.05
0.00
−0.05
−0.10
0.1
0.2
0.3
0.4
SNP effect on Selenium

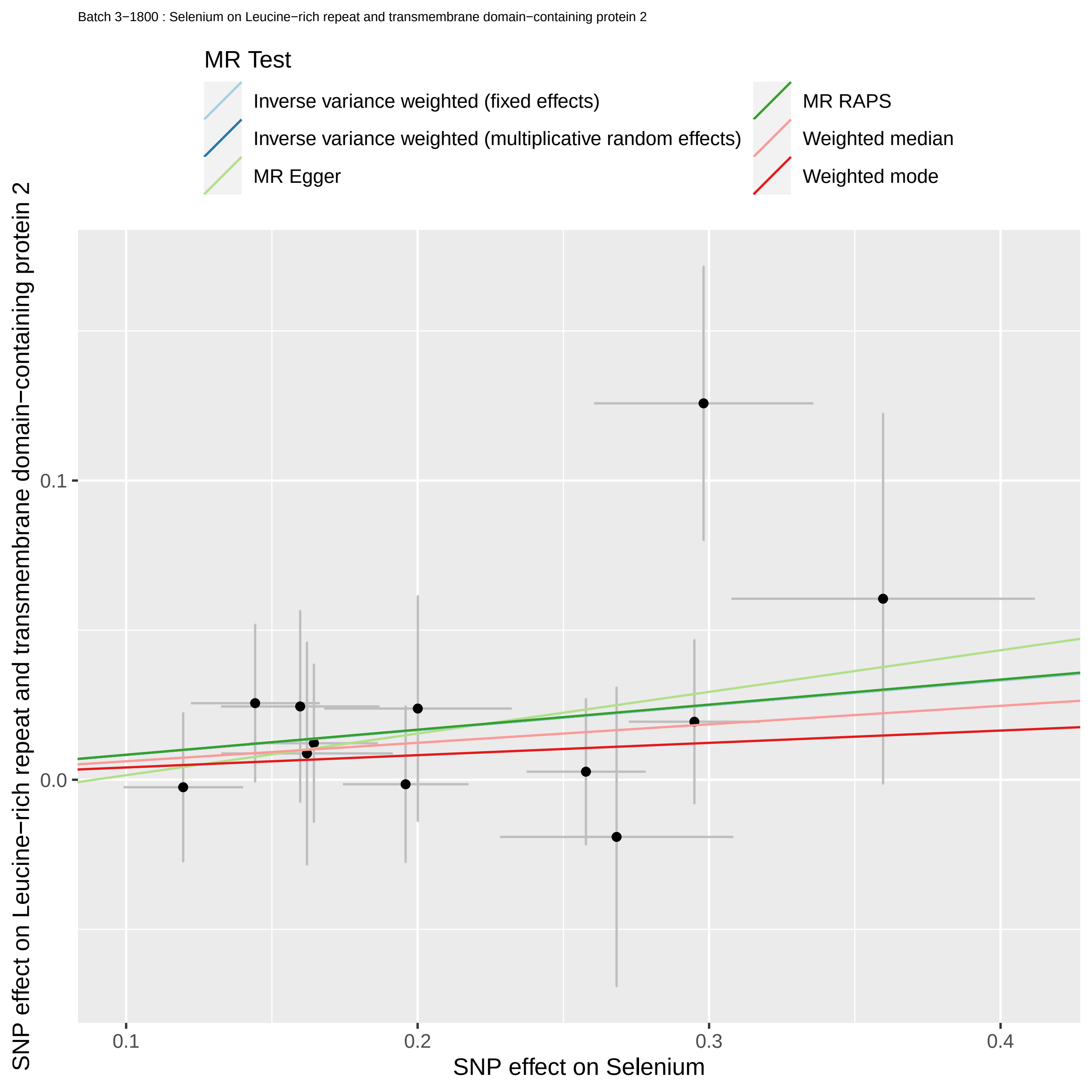

Batch 3−1800 : Selenium on Leucine−rich repeat and transmembrane domain−containing protein 2
MR Test
Inverse variance weighted (fixed effects)
Inverse variance weighted (multiplicative random effects)
MR Egger
MR RAPS
Weighted median
Weighted mode
0.1
0.0
0.1
0.2
0.3
0.4
SNP effect on Leucine−rich repeat and transmembrane domain−containing protein 2
SNP effect on Selenium

Batch 3−1801 : Selenium on Limbic system−associated membrane protein

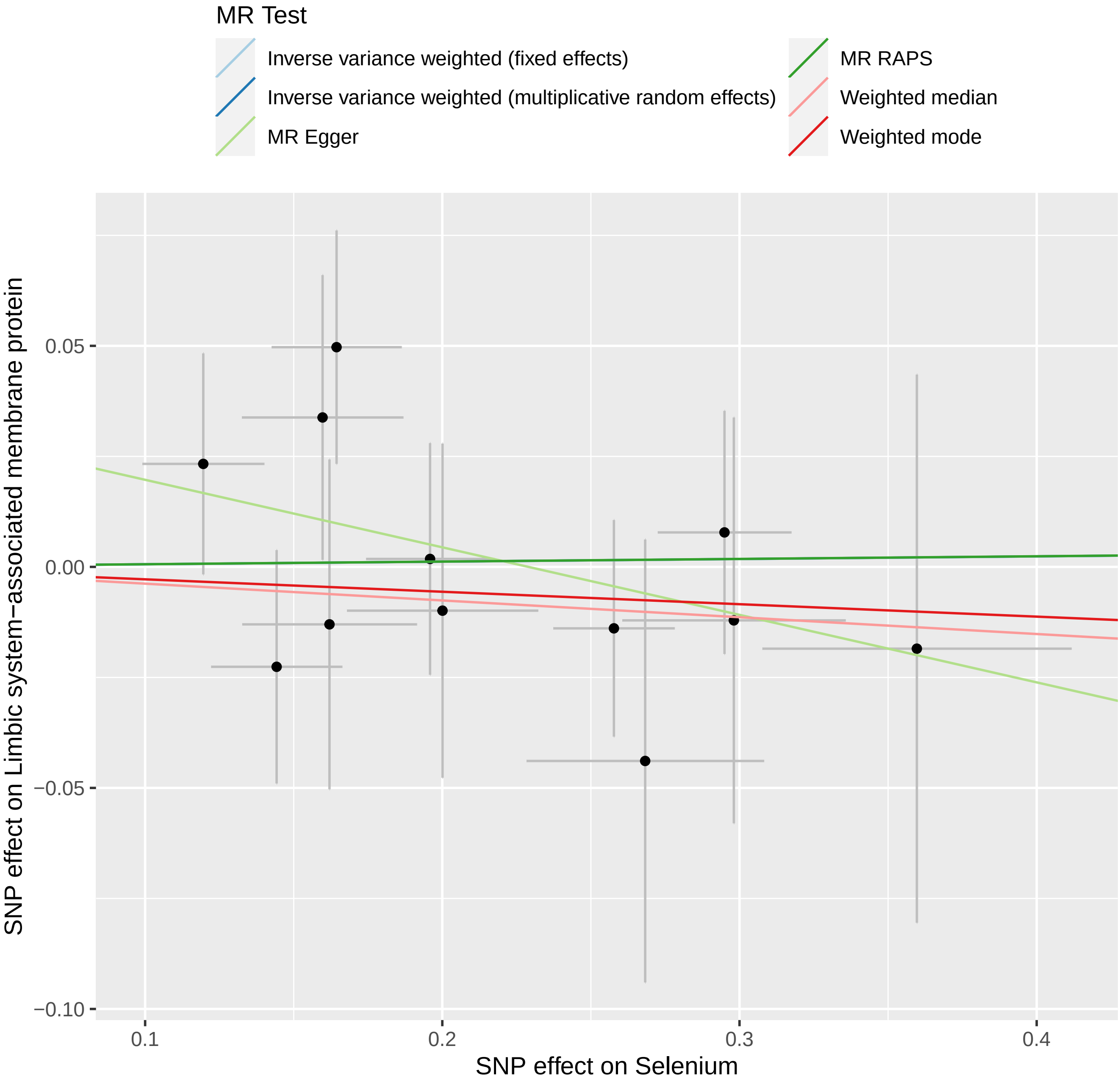

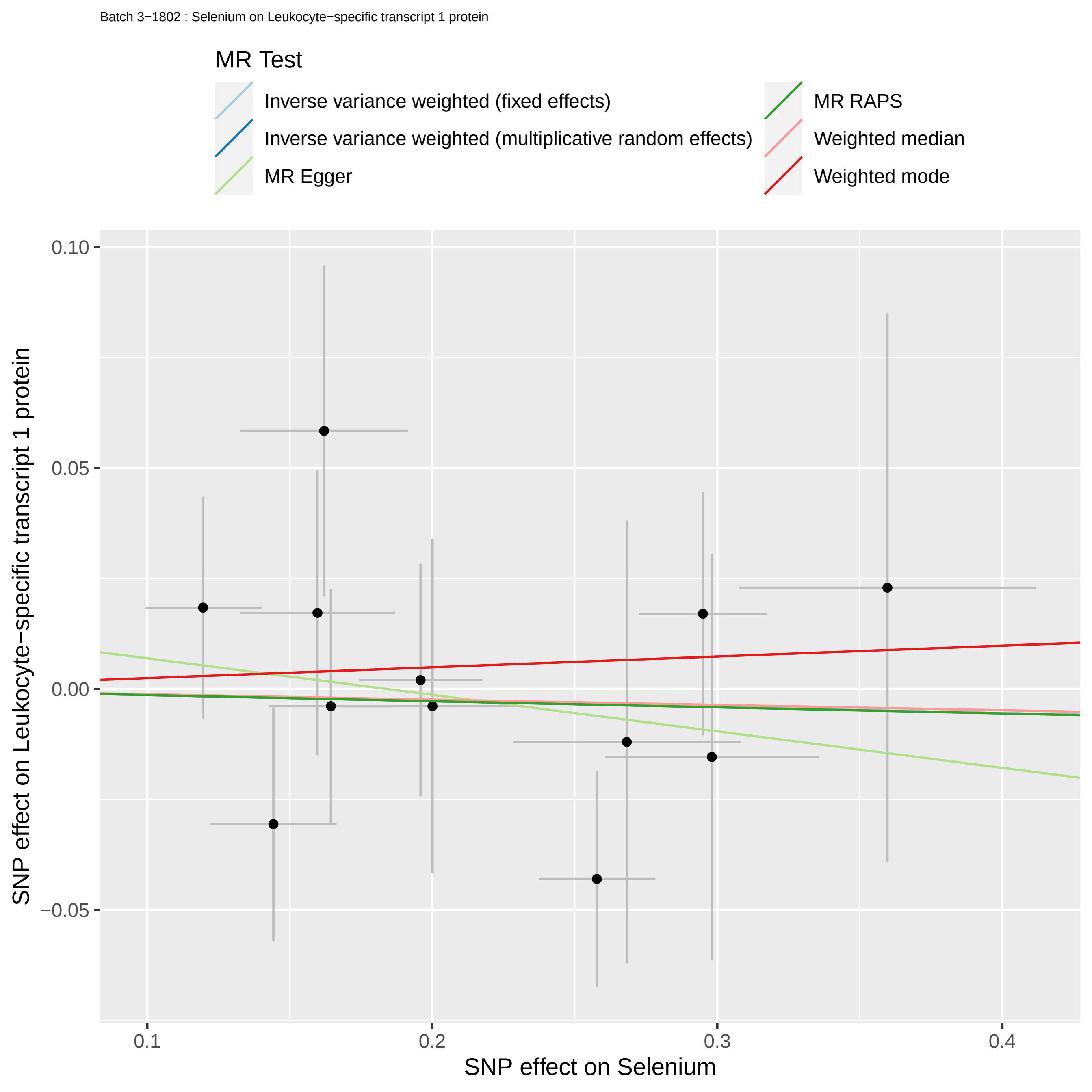

Batch 3−1802 : Selenium on Leukocyte−specific transcript 1 protein
MR Test
Inverse variance weighted (fixed effects)
Inverse variance weighted (multiplicative random effects)
MR Egger
MR RAPS
Weighted median
Weighted mode
0.10
0.05
0.00
−0.05
0.1
0.2
0.3
0.4
SNP effect on Selenium
SNP effect on Leukocyte−specific transcript 1 protein

Batch 3−1803 : Selenium on Lymphotoxin−alpha

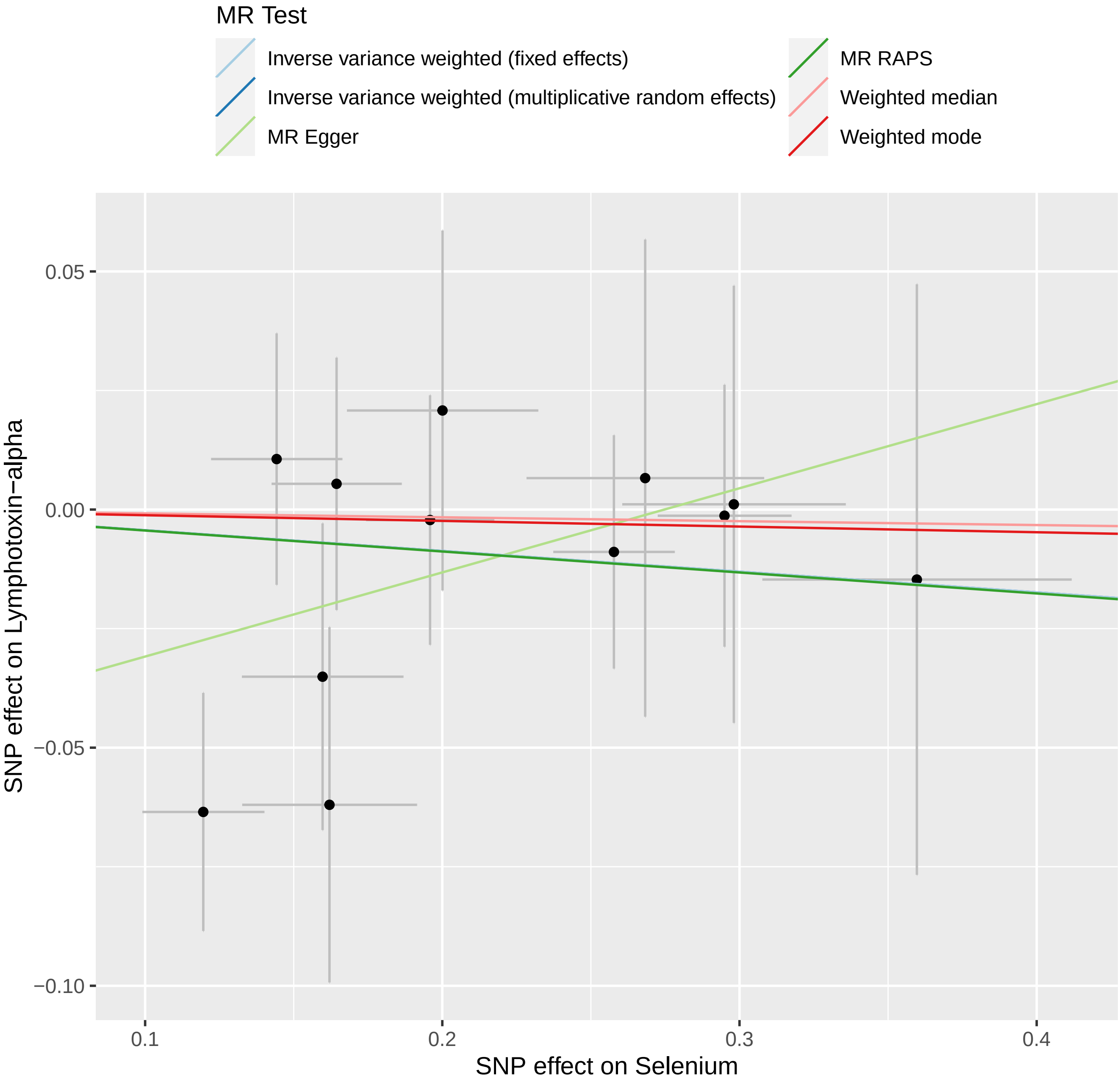

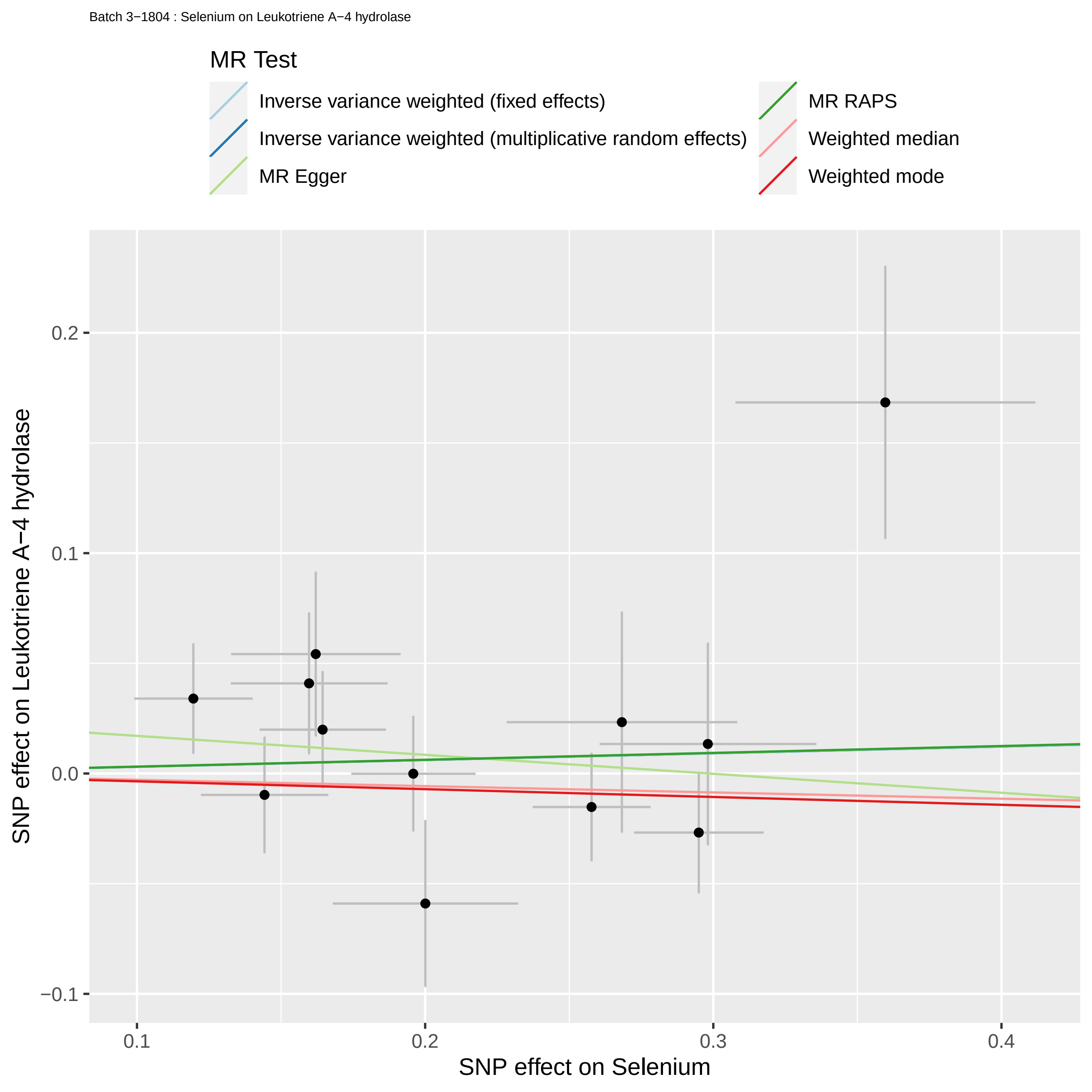

Batch 3−1804 : Selenium on Leukotriene A−4 hydrolase
MR Test
Inverse variance weighted (fixed effects)
Inverse variance weighted (multiplicative random effects)
MR Egger
MR RAPS
Weighted median
Weighted mode
SNP effect on Leukotriene A−4 hydrolase
0.2
0.1
0.0
−0.1
0.1
0.2
0.3
0.4
SNP effect on Selenium

Batch 3−1805 : Selenium on Lymphotoxin alpha2:beta1

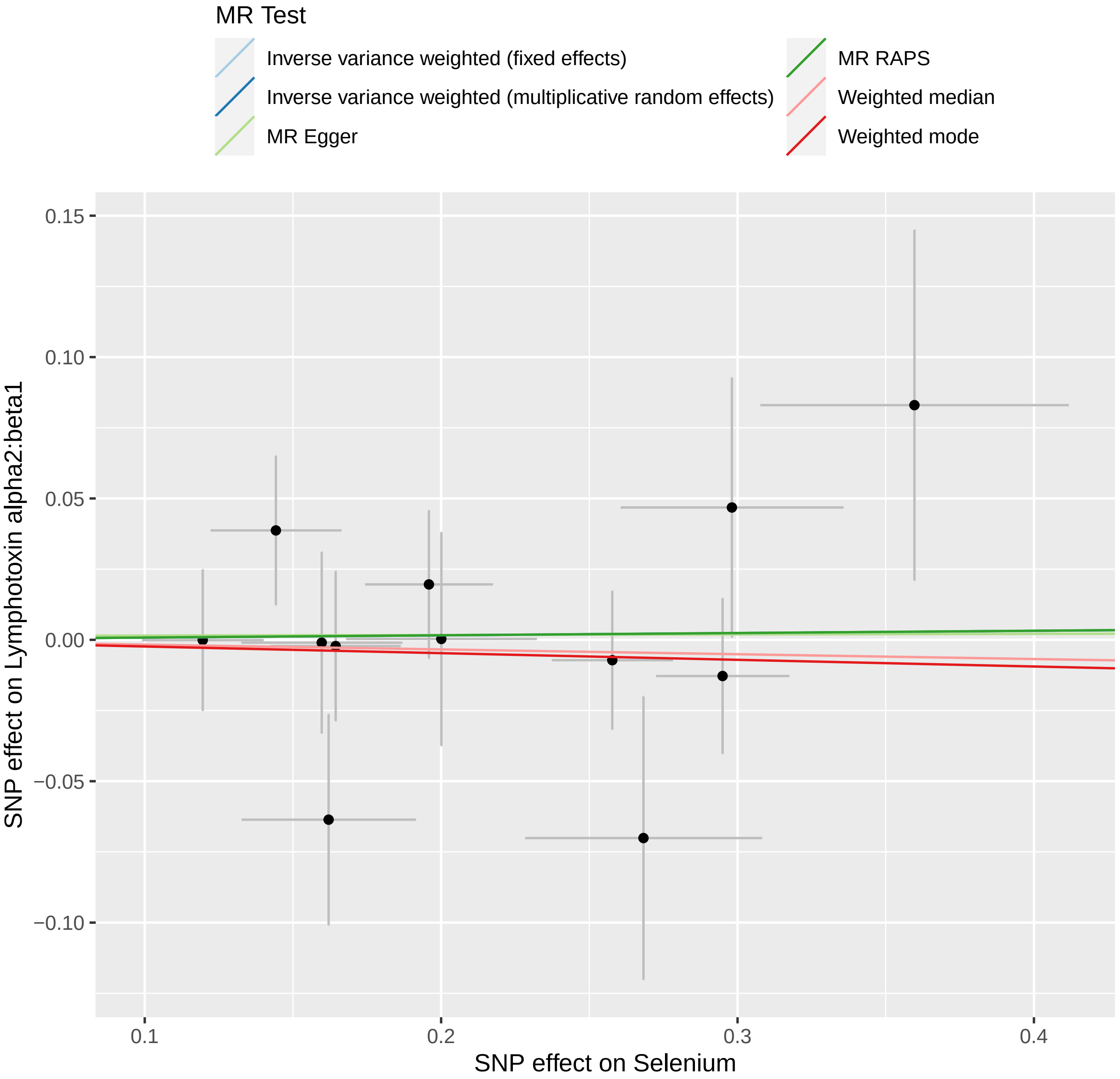

Batch 3−1806 : Selenium on Latent−transforming growth factor beta−binding protein 4

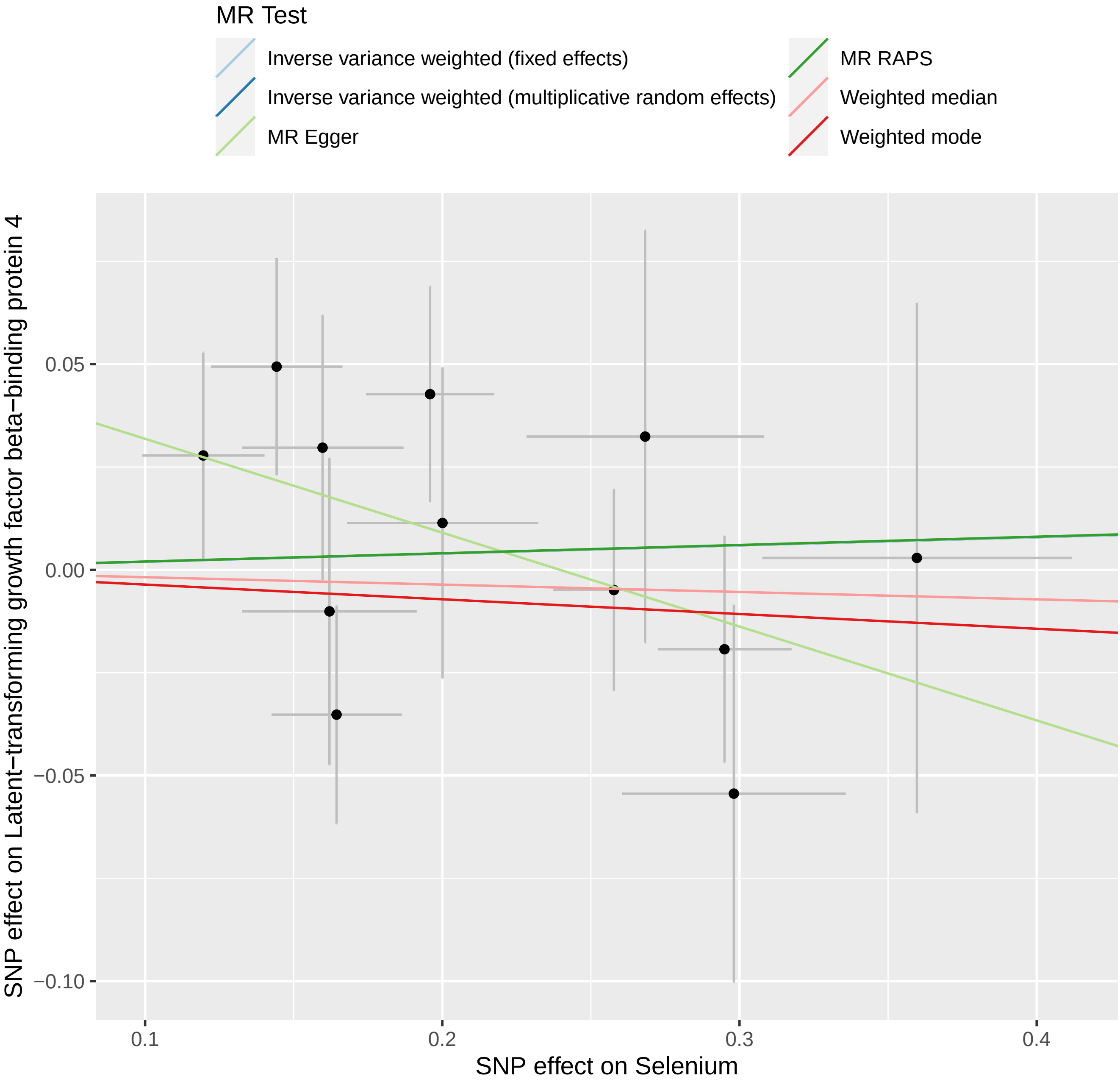

Batch 3−1807 : Selenium on Tumor necrosis factor receptor superfamily member 3

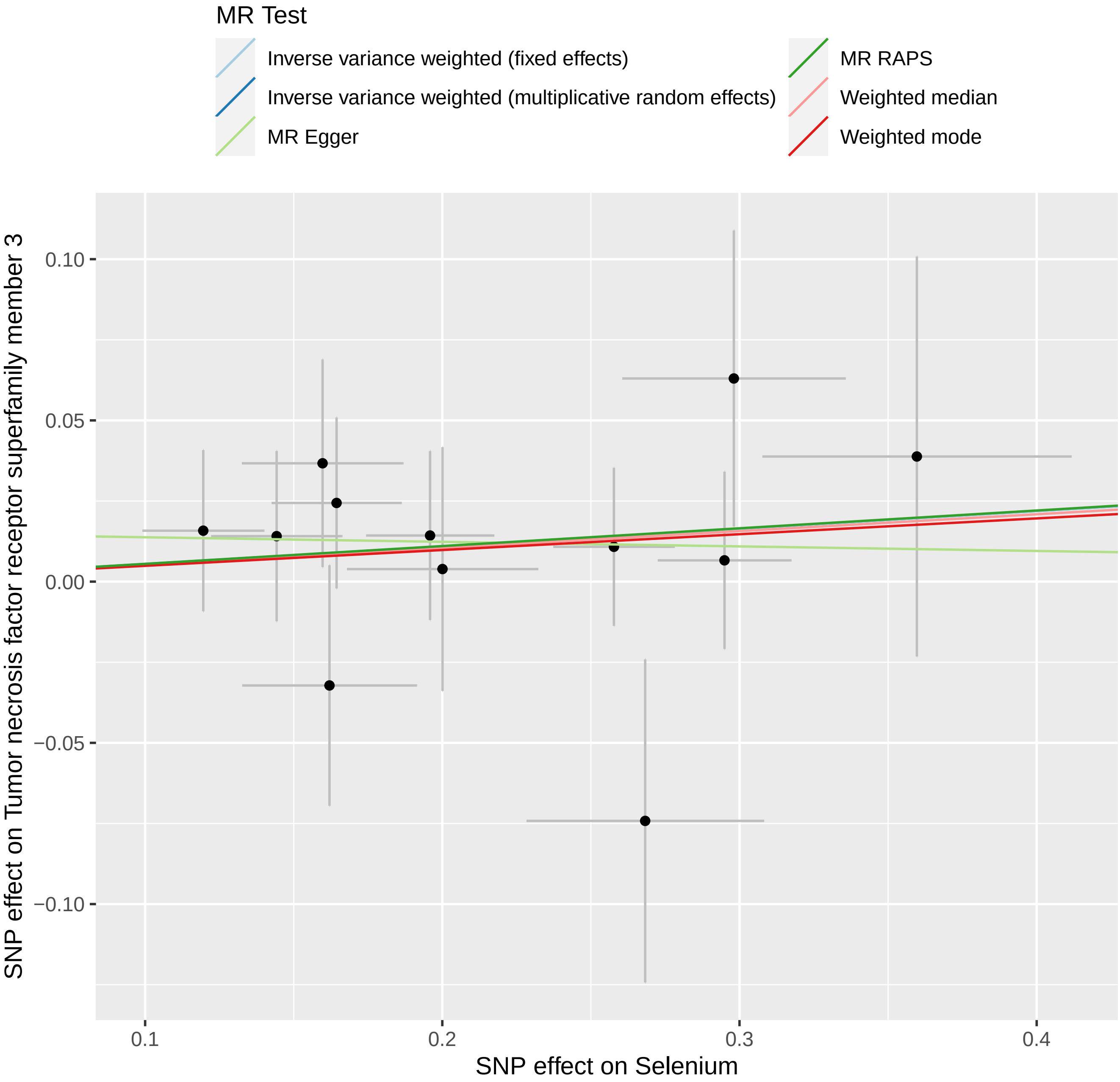

Batch 3−1808 : Selenium on Lactotransferrin

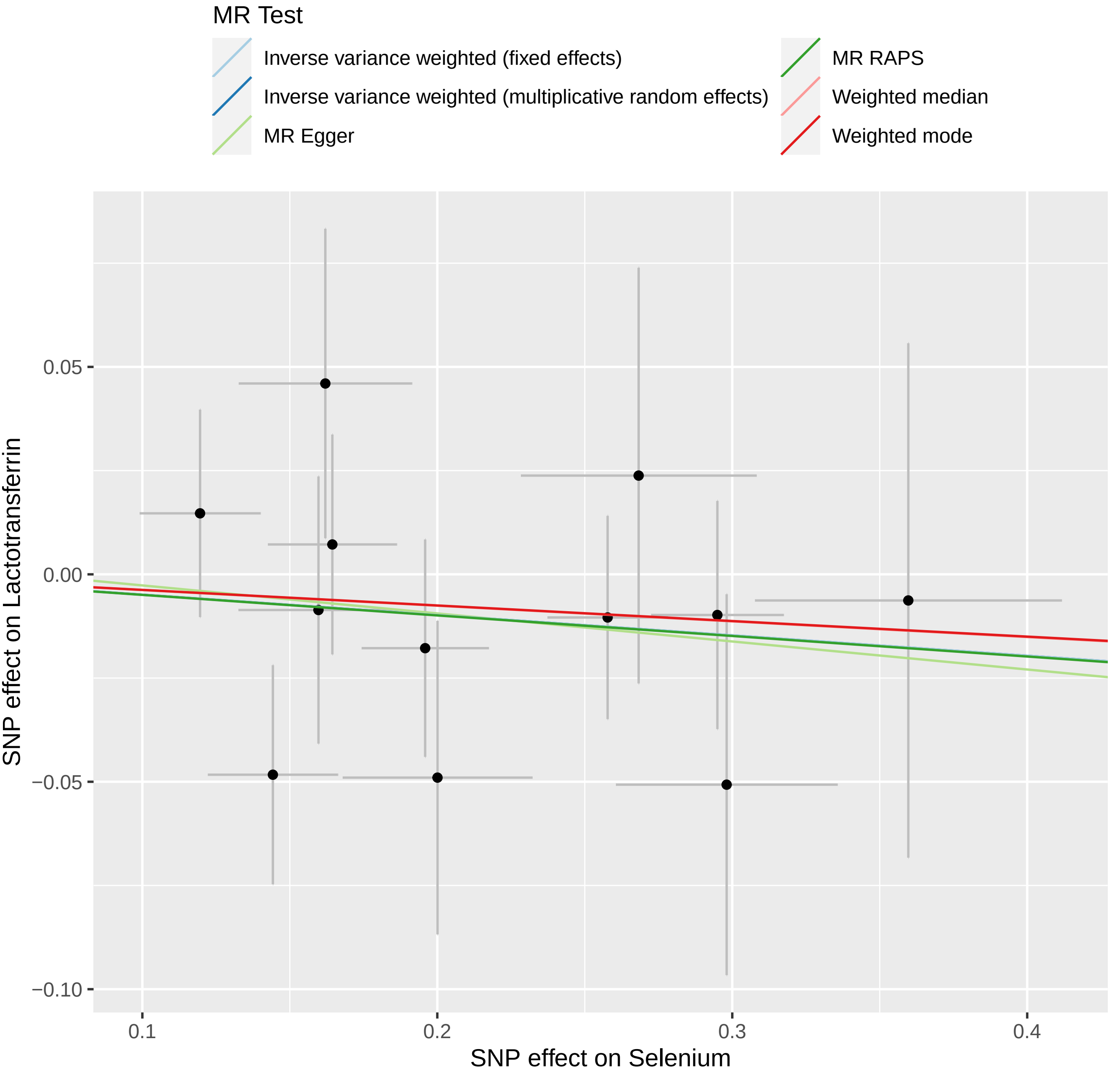

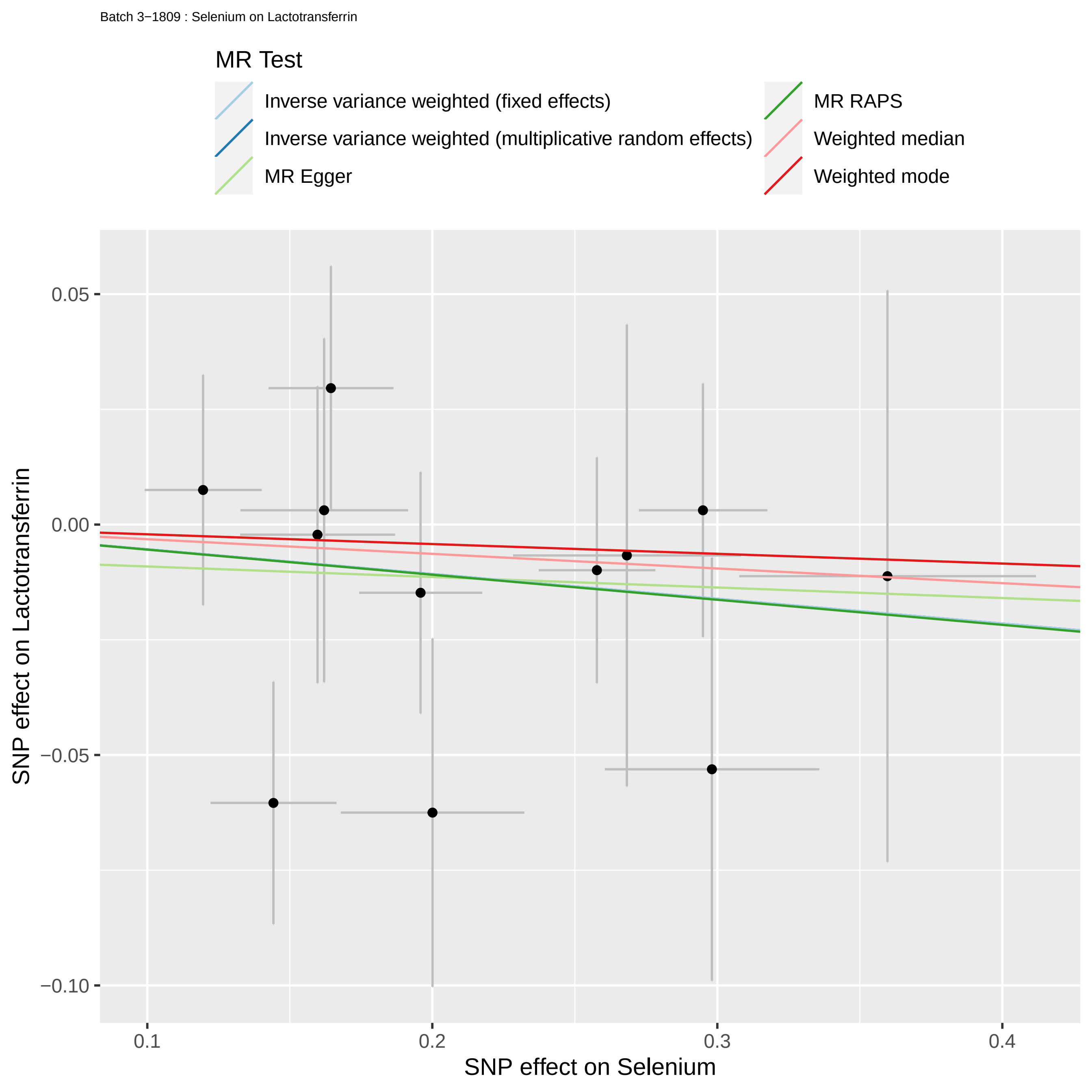

Batch 3−1809 : Selenium on Lactotransferrin
MR Test
Inverse variance weighted (fixed effects)
Inverse variance weighted (multiplicative random effects)
MR Egger
MR RAPS
Weighted median
Weighted mode
0.05
0.00
−0.05
−0.10
0.1
0.2
0.3
0.4
SNP effect on Lactotransferrin
SNP effect on Selenium

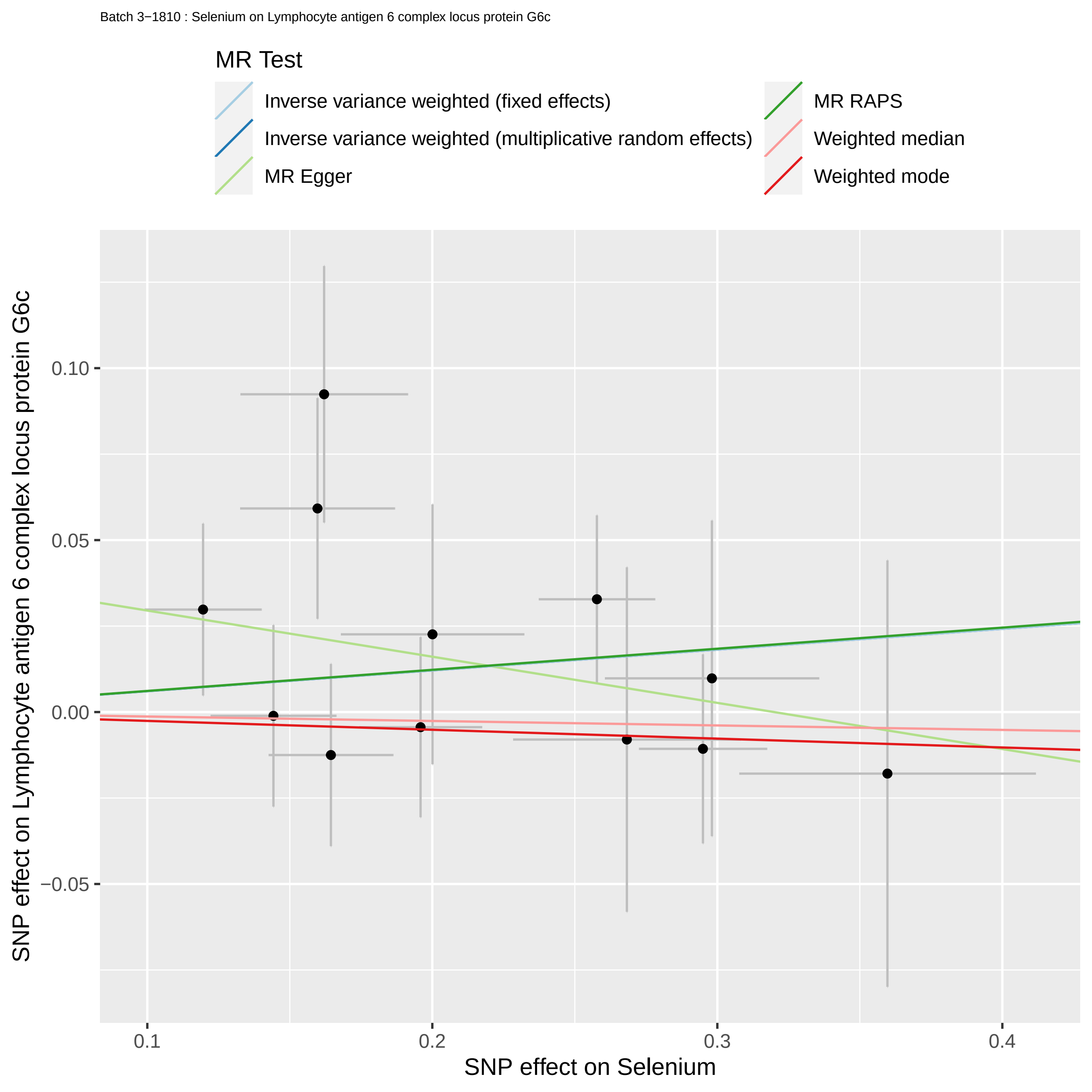

Batch 3−1810 : Selenium on Lymphocyte antigen 6 complex locus protein G6c
MR Test
Inverse variance weighted (fixed effects)
Inverse variance weighted (multiplicative random effects)
MR Egger
MR RAPS
Weighted median
Weighted mode
0.10
0.05
0.00
−0.05
0.1
0.2
0.3
0.4
SNP effect on Lymphocyte antigen 6 complex locus protein G6c
SNP effect on Selenium

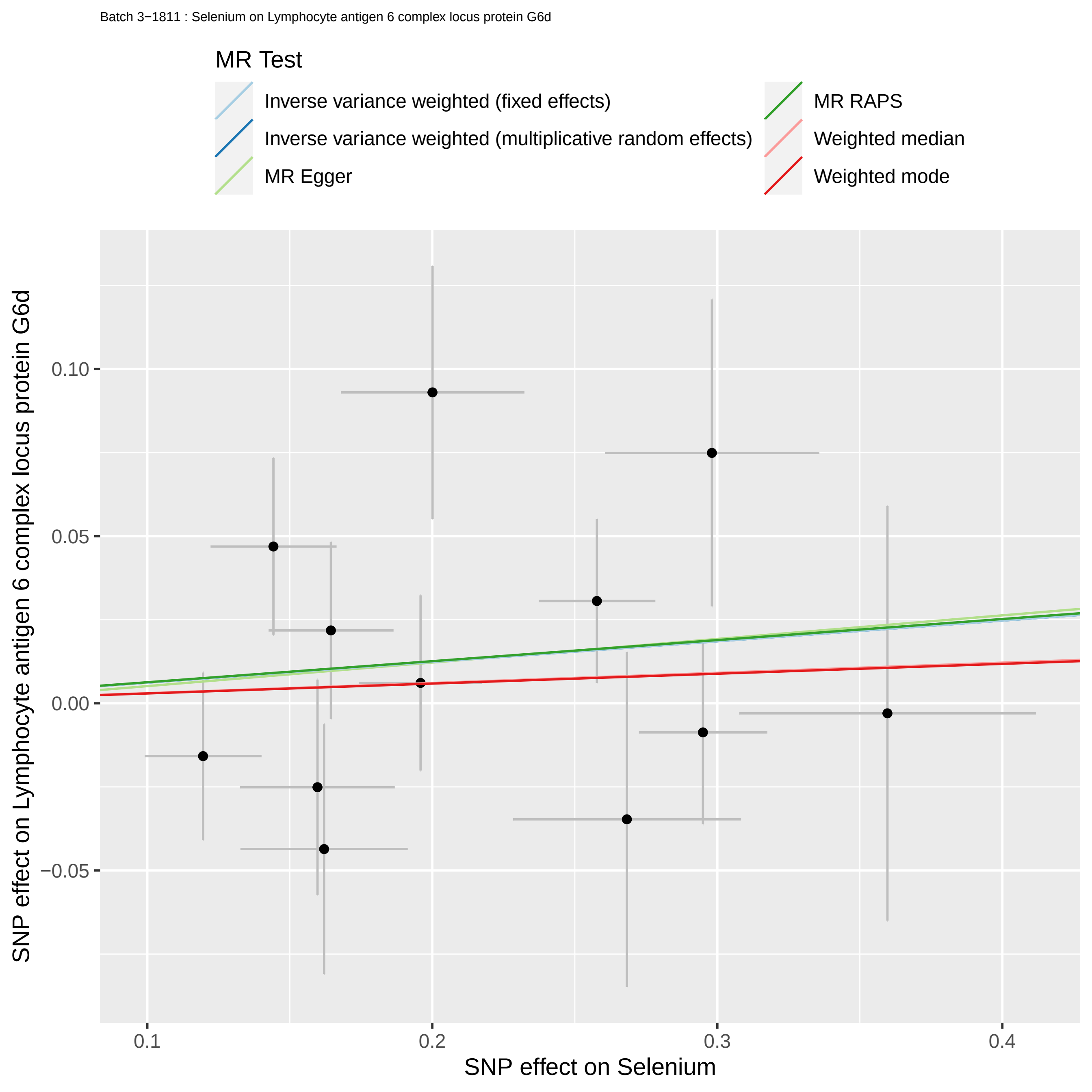
Batch 3−1811 : Selenium on Lymphocyte antigen 6 complex locus protein G6d
MR Test
Inverse variance weighted (fixed effects)
Inverse variance weighted (multiplicative random effects)
MR Egger
MR RAPS
Weighted median
Weighted mode
SNP effect on Lymphocyte antigen 6 complex locus protein G6d
0.10
0.05
0.00
−0.05
0.1
0.2
0.3
0.4
SNP effect on Selenium

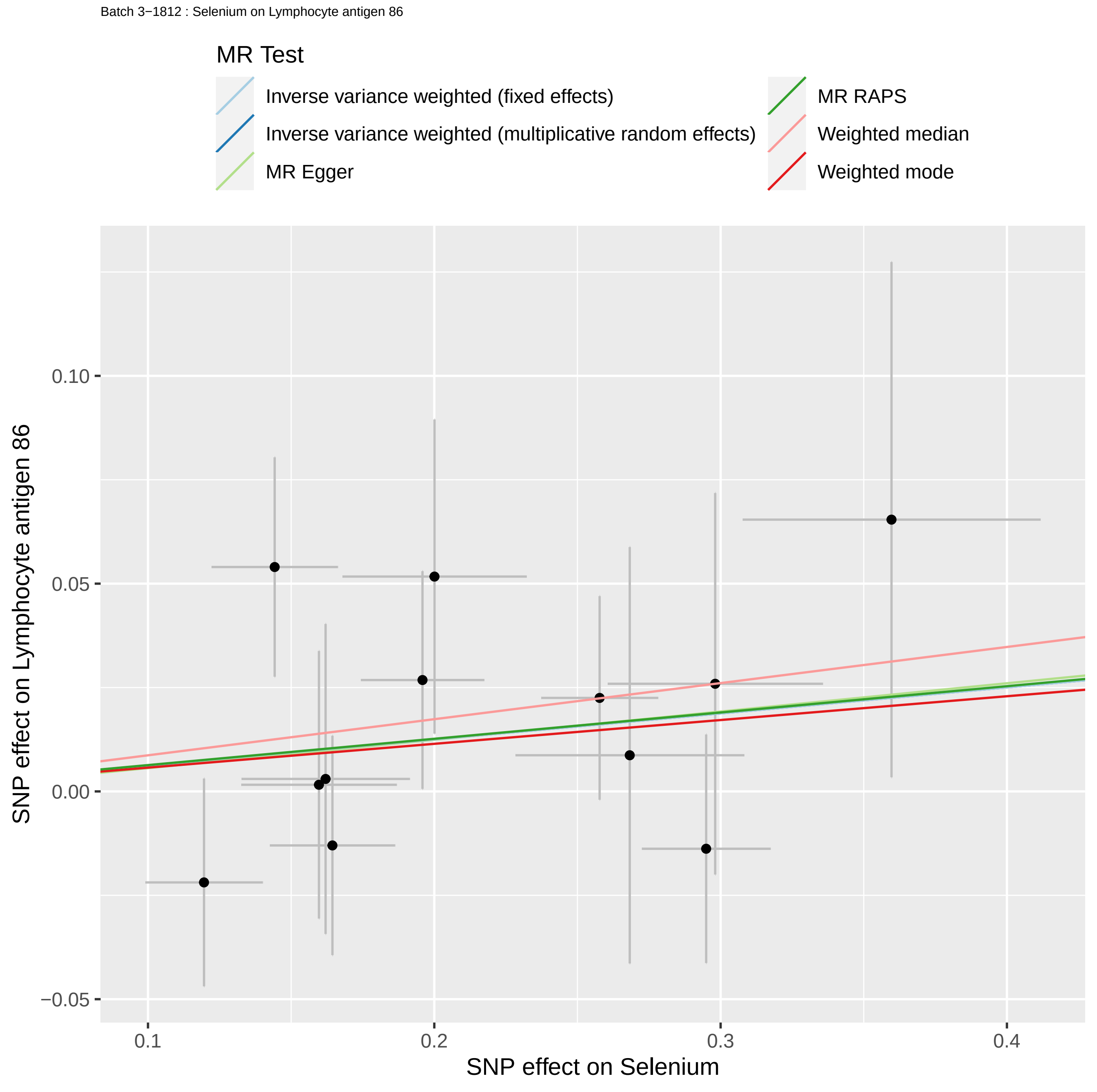

Batch 3−1812 : Selenium on Lymphocyte antigen 86
MR Test
Inverse variance weighted (fixed effects)
Inverse variance weighted (multiplicative random effects)
MR Egger
MR RAPS
Weighted median
Weighted mode
0.10
0.05
0.00
−0.05
0.1
0.2
0.3
0.4
SNP effect on Lymphocyte antigen 86
SNP effect on Selenium

Batch 3−1813 : Selenium on T−lymphocyte surface antigen Ly−9

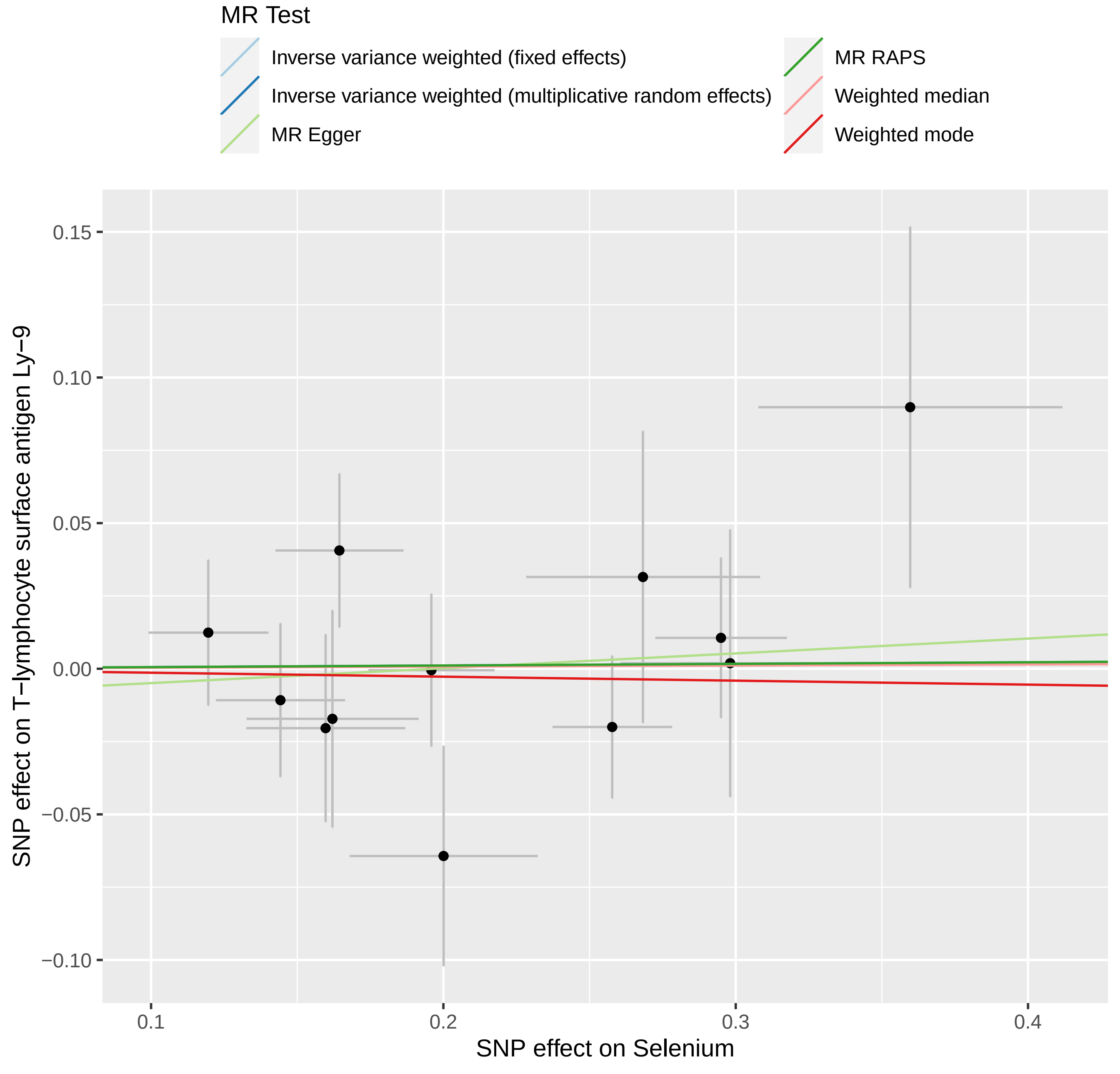

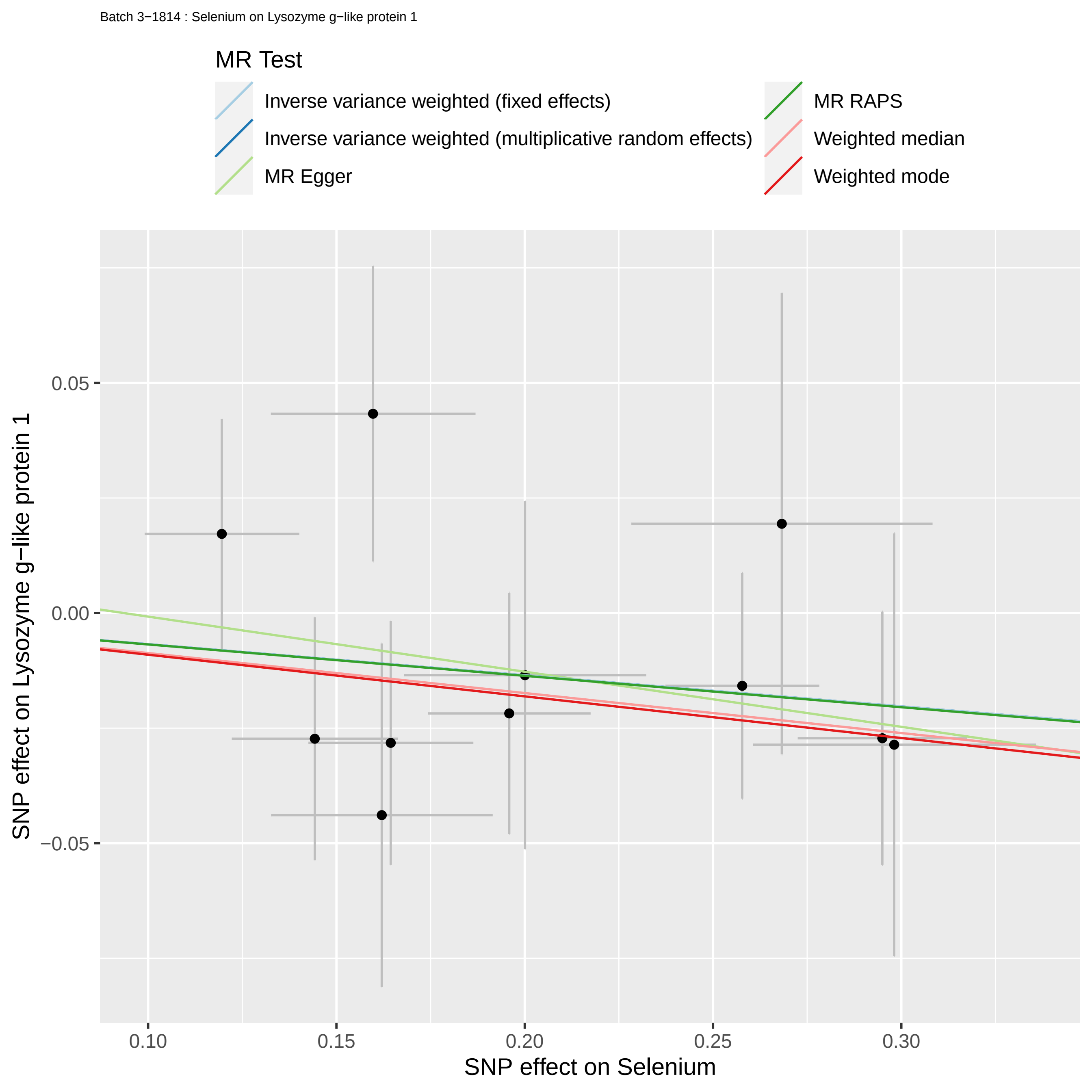
Batch 3−1814 : Selenium on Lysozyme g−like protein 1
MR Test
Inverse variance weighted (fixed effects)
Inverse variance weighted (multiplicative random effects)
MR Egger
MR RAPS
Weighted median
Weighted mode
0.05
0.00
−0.05
0.10
0.15
0.20
0.25
0.30
SNP effect on Lysozyme g−like protein 1
SNP effect on Selenium

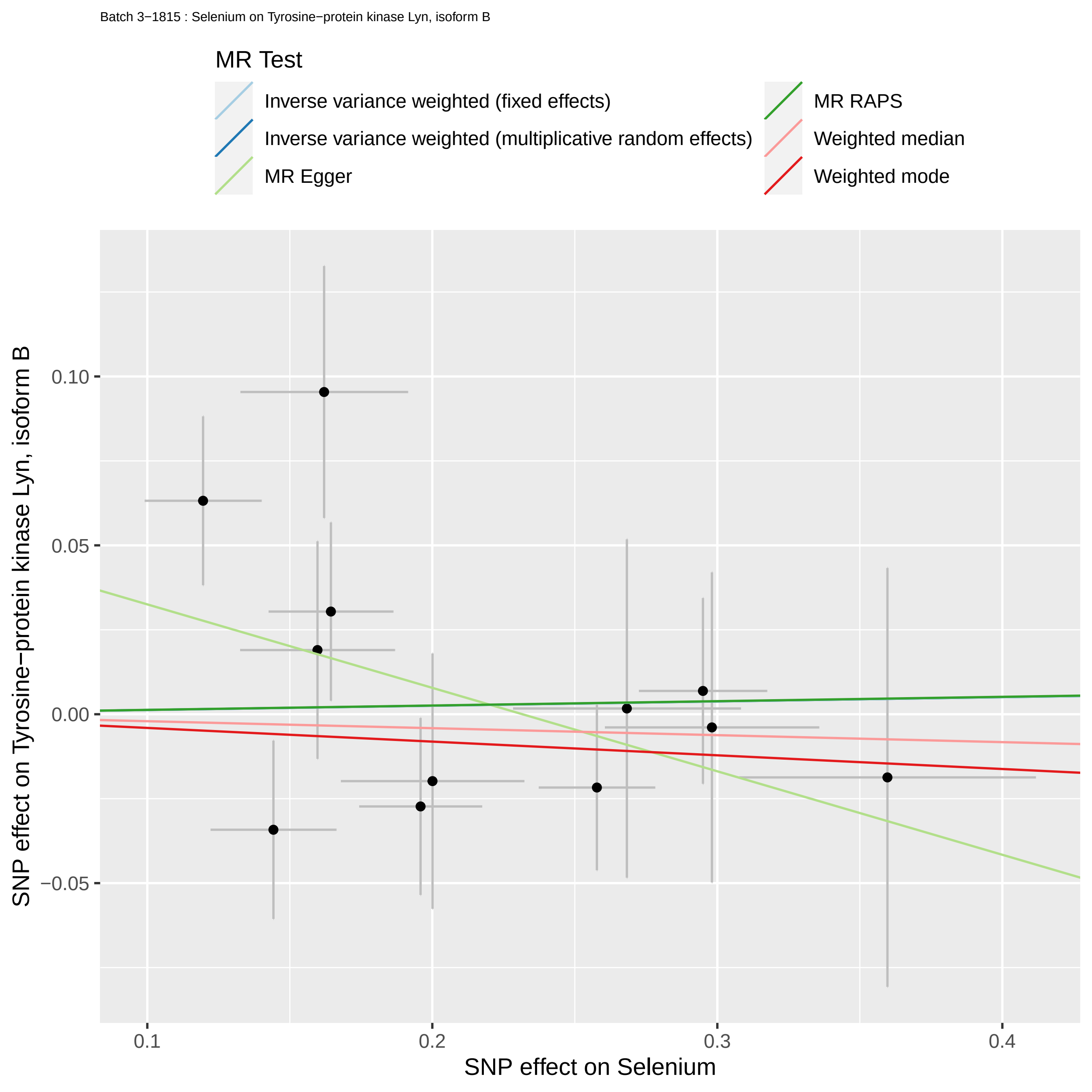

Batch 3−1815 : Selenium on Tyrosine−protein kinase Lyn, isoform B
MR Test
Inverse variance weighted (fixed effects)
Inverse variance weighted (multiplicative random effects)
MR Egger
MR RAPS
Weighted median
Weighted mode
SNP effect on Tyrosine−protein kinase Lyn, isoform B
0.10
0.05
0.00
−0.05
0.1
0.2
0.3
0.4
SNP effect on Selenium

Batch 3−1816 : Selenium on Tyrosine−protein kinase Lyn

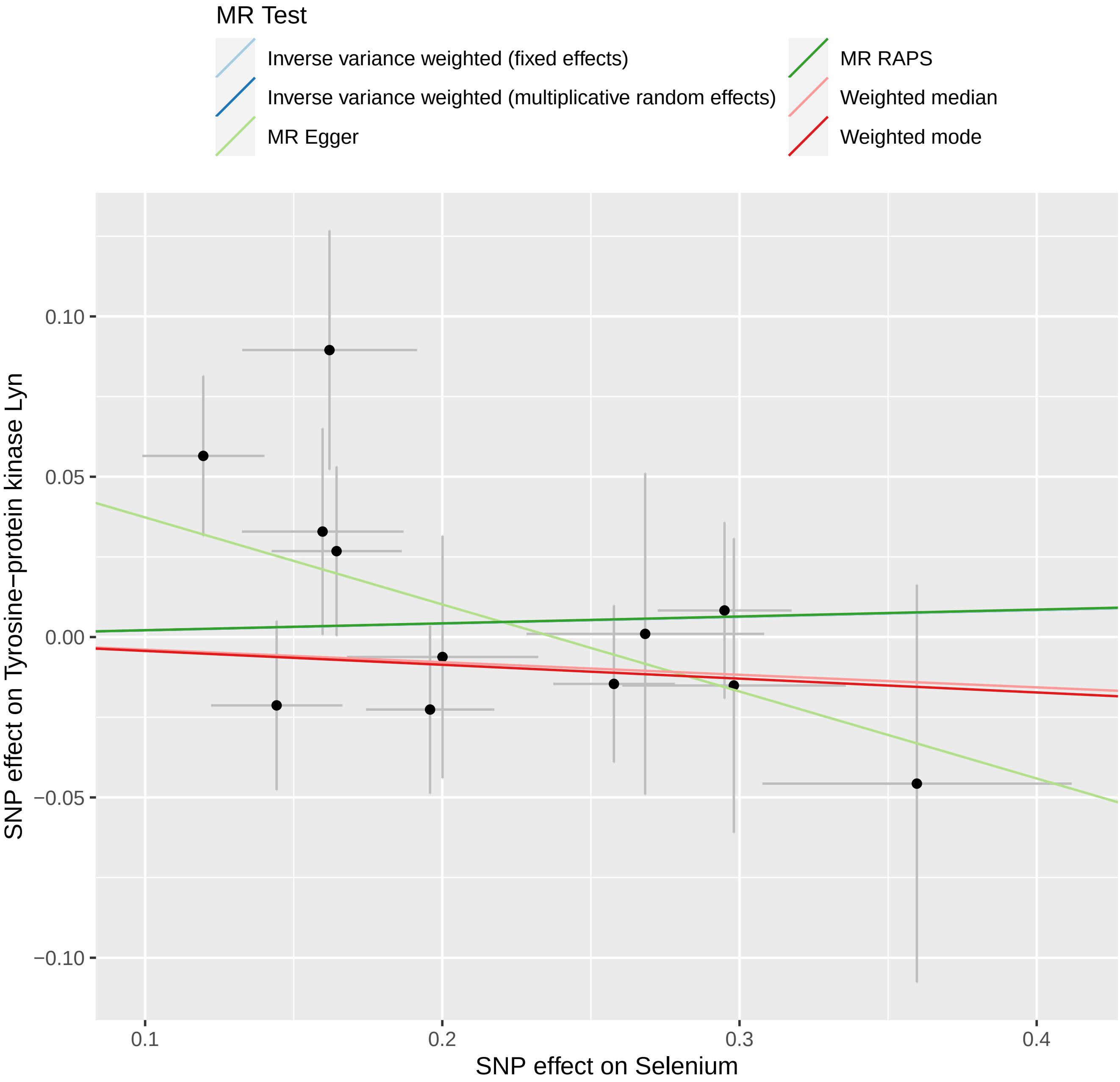

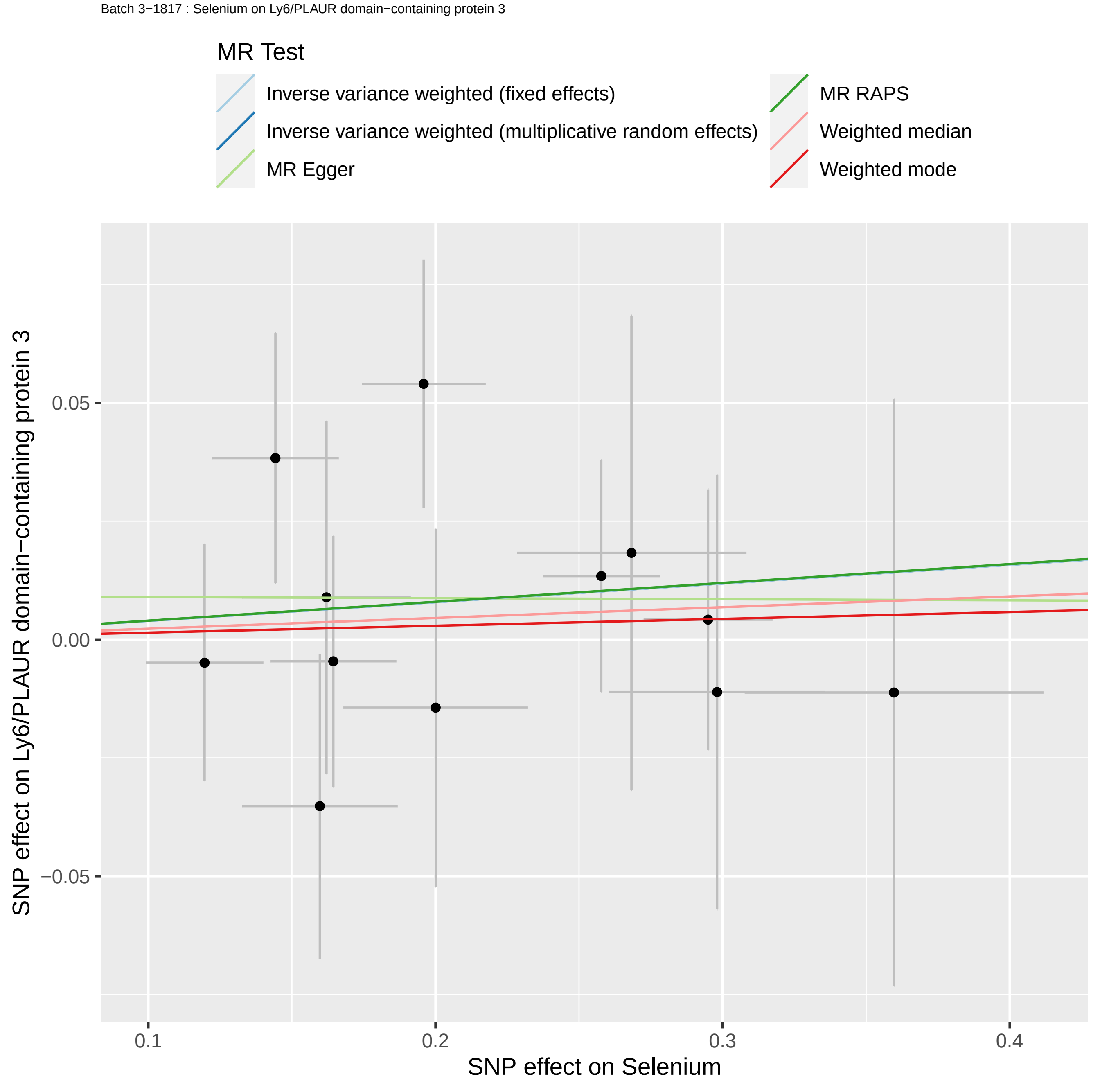

Batch 3−1817 : Selenium on Ly6/PLAUR domain−containing protein 3
MR Test
Inverse variance weighted (fixed effects)
Inverse variance weighted (multiplicative random effects)
MR Egger
MR RAPS
Weighted median
Weighted mode
0.05
0.00
−0.05
0.1
0.2
0.3
0.4
SNP effect on Selenium
SNP effect on Ly6/PLAUR domain−containing protein 3

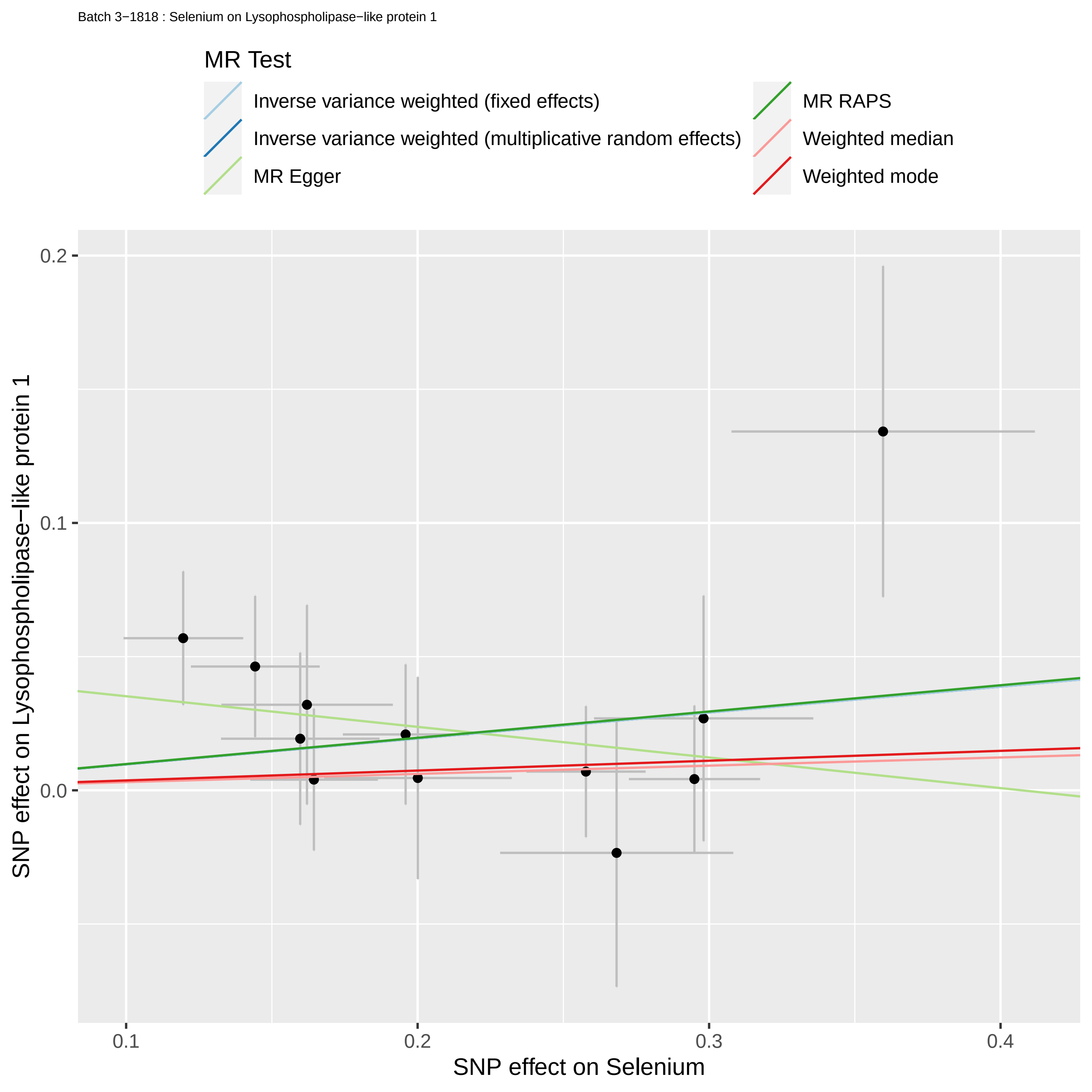

Batch 3−1818 : Selenium on Lysophospholipase−like protein 1
MR Test
Inverse variance weighted (fixed effects)
Inverse variance weighted (multiplicative random effects)
MR Egger
MR RAPS
Weighted median
Weighted mode
SNP effect on Lysophospholipase−like protein 1
SNP effect on Selenium
0.2
0.1
0.0
0.1
0.2
0.3
0.4

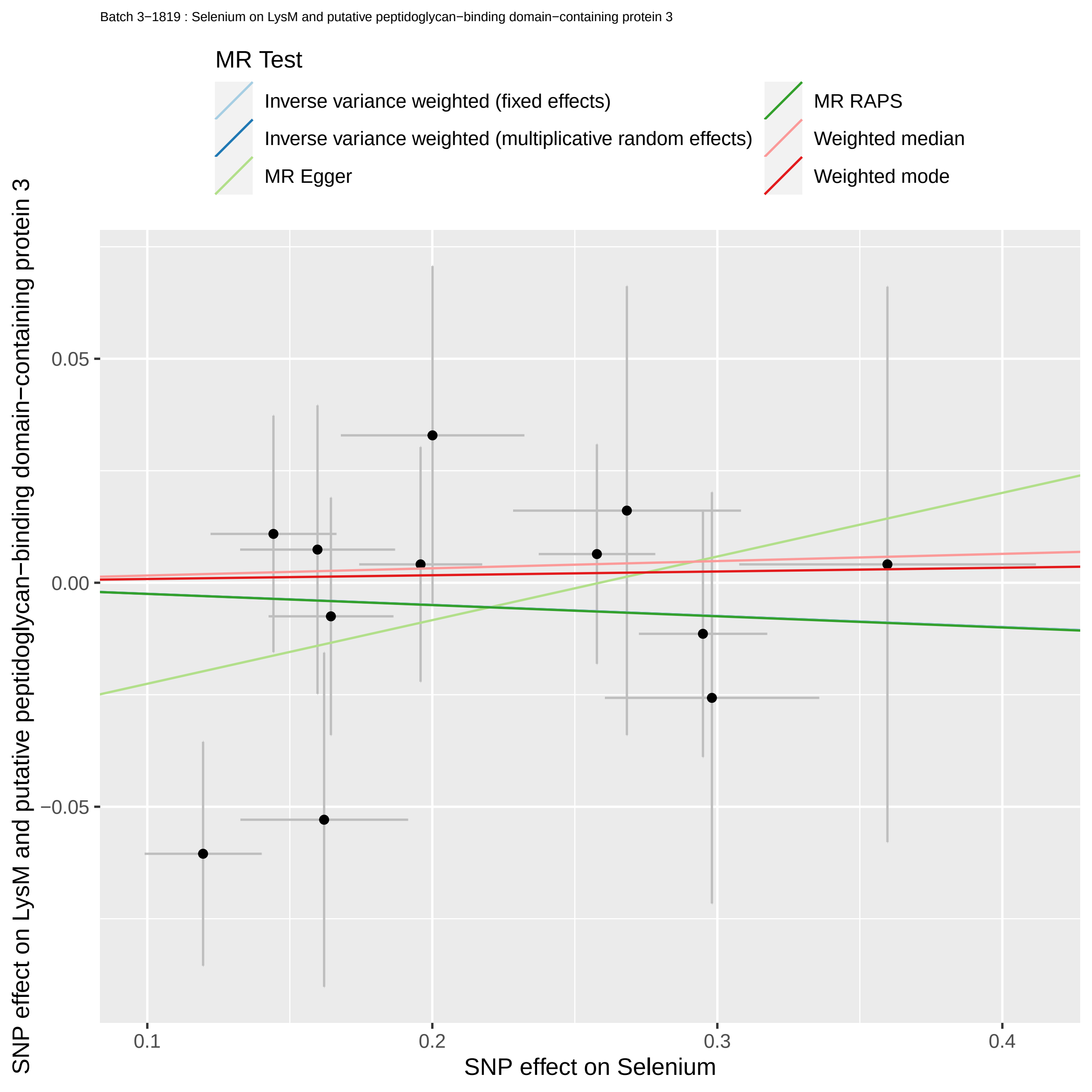
Batch 3−1819 : Selenium on LysM and putative peptidoglycan−binding domain−containing protein 3
MR Test
Inverse variance weighted (fixed effects)
Inverse variance weighted (multiplicative random effects)
MR Egger
MR RAPS
Weighted median
Weighted mode
SNP effect on LysM and putative peptidoglycan−binding domain−containing protein 3
0.05
0.00
−0.05
0.1
0.2
0.3
0.4
SNP effect on Selenium

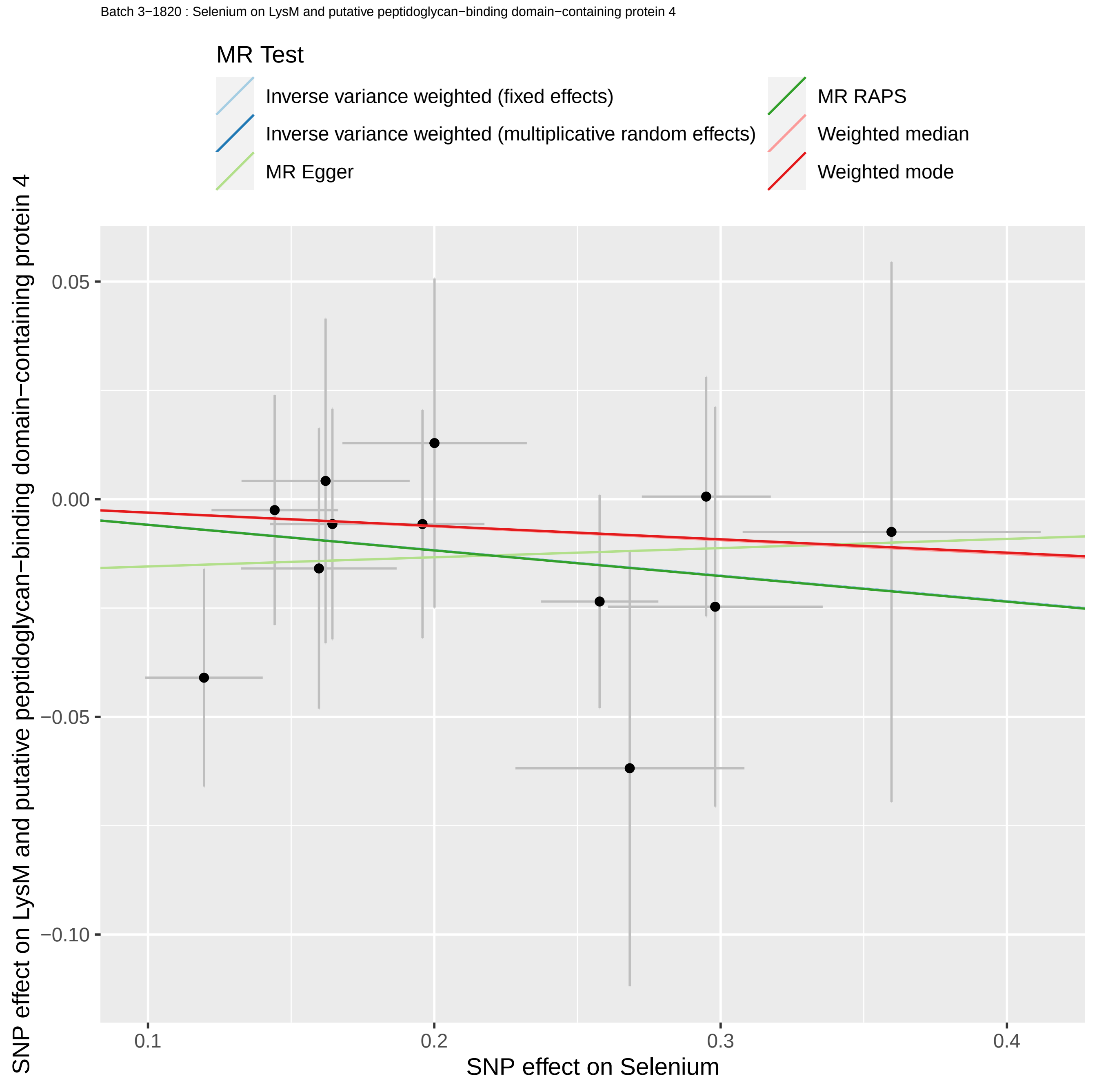
Batch 3−1820 : Selenium on LysM and putative peptidoglycan−binding domain−containing protein 4
MR Test
Inverse variance weighted (fixed effects)
Inverse variance weighted (multiplicative random effects)
MR Egger
MR RAPS
Weighted median
Weighted mode
SNP effect on LysM and putative peptidoglycan−binding domain−containing protein 4
0.05
0.00
−0.05
−0.10
0.1
0.2
0.3
0.4
SNP effect on Selenium

Batch 3−1821 : Selenium on Lysozyme C

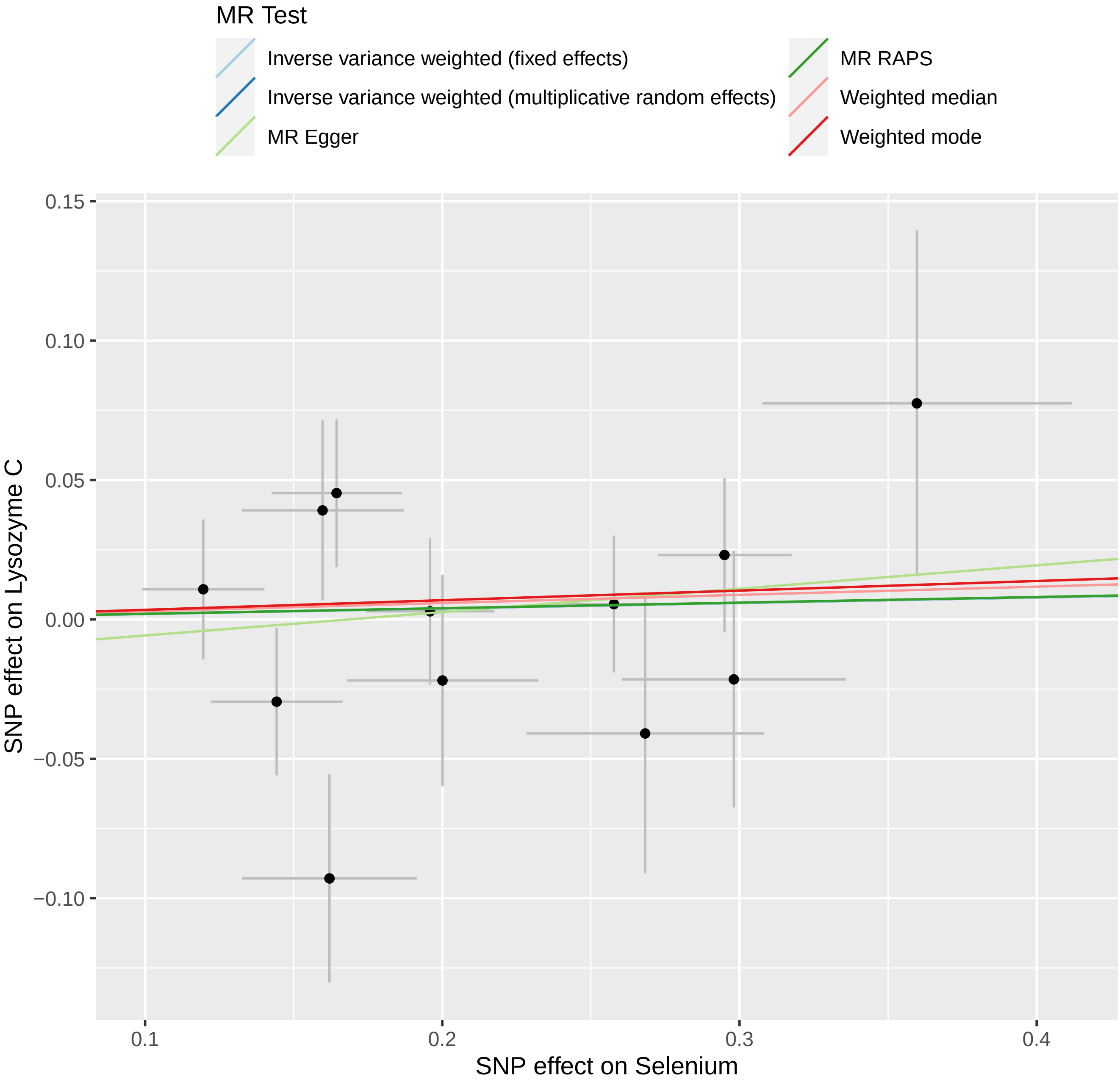

Batch 3−1822 : Selenium on Lysozyme−like protein 2

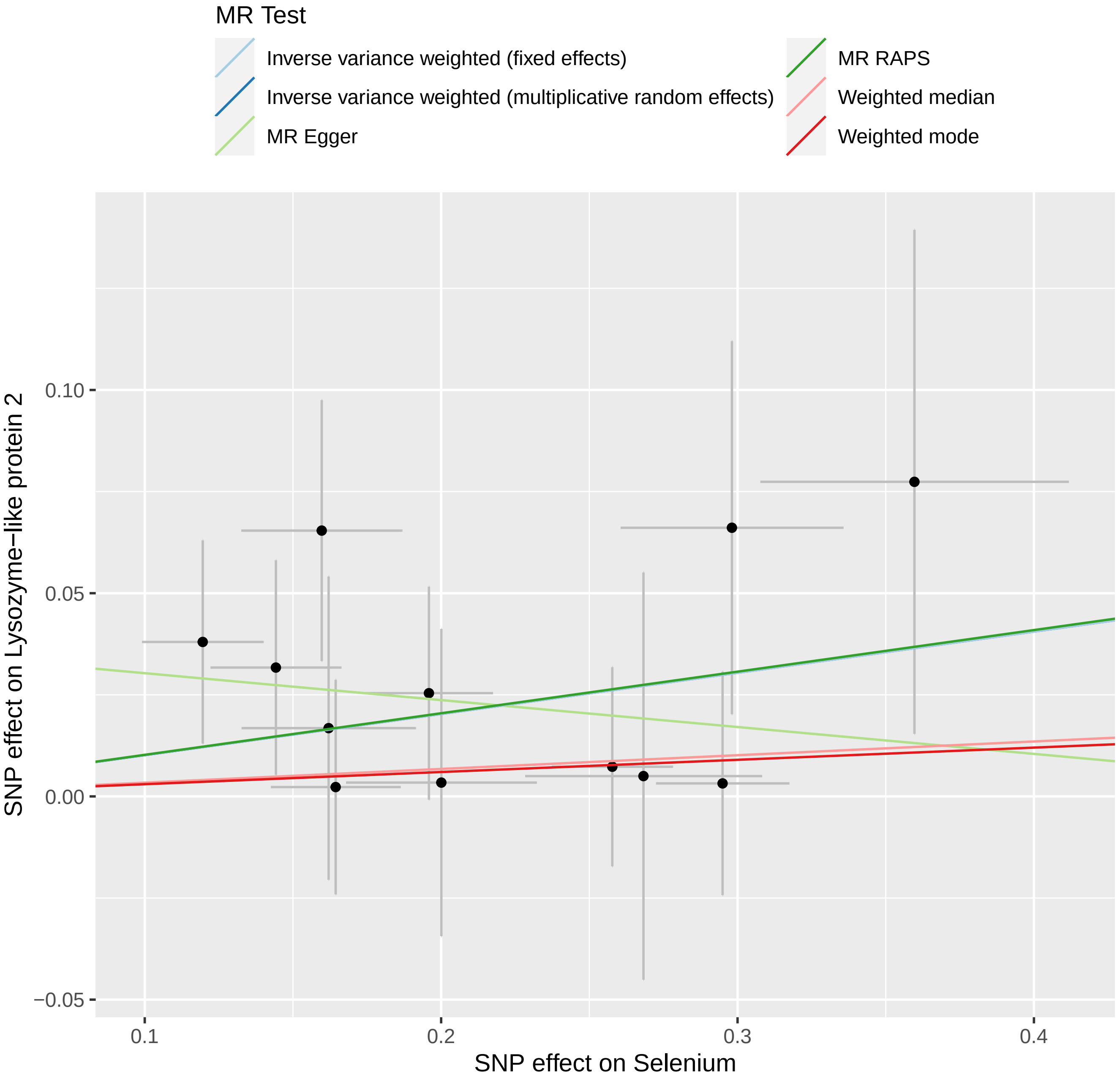

Batch 3−1823 : Selenium on Cation−dependent mannose−6−phosphate receptor

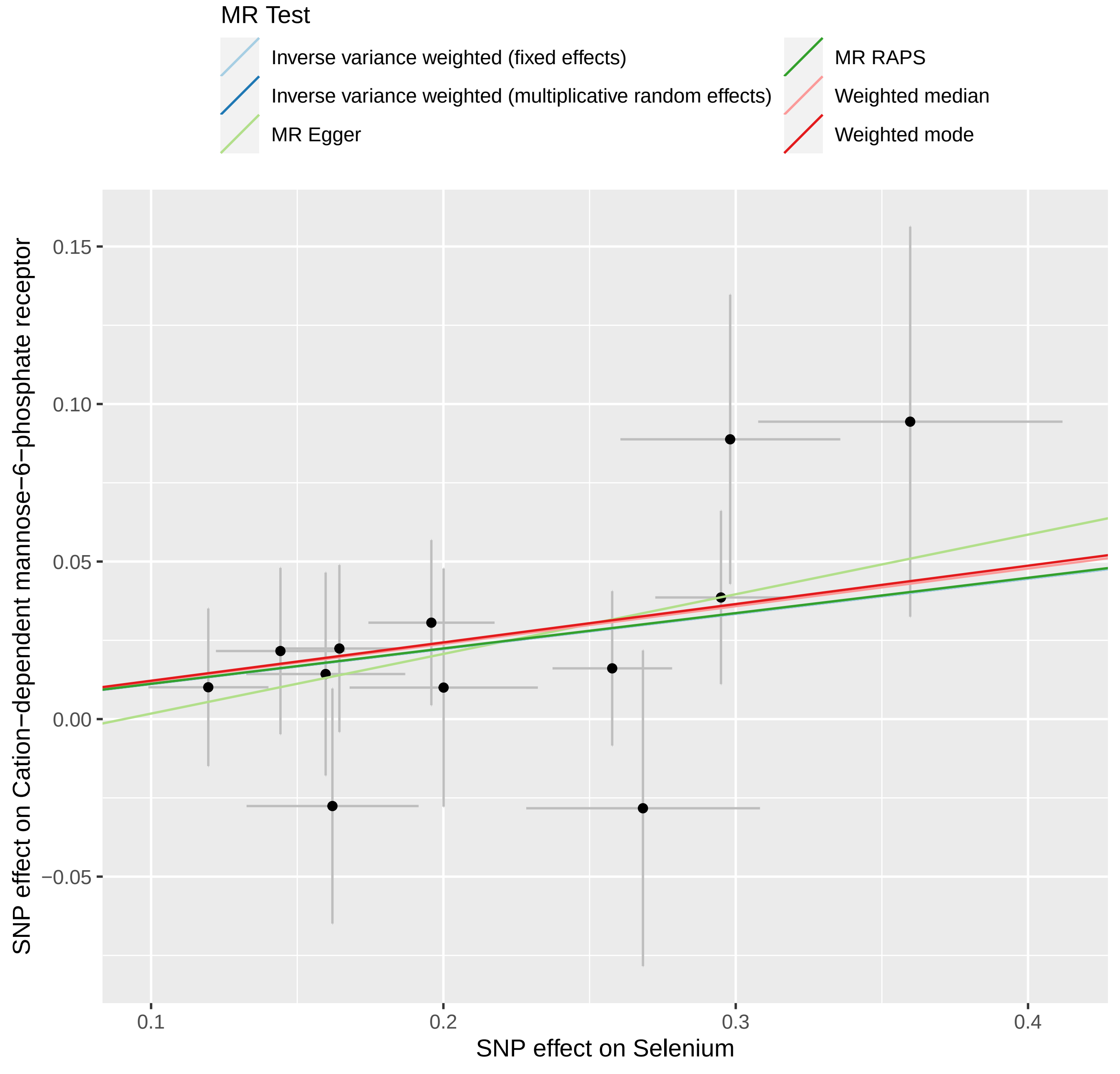

Batch 3−1824 : Selenium on O−acetyl−ADP−ribose deacetylase MACROD1

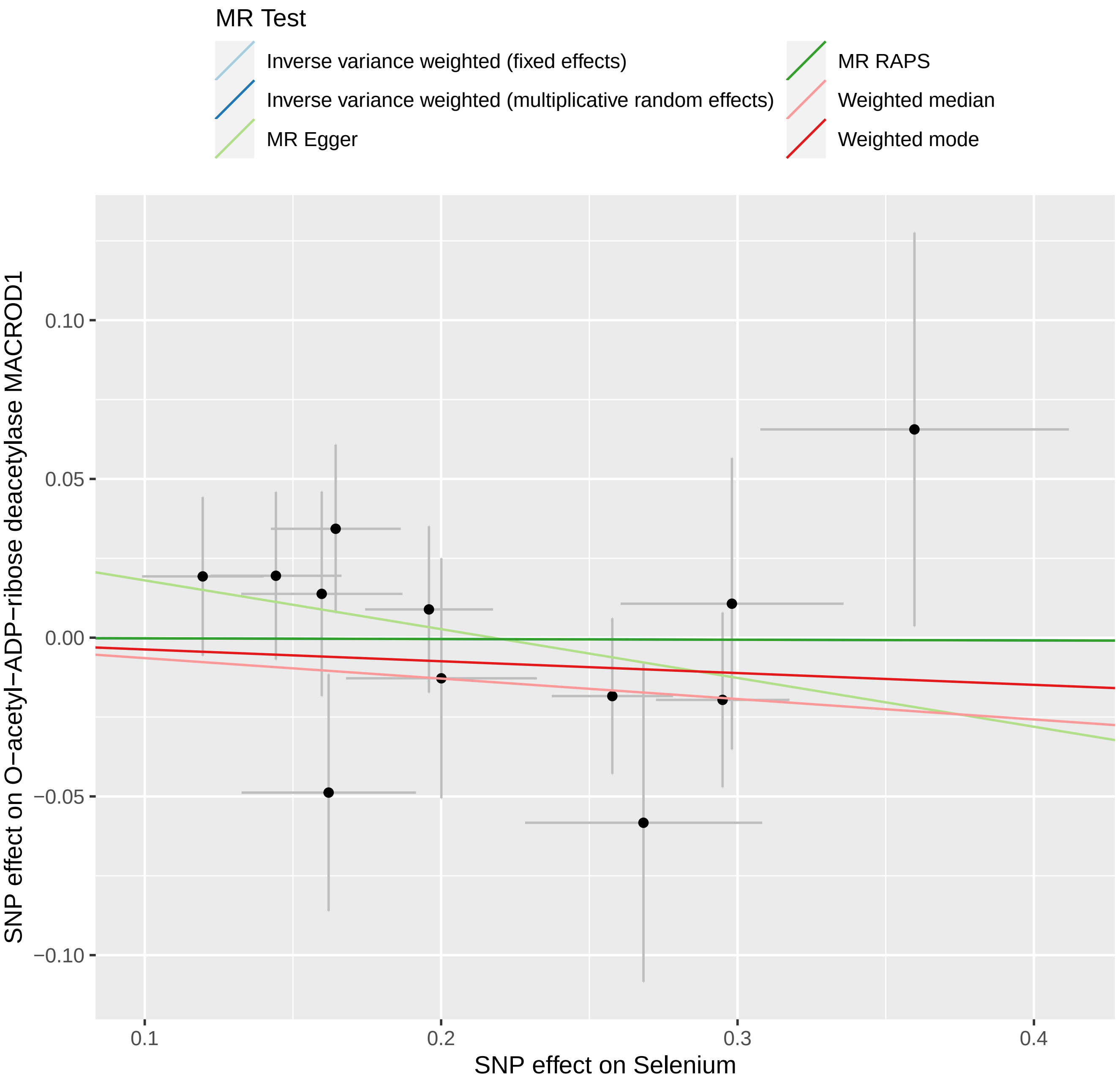

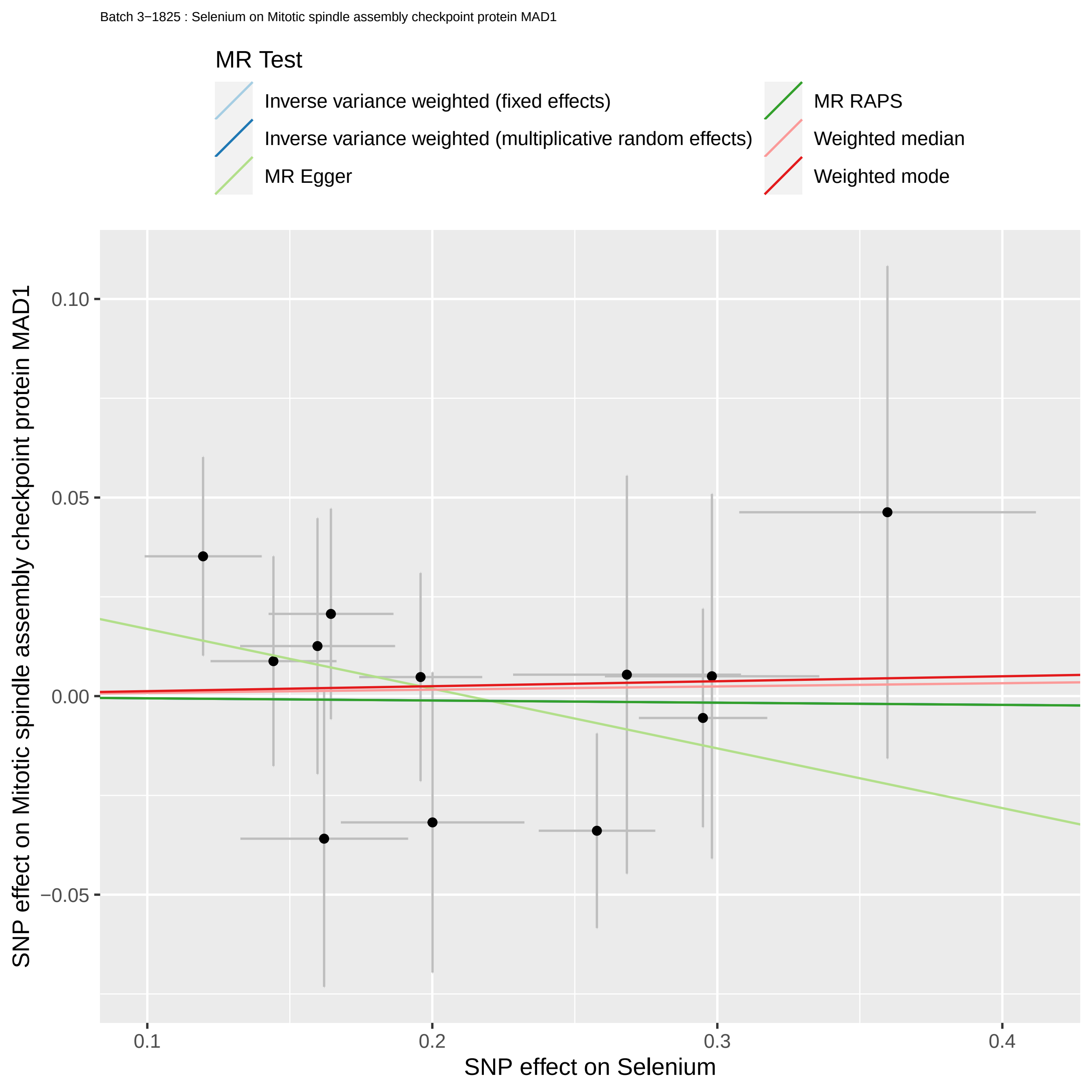

Batch 3−1825 : Selenium on Mitotic spindle assembly checkpoint protein MAD1
MR Test
Inverse variance weighted (fixed effects)
Inverse variance weighted (multiplicative random effects)
MR Egger
MR RAPS
Weighted median
Weighted mode
0.10
0.05
0.00
−0.05
0.1
0.2
0.3
0.4
SNP effect on Mitotic spindle assembly checkpoint protein MAD1
SNP effect on Selenium

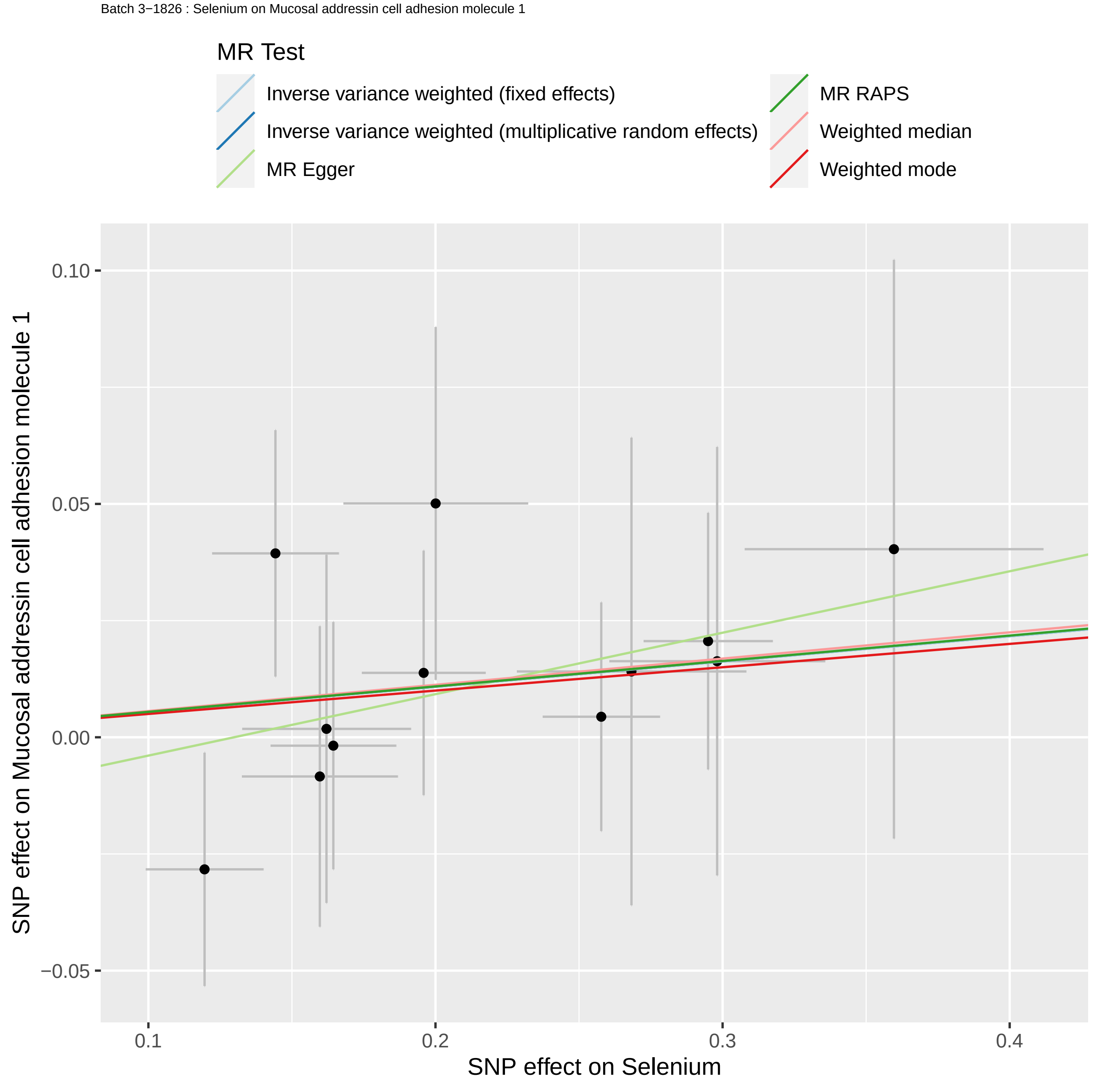
Batch 3−1826 : Selenium on Mucosal addressin cell adhesion molecule 1
MR Test
Inverse variance weighted (fixed effects)
Inverse variance weighted (multiplicative random effects)
MR Egger
MR RAPS
Weighted median
Weighted mode
0.10
0.05
0.00
−0.05
0.1
0.2
0.3
0.4
SNP effect on Selenium
SNP effect on Mucosal addressin cell adhesion molecule 1

Batch 3−1827 : Selenium on Melanoma−associated antigen 10

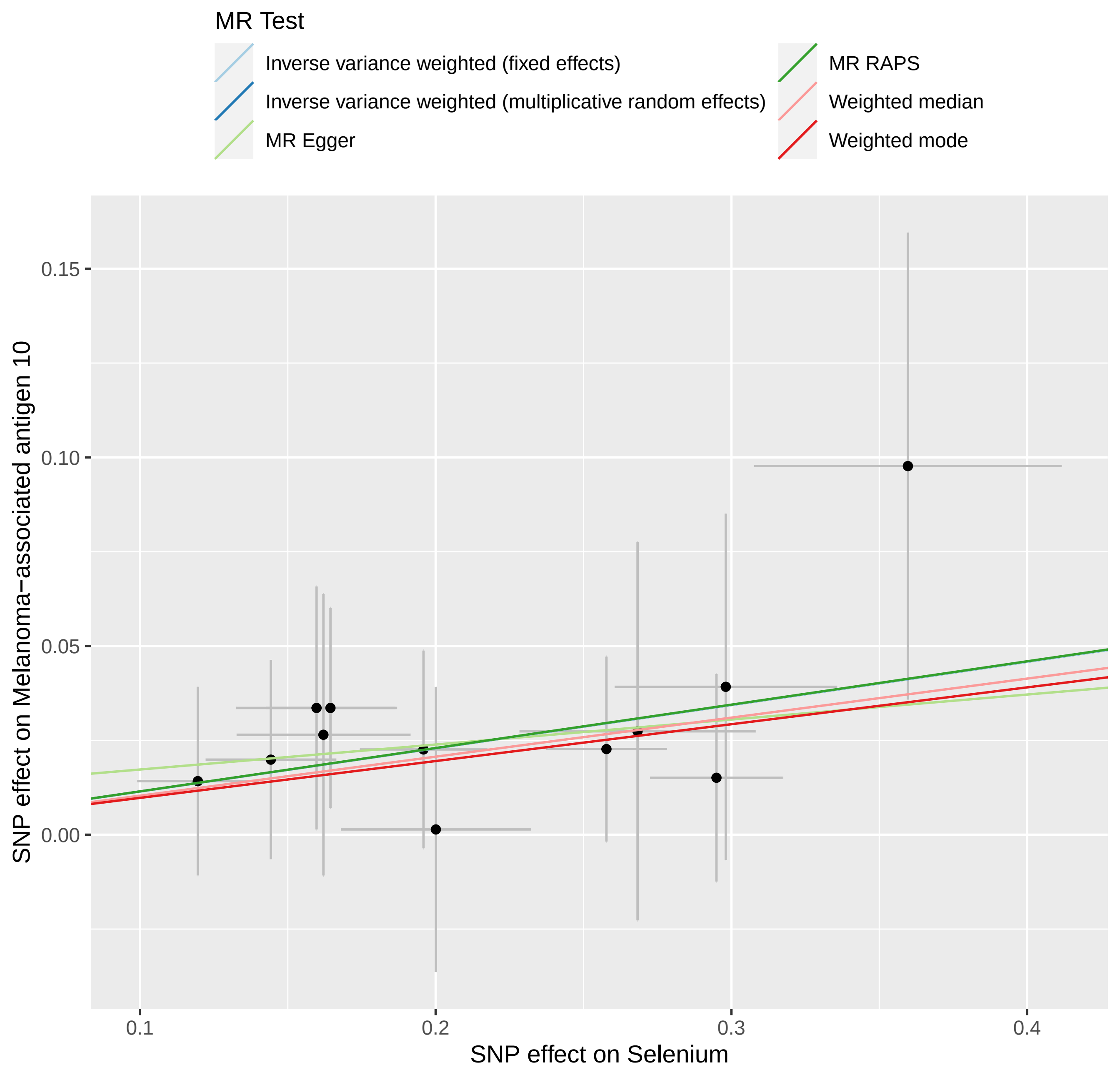

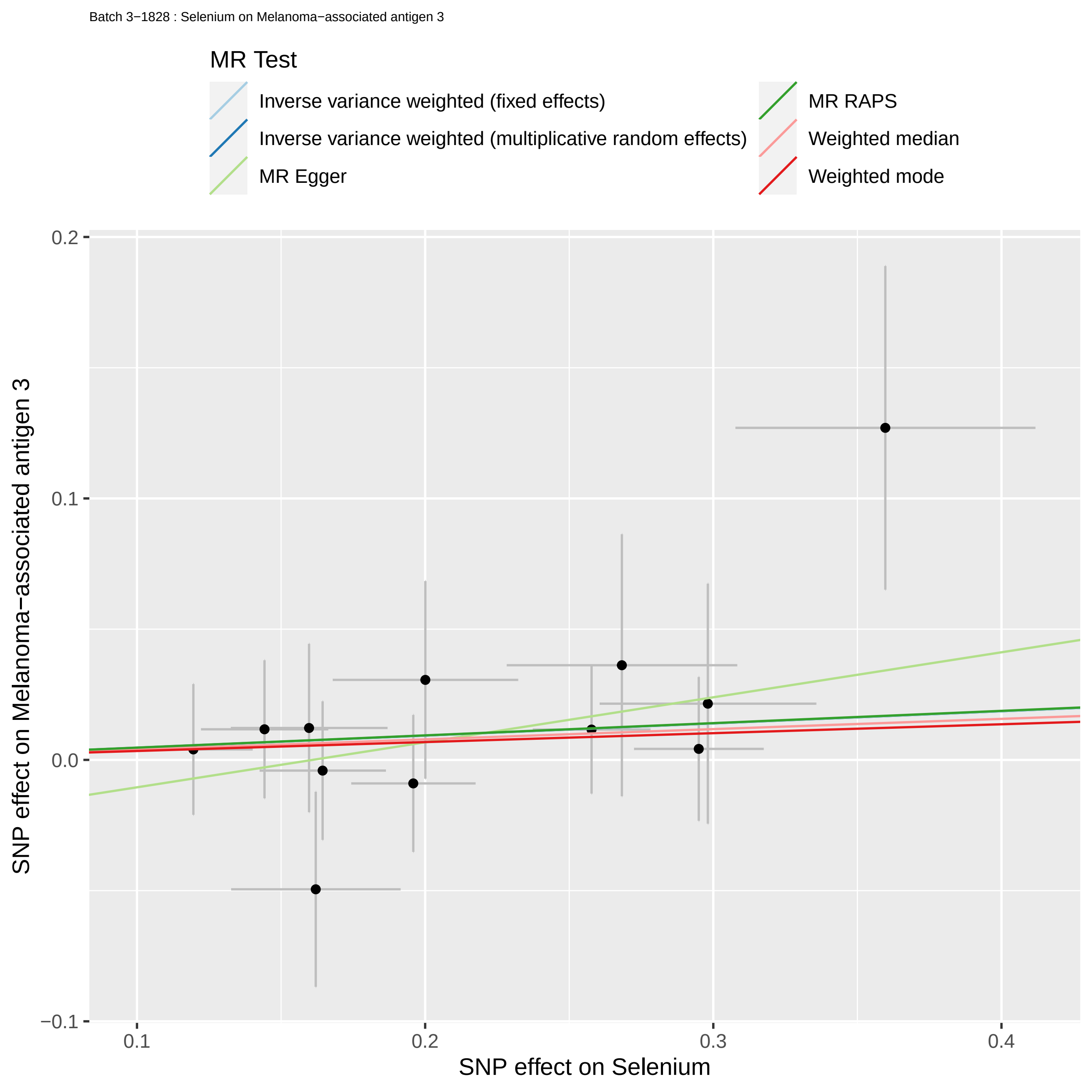
Batch 3−1828 : Selenium on Melanoma−associated antigen 3
MR Test
Inverse variance weighted (fixed effects)
Inverse variance weighted (multiplicative random effects)
MR Egger
MR RAPS
Weighted median
Weighted mode
0.2
0.1
0.0
−0.1
0.1
0.2
0.3
0.4
SNP effect on Melanoma−associated antigen 3
SNP effect on Selenium

Batch 3−1829 : Selenium on Melanoma−associated antigen B10

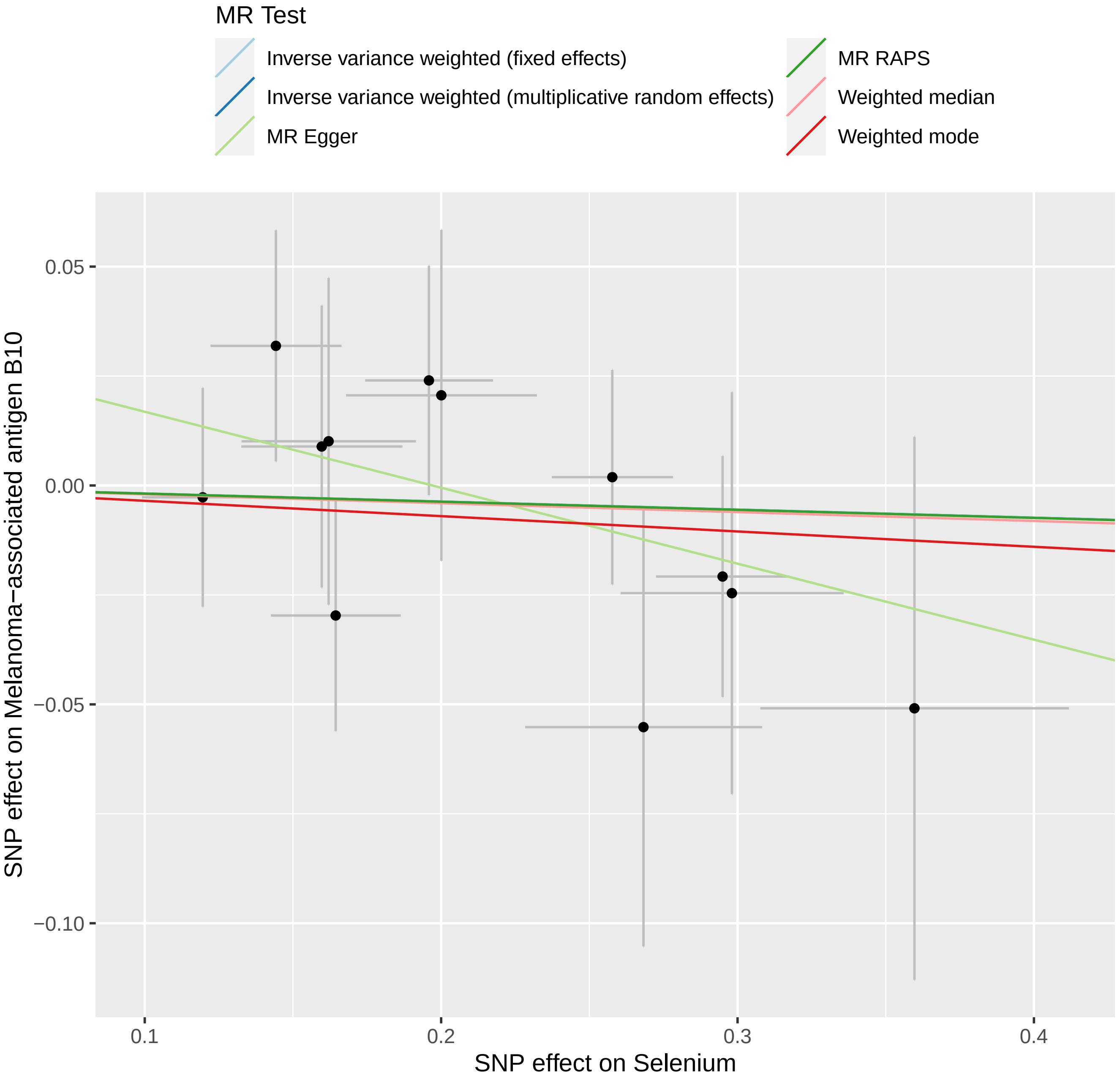

Batch 3−1830 : Selenium on Membrane−associated guanylate kinase, WW and PDZ domain−containing protein 2

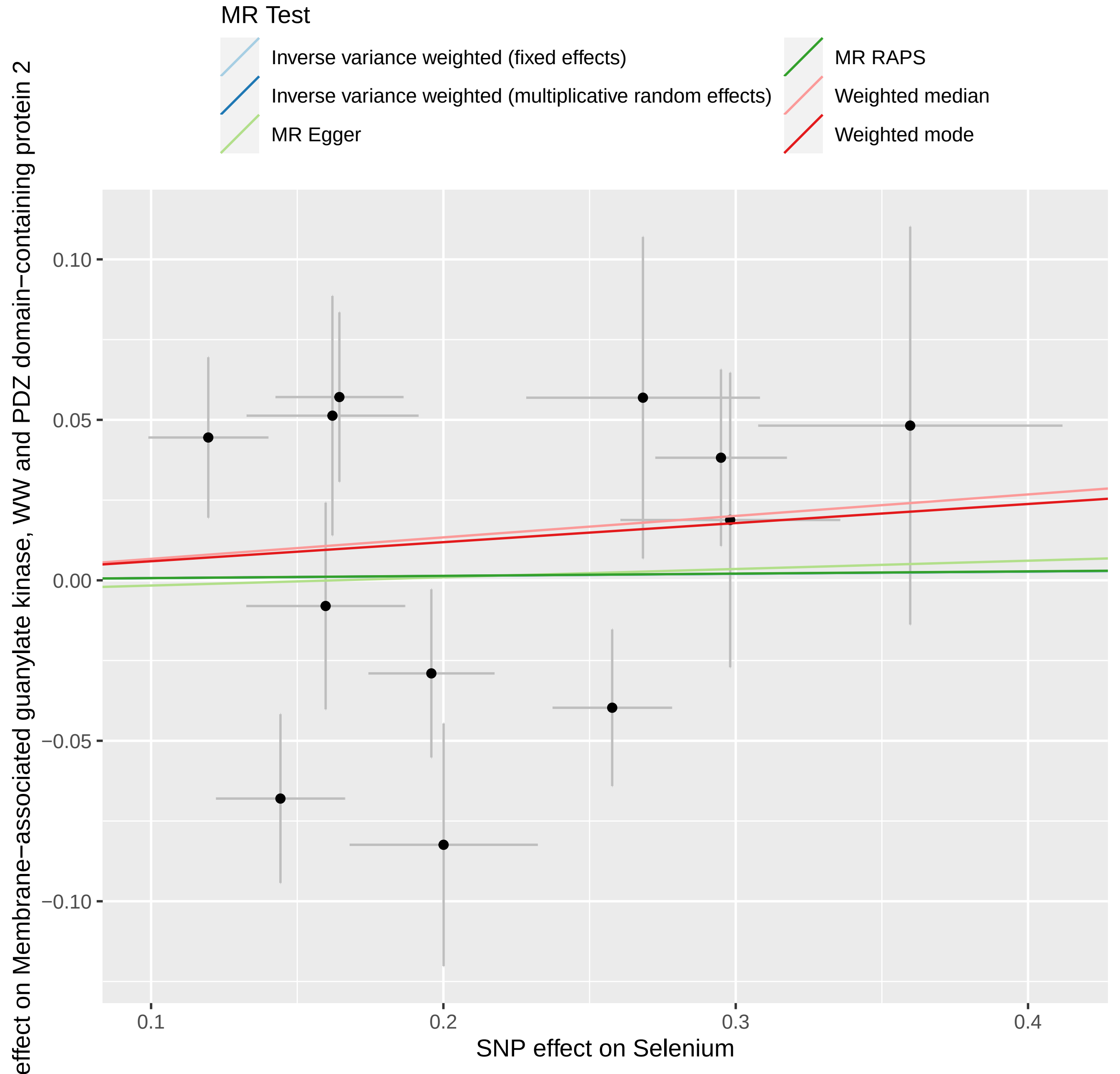

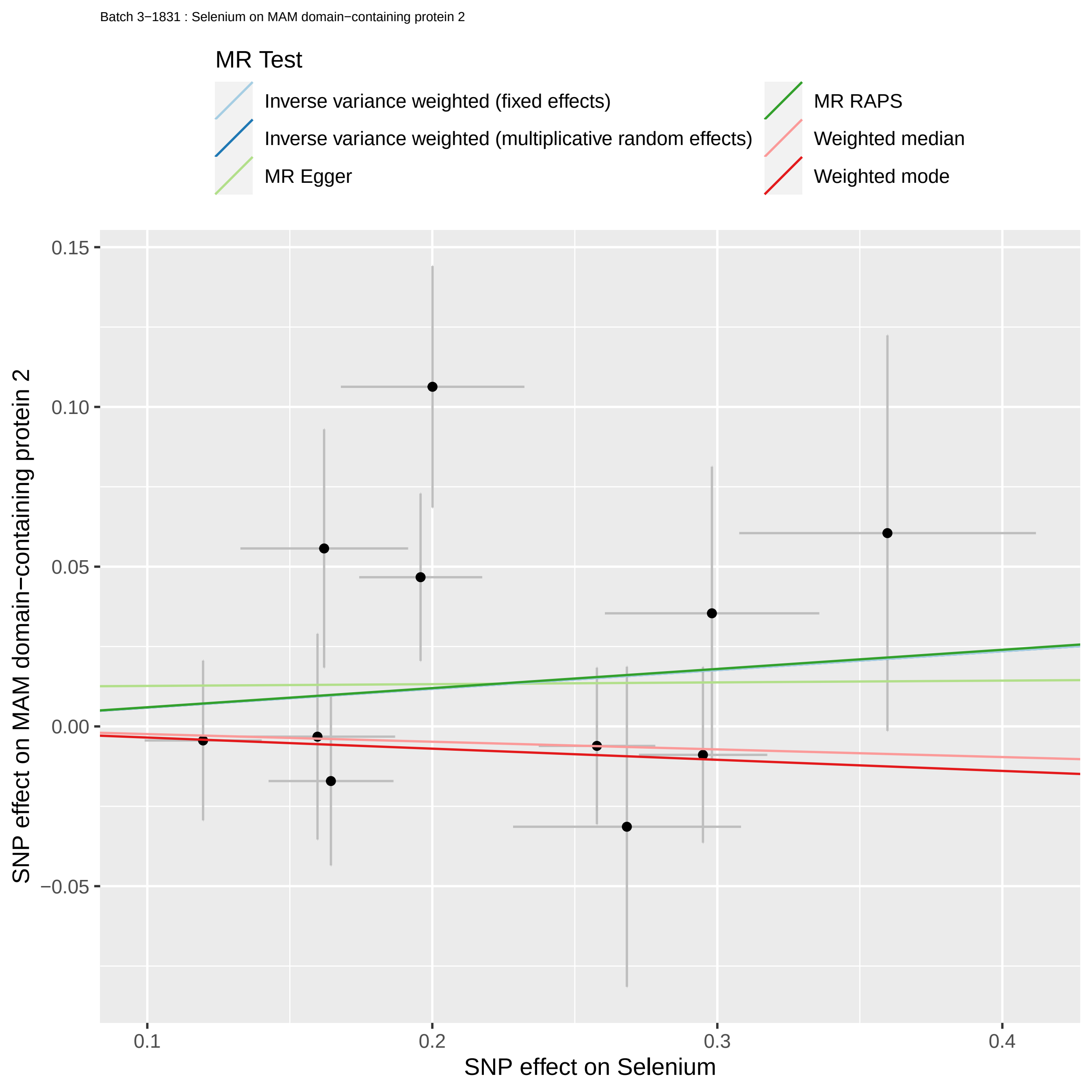
Batch 3−1831 : Selenium on MAM domain−containing protein 2
MR Test
Inverse variance weighted (fixed effects)
Inverse variance weighted (multiplicative random effects)
MR Egger
MR RAPS
Weighted median
Weighted mode
0.15
0.10
0.05
0.00
−0.05
0.1
0.2
0.3
0.4
SNP effect on MAM domain−containing protein 2
SNP effect on Selenium

Batch 3−1832 : Selenium on Mannosyl−oligosaccharide 1,2−alpha−mannosidase IA

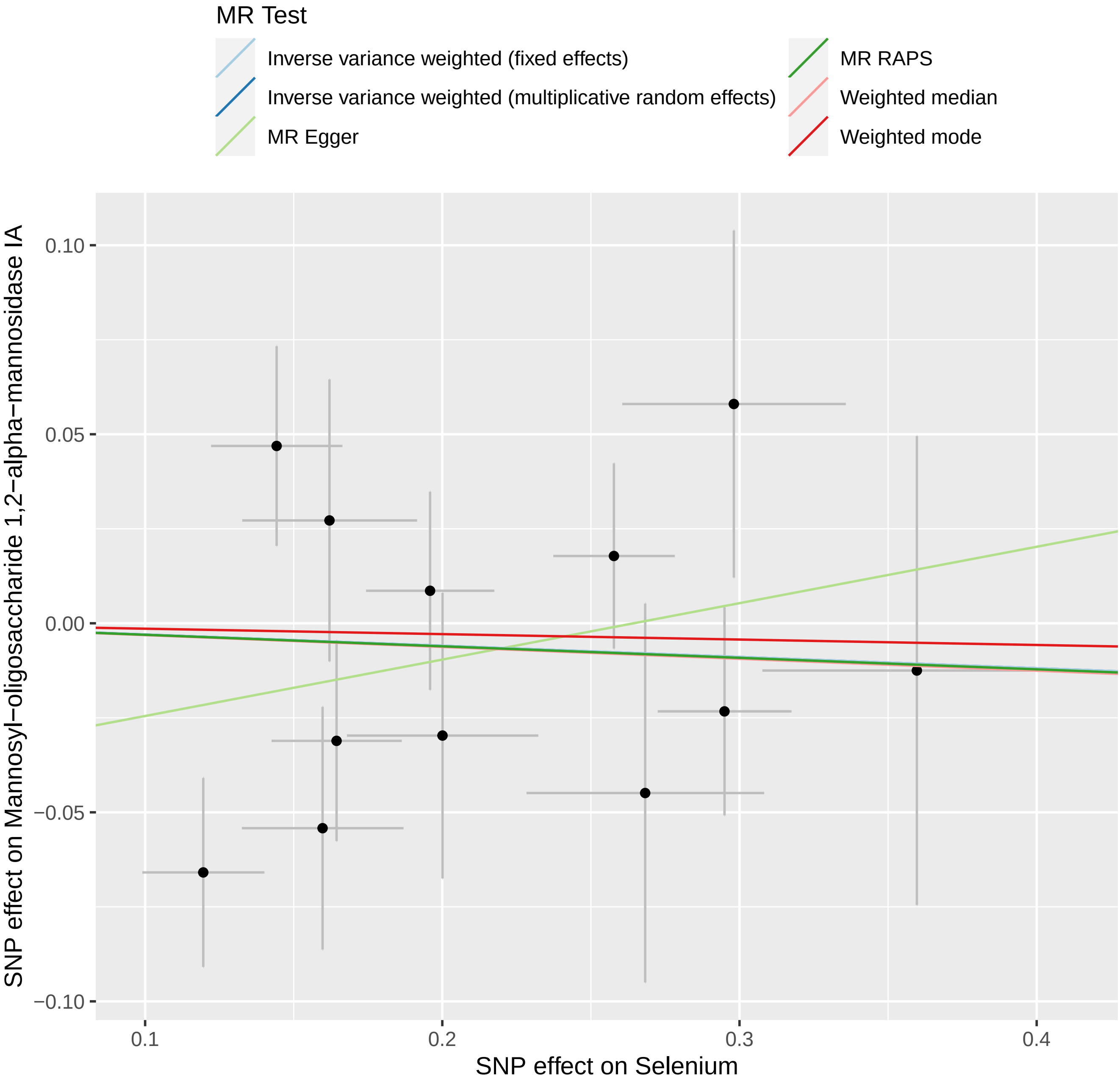

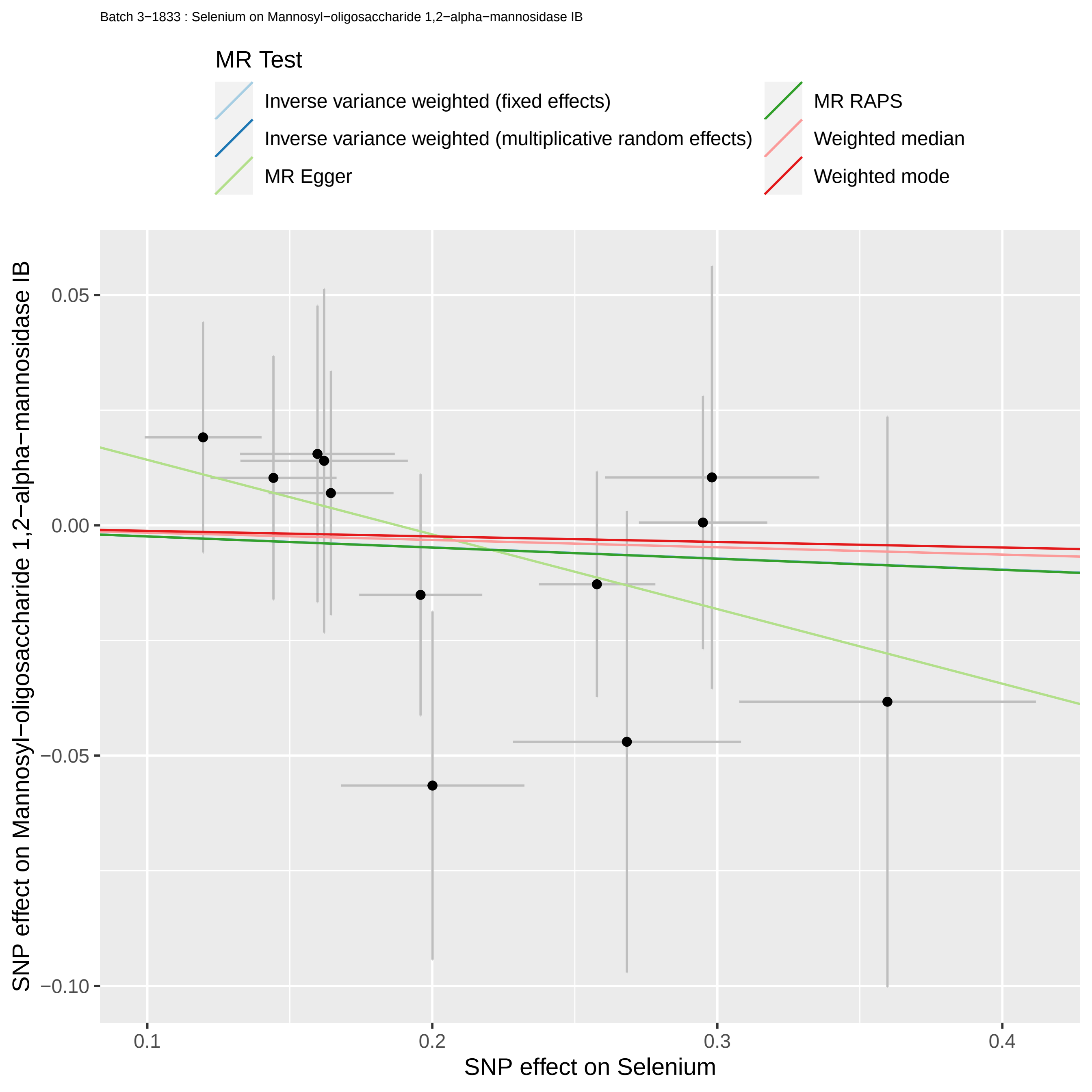

Batch 3−1833 : Selenium on Mannosyl−oligosaccharide 1,2−alpha−mannosidase IB
MR Test
Inverse variance weighted (fixed effects)
Inverse variance weighted (multiplicative random effects)
MR Egger
MR RAPS
Weighted median
Weighted mode
SNP effect on Mannosyl−oligosaccharide 1,2−alpha−mannosidase IB
0.05
0.00
−0.05
−0.10
0.1
0.2
0.3
0.4
SNP effect on Selenium

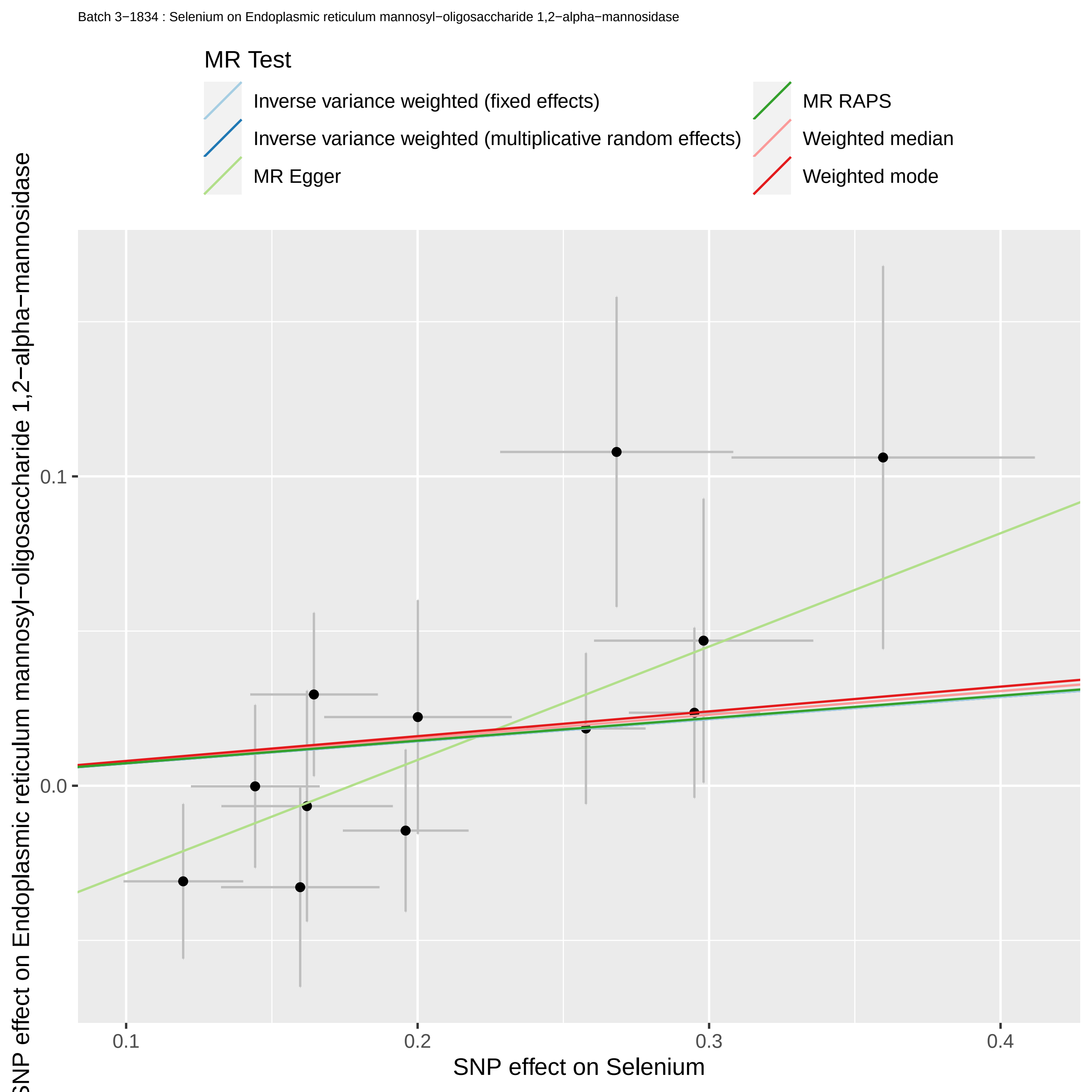

Batch 3−1834 : Selenium on Endoplasmic reticulum mannosyl−oligosaccharide 1,2−alpha−mannosidase
MR Test
Inverse variance weighted (fixed effects)
Inverse variance weighted (multiplicative random effects)
MR Egger
MR RAPS
Weighted median
Weighted mode
0.1
0.0
0.1
0.2
0.3
0.4
SNP effect on Selenium
SNP effect on Endoplasmic reticulum mannosyl−oligosaccharide 1,2−alpha−mannosidase

Batch 3−1835 : Selenium on Epididymis−specific alpha−mannosidase

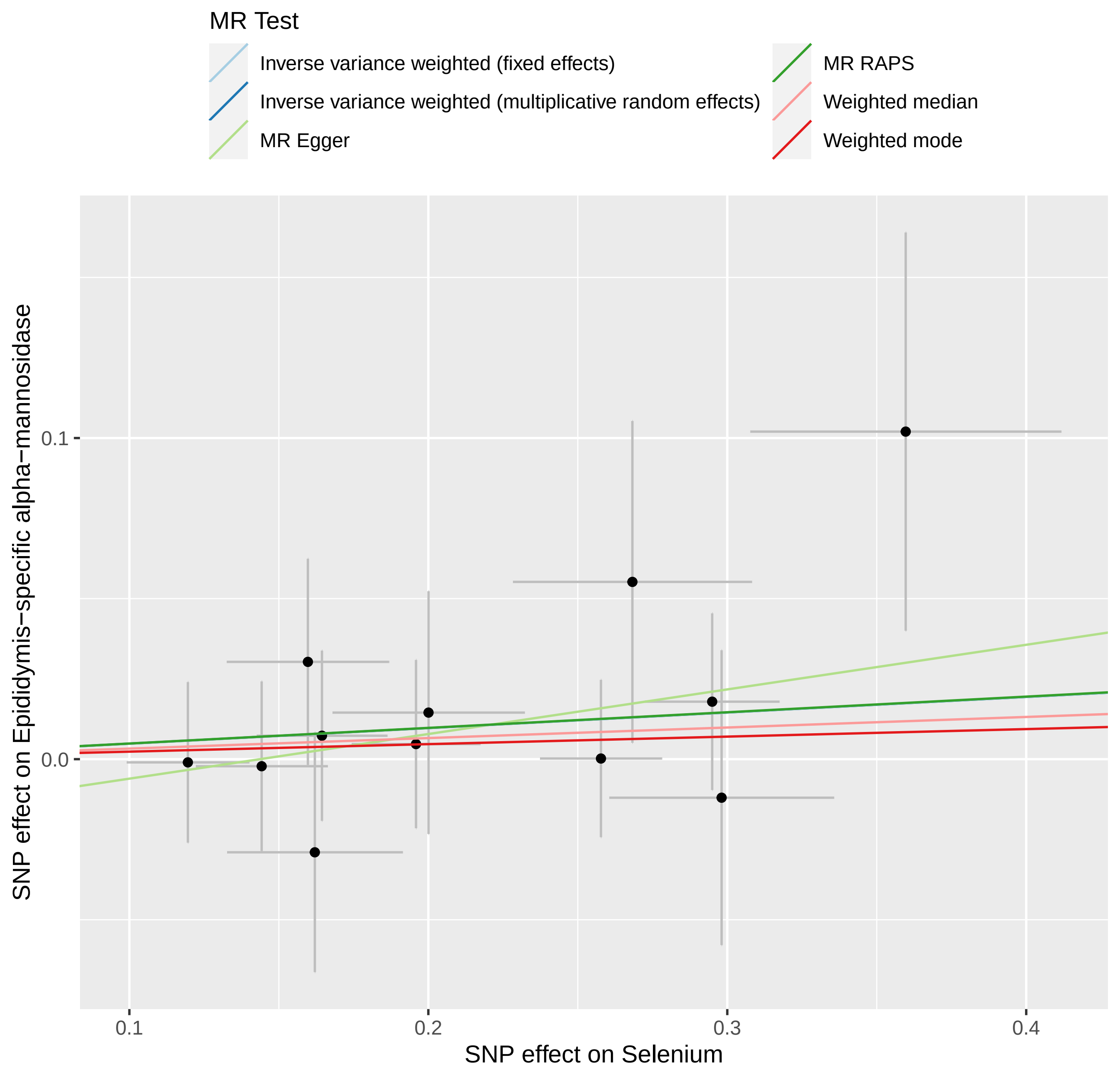

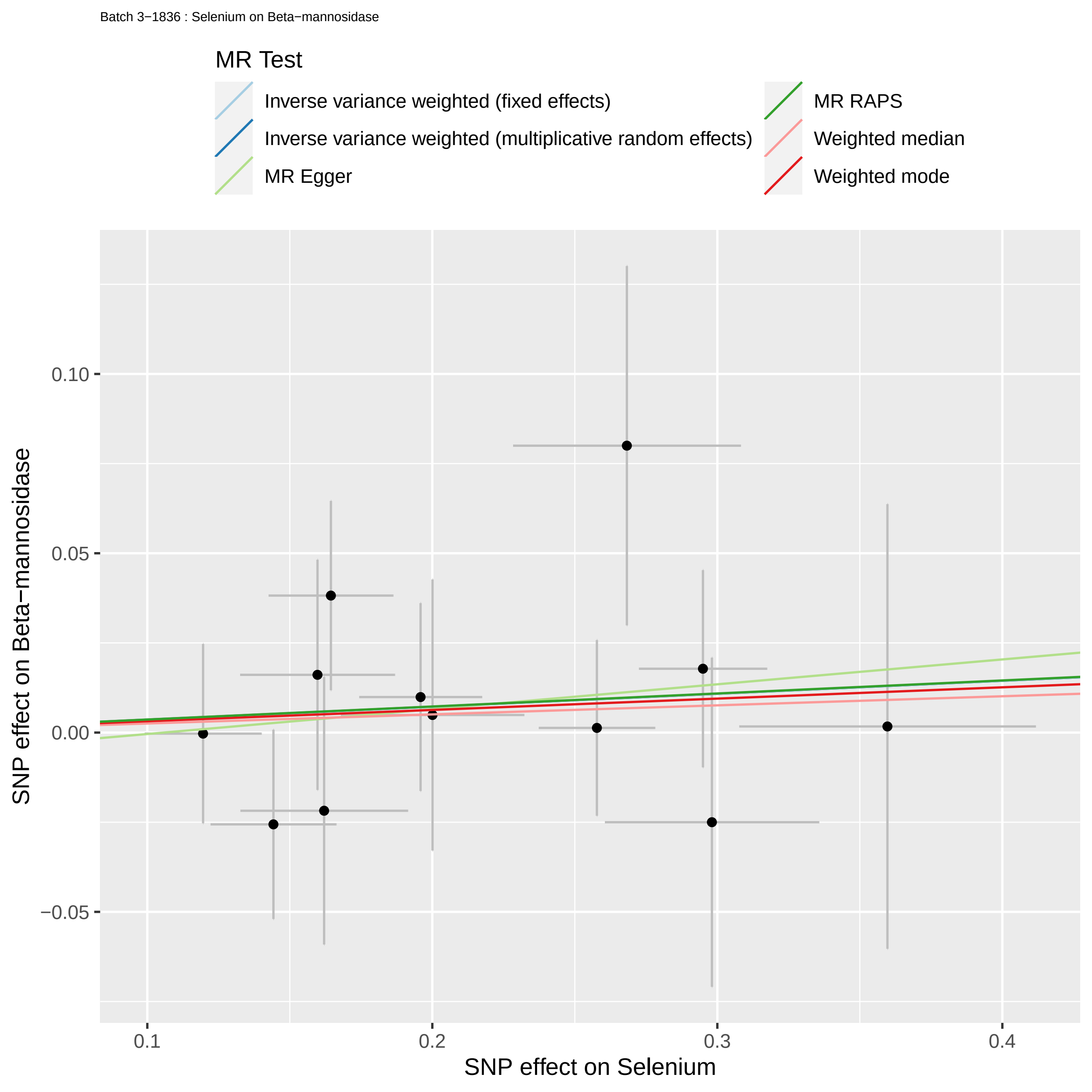

Batch 3−1836 : Selenium on Beta−mannosidase
MR Test
Inverse variance weighted (fixed effects)
Inverse variance weighted (multiplicative random effects)
MR Egger
MR RAPS
Weighted median
Weighted mode
0.10
0.05
0.00
−0.05
0.1
0.2
0.3
0.4
SNP effect on Beta−mannosidase
SNP effect on Selenium

Batch 3−1837 : Selenium on Glycoprotein endo−alpha−1,2−mannosidase

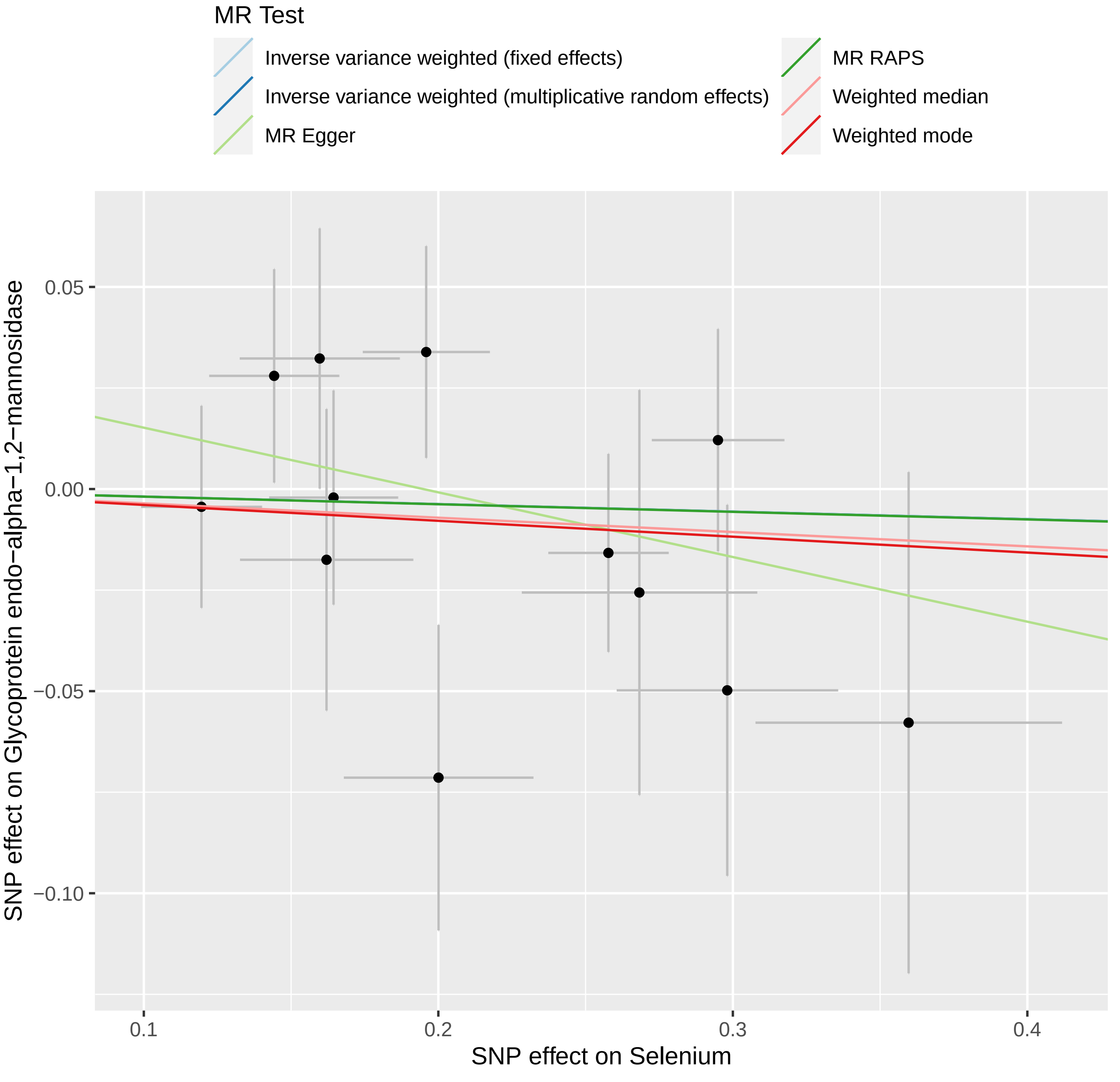

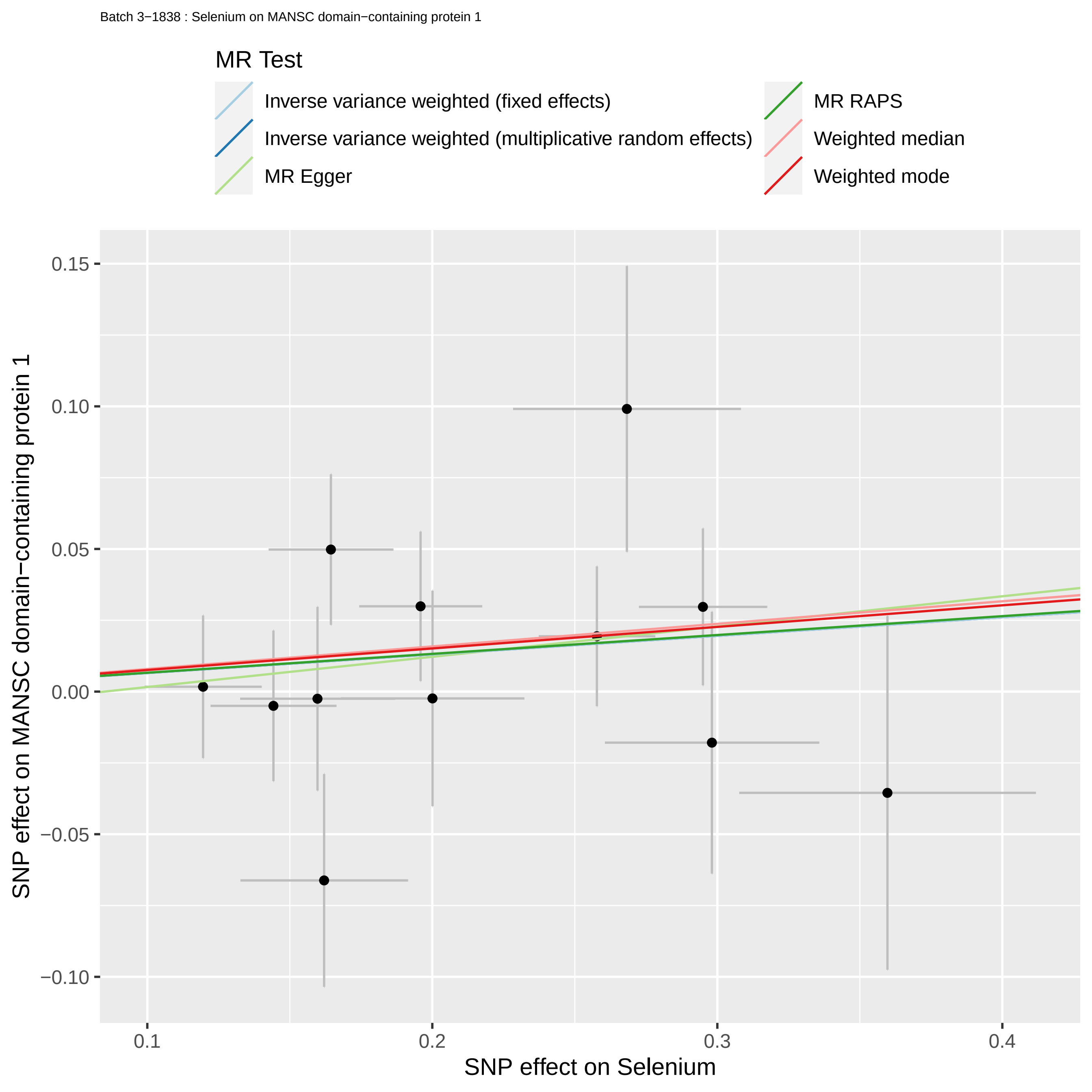

Batch 3−1838 : Selenium on MANSC domain−containing protein 1
MR Test
Inverse variance weighted (fixed effects)
Inverse variance weighted (multiplicative random effects)
MR Egger
MR RAPS
Weighted median
Weighted mode
SNP effect on MANSC domain−containing protein 1
0.15
0.10
0.05
0.00
−0.05
−0.10
0.1
0.2
0.3
0.4
SNP effect on Selenium

Batch 3−1839 : Selenium on MANSC domain−containing protein 4

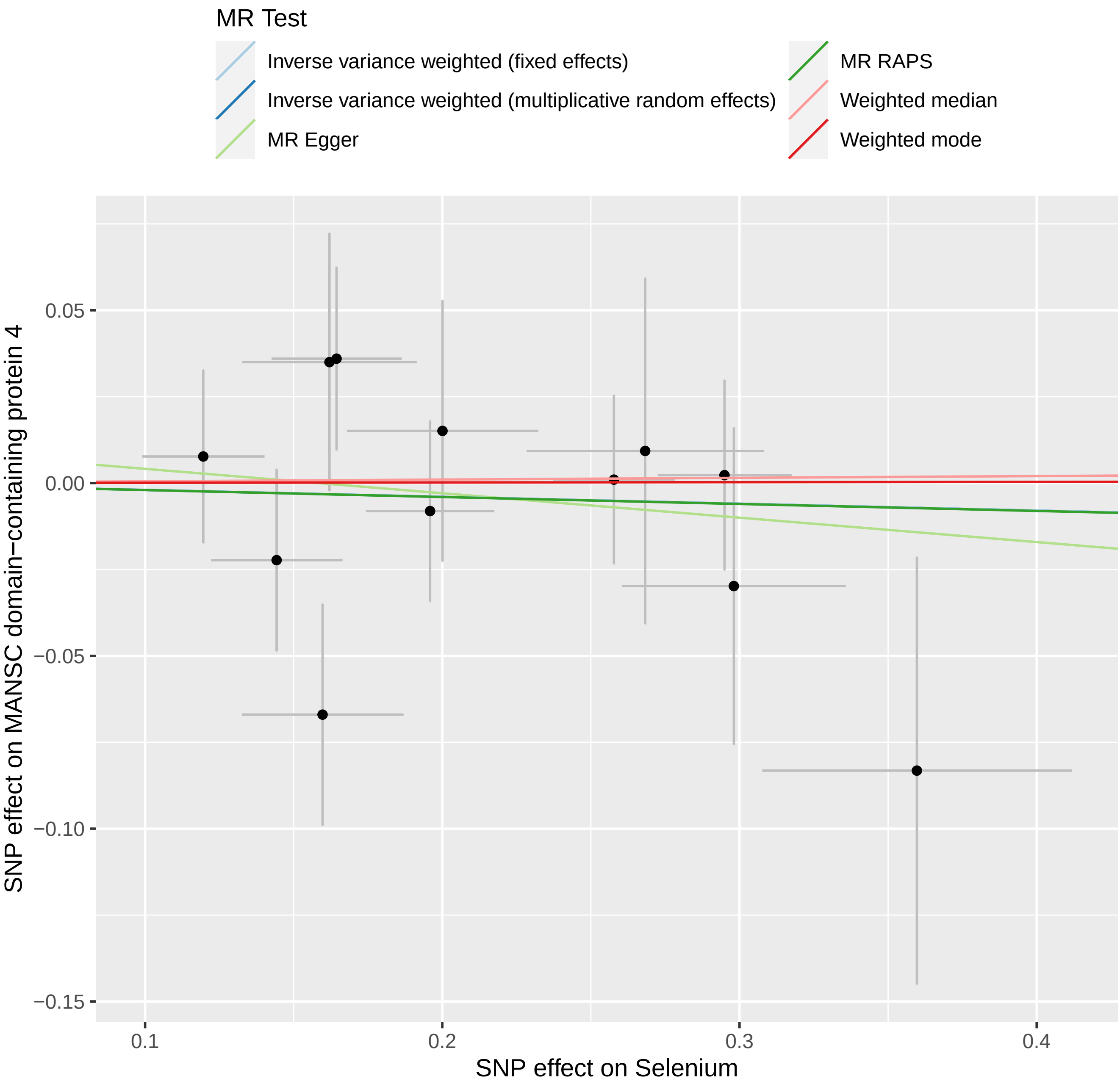

Batch 3−1840 : Selenium on Dual specificity mitogen−activated protein kinase kinase 1

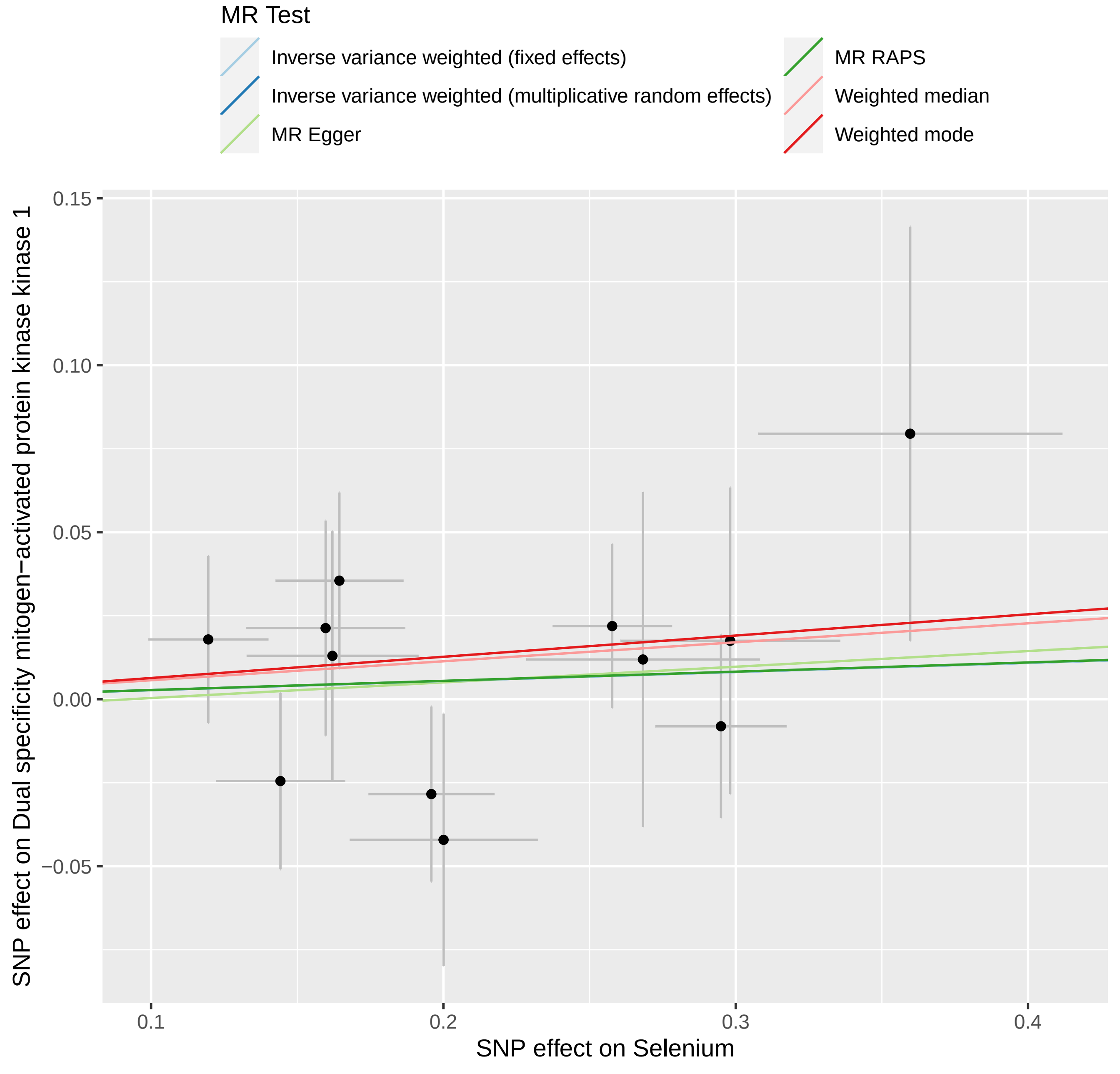

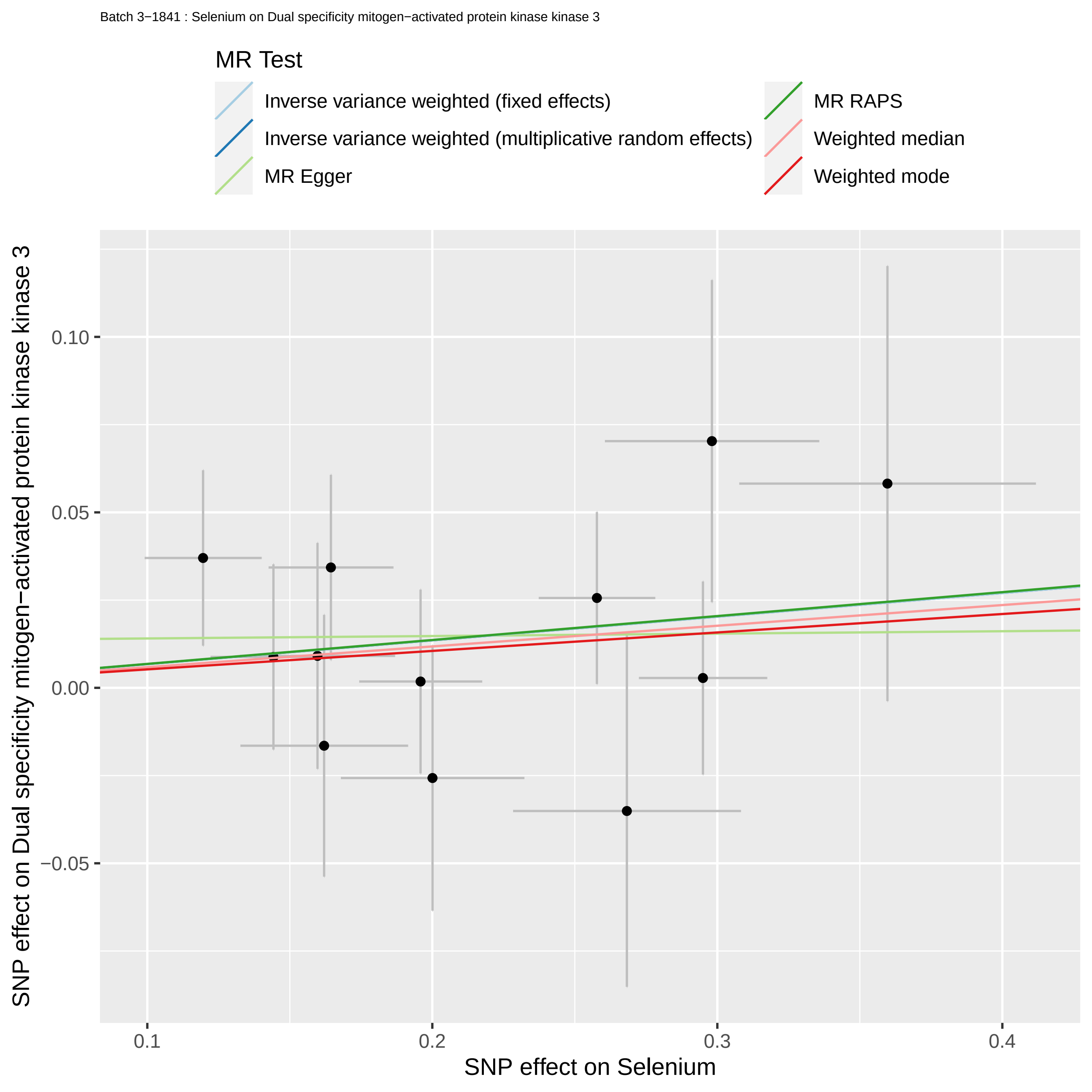
Batch 3−1841 : Selenium on Dual specificity mitogen−activated protein kinase kinase 3
MR Test
Inverse variance weighted (fixed effects)
Inverse variance weighted (multiplicative random effects)
MR Egger
MR RAPS
Weighted median
Weighted mode
SNP effect on Dual specificity mitogen−activated protein kinase kinase 3
0.10
0.05
0.00
−0.05
0.1
0.2
0.3
0.4
SNP effect on Selenium

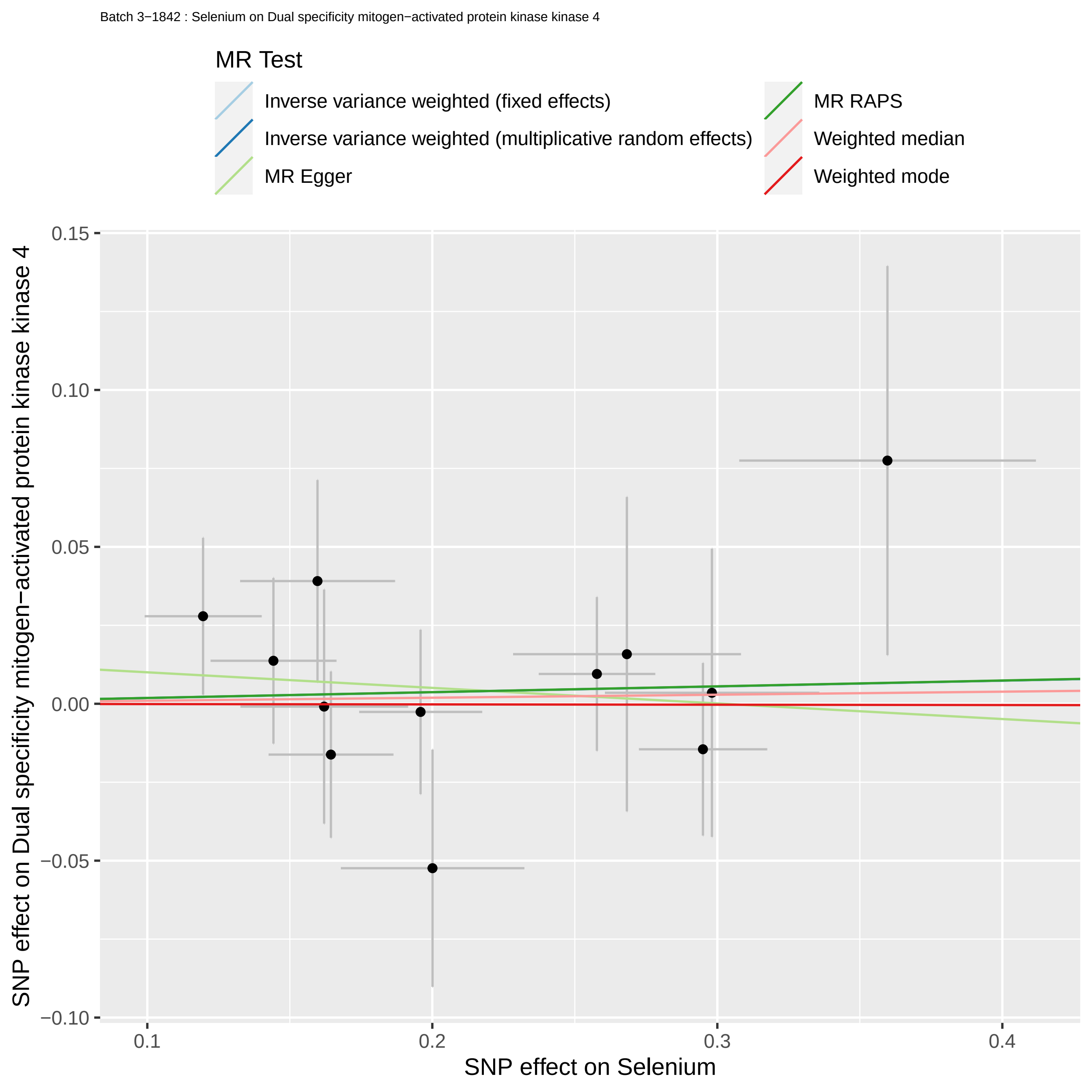
Batch 3−1842 : Selenium on Dual specificity mitogen−activated protein kinase kinase 4
MR Test
Inverse variance weighted (fixed effects)
Inverse variance weighted (multiplicative random effects)
MR Egger
MR RAPS
Weighted median
Weighted mode
SNP effect on Dual specificity mitogen−activated protein kinase kinase 4
0.15
0.10
0.05
0.00
−0.05
−0.10
0.1
0.2
0.3
0.4
SNP effect on Selenium

Batch 3−1843 : Selenium on Mitogen−activated protein kinase kinase kinase 3

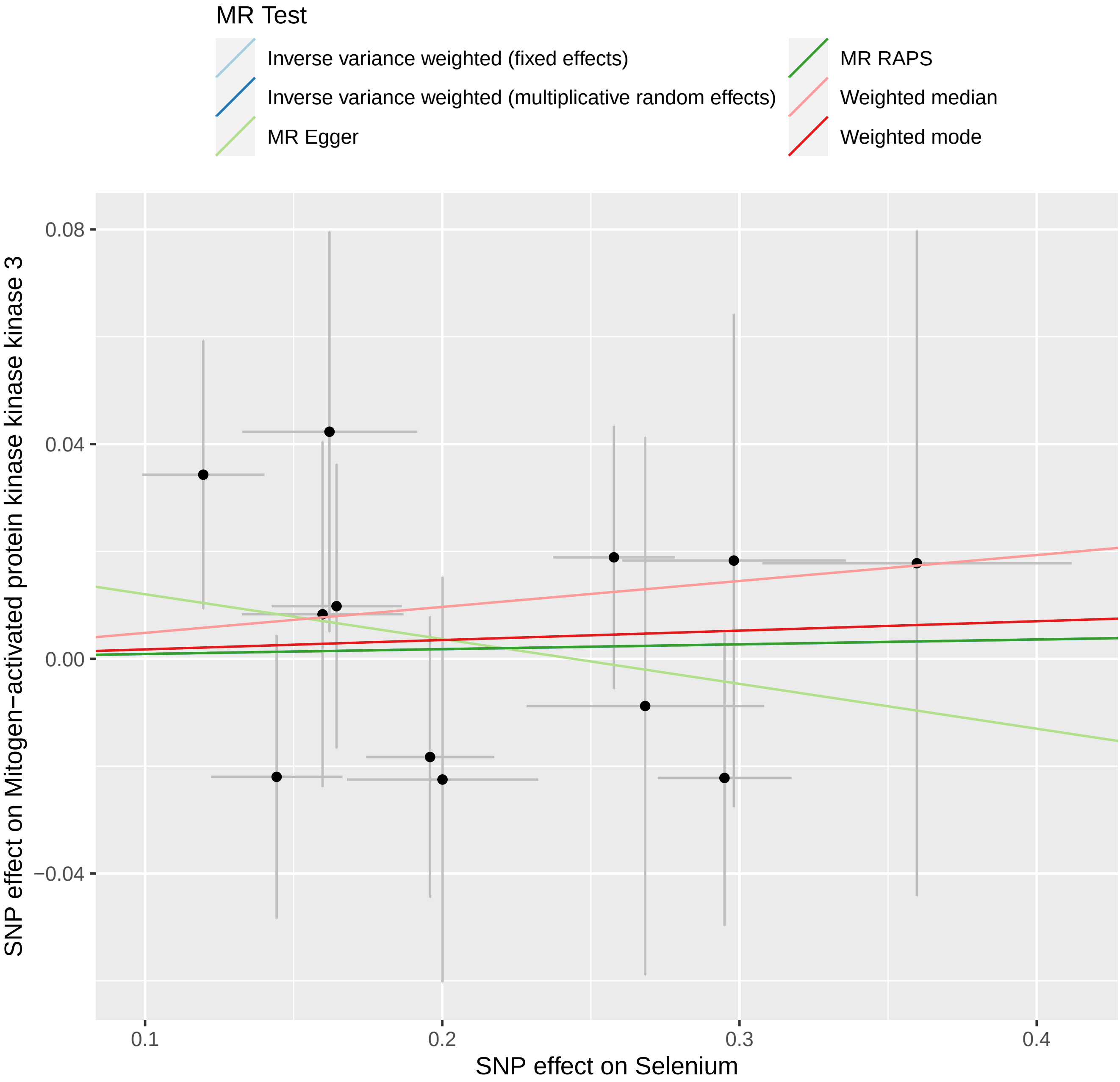

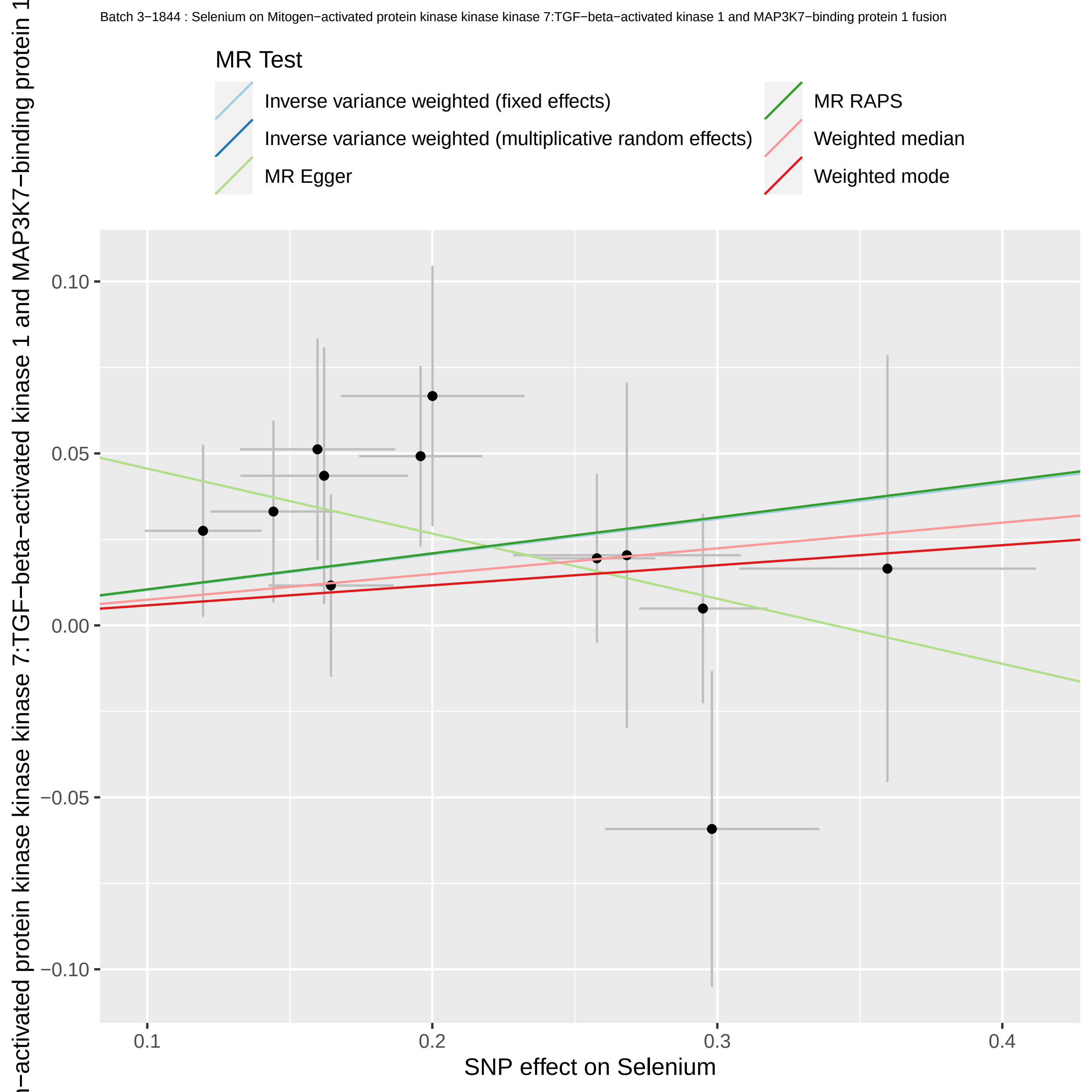

Batch 3−1844 : Selenium on Mitogen−activated protein kinase kinase kinase 7:TGF−beta−activated kinase 1 and MAP3K7−binding protein 1 fusion
MR Test
Inverse variance weighted (fixed effects)
Inverse variance weighted (multiplicative random effects)
MR Egger
MR RAPS
Weighted median
Weighted mode
0.10
0.05
0.00
−0.05
−0.10
0.1
0.2
0.3
0.4
SNP effect on Selenium
n−activated protein kinase kinase kinase 7:TGF−beta−activated kinase 1 and MAP3K7−binding protein 1

Batch 3−1845 : Selenium on Mitogen−activated protein kinase 1

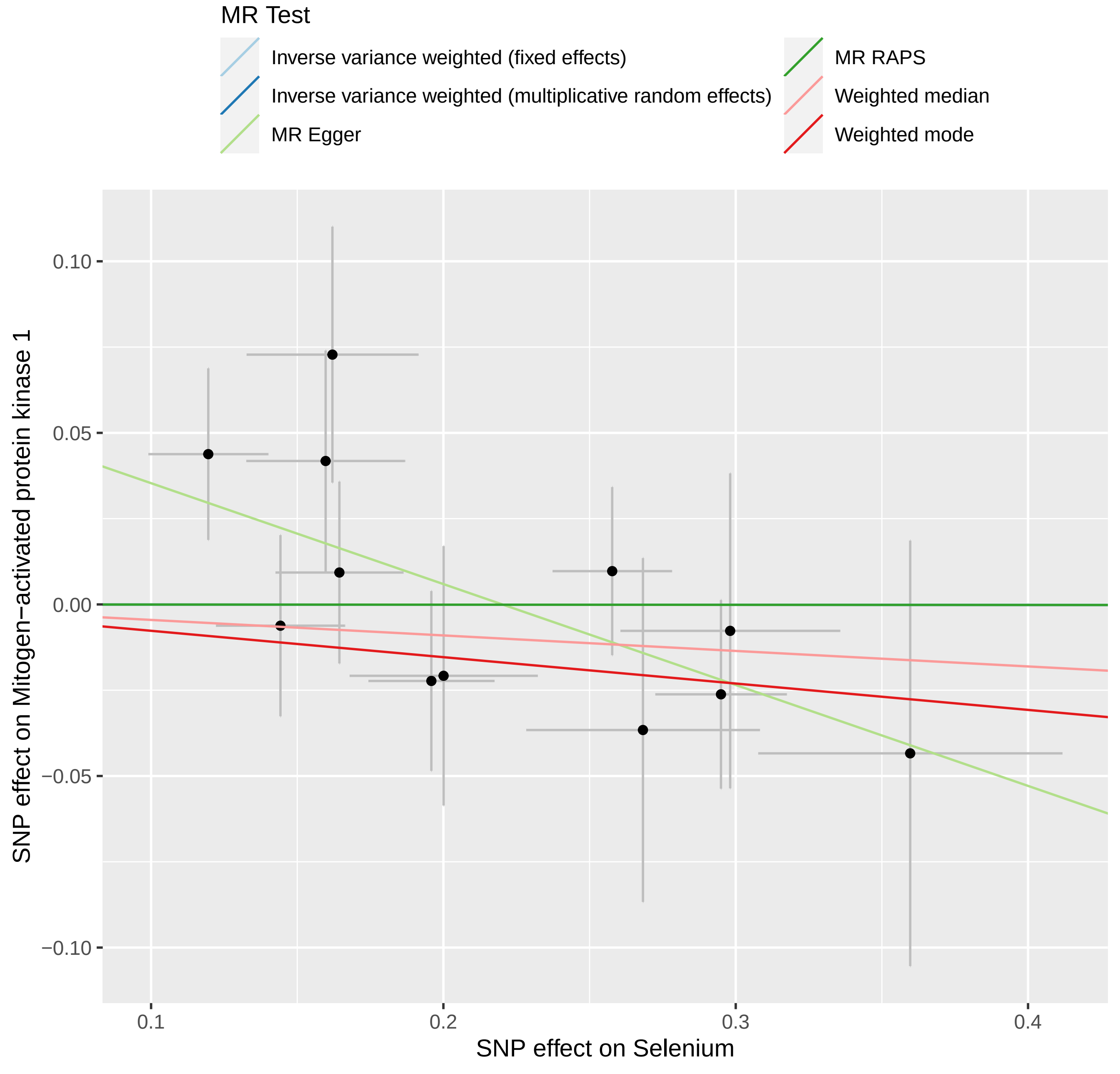

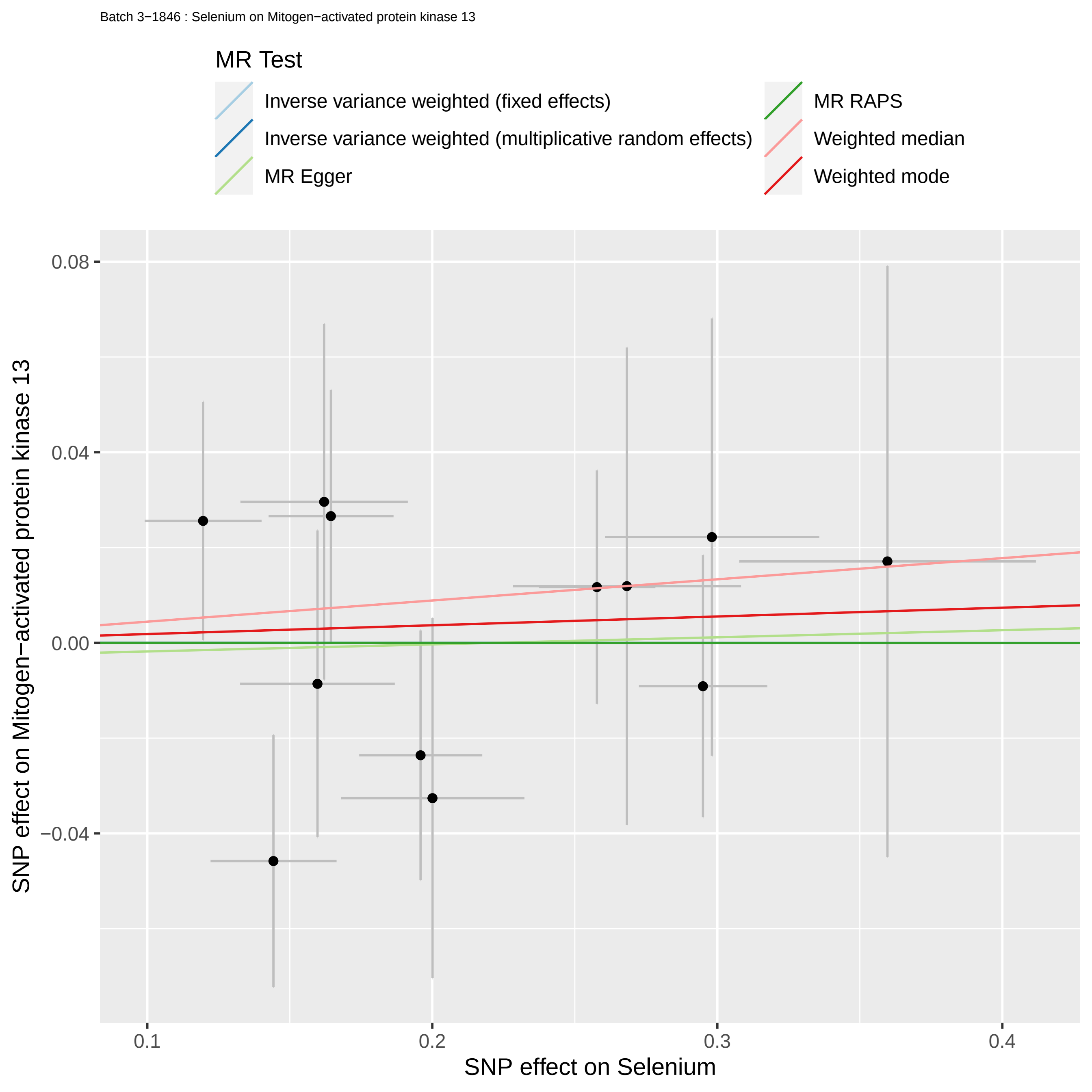

Batch 3−1846 : Selenium on Mitogen−activated protein kinase 13
MR Test
Inverse variance weighted (fixed effects)
Inverse variance weighted (multiplicative random effects)
MR Egger
MR RAPS
Weighted median
Weighted mode
0.08
0.04
0.00
−0.04
0.1
0.2
0.3
0.4
SNP effect on Selenium
SNP effect on Mitogen−activated protein kinase 13

Batch 3−1847 : Selenium on Mitogen−activated protein kinase 14

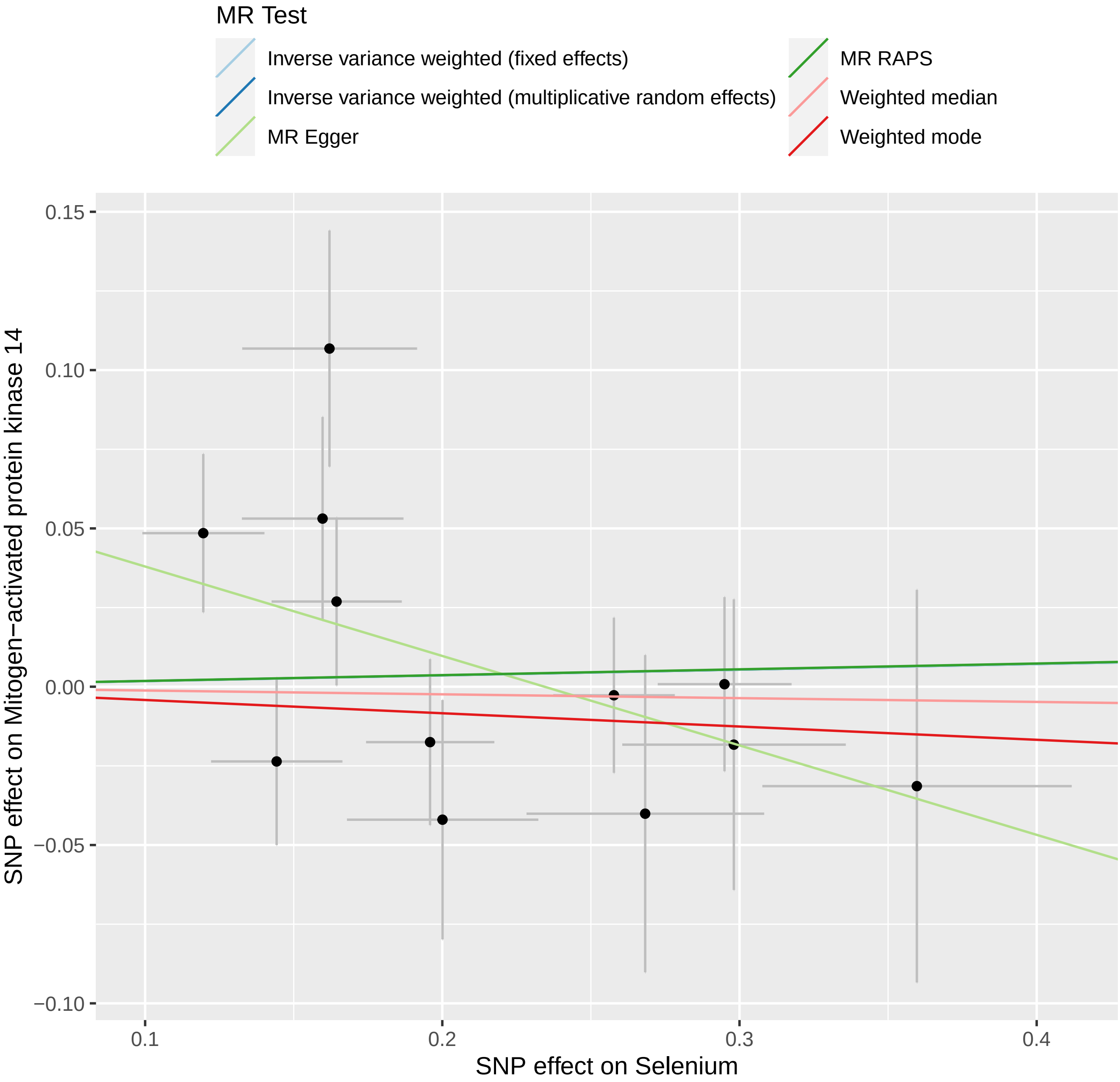

Batch 3−1848 : Selenium on Mitogen−activated protein kinase 3

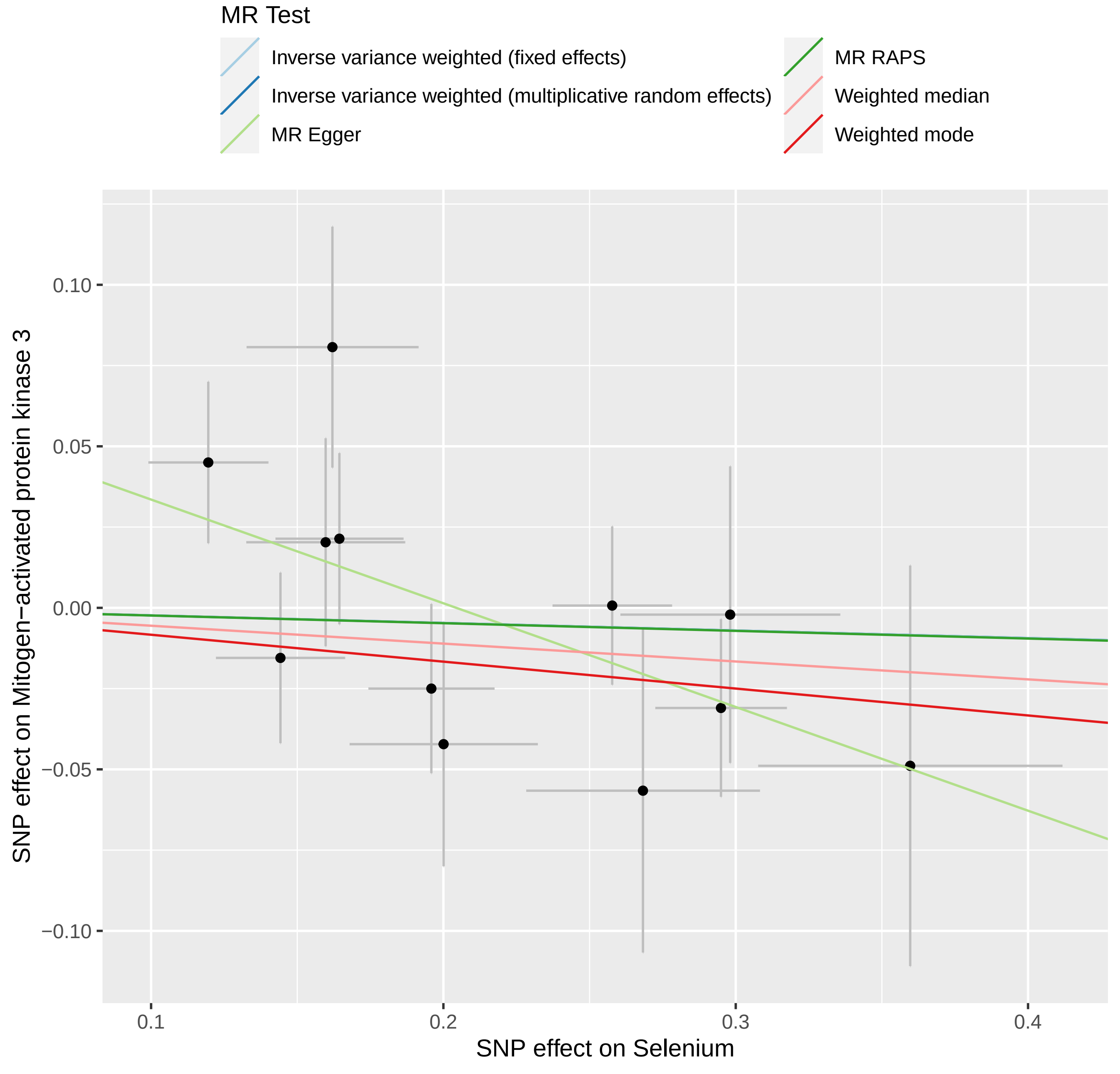

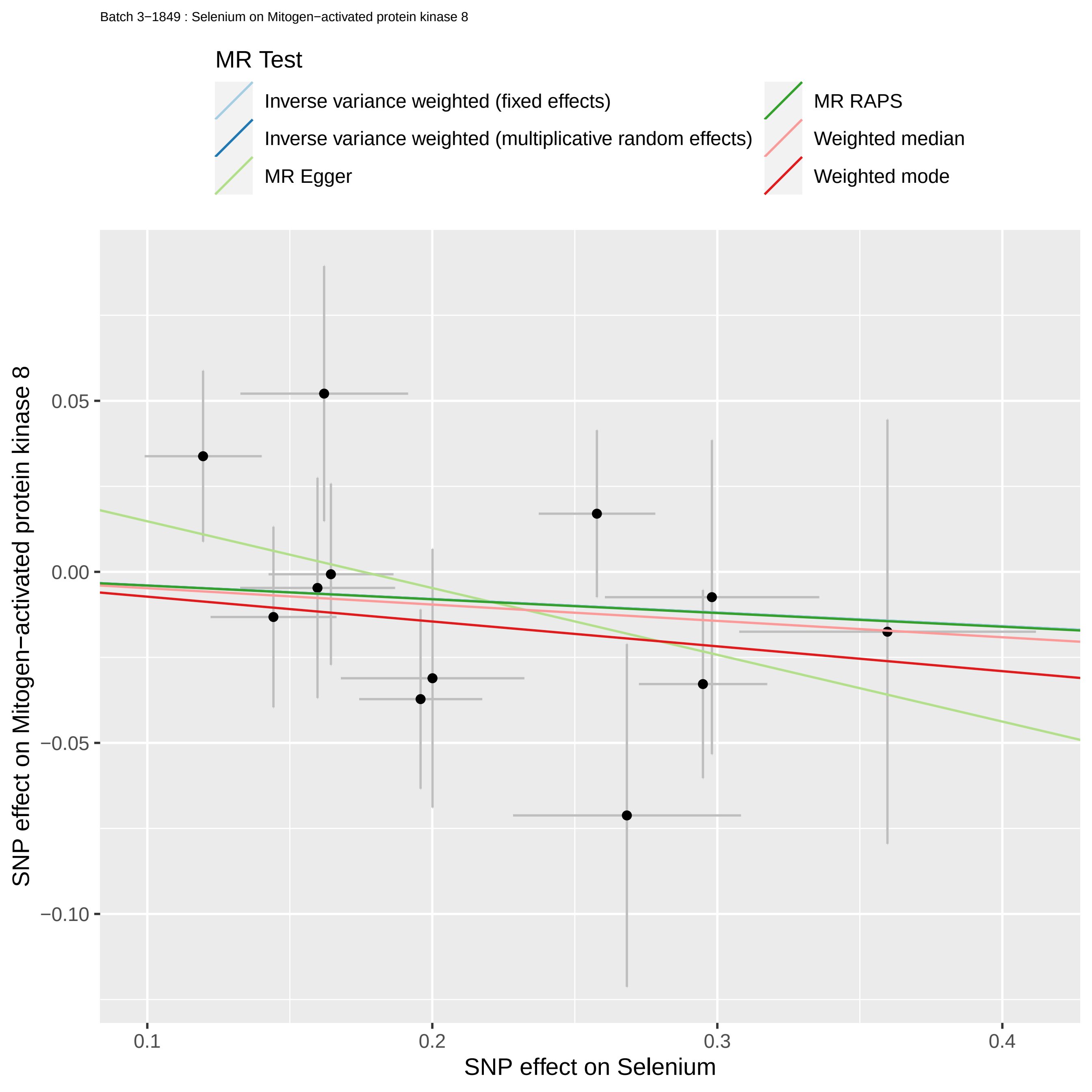

Batch 3−1849 : Selenium on Mitogen−activated protein kinase 8
MR Test
Inverse variance weighted (fixed effects)
Inverse variance weighted (multiplicative random effects)
MR Egger
MR RAPS
Weighted median
Weighted mode
0.05
0.00
−0.05
−0.10
0.1
0.2
0.3
0.4
SNP effect on Mitogen−activated protein kinase 8
SNP effect on Selenium

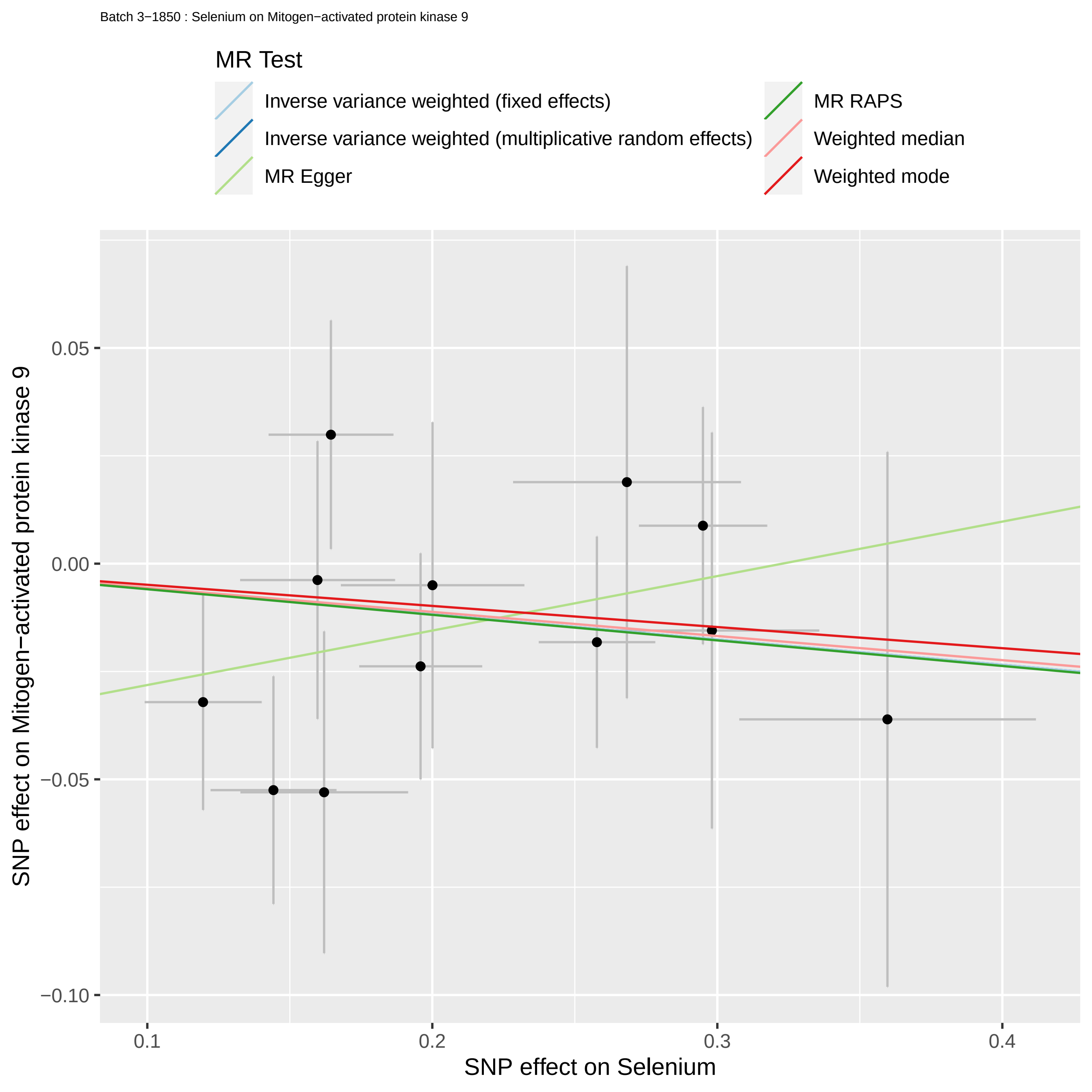

Batch 3−1850 : Selenium on Mitogen−activated protein kinase 9
MR Test
Inverse variance weighted (fixed effects)
Inverse variance weighted (multiplicative random effects)
MR Egger
MR RAPS
Weighted median
Weighted mode
0.05
0.00
−0.05
−0.10
0.1
0.2
0.3
0.4
SNP effect on Mitogen−activated protein kinase 9
SNP effect on Selenium

Batch 3−1851 : Selenium on MAP kinase−activated protein kinase 2

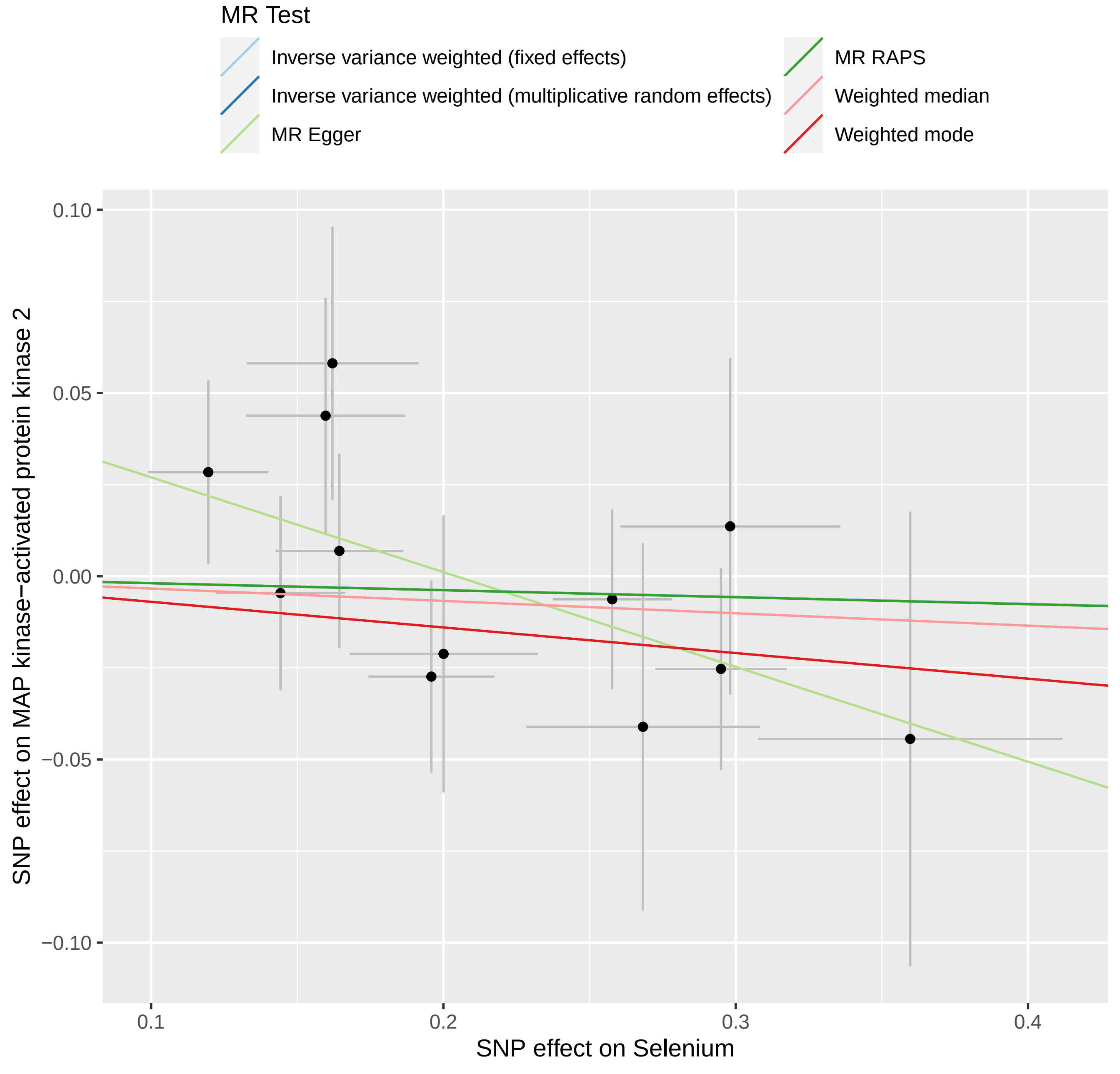

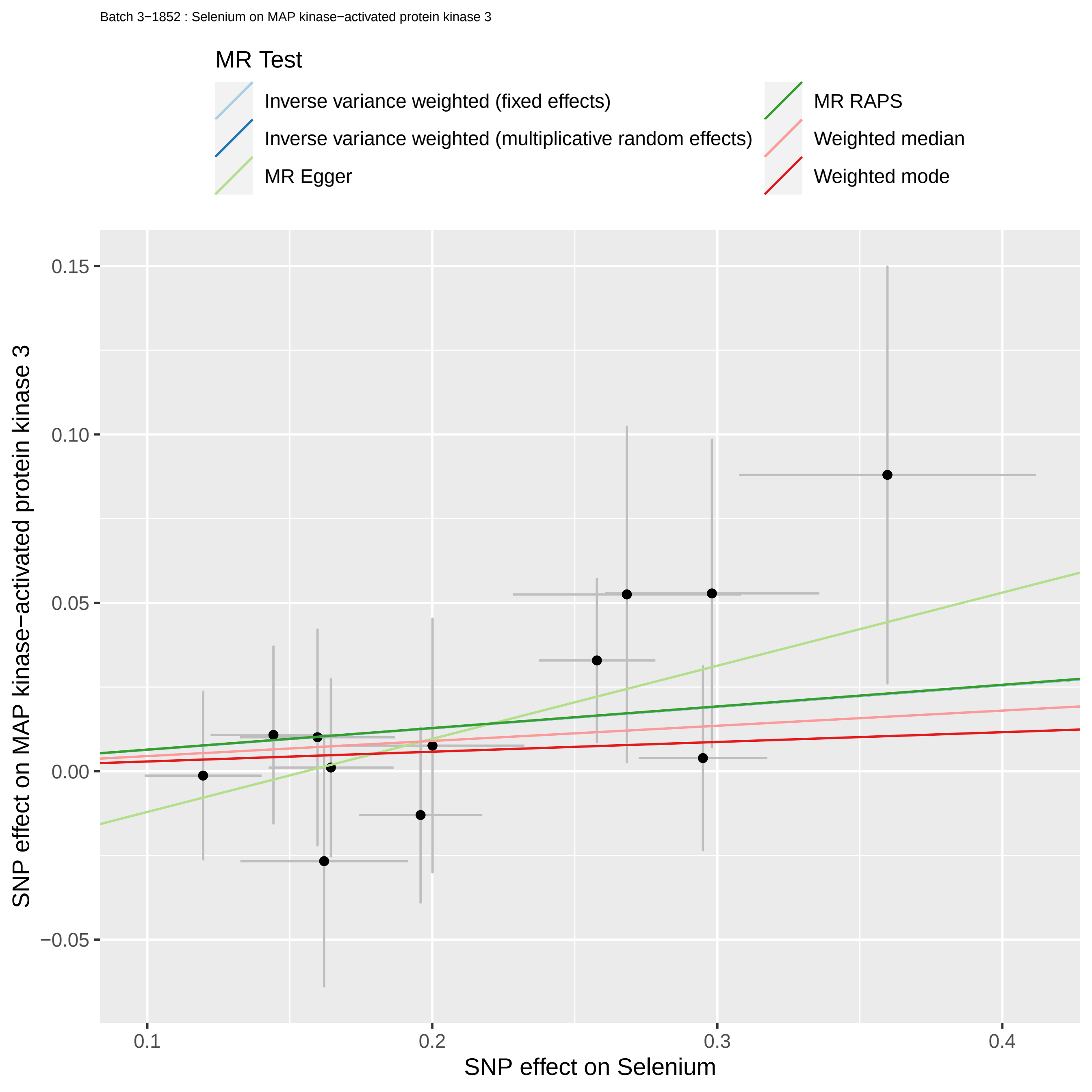

Batch 3−1852 : Selenium on MAP kinase−activated protein kinase 3
MR Test
Inverse variance weighted (fixed effects)
Inverse variance weighted (multiplicative random effects)
MR Egger
MR RAPS
Weighted median
Weighted mode
SNP effect on MAP kinase−activated protein kinase 3
0.15
0.10
0.05
0.00
−0.05
0.1
0.2
0.3
0.4
SNP effect on Selenium

Batch 3−1853 : Selenium on MAP kinase−activated protein kinase 5

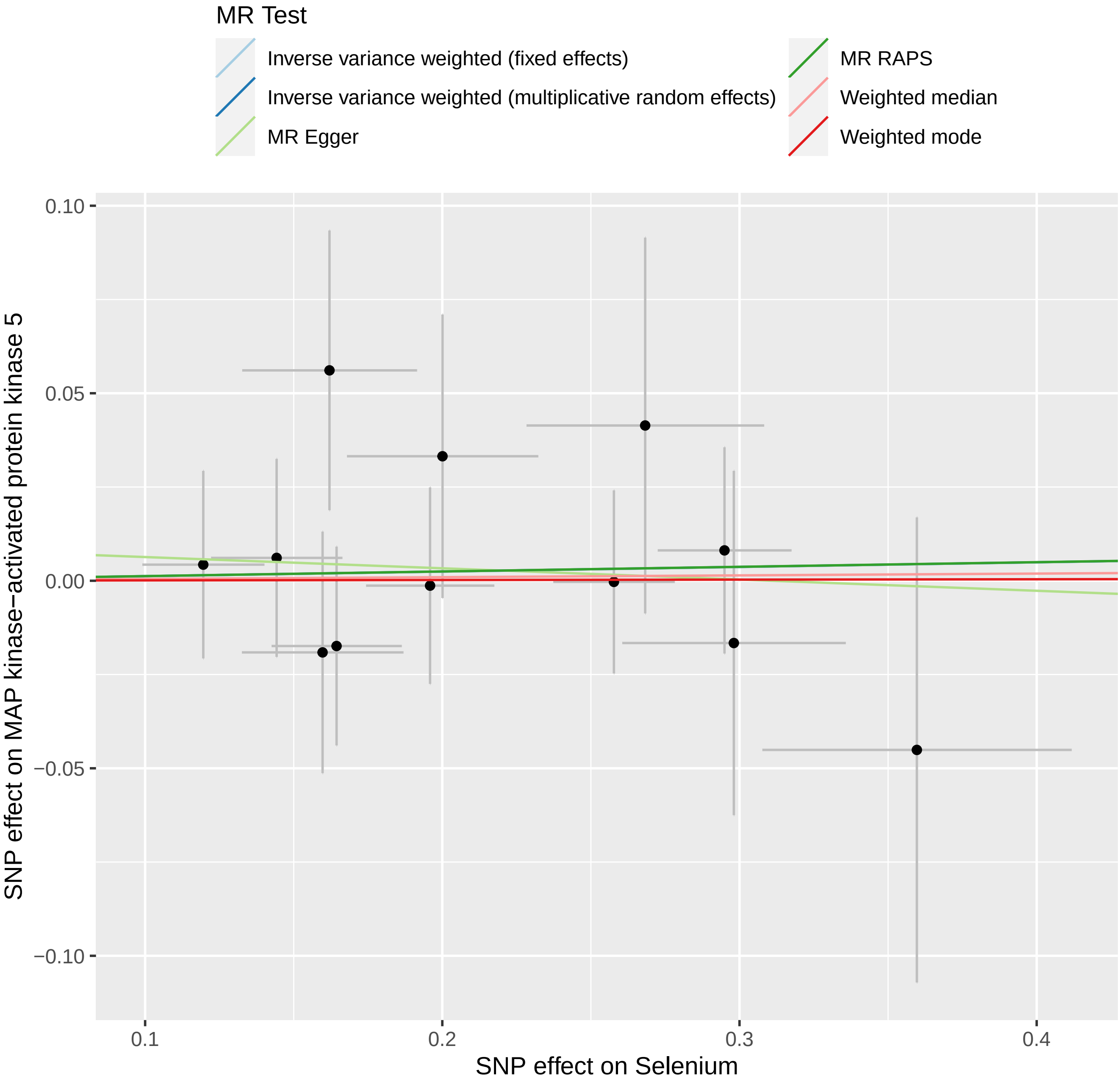

Batch 3−1854 : Selenium on MAP kinase−activated protein kinase 5

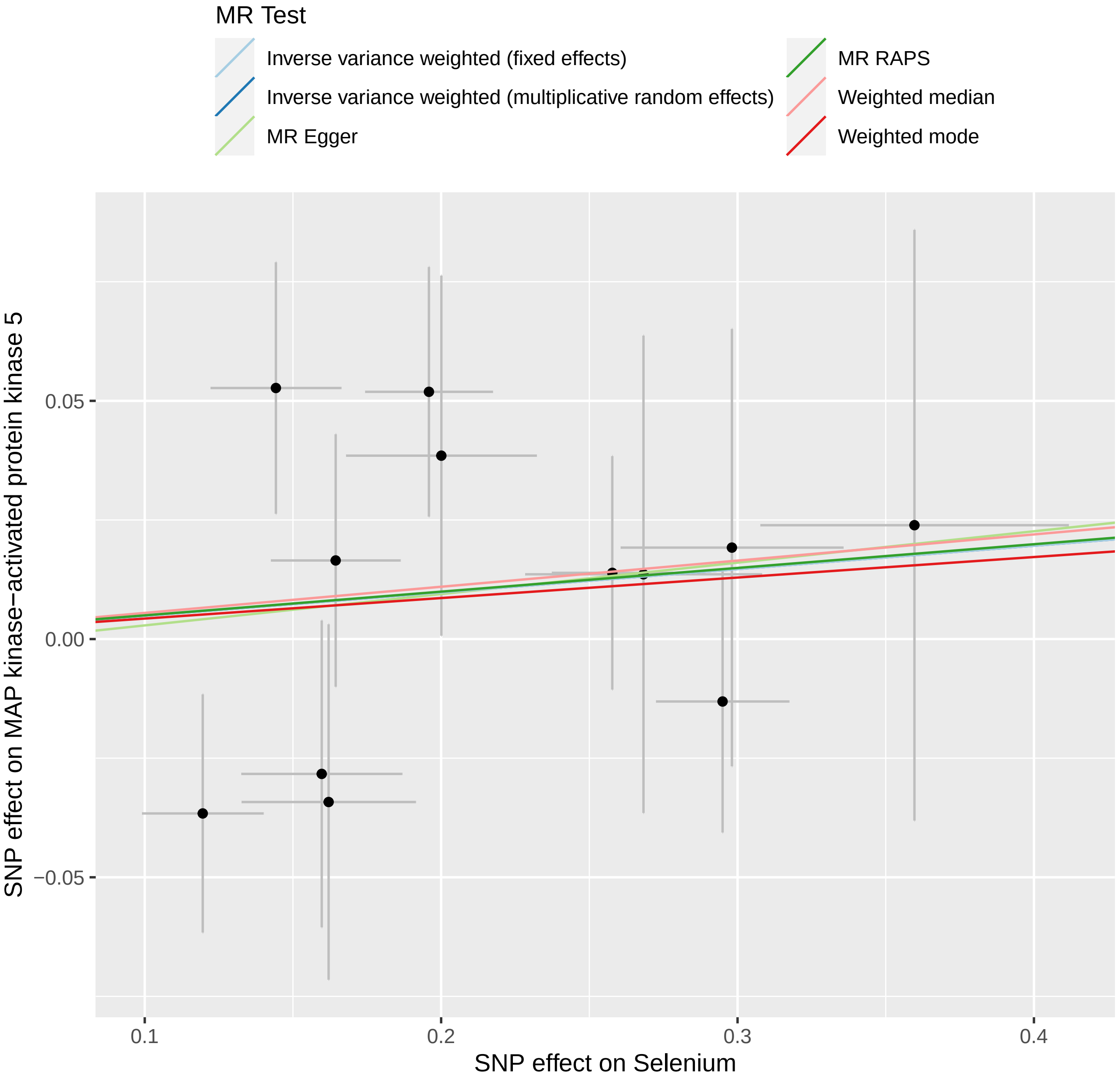

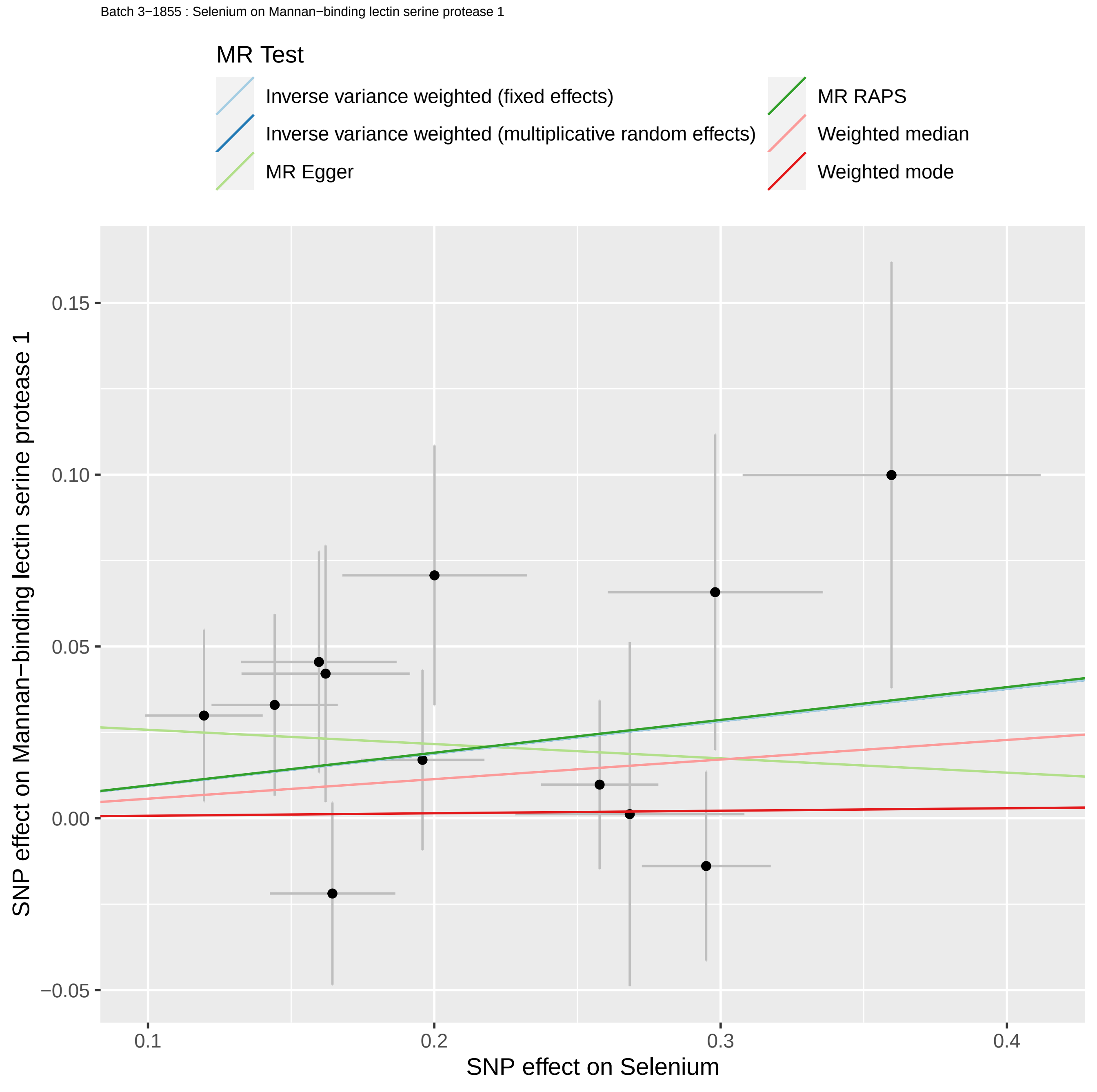

Batch 3−1855 : Selenium on Mannan−binding lectin serine protease 1
MR Test
Inverse variance weighted (fixed effects)
Inverse variance weighted (multiplicative random effects)
MR Egger
MR RAPS
Weighted median
Weighted mode
SNP effect on Mannan−binding lectin serine protease 1
0.15
0.10
0.05
0.00
−0.05
0.1
0.2
0.3
0.4
SNP effect on Selenium

Batch 3−1856 : Selenium on Megakaryocyte−associated tyrosine−protein kinase

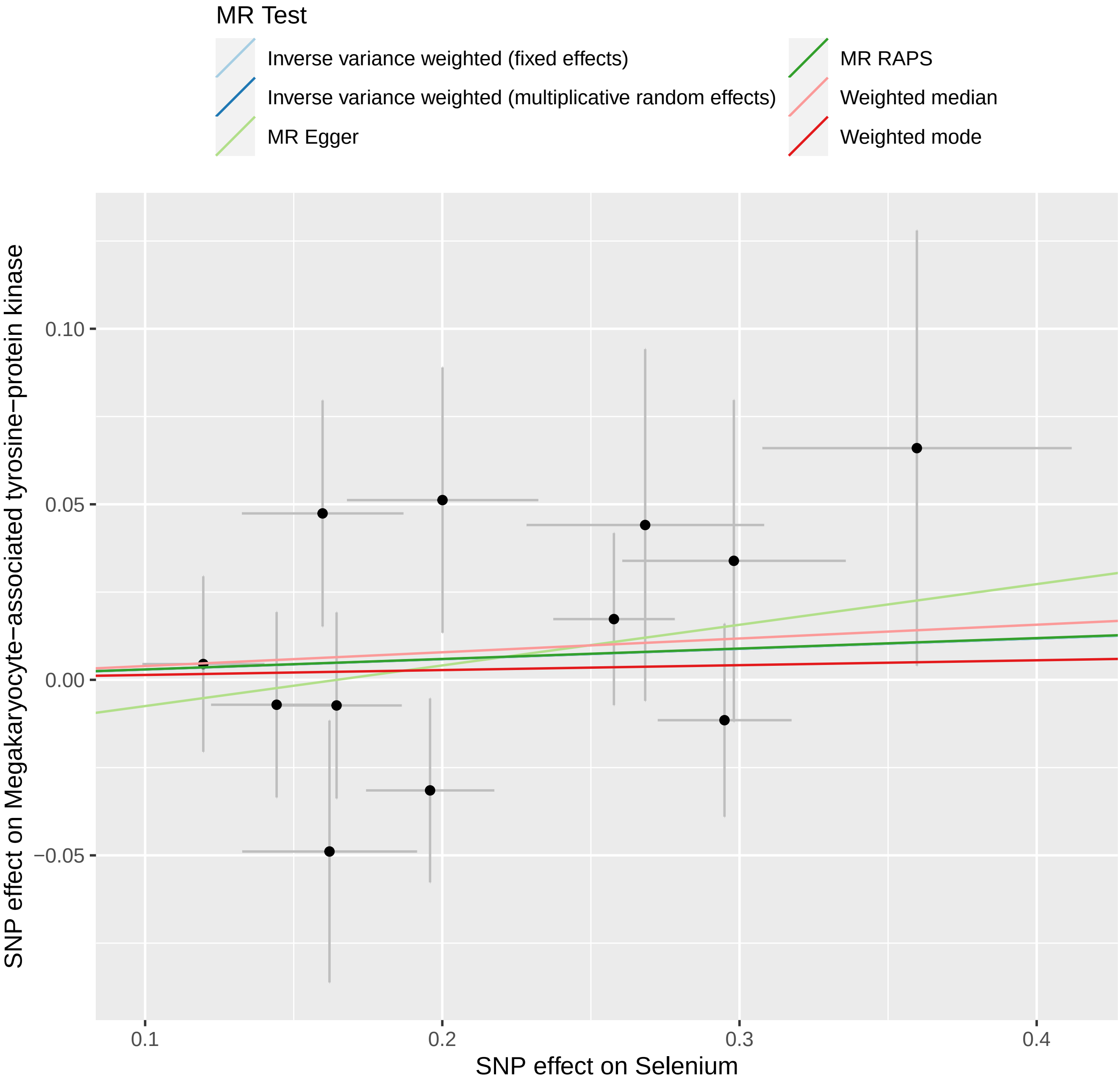

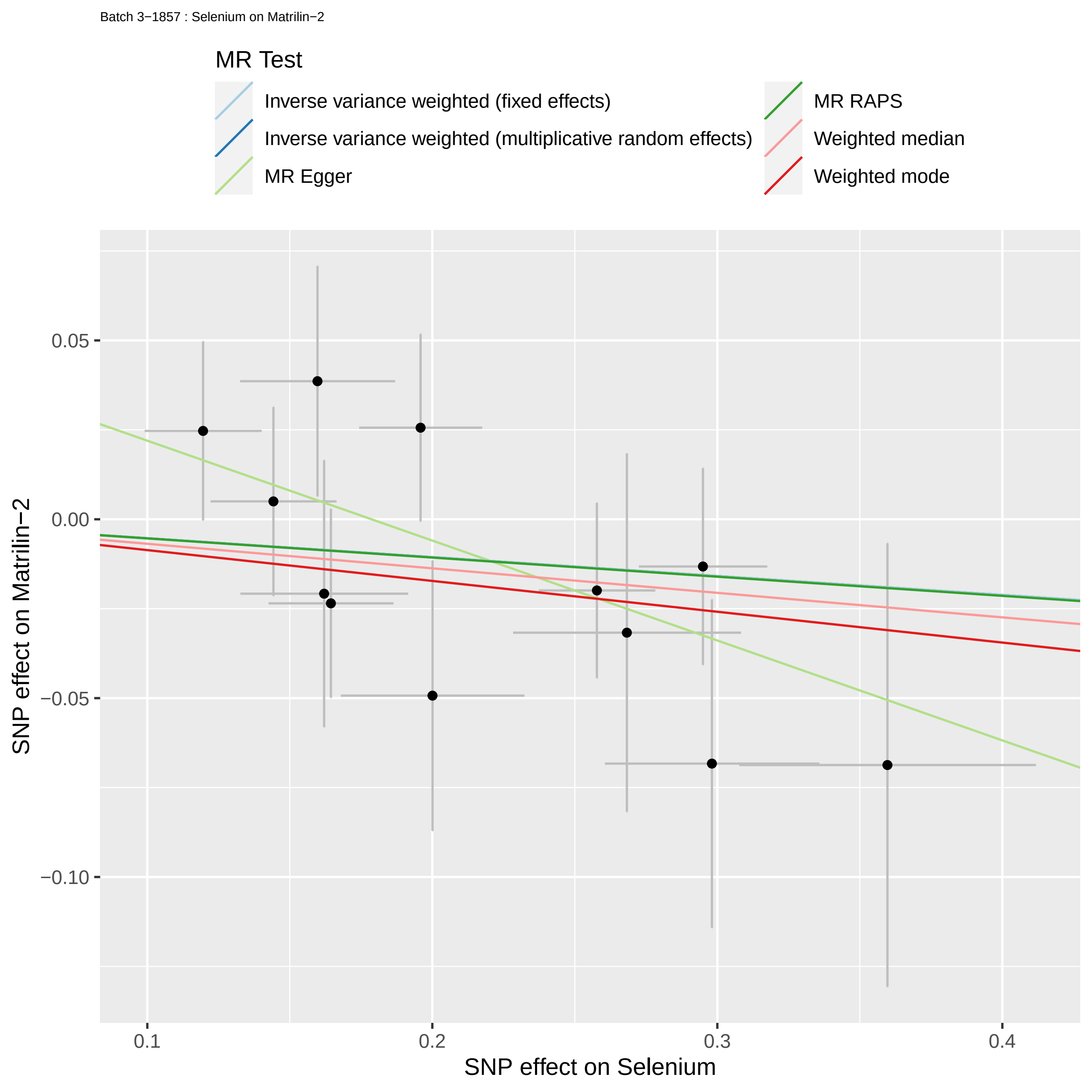

Batch 3−1857 : Selenium on Matrilin−2
MR Test
Inverse variance weighted (fixed effects)
Inverse variance weighted (multiplicative random effects)
MR Egger
MR RAPS
Weighted median
Weighted mode
0.05
0.00
−0.05
−0.10
0.1
0.2
0.3
0.4
SNP effect on Matrilin−2
SNP effect on Selenium

Batch 3−1858 : Selenium on Matrilin−3

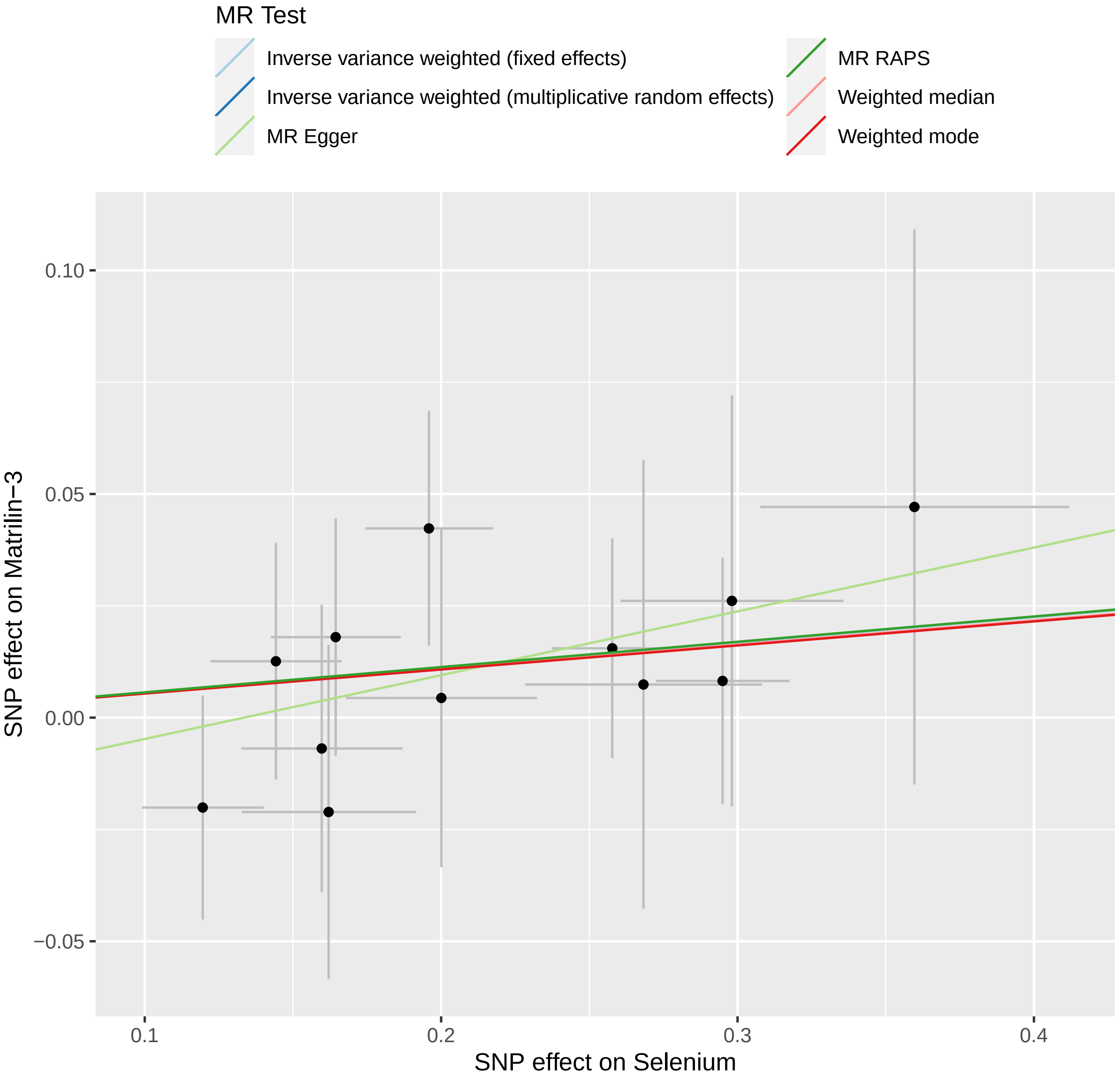

Batch 3−1859 : Selenium on Matrilin−4

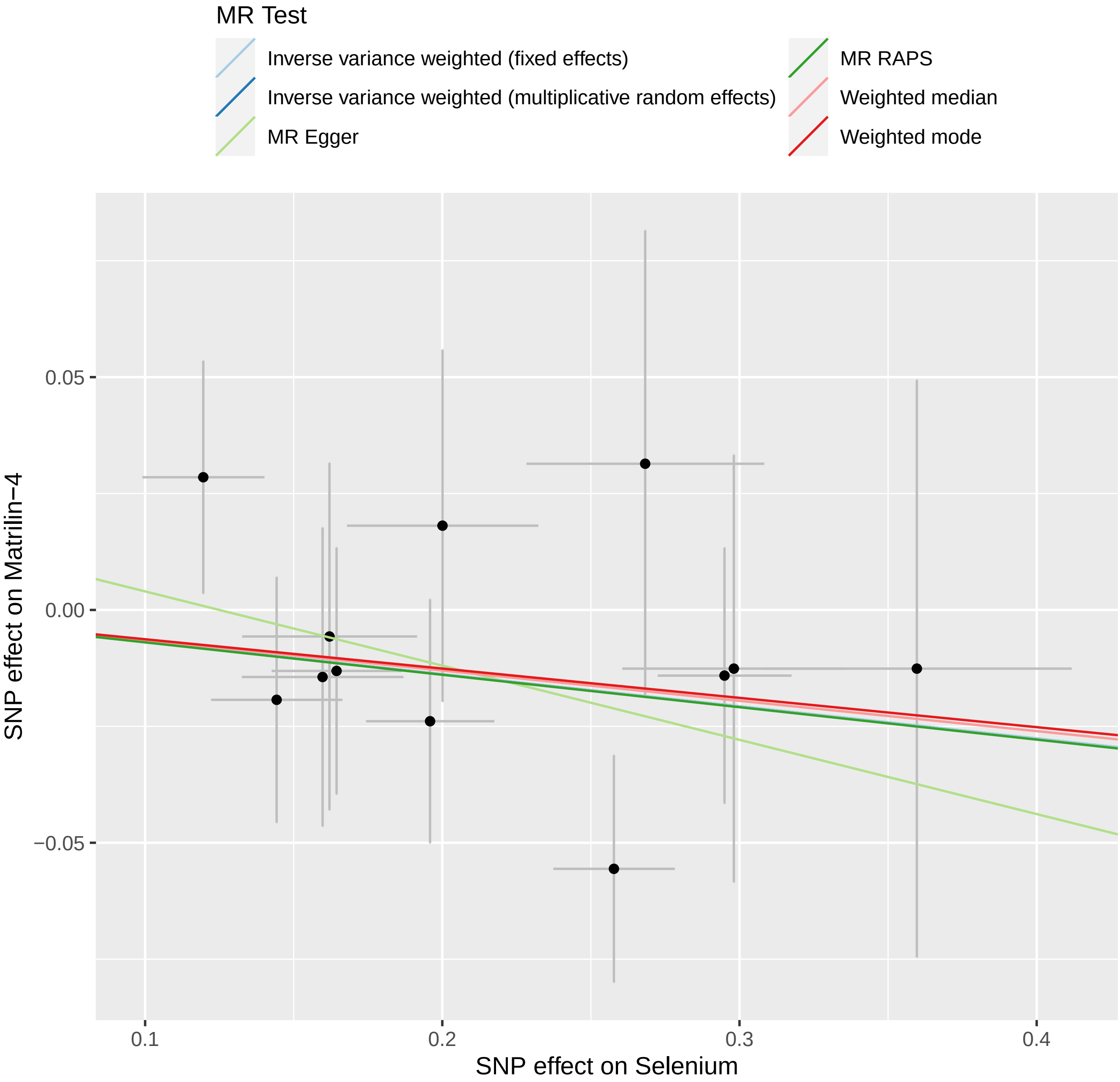

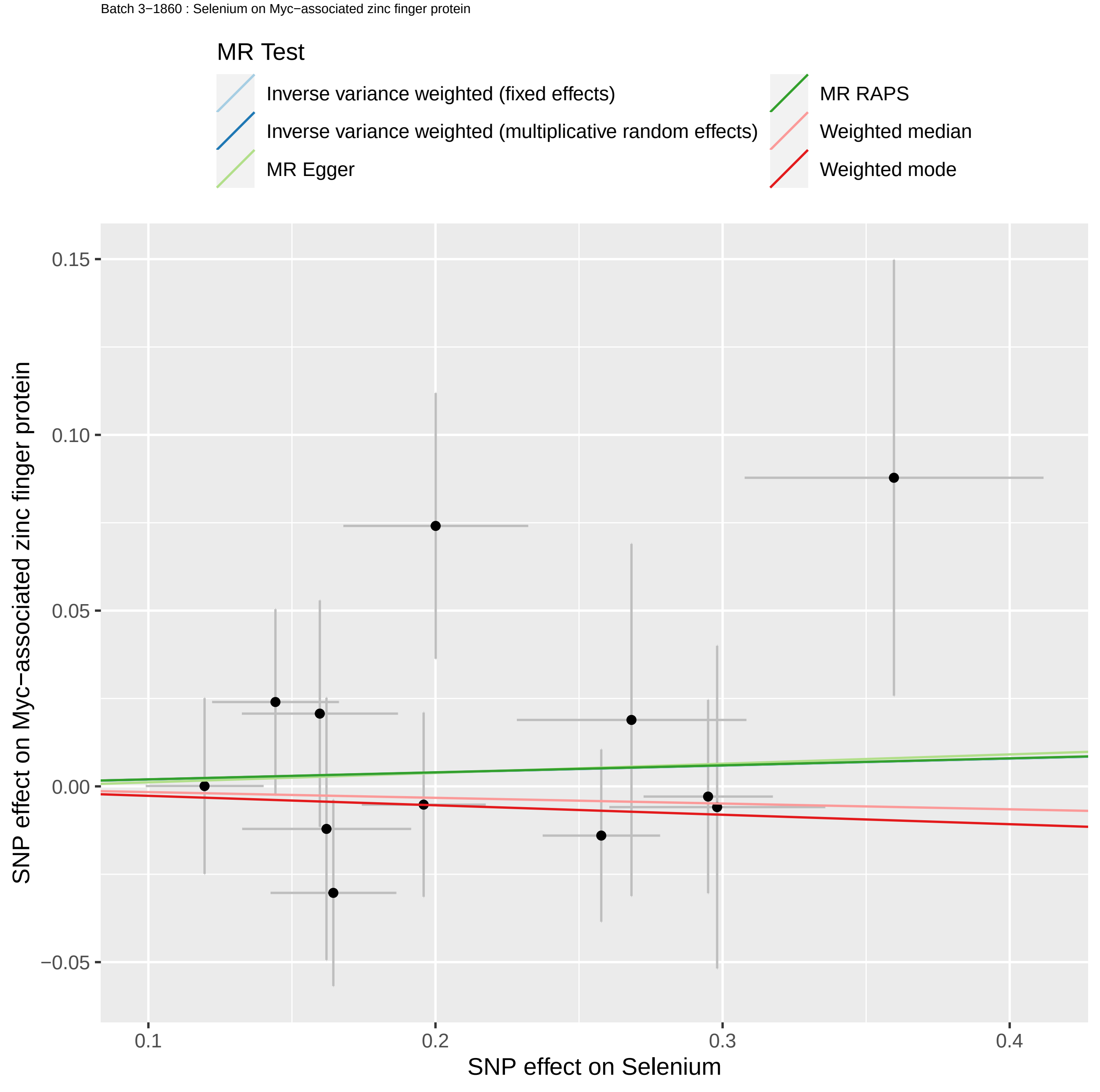
Batch 3−1860 : Selenium on Myc−associated zinc finger protein
MR Test
Inverse variance weighted (fixed effects)
Inverse variance weighted (multiplicative random effects)
MR Egger
MR RAPS
Weighted median
Weighted mode
0.15
0.10
0.05
0.00
−0.05
0.1
0.2
0.3
0.4
SNP effect on Myc−associated zinc finger protein
SNP effect on Selenium

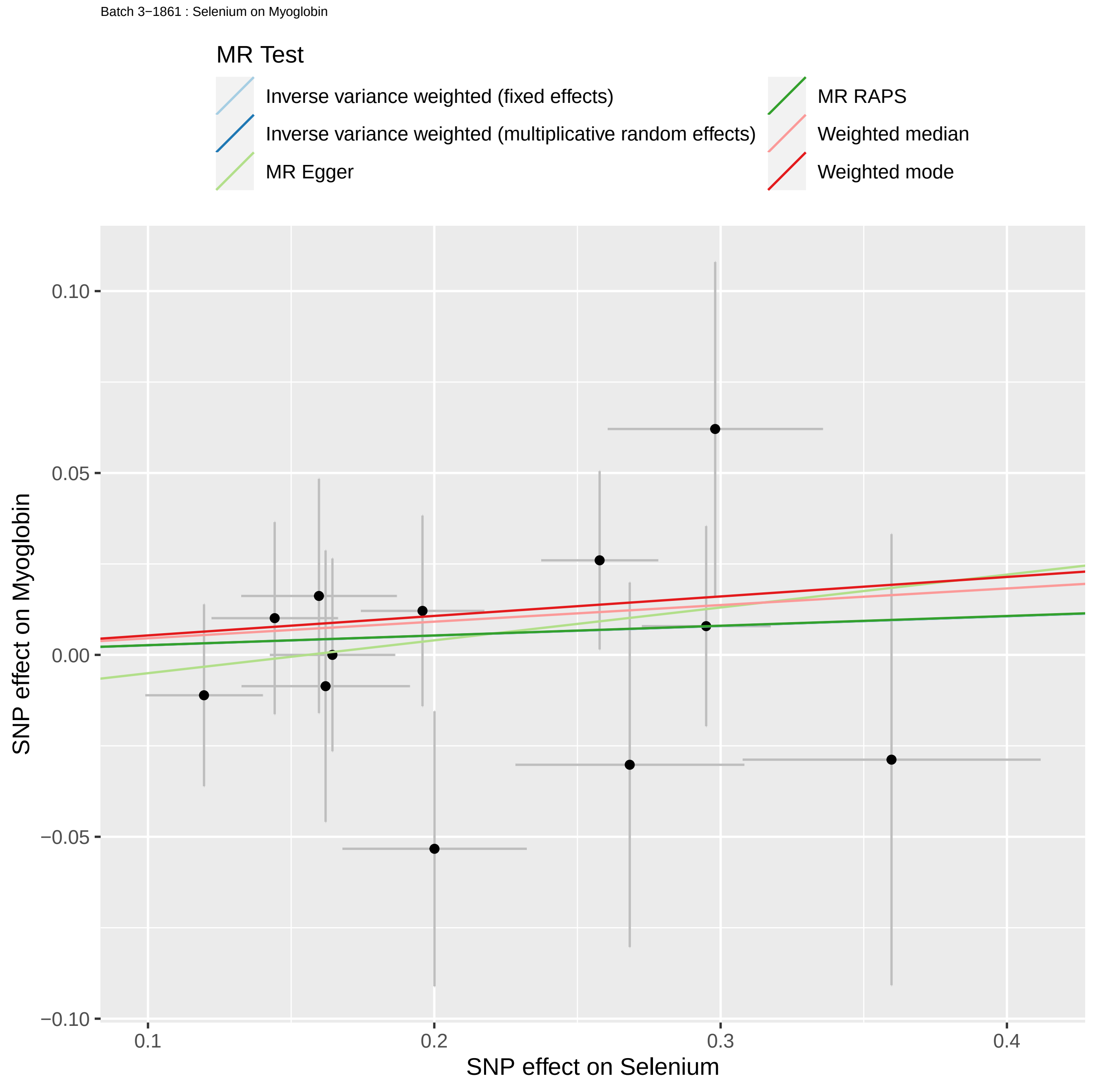
Batch 3−1861 : Selenium on Myoglobin
MR Test
Inverse variance weighted (fixed effects)
Inverse variance weighted (multiplicative random effects)
MR Egger
MR RAPS
Weighted median
Weighted mode
SNP effect on Myoglobin
SNP effect on Selenium
0.10
0.05
0.00
−0.05
−0.10
0.1
0.2
0.3
0.4

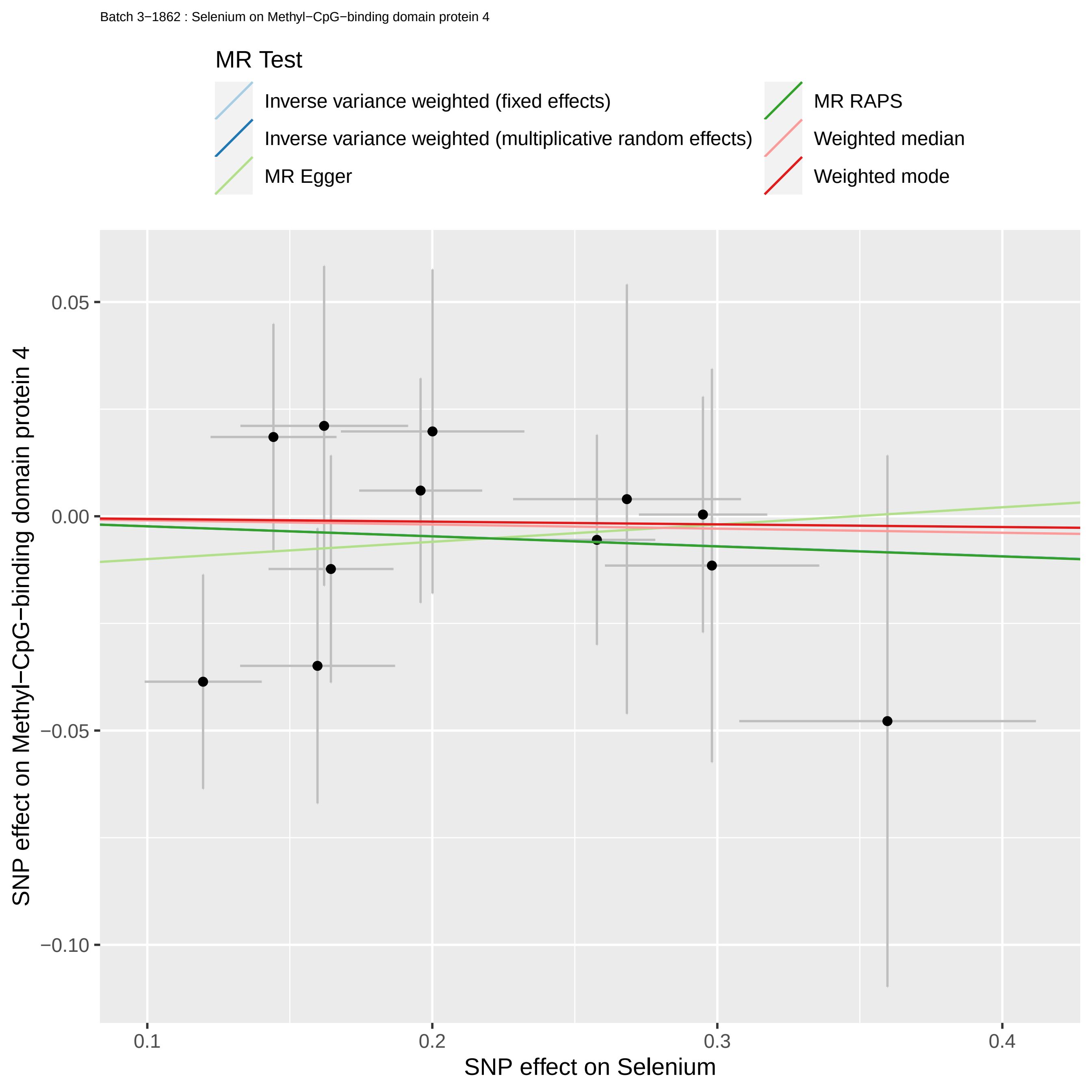

Batch 3−1862 : Selenium on Methyl−CpG−binding domain protein 4
MR Test
Inverse variance weighted (fixed effects)
Inverse variance weighted (multiplicative random effects)
MR Egger
MR RAPS
Weighted median
Weighted mode
0.05
0.00
−0.05
−0.10
0.1
0.2
0.3
0.4
SNP effect on Methyl−CpG−binding domain protein 4
SNP effect on Selenium

Batch 3−1863 : Selenium on Mannose−binding protein C

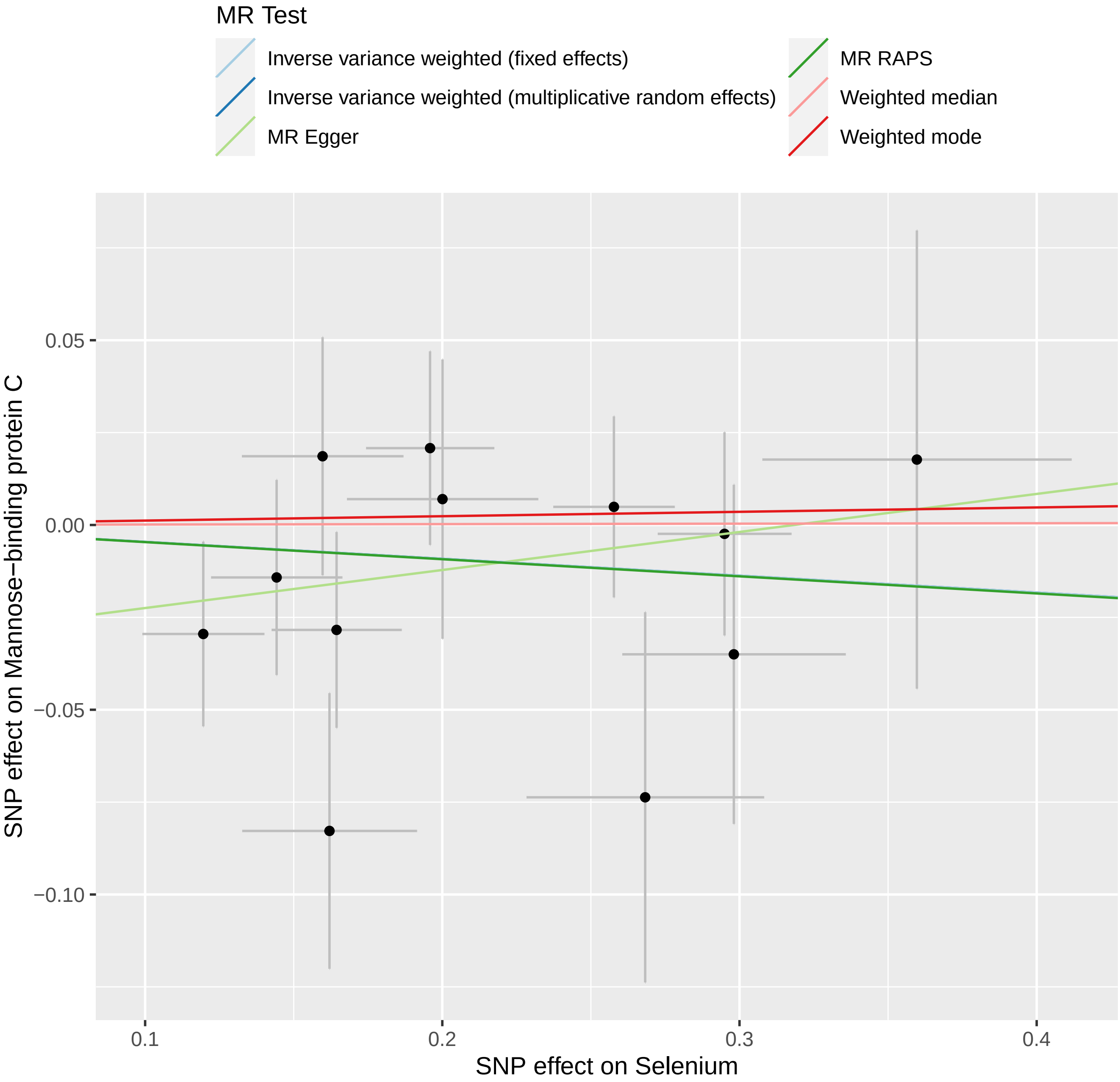

Batch 3−1864 : Selenium on Methylmalonyl−CoA epimerase, mitochondrial

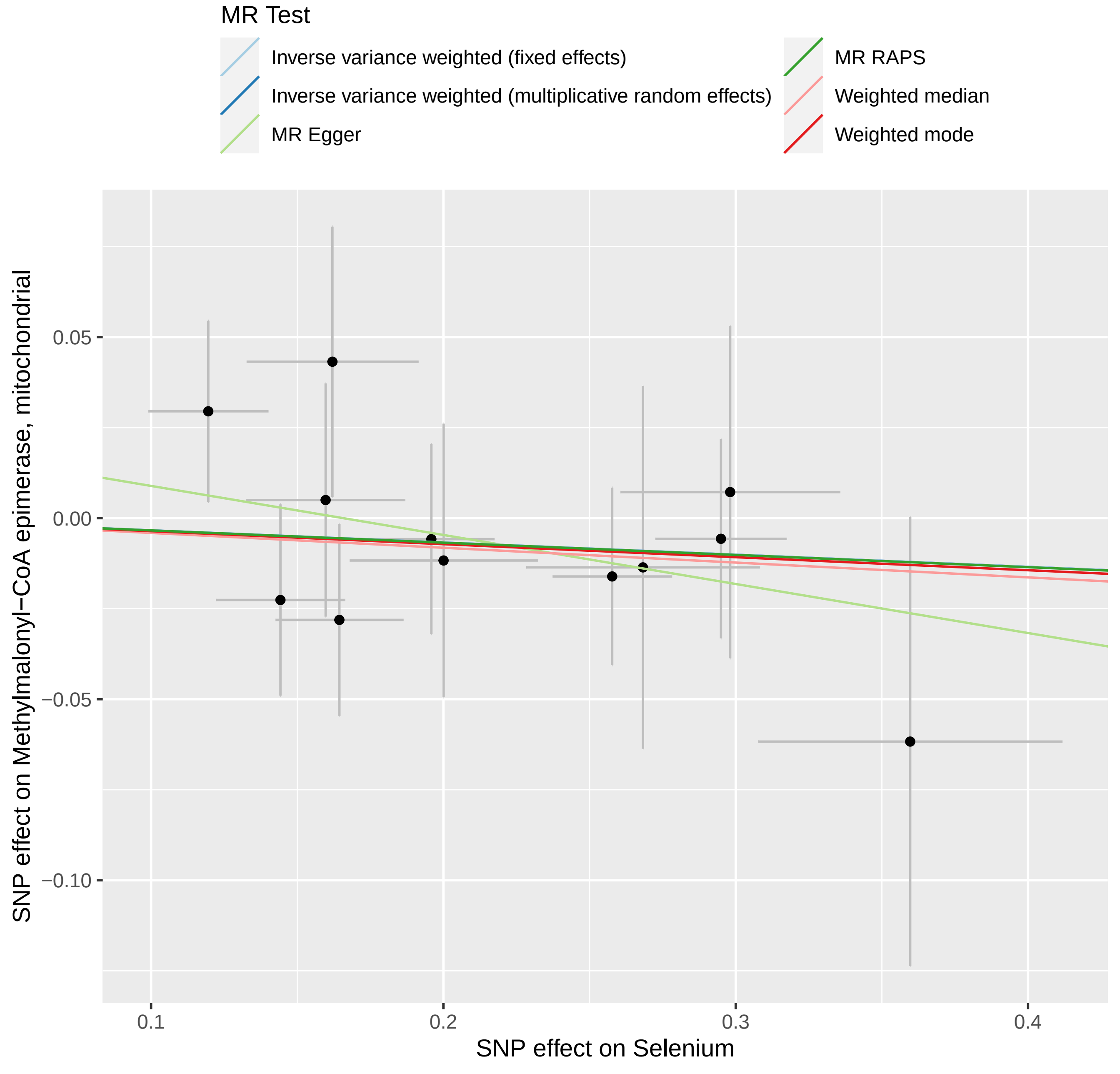

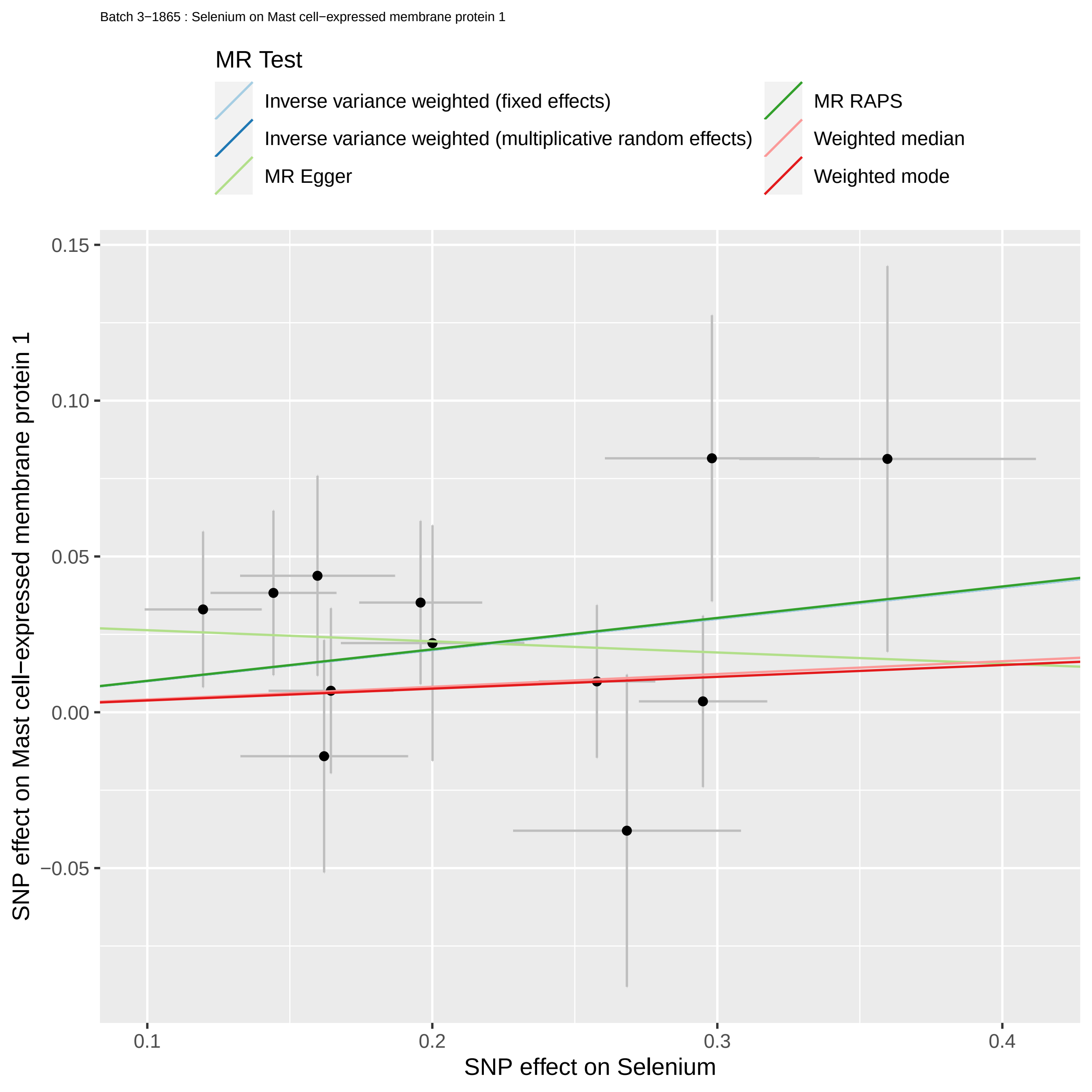

Batch 3−1865 : Selenium on Mast cell−expressed membrane protein 1
MR Test
Inverse variance weighted (fixed effects)
Inverse variance weighted (multiplicative random effects)
MR Egger
MR RAPS
Weighted median
Weighted mode
0.15
0.10
0.05
0.00
−0.05
0.1
0.2
0.3
0.4
SNP effect on Mast cell−expressed membrane protein 1
SNP effect on Selenium

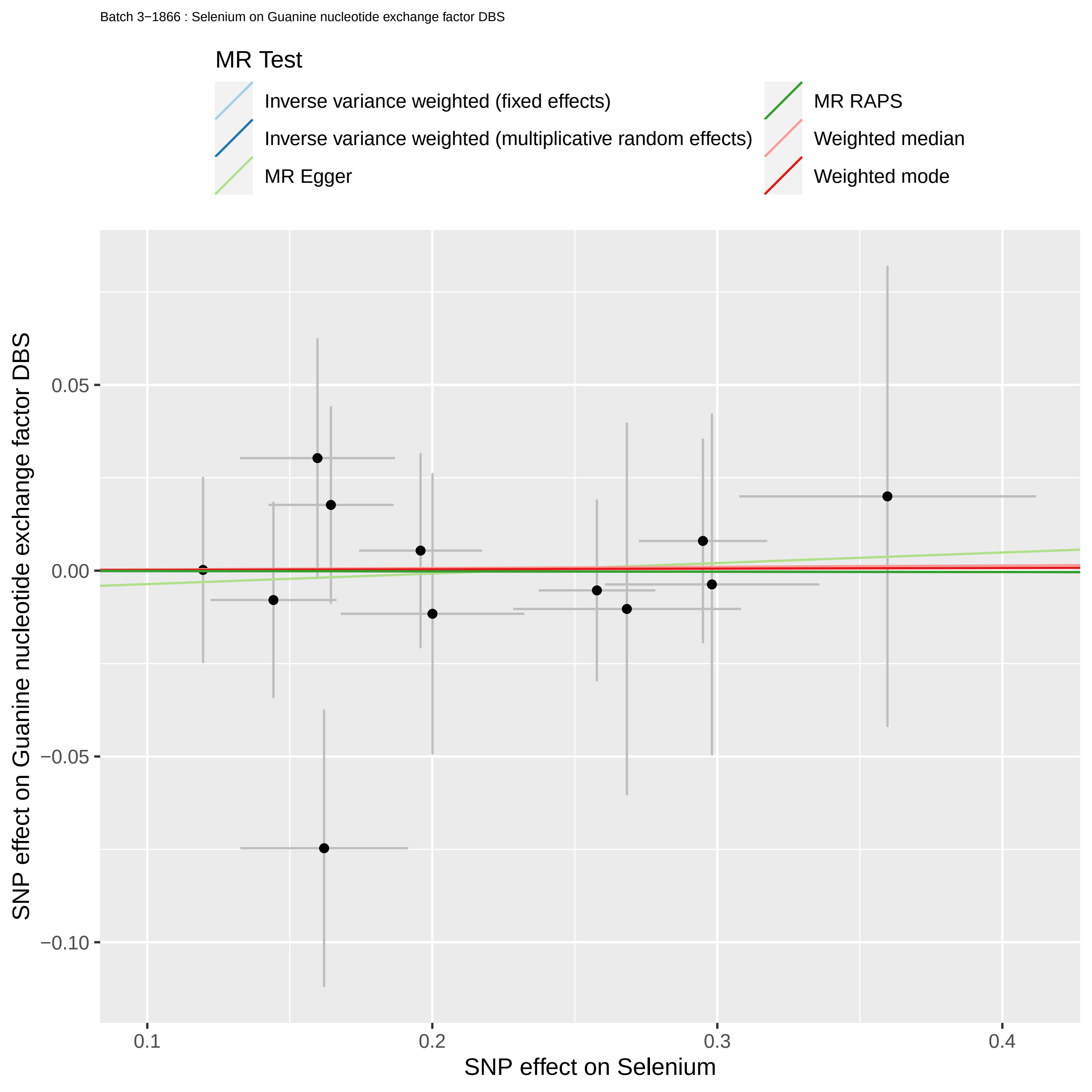
Batch 3−1866 : Selenium on Guanine nucleotide exchange factor DBS
MR Test
Inverse variance weighted (fixed effects)
Inverse variance weighted (multiplicative random effects)
MR Egger
MR RAPS
Weighted median
Weighted mode
0.05
0.00
−0.05
−0.10
0.1
0.2
0.3
0.4
SNP effect on Guanine nucleotide exchange factor DBS
SNP effect on Selenium

Batch 3−1867 : Selenium on Multiple coagulation factor deficiency protein 2

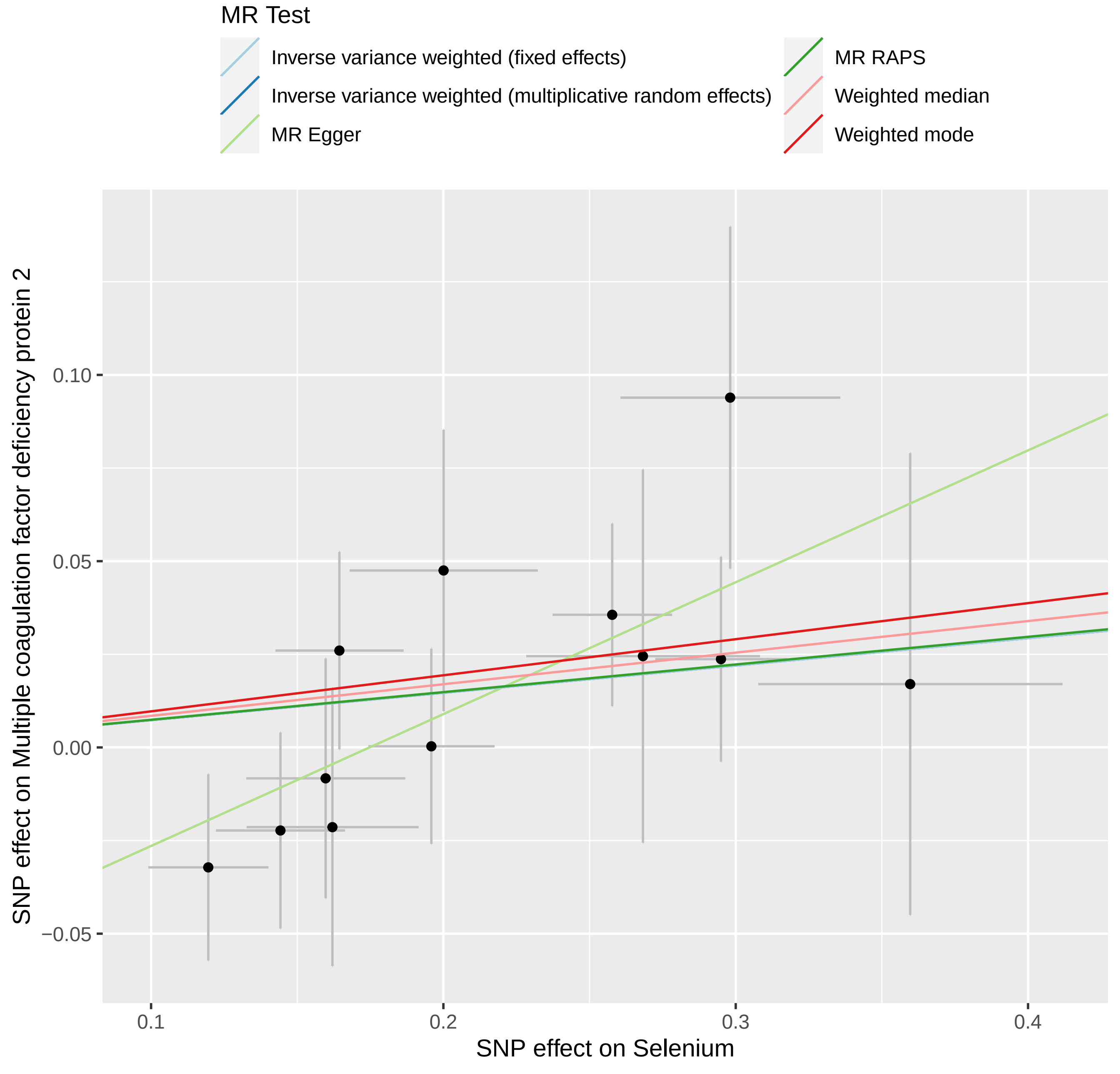

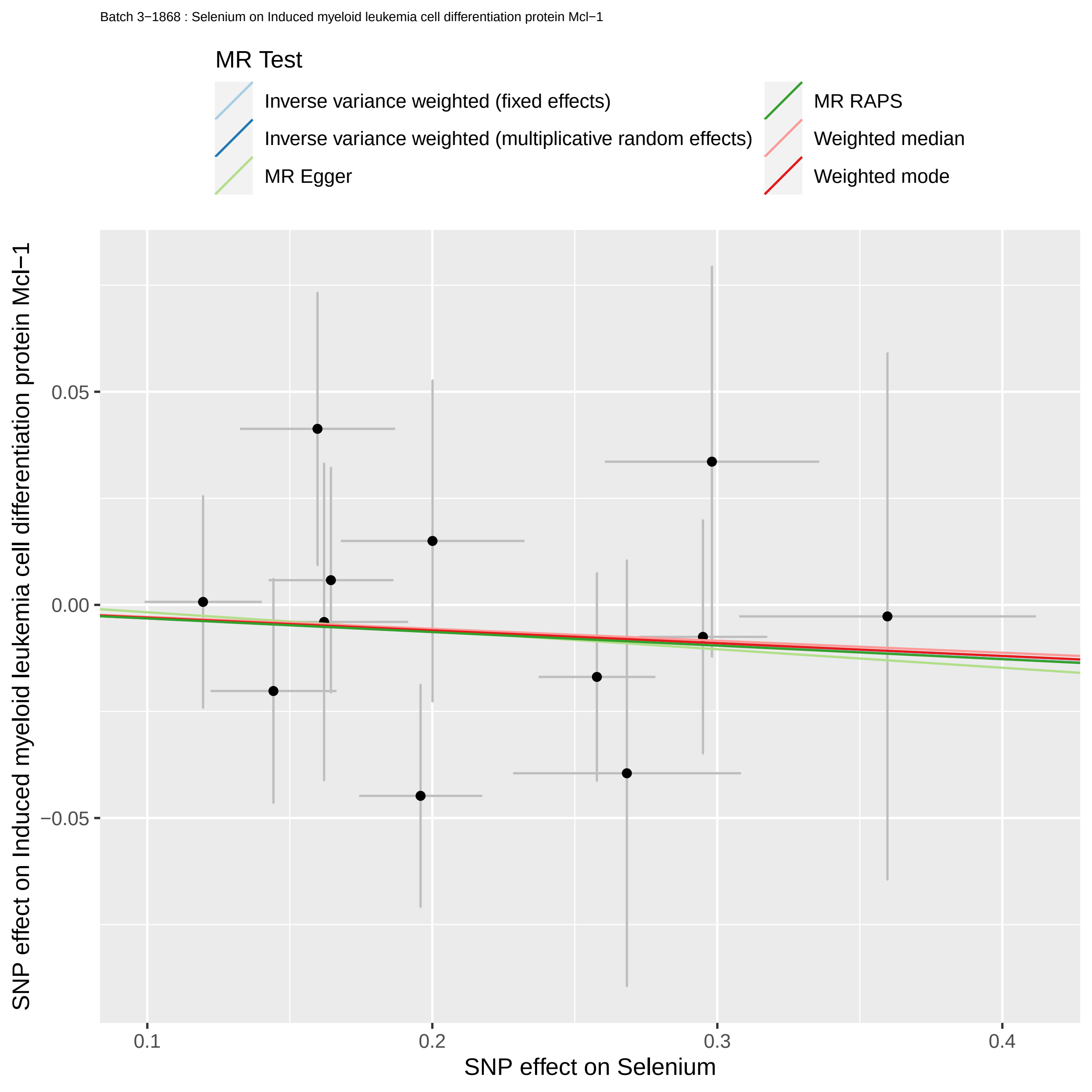
Batch 3−1868 : Selenium on Induced myeloid leukemia cell differentiation protein Mcl−1
MR Test
Inverse variance weighted (fixed effects)
Inverse variance weighted (multiplicative random effects)
MR Egger
MR RAPS
Weighted median
Weighted mode
SNP effect on Induced myeloid leukemia cell differentiation protein Mcl−1
0.05
0.00
−0.05
0.1
0.2
0.3
0.4
SNP effect on Selenium

Batch 3−1869 : Selenium on Malignant T−cell−amplified sequence 1

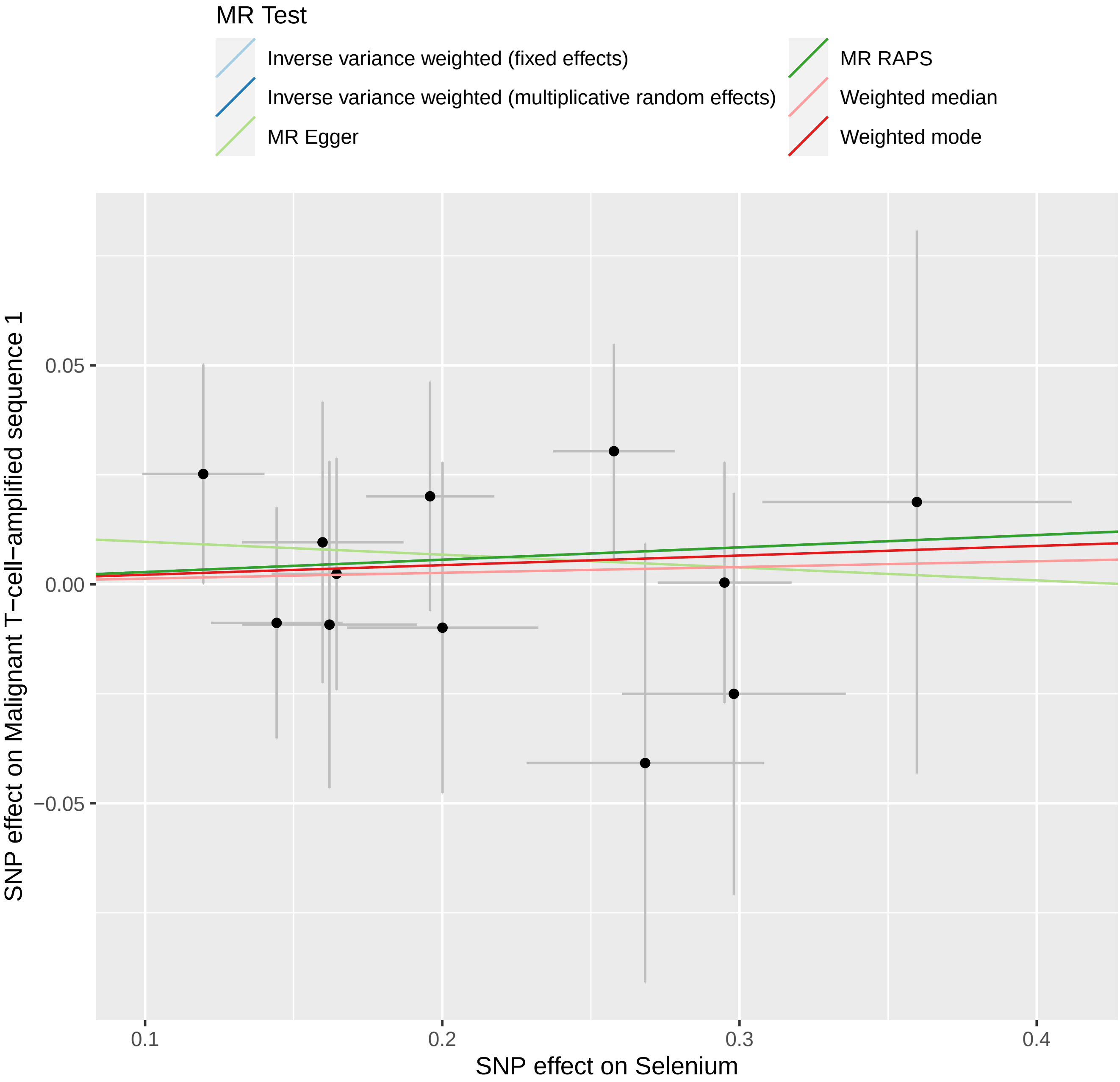

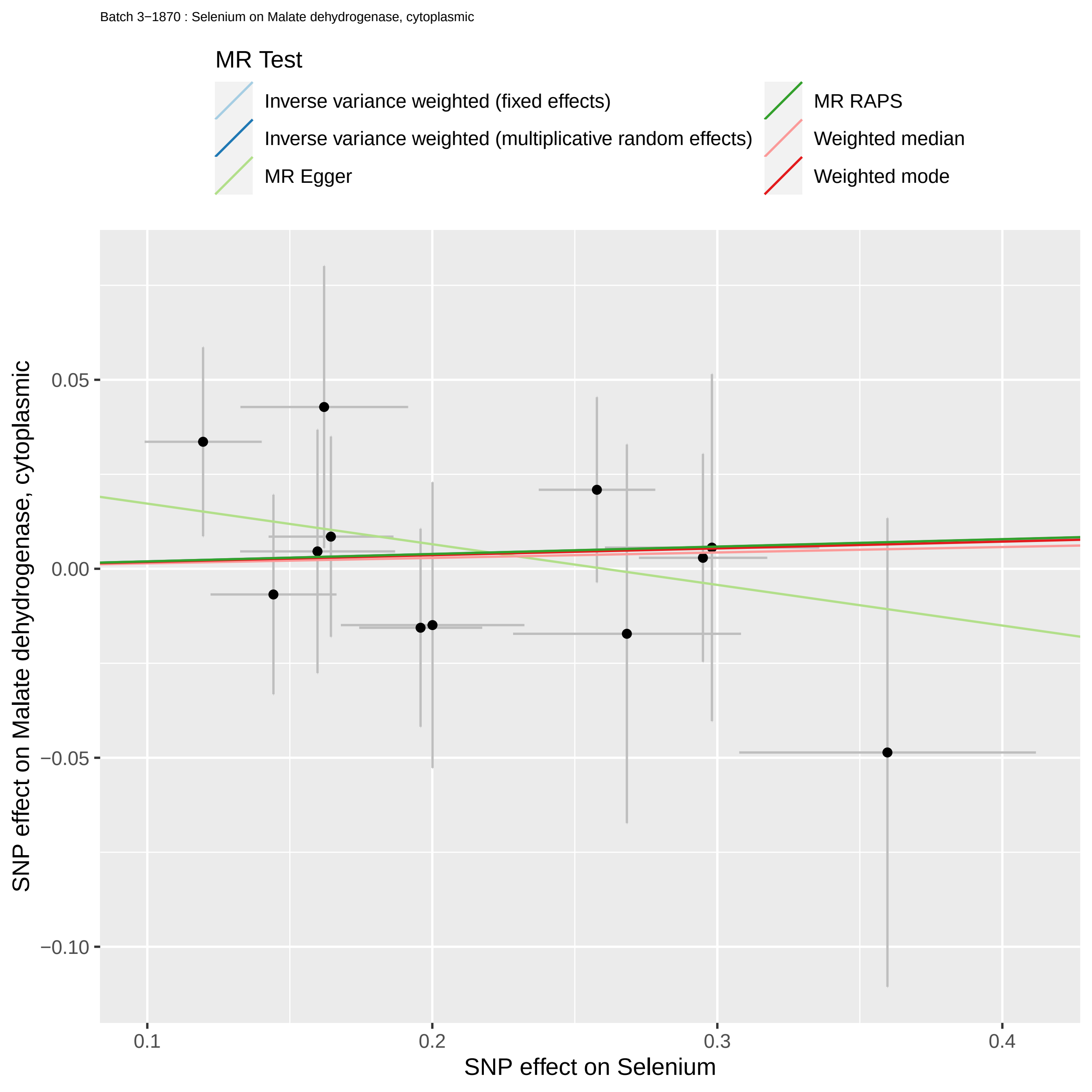
Batch 3−1870 : Selenium on Malate dehydrogenase, cytoplasmic
MR Test
Inverse variance weighted (fixed effects)
Inverse variance weighted (multiplicative random effects)
MR Egger
MR RAPS
Weighted median
Weighted mode
SNP effect on Malate dehydrogenase, cytoplasmic
0.05
0.00
−0.05
−0.10
0.1
0.2
0.3
0.4
SNP effect on Selenium

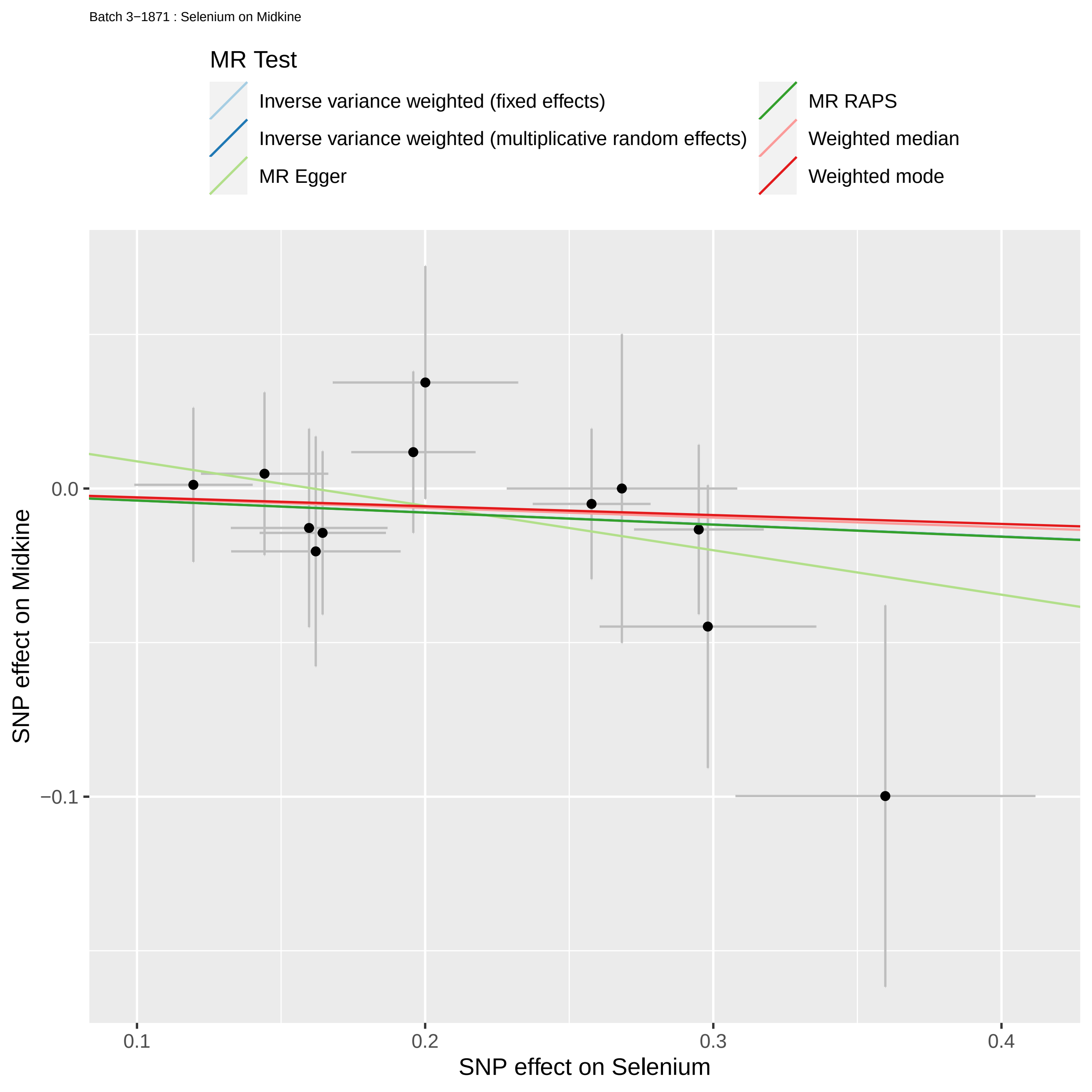
Batch 3−1871 : Selenium on Midkine
MR Test
Inverse variance weighted (fixed effects)
Inverse variance weighted (multiplicative random effects)
MR Egger
MR RAPS
Weighted median
Weighted mode
0.0
−0.1
0.1
0.2
0.3
0.4
SNP effect on Midkine
SNP effect on Selenium

Batch 3−1872 : Selenium on Nuclear protein MDM1

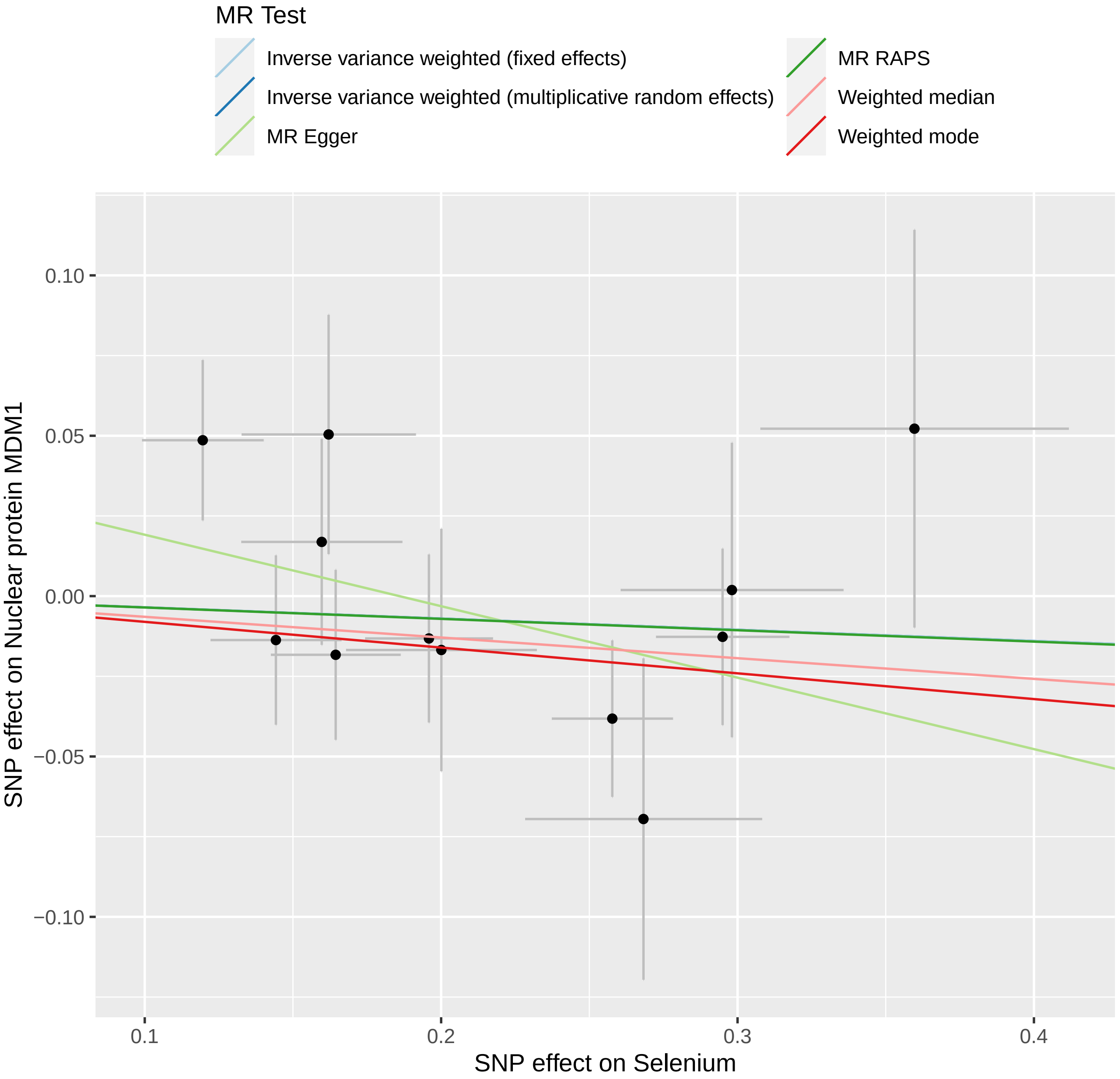

Batch 3−1873 : Selenium on E3 ubiquitin−protein ligase Mdm2

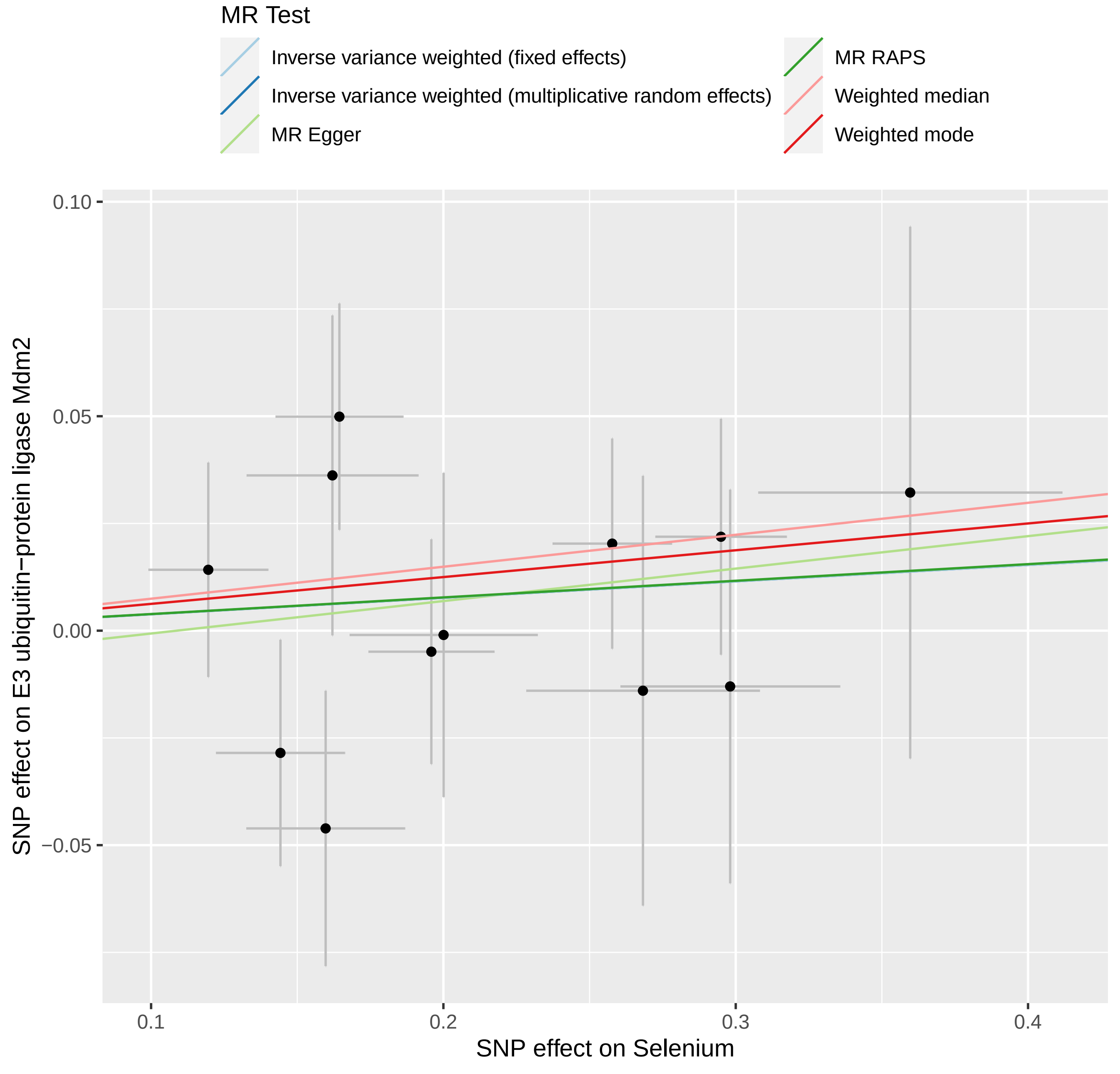

Batch 3−1874 : Selenium on Mediator of RNA polymerase II transcription subunit 1

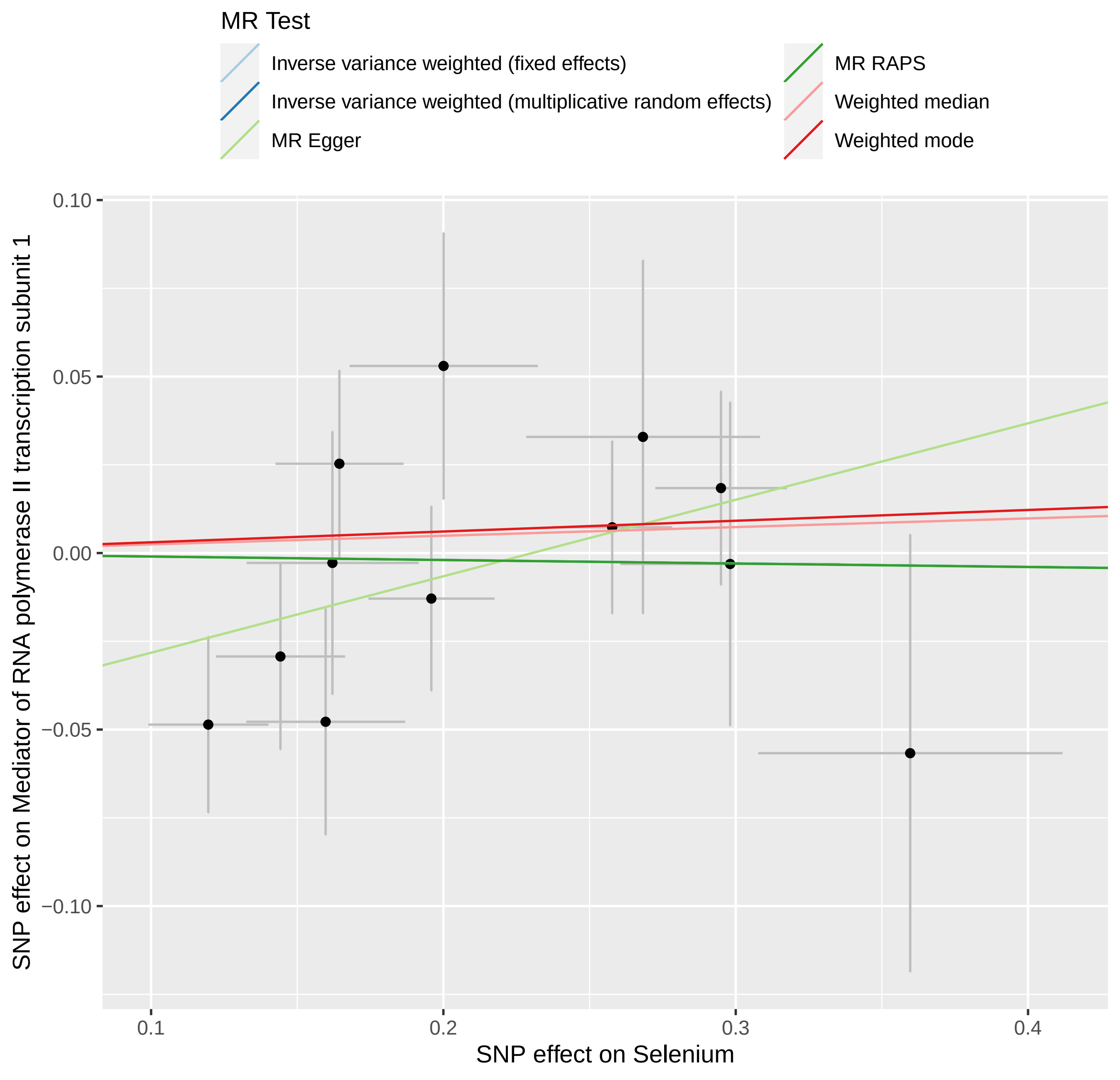

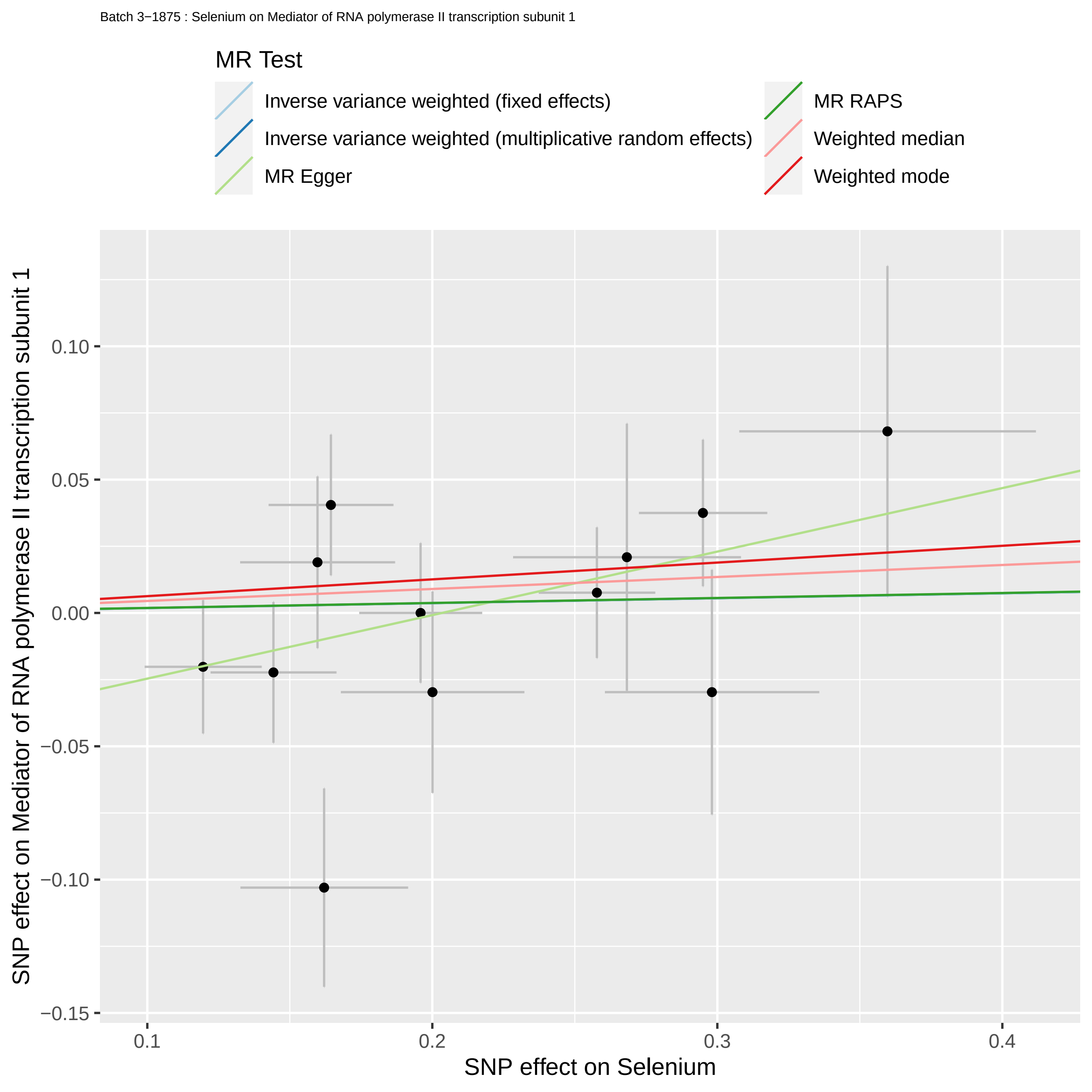

Batch 3−1875 : Selenium on Mediator of RNA polymerase II transcription subunit 1
MR Test
Inverse variance weighted (fixed effects)
Inverse variance weighted (multiplicative random effects)
MR Egger
MR RAPS
Weighted median
Weighted mode
SNP effect on Mediator of RNA polymerase II transcription subunit 1
0.10
0.05
0.00
−0.05
−0.10
−0.15
0.1
0.2
0.3
0.4
SNP effect on Selenium

Batch 3−1876 : Selenium on Mediator of RNA polymerase II transcription subunit 4

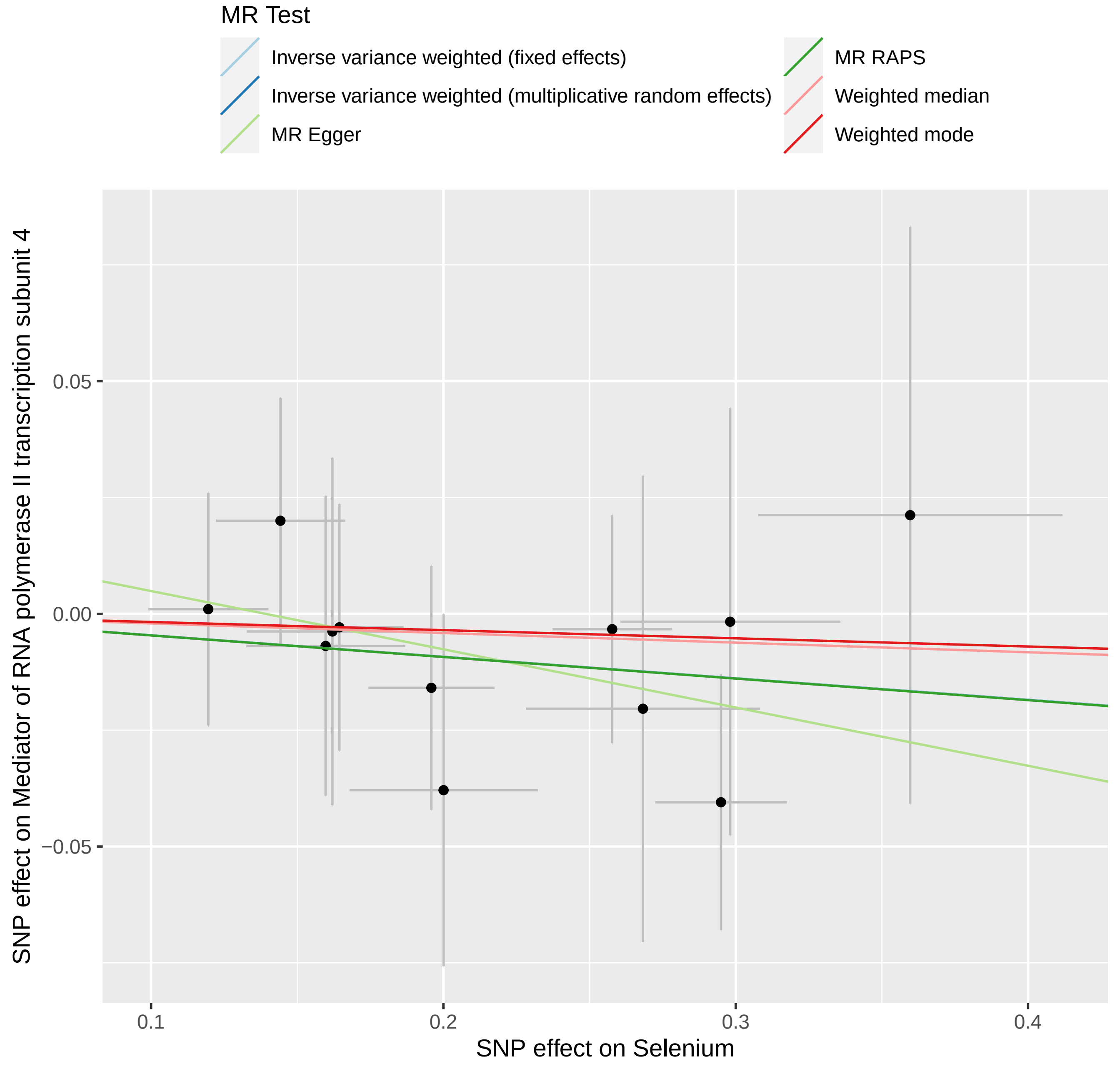

Batch 3−1877 : Selenium on Myocyte−specific enhancer factor 2C

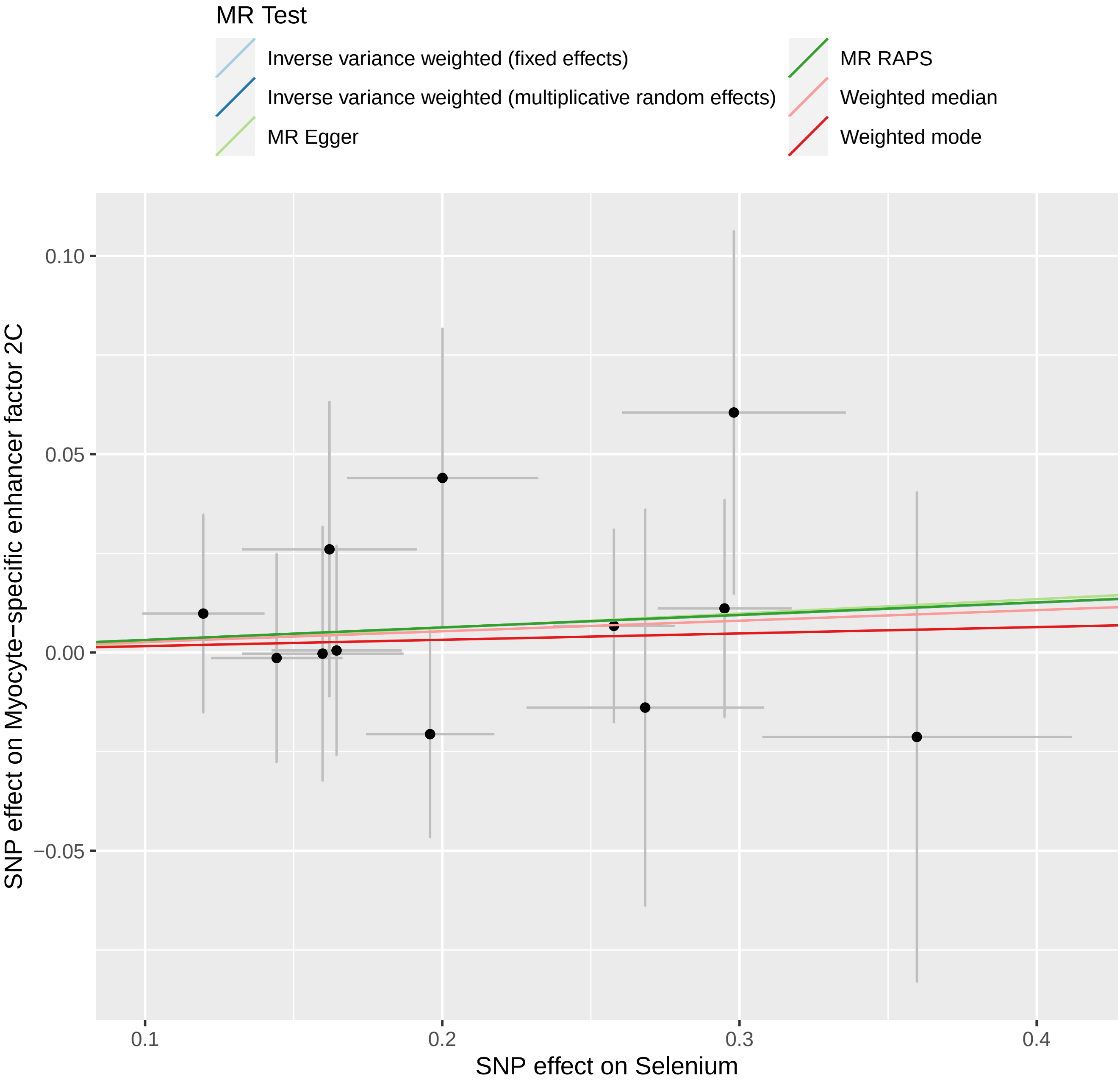

Batch 3−1878 : Selenium on Multiple epidermal growth factor−like domains protein 10

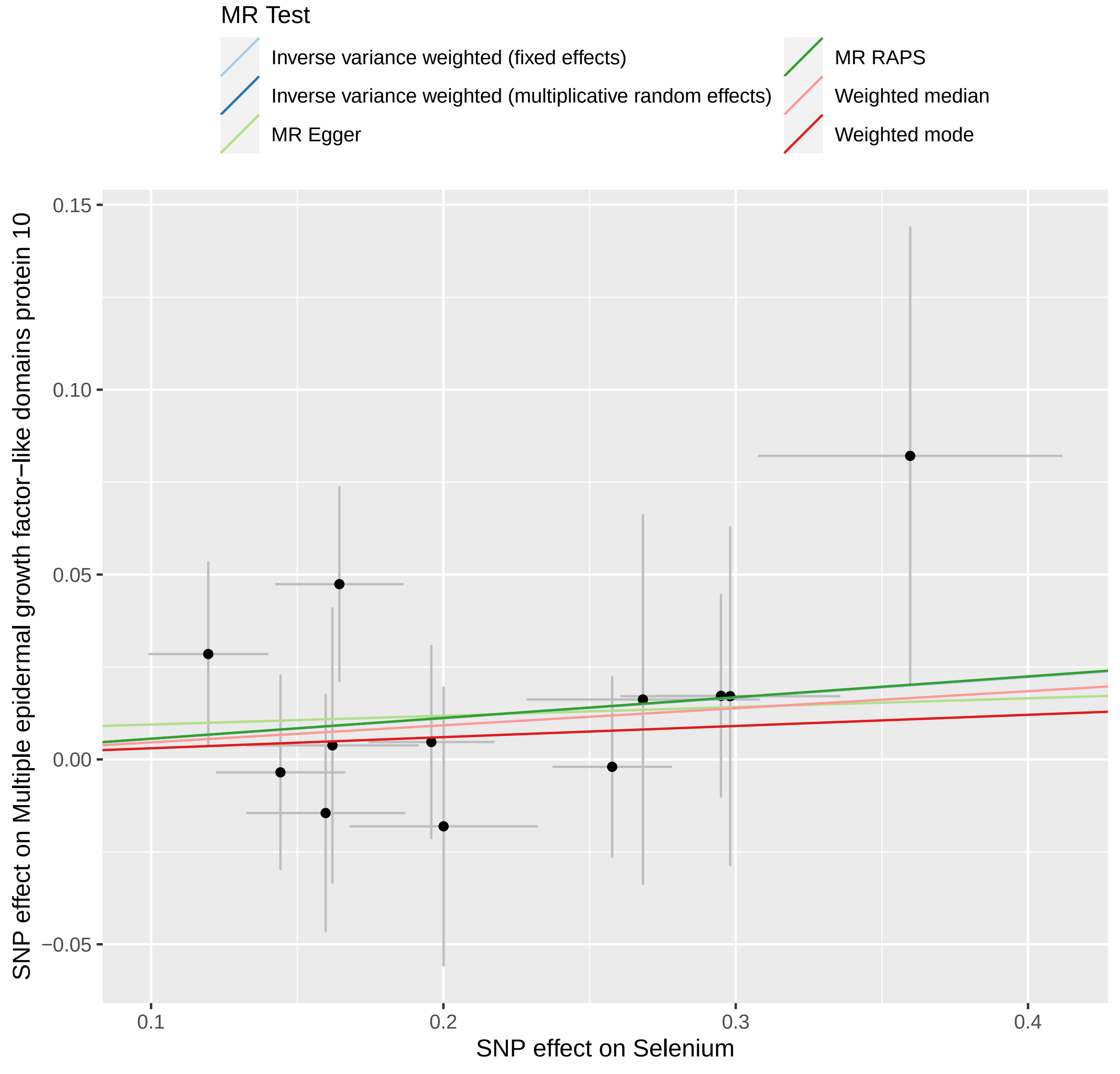

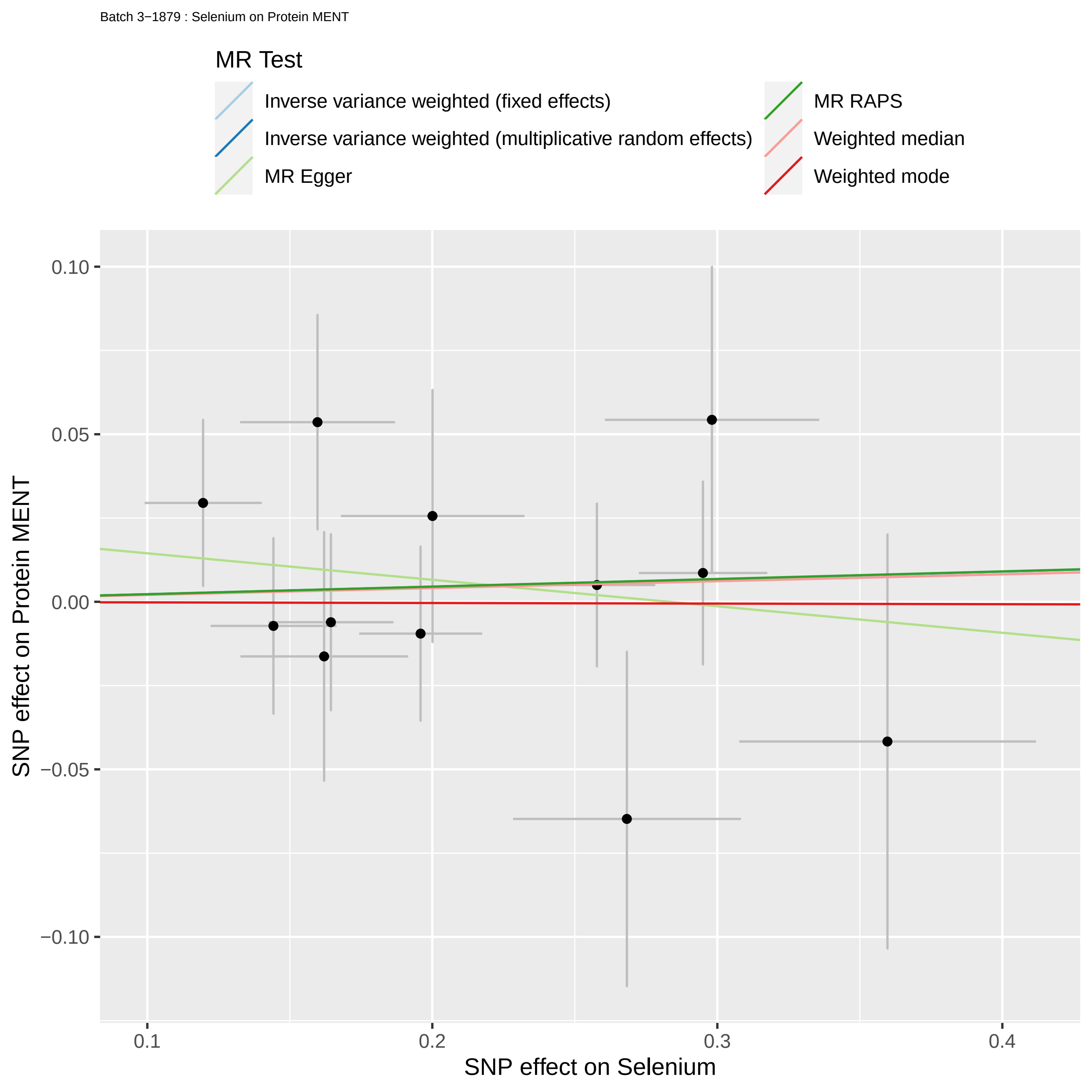
Batch 3−1879 : Selenium on Protein MENT
MR Test
Inverse variance weighted (fixed effects)
Inverse variance weighted (multiplicative random effects)
MR Egger
MR RAPS
Weighted median
Weighted mode
0.10
0.05
0.00
−0.05
−0.10
SNP effect on Protein MENT
0.1
0.2
0.3
0.4
SNP effect on Selenium

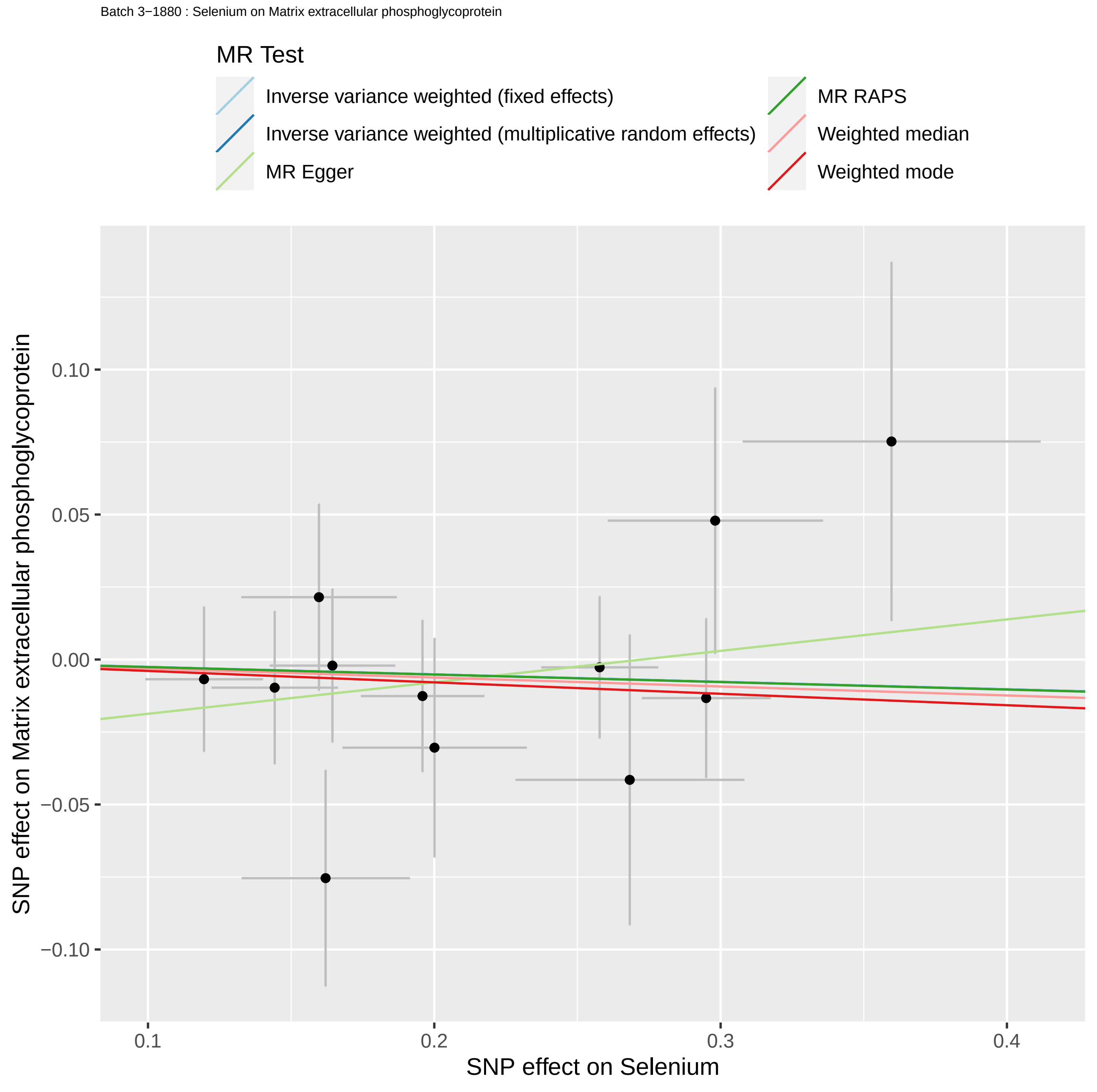

Batch 3−1880 : Selenium on Matrix extracellular phosphoglycoprotein
MR Test
Inverse variance weighted (fixed effects)
Inverse variance weighted (multiplicative random effects)
MR Egger
MR RAPS
Weighted median
Weighted mode
0.10
0.05
0.00
−0.05
−0.10
0.1
0.2
0.3
0.4
SNP effect on Matrix extracellular phosphoglycoprotein
SNP effect on Selenium

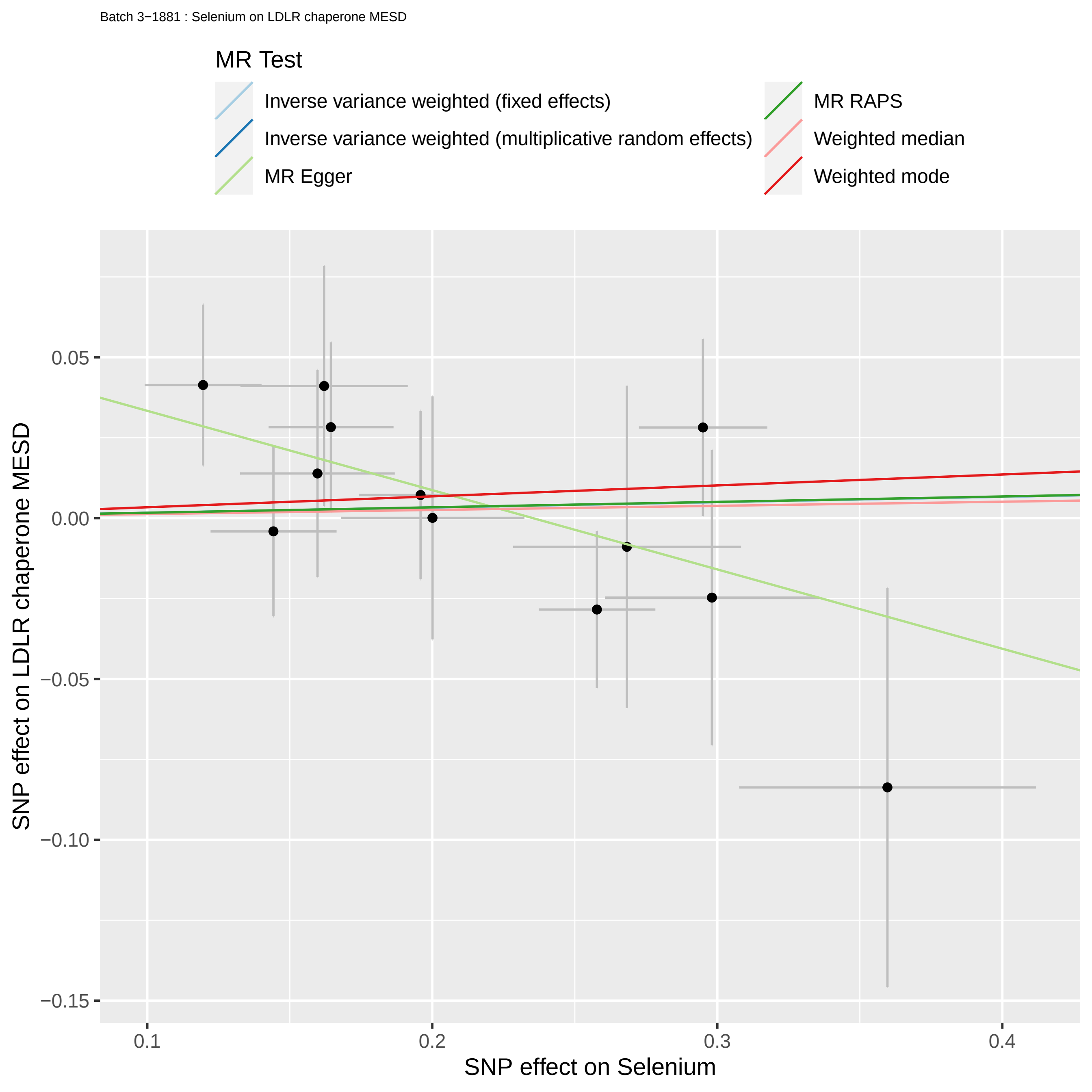
Batch 3−1881 : Selenium on LDLR chaperone MESD
MR Test
Inverse variance weighted (fixed effects)
Inverse variance weighted (multiplicative random effects)
MR Egger
MR RAPS
Weighted median
Weighted mode
SNP effect on LDLR chaperone MESD
0.05
0.00
−0.05
−0.10
−0.15
0.1
0.2
0.3
0.4
SNP effect on Selenium

Batch 3−1882 : Selenium on Hepatocyte growth factor receptor

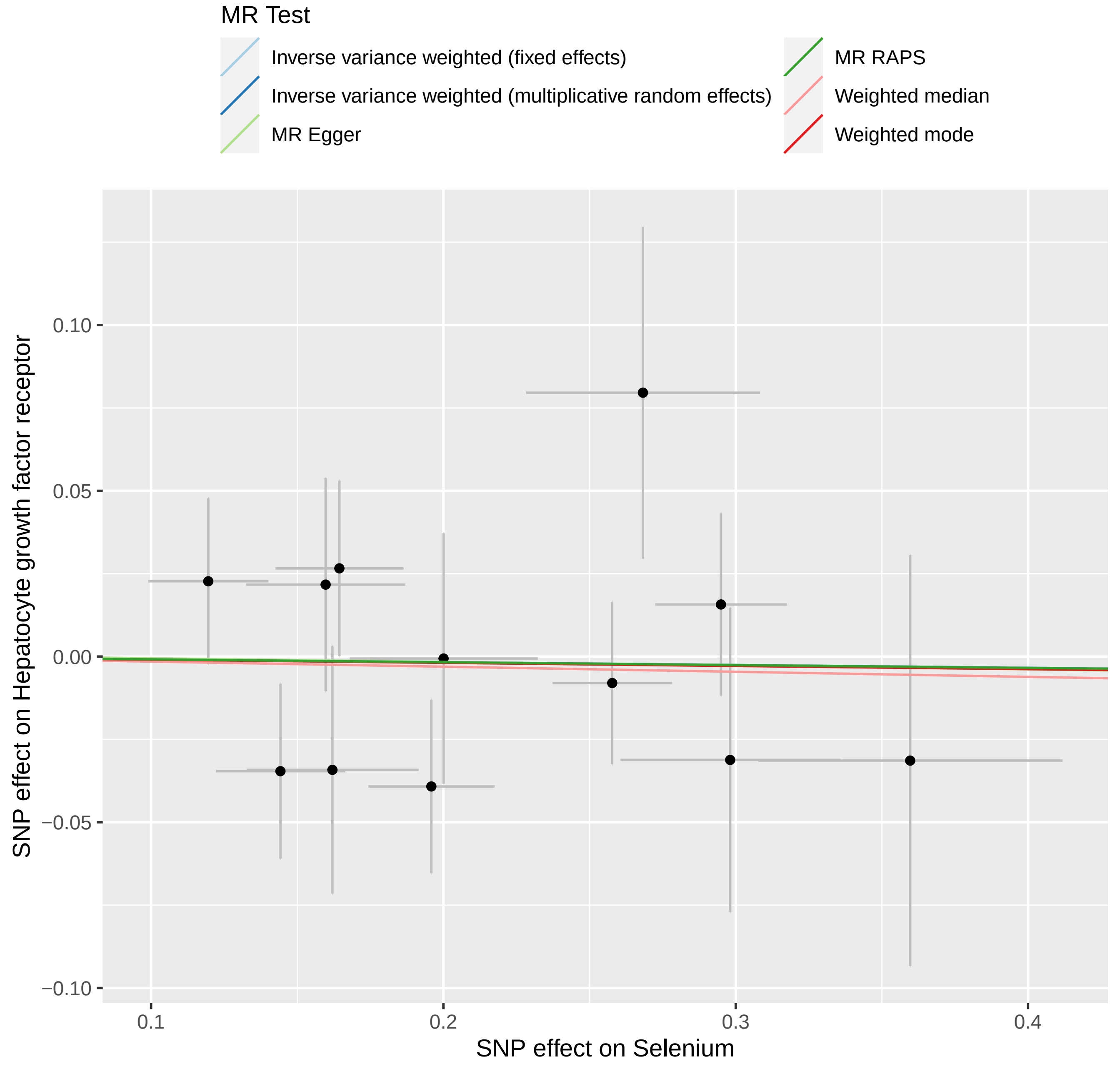

Batch 3−1883 : Selenium on tRNA (guanine−N(7)−)−methyltransferase

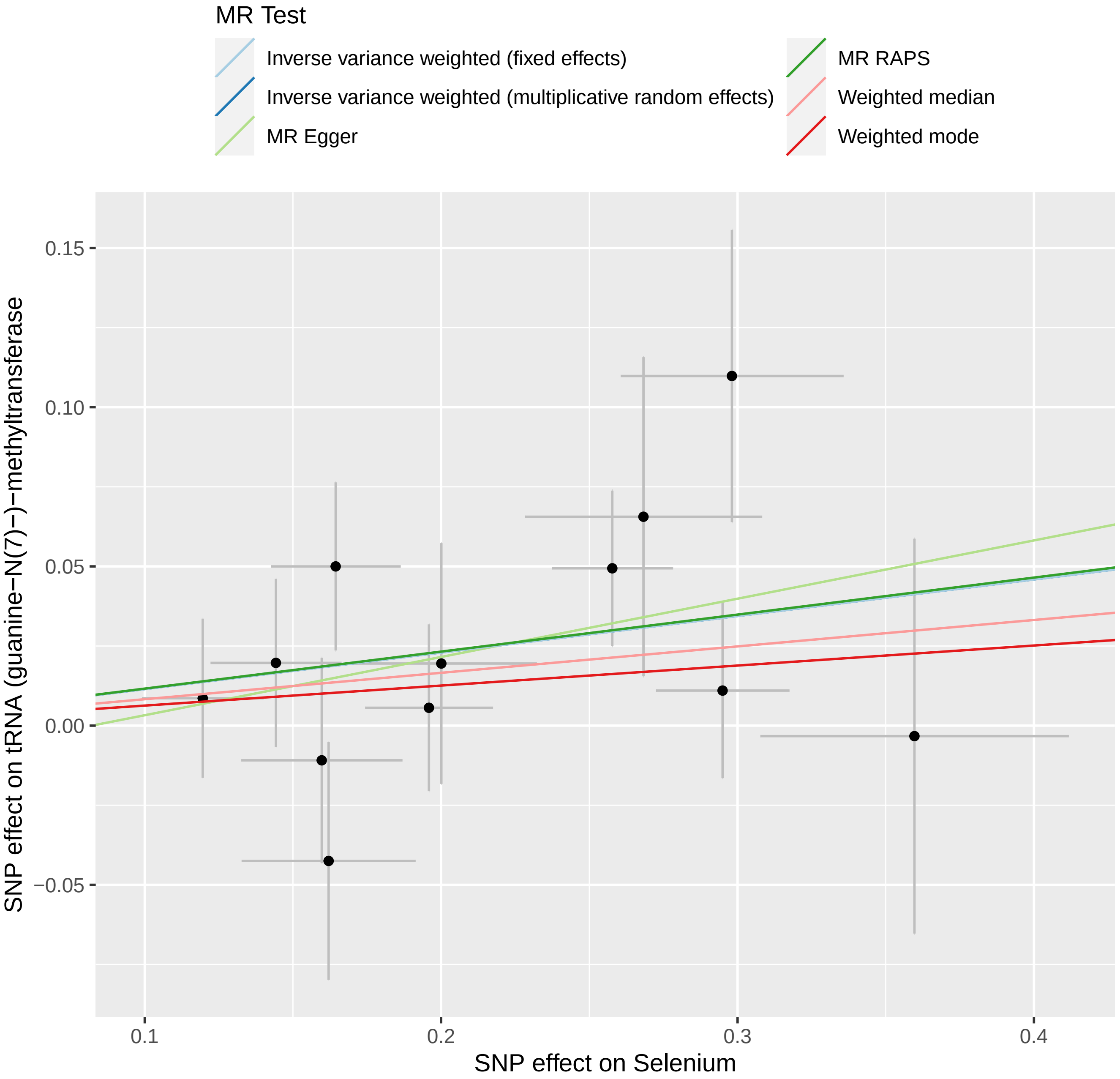

Batch 3−1884 : Selenium on Microfibrillar−associated protein 1

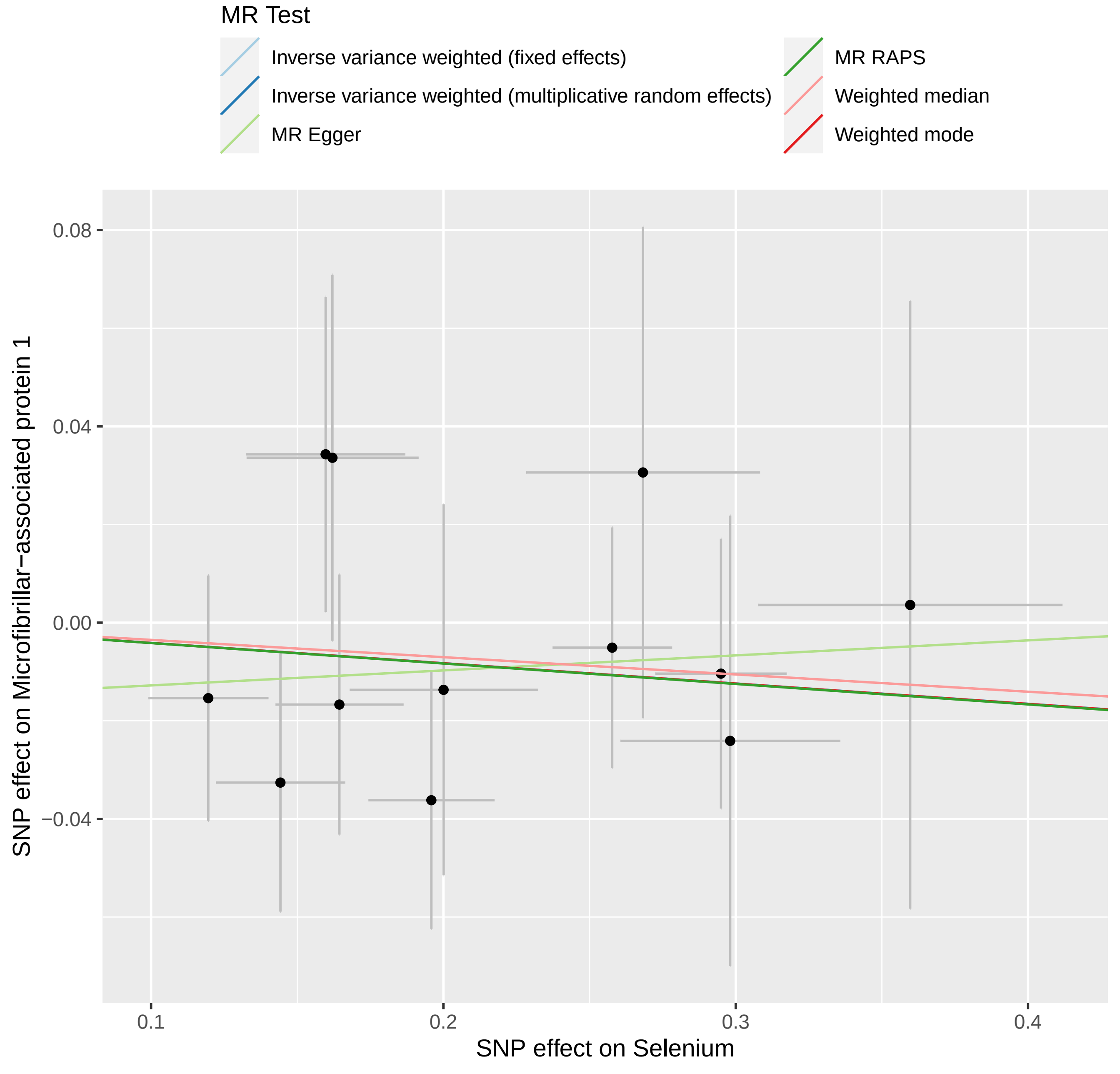

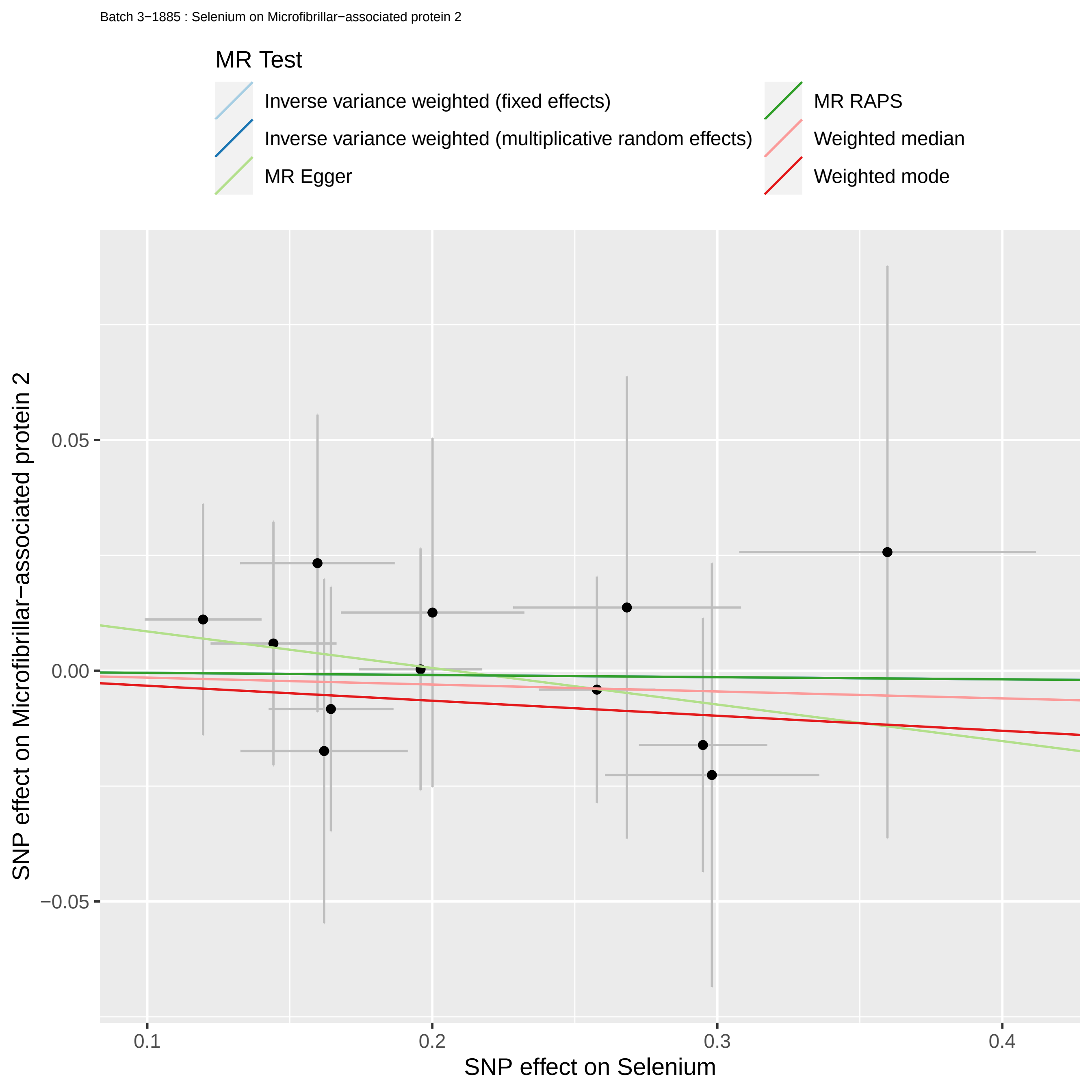

Batch 3−1885 : Selenium on Microfibrillar−associated protein 2
MR Test
Inverse variance weighted (fixed effects)
Inverse variance weighted (multiplicative random effects)
MR Egger
MR RAPS
Weighted median
Weighted mode
SNP effect on Microfibrillar−associated protein 2
0.05
0.00
−0.05
0.1
0.2
0.3
0.4
SNP effect on Selenium

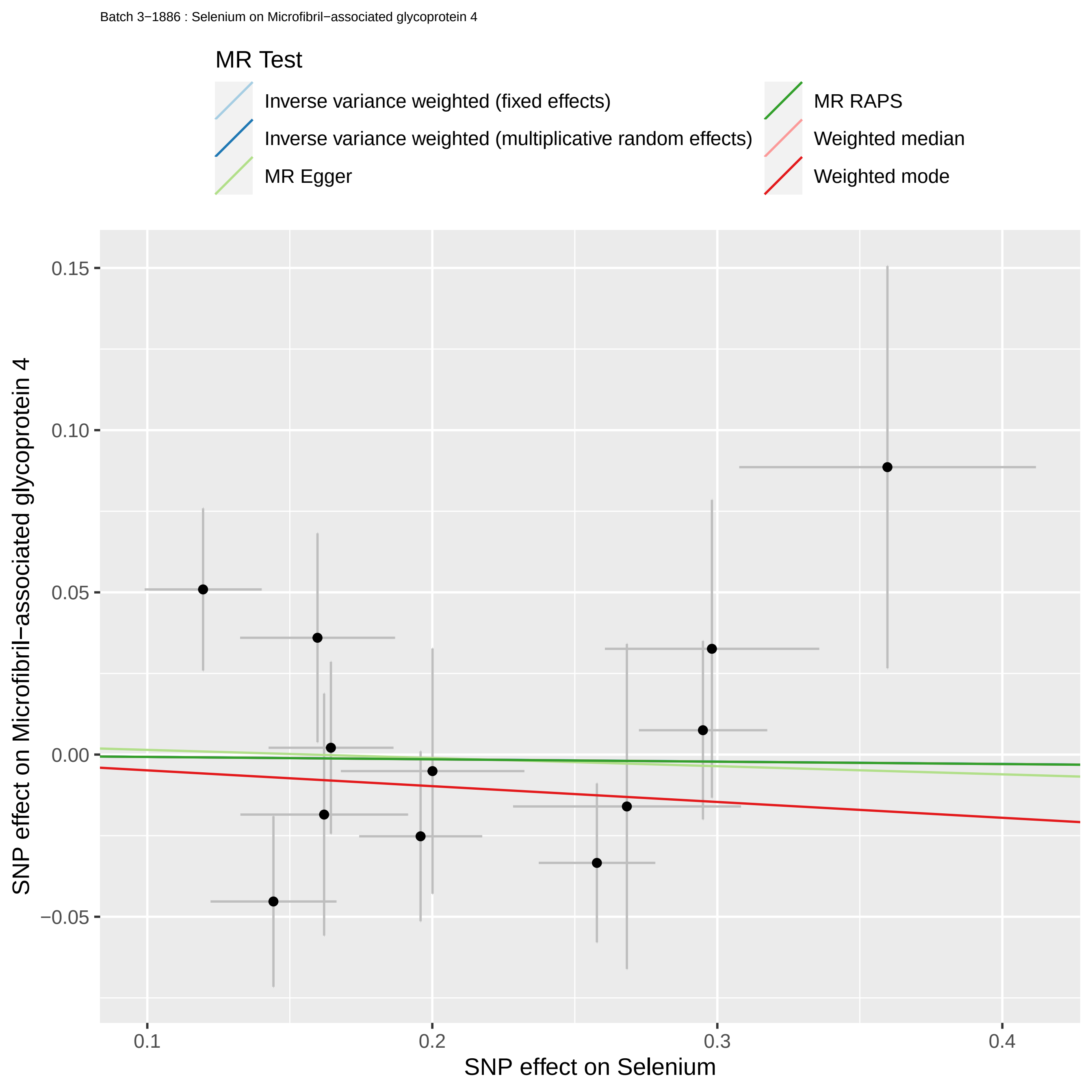
Batch 3−1886 : Selenium on Microfibril−associated glycoprotein 4
MR Test
Inverse variance weighted (fixed effects)
Inverse variance weighted (multiplicative random effects)
MR Egger
MR RAPS
Weighted median
Weighted mode
0.15
0.10
0.05
0.00
−0.05
0.1
0.2
0.3
0.4
SNP effect on Microfibril−associated glycoprotein 4
SNP effect on Selenium

Batch 3−1887 : Selenium on Microfibrillar−associated protein 5

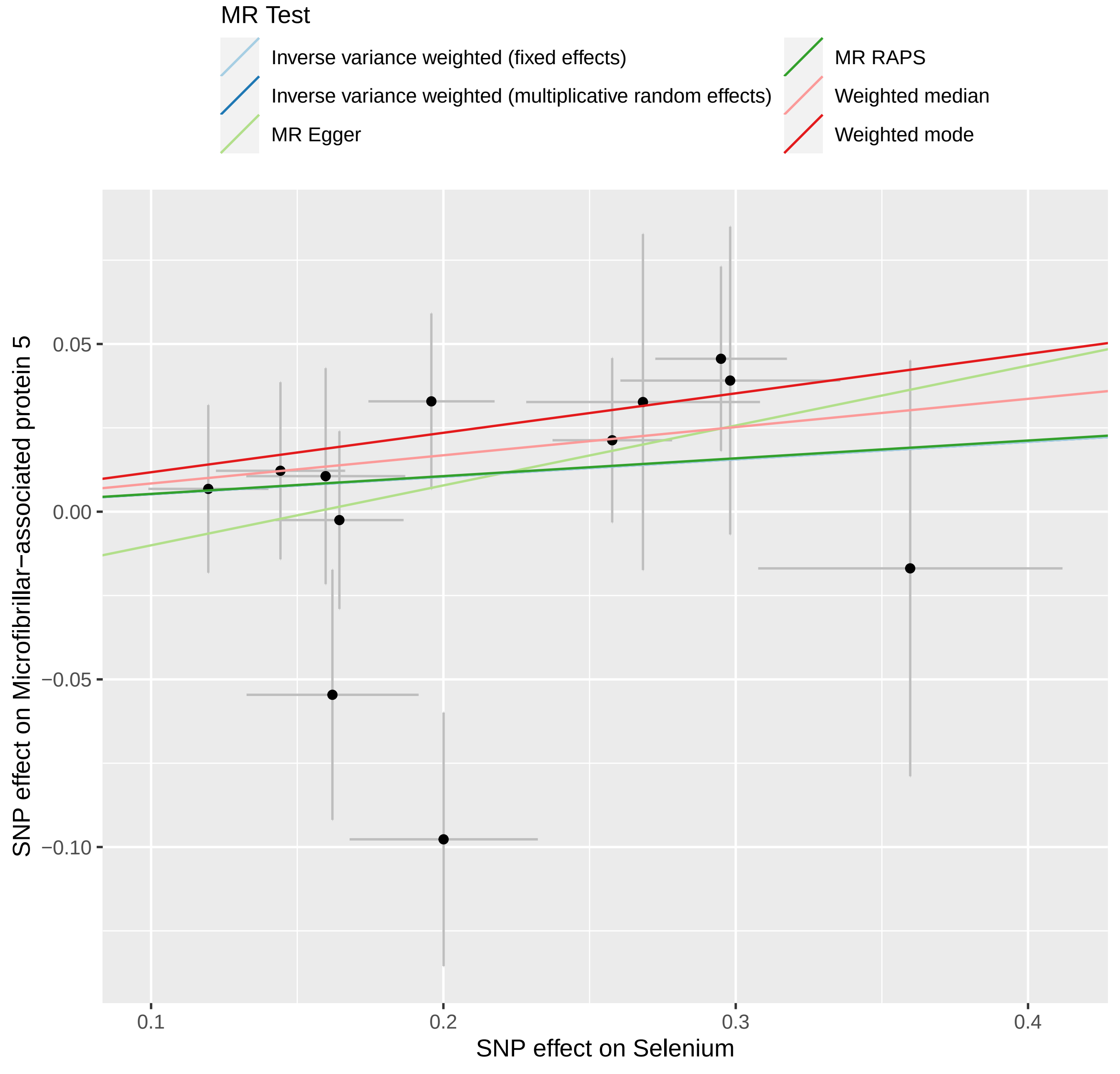

Batch 3−1888 : Selenium on Lactadherin

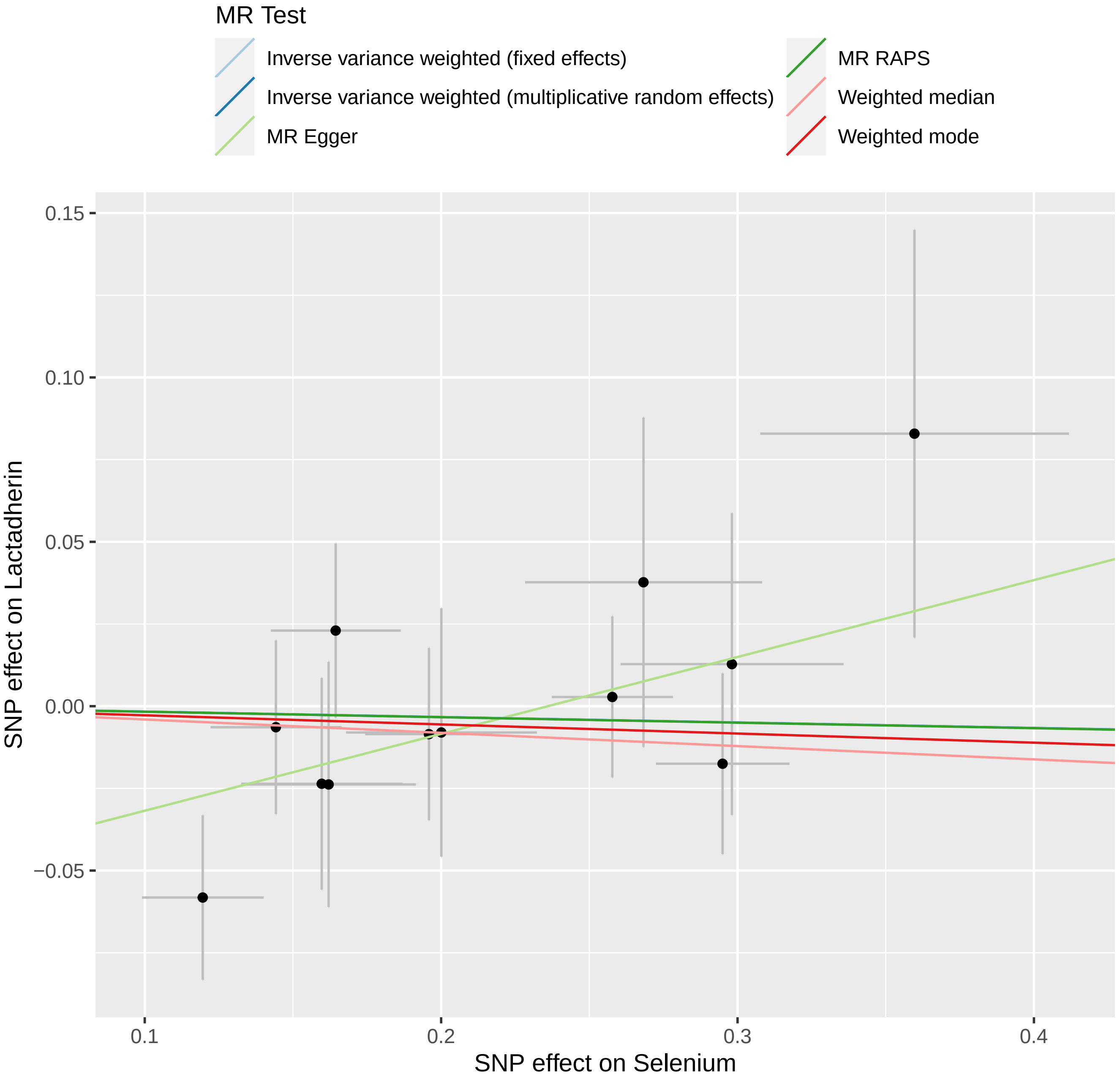

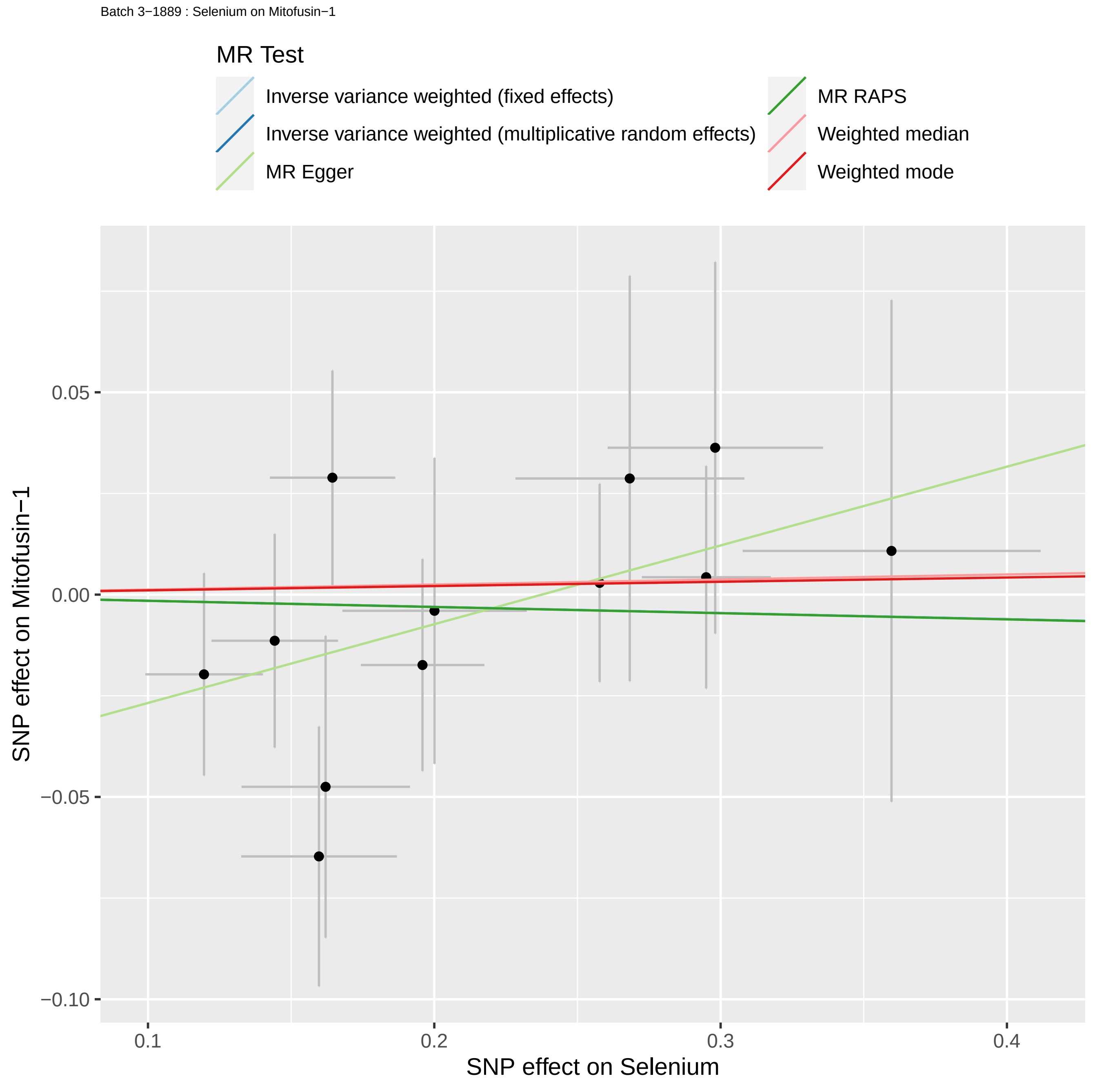

Batch 3−1889 : Selenium on Mitofusin−1
MR Test
Inverse variance weighted (fixed effects)
Inverse variance weighted (multiplicative random effects)
MR Egger
MR RAPS
Weighted median
Weighted mode
0.05
0.00
−0.05
−0.10
0.1
0.2
0.3
0.4
SNP effect on Mitofusin−1
SNP effect on Selenium

Batch 3−1890 : Selenium on Beta−1,3−N−acetylglucosaminyltransferase manic fringe

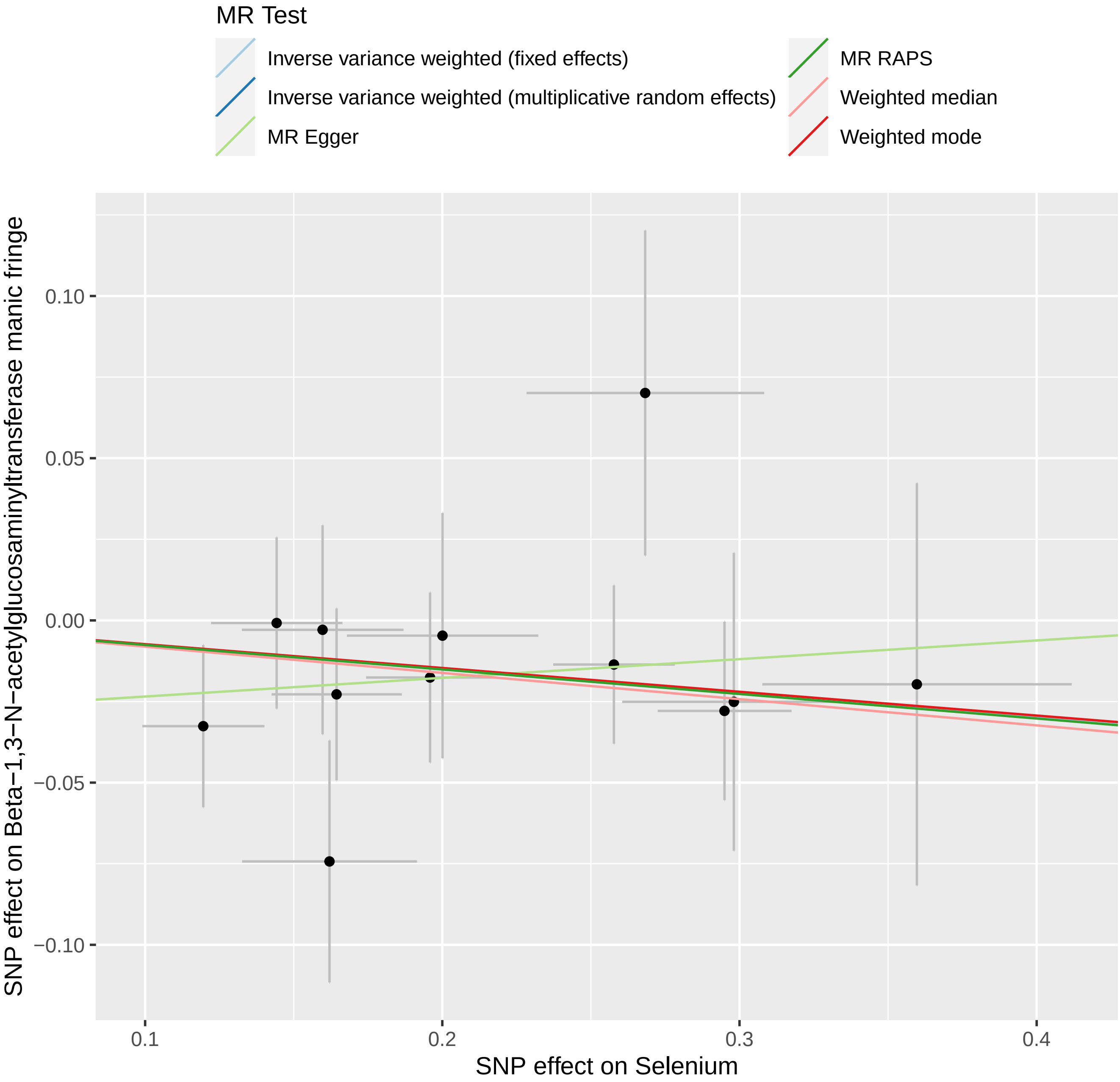

Batch 3−1891 : Selenium on MAX gene−associated protein

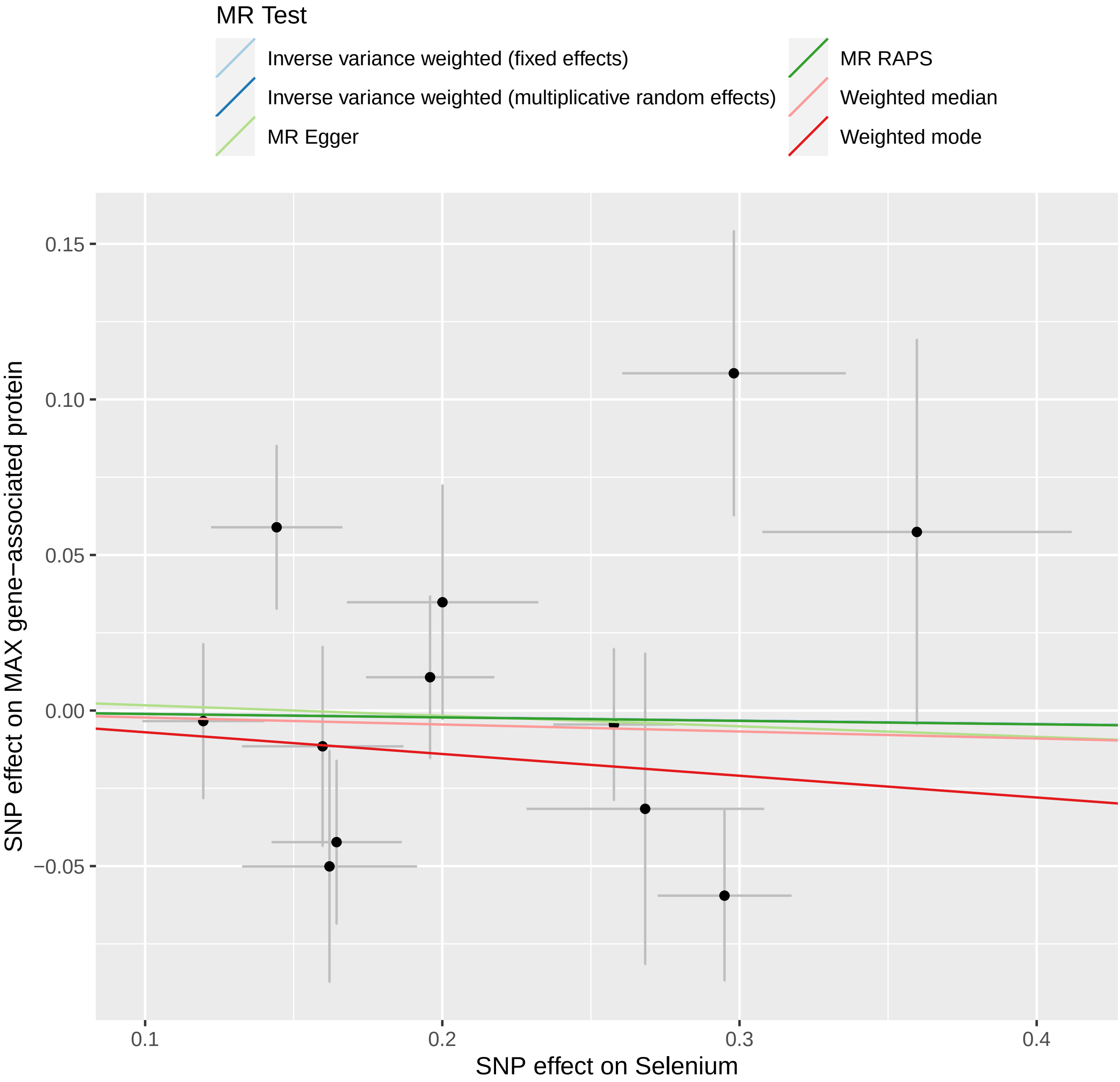

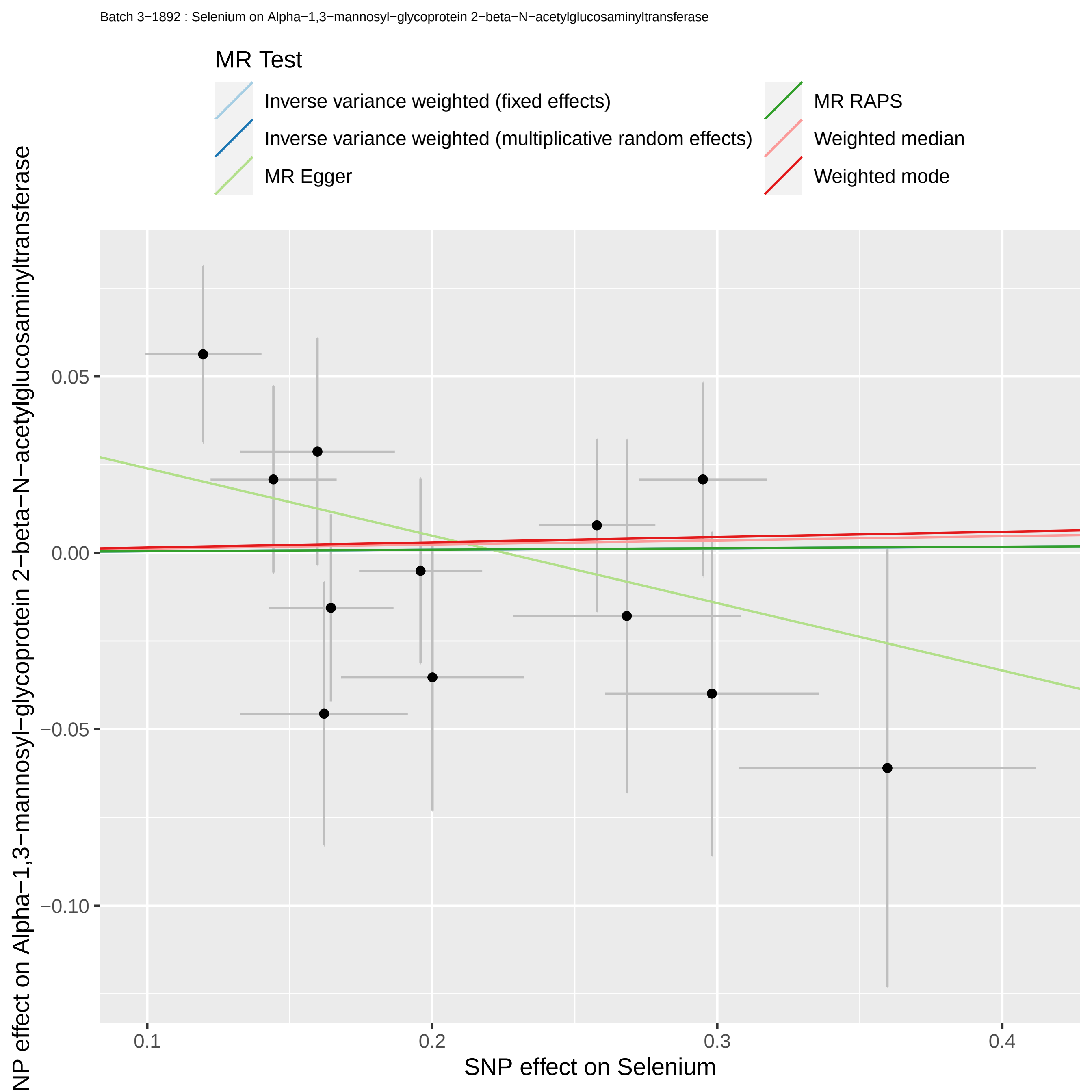

Batch 3−1892 : Selenium on Alpha−1,3−mannosyl−glycoprotein 2−beta−N−acetylglucosaminyltransferase
MR Test
Inverse variance weighted (fixed effects)
Inverse variance weighted (multiplicative random effects)
MR Egger
MR RAPS
Weighted median
Weighted mode
0.05
0.00
−0.05
−0.10
0.1
0.2
0.3
0.4
SNP effect on Selenium
SNP effect on Alpha−1,3−mannosyl−glycoprotein 2−beta−N−acetylglucosaminyltransferase

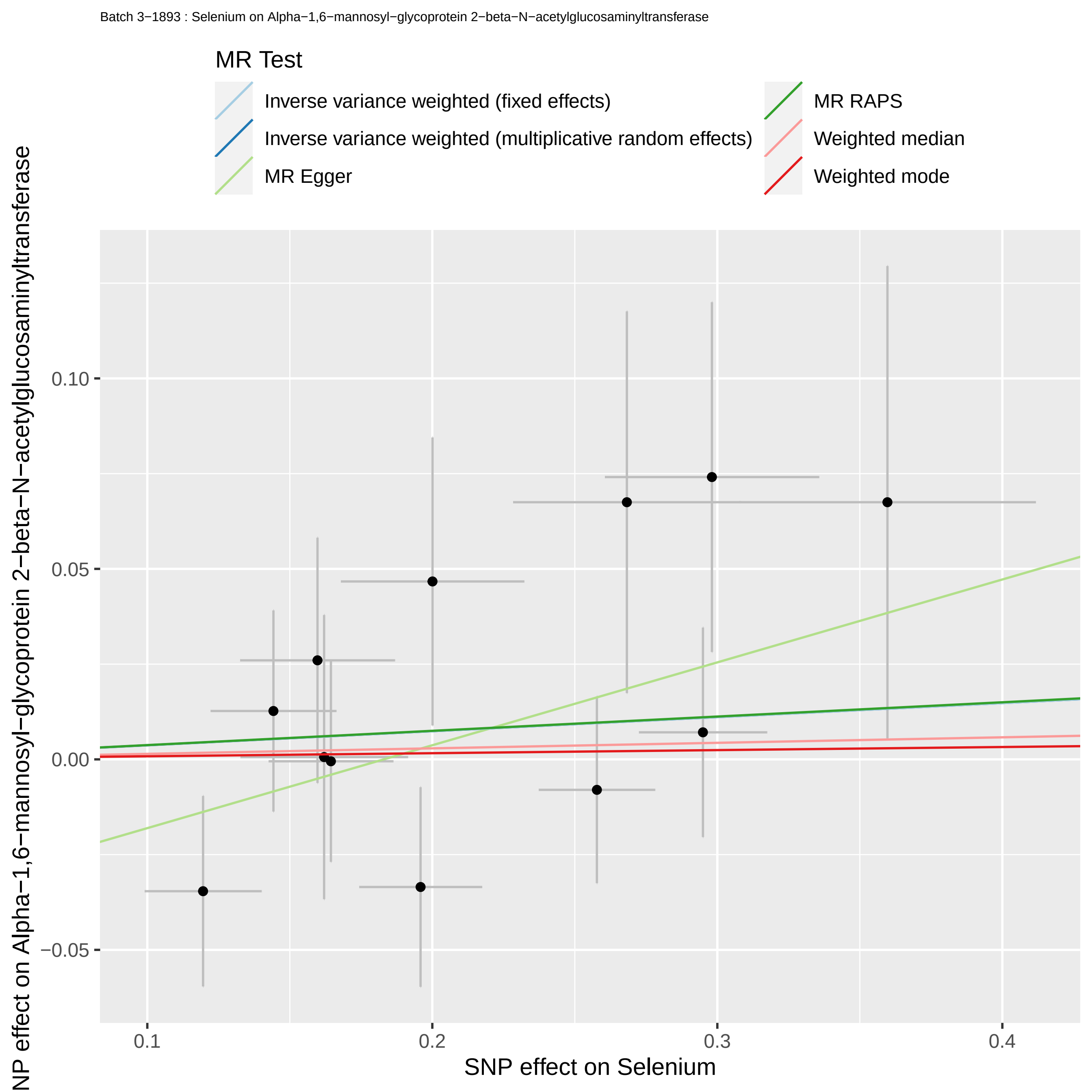
Batch 3−1893 : Selenium on Alpha−1,6−mannosyl−glycoprotein 2−beta−N−acetylglucosaminyltransferase
MR Test
Inverse variance weighted (fixed effects)
Inverse variance weighted (multiplicative random effects)
MR Egger
MR RAPS
Weighted median
Weighted mode
0.10
0.05
0.00
−0.05
0.1
0.2
0.3
0.4
SNP effect on Selenium
SNP effect on Alpha−1,6−mannosyl−glycoprotein 2−beta−N−acetylglucosaminyltransferase

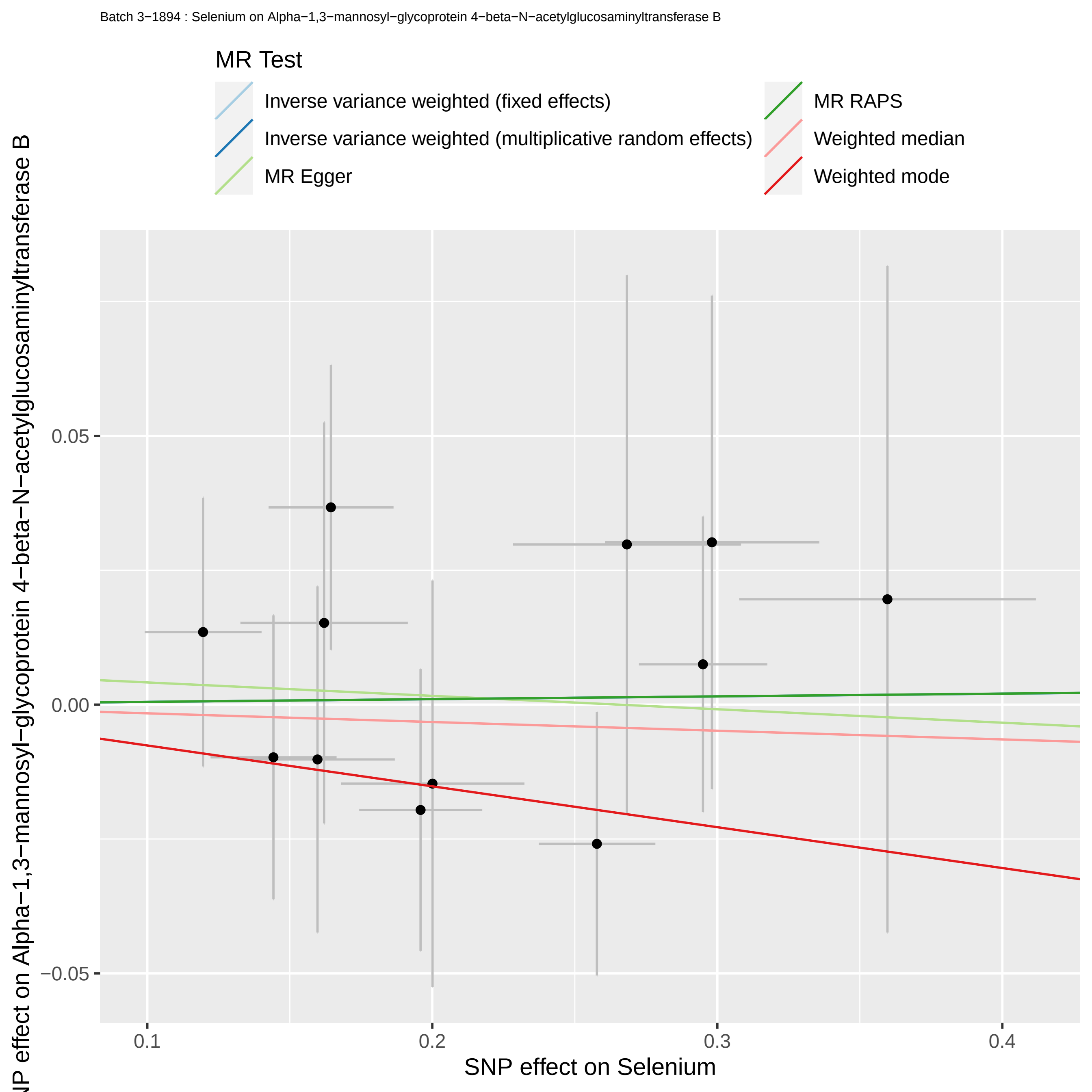

Batch 3−1894 : Selenium on Alpha−1,3−mannosyl−glycoprotein 4−beta−N−acetylglucosaminyltransferase B
MR Test
Inverse variance weighted (fixed effects)
Inverse variance weighted (multiplicative random effects)
MR Egger
MR RAPS
Weighted median
Weighted mode
0.05
0.00
−0.05
0.1
0.2
0.3
0.4
SNP effect on Selenium
NP effect on Alpha−1,3−mannosyl−glycoprotein 4−beta−N−acetylglucosaminyltransferase B

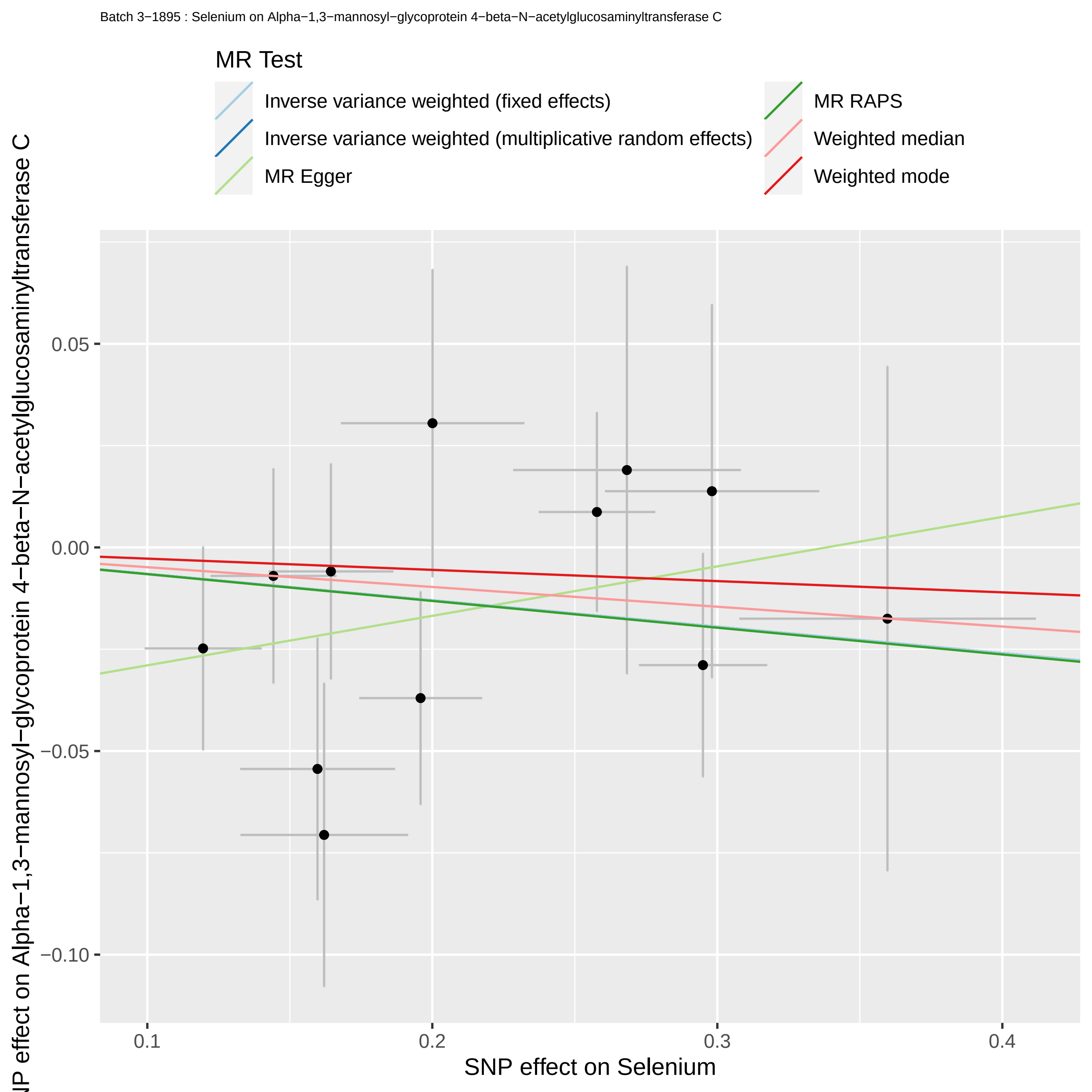
Batch 3−1895 : Selenium on Alpha−1,3−mannosyl−glycoprotein 4−beta−N−acetylglucosaminyltransferase C
MR Test
Inverse variance weighted (fixed effects)
Inverse variance weighted (multiplicative random effects)
MR Egger
MR RAPS
Weighted median
Weighted mode
0.05
0.00
−0.05
−0.10
0.1
0.2
0.3
0.4
SNP effect on Selenium
SNP effect on Alpha−1,3−mannosyl−glycoprotein 4−beta−N−acetylglucosaminyltransferase C

Batch 3−1896 : Selenium on Melanoma−derived growth regulatory protein

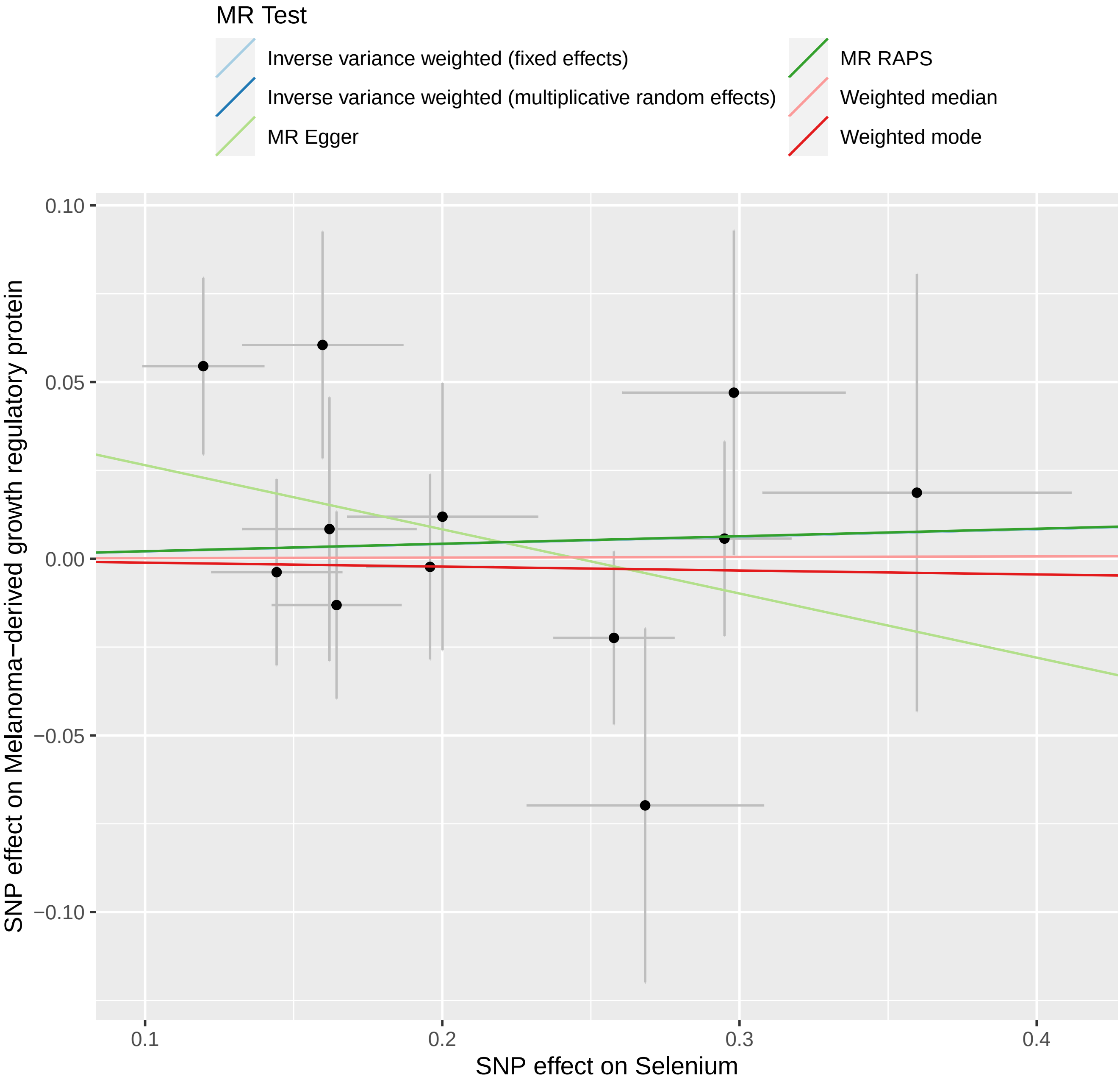

Batch 3−1897 : Selenium on MICAL−like protein 2

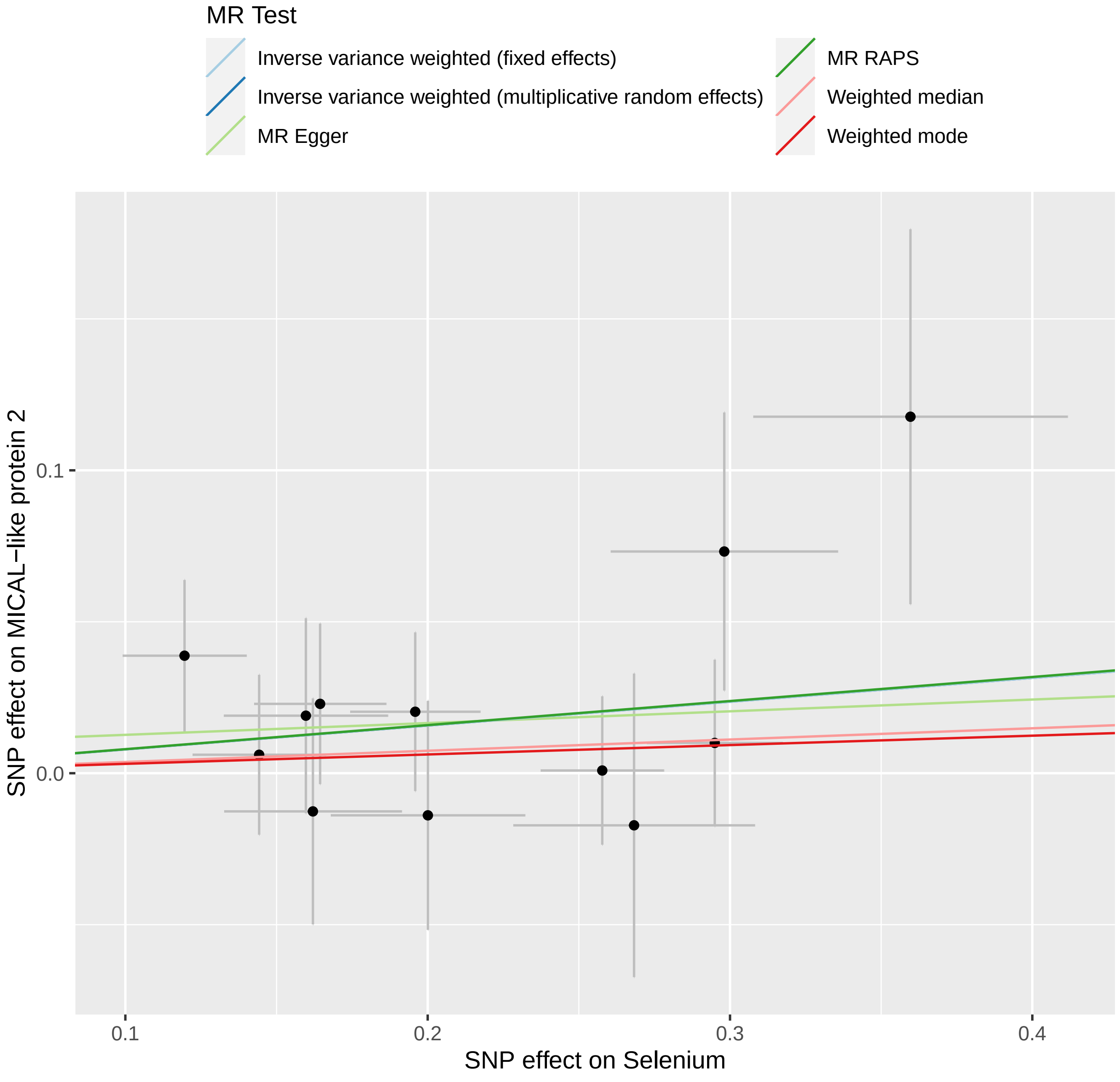

Batch 3−1898 : Selenium on MHC class I polypeptide−related sequence B

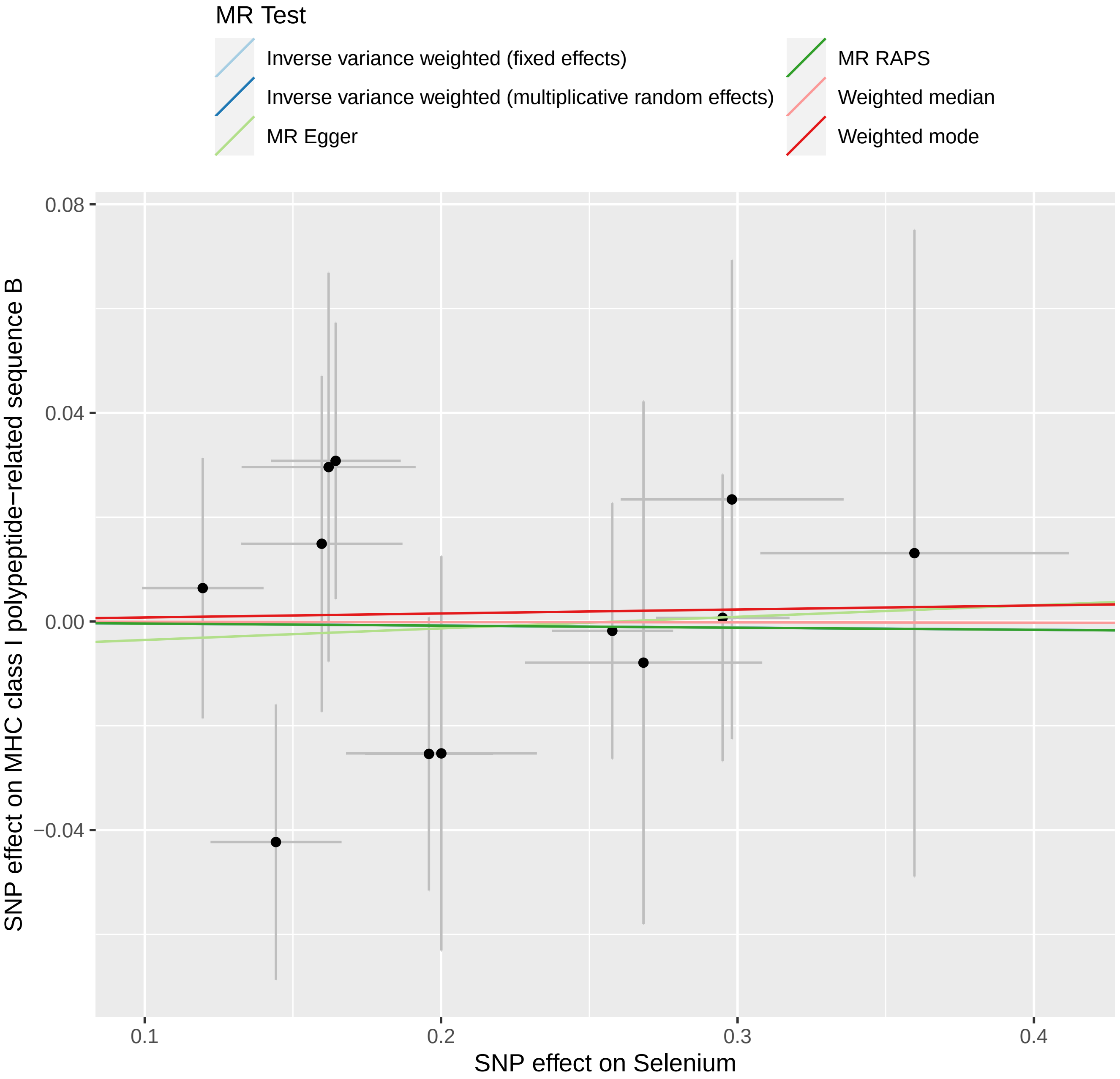

Batch 3−1899 : Selenium on Probable E3 ubiquitin−protein ligase MID2

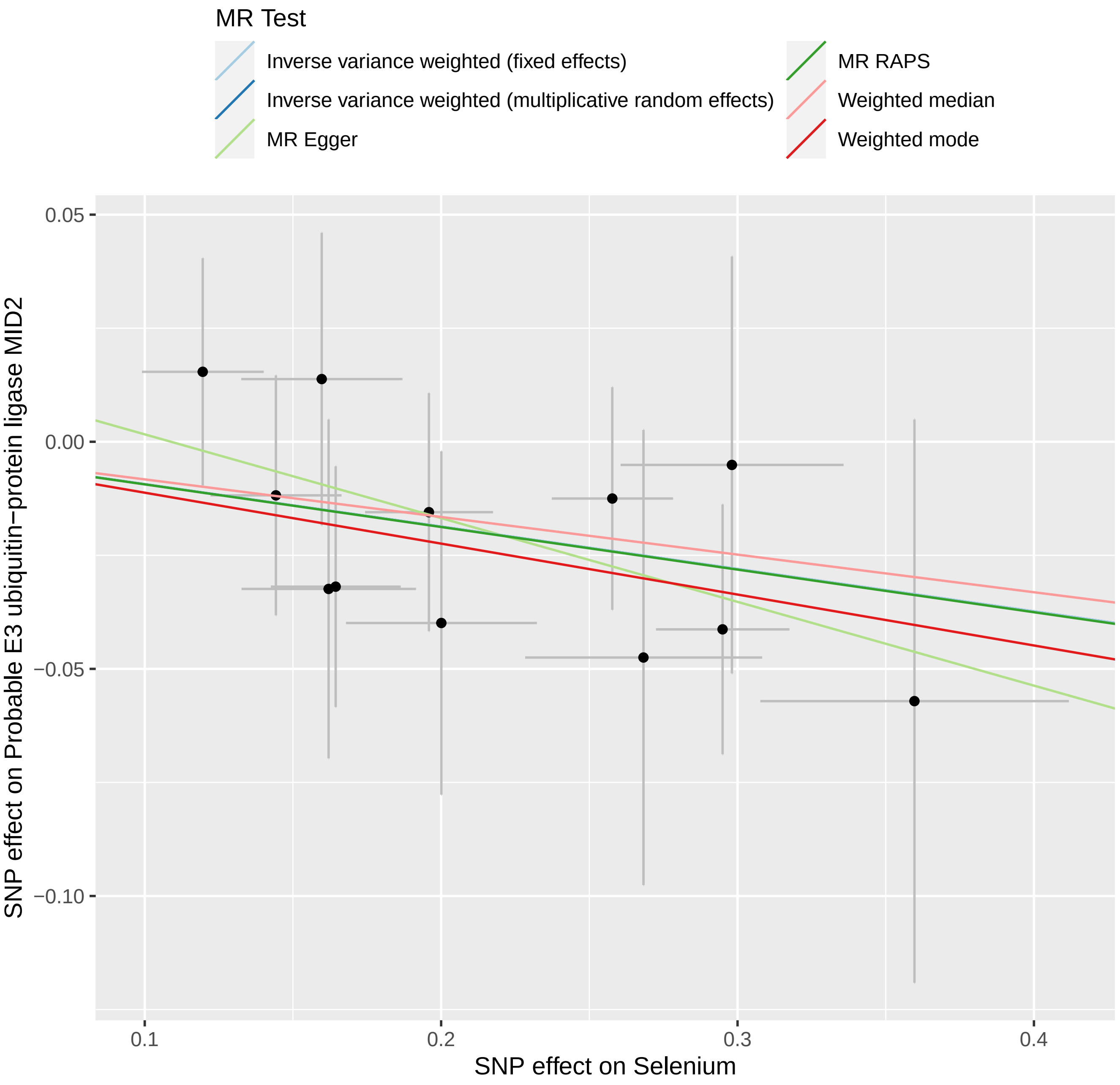

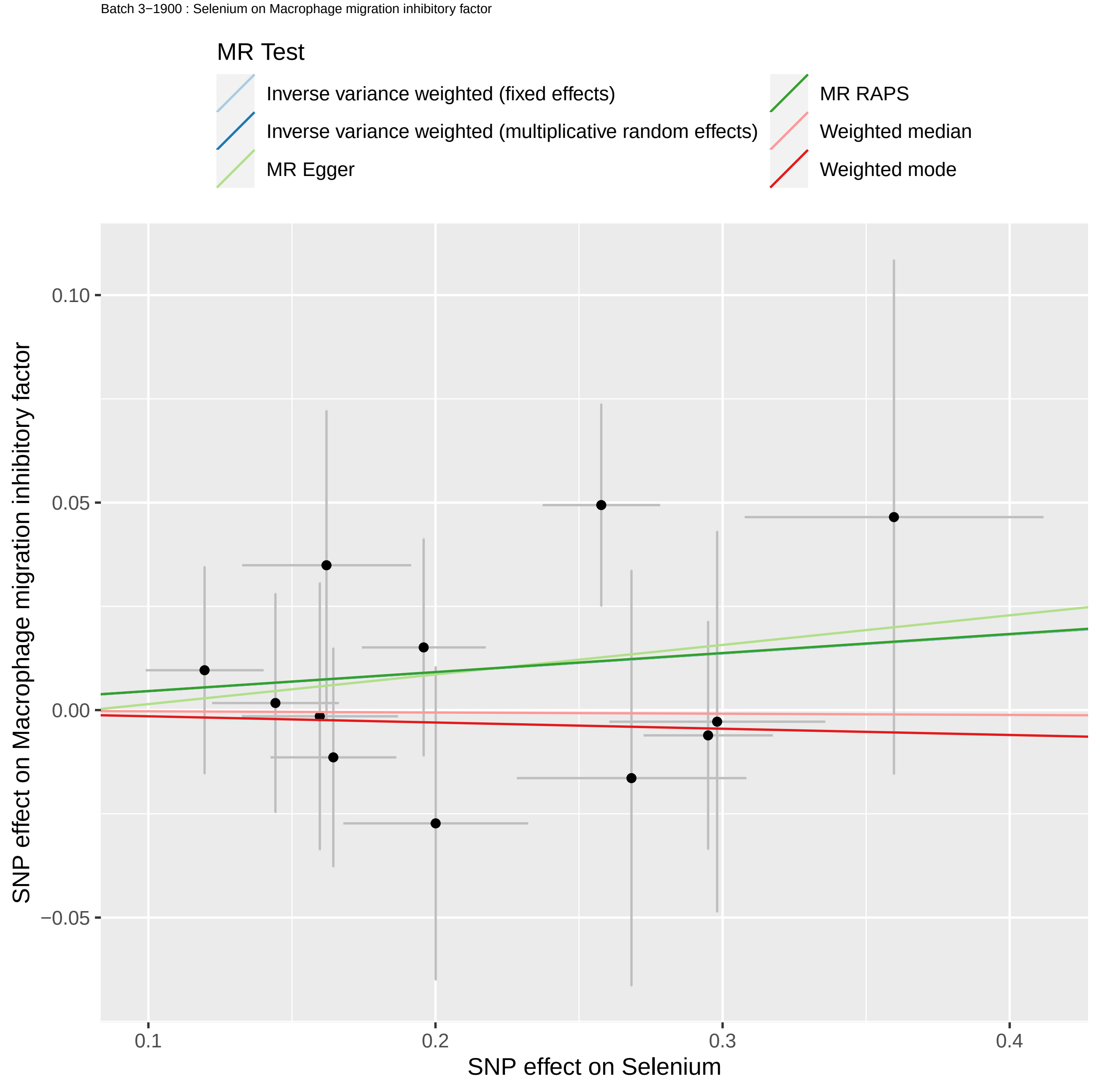
Batch 3−1900 : Selenium on Macrophage migration inhibitory factor
MR Test
Inverse variance weighted (fixed effects)
Inverse variance weighted (multiplicative random effects)
MR Egger
MR RAPS
Weighted median
Weighted mode
SNP effect on Macrophage migration inhibitory factor
0.10
0.05
0.00
−0.05
0.1
0.2
0.3
0.4
SNP effect on Selenium

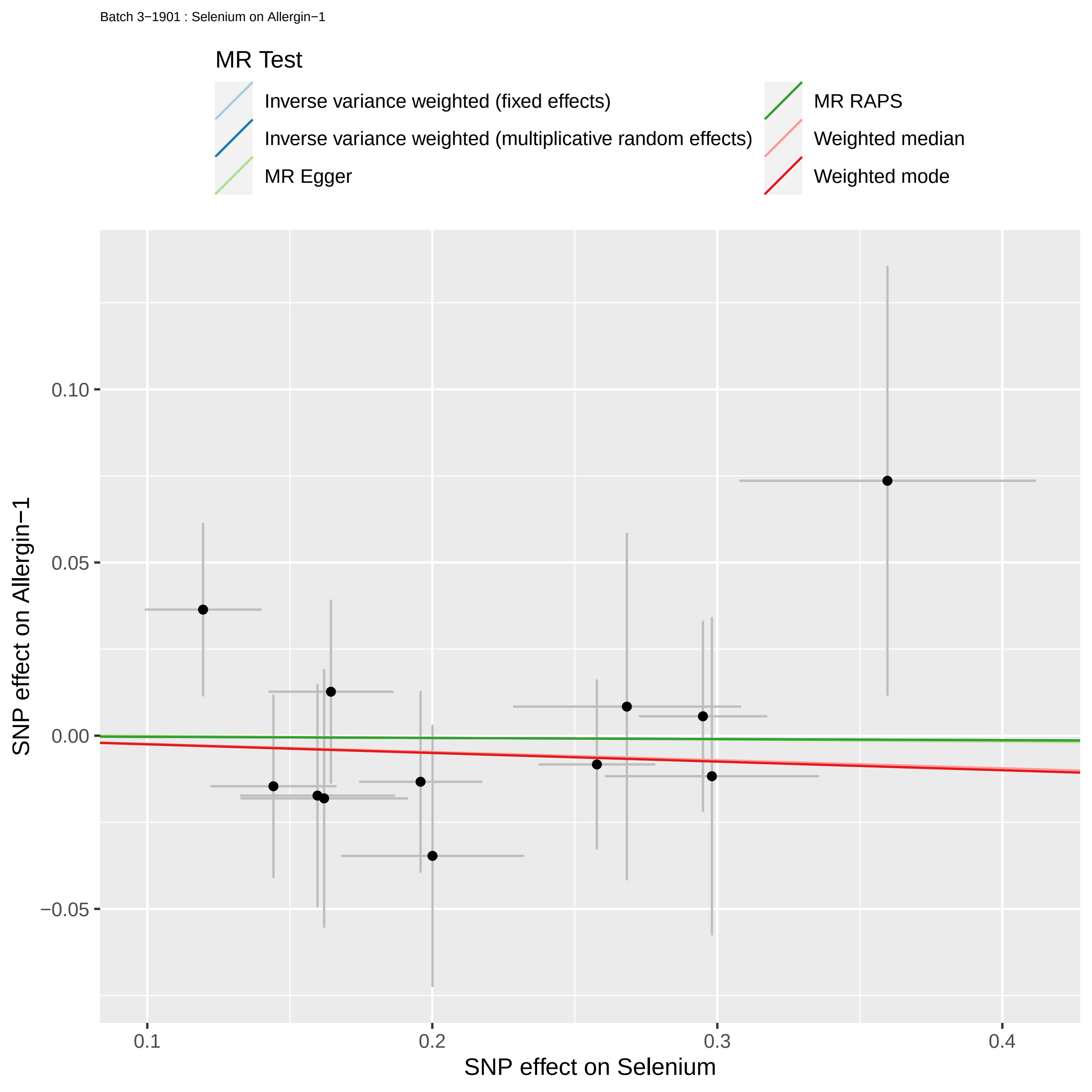
Batch 3−1901 : Selenium on Allergin−1
MR Test
Inverse variance weighted (fixed effects)
Inverse variance weighted (multiplicative random effects)
MR Egger
MR RAPS
Weighted median
Weighted mode
0.10
0.05
0.00
−0.05
0.1
0.2
0.3
0.4
SNP effect on Allergin−1
SNP effect on Selenium

Batch 3−1902 : Selenium on MICOS complex subunit MIC10

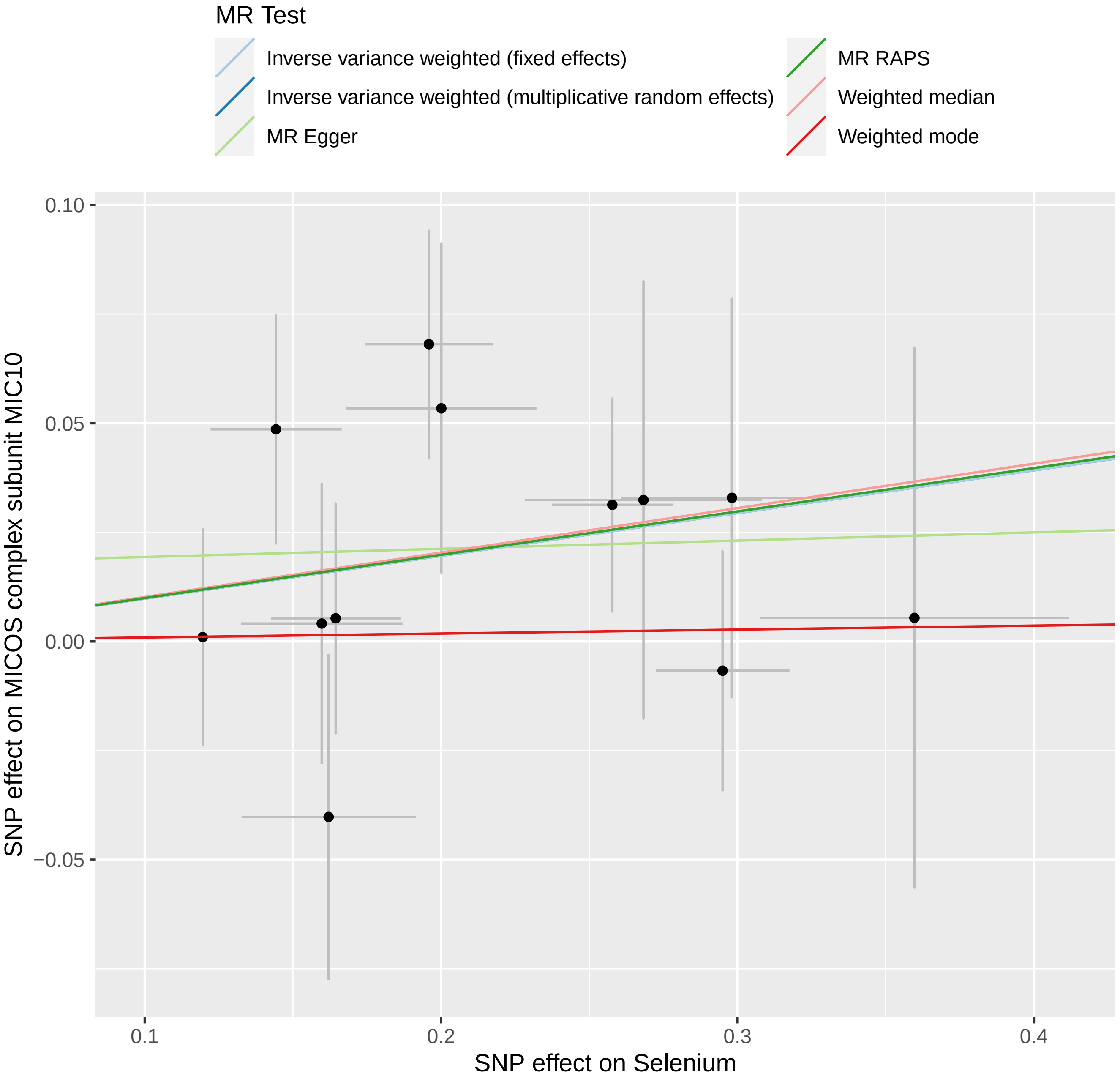

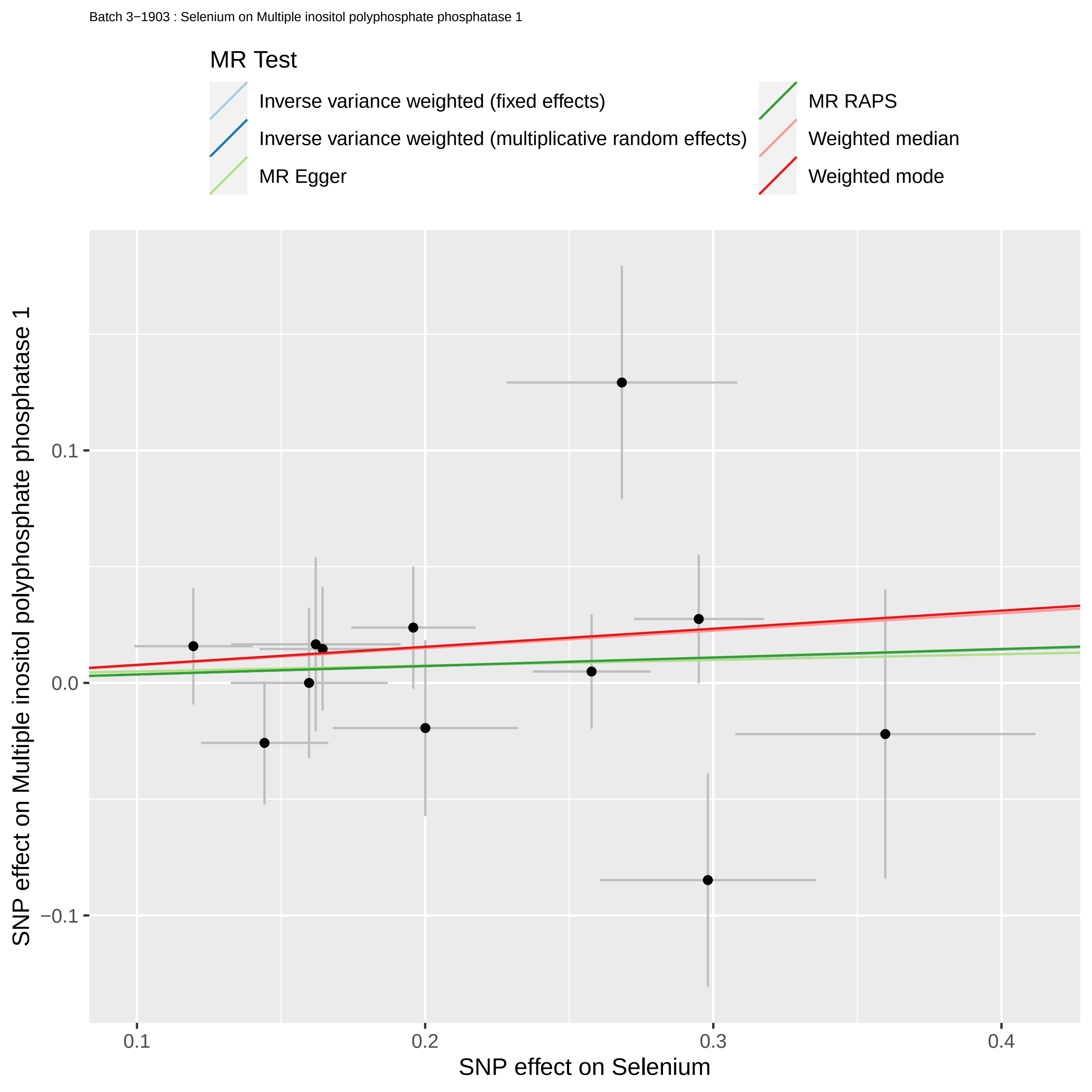
Batch 3−1903 : Selenium on Multiple inositol polyphosphate phosphatase 1
MR Test
Inverse variance weighted (fixed effects)
Inverse variance weighted (multiplicative random effects)
MR Egger
MR RAPS
Weighted median
Weighted mode
SNP effect on Multiple inositol polyphosphate phosphatase 1
0.1
0.0
−0.1
0.1
0.2
0.3
0.4
SNP effect on Selenium

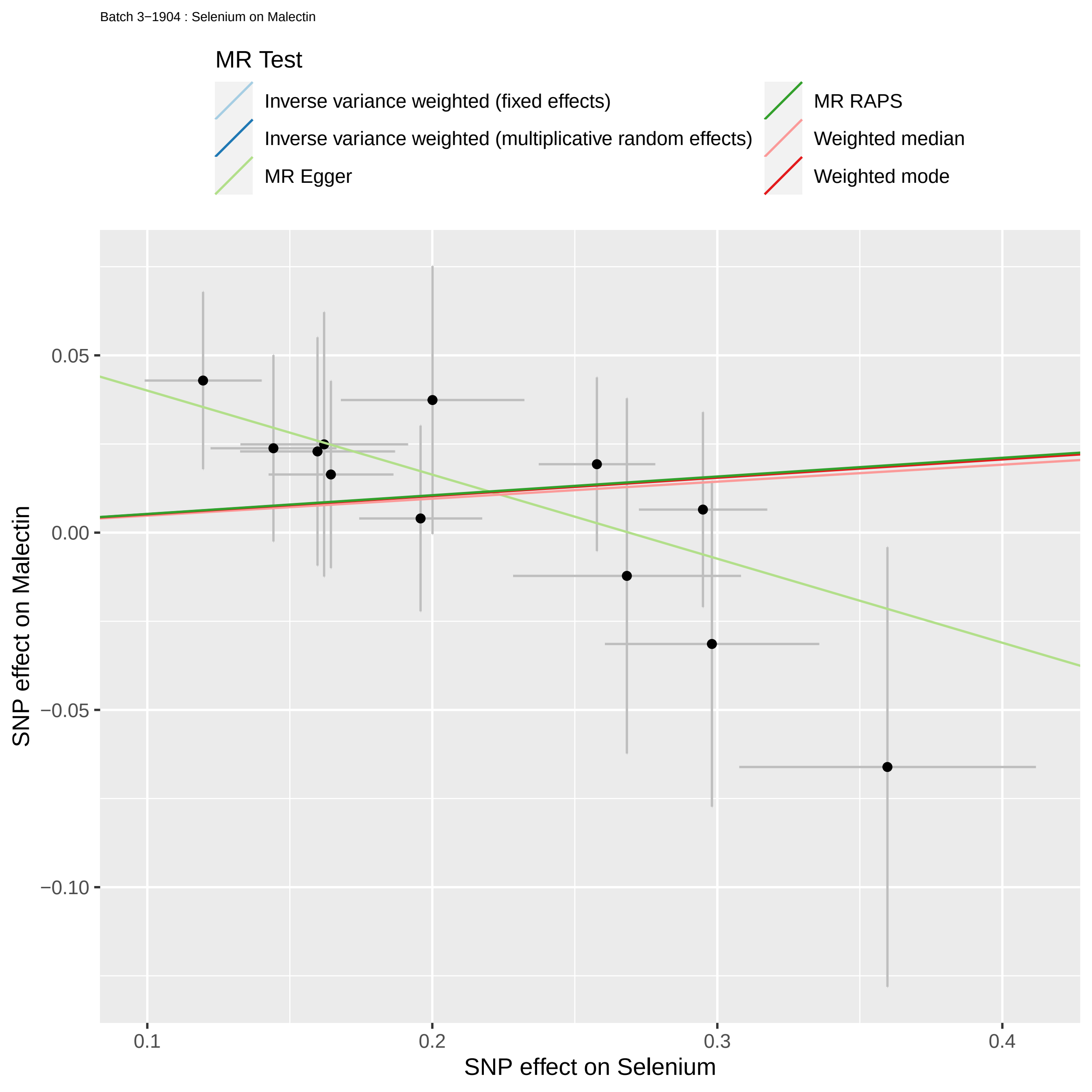

Batch 3−1904 : Selenium on Malectin
MR Test
Inverse variance weighted (fixed effects)
Inverse variance weighted (multiplicative random effects)
MR Egger
MR RAPS
Weighted median
Weighted mode
0.05
0.00
−0.05
−0.10
0.1
0.2
0.3
0.4
SNP effect on Malectin
SNP effect on Selenium

Batch 3−1905 : Selenium on Histone−lysine N−methyltransferase 2D

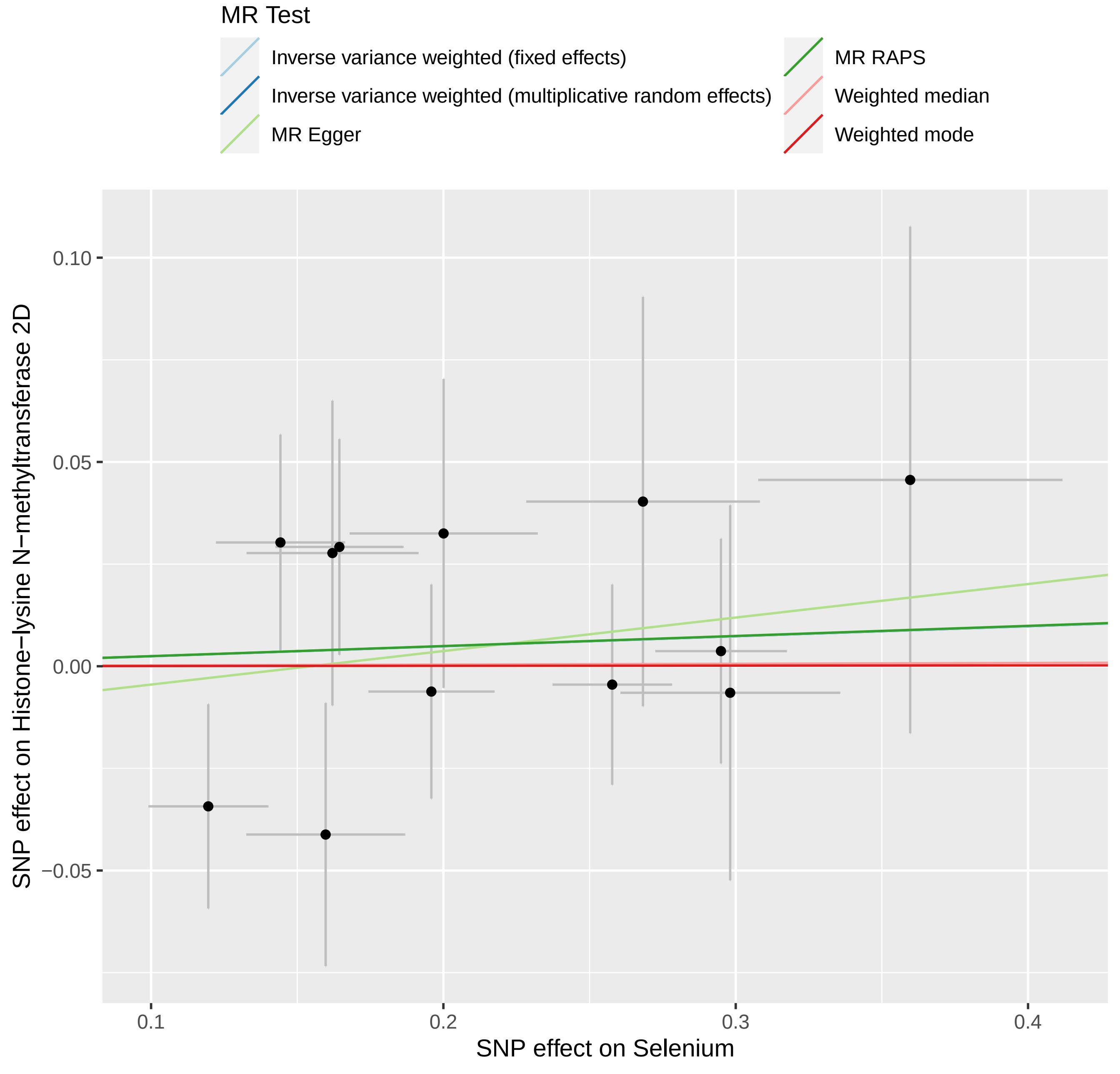

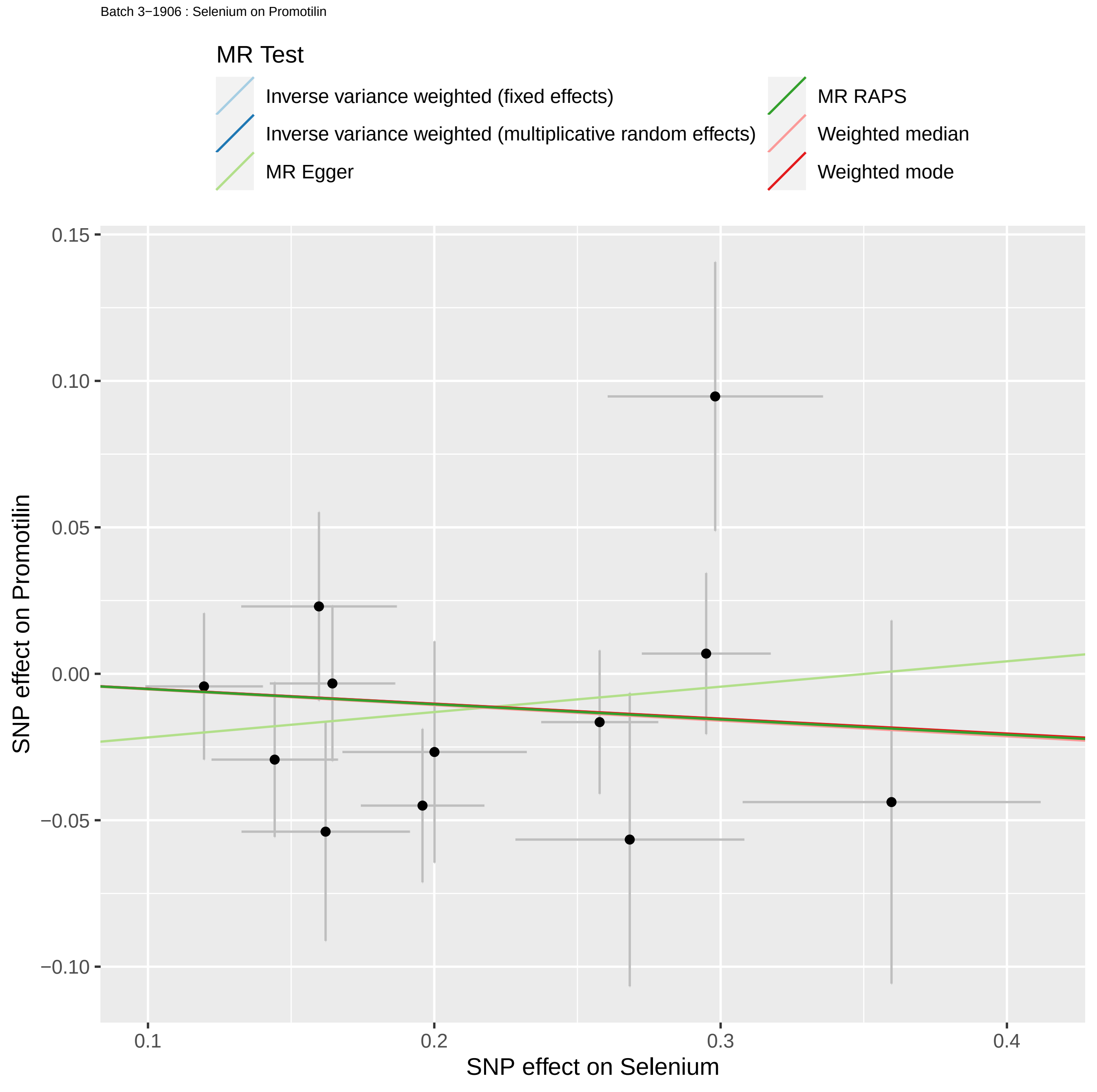

Batch 3−1906 : Selenium on Promotilin
MR Test
Inverse variance weighted (fixed effects)
Inverse variance weighted (multiplicative random effects)
MR Egger
MR RAPS
Weighted median
Weighted mode
0.15
0.10
0.05
0.00
−0.05
−0.10
0.1
0.2
0.3
0.4
SNP effect on Promotilin
SNP effect on Selenium

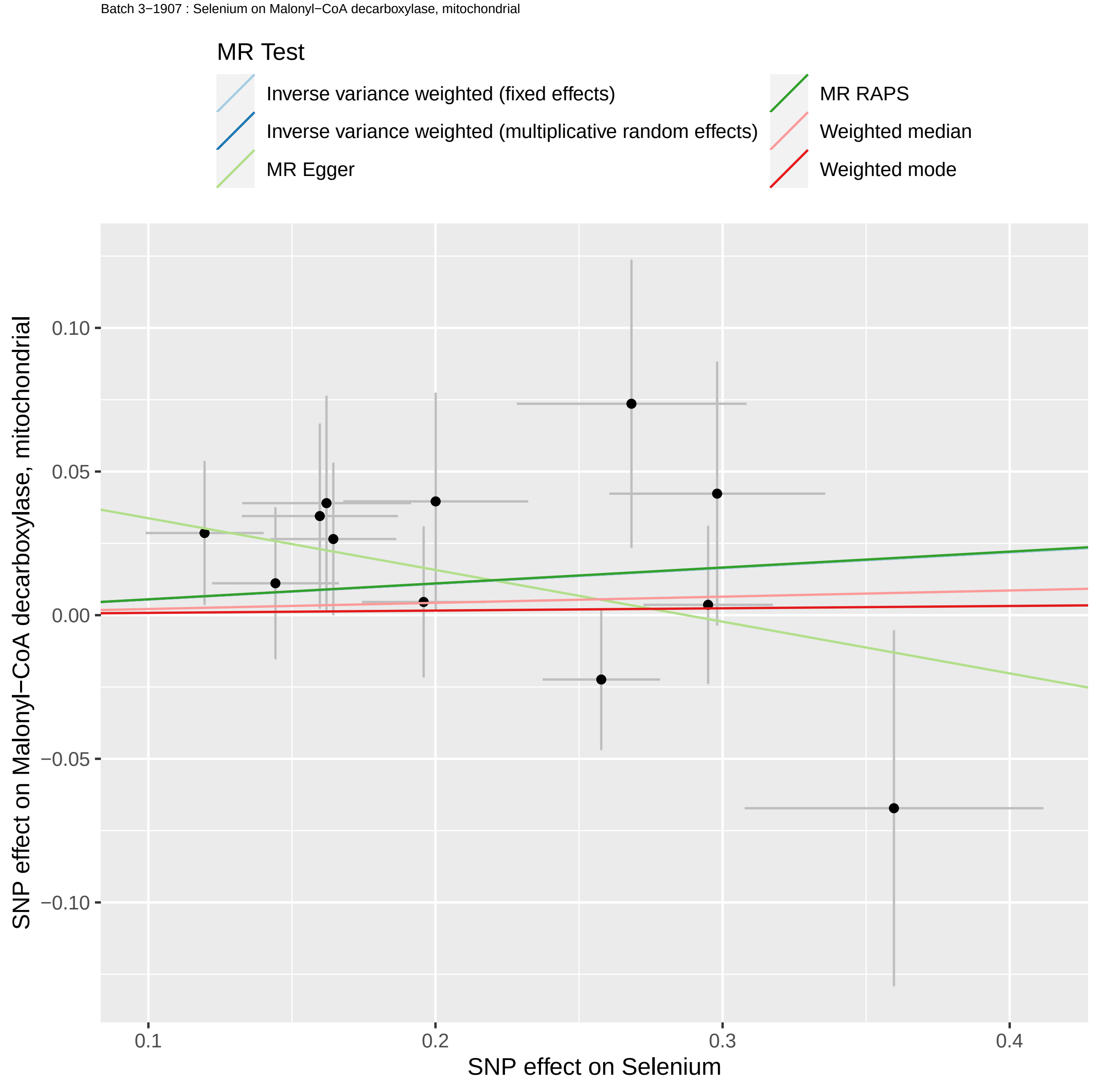
Batch 3−1907 : Selenium on Malonyl−CoA decarboxylase, mitochondrial
MR Test
Inverse variance weighted (fixed effects)
Inverse variance weighted (multiplicative random effects)
MR Egger
MR RAPS
Weighted median
Weighted mode
0.10
0.05
0.00
−0.05
−0.10
0.1
0.2
0.3
0.4
SNP effect on Selenium
SNP effect on Malonyl−CoA decarboxylase, mitochondrial

Batch 3−1908 : Selenium on Membrane metallo−endopeptidase−like 1

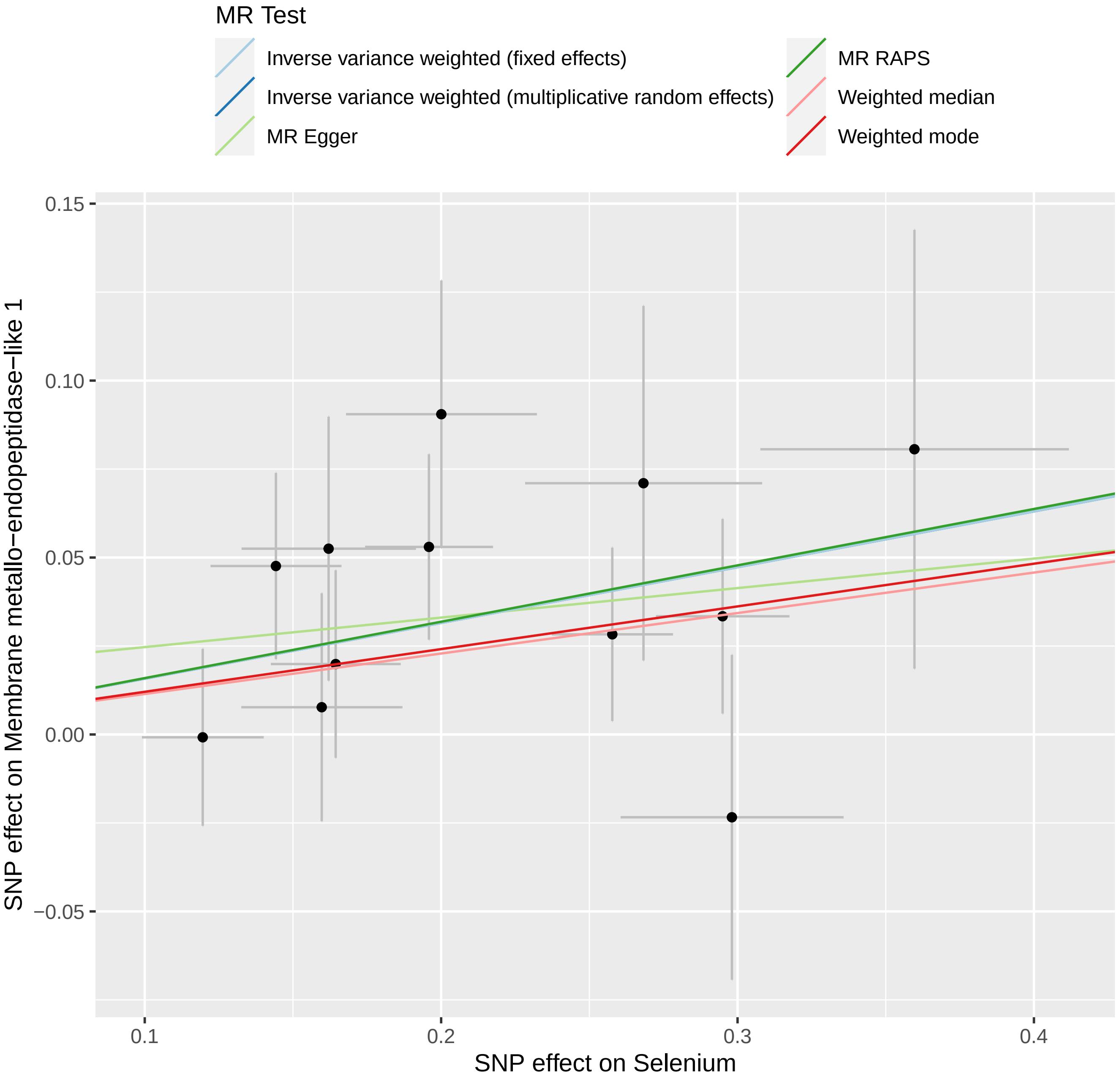

Batch 3−1909 : Selenium on Membrane magnesium transporter 1

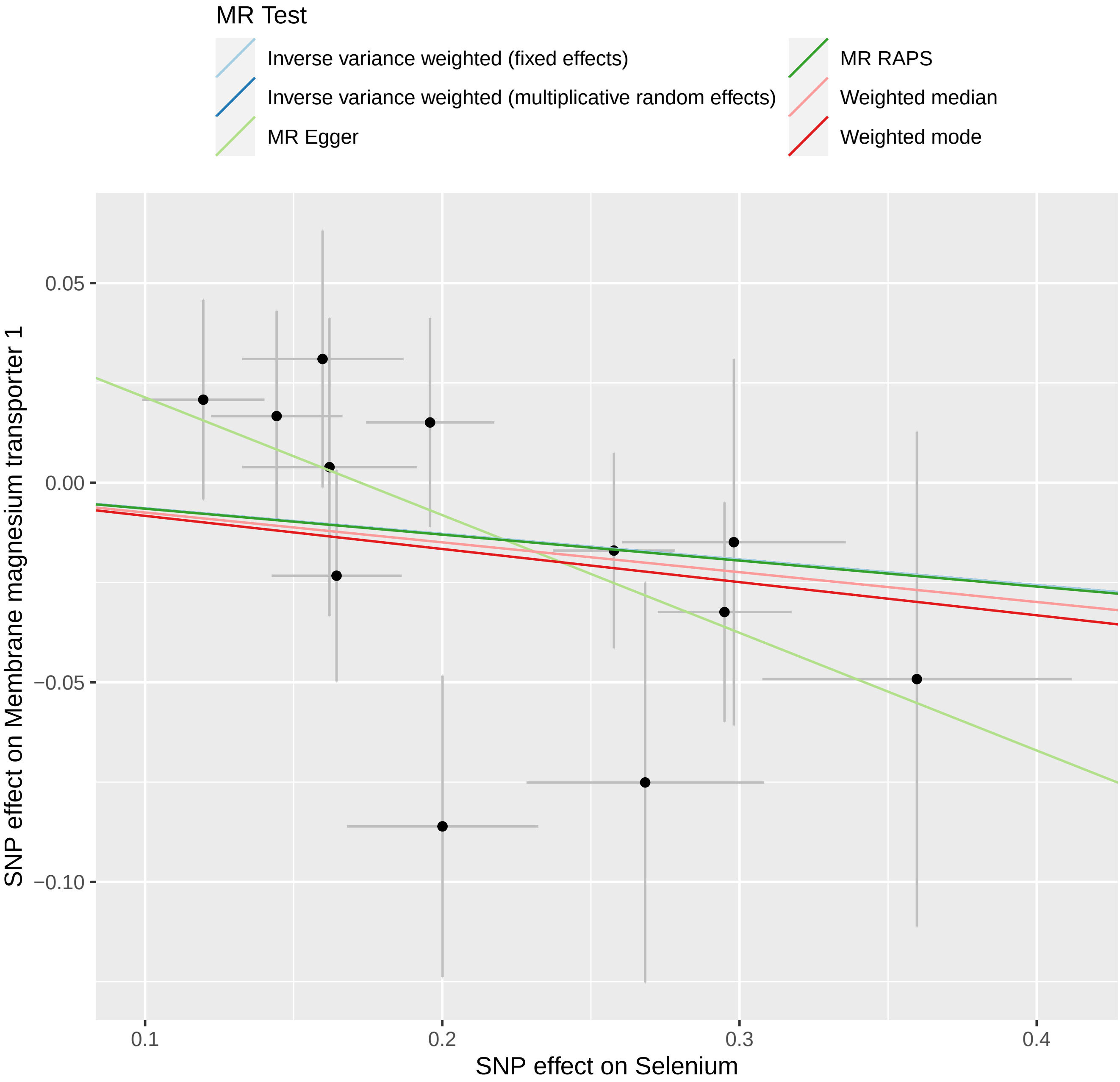

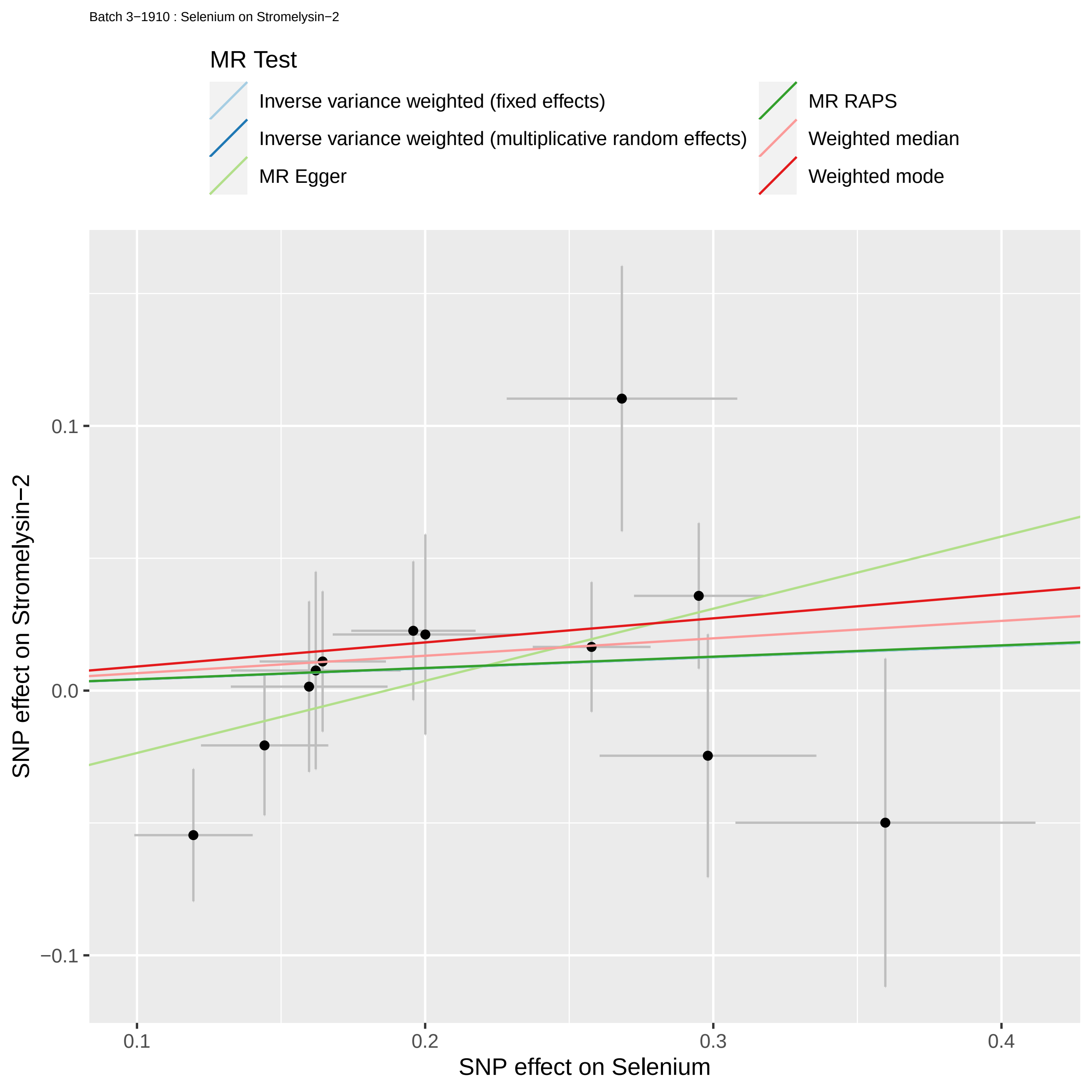

Batch 3−1910 : Selenium on Stromelysin−2
MR Test
Inverse variance weighted (fixed effects)
Inverse variance weighted (multiplicative random effects)
MR Egger
MR RAPS
Weighted median
Weighted mode
0.1
0.0
−0.1
0.1
0.2
0.3
0.4
SNP effect on Stromelysin−2
SNP effect on Selenium

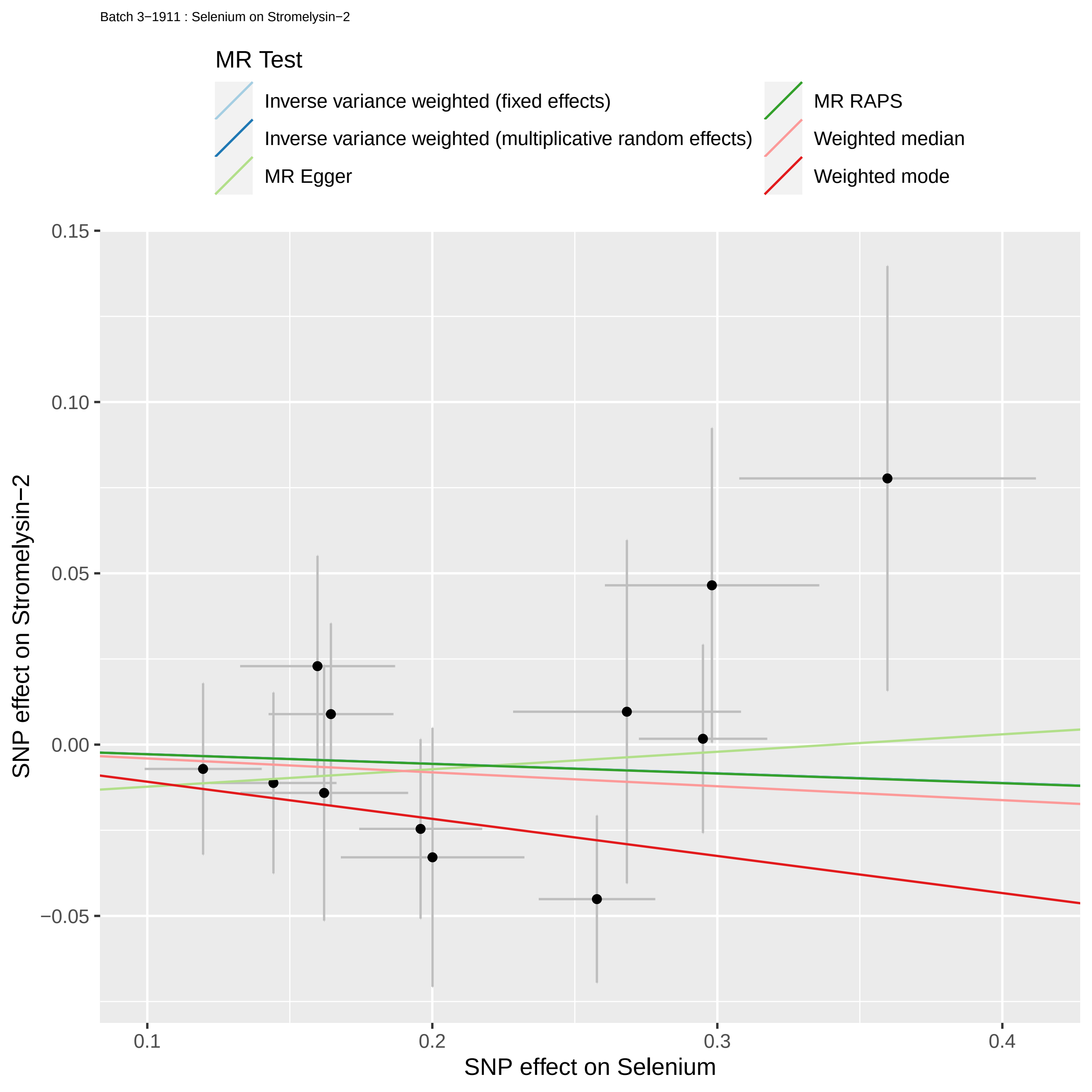

Batch 3−1911 : Selenium on Stromelysin−2
MR Test
Inverse variance weighted (fixed effects)
Inverse variance weighted (multiplicative random effects)
MR Egger
MR RAPS
Weighted median
Weighted mode
0.15
0.10
0.05
0.00
−0.05
0.1
0.2
0.3
0.4
SNP effect on Stromelysin−2
SNP effect on Selenium

Batch 3−1912 : Selenium on Macrophage metalloelastase

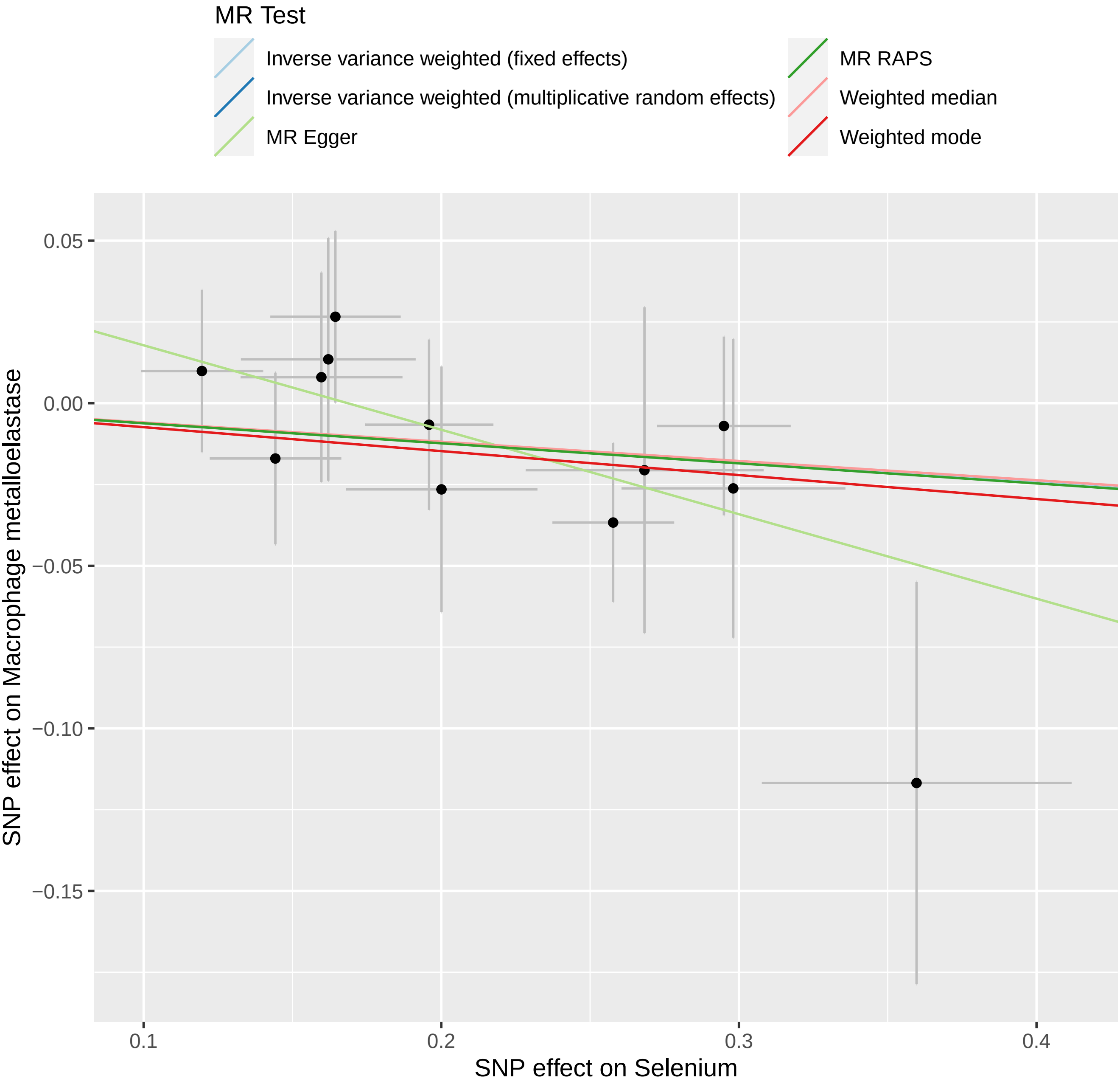

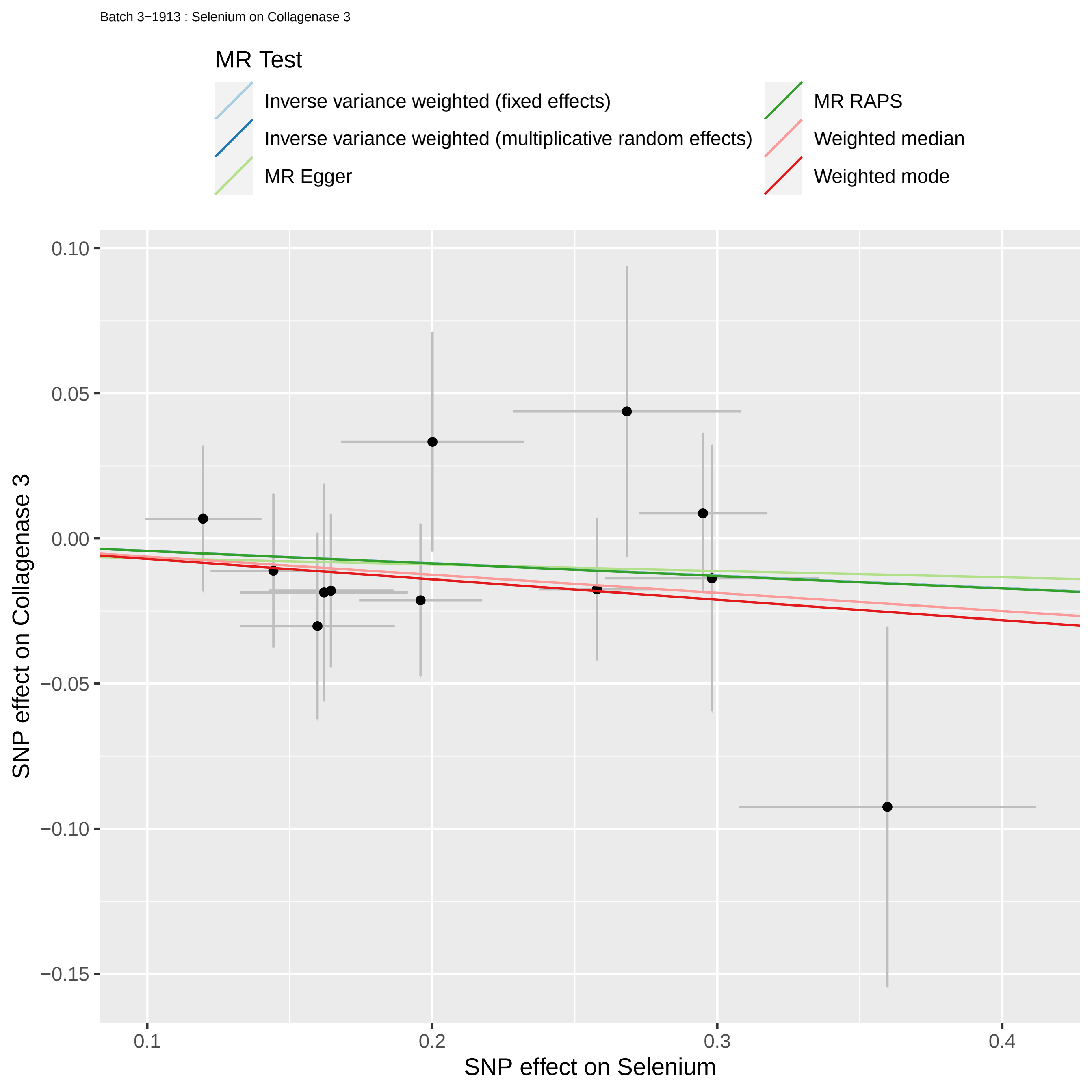
Batch 3−1913 : Selenium on Collagenase 3
MR Test
Inverse variance weighted (fixed effects)
Inverse variance weighted (multiplicative random effects)
MR Egger
MR RAPS
Weighted median
Weighted mode
0.10
0.05
0.00
−0.05
−0.10
−0.15
0.1
0.2
0.3
0.4
SNP effect on Collagenase 3
SNP effect on Selenium

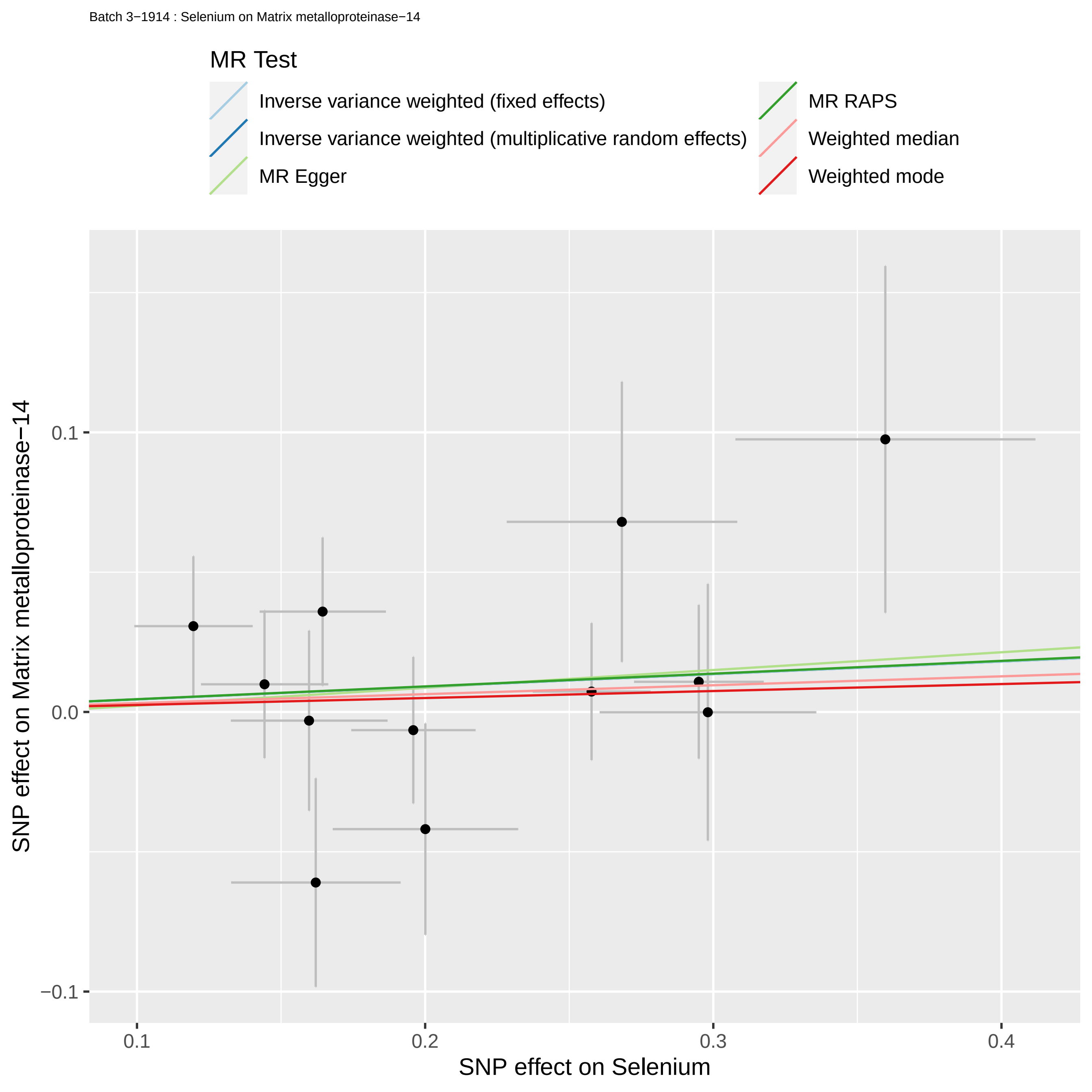
Batch 3−1914 : Selenium on Matrix metalloproteinase−14
MR Test
Inverse variance weighted (fixed effects)
Inverse variance weighted (multiplicative random effects)
MR Egger
MR RAPS
Weighted median
Weighted mode
SNP effect on Matrix metalloproteinase−14
0.1
0.0
−0.1
0.1
0.2
0.3
0.4
SNP effect on Selenium

Batch 3−1915 : Selenium on Matrix metalloproteinase−16

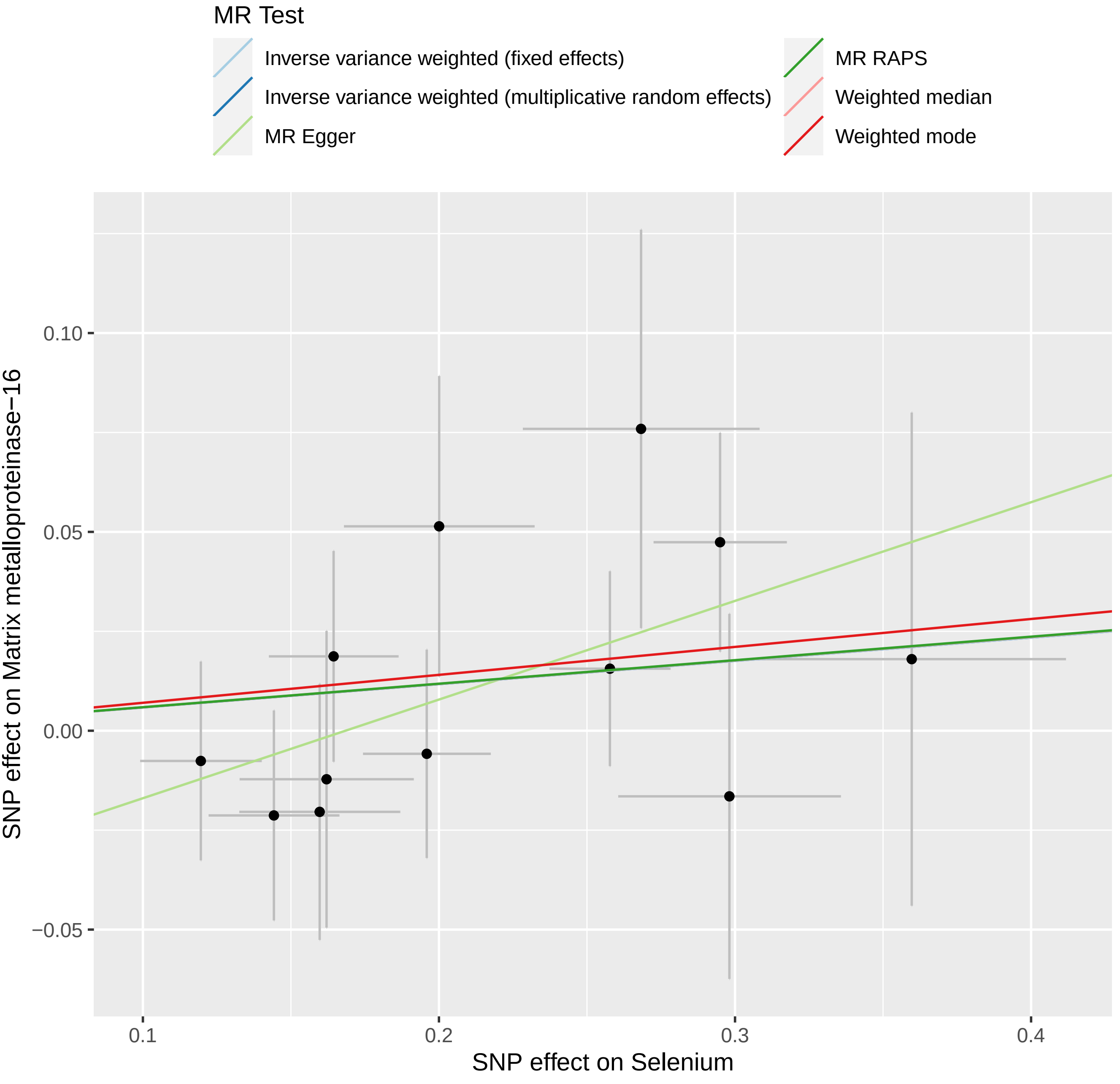

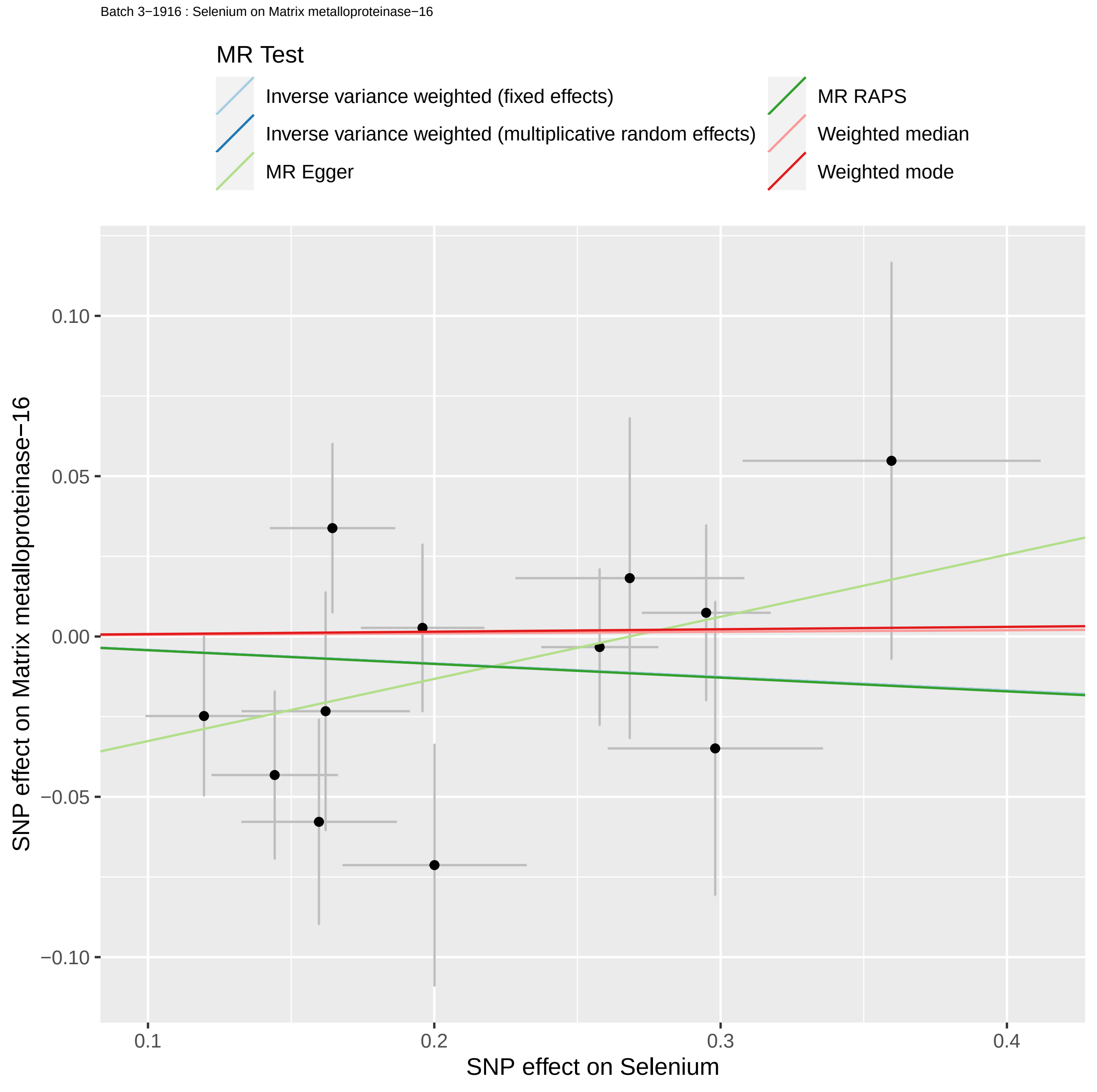
Batch 3−1916 : Selenium on Matrix metalloproteinase−16
MR Test
Inverse variance weighted (fixed effects)
Inverse variance weighted (multiplicative random effects)
MR Egger
MR RAPS
Weighted median
Weighted mode
0.10
0.05
0.00
−0.05
−0.10
0.1
0.2
0.3
0.4
SNP effect on Selenium
SNP effect on Matrix metalloproteinase−16

Batch 3−1917 : Selenium on Matrix metalloproteinase−17

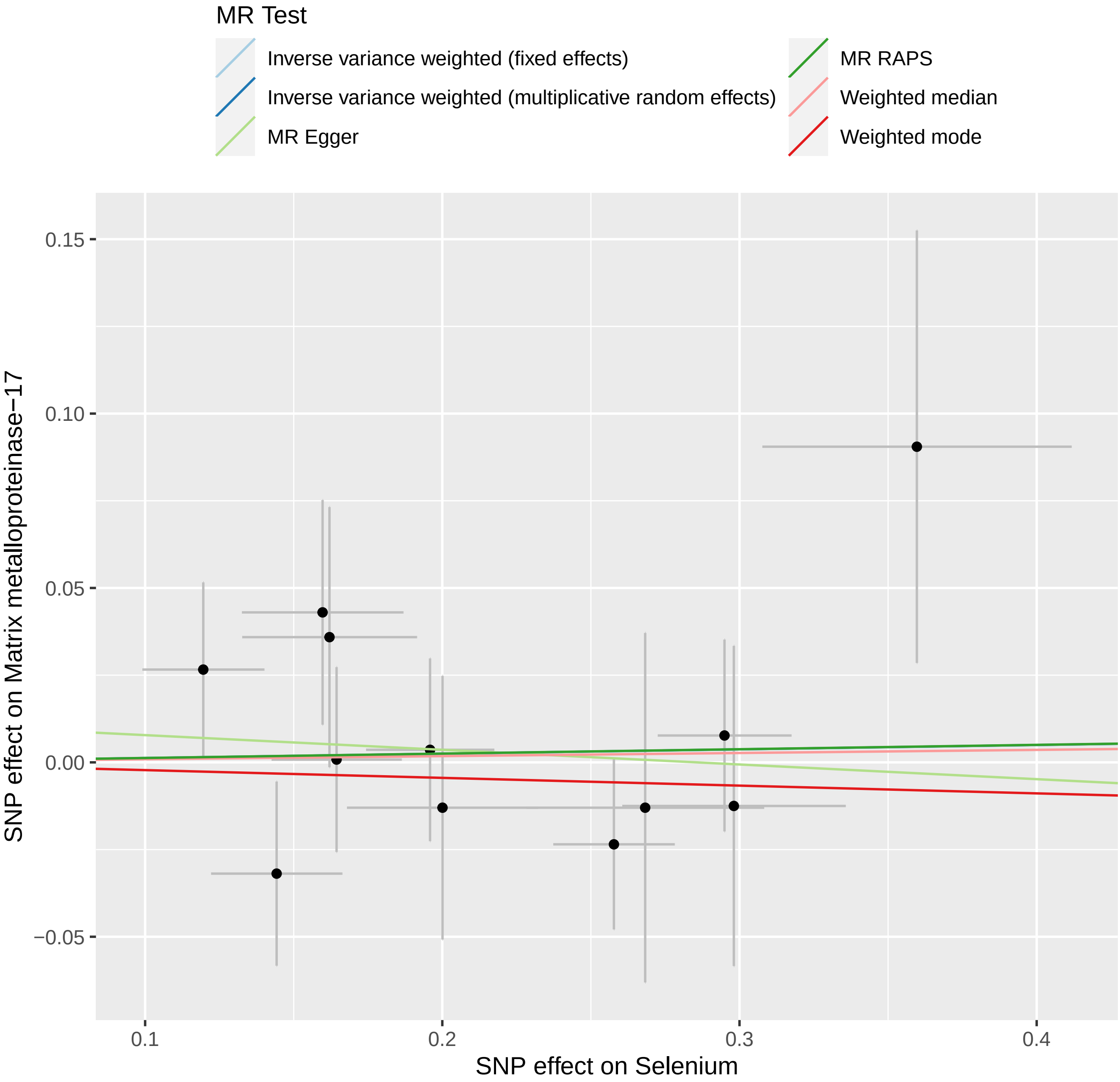

Batch 3−1918 : Selenium on 72 kDa type IV collagenase

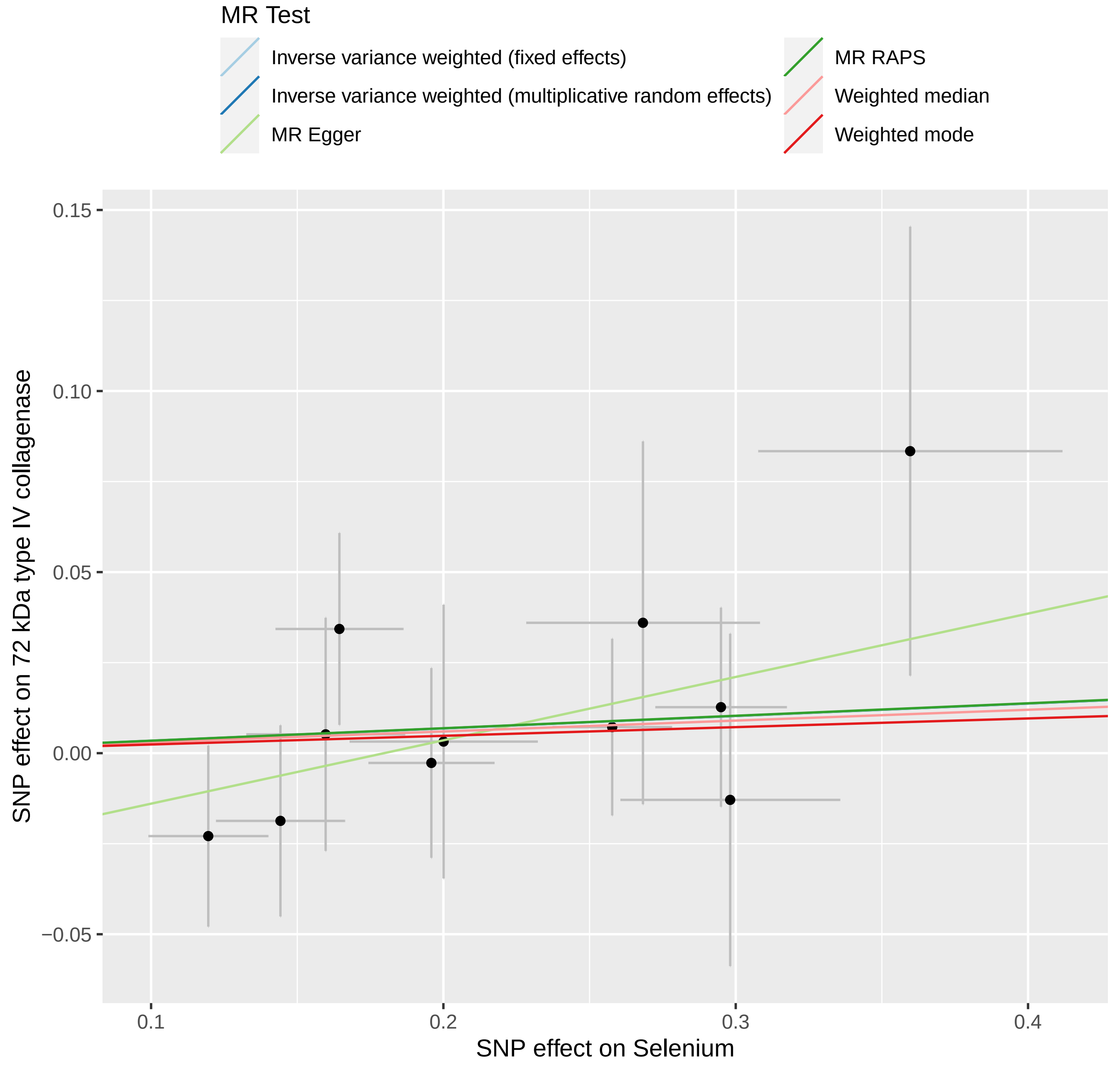

Batch 3−1919 : Selenium on Matrilysin

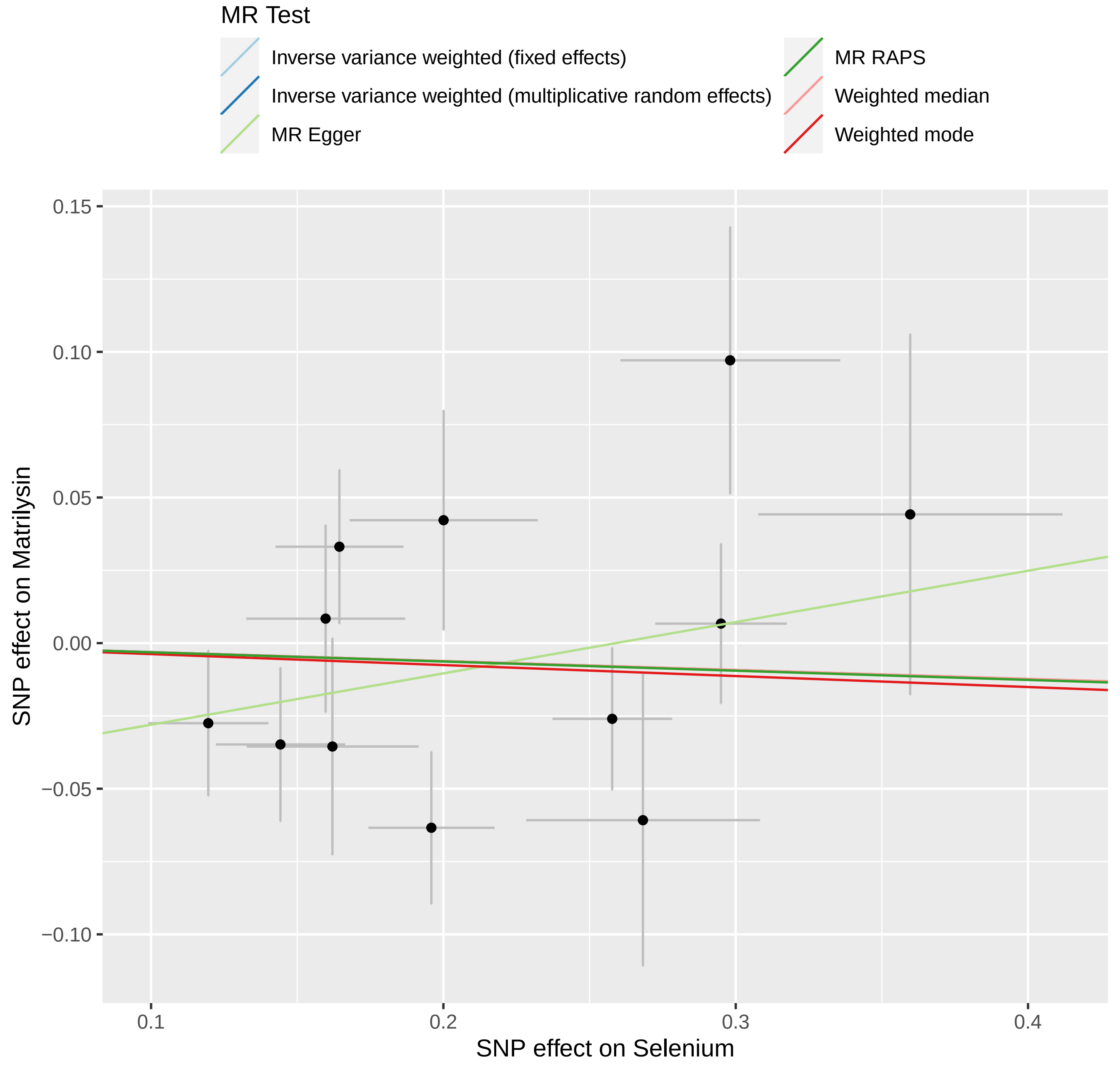

Batch 3−1920 : Selenium on Neutrophil collagenase

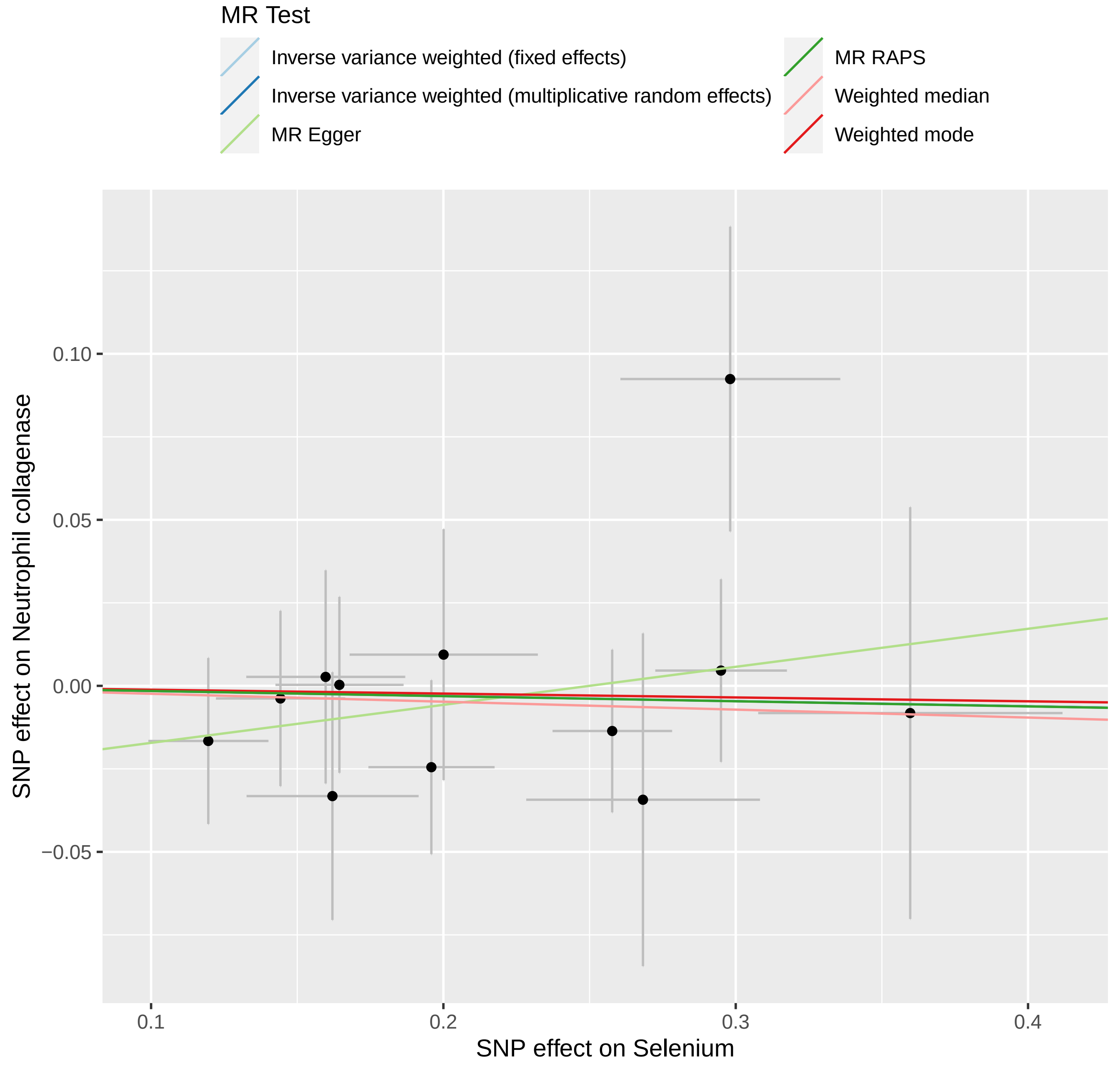

Batch 3−1921 : Selenium on Matrix metalloproteinase−9

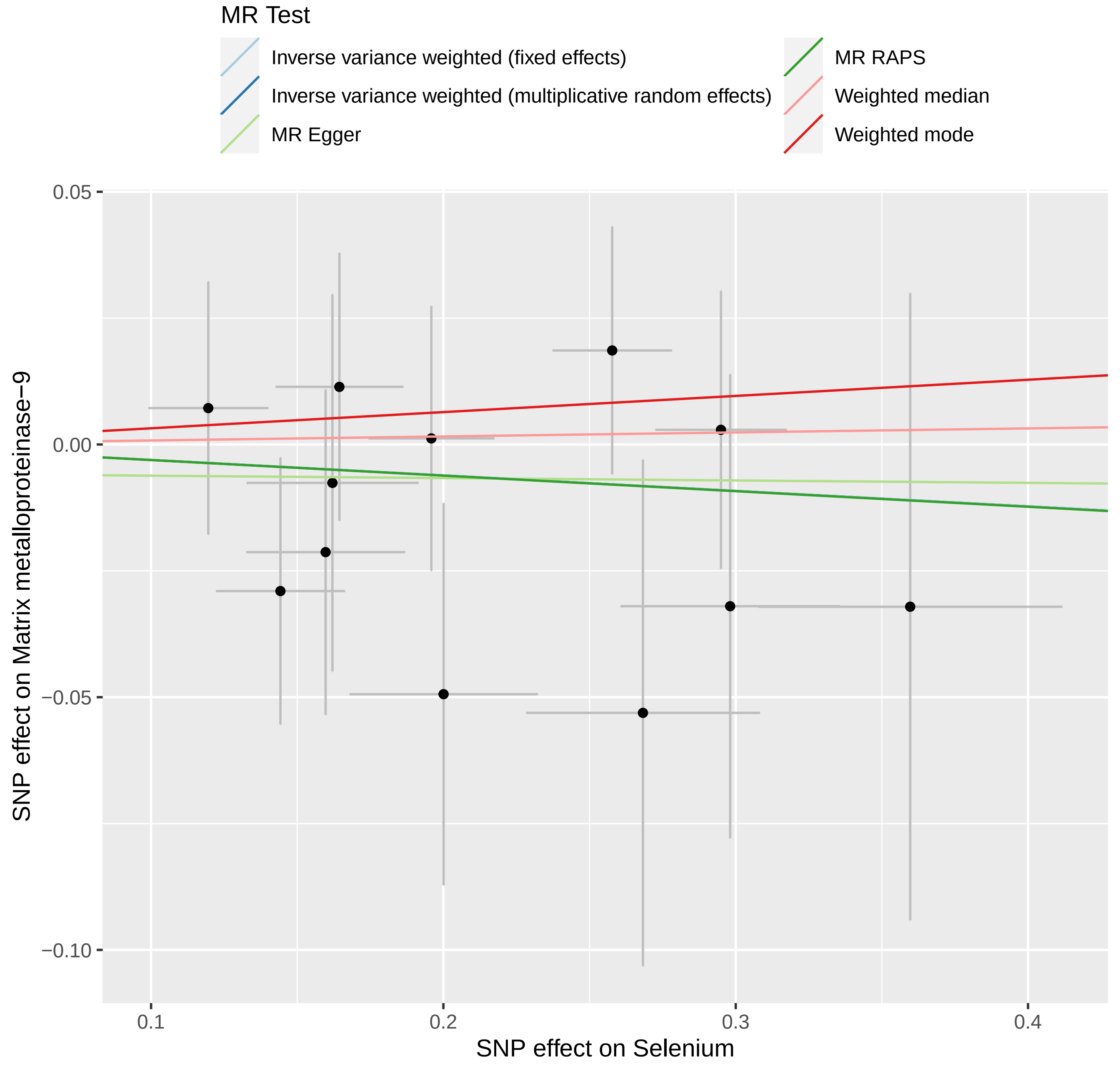

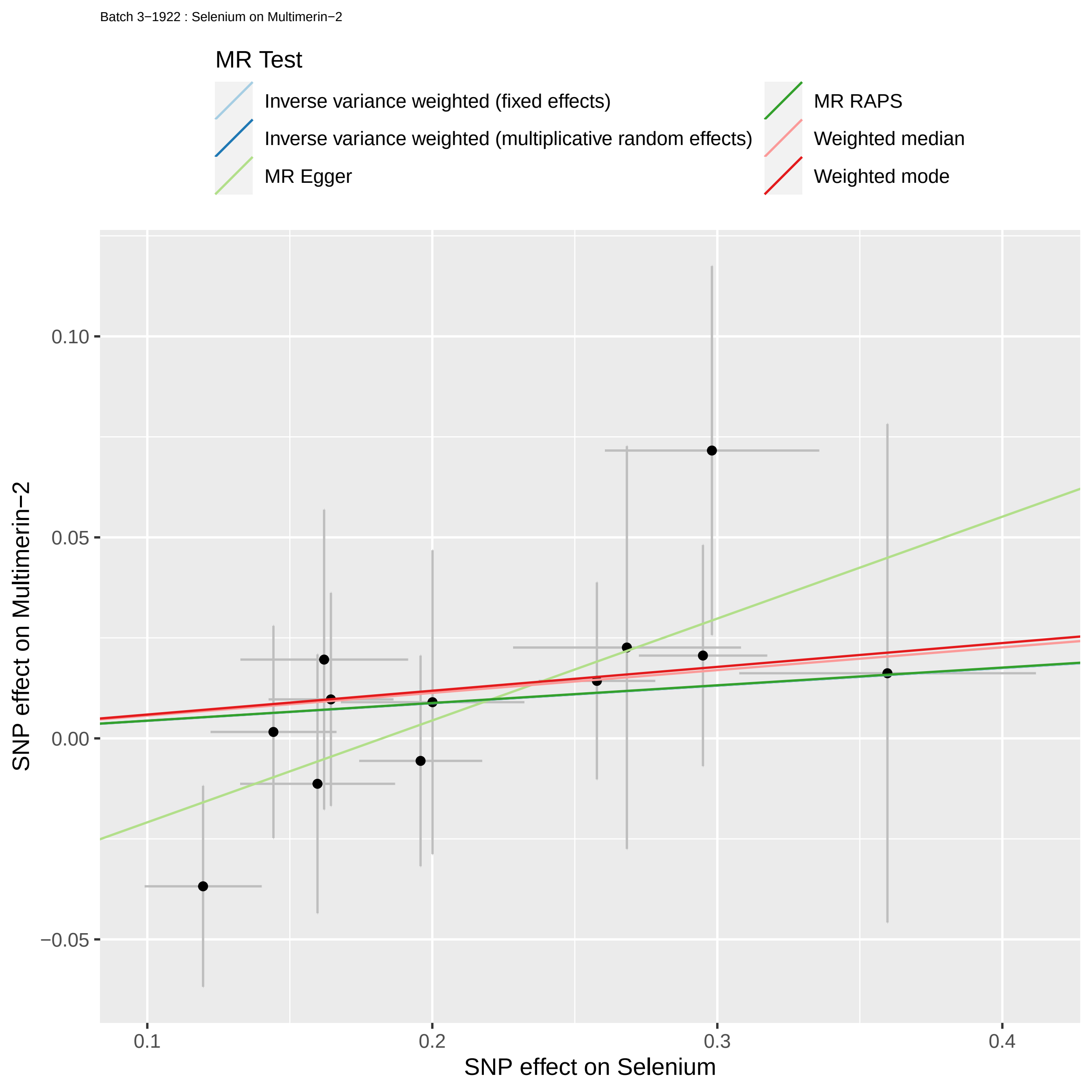
Batch 3−1922 : Selenium on Multimerin−2
MR Test
Inverse variance weighted (fixed effects)
Inverse variance weighted (multiplicative random effects)
MR Egger
MR RAPS
Weighted median
Weighted mode
0.10
0.05
0.00
−0.05
0.1
0.2
0.3
0.4
SNP effect on Selenium
SNP effect on Multimerin−2

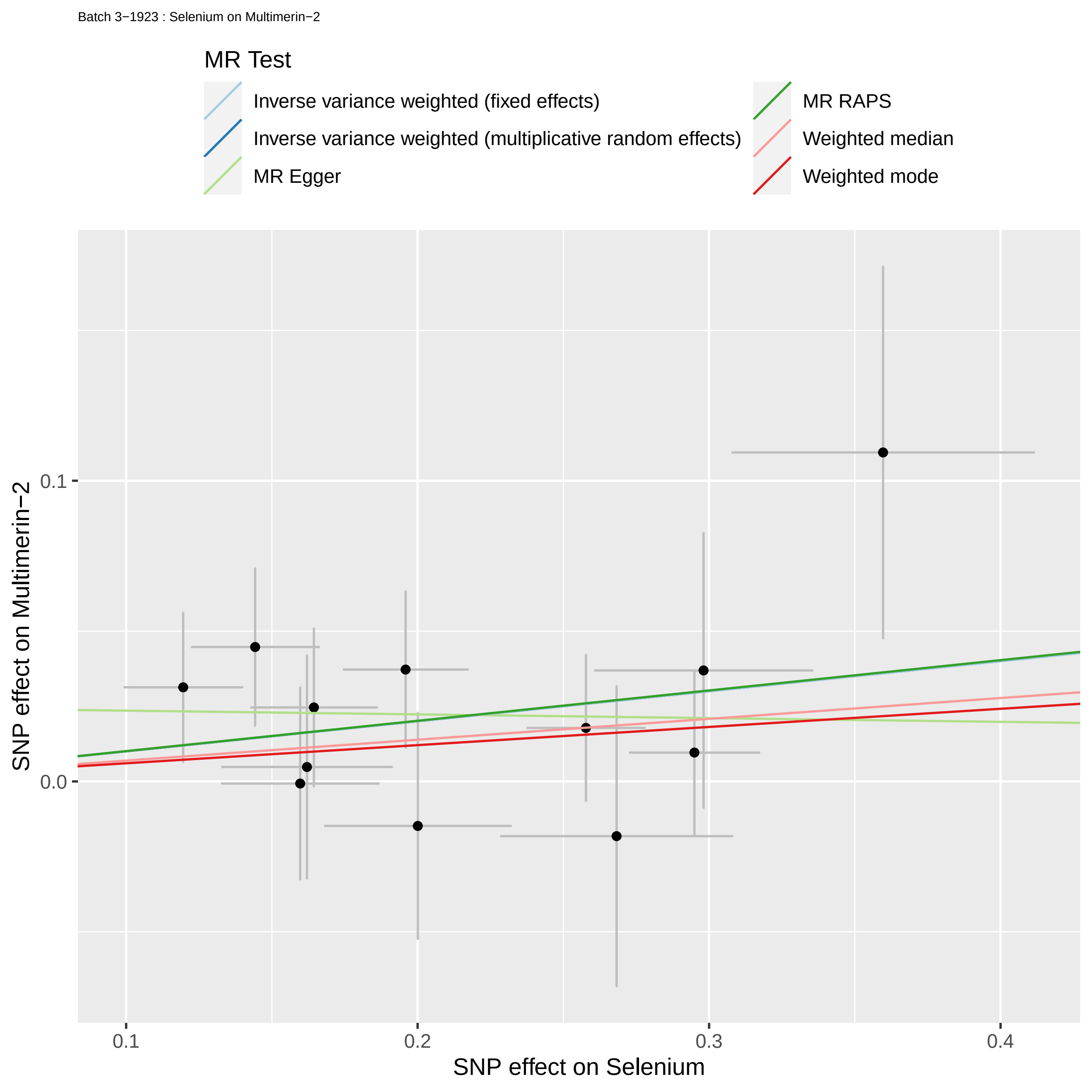
Batch 3−1923 : Selenium on Multimerin−2
MR Test
Inverse variance weighted (fixed effects)
Inverse variance weighted (multiplicative random effects)
MR Egger
MR RAPS
Weighted median
Weighted mode
SNP effect on Multimerin−2
0.1
0.0
0.1
0.2
0.3
0.4
SNP effect on Selenium

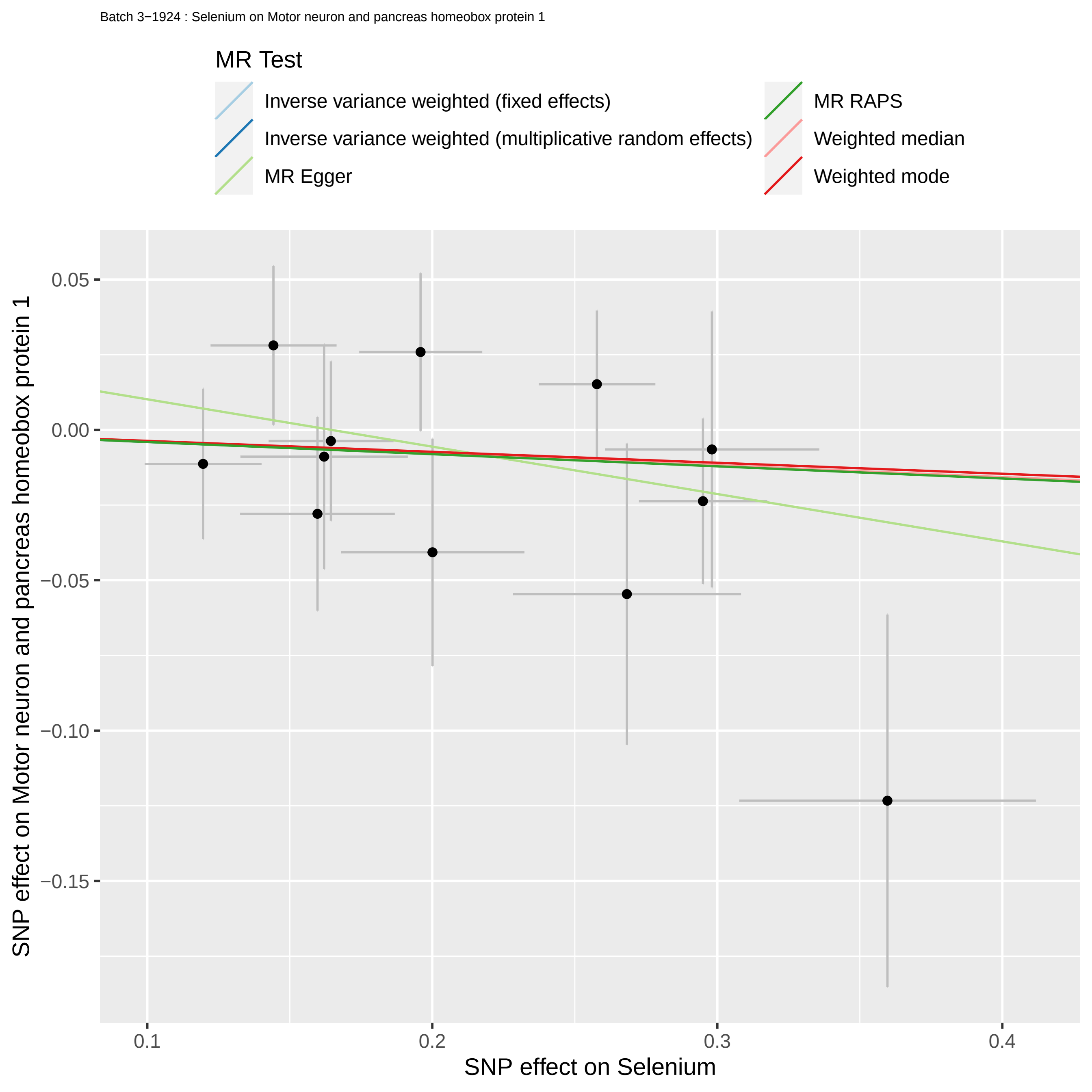
Batch 3−1924 : Selenium on Motor neuron and pancreas homeobox protein 1
MR Test
Inverse variance weighted (fixed effects)
Inverse variance weighted (multiplicative random effects)
MR Egger
MR RAPS
Weighted median
Weighted mode
SNP effect on Motor neuron and pancreas homeobox protein 1
0.05
0.00
−0.05
−0.10
−0.15
0.1
0.2
0.3
0.4
SNP effect on Selenium

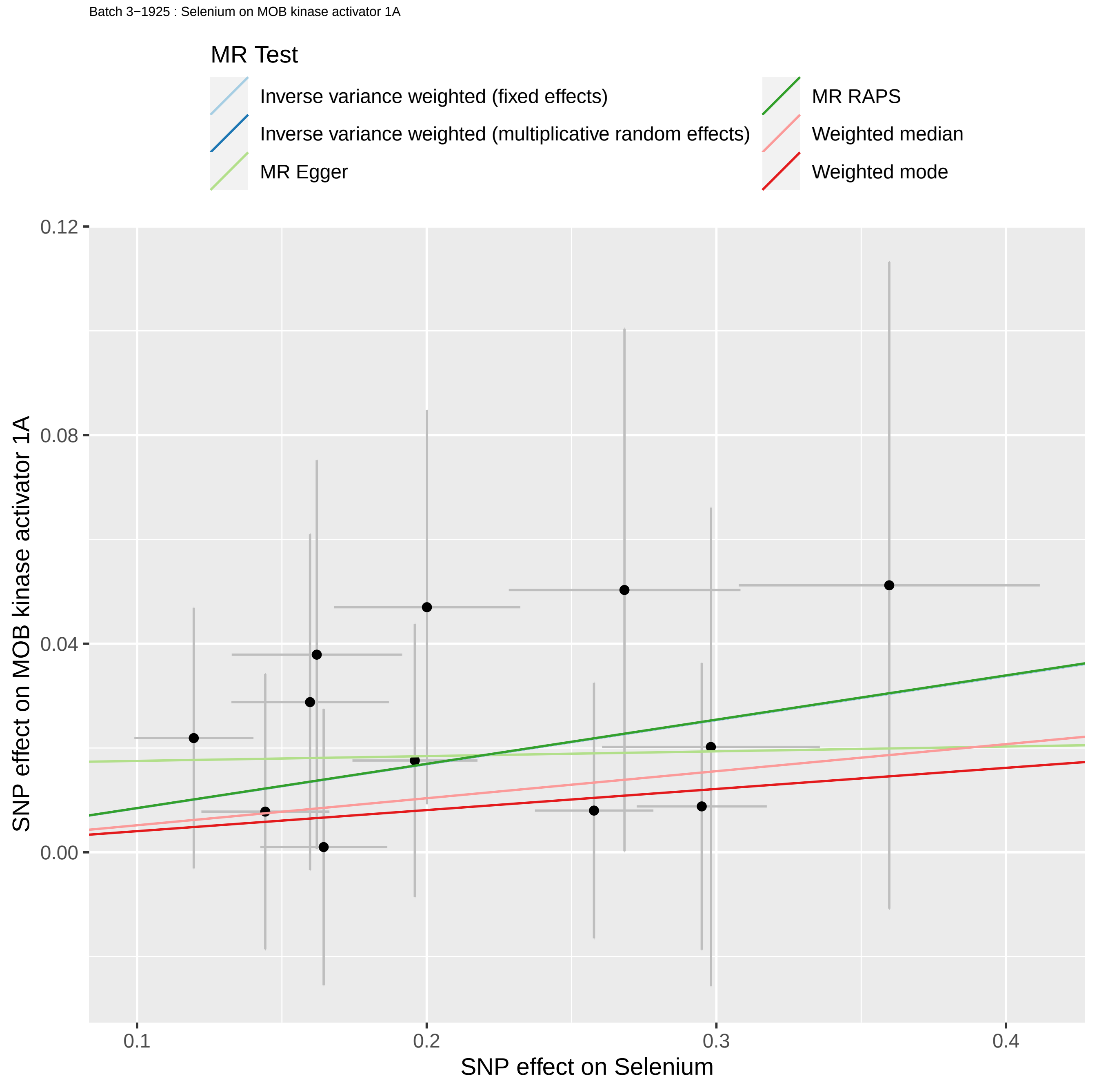

Batch 3−1925 : Selenium on MOB kinase activator 1A
MR Test
Inverse variance weighted (fixed effects)
Inverse variance weighted (multiplicative random effects)
MR Egger
MR RAPS
Weighted median
Weighted mode
0.12
0.08
0.04
0.00
0.1
0.2
0.3
0.4
SNP effect on MOB kinase activator 1A
SNP effect on Selenium

Batch 3−1926 : Selenium on Adenylyltransferase and sulfurtransferase MOCS3

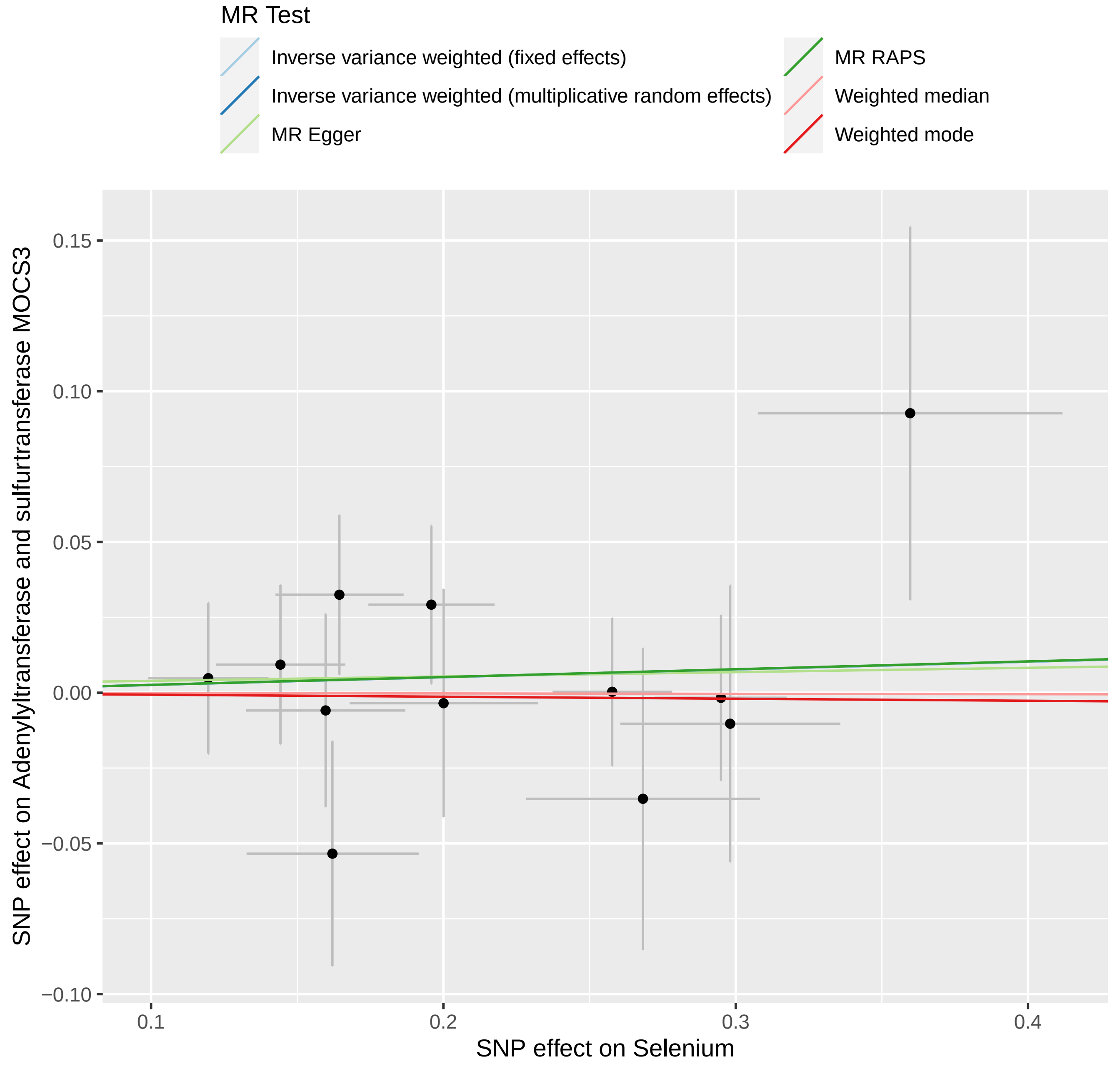

Batch 3−1927 : Selenium on Multiple PDZ domain protein

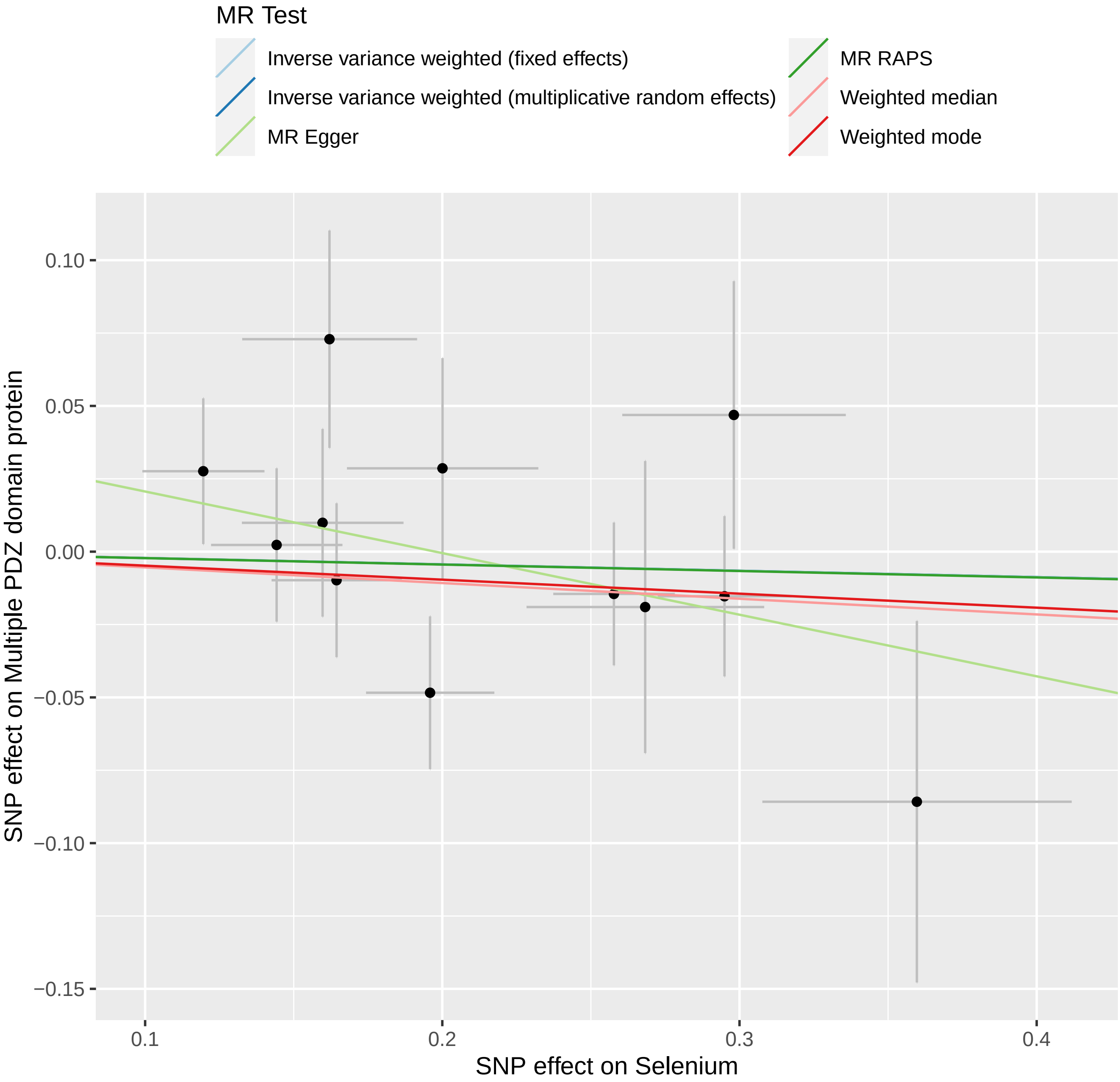

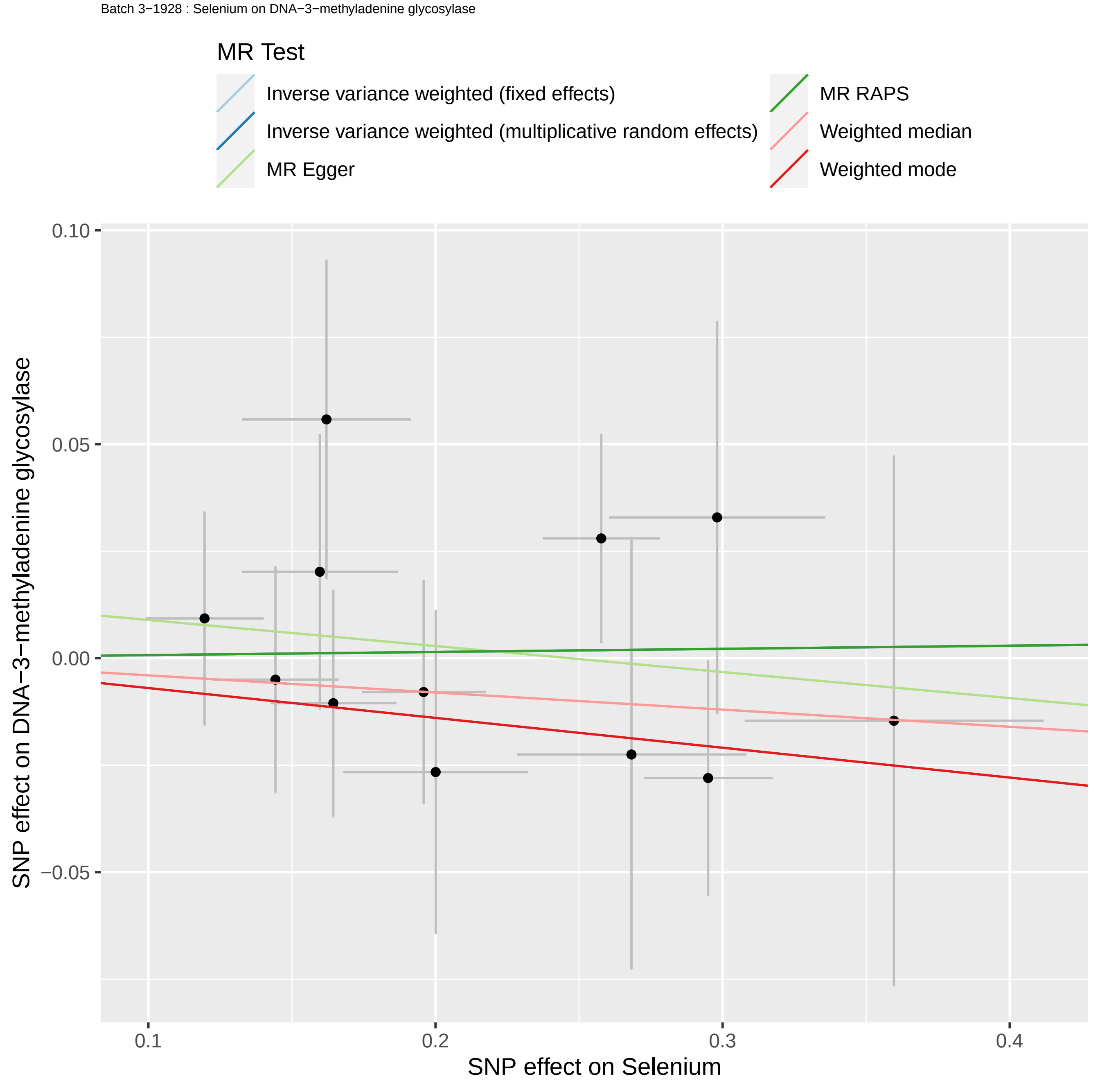

Batch 3−1928 : Selenium on DNA−3−methyladenine glycosylase
MR Test
Inverse variance weighted (fixed effects)
Inverse variance weighted (multiplicative random effects)
MR Egger
MR RAPS
Weighted median
Weighted mode
0.10
0.05
0.00
−0.05
0.1
0.2
0.3
0.4
SNP effect on DNA−3−methyladenine glycosylase
SNP effect on Selenium

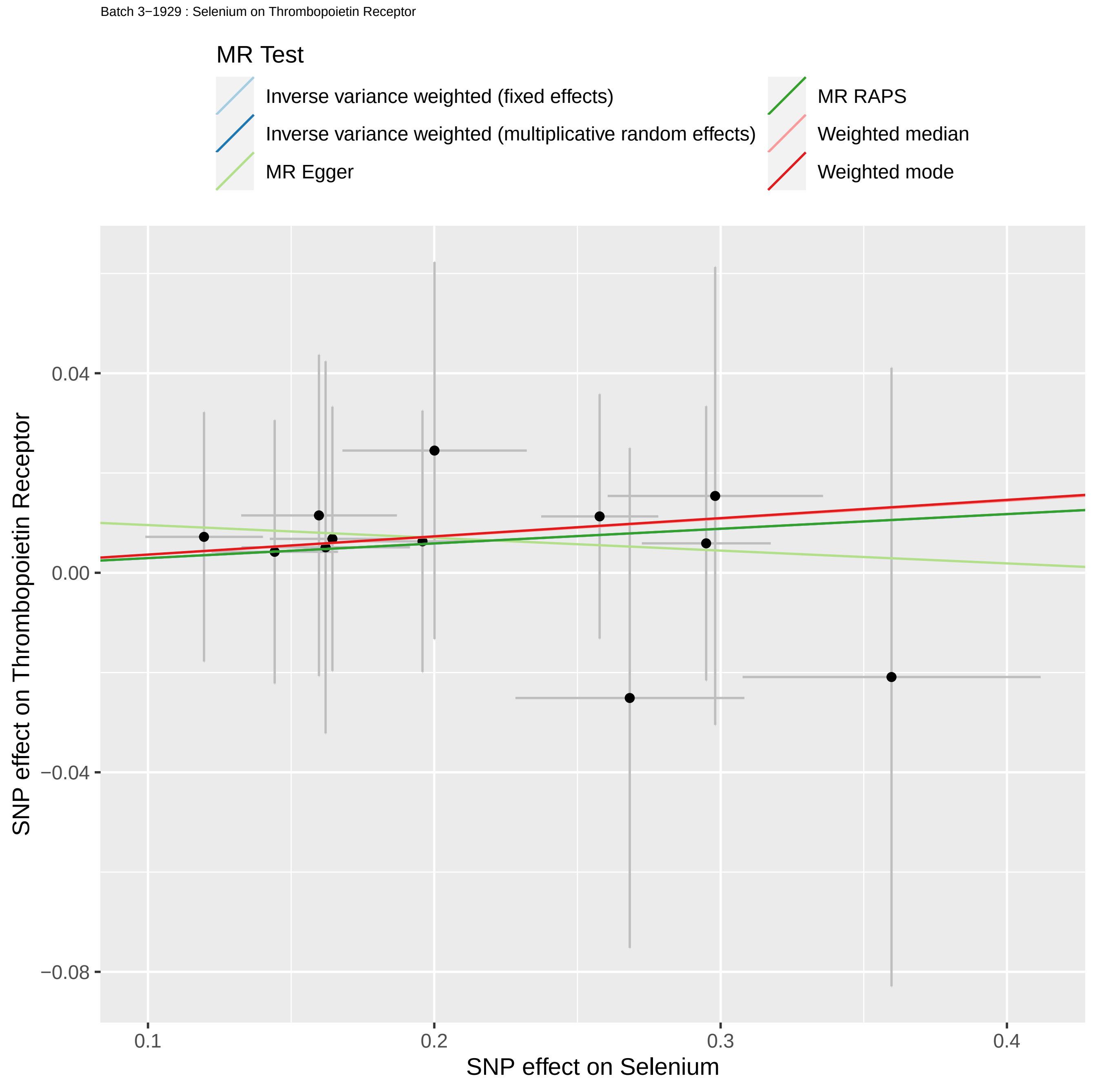
Batch 3−1929 : Selenium on Thrombopoietin Receptor
MR Test
Inverse variance weighted (fixed effects)
Inverse variance weighted (multiplicative random effects)
MR Egger
MR RAPS
Weighted median
Weighted mode
SNP effect on Thrombopoietin Receptor
0.04
0.00
−0.04
−0.08
0.1
0.2
0.3
0.4
SNP effect on Selenium

Batch 3−1930 : Selenium on Myeloperoxidase

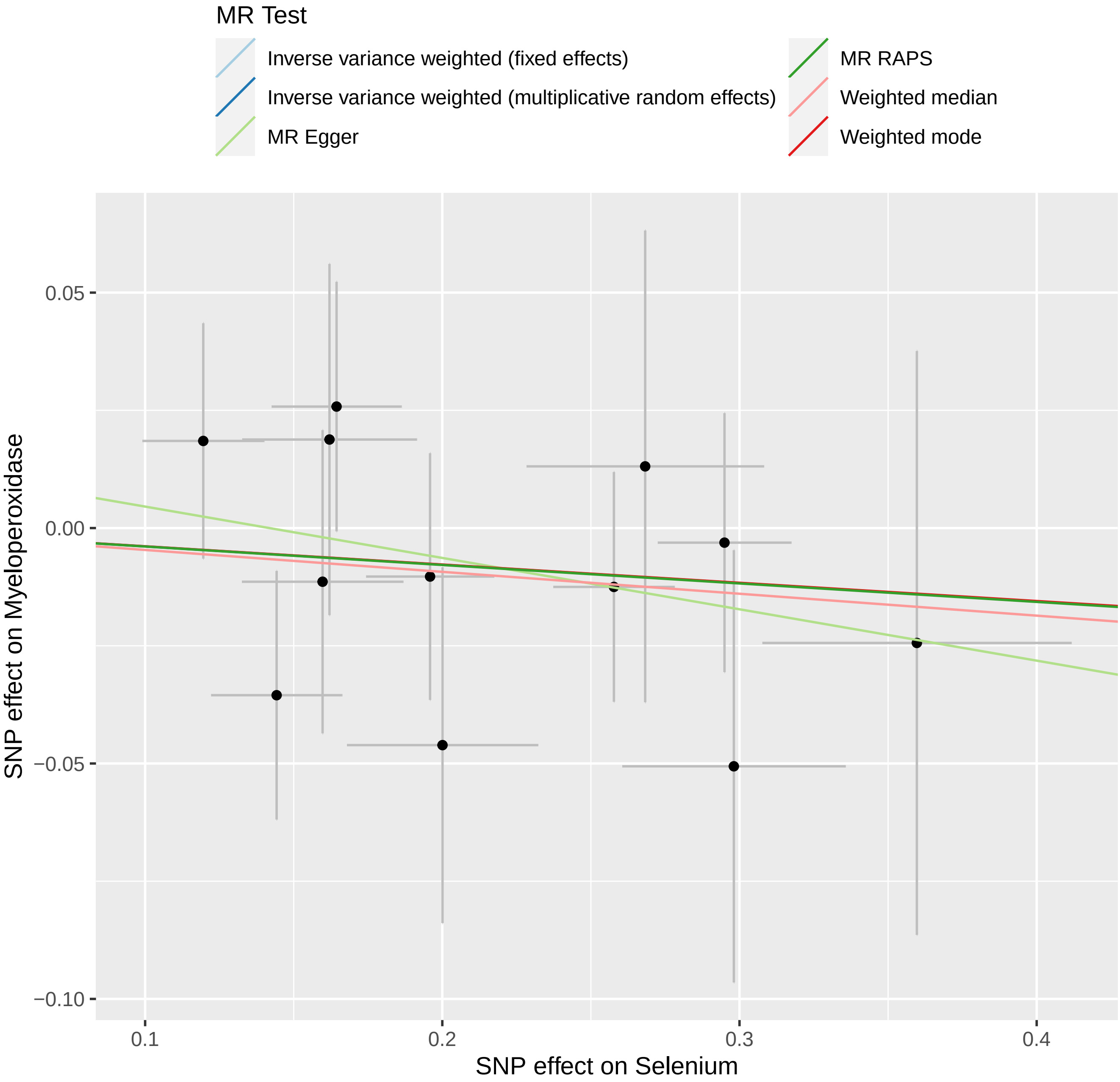

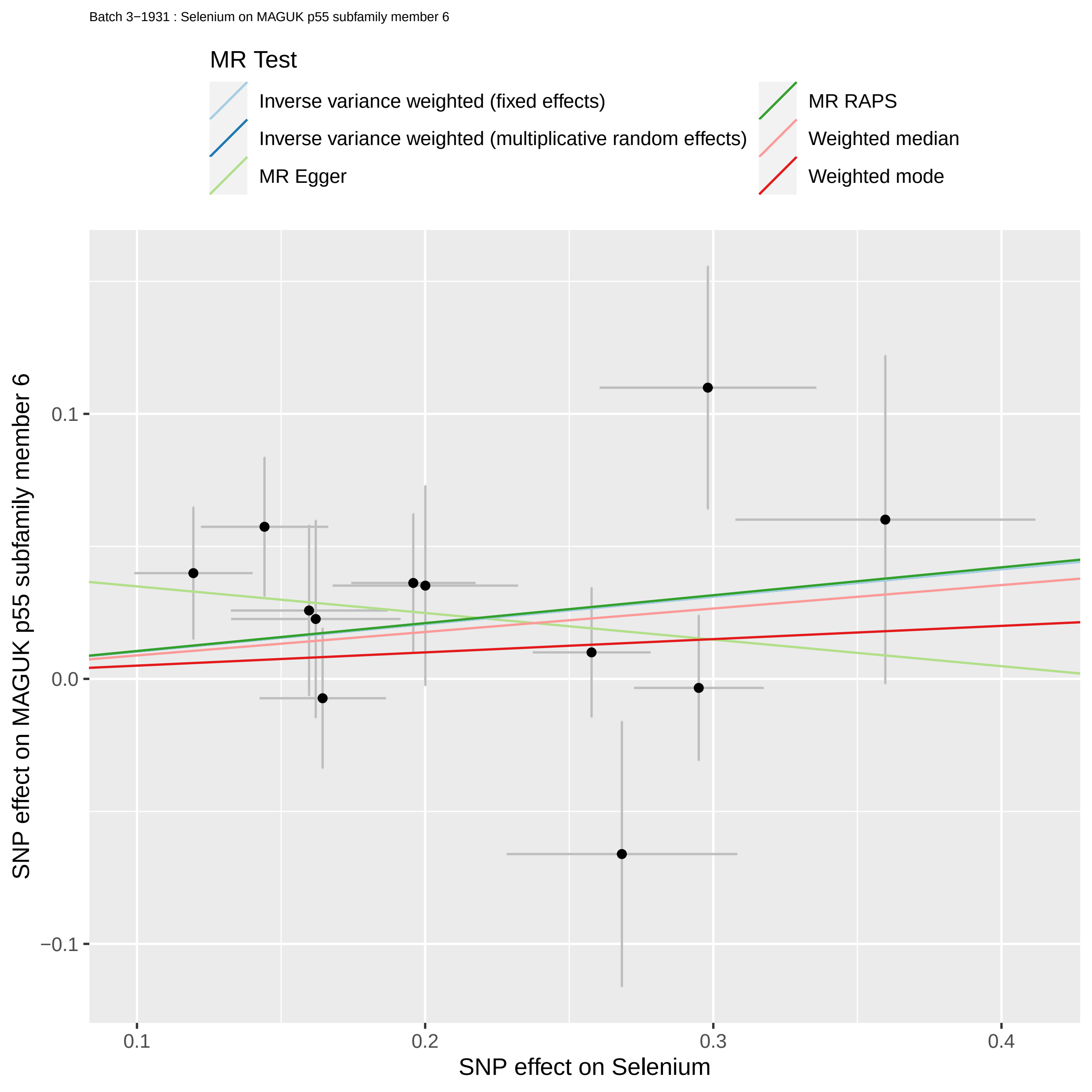
Batch 3−1931 : Selenium on MAGUK p55 subfamily member 6
MR Test
Inverse variance weighted (fixed effects)
Inverse variance weighted (multiplicative random effects)
MR Egger
MR RAPS
Weighted median
Weighted mode
SNP effect on MAGUK p55 subfamily member 6
0.1
0.0
−0.1
0.1
0.2
0.3
0.4
SNP effect on Selenium

Batch 3−1932 : Selenium on MAGUK p55 subfamily member 7

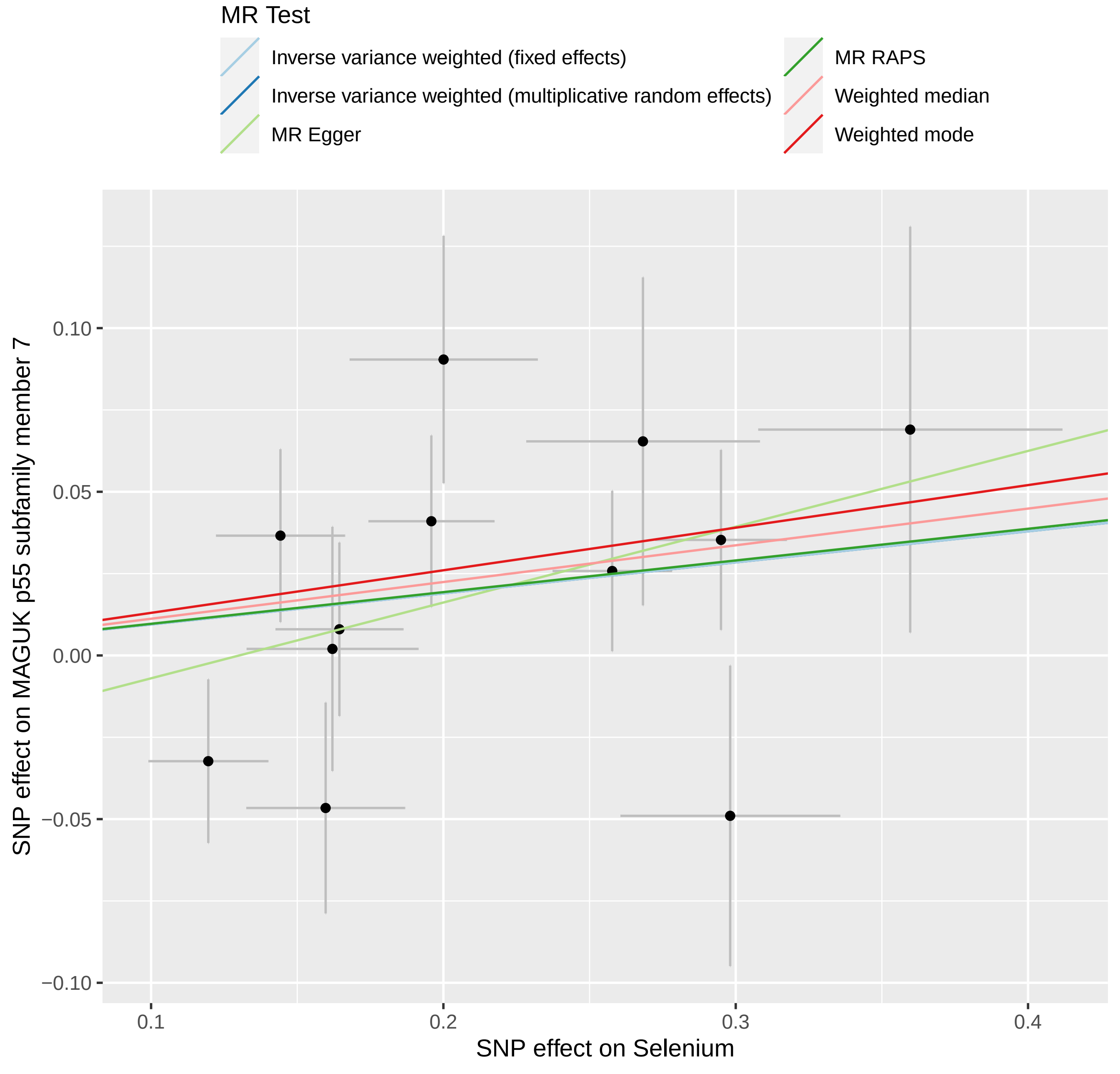

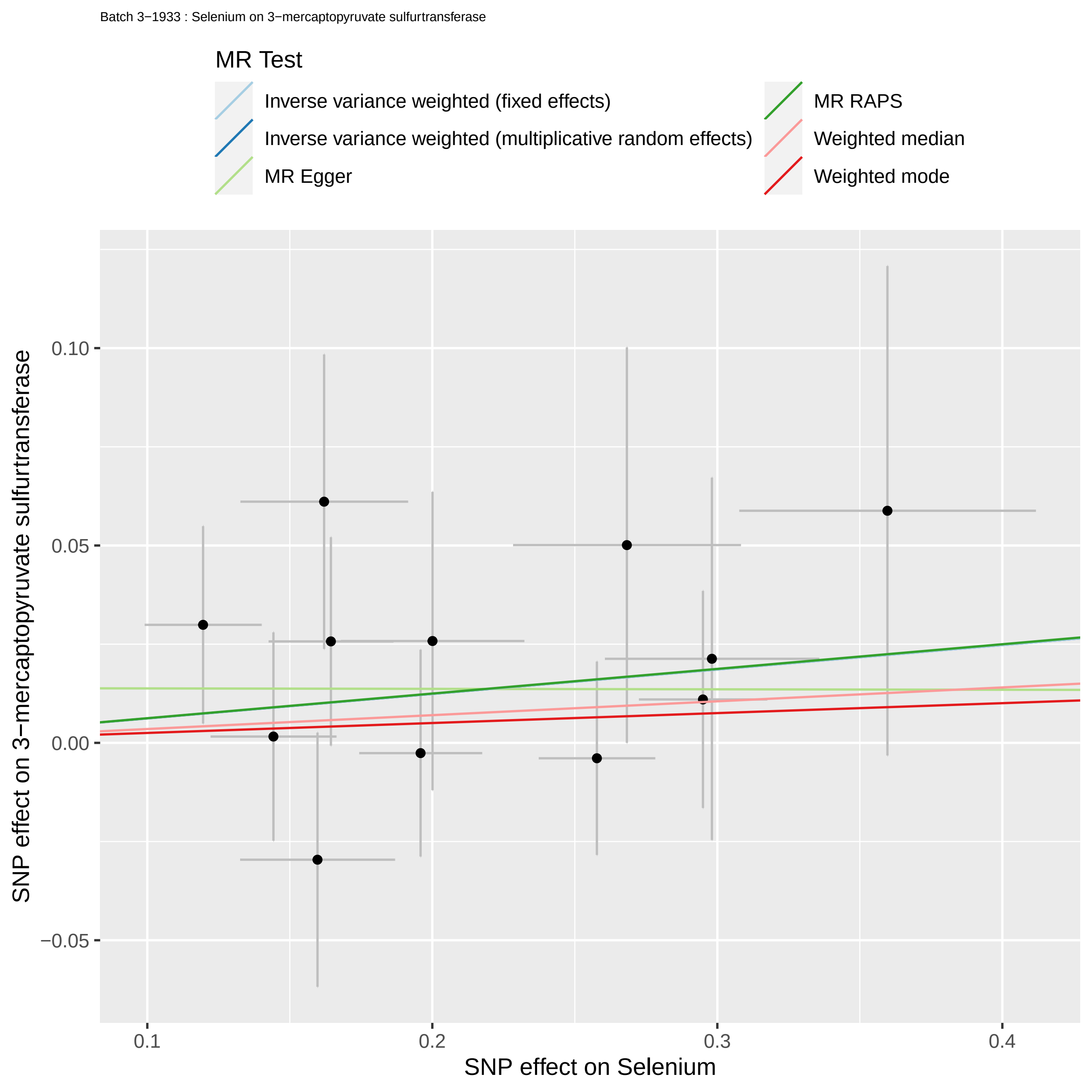

Batch 3−1933 : Selenium on 3−mercaptopyruvate sulfurtransferase
MR Test
Inverse variance weighted (fixed effects)
Inverse variance weighted (multiplicative random effects)
MR Egger
MR RAPS
Weighted median
Weighted mode
SNP effect on 3−mercaptopyruvate sulfurtransferase
0.10
0.05
0.00
−0.05
0.1
0.2
0.3
0.4
SNP effect on Selenium

Batch 3−1934 : Selenium on Myelin protein P0

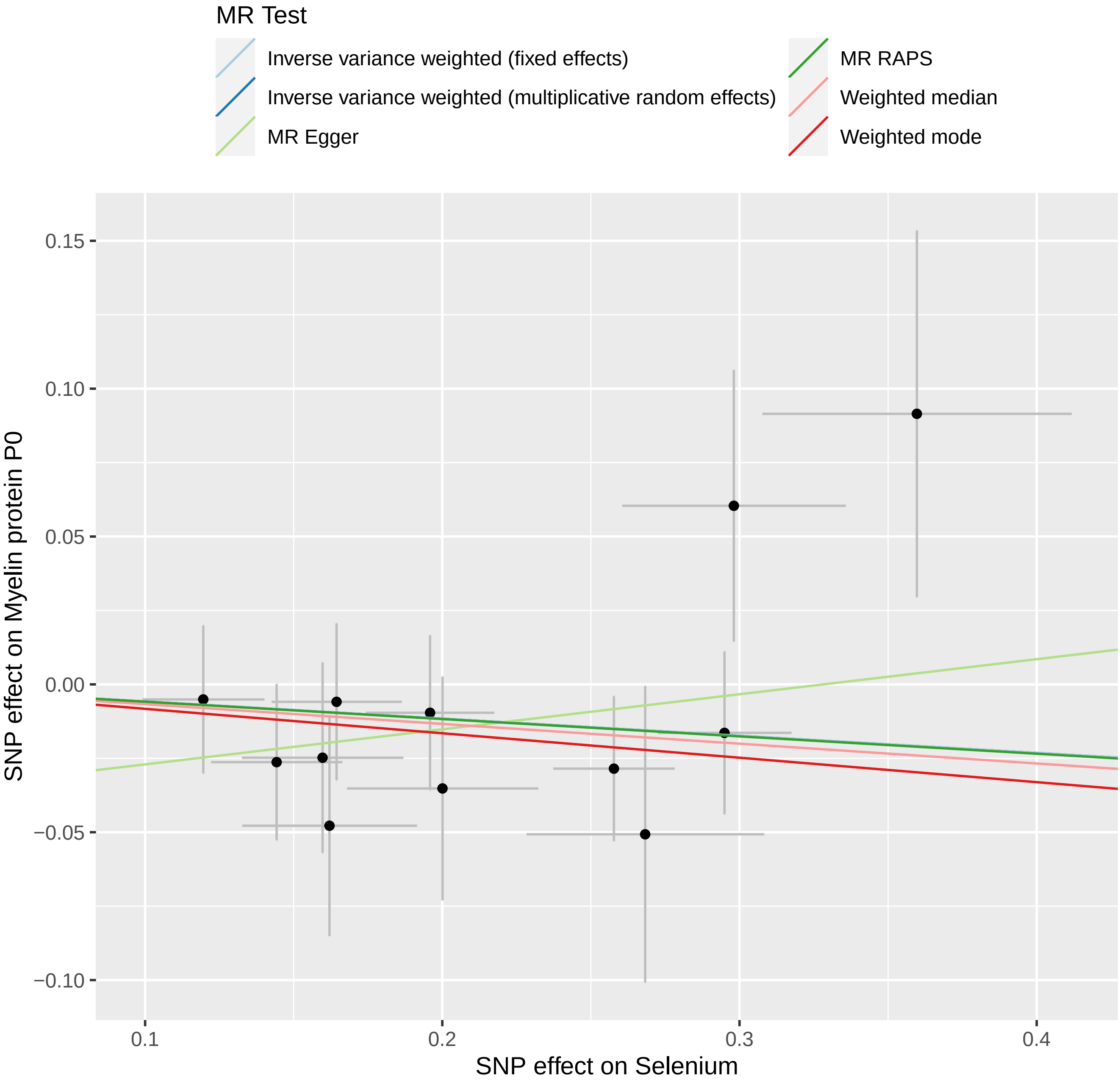

Batch 3−1935 : Selenium on Melanocortin−2 receptor accessory protein 2

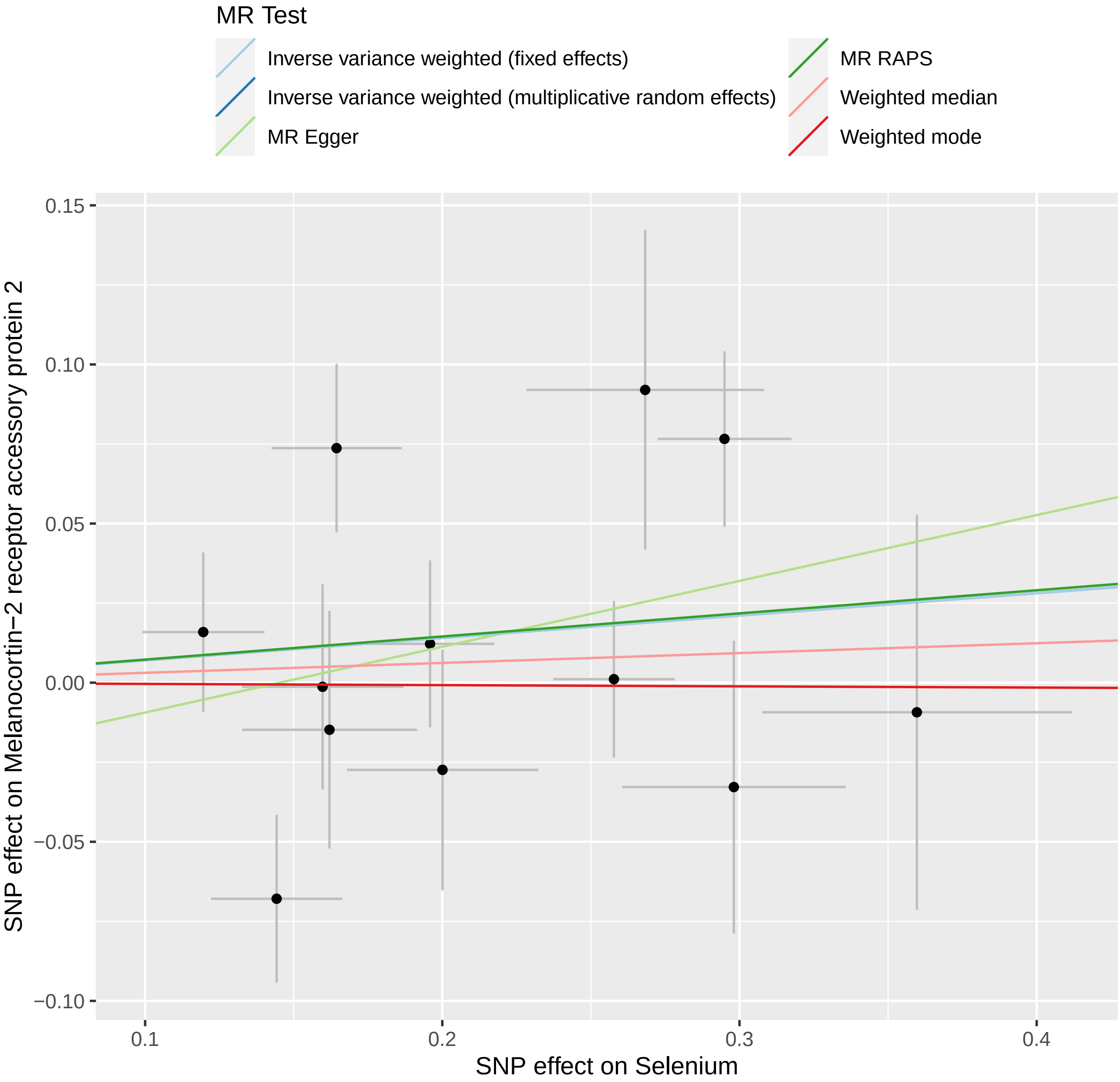

Batch 3−1936 : Selenium on Melanocortin−2 receptor accessory protein

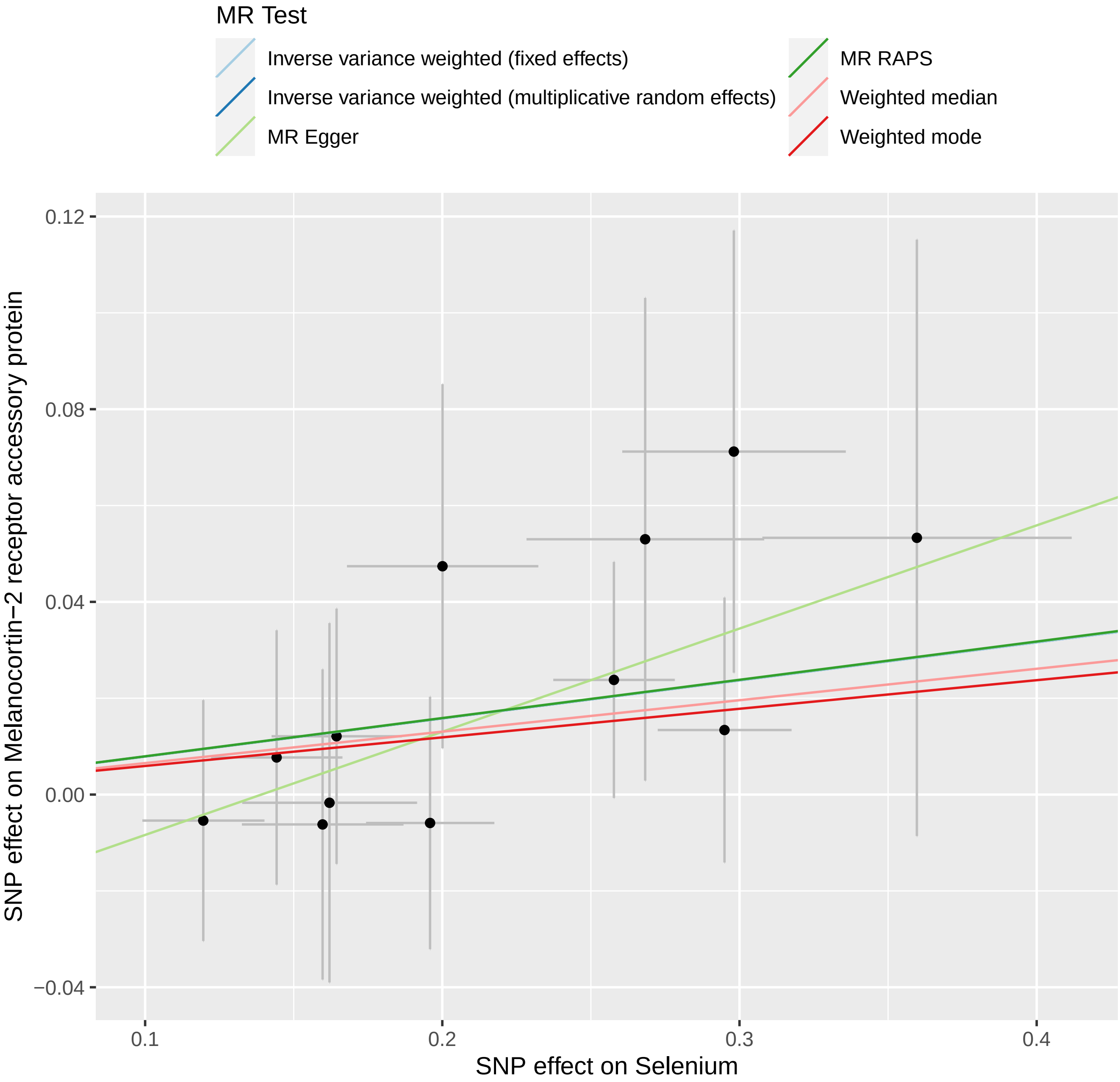

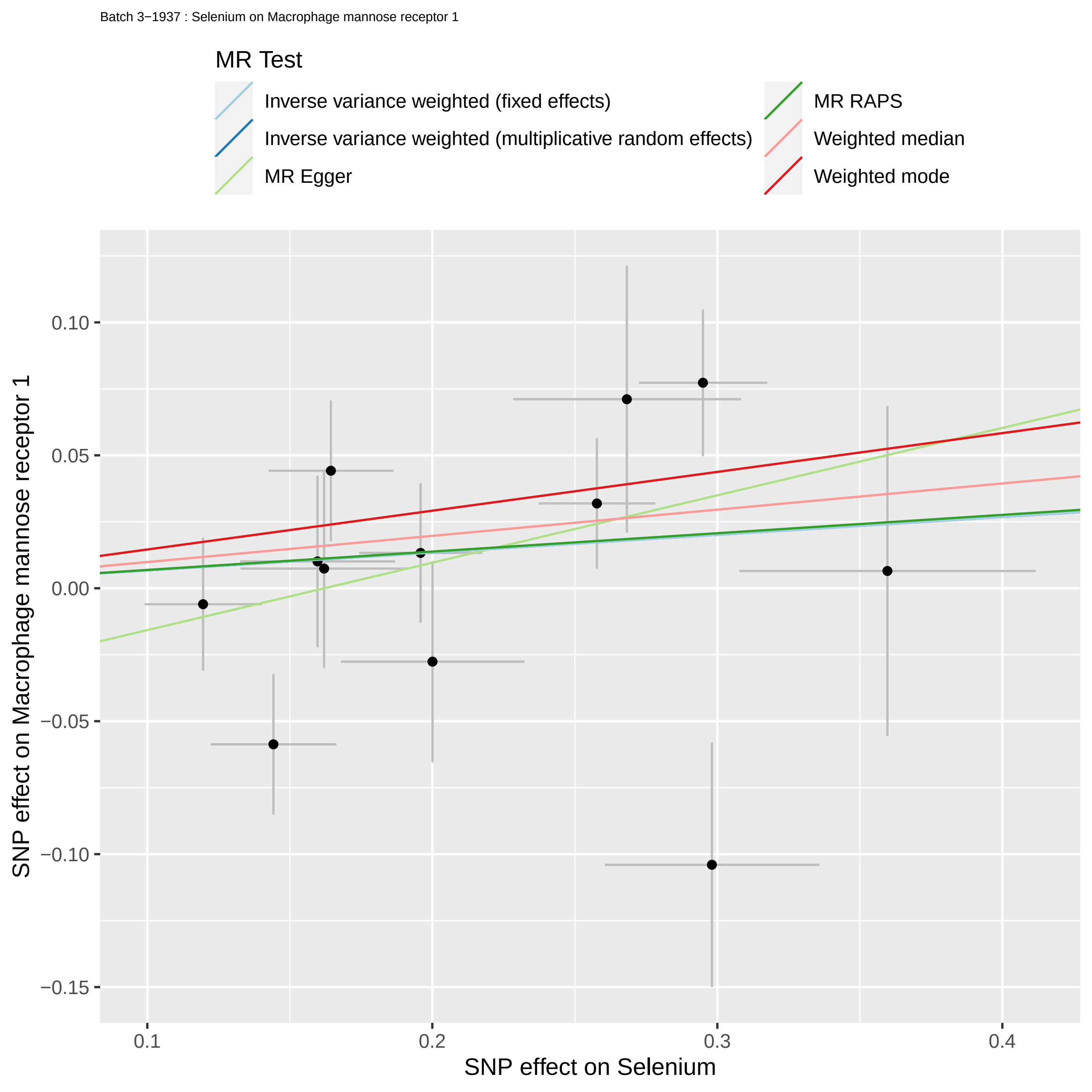

Batch 3−1937 : Selenium on Macrophage mannose receptor 1
MR Test
Inverse variance weighted (fixed effects)
Inverse variance weighted (multiplicative random effects)
MR Egger
MR RAPS
Weighted median
Weighted mode
SNP effect on Macrophage mannose receptor 1
0.10
0.05
0.00
−0.05
−0.10
−0.15
0.1
0.2
0.3
0.4
SNP effect on Selenium

Batch 3−1938 : Selenium on C−type mannose receptor 2

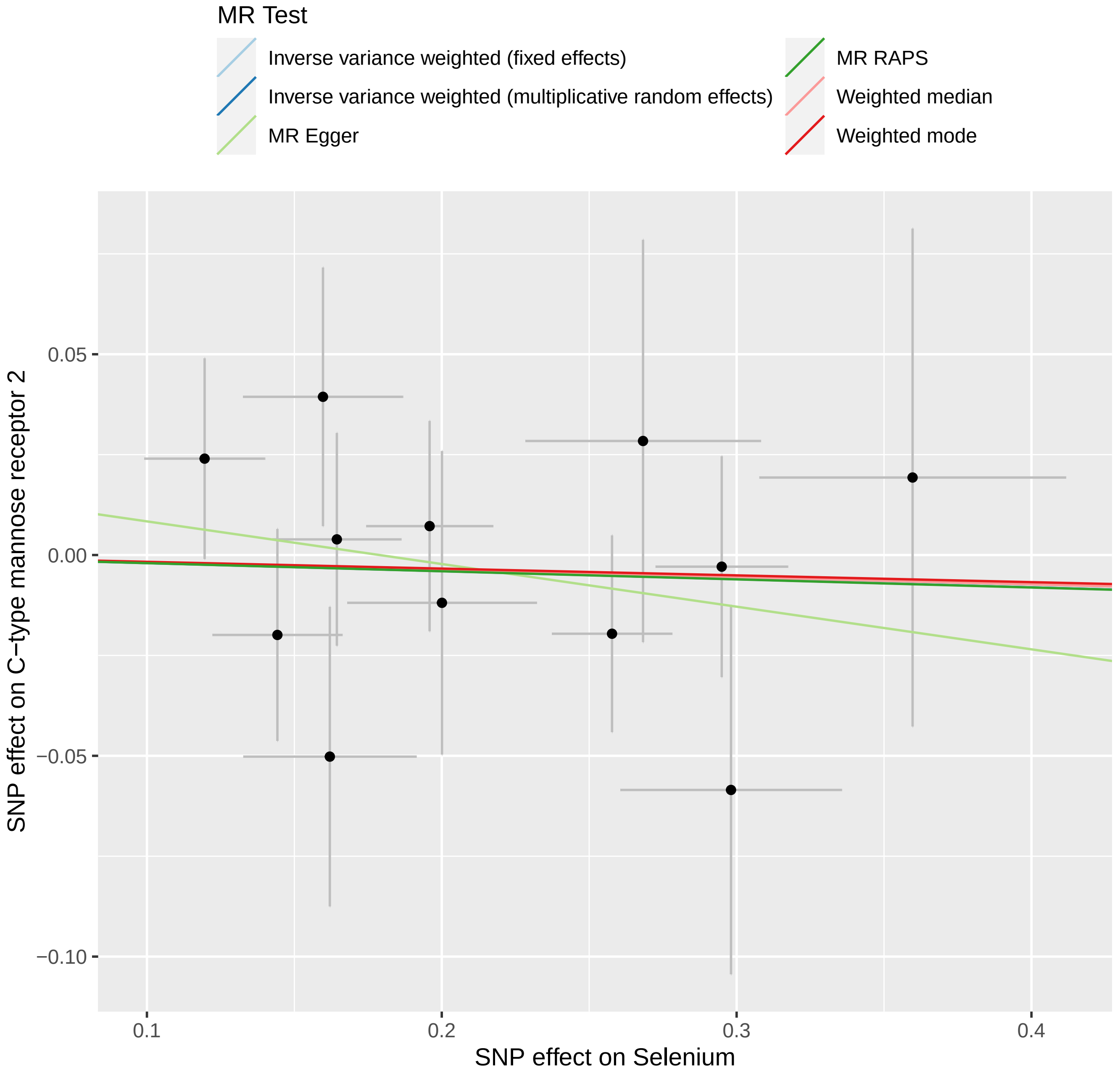

Batch 3−1939 : Selenium on Double−strand break repair protein MRE11A

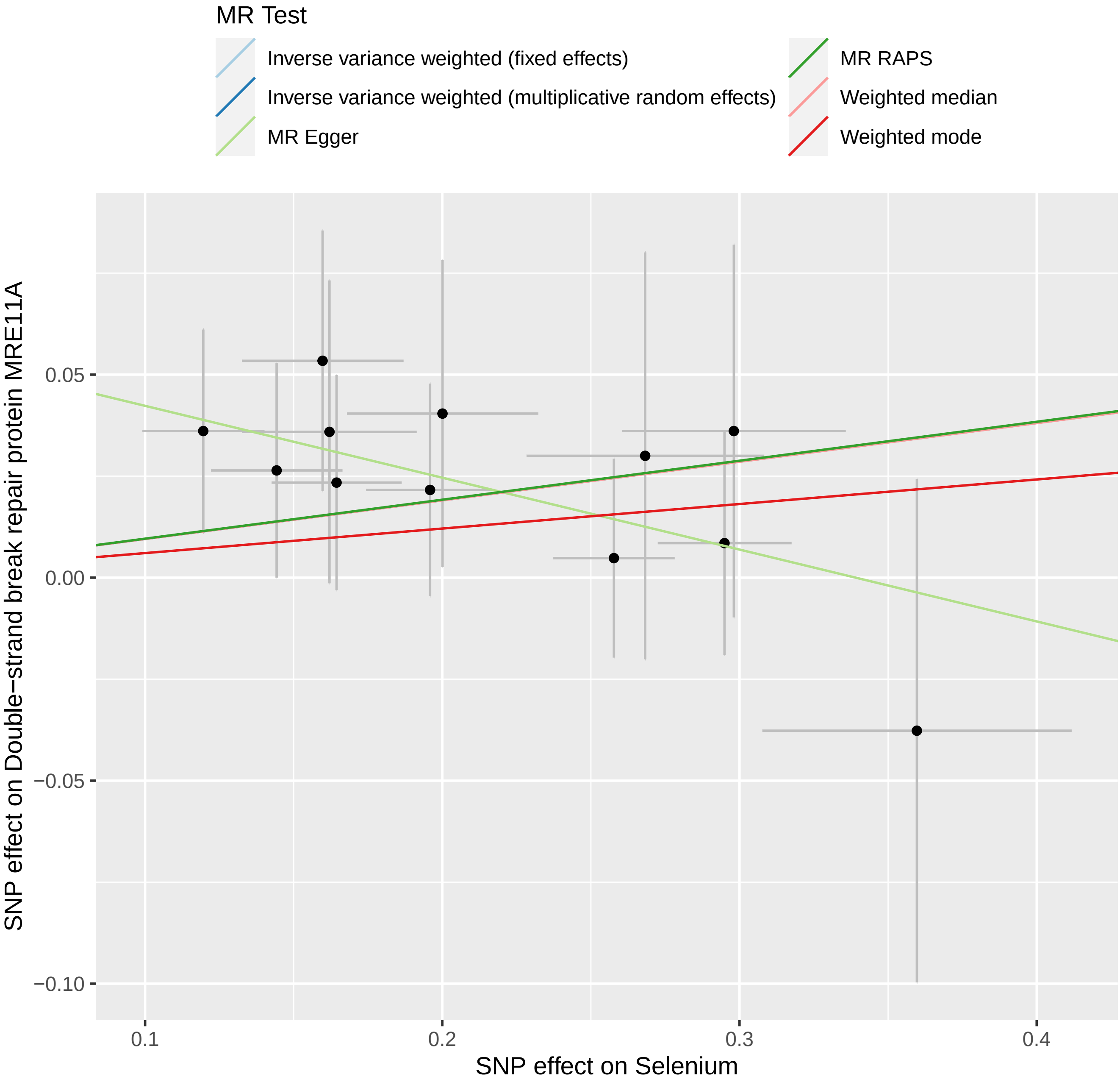

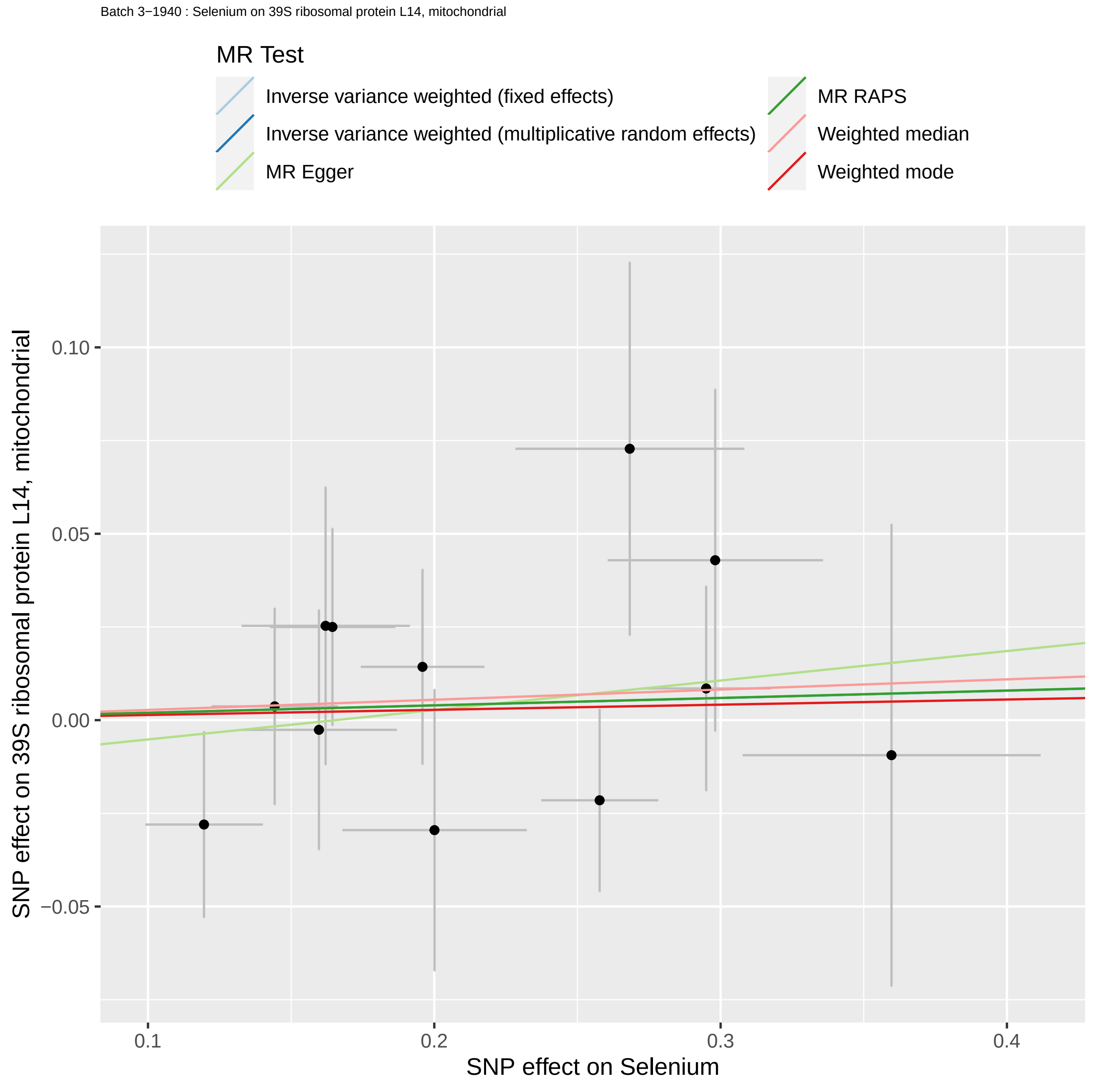
Batch 3−1940 : Selenium on 39S ribosomal protein L14, mitochondrial
MR Test
Inverse variance weighted (fixed effects)
Inverse variance weighted (multiplicative random effects)
MR Egger
MR RAPS
Weighted median
Weighted mode
SNP effect on 39S ribosomal protein L14, mitochondrial
0.10
0.05
0.00
−0.05
0.1
0.2
0.3
0.4
SNP effect on Selenium

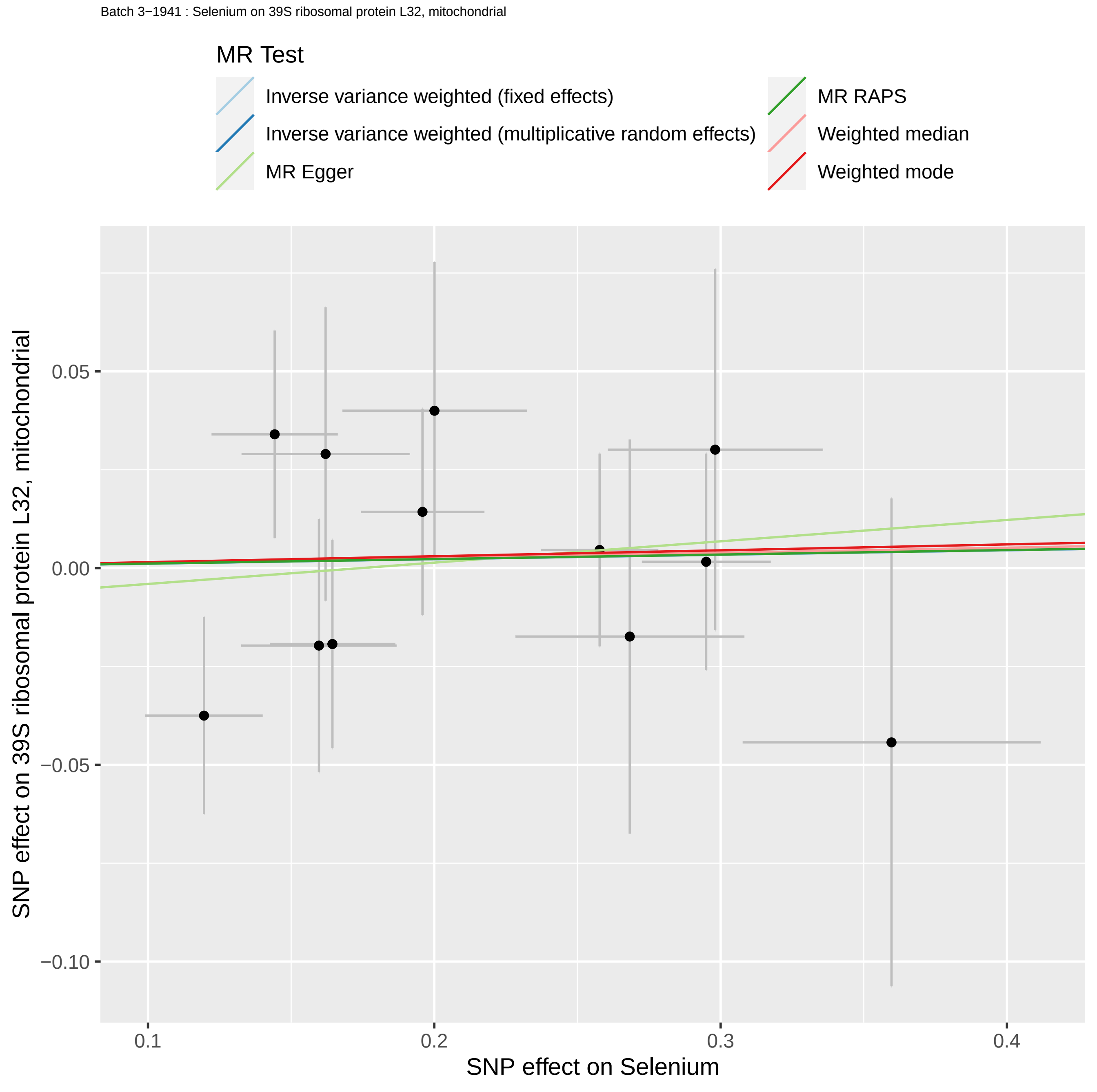
Batch 3−1941 : Selenium on 39S ribosomal protein L32, mitochondrial
MR Test
Inverse variance weighted (fixed effects)
Inverse variance weighted (multiplicative random effects)
MR Egger
MR RAPS
Weighted median
Weighted mode
SNP effect on 39S ribosomal protein L32, mitochondrial
0.05
0.00
−0.05
−0.10
0.1
0.2
0.3
0.4
SNP effect on Selenium

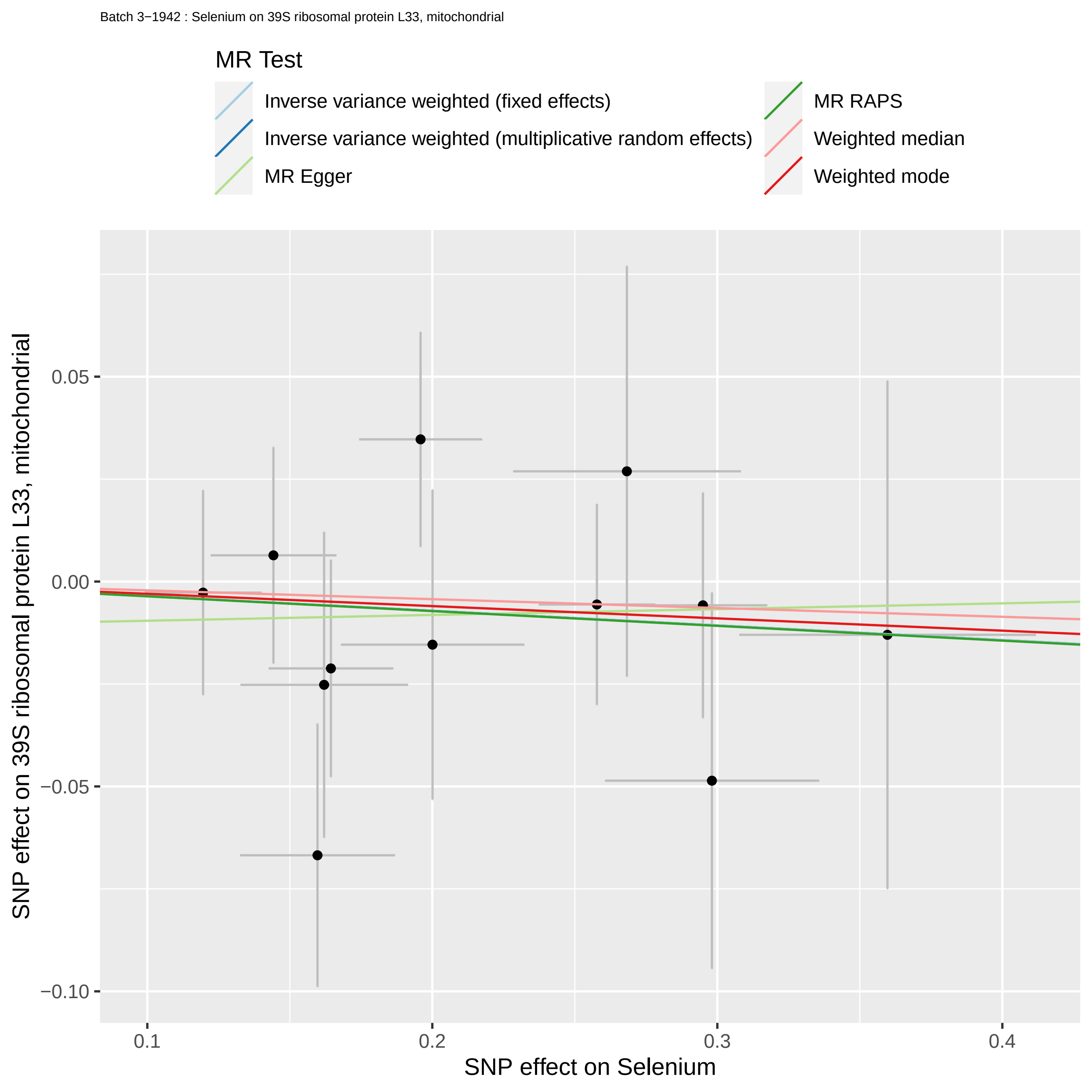

Batch 3−1942 : Selenium on 39S ribosomal protein L33, mitochondrial
MR Test
Inverse variance weighted (fixed effects)
Inverse variance weighted (multiplicative random effects)
MR Egger
MR RAPS
Weighted median
Weighted mode
SNP effect on 39S ribosomal protein L33, mitochondrial
0.05
0.00
−0.05
−0.10
0.1
0.2
0.3
0.4
SNP effect on Selenium

Batch 3−1943 : Selenium on 39S ribosomal protein L34, mitochondrial

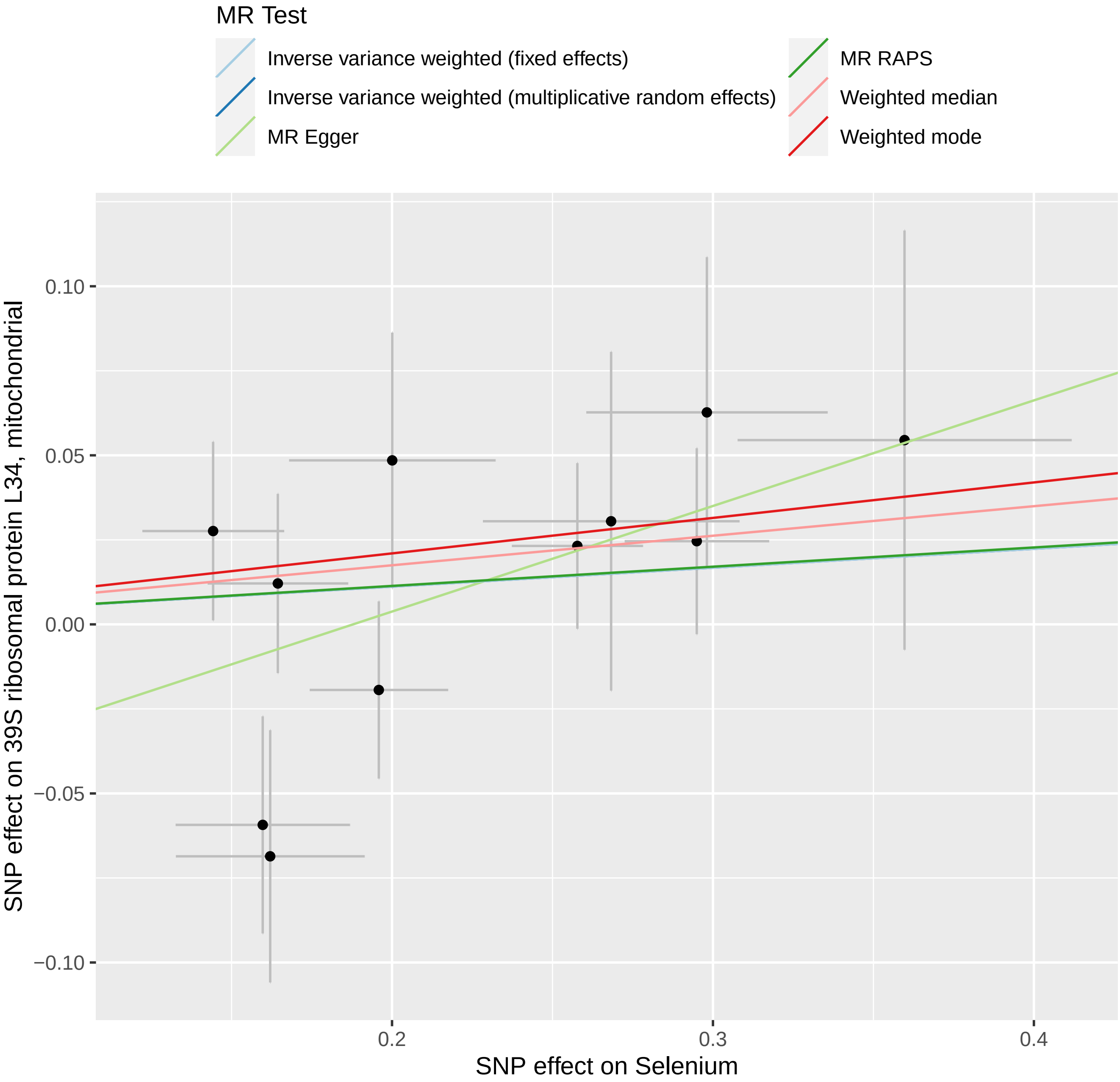

Batch 3−1944 : Selenium on 39S ribosomal protein L52, mitochondrial

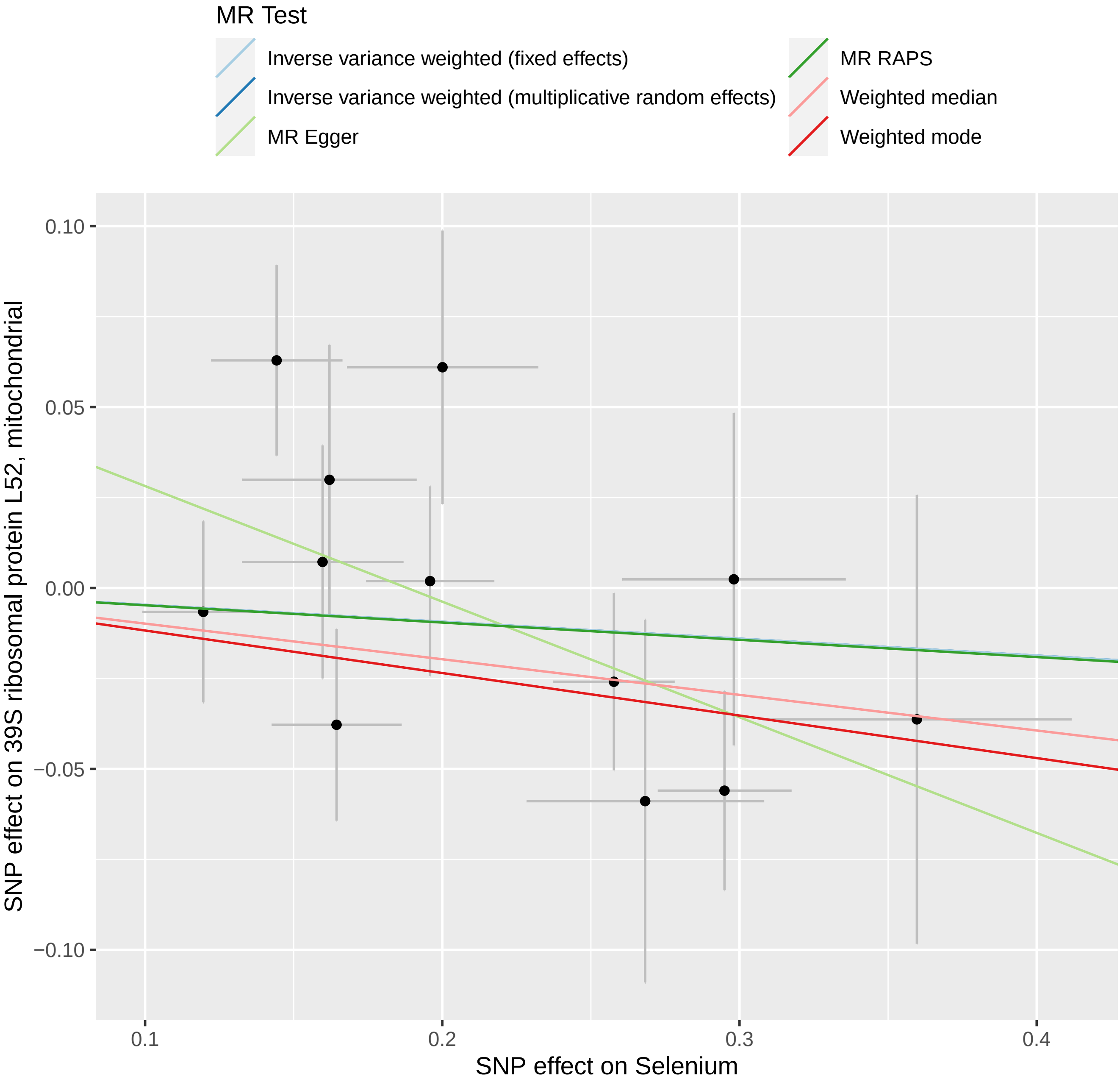

Batch 3−1945 : Selenium on Ribosome−recycling factor, mitochondrial

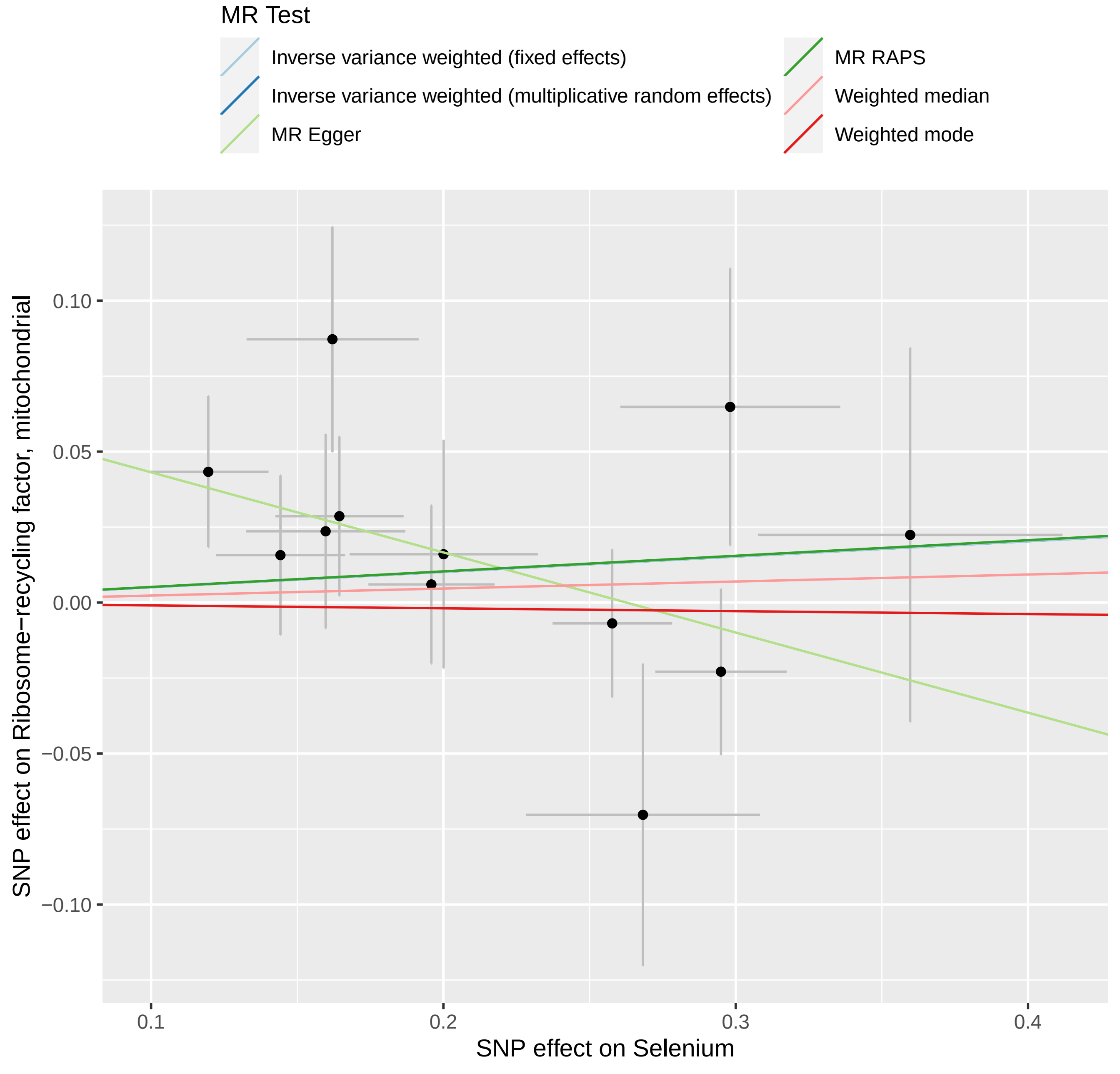

Batch 3−1946 : Selenium on Protein MRVI1

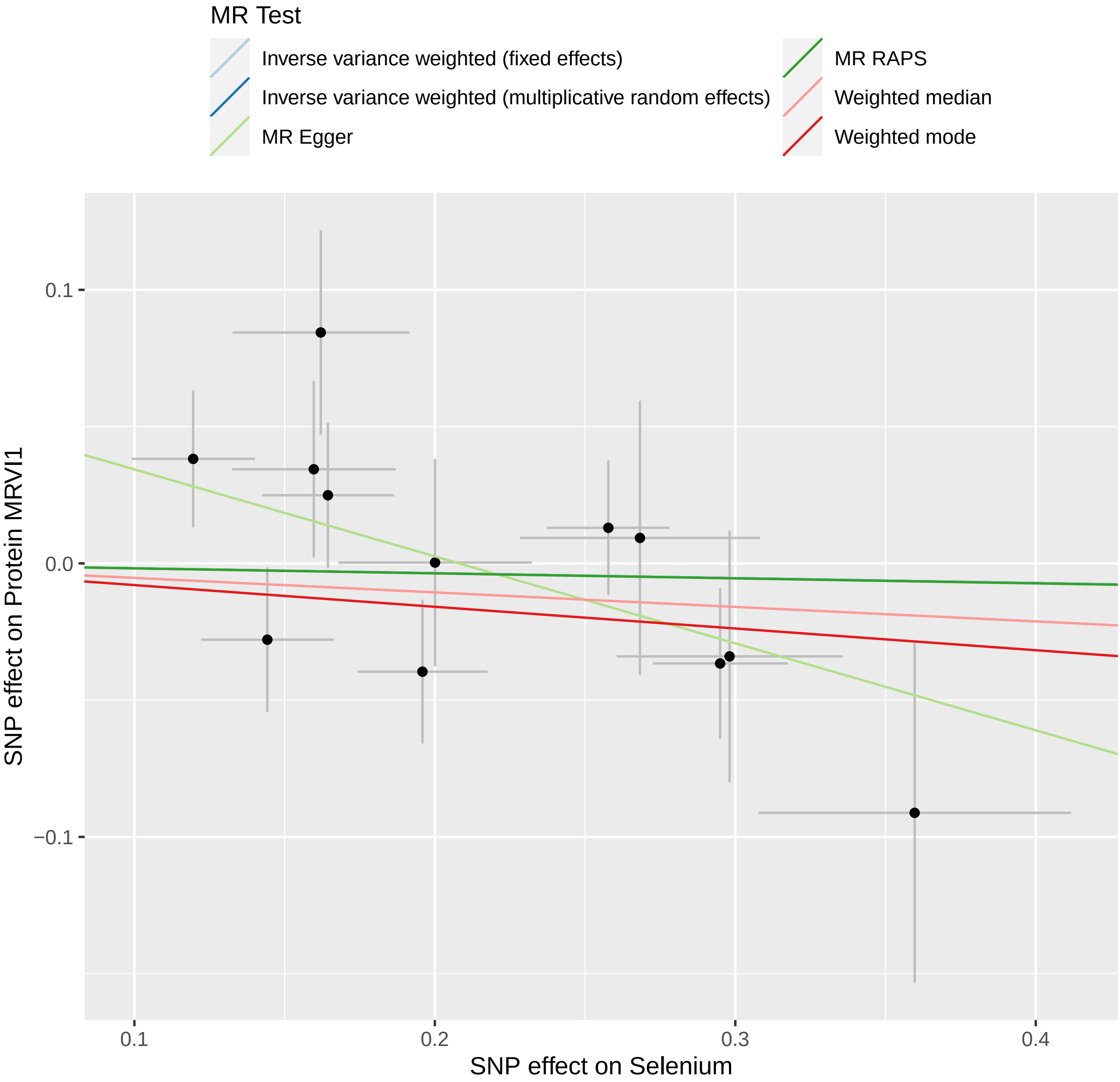

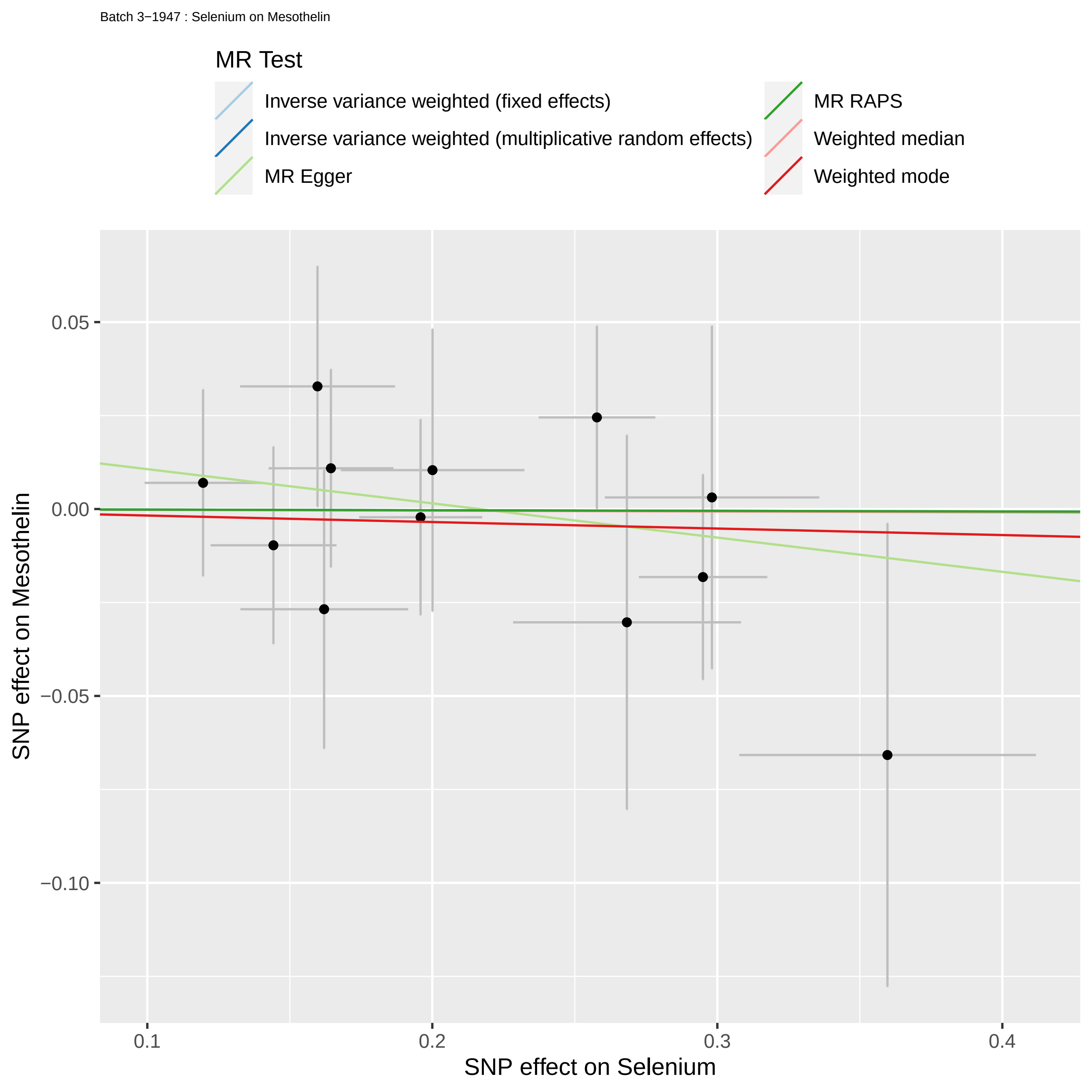
Batch 3−1947 : Selenium on Mesothelin
MR Test
Inverse variance weighted (fixed effects)
Inverse variance weighted (multiplicative random effects)
MR Egger
MR RAPS
Weighted median
Weighted mode
0.05
0.00
−0.05
−0.10
0.1
0.2
0.3
0.4
SNP effect on Mesothelin
SNP effect on Selenium

Batch 3−1948 : Selenium on Mesothelin

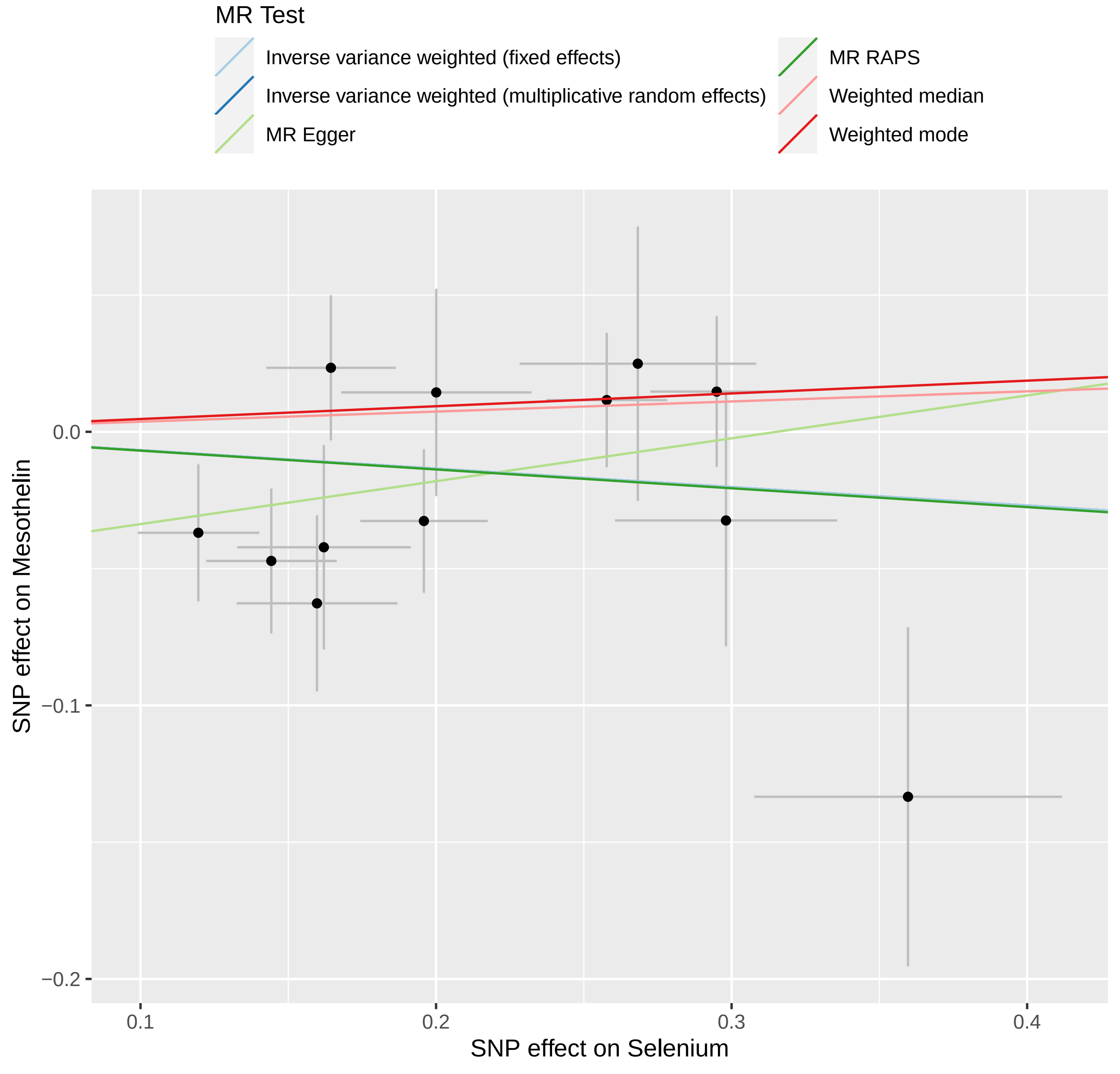

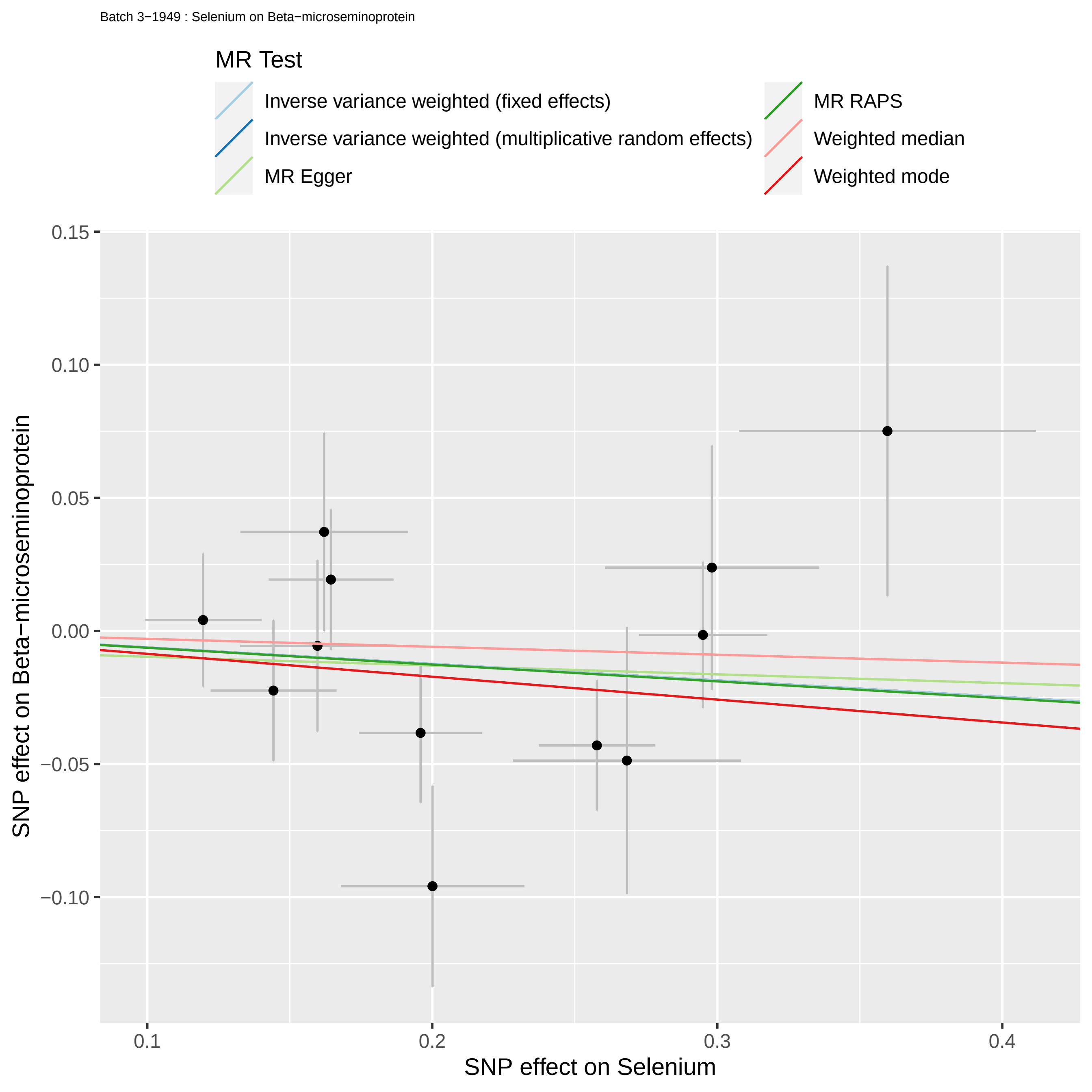

Batch 3−1949 : Selenium on Beta−microseminoprotein
MR Test
Inverse variance weighted (fixed effects)
Inverse variance weighted (multiplicative random effects)
MR Egger
MR RAPS
Weighted median
Weighted mode
0.15
0.10
0.05
0.00
−0.05
−0.10
0.1
0.2
0.3
0.4
SNP effect on Beta−microseminoprotein
SNP effect on Selenium

Batch 3−1950 : Selenium on Prostate−associated microseminoprotein

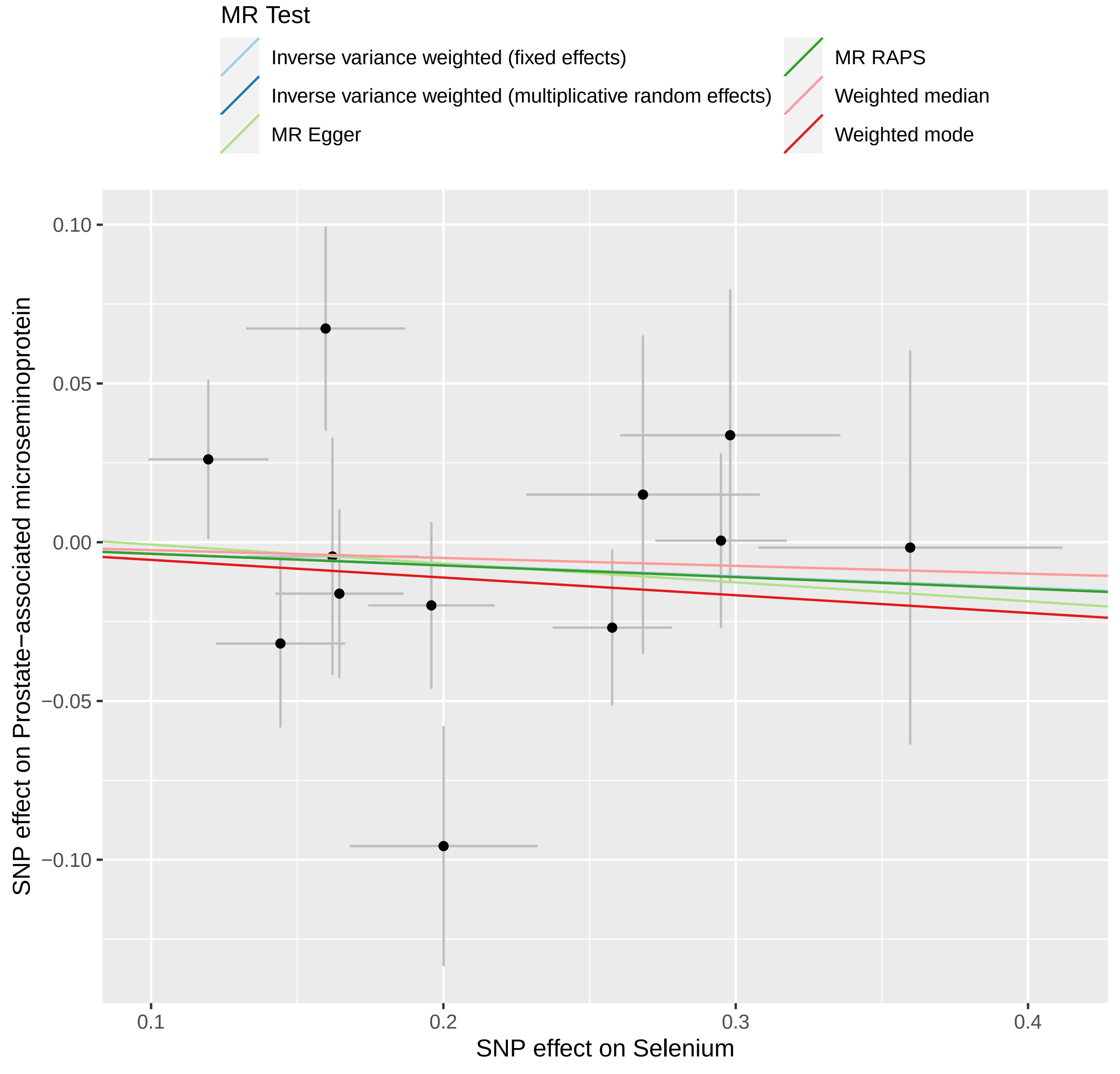

Batch 3−1951 : Selenium on Macrophage scavenger receptor types I and II

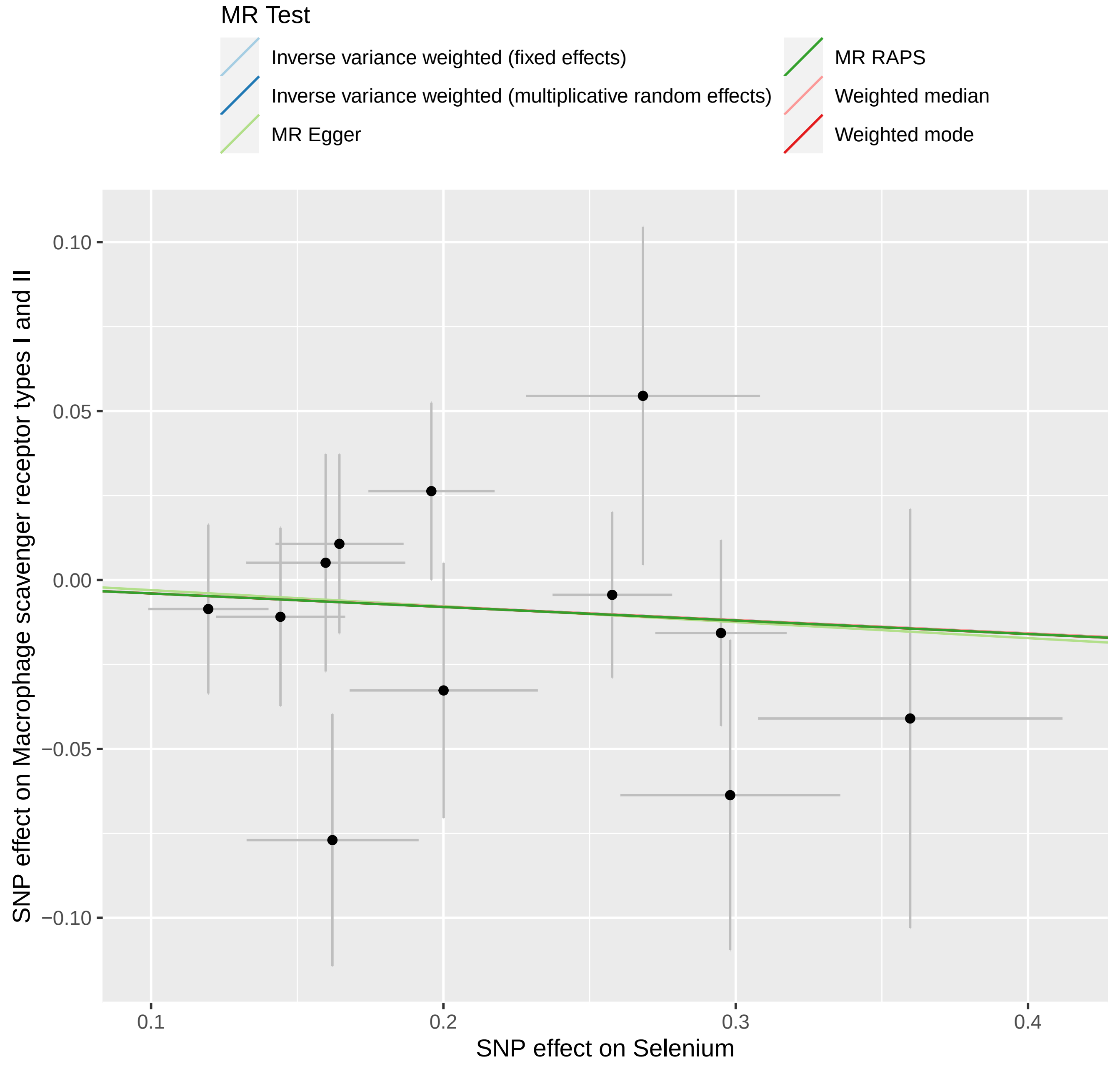

Batch 3−1952 : Selenium on Macrophage scavenger receptor types I and II

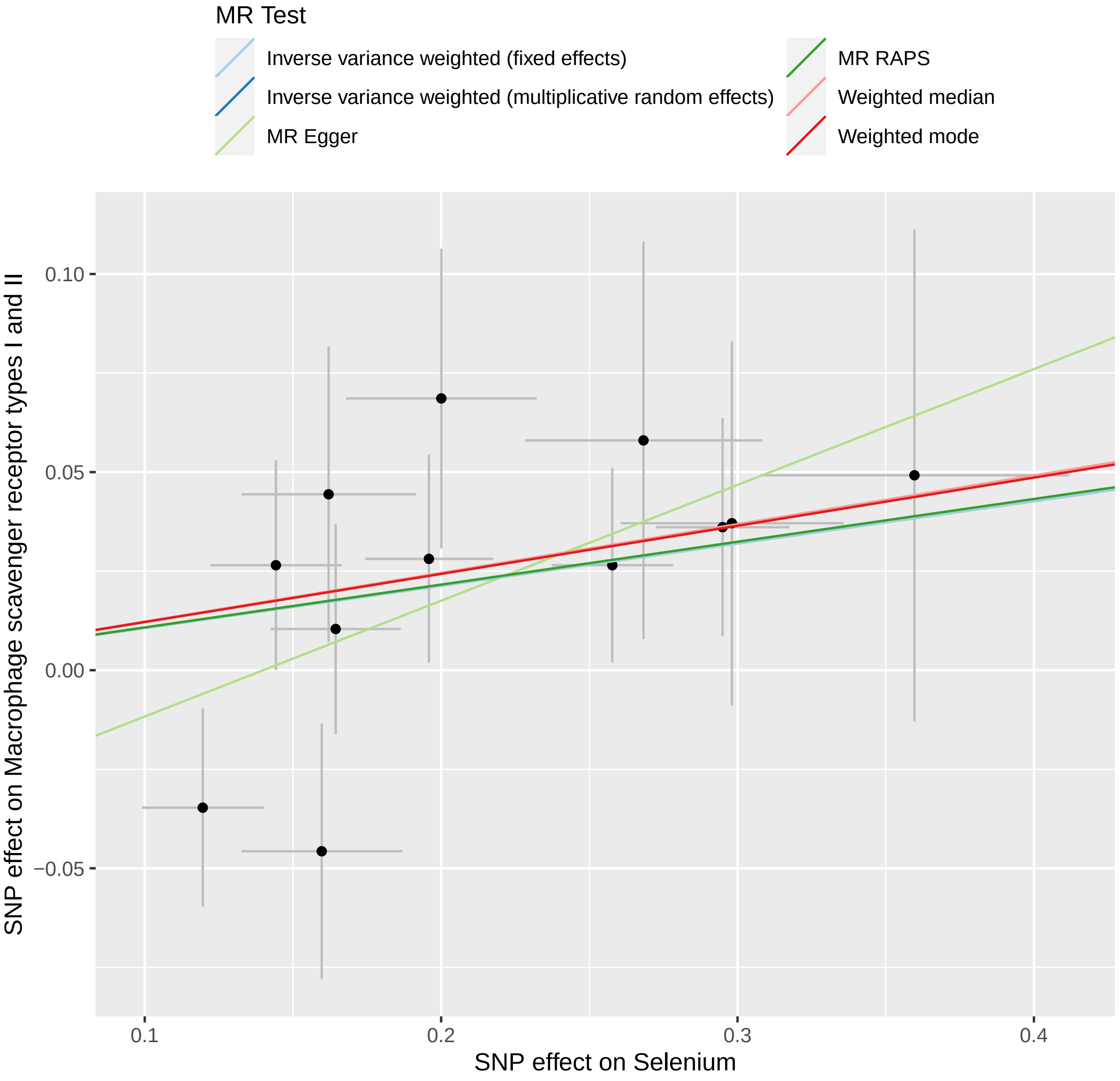

Batch 3−1953 : Selenium on Mitochondrial peptide methionine sulfoxide reductase

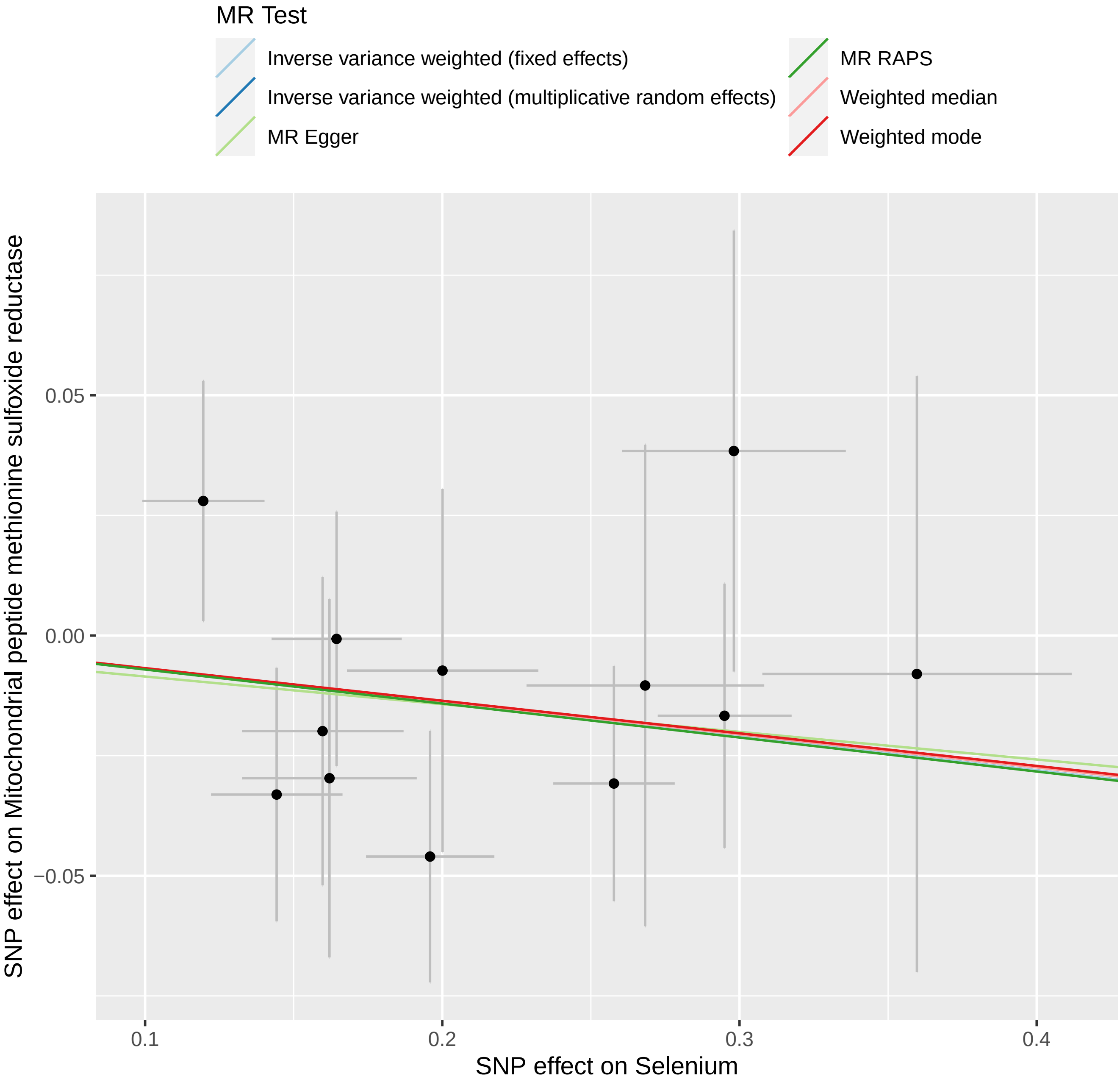

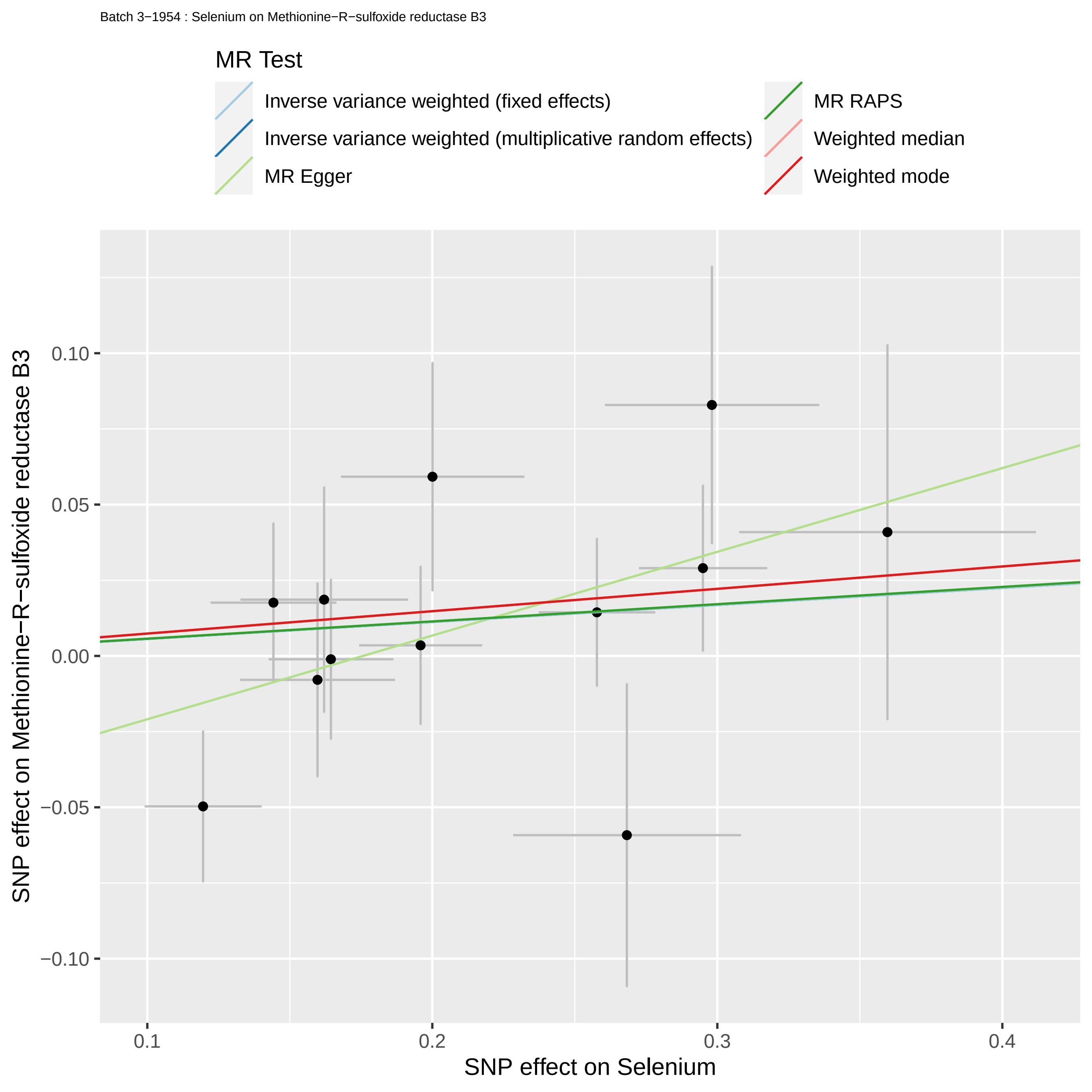

Batch 3−1954 : Selenium on Methionine−R−sulfoxide reductase B3
MR Test
Inverse variance weighted (fixed effects)
Inverse variance weighted (multiplicative random effects)
MR Egger
MR RAPS
Weighted median
Weighted mode
0.10
0.05
0.00
−0.05
−0.10
0.1
0.2
0.3
0.4
SNP effect on Selenium
SNP effect on Methionine−R−sulfoxide reductase B3

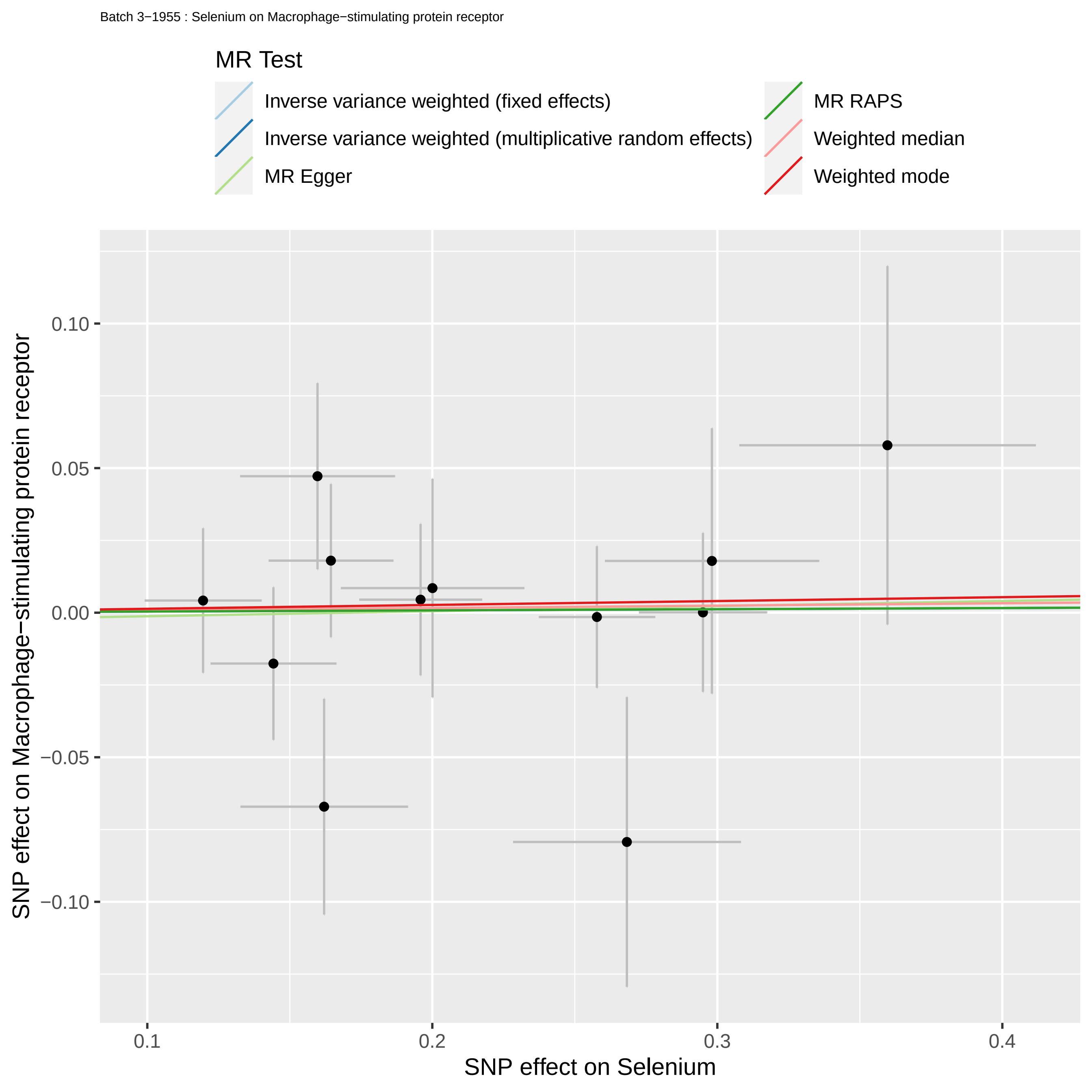

Batch 3−1955 : Selenium on Macrophage−stimulating protein receptor
MR Test
Inverse variance weighted (fixed effects)
Inverse variance weighted (multiplicative random effects)
MR Egger
MR RAPS
Weighted median
Weighted mode
SNP effect on Macrophage−stimulating protein receptor
0.10
0.05
0.00
−0.05
−0.10
0.1
0.2
0.3
0.4
SNP effect on Selenium

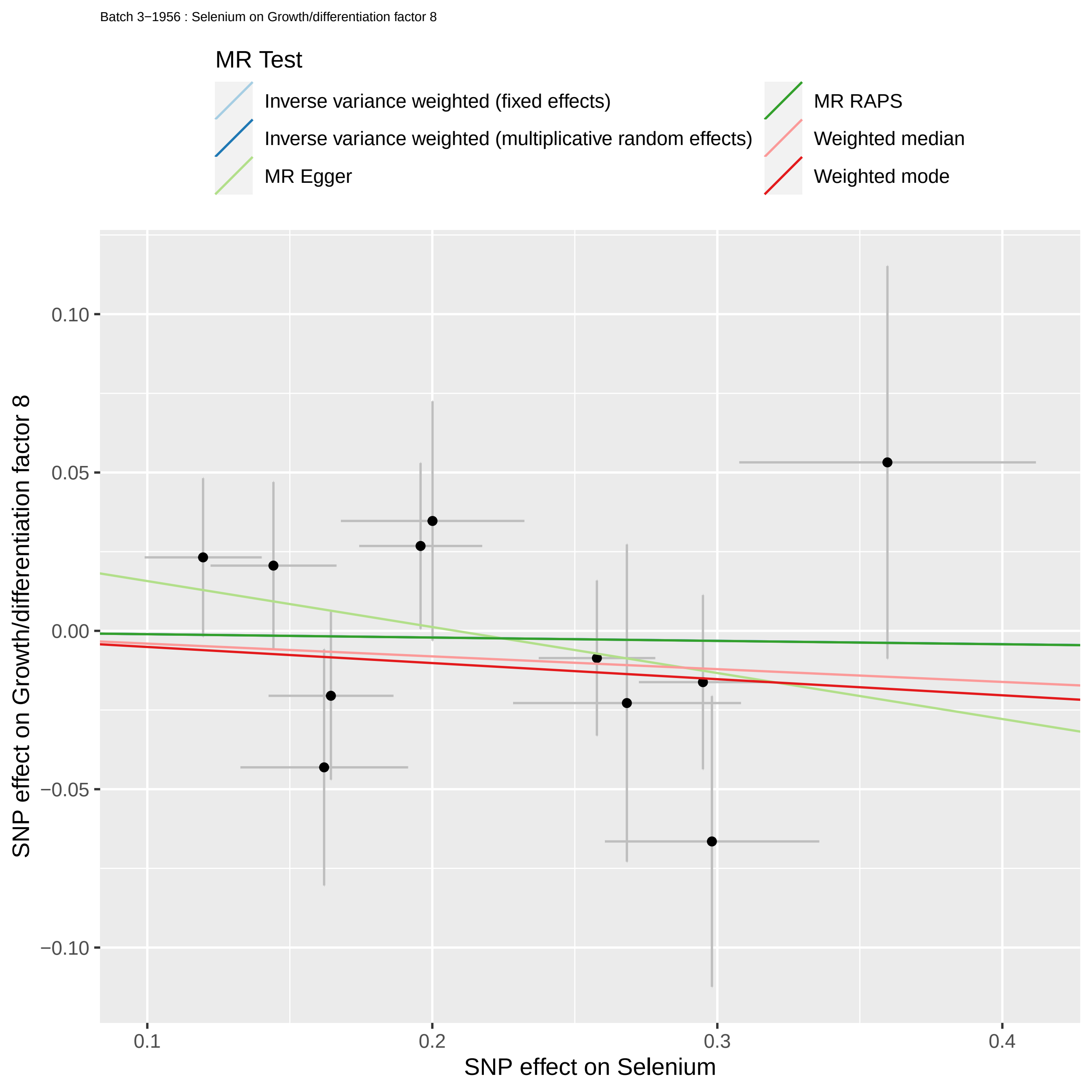

Batch 3−1956 : Selenium on Growth/differentiation factor 8
MR Test
Inverse variance weighted (fixed effects)
Inverse variance weighted (multiplicative random effects)
MR Egger
MR RAPS
Weighted median
Weighted mode
0.10
0.05
0.00
−0.05
−0.10
0.1
0.2
0.3
0.4
SNP effect on Growth/differentiation factor 8
SNP effect on Selenium

Batch 3−1957 : Selenium on Growth/differentiation factor 8

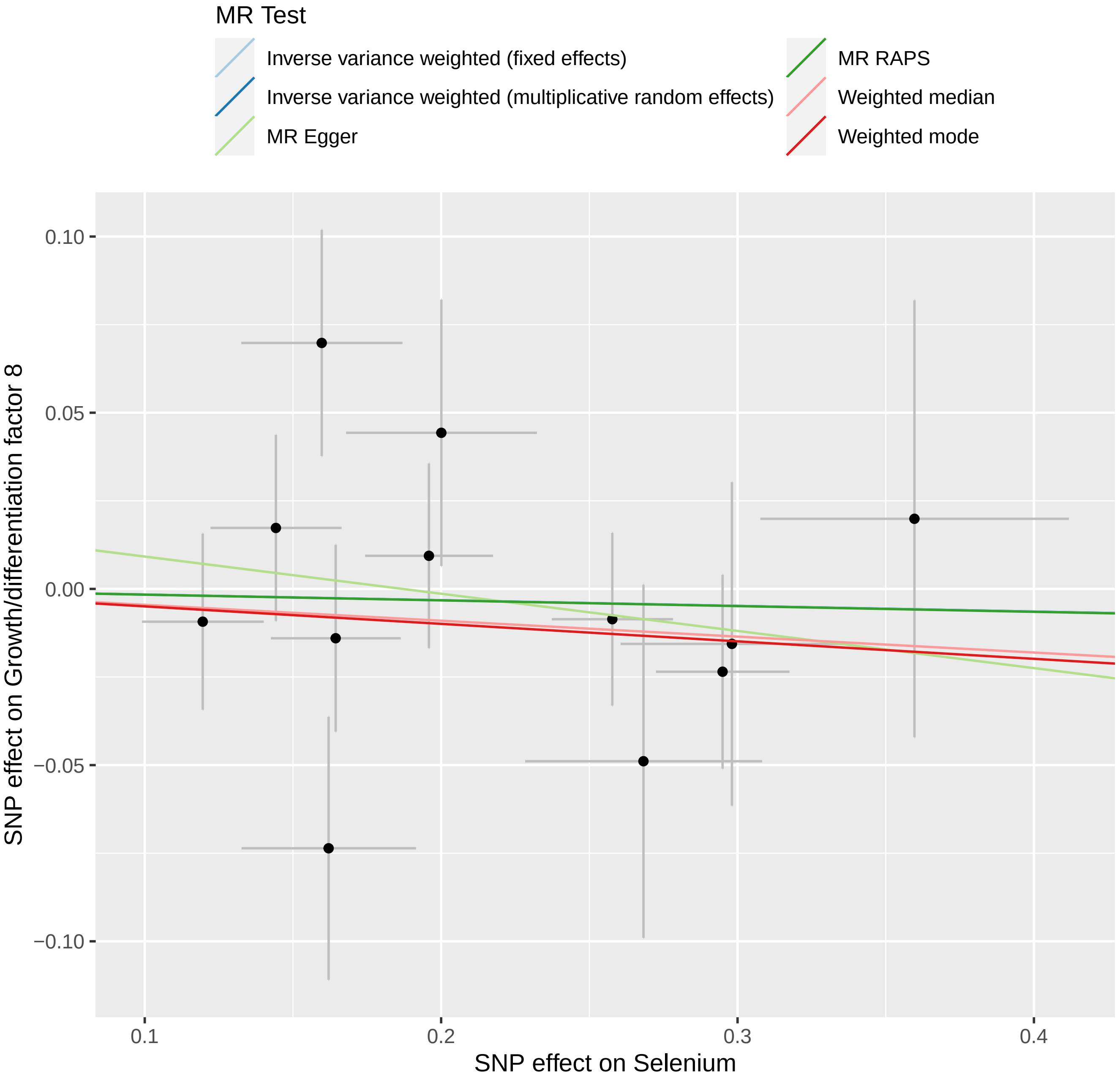

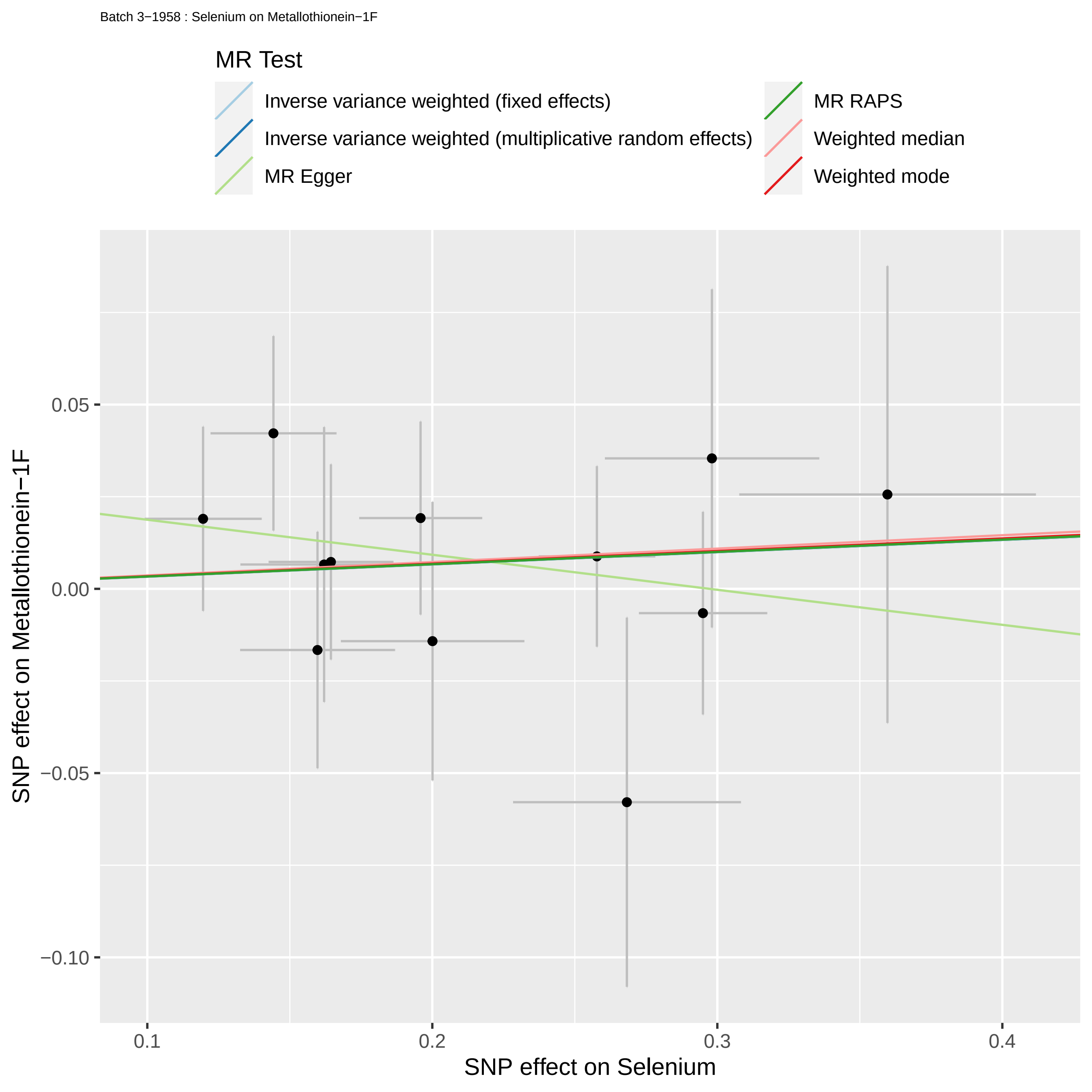

Batch 3−1958 : Selenium on Metallothionein−1F
MR Test
Inverse variance weighted (fixed effects)
Inverse variance weighted (multiplicative random effects)
MR Egger
MR RAPS
Weighted median
Weighted mode
0.05
0.00
−0.05
−0.10
0.1
0.2
0.3
0.4
SNP effect on Metallothionein−1F
SNP effect on Selenium

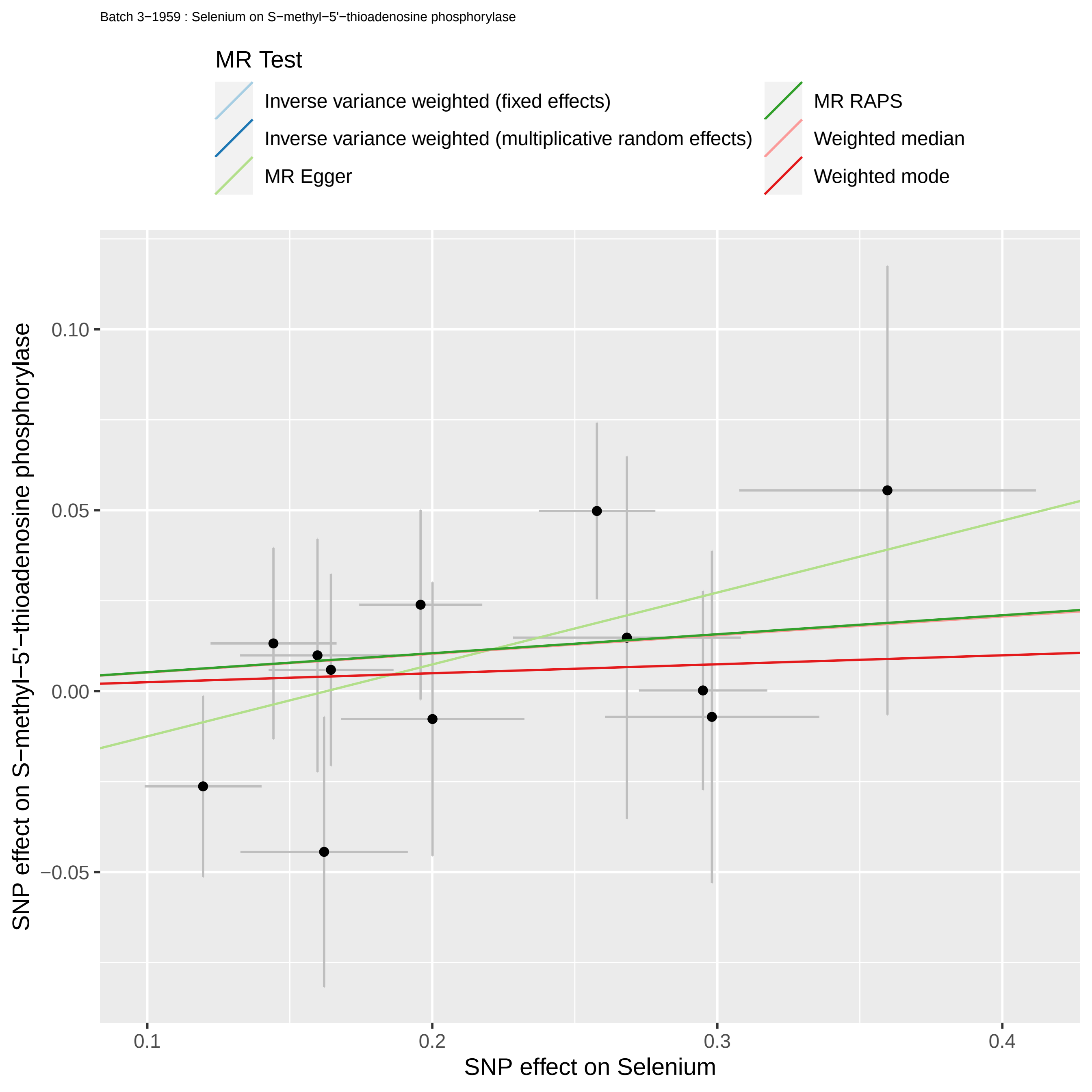
Batch 3−1959 : Selenium on S−methyl−5'−thioadenosine phosphorylase
MR Test
Inverse variance weighted (fixed effects)
Inverse variance weighted (multiplicative random effects)
MR Egger
MR RAPS
Weighted median
Weighted mode
0.10
0.05
0.00
−0.05
0.1
0.2
0.3
0.4
SNP effect on S−methyl−5'−thioadenosine phosphorylase
SNP effect on Selenium

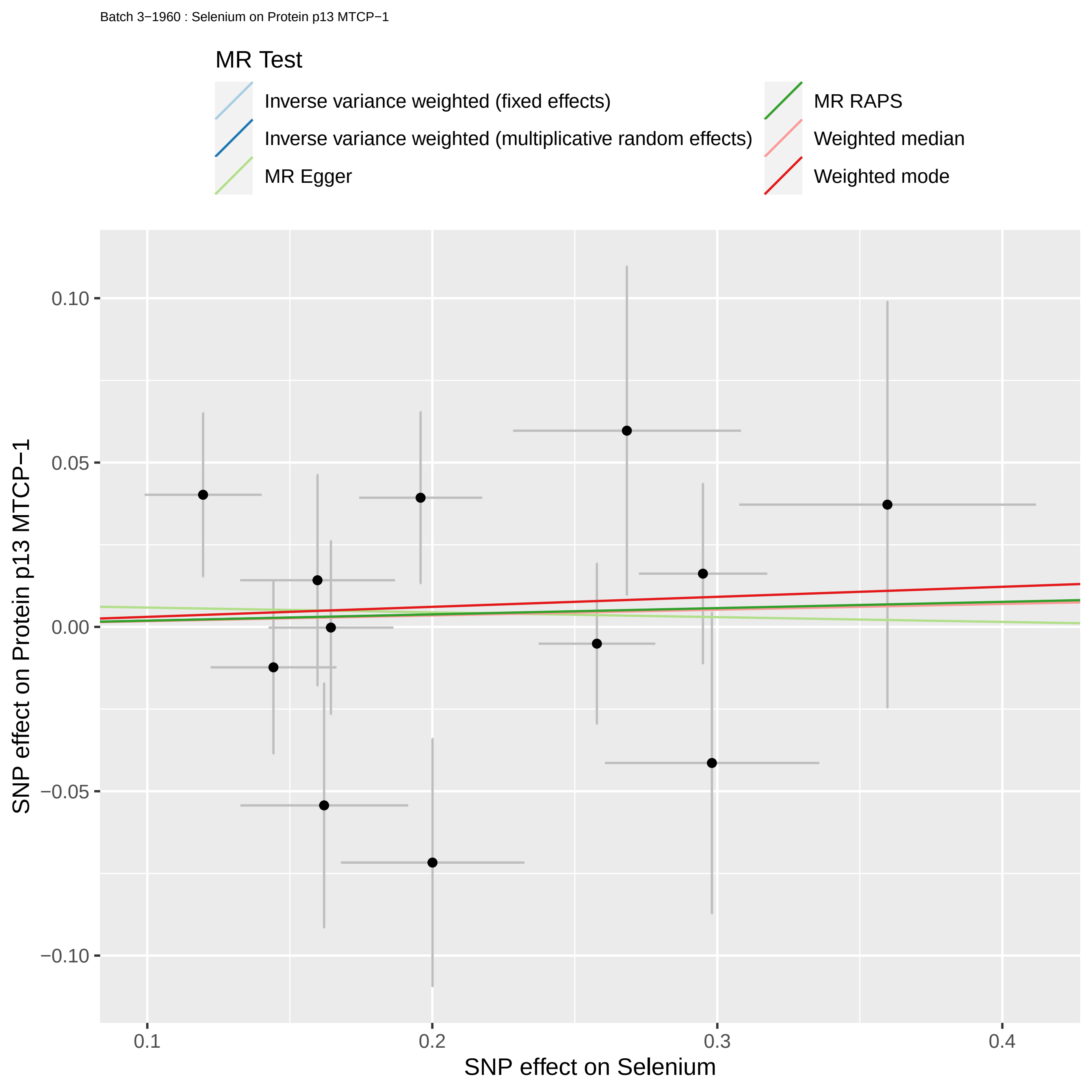
Batch 3−1960 : Selenium on Protein p13 MTCP−1
MR Test
Inverse variance weighted (fixed effects)
Inverse variance weighted (multiplicative random effects)
MR Egger
MR RAPS
Weighted median
Weighted mode
0.10
0.05
0.00
−0.05
−0.10
0.1
0.2
0.3
0.4
SNP effect on Protein p13 MTCP−1
SNP effect on Selenium

Batch 3−1961 : Selenium on Mitochondrial fission regulator 1

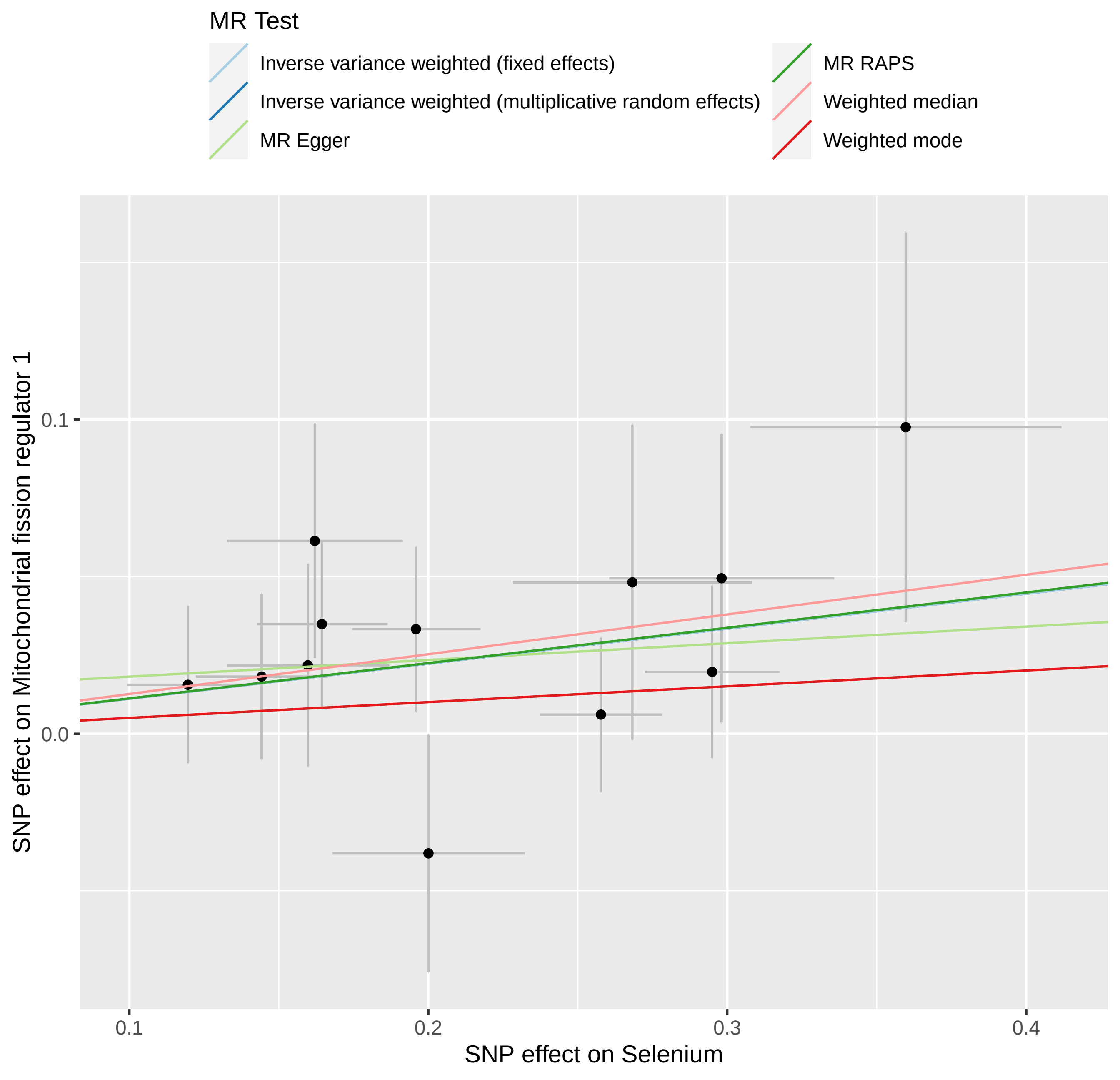

Batch 3−1962 : Selenium on 5−formyltetrahydrofolate cyclo−ligase

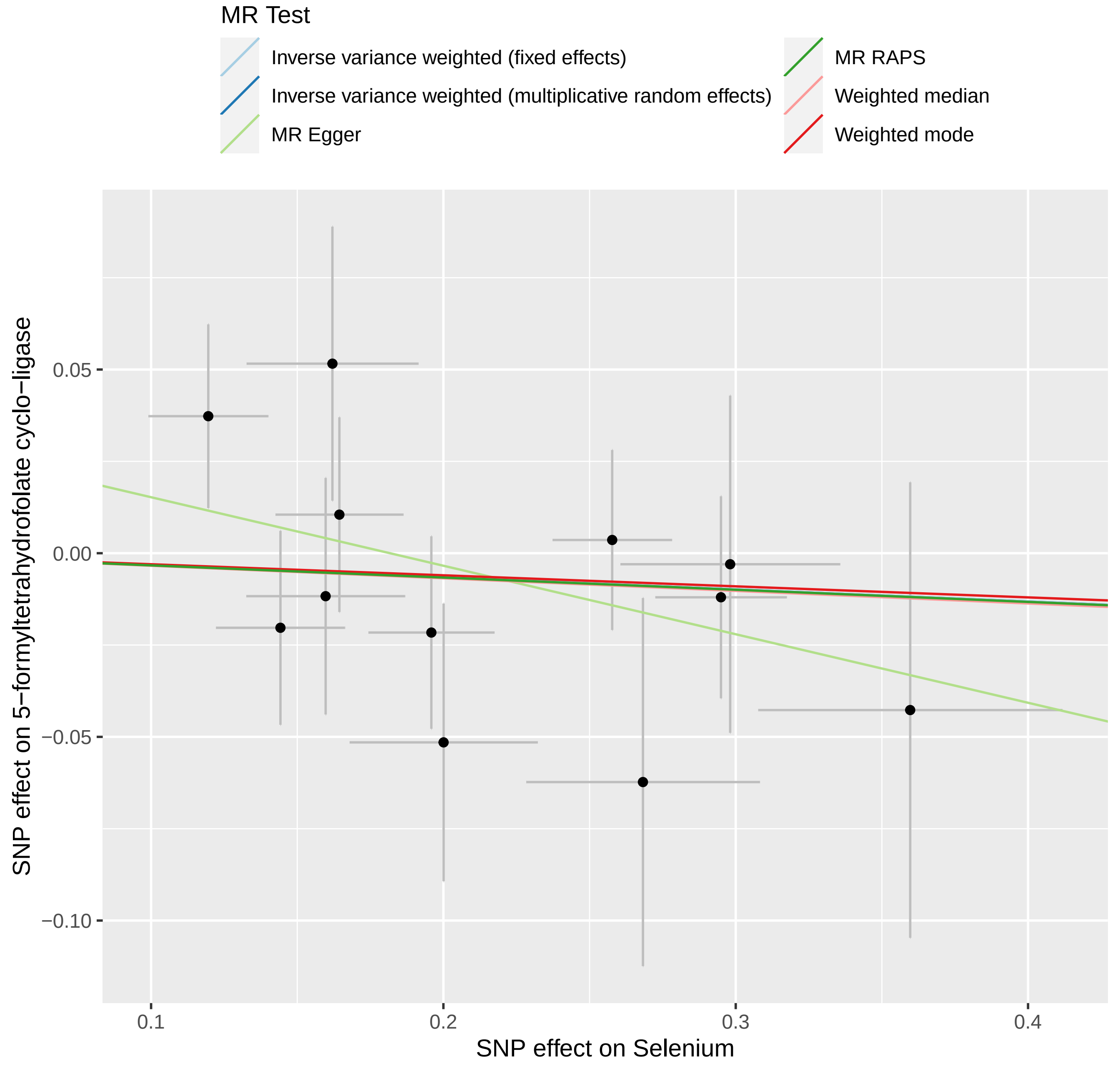

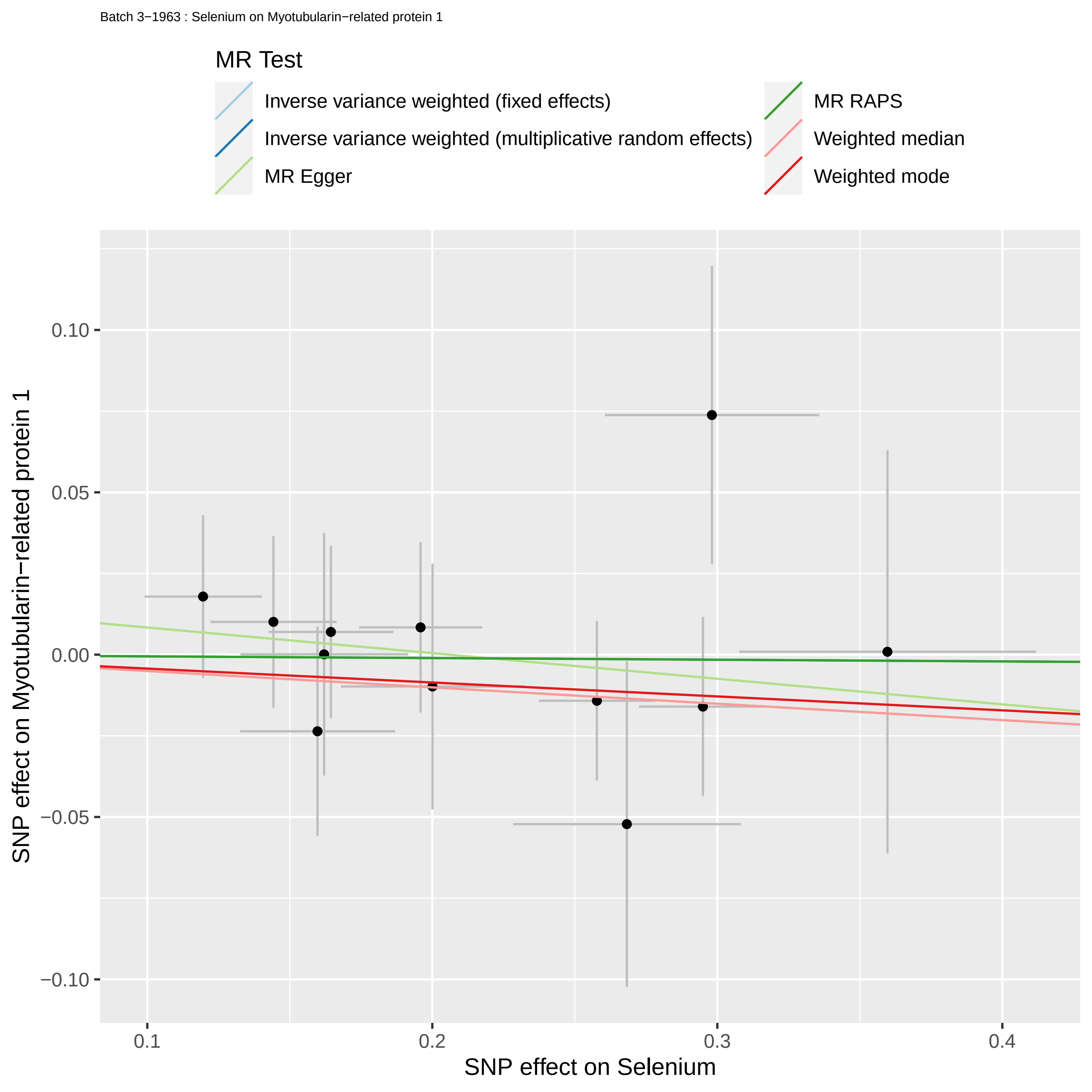
Batch 3−1963 : Selenium on Myotubularin−related protein 1
MR Test
Inverse variance weighted (fixed effects)
Inverse variance weighted (multiplicative random effects)
MR Egger
MR RAPS
Weighted median
Weighted mode
0.10
0.05
0.00
−0.05
−0.10
0.1
0.2
0.3
0.4
SNP effect on Myotubularin−related protein 1
SNP effect on Selenium

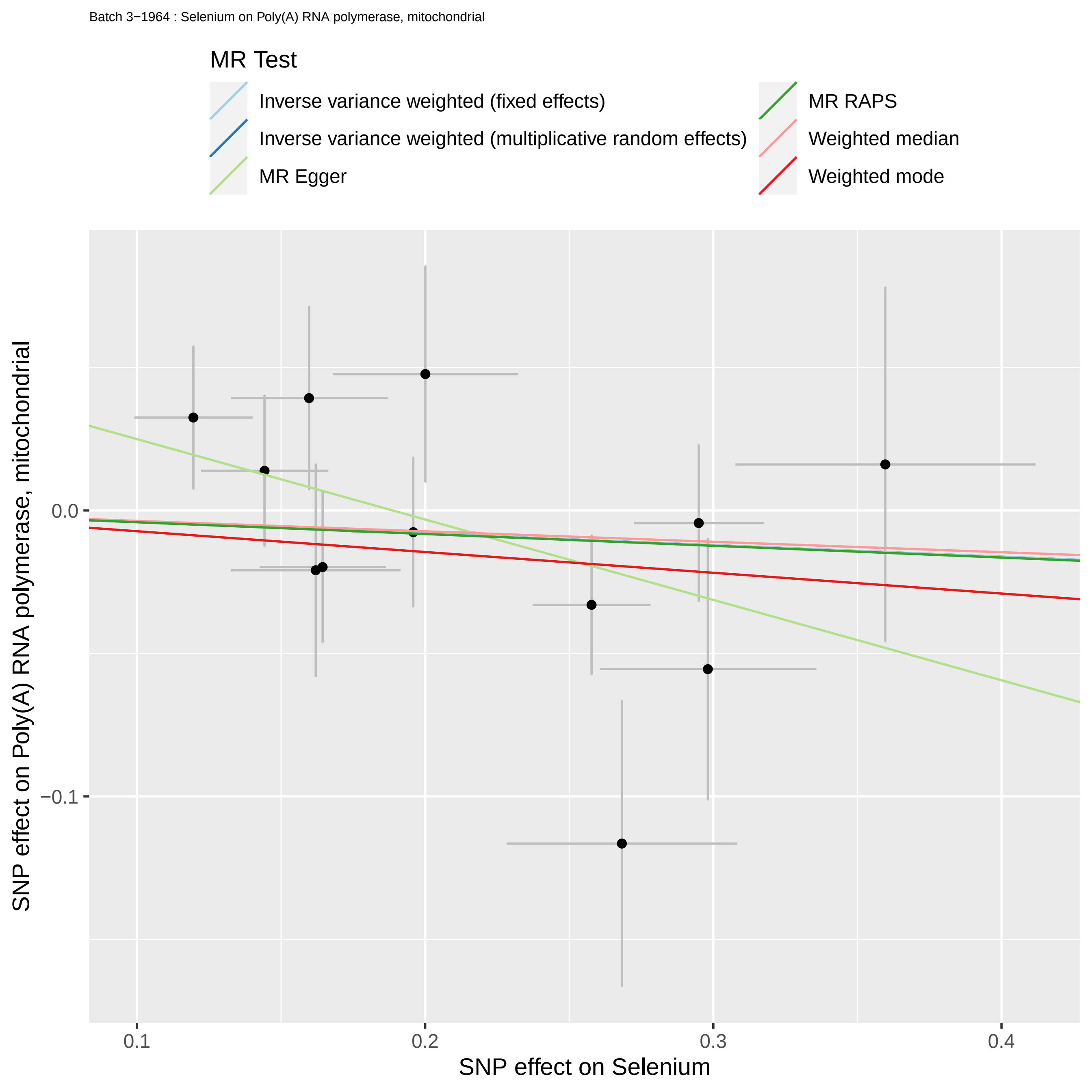

Batch 3−1964 : Selenium on Poly(A) RNA polymerase, mitochondrial
MR Test
Inverse variance weighted (fixed effects)
Inverse variance weighted (multiplicative random effects)
MR Egger
MR RAPS
Weighted median
Weighted mode
SNP effect on Poly(A) RNA polymerase, mitochondrial
0.0
−0.1
0.1
0.2
0.3
0.4
SNP effect on Selenium

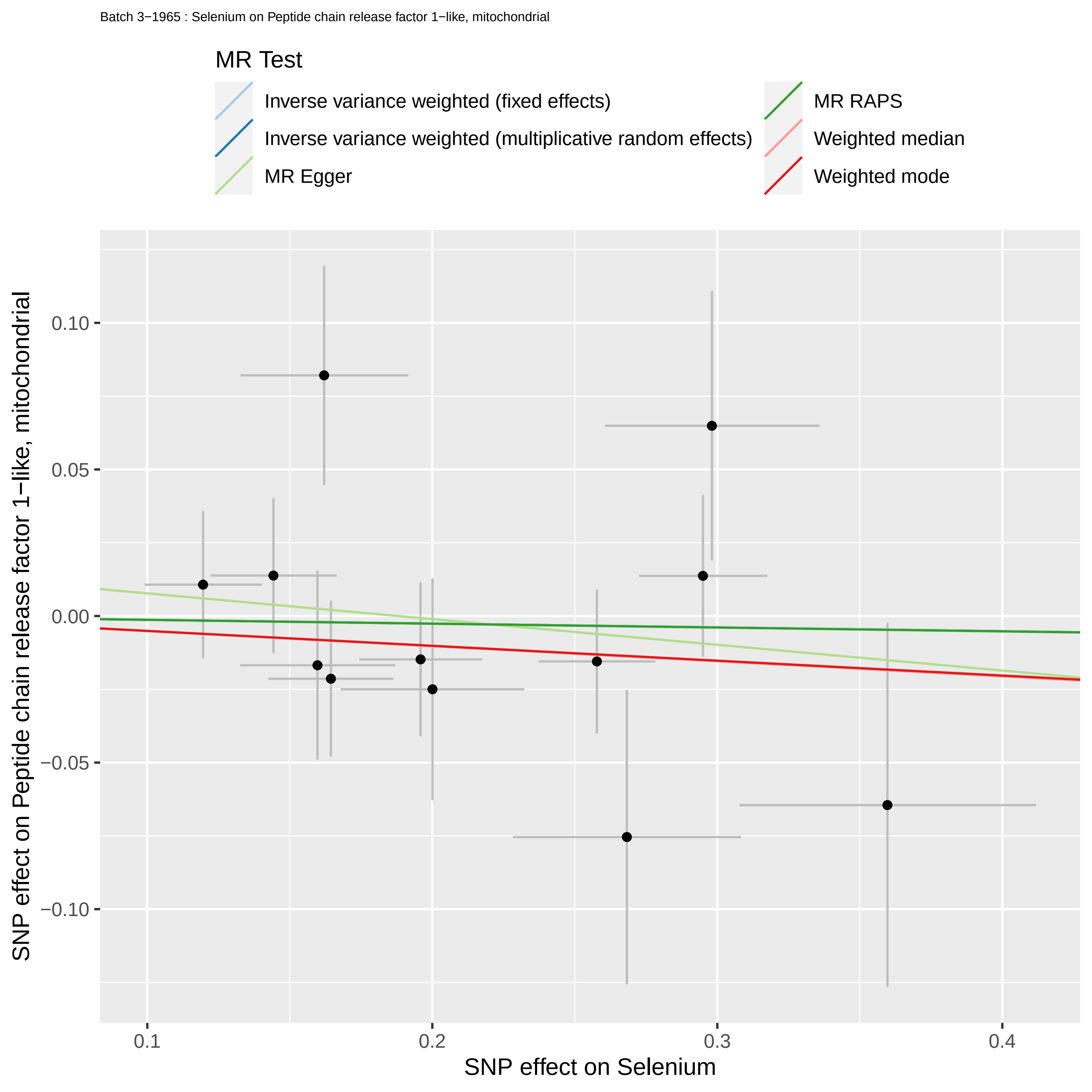

Batch 3−1965 : Selenium on Peptide chain release factor 1−like, mitochondrial
MR Test
Inverse variance weighted (fixed effects)
Inverse variance weighted (multiplicative random effects)
MR Egger
MR RAPS
Weighted median
Weighted mode
0.10
0.05
0.00
−0.05
−0.10
0.1
0.2
0.3
0.4
SNP effect on Selenium
SNP effect on Peptide chain release factor 1−like, mitochondrial

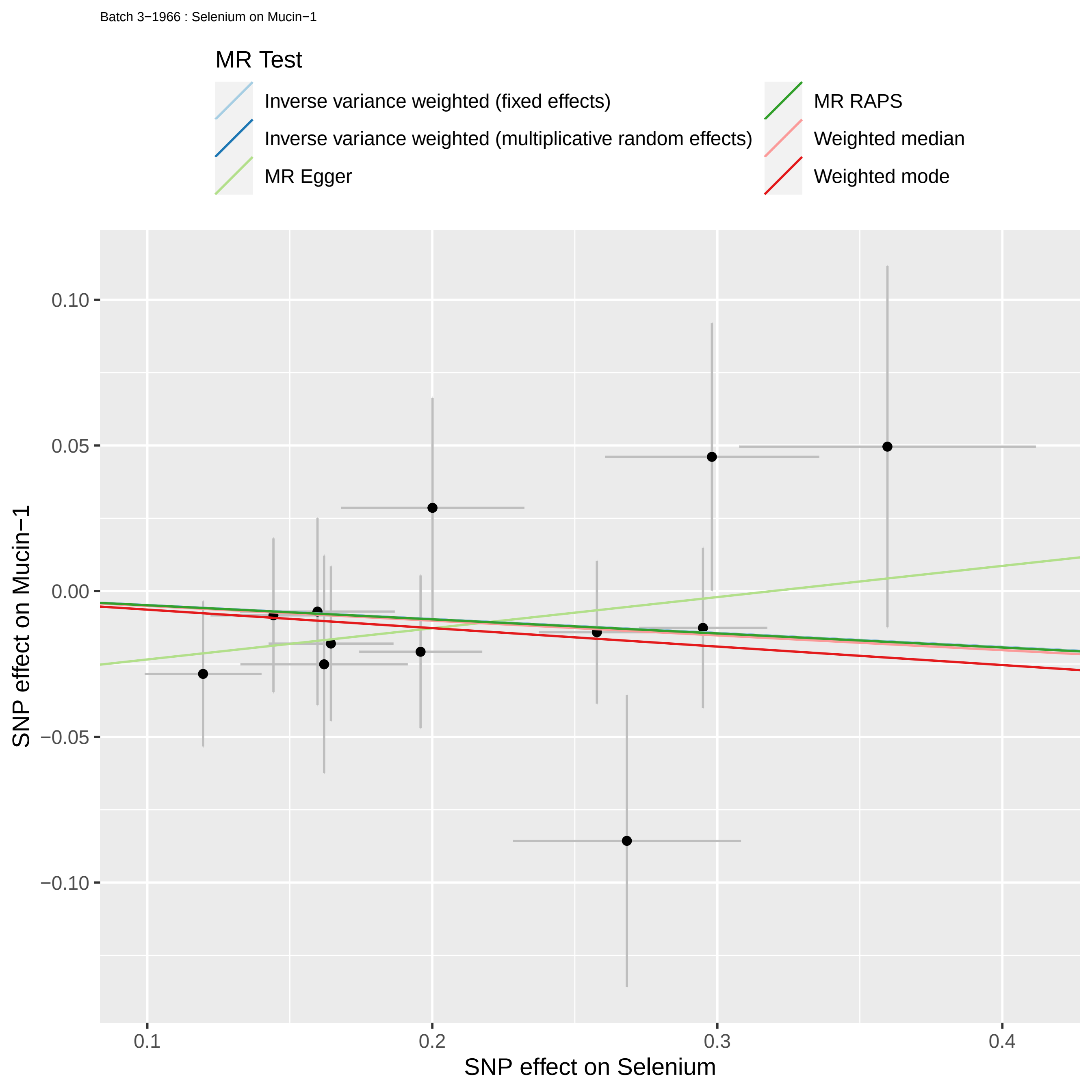

Batch 3−1966 : Selenium on Mucin−1
MR Test
Inverse variance weighted (fixed effects)
Inverse variance weighted (multiplicative random effects)
MR Egger
MR RAPS
Weighted median
Weighted mode
0.10
0.05
0.00
−0.05
−0.10
0.1
0.2
0.3
0.4
SNP effect on Mucin−1
SNP effect on Selenium

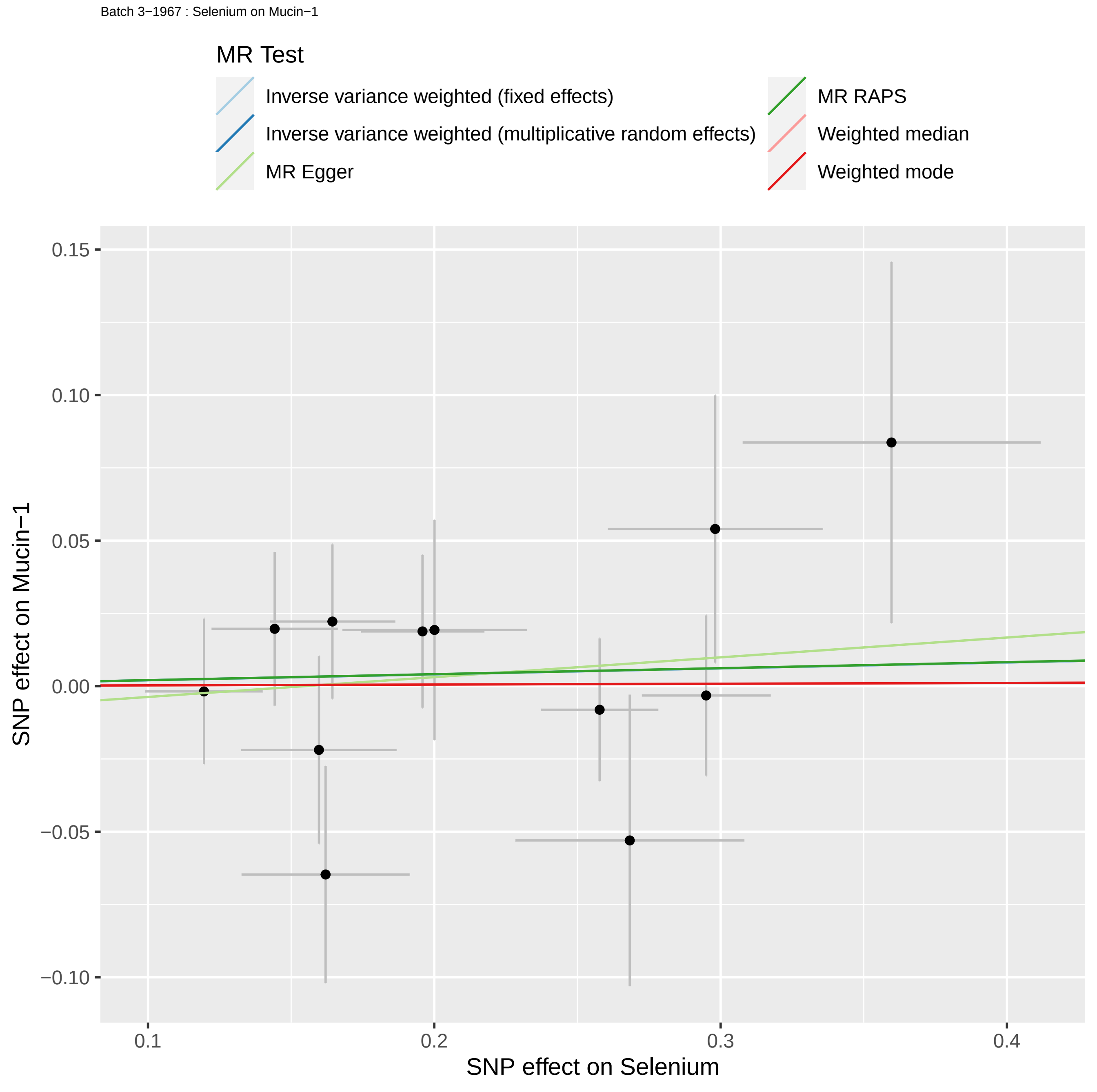

Batch 3−1967 : Selenium on Mucin−1
MR Test
Inverse variance weighted (fixed effects)
Inverse variance weighted (multiplicative random effects)
MR Egger
MR RAPS
Weighted median
Weighted mode
0.15
0.10
0.05
0.00
−0.05
−0.10
0.1
0.2
0.3
0.4
SNP effect on Mucin−1
SNP effect on Selenium

Batch 3−1968 : Selenium on Mucin−4

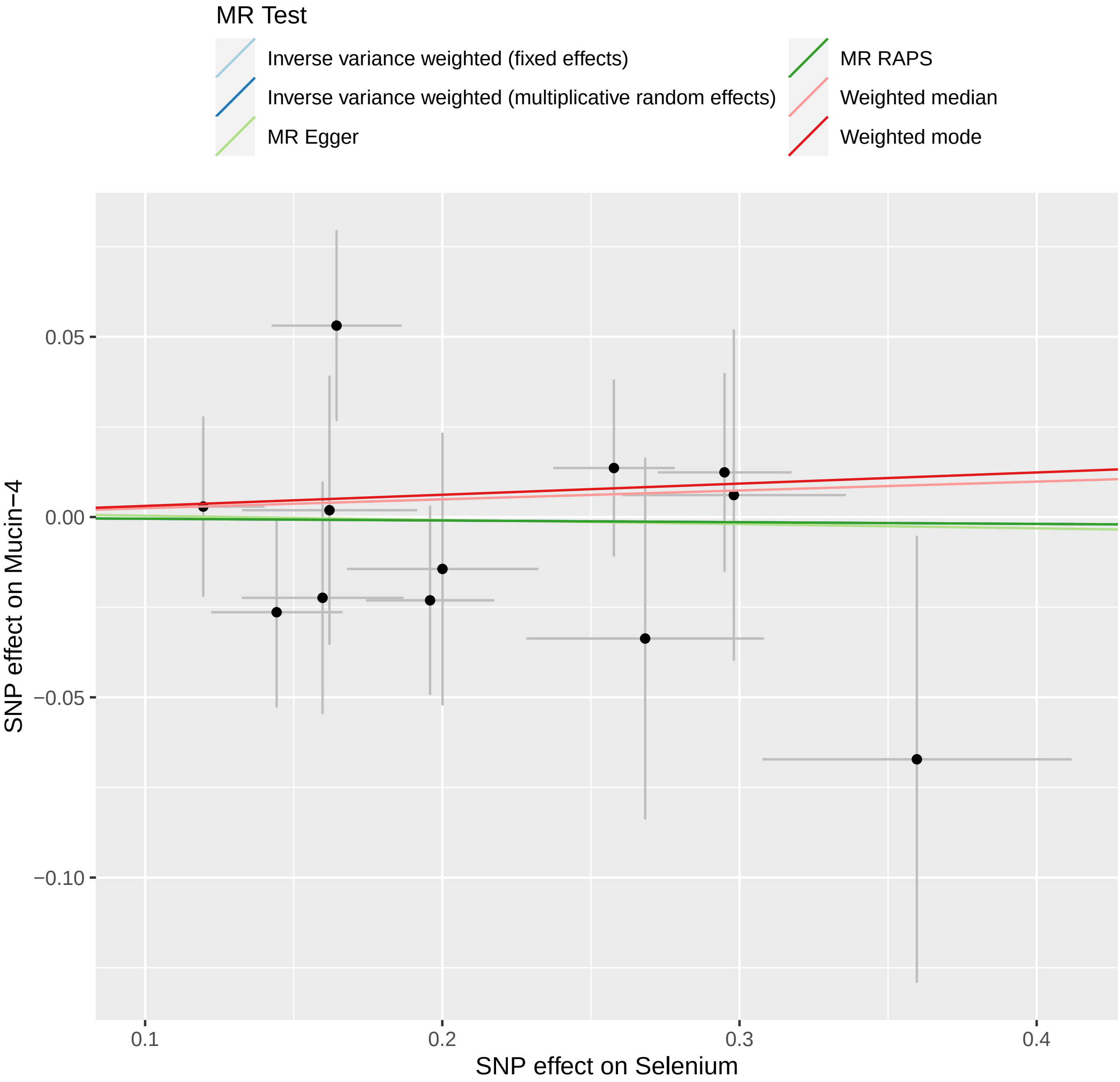

Batch 3−1969 : Selenium on Mitochondrial ubiquitin ligase activator of NFKB 1

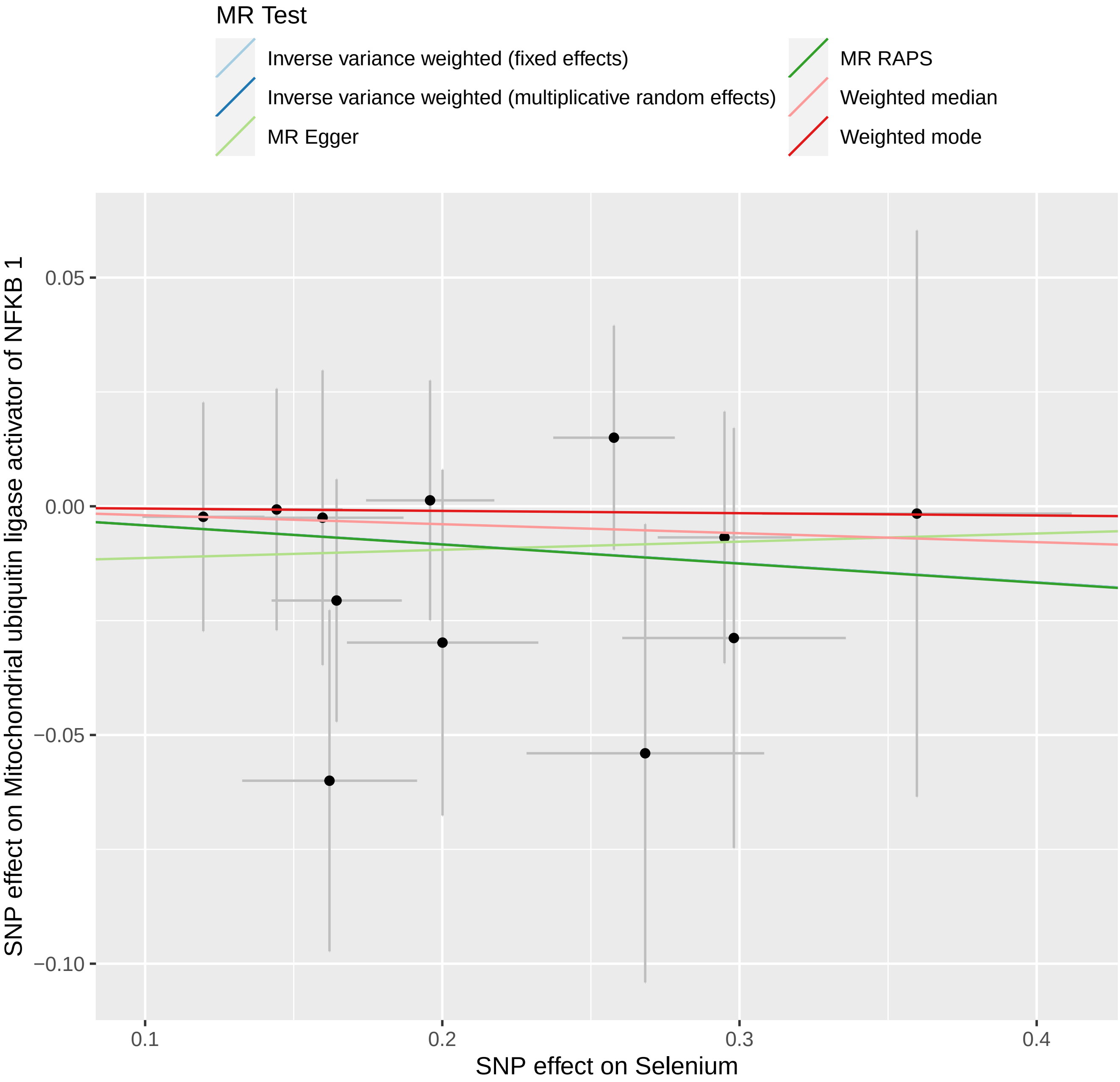

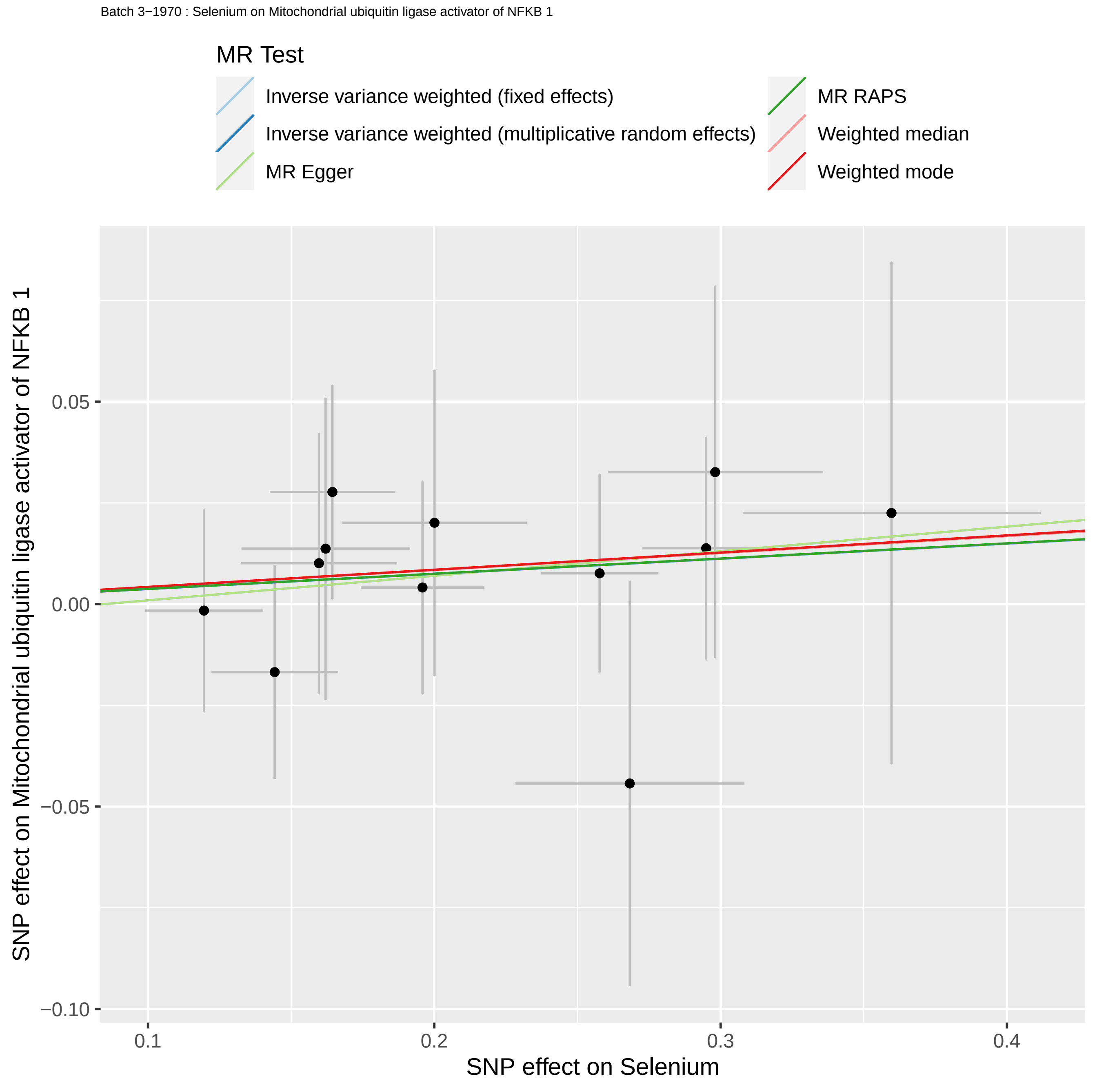

Batch 3−1970 : Selenium on Mitochondrial ubiquitin ligase activator of NFKB 1
MR Test
Inverse variance weighted (fixed effects)
Inverse variance weighted (multiplicative random effects)
MR Egger
MR RAPS
Weighted median
Weighted mode
SNP effect on Mitochondrial ubiquitin ligase activator of NFKB 1
0.05
0.00
−0.05
−0.10
0.1
0.2
0.3
0.4
SNP effect on Selenium

Batch 3−1971 : Selenium on Muscle, skeletal receptor tyrosine−protein kinase

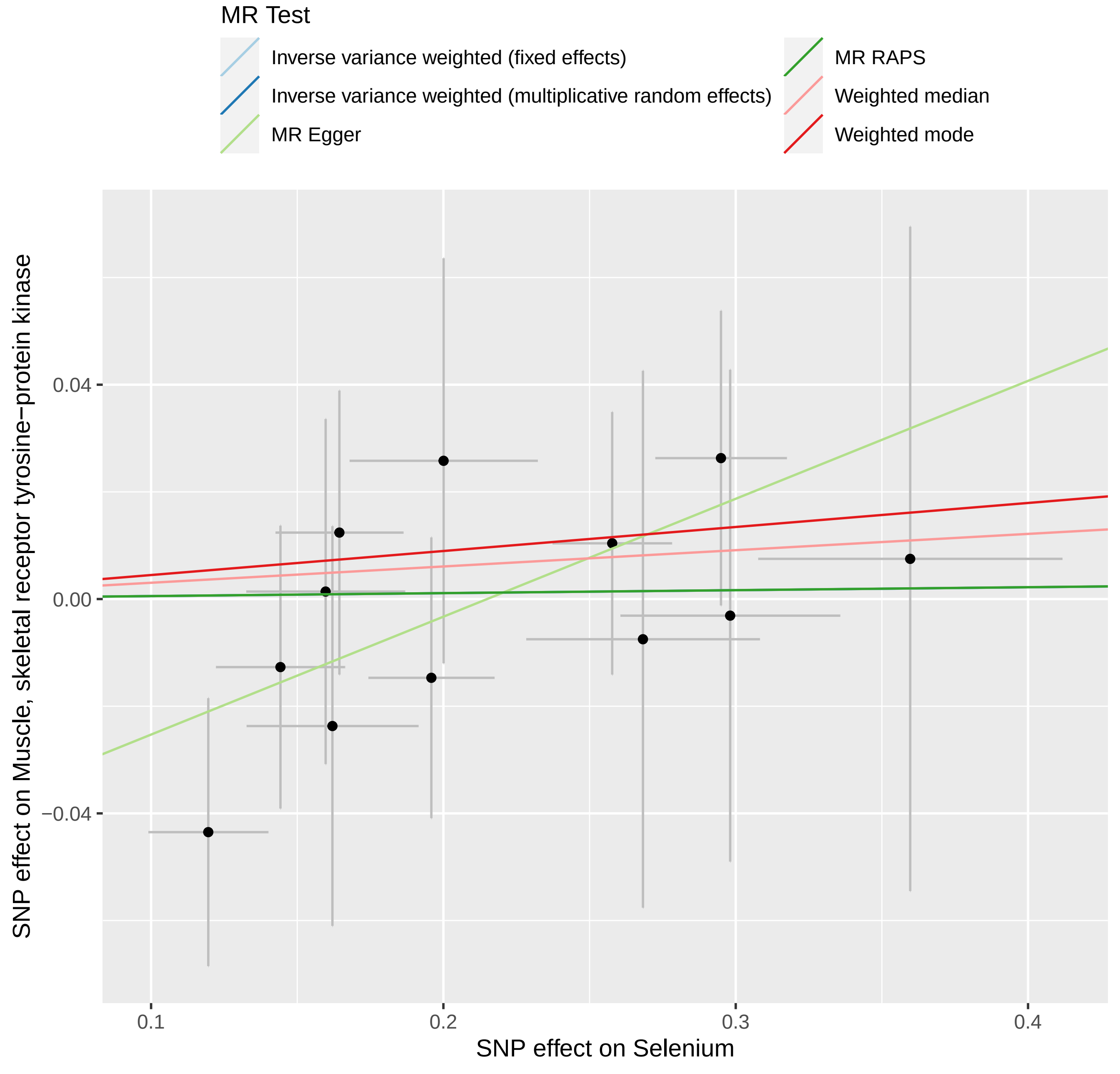

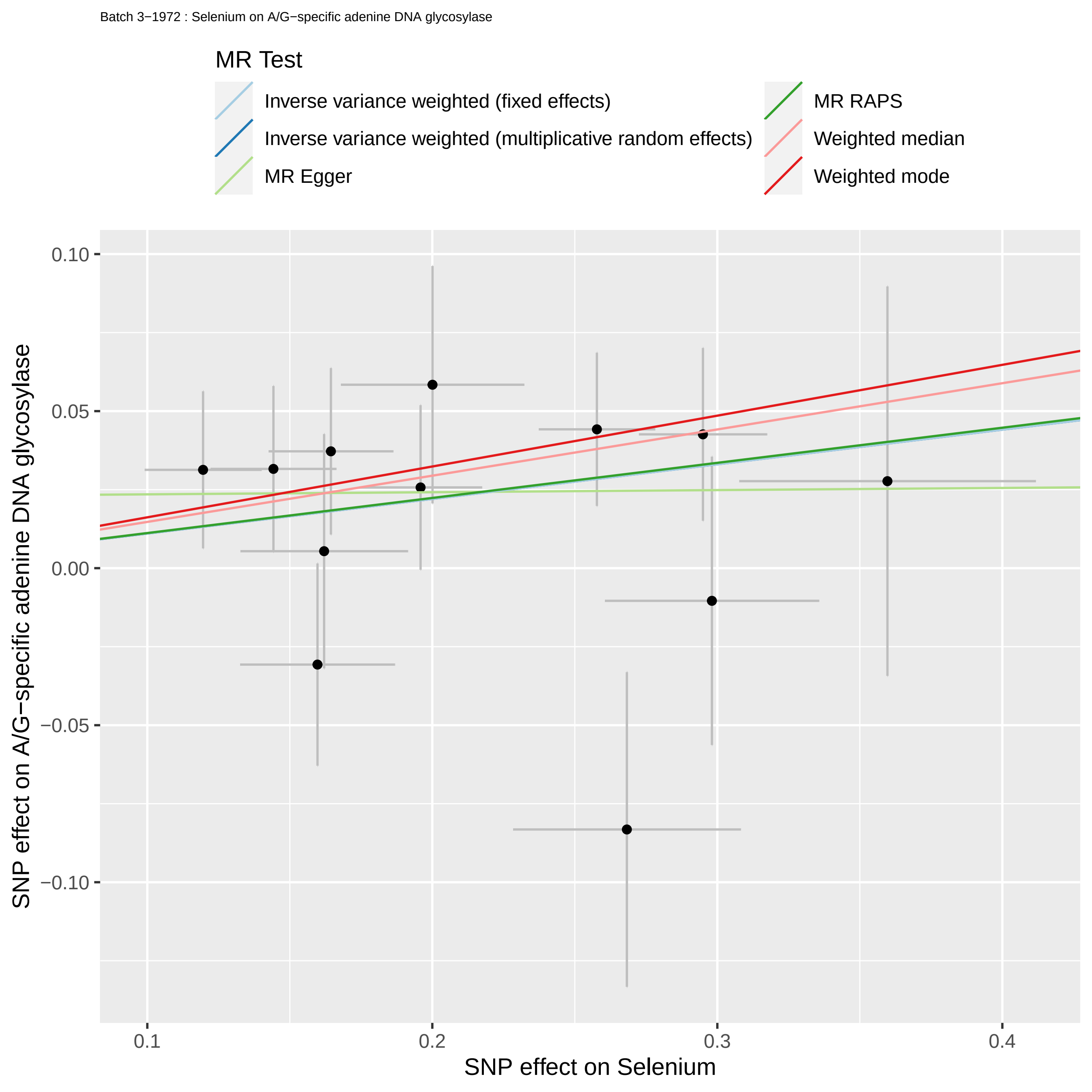
Batch 3−1972 : Selenium on A/G−specific adenine DNA glycosylase
MR Test
Inverse variance weighted (fixed effects)
Inverse variance weighted (multiplicative random effects)
MR Egger
MR RAPS
Weighted median
Weighted mode
0.10
0.05
0.00
−0.05
−0.10
0.1
0.2
0.3
0.4
SNP effect on Selenium
SNP effect on A/G−specific adenine DNA glycosylase

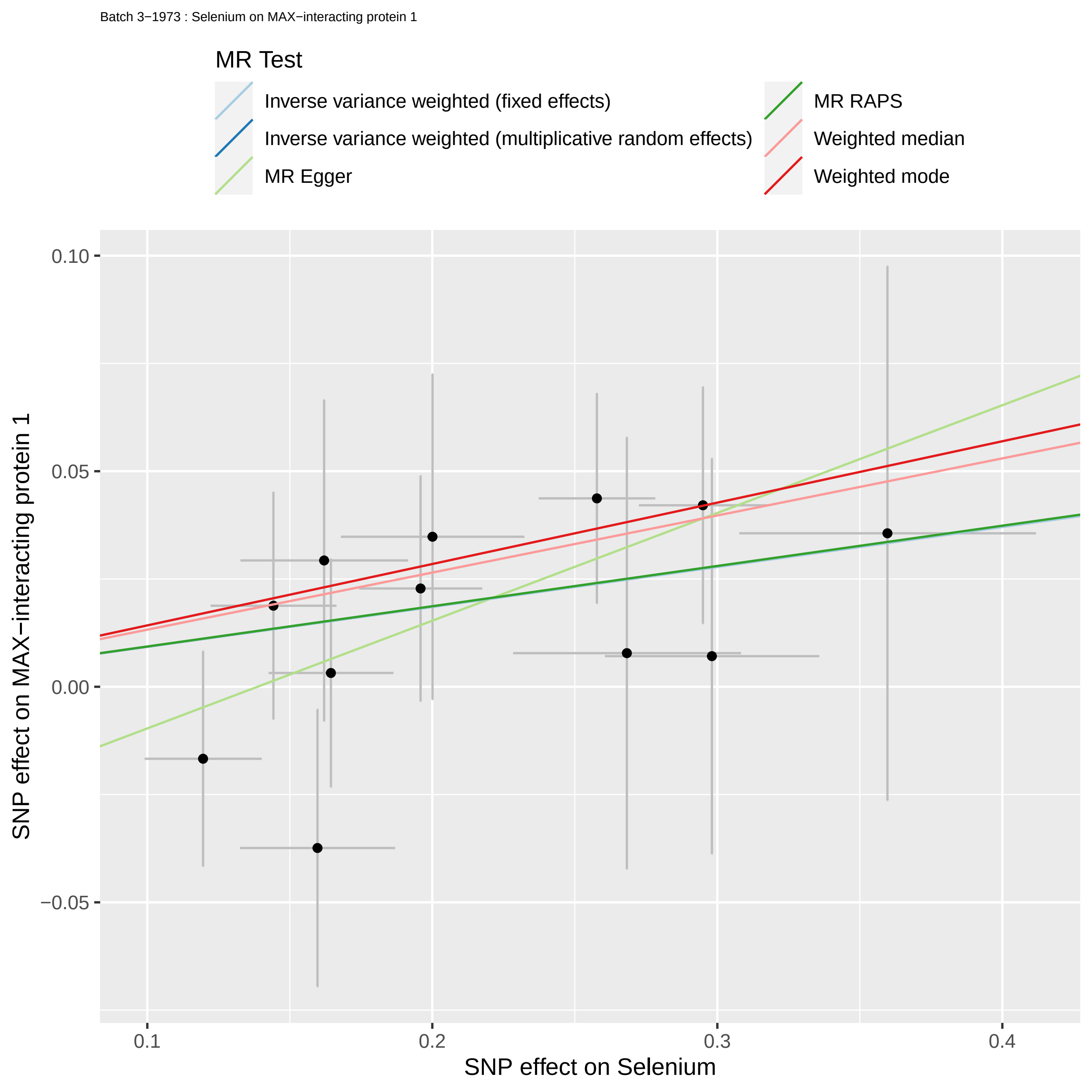
Batch 3−1973 : Selenium on MAX−interacting protein 1
MR Test
Inverse variance weighted (fixed effects)
Inverse variance weighted (multiplicative random effects)
MR Egger
MR RAPS
Weighted median
Weighted mode
0.10
0.05
0.00
−0.05
0.1
0.2
0.3
0.4
SNP effect on Selenium
SNP effect on MAX−interacting protein 1

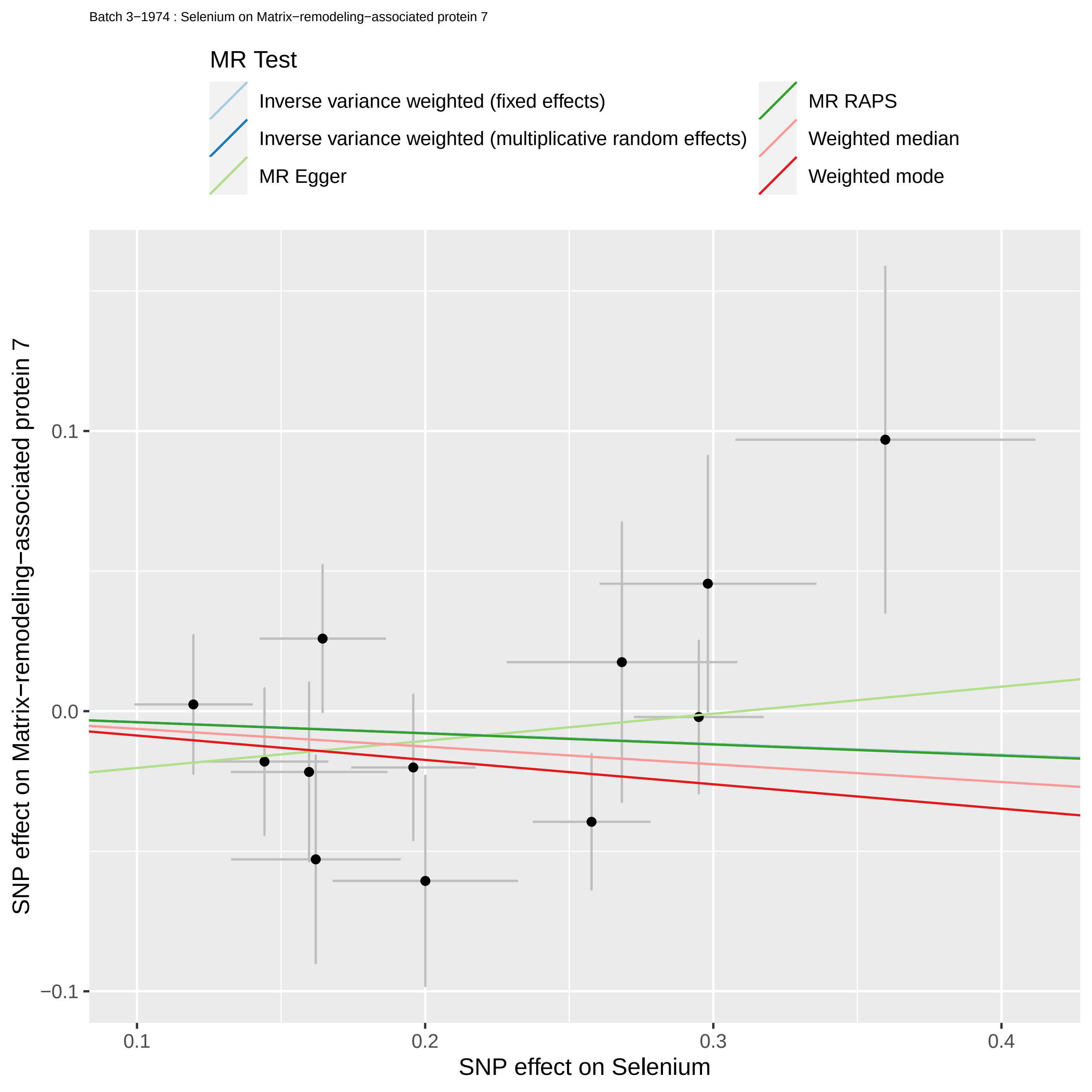
Batch 3−1974 : Selenium on Matrix−remodeling−associated protein 7
MR Test
Inverse variance weighted (fixed effects)
Inverse variance weighted (multiplicative random effects)
MR Egger
MR RAPS
Weighted median
Weighted mode
0.1
0.0
−0.1
0.1
0.2
0.3
0.4
SNP effect on Matrix−remodeling−associated protein 7
SNP effect on Selenium

Batch 3−1975 : Selenium on Matrix−remodeling−associated protein 8

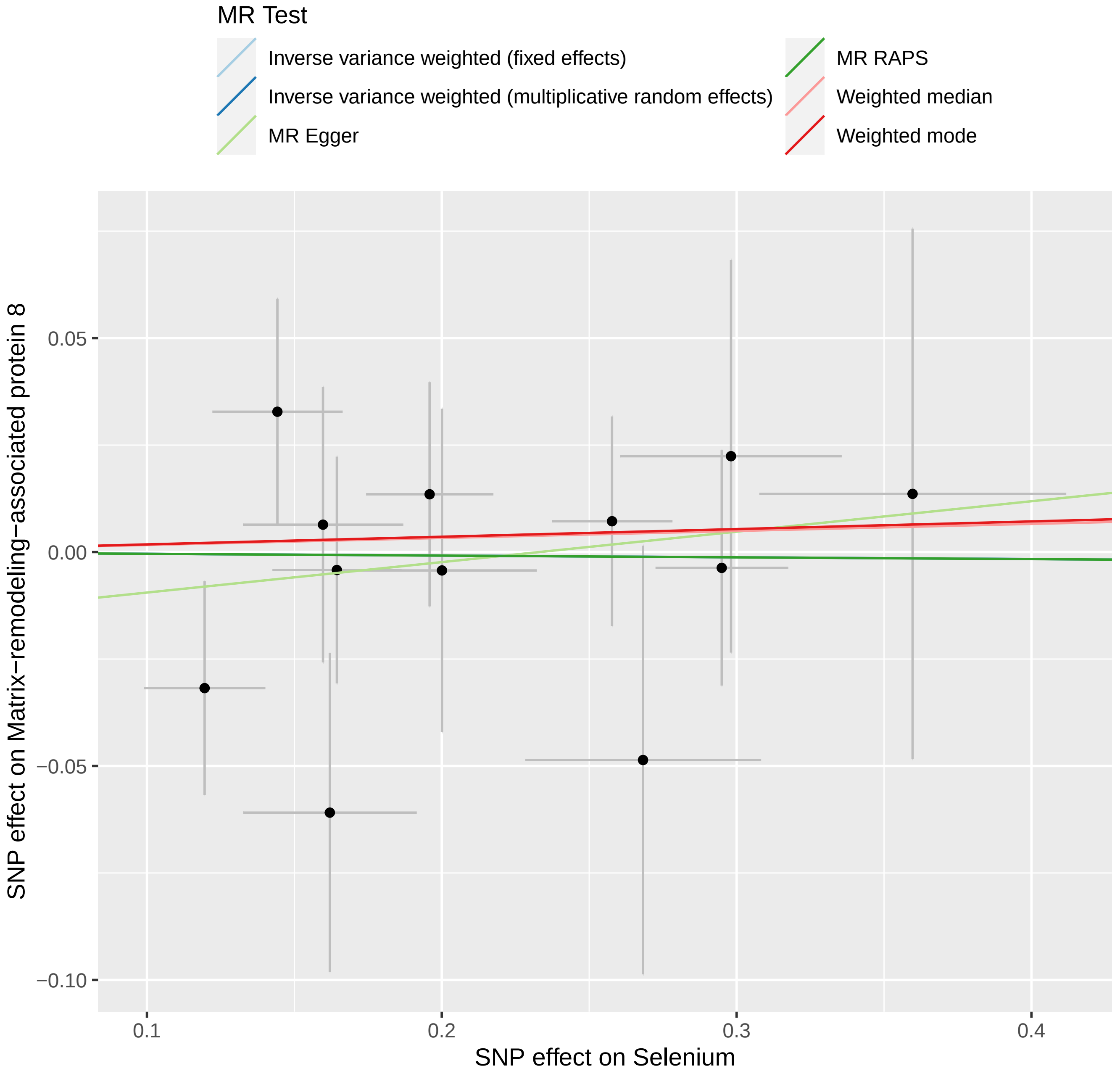

Batch 3−1976 : Selenium on Matrix−remodeling−associated protein 8

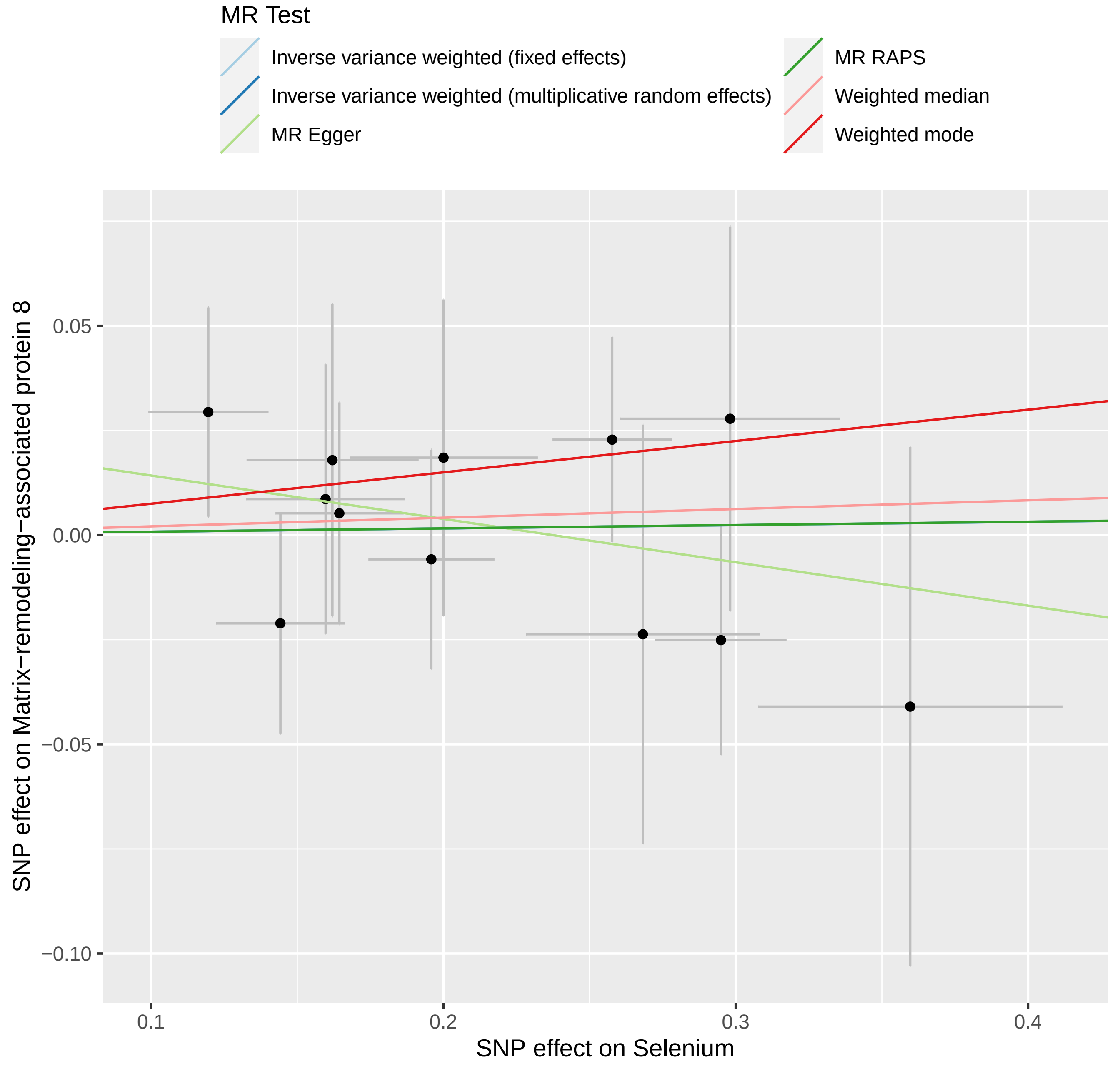

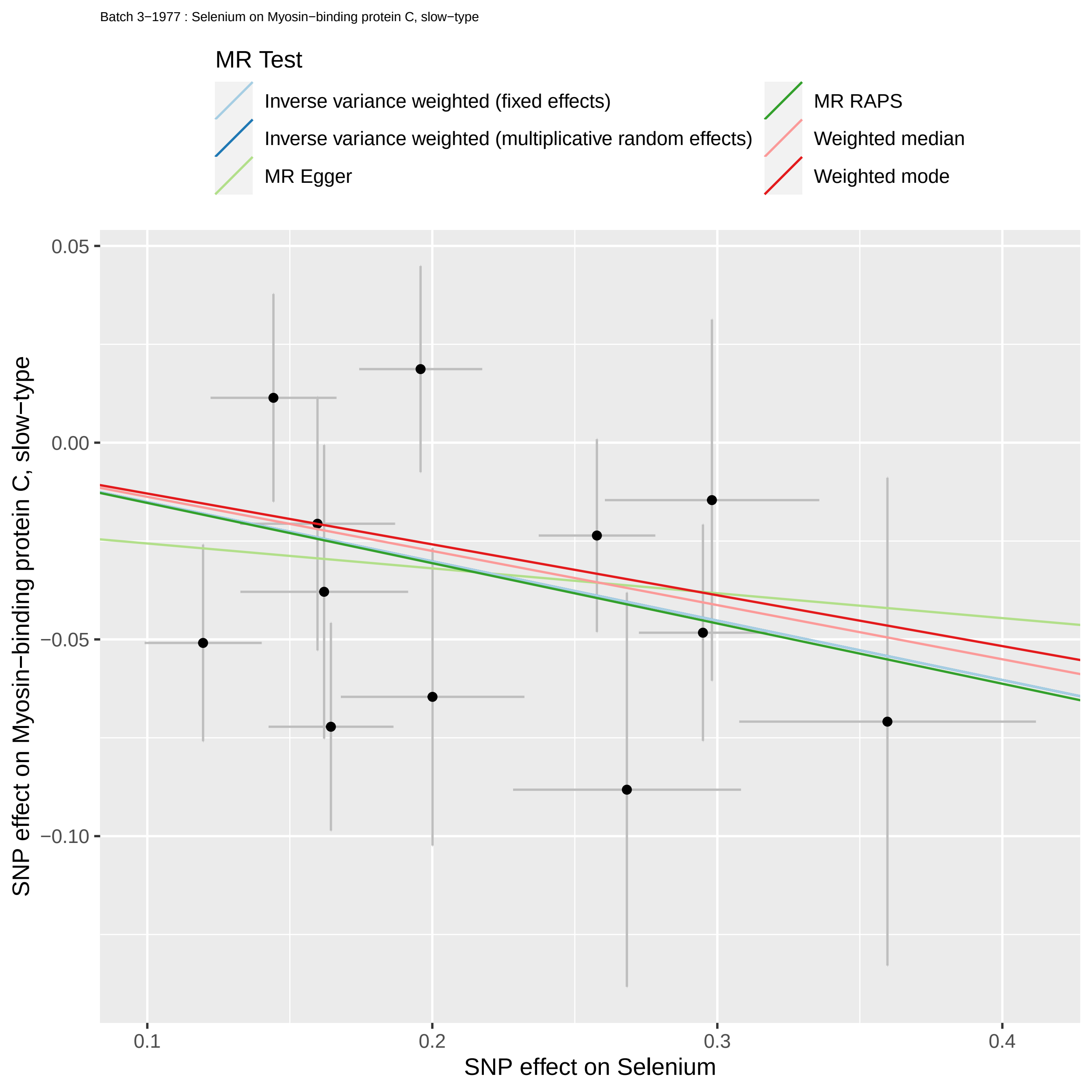

Batch 3−1977 : Selenium on Myosin−binding protein C, slow−type
MR Test
Inverse variance weighted (fixed effects)
Inverse variance weighted (multiplicative random effects)
MR Egger
MR RAPS
Weighted median
Weighted mode
0.05
0.00
−0.05
−0.10
0.1
0.2
0.3
0.4
SNP effect on Selenium
SNP effect on Myosin−binding protein C, slow−type

Batch 3−1978 : Selenium on Myc proto−oncogene protein

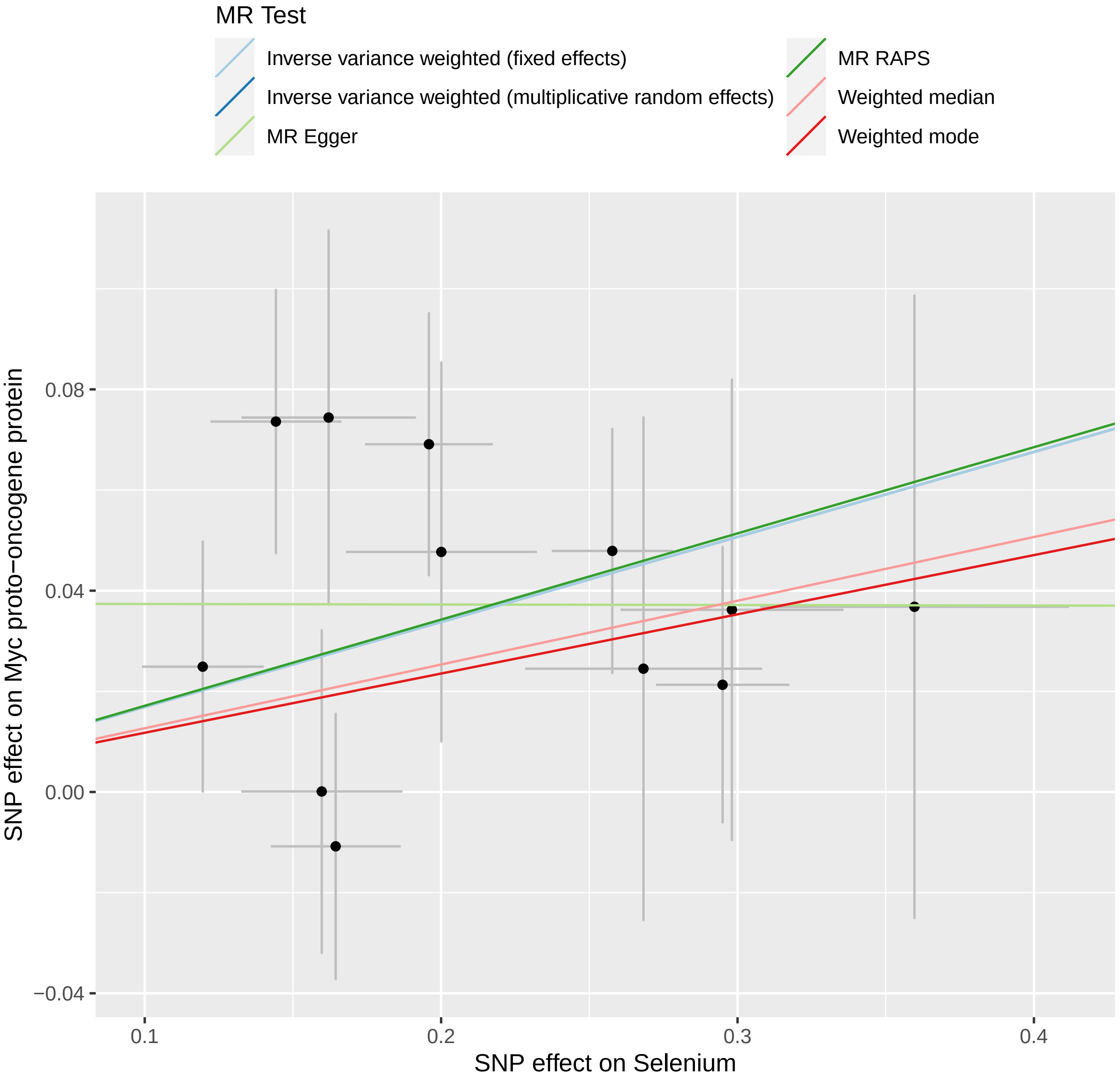

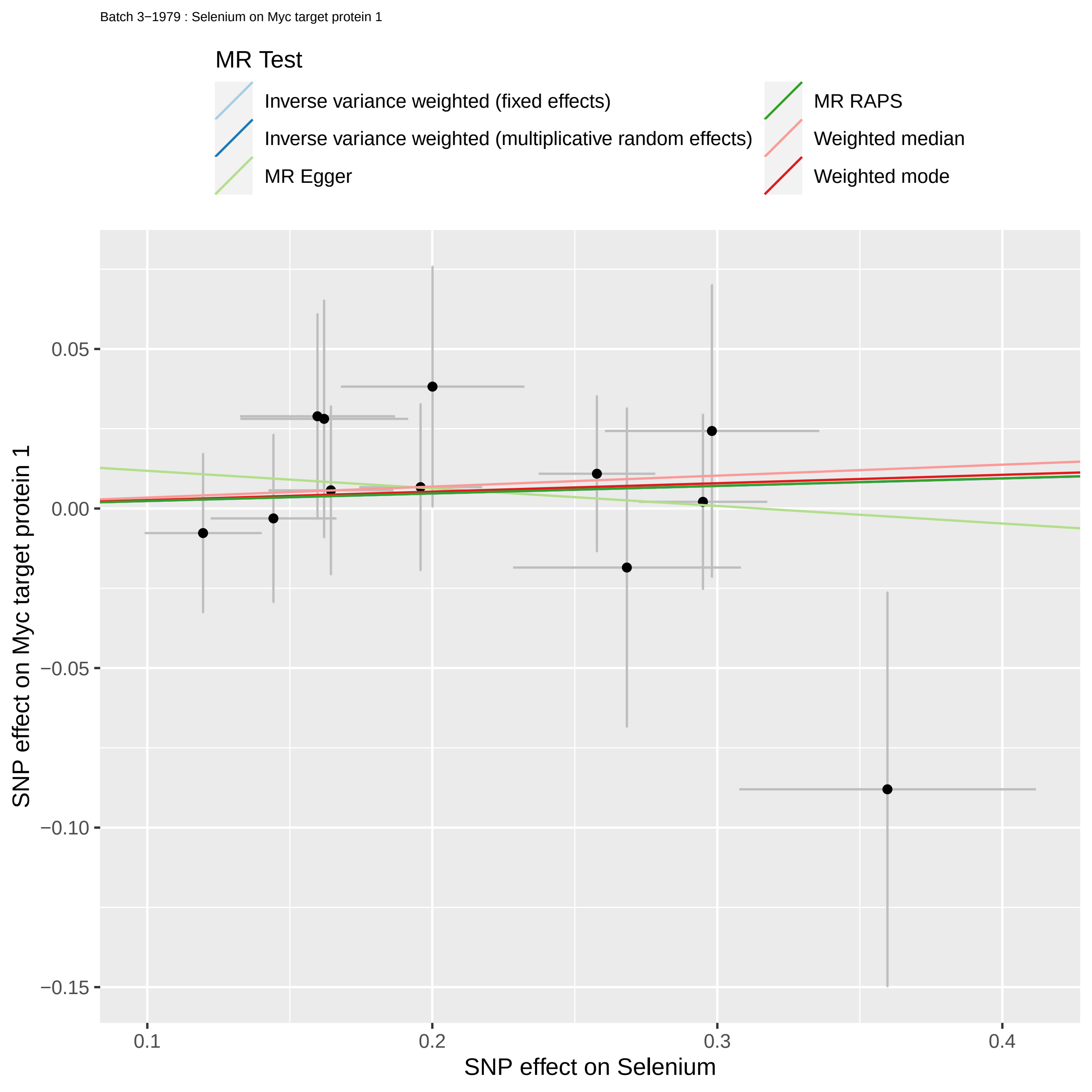
Batch 3−1979 : Selenium on Myc target protein 1
MR Test
Inverse variance weighted (fixed effects)
Inverse variance weighted (multiplicative random effects)
MR Egger
MR RAPS
Weighted median
Weighted mode
0.05
0.00
−0.05
−0.10
−0.15
0.1
0.2
0.3
0.4
SNP effect on Myc target protein 1
SNP effect on Selenium

Batch 3−1980 : Selenium on Unconventional myosin−VI

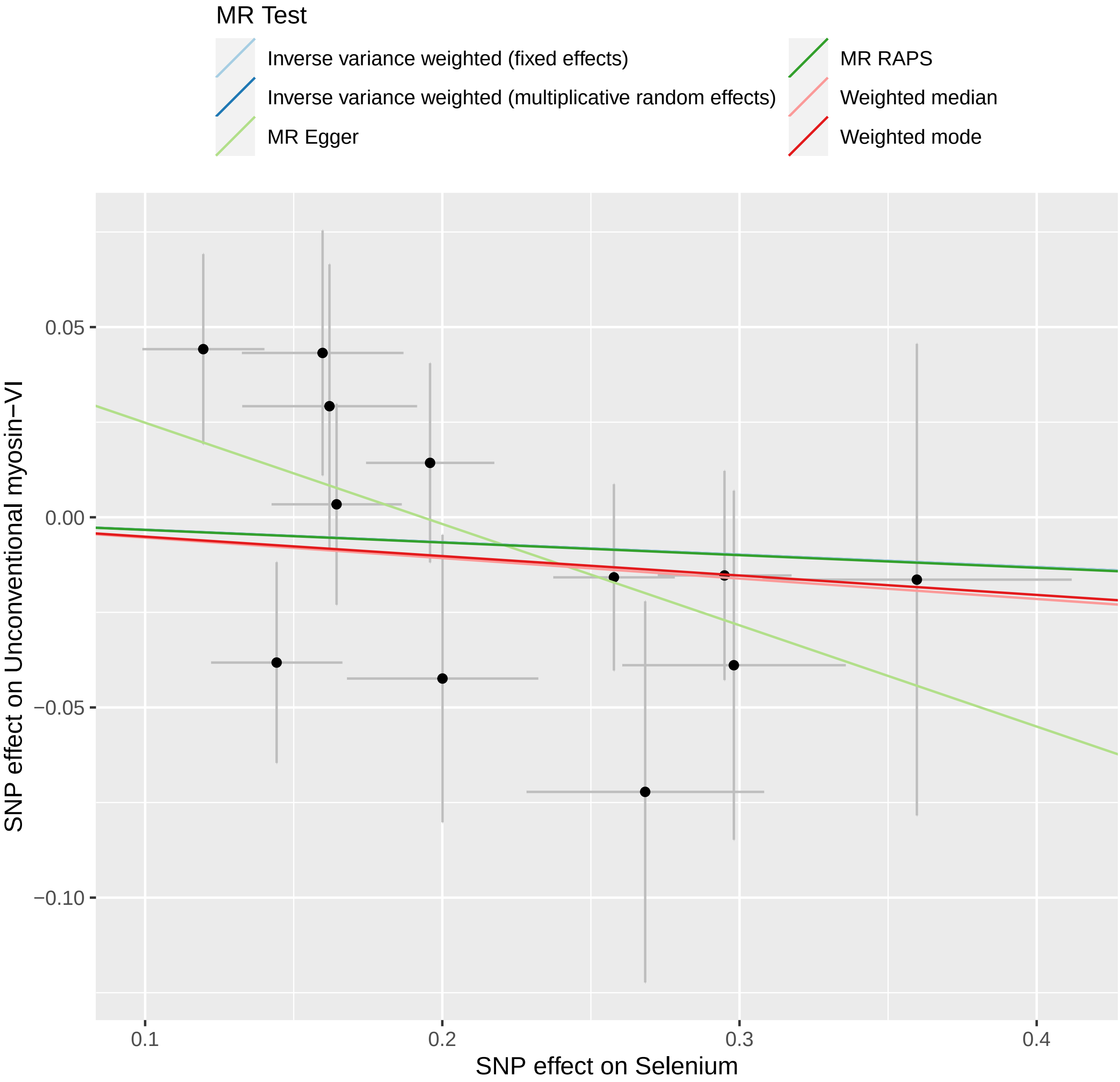

Batch 3−1981 : Selenium on Myomesin−2

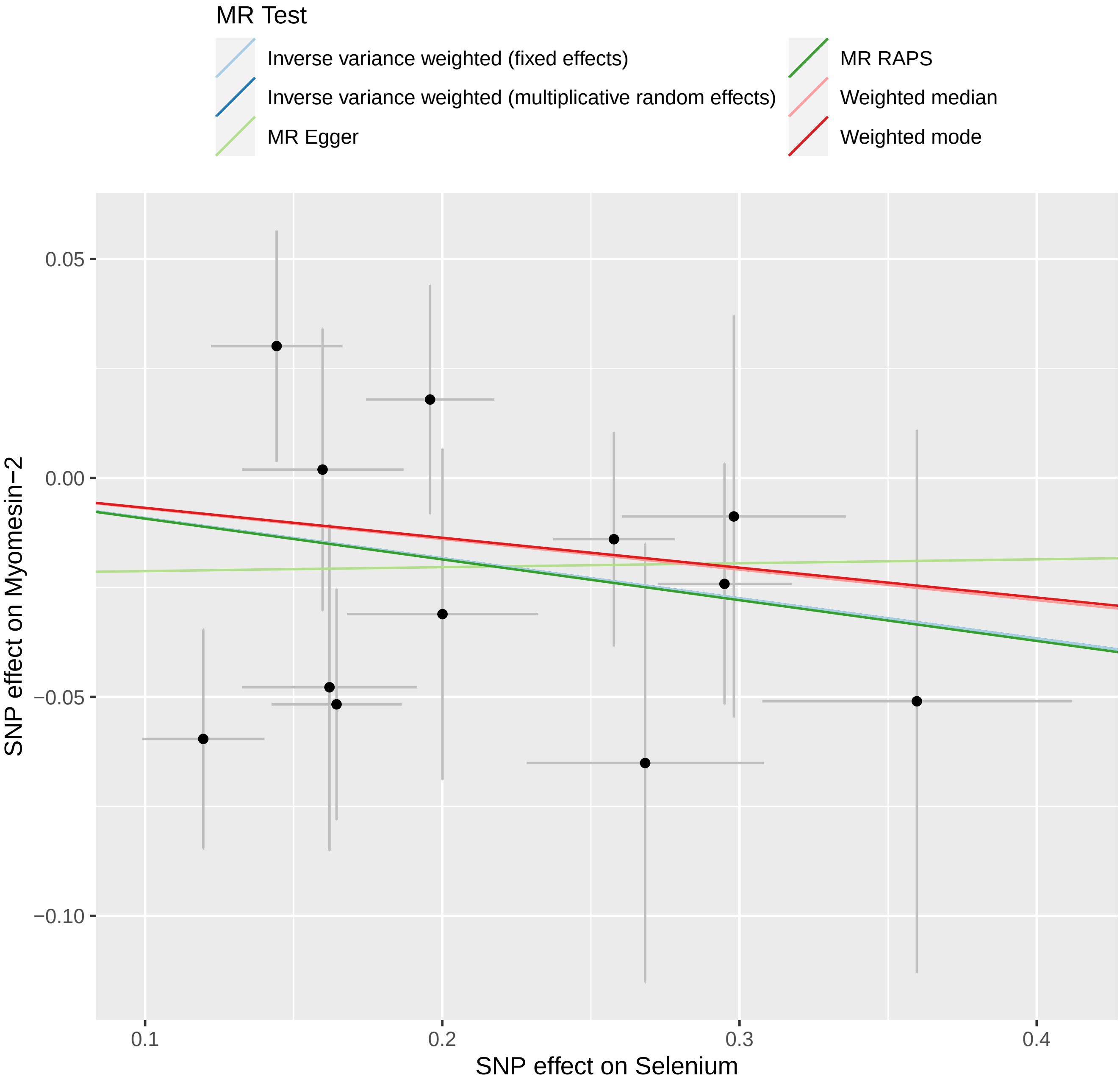

Batch 3−1982 : Selenium on Myelin regulatory factor

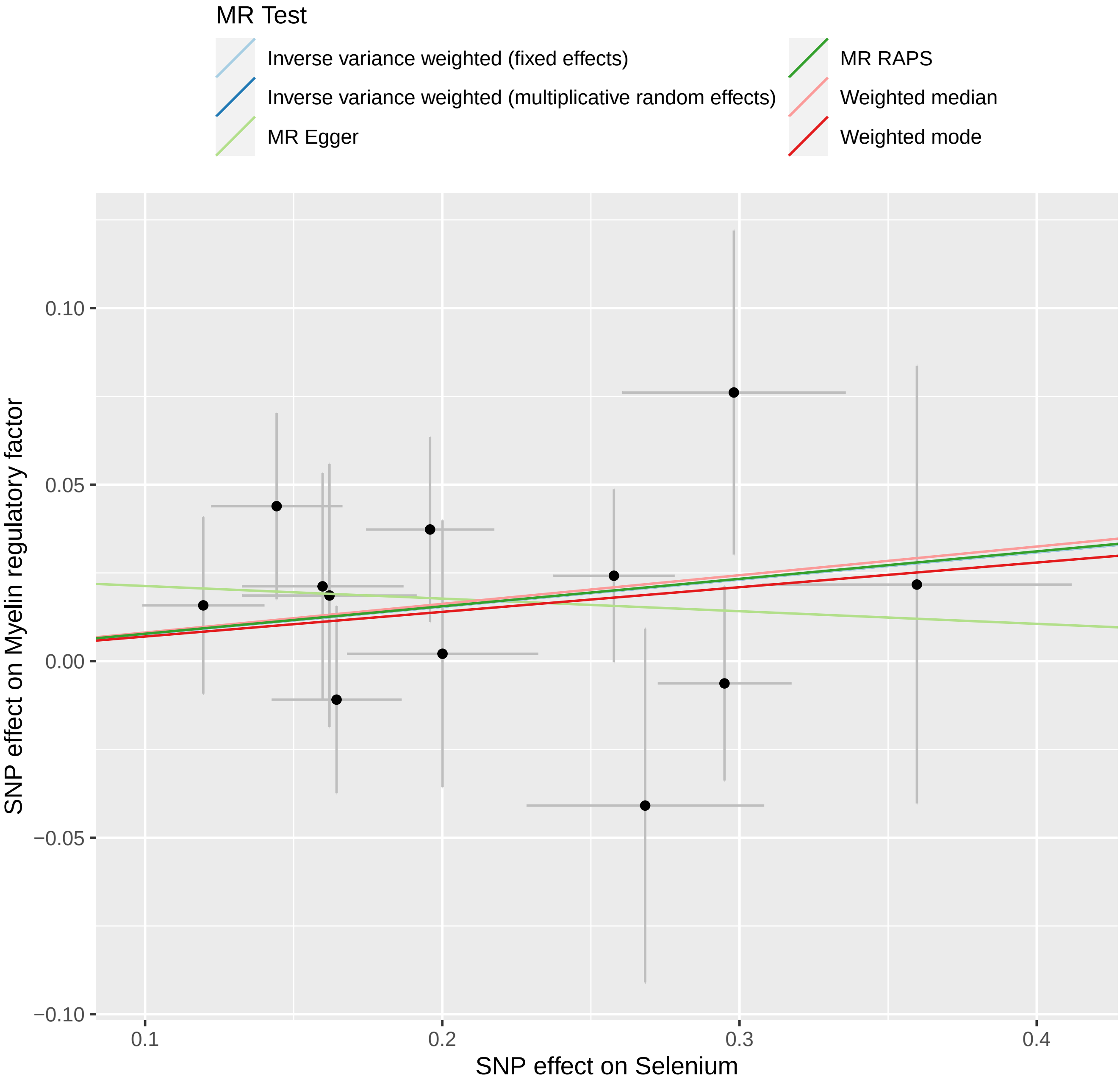

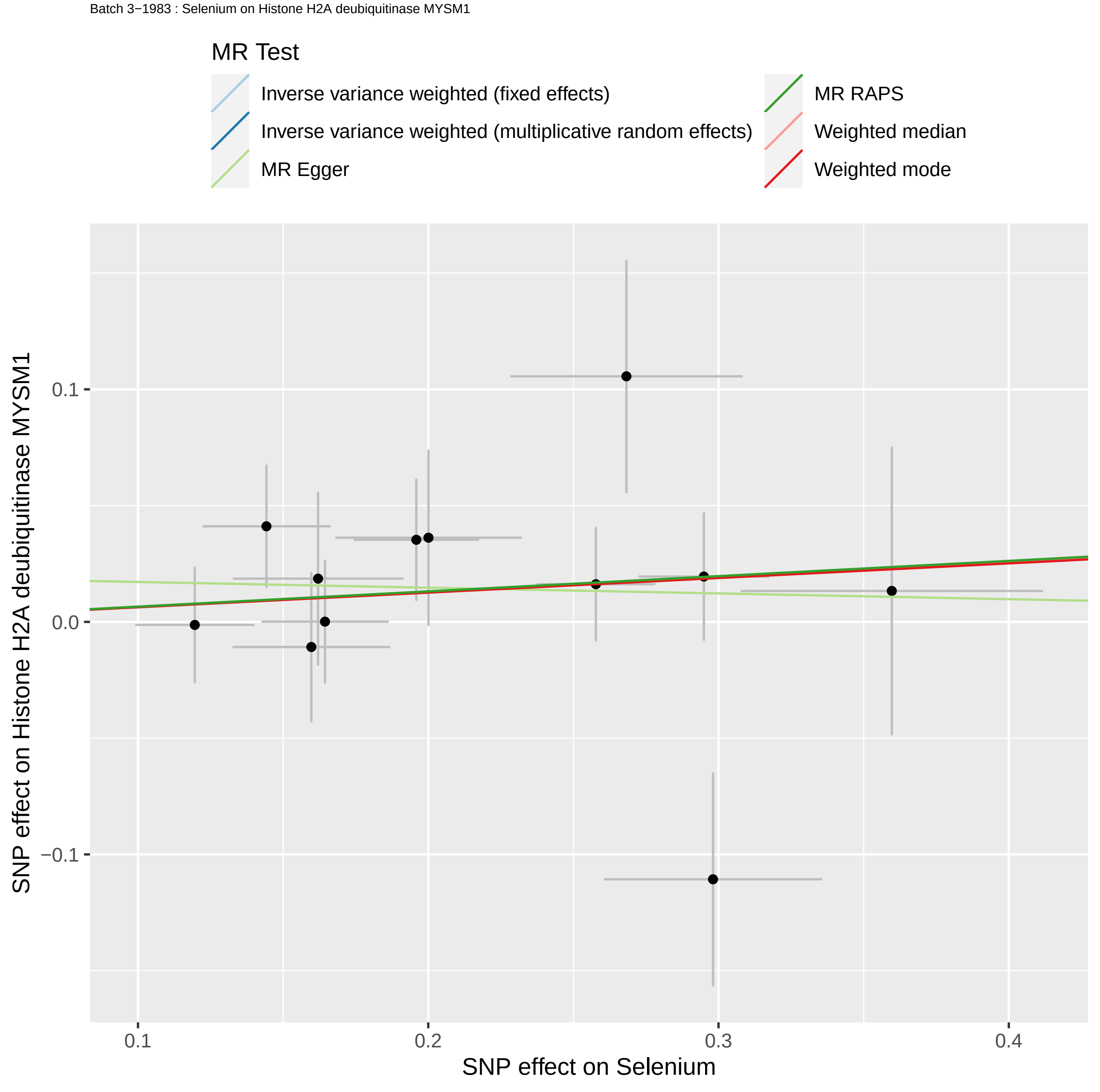
Batch 3−1983 : Selenium on Histone H2A deubiquitinase MYSM1
MR Test
Inverse variance weighted (fixed effects)
Inverse variance weighted (multiplicative random effects)
MR Egger
MR RAPS
Weighted median
Weighted mode
0.1
0.0
−0.1
0.1
0.2
0.3
0.4
SNP effect on Histone H2A deubiquitinase MYSM1
SNP effect on Selenium

Batch 3−1984 : Selenium on Myocardial zonula adherens protein

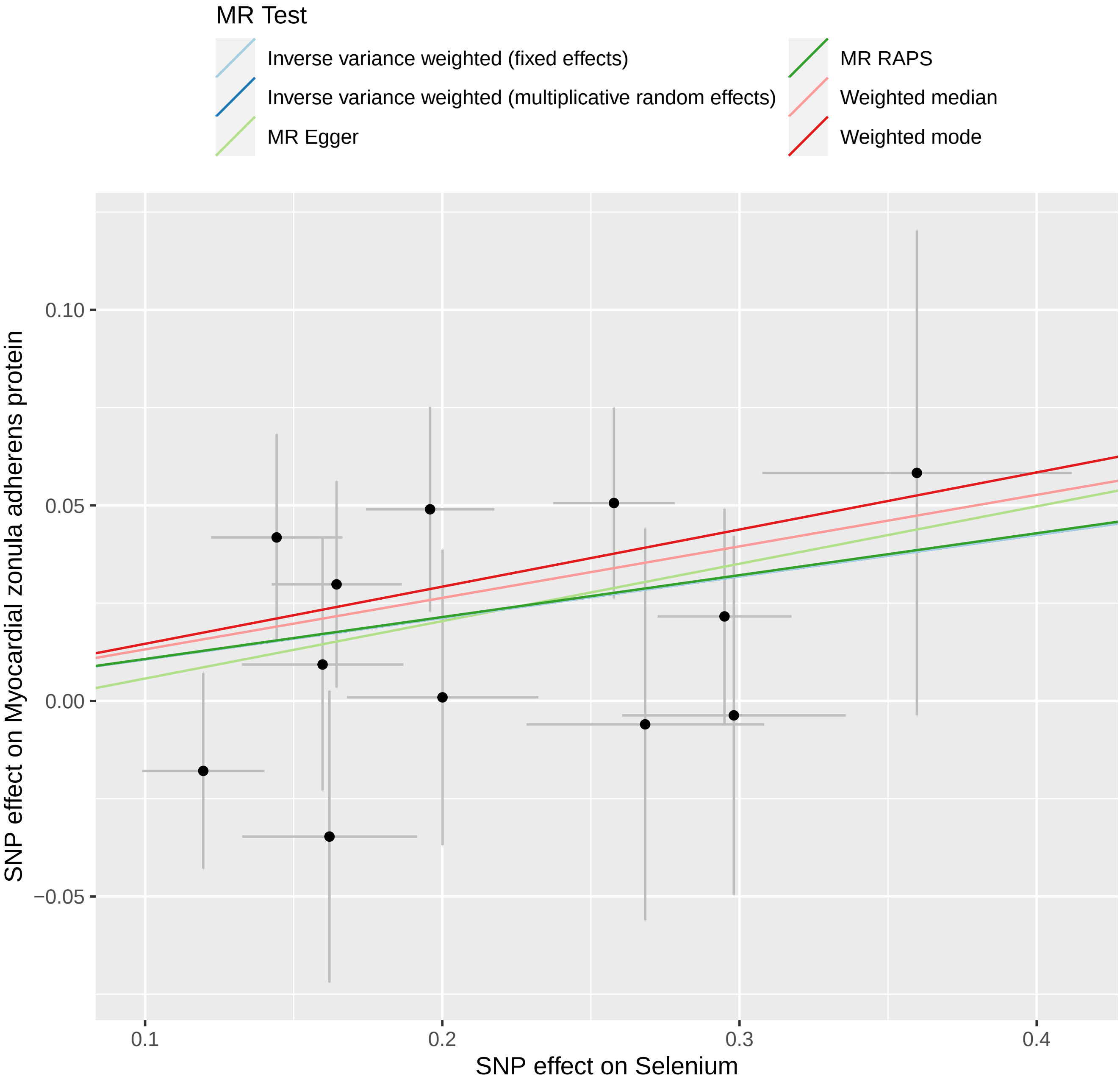

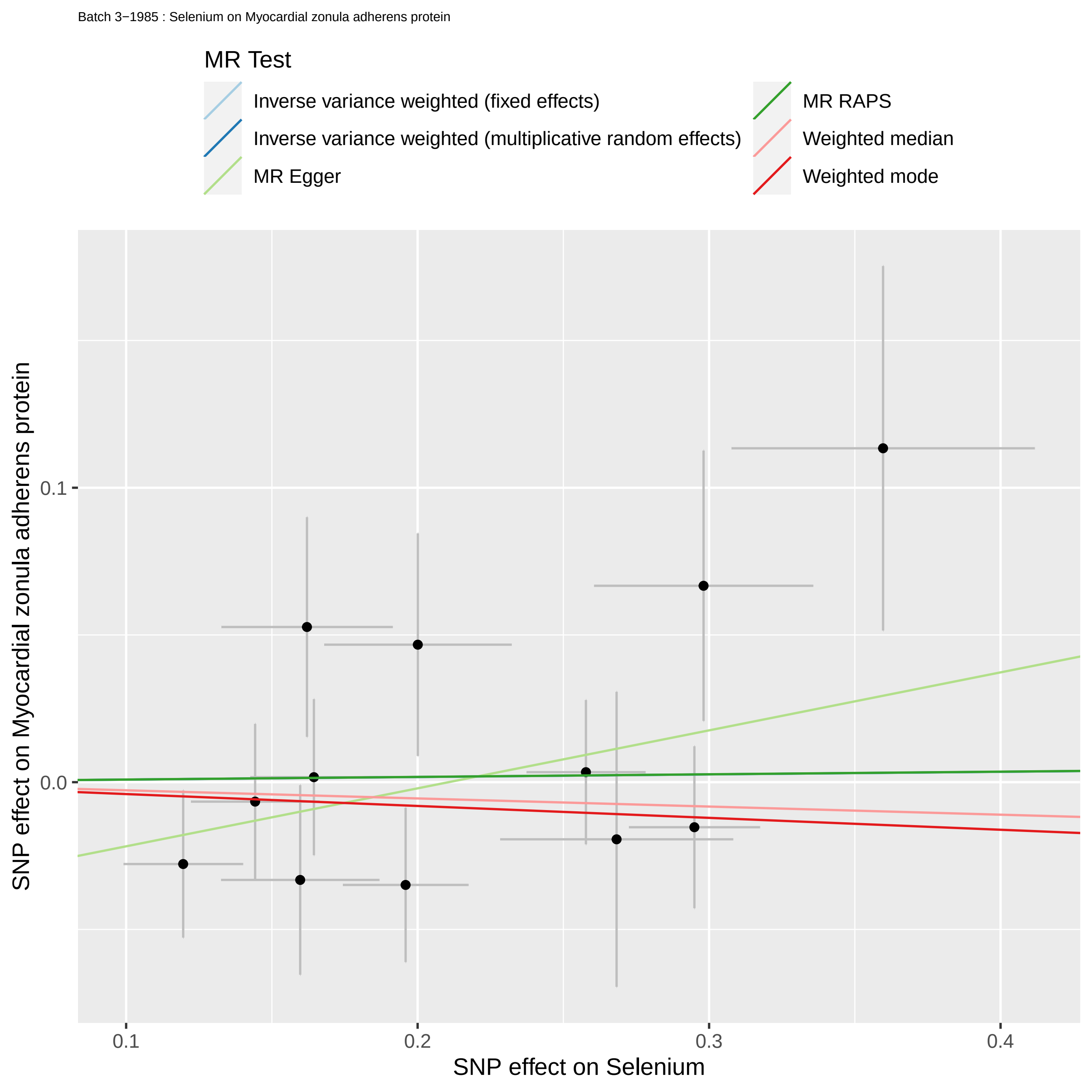

Batch 3−1985 : Selenium on Myocardial zonula adherens protein
MR Test
Inverse variance weighted (fixed effects)
Inverse variance weighted (multiplicative random effects)
MR Egger
MR RAPS
Weighted median
Weighted mode
SNP effect on Myocardial zonula adherens protein
0.1
0.0
0.1
0.2
0.3
0.4
SNP effect on Selenium

Batch 3−1986 : Selenium on Myocardial zonula adherens protein

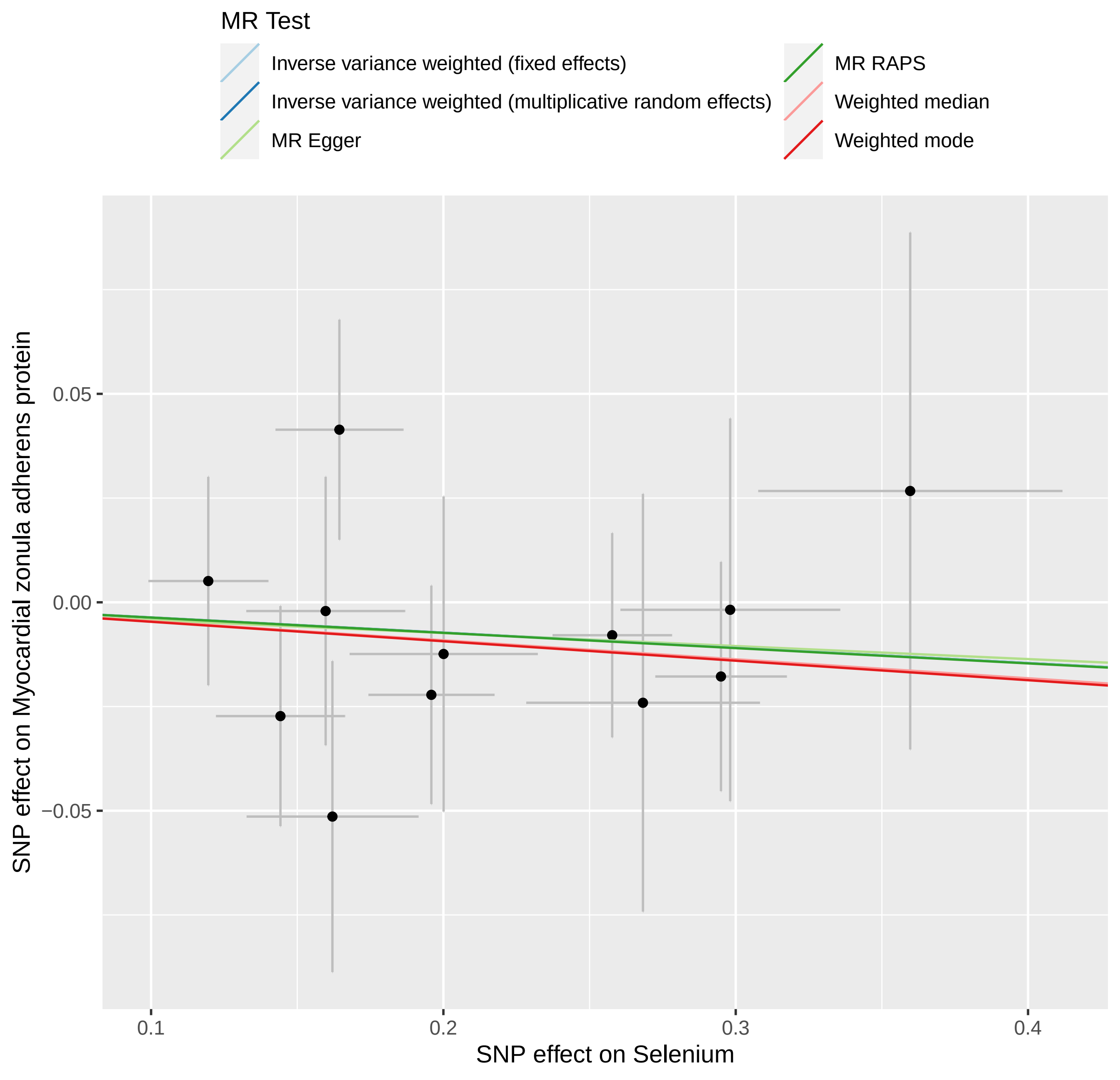

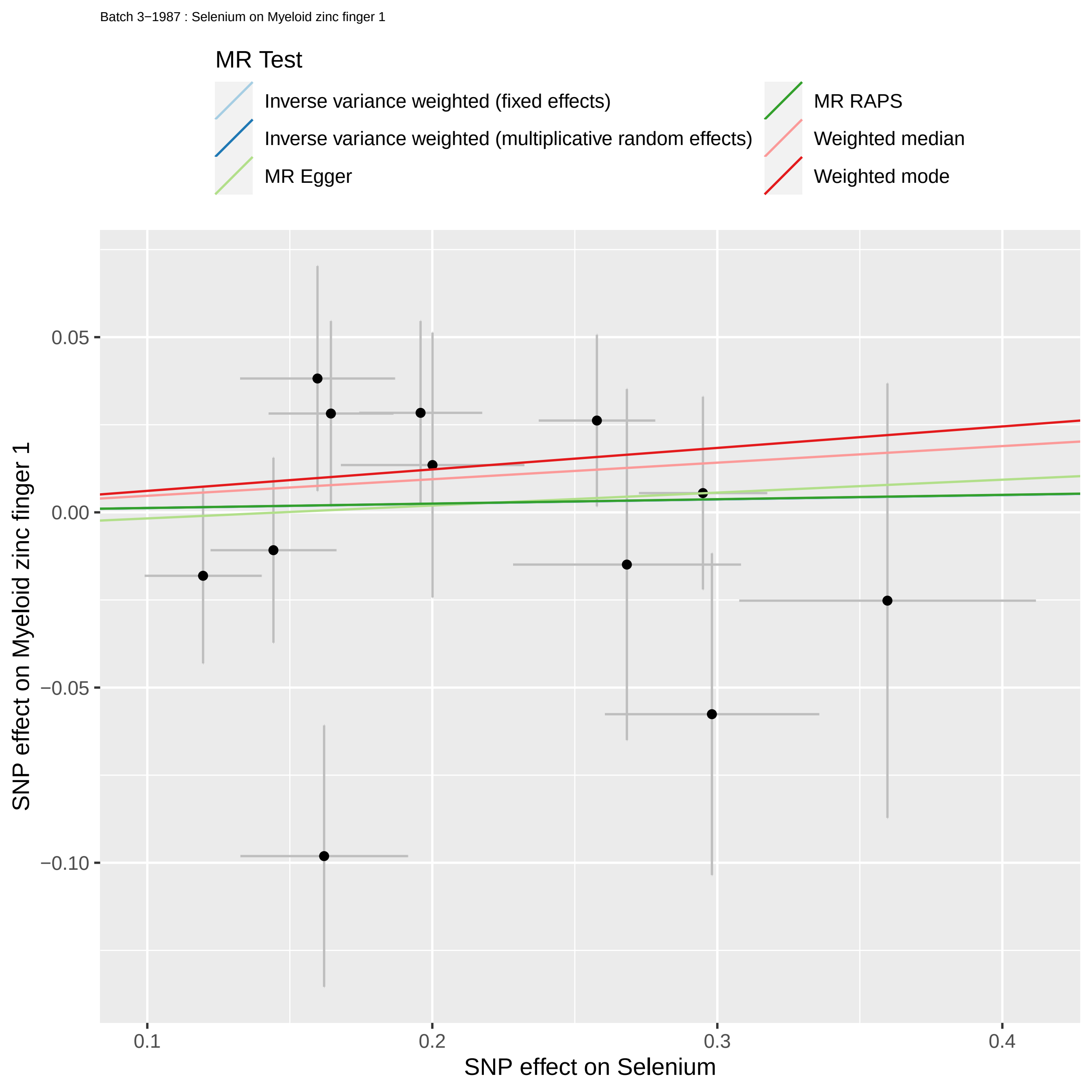

Batch 3−1987 : Selenium on Myeloid zinc finger 1
MR Test
Inverse variance weighted (fixed effects)
Inverse variance weighted (multiplicative random effects)
MR Egger
MR RAPS
Weighted median
Weighted mode
0.05
0.00
−0.05
−0.10
0.1
0.2
0.3
0.4
SNP effect on Myeloid zinc finger 1
SNP effect on Selenium

Batch 3−1988 : Selenium on Mitotic−spindle organizing protein 1

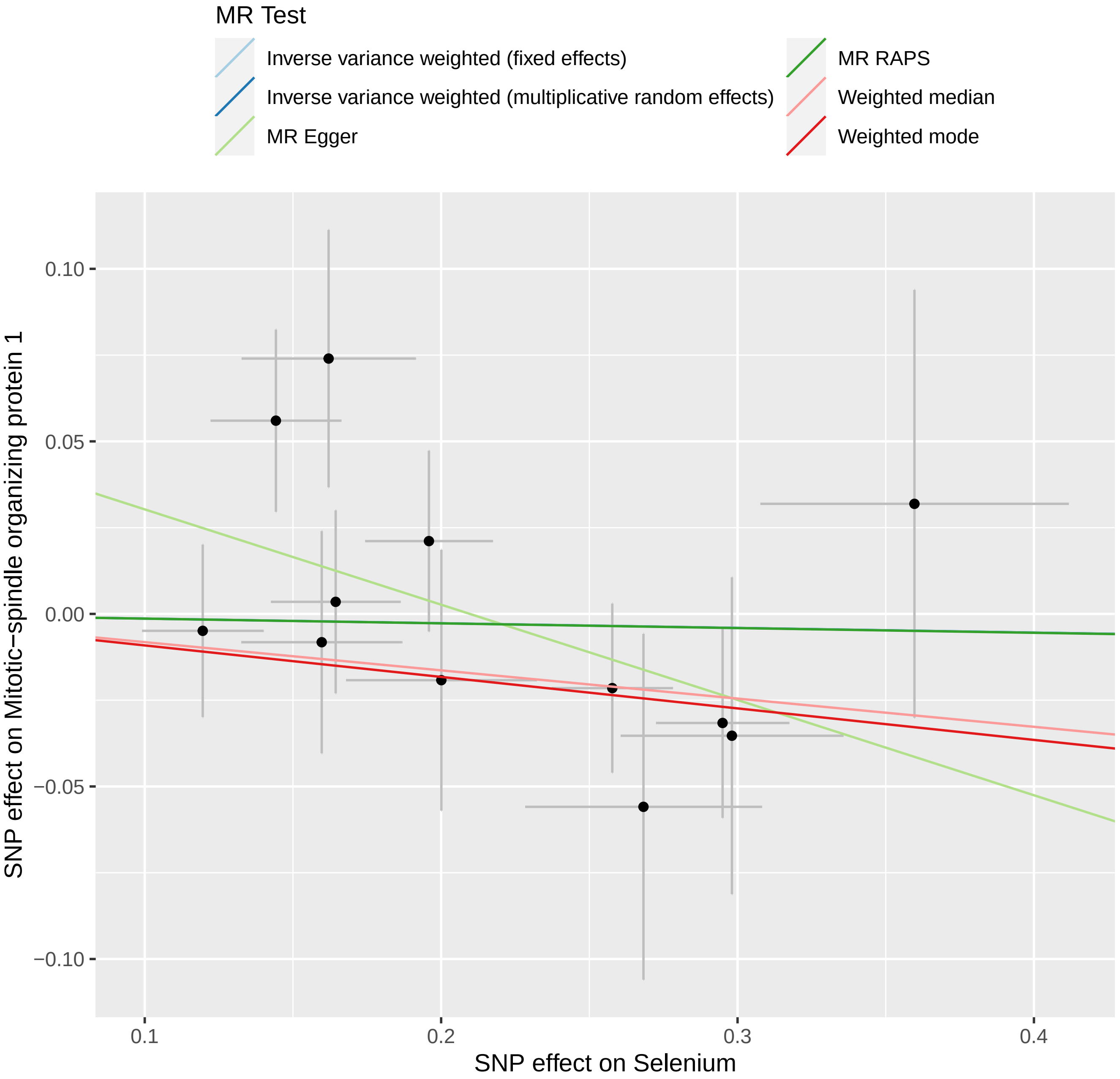

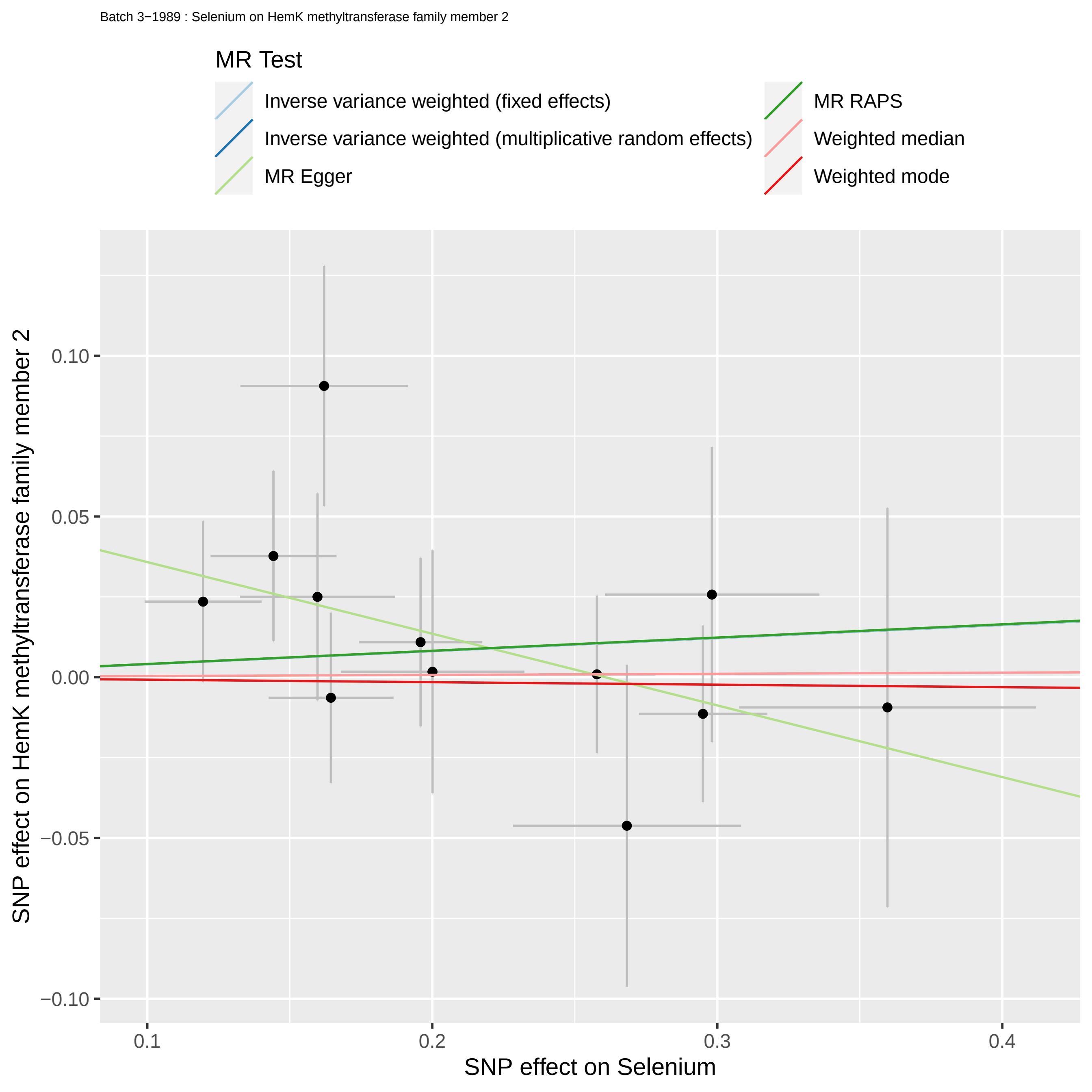

Batch 3−1989 : Selenium on HemK methyltransferase family member 2
MR Test
Inverse variance weighted (fixed effects)
Inverse variance weighted (multiplicative random effects)
MR Egger
MR RAPS
Weighted median
Weighted mode
0.10
0.05
0.00
−0.05
−0.10
0.1
0.2
0.3
0.4
SNP effect on HemK methyltransferase family member 2
SNP effect on Selenium

Batch 3−1990 : Selenium on N−acylethanolamine−hydrolyzing acid amidase

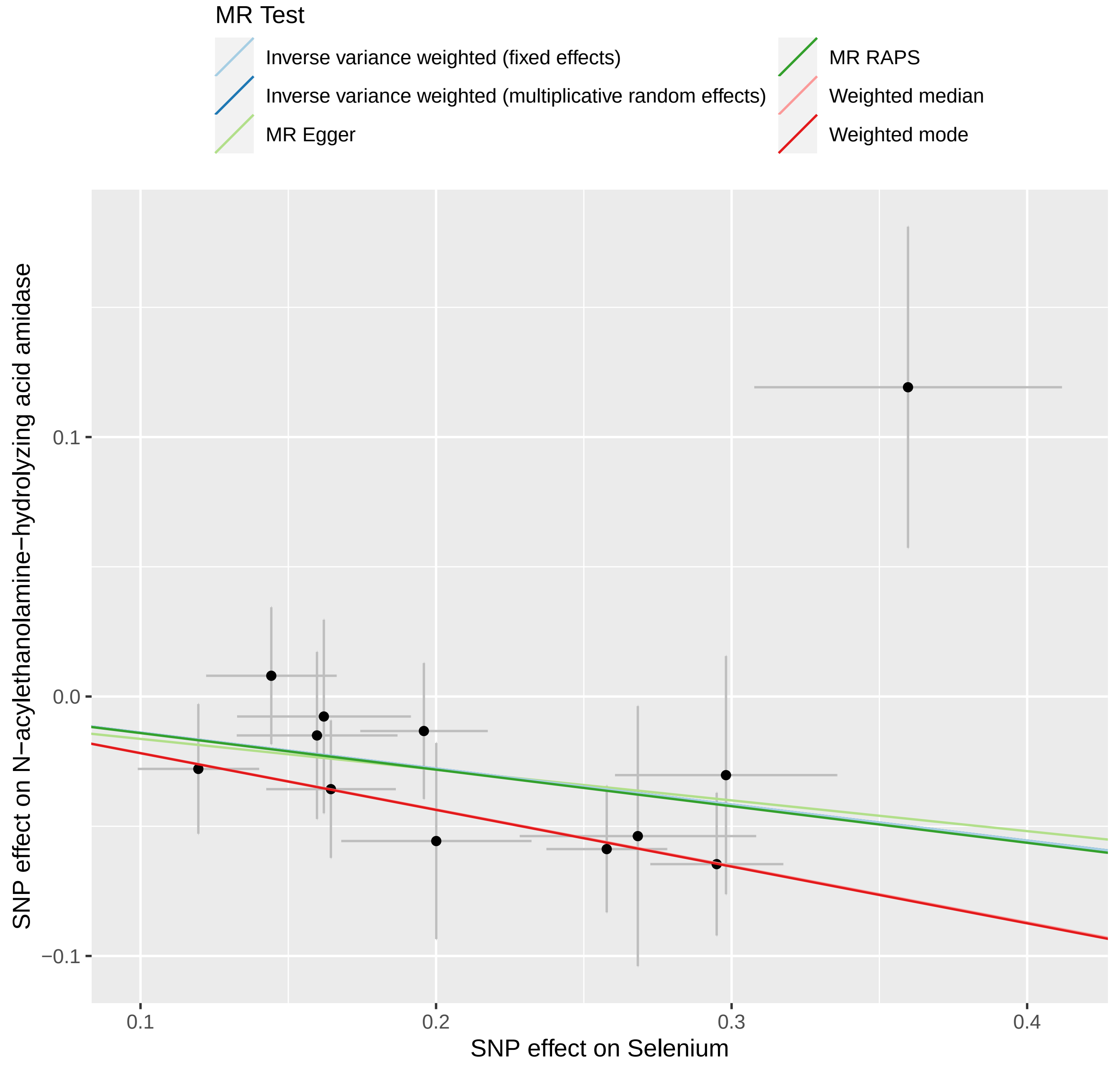

Batch 3−1991 : Selenium on N−acetylated−alpha−linked acidic dipeptidase 2

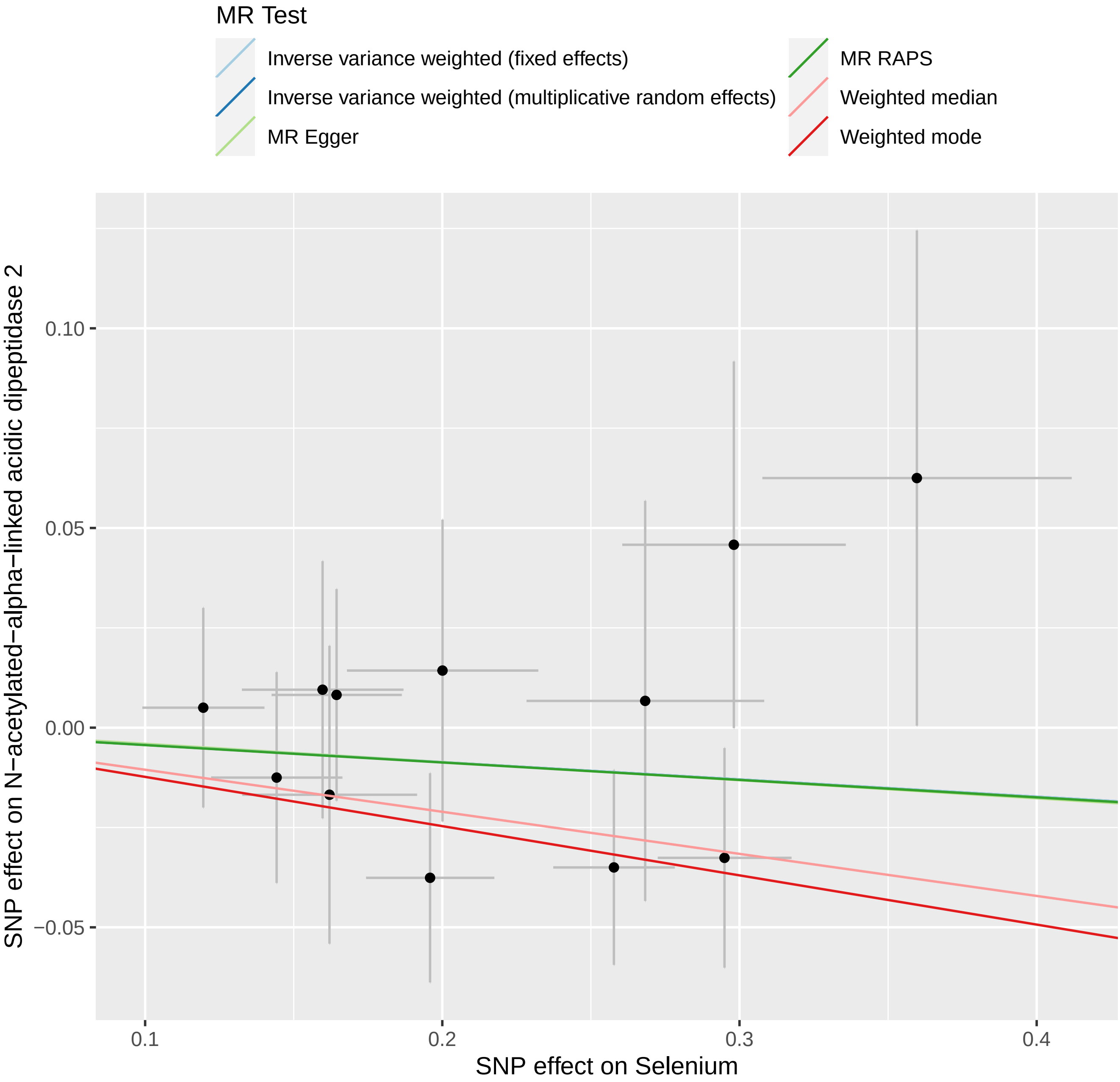

Batch 3−1992 : Selenium on NGFI−A−binding protein 1

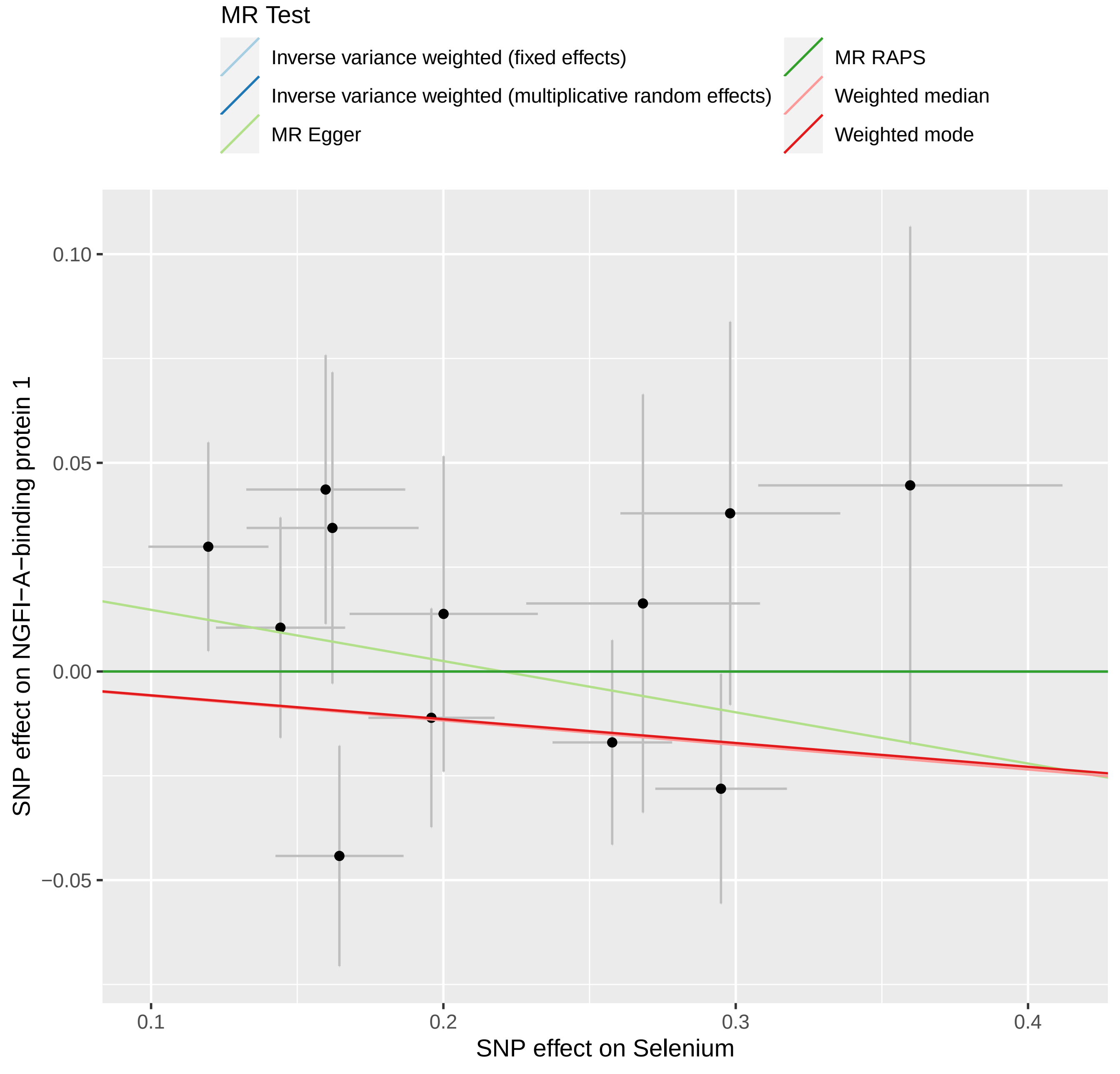

Batch 3−1993 : Selenium on NGFI−A−binding protein 2

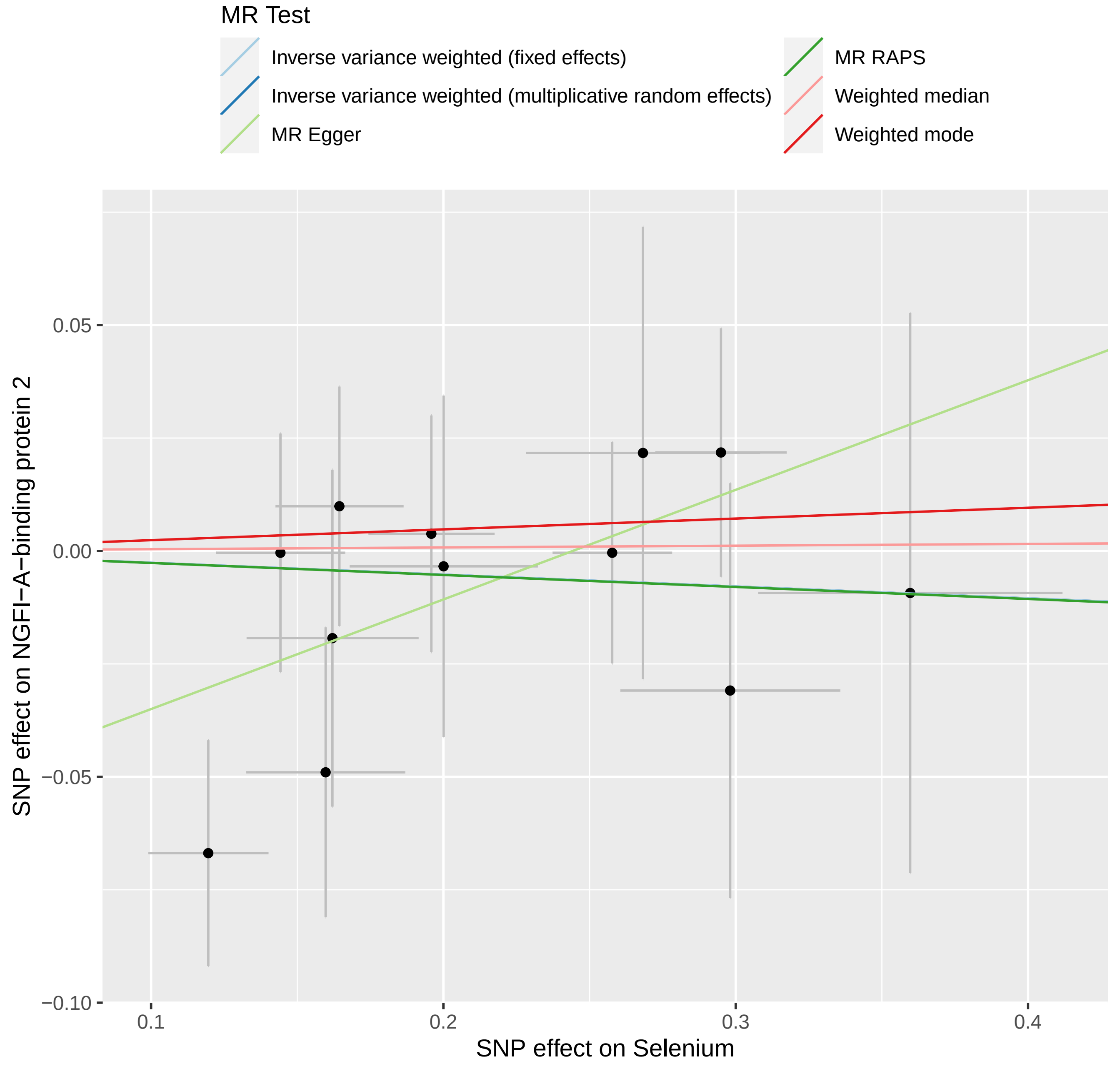

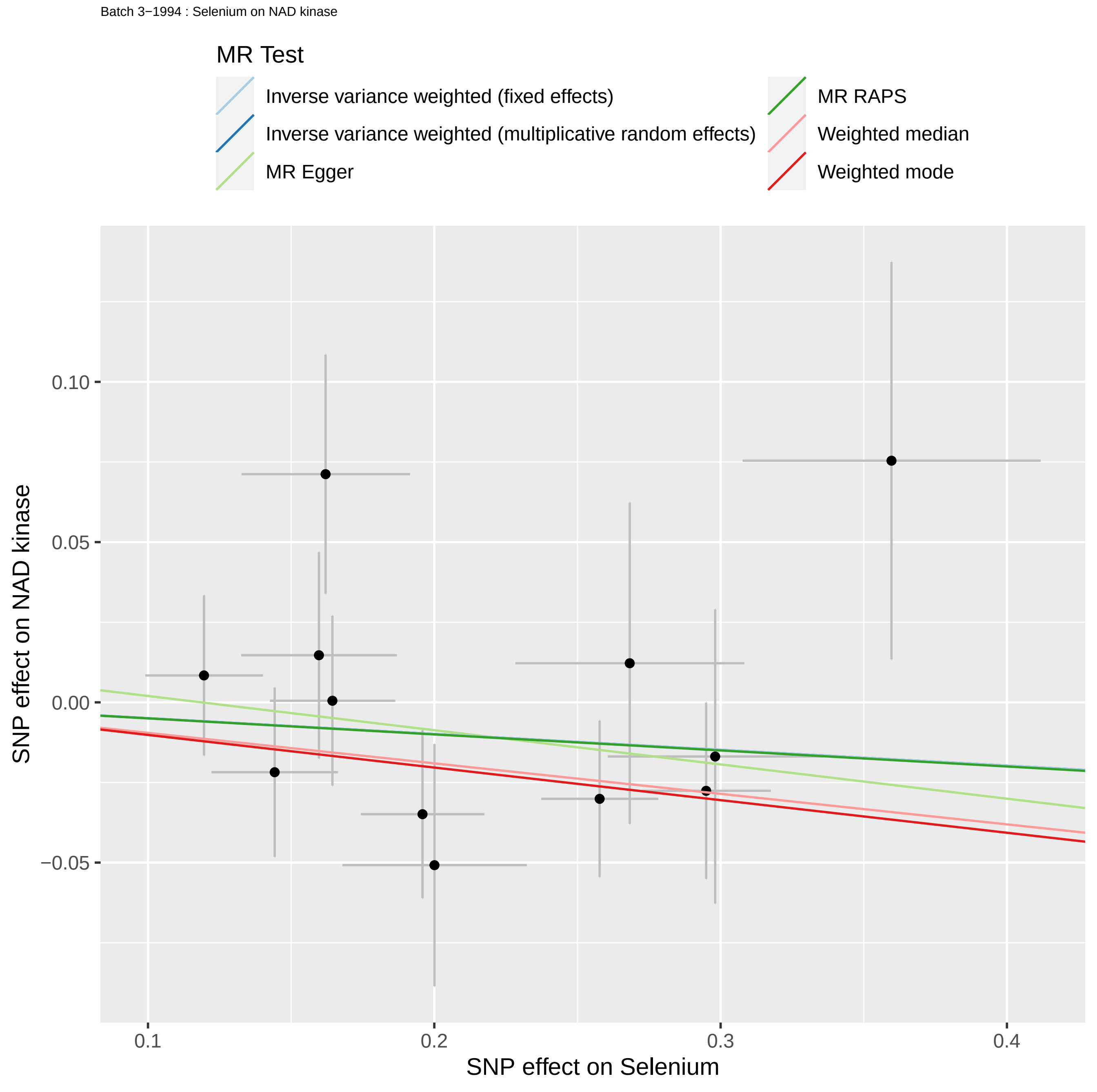

Batch 3−1994 : Selenium on NAD kinase
MR Test
Inverse variance weighted (fixed effects)
Inverse variance weighted (multiplicative random effects)
MR Egger
MR RAPS
Weighted median
Weighted mode
0.10
0.05
0.00
−0.05
0.1
0.2
0.3
0.4
SNP effect on NAD kinase
SNP effect on Selenium

Batch 3−1995 : Selenium on N−acetyl−D−glucosamine kinase

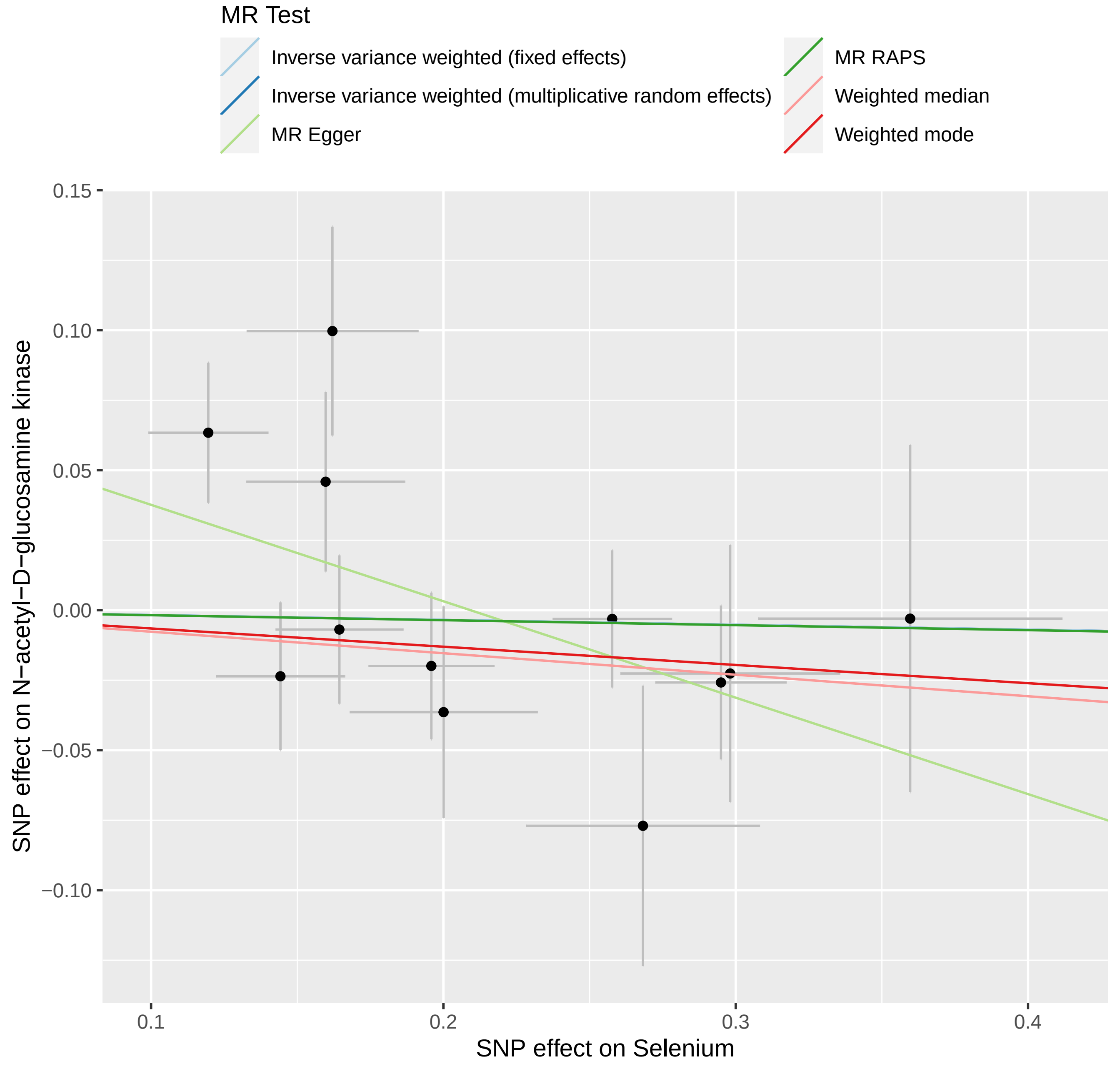

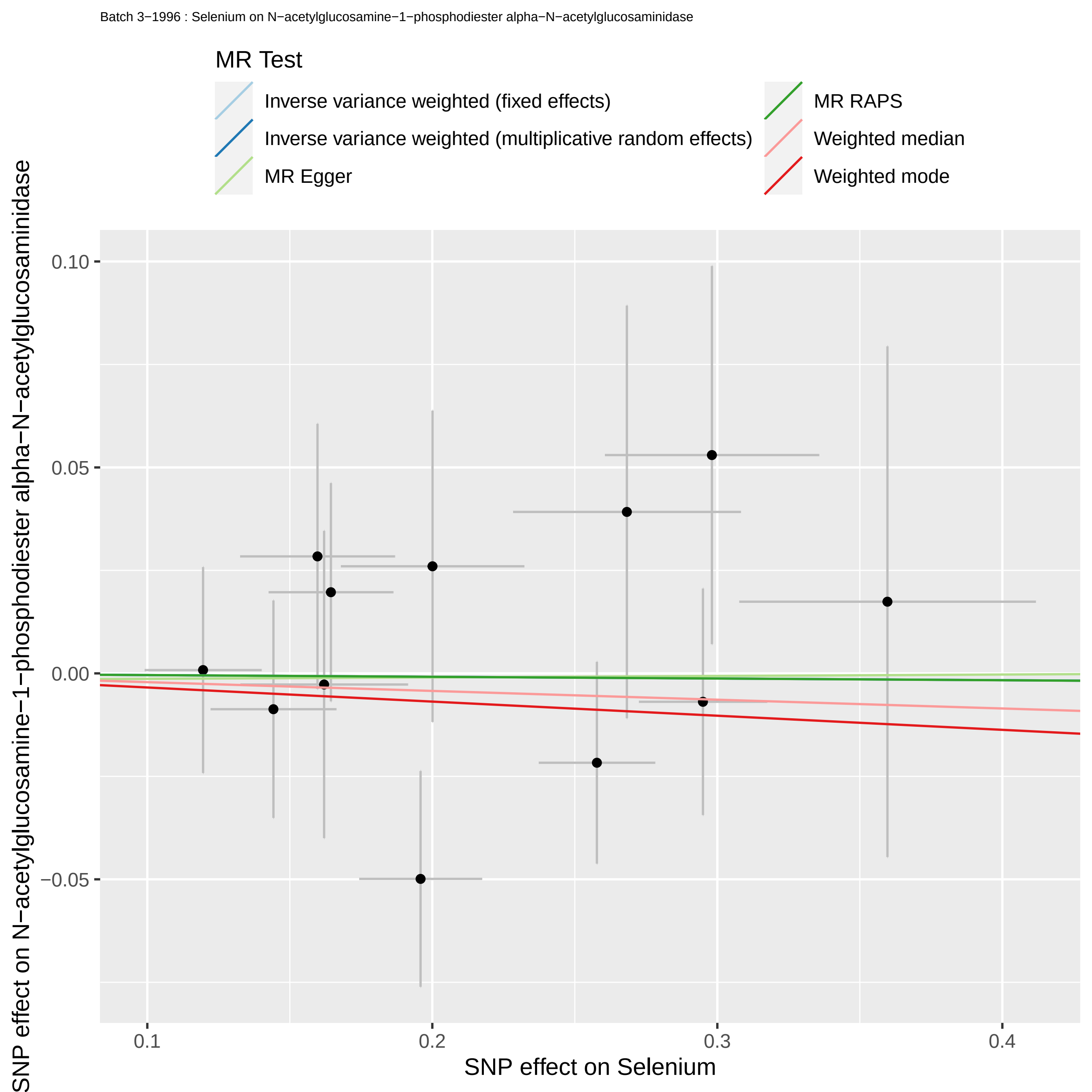

Batch 3−1996 : Selenium on N−acetylglucosamine−1−phosphodiester alpha−N−acetylglucosaminidase
MR Test
Inverse variance weighted (fixed effects)
Inverse variance weighted (multiplicative random effects)
MR Egger
MR RAPS
Weighted median
Weighted mode
0.10
0.05
0.00
−0.05
0.1
0.2
0.3
0.4
SNP effect on Selenium
SNP effect on N−acetylglucosamine−1−phosphodiester alpha−N−acetylglucosaminidase

Batch 3−1997 : Selenium on N−acetylglutamate synthase, mitochondrial

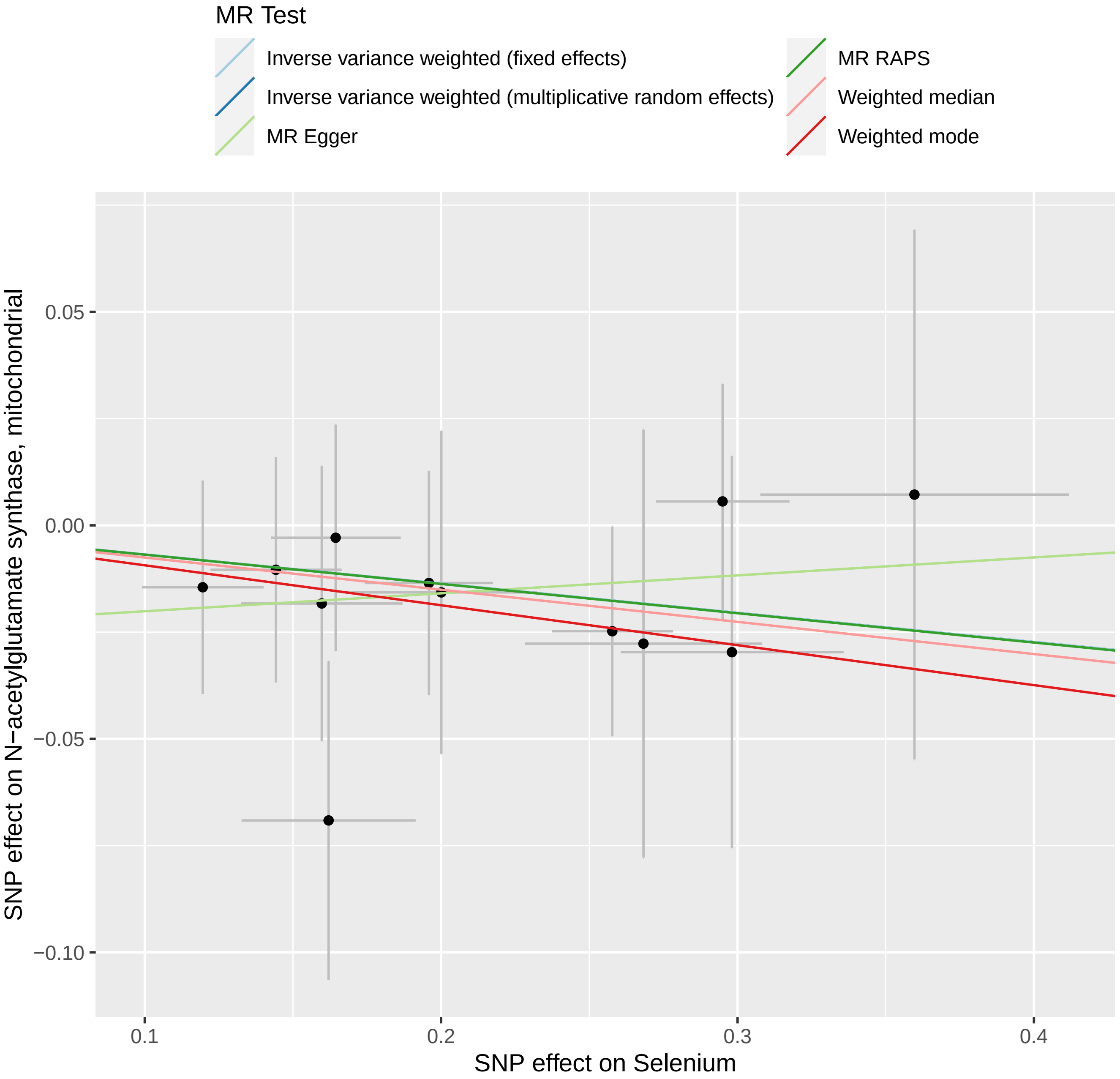

Batch 3−1998 : Selenium on Nicotinamide phosphoribosyltransferase

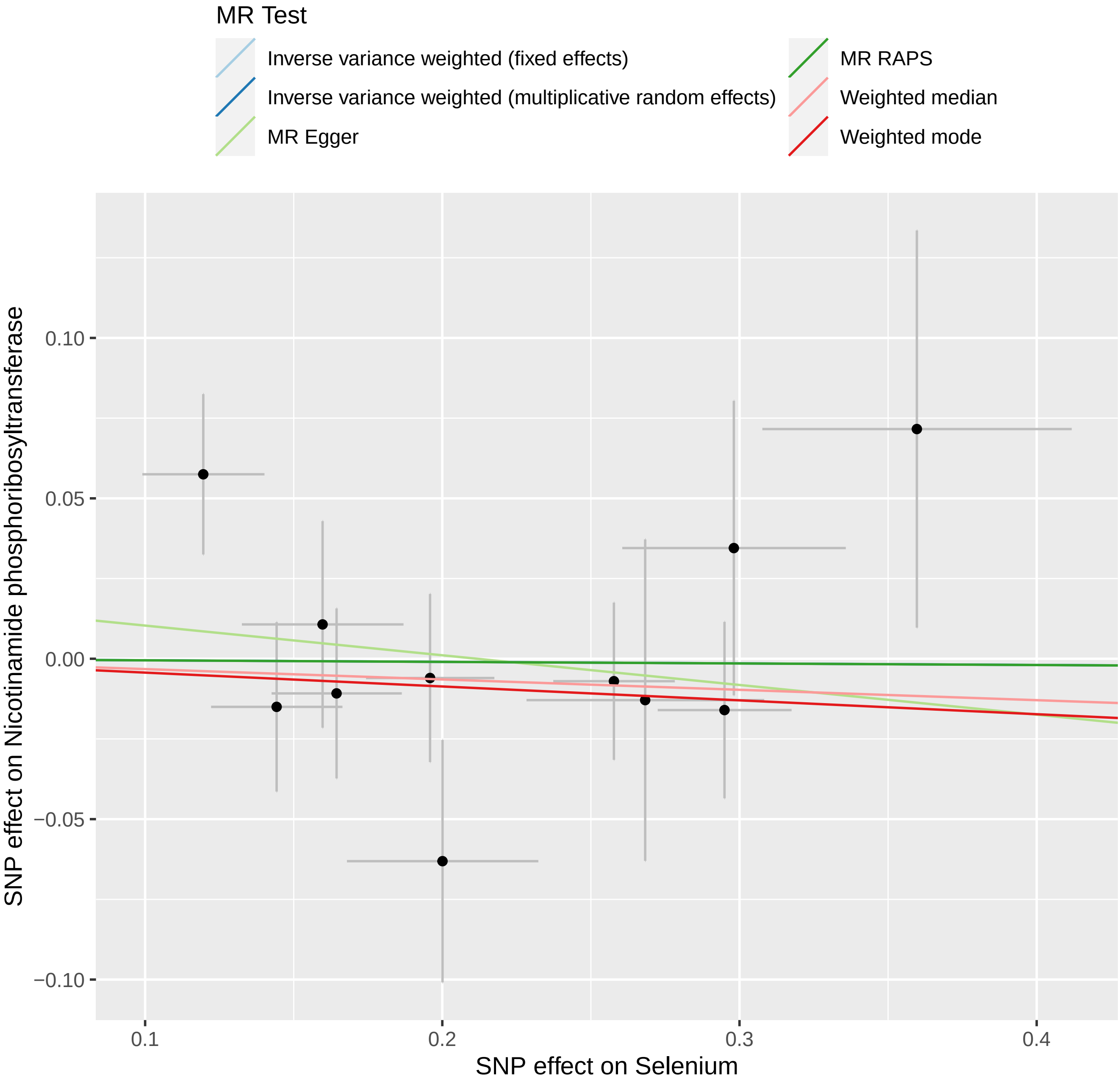

Batch 3−1999 : Selenium on Nucleosome assembly protein 1−like 2

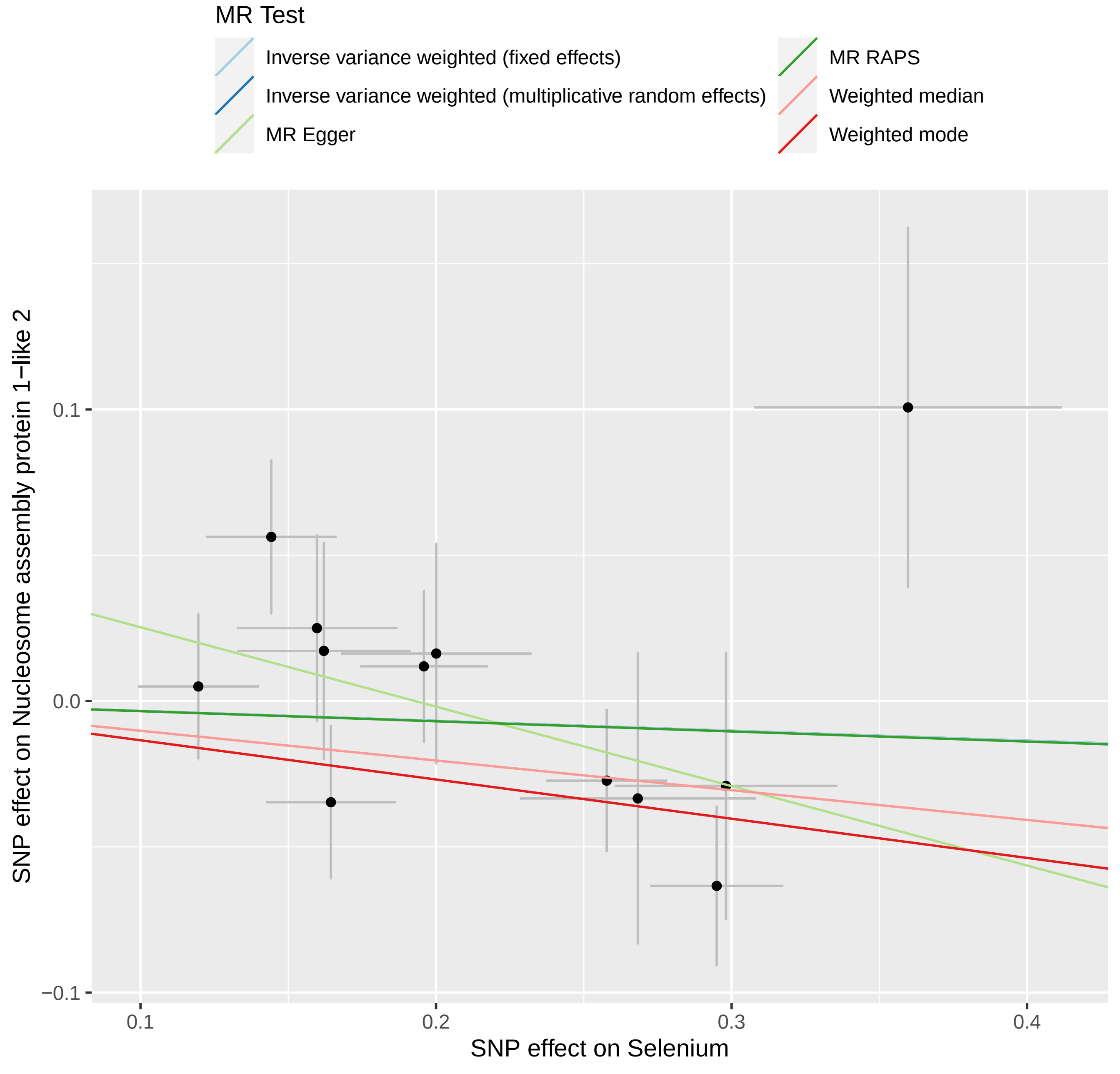

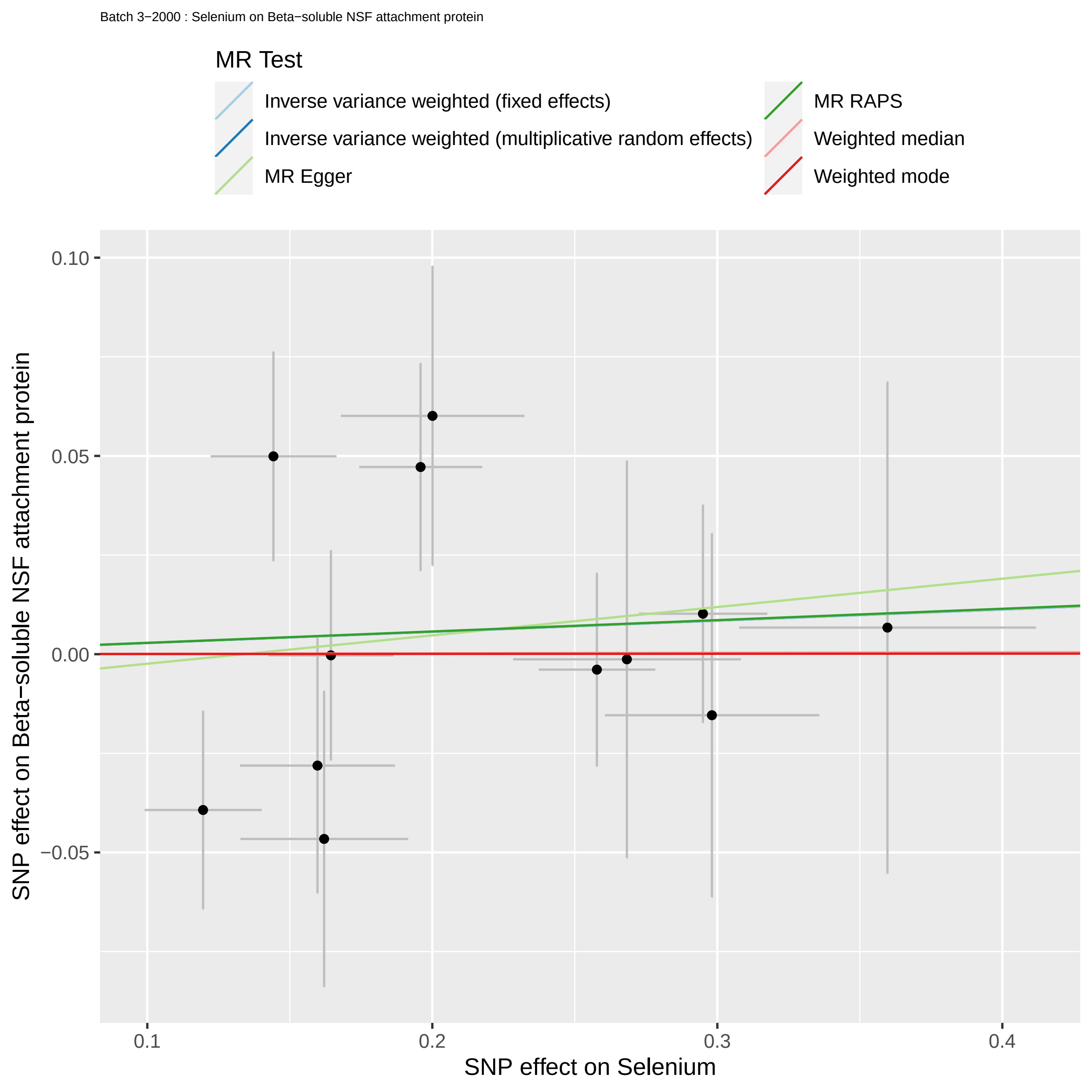

Batch 3−2000 : Selenium on Beta−soluble NSF attachment protein
MR Test
Inverse variance weighted (fixed effects)
Inverse variance weighted (multiplicative random effects)
MR Egger
MR RAPS
Weighted median
Weighted mode
0.10
0.05
0.00
−0.05
0.1
0.2
0.3
0.4
SNP effect on Beta−soluble NSF attachment protein
SNP effect on Selenium

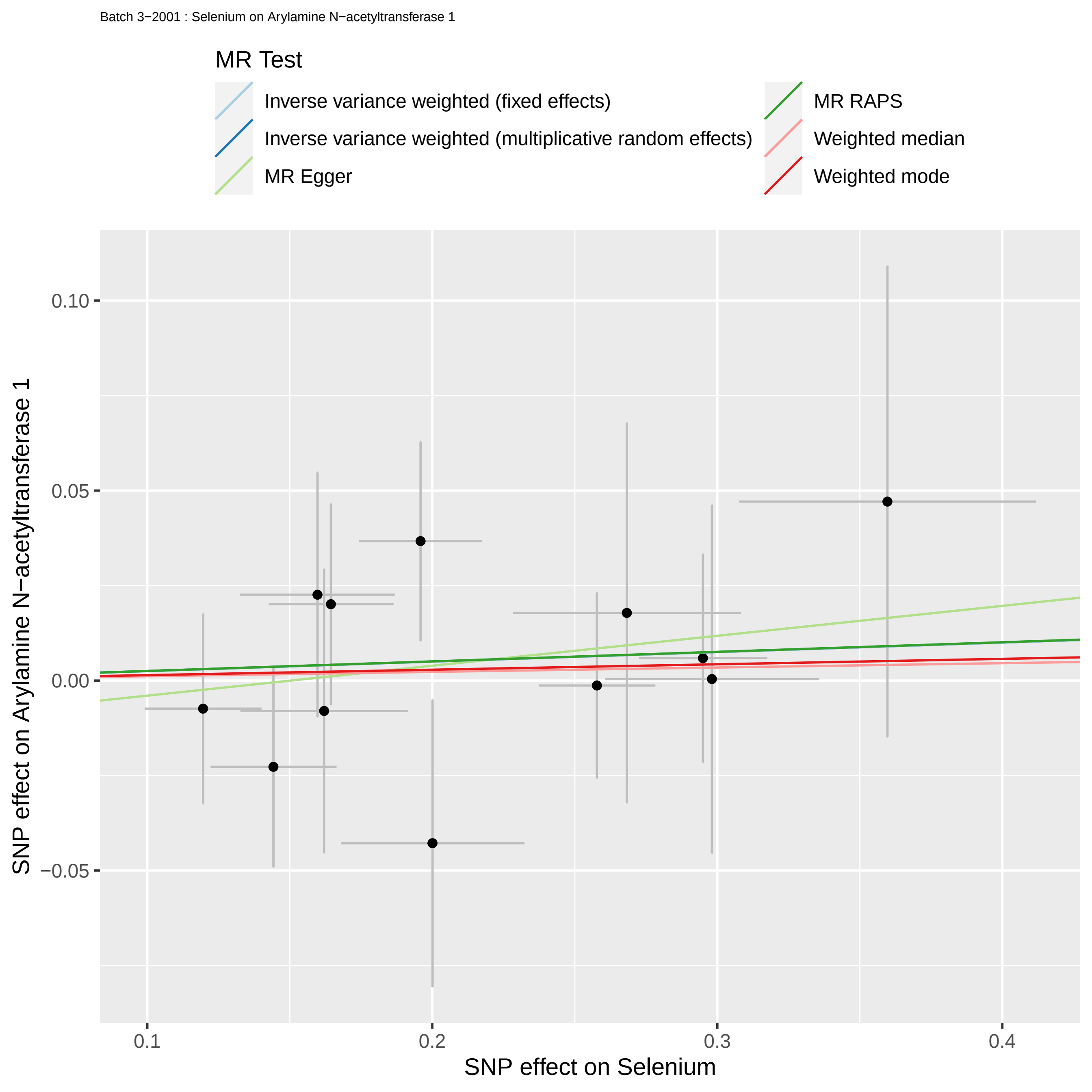

Batch 3−2001 : Selenium on Arylamine N−acetyltransferase 1
MR Test
Inverse variance weighted (fixed effects)
Inverse variance weighted (multiplicative random effects)
MR Egger
MR RAPS
Weighted median
Weighted mode
0.10
0.05
0.00
−0.05
0.1
0.2
0.3
0.4
SNP effect on Selenium
SNP effect on Arylamine N−acetyltransferase 1

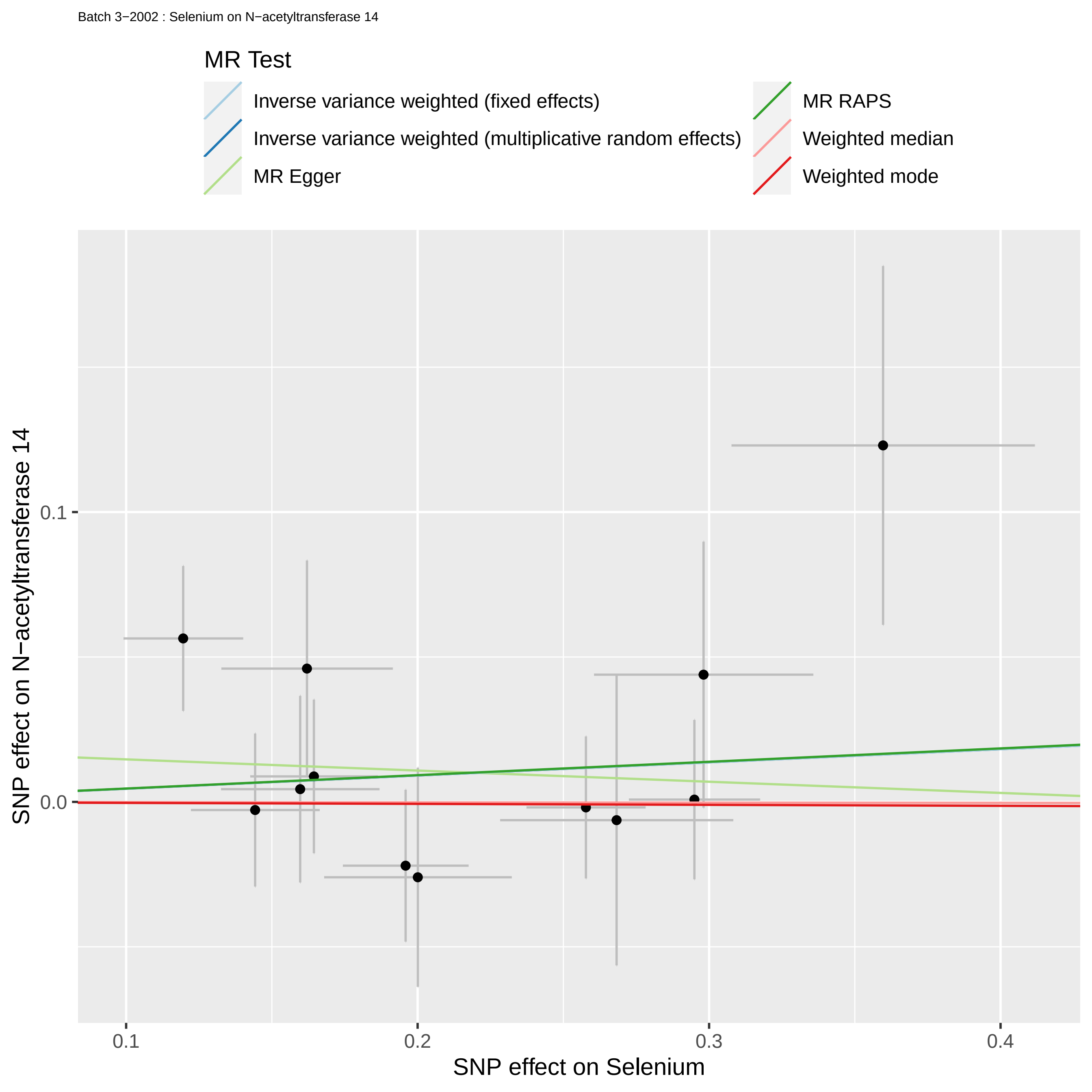

Batch 3−2002 : Selenium on N−acetyltransferase 14
MR Test
Inverse variance weighted (fixed effects)
Inverse variance weighted (multiplicative random effects)
MR Egger
MR RAPS
Weighted median
Weighted mode
SNP effect on N−acetyltransferase 14
0.1
0.0
0.1
0.2
0.3
0.4
SNP effect on Selenium

Batch 3−2003 : Selenium on Neuroblastoma suppressor of tumorigenicity 1

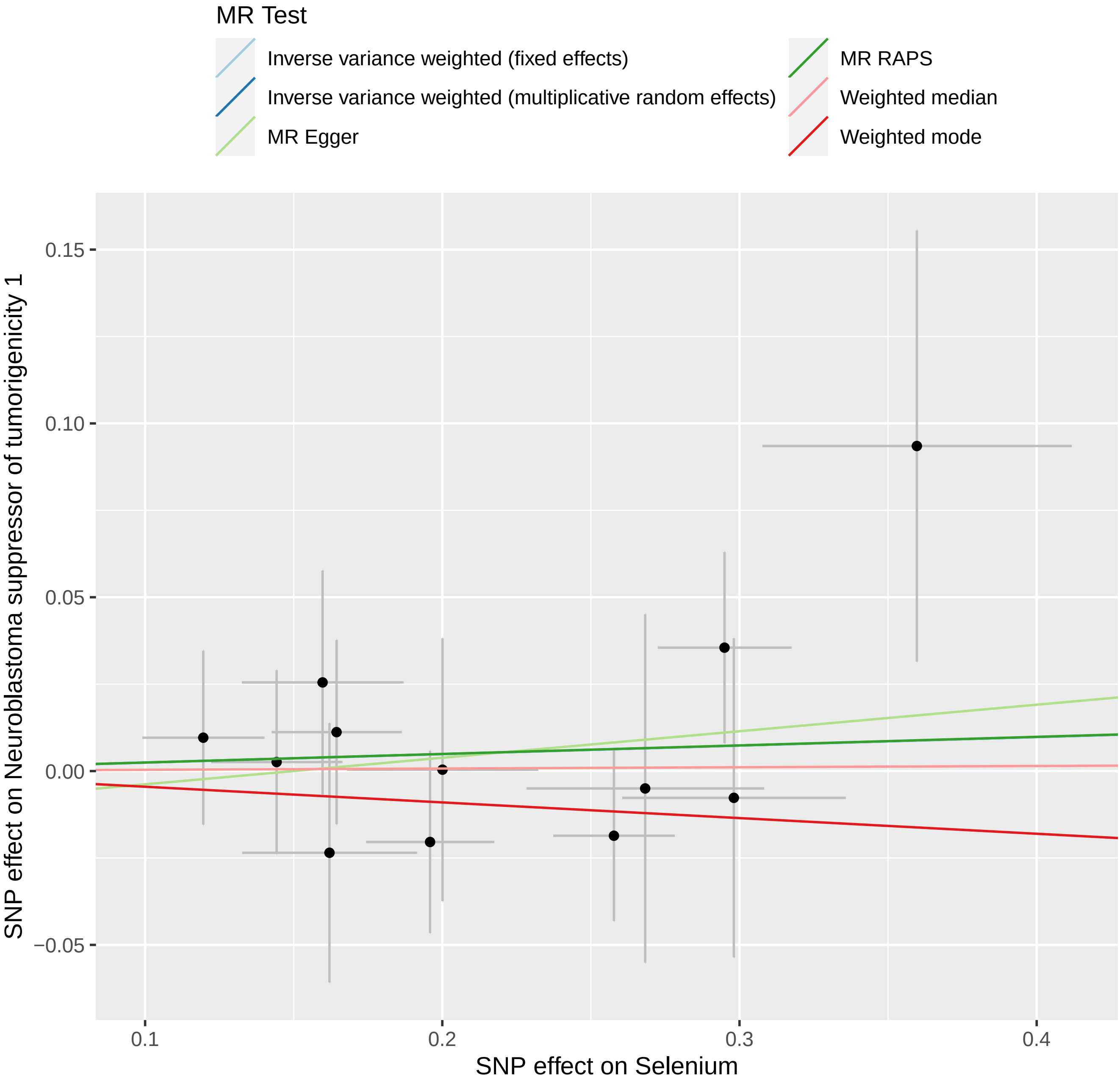

Batch 3−2004 : Selenium on Next to BRCA1 gene 1 protein

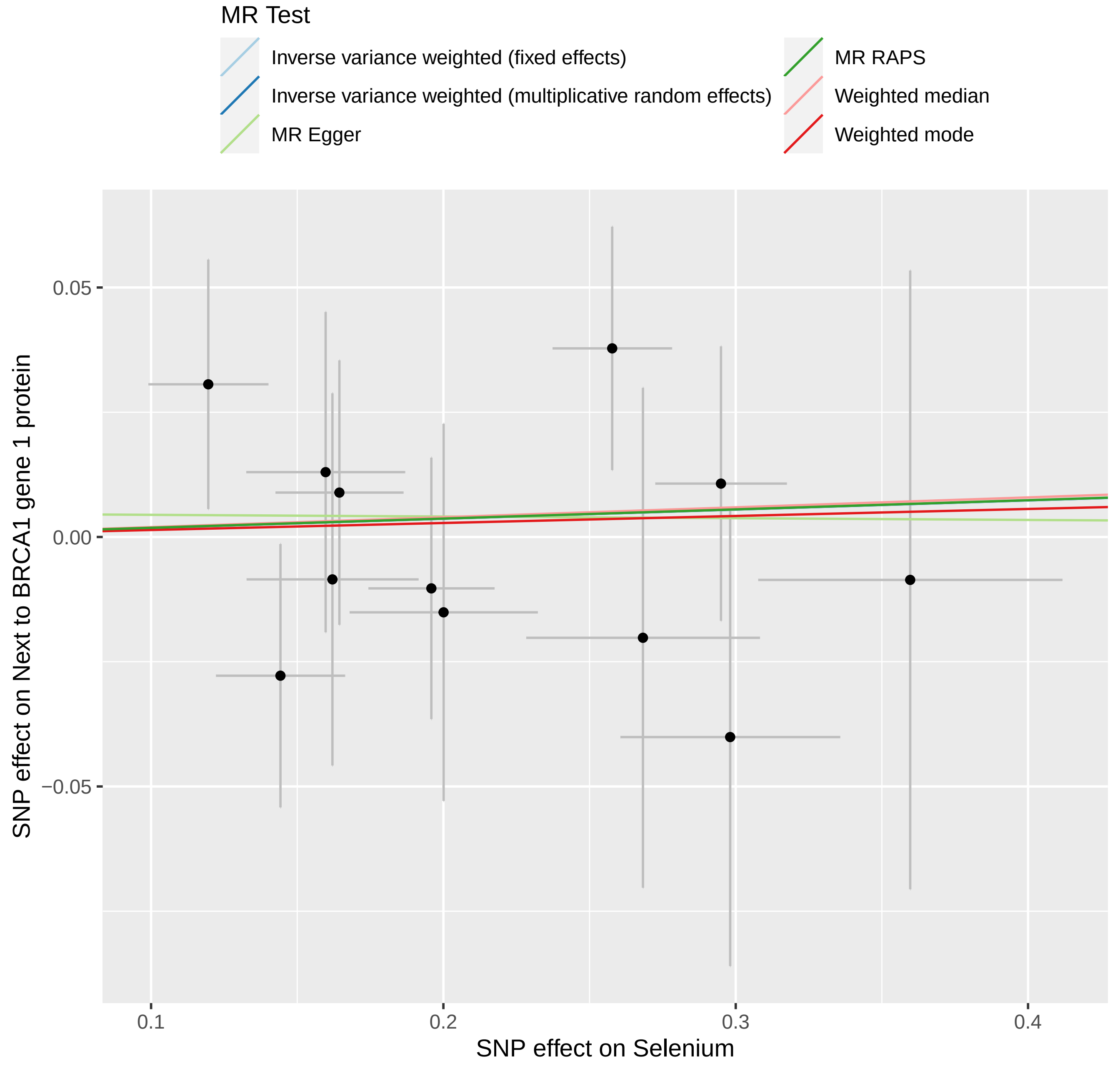

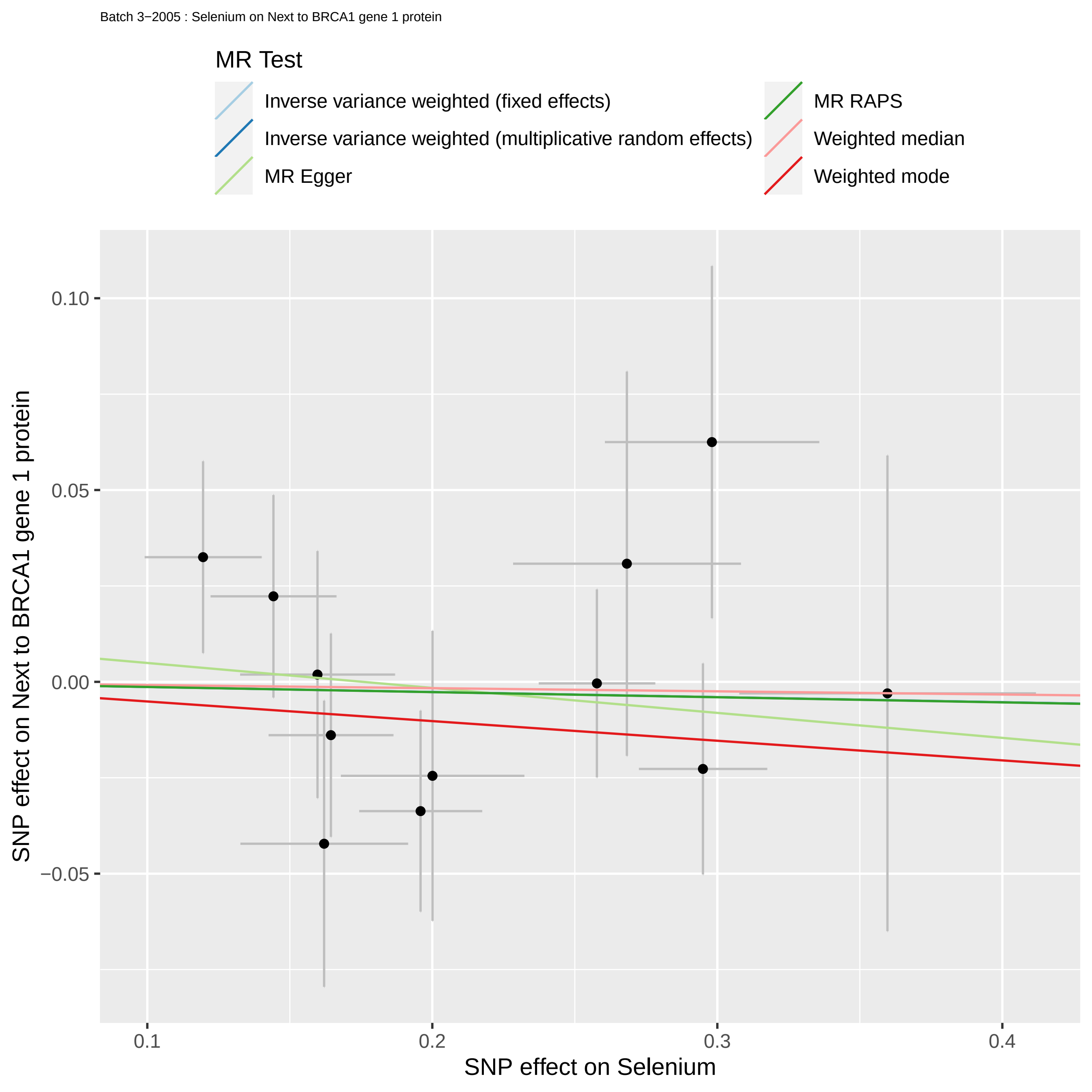

Batch 3−2005 : Selenium on Next to BRCA1 gene 1 protein
MR Test
Inverse variance weighted (fixed effects)
Inverse variance weighted (multiplicative random effects)
MR Egger
MR RAPS
Weighted median
Weighted mode
0.10
0.05
0.00
−0.05
0.1
0.2
0.3
0.4
SNP effect on Next to BRCA1 gene 1 protein
SNP effect on Selenium

Batch 3−2006 : Selenium on Neural cell adhesion molecule 1, 120 kDa isoform

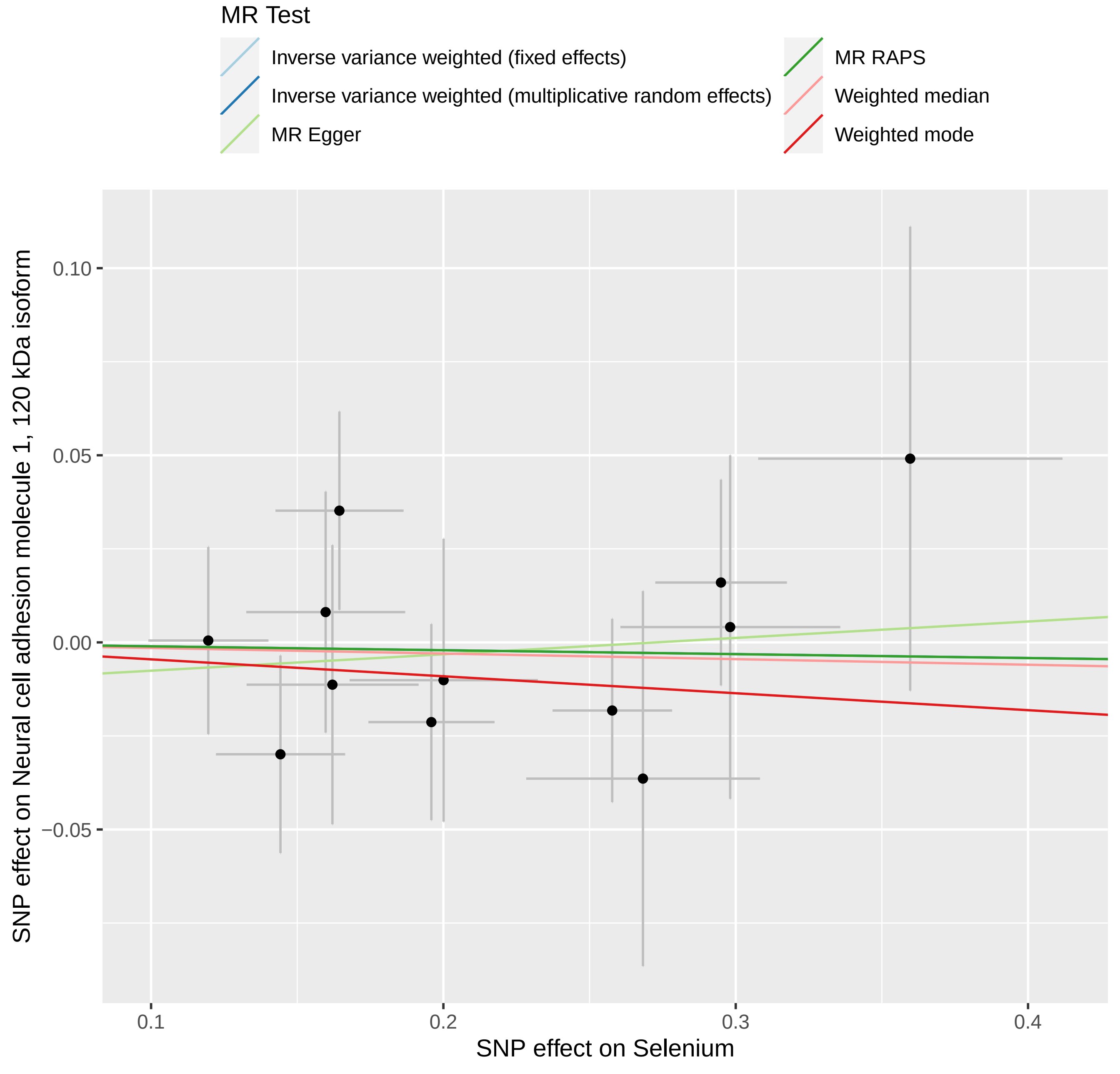

Batch 3−2007 : Selenium on Neural cell adhesion molecule 1

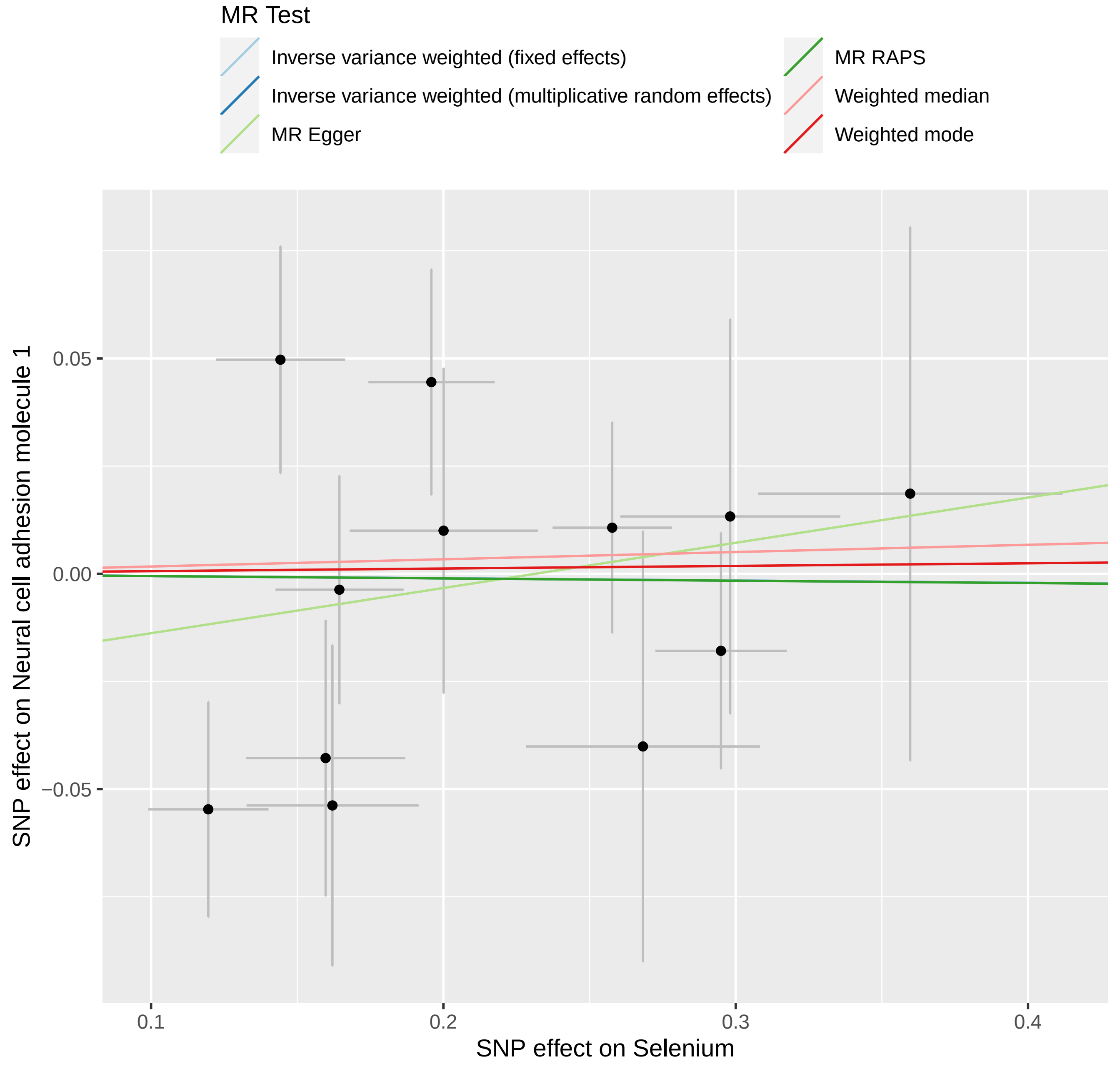

Batch 3−2008 : Selenium on Neural cell adhesion molecule 2

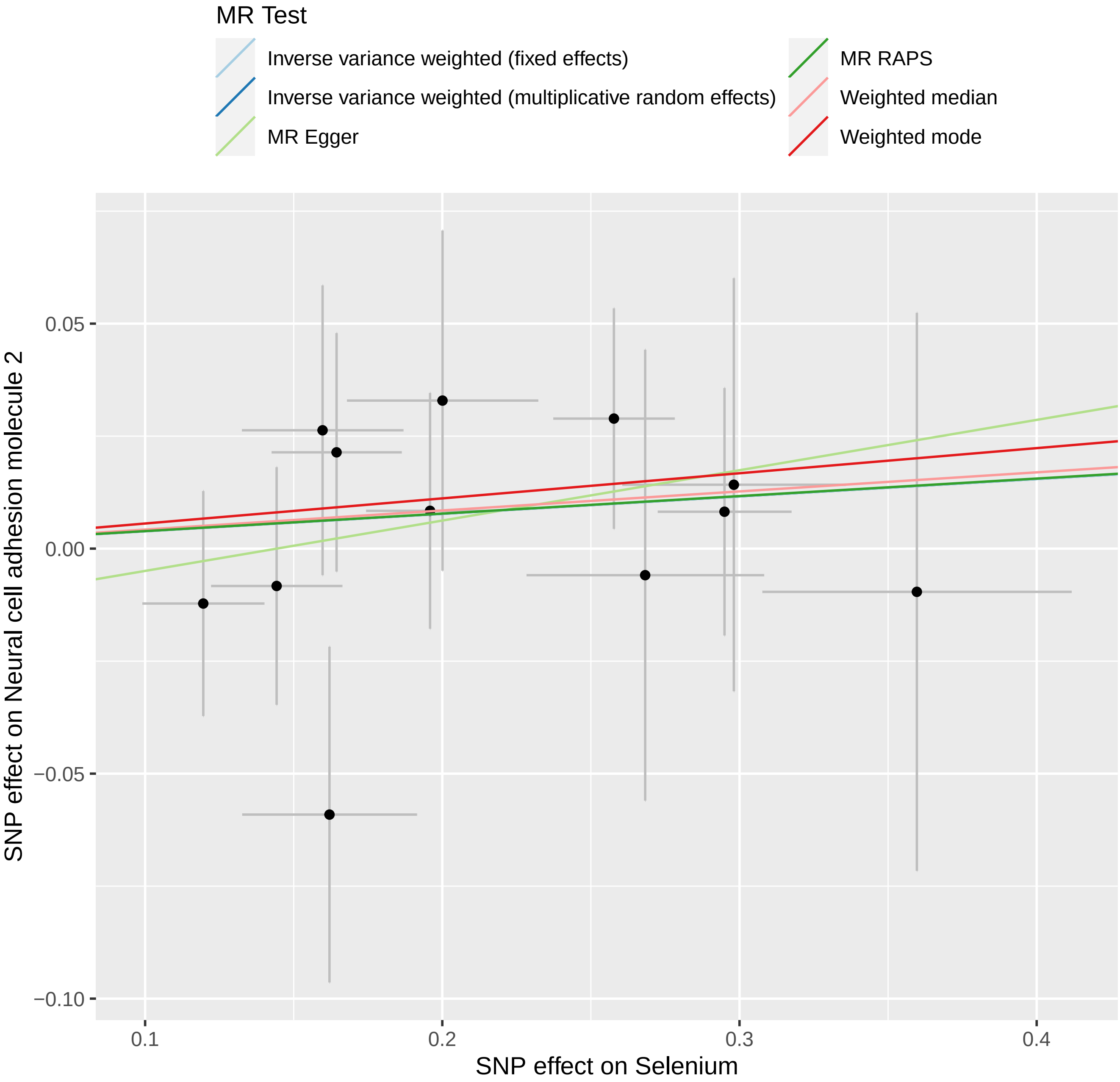

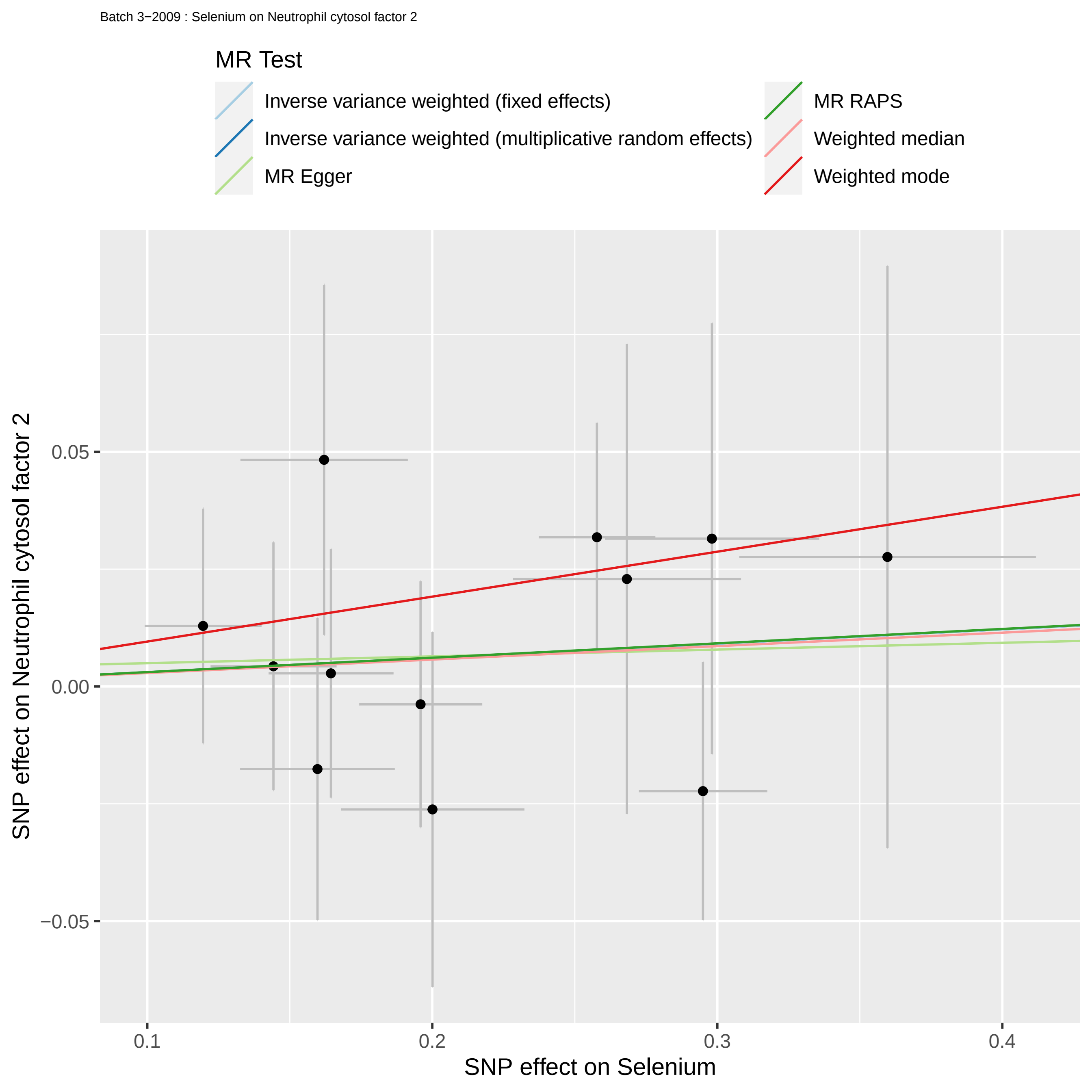

Batch 3−2009 : Selenium on Neutrophil cytosol factor 2
MR Test
Inverse variance weighted (fixed effects)
Inverse variance weighted (multiplicative random effects)
MR Egger
MR RAPS
Weighted median
Weighted mode
0.05
0.00
−0.05
0.1
0.2
0.3
0.4
SNP effect on Neutrophil cytosol factor 2
SNP effect on Selenium

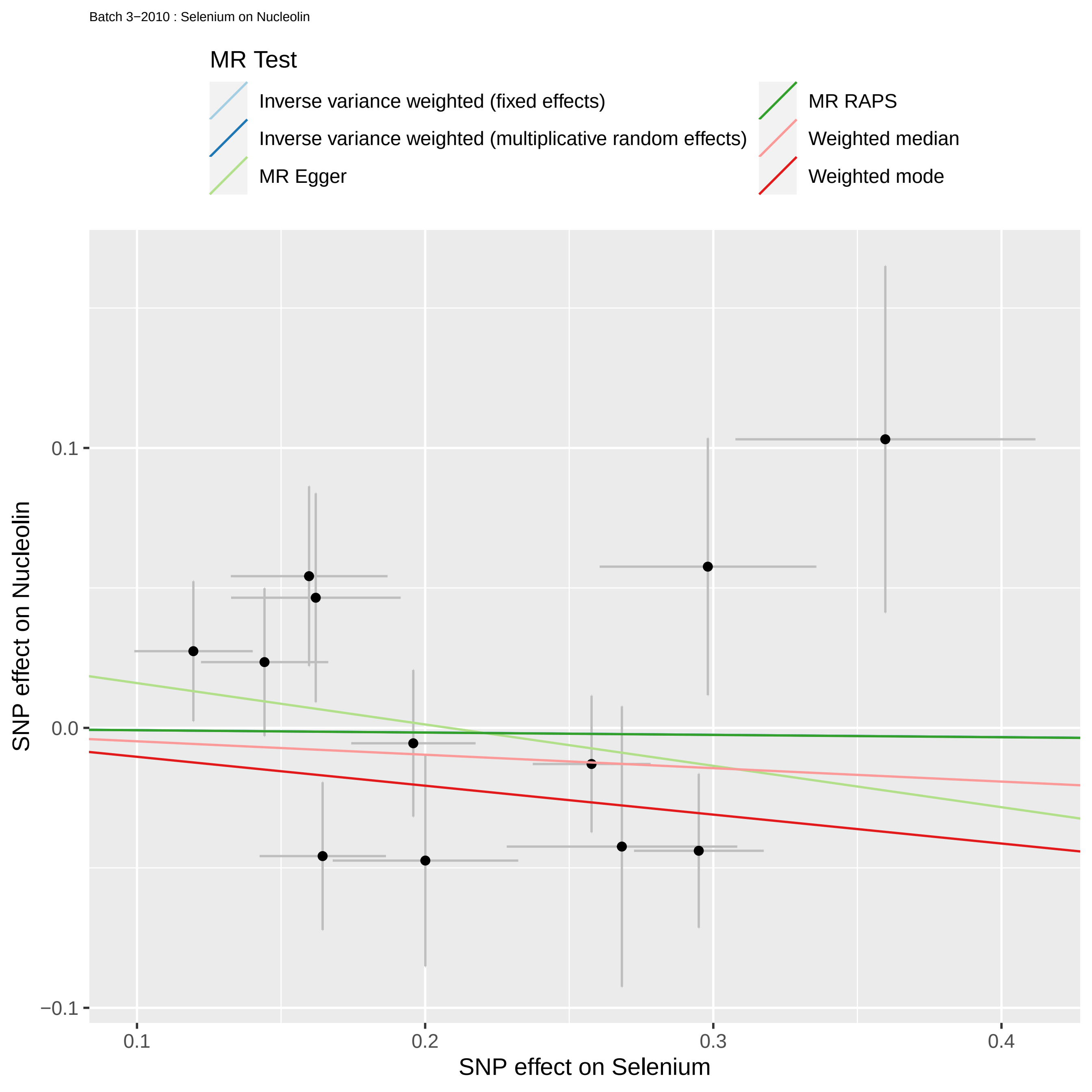

Batch 3−2010 : Selenium on Nucleolin
MR Test
Inverse variance weighted (fixed effects)
Inverse variance weighted (multiplicative random effects)
MR Egger
MR RAPS
Weighted median
Weighted mode
0.1
0.0
−0.1
SNP effect on Nucleolin
0.1
0.2
0.3
0.4
SNP effect on Selenium

Batch 3−2011 : Selenium on Noncompact myelin−associated protein

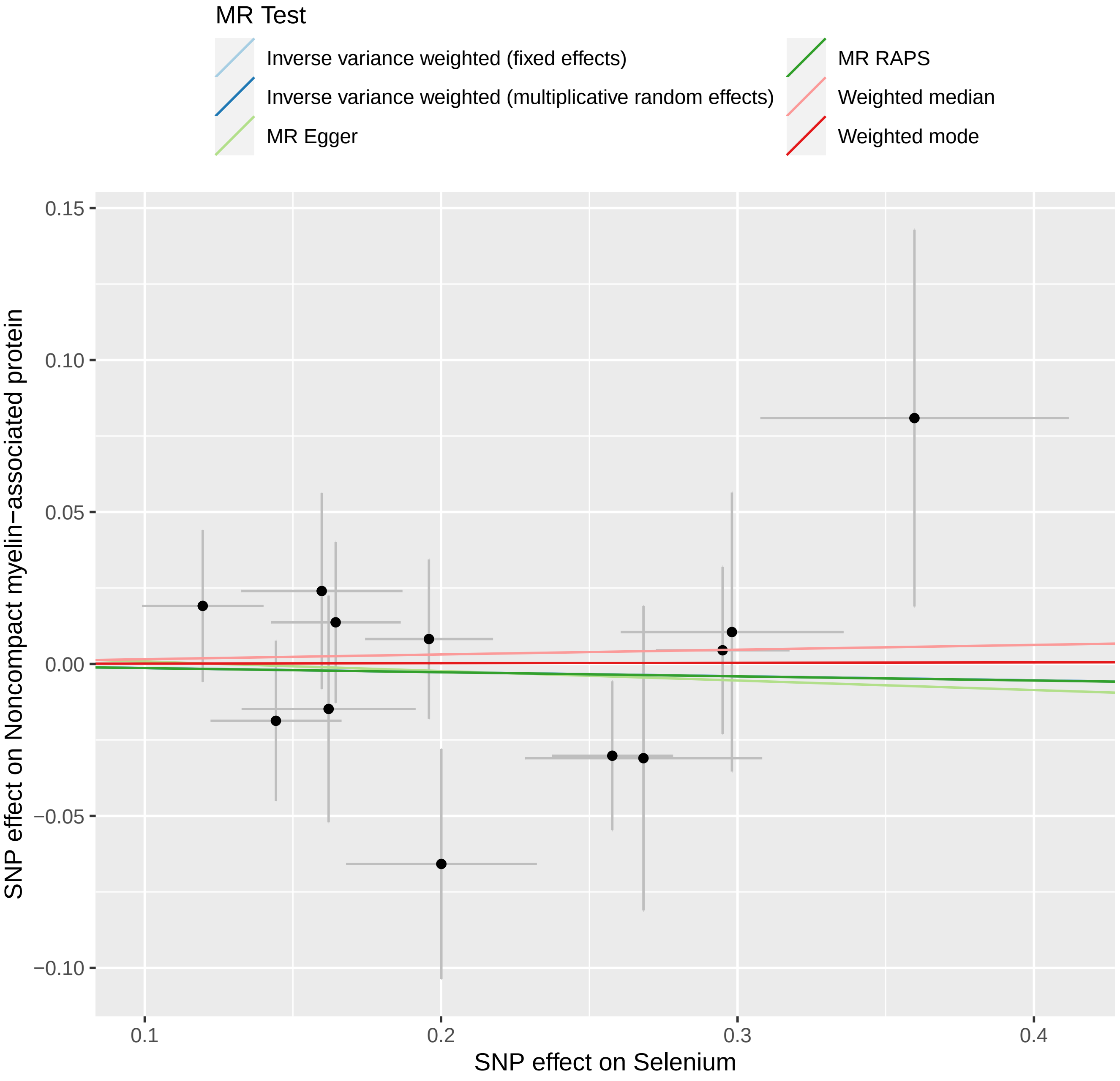

Batch 3−2012 : Selenium on Nuclear receptor coactivator 2

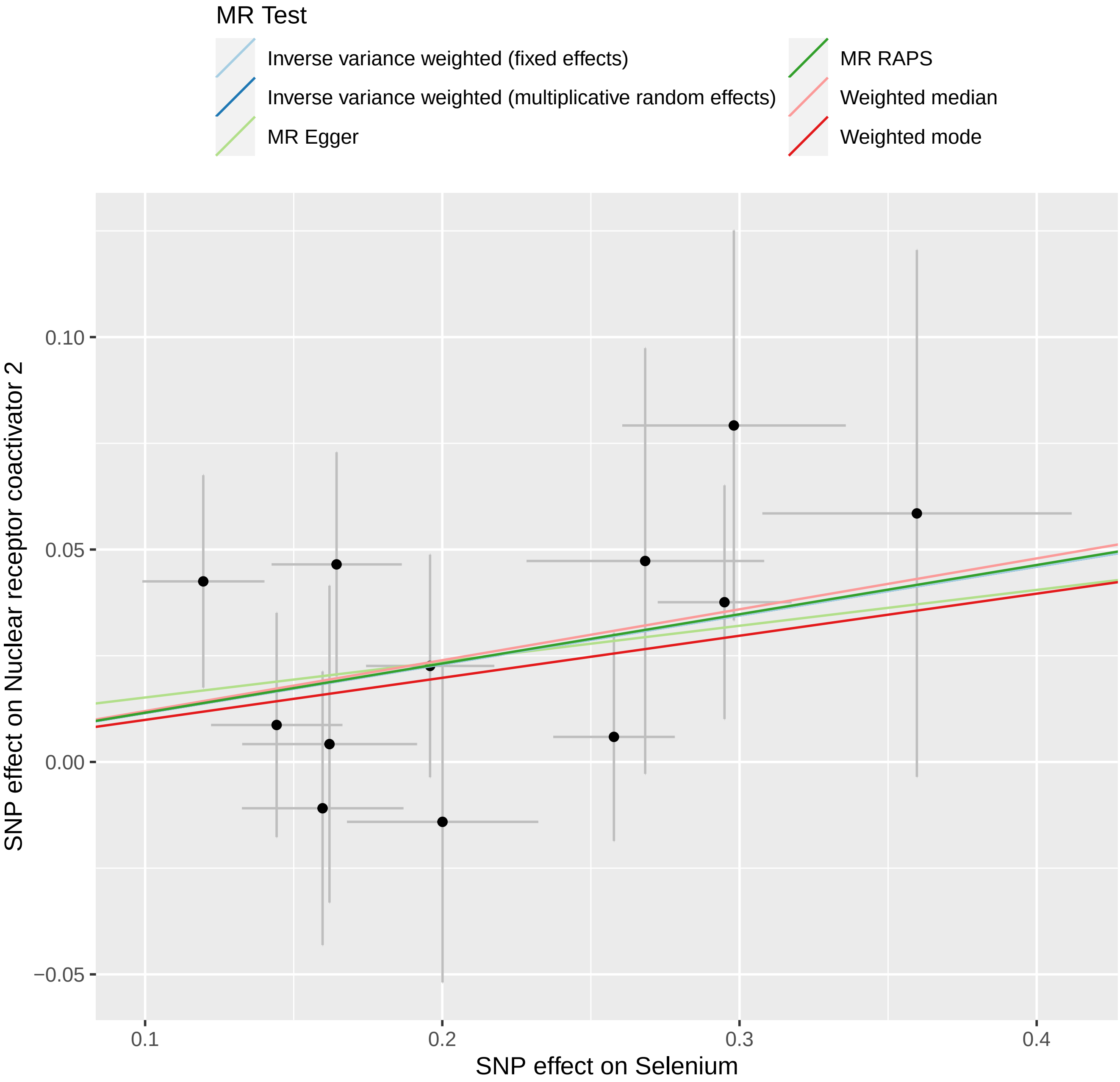

Batch 3−2013 : Selenium on Natural cytotoxicity triggering receptor 1

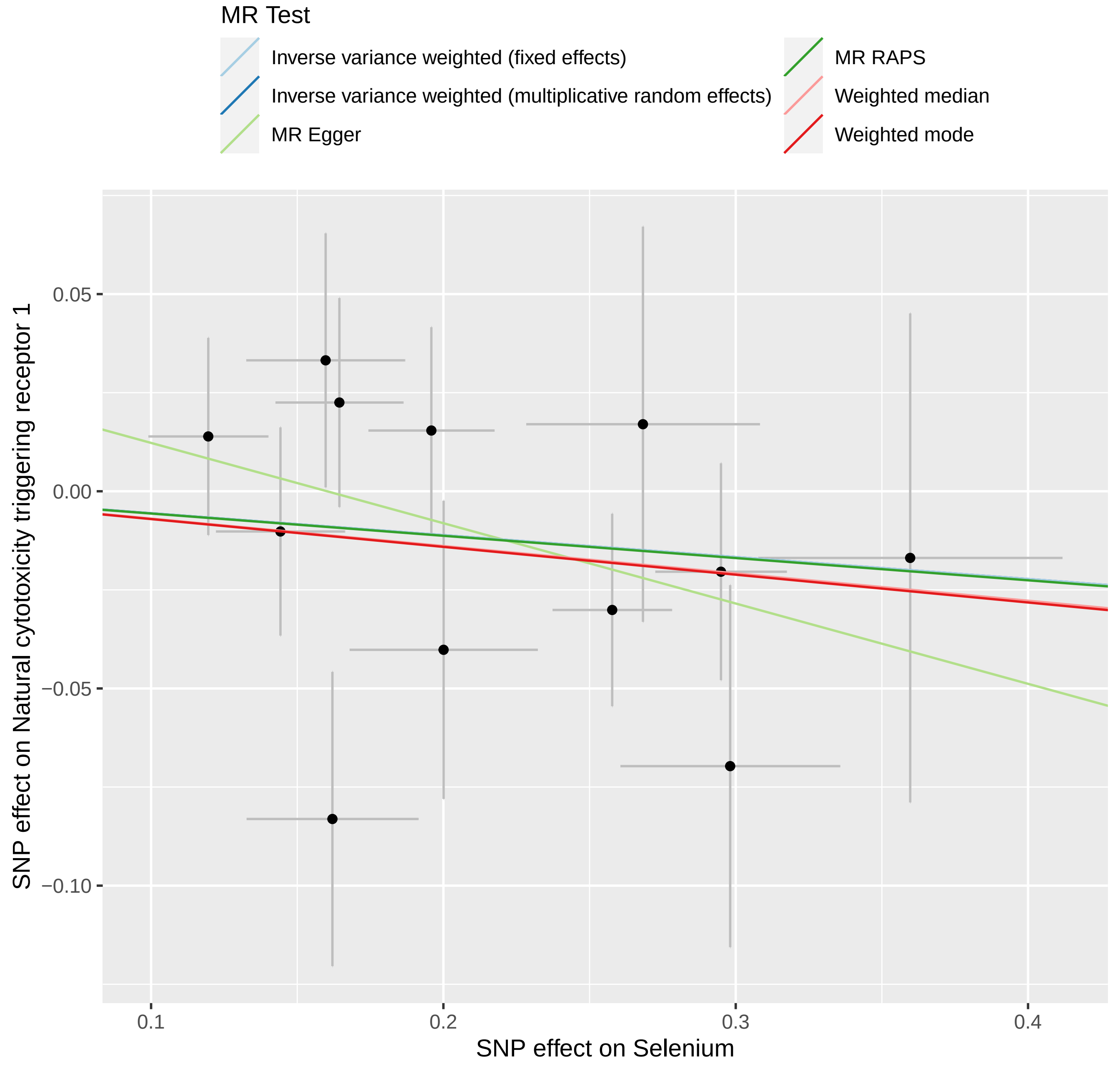

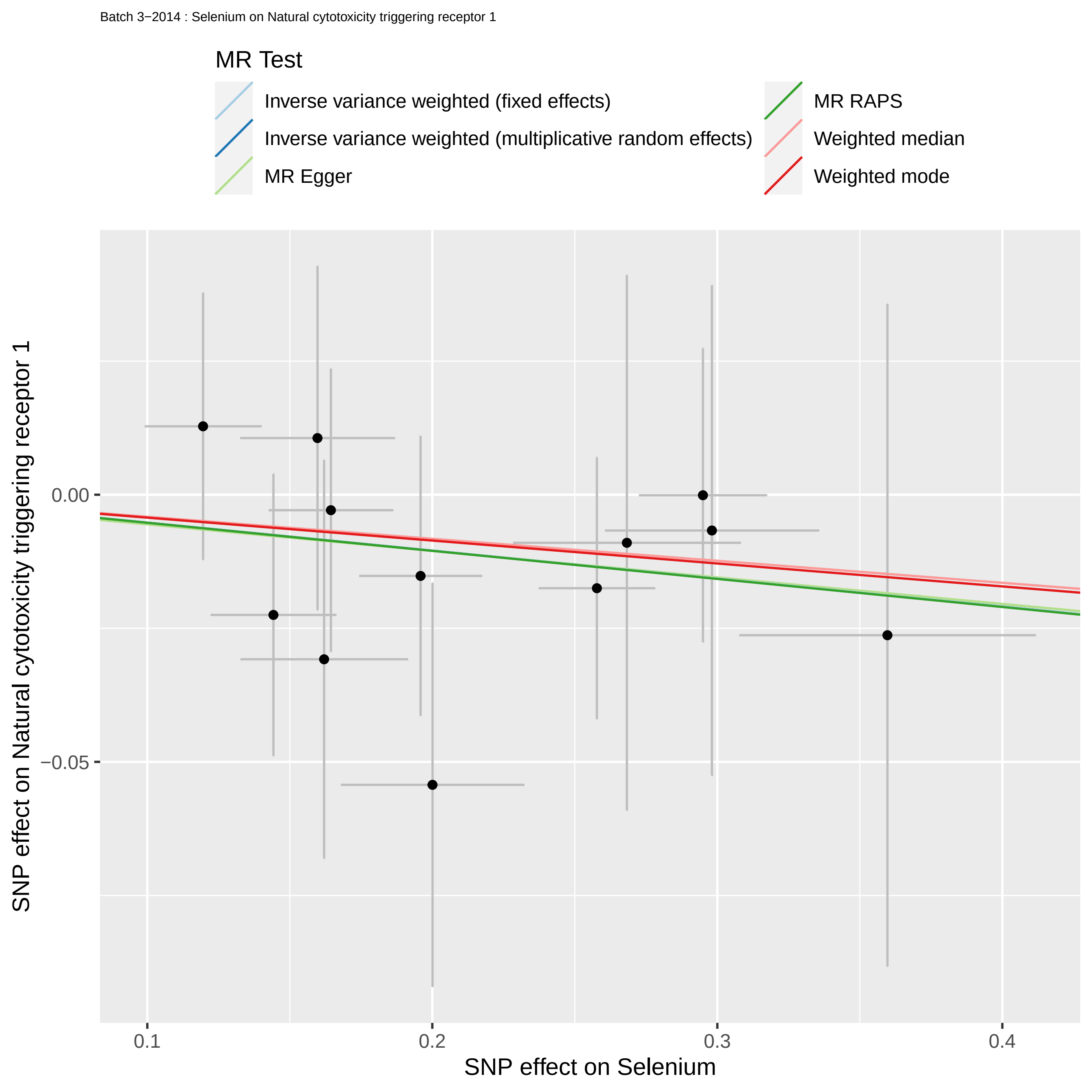
Batch 3−2014 : Selenium on Natural cytotoxicity triggering receptor 1
MR Test
Inverse variance weighted (fixed effects)
Inverse variance weighted (multiplicative random effects)
MR Egger
MR RAPS
Weighted median
Weighted mode
0.00
−0.05
0.1
0.2
0.3
0.4
SNP effect on Selenium
SNP effect on Natural cytotoxicity triggering receptor 1

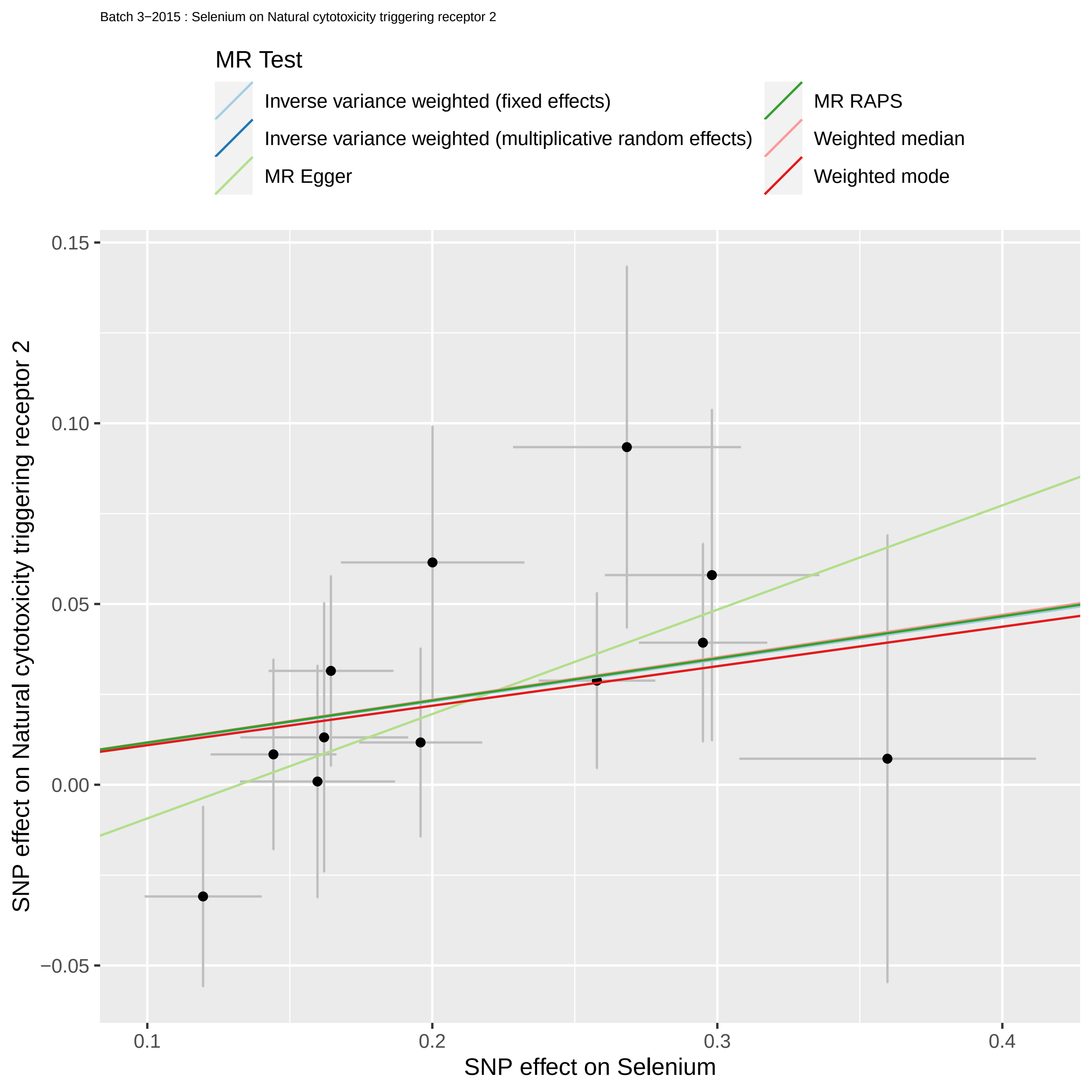

Batch 3−2015 : Selenium on Natural cytotoxicity triggering receptor 2
MR Test
Inverse variance weighted (fixed effects)
Inverse variance weighted (multiplicative random effects)
MR Egger
MR RAPS
Weighted median
Weighted mode
SNP effect on Natural cytotoxicity triggering receptor 2
SNP effect on Selenium
0.15
0.10
0.05
0.00
−0.05
0.1
0.2
0.3
0.4

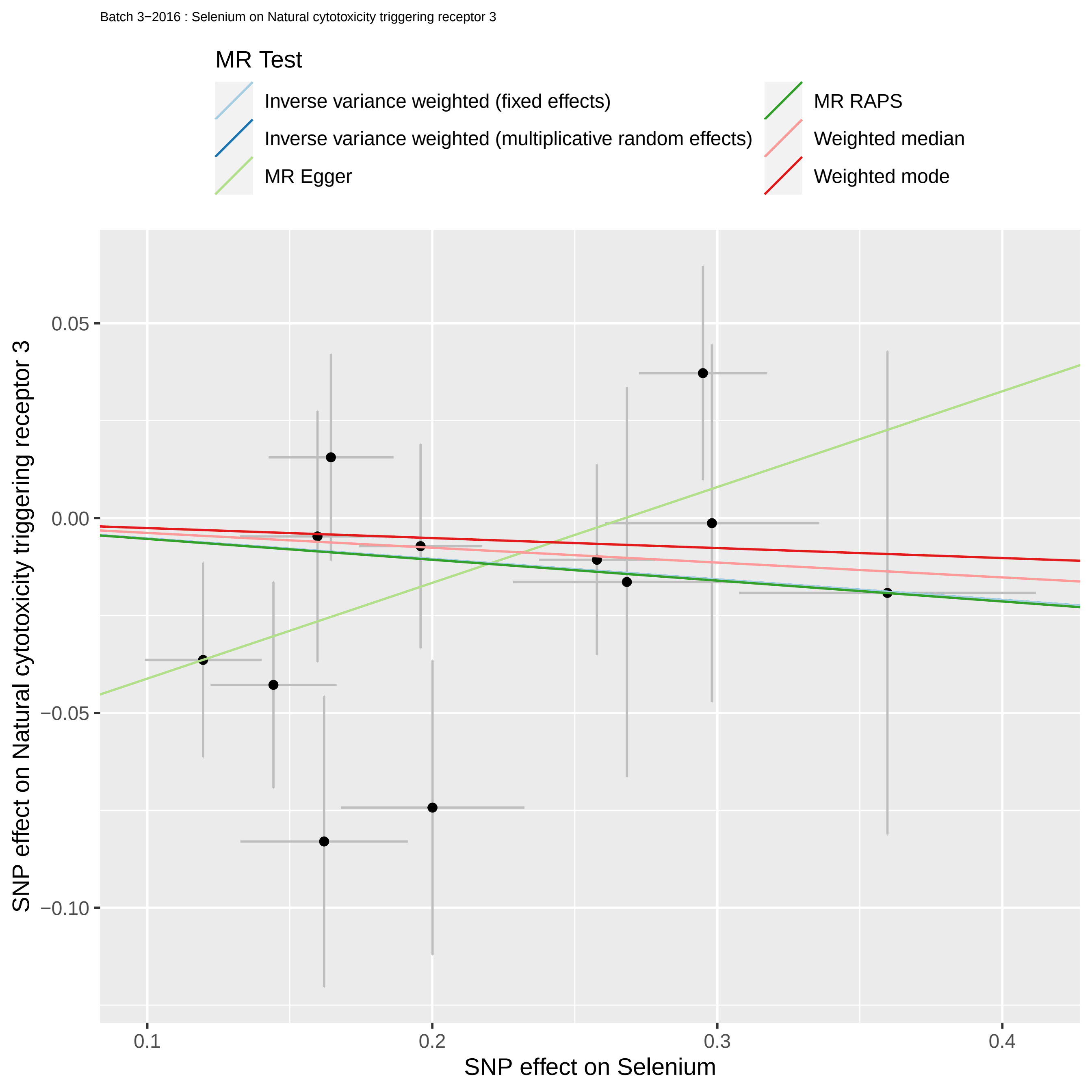

Batch 3−2016 : Selenium on Natural cytotoxicity triggering receptor 3
MR Test
Inverse variance weighted (fixed effects)
Inverse variance weighted (multiplicative random effects)
MR Egger
MR RAPS
Weighted median
Weighted mode
0.05
0.00
−0.05
−0.10
0.1
0.2
0.3
0.4
SNP effect on Natural cytotoxicity triggering receptor 3
SNP effect on Selenium

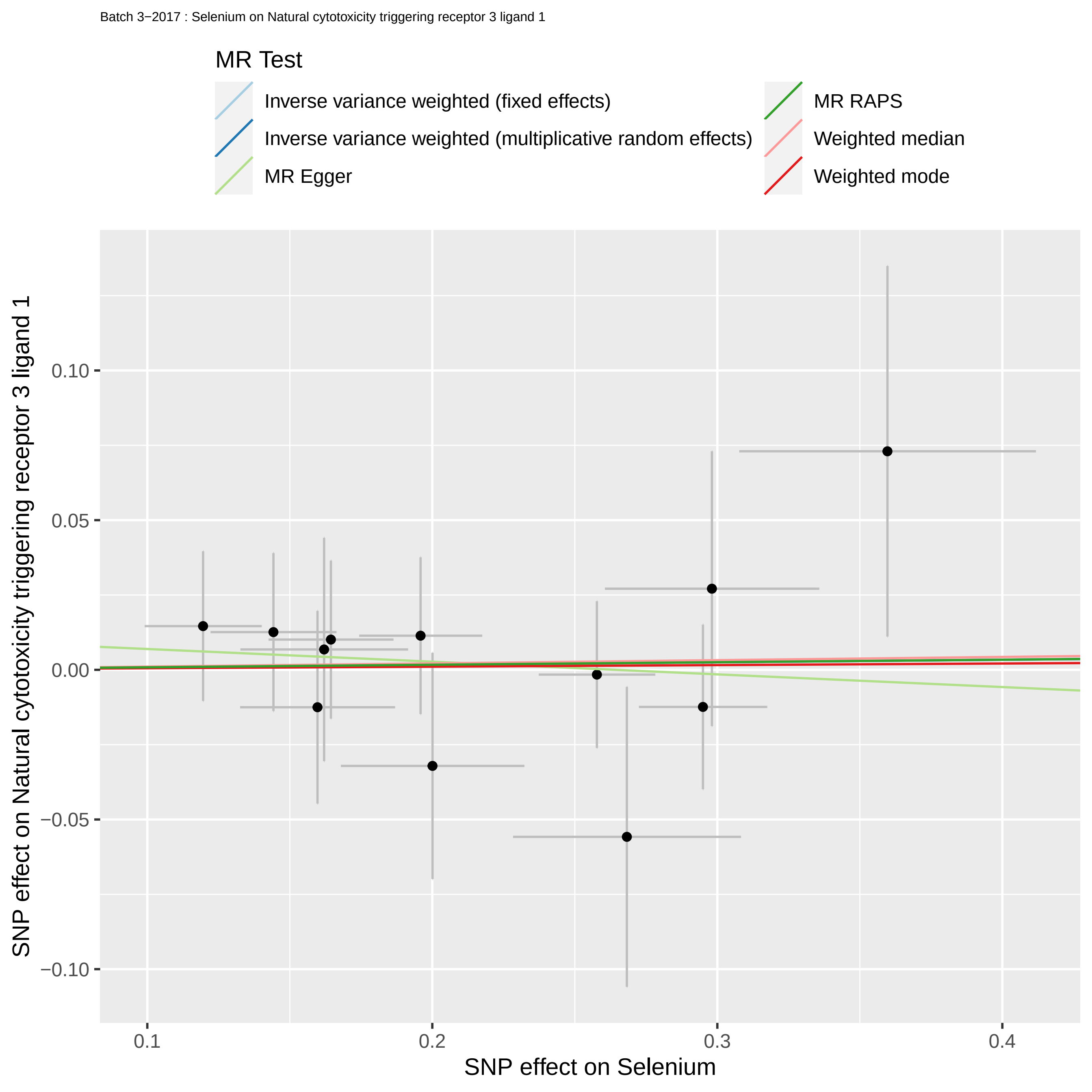

Batch 3−2017 : Selenium on Natural cytotoxicity triggering receptor 3 ligand 1
MR Test
Inverse variance weighted (fixed effects)
Inverse variance weighted (multiplicative random effects)
MR Egger
MR RAPS
Weighted median
Weighted mode
SNP effect on Natural cytotoxicity triggering receptor 3 ligand 1
0.10
0.05
0.00
−0.05
−0.10
0.1
0.2
0.3
0.4
SNP effect on Selenium

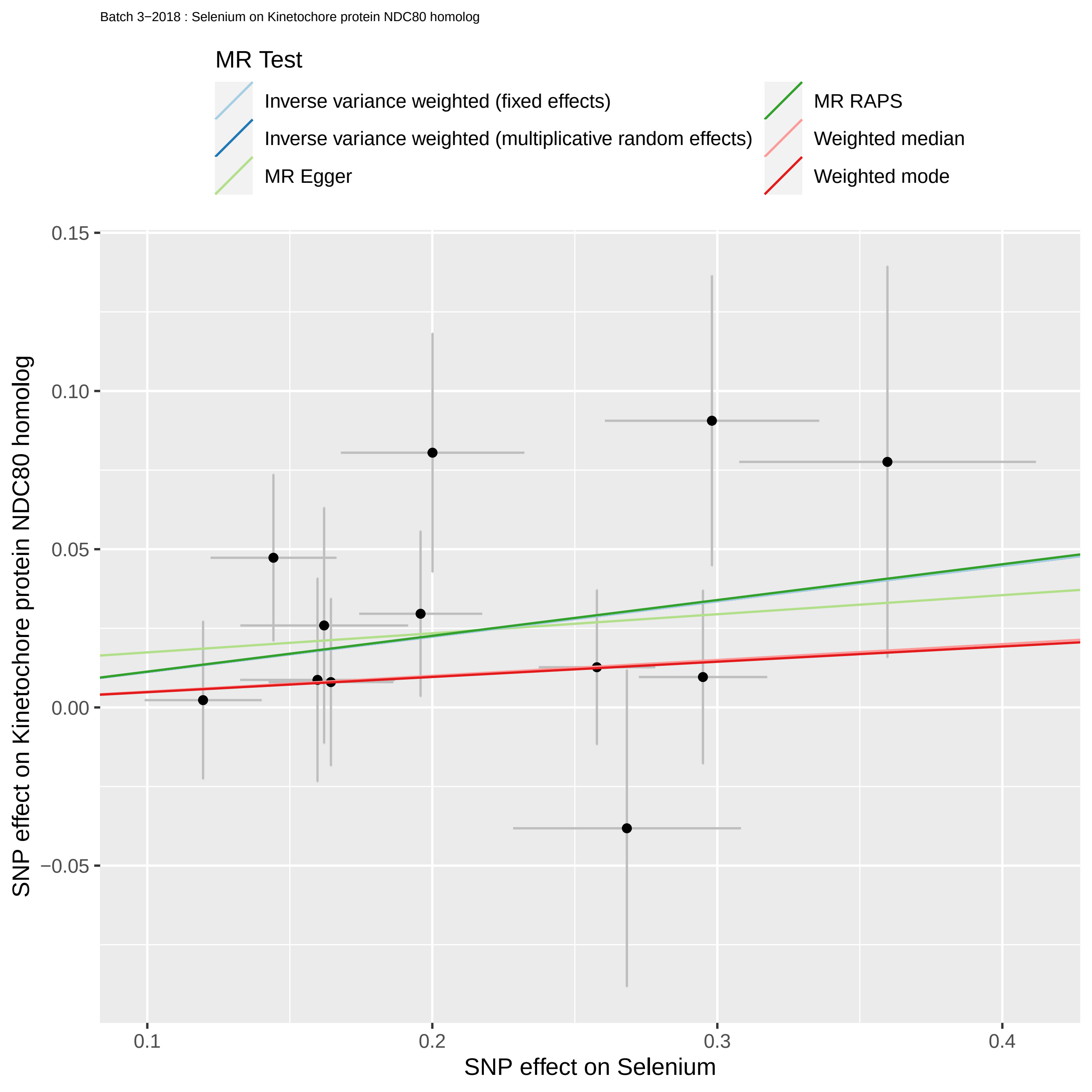
Batch 3−2018 : Selenium on Kinetochore protein NDC80 homolog
MR Test
Inverse variance weighted (fixed effects)
Inverse variance weighted (multiplicative random effects)
MR Egger
MR RAPS
Weighted median
Weighted mode
0.15
0.10
0.05
0.00
−0.05
0.1
0.2
0.3
0.4
SNP effect on Kinetochore protein NDC80 homolog
SNP effect on Selenium

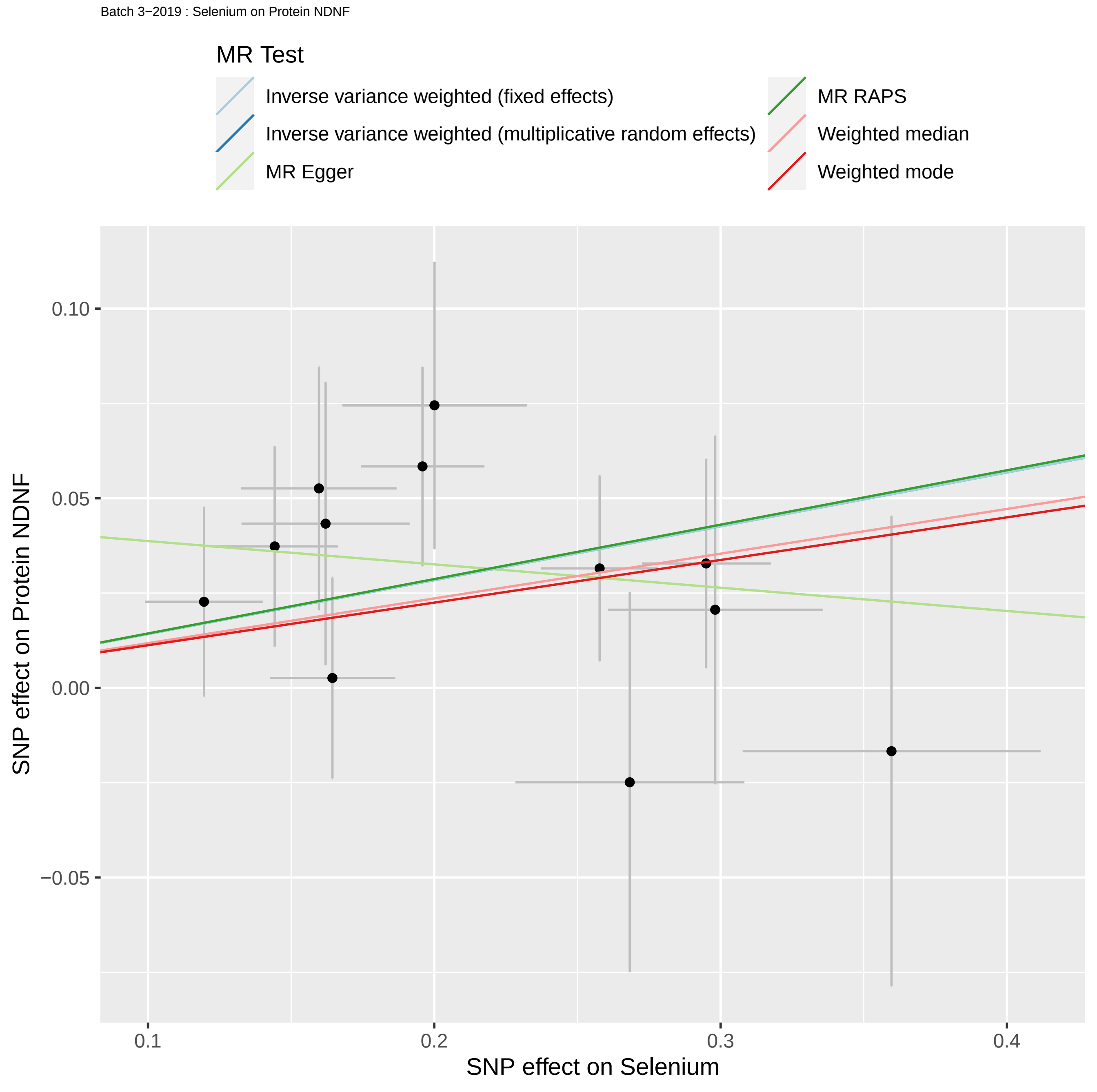
Batch 3−2019 : Selenium on Protein NDNF
MR Test
Inverse variance weighted (fixed effects)
Inverse variance weighted (multiplicative random effects)
MR Egger
MR RAPS
Weighted median
Weighted mode
0.10
0.05
0.00
−0.05
0.1
0.2
0.3
0.4
SNP effect on Protein NDNF
SNP effect on Selenium

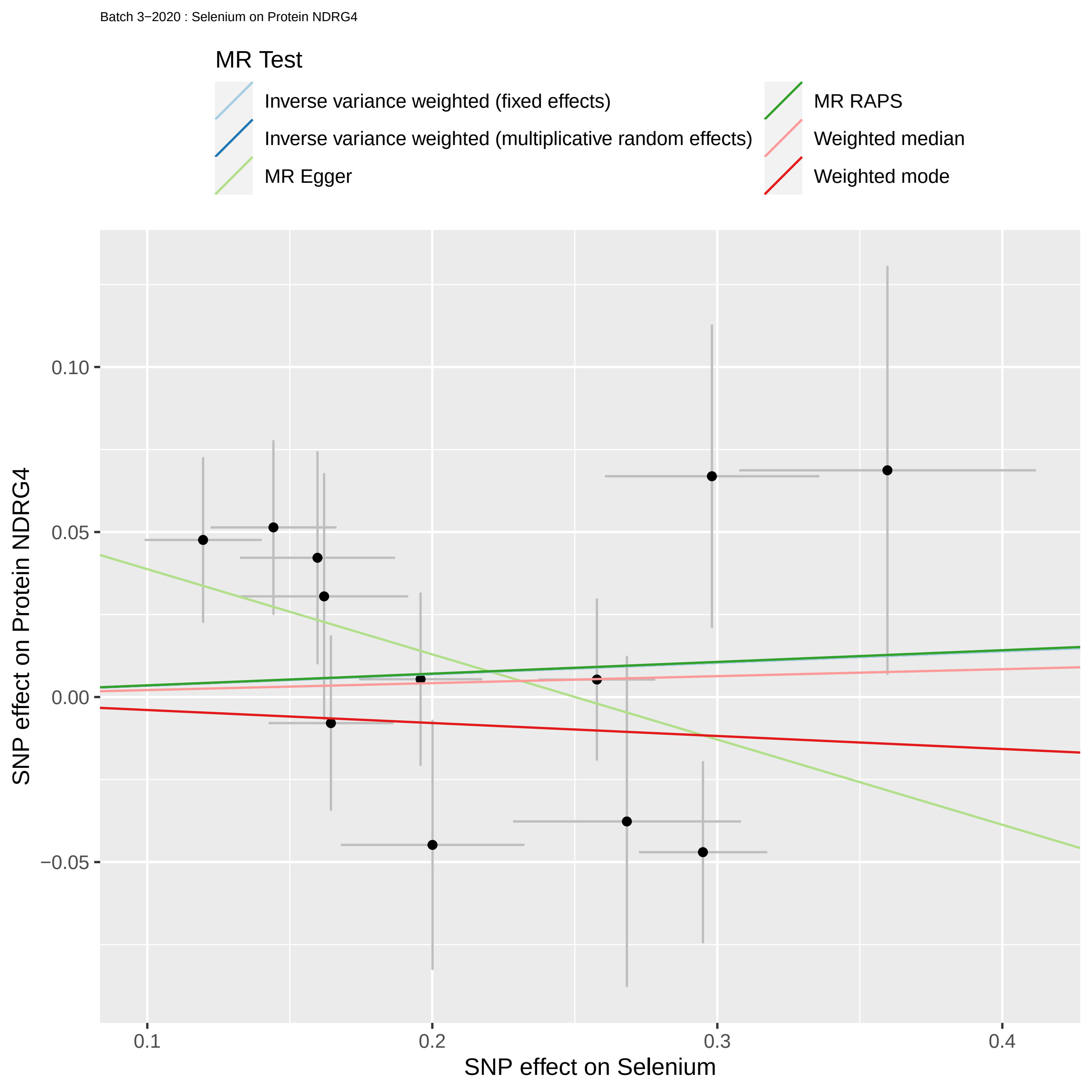
Batch 3−2020 : Selenium on Protein NDRG4
MR Test
Inverse variance weighted (fixed effects)
Inverse variance weighted (multiplicative random effects)
MR Egger
MR RAPS
Weighted median
Weighted mode
0.10
0.05
0.00
−0.05
0.1
0.2
0.3
0.4
SNP effect on Protein NDRG4
SNP effect on Selenium

Batch 3−2021 : Selenium on Bifunctional heparan sulfate N−deacetylase/N−sulfotransferase 1

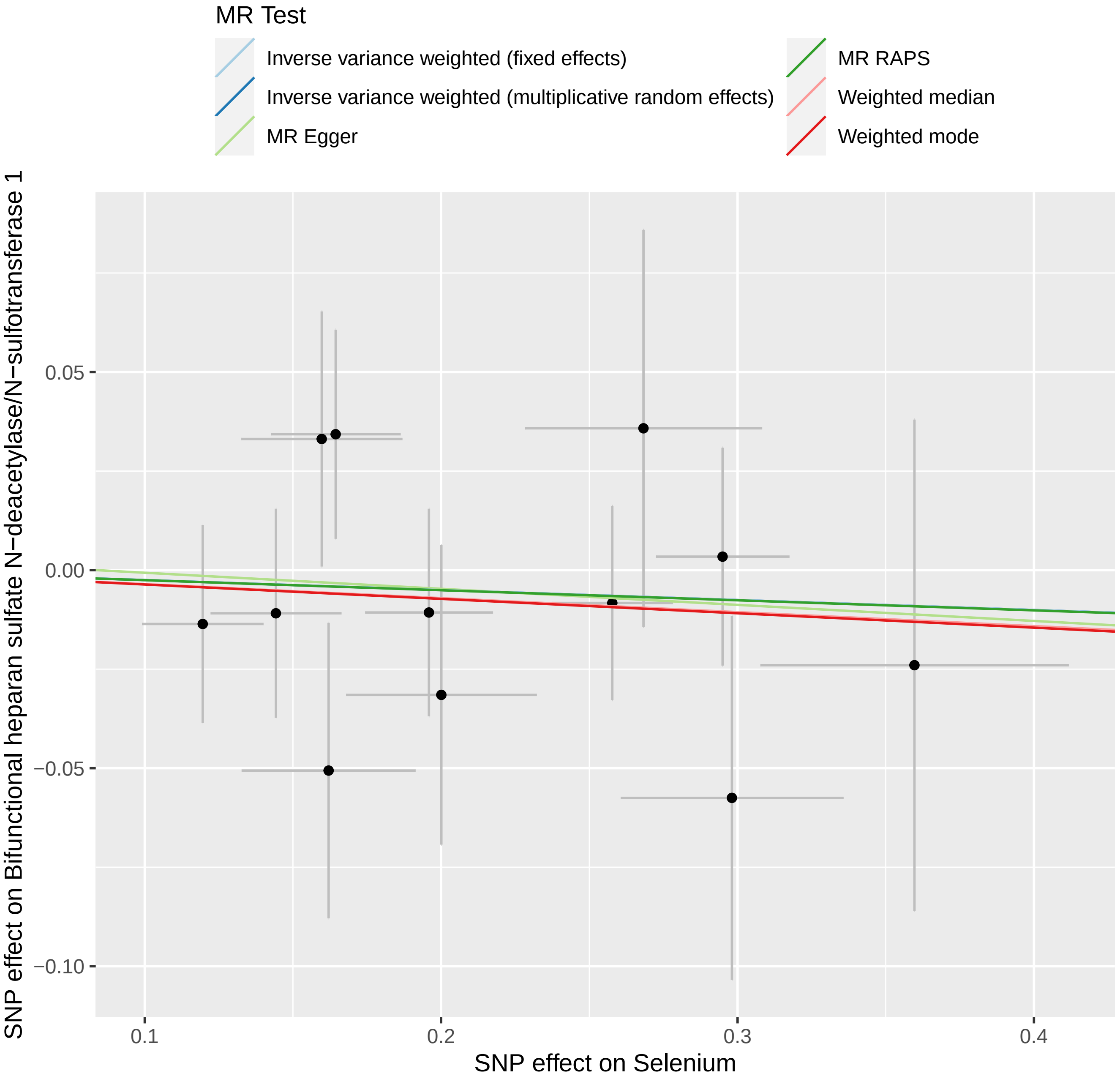

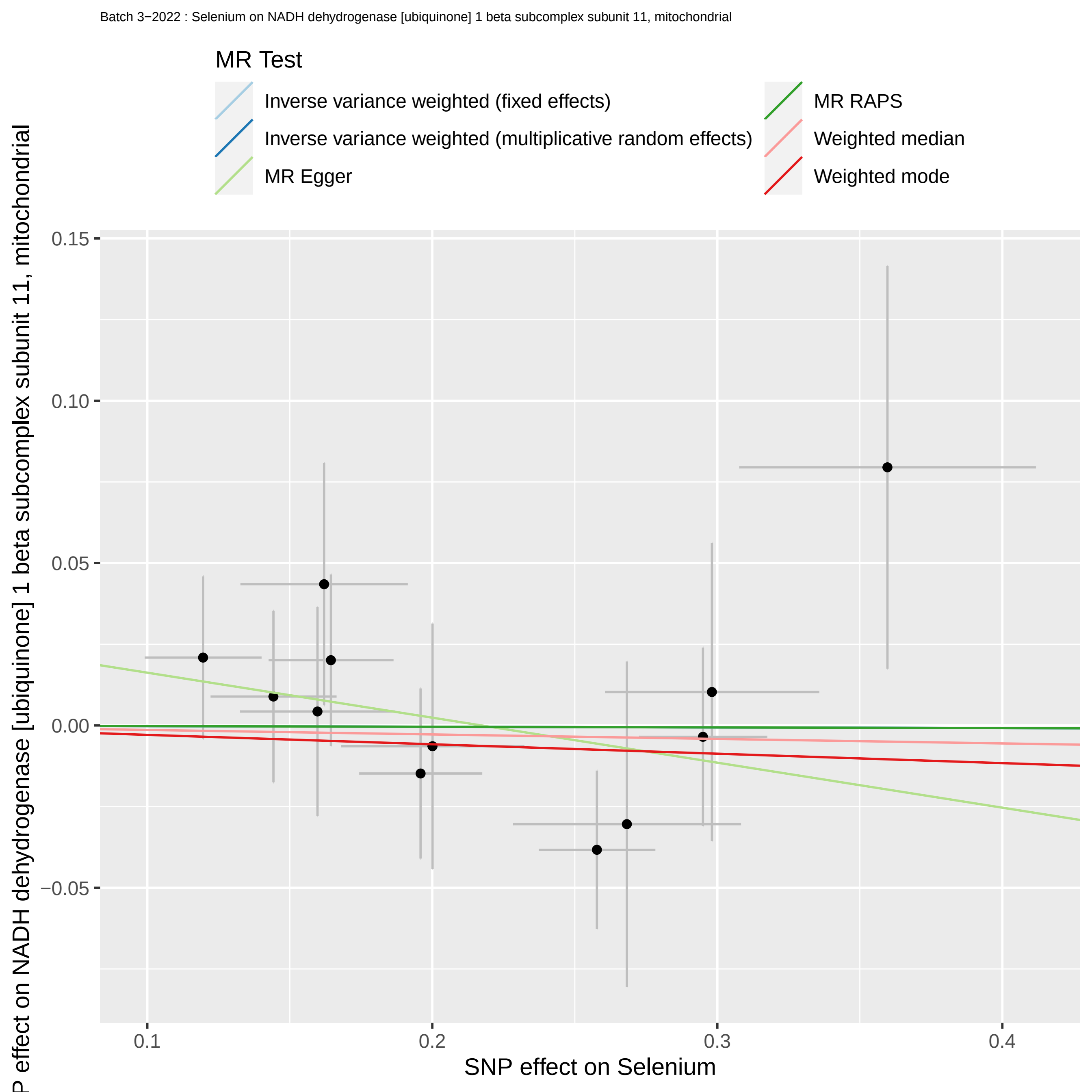

Batch 3−2022 : Selenium on NADH dehydrogenase [ubiquinone] 1 beta subcomplex subunit 11, mitochondrial
MR Test
Inverse variance weighted (fixed effects)
Inverse variance weighted (multiplicative random effects)
MR Egger
MR RAPS
Weighted median
Weighted mode
0.15
0.10
0.05
0.00
−0.05
0.1
0.2
0.3
0.4
SNP effect on Selenium
P effect on NADH dehydrogenase [ubiquinone] 1 beta subcomplex subunit 11, mitochondrial

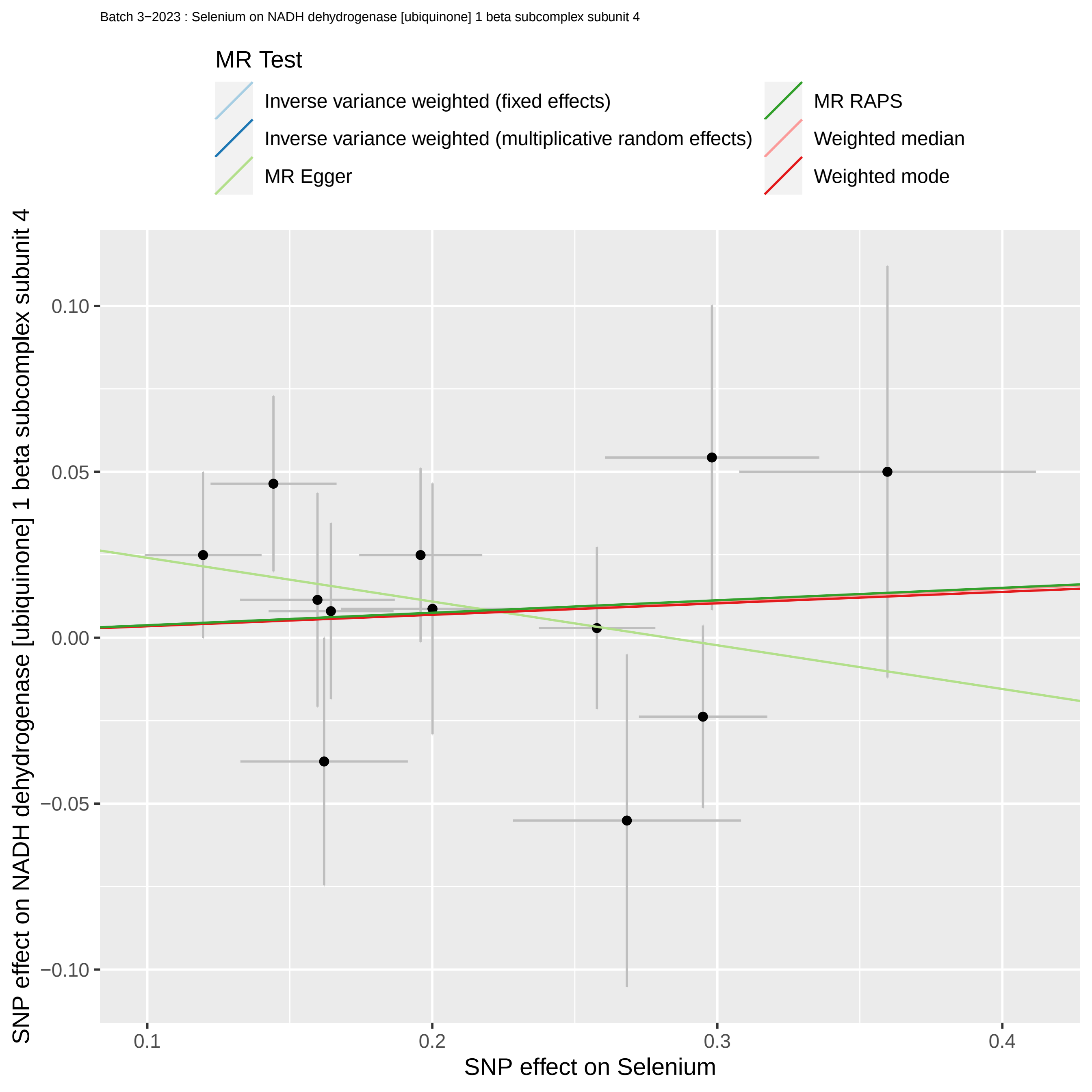
Batch 3−2023 : Selenium on NADH dehydrogenase [ubiquinone] 1 beta subcomplex subunit 4
MR Test
Inverse variance weighted (fixed effects)
Inverse variance weighted (multiplicative random effects)
MR Egger
MR RAPS
Weighted median
Weighted mode
0.10
0.05
0.00
−0.05
−0.10
0.1
0.2
0.3
0.4
SNP effect on NADH dehydrogenase [ubiquinone] 1 beta subcomplex subunit 4
SNP effect on Selenium

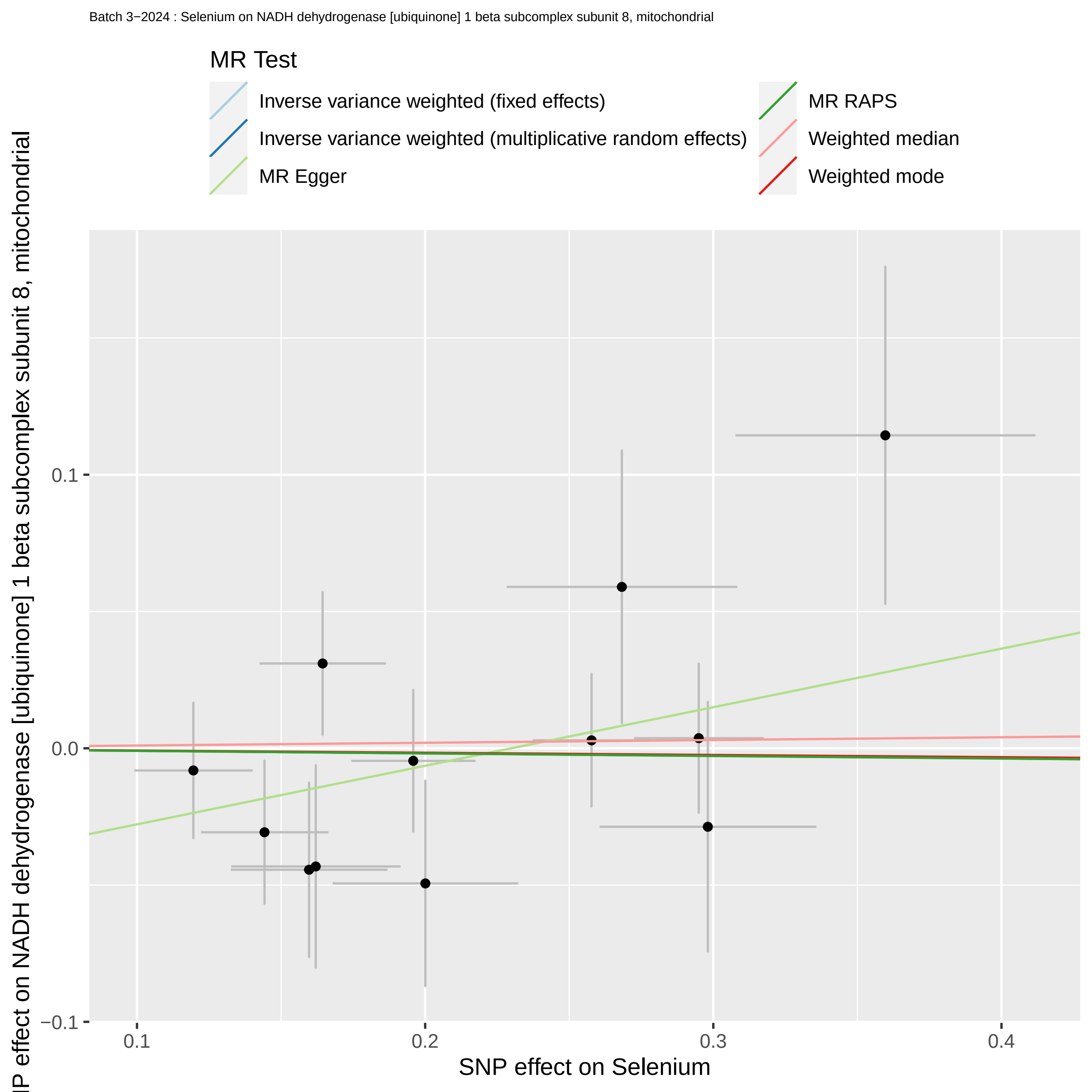
Batch 3−2024 : Selenium on NADH dehydrogenase [ubiquinone] 1 beta subcomplex subunit 8, mitochondrial
MR Test
Inverse variance weighted (fixed effects)
Inverse variance weighted (multiplicative random effects)
MR Egger
MR RAPS
Weighted median
Weighted mode
0.1
0.0
−0.1
0.1
0.2
0.3
0.4
SNP effect on Selenium
P effect on NADH dehydrogenase [ubiquinone] 1 beta subcomplex subunit 8, mitochondrial

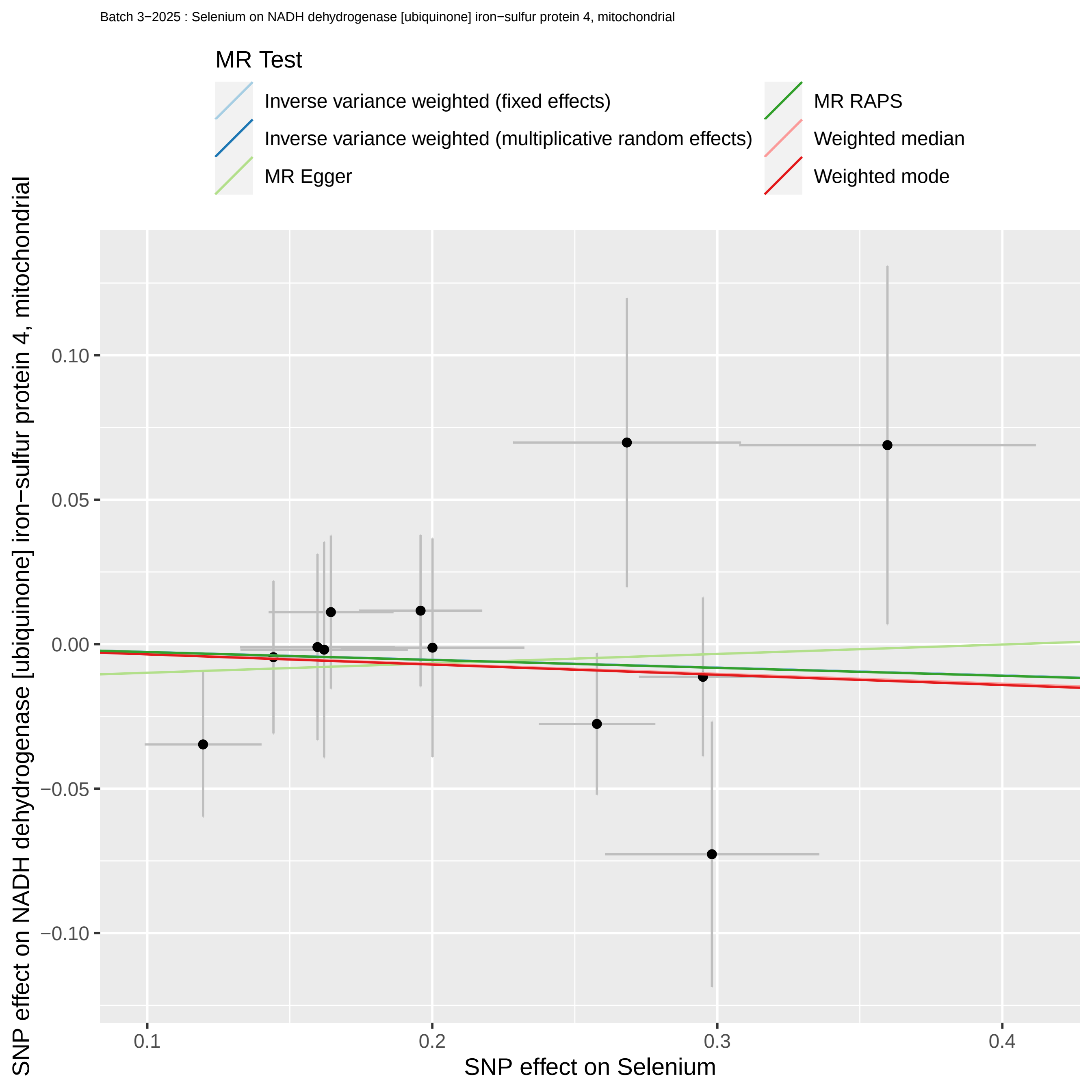
Batch 3−2025 : Selenium on NADH dehydrogenase [ubiquinone] iron−sulfur protein 4, mitochondrial
MR Test
Inverse variance weighted (fixed effects)
Inverse variance weighted (multiplicative random effects)
MR Egger
MR RAPS
Weighted median
Weighted mode
0.10
0.05
0.00
−0.05
−0.10
0.1
0.2
0.3
0.4
SNP effect on Selenium
SNP effect on NADH dehydrogenase [ubiquinone] iron−sulfur protein 4, mitochondrial

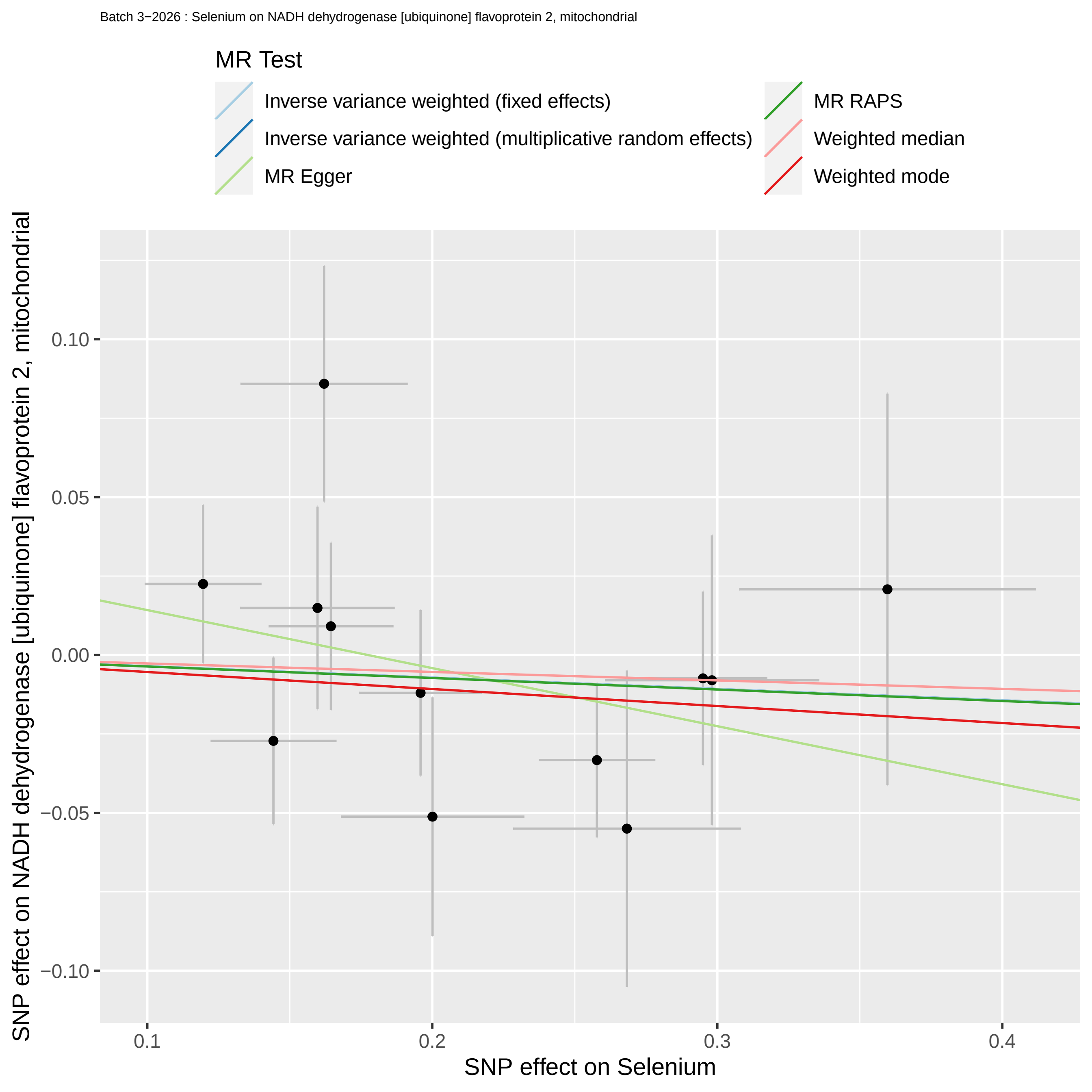
Batch 3−2026 : Selenium on NADH dehydrogenase [ubiquinone] flavoprotein 2, mitochondrial
MR Test
Inverse variance weighted (fixed effects)
Inverse variance weighted (multiplicative random effects)
MR Egger
MR RAPS
Weighted median
Weighted mode
SNP effect on NADH dehydrogenase [ubiquinone] flavoprotein 2, mitochondrial
0.10
0.05
0.00
−0.05
−0.10
0.1
0.2
0.3
0.4
SNP effect on Selenium

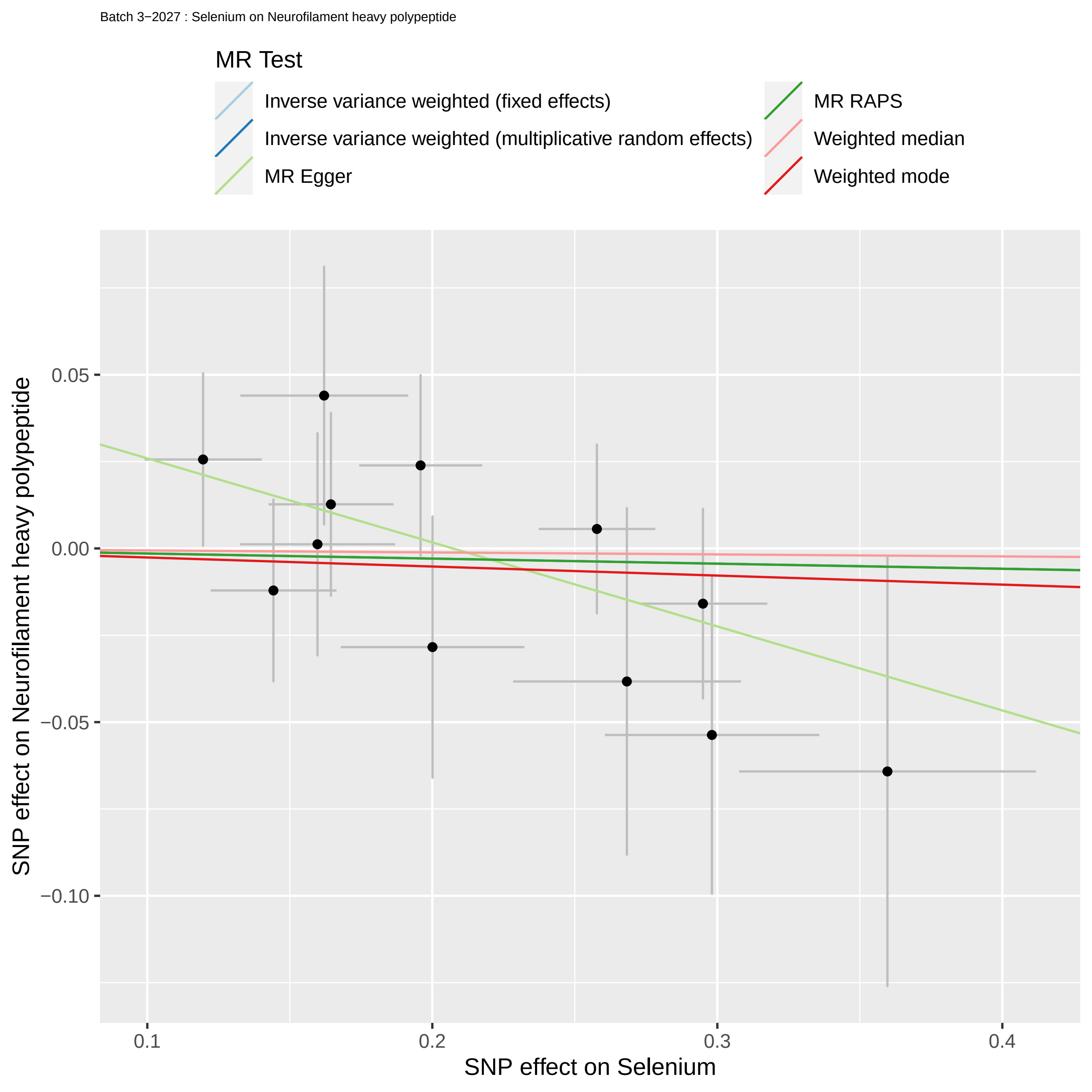
Batch 3−2027 : Selenium on Neurofilament heavy polypeptide
MR Test
Inverse variance weighted (fixed effects)
Inverse variance weighted (multiplicative random effects)
MR Egger
MR RAPS
Weighted median
Weighted mode
0.05
0.00
−0.05
−0.10
0.1
0.2
0.3
0.4
SNP effect on Neurofilament heavy polypeptide
SNP effect on Selenium

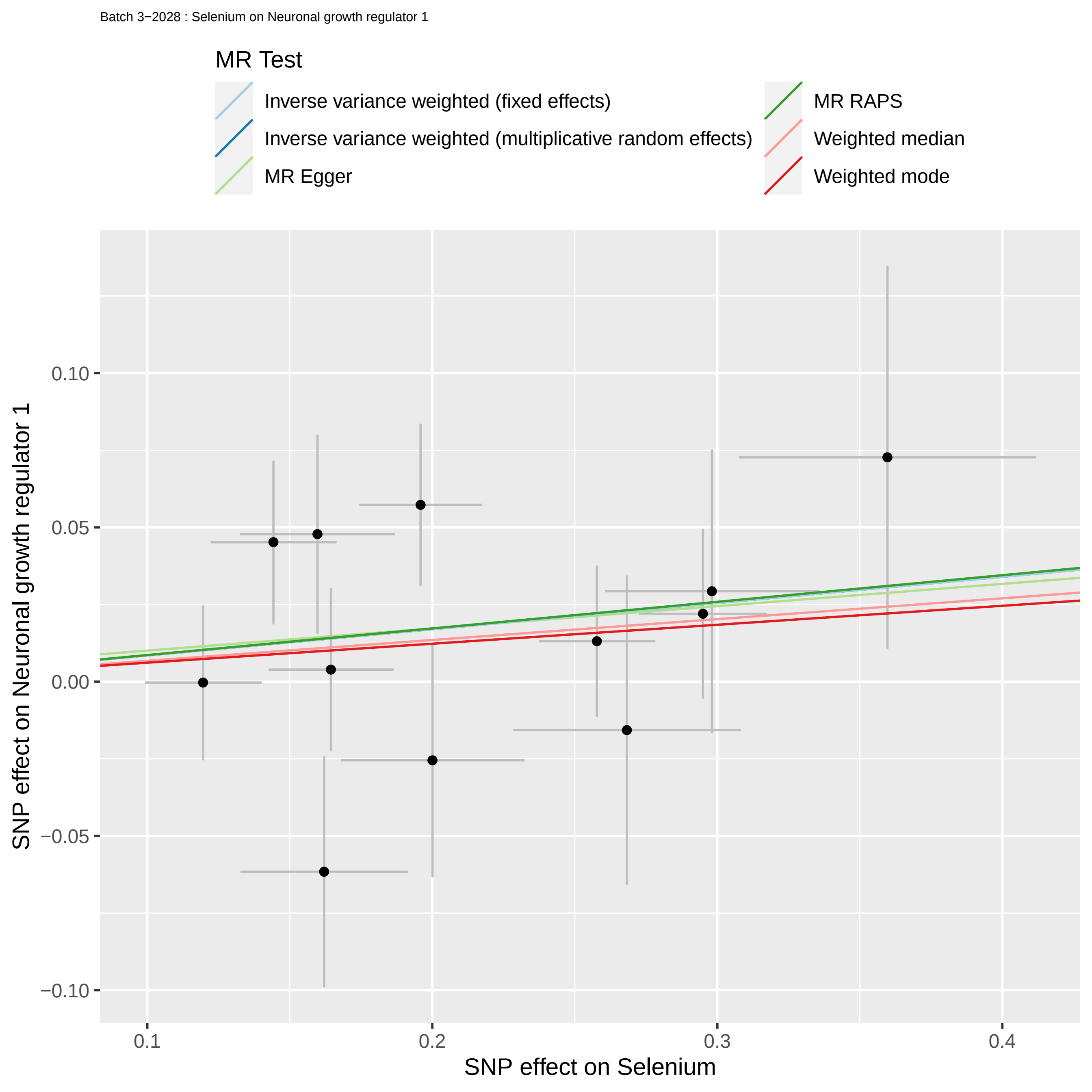
Batch 3−2028 : Selenium on Neuronal growth regulator 1
MR Test
Inverse variance weighted (fixed effects)
Inverse variance weighted (multiplicative random effects)
MR Egger
MR RAPS
Weighted median
Weighted mode
0.10
0.05
0.00
−0.05
−0.10
0.1
0.2
0.3
0.4
SNP effect on Neuronal growth regulator 1
SNP effect on Selenium

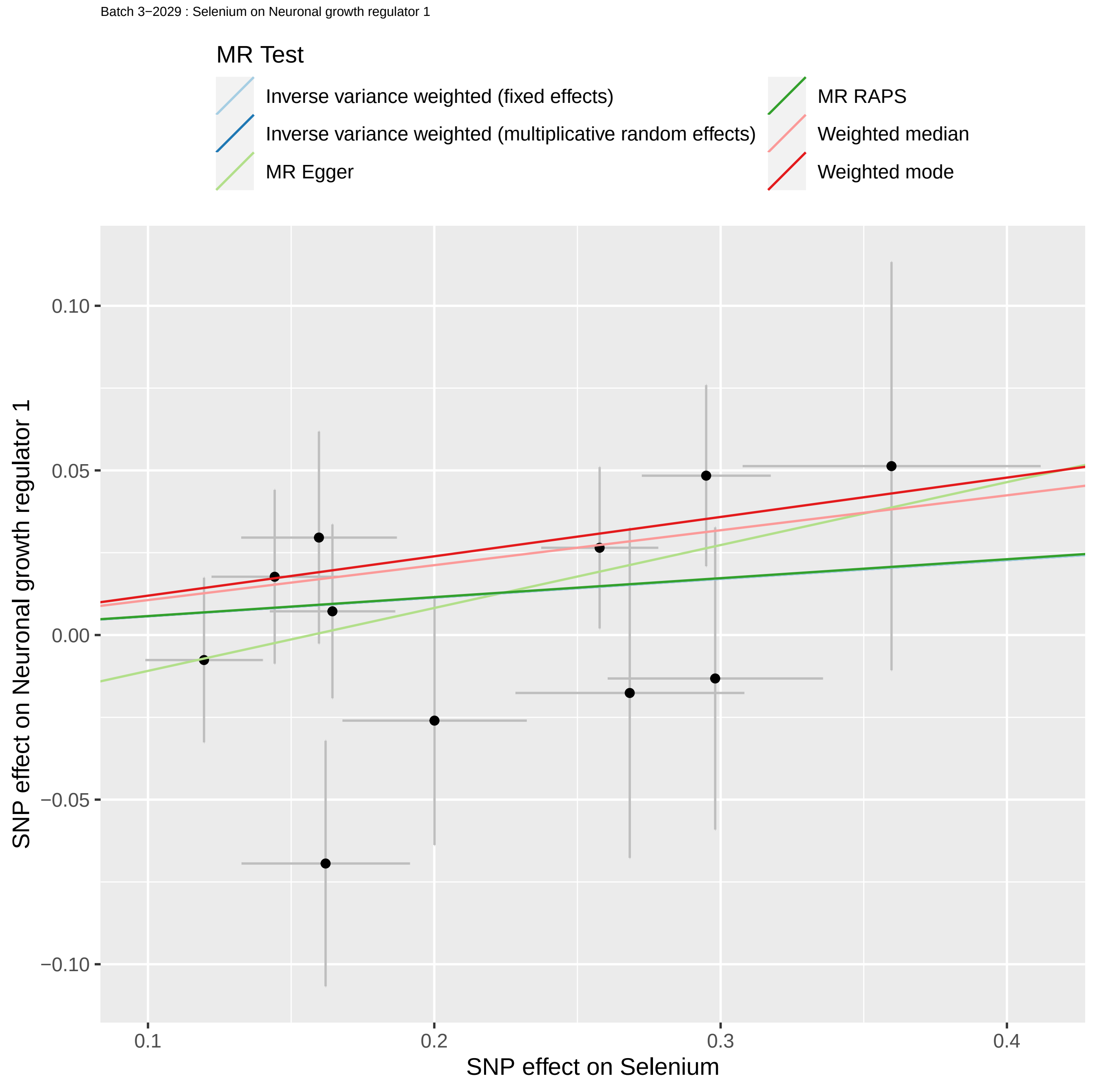
Batch 3−2029 : Selenium on Neuronal growth regulator 1
MR Test
Inverse variance weighted (fixed effects)
Inverse variance weighted (multiplicative random effects)
MR Egger
MR RAPS
Weighted median
Weighted mode
0.10
0.05
0.00
−0.05
−0.10
0.1
0.2
0.3
0.4
SNP effect on Selenium
SNP effect on Neuronal growth regulator 1

Batch 3−2030 : Selenium on Protein kinase C−binding protein NELL1

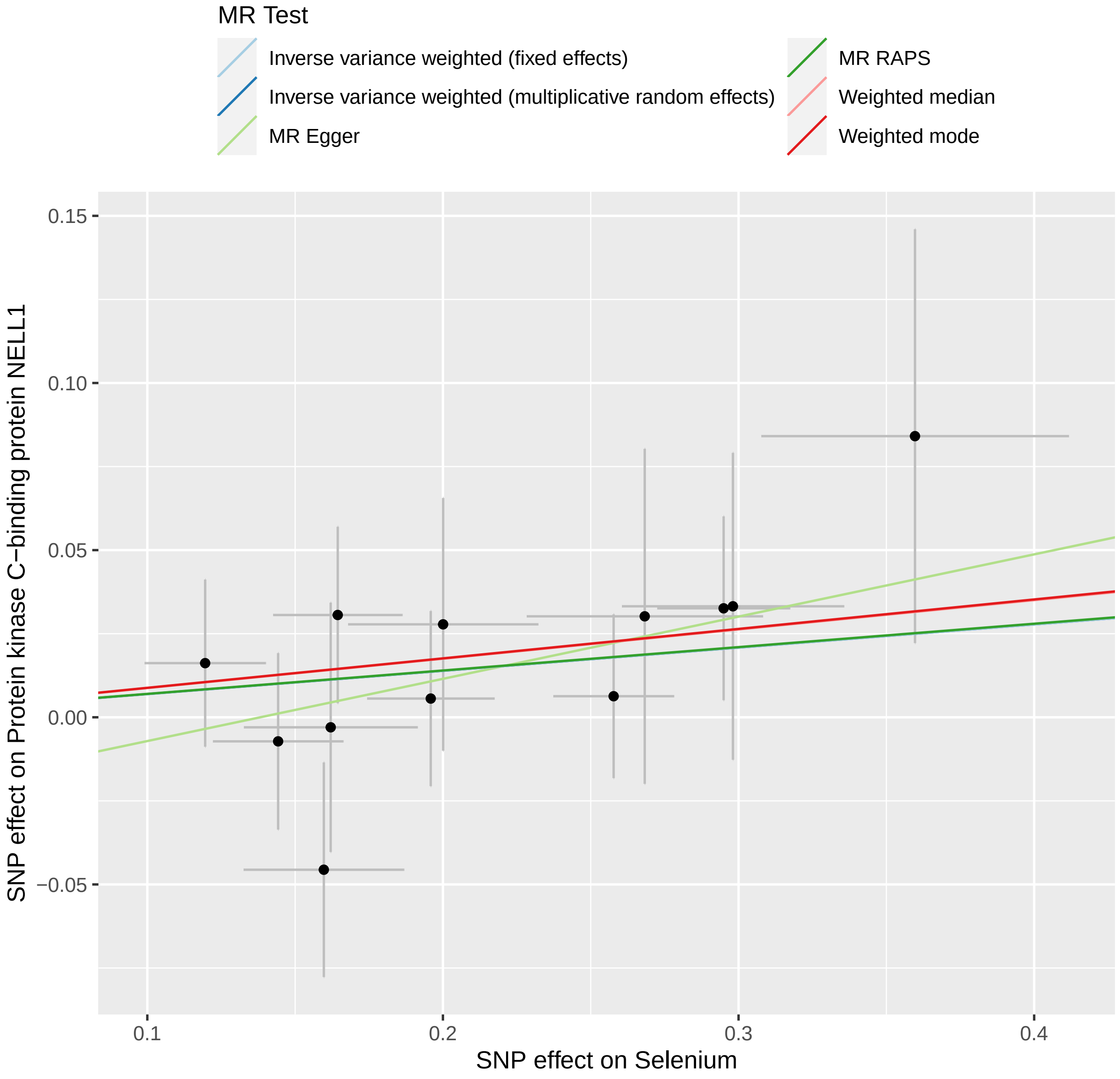

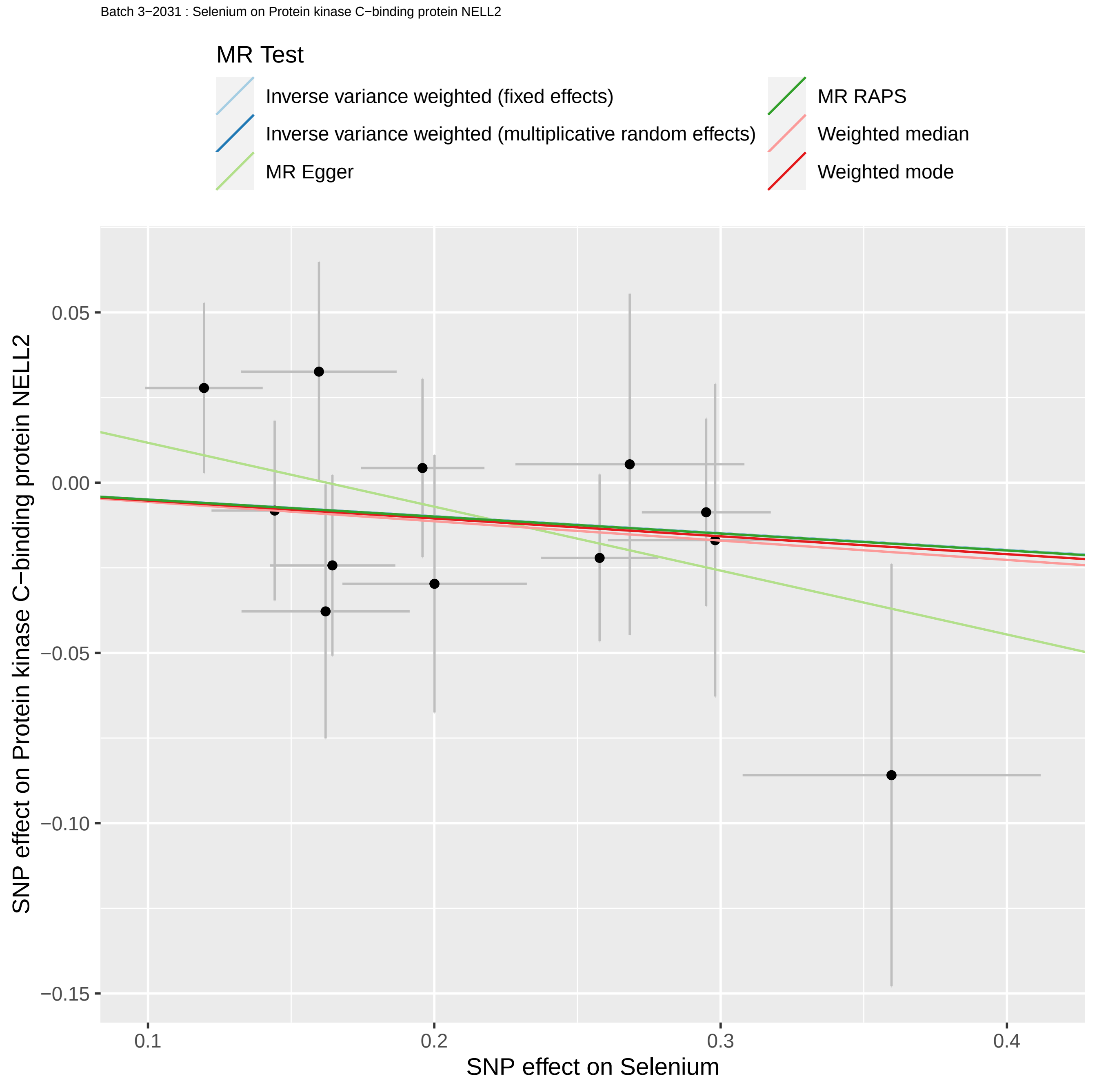
Batch 3−2031 : Selenium on Protein kinase C−binding protein NELL2
MR Test
Inverse variance weighted (fixed effects)
Inverse variance weighted (multiplicative random effects)
MR Egger
MR RAPS
Weighted median
Weighted mode
0.05
0.00
−0.05
−0.10
−0.15
0.1
0.2
0.3
0.4
SNP effect on Protein kinase C−binding protein NELL2
SNP effect on Selenium

Batch 3−2032 : Selenium on Neudesin

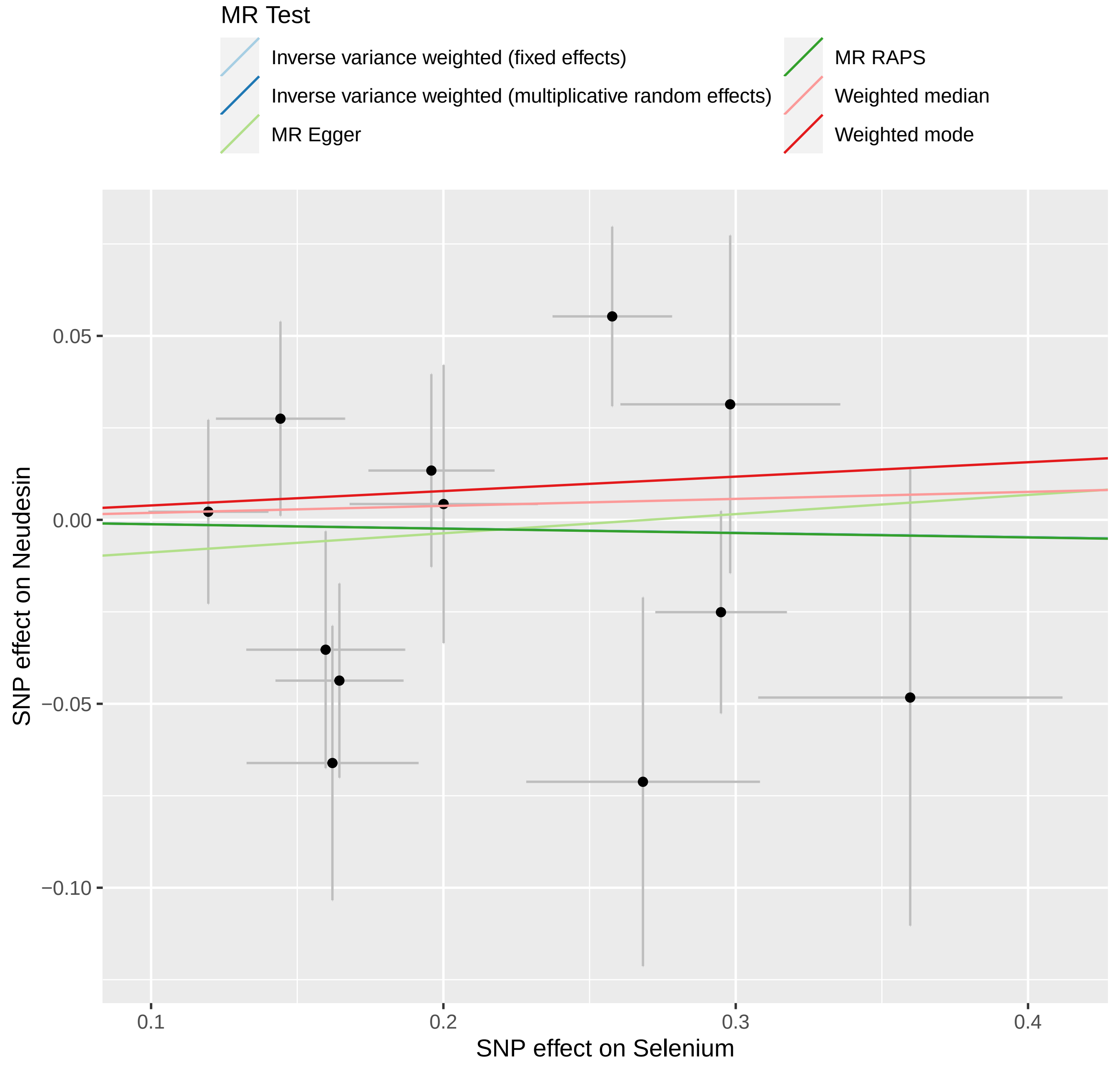

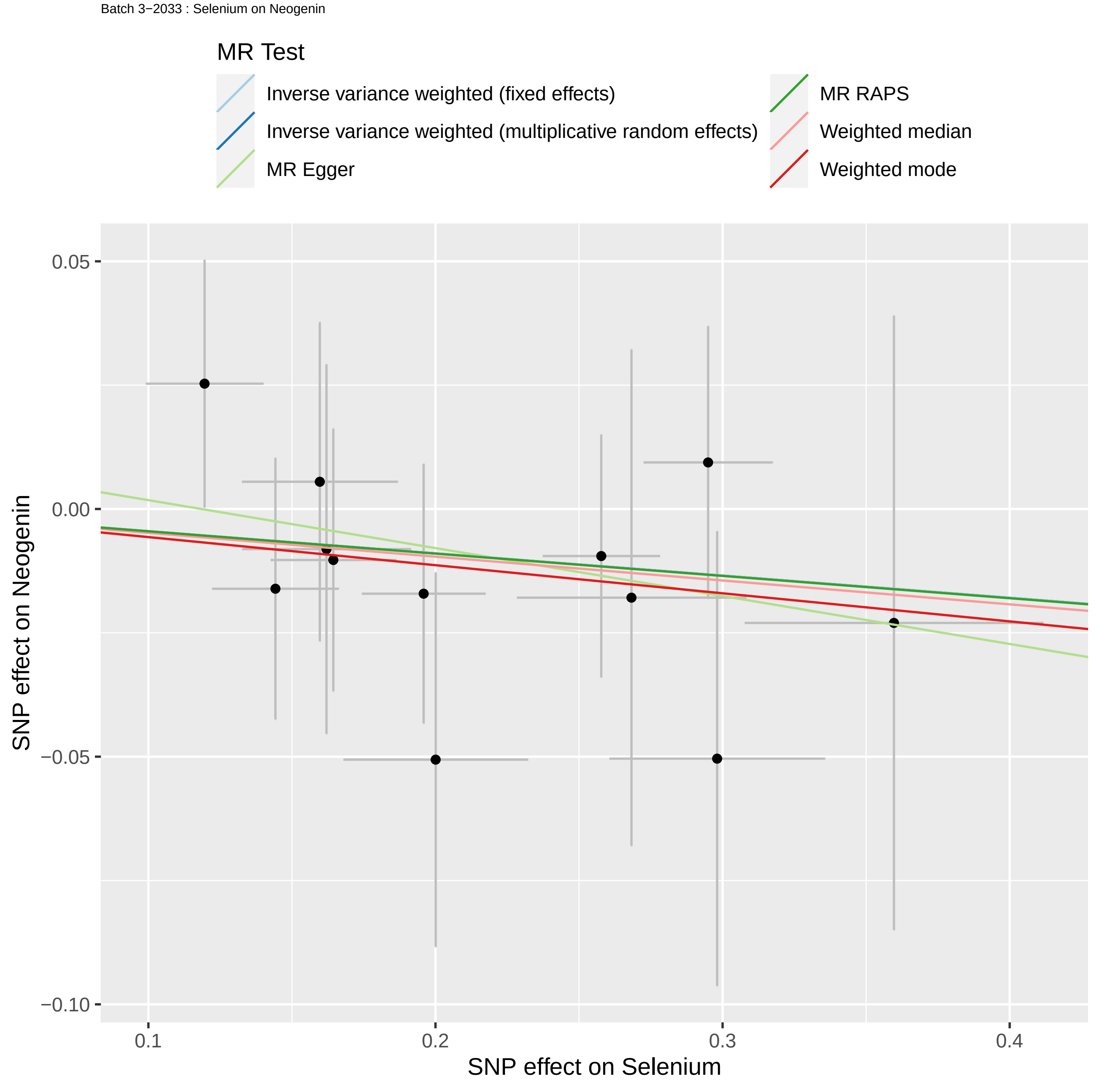

Batch 3−2033 : Selenium on Neogenin
MR Test
Inverse variance weighted (fixed effects)
Inverse variance weighted (multiplicative random effects)
MR Egger
MR RAPS
Weighted median
Weighted mode
0.05
0.00
−0.05
−0.10
0.1
0.2
0.3
0.4
SNP effect on Neogenin
SNP effect on Selenium

Batch 3−2034 : Selenium on Neuroepithelial cell−transforming gene 1 protein

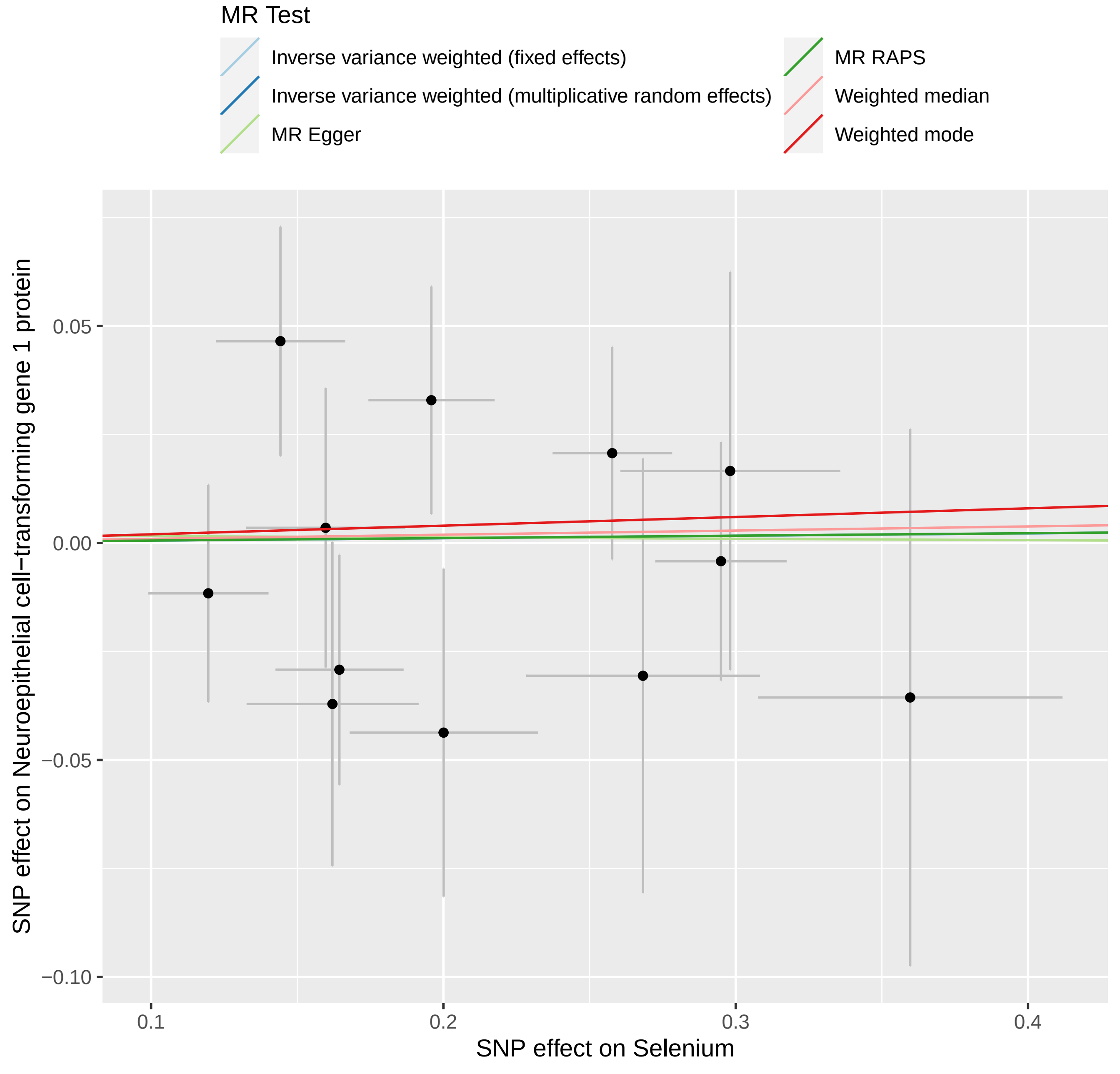

Batch 3−2035 : Selenium on Neuropilin and tolloid−like protein 1

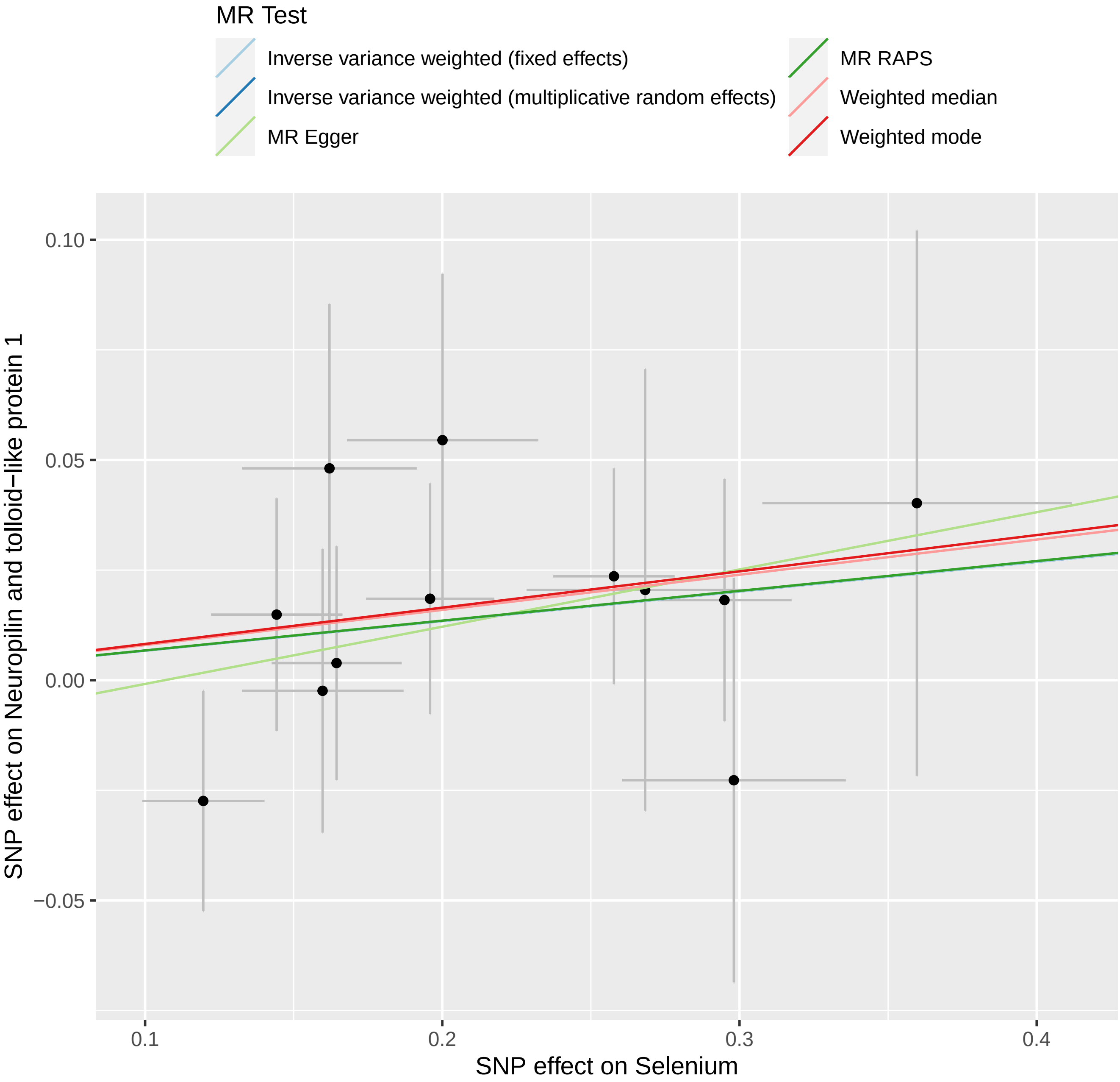

Batch 3−2036 : Selenium on Neuropilin and tolloid−like protein 2

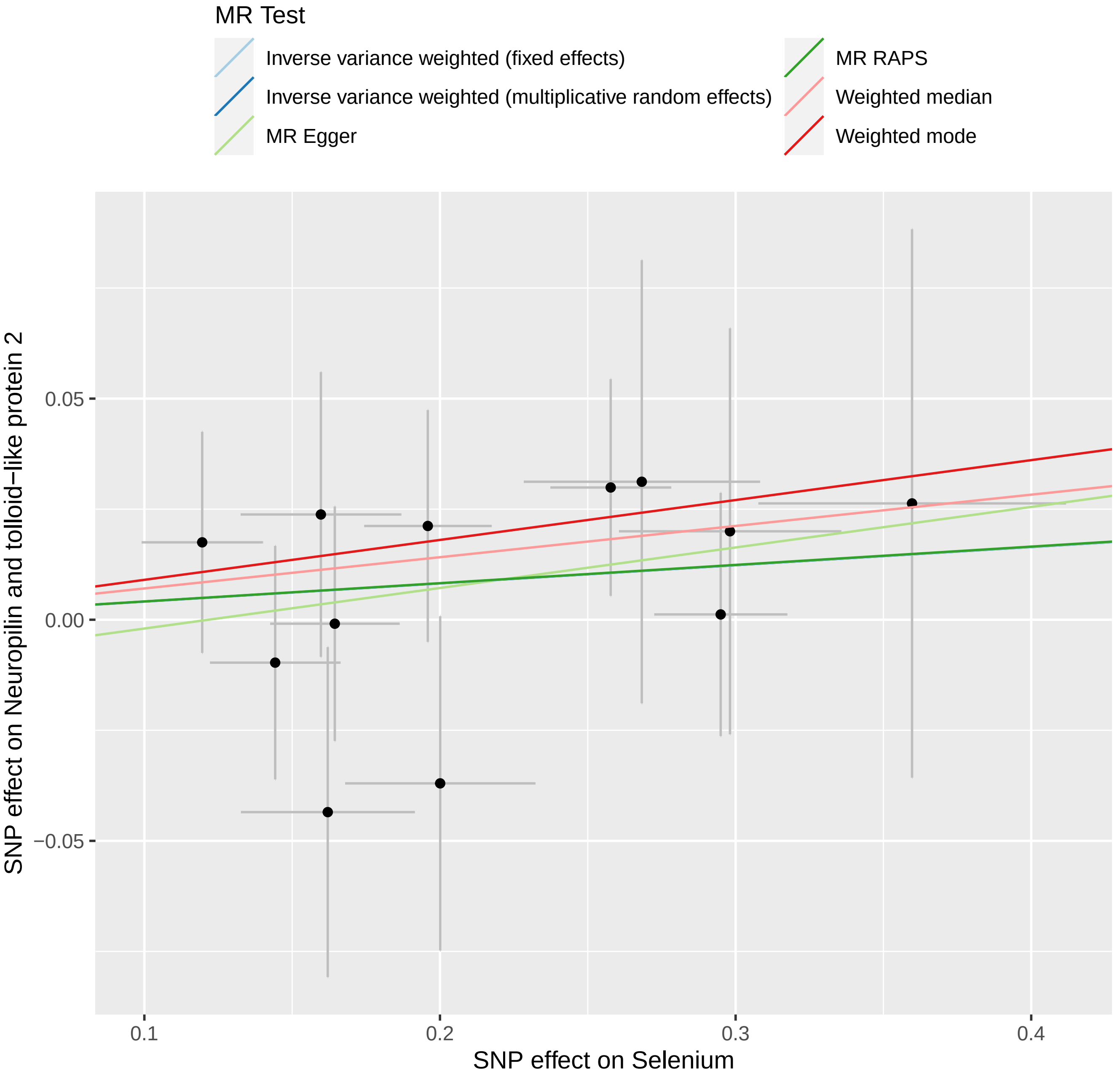

Batch 3−2037 : Selenium on E3 ubiquitin−protein ligase NEURL1

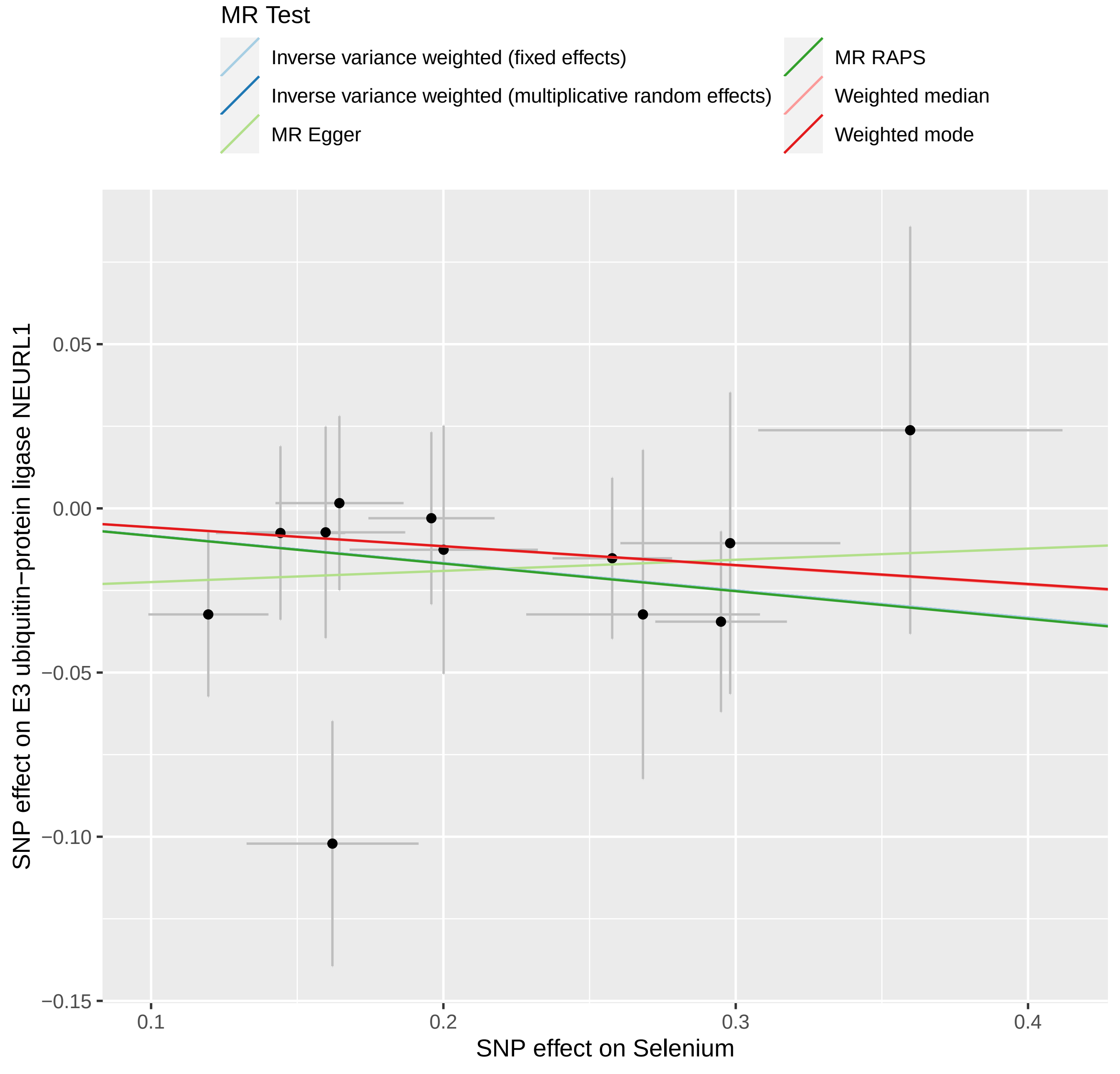

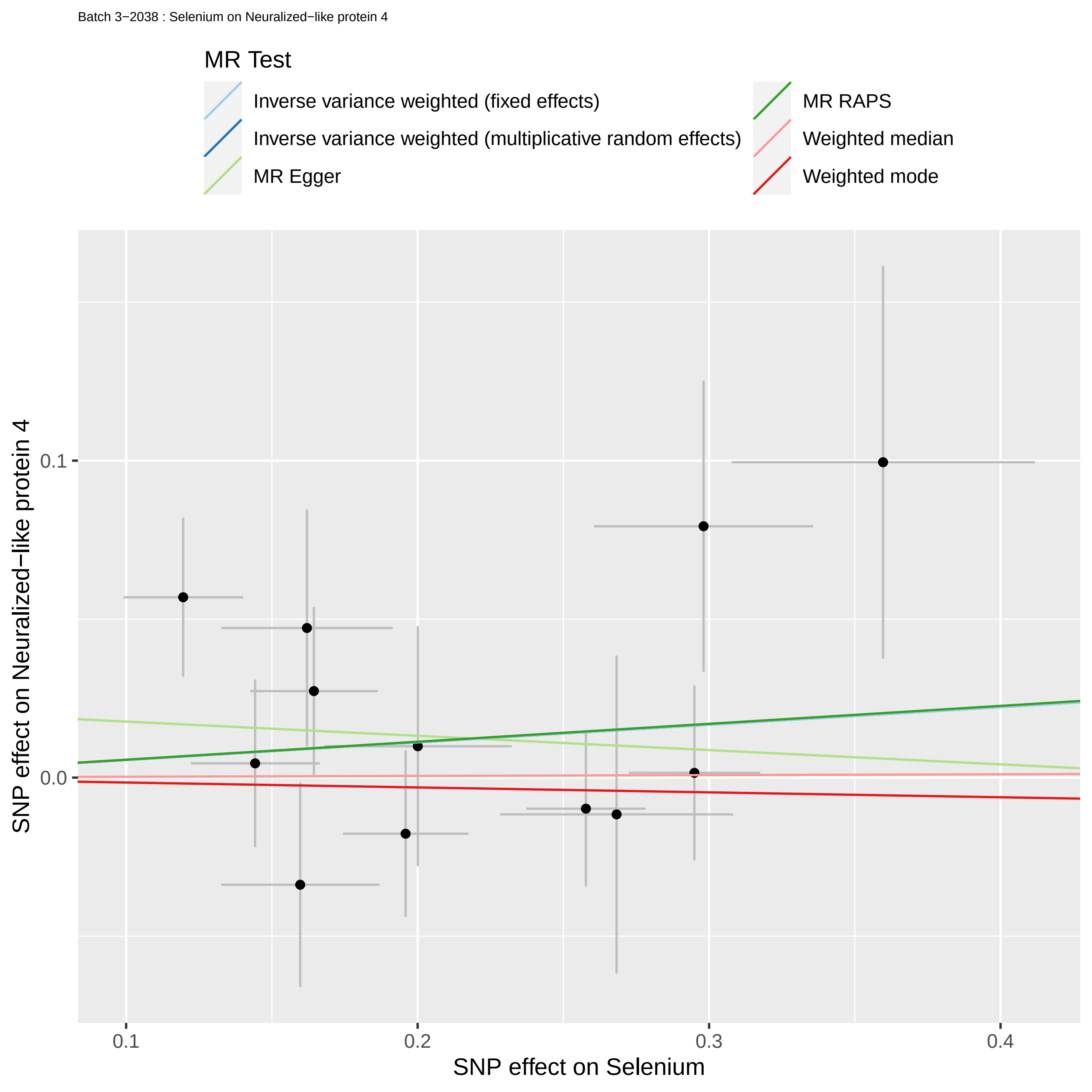
Batch 3−2038 : Selenium on Neuralized−like protein 4
MR Test
Inverse variance weighted (fixed effects)
Inverse variance weighted (multiplicative random effects)
MR Egger
MR RAPS
Weighted median
Weighted mode
0.1
0.0
0.1
0.2
0.3
0.4
SNP effect on Selenium
SNP effect on Neuralized−like protein 4

Batch 3−2039 : Selenium on Neurofascin

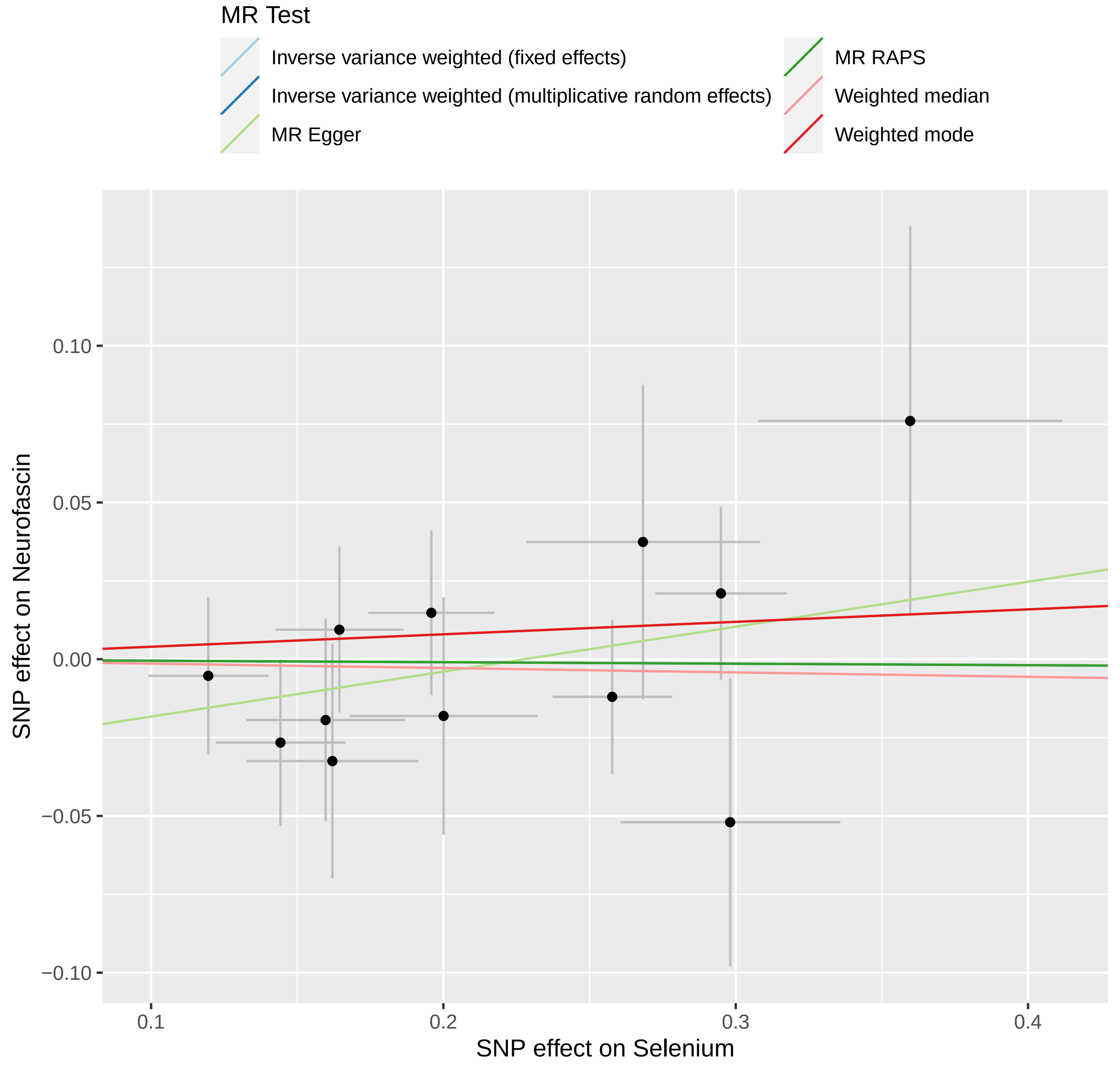

Batch 3−2040 : Selenium on Nuclear factor erythroid 2−related factor 1

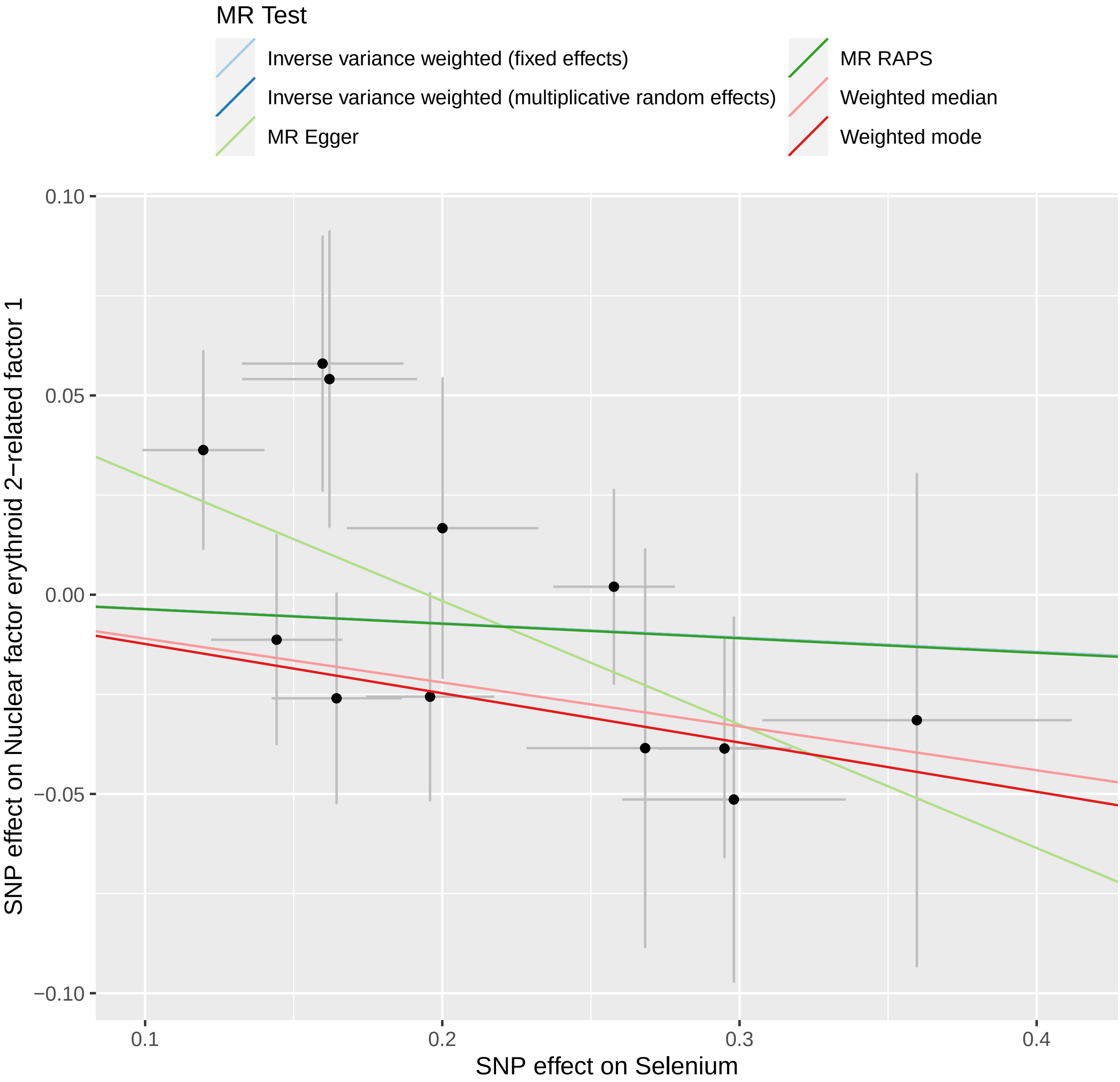

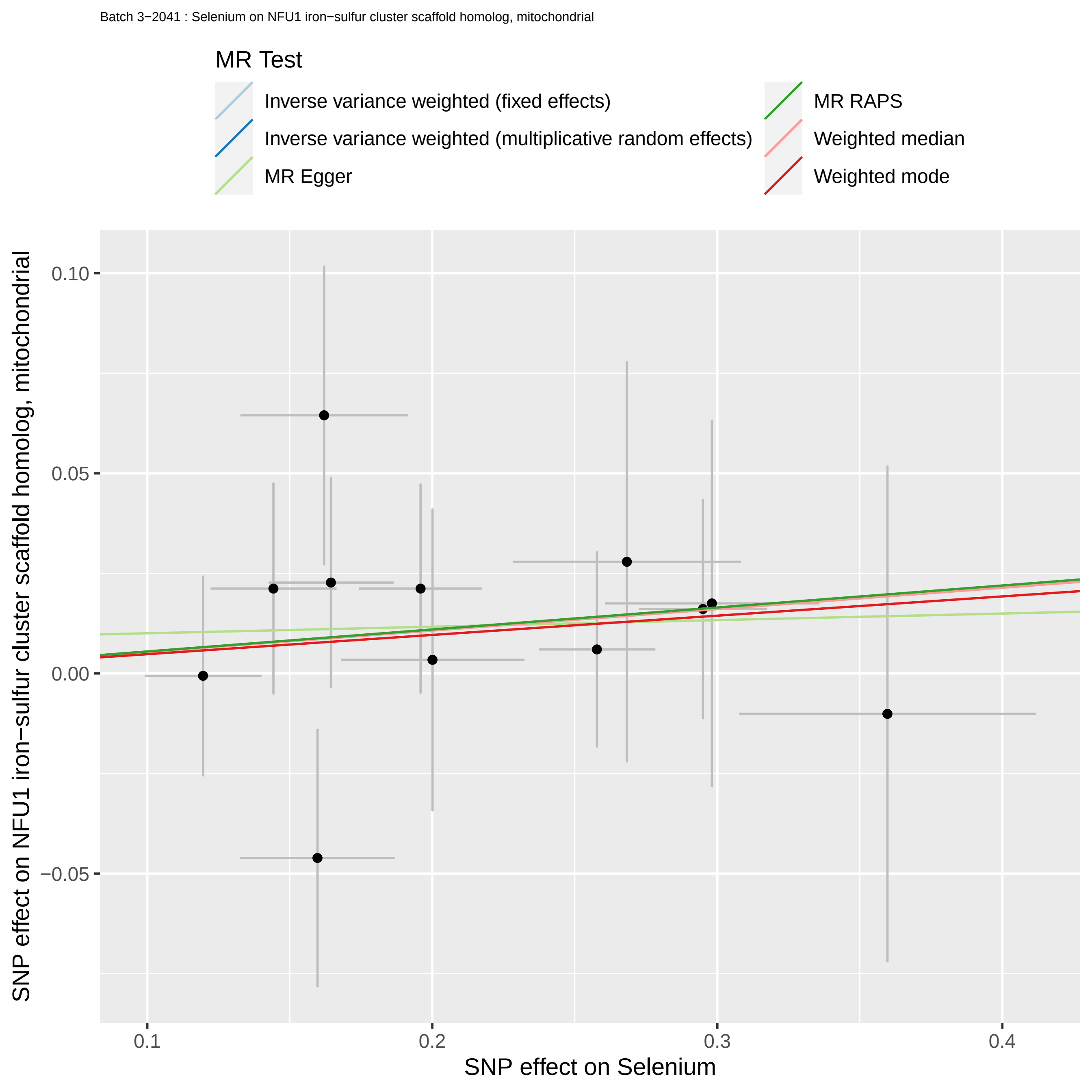
Batch 3−2041 : Selenium on NFU1 iron−sulfur cluster scaffold homolog, mitochondrial
MR Test
Inverse variance weighted (fixed effects)
Inverse variance weighted (multiplicative random effects)
MR Egger
MR RAPS
Weighted median
Weighted mode
0.10
0.05
0.00
−0.05
0.1
0.2
0.3
0.4
SNP effect on Selenium
SNP effect on NFU1 iron−sulfur cluster scaffold homolog, mitochondrial

Batch 3−2042 : Selenium on beta−nerve growth factor

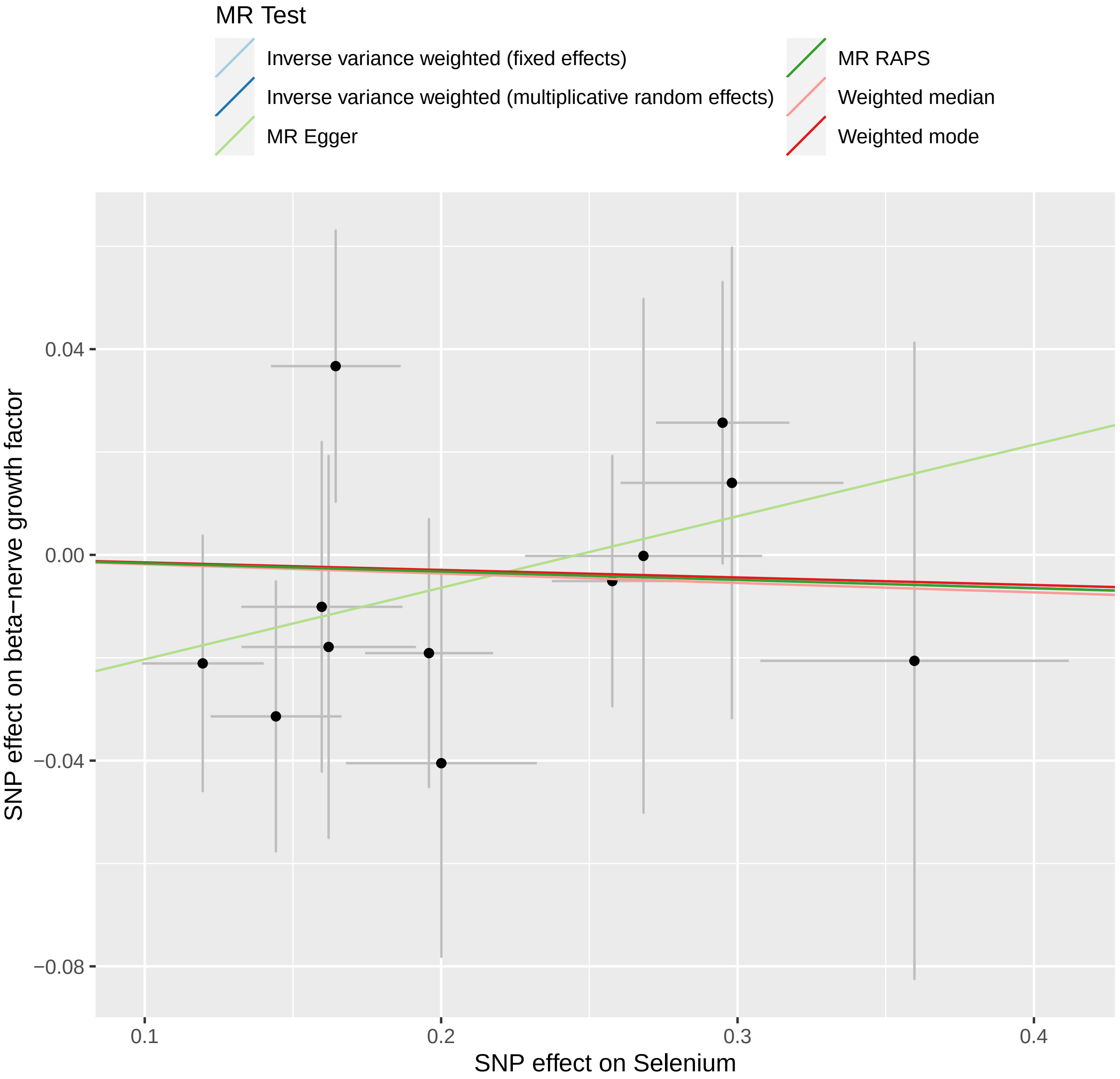

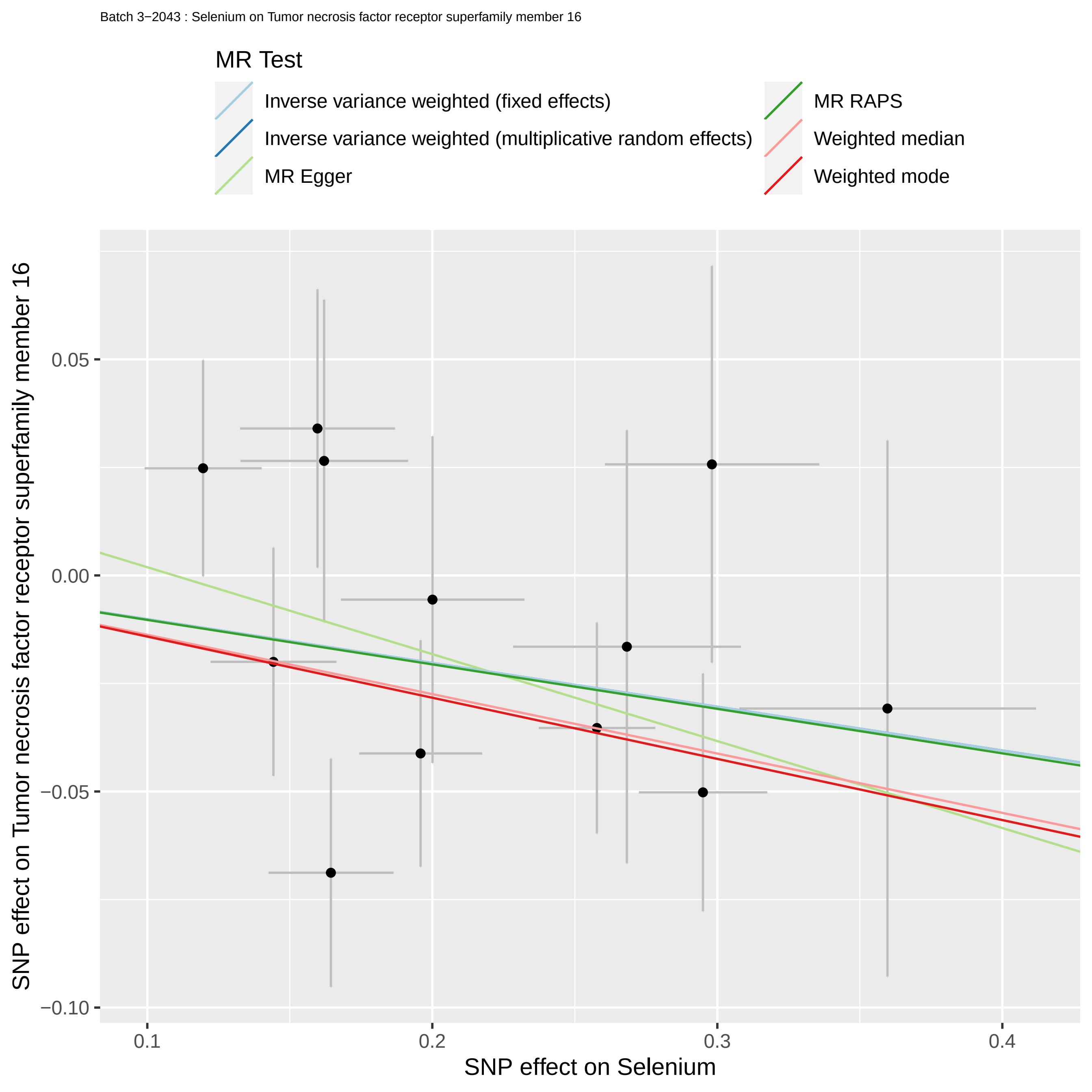
Batch 3−2043 : Selenium on Tumor necrosis factor receptor superfamily member 16
MR Test
Inverse variance weighted (fixed effects)
Inverse variance weighted (multiplicative random effects)
MR Egger
MR RAPS
Weighted median
Weighted mode
0.05
0.00
−0.05
−0.10
0.1
0.2
0.3
0.4
SNP effect on Tumor necrosis factor receptor superfamily member 16
SNP effect on Selenium

Batch 3−2044 : Selenium on Tumor necrosis factor receptor superfamily member 16

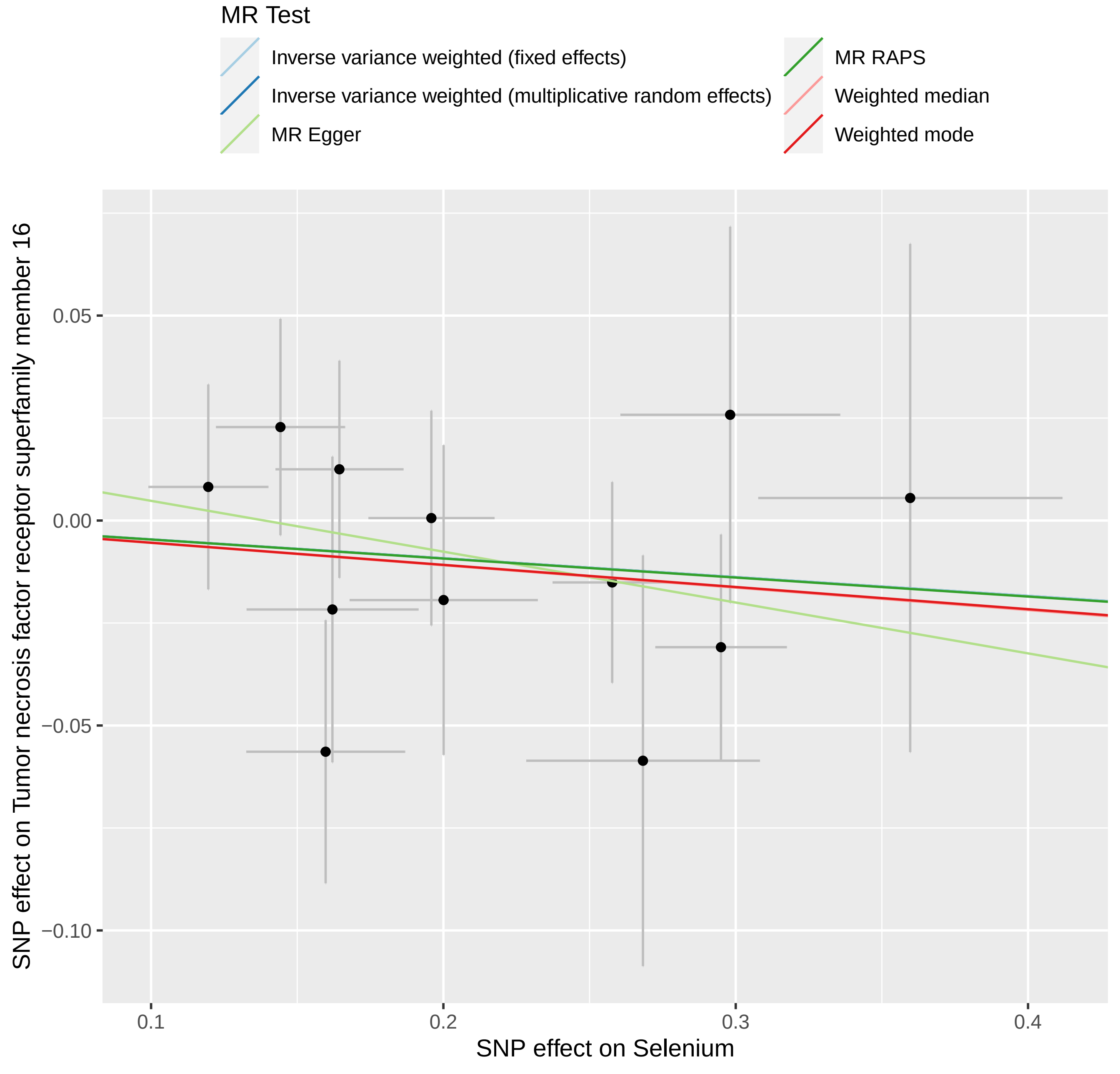

Batch 3−2045 : Selenium on Neugrin

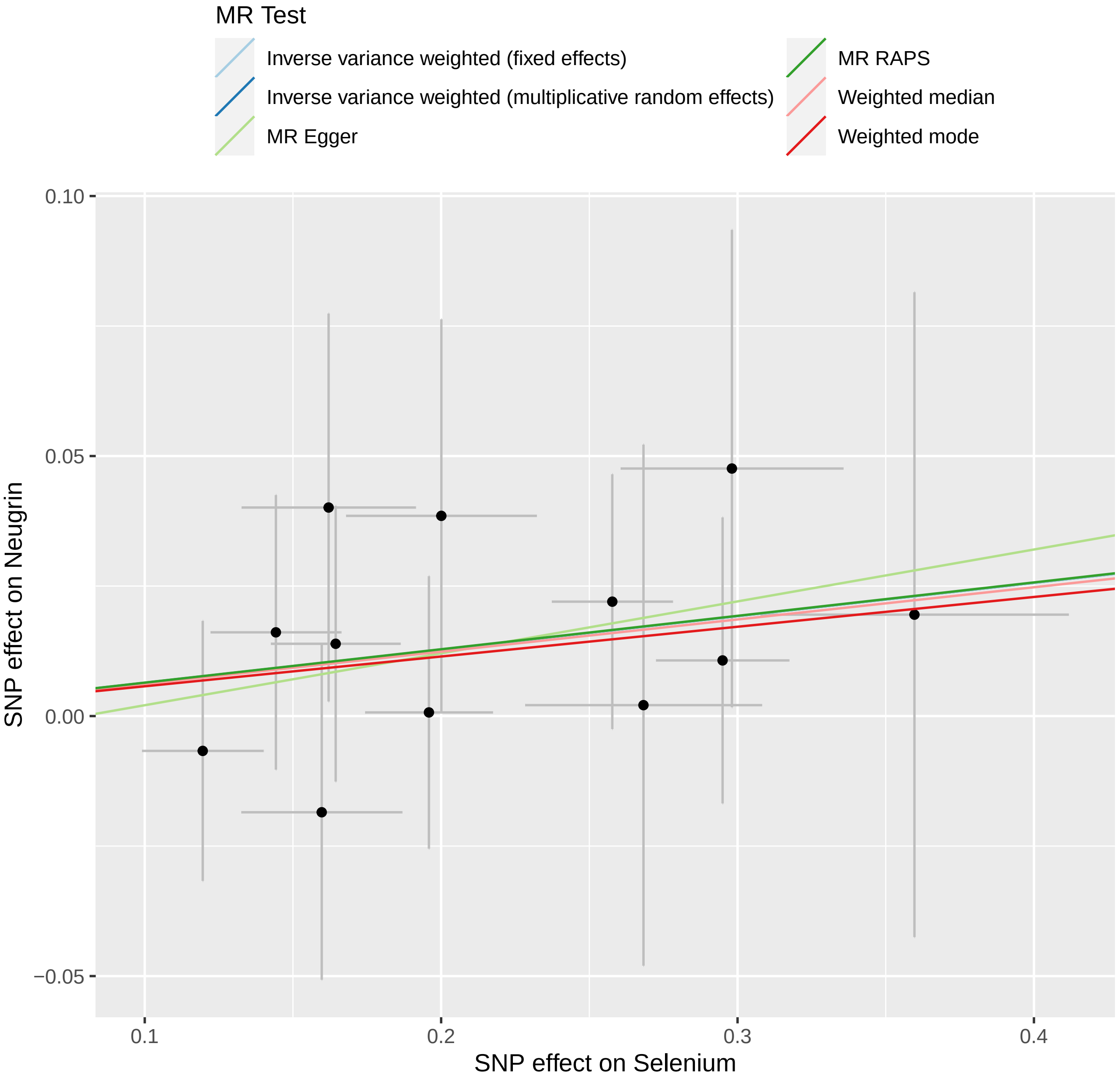

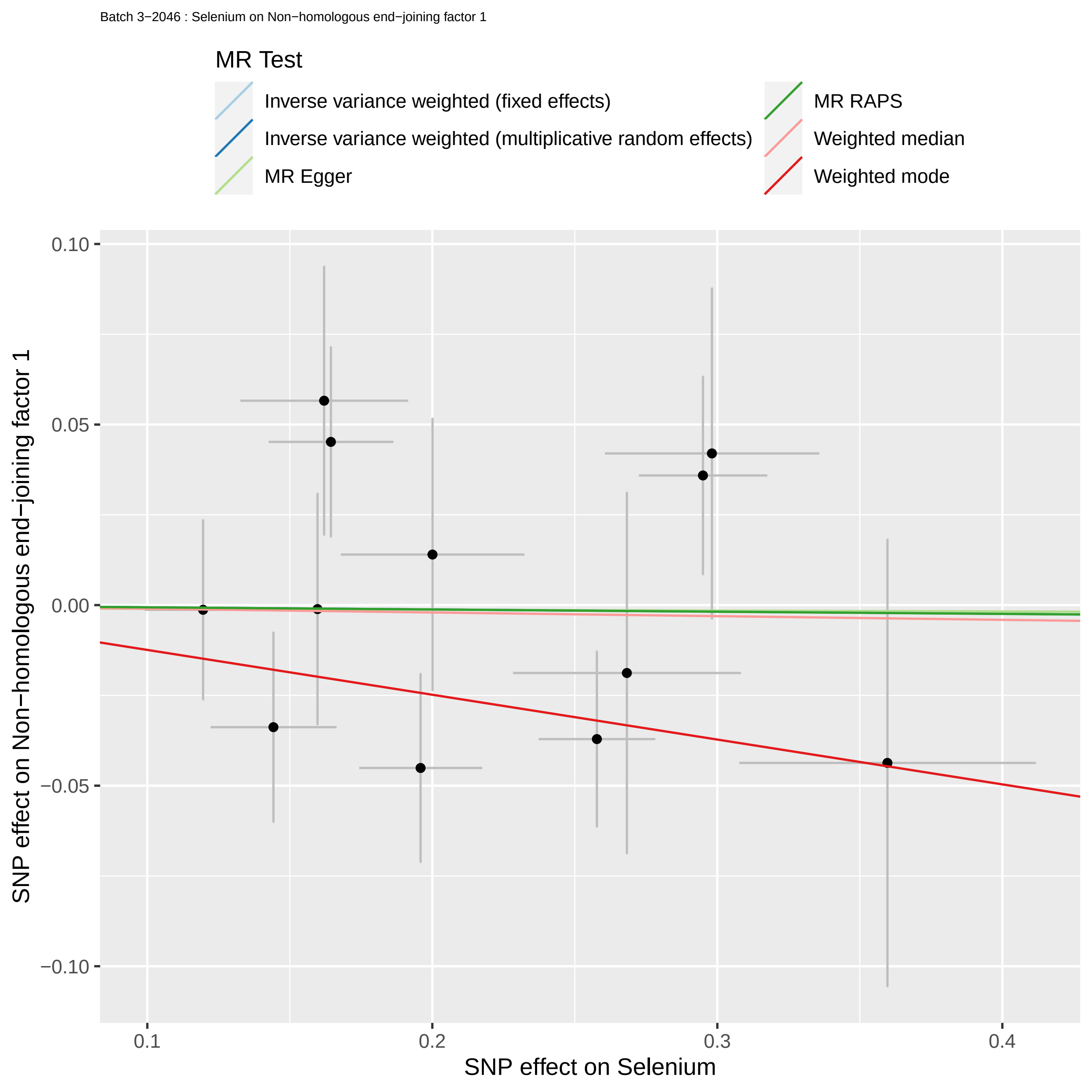

Batch 3−2046 : Selenium on Non−homologous end−joining factor 1
MR Test
Inverse variance weighted (fixed effects)
Inverse variance weighted (multiplicative random effects)
MR Egger
MR RAPS
Weighted median
Weighted mode
0.10
0.05
0.00
−0.05
−0.10
SNP effect on Non−homologous end−joining factor 1
0.1
0.2
0.3
0.4
SNP effect on Selenium

Batch 3−2047 : Selenium on NHL repeat−containing protein 3

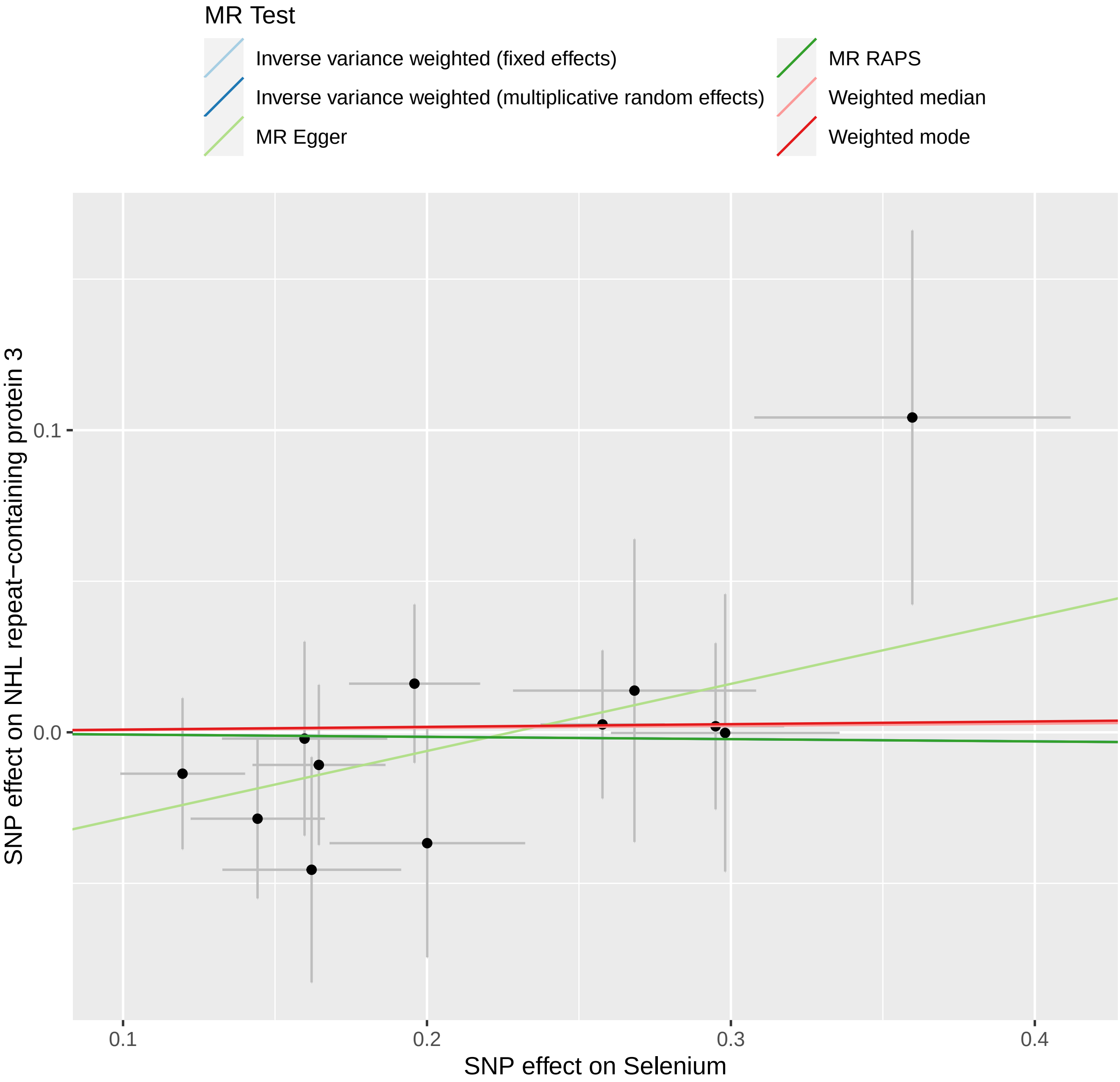

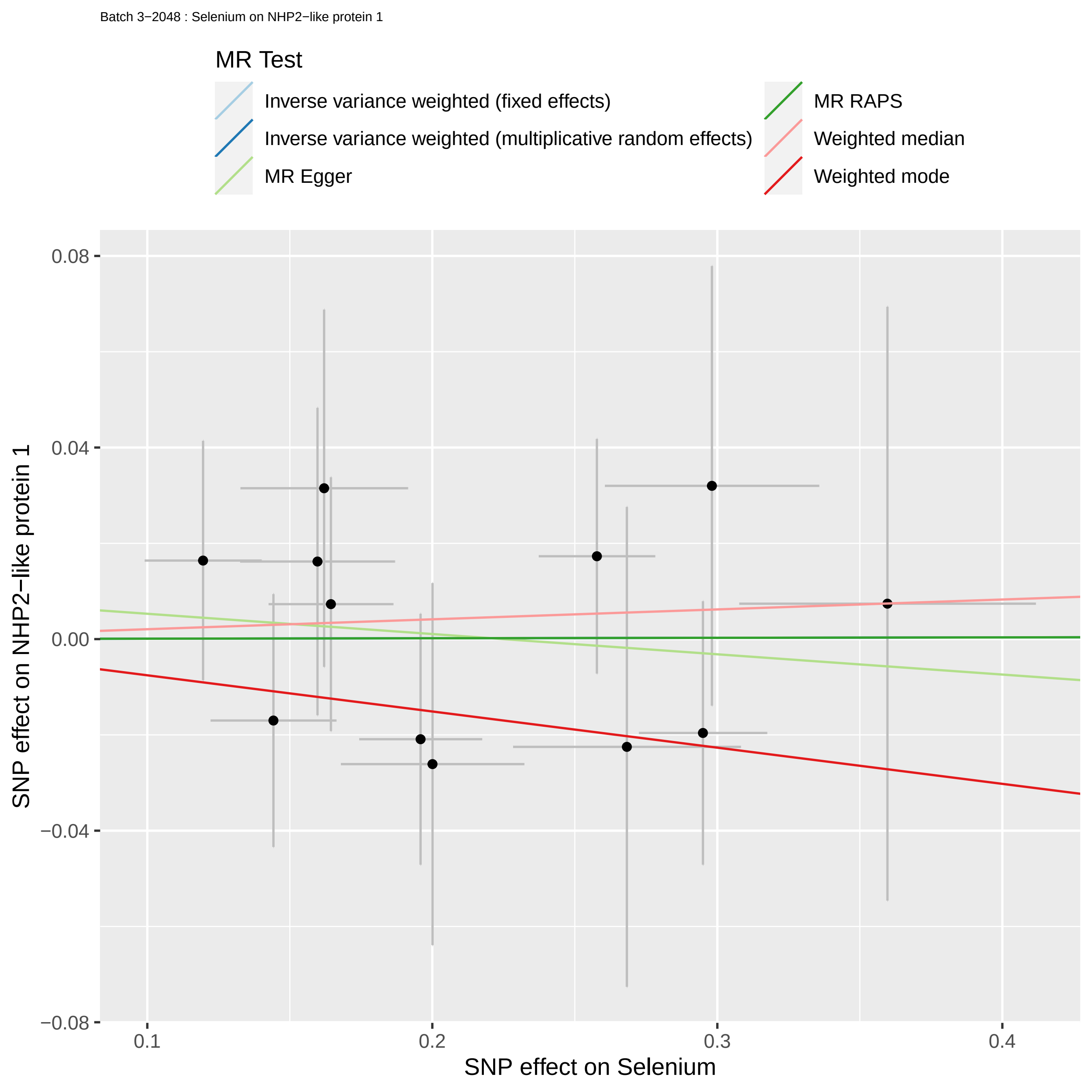

Batch 3−2048 : Selenium on NHP2−like protein 1
MR Test
Inverse variance weighted (fixed effects)
Inverse variance weighted (multiplicative random effects)
MR Egger
MR RAPS
Weighted median
Weighted mode
0.08
0.04
0.00
−0.04
−0.08
0.1
0.2
0.3
0.4
SNP effect on NHP2−like protein 1
SNP effect on Selenium

Batch 3−2049 : Selenium on Nidogen−1

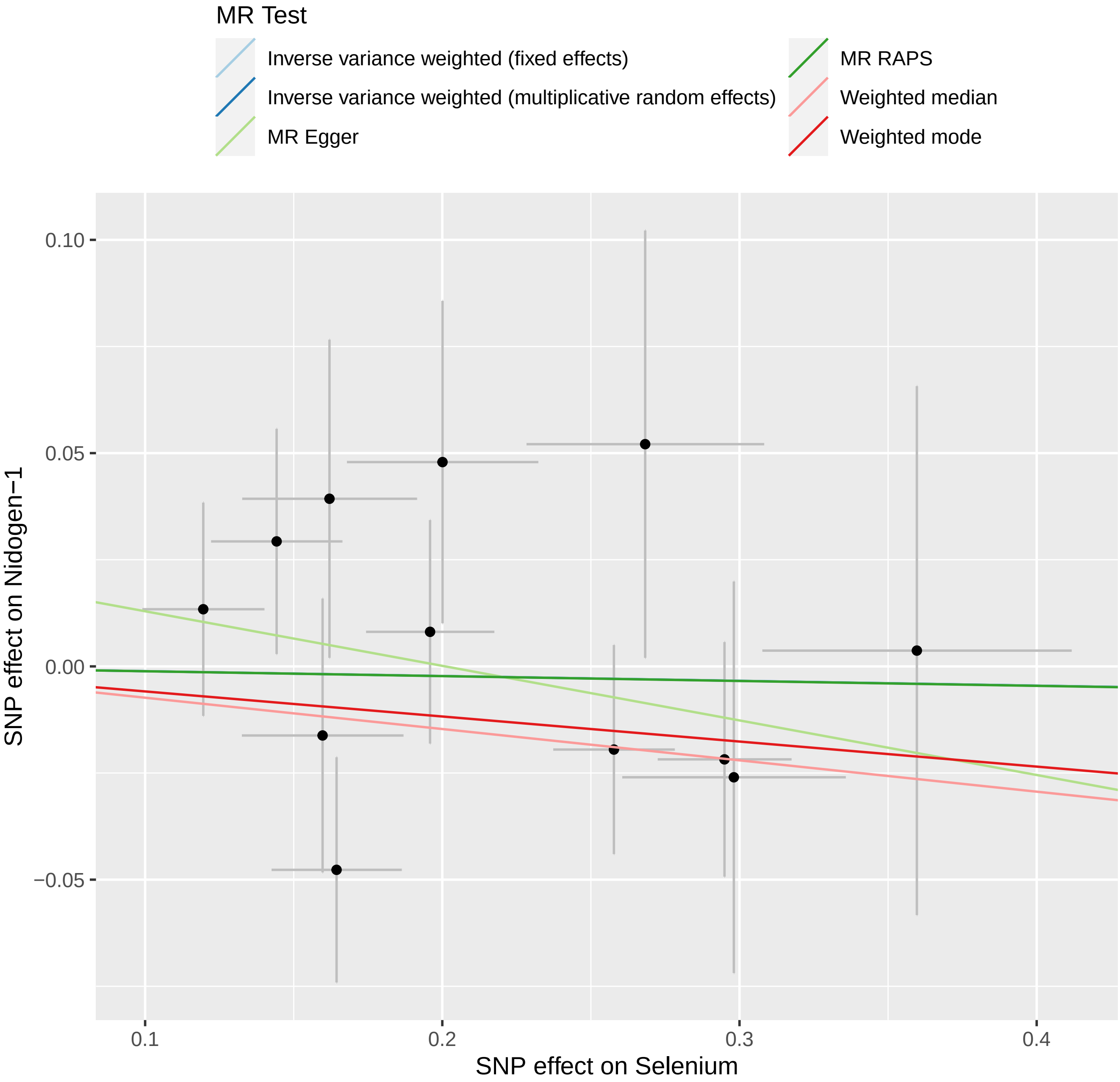

Batch 3−2050 : Selenium on Nidogen−2

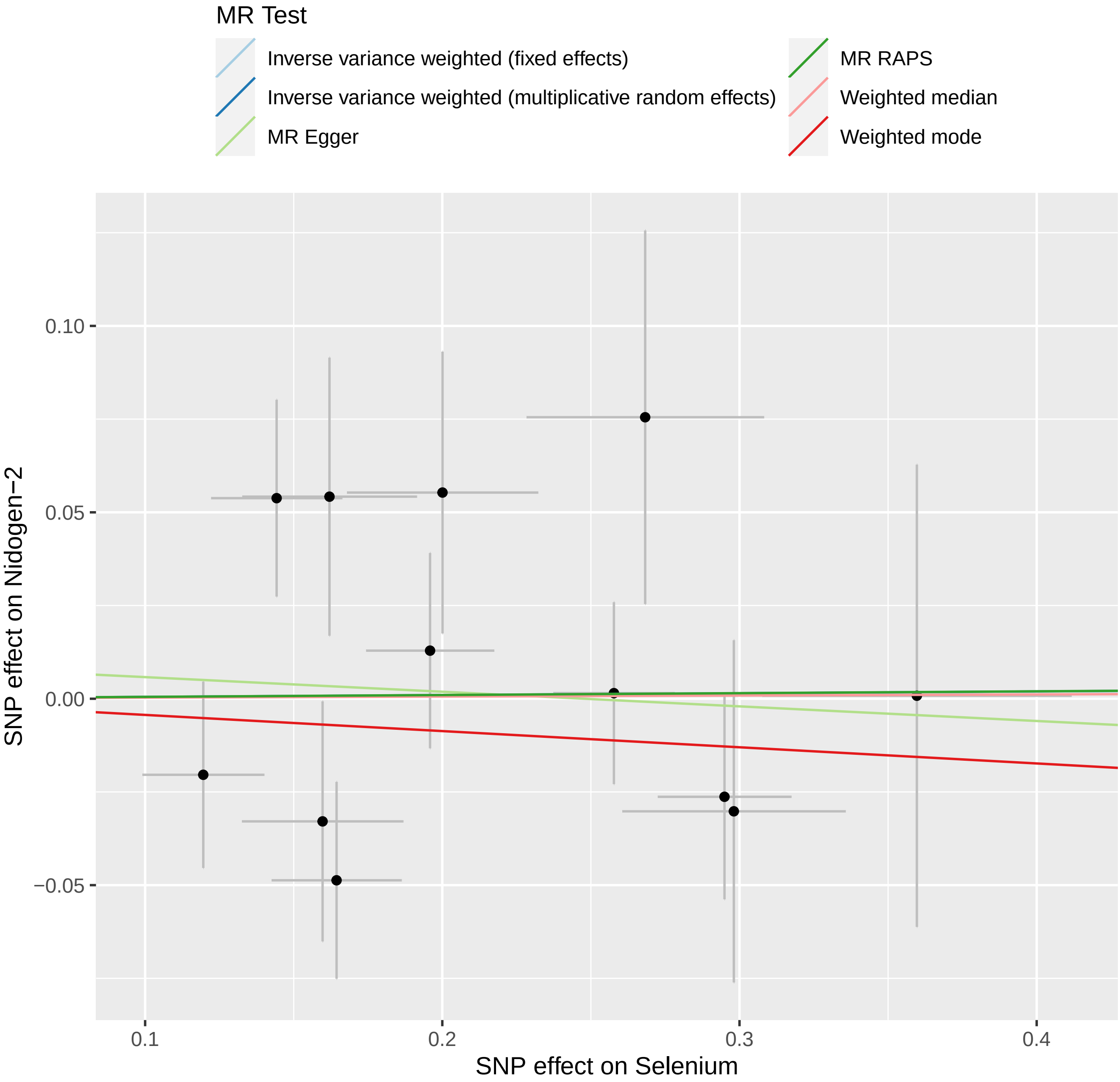

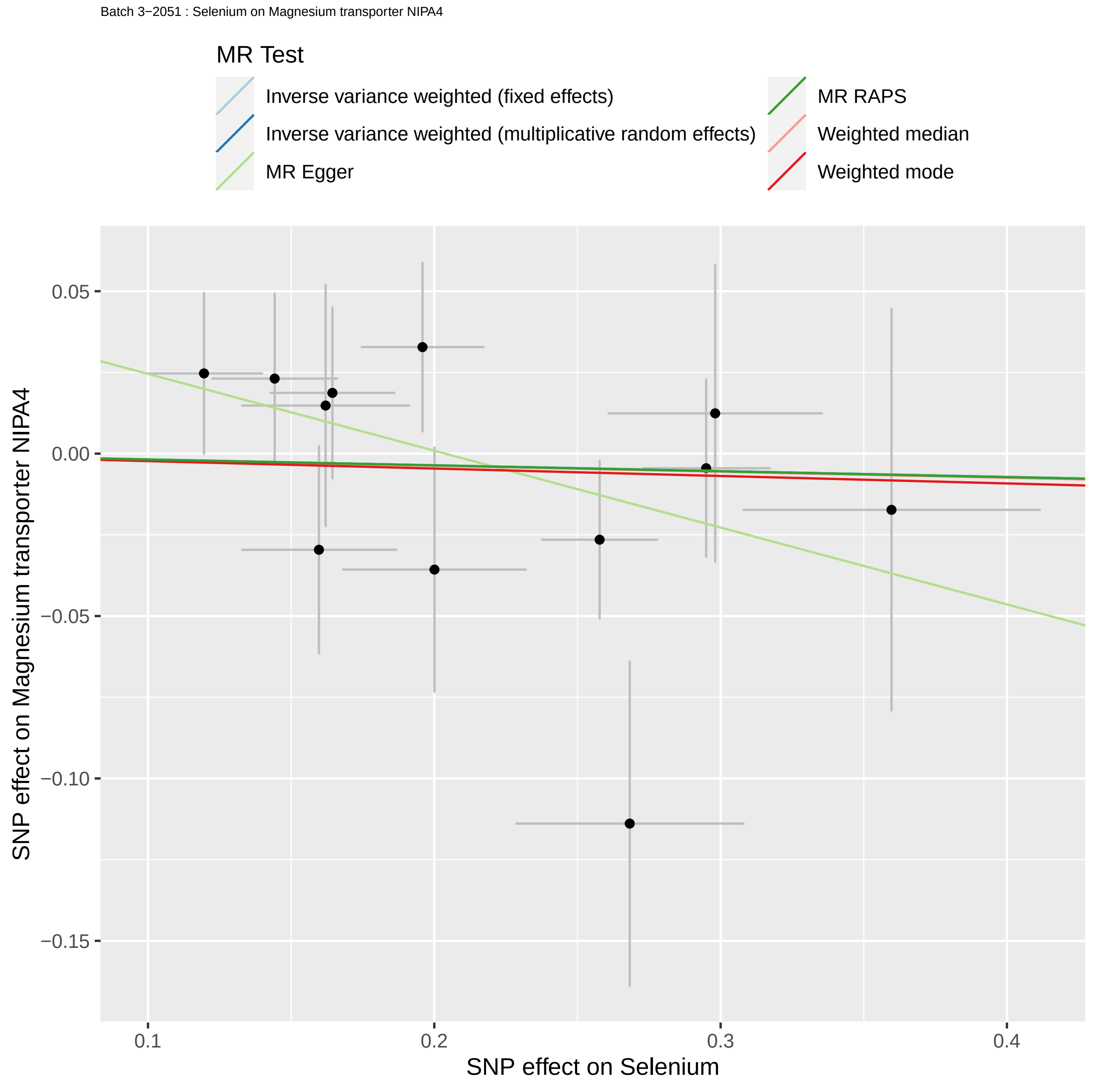

Batch 3−2051 : Selenium on Magnesium transporter NIPA4
MR Test
Inverse variance weighted (fixed effects)
Inverse variance weighted (multiplicative random effects)
MR Egger
MR RAPS
Weighted median
Weighted mode
0.05
0.00
−0.05
−0.10
−0.15
0.1
0.2
0.3
0.4
SNP effect on Magnesium transporter NIPA4
SNP effect on Selenium

Batch 3−2052 : Selenium on Nischarin

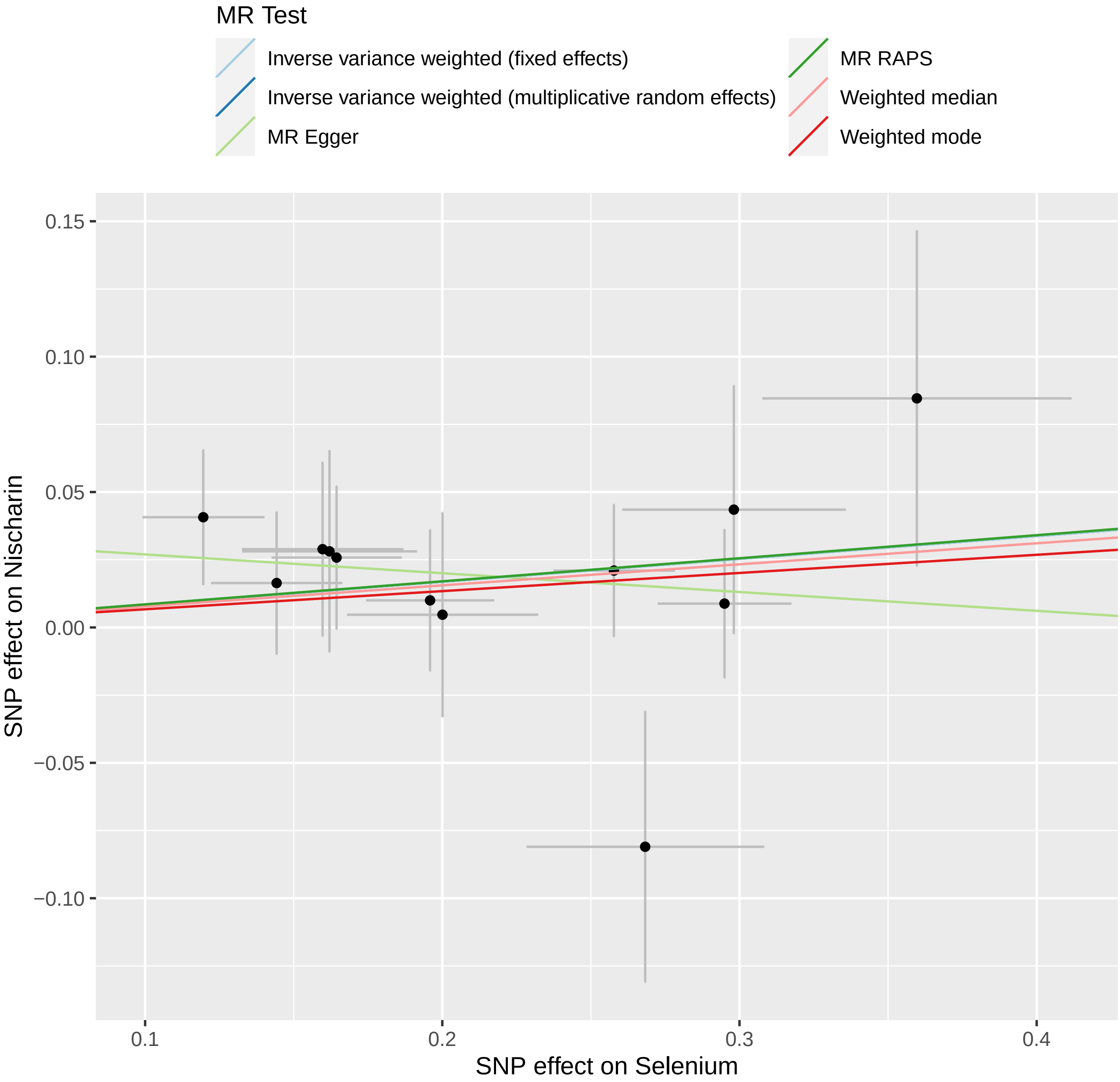

Batch 3−2053 : Selenium on Neuroligin−1

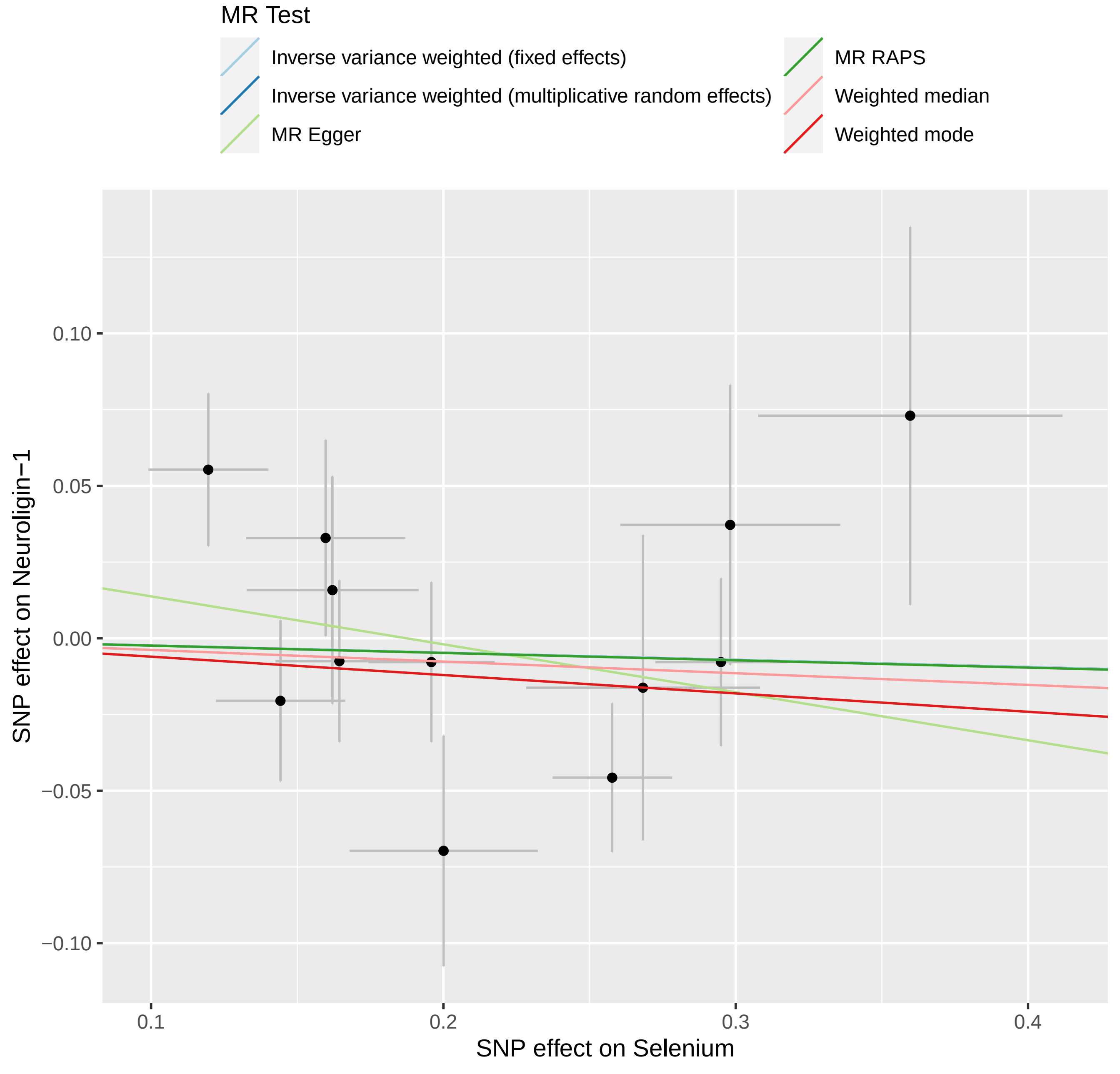

Batch 3−2054 : Selenium on Neuroligin−4, X−linked

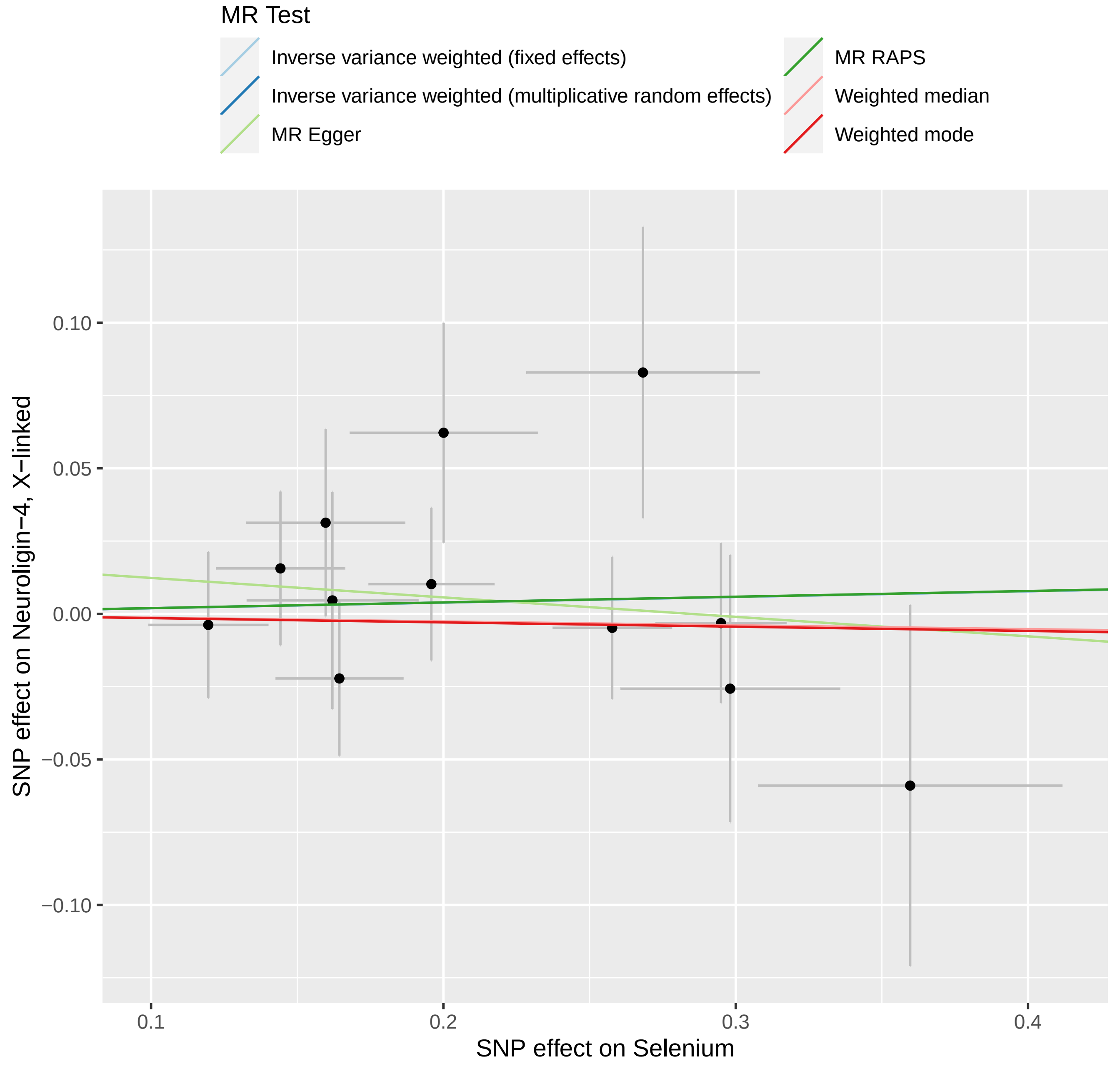

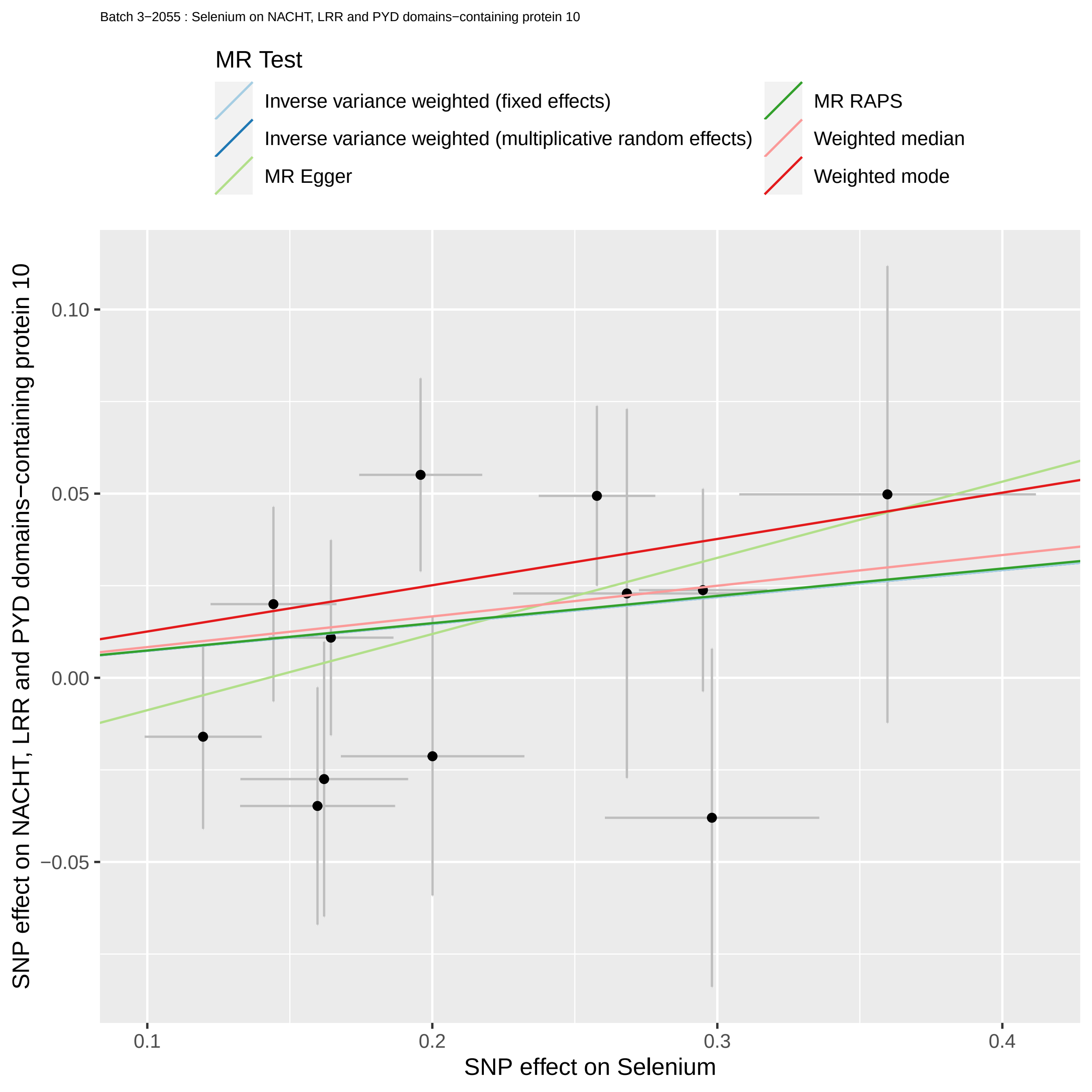
Batch 3−2055 : Selenium on NACHT, LRR and PYD domains−containing protein 10
MR Test
Inverse variance weighted (fixed effects)
Inverse variance weighted (multiplicative random effects)
MR Egger
MR RAPS
Weighted median
Weighted mode
SNP effect on NACHT, LRR and PYD domains−containing protein 10
0.10
0.05
0.00
−0.05
0.1
0.2
0.3
0.4
SNP effect on Selenium

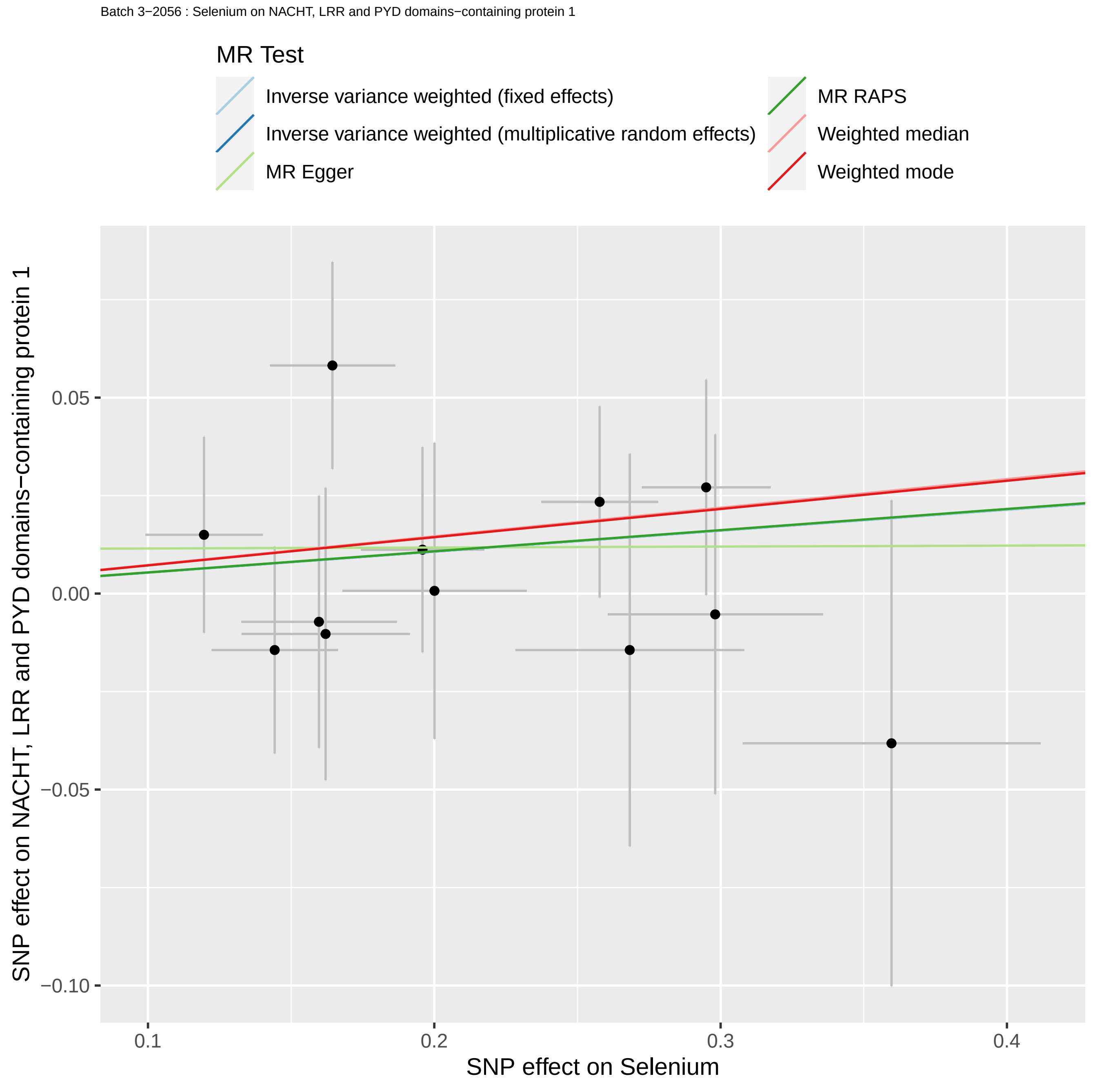

Batch 3−2056 : Selenium on NACHT, LRR and PYD domains−containing protein 1
MR Test
Inverse variance weighted (fixed effects)
Inverse variance weighted (multiplicative random effects)
MR Egger
MR RAPS
Weighted median
Weighted mode
SNP effect on NACHT, LRR and PYD domains−containing protein 1
0.05
0.00
−0.05
−0.10
0.1
0.2
0.3
0.4
SNP effect on Selenium

Batch 3−2057 : Selenium on NACHT, LRR and PYD domains−containing protein 4

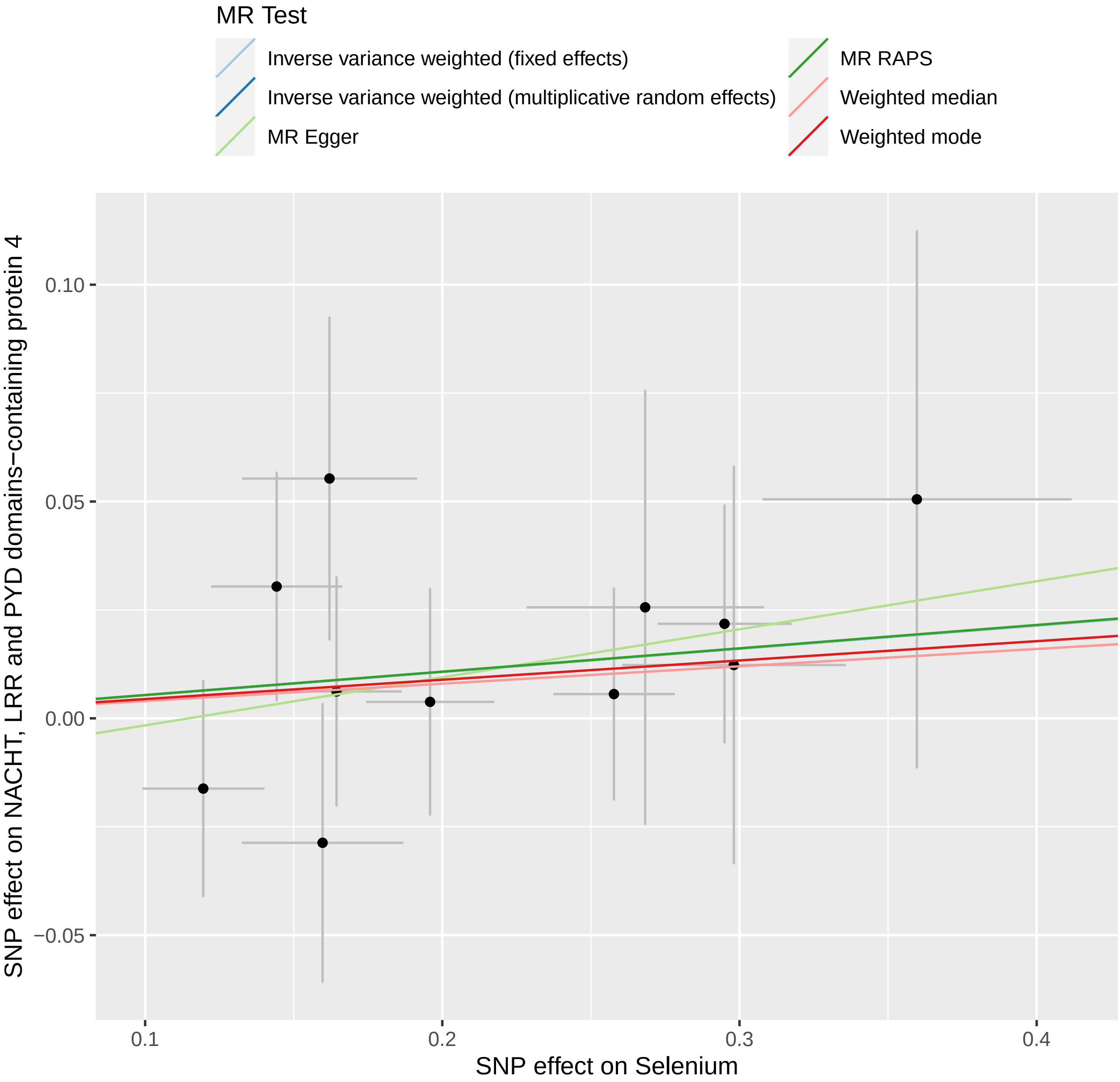

Batch 3−2058 : Selenium on Neuromedin−B

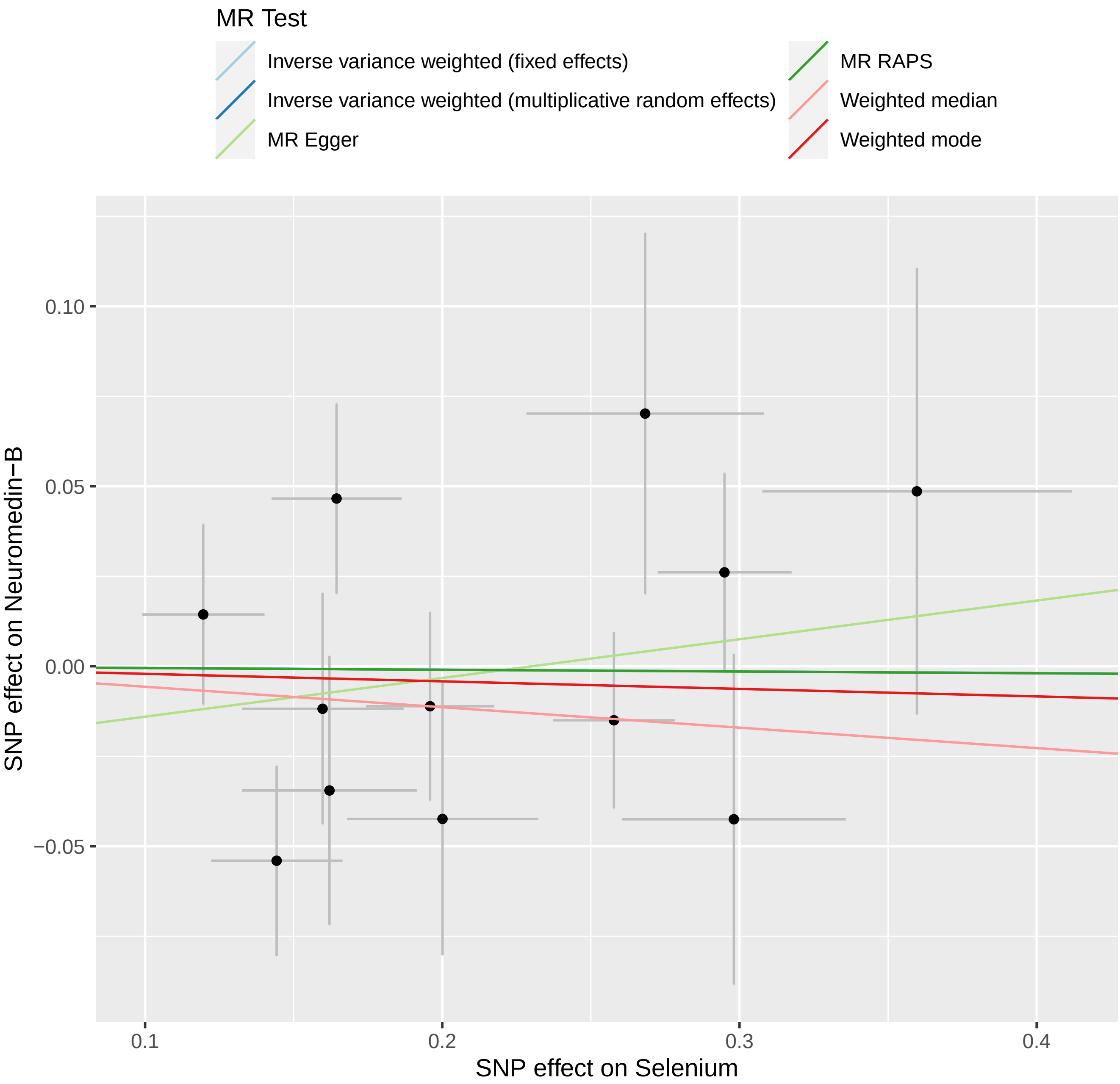

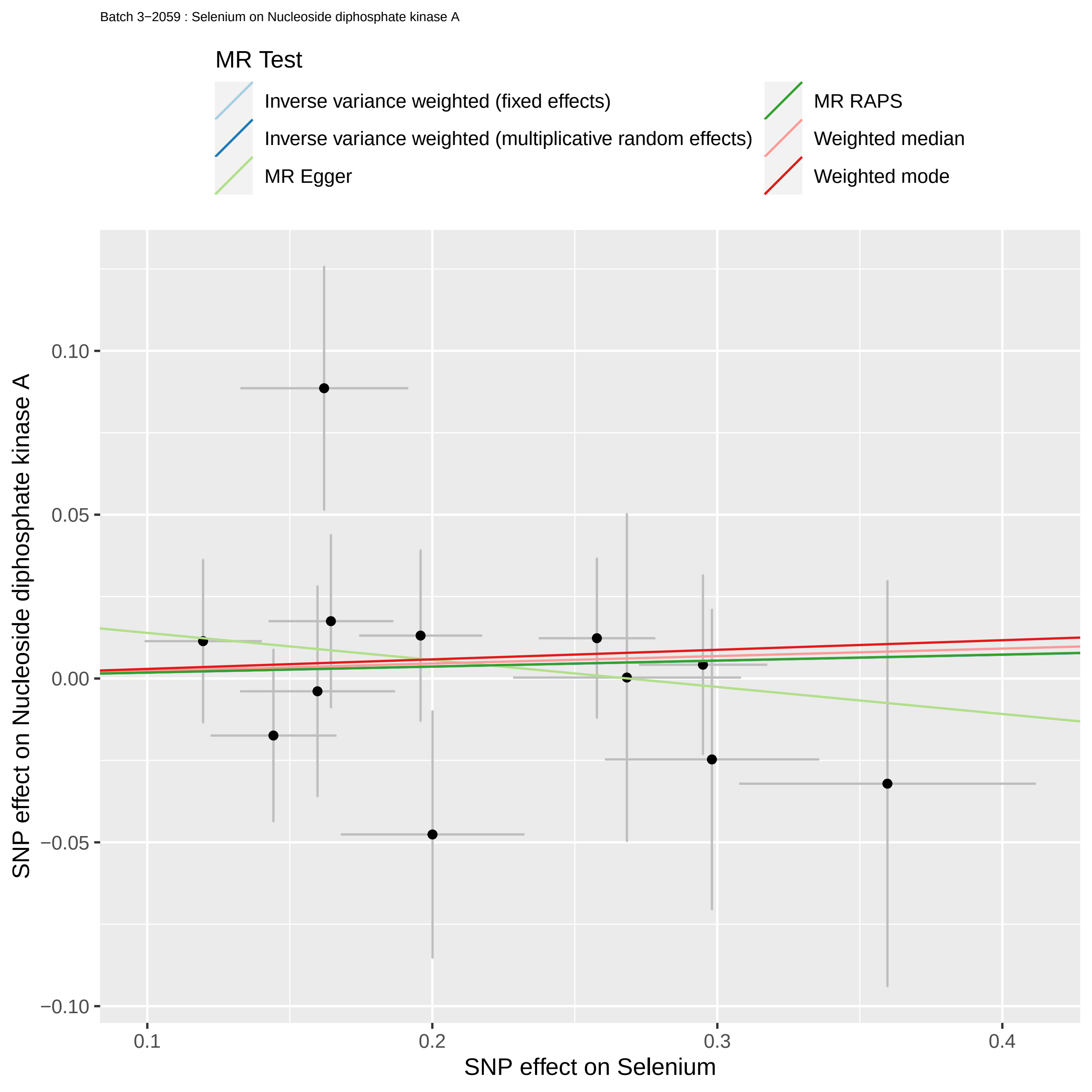
Batch 3−2059 : Selenium on Nucleoside diphosphate kinase A
MR Test
Inverse variance weighted (fixed effects)
Inverse variance weighted (multiplicative random effects)
MR Egger
MR RAPS
Weighted median
Weighted mode
0.10
0.05
0.00
−0.05
−0.10
0.1
0.2
0.3
0.4
SNP effect on Nucleoside diphosphate kinase A
SNP effect on Selenium

Batch 3−2060 : Selenium on Nucleoside diphosphate kinase B

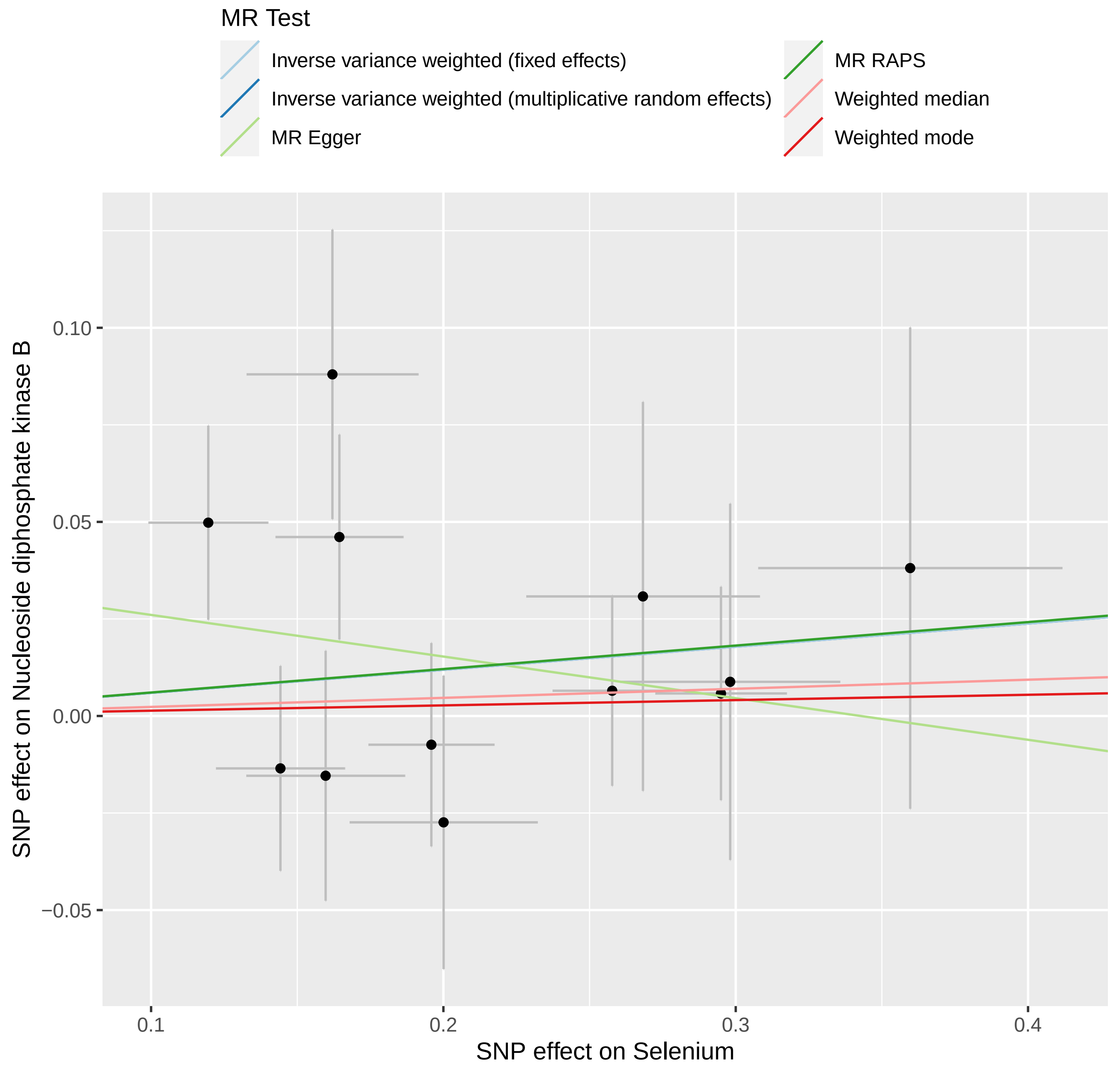

Batch 3−2061 : Selenium on Normal mucosa of esophagus−specific gene 1 protein

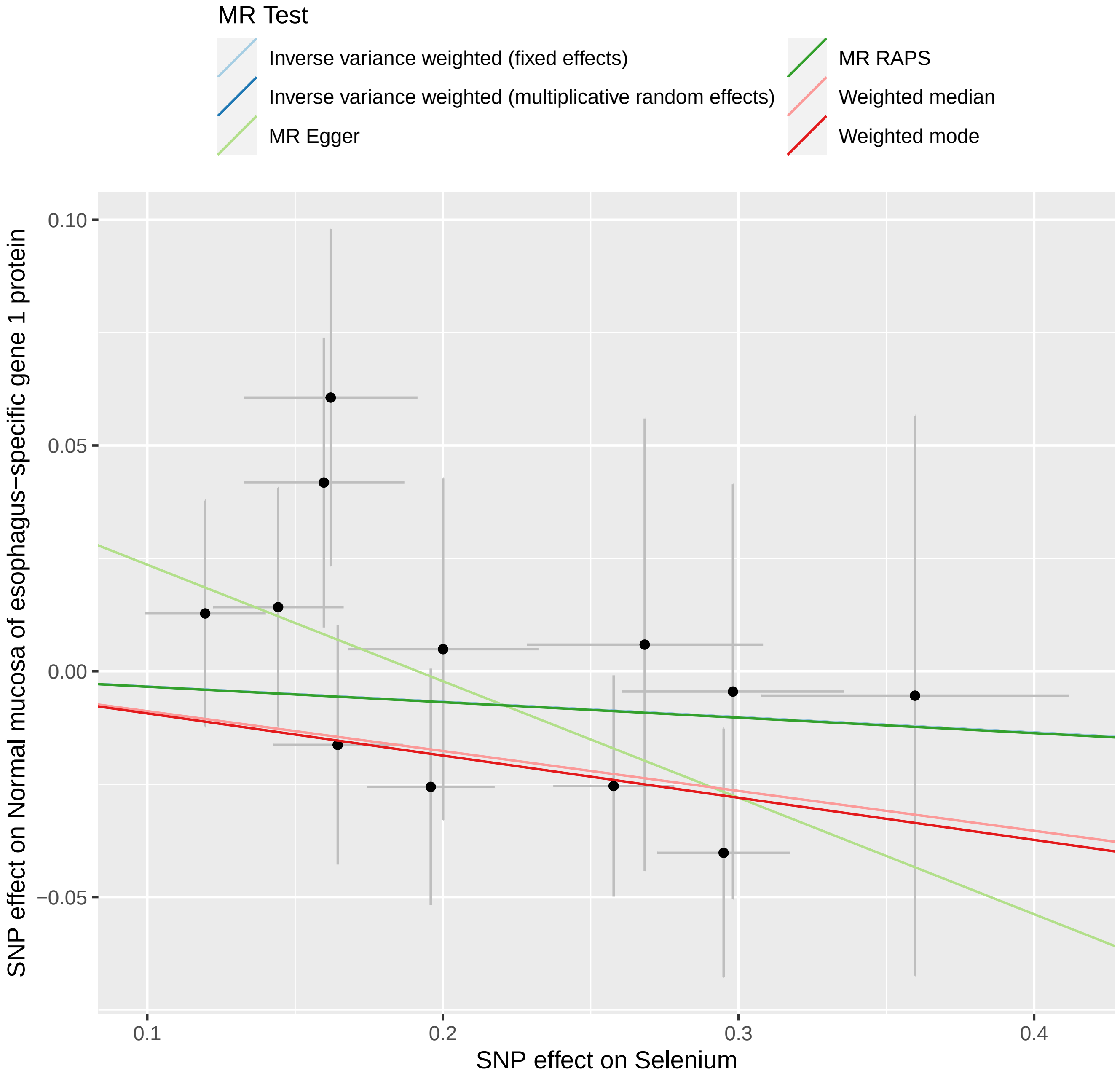

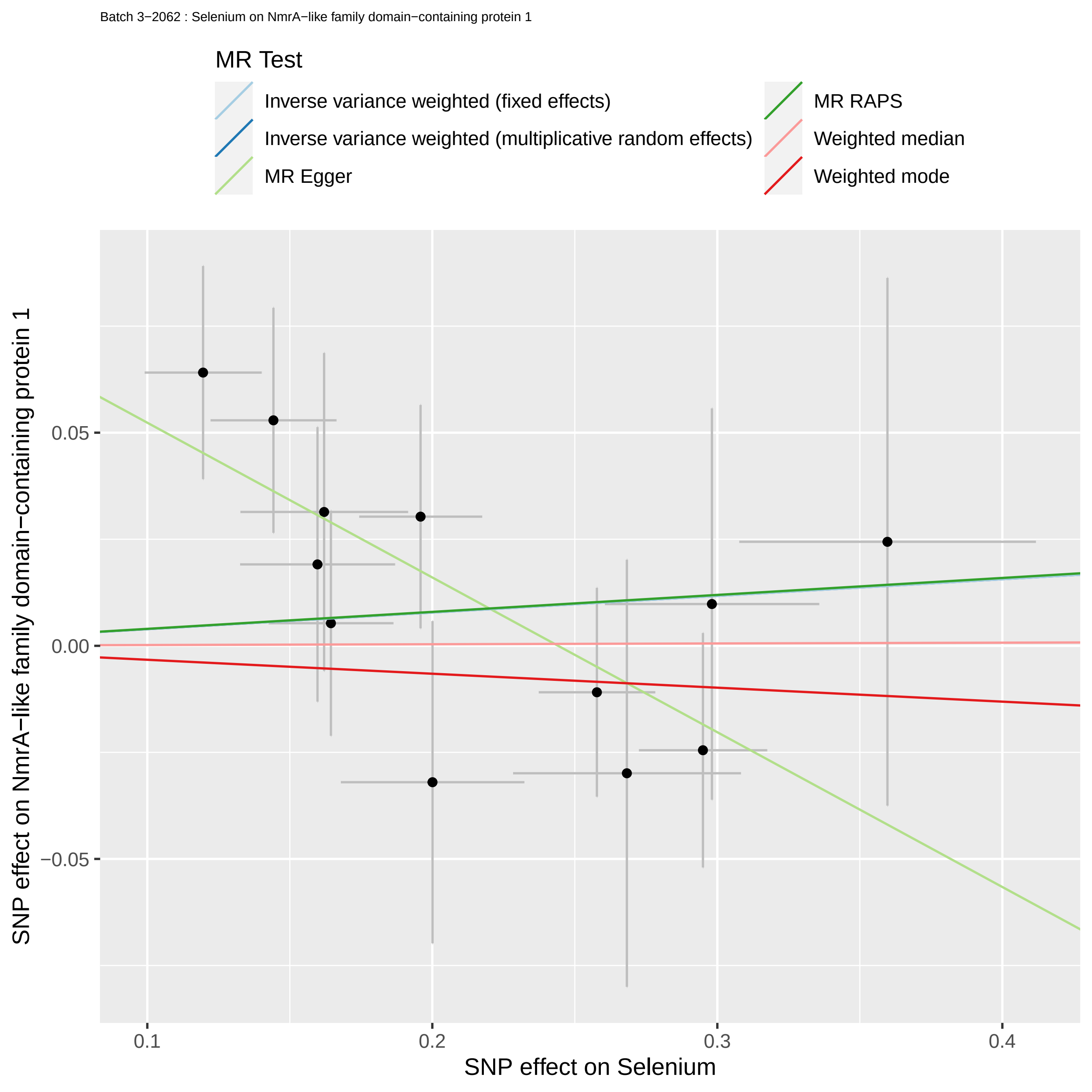

Batch 3−2062 : Selenium on NmrA−like family domain−containing protein 1
MR Test
Inverse variance weighted (fixed effects)
Inverse variance weighted (multiplicative random effects)
MR Egger
MR RAPS
Weighted median
Weighted mode
0.05
0.00
−0.05
0.1
0.2
0.3
0.4
SNP effect on NmrA−like family domain−containing protein 1
SNP effect on Selenium

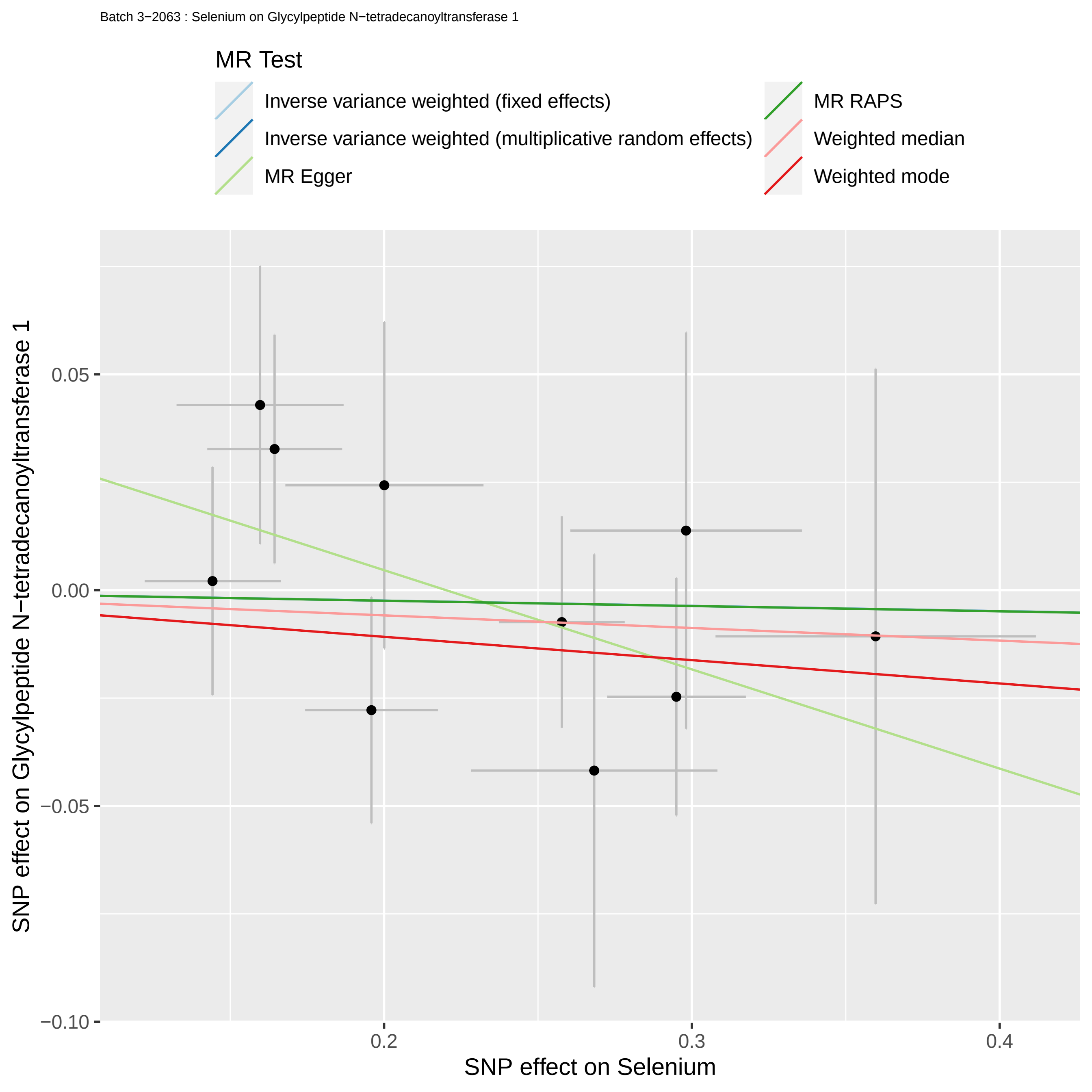

Batch 3−2063 : Selenium on Glycylpeptide N−tetradecanoyltransferase 1
MR Test
Inverse variance weighted (fixed effects)
Inverse variance weighted (multiplicative random effects)
MR Egger
MR RAPS
Weighted median
Weighted mode
SNP effect on Glycylpeptide N−tetradecanoyltransferase 1
0.05
0.00
−0.05
−0.10
0.2
0.3
0.4
SNP effect on Selenium

Batch 3−2064 : Selenium on Noggin

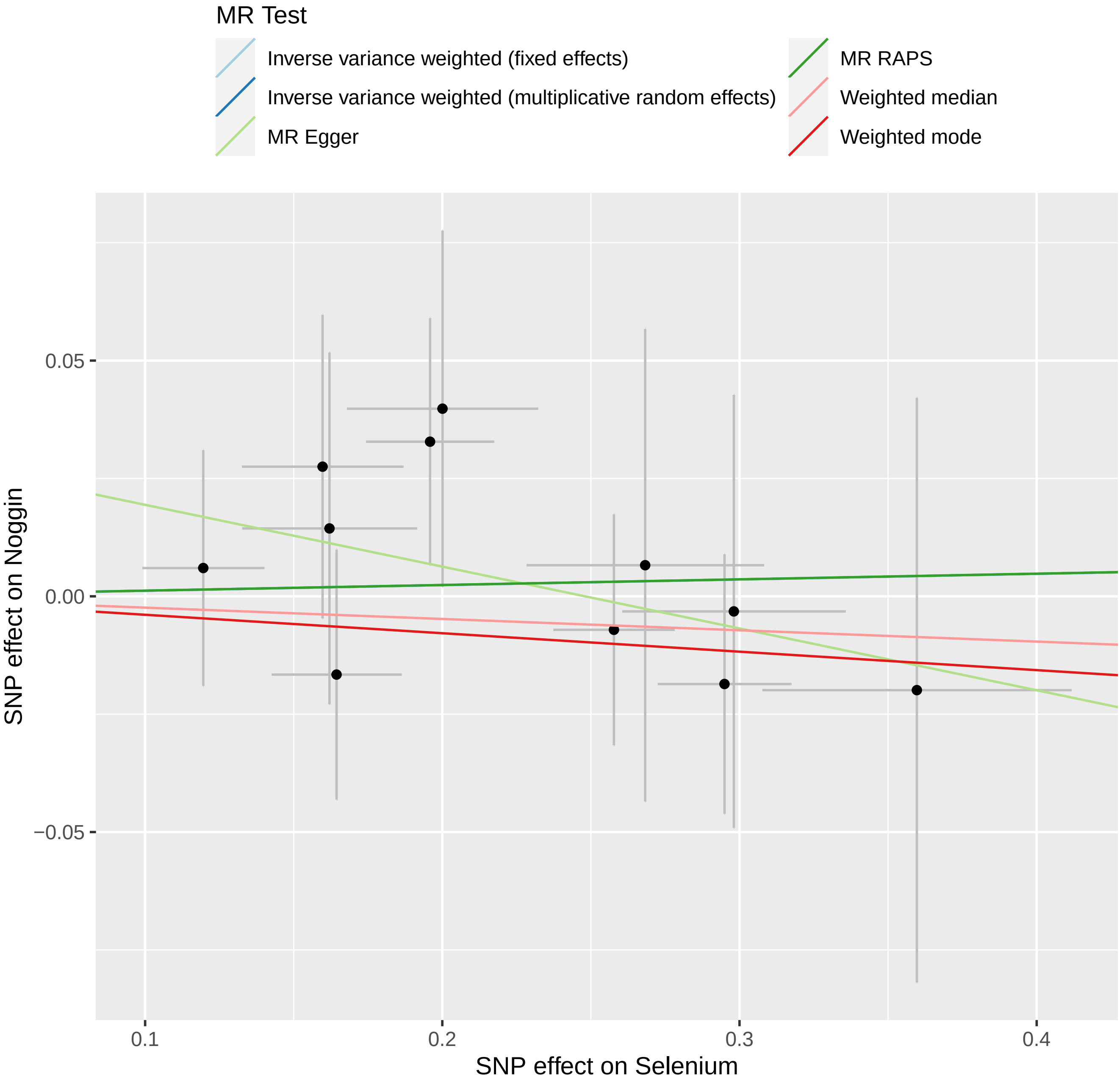

Batch 3−2065 : Selenium on NoneX

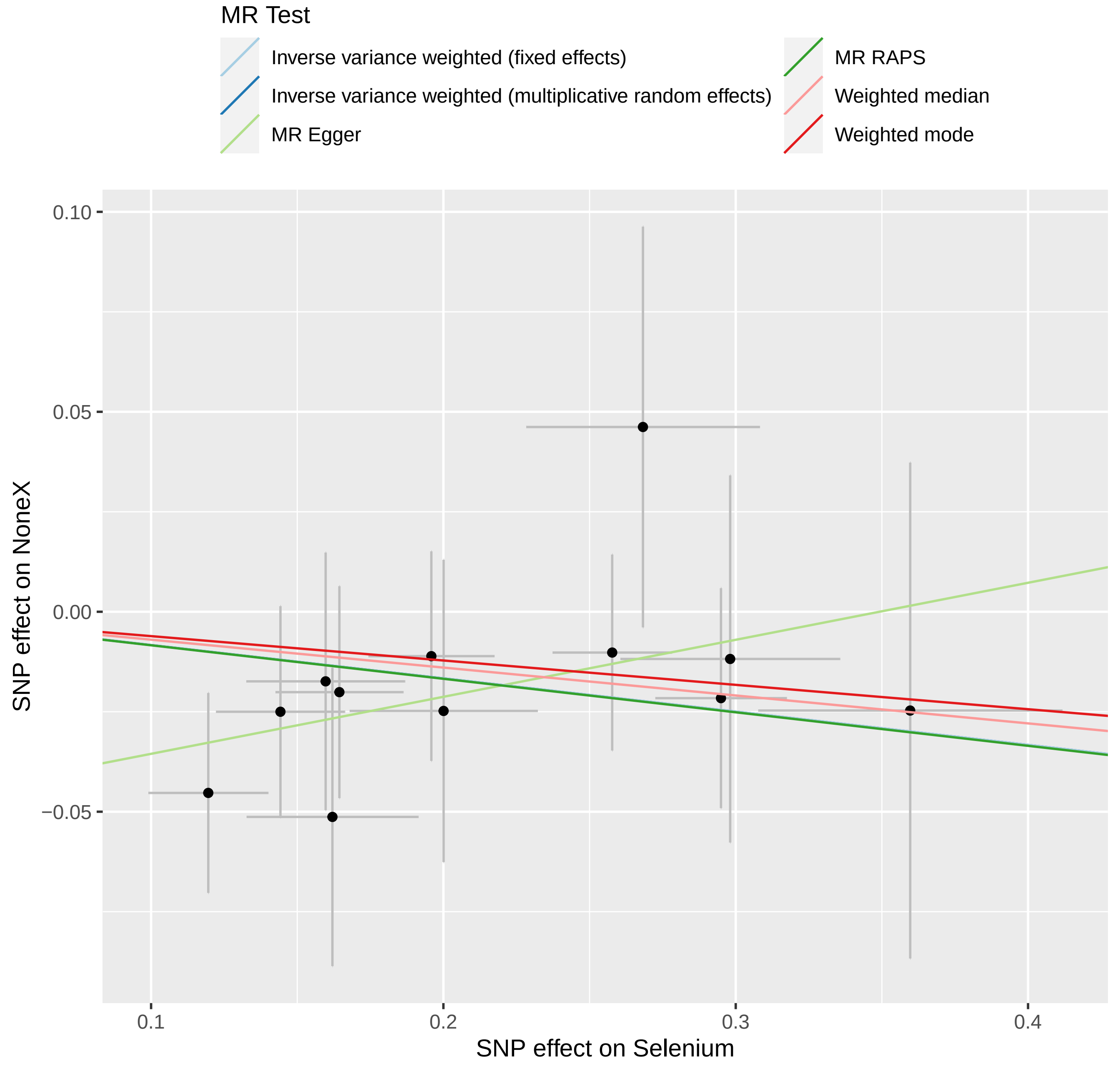

Batch 3−2066 : Selenium on NoneX

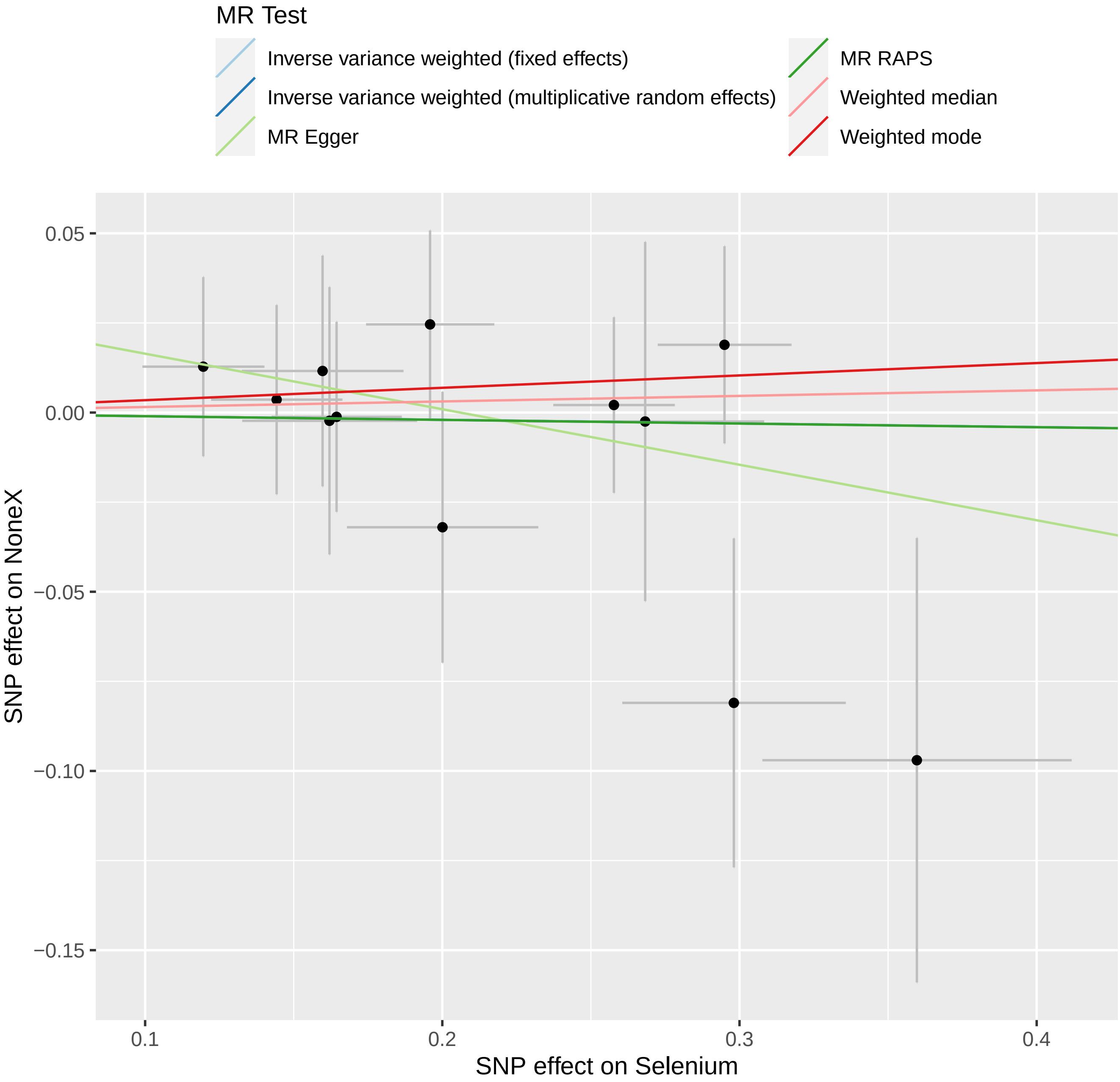

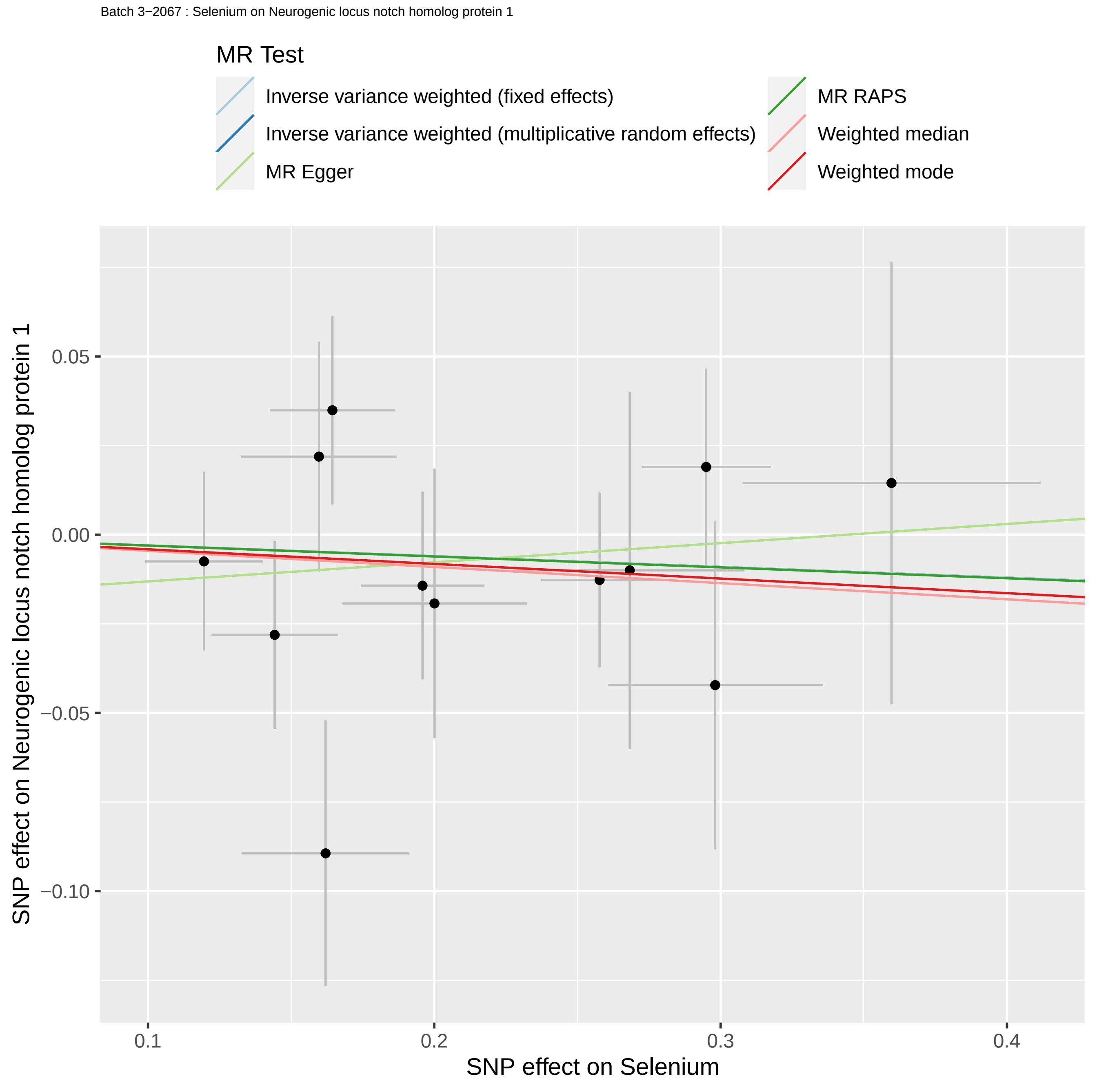

Batch 3−2067 : Selenium on Neurogenic locus notch homolog protein 1
MR Test
Inverse variance weighted (fixed effects)
Inverse variance weighted (multiplicative random effects)
MR Egger
MR RAPS
Weighted median
Weighted mode
0.05
0.00
−0.05
−0.10
0.1
0.2
0.3
0.4
SNP effect on Neurogenic locus notch homolog protein 1
SNP effect on Selenium

Batch 3−2068 : Selenium on Palmitoleoyl−protein carboxylesterase NOTUM

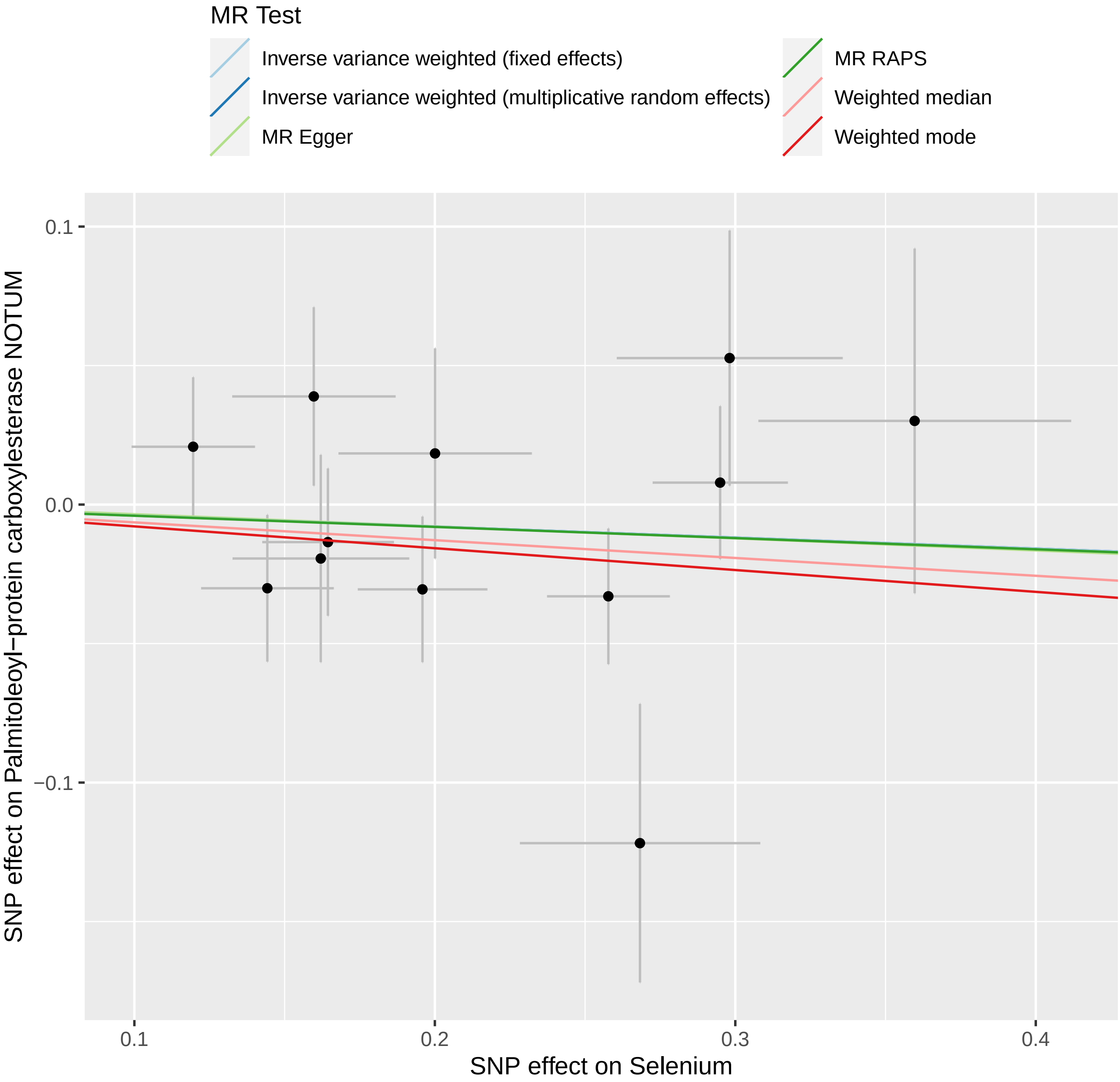

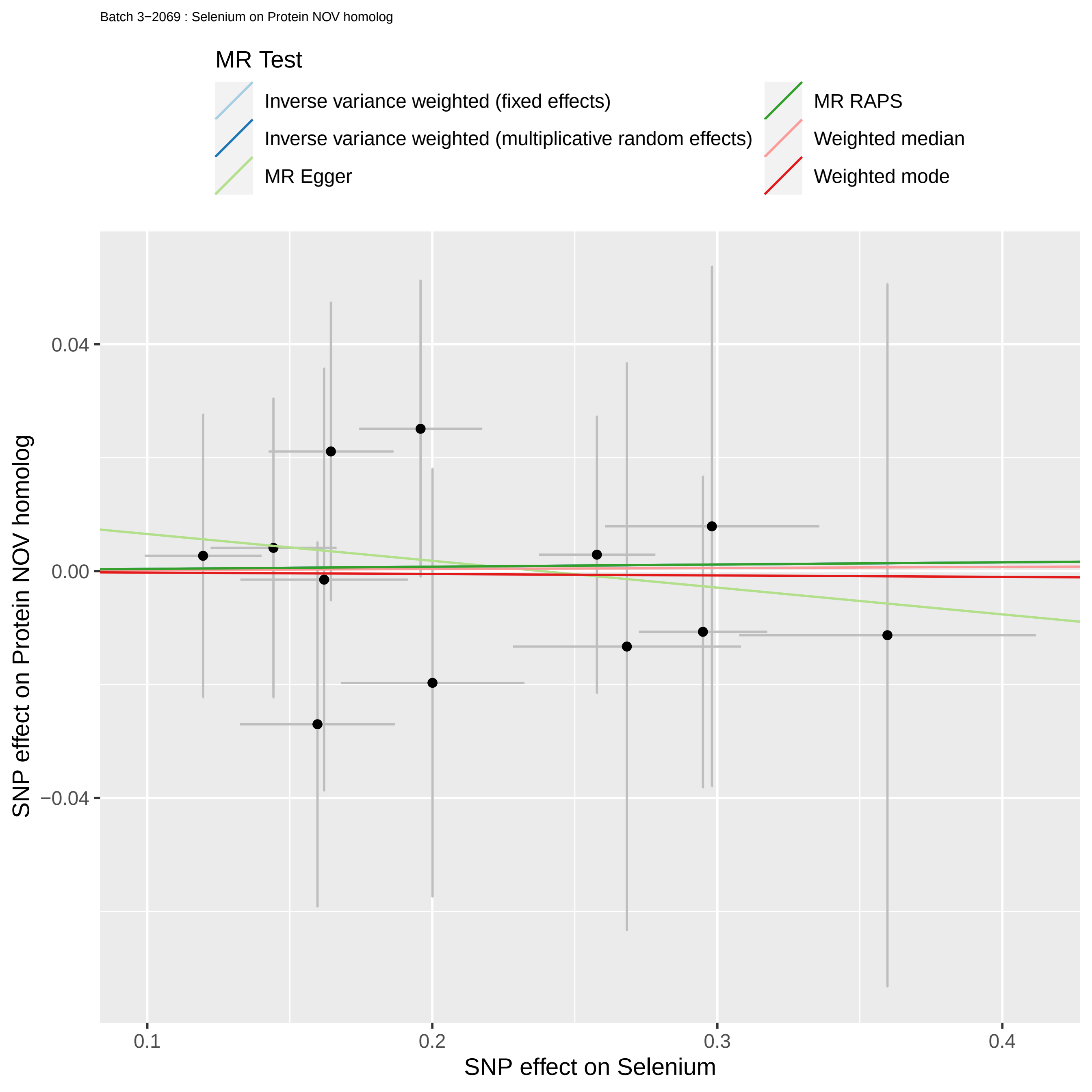

Batch 3−2069 : Selenium on Protein NOV homolog
MR Test
Inverse variance weighted (fixed effects)
Inverse variance weighted (multiplicative random effects)
MR Egger
MR RAPS
Weighted median
Weighted mode
0.04
0.00
−0.04
0.1
0.2
0.3
0.4
SNP effect on Selenium
SNP effect on Protein NOV homolog

Batch 3−2070 : Selenium on RNA−binding protein Nova−1

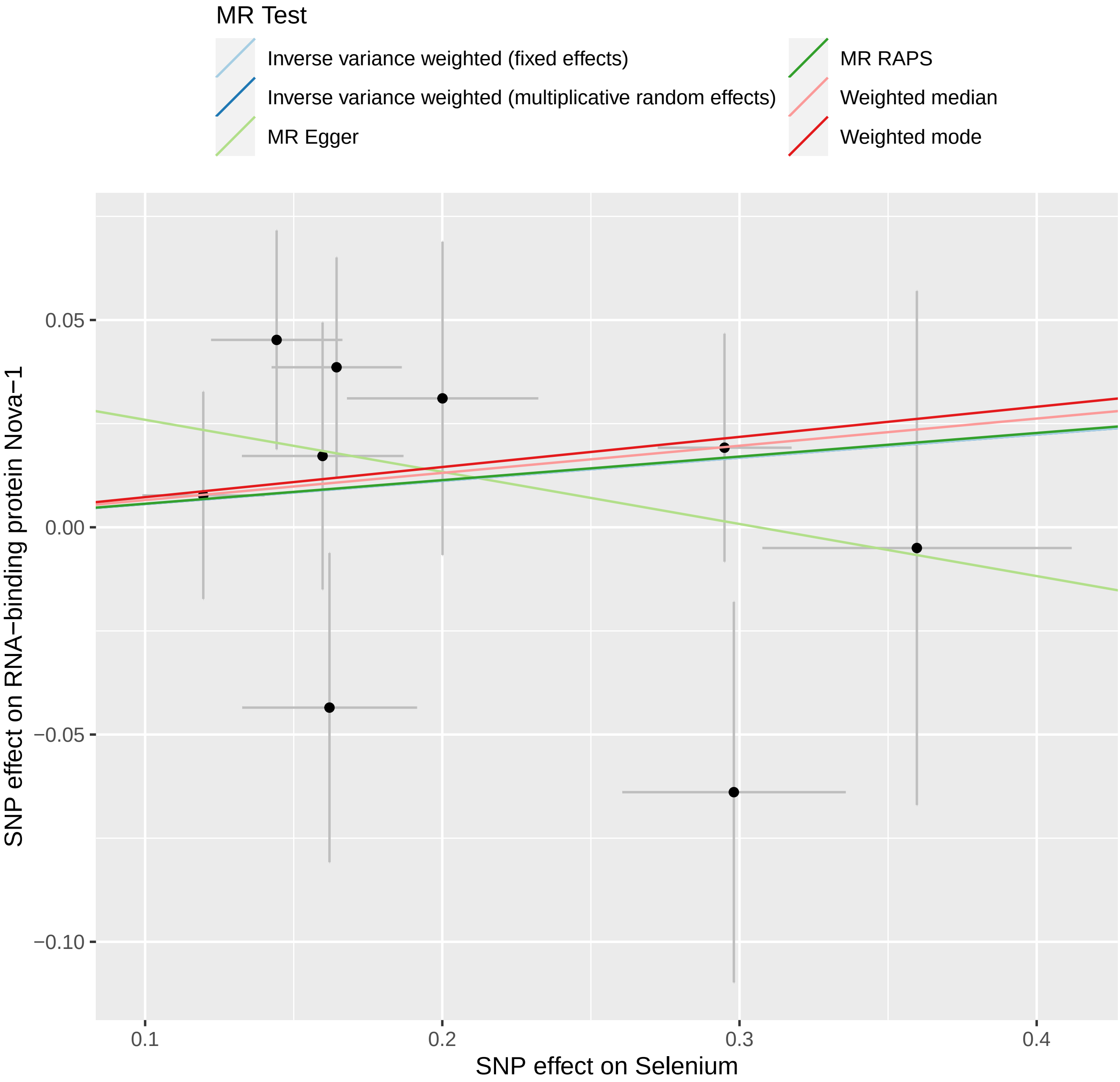

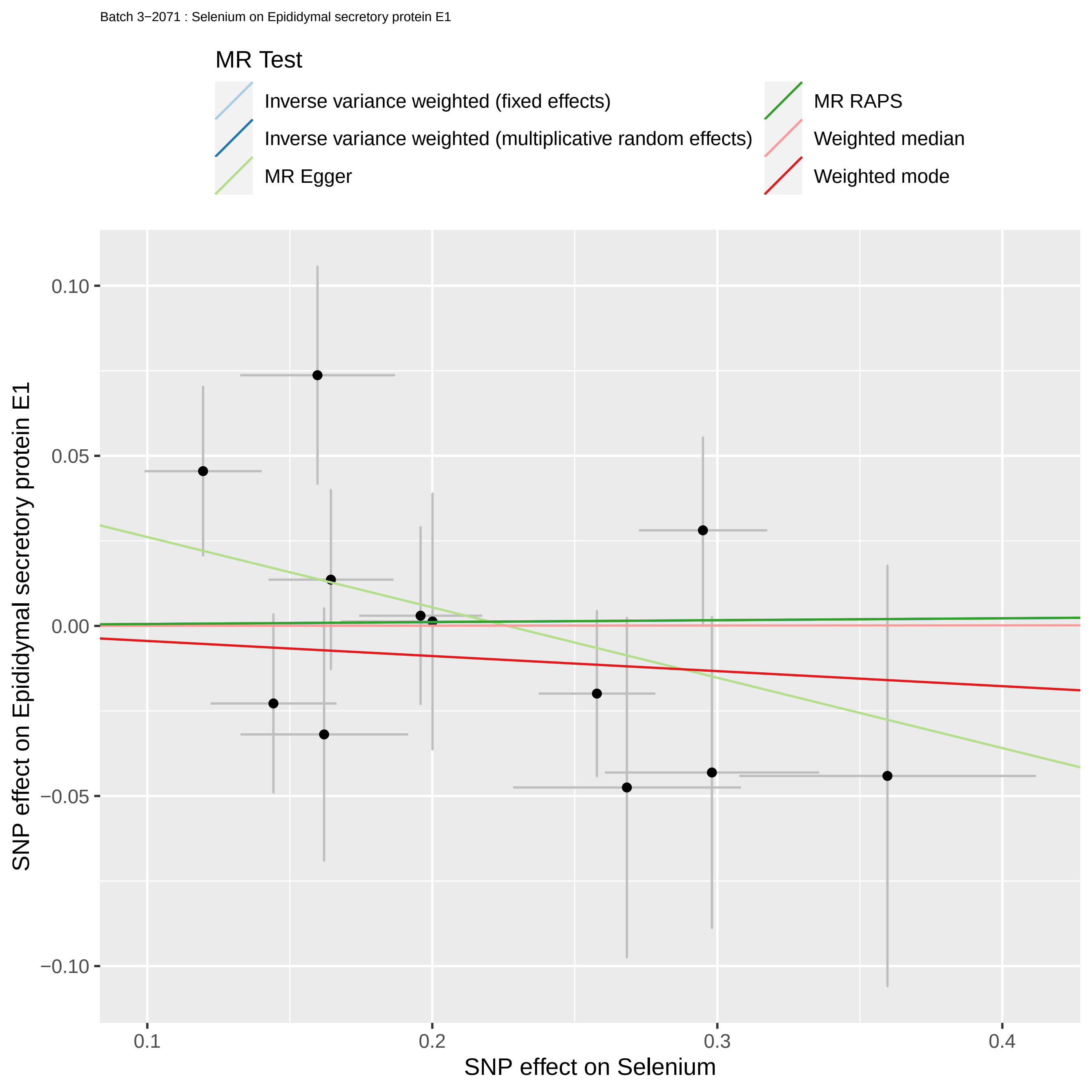

Batch 3−2071 : Selenium on Epididymal secretory protein E1
MR Test
Inverse variance weighted (fixed effects)
Inverse variance weighted (multiplicative random effects)
MR Egger
MR RAPS
Weighted median
Weighted mode
SNP effect on Epididymal secretory protein E1
0.10
0.05
0.00
−0.05
−0.10
0.1
0.2
0.3
0.4
SNP effect on Selenium

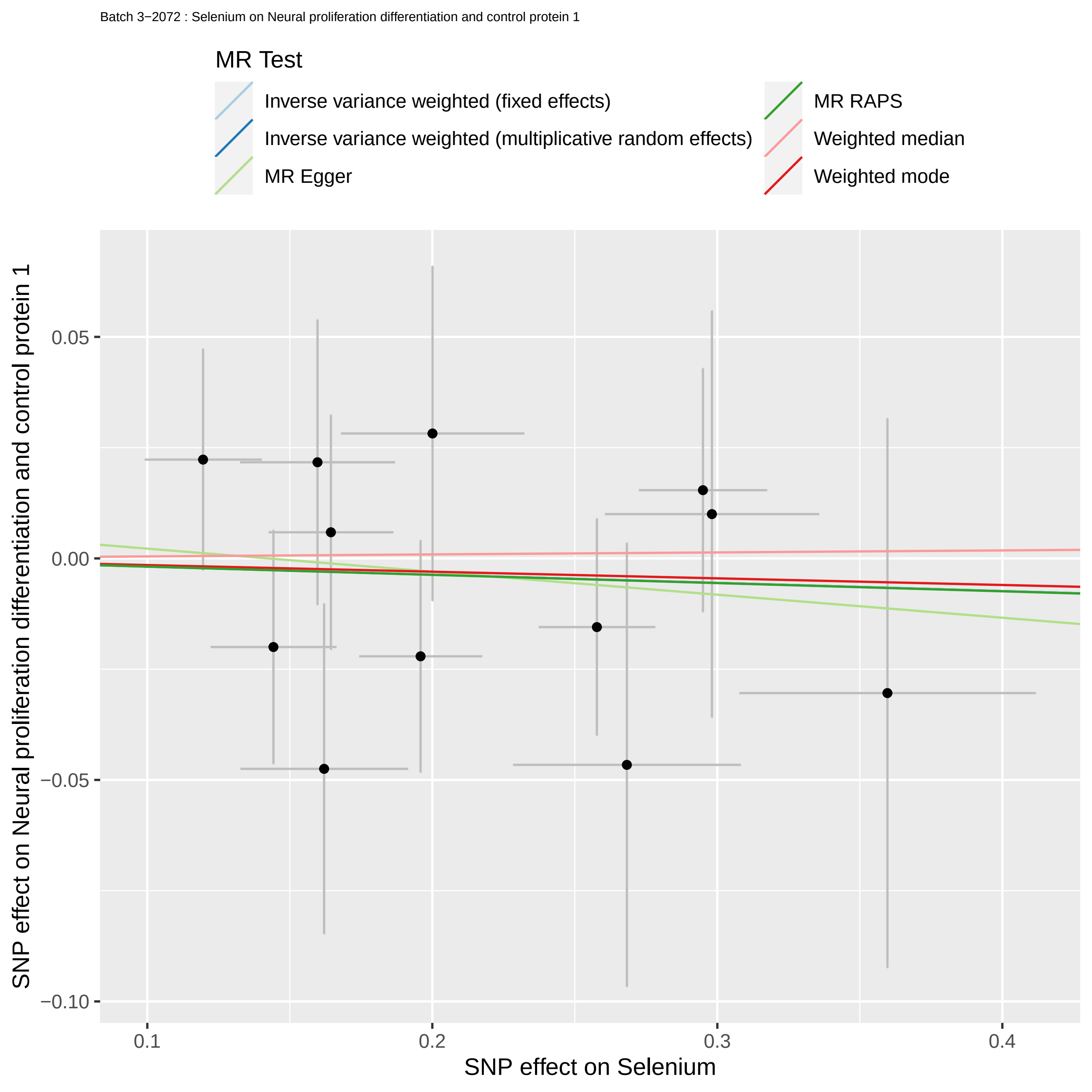
Batch 3−2072 : Selenium on Neural proliferation differentiation and control protein 1
MR Test
Inverse variance weighted (fixed effects)
Inverse variance weighted (multiplicative random effects)
MR Egger
MR RAPS
Weighted median
Weighted mode
SNP effect on Neural proliferation differentiation and control protein 1
0.05
0.00
−0.05
−0.10
0.1
0.2
0.3
0.4
SNP effect on Selenium

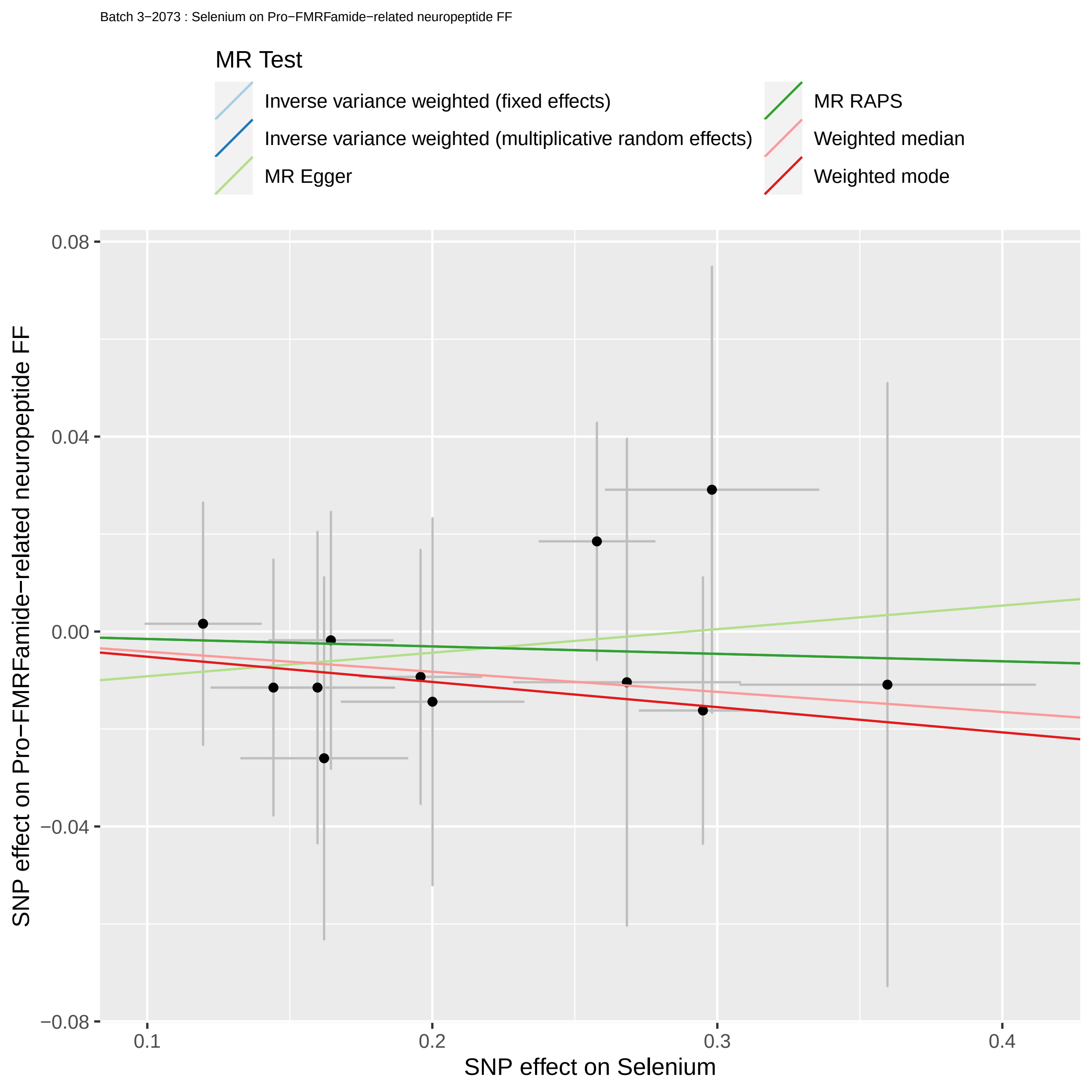
Batch 3−2073 : Selenium on Pro−FMRFamide−related neuropeptide FF
MR Test
Inverse variance weighted (fixed effects)
Inverse variance weighted (multiplicative random effects)
MR Egger
MR RAPS
Weighted median
Weighted mode
0.08
0.04
0.00
−0.04
−0.08
0.1
0.2
0.3
0.4
SNP effect on Pro−FMRFamide−related neuropeptide FF
SNP effect on Selenium

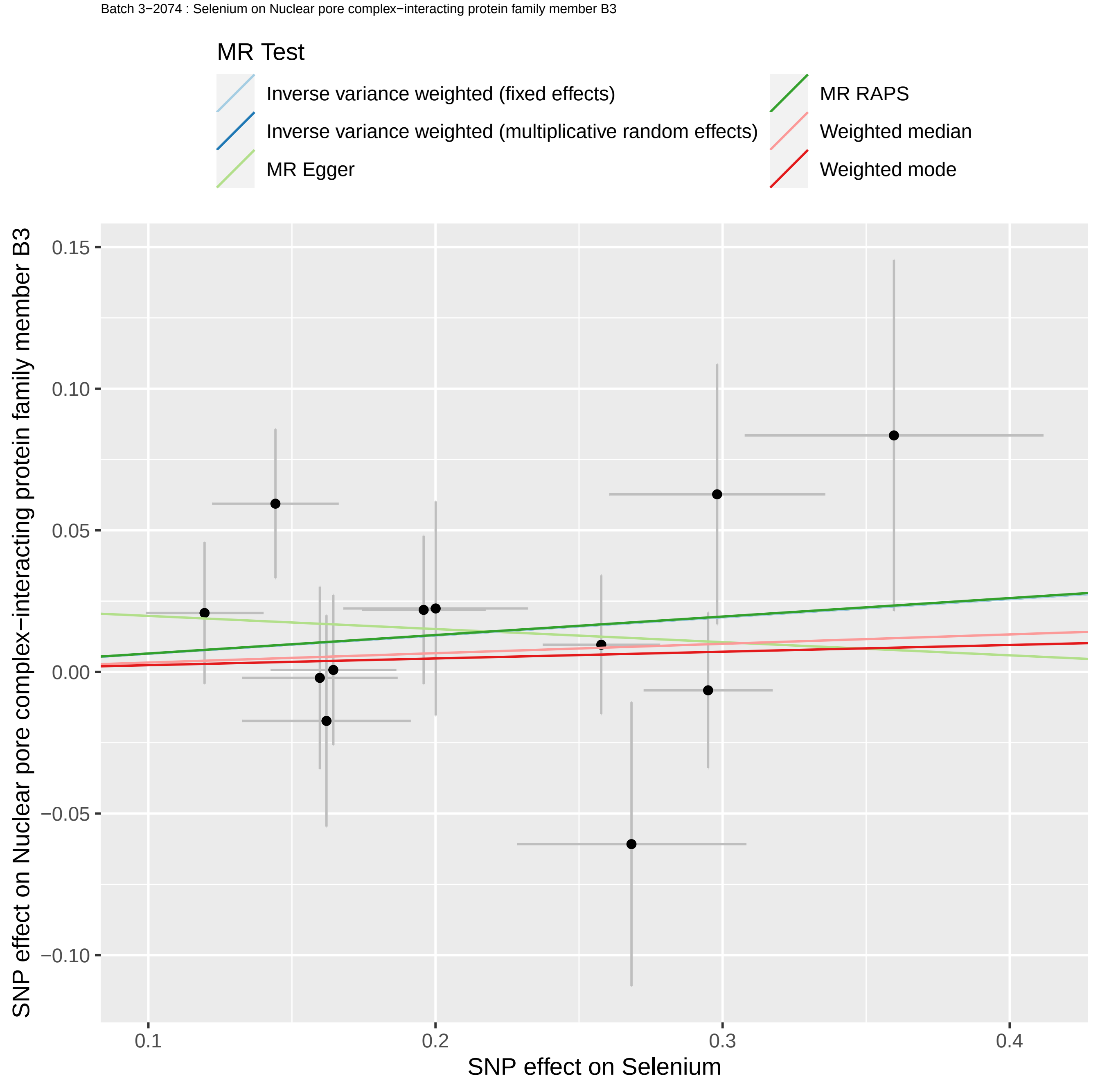
Batch 3−2074 : Selenium on Nuclear pore complex−interacting protein family member B3
MR Test
Inverse variance weighted (fixed effects)
Inverse variance weighted (multiplicative random effects)
MR Egger
MR RAPS
Weighted median
Weighted mode
SNP effect on Nuclear pore complex−interacting protein family member B3
0.15
0.10
0.05
0.00
−0.05
−0.10
0.1
0.2
0.3
0.4
SNP effect on Selenium

Batch 3−2075 : Selenium on Nuclear protein localization protein 4 homolog

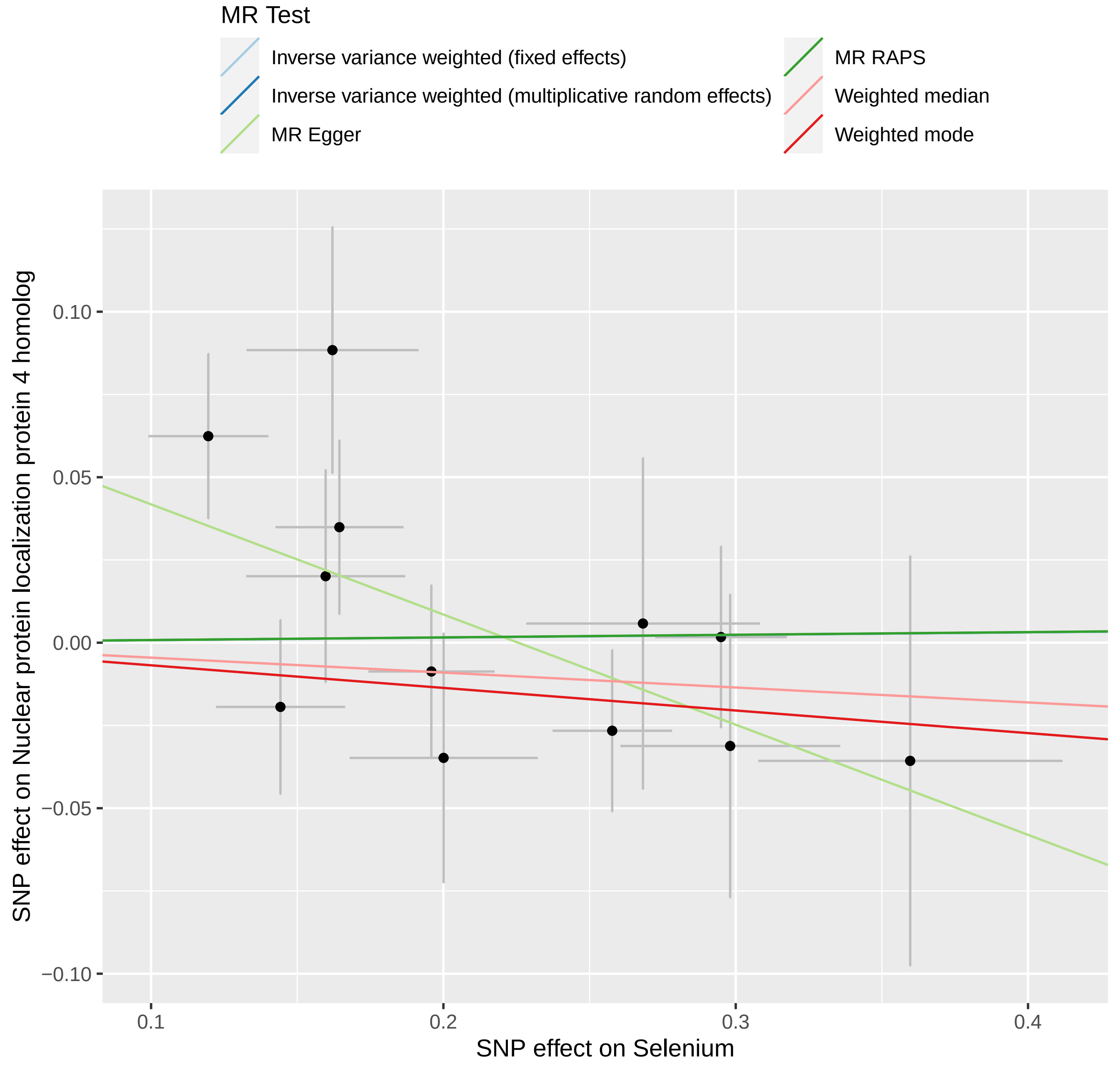

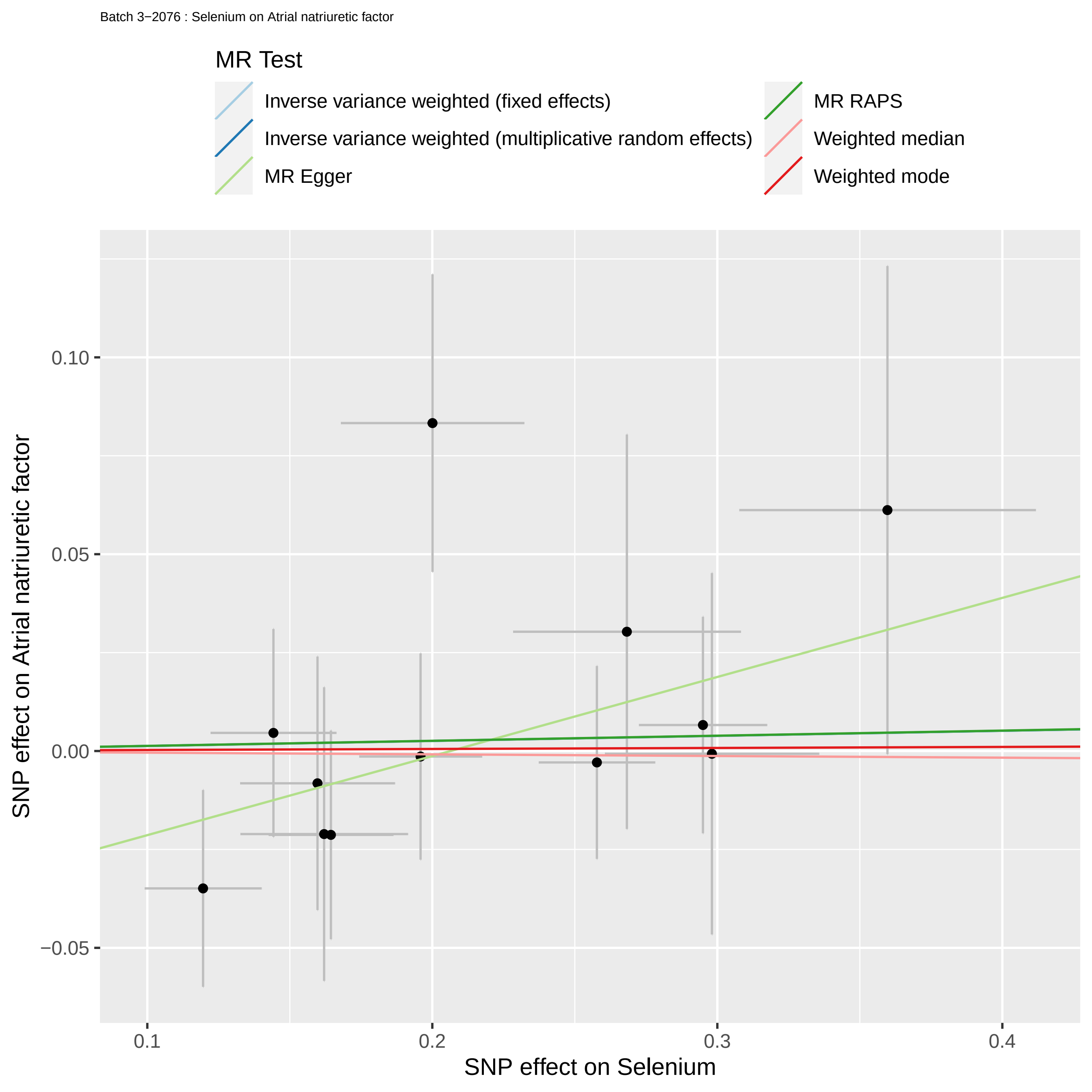
Batch 3−2076 : Selenium on Atrial natriuretic factor
MR Test
Inverse variance weighted (fixed effects)
Inverse variance weighted (multiplicative random effects)
MR Egger
MR RAPS
Weighted median
Weighted mode
0.10
0.05
0.00
−0.05
0.1
0.2
0.3
0.4
SNP effect on Atrial natriuretic factor
SNP effect on Selenium

Batch 3−2077 : Selenium on Brain natriuretic peptide 32

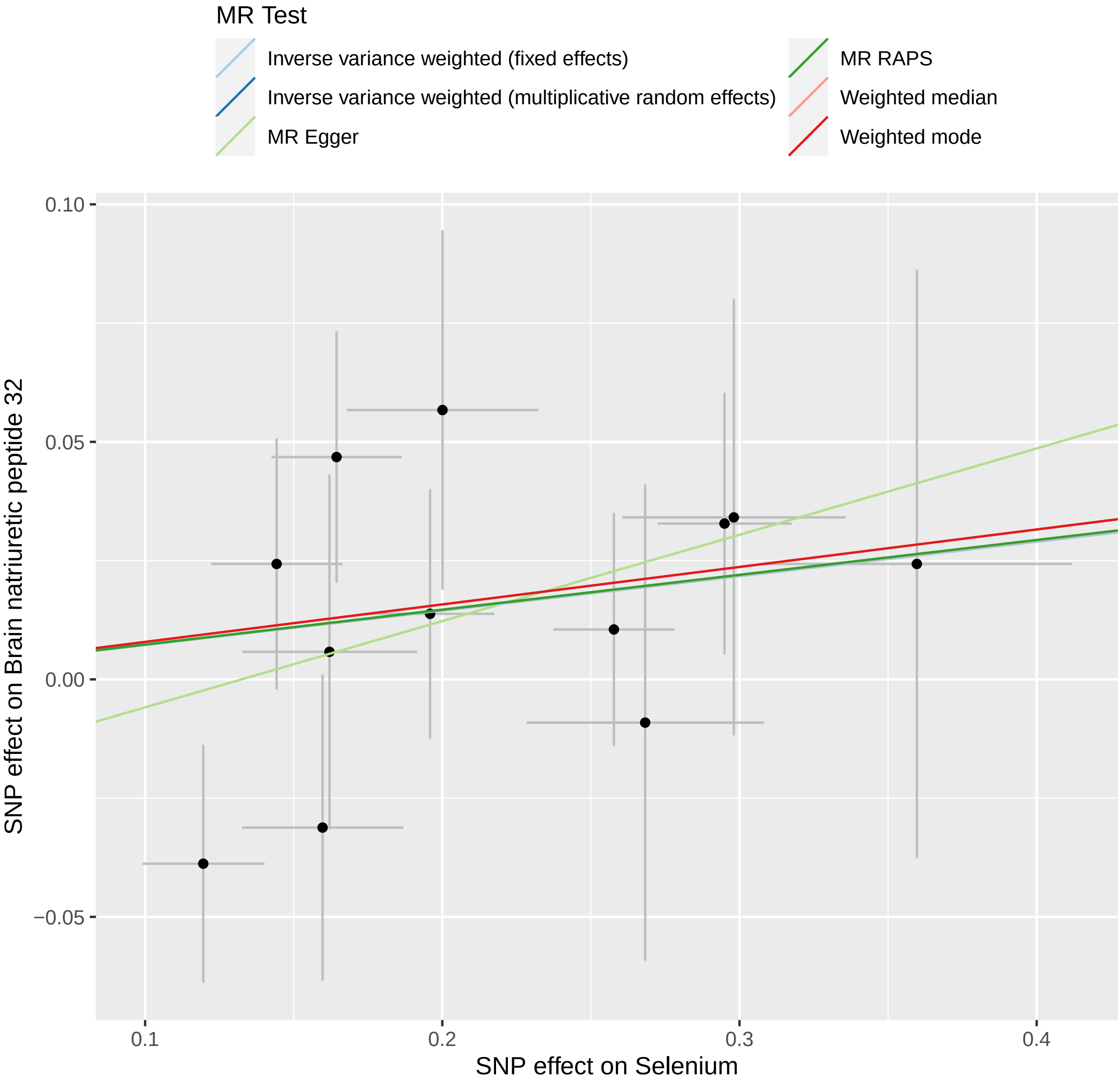

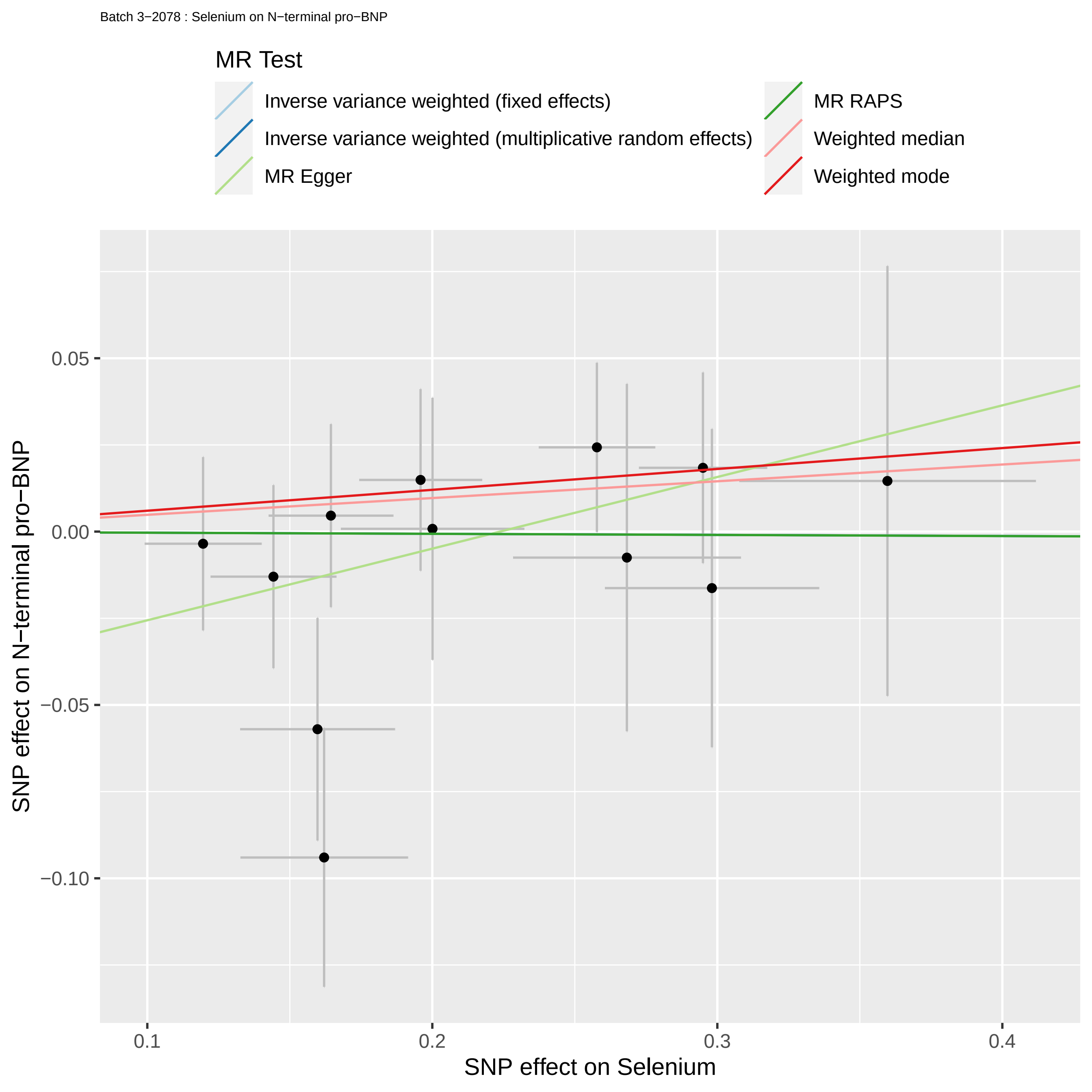
Batch 3−2078 : Selenium on N−terminal pro−BNP
MR Test
Inverse variance weighted (fixed effects)
Inverse variance weighted (multiplicative random effects)
MR Egger
MR RAPS
Weighted median
Weighted mode
0.05
0.00
−0.05
−0.10
0.1
0.2
0.3
0.4
SNP effect on N−terminal pro−BNP
SNP effect on Selenium

Batch 3−2079 : Selenium on Neuropeptide S

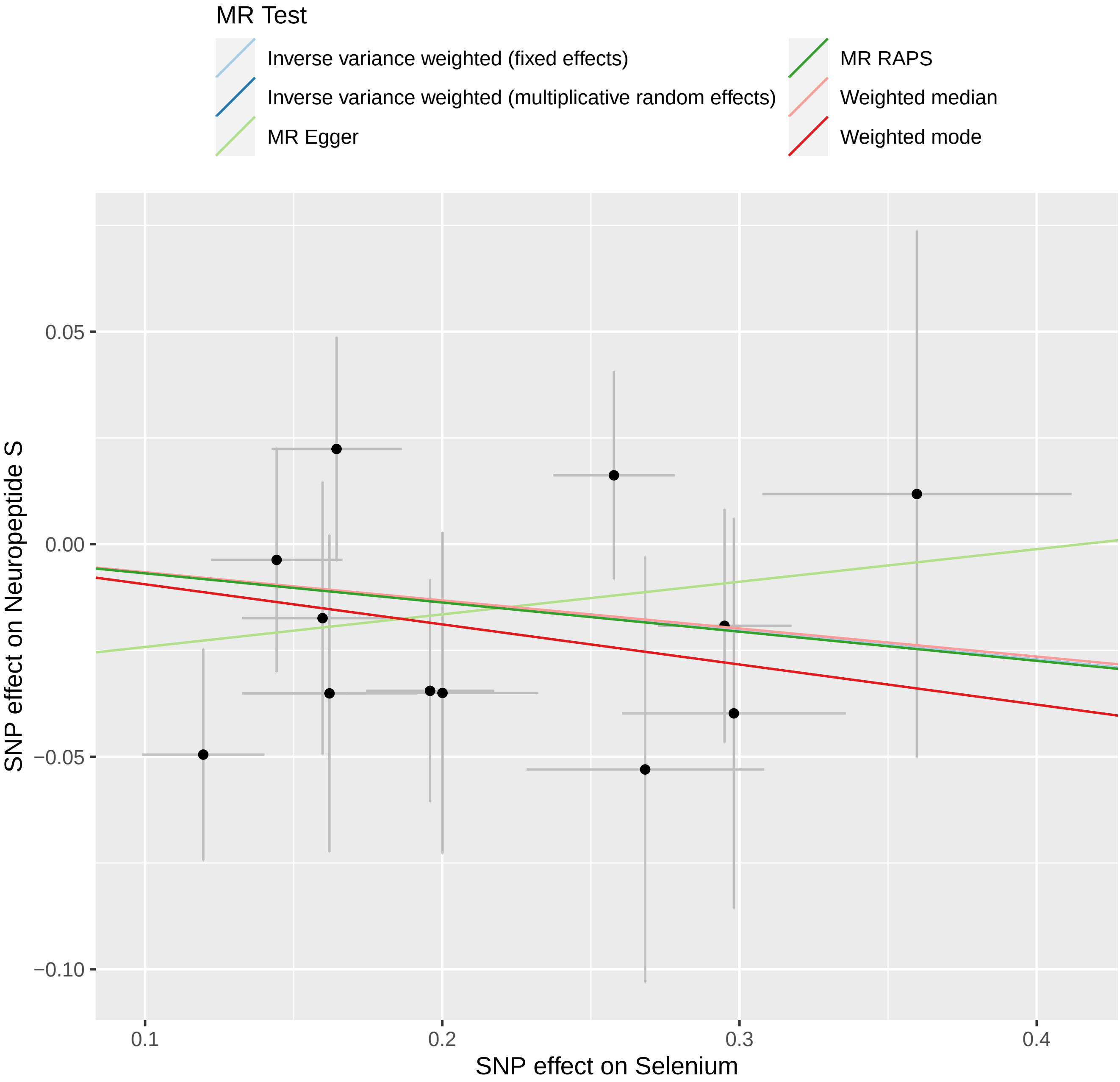

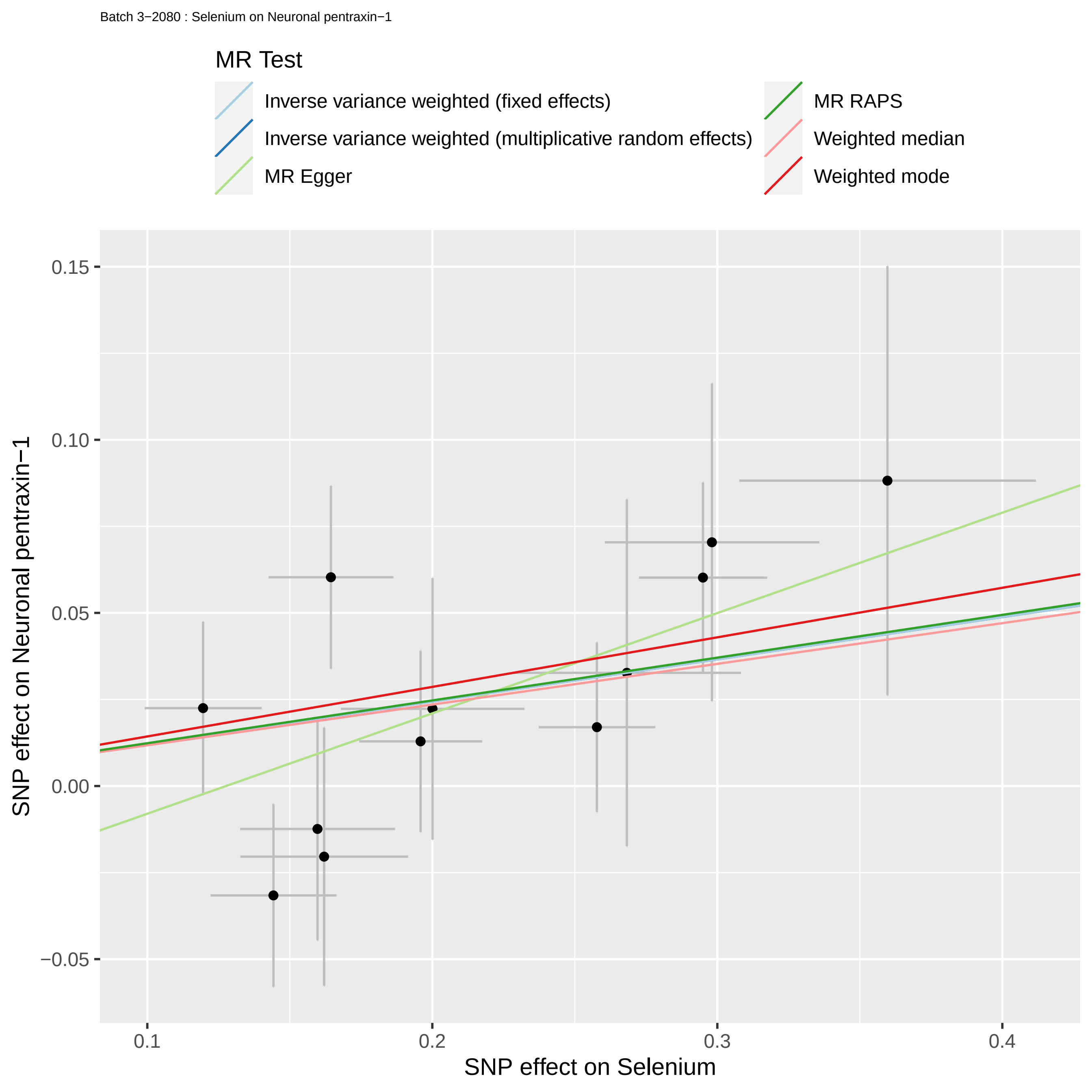

Batch 3−2080 : Selenium on Neuronal pentraxin−1
MR Test
Inverse variance weighted (fixed effects)
Inverse variance weighted (multiplicative random effects)
MR Egger
MR RAPS
Weighted median
Weighted mode
0.15
0.10
0.05
0.00
−0.05
0.1
0.2
0.3
0.4
SNP effect on Neuronal pentraxin−1
SNP effect on Selenium

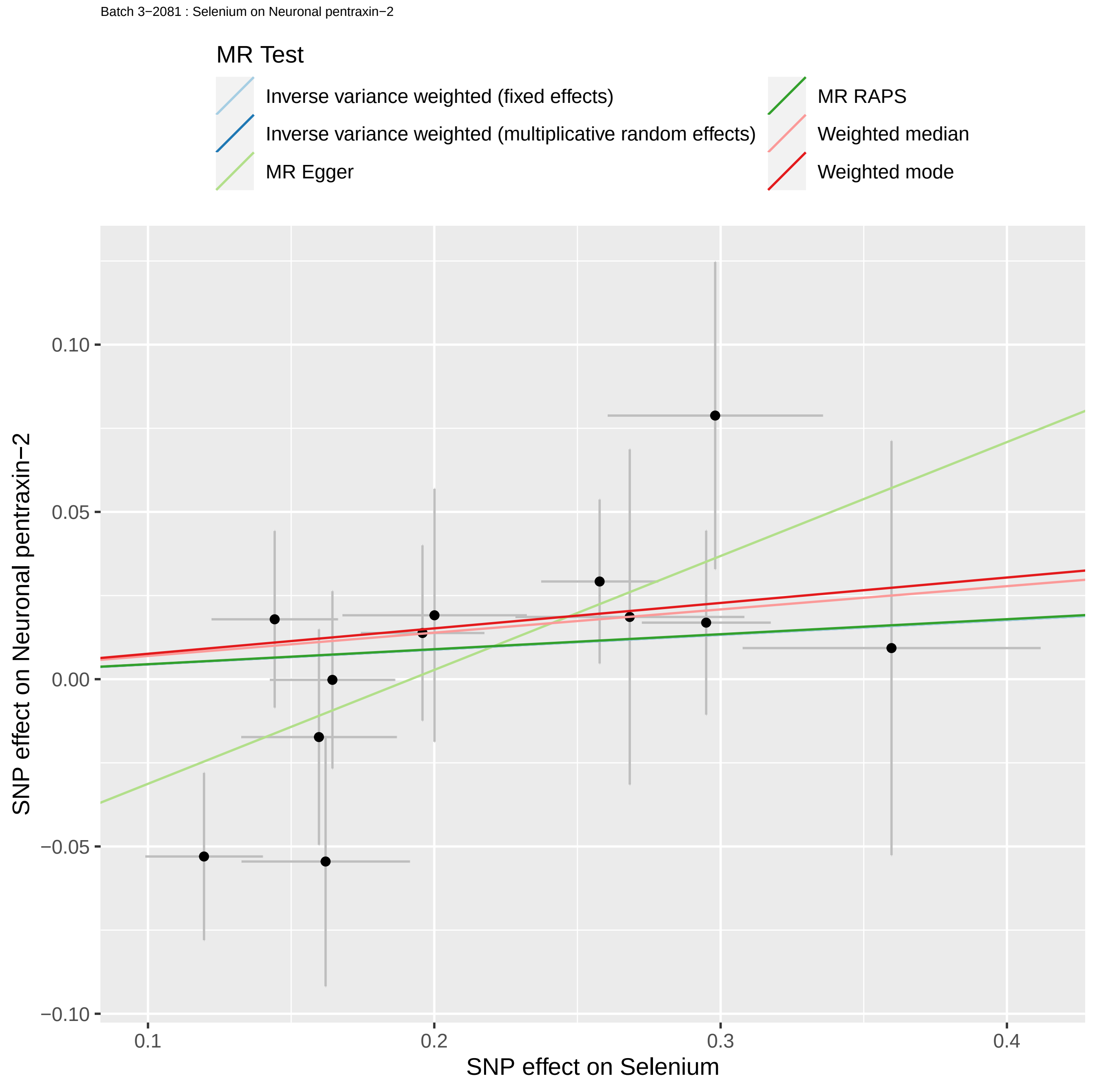

Batch 3−2081 : Selenium on Neuronal pentraxin−2
MR Test
Inverse variance weighted (fixed effects)
Inverse variance weighted (multiplicative random effects)
MR Egger
MR RAPS
Weighted median
Weighted mode
0.10
0.05
0.00
−0.05
−0.10
0.1
0.2
0.3
0.4
SNP effect on Neuronal pentraxin−2
SNP effect on Selenium

Batch 3−2082 : Selenium on Neuropeptide W

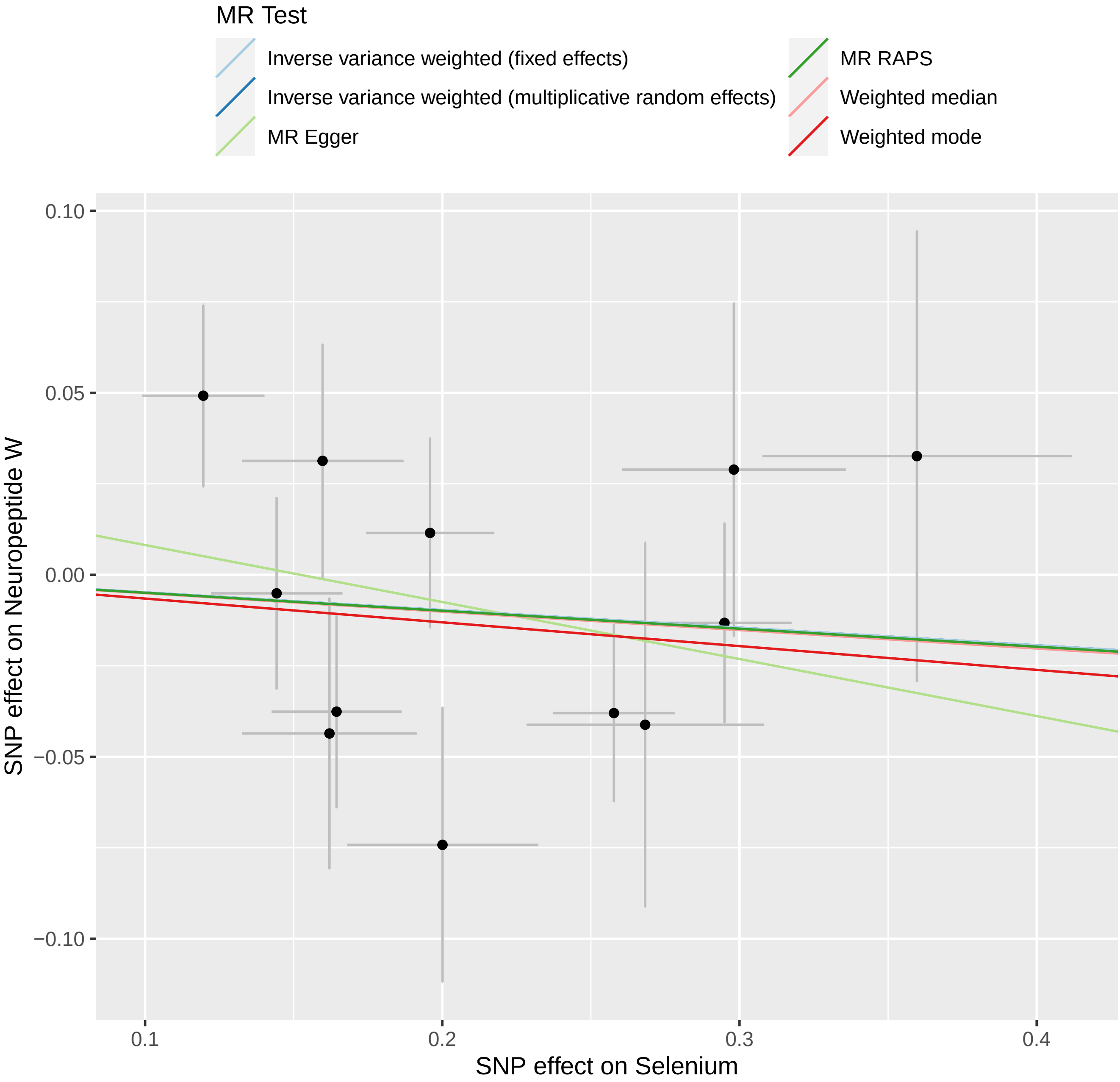

Batch 3−2083 : Selenium on NAD(P)H dehydrogenase [quinone] 1

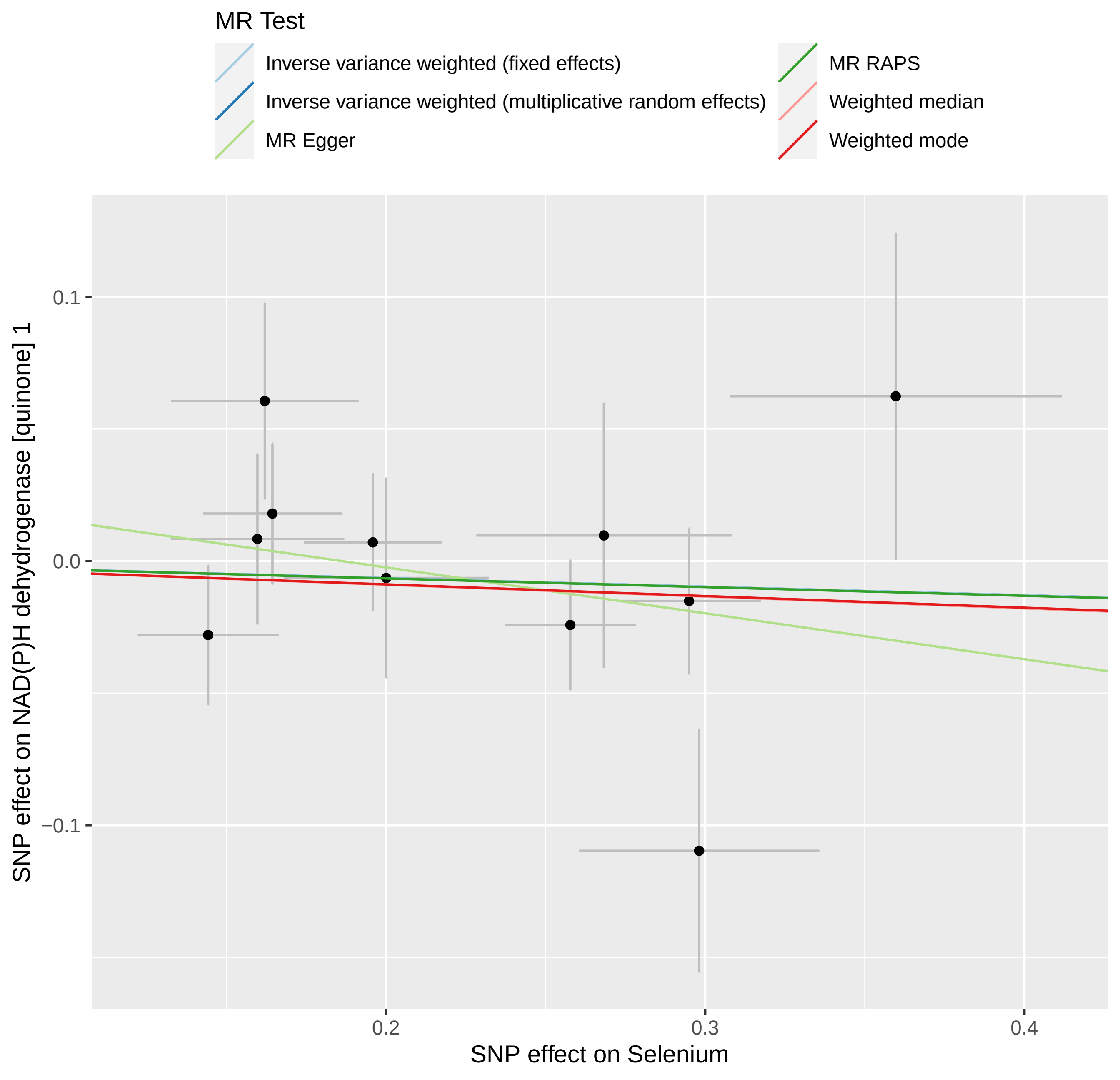

Batch 3−2084 : Selenium on Ribosyldihydronicotinamide dehydrogenase [quinone]

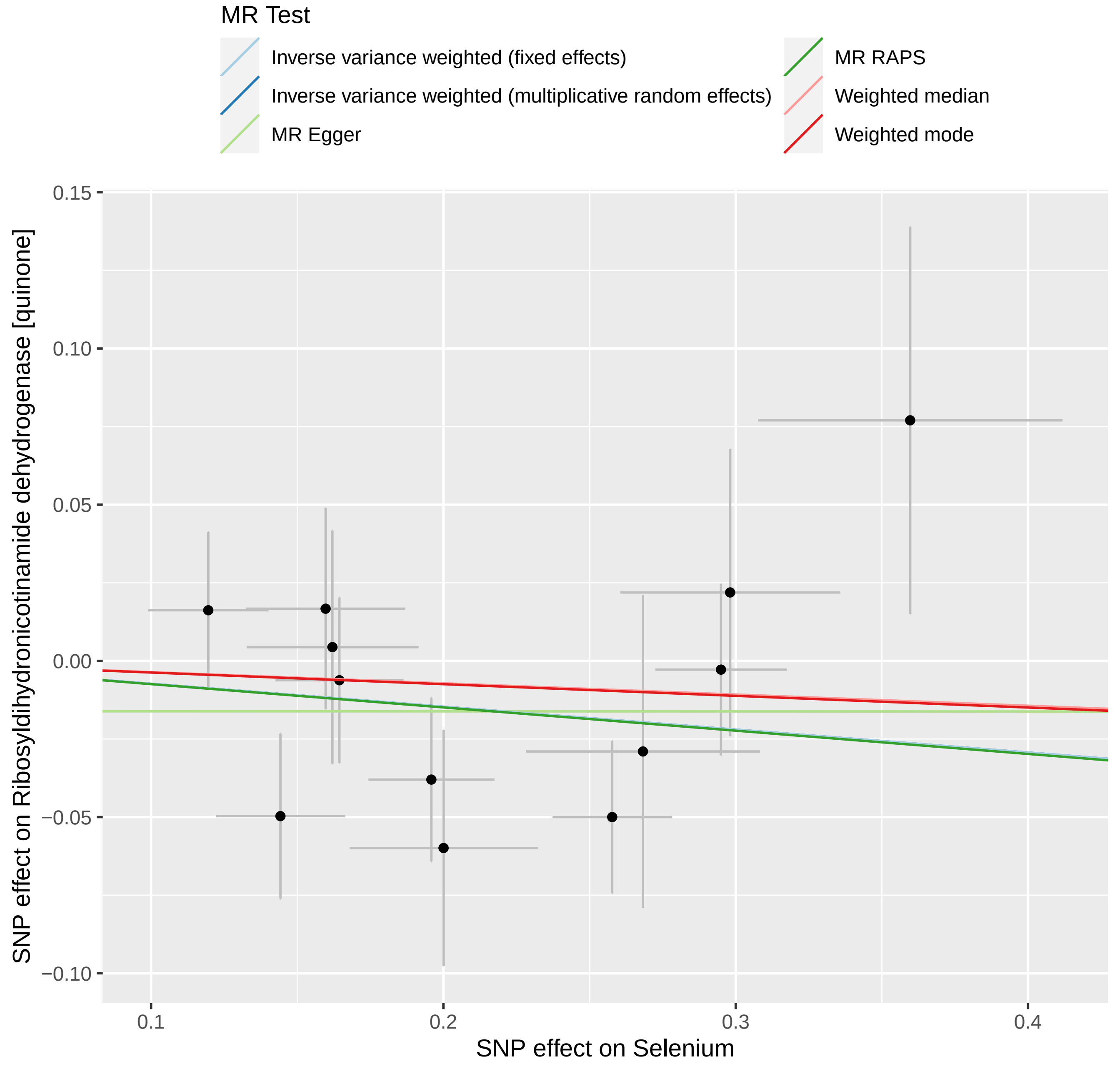

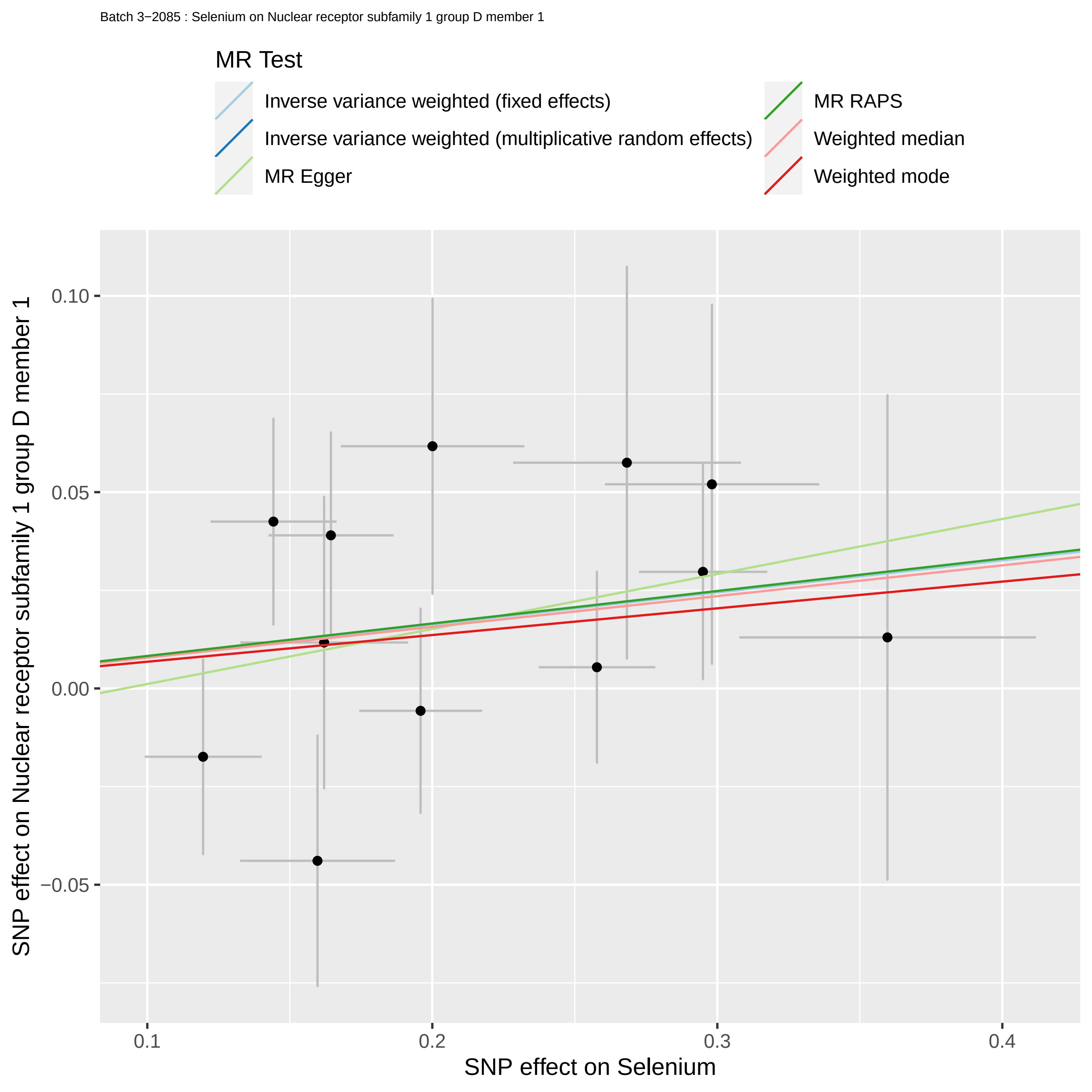

Batch 3−2085 : Selenium on Nuclear receptor subfamily 1 group D member 1
MR Test
Inverse variance weighted (fixed effects)
Inverse variance weighted (multiplicative random effects)
MR Egger
MR RAPS
Weighted median
Weighted mode
SNP effect on Nuclear receptor subfamily 1 group D member 1
0.10
0.05
0.00
−0.05
0.1
0.2
0.3
0.4
SNP effect on Selenium

Batch 3−2086 : Selenium on Nuclear receptor subfamily 1 group D member 2

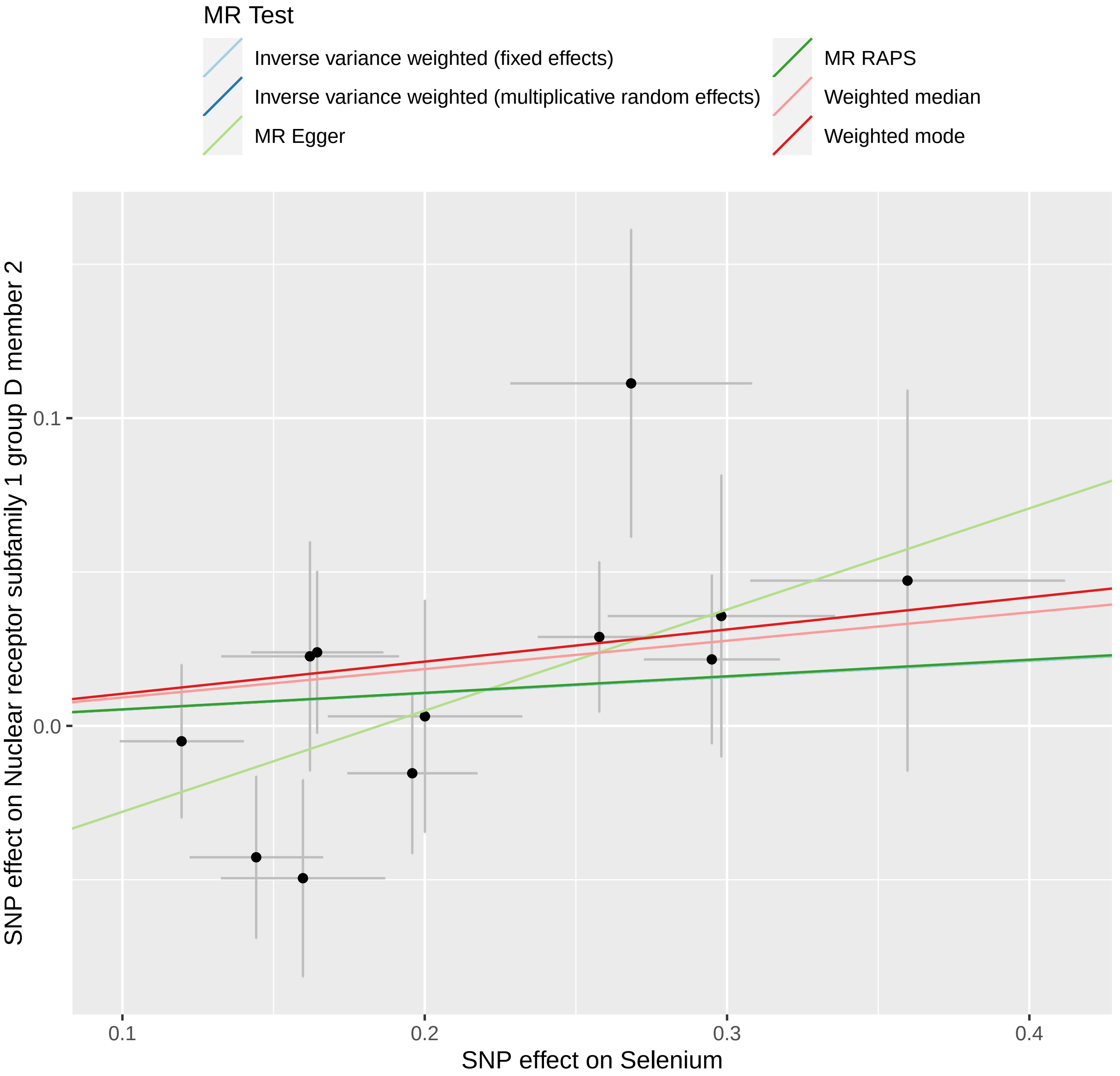

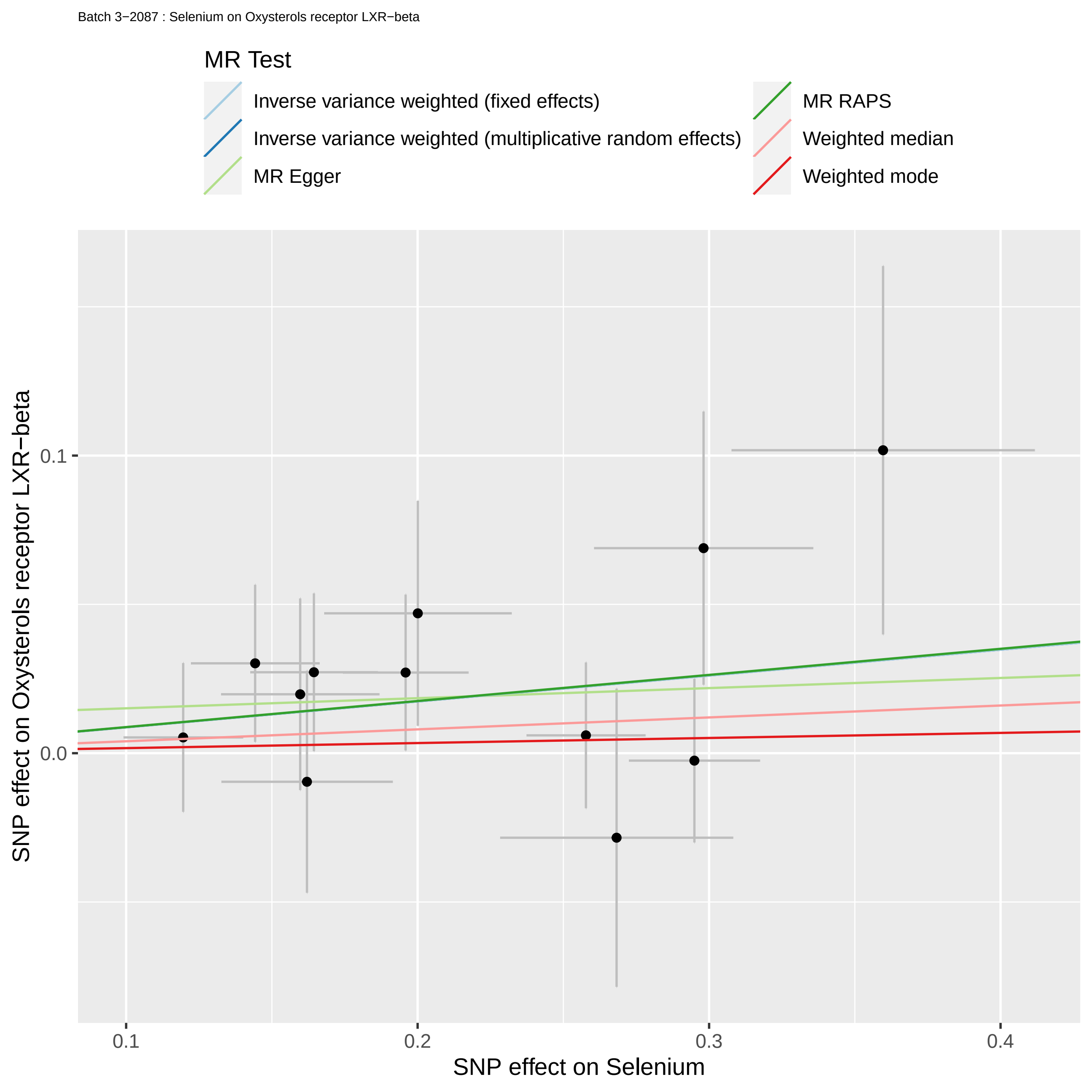

Batch 3−2087 : Selenium on Oxysterols receptor LXR−beta
MR Test
Inverse variance weighted (fixed effects)
Inverse variance weighted (multiplicative random effects)
MR Egger
MR RAPS
Weighted median
Weighted mode
SNP effect on Oxysterols receptor LXR−beta
0.1
0.0
0.1
0.2
0.3
0.4
SNP effect on Selenium

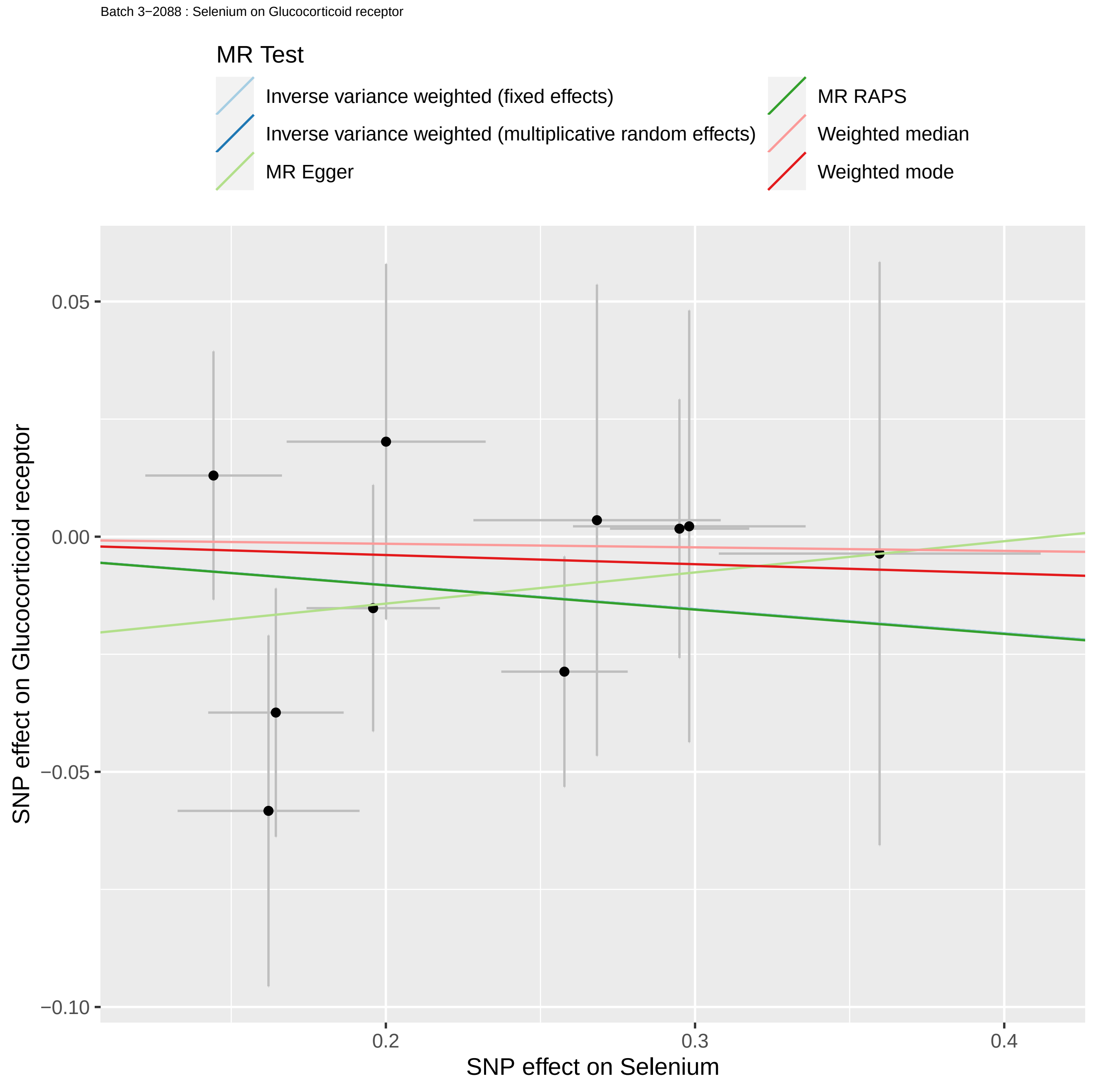

Batch 3−2088 : Selenium on Glucocorticoid receptor
MR Test
Inverse variance weighted (fixed effects)
Inverse variance weighted (multiplicative random effects)
MR Egger
MR RAPS
Weighted median
Weighted mode
0.05
0.00
−0.05
−0.10
0.2
0.3
0.4
SNP effect on Glucocorticoid receptor
SNP effect on Selenium

Batch 3−2089 : Selenium on Mineralocorticoid receptor

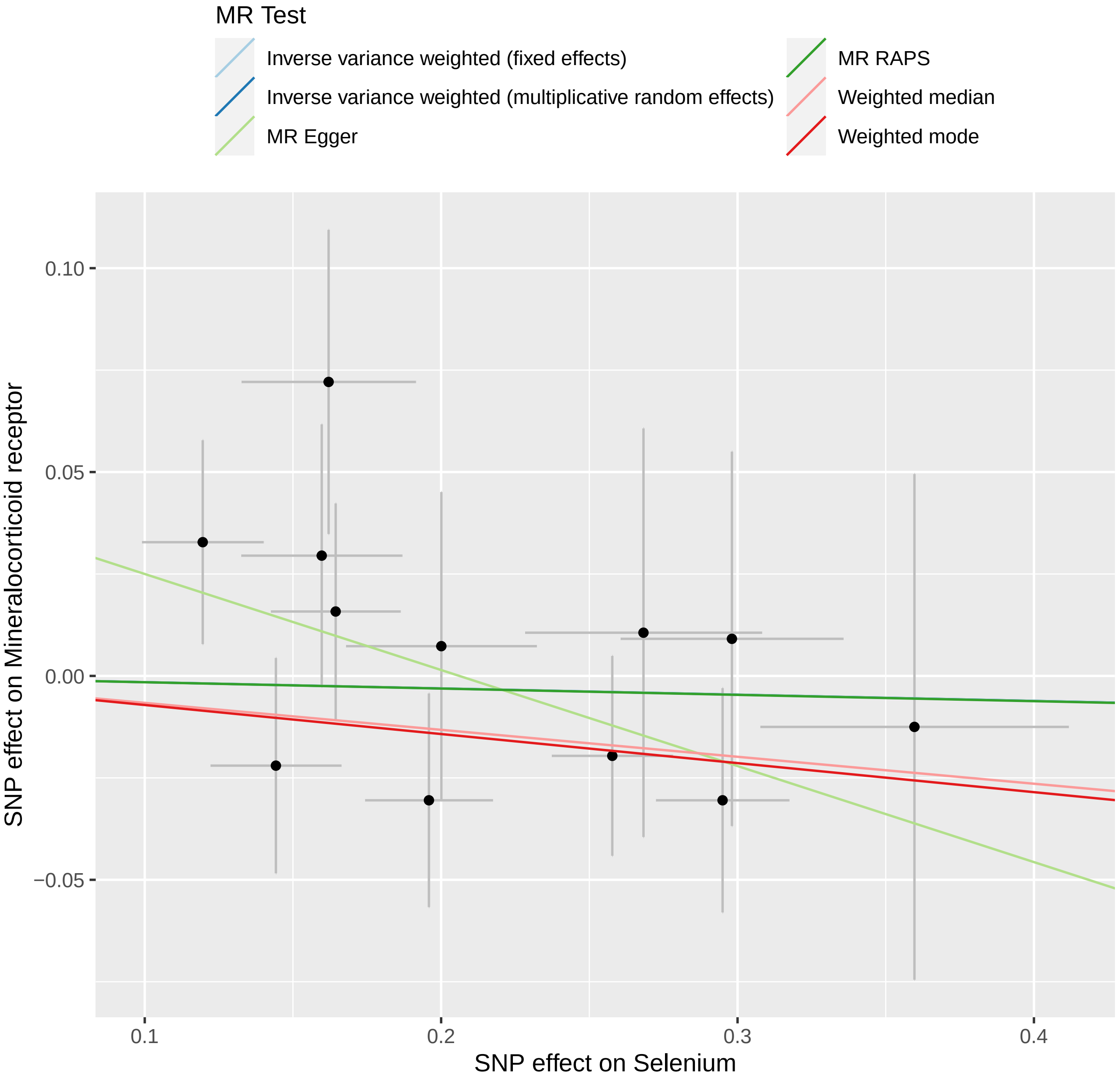

Batch 3−2090 : Selenium on Nuclear receptor subfamily 4 group A member 1

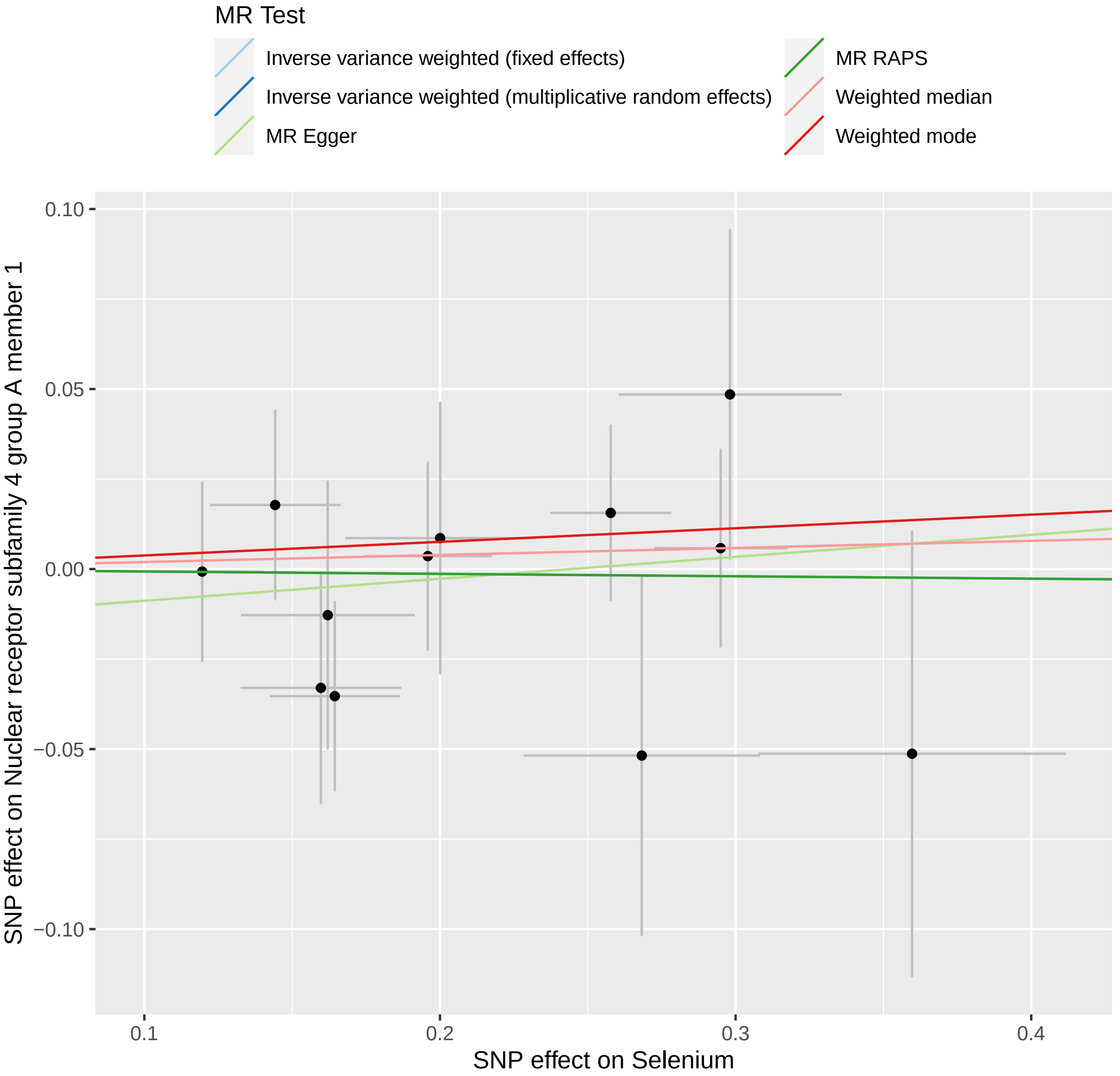

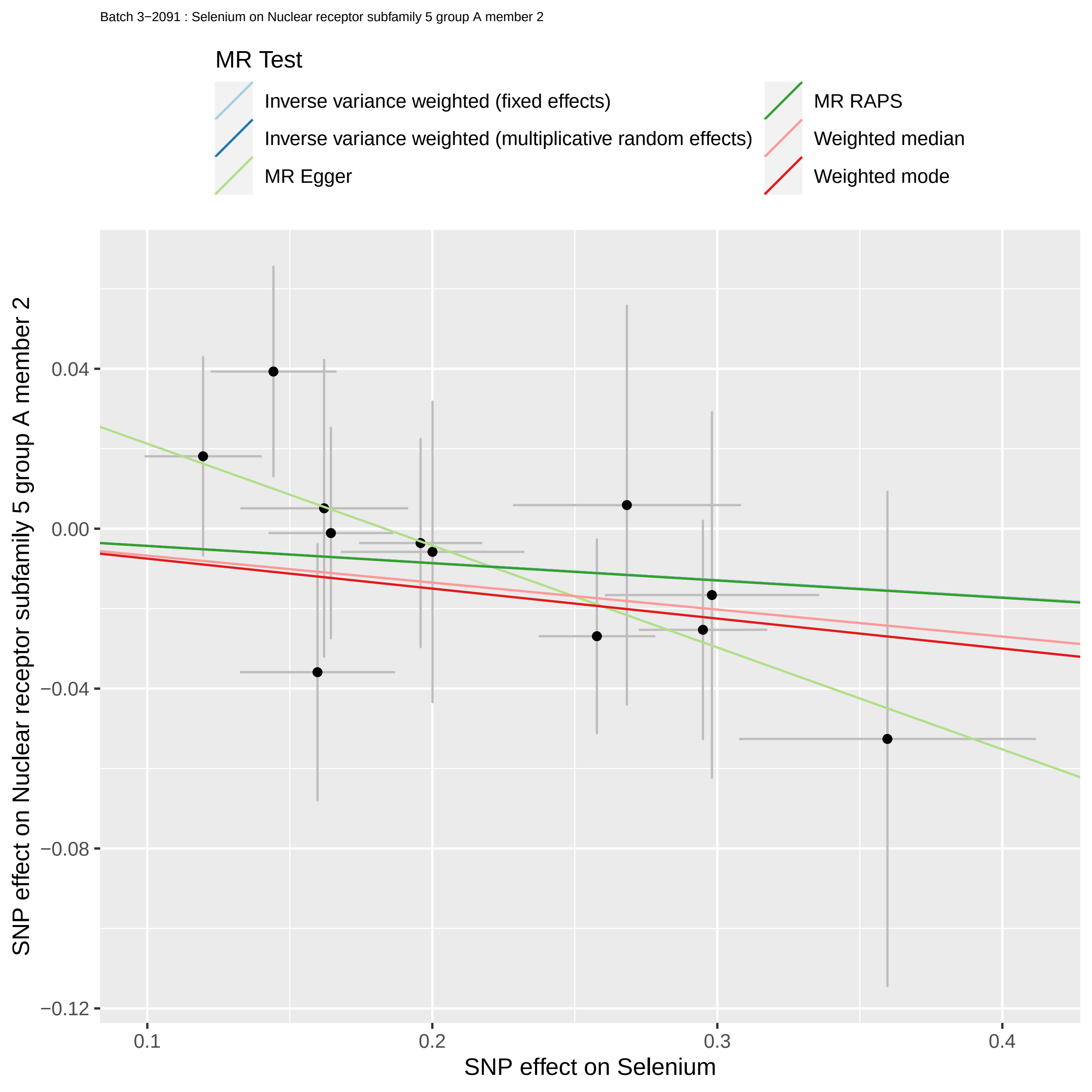

Batch 3−2091 : Selenium on Nuclear receptor subfamily 5 group A member 2
MR Test
Inverse variance weighted (fixed effects)
Inverse variance weighted (multiplicative random effects)
MR Egger
MR RAPS
Weighted median
Weighted mode
0.04
0.00
−0.04
−0.08
−0.12
0.1
0.2
0.3
0.4
SNP effect on Selenium
SNP effect on Nuclear receptor subfamily 5 group A member 2

Batch 3−2092 : Selenium on Nutritionally−regulated adipose and cardiac enriched protein homolog

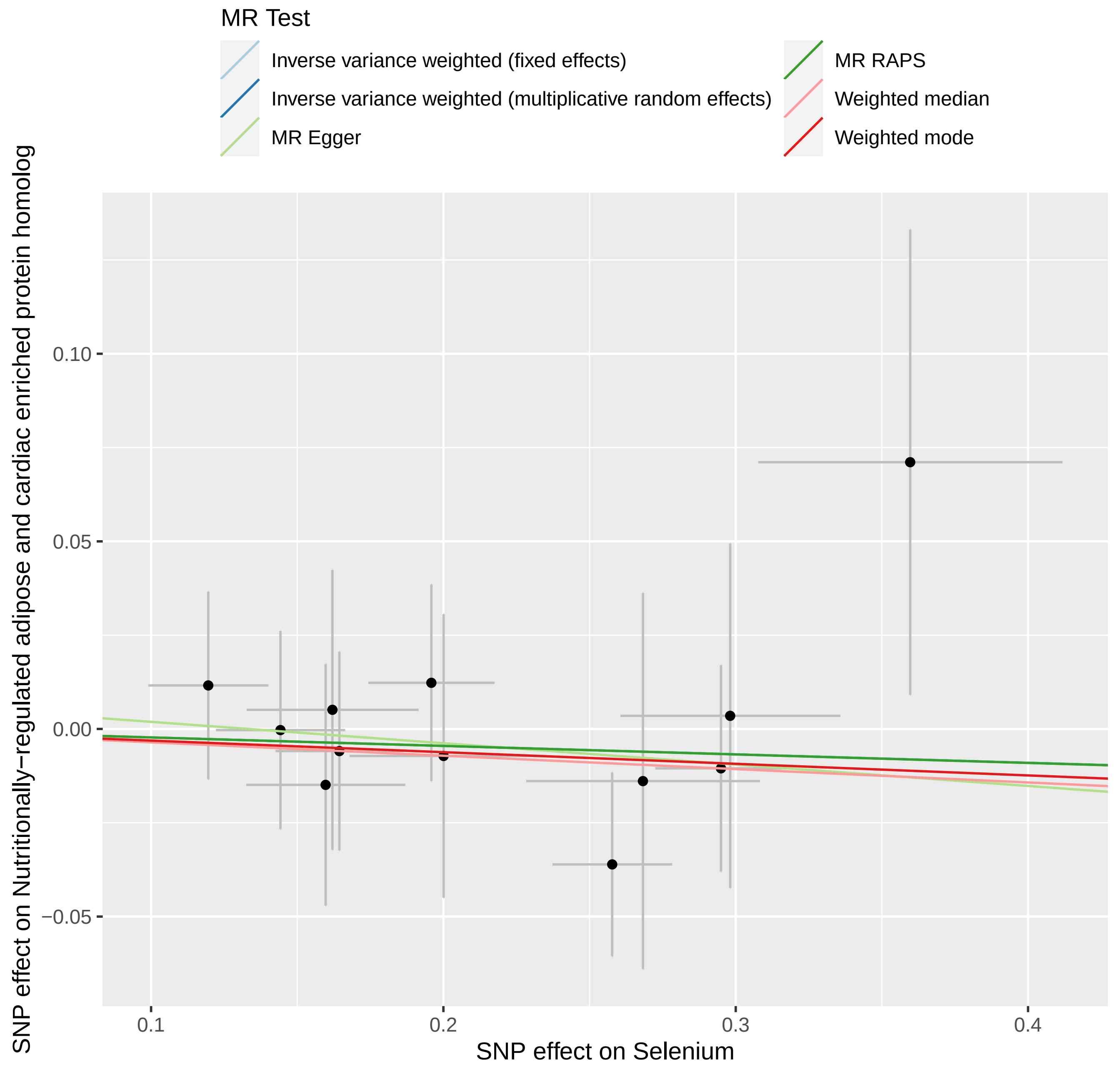

Batch 3−2093 : Selenium on Nuclear receptor−binding protein

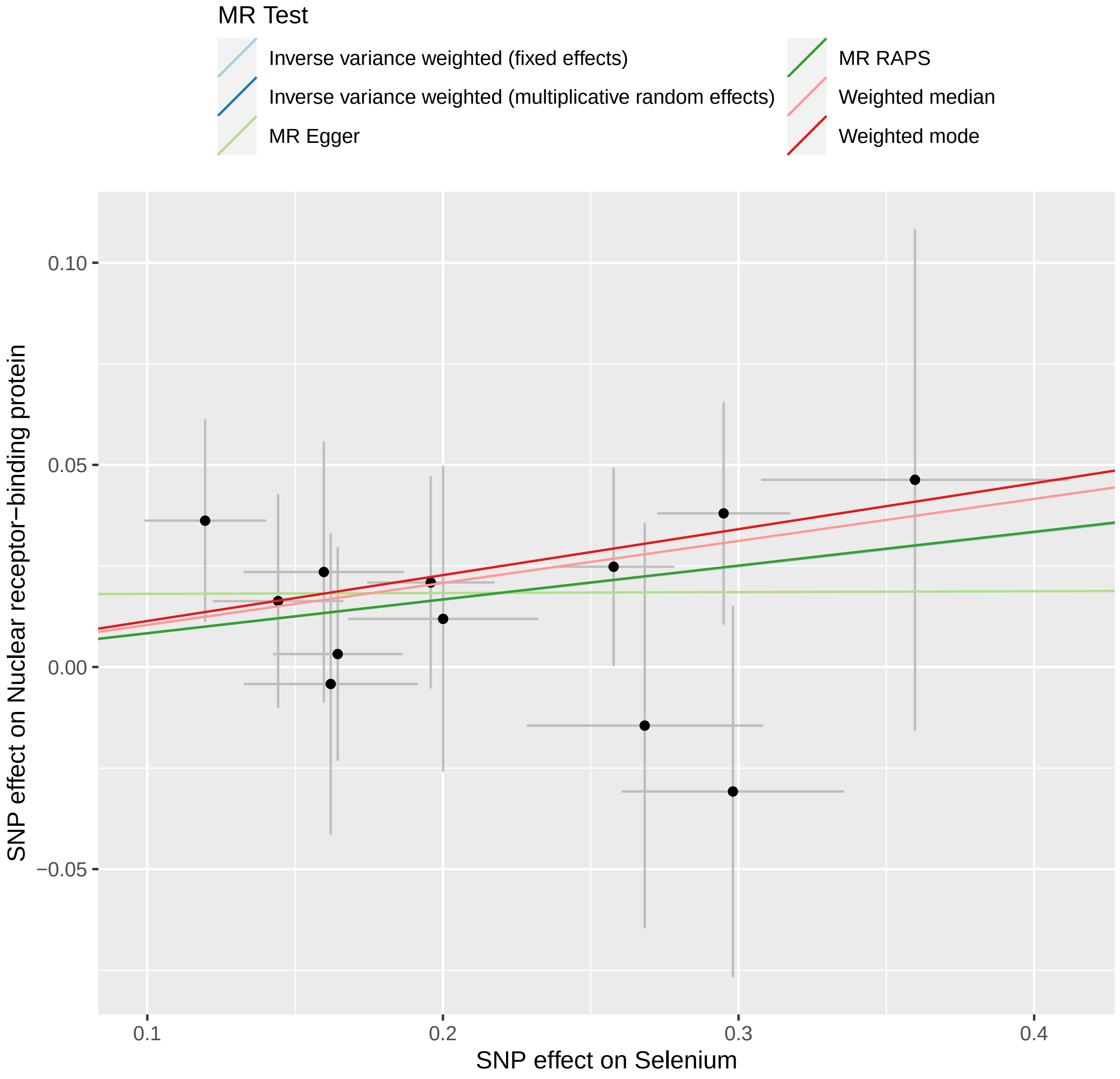

Batch 3−2094 : Selenium on Neuronal cell adhesion molecule

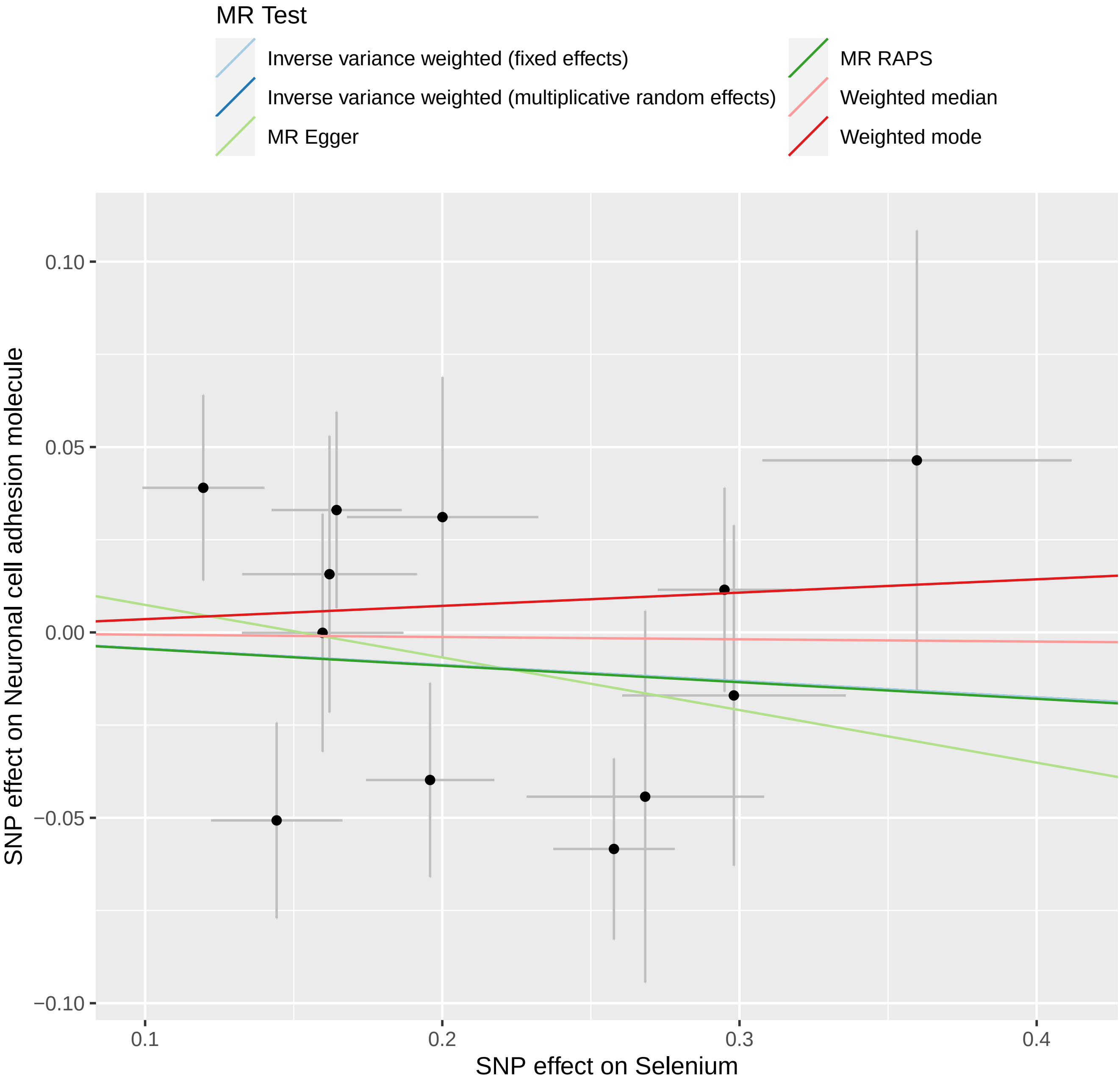

Batch 3−2095 : Selenium on Neuregulin−1

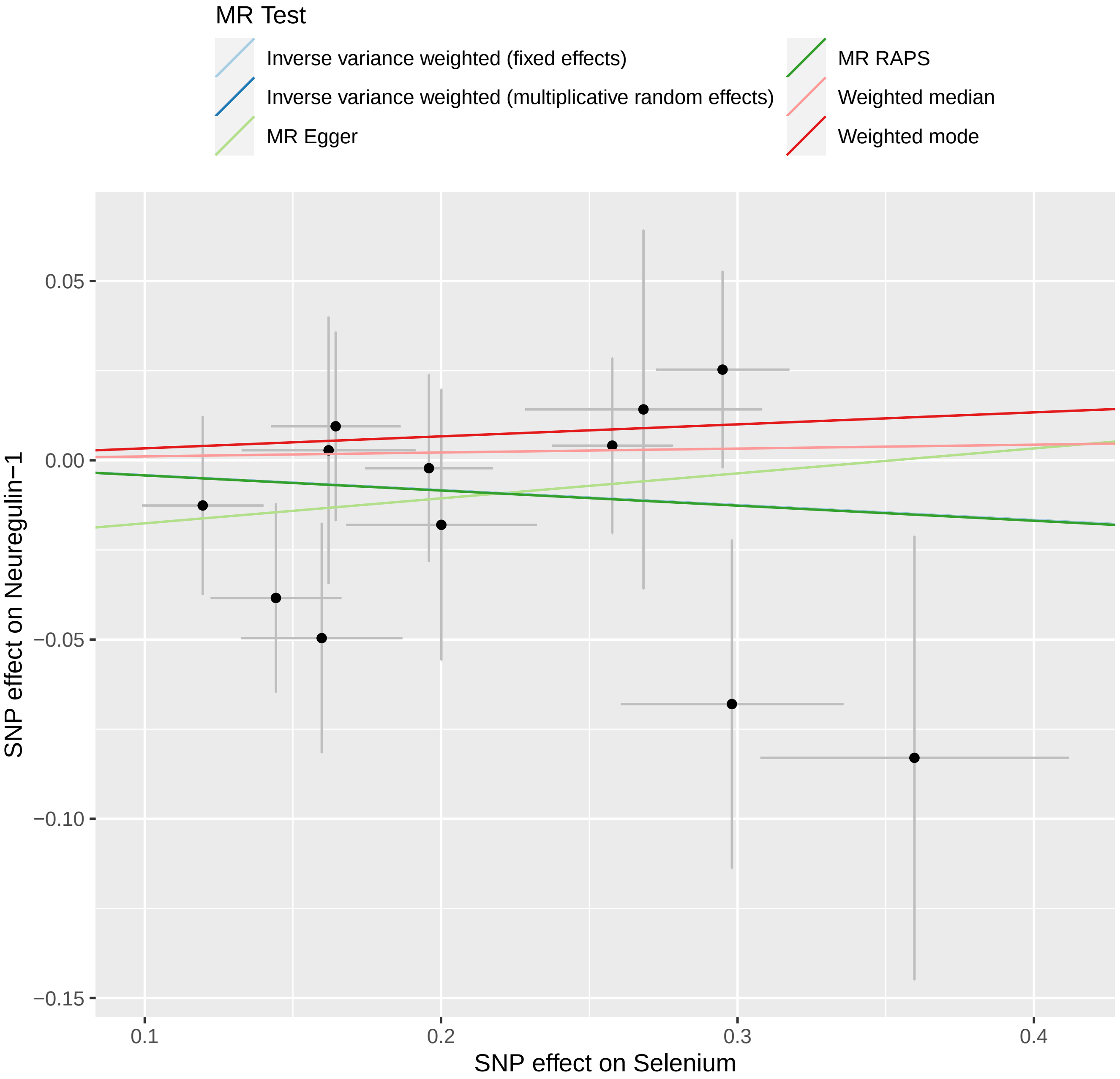

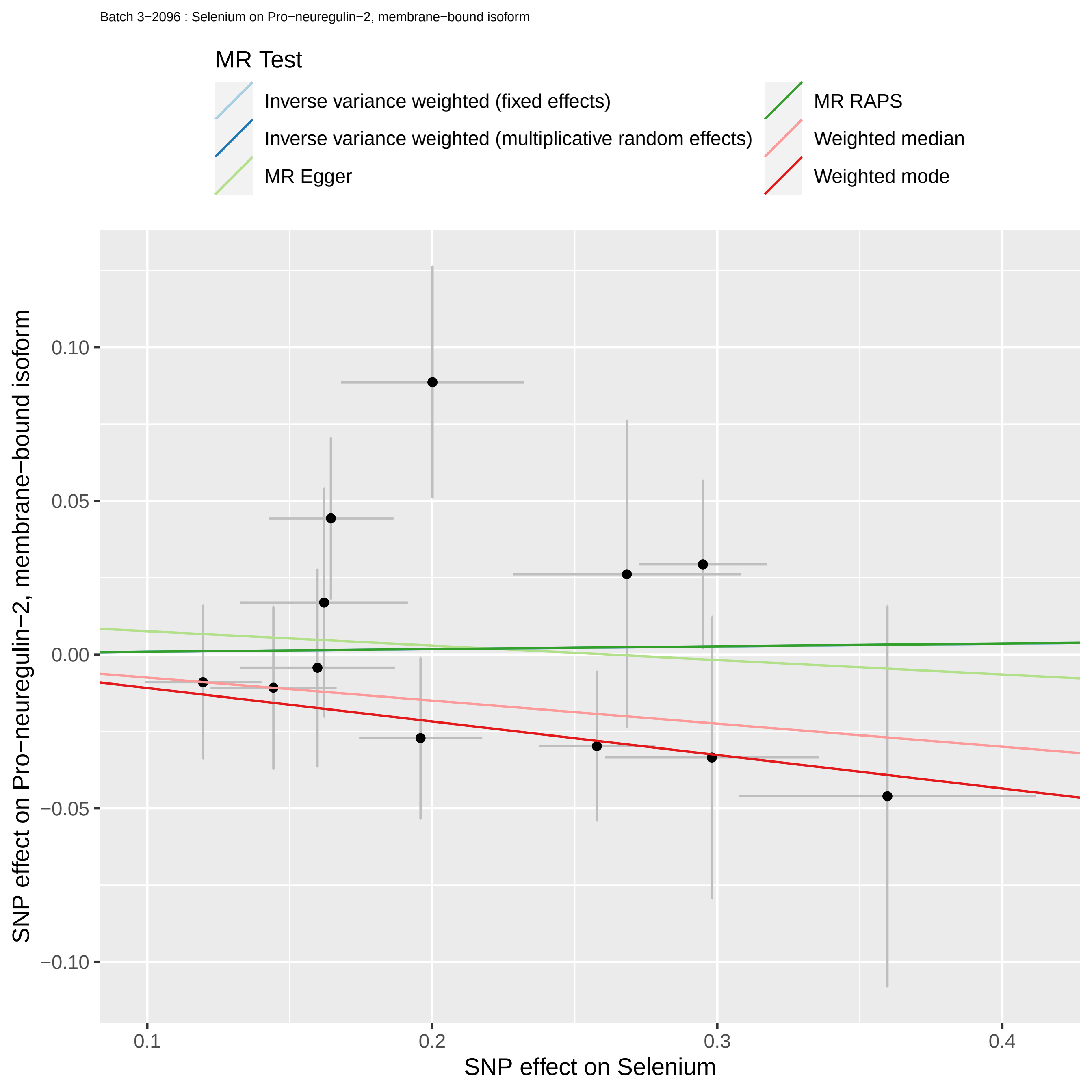

Batch 3−2096 : Selenium on Pro−neuregulin−2, membrane−bound isoform
MR Test
Inverse variance weighted (fixed effects)
Inverse variance weighted (multiplicative random effects)
MR Egger
MR RAPS
Weighted median
Weighted mode
0.10
0.05
0.00
−0.05
−0.10
0.1
0.2
0.3
0.4
SNP effect on Pro−neuregulin−2, membrane−bound isoform
SNP effect on Selenium

Batch 3−2097 : Selenium on Neuregulin−4

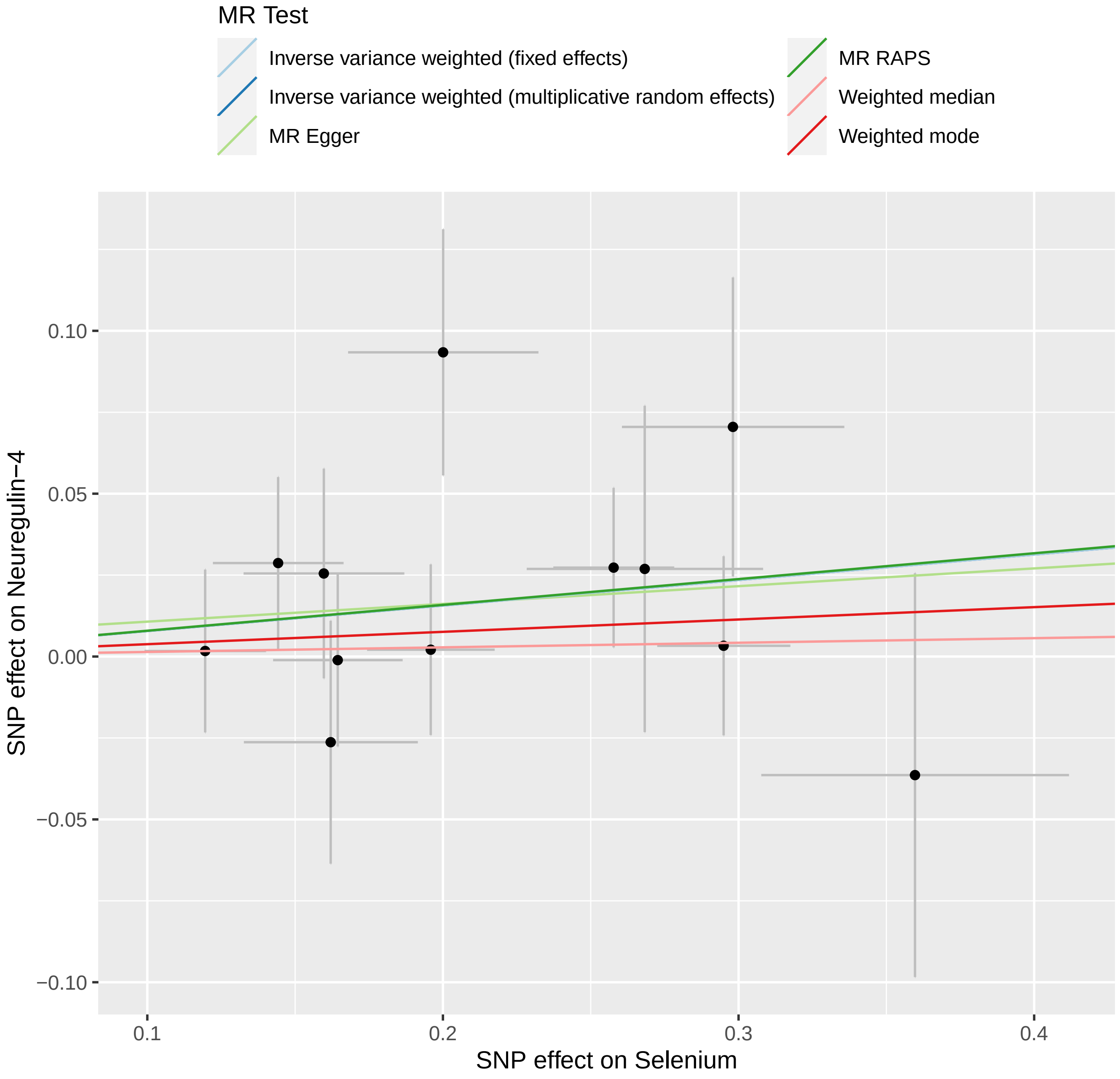

Batch 3−2098 : Selenium on Neuritin

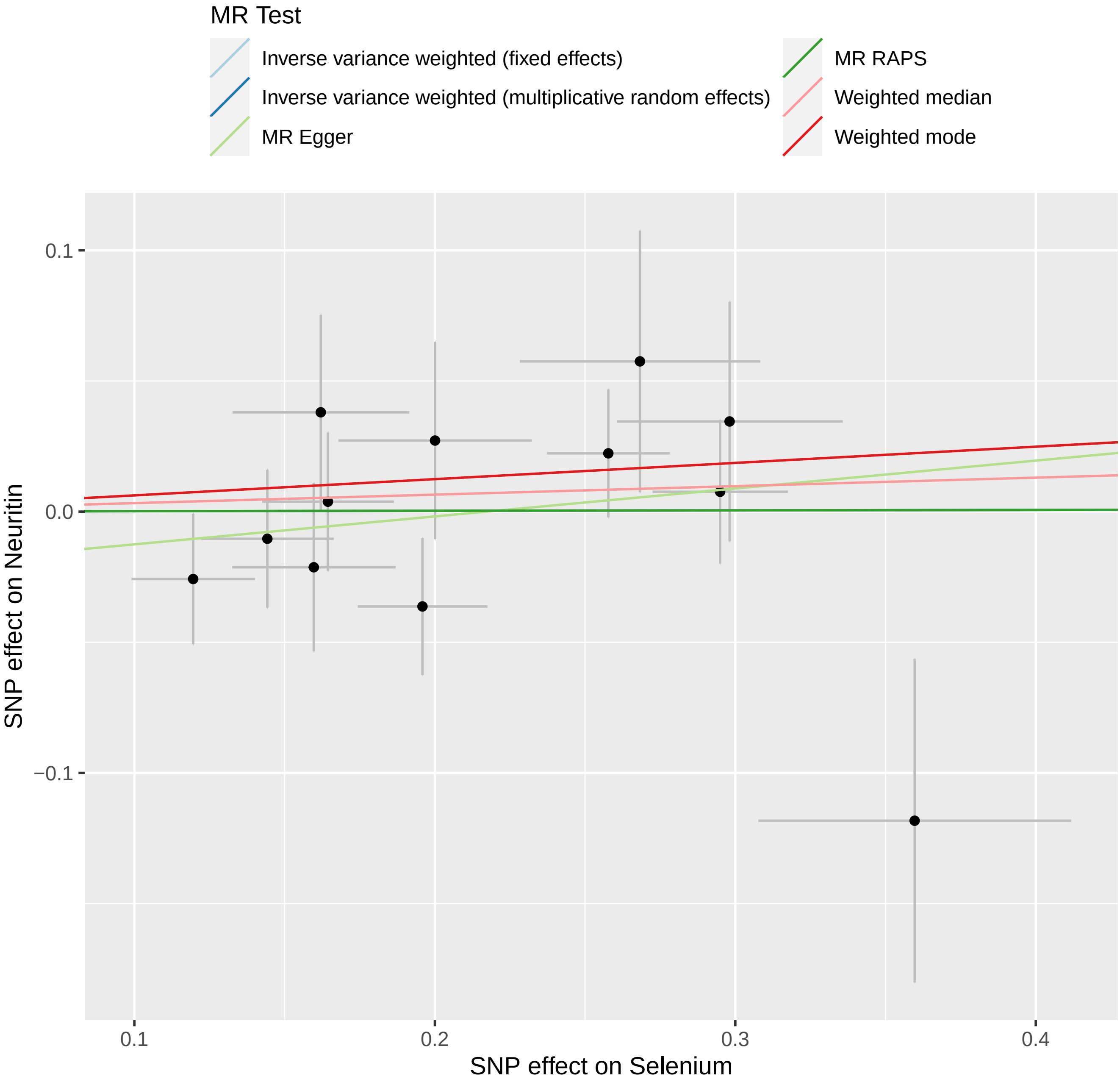

Batch 3−2099 : Selenium on Neuropilin−1

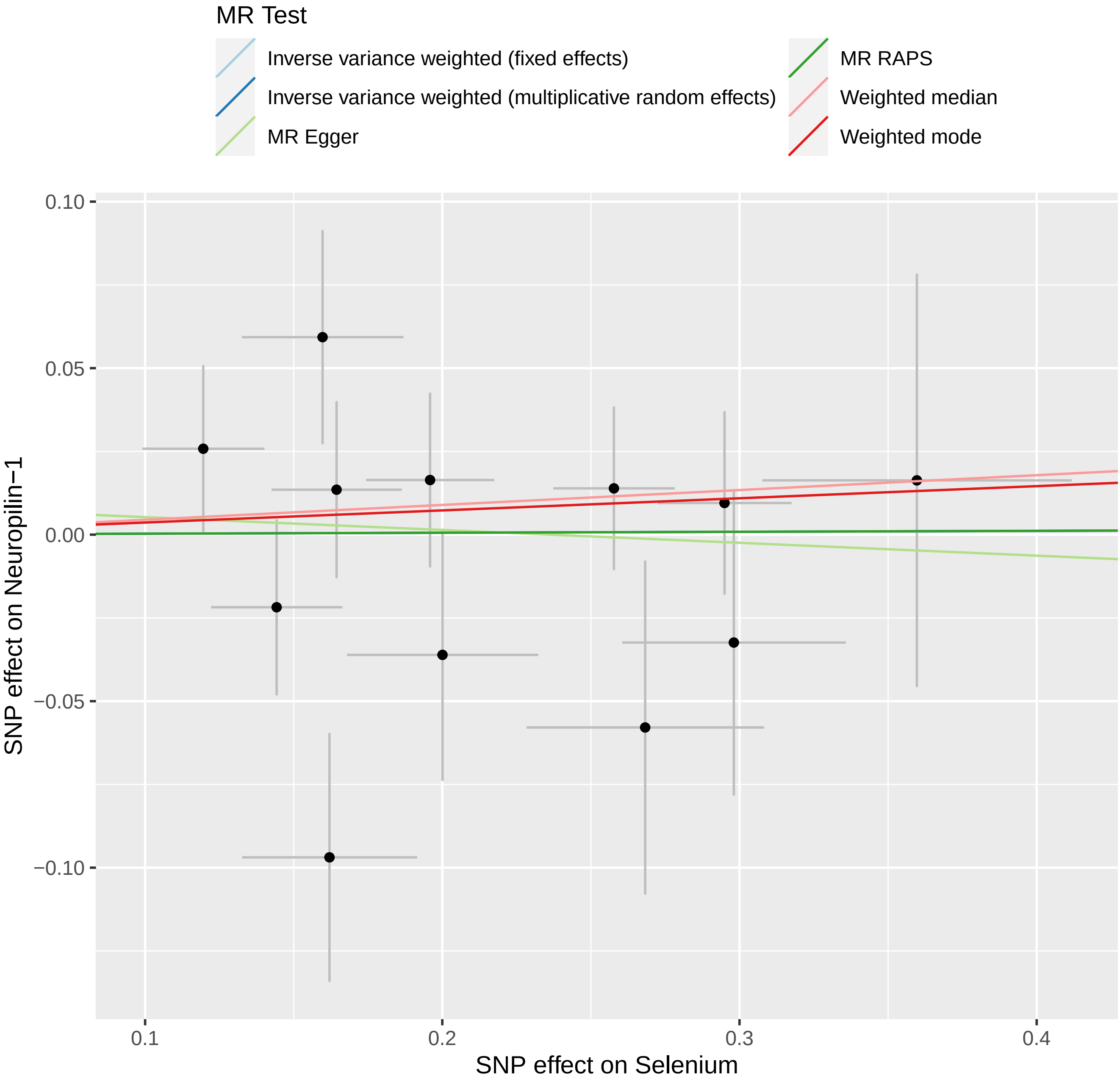

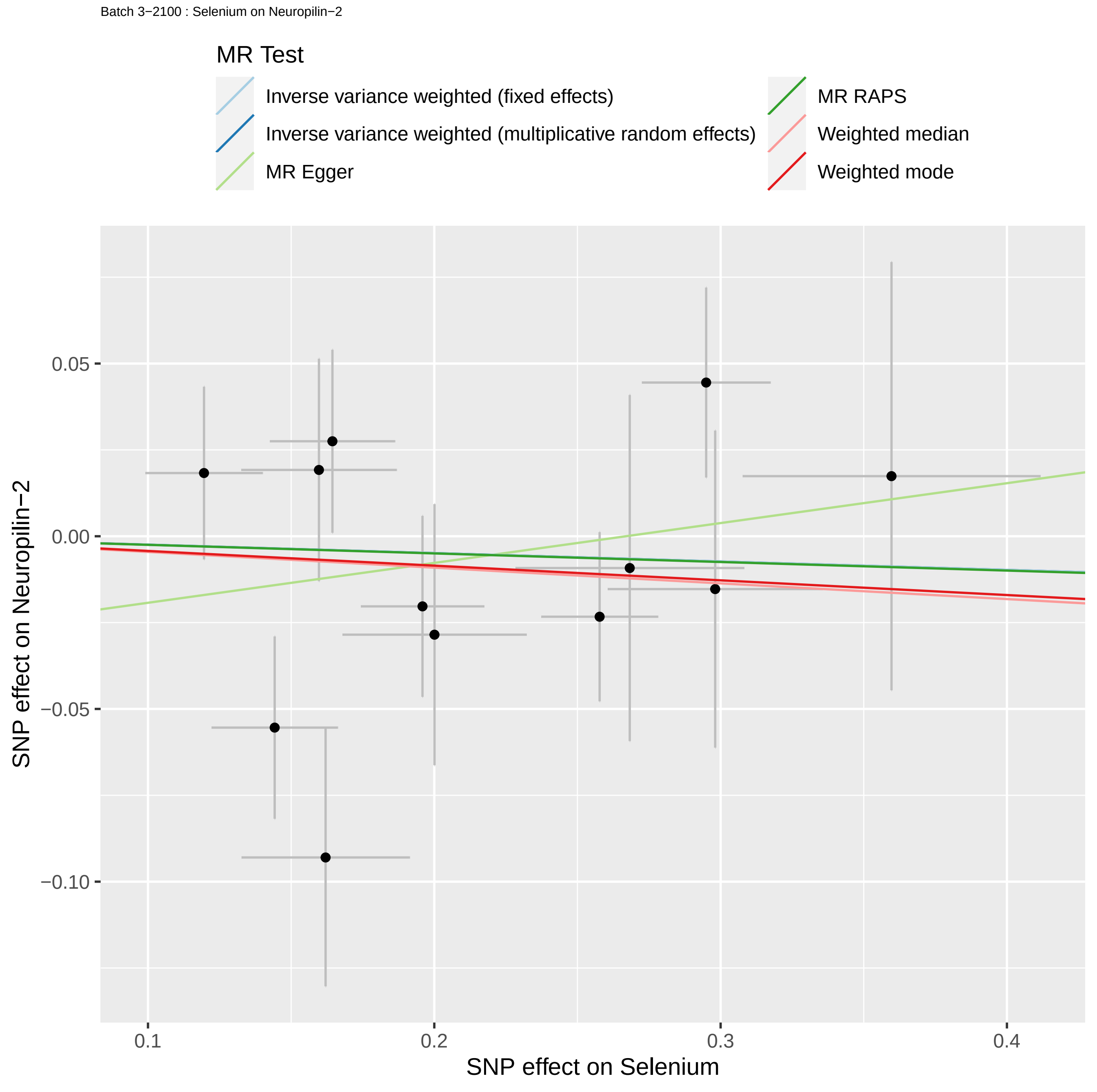
Batch 3−2100 : Selenium on Neuropilin−2
MR Test
Inverse variance weighted (fixed effects)
Inverse variance weighted (multiplicative random effects)
MR Egger
MR RAPS
Weighted median
Weighted mode
SNP effect on Neuropilin−2
0.05
0.00
−0.05
−0.10
0.1
0.2
0.3
0.4
SNP effect on Selenium

Batch 3−2101 : Selenium on Neurensin−1

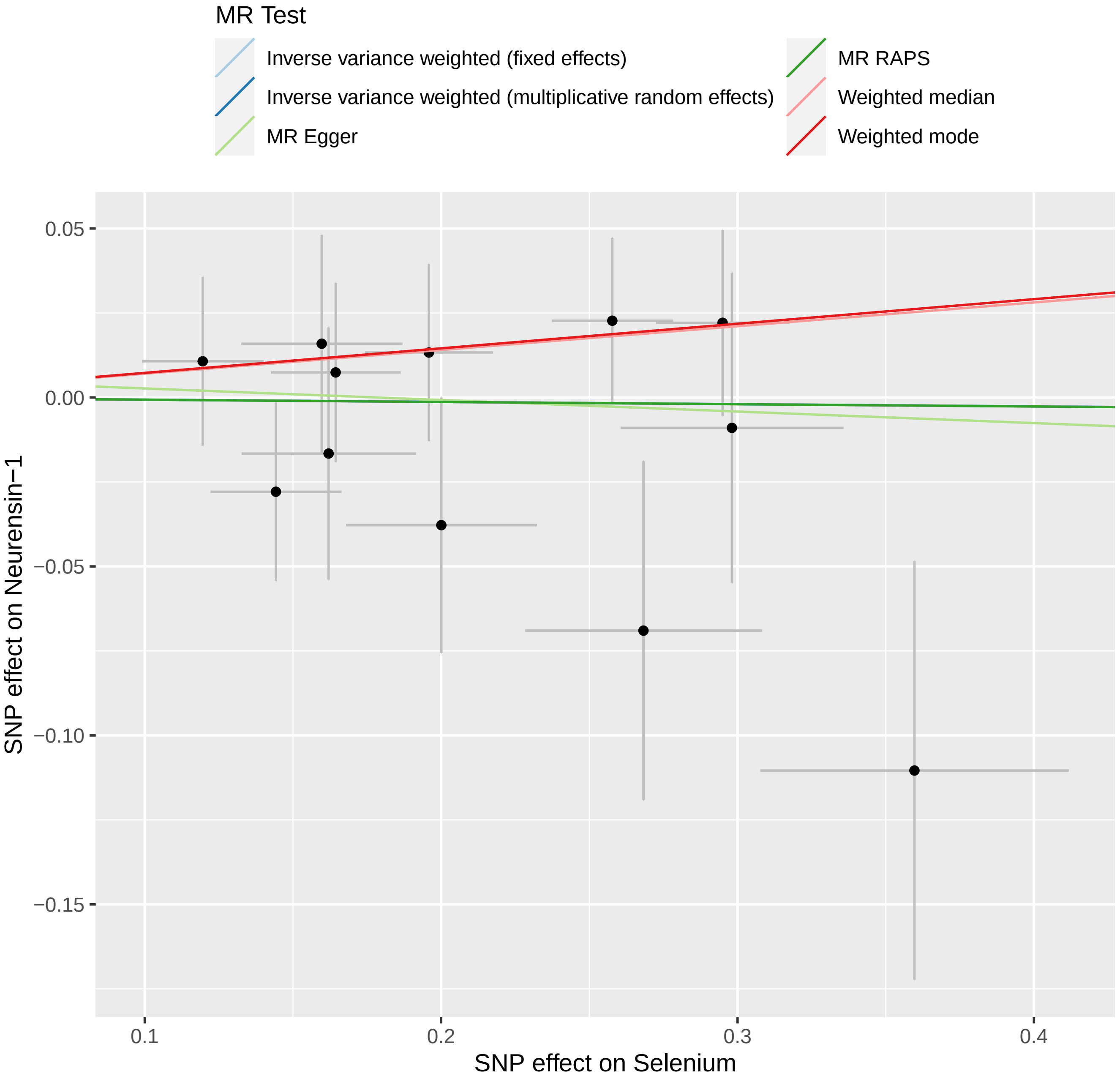

Batch 3−2102 : Selenium on Neurexin−1−beta

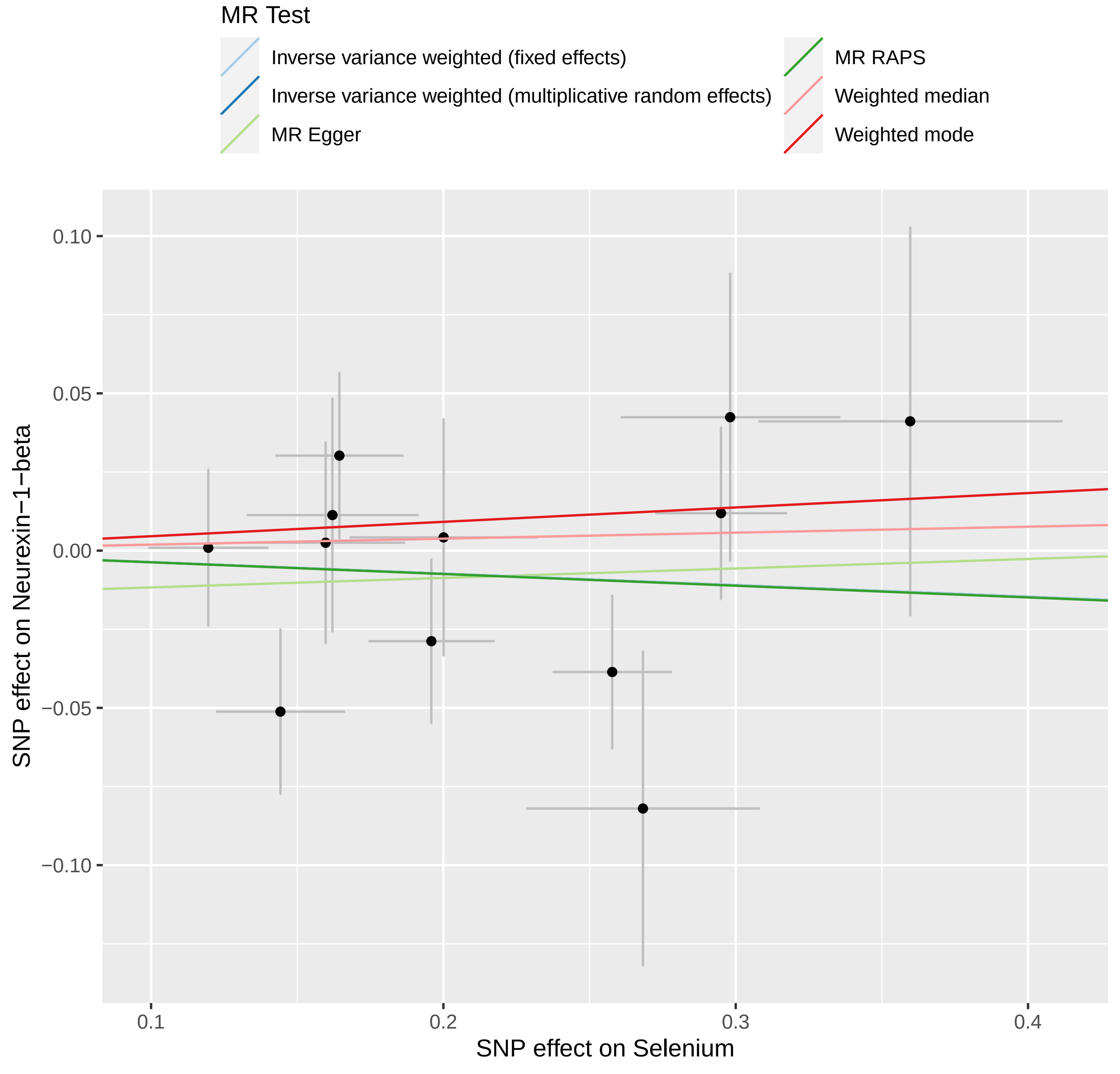

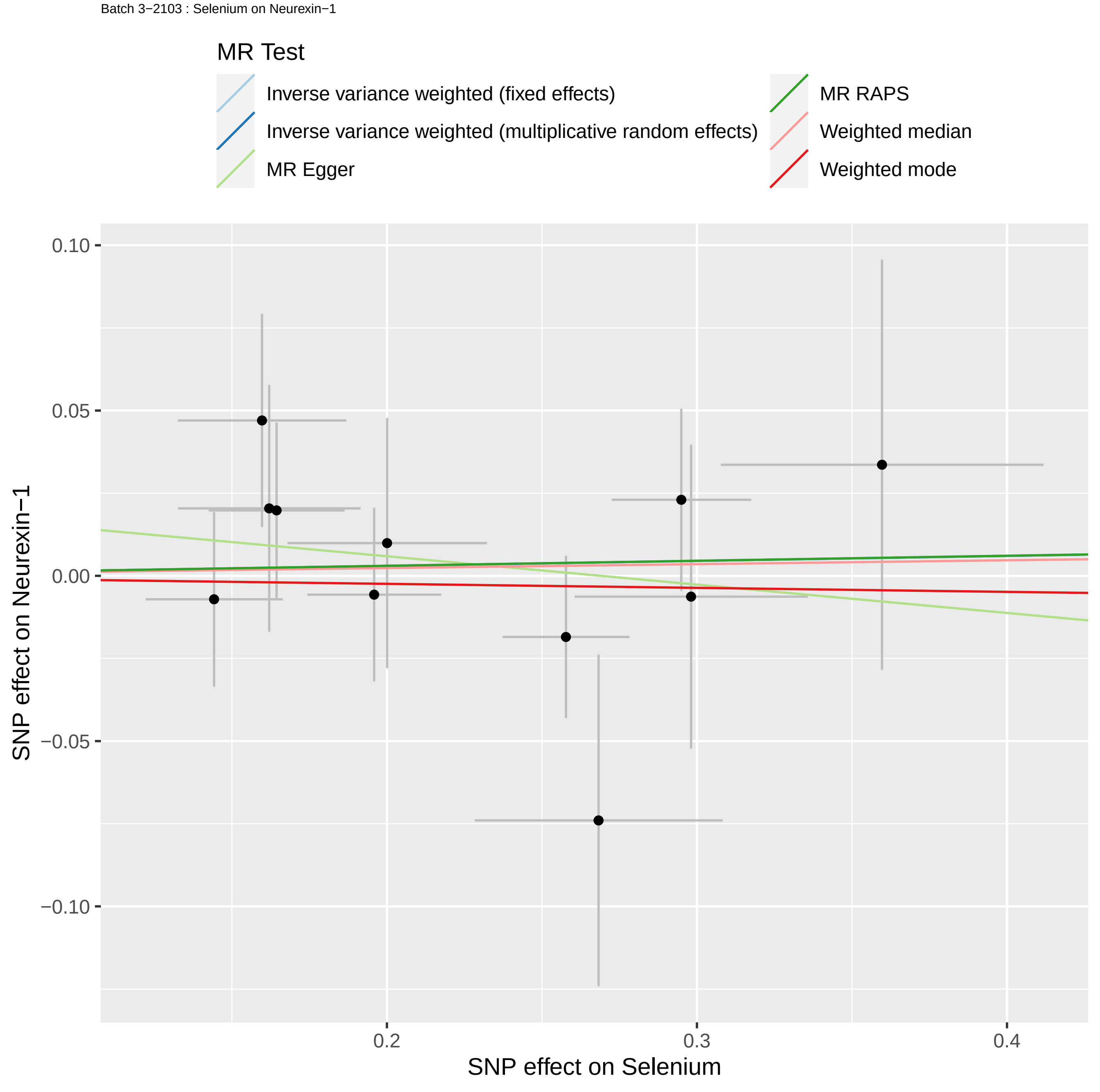
Batch 3−2103 : Selenium on Neurexin−1
MR Test
Inverse variance weighted (fixed effects)
Inverse variance weighted (multiplicative random effects)
MR Egger
MR RAPS
Weighted median
Weighted mode
0.10
0.05
0.00
−0.05
−0.10
0.2
0.3
0.4
SNP effect on Neurexin−1
SNP effect on Selenium

Batch 3−2104 : Selenium on Neurexin−2−beta

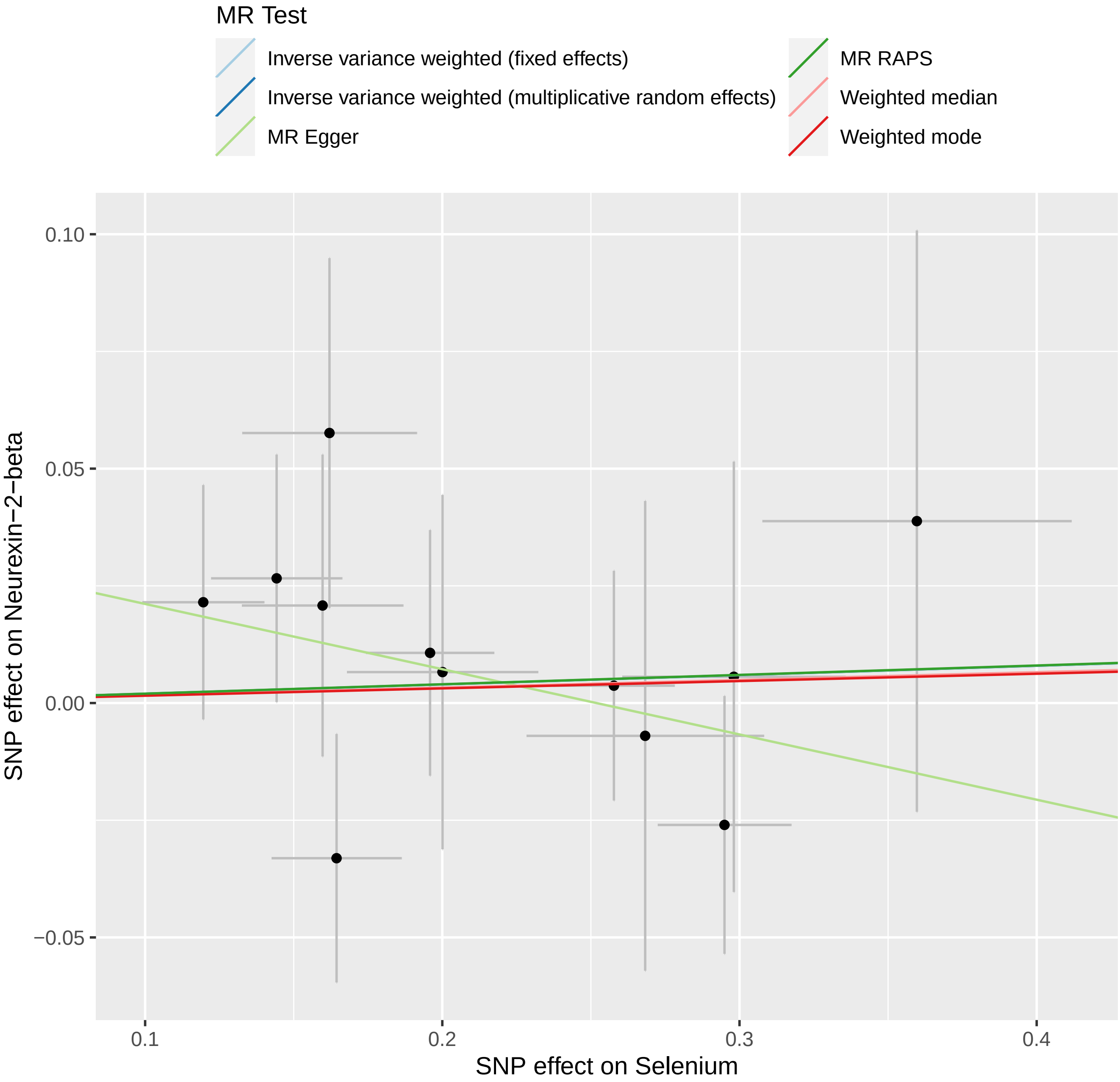

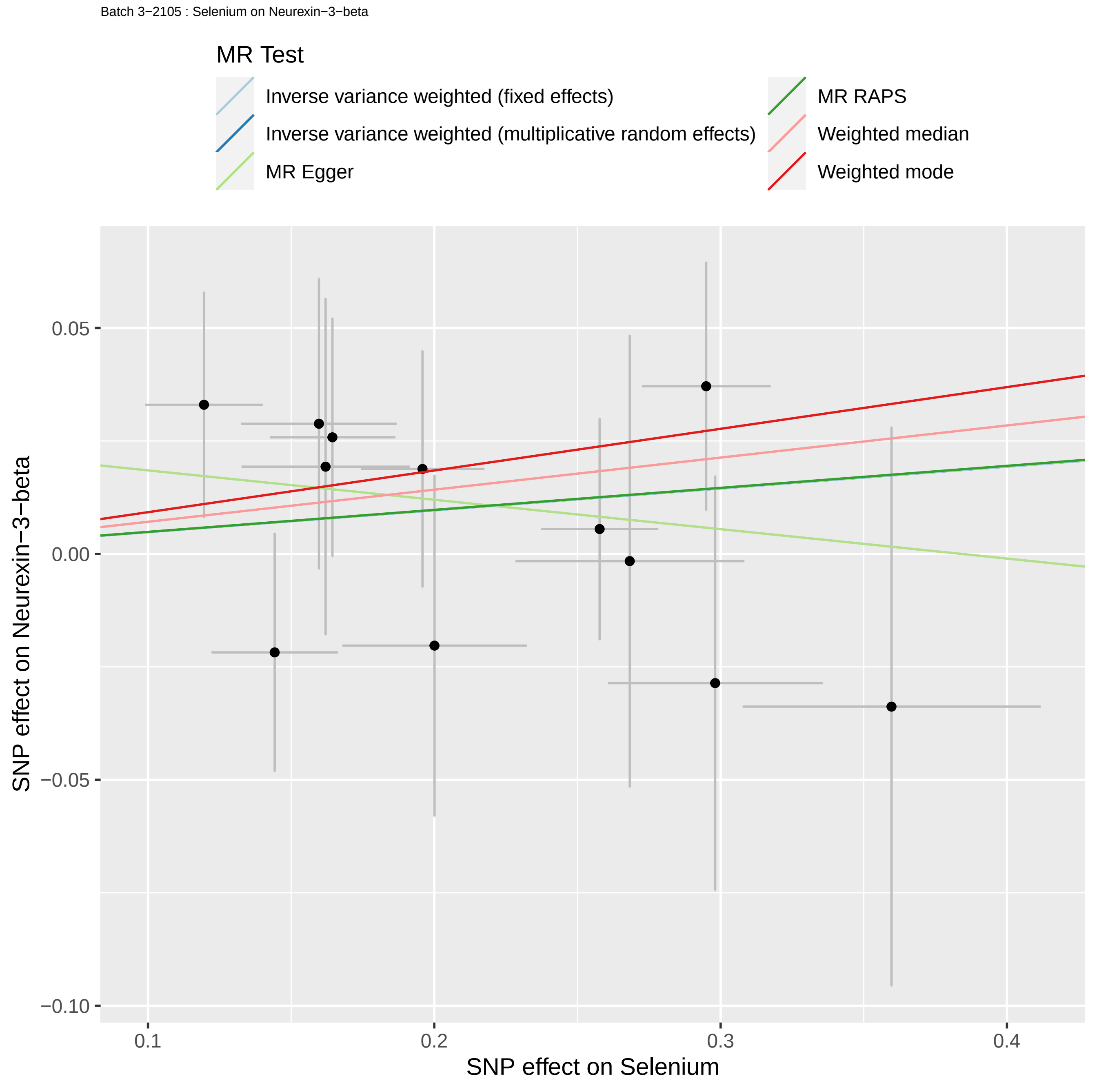

Batch 3−2105 : Selenium on Neurexin−3−beta
MR Test
Inverse variance weighted (fixed effects)
Inverse variance weighted (multiplicative random effects)
MR Egger
MR RAPS
Weighted median
Weighted mode
SNP effect on Neurexin−3−beta
0.05
0.00
−0.05
−0.10
0.1
0.2
0.3
0.4
SNP effect on Selenium

Batch 3−2106 : Selenium on Neurexin−3−beta

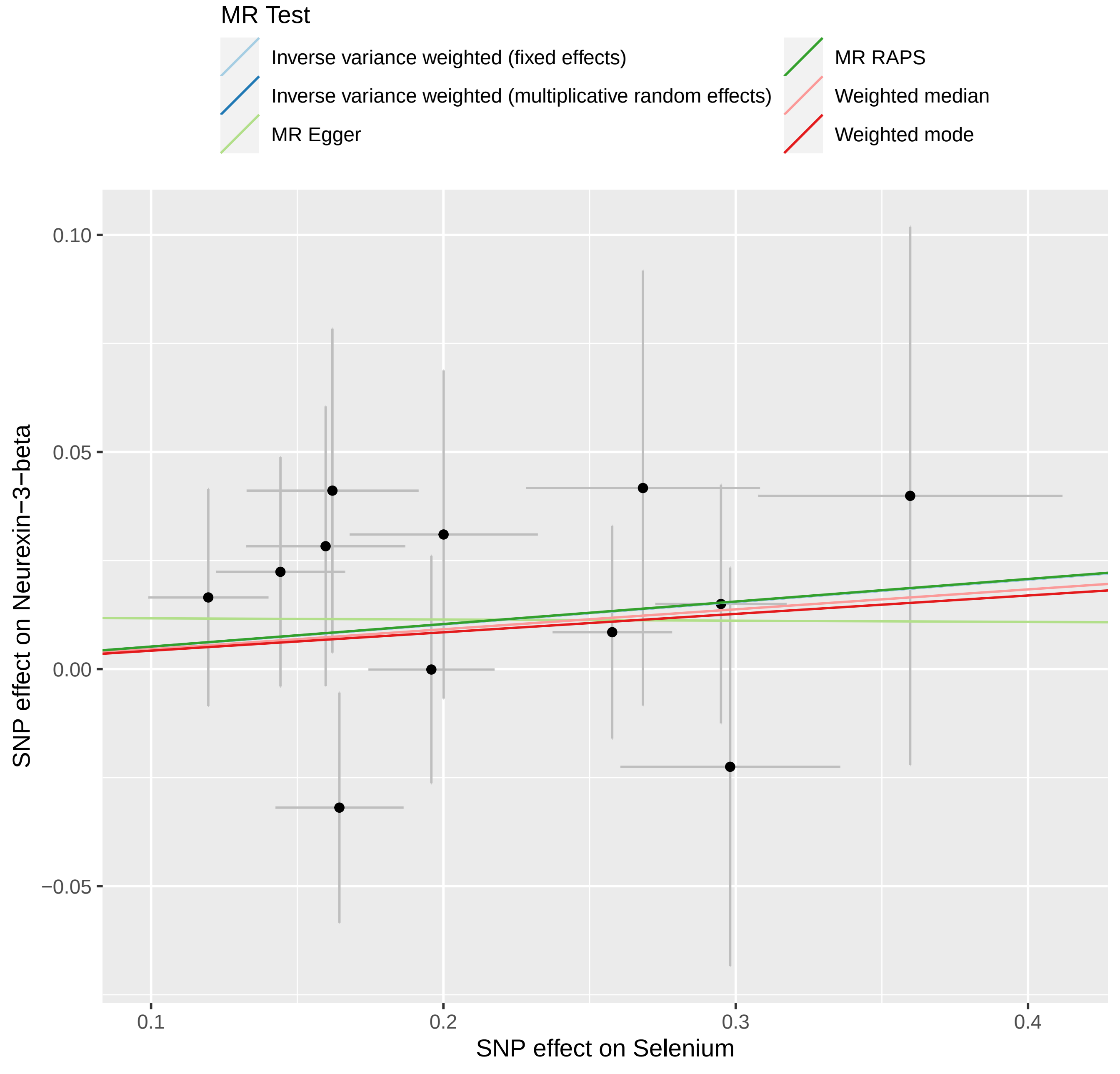

Batch 3−2107 : Selenium on Neurexin−3−beta

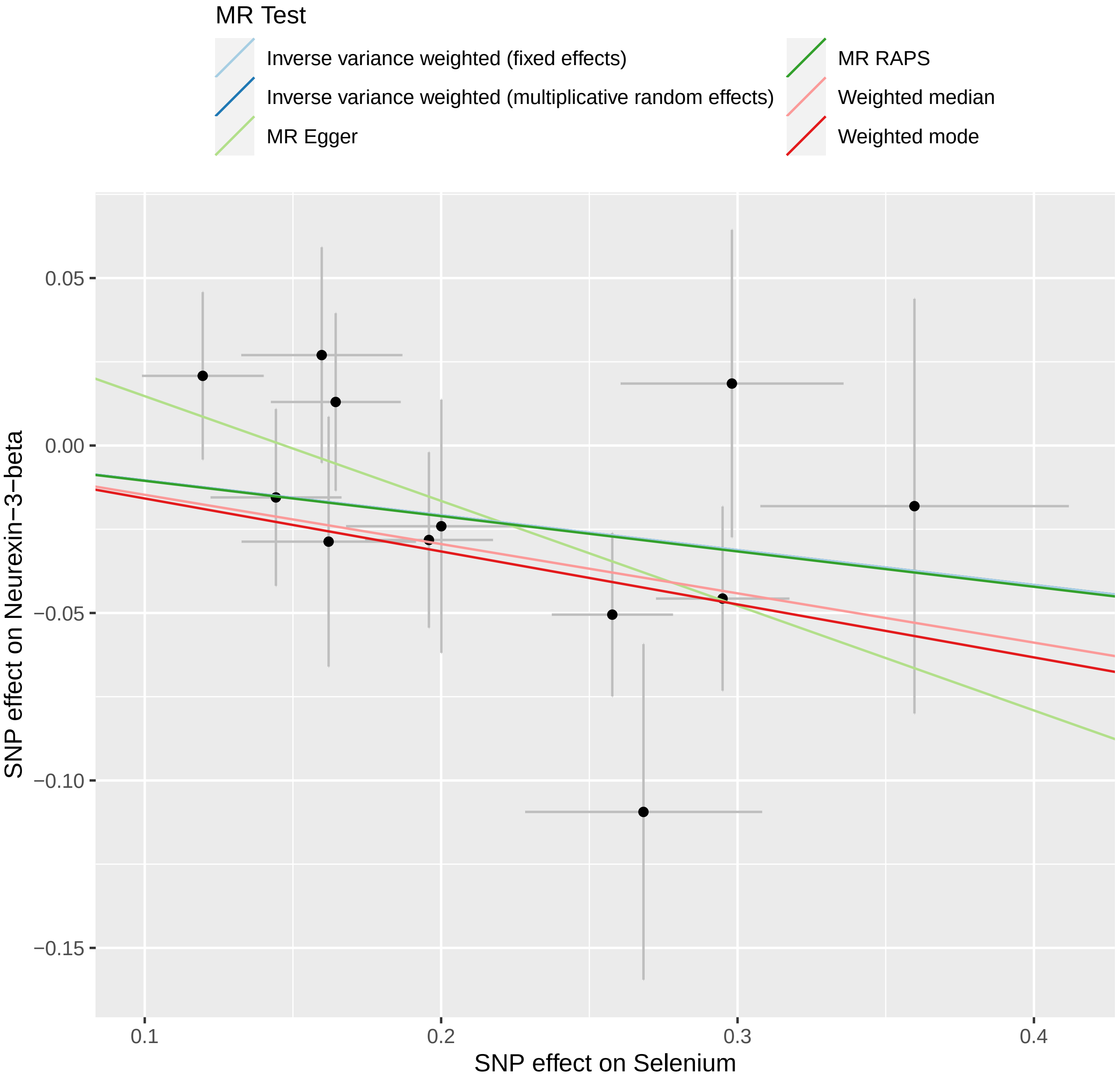

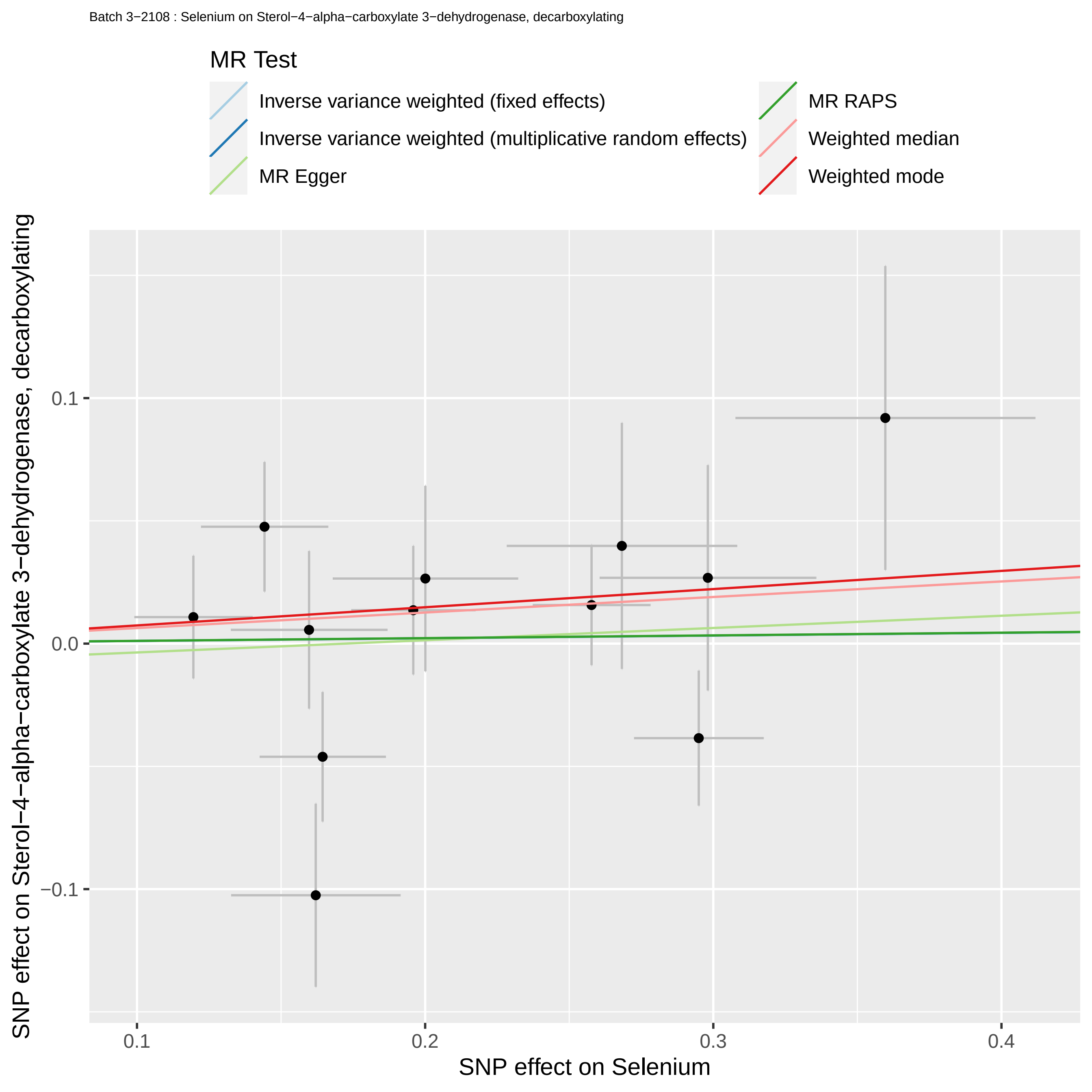

Batch 3−2108 : Selenium on Sterol−4−alpha−carboxylate 3−dehydrogenase, decarboxylating
MR Test
Inverse variance weighted (fixed effects)
Inverse variance weighted (multiplicative random effects)
MR Egger
MR RAPS
Weighted median
Weighted mode
SNP effect on Sterol−4−alpha−carboxylate 3−dehydrogenase, decarboxylating
0.1
0.0
−0.1
0.1
0.2
0.3
0.4
SNP effect on Selenium

Batch 3−2109 : Selenium on Vesicle−fusing ATPase

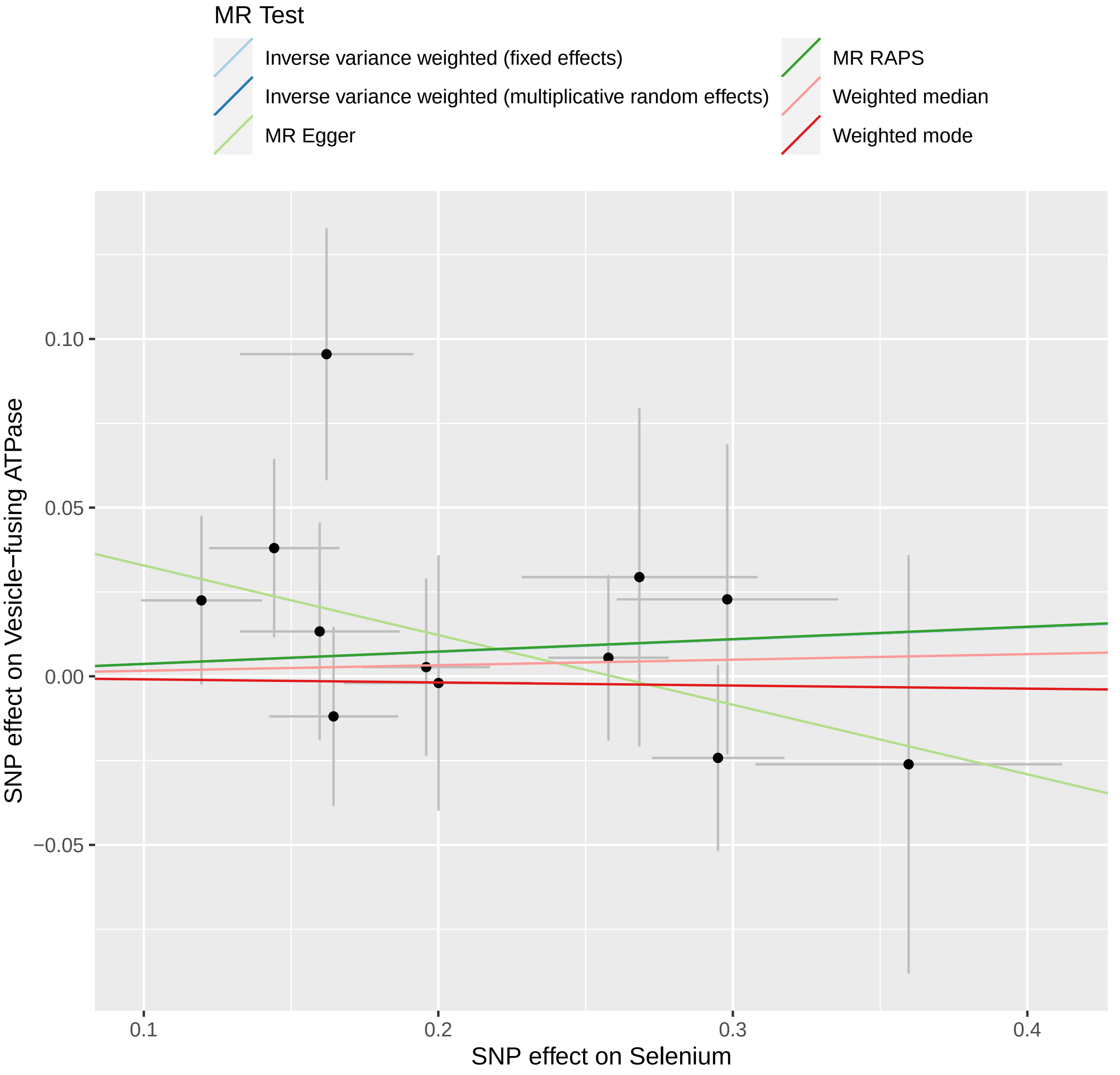

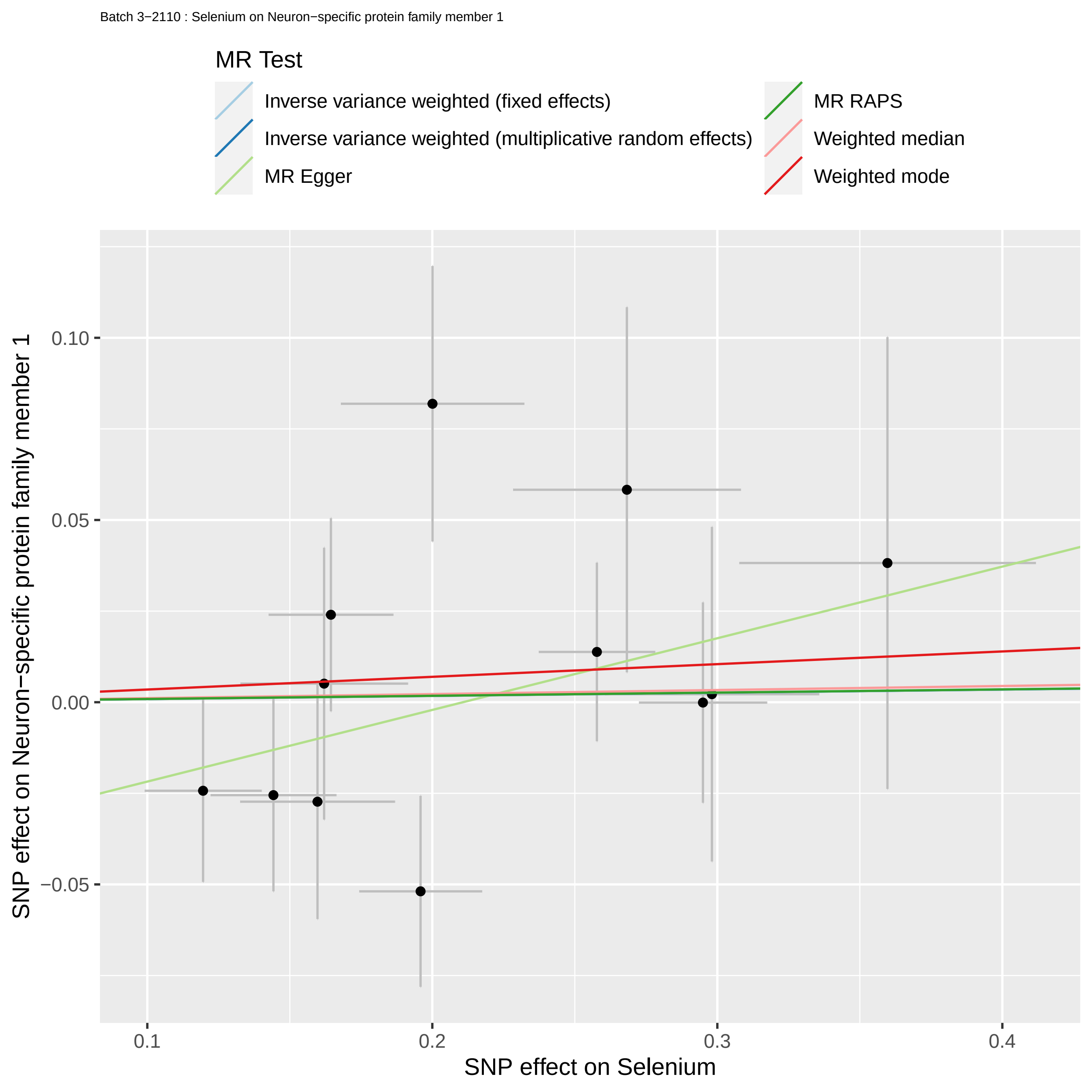

Batch 3−2110 : Selenium on Neuron−specific protein family member 1
MR Test
Inverse variance weighted (fixed effects)
Inverse variance weighted (multiplicative random effects)
MR Egger
MR RAPS
Weighted median
Weighted mode
0.10
0.05
0.00
−0.05
0.1
0.2
0.3
0.4
SNP effect on Neuron−specific protein family member 1
SNP effect on Selenium

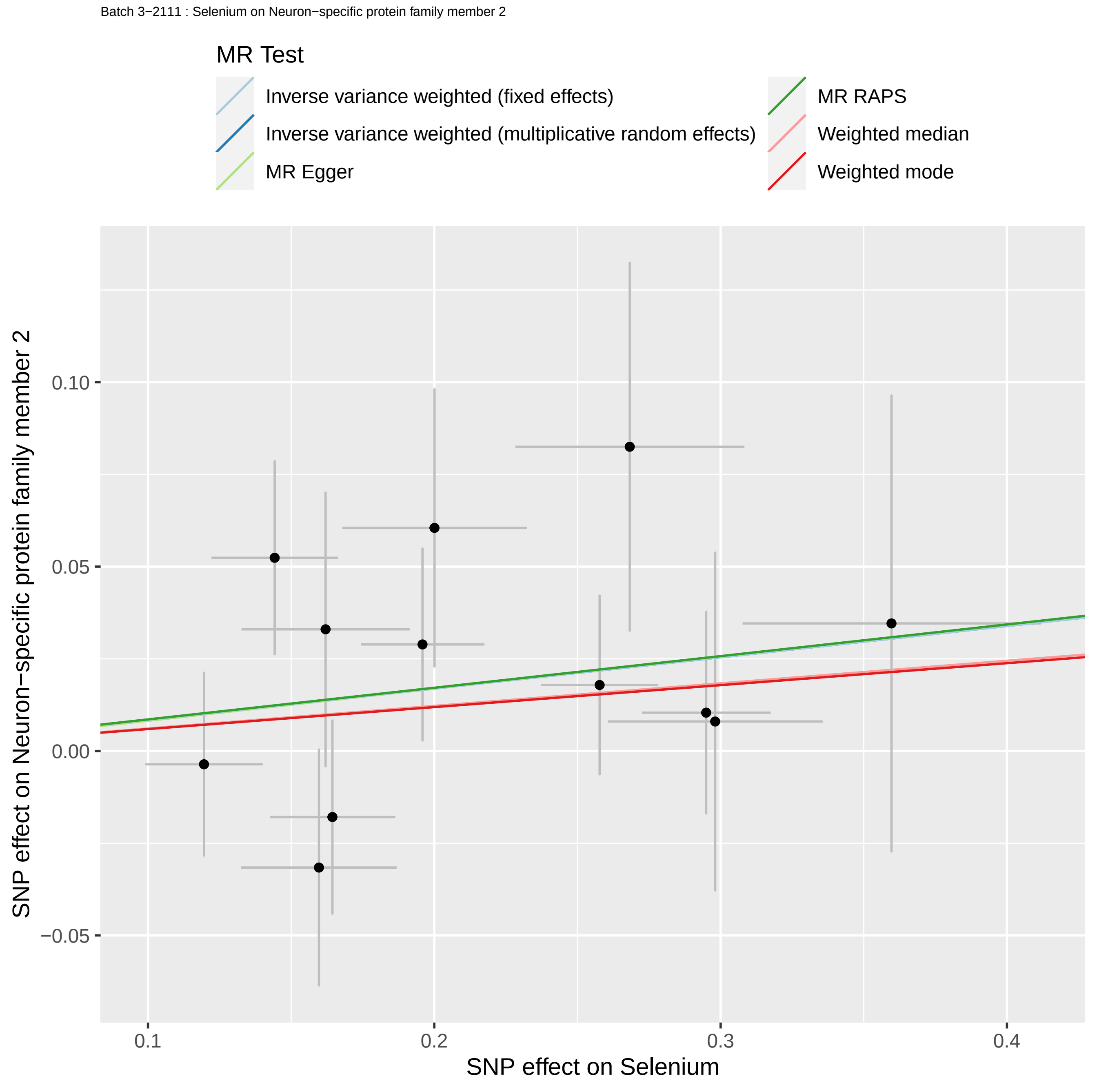
Batch 3−2111 : Selenium on Neuron−specific protein family member 2
MR Test
Inverse variance weighted (fixed effects)
Inverse variance weighted (multiplicative random effects)
MR Egger
MR RAPS
Weighted median
Weighted mode
SNP effect on Neuron−specific protein family member 2
0.10
0.05
0.00
−0.05
0.1
0.2
0.3
0.4
SNP effect on Selenium

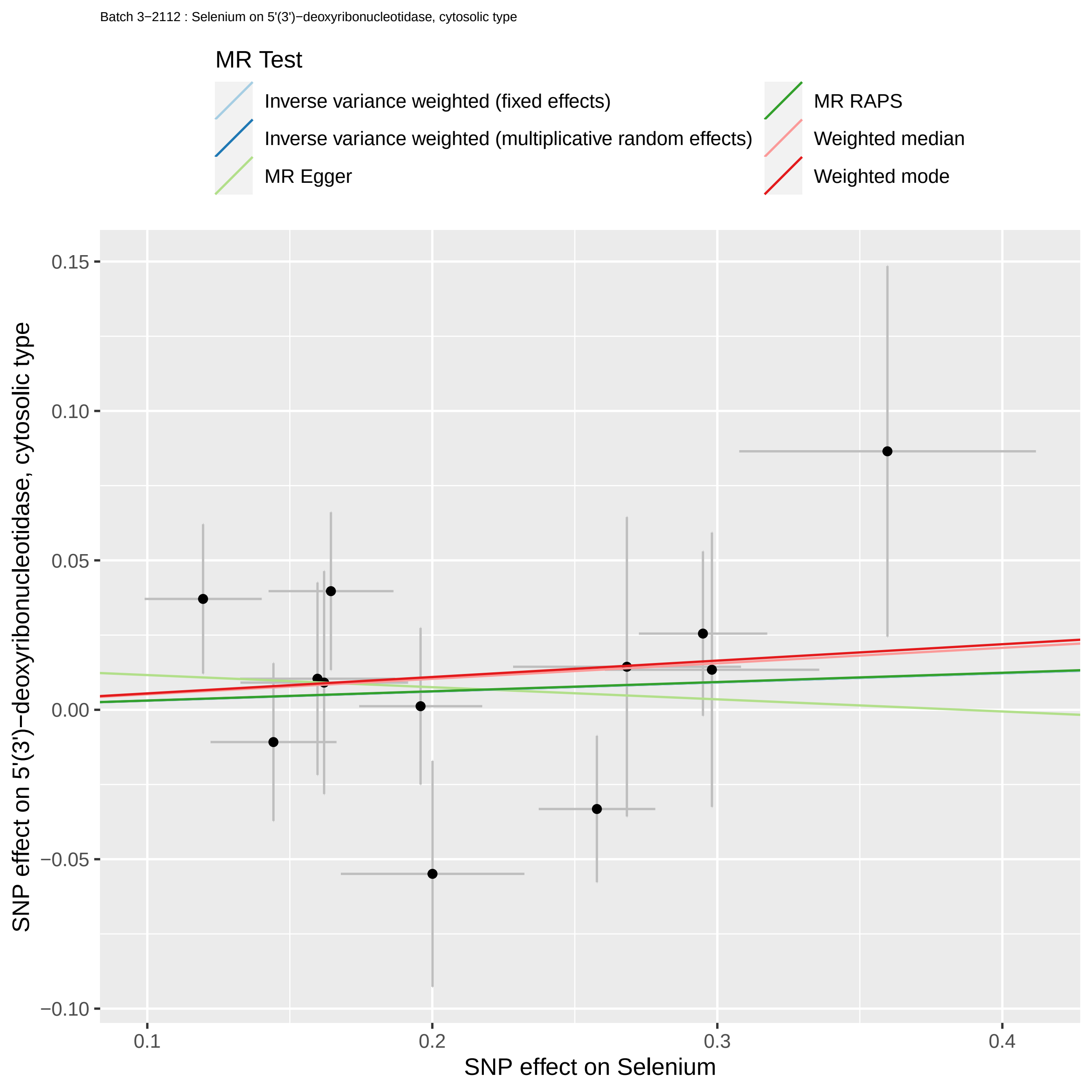
Batch 3−2112 : Selenium on 5'(3')−deoxyribonucleotidase, cytosolic type
MR Test
Inverse variance weighted (fixed effects)
Inverse variance weighted (multiplicative random effects)
MR Egger
MR RAPS
Weighted median
Weighted mode
0.15
0.10
0.05
0.00
−0.05
−0.10
0.1
0.2
0.3
0.4
SNP effect on 5'(3')−deoxyribonucleotidase, cytosolic type
SNP effect on Selenium

Batch 3−2113 : Selenium on 5'−Nucleotidase

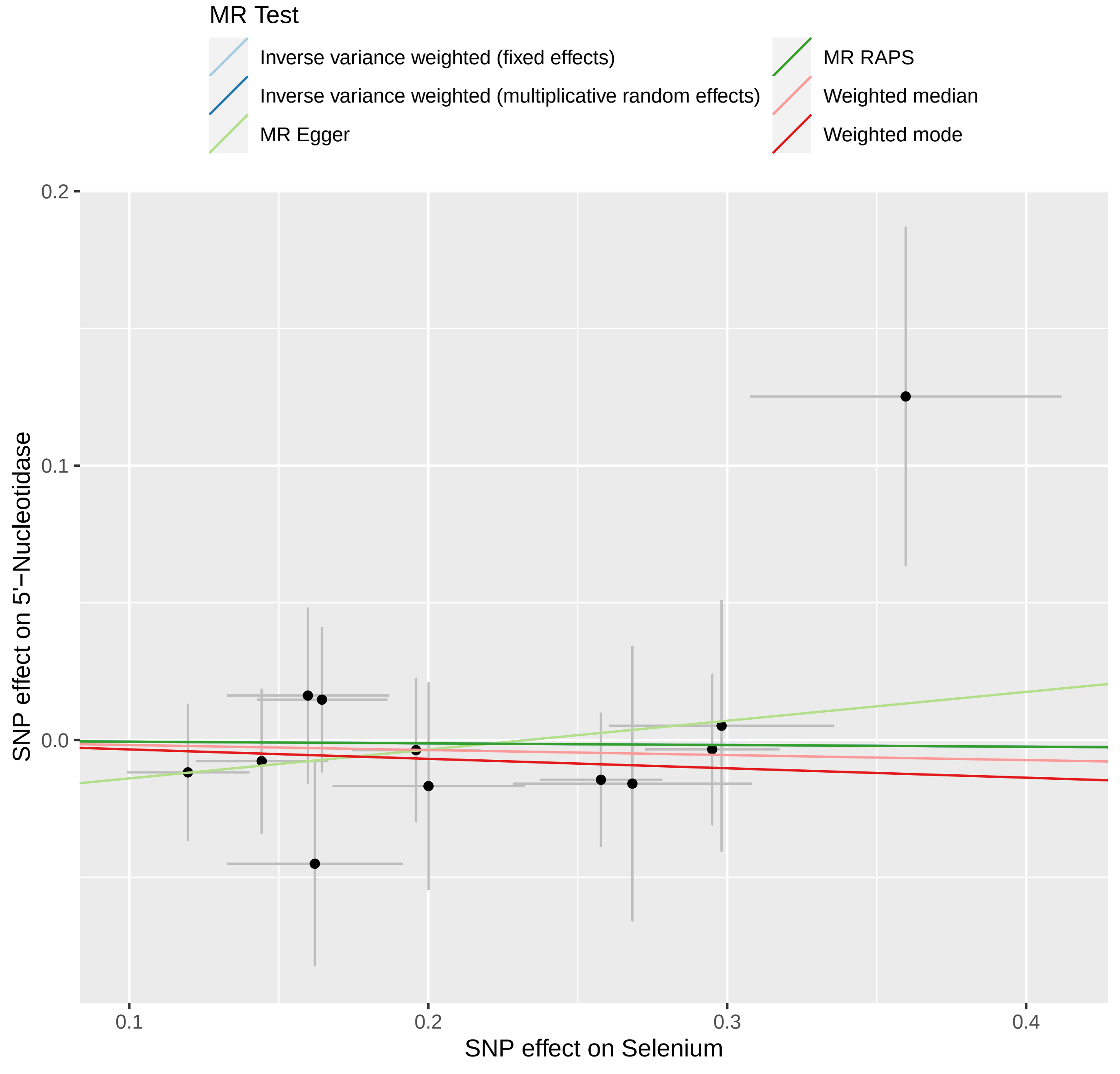

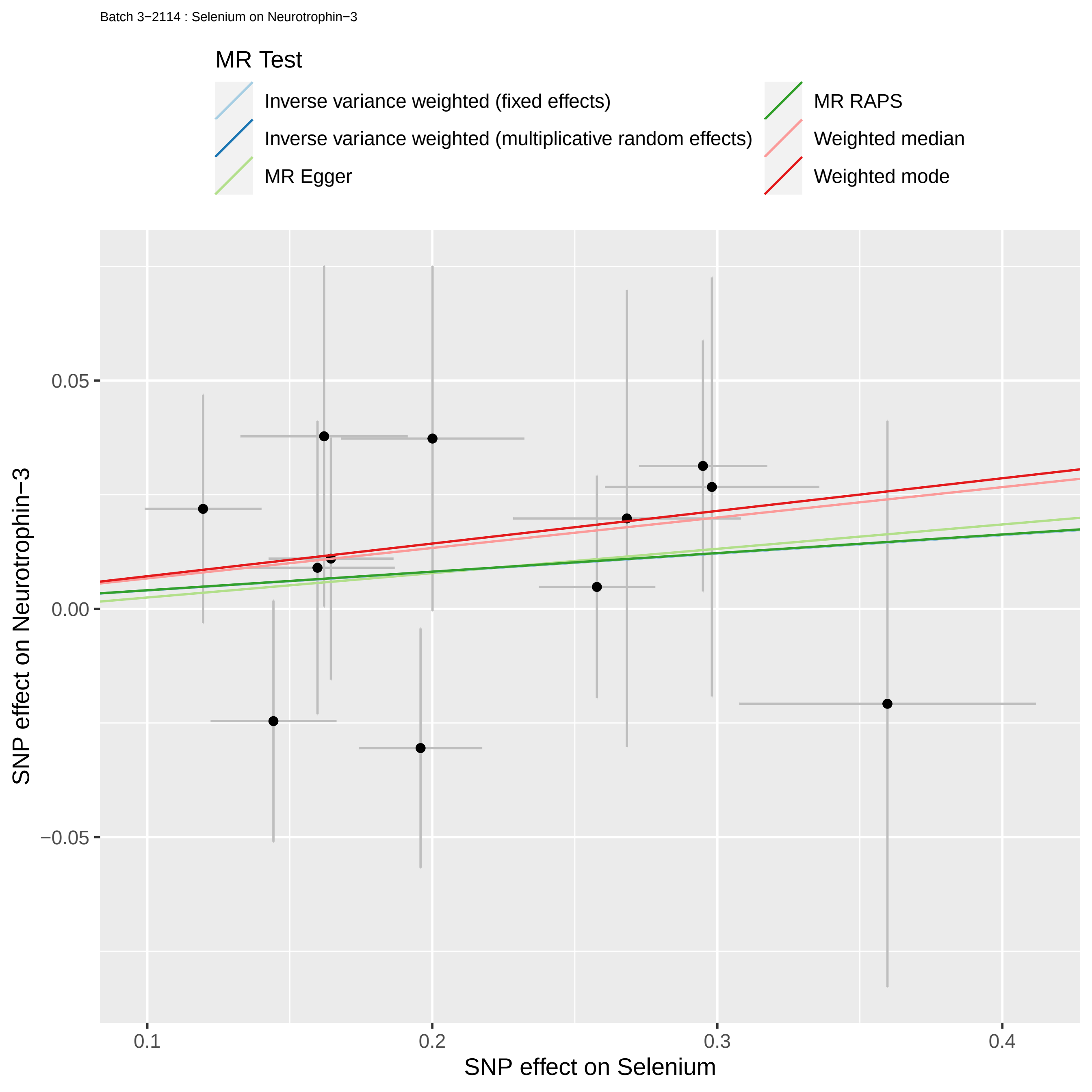
Batch 3−2114 : Selenium on Neurotrophin−3
MR Test
Inverse variance weighted (fixed effects)
Inverse variance weighted (multiplicative random effects)
MR Egger
MR RAPS
Weighted median
Weighted mode
SNP effect on Neurotrophin−3
0.05
0.00
−0.05
0.1
0.2
0.3
0.4
SNP effect on Selenium

Batch 3−2115 : Selenium on Neurotrophin−4

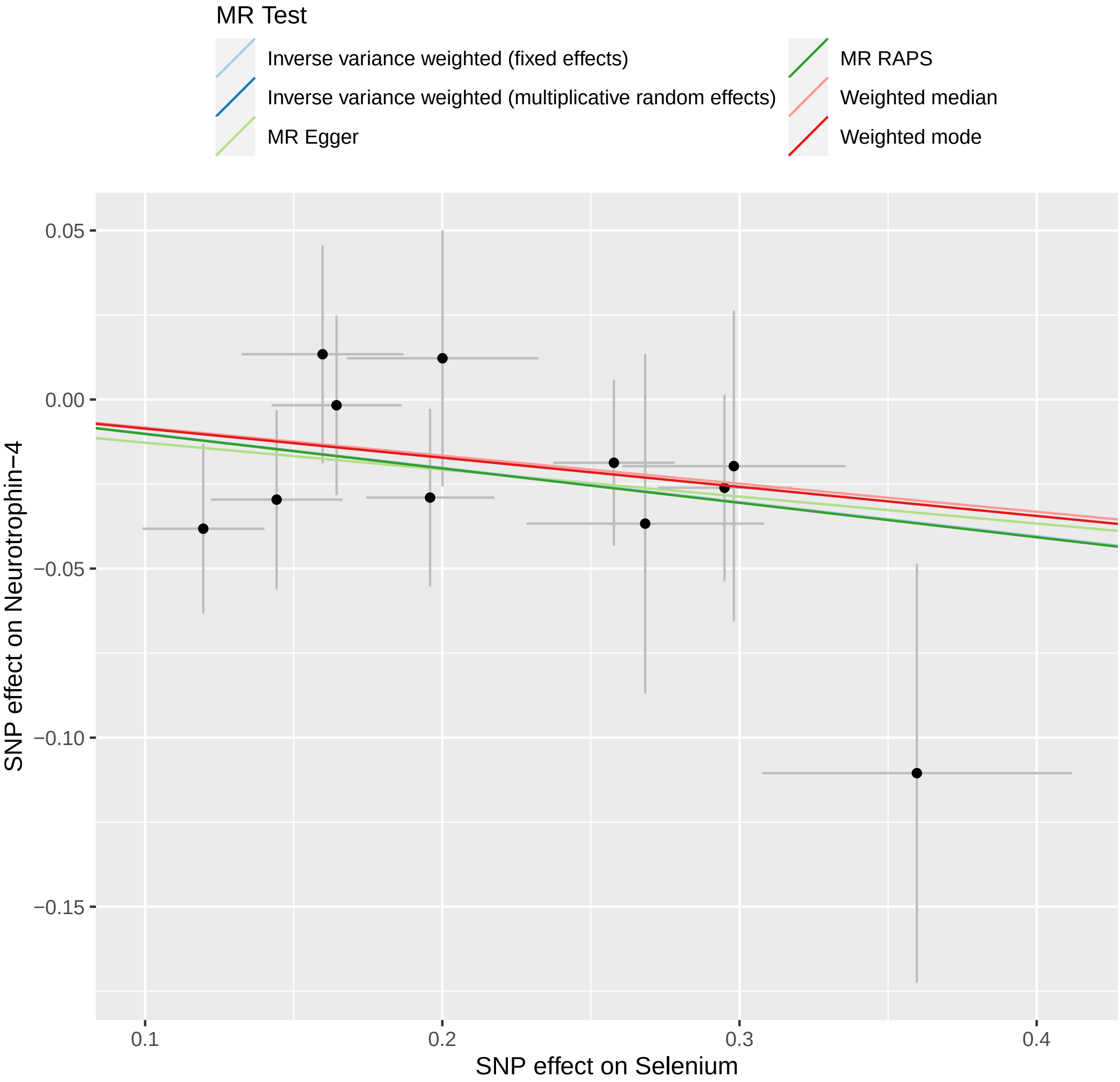

Batch 3−2116 : Selenium on N−terminal Xaa−Pro−Lys N−methyltransferase 1

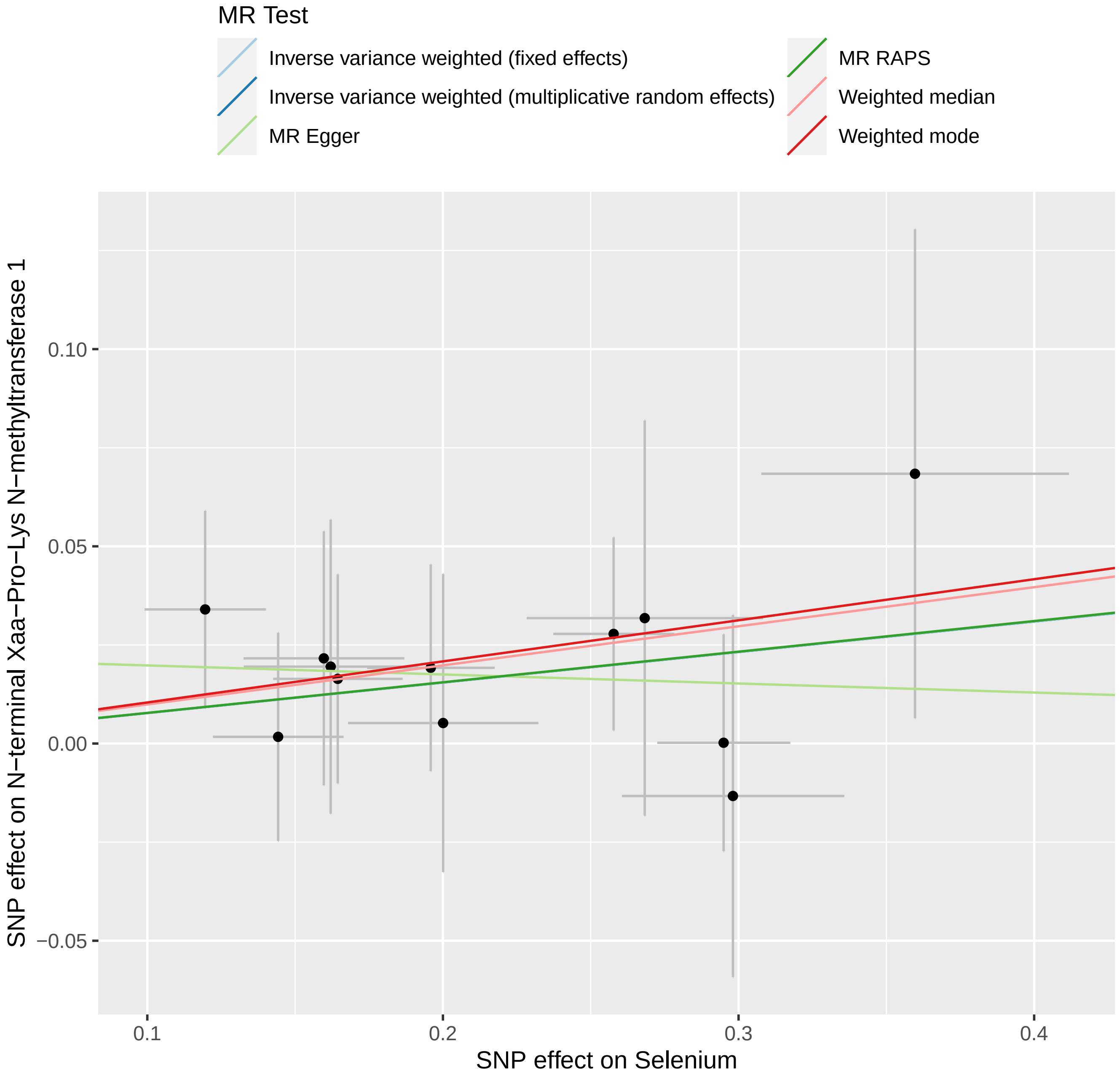

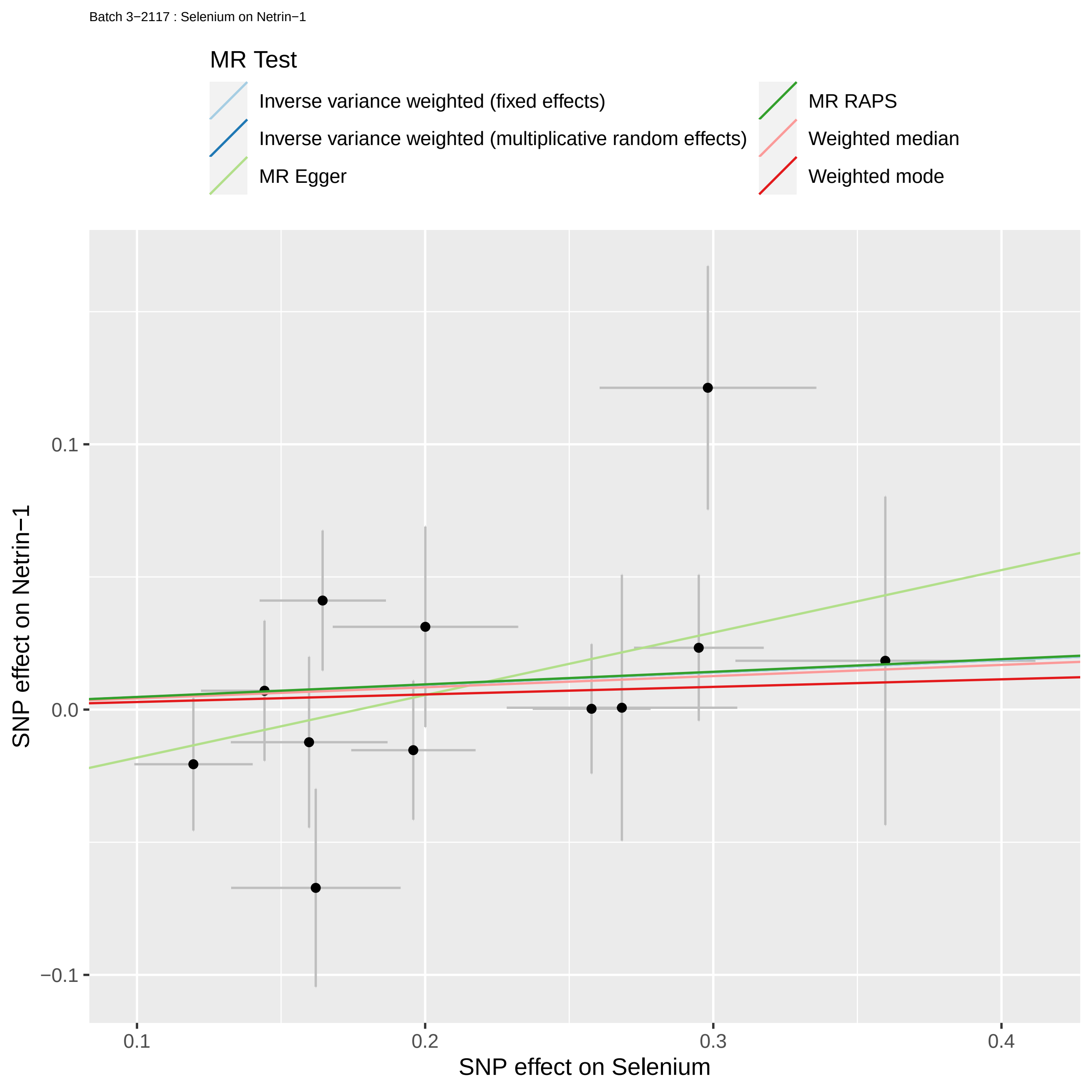

Batch 3−2117 : Selenium on Netrin−1
MR Test
Inverse variance weighted (fixed effects)
Inverse variance weighted (multiplicative random effects)
MR Egger
MR RAPS
Weighted median
Weighted mode
SNP effect on Netrin−1
0.1
0.0
−0.1
0.1
0.2
0.3
0.4
SNP effect on Selenium

Batch 3−2118 : Selenium on Netrin−1

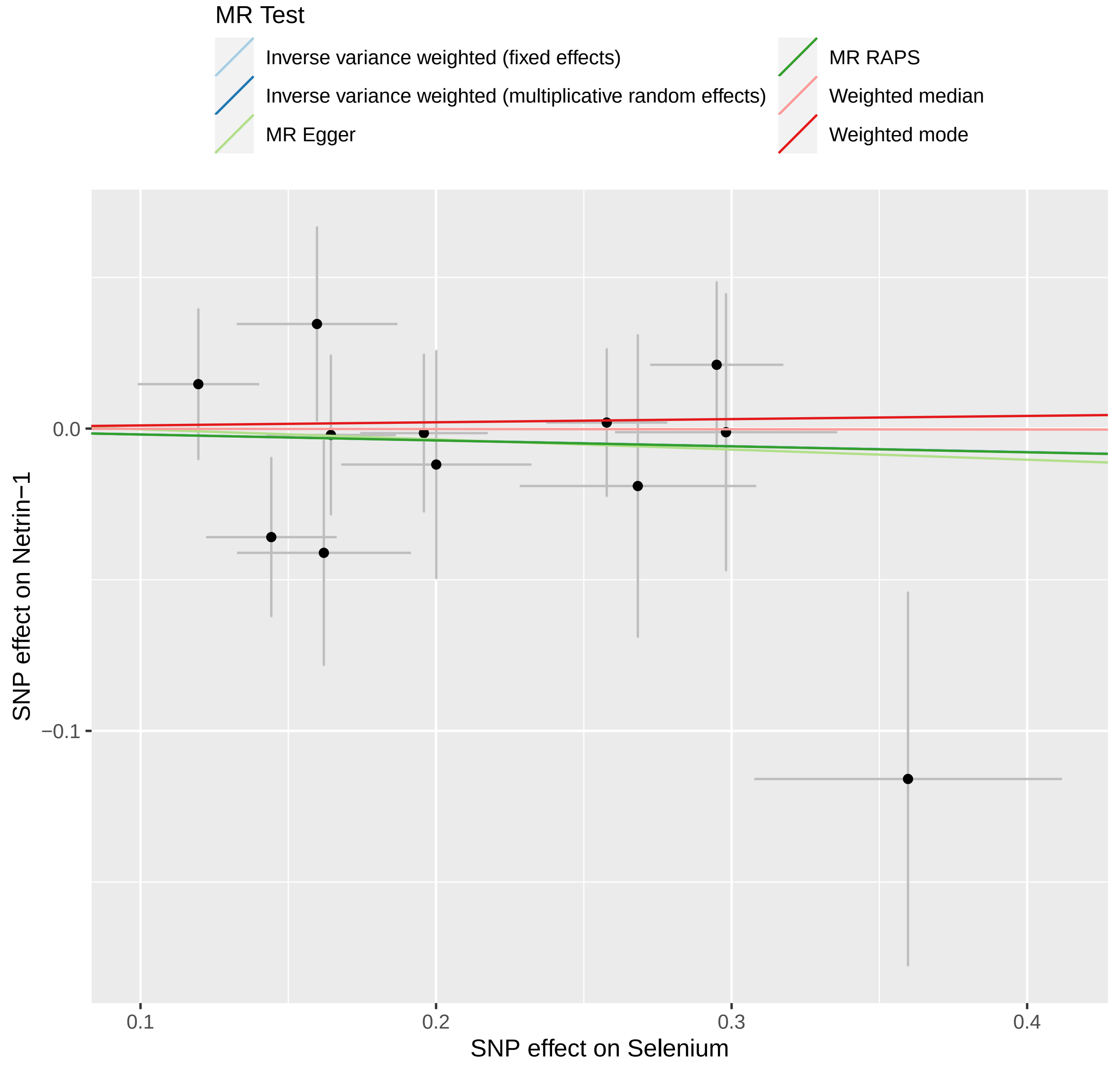

Batch 3−2119 : Selenium on Netrin−4

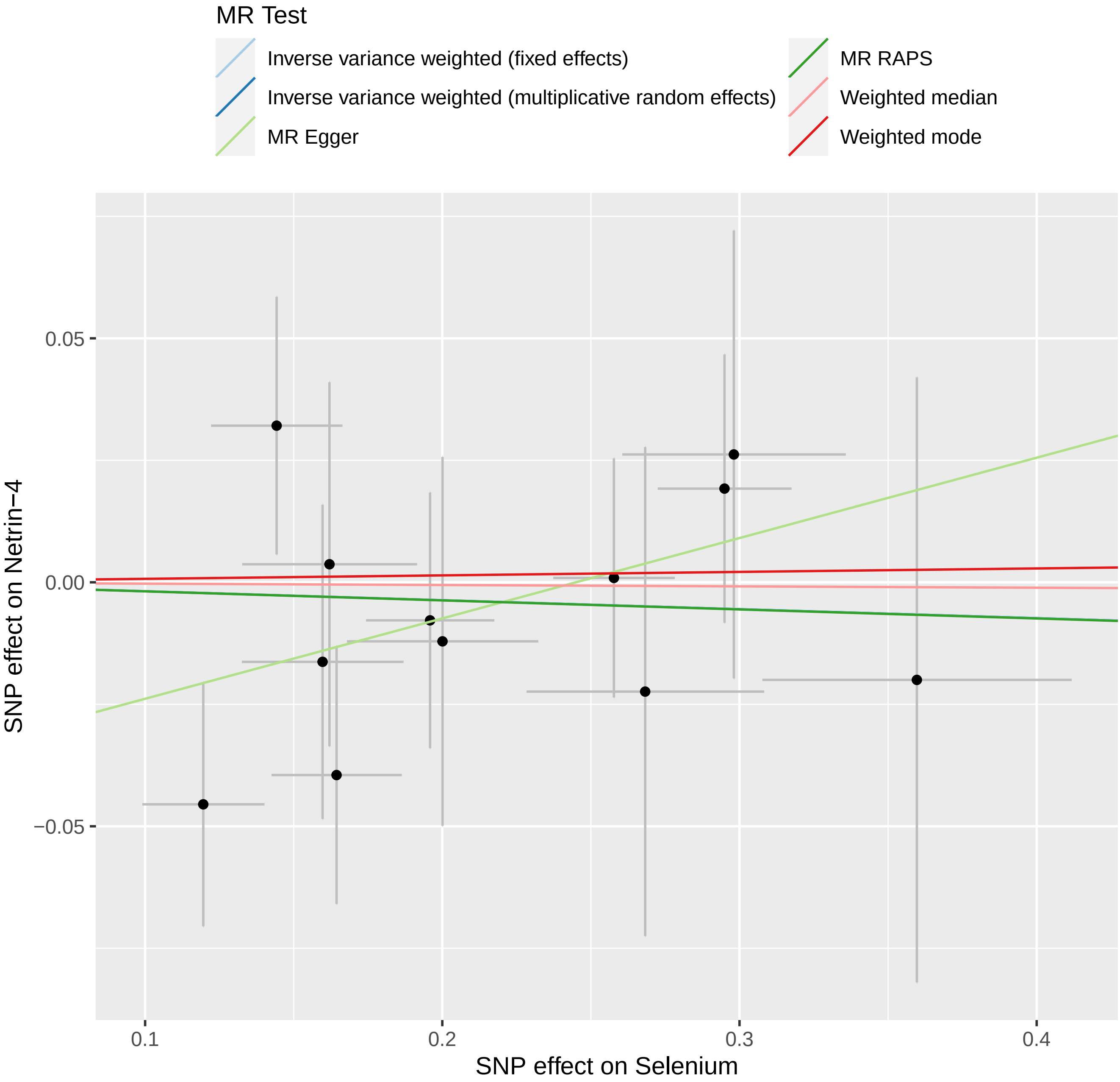

Batch 3−2120 : Selenium on Netrin−G1

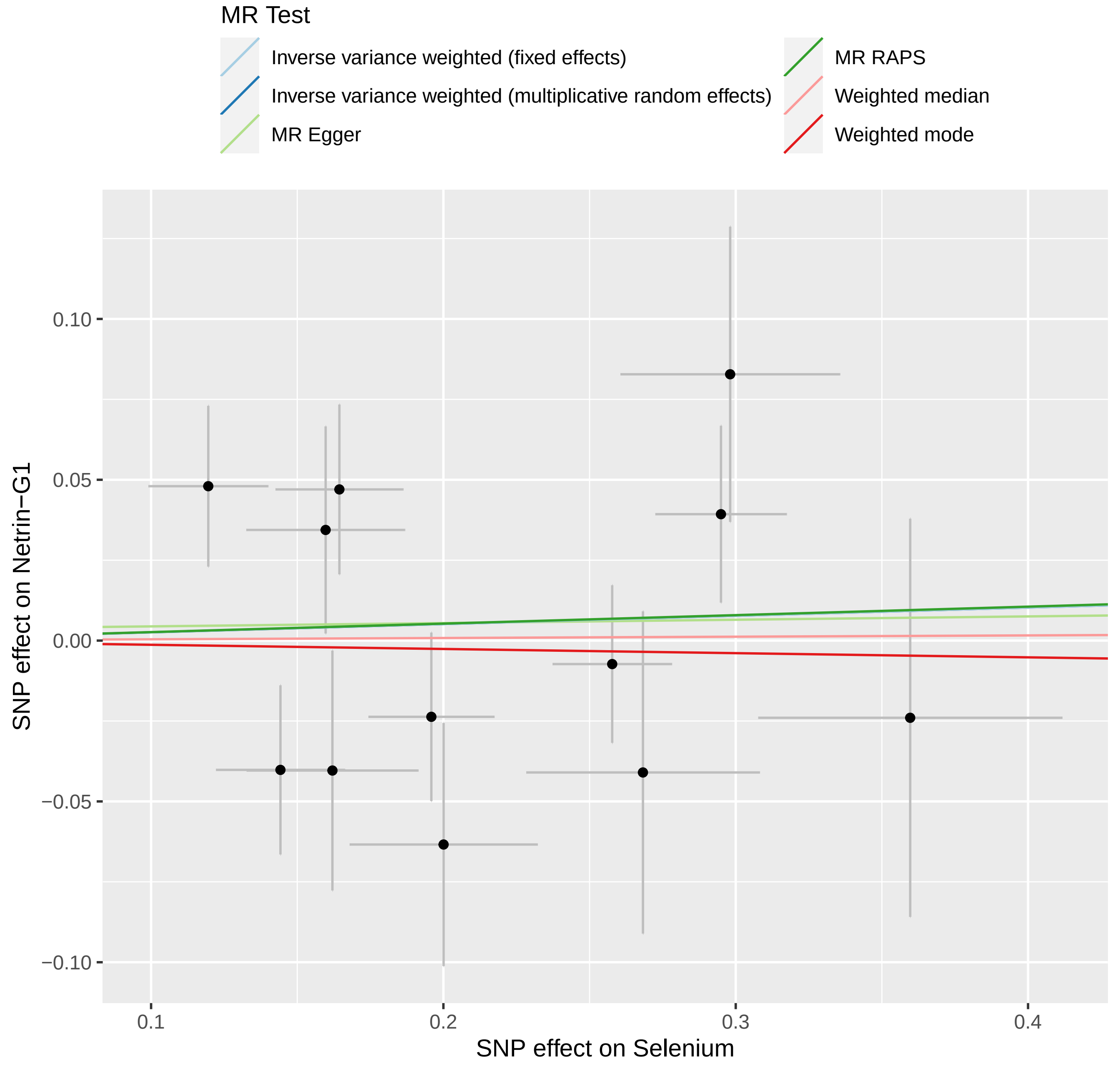

Batch 3−2121 : Selenium on High affinity nerve growth factor receptor

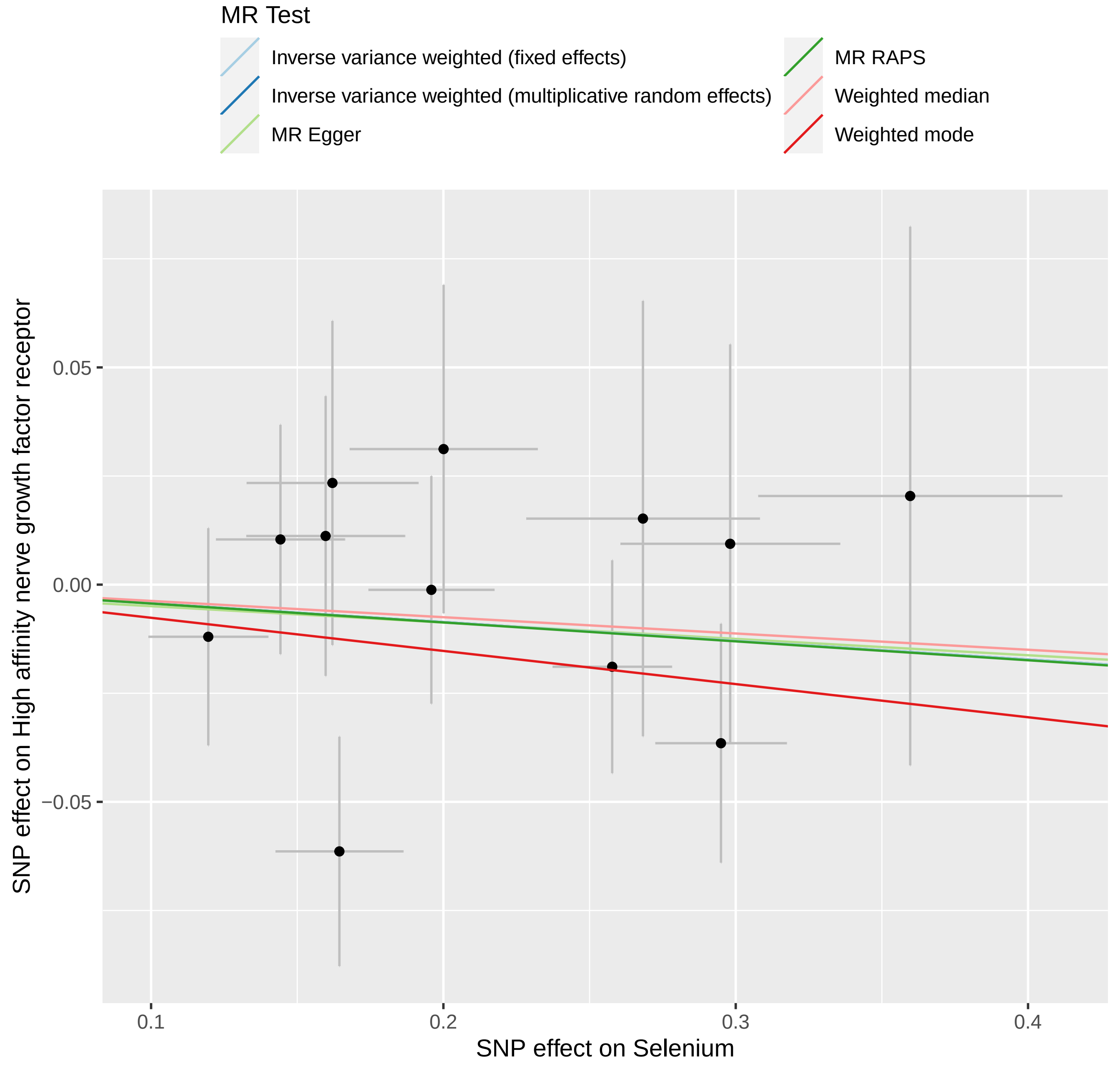

Batch 3−2122 : Selenium on BDNF/NT−3 growth factors receptor

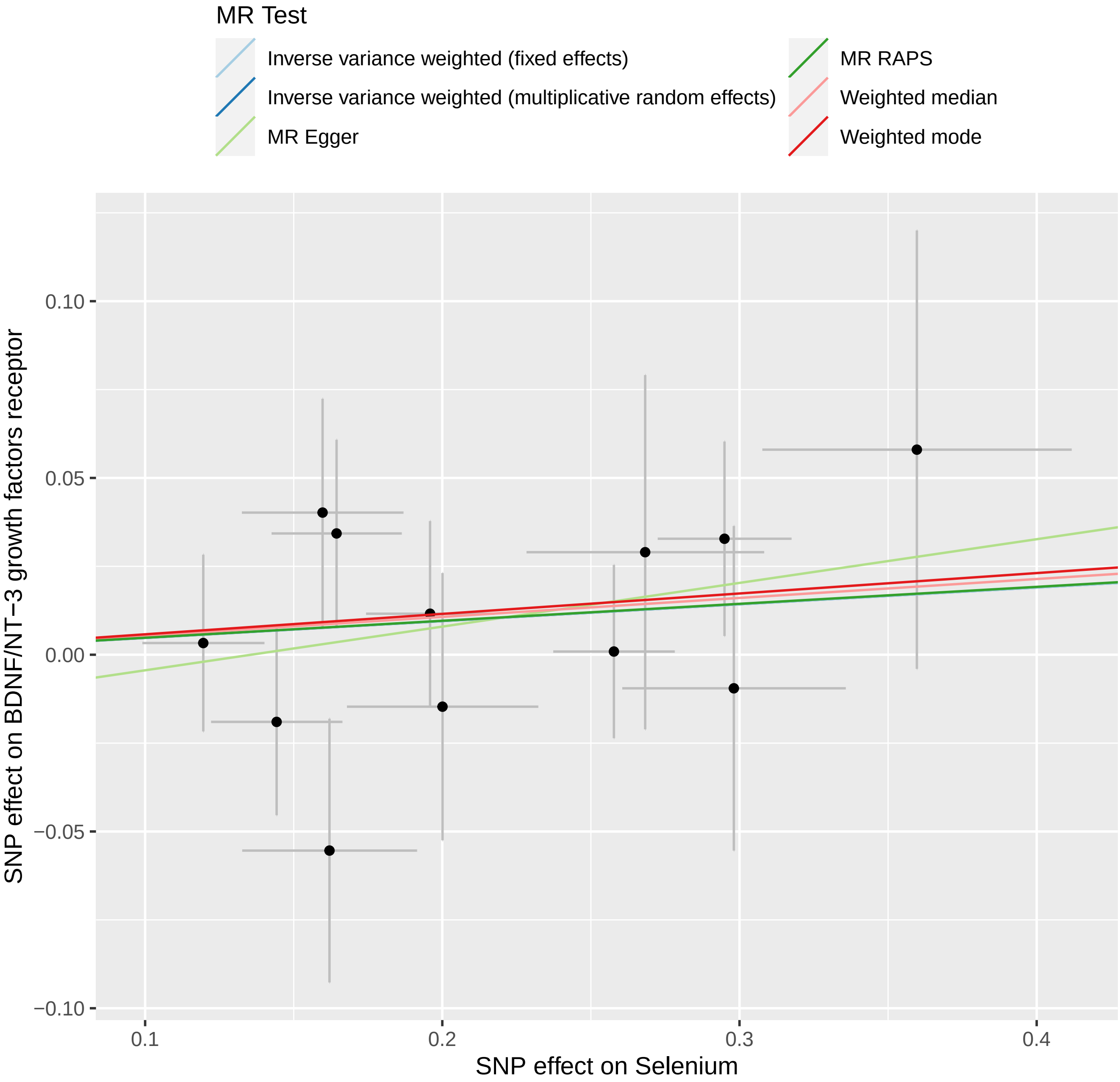

Batch 3−2123 : Selenium on NT−3 growth factor receptor

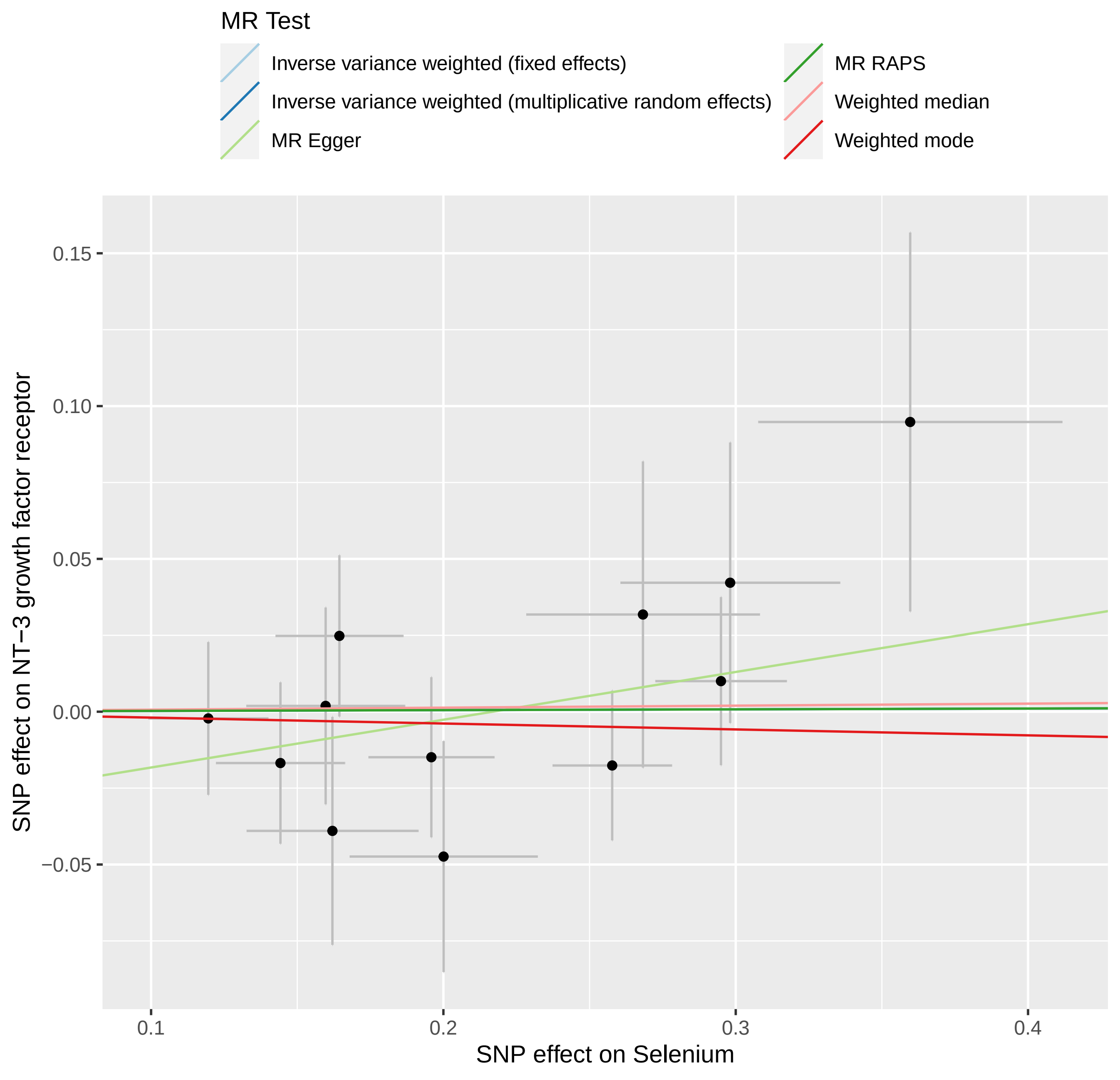

Batch 3−2124 : Selenium on Neurotensin/neuromedin N

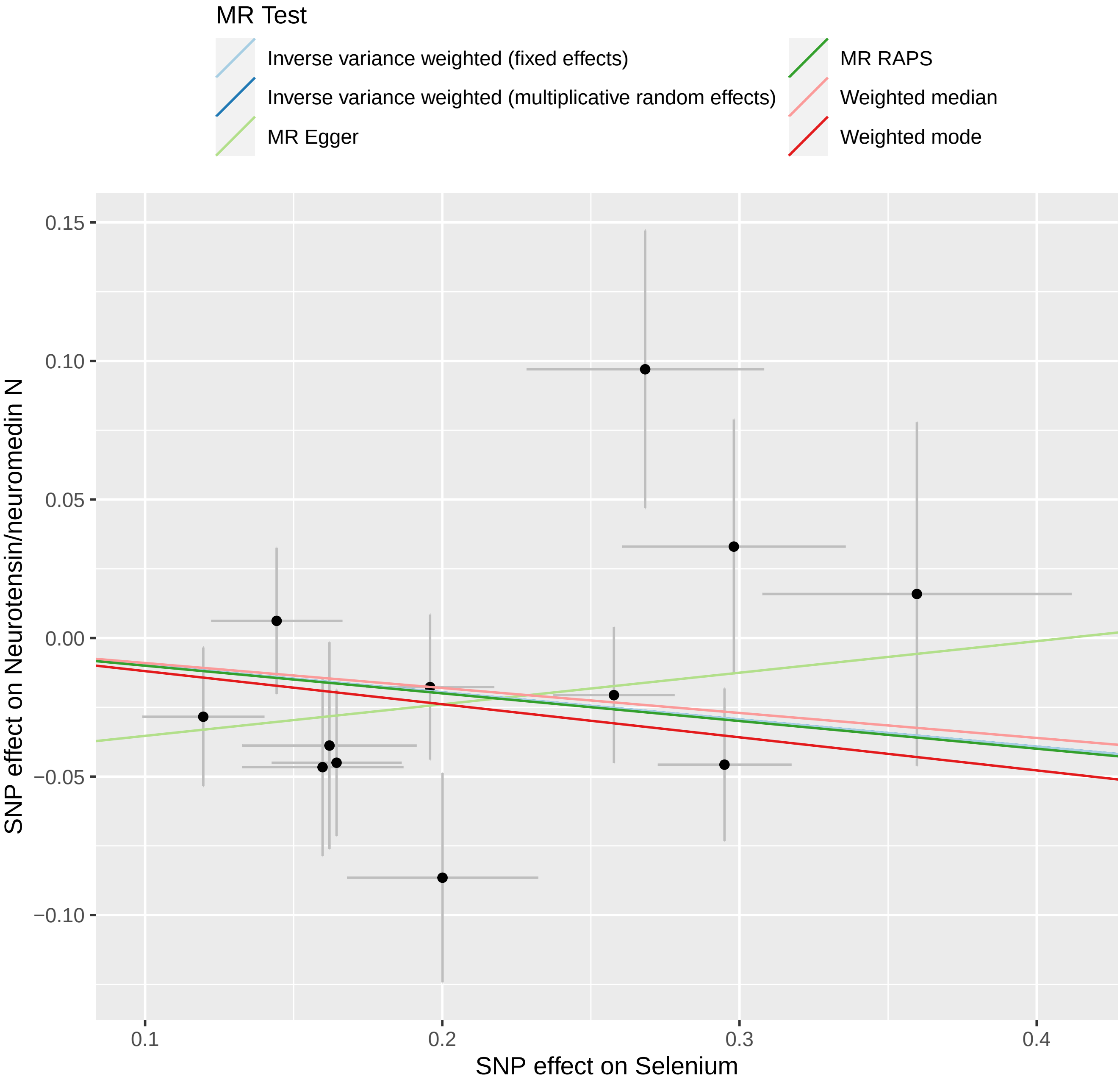

Batch 3−2125 : Selenium on Nuclear migration protein nudC

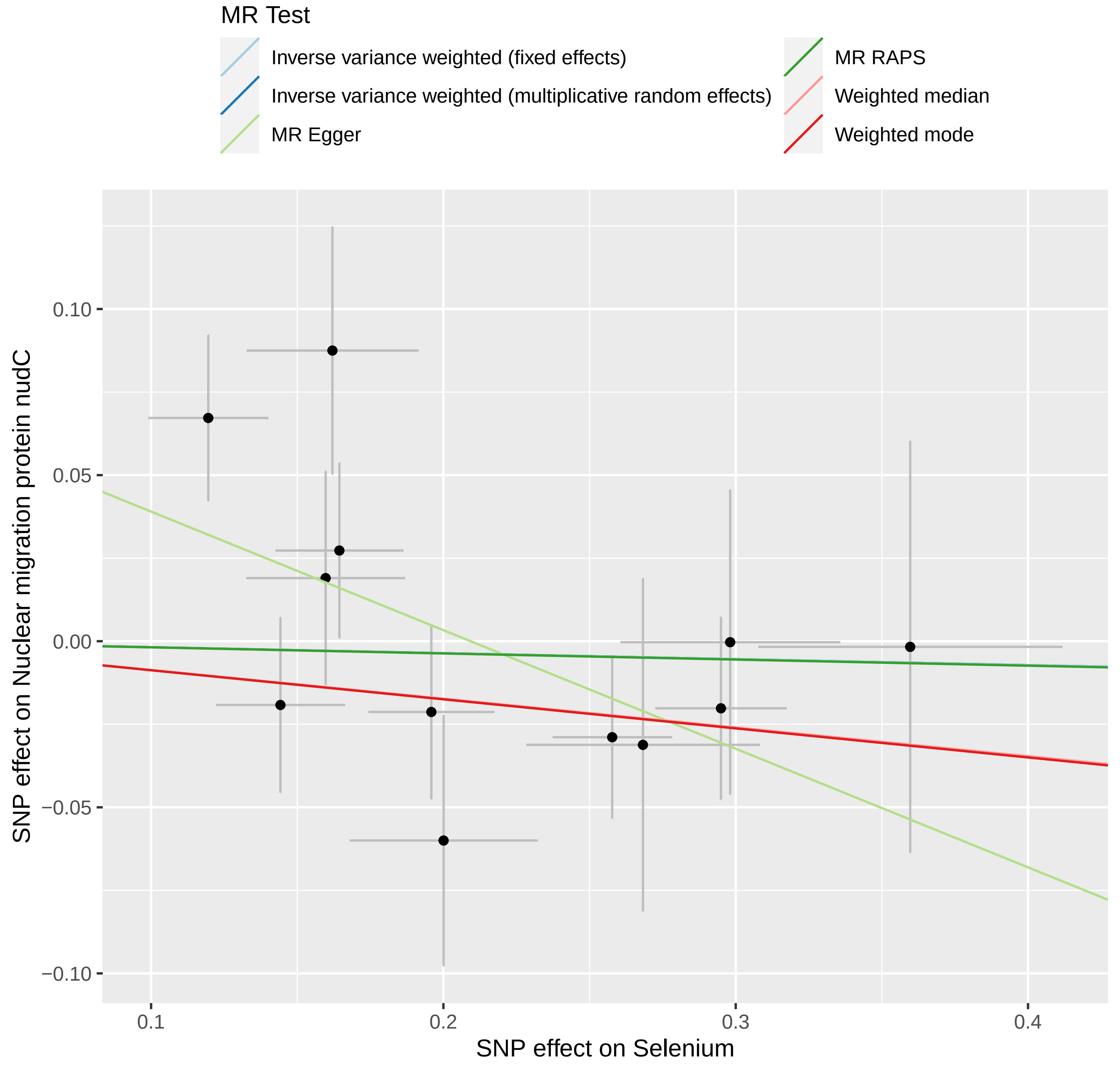

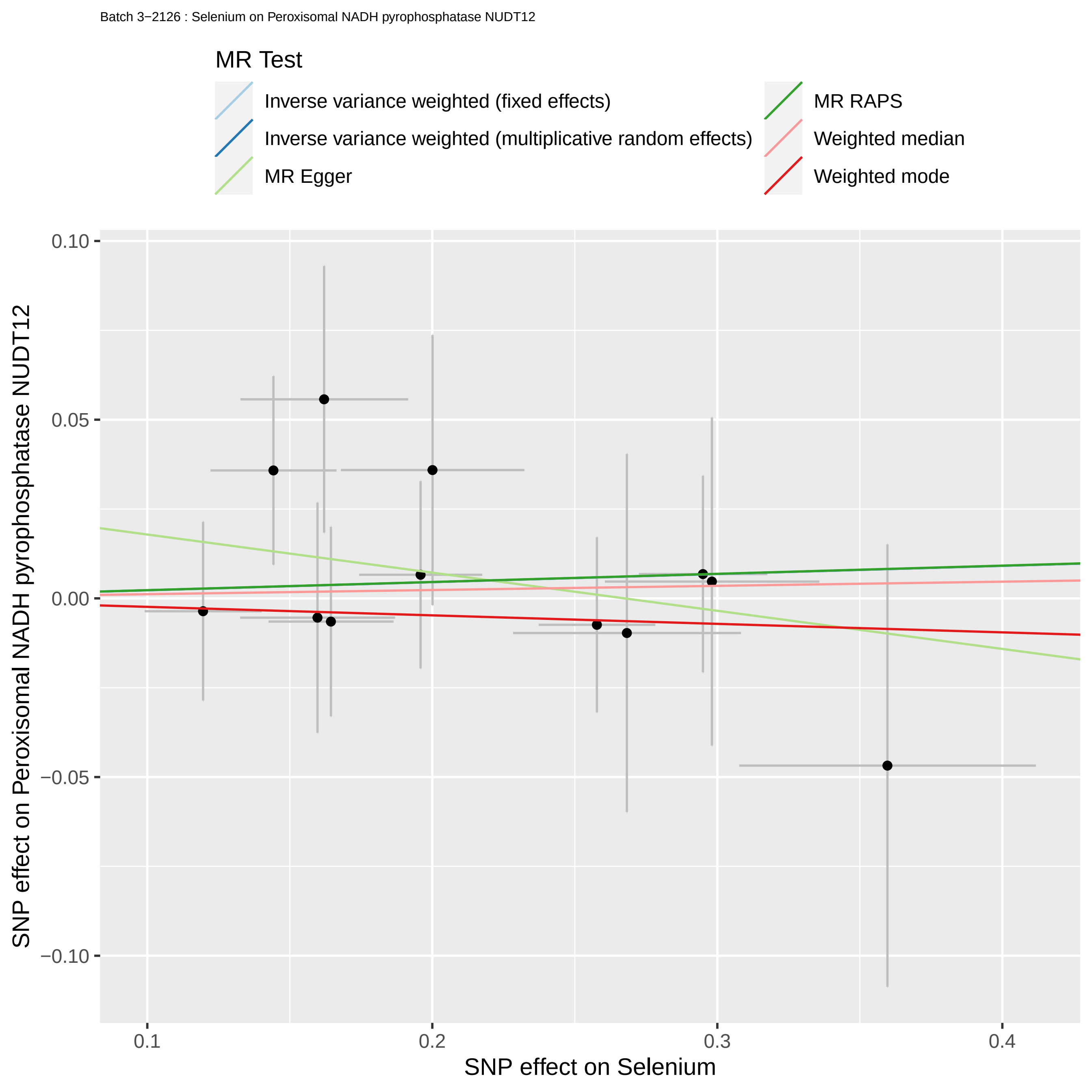

Batch 3−2126 : Selenium on Peroxisomal NADH pyrophosphatase NUDT12
MR Test
Inverse variance weighted (fixed effects)
Inverse variance weighted (multiplicative random effects)
MR Egger
MR RAPS
Weighted median
Weighted mode
0.10
0.05
0.00
−0.05
−0.10
0.1
0.2
0.3
0.4
SNP effect on Peroxisomal NADH pyrophosphatase NUDT12
SNP effect on Selenium

Batch 3−2127 : Selenium on Protein syndesmos

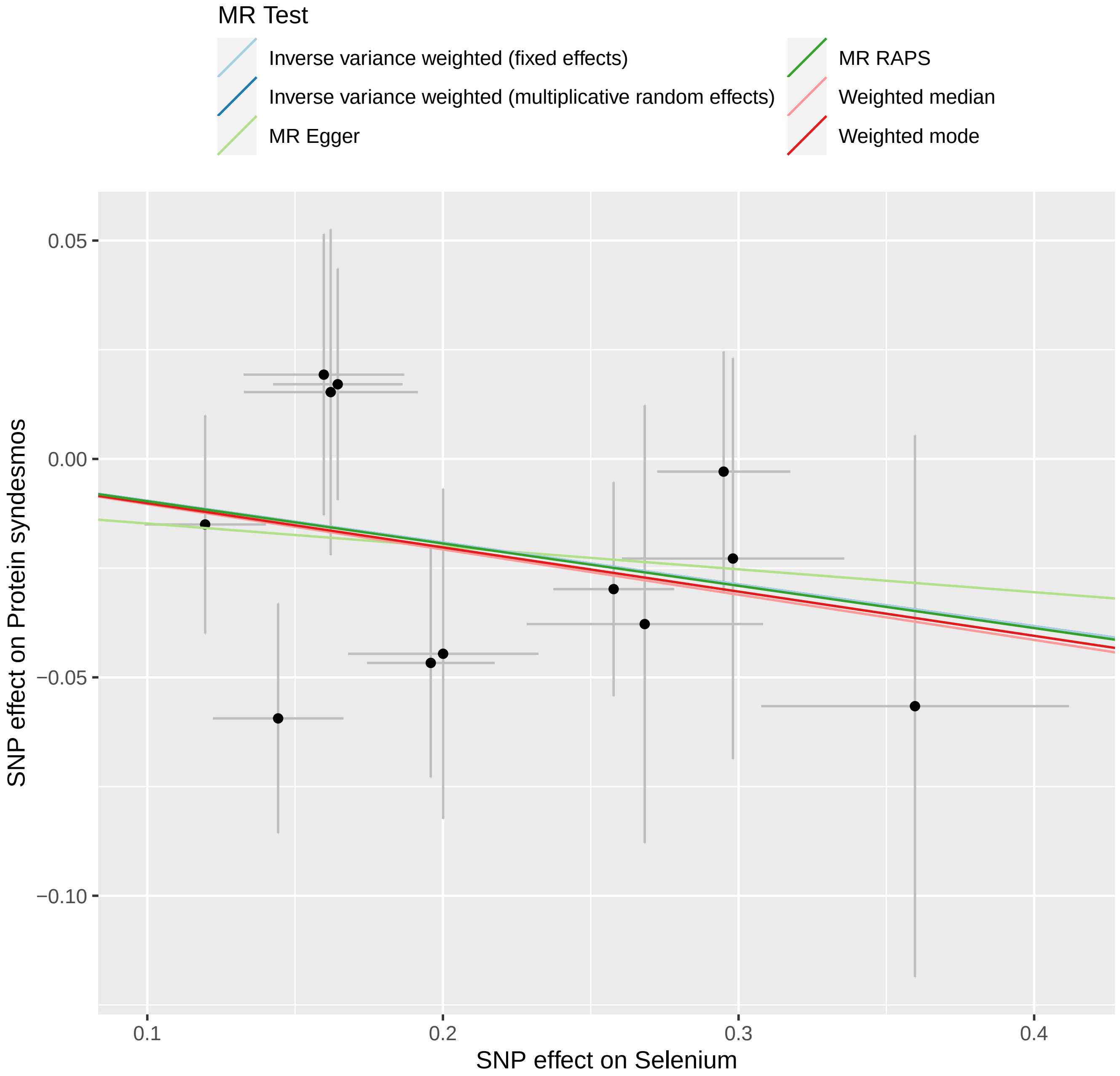

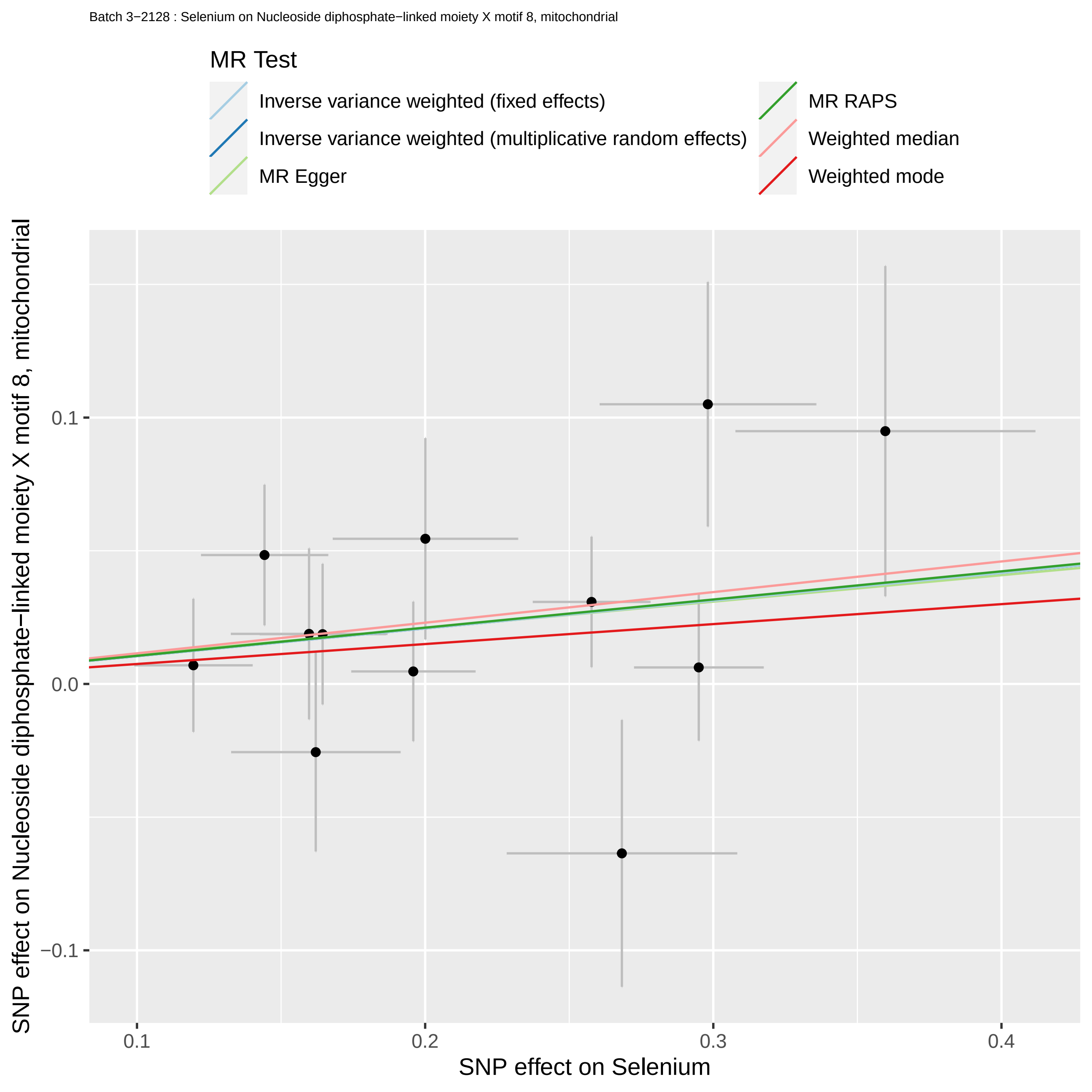
Batch 3−2128 : Selenium on Nucleoside diphosphate−linked moiety X motif 8, mitochondrial
MR Test
Inverse variance weighted (fixed effects)
Inverse variance weighted (multiplicative random effects)
MR Egger
MR RAPS
Weighted median
Weighted mode
SNP effect on Nucleoside diphosphate−linked moiety X motif 8, mitochondrial
0.1
0.0
−0.1
0.1
0.2
0.3
0.4
SNP effect on Selenium

Batch 3−2129 : Selenium on ADP−ribose pyrophosphatase, mitochondrial

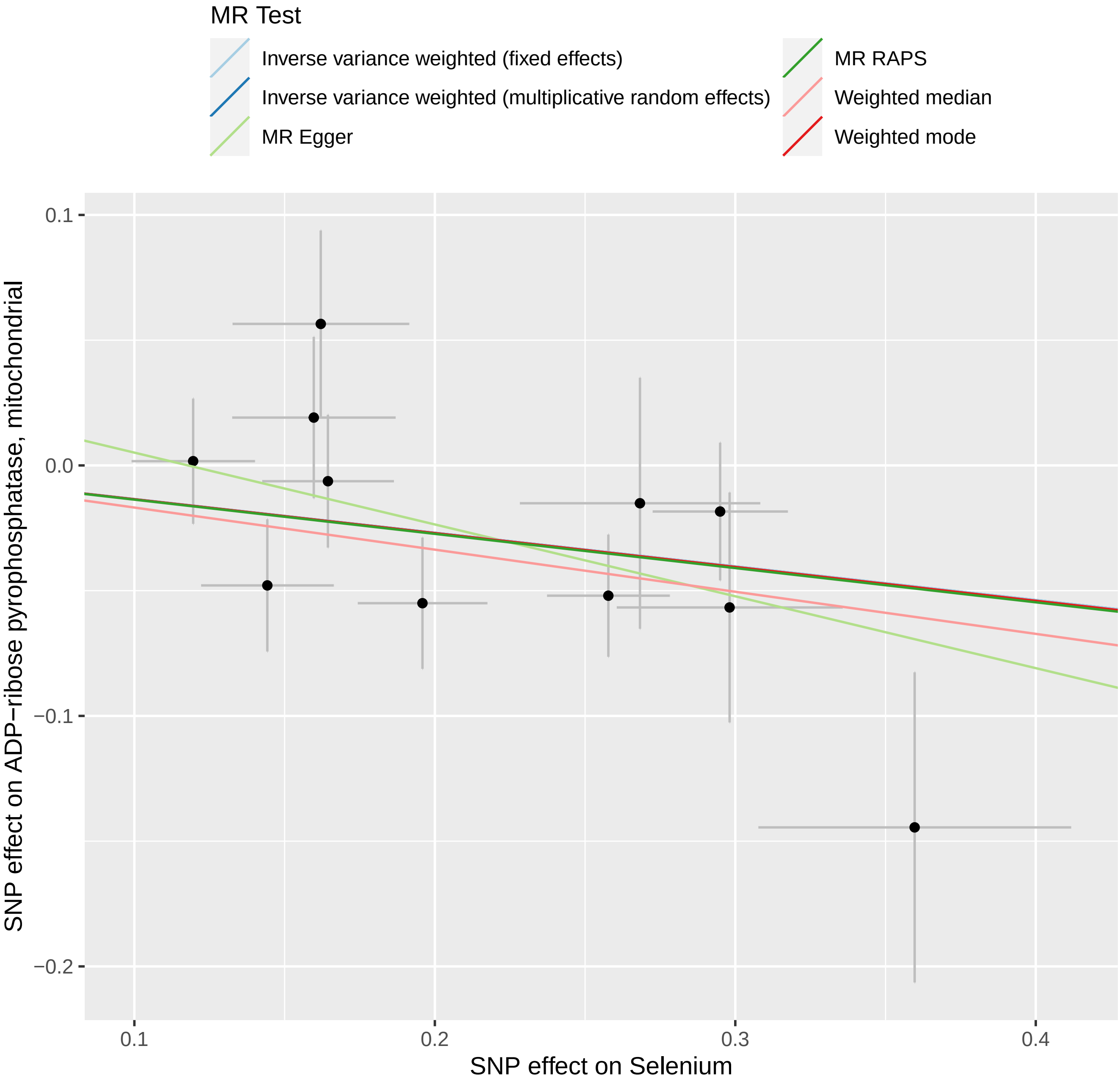

Batch 3−2130 : Selenium on Nuclear pore membrane glycoprotein 210−like

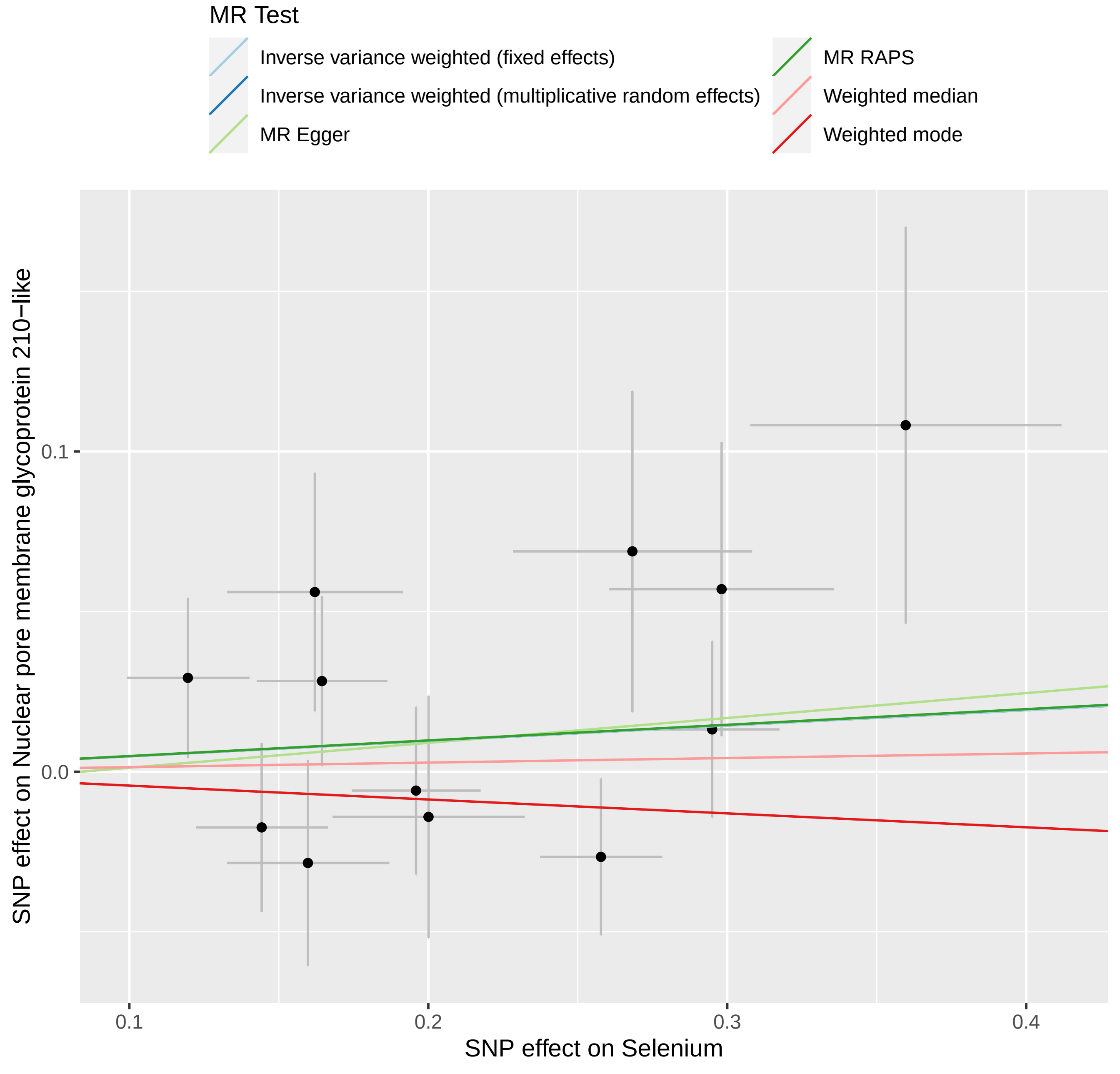

Batch 3−2131 : Selenium on Nuclear RNA export factor 1

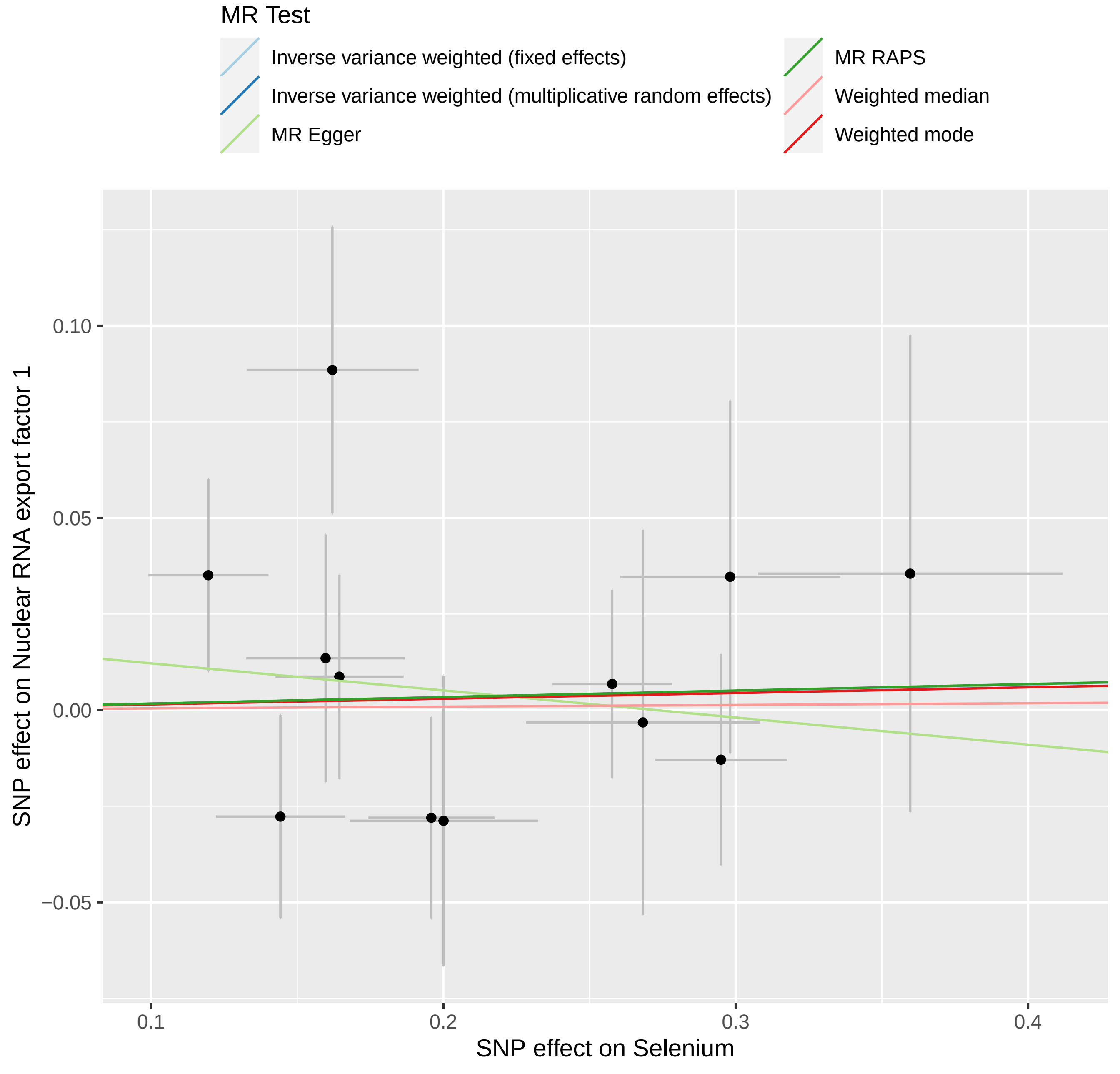

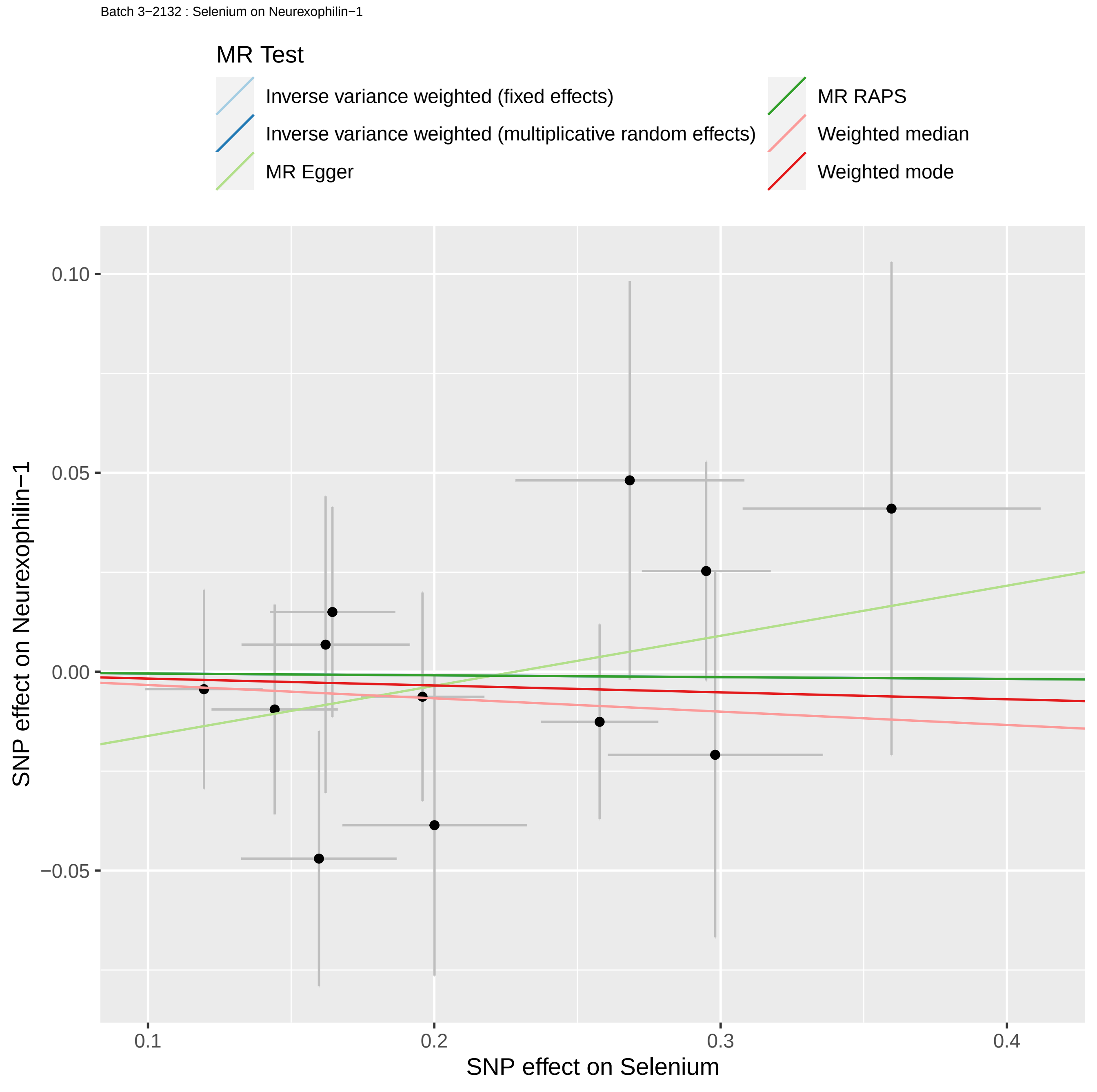

Batch 3−2132 : Selenium on Neurexophilin−1
MR Test
Inverse variance weighted (fixed effects)
Inverse variance weighted (multiplicative random effects)
MR Egger
MR RAPS
Weighted median
Weighted mode
0.10
0.05
0.00
−0.05
0.1
0.2
0.3
0.4
SNP effect on Neurexophilin−1
SNP effect on Selenium

Batch 3−2133 : Selenium on Neurexophilin−3

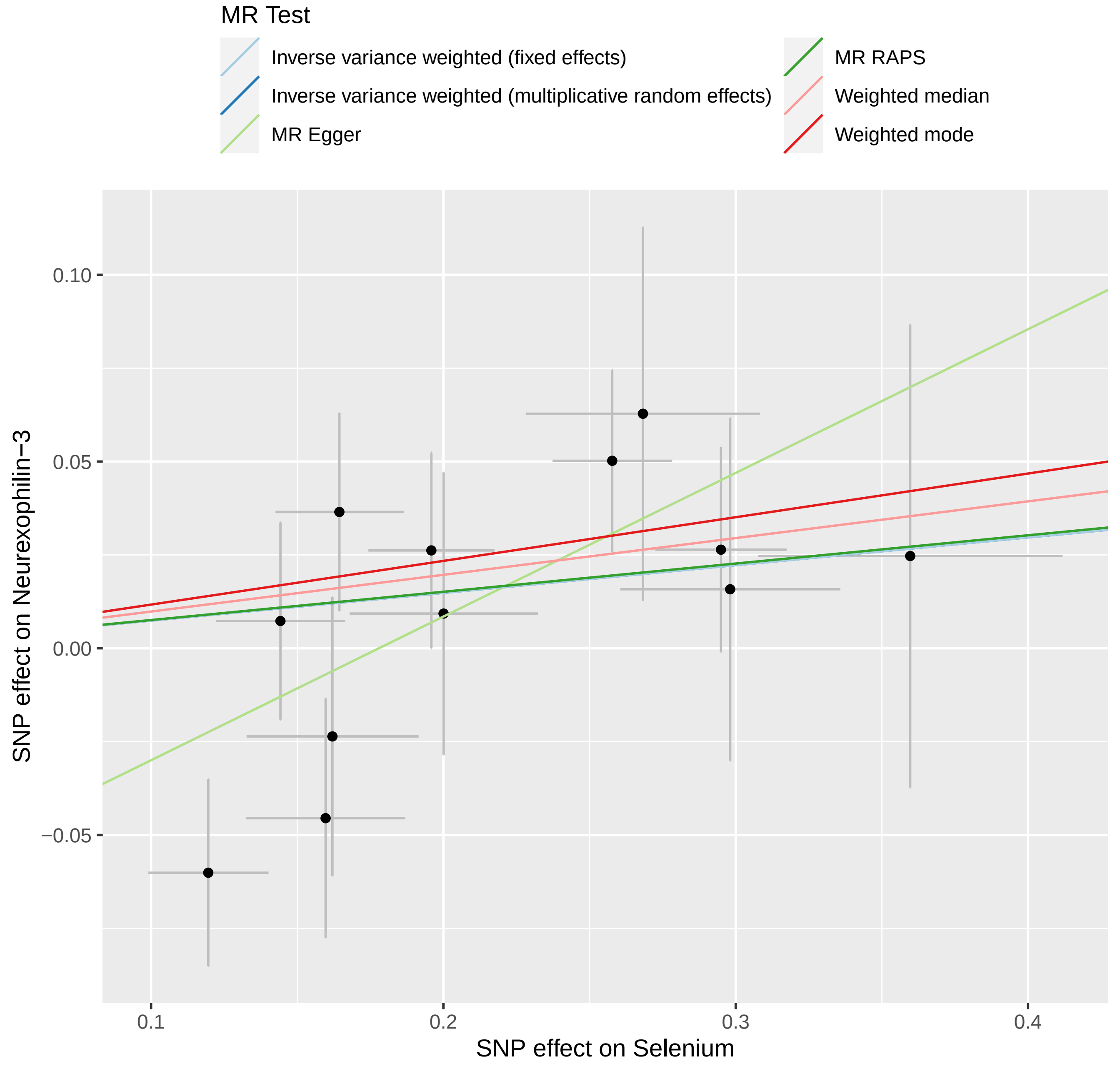

Batch 3−2134 : Selenium on NTF2−related export protein 1

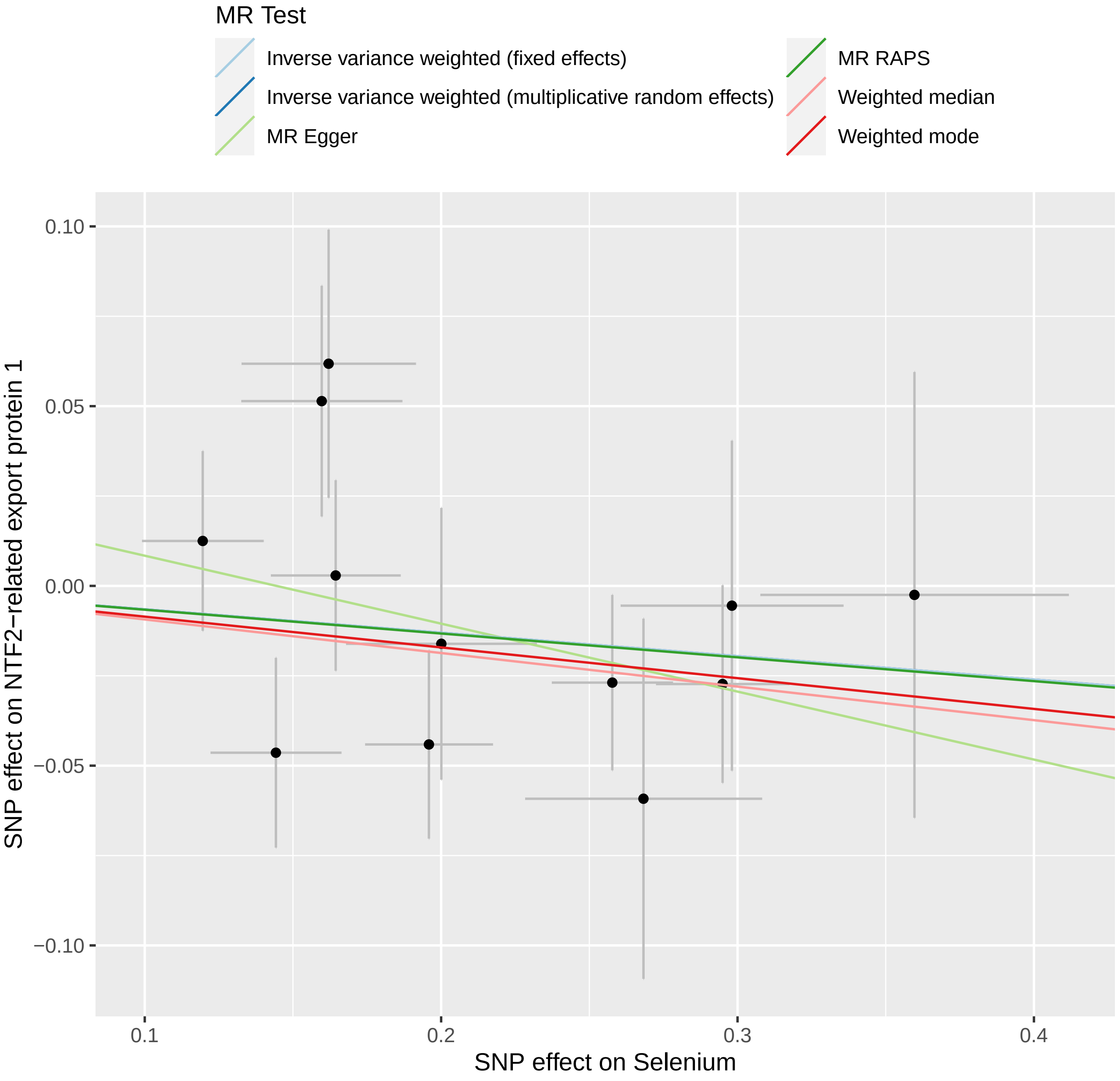

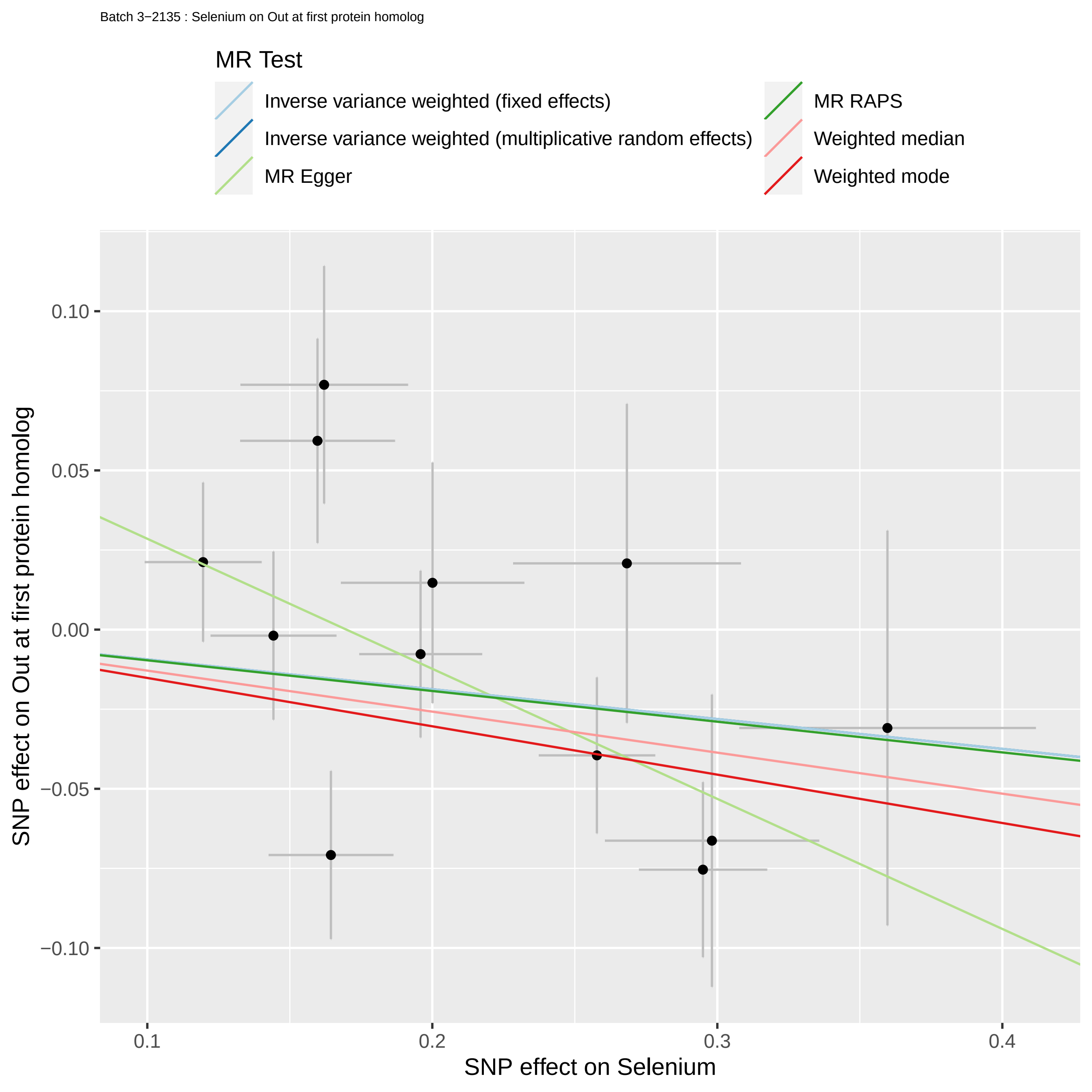

Batch 3−2135 : Selenium on Out at first protein homolog
MR Test
Inverse variance weighted (fixed effects)
Inverse variance weighted (multiplicative random effects)
MR Egger
MR RAPS
Weighted median
Weighted mode
0.10
0.05
0.00
−0.05
−0.10
0.1
0.2
0.3
0.4
SNP effect on Out at first protein homolog
SNP effect on Selenium

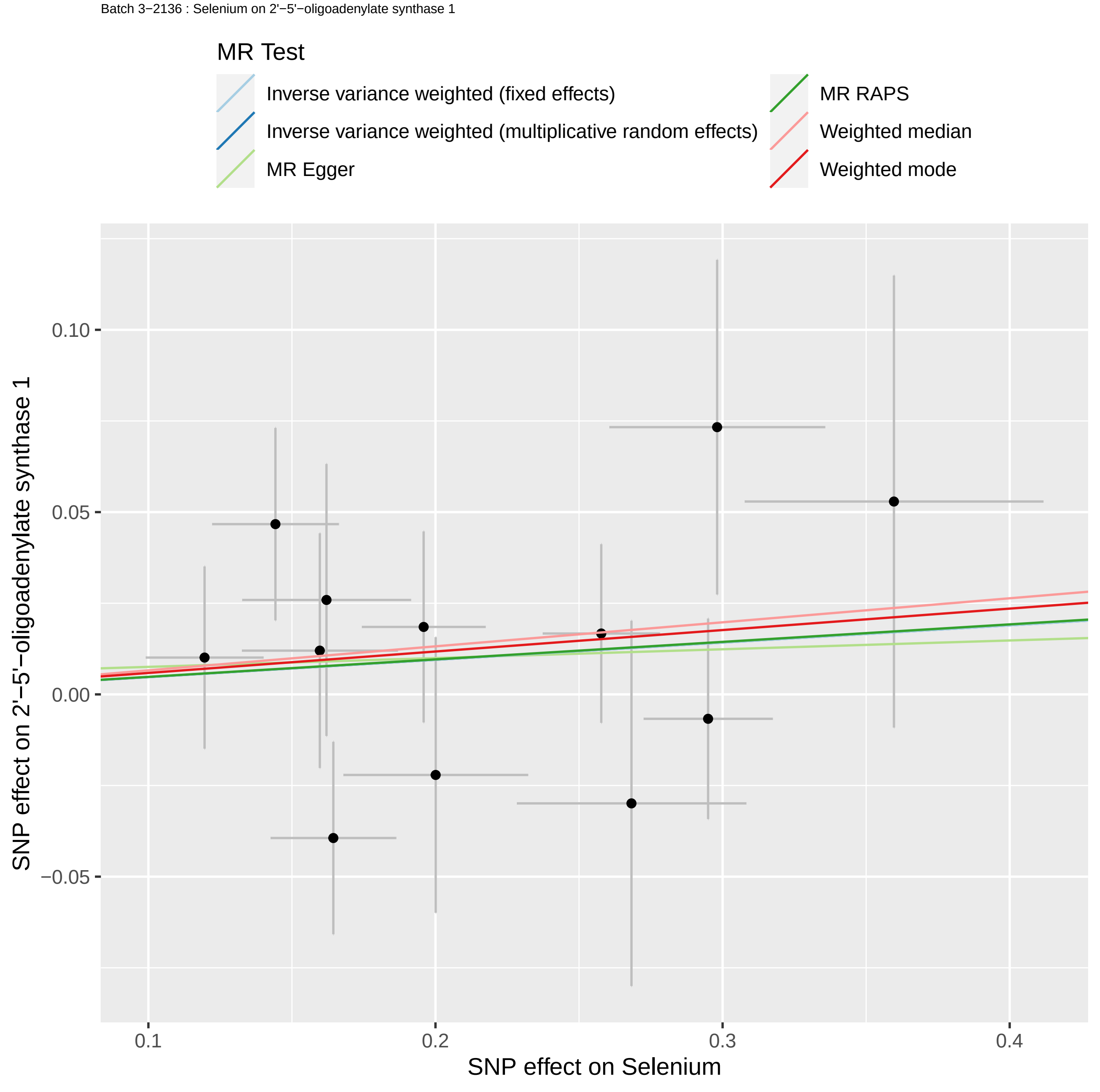

Batch 3−2136 : Selenium on 2'−5'−oligoadenylate synthase 1
MR Test
Inverse variance weighted (fixed effects)
Inverse variance weighted (multiplicative random effects)
MR Egger
MR RAPS
Weighted median
Weighted mode
0.10
0.05
0.00
−0.05
0.1
0.2
0.3
0.4
SNP effect on 2'−5'−oligoadenylate synthase 1
SNP effect on Selenium

Batch 3−2137 : Selenium on Ornithine decarboxylase antizyme 1

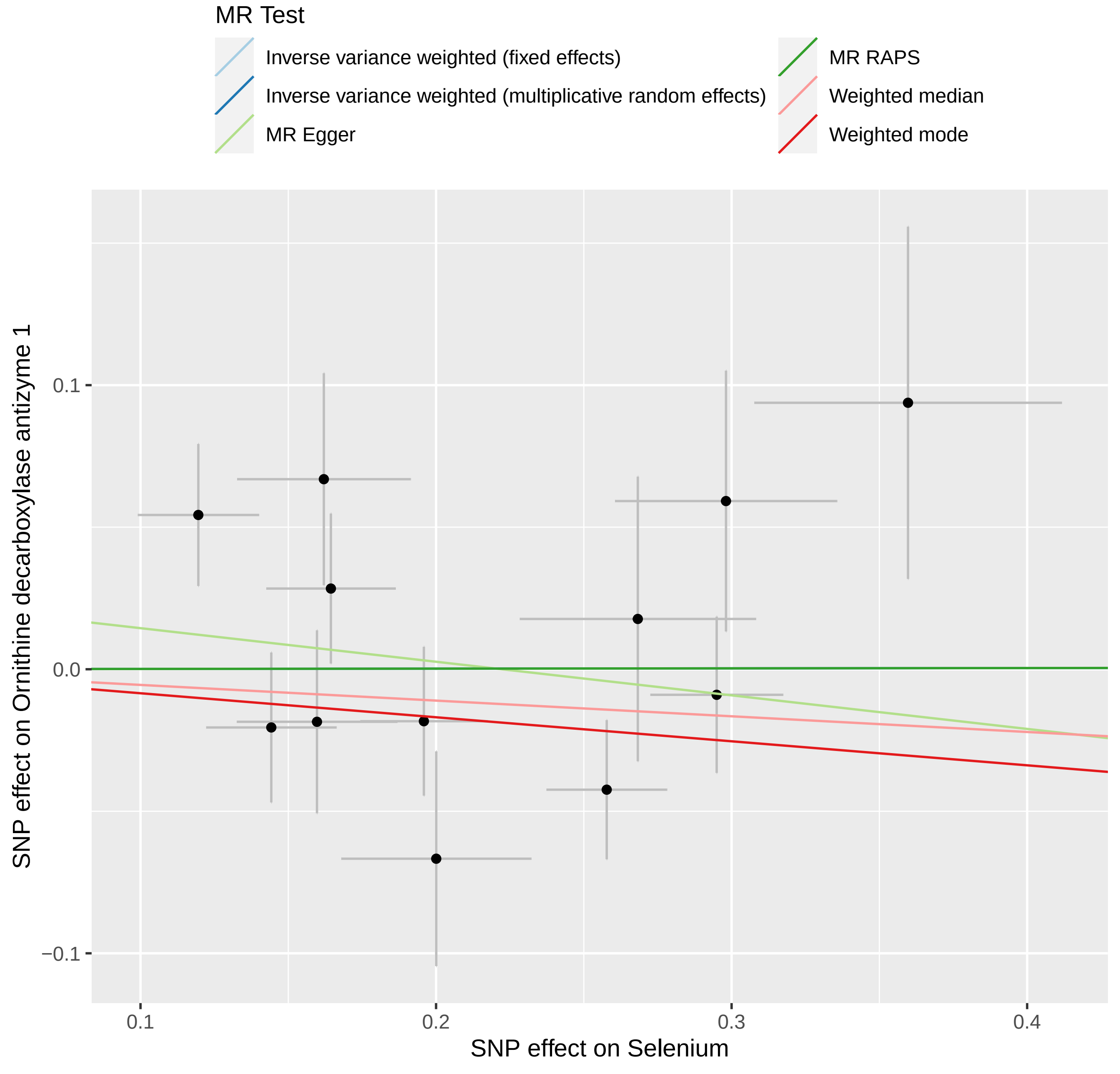

Batch 3−2138 : Selenium on Odorant−binding protein 2a

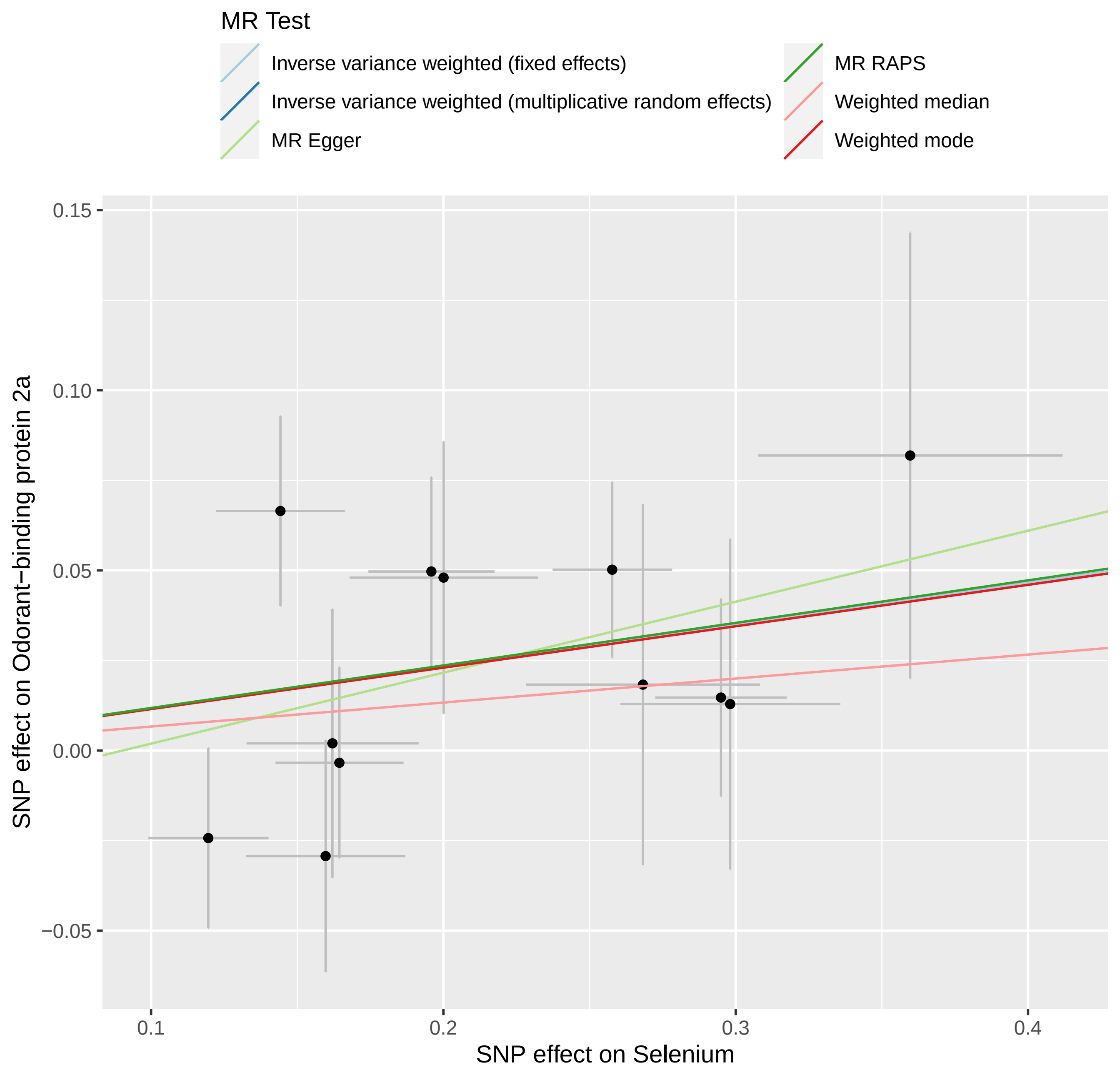

Batch 3−2139 : Selenium on Odorant−binding protein 2b

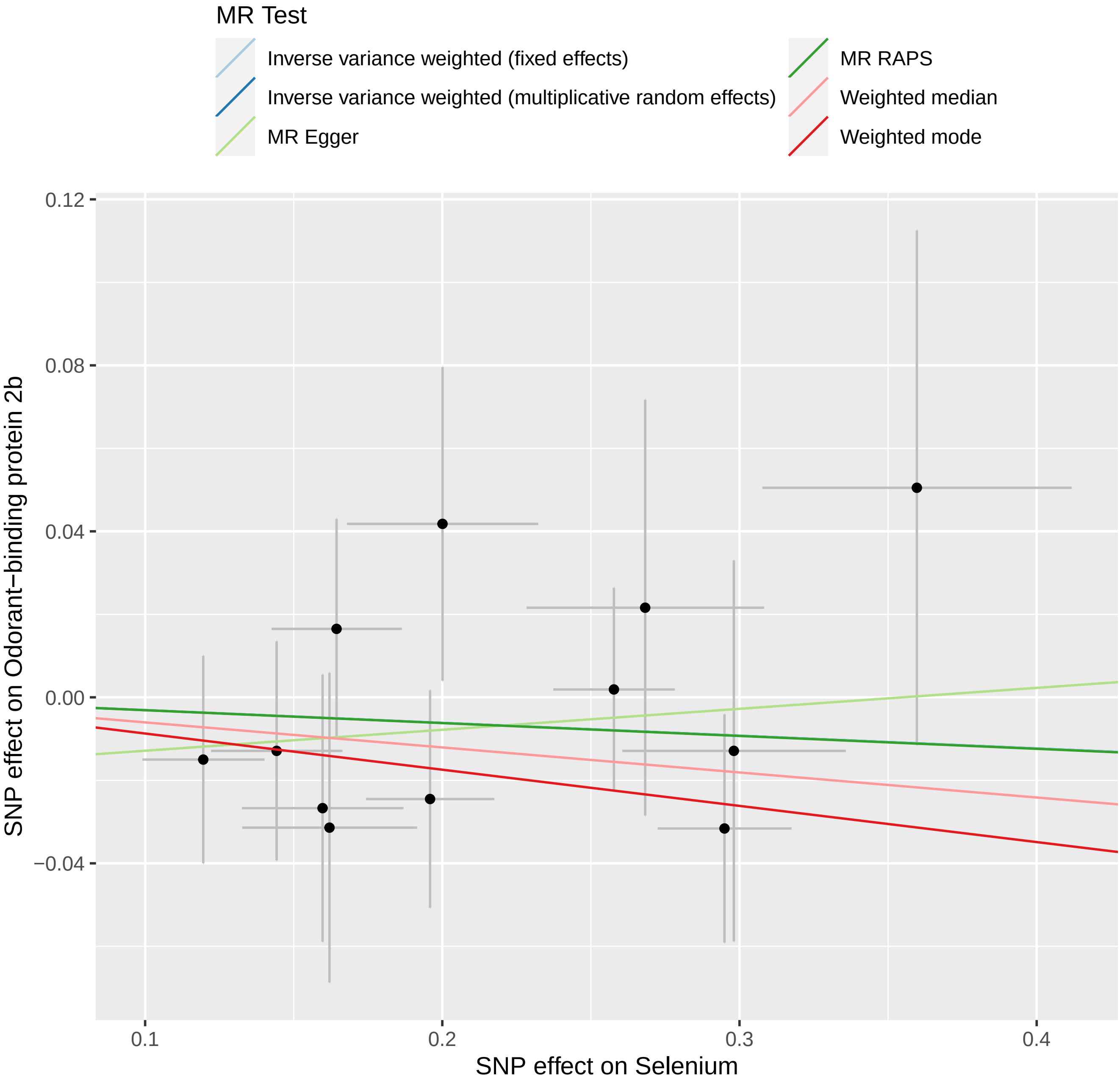

Batch 3−2140 : Selenium on OCIA domain−containing protein 1

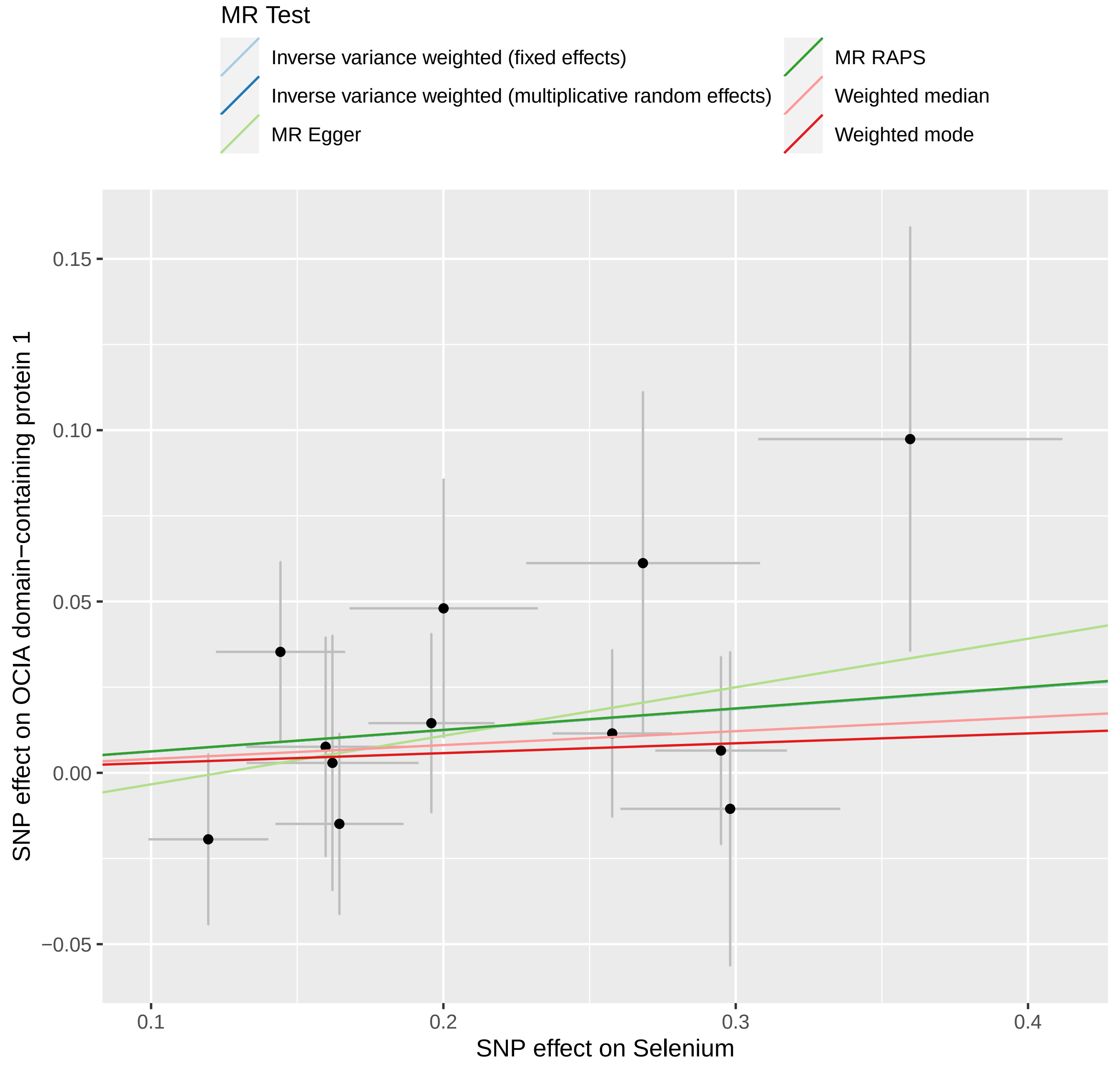

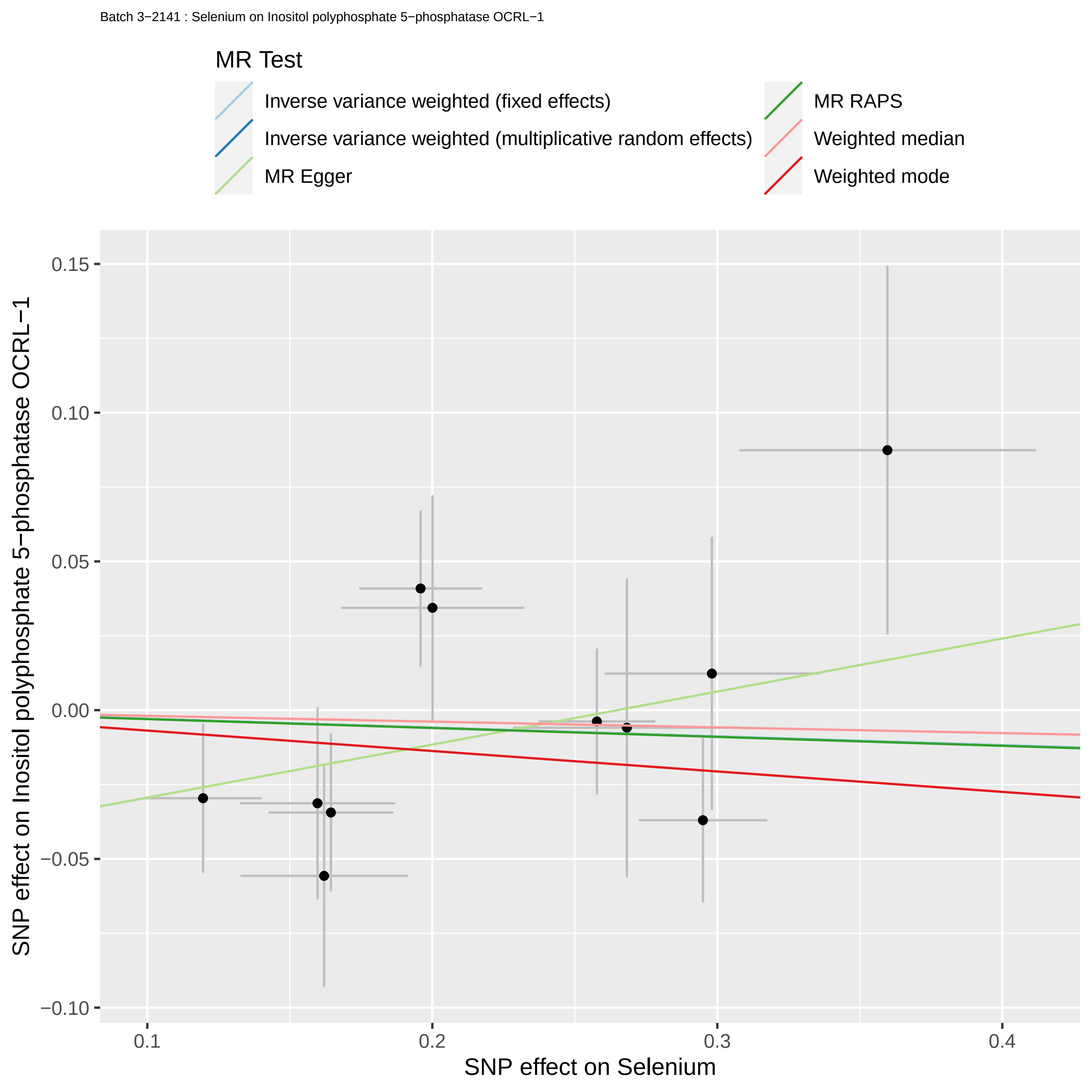

Batch 3−2141 : Selenium on Inositol polyphosphate 5−phosphatase OCRL−1
MR Test
Inverse variance weighted (fixed effects)
Inverse variance weighted (multiplicative random effects)
MR Egger
MR RAPS
Weighted median
Weighted mode
SNP effect on Inositol polyphosphate 5−phosphatase OCRL−1
0.15
0.10
0.05
0.00
−0.05
−0.10
0.1
0.2
0.3
0.4
SNP effect on Selenium

Batch 3−2142 : Selenium on Ornithine decarboxylase

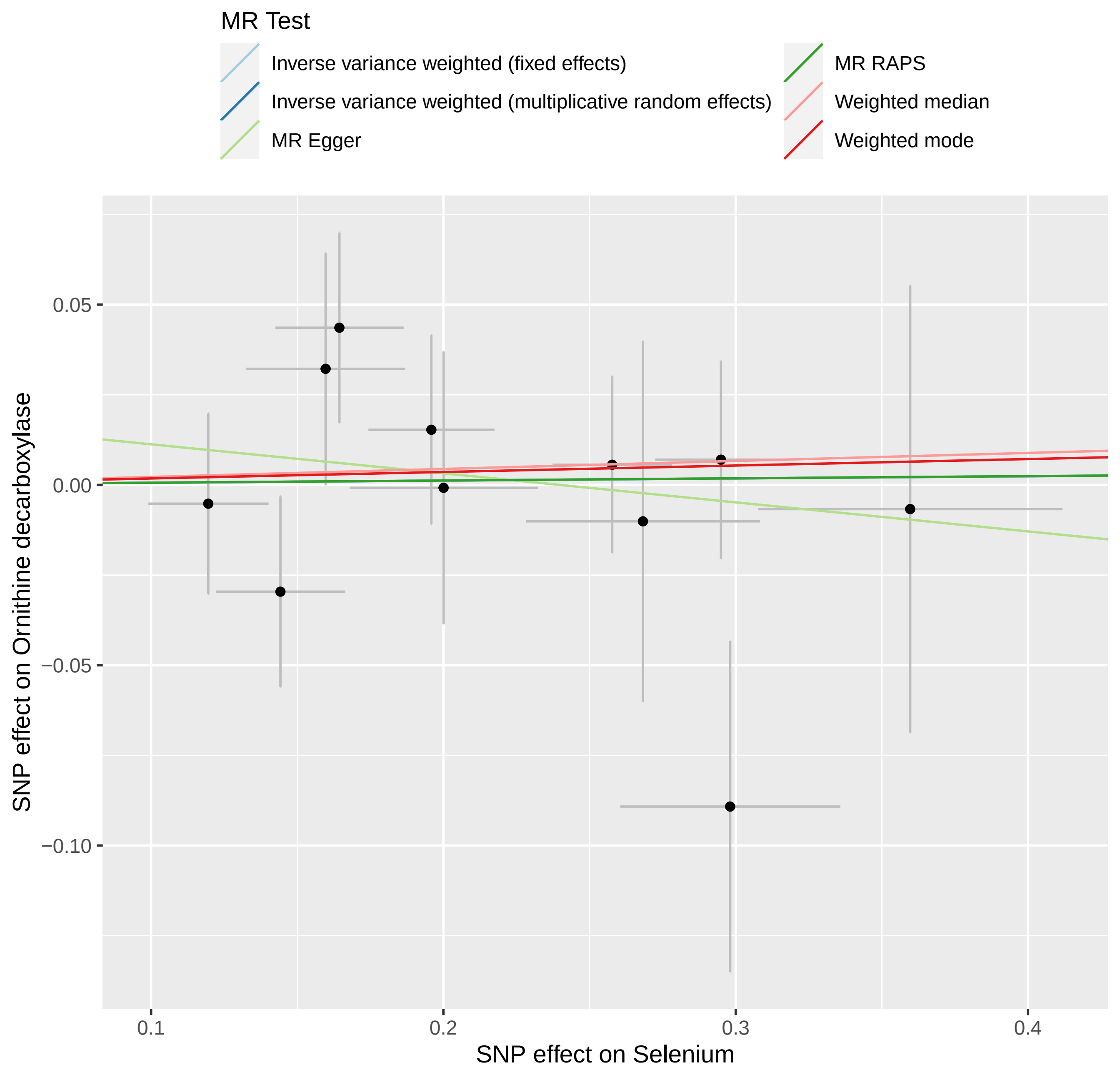

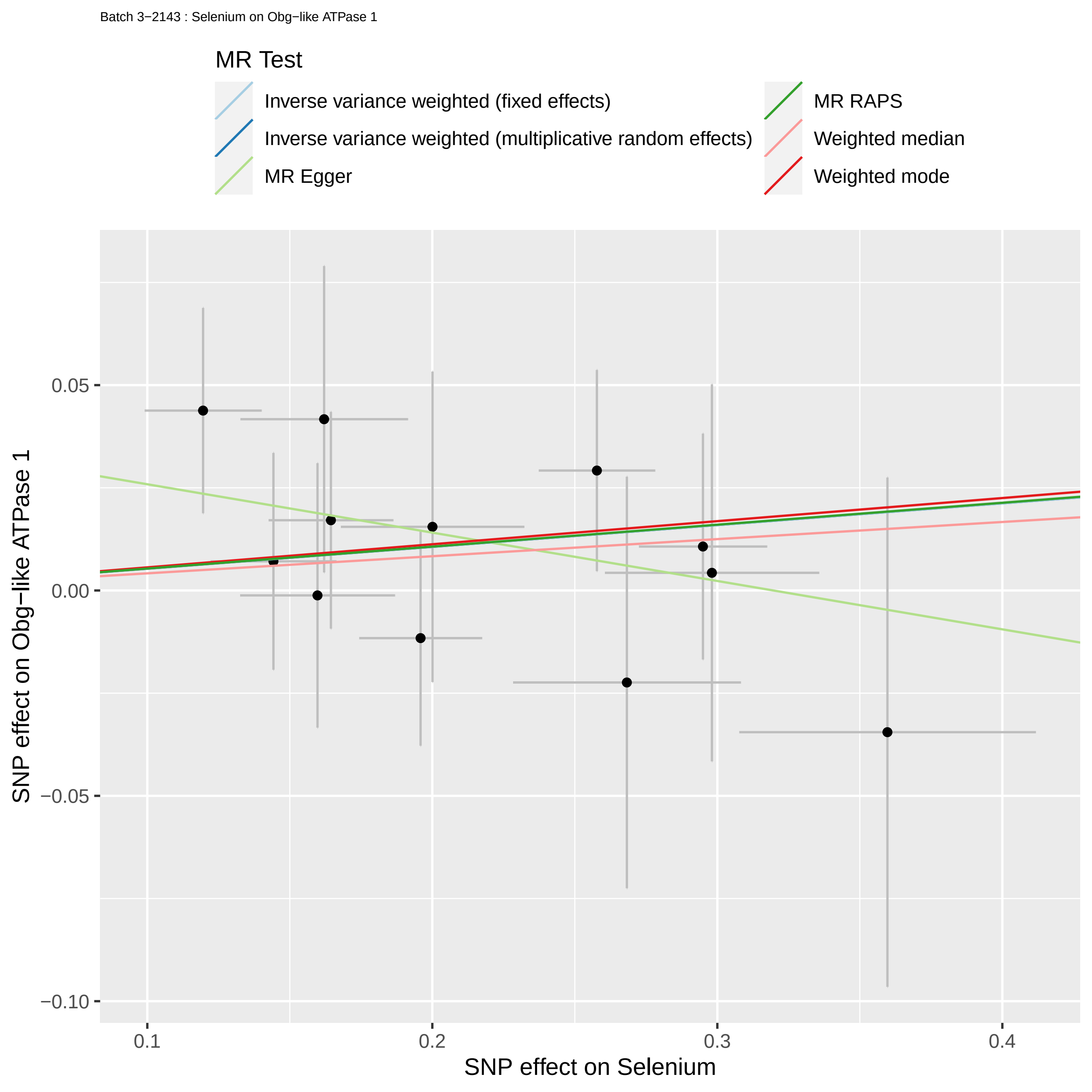

Batch 3−2143 : Selenium on Obg−like ATPase 1
MR Test
Inverse variance weighted (fixed effects)
Inverse variance weighted (multiplicative random effects)
MR Egger
MR RAPS
Weighted median
Weighted mode
0.05
0.00
−0.05
−0.10
0.1
0.2
0.3
0.4
SNP effect on Obg−like ATPase 1
SNP effect on Selenium

Batch 3−2144 : Selenium on Olfactomedin−like protein 3

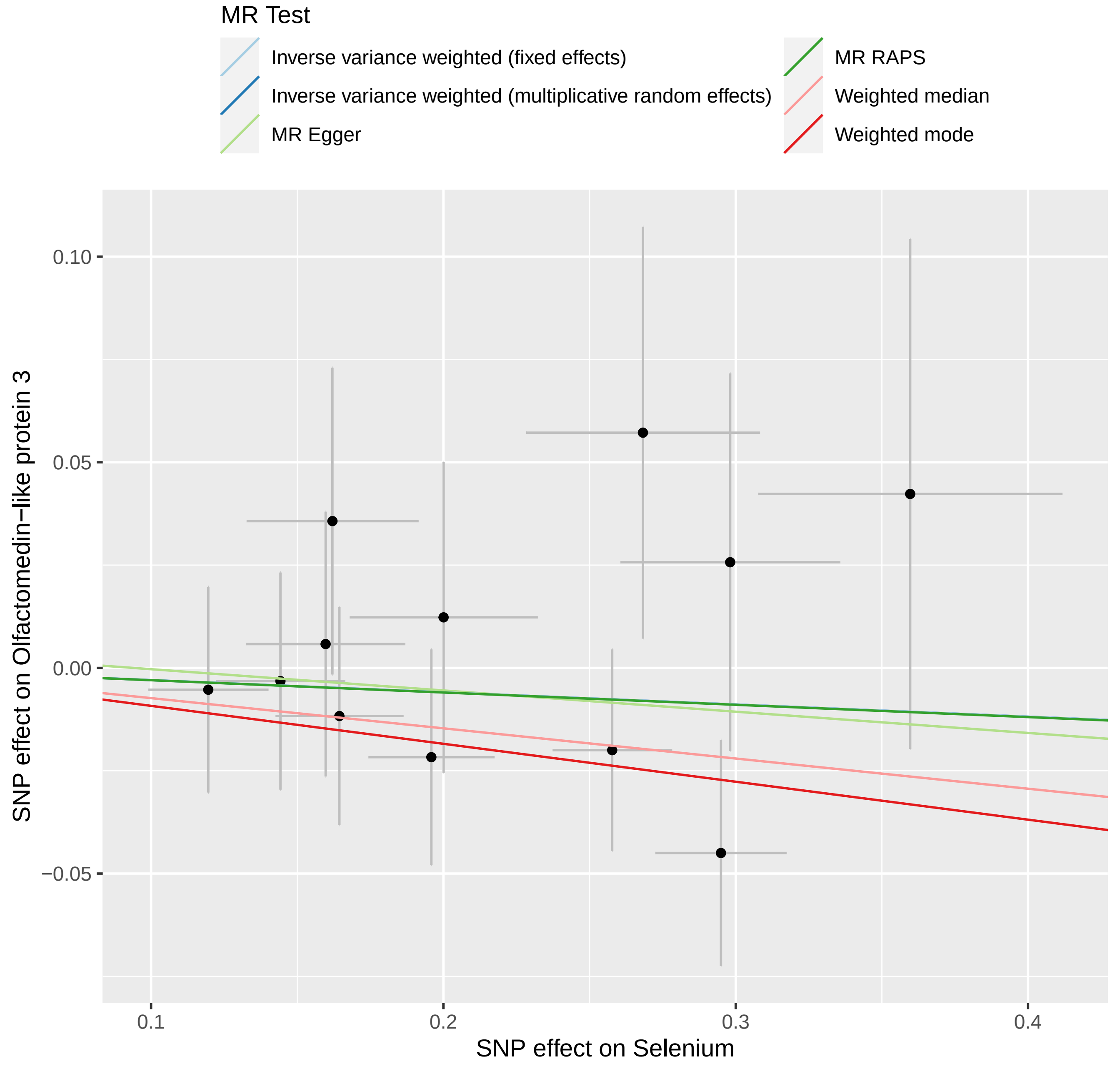

Batch 3−2145 : Selenium on Olfactomedin−like protein 3

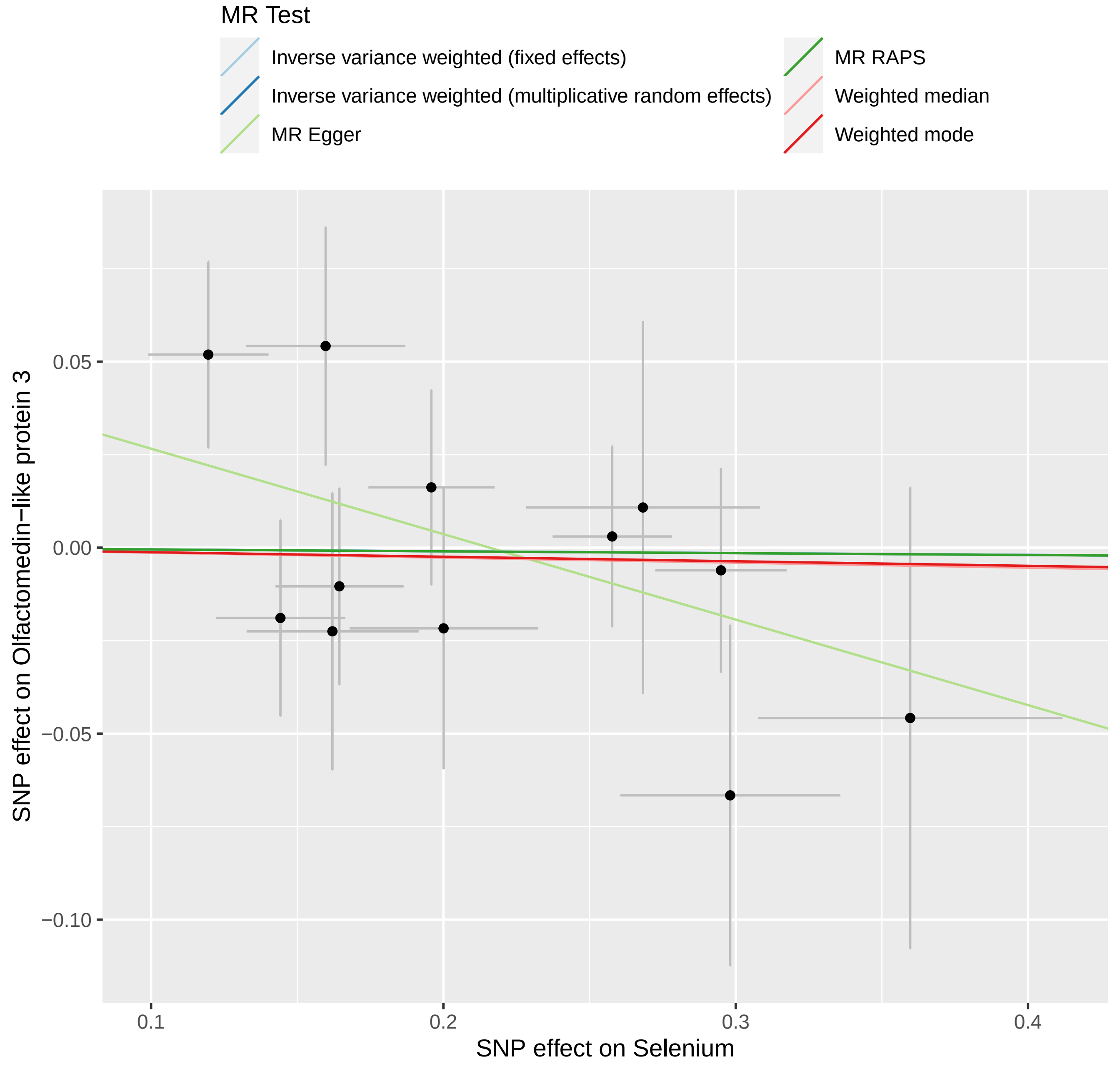

Batch 3−2146 : Selenium on Oxidized low−density lipoprotein receptor 1

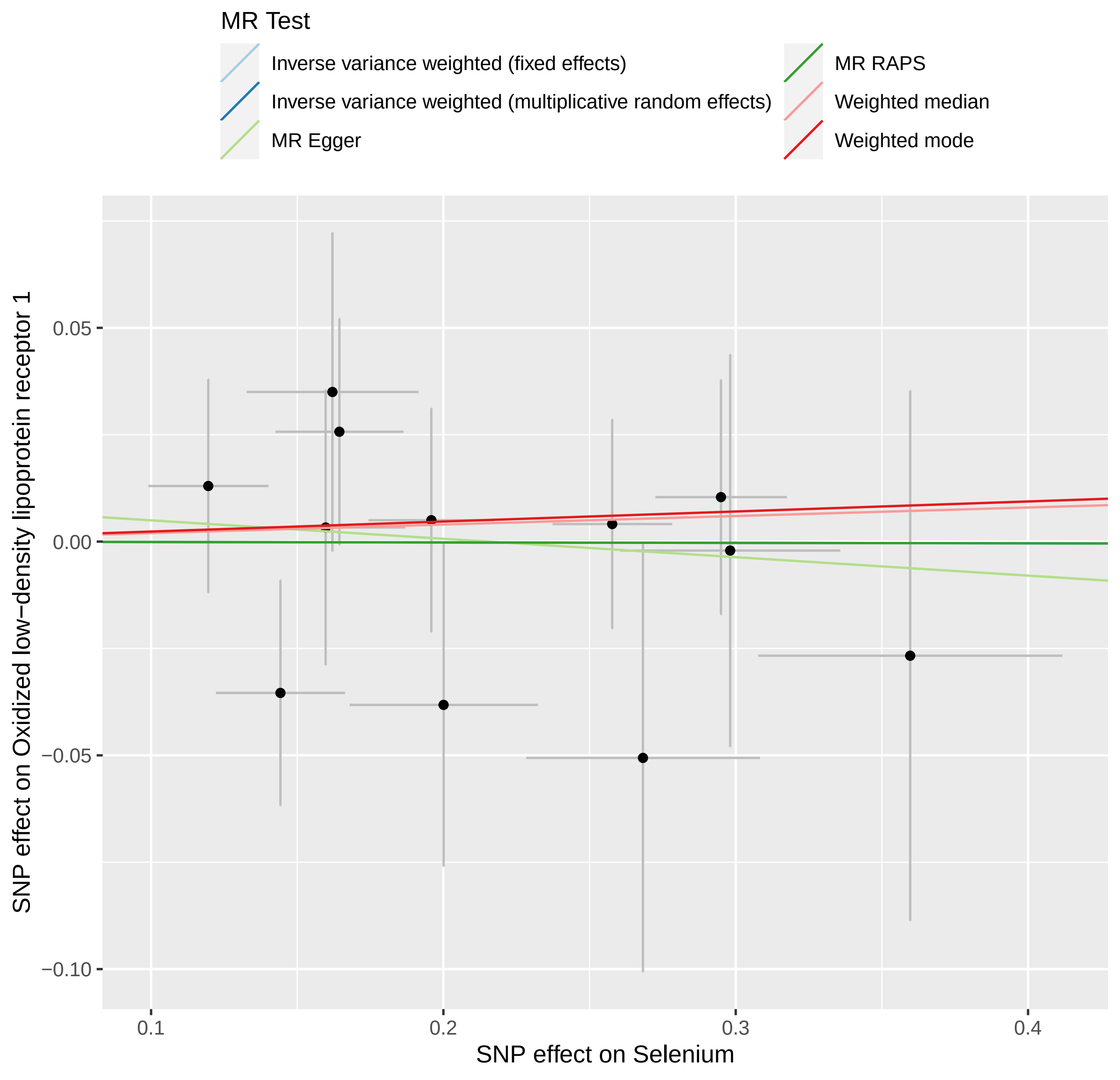

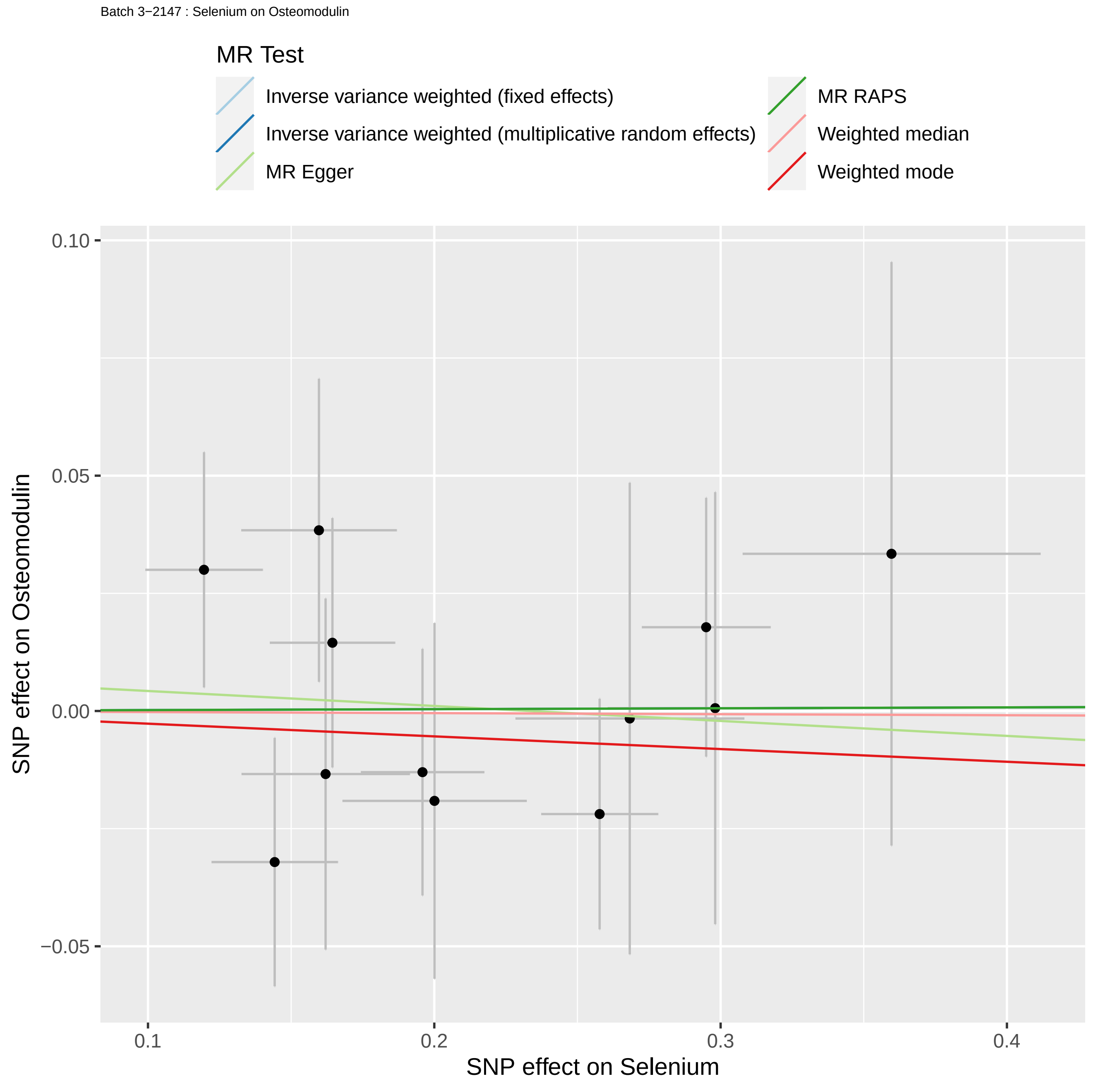
Batch 3−2147 : Selenium on Osteomodulin
MR Test
Inverse variance weighted (fixed effects)
Inverse variance weighted (multiplicative random effects)
MR Egger
MR RAPS
Weighted median
Weighted mode
0.10
0.05
0.00
−0.05
0.1
0.2
0.3
0.4
SNP effect on Osteomodulin
SNP effect on Selenium

Batch 3−2148 : Selenium on Opalin

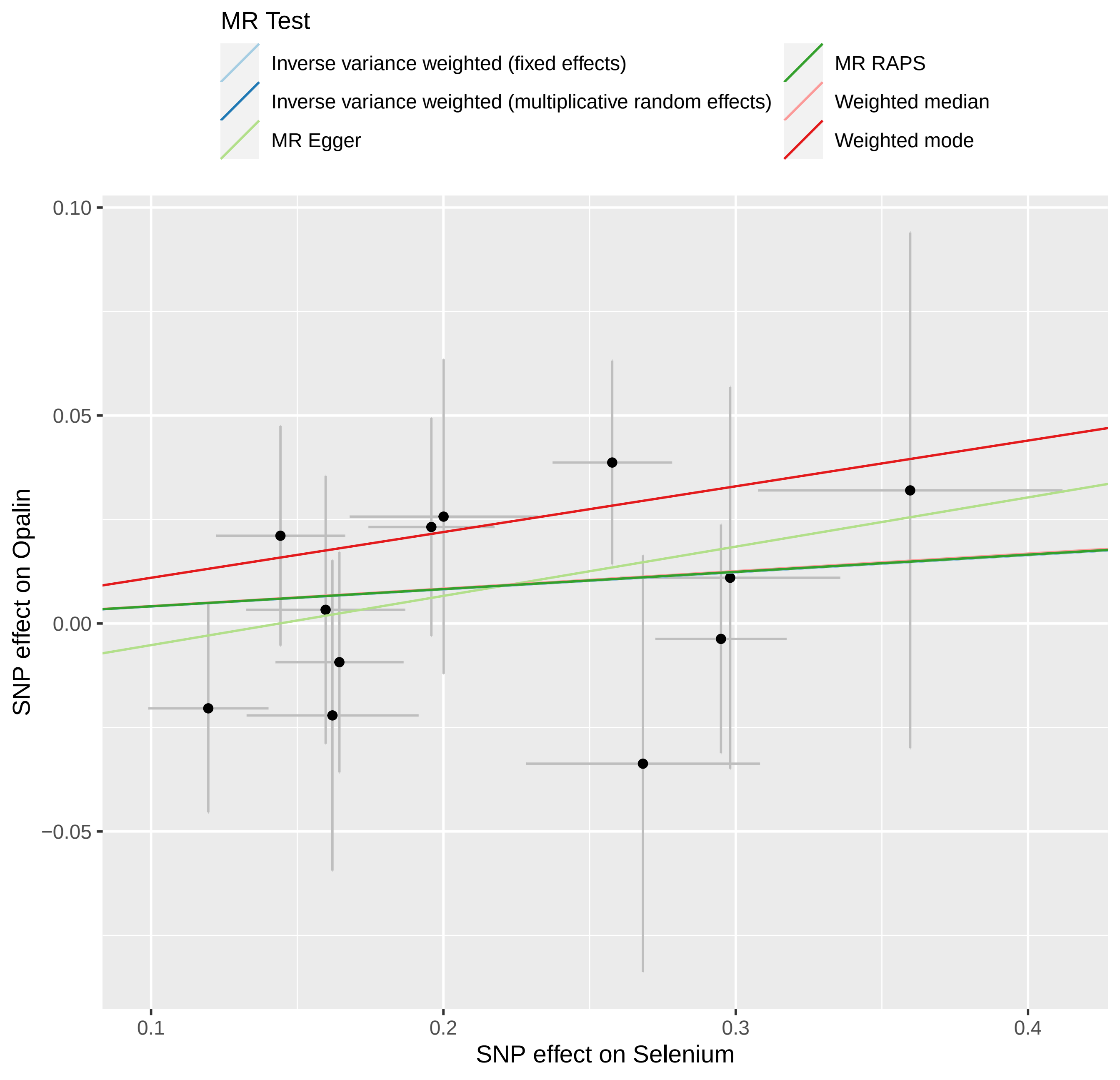

Batch 3−2149 : Selenium on Opioid−binding protein/cell adhesion molecule

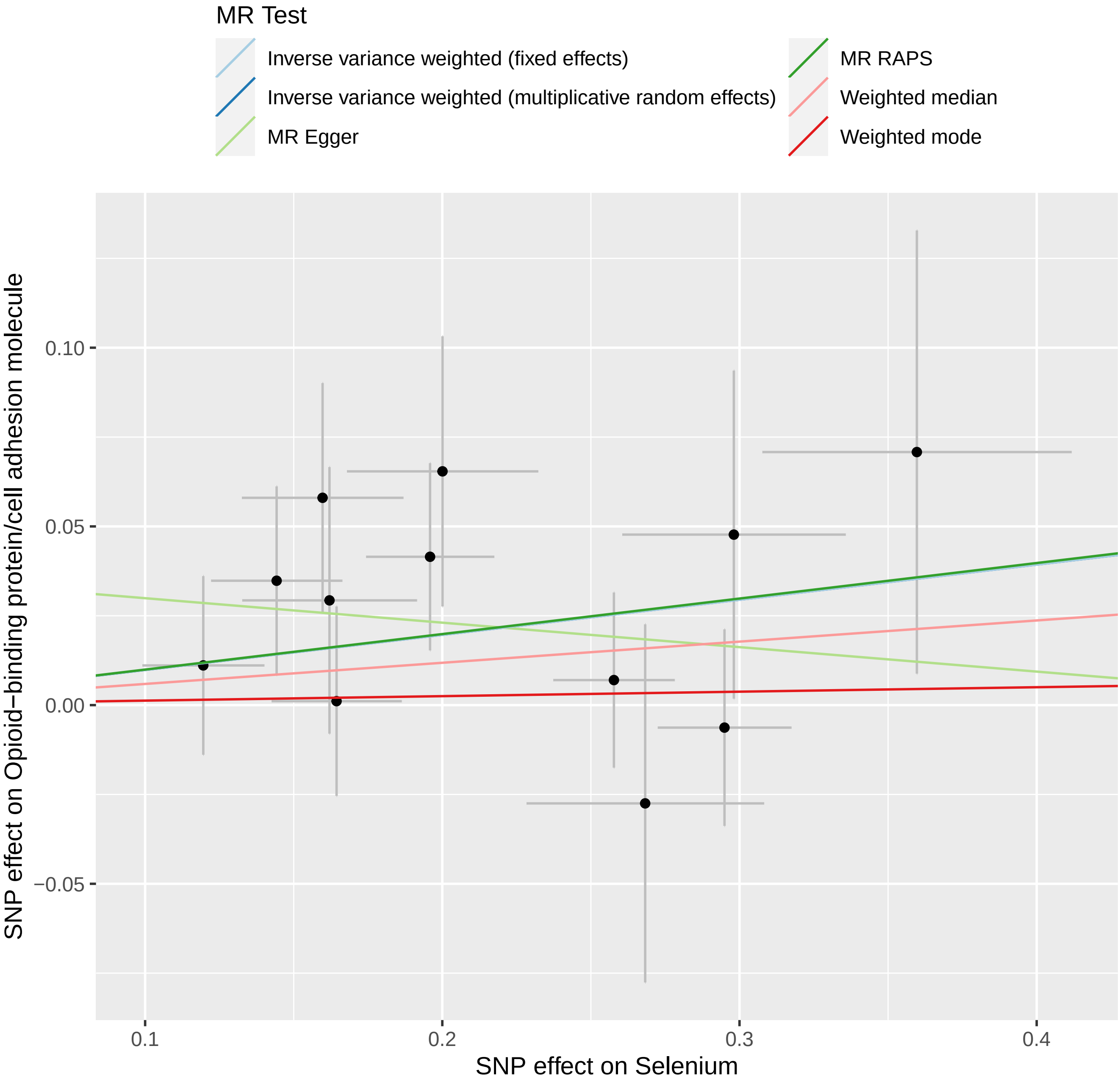

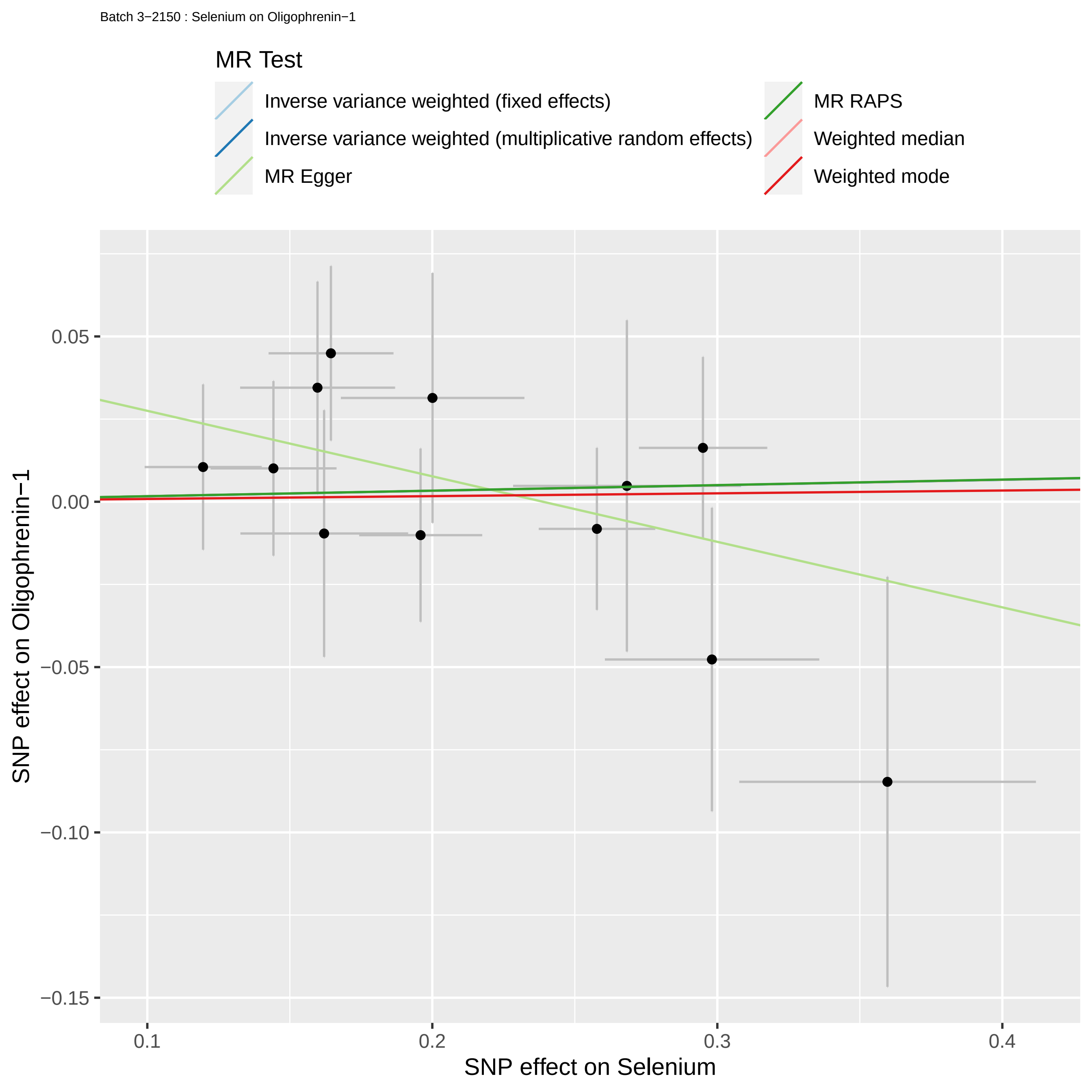

Batch 3−2150 : Selenium on Oligophrenin−1
MR Test
Inverse variance weighted (fixed effects)
Inverse variance weighted (multiplicative random effects)
MR Egger
MR RAPS
Weighted median
Weighted mode
0.05
0.00
−0.05
−0.10
−0.15
0.1
0.2
0.3
0.4
SNP effect on Oligophrenin−1
SNP effect on Selenium

Batch 3−2151 : Selenium on Origin recognition complex subunit 6

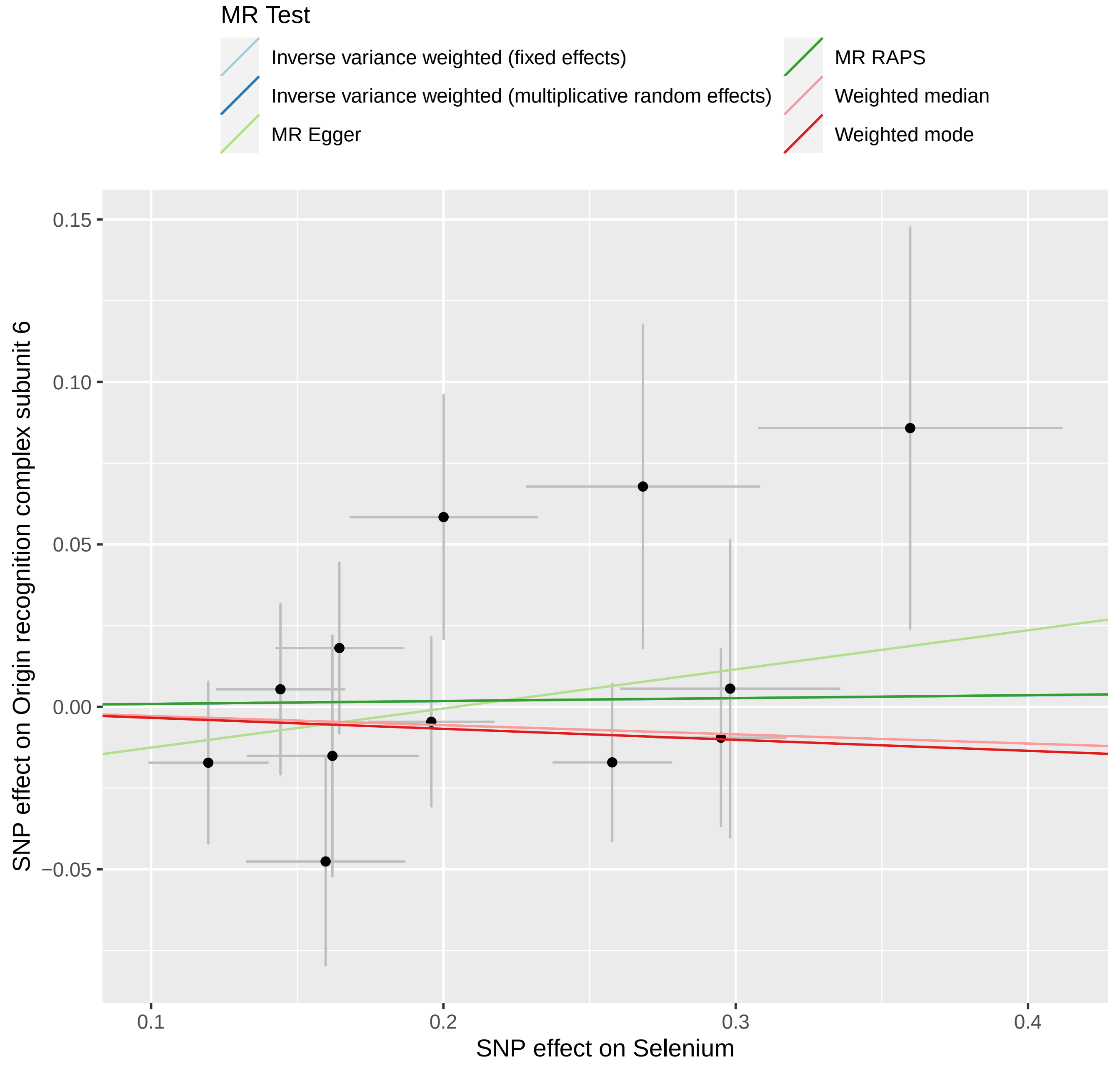

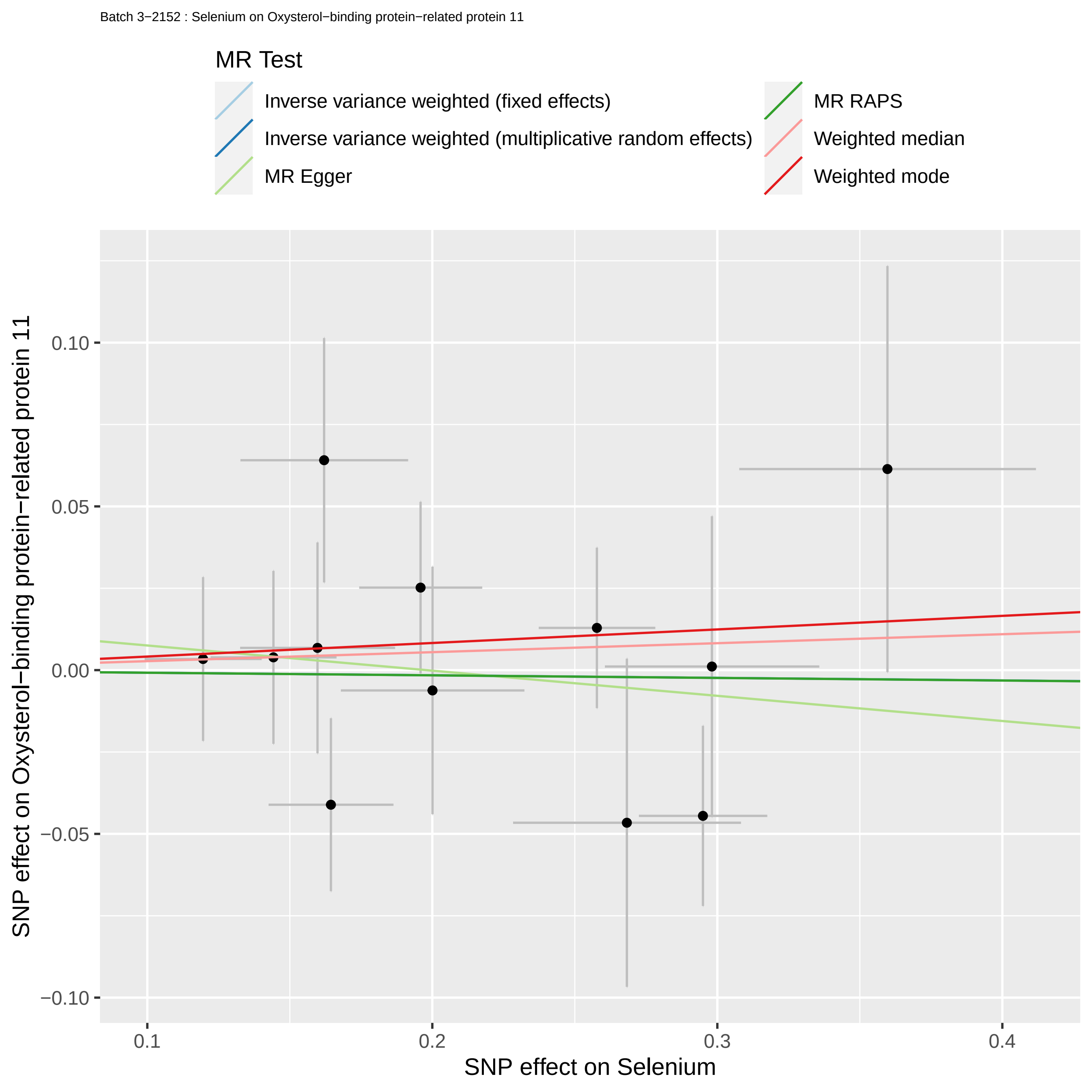
Batch 3−2152 : Selenium on Oxysterol−binding protein−related protein 11
MR Test
Inverse variance weighted (fixed effects)
Inverse variance weighted (multiplicative random effects)
MR Egger
MR RAPS
Weighted median
Weighted mode
SNP effect on Oxysterol−binding protein−related protein 11
0.10
0.05
0.00
−0.05
−0.10
0.1
0.2
0.3
0.4
SNP effect on Selenium

Batch 3−2153 : Selenium on Osteoclast−associated immunoglobulin−like receptor

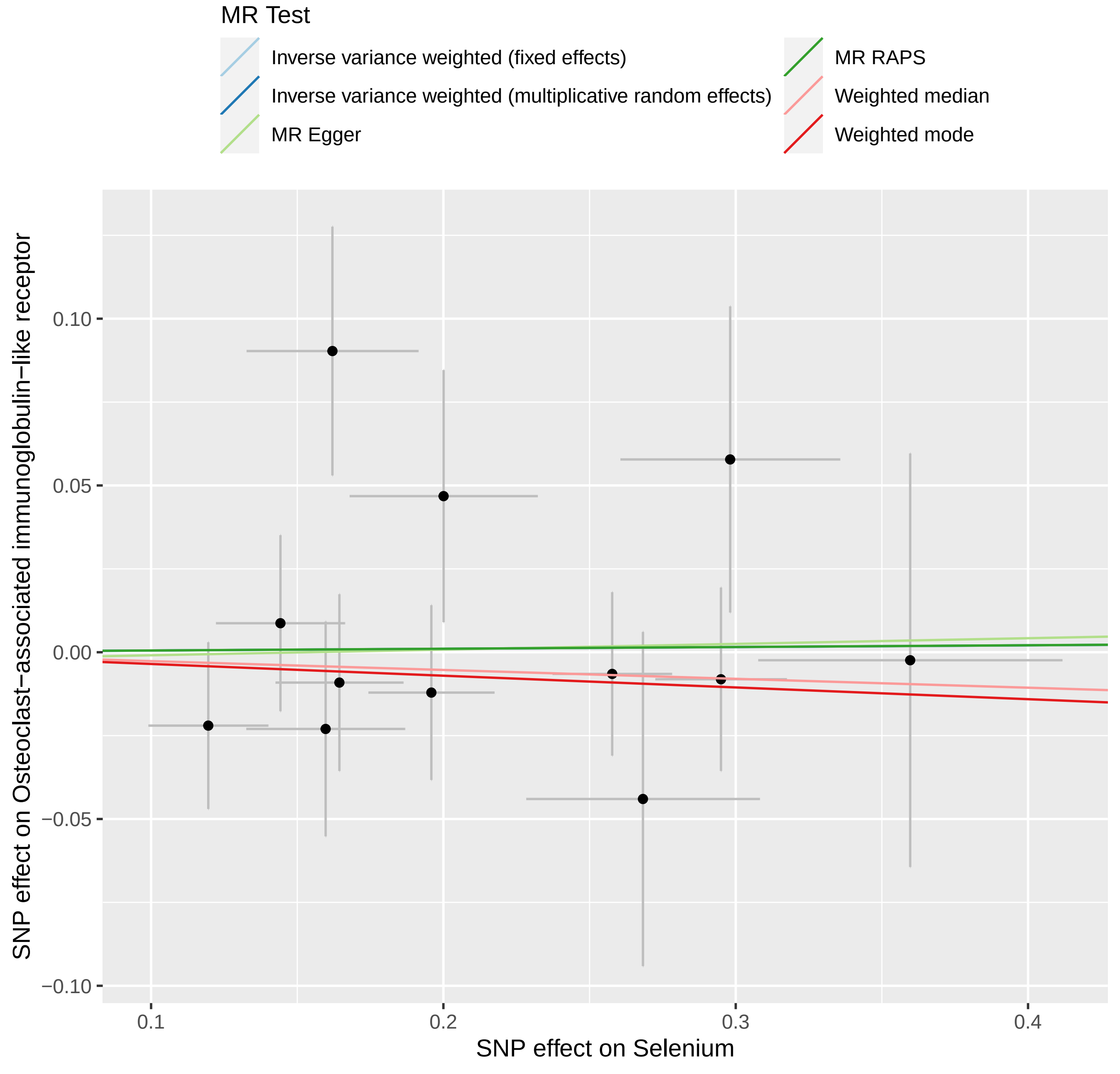

Batch 3−2154 : Selenium on Oncostatin−M

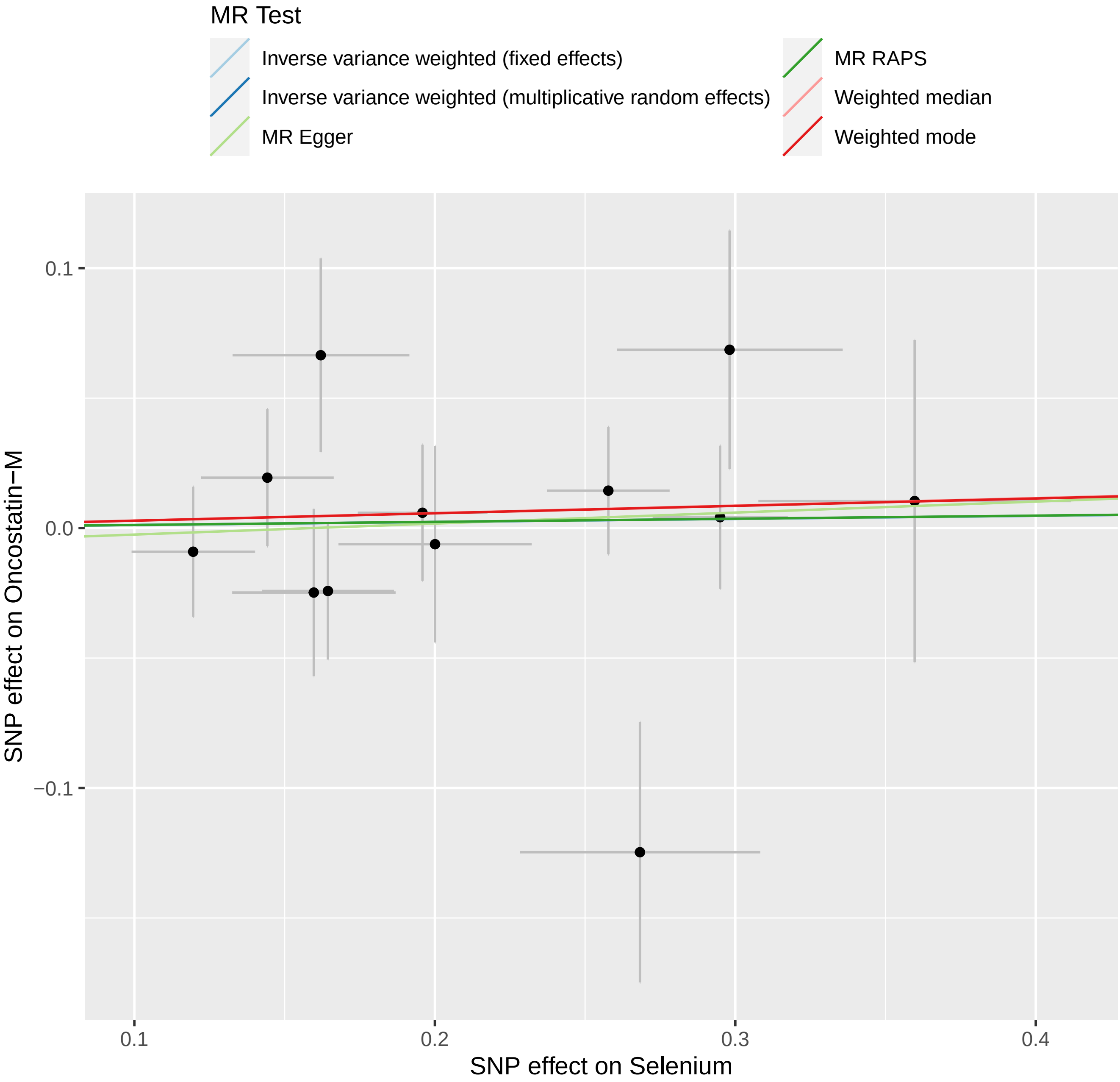

Batch 3−2155 : Selenium on Oncostatin−M

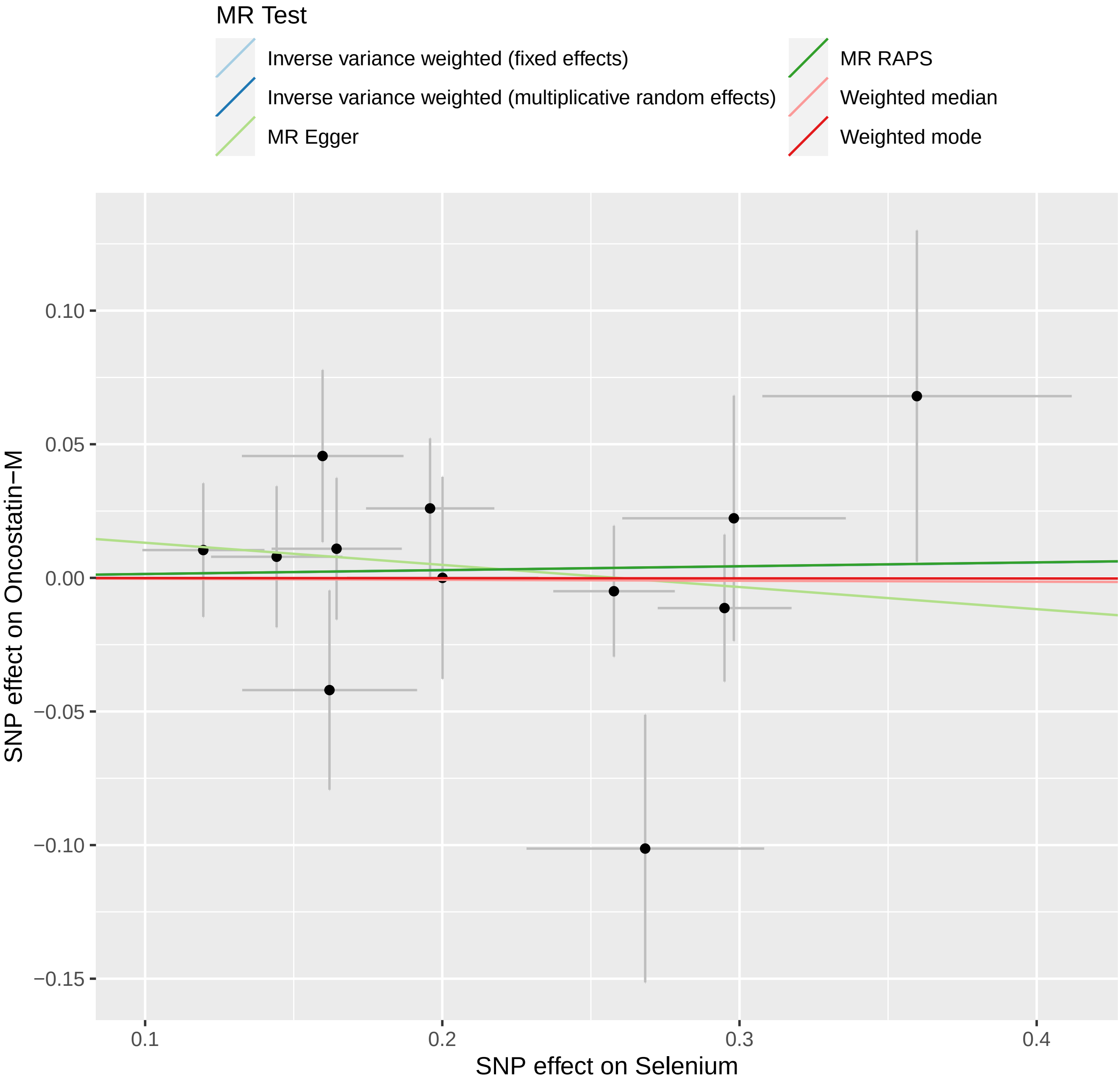

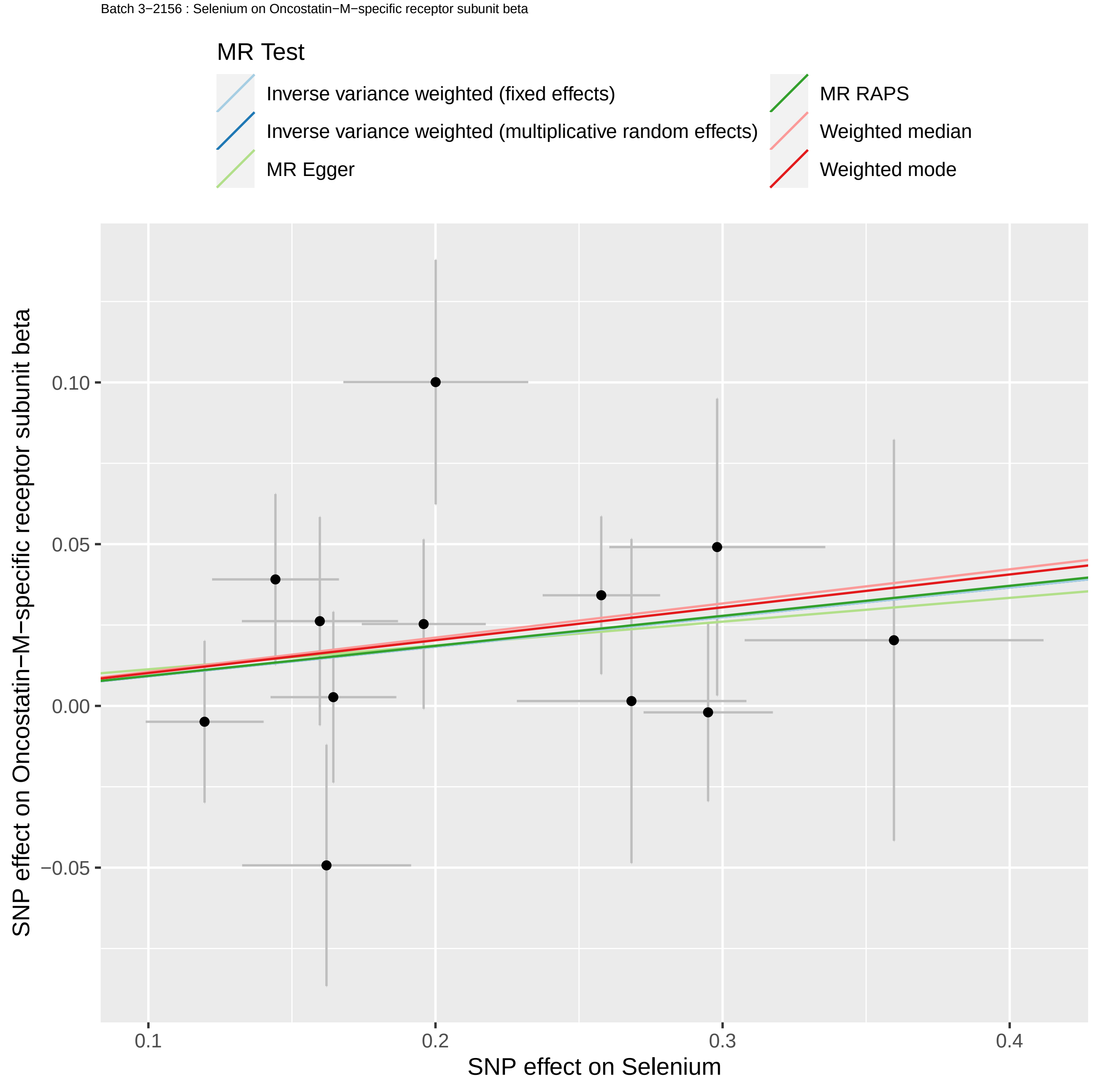

Batch 3−2156 : Selenium on Oncostatin−M−specific receptor subunit beta
MR Test
Inverse variance weighted (fixed effects)
Inverse variance weighted (multiplicative random effects)
MR Egger
MR RAPS
Weighted median
Weighted mode
SNP effect on Oncostatin−M−specific receptor subunit beta
0.10
0.05
0.00
−0.05
0.1
0.2
0.3
0.4
SNP effect on Selenium

Batch 3−2157 : Selenium on Osteocrin

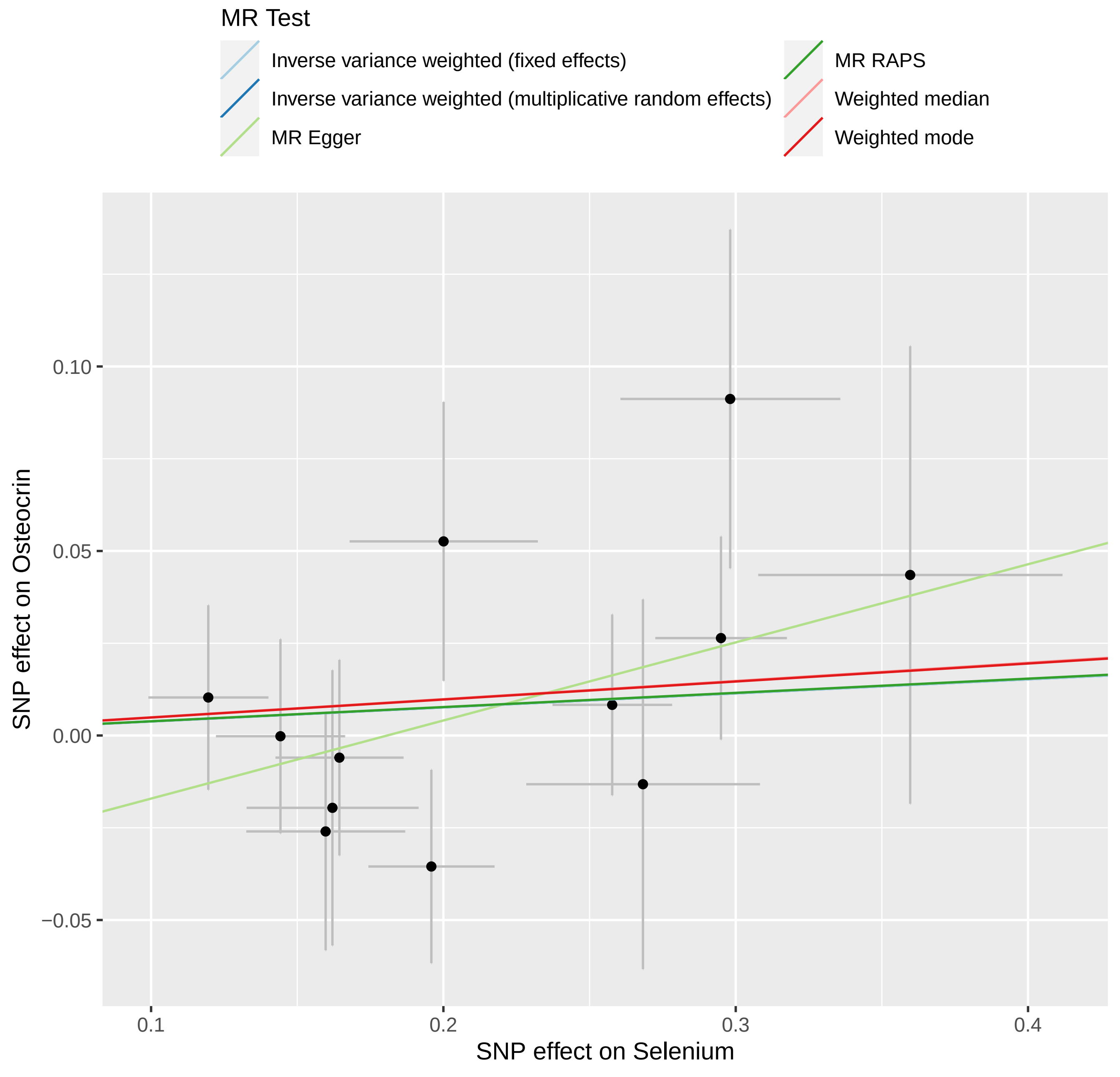

Batch 3−2158 : Selenium on Ubiquitin thioesterase OTUB2

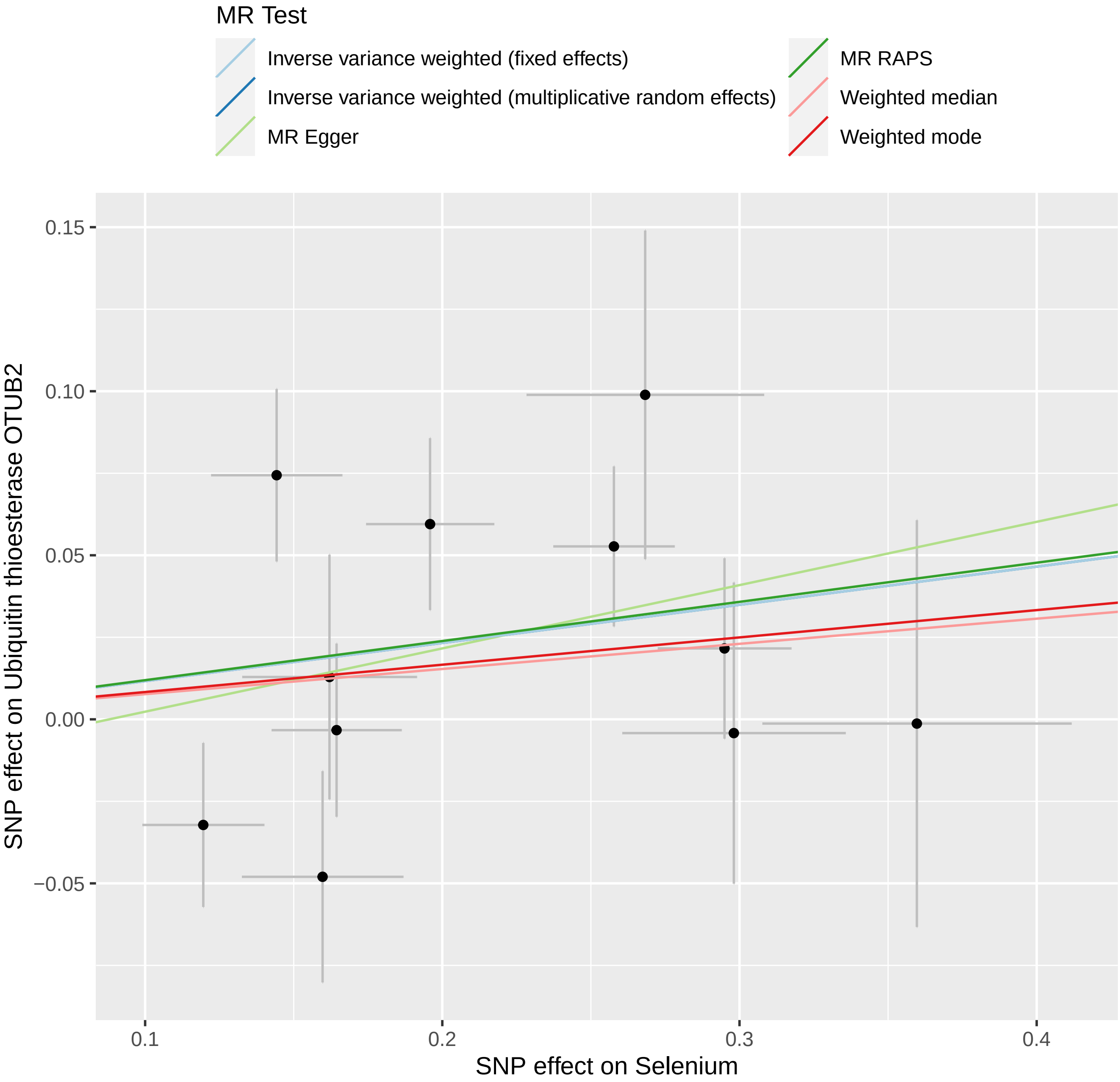

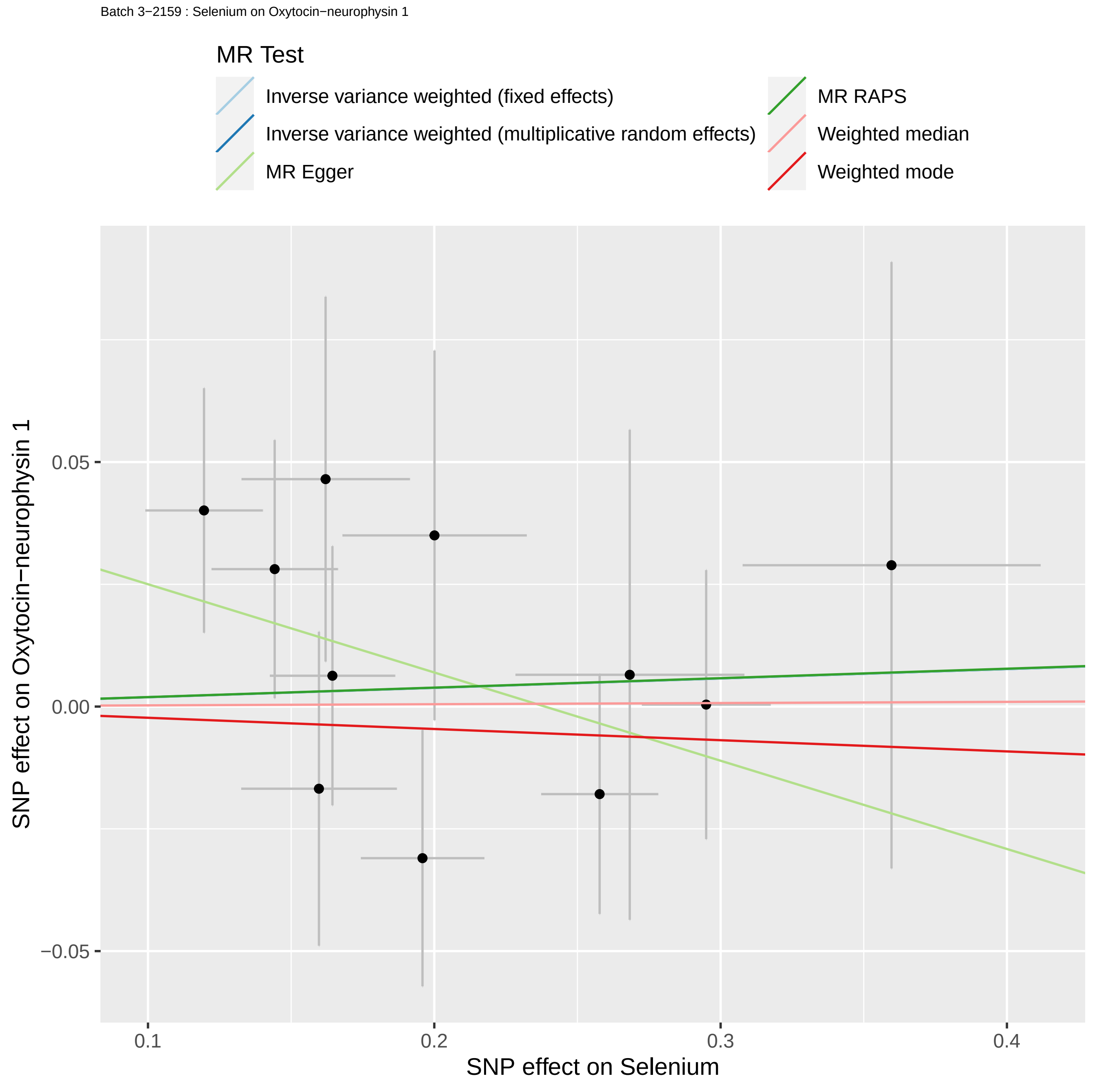

Batch 3−2159 : Selenium on Oxytocin−neurophysin 1
MR Test
Inverse variance weighted (fixed effects)
Inverse variance weighted (multiplicative random effects)
MR Egger
MR RAPS
Weighted median
Weighted mode
SNP effect on Oxytocin−neurophysin 1
0.05
0.00
−0.05
0.1
0.2
0.3
0.4
SNP effect on Selenium

Batch 3−2160 : Selenium on P2X purinoceptor 6

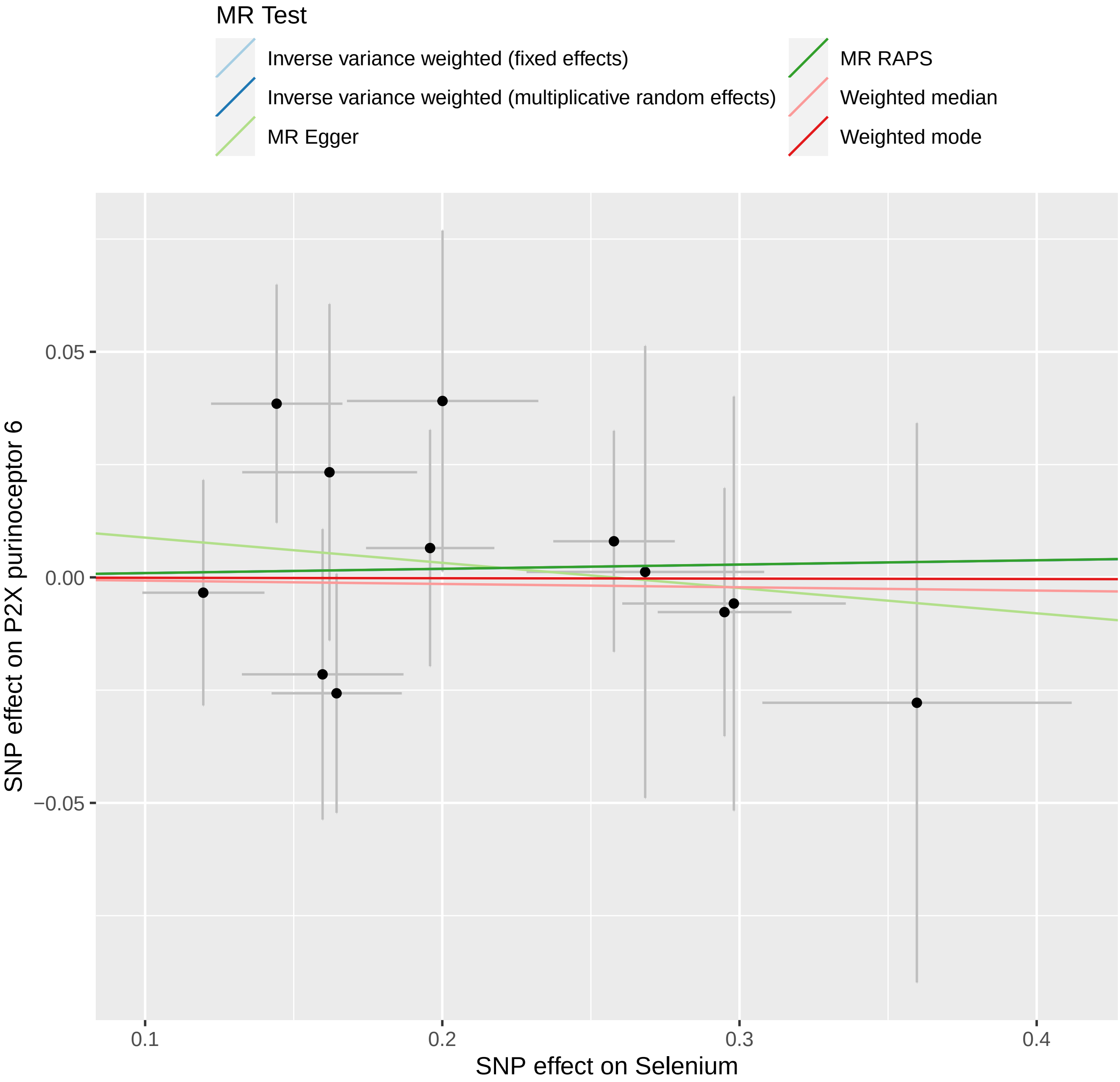

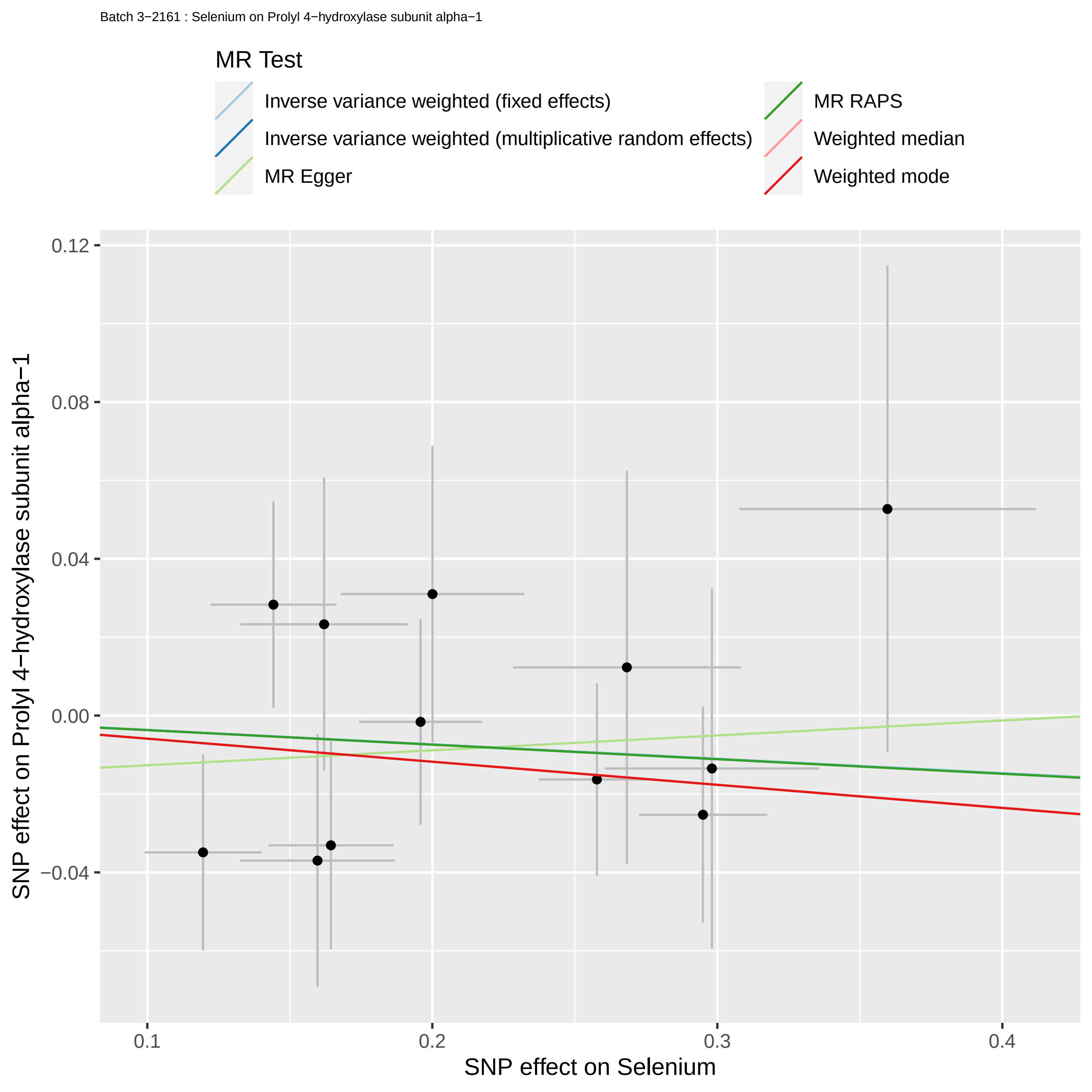
Batch 3−2161 : Selenium on Prolyl 4−hydroxylase subunit alpha−1
MR Test
Inverse variance weighted (fixed effects)
Inverse variance weighted (multiplicative random effects)
MR Egger
MR RAPS
Weighted median
Weighted mode
0.12
0.08
0.04
0.00
−0.04
0.1
0.2
0.3
0.4
SNP effect on Prolyl 4−hydroxylase subunit alpha−1
SNP effect on Selenium

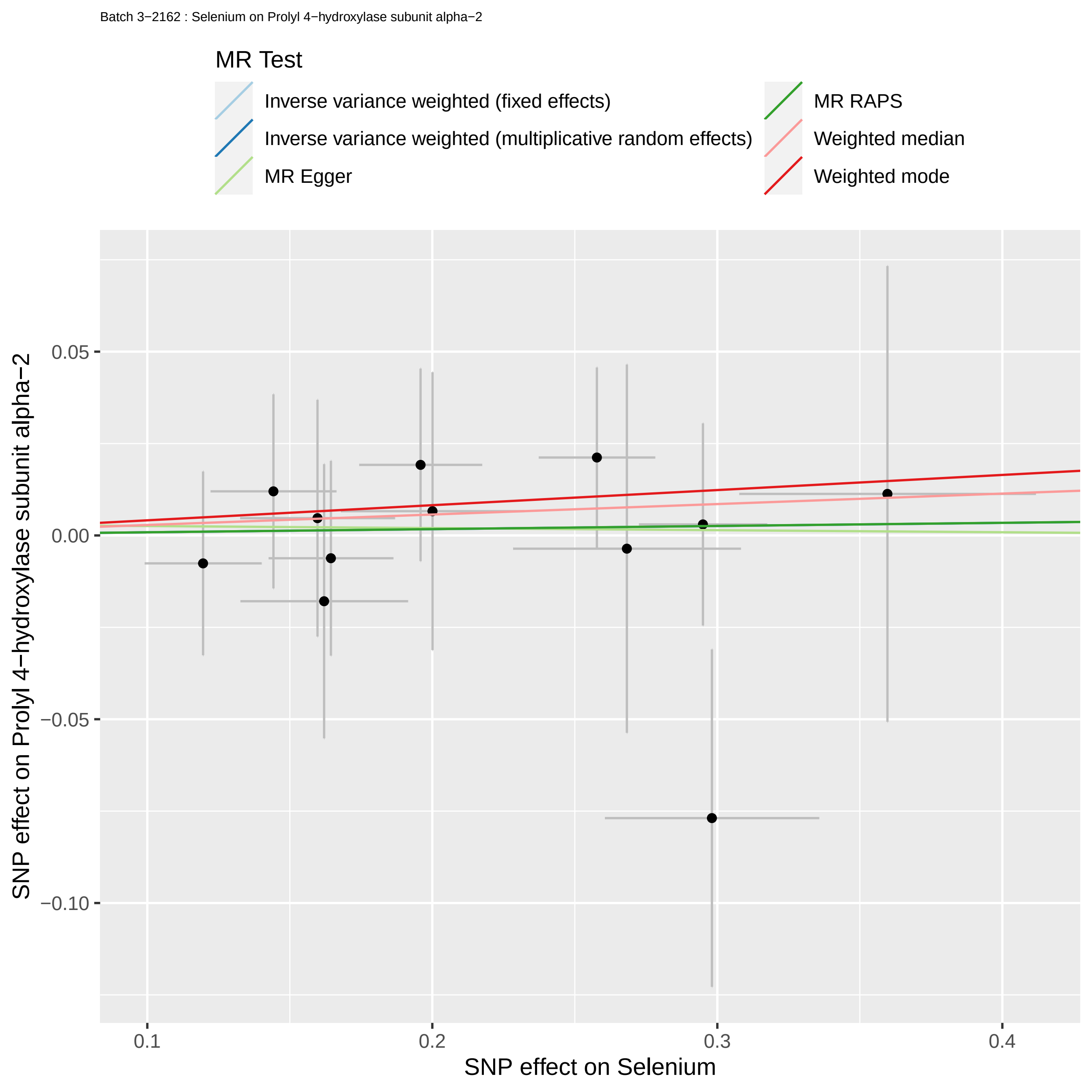
Batch 3−2162 : Selenium on Prolyl 4−hydroxylase subunit alpha−2
MR Test
Inverse variance weighted (fixed effects)
Inverse variance weighted (multiplicative random effects)
MR Egger
MR RAPS
Weighted median
Weighted mode
0.05
0.00
−0.05
−0.10
0.1
0.2
0.3
0.4
SNP effect on Prolyl 4−hydroxylase subunit alpha−2
SNP effect on Selenium

Batch 3−2163 : Selenium on Polyadenylate−binding protein 3

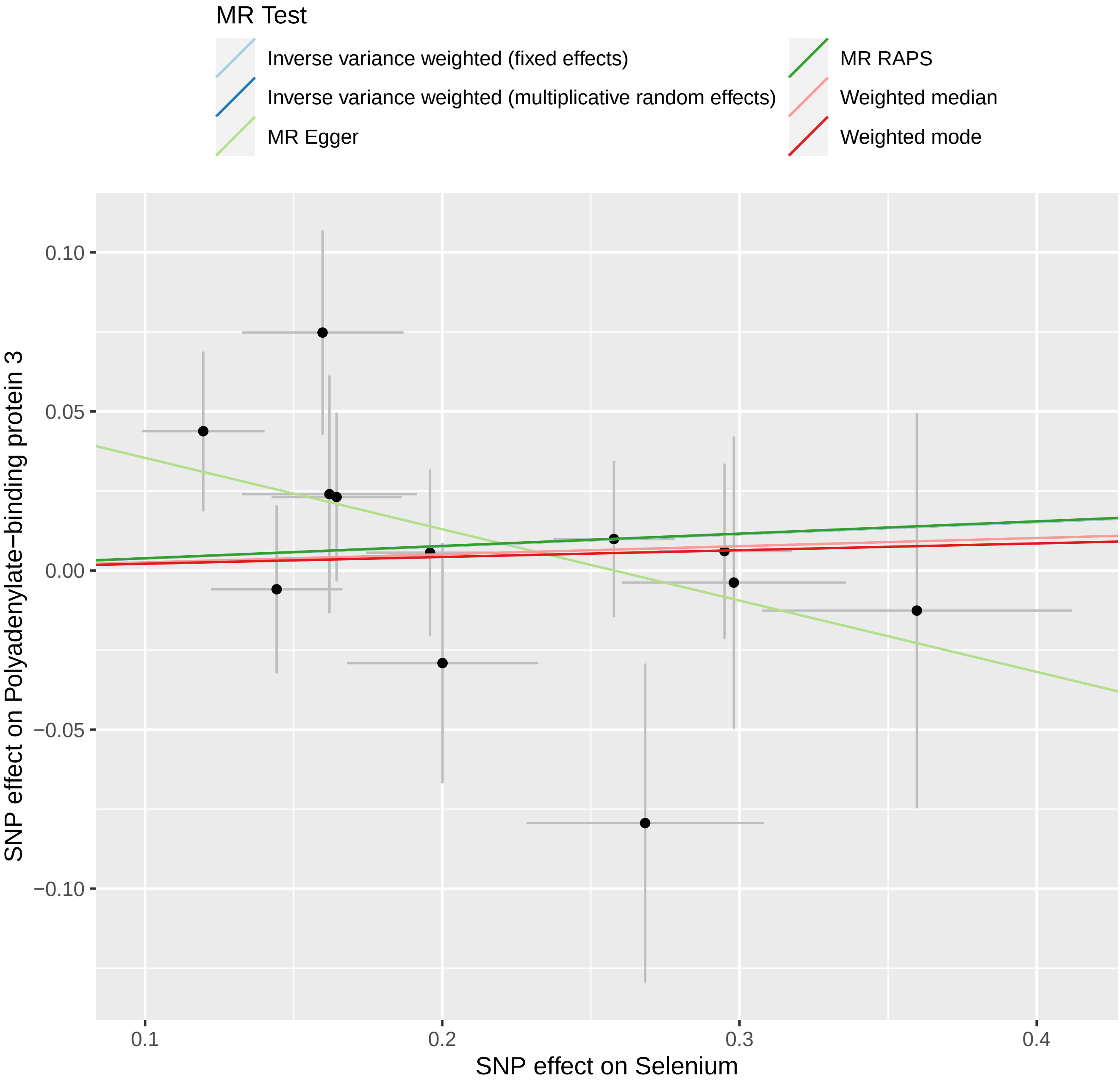

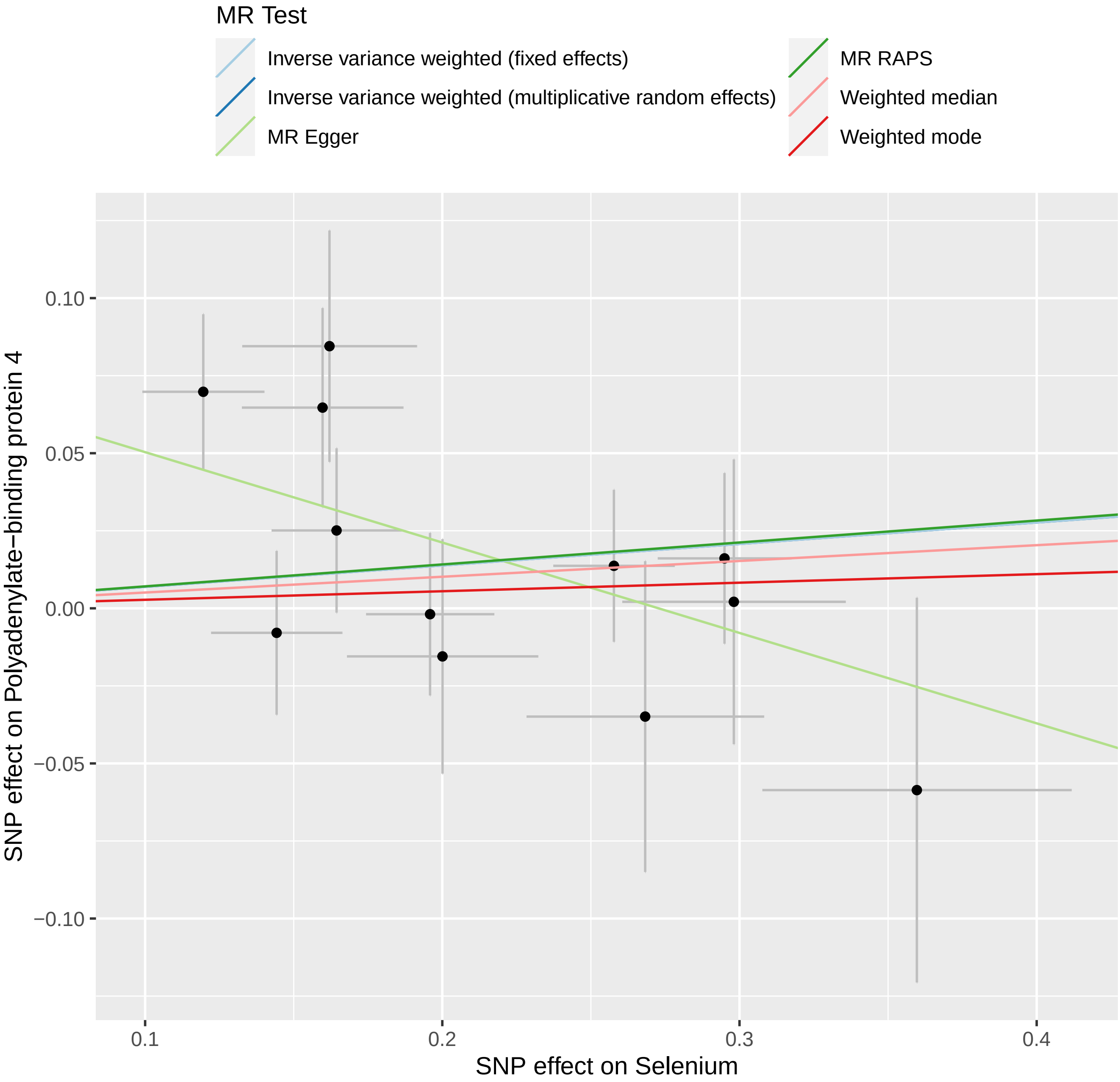
MR Test
Inverse variance weighted (fixed effects)
Inverse variance weighted (multiplicative random effects)
MR Egger
MR RAPS
Weighted median
Weighted mode
SNP effect on Polyadenylate−binding protein 4
0.10
0.05
0.00
−0.05
−0.10
0.1
0.2
0.3
0.4
SNP effect on Selenium

Batch 3−2165 : Selenium on Marginal zone B− and B1−cell−specific protein

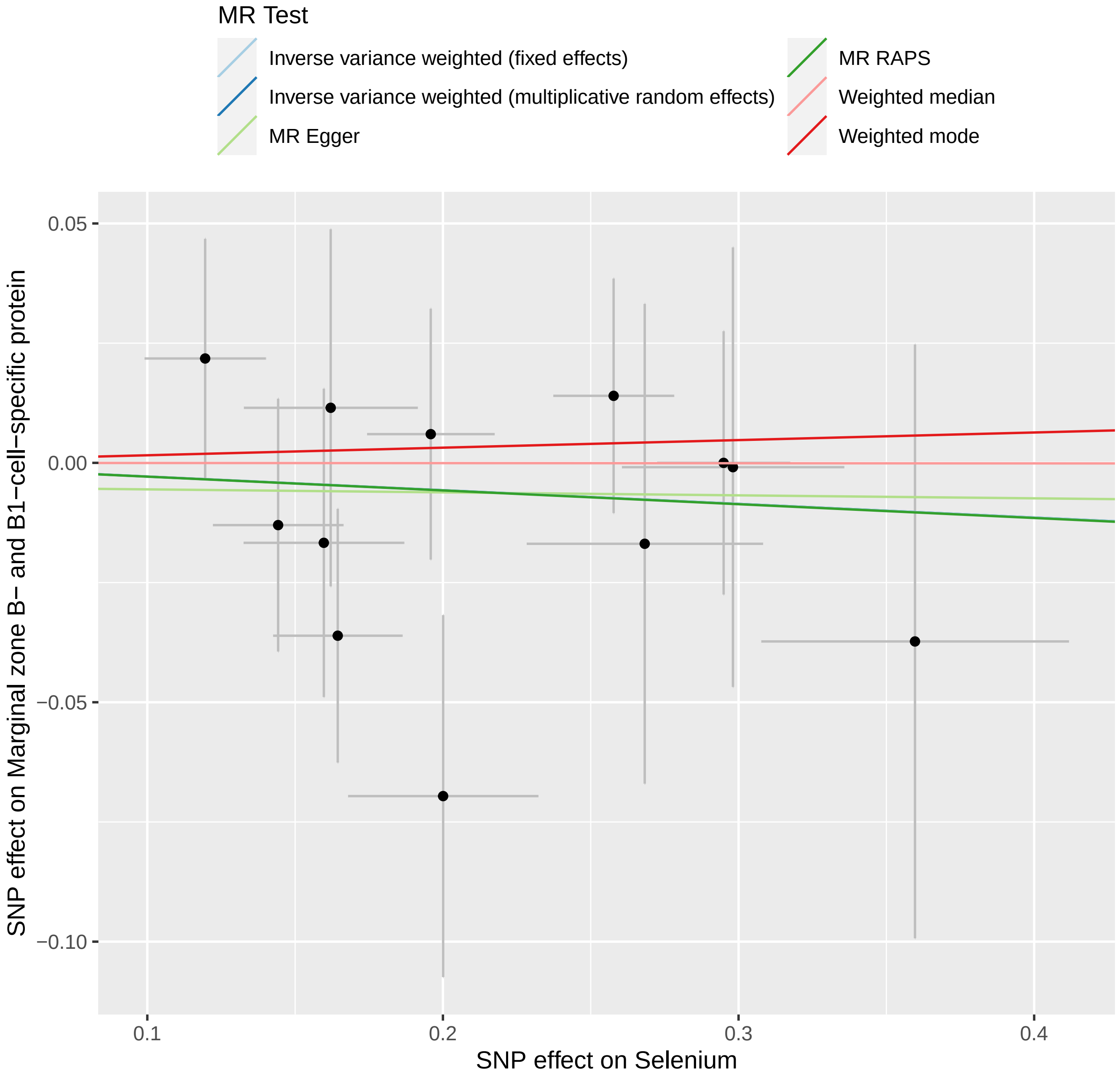

Batch 3−2166 : Selenium on Platelet−activating factor acetylhydrolase IB subunit beta

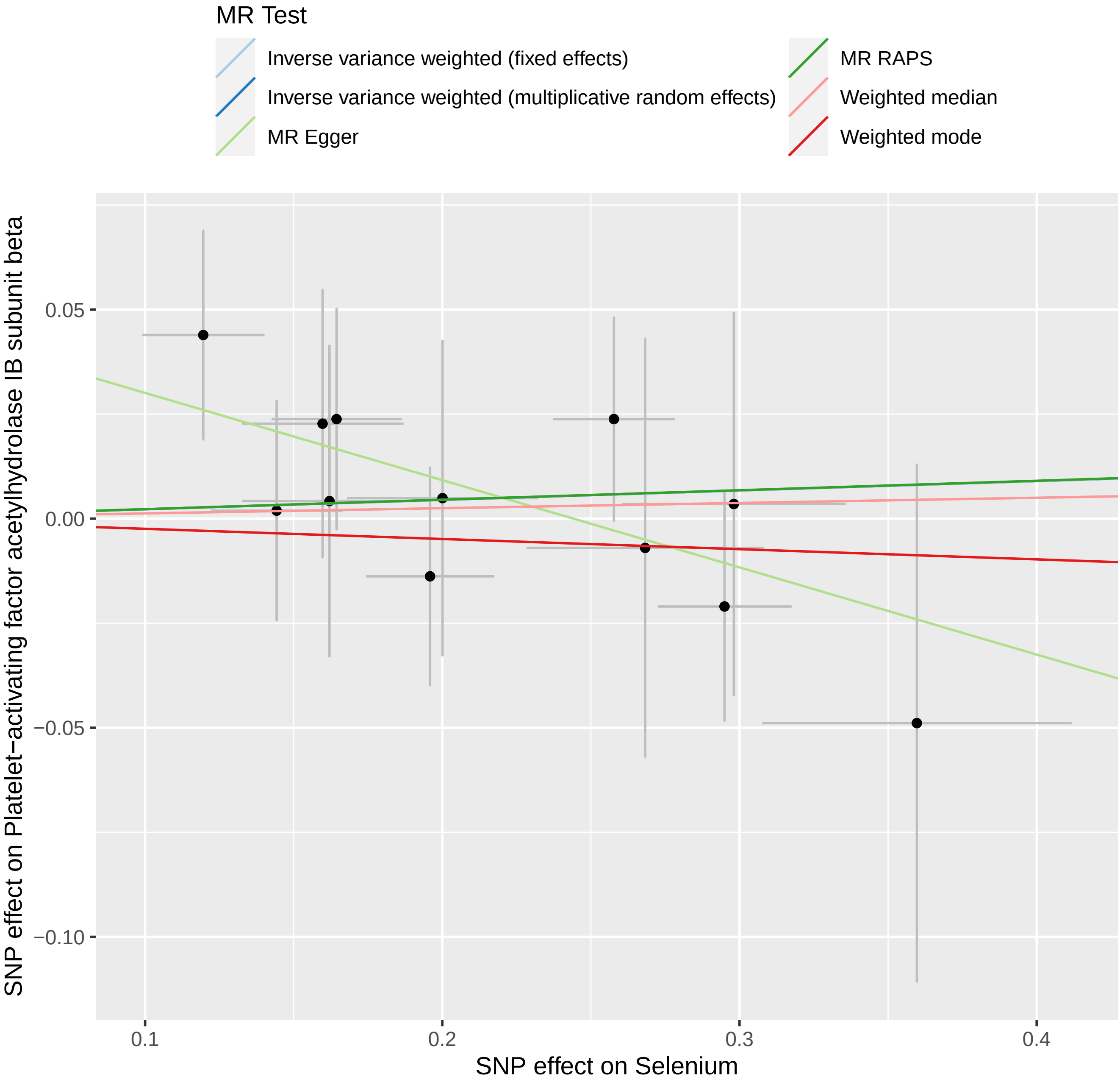

Batch 3−2167 : Selenium on Multifunctional protein ADE2

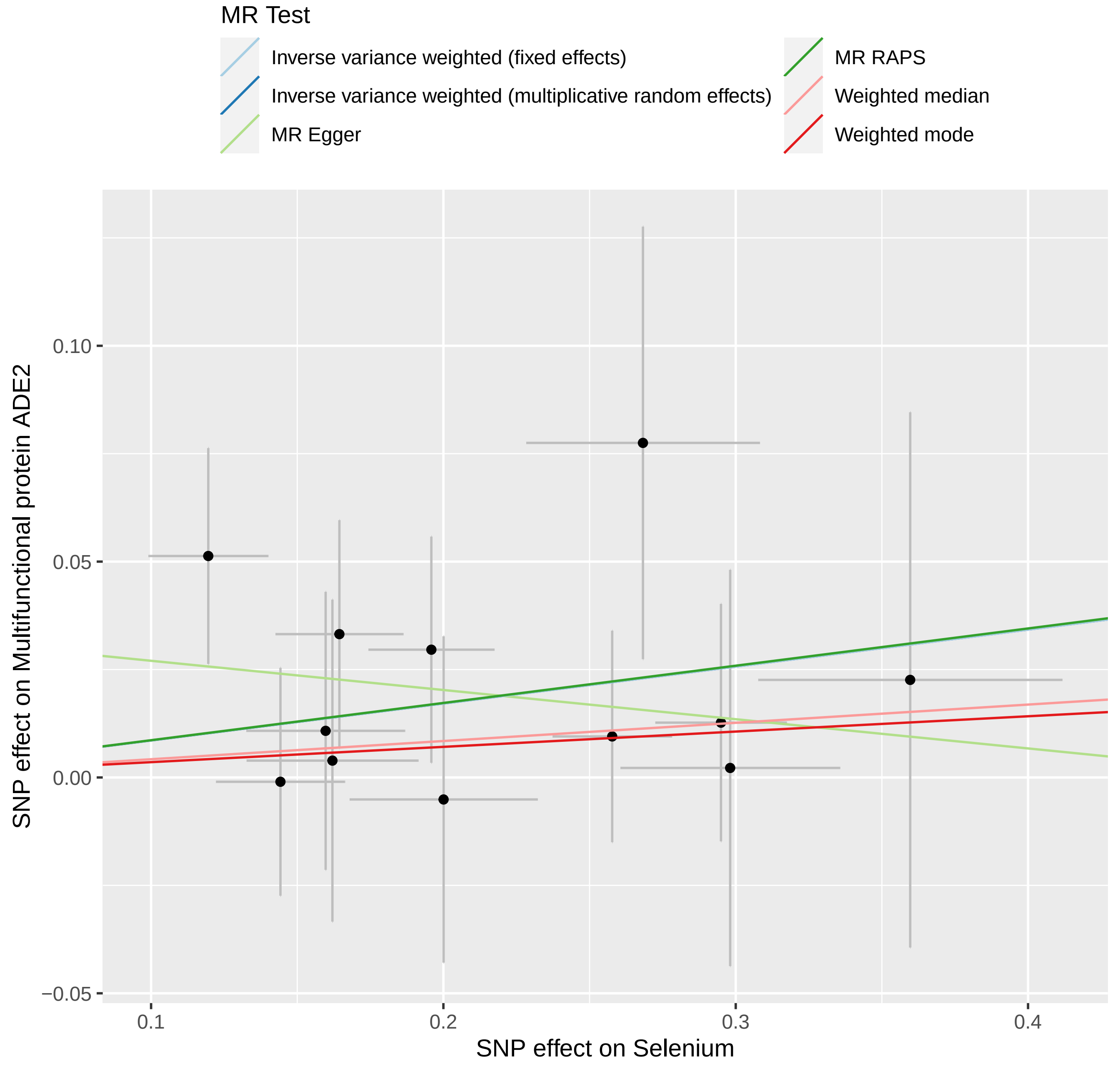

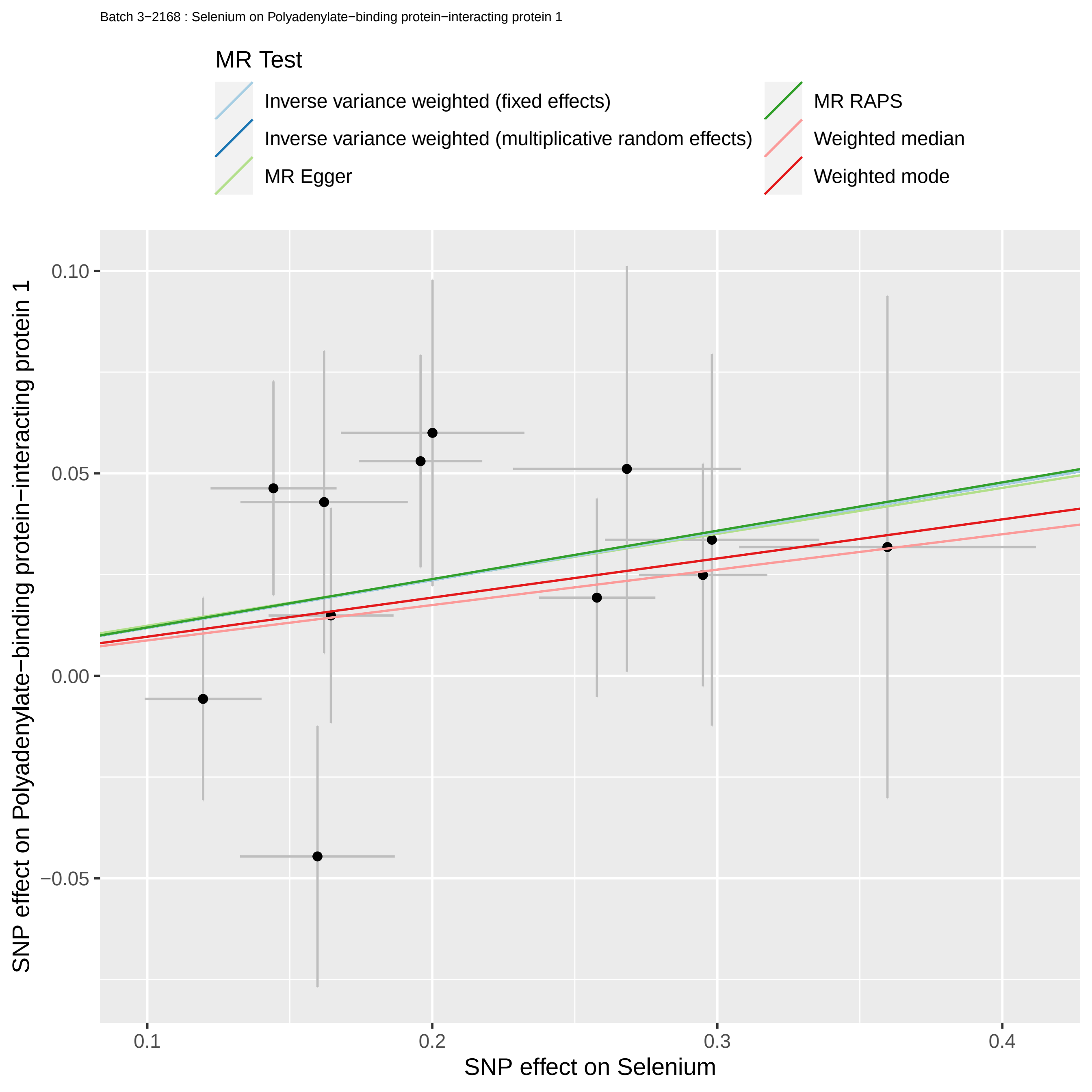

Batch 3−2168 : Selenium on Polyadenylate−binding protein−interacting protein 1
MR Test
Inverse variance weighted (fixed effects)
Inverse variance weighted (multiplicative random effects)
MR Egger
MR RAPS
Weighted median
Weighted mode
0.10
0.05
0.00
−0.05
0.1
0.2
0.3
0.4
SNP effect on Polyadenylate−binding protein−interacting protein 1
SNP effect on Selenium

Batch 3−2169 : Selenium on Serine/threonine−protein kinase PAK 3

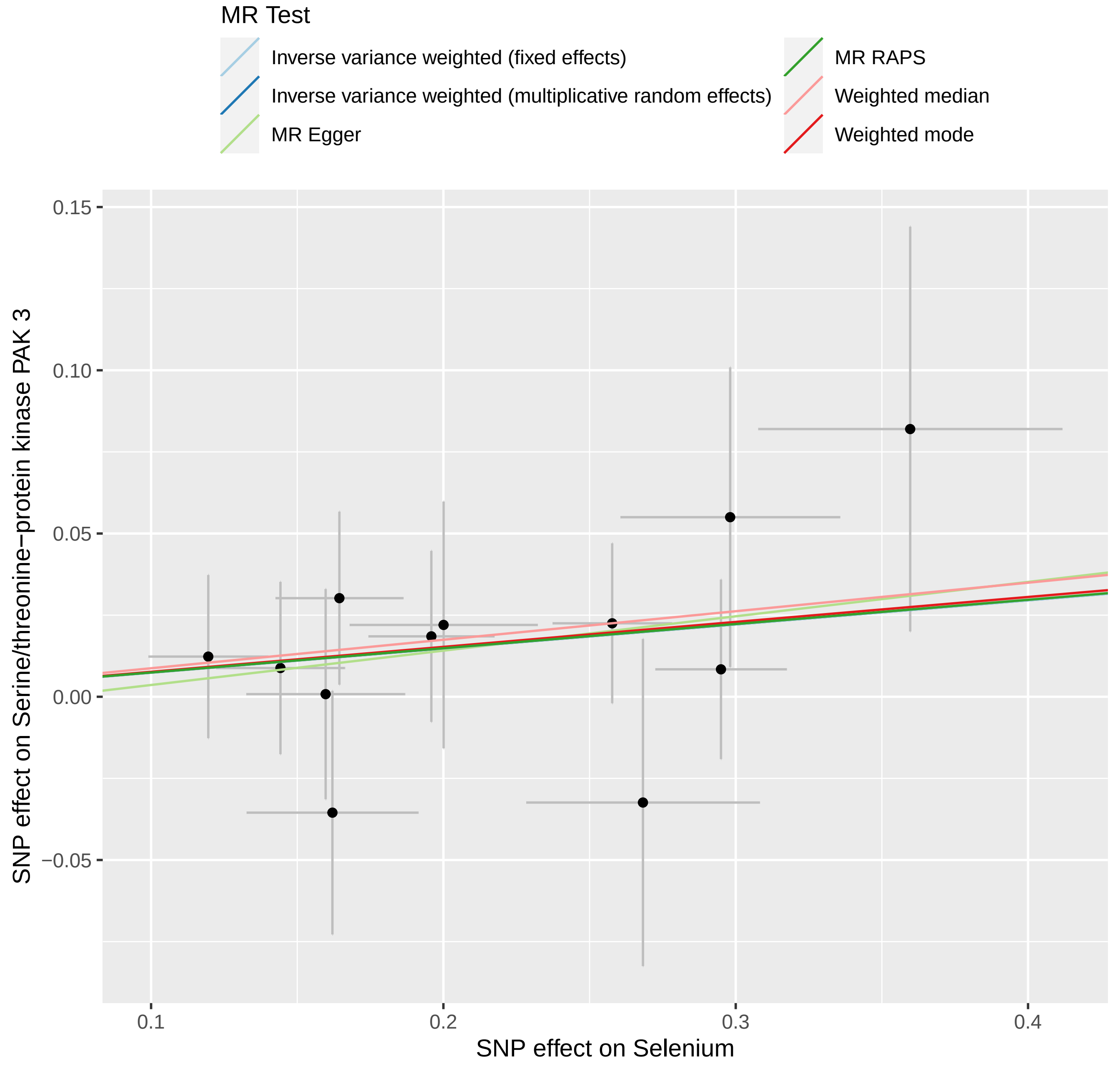

Batch 3−2170 : Selenium on Serine/threonine−protein kinase PAK 4

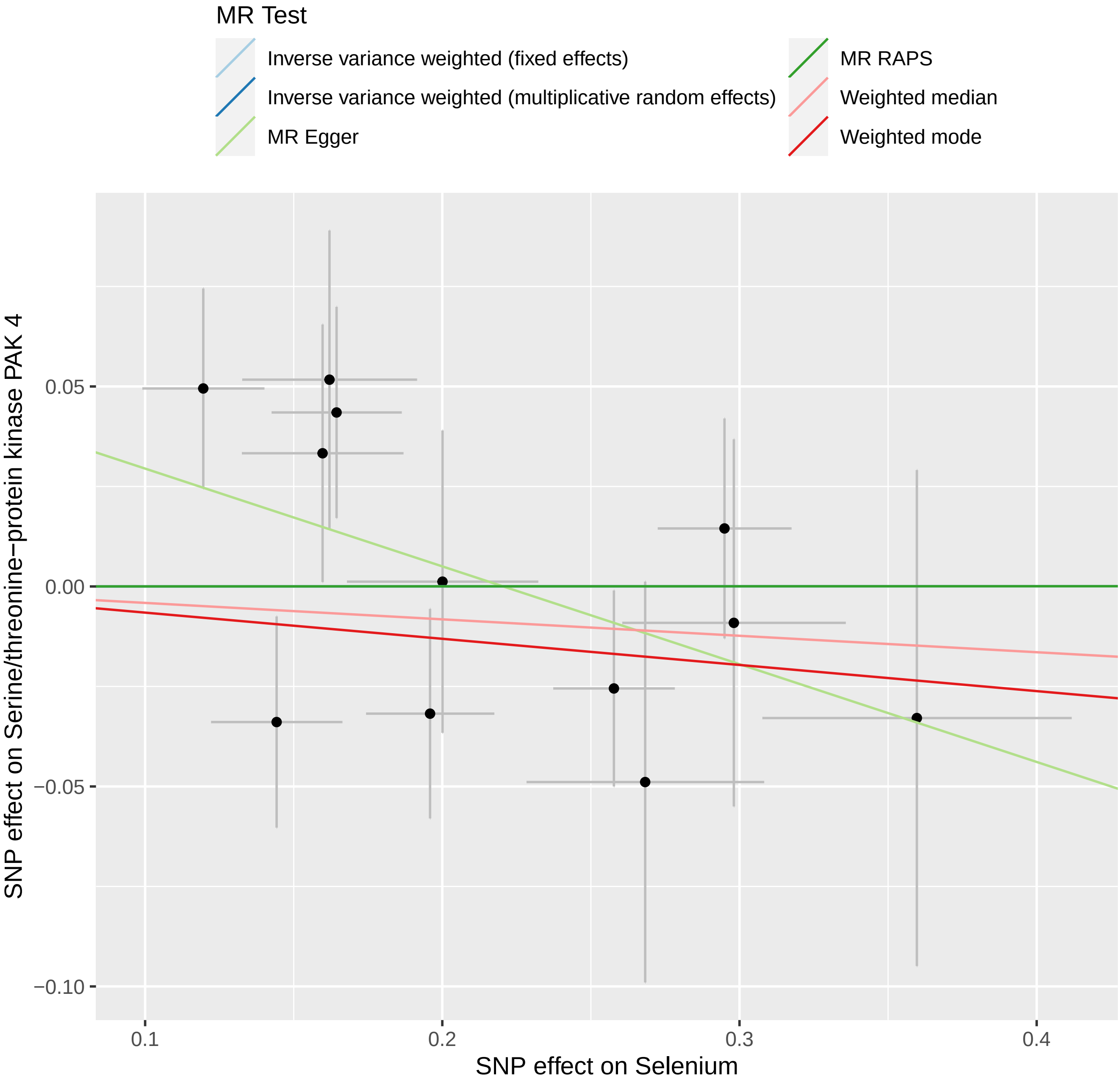

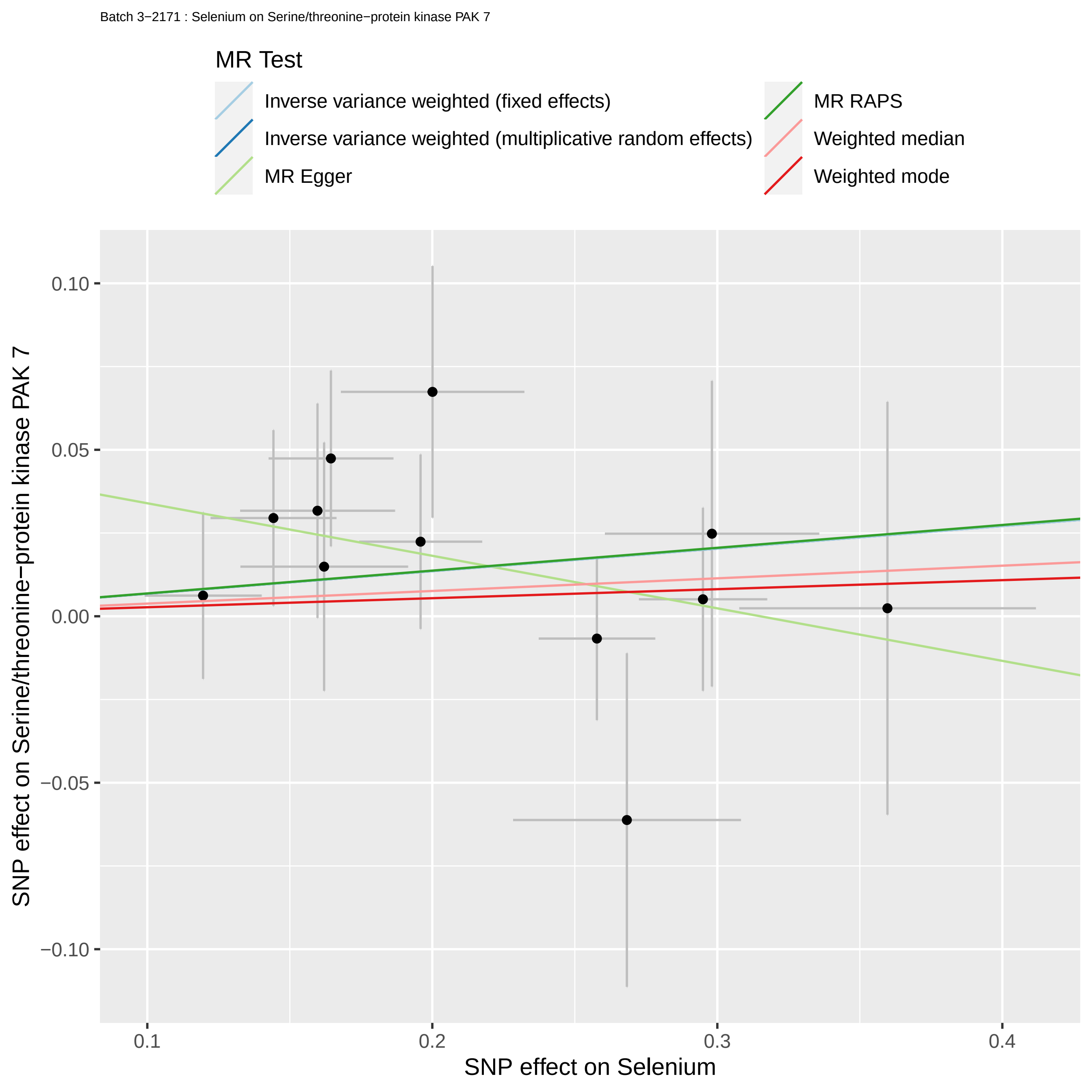

Batch 3−2171 : Selenium on Serine/threonine−protein kinase PAK 7
MR Test
Inverse variance weighted (fixed effects)
Inverse variance weighted (multiplicative random effects)
MR Egger
MR RAPS
Weighted median
Weighted mode
0.10
0.05
0.00
−0.05
−0.10
0.1
0.2
0.3
0.4
SNP effect on Serine/threonine−protein kinase PAK 7
SNP effect on Selenium

Batch 3−2172 : Selenium on Peptidyl−glycine alpha−amidating monooxygenase

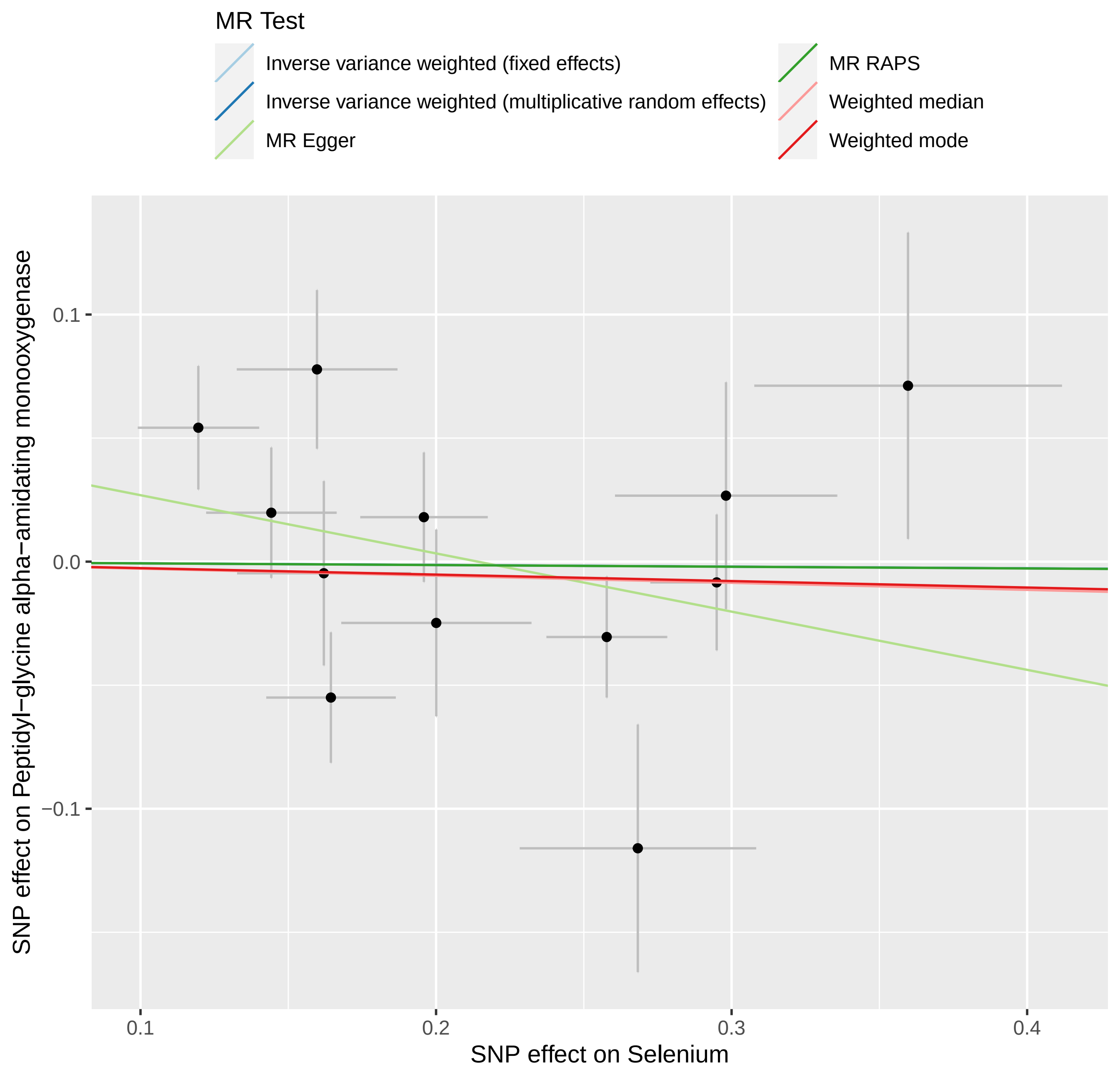

Batch 3−2173 : Selenium on Pantothenate kinase 3

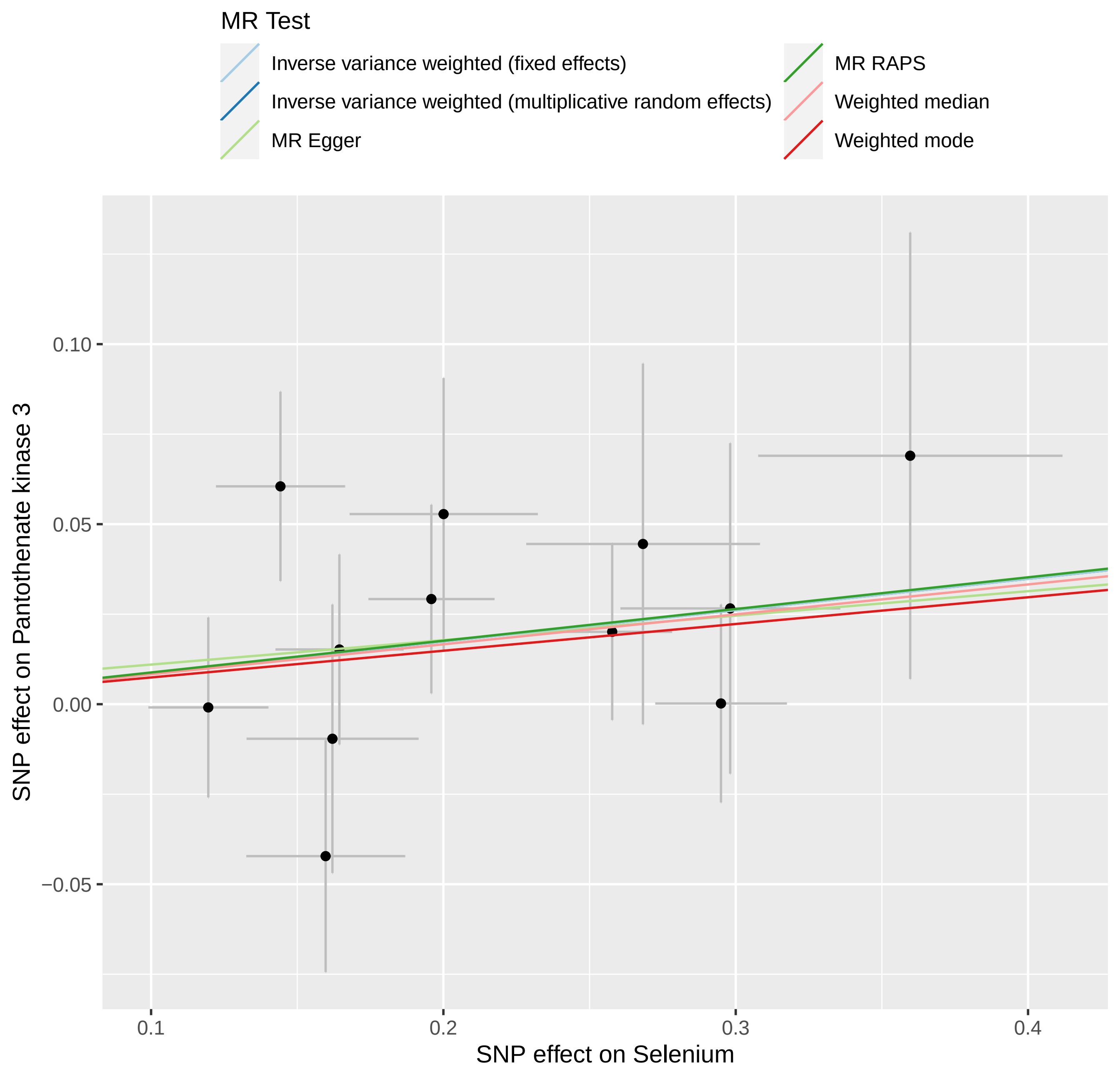

Batch 3−2174 : Selenium on Papilin

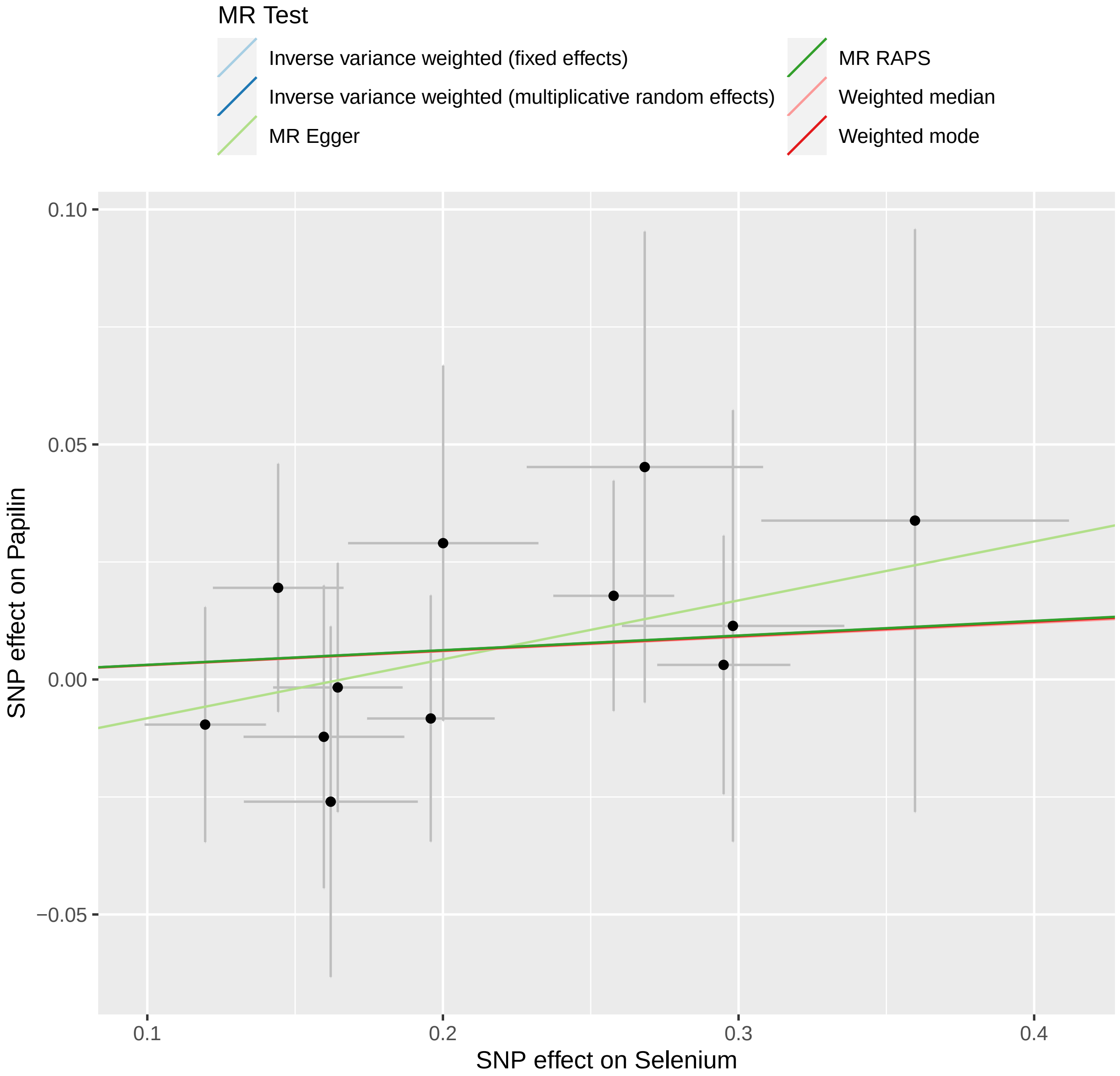

Batch 3−2175 : Selenium on Poly(A) polymerase gamma

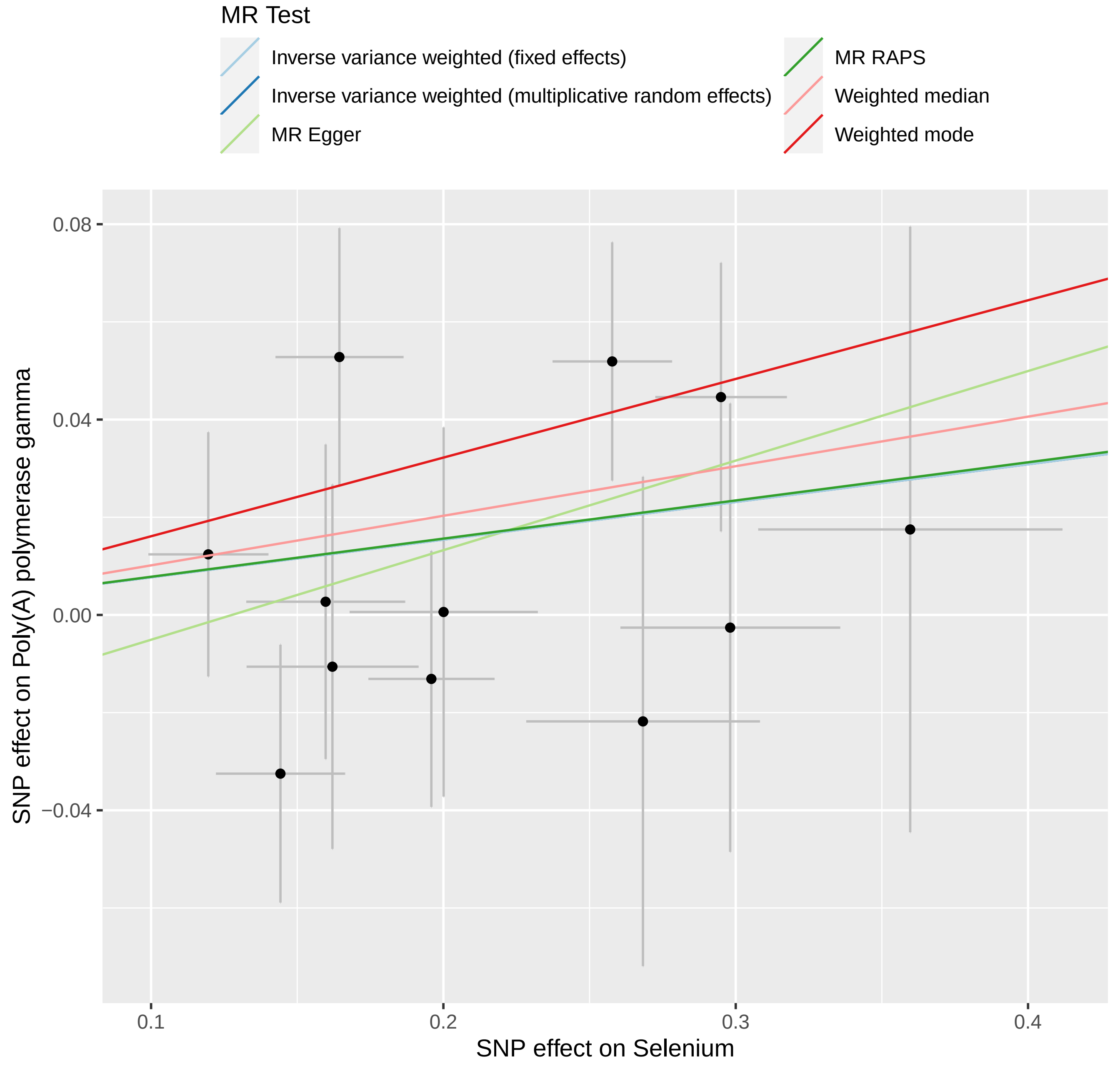

Batch 3−2176 : Selenium on Pappalysin−2

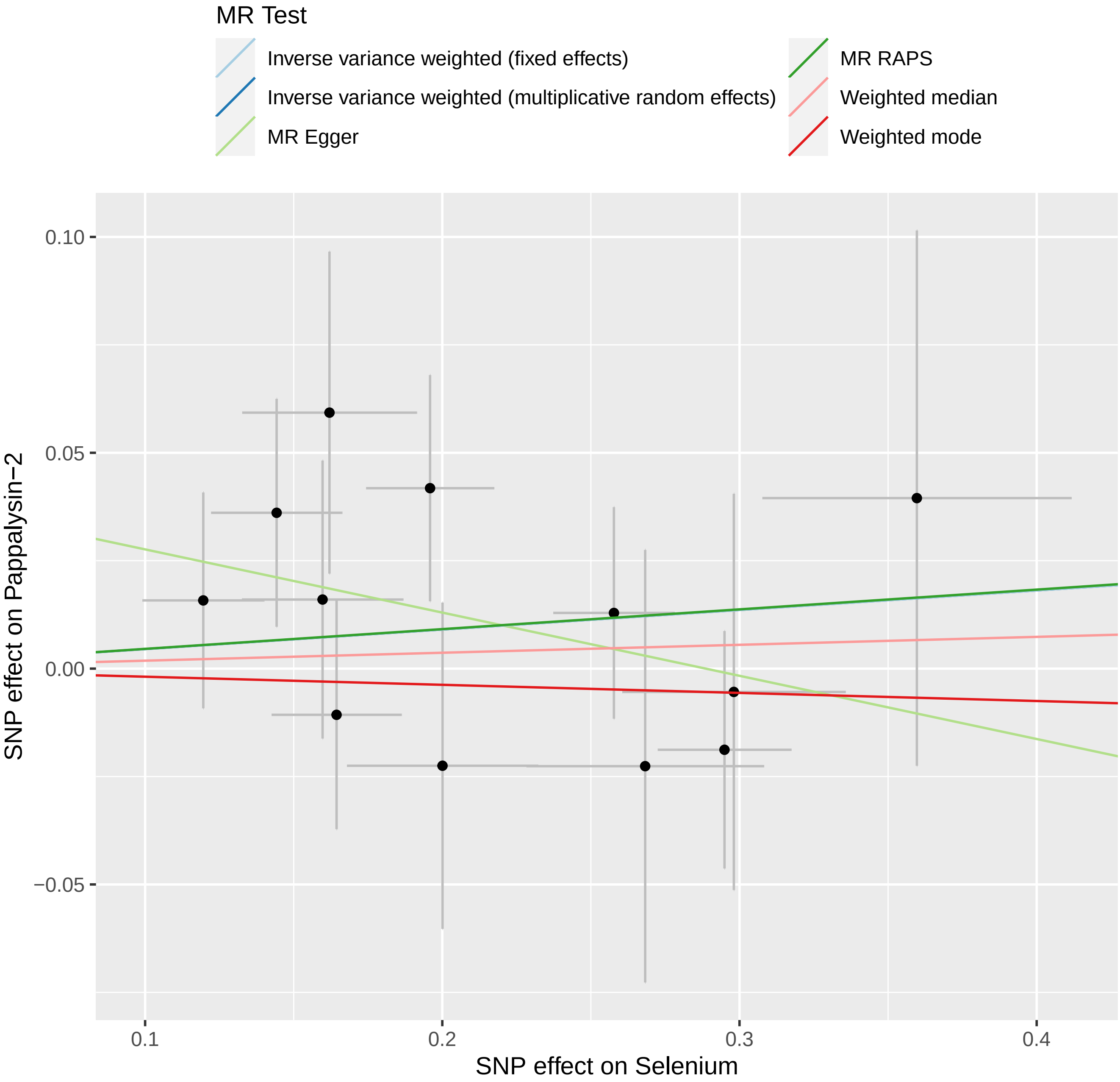

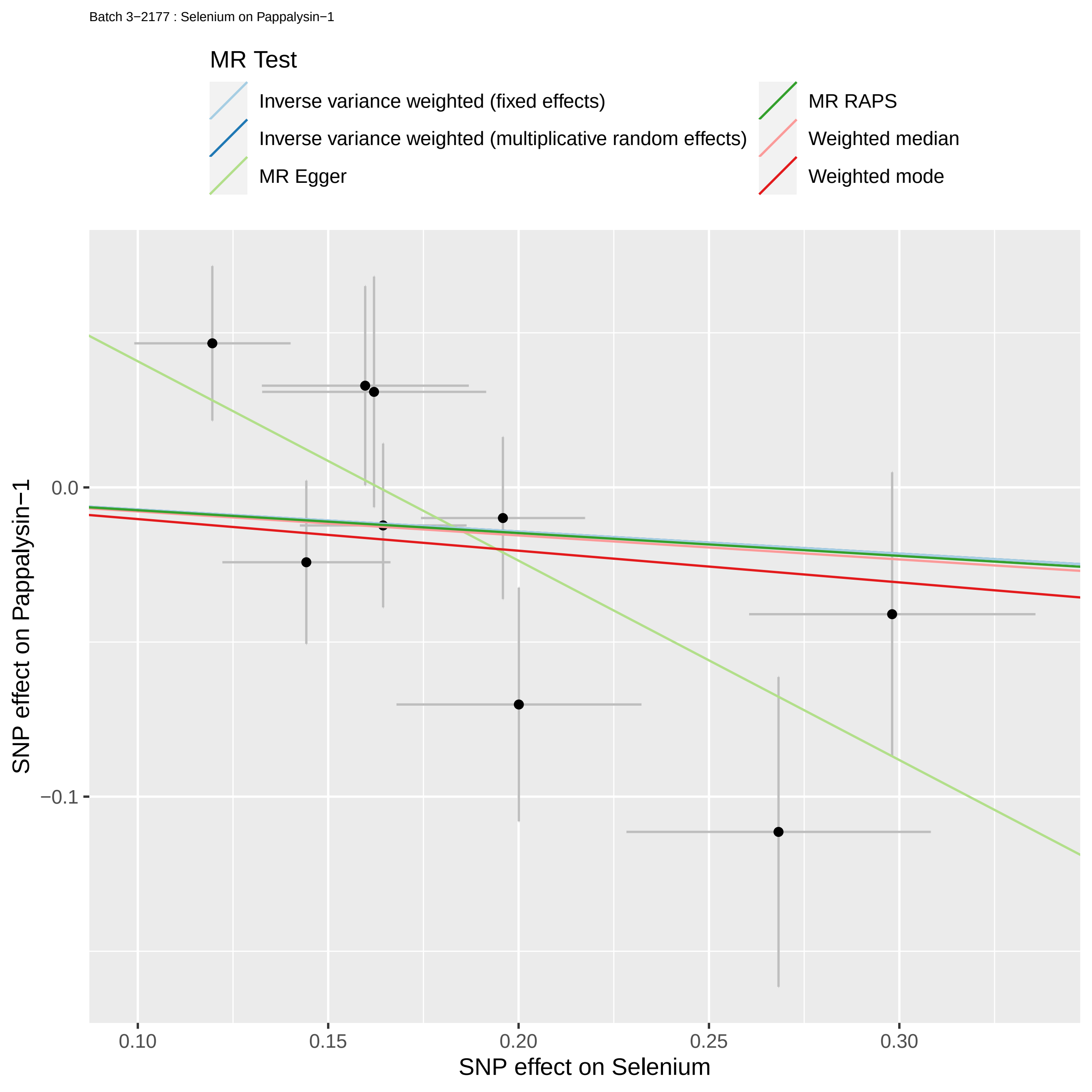
Batch 3−2177 : Selenium on Pappalysin−1
MR Test
Inverse variance weighted (fixed effects)
Inverse variance weighted (multiplicative random effects)
MR Egger
MR RAPS
Weighted median
Weighted mode
SNP effect on Pappalysin−1
0.0
−0.1
0.10
0.15
0.20
0.25
0.30
SNP effect on Selenium

Batch 3−2178 : Selenium on Bifunctional 3'−phosphoadenosine 5'−phosphosulfate synthase 1

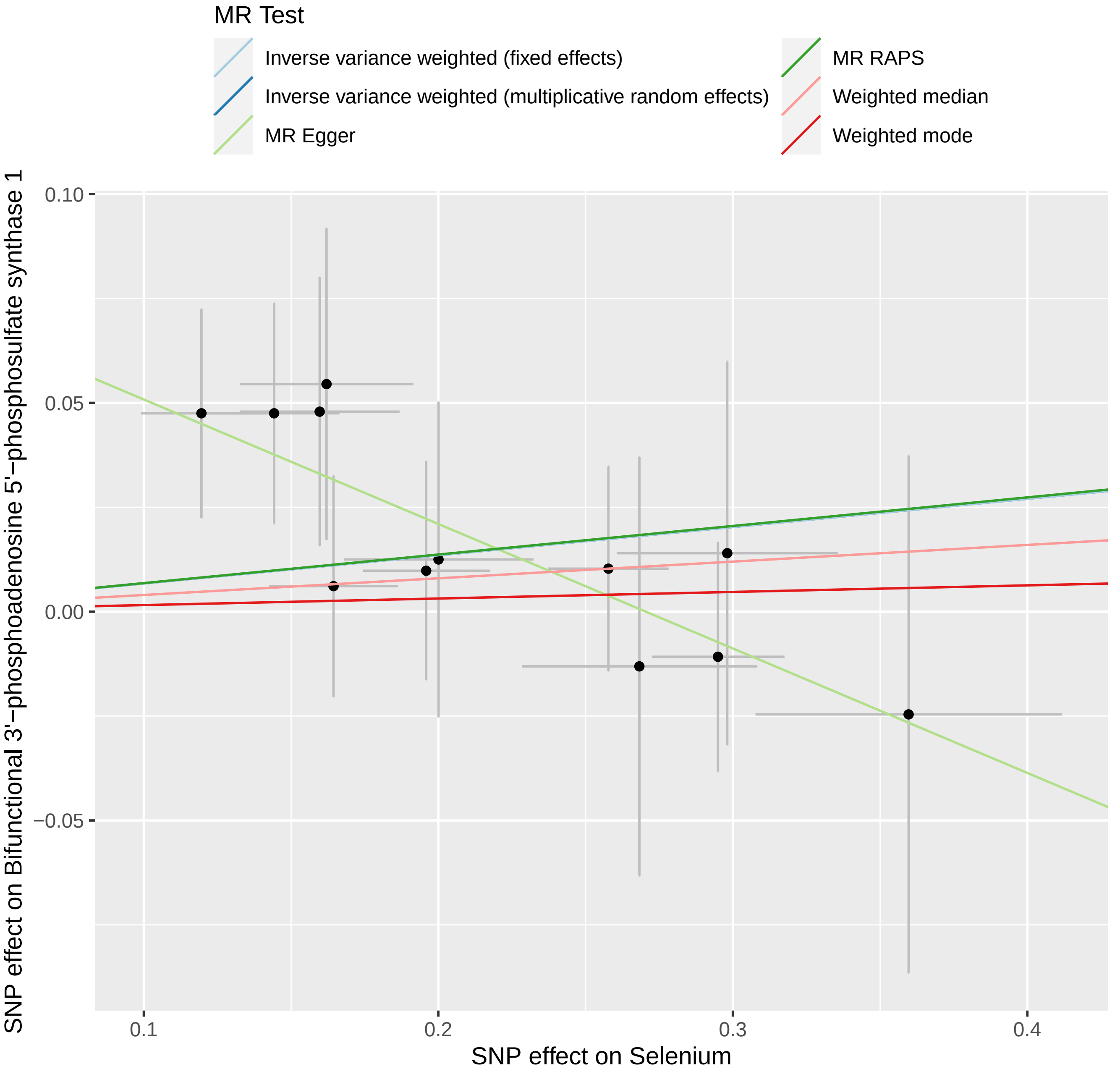

Batch 3−2179 : Selenium on E3 ubiquitin−protein ligase parkin

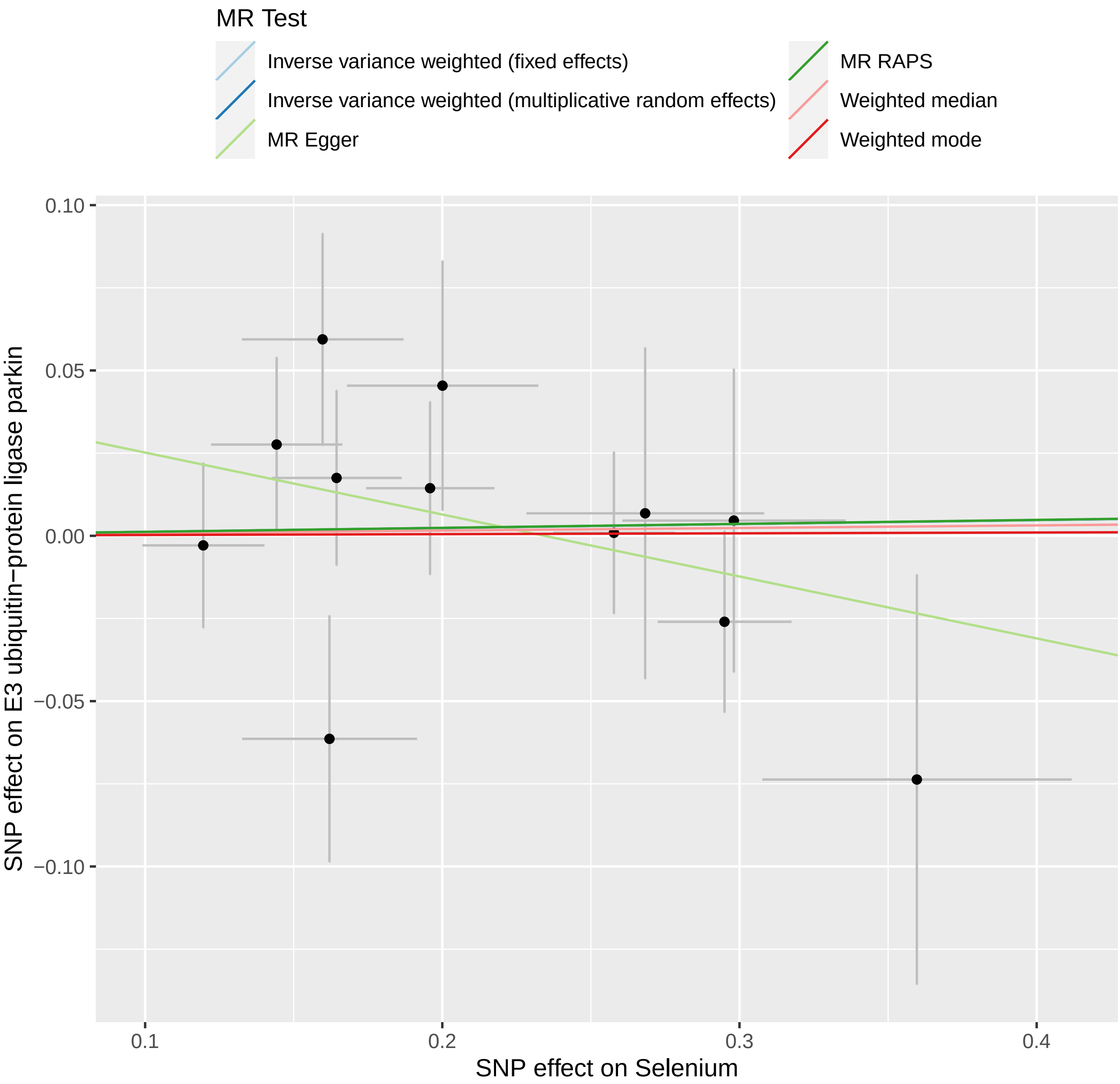

Batch 3−2180 : Selenium on Protein deglycase DJ−1

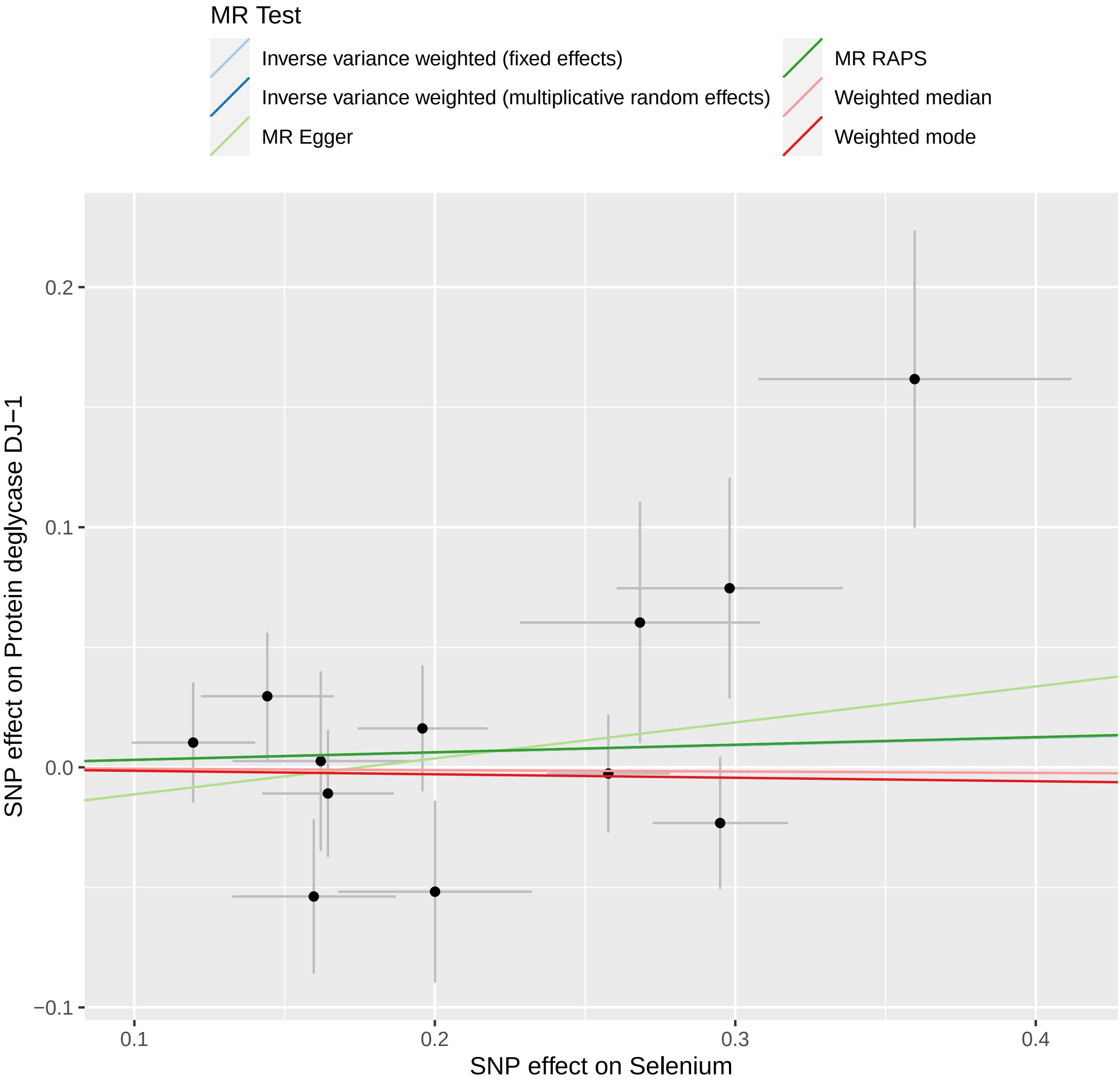

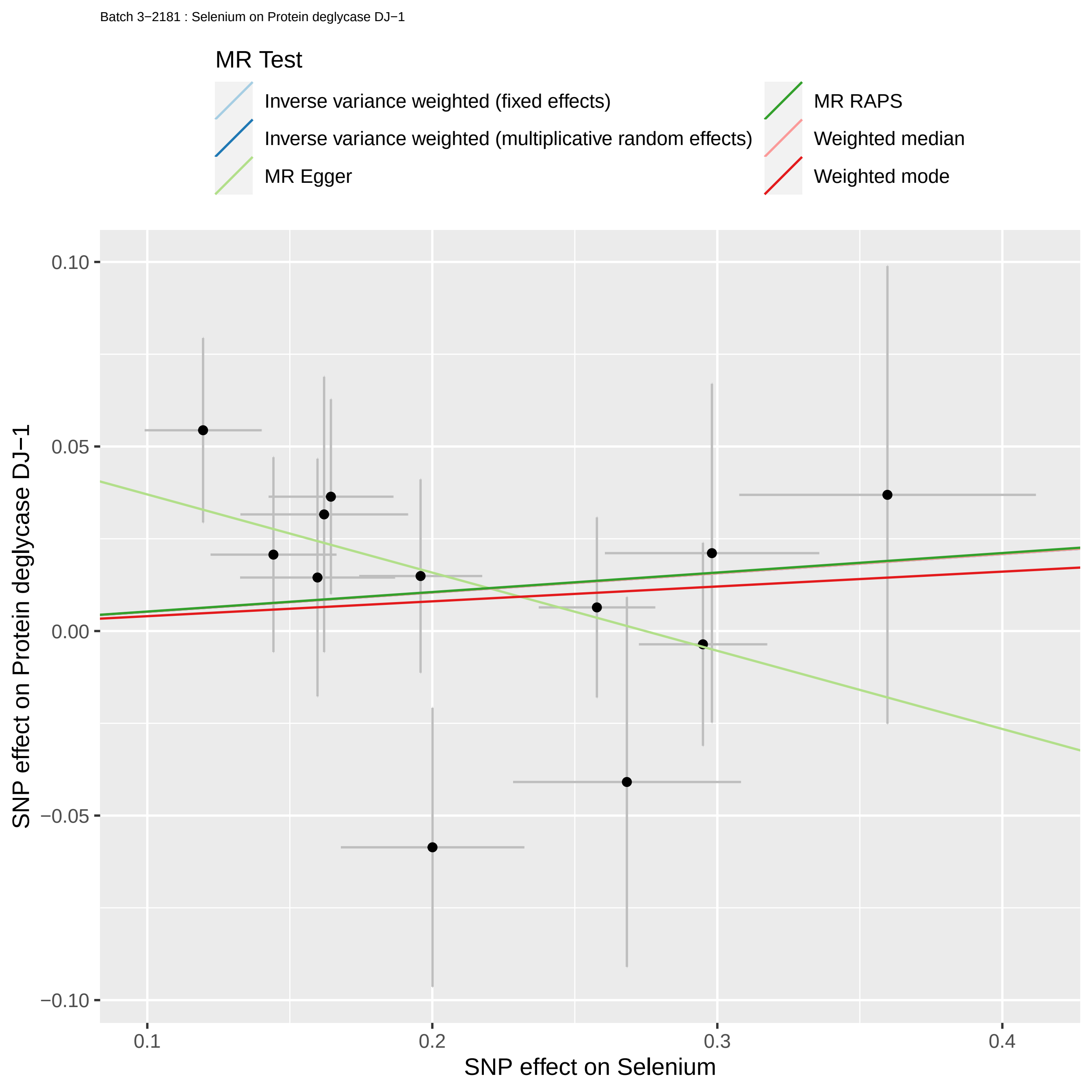

Batch 3−2181 : Selenium on Protein deglycase DJ−1
MR Test
Inverse variance weighted (fixed effects)
Inverse variance weighted (multiplicative random effects)
MR Egger
MR RAPS
Weighted median
Weighted mode
0.10
0.05
0.00
−0.05
−0.10
0.1
0.2
0.3
0.4
SNP effect on Selenium
SNP effect on Protein deglycase DJ−1

Batch 3−2182 : Selenium on Poly [ADP−ribose] polymerase 1

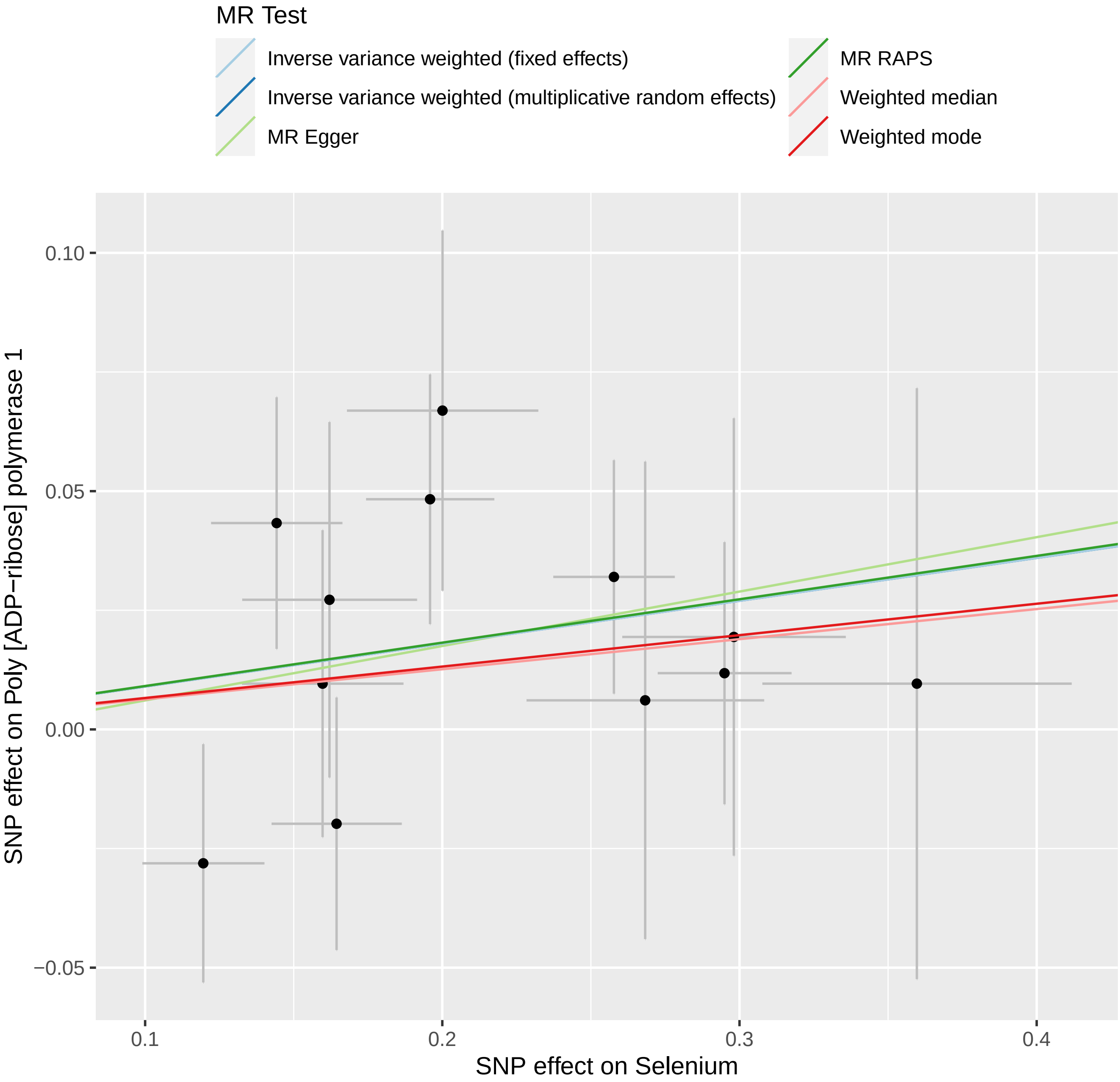

Batch 3−2183 : Selenium on Poly [ADP−ribose] polymerase 11

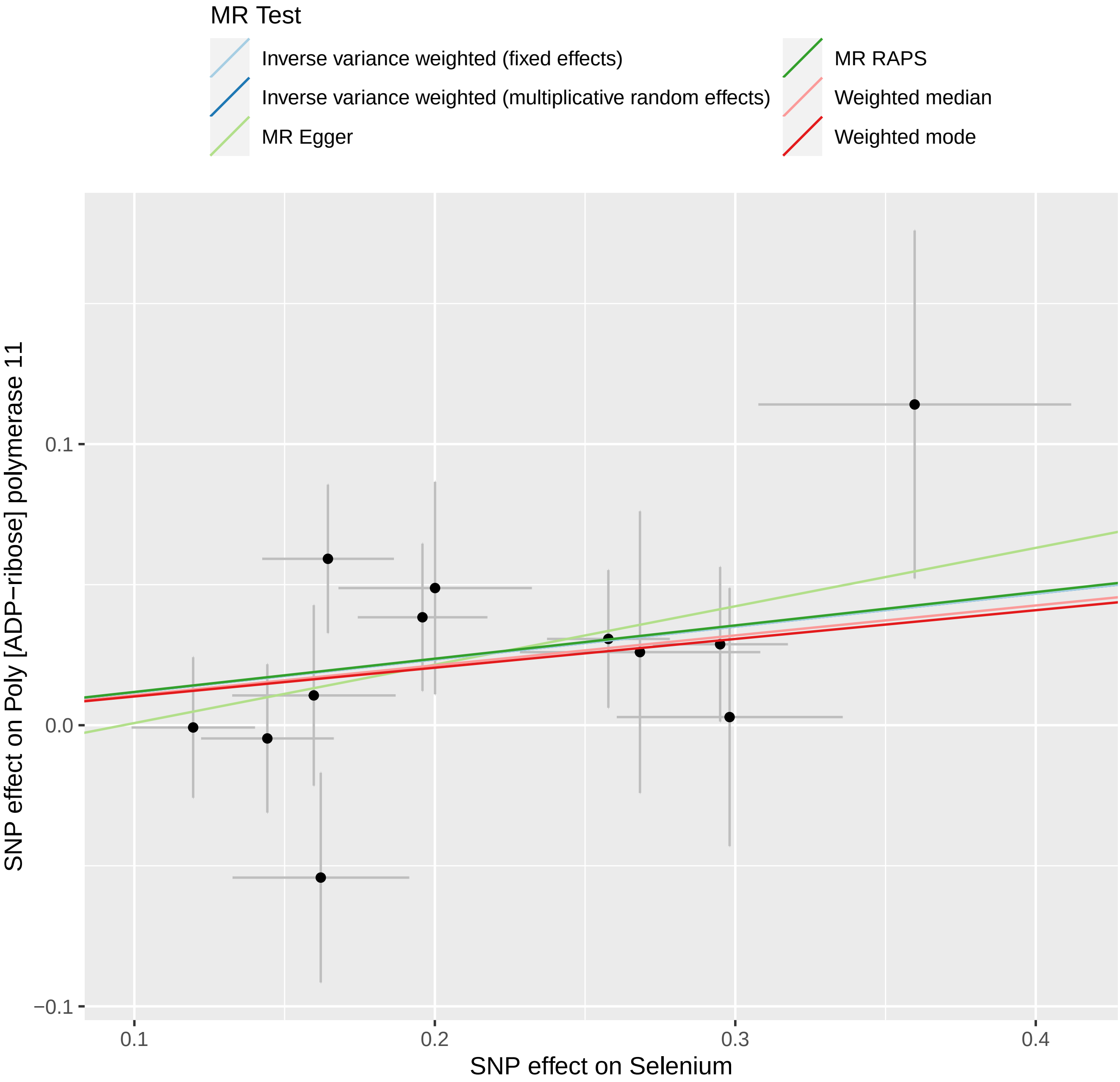

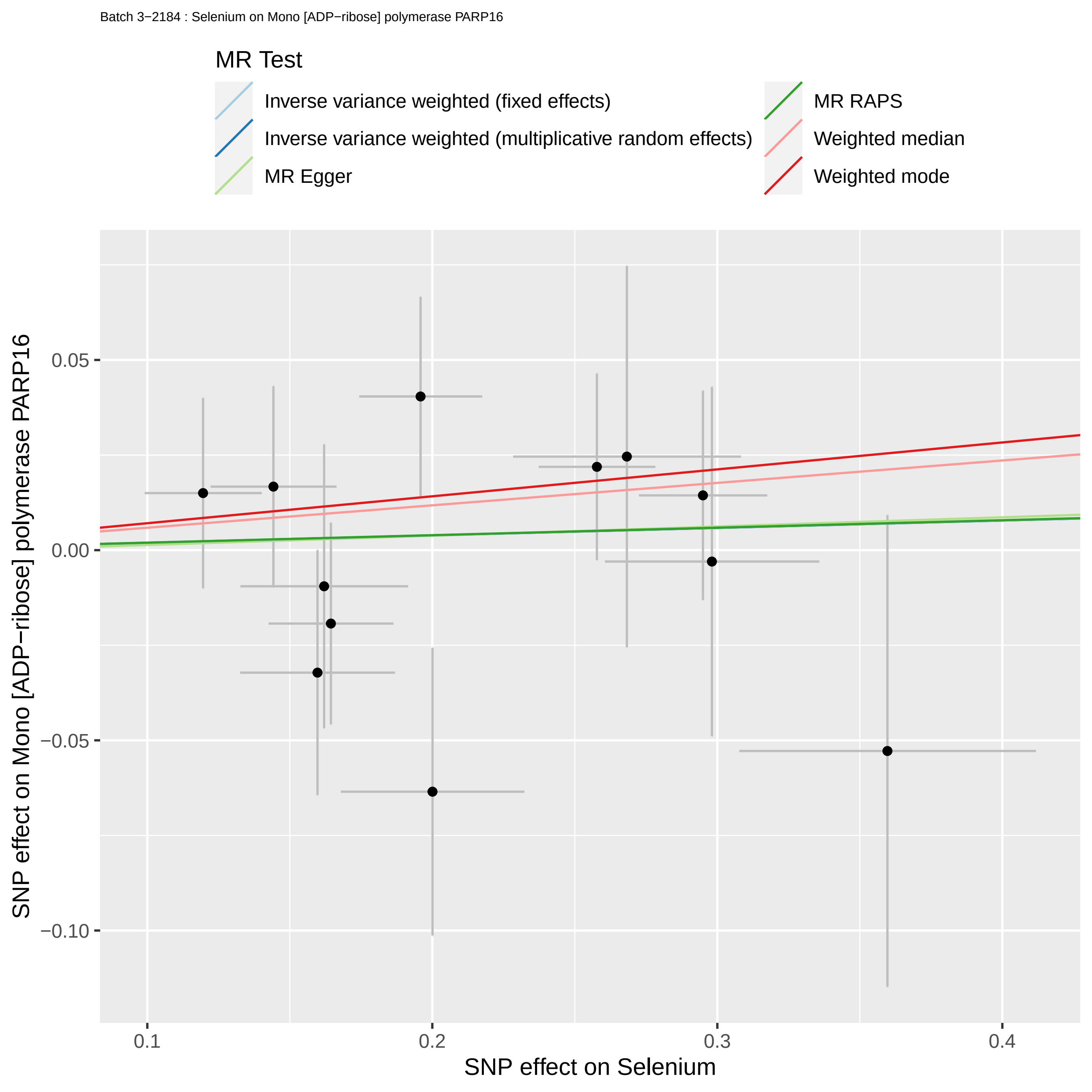

Batch 3−2184 : Selenium on Mono [ADP−ribose] polymerase PARP16
MR Test
Inverse variance weighted (fixed effects)
Inverse variance weighted (multiplicative random effects)
MR Egger
MR RAPS
Weighted median
Weighted mode
SNP effect on Mono [ADP−ribose] polymerase PARP16
0.05
0.00
−0.05
−0.10
0.1
0.2
0.3
0.4
SNP effect on Selenium

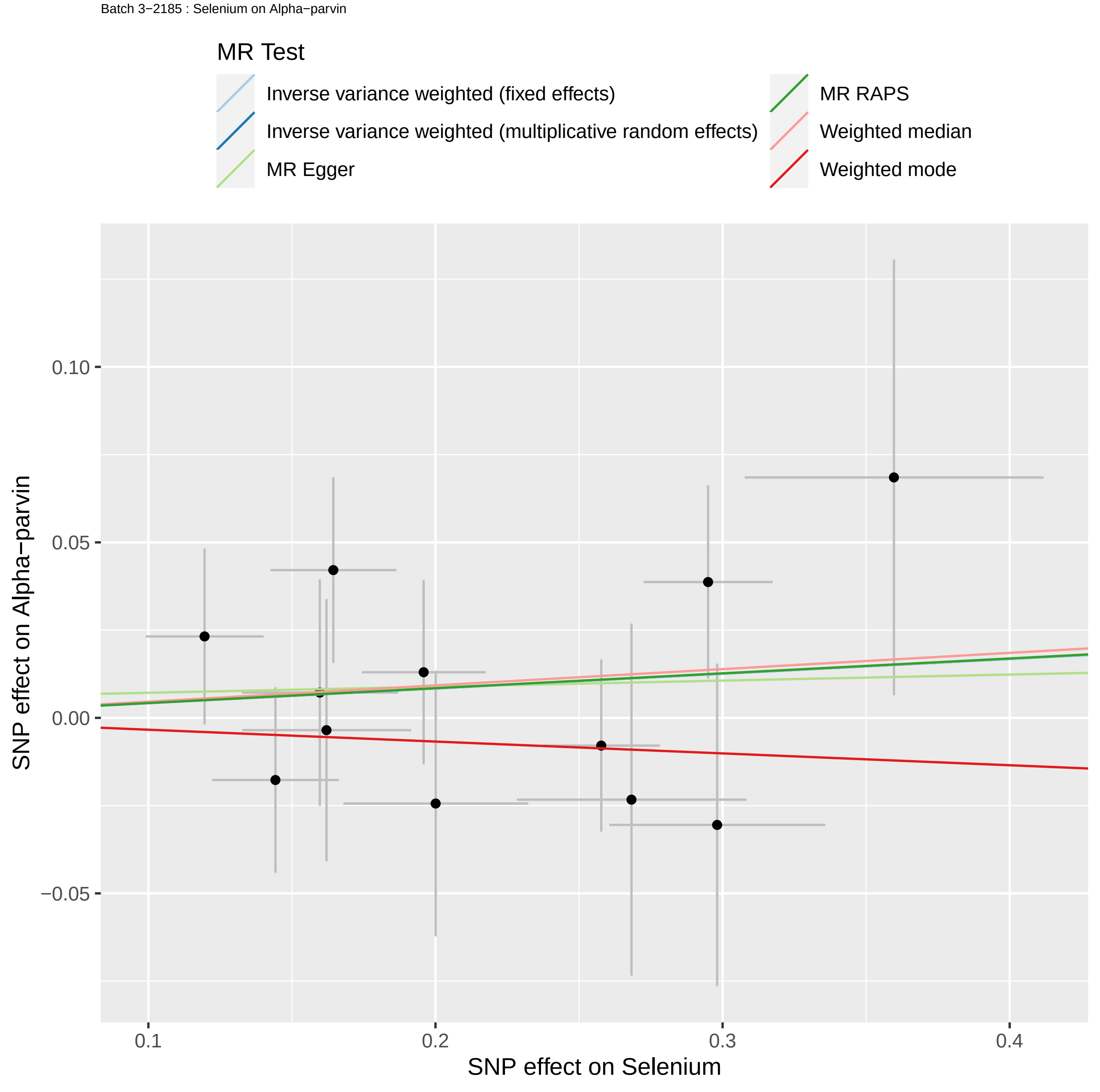
Batch 3−2185 : Selenium on Alpha−parvin
MR Test
Inverse variance weighted (fixed effects)
Inverse variance weighted (multiplicative random effects)
MR Egger
MR RAPS
Weighted median
Weighted mode
0.10
0.05
0.00
−0.05
0.1
0.2
0.3
0.4
SNP effect on Selenium
SNP effect on Alpha−parvin

Batch 3−2186 : Selenium on Prostate and testis expressed protein 1

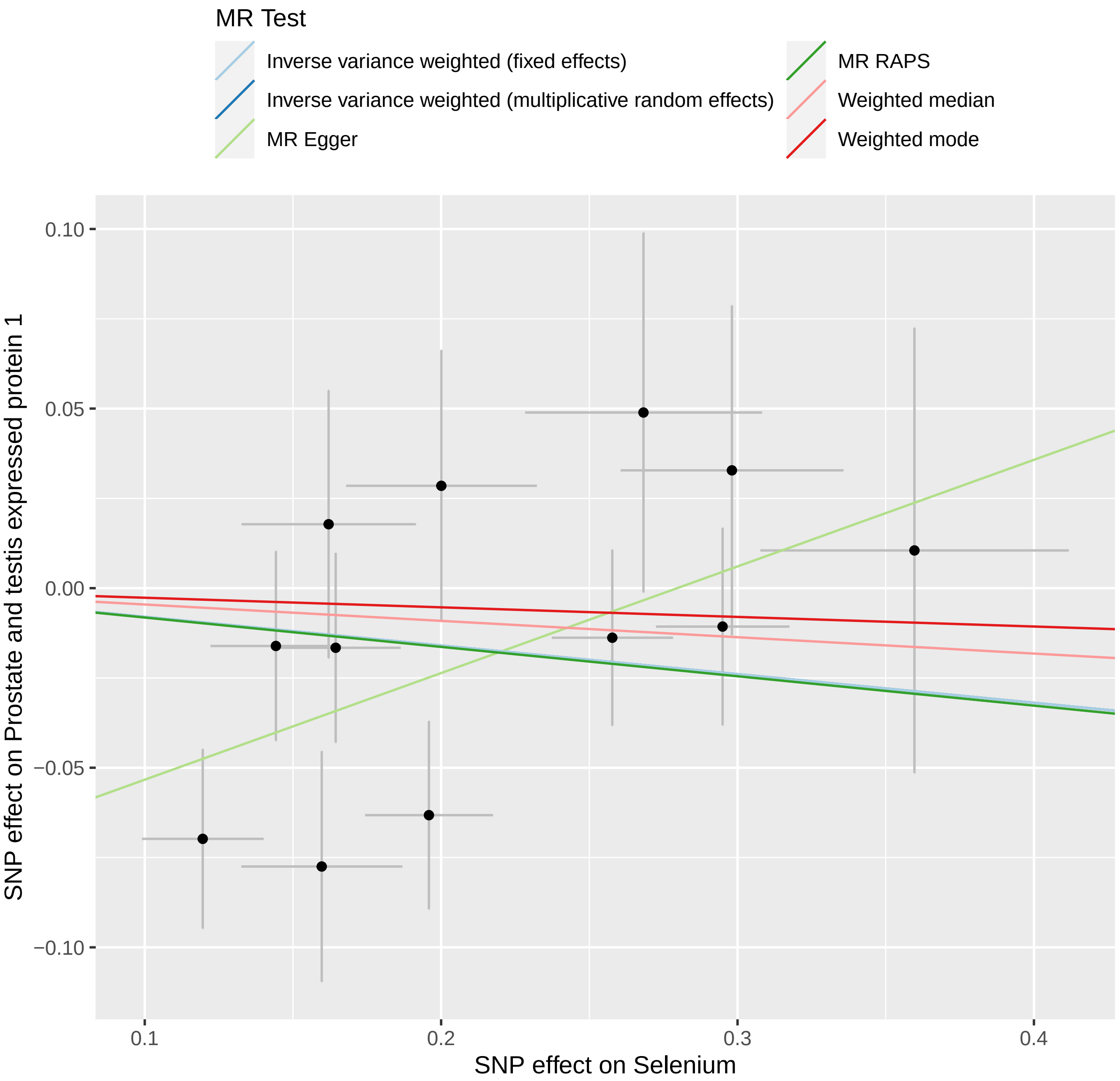

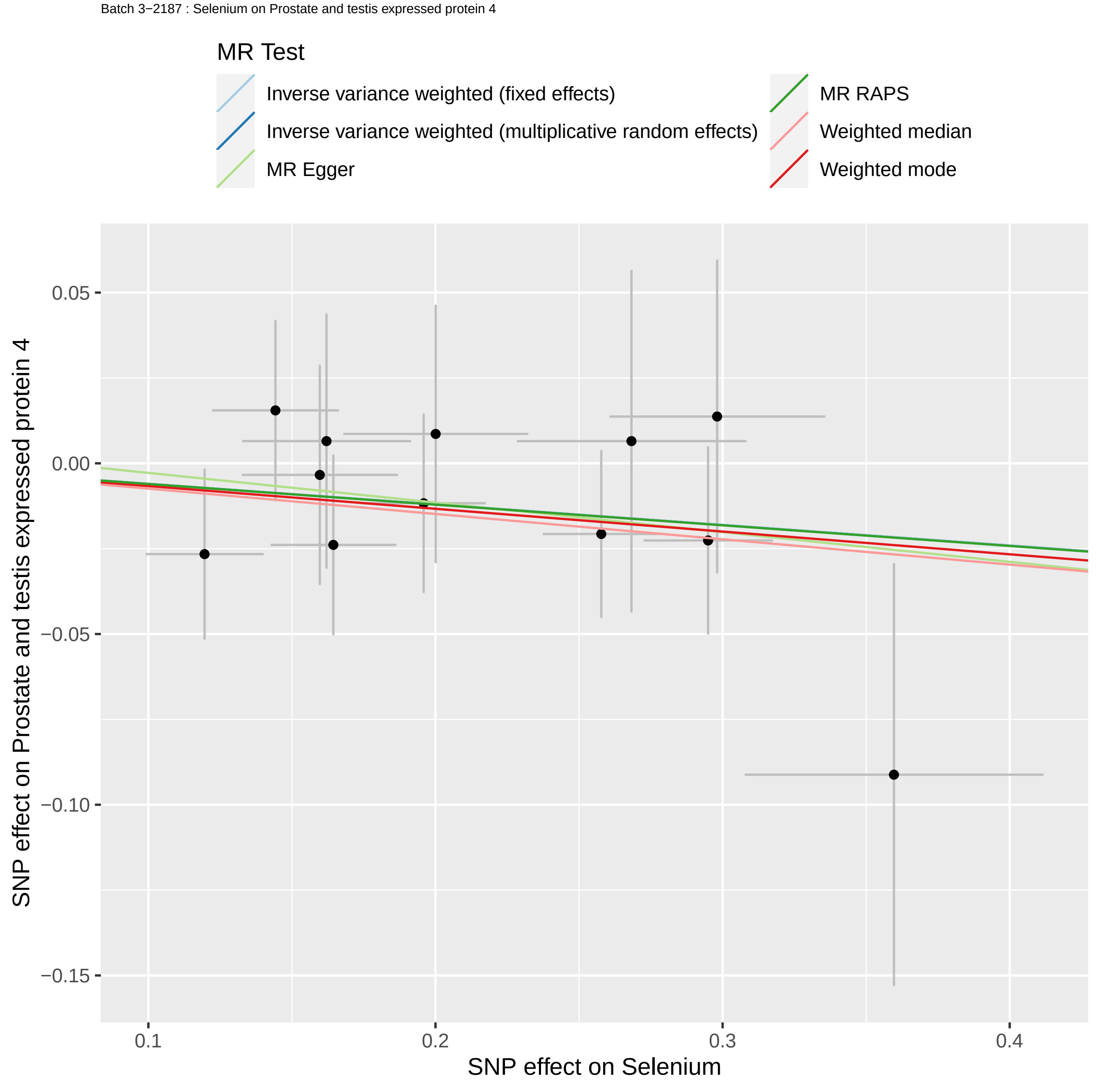

Batch 3−2187 : Selenium on Prostate and testis expressed protein 4
MR Test
Inverse variance weighted (fixed effects)
Inverse variance weighted (multiplicative random effects)
MR Egger
MR RAPS
Weighted median
Weighted mode
0.05
0.00
−0.05
−0.10
−0.15
0.1
0.2
0.3
0.4
SNP effect on Prostate and testis expressed protein 4
SNP effect on Selenium

Batch 3−2188 : Selenium on Paired box protein Pax−4

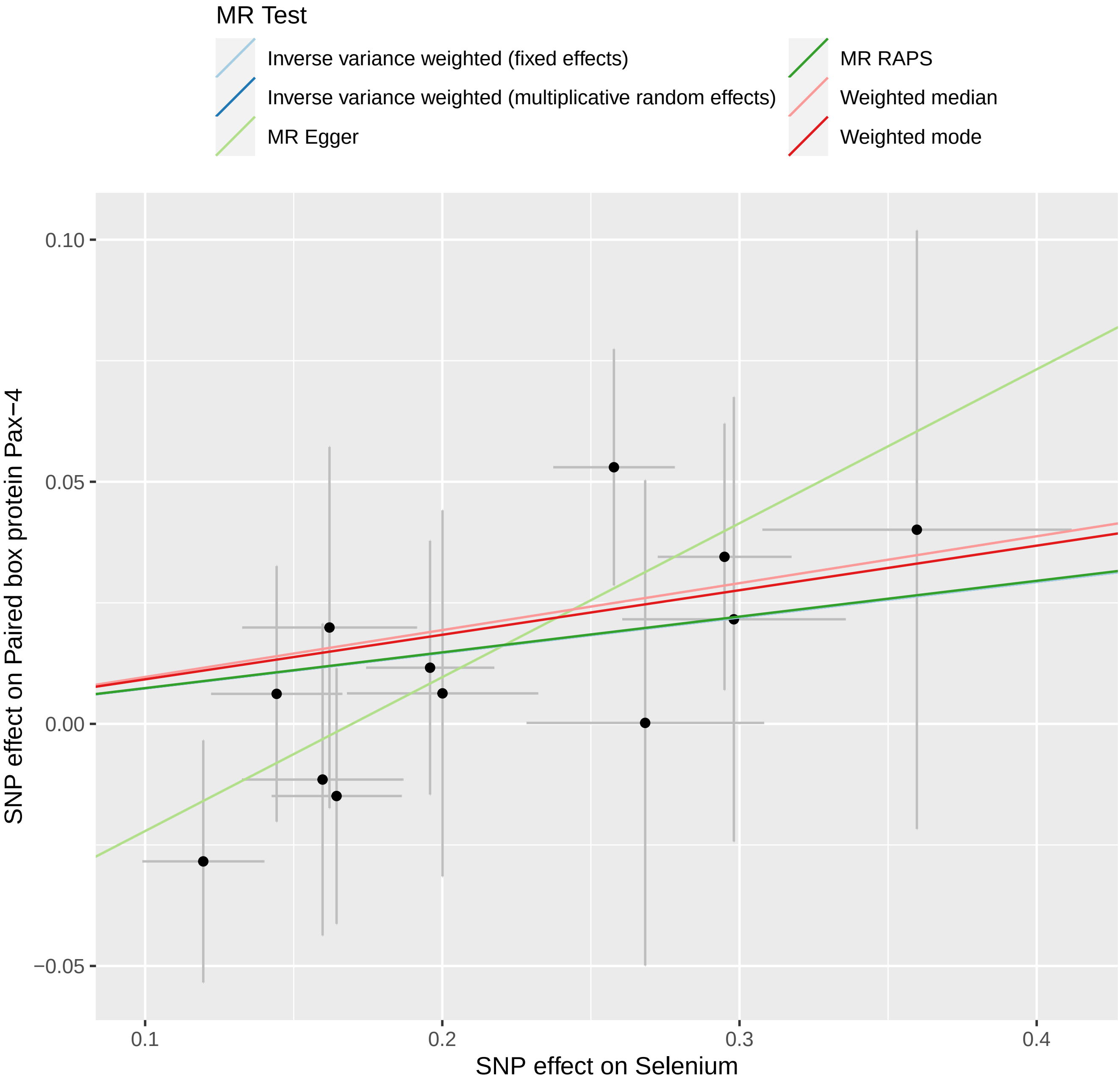

Batch 3−2189 : Selenium on PAX−interacting protein 1

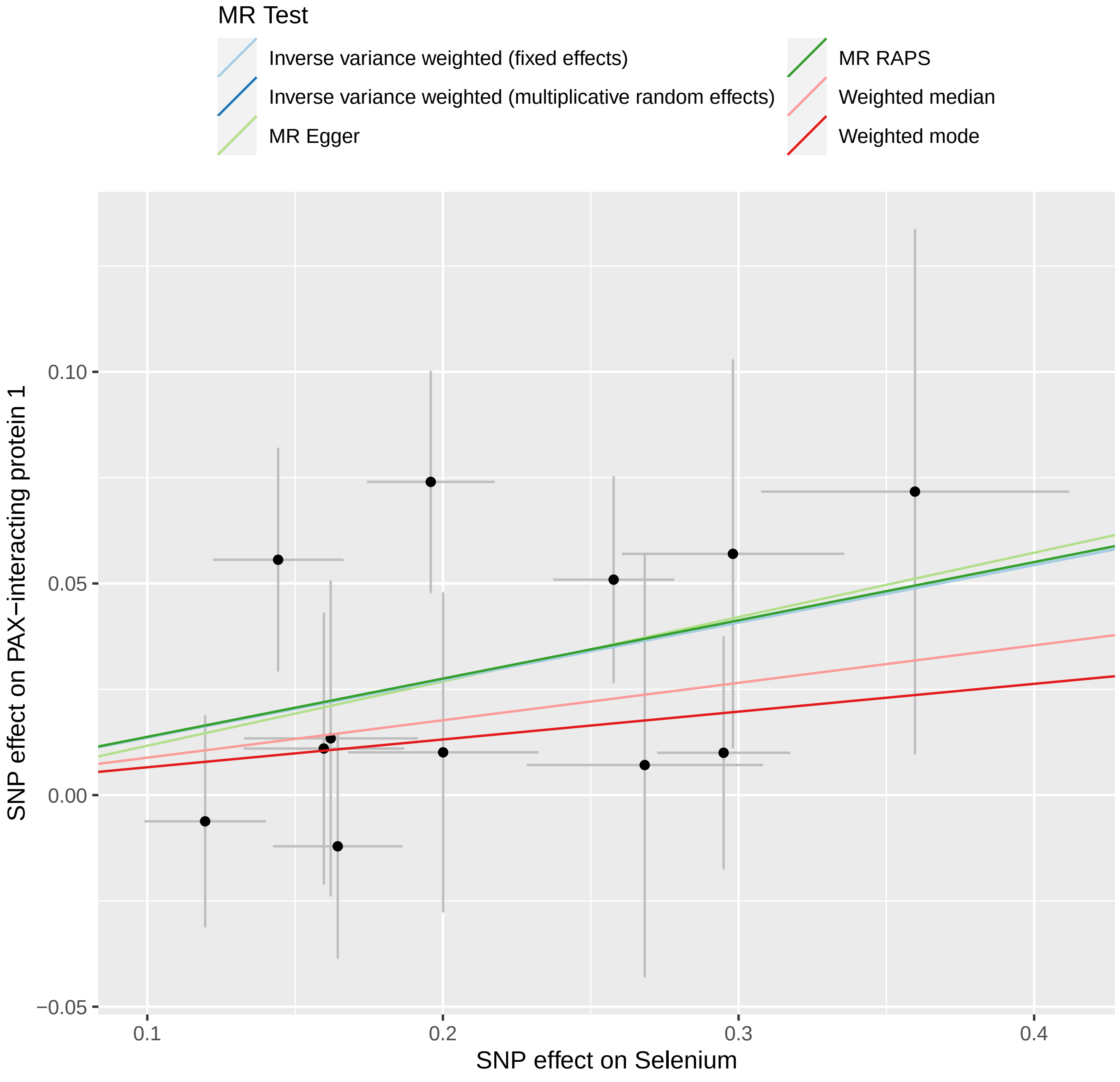

Batch 3−2190 : Selenium on Pyruvate carboxylase, mitochondrial

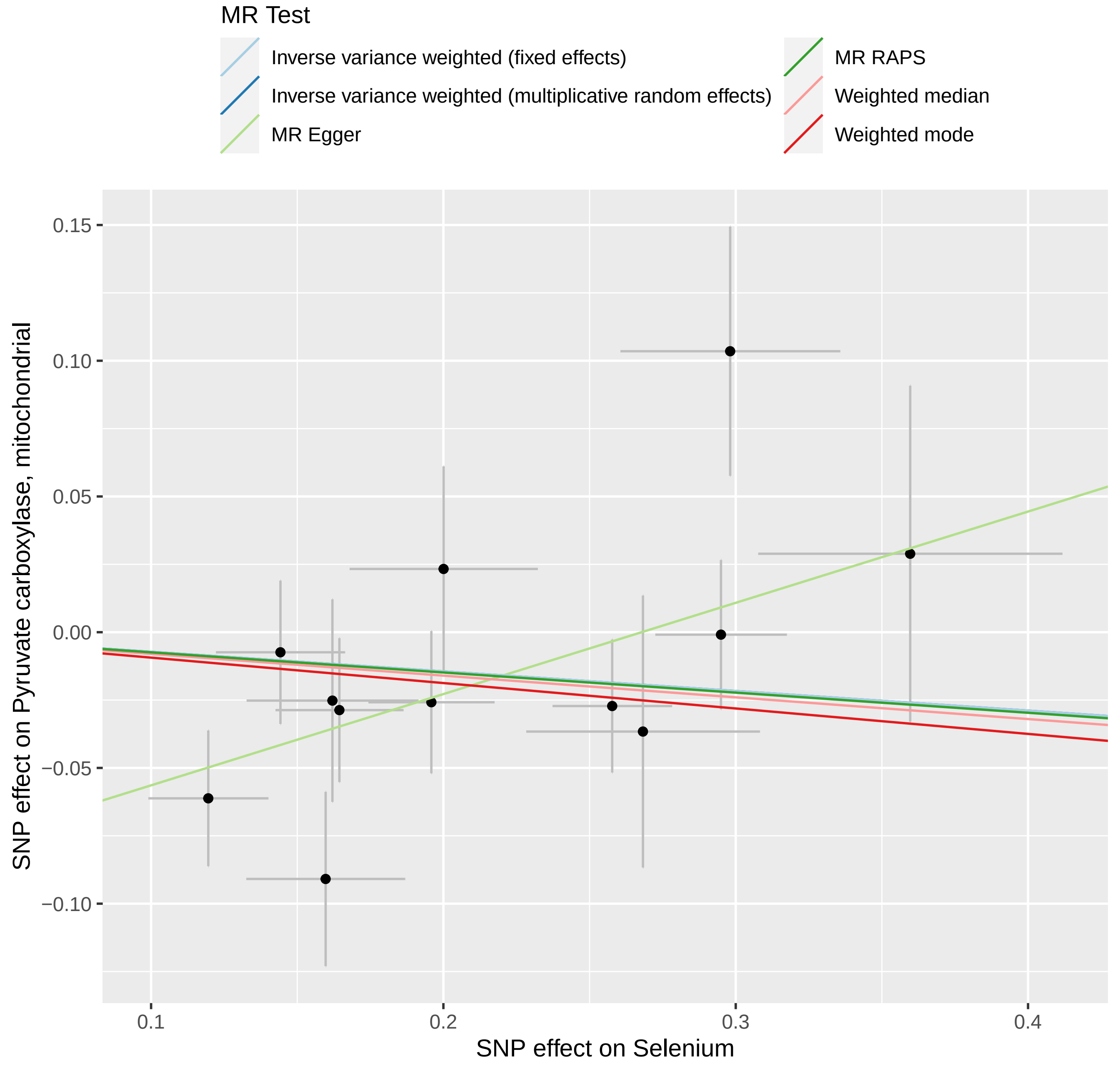

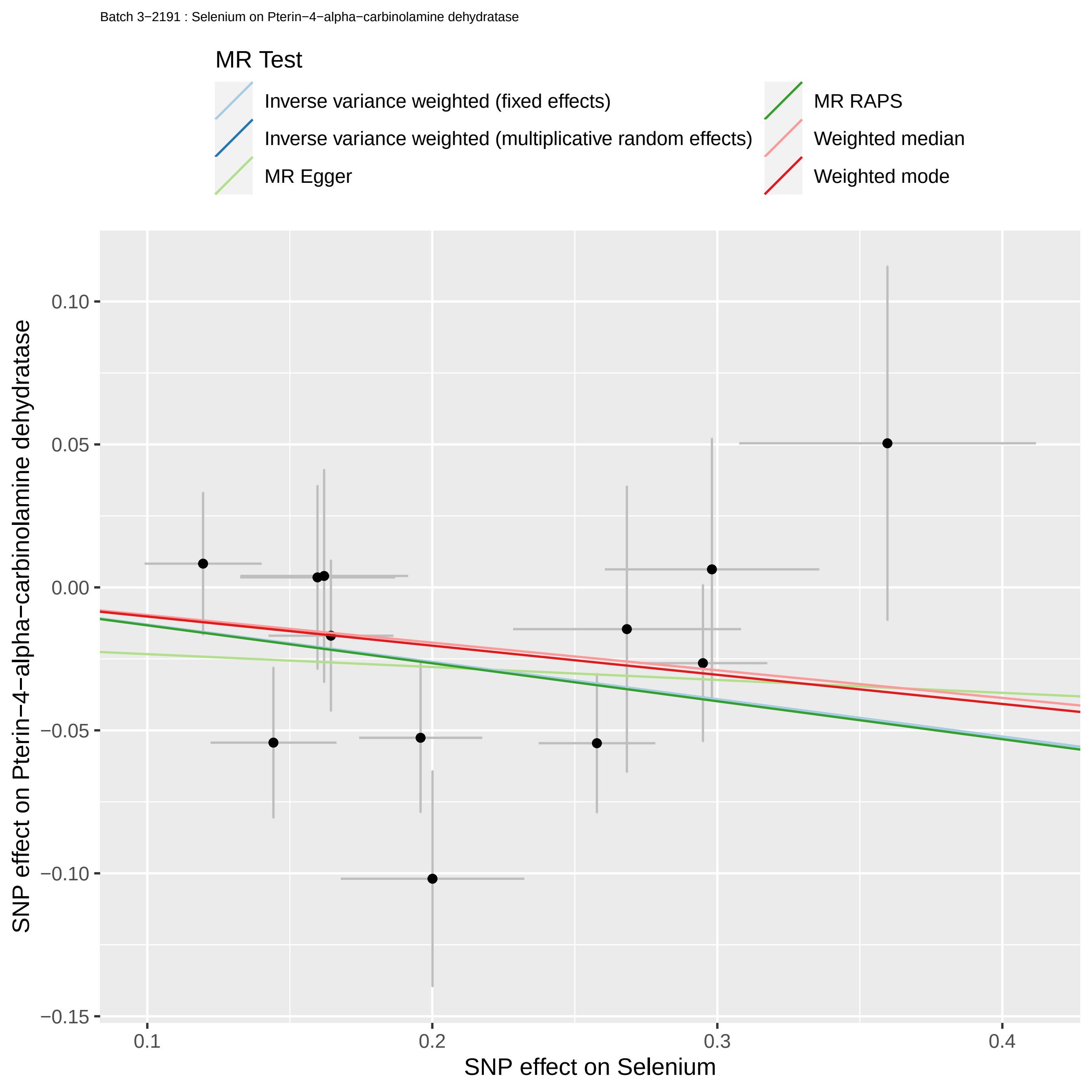
Batch 3−2191 : Selenium on Pterin−4−alpha−carbinolamine dehydratase
MR Test
Inverse variance weighted (fixed effects)
Inverse variance weighted (multiplicative random effects)
MR Egger
MR RAPS
Weighted median
Weighted mode
0.10
0.05
0.00
−0.05
−0.10
−0.15
0.1
0.2
0.3
0.4
SNP effect on Pterin−4−alpha−carbinolamine dehydratase
SNP effect on Selenium

Batch 3−2192 : Selenium on Pterin−4−alpha−carbinolamine dehydratase 2

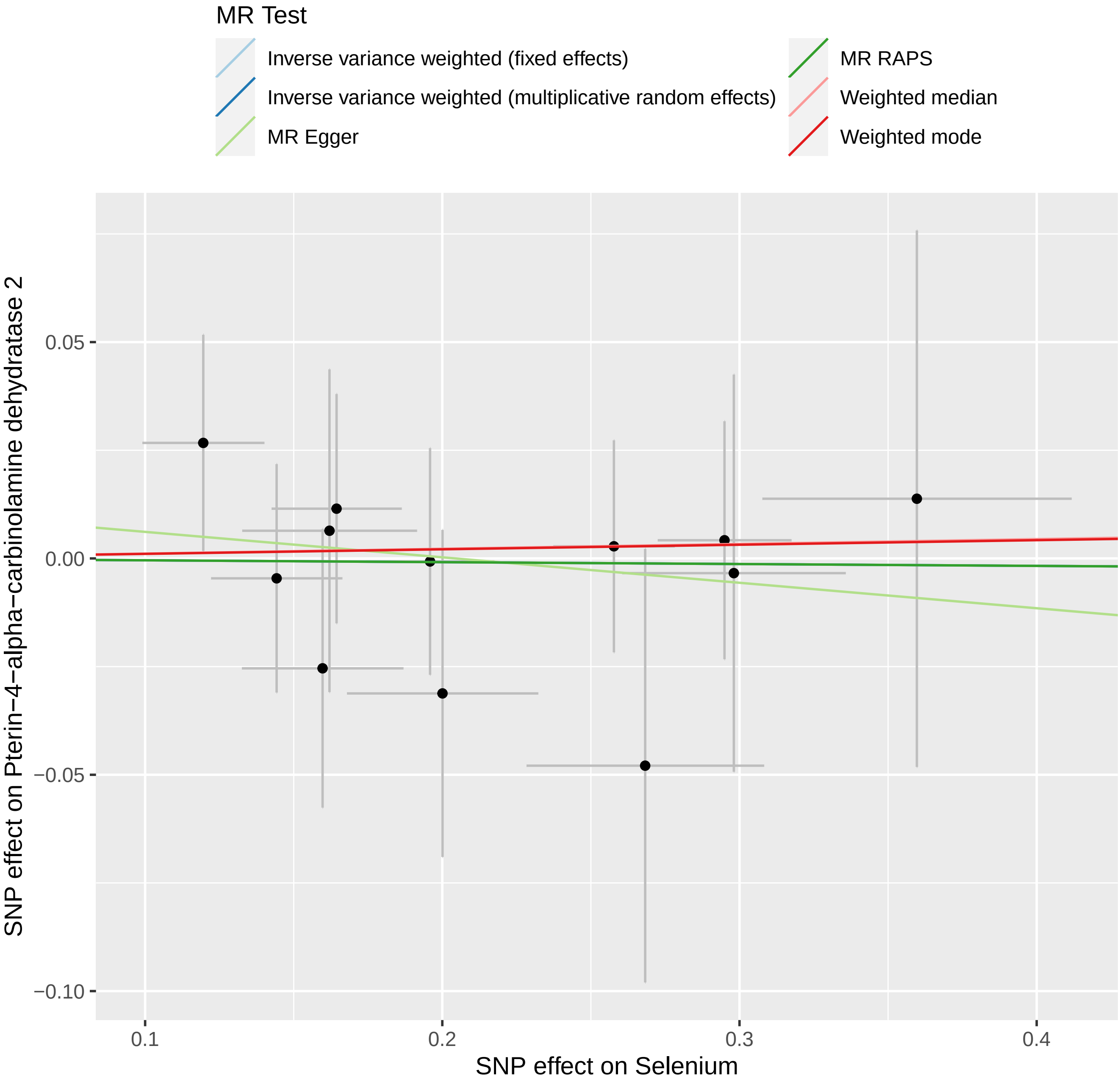

Batch 3−2193 : Selenium on Poly(rC)−binding protein 1

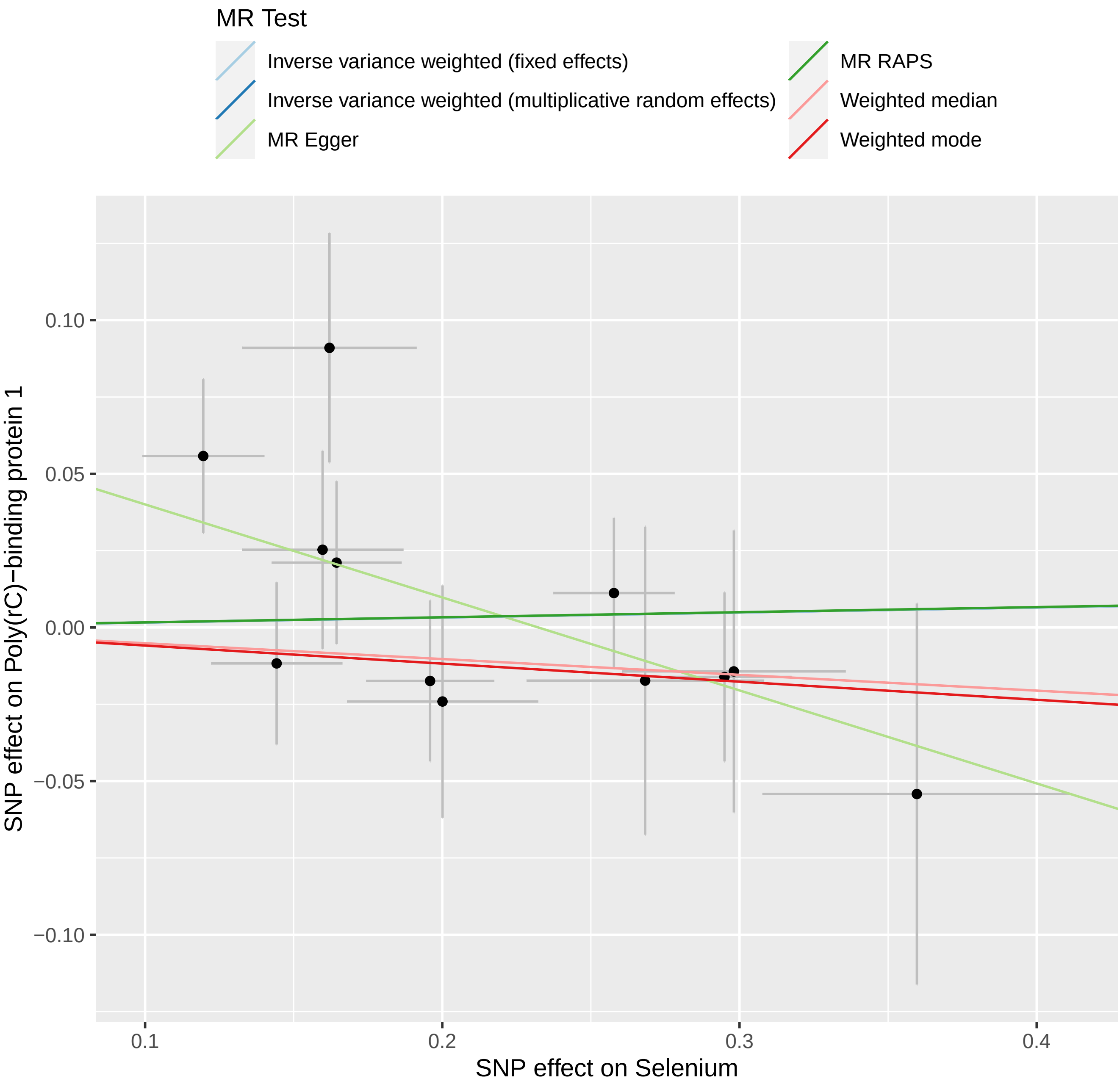

Batch 3−2194 : Selenium on Protocadherin−10

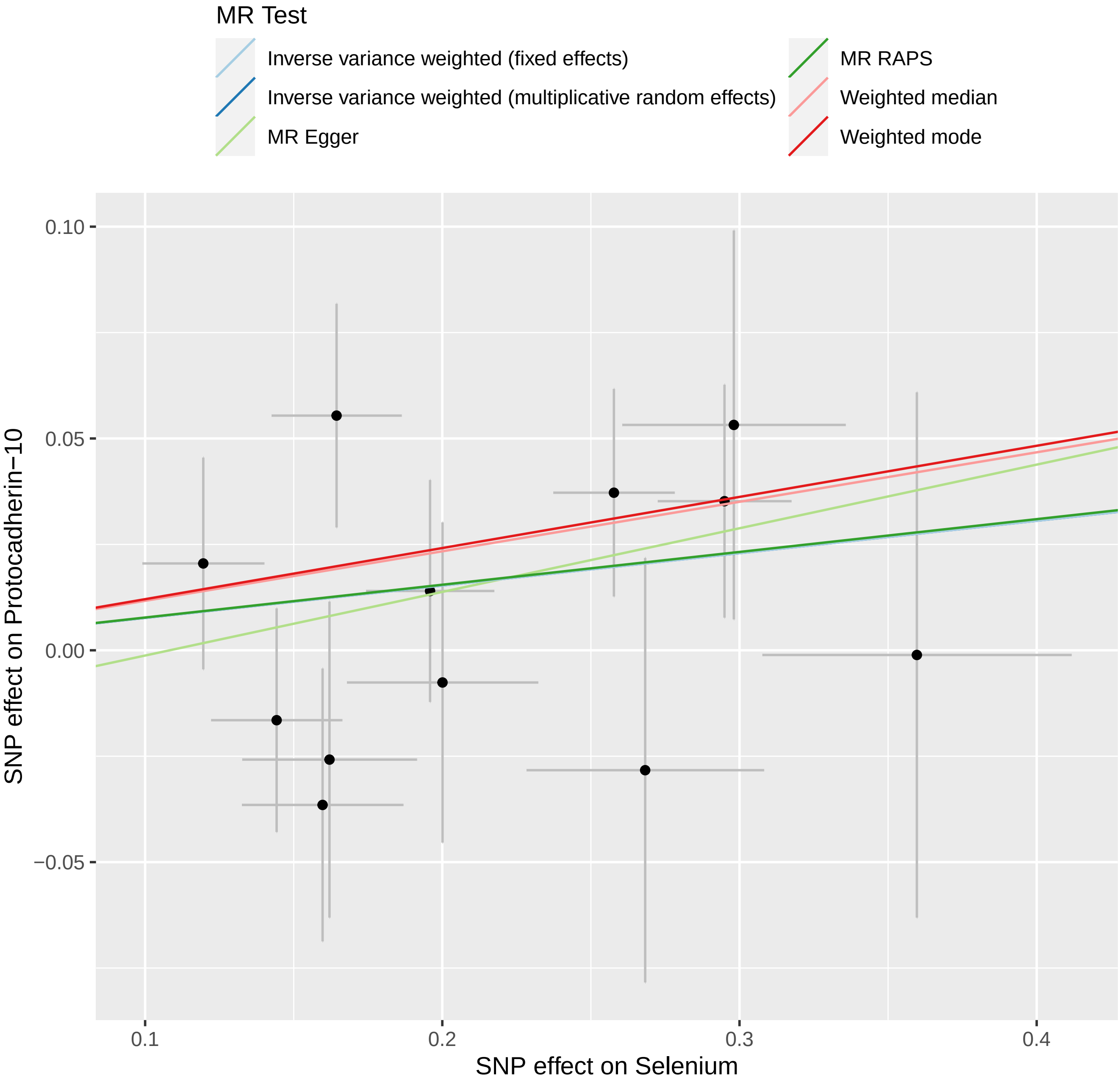

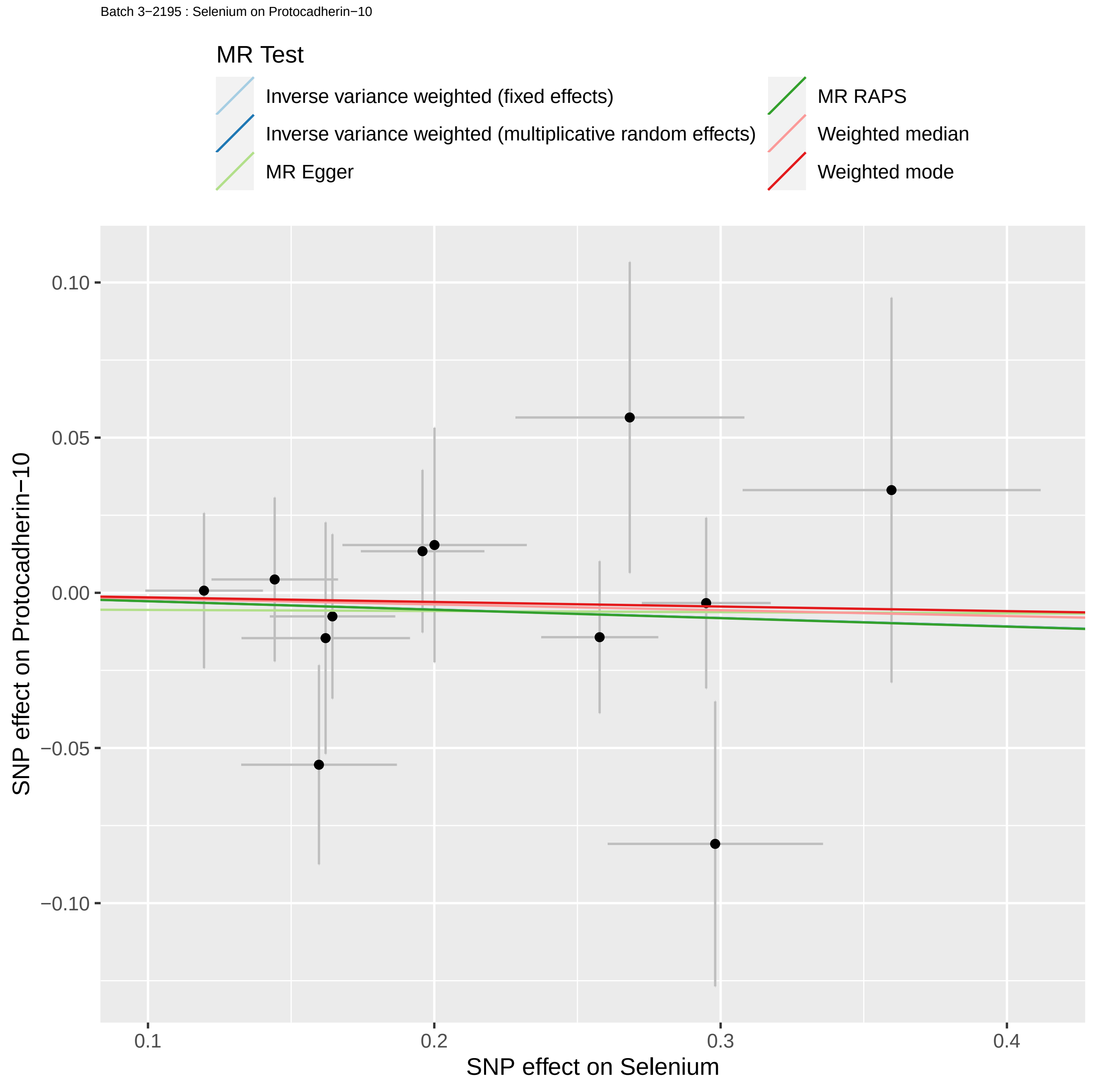
Batch 3−2195 : Selenium on Protocadherin−10
MR Test
Inverse variance weighted (fixed effects)
Inverse variance weighted (multiplicative random effects)
MR Egger
MR RAPS
Weighted median
Weighted mode
0.10
0.05
0.00
−0.05
−0.10
SNP effect on Protocadherin−10
0.1
0.2
0.3
0.4
SNP effect on Selenium

Batch 3−2196 : Selenium on Protocadherin−15

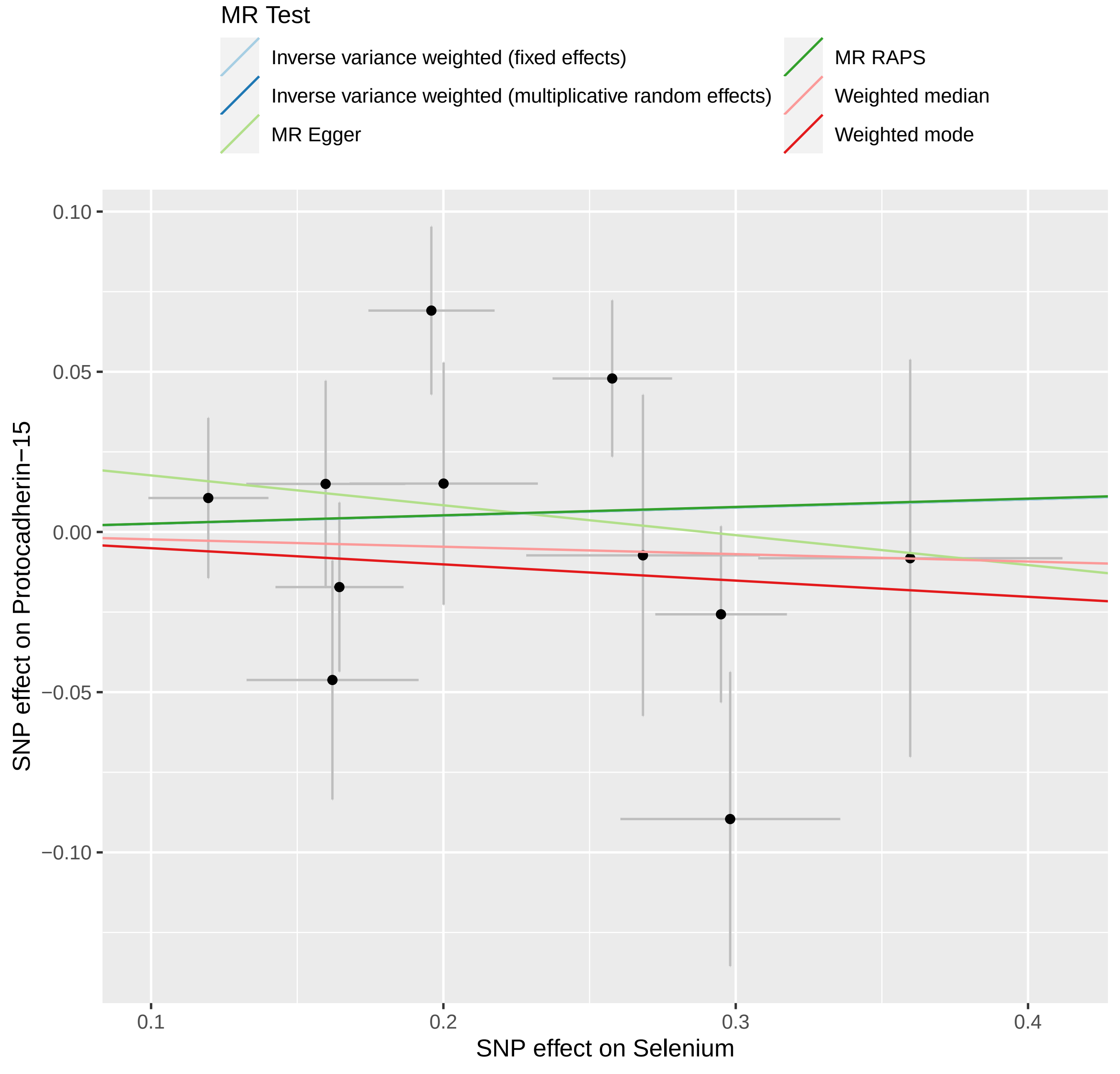

Batch 3−2197 : Selenium on Protocadherin−9

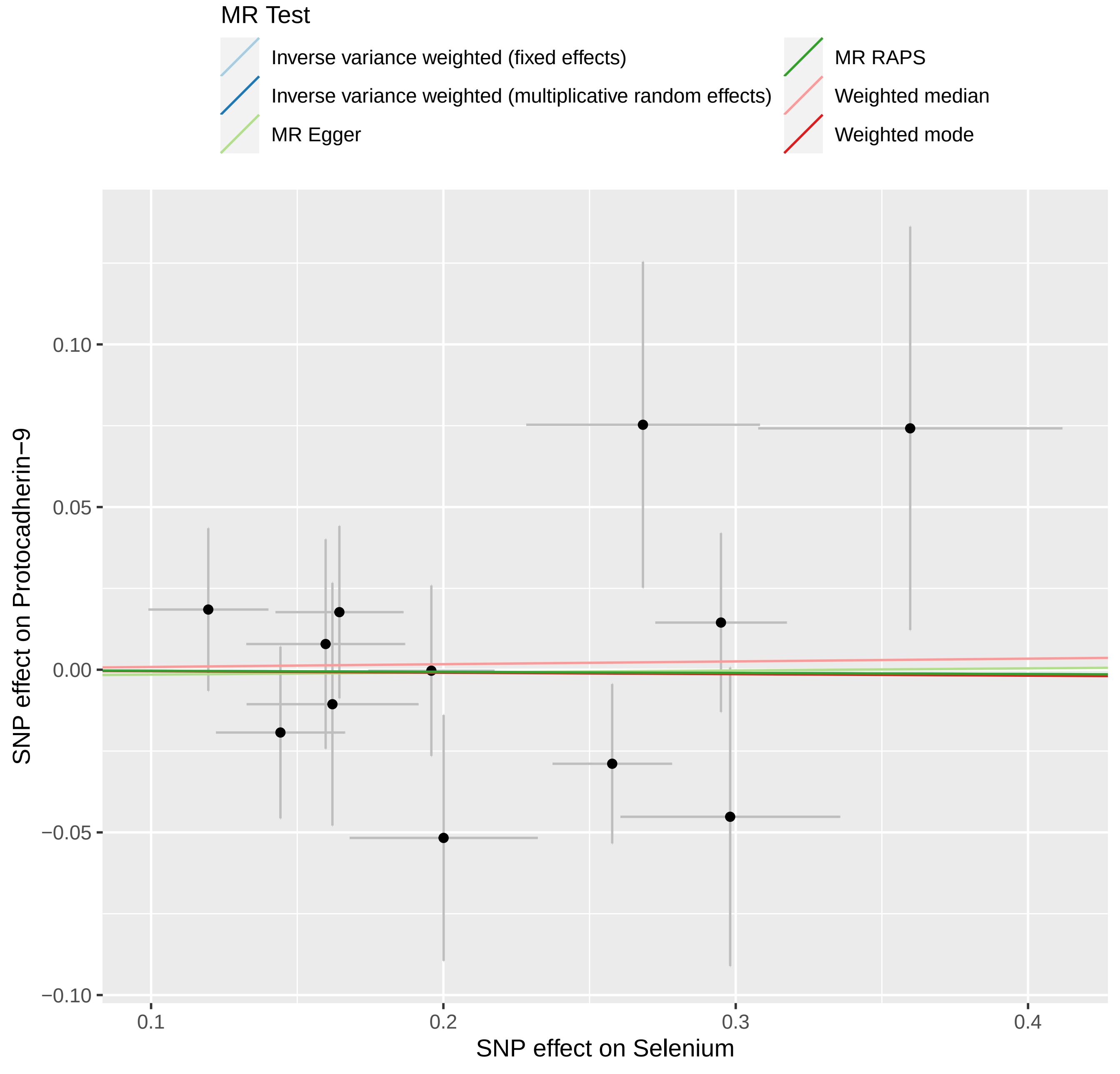

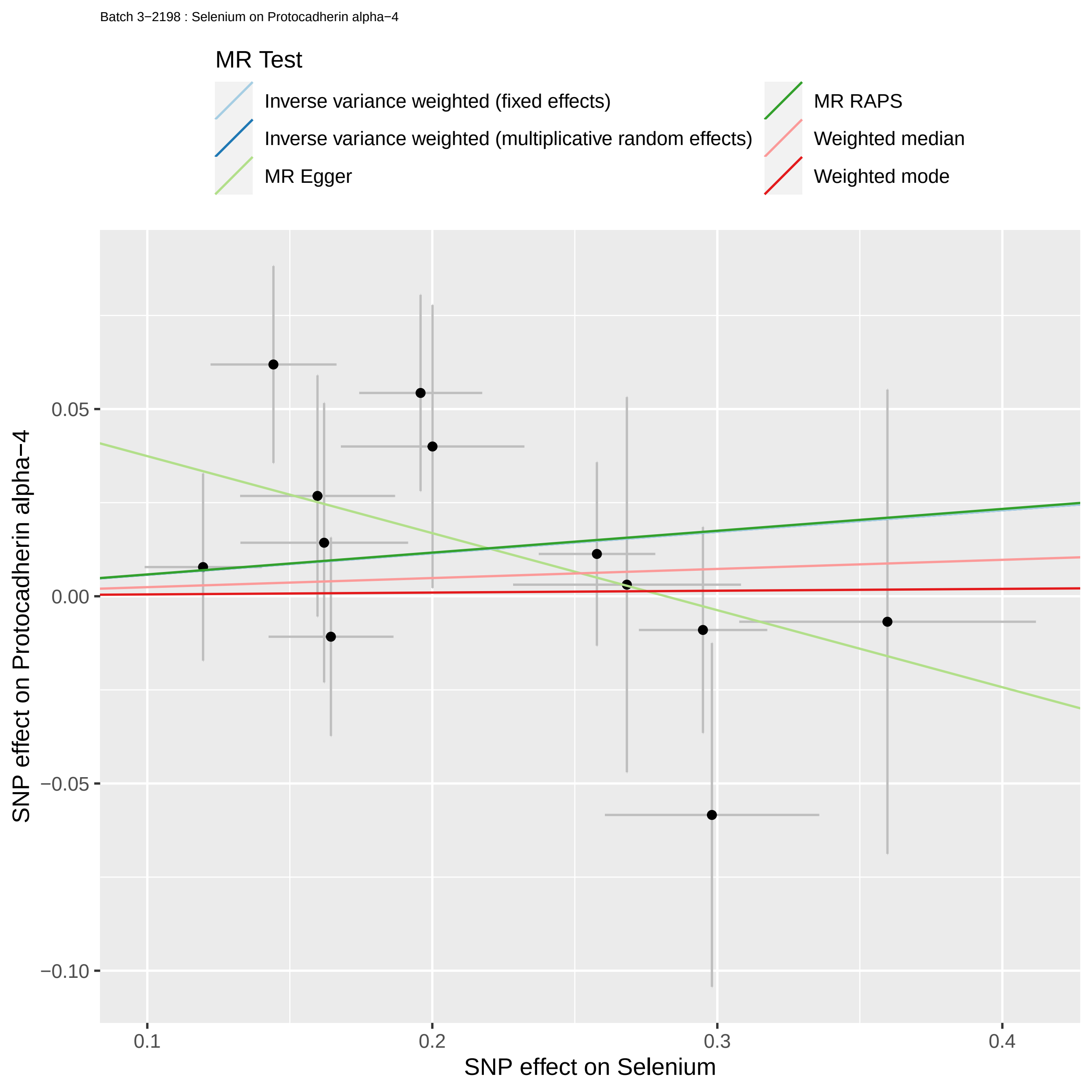
Batch 3−2198 : Selenium on Protocadherin alpha−4
MR Test
Inverse variance weighted (fixed effects)
Inverse variance weighted (multiplicative random effects)
MR Egger
MR RAPS
Weighted median
Weighted mode
0.05
0.00
−0.05
−0.10
0.1
0.2
0.3
0.4
SNP effect on Protocadherin alpha−4
SNP effect on Selenium

Batch 3−2199 : Selenium on Protocadherin alpha−4

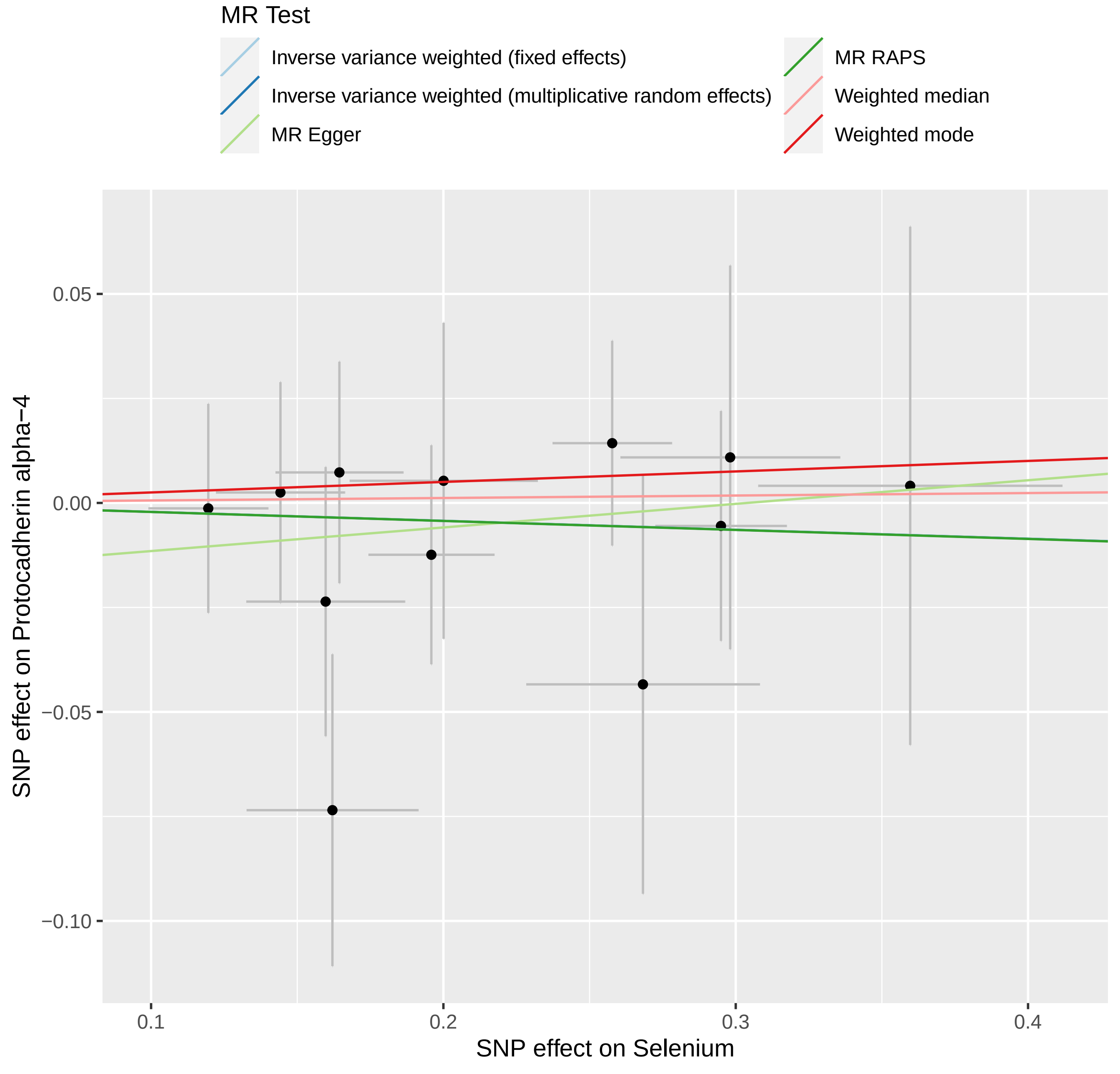

Batch 3−2200 : Selenium on Protocadherin alpha−7

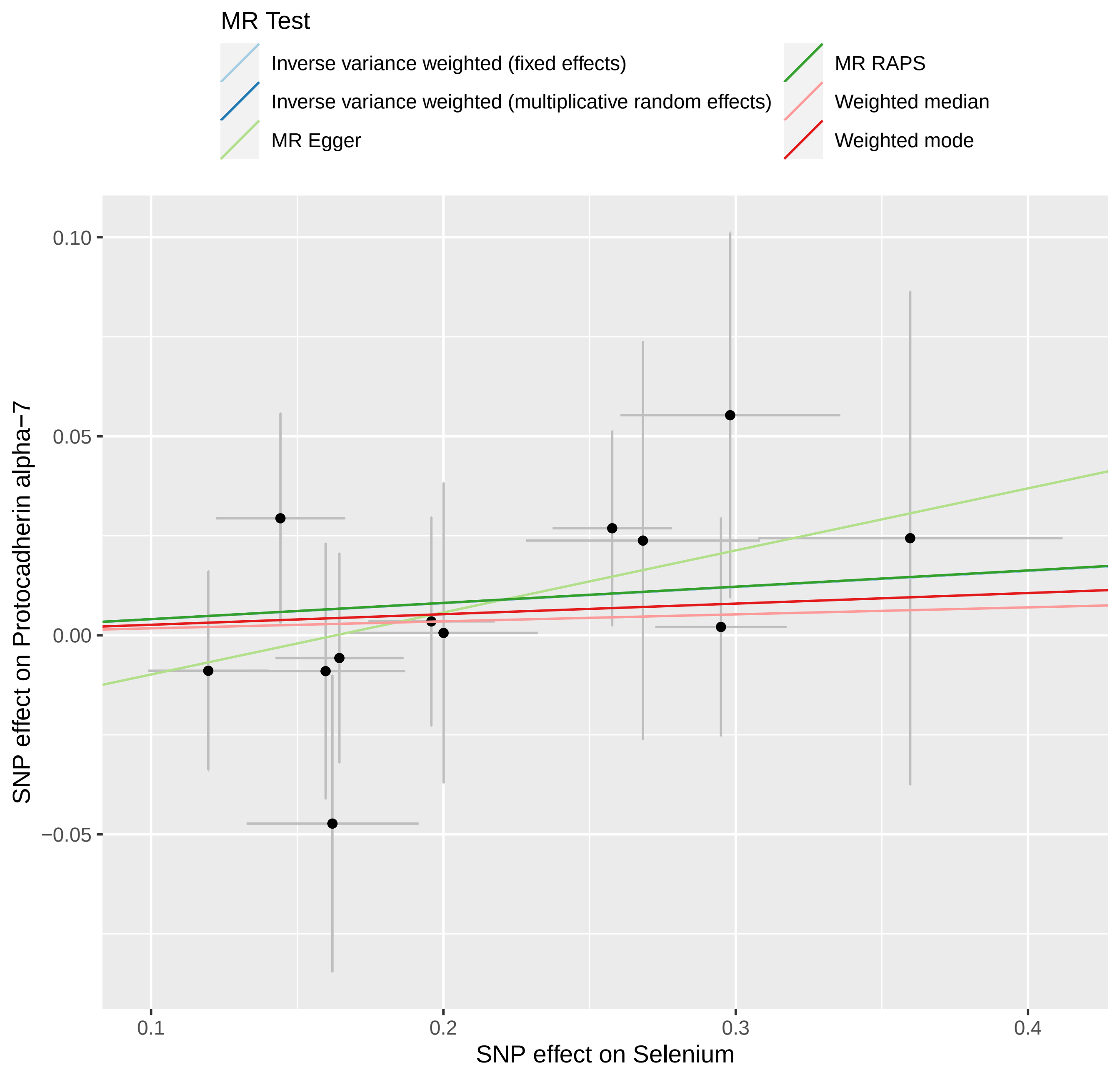

Batch 3−2201 : Selenium on Protocadherin alpha−C2

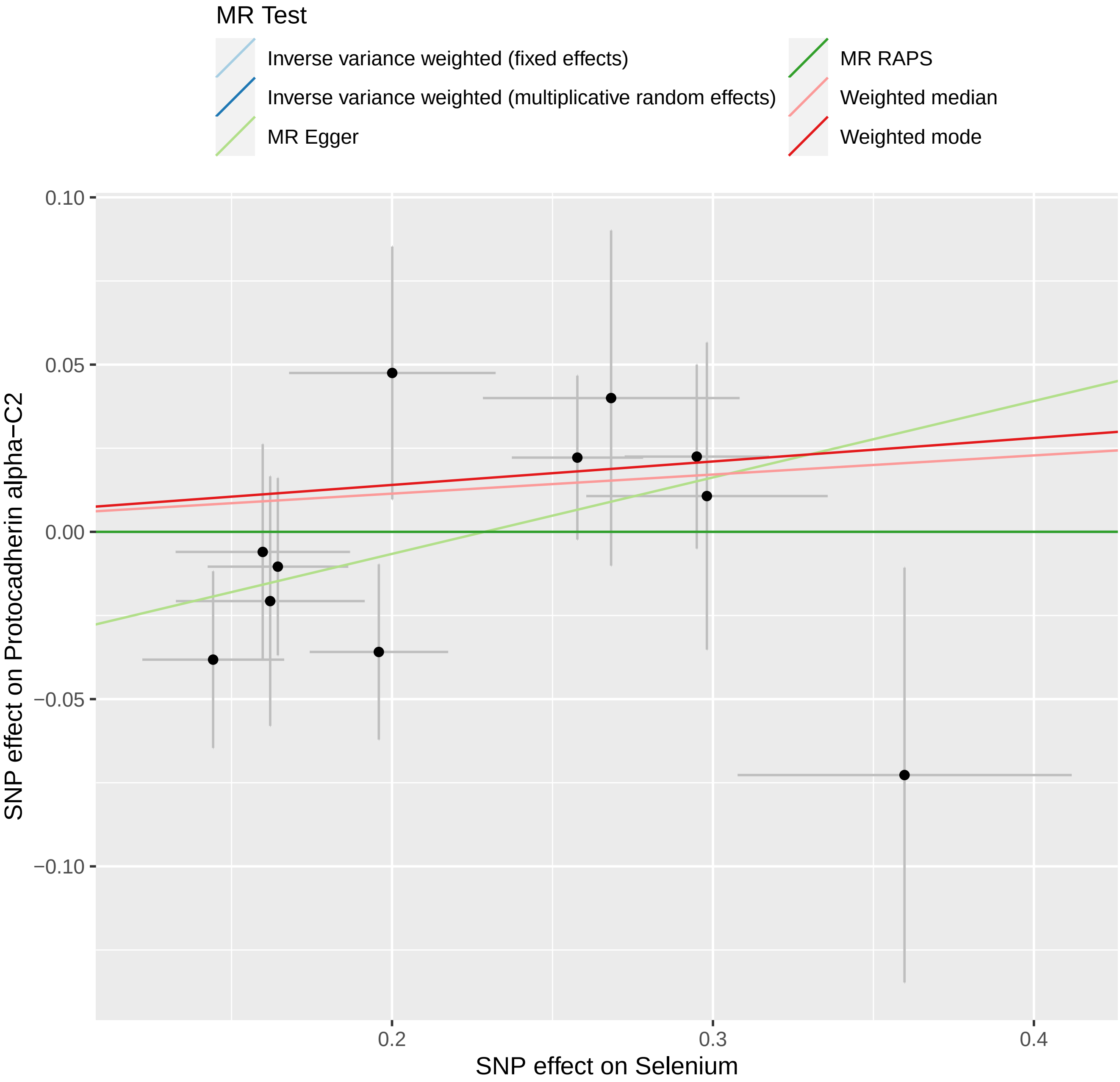

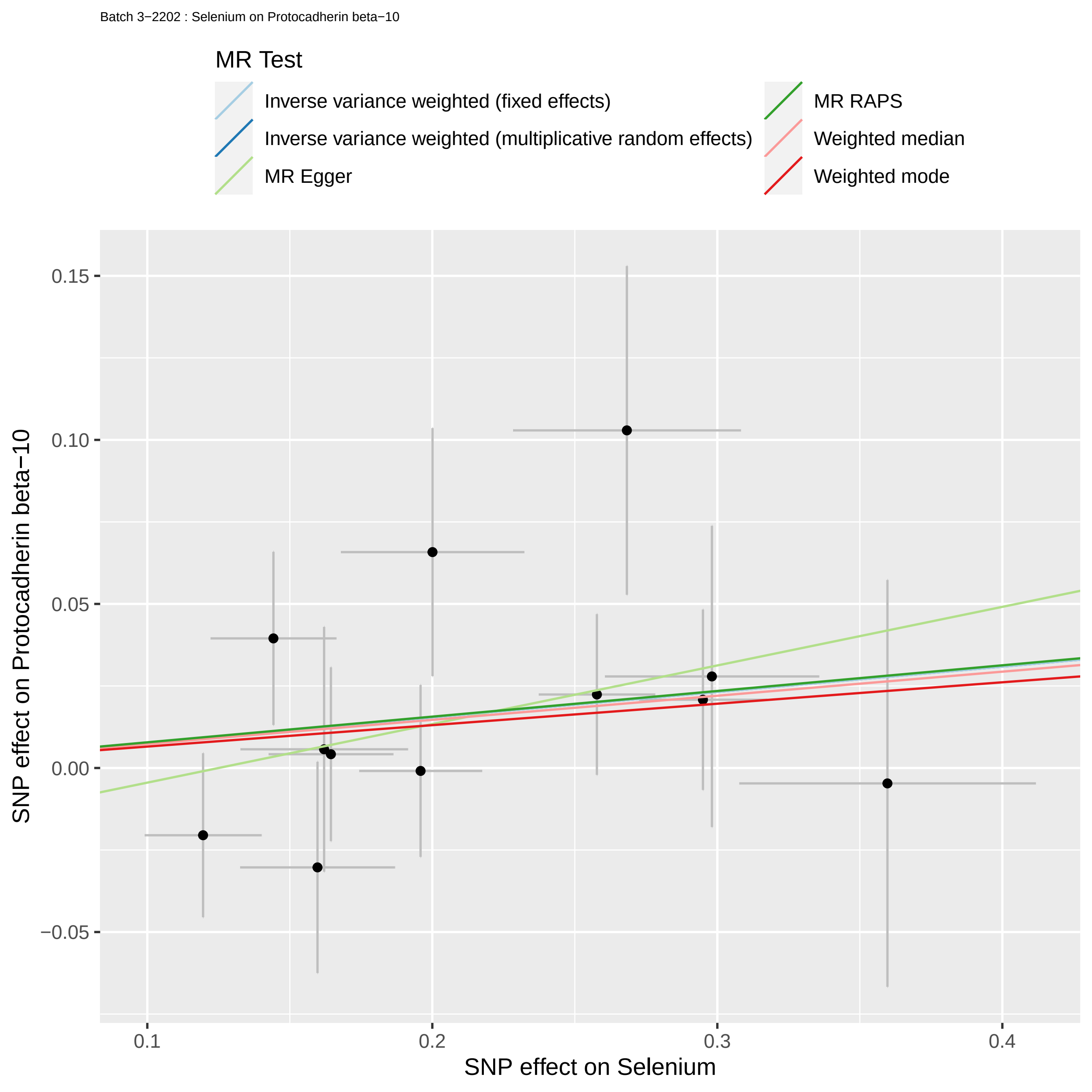
Batch 3−2202 : Selenium on Protocadherin beta−10
MR Test
Inverse variance weighted (fixed effects)
Inverse variance weighted (multiplicative random effects)
MR Egger
MR RAPS
Weighted median
Weighted mode
0.15
0.10
0.05
0.00
−0.05
0.1
0.2
0.3
0.4
SNP effect on Selenium
SNP effect on Protocadherin beta−10

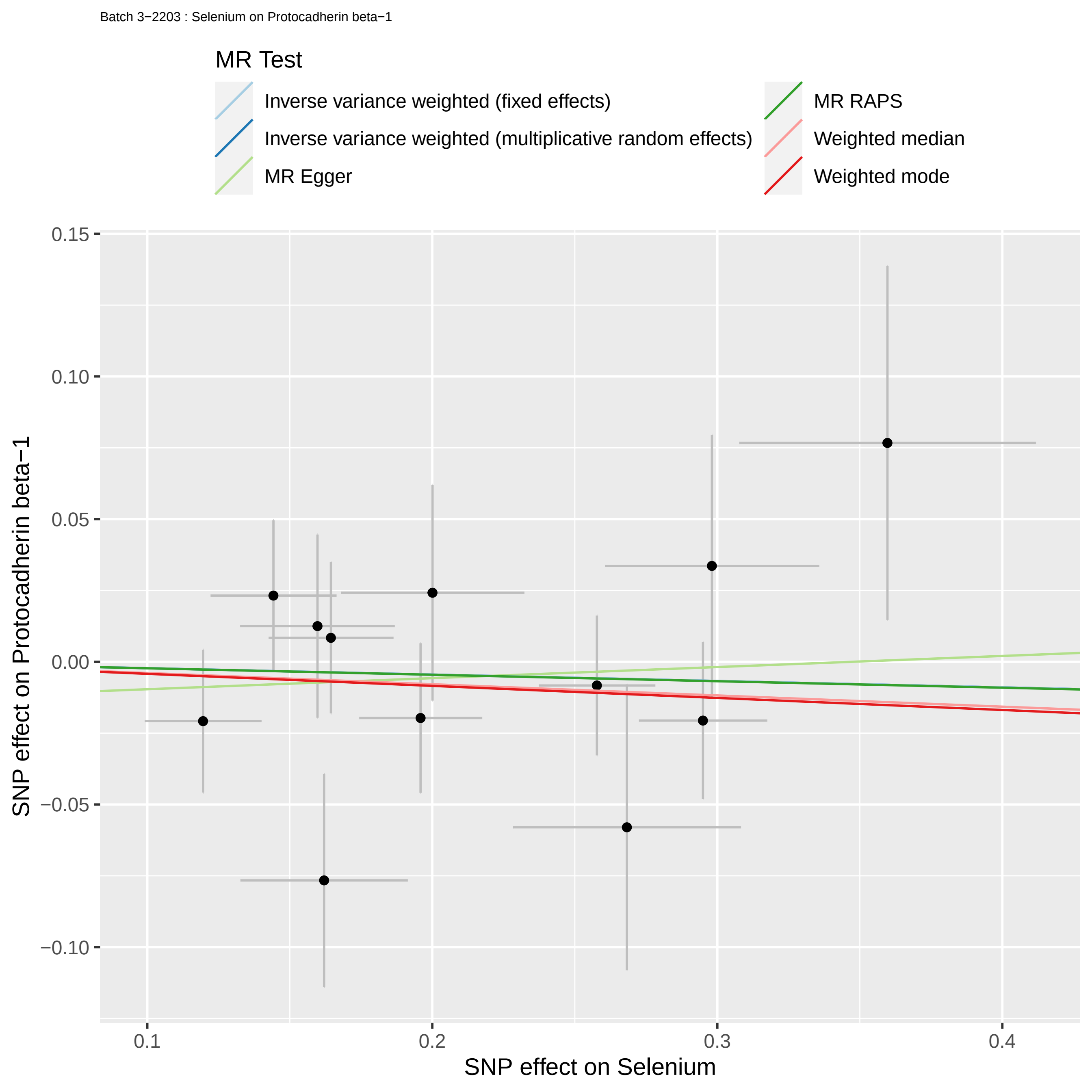

Batch 3−2203 : Selenium on Protocadherin beta−1
MR Test
Inverse variance weighted (fixed effects)
Inverse variance weighted (multiplicative random effects)
MR Egger
MR RAPS
Weighted median
Weighted mode
0.15
0.10
0.05
0.00
−0.05
−0.10
SNP effect on Protocadherin beta−1
0.1
0.2
0.3
0.4
SNP effect on Selenium

Batch 3−2204 : Selenium on Protocadherin beta−2

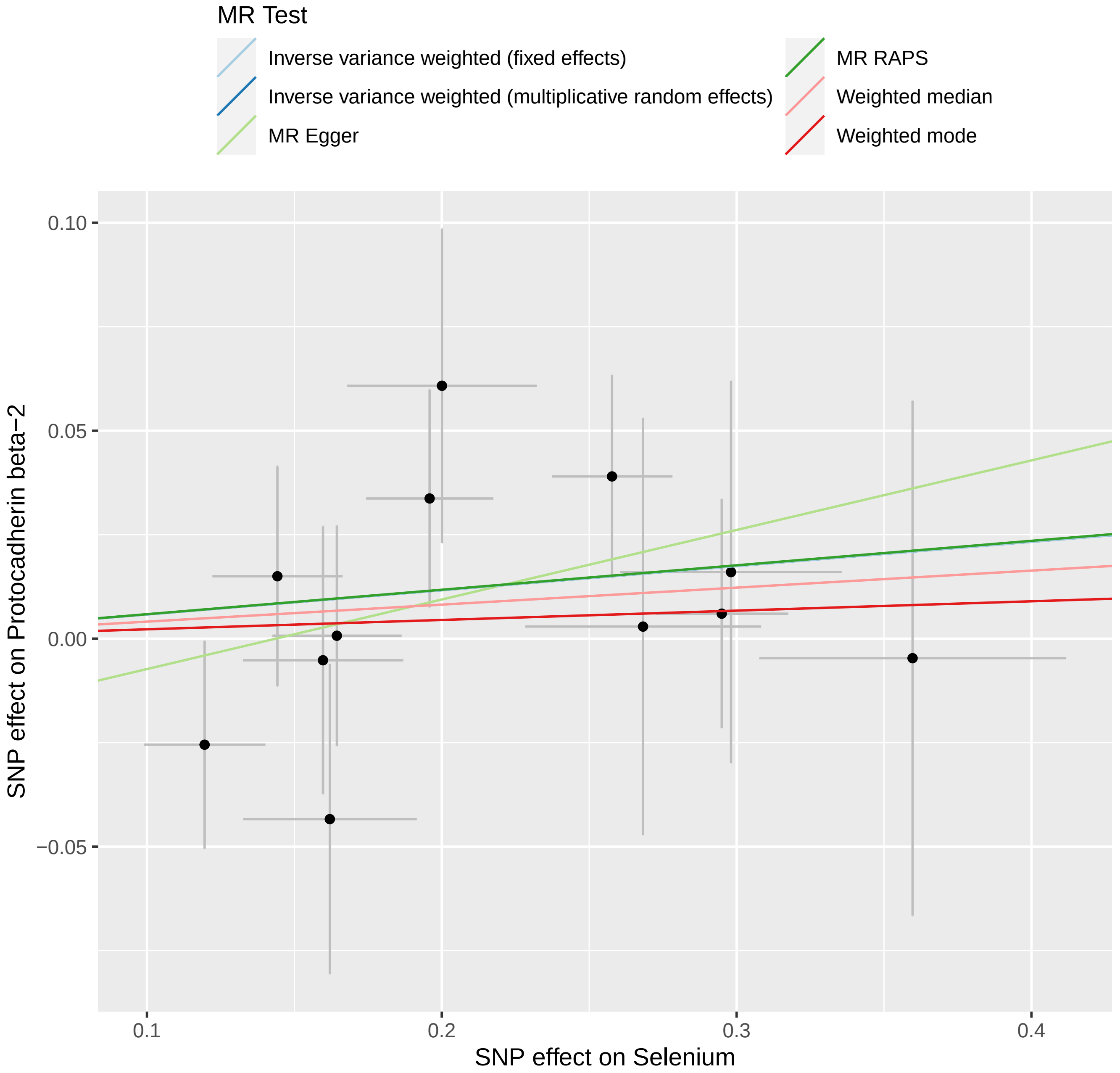

Batch 3−2205 : Selenium on Protocadherin beta−4

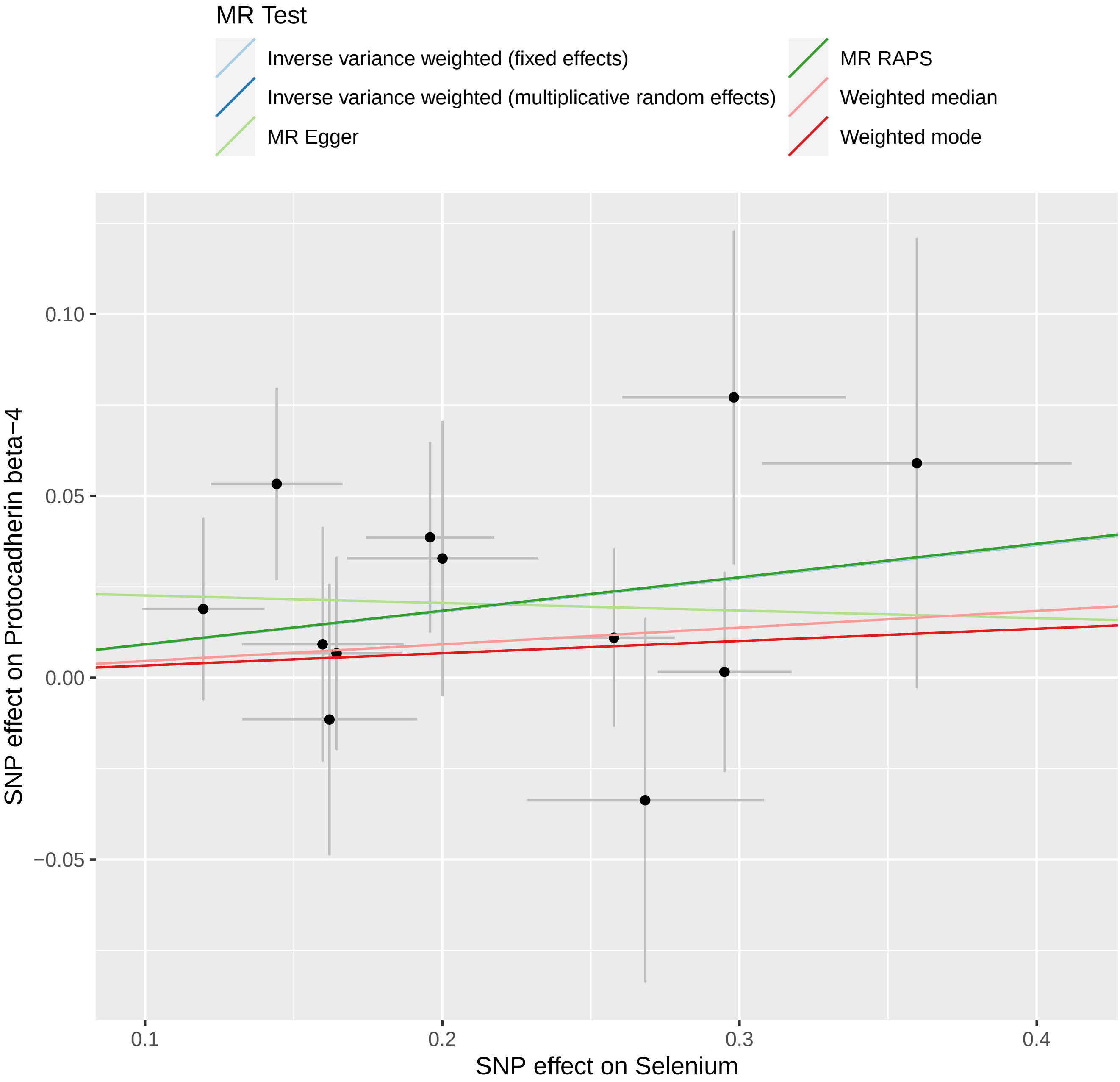

Batch 3−2206 : Selenium on Protocadherin gamma−A8

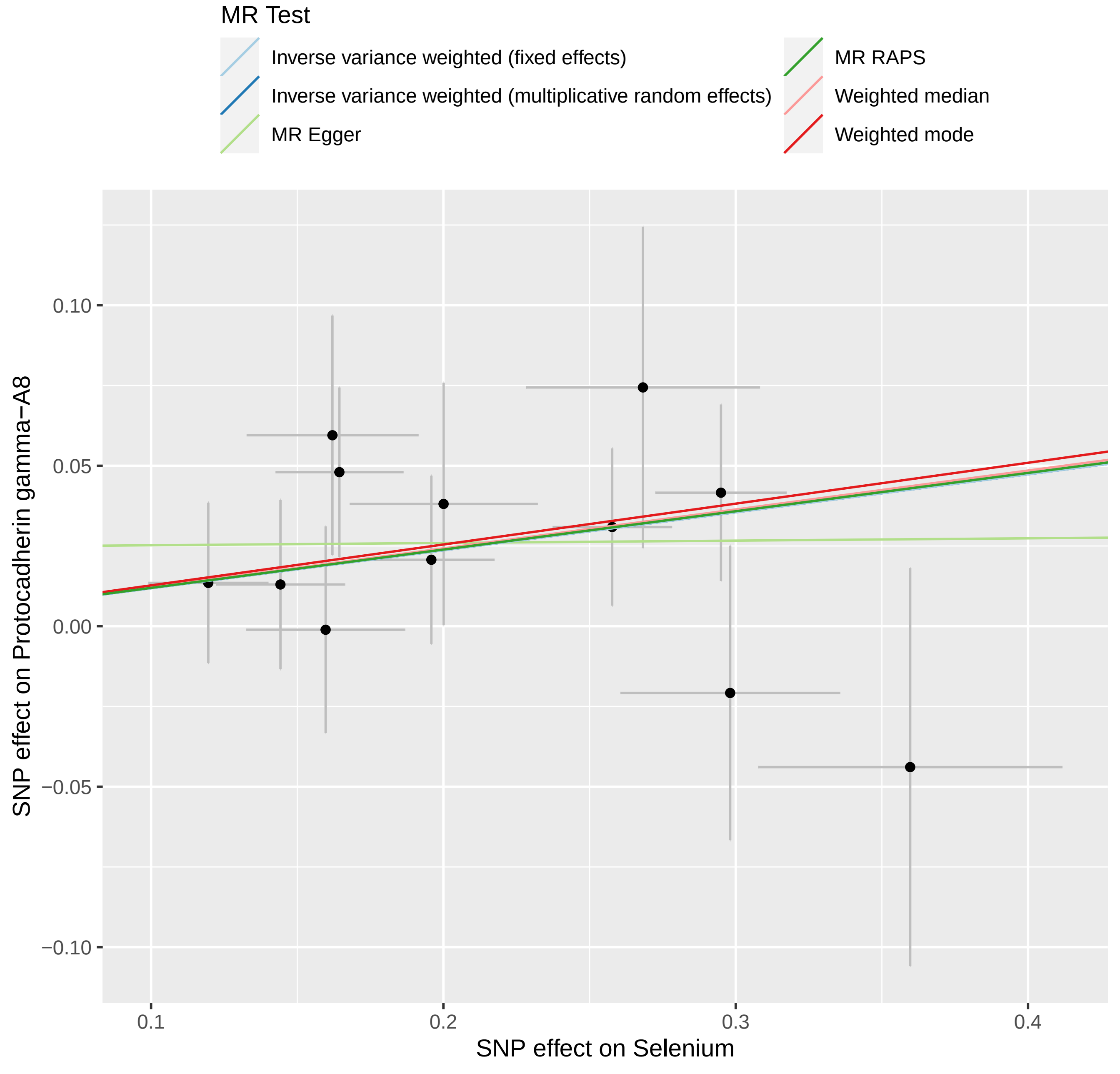

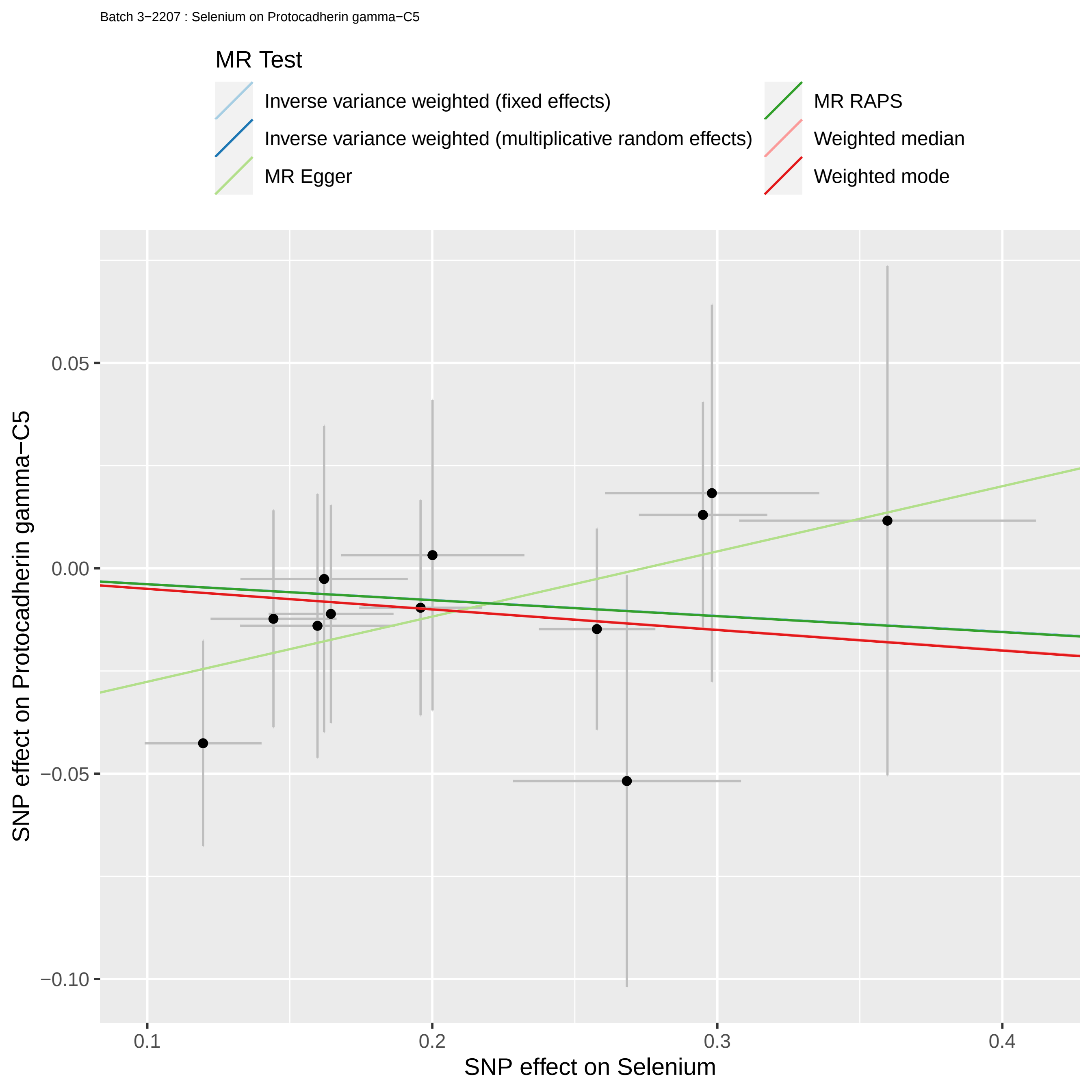

Batch 3−2207 : Selenium on Protocadherin gamma−C5
MR Test
Inverse variance weighted (fixed effects)
Inverse variance weighted (multiplicative random effects)
MR Egger
MR RAPS
Weighted median
Weighted mode
0.05
0.00
−0.05
−0.10
0.1
0.2
0.3
0.4
SNP effect on Protocadherin gamma−C5
SNP effect on Selenium

Batch 3−2208 : Selenium on Proliferating cell nuclear antigen

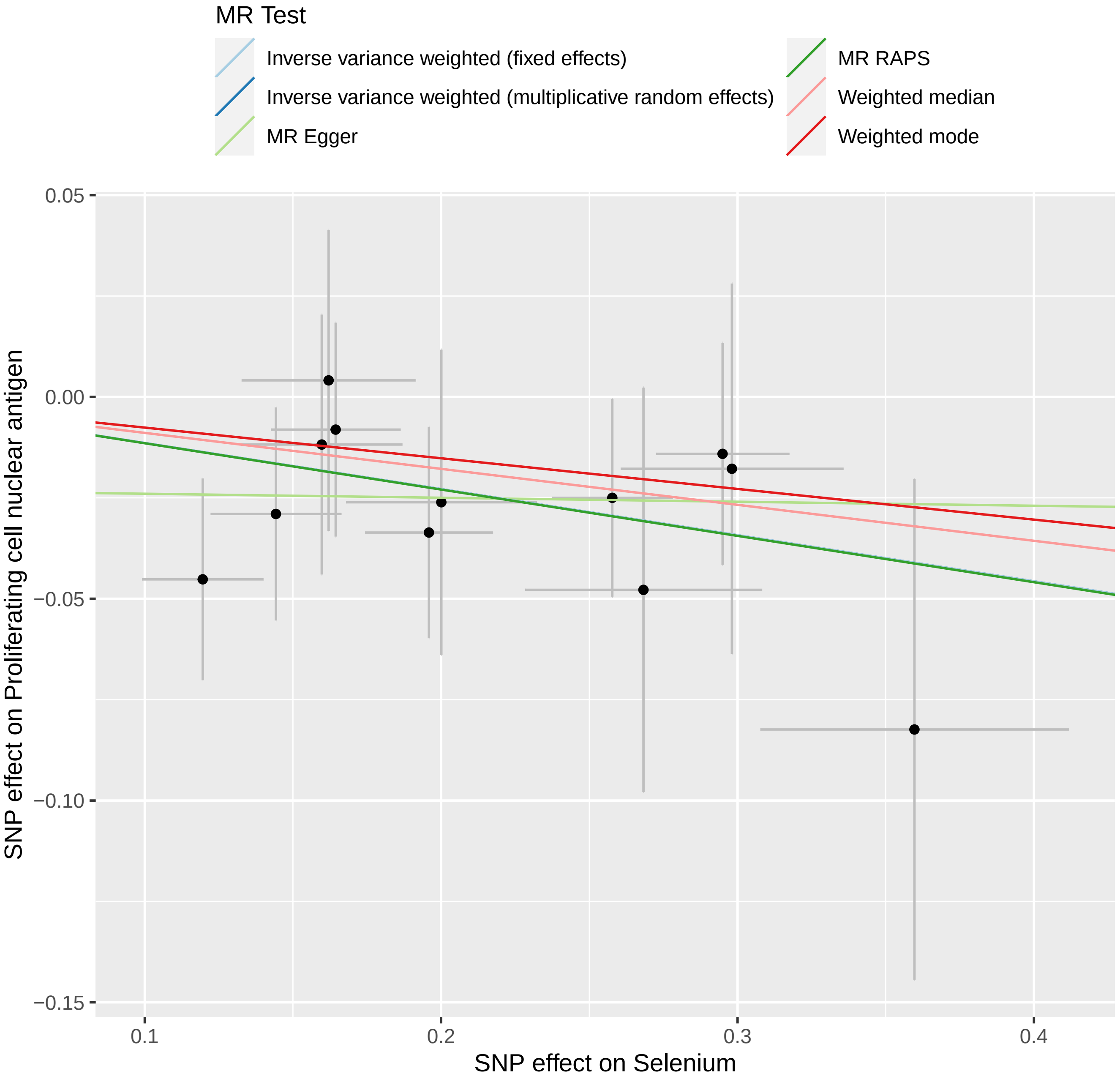

Batch 3−2209 : Selenium on Procollagen C−endopeptidase enhancer 1

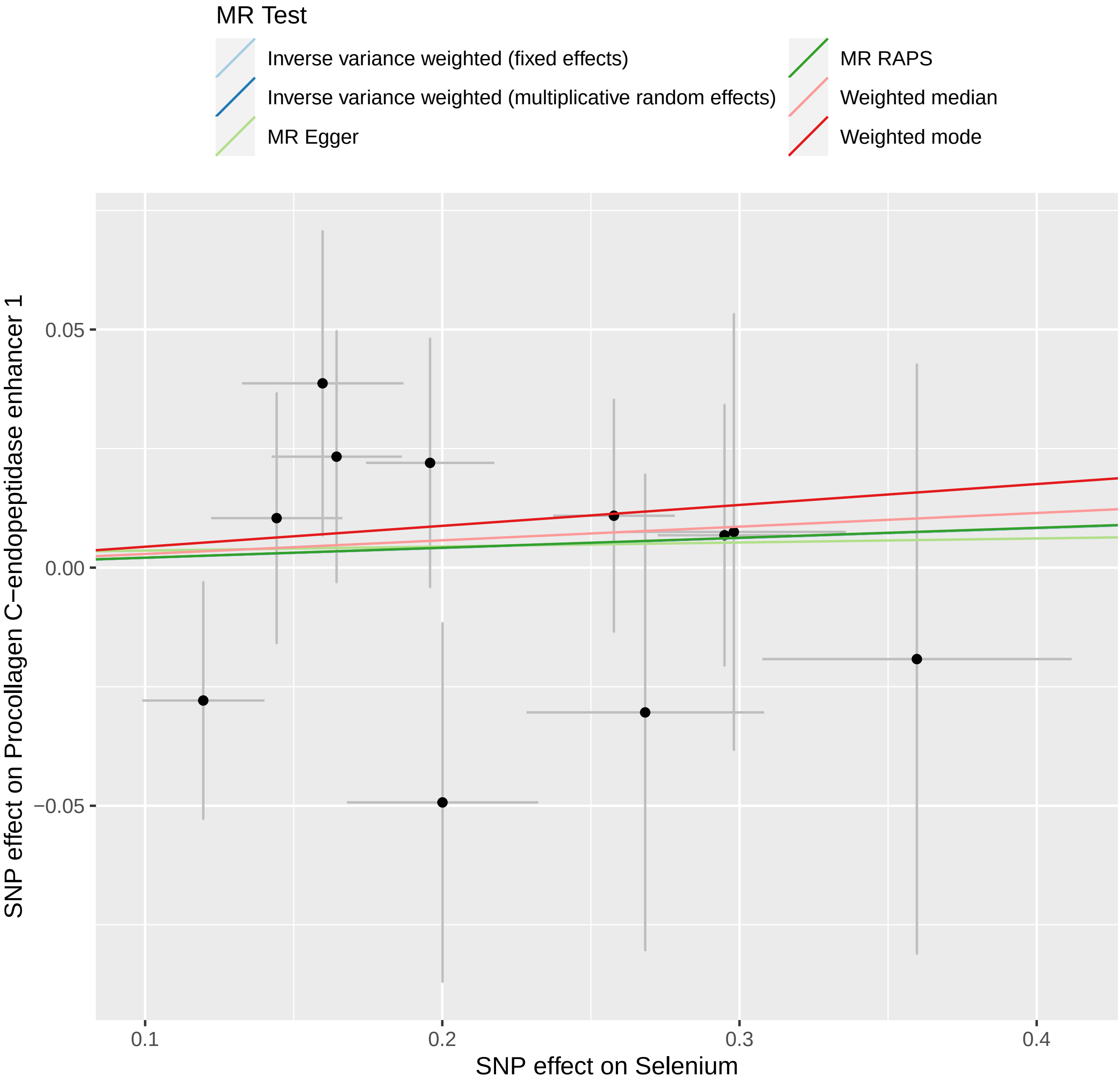

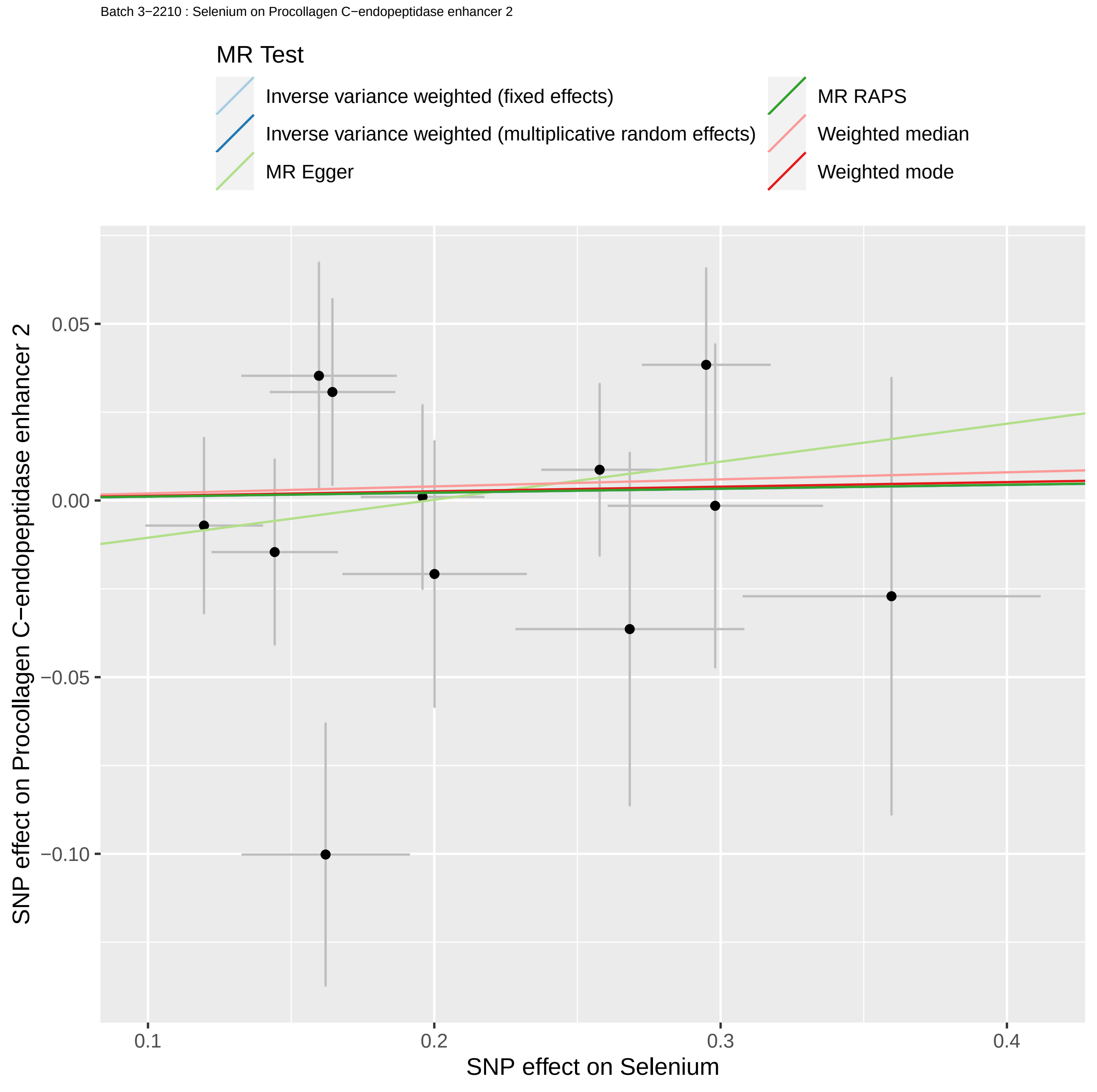
Batch 3−2210 : Selenium on Procollagen C−endopeptidase enhancer 2
MR Test
Inverse variance weighted (fixed effects)
Inverse variance weighted (multiplicative random effects)
MR Egger
MR RAPS
Weighted median
Weighted mode
0.05
0.00
−0.05
−0.10
0.1
0.2
0.3
0.4
SNP effect on Selenium
SNP effect on Procollagen C−endopeptidase enhancer 2

Batch 3−2211 : Selenium on Neuroendocrine convertase 1

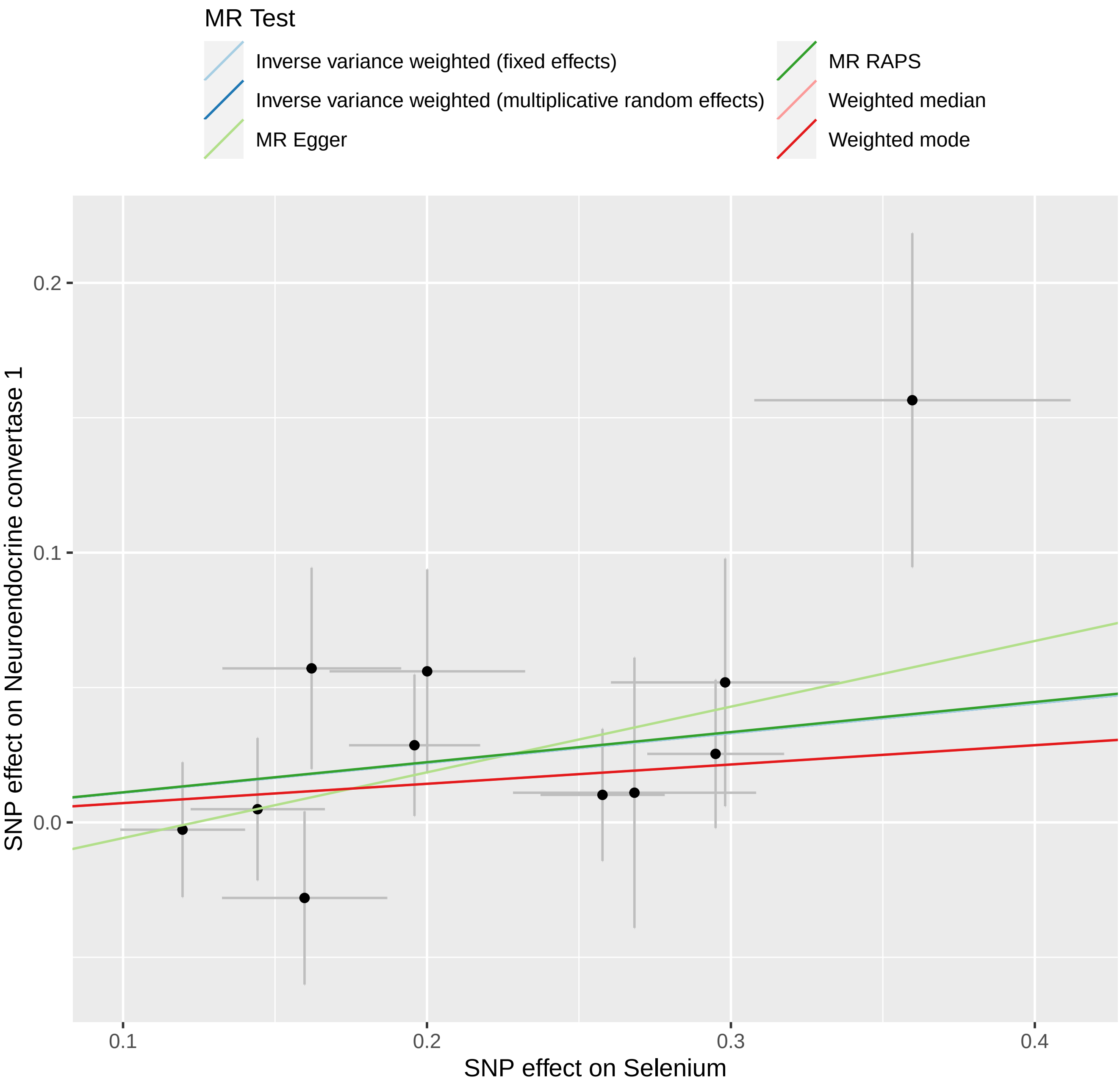

Batch 3−2212 : Selenium on Proprotein convertase subtilisin/kexin type 7

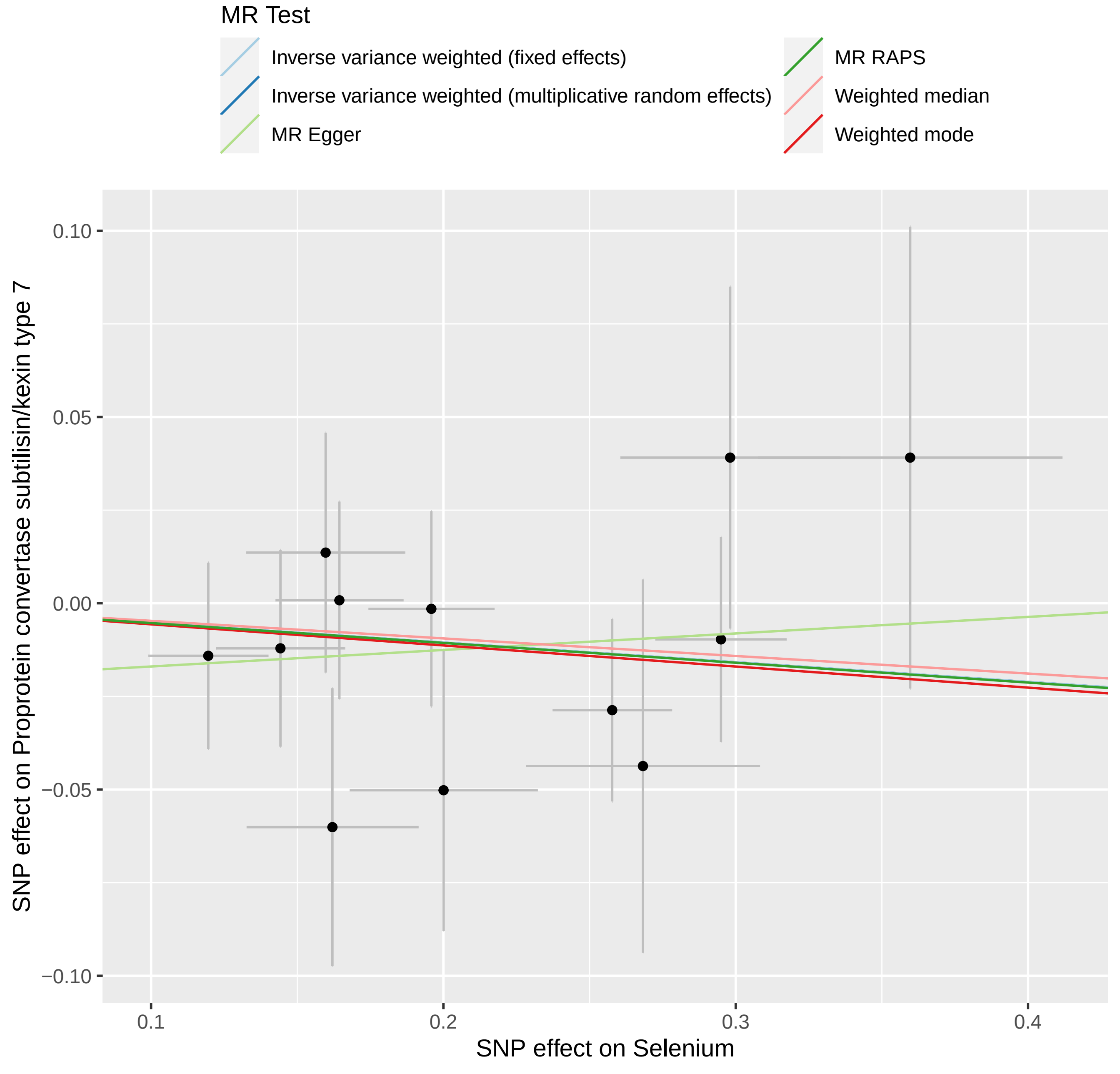

Batch 3−2213 : Selenium on Prenylcysteine oxidase−like

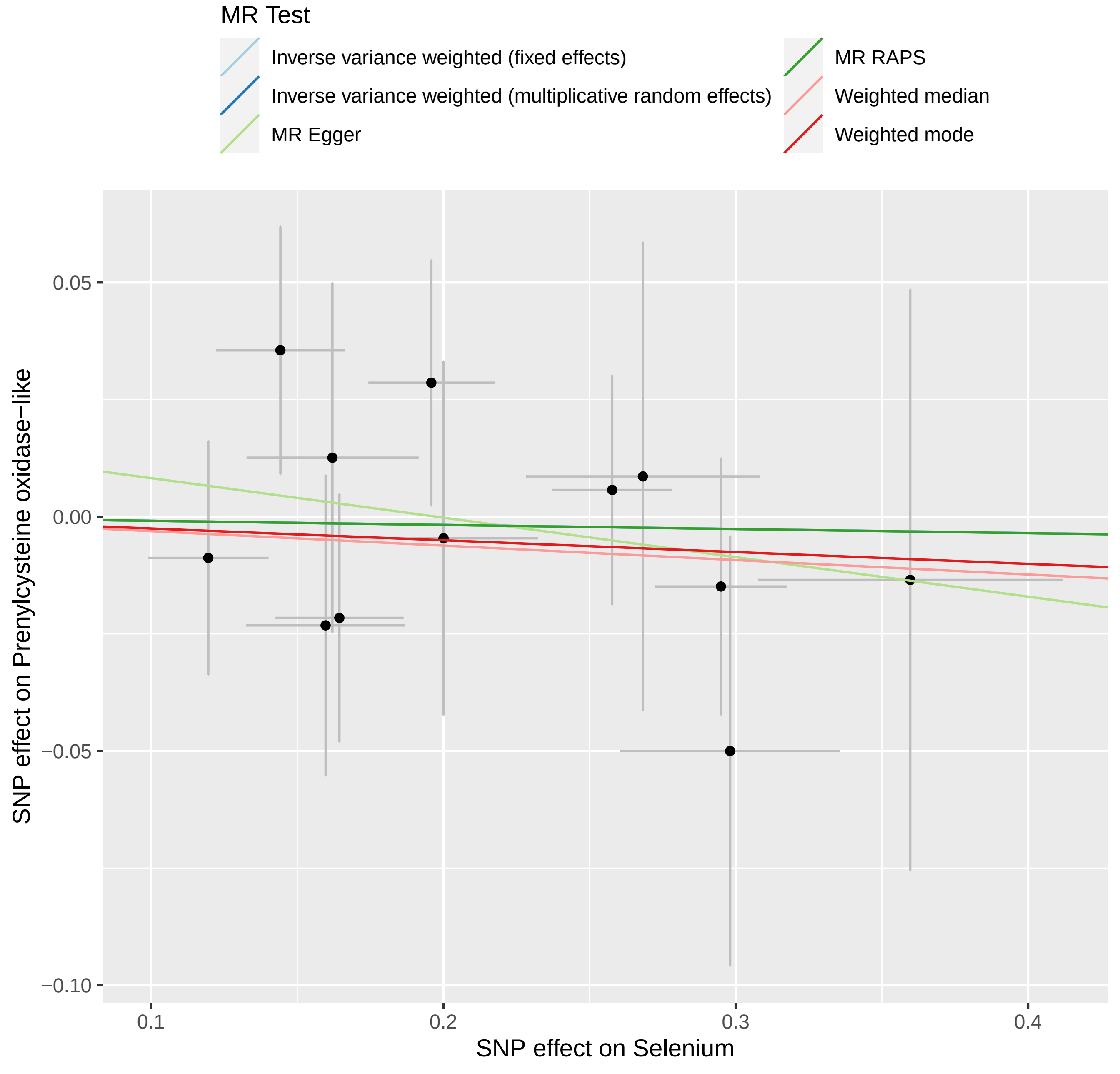

Batch 3−2214 : Selenium on Programmed cell death protein 1

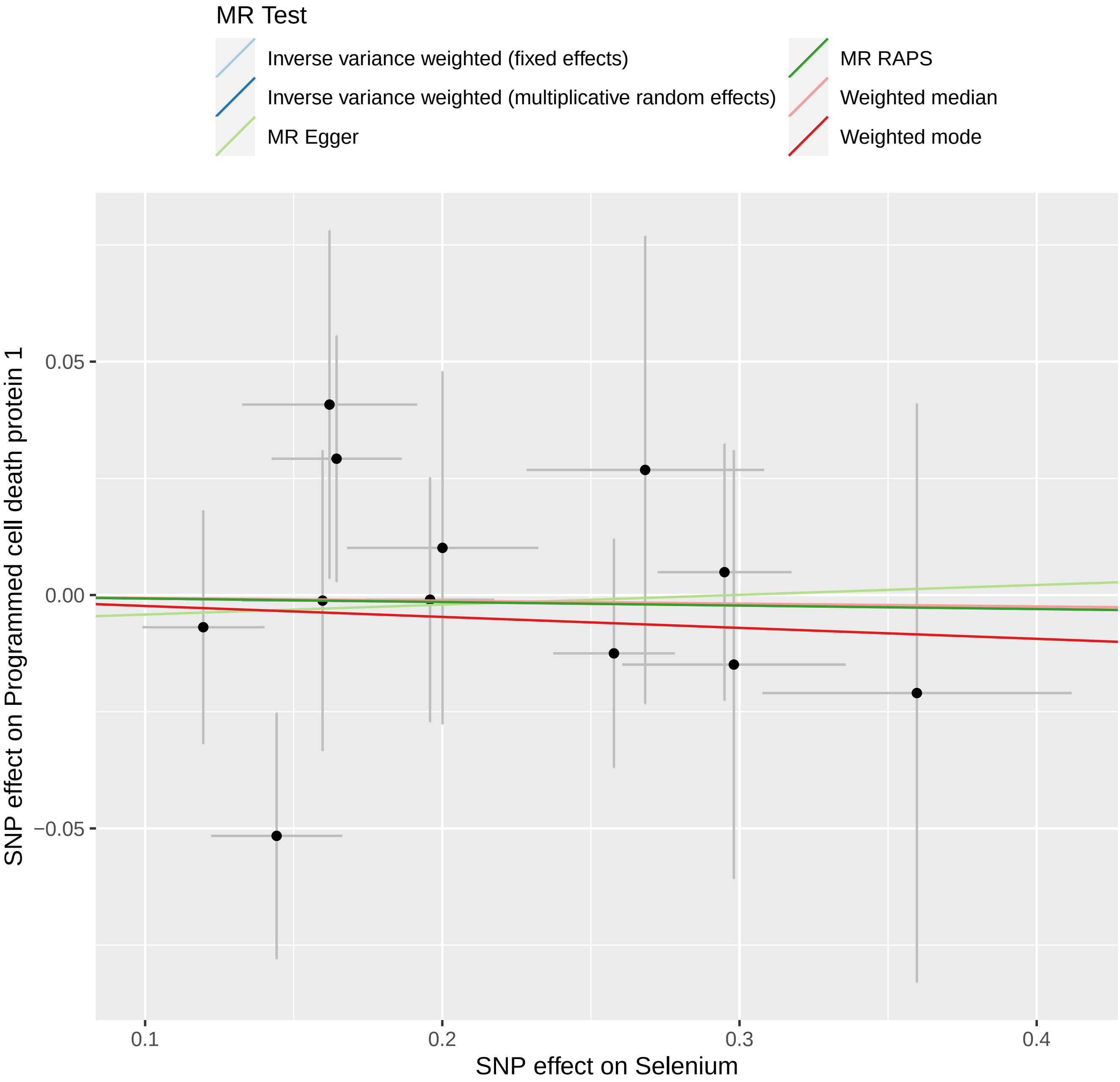

Batch 3−2215 : Selenium on Programmed cell death 1 ligand 2

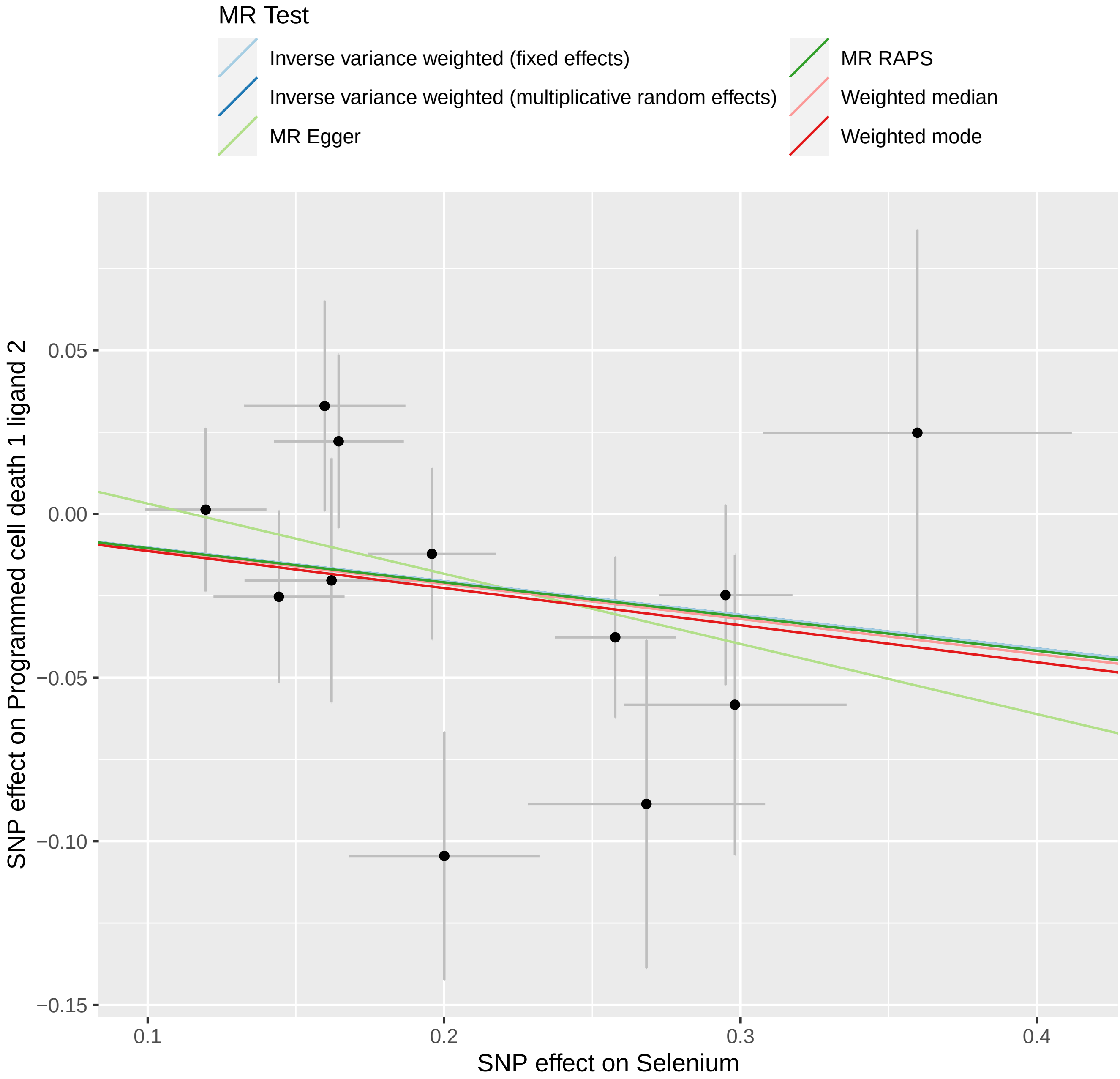

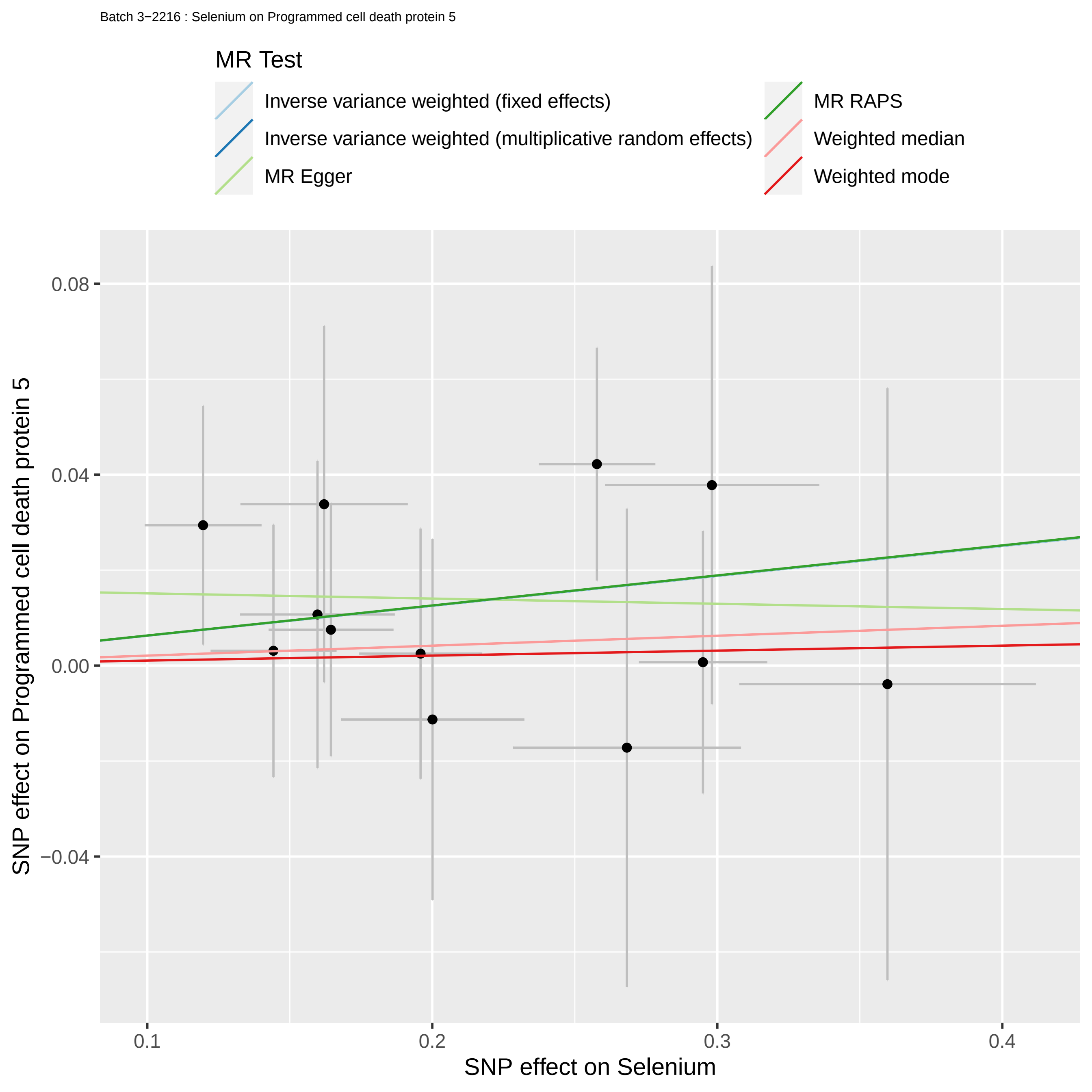
Batch 3−2216 : Selenium on Programmed cell death protein 5
MR Test
Inverse variance weighted (fixed effects)
Inverse variance weighted (multiplicative random effects)
MR Egger
MR RAPS
Weighted median
Weighted mode
SNP effect on Programmed cell death protein 5
0.08
0.04
0.00
−0.04
0.1
0.2
0.3
0.4
SNP effect on Selenium

Batch 3−2217 : Selenium on Phosducin−like protein 2

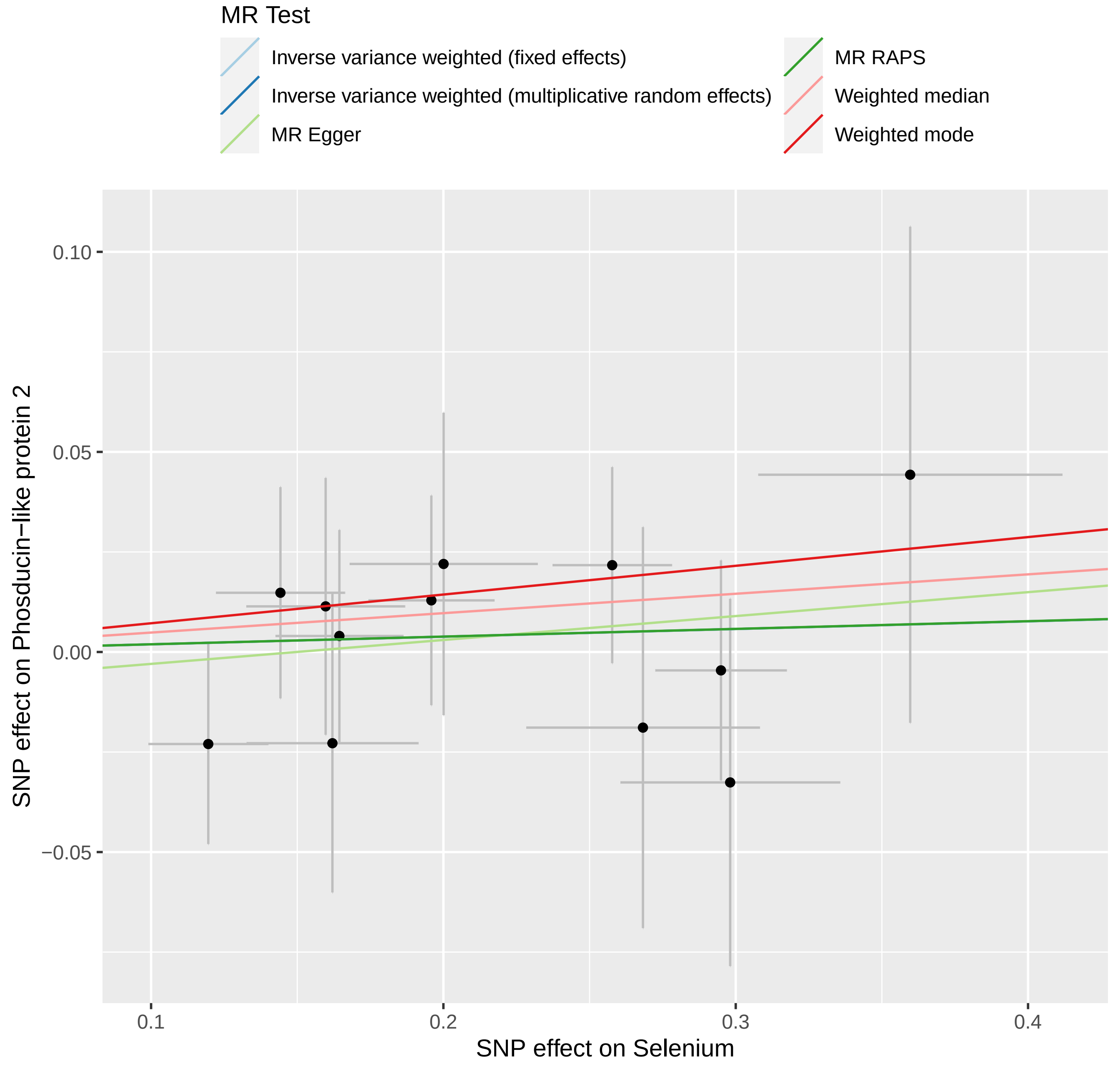

Batch 3−2218 : Selenium on Calcium/calmodulin−dependent 3',5'−cyclic nucleotide phosphodiesterase 1A

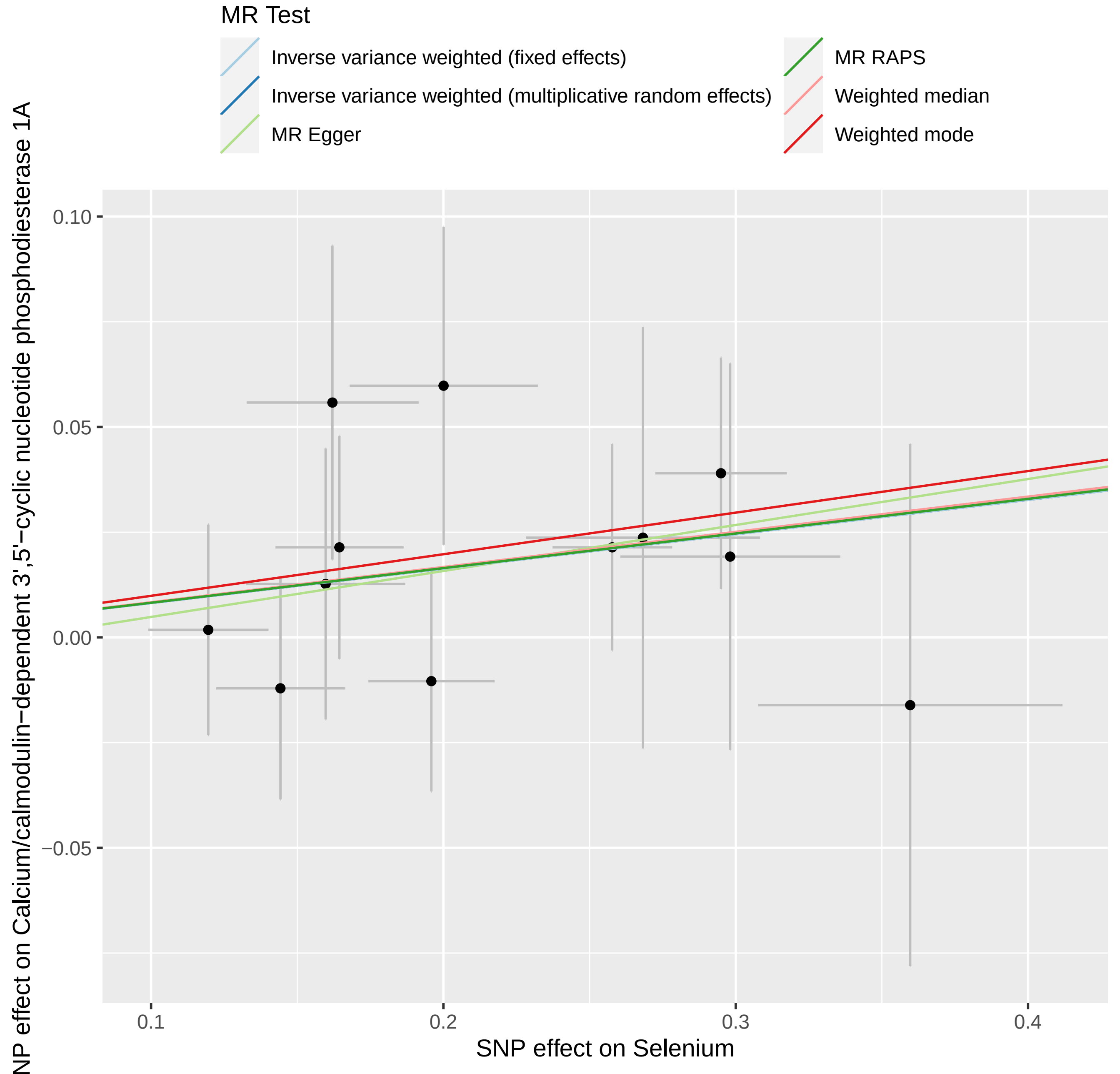

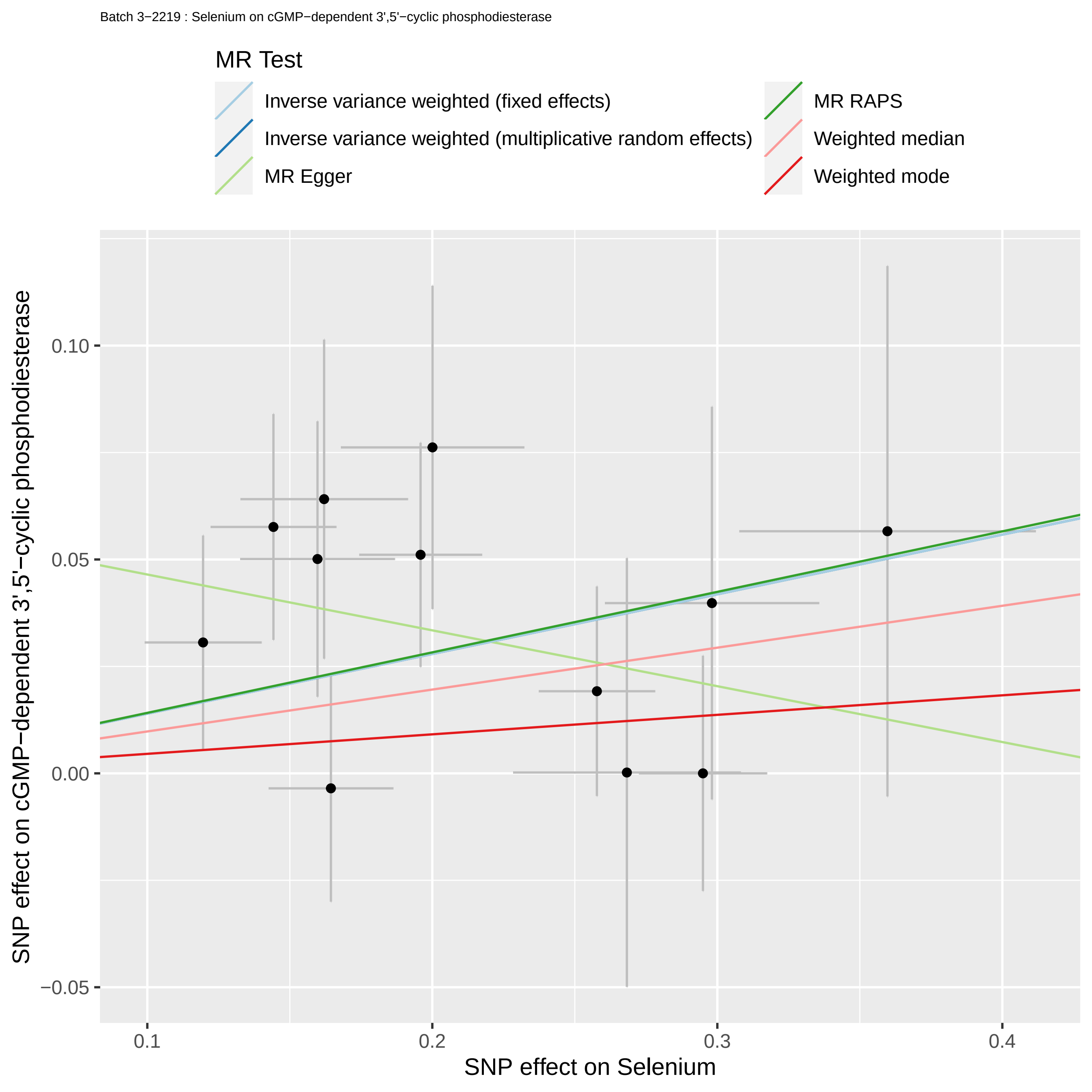
Batch 3−2219 : Selenium on cGMP−dependent 3',5'−cyclic phosphodiesterase
MR Test
Inverse variance weighted (fixed effects)
Inverse variance weighted (multiplicative random effects)
MR Egger
MR RAPS
Weighted median
Weighted mode
SNP effect on cGMP−dependent 3',5'−cyclic phosphodiesterase
0.10
0.05
0.00
−0.05
0.1
0.2
0.3
0.4
SNP effect on Selenium

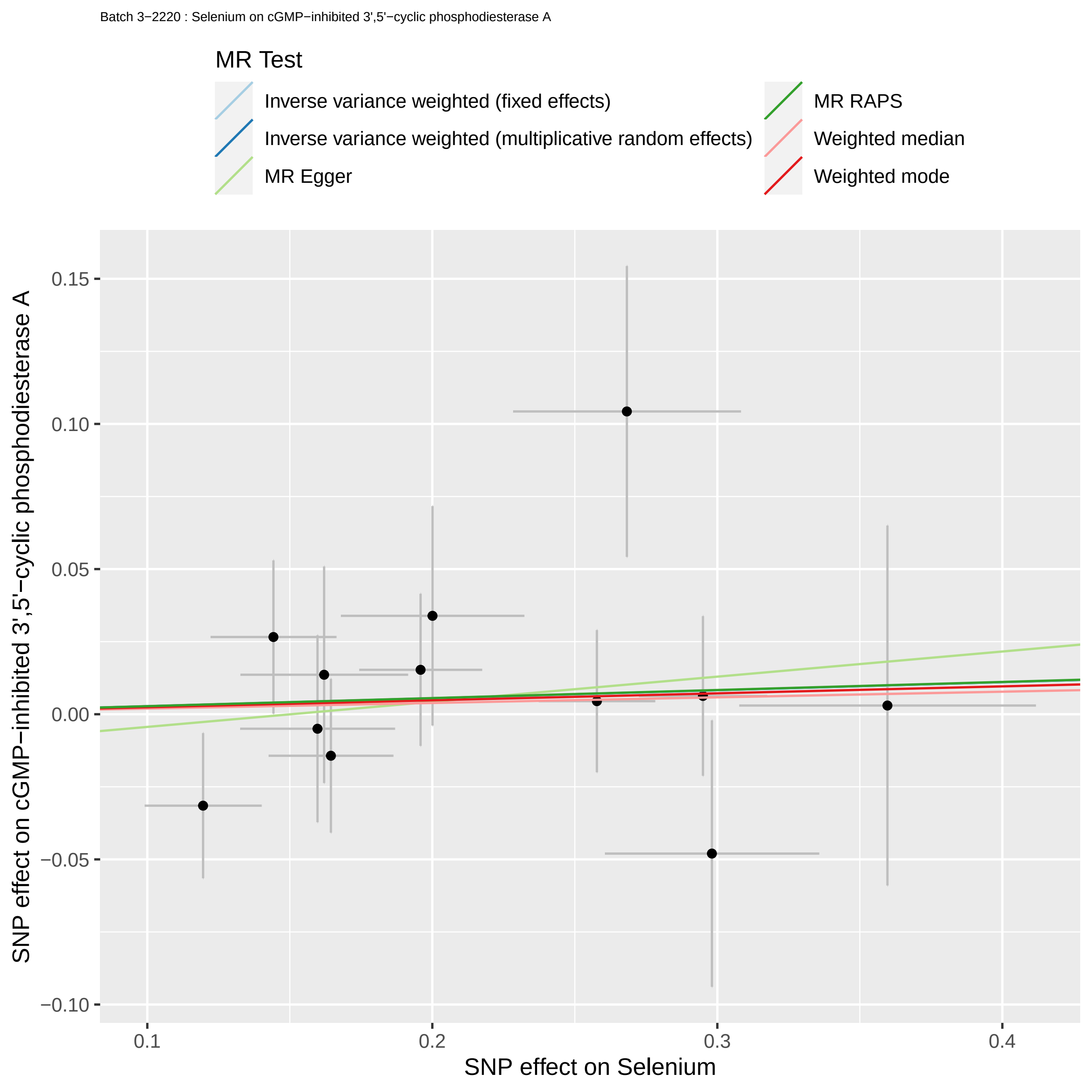
Batch 3−2220 : Selenium on cGMP−inhibited 3',5'−cyclic phosphodiesterase A
MR Test
Inverse variance weighted (fixed effects)
Inverse variance weighted (multiplicative random effects)
MR Egger
MR RAPS
Weighted median
Weighted mode
0.15
0.10
0.05
0.00
−0.05
−0.10
0.1
0.2
0.3
0.4
SNP effect on cGMP−inhibited 3',5'−cyclic phosphodiesterase A
SNP effect on Selenium

Batch 3−2221 : Selenium on cAMP−specific 3',5'−cyclic phosphodiesterase 4D

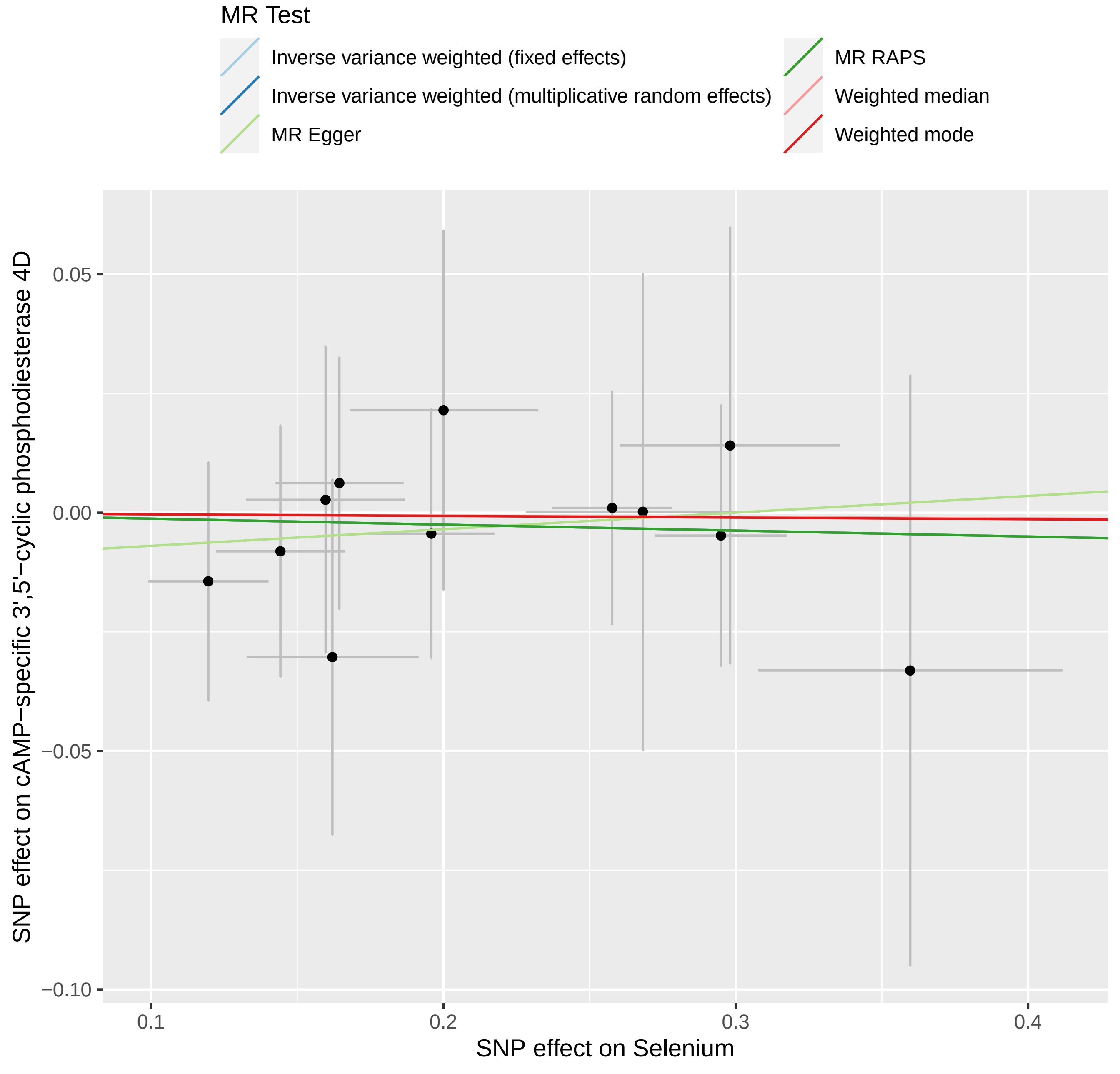

Batch 3−2222 : Selenium on Retinal rod rhodopsin−sensitive cGMP 3',5'−cyclic phosphodiesterase subunit delta

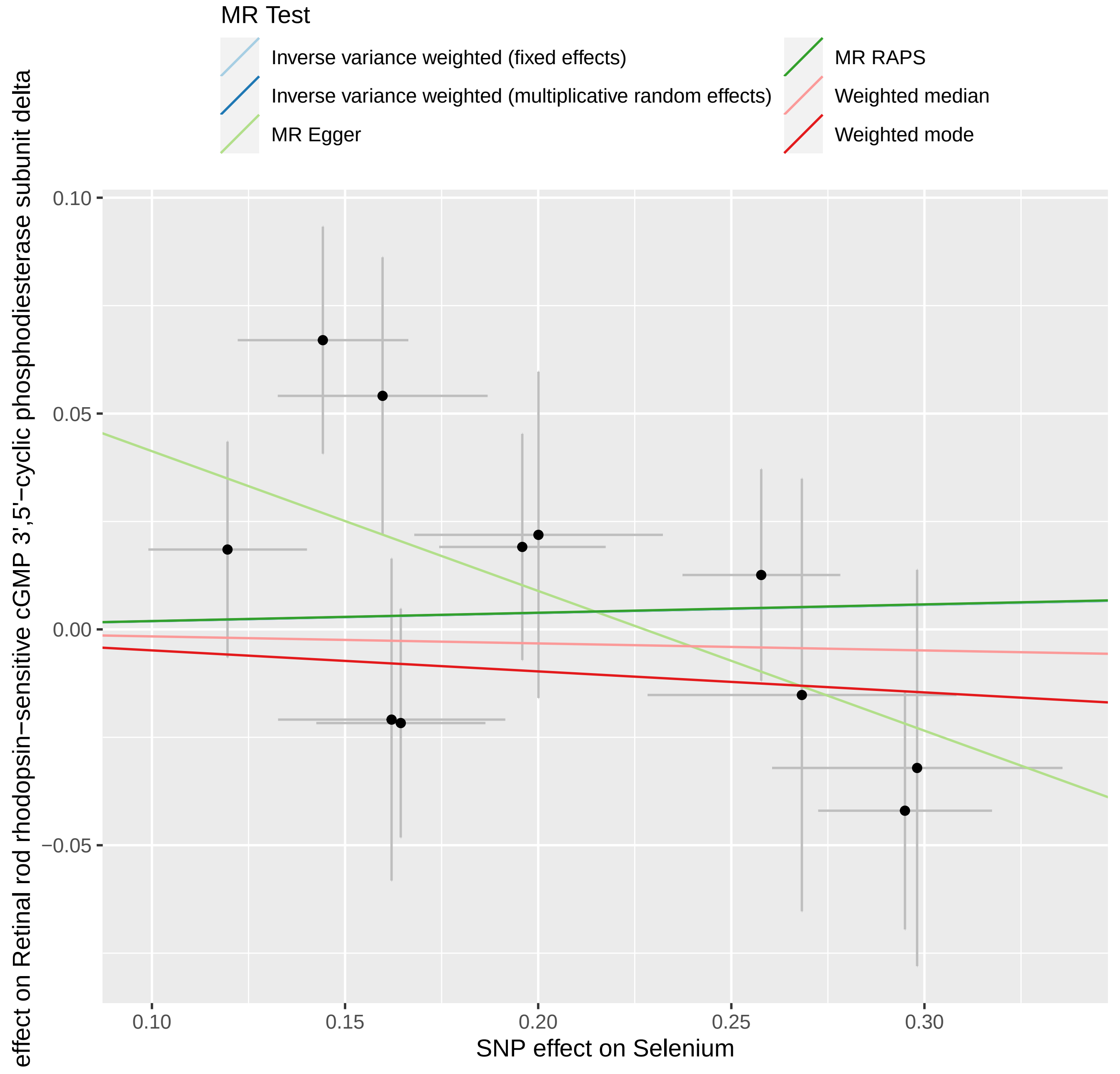

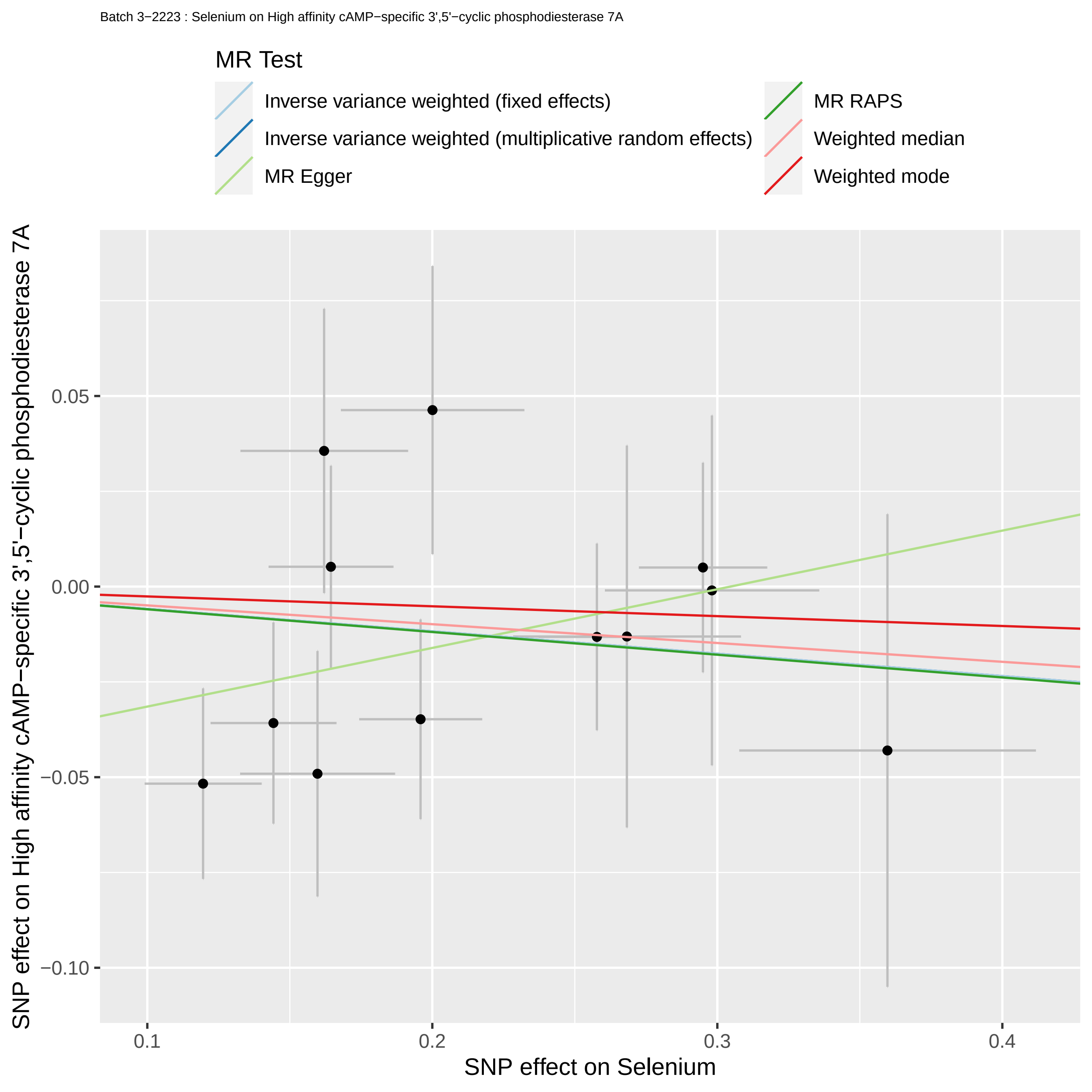
Batch 3−2223 : Selenium on High affinity cAMP−specific 3',5'−cyclic phosphodiesterase 7A
MR Test
Inverse variance weighted (fixed effects)
Inverse variance weighted (multiplicative random effects)
MR Egger
MR RAPS
Weighted median
Weighted mode
SNP effect on High affinity cAMP−specific 3',5'−cyclic phosphodiesterase 7A
0.05
0.00
−0.05
−0.10
0.1
0.2
0.3
0.4
SNP effect on Selenium

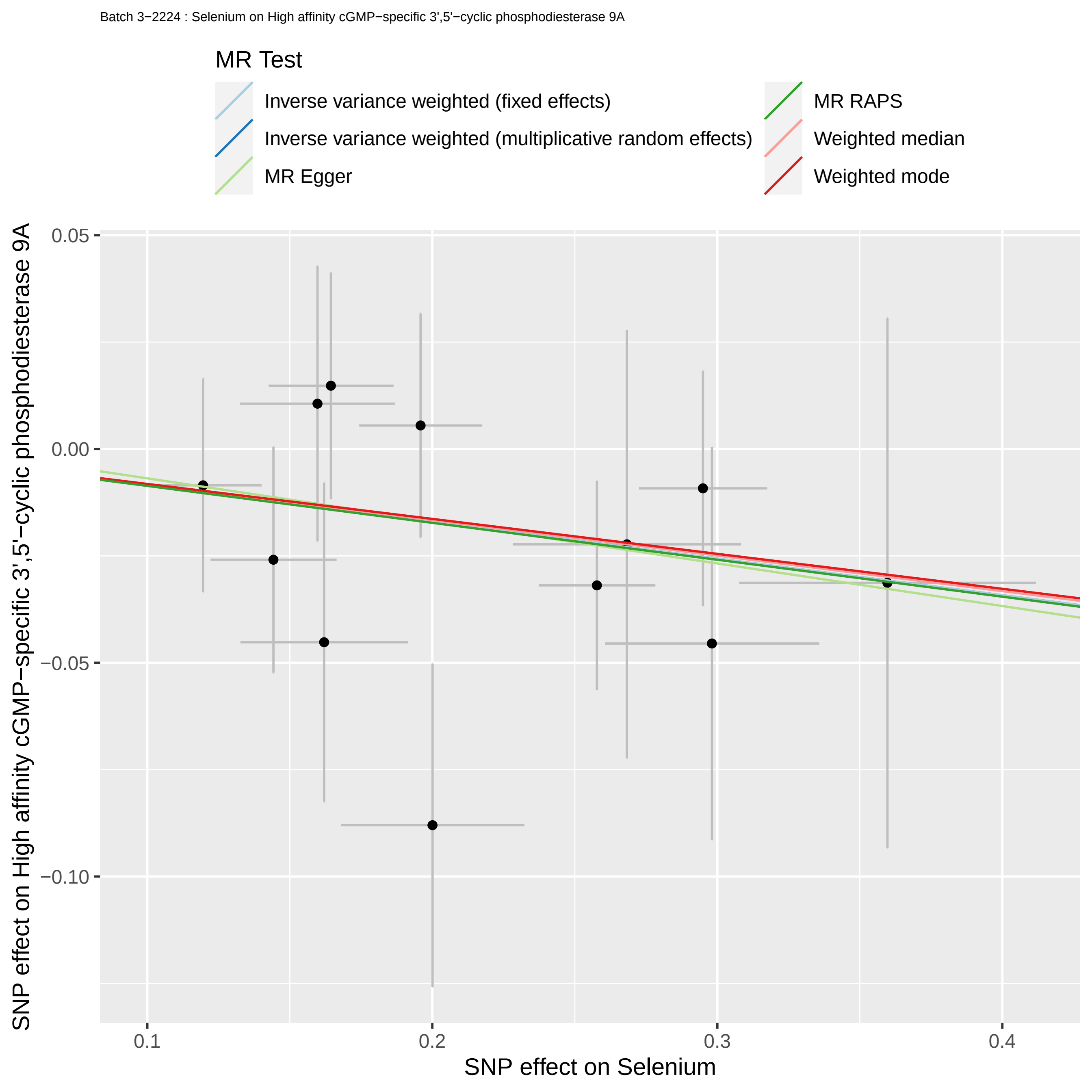
Batch 3−2224 : Selenium on High affinity cGMP−specific 3',5'−cyclic phosphodiesterase 9A
MR Test
Inverse variance weighted (fixed effects)
Inverse variance weighted (multiplicative random effects)
MR Egger
MR RAPS
Weighted median
Weighted mode
0.05
0.00
−0.05
−0.10
0.1
0.2
0.3
0.4
SNP effect on High affinity cGMP−specific 3',5'−cyclic phosphodiesterase 9A
SNP effect on Selenium

Batch 3−2225 : Selenium on Platelet−derived growth factor subunit A

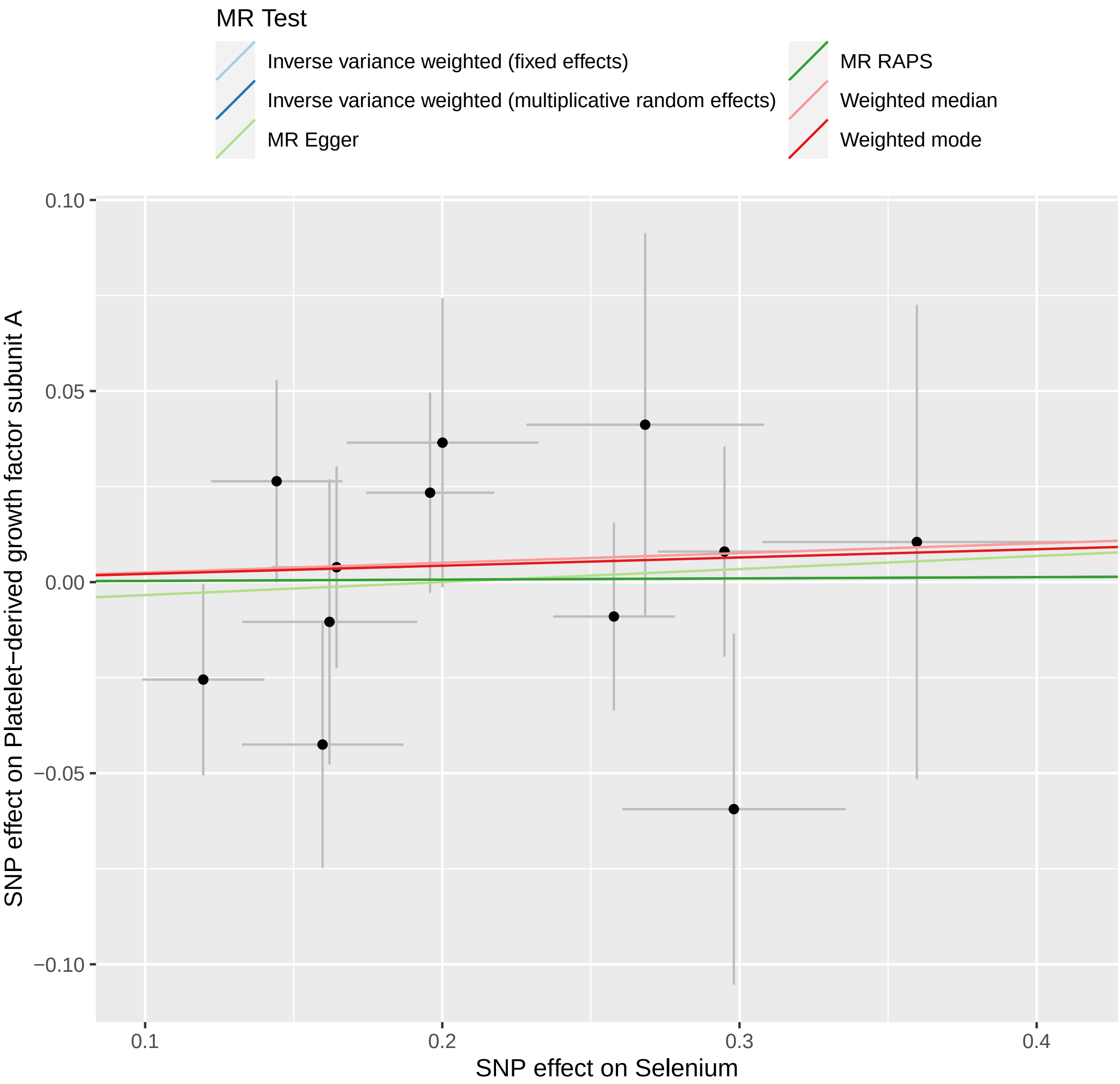

Batch 3−2226 : Selenium on Platelet−derived growth factor subunit B

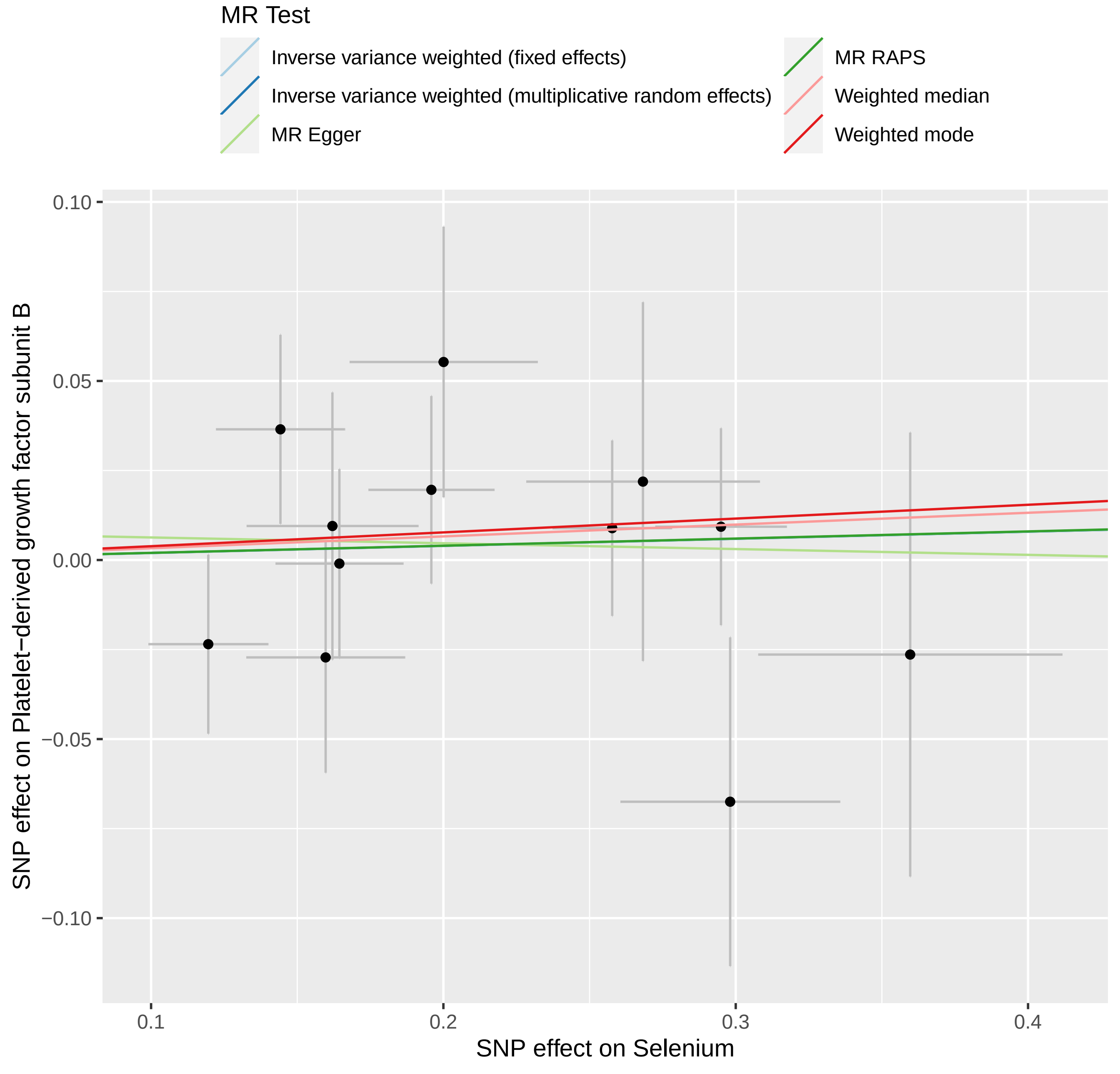

Batch 3−2227 : Selenium on Platelet−derived growth factor C

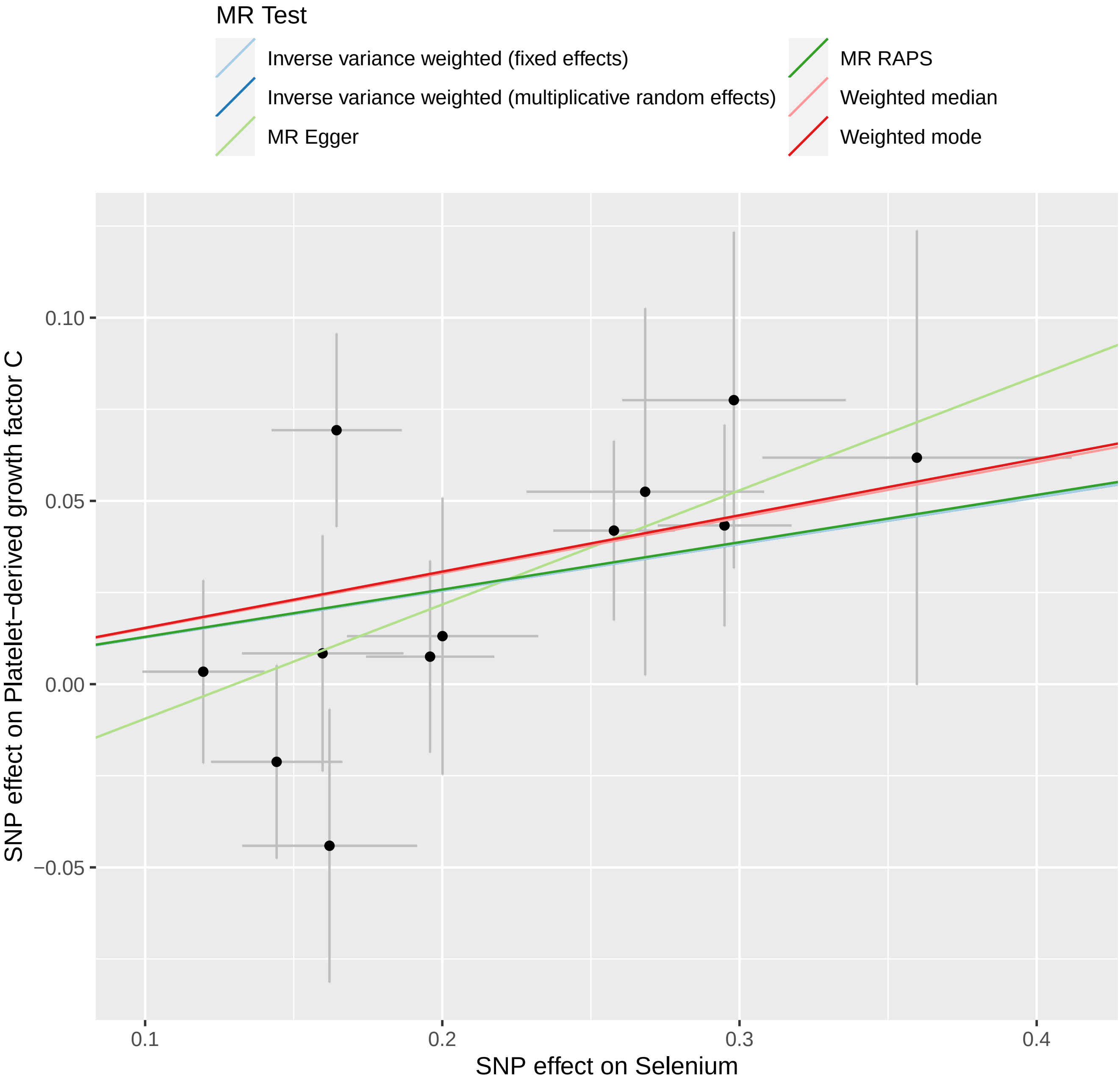

Batch 3−2228 : Selenium on Platelet−derived growth factor D

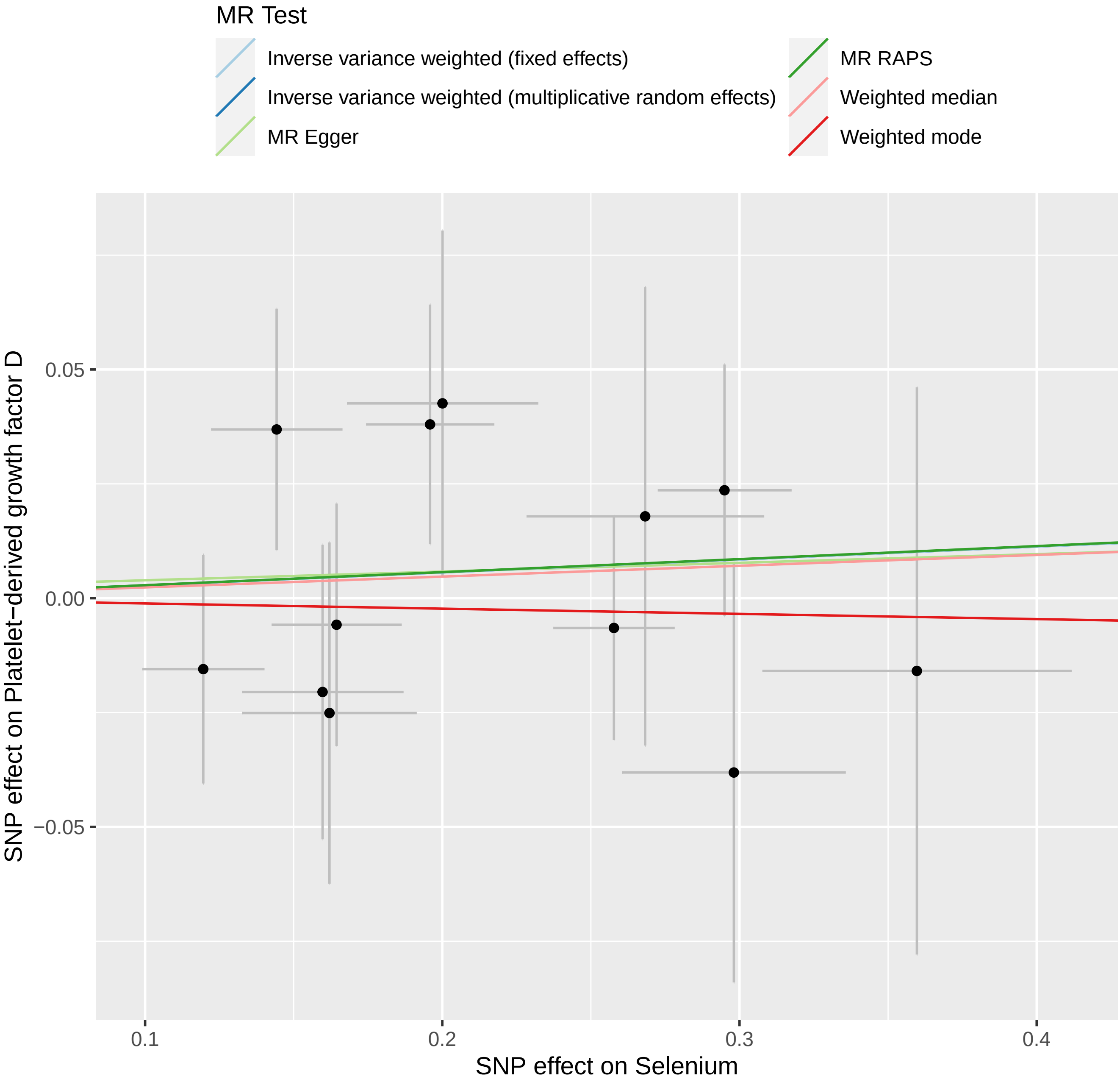

Batch 3−2229 : Selenium on Platelet−derived growth factor receptor alpha

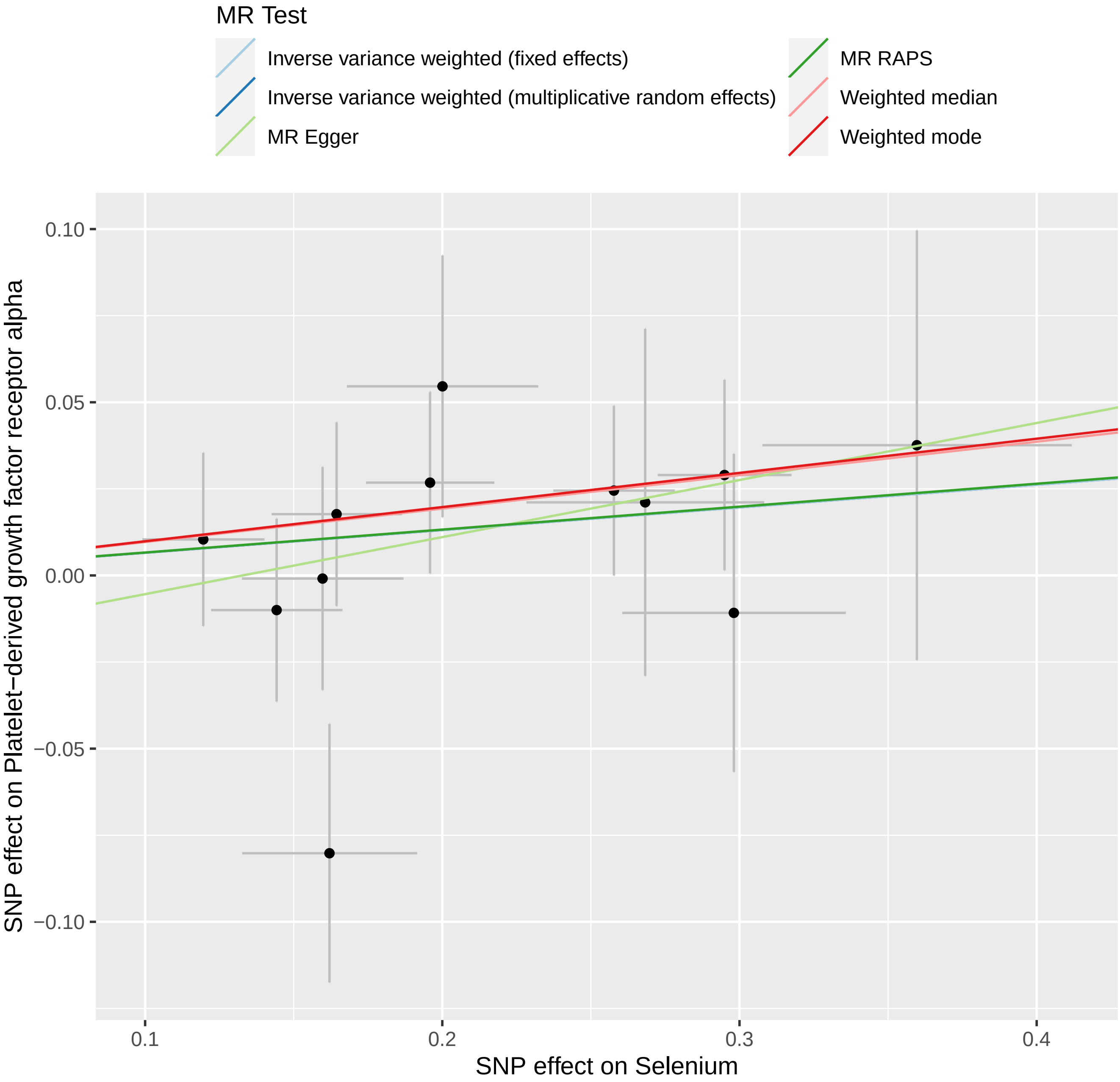

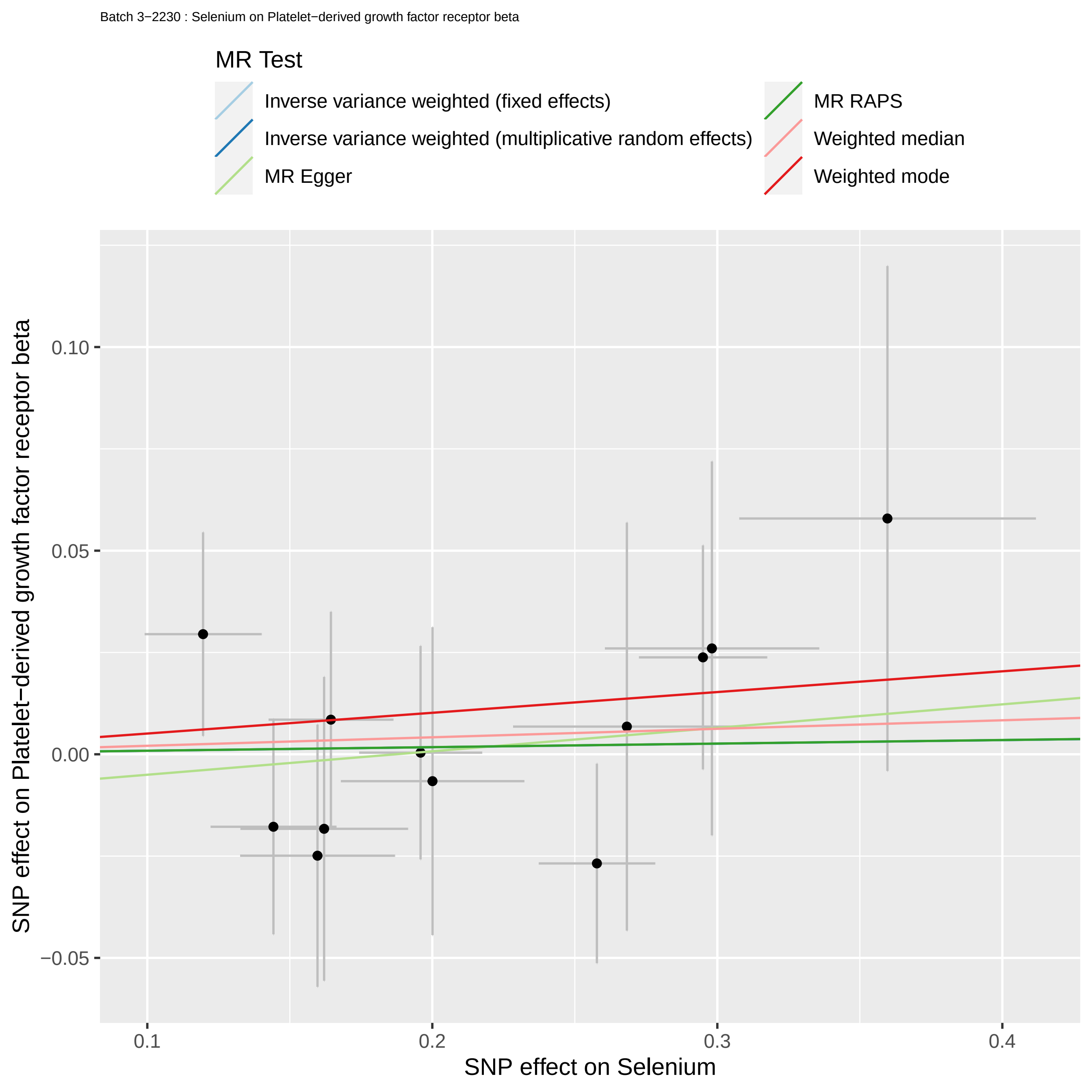

Batch 3−2230 : Selenium on Platelet−derived growth factor receptor beta
MR Test
Inverse variance weighted (fixed effects)
Inverse variance weighted (multiplicative random effects)
MR Egger
MR RAPS
Weighted median
Weighted mode
0.10
0.05
0.00
−0.05
0.1
0.2
0.3
0.4
SNP effect on Platelet−derived growth factor receptor beta
SNP effect on Selenium

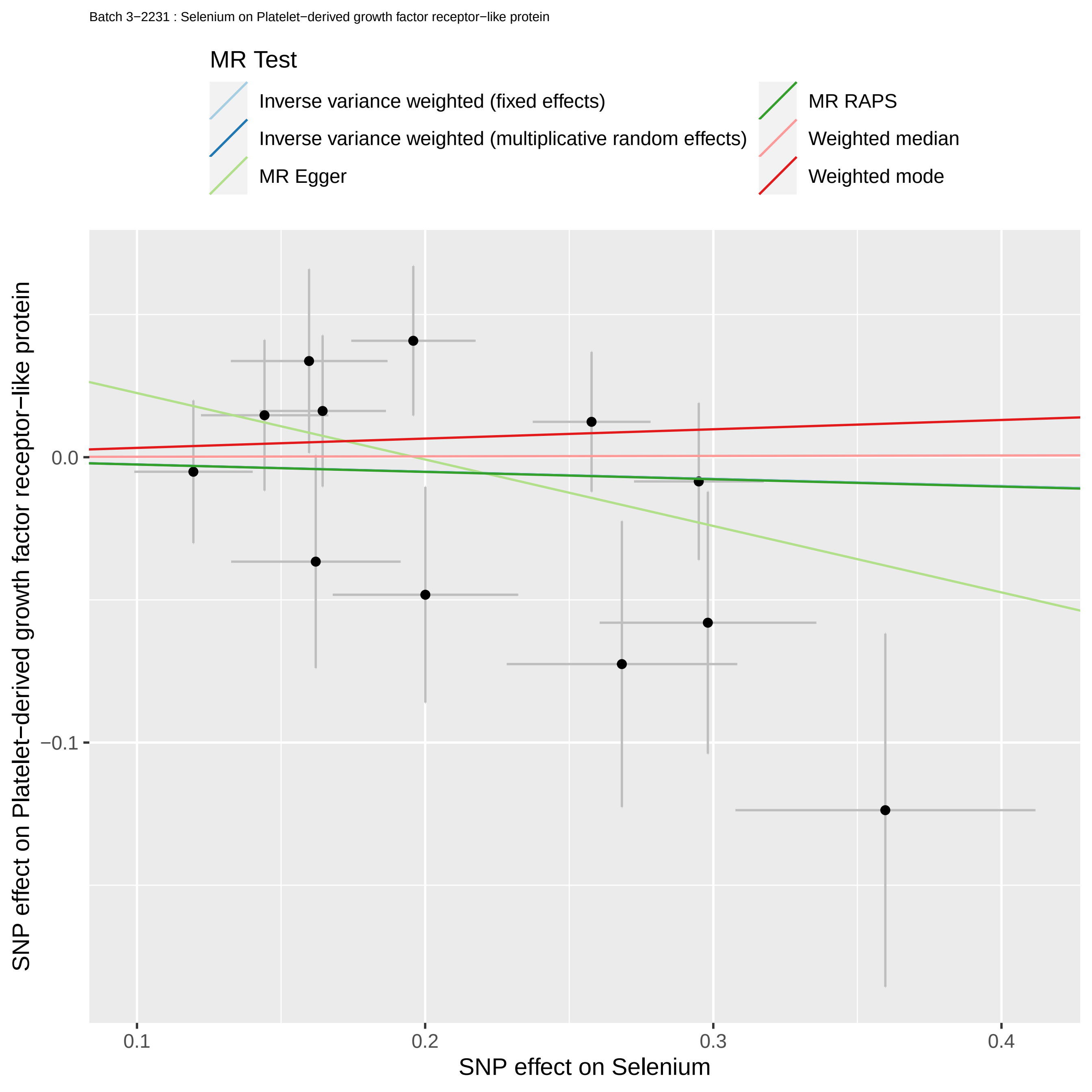
Batch 3−2231 : Selenium on Platelet−derived growth factor receptor−like protein
MR Test
Inverse variance weighted (fixed effects)
Inverse variance weighted (multiplicative random effects)
MR Egger
MR RAPS
Weighted median
Weighted mode
SNP effect on Platelet−derived growth factor receptor−like protein
0.0
−0.1
0.1
0.2
0.3
0.4
SNP effect on Selenium

Batch 3−2232 : Selenium on Protein disulfide−isomerase A3

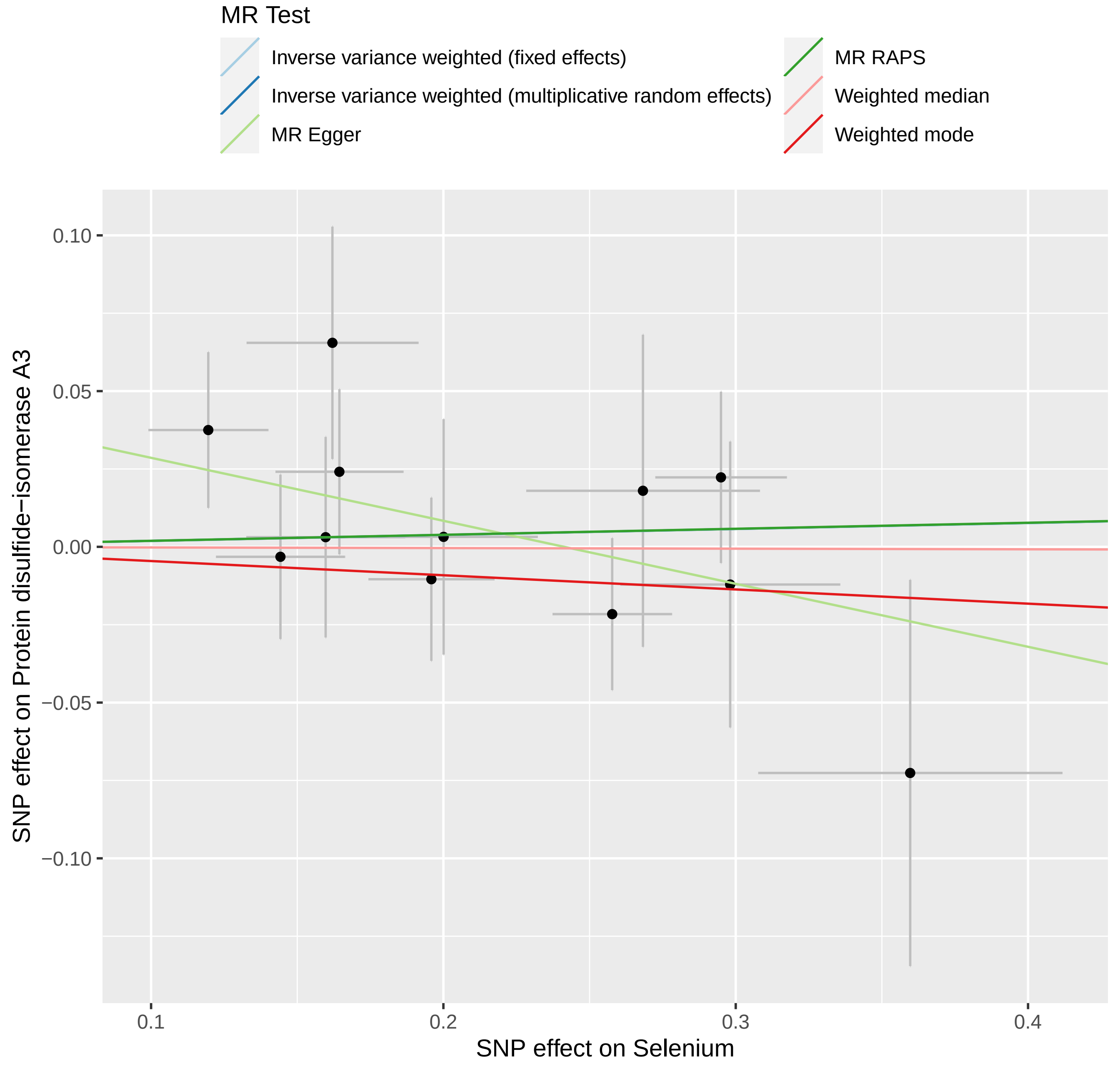

Batch 3−2233 : Selenium on Protein disulfide−isomerase A5

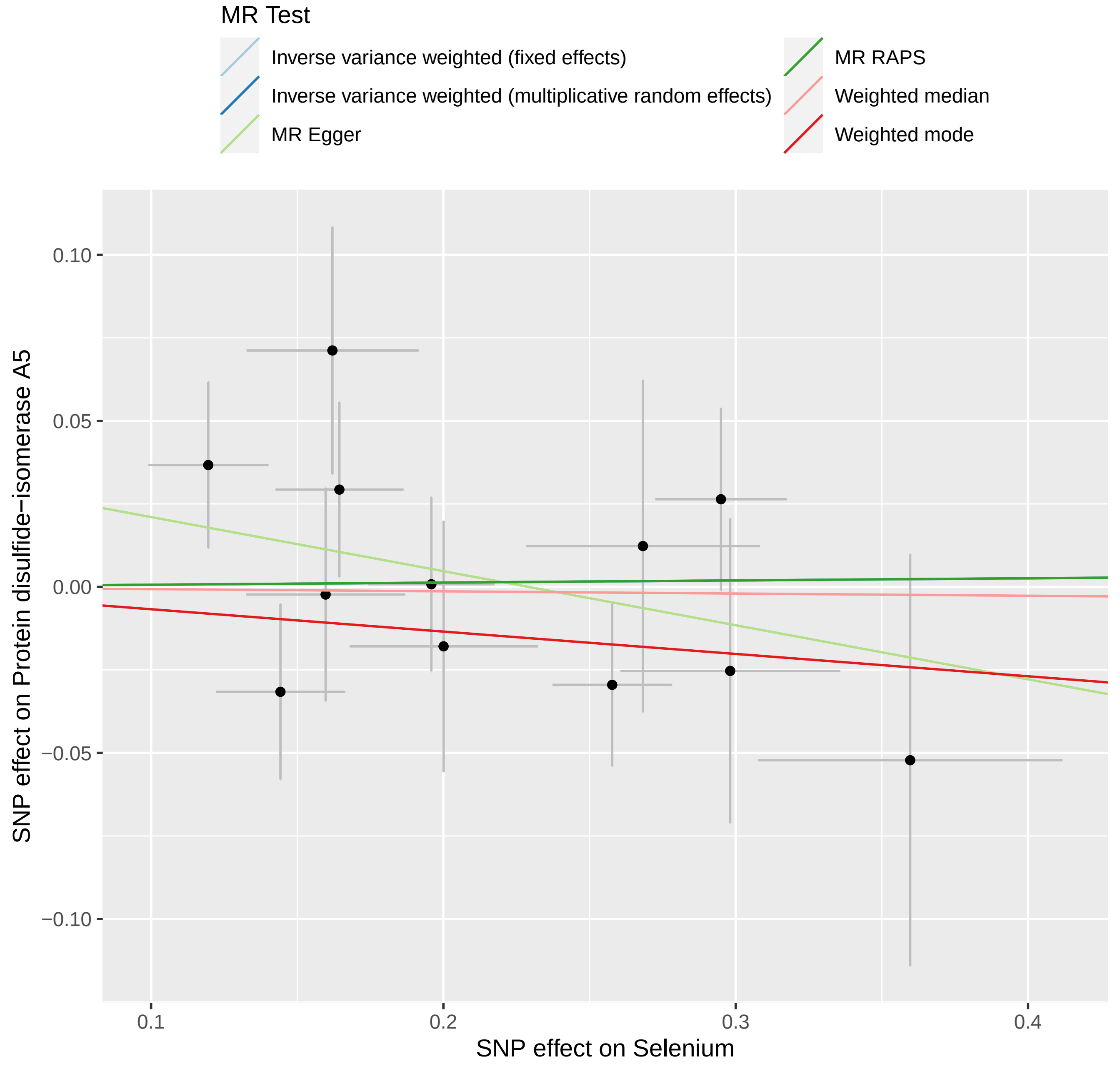

Batch 3−2234 : Selenium on Protein disulfide−isomerase−like protein of the testis

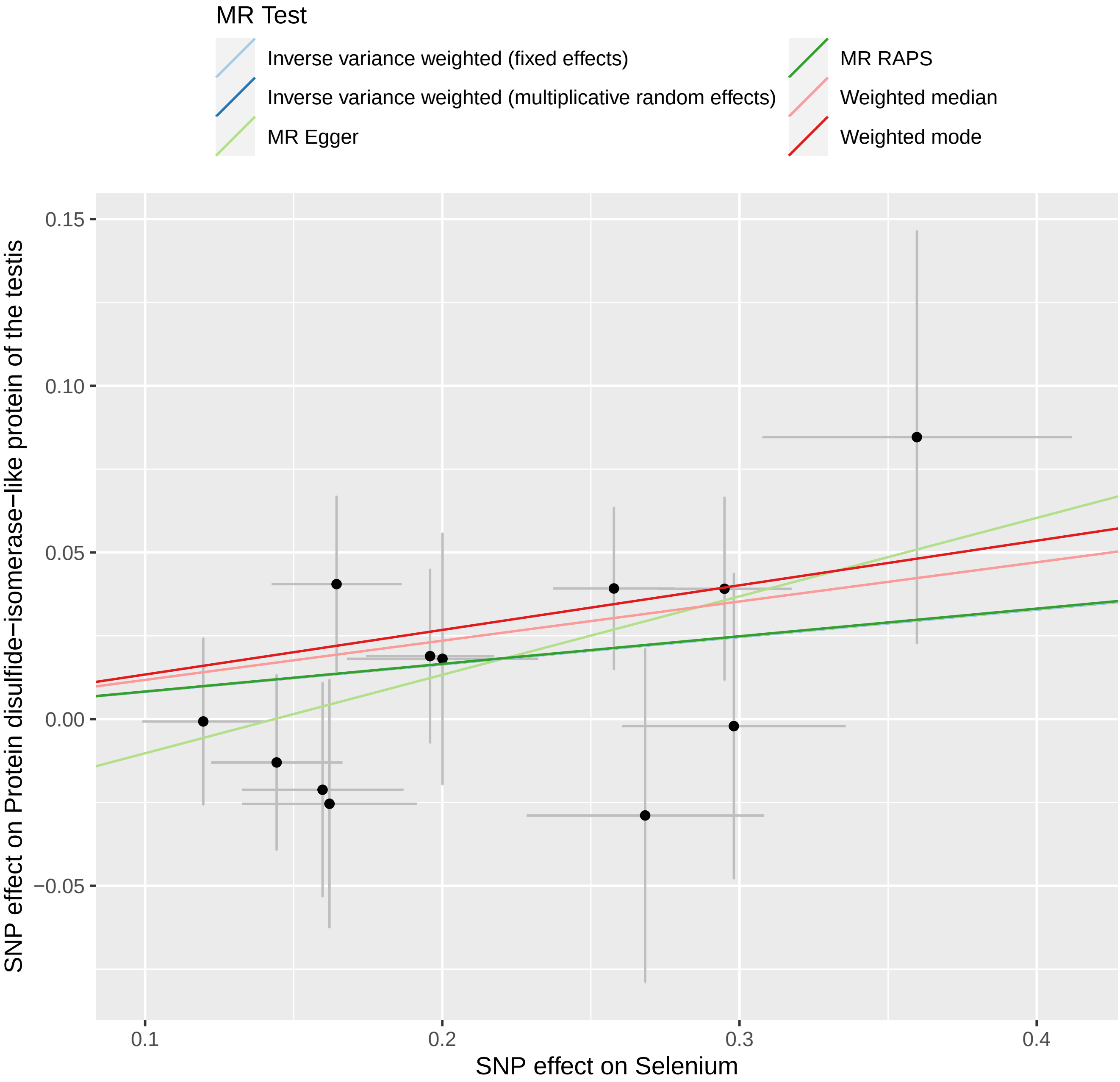

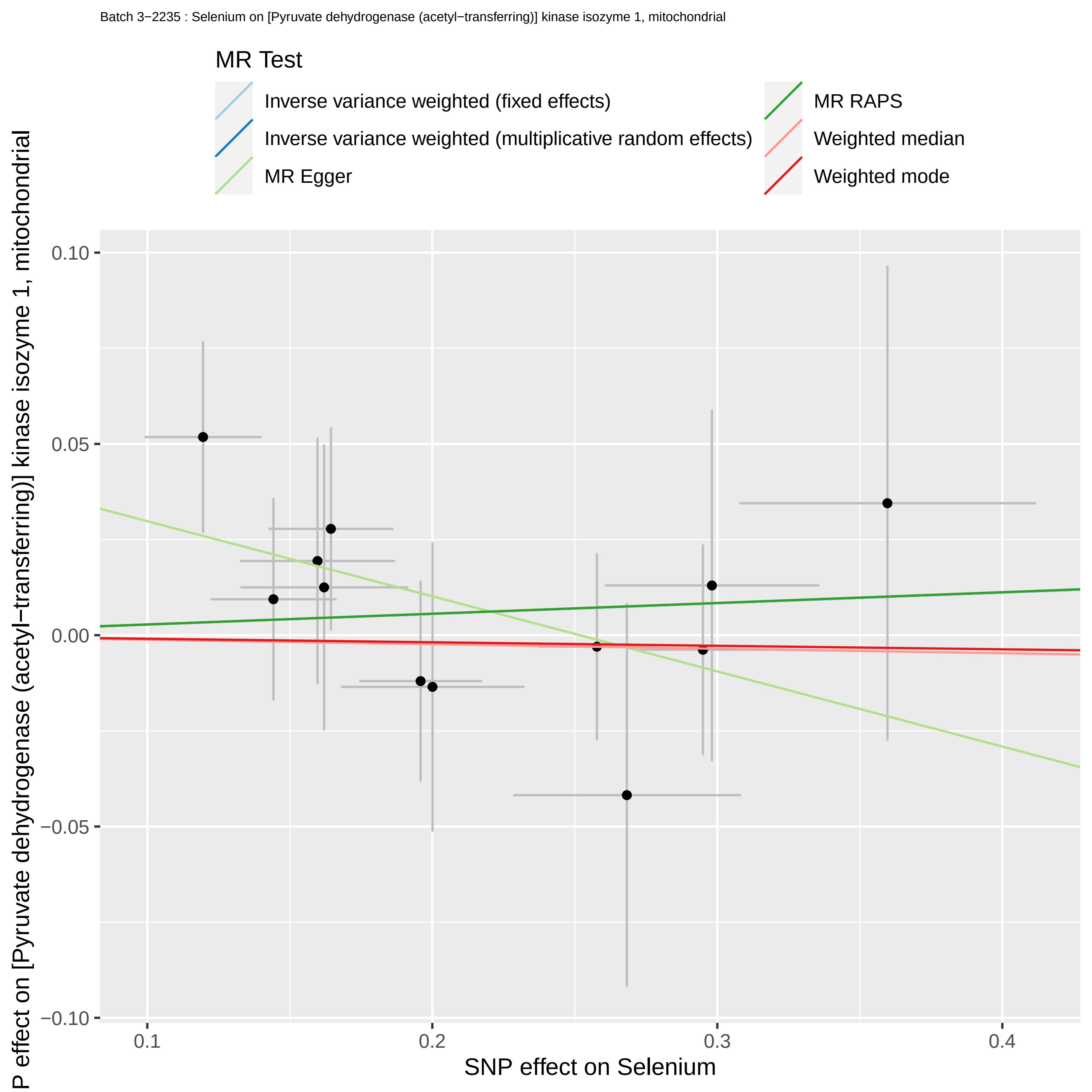

Batch 3−2235 : Selenium on [Pyruvate dehydrogenase (acetyl−transferring)] kinase isozyme 1, mitochondrial
MR Test
Inverse variance weighted (fixed effects)
Inverse variance weighted (multiplicative random effects)
MR Egger
MR RAPS
Weighted median
Weighted mode
0.10
0.05
0.00
−0.05
−0.10
0.1
0.2
0.3
0.4
SNP effect on Selenium
P effect on [Pyruvate dehydrogenase (acetyl−transferring)] kinase isozyme 1, mitochondrial

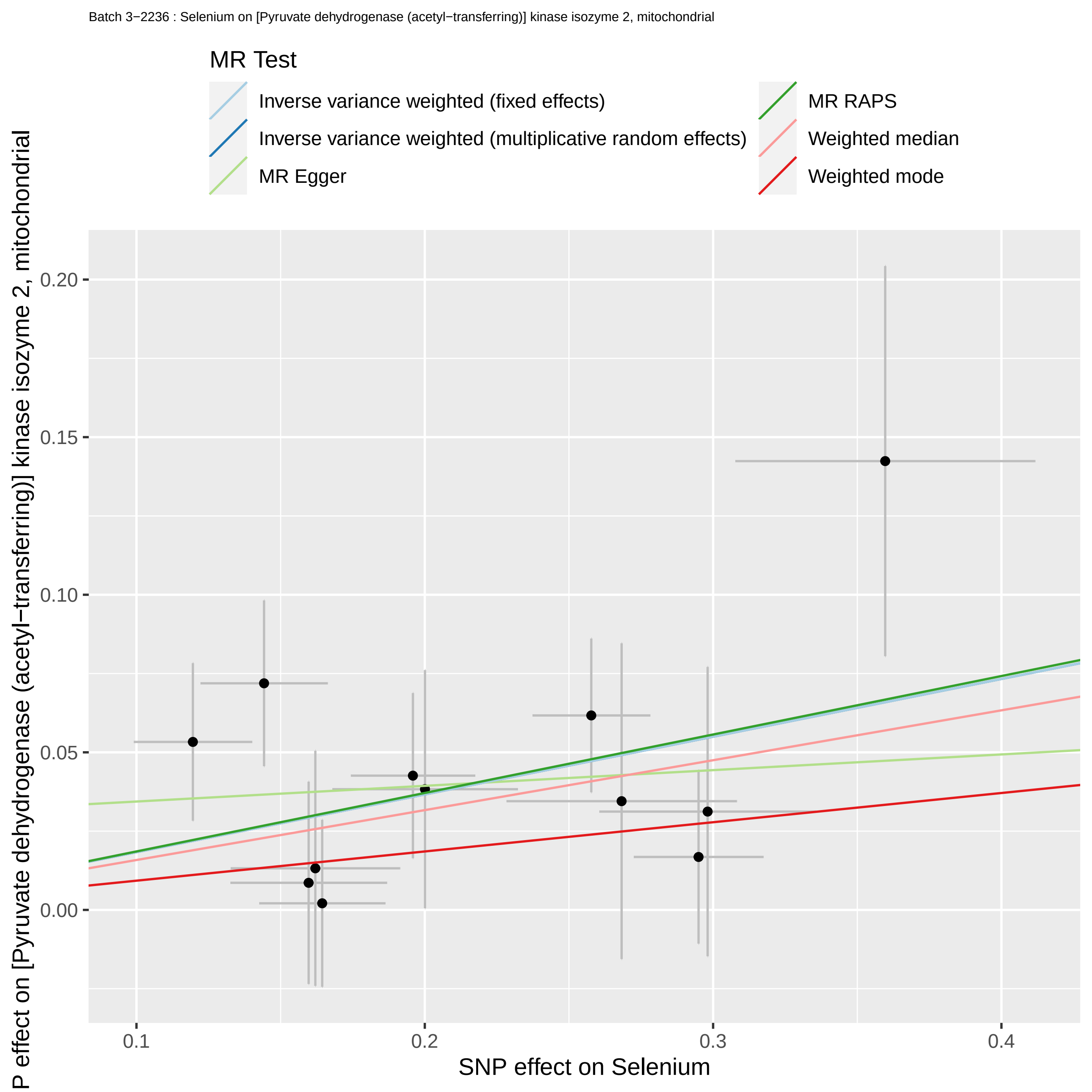

Batch 3−2236 : Selenium on [Pyruvate dehydrogenase (acetyl−transferring)] kinase isozyme 2, mitochondrial
MR Test
Inverse variance weighted (fixed effects)
Inverse variance weighted (multiplicative random effects)
MR Egger
MR RAPS
Weighted median
Weighted mode
0.20
0.15
0.10
0.05
0.00
0.1
0.2
0.3
0.4
SNP effect on Selenium
P effect on [Pyruvate dehydrogenase (acetyl−transferring)] kinase isozyme 2, mitochondrial

Batch 3−2237 : Selenium on PDZ and LIM domain protein 4

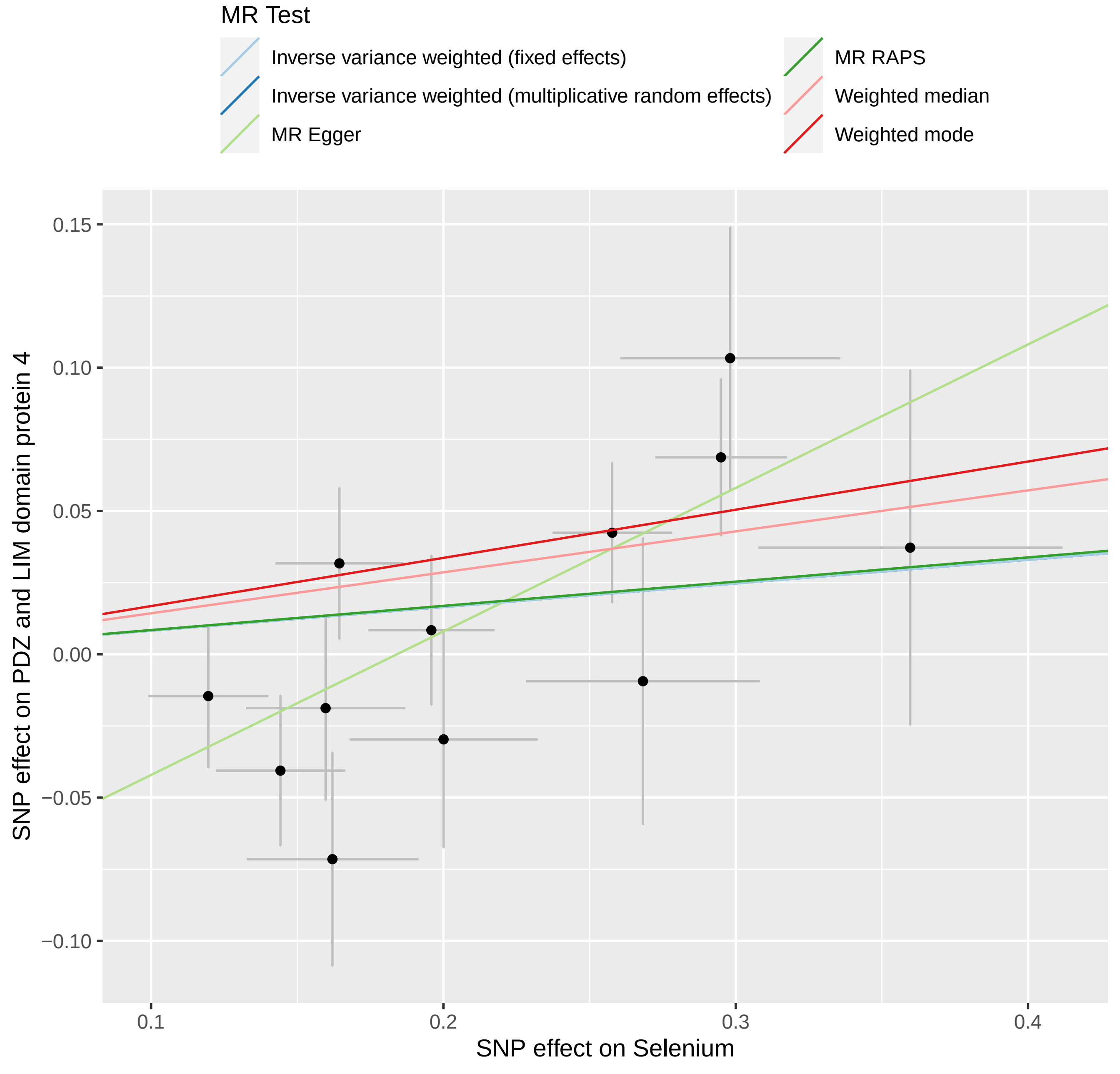

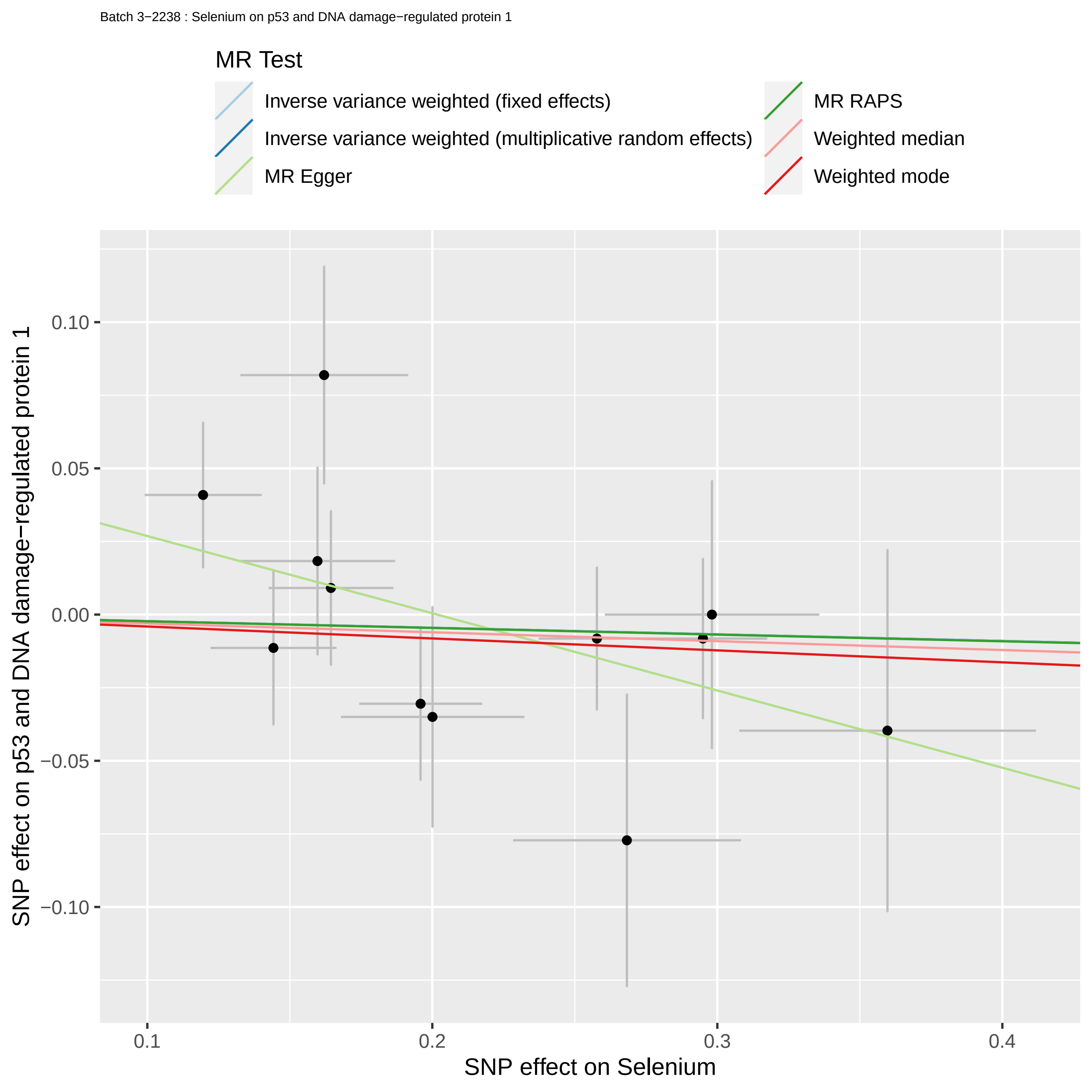
Batch 3−2238 : Selenium on p53 and DNA damage−regulated protein 1
MR Test
Inverse variance weighted (fixed effects)
Inverse variance weighted (multiplicative random effects)
MR Egger
MR RAPS
Weighted median
Weighted mode
0.10
0.05
0.00
−0.05
−0.10
0.1
0.2
0.3
0.4
SNP effect on p53 and DNA damage−regulated protein 1
SNP effect on Selenium

Batch 3−2239 : Selenium on Pyridoxal kinase

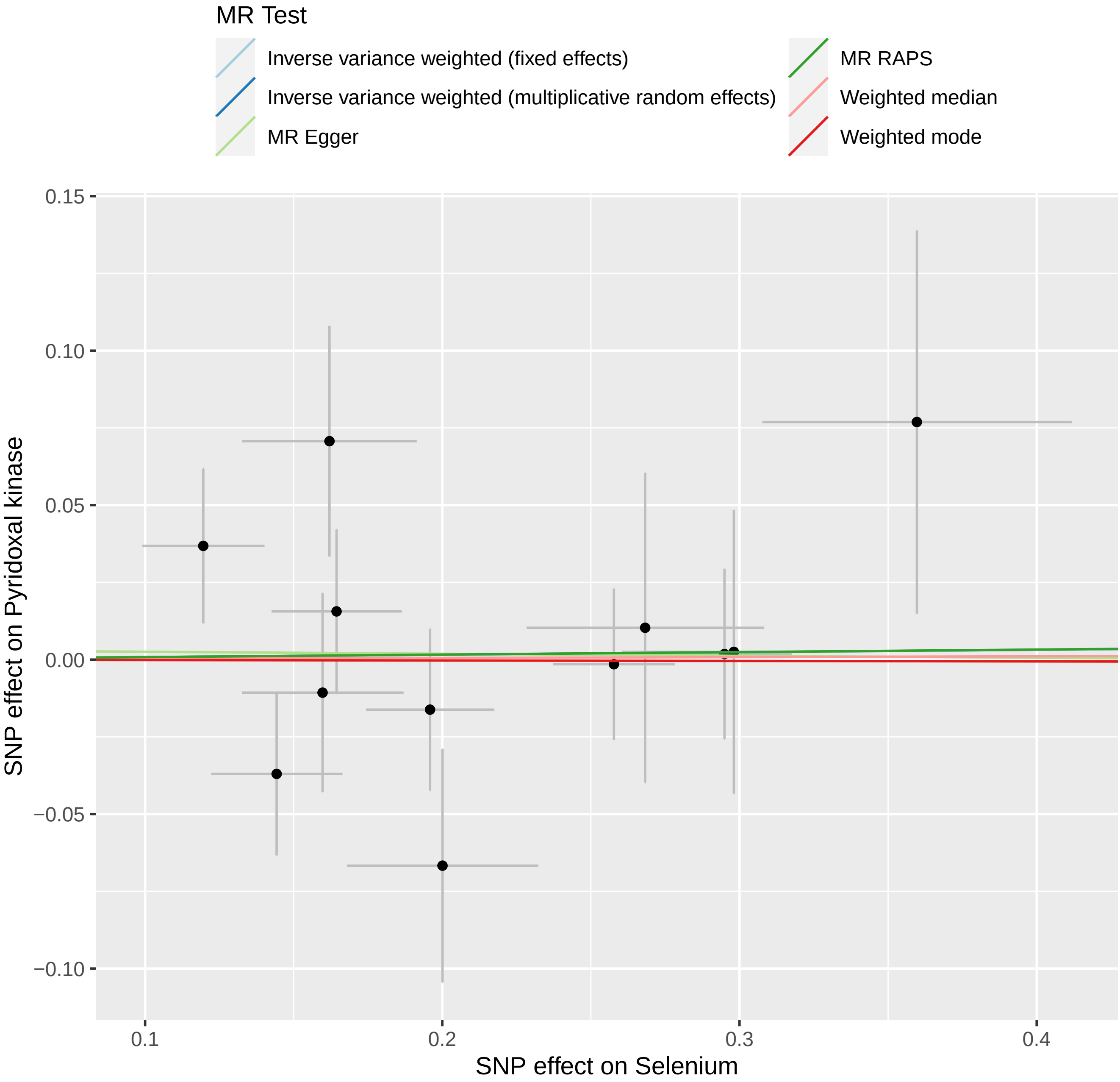

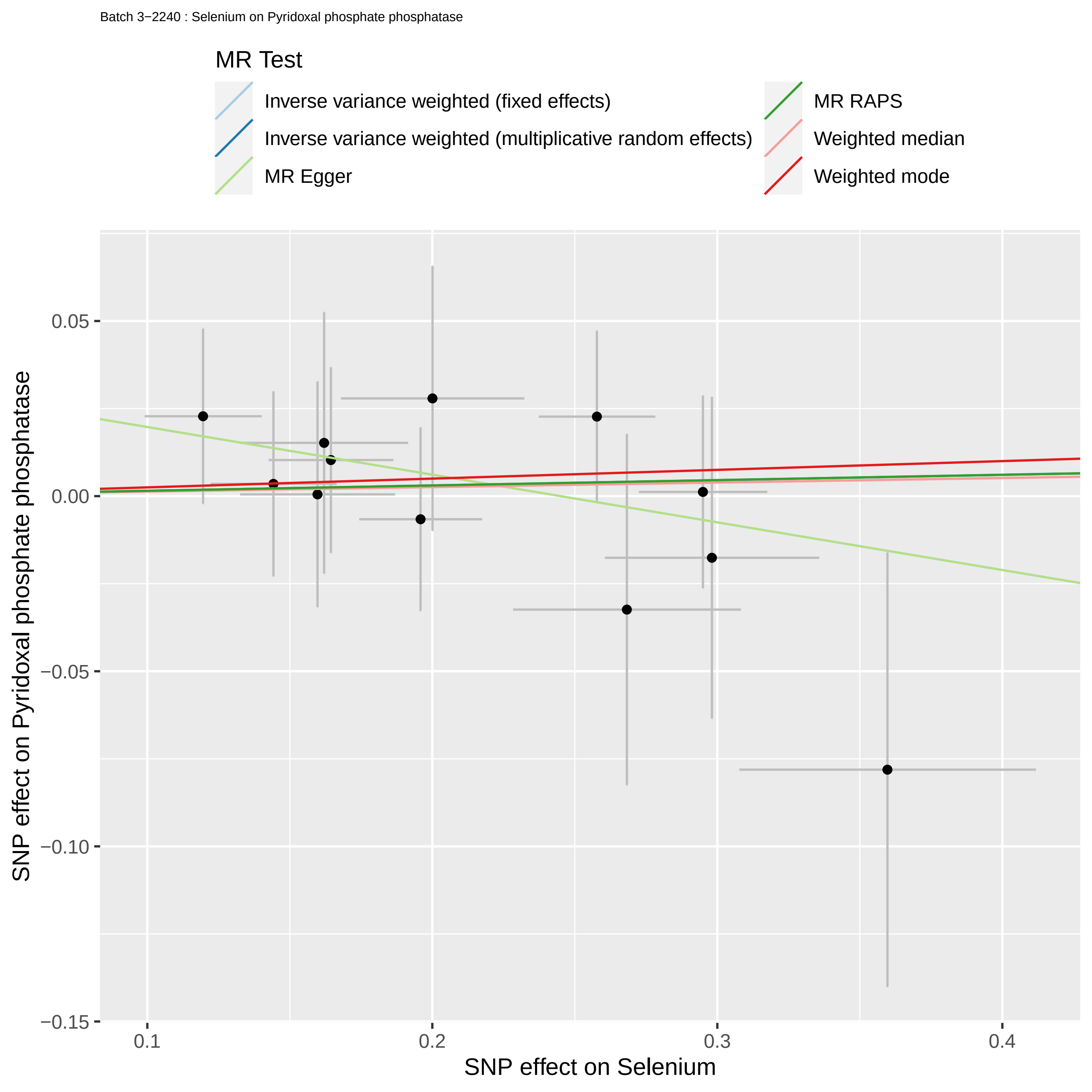

Batch 3−2240 : Selenium on Pyridoxal phosphate phosphatase
MR Test
Inverse variance weighted (fixed effects)
Inverse variance weighted (multiplicative random effects)
MR Egger
MR RAPS
Weighted median
Weighted mode
0.05
0.00
−0.05
−0.10
−0.15
0.1
0.2
0.3
0.4
SNP effect on Pyridoxal phosphate phosphatase
SNP effect on Selenium

Batch 3−2241 : Selenium on PDZ domain−containing protein 11

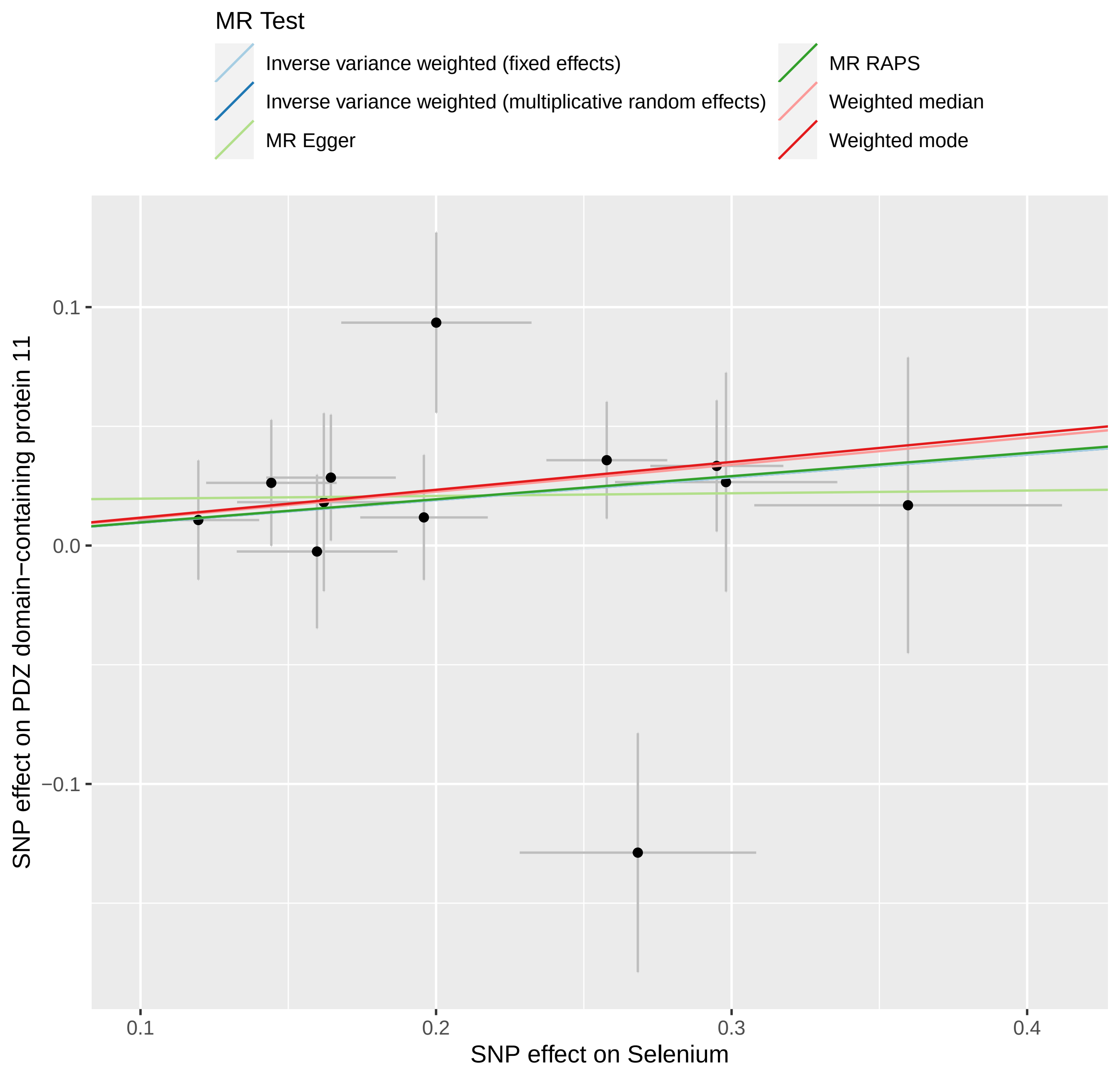

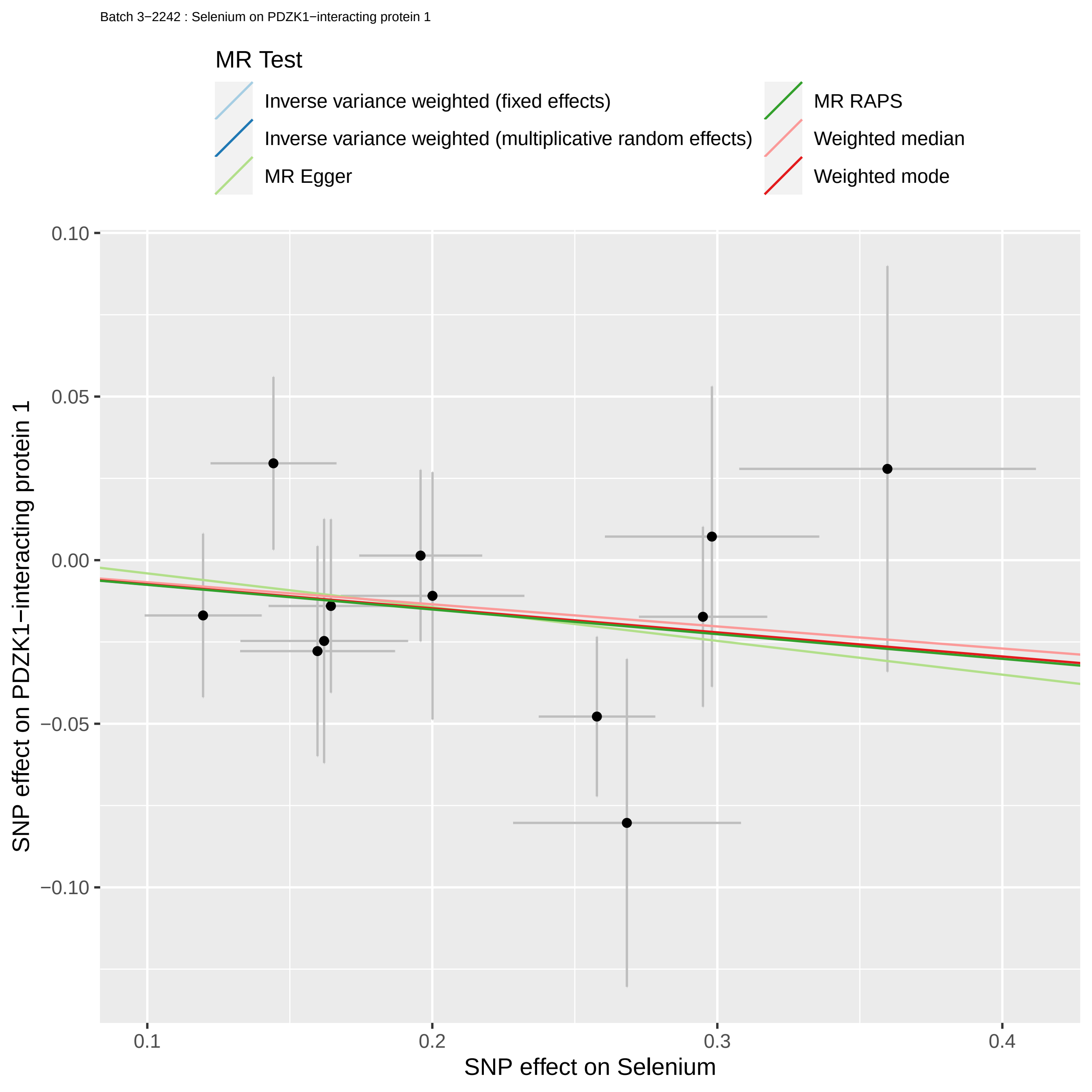
Batch 3−2242 : Selenium on PDZK1−interacting protein 1
MR Test
Inverse variance weighted (fixed effects)
Inverse variance weighted (multiplicative random effects)
MR Egger
MR RAPS
Weighted median
Weighted mode
0.10
0.05
0.00
−0.05
−0.10
0.1
0.2
0.3
0.4
SNP effect on PDZK1−interacting protein 1
SNP effect on Selenium

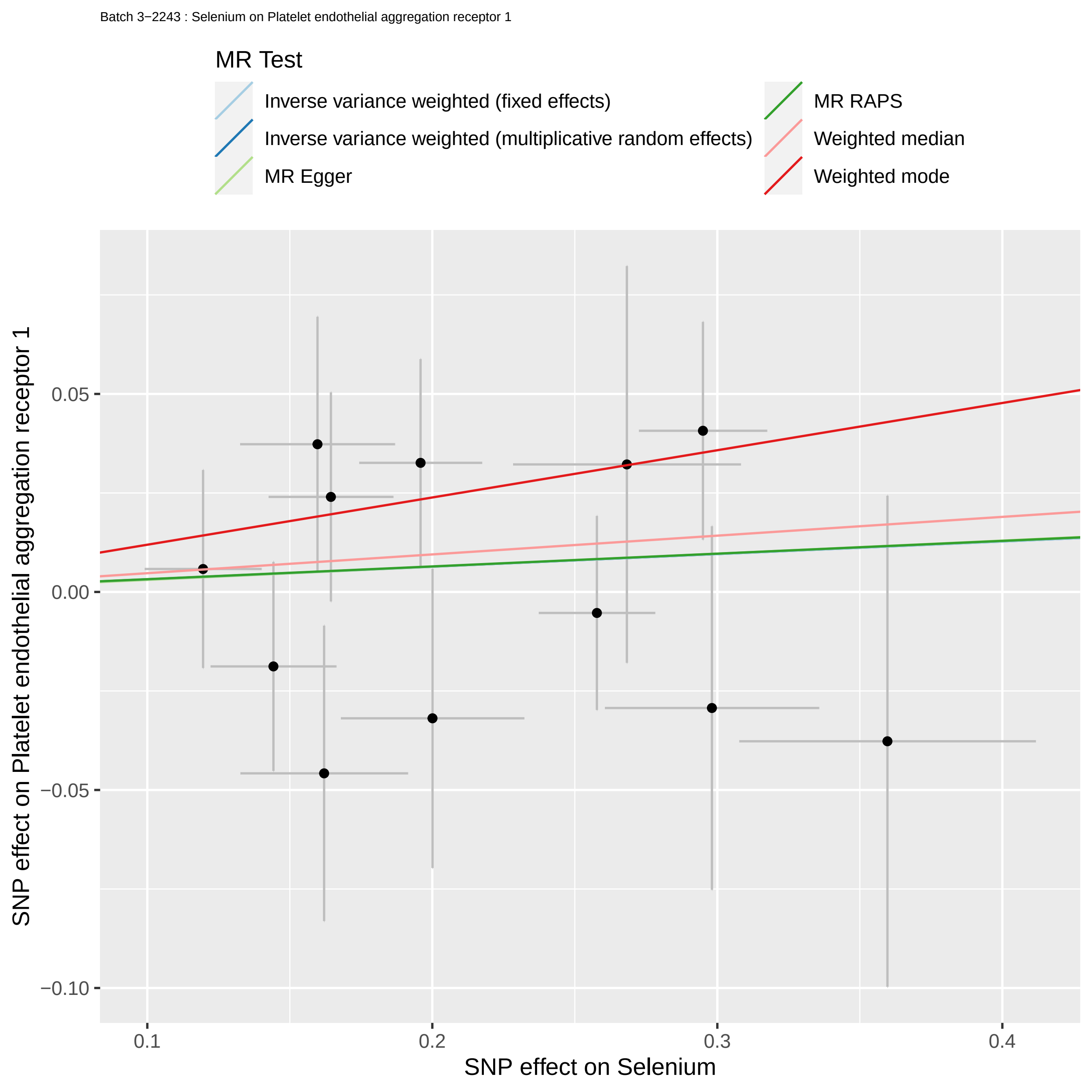
Batch 3−2243 : Selenium on Platelet endothelial aggregation receptor 1
MR Test
Inverse variance weighted (fixed effects)
Inverse variance weighted (multiplicative random effects)
MR Egger
MR RAPS
Weighted median
Weighted mode
SNP effect on Platelet endothelial aggregation receptor 1
0.05
0.00
−0.05
−0.10
0.1
0.2
0.3
0.4
SNP effect on Selenium

Batch 3−2244 : Selenium on Platelet endothelial aggregation receptor 1

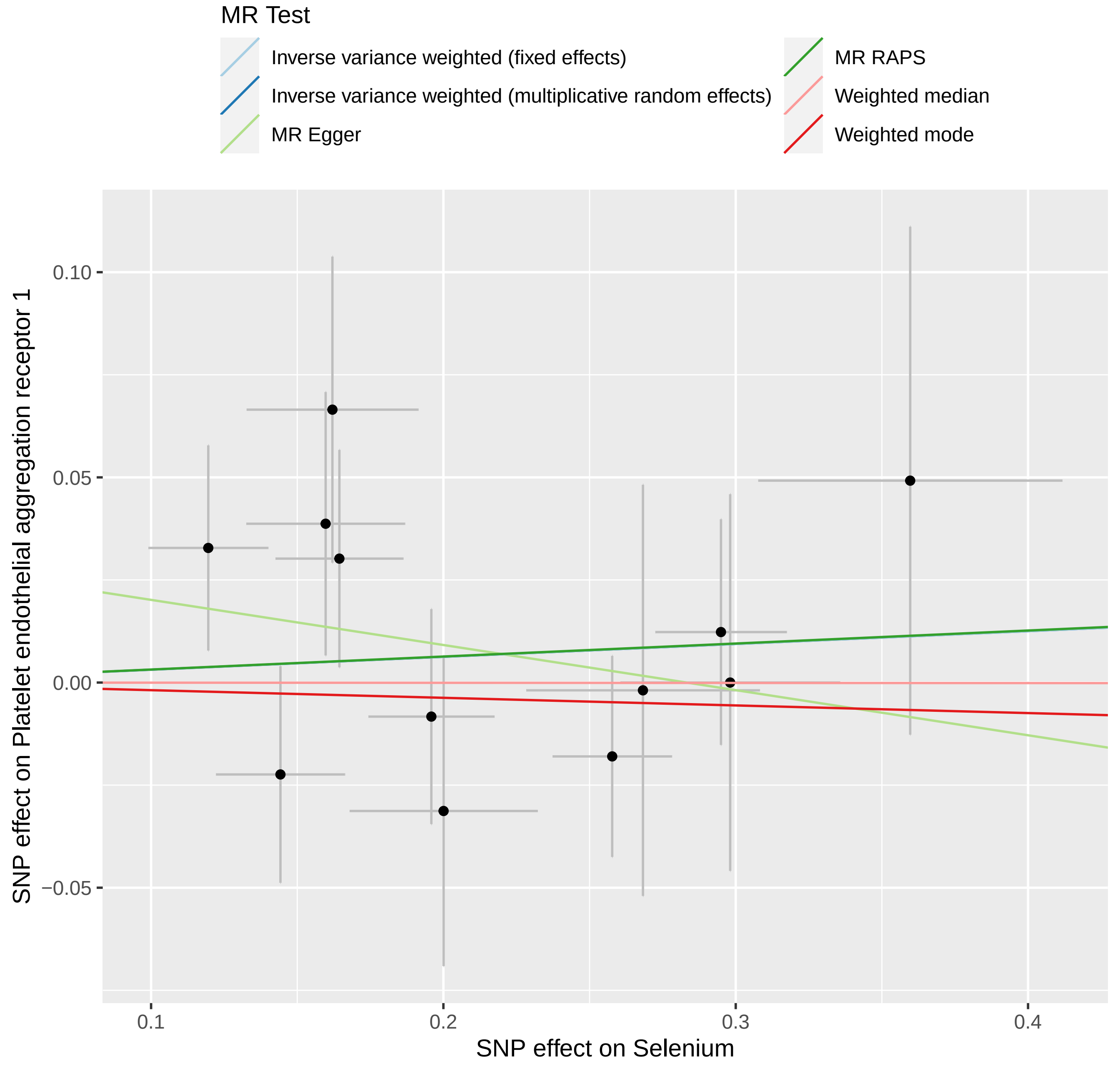

Batch 3−2245 : Selenium on Platelet endothelial cell adhesion molecule

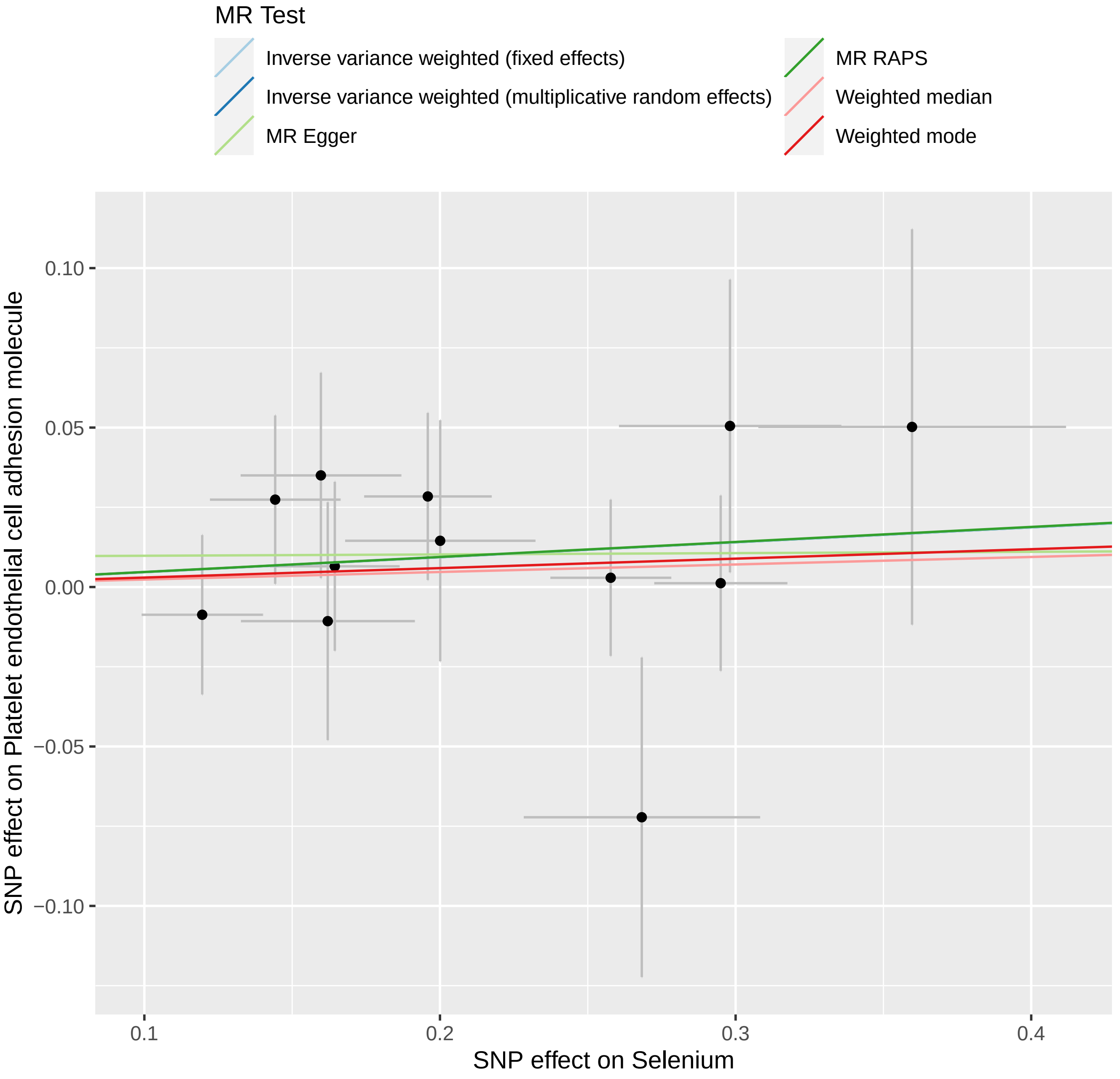

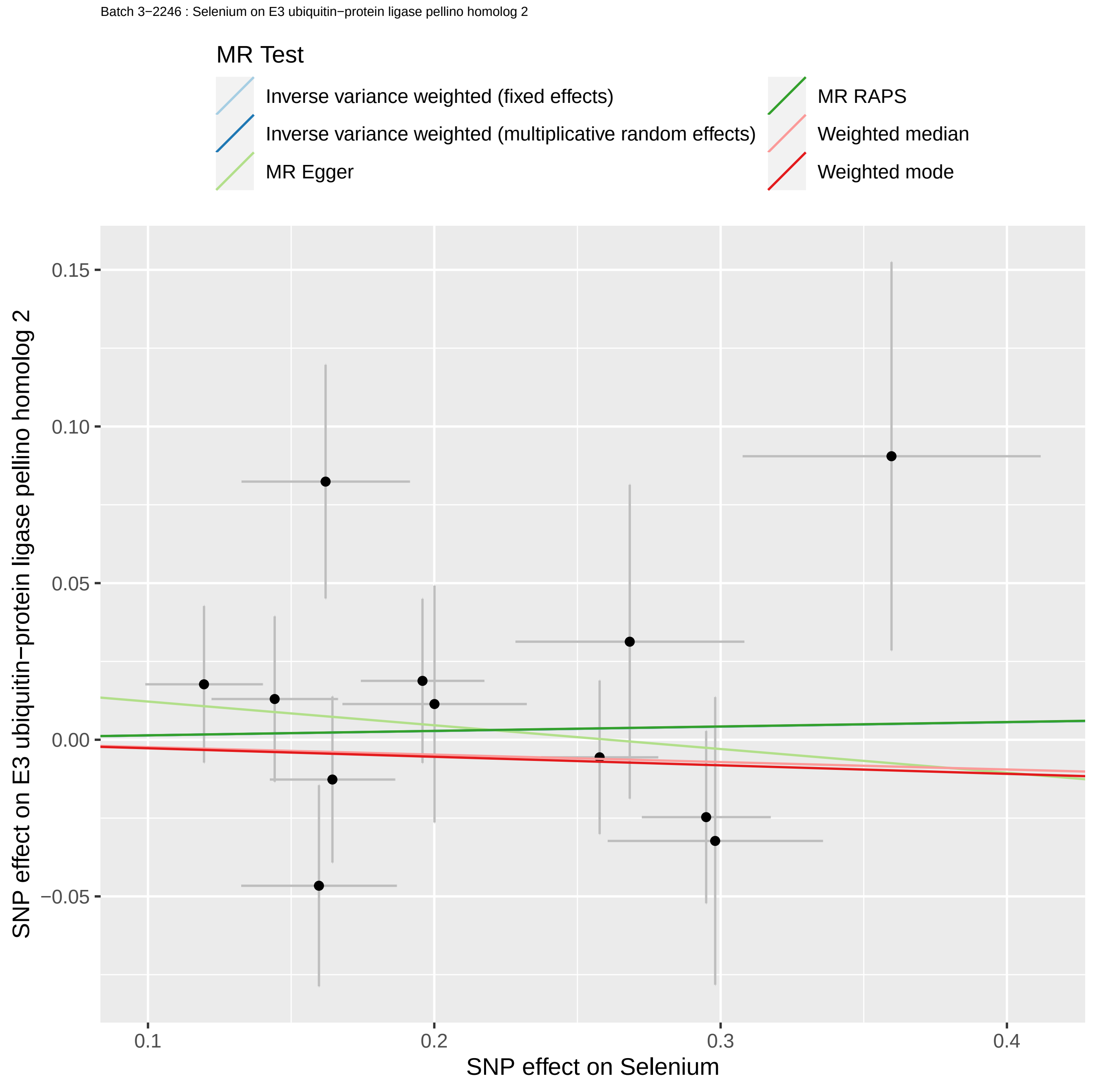
Batch 3−2246 : Selenium on E3 ubiquitin−protein ligase pellino homolog 2
MR Test
Inverse variance weighted (fixed effects)
Inverse variance weighted (multiplicative random effects)
MR Egger
MR RAPS
Weighted median
Weighted mode
SNP effect on E3 ubiquitin−protein ligase pellino homolog 2
0.15
0.10
0.05
0.00
−0.05
0.1
0.2
0.3
0.4
SNP effect on Selenium

Batch 3−2247 : Selenium on Proenkephalin−A

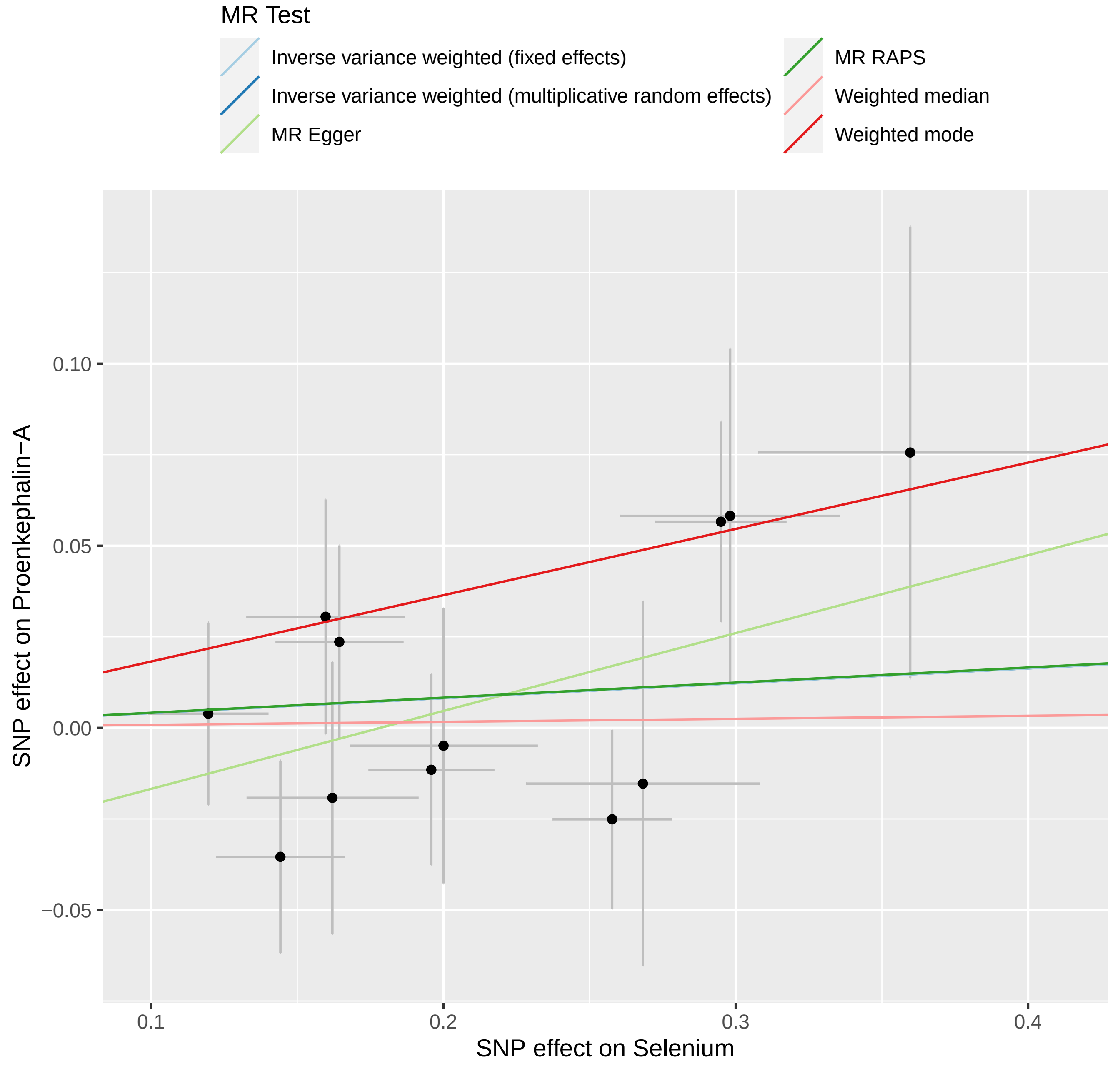

Batch 3−2248 : Selenium on Pescadillo homolog

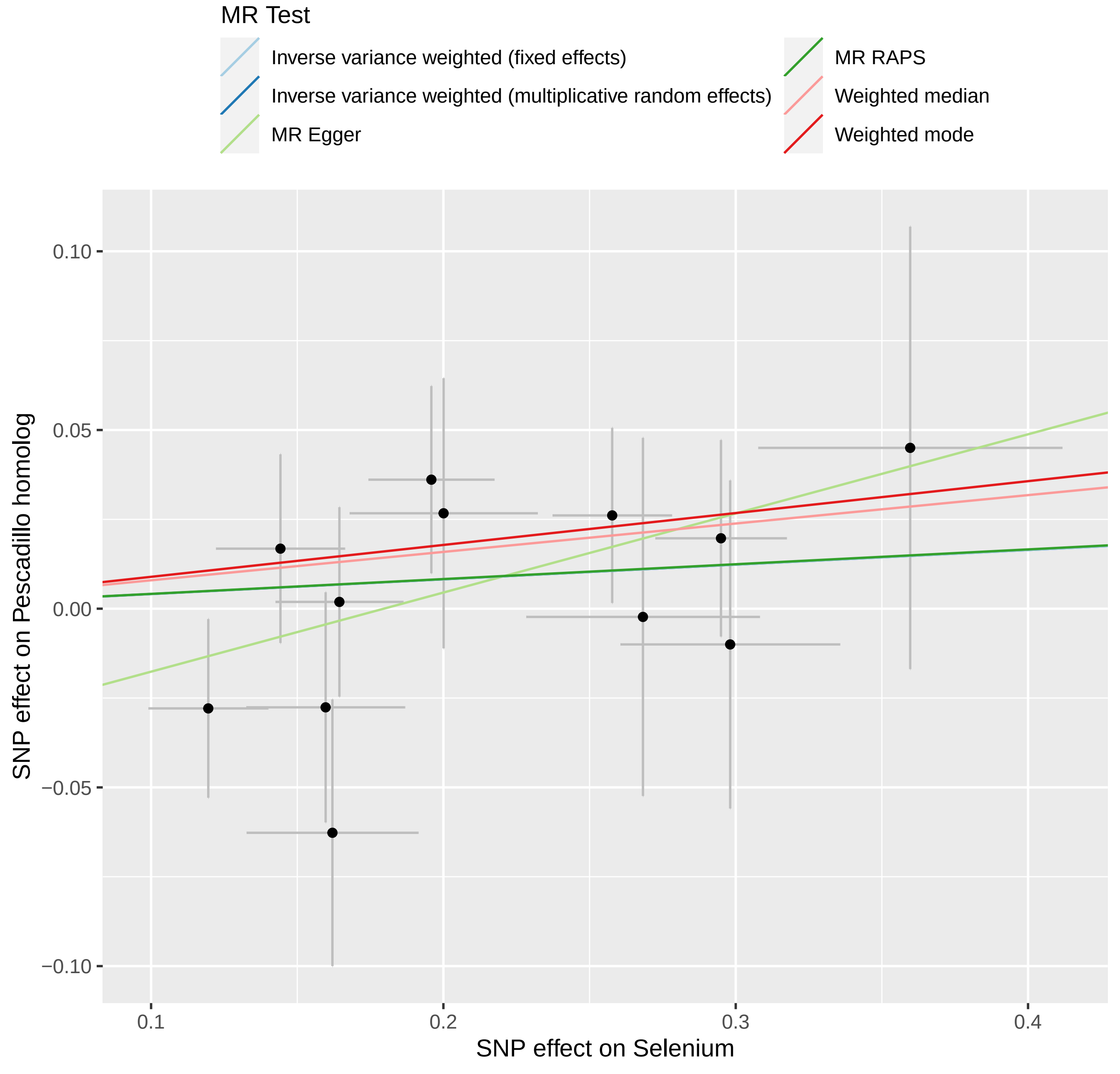

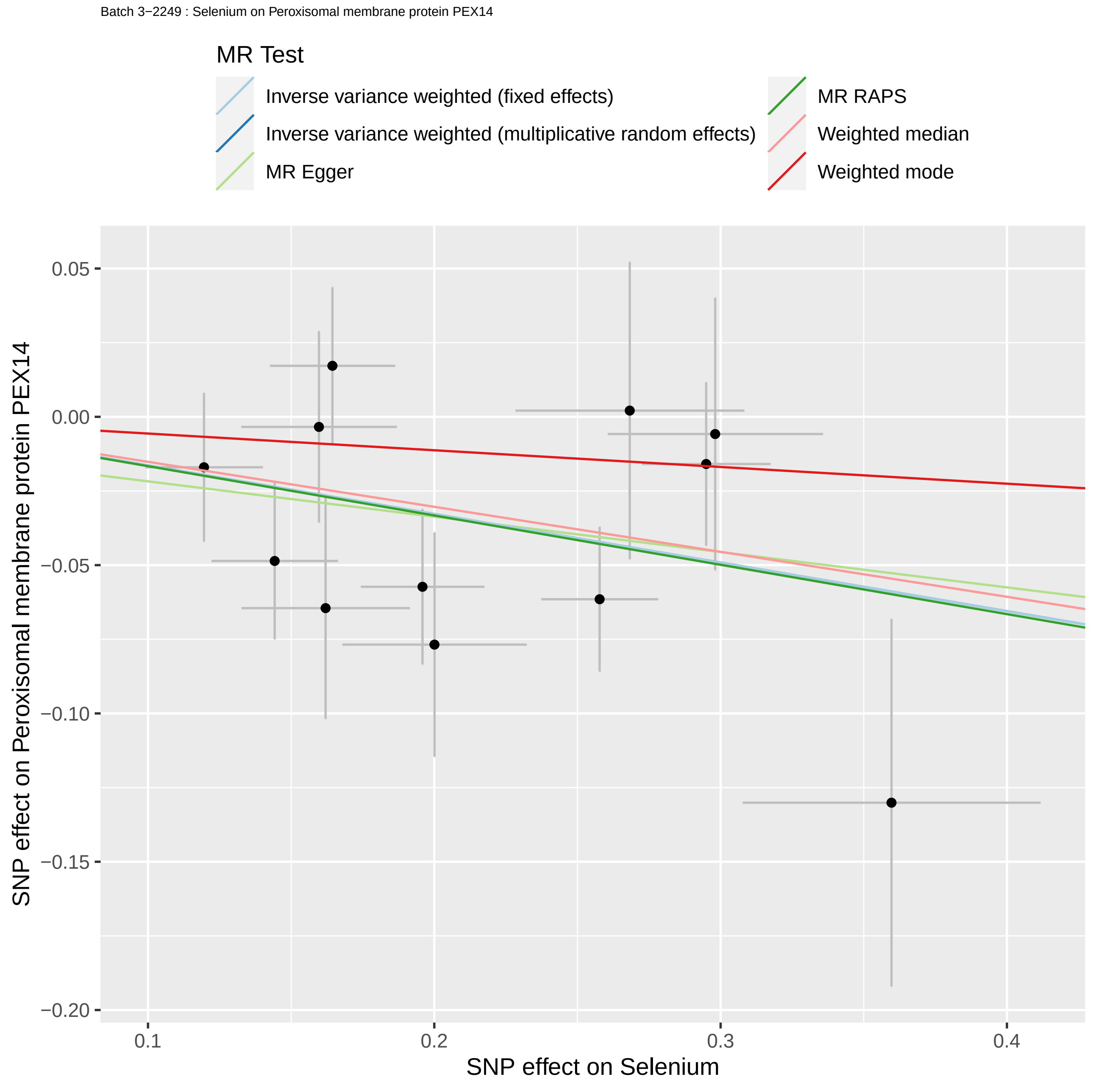
Batch 3−2249 : Selenium on Peroxisomal membrane protein PEX14
MR Test
Inverse variance weighted (fixed effects)
Inverse variance weighted (multiplicative random effects)
MR Egger
MR RAPS
Weighted median
Weighted mode
SNP effect on Peroxisomal membrane protein PEX14
0.05
0.00
−0.05
−0.10
−0.15
−0.20
0.1
0.2
0.3
0.4
SNP effect on Selenium

Batch 3−2250 : Selenium on Peroxisomal targeting signal 1 receptor

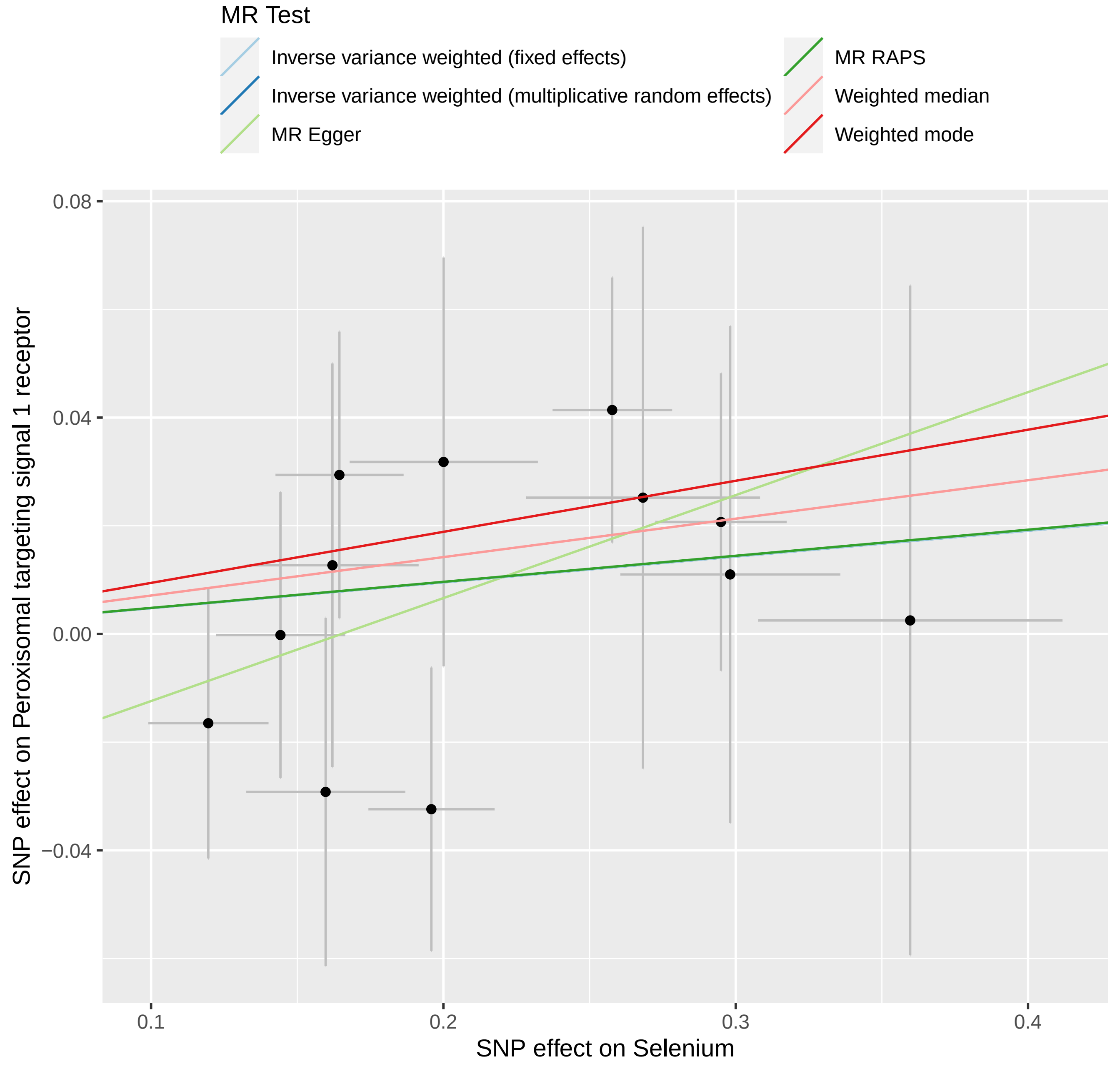

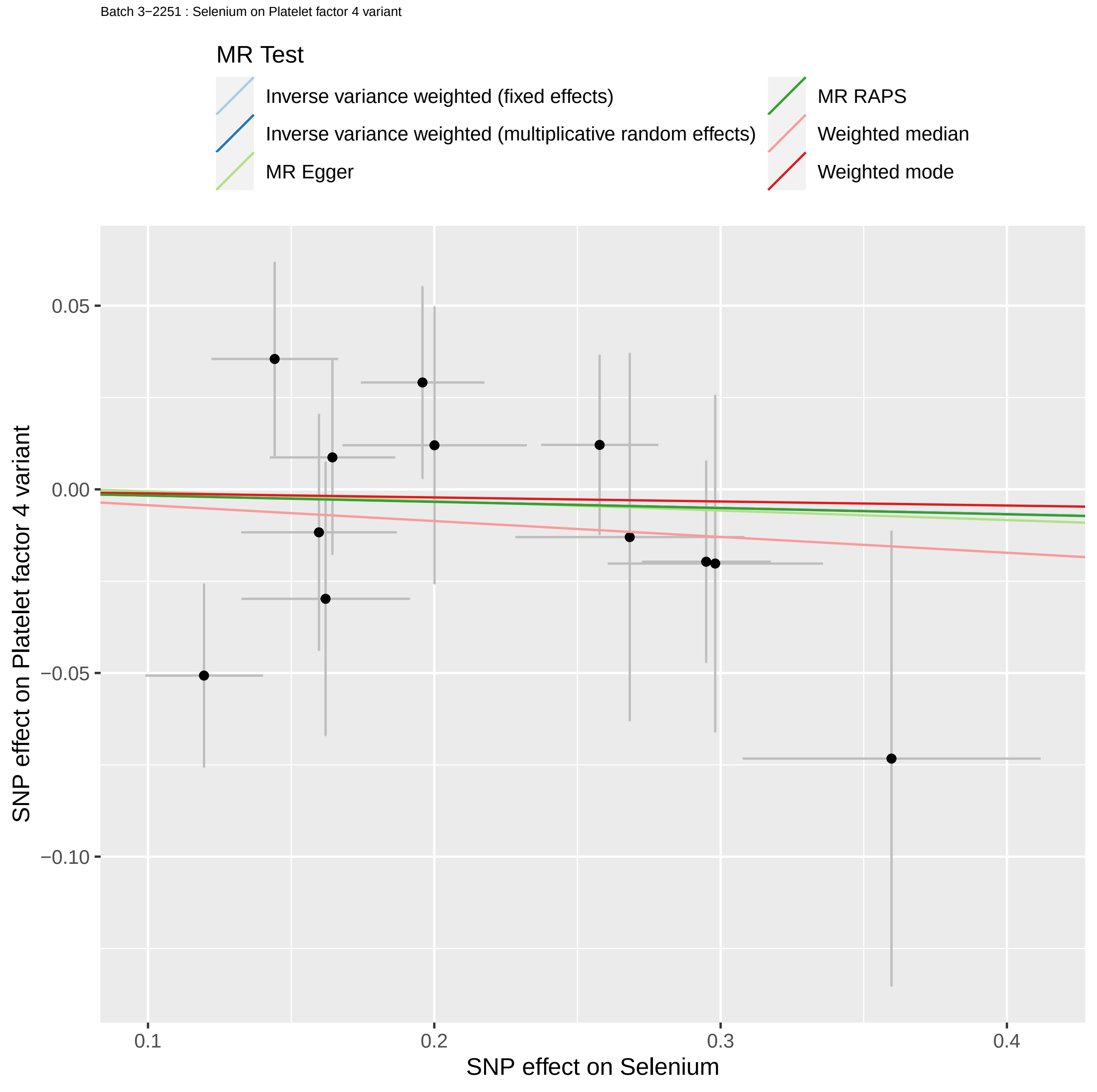
Batch 3−2251 : Selenium on Platelet factor 4 variant
MR Test
Inverse variance weighted (fixed effects)
Inverse variance weighted (multiplicative random effects)
MR Egger
MR RAPS
Weighted median
Weighted mode
0.05
0.00
−0.05
−0.10
0.1
0.2
0.3
0.4
SNP effect on Platelet factor 4 variant
SNP effect on Selenium

Batch 3−2252 : Selenium on Prefoldin subunit 5

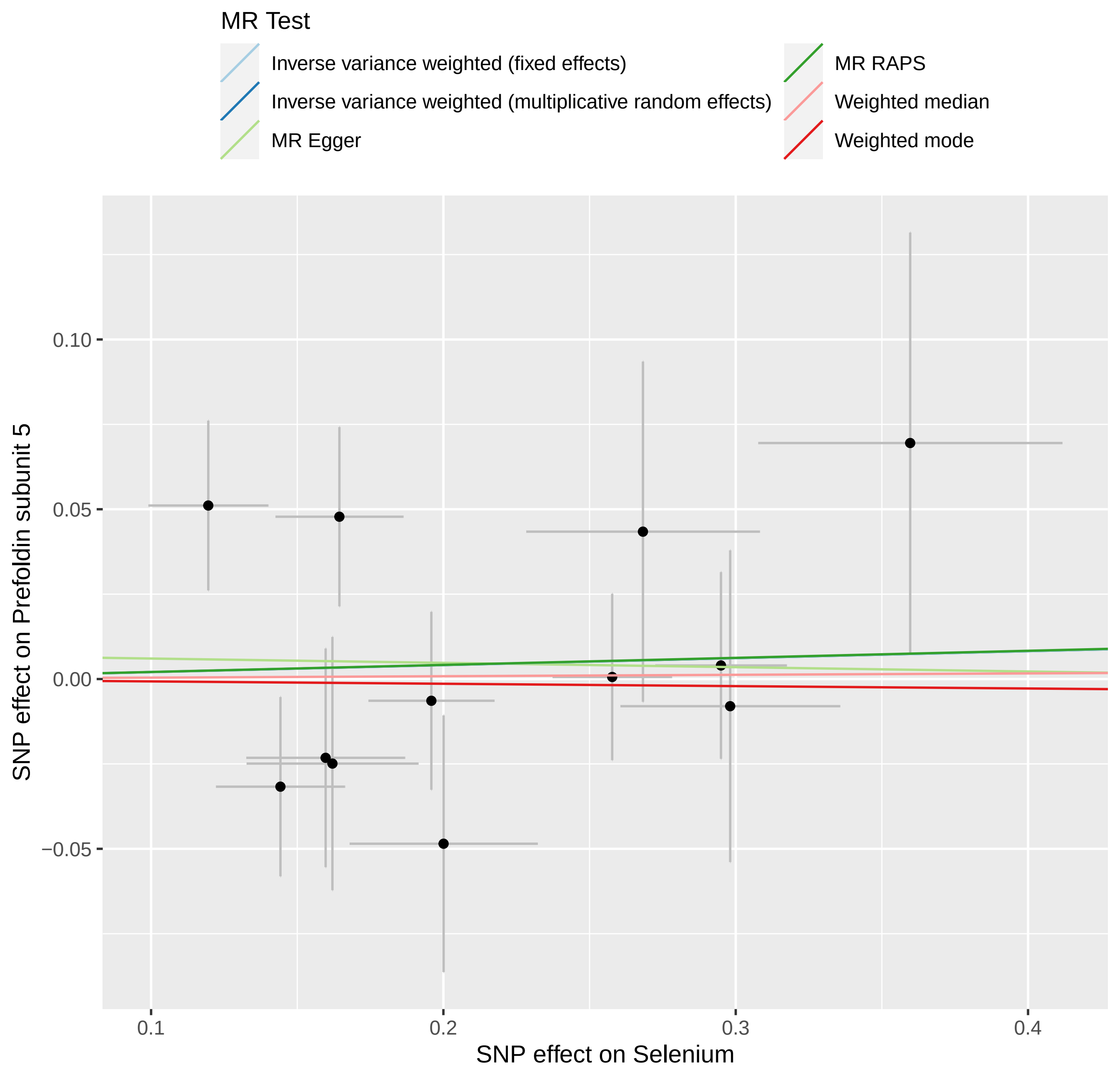

Batch 3−2253 : Selenium on Profilin−2

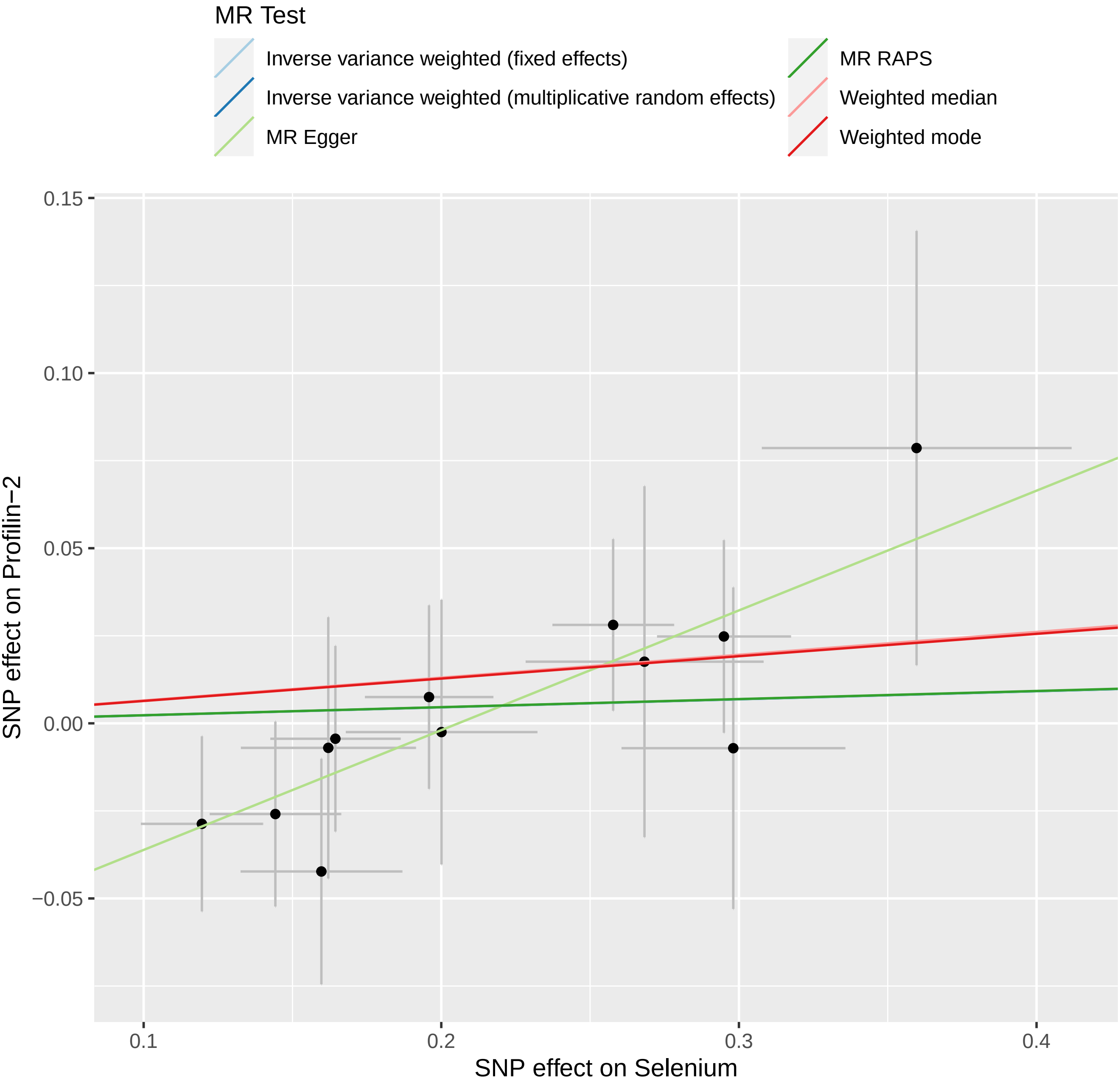

Batch 3−2254 : Selenium on Placenta growth factor

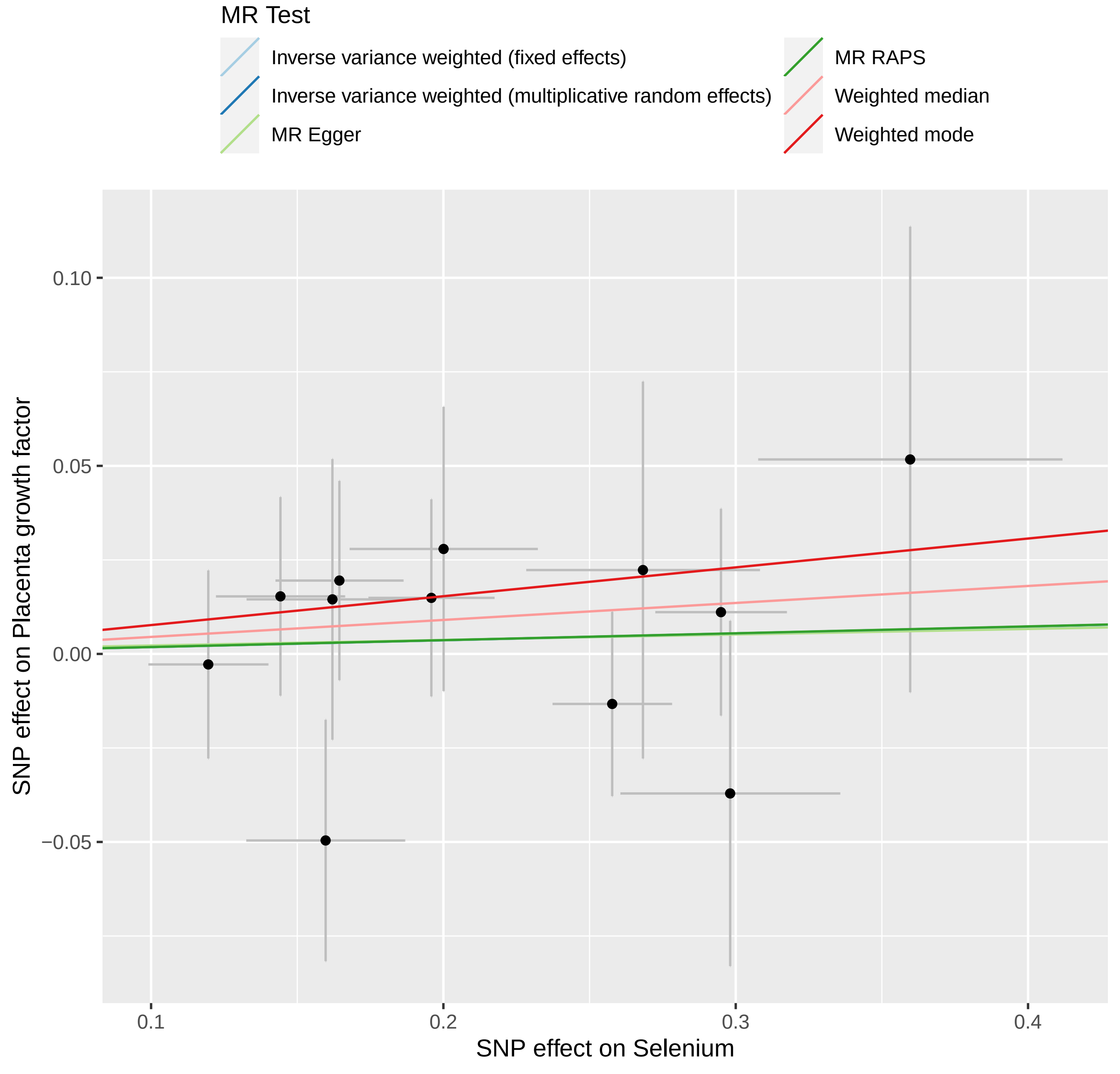

Batch 3−2255 : Selenium on Phosphoglycerate kinase 1

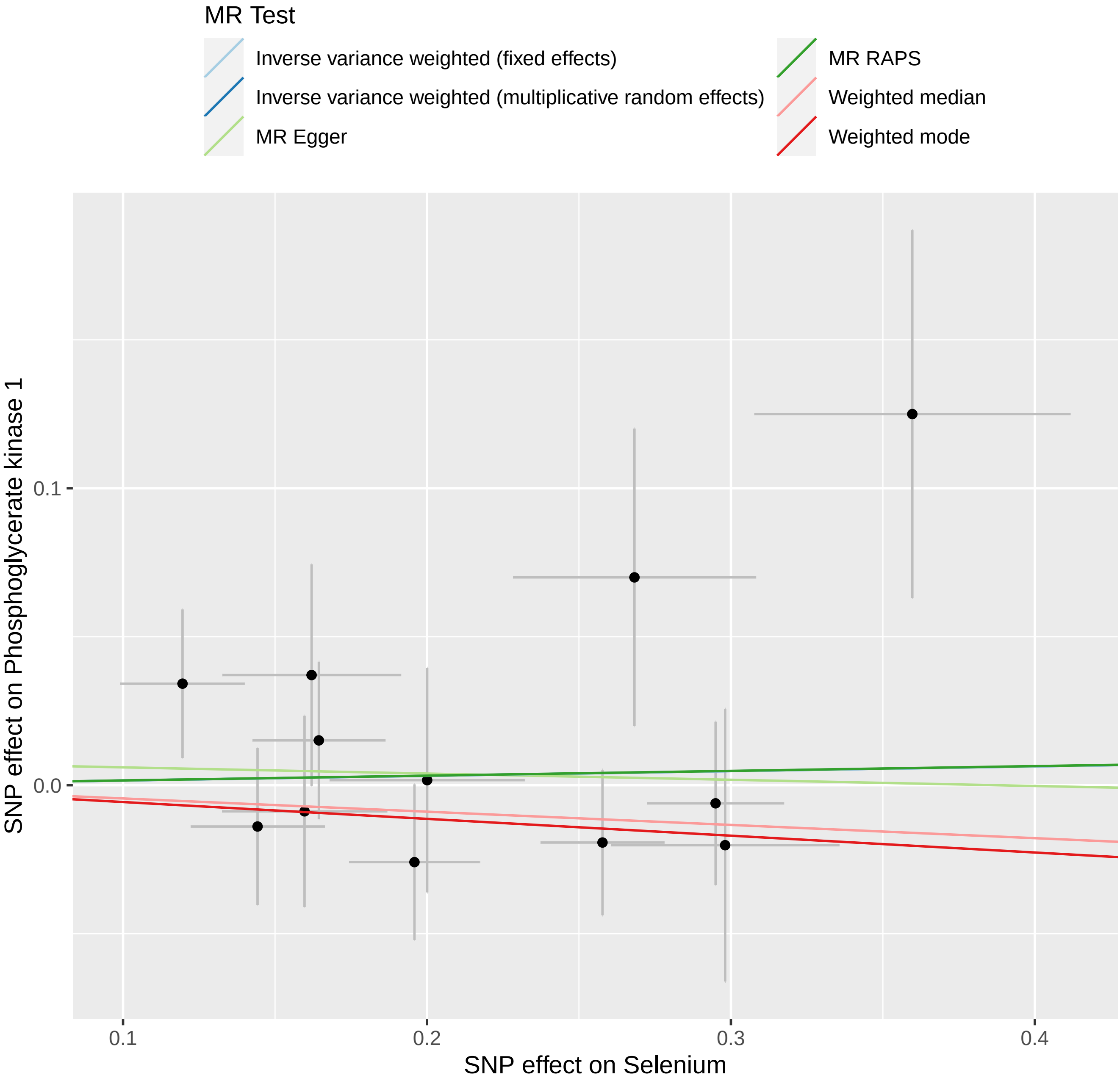

Batch 3−2256 : Selenium on Phosphoglycerate kinase 2

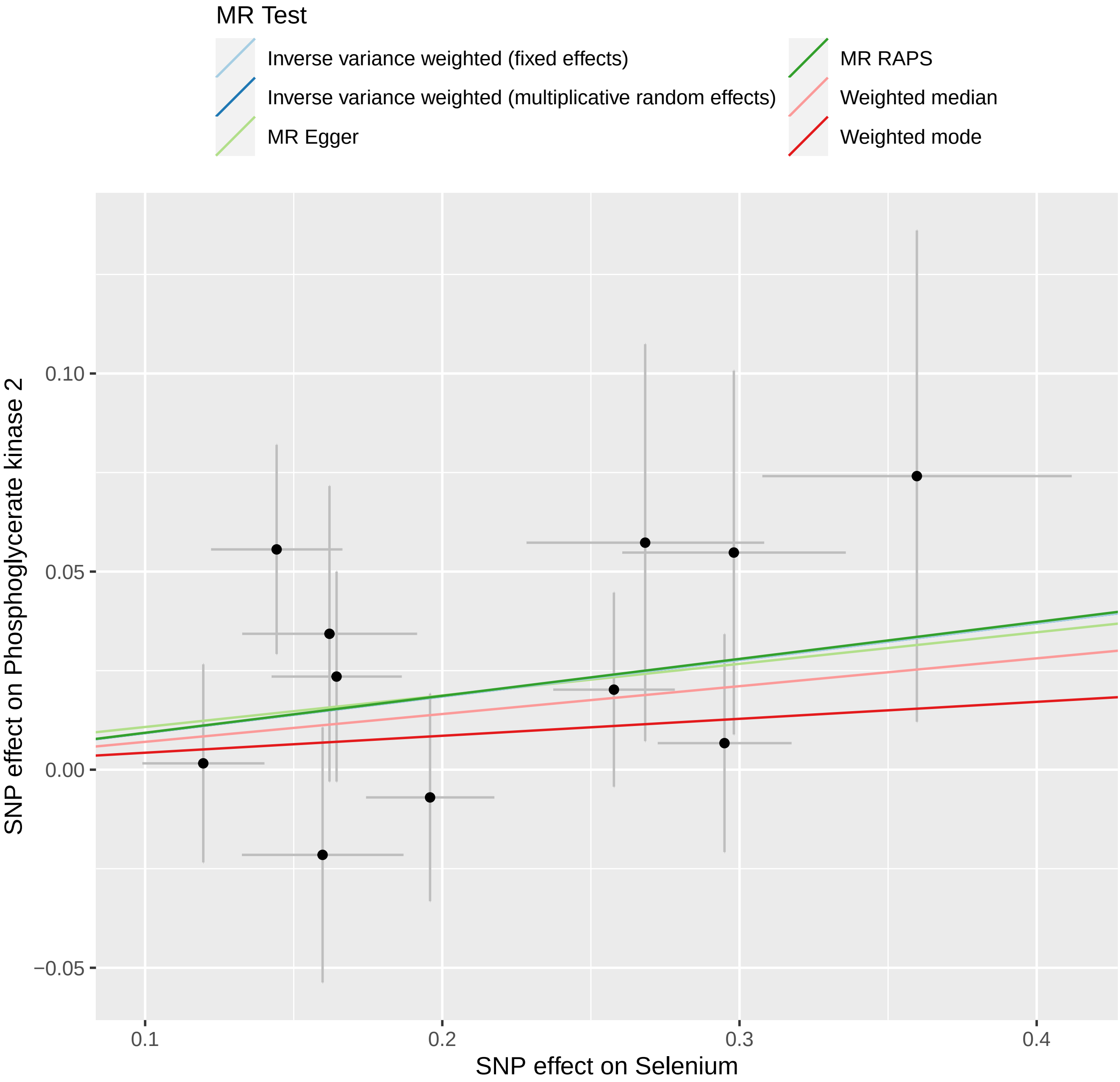

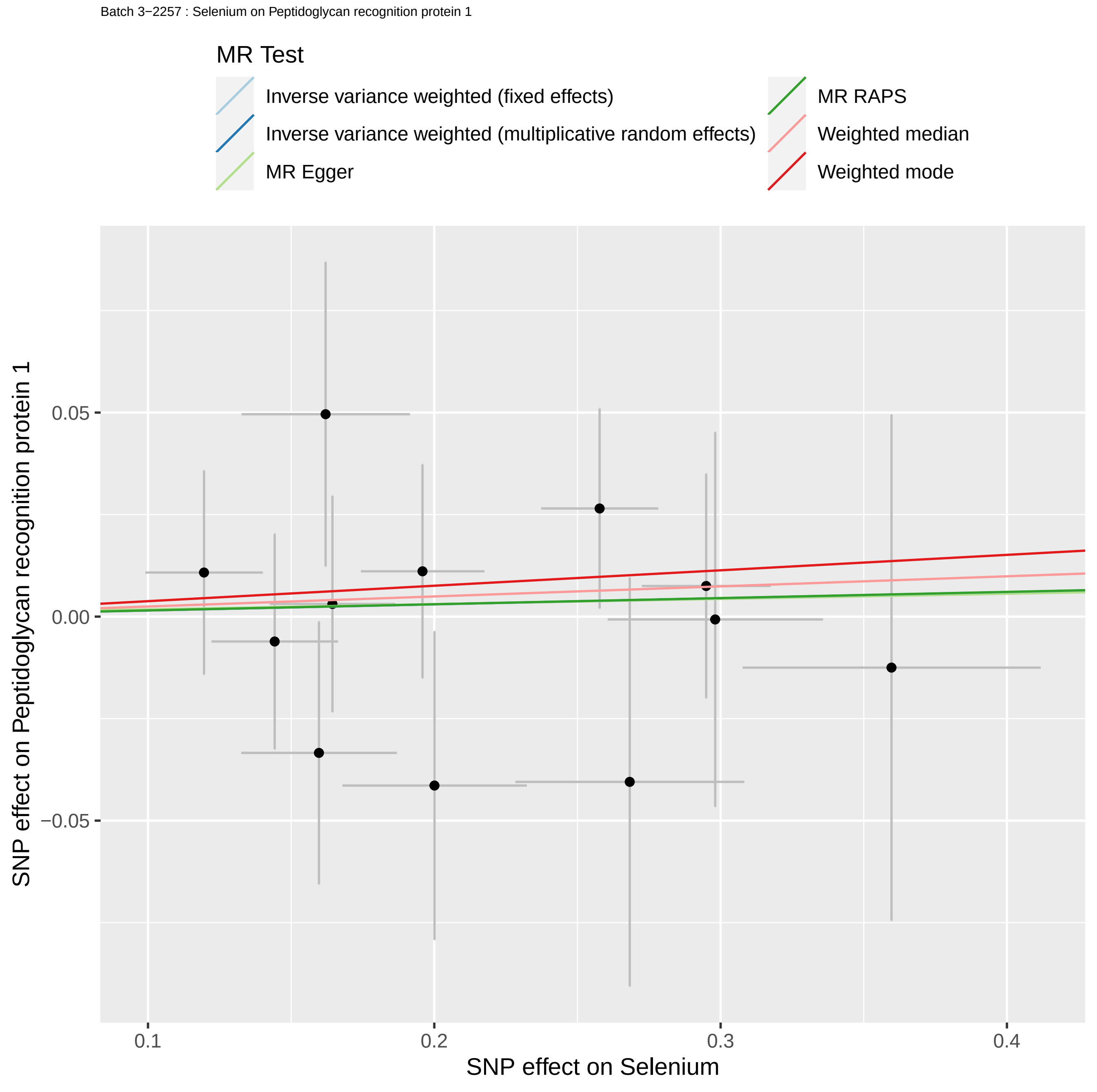

Batch 3−2257 : Selenium on Peptidoglycan recognition protein 1
MR Test
Inverse variance weighted (fixed effects)
Inverse variance weighted (multiplicative random effects)
MR Egger
MR RAPS
Weighted median
Weighted mode
SNP effect on Peptidoglycan recognition protein 1
0.05
0.00
−0.05
0.1
0.2
0.3
0.4
SNP effect on Selenium

Batch 3−2258 : Selenium on Phosphoglucomutase−1

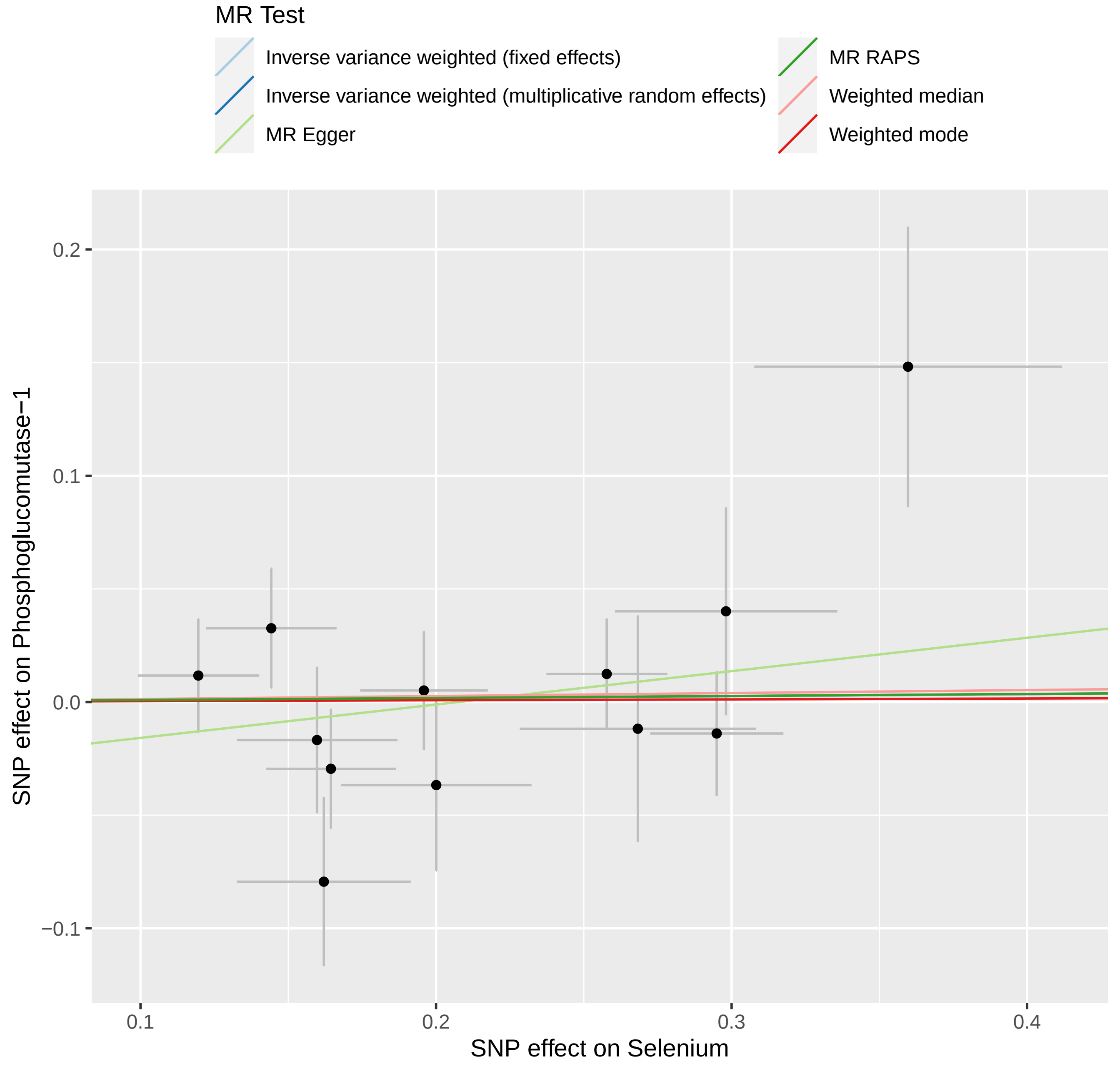

Batch 3−2259 : Selenium on Membrane−associated progesterone receptor component 1

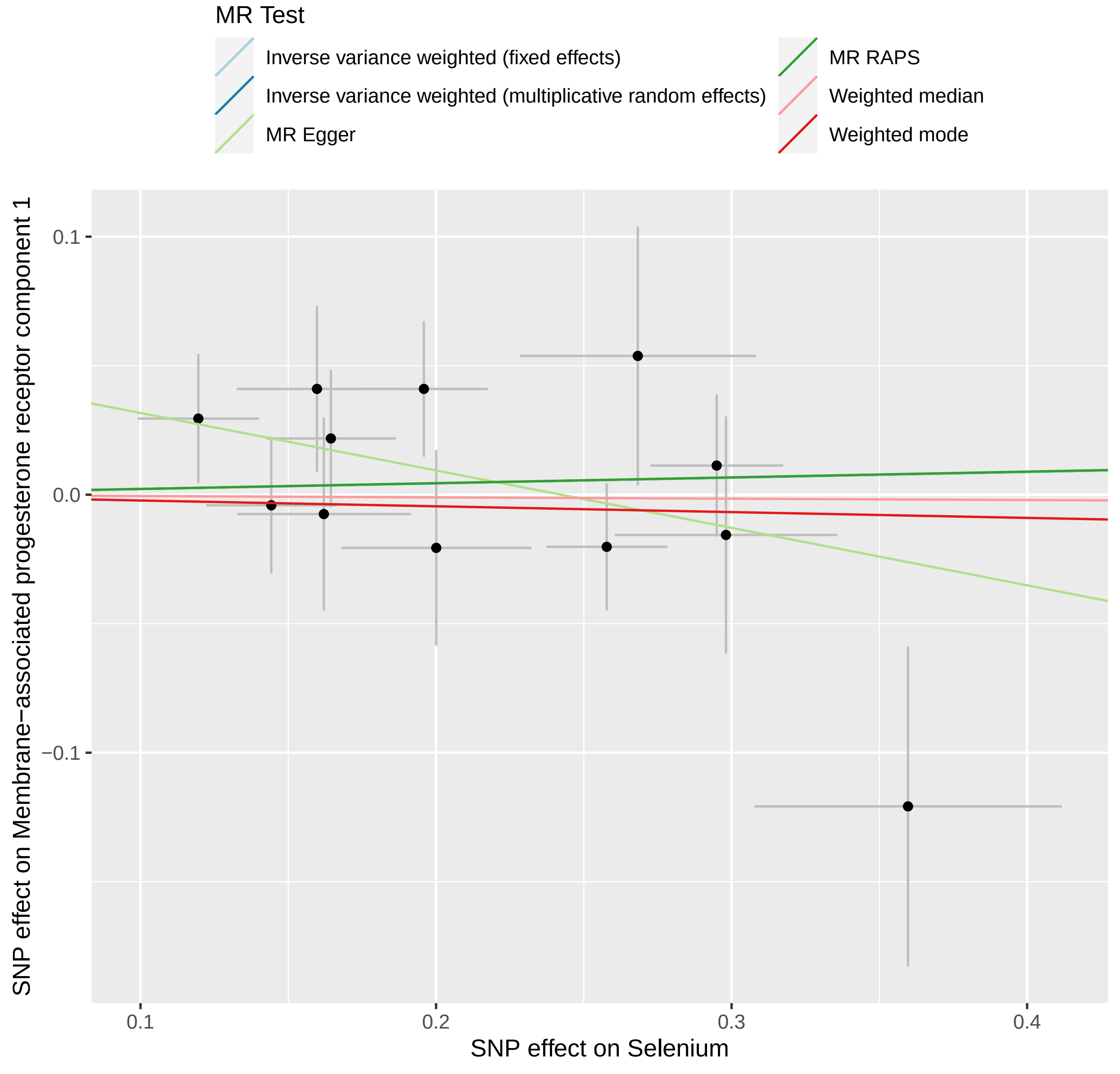

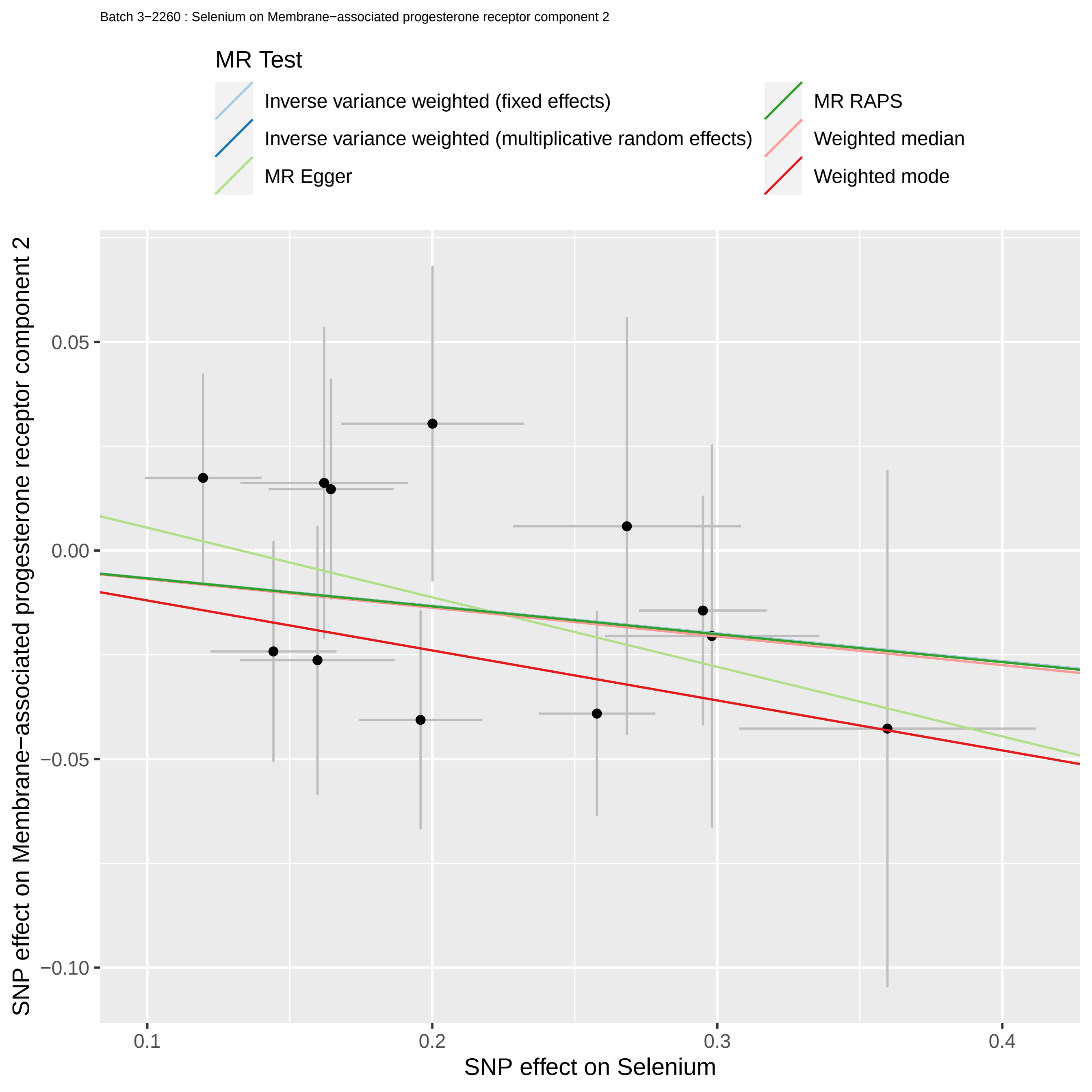
Batch 3−2260 : Selenium on Membrane−associated progesterone receptor component 2
MR Test
Inverse variance weighted (fixed effects)
Inverse variance weighted (multiplicative random effects)
MR Egger
MR RAPS
Weighted median
Weighted mode
SNP effect on Membrane−associated progesterone receptor component 2
0.05
0.00
−0.05
−0.10
0.1
0.2
0.3
0.4
SNP effect on Selenium

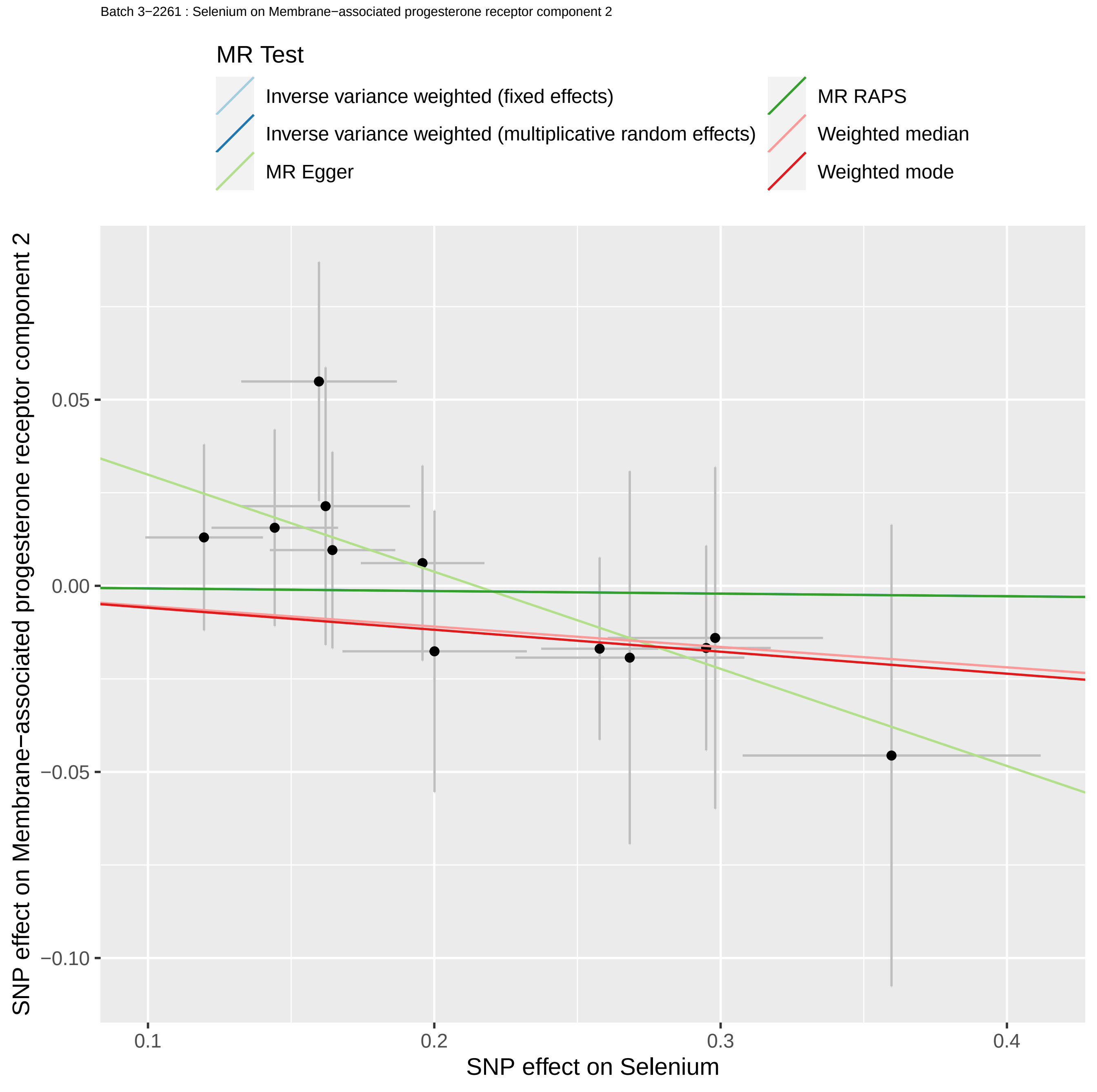
Batch 3−2261 : Selenium on Membrane−associated progesterone receptor component 2
MR Test
Inverse variance weighted (fixed effects)
Inverse variance weighted (multiplicative random effects)
MR Egger
MR RAPS
Weighted median
Weighted mode
SNP effect on Membrane−associated progesterone receptor component 2
0.05
0.00
−0.05
−0.10
0.1
0.2
0.3
0.4
SNP effect on Selenium

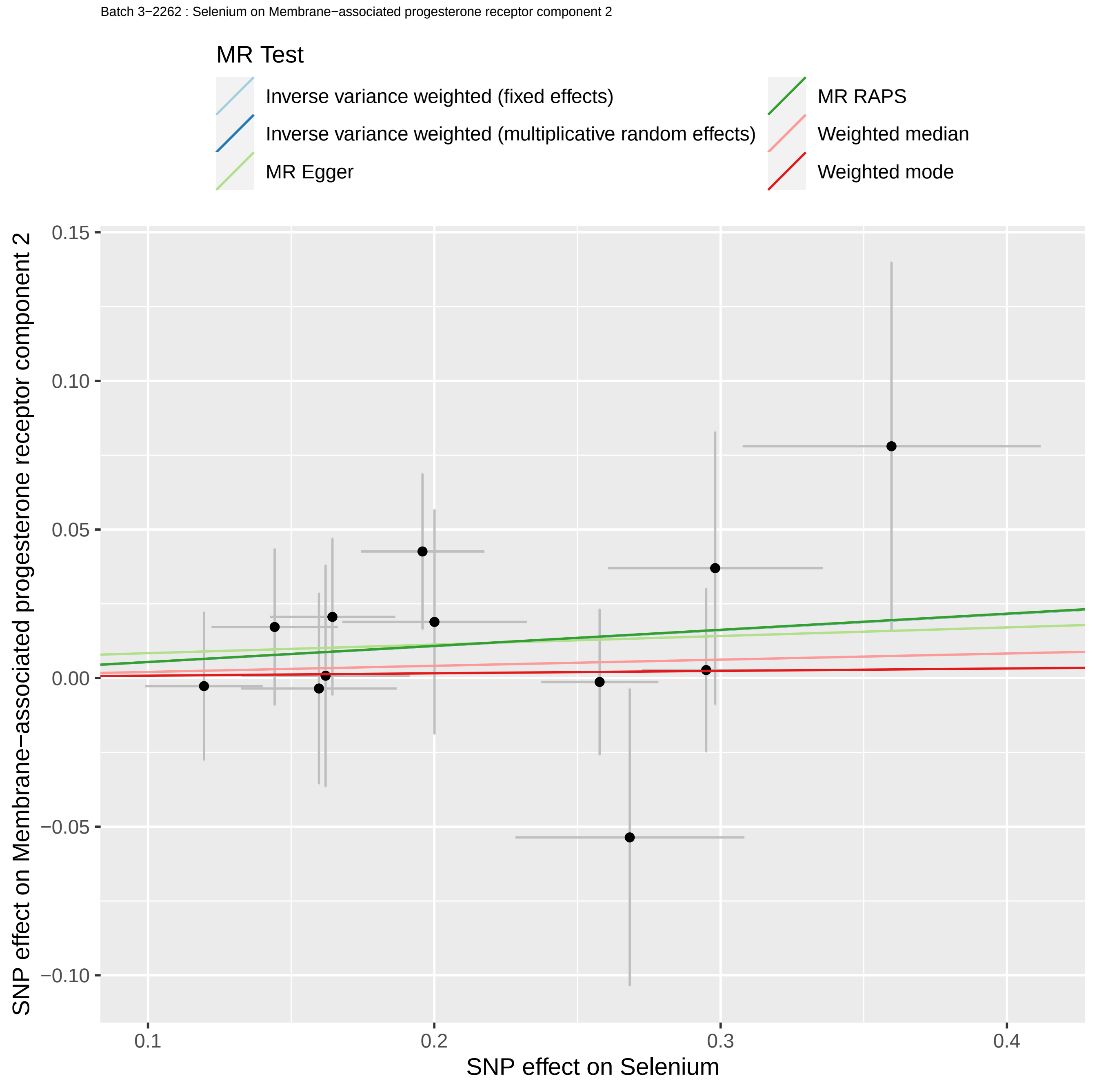

Batch 3−2262 : Selenium on Membrane−associated progesterone receptor component 2
MR Test
Inverse variance weighted (fixed effects)
Inverse variance weighted (multiplicative random effects)
MR Egger
MR RAPS
Weighted median
Weighted mode
0.15
0.10
0.05
0.00
−0.05
−0.10
0.1
0.2
0.3
0.4
SNP effect on Membrane−associated progesterone receptor component 2
SNP effect on Selenium

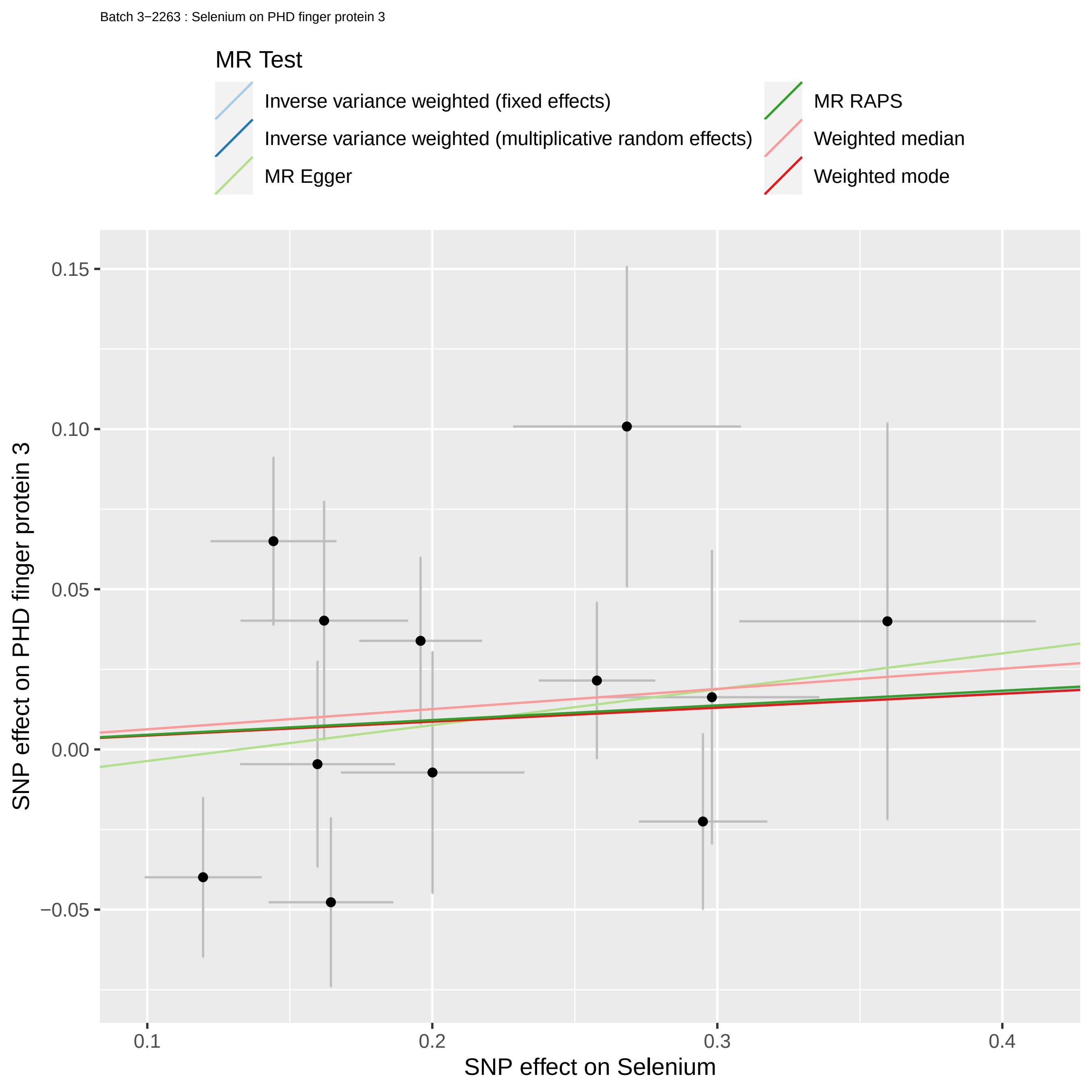
Batch 3−2263 : Selenium on PHD finger protein 3
MR Test
Inverse variance weighted (fixed effects)
Inverse variance weighted (multiplicative random effects)
MR Egger
MR RAPS
Weighted median
Weighted mode
0.15
0.10
0.05
0.00
−0.05
SNP effect on PHD finger protein 3
0.1
0.2
0.3
0.4
SNP effect on Selenium

Batch 3−2264 : Selenium on Peptidase inhibitor 15

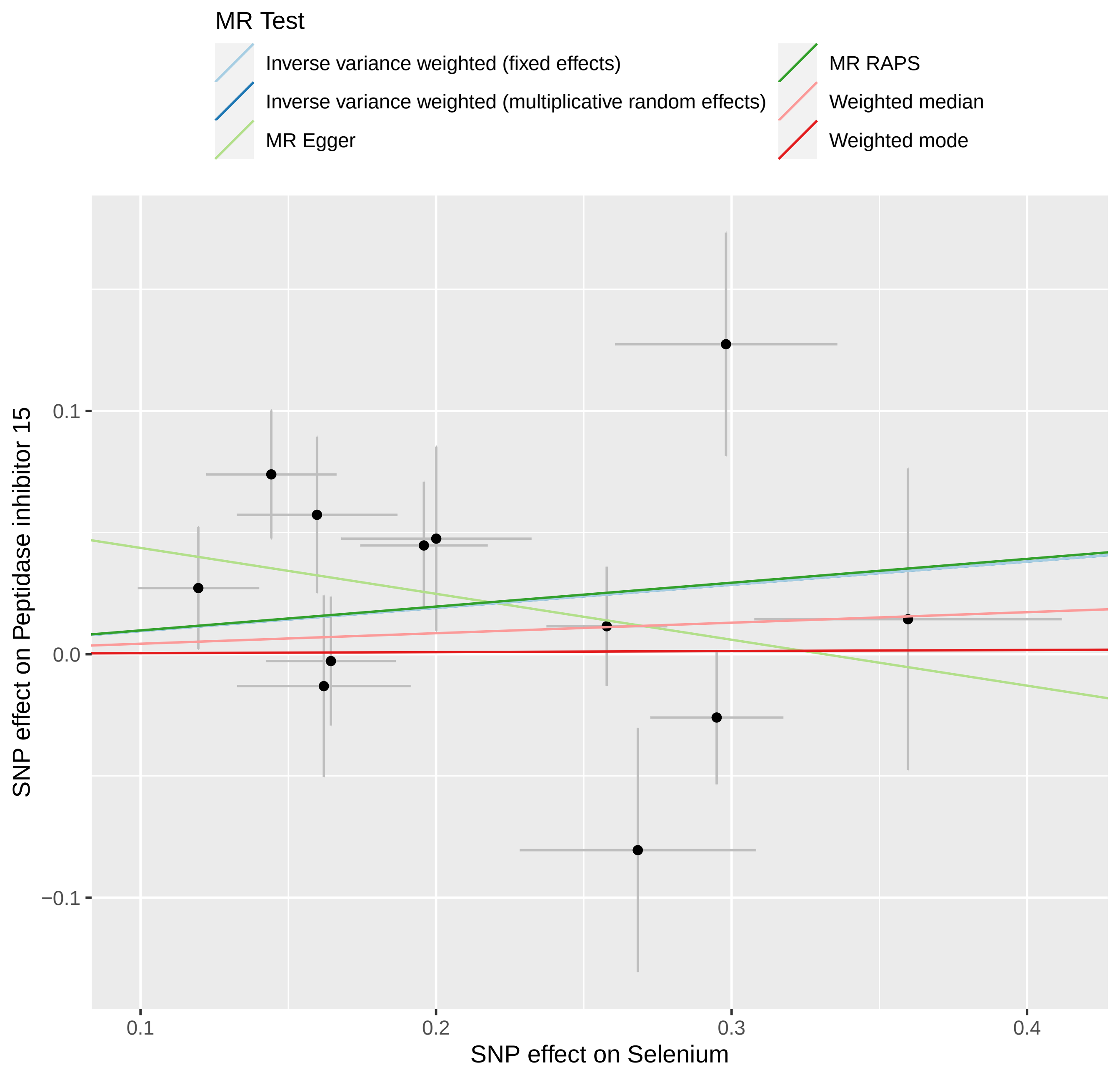

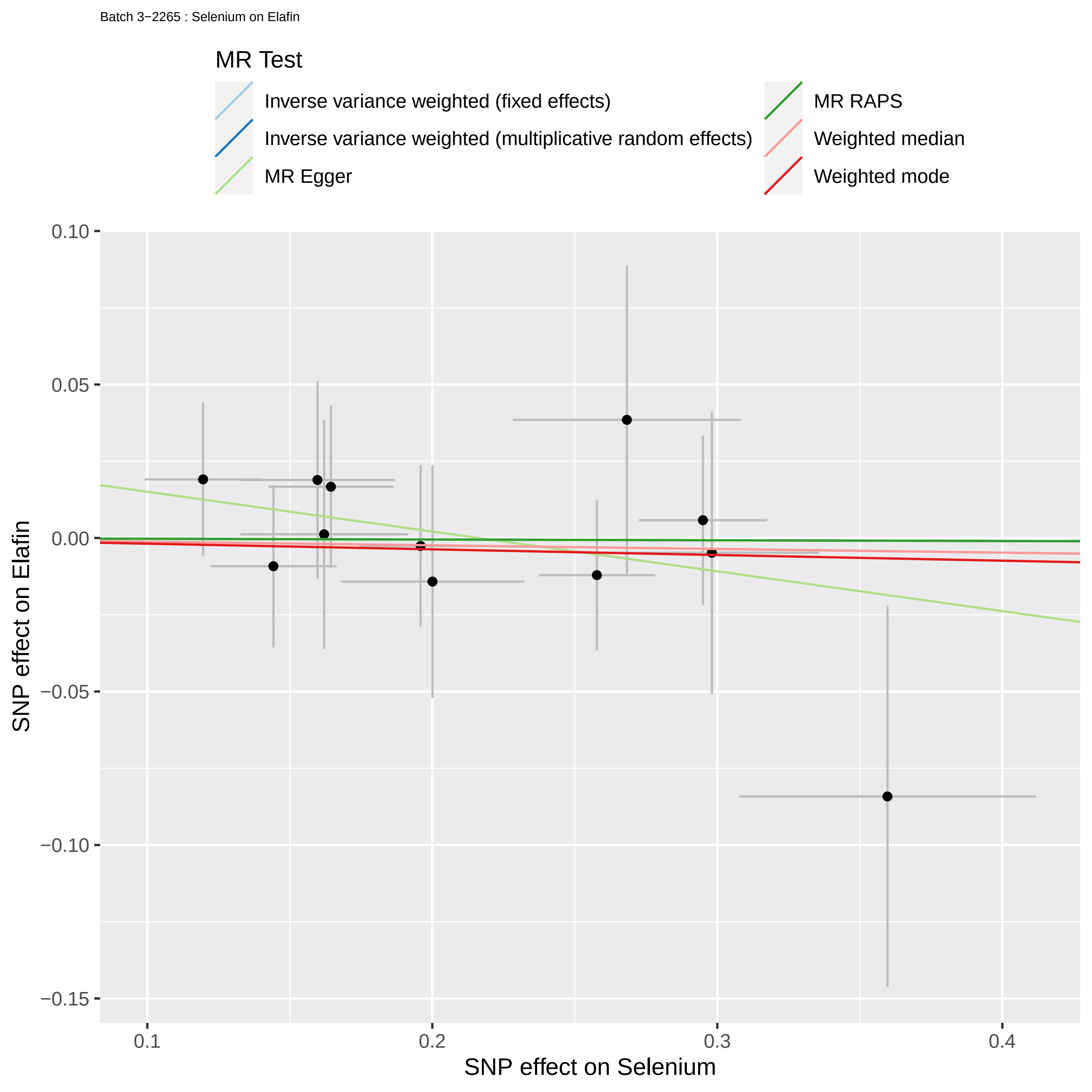
Batch 3−2265 : Selenium on Elafin
MR Test
Inverse variance weighted (fixed effects)
Inverse variance weighted (multiplicative random effects)
MR Egger
MR RAPS
Weighted median
Weighted mode
0.10
0.05
0.00
−0.05
−0.10
−0.15
0.1
0.2
0.3
0.4
SNP effect on Elafin
SNP effect on Selenium

Batch 3−2266 : Selenium on PILR alpha−associated neural protein

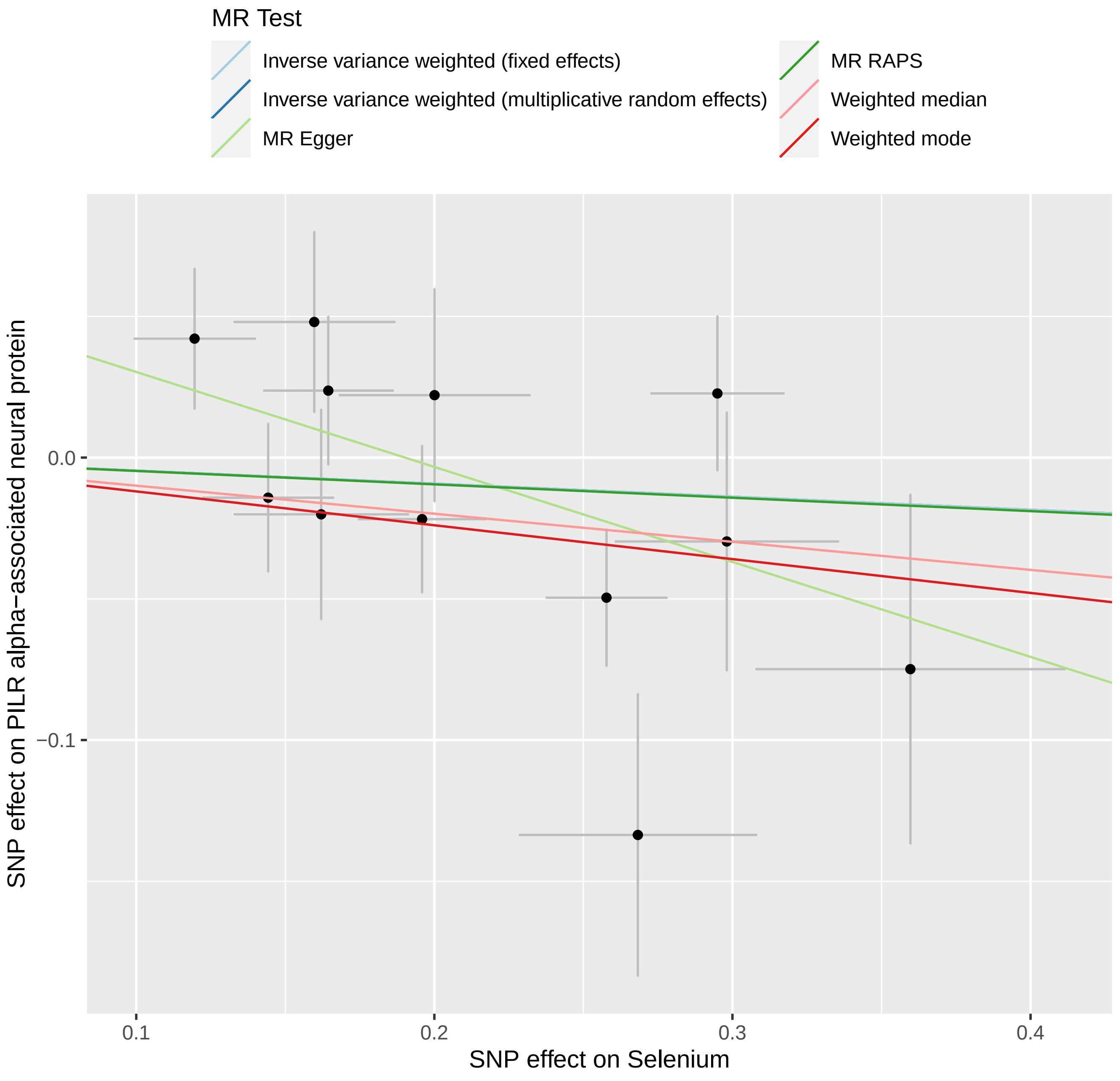

Batch 3−2267 : Selenium on PILR alpha−associated neural protein

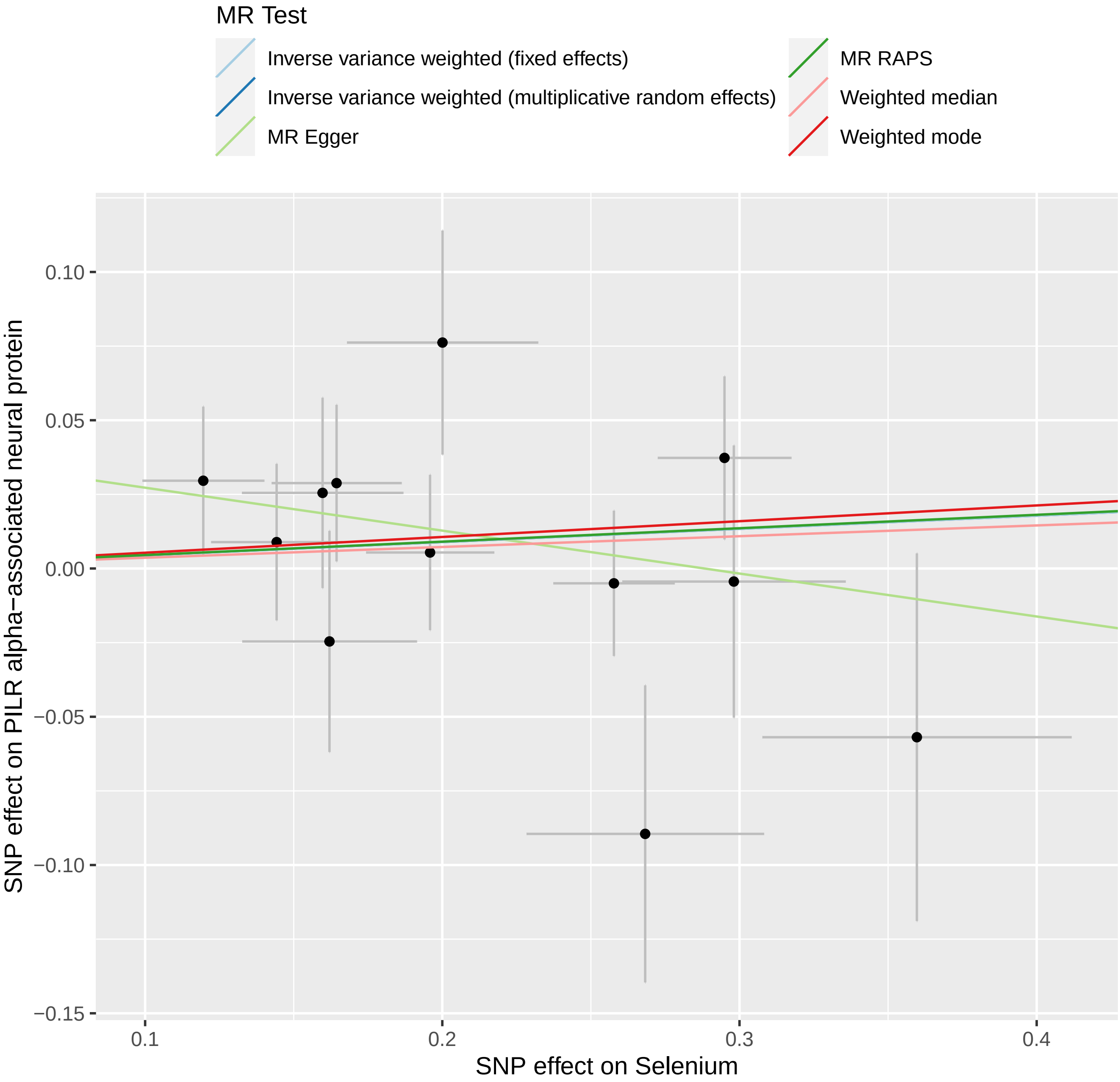

Batch 3−2268 : Selenium on Polymeric immunoglobulin receptor

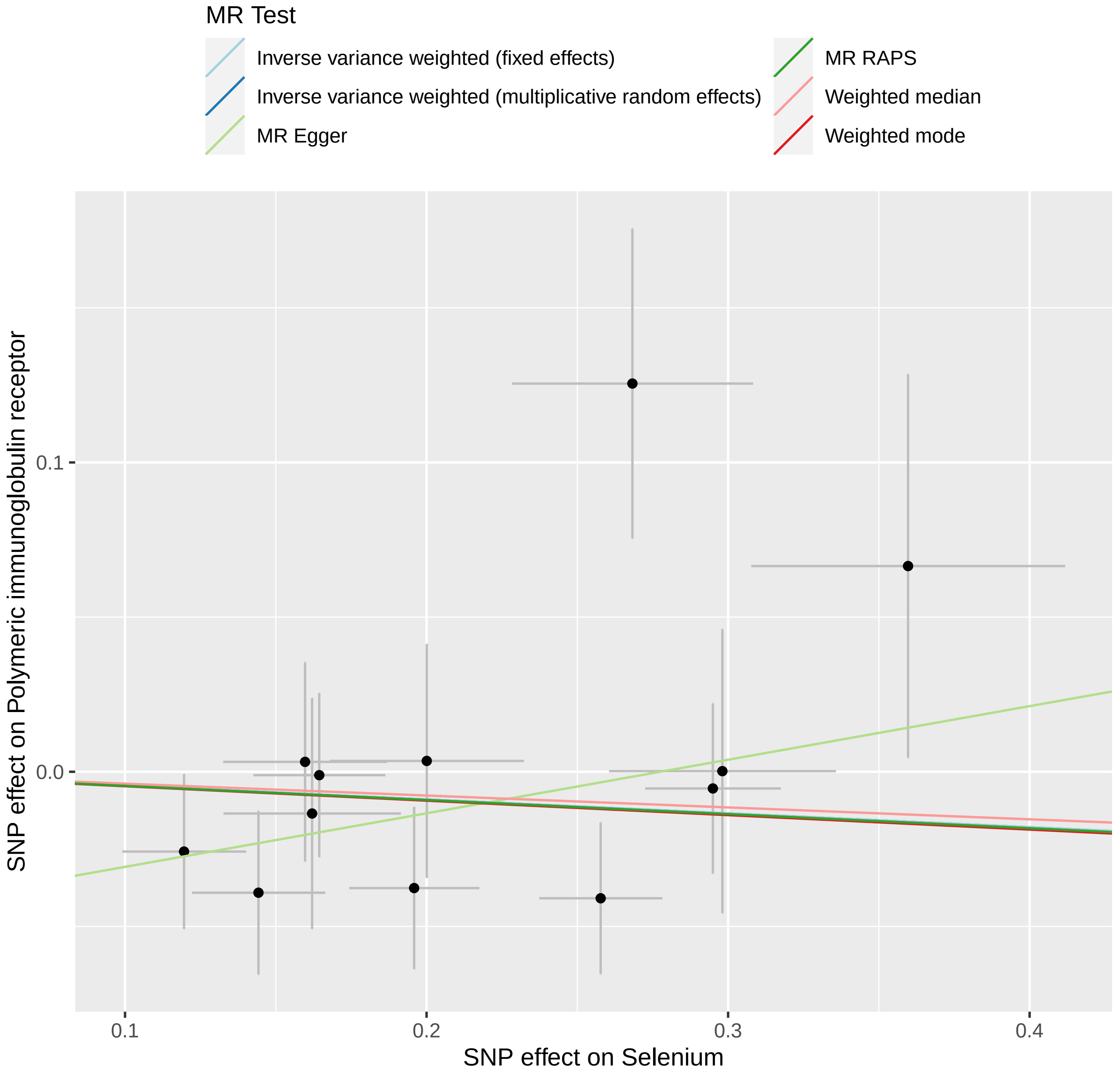

Batch 3−2269 : Selenium on Phosphatidylinositol 4−phosphate 3−kinase C2 domain−containing subunit alpha

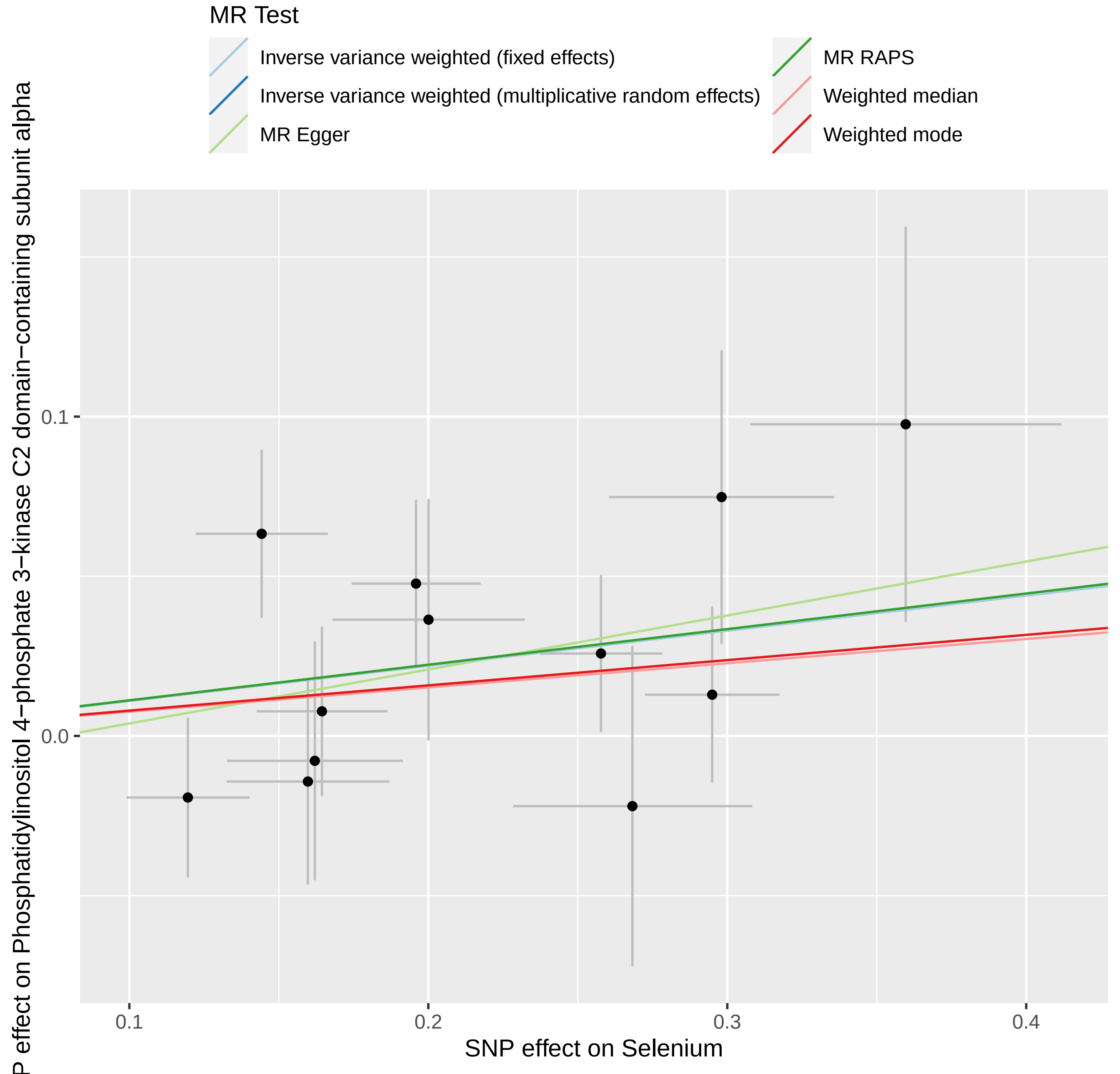

Batch 3−2270 : Selenium on PIK3CA/PIK3R1

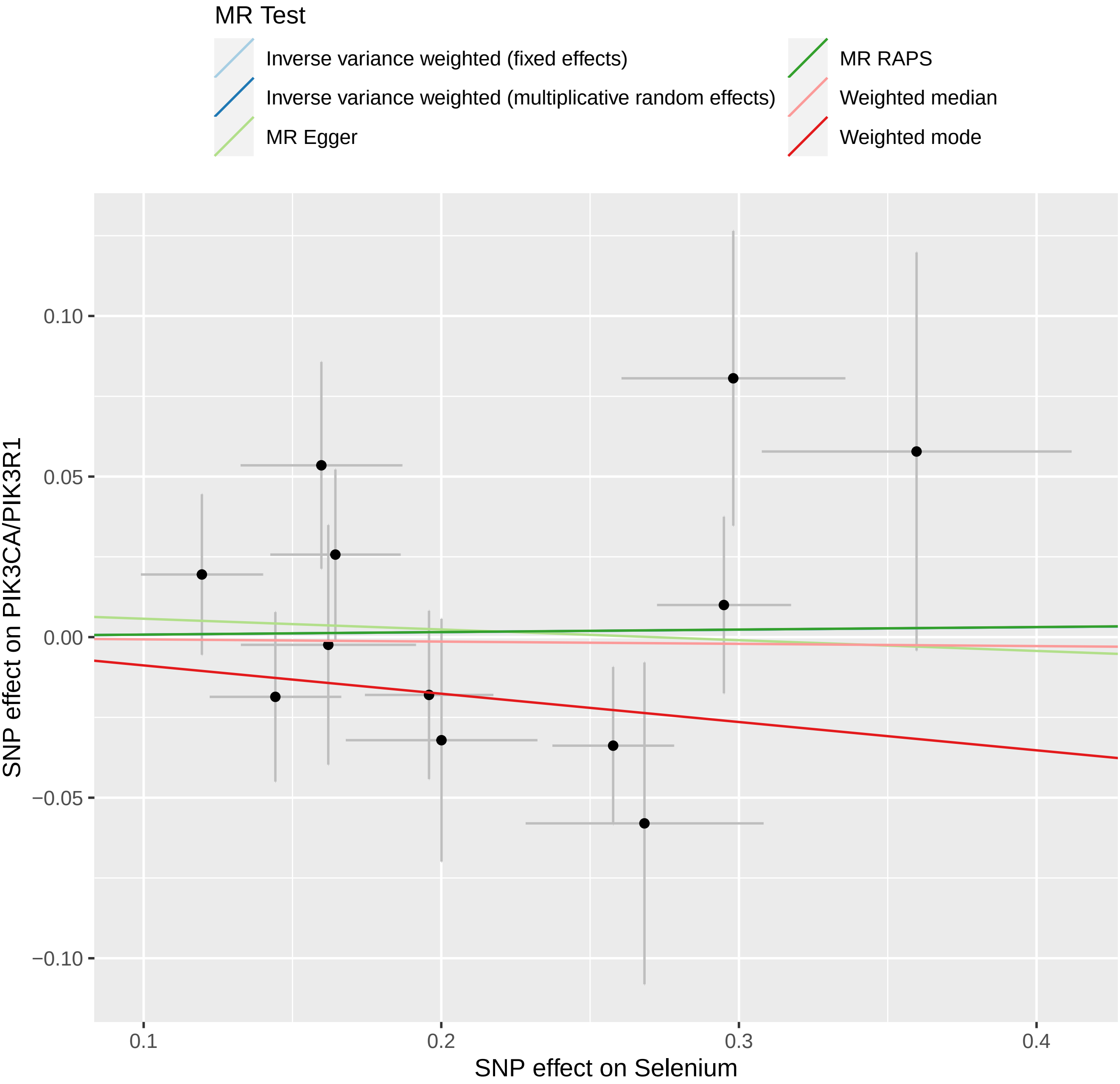

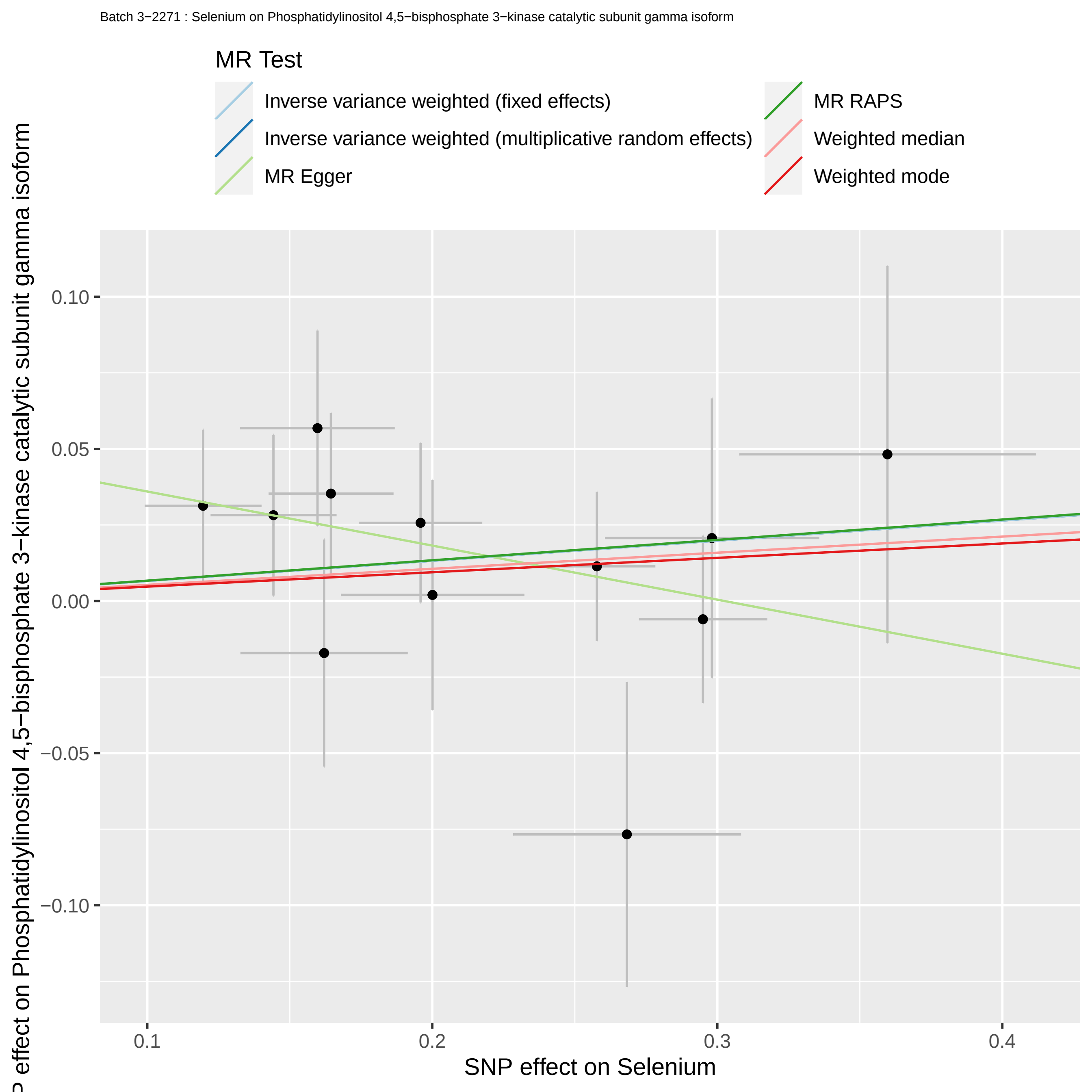

Batch 3−2271 : Selenium on Phosphatidylinositol 4,5−bisphosphate 3−kinase catalytic subunit gamma isoform
MR Test
Inverse variance weighted (fixed effects)
Inverse variance weighted (multiplicative random effects)
MR Egger
MR RAPS
Weighted median
Weighted mode
0.10
0.05
0.00
−0.05
−0.10
0.1
0.2
0.3
0.4
SNP effect on Selenium
P effect on Phosphatidylinositol 4,5−bisphosphate 3−kinase catalytic subunit gamma isoform

Batch 3−2272 : Selenium on Paired immunoglobulin−like type 2 receptor alpha

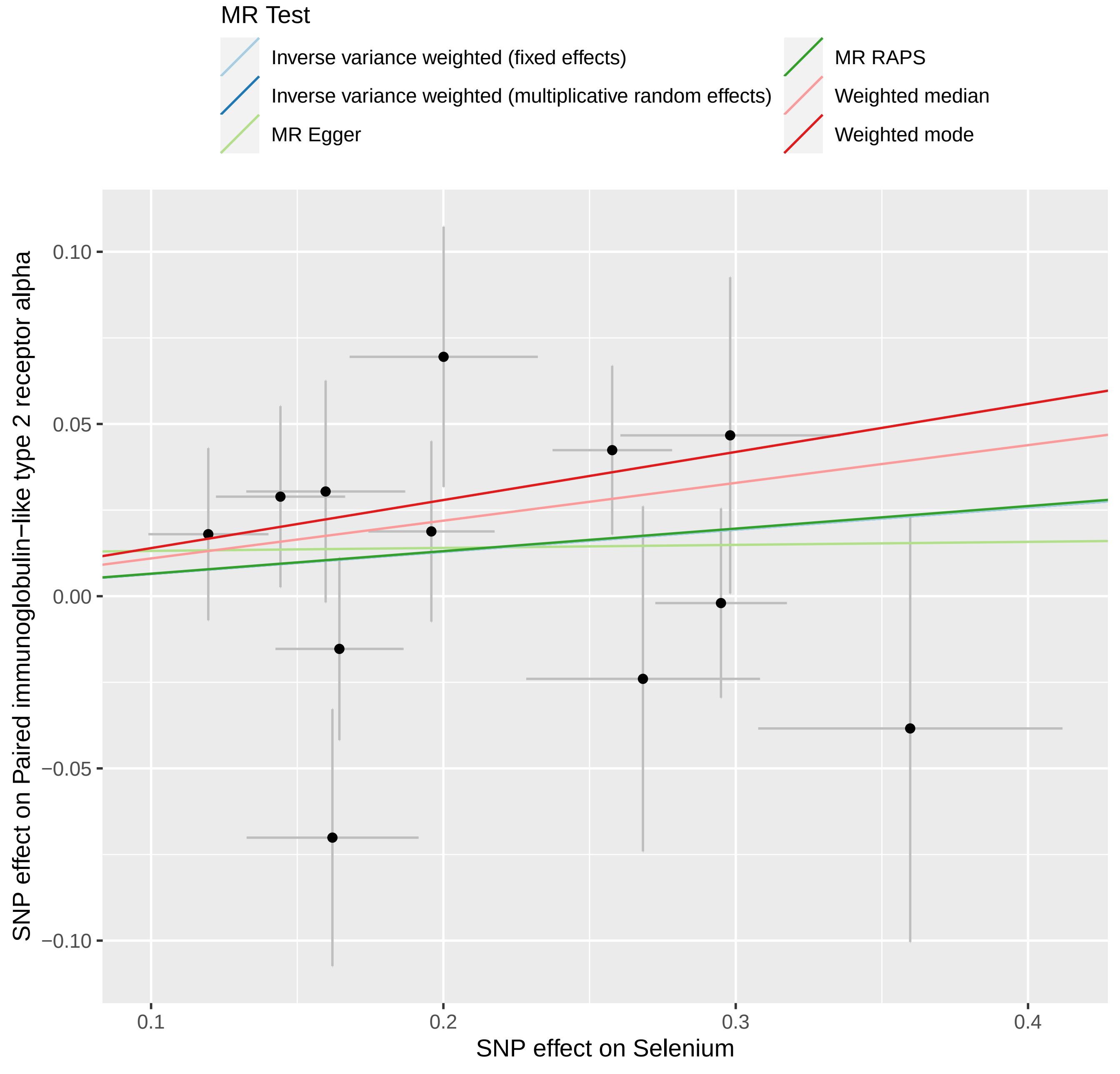

Batch 3−2273 : Selenium on Paired immunoglobulin−like type 2 receptor alpha

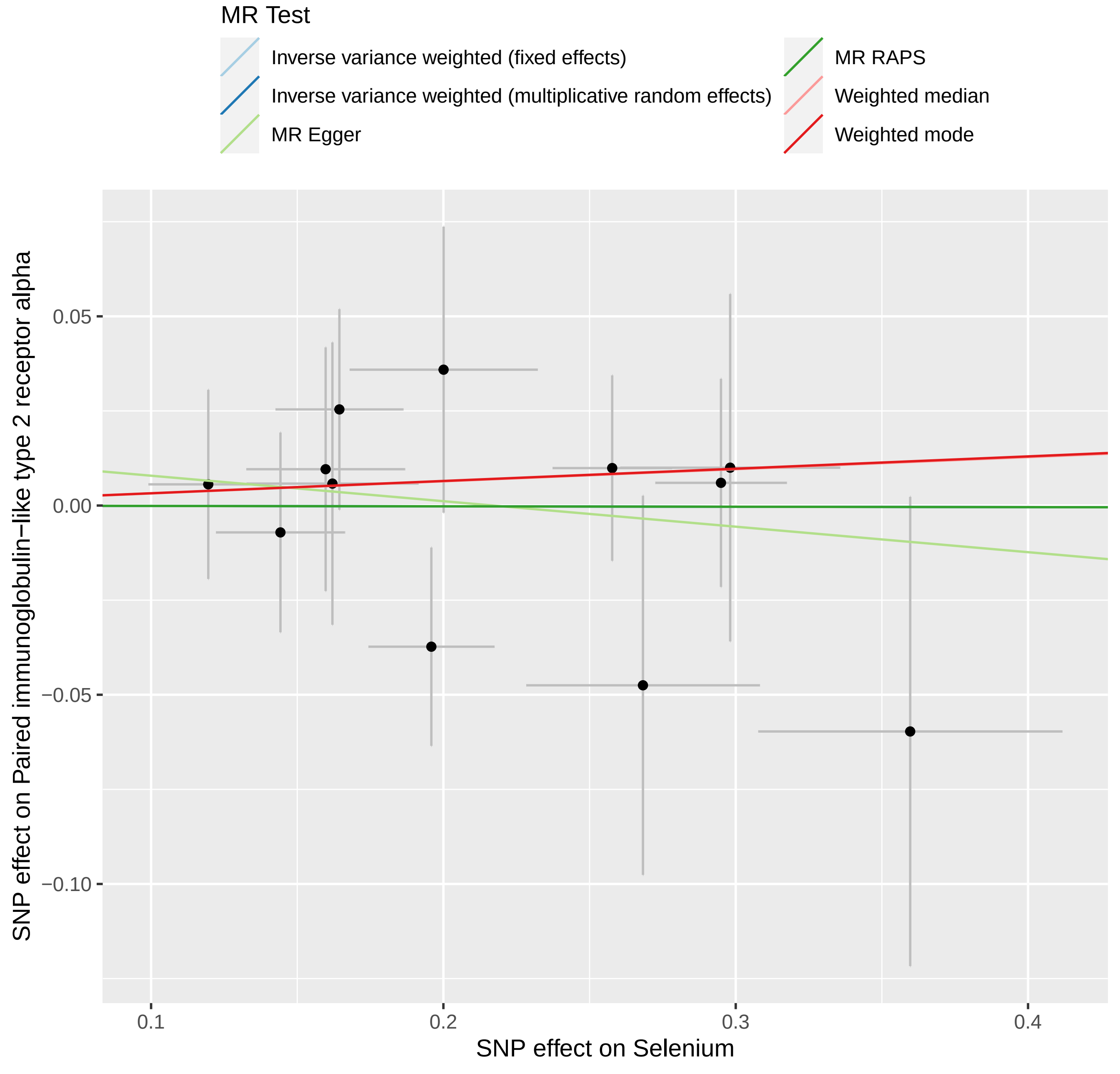

Batch 3−2274 : Selenium on Serine/threonine−protein kinase pim−1

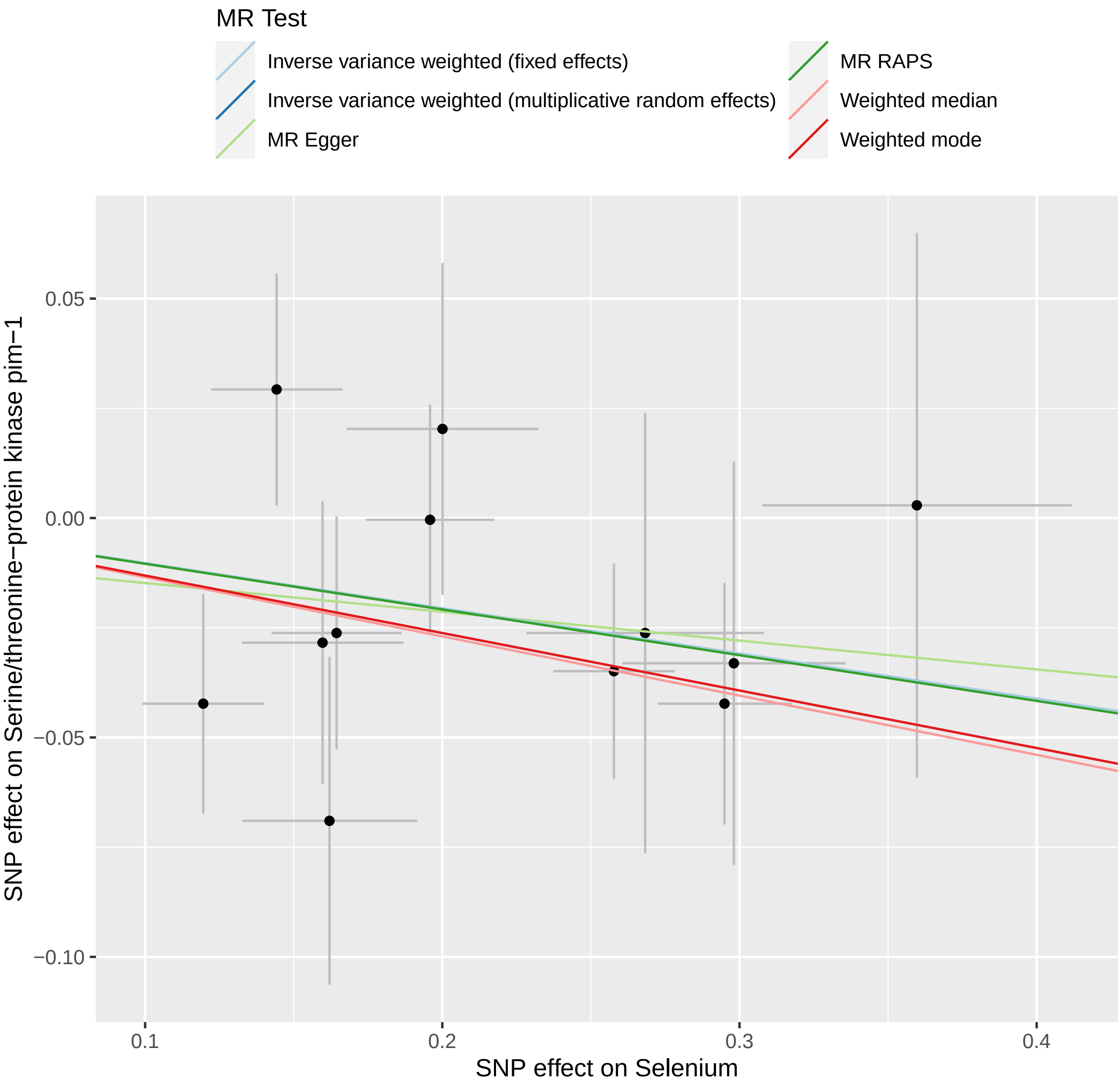

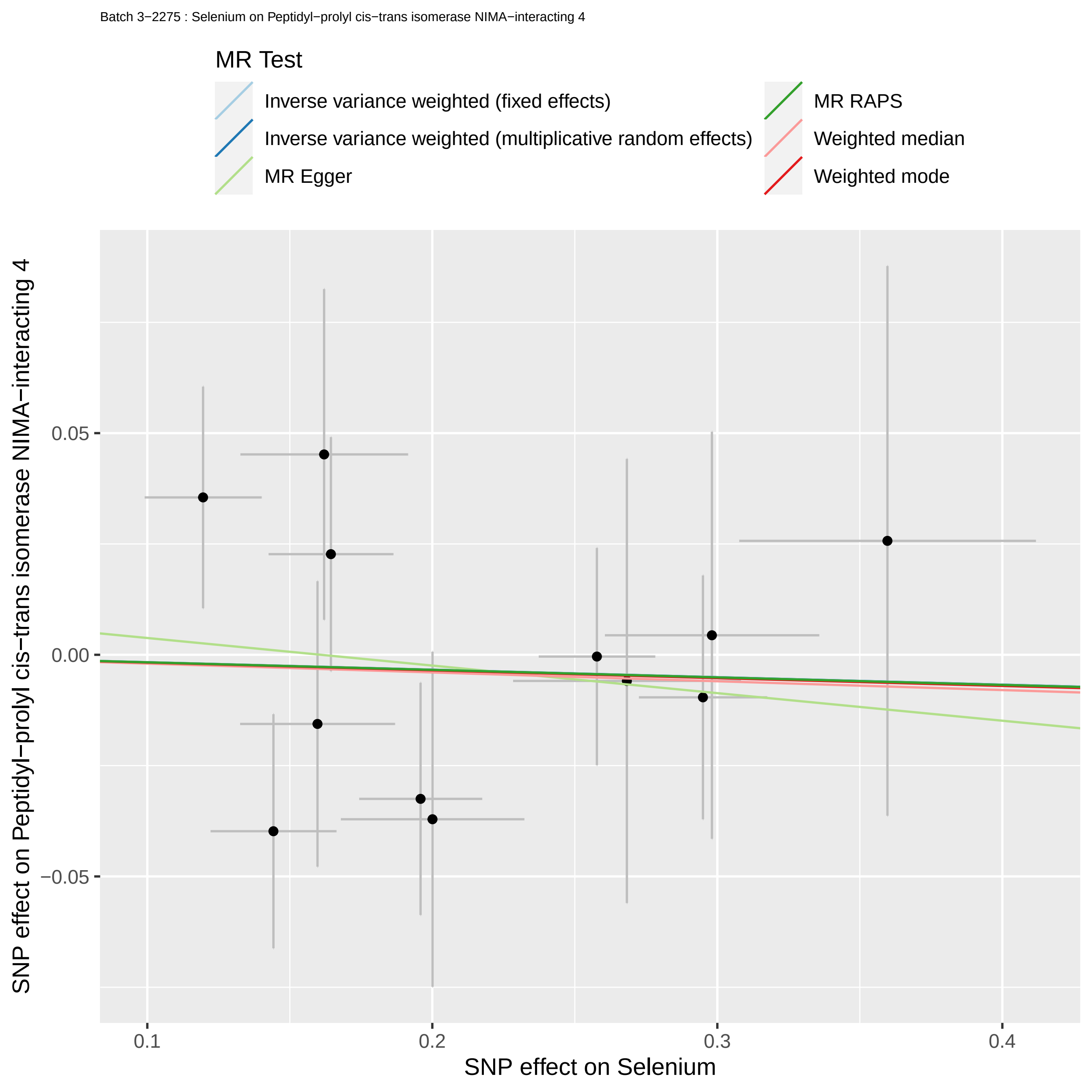
Batch 3−2275 : Selenium on Peptidyl−prolyl cis−trans isomerase NIMA−interacting 4
MR Test
Inverse variance weighted (fixed effects)
Inverse variance weighted (multiplicative random effects)
MR Egger
MR RAPS
Weighted median
Weighted mode
SNP effect on Peptidyl−prolyl cis−trans isomerase NIMA−interacting 4
0.05
0.00
−0.05
0.1
0.2
0.3
0.4
SNP effect on Selenium

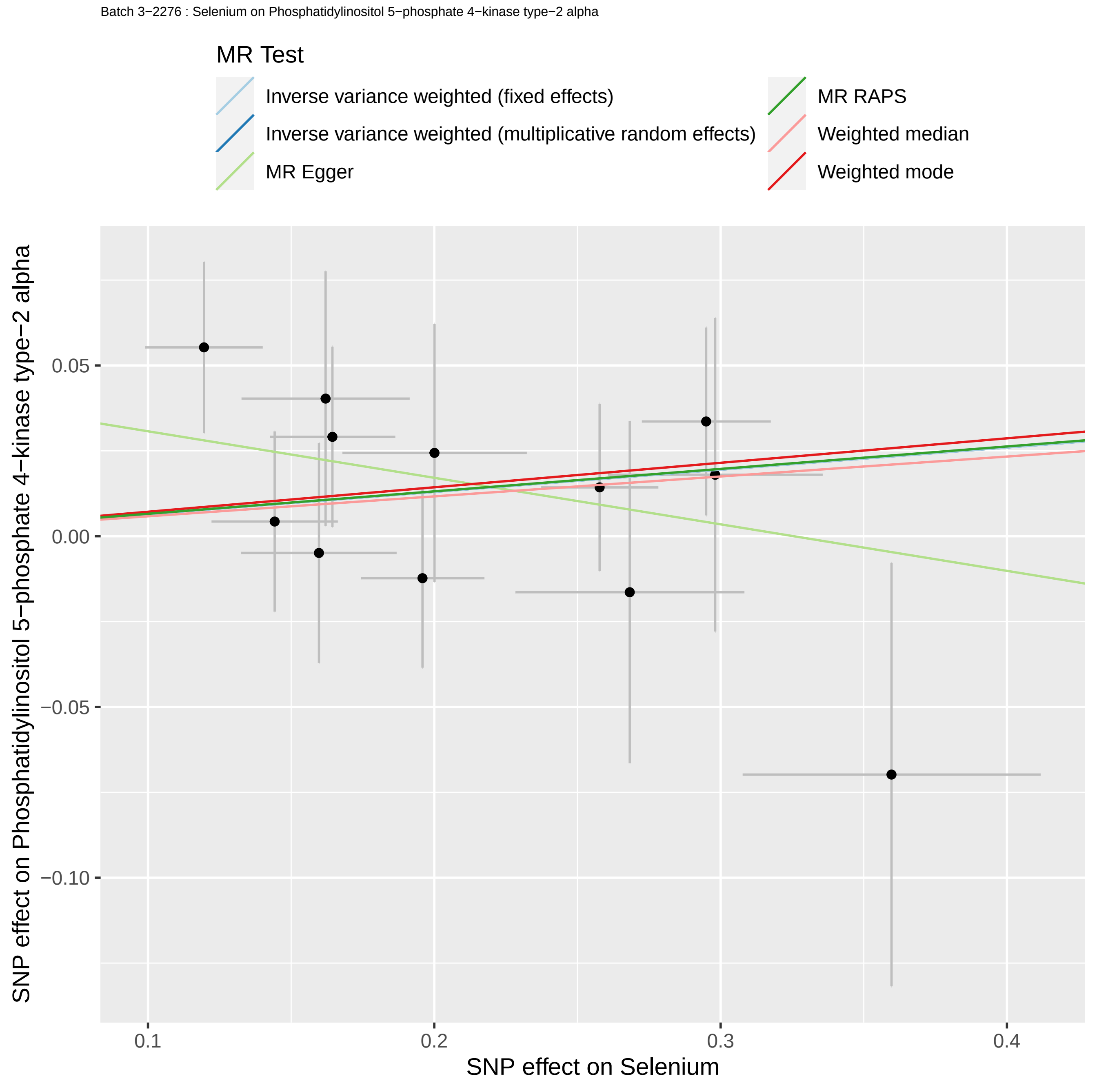
Batch 3−2276 : Selenium on Phosphatidylinositol 5−phosphate 4−kinase type−2 alpha
MR Test
Inverse variance weighted (fixed effects)
Inverse variance weighted (multiplicative random effects)
MR Egger
MR RAPS
Weighted median
Weighted mode
SNP effect on Phosphatidylinositol 5−phosphate 4−kinase type−2 alpha
0.05
0.00
−0.05
−0.10
0.1
0.2
0.3
0.4
SNP effect on Selenium

Batch 3−2277 : Selenium on Pirin

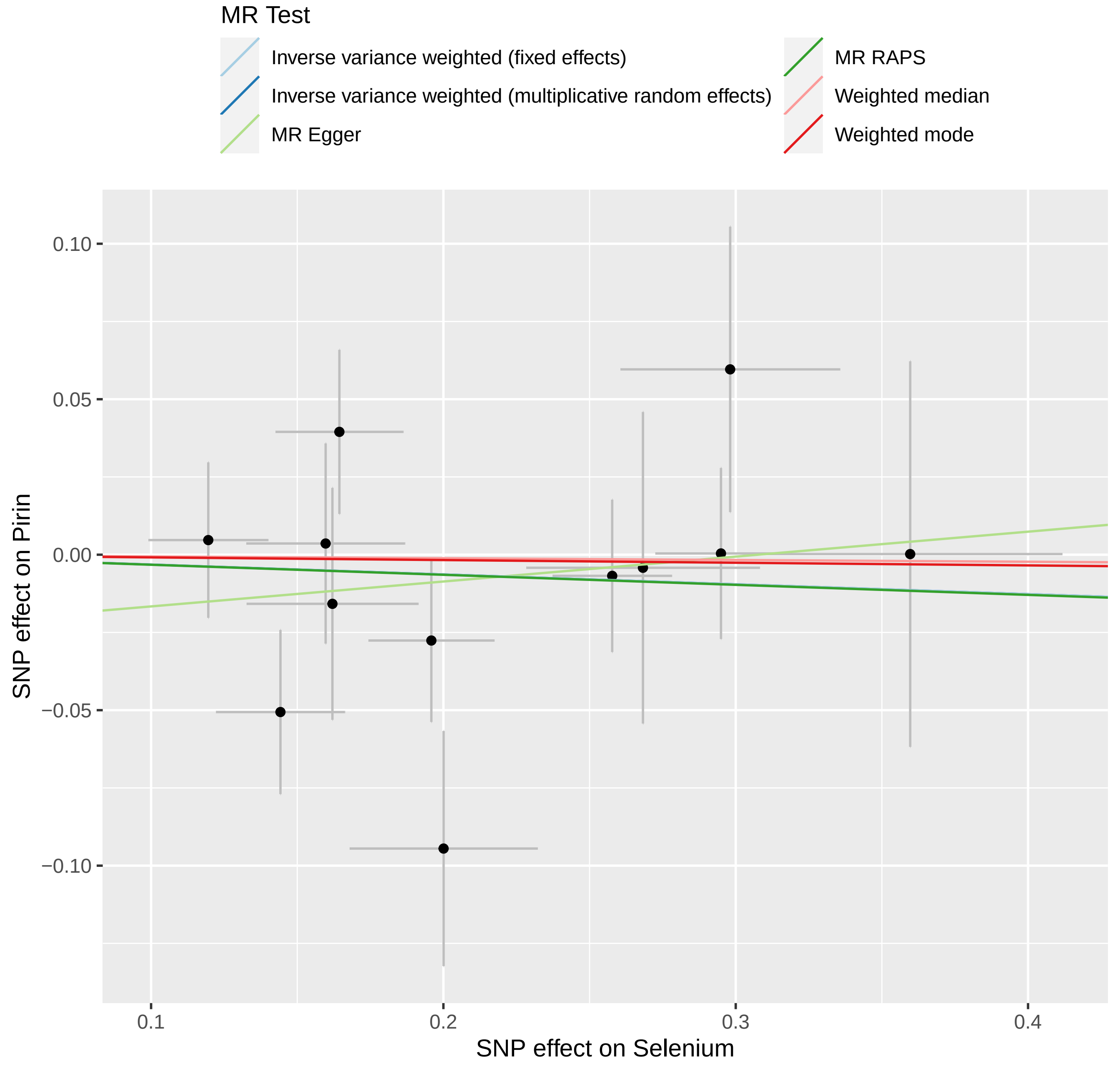

Batch 3−2278 : Selenium on Phosphatidylinositol transfer protein alpha isoform

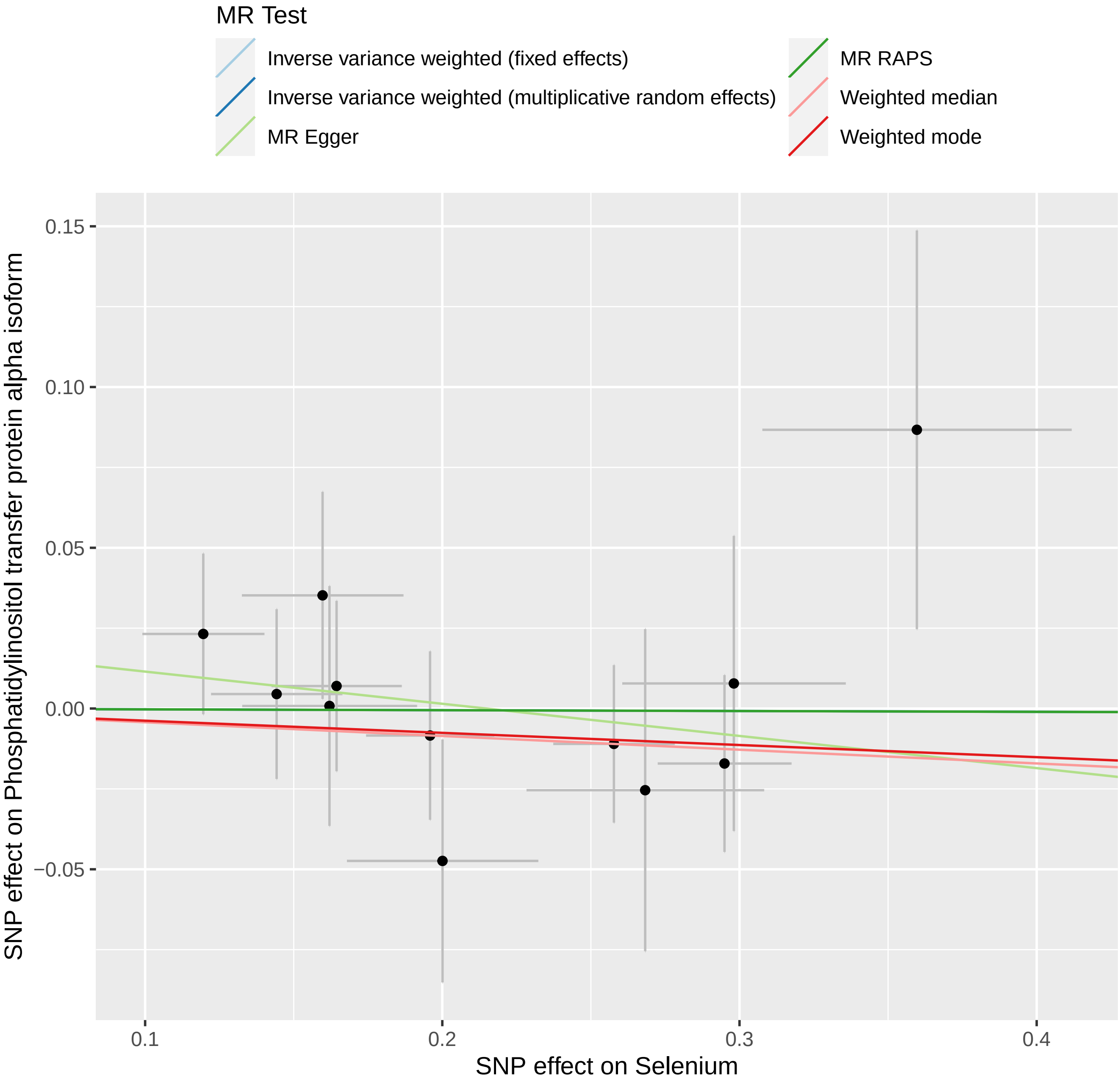

Batch 3−2279 : Selenium on Phosphatidylinositol transfer protein beta isoform

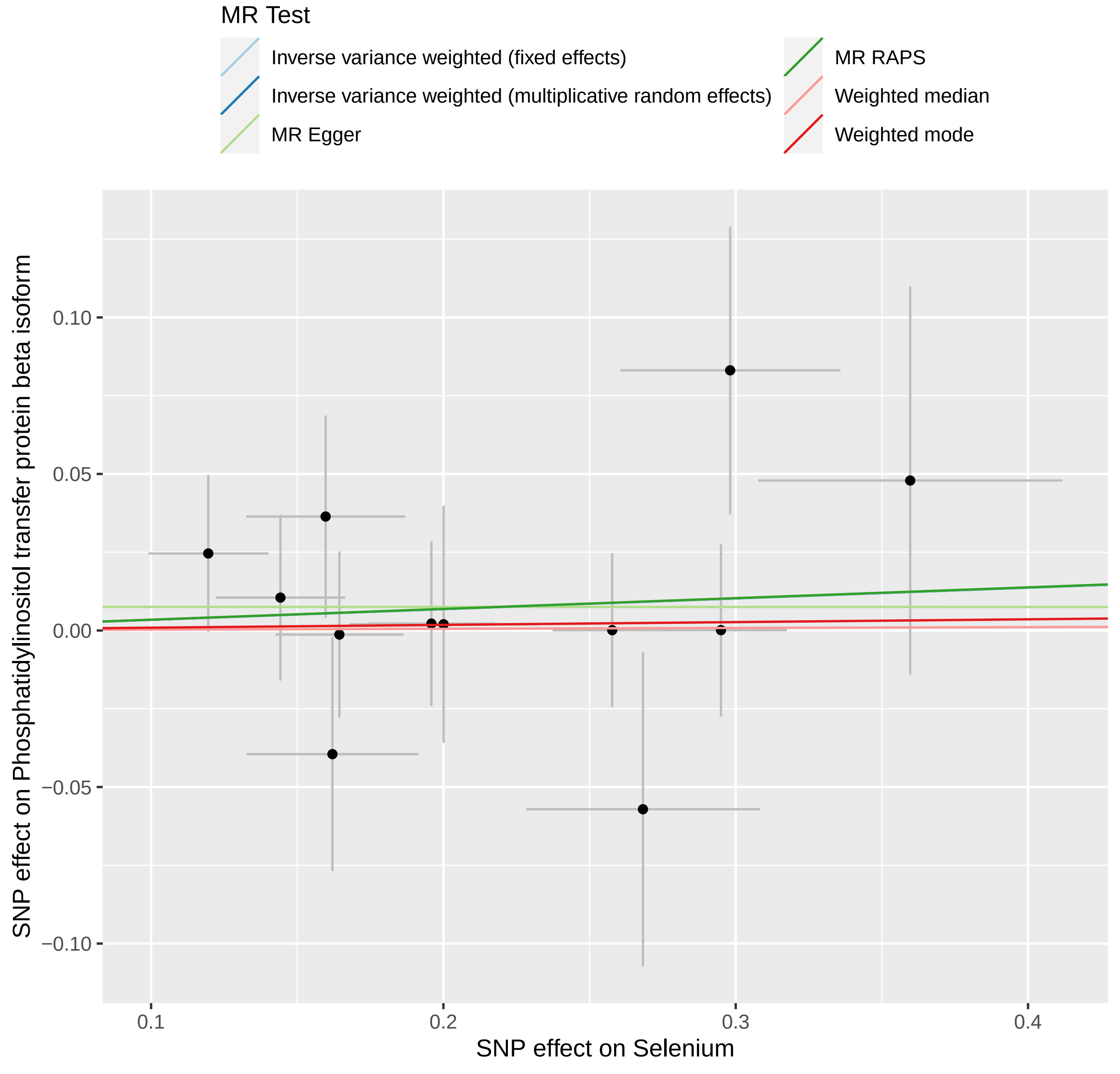

Batch 3−2280 : Selenium on Piwi−like protein 1

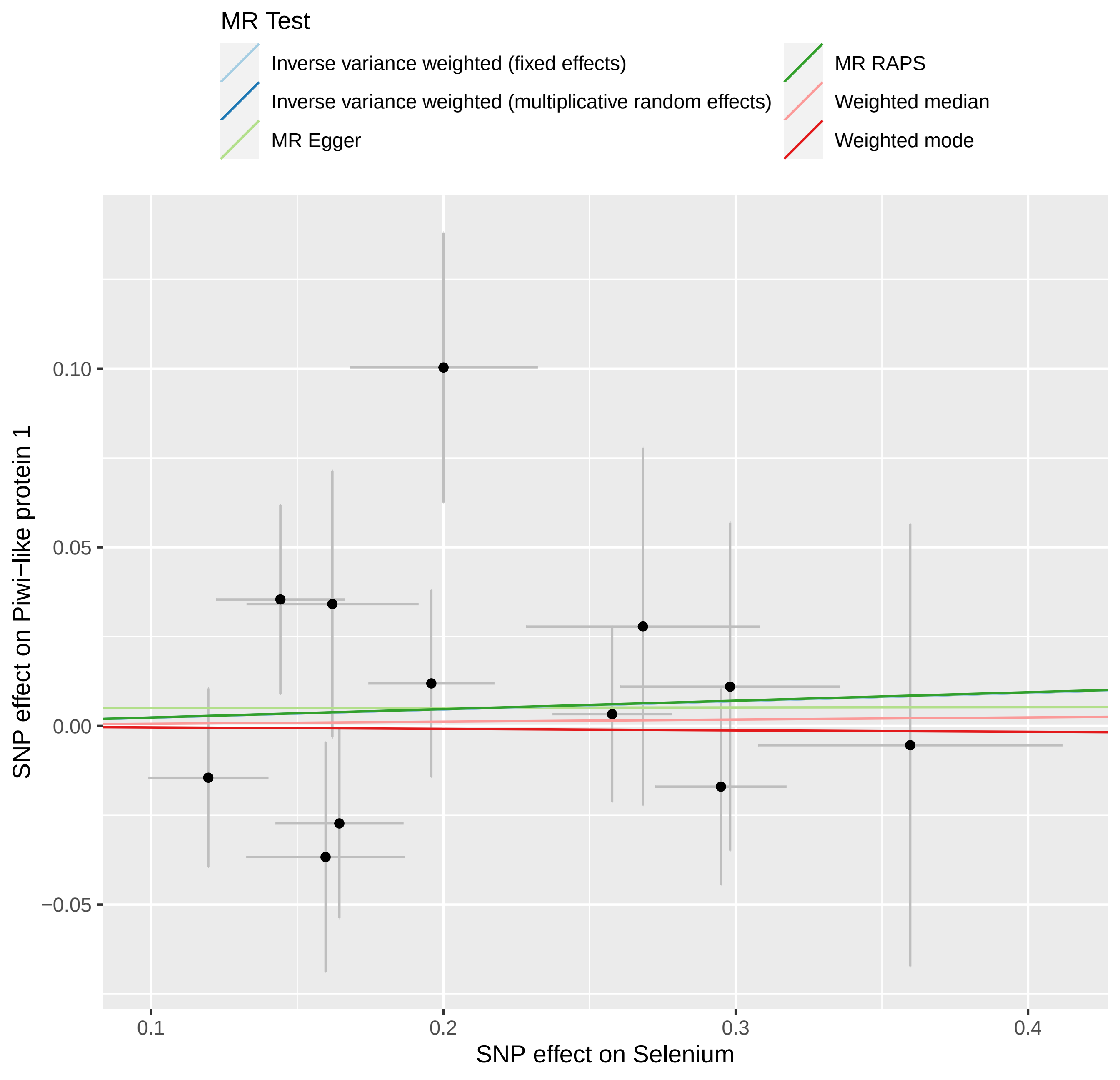

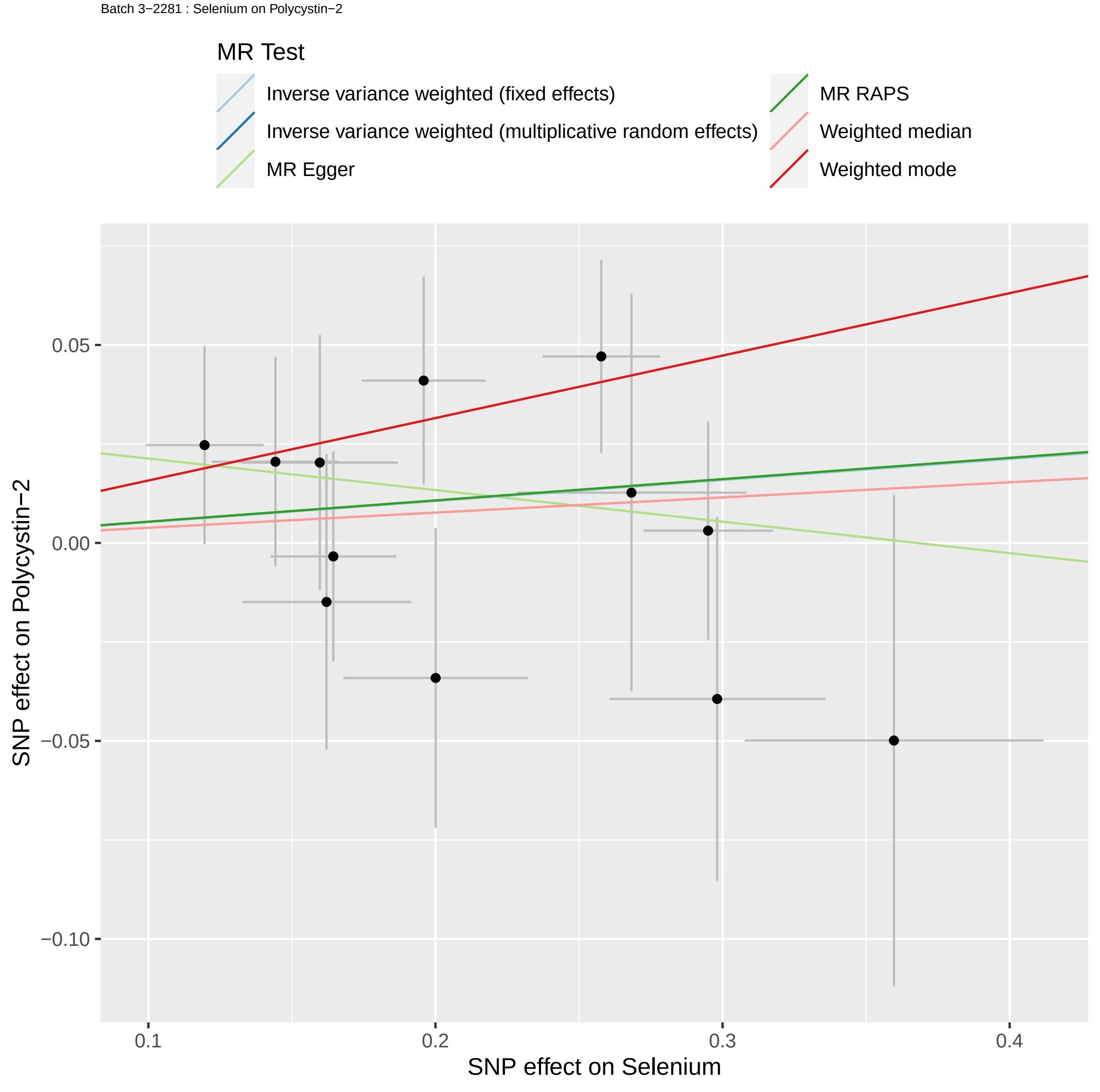

Batch 3−2281 : Selenium on Polycystin−2
MR Test
Inverse variance weighted (fixed effects)
Inverse variance weighted (multiplicative random effects)
MR Egger
MR RAPS
Weighted median
Weighted mode
0.05
0.00
−0.05
−0.10
0.1
0.2
0.3
0.4
SNP effect on Polycystin−2
SNP effect on Selenium

Batch 3−2282 : Selenium on Pyruvate kinase PKLR

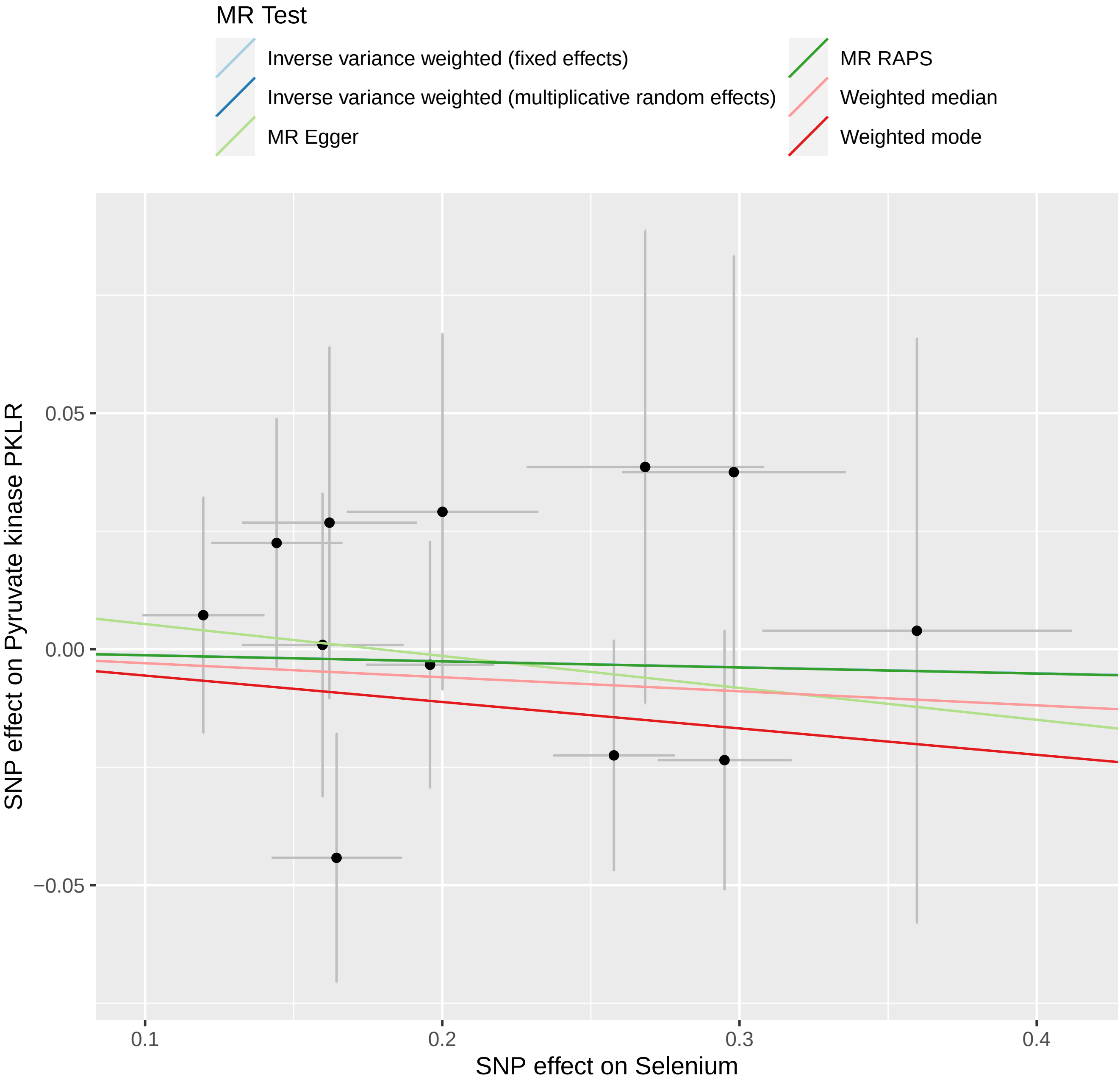

Batch 3−2283 : Selenium on Group 10 secretory phospholipase A2

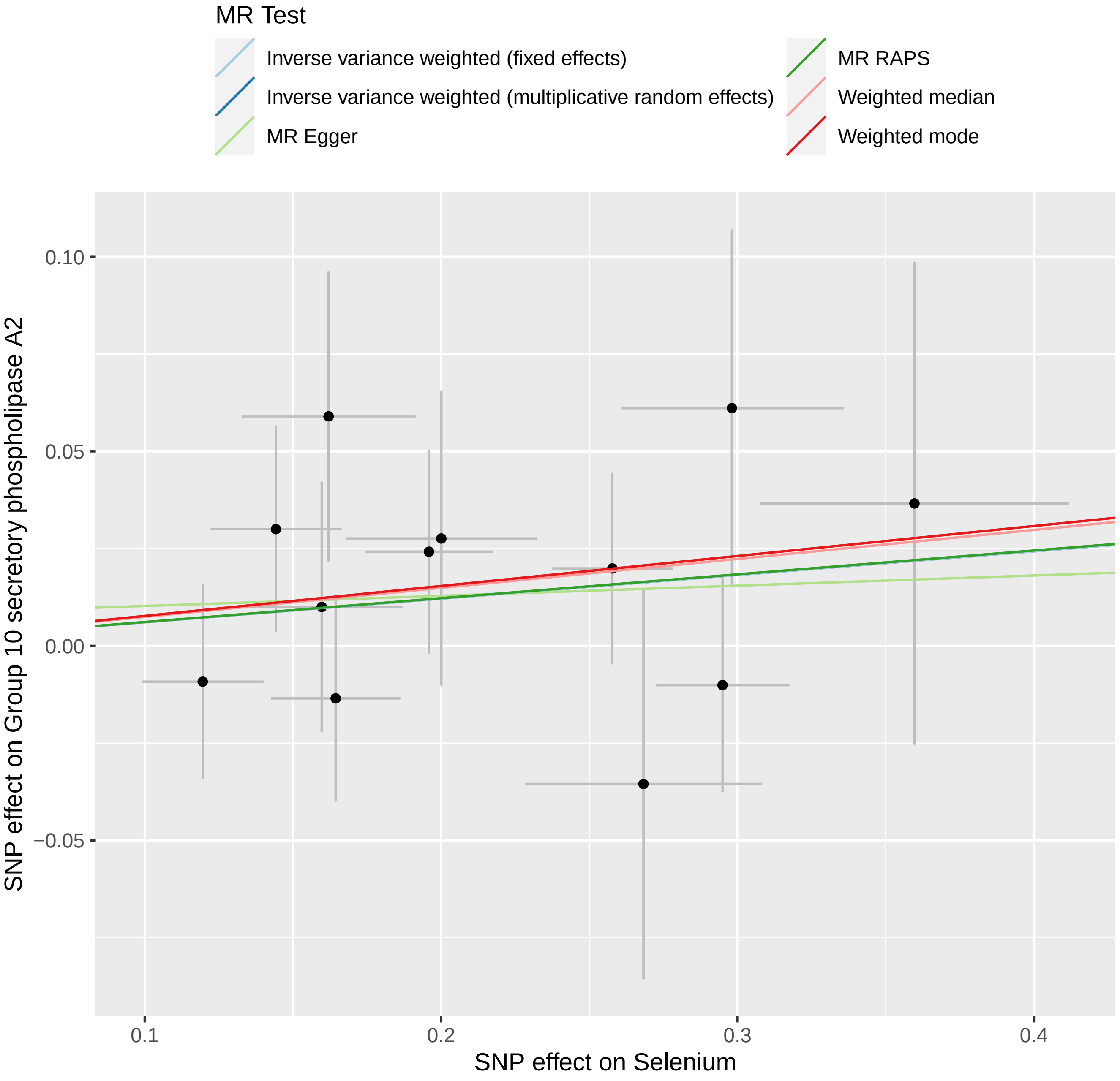

Batch 3−2284 : Selenium on Group XIIB secretory phospholipase A2−like protein

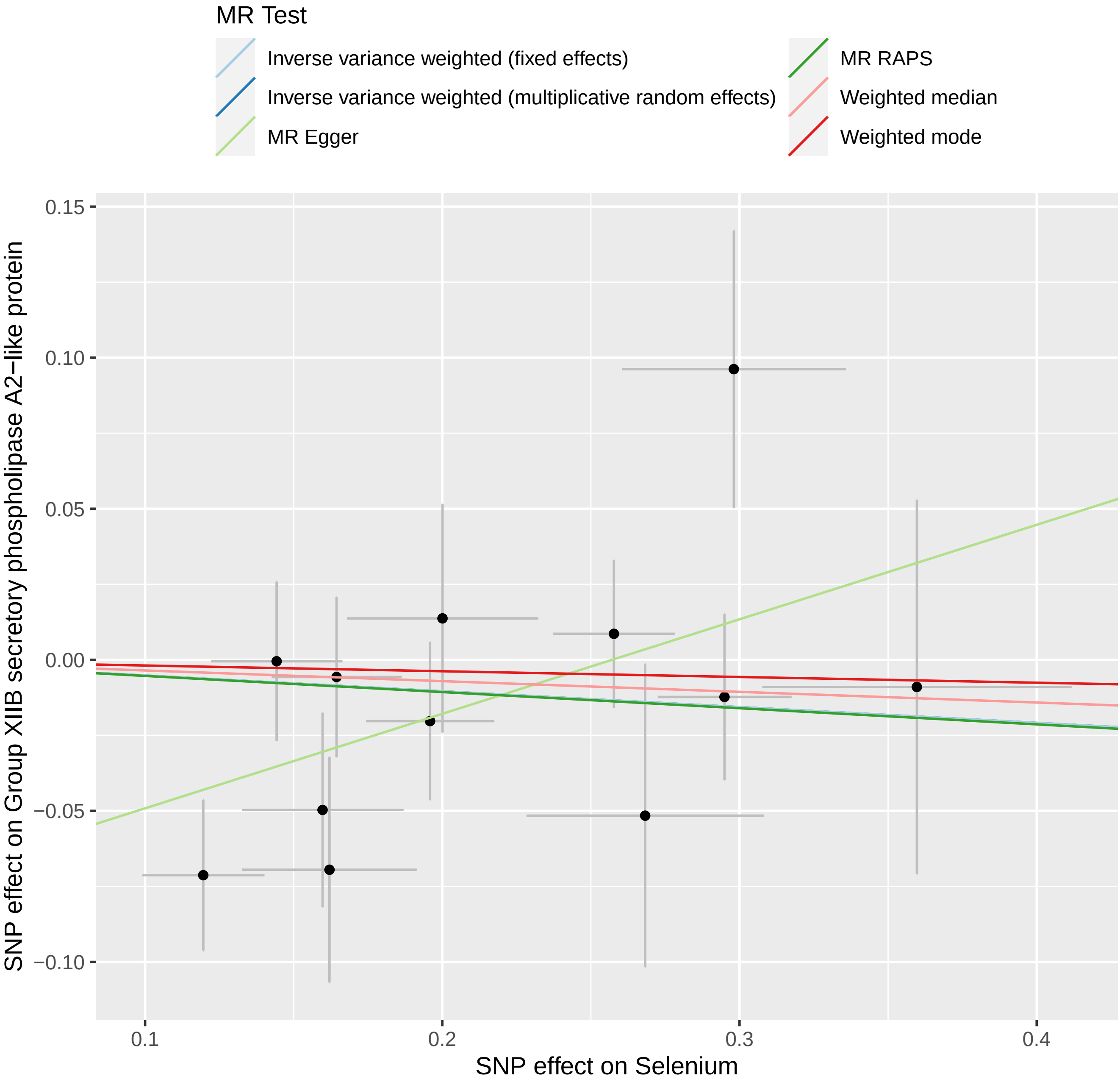

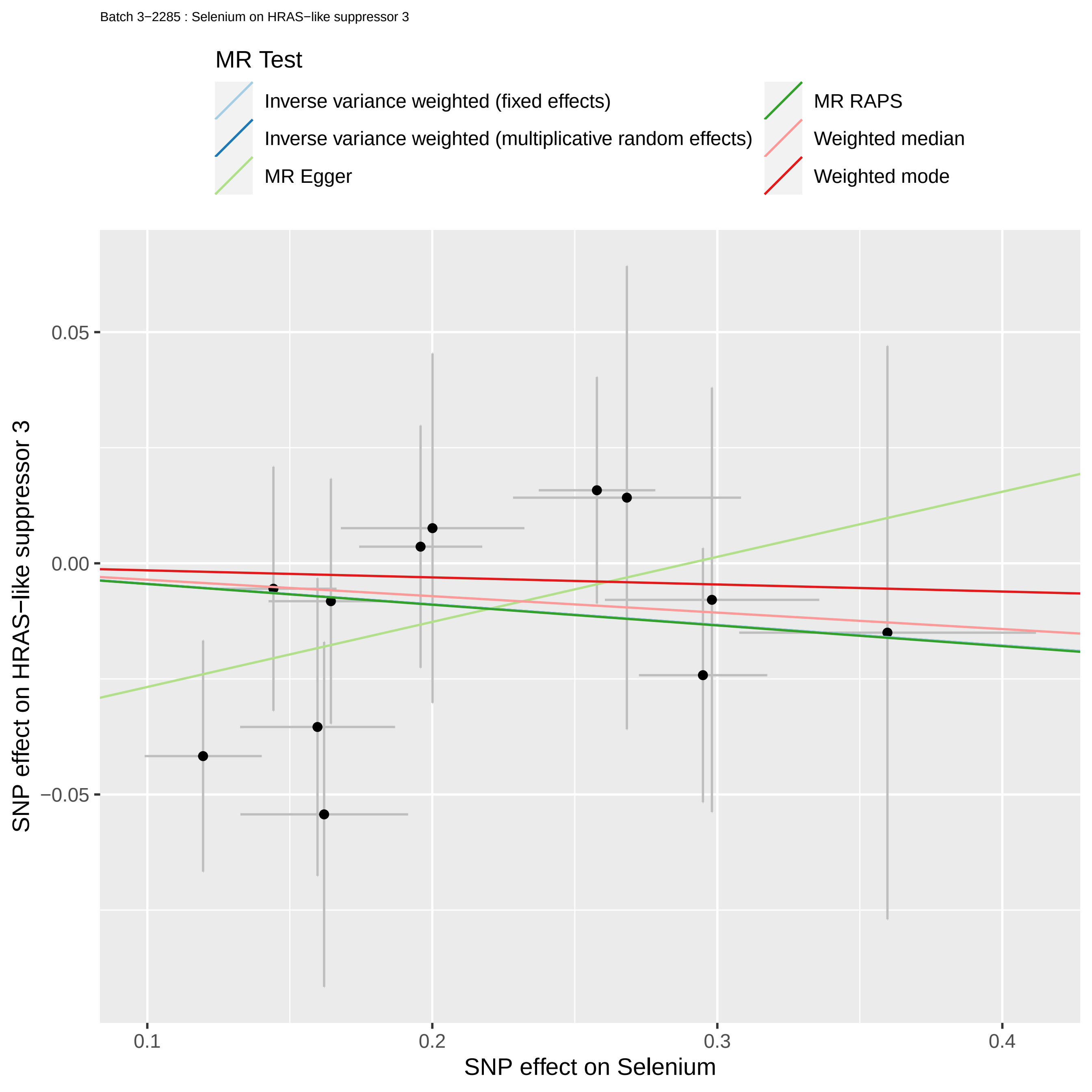
Batch 3−2285 : Selenium on HRAS−like suppressor 3
MR Test
Inverse variance weighted (fixed effects)
Inverse variance weighted (multiplicative random effects)
MR Egger
MR RAPS
Weighted median
Weighted mode
SNP effect on HRAS−like suppressor 3
0.05
0.00
−0.05
0.1
0.2
0.3
0.4
SNP effect on Selenium

Batch 3−2286 : Selenium on Phospholipase A2, membrane associated

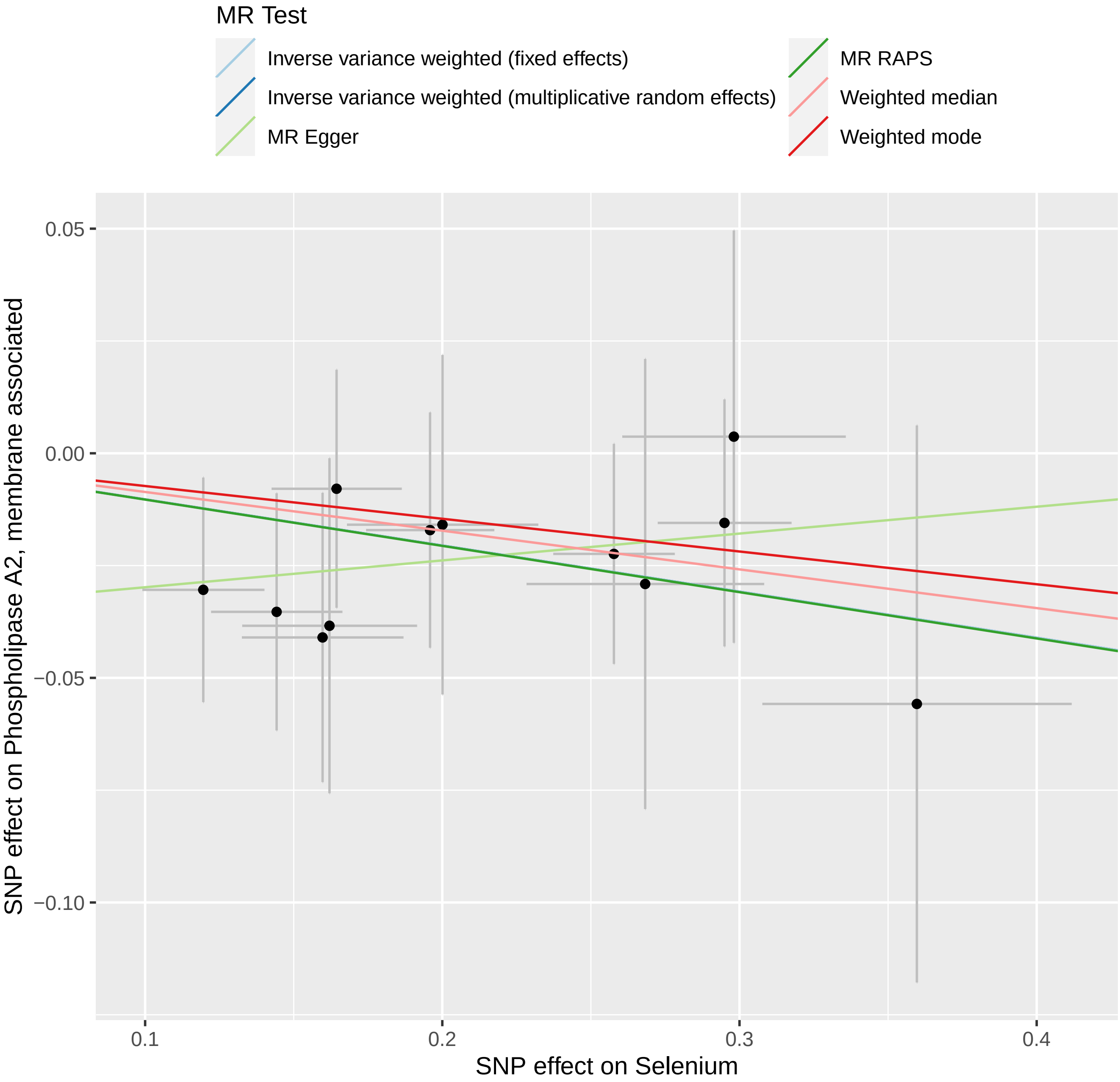

Batch 3−2287 : Selenium on Putative inactive group IIC secretory phospholipase A2

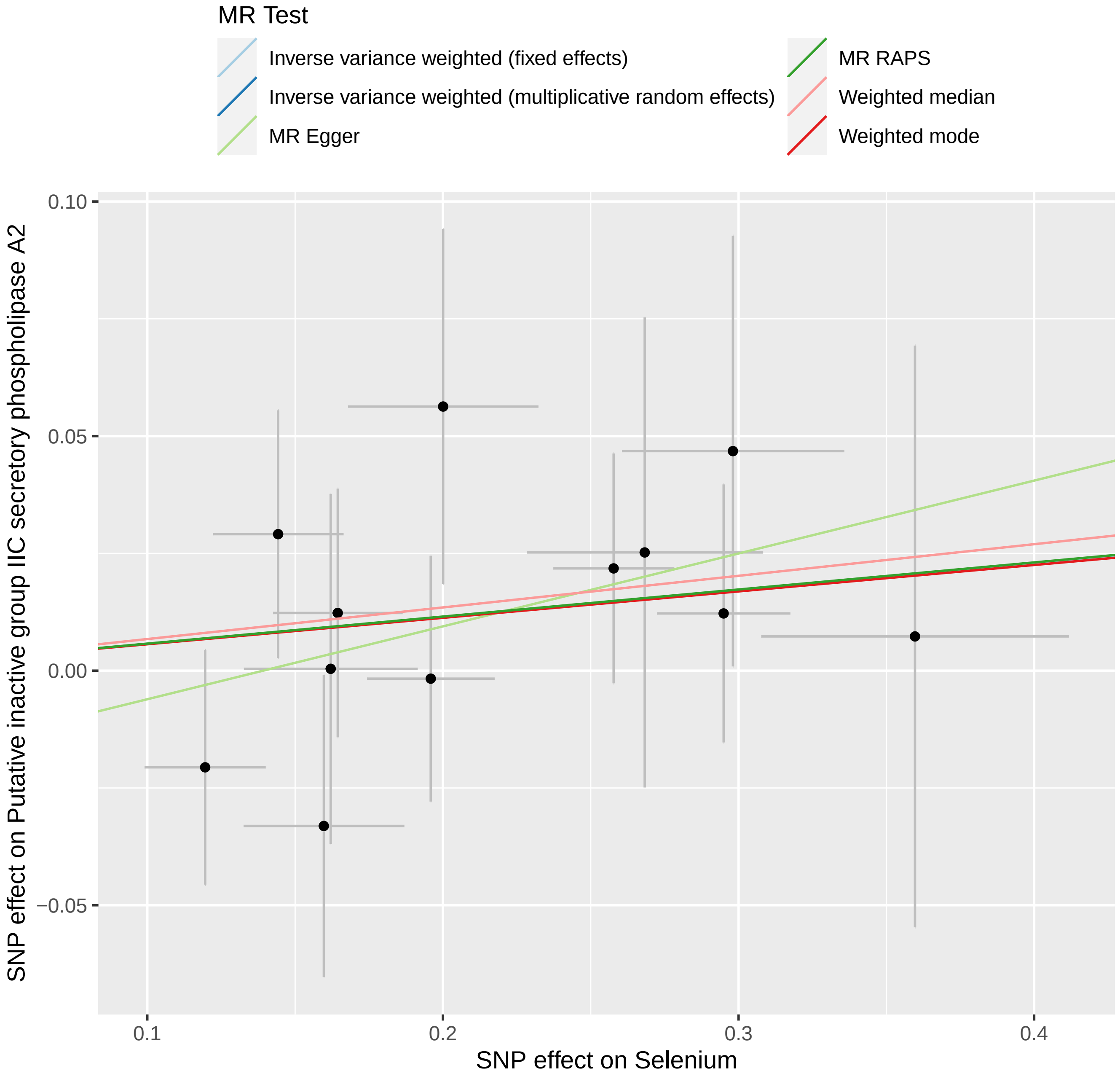

Batch 3−2288 : Selenium on Group IIE secretory phospholipase A2

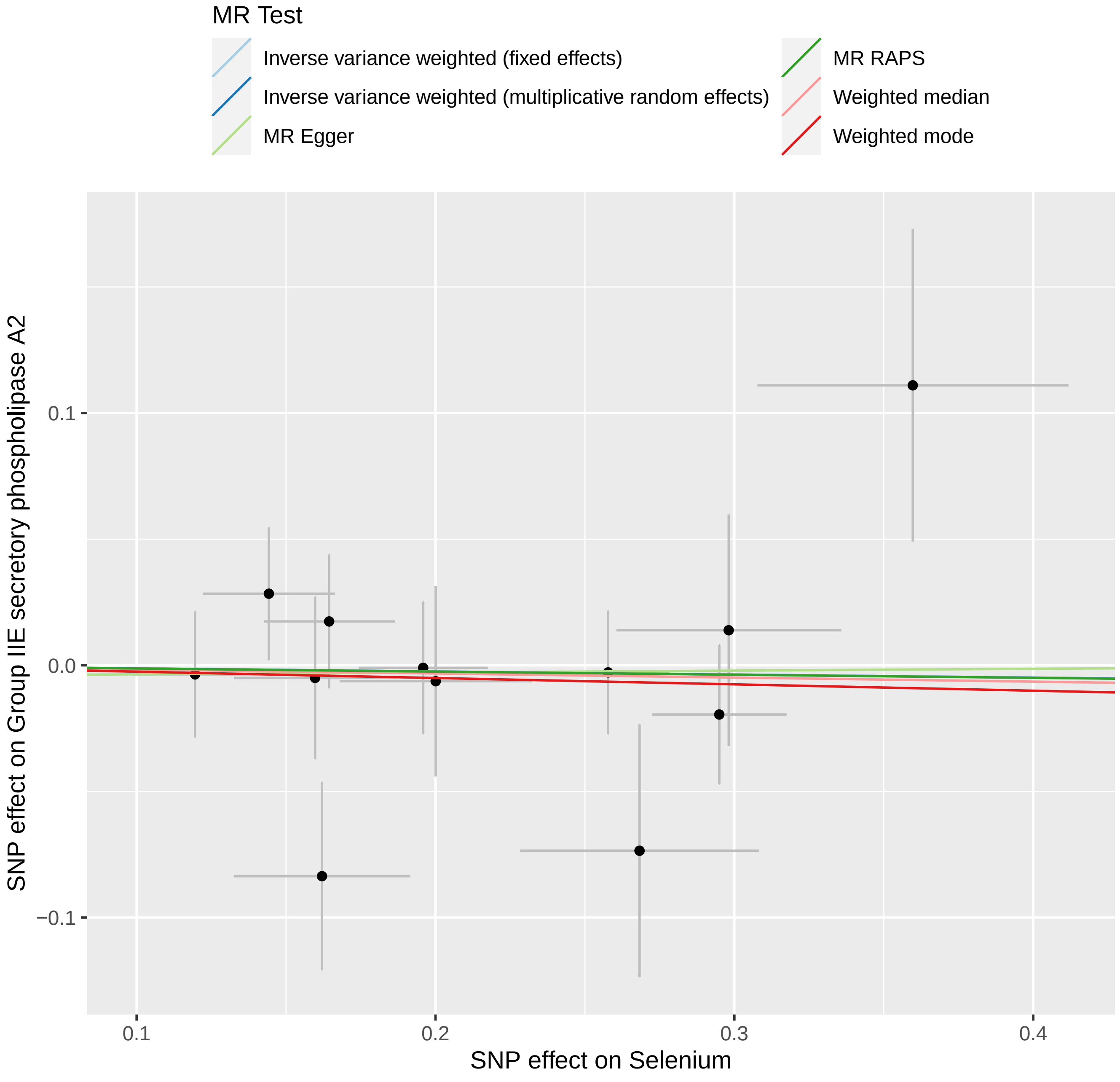

Batch 3−2289 : Selenium on Group IIE secretory phospholipase A2

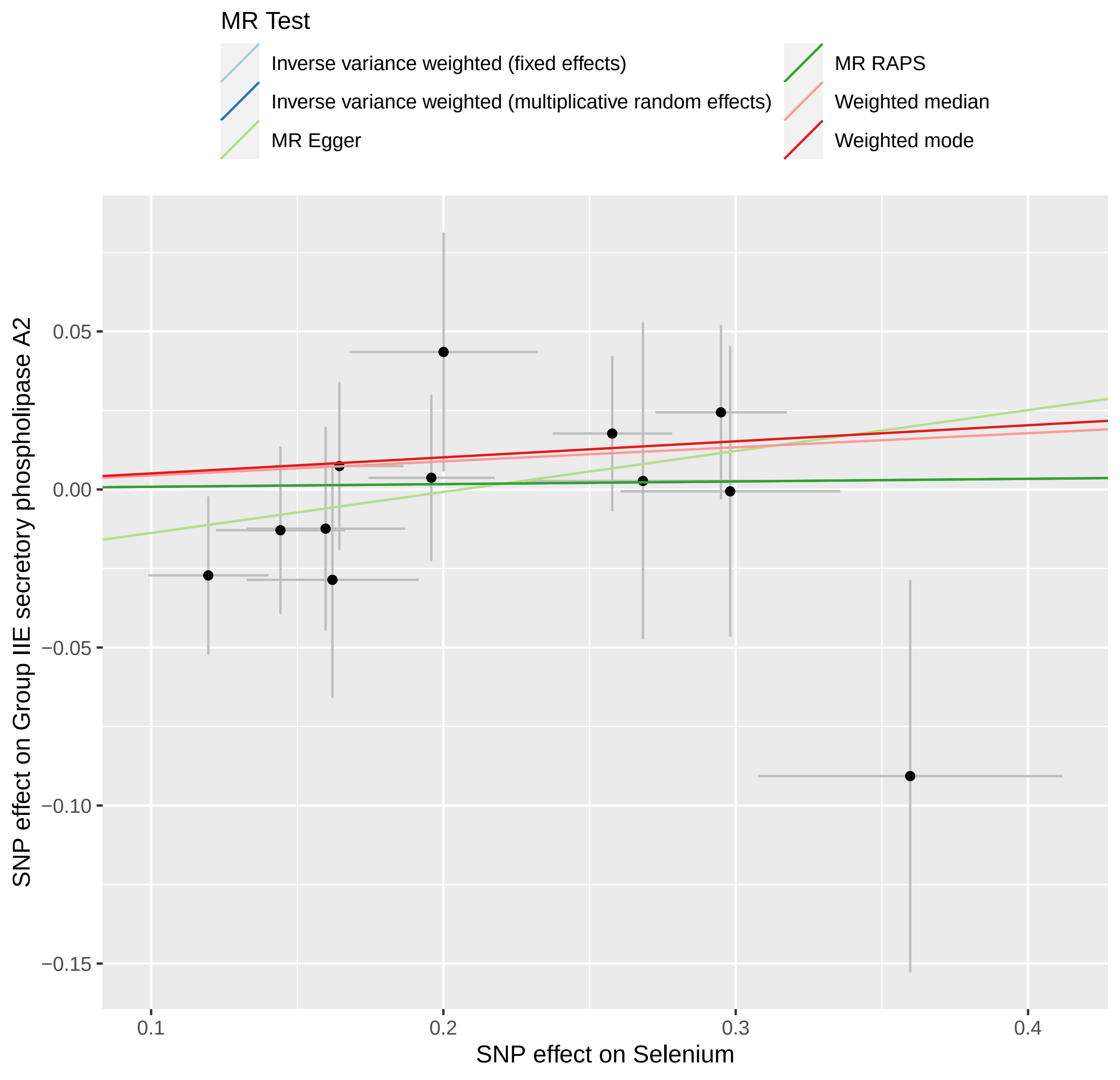

Batch 3−2290 : Selenium on Calcium−dependent phospholipase A2

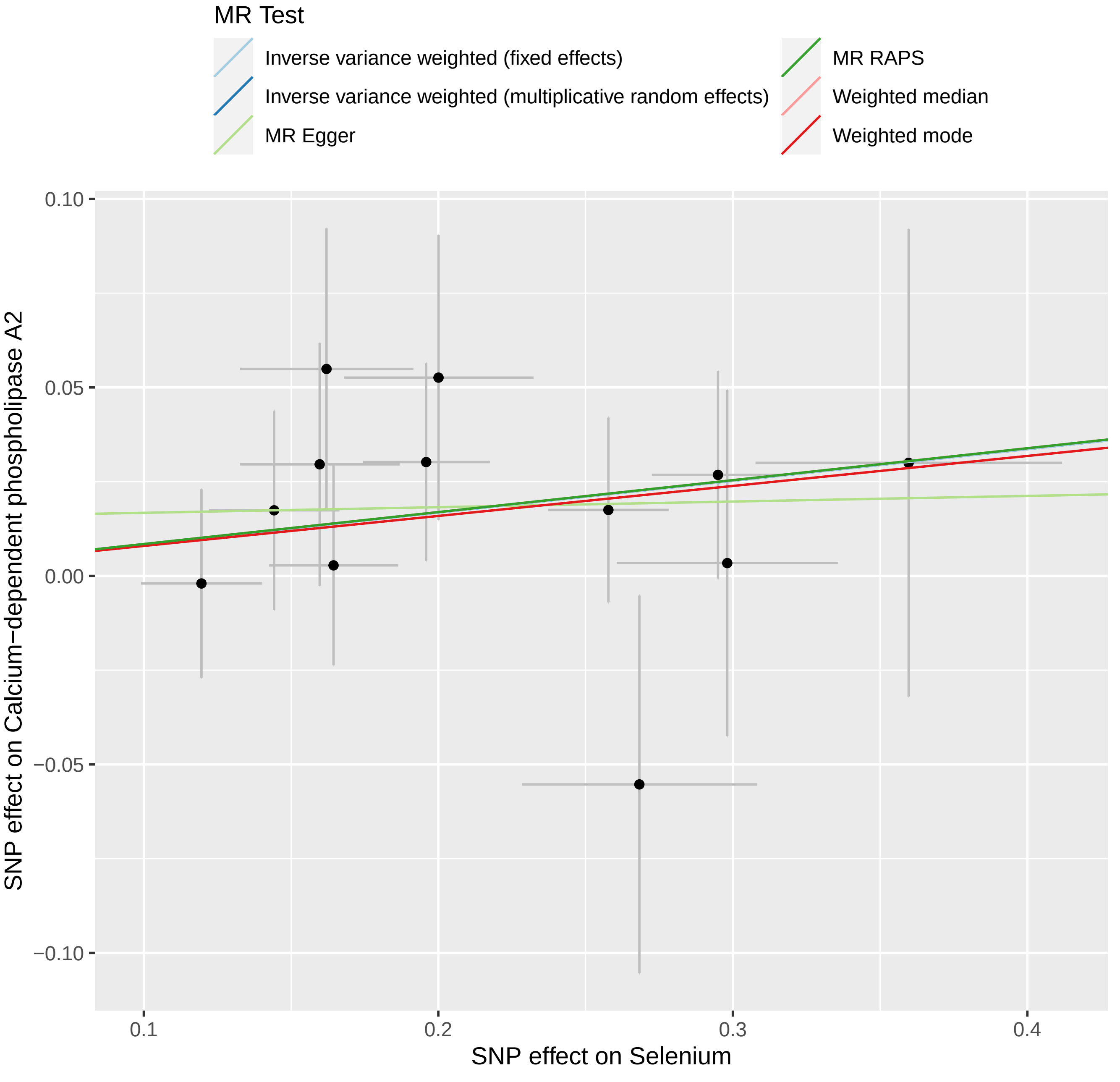

Batch 3−2291 : Selenium on Secretory phospholipase A2 receptor

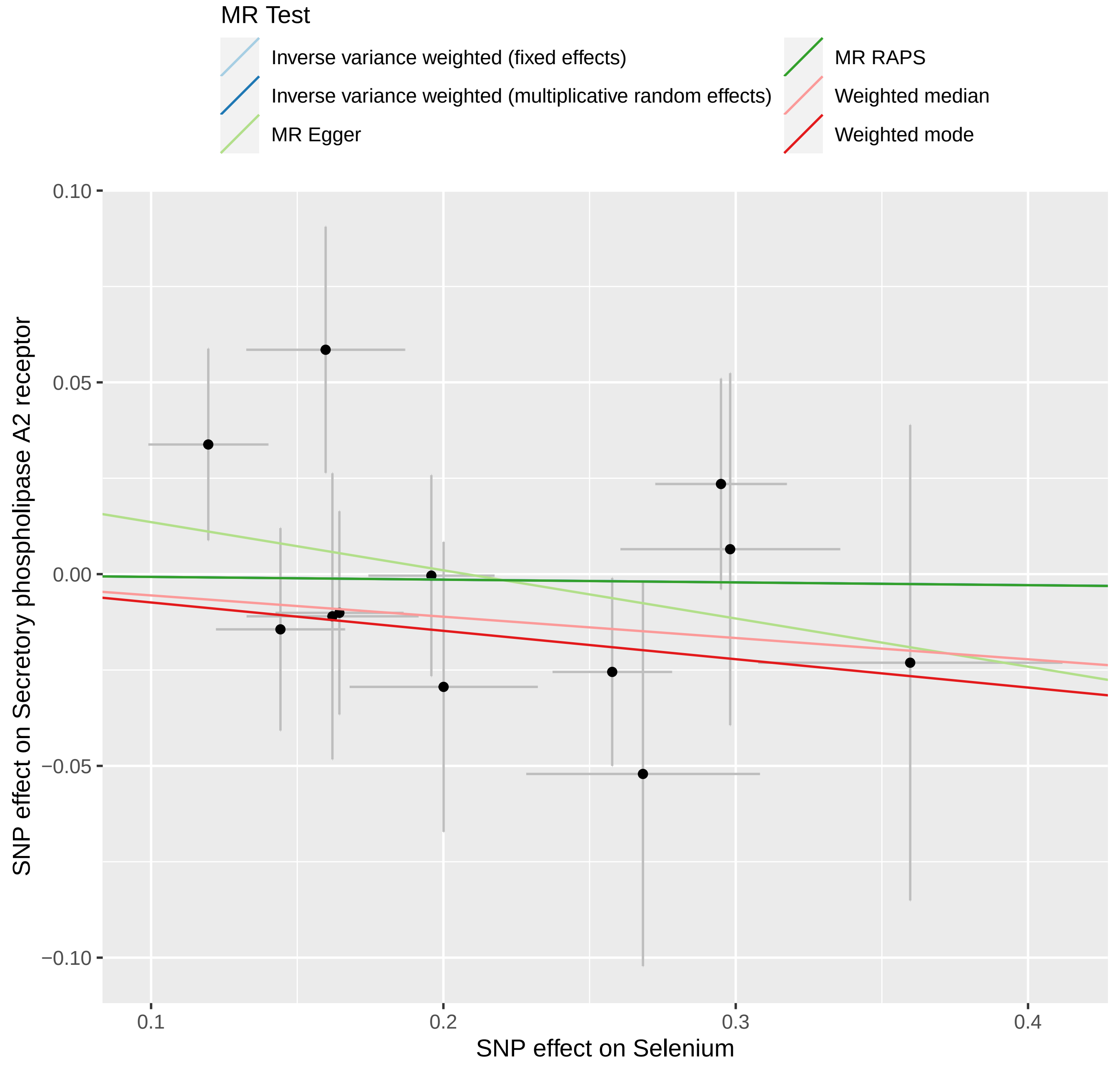

Batch 3−2292 : Selenium on Urokinase−type plasminogen activator

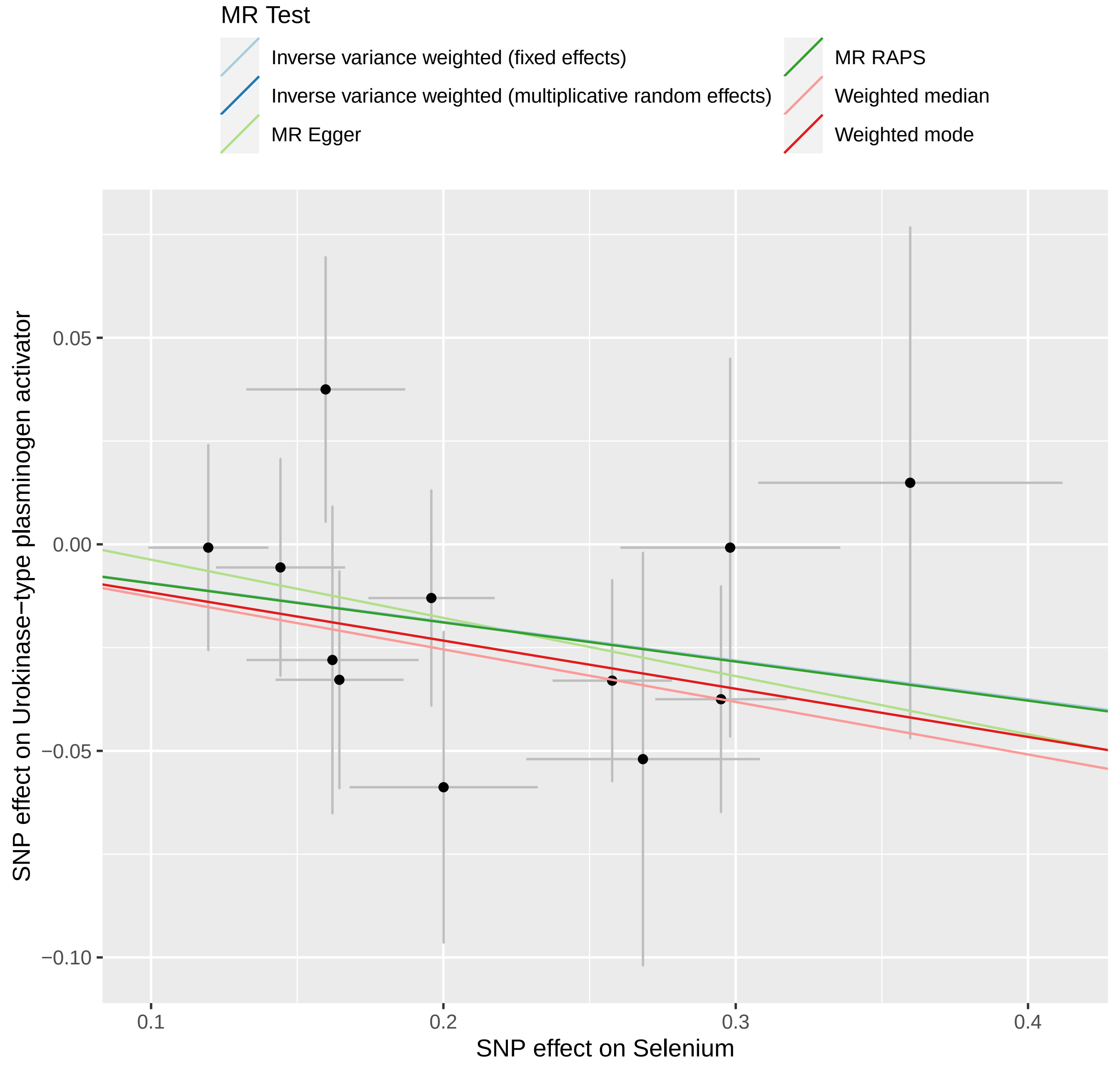

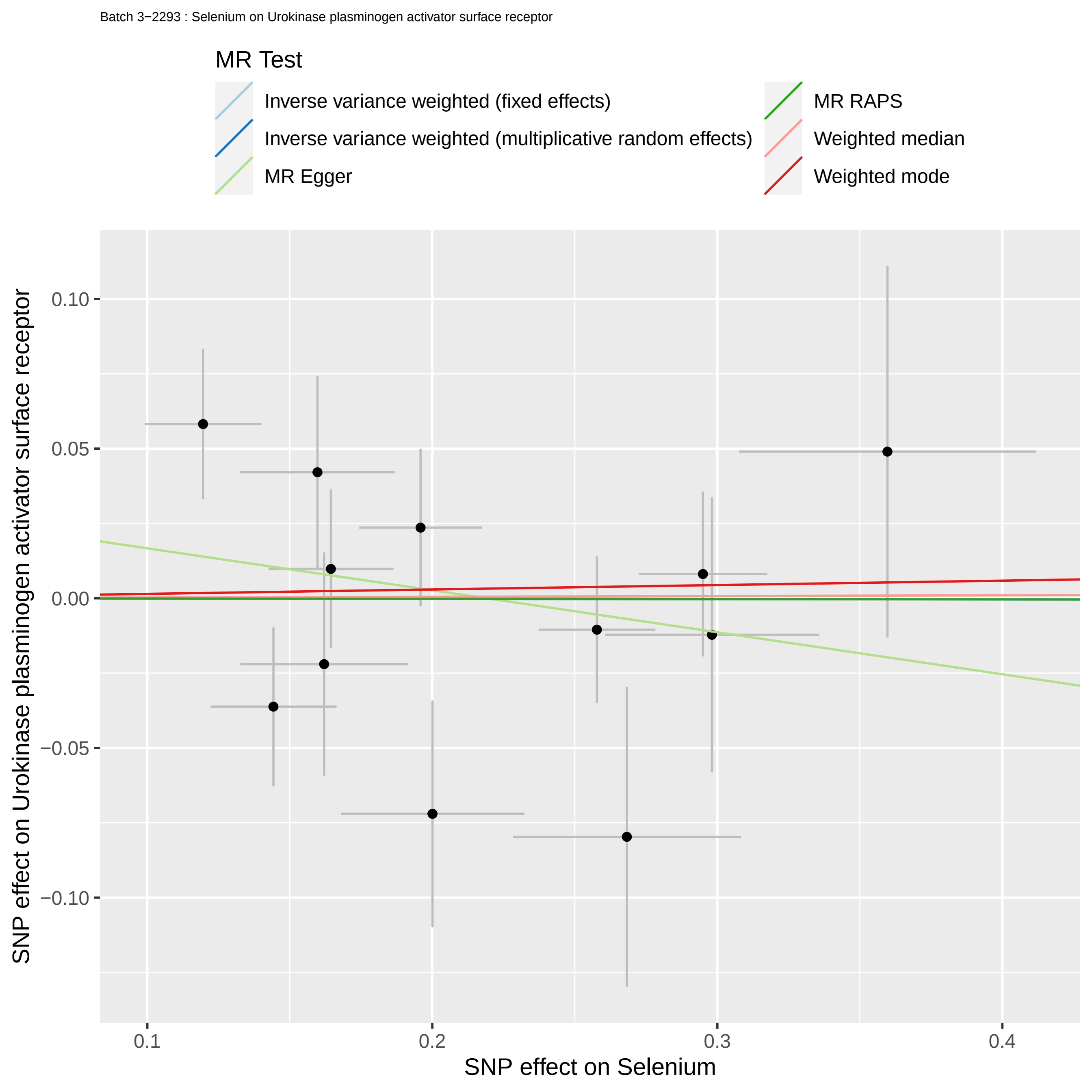
Batch 3−2293 : Selenium on Urokinase plasminogen activator surface receptor
MR Test
Inverse variance weighted (fixed effects)
Inverse variance weighted (multiplicative random effects)
MR Egger
MR RAPS
Weighted median
Weighted mode
0.10
0.05
0.00
−0.05
−0.10
0.1
0.2
0.3
0.4
SNP effect on Selenium
SNP effect on Urokinase plasminogen activator surface receptor

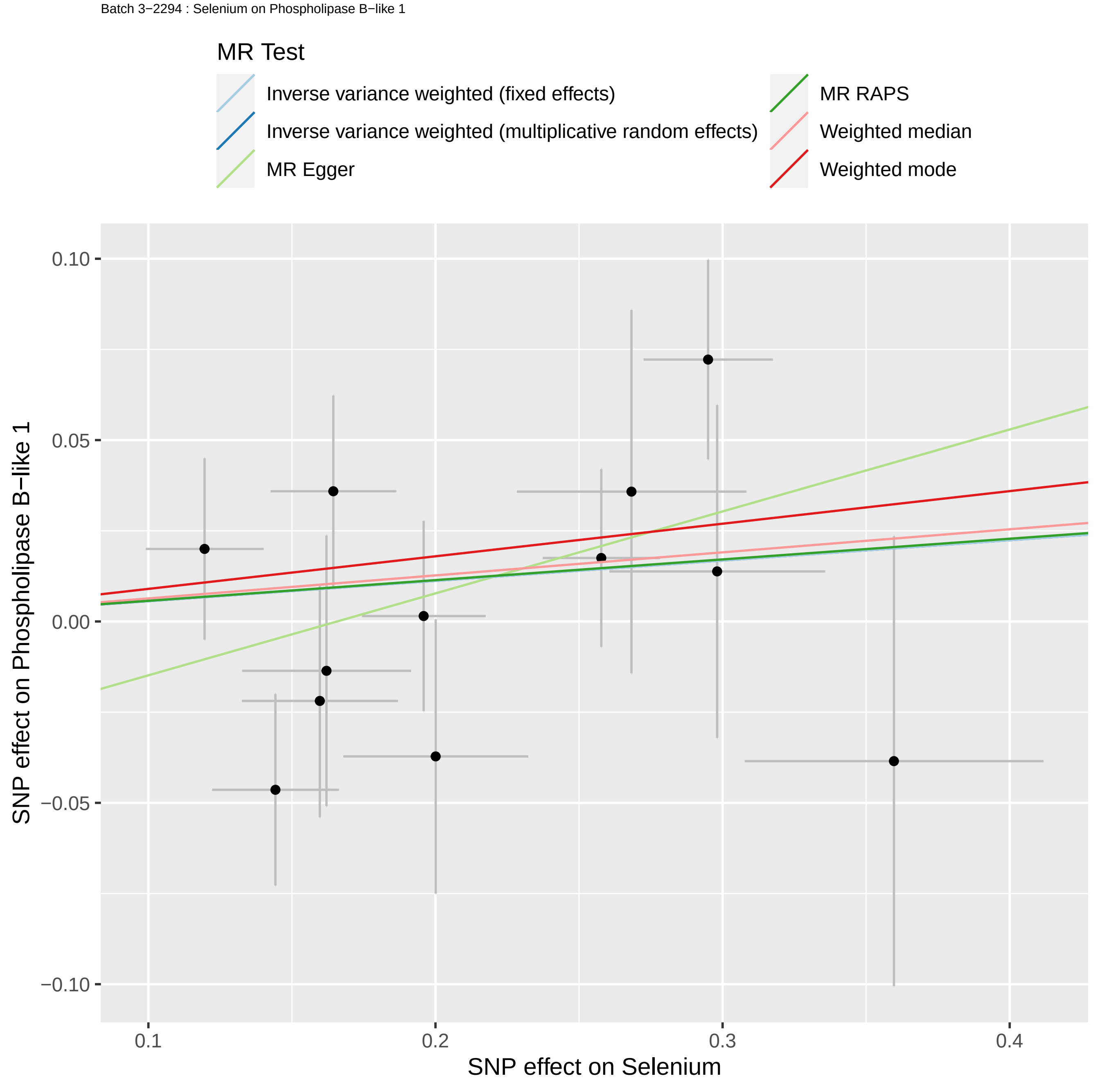

Batch 3−2294 : Selenium on Phospholipase B−like 1
MR Test
Inverse variance weighted (fixed effects)
Inverse variance weighted (multiplicative random effects)
MR Egger
MR RAPS
Weighted median
Weighted mode
SNP effect on Phospholipase B−like 1
0.10
0.05
0.00
−0.05
−0.10
SNP effect on Selenium
0.1
0.2
0.3
0.4

Batch 3−2295 : Selenium on Putative phospholipase B−like 2

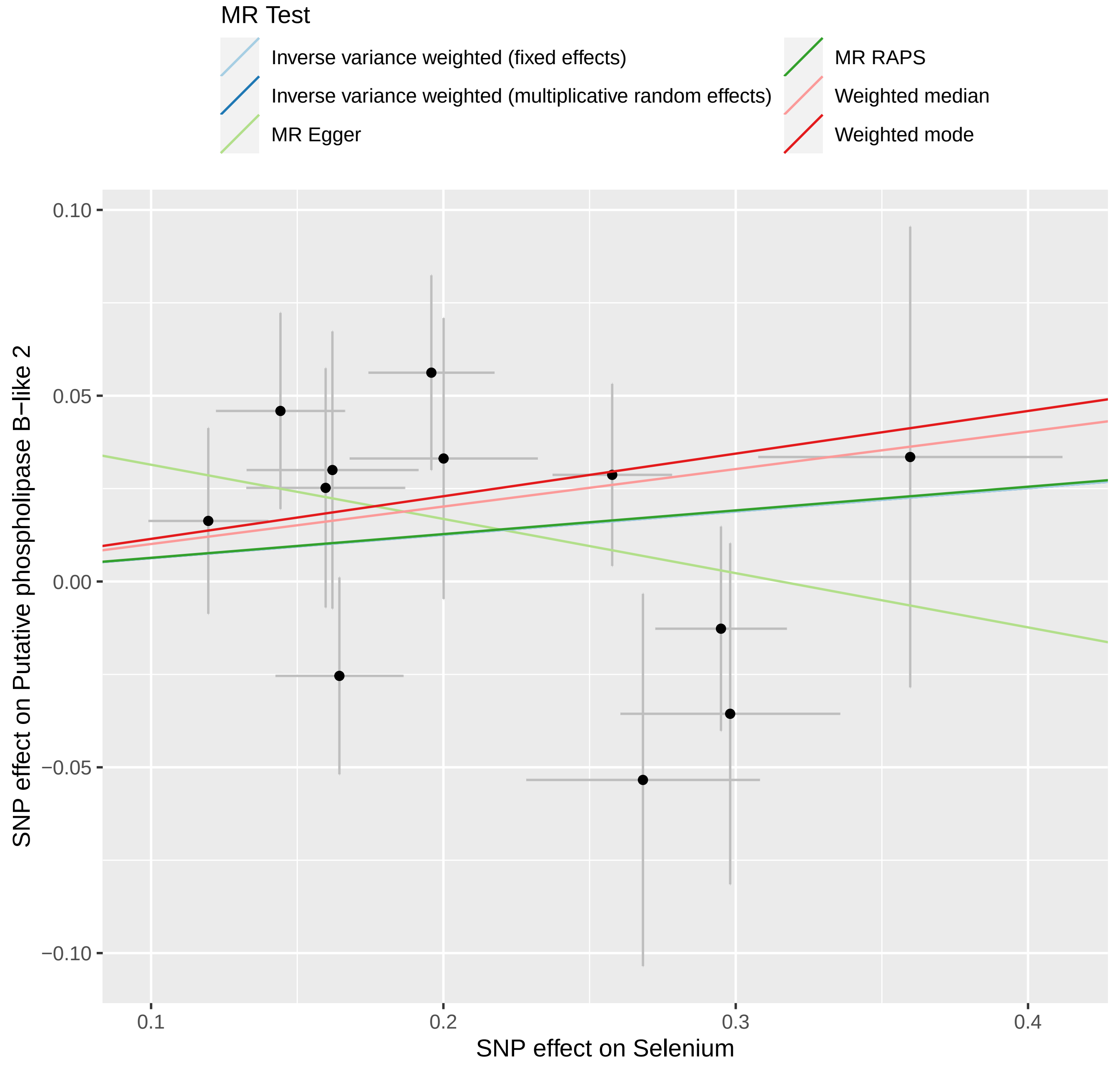

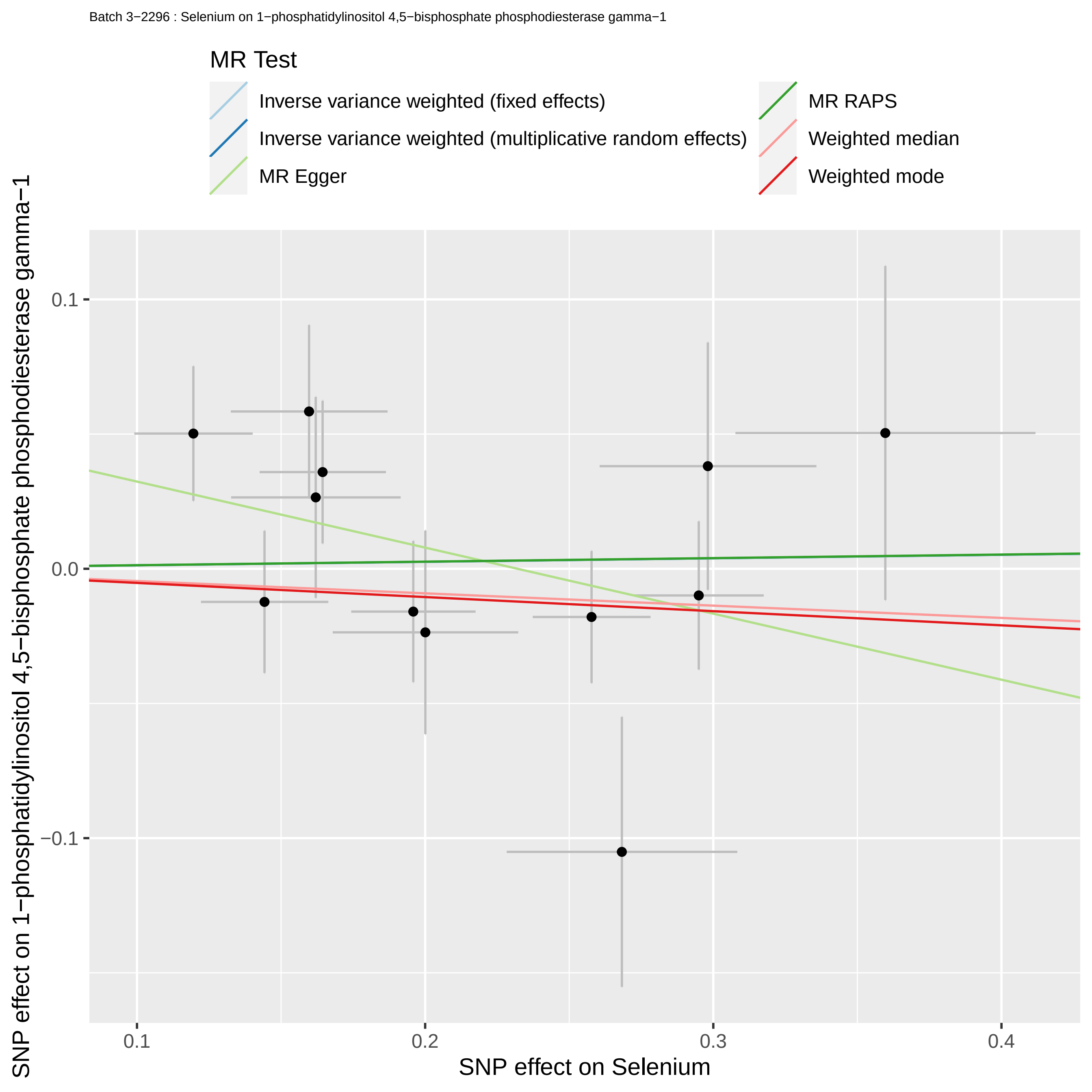

Batch 3−2296 : Selenium on 1−phosphatidylinositol 4,5−bisphosphate phosphodiesterase gamma−1
MR Test
Inverse variance weighted (fixed effects)
Inverse variance weighted (multiplicative random effects)
MR Egger
MR RAPS
Weighted median
Weighted mode
SNP effect on 1−phosphatidylinositol 4,5−bisphosphate phosphodiesterase gamma−1
0.1
0.0
−0.1
0.1
0.2
0.3
0.4
SNP effect on Selenium

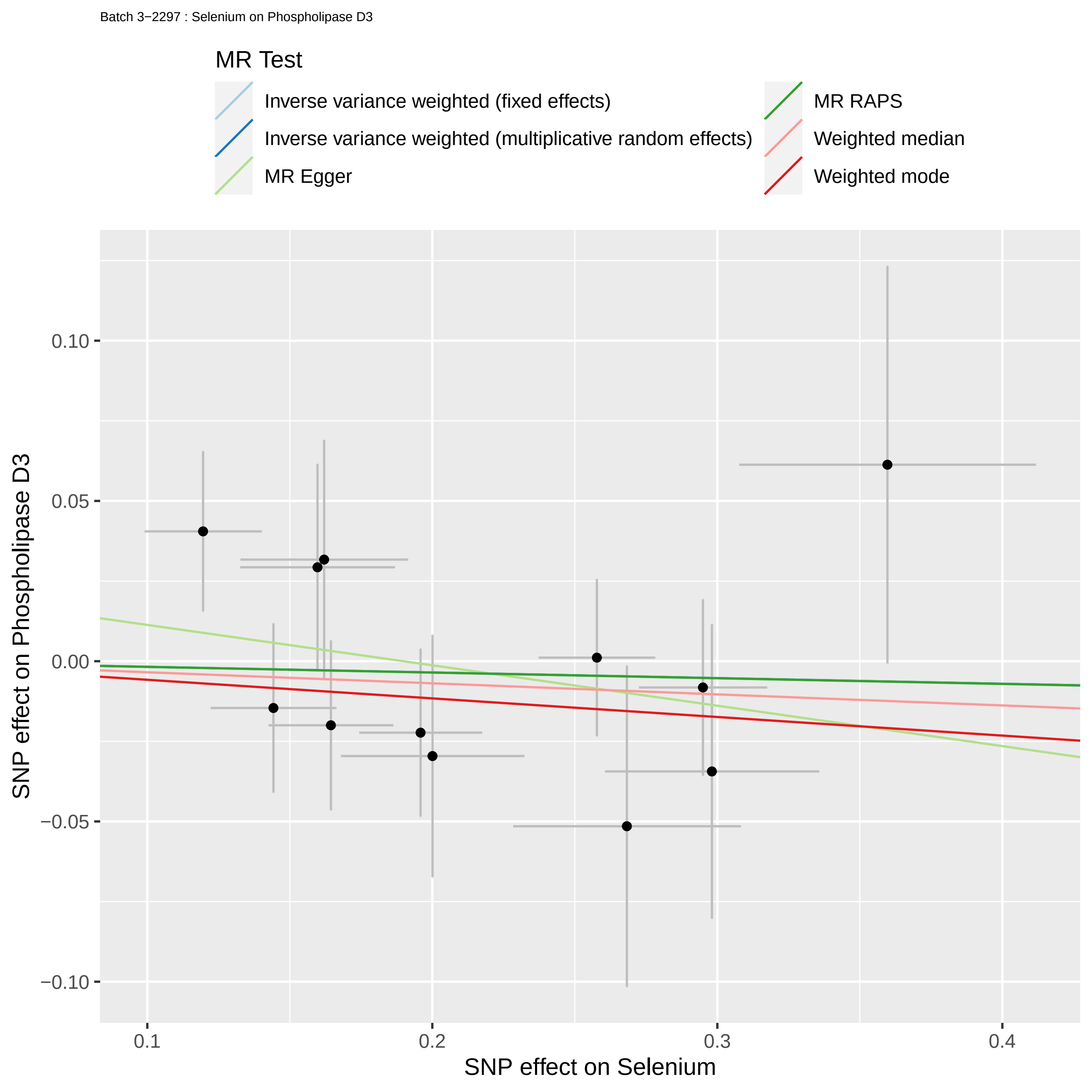
Batch 3−2297 : Selenium on Phospholipase D3
MR Test
Inverse variance weighted (fixed effects)
Inverse variance weighted (multiplicative random effects)
MR Egger
MR RAPS
Weighted median
Weighted mode
0.10
0.05
0.00
−0.05
−0.10
0.1
0.2
0.3
0.4
SNP effect on Phospholipase D3
SNP effect on Selenium

Batch 3−2298 : Selenium on Inactive phospholipase D5

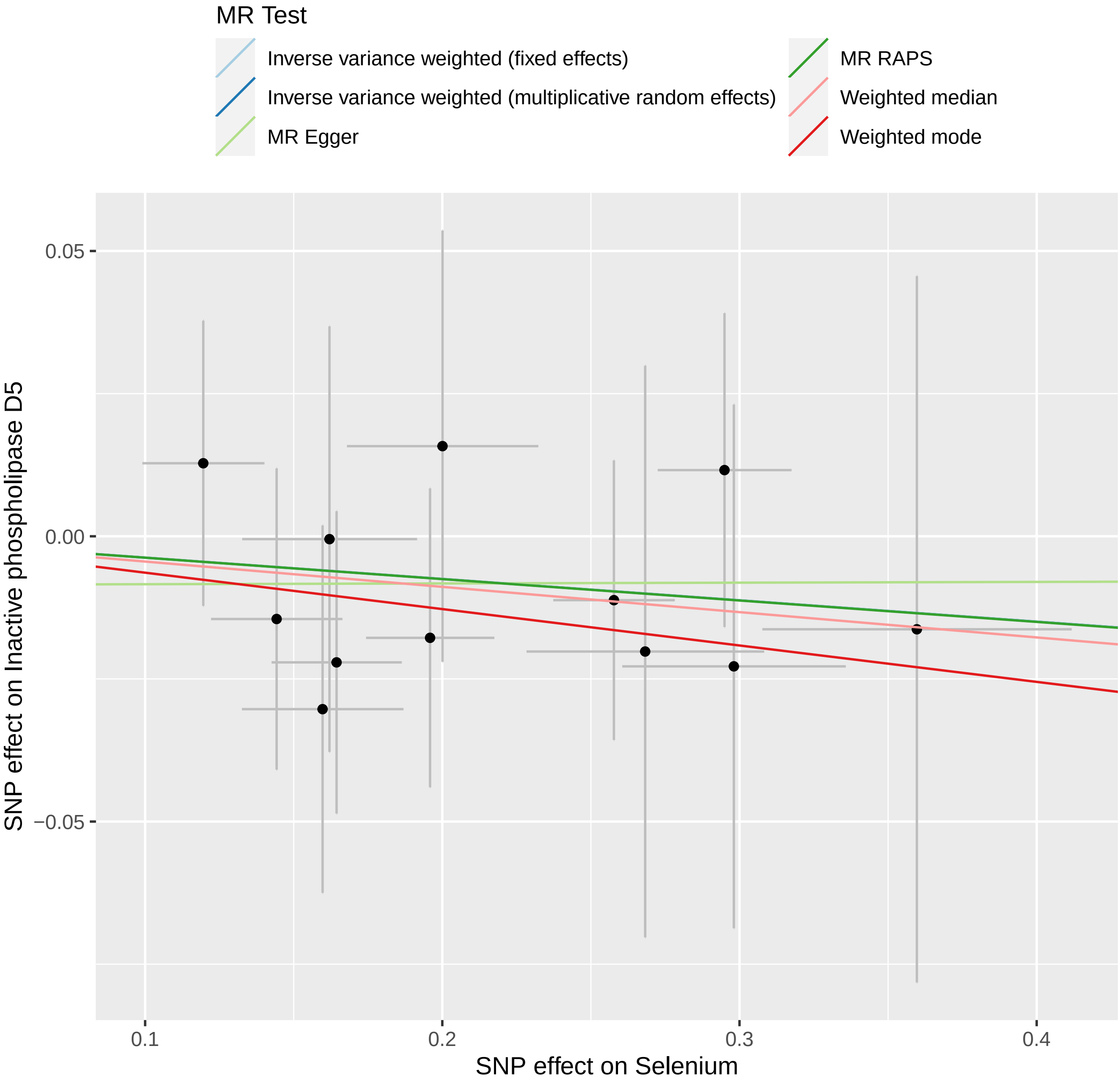

Batch 3−2299 : Selenium on Pleckstrin homology domain−containing family A member 1

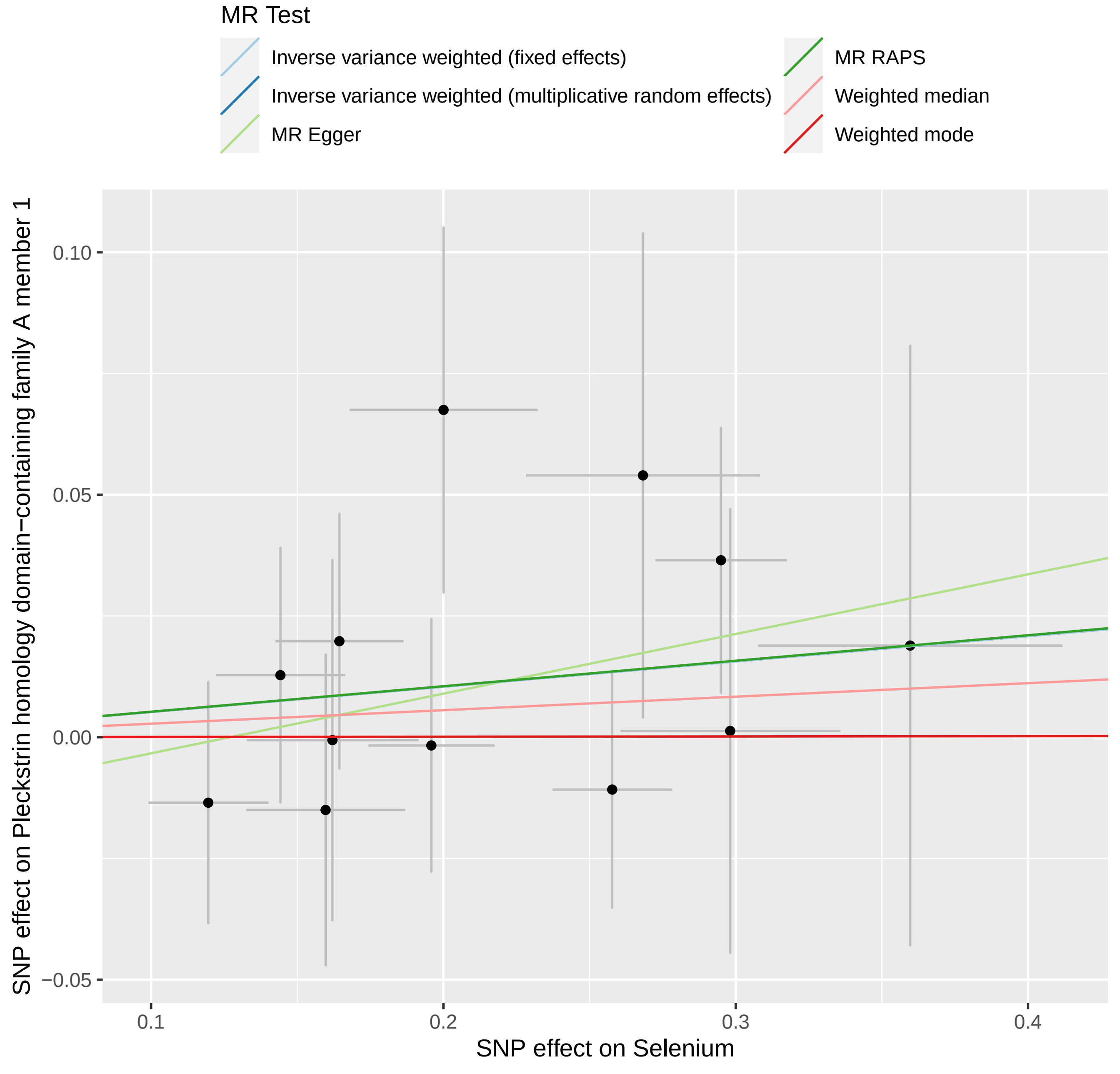

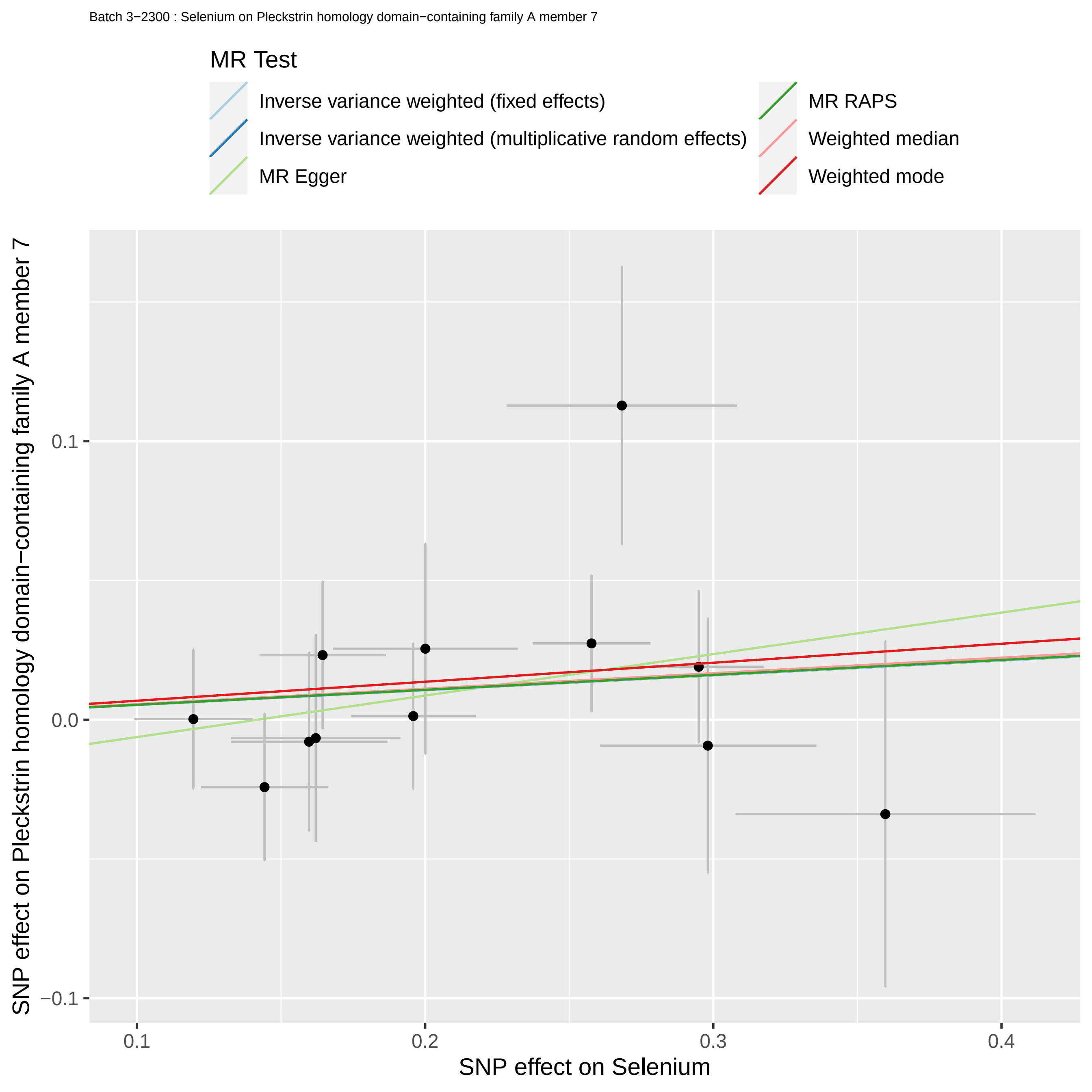

Batch 3−2300 : Selenium on Pleckstrin homology domain−containing family A member 7
MR Test
Inverse variance weighted (fixed effects)
Inverse variance weighted (multiplicative random effects)
MR Egger
MR RAPS
Weighted median
Weighted mode
SNP effect on Pleckstrin homology domain−containing family A member 7
0.1
0.0
−0.1
0.1
0.2
0.3
0.4
SNP effect on Selenium

Batch 3−2301 : Selenium on Angiostatin

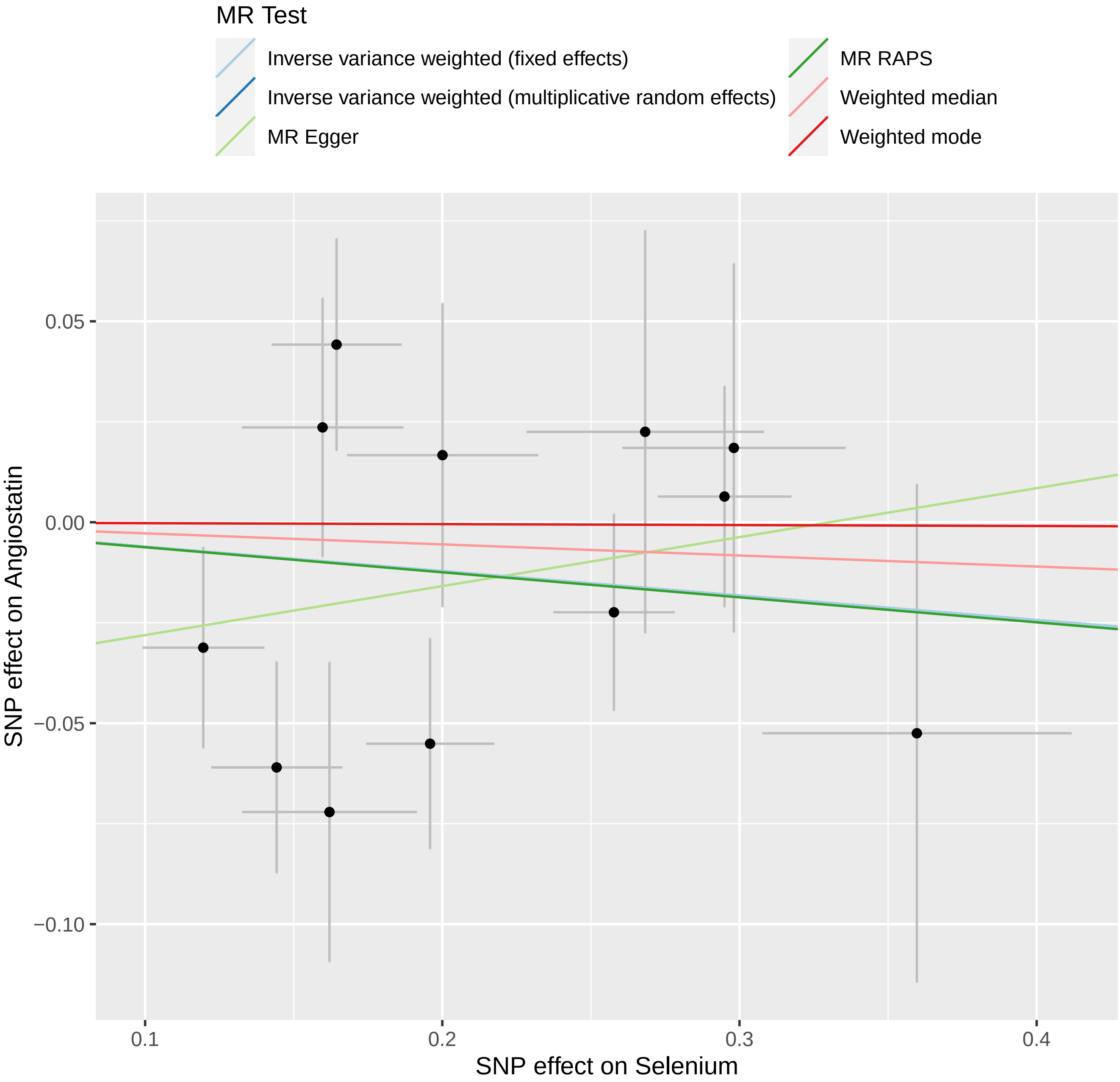

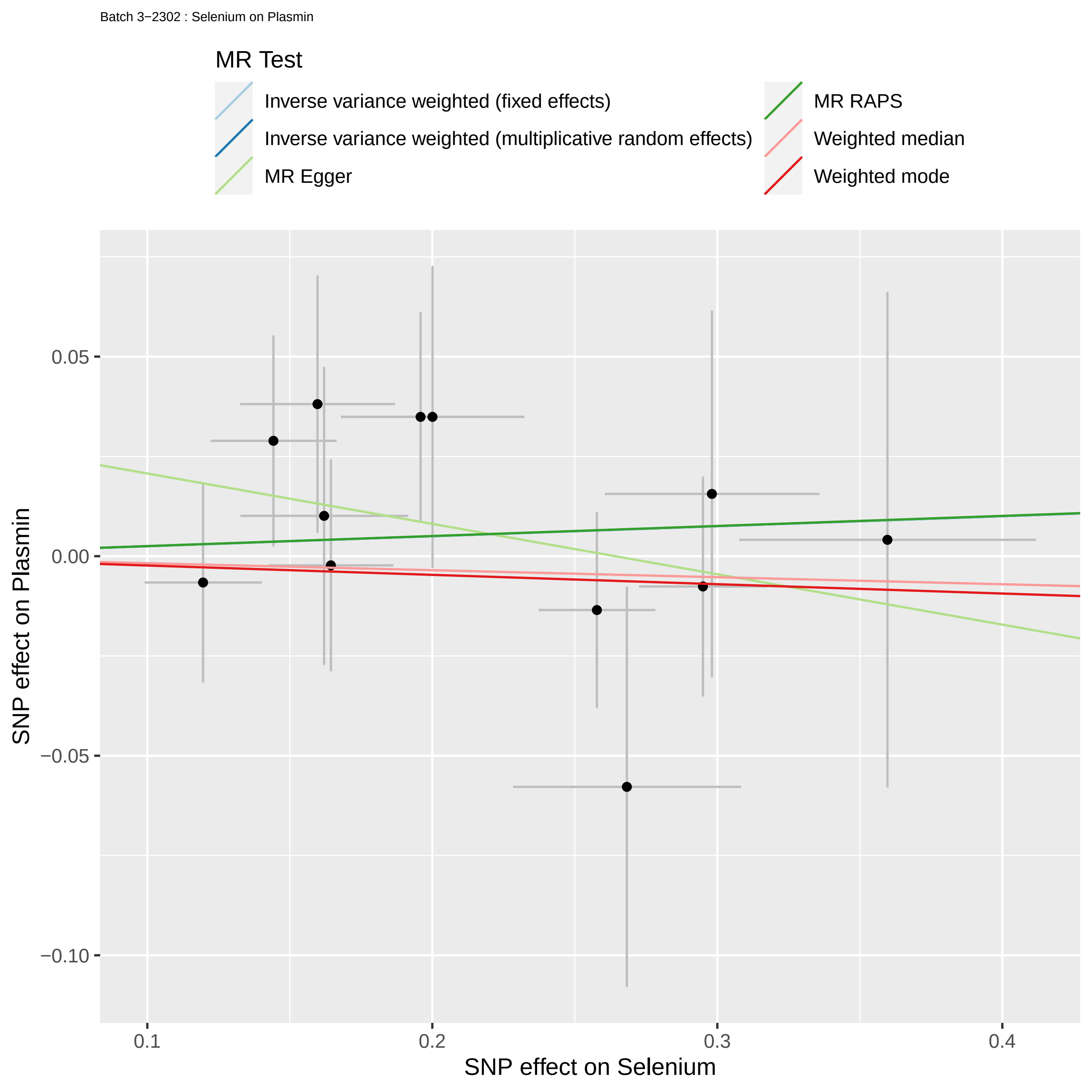
Batch 3−2302 : Selenium on Plasmin
MR Test
Inverse variance weighted (fixed effects)
Inverse variance weighted (multiplicative random effects)
MR Egger
MR RAPS
Weighted median
Weighted mode
0.05
0.00
−0.05
−0.10
0.1
0.2
0.3
0.4
SNP effect on Plasmin
SNP effect on Selenium

Batch 3−2303 : Selenium on Serine/threonine−protein kinase PLK1

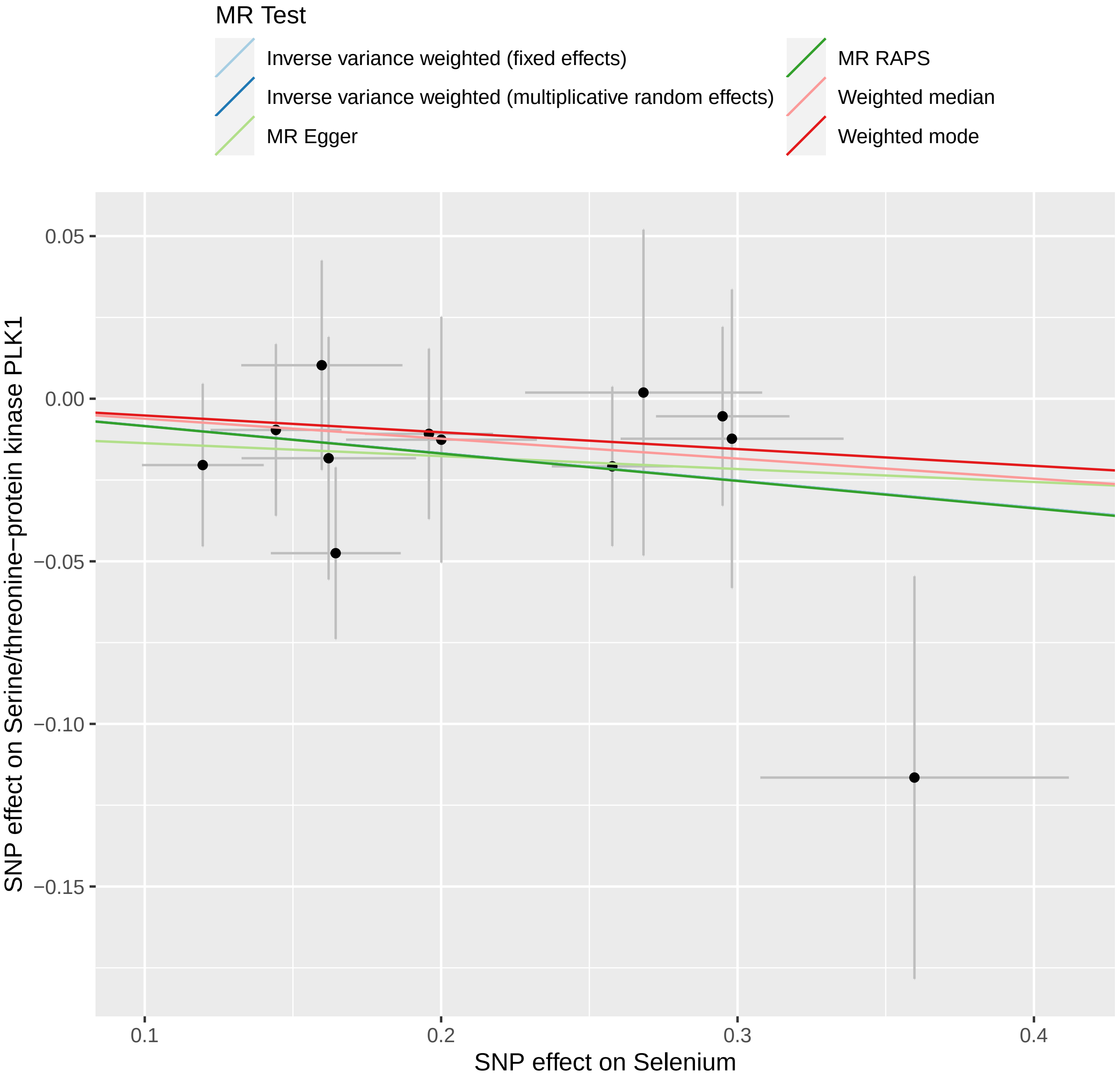

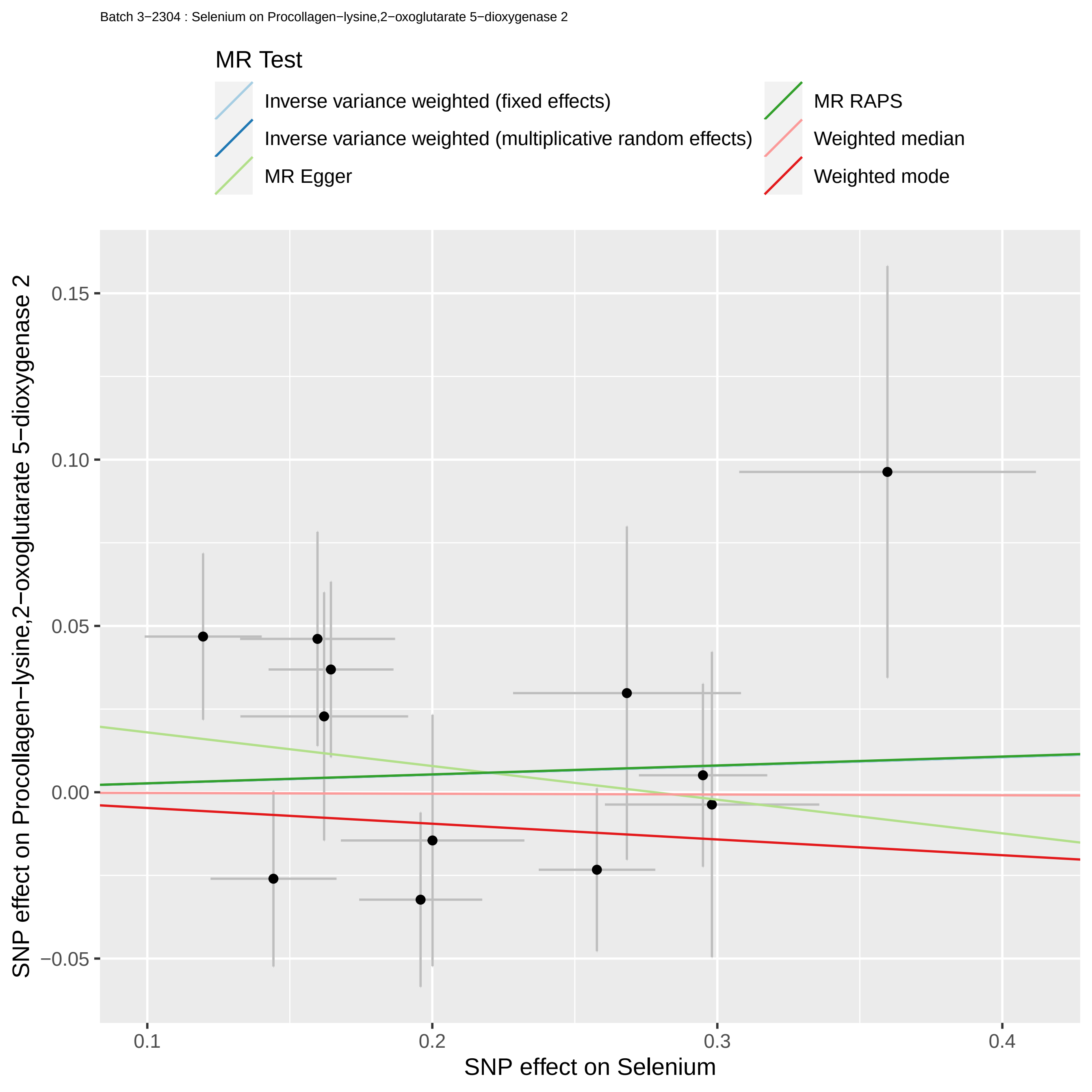
Batch 3−2304 : Selenium on Procollagen−lysine,2−oxoglutarate 5−dioxygenase 2
MR Test
Inverse variance weighted (fixed effects)
Inverse variance weighted (multiplicative random effects)
MR Egger
MR RAPS
Weighted median
Weighted mode
0.15
0.10
0.05
0.00
−0.05
0.1
0.2
0.3
0.4
SNP effect on Procollagen−lysine,2−oxoglutarate 5−dioxygenase 2
SNP effect on Selenium

Batch 3−2305 : Selenium on Procollagen−lysine,2−oxoglutarate 5−dioxygenase 3

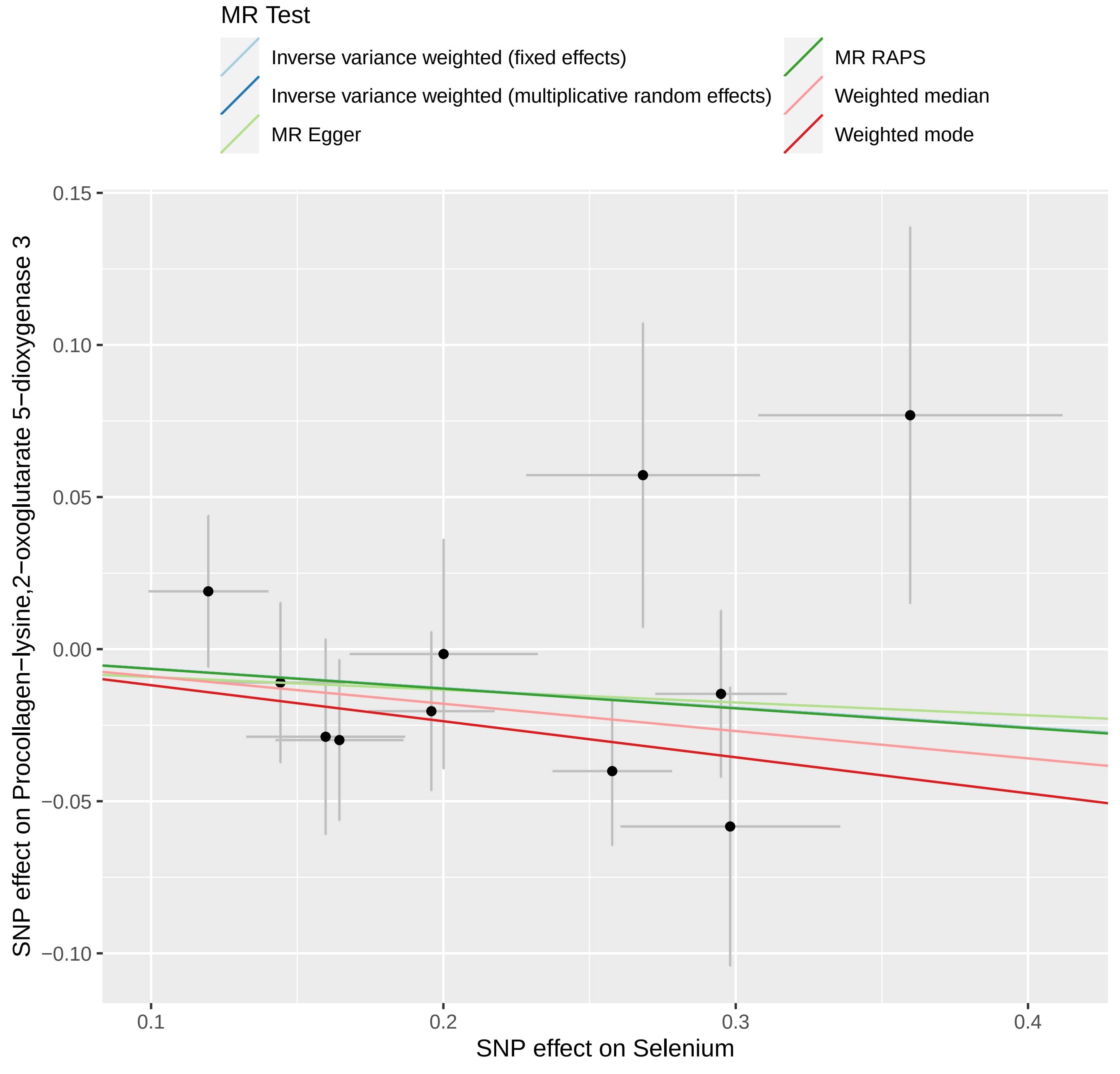

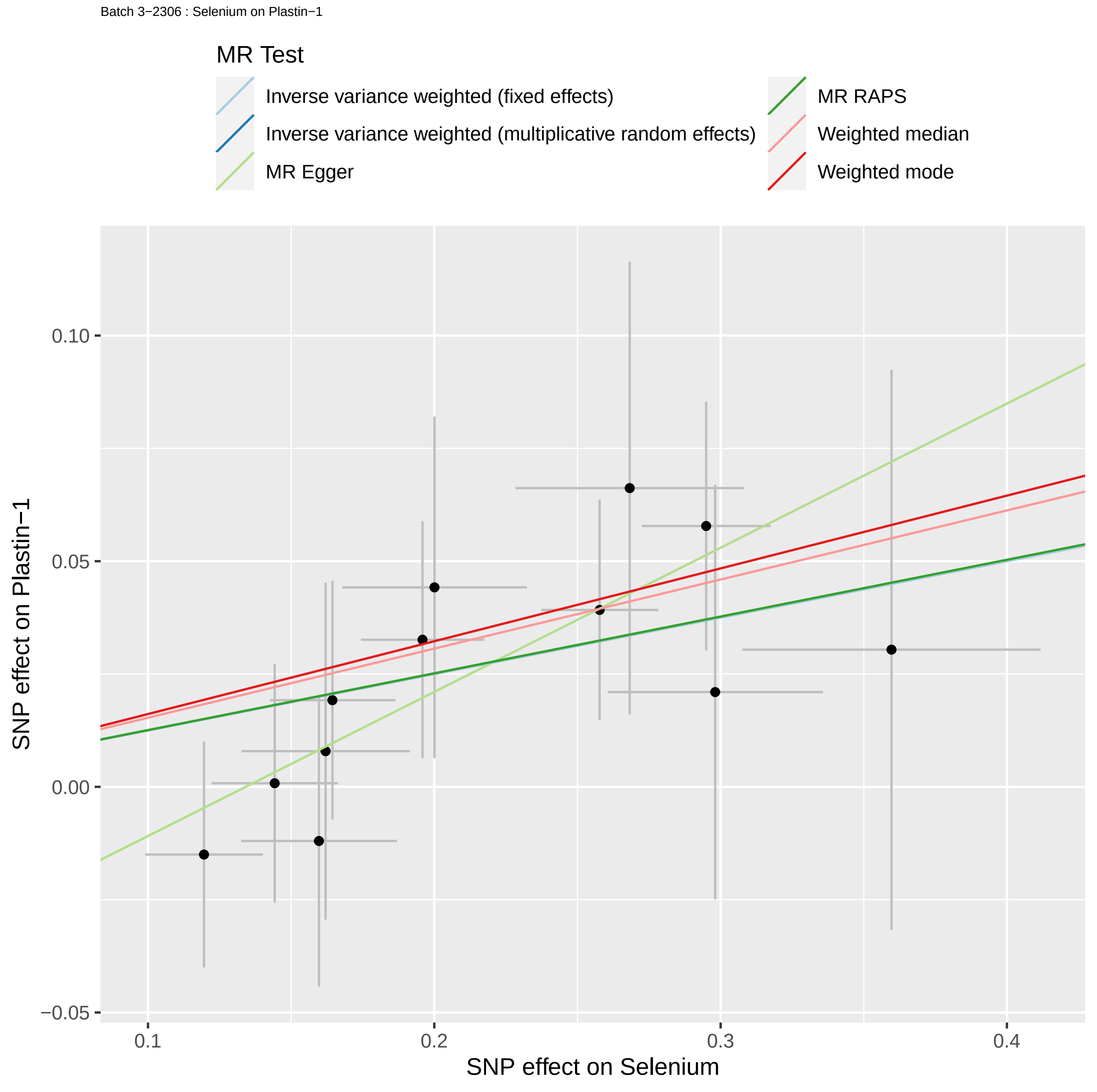

Batch 3−2306 : Selenium on Plastin−1
MR Test
Inverse variance weighted (fixed effects)
Inverse variance weighted (multiplicative random effects)
MR Egger
MR RAPS
Weighted median
Weighted mode
0.10
0.05
0.00
−0.05
0.1
0.2
0.3
0.4
SNP effect on Plastin−1
SNP effect on Selenium

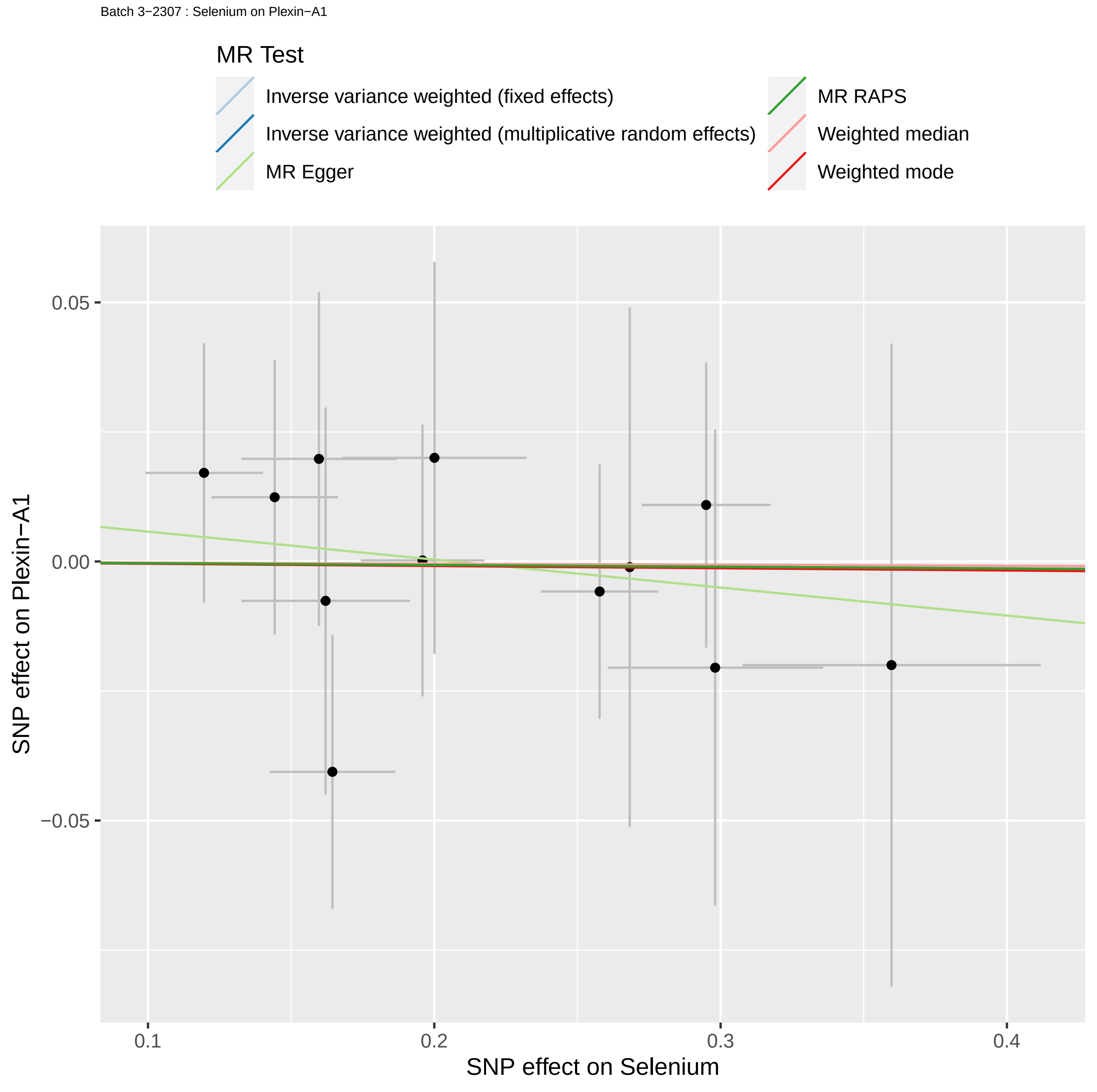

Batch 3−2307 : Selenium on Plexin−A1
MR Test
Inverse variance weighted (fixed effects)
Inverse variance weighted (multiplicative random effects)
MR Egger
MR RAPS
Weighted median
Weighted mode
0.05
0.00
−0.05
0.1
0.2
0.3
0.4
SNP effect on Plexin−A1
SNP effect on Selenium

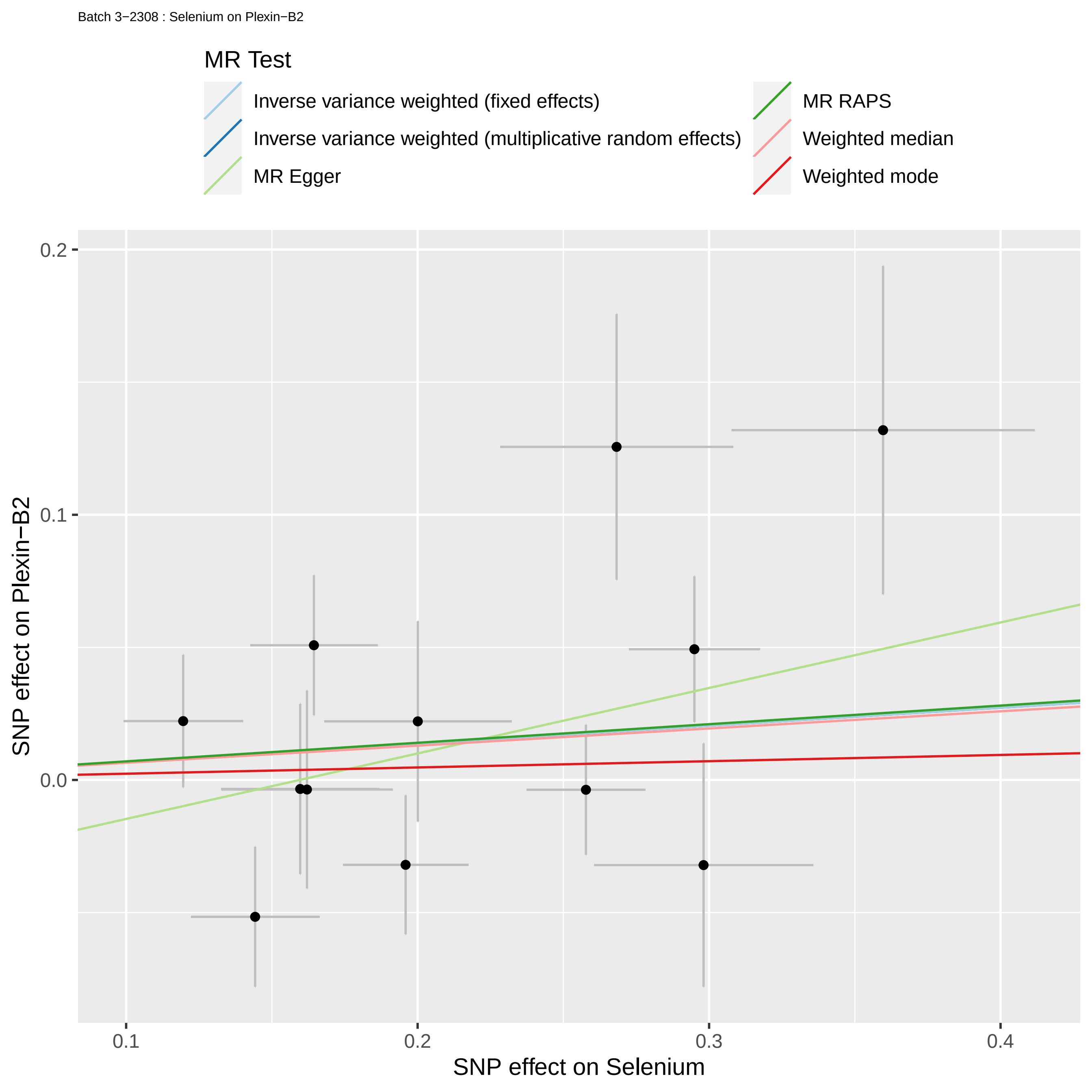
Batch 3−2308 : Selenium on Plexin−B2
MR Test
Inverse variance weighted (fixed effects)
Inverse variance weighted (multiplicative random effects)
MR Egger
MR RAPS
Weighted median
Weighted mode
0.2
0.1
0.0
0.1
0.2
0.3
0.4
SNP effect on Plexin−B2
SNP effect on Selenium

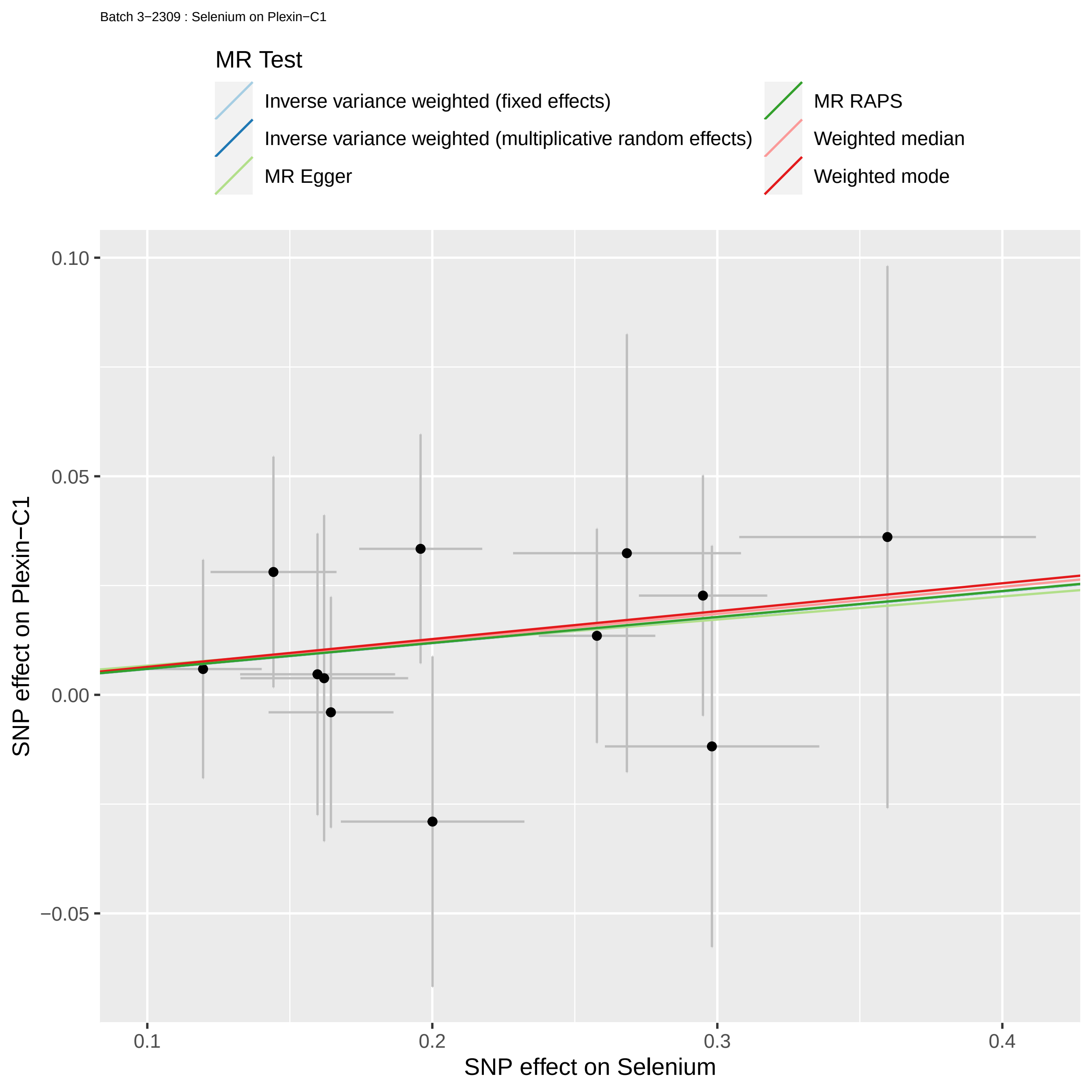
Batch 3−2309 : Selenium on Plexin−C1
MR Test
Inverse variance weighted (fixed effects)
Inverse variance weighted (multiplicative random effects)
MR Egger
MR RAPS
Weighted median
Weighted mode
0.10
0.05
0.00
−0.05
SNP effect on Plexin−C1
0.1
0.2
0.3
0.4
SNP effect on Selenium

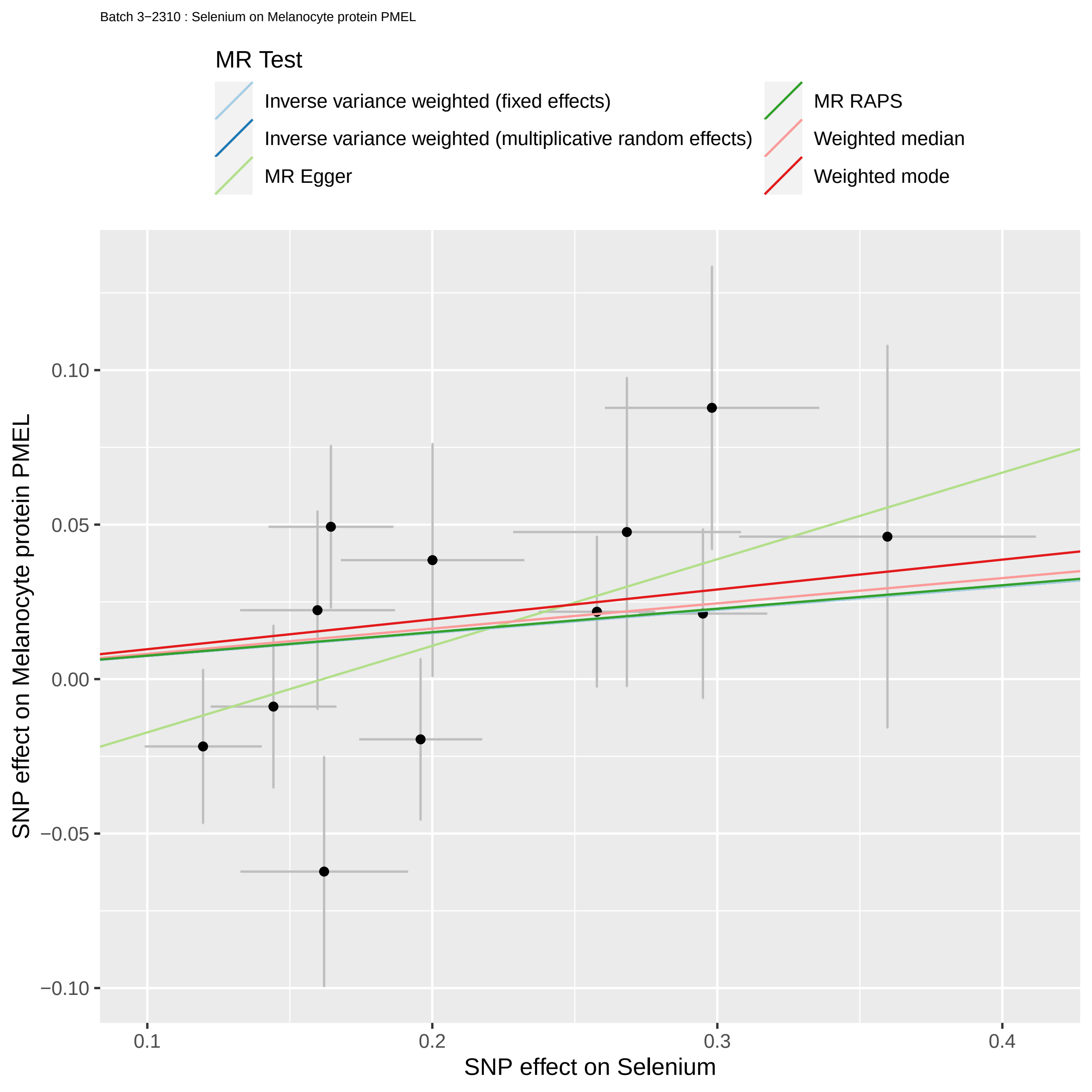

Batch 3−2310 : Selenium on Melanocyte protein PMEL
MR Test
Inverse variance weighted (fixed effects)
Inverse variance weighted (multiplicative random effects)
MR Egger
MR RAPS
Weighted median
Weighted mode
SNP effect on Melanocyte protein PMEL
0.10
0.05
0.00
−0.05
−0.10
0.1
0.2
0.3
0.4
SNP effect on Selenium

Batch 3−2311 : Selenium on Protein TMEPAI

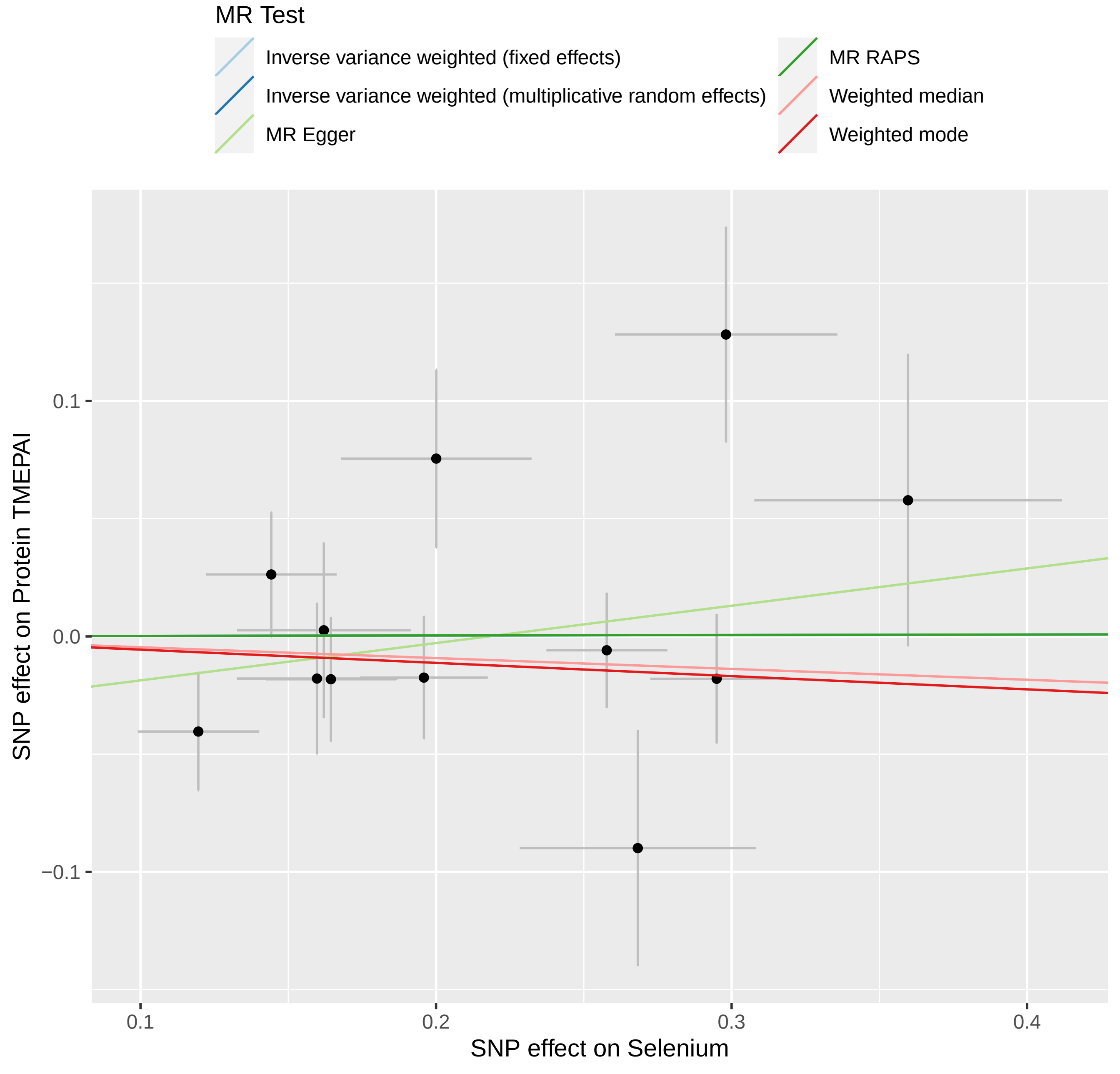

Batch 3−2312 : Selenium on Mismatch repair endonuclease PMS2

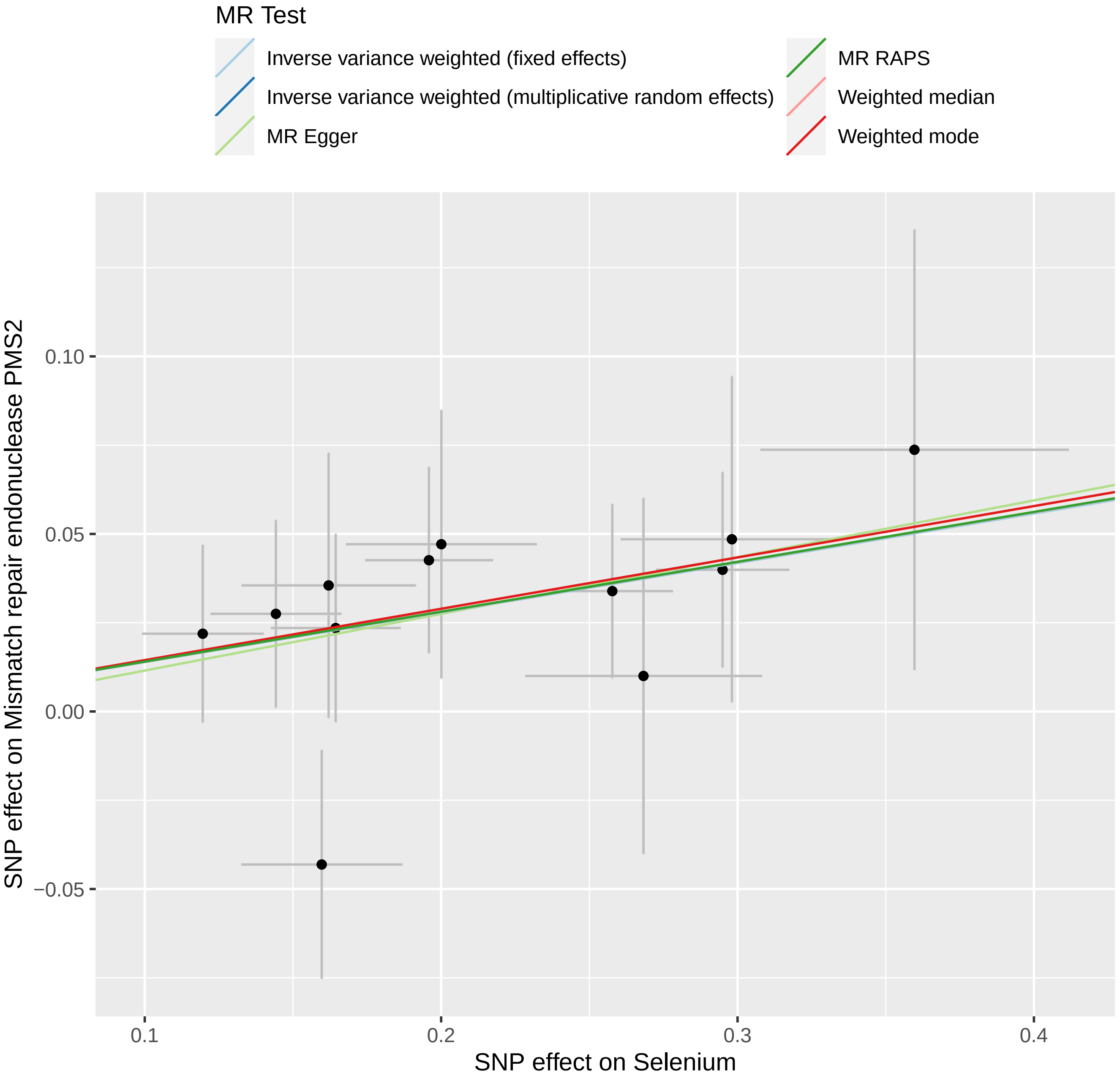

Batch 3−2313 : Selenium on Phosphomevalonate kinase

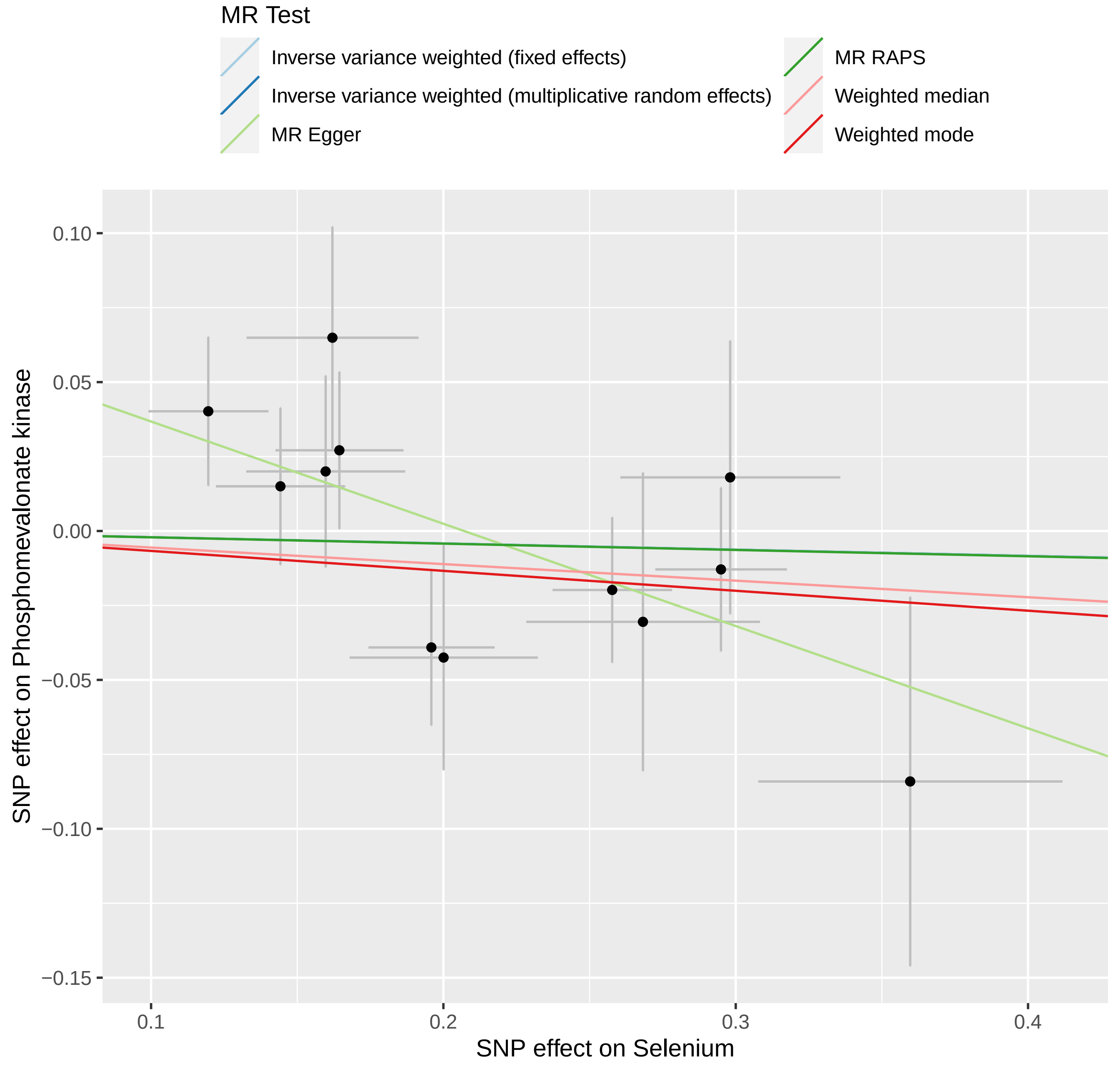

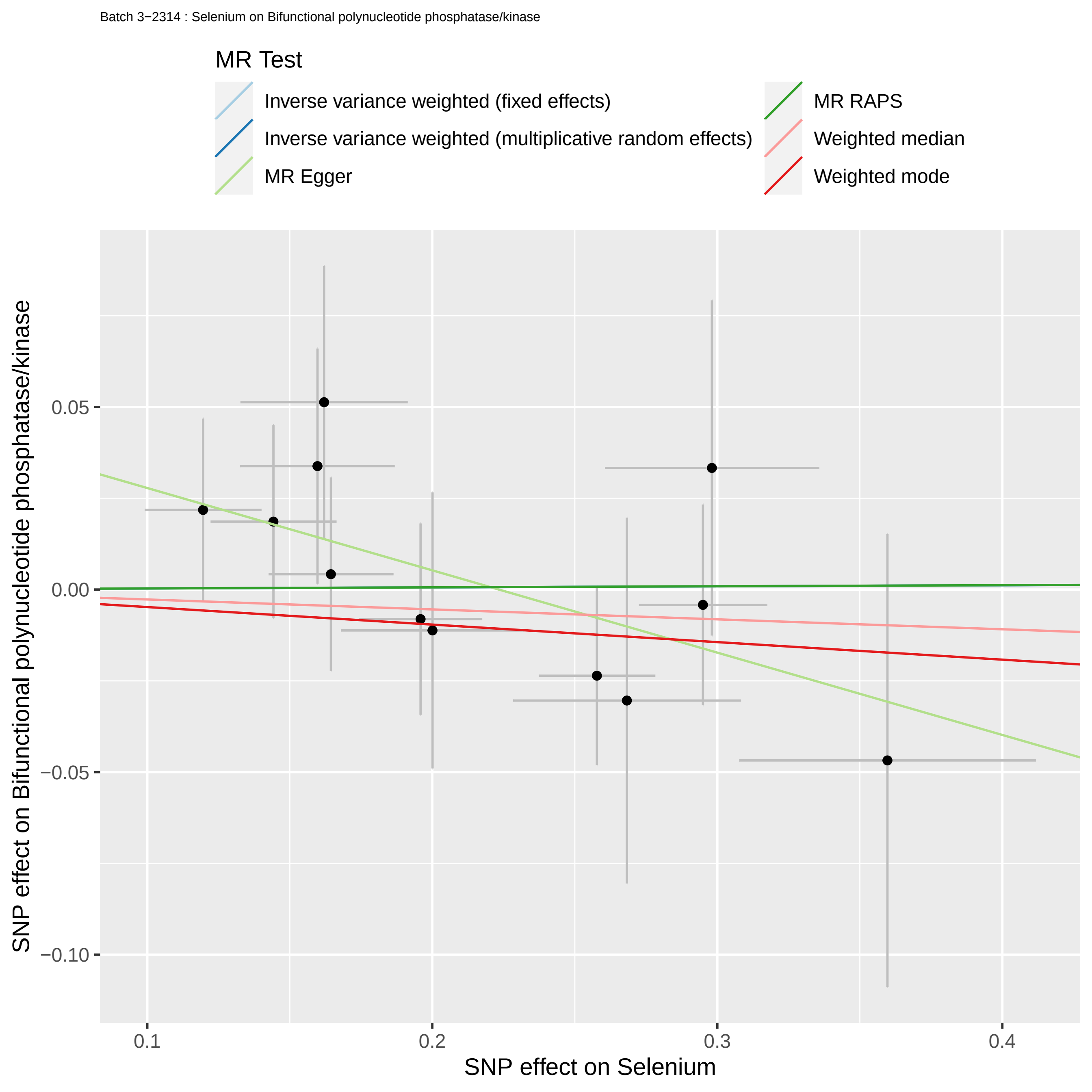

Batch 3−2314 : Selenium on Bifunctional polynucleotide phosphatase/kinase
MR Test
Inverse variance weighted (fixed effects)
Inverse variance weighted (multiplicative random effects)
MR Egger
MR RAPS
Weighted median
Weighted mode
0.05
0.00
−0.05
−0.10
0.1
0.2
0.3
0.4
SNP effect on Bifunctional polynucleotide phosphatase/kinase
SNP effect on Selenium

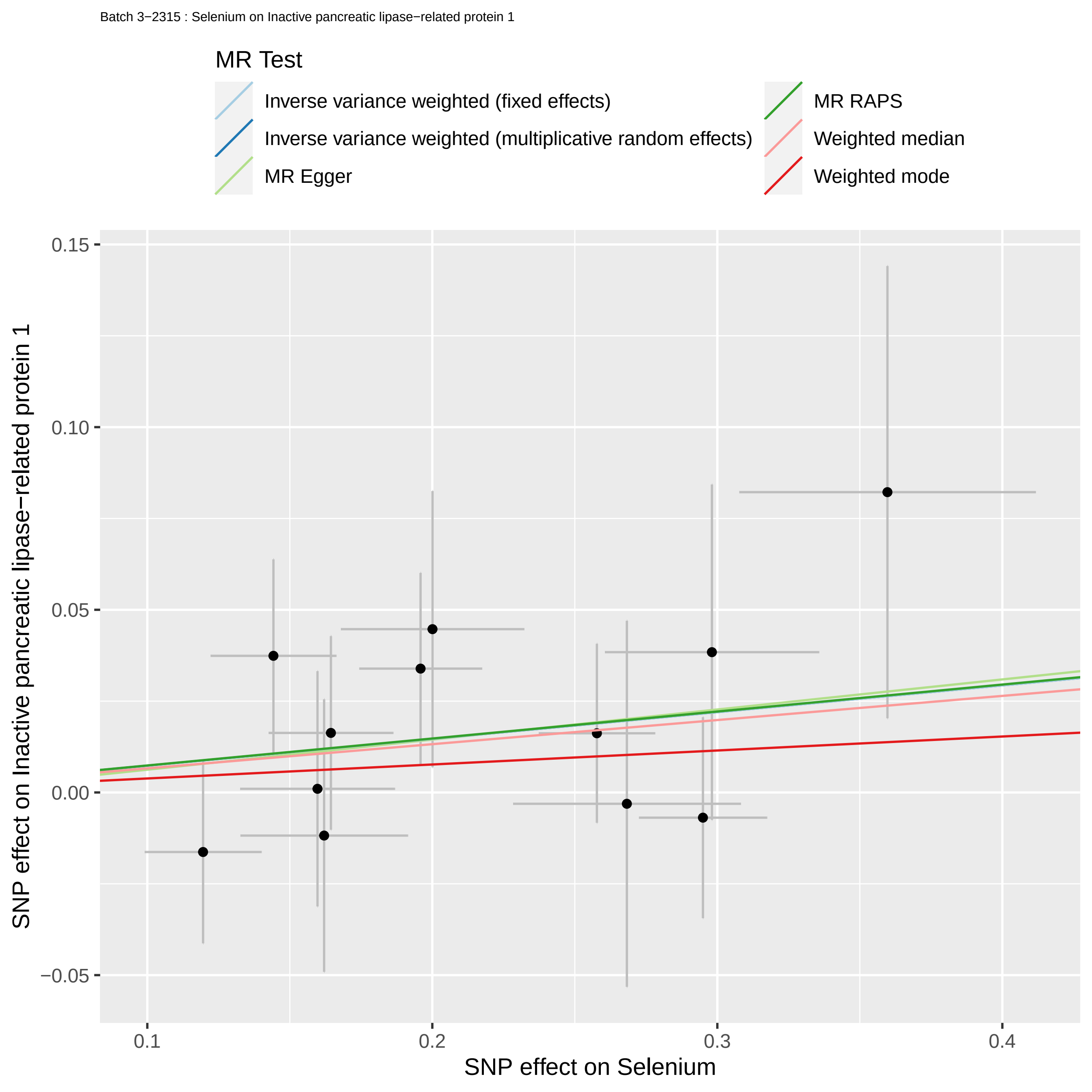

Batch 3−2315 : Selenium on Inactive pancreatic lipase−related protein 1
MR Test
Inverse variance weighted (fixed effects)
Inverse variance weighted (multiplicative random effects)
MR Egger
MR RAPS
Weighted median
Weighted mode
0.15
0.10
0.05
0.00
−0.05
0.1
0.2
0.3
0.4
SNP effect on Inactive pancreatic lipase−related protein 1
SNP effect on Selenium

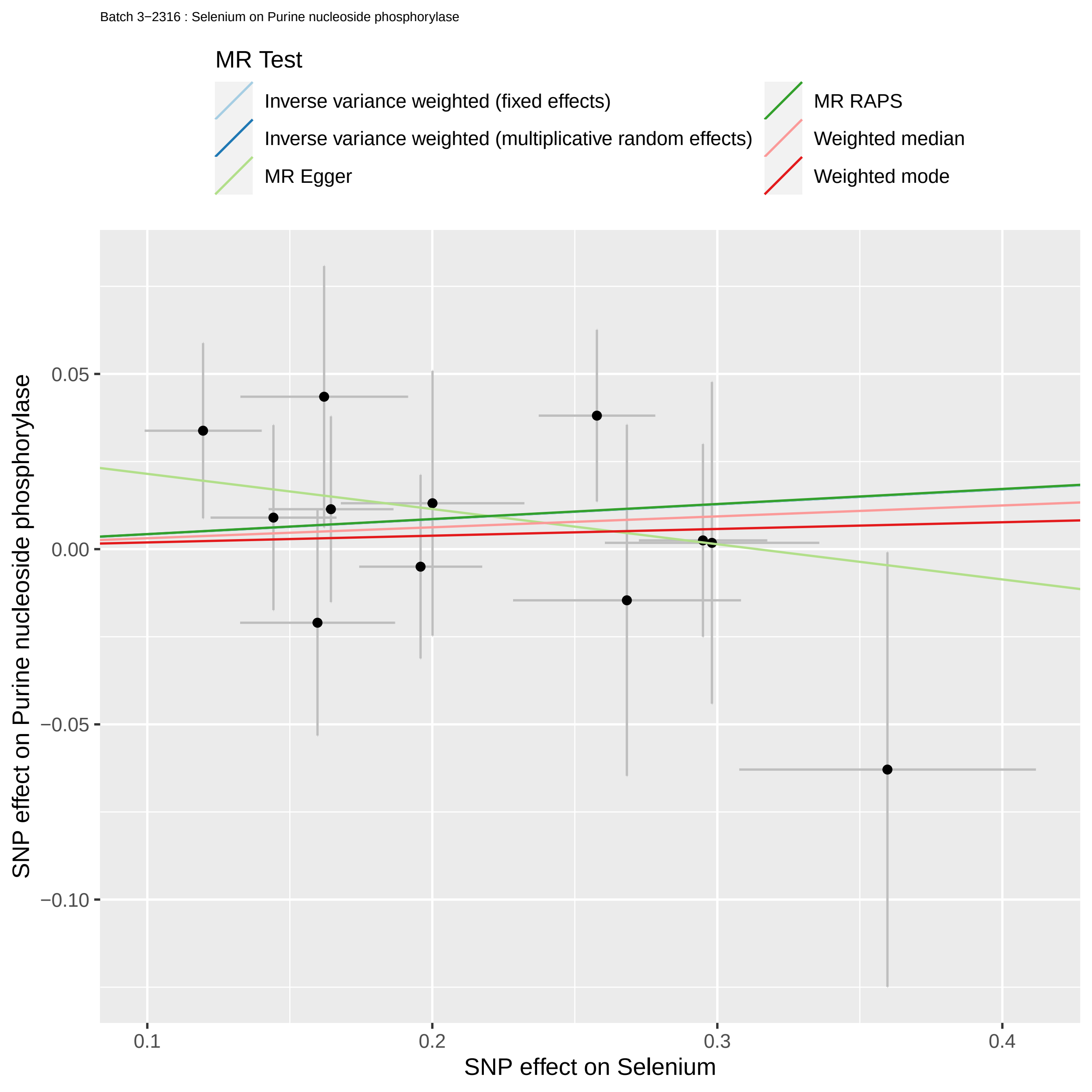
Batch 3−2316 : Selenium on Purine nucleoside phosphorylase
MR Test
Inverse variance weighted (fixed effects)
Inverse variance weighted (multiplicative random effects)
MR Egger
MR RAPS
Weighted median
Weighted mode
SNP effect on Purine nucleoside phosphorylase
0.05
0.00
−0.05
−0.10
0.1
0.2
0.3
0.4
SNP effect on Selenium

Batch 3−2317 : Selenium on Podocalyxin

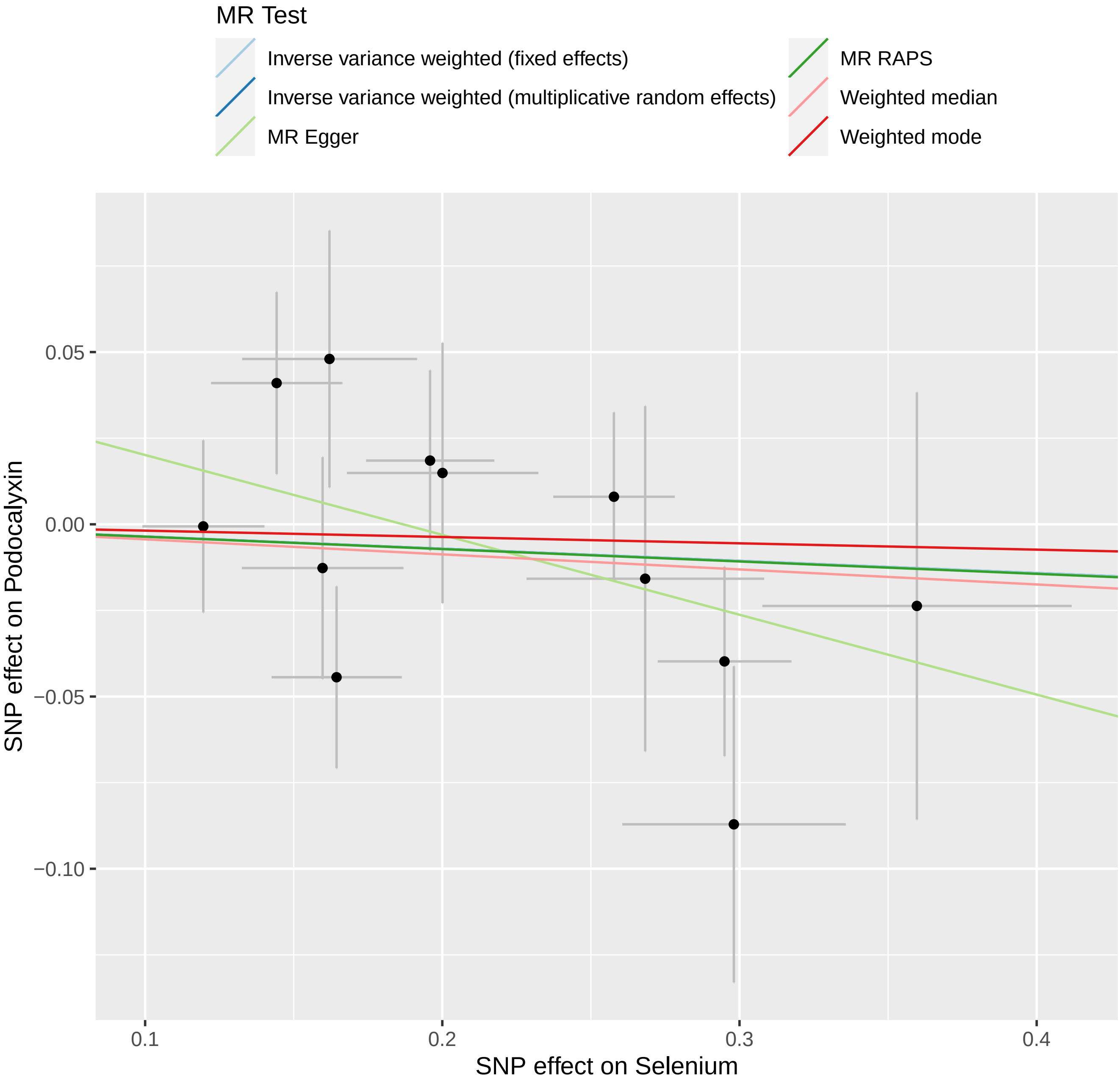

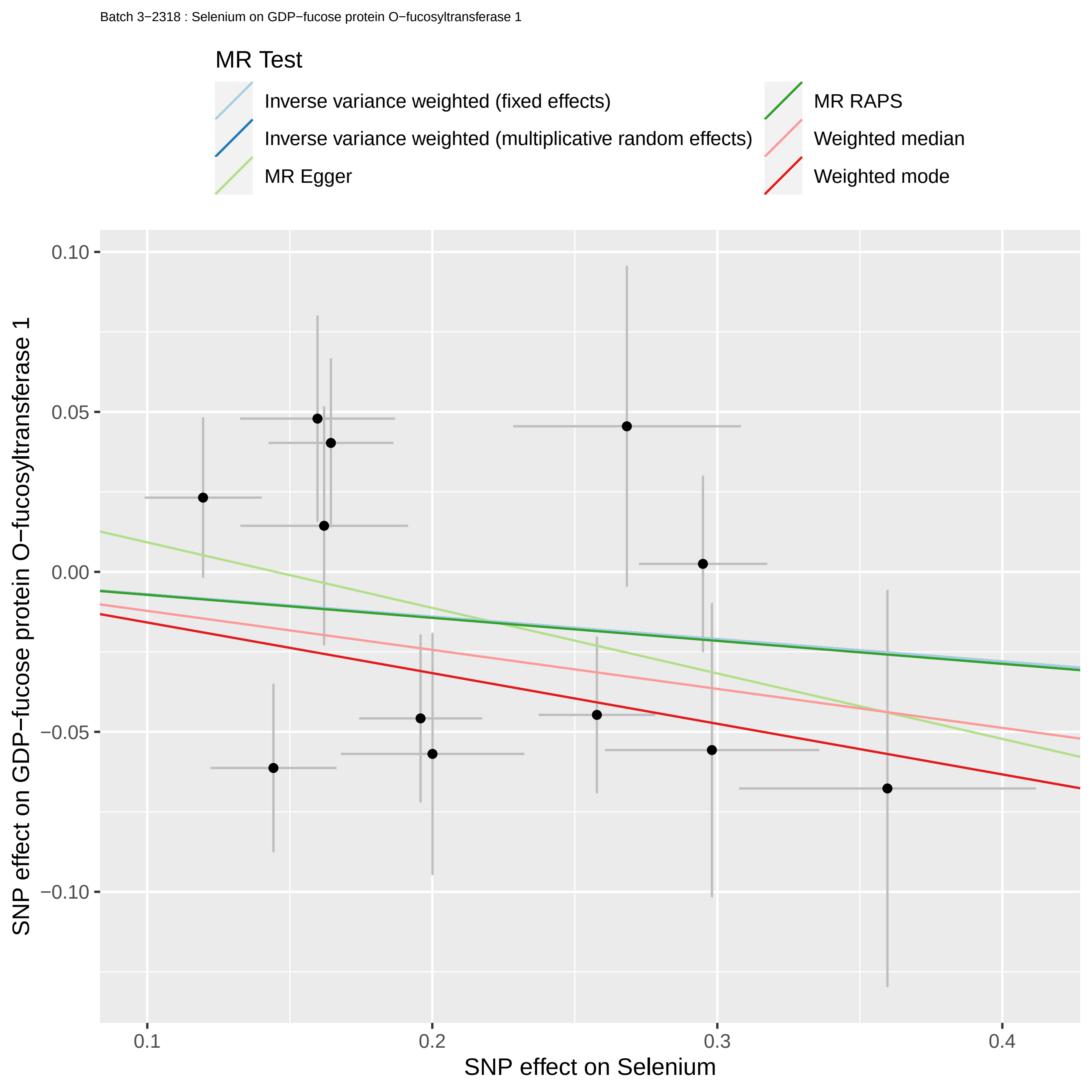

Batch 3−2318 : Selenium on GDP−fucose protein O−fucosyltransferase 1
MR Test
Inverse variance weighted (fixed effects)
Inverse variance weighted (multiplicative random effects)
MR Egger
MR RAPS
Weighted median
Weighted mode
0.10
0.05
0.00
−0.05
−0.10
0.1
0.2
0.3
0.4
SNP effect on GDP−fucose protein O−fucosyltransferase 1
SNP effect on Selenium

Batch 3−2319 : Selenium on GDP−fucose protein O−fucosyltransferase 2

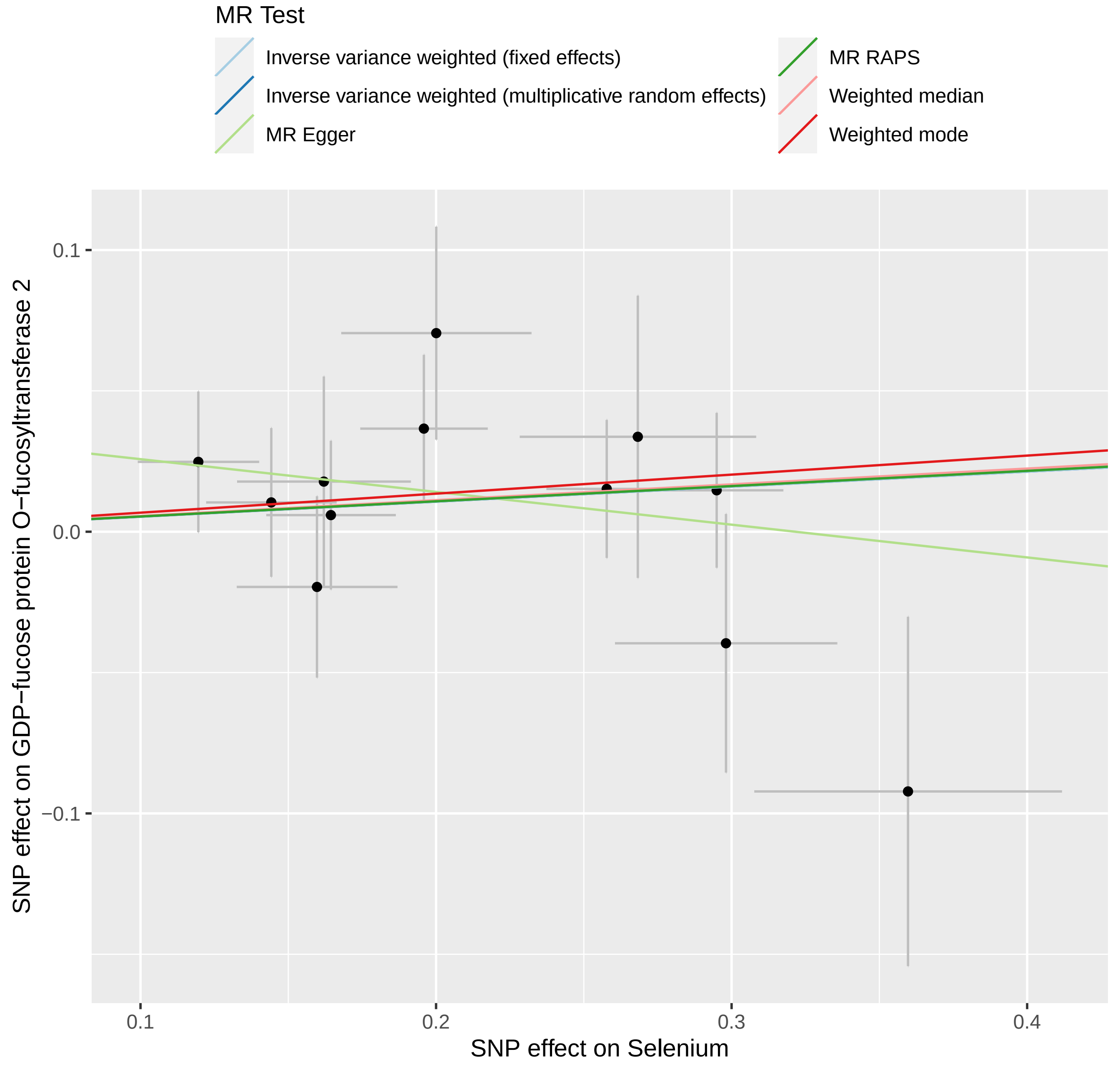

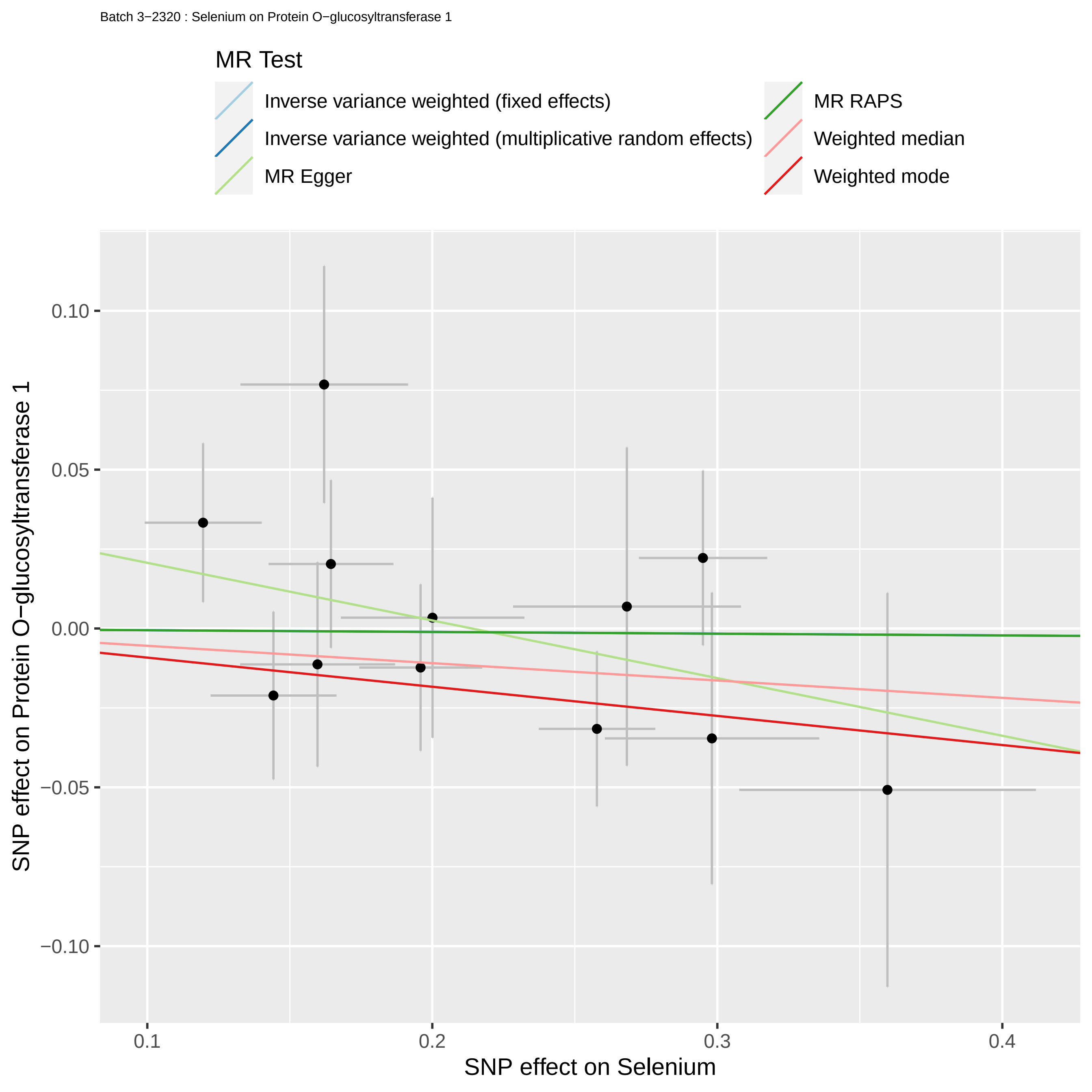

Batch 3−2320 : Selenium on Protein O−glucosyltransferase 1
MR Test
Inverse variance weighted (fixed effects)
Inverse variance weighted (multiplicative random effects)
MR Egger
MR RAPS
Weighted median
Weighted mode
SNP effect on Protein O−glucosyltransferase 1
0.10
0.05
0.00
−0.05
−0.10
0.1
0.2
0.3
0.4
SNP effect on Selenium

Batch 3−2321 : Selenium on DNA polymerase eta

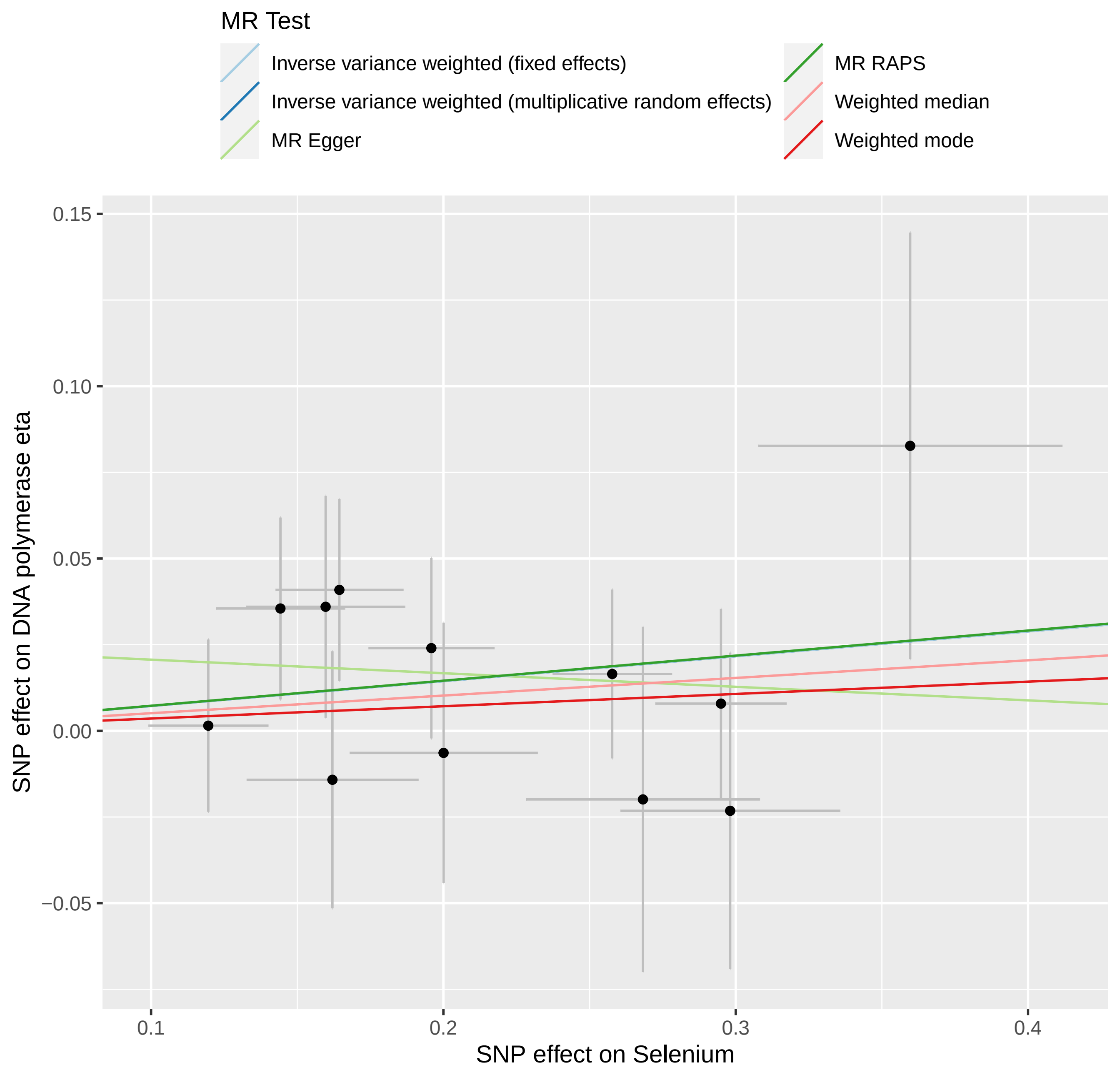

Batch 3−2322 : Selenium on DNA polymerase iota

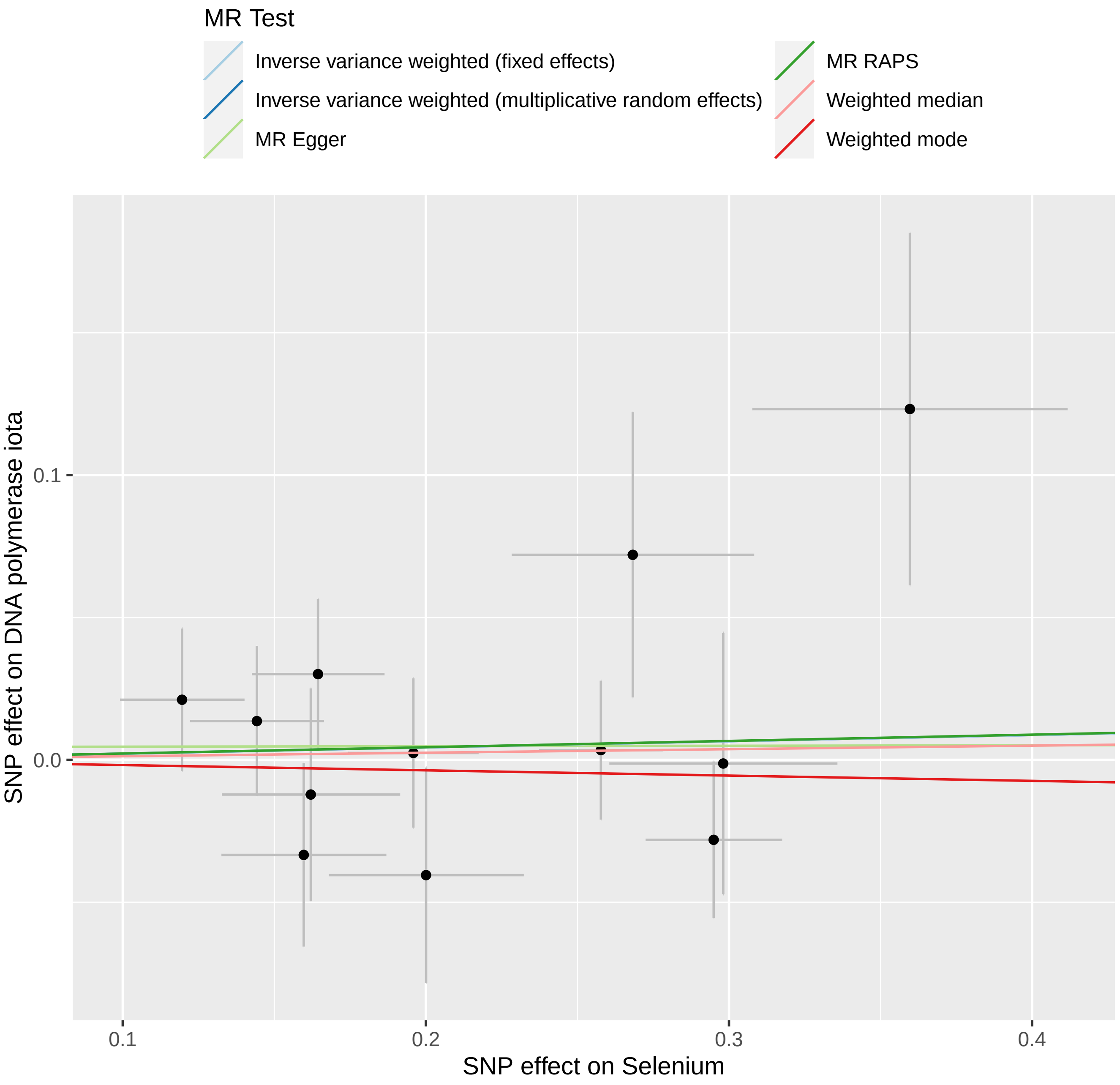

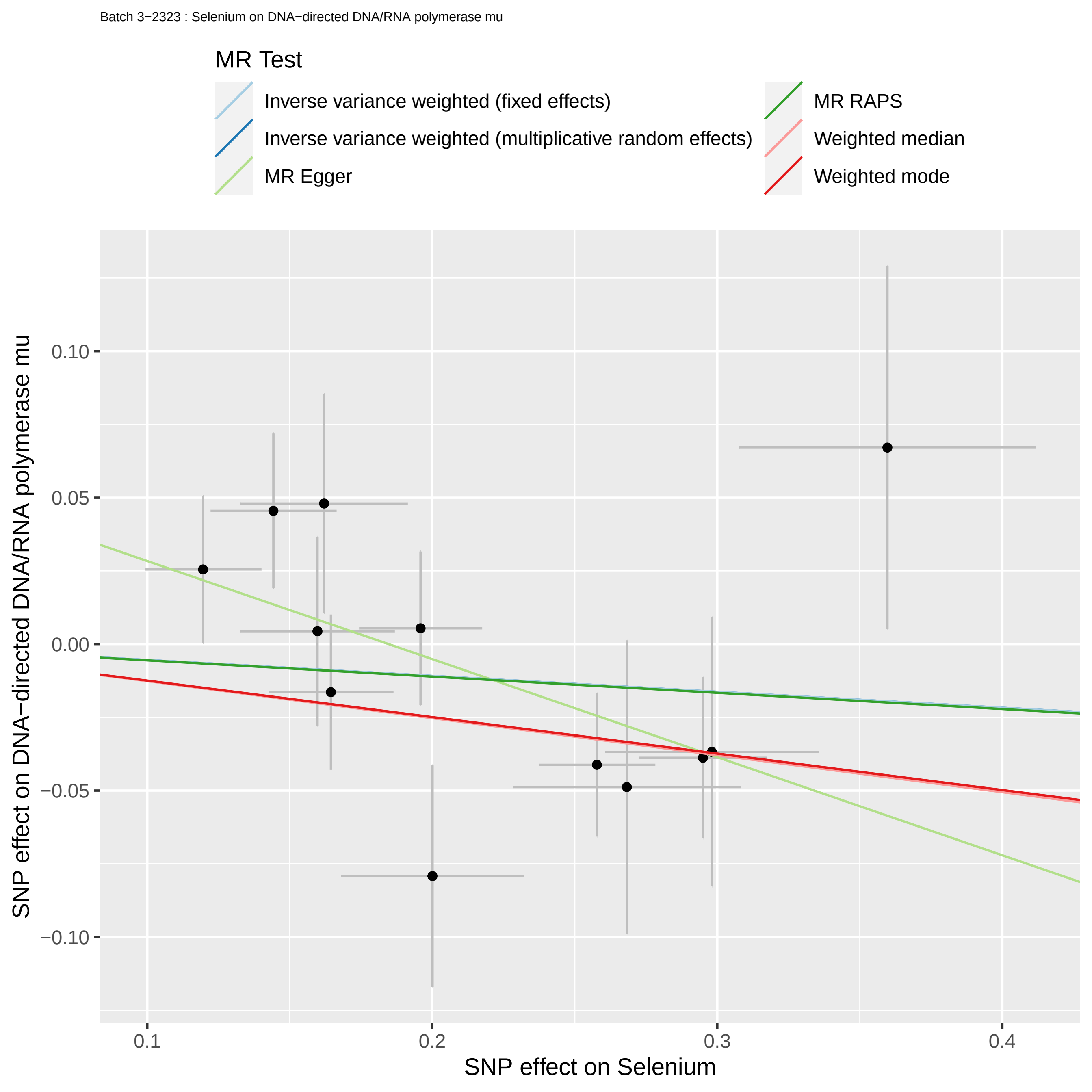

Batch 3−2323 : Selenium on DNA−directed DNA/RNA polymerase mu
MR Test
Inverse variance weighted (fixed effects)
Inverse variance weighted (multiplicative random effects)
MR Egger
MR RAPS
Weighted median
Weighted mode
SNP effect on DNA−directed DNA/RNA polymerase mu
0.10
0.05
0.00
−0.05
−0.10
0.1
0.2
0.3
0.4
SNP effect on Selenium

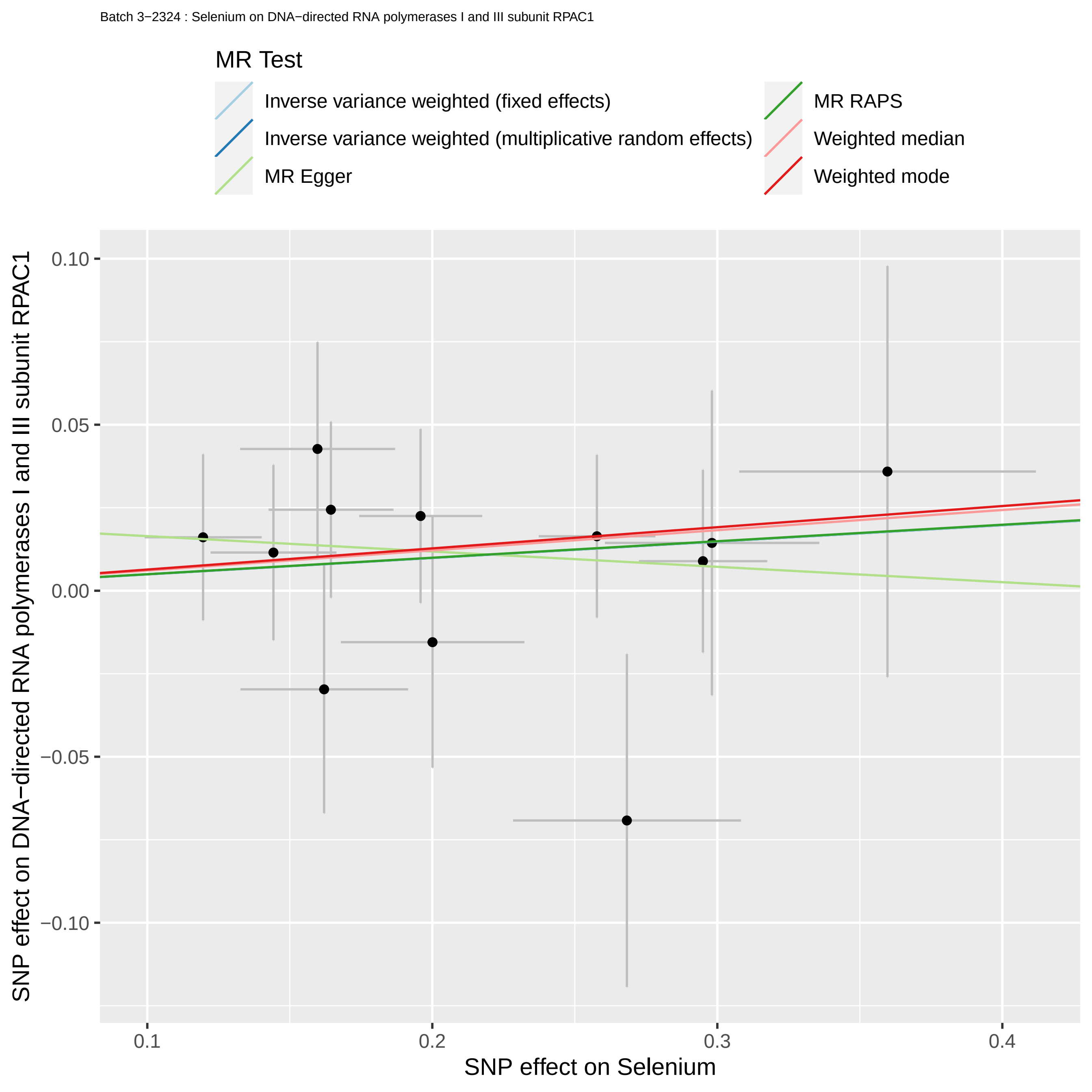
Batch 3−2324 : Selenium on DNA−directed RNA polymerases I and III subunit RPAC1
MR Test
Inverse variance weighted (fixed effects)
Inverse variance weighted (multiplicative random effects)
MR Egger
MR RAPS
Weighted median
Weighted mode
0.10
0.05
0.00
−0.05
−0.10
0.1
0.2
0.3
0.4
SNP effect on DNA−directed RNA polymerases I and III subunit RPAC1
SNP effect on Selenium

Batch 3−2325 : Selenium on Beta−endorphin

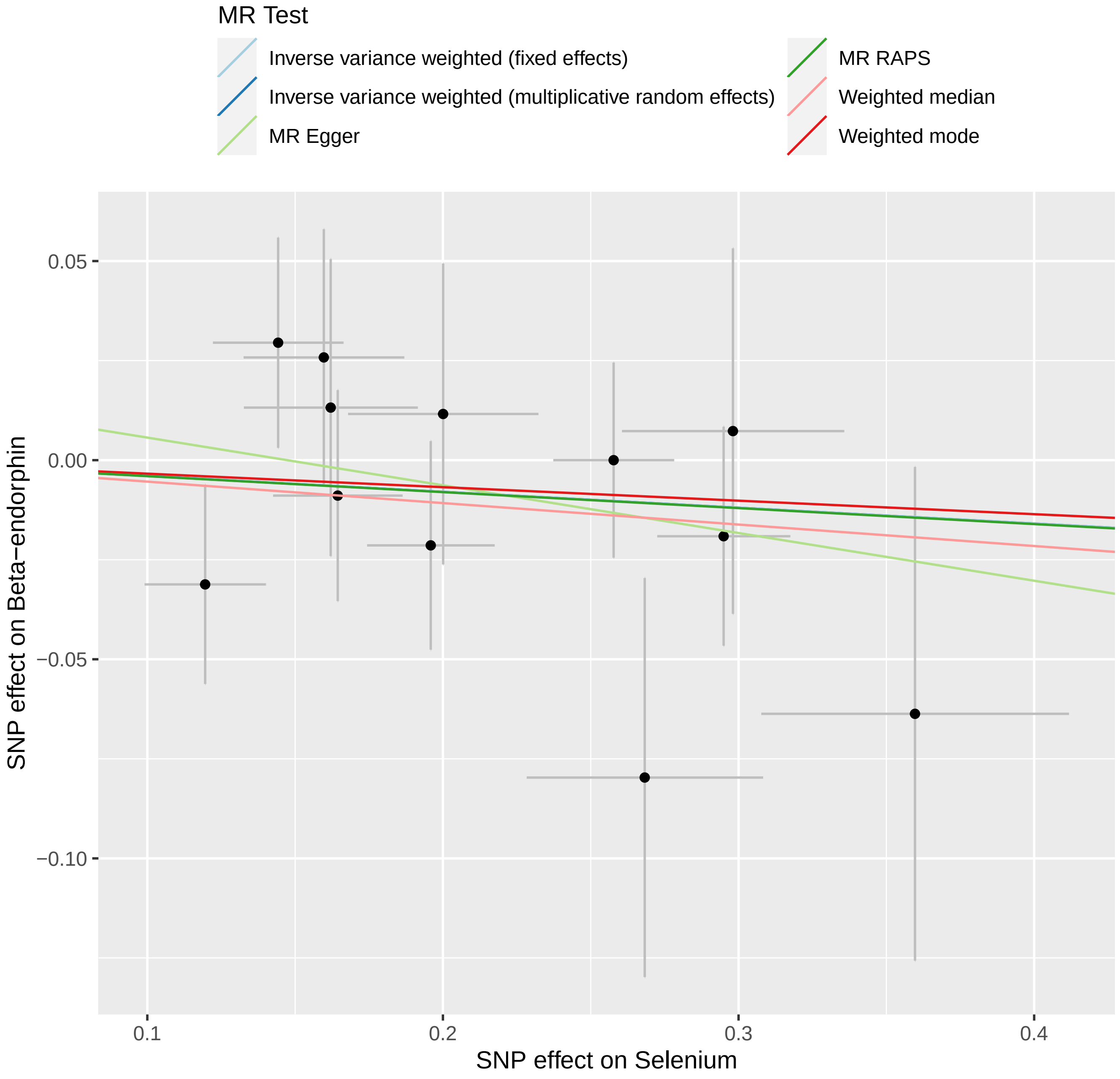

Batch 3−2326 : Selenium on Corticotropin

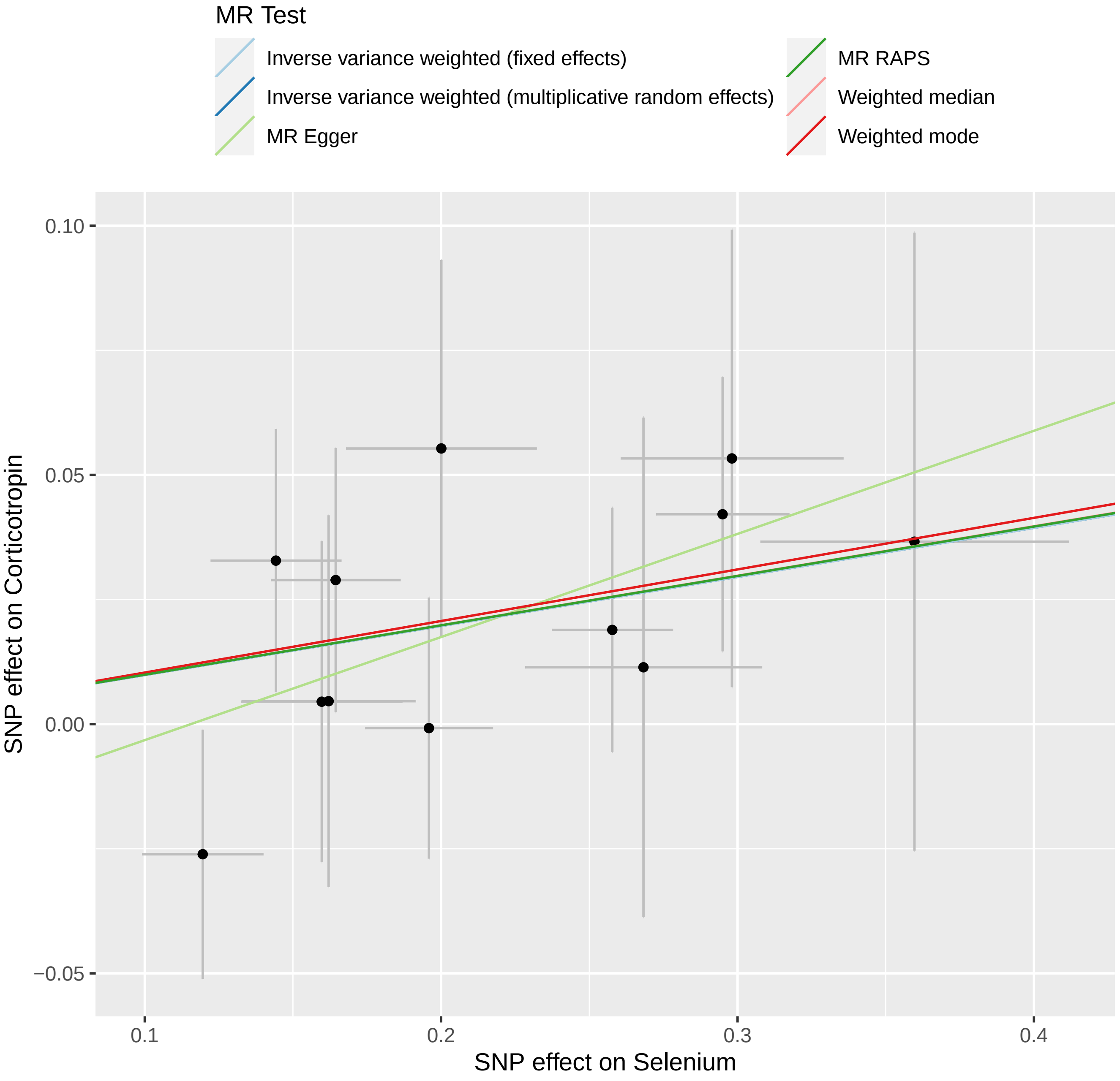

Batch 3−2327 : Selenium on Pro−opiomelanocortin

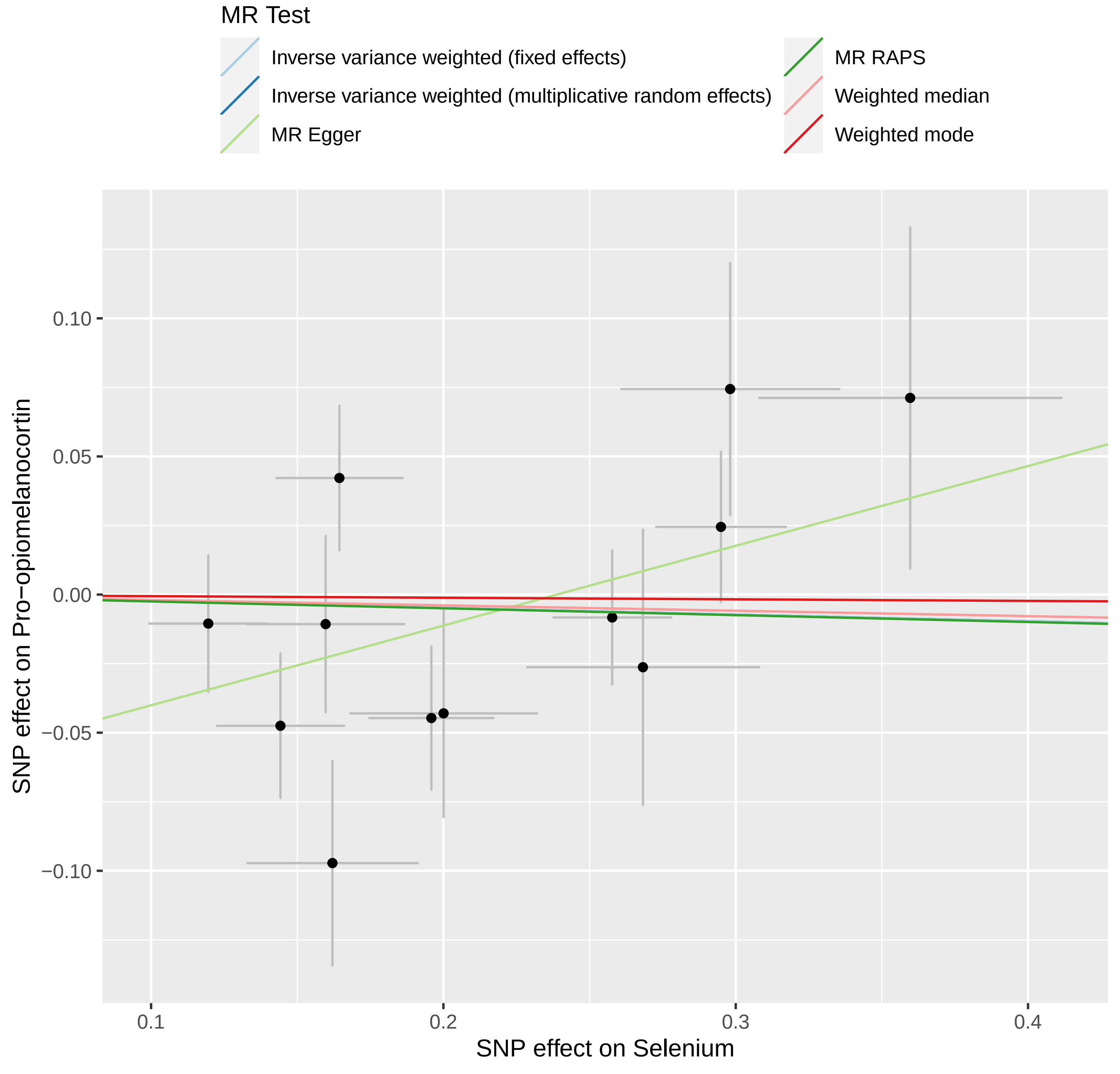

Batch 3−2328 : Selenium on Protein O−linked−mannose beta−1,2−N−acetylglucosaminyltransferase 1

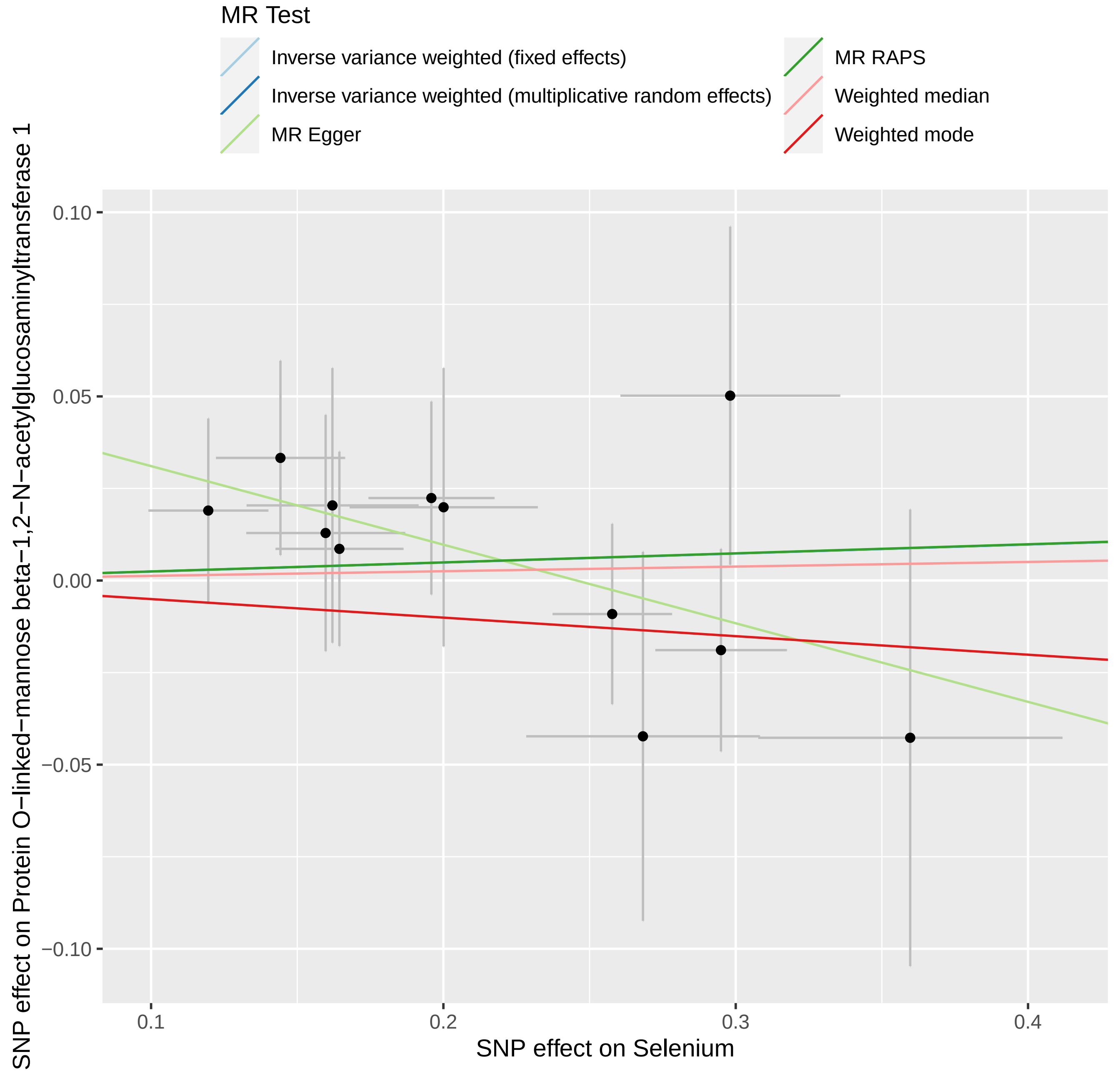

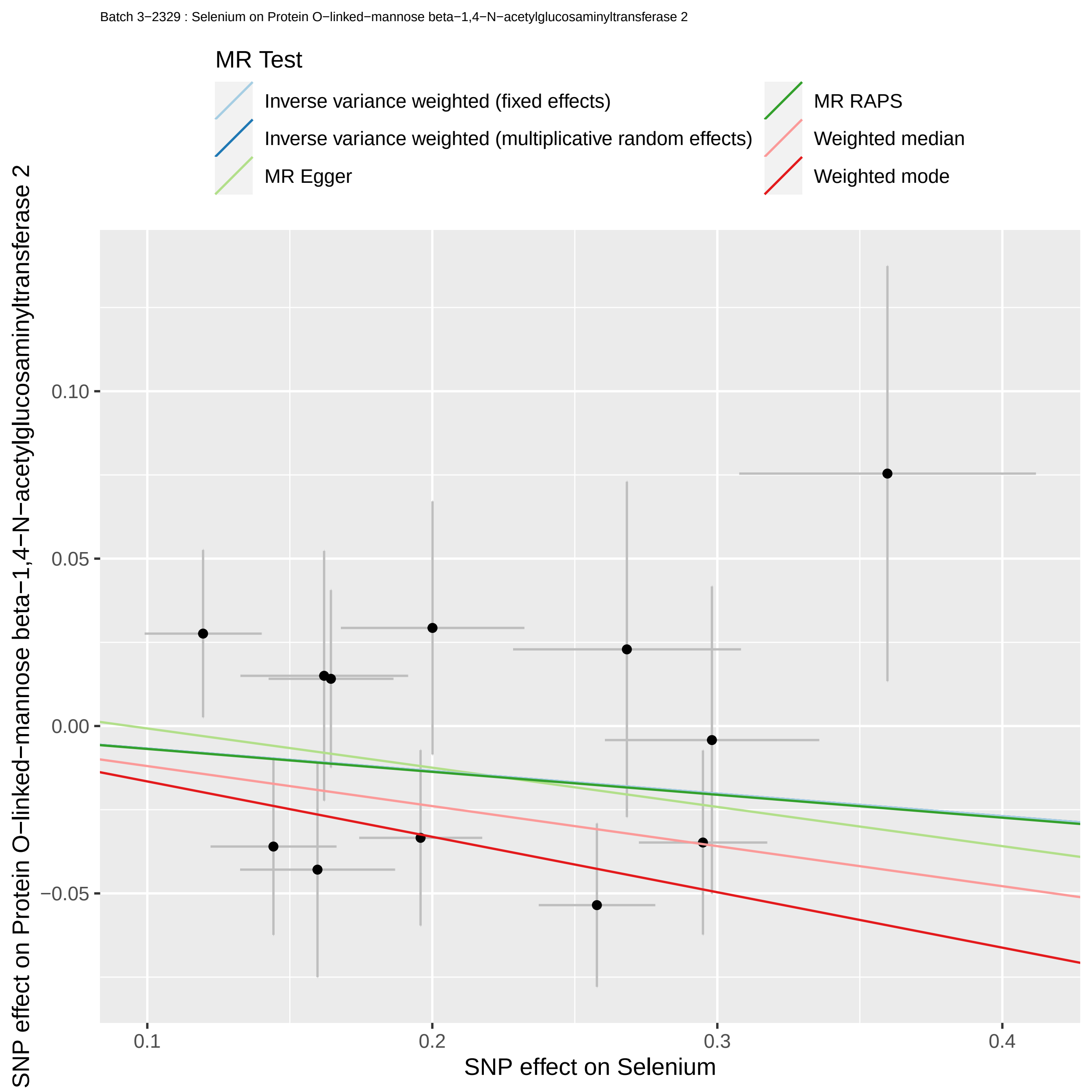

Batch 3−2329 : Selenium on Protein O−linked−mannose beta−1,4−N−acetylglucosaminyltransferase 2
MR Test
Inverse variance weighted (fixed effects)
Inverse variance weighted (multiplicative random effects)
MR Egger
MR RAPS
Weighted median
Weighted mode
0.10
0.05
0.00
−0.05
0.1
0.2
0.3
0.4
SNP effect on Selenium
SNP effect on Protein O−linked−mannose beta−1,4−N−acetylglucosaminyltransferase 2

Batch 3−2330 : Selenium on NADPH−−cytochrome P450 reductase

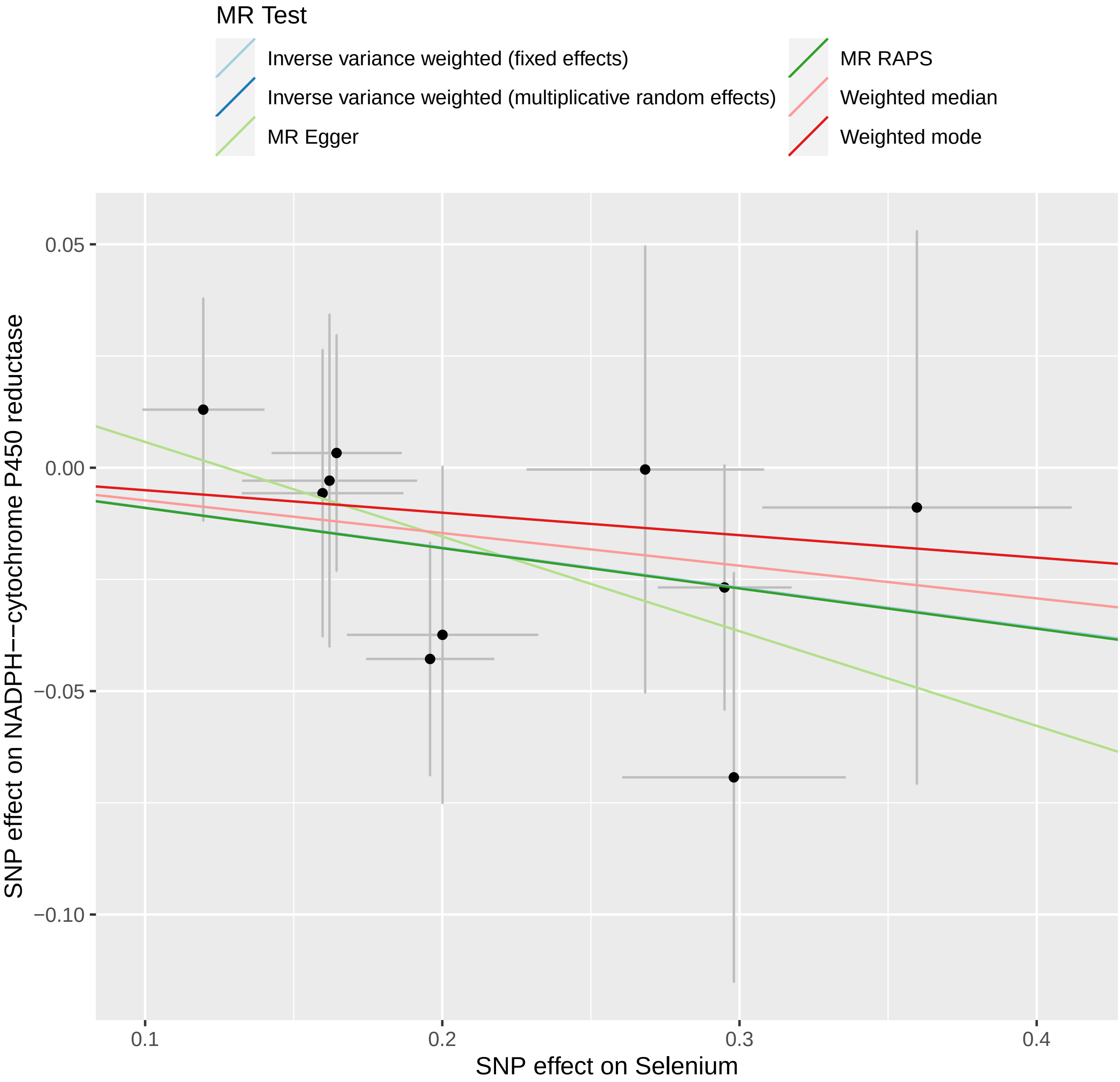

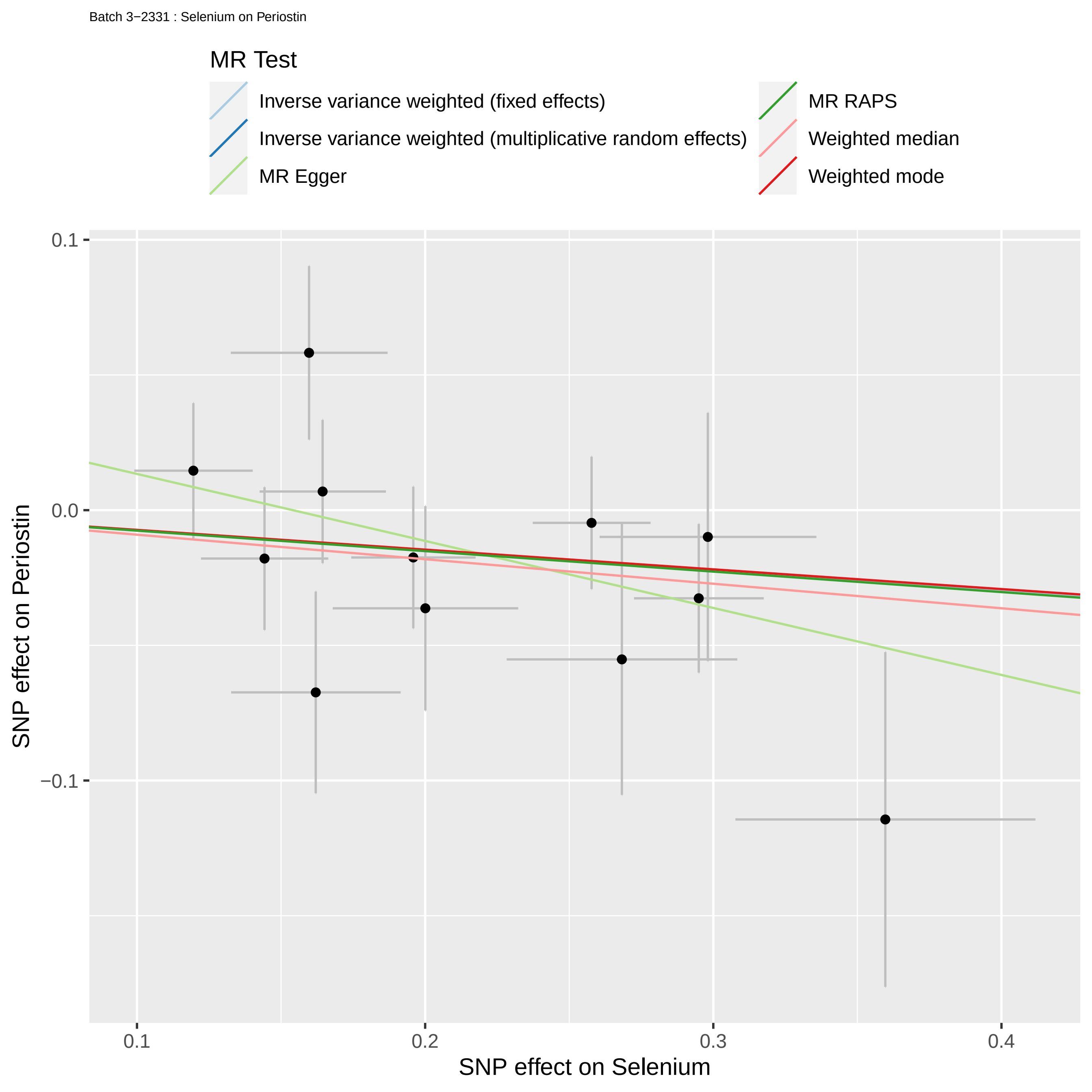

Batch 3−2331 : Selenium on Periostin
MR Test
Inverse variance weighted (fixed effects)
Inverse variance weighted (multiplicative random effects)
MR Egger
MR RAPS
Weighted median
Weighted mode
0.1
0.0
−0.1
0.1
0.2
0.3
0.4
SNP effect on Periostin
SNP effect on Selenium

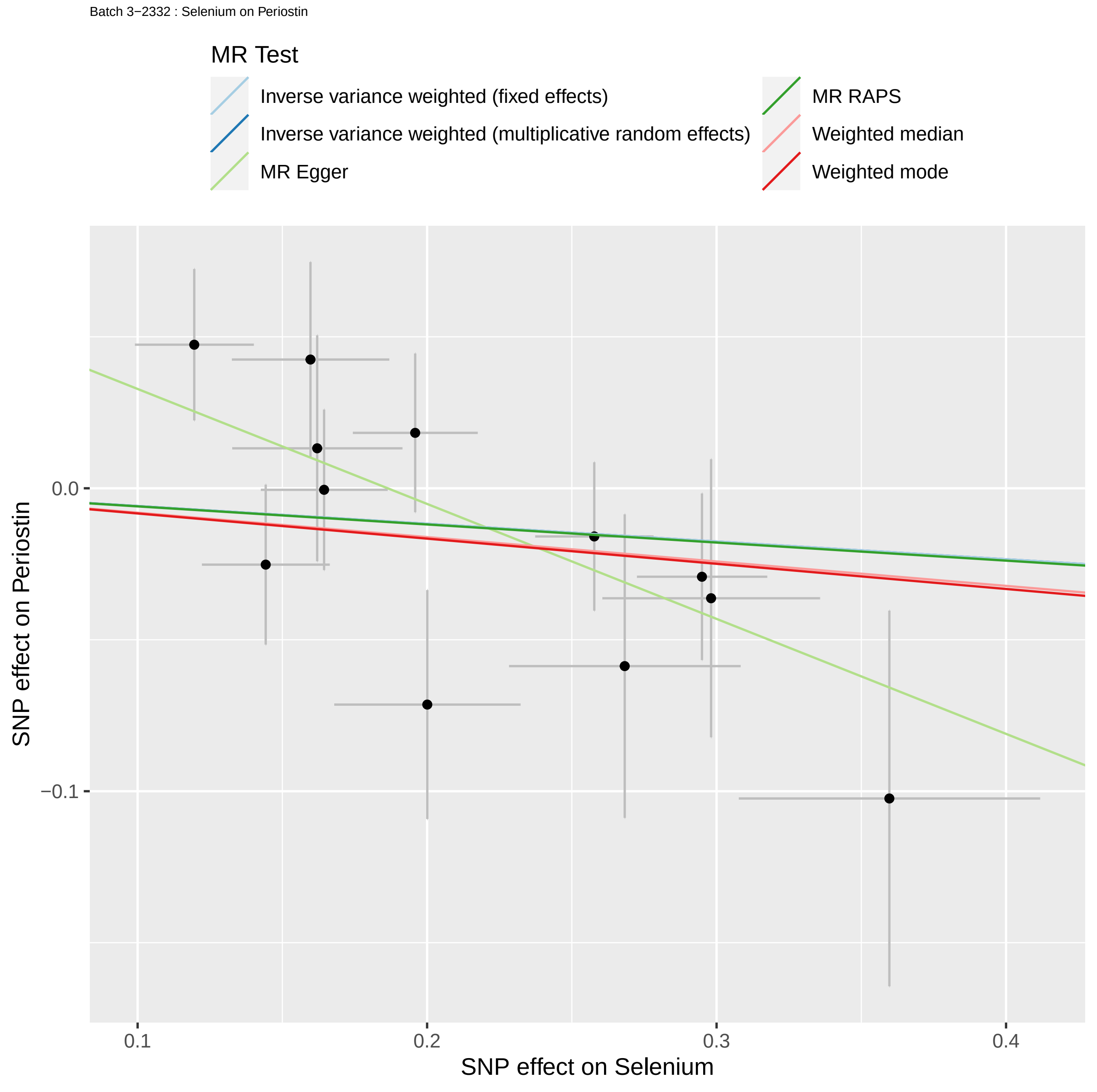
Batch 3−2332 : Selenium on Periostin
MR Test
Inverse variance weighted (fixed effects)
Inverse variance weighted (multiplicative random effects)
MR Egger
MR RAPS
Weighted median
Weighted mode
0.0
−0.1
0.1
0.2
0.3
0.4
SNP effect on Selenium
SNP effect on Periostin

Batch 3−2333 : Selenium on Periostin

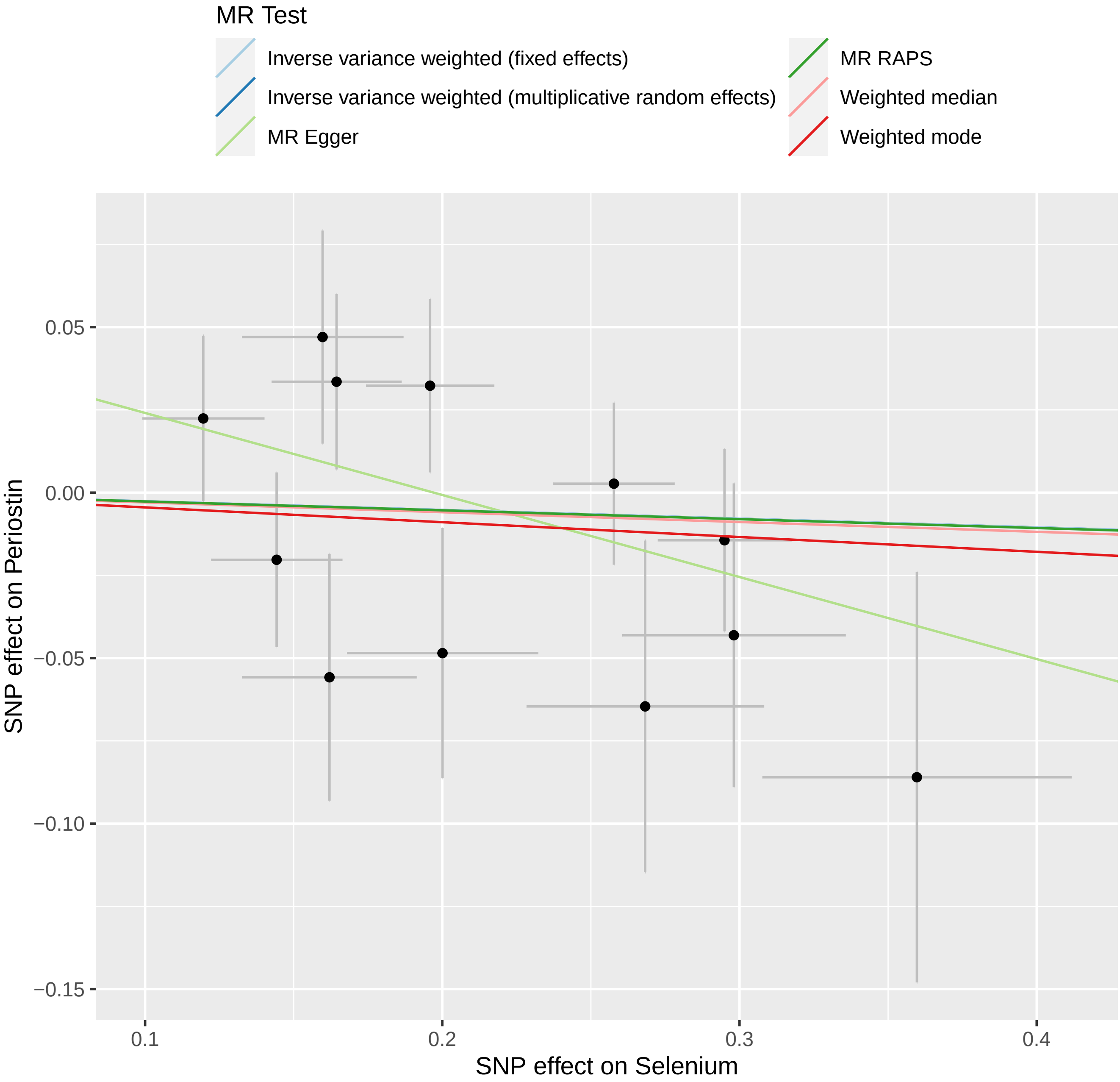

Batch 3−2334 : Selenium on POU domain, class 2, transcription factor 1

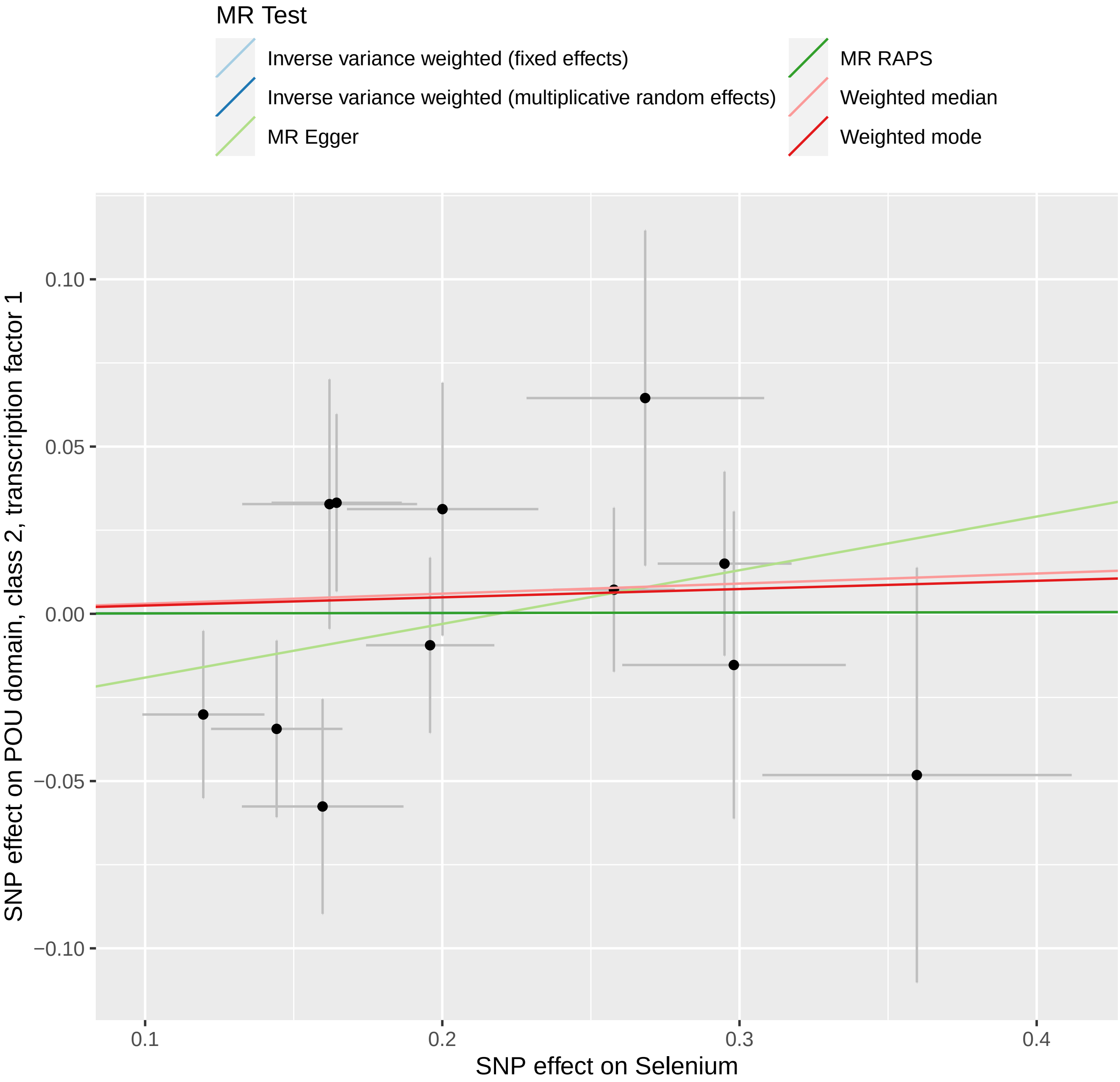

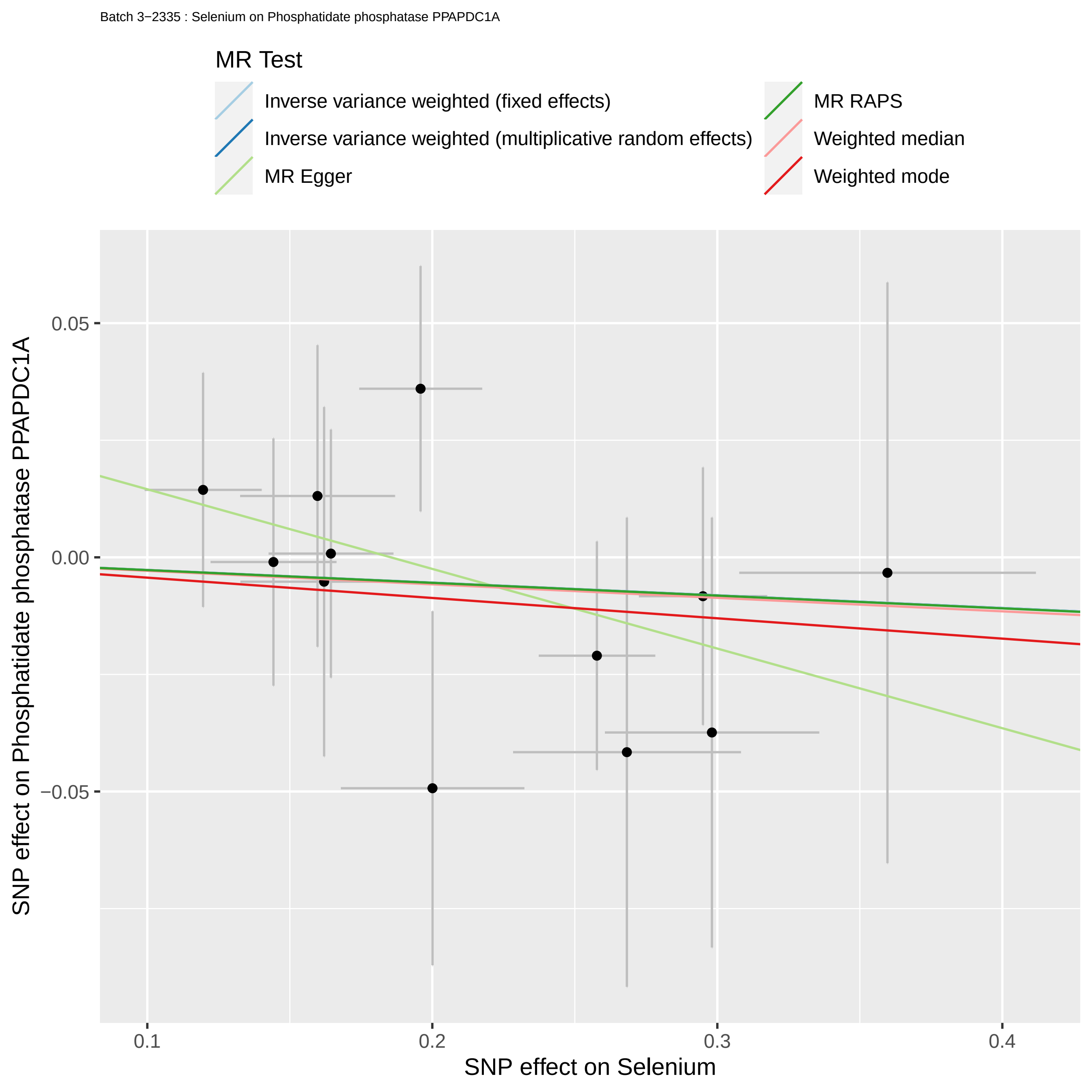

Batch 3−2335 : Selenium on Phosphatidate phosphatase PPAPDC1A
MR Test
Inverse variance weighted (fixed effects)
Inverse variance weighted (multiplicative random effects)
MR Egger
MR RAPS
Weighted median
Weighted mode
0.05
0.00
−0.05
0.1
0.2
0.3
0.4
SNP effect on Phosphatidate phosphatase PPAPDC1A
SNP effect on Selenium

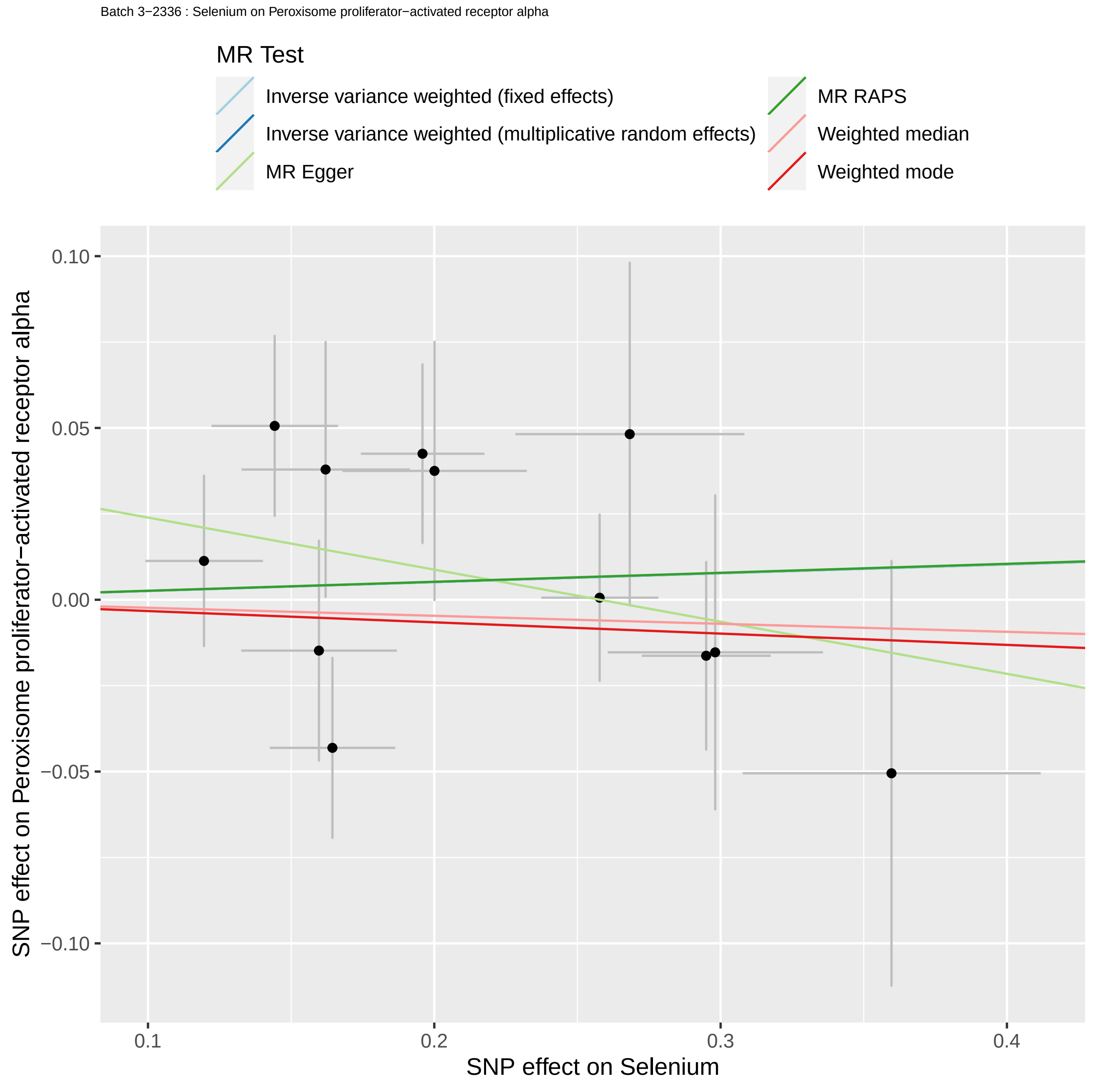
Batch 3−2336 : Selenium on Peroxisome proliferator−activated receptor alpha
MR Test
Inverse variance weighted (fixed effects)
Inverse variance weighted (multiplicative random effects)
MR Egger
MR RAPS
Weighted median
Weighted mode
0.10
0.05
0.00
−0.05
−0.10
0.1
0.2
0.3
0.4
SNP effect on Selenium
SNP effect on Peroxisome proliferator−activated receptor alpha

Batch 3−2337 : Selenium on Neutrophil−activating peptide 2

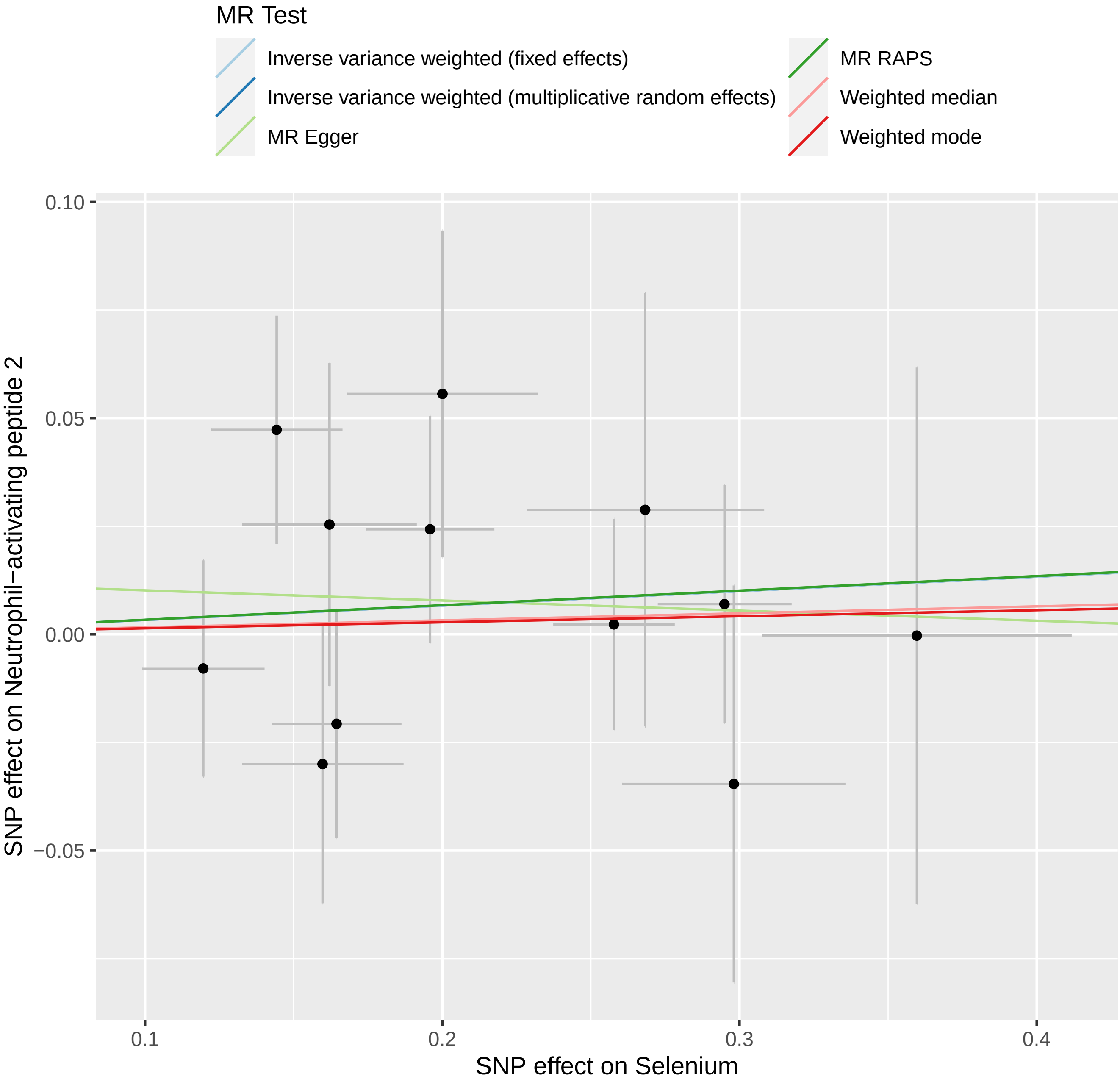

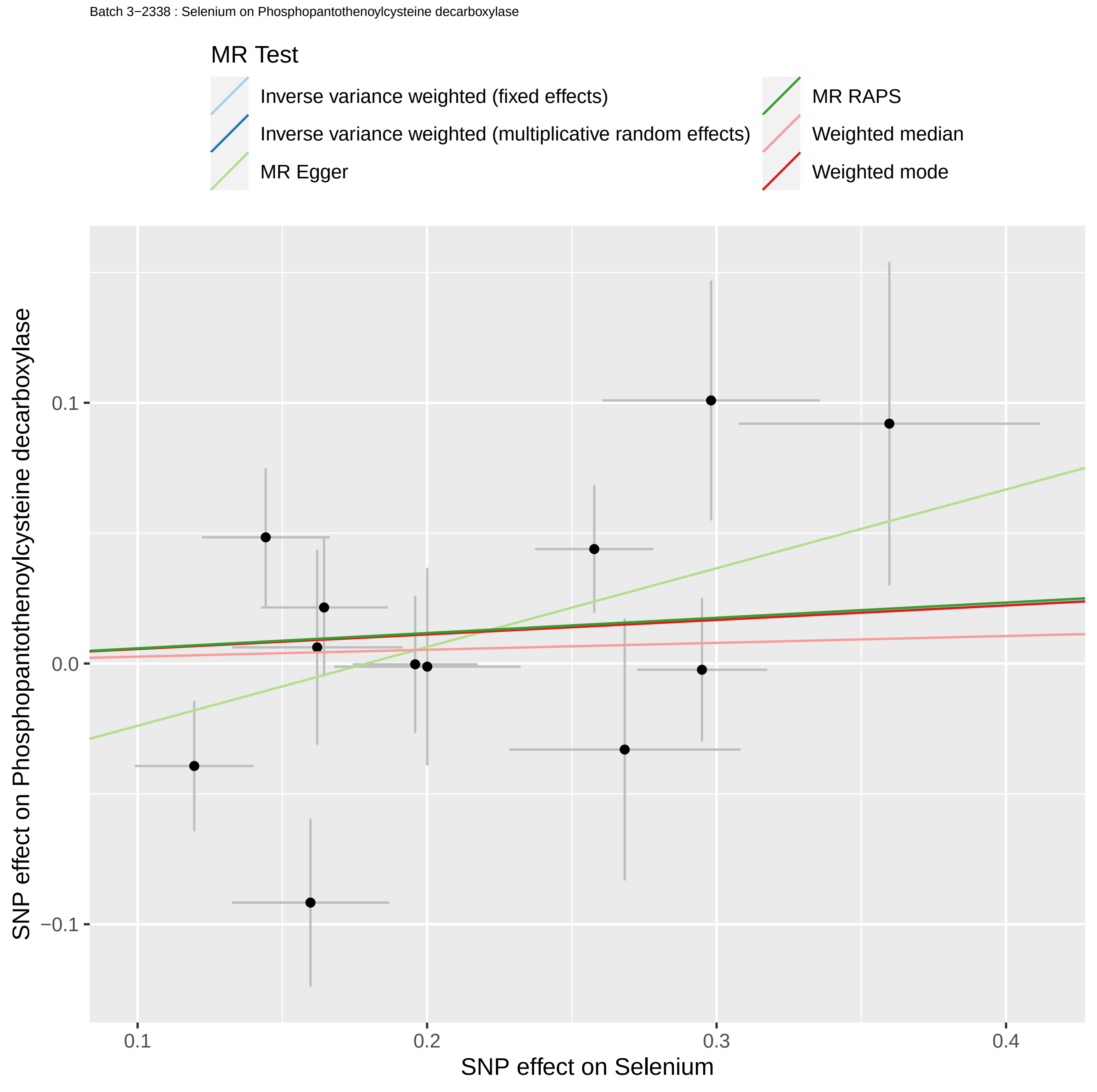

Batch 3−2338 : Selenium on Phosphopantothenoylcysteine decarboxylase
MR Test
Inverse variance weighted (fixed effects)
Inverse variance weighted (multiplicative random effects)
MR Egger
MR RAPS
Weighted median
Weighted mode
SNP effect on Phosphopantothenoylcysteine decarboxylase
0.1
0.0
−0.1
0.1
0.2
0.3
0.4
SNP effect on Selenium

Batch 3−2339 : Selenium on Peptidyl−prolyl cis−trans isomerase A

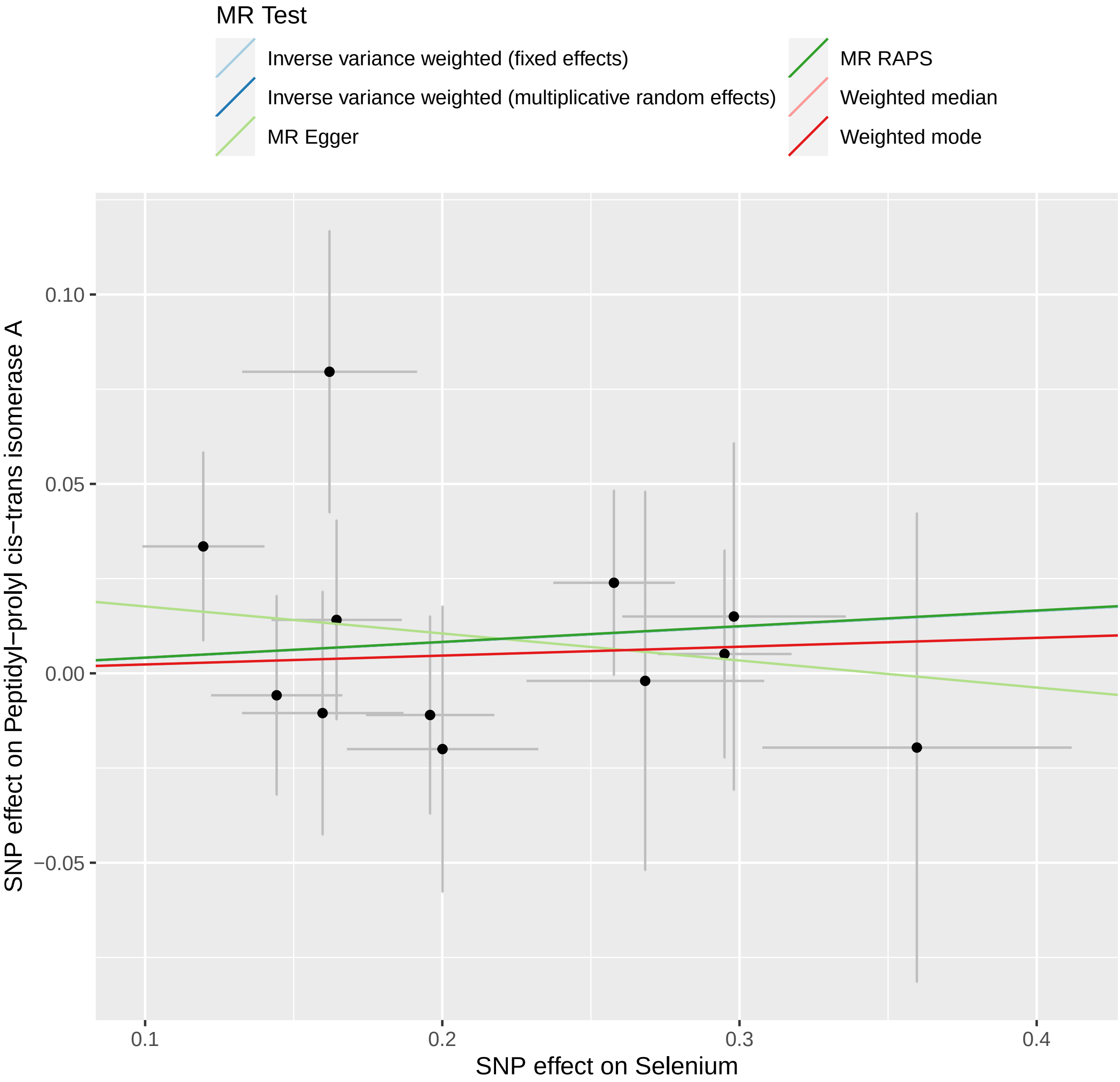

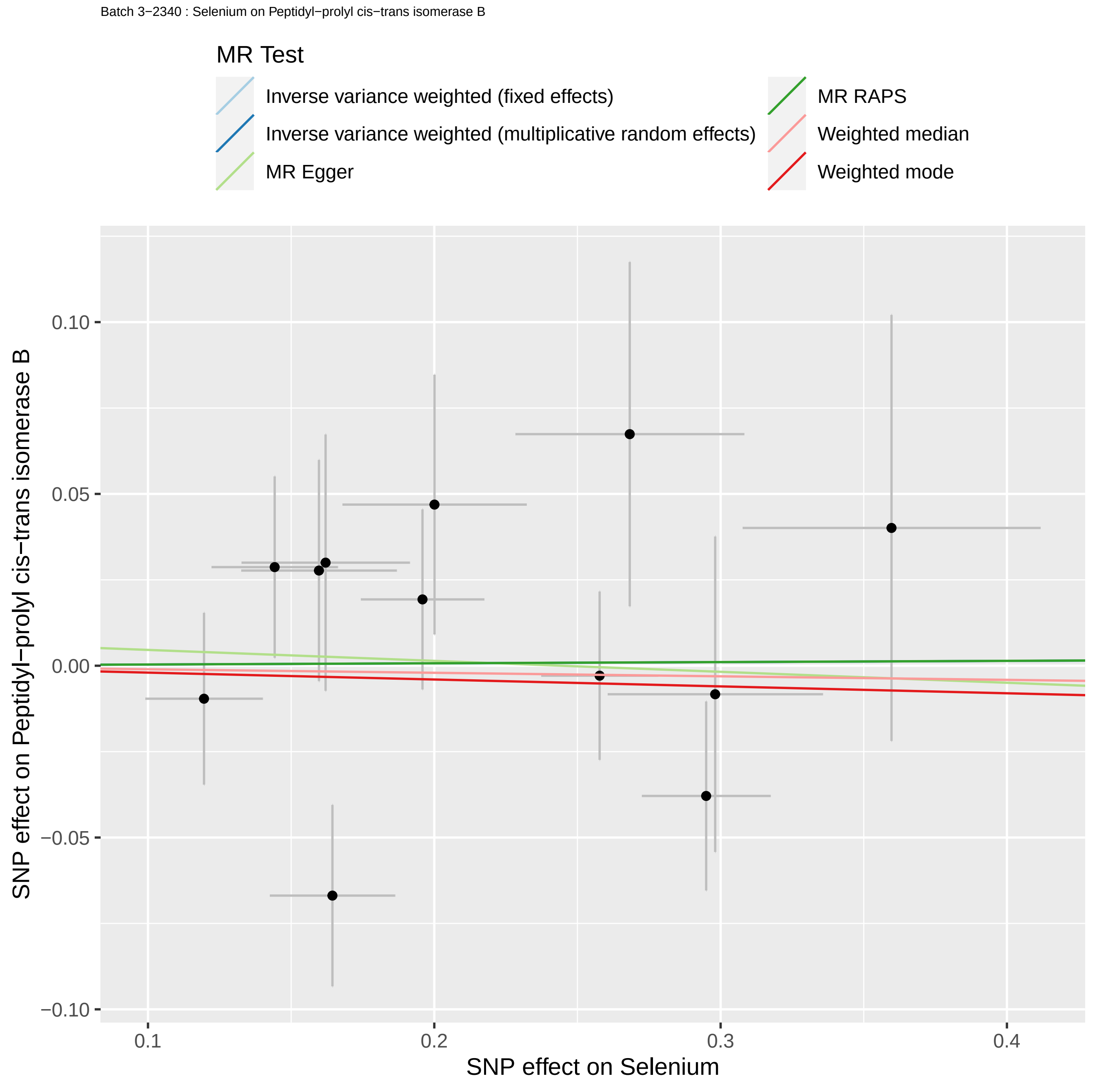
Batch 3−2340 : Selenium on Peptidyl−prolyl cis−trans isomerase B
MR Test
Inverse variance weighted (fixed effects)
Inverse variance weighted (multiplicative random effects)
MR Egger
MR RAPS
Weighted median
Weighted mode
0.10
0.05
0.00
−0.05
−0.10
0.1
0.2
0.3
0.4
SNP effect on Selenium
SNP effect on Peptidyl−prolyl cis−trans isomerase B

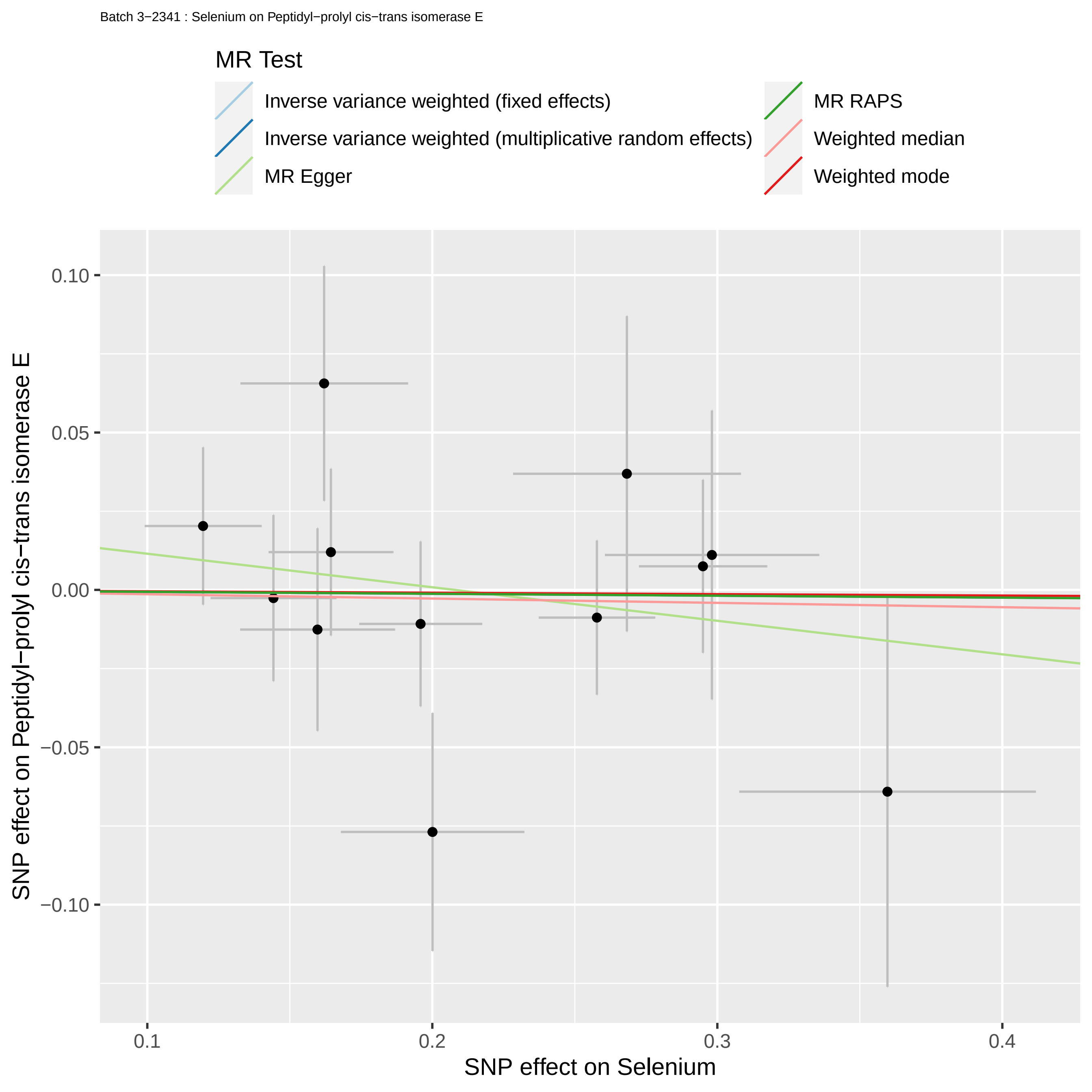
Batch 3−2341 : Selenium on Peptidyl−prolyl cis−trans isomerase E
MR Test
Inverse variance weighted (fixed effects)
Inverse variance weighted (multiplicative random effects)
MR Egger
MR RAPS
Weighted median
Weighted mode
SNP effect on Peptidyl−prolyl cis−trans isomerase E
0.10
0.05
0.00
−0.05
−0.10
0.1
0.2
0.3
0.4
SNP effect on Selenium

Batch 3−2342 : Selenium on Peptidyl−prolyl cis−trans isomerase H

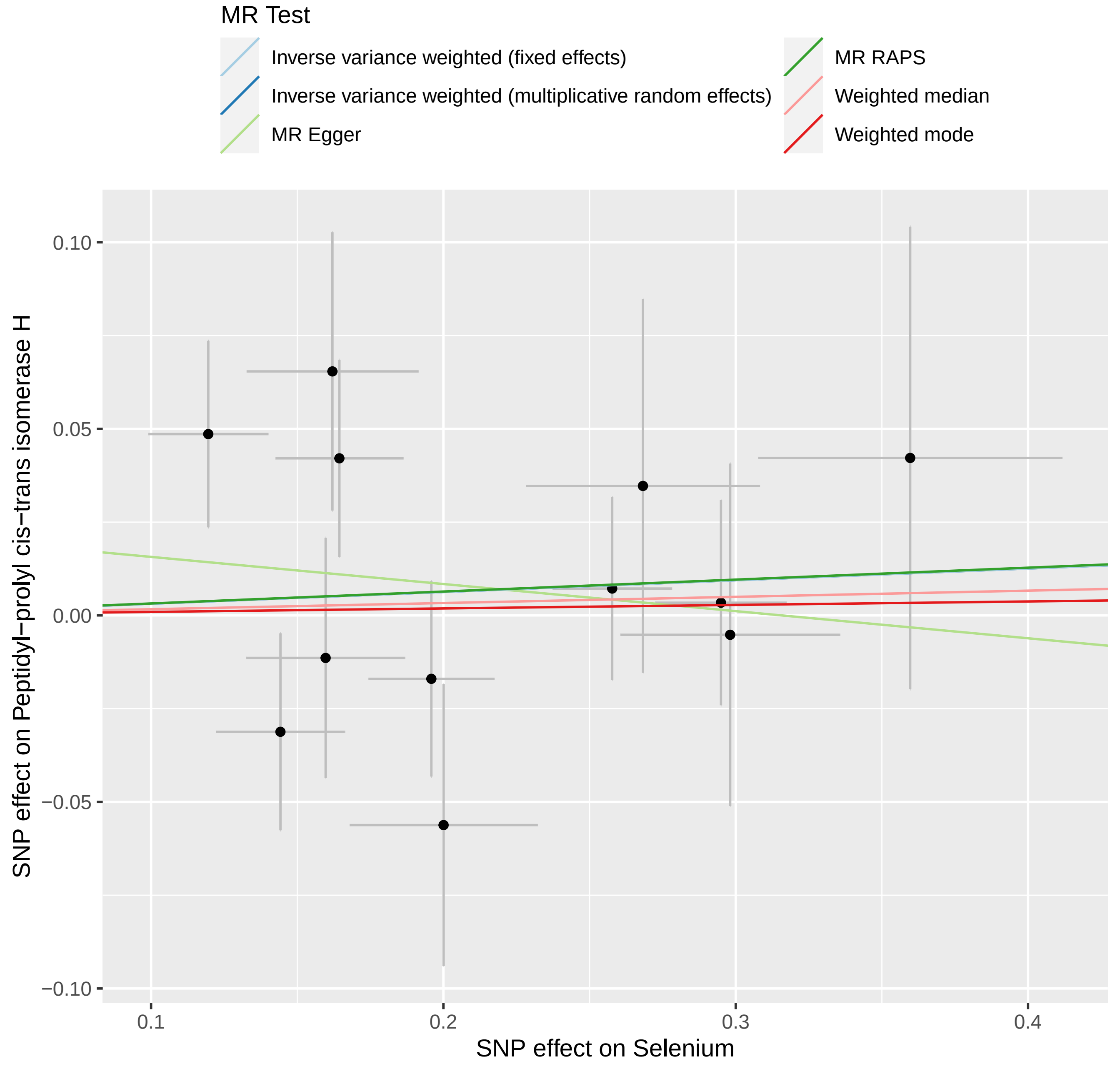

Batch 3−2343 : Selenium on Peptidyl−prolyl cis−trans isomerase−like 1

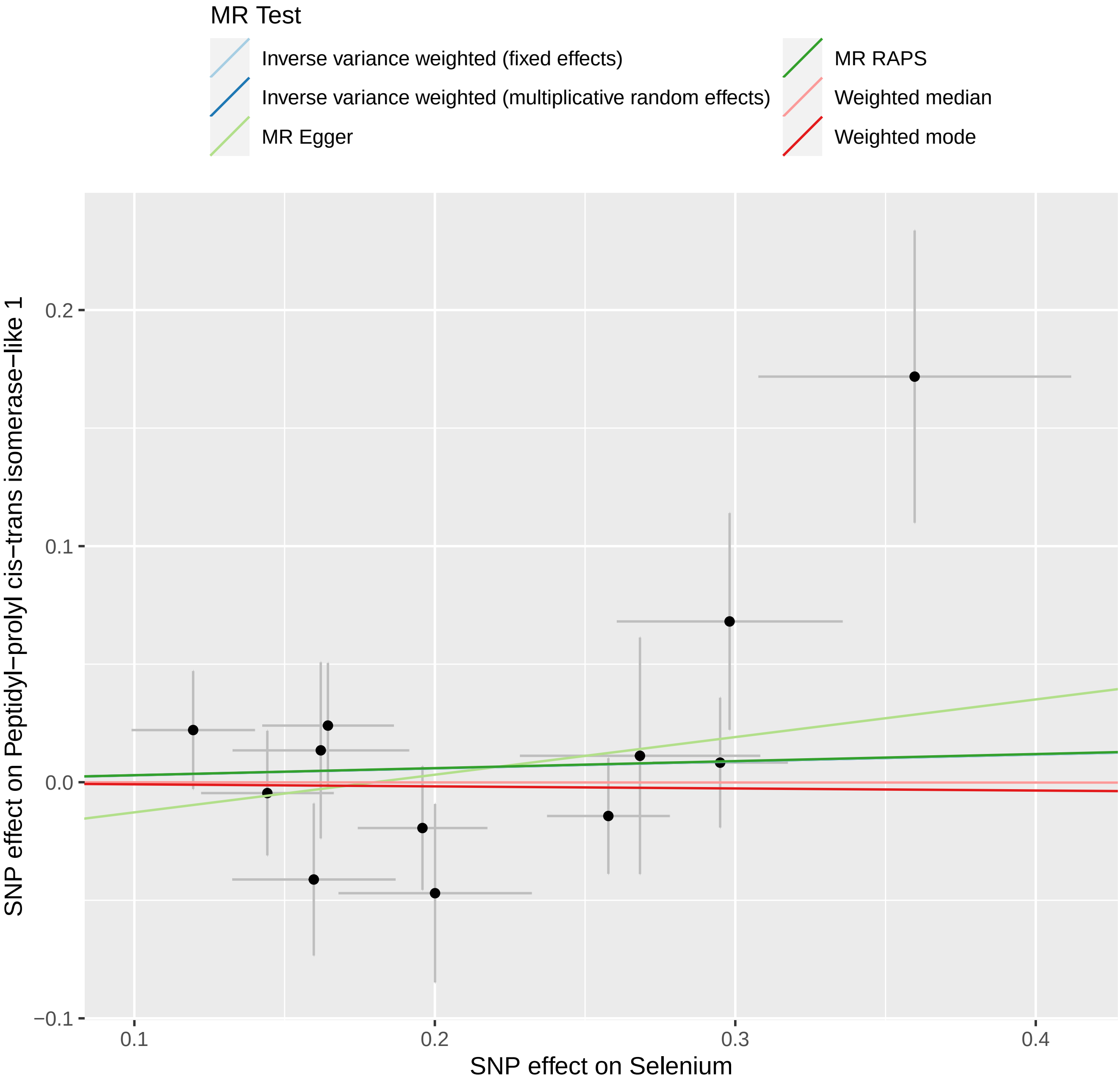

Batch 3−2344 : Selenium on Peptidyl−prolyl cis−trans isomerase−like 2

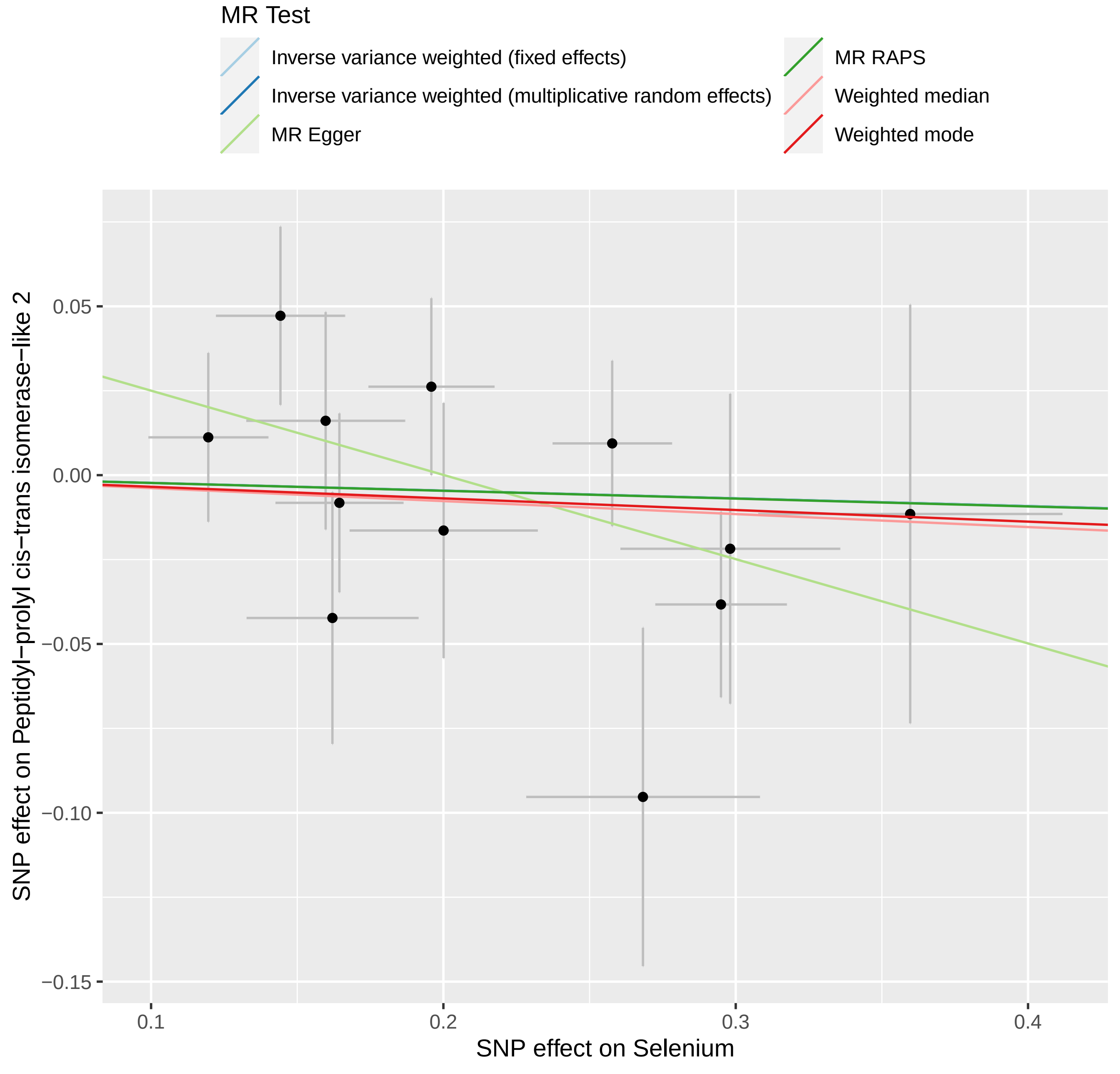

Batch 3−2345 : Selenium on Protein phosphatase 1A

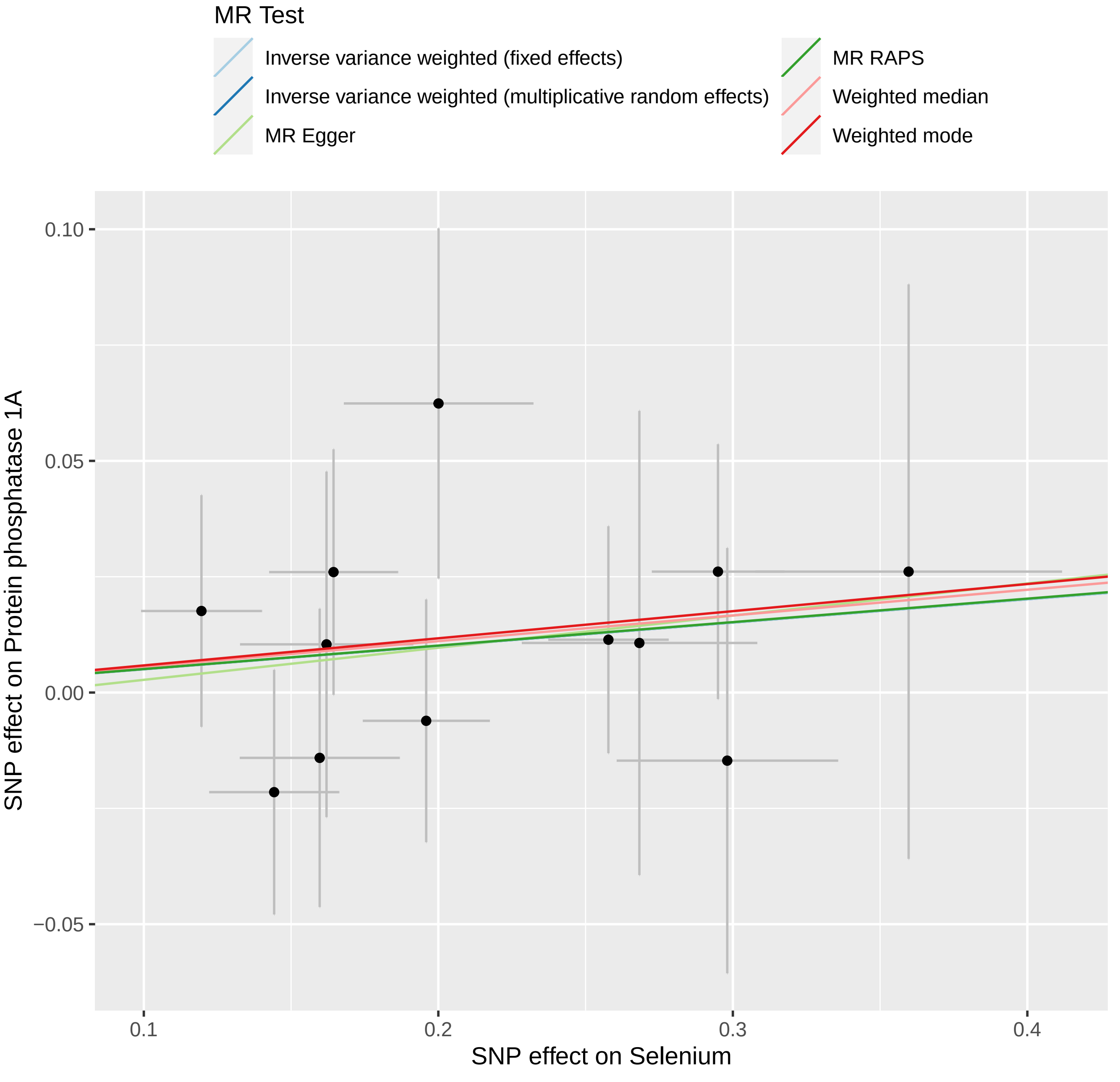

Batch 3−2346 : Selenium on Protein phosphatase 1L

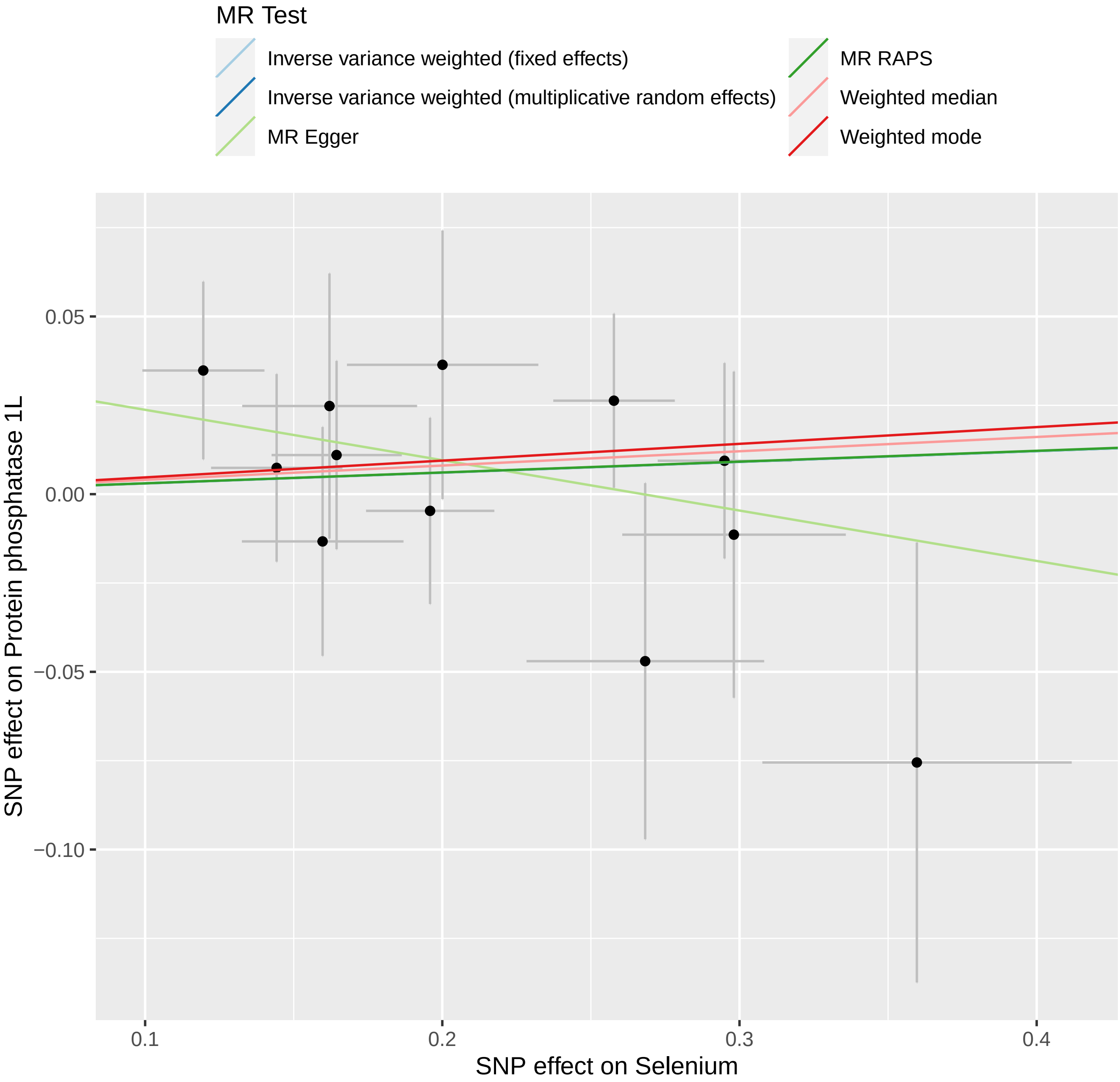

Batch 3−2347 : Selenium on Protein phosphatase 1 regulatory subunit 3B

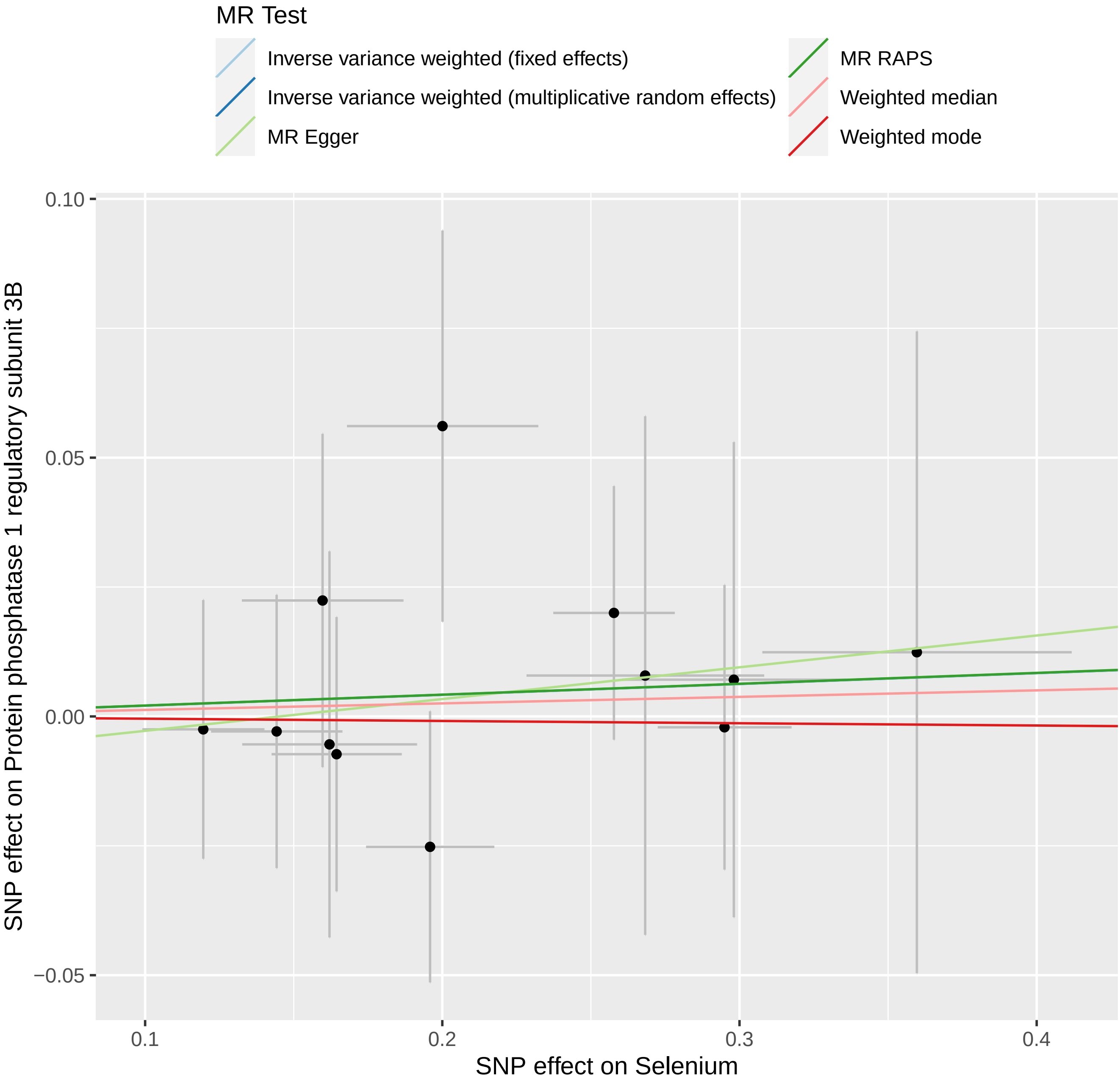

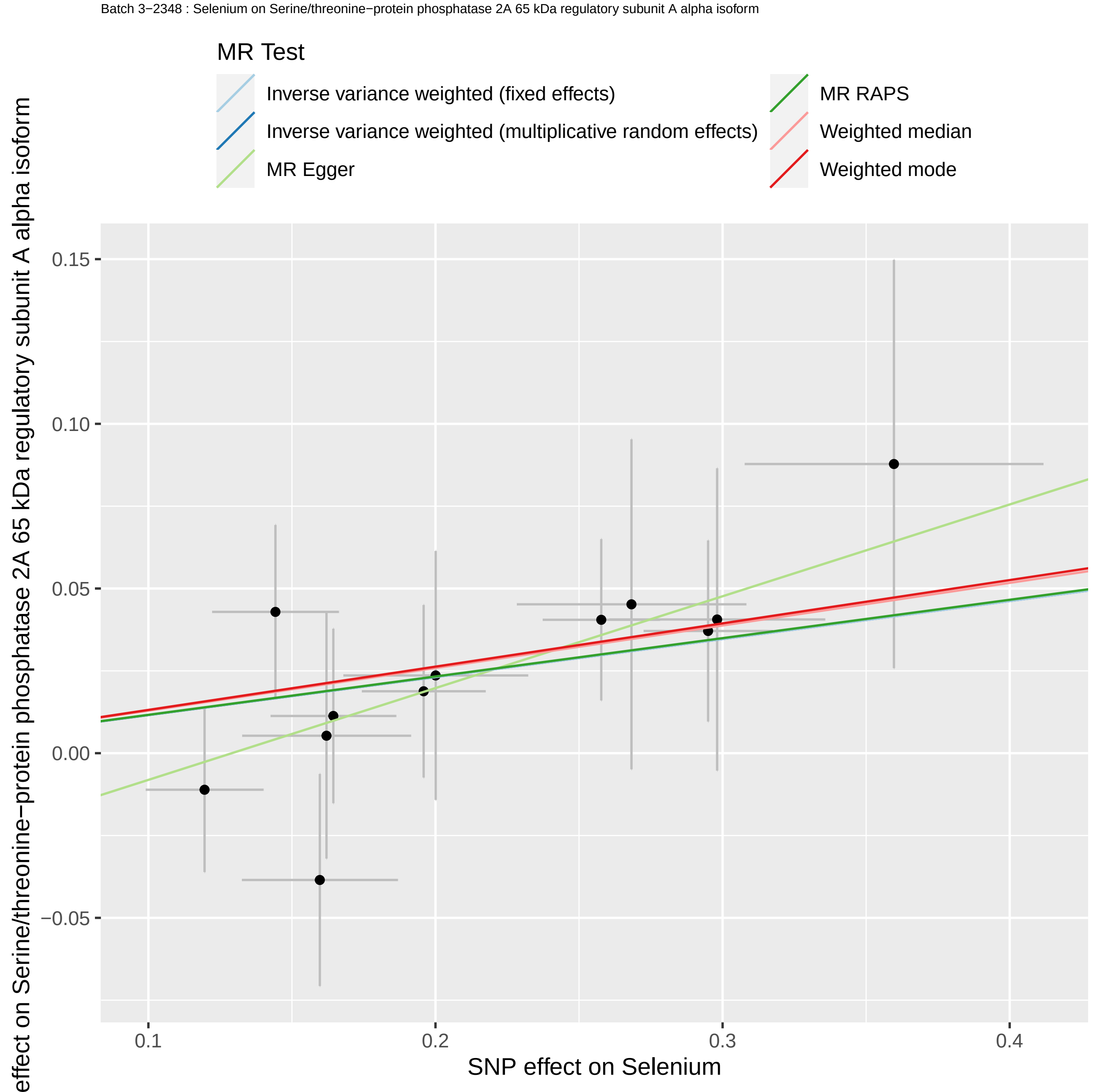
Batch 3−2348 : Selenium on Serine/threonine−protein phosphatase 2A 65 kDa regulatory subunit A alpha isoform
MR Test
Inverse variance weighted (fixed effects)
Inverse variance weighted (multiplicative random effects)
MR Egger
MR RAPS
Weighted median
Weighted mode
0.15
0.10
0.05
0.00
−0.05
0.1
0.2
0.3
0.4
SNP effect on Selenium
effect on Serine/threonine−protein phosphatase 2A 65 kDa regulatory subunit A alpha isoform

Batch 3−2349 : Selenium on Serine/threonine−protein phosphatase 2A regulatory subunit B" subunit alpha

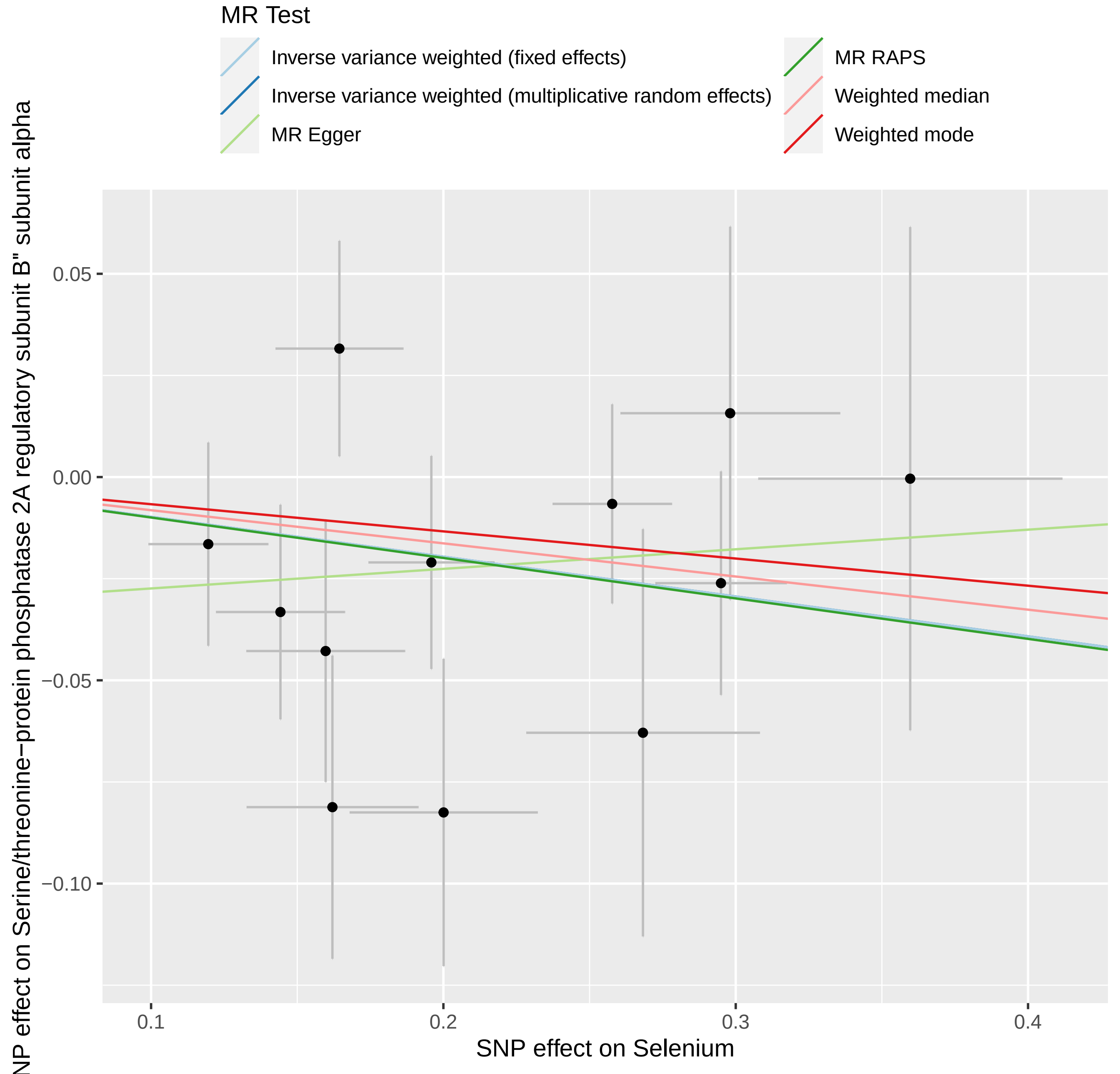

Batch 3−2350 : Selenium on Calcineurin subunit B type 1

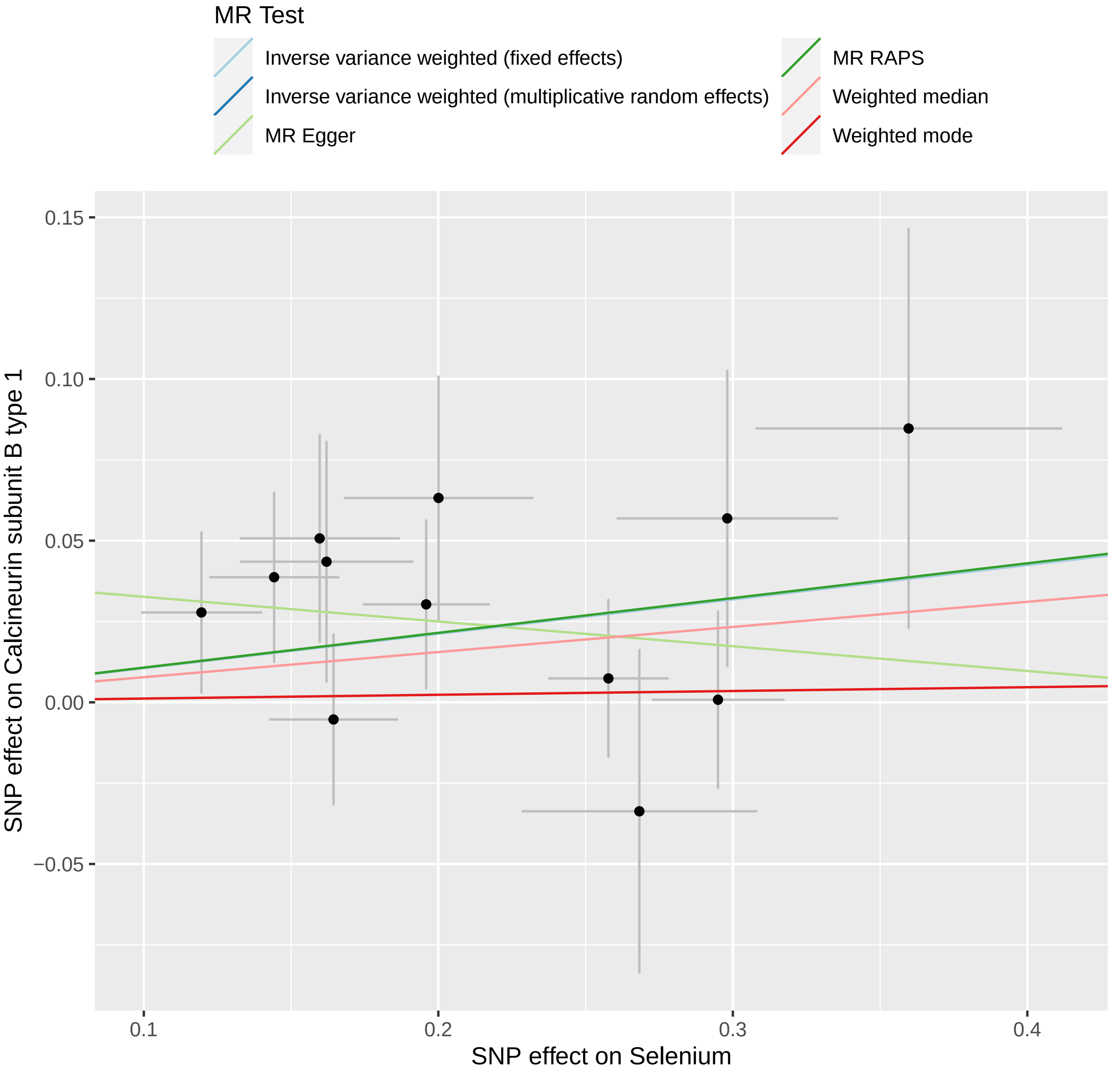

Batch 3−2351 : Selenium on Palmitoyl−protein thioesterase 1

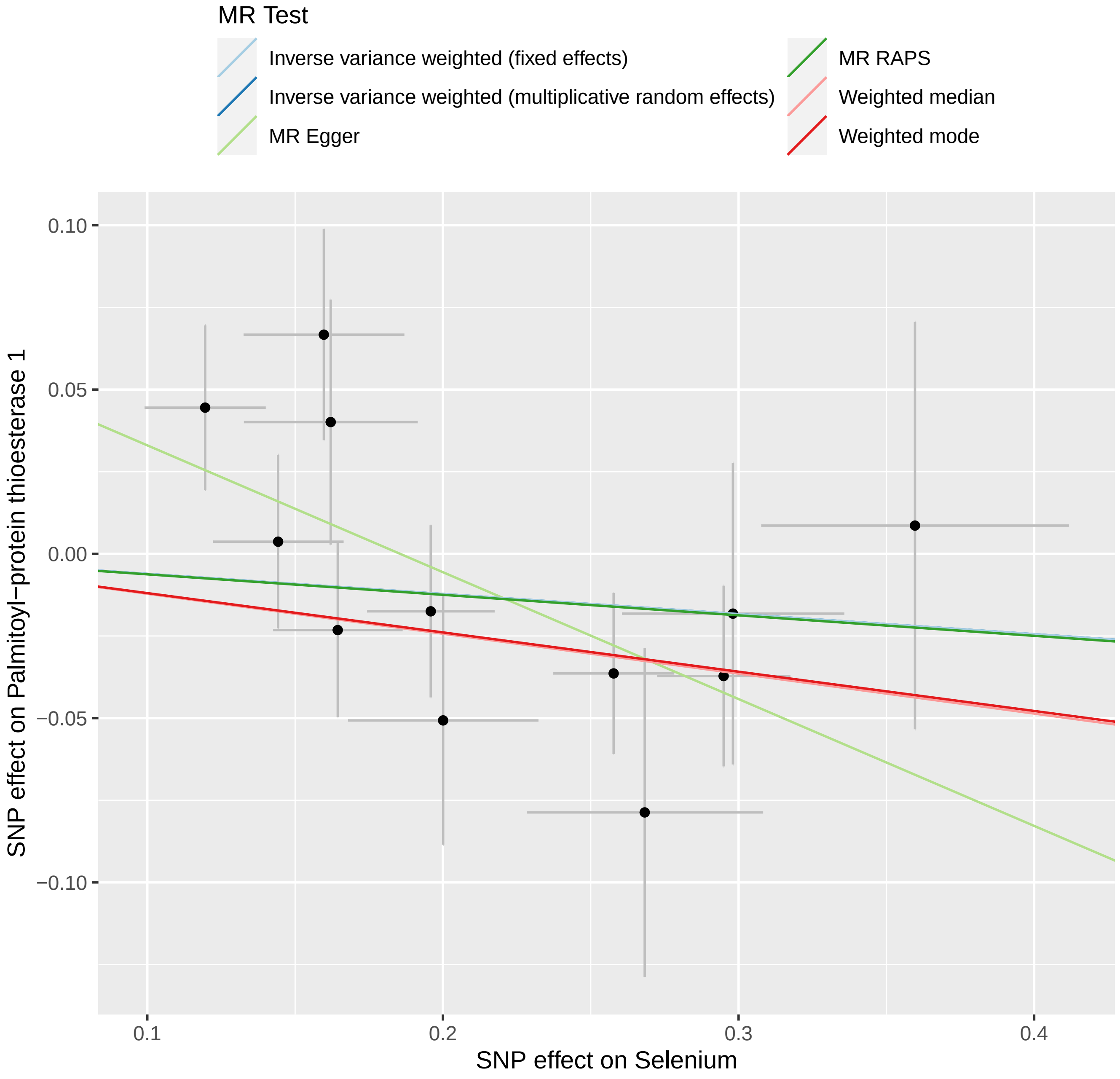

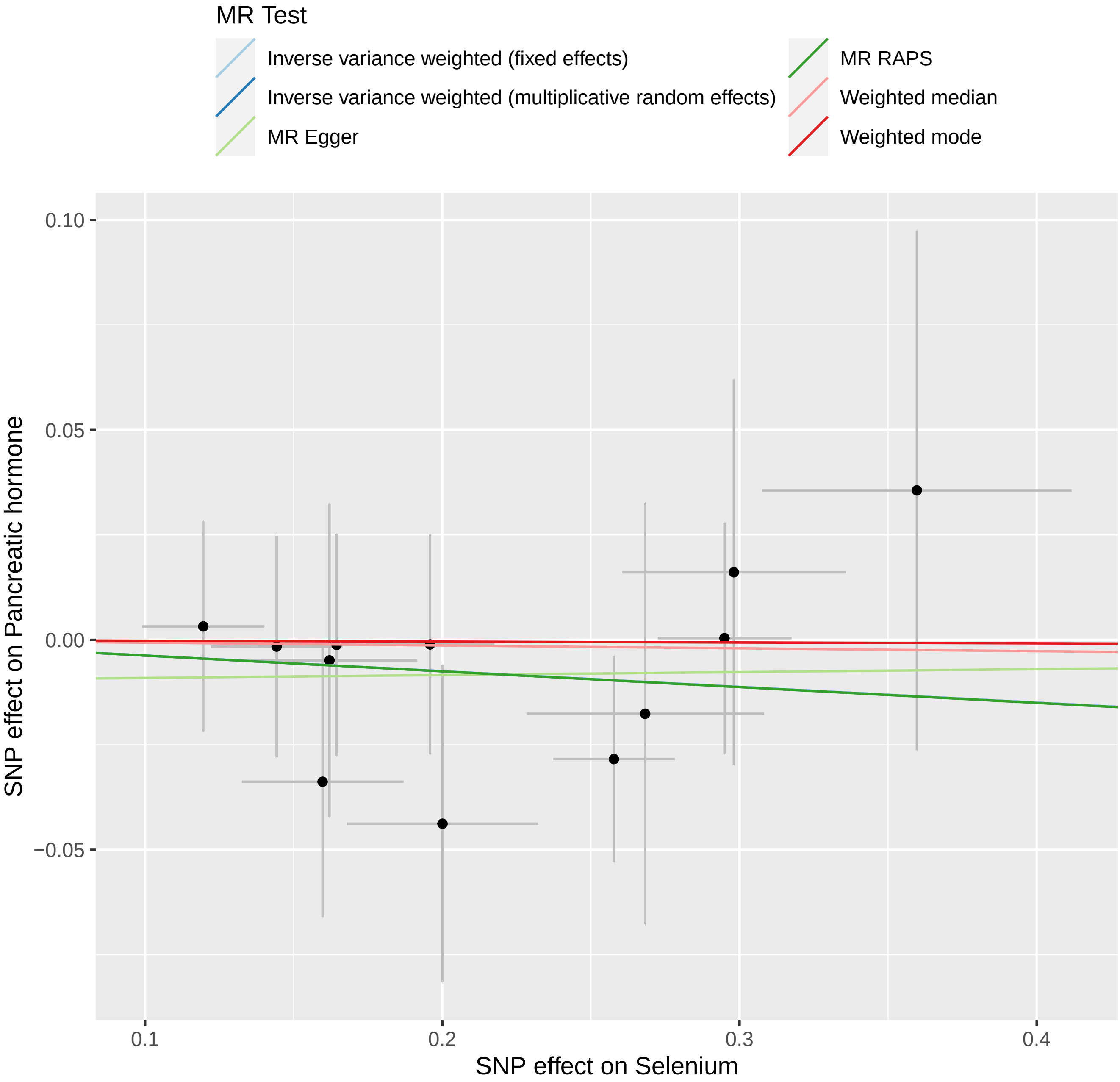
MR Test
Inverse variance weighted (fixed effects)
Inverse variance weighted (multiplicative random effects)
MR Egger
MR RAPS
Weighted median
Weighted mode
0.10
0.05
0.00
−0.05
0.1
0.2
0.3
0.4
SNP effect on Pancreatic hormone
SNP effect on Selenium

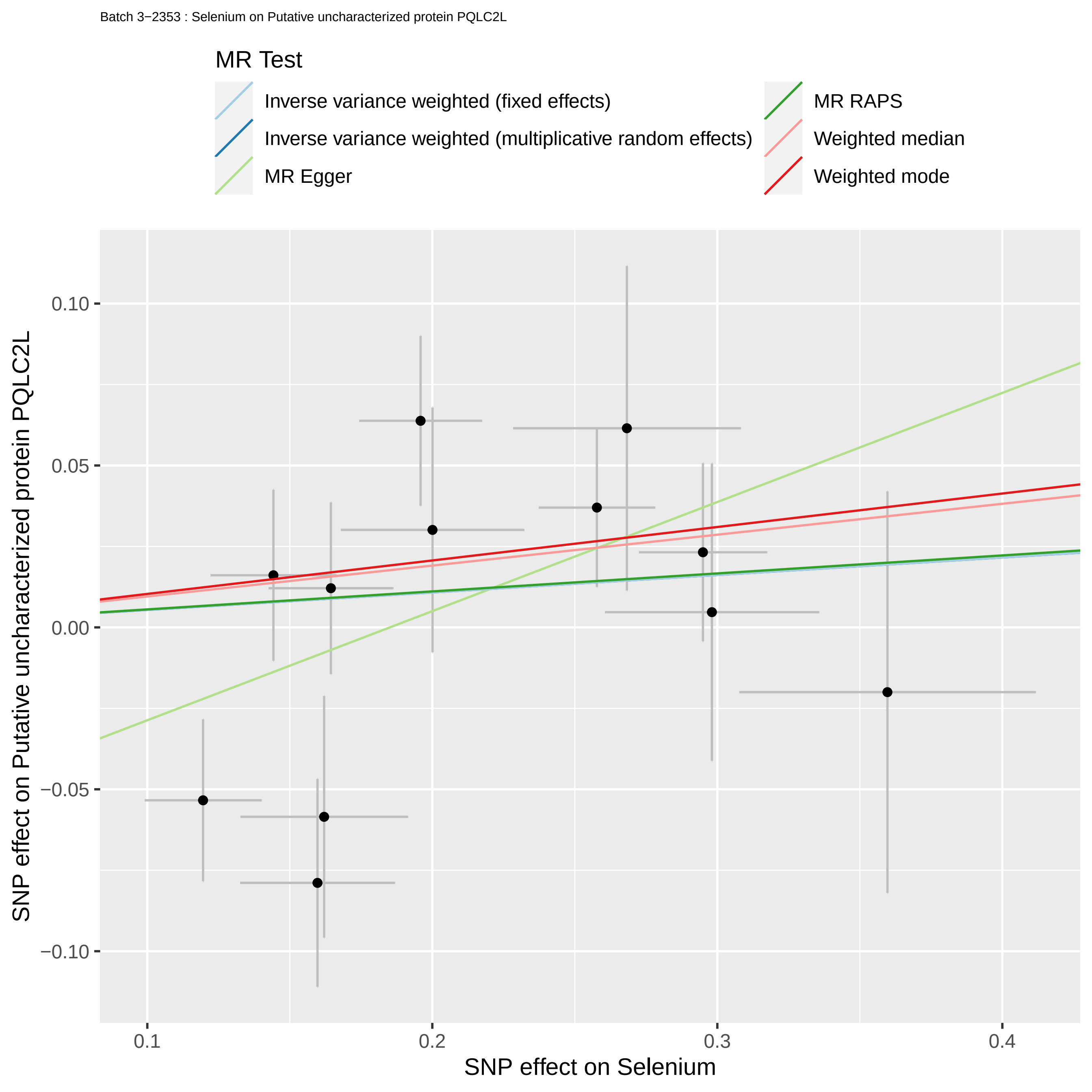

Batch 3−2353 : Selenium on Putative uncharacterized protein PQLC2L
MR Test
Inverse variance weighted (fixed effects)
Inverse variance weighted (multiplicative random effects)
MR Egger
MR RAPS
Weighted median
Weighted mode
0.10
0.05
0.00
−0.05
−0.10
0.1
0.2
0.3
0.4
SNP effect on Putative uncharacterized protein PQLC2L
SNP effect on Selenium

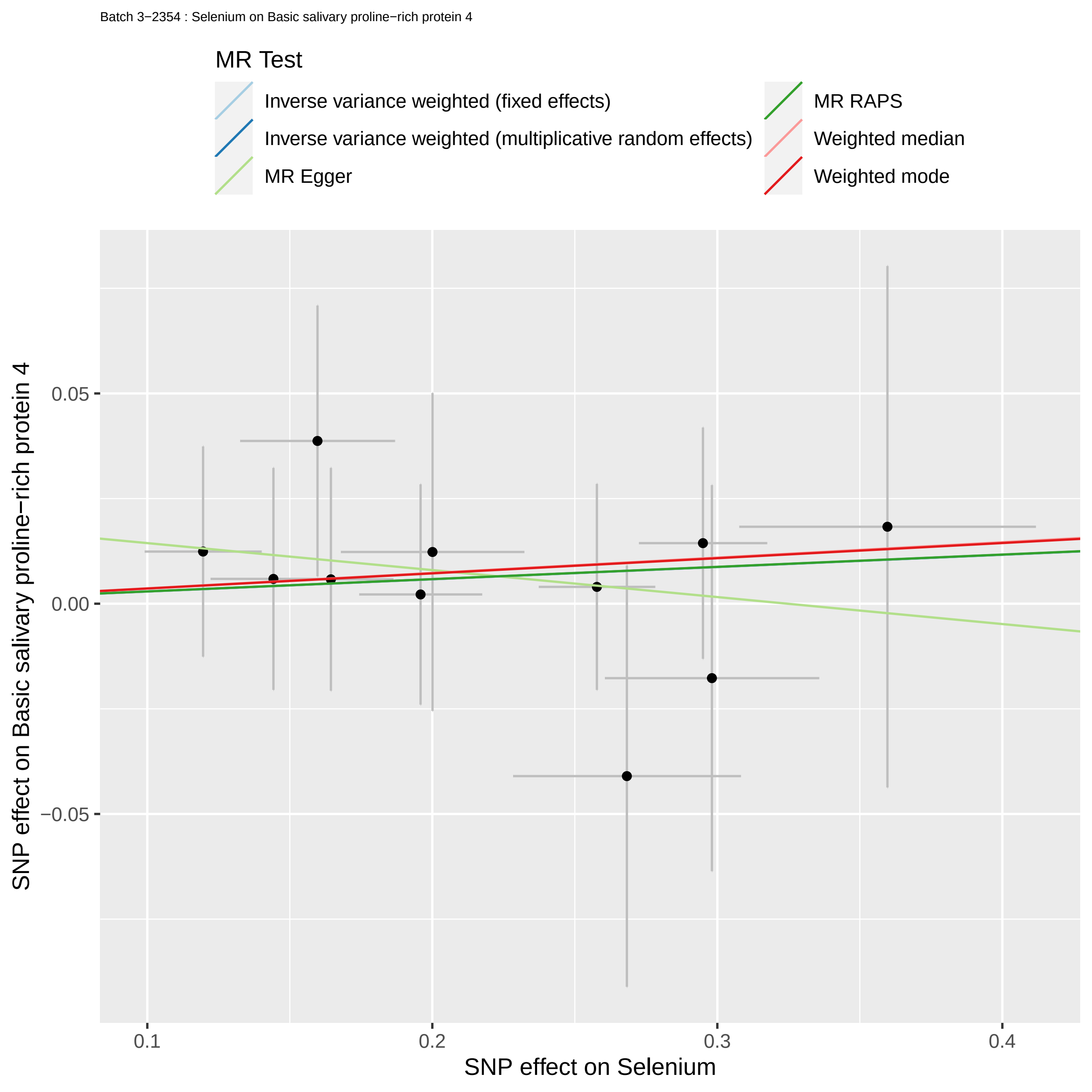

Batch 3−2354 : Selenium on Basic salivary proline−rich protein 4
MR Test
Inverse variance weighted (fixed effects)
Inverse variance weighted (multiplicative random effects)
MR Egger
MR RAPS
Weighted median
Weighted mode
0.05
0.00
−0.05
0.1
0.2
0.3
0.4
SNP effect on Basic salivary proline−rich protein 4
SNP effect on Selenium

Batch 3−2355 : Selenium on Protein regulator of cytokinesis 1

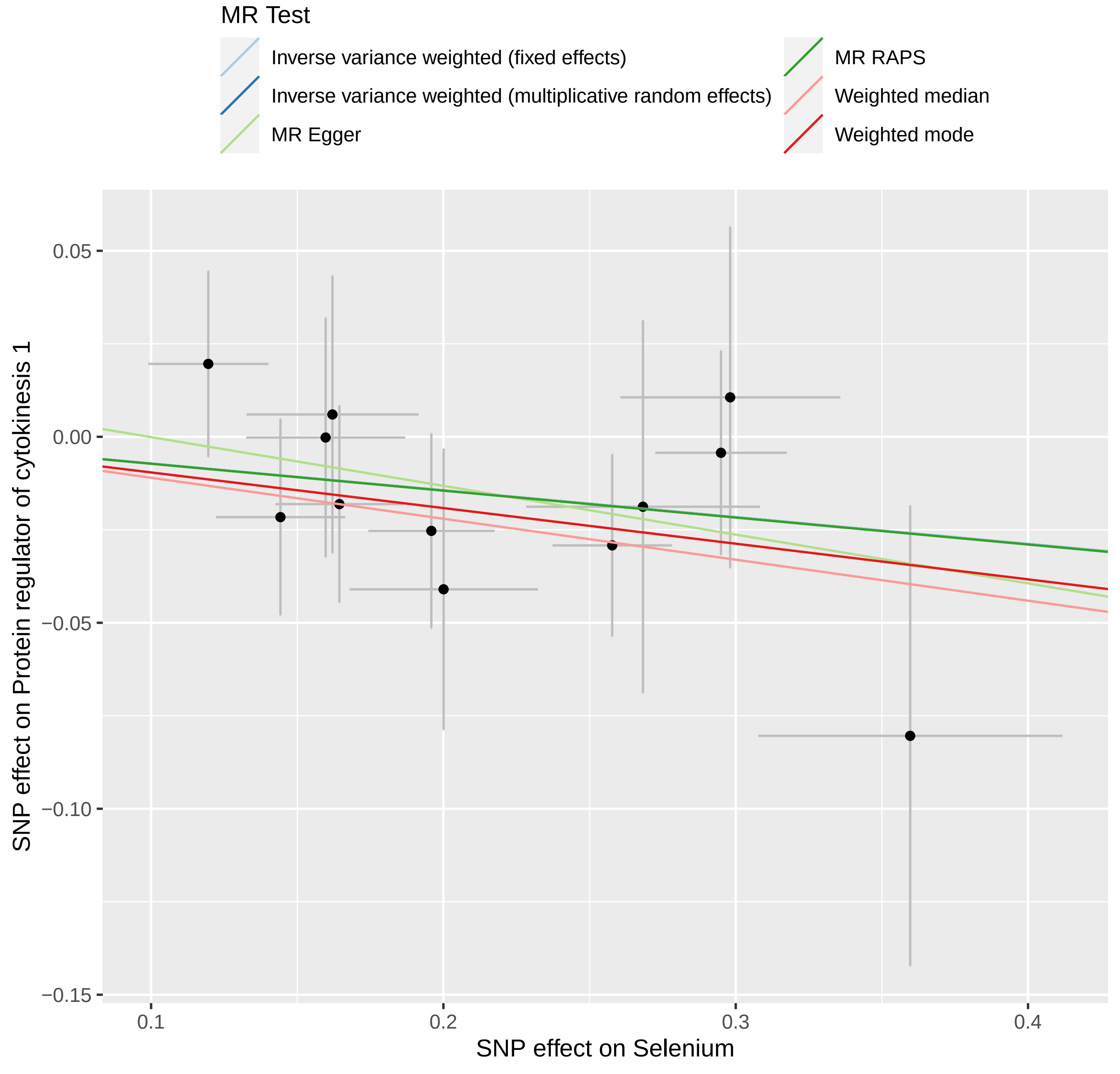

Batch 3−2356 : Selenium on Lysosomal Pro−X carboxypeptidase

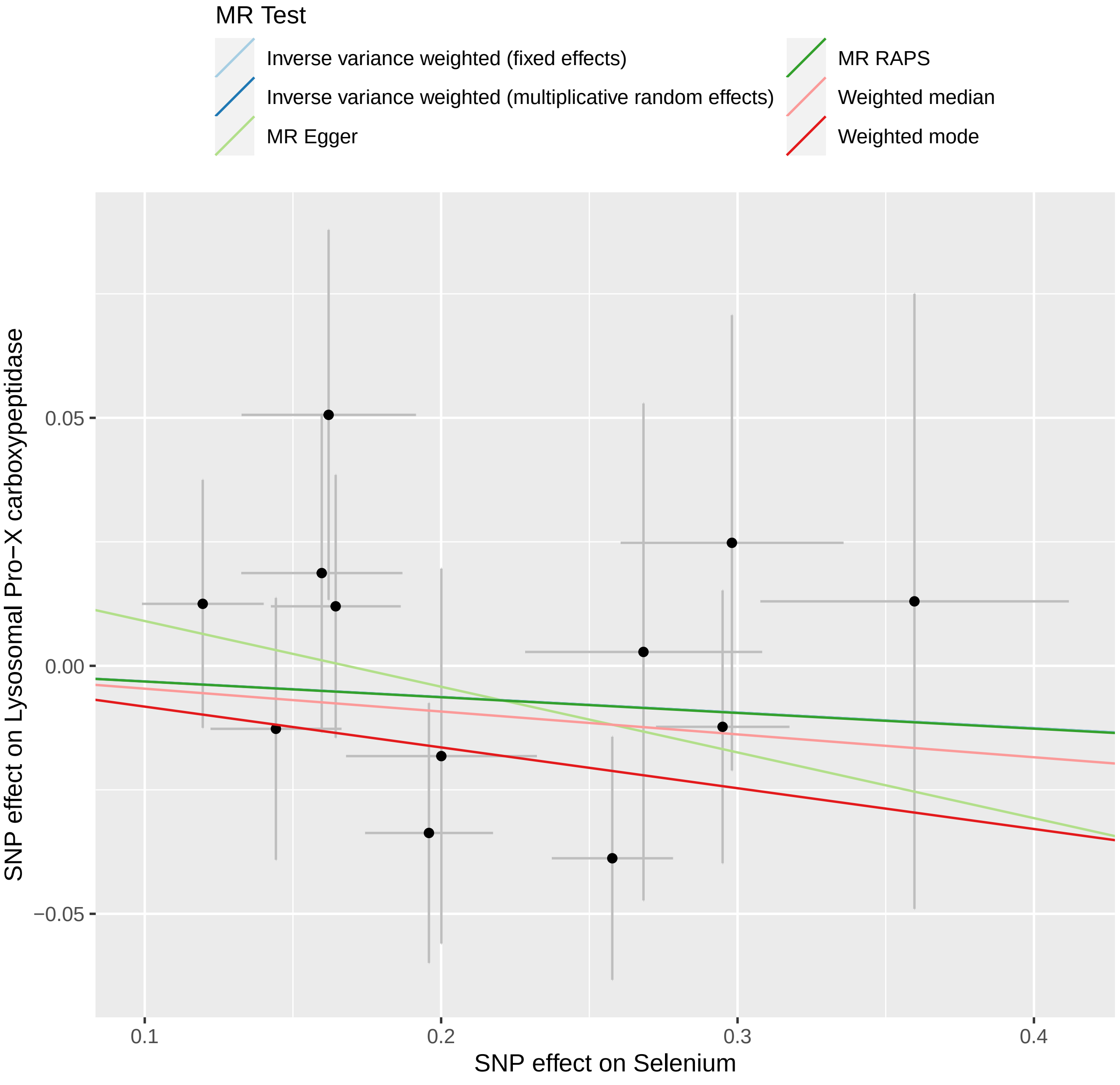

Batch 3−2357 : Selenium on PR domain zinc finger protein 1

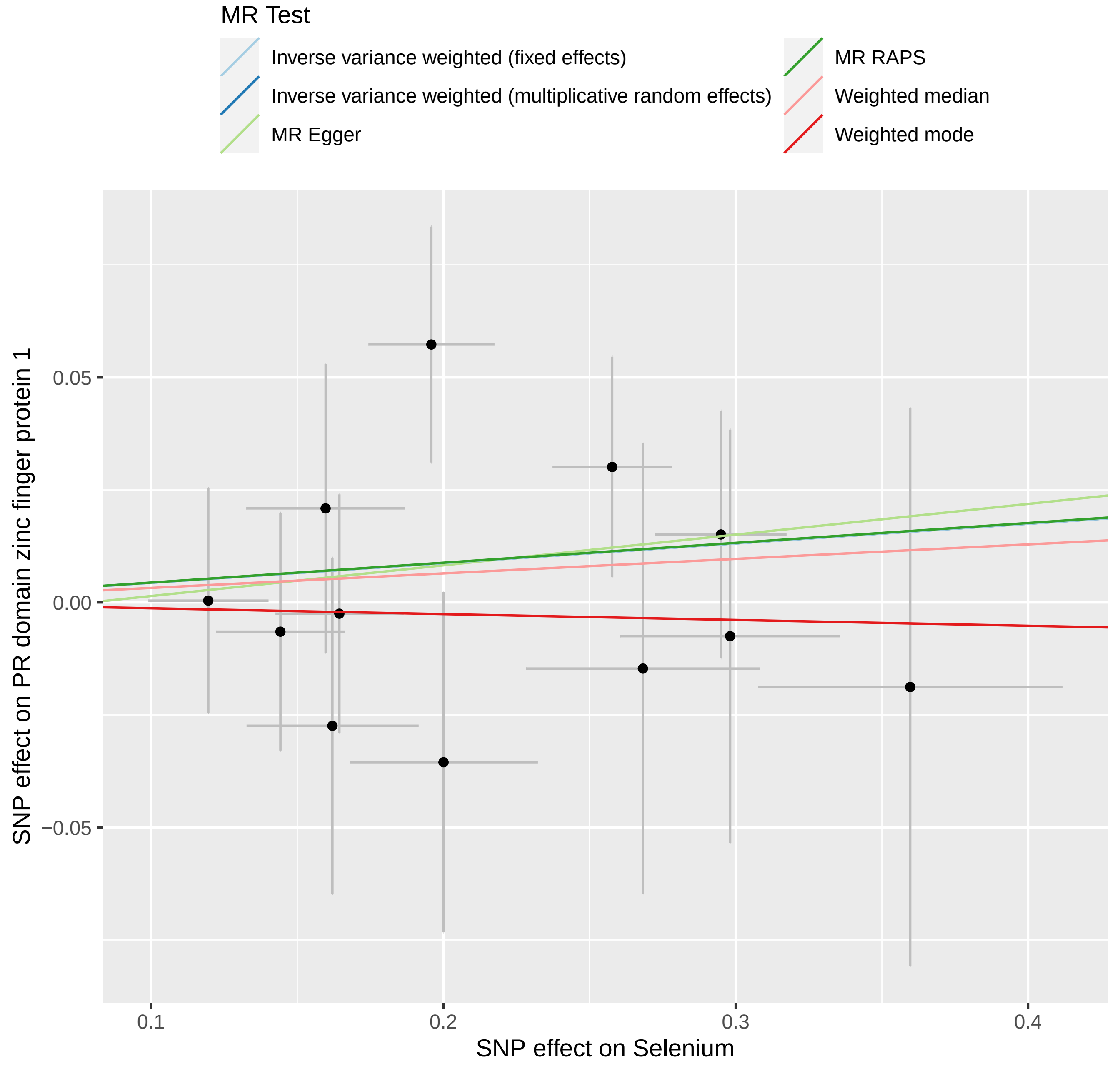

Batch 3−2358 : Selenium on PR domain zinc finger protein 4

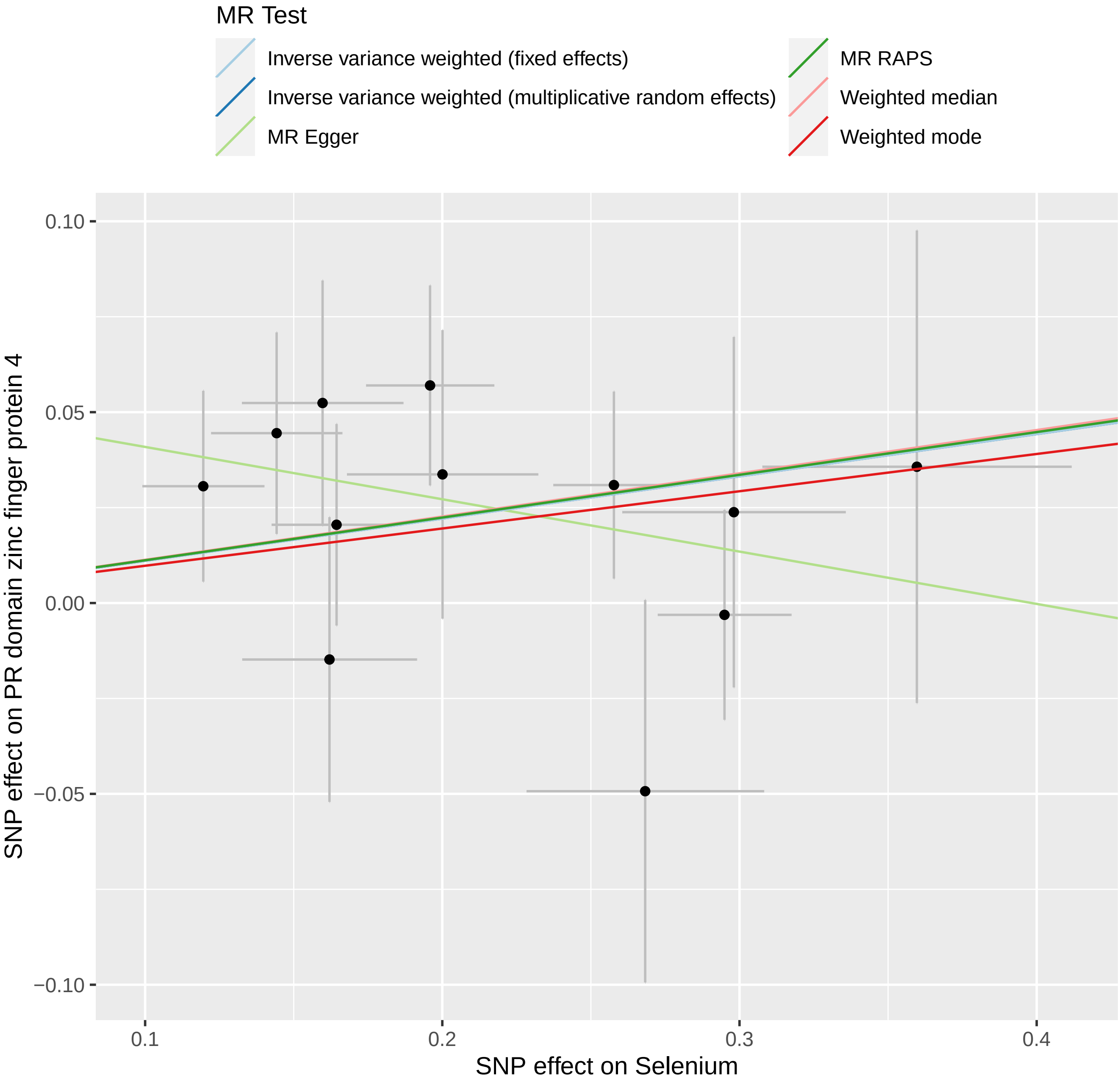

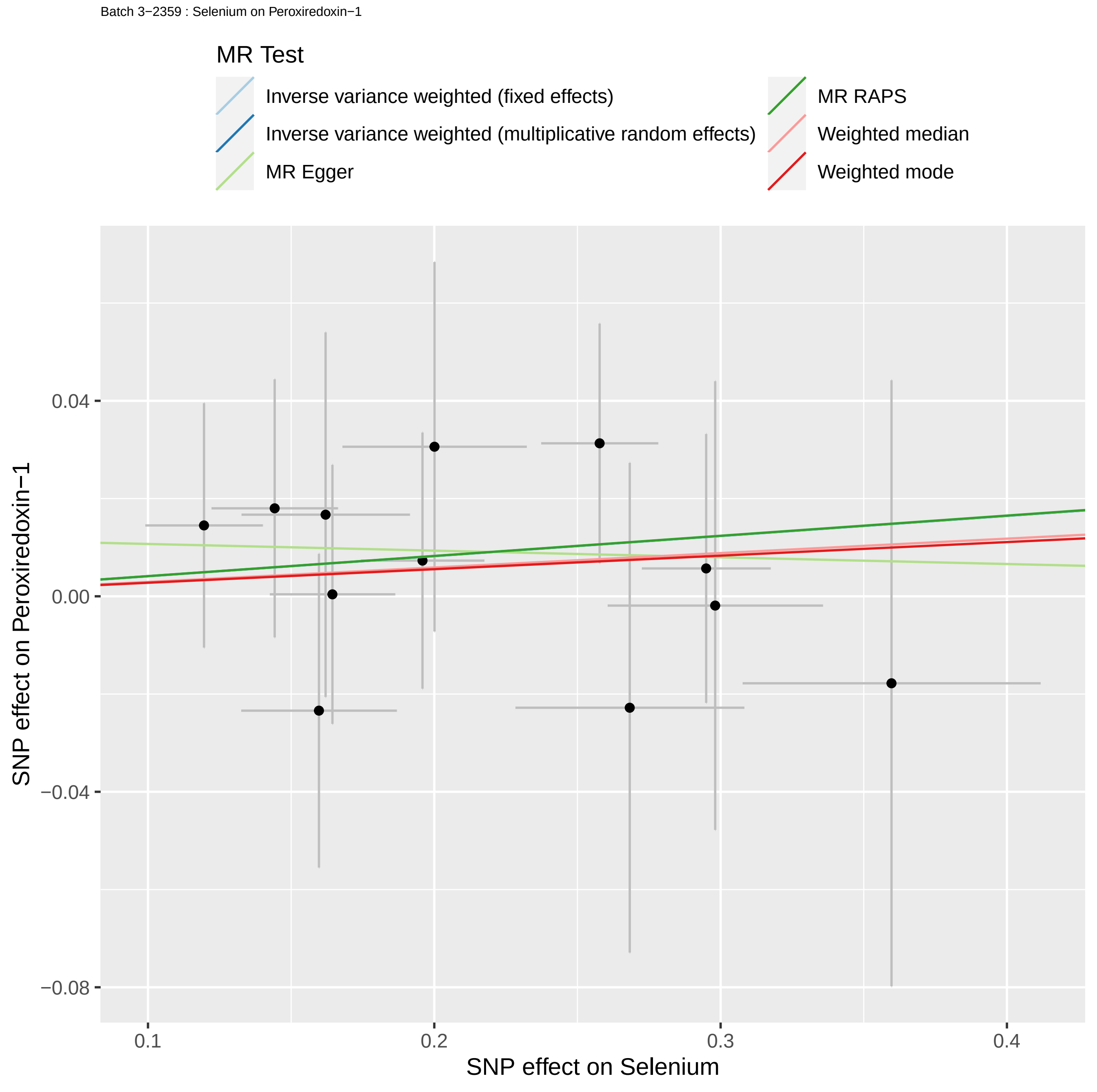
Batch 3−2359 : Selenium on Peroxiredoxin−1
MR Test
Inverse variance weighted (fixed effects)
Inverse variance weighted (multiplicative random effects)
MR Egger
MR RAPS
Weighted median
Weighted mode
SNP effect on Peroxiredoxin−1
0.04
0.00
−0.04
−0.08
0.1
0.2
0.3
0.4
SNP effect on Selenium

Batch 3−2360 : Selenium on Peroxiredoxin−4

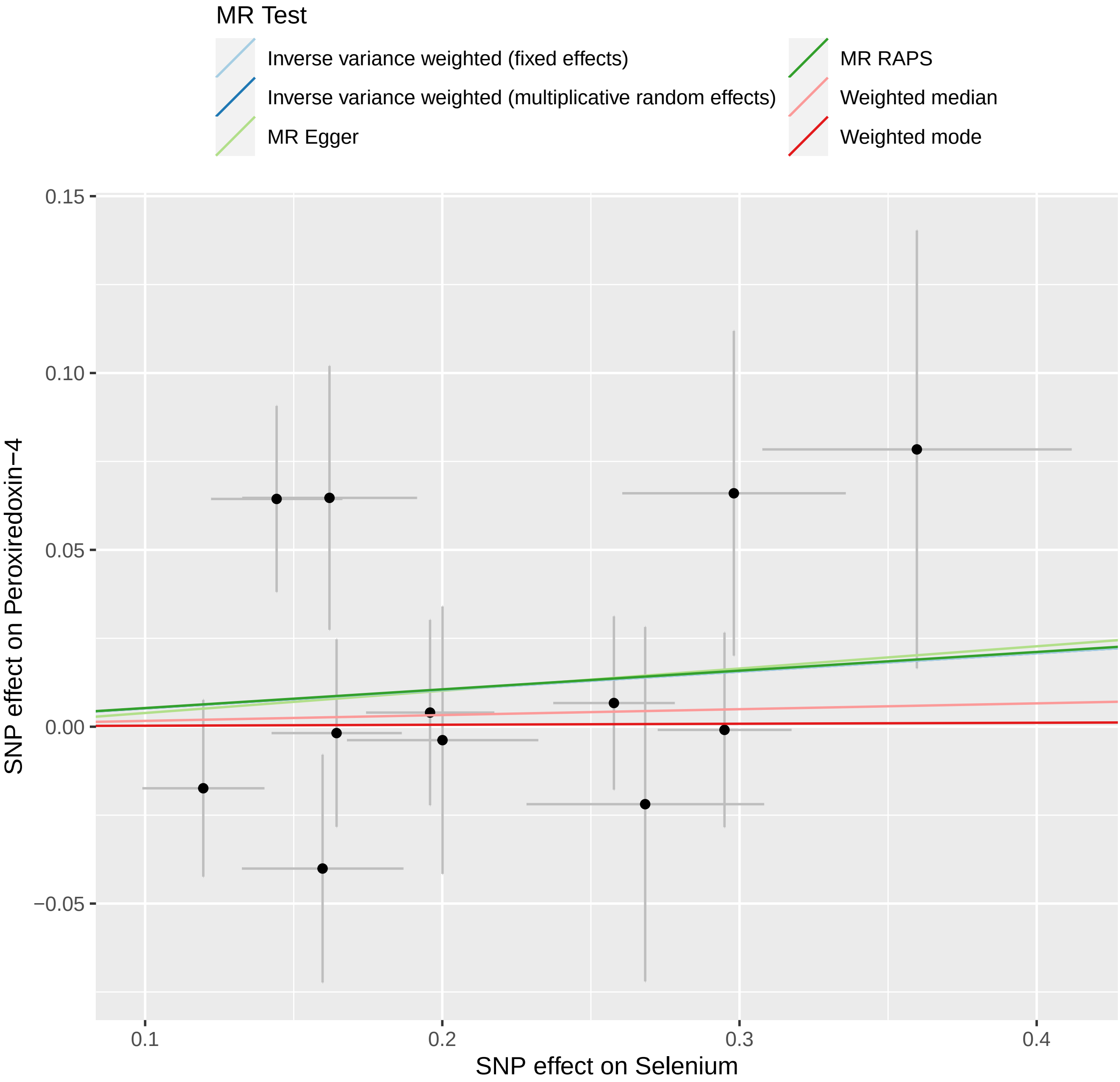

Batch 3−2361 : Selenium on Prolargin

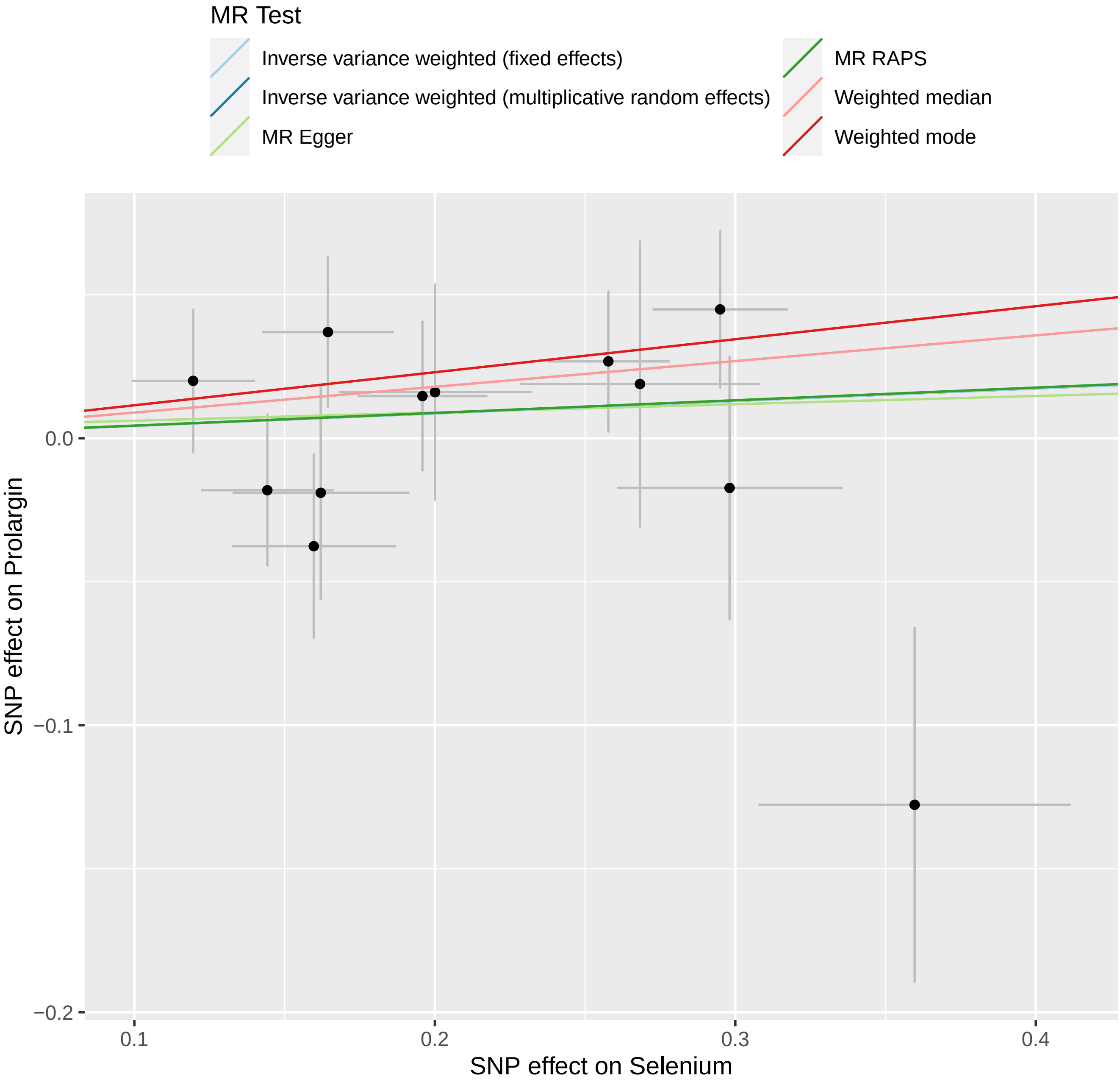

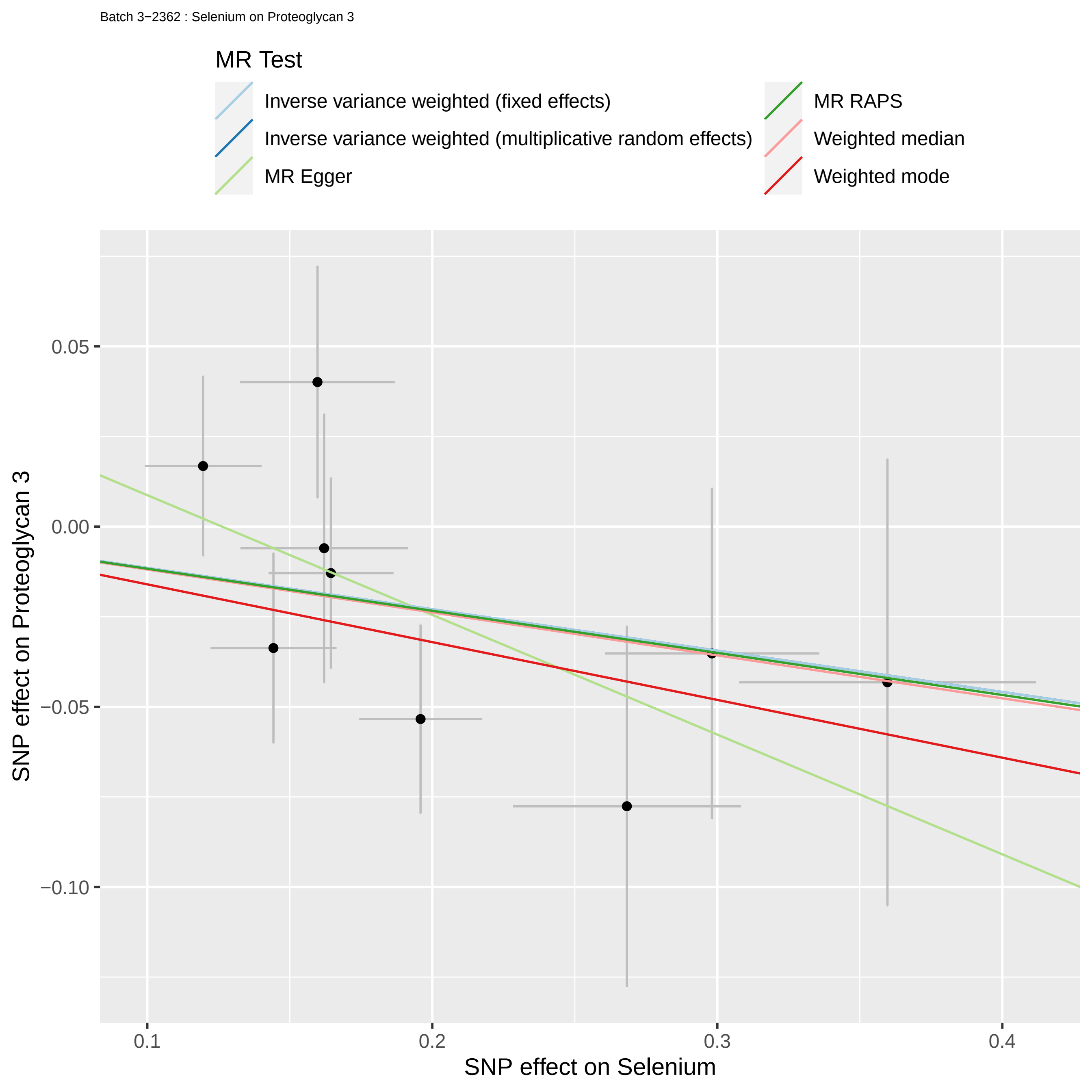
Batch 3−2362 : Selenium on Proteoglycan 3
MR Test
Inverse variance weighted (fixed effects)
Inverse variance weighted (multiplicative random effects)
MR Egger
MR RAPS
Weighted median
Weighted mode
0.05
0.00
−0.05
−0.10
0.1
0.2
0.3
0.4
SNP effect on Proteoglycan 3
SNP effect on Selenium

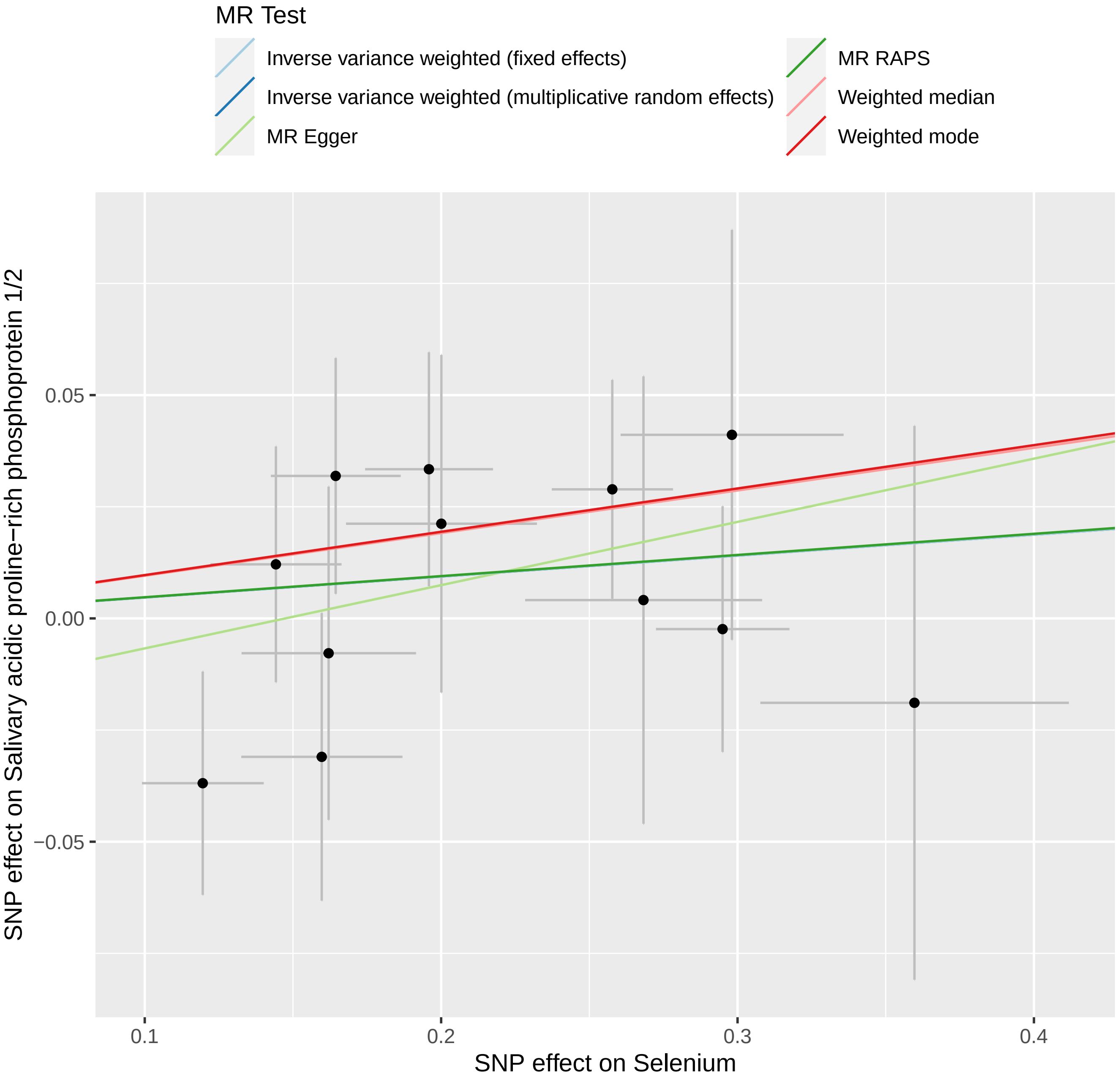
MR Test
Inverse variance weighted (fixed effects)
Inverse variance weighted (multiplicative random effects)
MR Egger
MR RAPS
Weighted median
Weighted mode
0.05
0.00
−0.05
0.1
0.2
0.3
0.4
SNP effect on Selenium
SNP effect on Salivary acidic proline−rich phosphoprotein 1/2

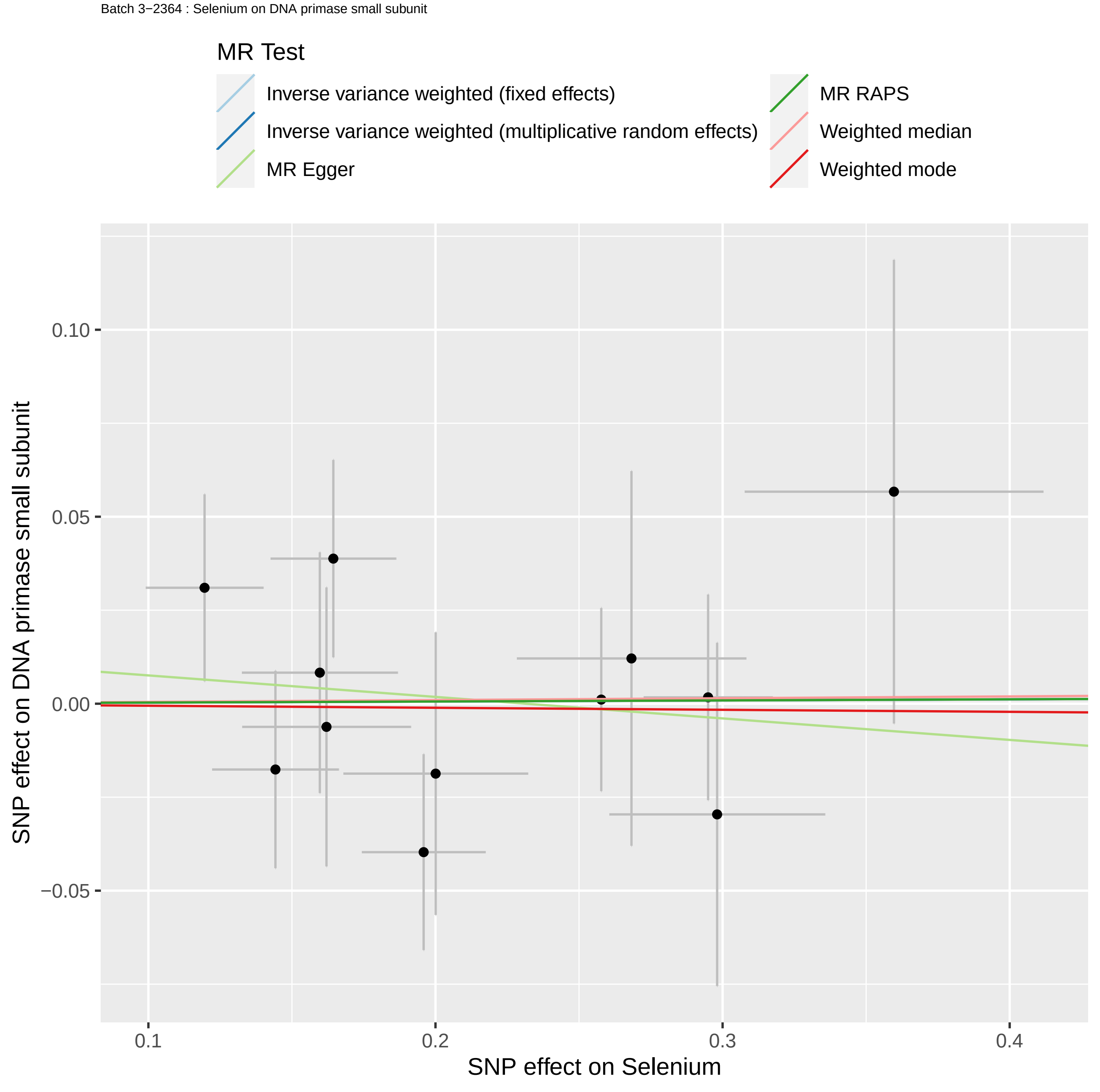
Batch 3−2364 : Selenium on DNA primase small subunit
MR Test
Inverse variance weighted (fixed effects)
Inverse variance weighted (multiplicative random effects)
MR Egger
MR RAPS
Weighted median
Weighted mode
0.10
0.05
0.00
−0.05
0.1
0.2
0.3
0.4
SNP effect on Selenium
SNP effect on DNA primase small subunit

Batch 3−2365 : Selenium on AMP Kinase (alpha1beta1gamma1)

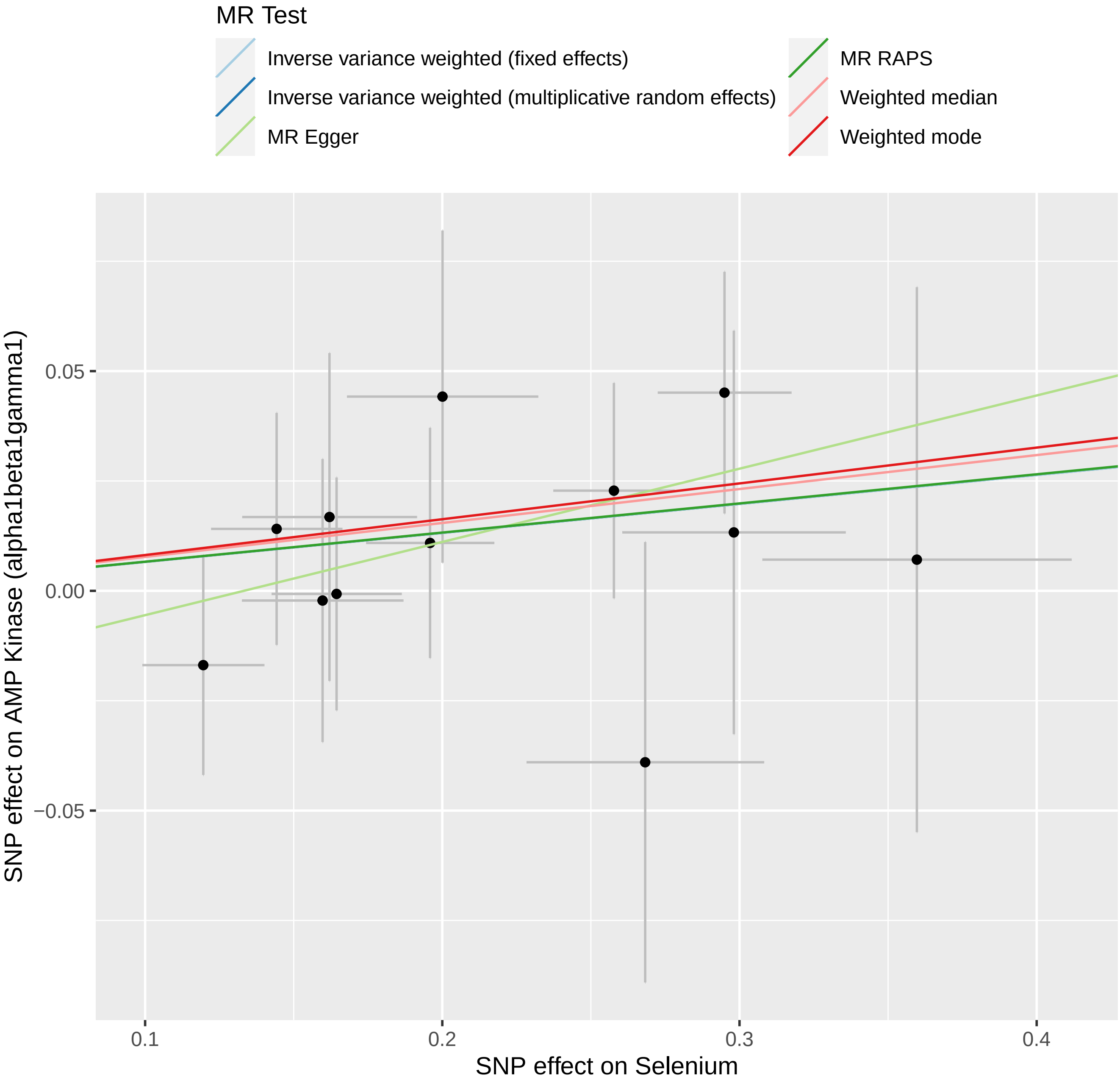

Batch 3−2366 : Selenium on AMP Kinase (alpha2beta2gamma1)

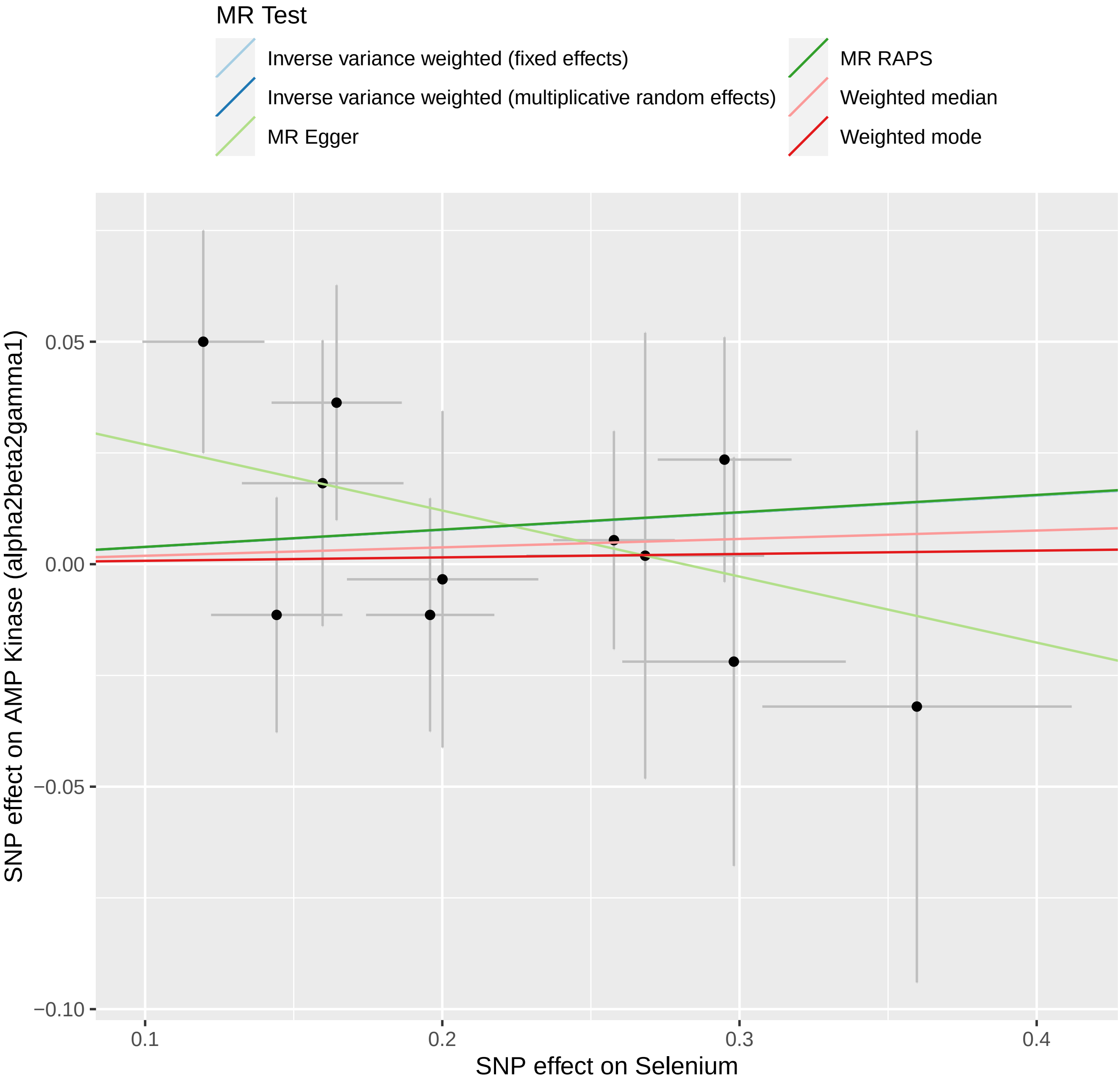

Batch 3−2367 : Selenium on cAMP−dependent protein kinase catalytic subunit alpha

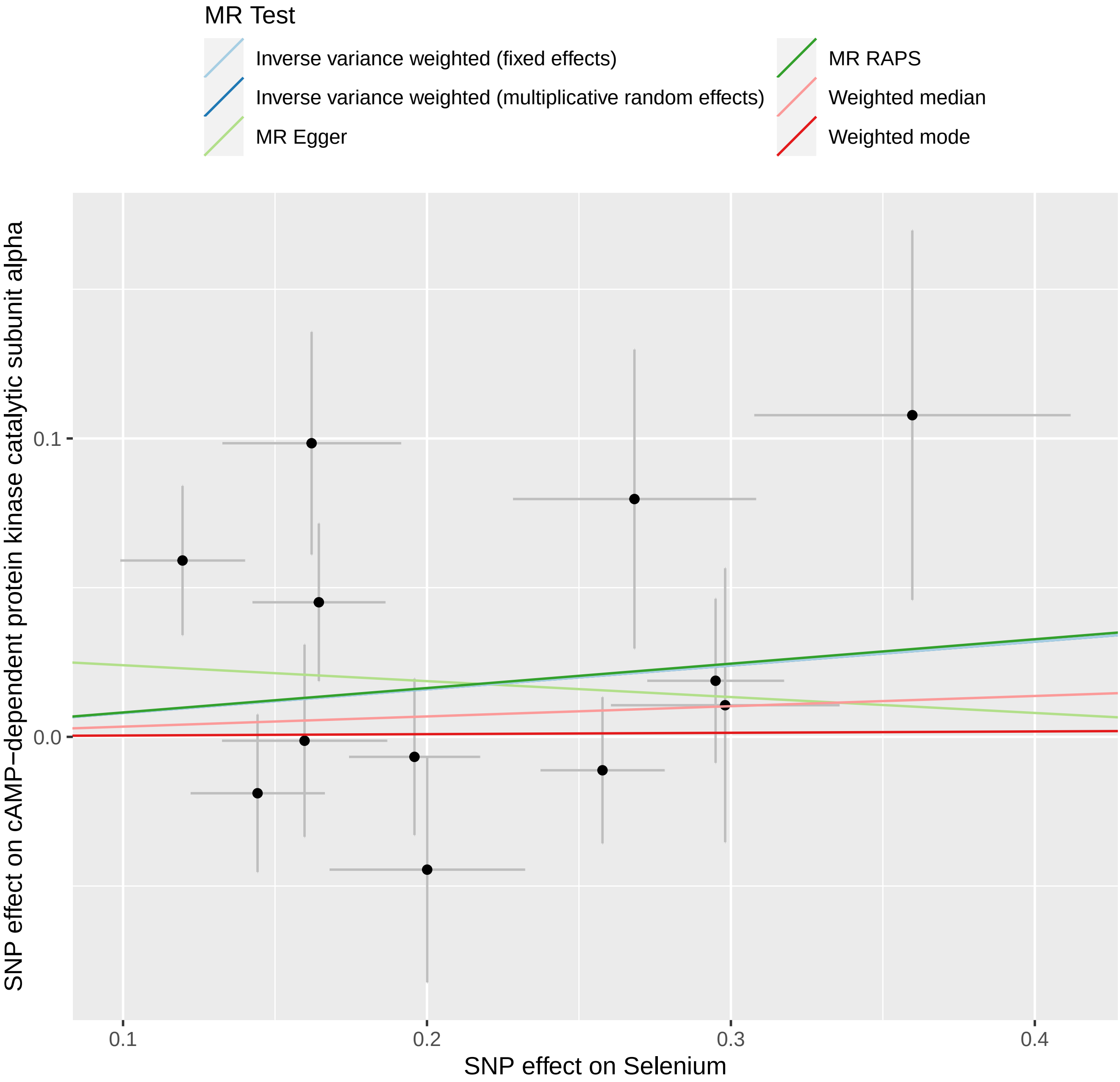

Batch 3−2368 : Selenium on cAMP−dependent protein kinase type I−beta regulatory subunit

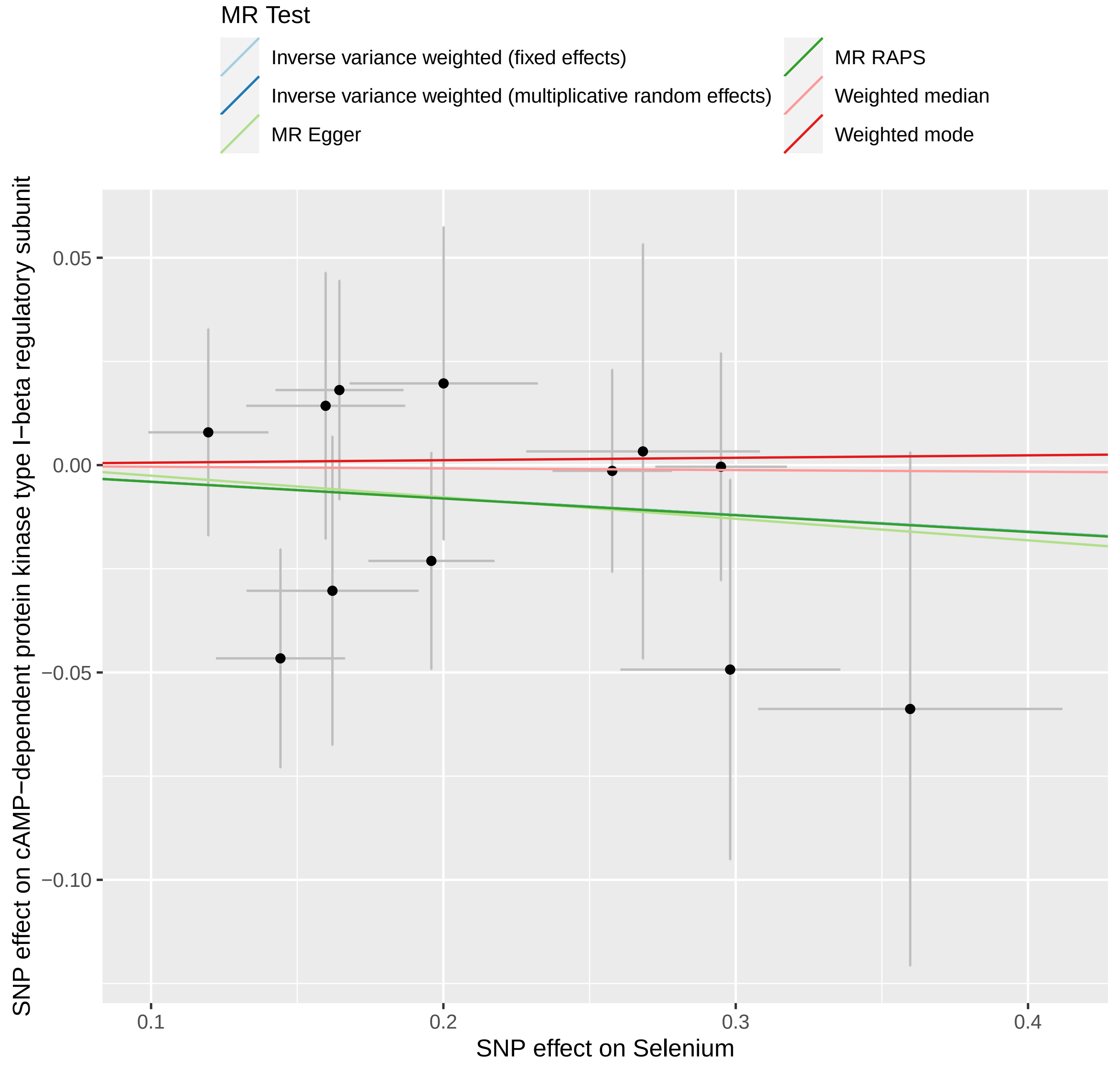

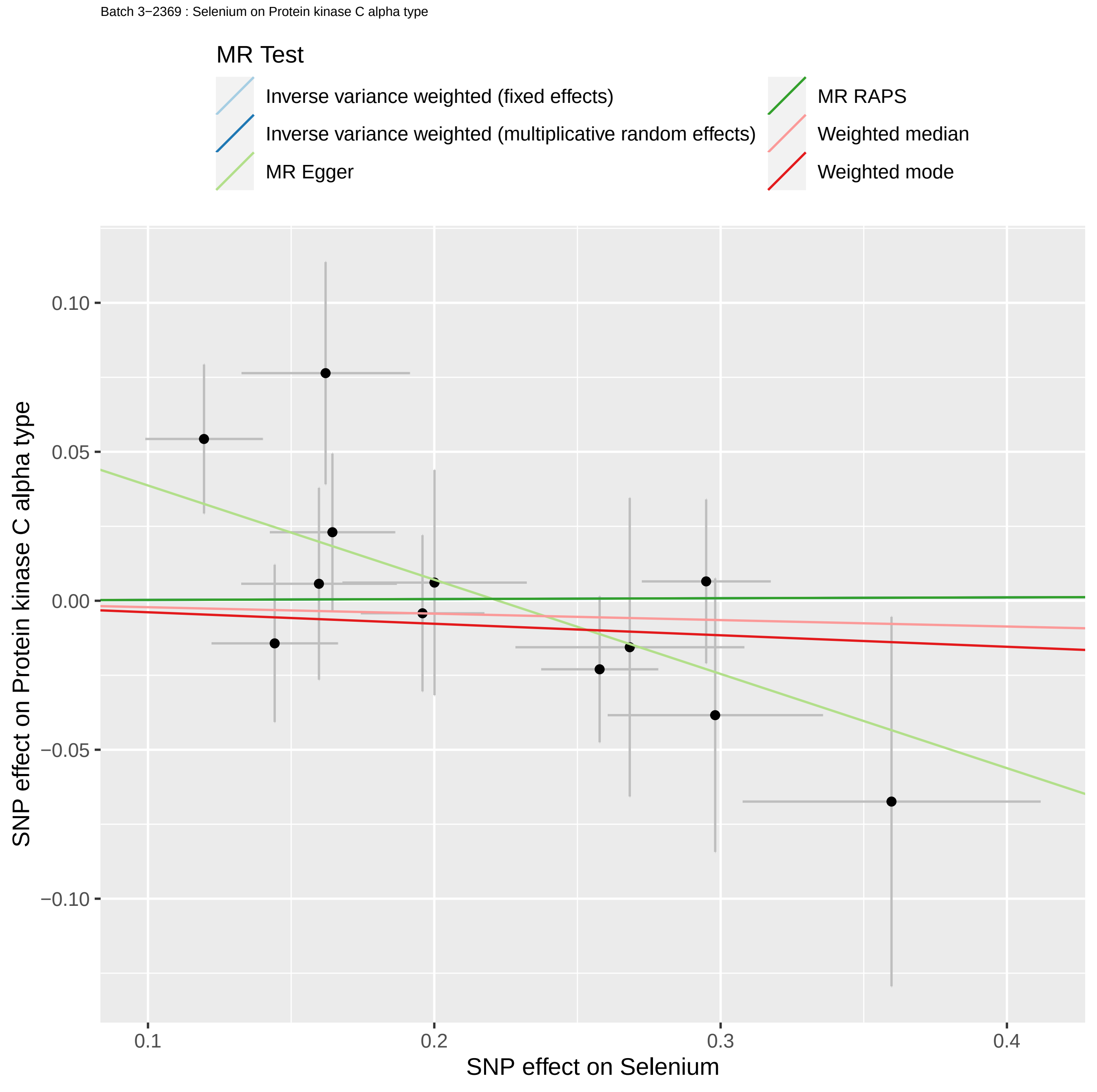

Batch 3−2369 : Selenium on Protein kinase C alpha type
MR Test
Inverse variance weighted (fixed effects)
Inverse variance weighted (multiplicative random effects)
MR Egger
MR RAPS
Weighted median
Weighted mode
0.10
0.05
0.00
−0.05
−0.10
0.1
0.2
0.3
0.4
SNP effect on Selenium
SNP effect on Protein kinase C alpha type

Batch 3−2370 : Selenium on Protein kinase C beta type (splice variant beta−II)

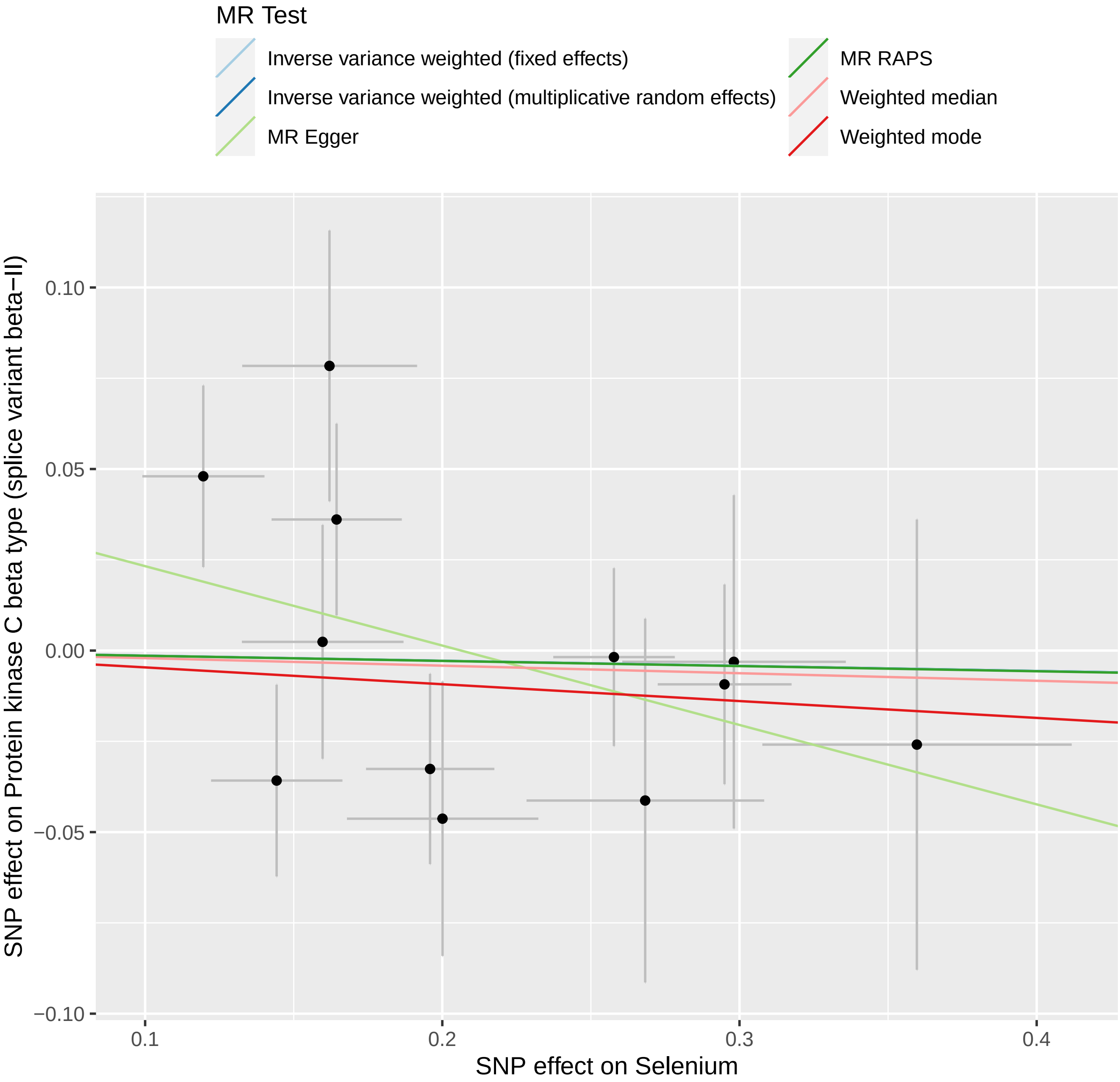

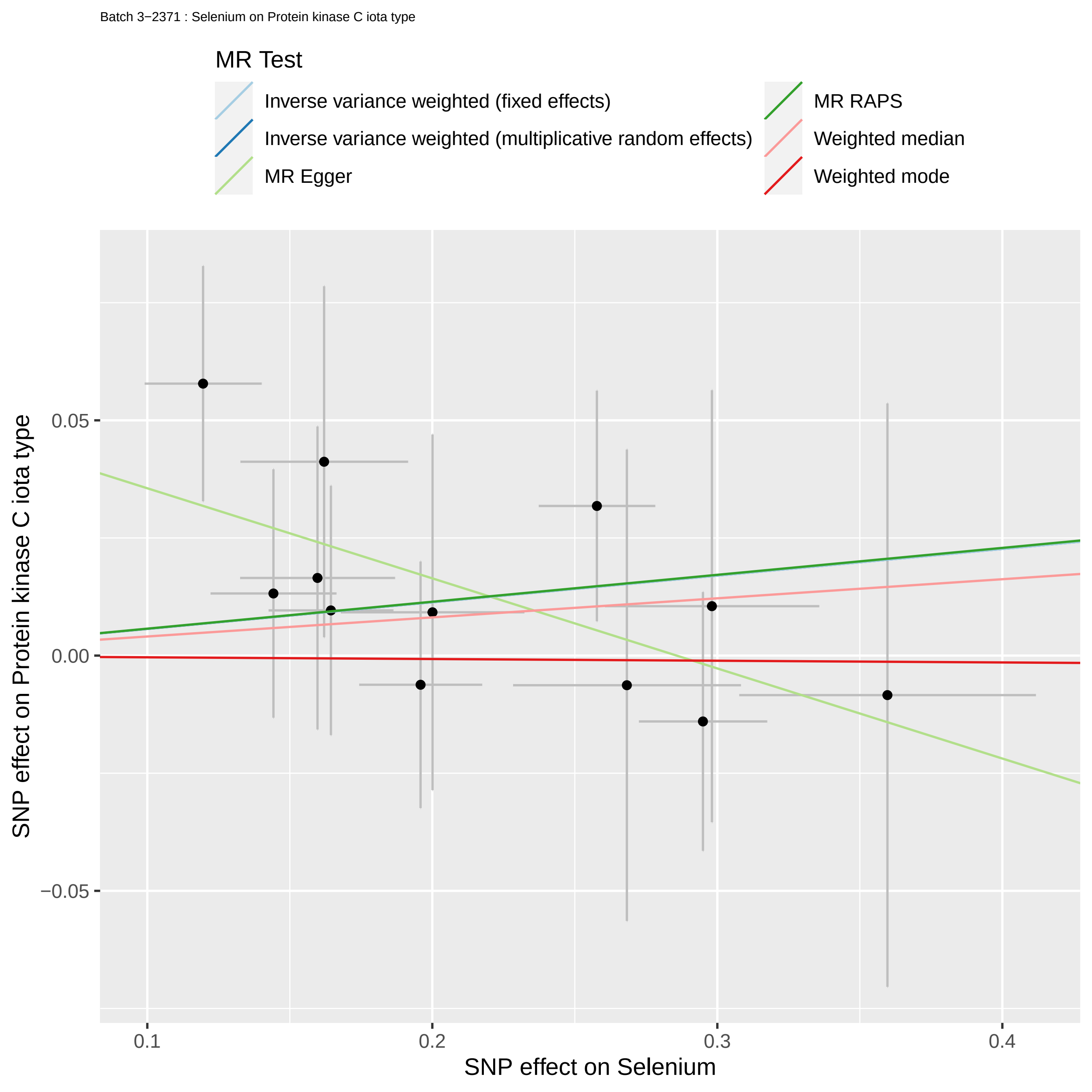
Batch 3−2371 : Selenium on Protein kinase C iota type
MR Test
Inverse variance weighted (fixed effects)
Inverse variance weighted (multiplicative random effects)
MR Egger
MR RAPS
Weighted median
Weighted mode
0.05
0.00
−0.05
0.1
0.2
0.3
0.4
SNP effect on Protein kinase C iota type
SNP effect on Selenium

Batch 3−2372 : Selenium on Glucosidase 2 subunit beta

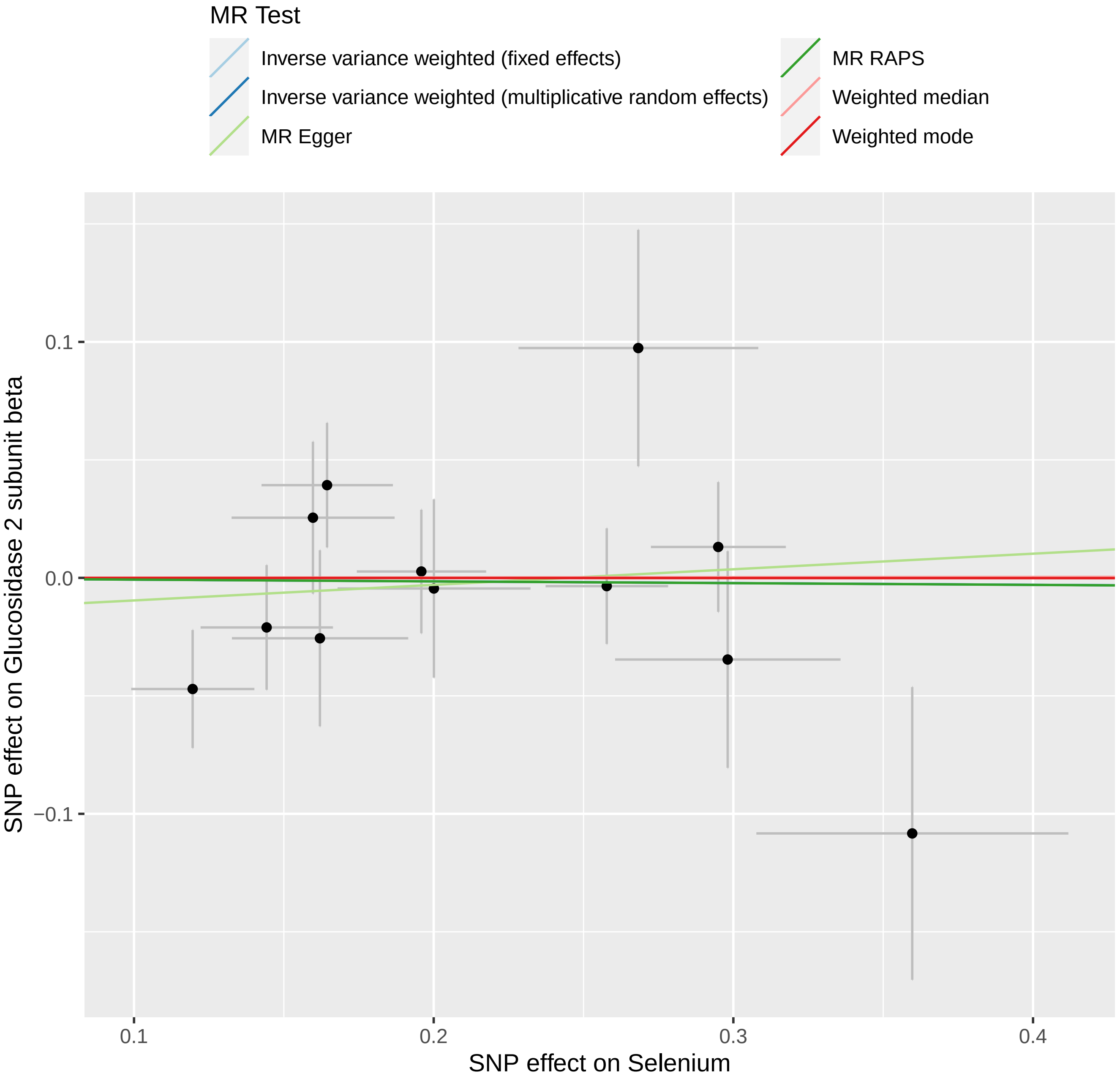

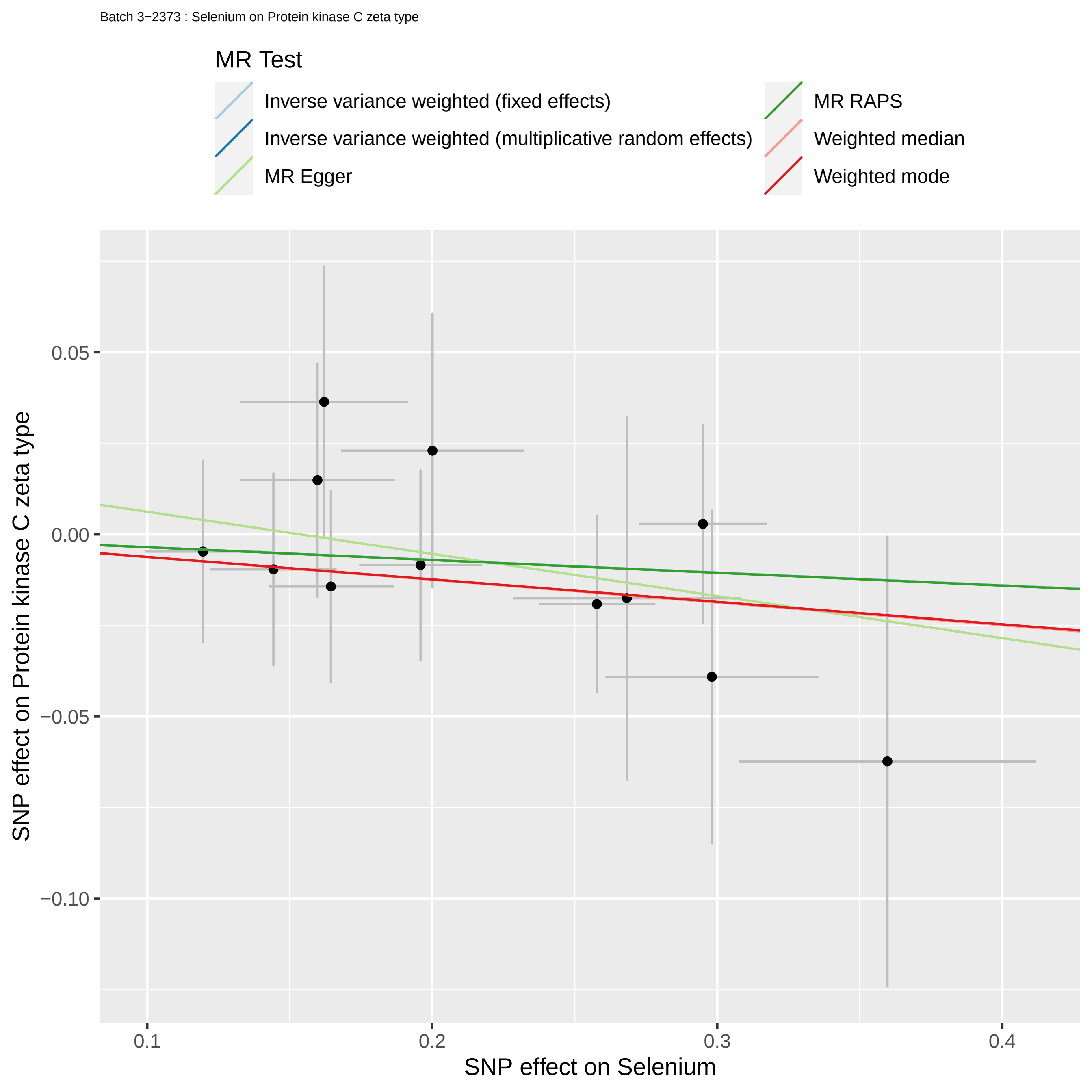
Batch 3−2373 : Selenium on Protein kinase C zeta type
MR Test
Inverse variance weighted (fixed effects)
Inverse variance weighted (multiplicative random effects)
MR Egger
MR RAPS
Weighted median
Weighted mode
0.05
0.00
−0.05
−0.10
0.1
0.2
0.3
0.4
SNP effect on Protein kinase C zeta type
SNP effect on Selenium

Batch 3−2374 : Selenium on cGMP−dependent protein kinase 1, beta isozyme

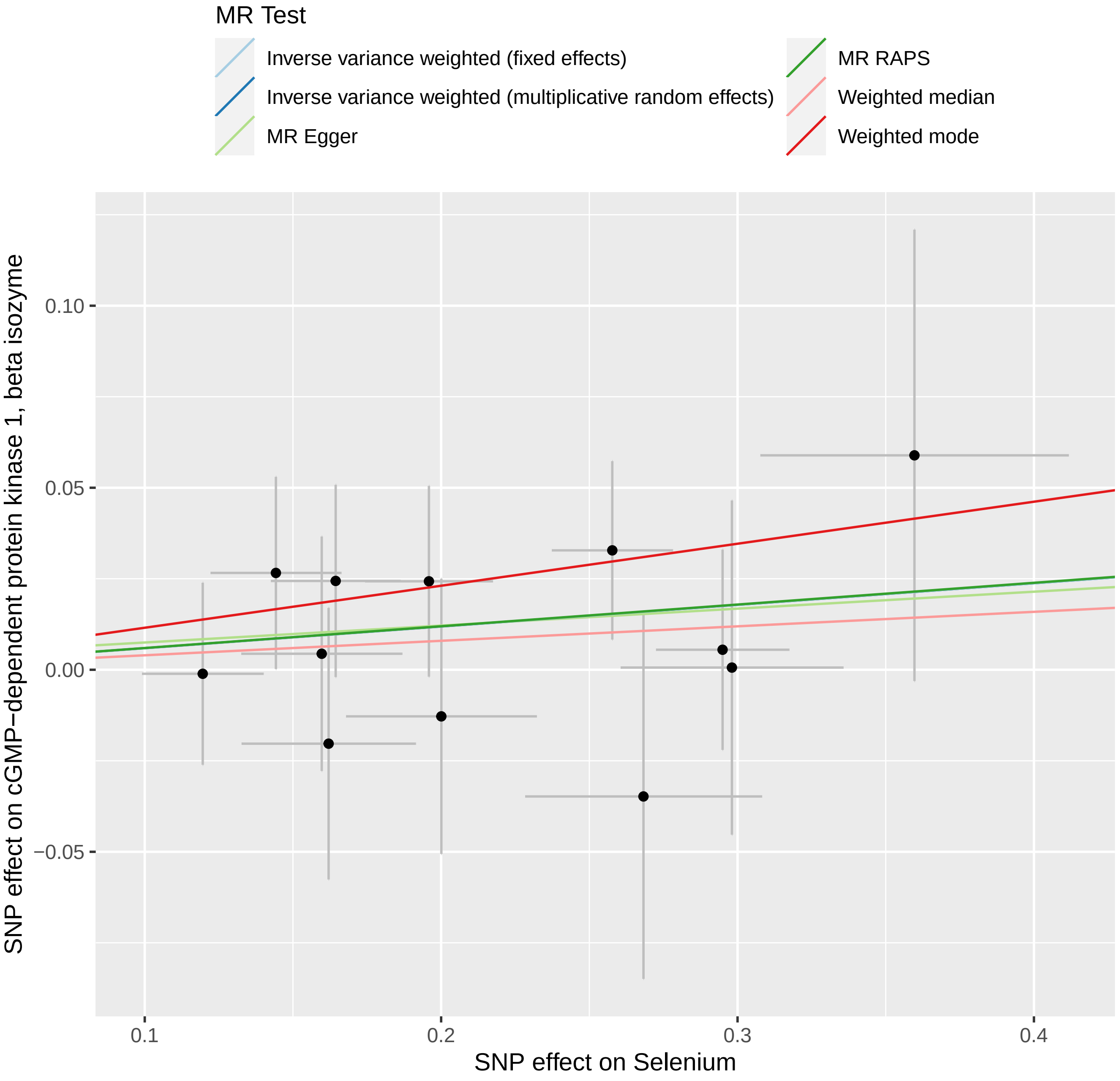

Batch 3−2375 : Selenium on Prolactin

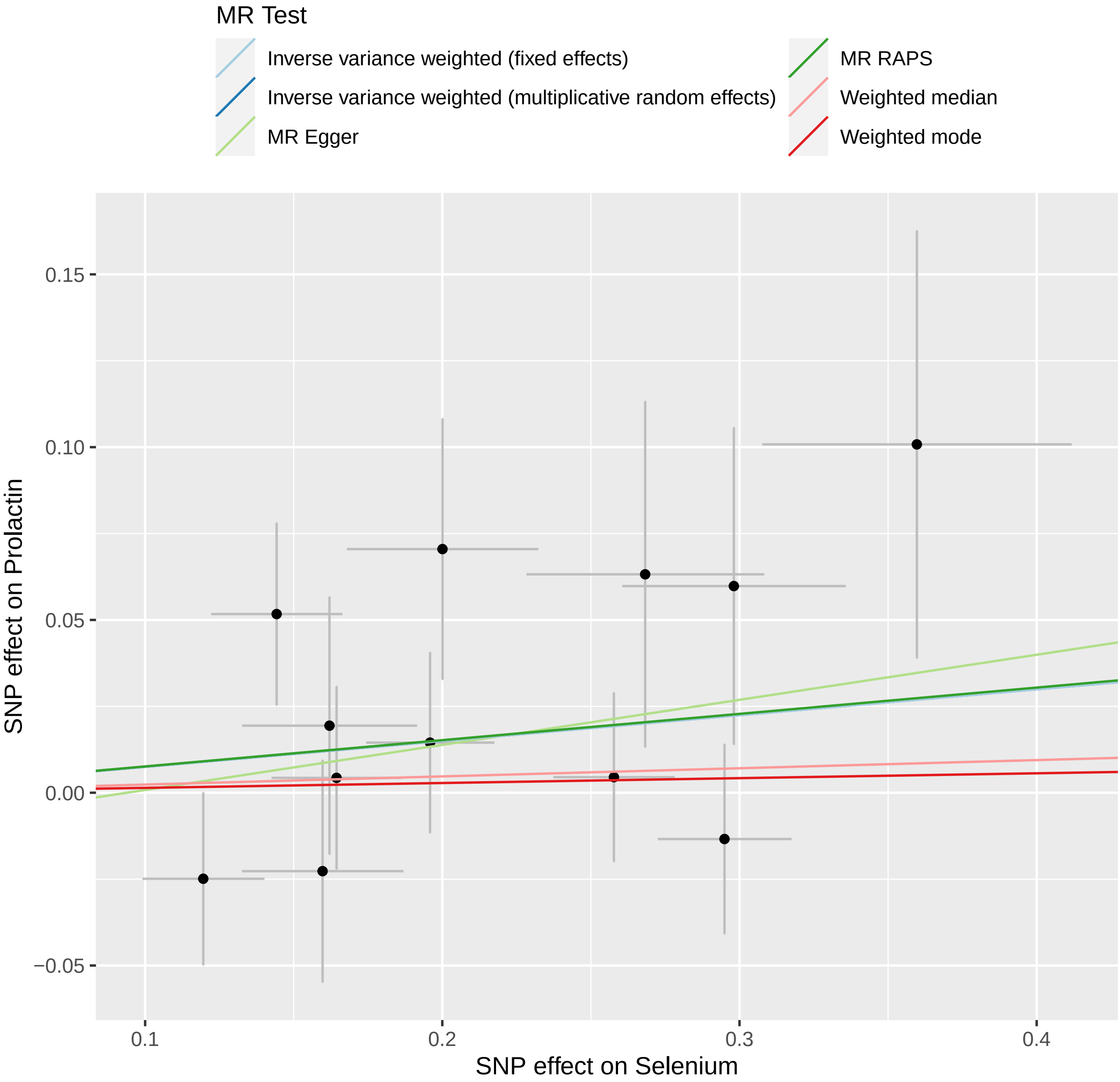

Batch 3−2376 : Selenium on Prolactin−releasing peptide

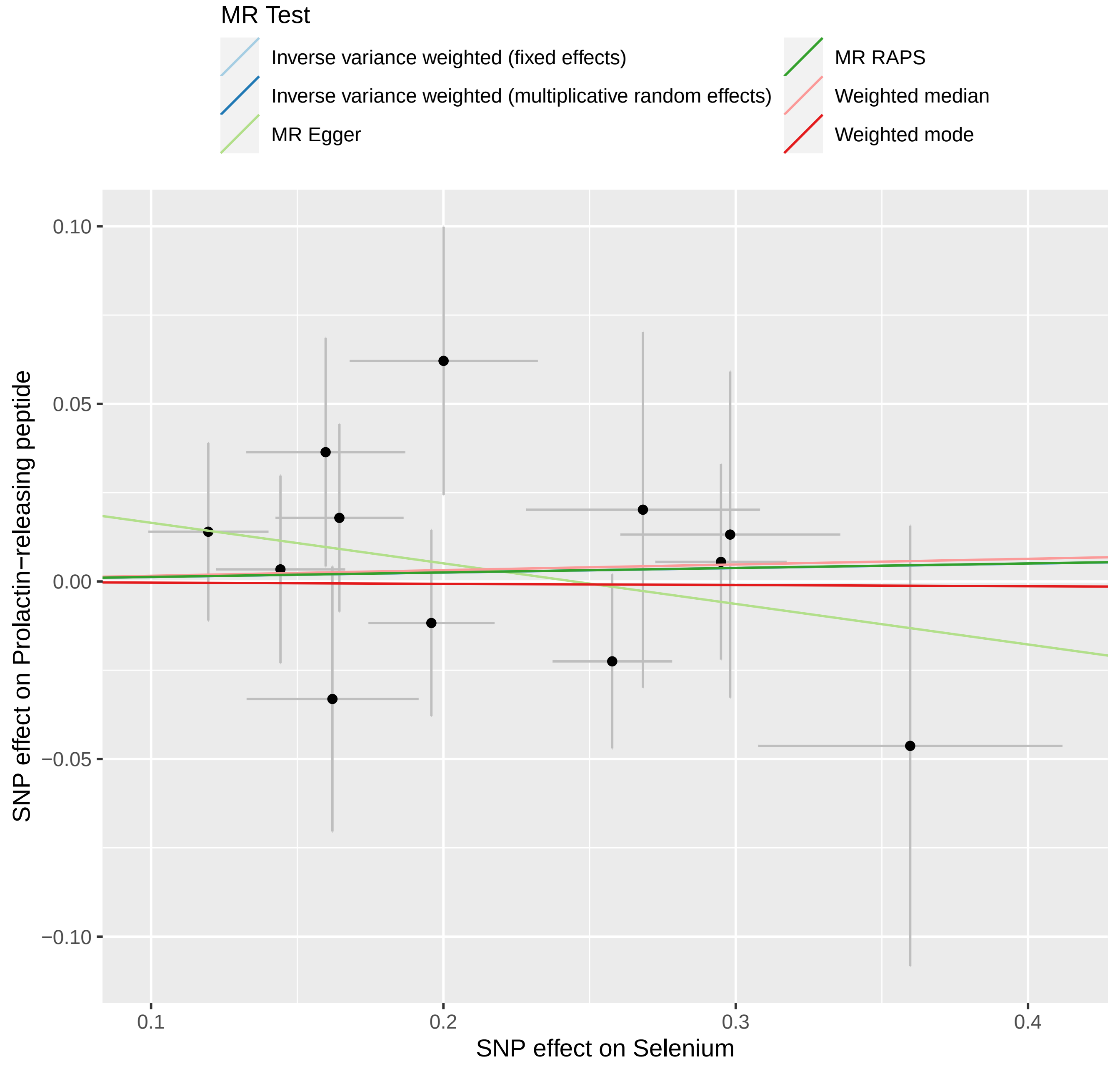

Batch 3−2377 : Selenium on Prolactin receptor

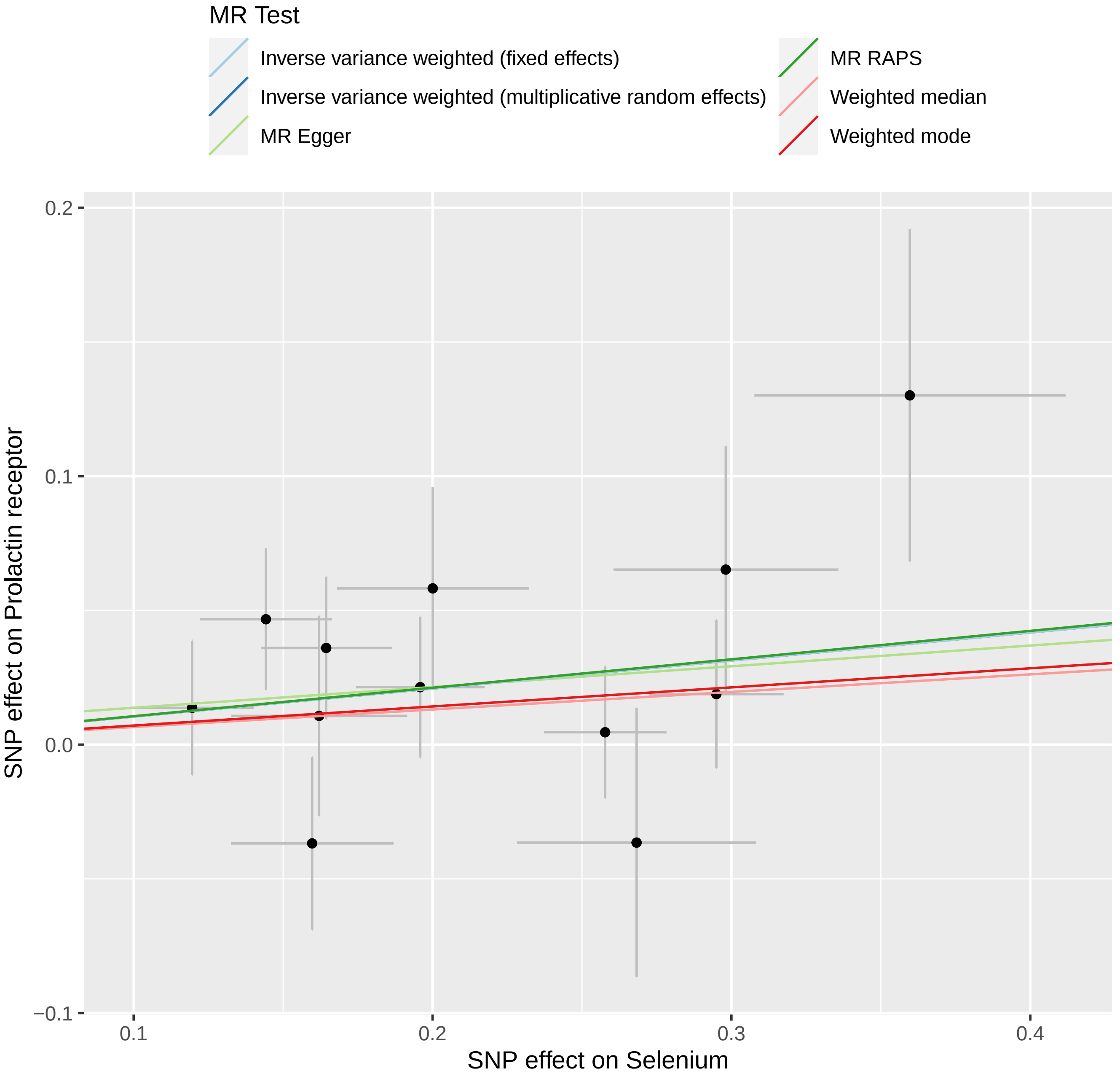

Batch 3−2378 : Selenium on Protein arginine N−methyltransferase 3

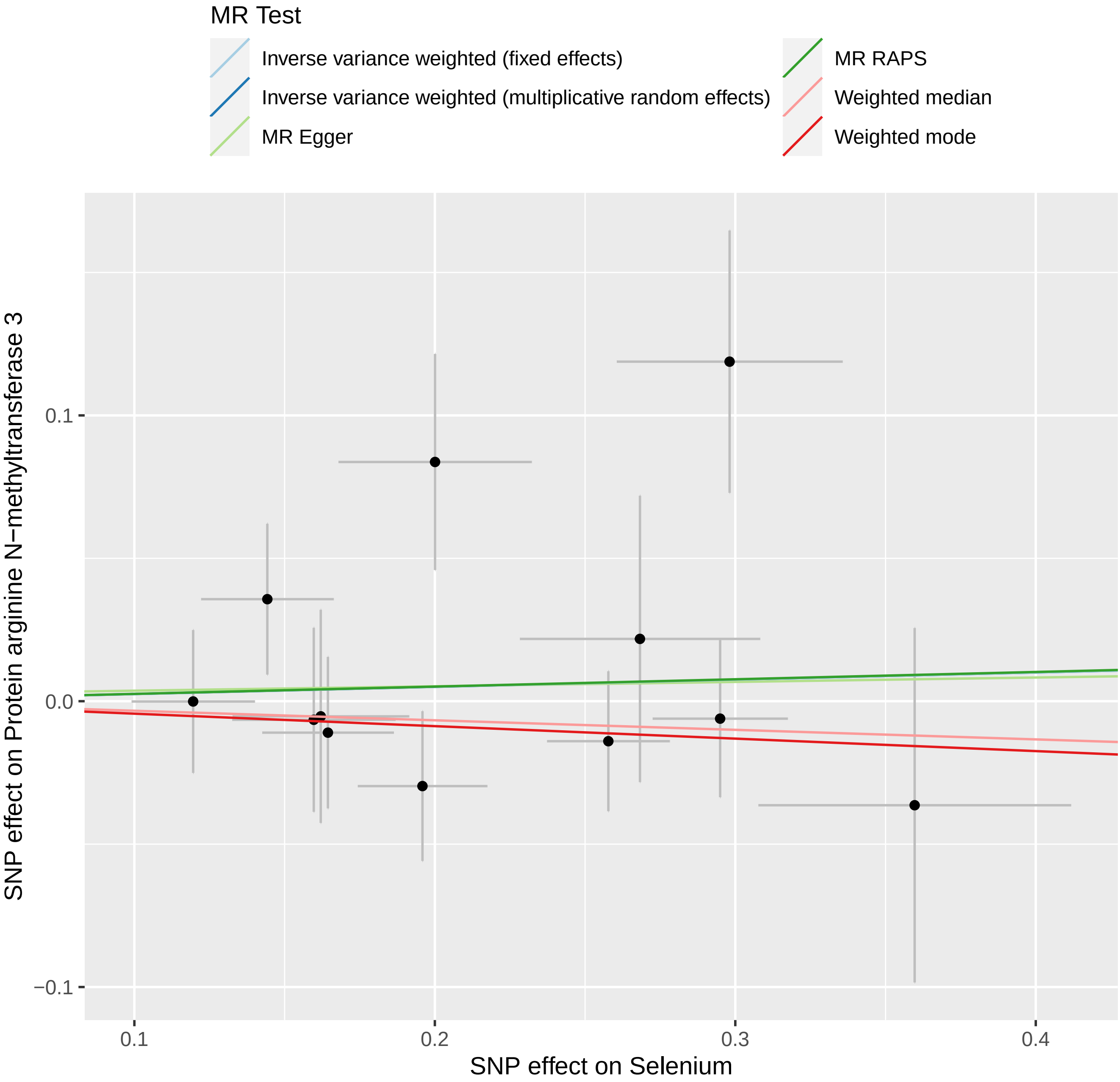

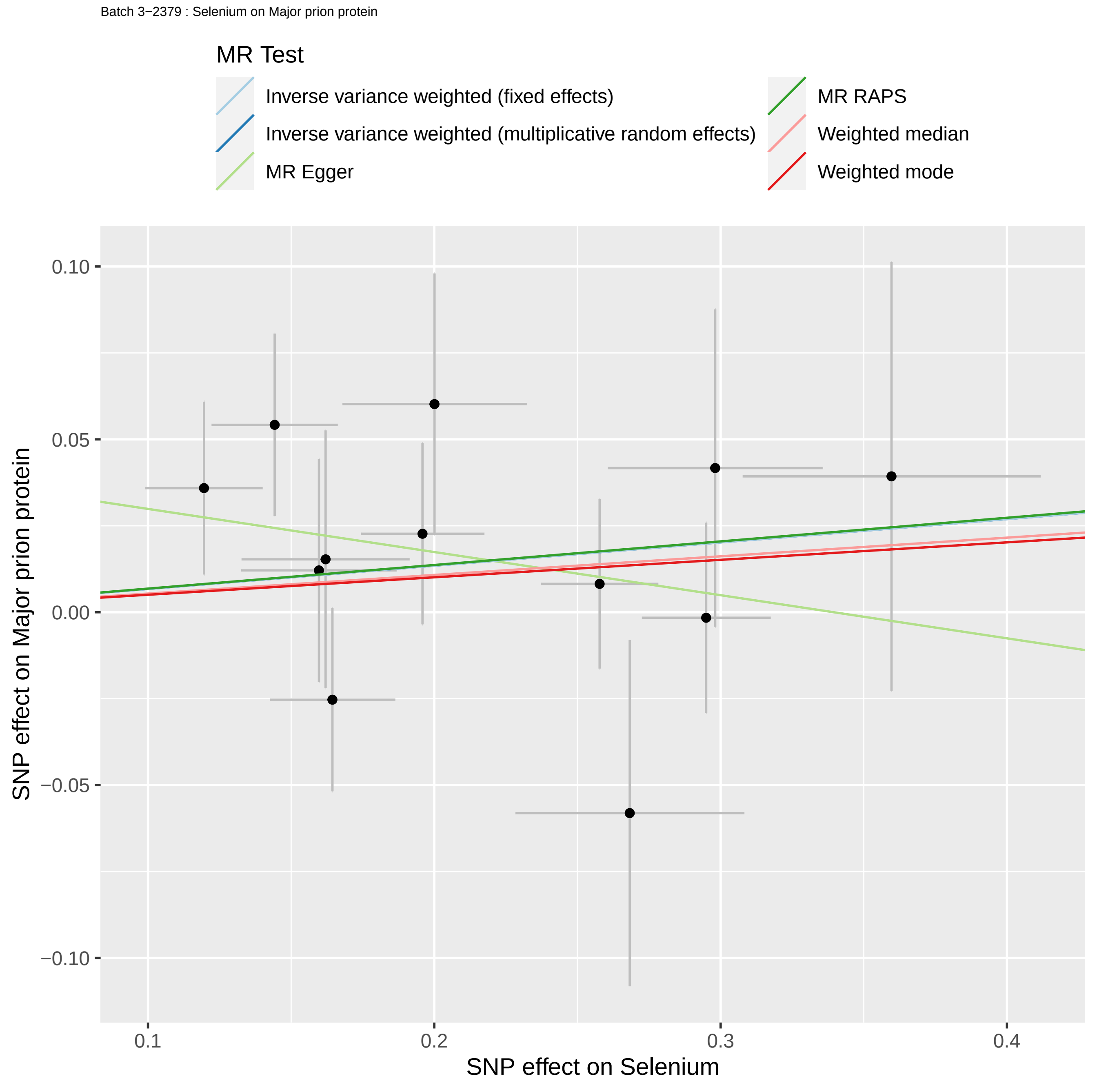

Batch 3−2379 : Selenium on Major prion protein
MR Test
Inverse variance weighted (fixed effects)
Inverse variance weighted (multiplicative random effects)
MR Egger
MR RAPS
Weighted median
Weighted mode
0.10
0.05
0.00
−0.05
−0.10
0.1
0.2
0.3
0.4
SNP effect on Major prion protein
SNP effect on Selenium

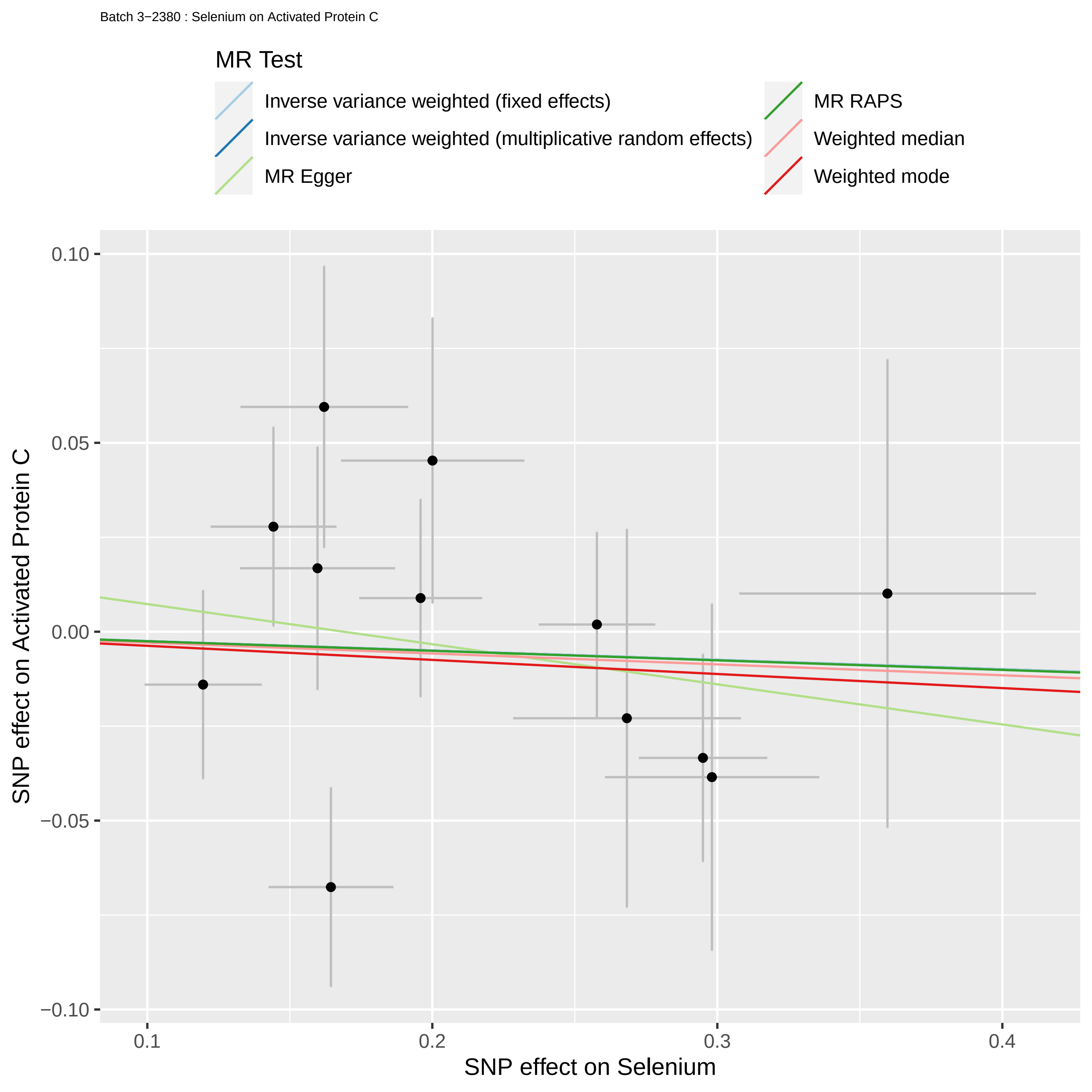

Batch 3−2380 : Selenium on Activated Protein C
MR Test
Inverse variance weighted (fixed effects)
Inverse variance weighted (multiplicative random effects)
MR Egger
MR RAPS
Weighted median
Weighted mode
0.10
0.05
0.00
−0.05
−0.10
0.1
0.2
0.3
0.4
SNP effect on Activated Protein C
SNP effect on Selenium

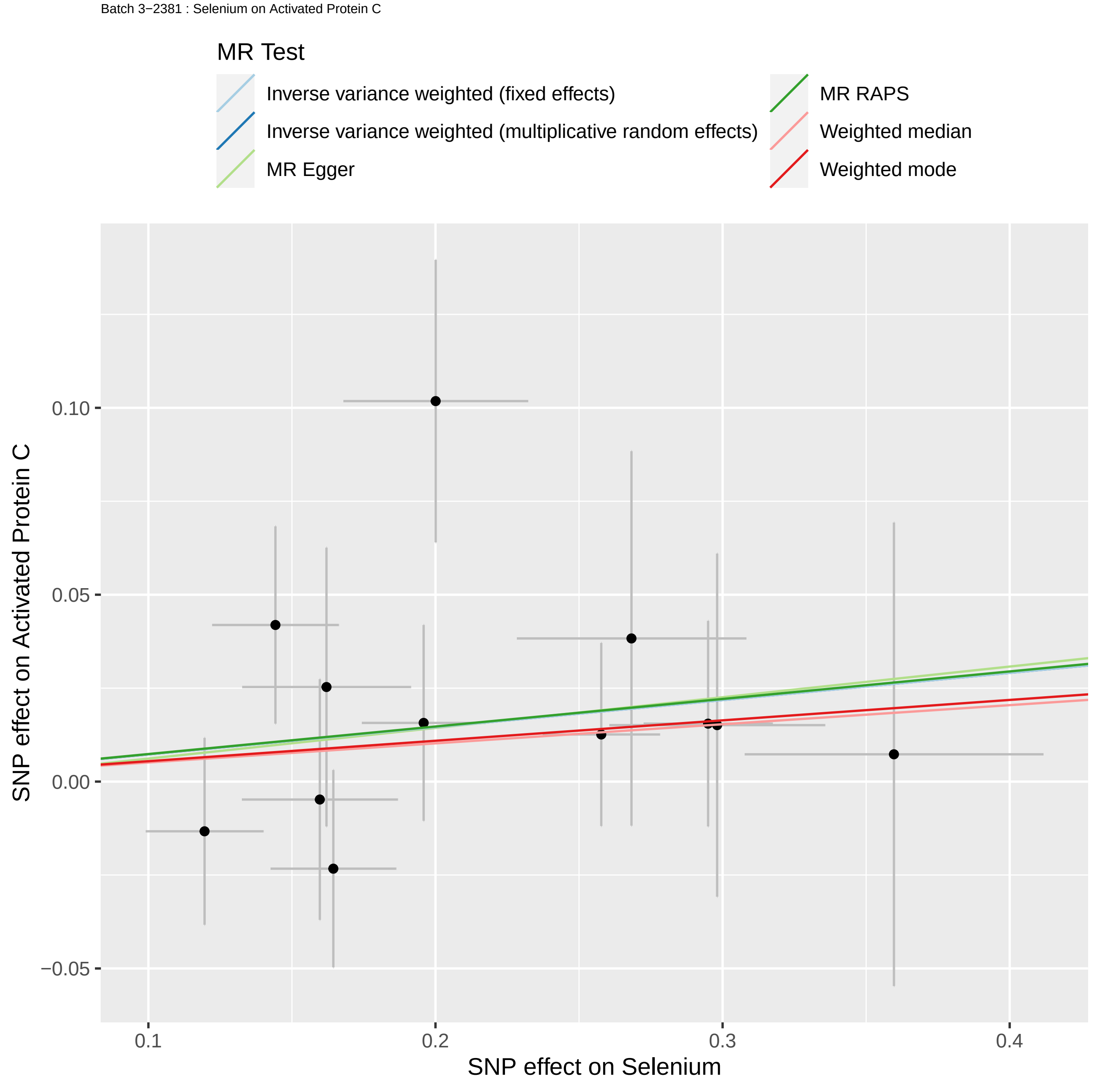
Batch 3−2381 : Selenium on Activated Protein C
MR Test
Inverse variance weighted (fixed effects)
Inverse variance weighted (multiplicative random effects)
MR Egger
MR RAPS
Weighted median
Weighted mode
SNP effect on Activated Protein C
0.10
0.05
0.00
−0.05
0.1
0.2
0.3
0.4
SNP effect on Selenium

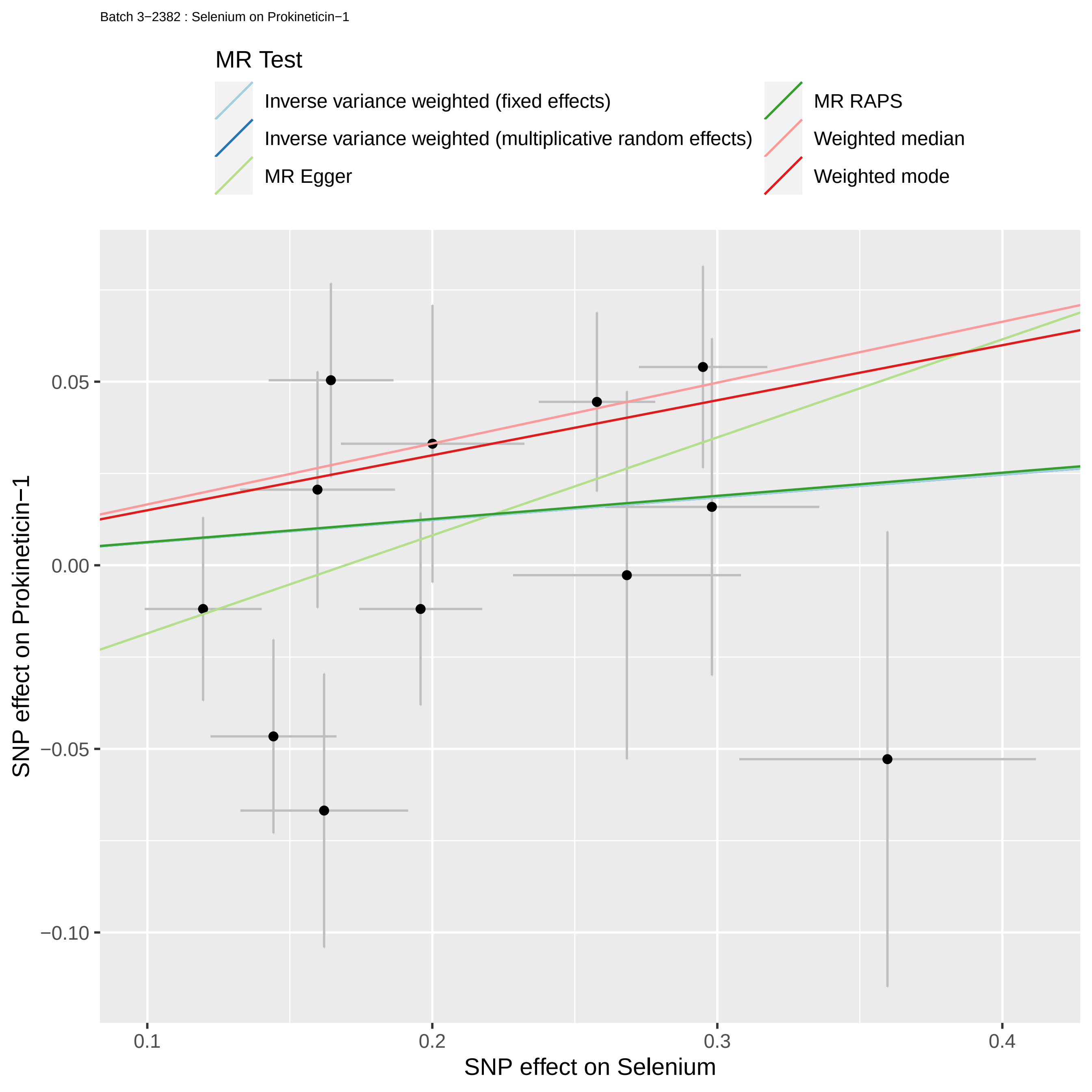
Batch 3−2382 : Selenium on Prokineticin−1
MR Test
Inverse variance weighted (fixed effects)
Inverse variance weighted (multiplicative random effects)
MR Egger
MR RAPS
Weighted median
Weighted mode
0.05
0.00
−0.05
−0.10
0.1
0.2
0.3
0.4
SNP effect on Prokineticin−1
SNP effect on Selenium

Batch 3−2383 : Selenium on Proline−rich protein 1

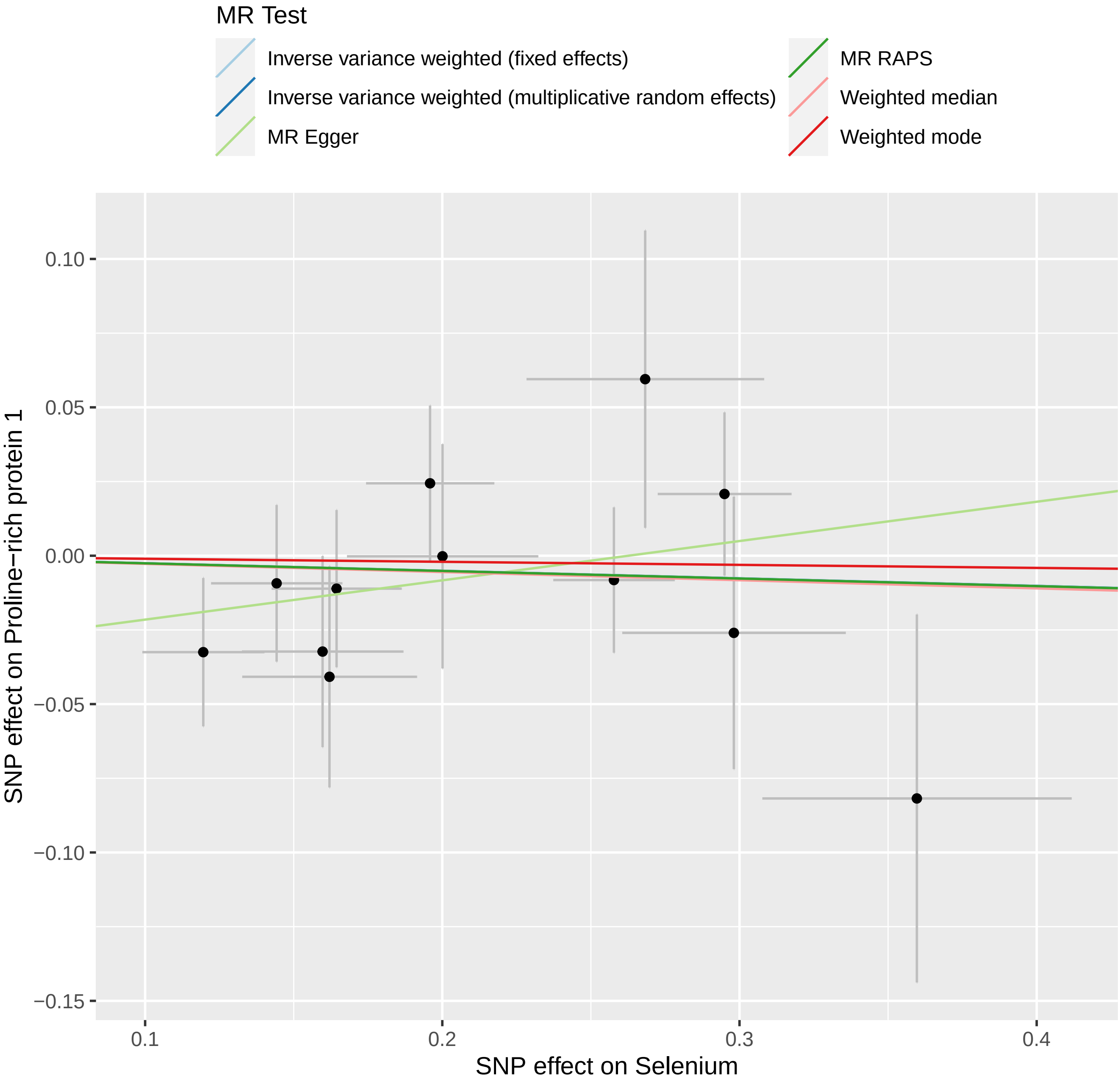

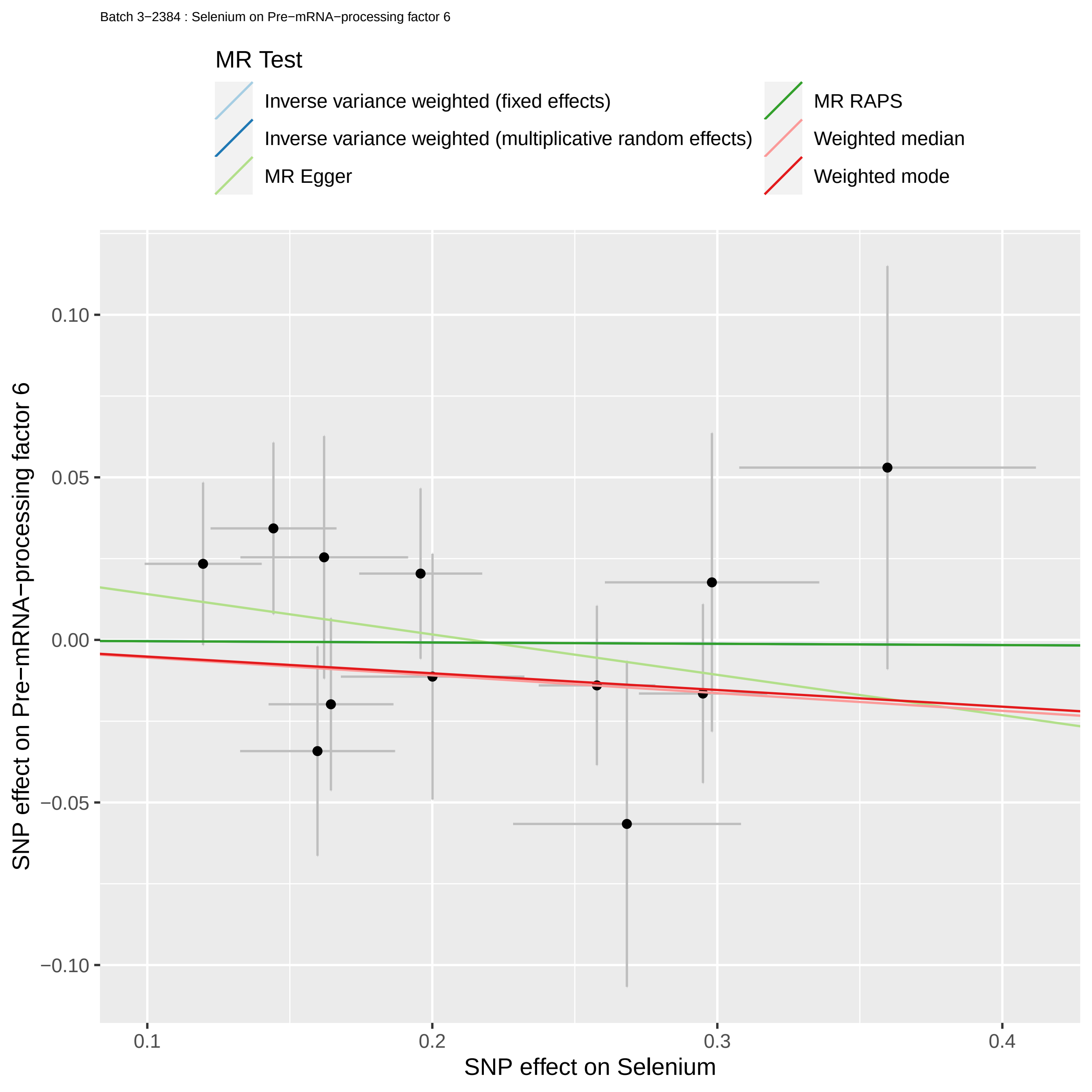

Batch 3−2384 : Selenium on Pre−mRNA−processing factor 6
MR Test
Inverse variance weighted (fixed effects)
Inverse variance weighted (multiplicative random effects)
MR Egger
MR RAPS
Weighted median
Weighted mode
SNP effect on Pre−mRNA−processing factor 6
0.10
0.05
0.00
−0.05
−0.10
0.1
0.2
0.3
0.4
SNP effect on Selenium

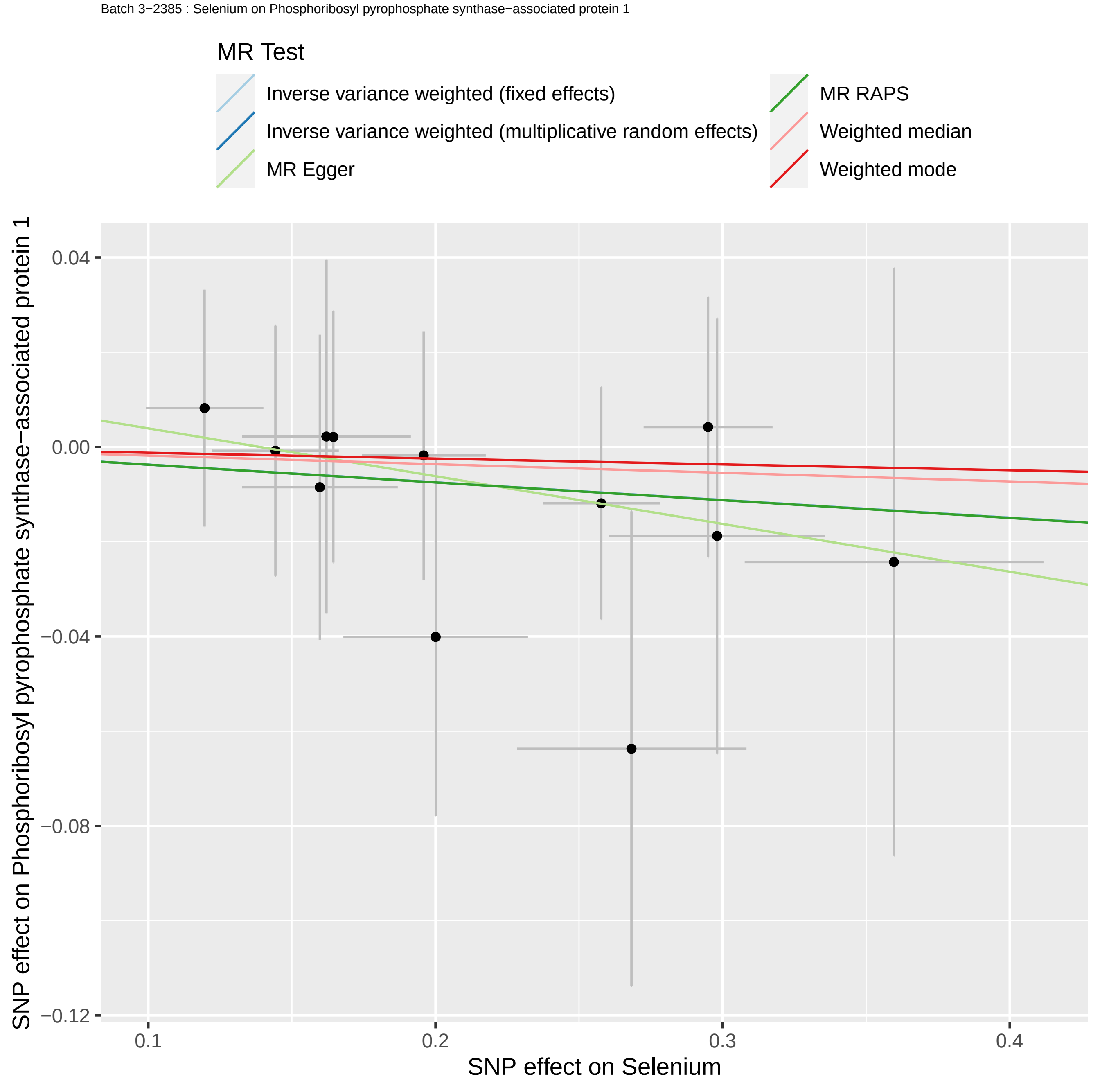
Batch 3−2385 : Selenium on Phosphoribosyl pyrophosphate synthase−associated protein 1
MR Test
Inverse variance weighted (fixed effects)
Inverse variance weighted (multiplicative random effects)
MR Egger
MR RAPS
Weighted median
Weighted mode
SNP effect on Phosphoribosyl pyrophosphate synthase−associated protein 1
0.04
0.00
−0.04
−0.08
−0.12
0.1
0.2
0.3
0.4
SNP effect on Selenium

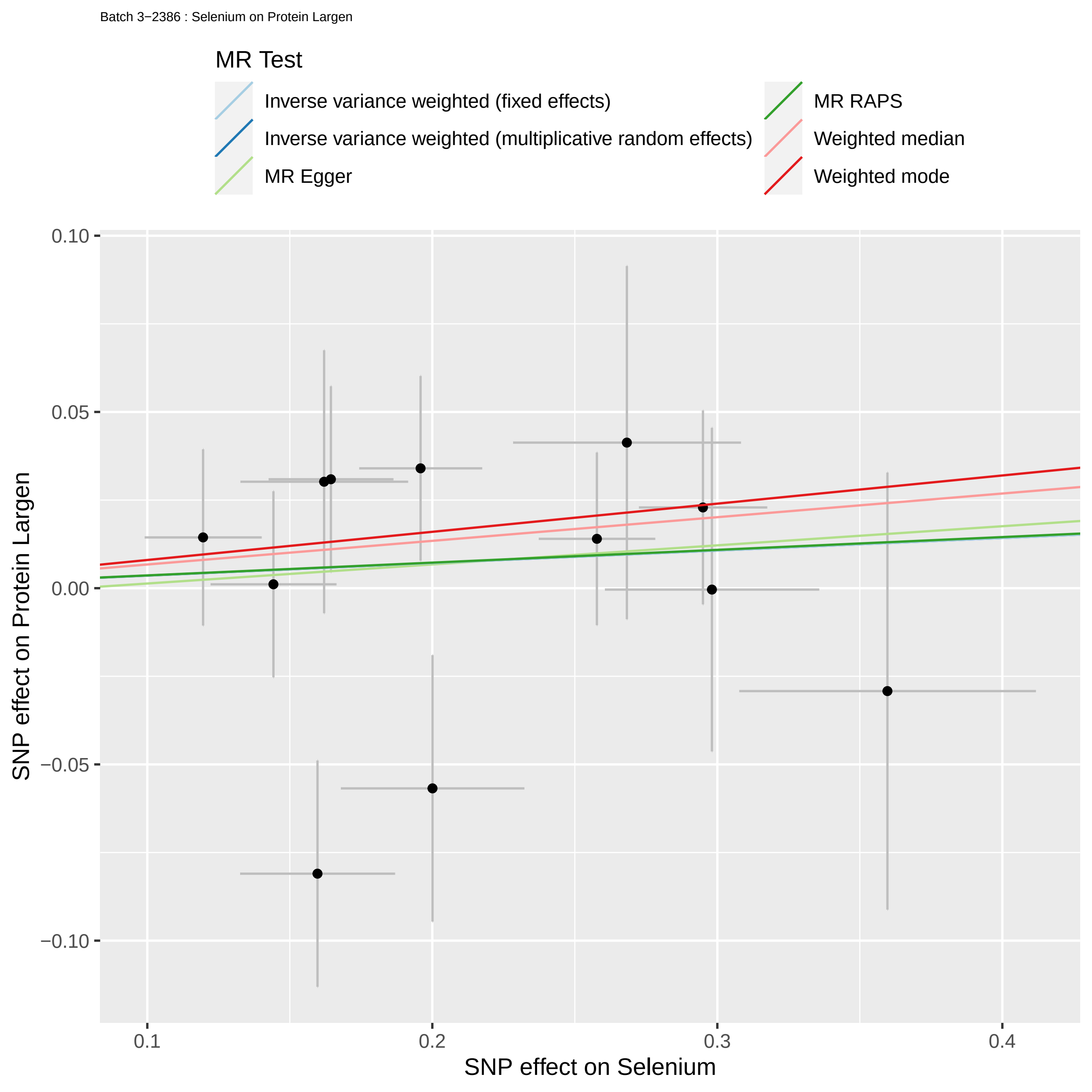

Batch 3−2386 : Selenium on Protein Largen
MR Test
Inverse variance weighted (fixed effects)
Inverse variance weighted (multiplicative random effects)
MR Egger
MR RAPS
Weighted median
Weighted mode
0.10
0.05
0.00
−0.05
−0.10
SNP effect on Protein Largen
0.1
0.2
0.3
0.4
SNP effect on Selenium

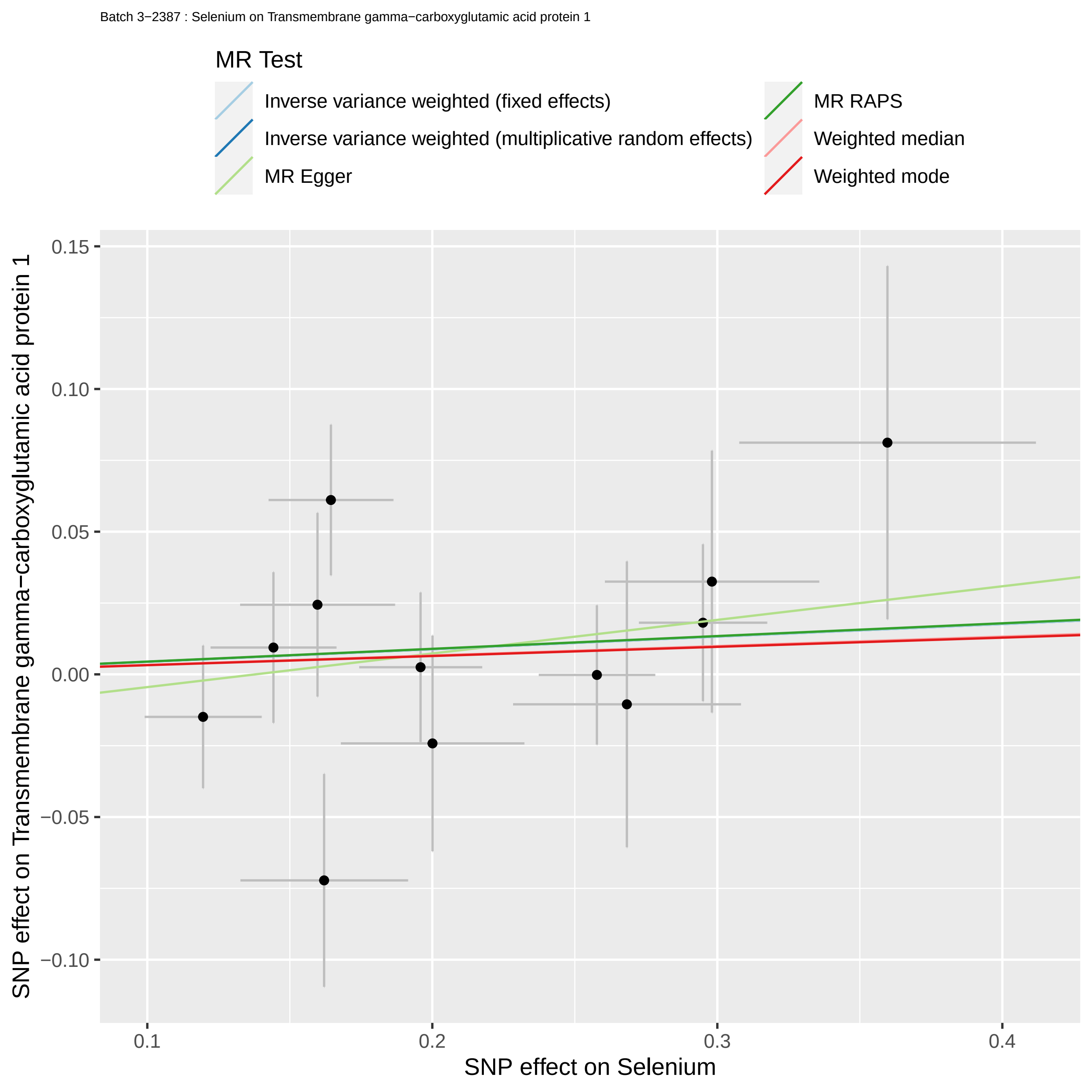
Batch 3−2387 : Selenium on Transmembrane gamma−carboxyglutamic acid protein 1
MR Test
Inverse variance weighted (fixed effects)
Inverse variance weighted (multiplicative random effects)
MR Egger
MR RAPS
Weighted median
Weighted mode
SNP effect on Transmembrane gamma−carboxyglutamic acid protein 1
0.15
0.10
0.05
0.00
−0.05
−0.10
0.1
0.2
0.3
0.4
SNP effect on Selenium

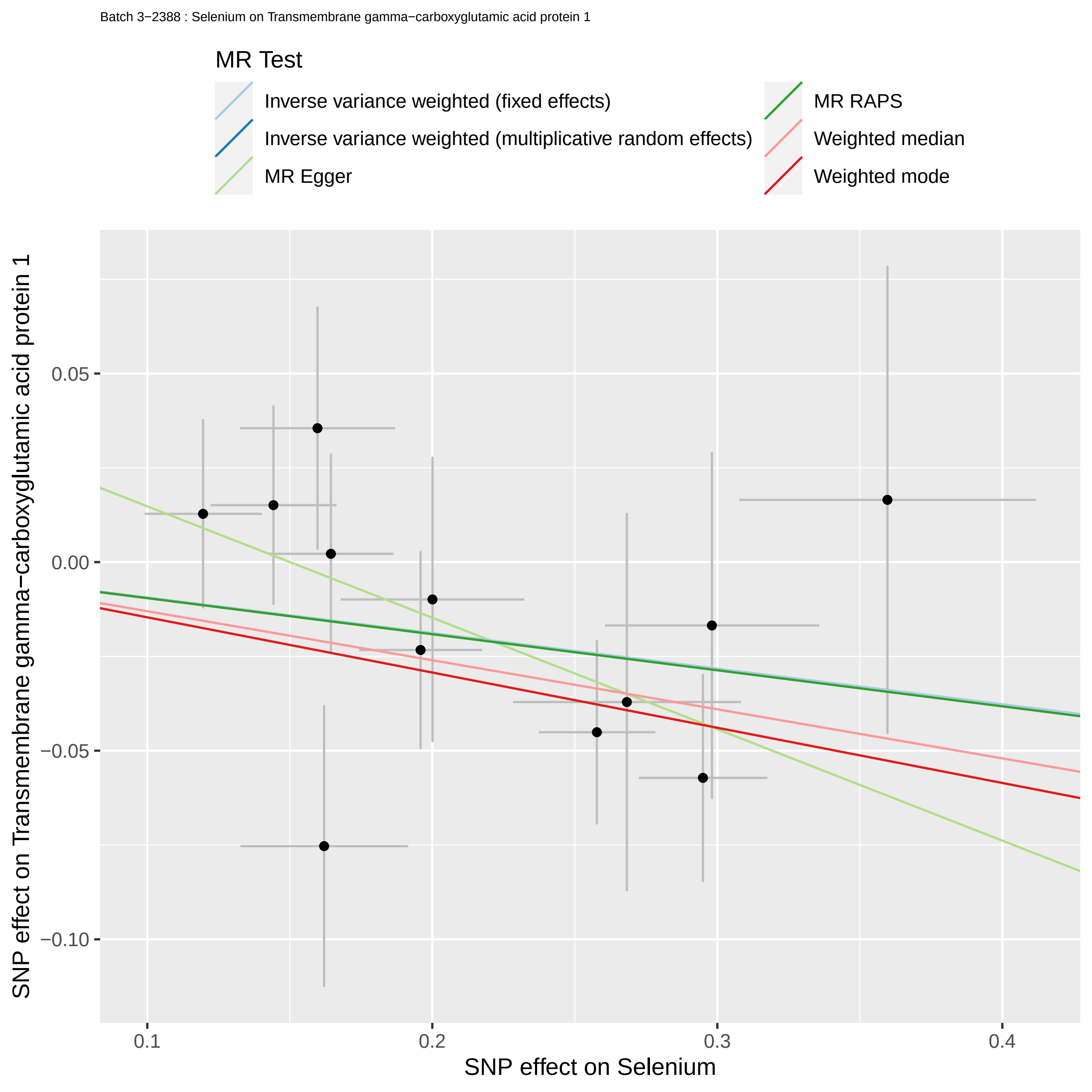
Batch 3−2388 : Selenium on Transmembrane gamma−carboxyglutamic acid protein 1
MR Test
Inverse variance weighted (fixed effects)
Inverse variance weighted (multiplicative random effects)
MR Egger
MR RAPS
Weighted median
Weighted mode
SNP effect on Transmembrane gamma−carboxyglutamic acid protein 1
0.05
0.00
−0.05
−0.10
0.1
0.2
0.3
0.4
SNP effect on Selenium

Batch 3−2389 : Selenium on Transmembrane gamma−carboxyglutamic acid protein 4

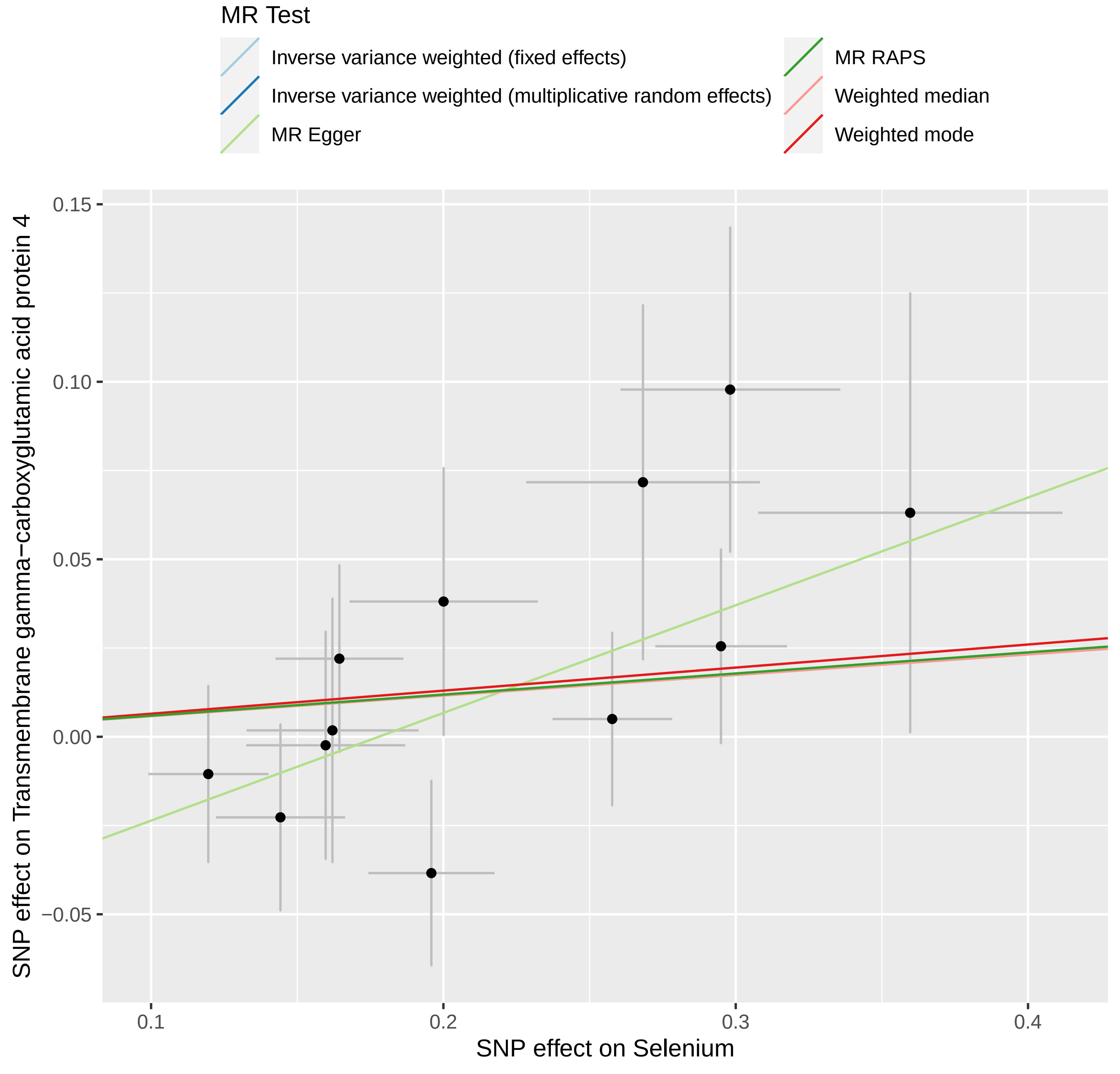

Batch 3−2390 : Selenium on Trypsin−1

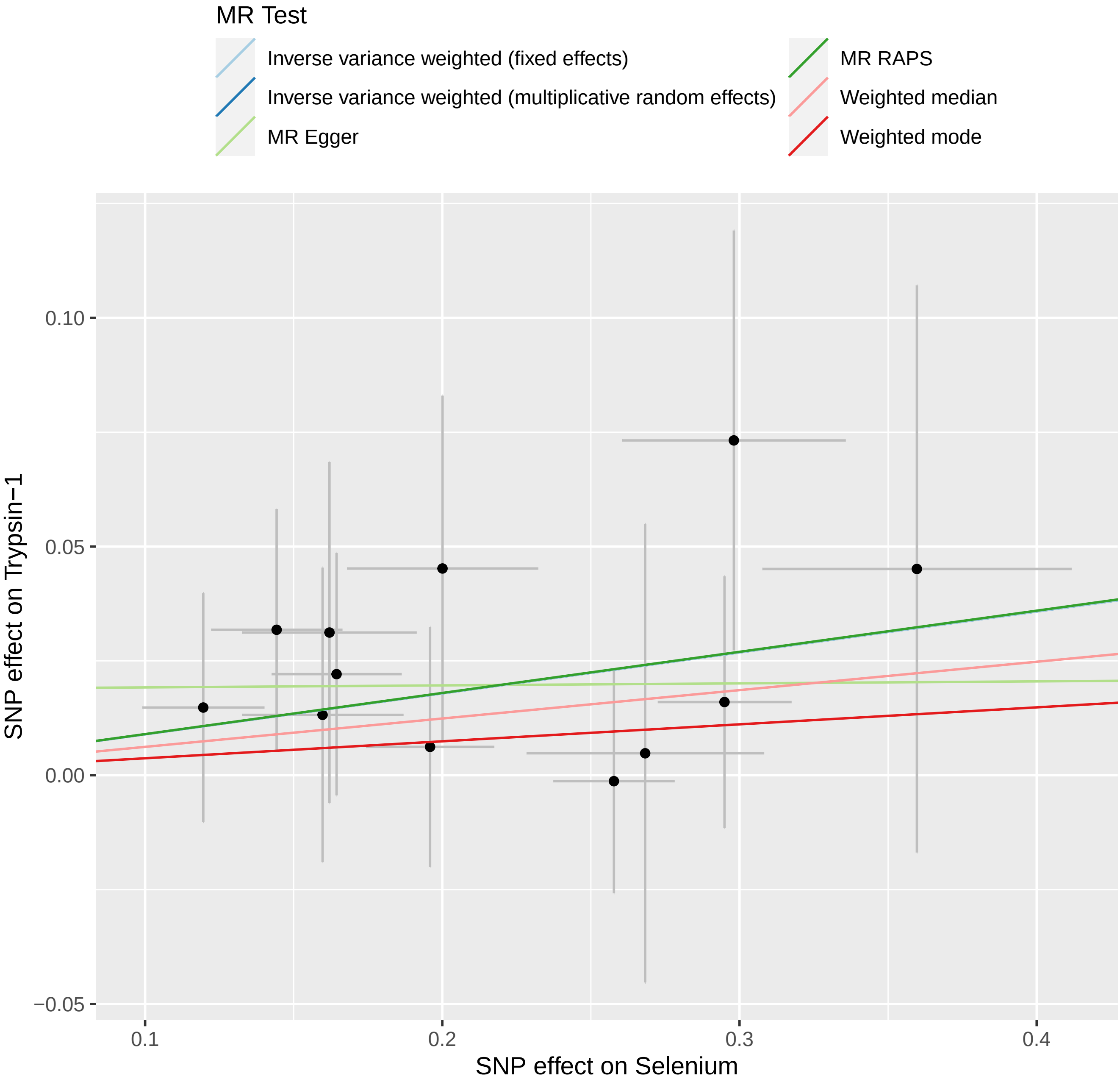

Batch 3−2391 : Selenium on Brain−specific serine protease 4

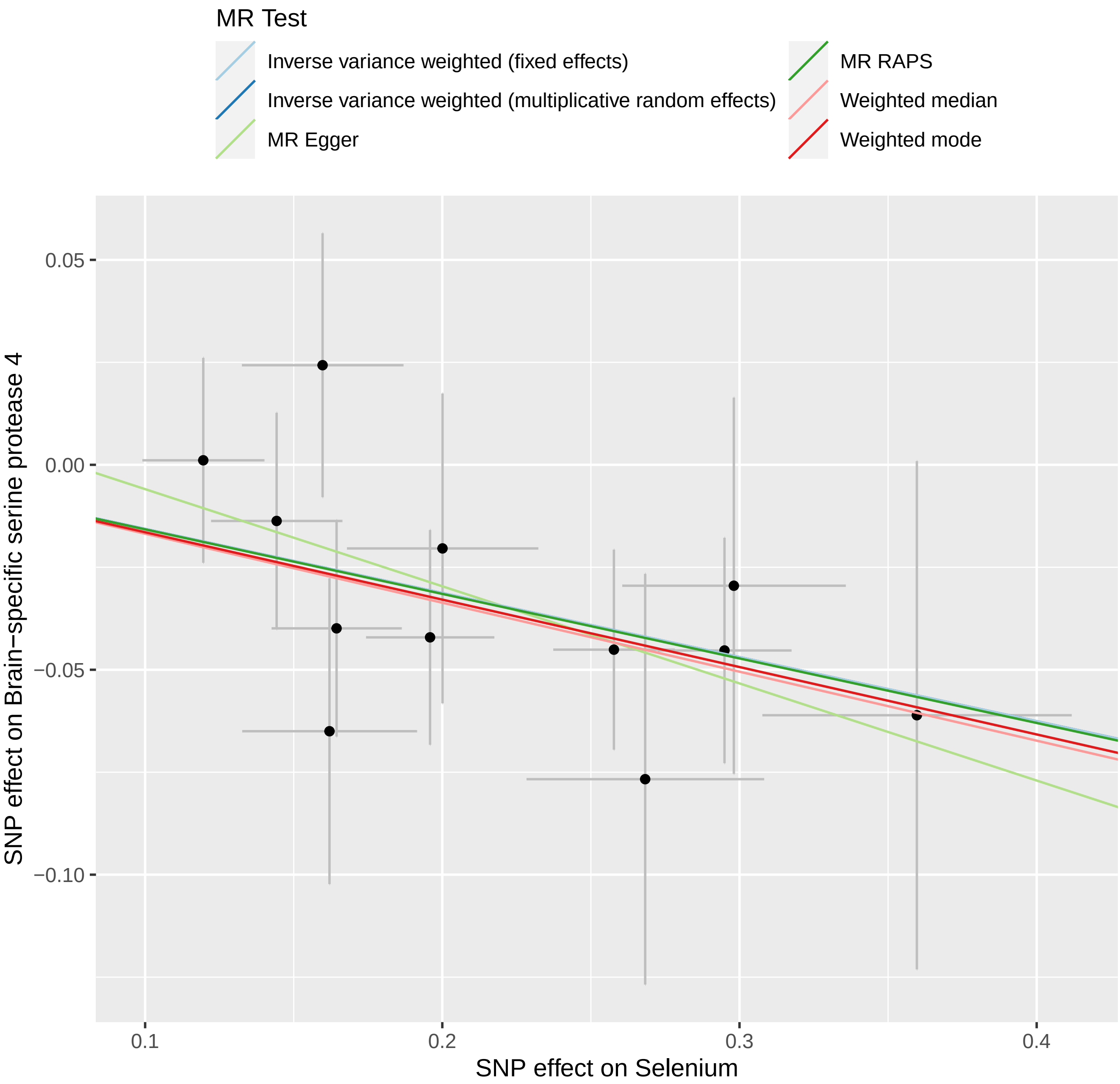

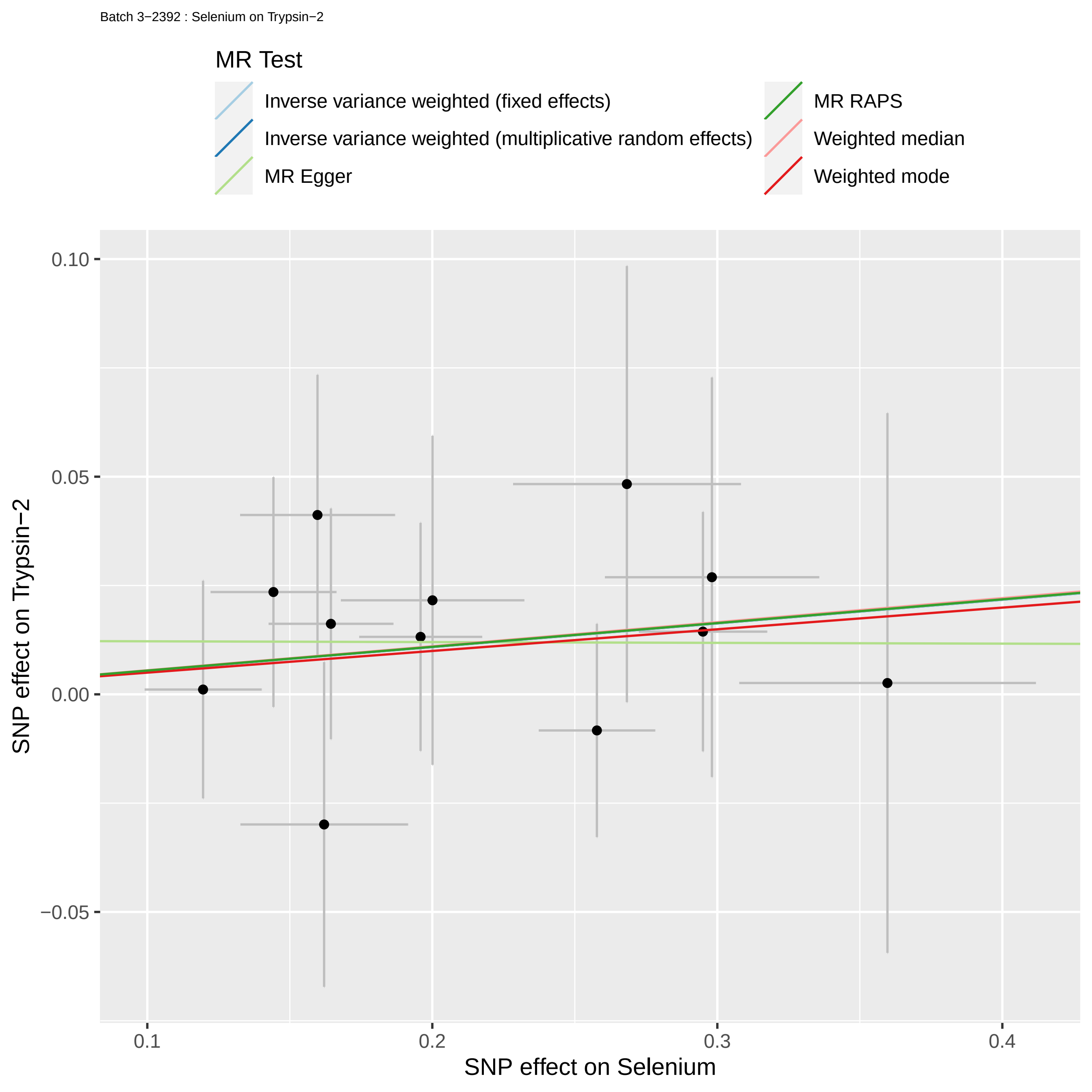
Batch 3−2392 : Selenium on Trypsin−2
MR Test
Inverse variance weighted (fixed effects)
Inverse variance weighted (multiplicative random effects)
MR Egger
MR RAPS
Weighted median
Weighted mode
0.10
0.05
0.00
−0.05
0.1
0.2
0.3
0.4
SNP effect on Selenium
SNP effect on Trypsin−2

Batch 3−2393 : Selenium on Trypsin−3

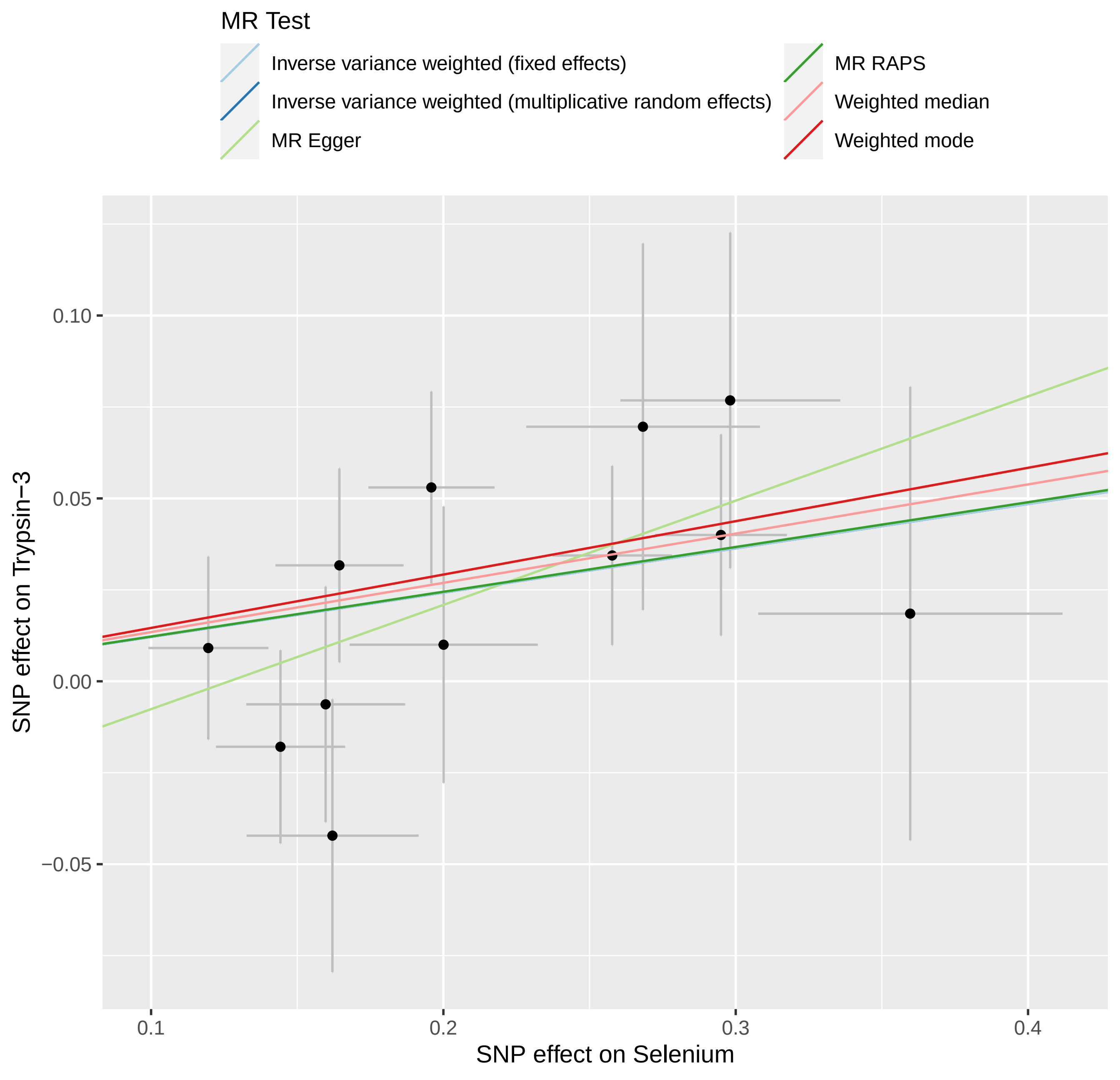

Batch 3−2394 : Selenium on Probable inactive serine protease 37

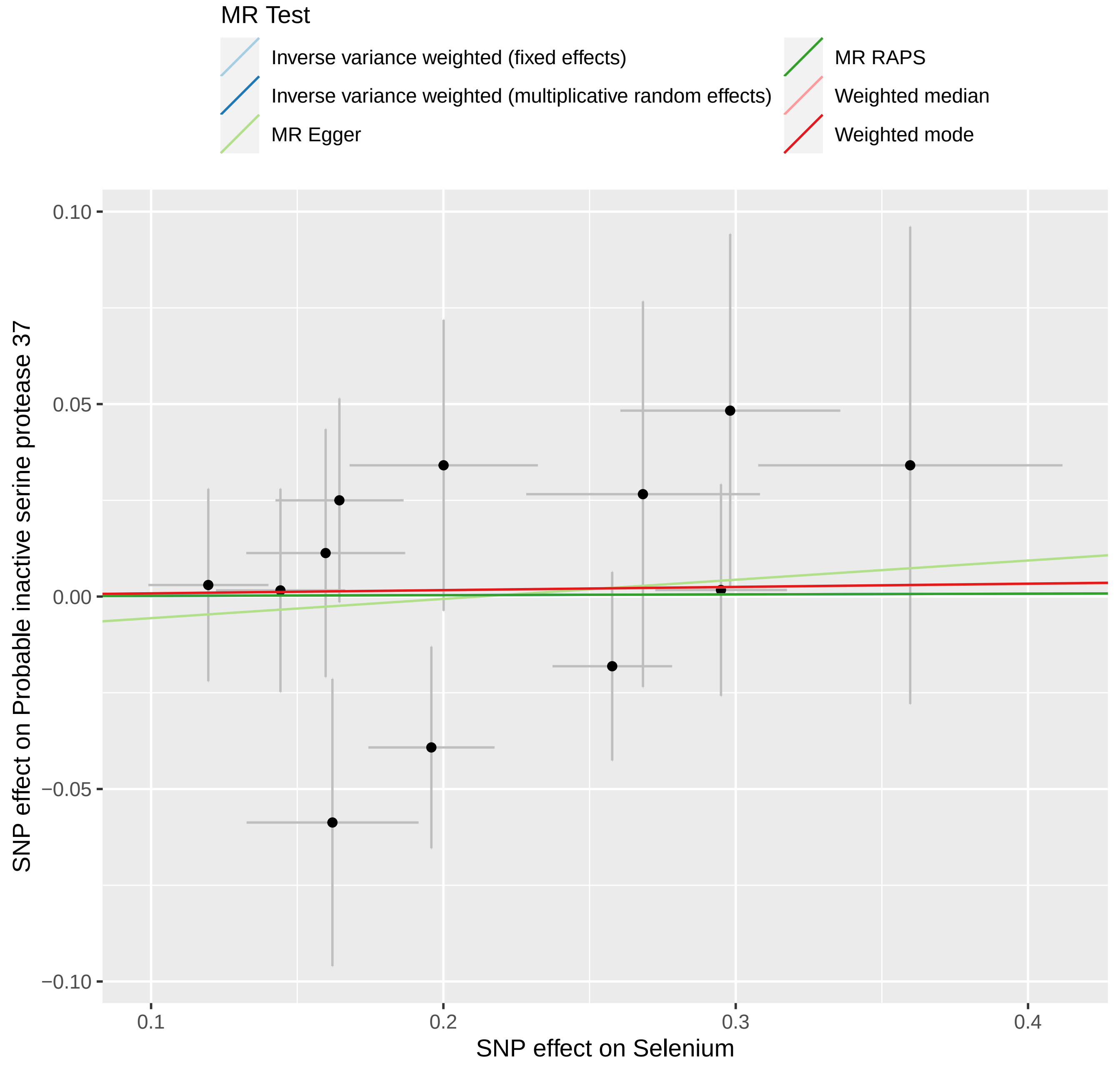

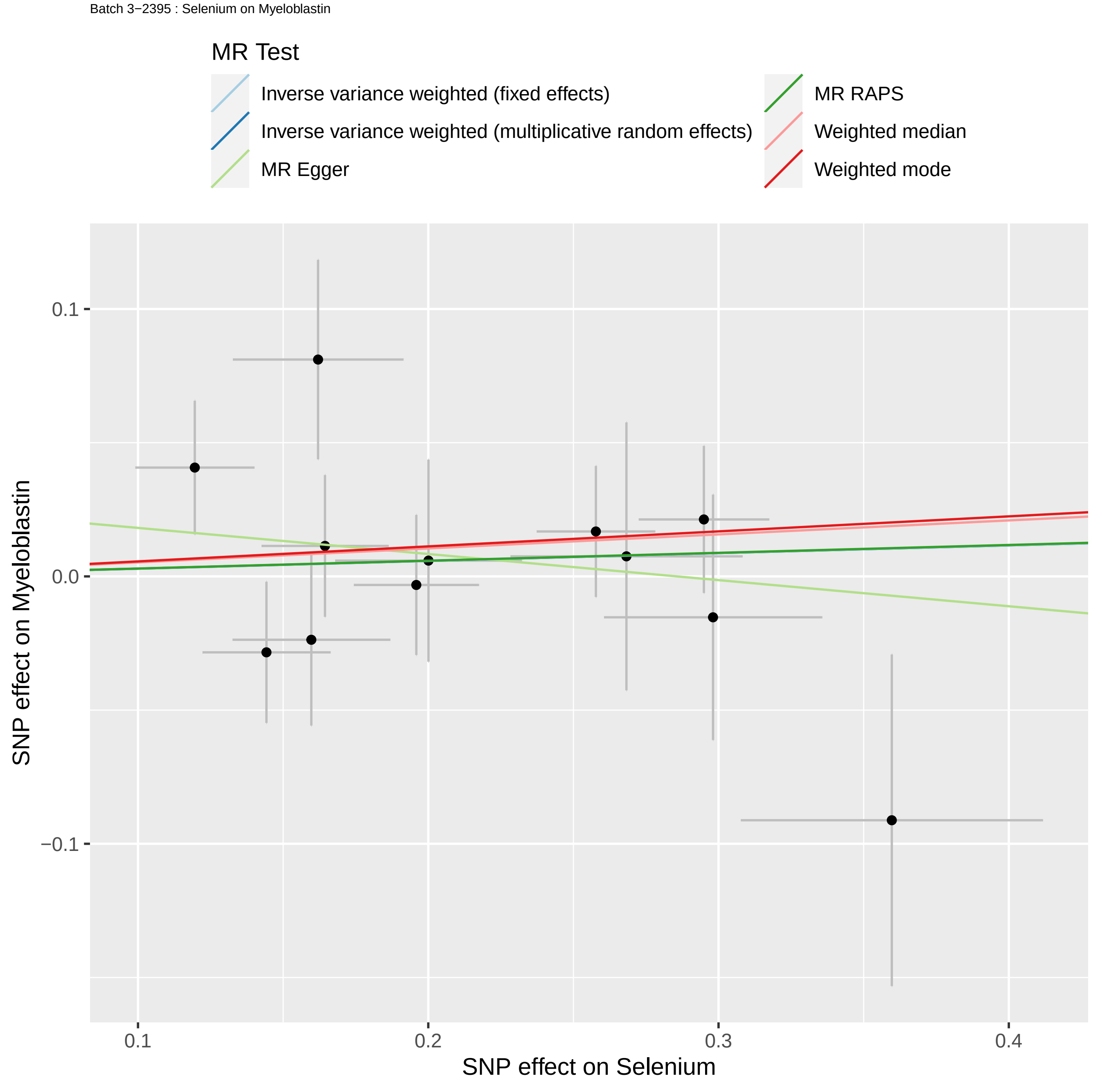
Batch 3−2395 : Selenium on Myeloblastin
MR Test
Inverse variance weighted (fixed effects)
Inverse variance weighted (multiplicative random effects)
MR Egger
MR RAPS
Weighted median
Weighted mode
0.1
0.0
−0.1
0.1
0.2
0.3
0.4
SNP effect on Myeloblastin
SNP effect on Selenium

Batch 3−2396 : Selenium on Myeloblastin

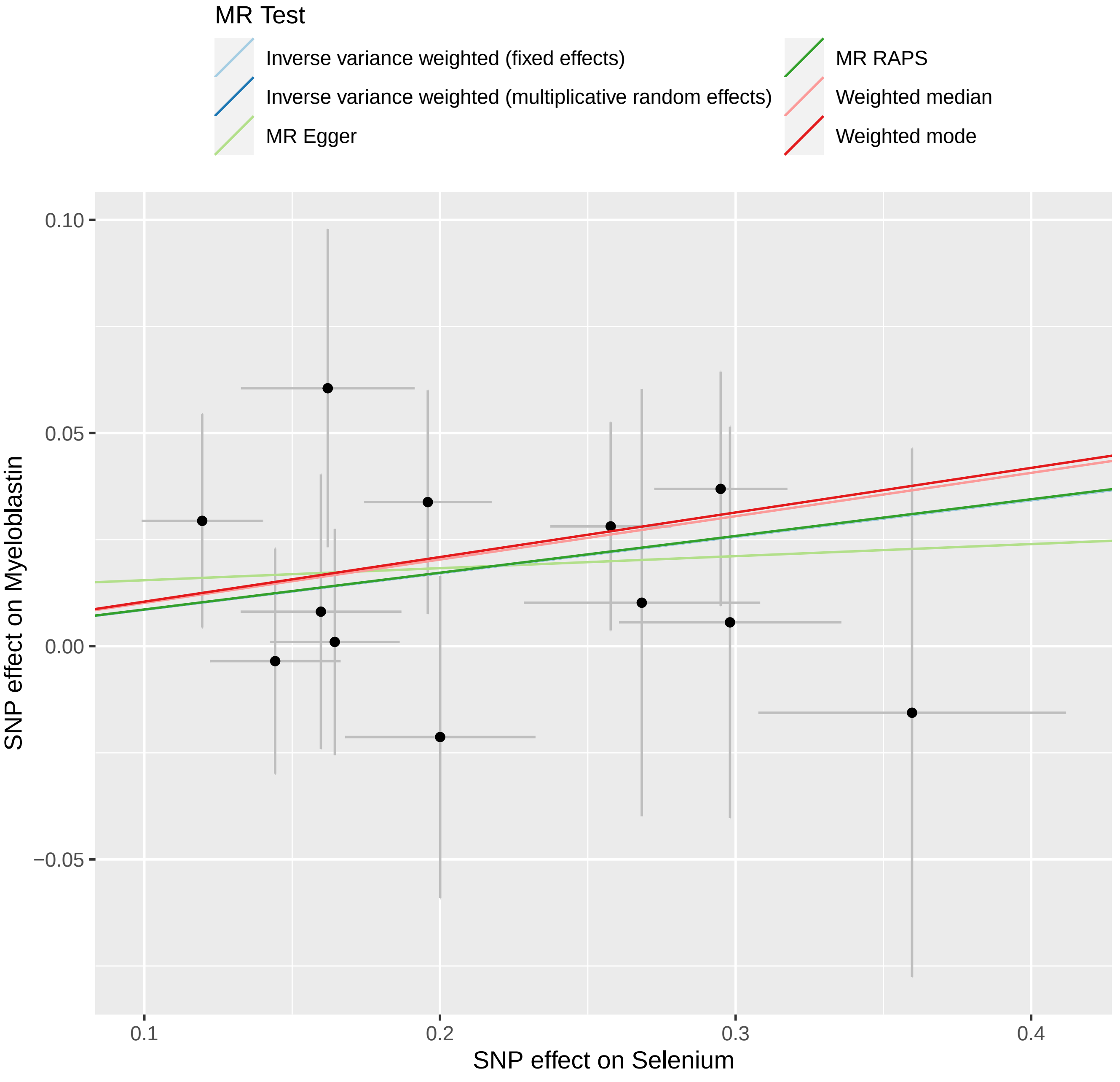

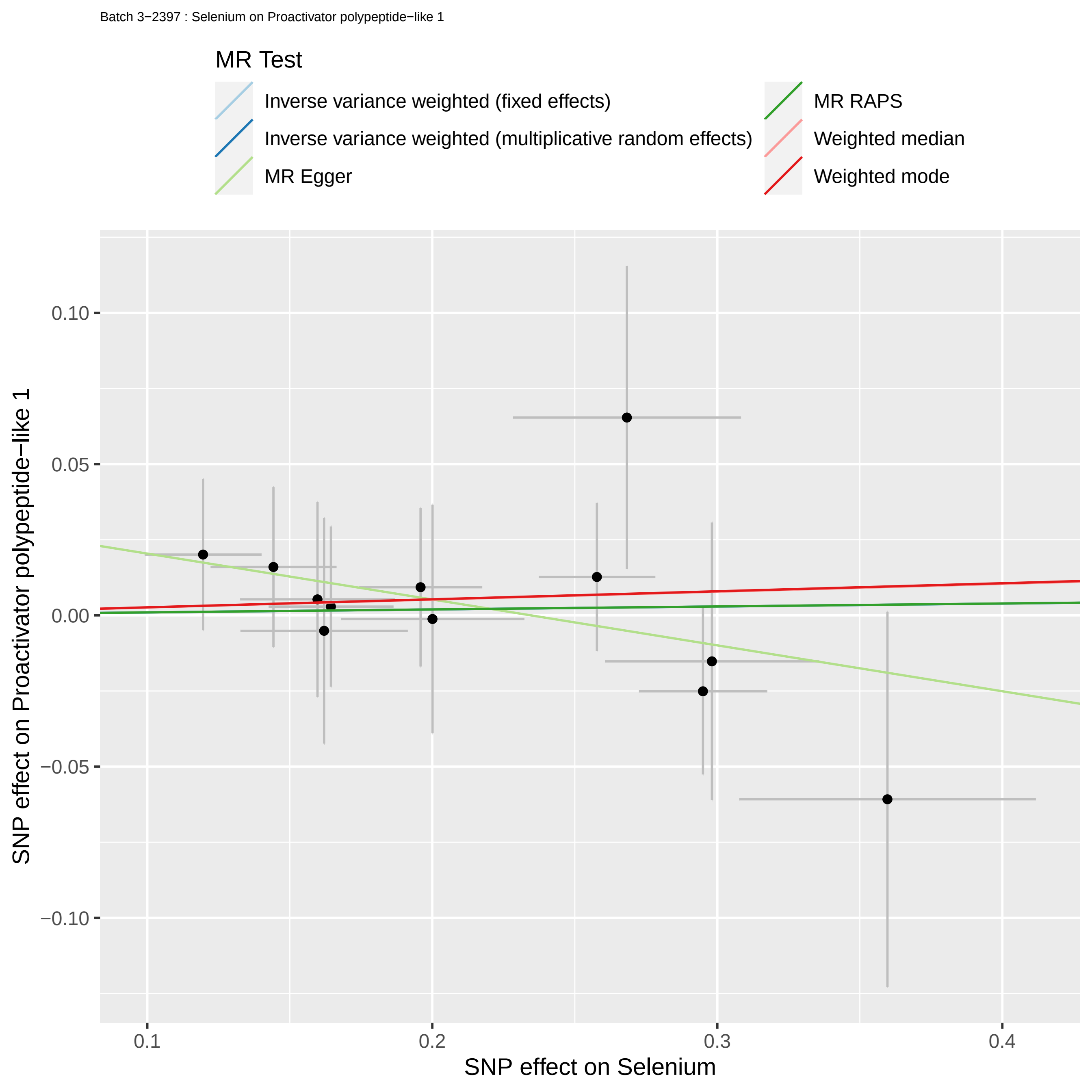
Batch 3−2397 : Selenium on Proactivator polypeptide−like 1
MR Test
Inverse variance weighted (fixed effects)
Inverse variance weighted (multiplicative random effects)
MR Egger
MR RAPS
Weighted median
Weighted mode
0.10
0.05
0.00
−0.05
−0.10
0.1
0.2
0.3
0.4
SNP effect on Proactivator polypeptide−like 1
SNP effect on Selenium

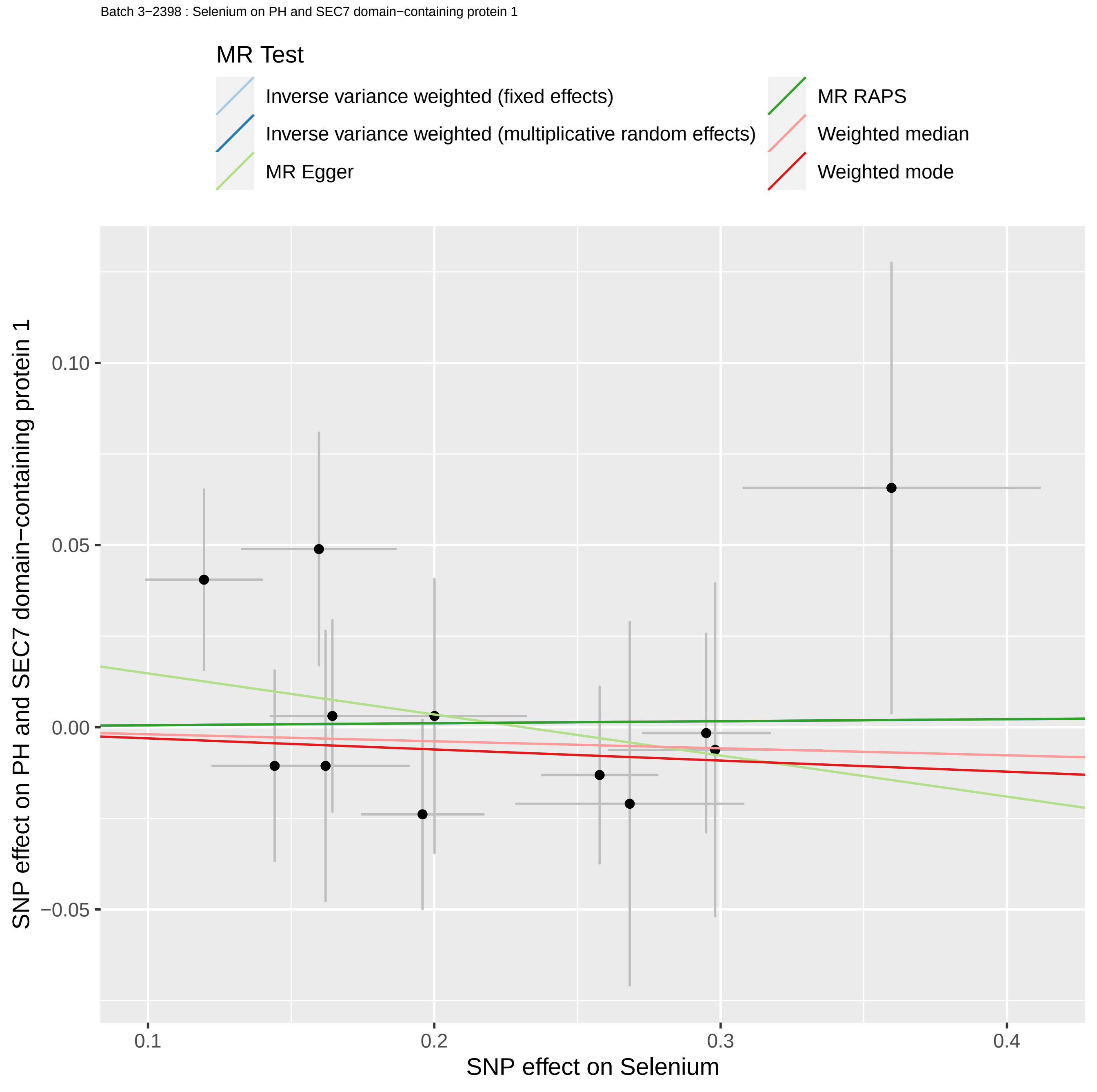

Batch 3−2398 : Selenium on PH and SEC7 domain−containing protein 1
MR Test
Inverse variance weighted (fixed effects)
Inverse variance weighted (multiplicative random effects)
MR Egger
MR RAPS
Weighted median
Weighted mode
0.10
0.05
0.00
−0.05
0.1
0.2
0.3
0.4
SNP effect on PH and SEC7 domain−containing protein 1
SNP effect on Selenium

Batch 3−2399 : Selenium on PH and SEC7 domain−containing protein 2

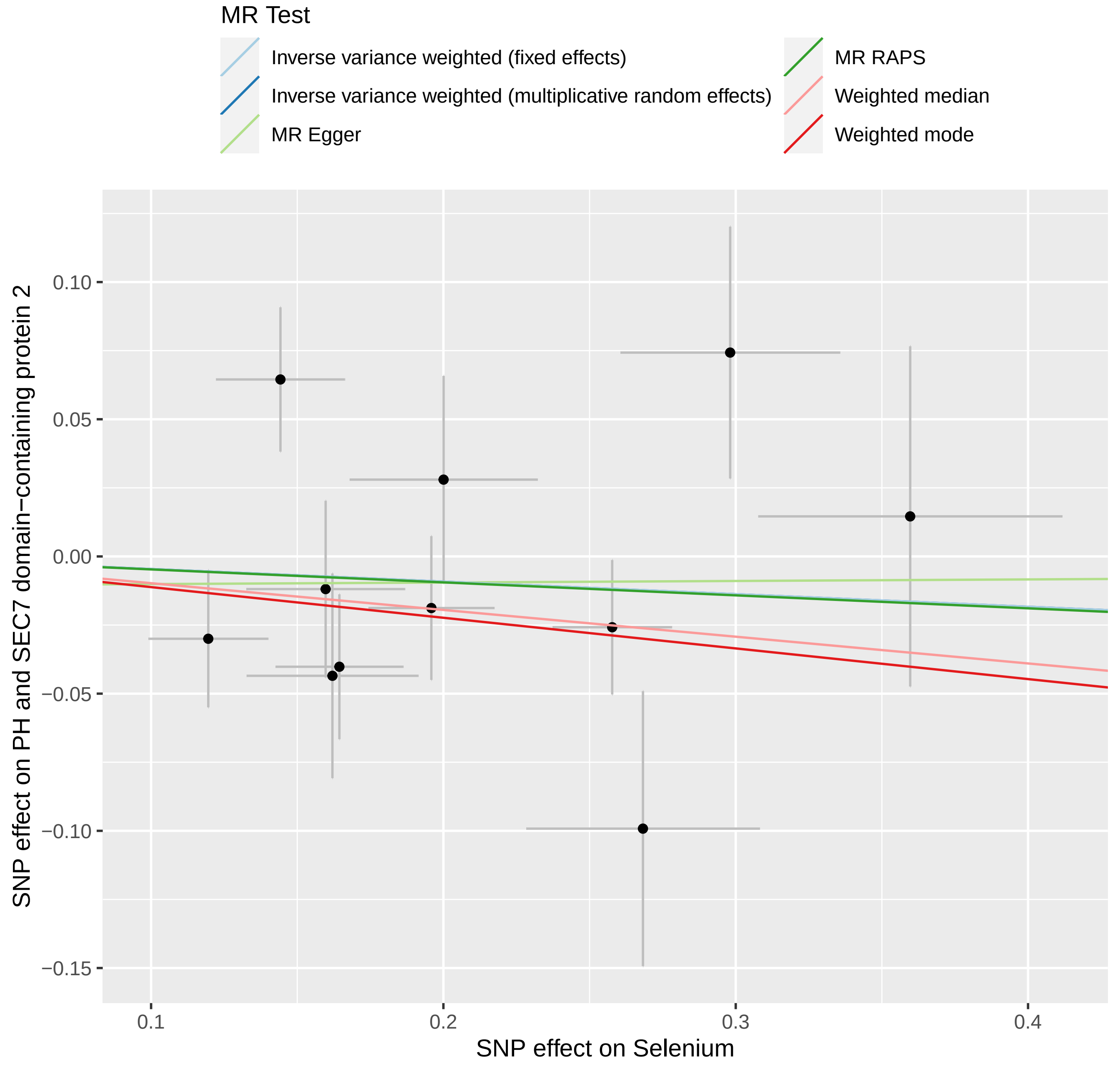

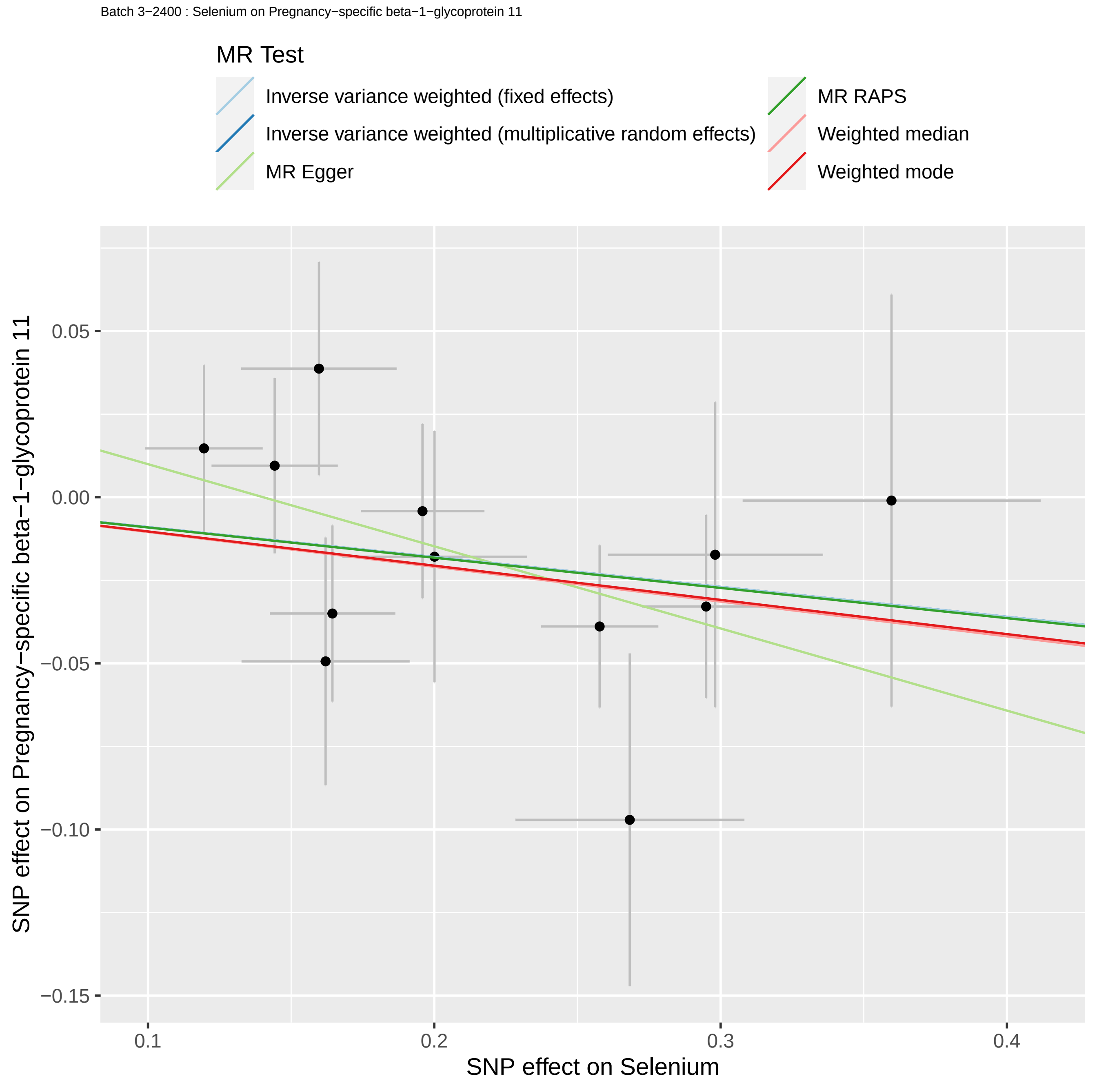

Batch 3−2400 : Selenium on Pregnancy−specific beta−1−glycoprotein 11
MR Test
Inverse variance weighted (fixed effects)
Inverse variance weighted (multiplicative random effects)
MR Egger
MR RAPS
Weighted median
Weighted mode
0.05
0.00
−0.05
−0.10
−0.15
0.1
0.2
0.3
0.4
SNP effect on Pregnancy−specific beta−1−glycoprotein 11
SNP effect on Selenium

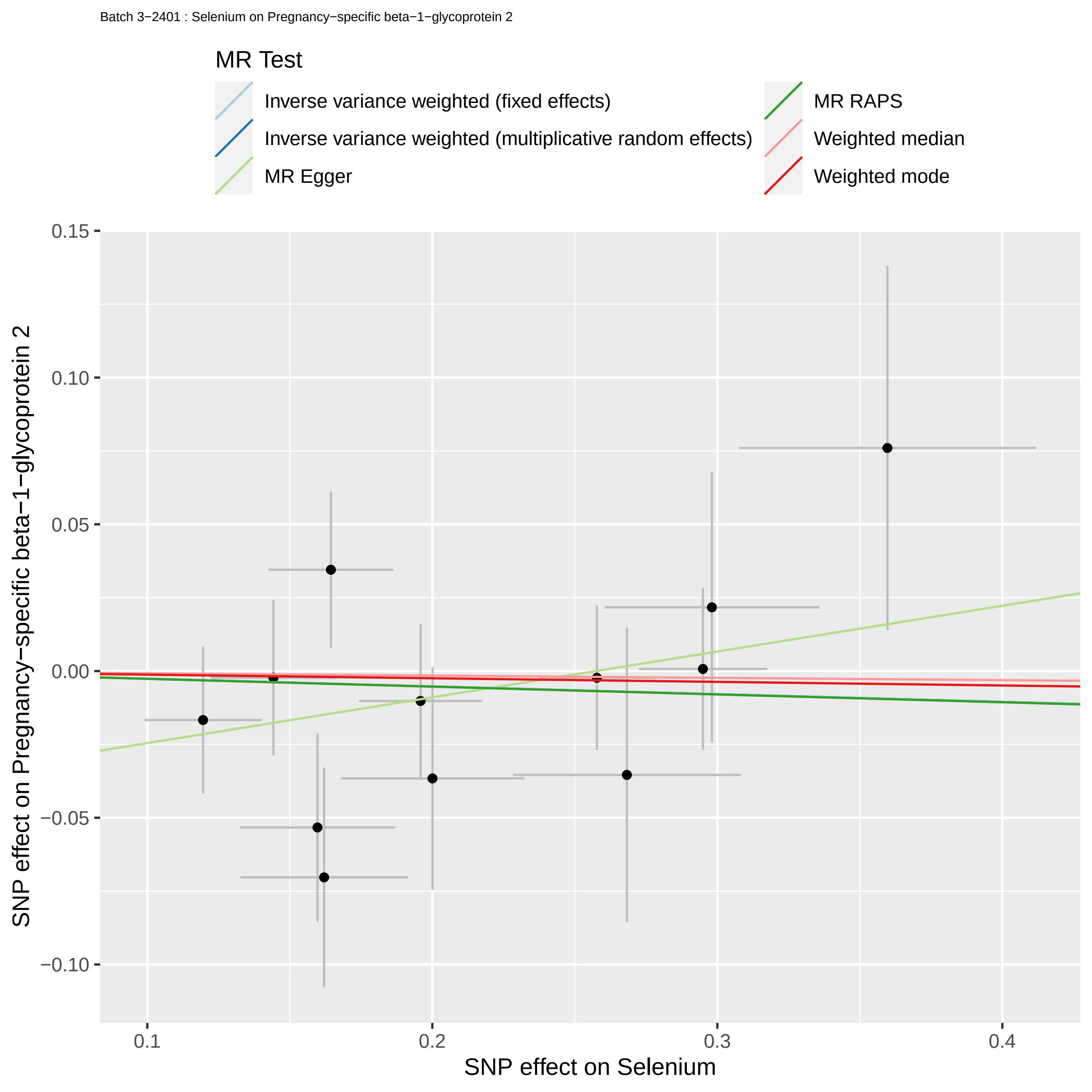
Batch 3−2401 : Selenium on Pregnancy−specific beta−1−glycoprotein 2
MR Test
Inverse variance weighted (fixed effects)
Inverse variance weighted (multiplicative random effects)
MR Egger
MR RAPS
Weighted median
Weighted mode
0.15
0.10
0.05
0.00
−0.05
−0.10
0.1
0.2
0.3
0.4
SNP effect on Pregnancy−specific beta−1−glycoprotein 2
SNP effect on Selenium

Batch 3−2402 : Selenium on Pregnancy−specific beta−1−glycoprotein 3

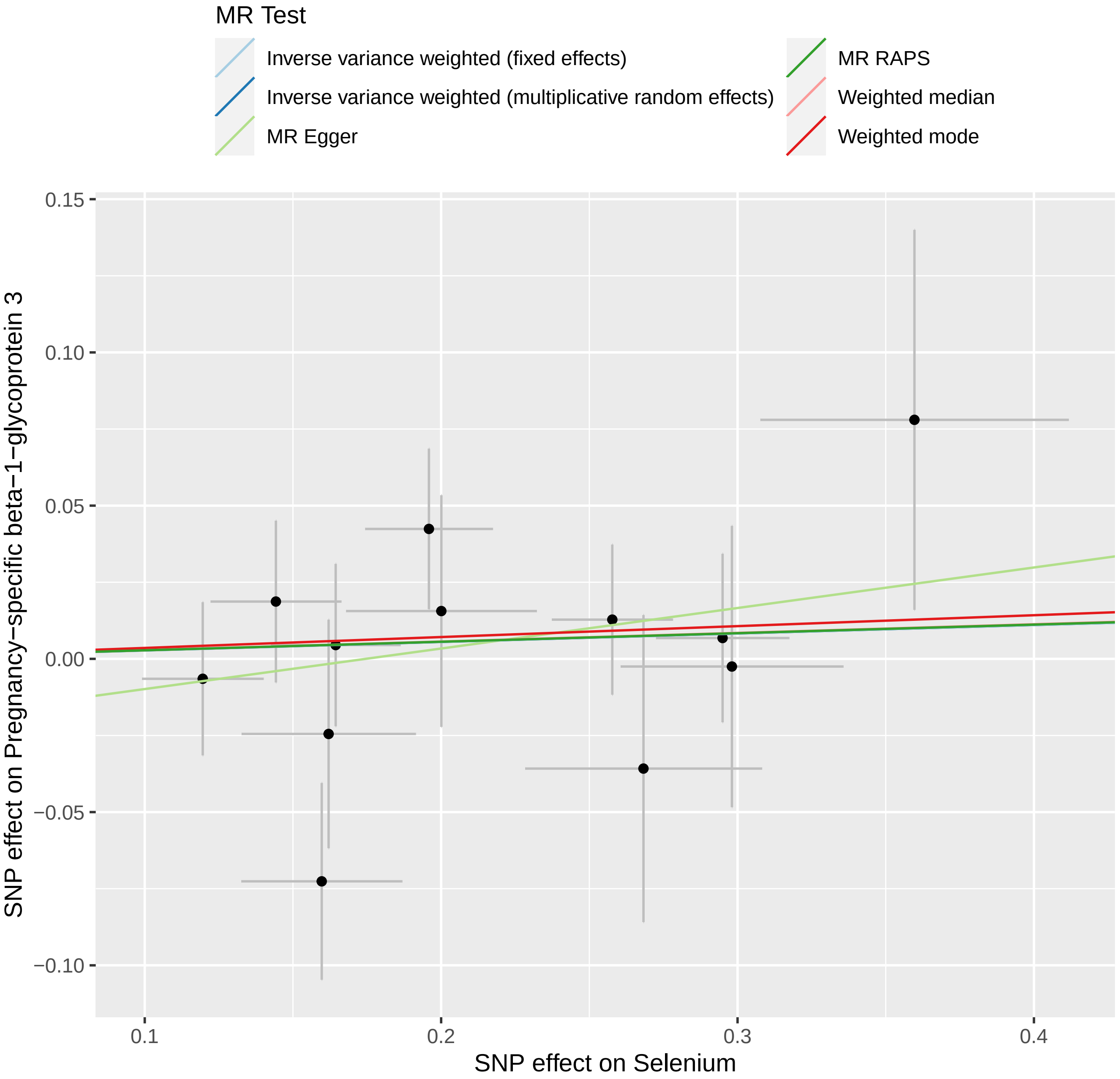

Batch 3−2403 : Selenium on Pregnancy−specific beta−1−glycoprotein 4

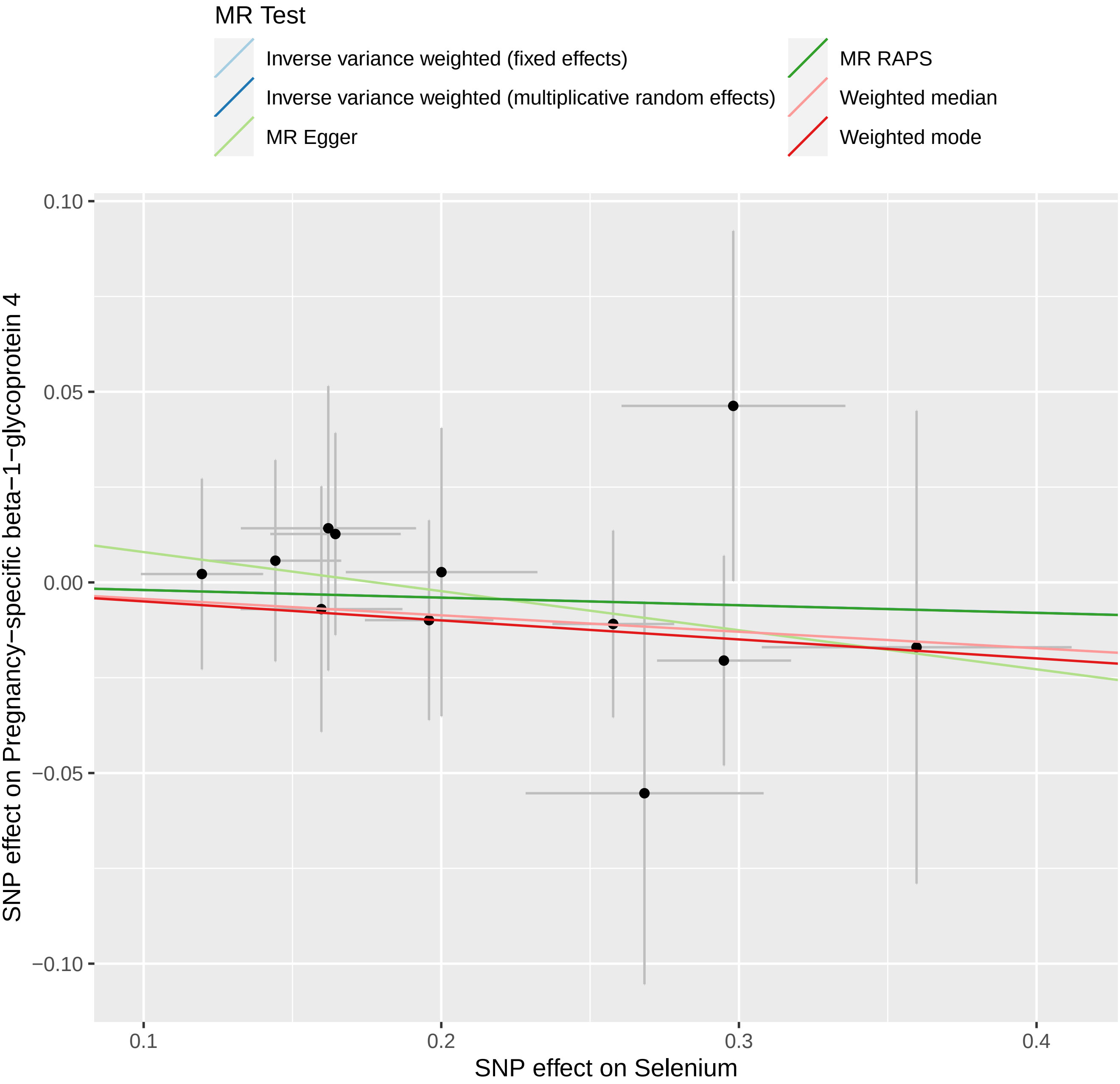

Batch 3−2404 : Selenium on Pregnancy−specific beta−1−glycoprotein 5

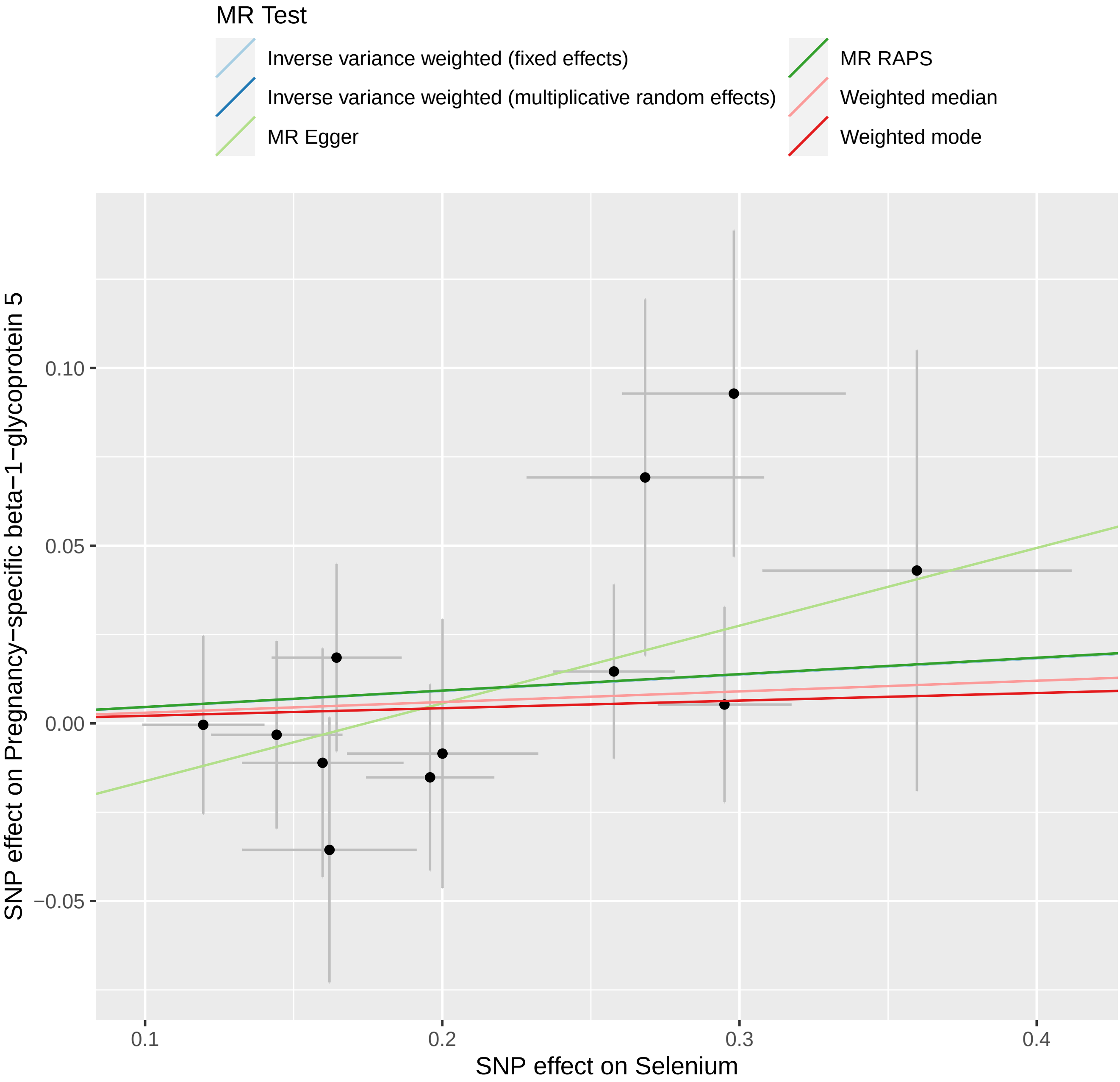

Batch 3−2405 : Selenium on Pregnancy−specific beta−1−glycoprotein 6

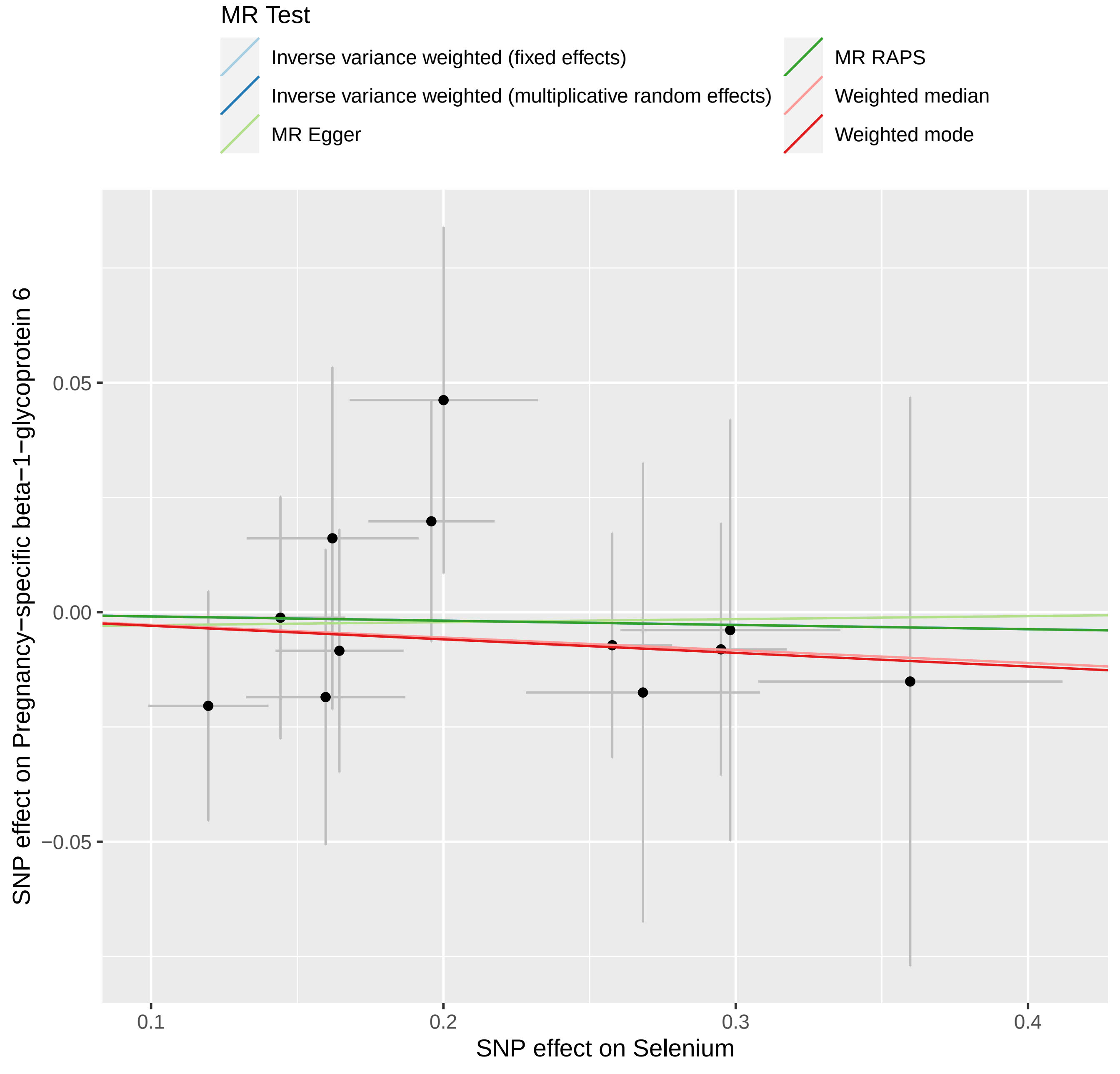

Batch 3−2406 : Selenium on Putative pregnancy−specific beta−1−glycoprotein 7

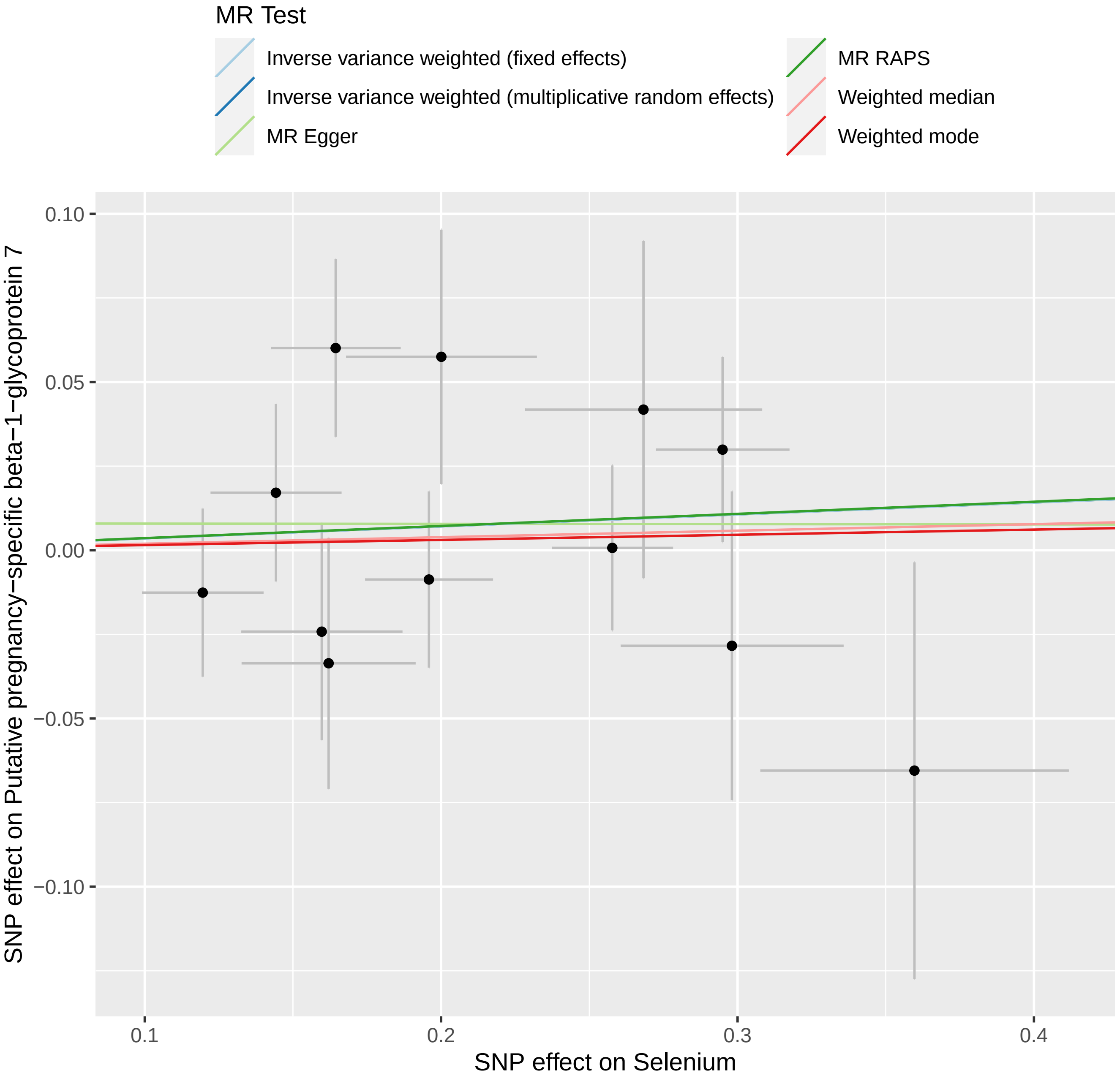

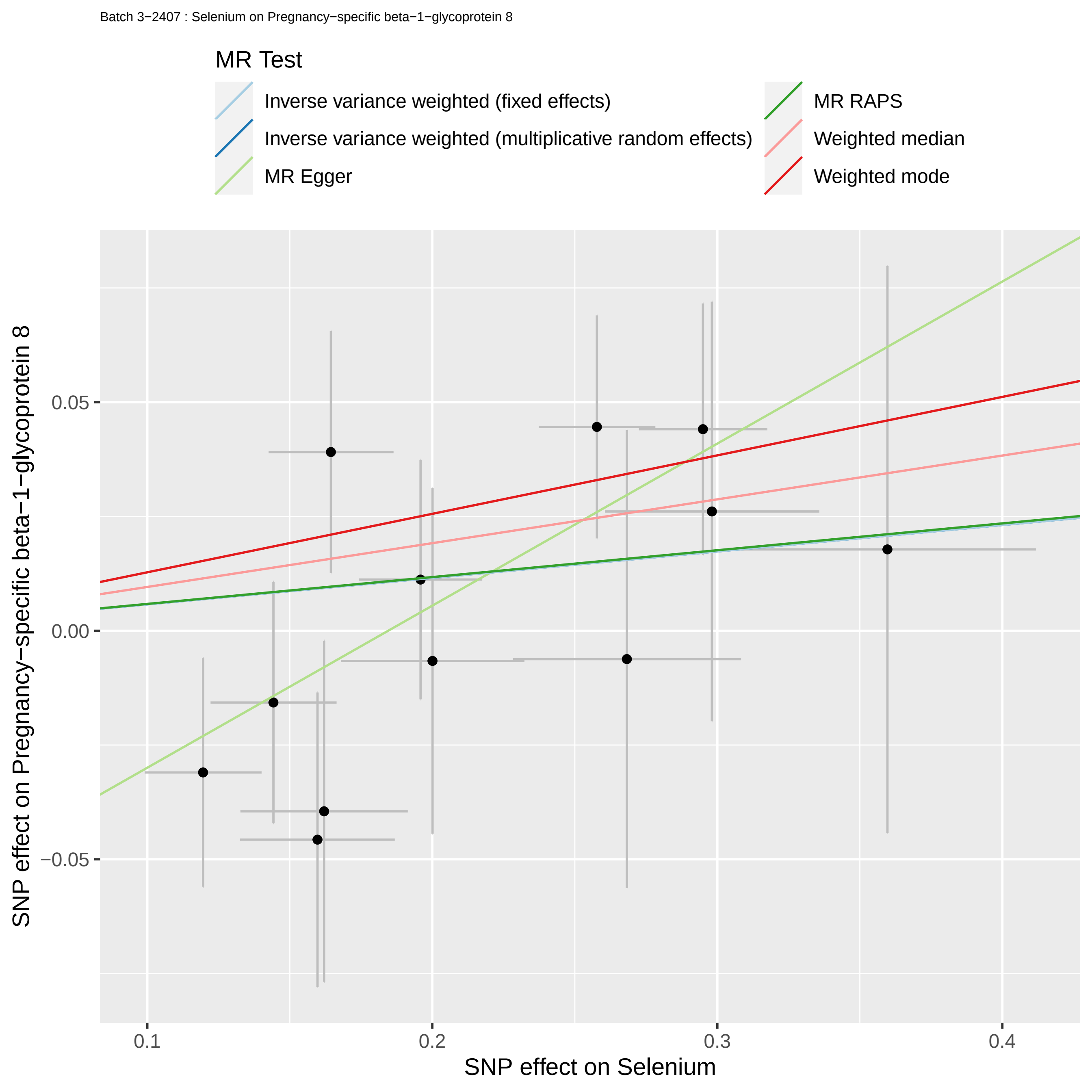
Batch 3−2407 : Selenium on Pregnancy−specific beta−1−glycoprotein 8
MR Test
Inverse variance weighted (fixed effects)
Inverse variance weighted (multiplicative random effects)
MR Egger
MR RAPS
Weighted median
Weighted mode
SNP effect on Pregnancy−specific beta−1−glycoprotein 8
0.05
0.00
−0.05
0.1
0.2
0.3
0.4
SNP effect on Selenium

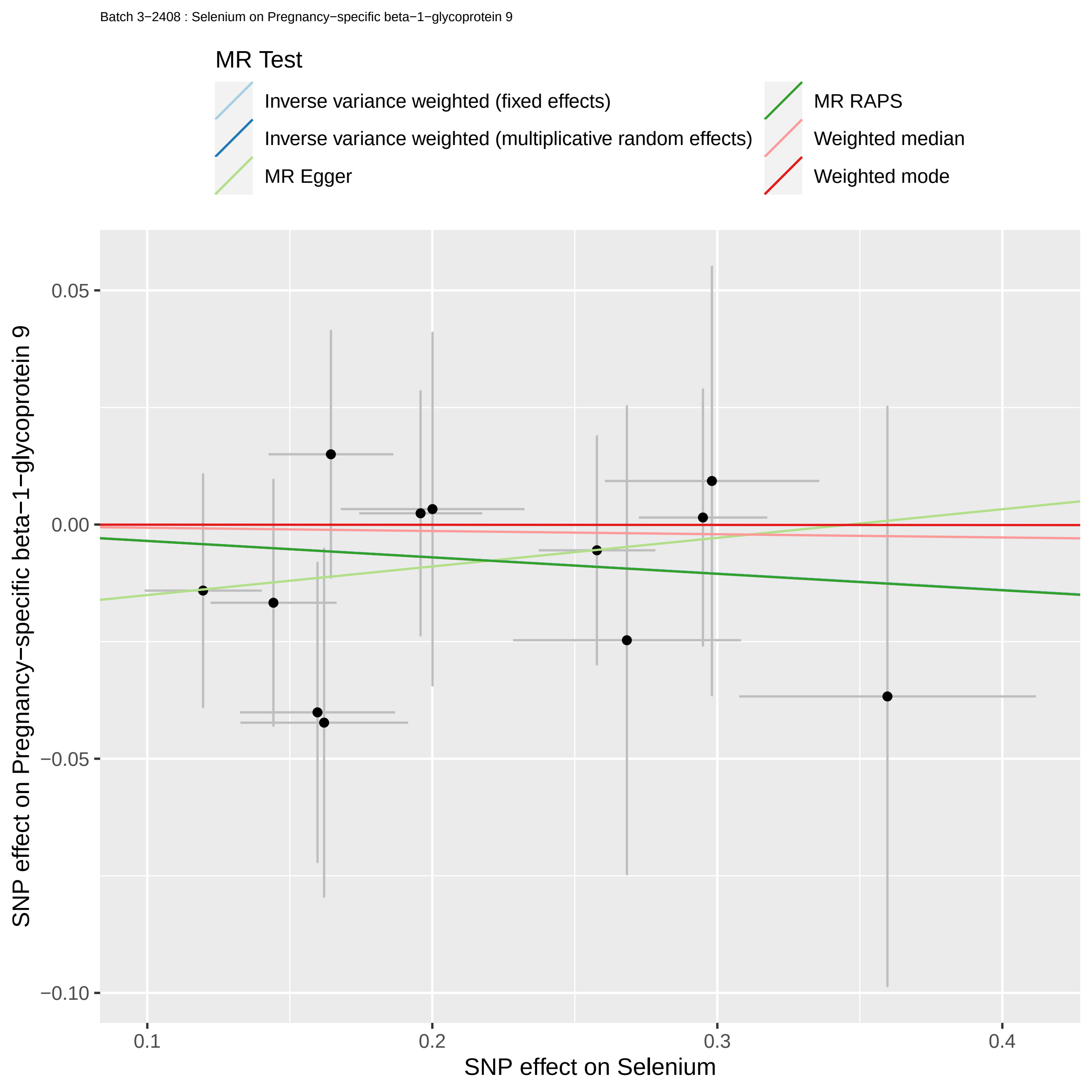

Batch 3−2408 : Selenium on Pregnancy−specific beta−1−glycoprotein 9
MR Test
Inverse variance weighted (fixed effects)
Inverse variance weighted (multiplicative random effects)
MR Egger
MR RAPS
Weighted median
Weighted mode
0.05
0.00
−0.05
−0.10
0.1
0.2
0.3
0.4
SNP effect on Selenium
SNP effect on Pregnancy−specific beta−1−glycoprotein 9

Batch 3−2409 : Selenium on Proteasome subunit alpha type−1

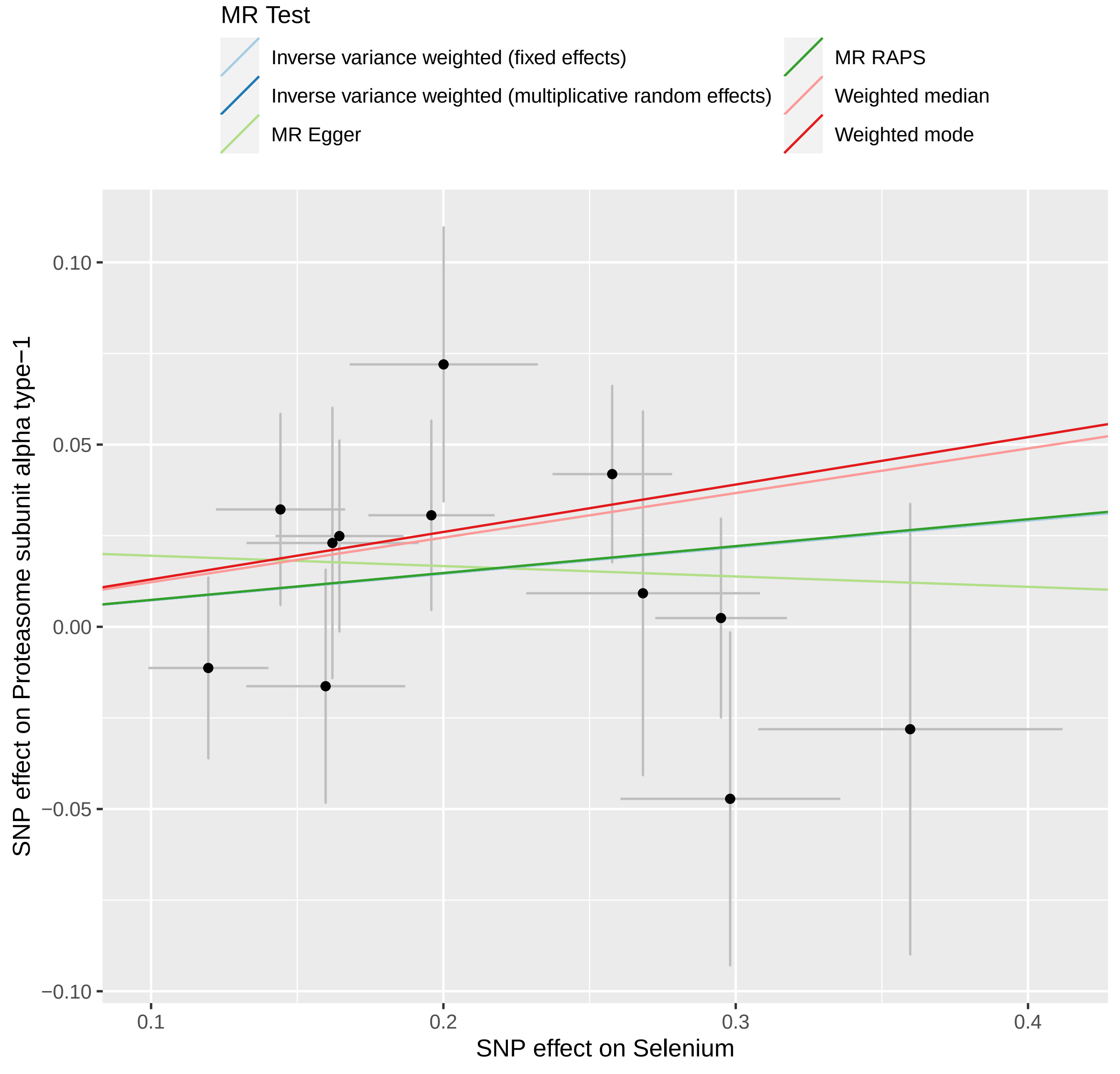

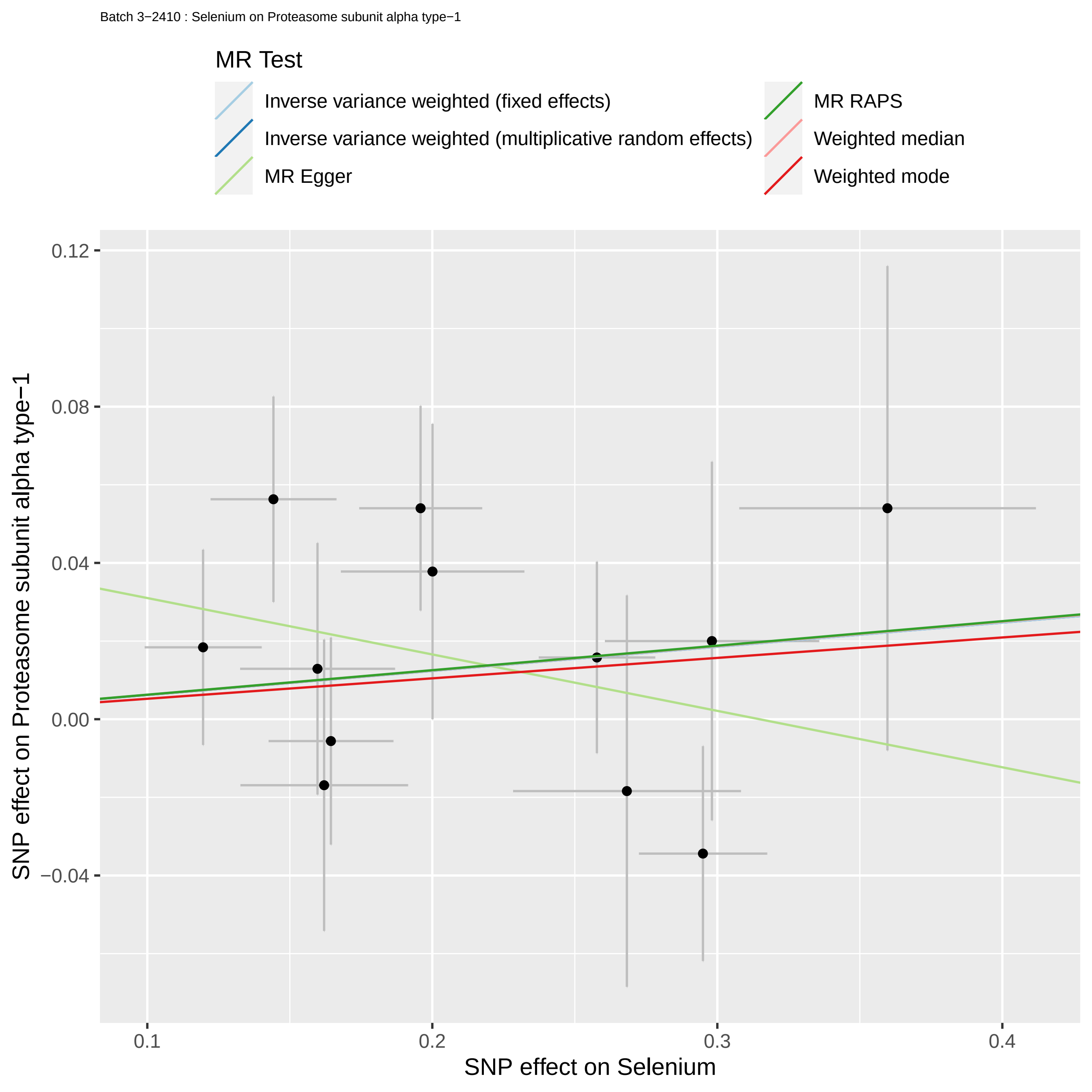

Batch 3−2410 : Selenium on Proteasome subunit alpha type−1
MR Test
Inverse variance weighted (fixed effects)
Inverse variance weighted (multiplicative random effects)
MR Egger
MR RAPS
Weighted median
Weighted mode
0.12
0.08
0.04
0.00
−0.04
0.1
0.2
0.3
0.4
SNP effect on Proteasome subunit alpha type−1
SNP effect on Selenium

Batch 3−2411 : Selenium on Proteasome subunit alpha type−2

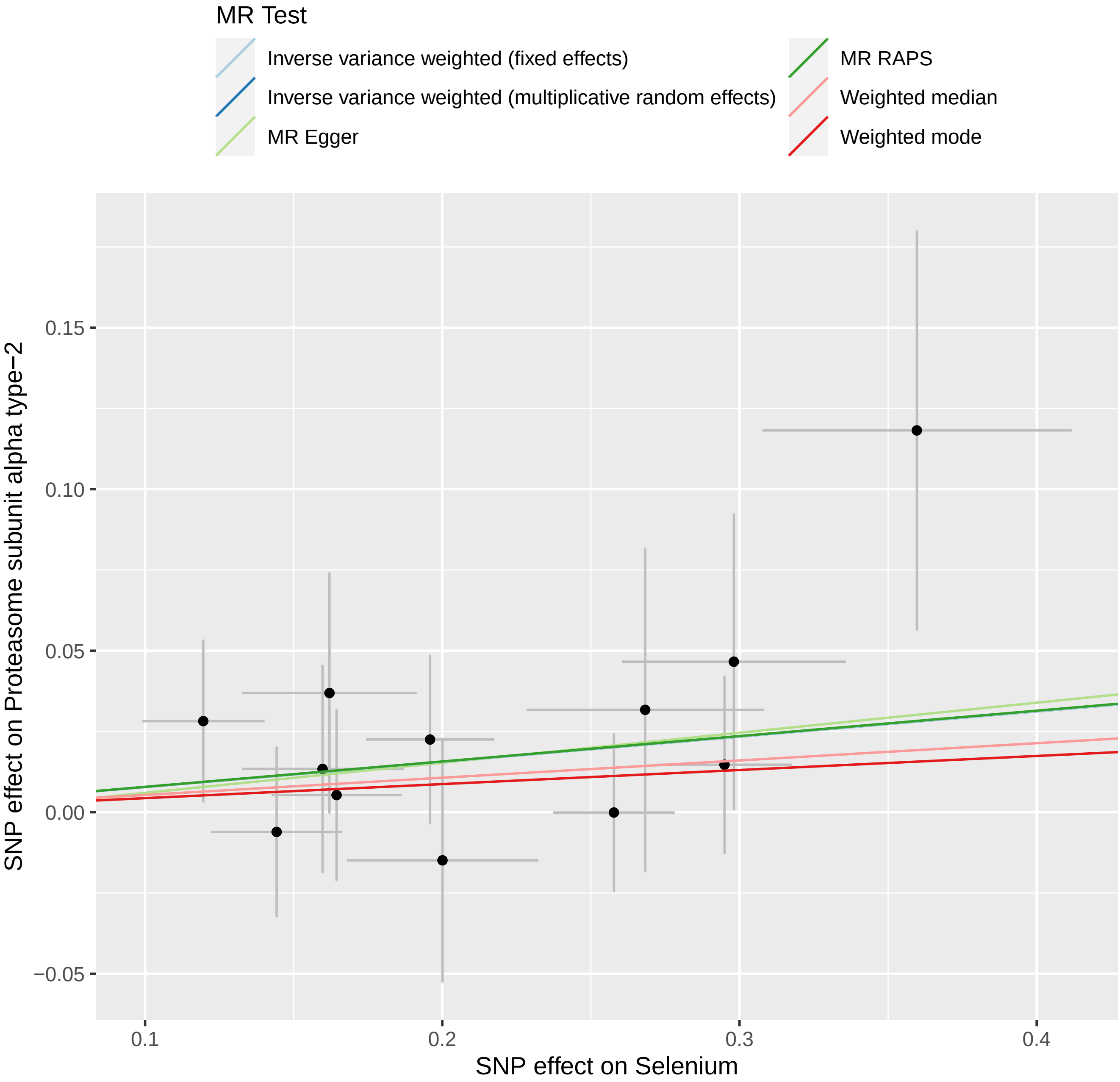

Batch 3−2412 : Selenium on Proteasome subunit alpha type−4

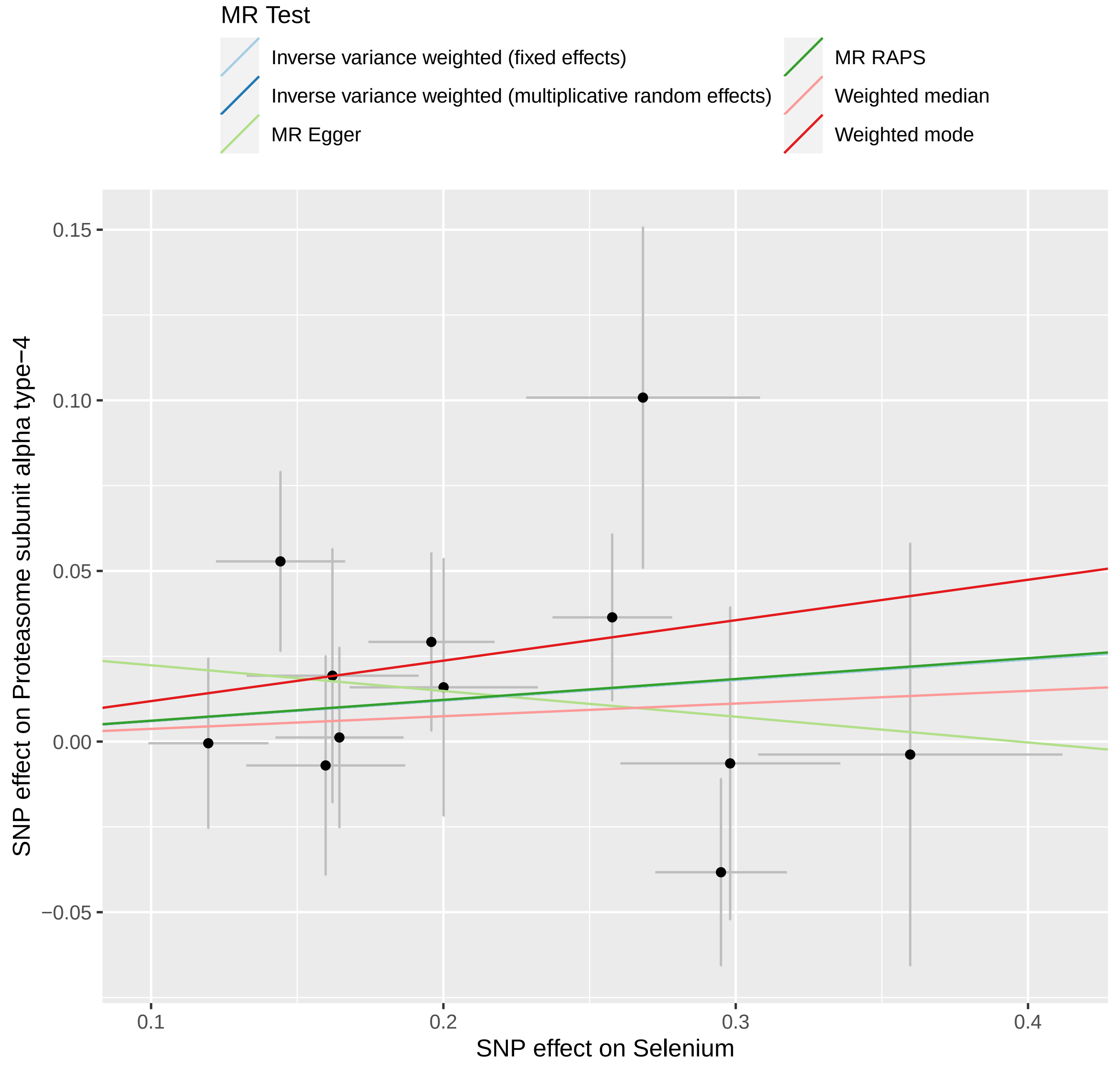

Batch 3−2413 : Selenium on Proteasome subunit alpha type−6

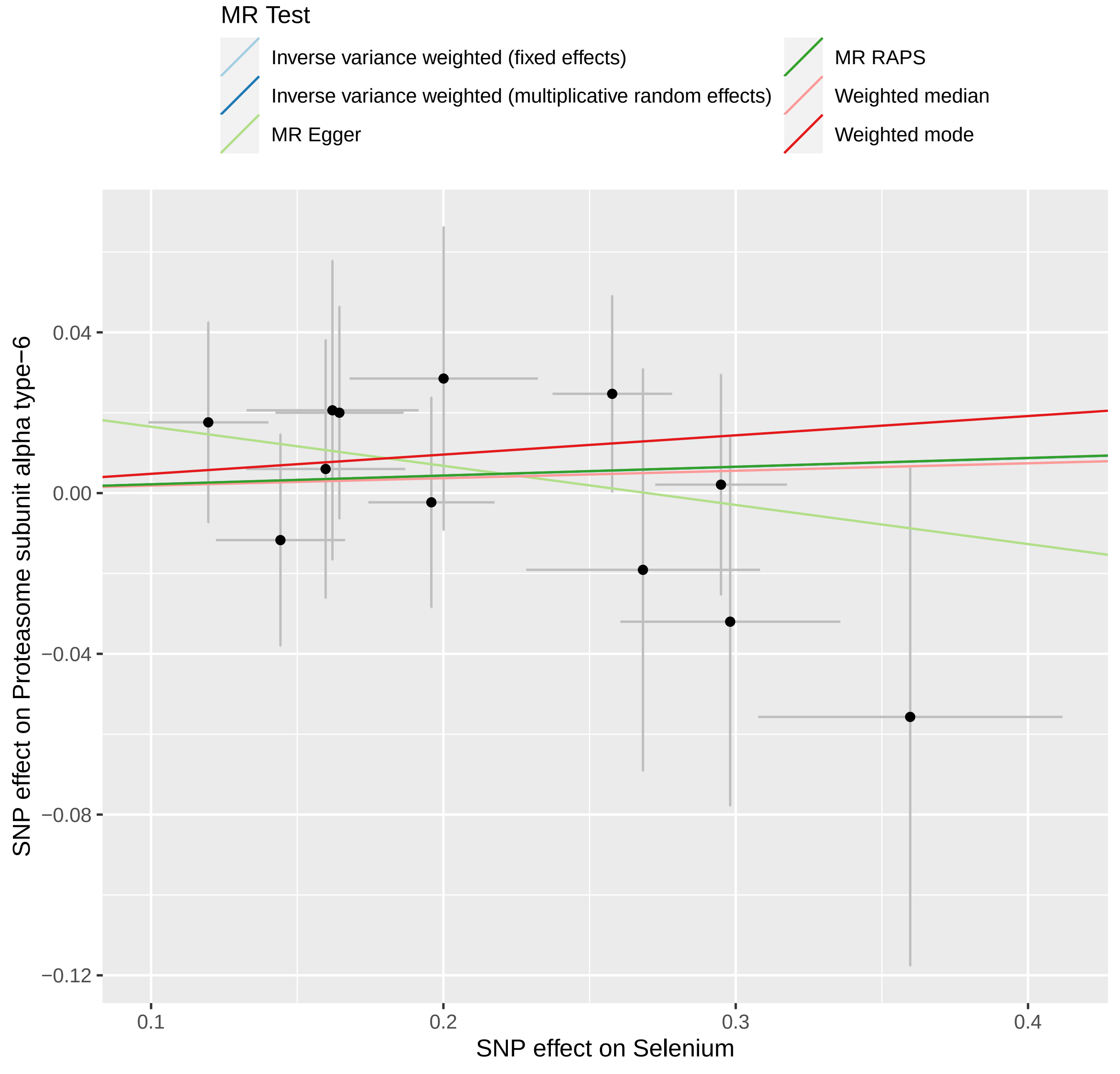

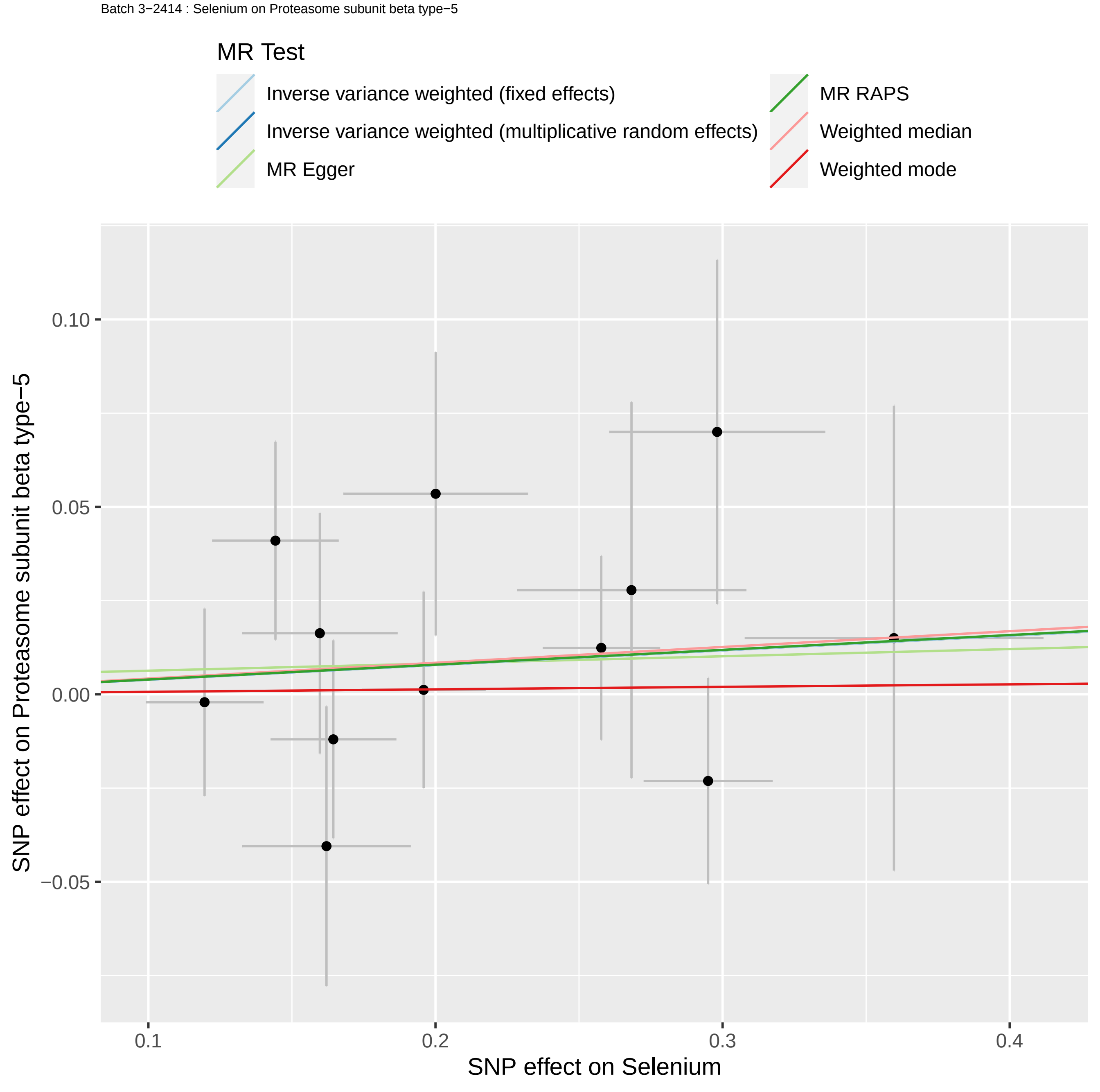

Batch 3−2414 : Selenium on Proteasome subunit beta type−5
MR Test
Inverse variance weighted (fixed effects)
Inverse variance weighted (multiplicative random effects)
MR Egger
MR RAPS
Weighted median
Weighted mode
0.10
0.05
0.00
−0.05
0.1
0.2
0.3
0.4
SNP effect on Proteasome subunit beta type−5
SNP effect on Selenium

Batch 3−2415 : Selenium on 26S proteasome non−ATPase regulatory subunit 11

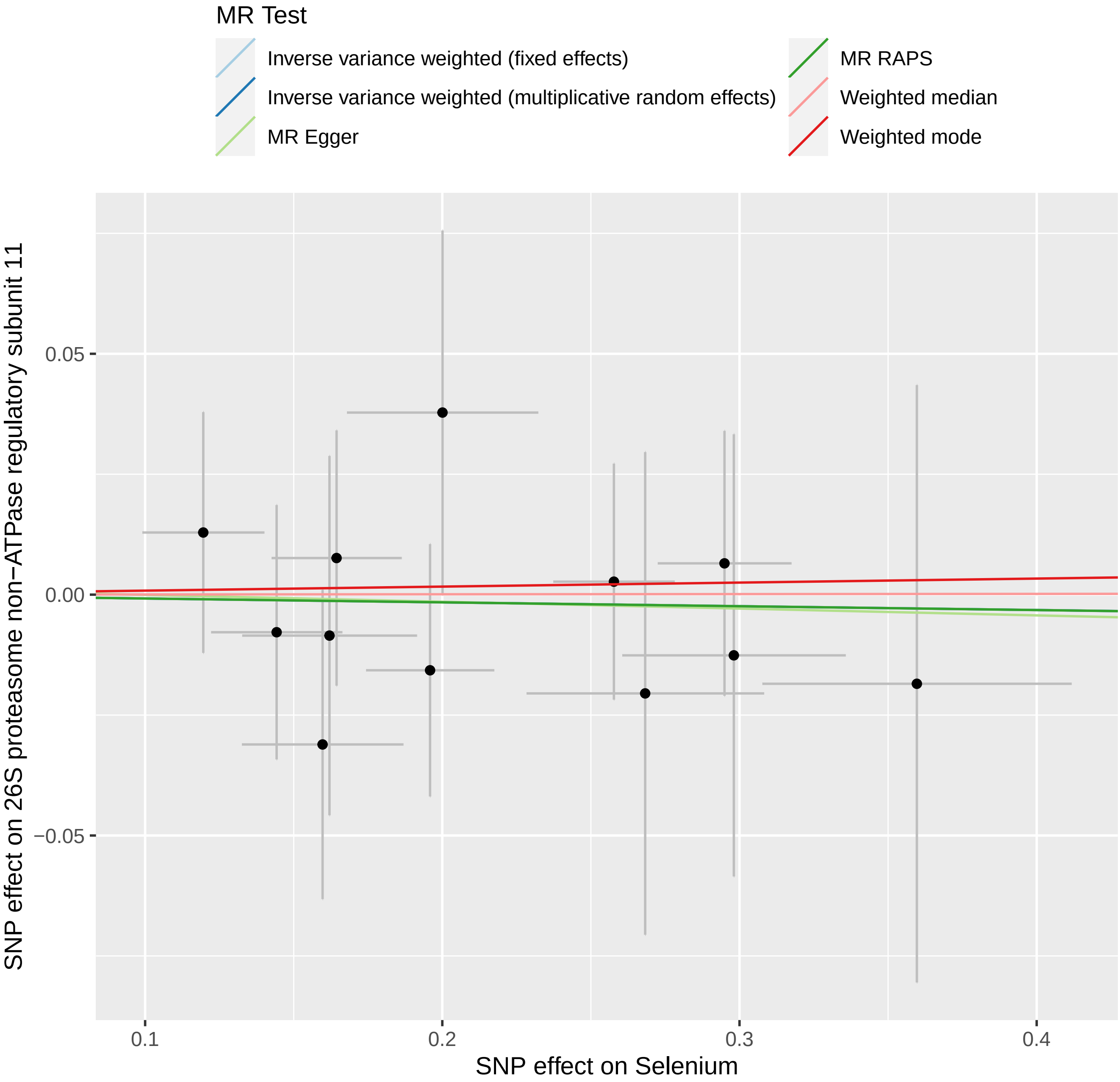

Batch 3−2416 : Selenium on 26S proteasome non−ATPase regulatory subunit 1

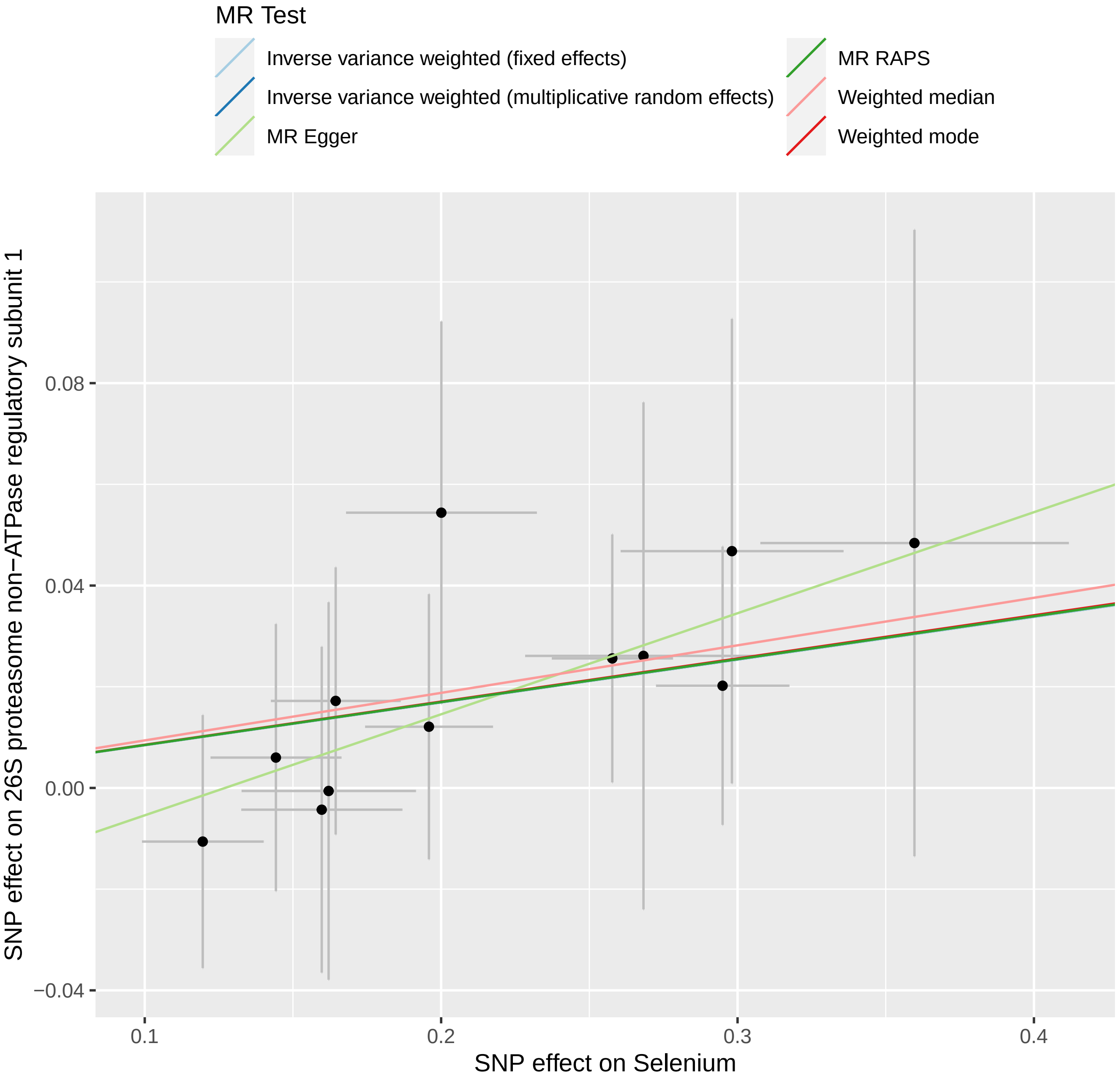

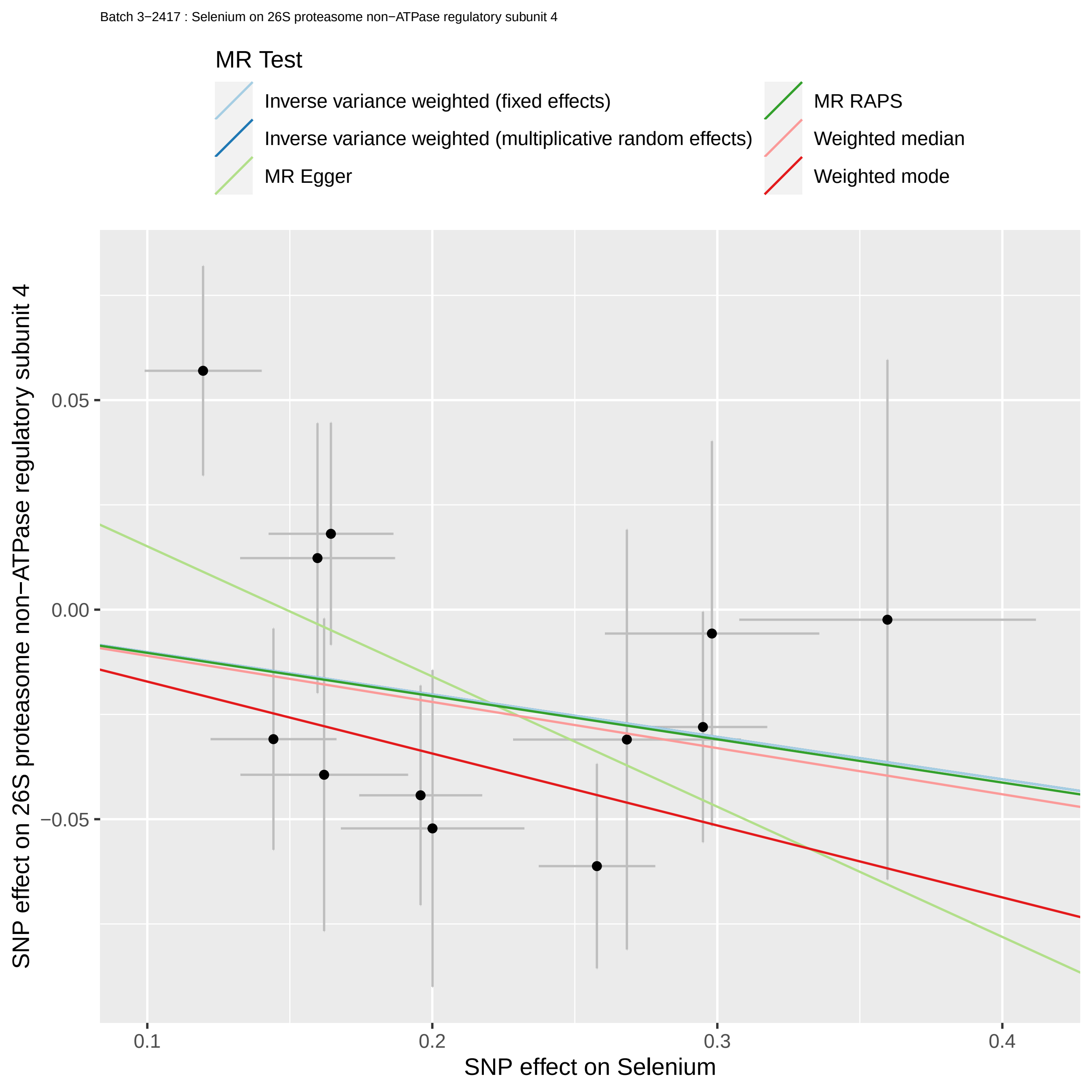

Batch 3−2417 : Selenium on 26S proteasome non−ATPase regulatory subunit 4
MR Test
Inverse variance weighted (fixed effects)
Inverse variance weighted (multiplicative random effects)
MR Egger
MR RAPS
Weighted median
Weighted mode
0.05
0.00
−0.05
0.1
0.2
0.3
0.4
SNP effect on Selenium
SNP effect on 26S proteasome non−ATPase regulatory subunit 4

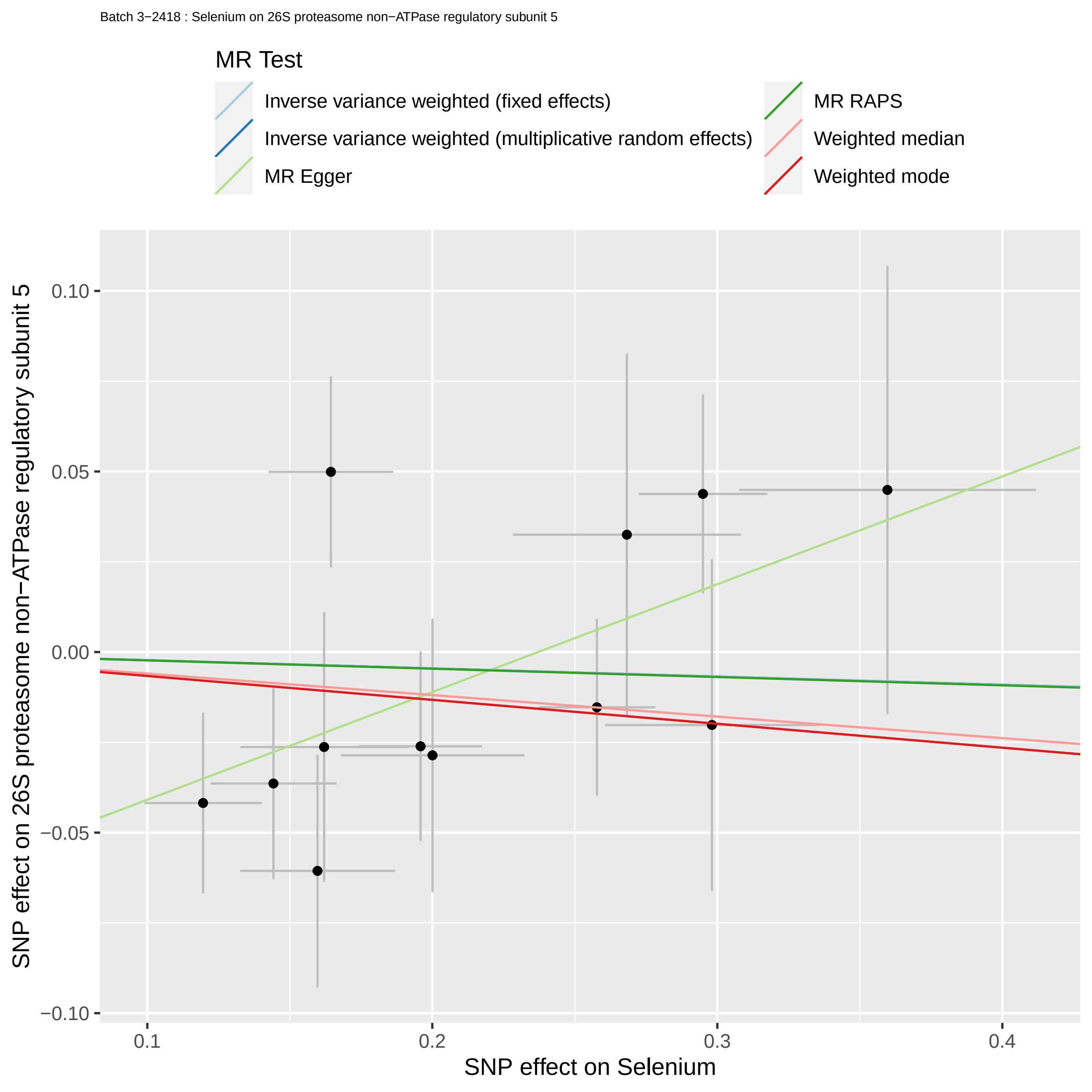

Batch 3−2418 : Selenium on 26S proteasome non−ATPase regulatory subunit 5
MR Test
Inverse variance weighted (fixed effects)
Inverse variance weighted (multiplicative random effects)
MR Egger
MR RAPS
Weighted median
Weighted mode
SNP effect on 26S proteasome non−ATPase regulatory subunit 5
0.10
0.05
0.00
−0.05
−0.10
0.1
0.2
0.3
0.4
SNP effect on Selenium

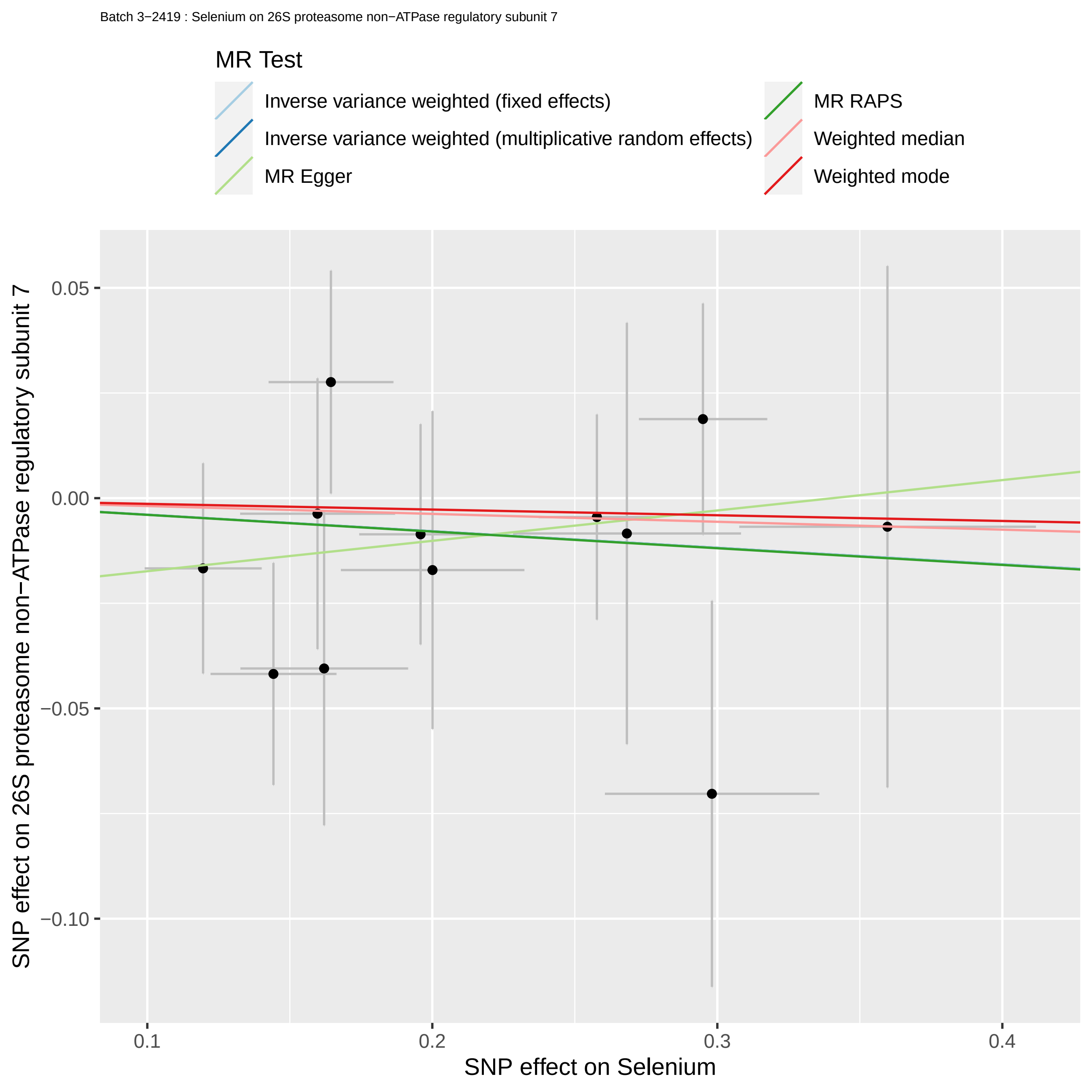

Batch 3−2419 : Selenium on 26S proteasome non−ATPase regulatory subunit 7
MR Test
Inverse variance weighted (fixed effects)
Inverse variance weighted (multiplicative random effects)
MR Egger
MR RAPS
Weighted median
Weighted mode
0.05
0.00
−0.05
−0.10
0.1
0.2
0.3
0.4
SNP effect on 26S proteasome non−ATPase regulatory subunit 7
SNP effect on Selenium

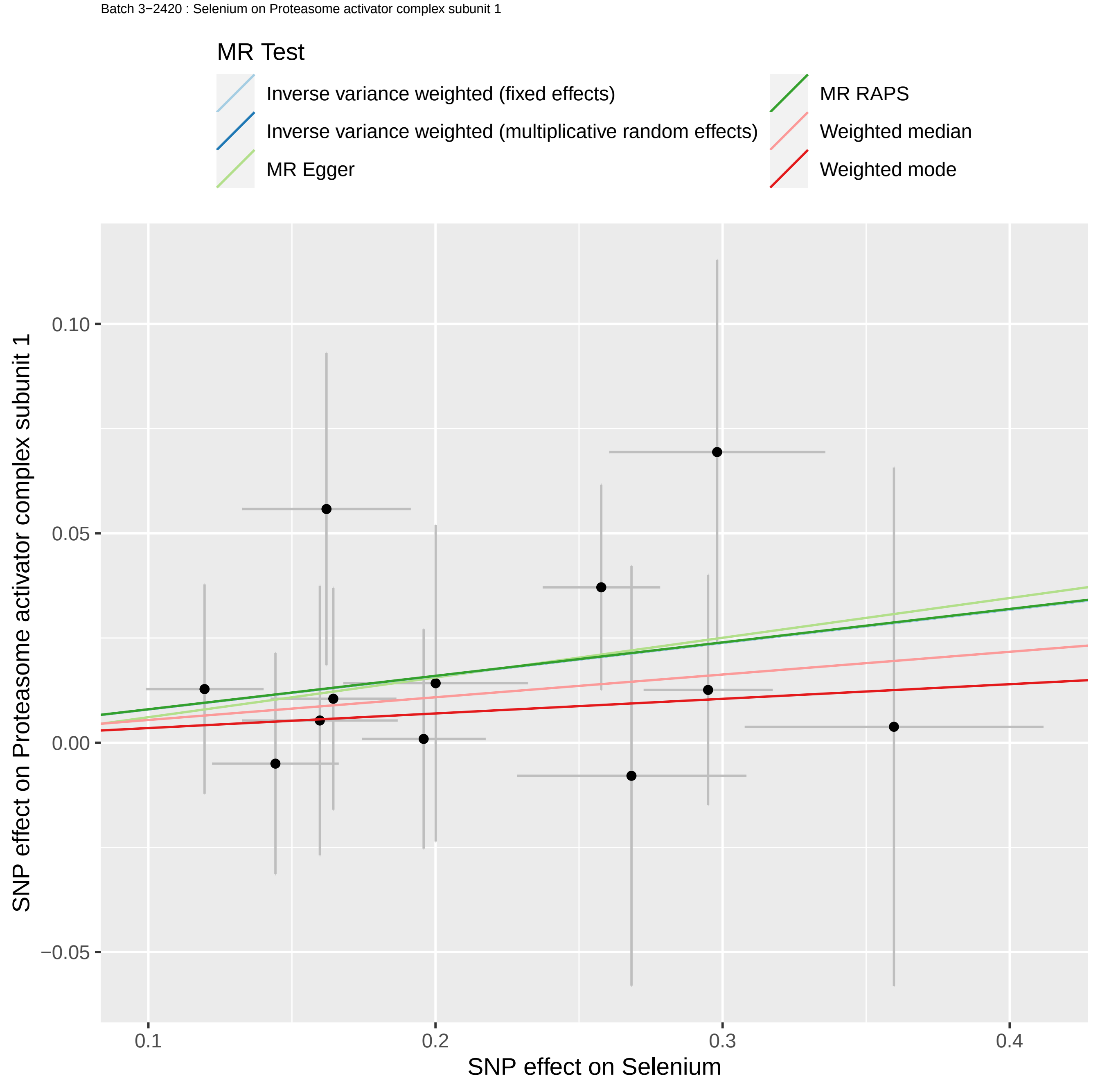

Batch 3−2420 : Selenium on Proteasome activator complex subunit 1
MR Test
Inverse variance weighted (fixed effects)
Inverse variance weighted (multiplicative random effects)
MR Egger
MR RAPS
Weighted median
Weighted mode
SNP effect on Proteasome activator complex subunit 1
0.10
0.05
0.00
−0.05
0.1
0.2
0.3
0.4
SNP effect on Selenium

Batch 3−2421 : Selenium on Proteasome activator complex subunit 3

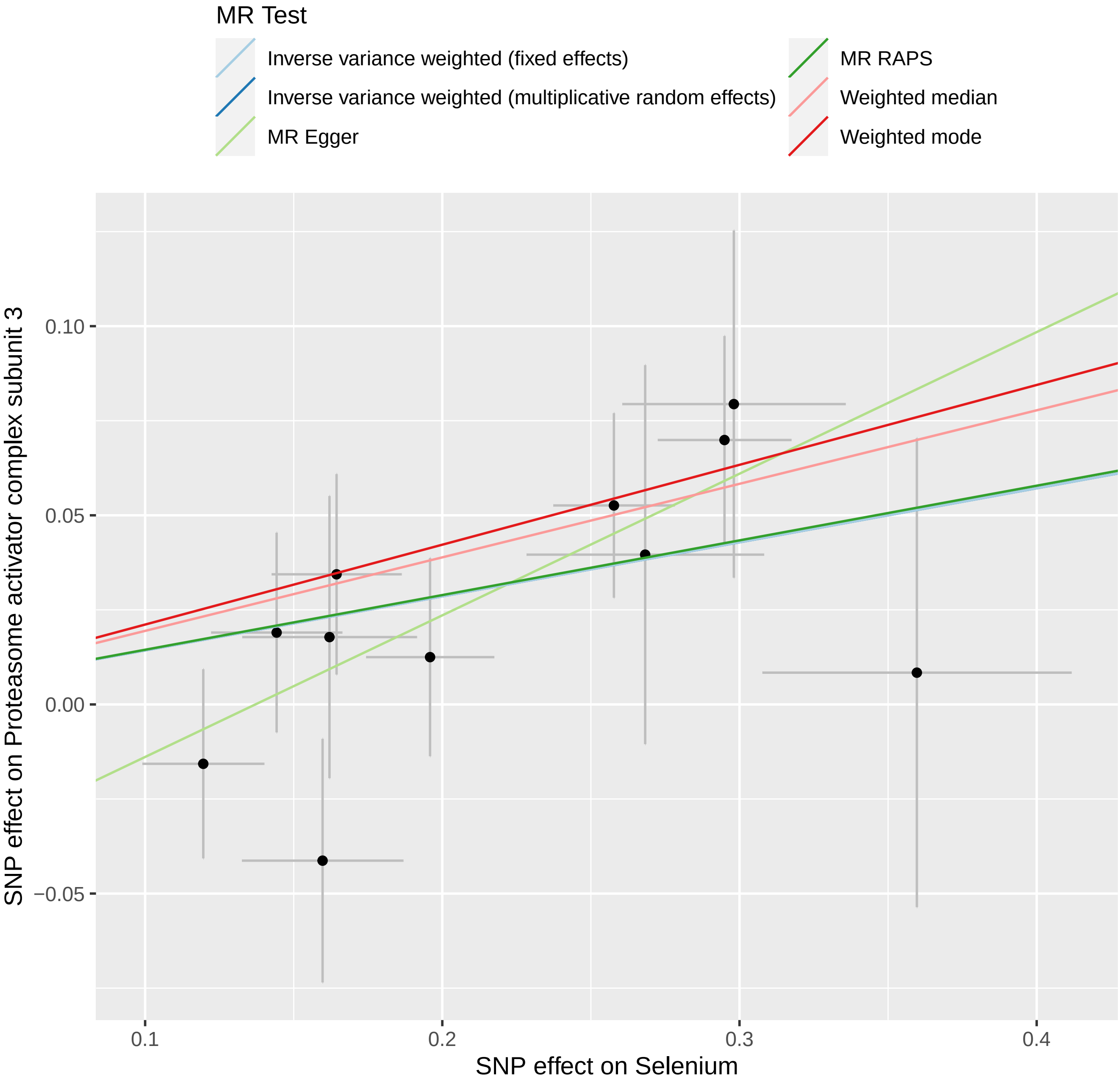

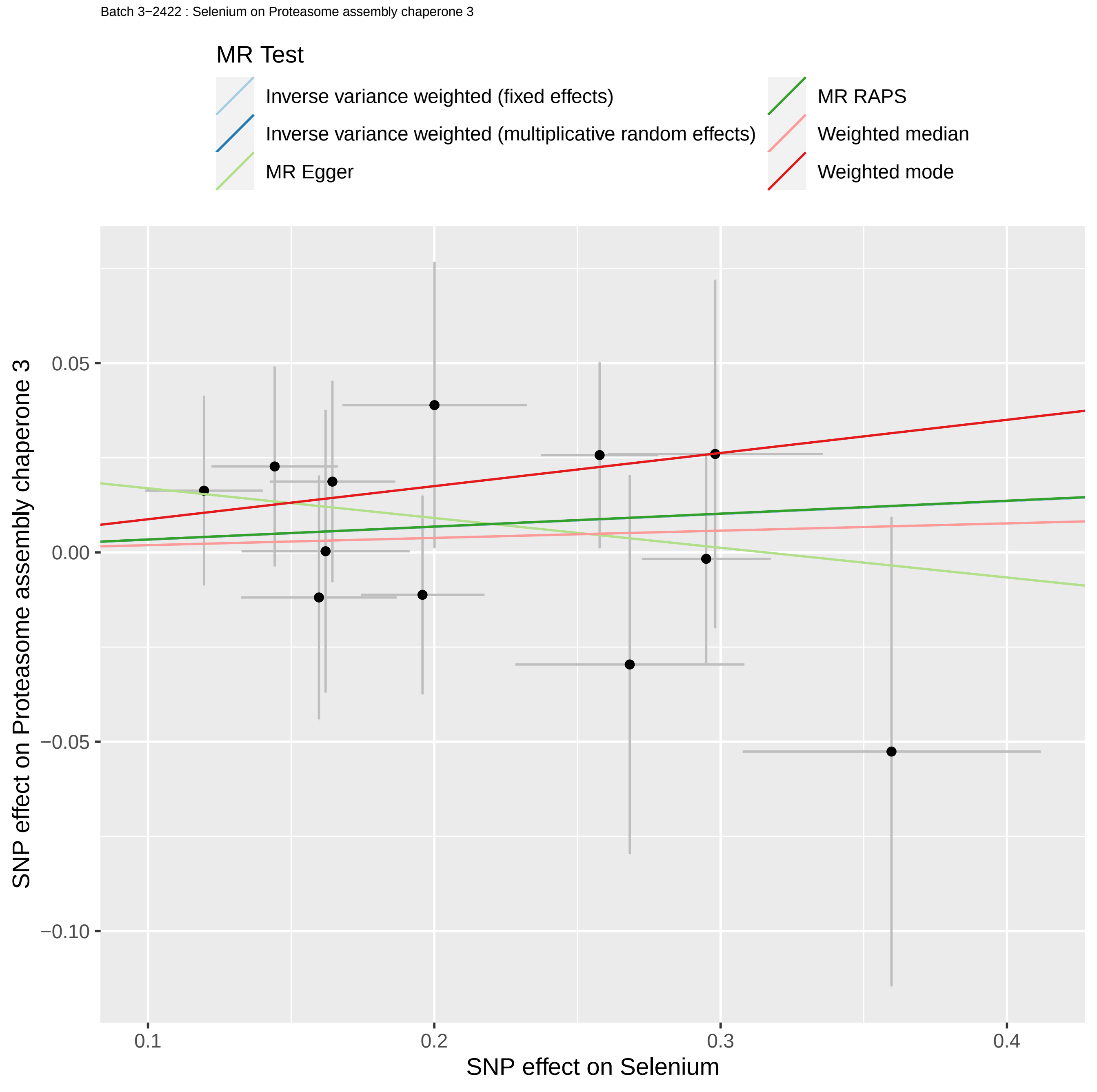
Batch 3−2422 : Selenium on Proteasome assembly chaperone 3
MR Test
Inverse variance weighted (fixed effects)
Inverse variance weighted (multiplicative random effects)
MR Egger
MR RAPS
Weighted median
Weighted mode
SNP effect on Proteasome assembly chaperone 3
0.05
0.00
−0.05
−0.10
0.1
0.2
0.3
0.4
SNP effect on Selenium

Batch 3−2423 : Selenium on Persephin

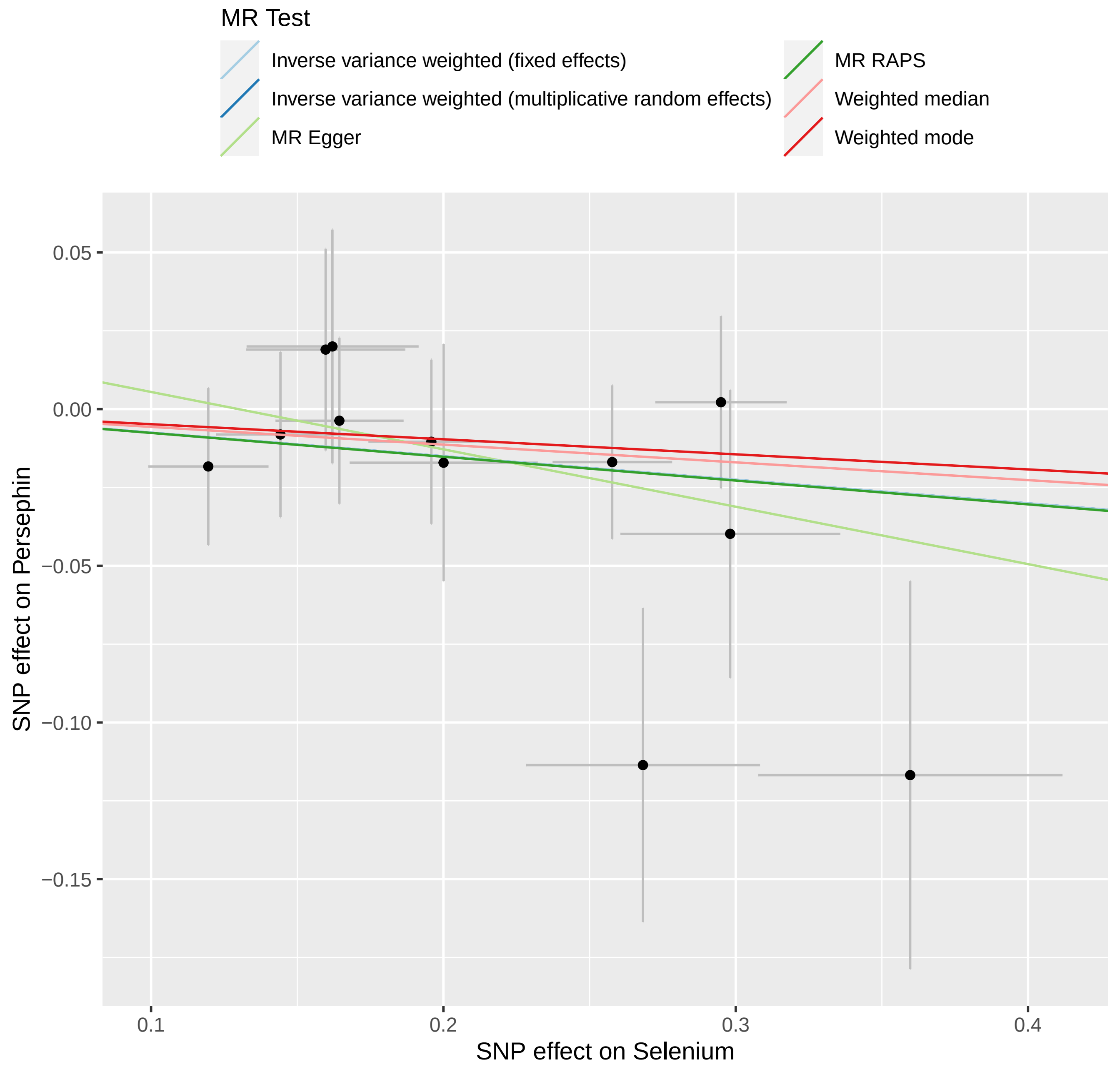

Batch 3−2424 : Selenium on Patched domain−containing protein 3

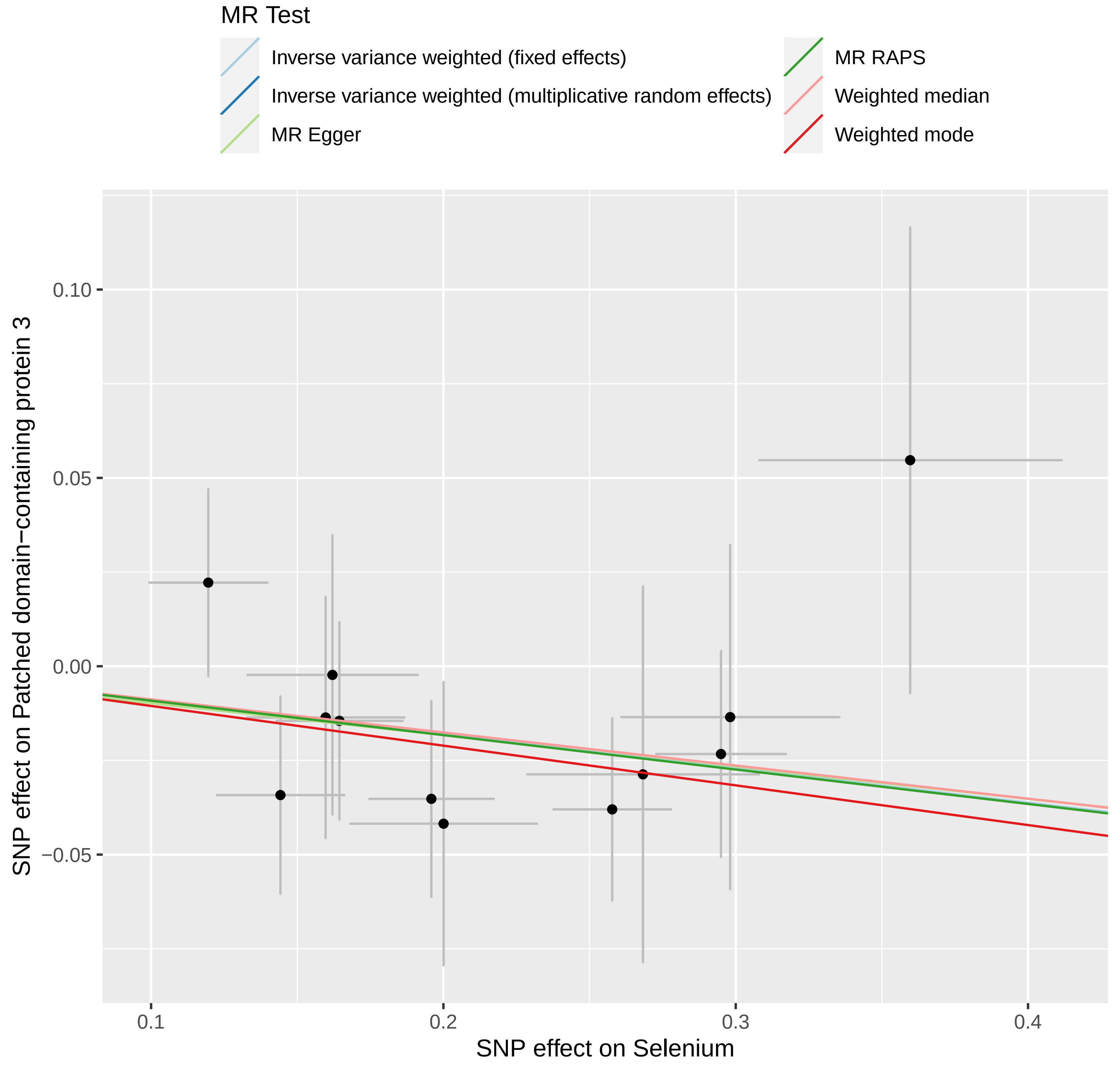

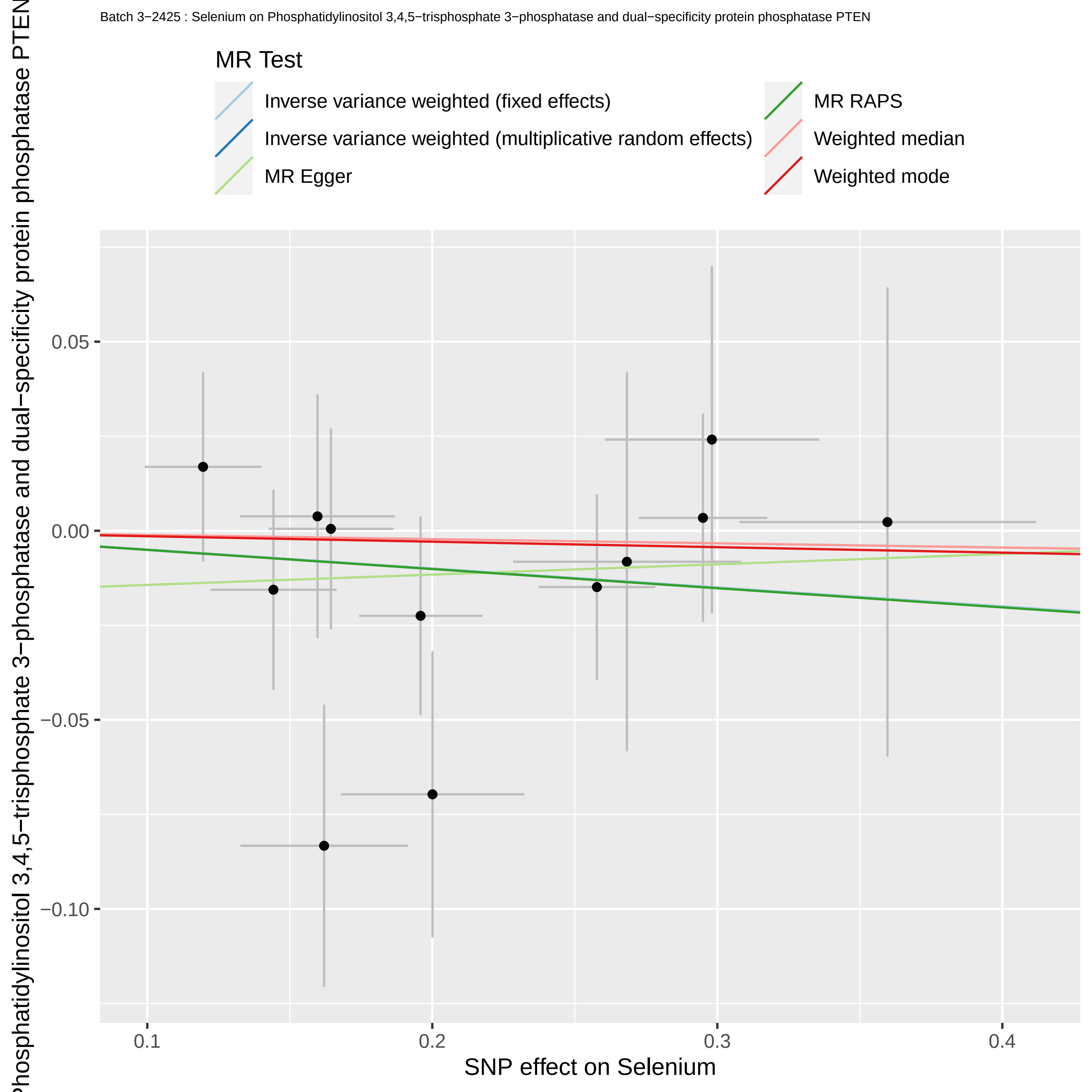
Batch 3−2425 : Selenium on Phosphatidylinositol 3,4,5−trisphosphate 3−phosphatase and dual−specificity protein phosphatase PTEN
MR Test
Inverse variance weighted (fixed effects)
Inverse variance weighted (multiplicative random effects)
MR Egger
MR RAPS
Weighted median
Weighted mode
0.05
0.00
−0.05
−0.10
0.1
0.2
0.3
0.4
SNP effect on Selenium
Phosphatidylinositol 3,4,5−trisphosphate 3−phosphatase and dual−specificity protein phosphatase PTEN

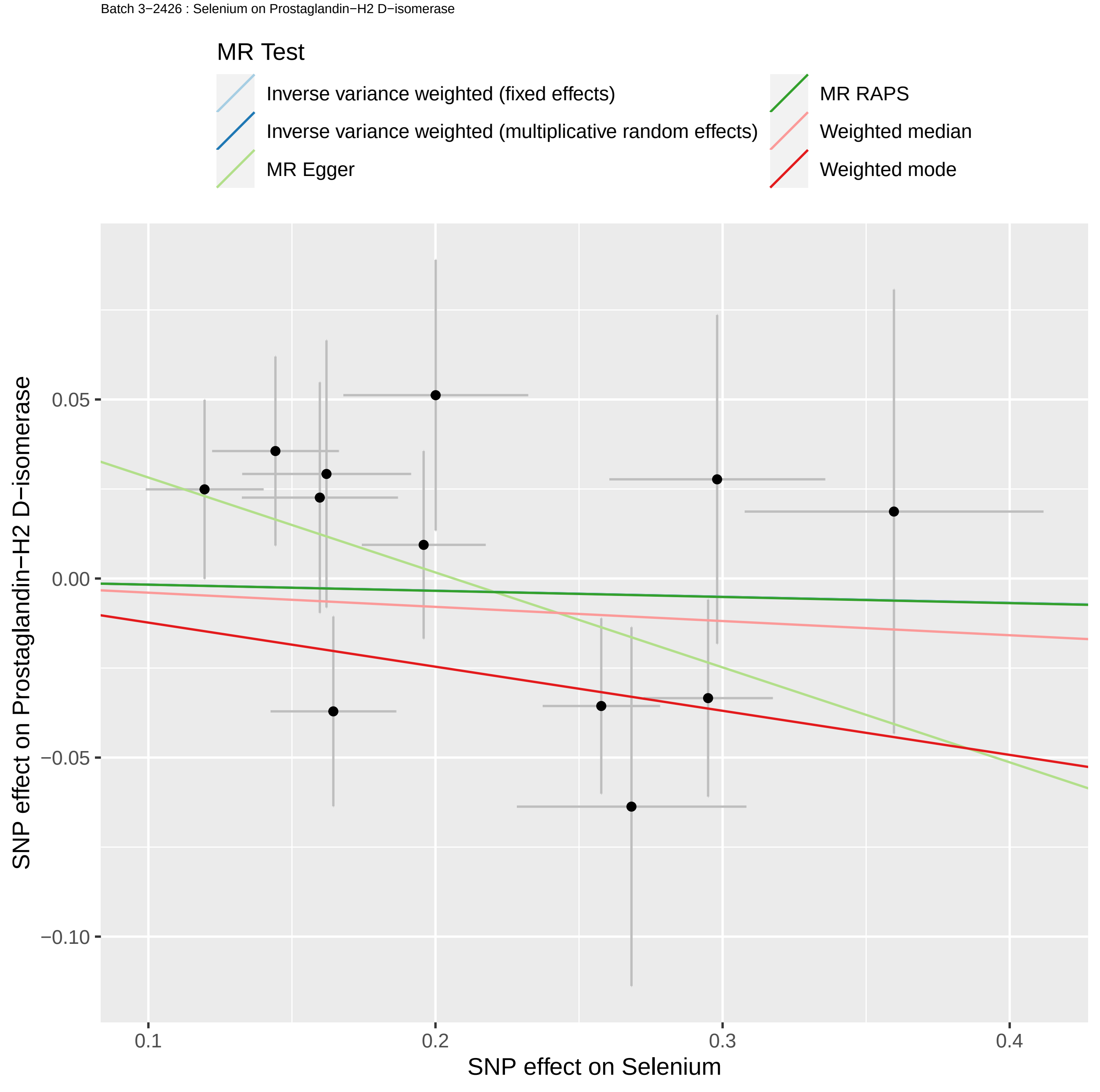
Batch 3−2426 : Selenium on Prostaglandin−H2 D−isomerase
MR Test
Inverse variance weighted (fixed effects)
Inverse variance weighted (multiplicative random effects)
MR Egger
MR RAPS
Weighted median
Weighted mode
SNP effect on Prostaglandin−H2 D−isomerase
0.05
0.00
−0.05
−0.10
0.1
0.2
0.3
0.4
SNP effect on Selenium

Batch 3−2427 : Selenium on Prostaglandin F2 receptor negative regulator

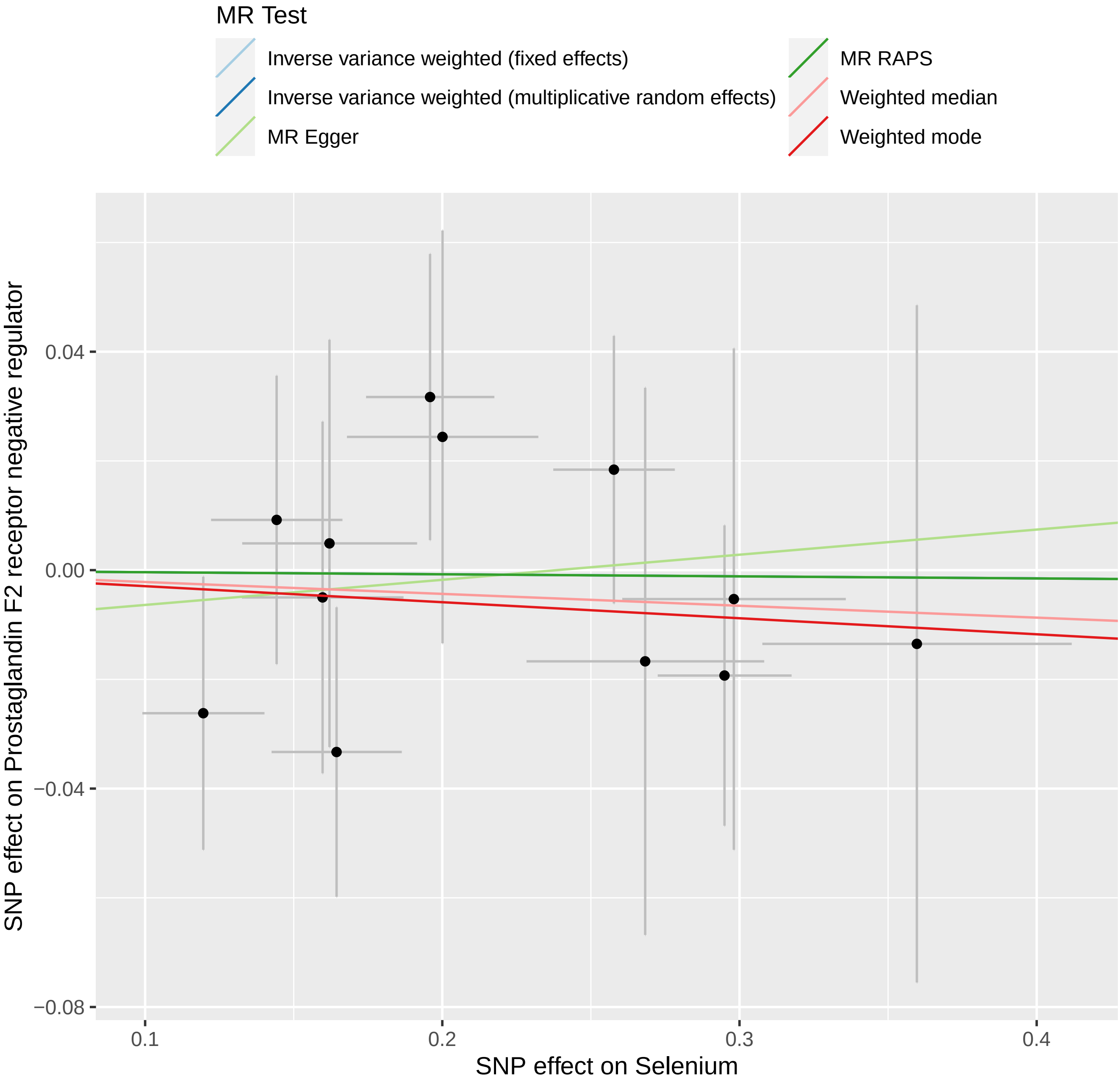

Batch 3−2428 : Selenium on Prostaglandin reductase 1

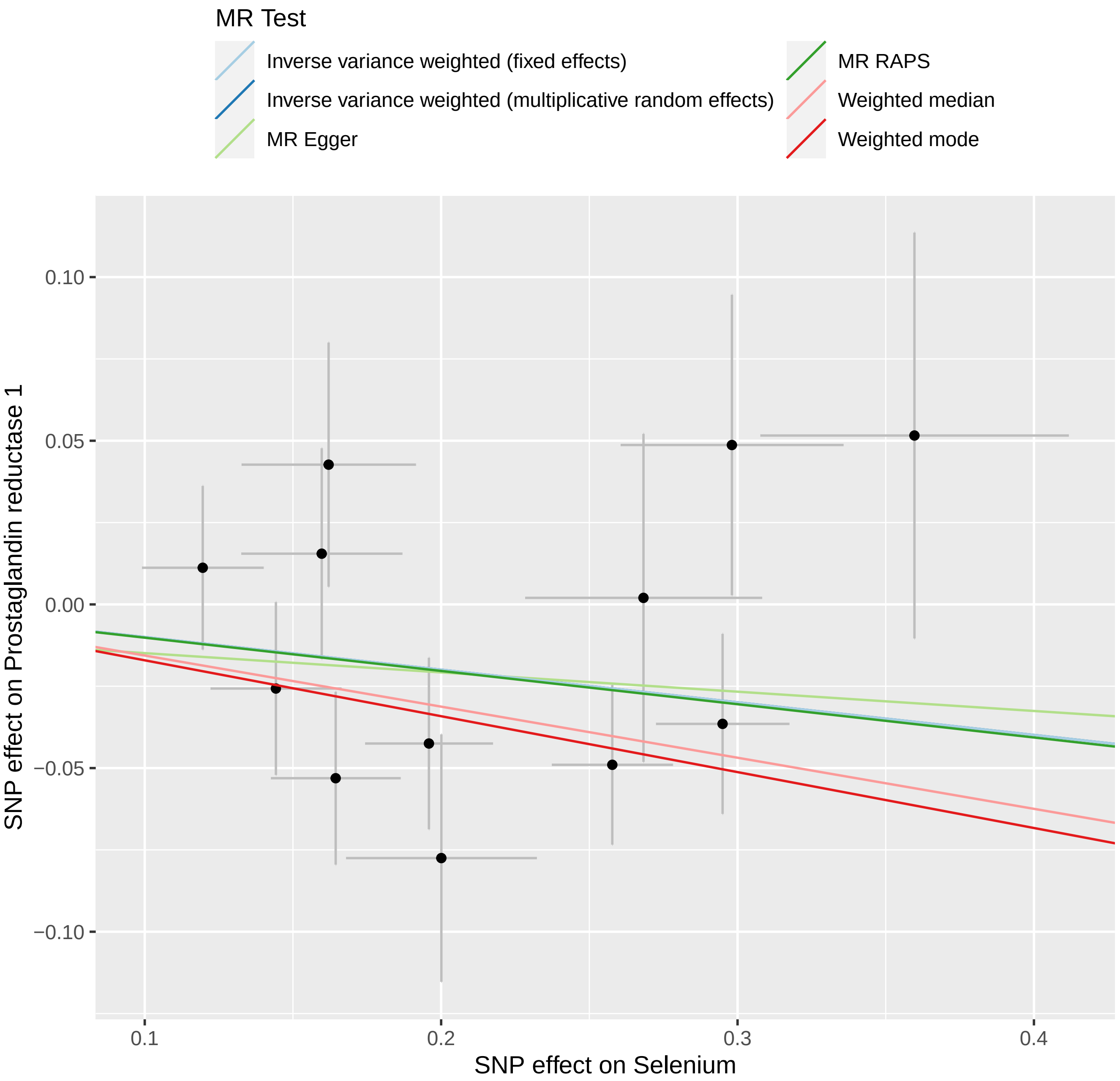

Batch 3−2429 : Selenium on Parathyroid hormone/parathyroid hormone−related peptide receptor

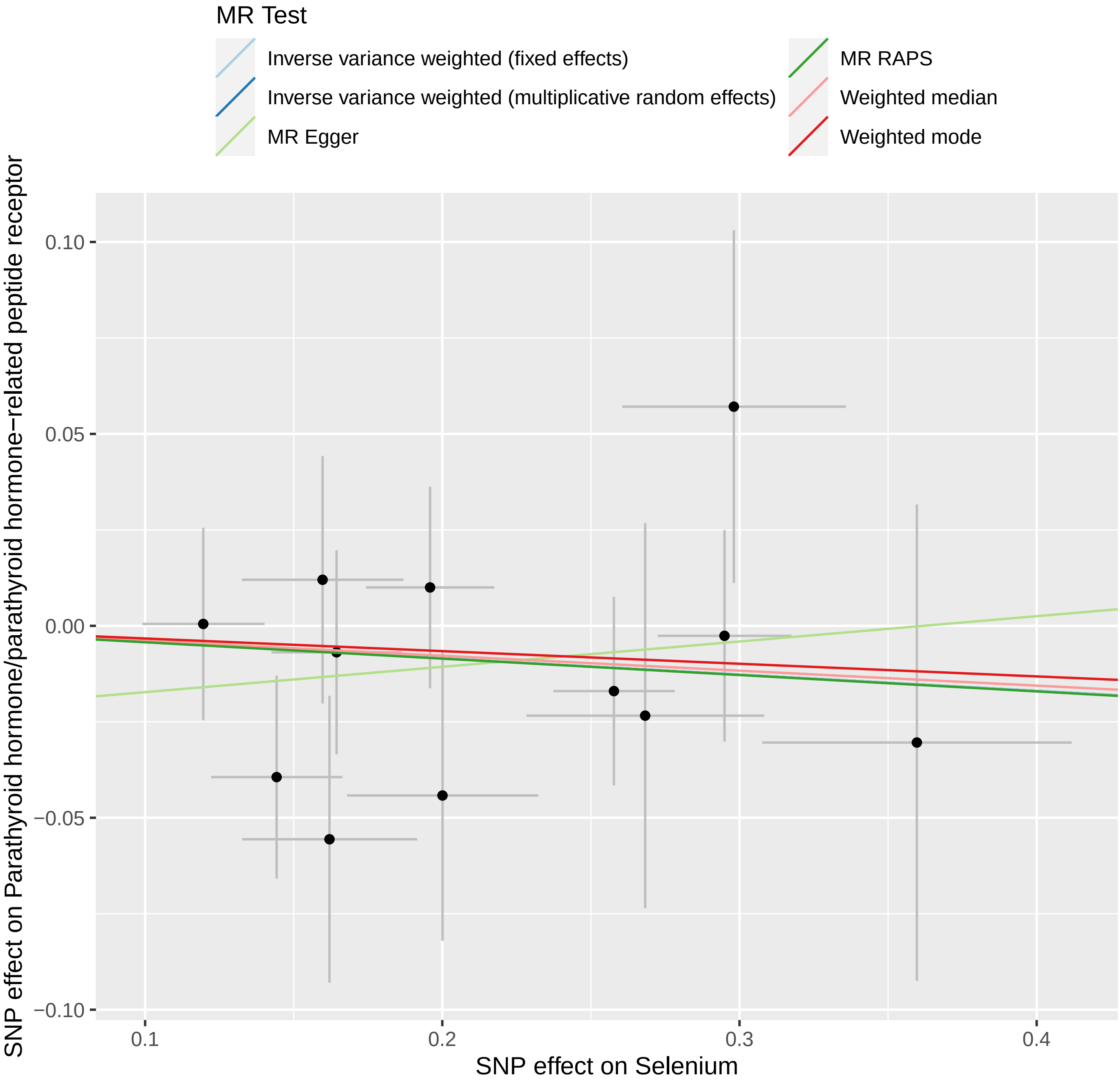

Batch 3−2430 : Selenium on Tuberoinfundibular peptide of 39 residues

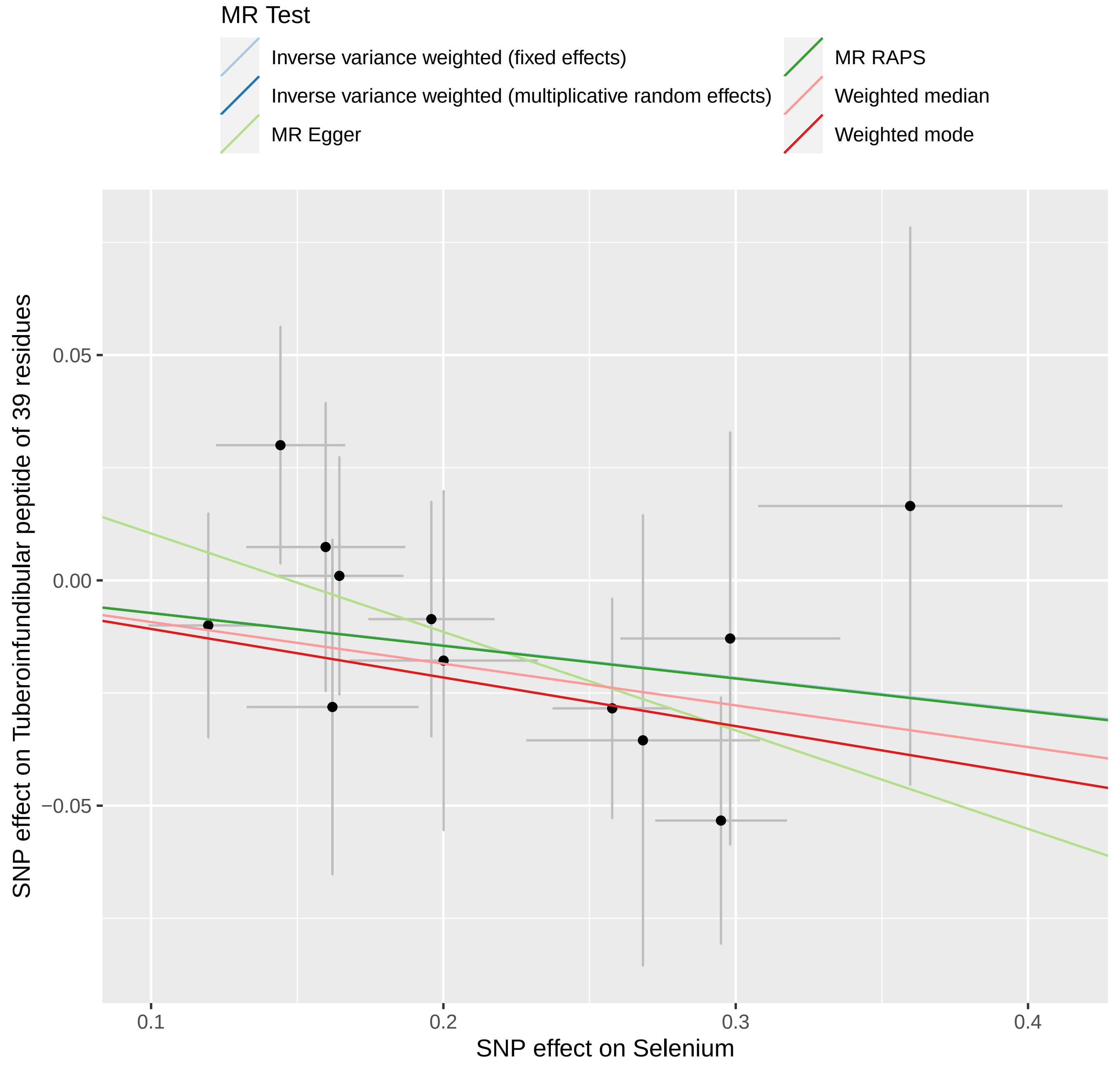

Batch 3−2431 : Selenium on Parathyroid hormone

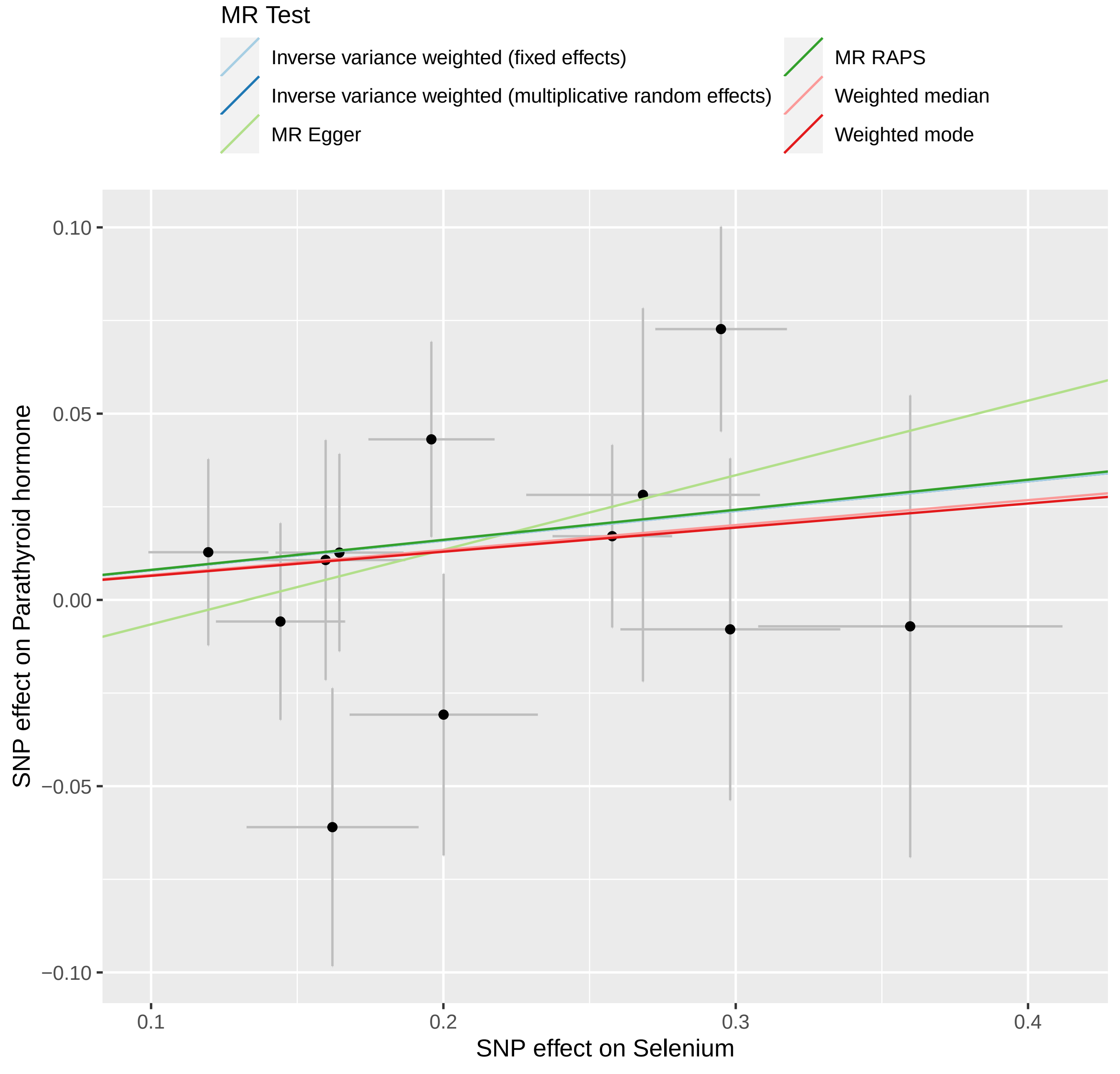

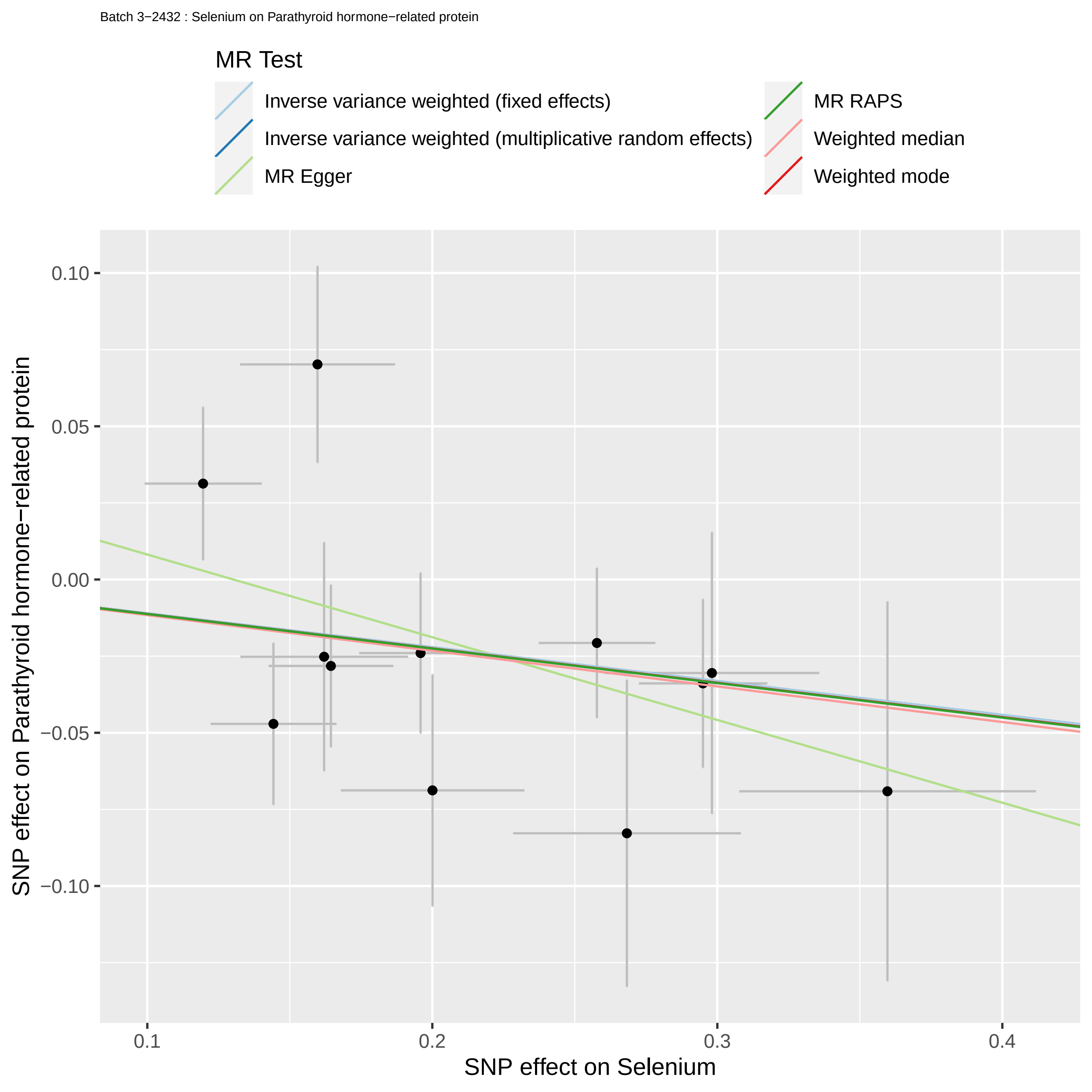

Batch 3−2432 : Selenium on Parathyroid hormone−related protein
MR Test
Inverse variance weighted (fixed effects)
Inverse variance weighted (multiplicative random effects)
MR Egger
MR RAPS
Weighted median
Weighted mode
SNP effect on Parathyroid hormone−related protein
0.10
0.05
0.00
−0.05
−0.10
0.1
0.2
0.3
0.4
SNP effect on Selenium

Batch 3−2433 : Selenium on Protein−tyrosine kinase 2−beta

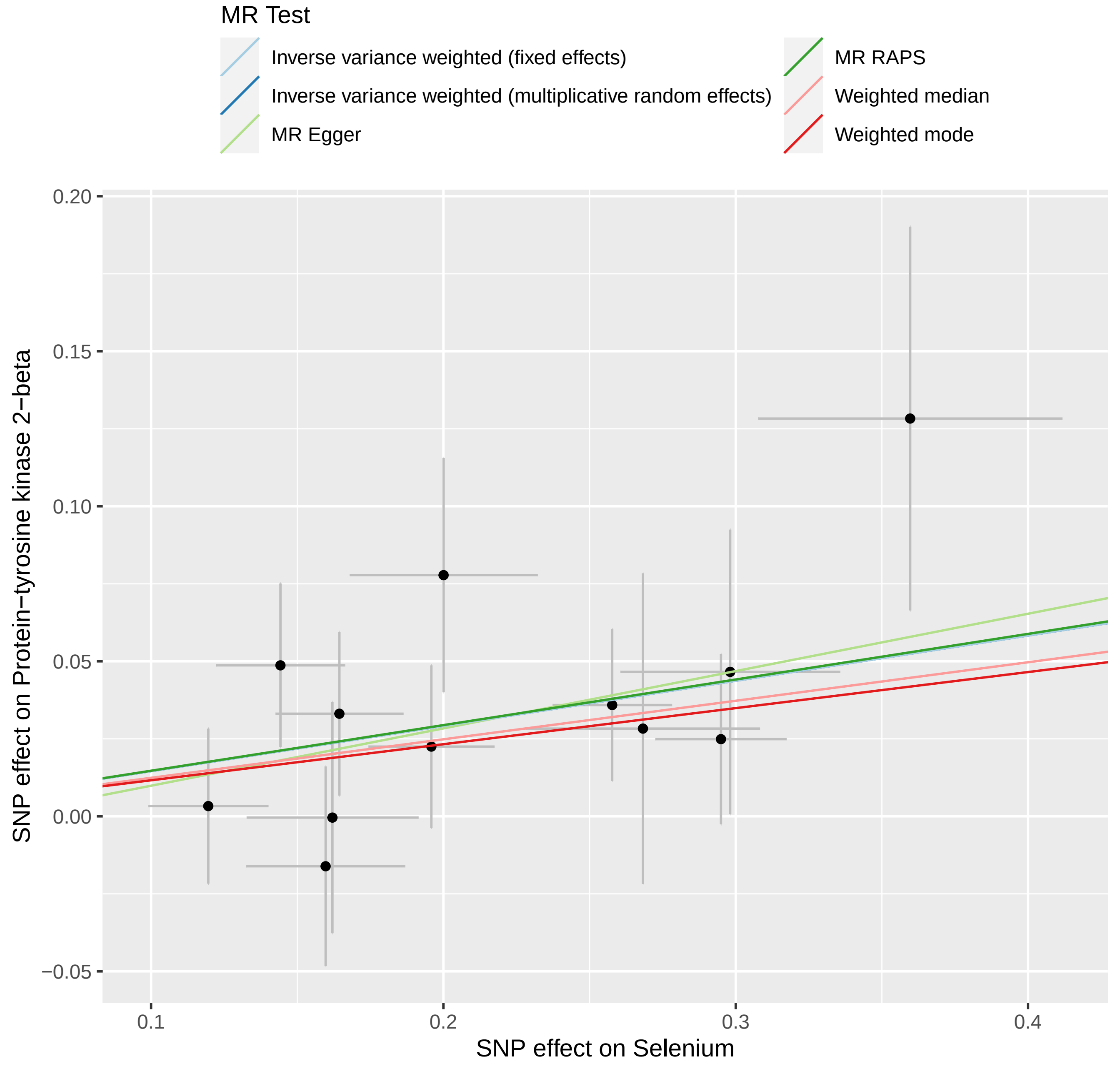

Batch 3−2434 : Selenium on Protein−tyrosine kinase 6

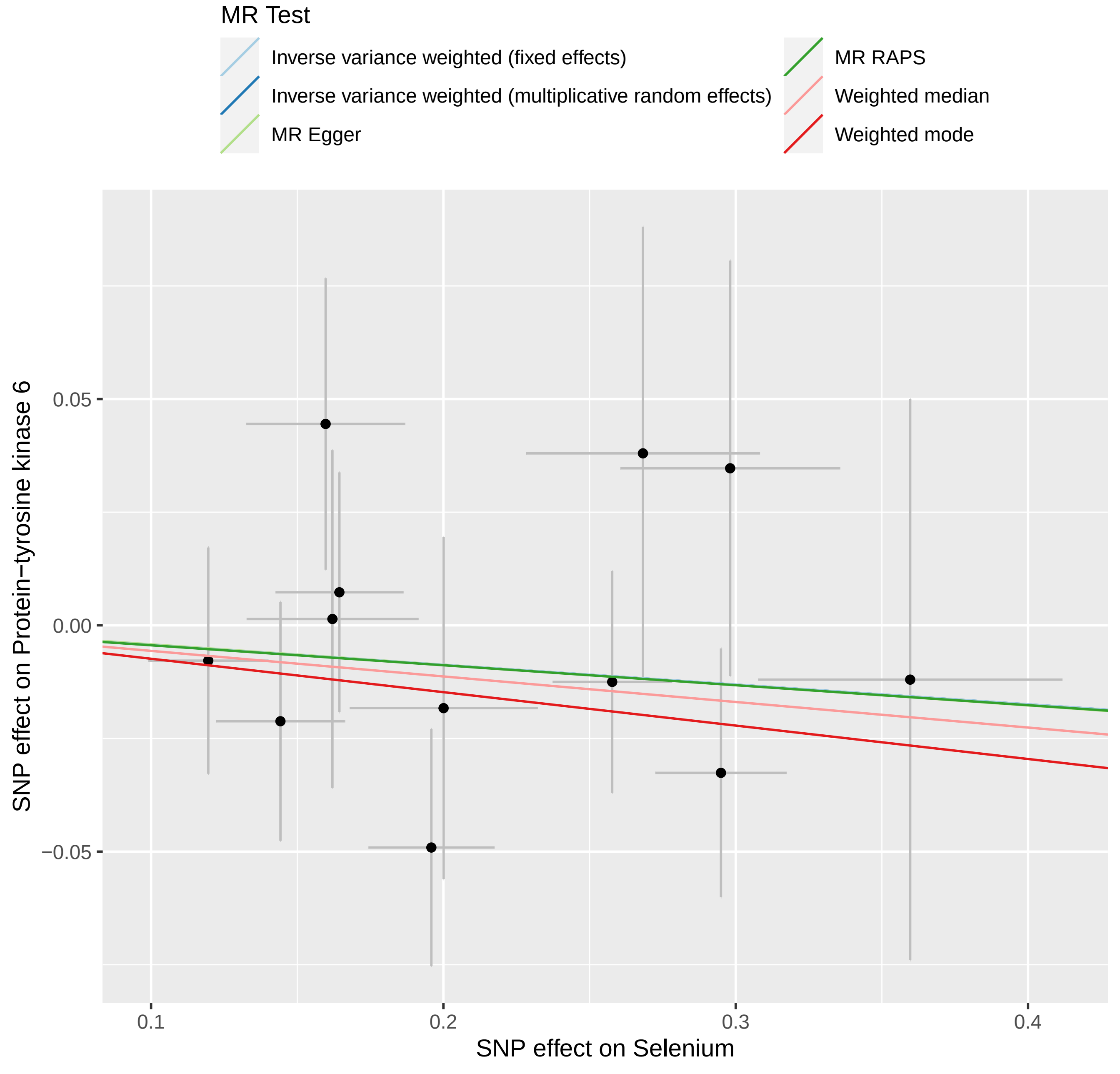

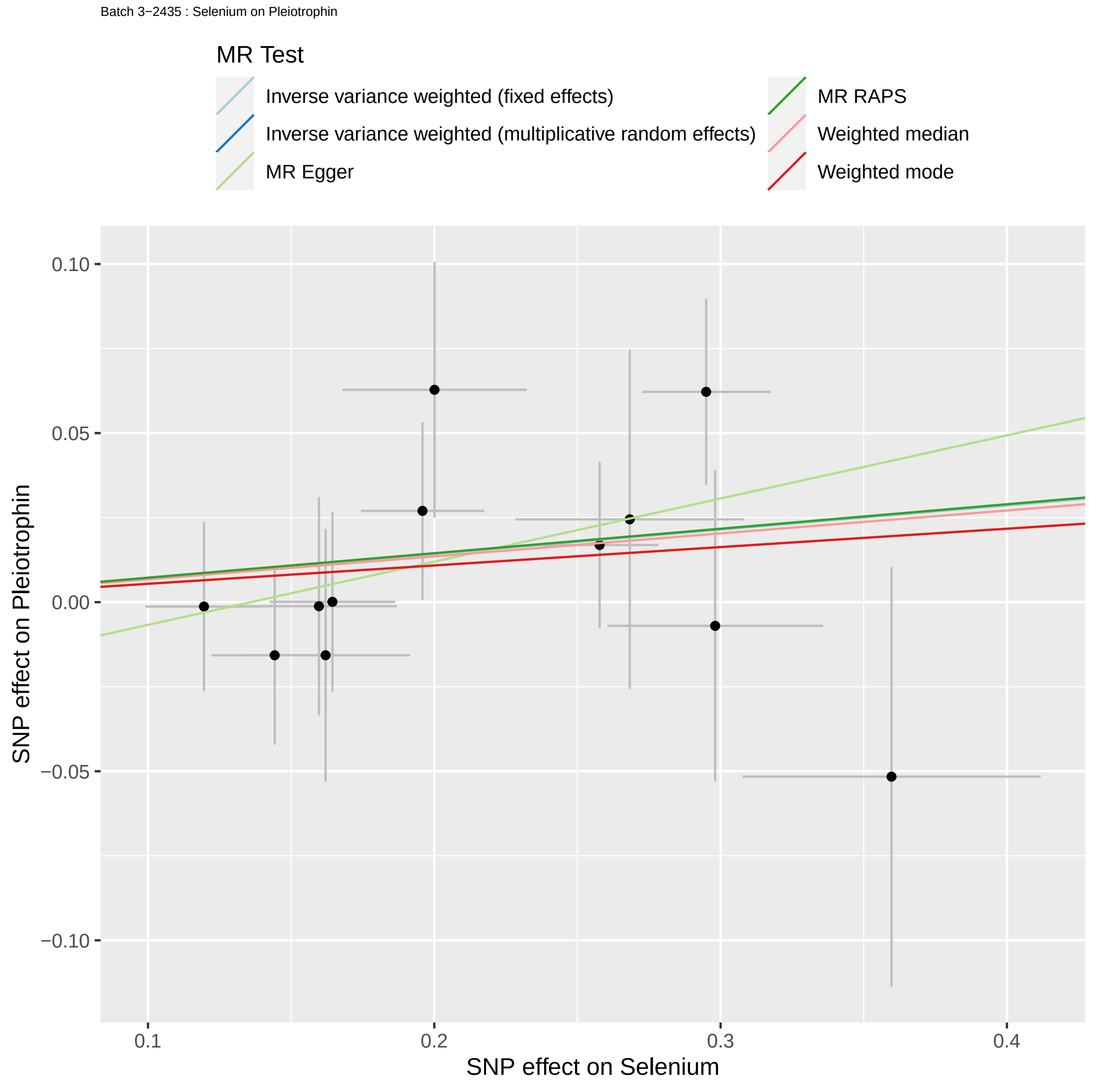
Batch 3−2435 : Selenium on Pleiotrophin
MR Test
Inverse variance weighted (fixed effects)
Inverse variance weighted (multiplicative random effects)
MR Egger
MR RAPS
Weighted median
Weighted mode
0.10
0.05
0.00
−0.05
−0.10
0.1
0.2
0.3
0.4
SNP effect on Pleiotrophin
SNP effect on Selenium

Batch 3−2436 : Selenium on Protein tyrosine phosphatase type IVA 2

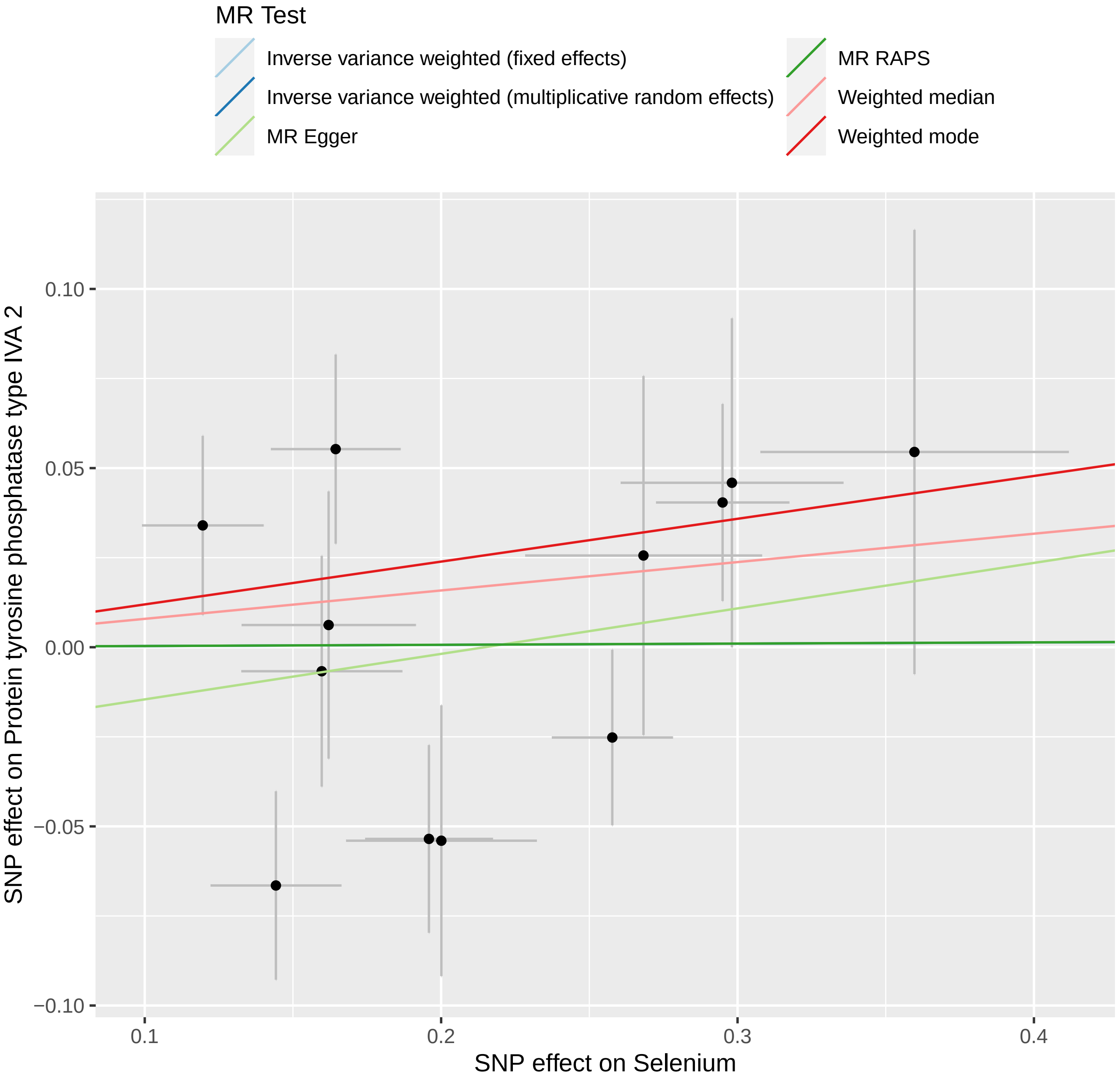

Batch 3−2437 : Selenium on Tyrosine−protein phosphatase non−receptor type 11

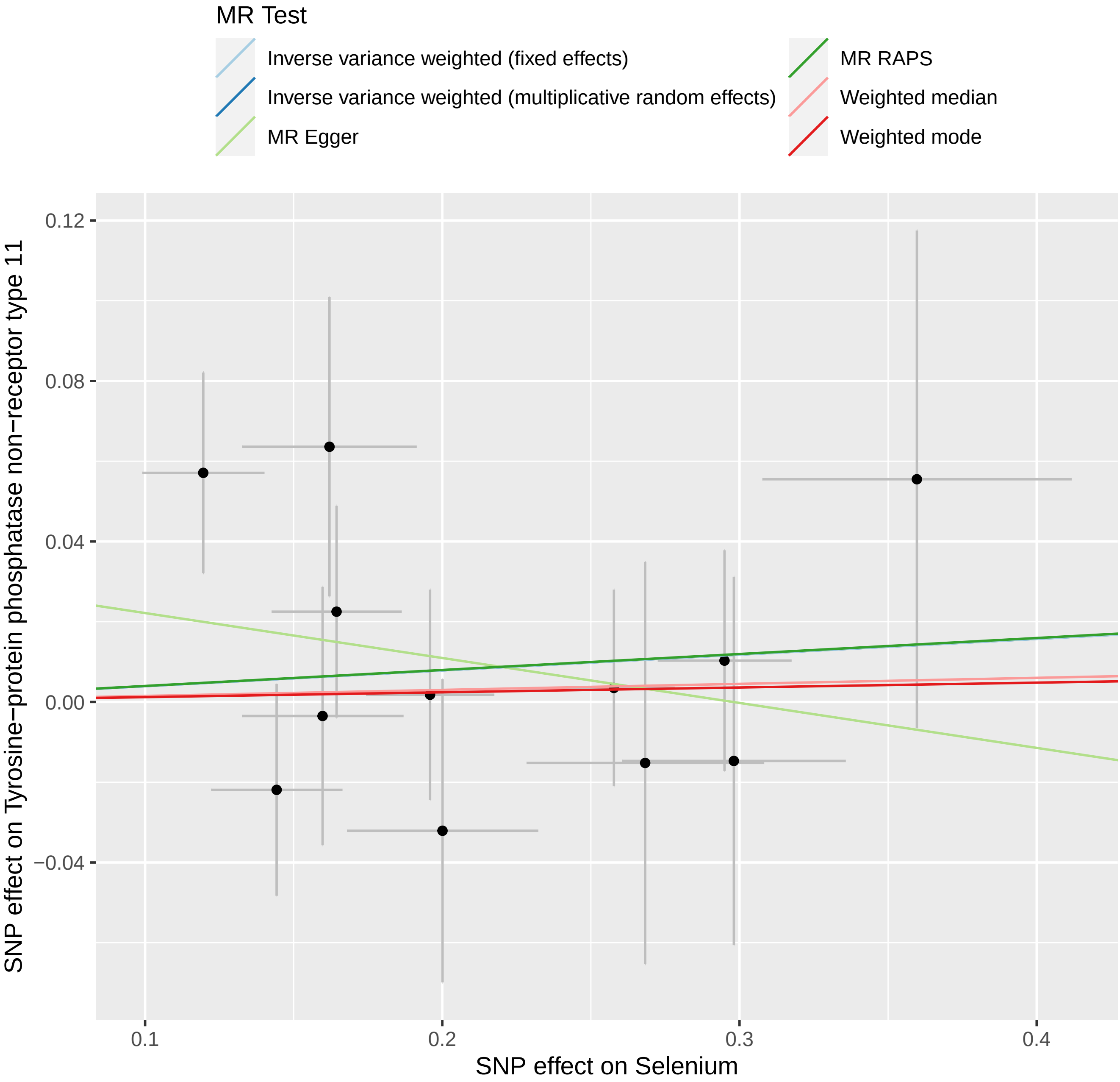

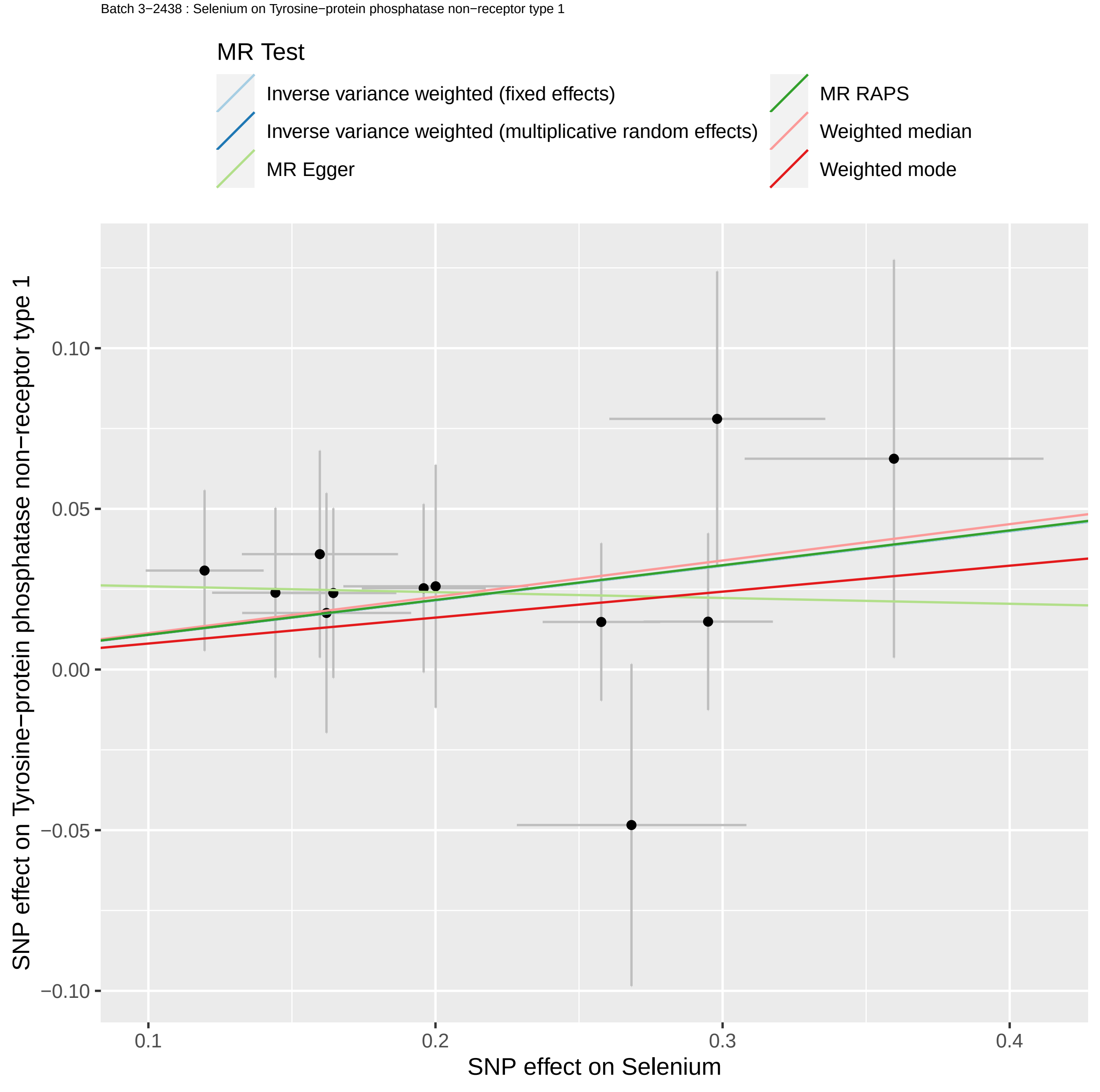
Batch 3−2438 : Selenium on Tyrosine−protein phosphatase non−receptor type 1
MR Test
Inverse variance weighted (fixed effects)
Inverse variance weighted (multiplicative random effects)
MR Egger
MR RAPS
Weighted median
Weighted mode
SNP effect on Tyrosine−protein phosphatase non−receptor type 1
0.10
0.05
0.00
−0.05
−0.10
0.1
0.2
0.3
0.4
SNP effect on Selenium

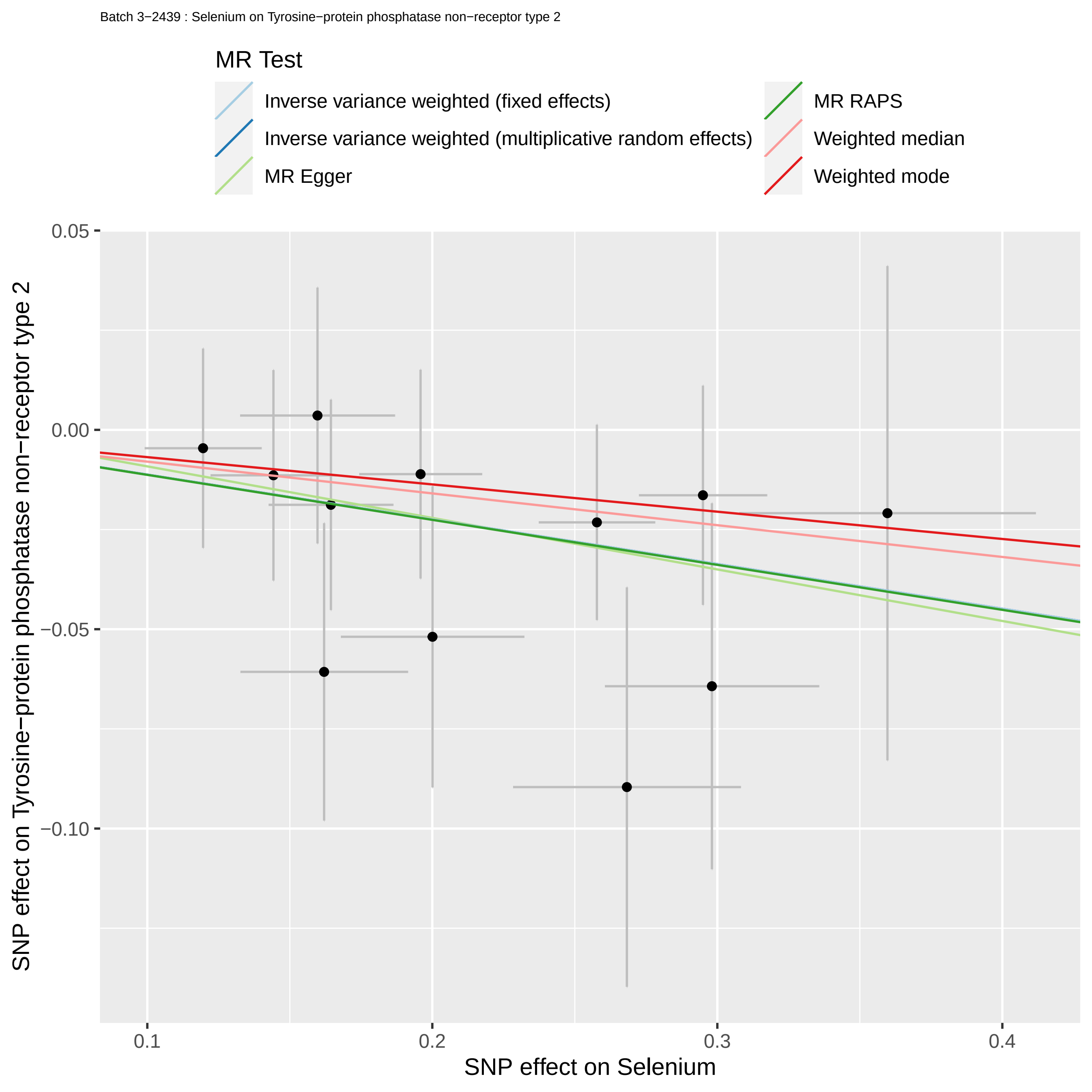

Batch 3−2439 : Selenium on Tyrosine−protein phosphatase non−receptor type 2
MR Test
Inverse variance weighted (fixed effects)
Inverse variance weighted (multiplicative random effects)
MR Egger
MR RAPS
Weighted median
Weighted mode
0.05
0.00
−0.05
−0.10
0.1
0.2
0.3
0.4
SNP effect on Tyrosine−protein phosphatase non−receptor type 2
SNP effect on Selenium

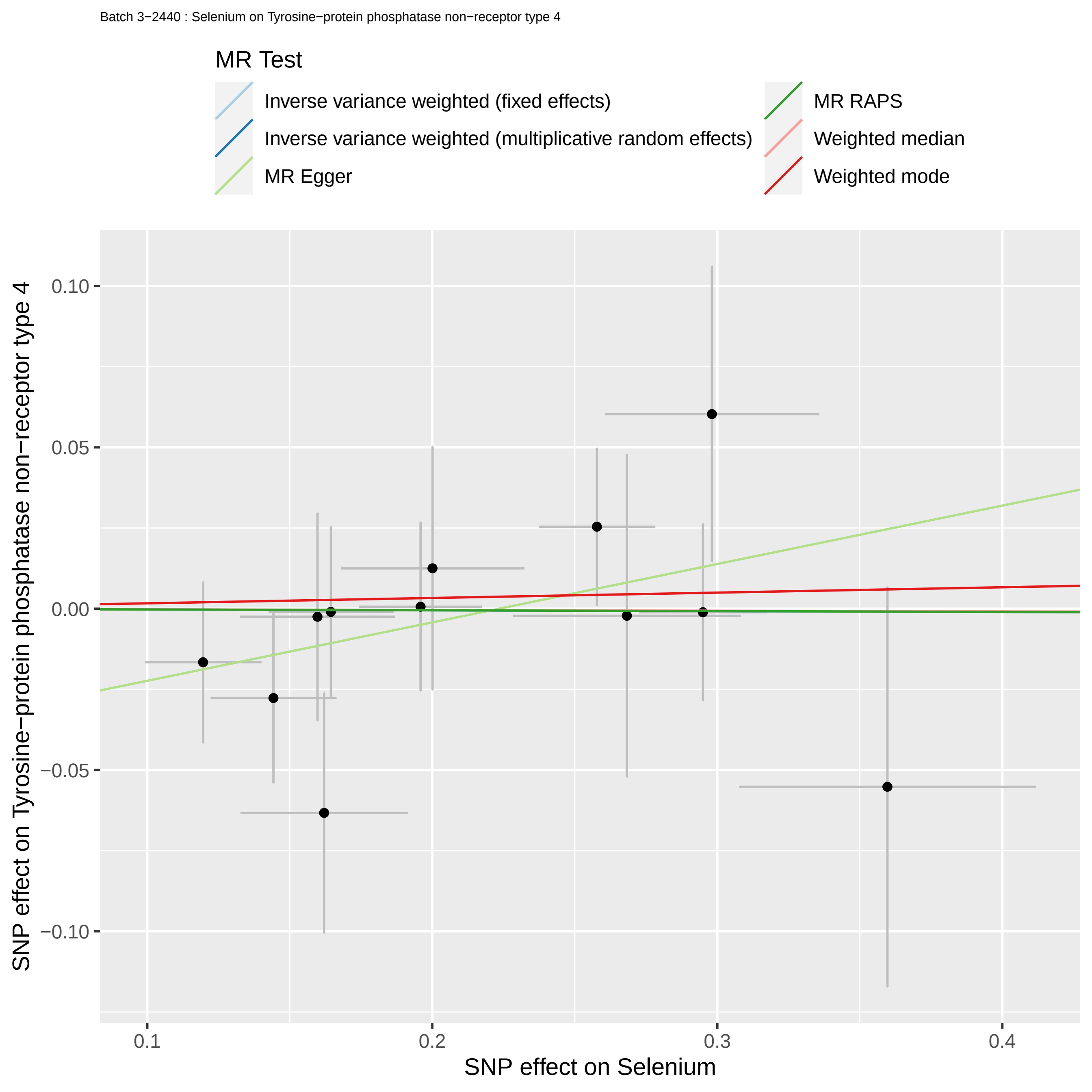

Batch 3−2440 : Selenium on Tyrosine−protein phosphatase non−receptor type 4
MR Test
Inverse variance weighted (fixed effects)
Inverse variance weighted (multiplicative random effects)
MR Egger
MR RAPS
Weighted median
Weighted mode
0.10
0.05
0.00
−0.05
−0.10
0.1
0.2
0.3
0.4
SNP effect on Tyrosine−protein phosphatase non−receptor type 4
SNP effect on Selenium

Batch 3−2441 : Selenium on Tyrosine−protein phosphatase non−receptor type 7

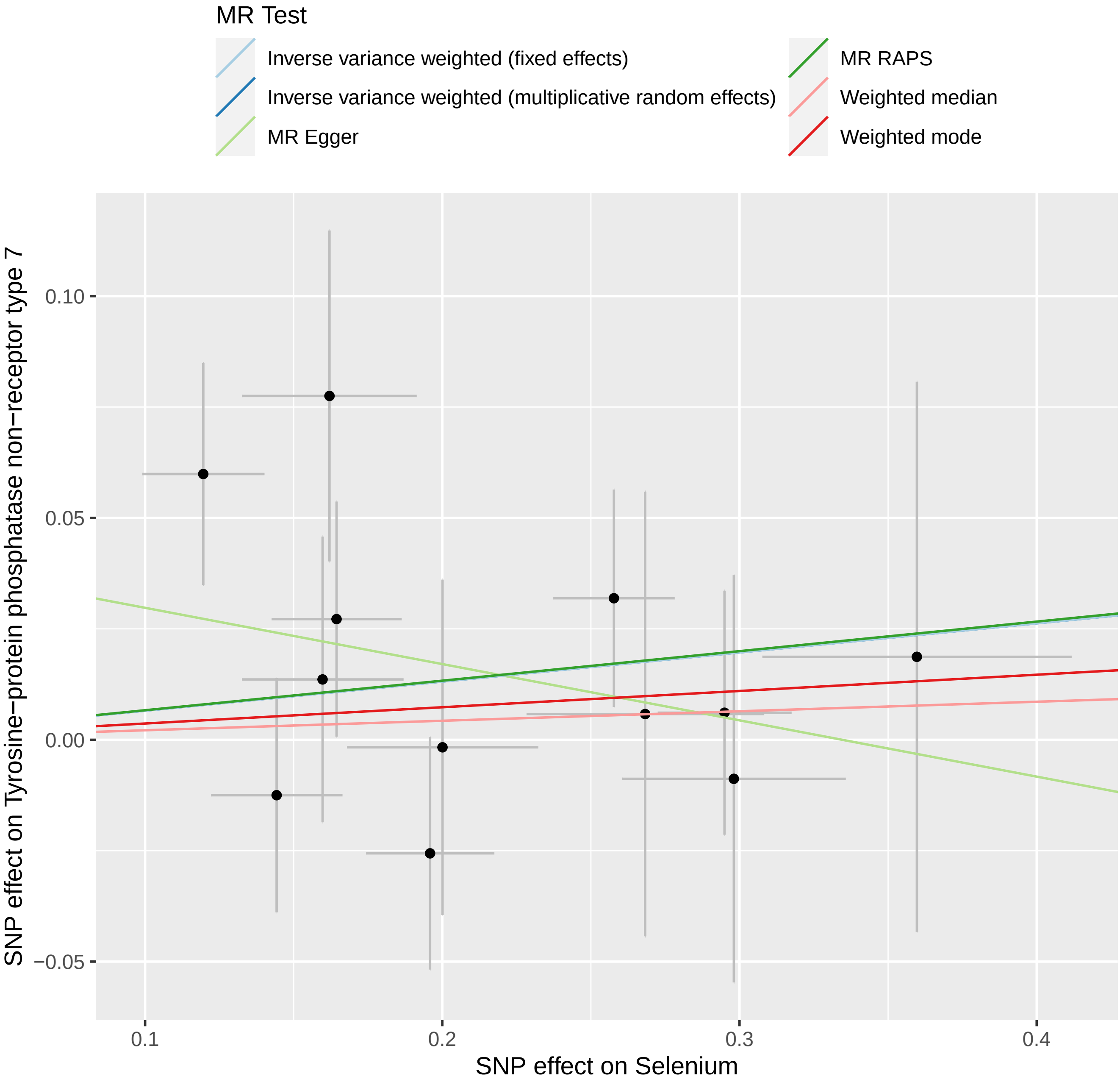

Batch 3−2442 : Selenium on Tyrosine−protein phosphatase non−receptor type 9

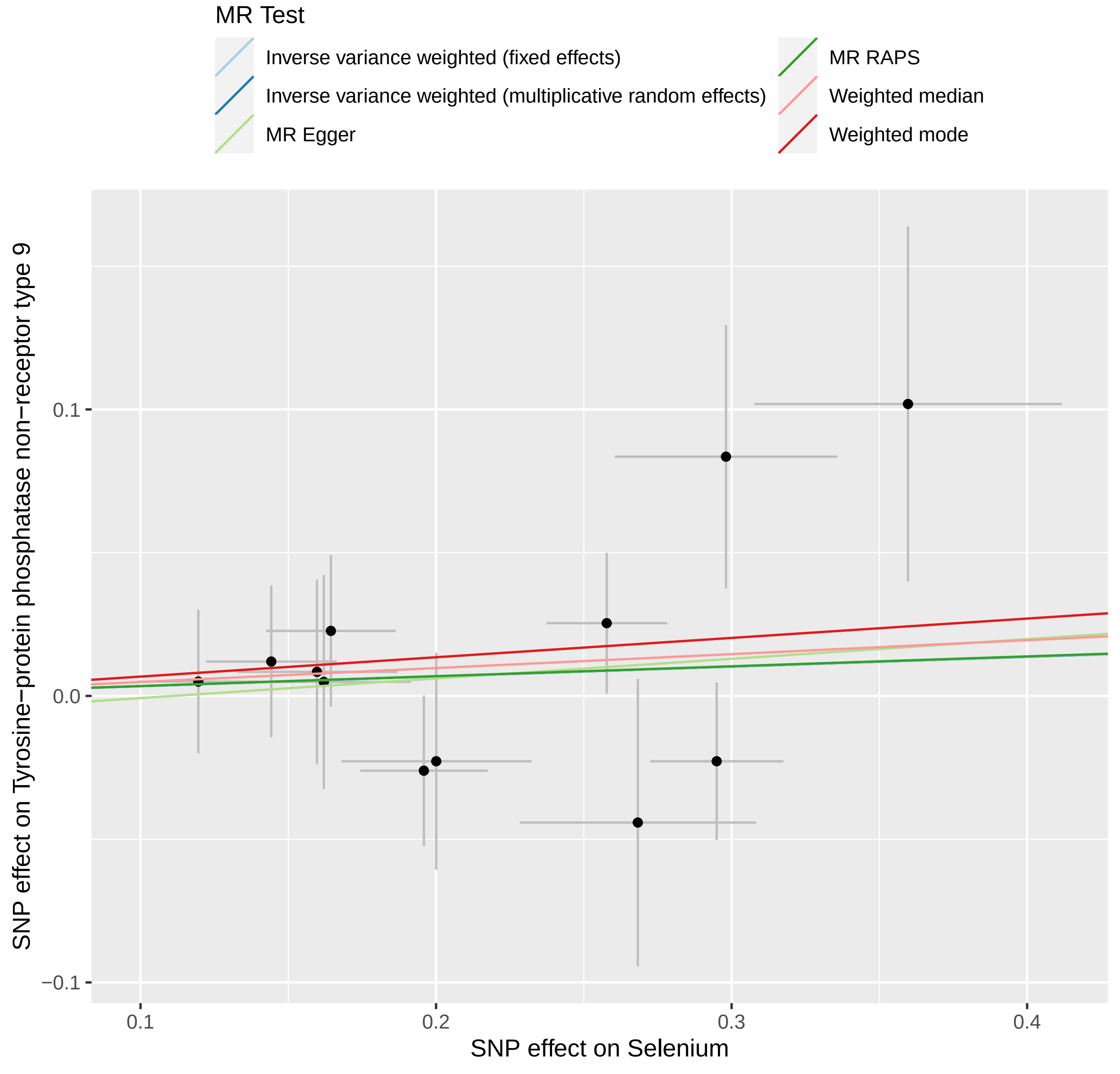

Batch 3−2443 : Selenium on Receptor−type tyrosine−protein phosphatase delta

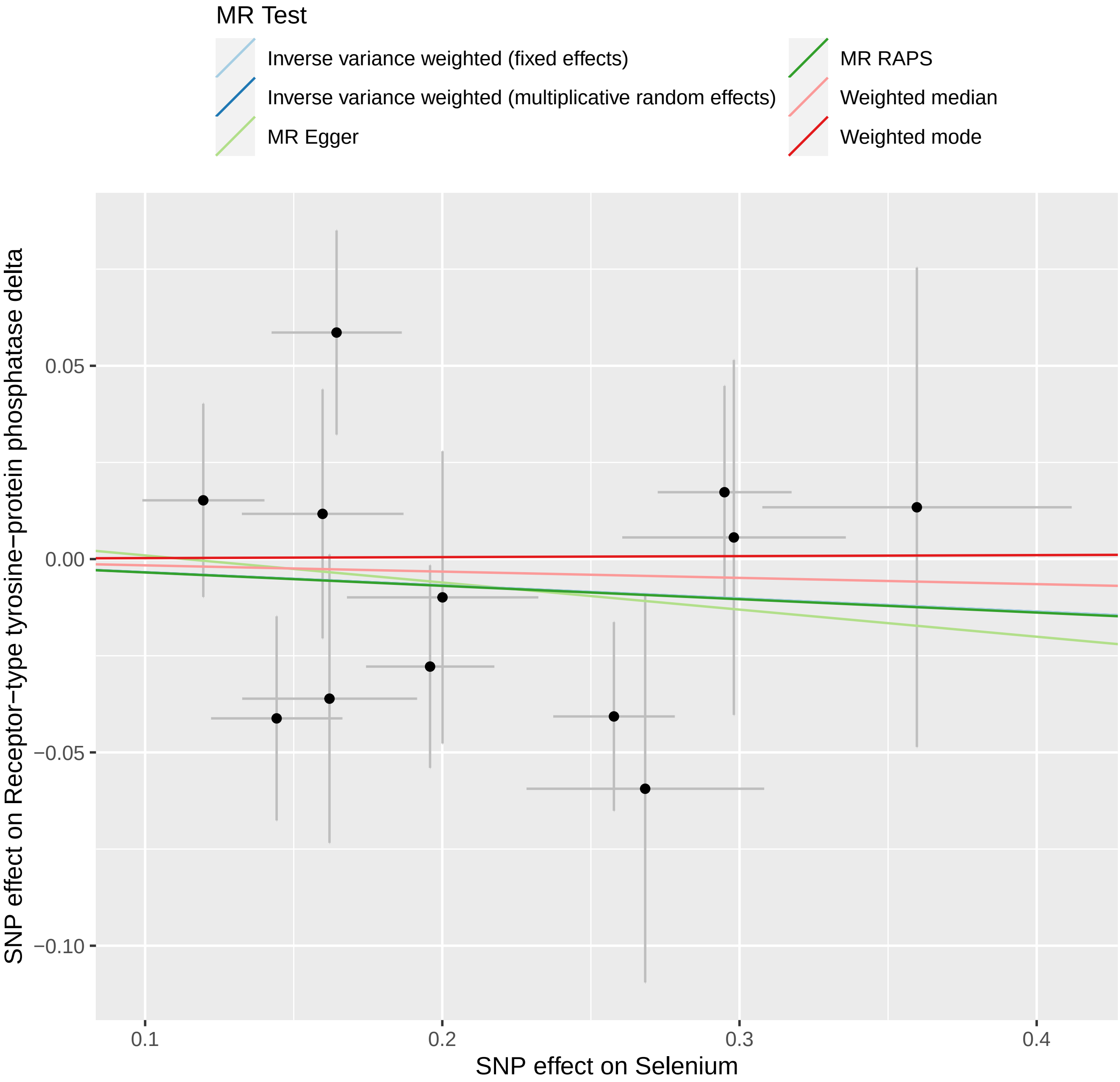

Batch 3−2444 : Selenium on Receptor−type tyrosine−protein phosphatase H

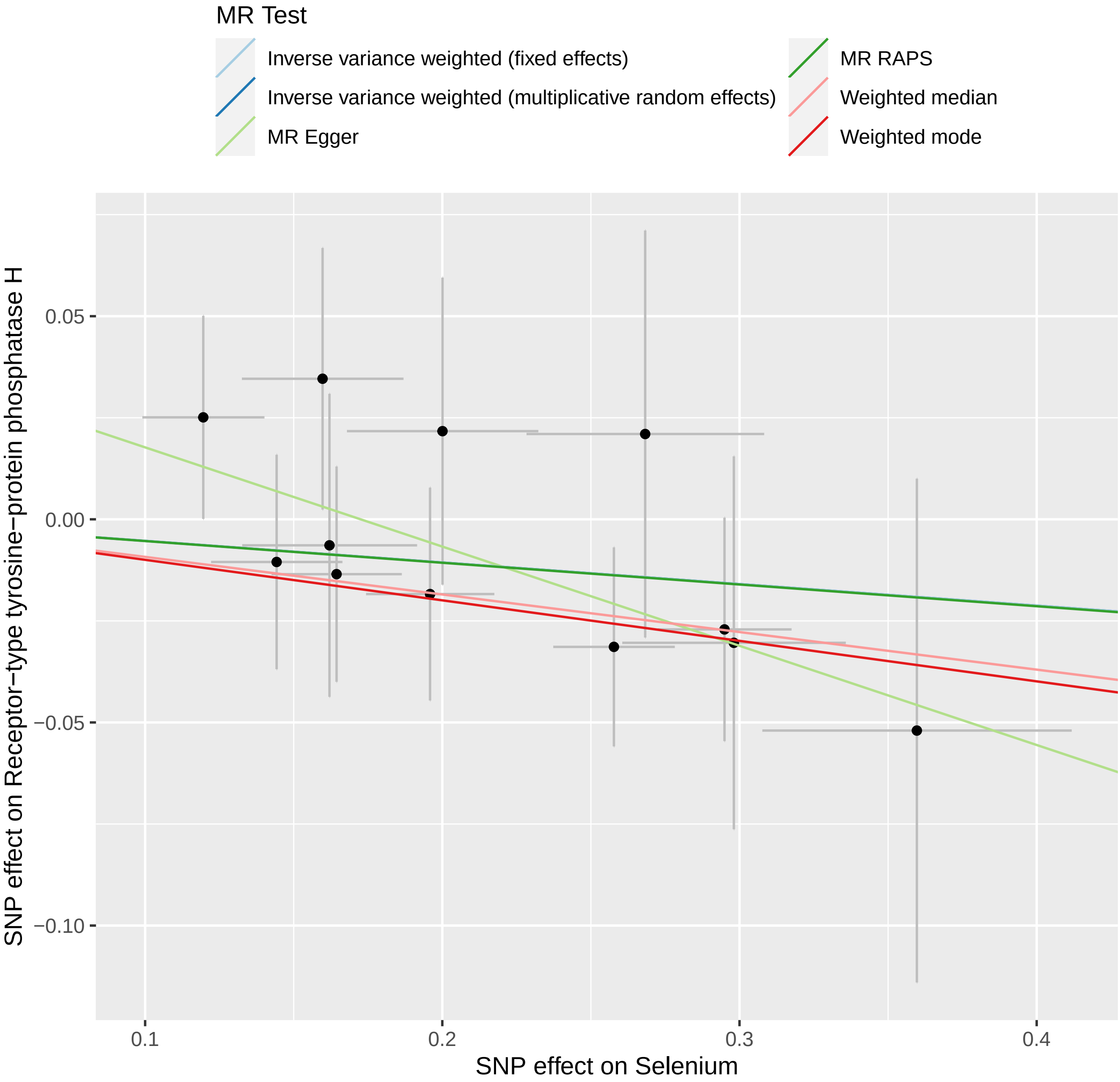

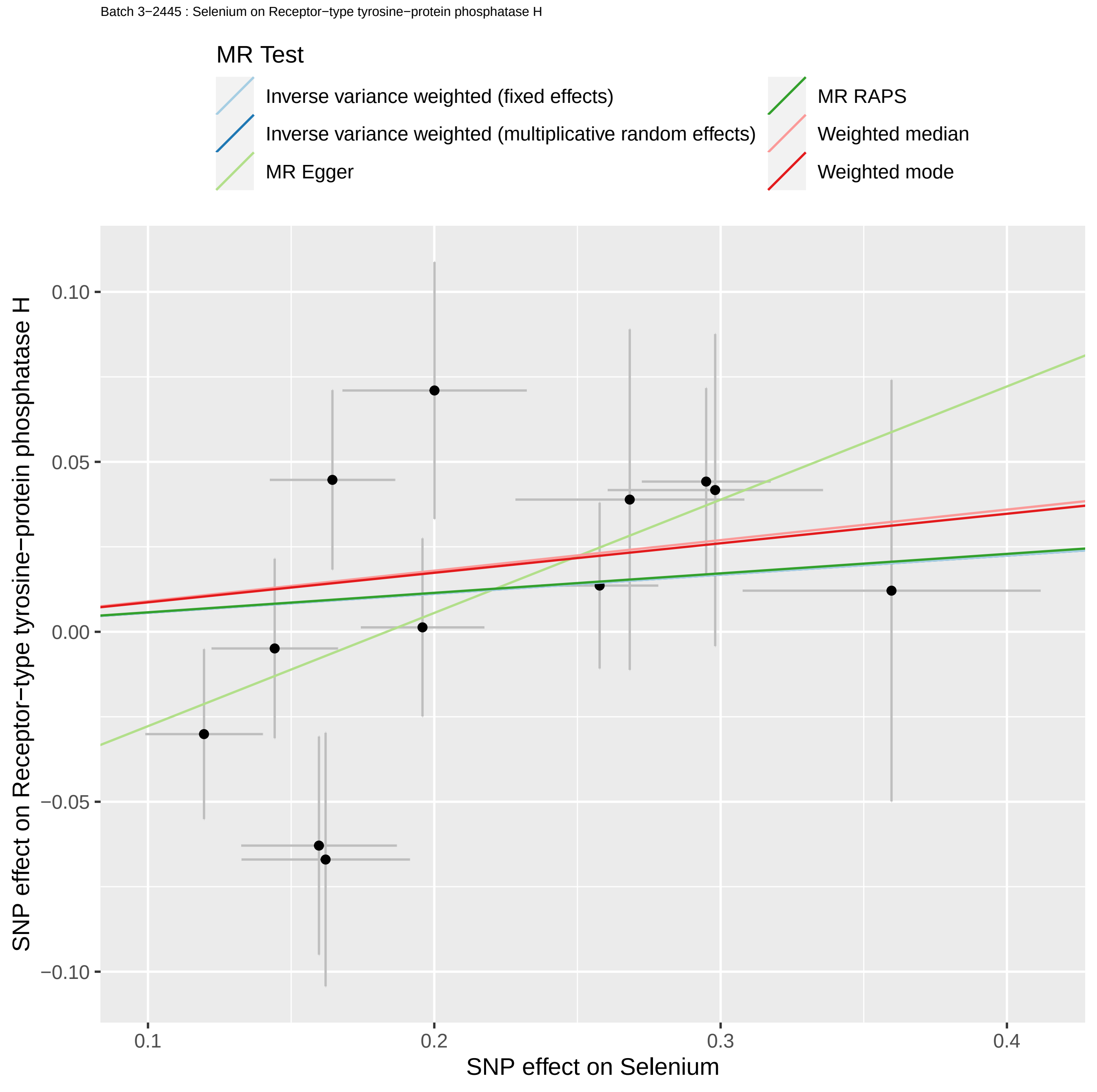

Batch 3−2445 : Selenium on Receptor−type tyrosine−protein phosphatase H
MR Test
Inverse variance weighted (fixed effects)
Inverse variance weighted (multiplicative random effects)
MR Egger
MR RAPS
Weighted median
Weighted mode
0.10
0.05
0.00
−0.05
−0.10
0.1
0.2
0.3
0.4
SNP effect on Receptor−type tyrosine−protein phosphatase H
SNP effect on Selenium

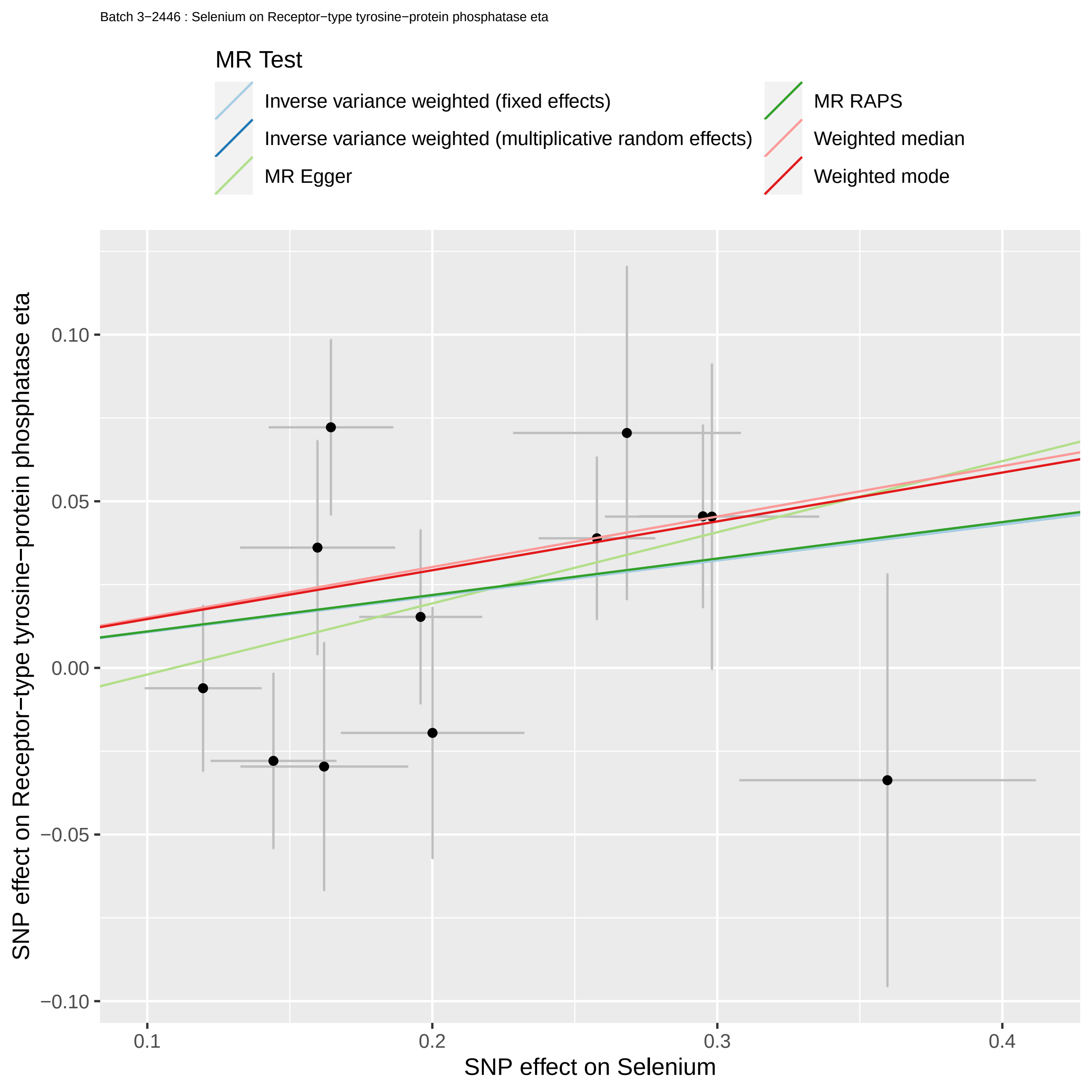

Batch 3−2446 : Selenium on Receptor−type tyrosine−protein phosphatase eta
MR Test
Inverse variance weighted (fixed effects)
Inverse variance weighted (multiplicative random effects)
MR Egger
MR RAPS
Weighted median
Weighted mode
0.10
0.05
0.00
−0.05
−0.10
0.1
0.2
0.3
0.4
SNP effect on Receptor−type tyrosine−protein phosphatase eta
SNP effect on Selenium

Batch 3−2447 : Selenium on Receptor−type tyrosine−protein phosphatase R

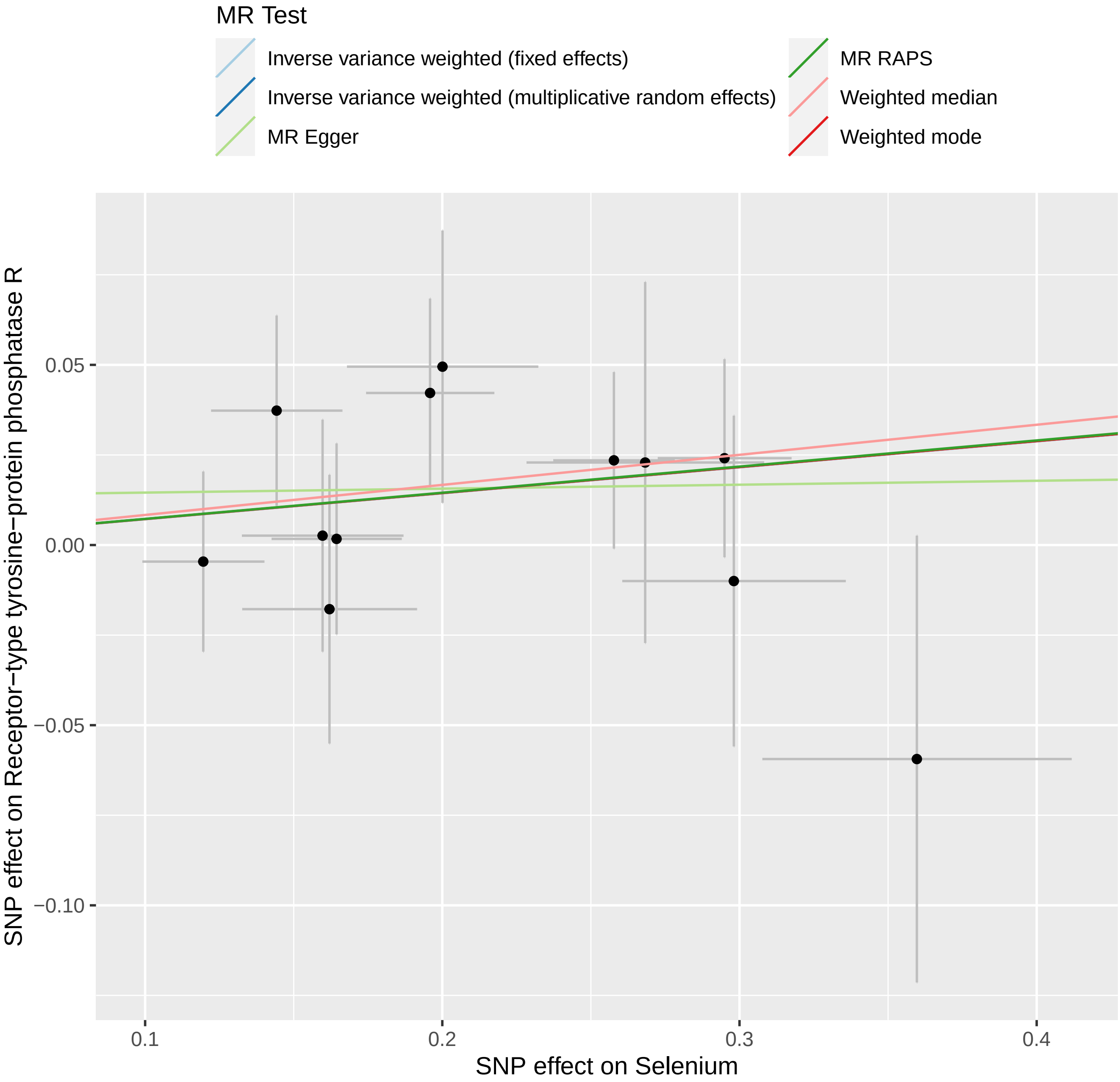

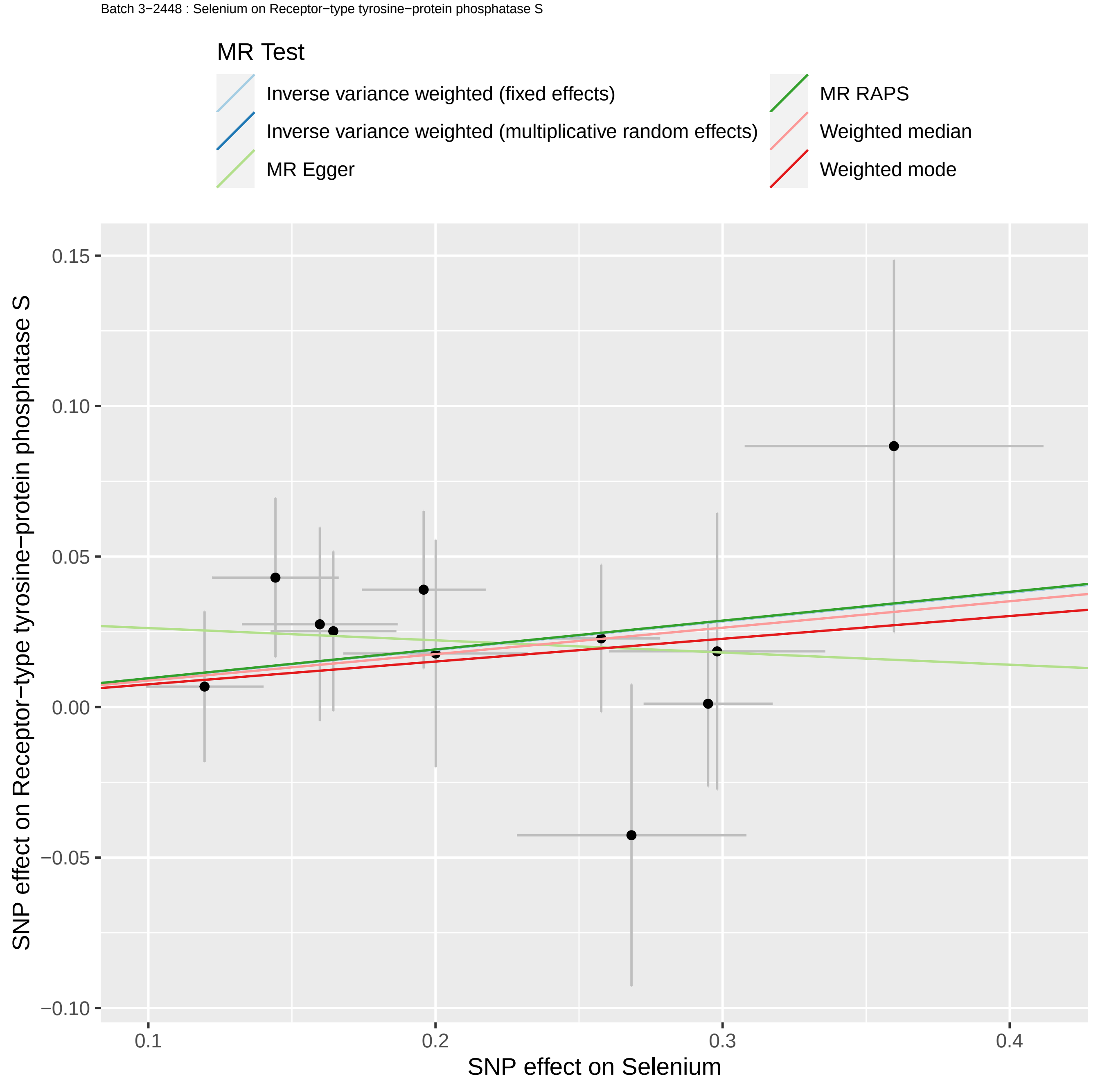

Batch 3−2448 : Selenium on Receptor−type tyrosine−protein phosphatase S
MR Test
Inverse variance weighted (fixed effects)
Inverse variance weighted (multiplicative random effects)
MR Egger
MR RAPS
Weighted median
Weighted mode
SNP effect on Receptor−type tyrosine−protein phosphatase S
0.15
0.10
0.05
0.00
−0.05
−0.10
0.1
0.2
0.3
0.4
SNP effect on Selenium

Batch 3−2449 : Selenium on Receptor−type tyrosine−protein phosphatase U

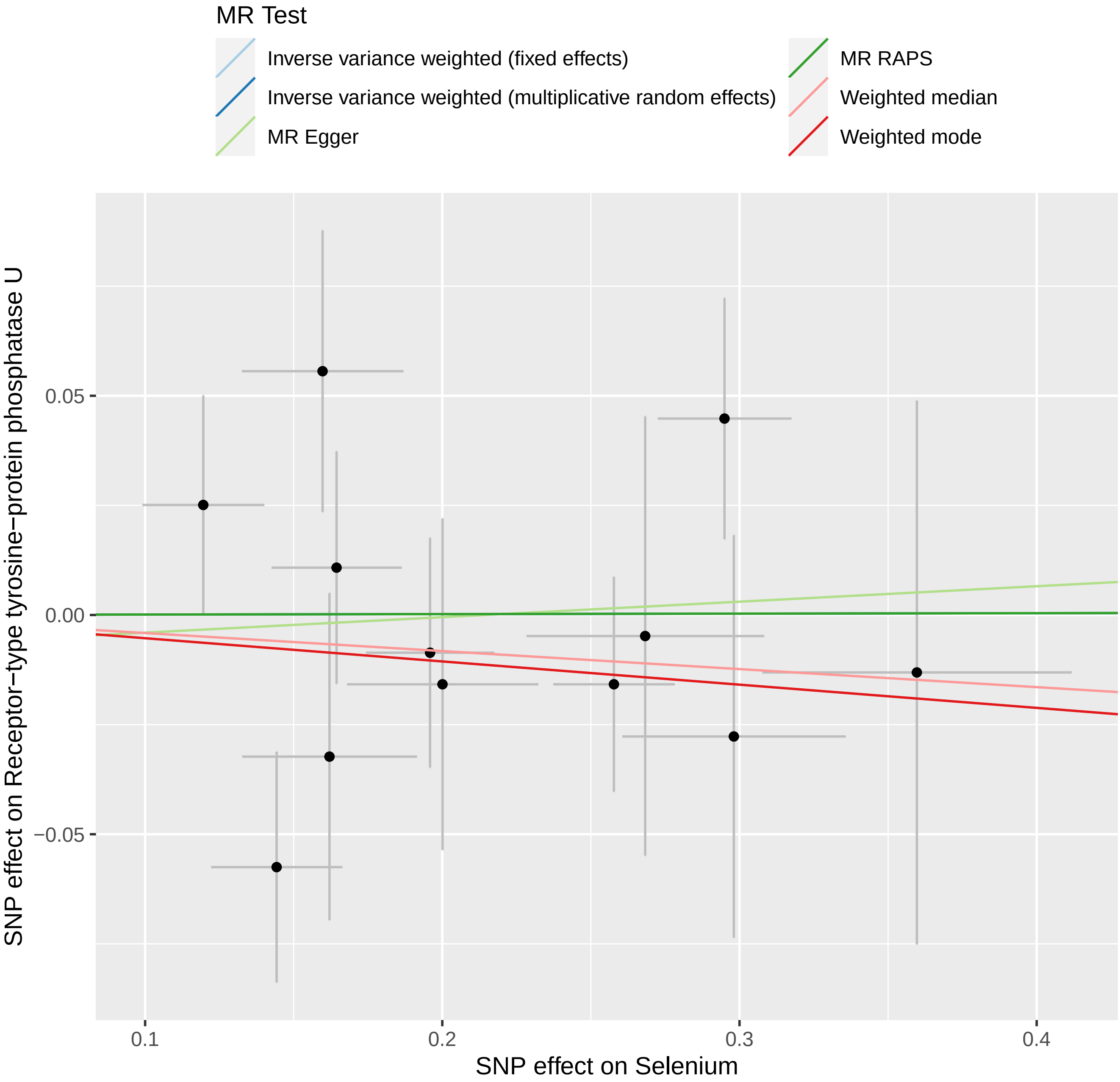

Batch 3−2450 : Selenium on 6−pyruvoyl tetrahydrobiopterin synthase

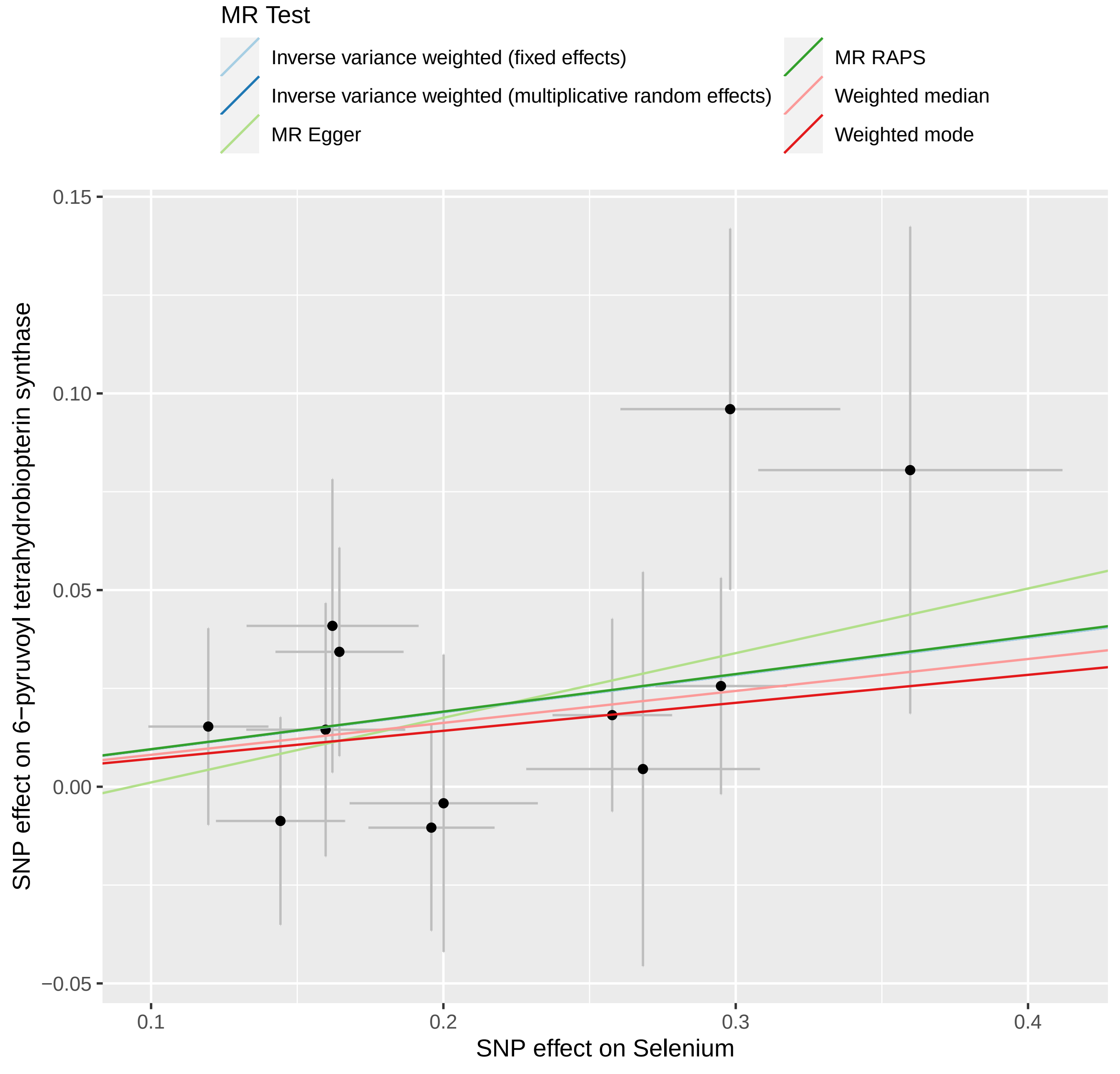

Batch 3−2451 : Selenium on Pentraxin−related protein PTX3

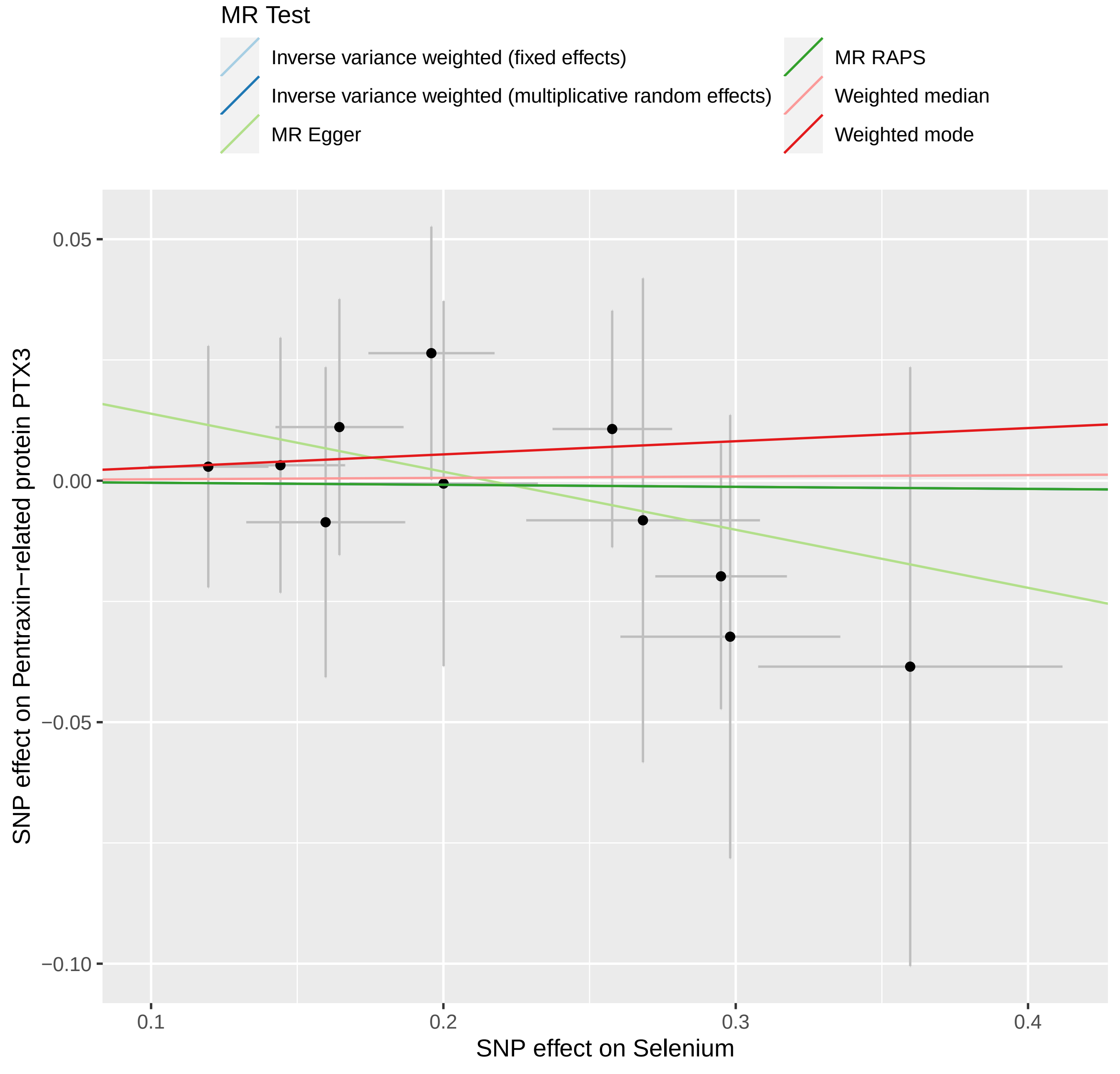

Batch 3−2452 : Selenium on Poly(U)−binding−splicing factor PUF60

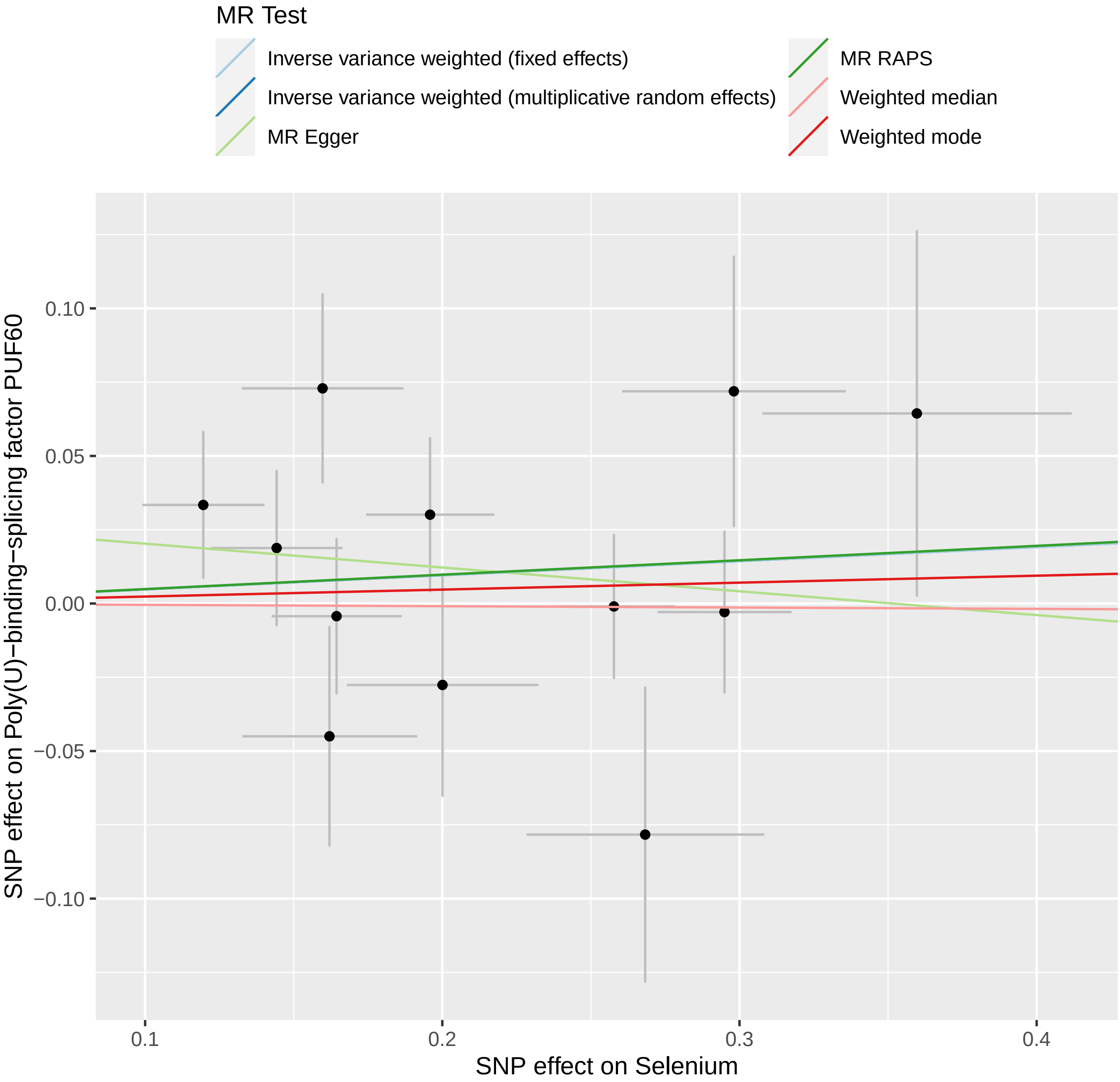

Batch 3−2453 : Selenium on Transcriptional activator protein Pur−alpha

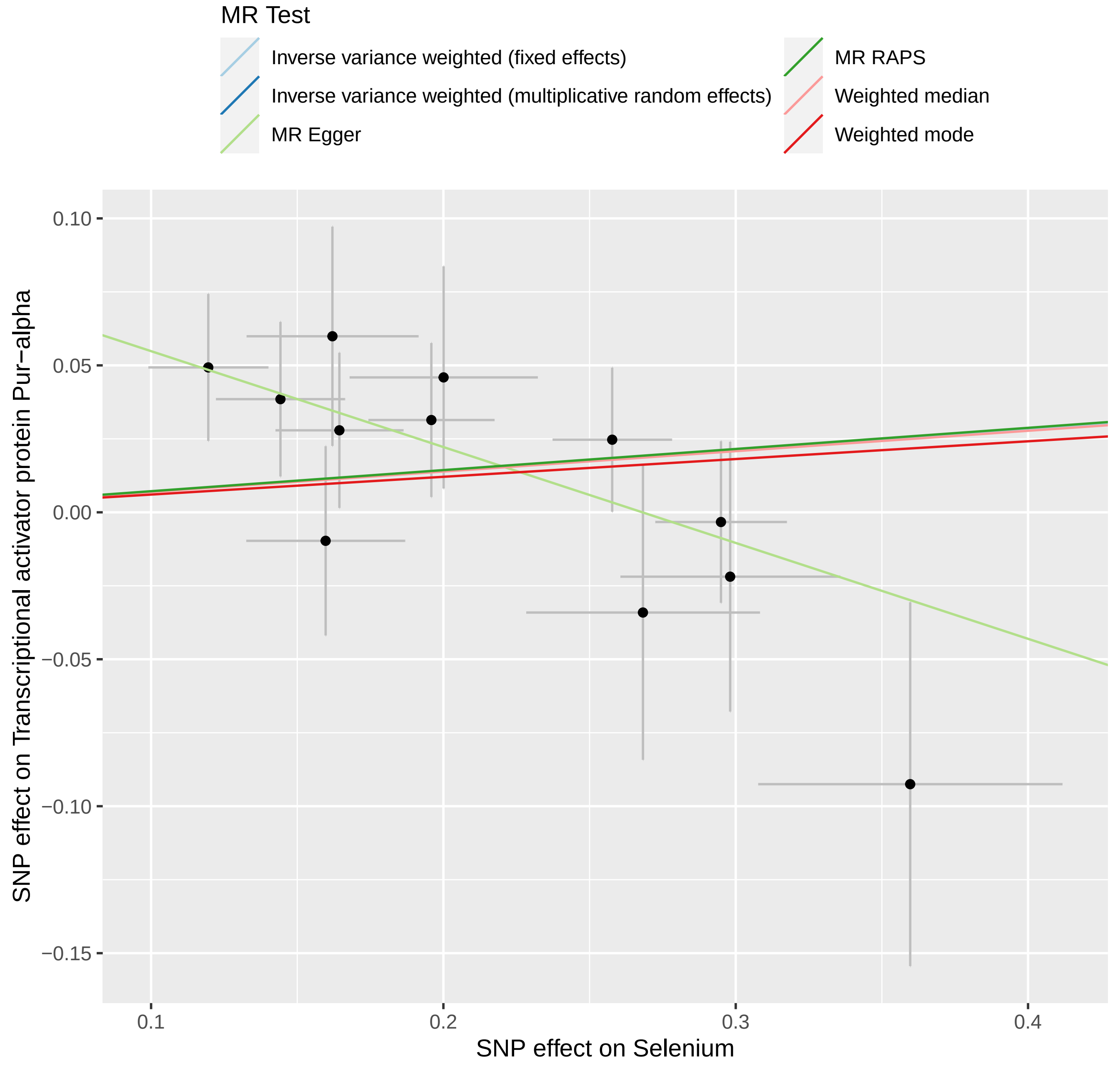

Batch 3−2454 : Selenium on tRNA pseudouridine synthase A, mitochondrial

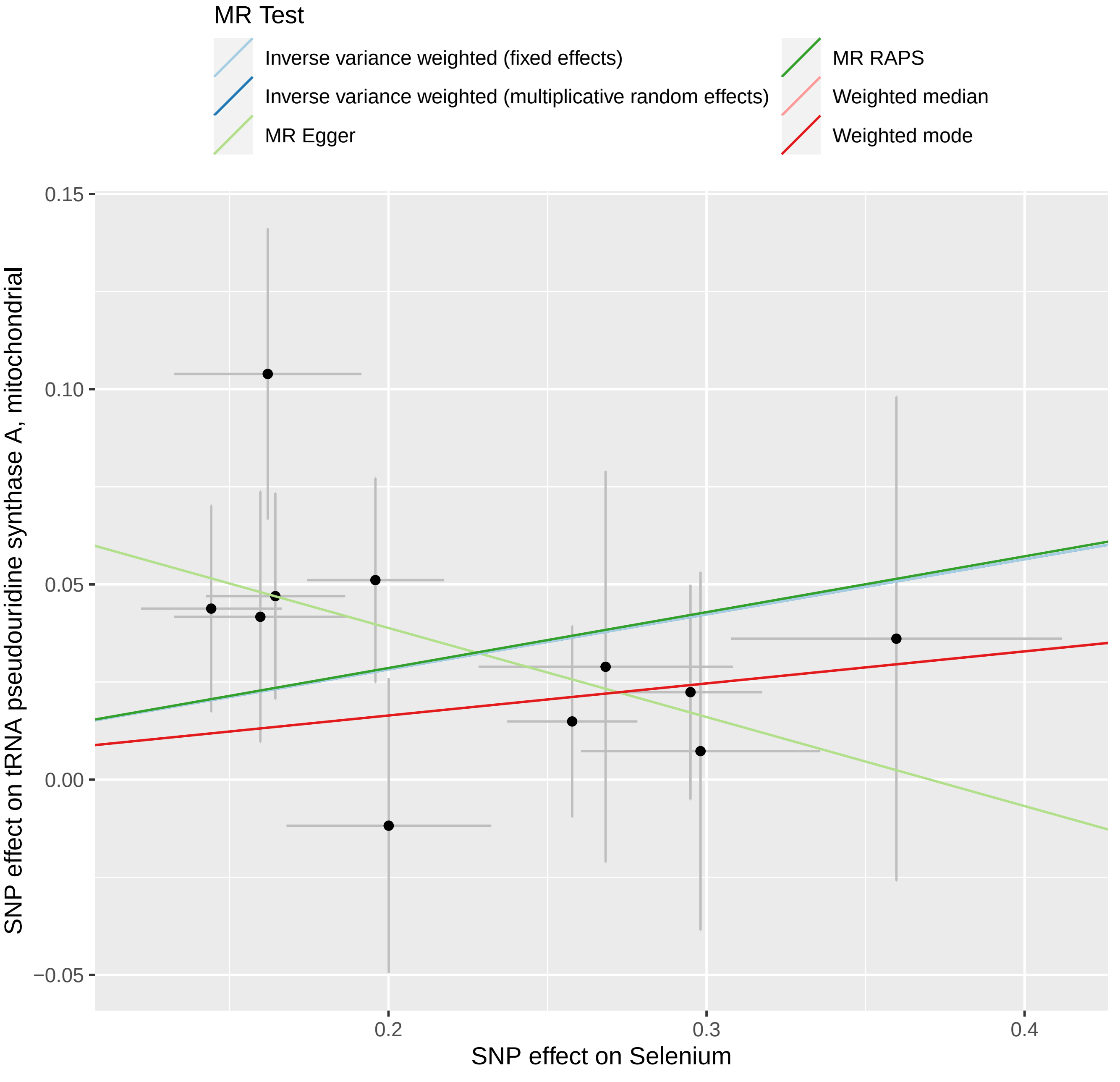

Batch 3−2455 : Selenium on Poliovirus receptor

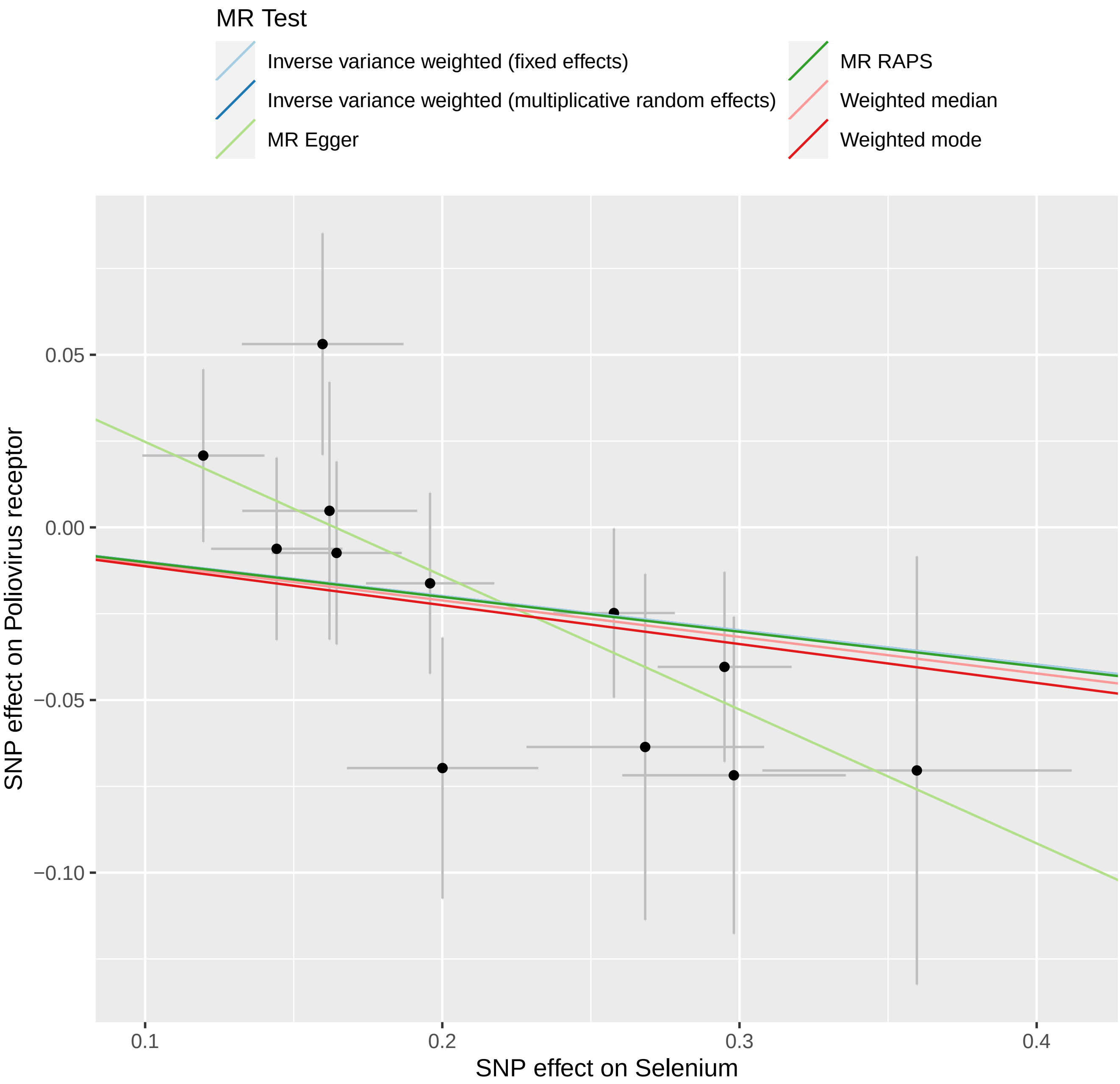

Batch 3−2456 : Selenium on Transmembrane protein PVRIG

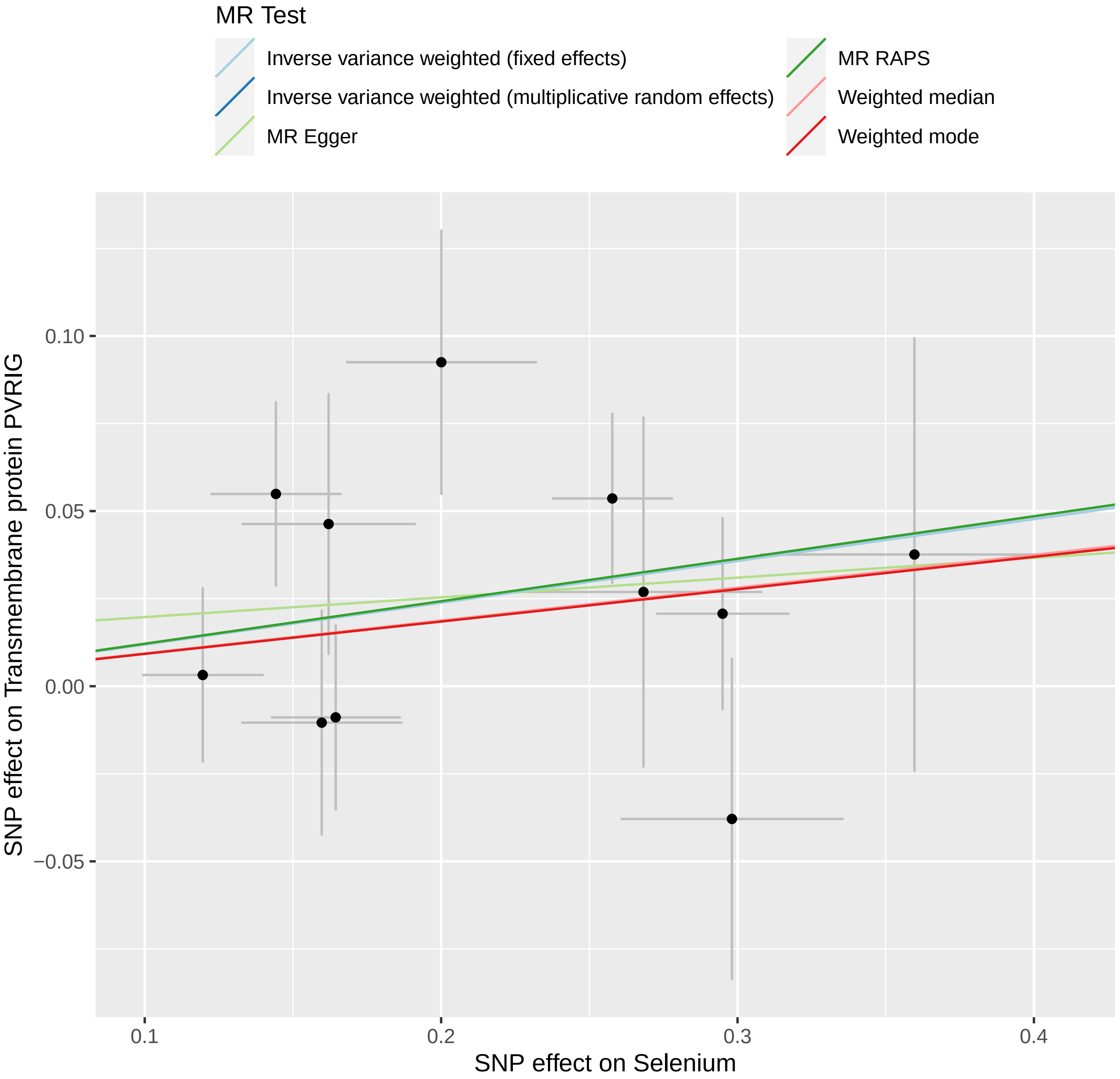

Batch 3−2457 : Selenium on Nectin−2

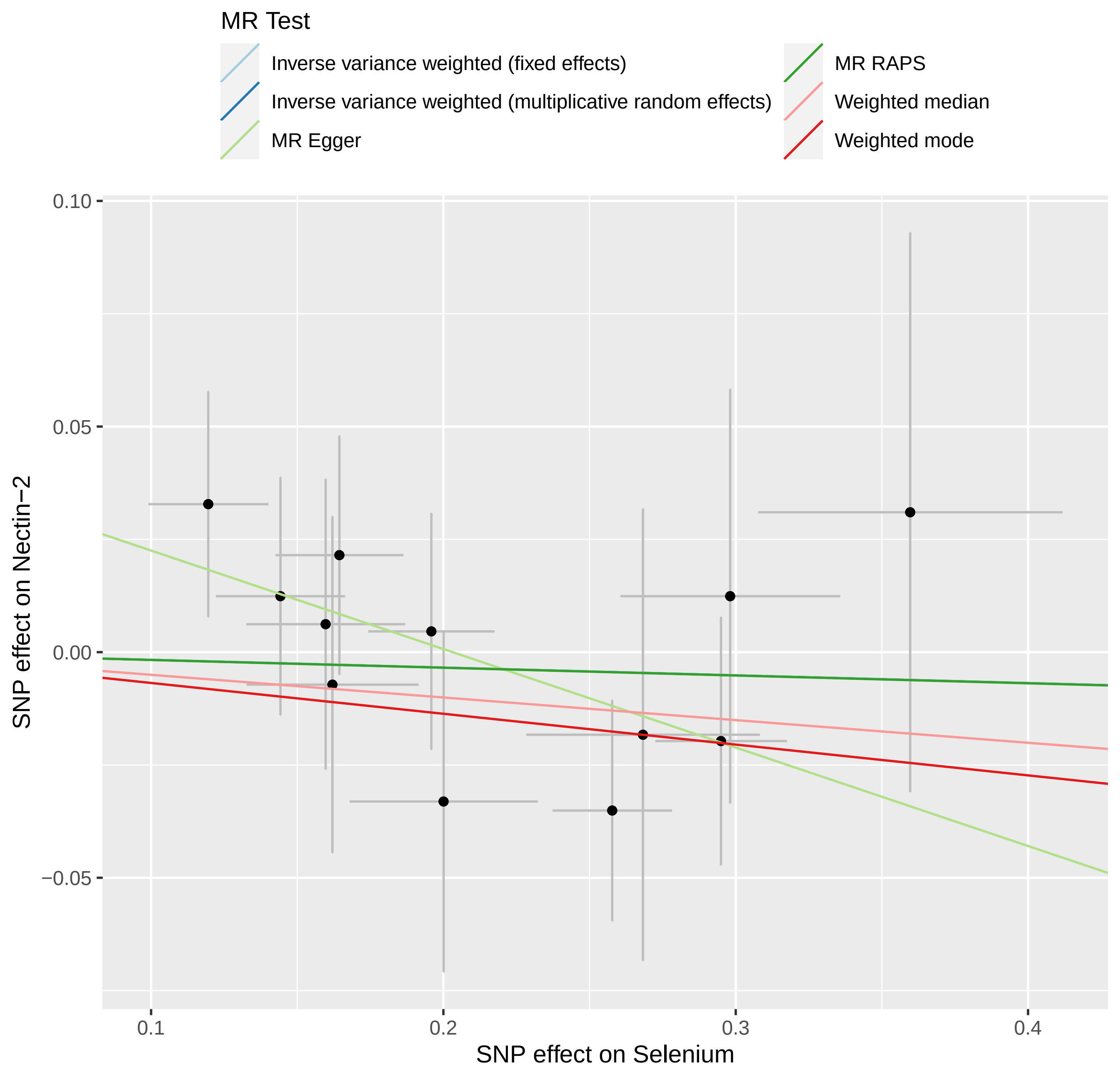

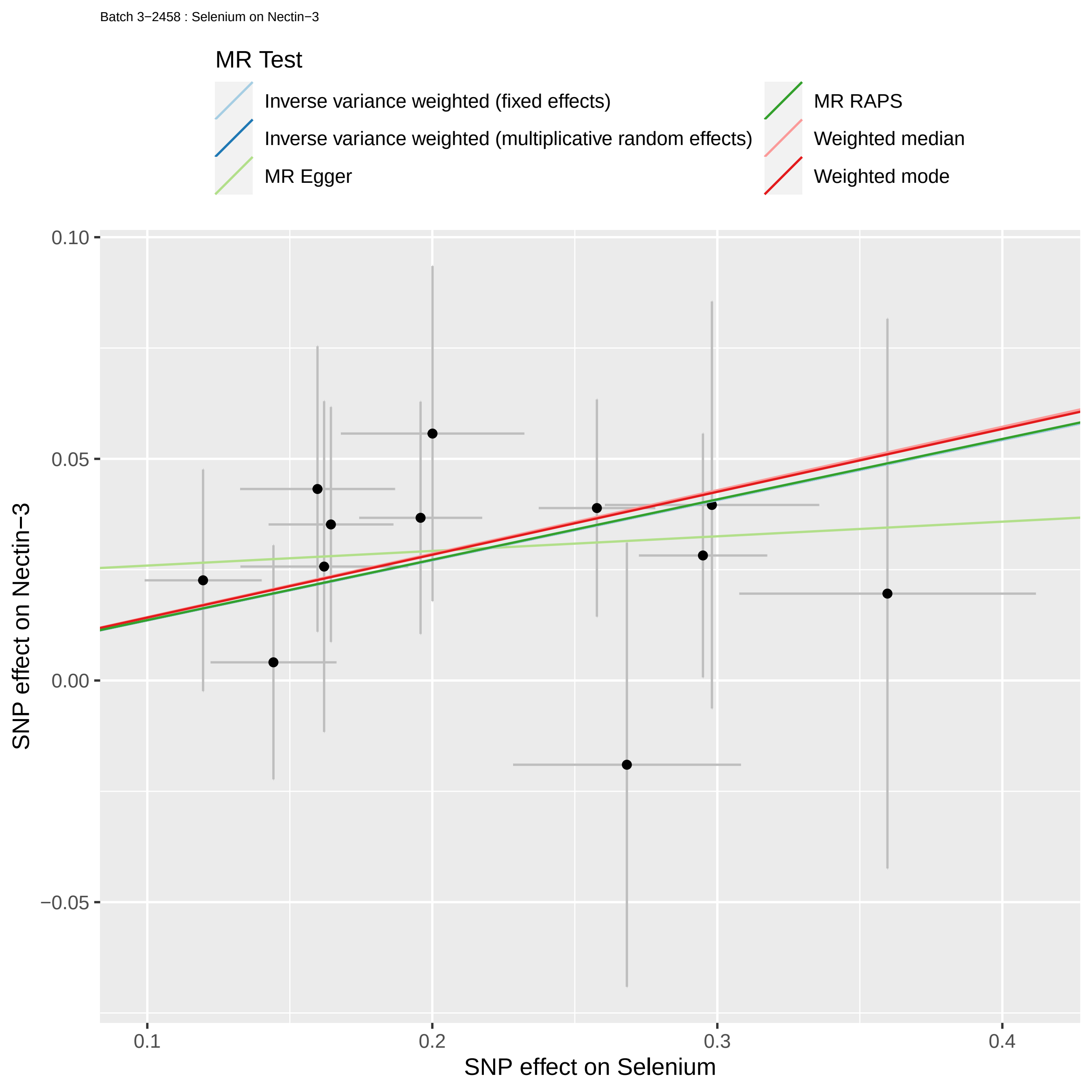

Batch 3−2458 : Selenium on Nectin−3
MR Test
Inverse variance weighted (fixed effects)
Inverse variance weighted (multiplicative random effects)
MR Egger
MR RAPS
Weighted median
Weighted mode
0.10
0.05
0.00
−0.05
0.1
0.2
0.3
0.4
SNP effect on Nectin−3
SNP effect on Selenium

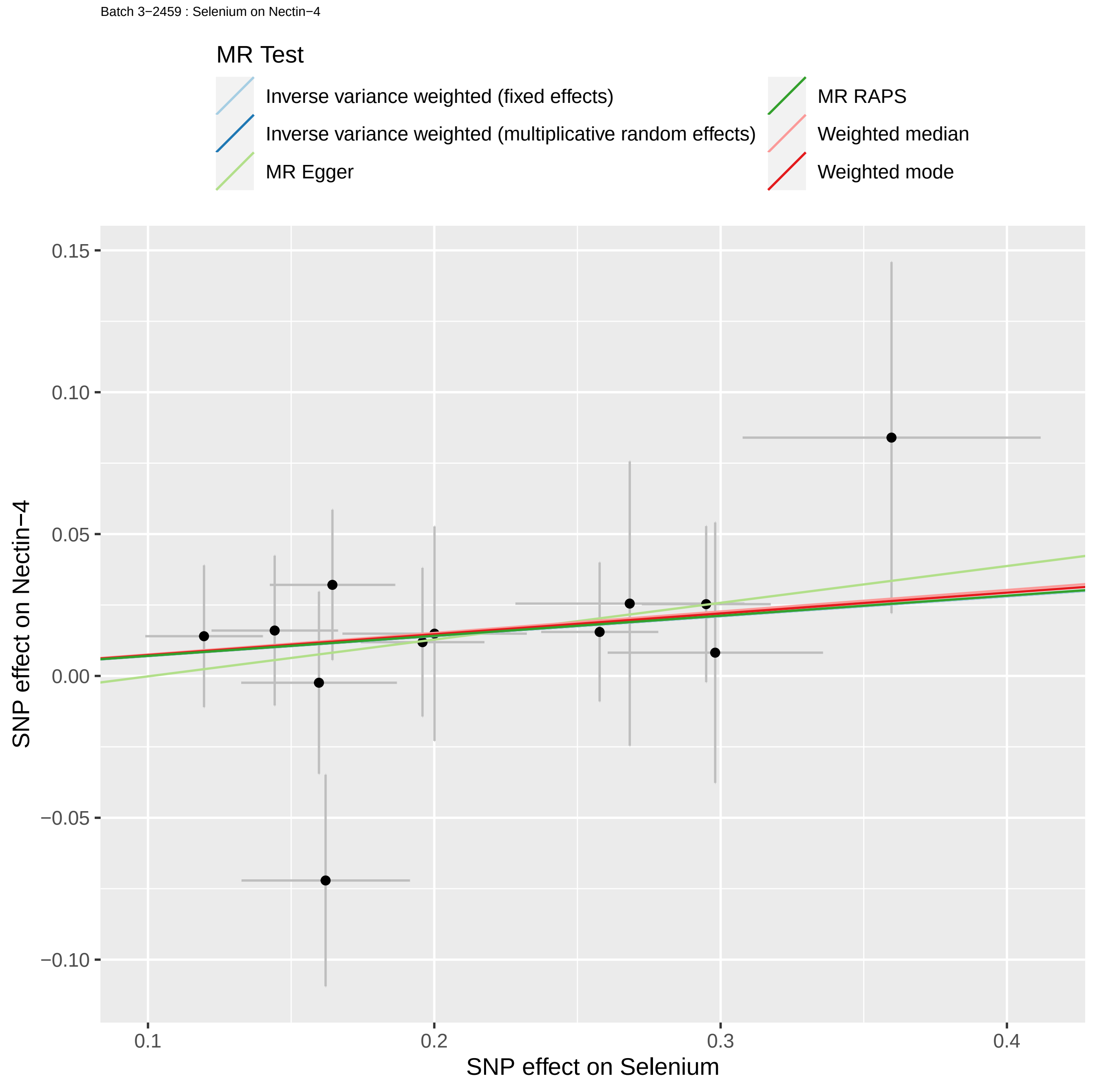

Batch 3−2459 : Selenium on Nectin−4
MR Test
Inverse variance weighted (fixed effects)
Inverse variance weighted (multiplicative random effects)
MR Egger
MR RAPS
Weighted median
Weighted mode
0.15
0.10
0.05
0.00
−0.05
−0.10
0.1
0.2
0.3
0.4
SNP effect on Nectin−4
SNP effect on Selenium

Batch 3−2460 : Selenium on Peroxidasin−like protein

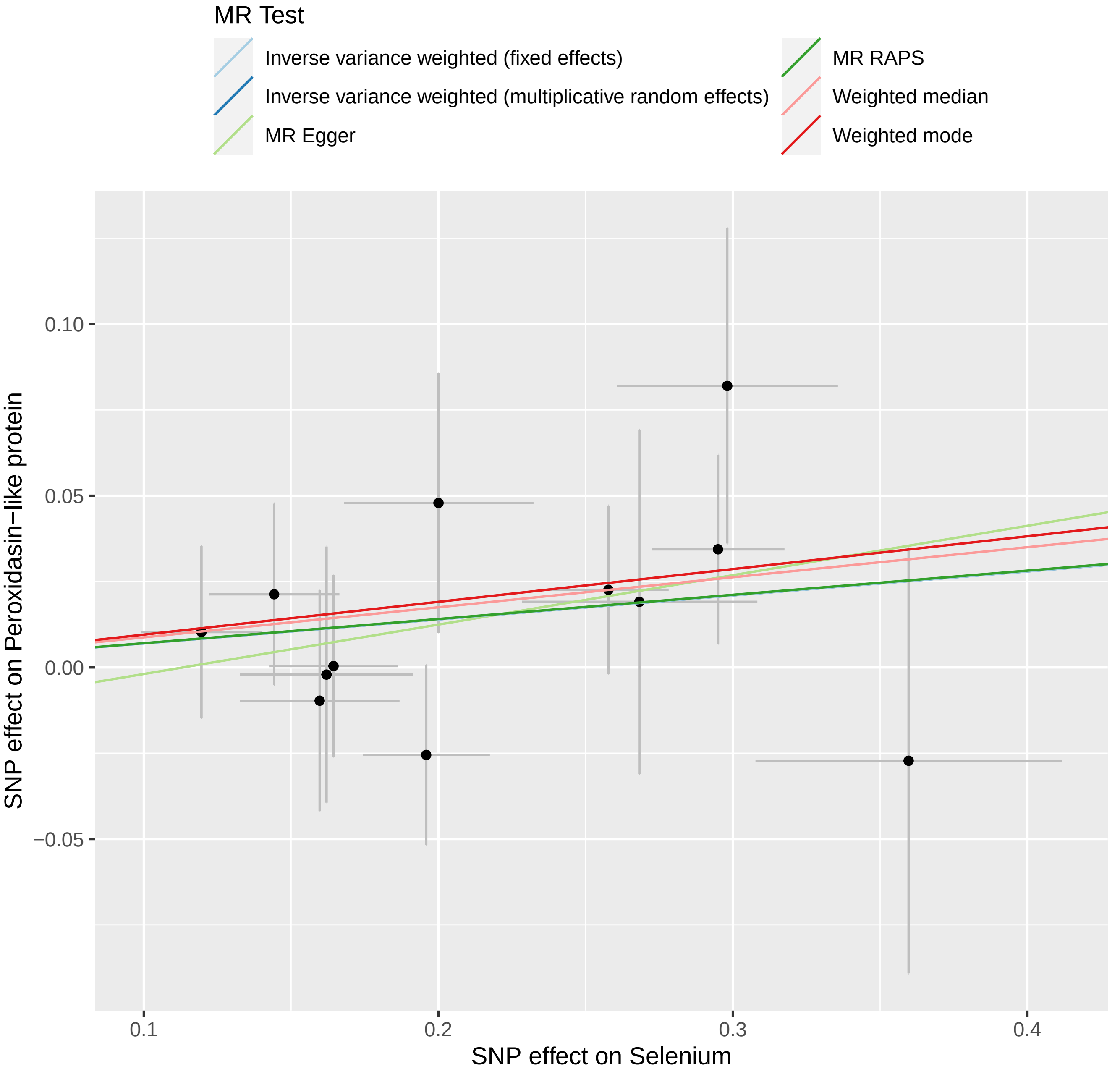

Batch 3−2461 : Selenium on Pyrin domain−containing protein 1

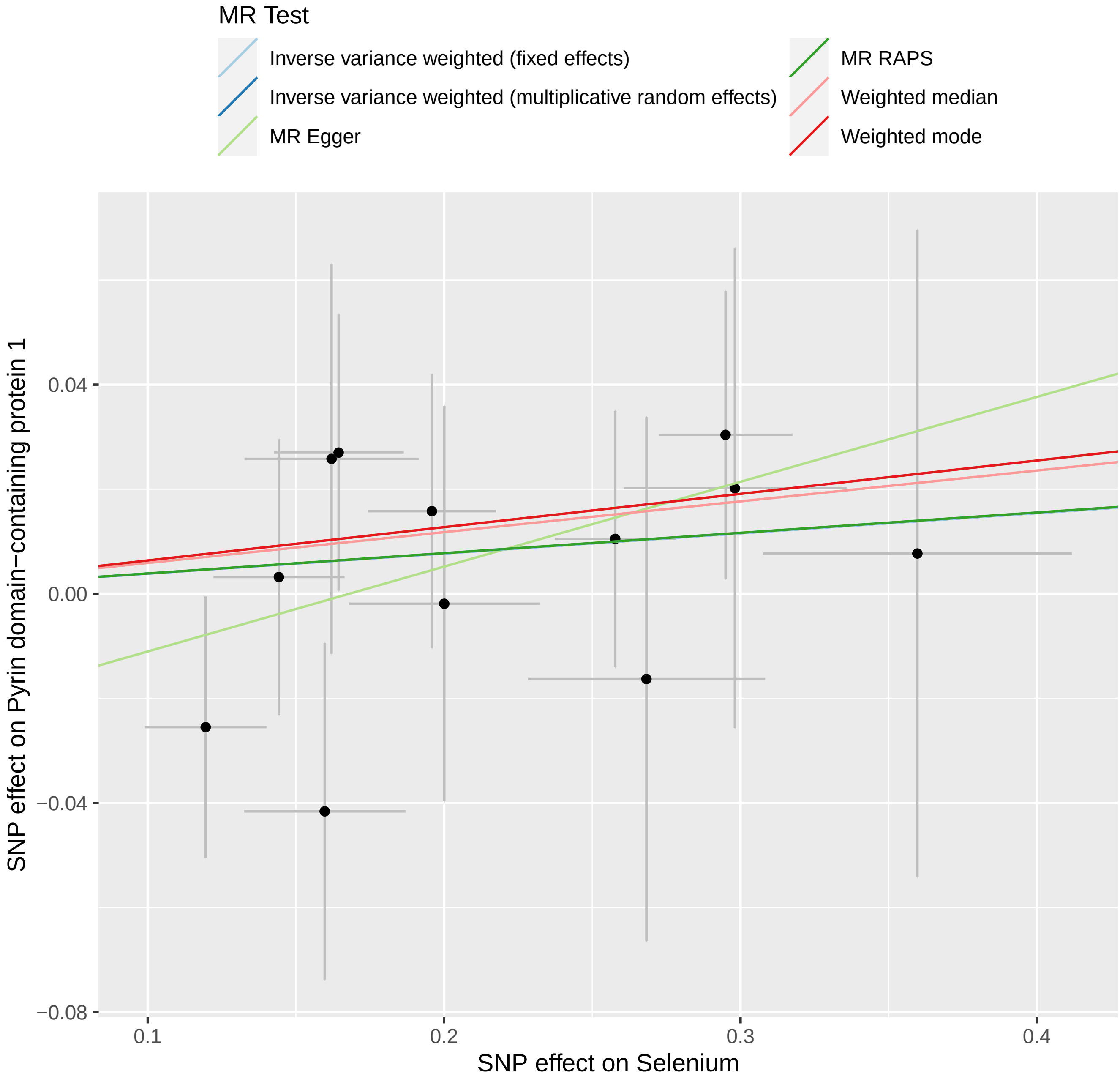

Batch 3−2462 : Selenium on Glycogen phosphorylase, liver form

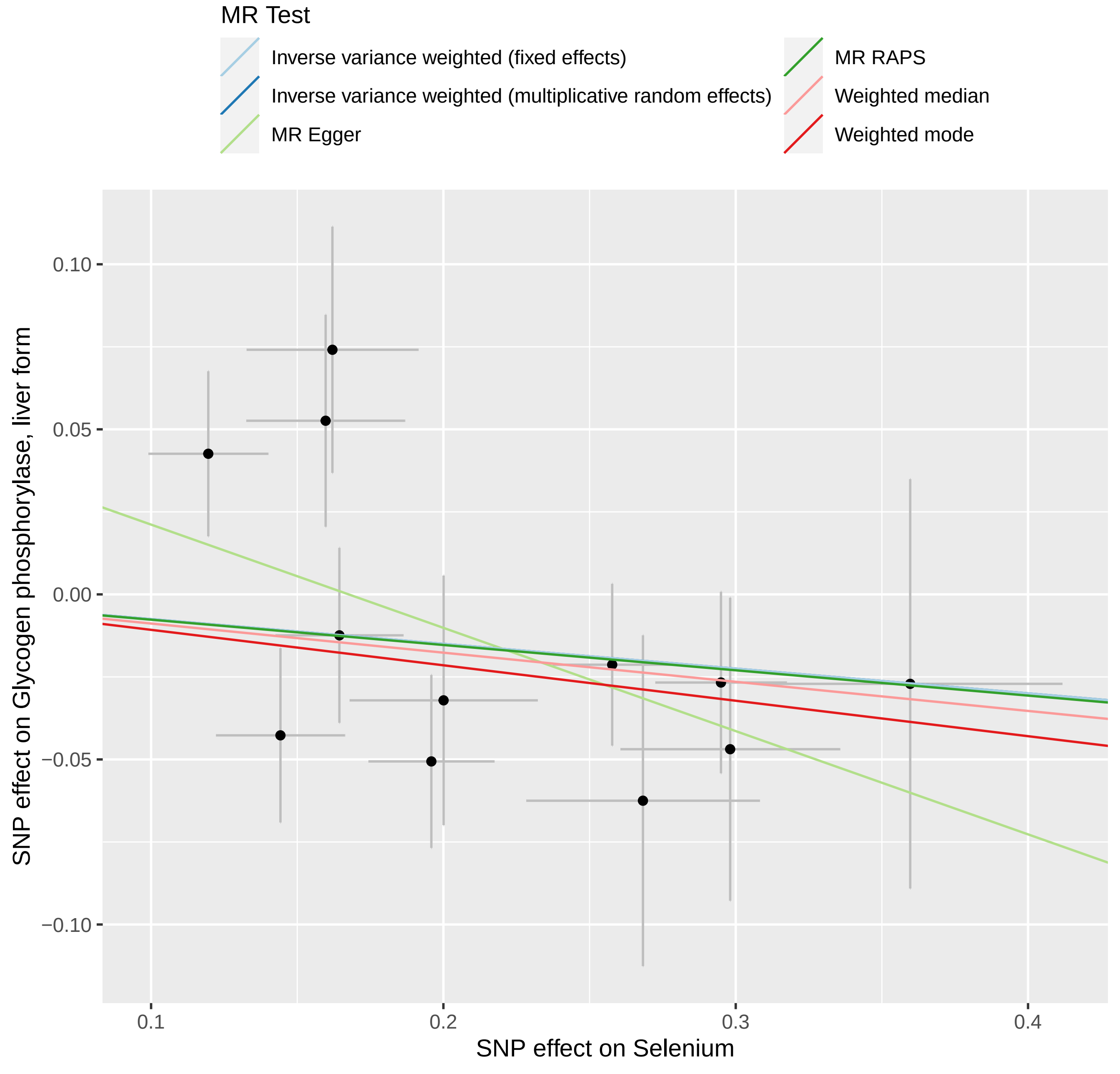

Batch 3−2463 : Selenium on Peptide YY

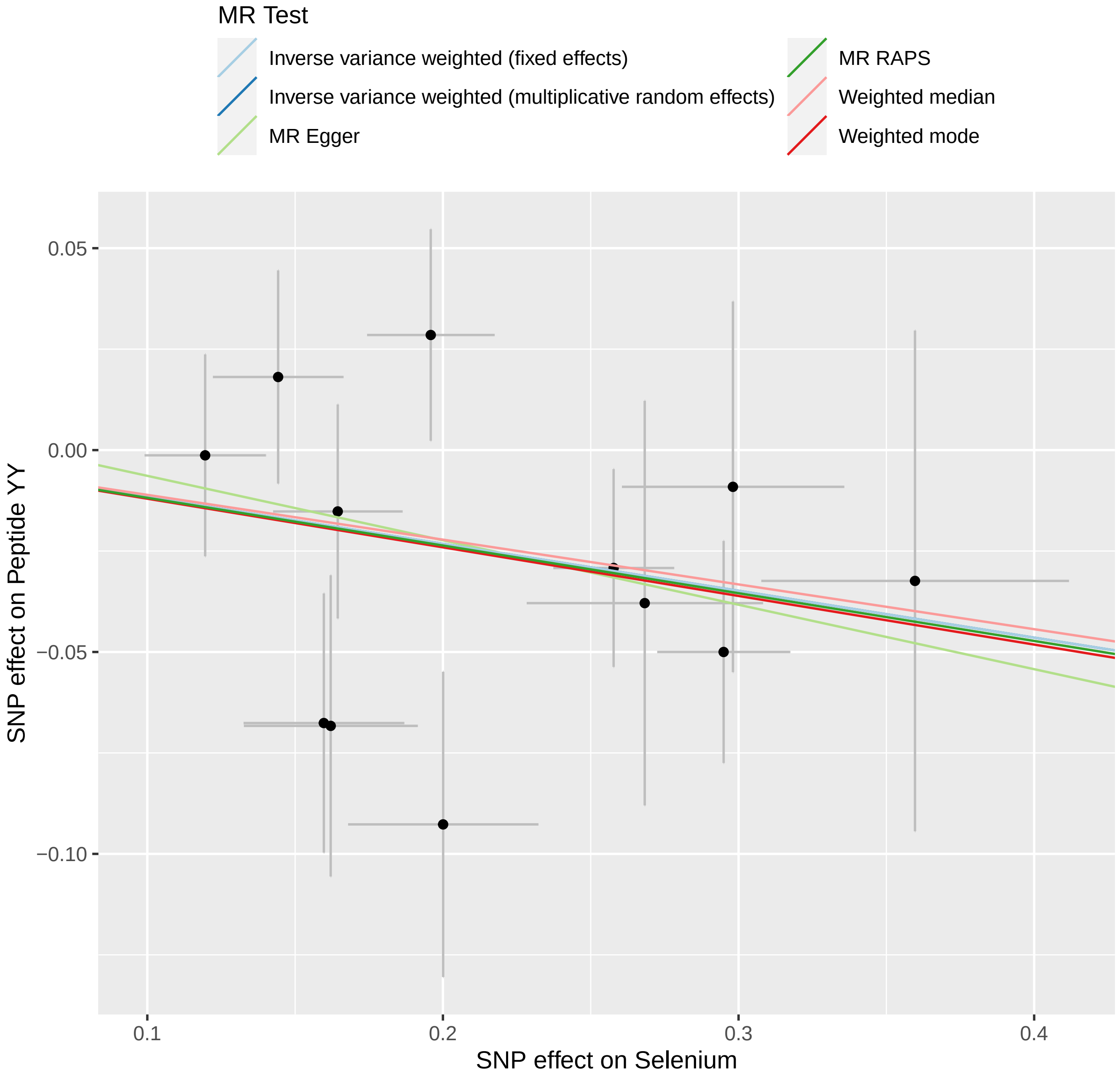

Batch 3−2464 : Selenium on Dihydropteridine reductase

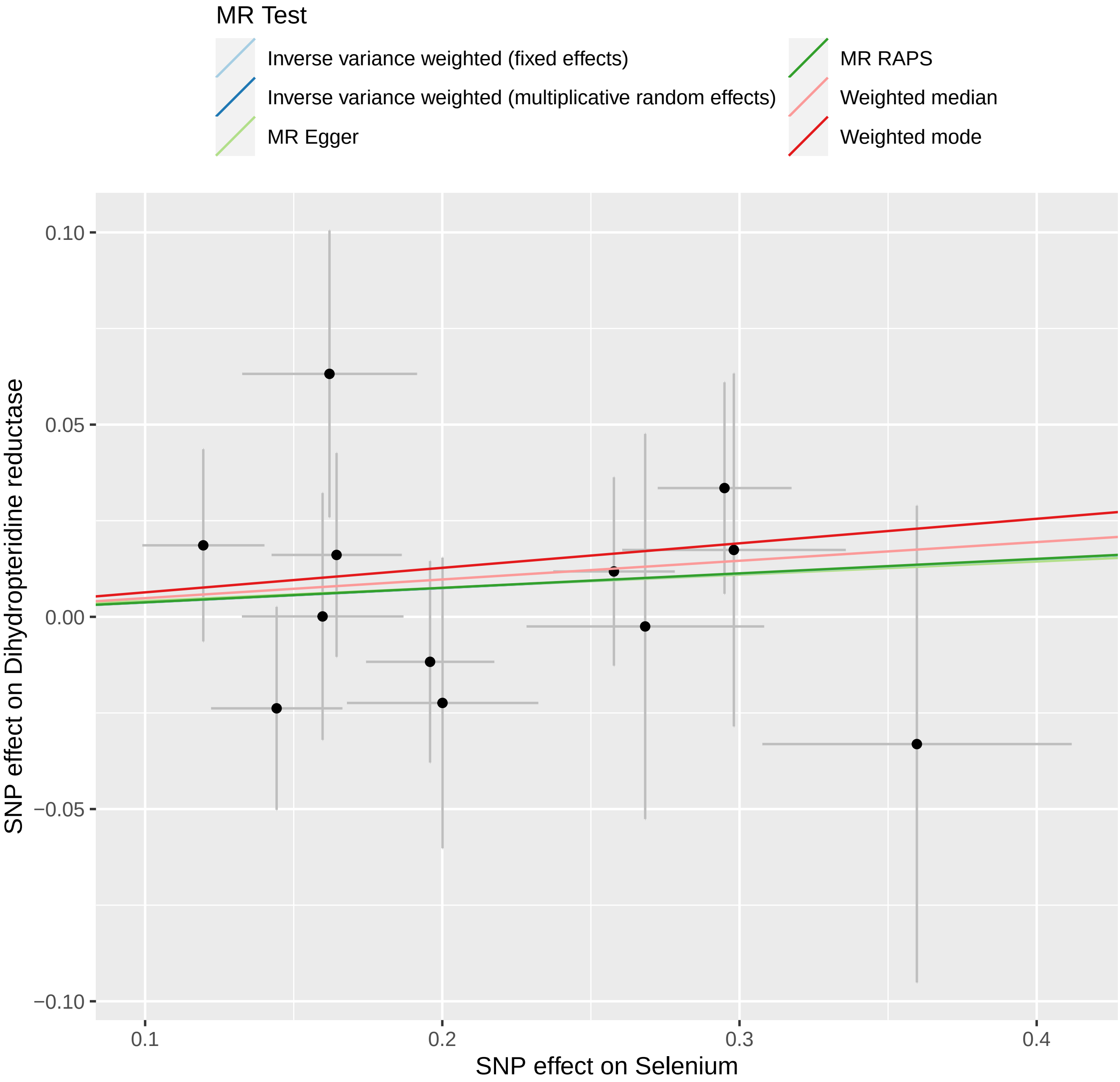

Batch 3−2465 : Selenium on Glutaminyl−peptide cyclotransferase

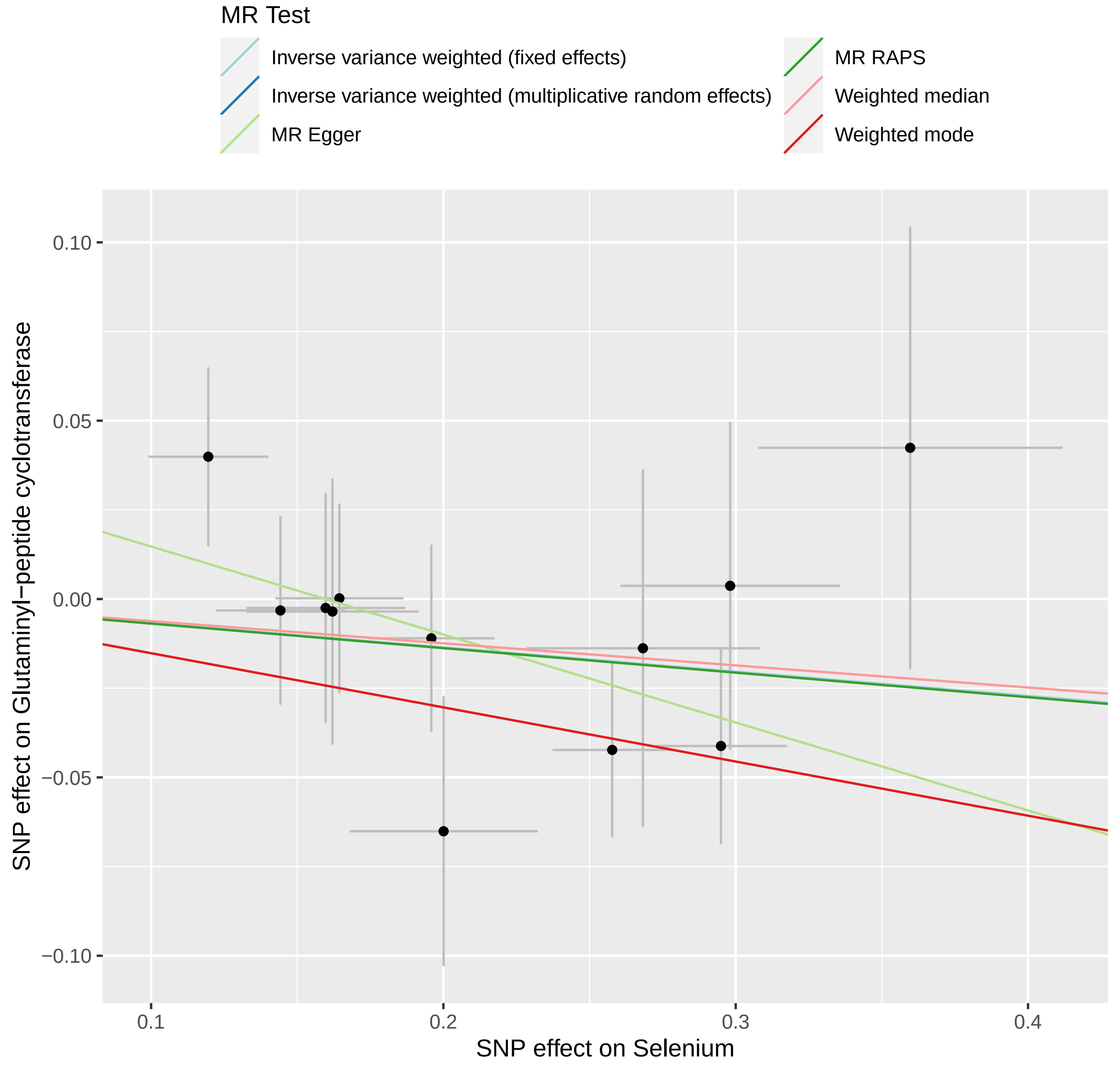

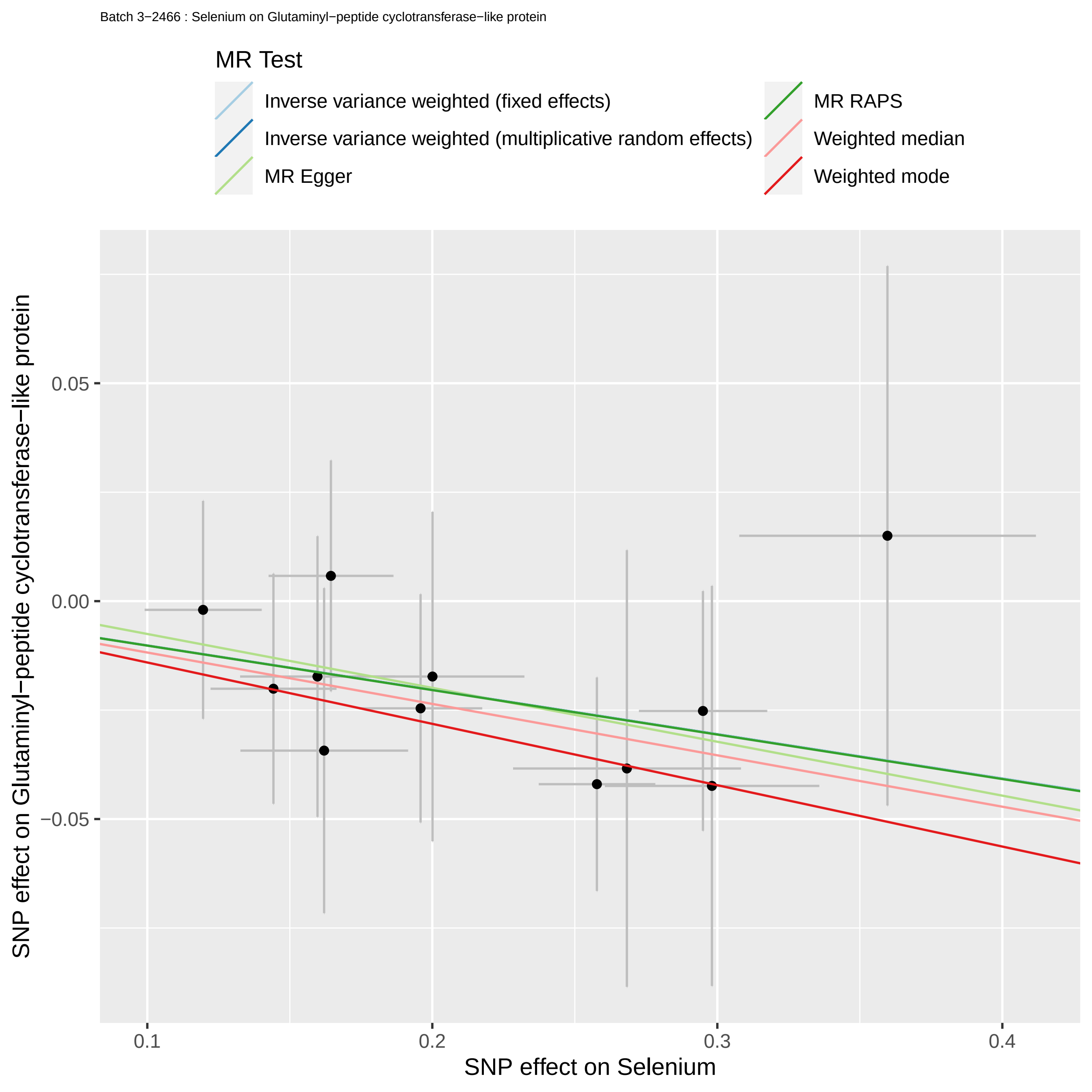
Batch 3−2466 : Selenium on Glutaminyl−peptide cyclotransferase−like protein
MR Test
Inverse variance weighted (fixed effects)
Inverse variance weighted (multiplicative random effects)
MR Egger
MR RAPS
Weighted median
Weighted mode
0.05
0.00
−0.05
0.1
0.2
0.3
0.4
SNP effect on Glutaminyl−peptide cyclotransferase−like protein
SNP effect on Selenium

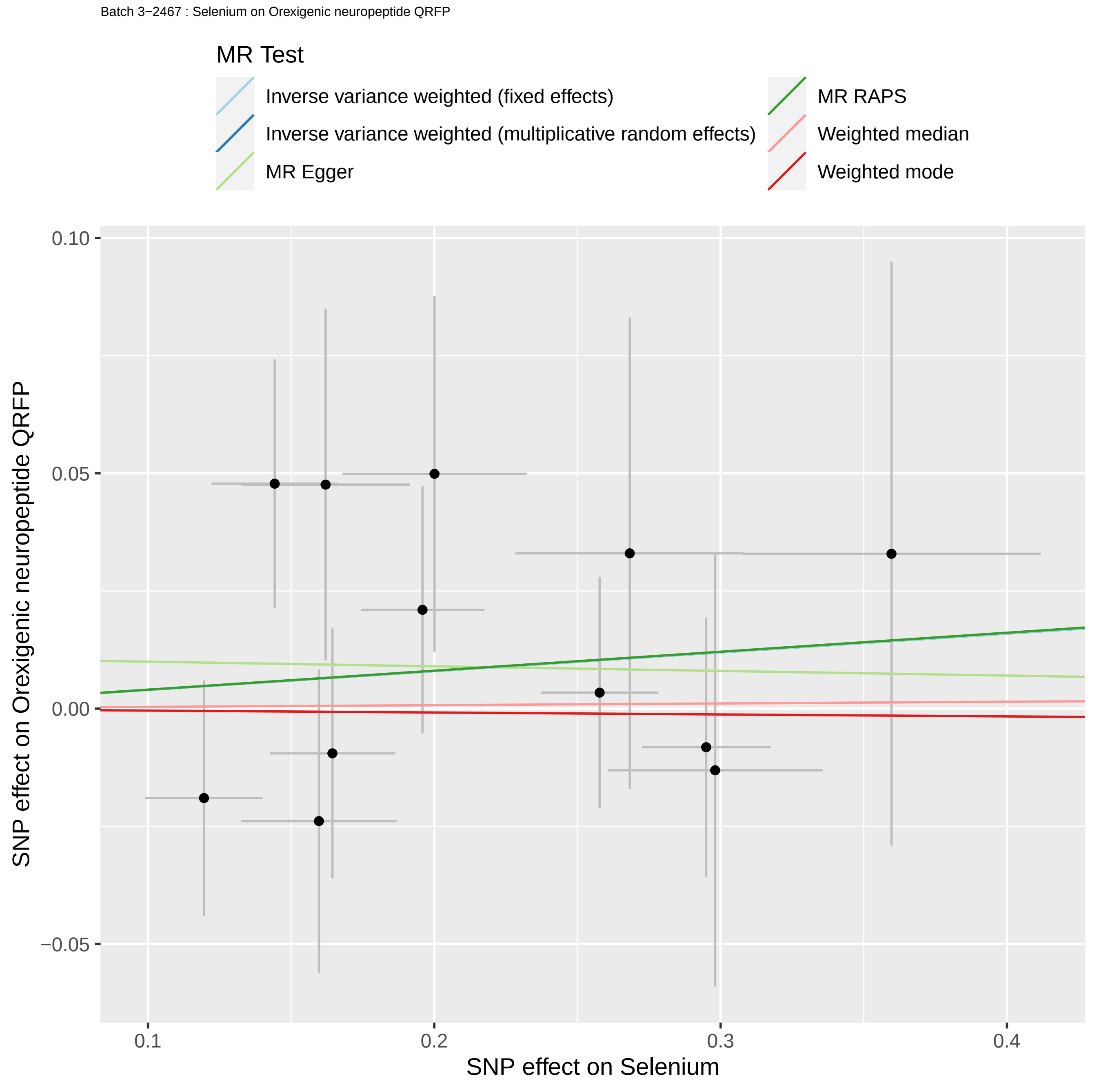
Batch 3−2467 : Selenium on Orexigenic neuropeptide QRFP
MR Test
Inverse variance weighted (fixed effects)
Inverse variance weighted (multiplicative random effects)
MR Egger
MR RAPS
Weighted median
Weighted mode
0.10
0.05
0.00
−0.05
0.1
0.2
0.3
0.4
SNP effect on Selenium
SNP effect on Orexigenic neuropeptide QRFP

Batch 3−2468 : Selenium on Sulfhydryl oxidase 1

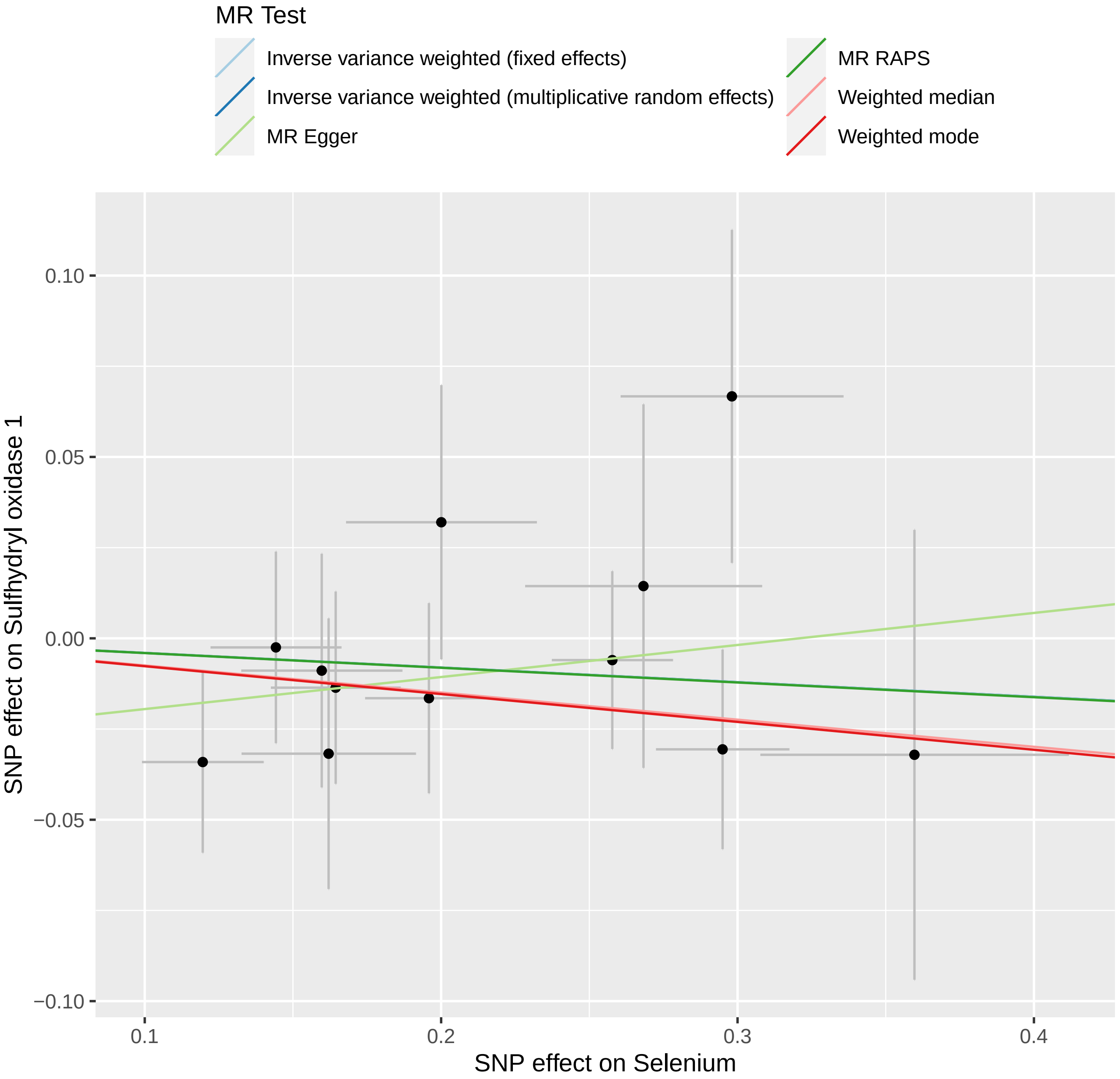

Batch 3−2469 : Selenium on Sulfhydryl oxidase 1

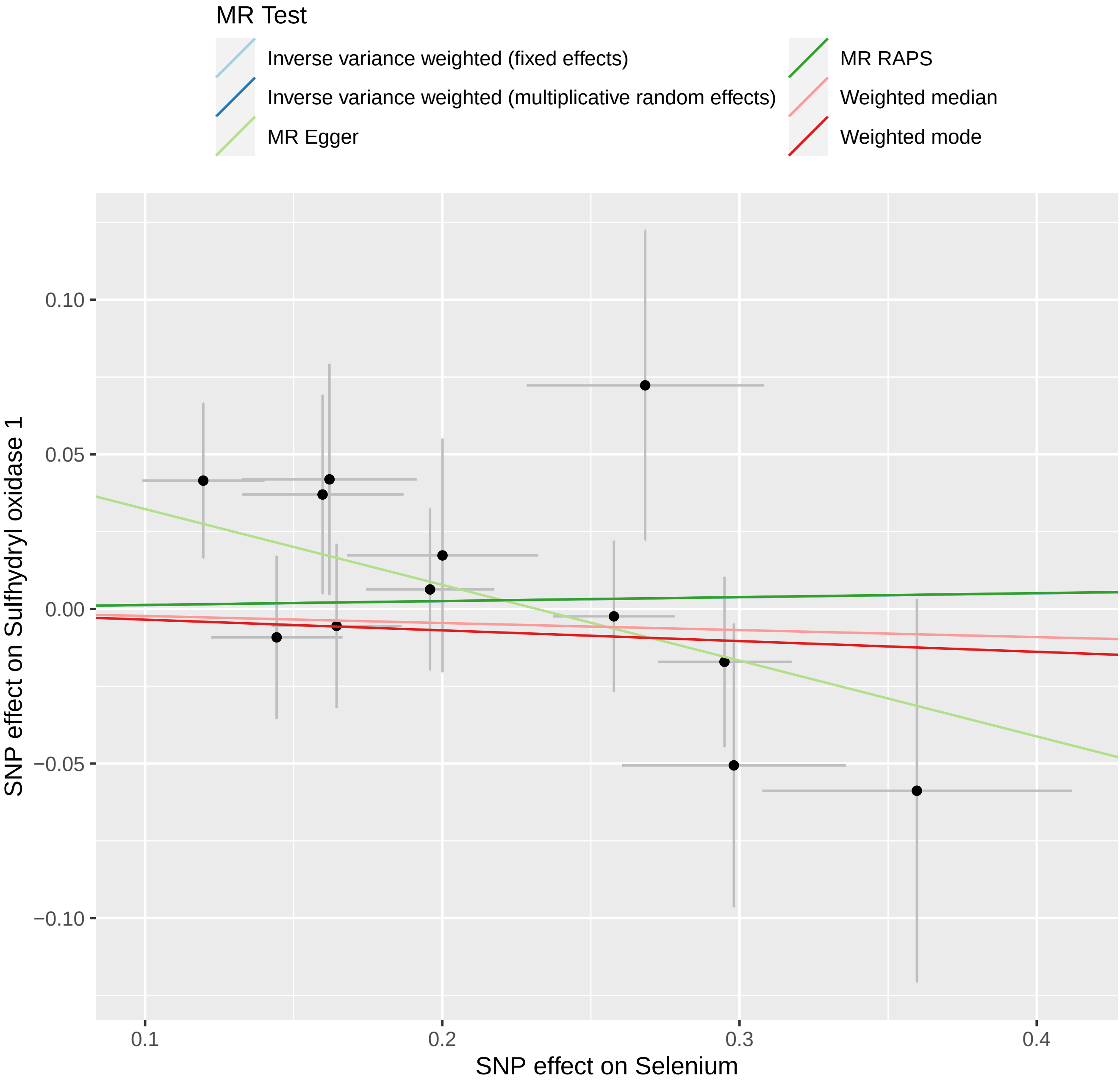

Batch 3−2470 : Selenium on Sulfhydryl oxidase 2

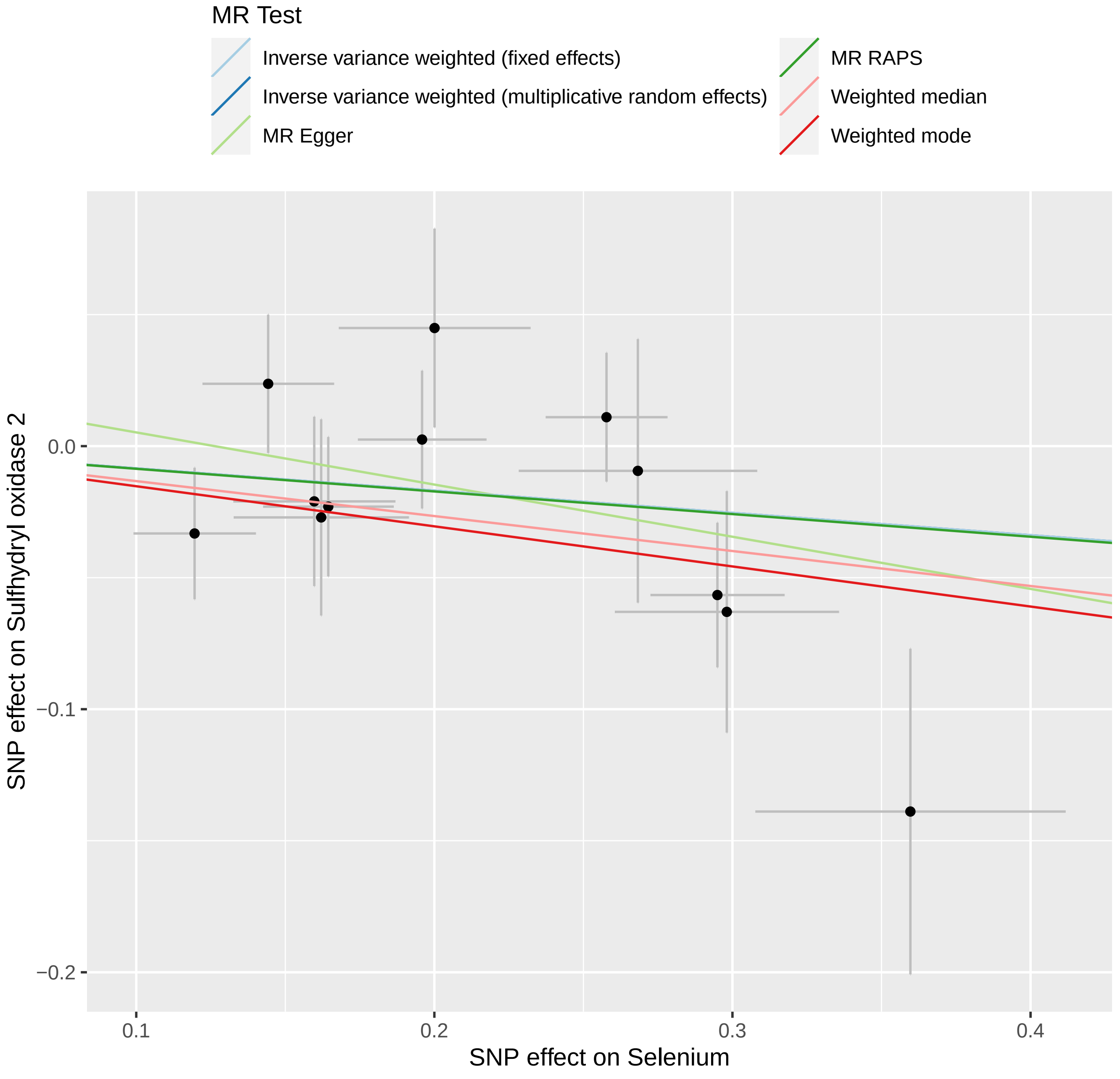

Batch 3−2471 : Selenium on Ras−related protein Rab−14

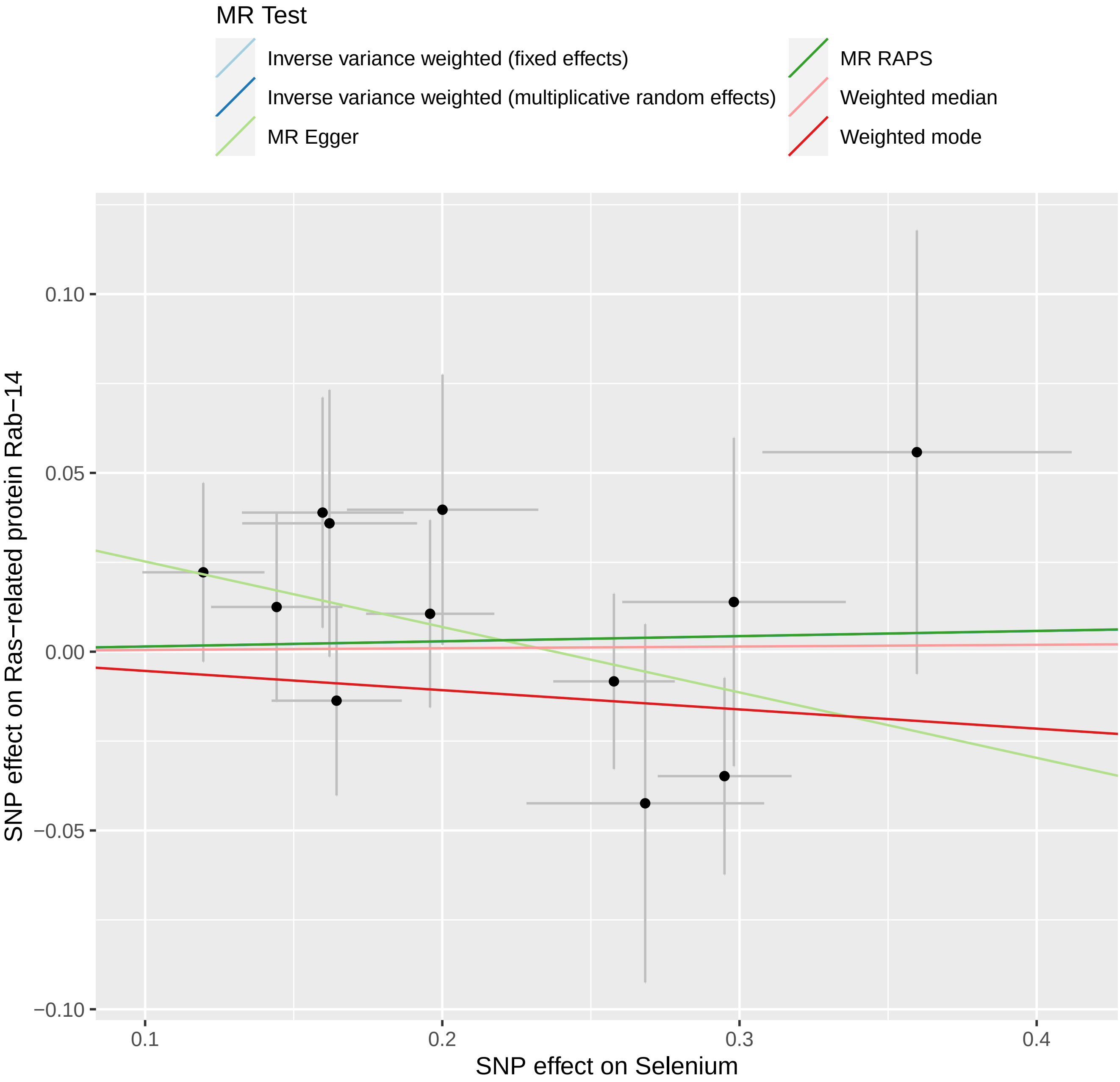

Batch 3−2472 : Selenium on Ras−related protein Rab−22A

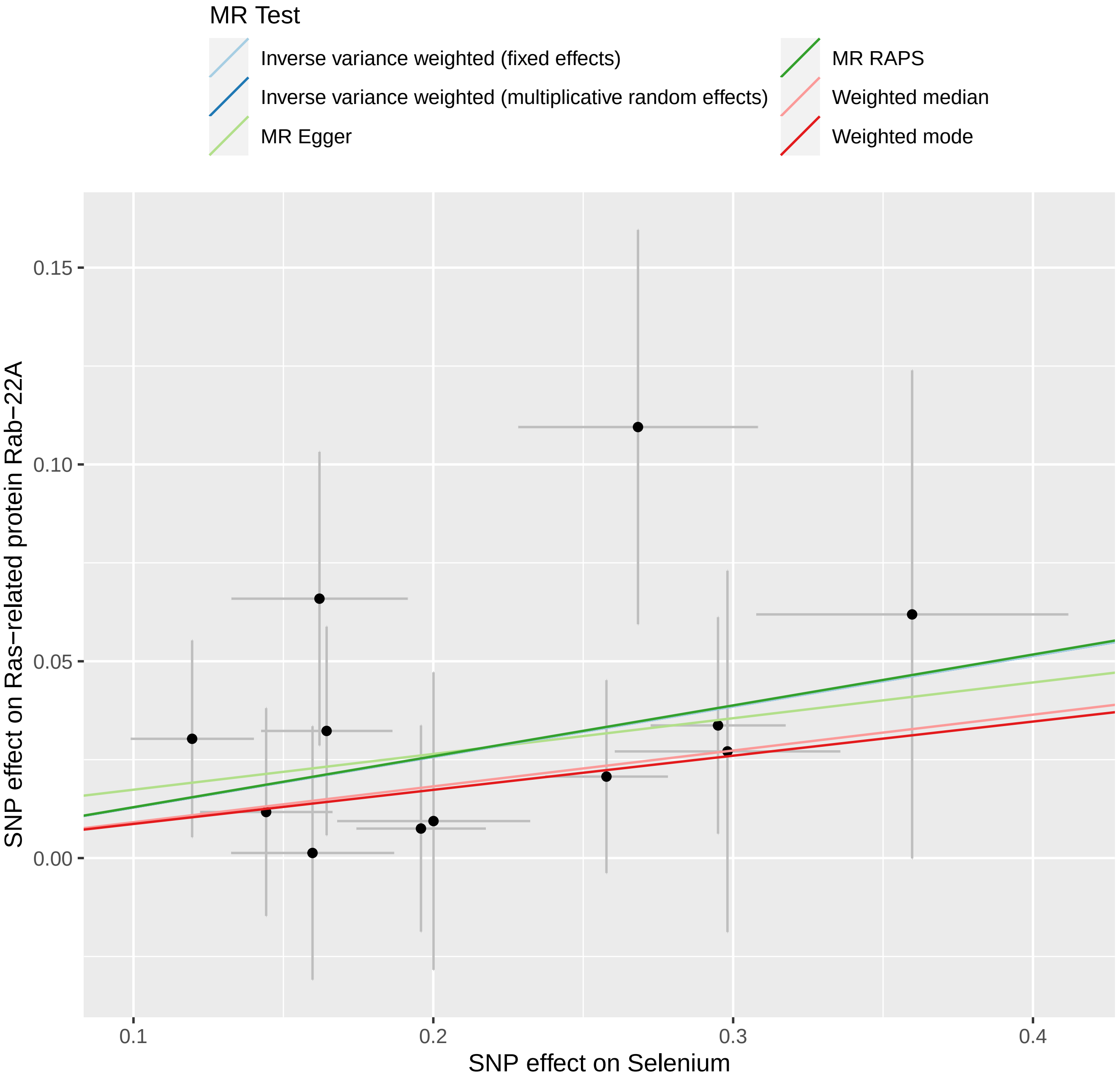

Batch 3−2473 : Selenium on Ras−related protein Rab−26

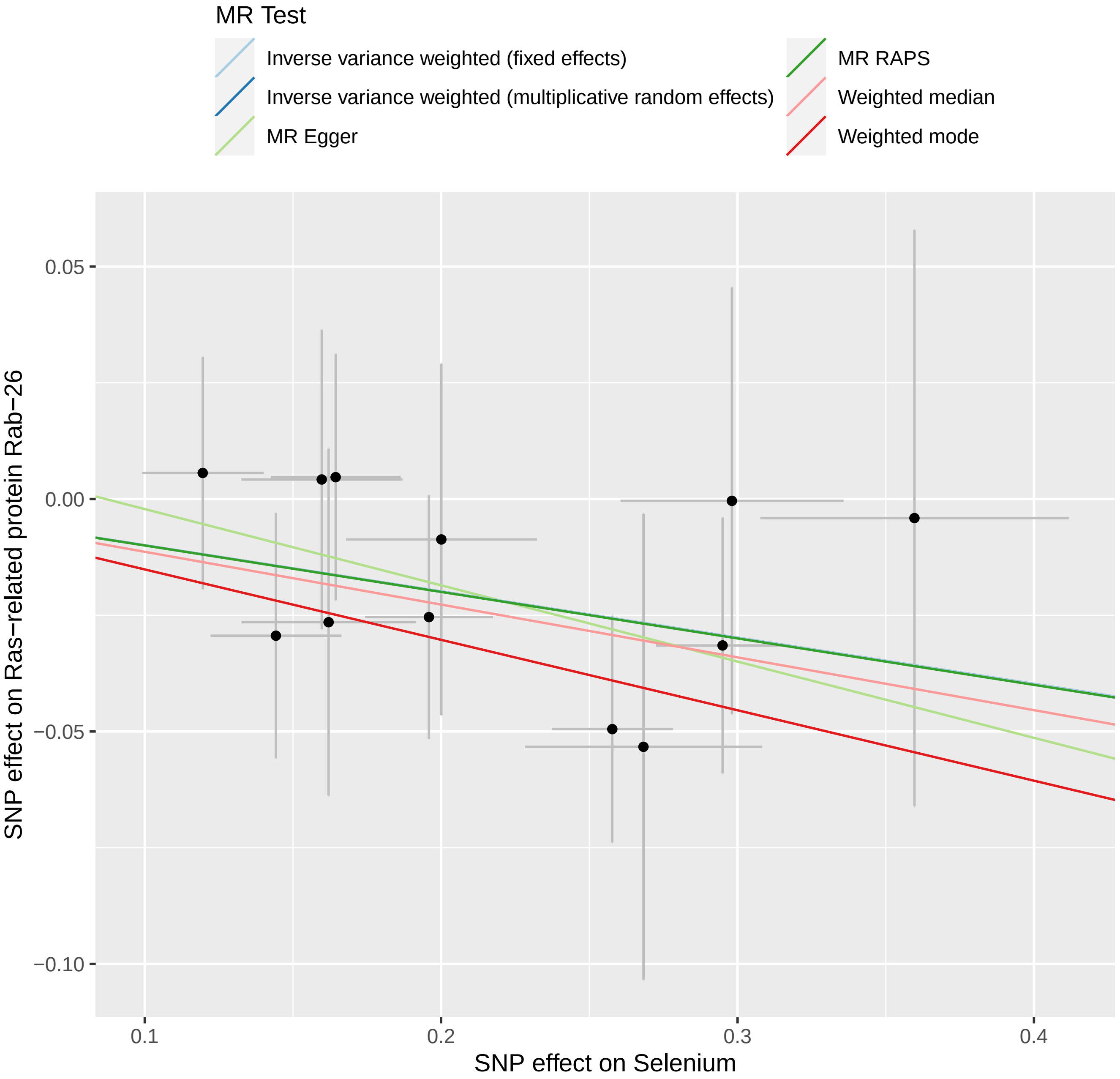

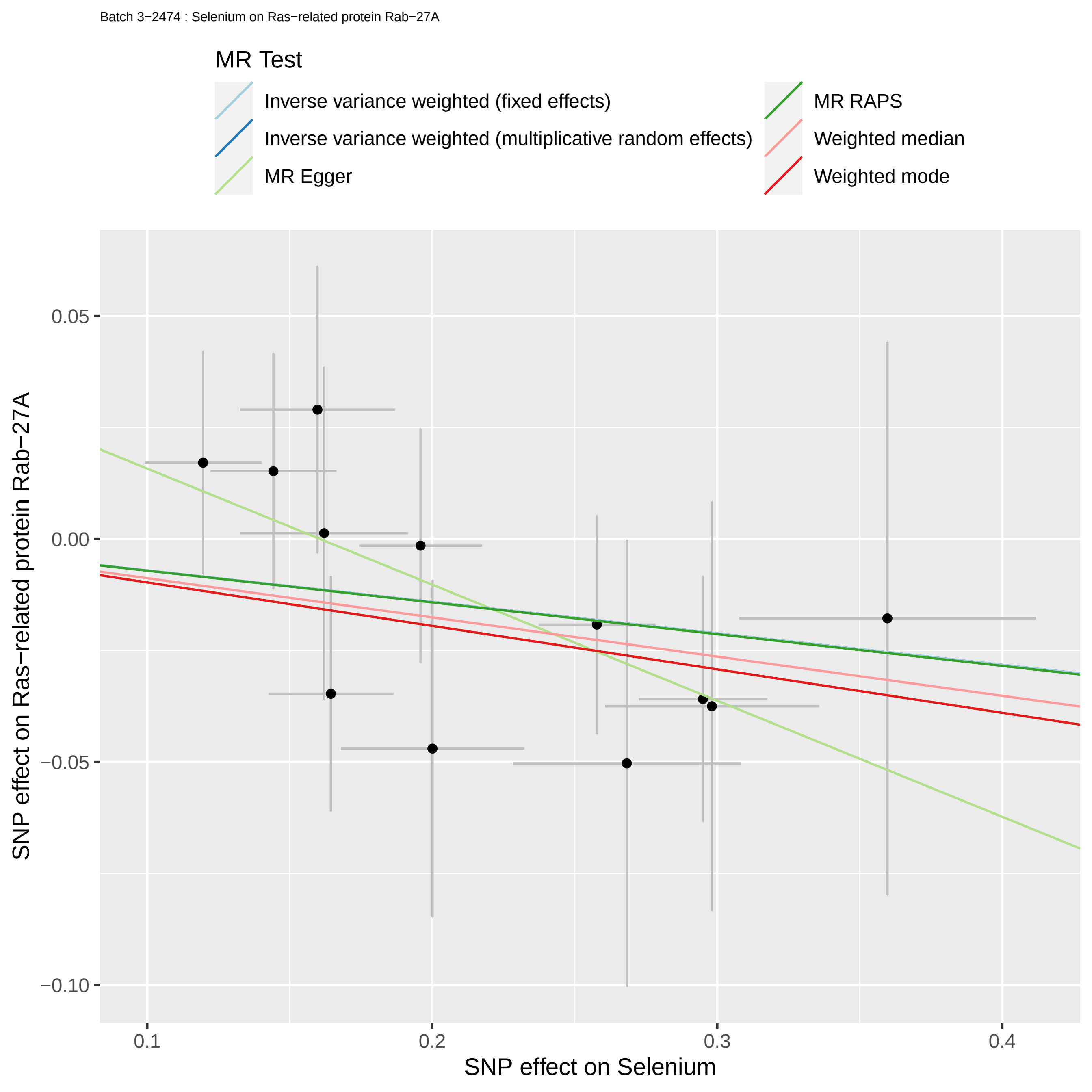
Batch 3−2474 : Selenium on Ras−related protein Rab−27A
MR Test
Inverse variance weighted (fixed effects)
Inverse variance weighted (multiplicative random effects)
MR Egger
MR RAPS
Weighted median
Weighted mode
SNP effect on Ras−related protein Rab−27A
SNP effect on Selenium
0.05
0.00
−0.05
−0.10
0.1
0.2
0.3
0.4

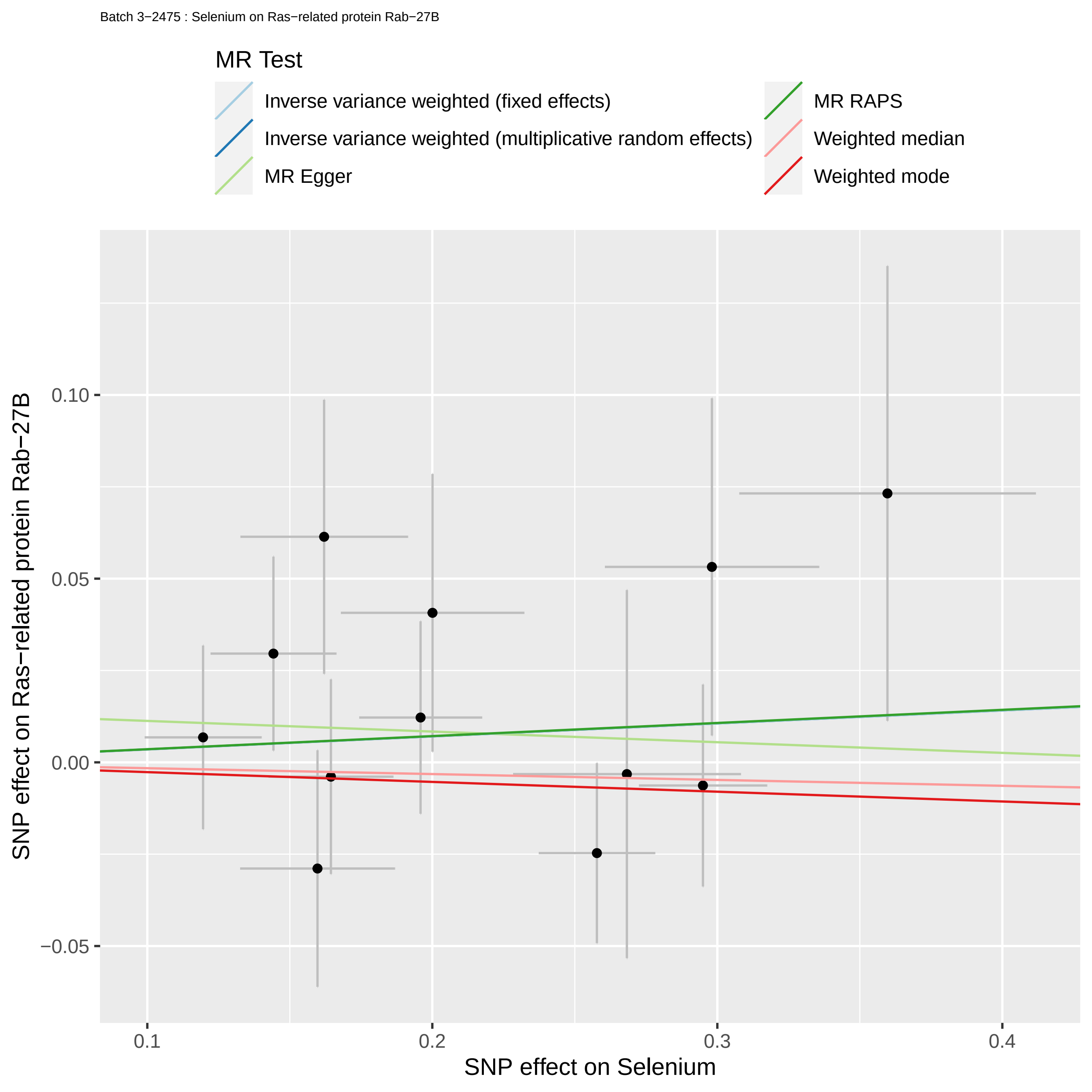

Batch 3−2475 : Selenium on Ras−related protein Rab−27B
MR Test
Inverse variance weighted (fixed effects)
Inverse variance weighted (multiplicative random effects)
MR Egger
MR RAPS
Weighted median
Weighted mode
0.10
0.05
0.00
−0.05
0.1
0.2
0.3
0.4
SNP effect on Selenium
SNP effect on Ras−related protein Rab−27B

Batch 3−2476 : Selenium on Ras−related protein Rab−35

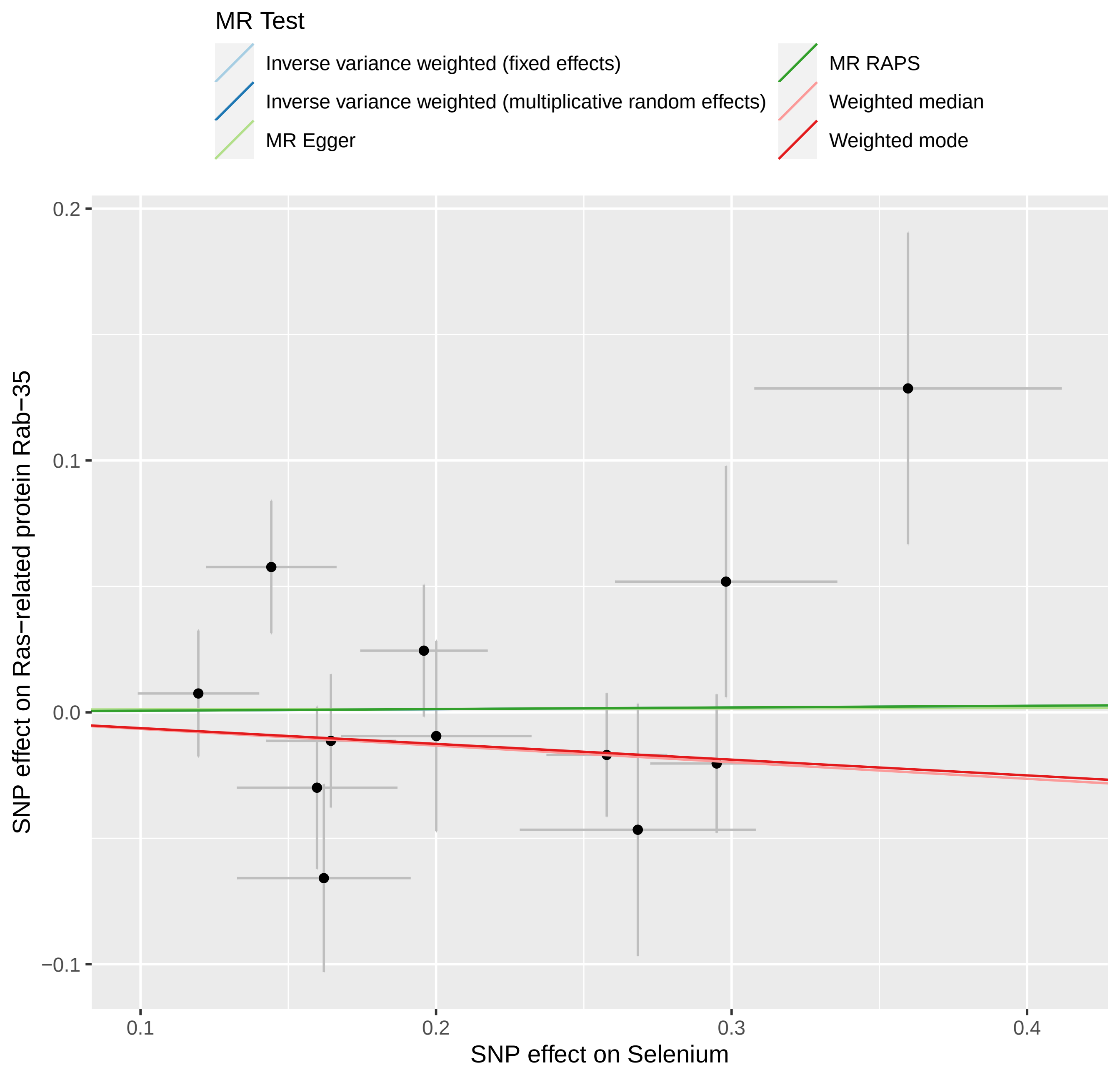

Batch 3−2477 : Selenium on Ras−related protein Rab−39B

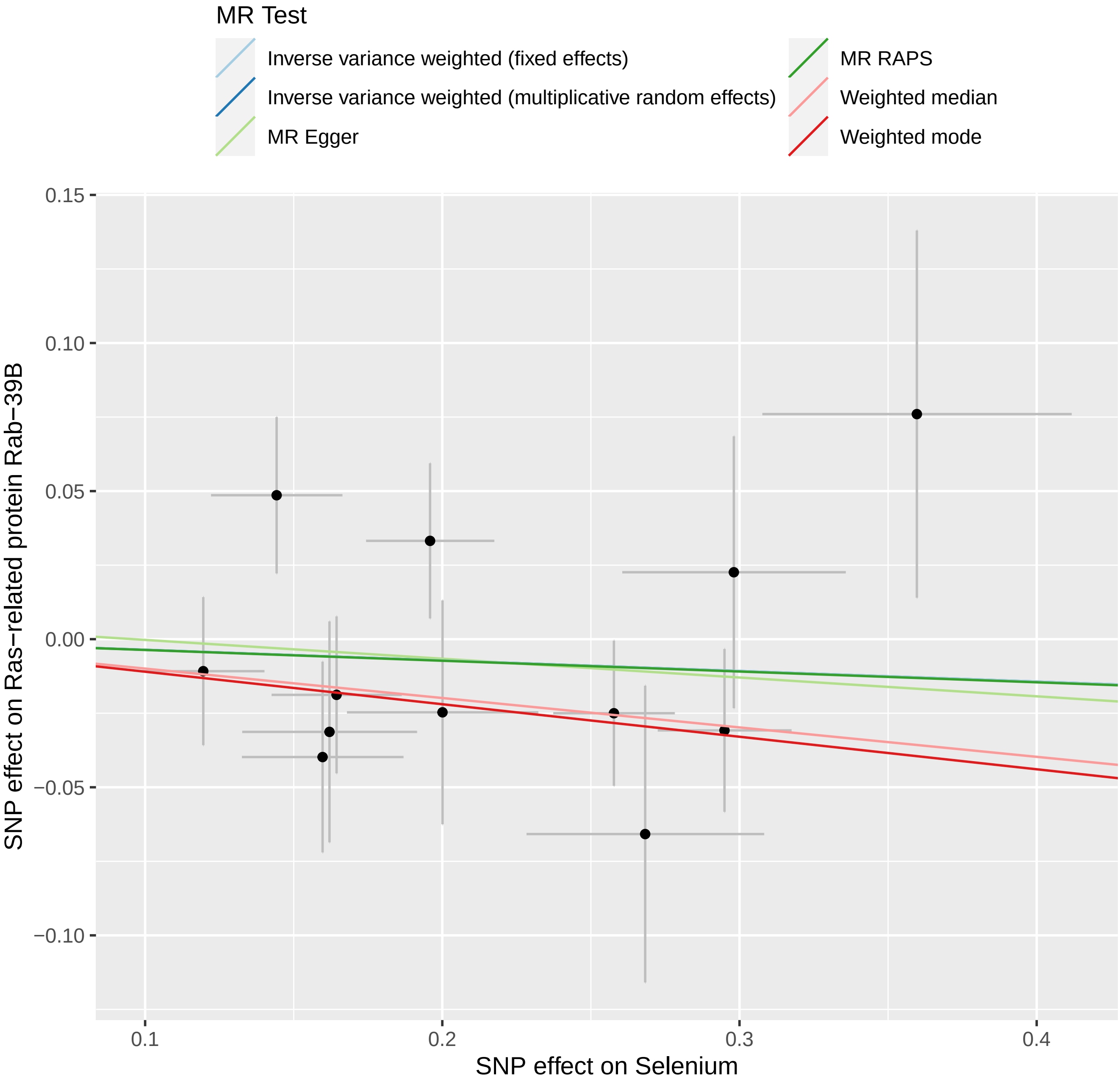

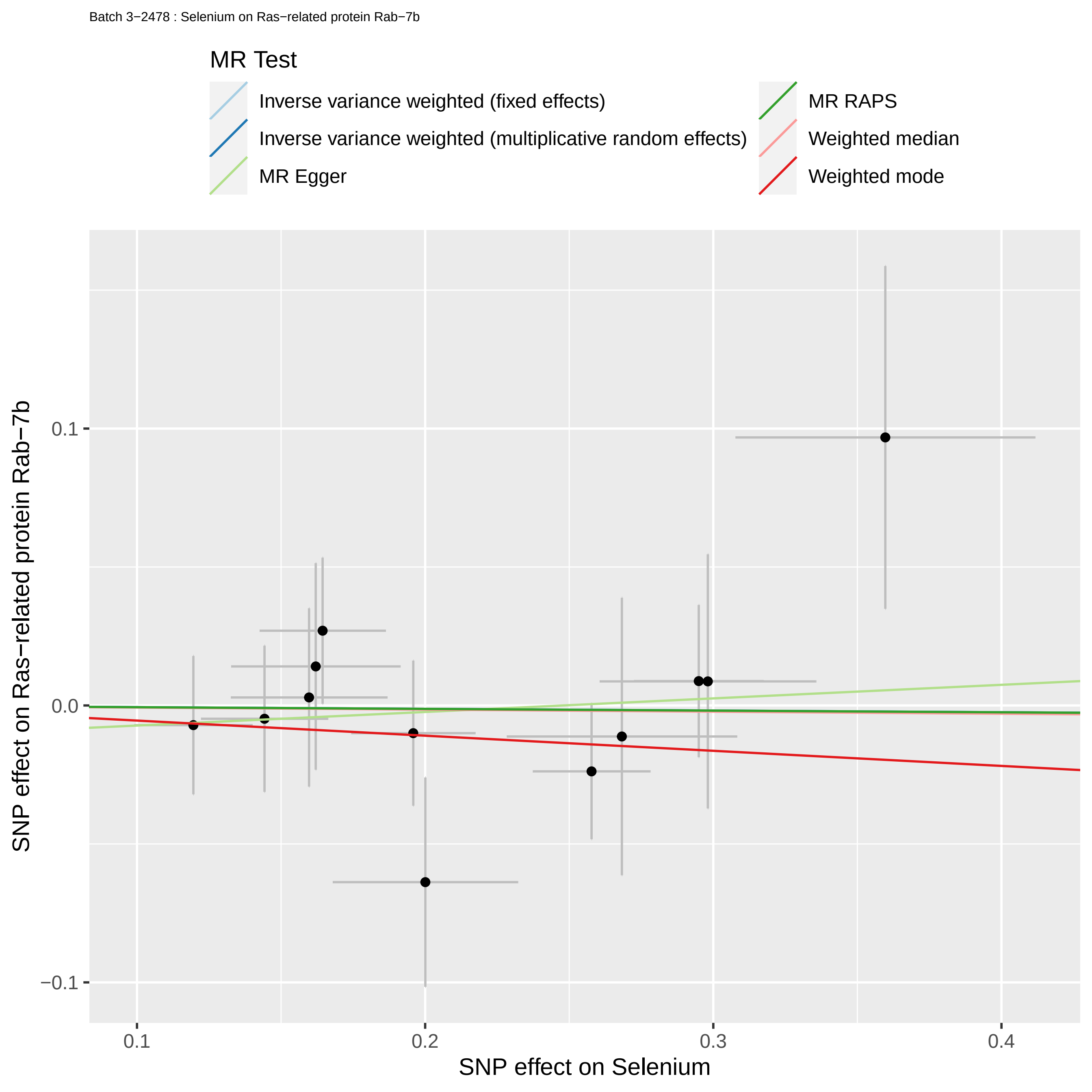
Batch 3−2478 : Selenium on Ras−related protein Rab−7b
MR Test
Inverse variance weighted (fixed effects)
Inverse variance weighted (multiplicative random effects)
MR Egger
MR RAPS
Weighted median
Weighted mode
SNP effect on Ras−related protein Rab−7b
0.1
0.0
−0.1
0.1
0.2
0.3
0.4
SNP effect on Selenium

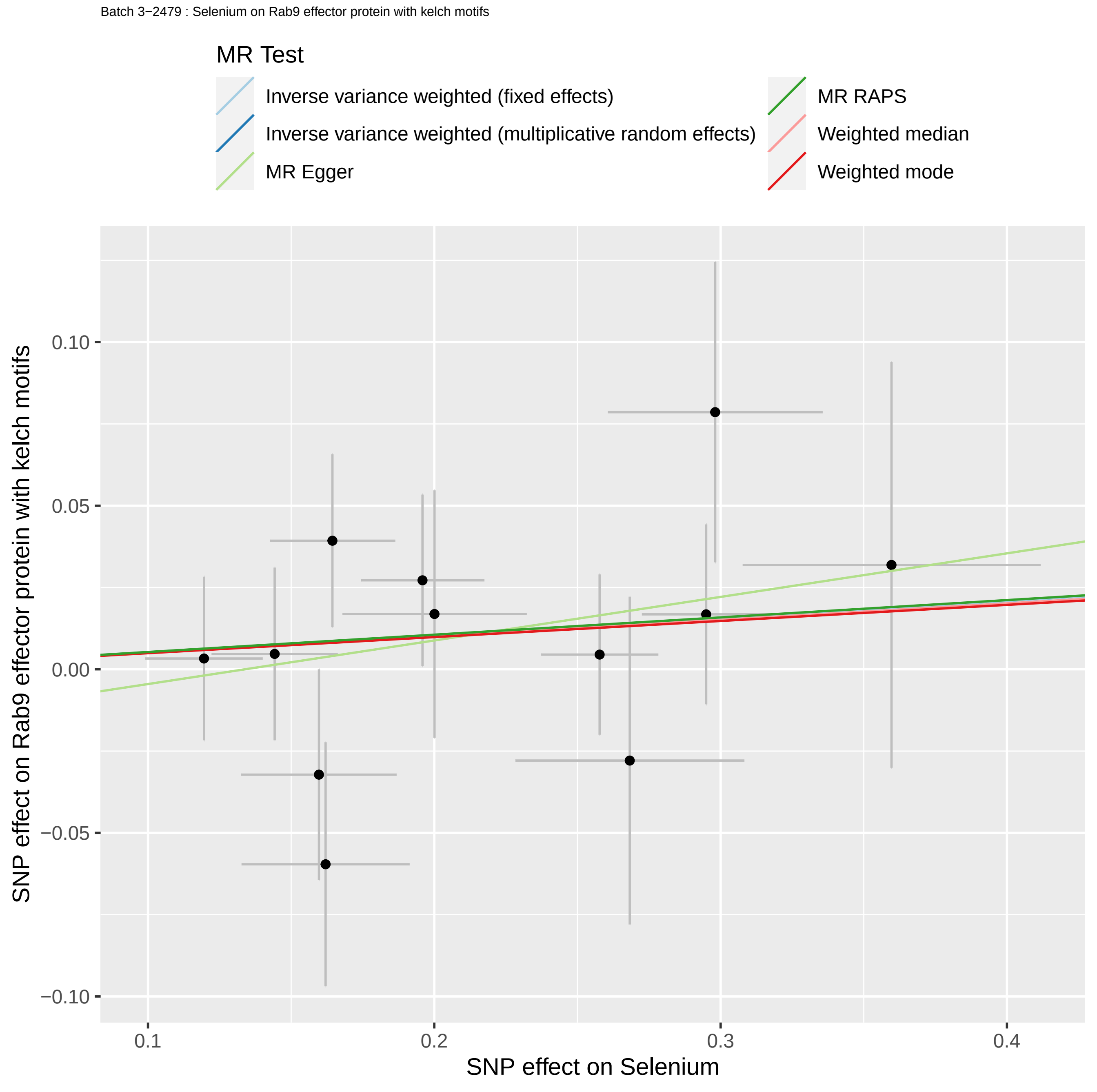

Batch 3−2479 : Selenium on Rab9 effector protein with kelch motifs
MR Test
Inverse variance weighted (fixed effects)
Inverse variance weighted (multiplicative random effects)
MR Egger
MR RAPS
Weighted median
Weighted mode
0.10
0.05
0.00
−0.05
−0.10
0.1
0.2
0.3
0.4
SNP effect on Rab9 effector protein with kelch motifs
SNP effect on Selenium

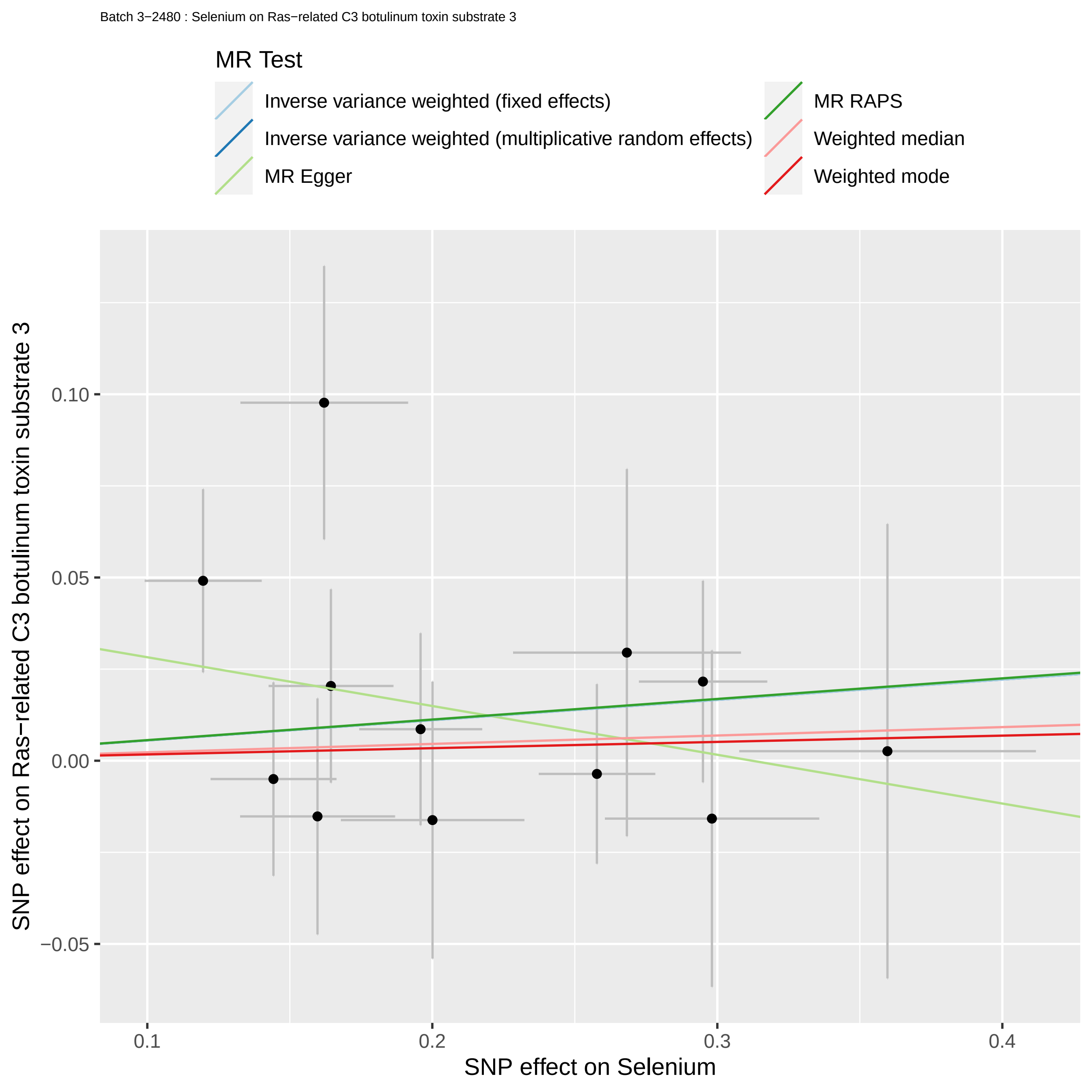
Batch 3−2480 : Selenium on Ras−related C3 botulinum toxin substrate 3
MR Test
Inverse variance weighted (fixed effects)
Inverse variance weighted (multiplicative random effects)
MR Egger
MR RAPS
Weighted median
Weighted mode
SNP effect on Ras−related C3 botulinum toxin substrate 3
0.10
0.05
0.00
−0.05
0.1
0.2
0.3
0.4
SNP effect on Selenium

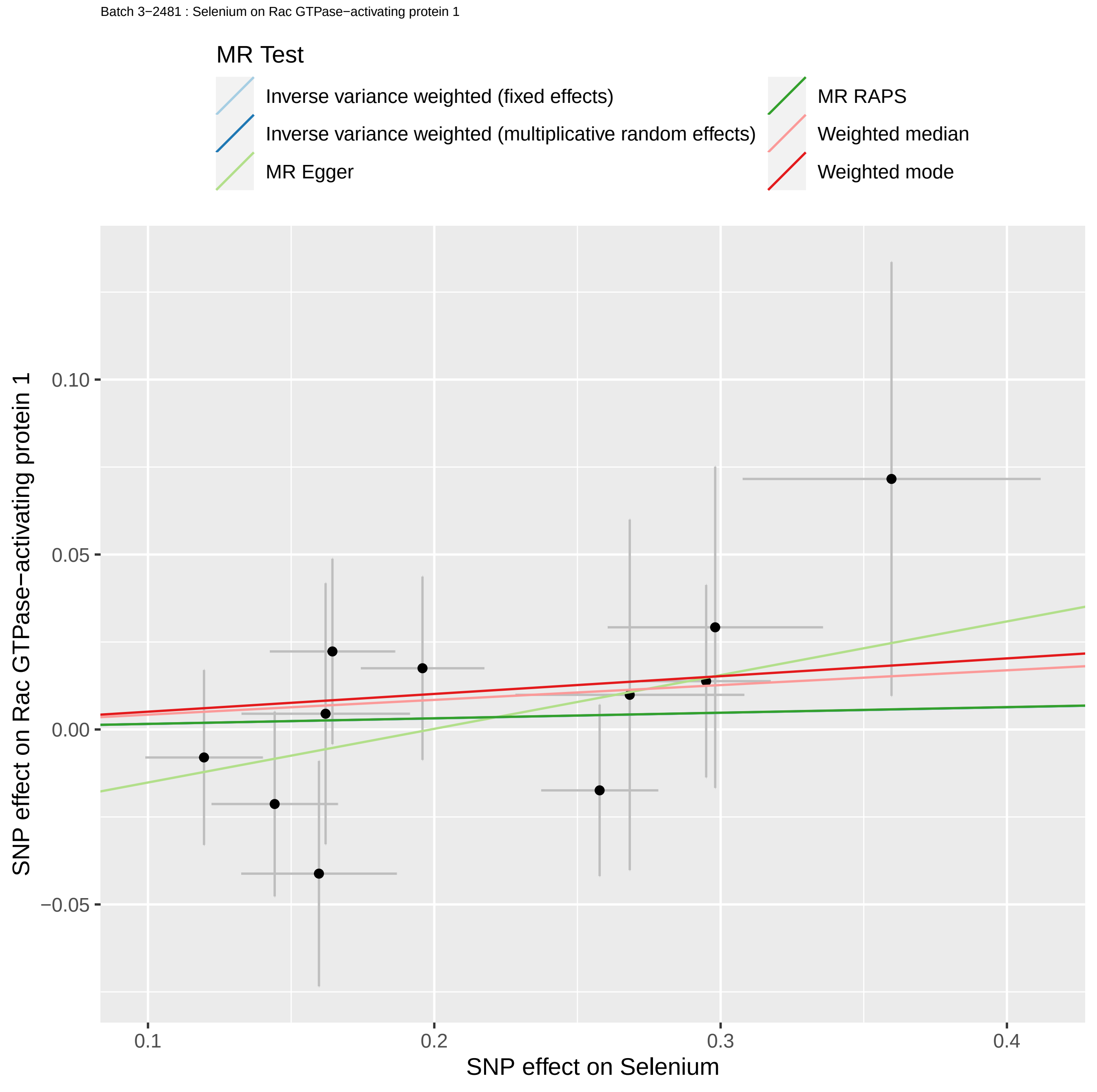
Batch 3−2481 : Selenium on Rac GTPase−activating protein 1
MR Test
Inverse variance weighted (fixed effects)
Inverse variance weighted (multiplicative random effects)
MR Egger
MR RAPS
Weighted median
Weighted mode
SNP effect on Rac GTPase−activating protein 1
0.10
0.05
0.00
−0.05
0.1
0.2
0.3
0.4
SNP effect on Selenium

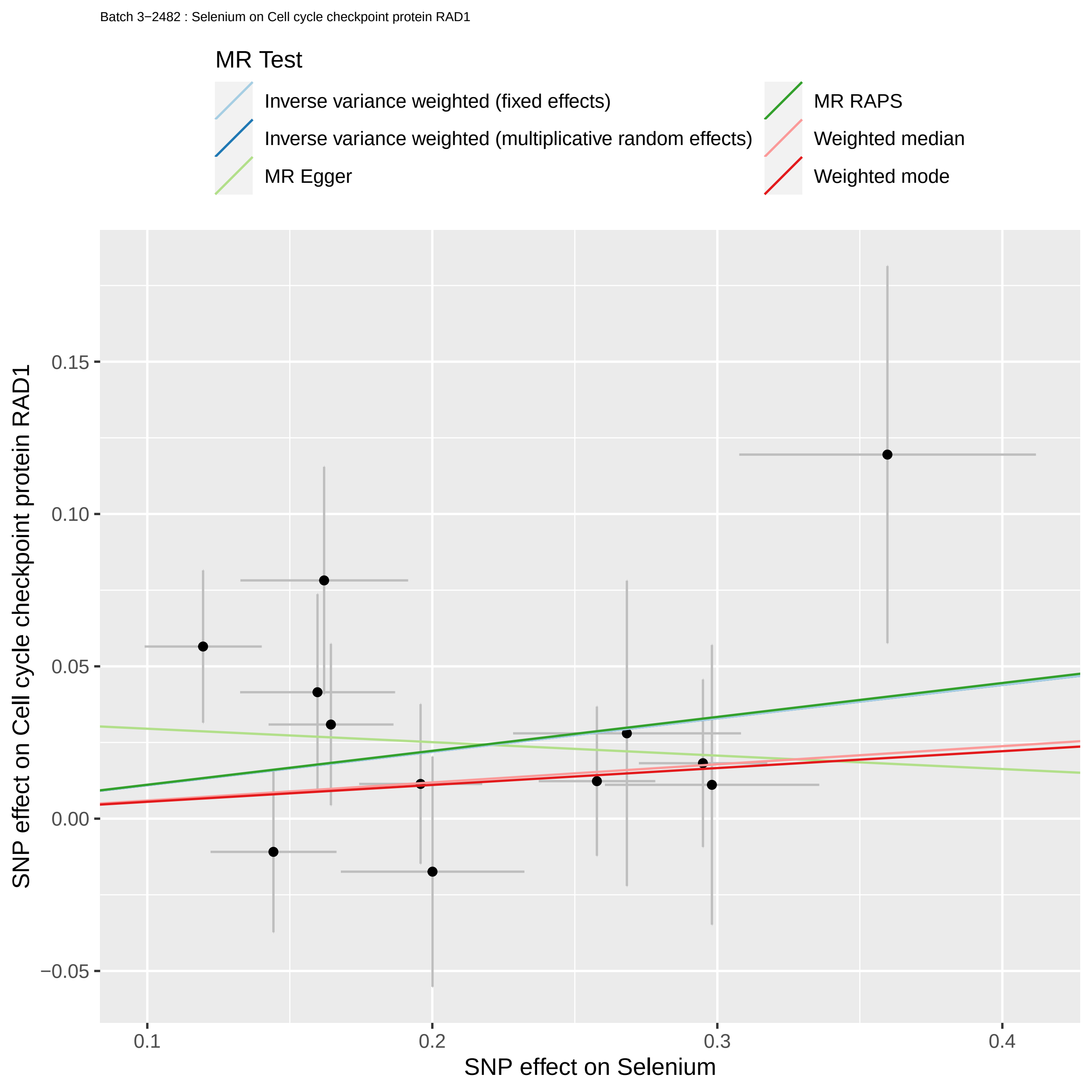
Batch 3−2482 : Selenium on Cell cycle checkpoint protein RAD1
MR Test
Inverse variance weighted (fixed effects)
Inverse variance weighted (multiplicative random effects)
MR Egger
MR RAPS
Weighted median
Weighted mode
SNP effect on Cell cycle checkpoint protein RAD1
0.15
0.10
0.05
0.00
−0.05
0.1
0.2
0.3
0.4
SNP effect on Selenium

Batch 3−2483 : Selenium on UV excision repair protein RAD23 homolog A

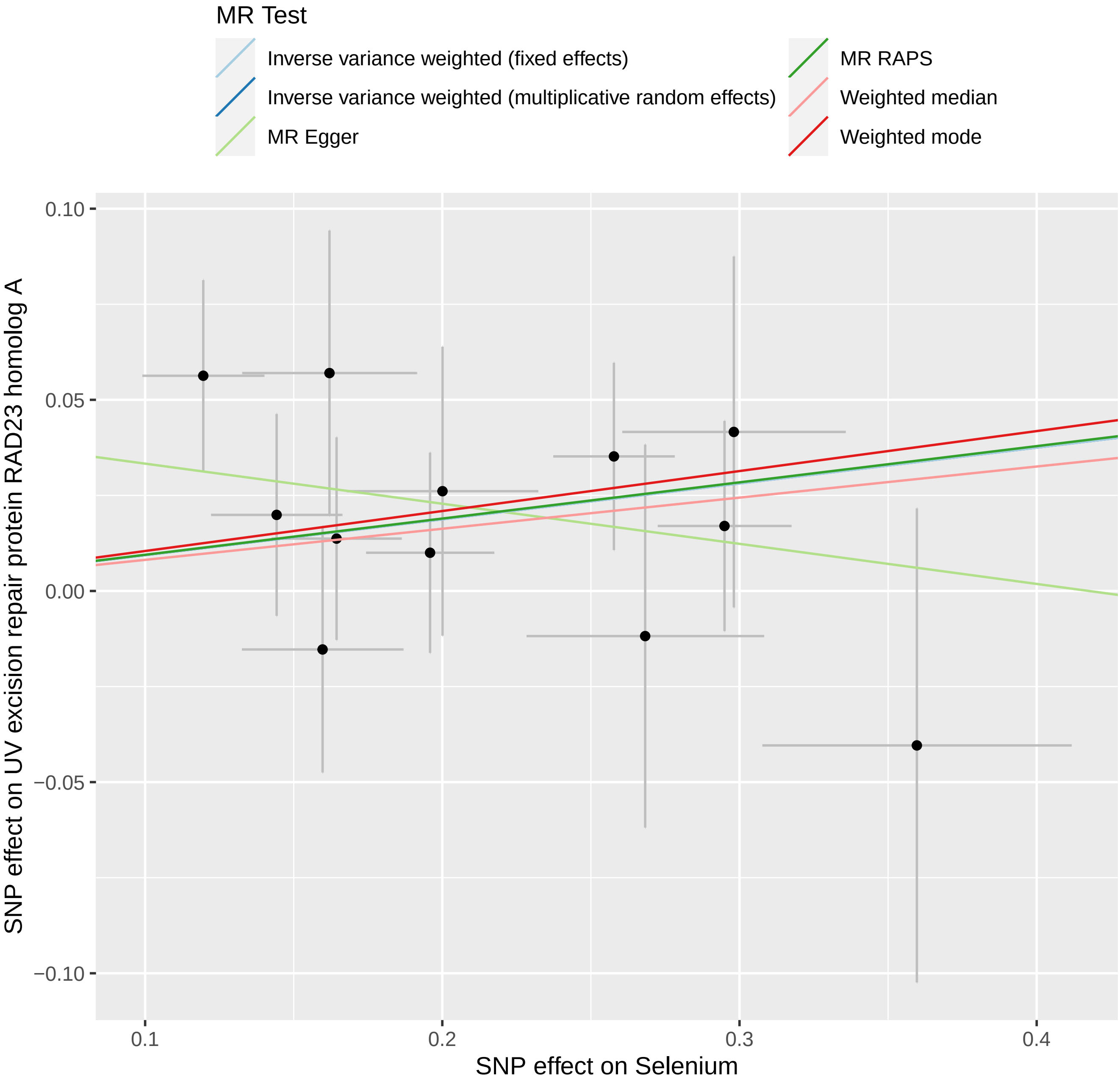

Batch 3−2484 : Selenium on UV excision repair protein RAD23 homolog B

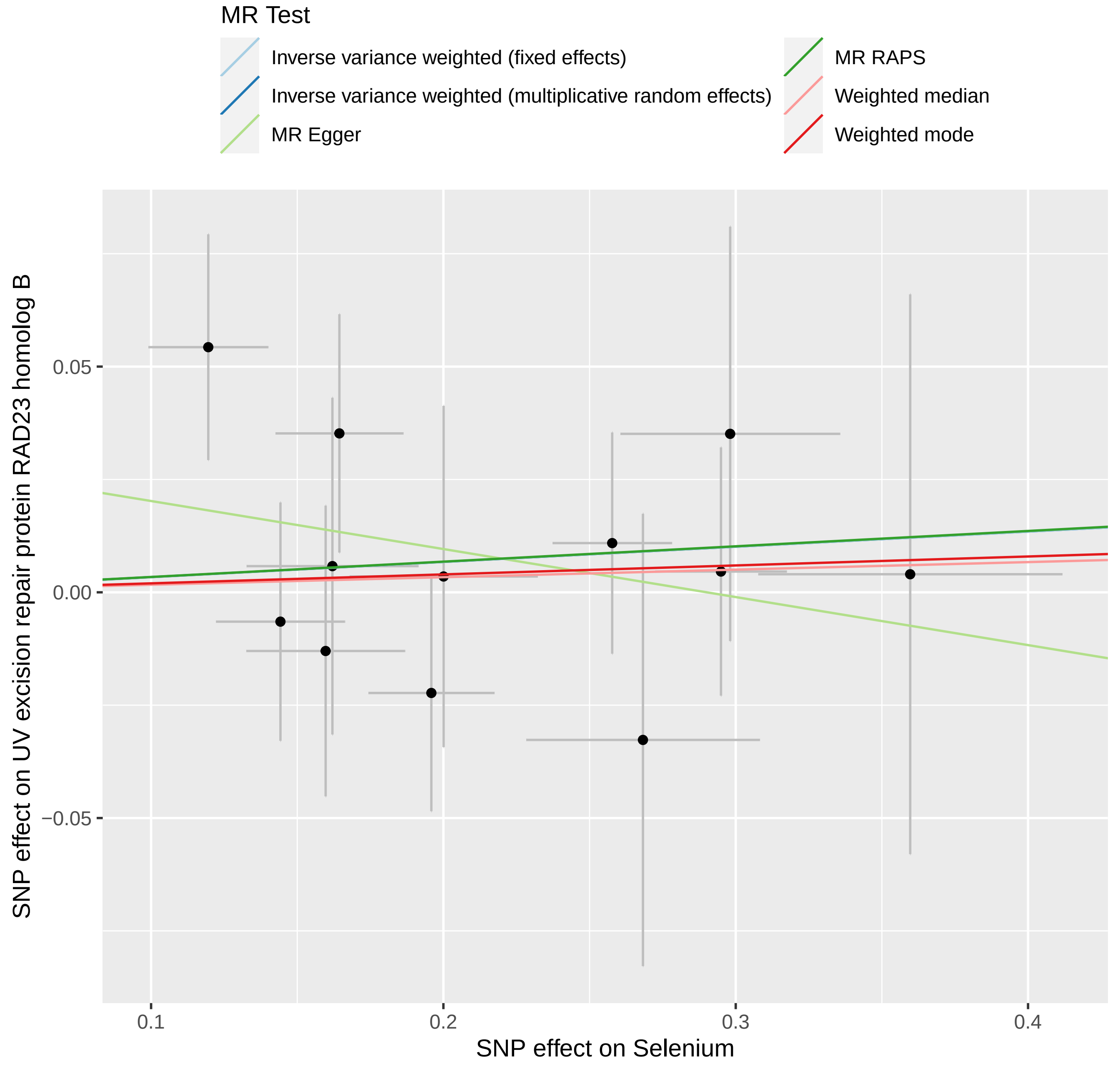

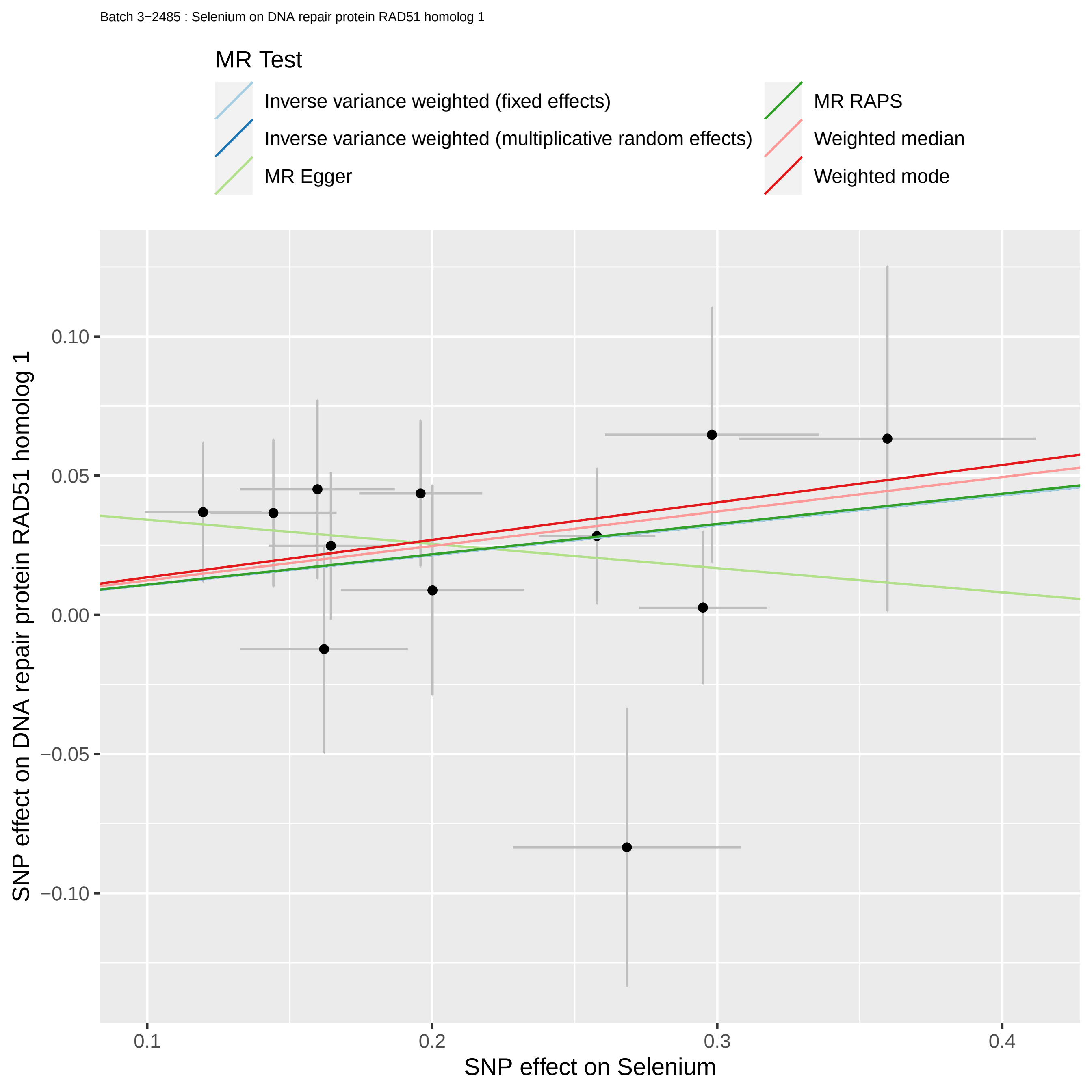
Batch 3−2485 : Selenium on DNA repair protein RAD51 homolog 1
MR Test
Inverse variance weighted (fixed effects)
Inverse variance weighted (multiplicative random effects)
MR Egger
MR RAPS
Weighted median
Weighted mode
0.10
0.05
0.00
−0.05
−0.10
0.1
0.2
0.3
0.4
SNP effect on Selenium
SNP effect on DNA repair protein RAD51 homolog 1

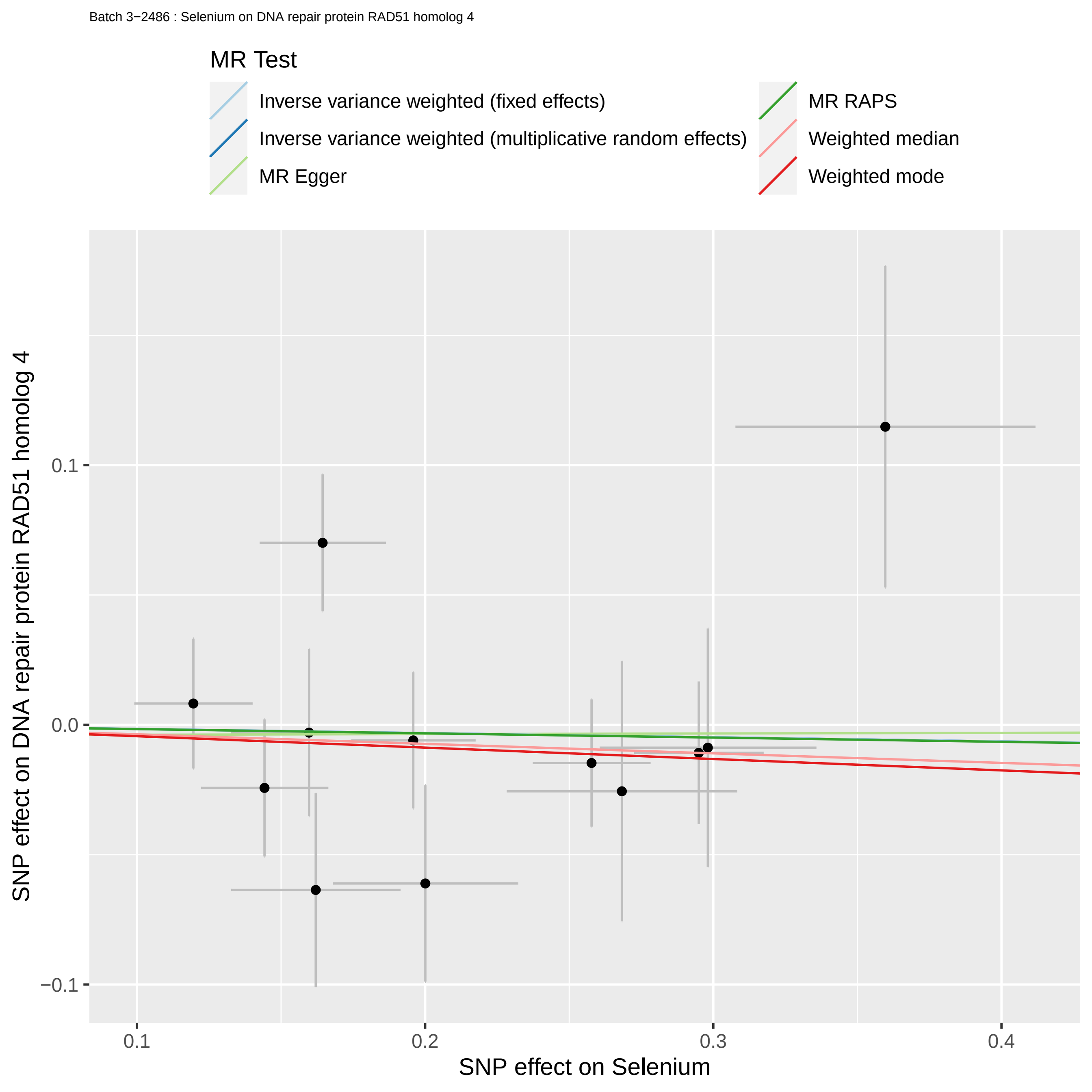
Batch 3−2486 : Selenium on DNA repair protein RAD51 homolog 4
MR Test
Inverse variance weighted (fixed effects)
Inverse variance weighted (multiplicative random effects)
MR Egger
MR RAPS
Weighted median
Weighted mode
0.1
0.0
−0.1
0.1
0.2
0.3
0.4
SNP effect on DNA repair protein RAD51 homolog 4
SNP effect on Selenium

Batch 3−2487 : Selenium on NKG2D ligand 4

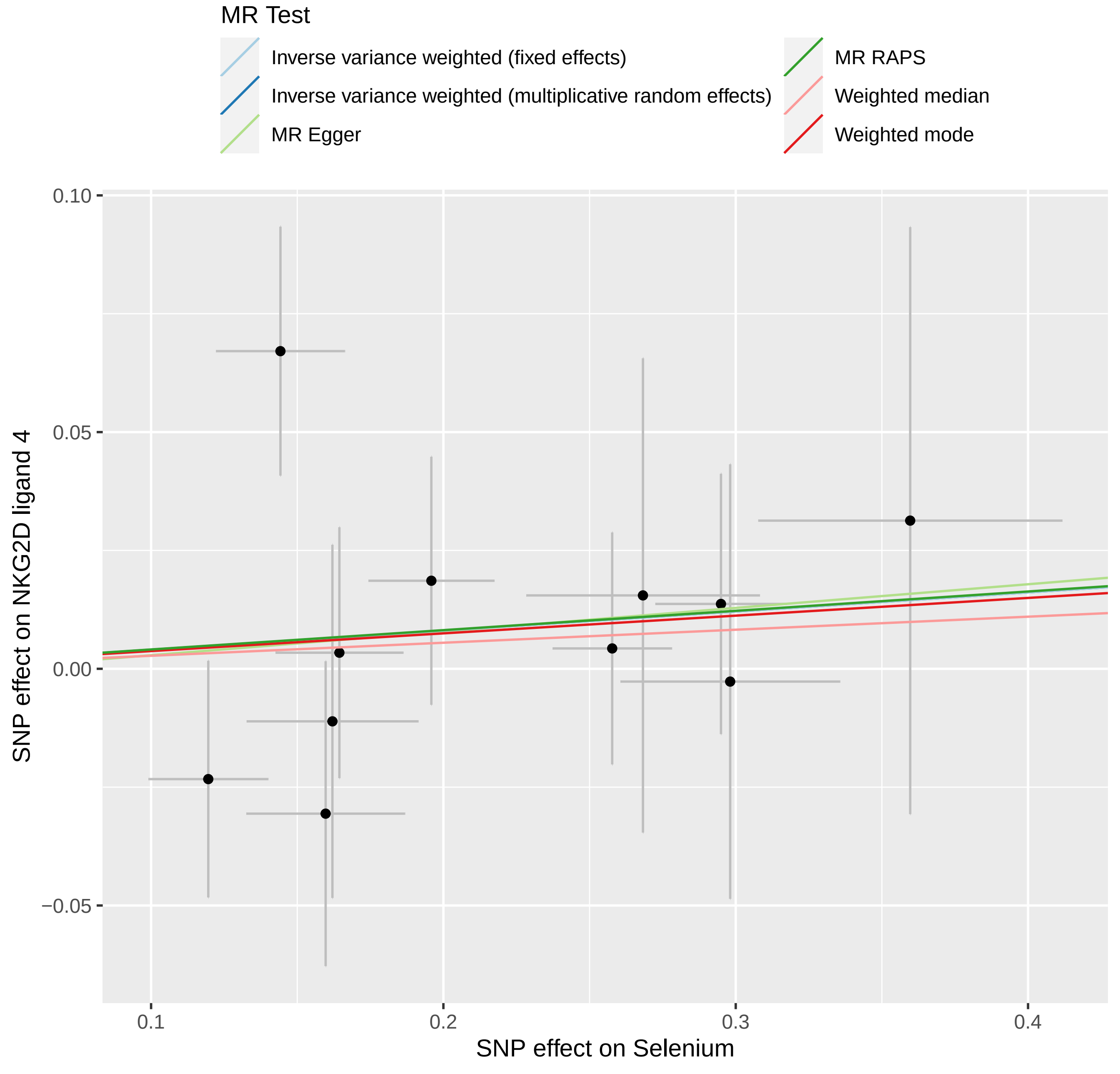

Batch 3−2488 : Selenium on Ran−binding protein 3

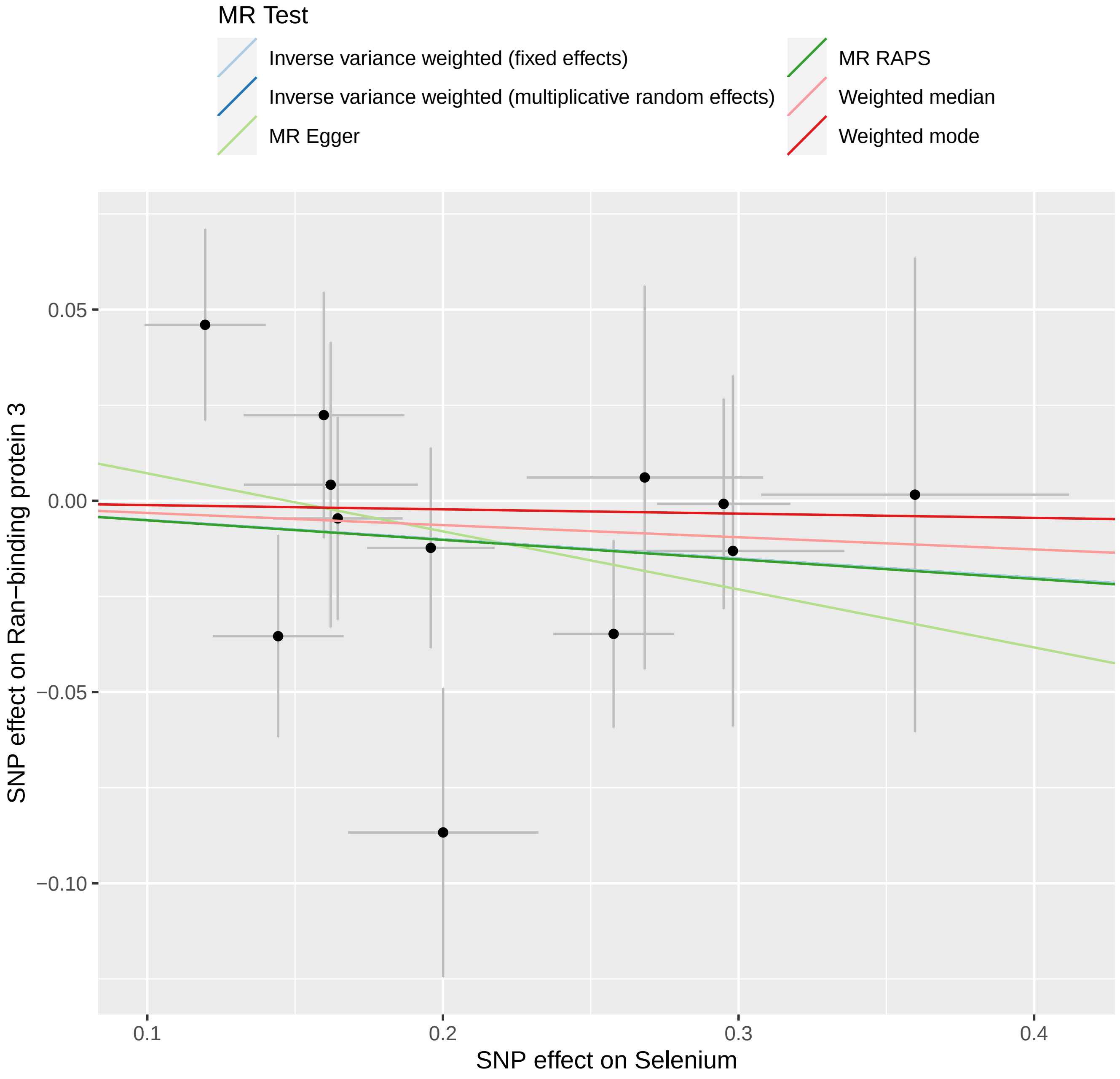

Batch 3−2489 : Selenium on Rap1 GTPase−activating protein 1

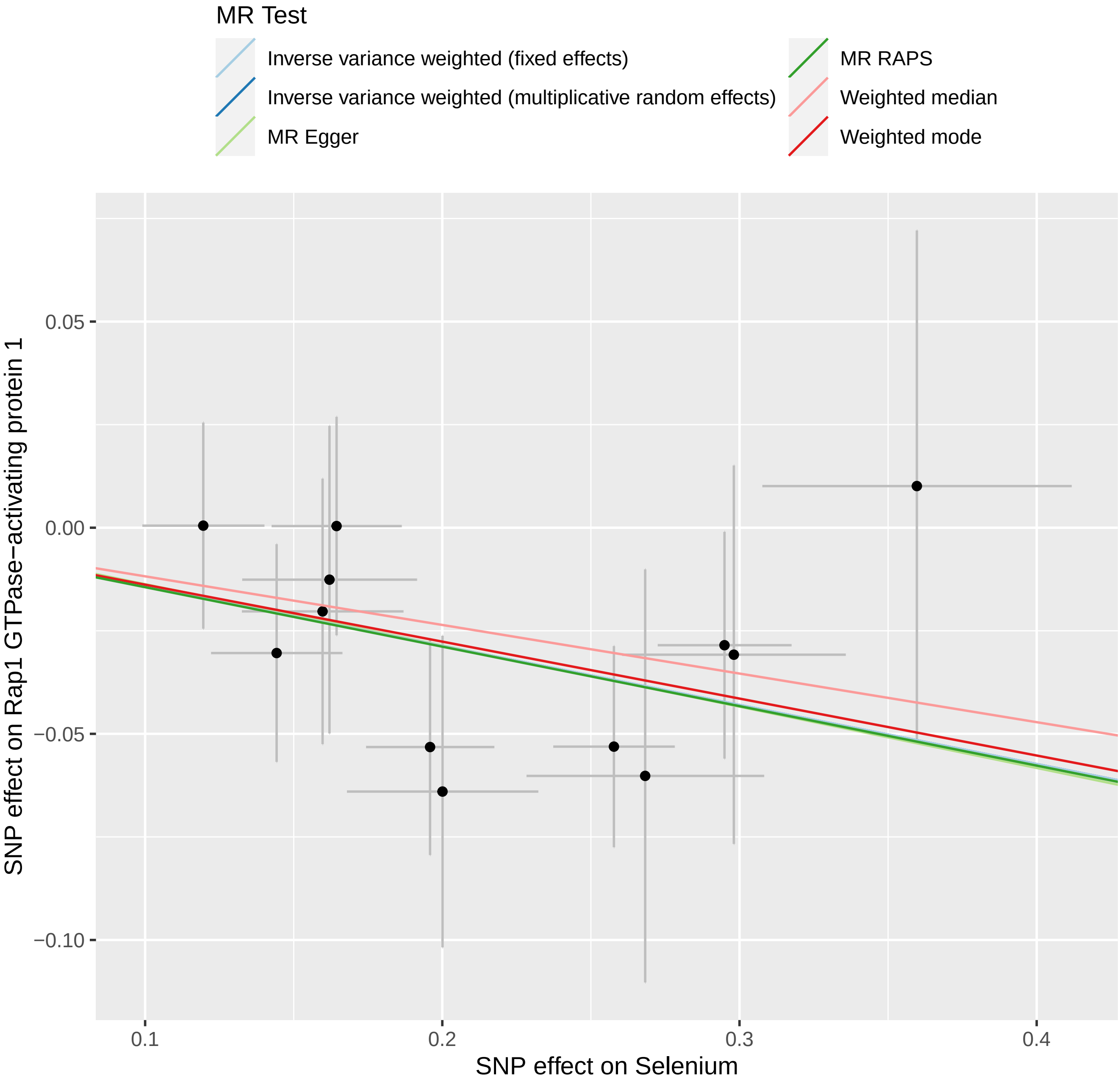

Batch 3−2490 : Selenium on Rap1 GTPase−GDP dissociation stimulator 1

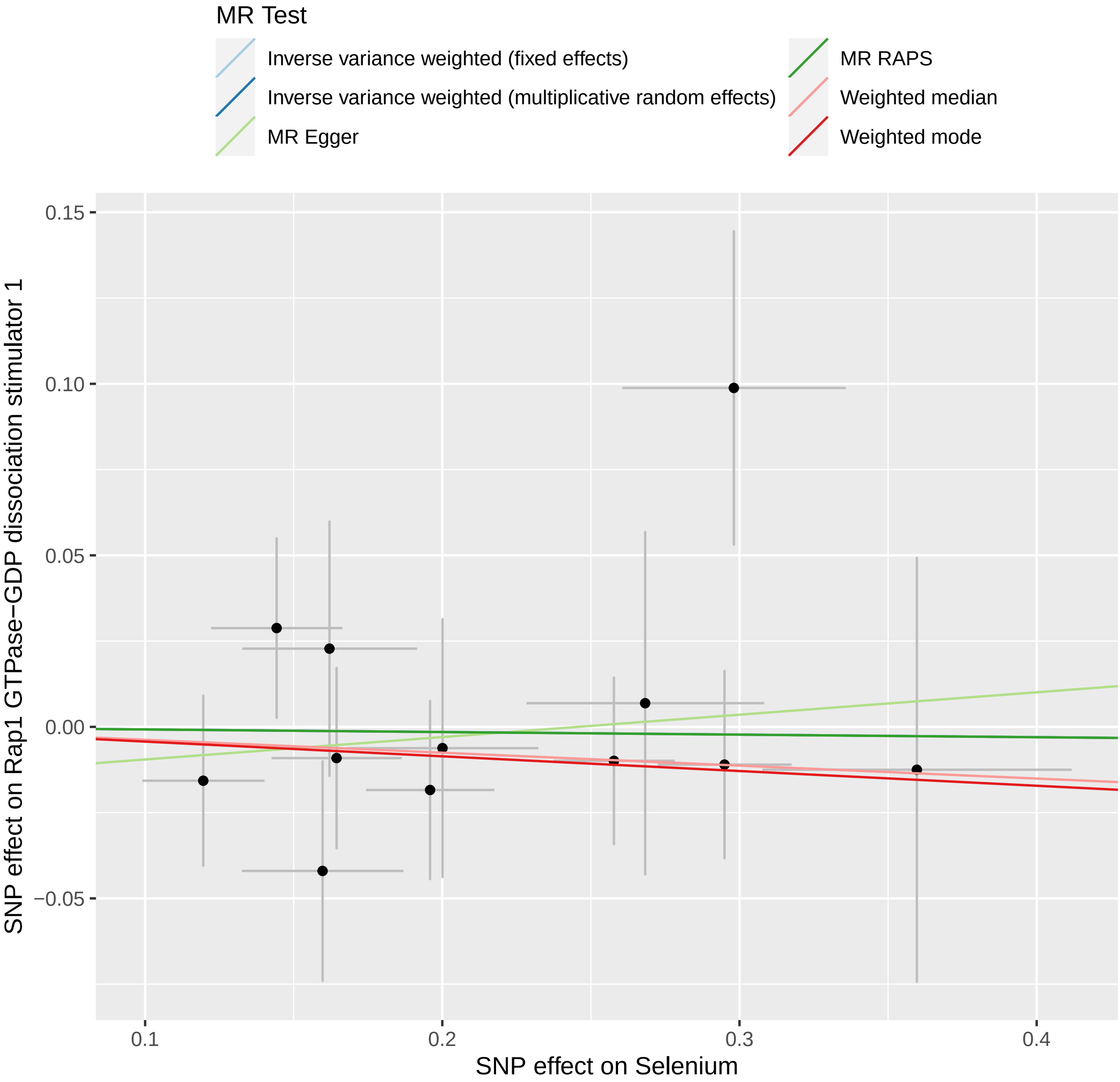

Batch 3−2491 : Selenium on Ras−related protein Rap−2a

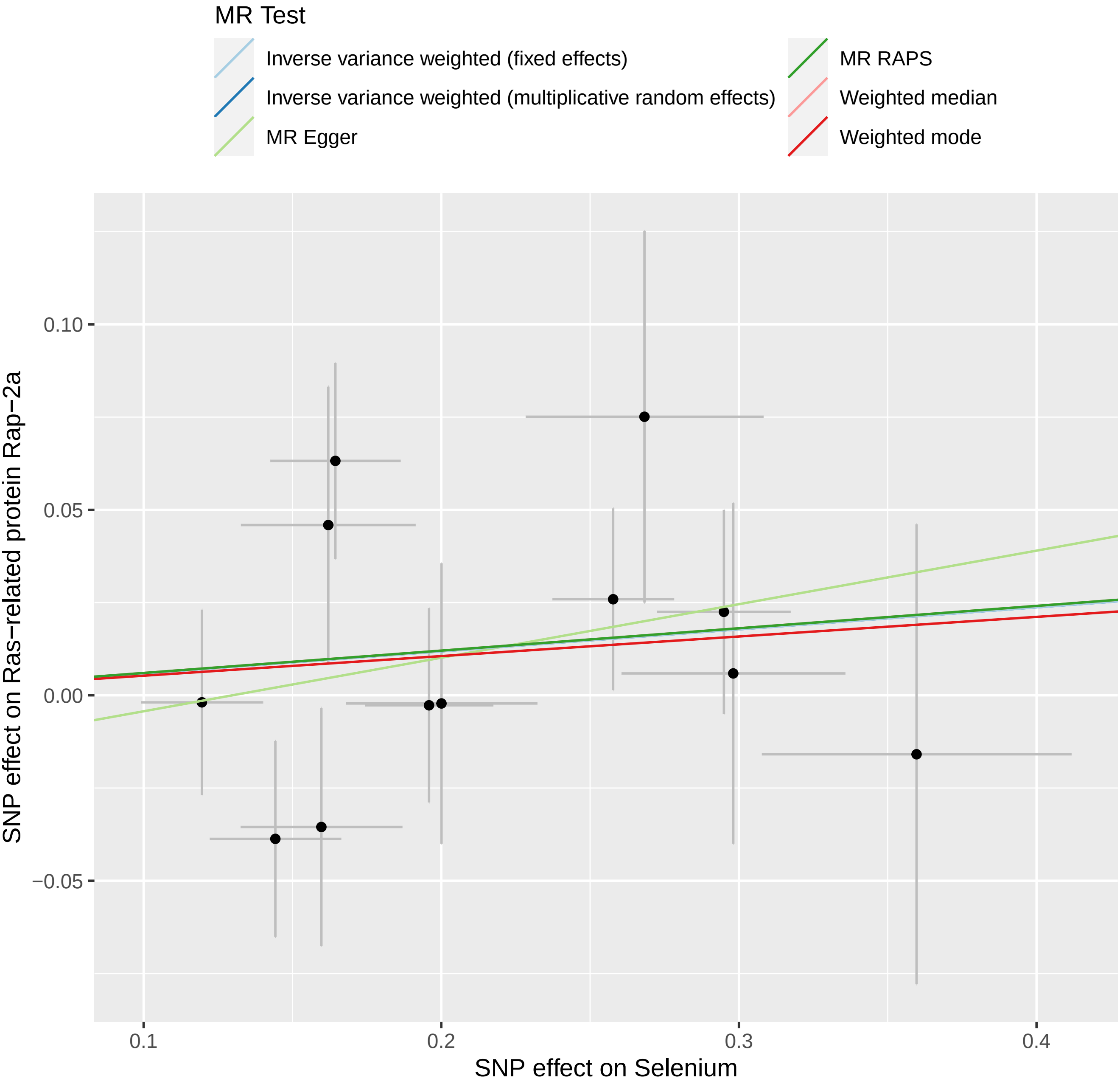

Batch 3−2492 : Selenium on Rap guanine nucleotide exchange factor 5

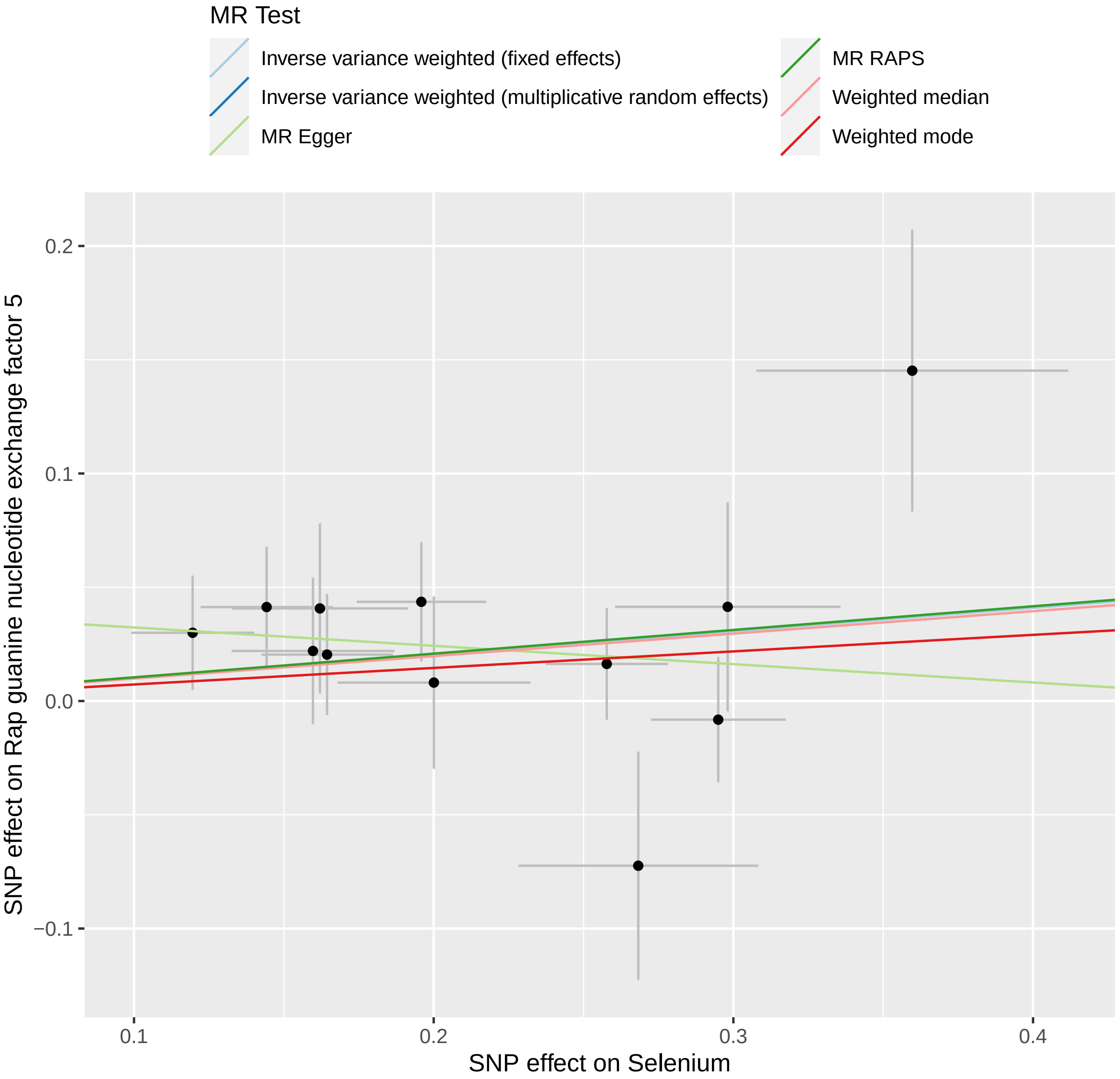

Batch 3−2493 : Selenium on Retinoic acid receptor responder protein 1

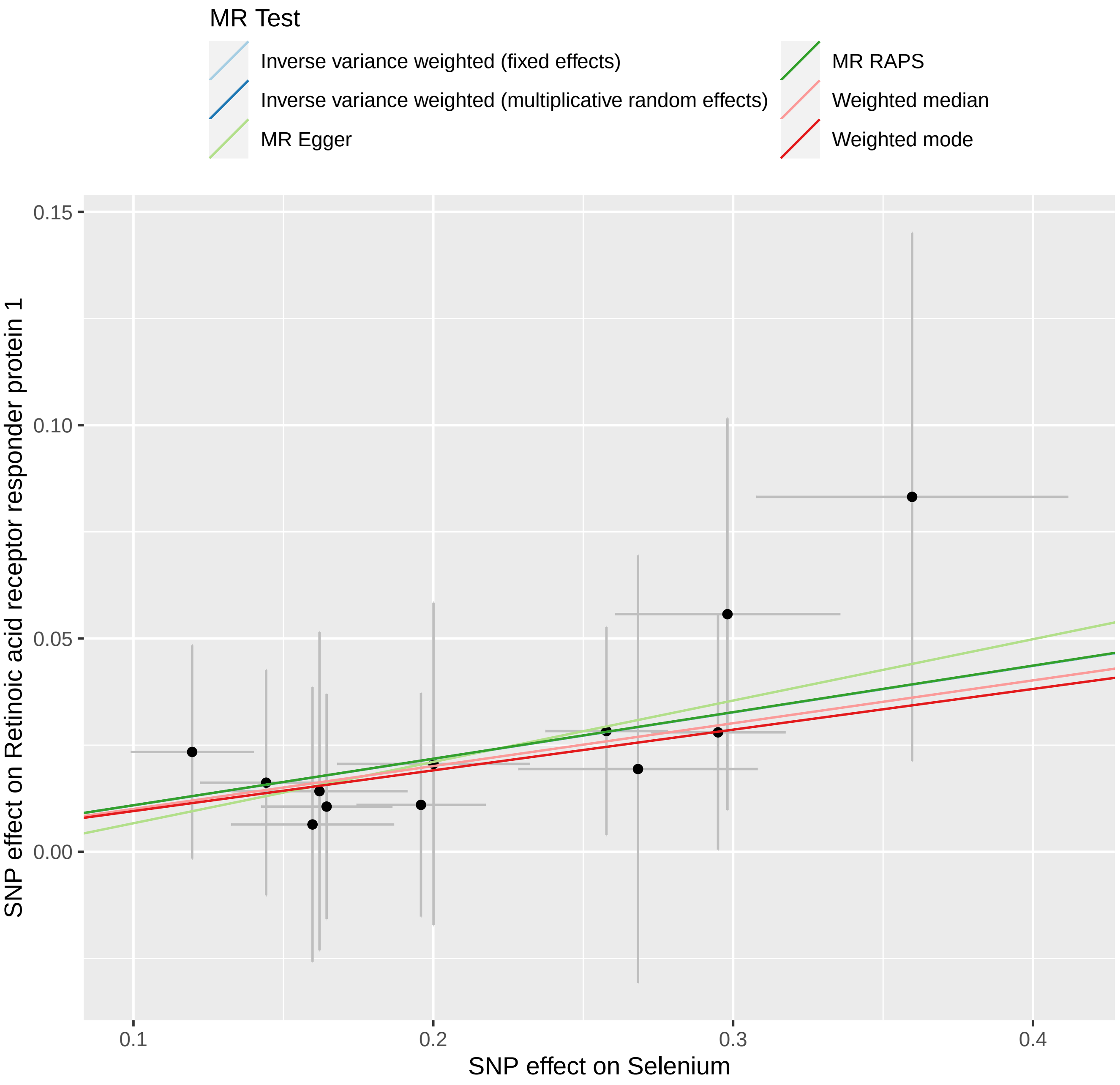

Batch 3−2494 : Selenium on Retinoic acid receptor responder protein 3

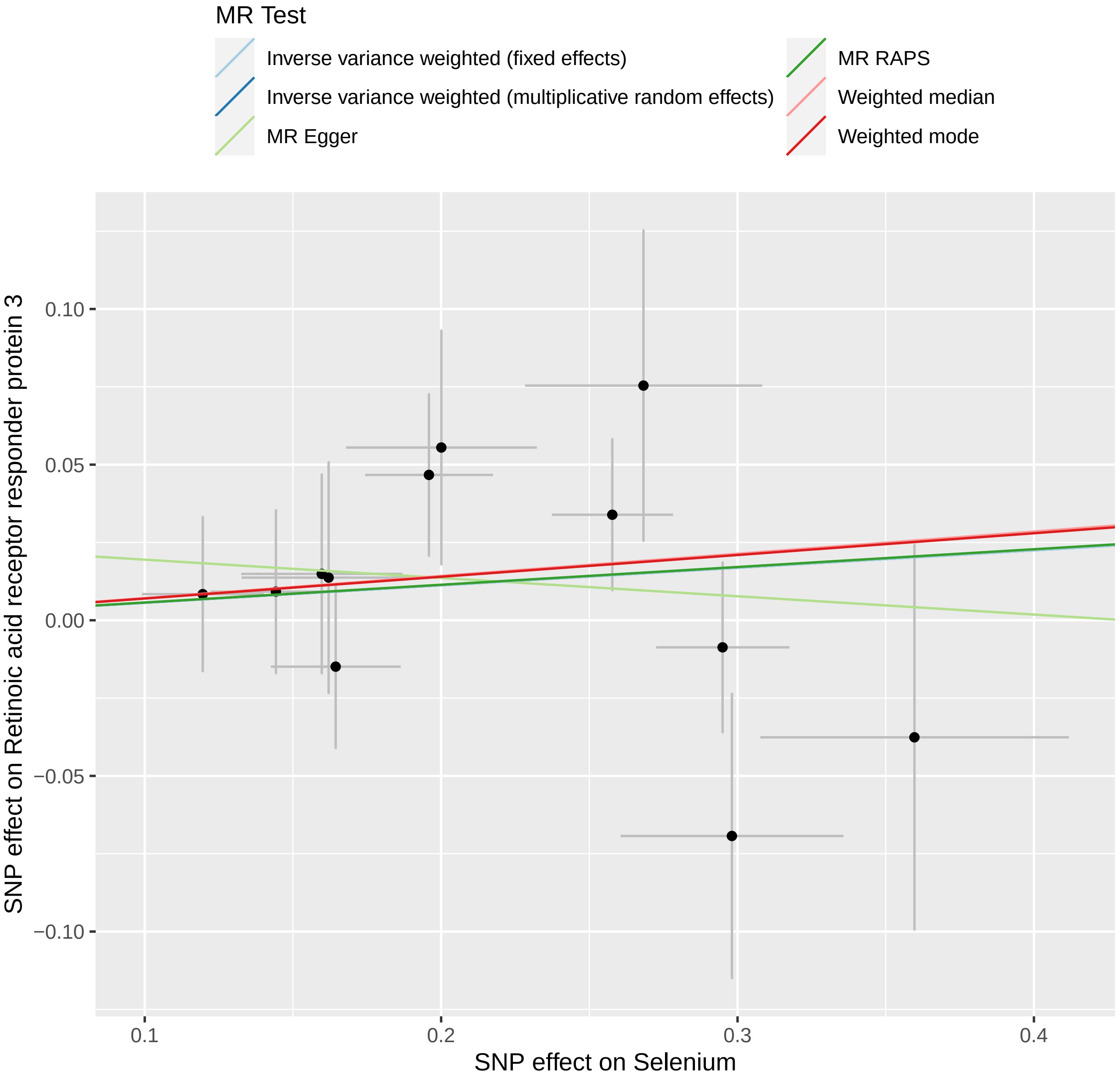

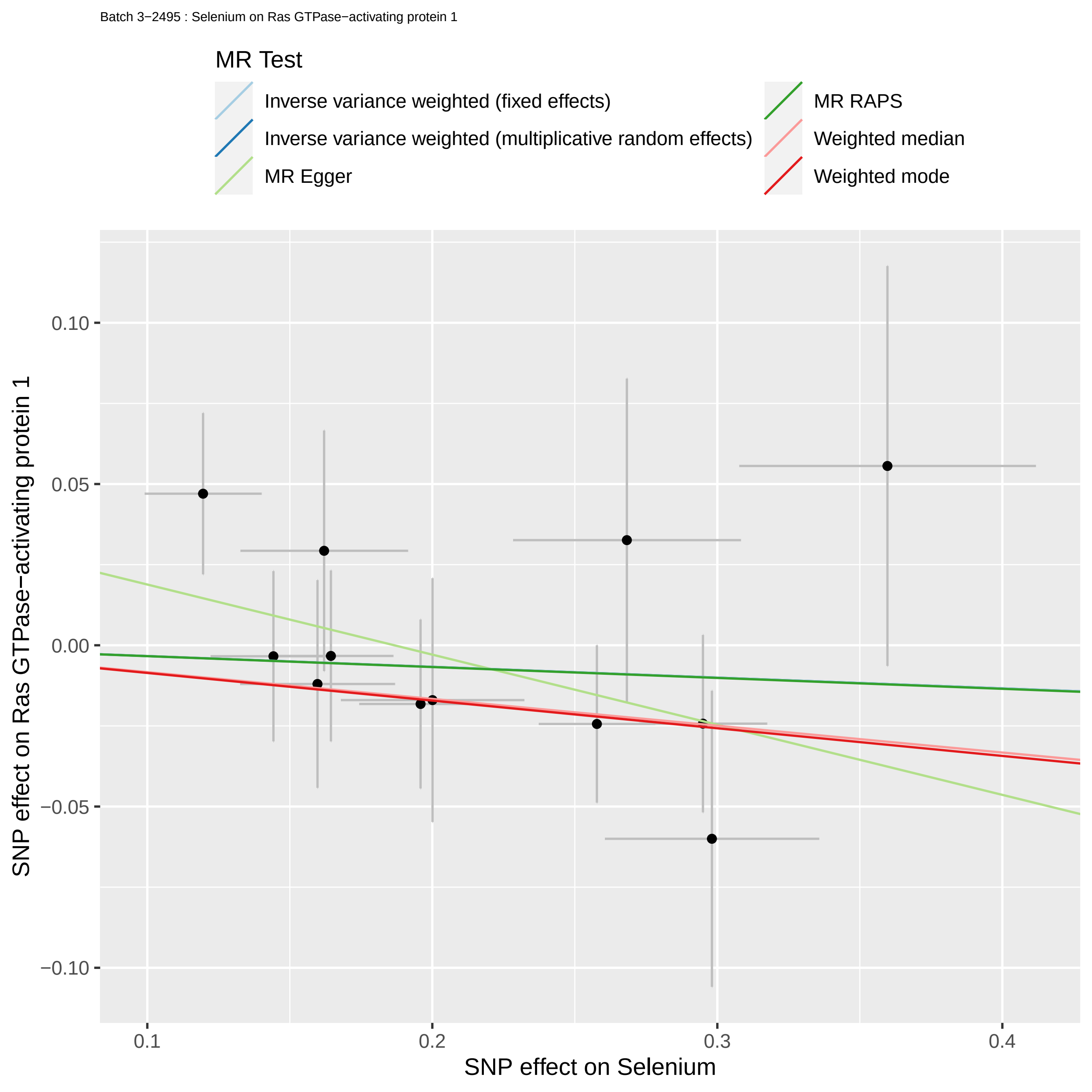

Batch 3−2495 : Selenium on Ras GTPase−activating protein 1
MR Test
Inverse variance weighted (fixed effects)
Inverse variance weighted (multiplicative random effects)
MR Egger
MR RAPS
Weighted median
Weighted mode
0.10
0.05
0.00
−0.05
−0.10
0.1
0.2
0.3
0.4
SNP effect on Ras GTPase−activating protein 1
SNP effect on Selenium

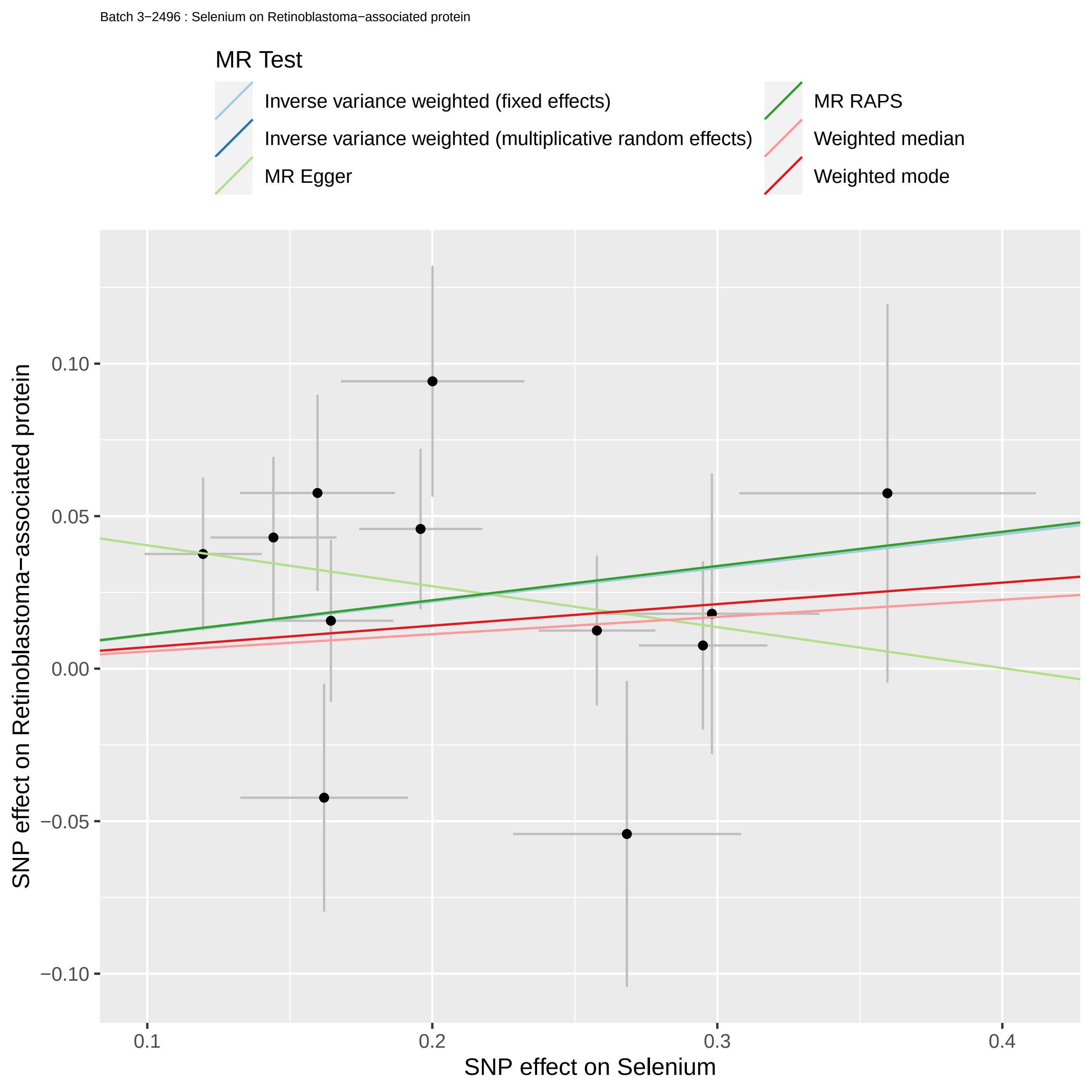

Batch 3−2496 : Selenium on Retinoblastoma−associated protein
MR Test
Inverse variance weighted (fixed effects)
Inverse variance weighted (multiplicative random effects)
MR Egger
MR RAPS
Weighted median
Weighted mode
SNP effect on Retinoblastoma−associated protein
SNP effect on Selenium
0.10
0.05
0.00
−0.05
−0.10
0.1
0.2
0.3
0.4

Batch 3−2497 : Selenium on Retinoblastoma−binding protein 5

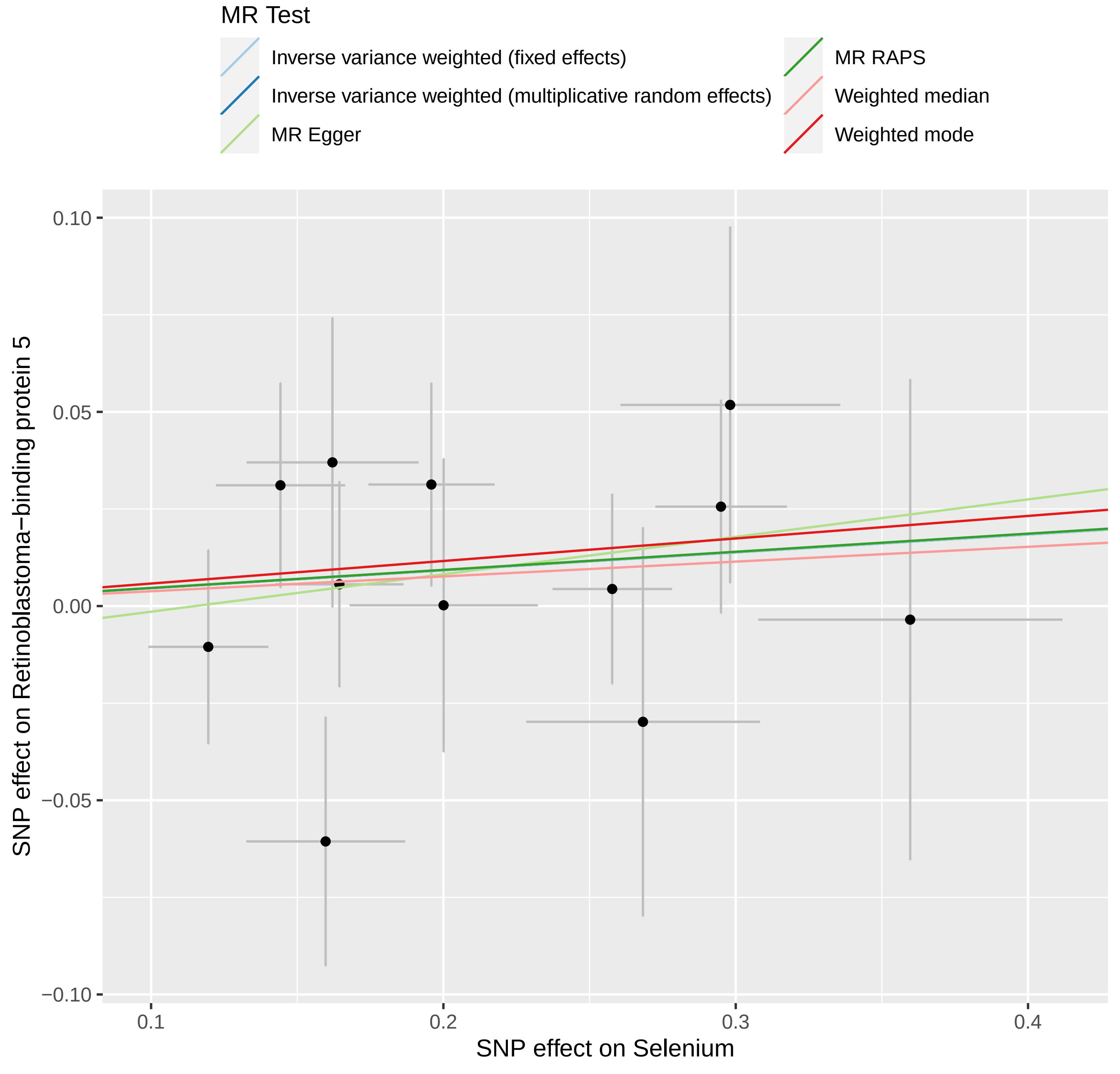

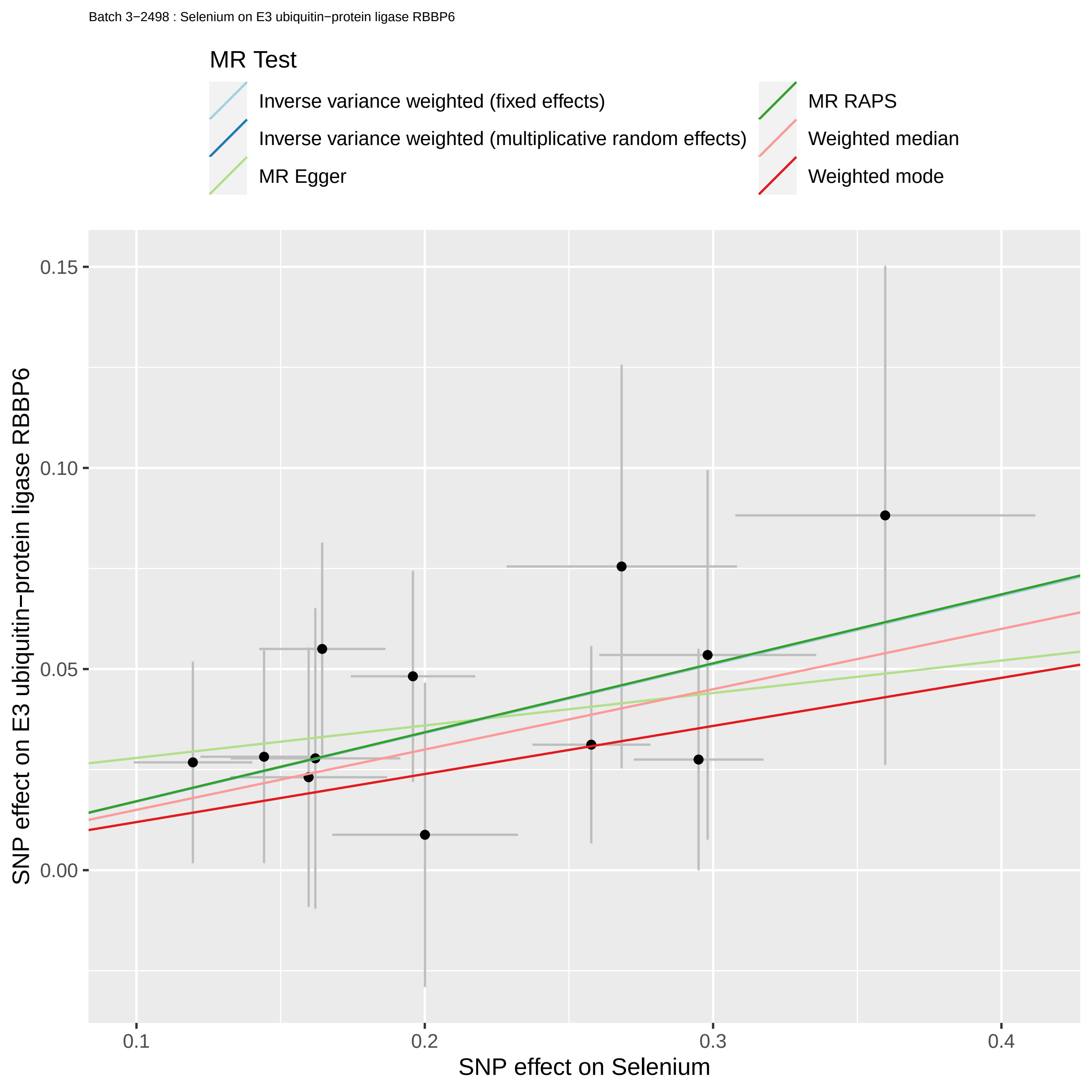
Batch 3−2498 : Selenium on E3 ubiquitin−protein ligase RBBP6
MR Test
Inverse variance weighted (fixed effects)
Inverse variance weighted (multiplicative random effects)
MR Egger
MR RAPS
Weighted median
Weighted mode
SNP effect on E3 ubiquitin−protein ligase RBBP6
0.15
0.10
0.05
0.00
0.1
0.2
0.3
0.4
SNP effect on Selenium

Batch 3−2499 : Selenium on RNA binding protein fox−1 homolog 2

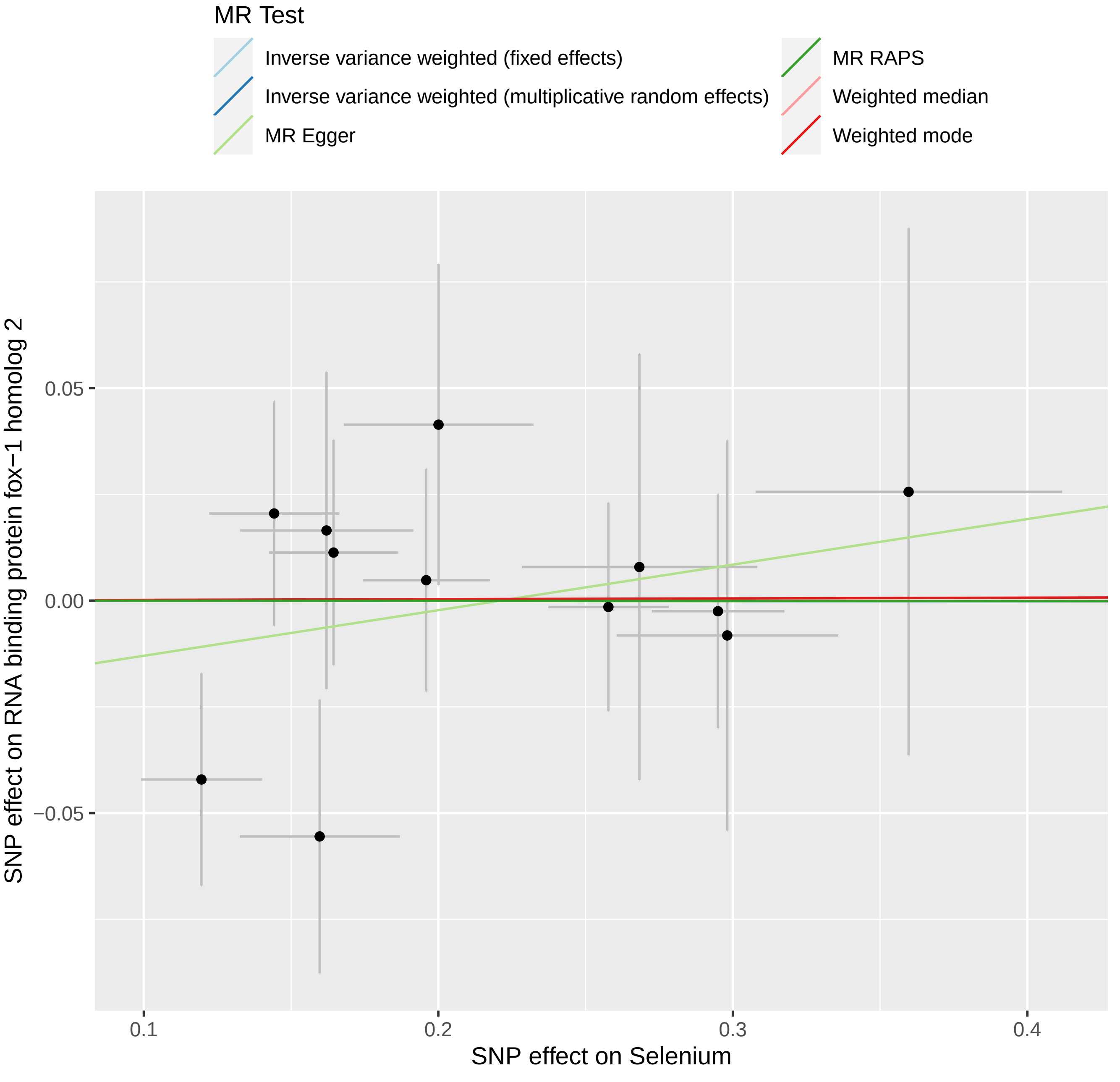

Batch 3−2500 : Selenium on Retinoblastoma−like protein 1

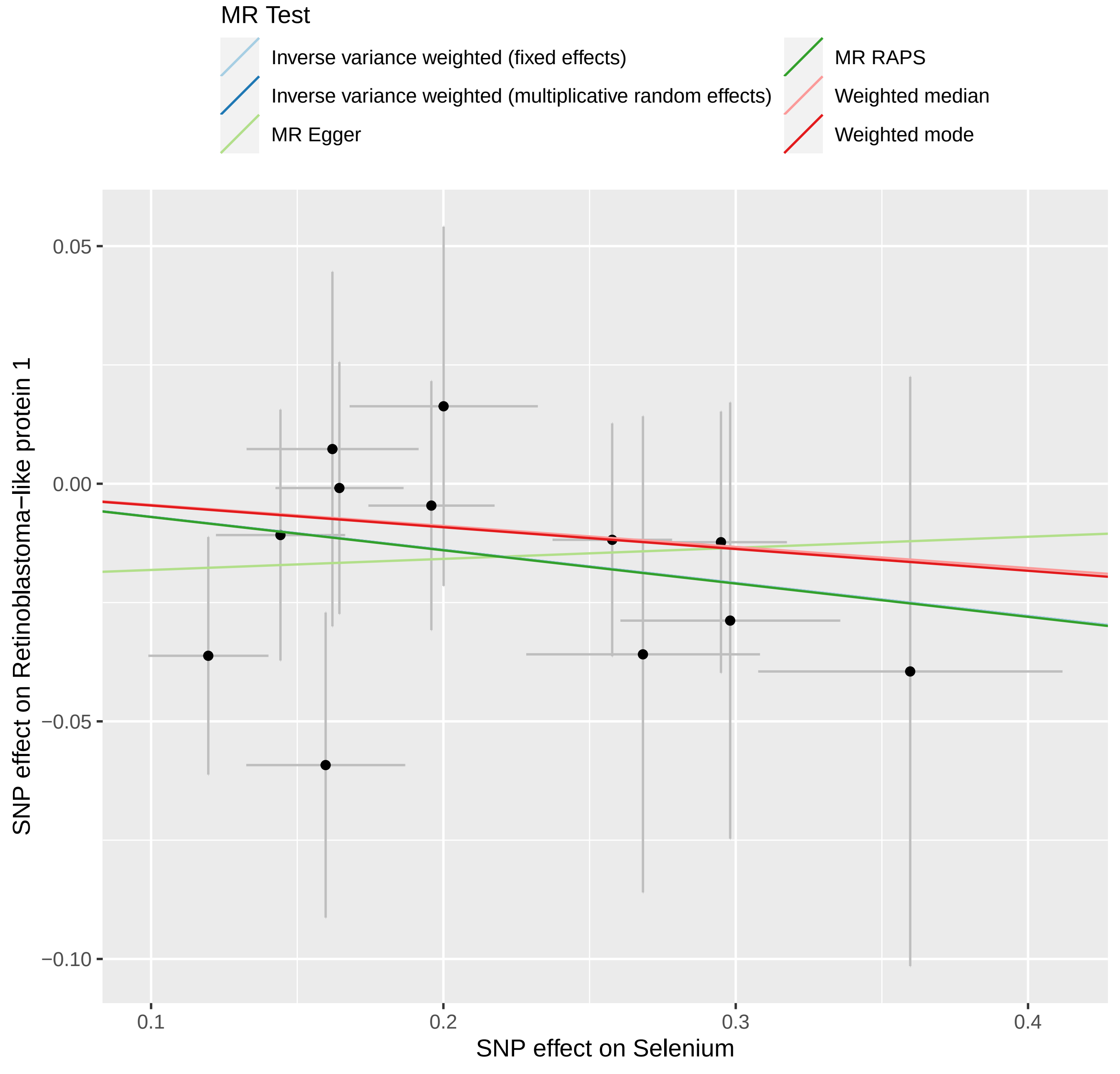

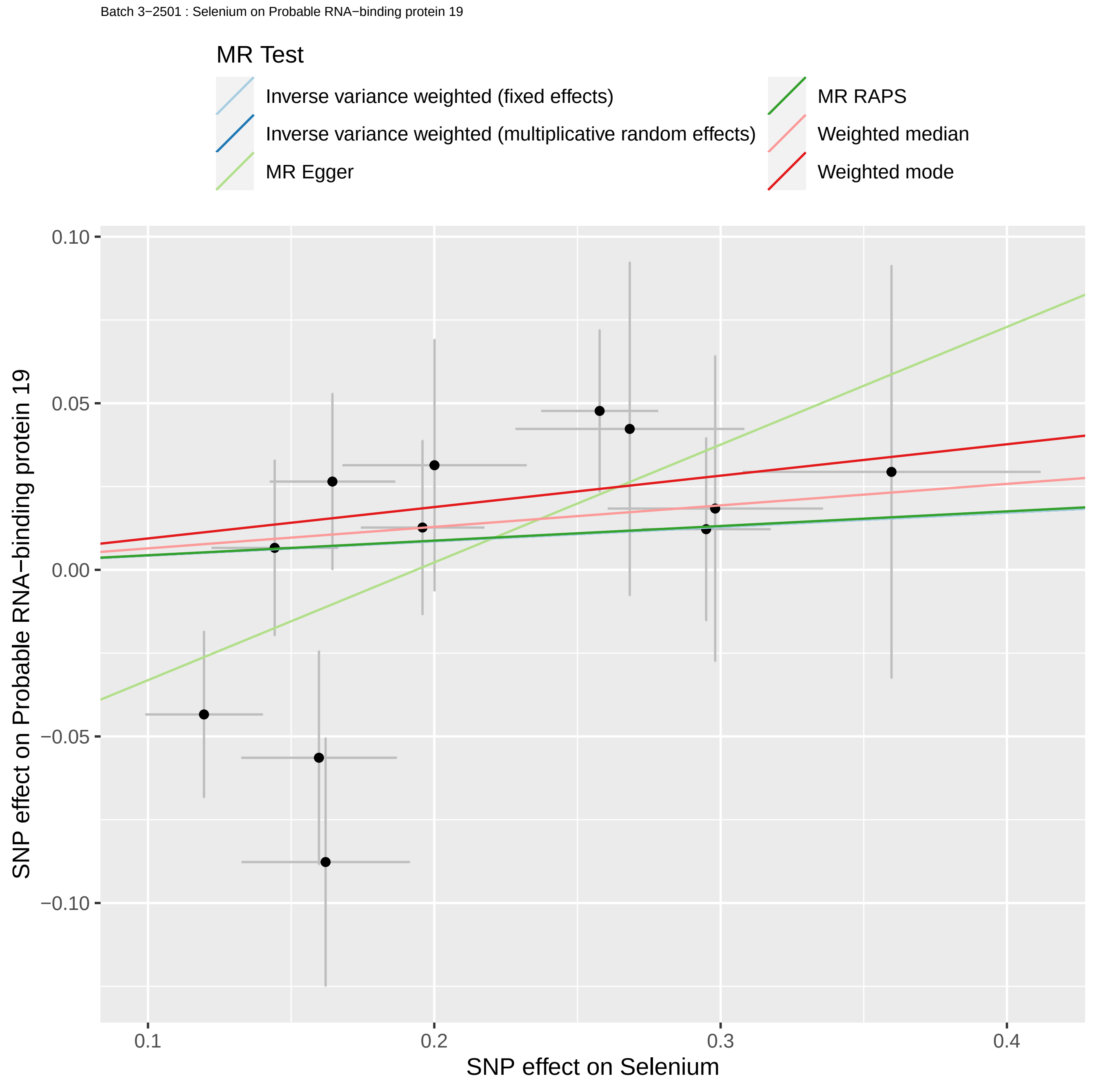

Batch 3−2501 : Selenium on Probable RNA−binding protein 19
MR Test
Inverse variance weighted (fixed effects)
Inverse variance weighted (multiplicative random effects)
MR Egger
MR RAPS
Weighted median
Weighted mode
0.10
0.05
0.00
−0.05
−0.10
SNP effect on Probable RNA−binding protein 19
0.1
0.2
0.3
0.4
SNP effect on Selenium

Batch 3−2502 : Selenium on Probable RNA−binding protein 23

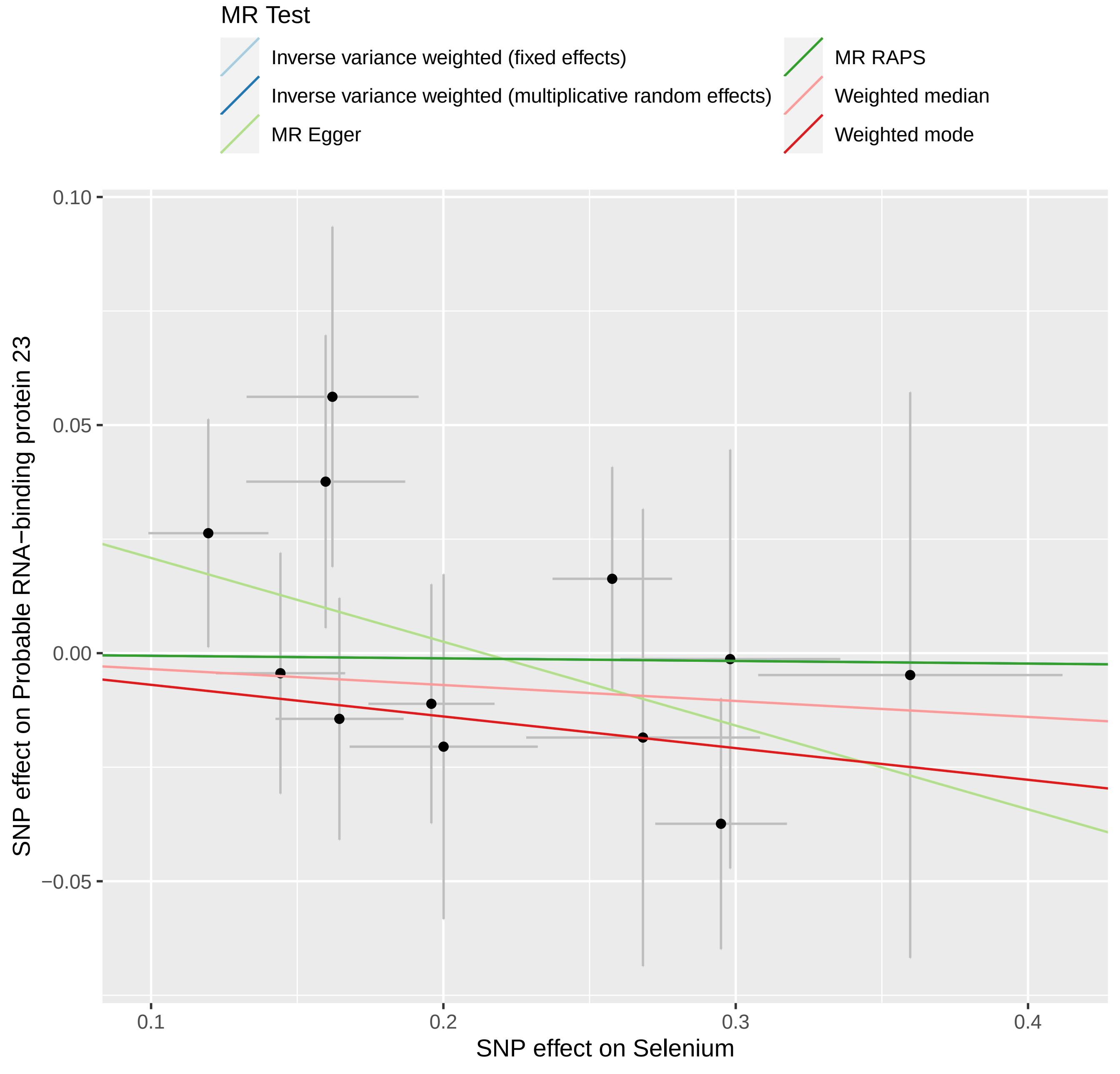

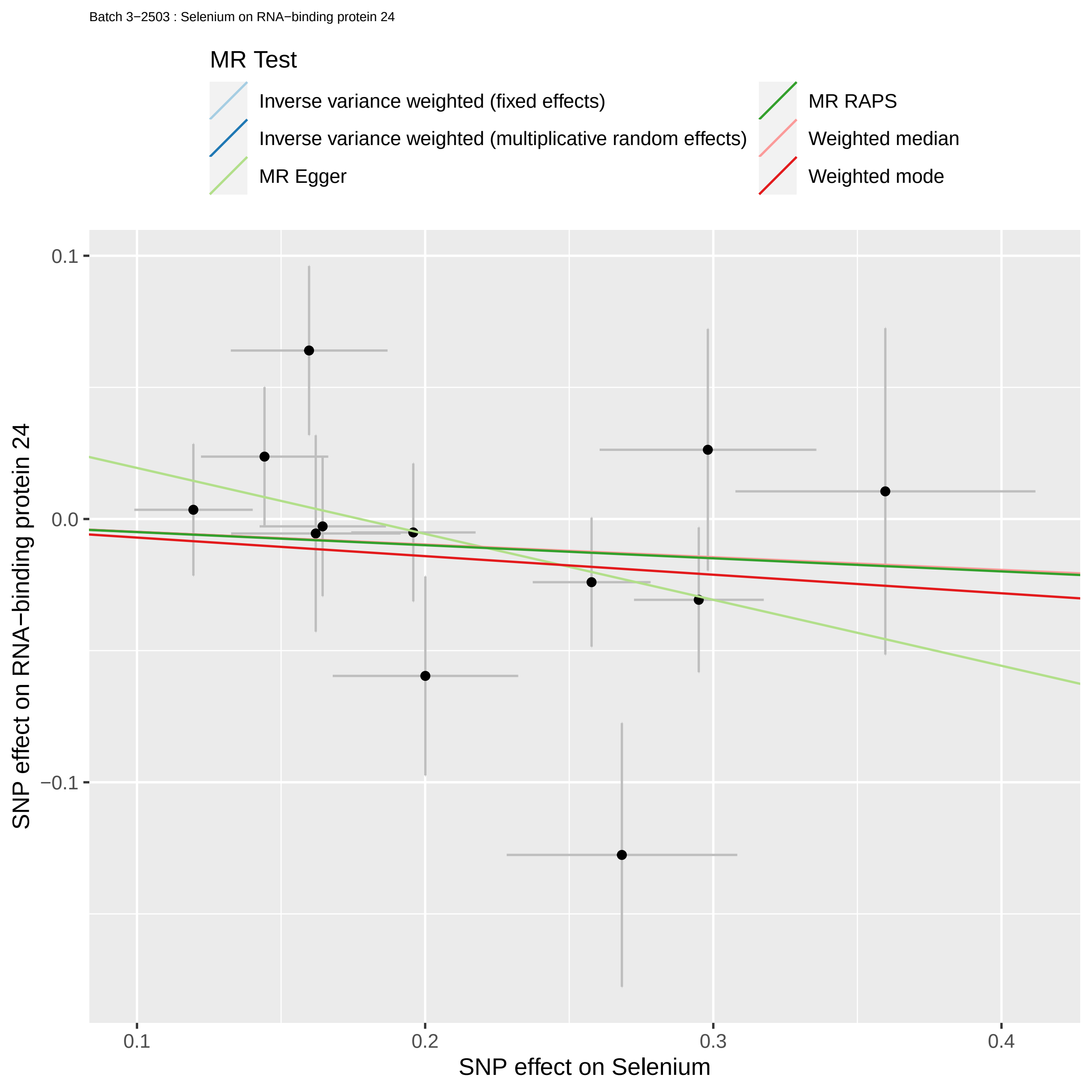

Batch 3−2503 : Selenium on RNA−binding protein 24
MR Test
Inverse variance weighted (fixed effects)
Inverse variance weighted (multiplicative random effects)
MR Egger
MR RAPS
Weighted median
Weighted mode
0.1
0.0
−0.1
0.1
0.2
0.3
0.4
SNP effect on RNA−binding protein 24
SNP effect on Selenium

Batch 3−2504 : Selenium on RNA−binding protein 28

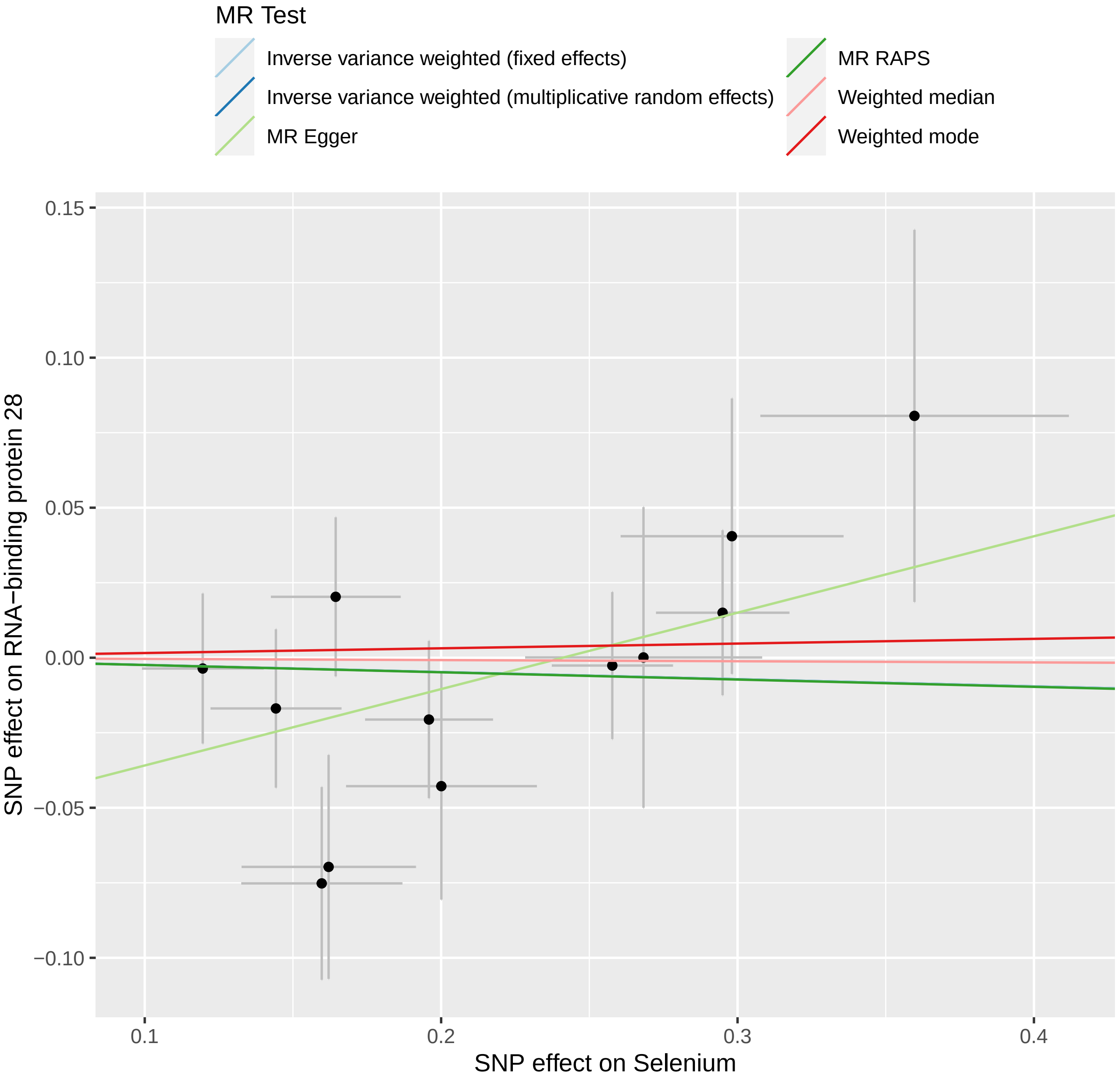

Batch 3−2505 : Selenium on RNA−binding protein 3

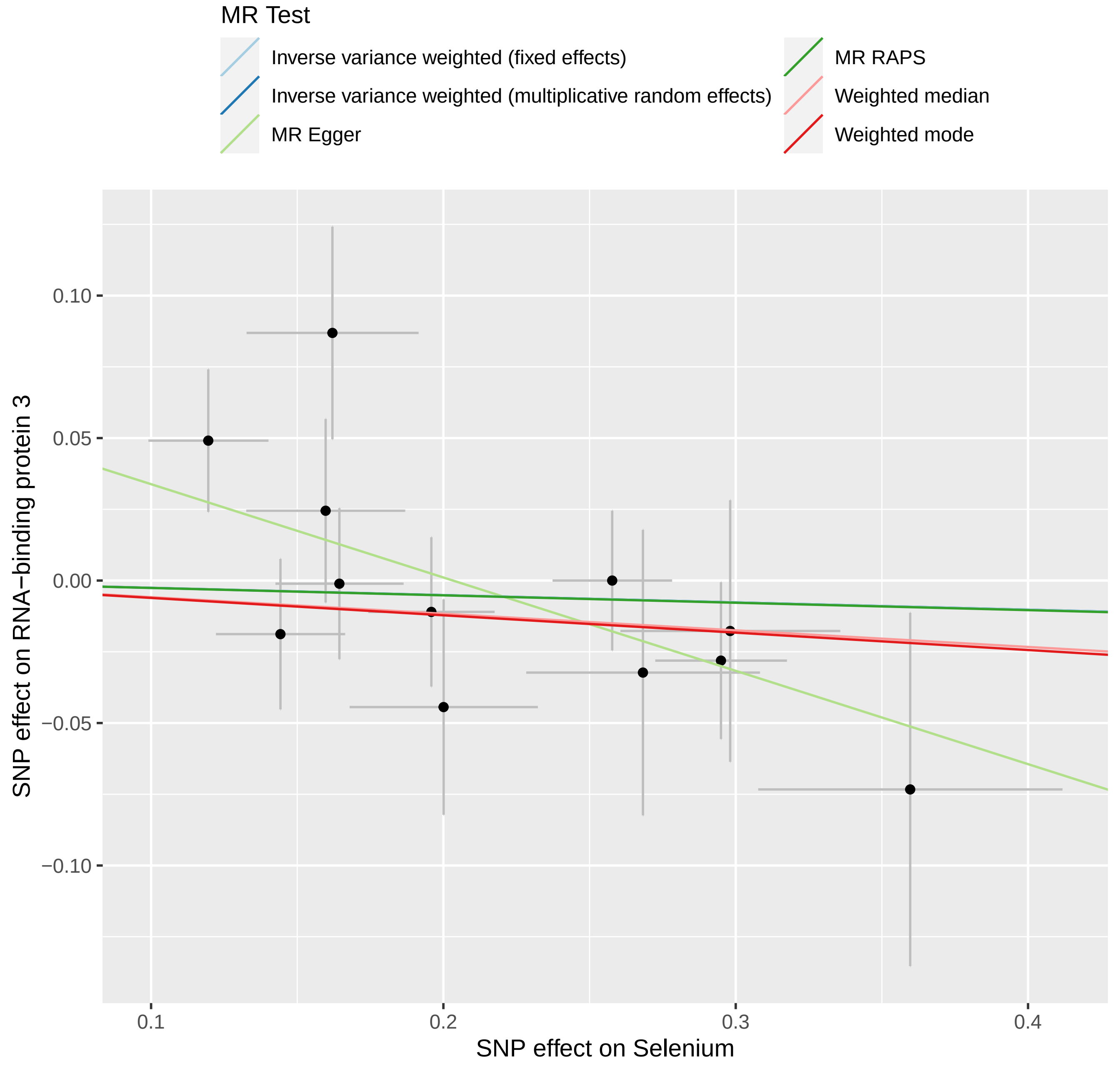

Batch 3−2506 : Selenium on RNA−binding protein 39

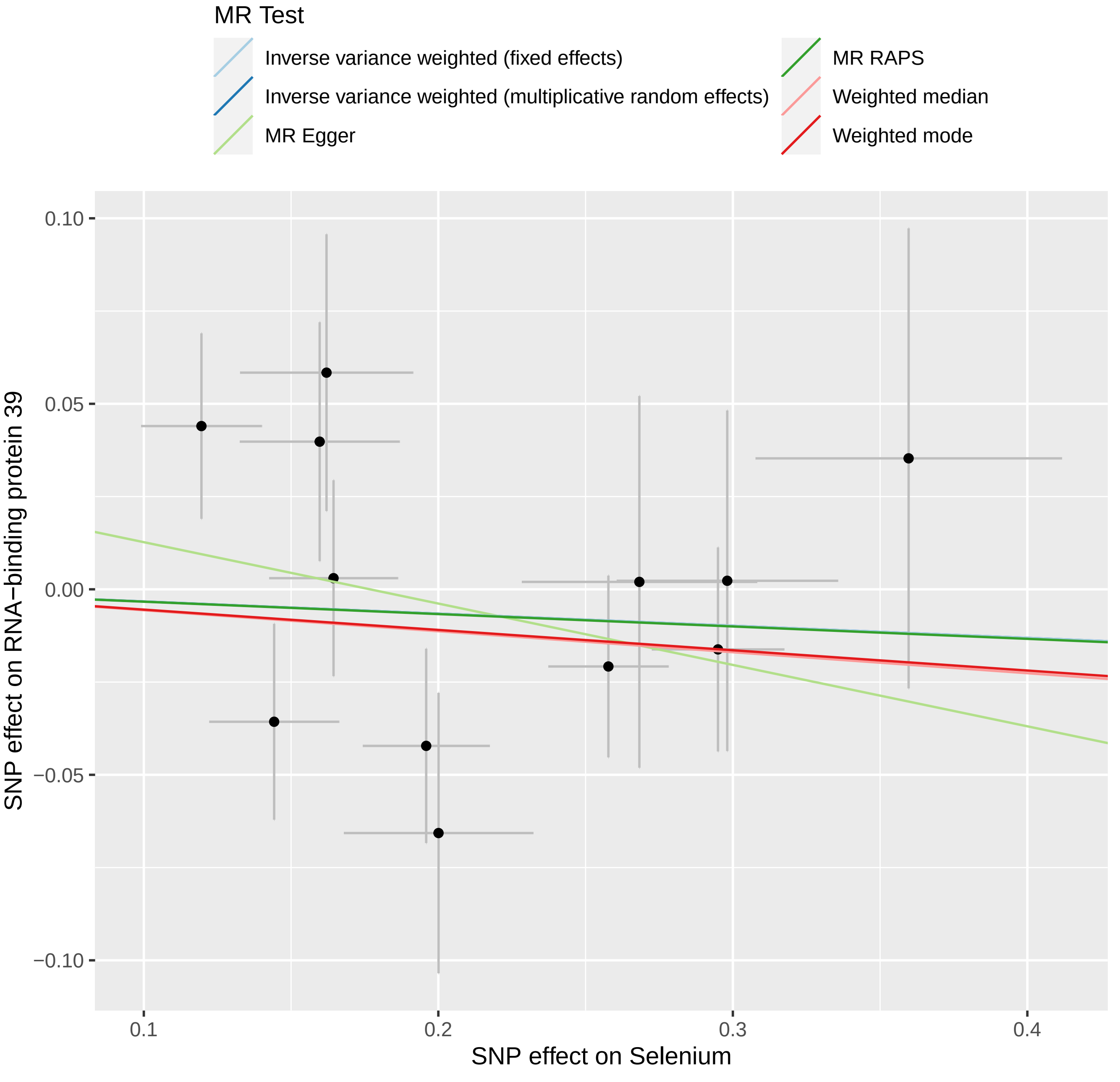

Batch 3−2507 : Selenium on Retinol−binding protein 4

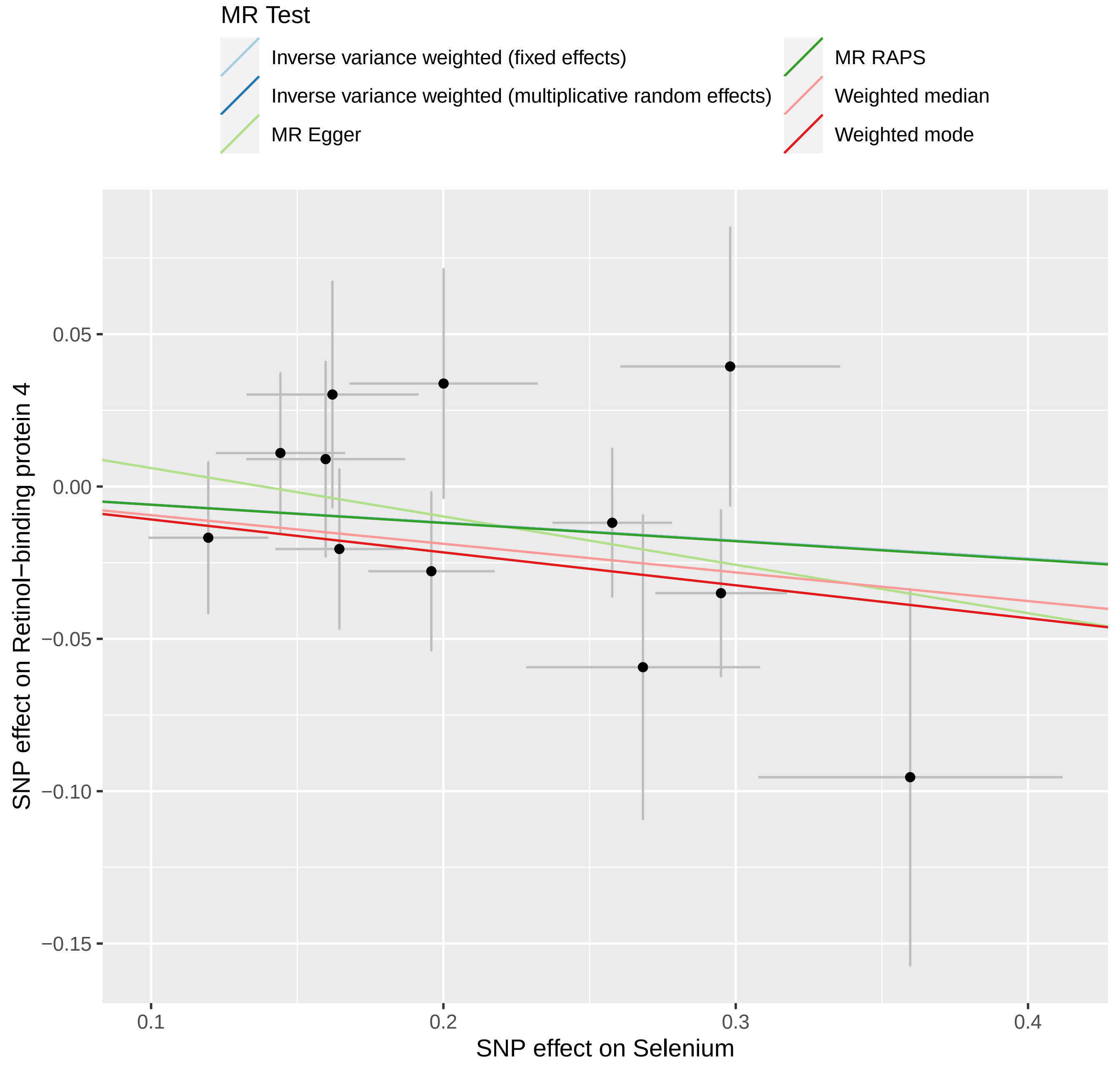

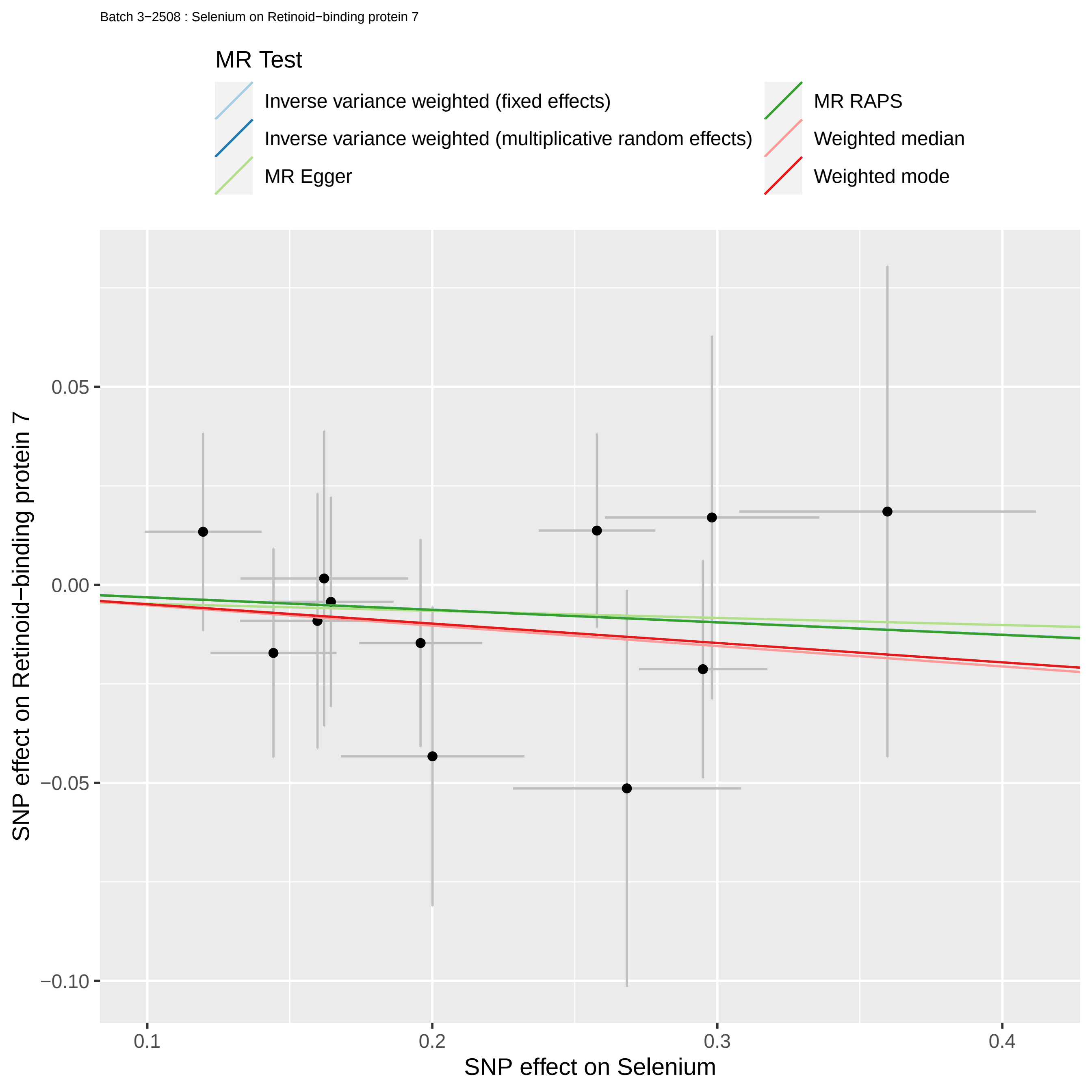
Batch 3−2508 : Selenium on Retinoid−binding protein 7
MR Test
Inverse variance weighted (fixed effects)
Inverse variance weighted (multiplicative random effects)
MR Egger
MR RAPS
Weighted median
Weighted mode
0.05
0.00
−0.05
−0.10
0.1
0.2
0.3
0.4
SNP effect on Retinoid−binding protein 7
SNP effect on Selenium

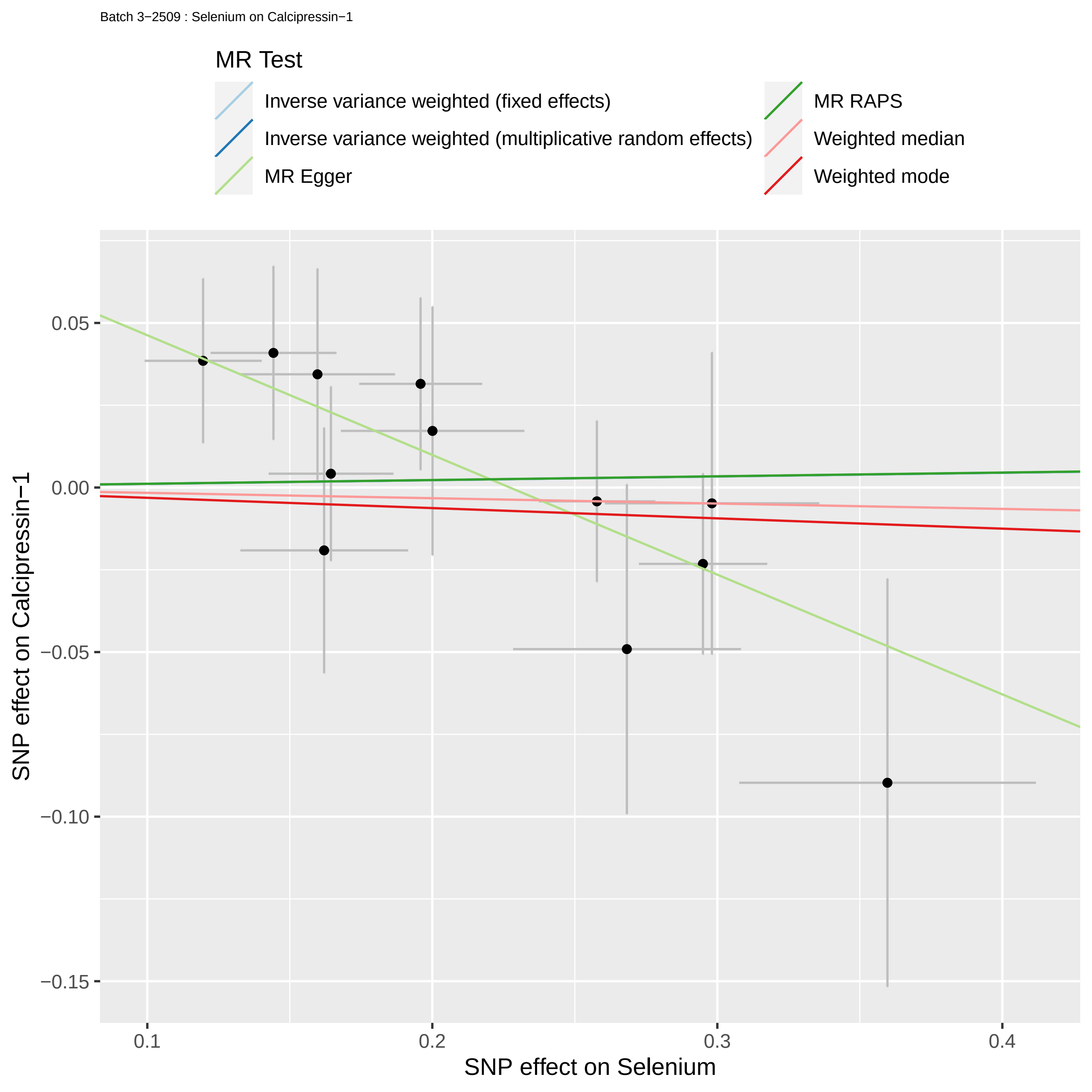

Batch 3−2509 : Selenium on Calcipressin−1
MR Test
Inverse variance weighted (fixed effects)
Inverse variance weighted (multiplicative random effects)
MR Egger
MR RAPS
Weighted median
Weighted mode
0.05
0.00
−0.05
−0.10
−0.15
0.1
0.2
0.3
0.4
SNP effect on Calcipressin−1
SNP effect on Selenium

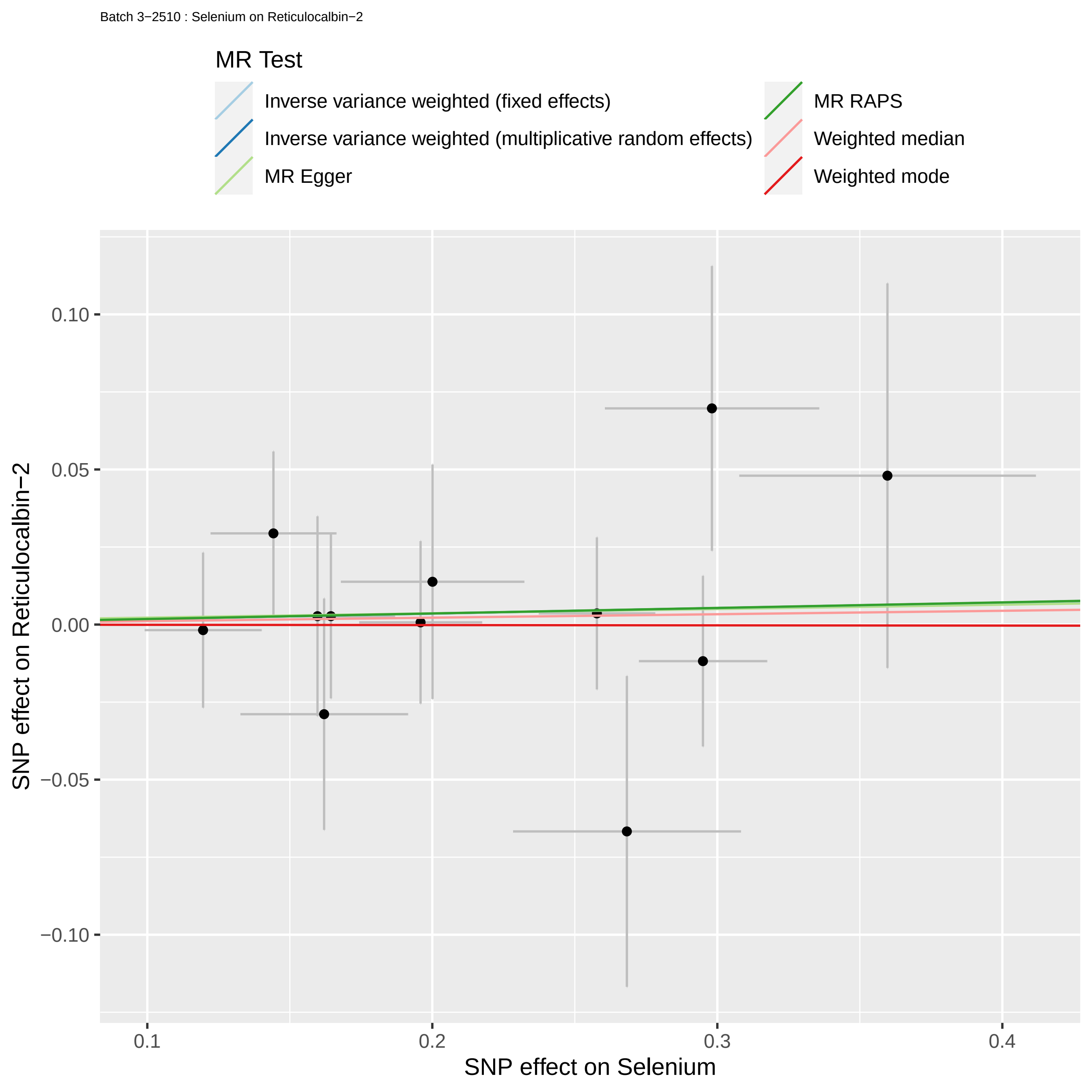
Batch 3−2510 : Selenium on Reticulocalbin−2
MR Test
Inverse variance weighted (fixed effects)
Inverse variance weighted (multiplicative random effects)
MR Egger
MR RAPS
Weighted median
Weighted mode
SNP effect on Reticulocalbin−2
0.10
0.05
0.00
−0.05
−0.10
0.1
0.2
0.3
0.4
SNP effect on Selenium

Batch 3−2511 : Selenium on Recoverin

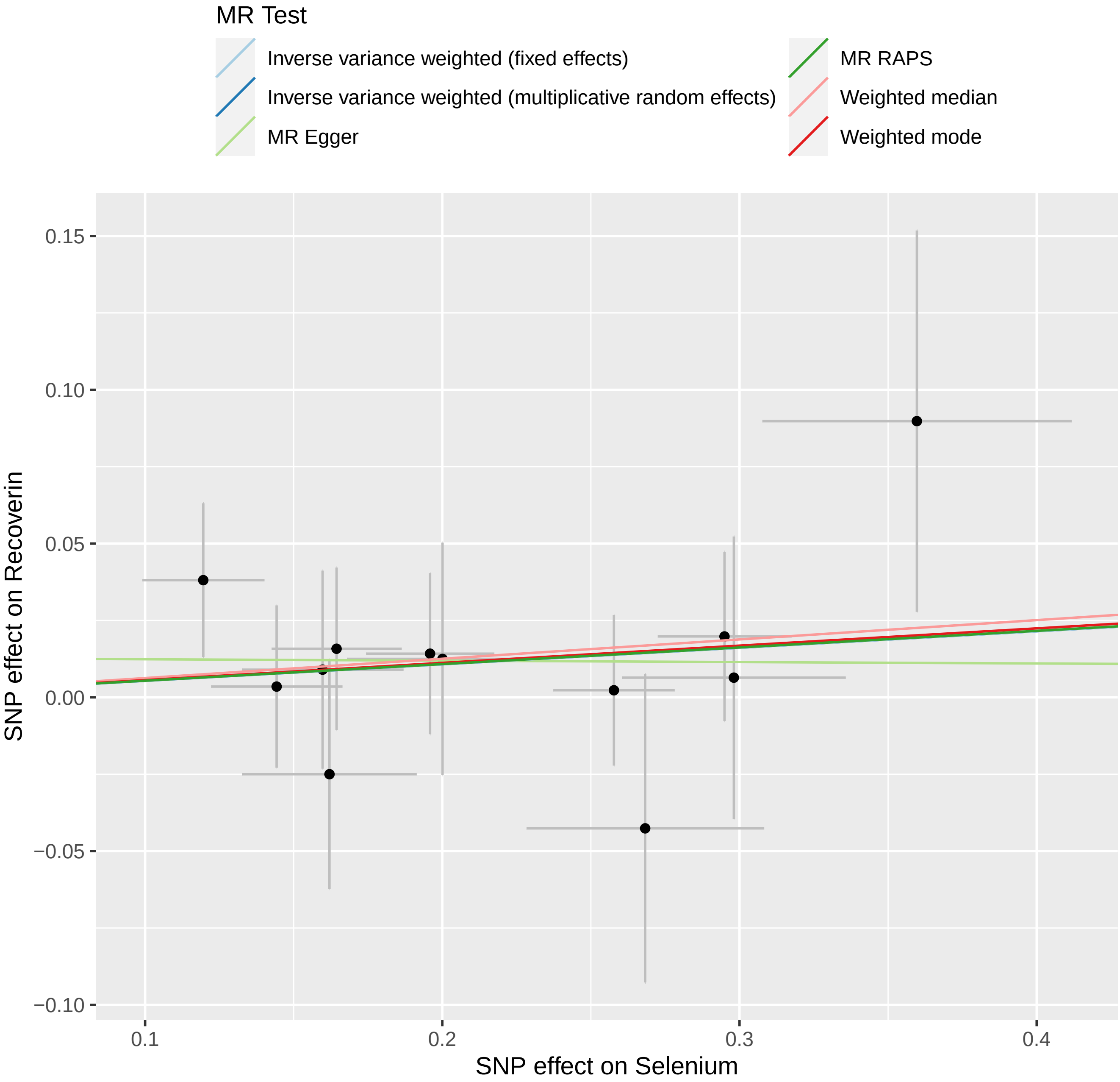

Batch 3−2512 : Selenium on Retinol dehydrogenase 16

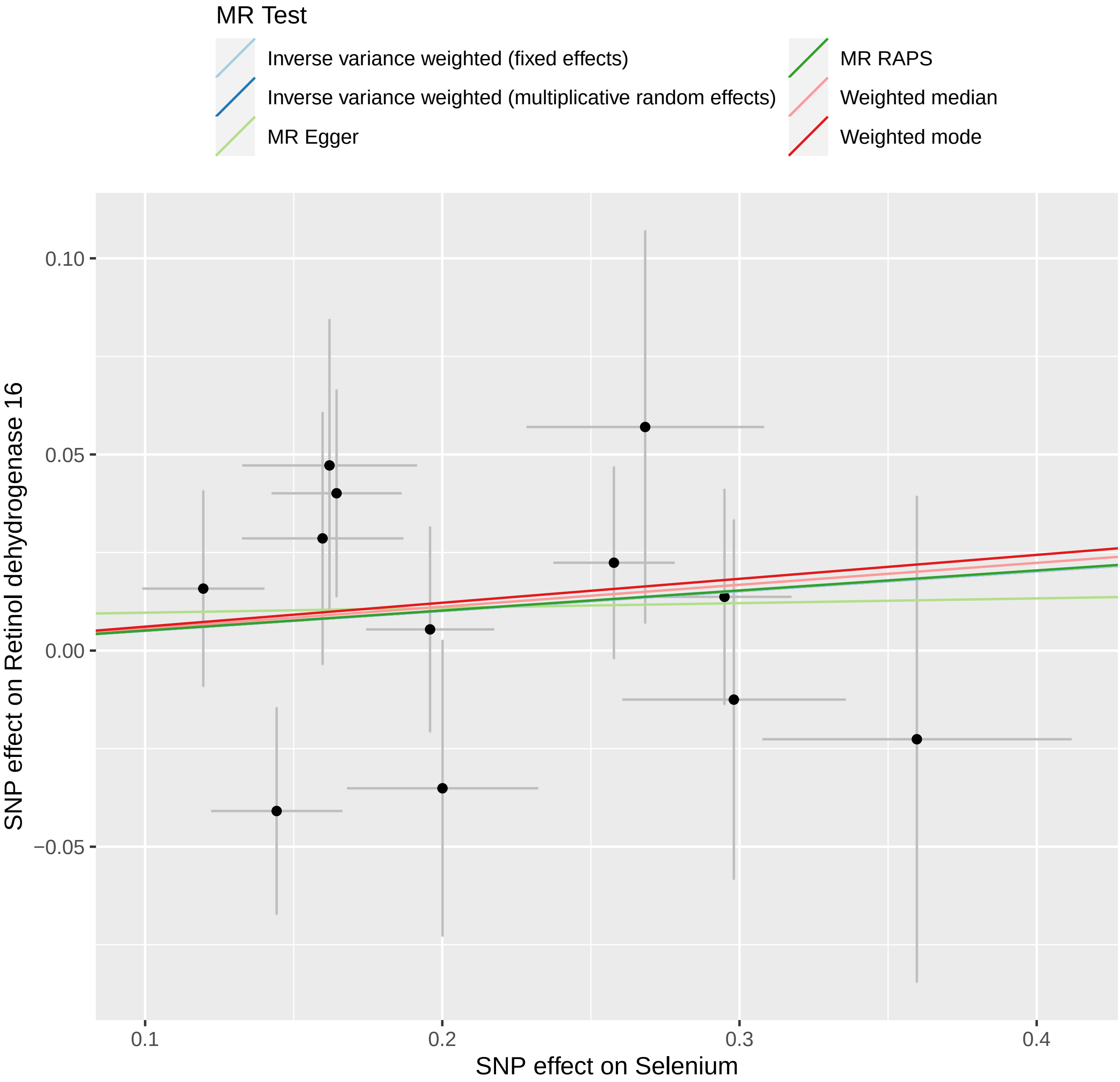

Batch 3−2513 : Selenium on ATP−dependent DNA helicase Q1

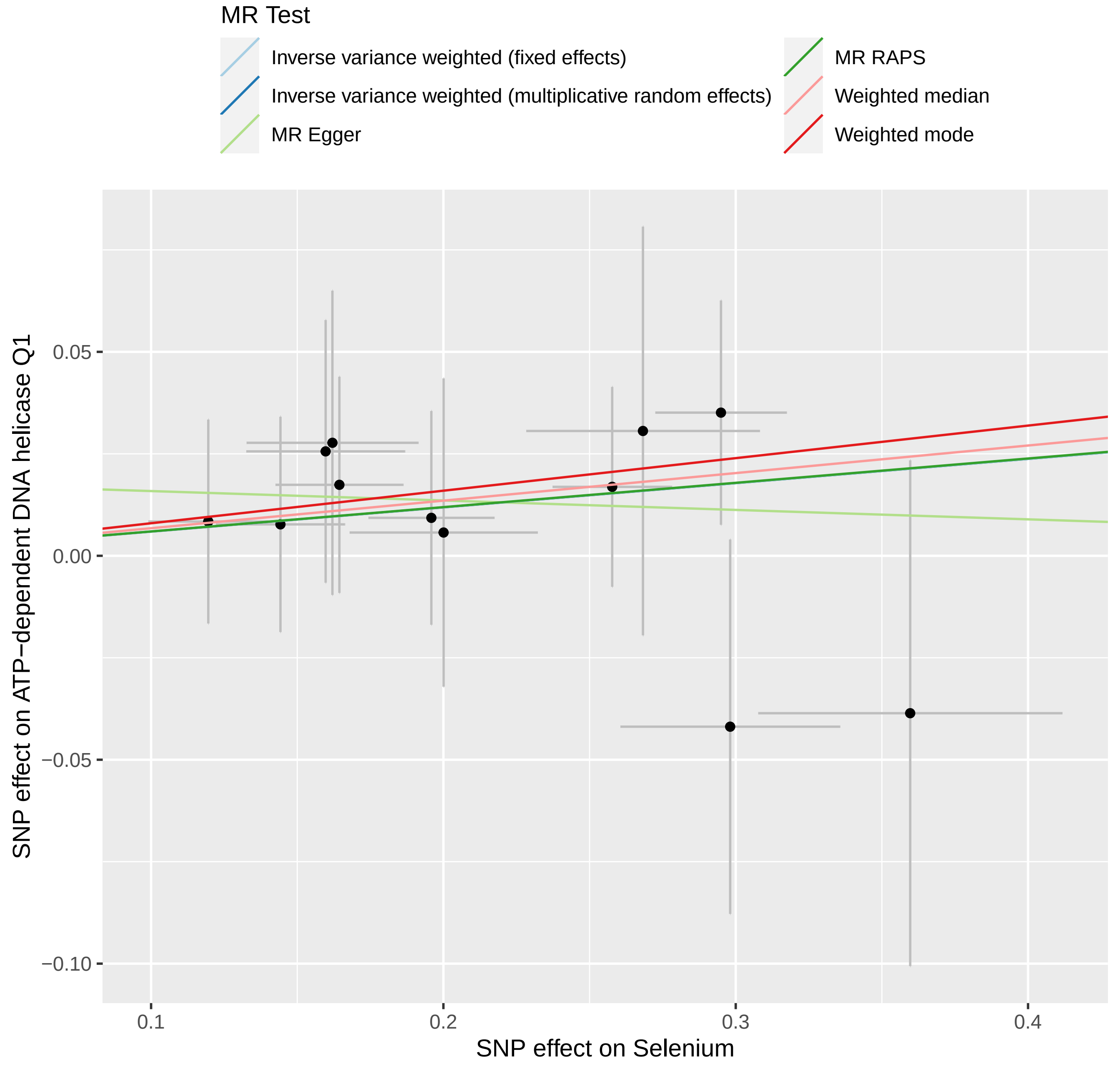

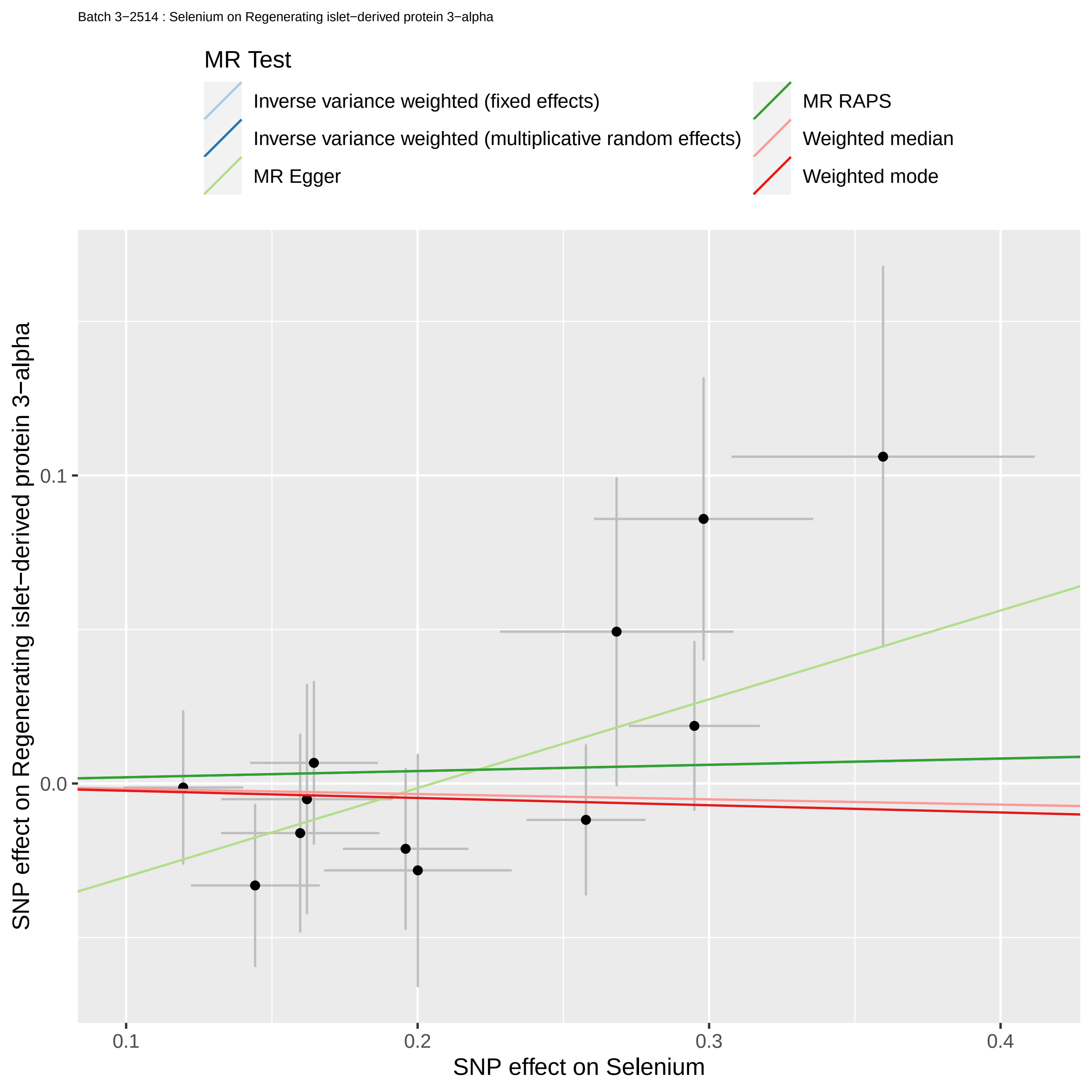
Batch 3−2514 : Selenium on Regenerating islet−derived protein 3−alpha
MR Test
Inverse variance weighted (fixed effects)
Inverse variance weighted (multiplicative random effects)
MR Egger
MR RAPS
Weighted median
Weighted mode
0.1
0.0
0.1
0.2
0.3
0.4
SNP effect on Selenium
SNP effect on Regenerating islet−derived protein 3−alpha

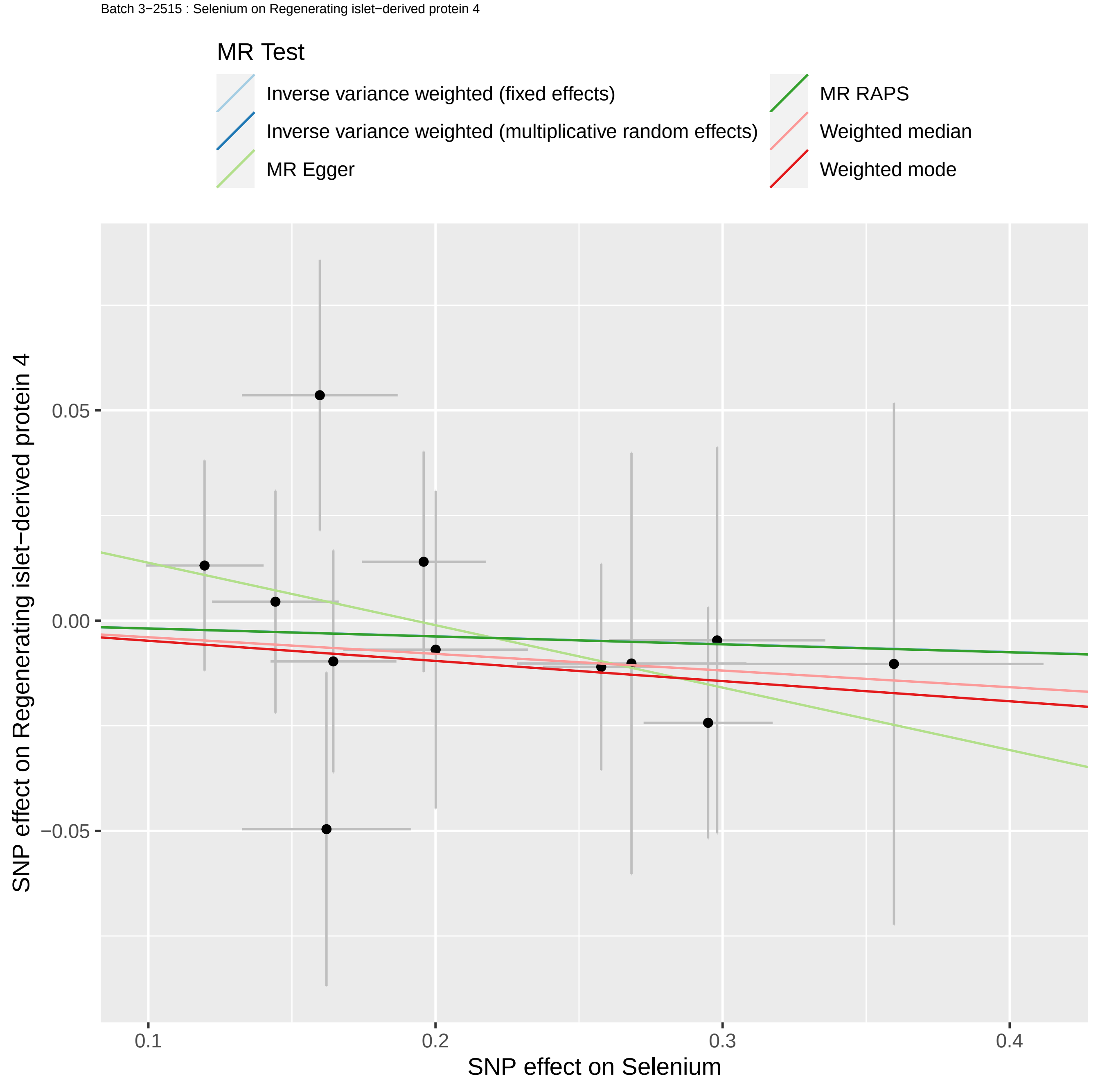

Batch 3−2515 : Selenium on Regenerating islet−derived protein 4
MR Test
Inverse variance weighted (fixed effects)
Inverse variance weighted (multiplicative random effects)
MR Egger
MR RAPS
Weighted median
Weighted mode
0.05
0.00
−0.05
0.1
0.2
0.3
0.4
SNP effect on Regenerating islet−derived protein 4
SNP effect on Selenium

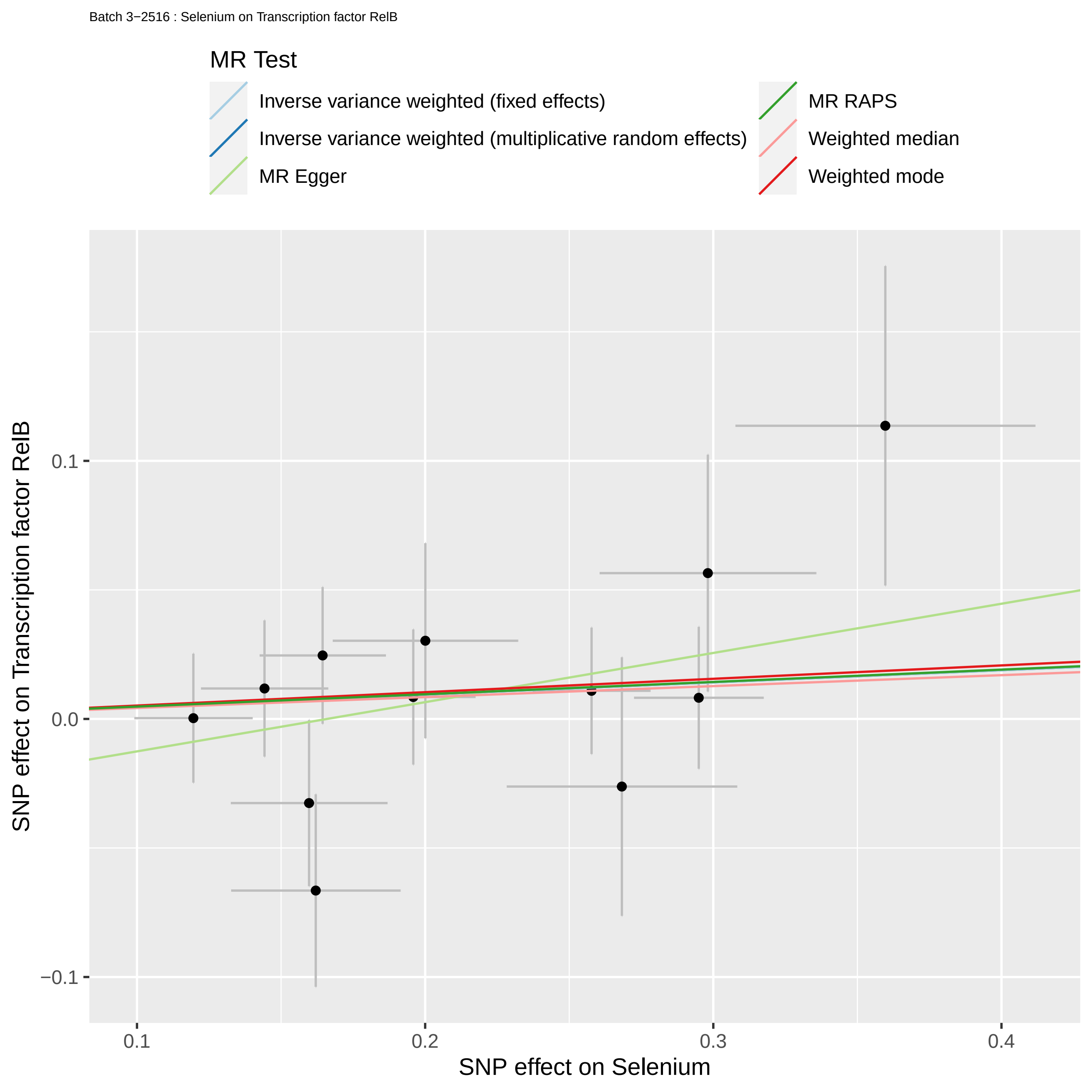

Batch 3−2516 : Selenium on Transcription factor RelB
MR Test
Inverse variance weighted (fixed effects)
Inverse variance weighted (multiplicative random effects)
MR Egger
MR RAPS
Weighted median
Weighted mode
SNP effect on Transcription factor RelB
0.1
0.0
−0.1
0.1
0.2
0.3
0.4
SNP effect on Selenium

Batch 3−2517 : Selenium on RELT−like protein 1

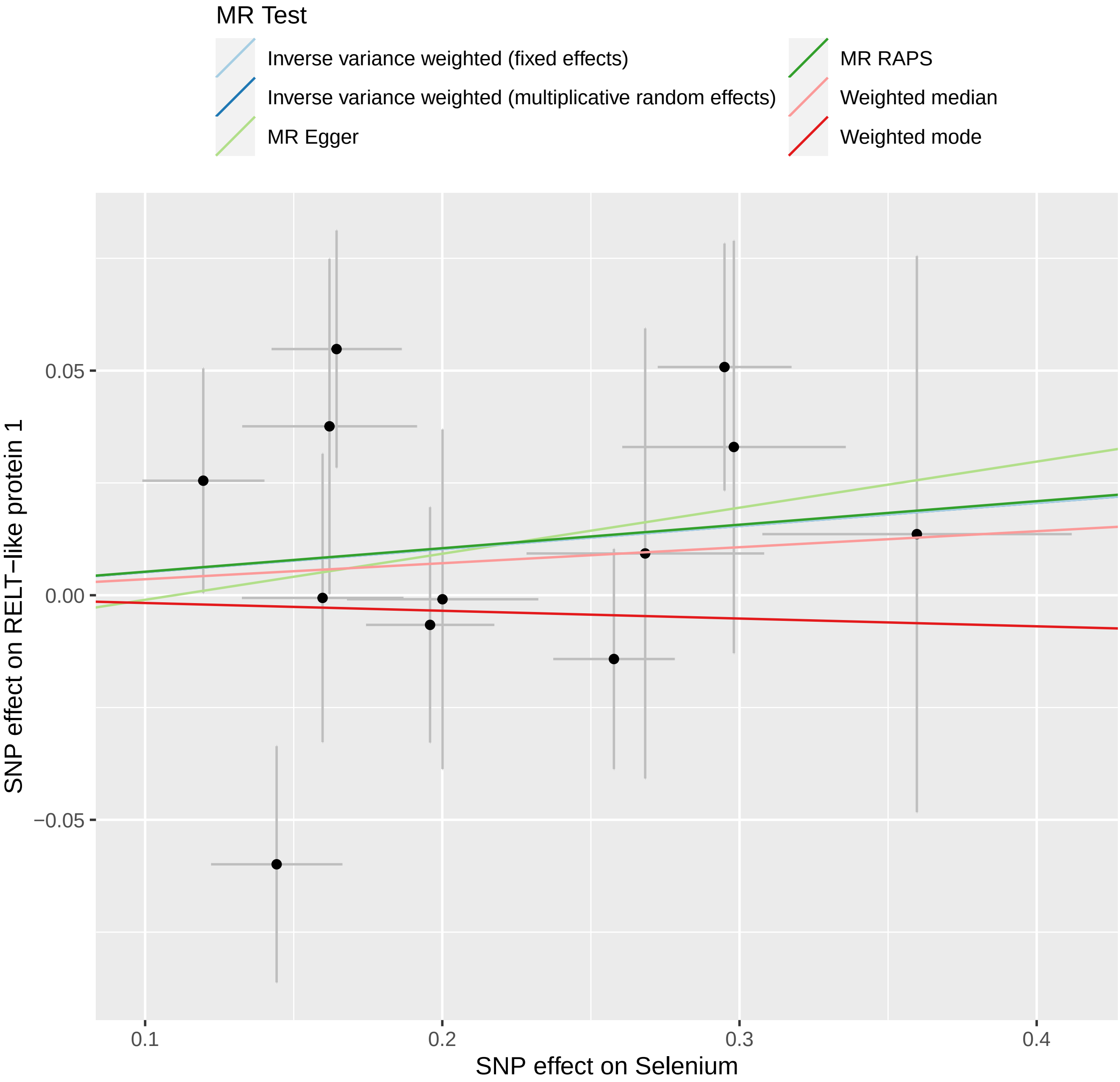

Batch 3−2518 : Selenium on RELT−like protein 2

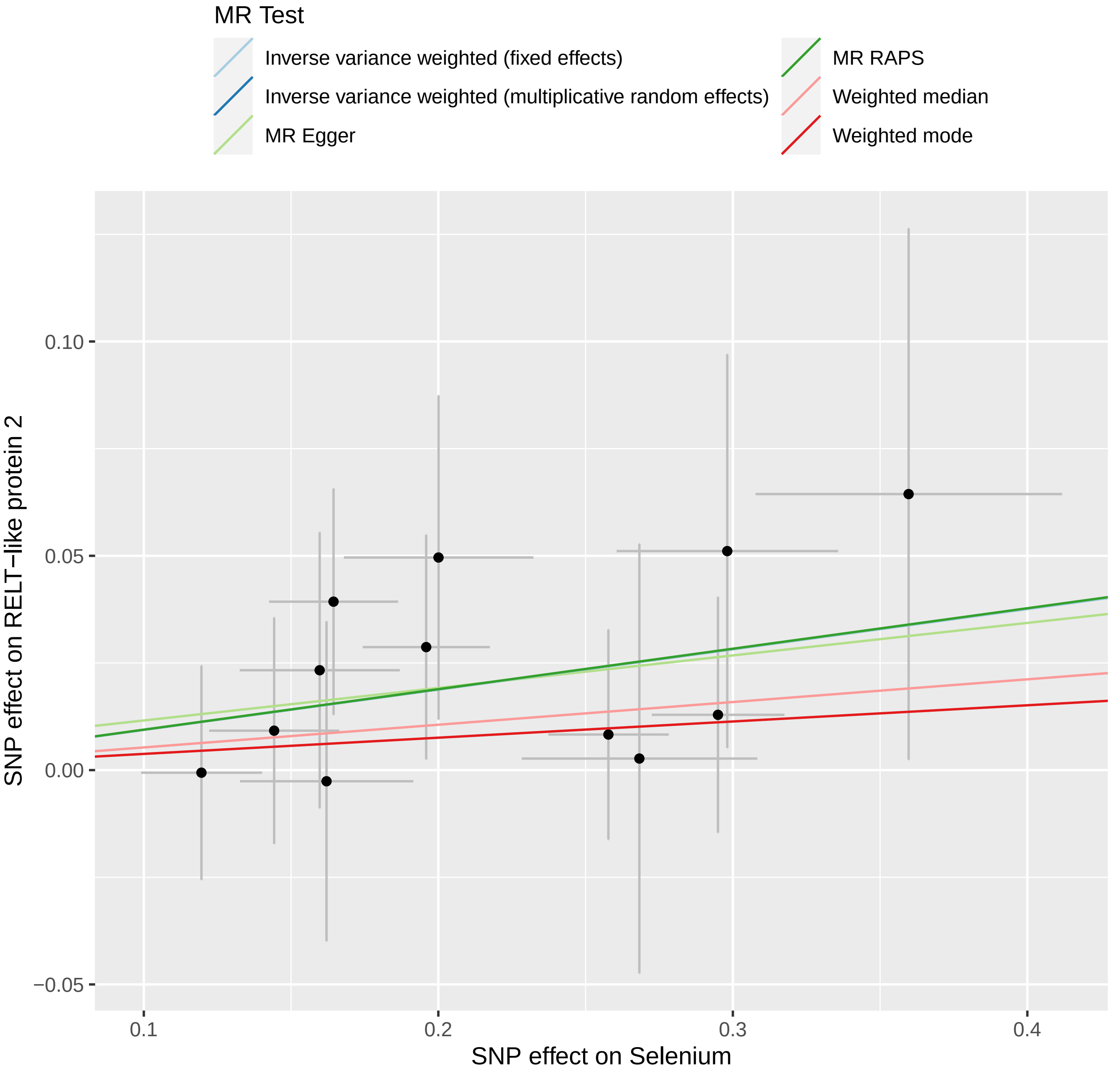

Batch 3−2519 : Selenium on Tumor necrosis factor receptor superfamily member 19L

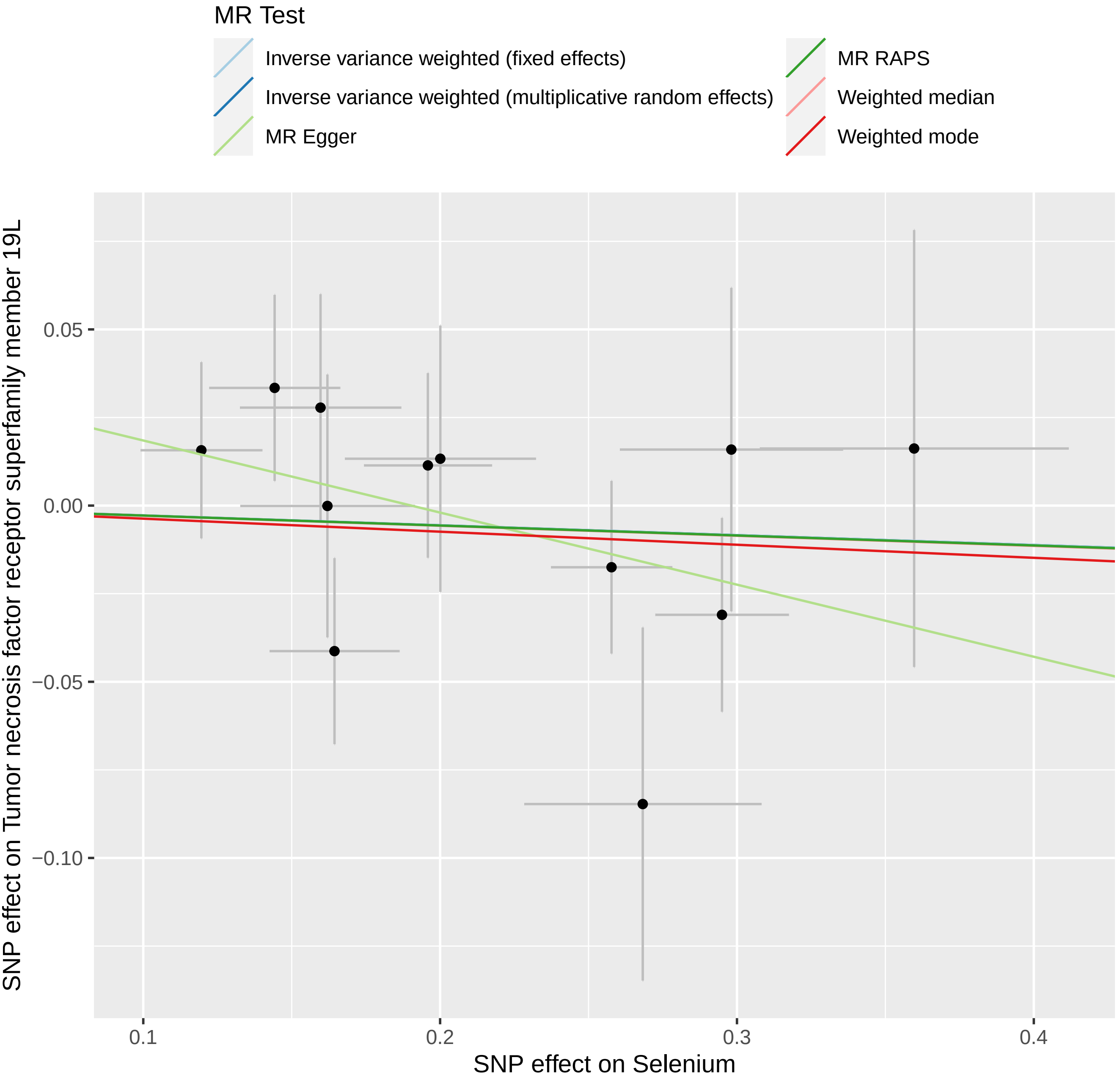

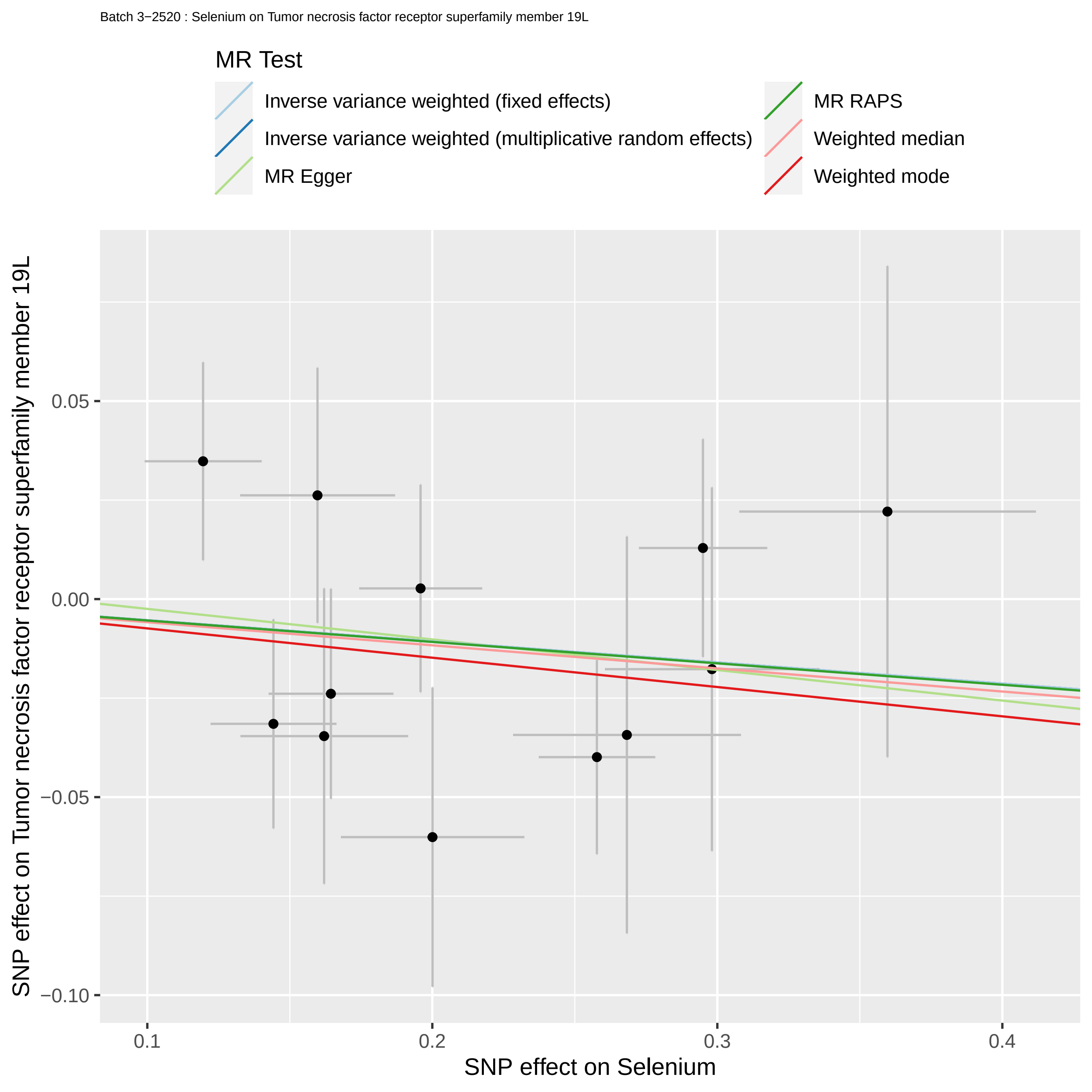
Batch 3−2520 : Selenium on Tumor necrosis factor receptor superfamily member 19L
MR Test
Inverse variance weighted (fixed effects)
Inverse variance weighted (multiplicative random effects)
MR Egger
MR RAPS
Weighted median
Weighted mode
SNP effect on Tumor necrosis factor receptor superfamily member 19L
0.05
0.00
−0.05
−0.10
0.1
0.2
0.3
0.4
SNP effect on Selenium

Batch 3−2521 : Selenium on Renin

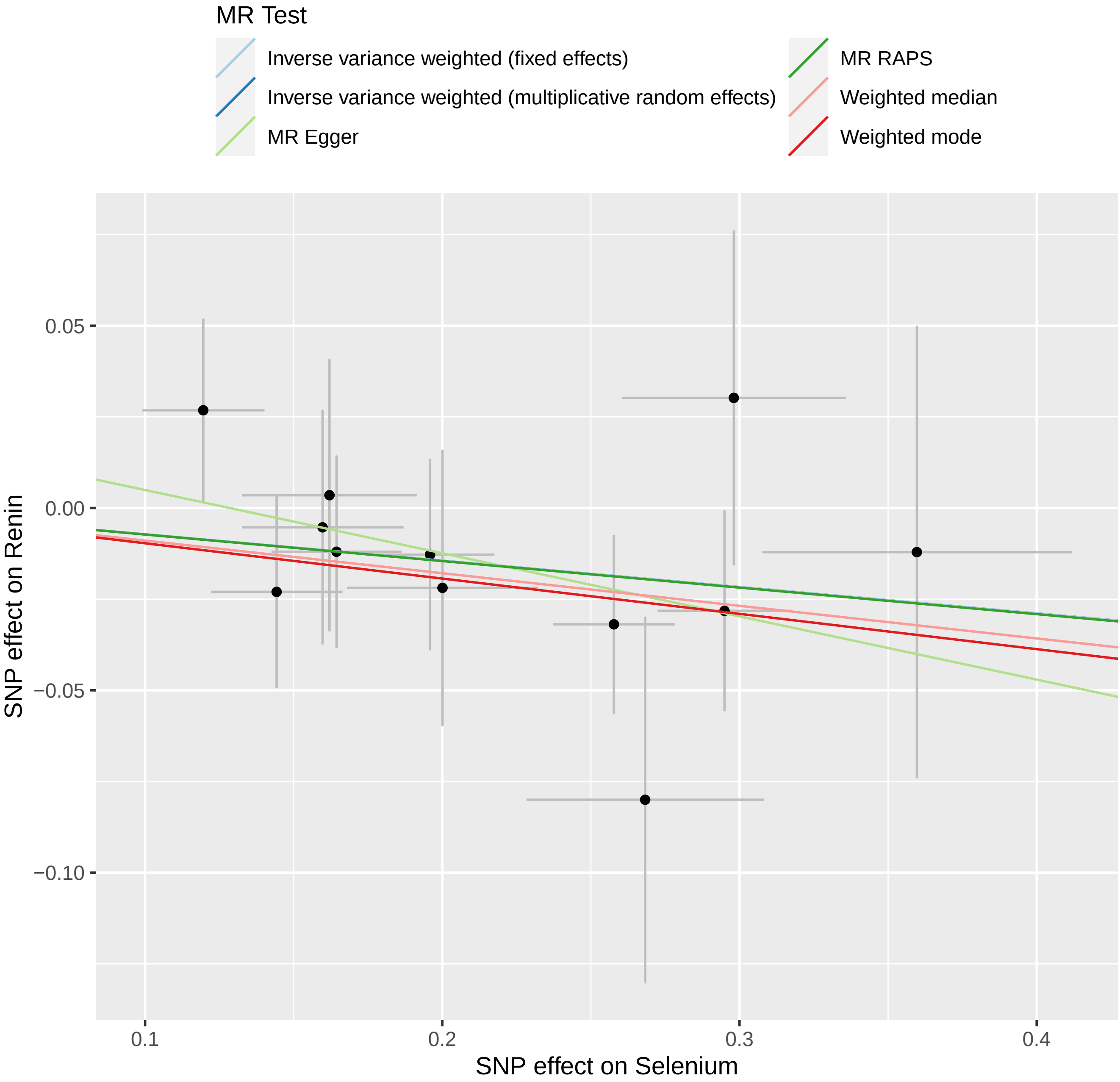

Batch 3−2522 : Selenium on Replication initiator 1

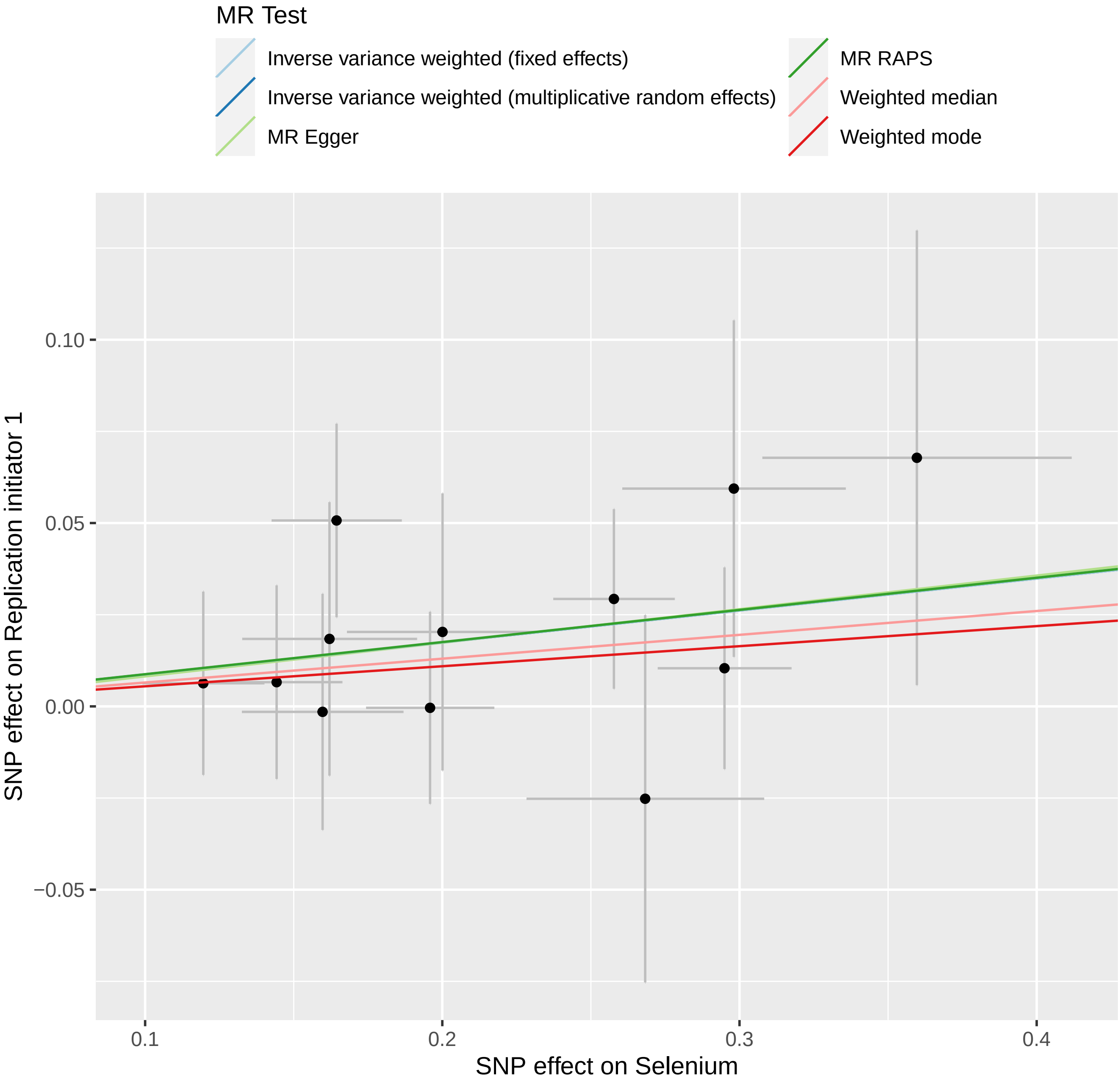

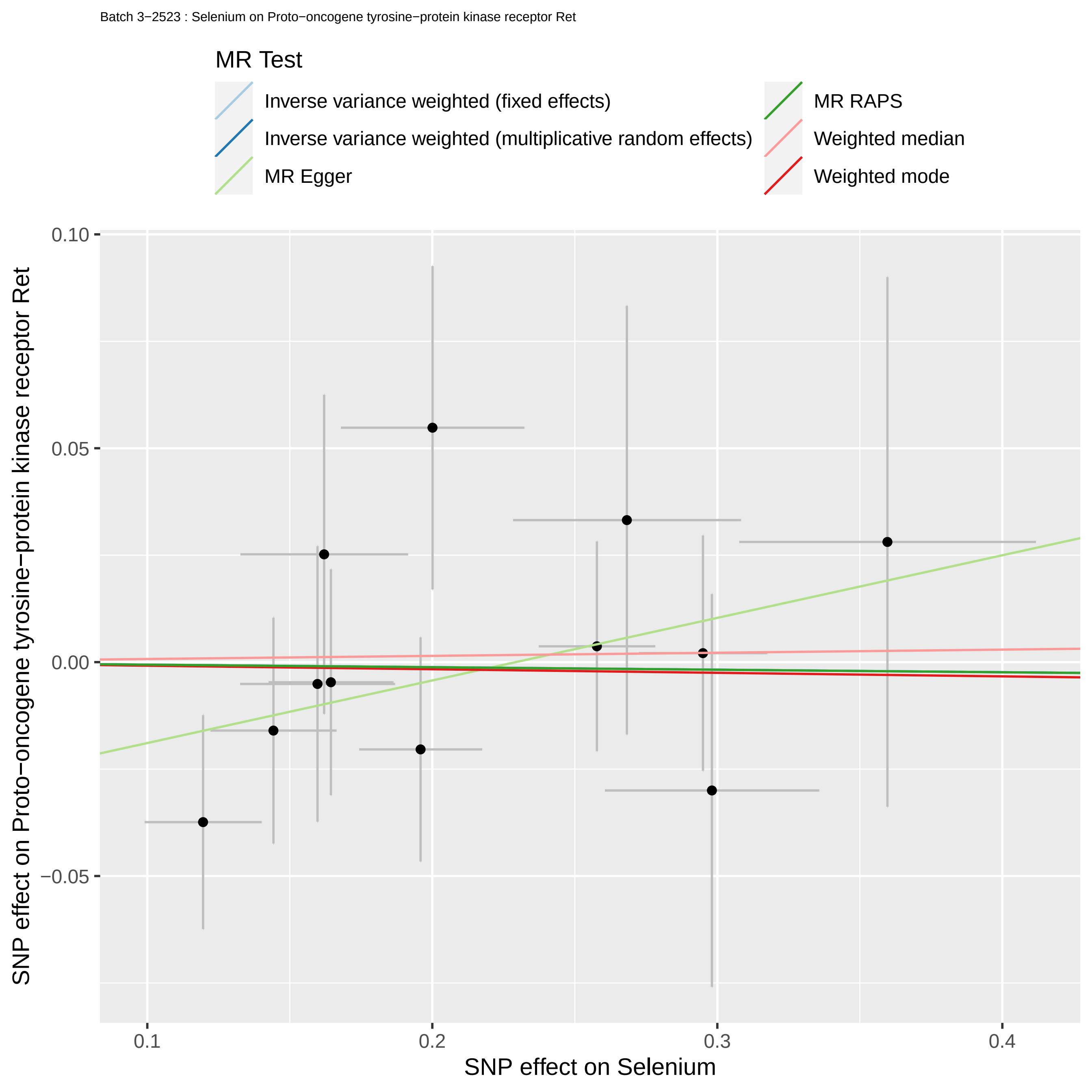

Batch 3−2523 : Selenium on Proto−oncogene tyrosine−protein kinase receptor Ret
MR Test
Inverse variance weighted (fixed effects)
Inverse variance weighted (multiplicative random effects)
MR Egger
MR RAPS
Weighted median
Weighted mode
0.10
0.05
0.00
−0.05
0.1
0.2
0.3
0.4
SNP effect on Proto−oncogene tyrosine−protein kinase receptor Ret
SNP effect on Selenium

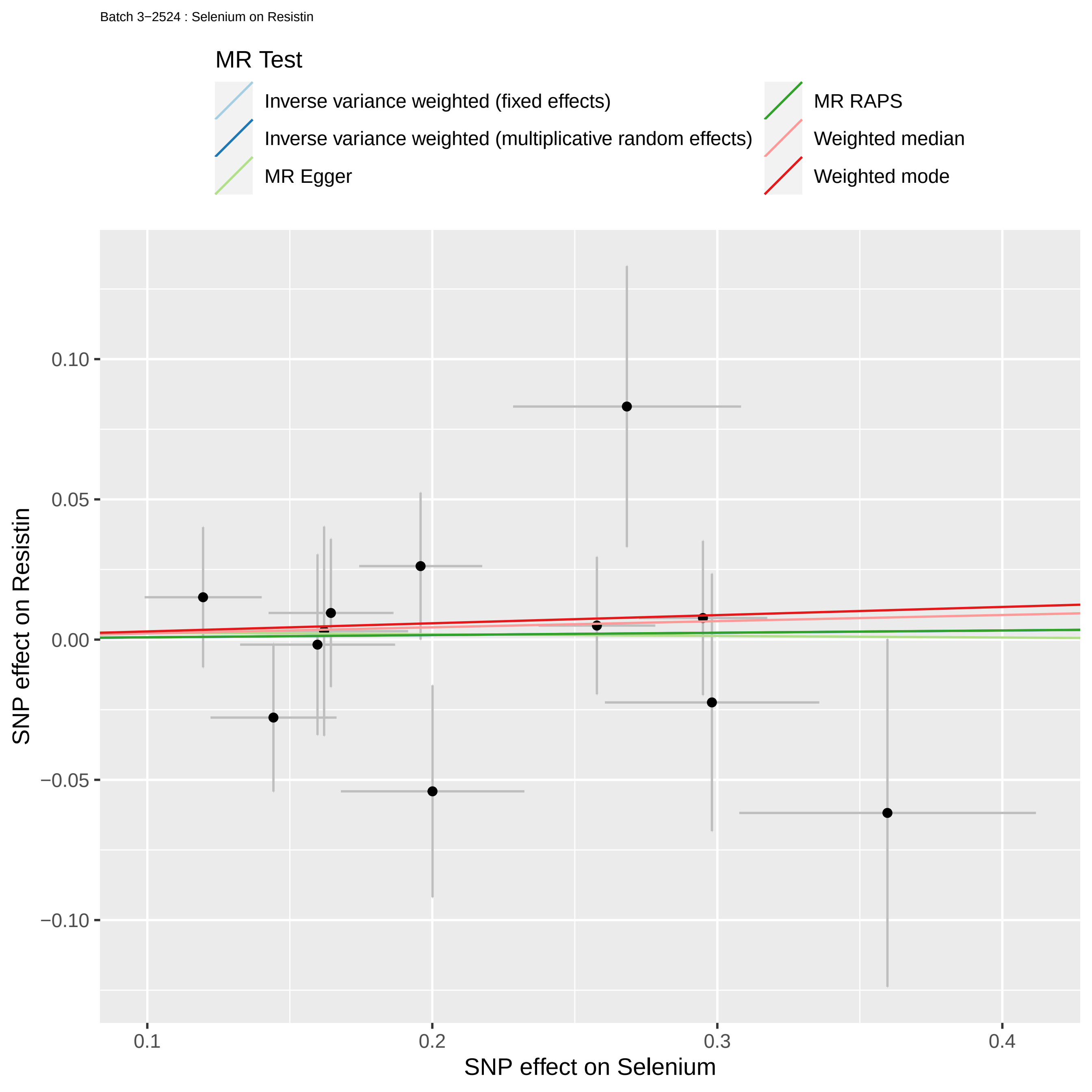

Batch 3−2524 : Selenium on Resistin
MR Test
Inverse variance weighted (fixed effects)
Inverse variance weighted (multiplicative random effects)
MR Egger
MR RAPS
Weighted median
Weighted mode
SNP effect on Resistin
0.10
0.05
0.00
−0.05
−0.10
0.1
0.2
0.3
0.4
SNP effect on Selenium

Batch 3−2525 : Selenium on All−trans−retinol 13,14−reductase

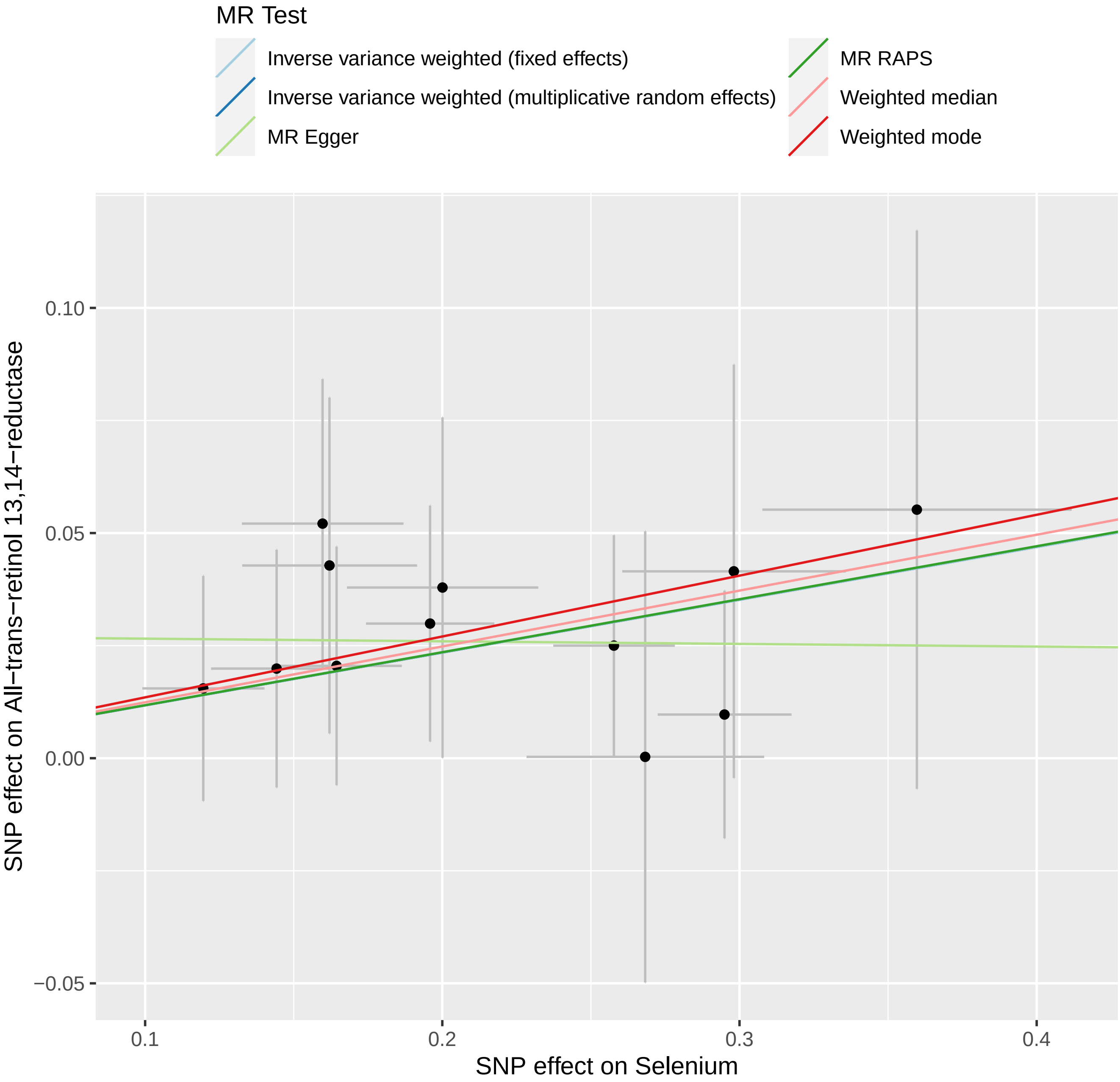

Batch 3−2526 : Selenium on Oligoribonuclease, mitochondrial

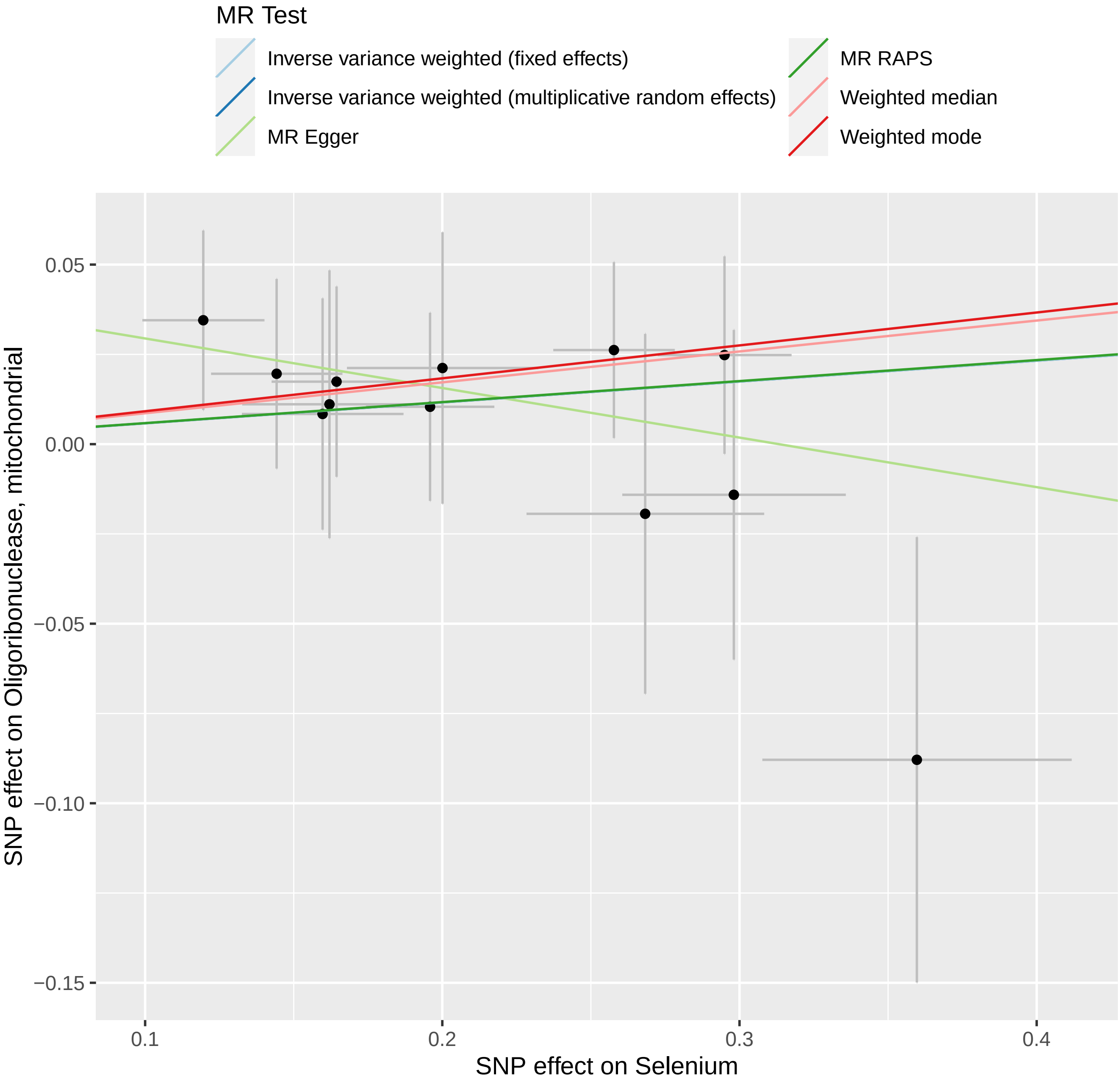

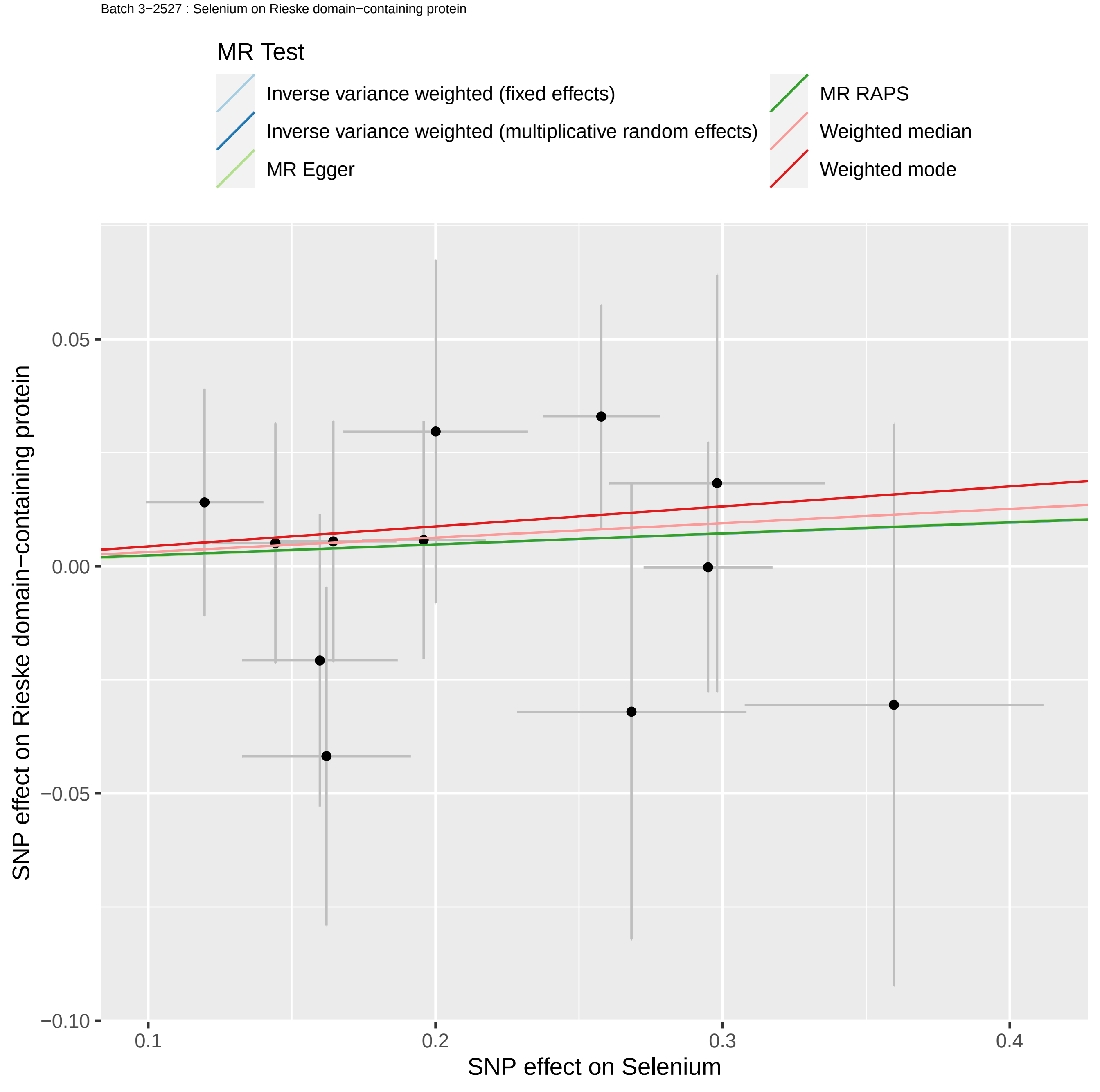
Batch 3−2527 : Selenium on Rieske domain−containing protein
MR Test
Inverse variance weighted (fixed effects)
Inverse variance weighted (multiplicative random effects)
MR Egger
MR RAPS
Weighted median
Weighted mode
SNP effect on Rieske domain−containing protein
0.05
0.00
−0.05
−0.10
0.1
0.2
0.3
0.4
SNP effect on Selenium

Batch 3−2528 : Selenium on E3 ubiquitin−protein ligase rififylin

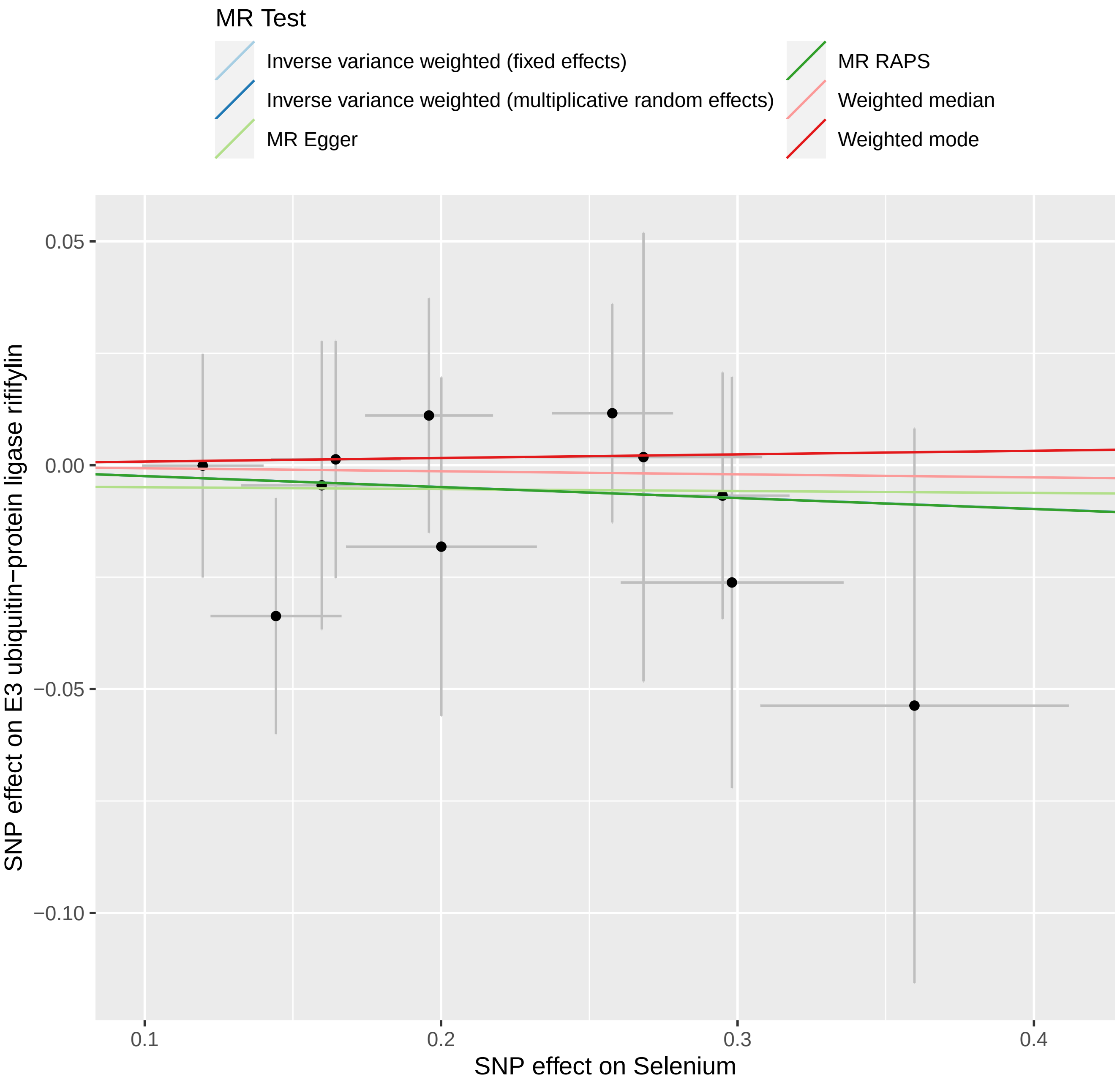

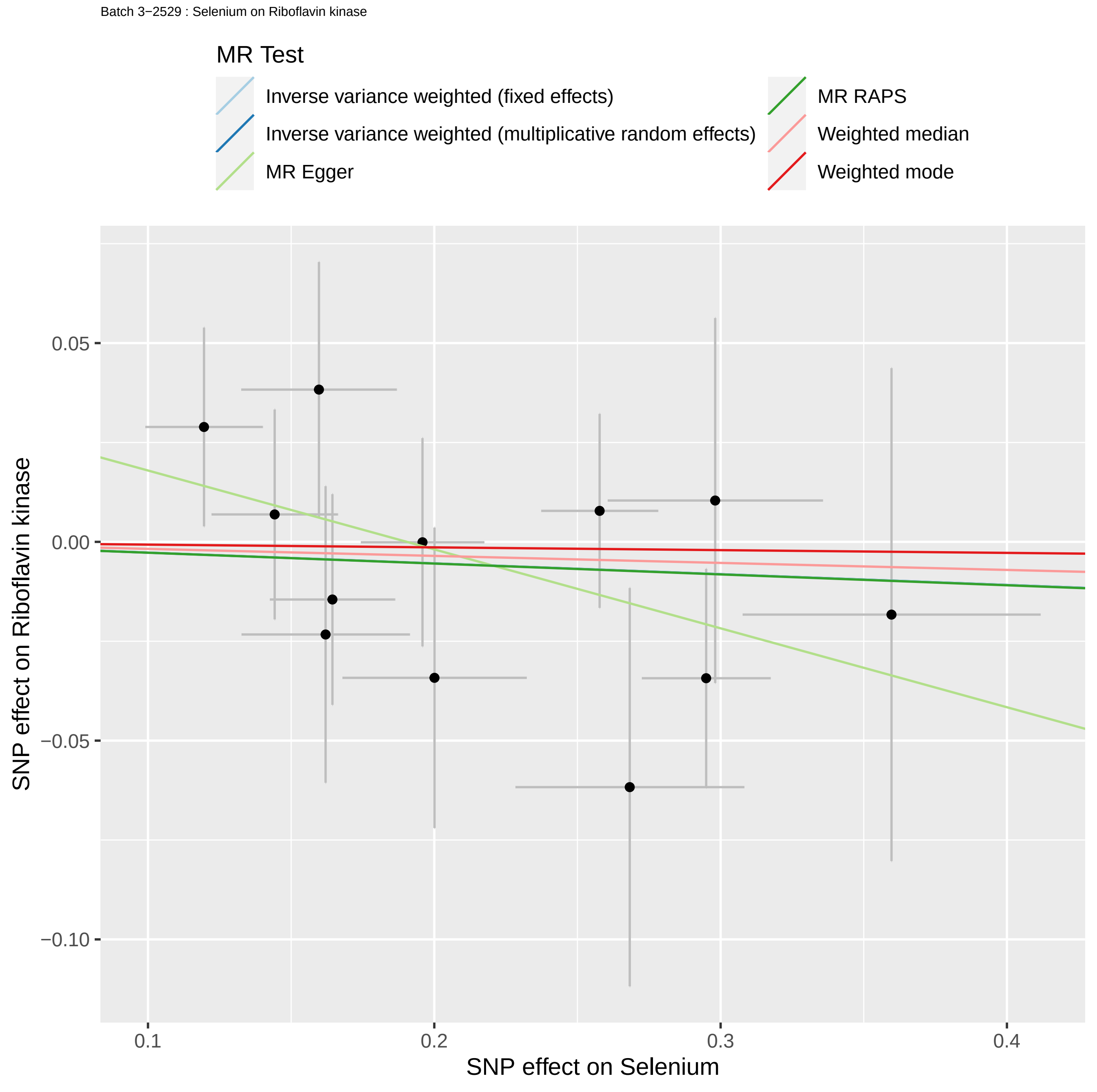

Batch 3−2529 : Selenium on Riboflavin kinase
MR Test
Inverse variance weighted (fixed effects)
Inverse variance weighted (multiplicative random effects)
MR Egger
MR RAPS
Weighted median
Weighted mode
0.05
0.00
−0.05
−0.10
0.1
0.2
0.3
0.4
SNP effect on Riboflavin kinase
SNP effect on Selenium

Batch 3−2530 : Selenium on Beta−1,3−N−acetylglucosaminyltransferase radical fringe

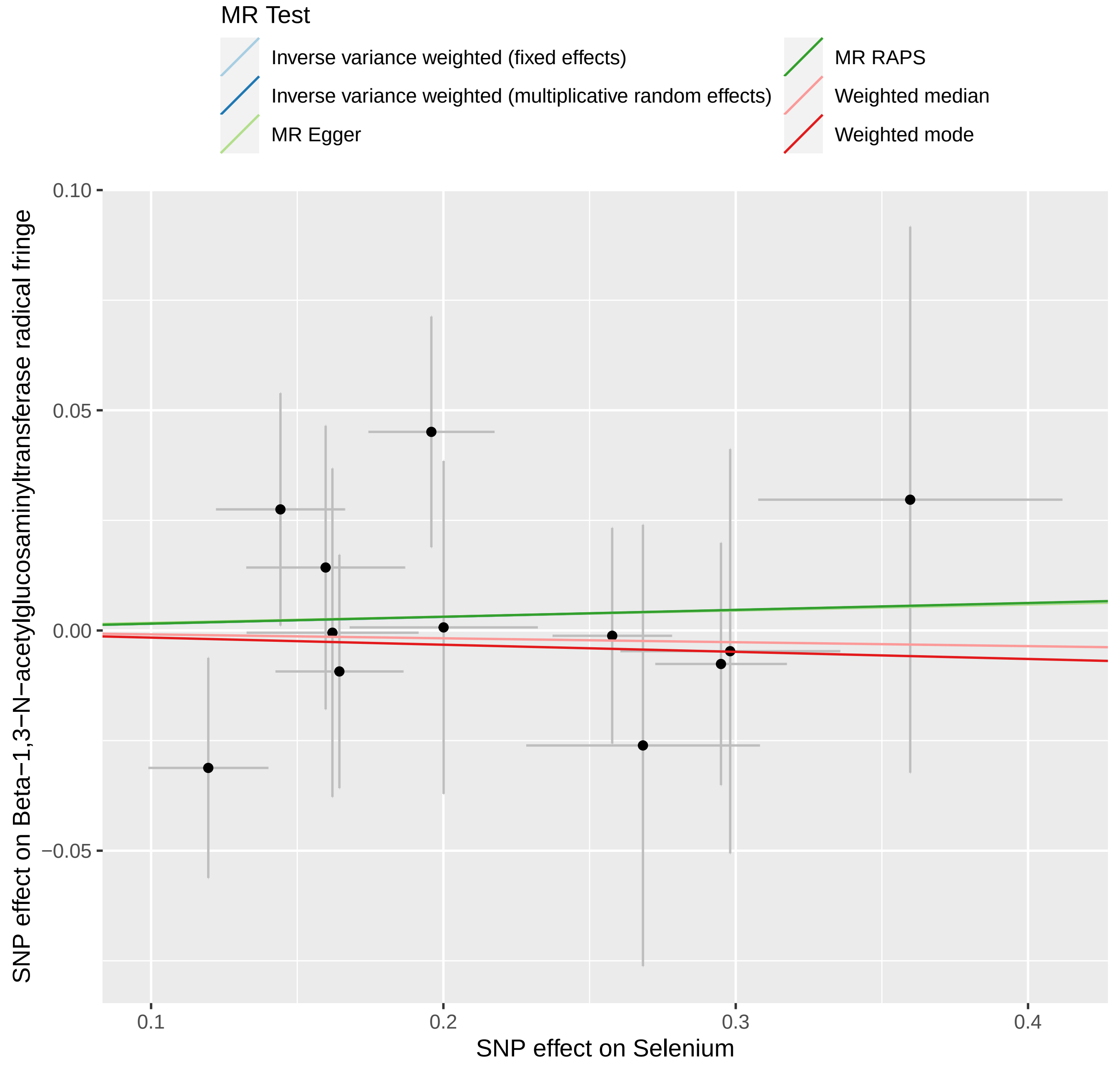

Batch 3−2531 : Selenium on Regulatory factor X−associated protein

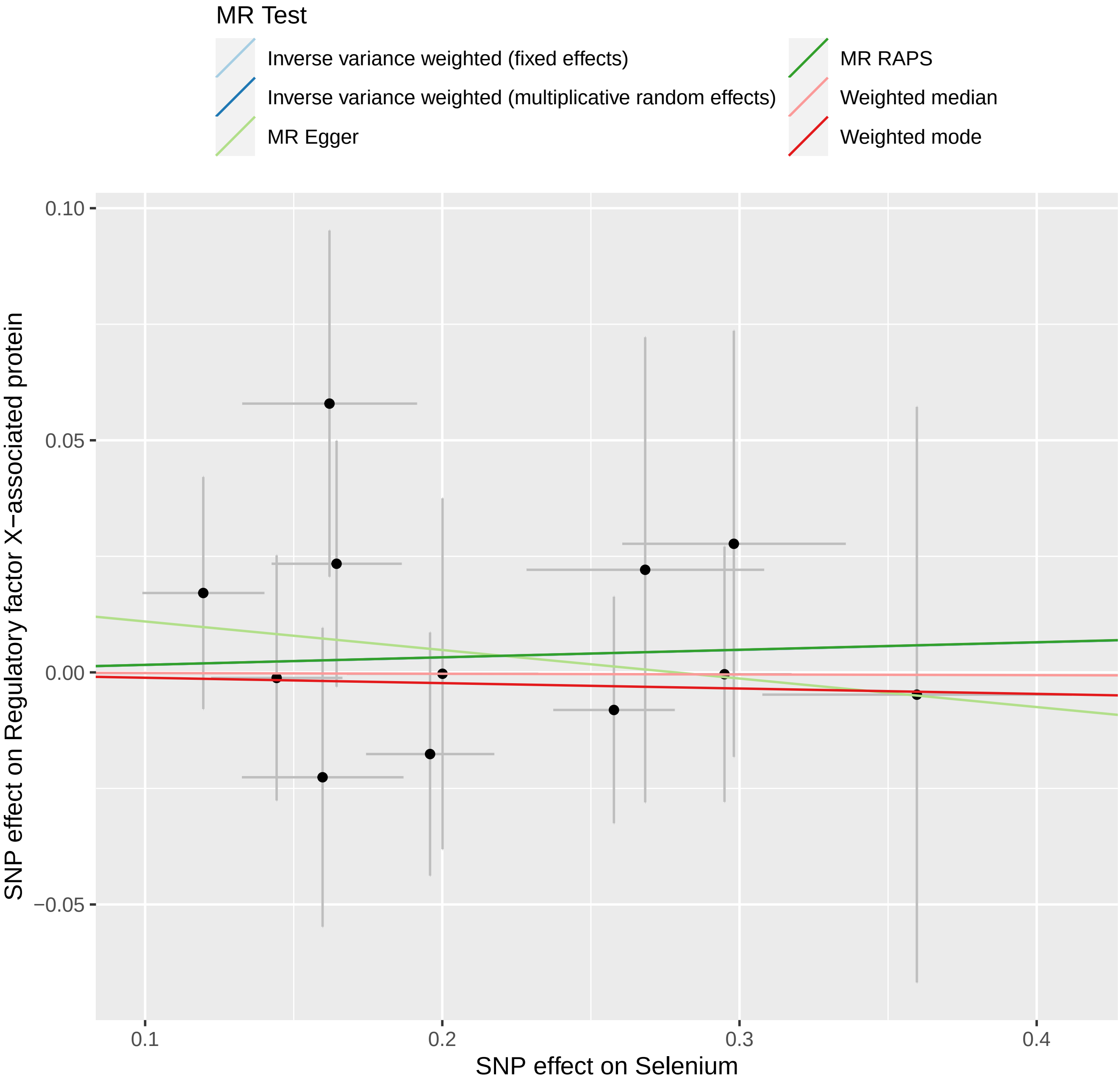

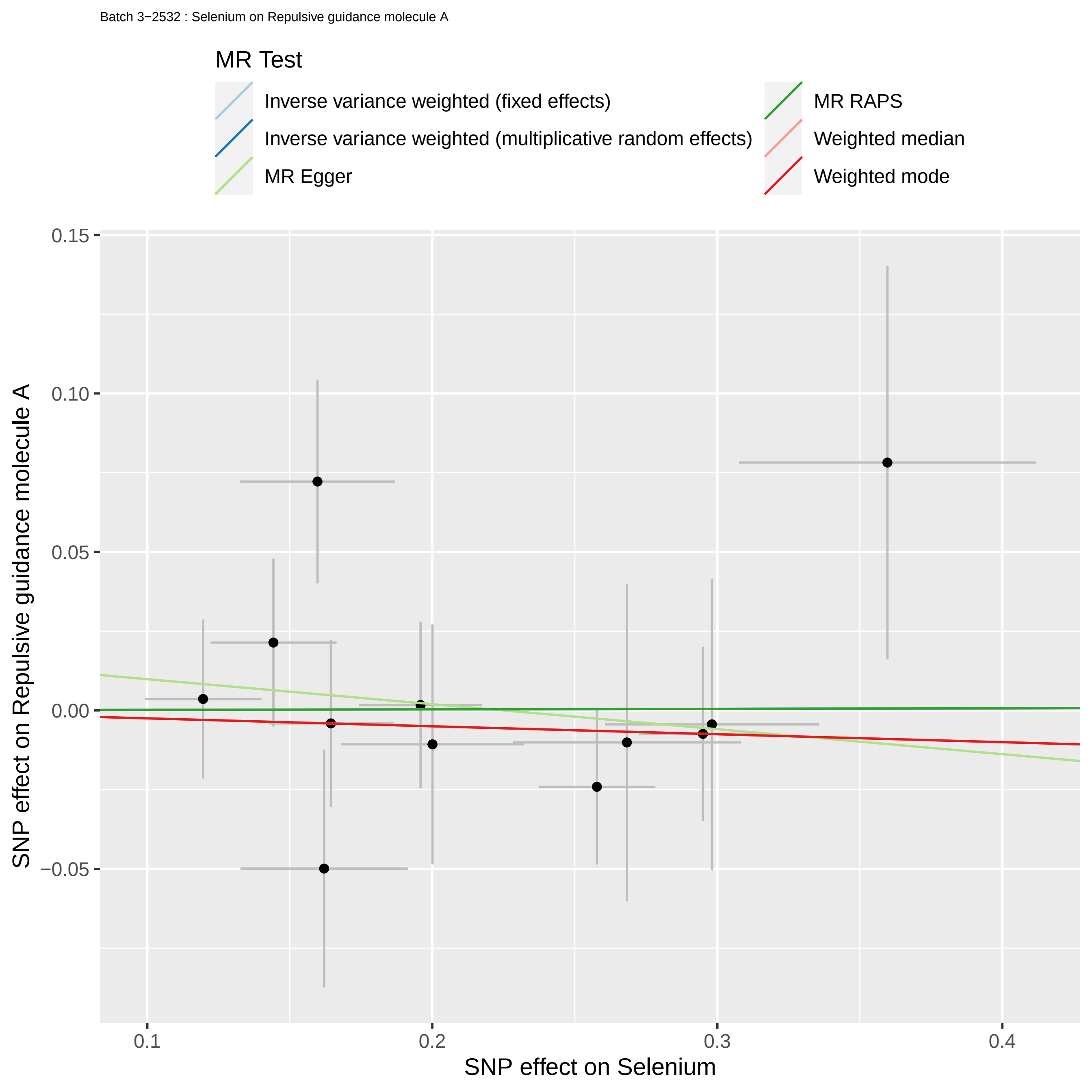

Batch 3−2532 : Selenium on Repulsive guidance molecule A
MR Test
Inverse variance weighted (fixed effects)
Inverse variance weighted (multiplicative random effects)
MR Egger
MR RAPS
Weighted median
Weighted mode
0.15
0.10
0.05
0.00
−0.05
0.1
0.2
0.3
0.4
SNP effect on Repulsive guidance molecule A
SNP effect on Selenium

Batch 3−2533 : Selenium on RGM domain family member B

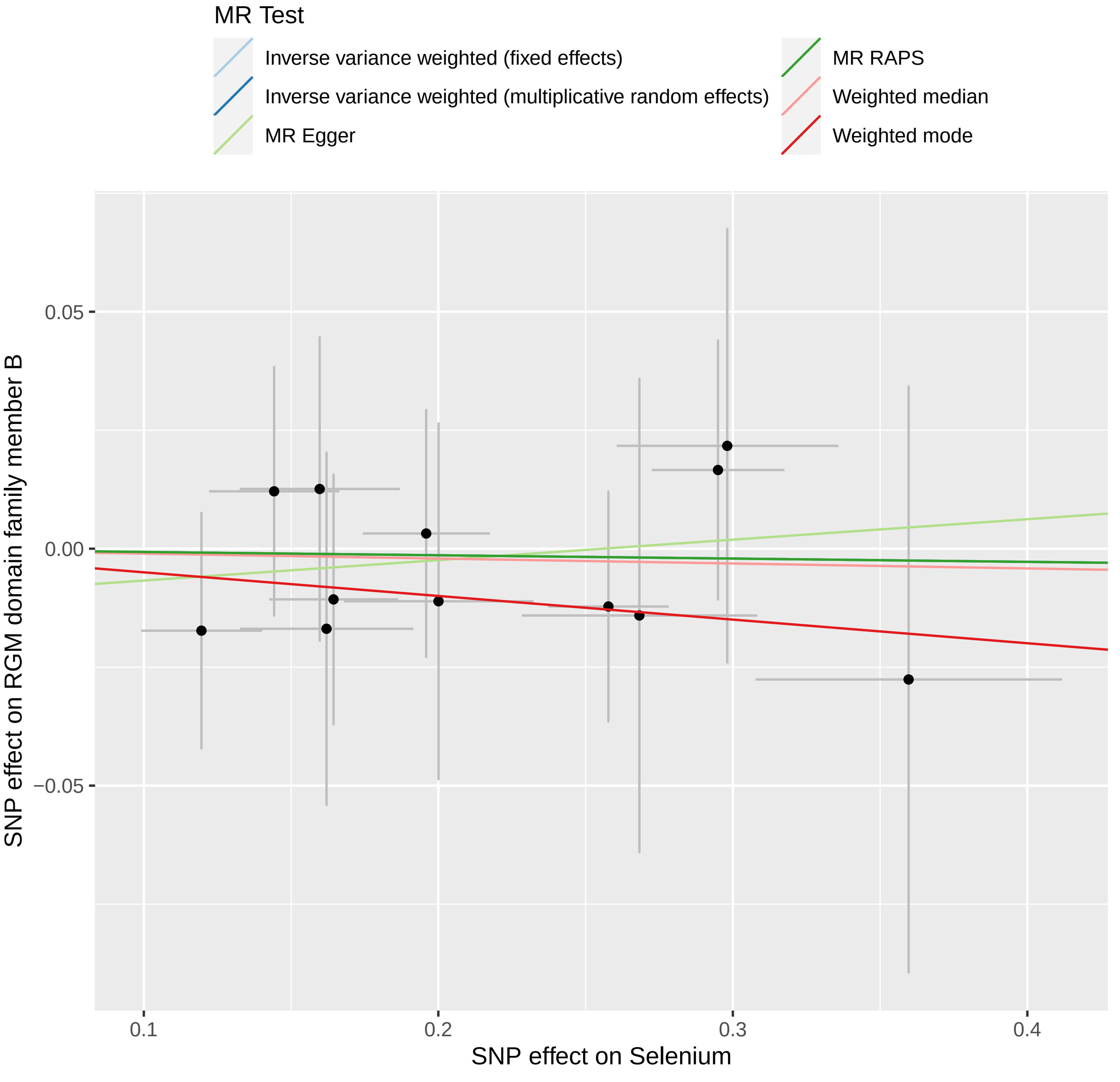

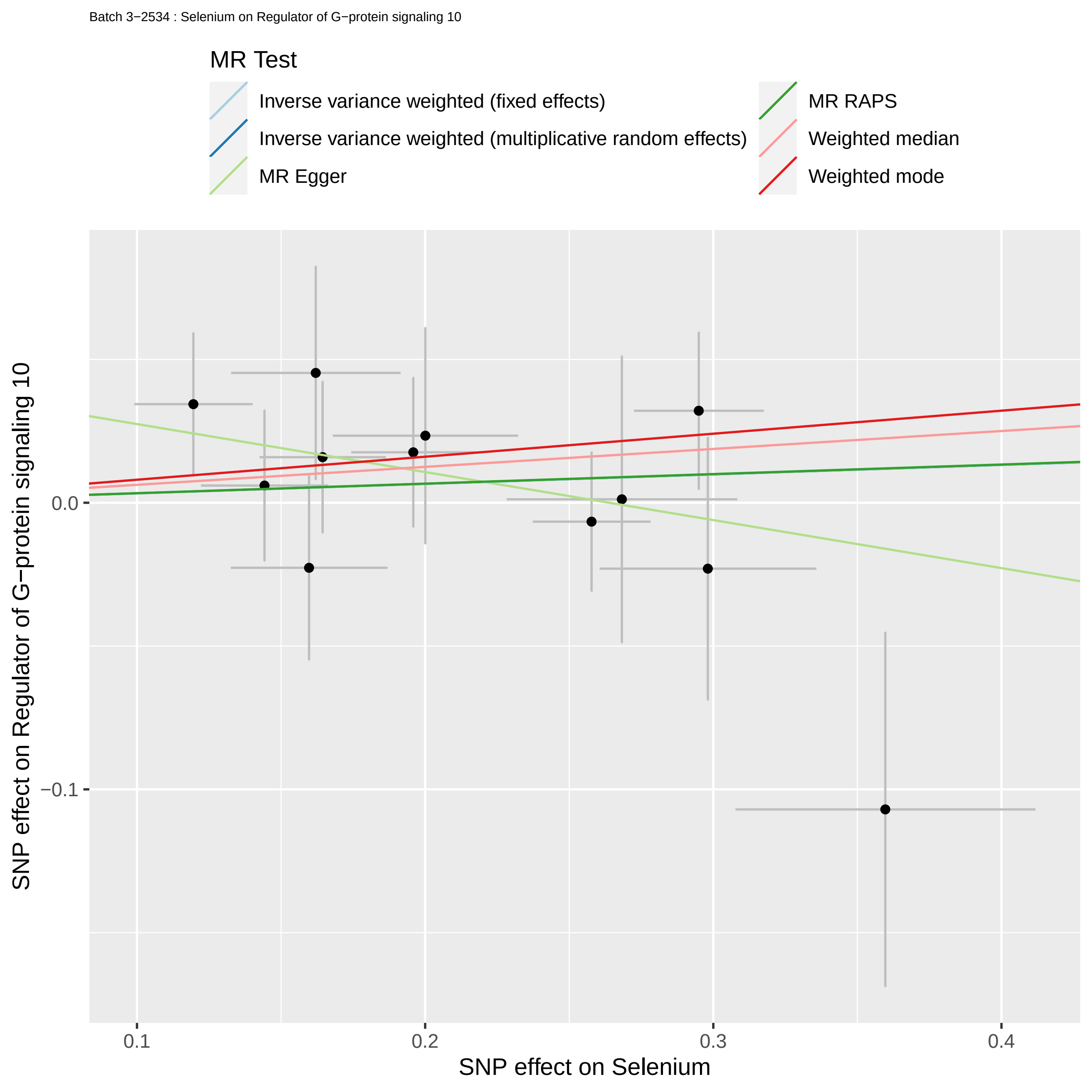

Batch 3−2534 : Selenium on Regulator of G−protein signaling 10
MR Test
Inverse variance weighted (fixed effects)
Inverse variance weighted (multiplicative random effects)
MR Egger
MR RAPS
Weighted median
Weighted mode
SNP effect on Regulator of G−protein signaling 10
0.0
−0.1
0.1
0.2
0.3
0.4
SNP effect on Selenium

Batch 3−2535 : Selenium on Regulator of G−protein signaling 18

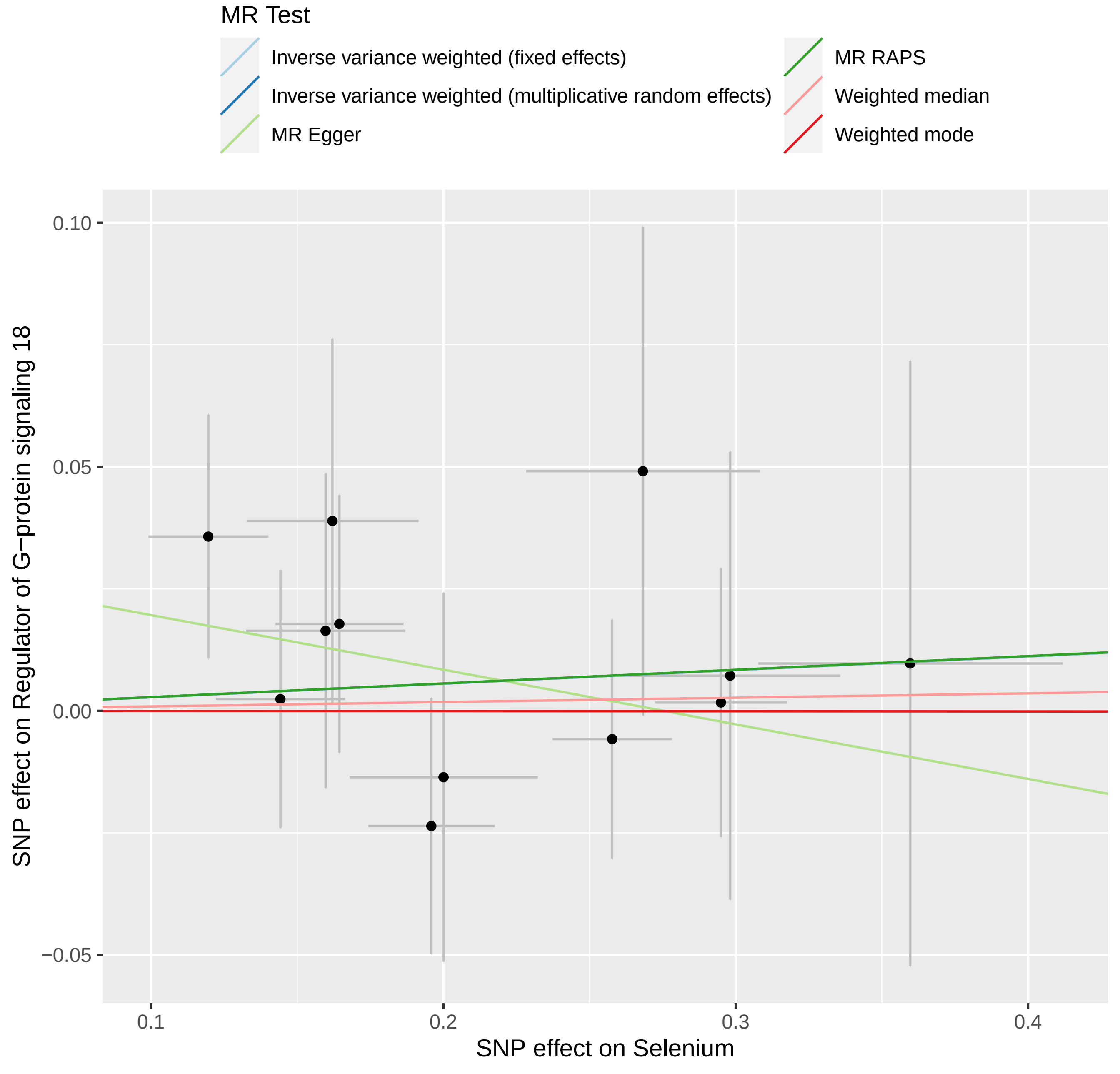

Batch 3−2536 : Selenium on Regulator of G−protein signaling 19

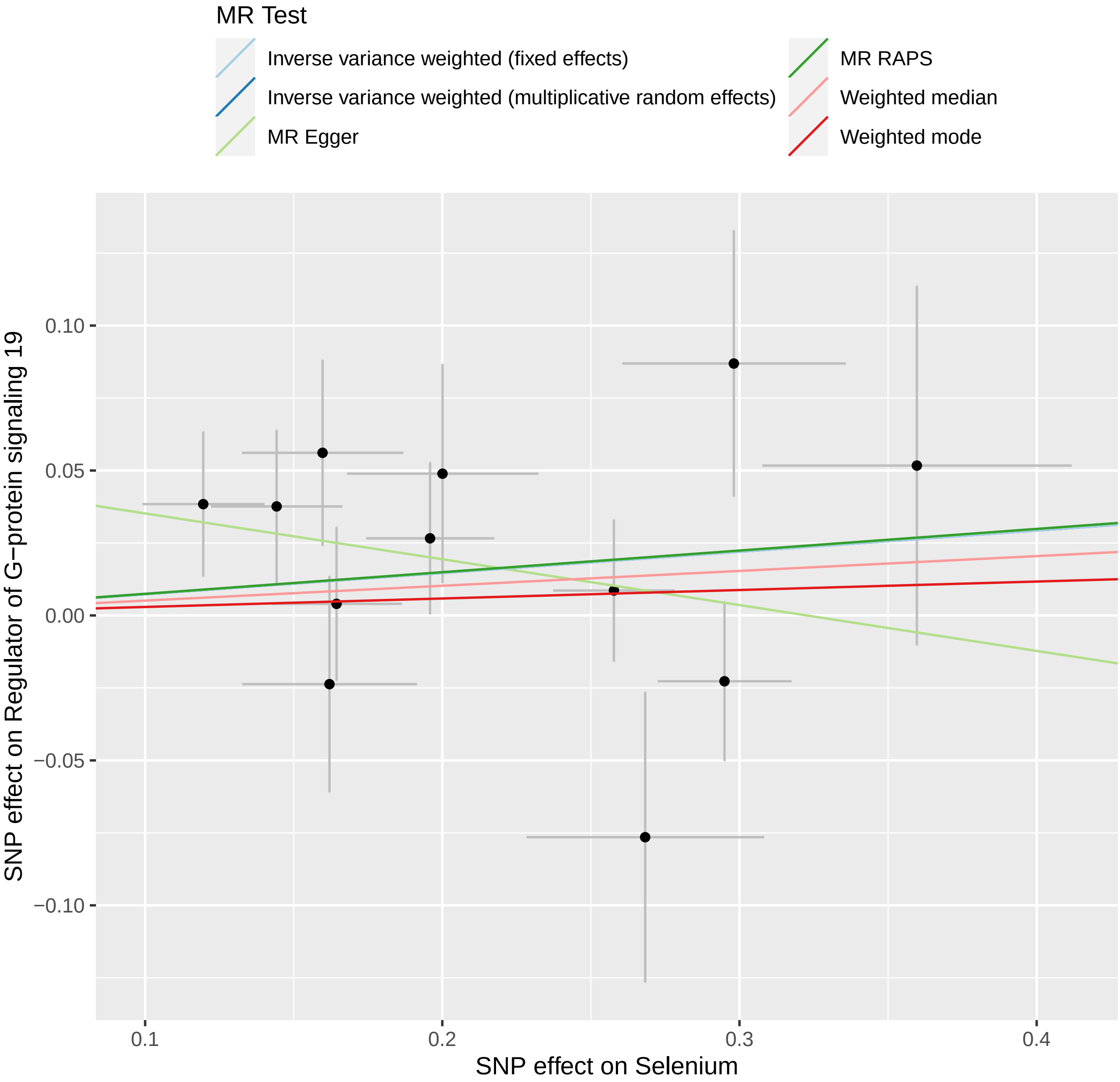

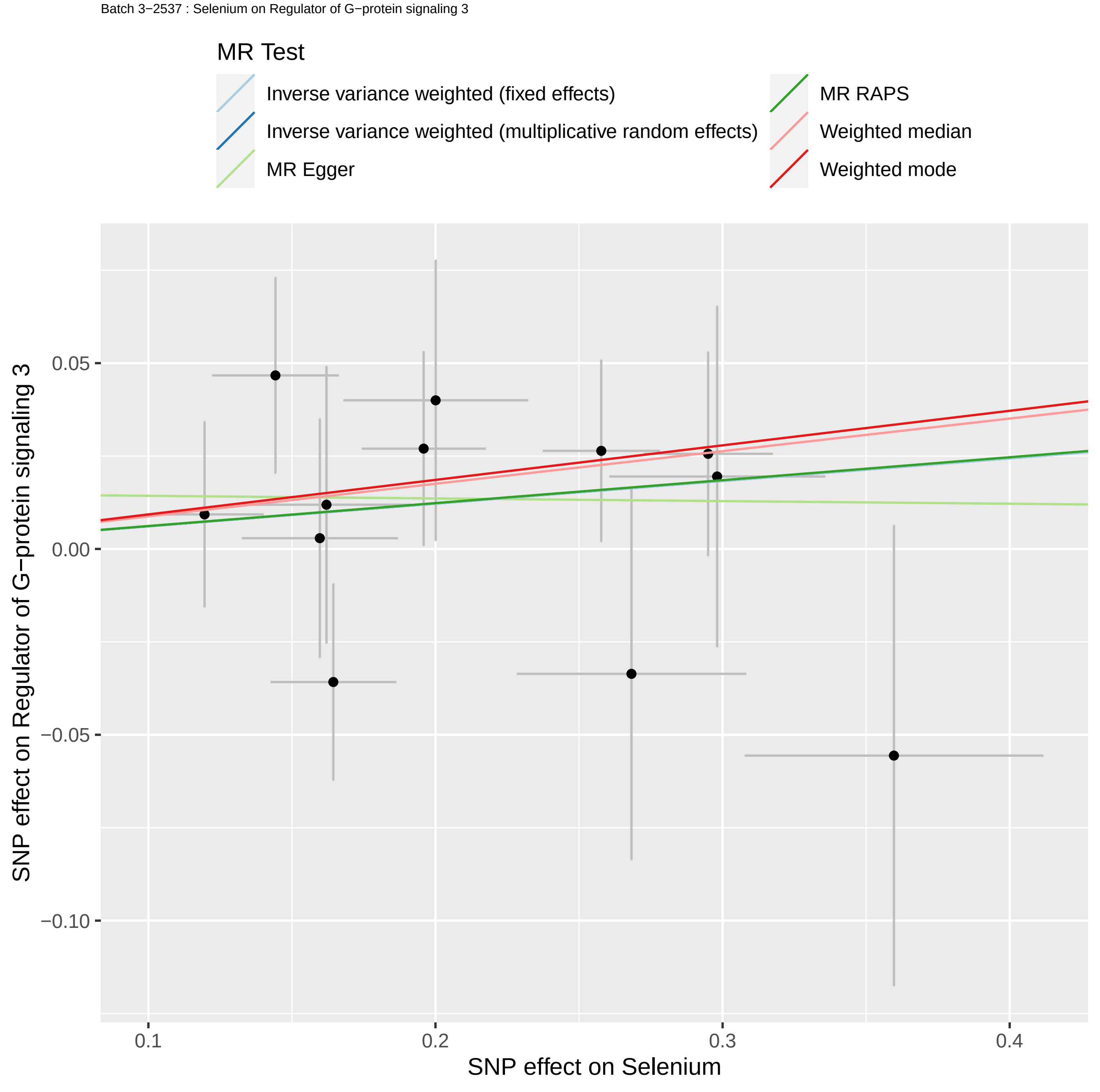

Batch 3−2537 : Selenium on Regulator of G−protein signaling 3
MR Test
Inverse variance weighted (fixed effects)
Inverse variance weighted (multiplicative random effects)
MR Egger
MR RAPS
Weighted median
Weighted mode
SNP effect on Regulator of G−protein signaling 3
0.05
0.00
−0.05
−0.10
0.1
0.2
0.3
0.4
SNP effect on Selenium

Batch 3−2538 : Selenium on Regulator of G−protein signaling 8

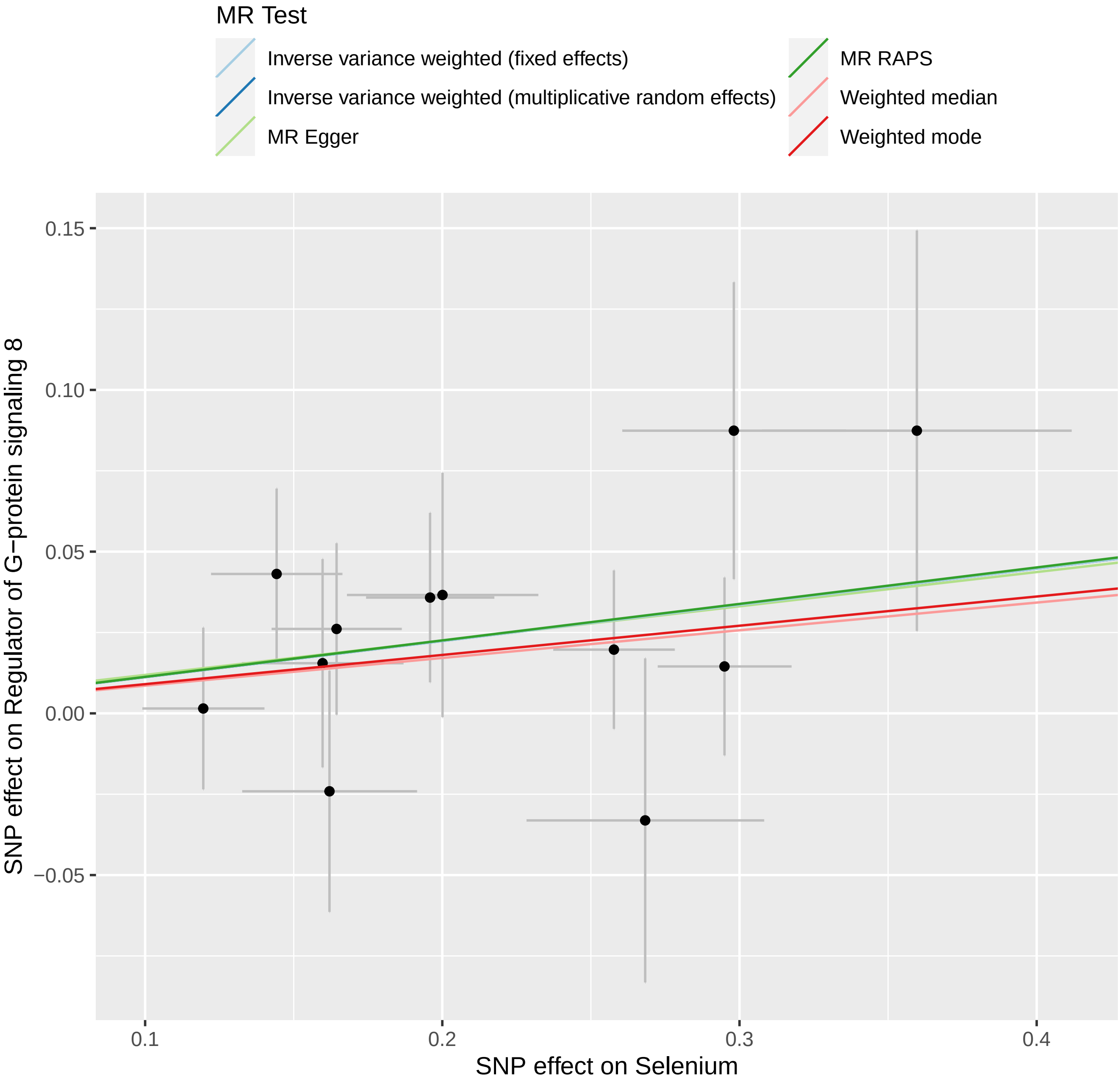

Batch 3−2539 : Selenium on Transforming protein RhoA

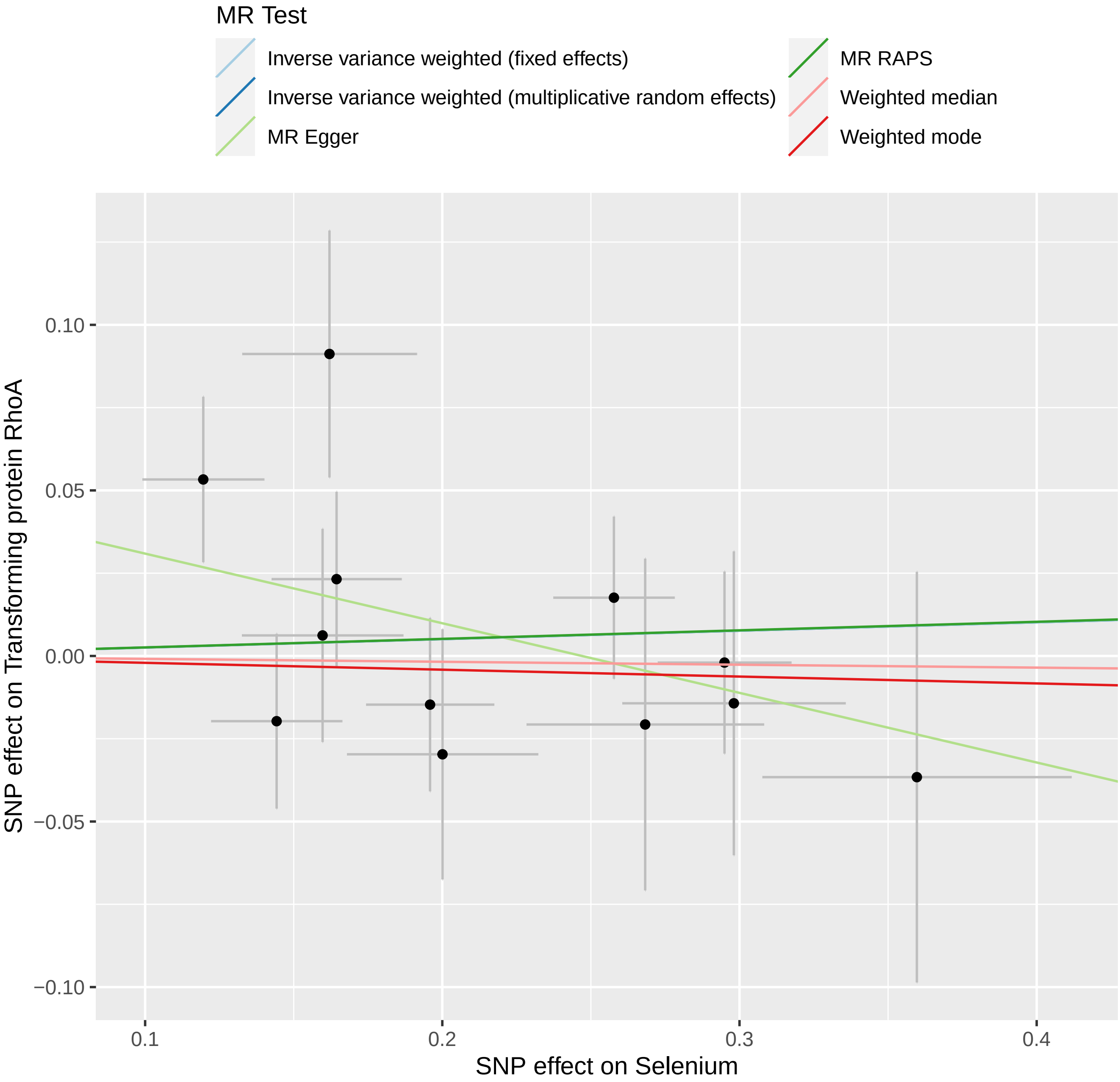

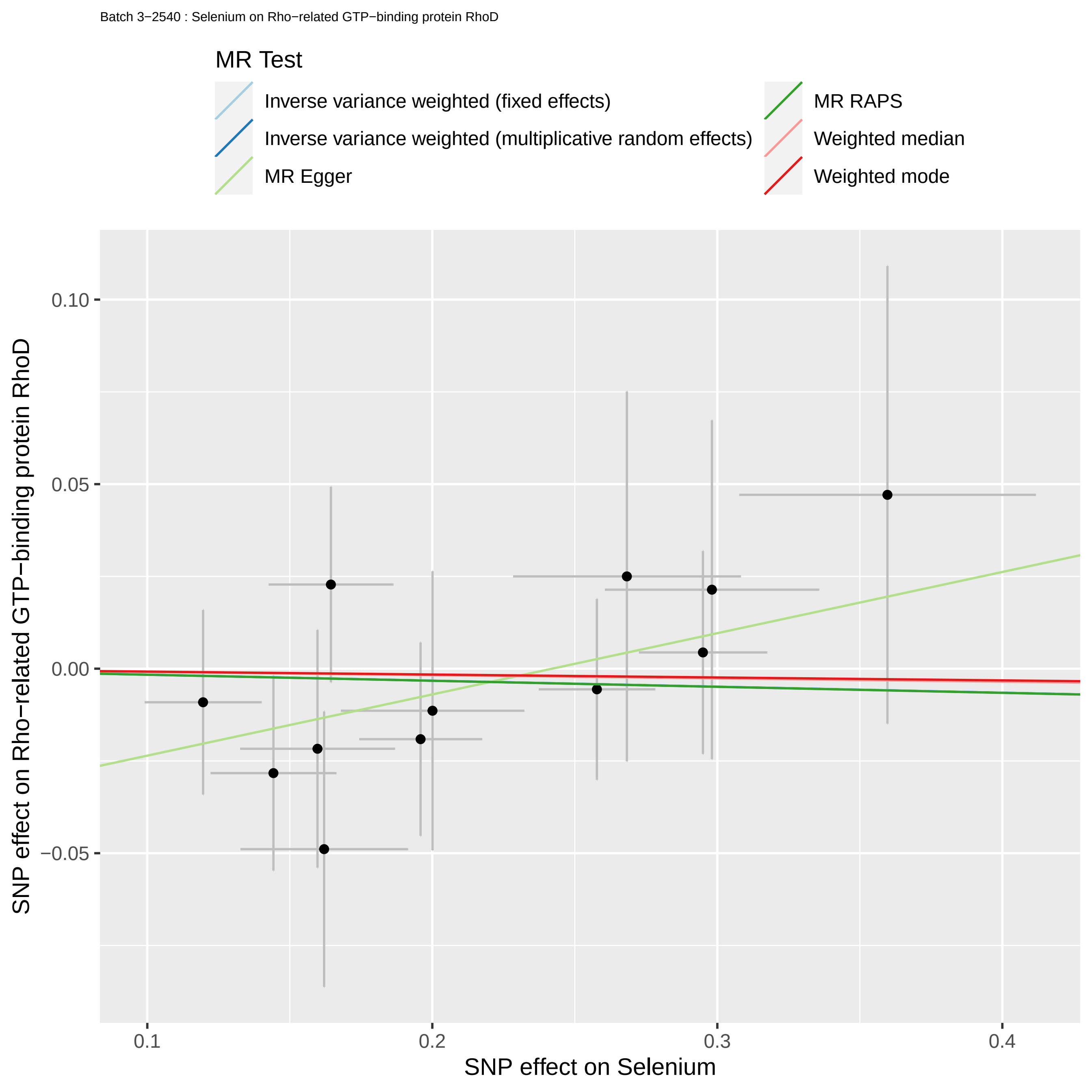

Batch 3−2540 : Selenium on Rho−related GTP−binding protein RhoD
MR Test
Inverse variance weighted (fixed effects)
Inverse variance weighted (multiplicative random effects)
MR Egger
MR RAPS
Weighted median
Weighted mode
0.10
0.05
0.00
−0.05
0.1
0.2
0.3
0.4
SNP effect on Selenium
SNP effect on Rho−related GTP−binding protein RhoD

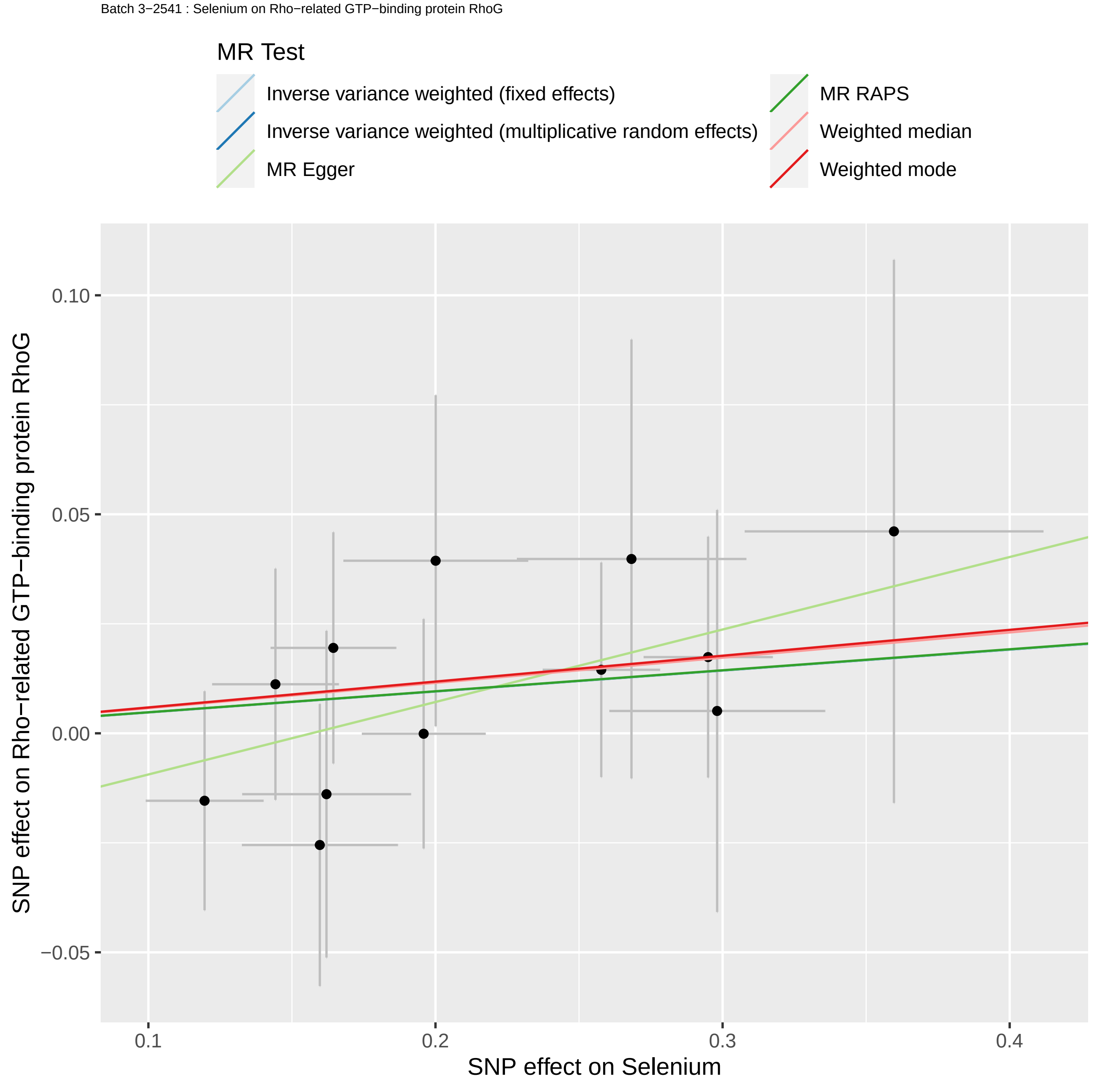
Batch 3−2541 : Selenium on Rho−related GTP−binding protein RhoG
MR Test
Inverse variance weighted (fixed effects)
Inverse variance weighted (multiplicative random effects)
MR Egger
MR RAPS
Weighted median
Weighted mode
0.10
0.05
0.00
−0.05
0.1
0.2
0.3
0.4
SNP effect on Rho−related GTP−binding protein RhoG
SNP effect on Selenium

Batch 3−2542 : Selenium on Rhophilin−2

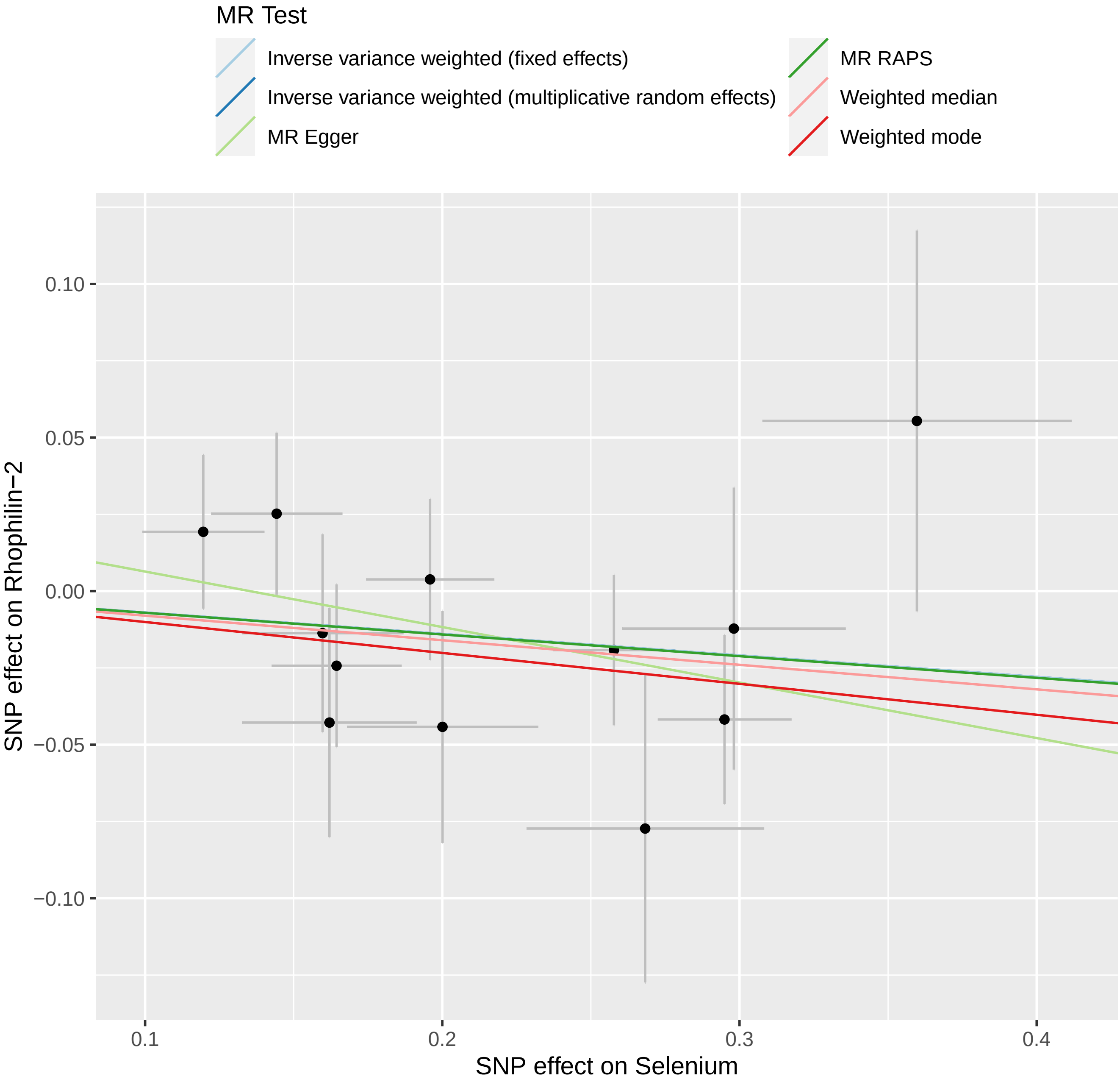

Batch 3−2543 : Selenium on Protein RIC−3

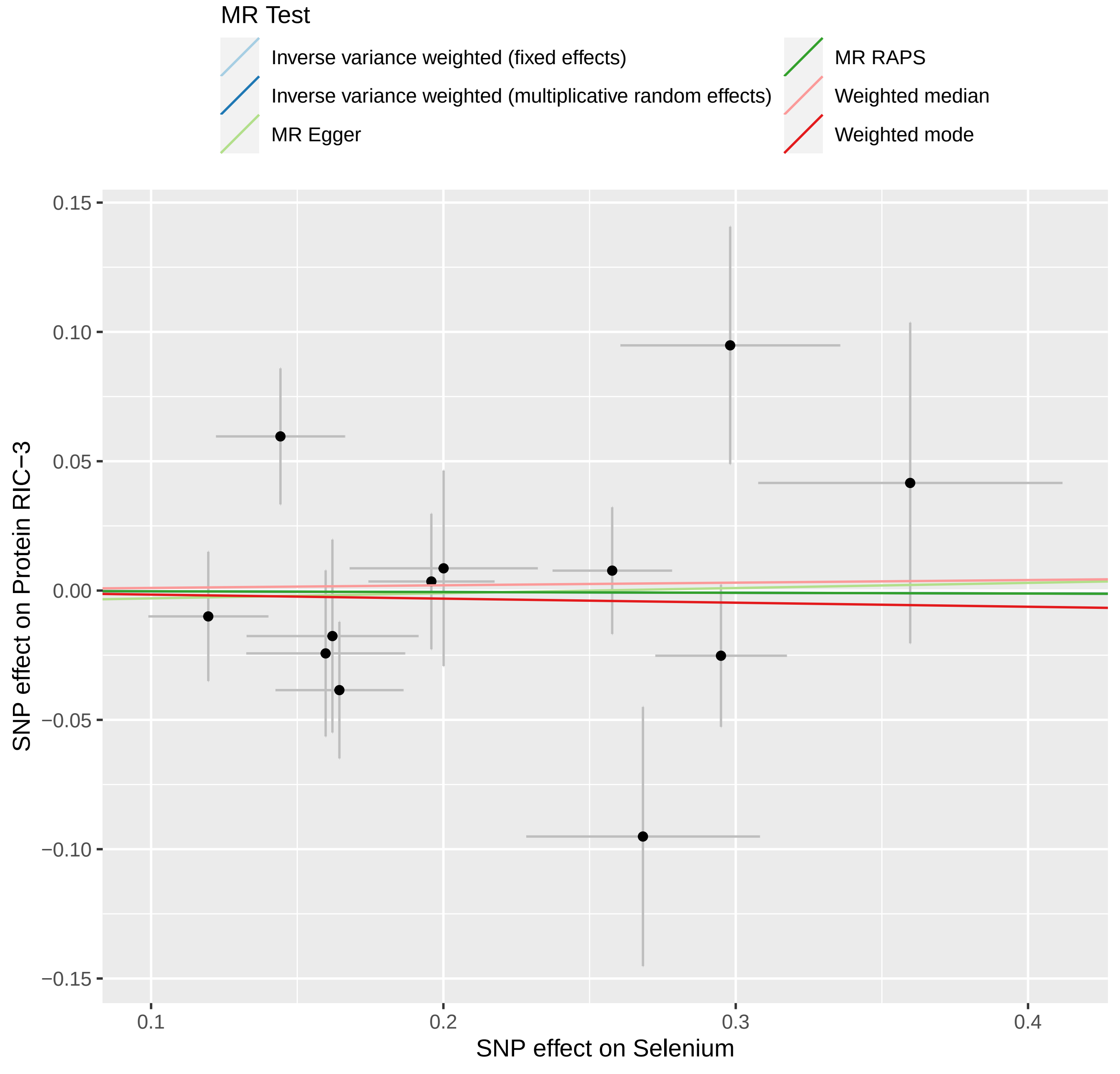

Batch 3−2544 : Selenium on Protein RIC−3

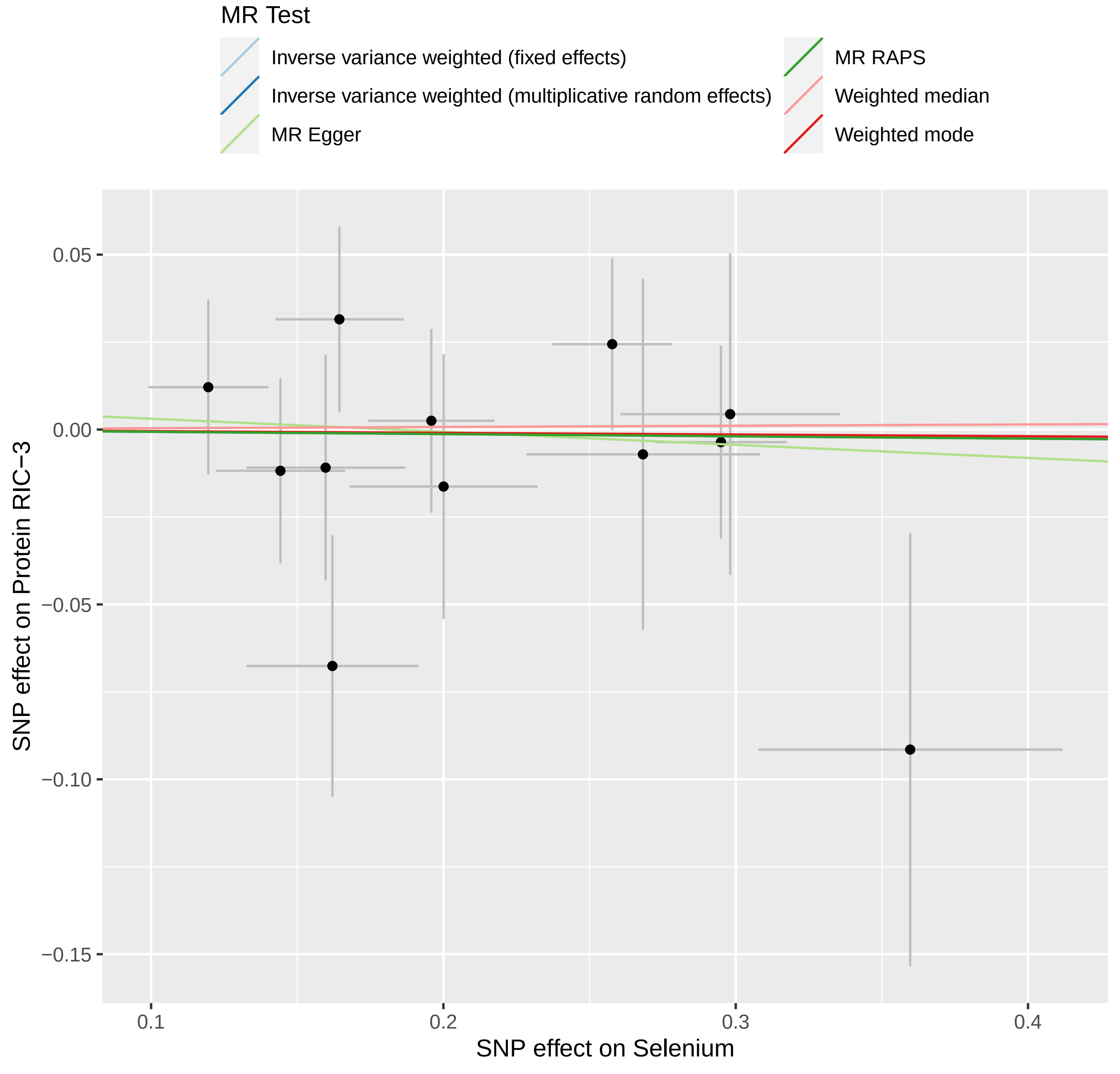

Batch 3−2545 : Selenium on Receptor−interacting serine/threonine−protein kinase 2

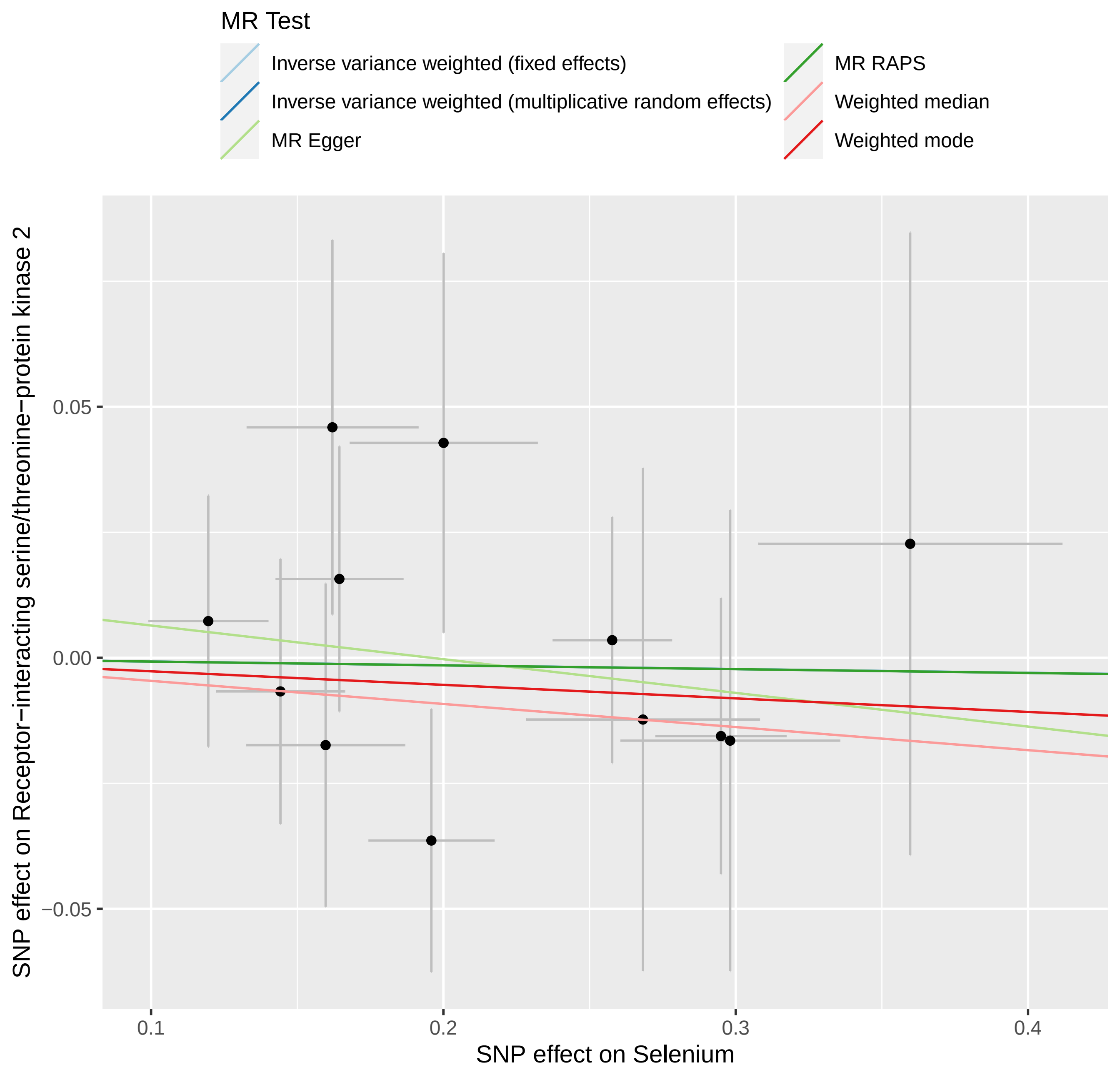

Batch 3−2546 : Selenium on Receptor−interacting serine/threonine−protein kinase 2

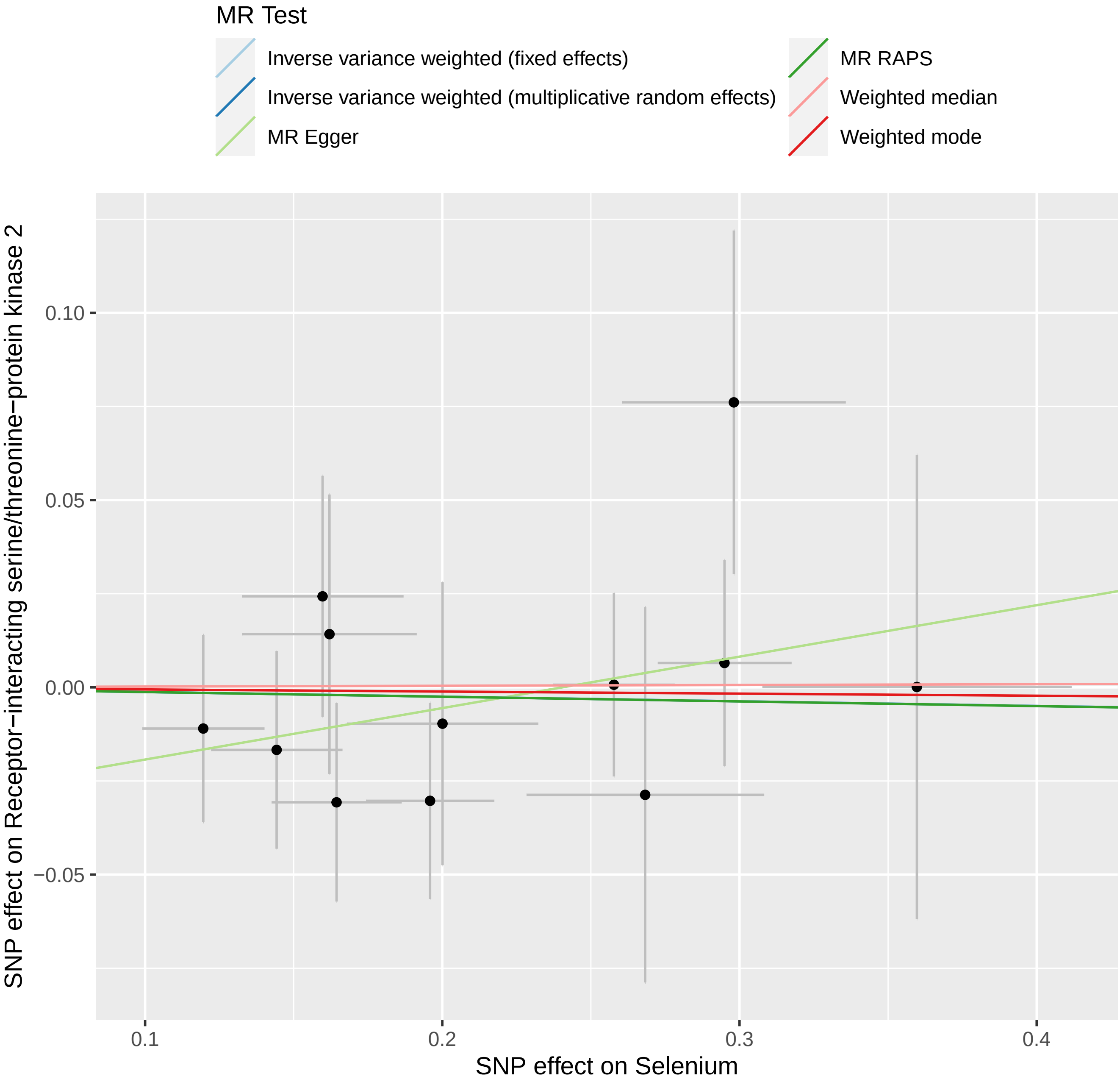

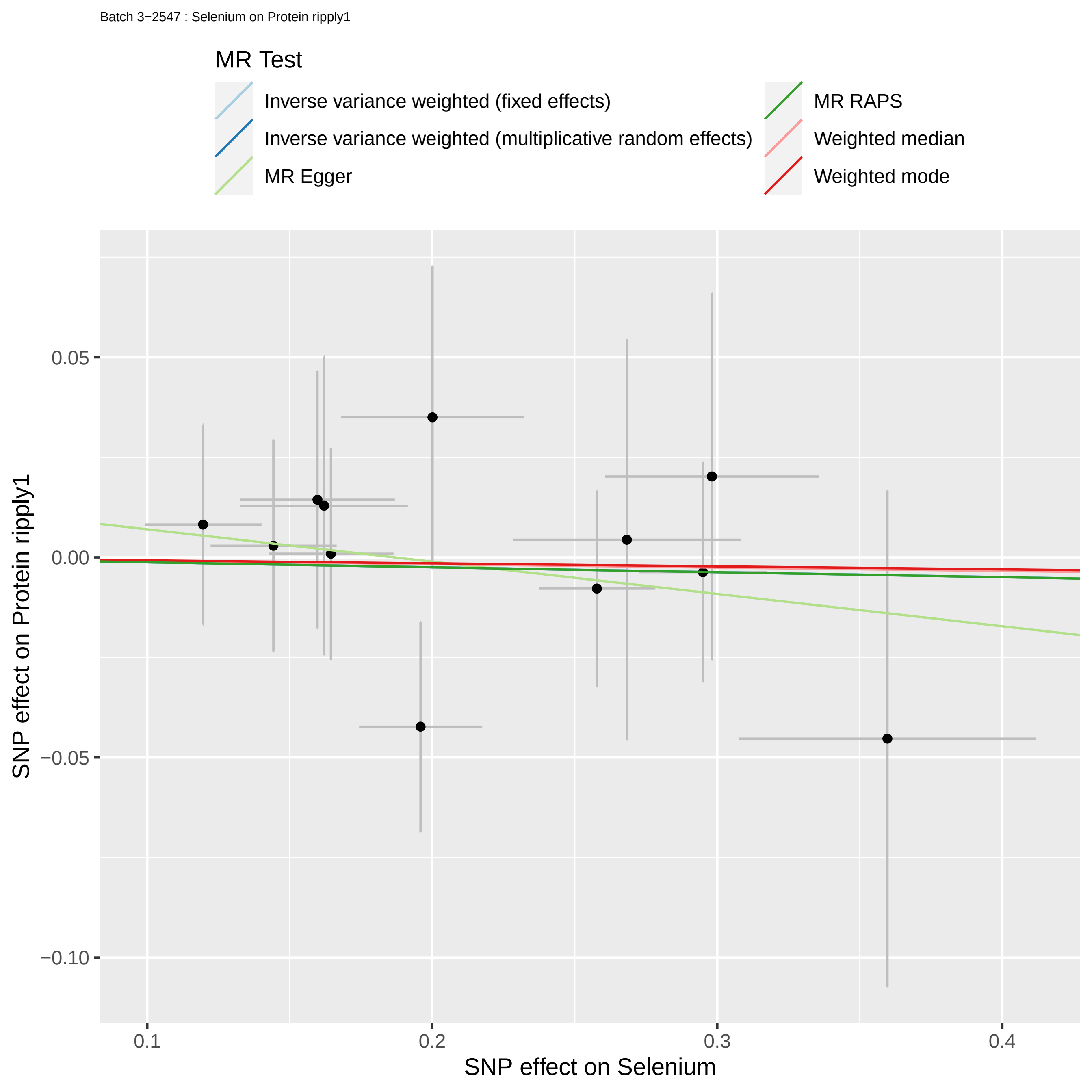
Batch 3−2547 : Selenium on Protein ripply1
MR Test
Inverse variance weighted (fixed effects)
Inverse variance weighted (multiplicative random effects)
MR Egger
MR RAPS
Weighted median
Weighted mode
SNP effect on Protein ripply1
0.05
0.00
−0.05
−0.10
0.1
0.2
0.3
0.4
SNP effect on Selenium

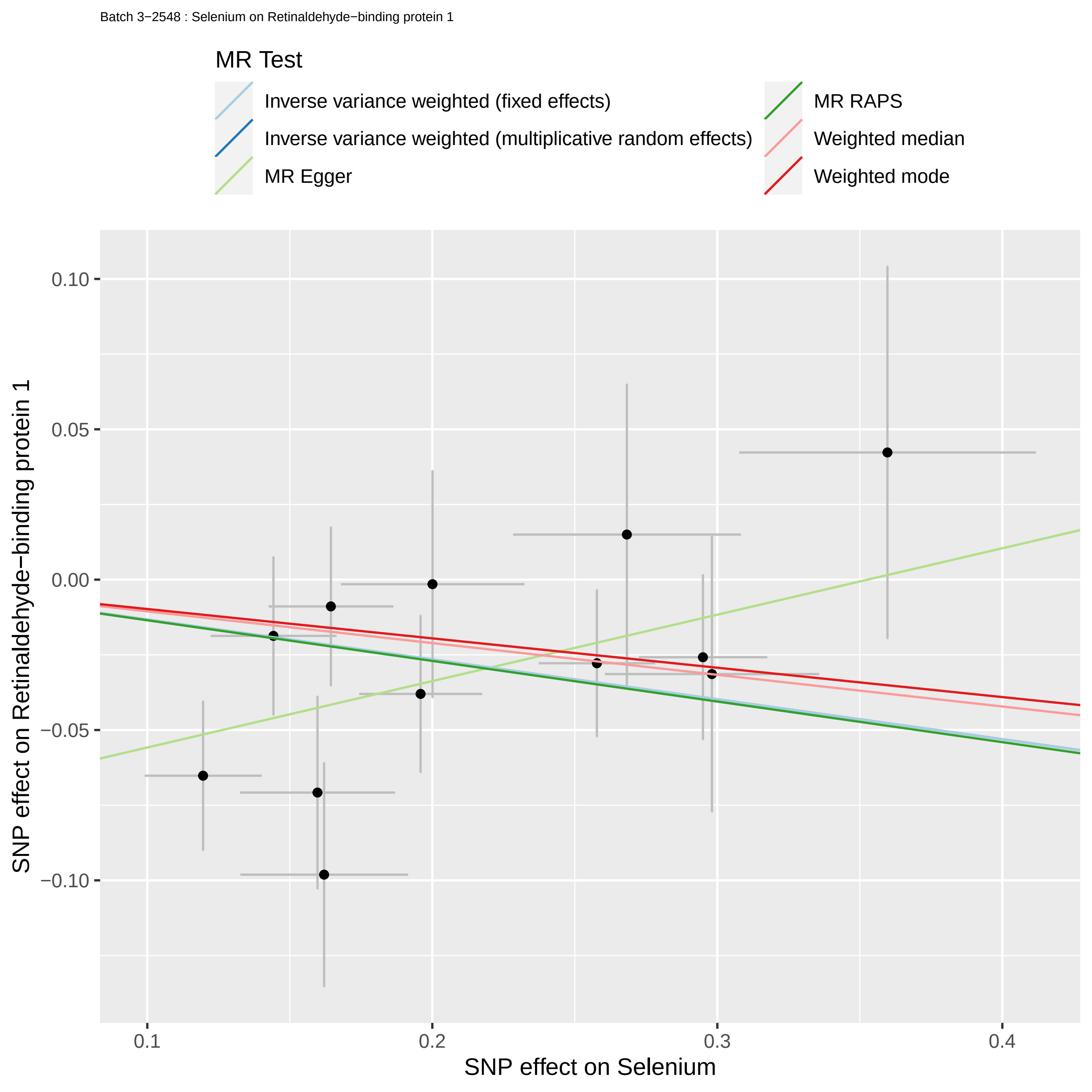
Batch 3−2548 : Selenium on Retinaldehyde−binding protein 1
MR Test
Inverse variance weighted (fixed effects)
Inverse variance weighted (multiplicative random effects)
MR Egger
MR RAPS
Weighted median
Weighted mode
0.10
0.05
0.00
−0.05
−0.10
0.1
0.2
0.3
0.4
SNP effect on Retinaldehyde−binding protein 1
SNP effect on Selenium

Batch 3−2549 : Selenium on Regulator of microtubule dynamics protein 1

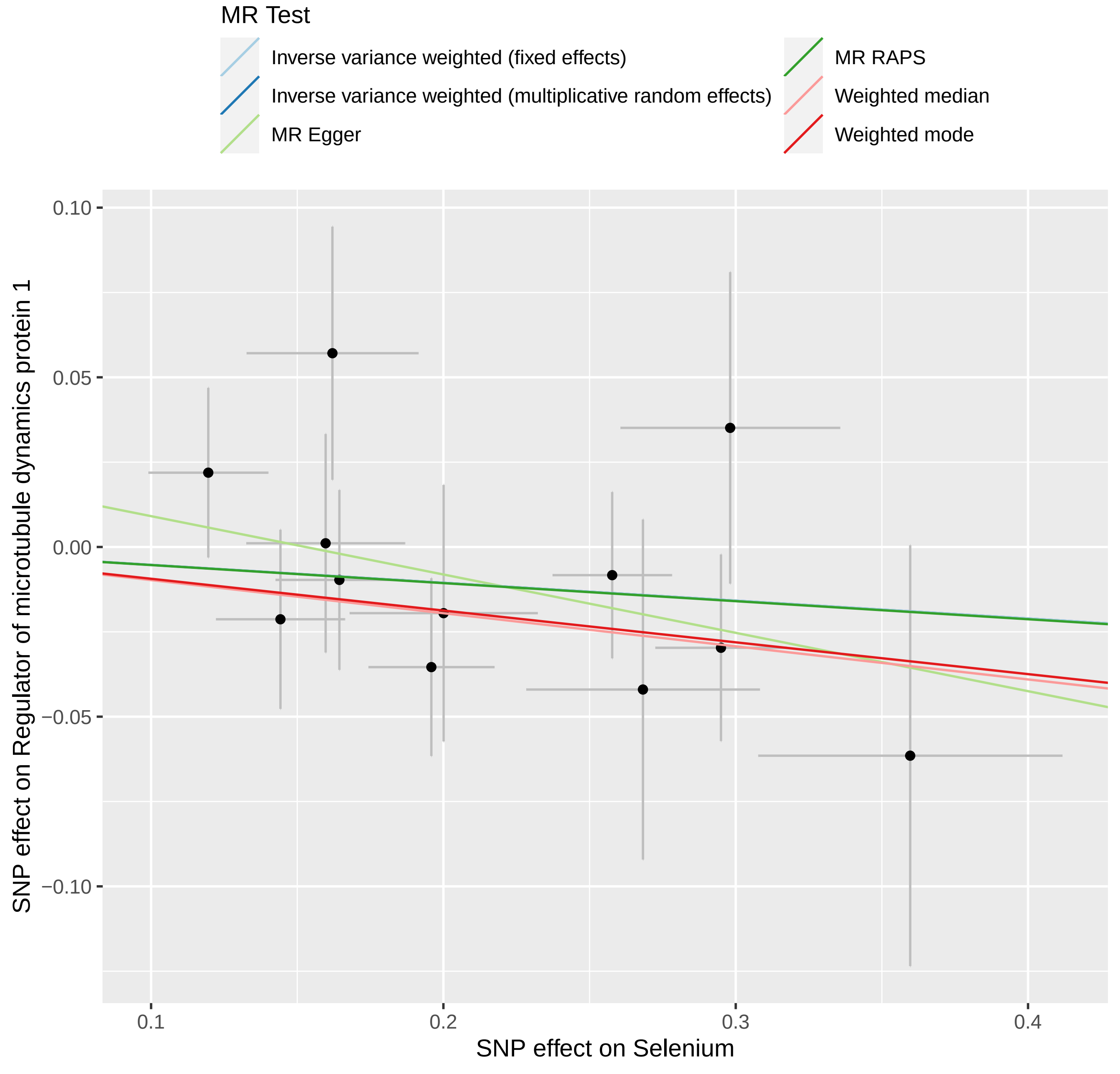

Batch 3−2550 : Selenium on Regulator of microtubule dynamics protein 3

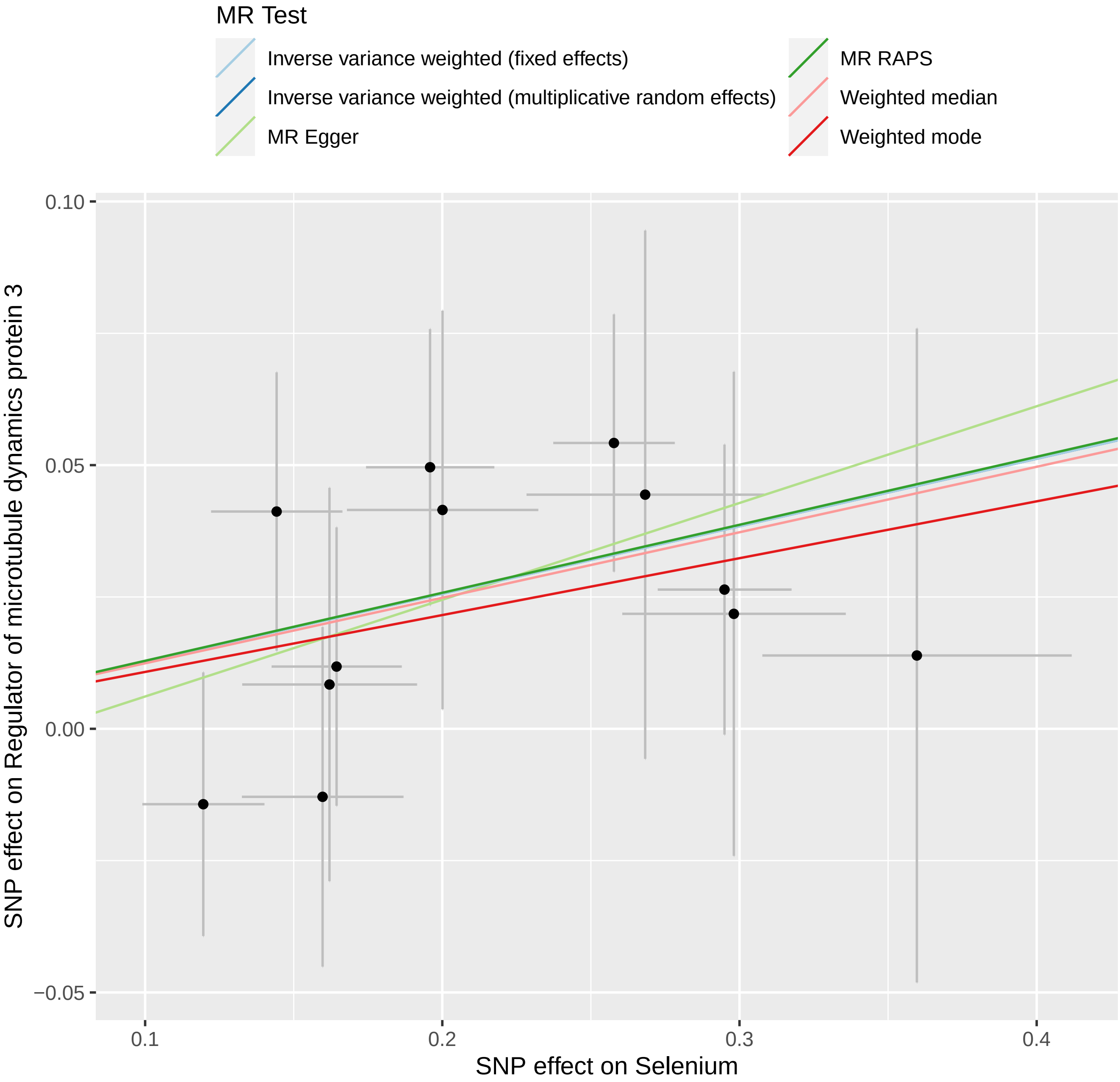

Batch 3−2551 : Selenium on RecQ−mediated genome instability protein 1

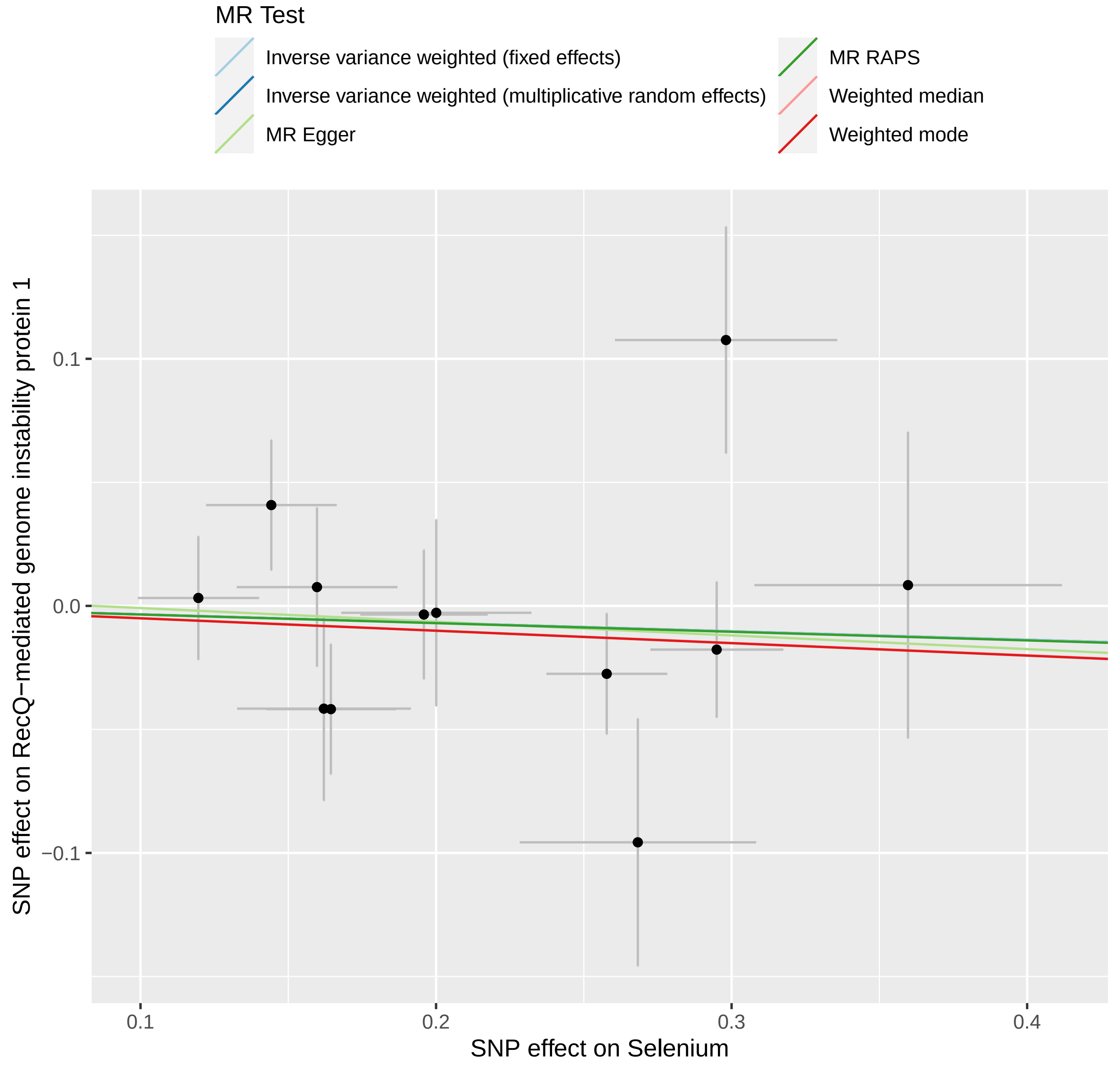

Batch 3−2552 : Selenium on Inactive ribonuclease−like protein 10

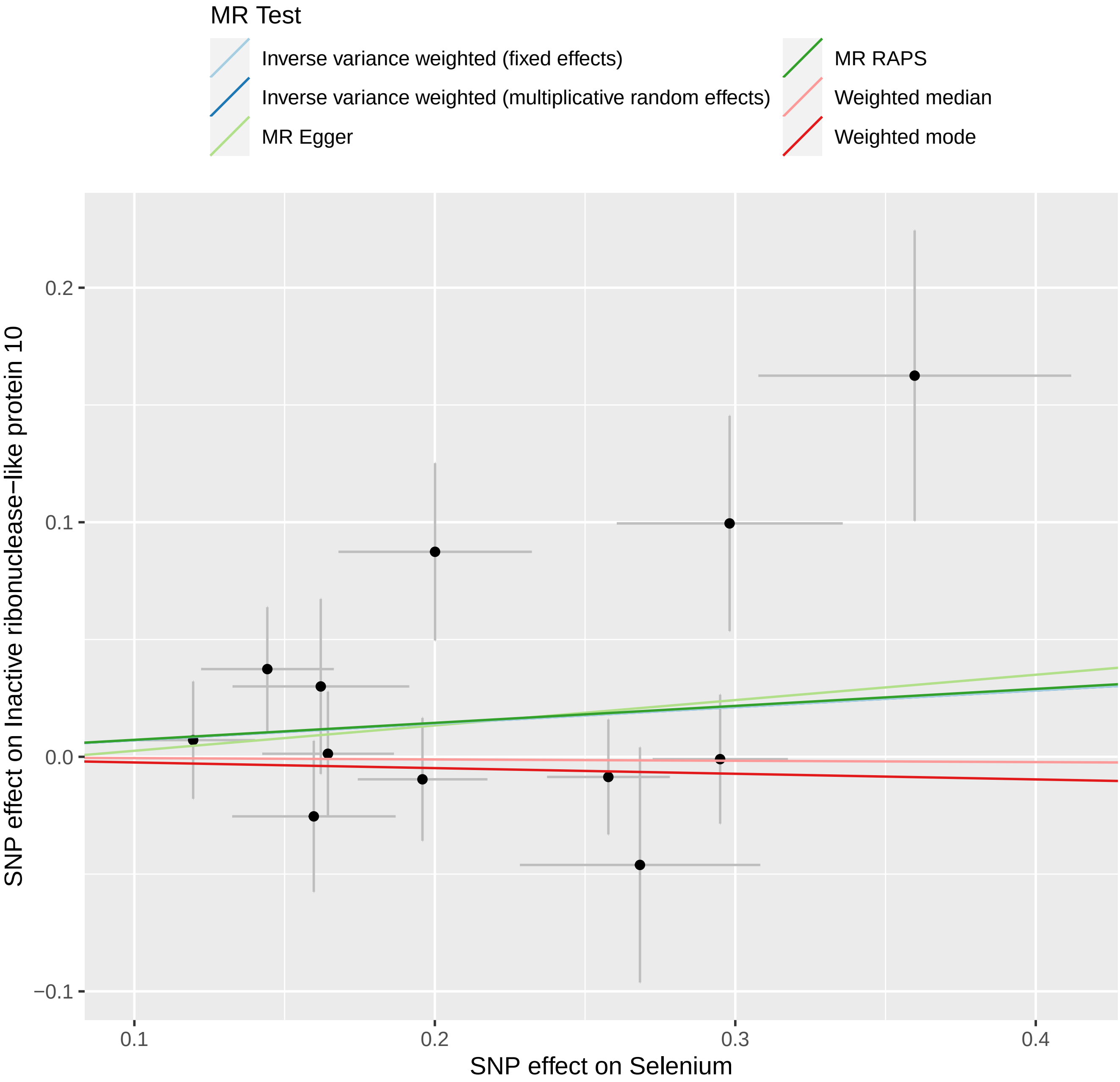

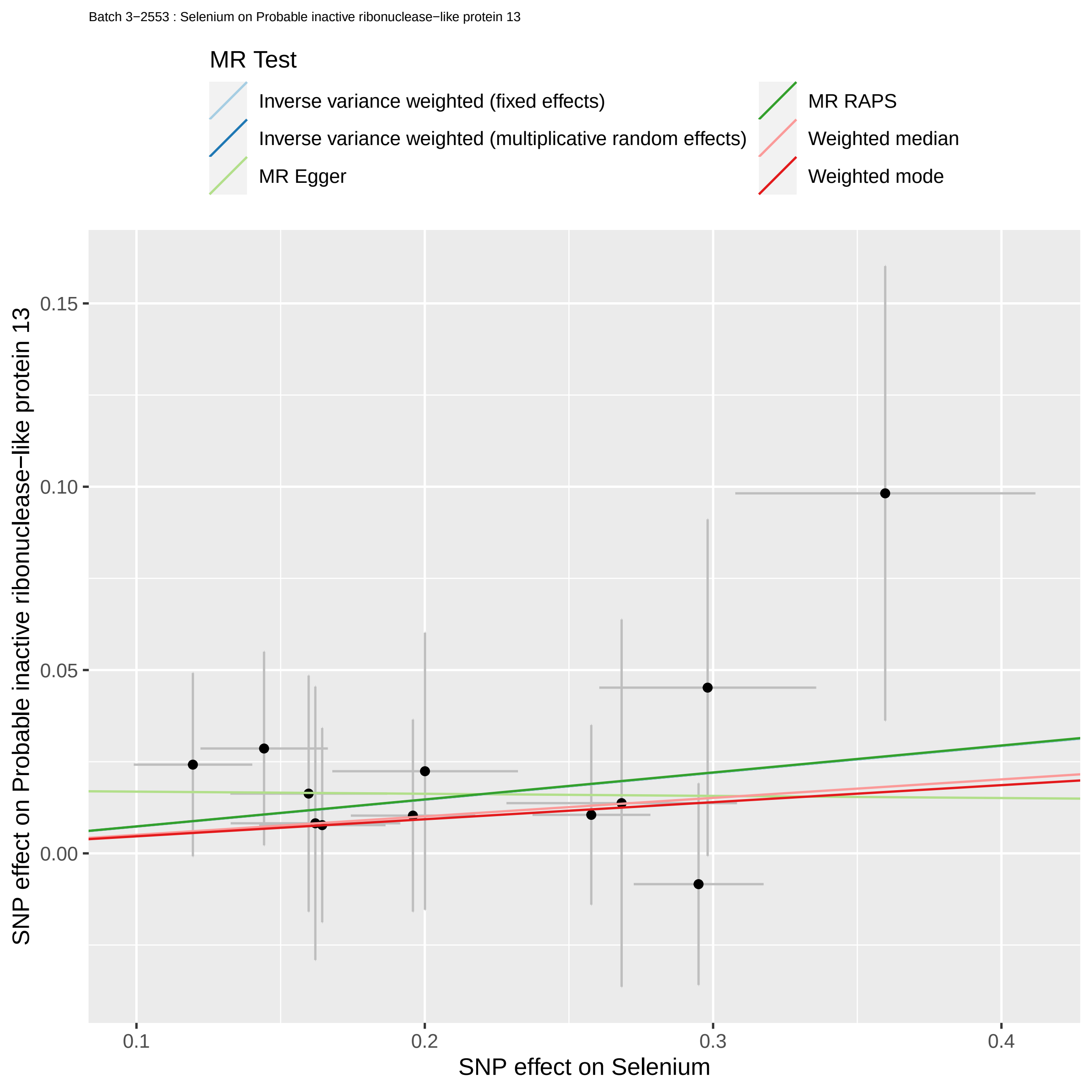

Batch 3−2553 : Selenium on Probable inactive ribonuclease−like protein 13
MR Test
Inverse variance weighted (fixed effects)
Inverse variance weighted (multiplicative random effects)
MR Egger
MR RAPS
Weighted median
Weighted mode
SNP effect on Probable inactive ribonuclease−like protein 13
0.15
0.10
0.05
0.00
0.1
0.2
0.3
0.4
SNP effect on Selenium

Batch 3−2554 : Selenium on Ribonuclease pancreatic

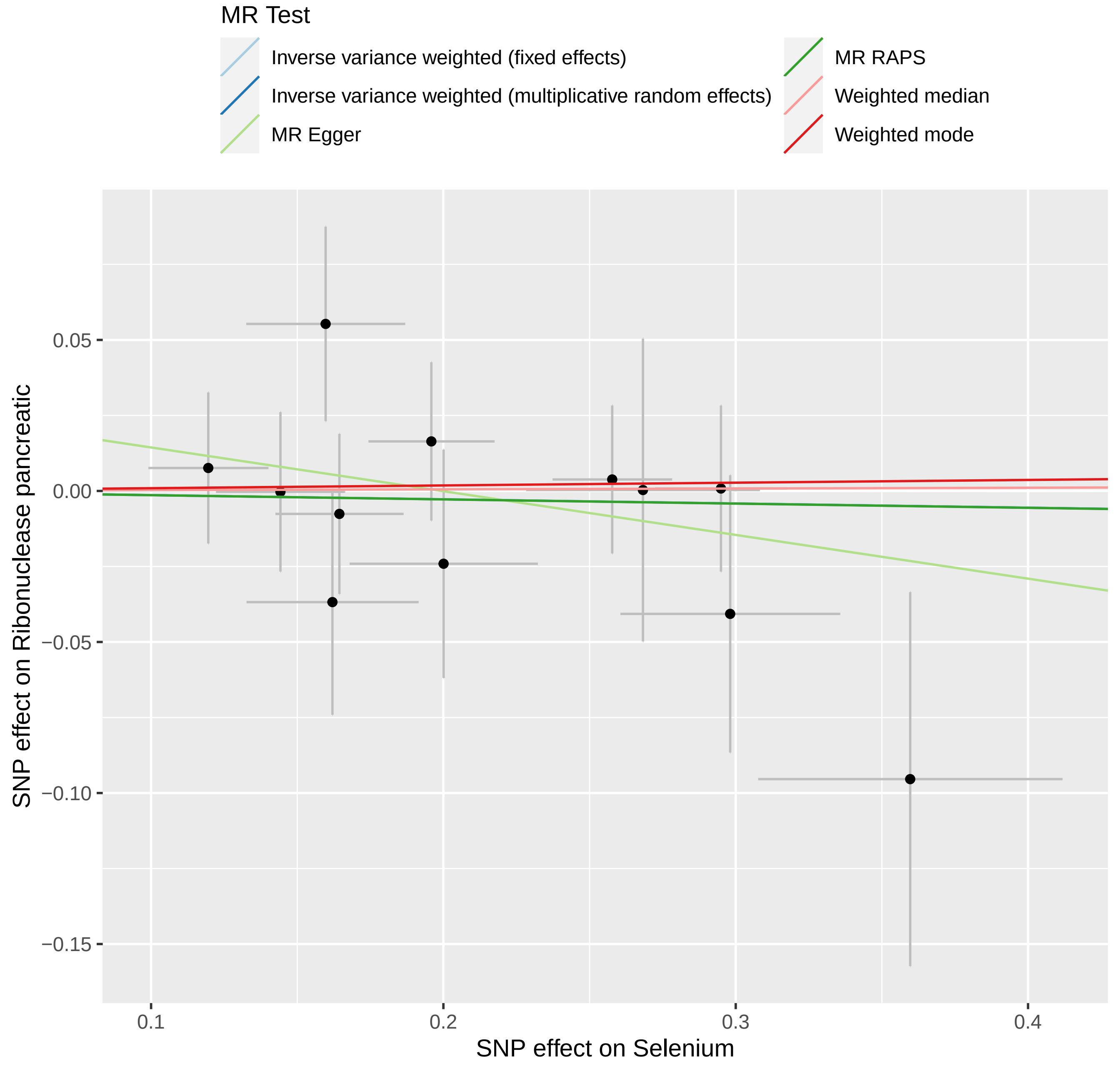

Batch 3−2555 : Selenium on Non−secretory ribonuclease

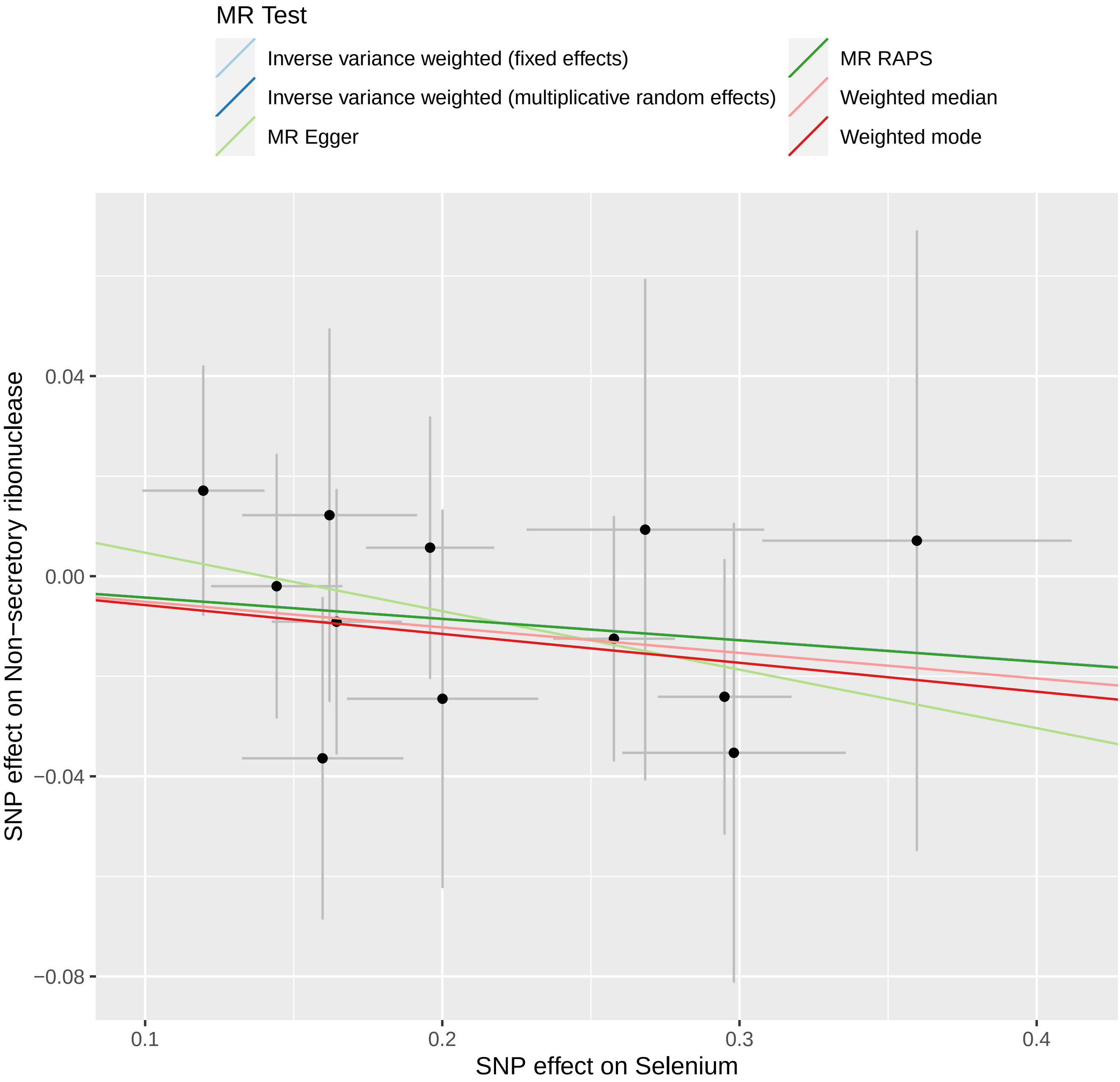

Batch 3−2556 : Selenium on Ribonuclease 4

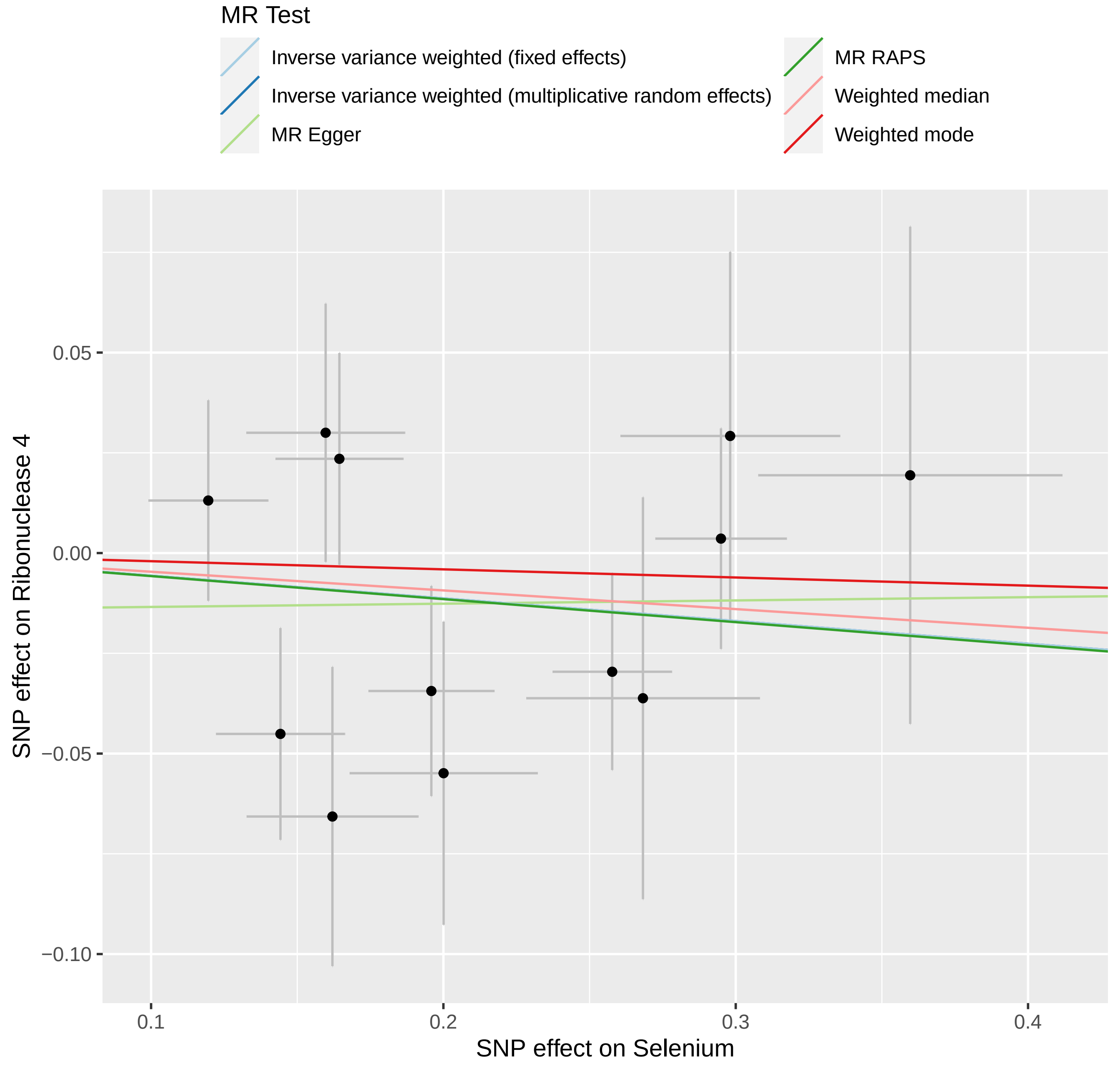

Batch 3−2557 : Selenium on Ribonuclease K6

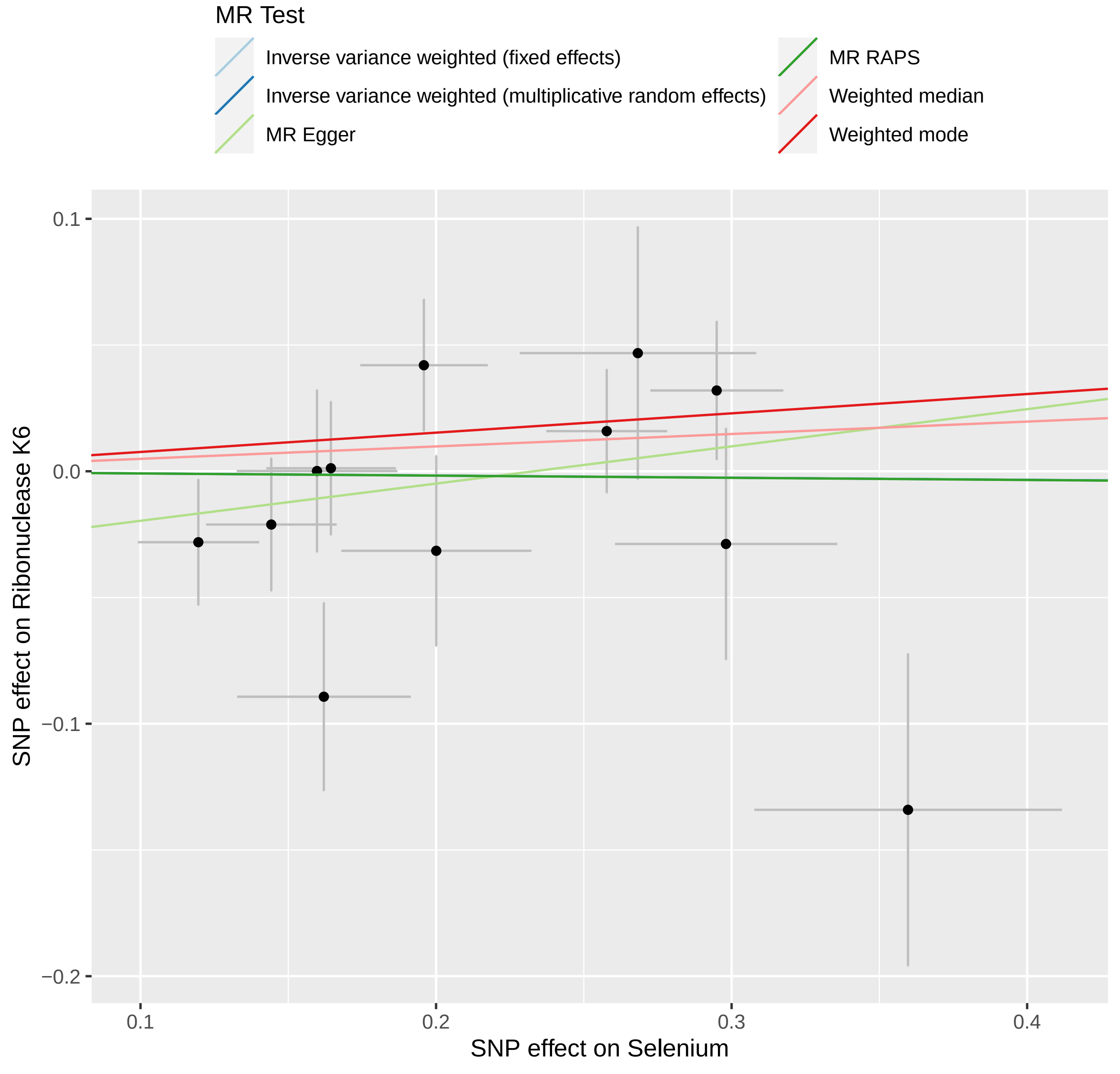

Batch 3−2558 : Selenium on Ribonuclease H1

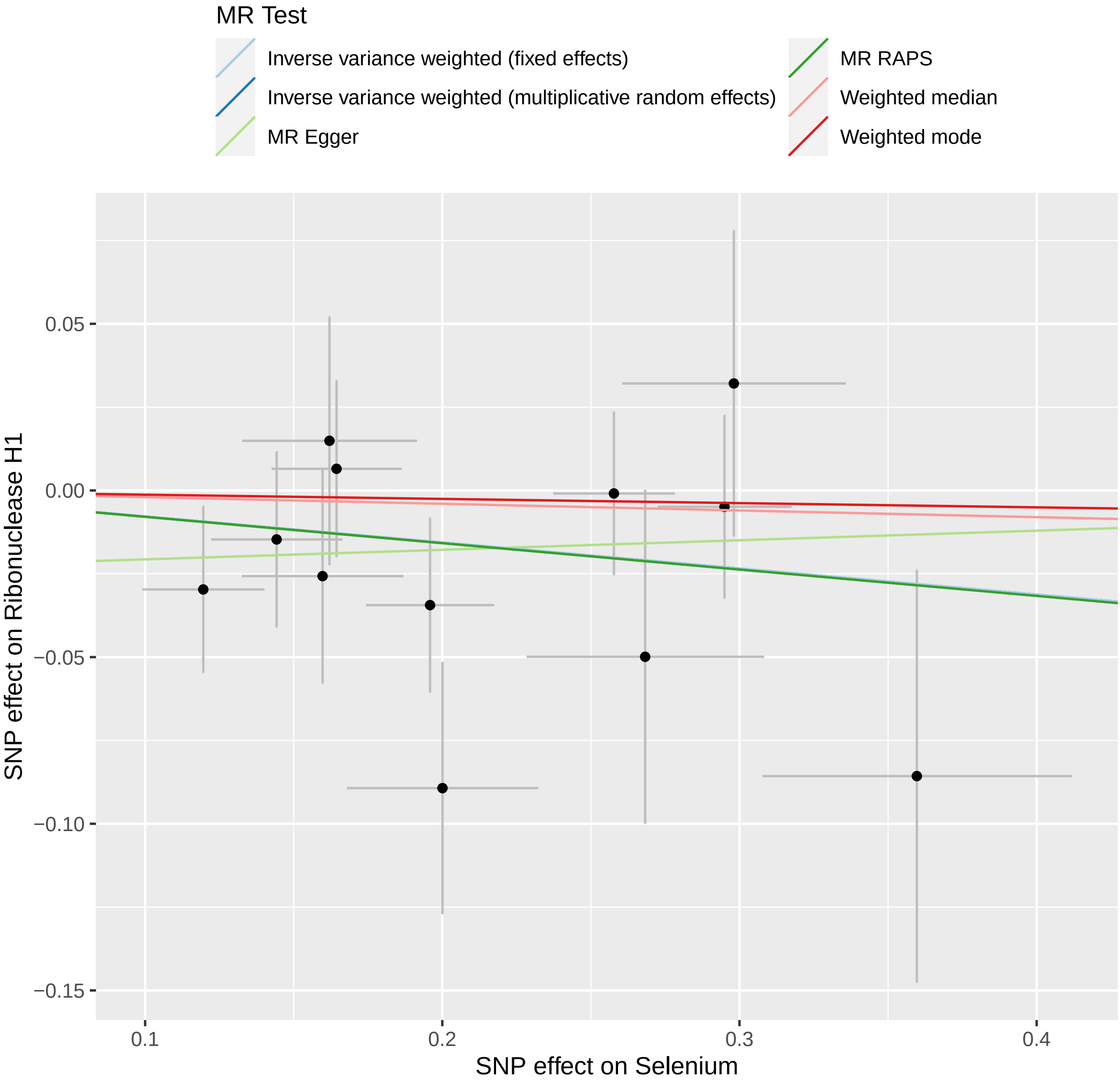

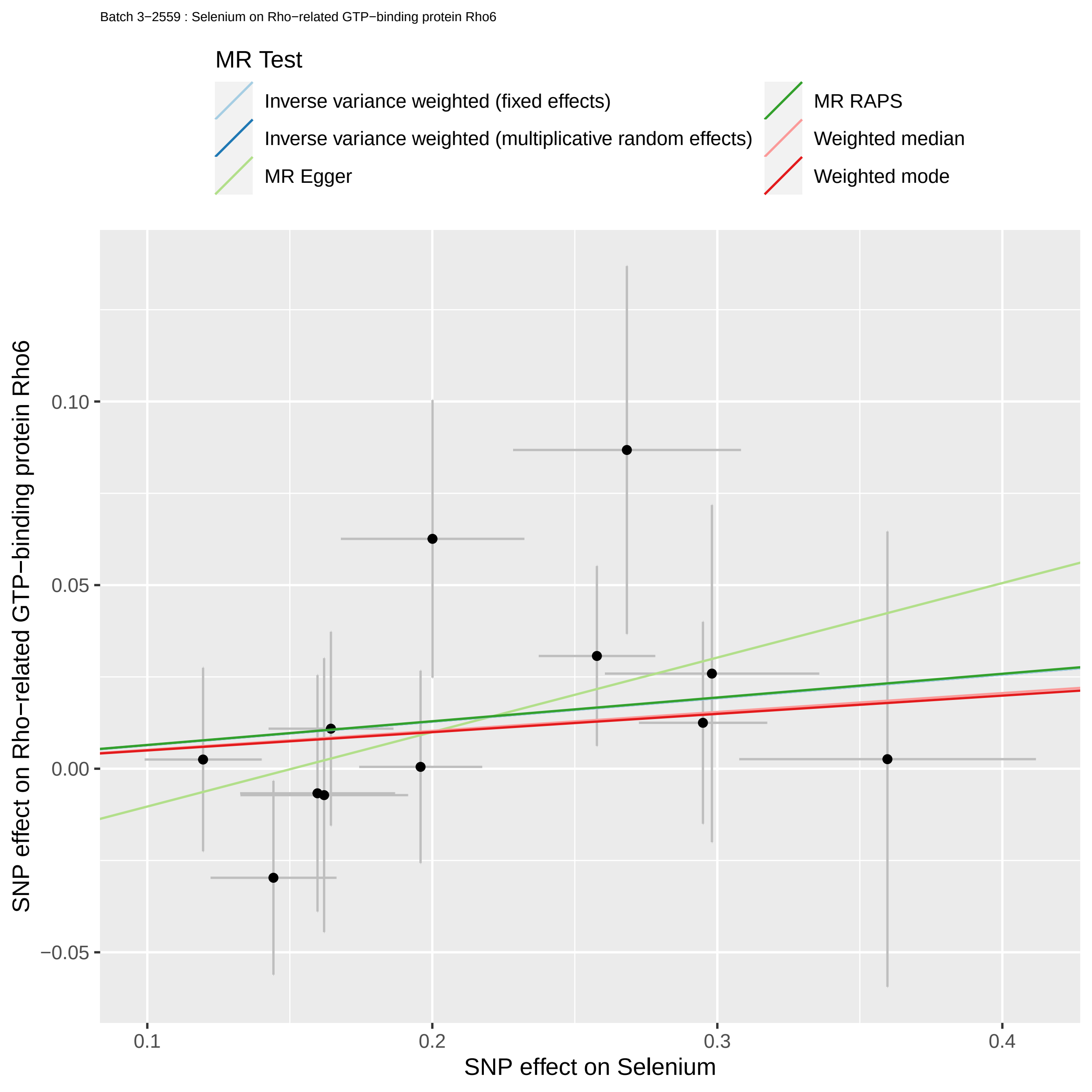
Batch 3−2559 : Selenium on Rho−related GTP−binding protein Rho6
MR Test
Inverse variance weighted (fixed effects)
Inverse variance weighted (multiplicative random effects)
MR Egger
MR RAPS
Weighted median
Weighted mode
SNP effect on Rho−related GTP−binding protein Rho6
0.10
0.05
0.00
−0.05
0.1
0.2
0.3
0.4
SNP effect on Selenium

Batch 3−2560 : Selenium on E3 ubiquitin−protein ligase RNF114

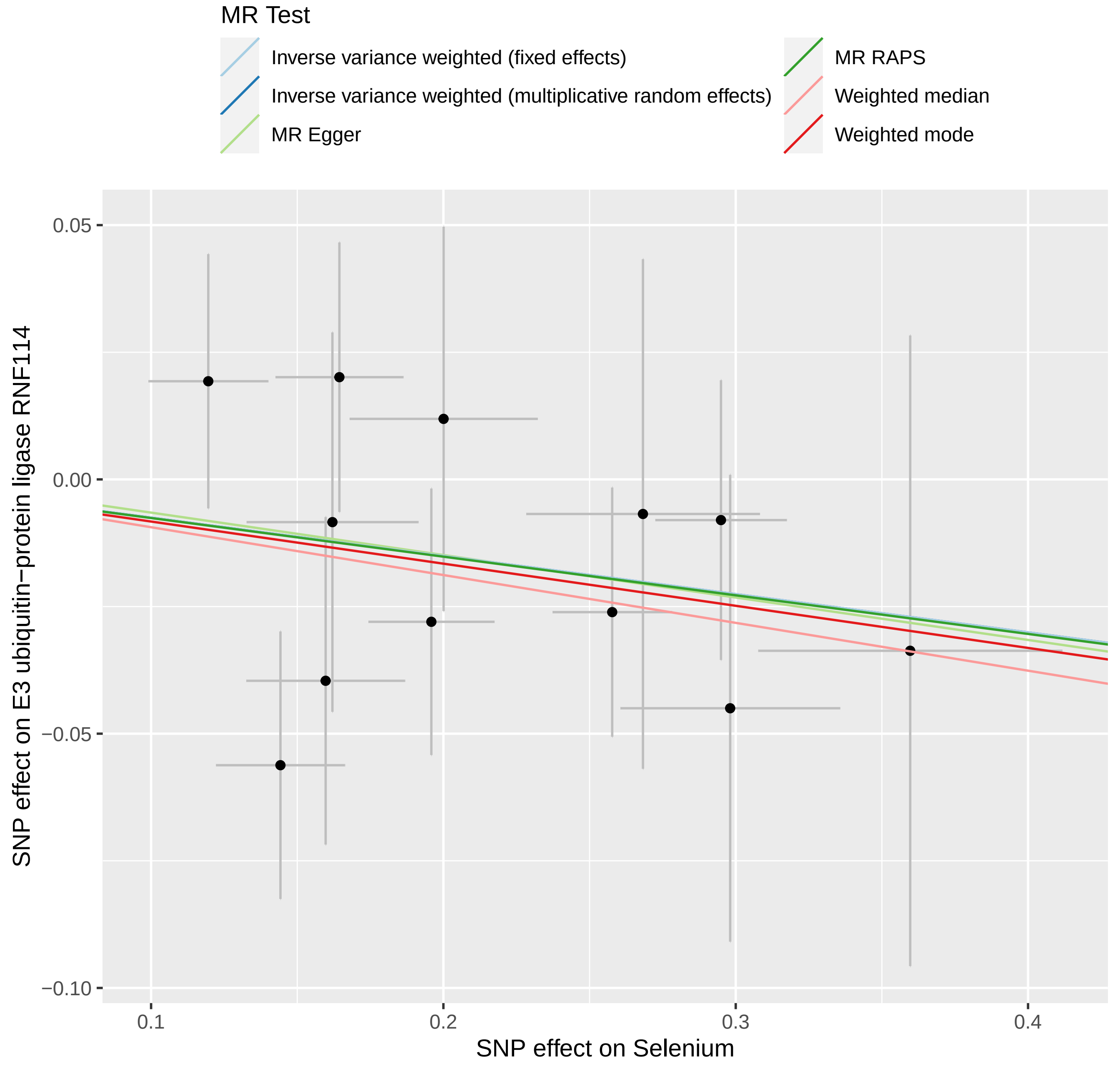

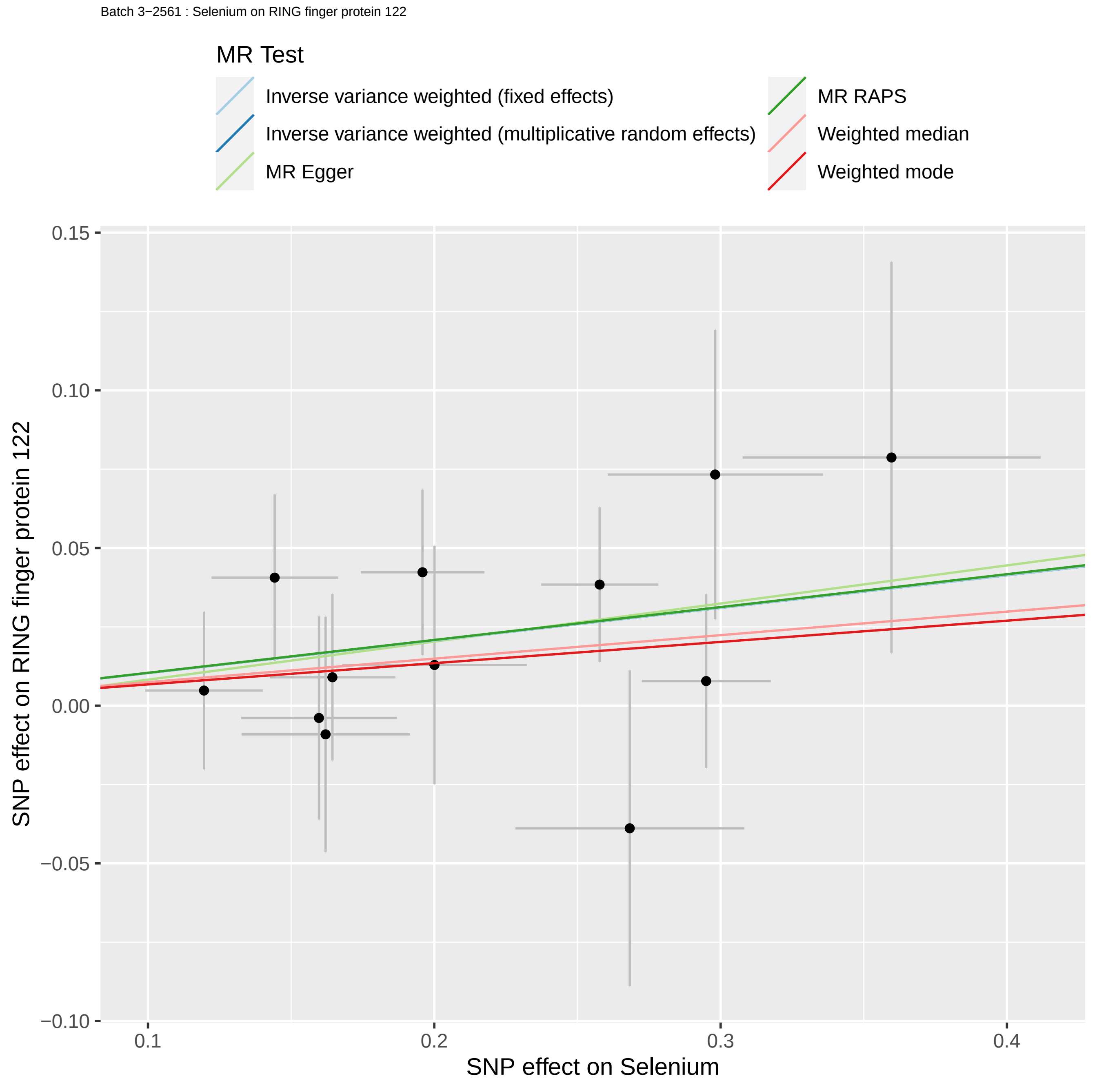

Batch 3−2561 : Selenium on RING finger protein 122
MR Test
Inverse variance weighted (fixed effects)
Inverse variance weighted (multiplicative random effects)
MR Egger
MR RAPS
Weighted median
Weighted mode
0.15
0.10
0.05
0.00
−0.05
−0.10
0.1
0.2
0.3
0.4
SNP effect on RING finger protein 122
SNP effect on Selenium

Batch 3−2562 : Selenium on E3 ubiquitin−protein ligase RNF128

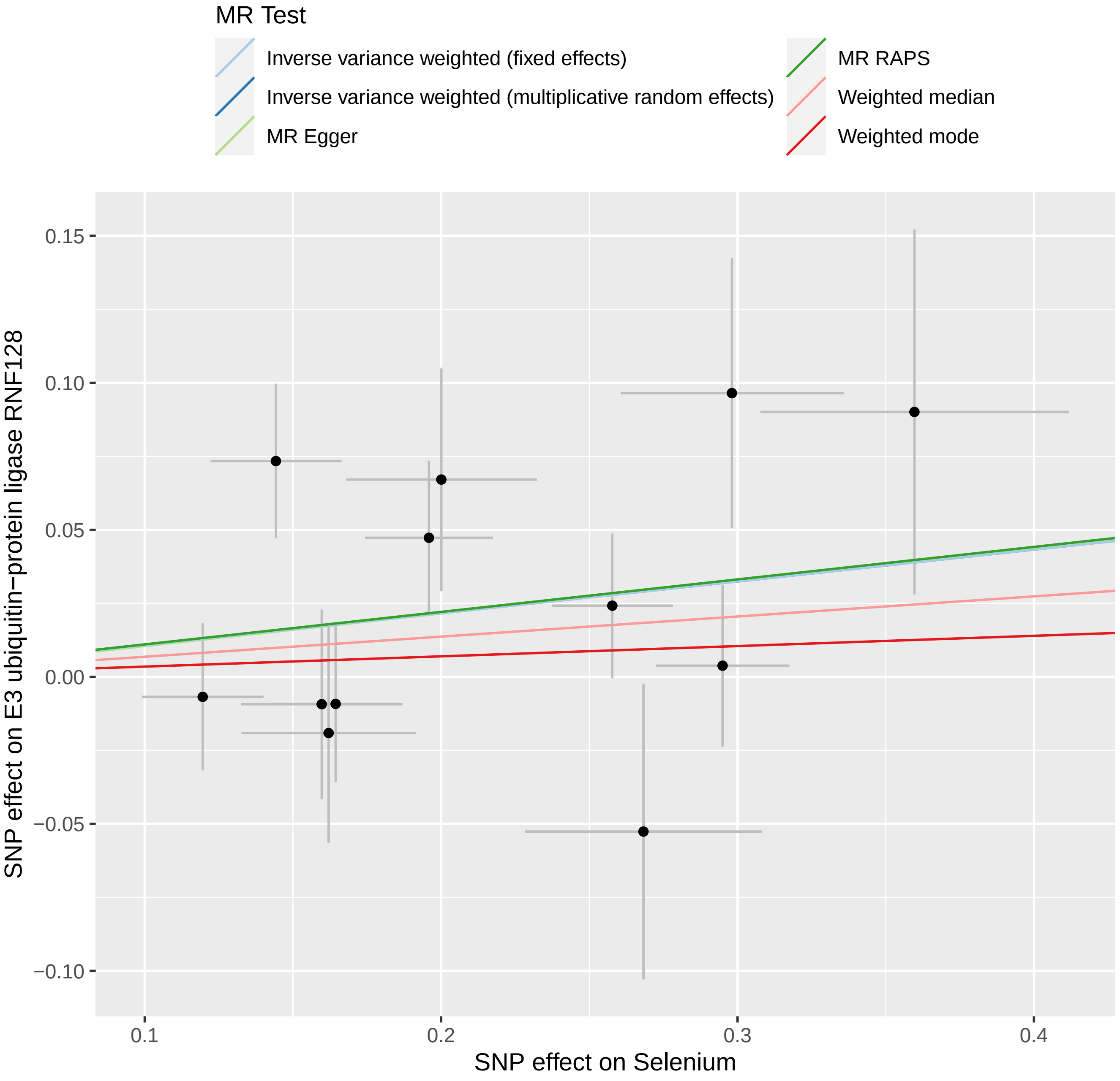

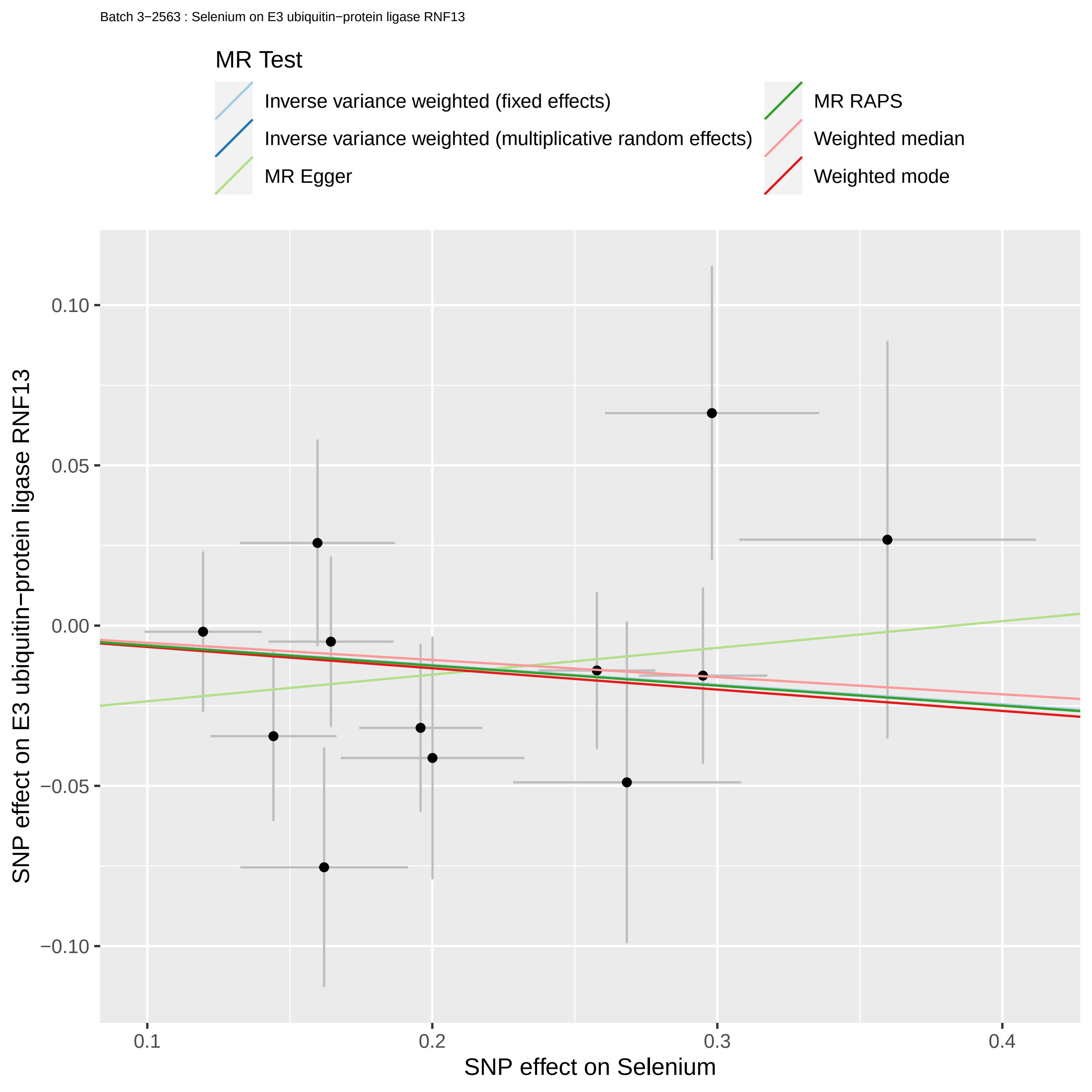
Batch 3−2563 : Selenium on E3 ubiquitin−protein ligase RNF13
MR Test
Inverse variance weighted (fixed effects)
Inverse variance weighted (multiplicative random effects)
MR Egger
MR RAPS
Weighted median
Weighted mode
0.10
0.05
0.00
−0.05
−0.10
0.1
0.2
0.3
0.4
SNP effect on Selenium
SNP effect on E3 ubiquitin−protein ligase RNF13

Batch 3−2564 : Selenium on RING finger protein 148

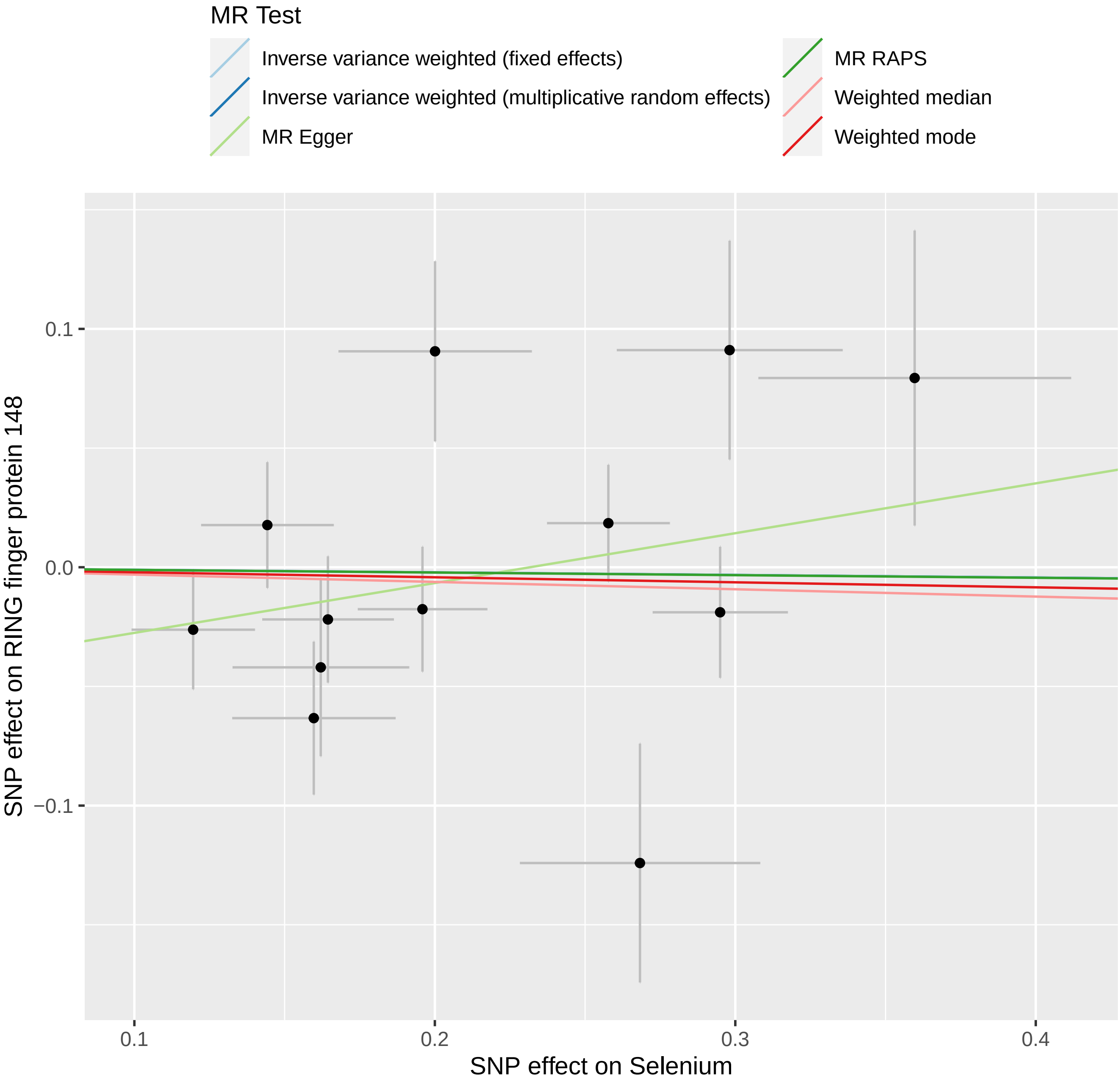

Batch 3−2565 : Selenium on RING finger protein 148

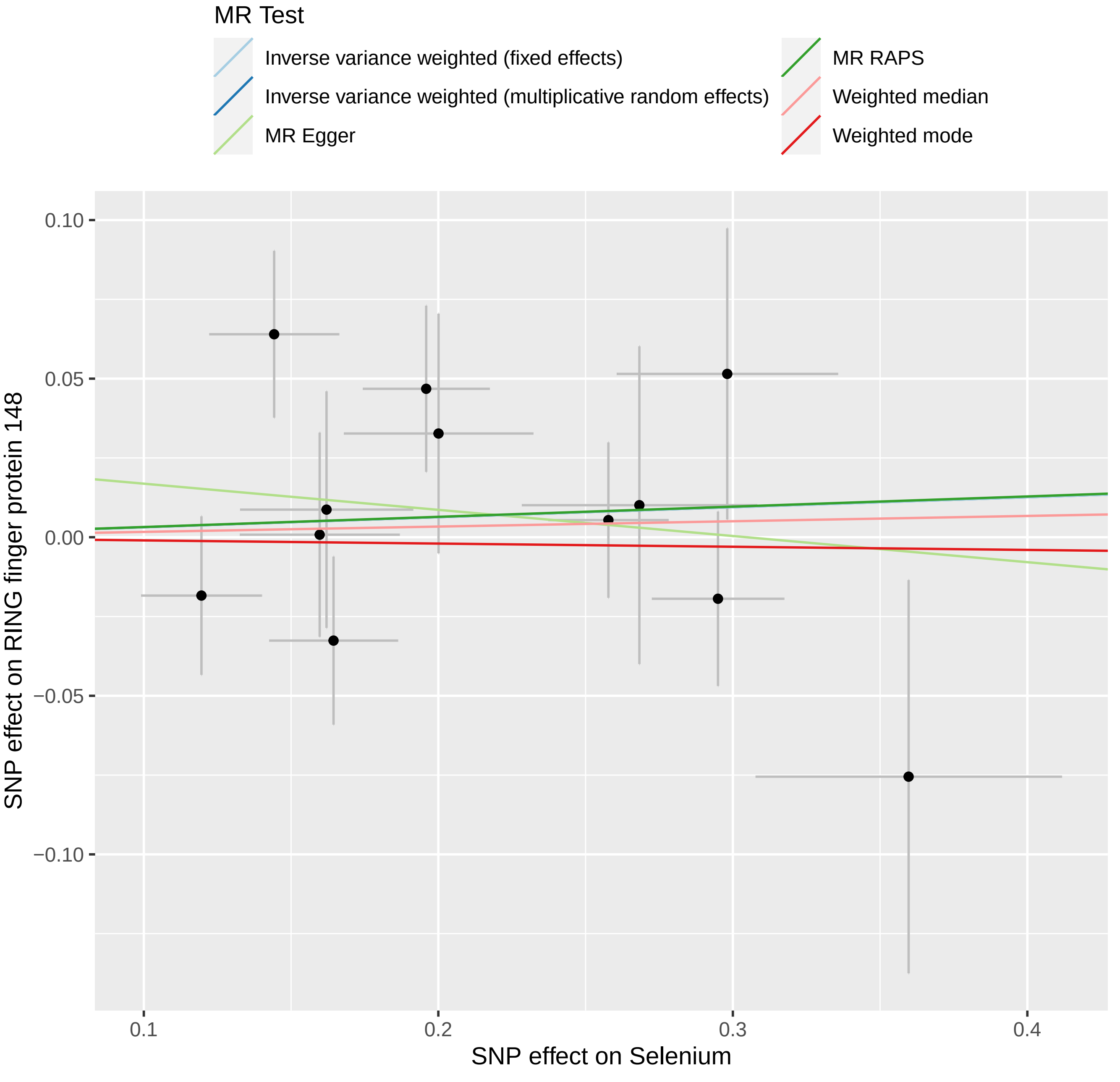

Batch 3−2566 : Selenium on E3 ubiquitin−protein ligase RNF149

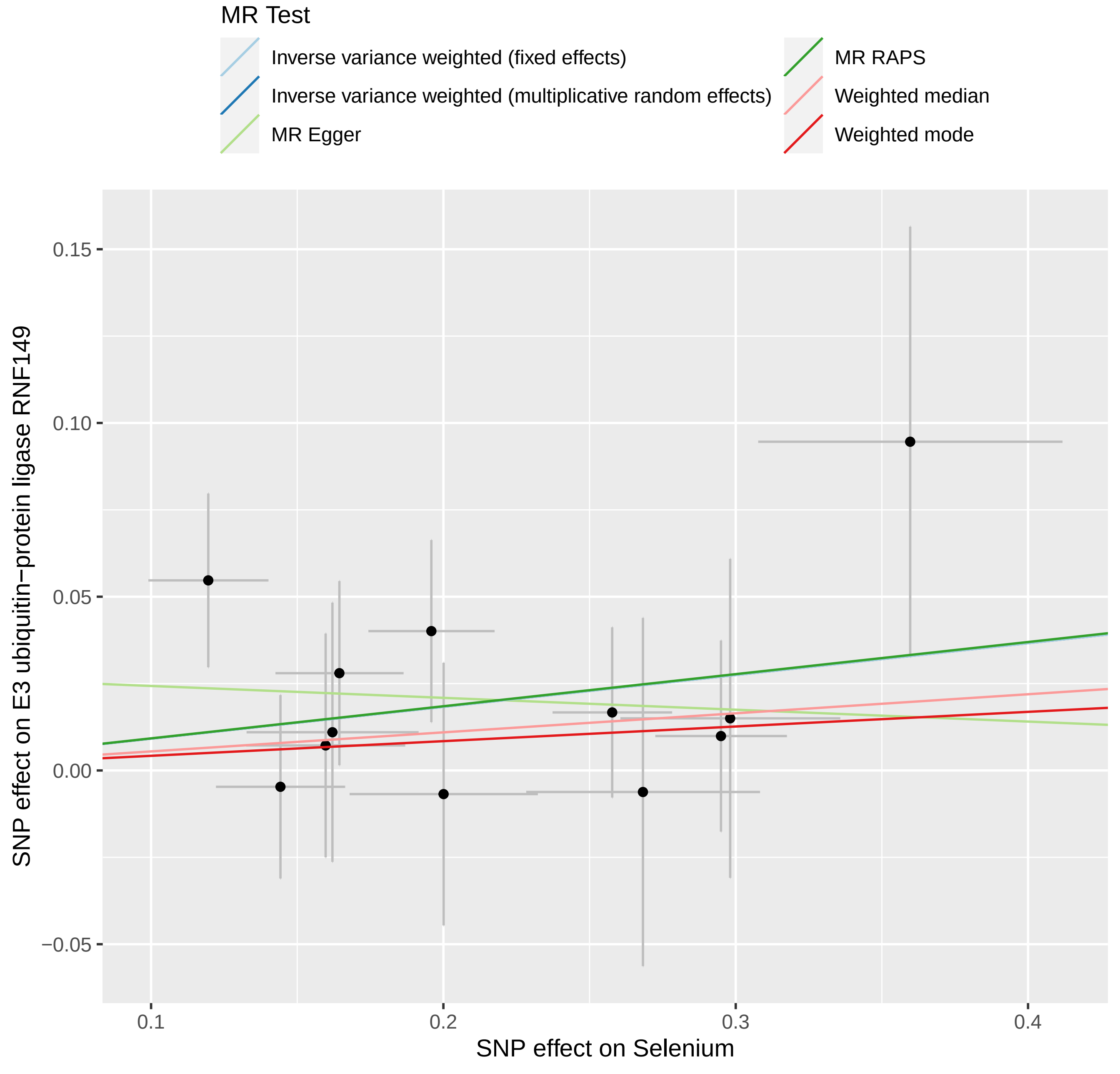

Batch 3−2567 : Selenium on RING finger protein 150

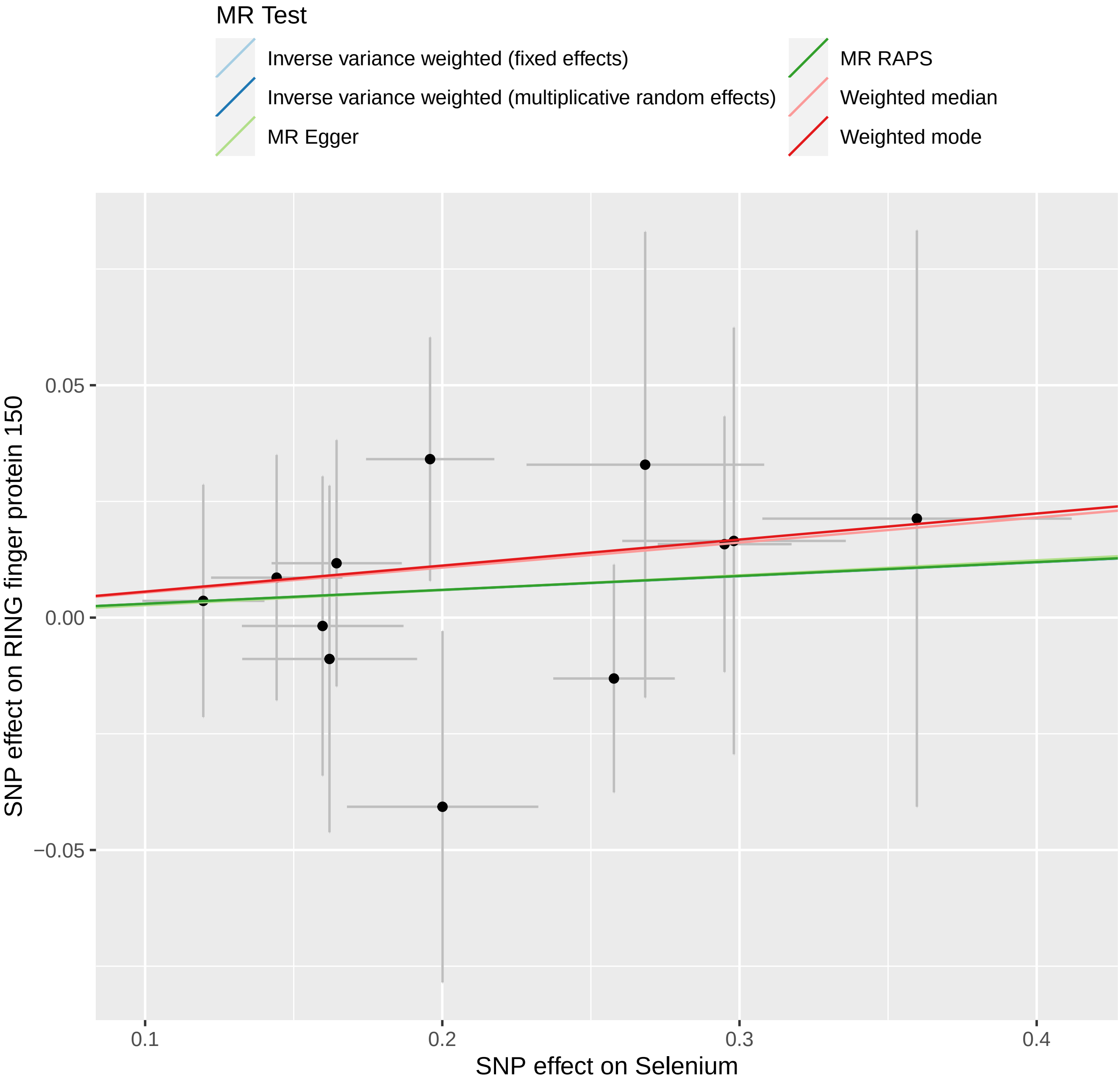

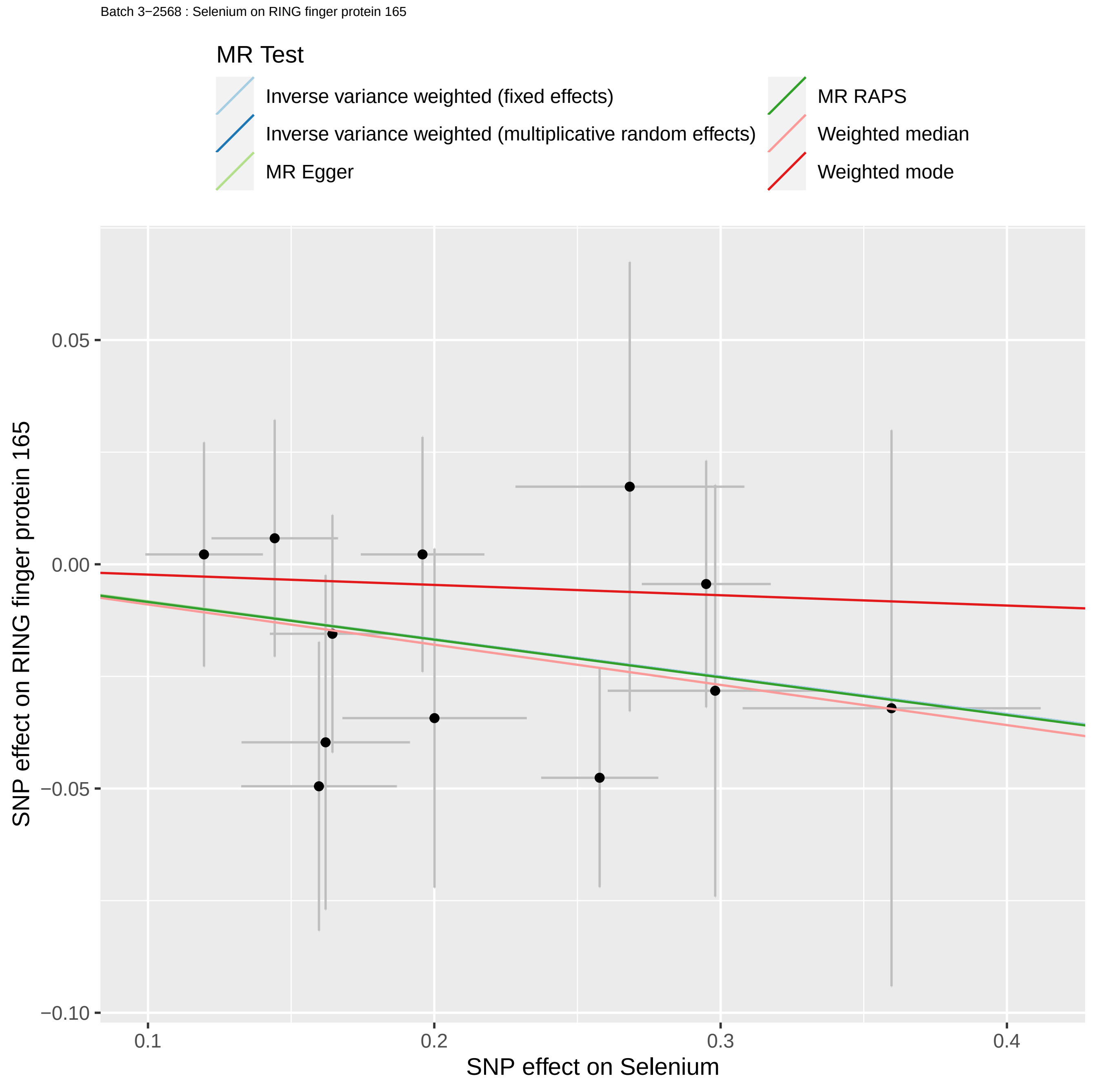

Batch 3−2568 : Selenium on RING finger protein 165
MR Test
Inverse variance weighted (fixed effects)
Inverse variance weighted (multiplicative random effects)
MR Egger
MR RAPS
Weighted median
Weighted mode
0.05
0.00
−0.05
−0.10
0.1
0.2
0.3
0.4
SNP effect on RING finger protein 165
SNP effect on Selenium

Batch 3−2569 : Selenium on RING finger protein 215

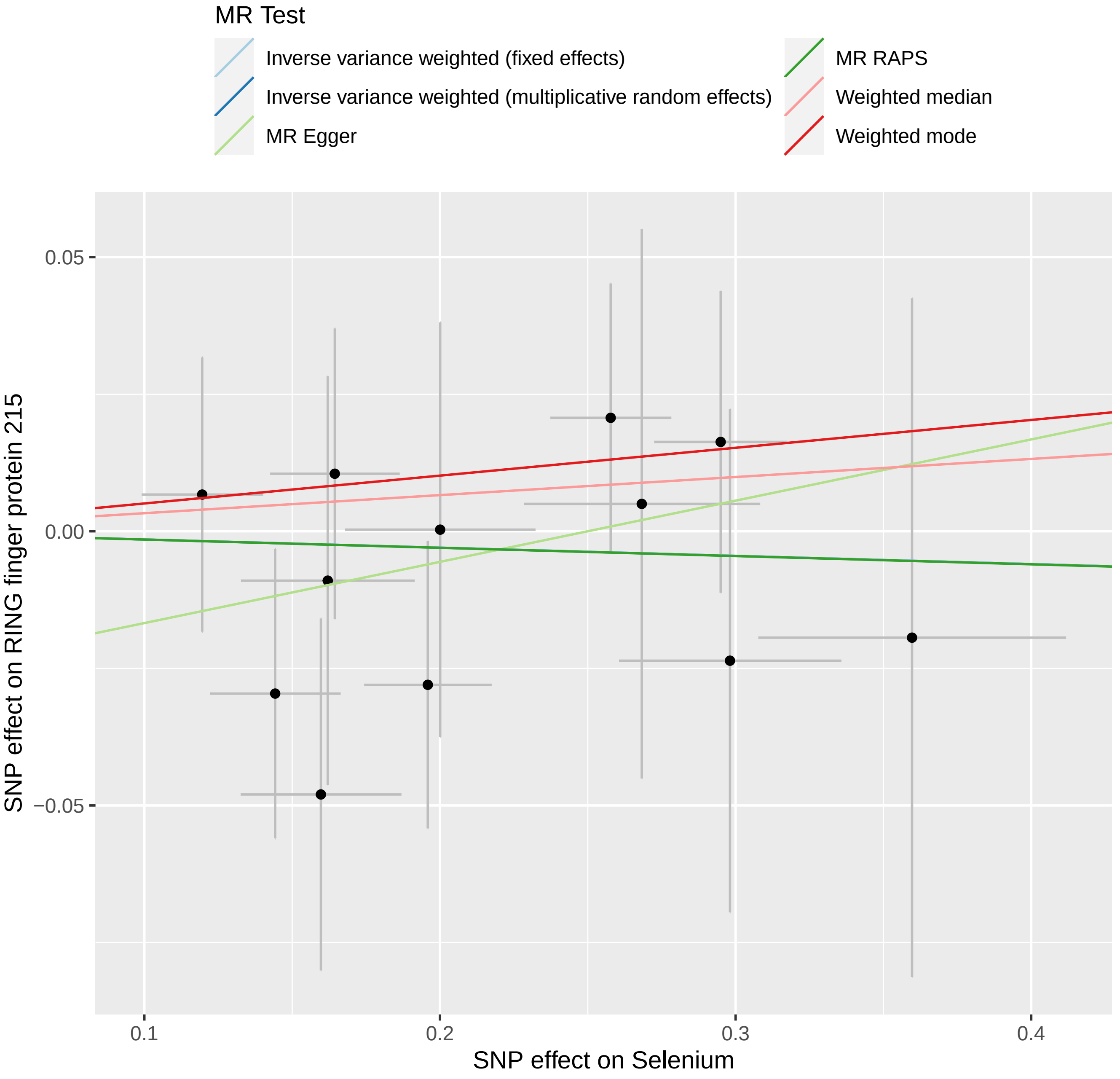

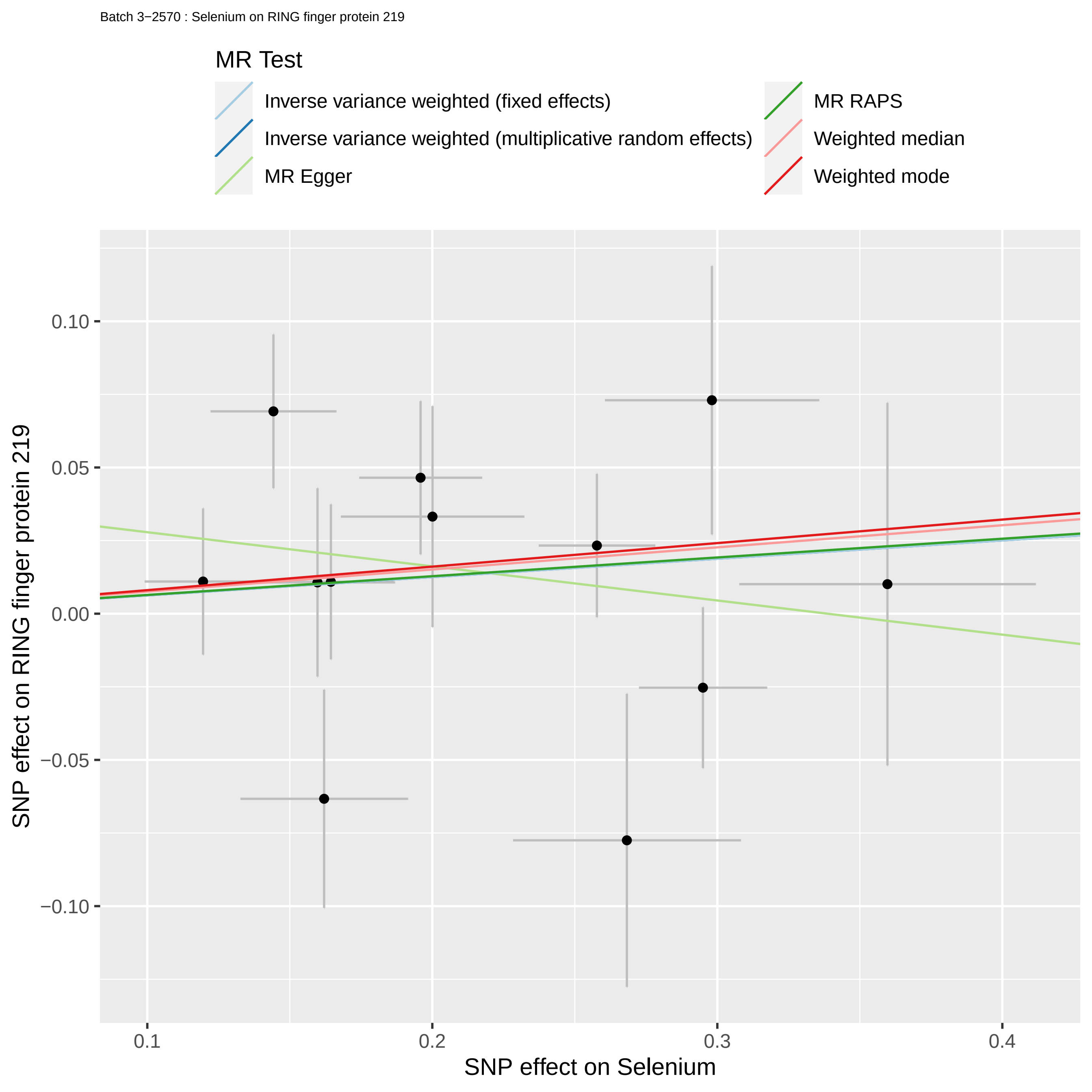

Batch 3−2570 : Selenium on RING finger protein 219
MR Test
Inverse variance weighted (fixed effects)
Inverse variance weighted (multiplicative random effects)
MR Egger
MR RAPS
Weighted median
Weighted mode
0.10
0.05
0.00
−0.05
−0.10
0.1
0.2
0.3
0.4
SNP effect on RING finger protein 219
SNP effect on Selenium

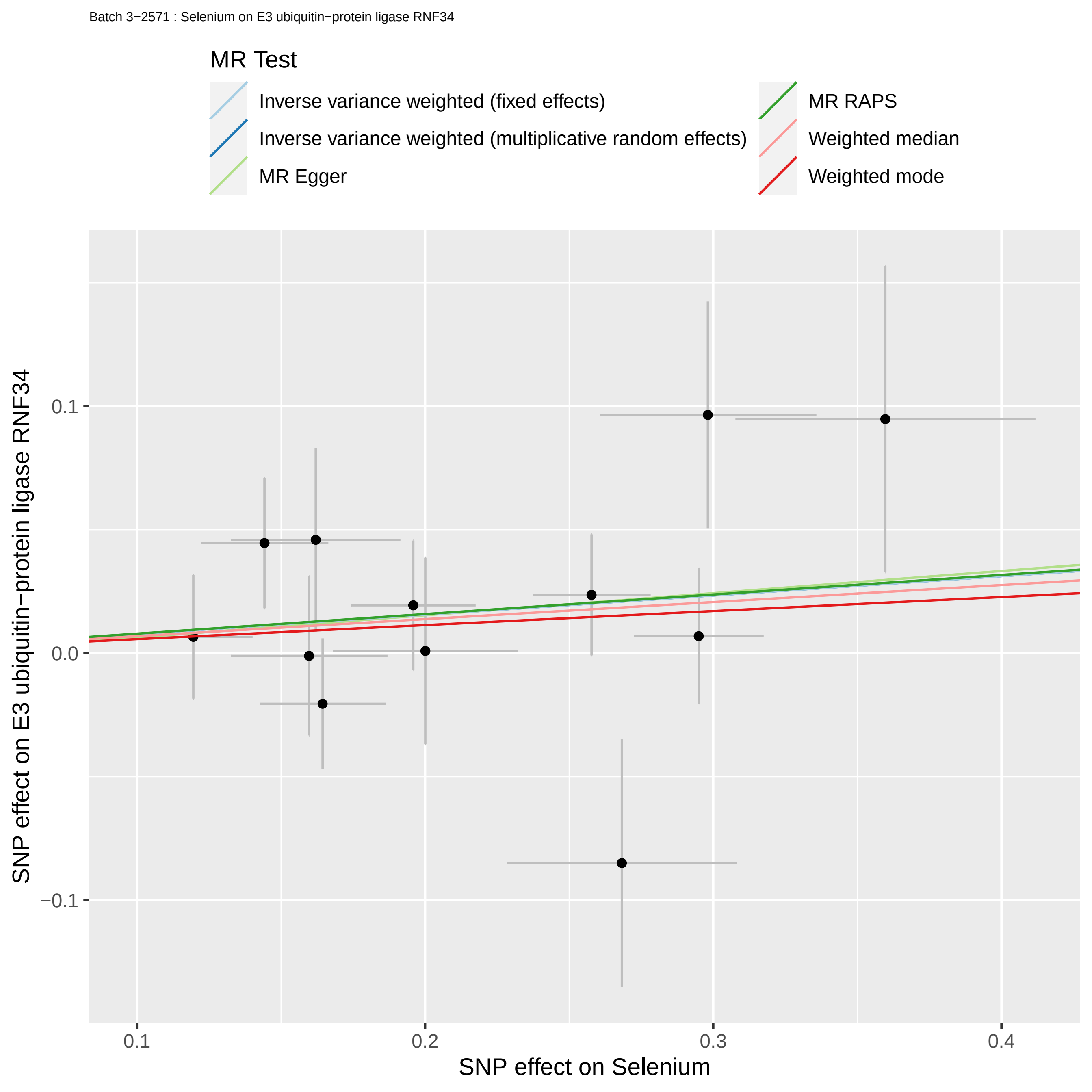
Batch 3−2571 : Selenium on E3 ubiquitin−protein ligase RNF34
MR Test
Inverse variance weighted (fixed effects)
Inverse variance weighted (multiplicative random effects)
MR Egger
MR RAPS
Weighted median
Weighted mode
SNP effect on E3 ubiquitin−protein ligase RNF34
0.1
0.0
−0.1
0.1
0.2
0.3
0.4
SNP effect on Selenium

Batch 3−2572 : Selenium on E3 ubiquitin−protein ligase RNF43

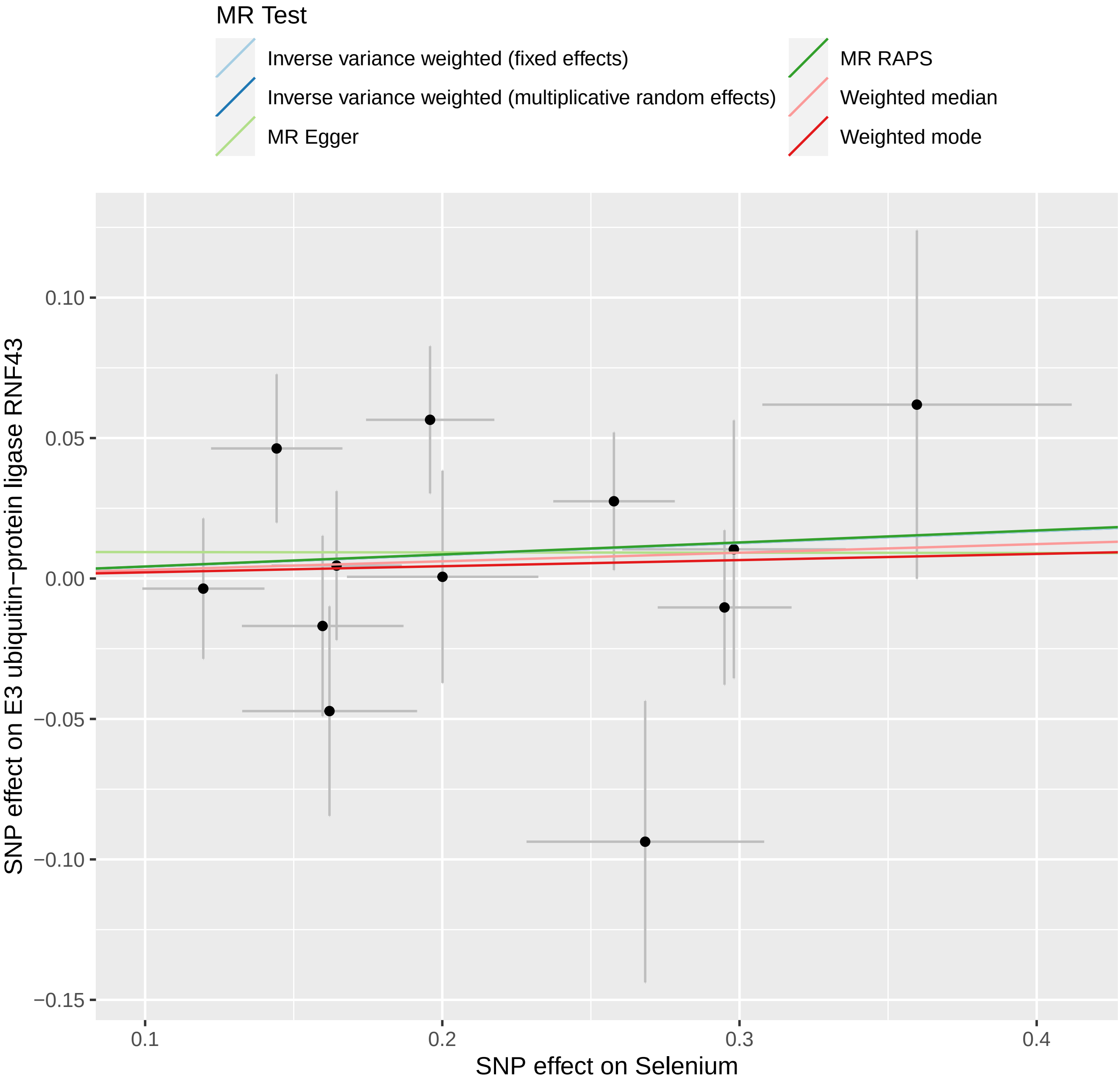

Batch 3−2573 : Selenium on E3 ubiquitin−protein ligase RNF8

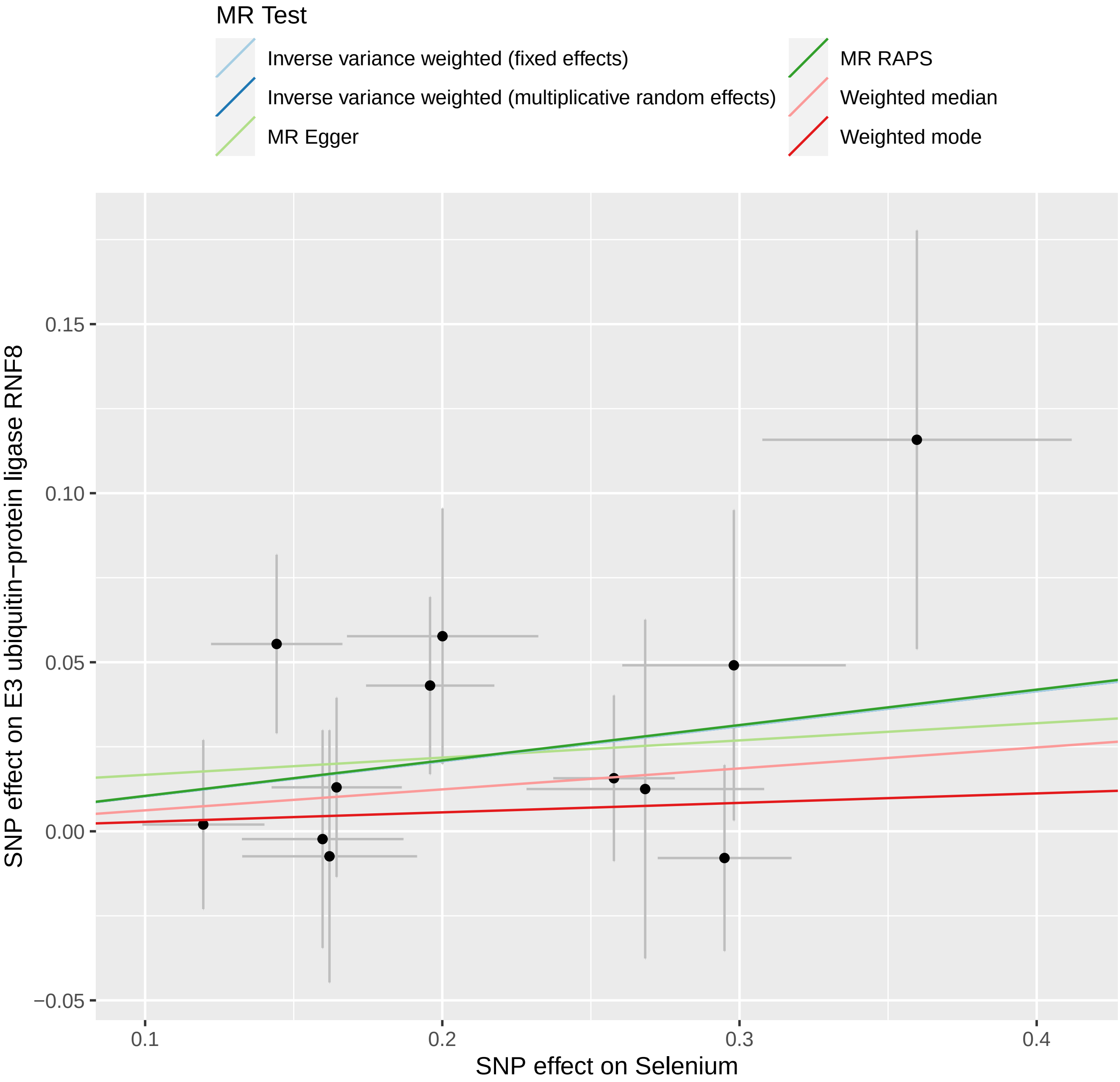

Batch 3−2574 : Selenium on mRNA−capping enzyme

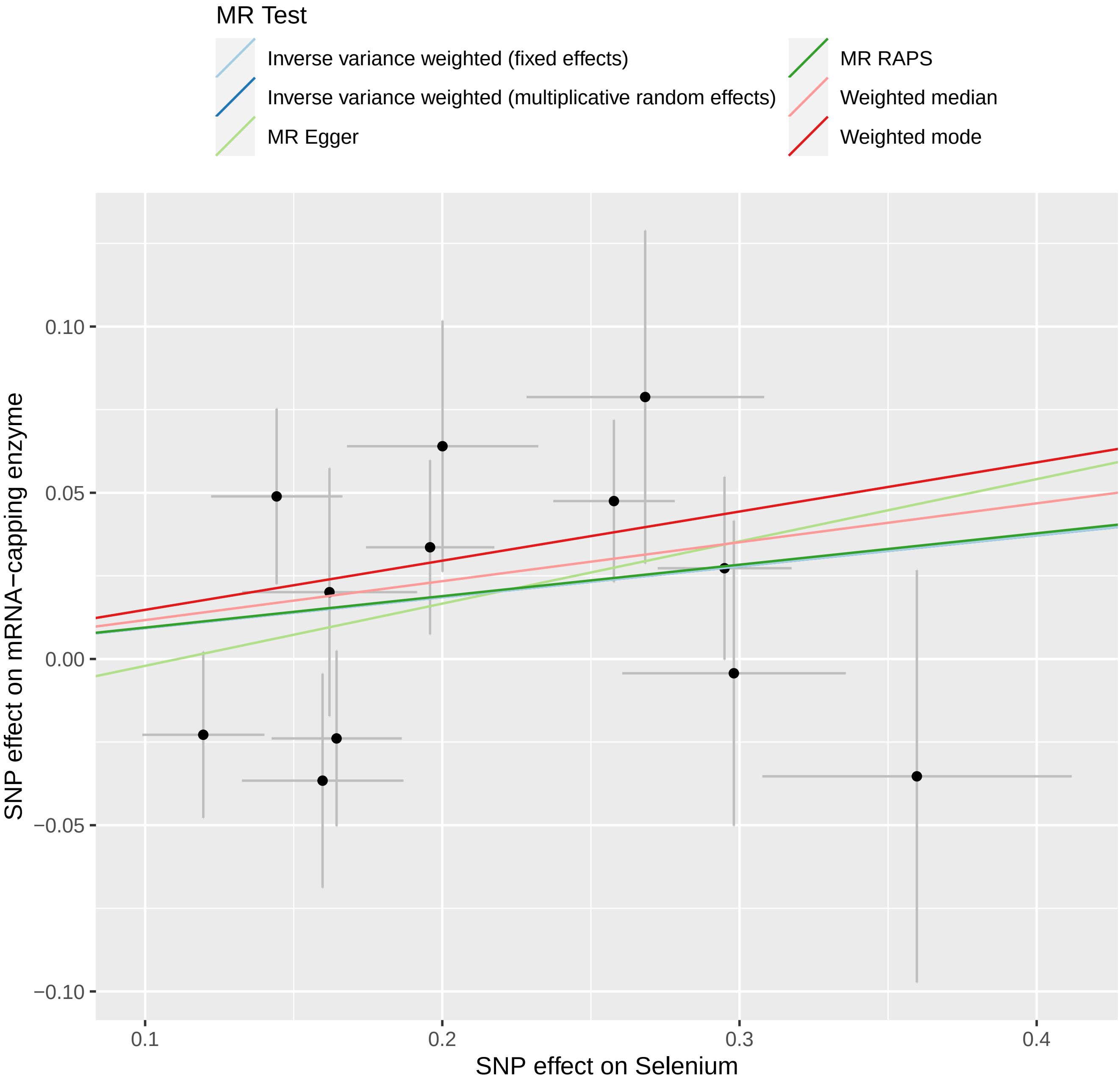

Batch 3−2575 : Selenium on rRNA methyltransferase 3, mitochondrial

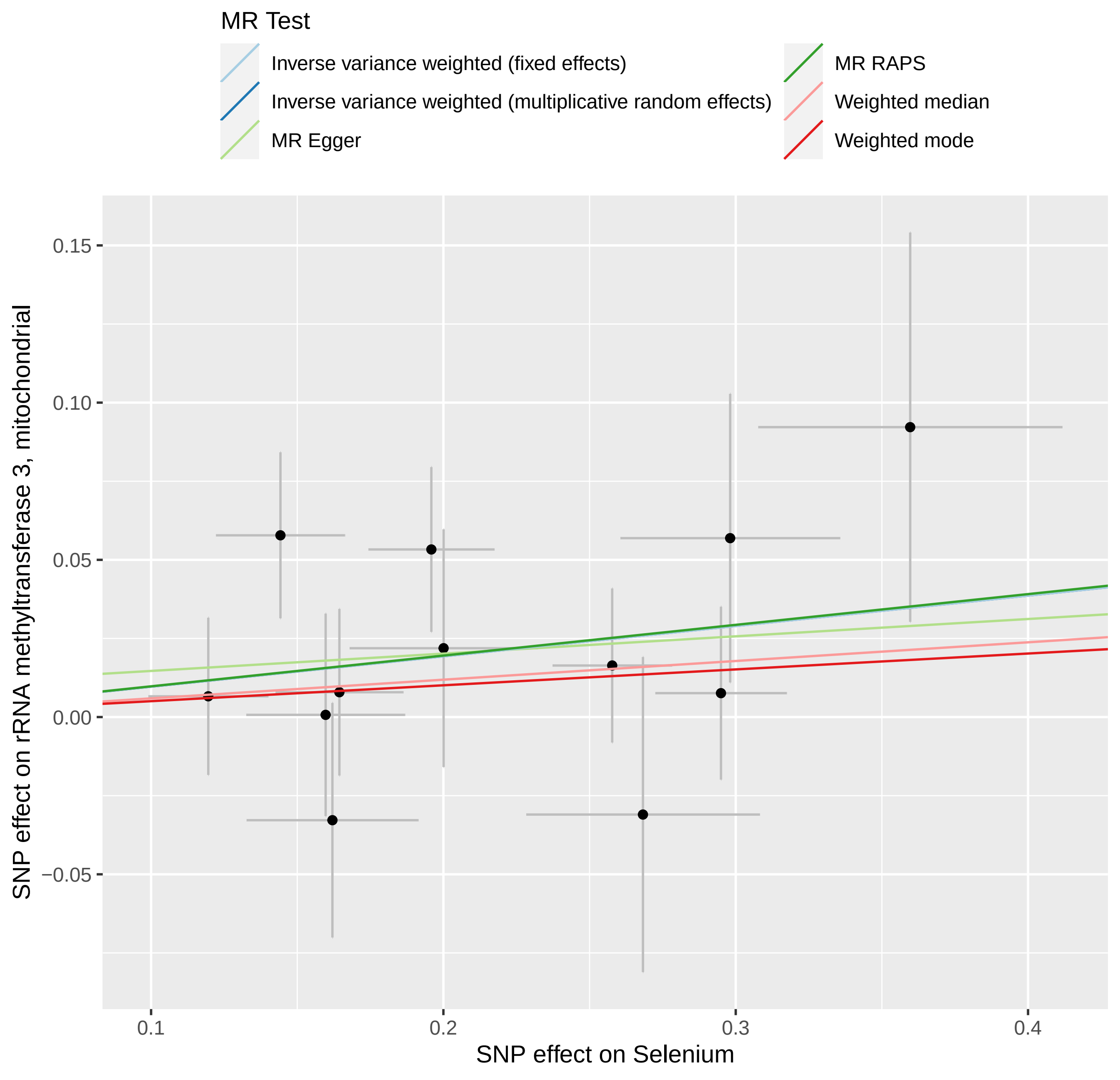

Batch 3−2576 : Selenium on RNA−binding protein 40

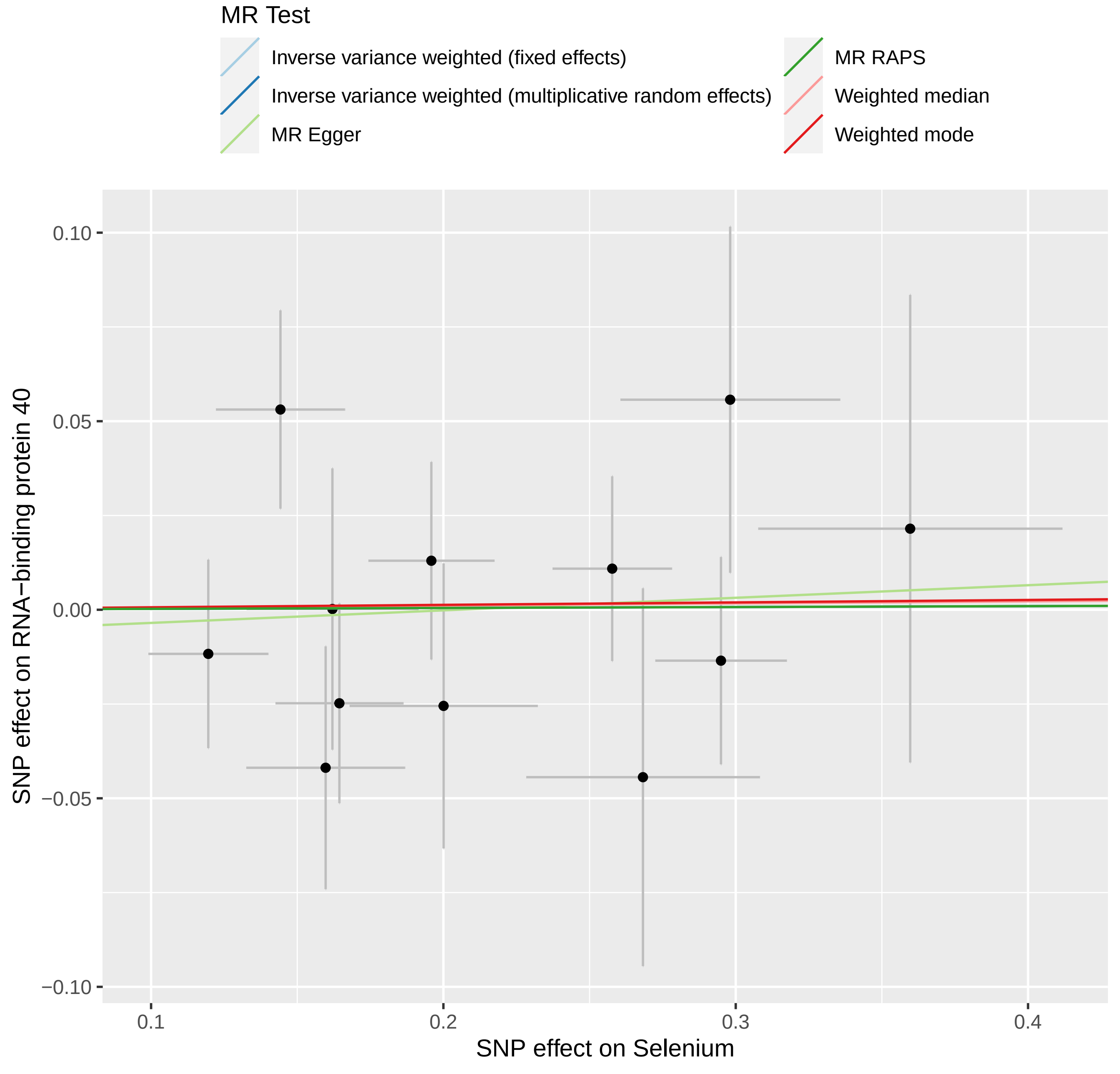

Batch 3−2577 : Selenium on Roundabout homolog 1

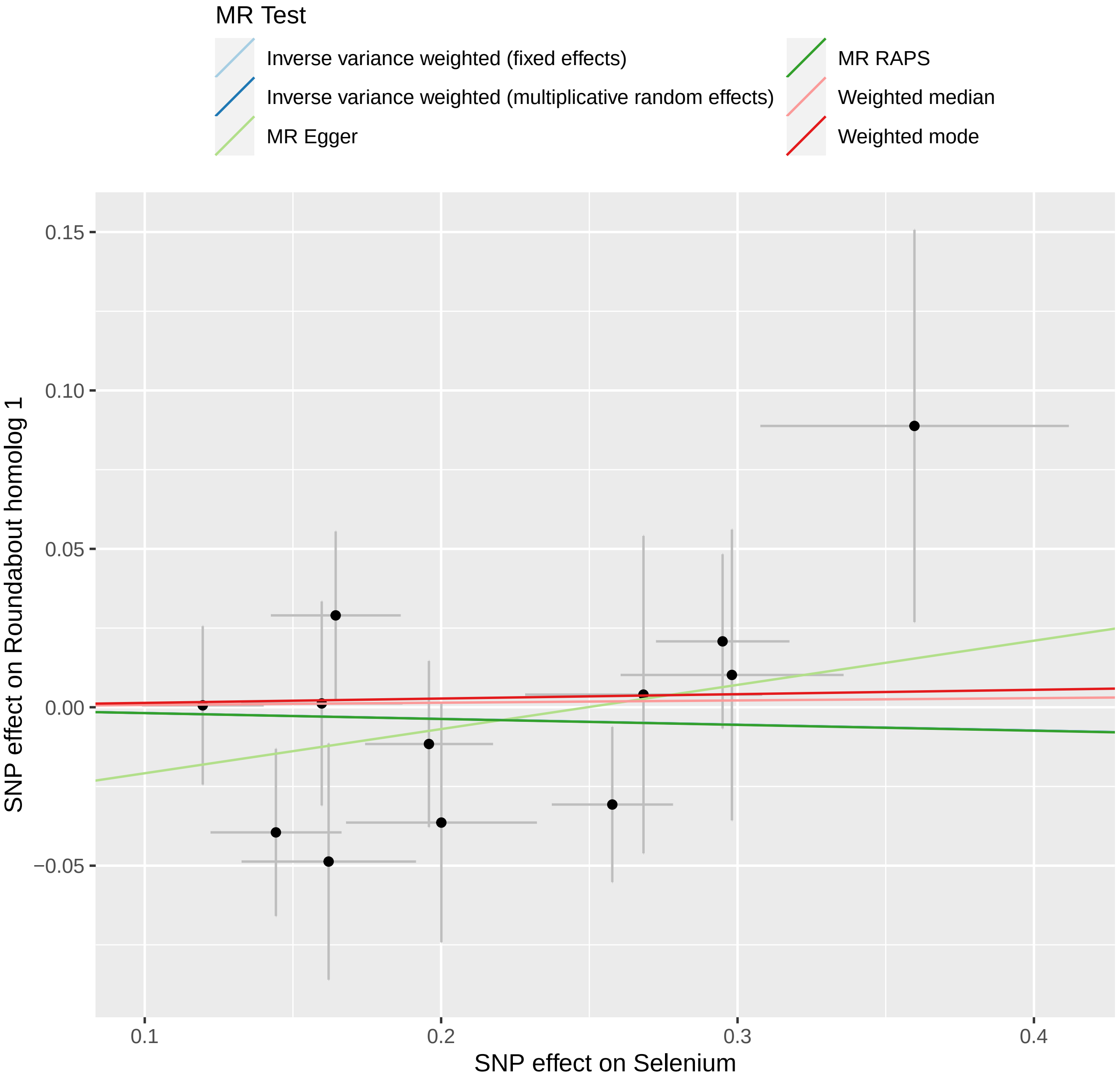

Batch 3−2578 : Selenium on Roundabout homolog 2

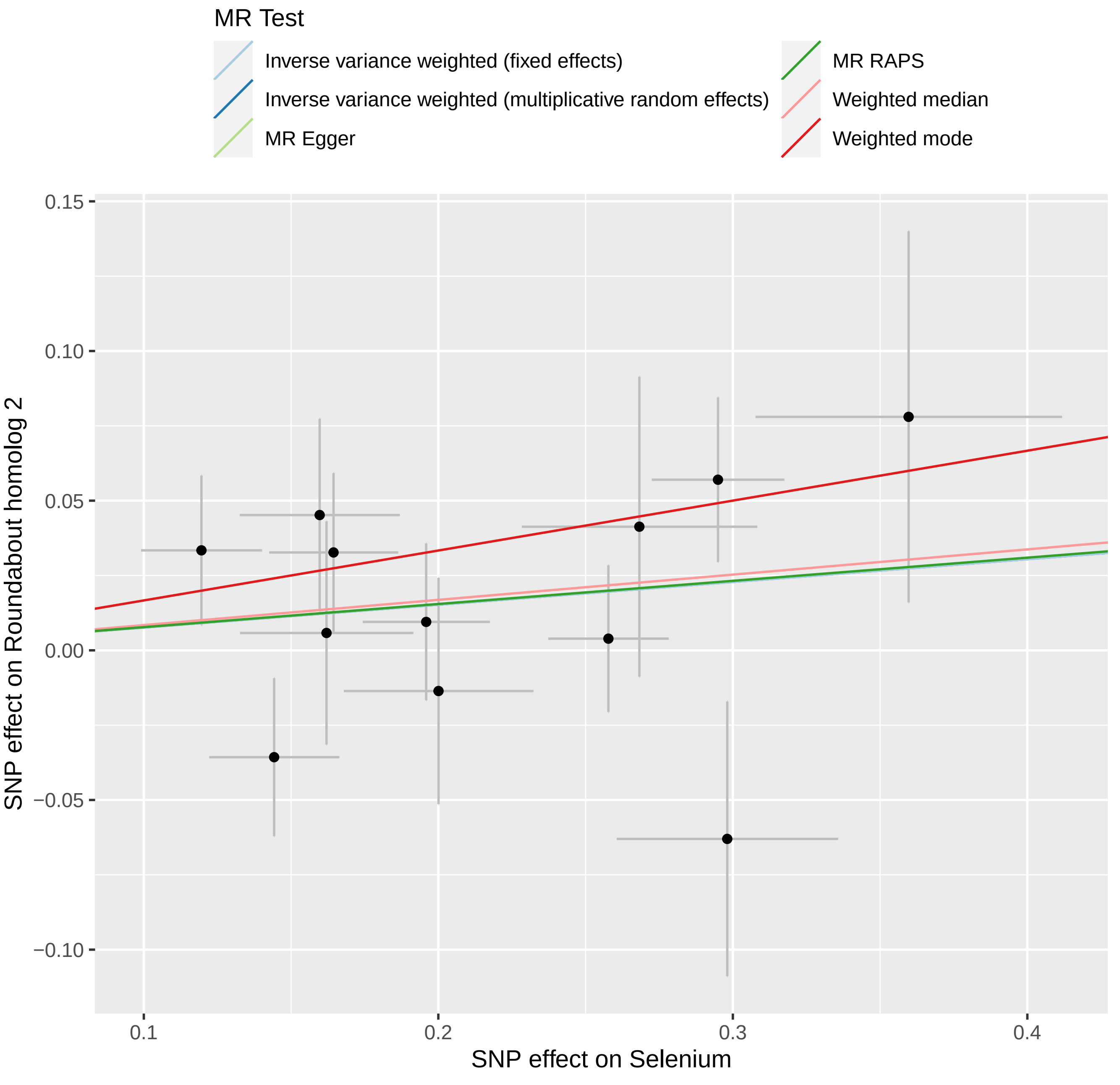

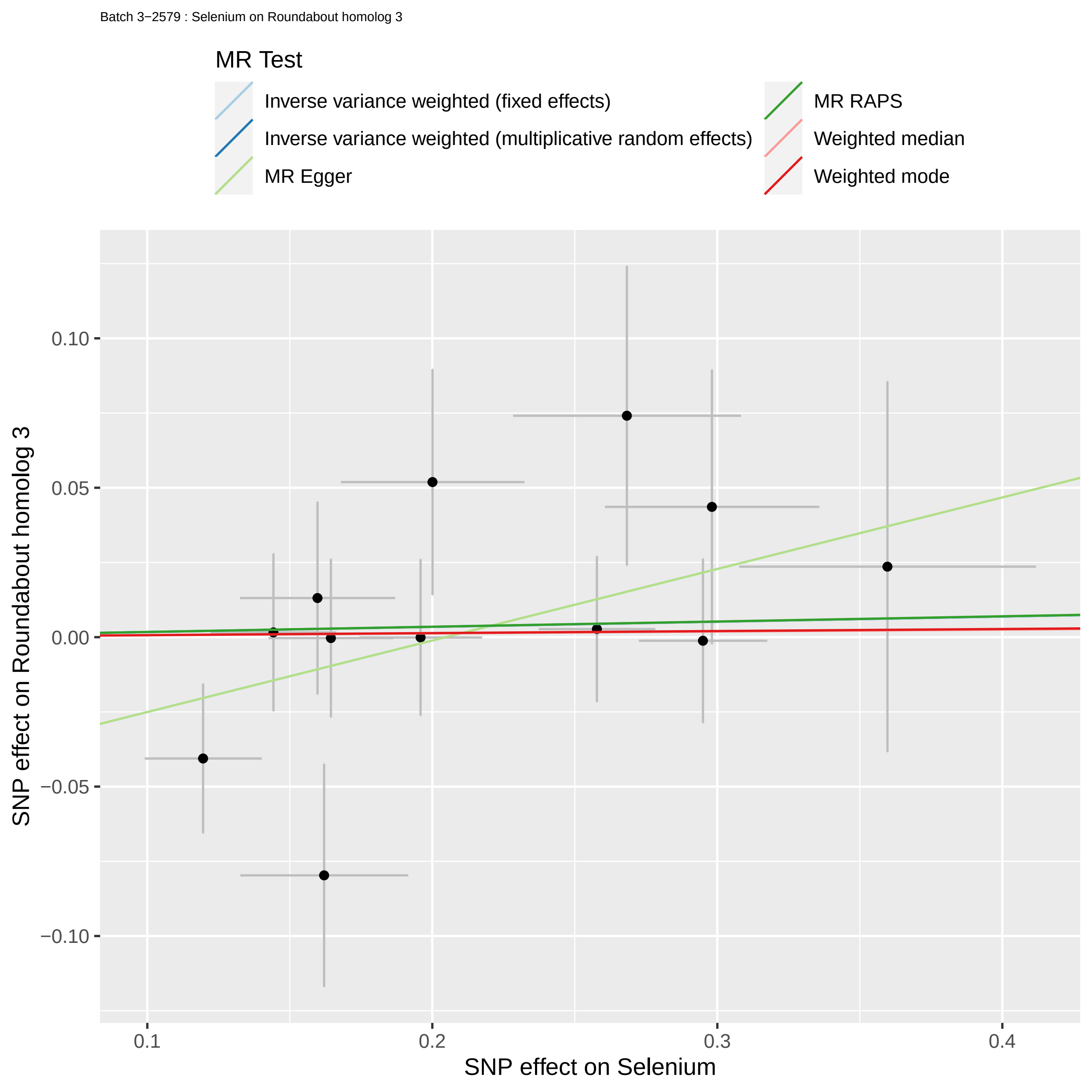
Batch 3−2579 : Selenium on Roundabout homolog 3
MR Test
Inverse variance weighted (fixed effects)
Inverse variance weighted (multiplicative random effects)
MR Egger
MR RAPS
Weighted median
Weighted mode
SNP effect on Roundabout homolog 3
0.10
0.05
0.00
−0.05
−0.10
0.1
0.2
0.3
0.4
SNP effect on Selenium

Batch 3−2580 : Selenium on Rho−associated protein kinase 2

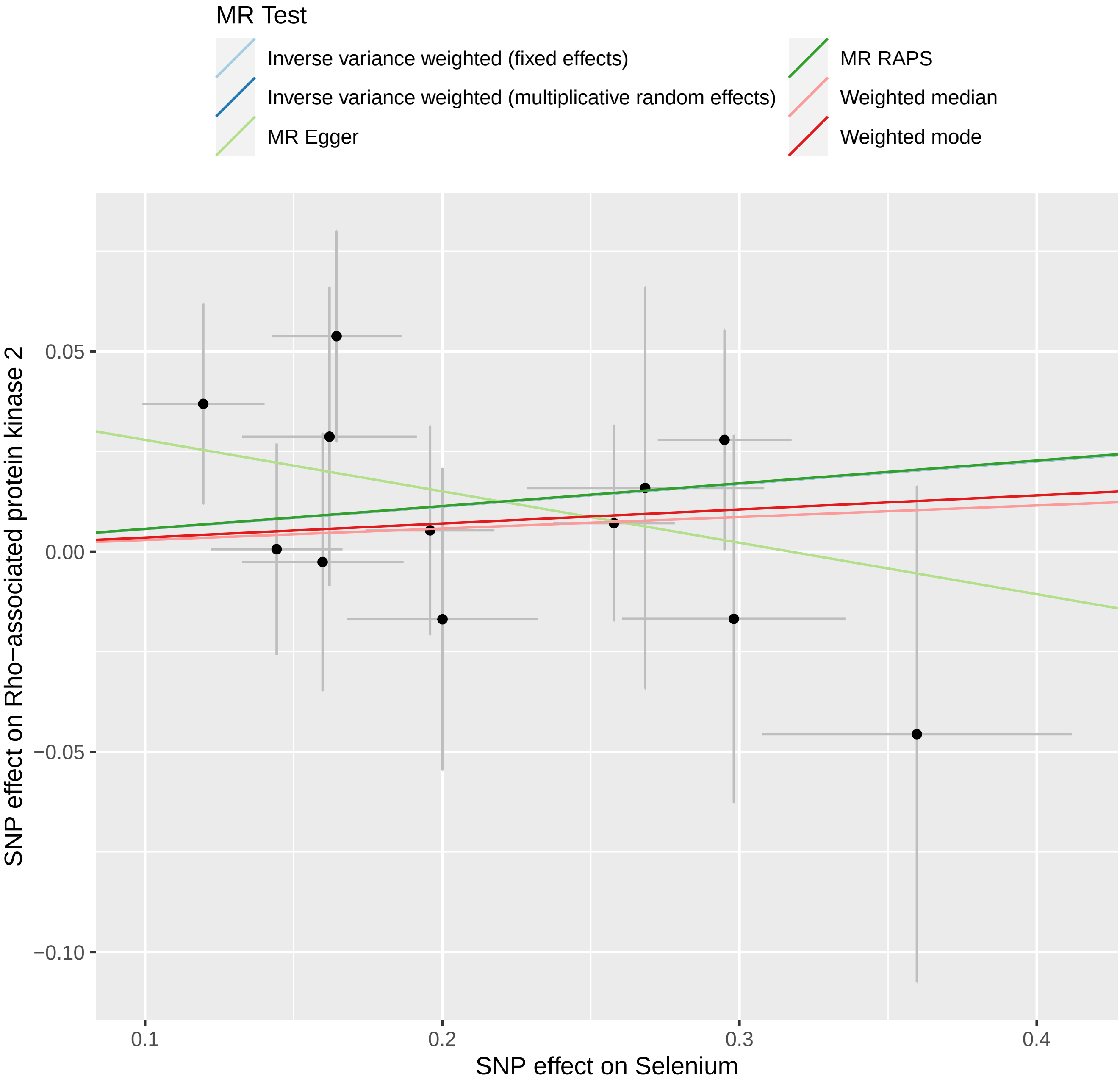

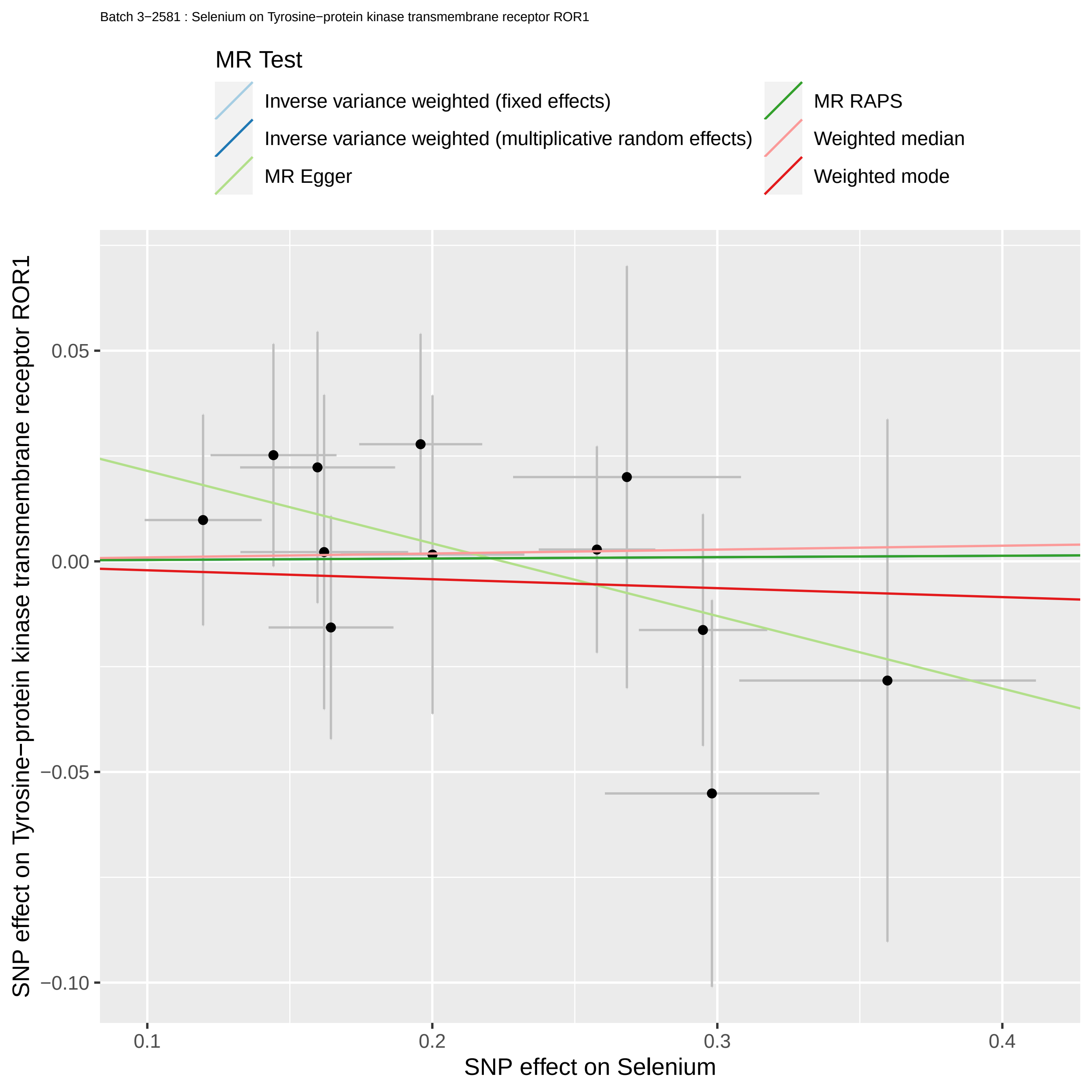
Batch 3−2581 : Selenium on Tyrosine−protein kinase transmembrane receptor ROR1
MR Test
Inverse variance weighted (fixed effects)
Inverse variance weighted (multiplicative random effects)
MR Egger
MR RAPS
Weighted median
Weighted mode
SNP effect on Tyrosine−protein kinase transmembrane receptor ROR1
0.05
0.00
−0.05
−0.10
0.1
0.2
0.3
0.4
SNP effect on Selenium

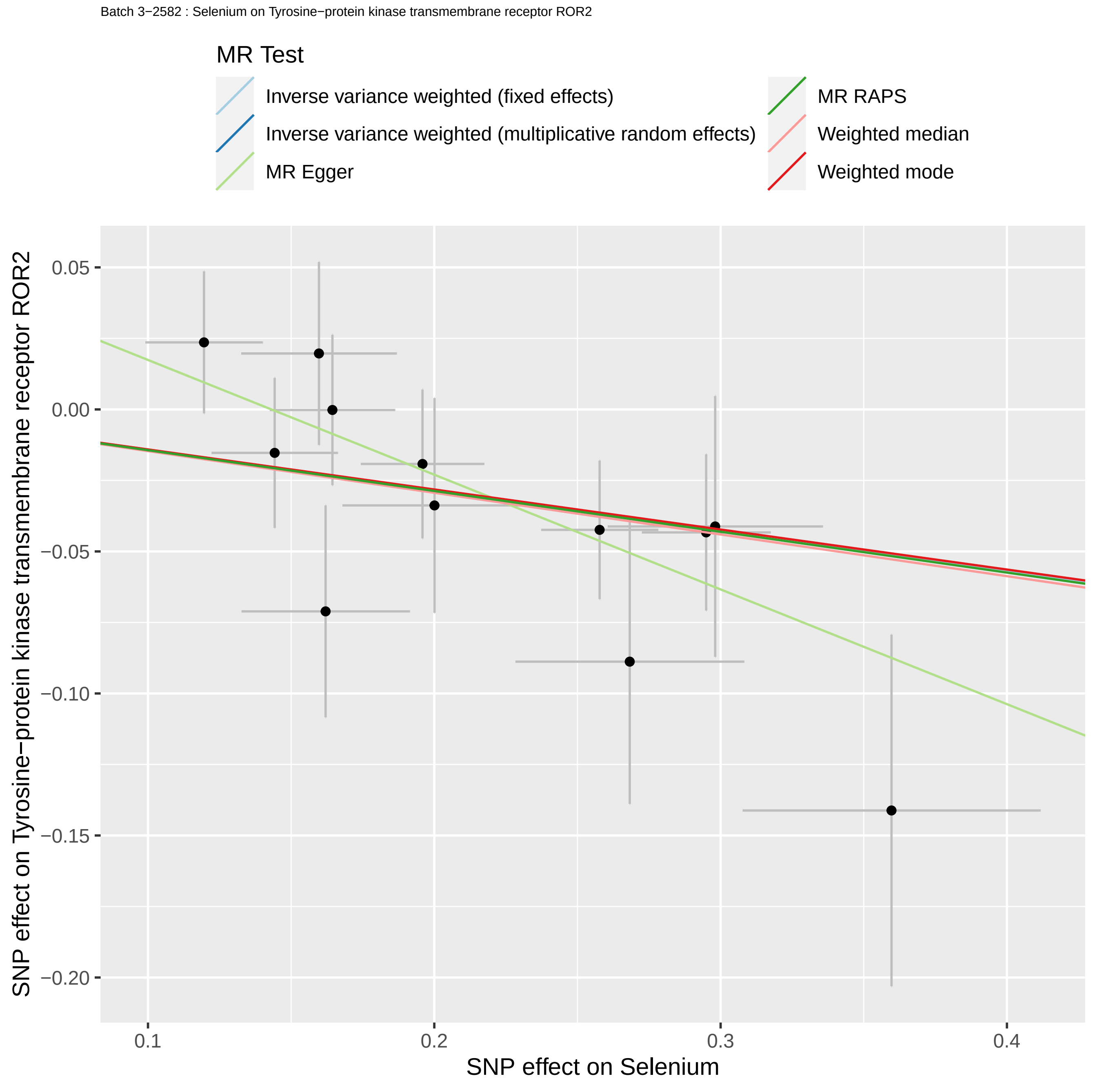

Batch 3−2582 : Selenium on Tyrosine−protein kinase transmembrane receptor ROR2
MR Test
Inverse variance weighted (fixed effects)
Inverse variance weighted (multiplicative random effects)
MR Egger
MR RAPS
Weighted median
Weighted mode
SNP effect on Tyrosine−protein kinase transmembrane receptor ROR2
0.05
0.00
−0.05
−0.10
−0.15
−0.20
0.1
0.2
0.3
0.4
SNP effect on Selenium

Batch 3−2583 : Selenium on Nuclear receptor ROR−beta

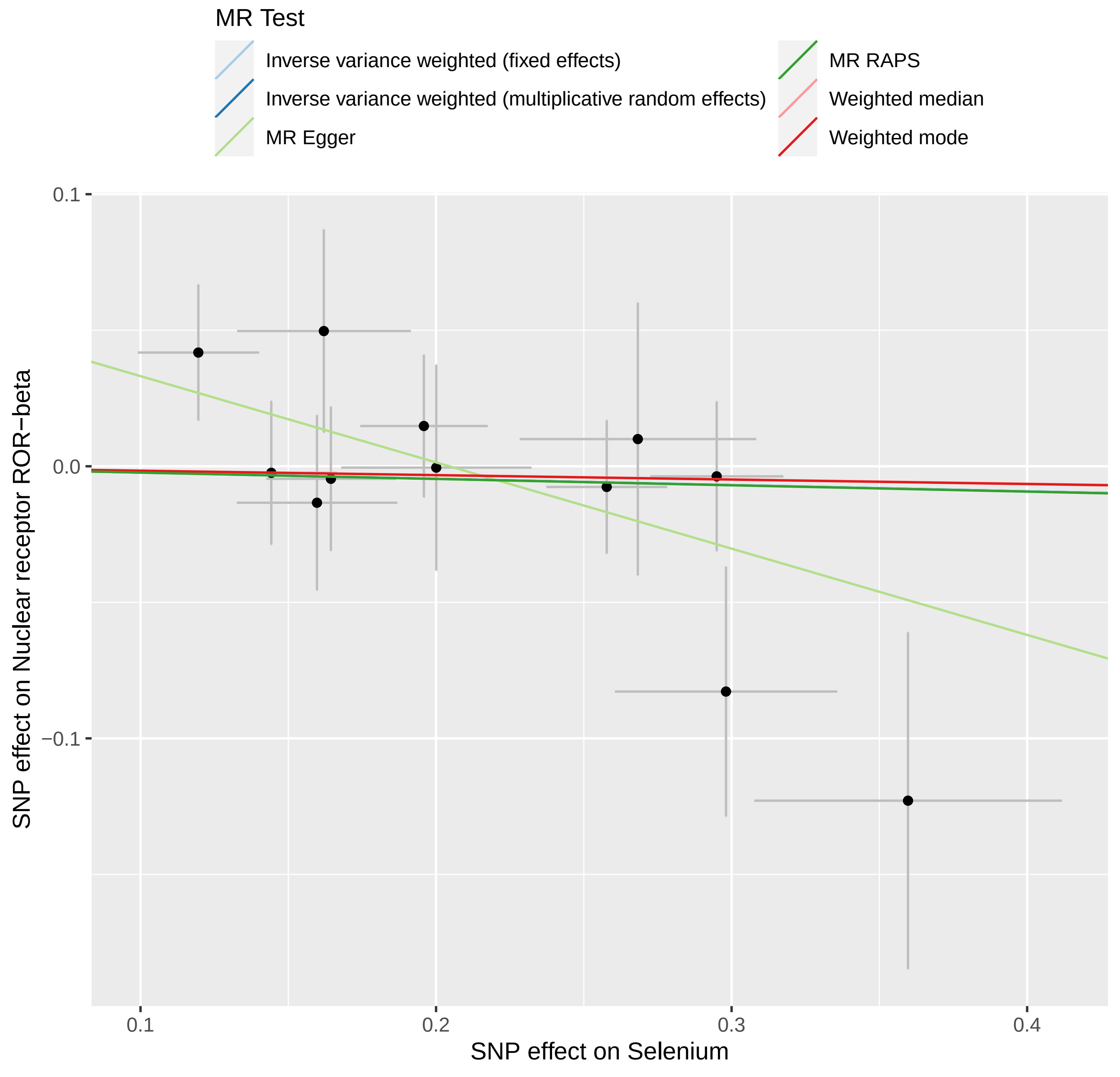

Batch 3−2584 : Selenium on Ribulose−phosphate 3−epimerase

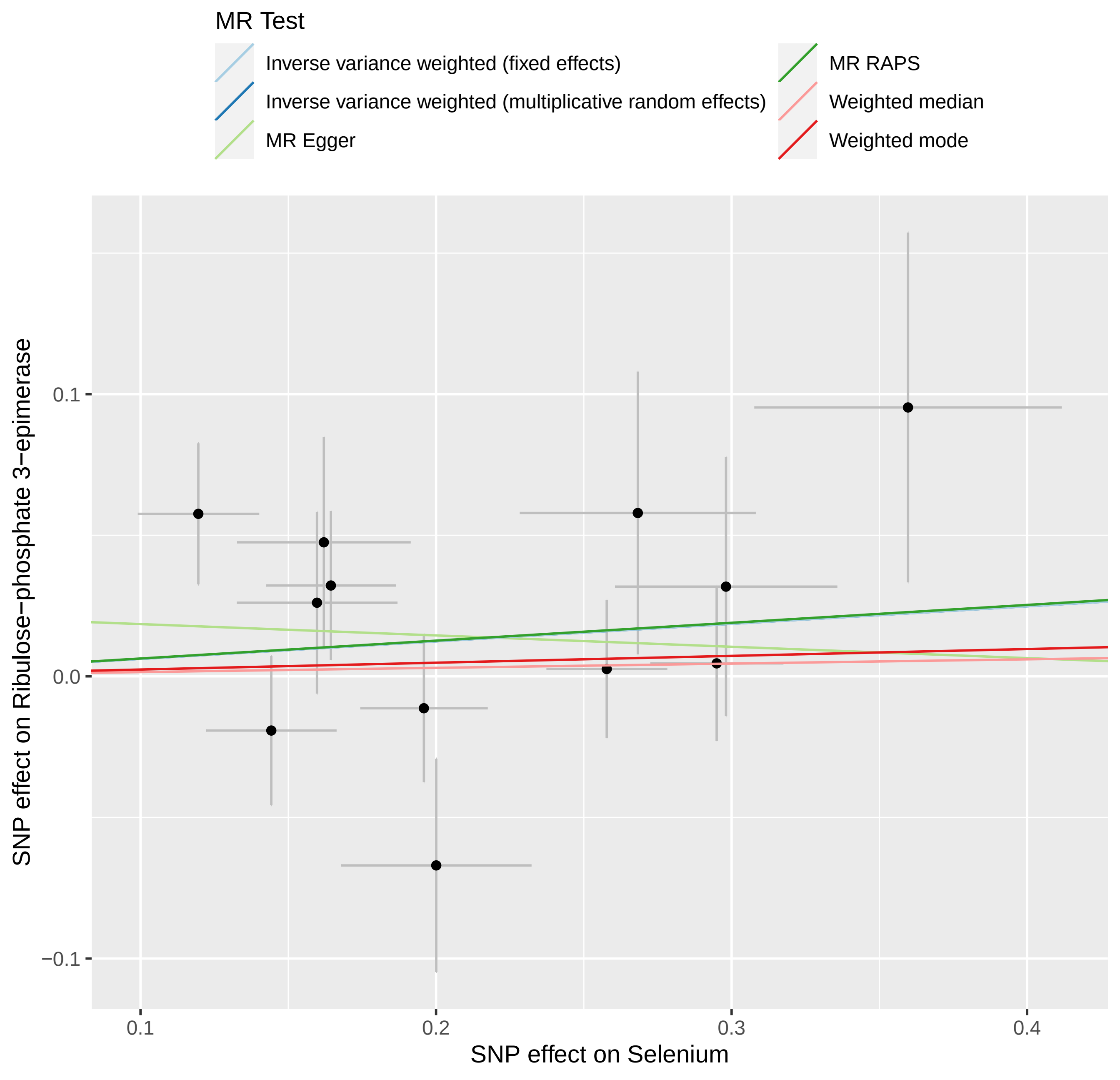

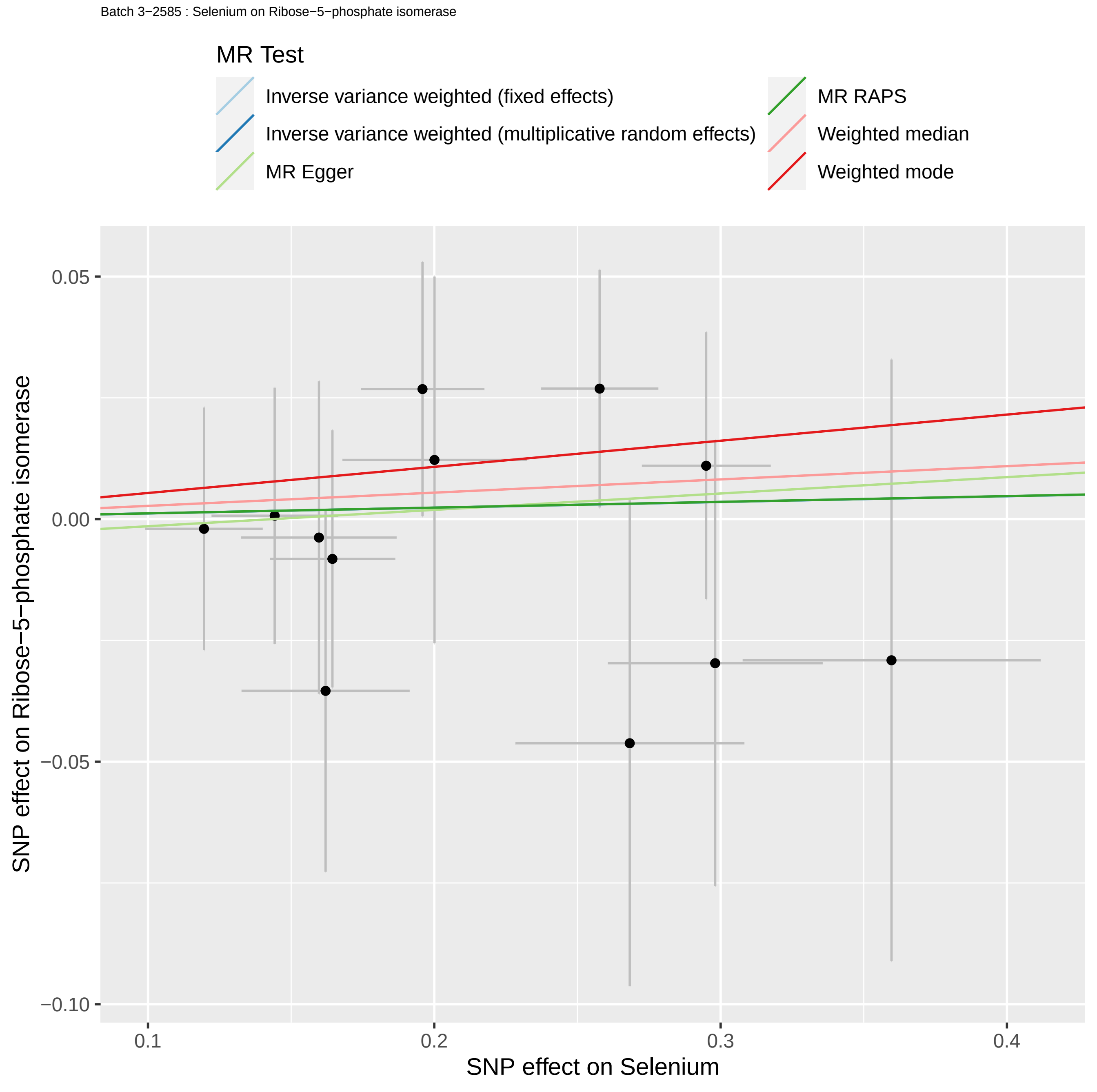

Batch 3−2585 : Selenium on Ribose−5−phosphate isomerase
MR Test
Inverse variance weighted (fixed effects)
Inverse variance weighted (multiplicative random effects)
MR Egger
MR RAPS
Weighted median
Weighted mode
0.05
0.00
−0.05
−0.10
0.1
0.2
0.3
0.4
SNP effect on Selenium
SNP effect on Ribose−5−phosphate isomerase

Batch 3−2586 : Selenium on 60S ribosomal protein L30

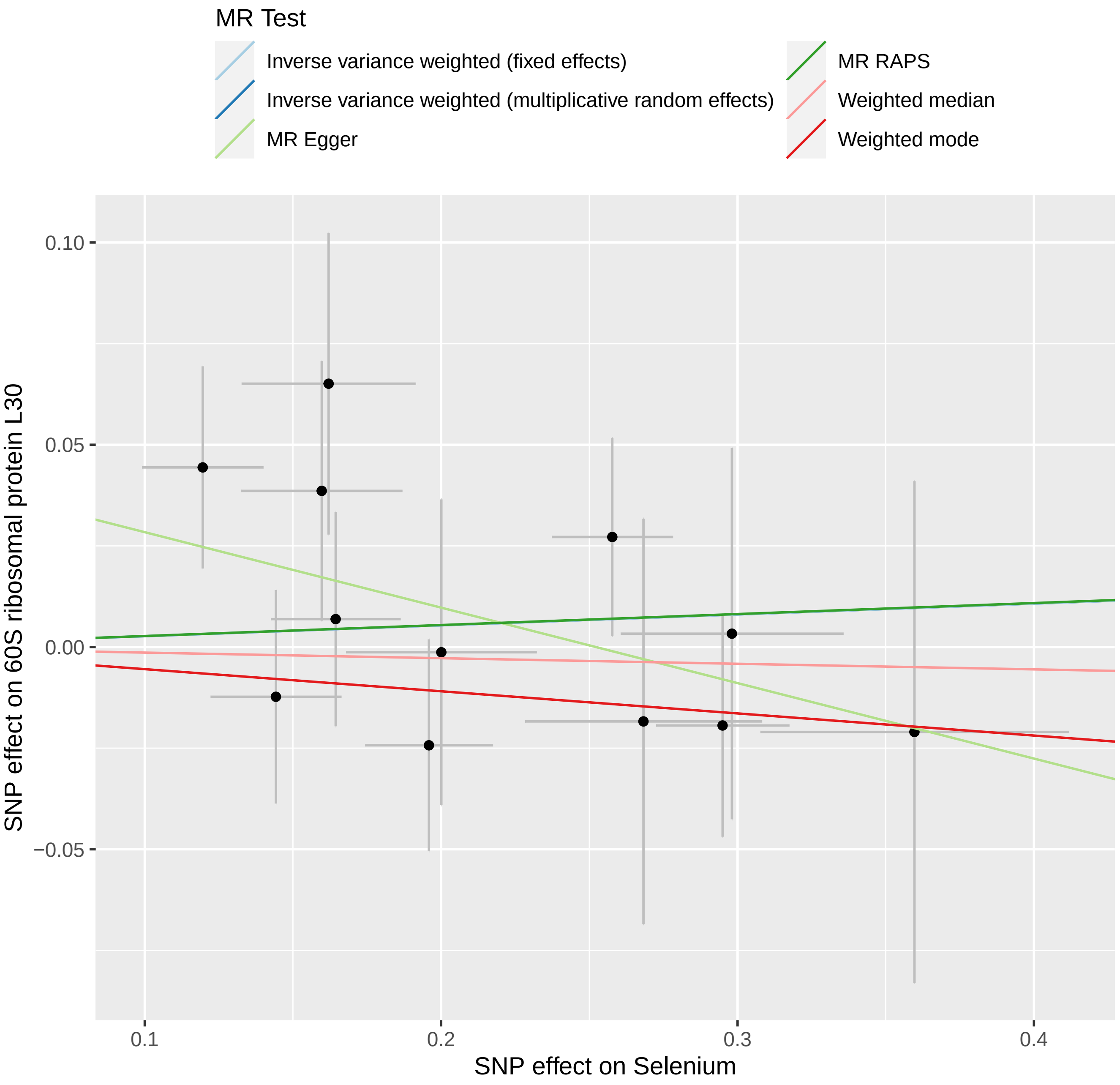

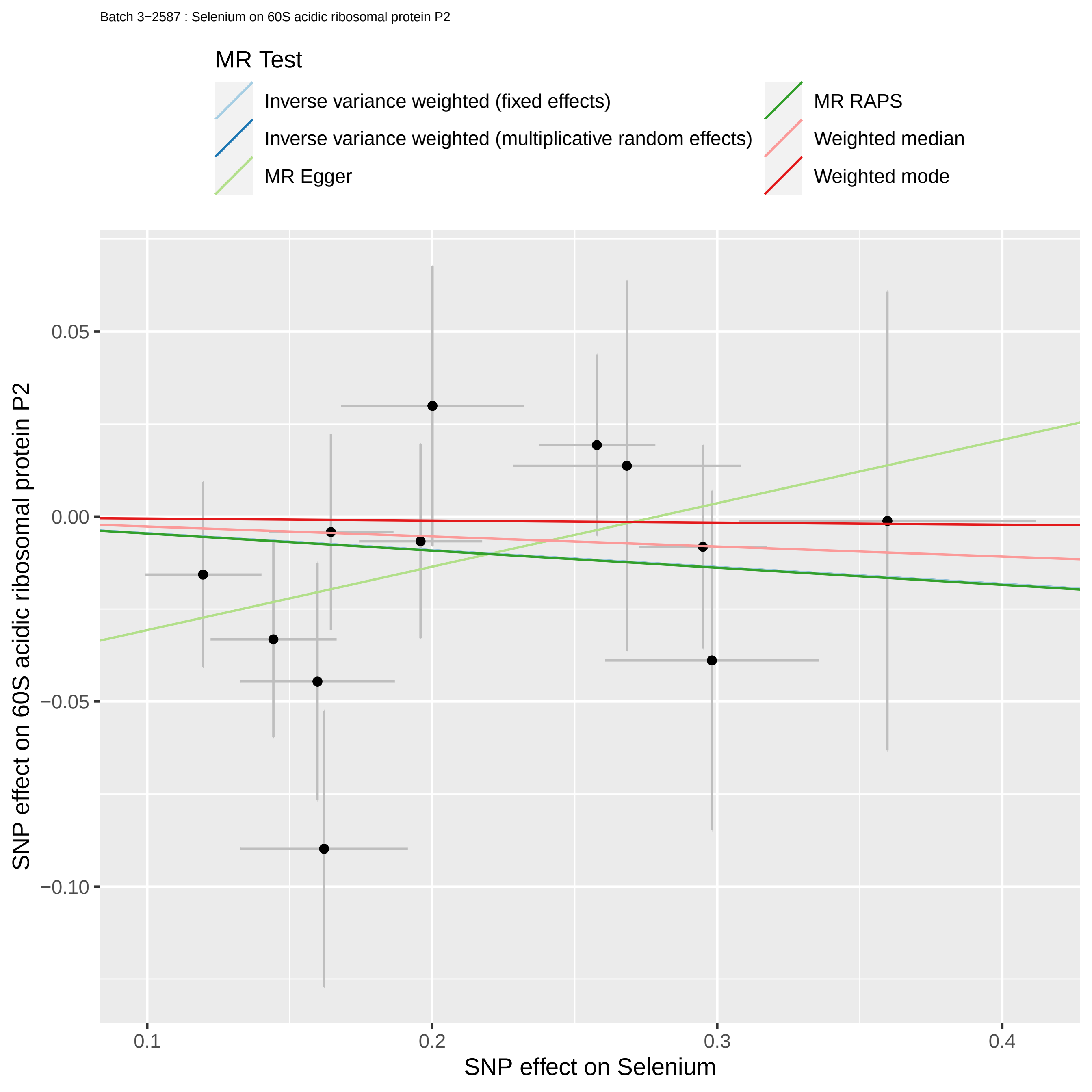
Batch 3−2587 : Selenium on 60S acidic ribosomal protein P2
MR Test
Inverse variance weighted (fixed effects)
Inverse variance weighted (multiplicative random effects)
MR Egger
MR RAPS
Weighted median
Weighted mode
SNP effect on 60S acidic ribosomal protein P2
0.05
0.00
−0.05
−0.10
0.1
0.2
0.3
0.4
SNP effect on Selenium

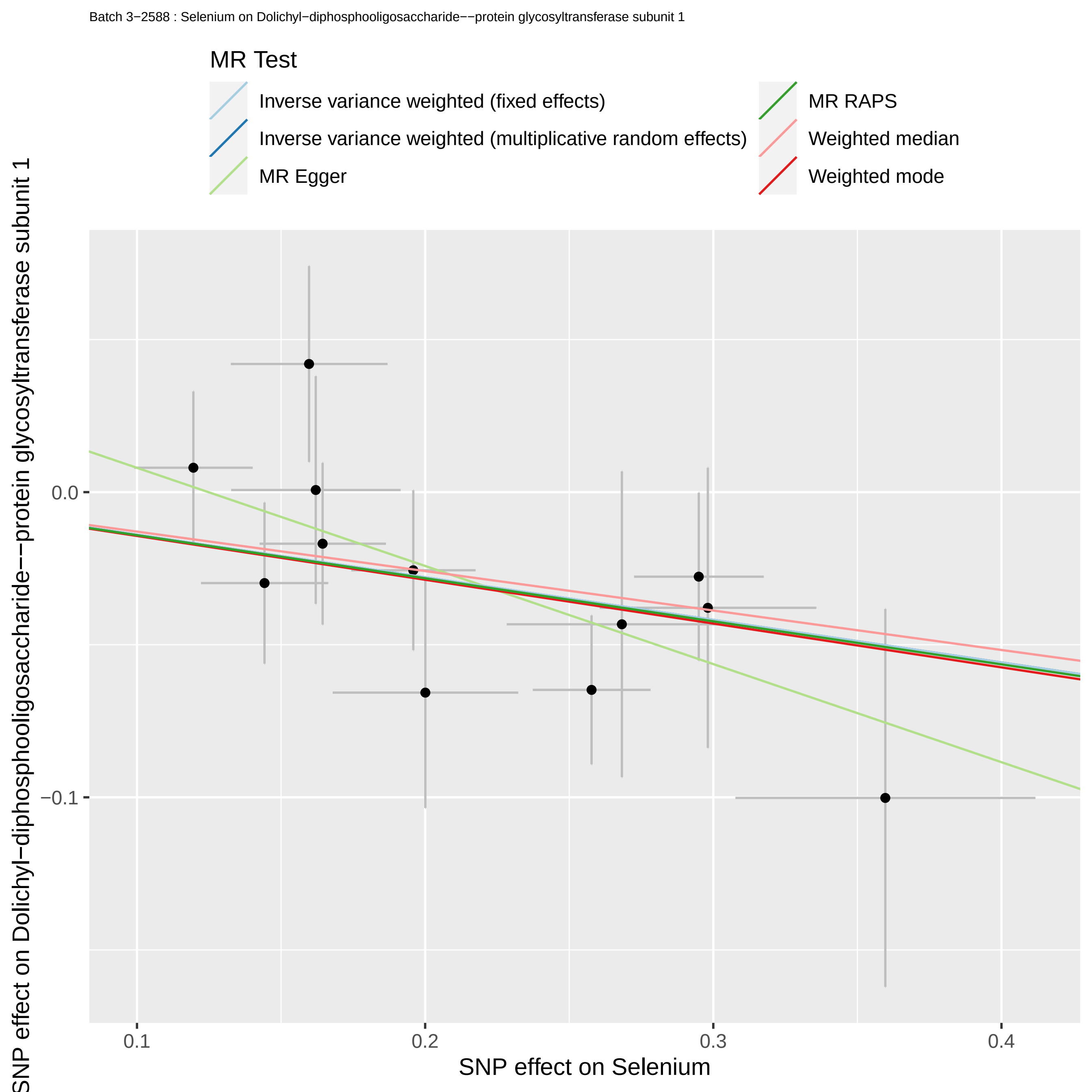

Batch 3−2588 : Selenium on Dolichyl−diphosphooligosaccharide−−protein glycosyltransferase subunit 1
MR Test
Inverse variance weighted (fixed effects)
Inverse variance weighted (multiplicative random effects)
MR Egger
MR RAPS
Weighted median
Weighted mode
SNP effect on Dolichyl−diphosphooligosaccharide−−protein glycosyltransferase subunit 1
0.0
−0.1
0.1
0.2
0.3
0.4
SNP effect on Selenium

Batch 3−2589 : Selenium on Dolichyl−diphosphooligosaccharide−−protein glycosyltransferase subunit 1

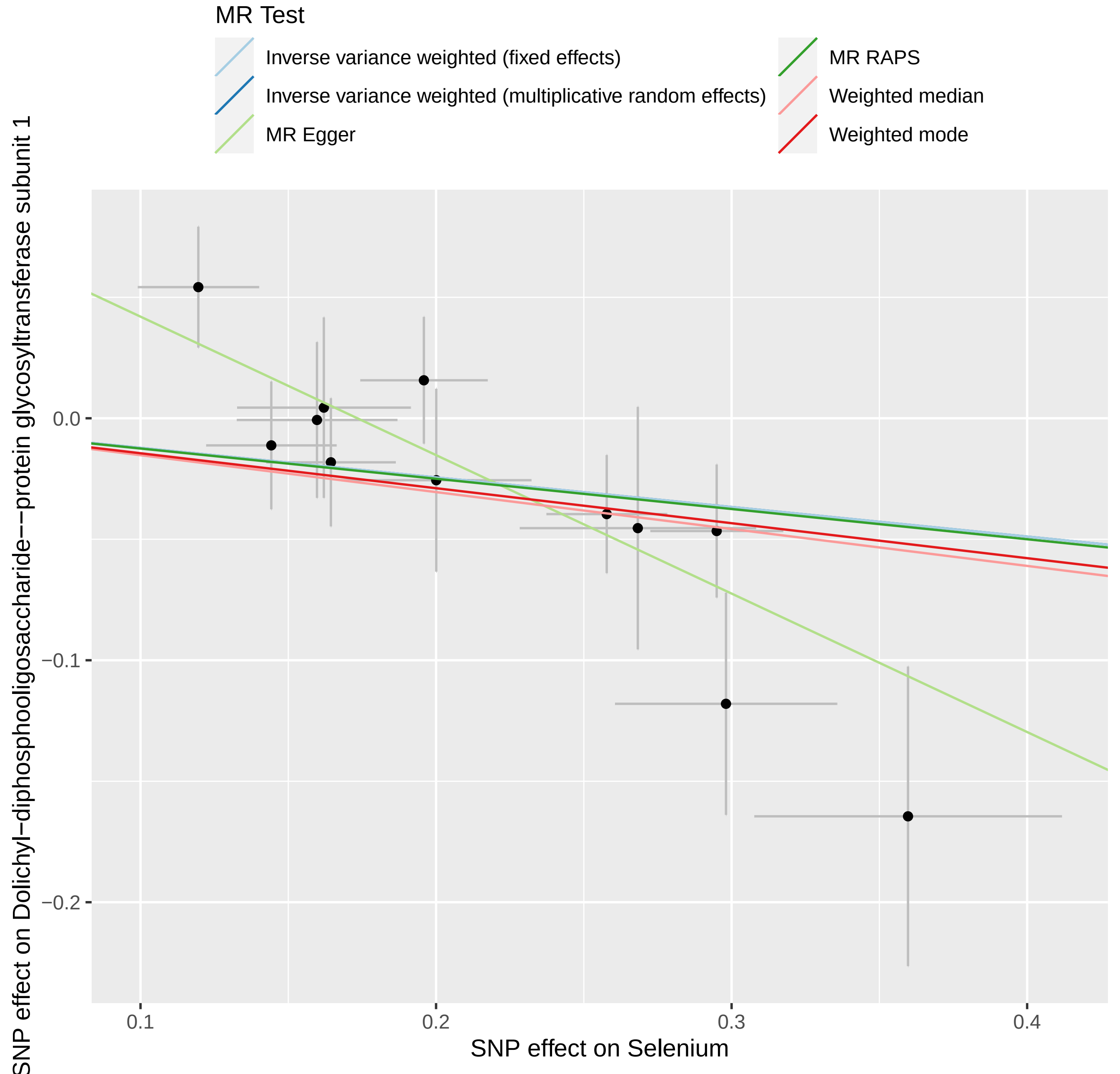

Batch 3−2590 : Selenium on Regulation of nuclear pre−mRNA domain−containing protein 1A

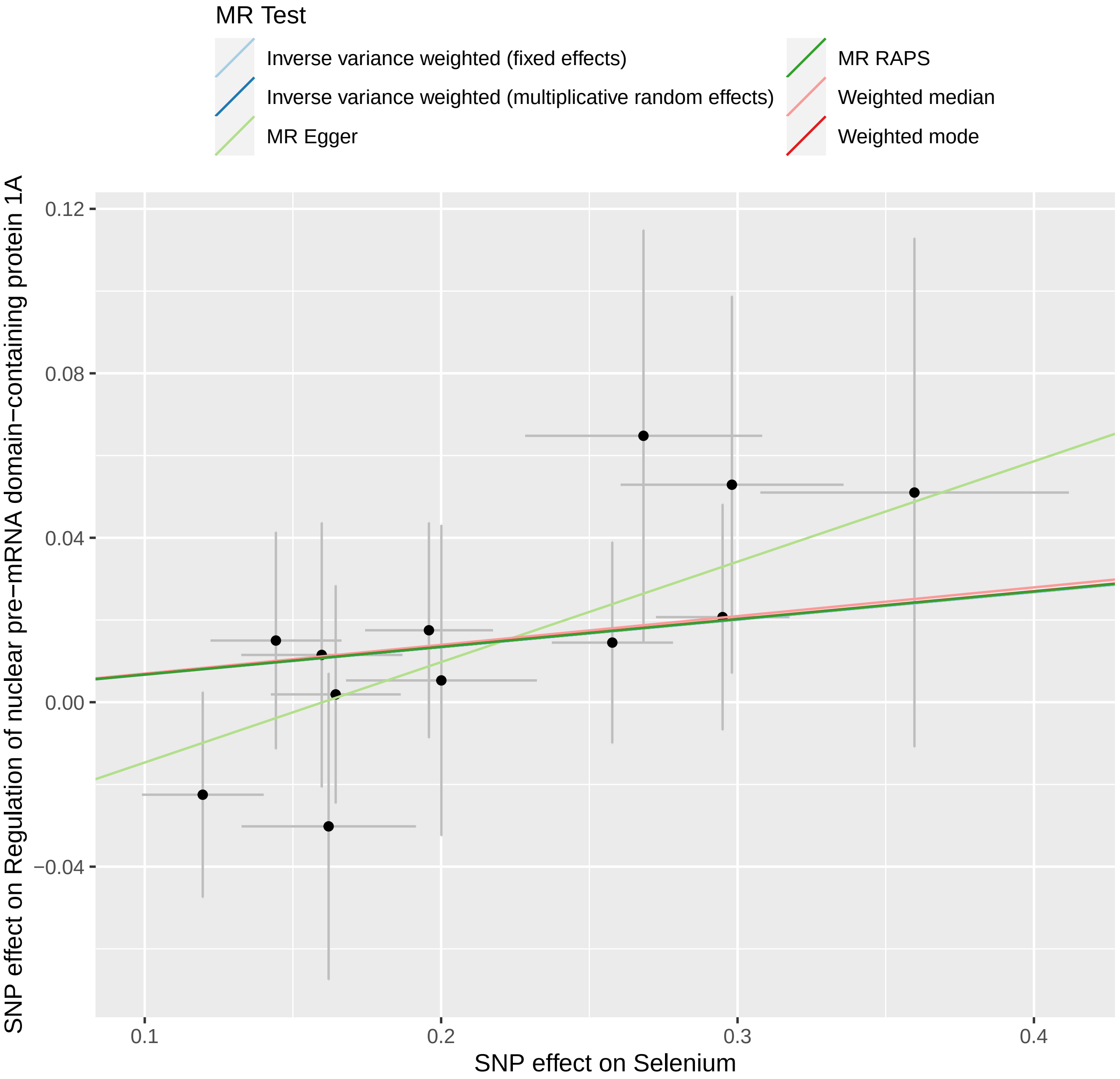

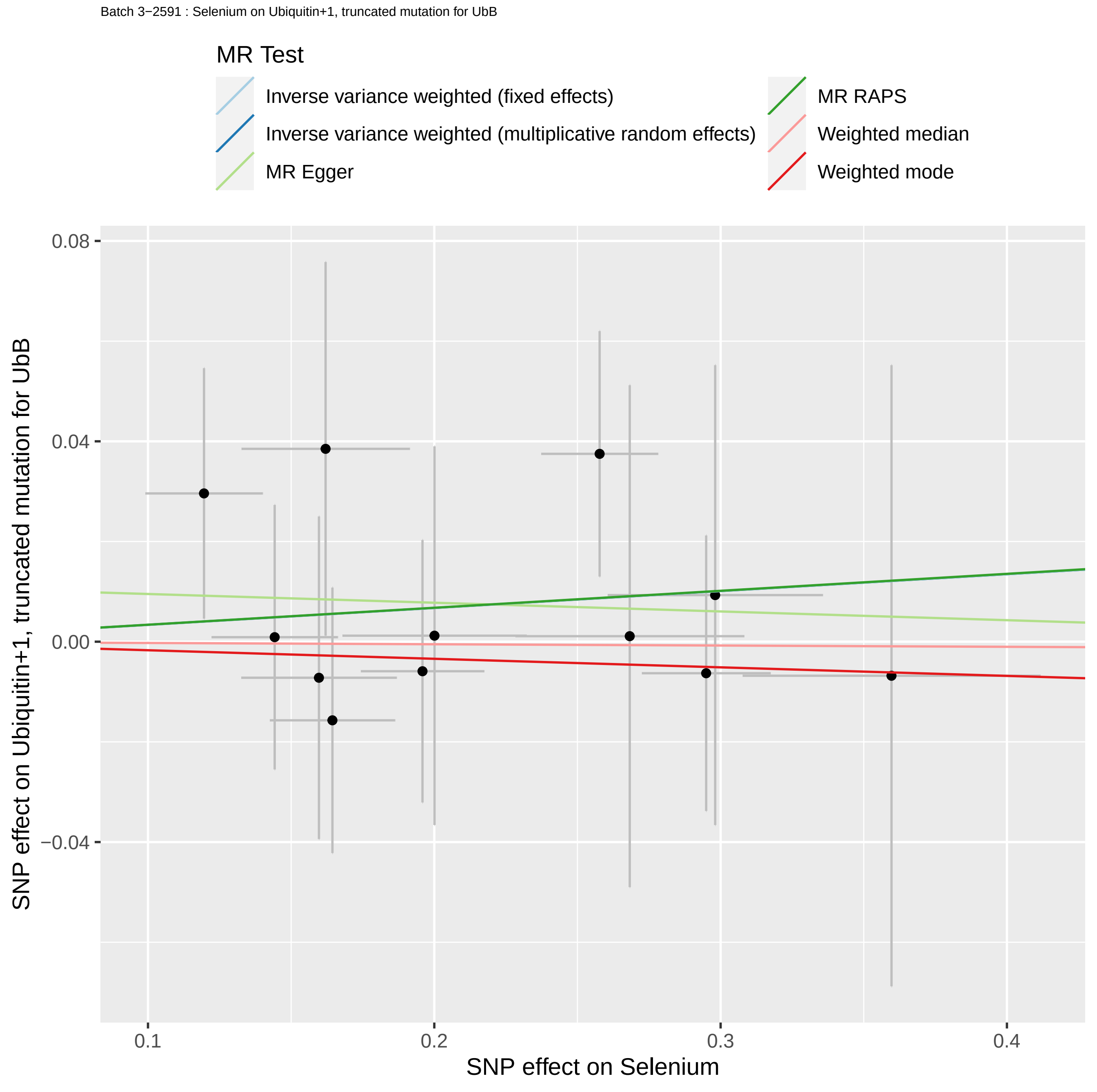

Batch 3−2591 : Selenium on Ubiquitin+1, truncated mutation for UbB
MR Test
Inverse variance weighted (fixed effects)
Inverse variance weighted (multiplicative random effects)
MR Egger
MR RAPS
Weighted median
Weighted mode
0.08
0.04
0.00
−0.04
0.1
0.2
0.3
0.4
SNP effect on Selenium
SNP effect on Ubiquitin+1, truncated mutation for UbB

Batch 3−2592 : Selenium on Ubiquitin

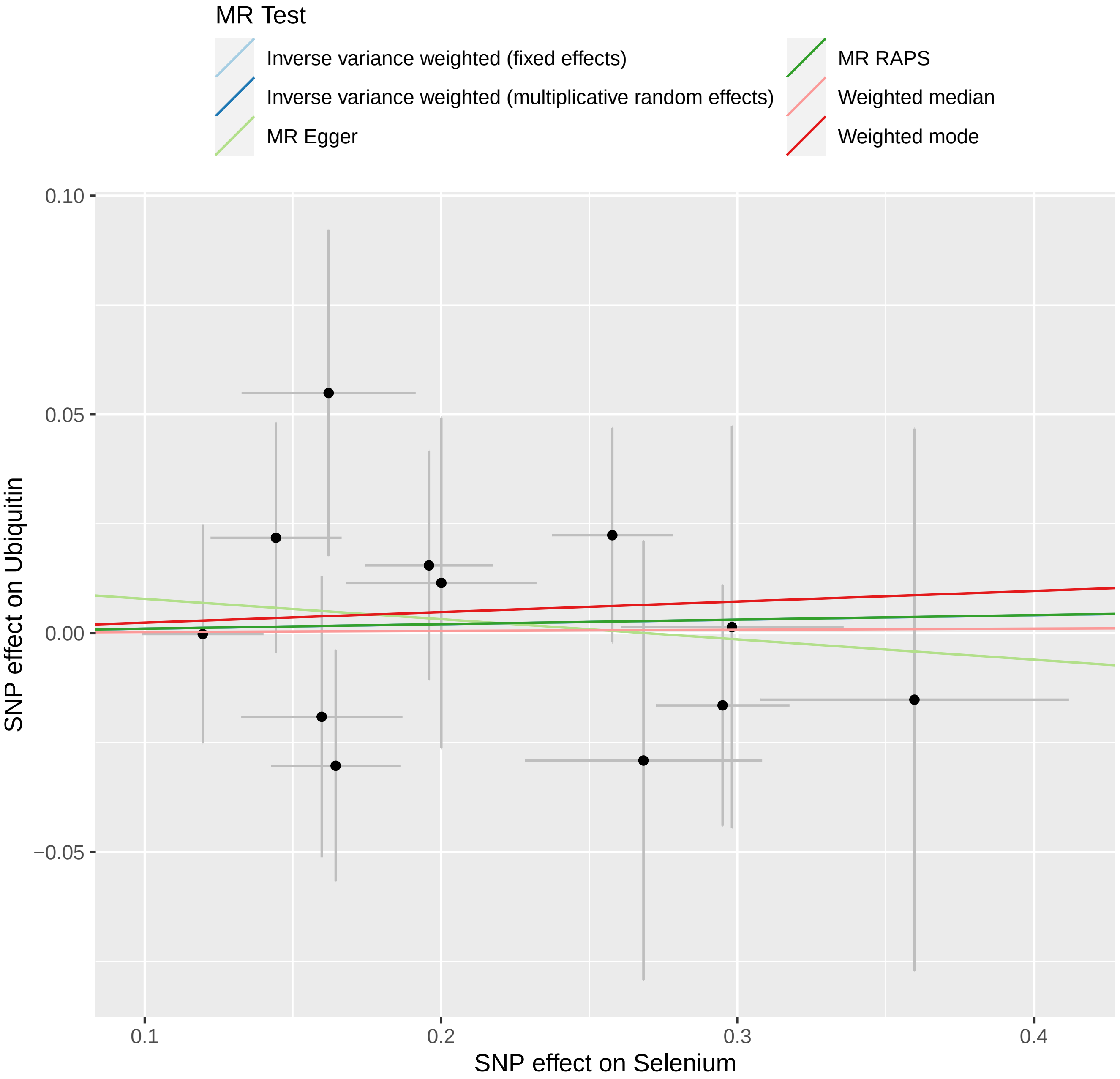

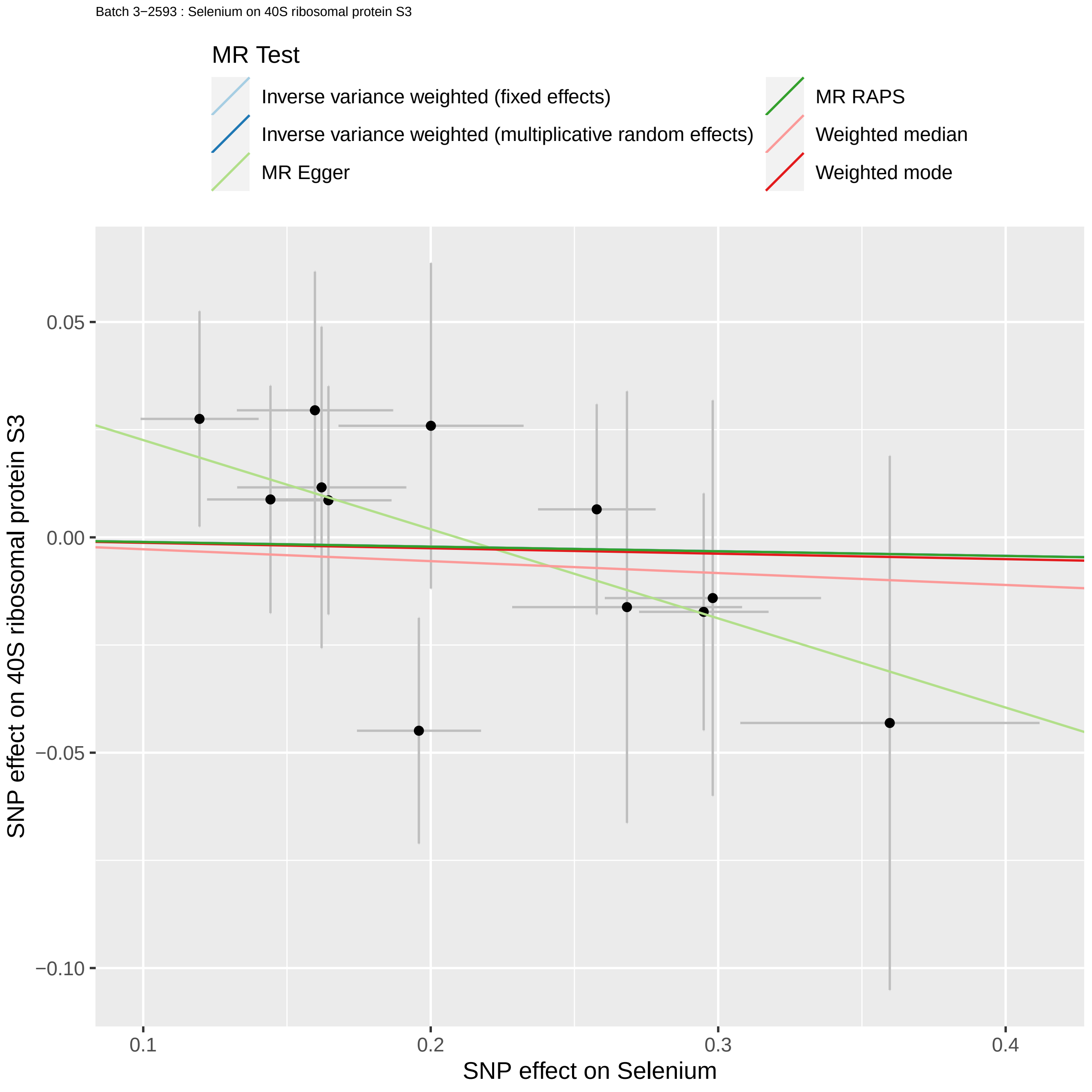
Batch 3−2593 : Selenium on 40S ribosomal protein S3
MR Test
Inverse variance weighted (fixed effects)
Inverse variance weighted (multiplicative random effects)
MR Egger
MR RAPS
Weighted median
Weighted mode
0.05
0.00
−0.05
−0.10
0.1
0.2
0.3
0.4
SNP effect on 40S ribosomal protein S3
SNP effect on Selenium

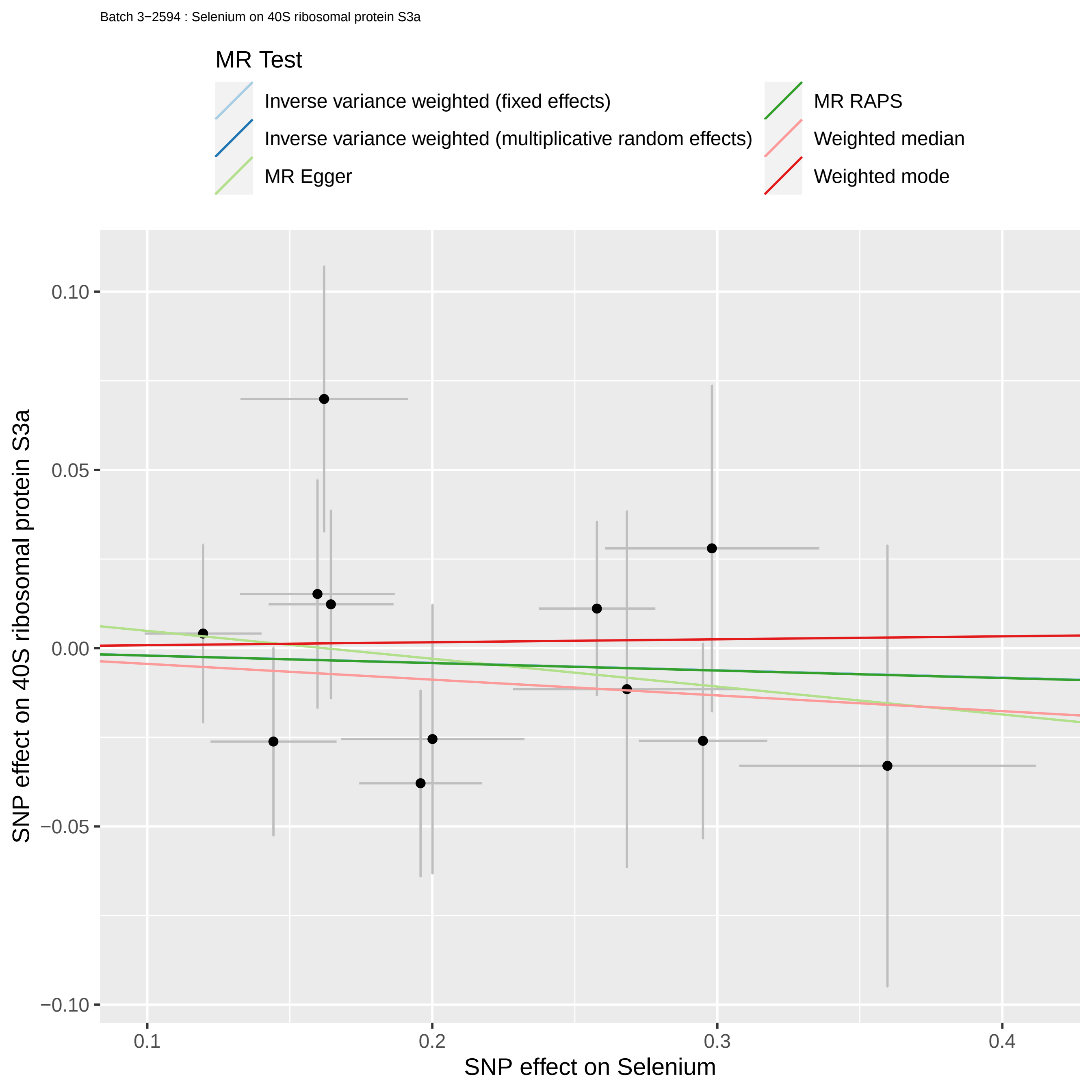
Batch 3−2594 : Selenium on 40S ribosomal protein S3a
MR Test
Inverse variance weighted (fixed effects)
Inverse variance weighted (multiplicative random effects)
MR Egger
MR RAPS
Weighted median
Weighted mode
0.10
0.05
0.00
−0.05
−0.10
0.1
0.2
0.3
0.4
SNP effect on 40S ribosomal protein S3a
SNP effect on Selenium

Batch 3−2595 : Selenium on 40S ribosomal protein S4, X isoform

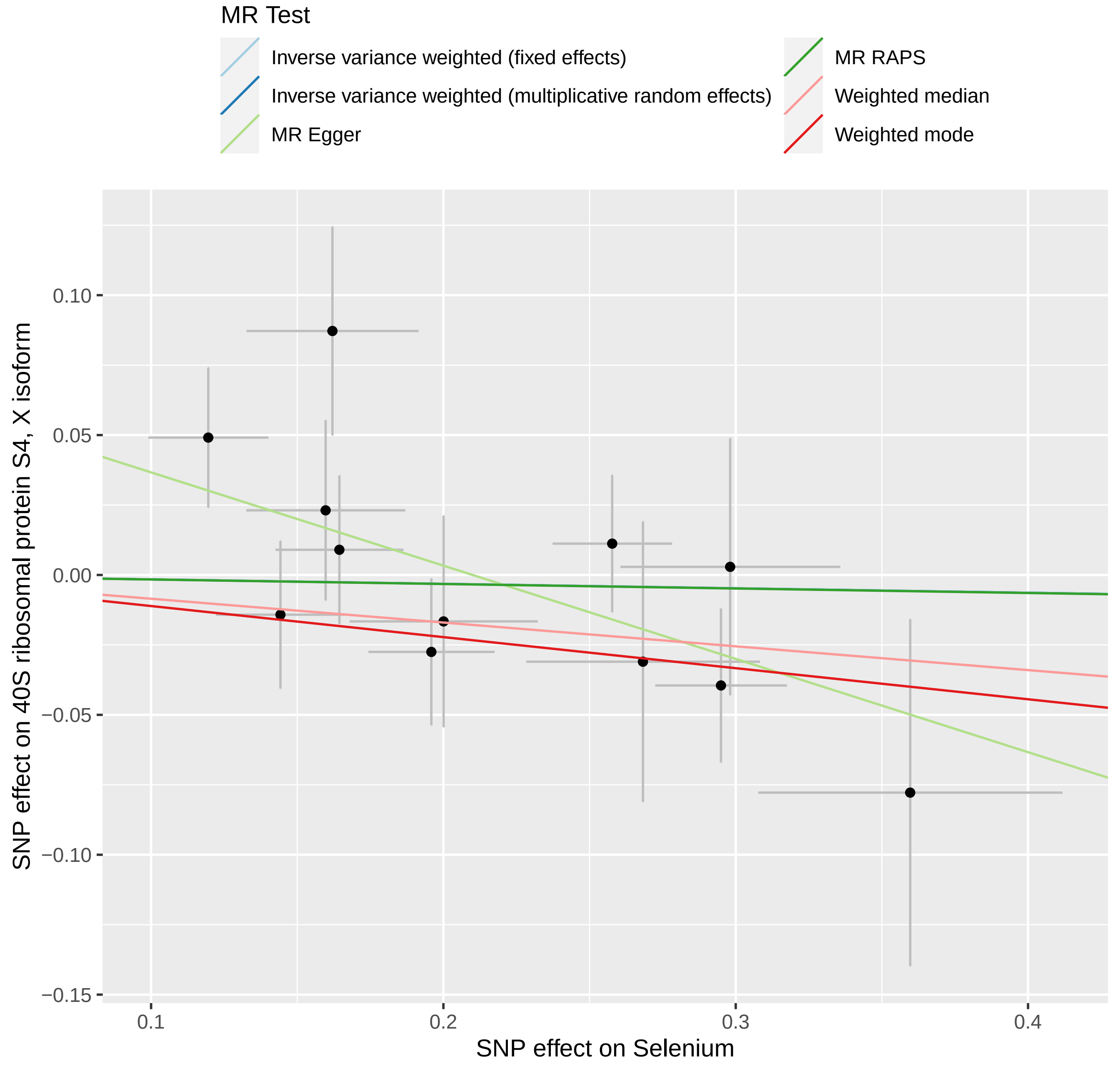

Batch 3−2596 : Selenium on Ribosomal protein S6 kinase alpha−6

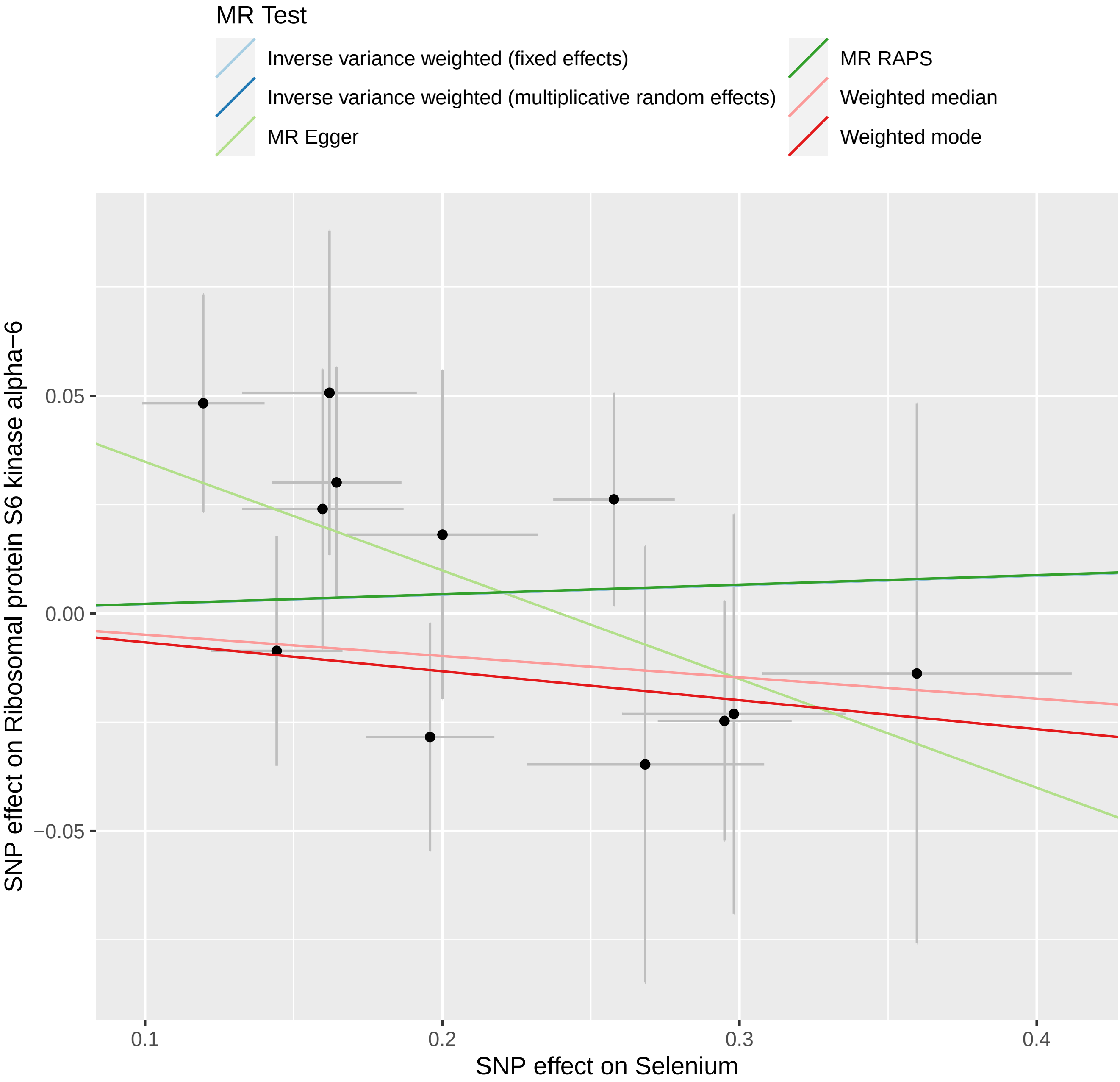

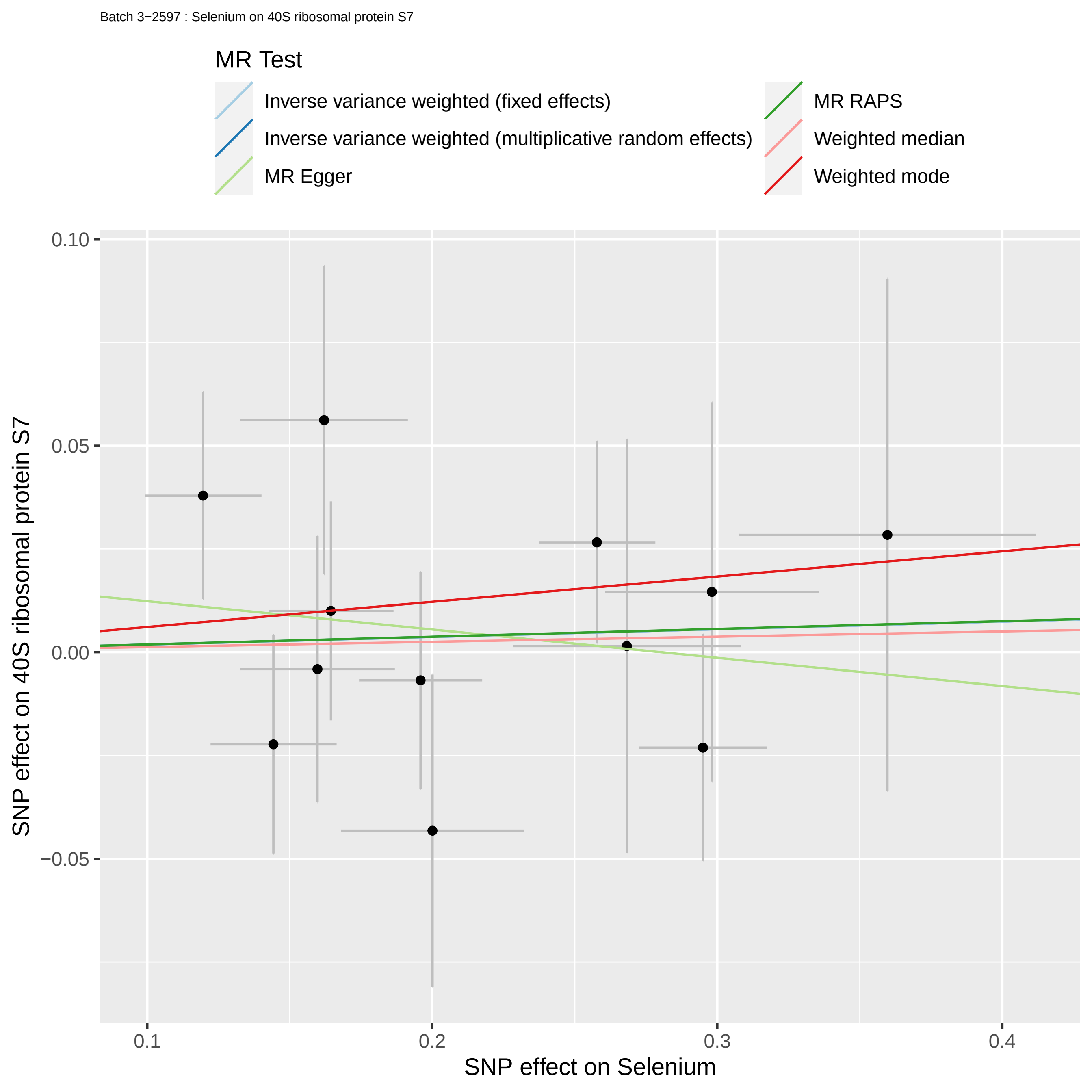

Batch 3−2597 : Selenium on 40S ribosomal protein S7
MR Test
Inverse variance weighted (fixed effects)
Inverse variance weighted (multiplicative random effects)
MR Egger
MR RAPS
Weighted median
Weighted mode
0.10
0.05
0.00
−0.05
0.1
0.2
0.3
0.4
SNP effect on Selenium
SNP effect on 40S ribosomal protein S7

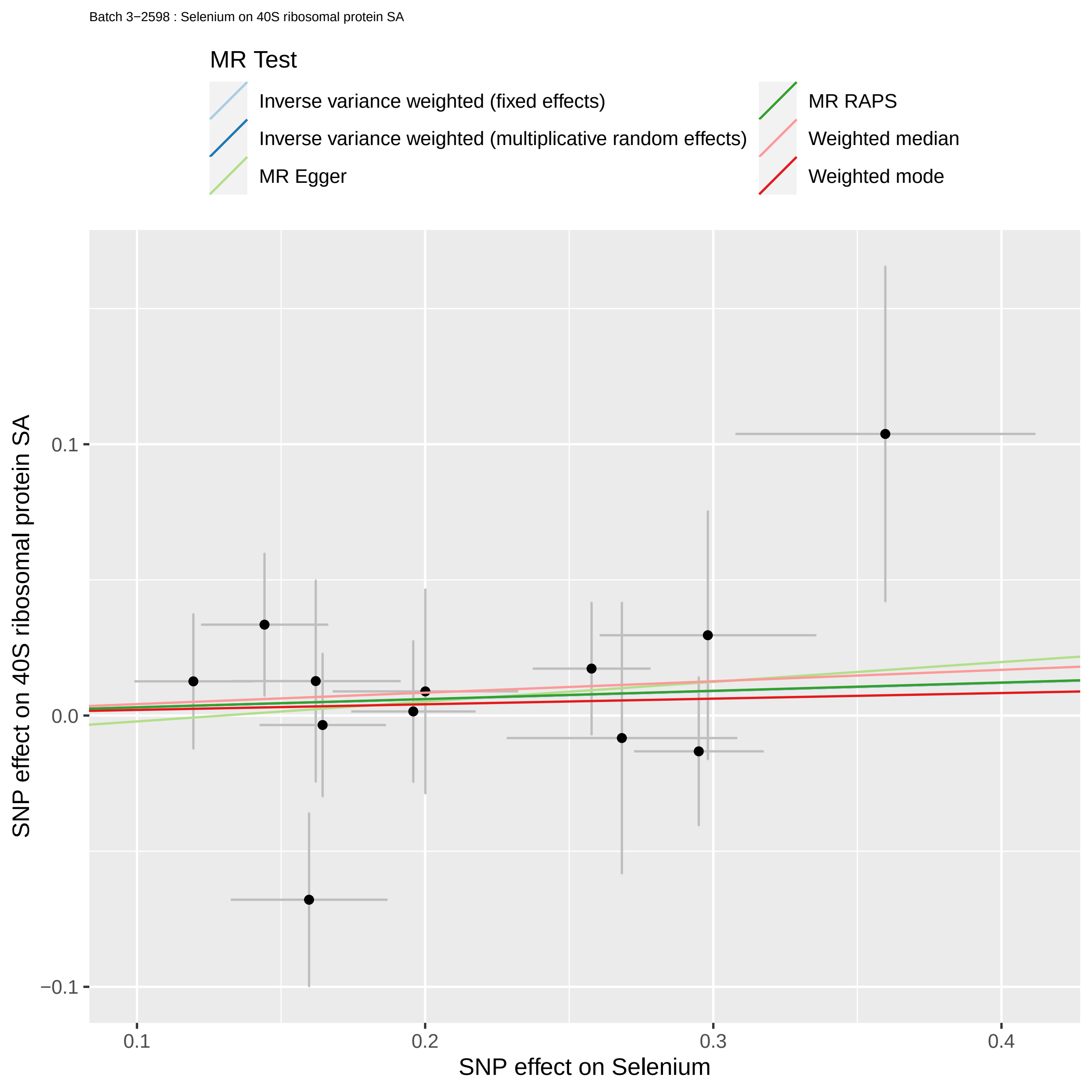
Batch 3−2598 : Selenium on 40S ribosomal protein SA
MR Test
Inverse variance weighted (fixed effects)
Inverse variance weighted (multiplicative random effects)
MR Egger
MR RAPS
Weighted median
Weighted mode
SNP effect on 40S ribosomal protein SA
0.1
0.0
−0.1
0.1
0.2
0.3
0.4
SNP effect on Selenium

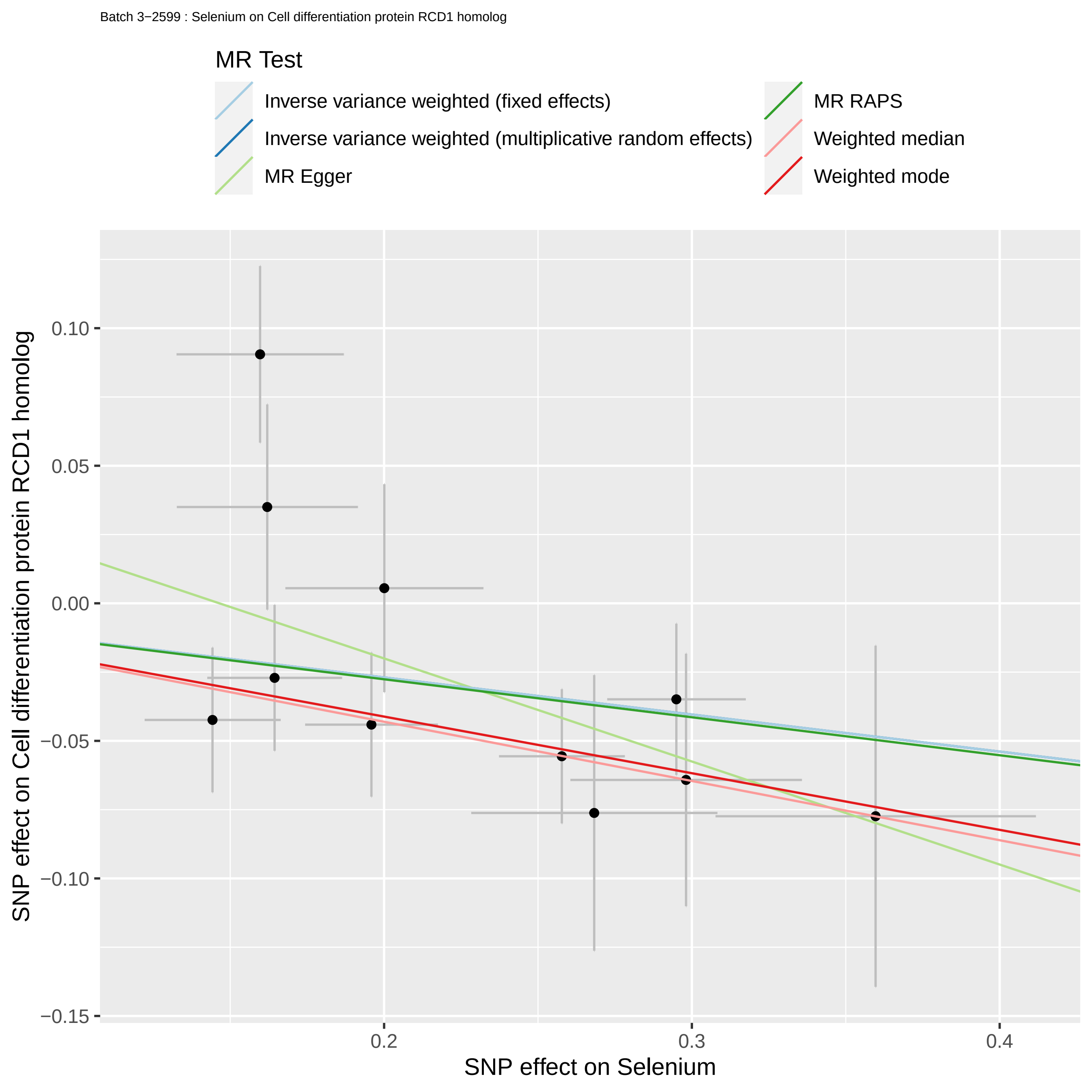

Batch 3−2599 : Selenium on Cell differentiation protein RCD1 homolog
MR Test
Inverse variance weighted (fixed effects)
Inverse variance weighted (multiplicative random effects)
MR Egger
MR RAPS
Weighted median
Weighted mode
0.10
0.05
0.00
−0.05
−0.10
−0.15
0.2
0.3
0.4
SNP effect on Cell differentiation protein RCD1 homolog
SNP effect on Selenium

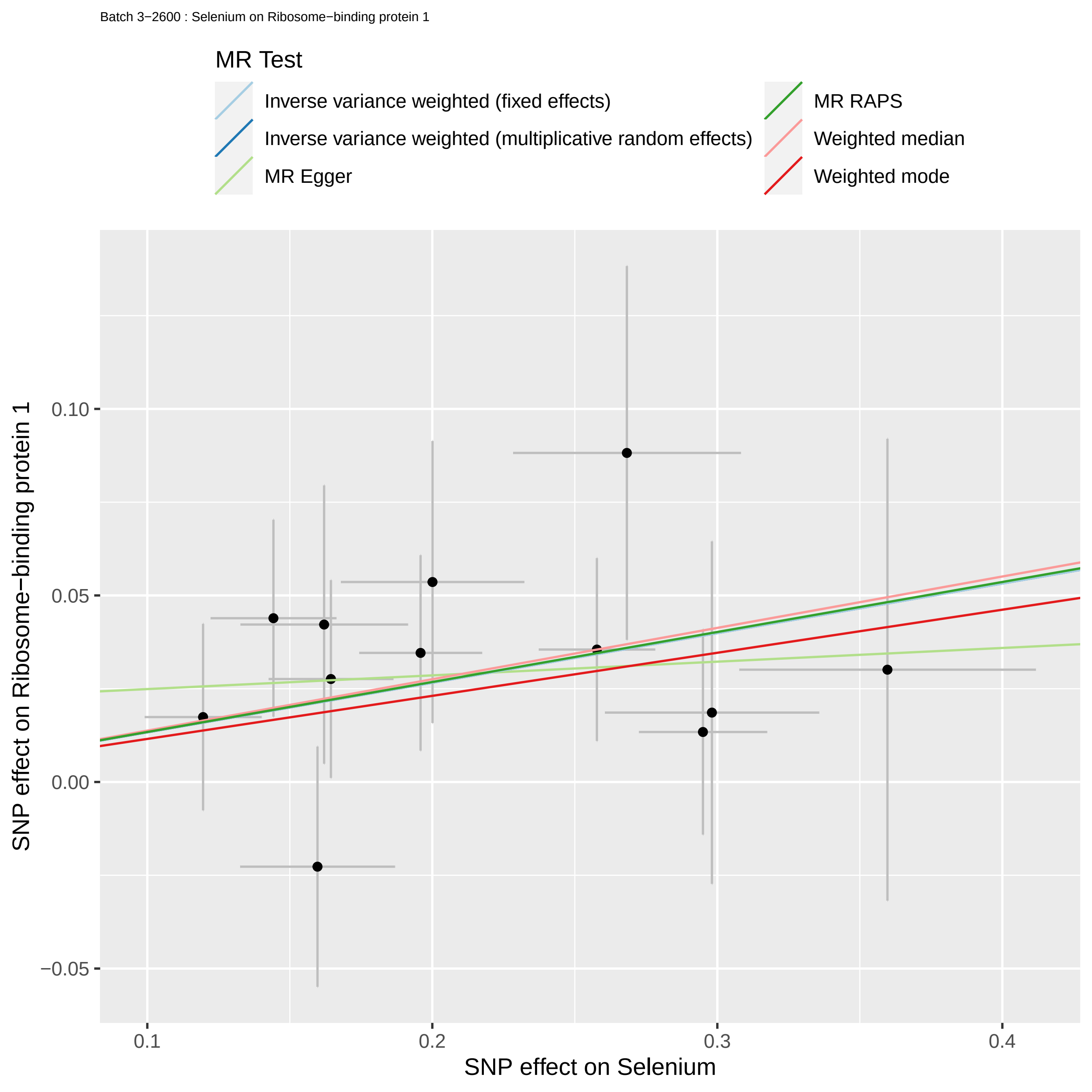
Batch 3−2600 : Selenium on Ribosome−binding protein 1
MR Test
Inverse variance weighted (fixed effects)
Inverse variance weighted (multiplicative random effects)
MR Egger
MR RAPS
Weighted median
Weighted mode
SNP effect on Ribosome−binding protein 1
0.10
0.05
0.00
−0.05
0.1
0.2
0.3
0.4
SNP effect on Selenium

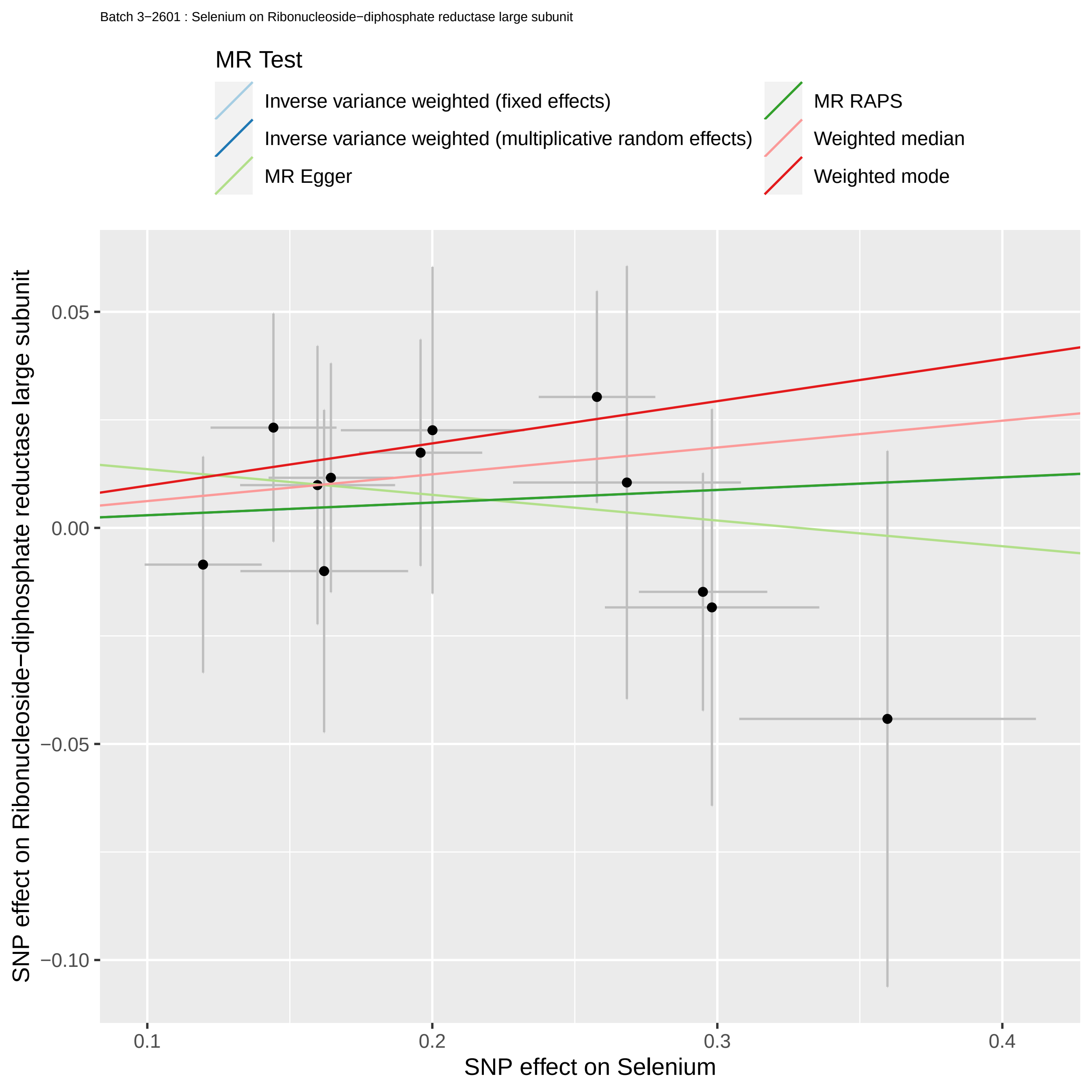

Batch 3−2601 : Selenium on Ribonucleoside−diphosphate reductase large subunit
MR Test
Inverse variance weighted (fixed effects)
Inverse variance weighted (multiplicative random effects)
MR Egger
MR RAPS
Weighted median
Weighted mode
0.05
0.00
−0.05
−0.10
0.1
0.2
0.3
0.4
SNP effect on Selenium
SNP effect on Ribonucleoside−diphosphate reductase large subunit

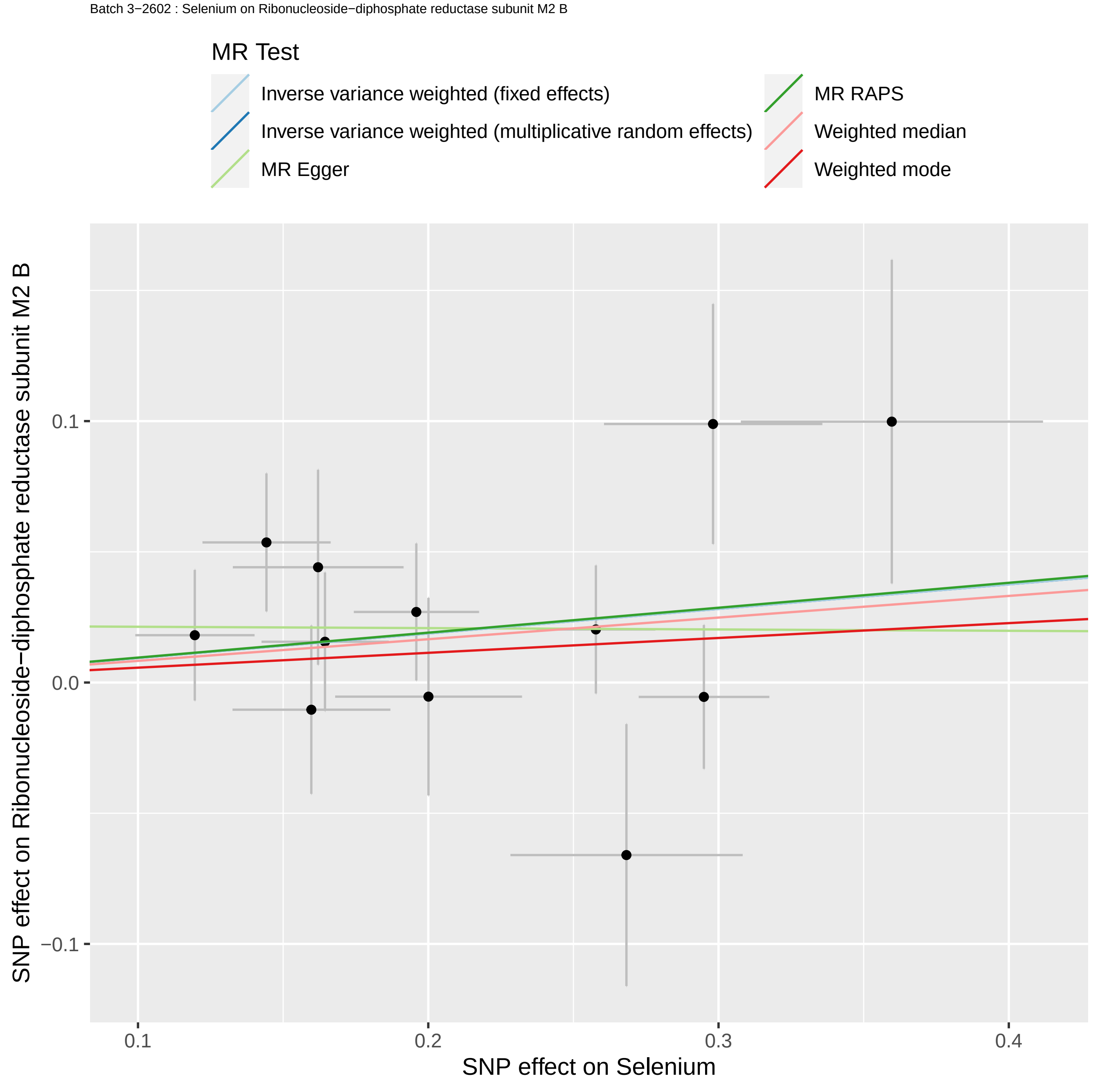
Batch 3−2602 : Selenium on Ribonucleoside−diphosphate reductase subunit M2 B
MR Test
Inverse variance weighted (fixed effects)
Inverse variance weighted (multiplicative random effects)
MR Egger
MR RAPS
Weighted median
Weighted mode
SNP effect on Ribonucleoside−diphosphate reductase subunit M2 B
0.1
0.0
−0.1
0.1
0.2
0.3
0.4
SNP effect on Selenium

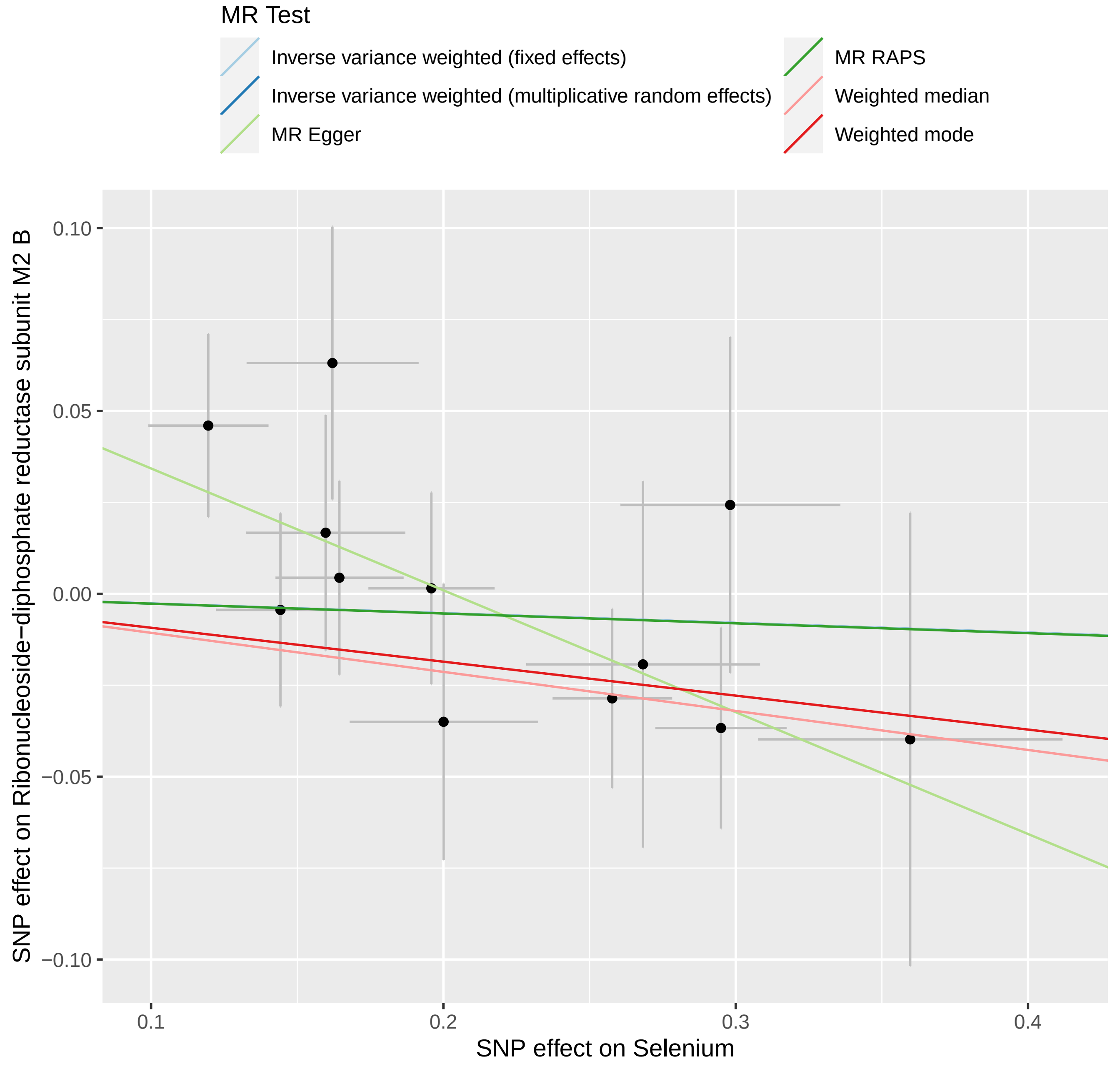
MR Test
Inverse variance weighted (fixed effects)
Inverse variance weighted (multiplicative random effects)
MR Egger
MR RAPS
Weighted median
Weighted mode
SNP effect on Ribonucleoside−diphosphate reductase subunit M2 B
0.10
0.05
0.00
−0.05
−0.10
0.1
0.2
0.3
0.4
SNP effect on Selenium

Batch 3−2604 : Selenium on Retinoschisin

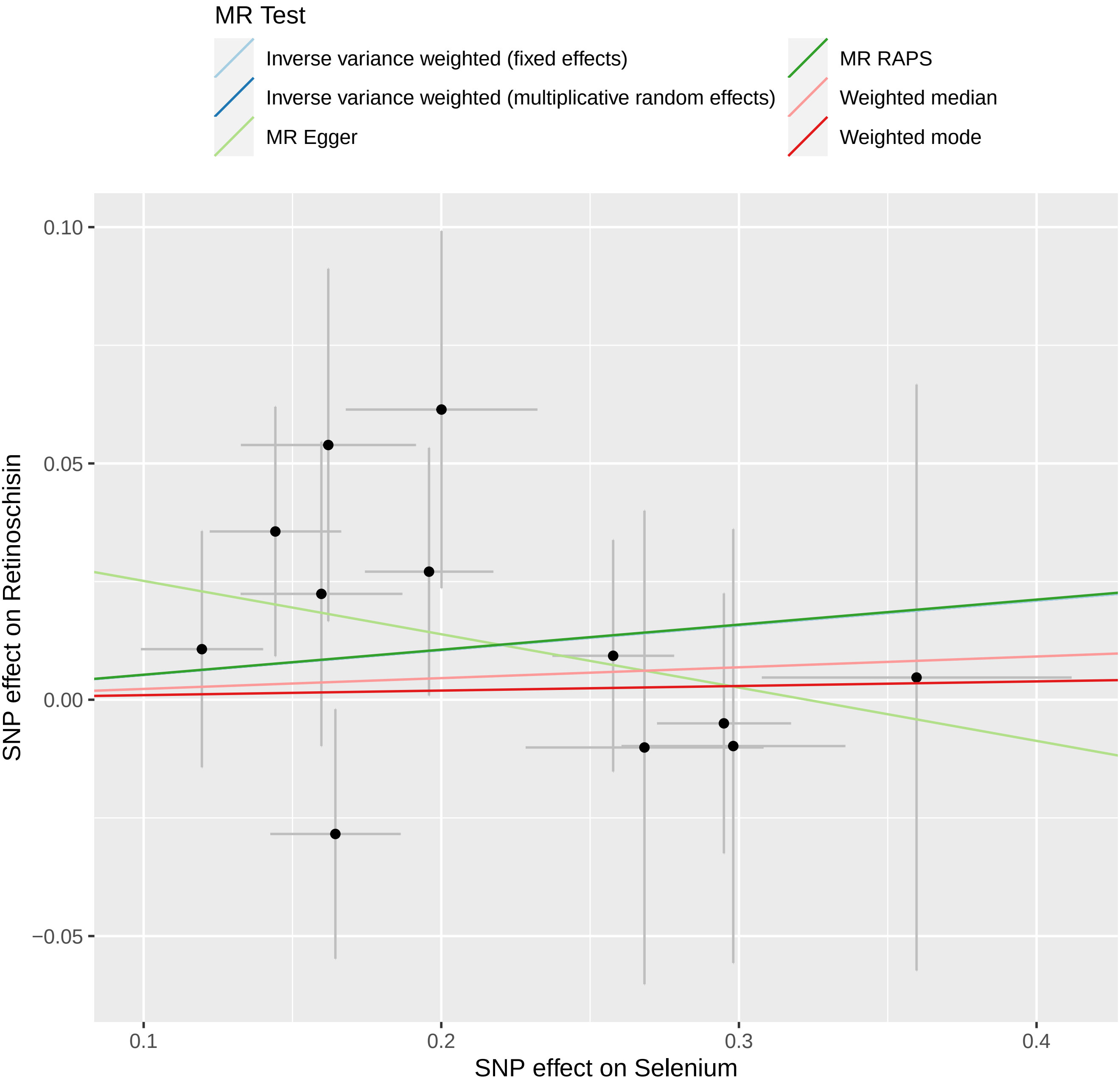

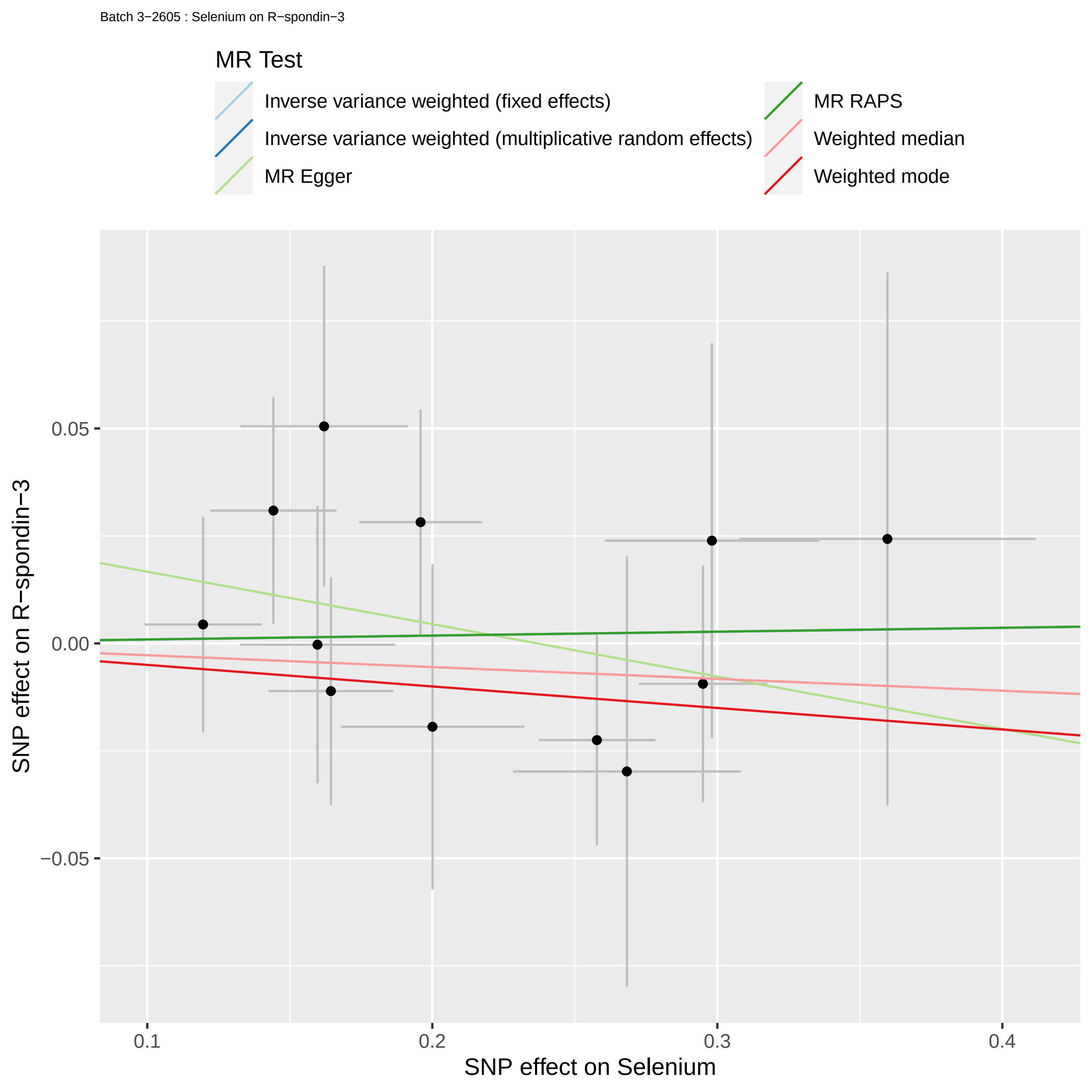

Batch 3−2605 : Selenium on R−spondin−3
MR Test
Inverse variance weighted (fixed effects)
Inverse variance weighted (multiplicative random effects)
MR Egger
MR RAPS
Weighted median
Weighted mode
0.05
0.00
−0.05
0.1
0.2
0.3
0.4
SNP effect on R−spondin−3
SNP effect on Selenium

Batch 3−2606 : Selenium on R−spondin−4

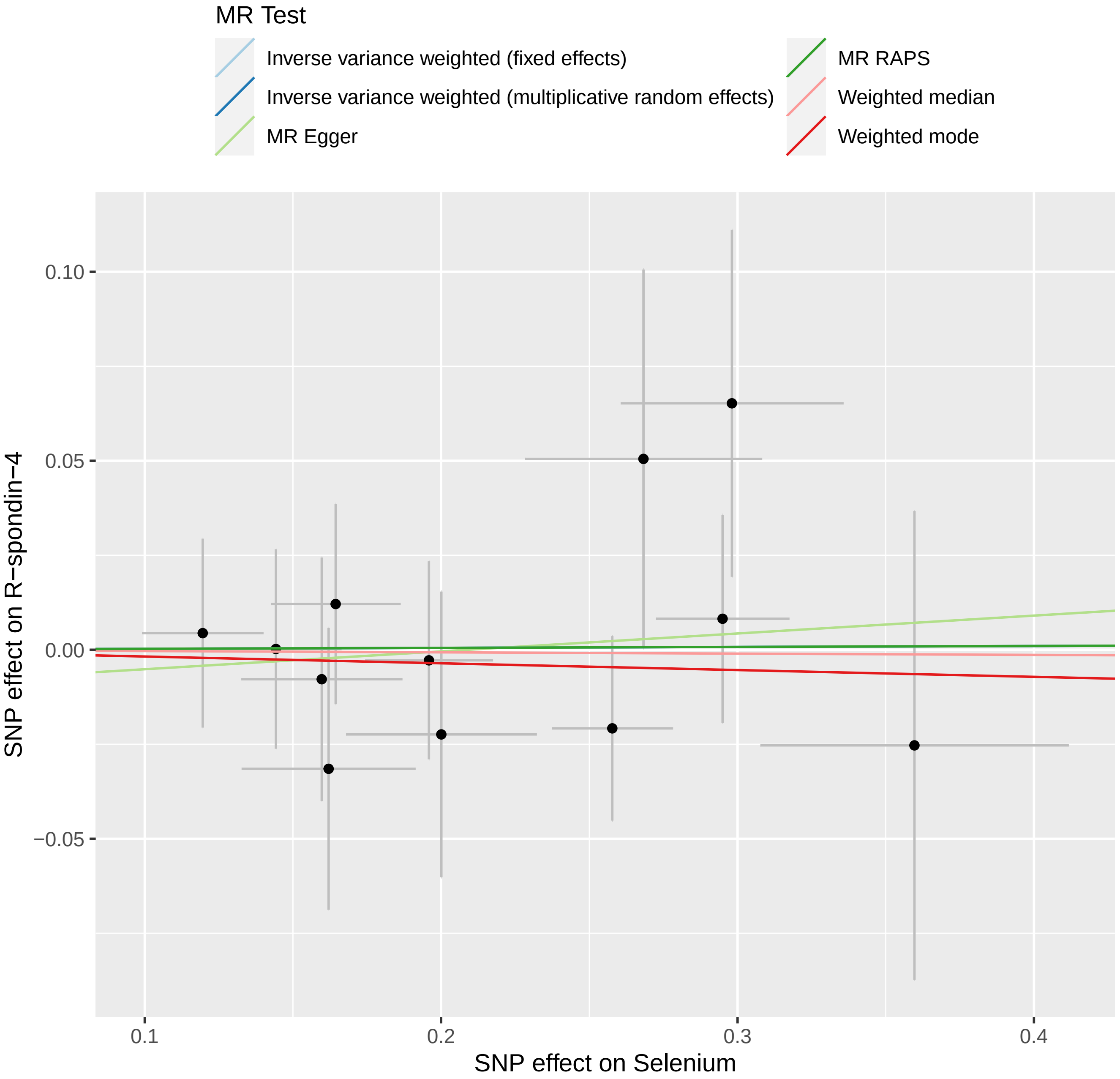

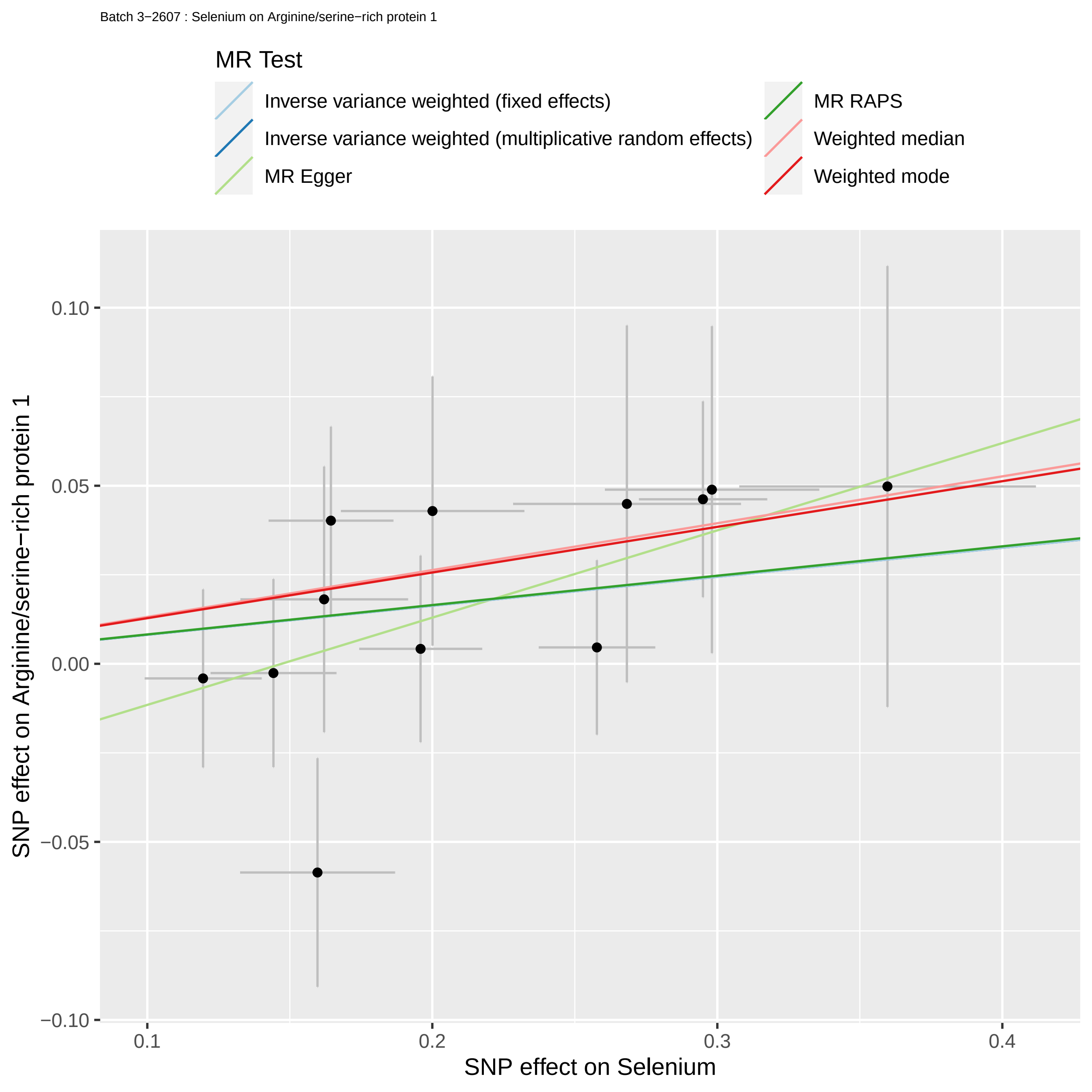

Batch 3−2607 : Selenium on Arginine/serine−rich protein 1
MR Test
Inverse variance weighted (fixed effects)
Inverse variance weighted (multiplicative random effects)
MR Egger
MR RAPS
Weighted median
Weighted mode
0.10
0.05
0.00
−0.05
−0.10
0.1
0.2
0.3
0.4
SNP effect on Arginine/serine−rich protein 1
SNP effect on Selenium

Batch 3−2608 : Selenium on Retbindin

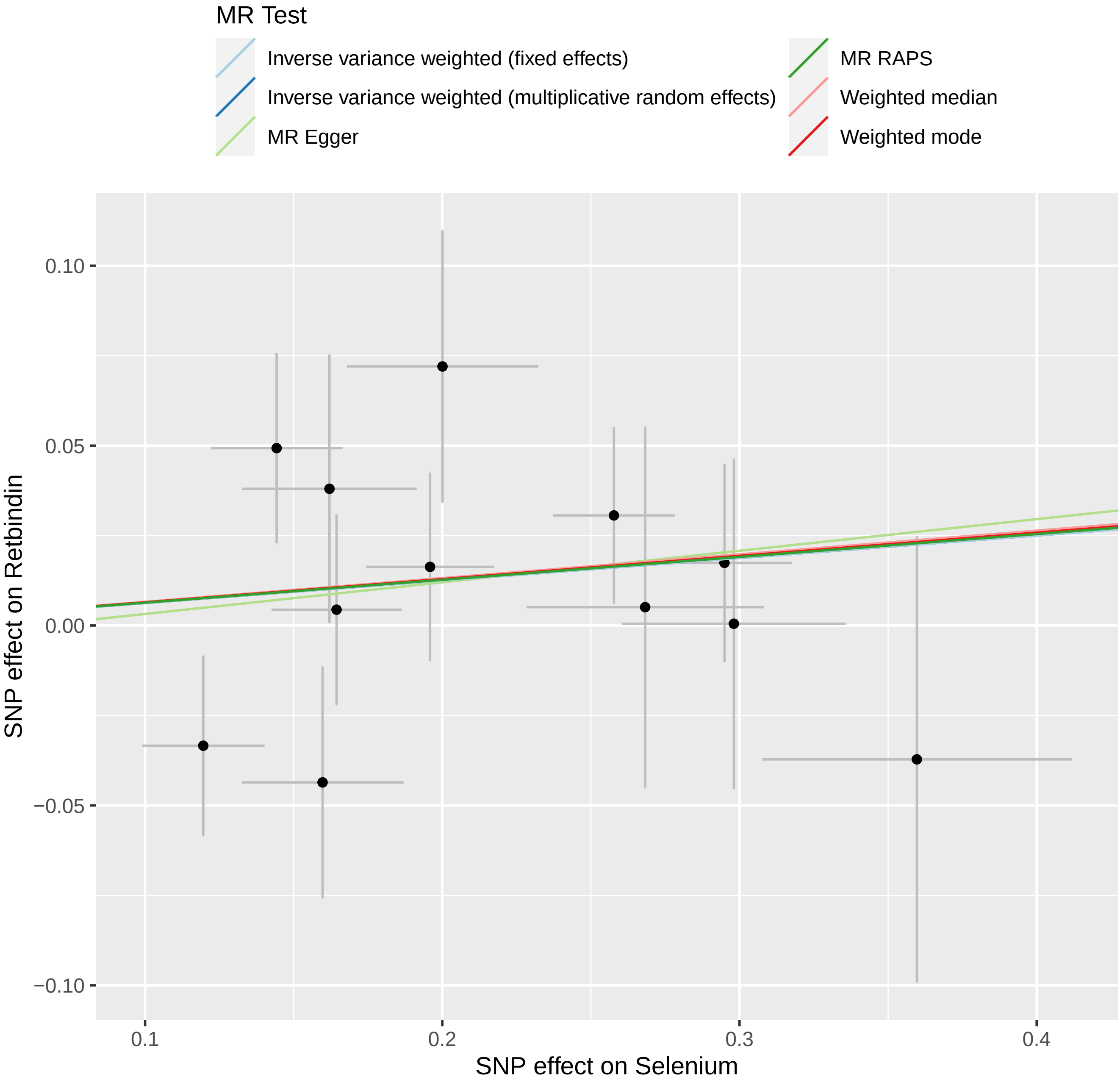

Batch 3−2609 : Selenium on Reticulon−4 receptor

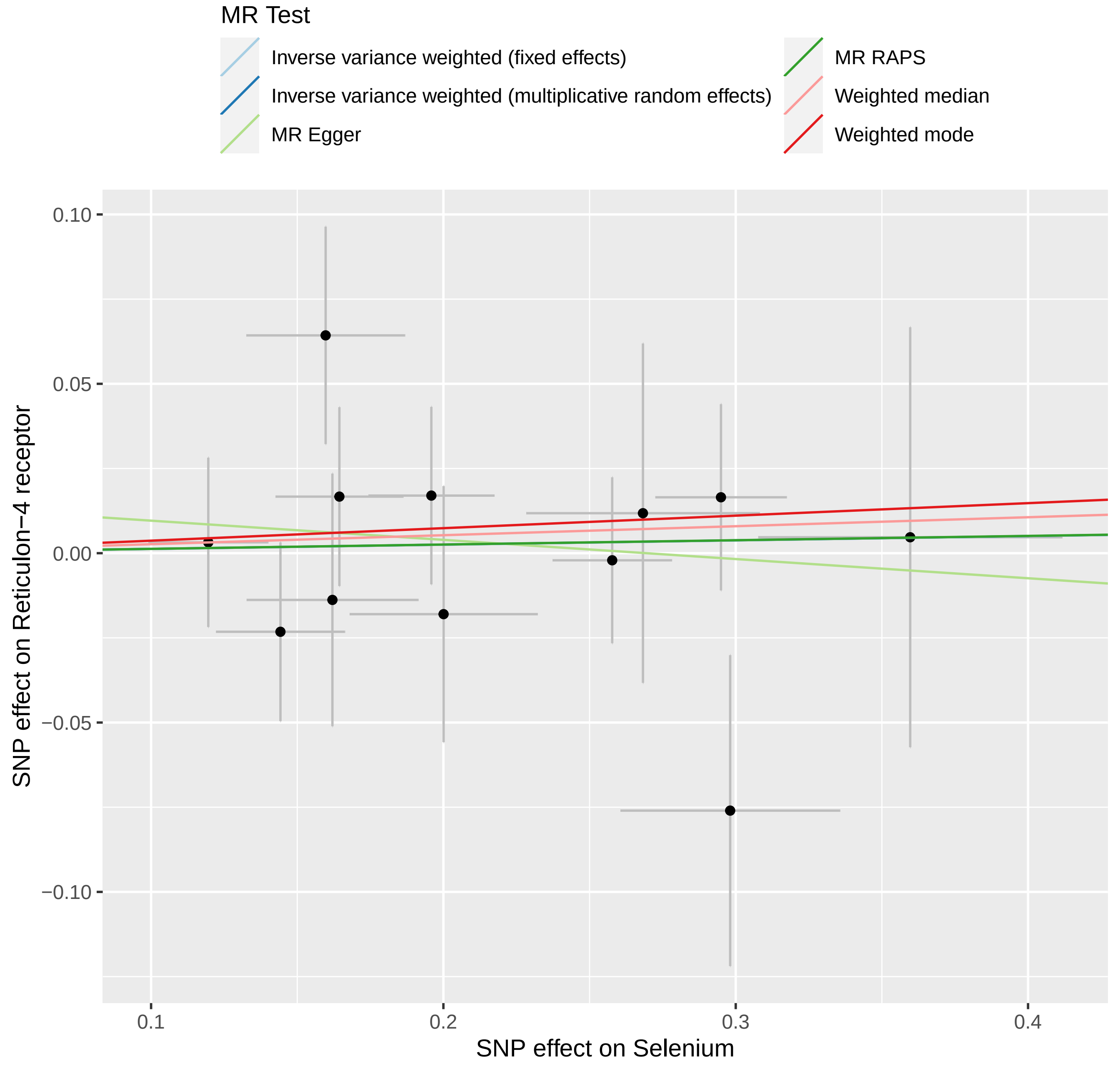

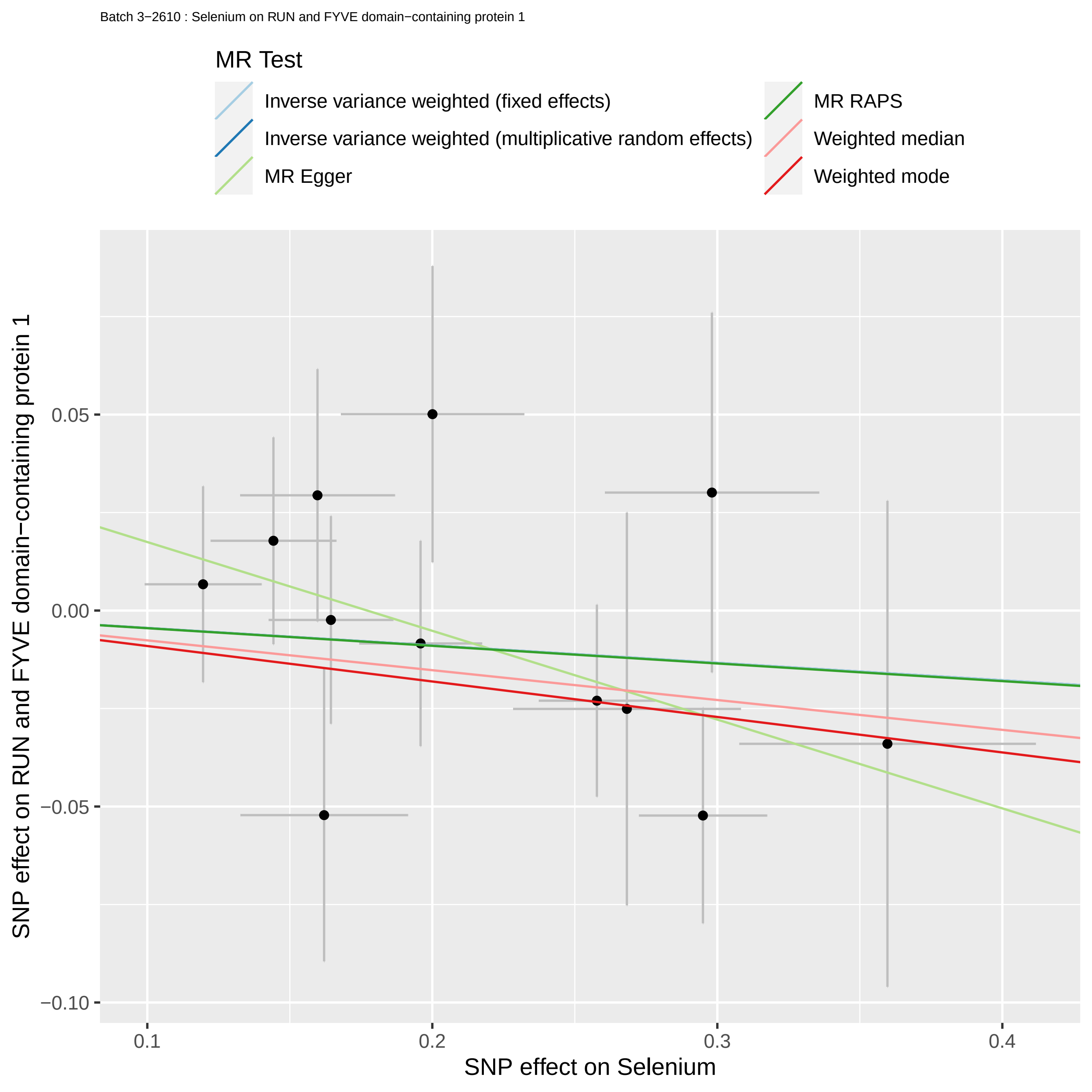

Batch 3−2610 : Selenium on RUN and FYVE domain−containing protein 1
MR Test
Inverse variance weighted (fixed effects)
Inverse variance weighted (multiplicative random effects)
MR Egger
MR RAPS
Weighted median
Weighted mode
0.05
0.00
−0.05
−0.10
0.1
0.2
0.3
0.4
SNP effect on Selenium
SNP effect on RUN and FYVE domain−containing protein 1

Batch 3−2611 : Selenium on Runt−related transcription factor 3

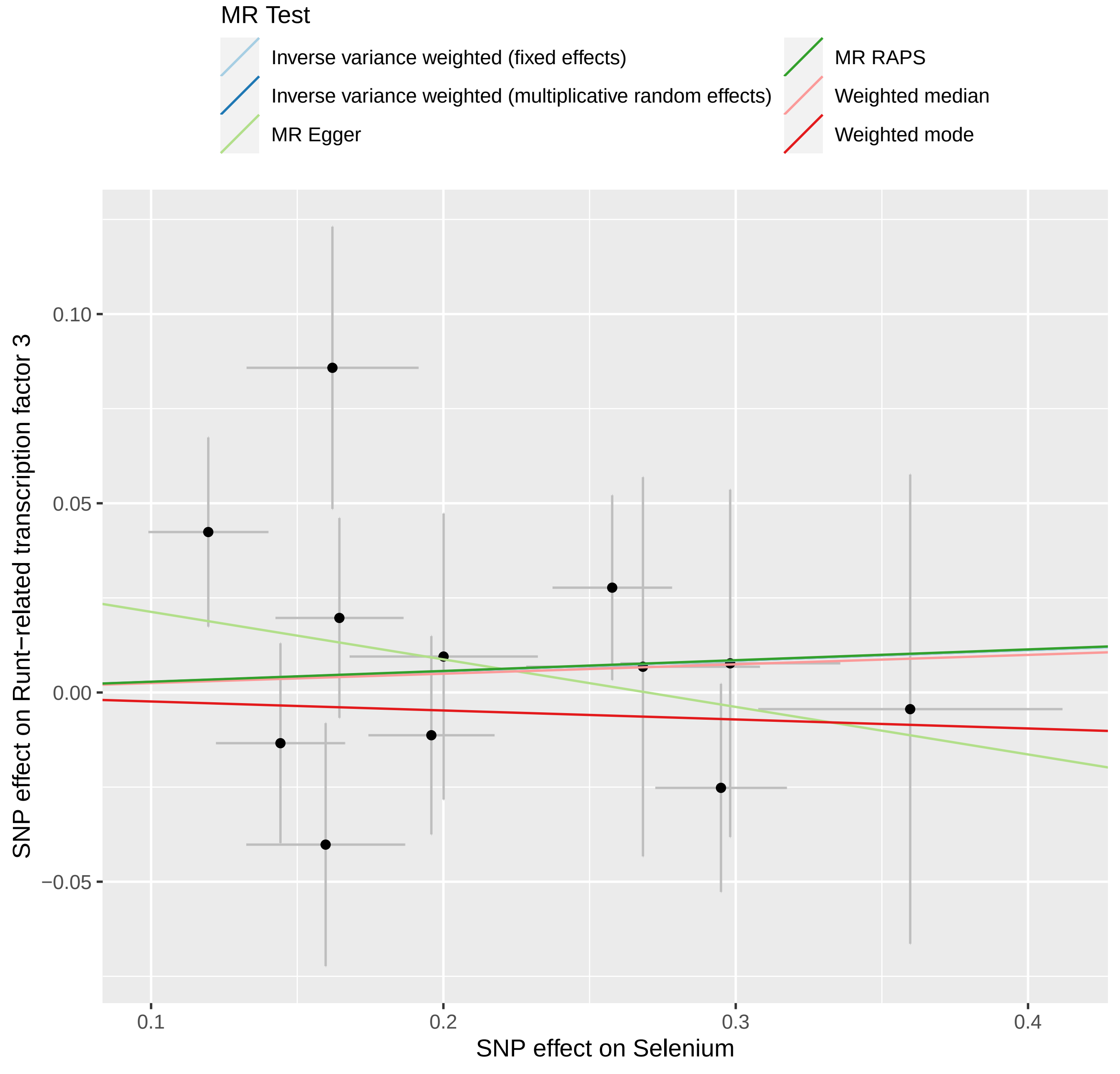

Batch 3−2612 : Selenium on Relaxin receptor 1

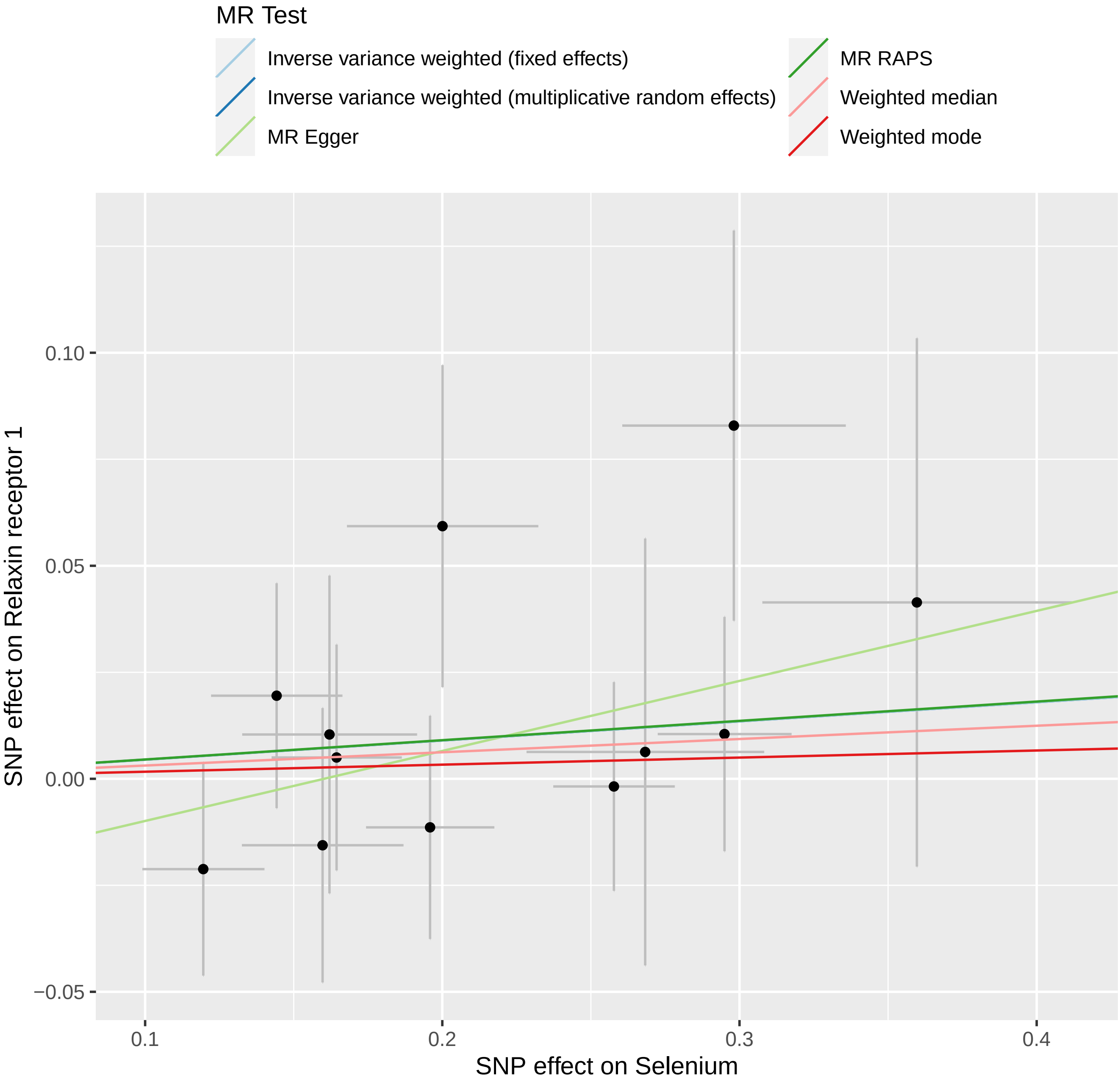

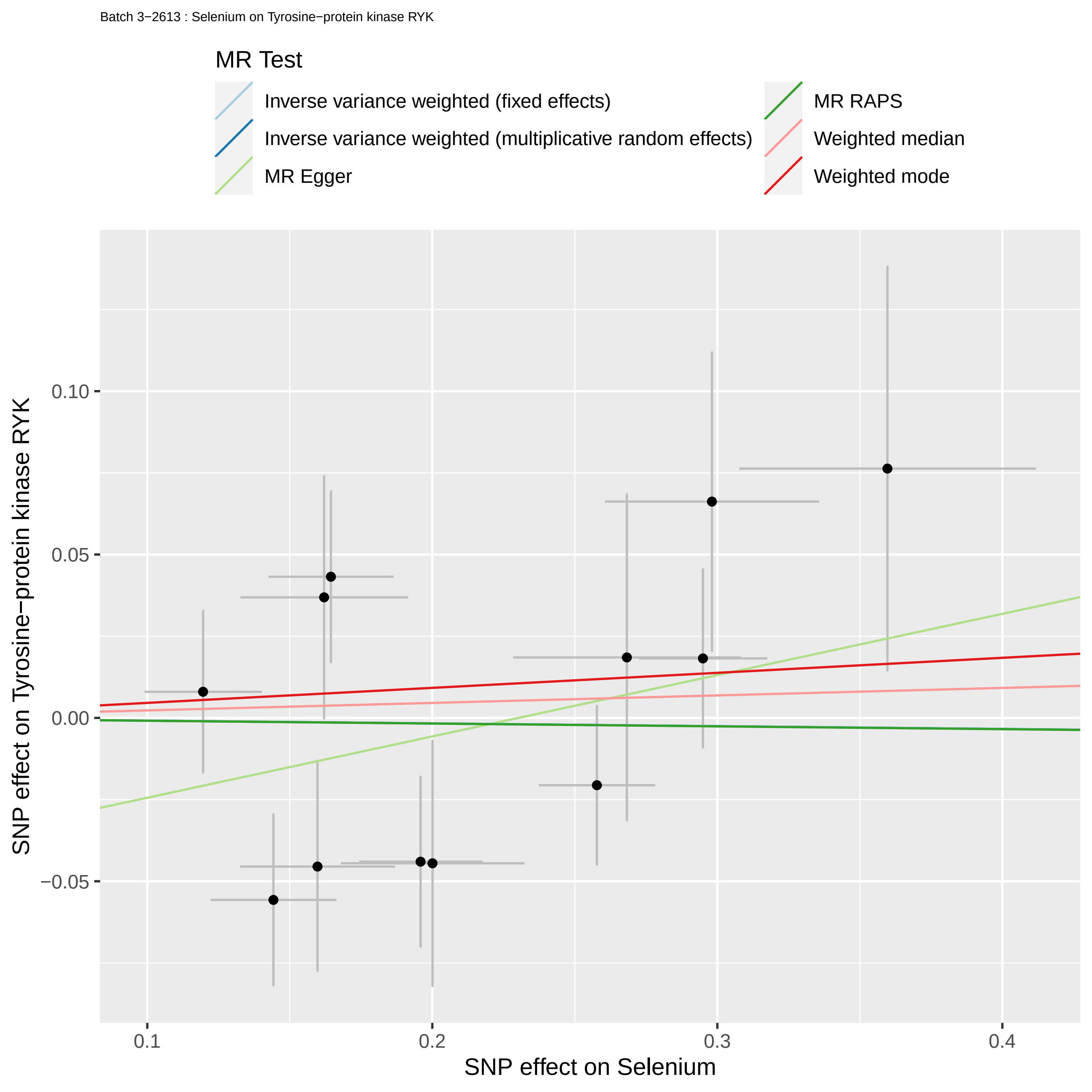

Batch 3−2613 : Selenium on Tyrosine−protein kinase RYK
MR Test
Inverse variance weighted (fixed effects)
Inverse variance weighted (multiplicative random effects)
MR Egger
MR RAPS
Weighted median
Weighted mode
0.10
0.05
0.00
−0.05
0.1
0.2
0.3
0.4
SNP effect on Tyrosine−protein kinase RYK
SNP effect on Selenium

Batch 3−2614 : Selenium on Protein S100−A11

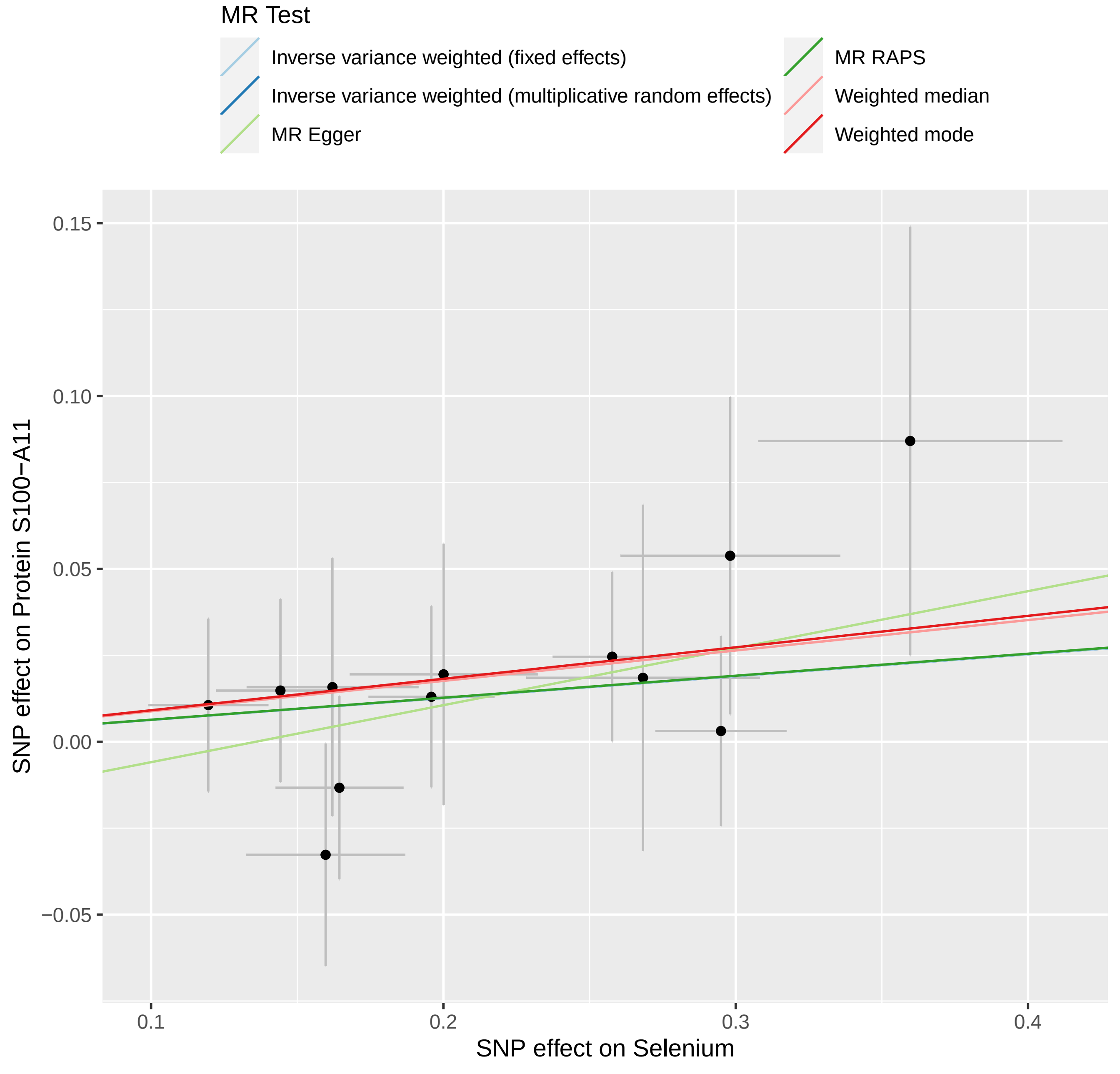

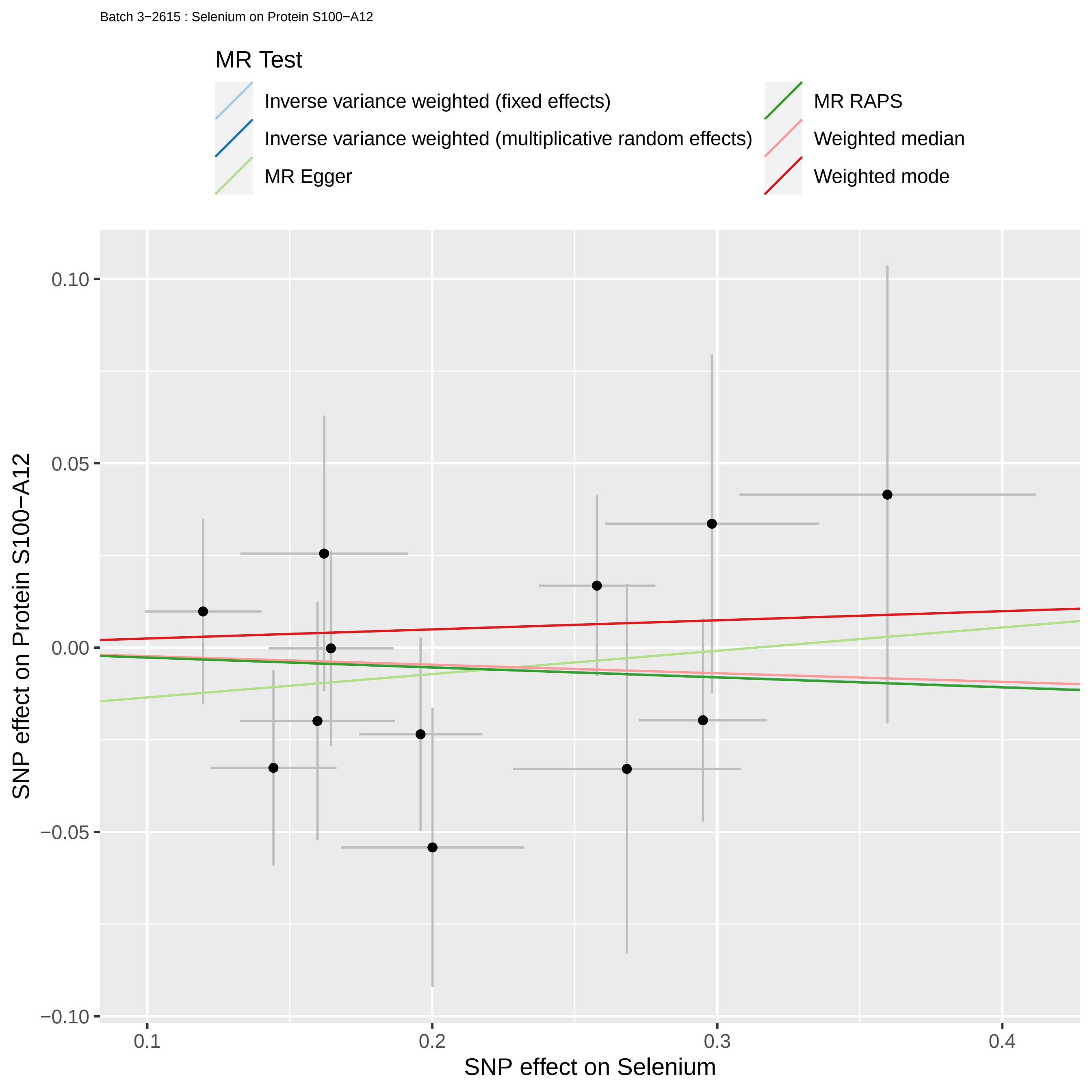

Batch 3−2615 : Selenium on Protein S100−A12
MR Test
Inverse variance weighted (fixed effects)
Inverse variance weighted (multiplicative random effects)
MR Egger
MR RAPS
Weighted median
Weighted mode
0.10
0.05
0.00
−0.05
−0.10
0.1
0.2
0.3
0.4
SNP effect on Protein S100−A12
SNP effect on Selenium

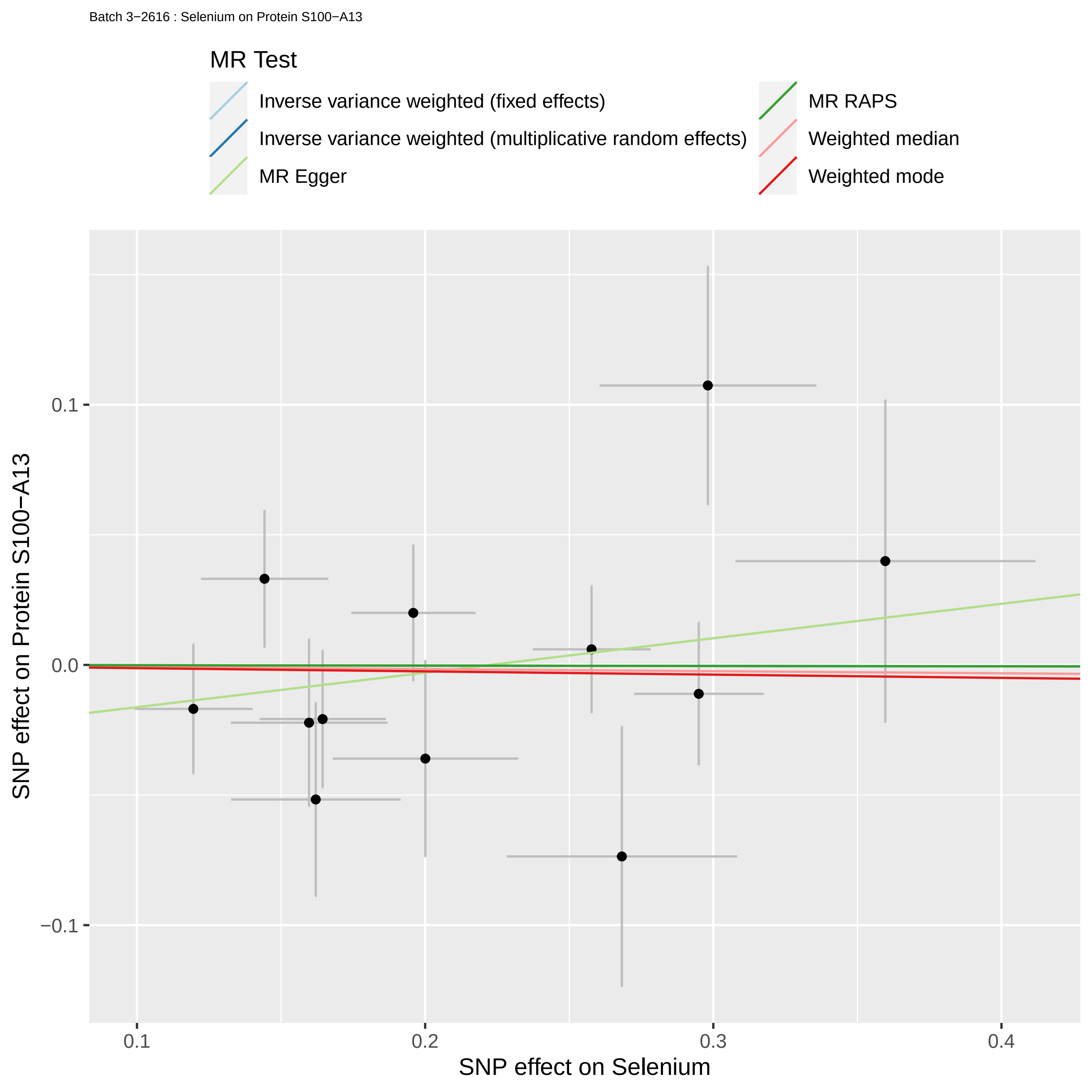

Batch 3−2616 : Selenium on Protein S100−A13
MR Test
Inverse variance weighted (fixed effects)
Inverse variance weighted (multiplicative random effects)
MR Egger
MR RAPS
Weighted median
Weighted mode
SNP effect on Protein S100−A13
0.1
0.0
−0.1
0.1
0.2
0.3
0.4
SNP effect on Selenium

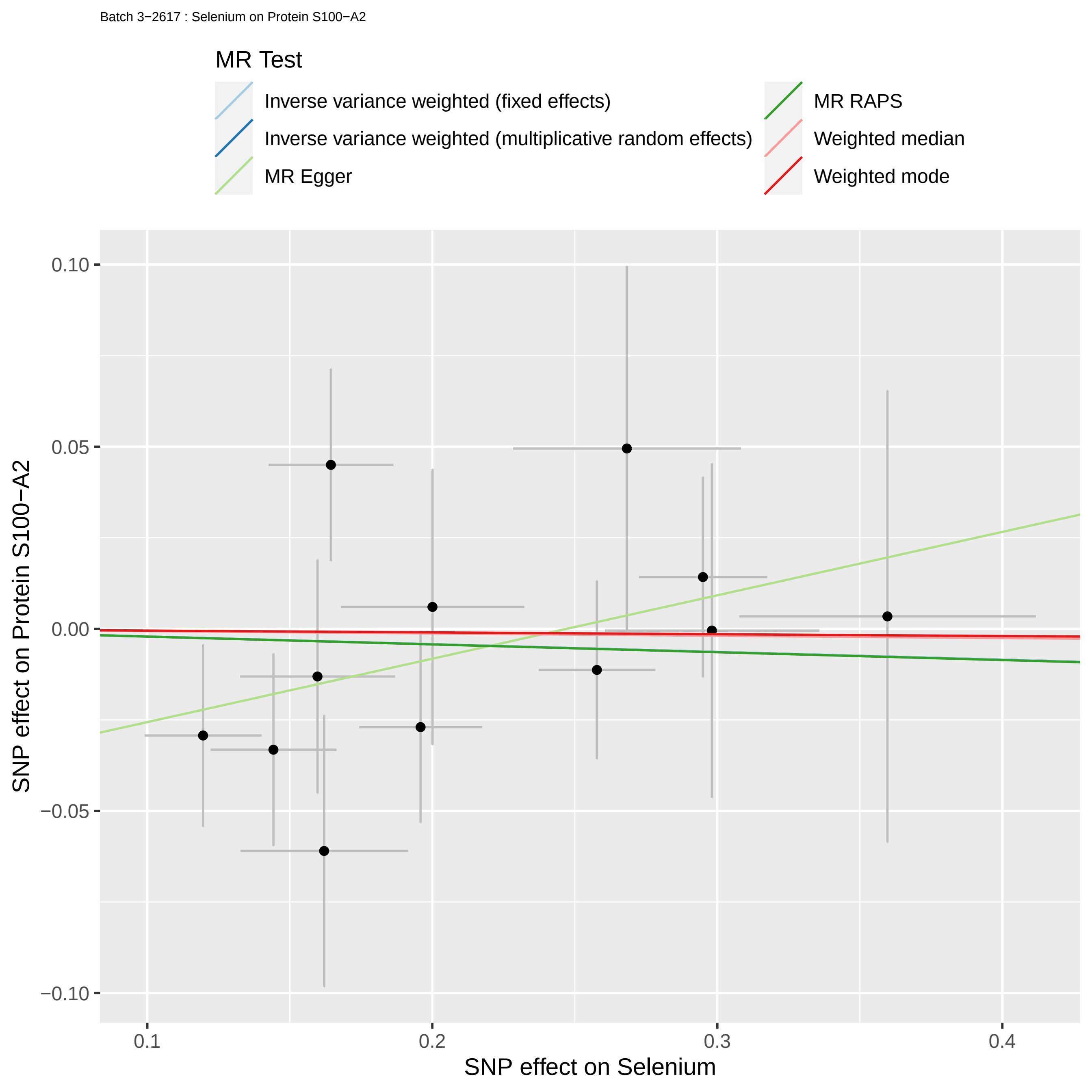

Batch 3−2617 : Selenium on Protein S100−A2
MR Test
Inverse variance weighted (fixed effects)
Inverse variance weighted (multiplicative random effects)
MR Egger
MR RAPS
Weighted median
Weighted mode
0.10
0.05
0.00
−0.05
−0.10
0.1
0.2
0.3
0.4
SNP effect on Protein S100−A2
SNP effect on Selenium

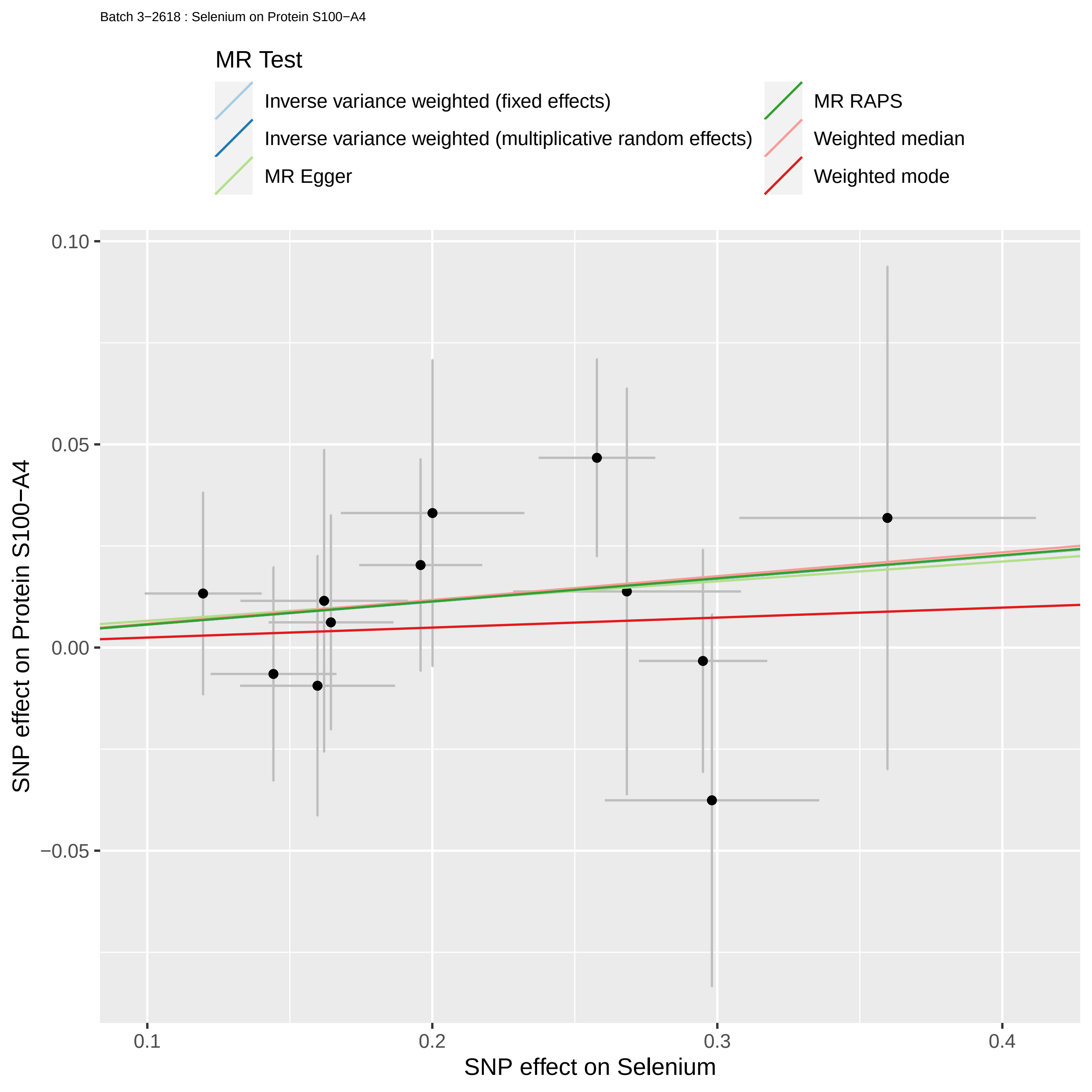

Batch 3−2618 : Selenium on Protein S100−A4
MR Test
Inverse variance weighted (fixed effects)
Inverse variance weighted (multiplicative random effects)
MR Egger
MR RAPS
Weighted median
Weighted mode
0.10
0.05
0.00
−0.05
SNP effect on Protein S100−A4
0.1
0.2
0.3
0.4
SNP effect on Selenium

Batch 3−2619 : Selenium on Protein S100−A5

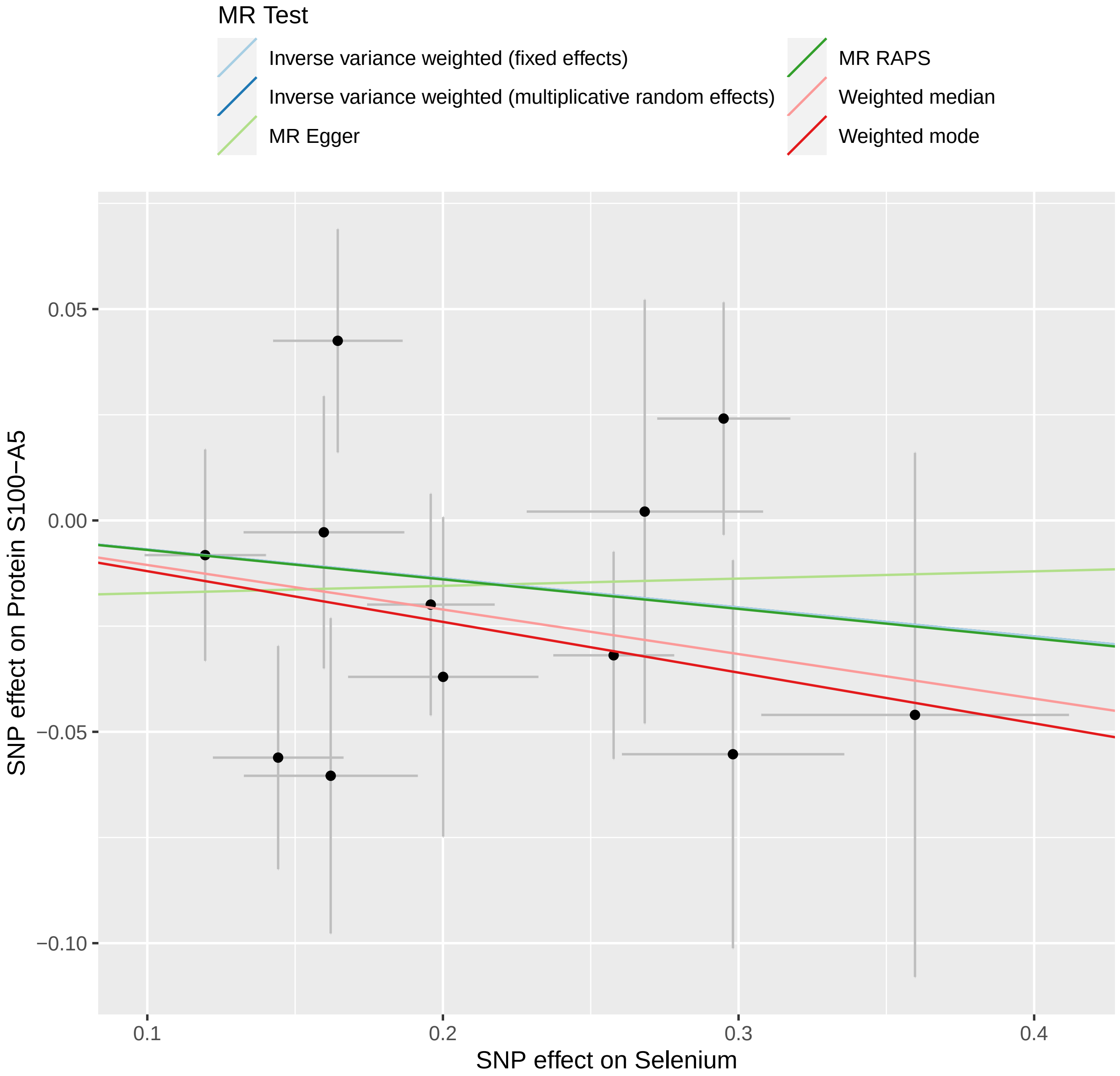

Batch 3−2620 : Selenium on Protein S100−A6

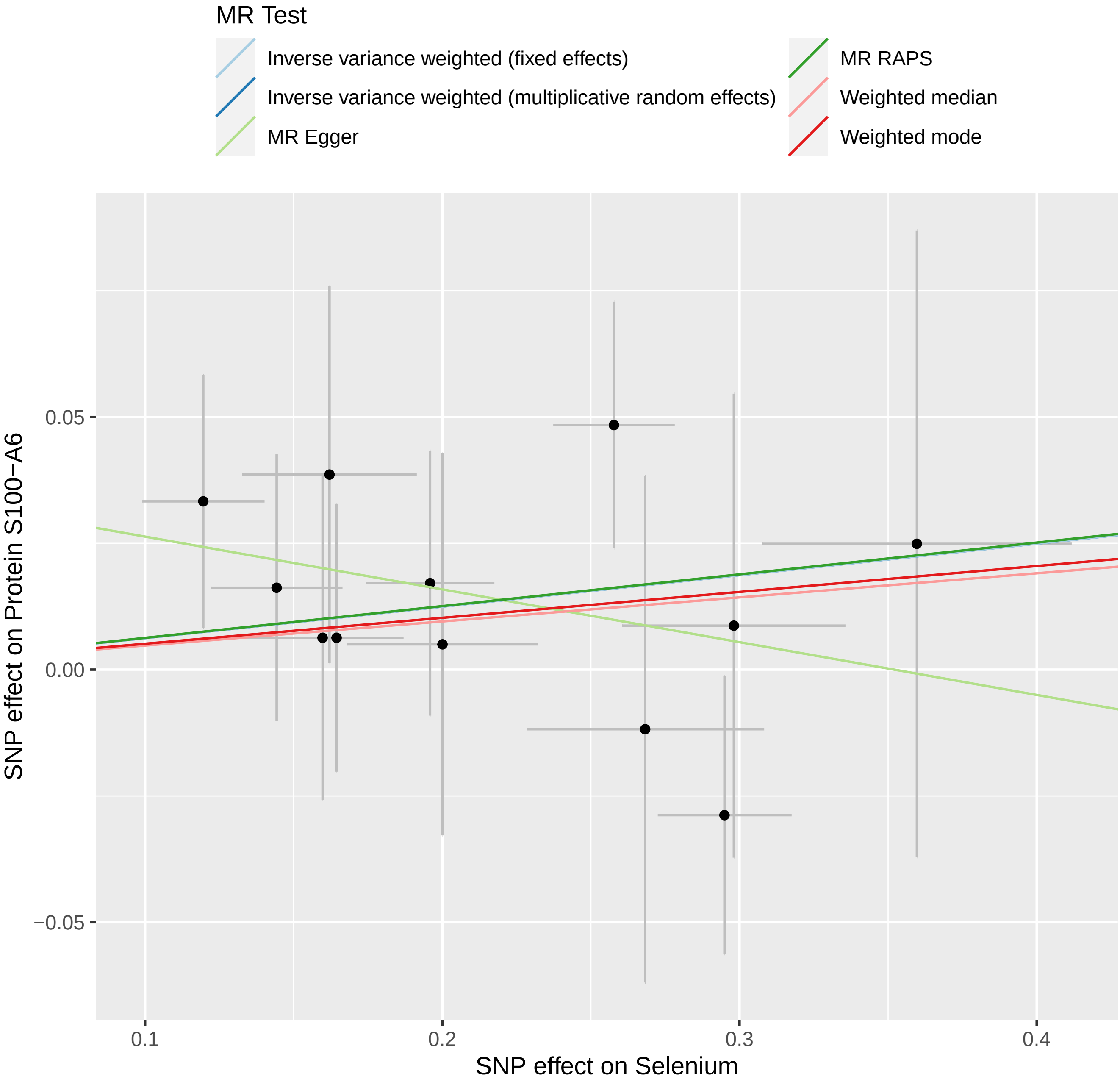

Batch 3−2621 : Selenium on Protein S100−A7

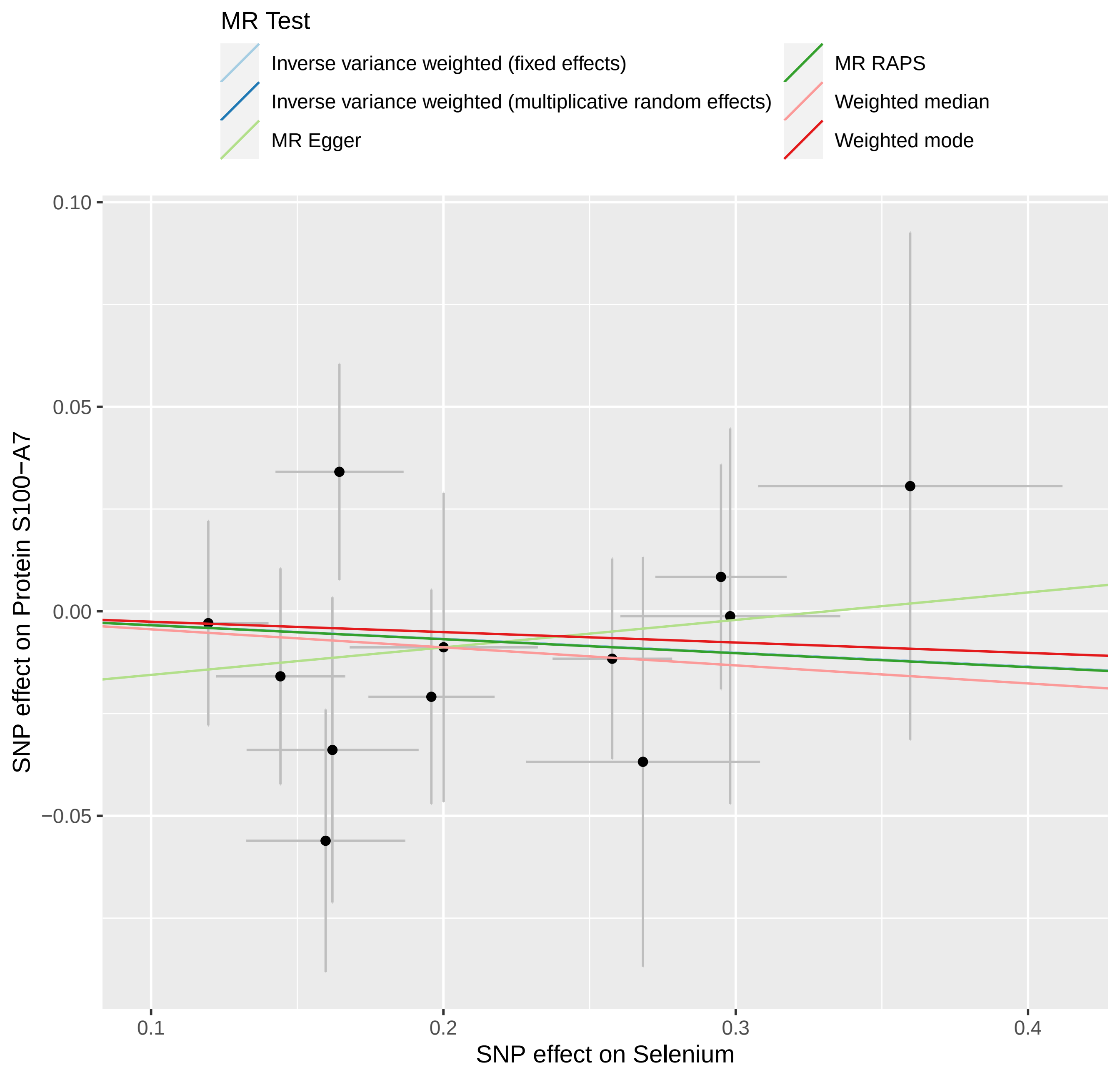

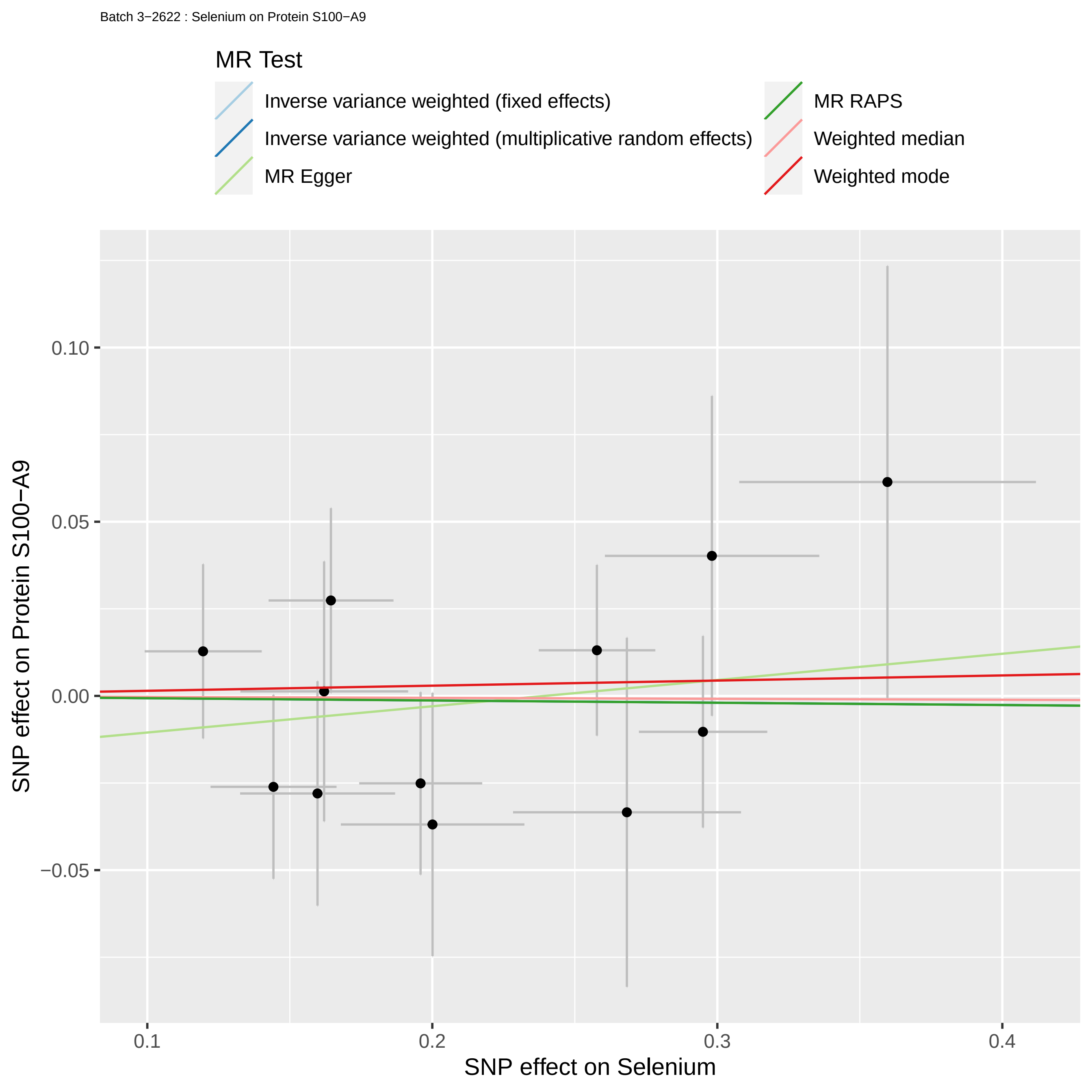
Batch 3−2622 : Selenium on Protein S100−A9
MR Test
Inverse variance weighted (fixed effects)
Inverse variance weighted (multiplicative random effects)
MR Egger
MR RAPS
Weighted median
Weighted mode
0.10
0.05
0.00
−0.05
0.1
0.2
0.3
0.4
SNP effect on Protein S100−A9
SNP effect on Selenium

Batch 3−2623 : Selenium on Serum amyloid A−1 protein

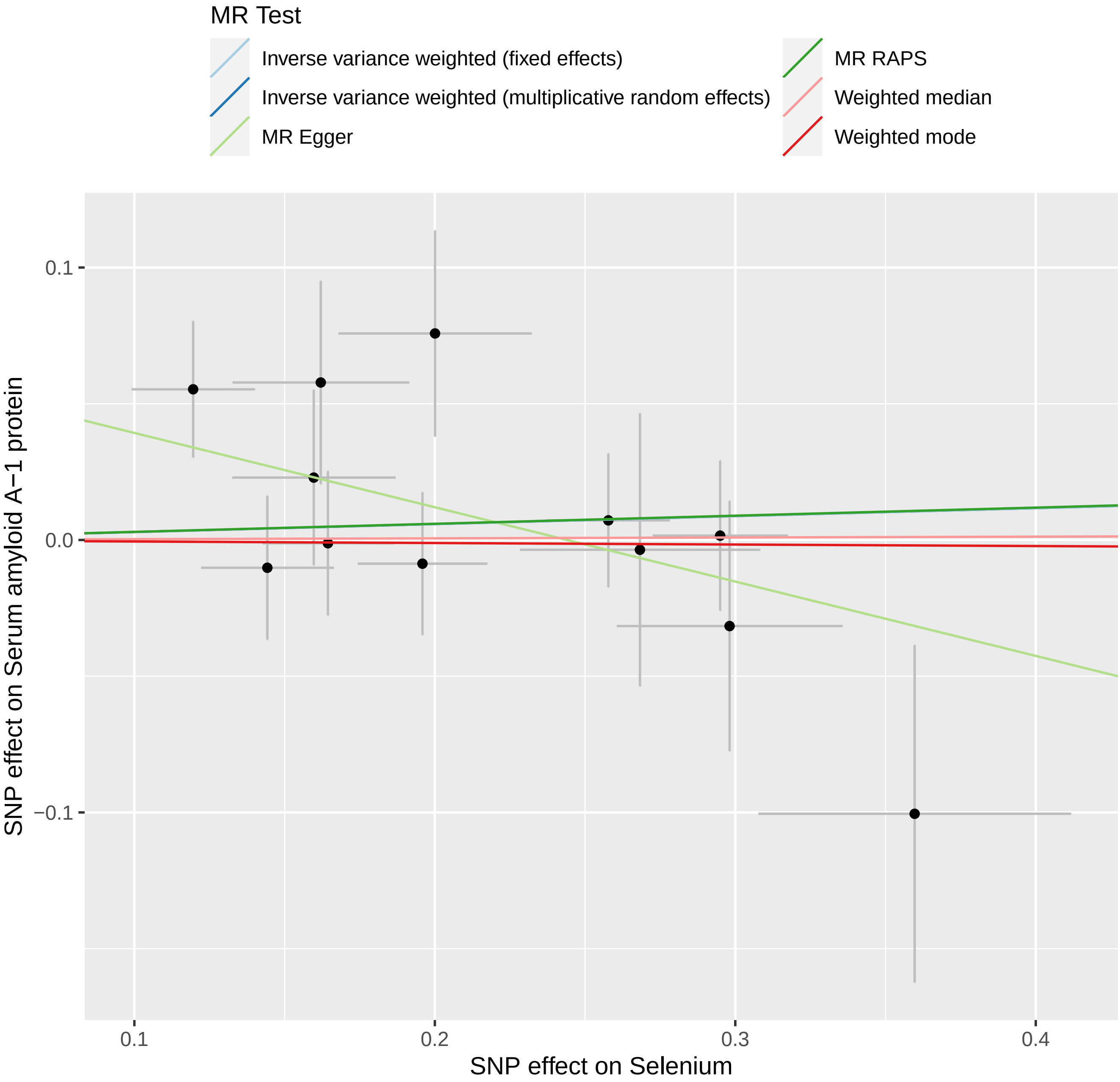

Batch 3−2624 : Selenium on Deoxynucleoside triphosphate triphosphohydrolase SAMHD1

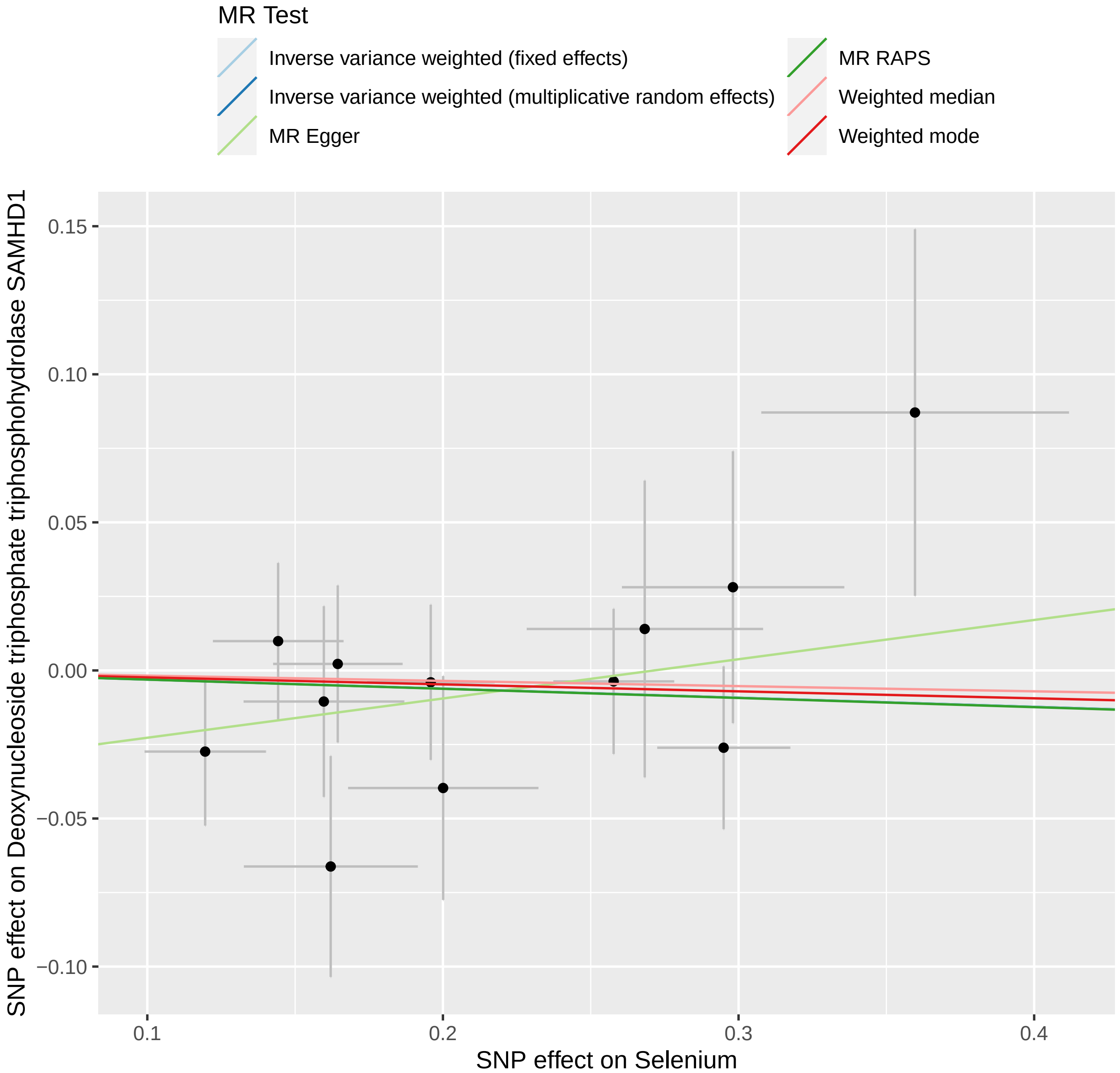

Batch 3−2625 : Selenium on Histone deacetylase complex subunit SAP18

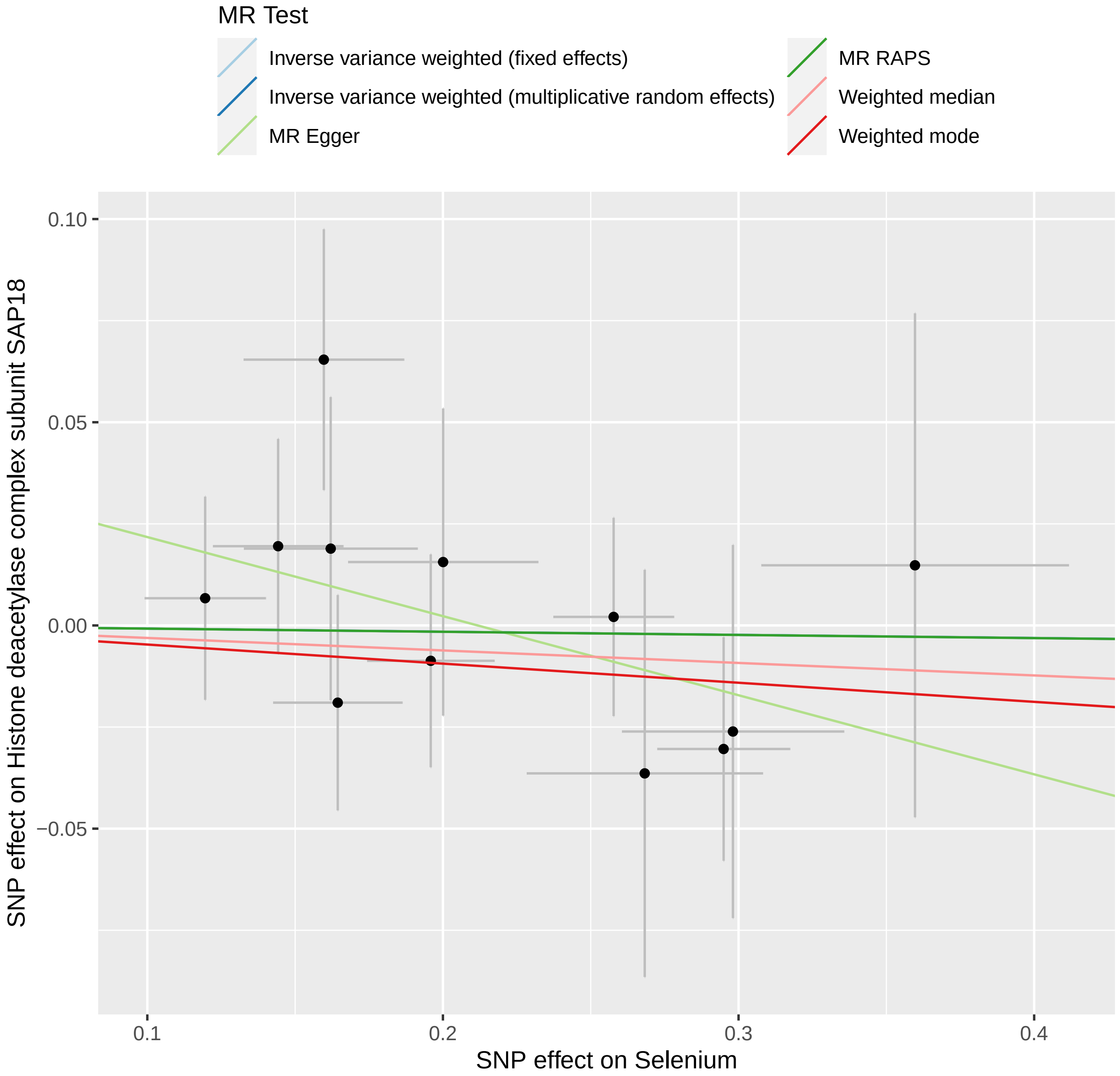

Batch 3−2626 : Selenium on Histone deacetylase complex subunit SAP30

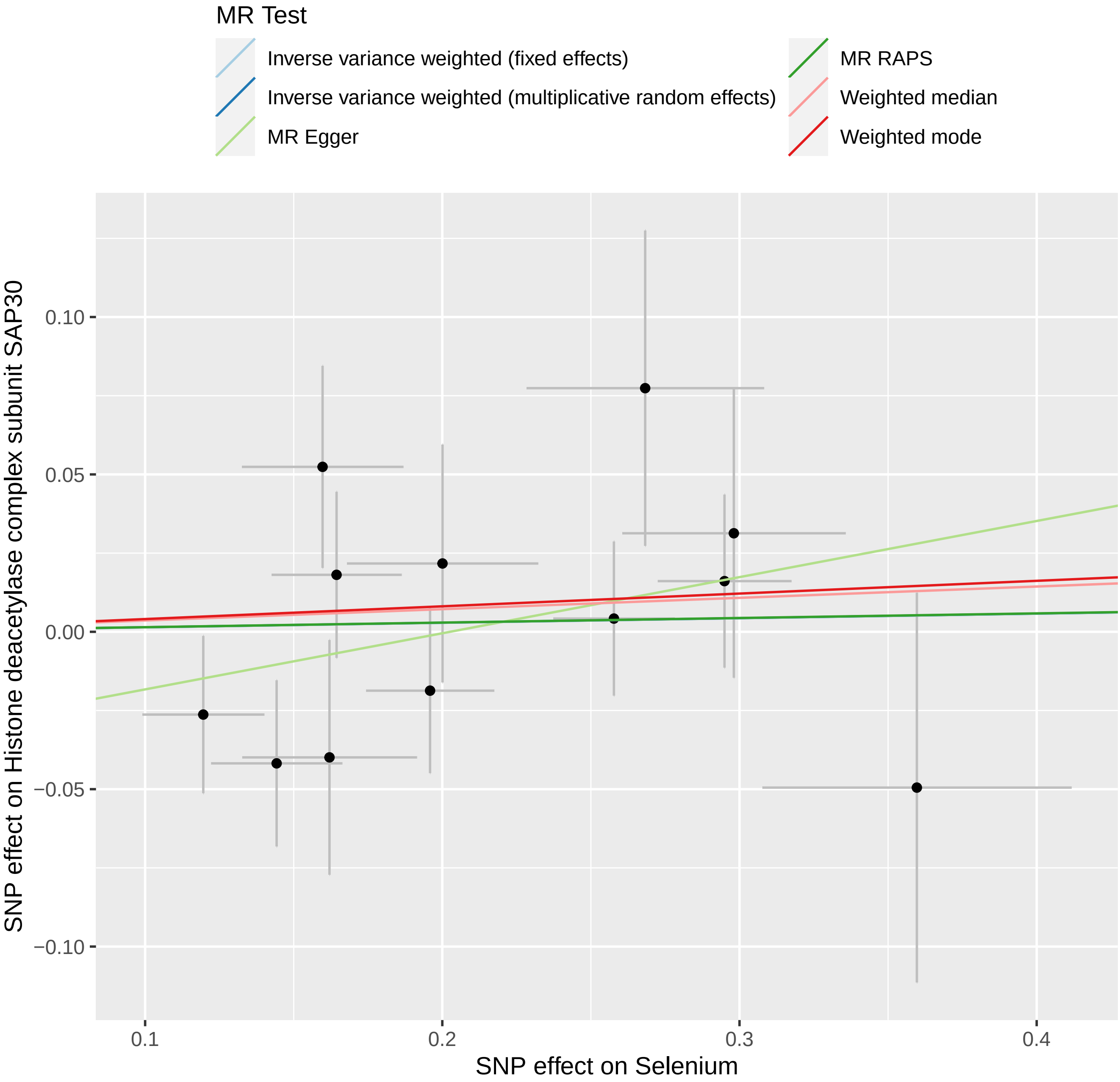

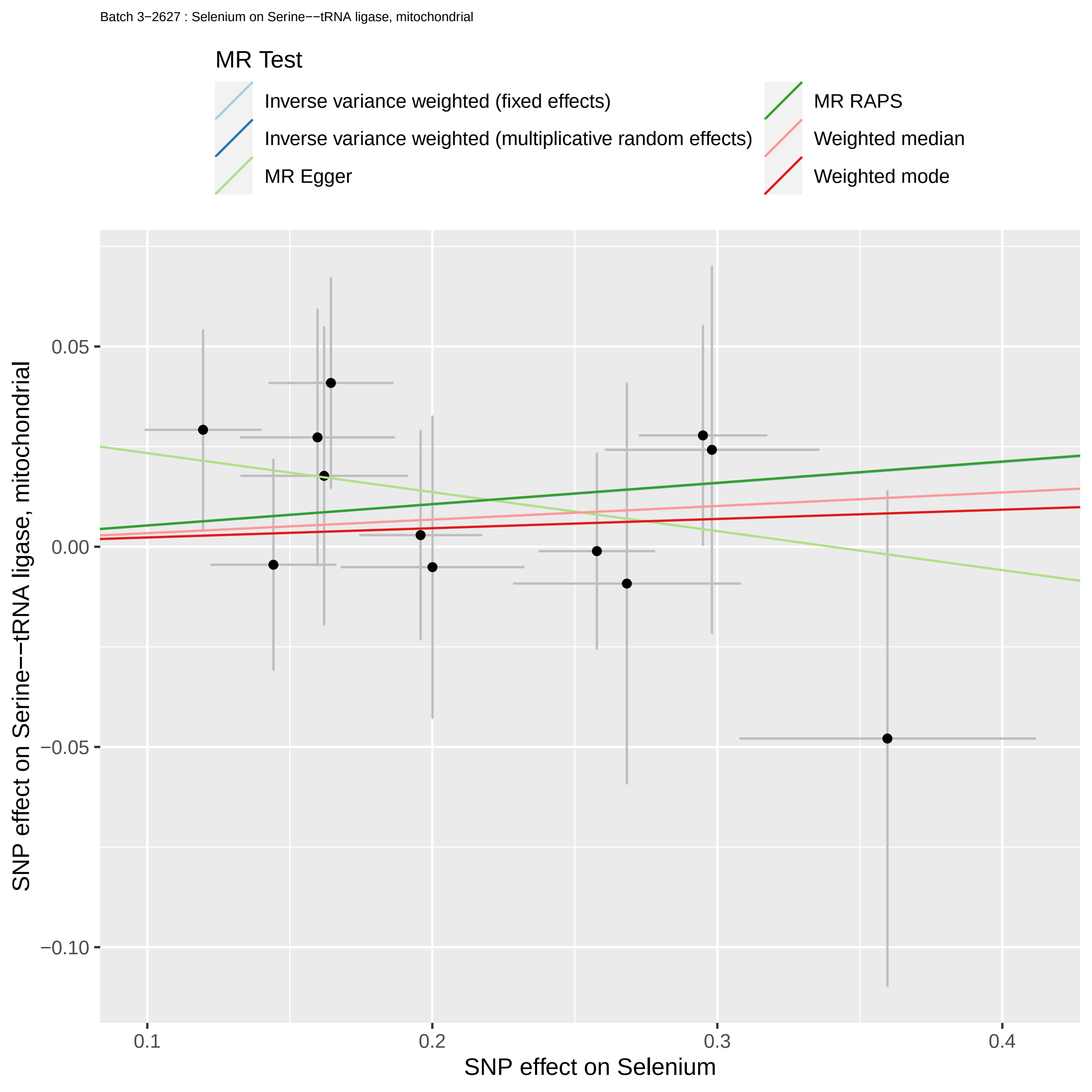

Batch 3−2627 : Selenium on Serine−−tRNA ligase, mitochondrial
MR Test
Inverse variance weighted (fixed effects)
Inverse variance weighted (multiplicative random effects)
MR Egger
MR RAPS
Weighted median
Weighted mode
0.05
0.00
−0.05
−0.10
0.1
0.2
0.3
0.4
SNP effect on Serine−−tRNA ligase, mitochondrial
SNP effect on Selenium

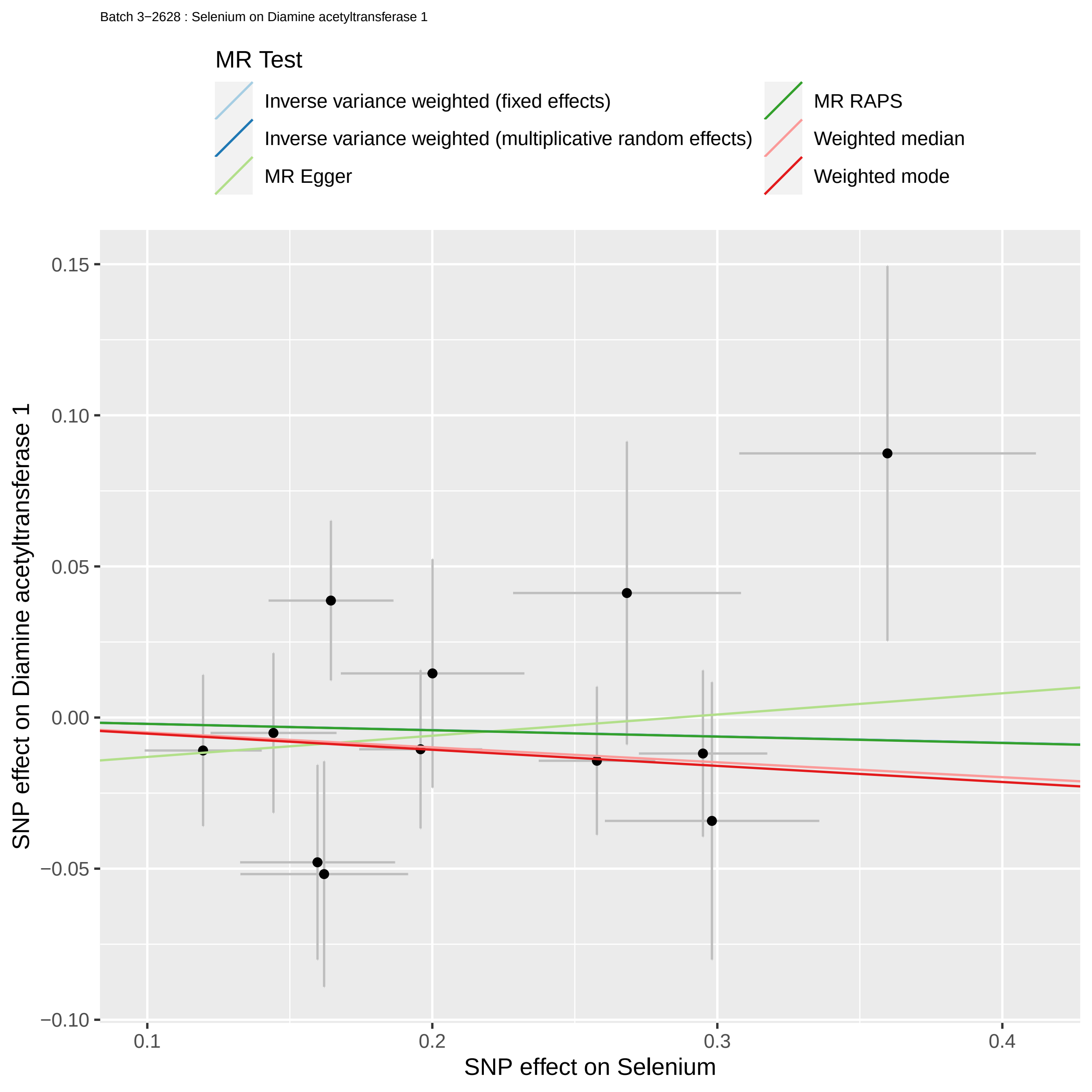

Batch 3−2628 : Selenium on Diamine acetyltransferase 1
MR Test
Inverse variance weighted (fixed effects)
Inverse variance weighted (multiplicative random effects)
MR Egger
MR RAPS
Weighted median
Weighted mode
0.15
0.10
0.05
0.00
−0.05
−0.10
0.1
0.2
0.3
0.4
SNP effect on Diamine acetyltransferase 1
SNP effect on Selenium

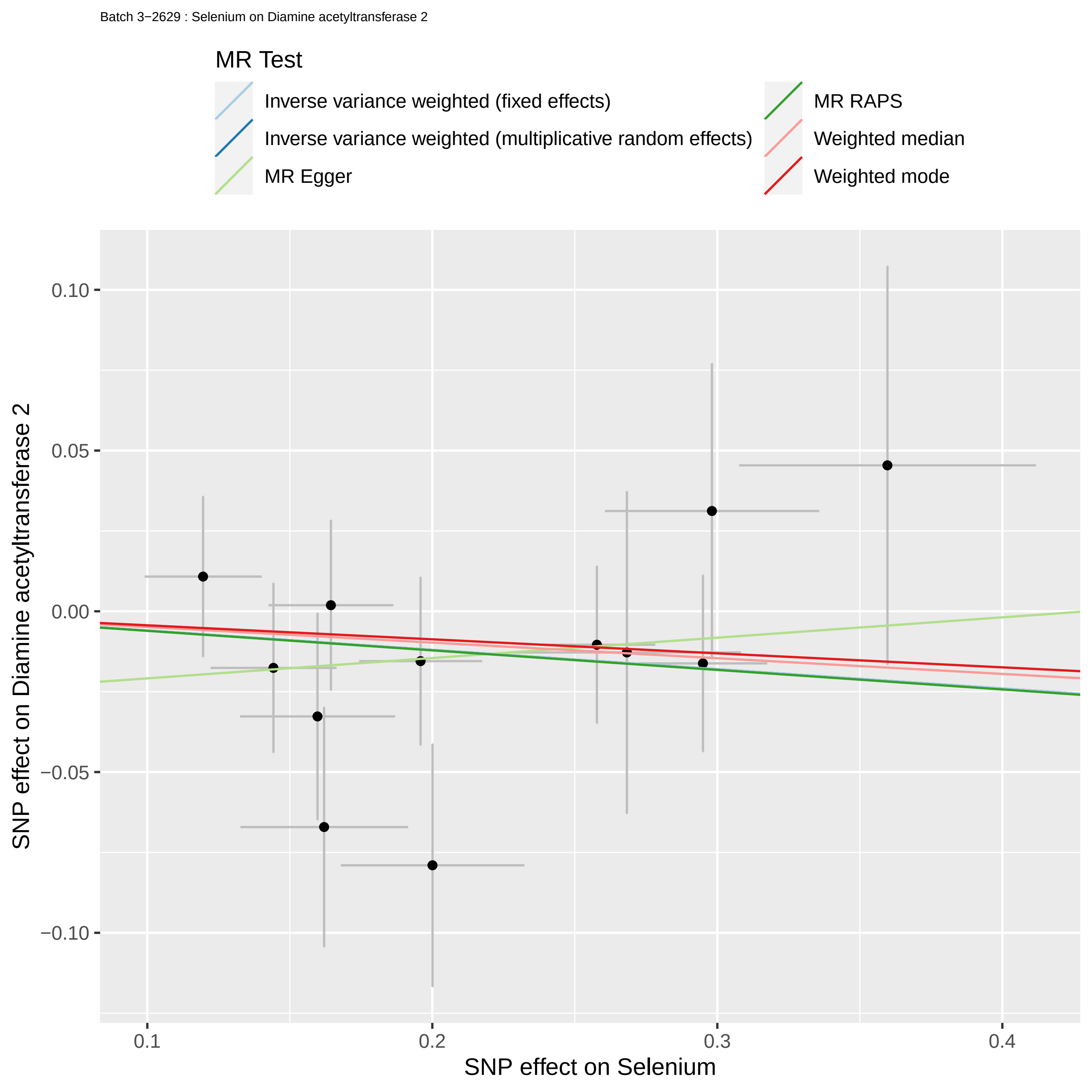

Batch 3−2629 : Selenium on Diamine acetyltransferase 2
MR Test
Inverse variance weighted (fixed effects)
Inverse variance weighted (multiplicative random effects)
MR Egger
MR RAPS
Weighted median
Weighted mode
SNP effect on Diamine acetyltransferase 2
0.10
0.05
0.00
−0.05
−0.10
0.1
0.2
0.3
0.4
SNP effect on Selenium

Batch 3−2630 : Selenium on DNA−binding protein SATB1

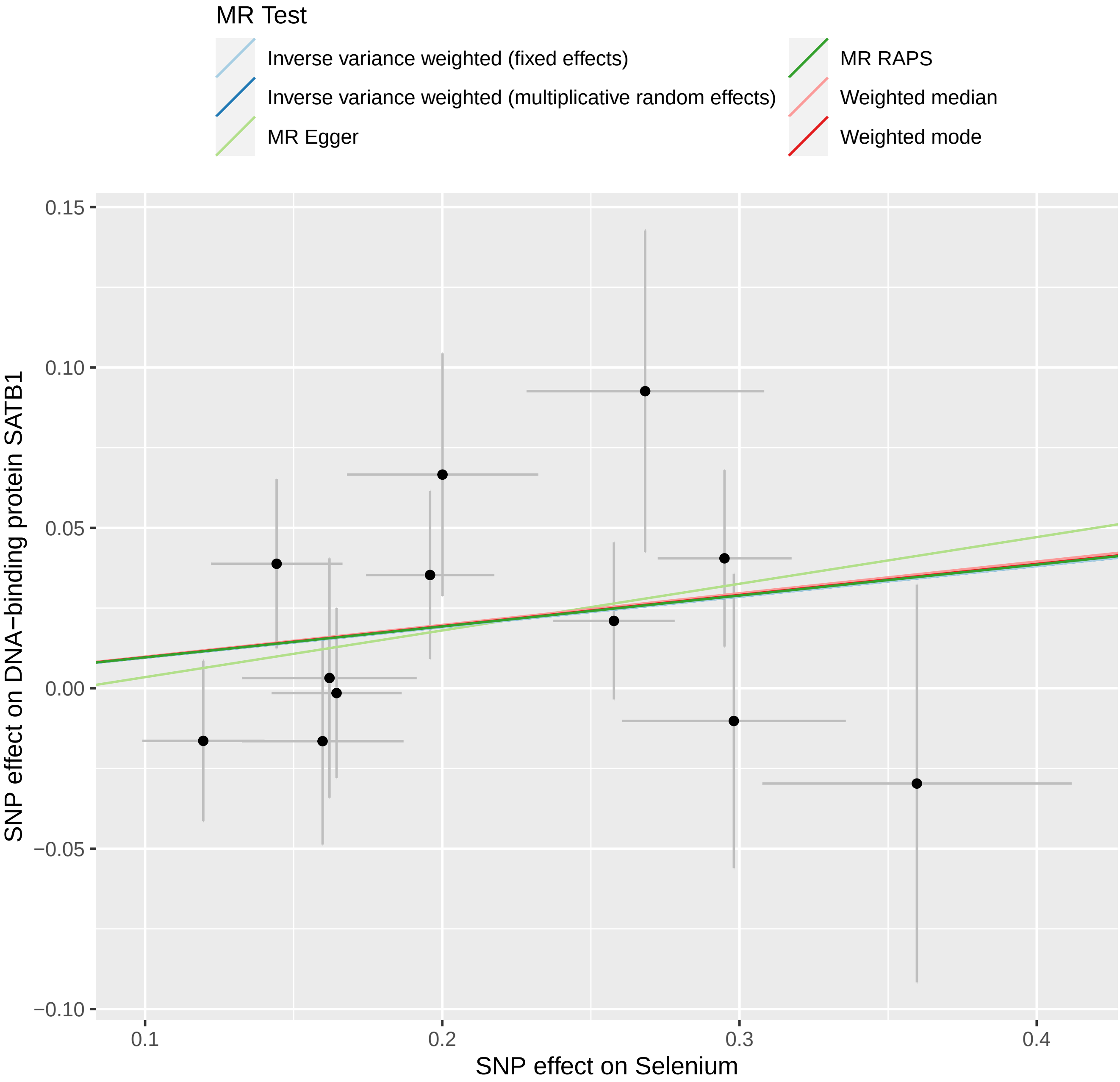

Batch 3−2631 : Selenium on DNA−binding protein SATB2

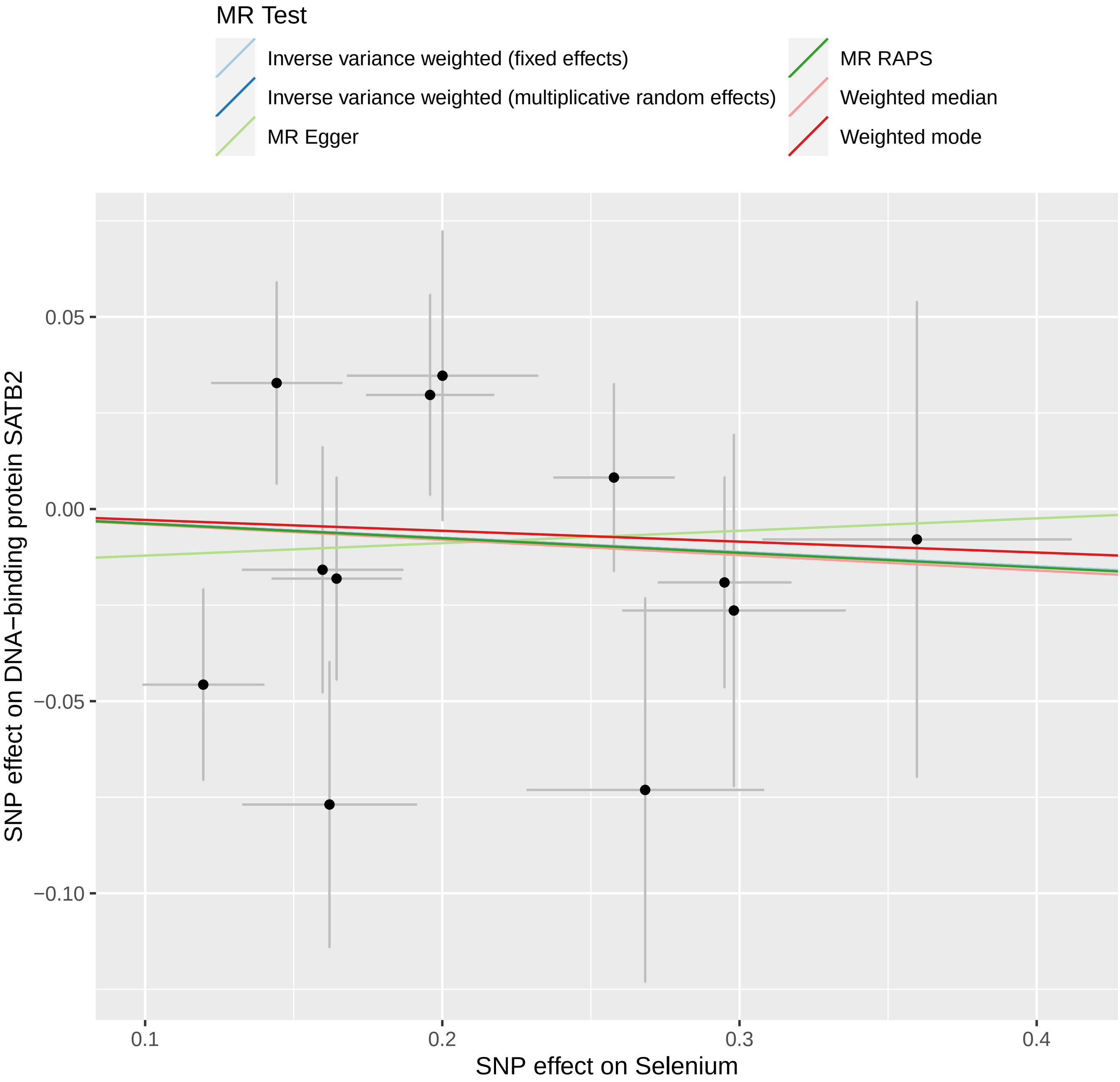

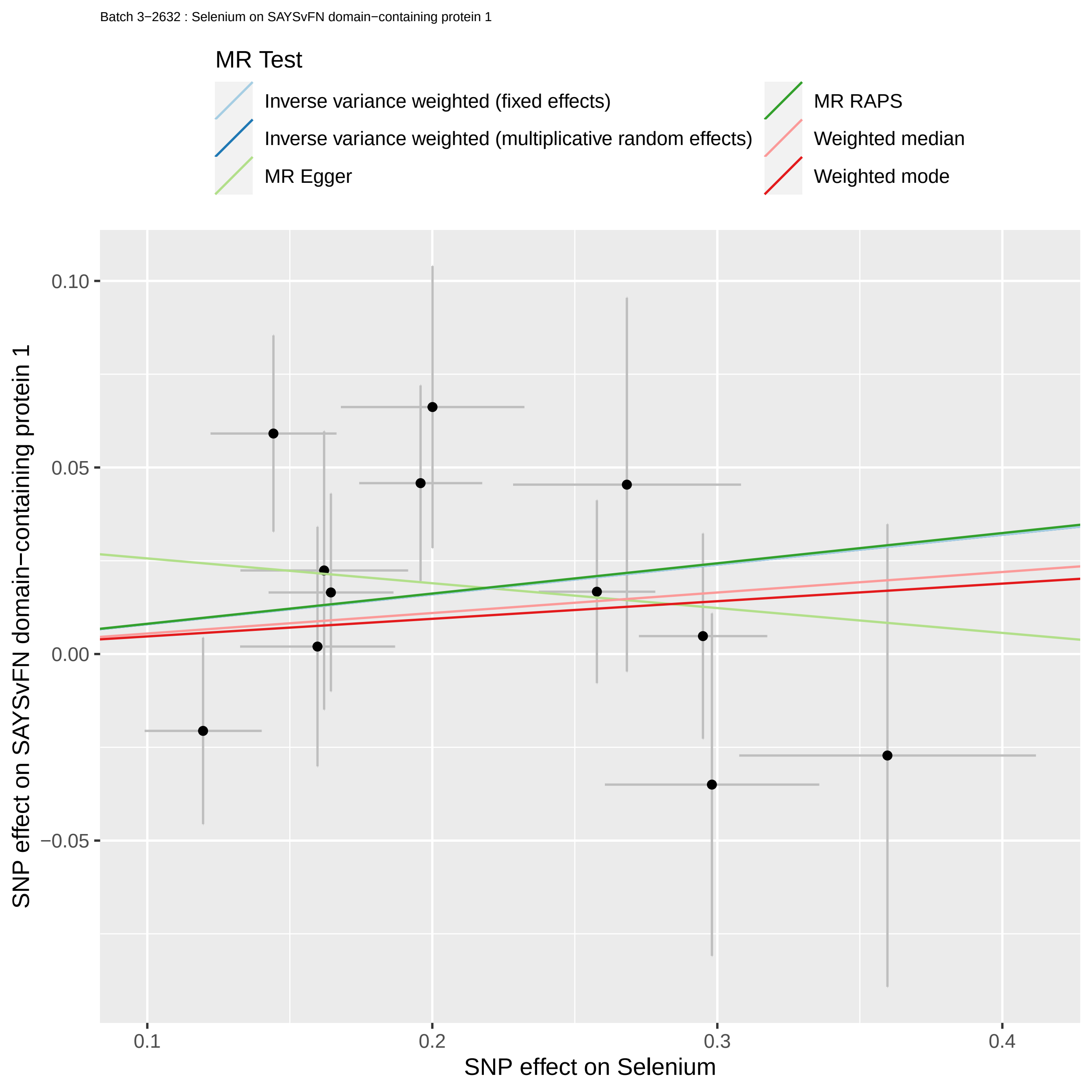
Batch 3−2632 : Selenium on SAYSvFN domain−containing protein 1
MR Test
Inverse variance weighted (fixed effects)
Inverse variance weighted (multiplicative random effects)
MR Egger
MR RAPS
Weighted median
Weighted mode
0.10
0.05
0.00
−0.05
0.1
0.2
0.3
0.4
SNP effect on SAYSvFN domain−containing protein 1
SNP effect on Selenium

Batch 3−2633 : Selenium on Suprabasin

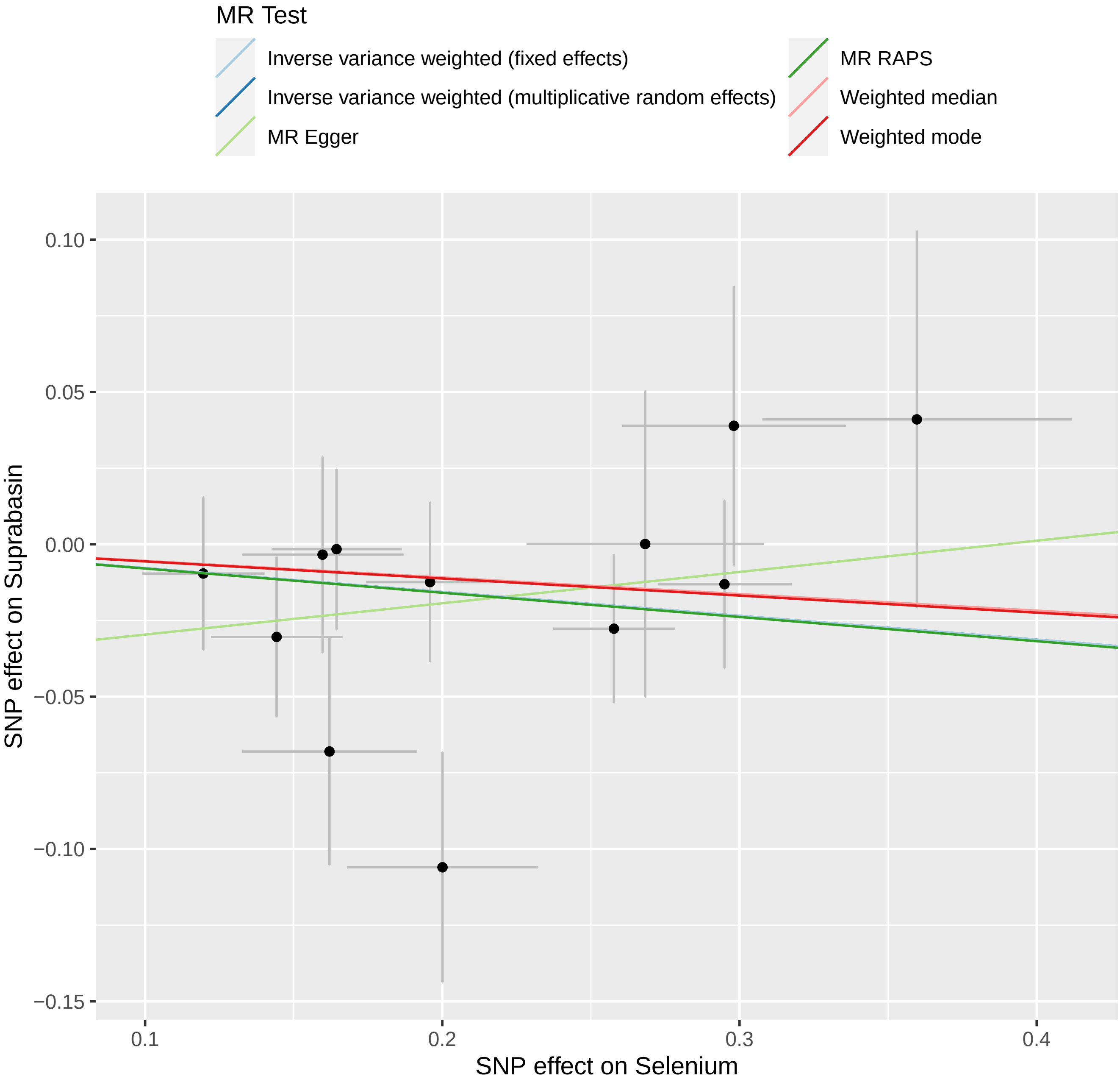

Batch 3−2634 : Selenium on Secretory carrier−associated membrane protein 5

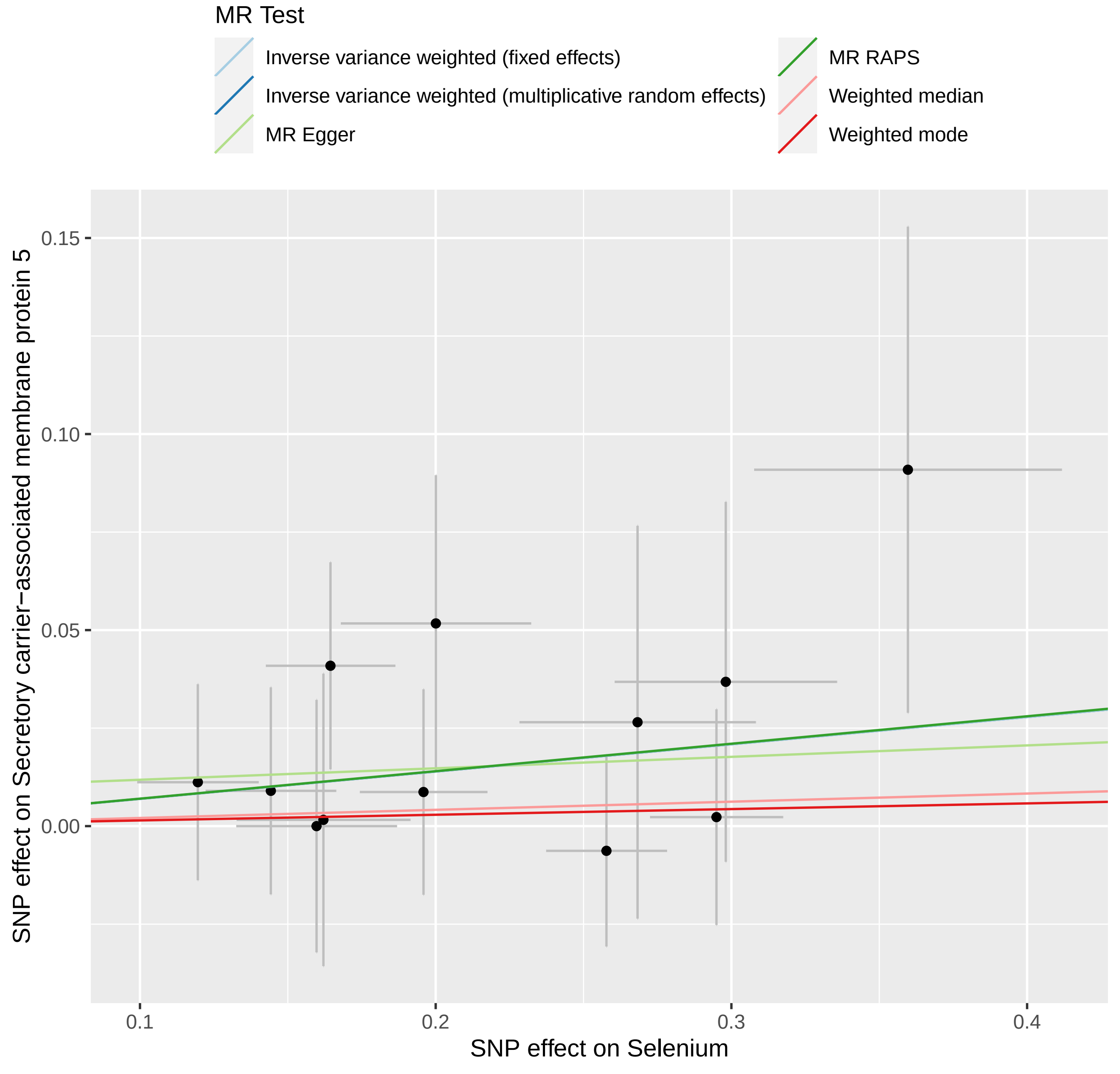

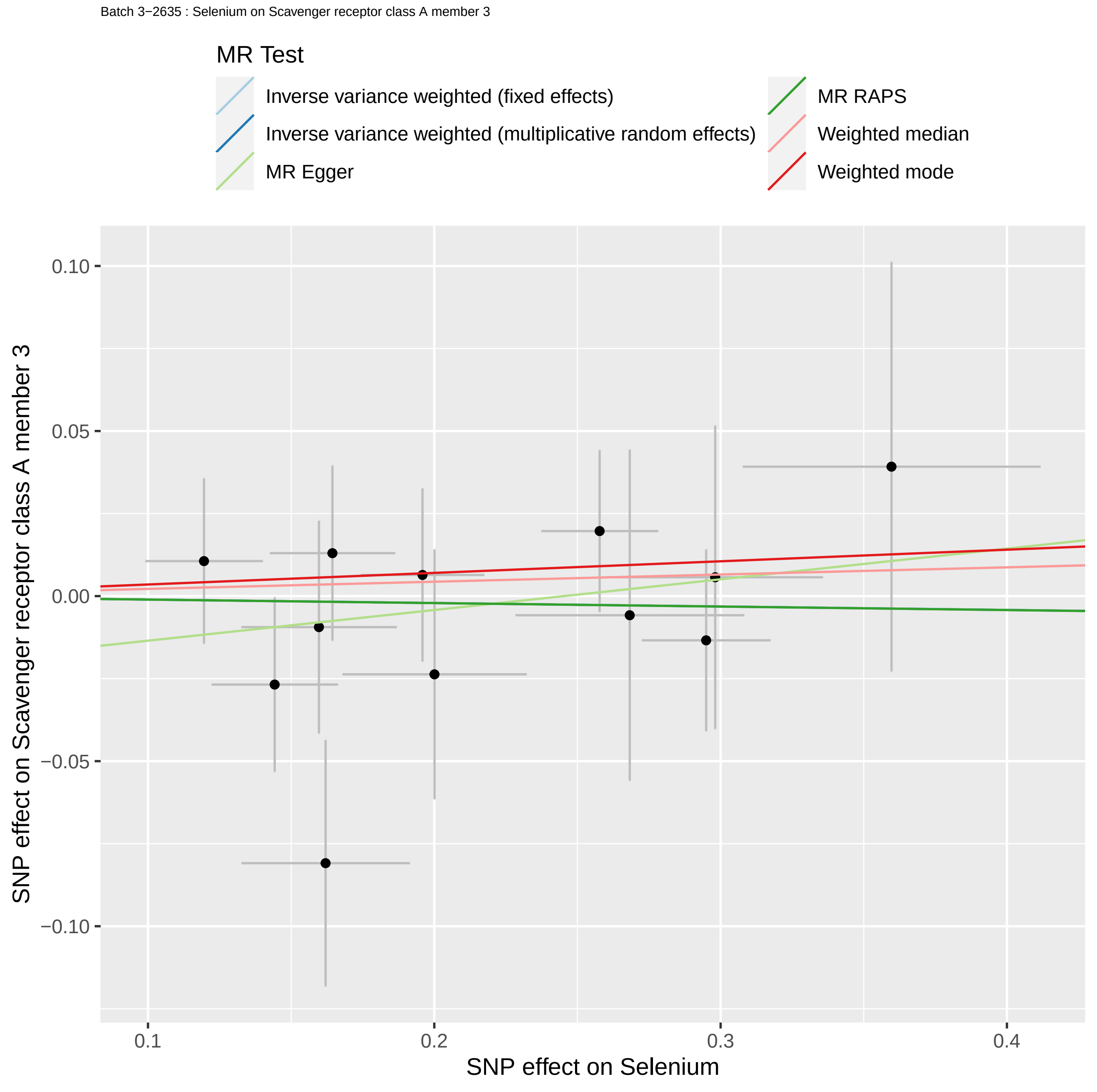
Batch 3−2635 : Selenium on Scavenger receptor class A member 3
MR Test
Inverse variance weighted (fixed effects)
Inverse variance weighted (multiplicative random effects)
MR Egger
MR RAPS
Weighted median
Weighted mode
0.10
0.05
0.00
−0.05
−0.10
0.1
0.2
0.3
0.4
SNP effect on Scavenger receptor class A member 3
SNP effect on Selenium

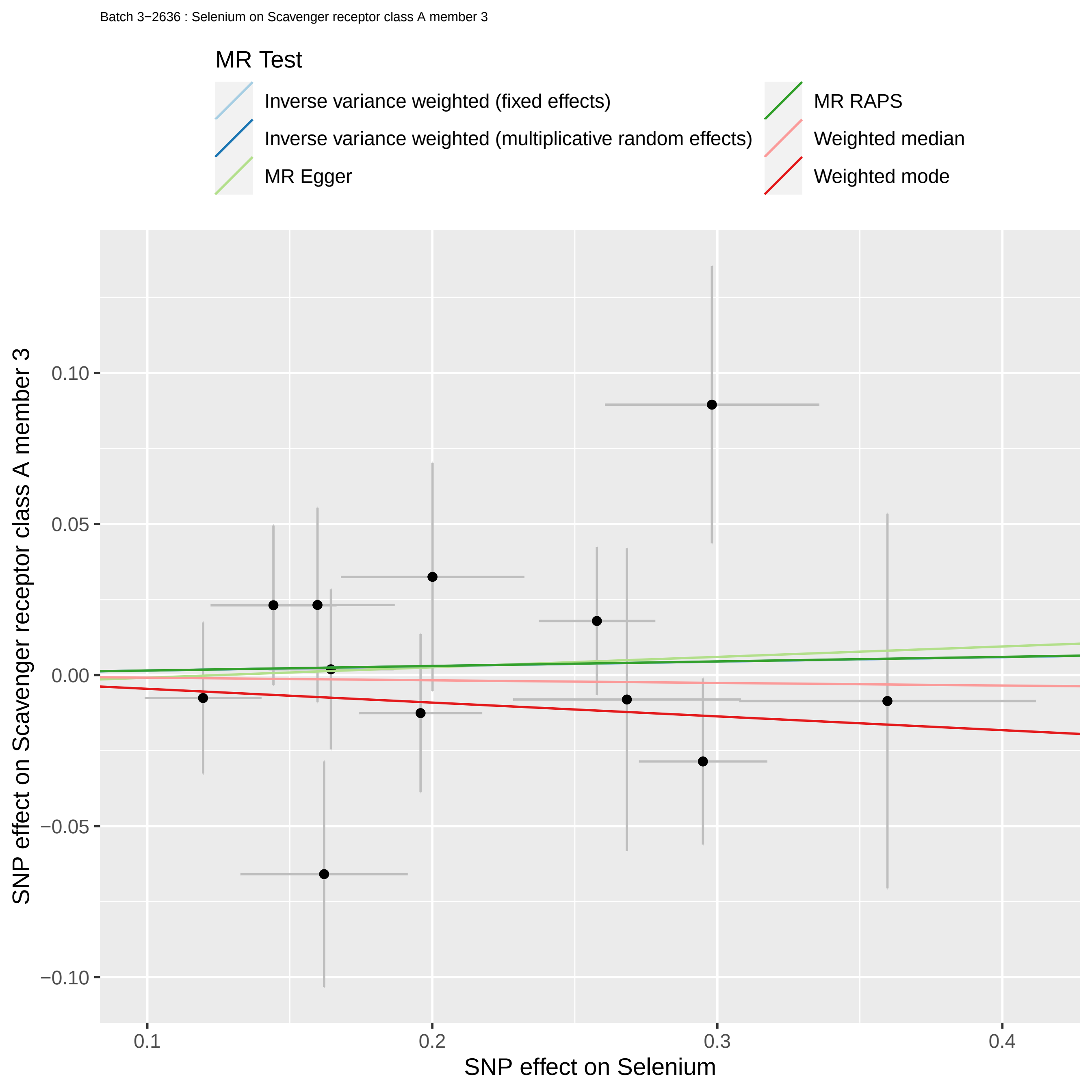

Batch 3−2636 : Selenium on Scavenger receptor class A member 3
MR Test
Inverse variance weighted (fixed effects)
Inverse variance weighted (multiplicative random effects)
MR Egger
MR RAPS
Weighted median
Weighted mode
SNP effect on Scavenger receptor class A member 3
0.10
0.05
0.00
−0.05
−0.10
0.1
0.2
0.3
0.4
SNP effect on Selenium

Batch 3−2637 : Selenium on Scavenger receptor class A member 3

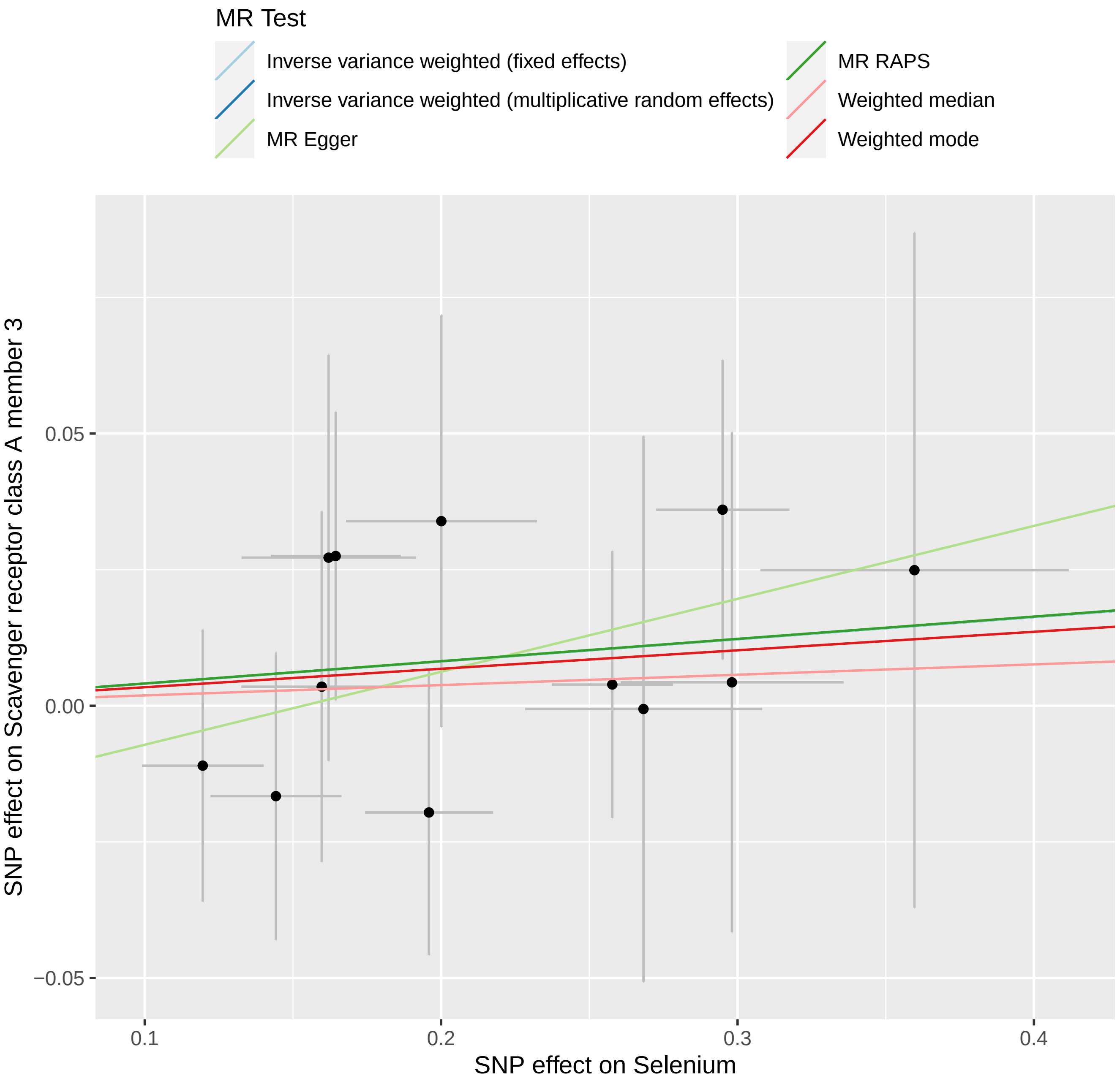

Batch 3−2638 : Selenium on Scavenger receptor class A member 3

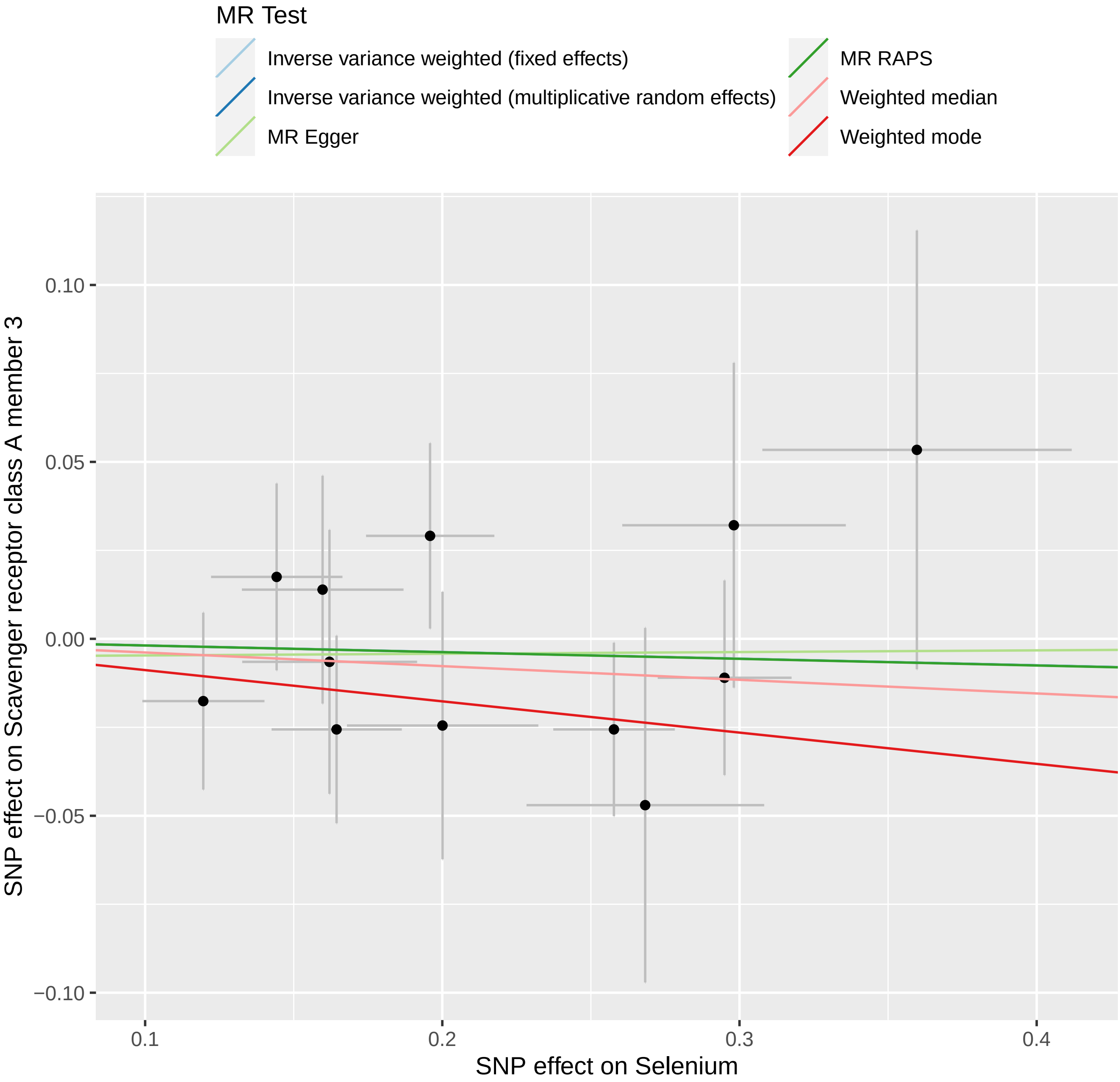

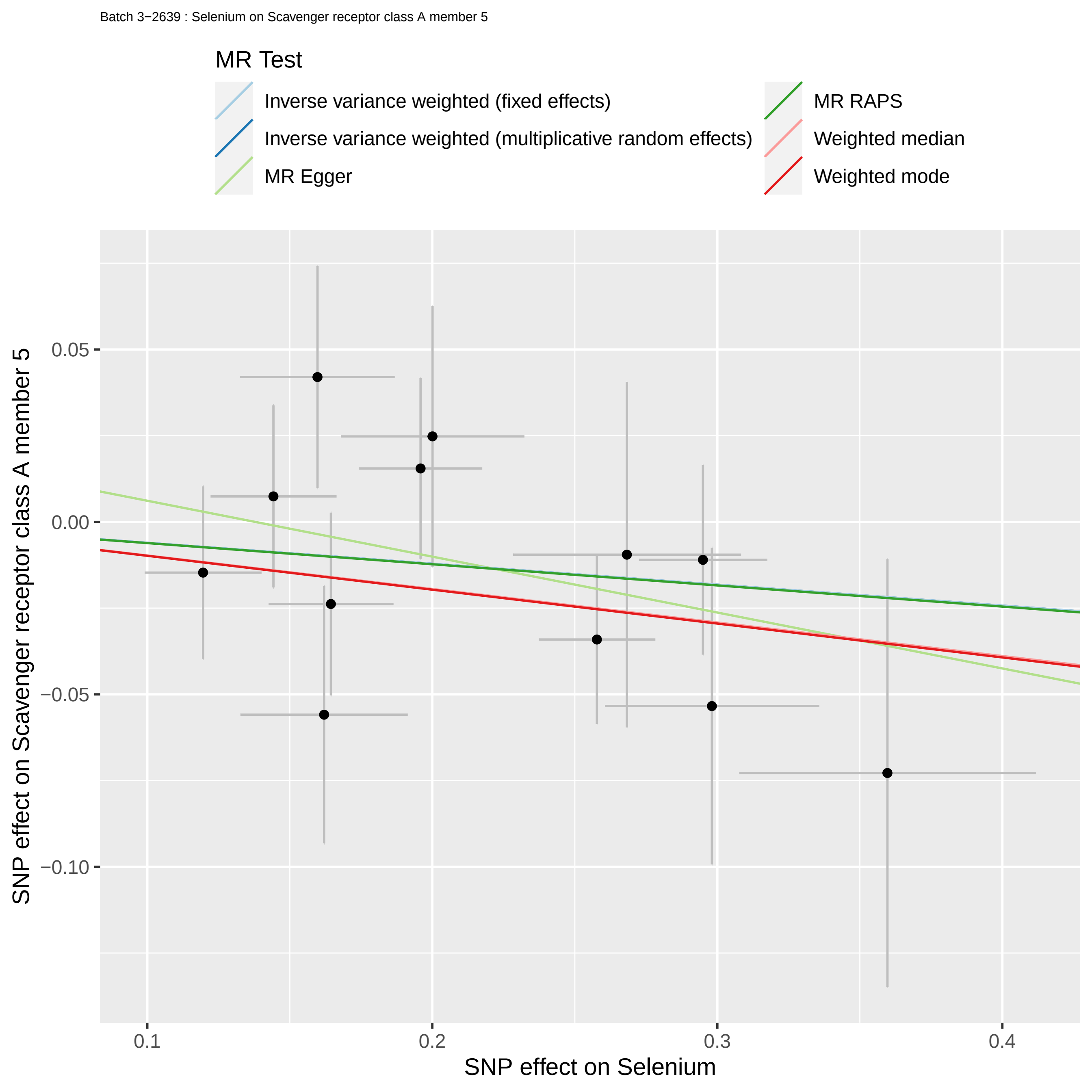

Batch 3−2639 : Selenium on Scavenger receptor class A member 5
MR Test
Inverse variance weighted (fixed effects)
Inverse variance weighted (multiplicative random effects)
MR Egger
MR RAPS
Weighted median
Weighted mode
0.05
0.00
−0.05
−0.10
0.1
0.2
0.3
0.4
SNP effect on Selenium
SNP effect on Scavenger receptor class A member 5

Batch 3−2640 : Selenium on Lysosome membrane protein 2

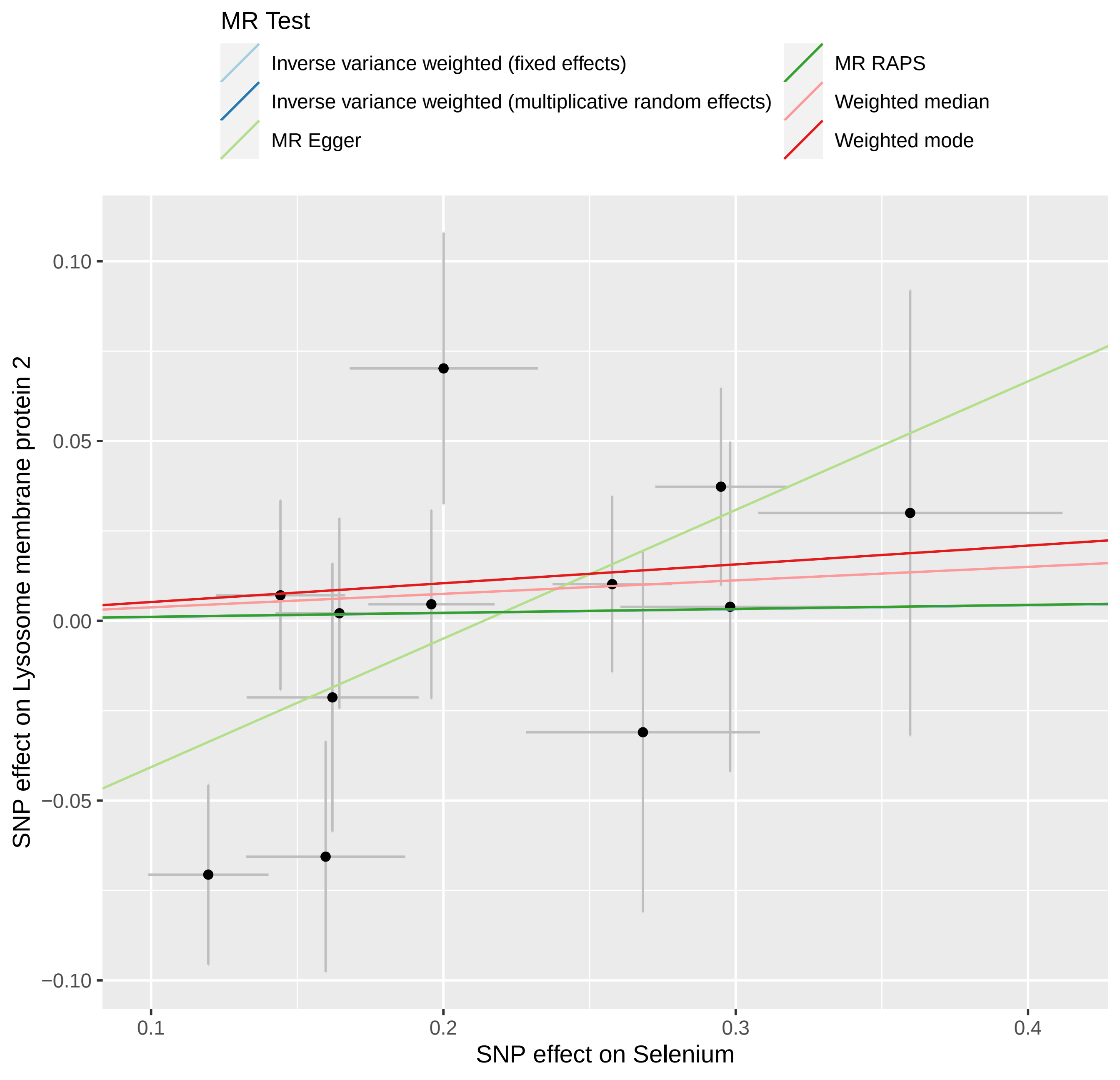

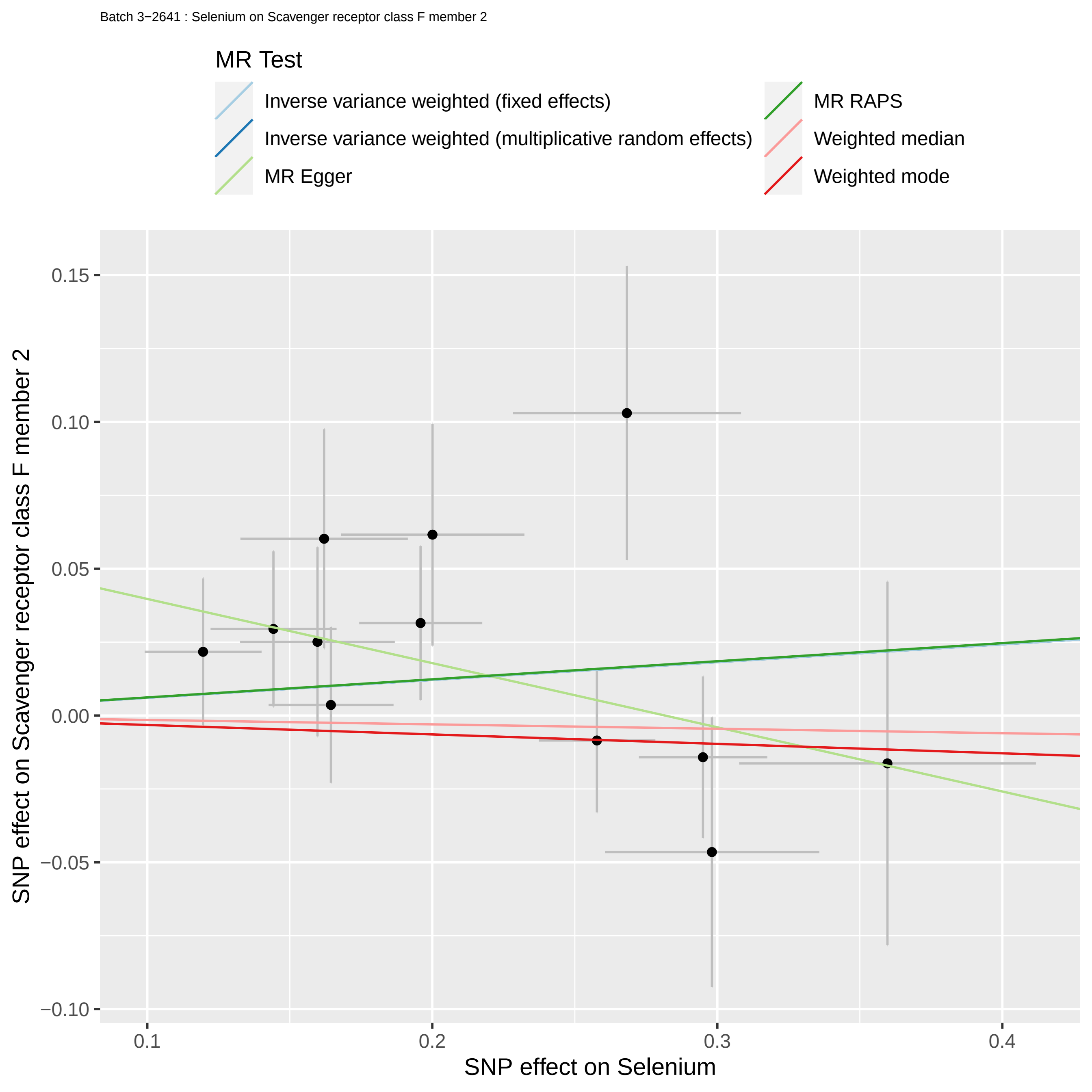
Batch 3−2641 : Selenium on Scavenger receptor class F member 2
MR Test
Inverse variance weighted (fixed effects)
Inverse variance weighted (multiplicative random effects)
MR Egger
MR RAPS
Weighted median
Weighted mode
0.15
0.10
0.05
0.00
−0.05
−0.10
0.1
0.2
0.3
0.4
SNP effect on Scavenger receptor class F member 2
SNP effect on Selenium

Batch 3−2642 : Selenium on Scavenger receptor class F member 2

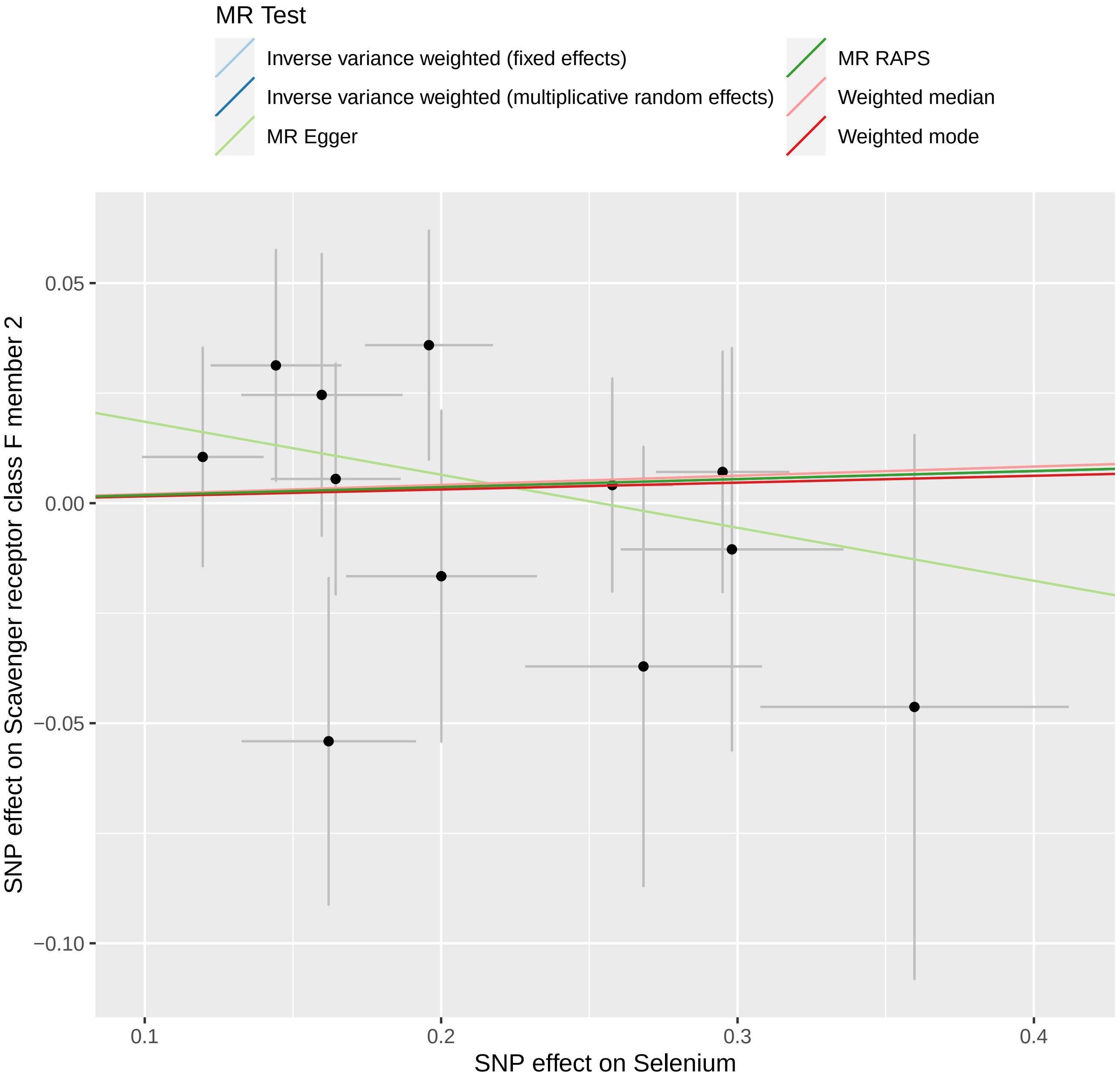

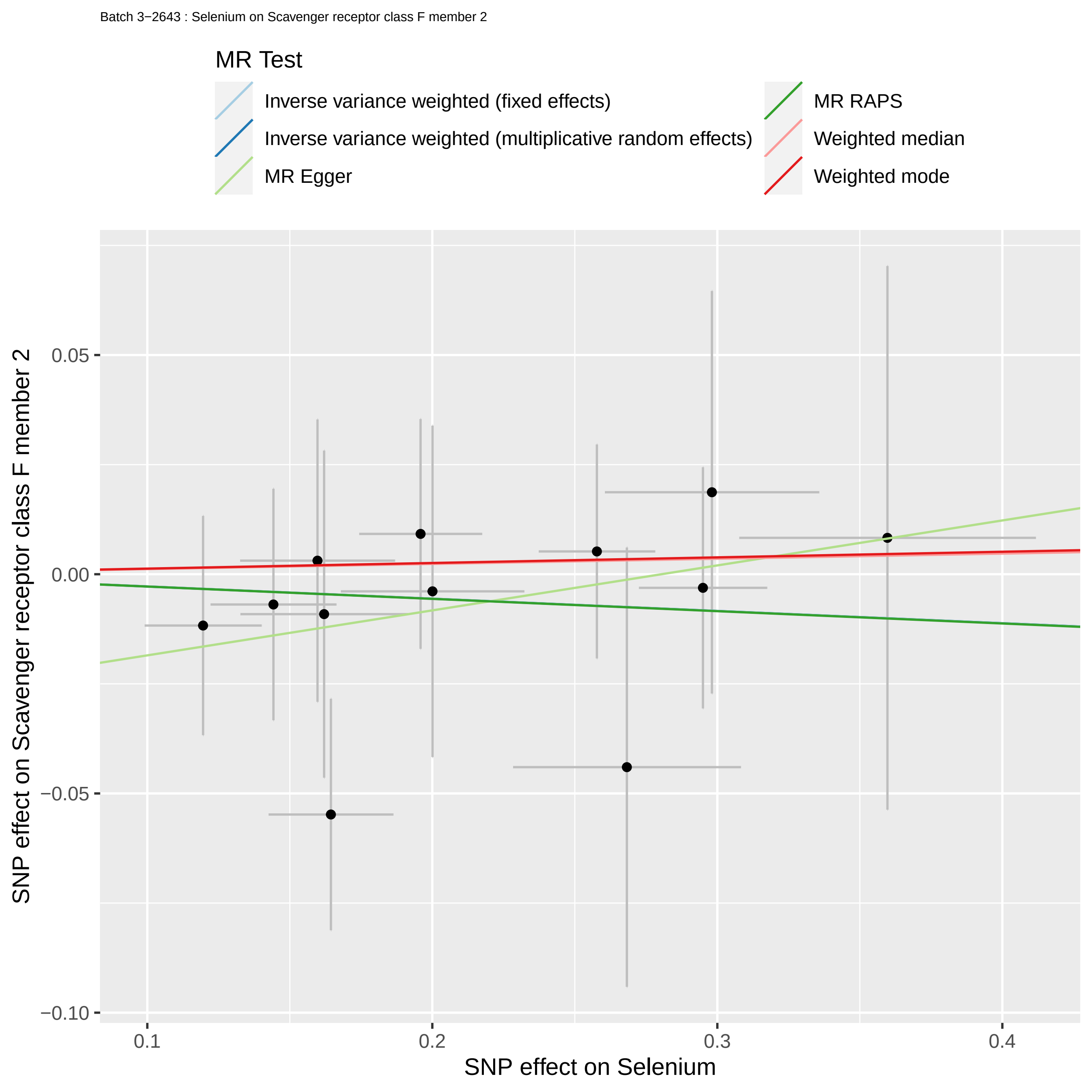

Batch 3−2643 : Selenium on Scavenger receptor class F member 2
MR Test
Inverse variance weighted (fixed effects)
Inverse variance weighted (multiplicative random effects)
MR Egger
MR RAPS
Weighted median
Weighted mode
0.05
0.00
−0.05
−0.10
0.1
0.2
0.3
0.4
SNP effect on Scavenger receptor class F member 2
SNP effect on Selenium

Batch 3−2644 : Selenium on Secretogranin−2

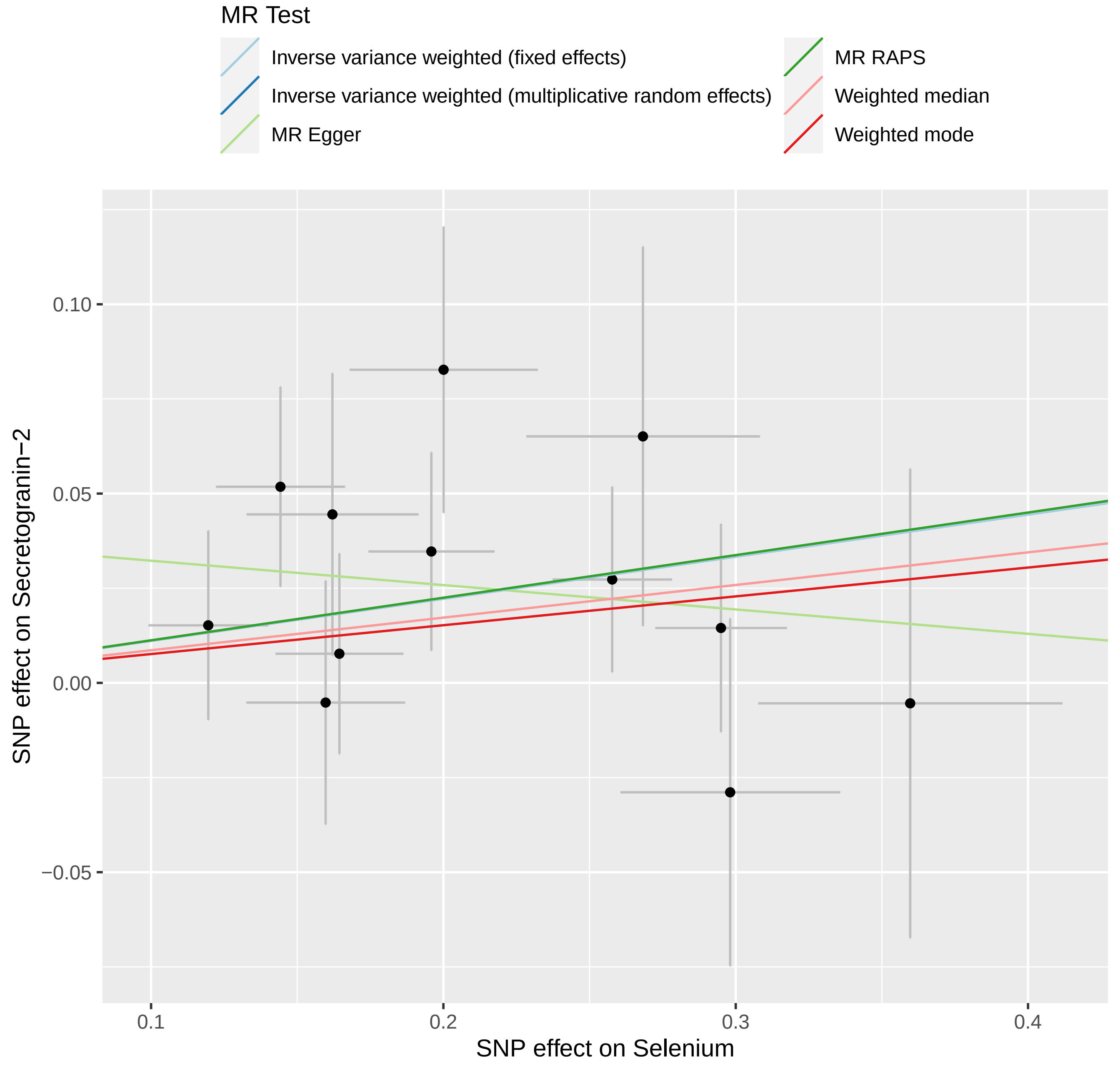

Batch 3−2645 : Selenium on Secretogranin−3

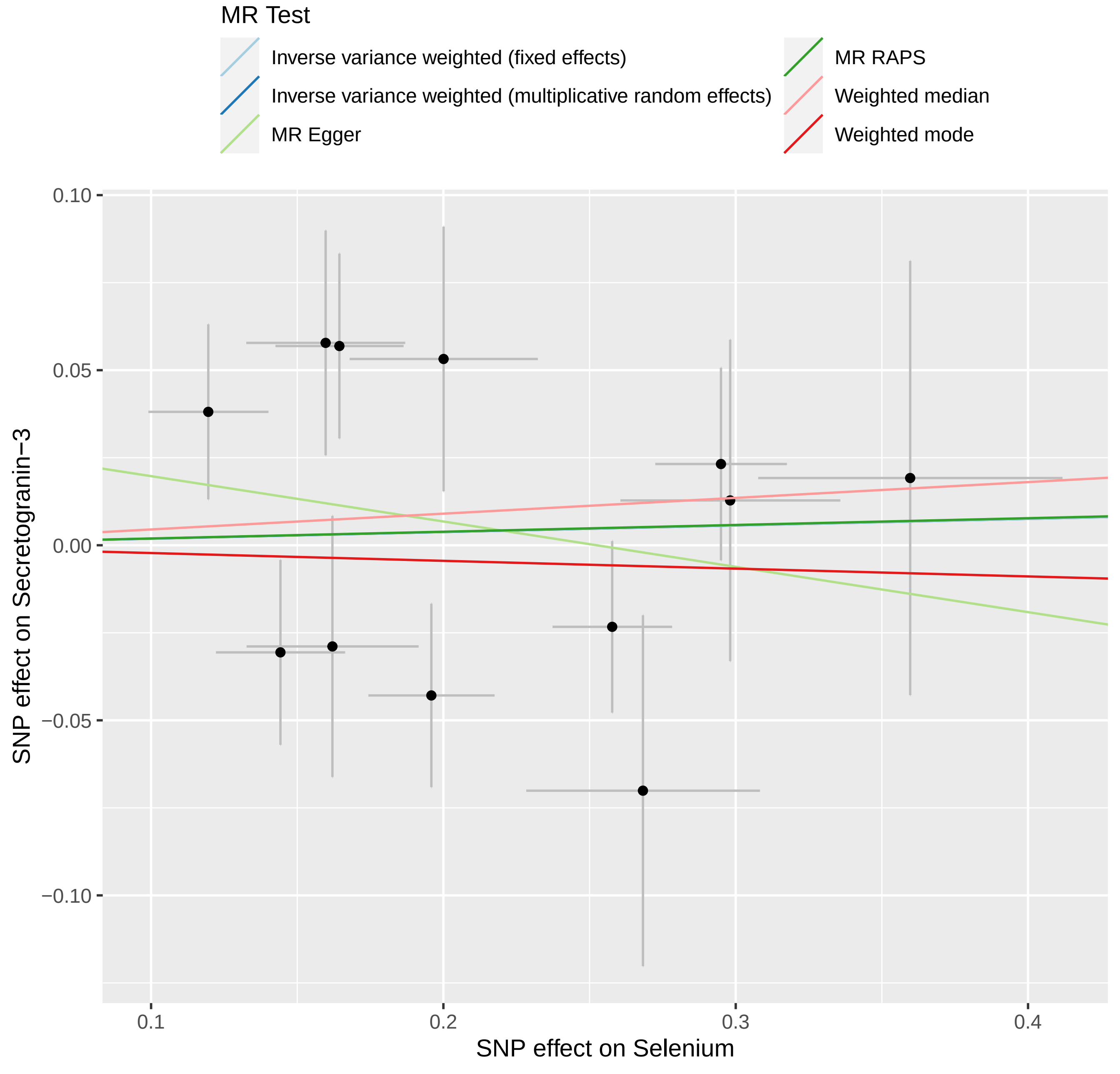

Batch 3−2646 : Selenium on Secretoglobin family 1C member 1

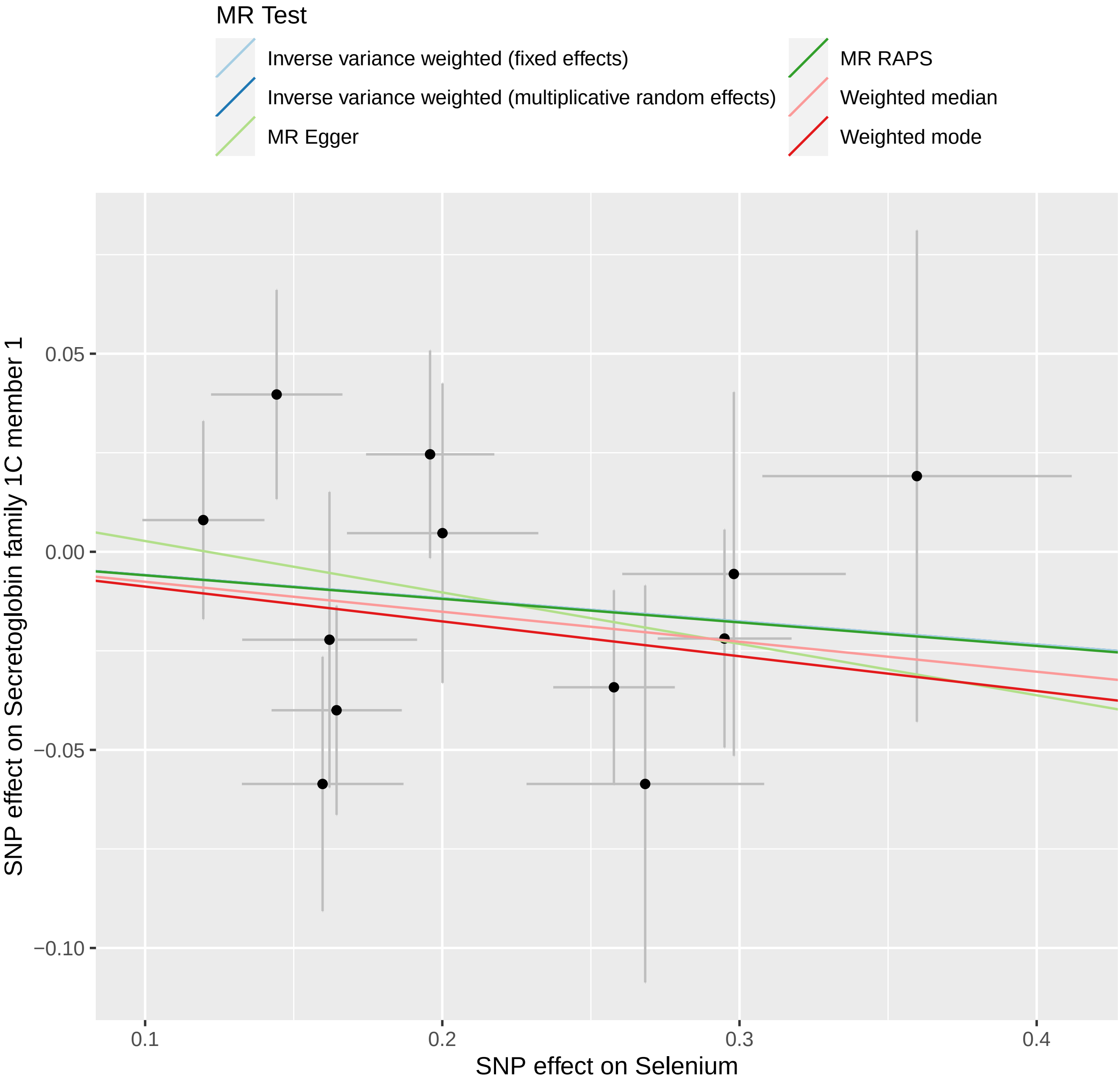

Batch 3−2647 : Selenium on Secretoglobin family 1D member 2

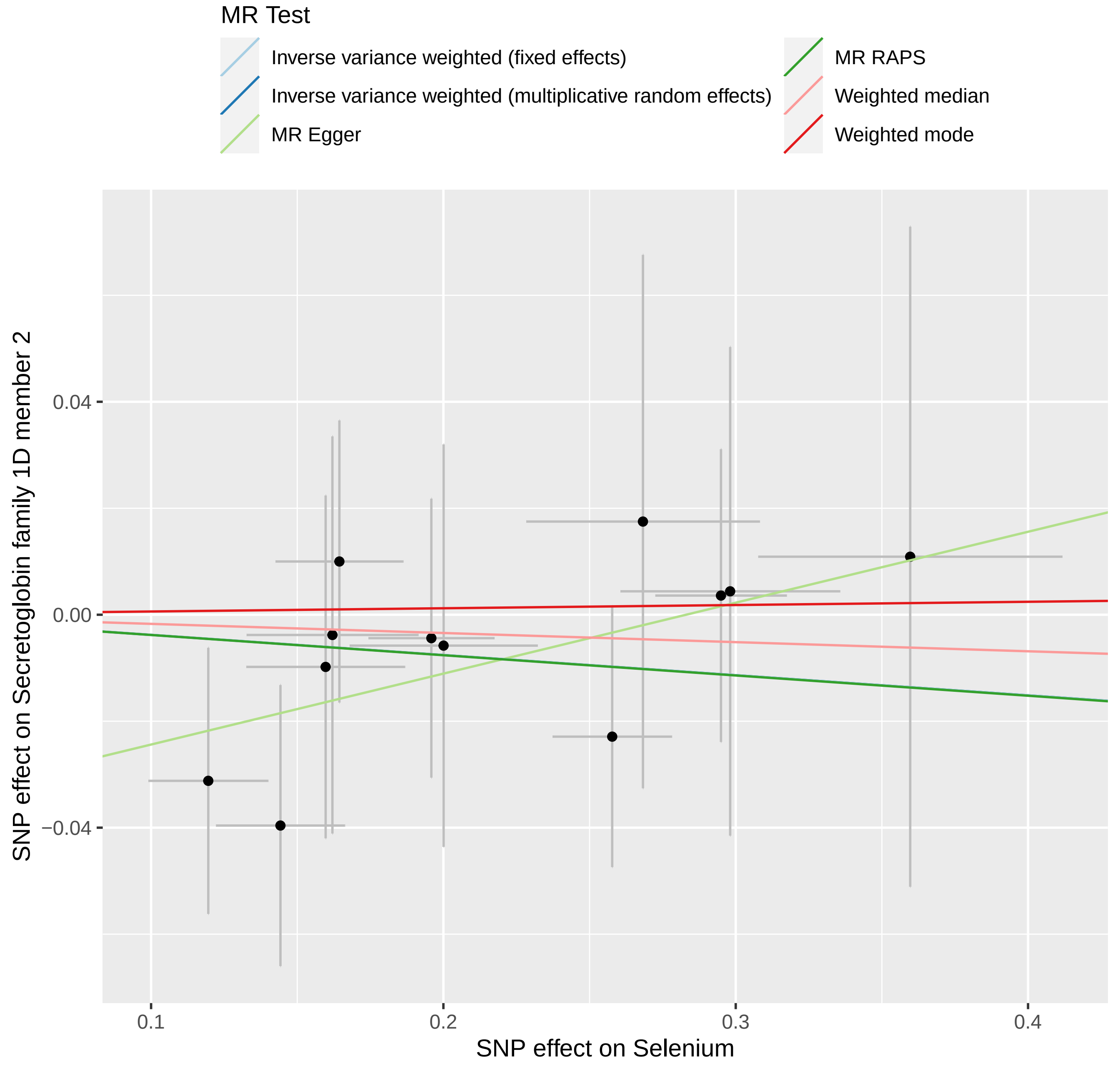

Batch 3−2648 : Selenium on Mammaglobin−B

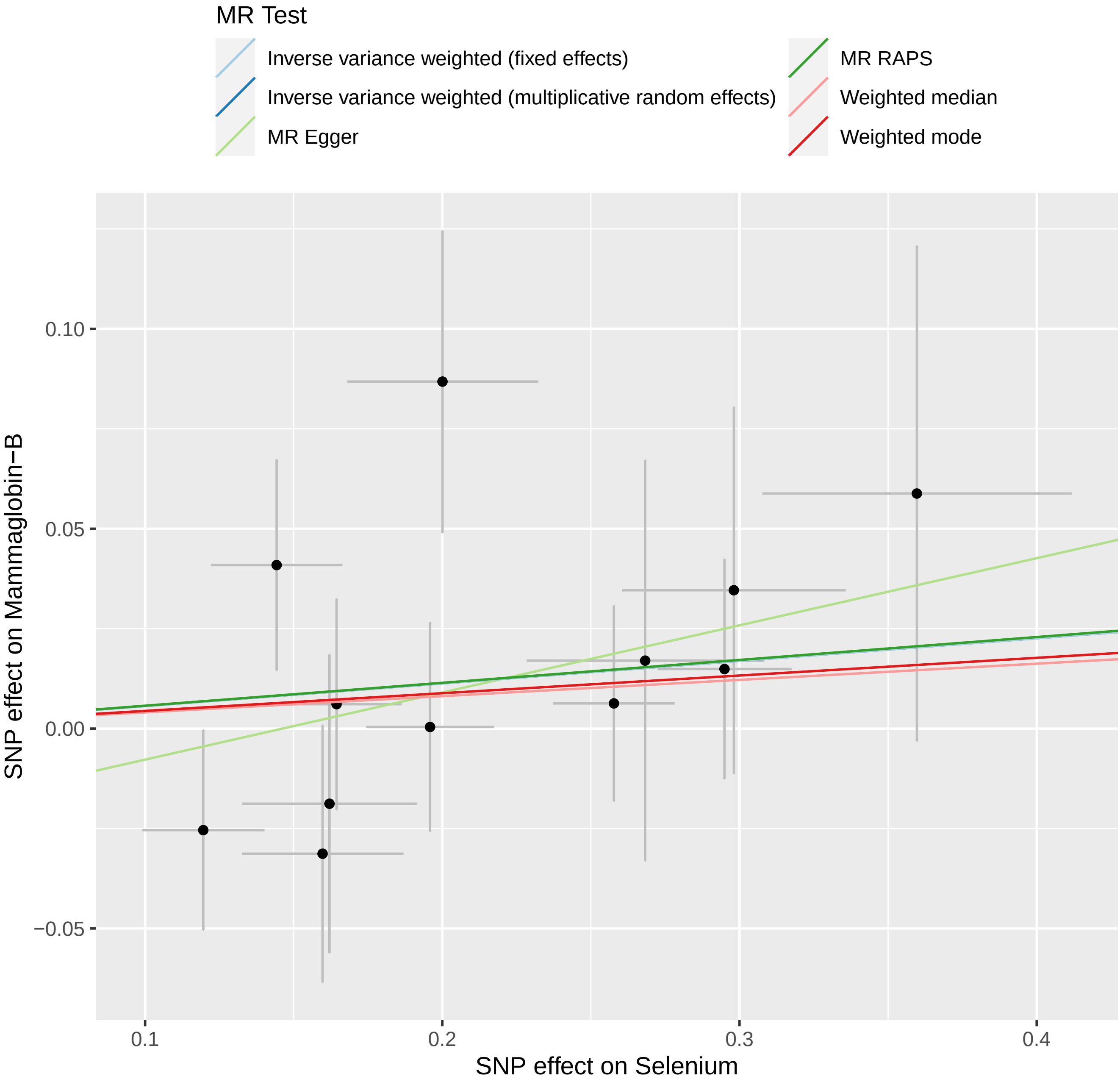

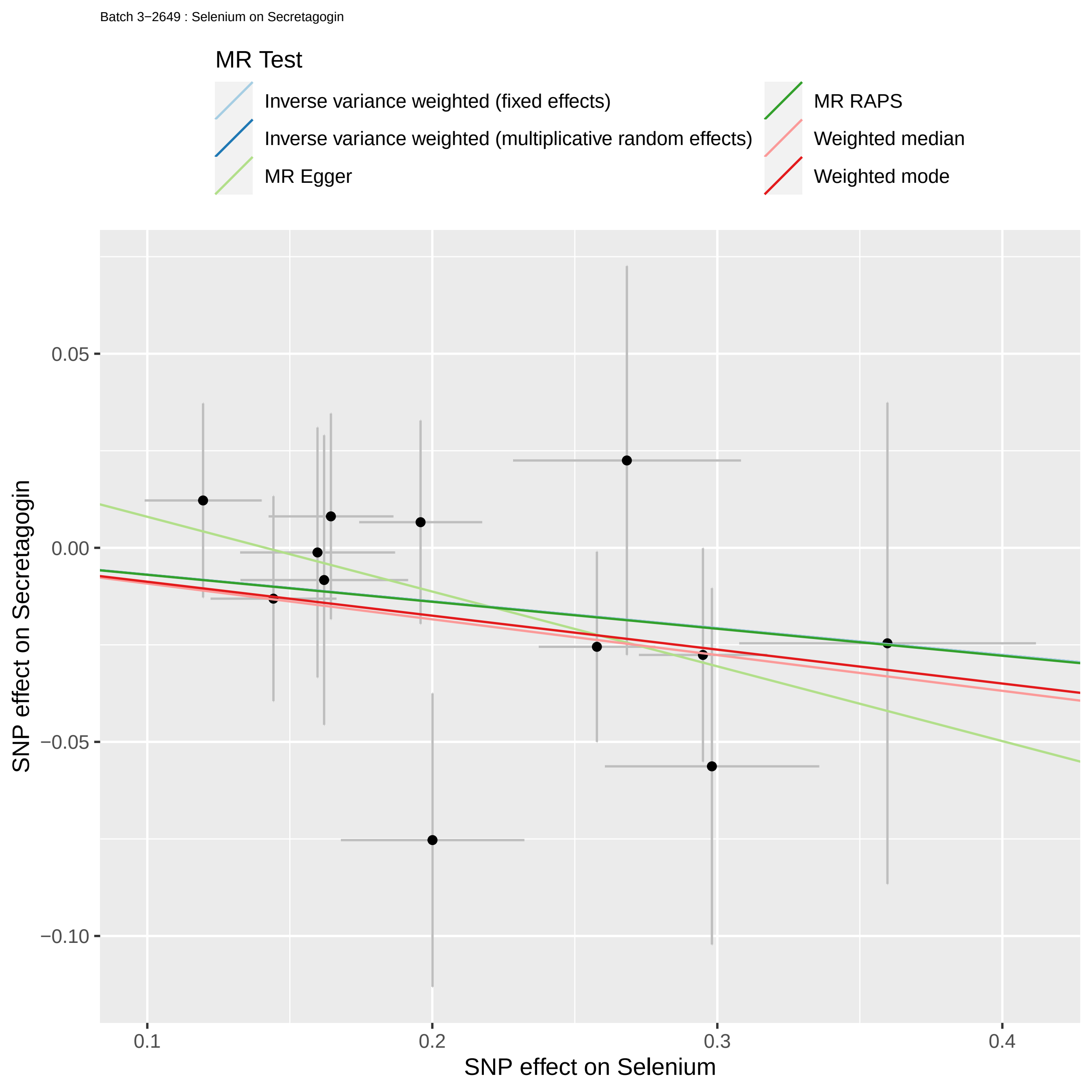
Batch 3−2649 : Selenium on Secretagogin
MR Test
Inverse variance weighted (fixed effects)
Inverse variance weighted (multiplicative random effects)
MR Egger
MR RAPS
Weighted median
Weighted mode
SNP effect on Secretagogin
0.05
0.00
−0.05
−0.10
0.1
0.2
0.3
0.4
SNP effect on Selenium

Batch 3−2650 : Selenium on Adseverin

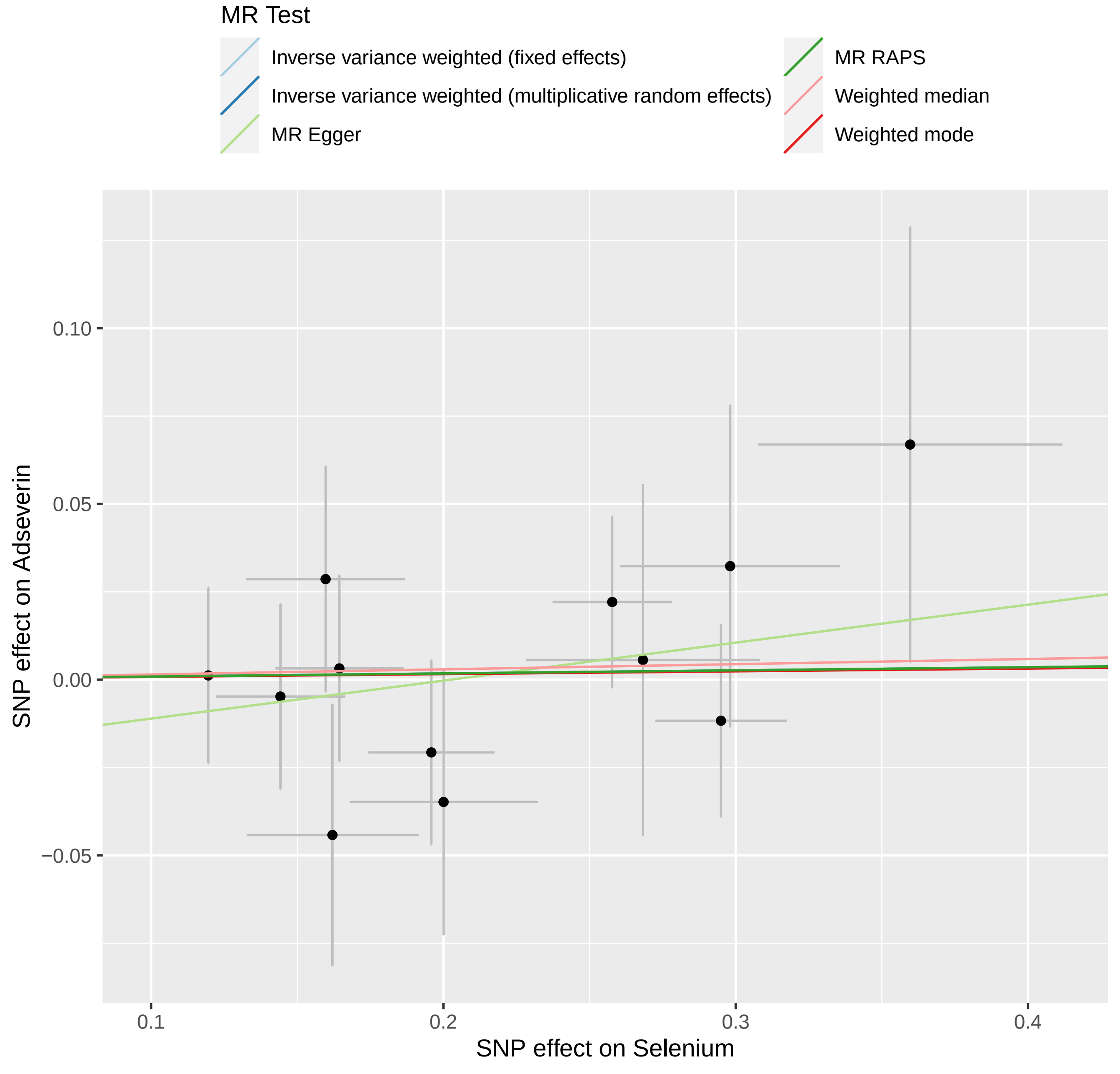

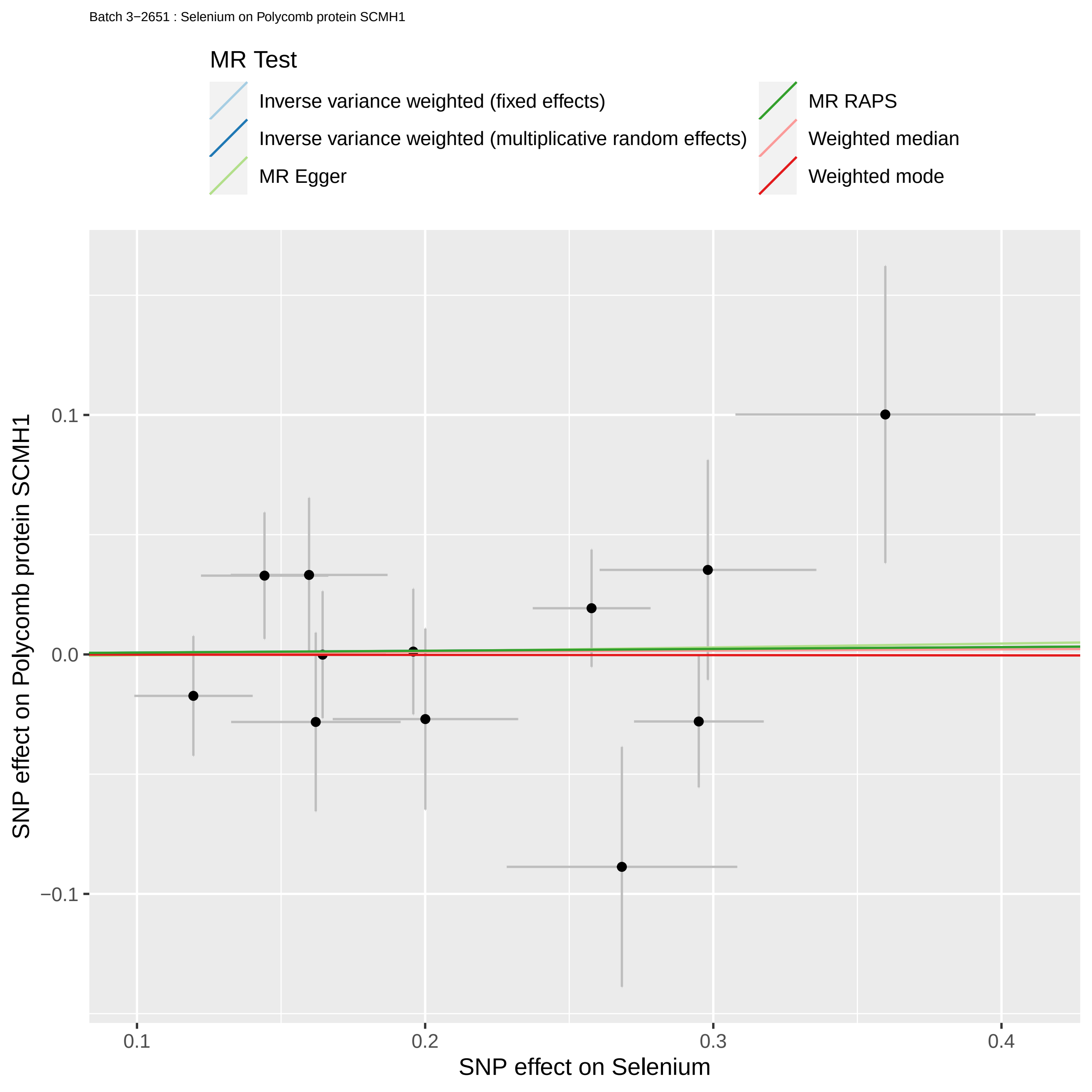

Batch 3−2651 : Selenium on Polycomb protein SCMH1
MR Test
Inverse variance weighted (fixed effects)
Inverse variance weighted (multiplicative random effects)
MR Egger
MR RAPS
Weighted median
Weighted mode
0.1
0.0
−0.1
0.1
0.2
0.3
0.4
SNP effect on Polycomb protein SCMH1
SNP effect on Selenium

Batch 3−2652 : Selenium on Sodium channel subunit beta−2

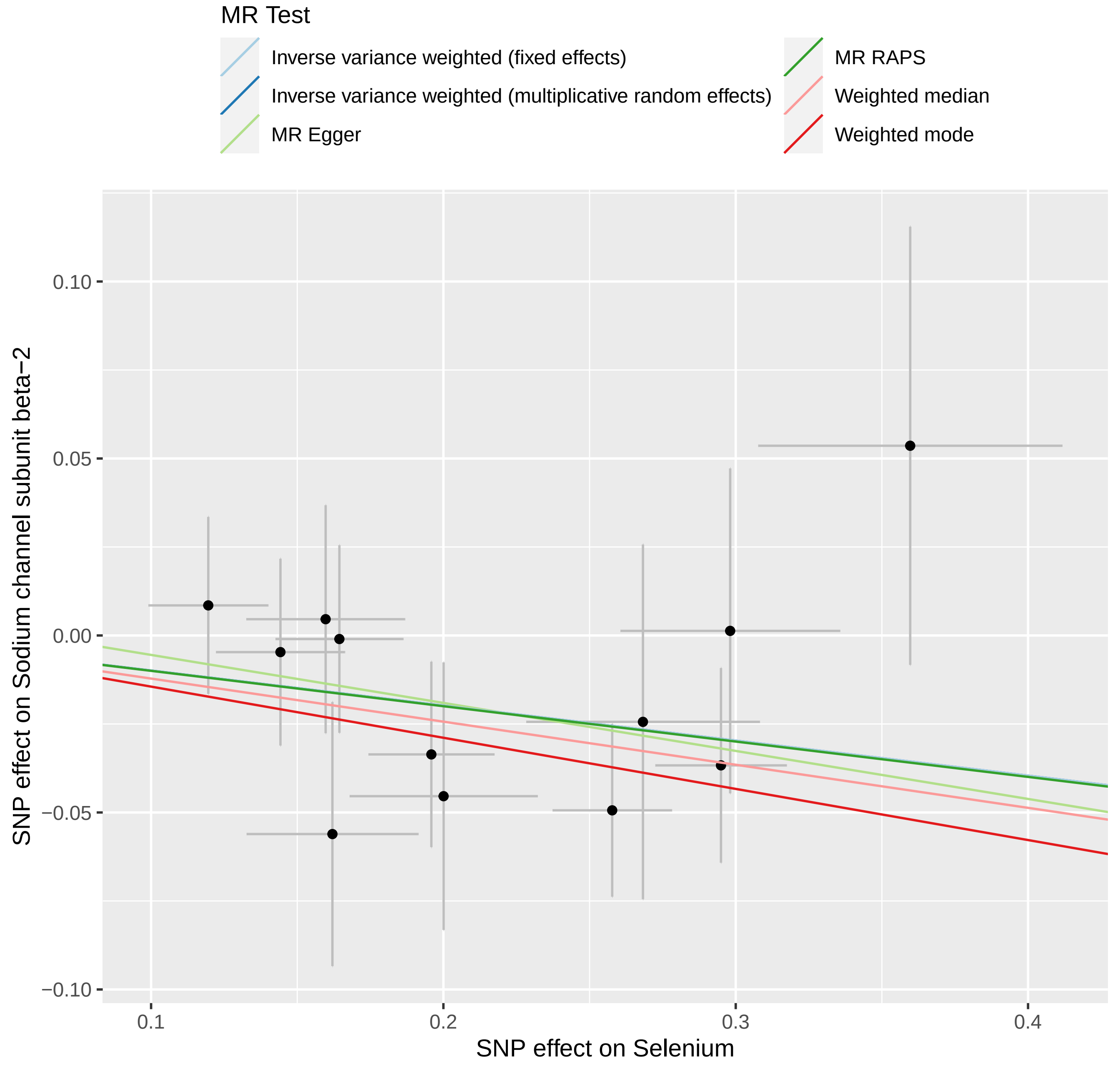

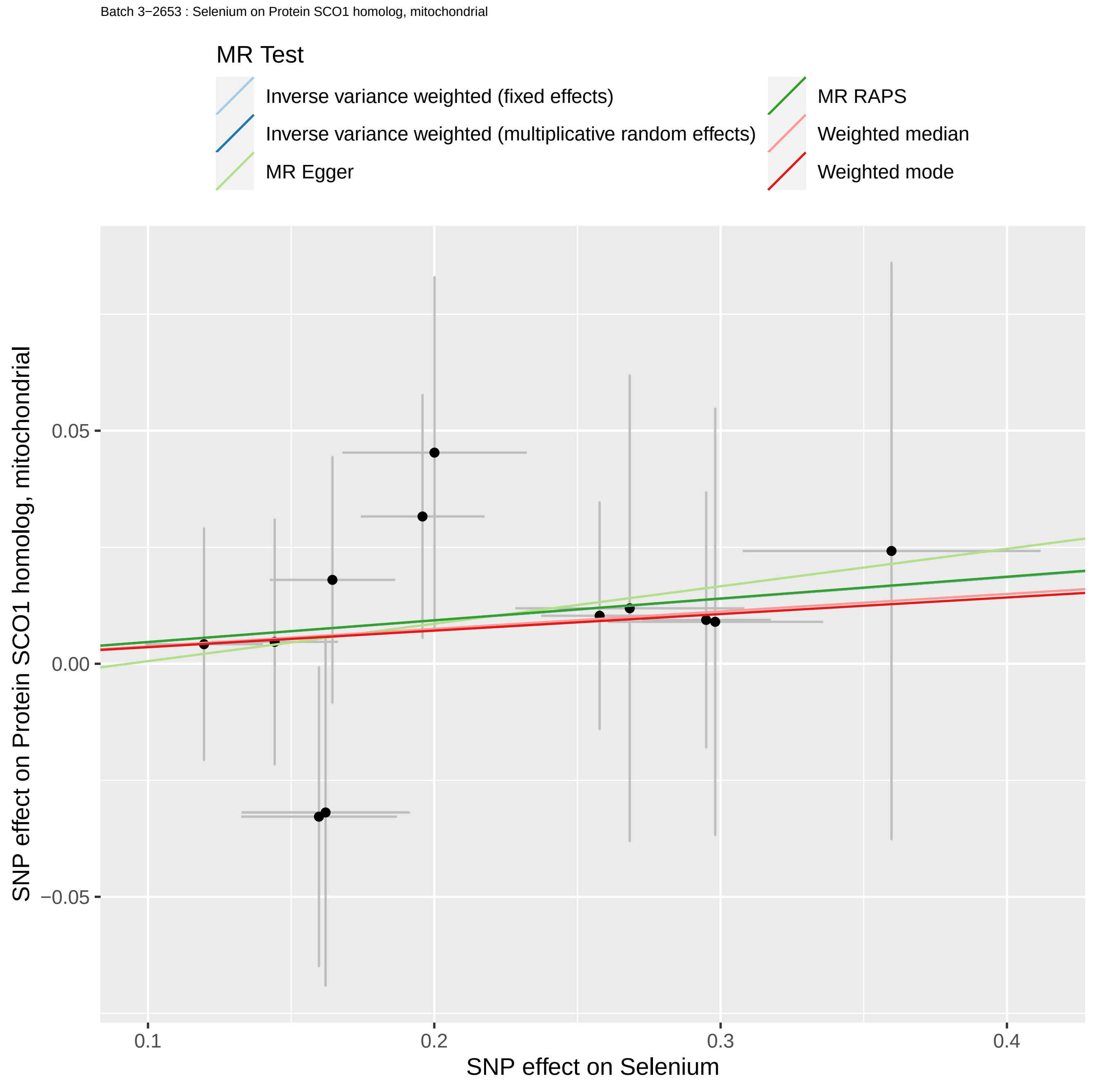

Batch 3−2653 : Selenium on Protein SCO1 homolog, mitochondrial
MR Test
Inverse variance weighted (fixed effects)
Inverse variance weighted (multiplicative random effects)
MR Egger
MR RAPS
Weighted median
Weighted mode
0.05
0.00
−0.05
0.1
0.2
0.3
0.4
SNP effect on Selenium
SNP effect on Protein SCO1 homolog, mitochondrial

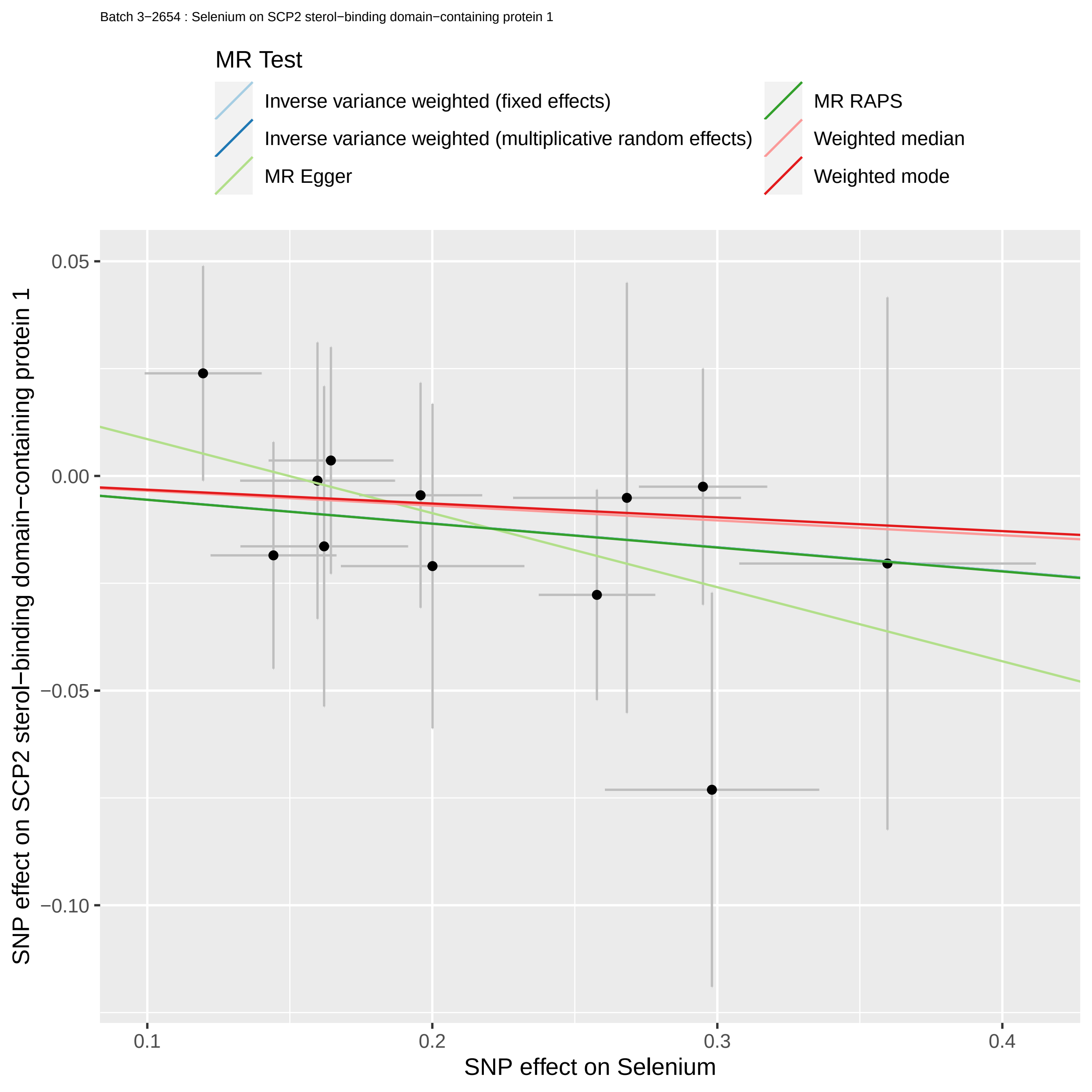

Batch 3−2654 : Selenium on SCP2 sterol−binding domain−containing protein 1
MR Test
Inverse variance weighted (fixed effects)
Inverse variance weighted (multiplicative random effects)
MR Egger
MR RAPS
Weighted median
Weighted mode
0.05
0.00
−0.05
−0.10
0.1
0.2
0.3
0.4
SNP effect on SCP2 sterol−binding domain−containing protein 1
SNP effect on Selenium

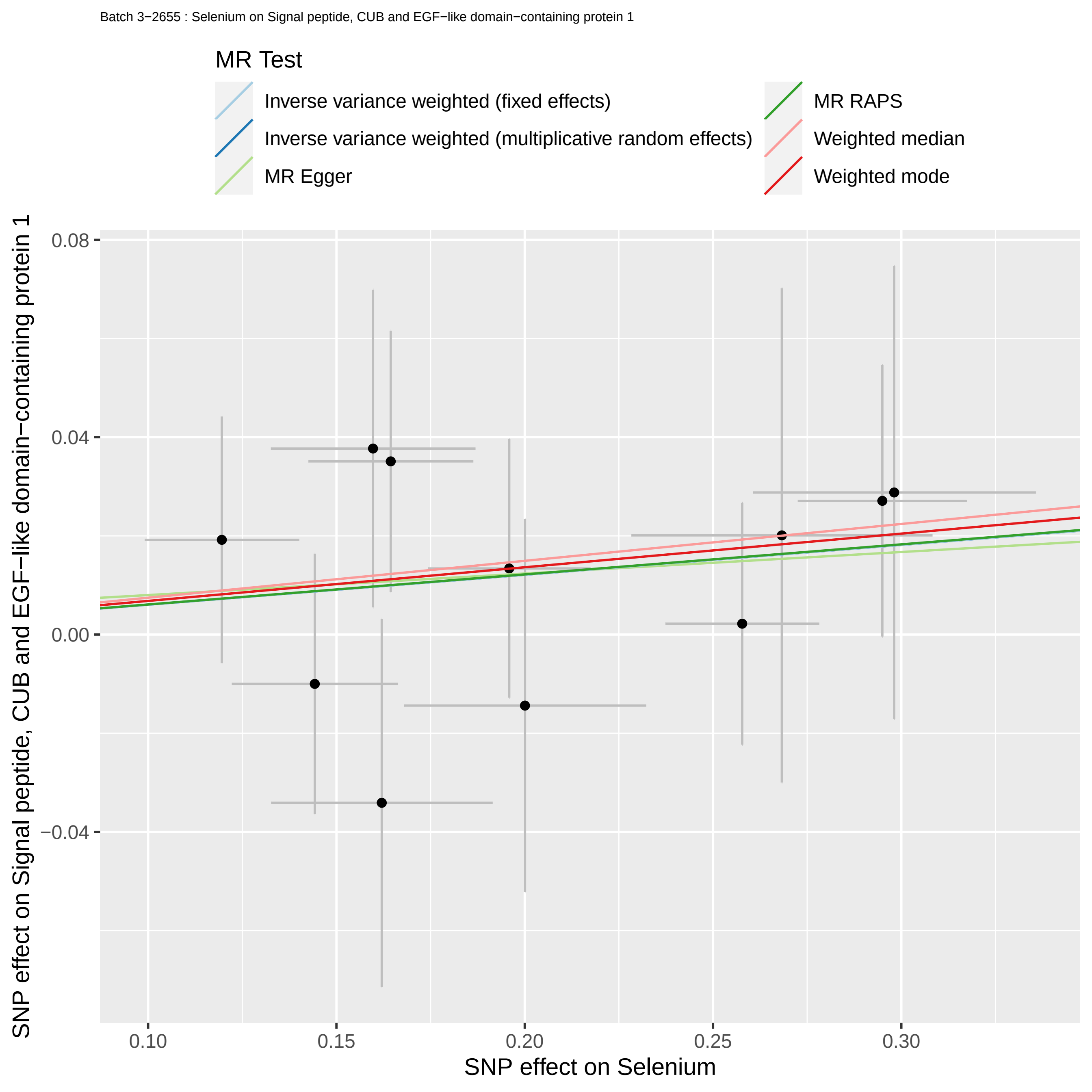
Batch 3−2655 : Selenium on Signal peptide, CUB and EGF−like domain−containing protein 1
MR Test
Inverse variance weighted (fixed effects)
Inverse variance weighted (multiplicative random effects)
MR Egger
MR RAPS
Weighted median
Weighted mode
SNP effect on Signal peptide, CUB and EGF−like domain−containing protein 1
0.08
0.04
0.00
−0.04
0.10
0.15
0.20
0.25
0.30
SNP effect on Selenium

Batch 3−2656 : Selenium on Stromal cell−derived factor 2−like protein 1

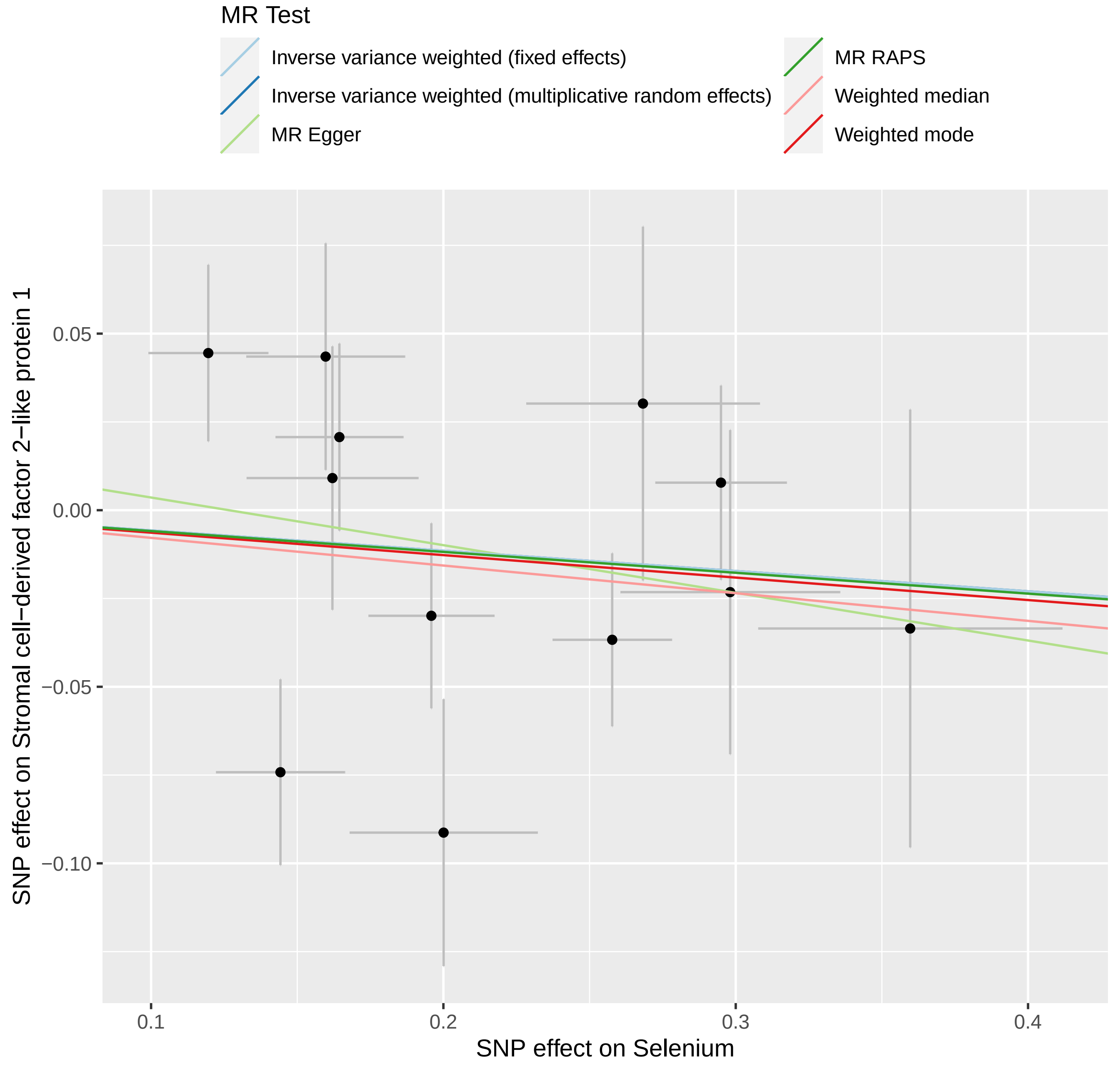

Batch 3−2657 : Selenium on Succinate dehydrogenase assembly factor 2, mitochondrial

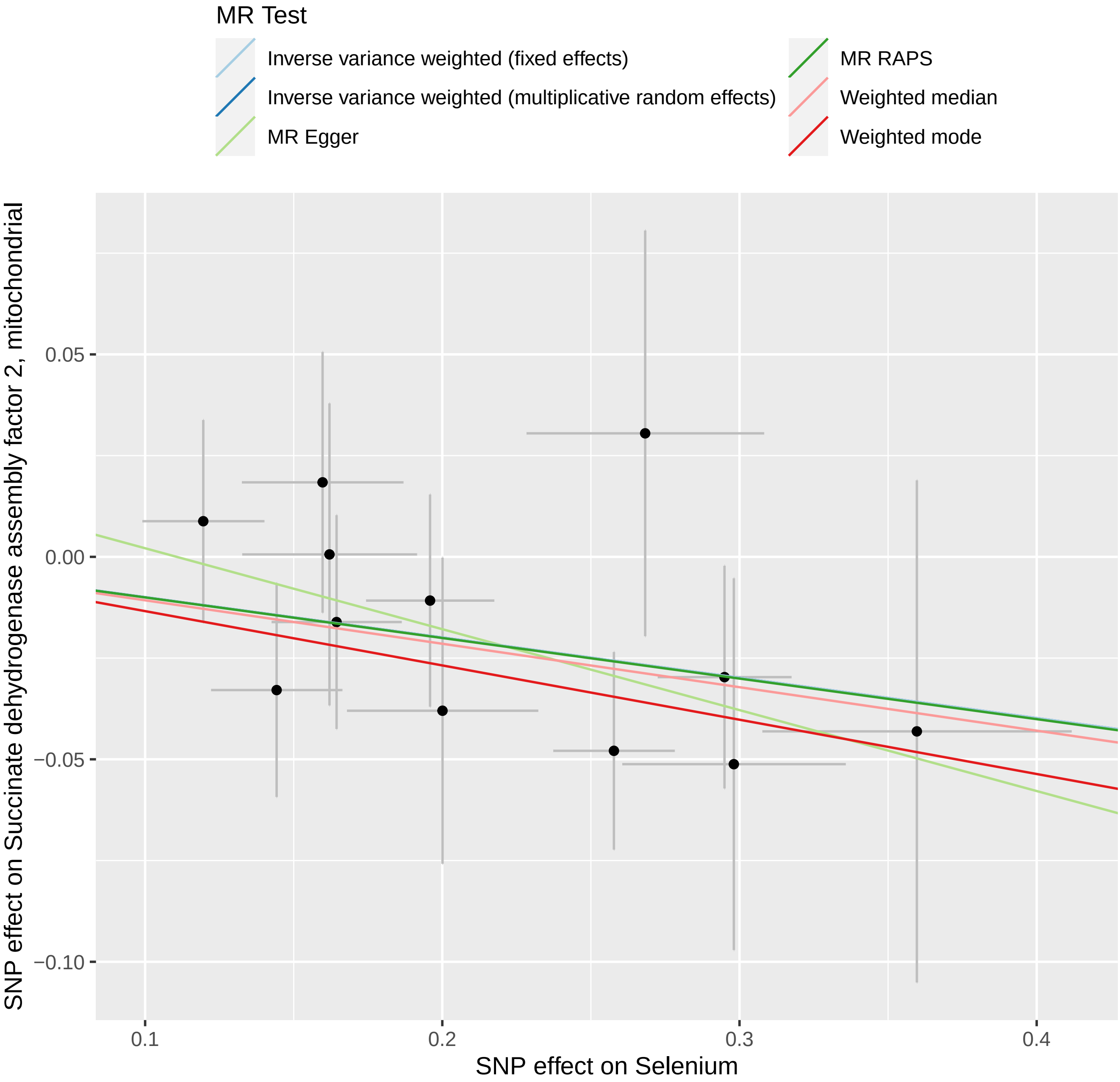

Batch 3−2658 : Selenium on Signal peptidase complex catalytic subunit SEC11C

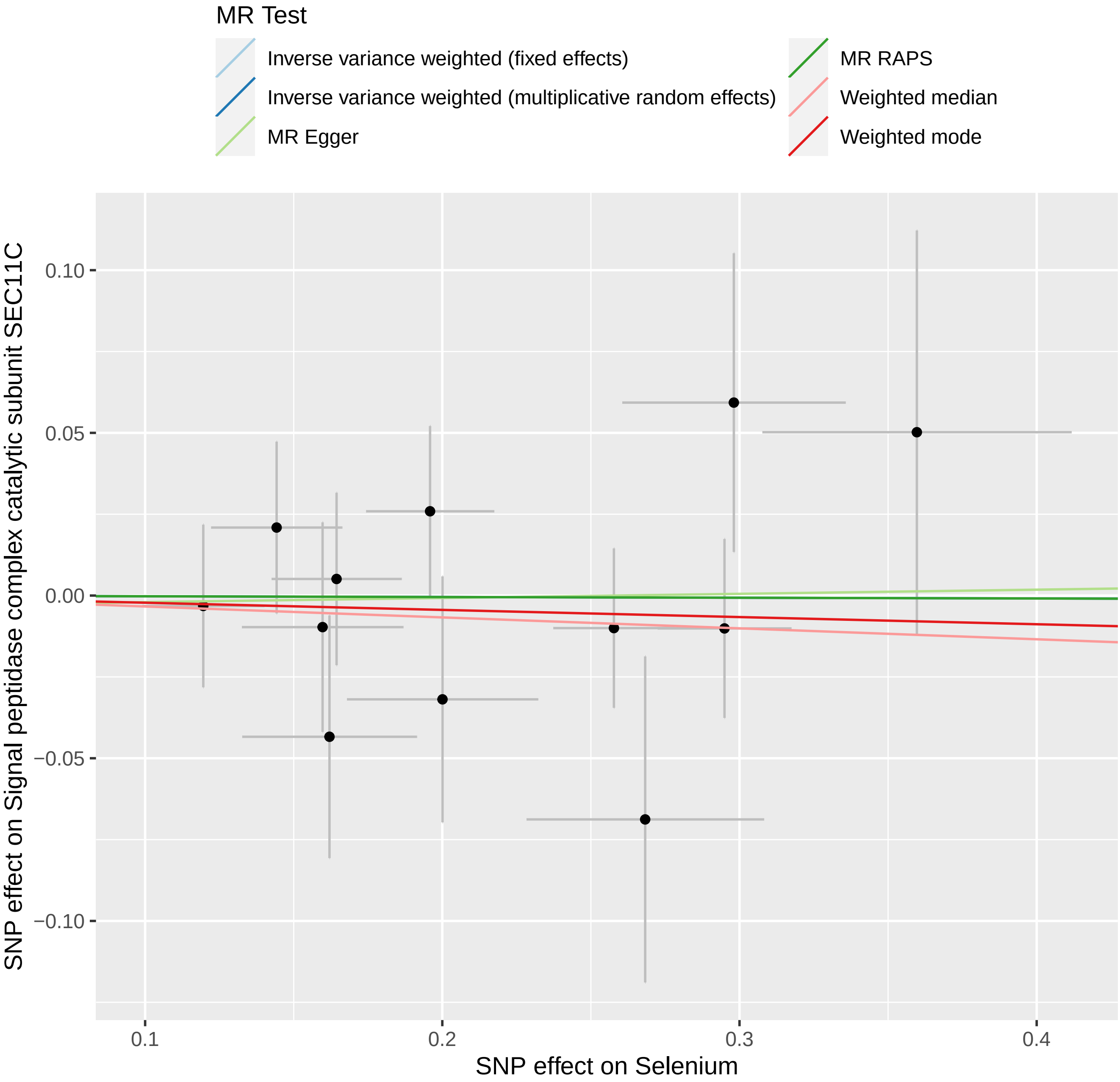

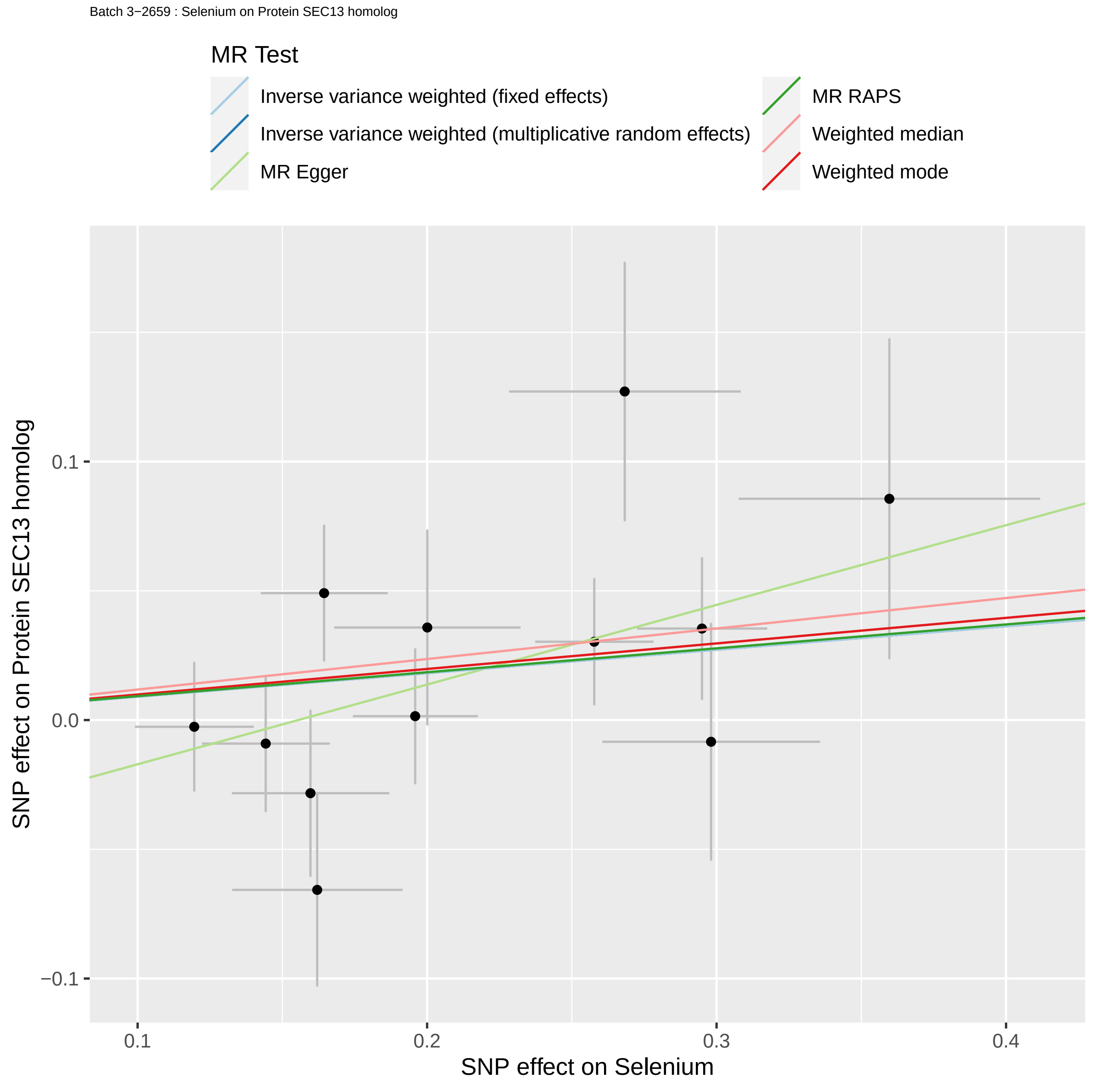
Batch 3−2659 : Selenium on Protein SEC13 homolog
MR Test
Inverse variance weighted (fixed effects)
Inverse variance weighted (multiplicative random effects)
MR Egger
MR RAPS
Weighted median
Weighted mode
SNP effect on Protein SEC13 homolog
0.1
0.0
−0.1
0.1
0.2
0.3
0.4
SNP effect on Selenium

Batch 3−2660 : Selenium on Vesicle−trafficking protein SEC22a

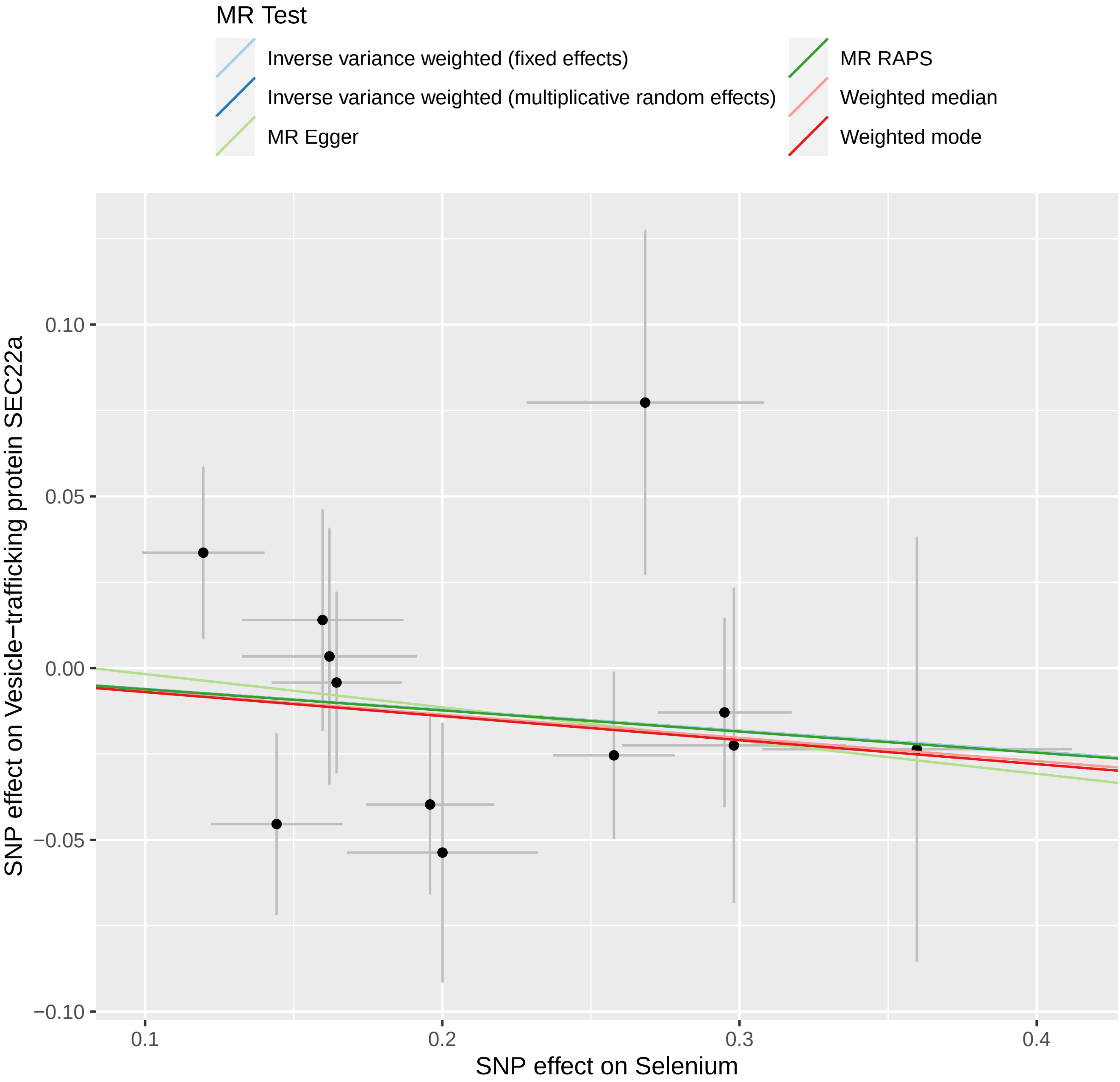

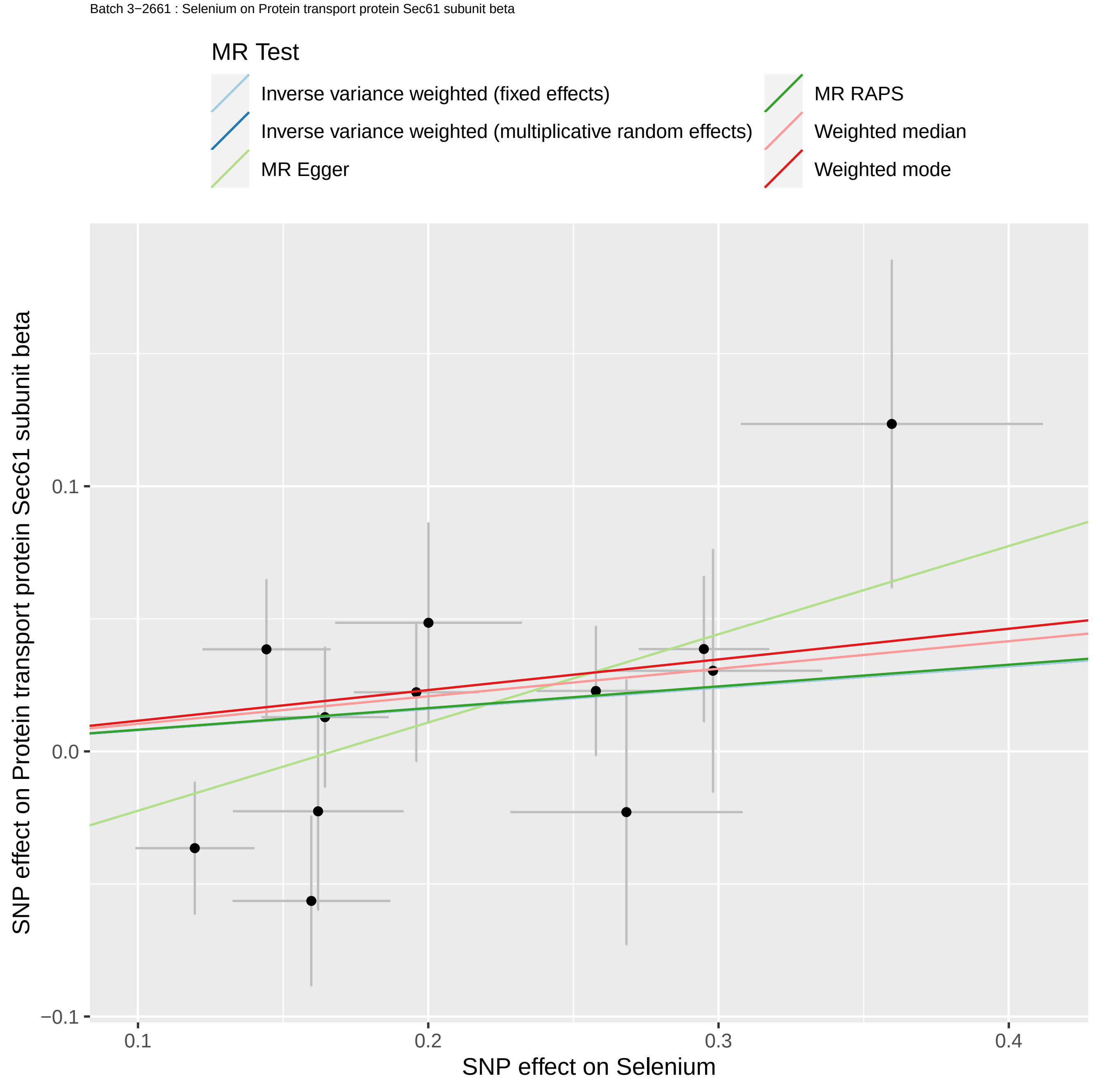
Batch 3−2661 : Selenium on Protein transport protein Sec61 subunit beta
MR Test
Inverse variance weighted (fixed effects)
Inverse variance weighted (multiplicative random effects)
MR Egger
MR RAPS
Weighted median
Weighted mode
SNP effect on Protein transport protein Sec61 subunit beta
0.1
0.0
−0.1
0.1
0.2
0.3
0.4
SNP effect on Selenium

Batch 3−2662 : Selenium on Protein transport protein Sec61 subunit gamma

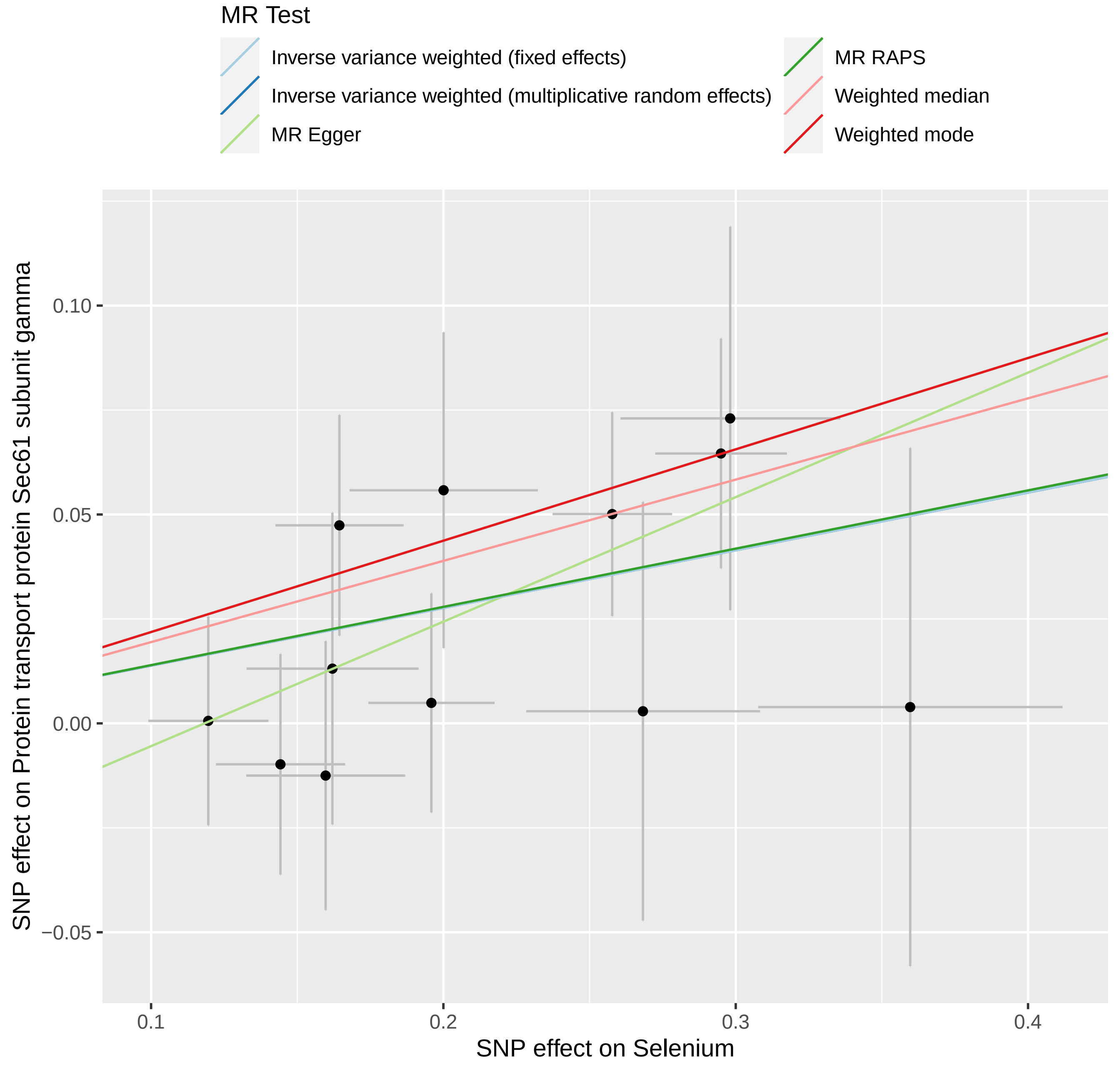

Batch 3−2663 : Selenium on Secreted and transmembrane protein 1

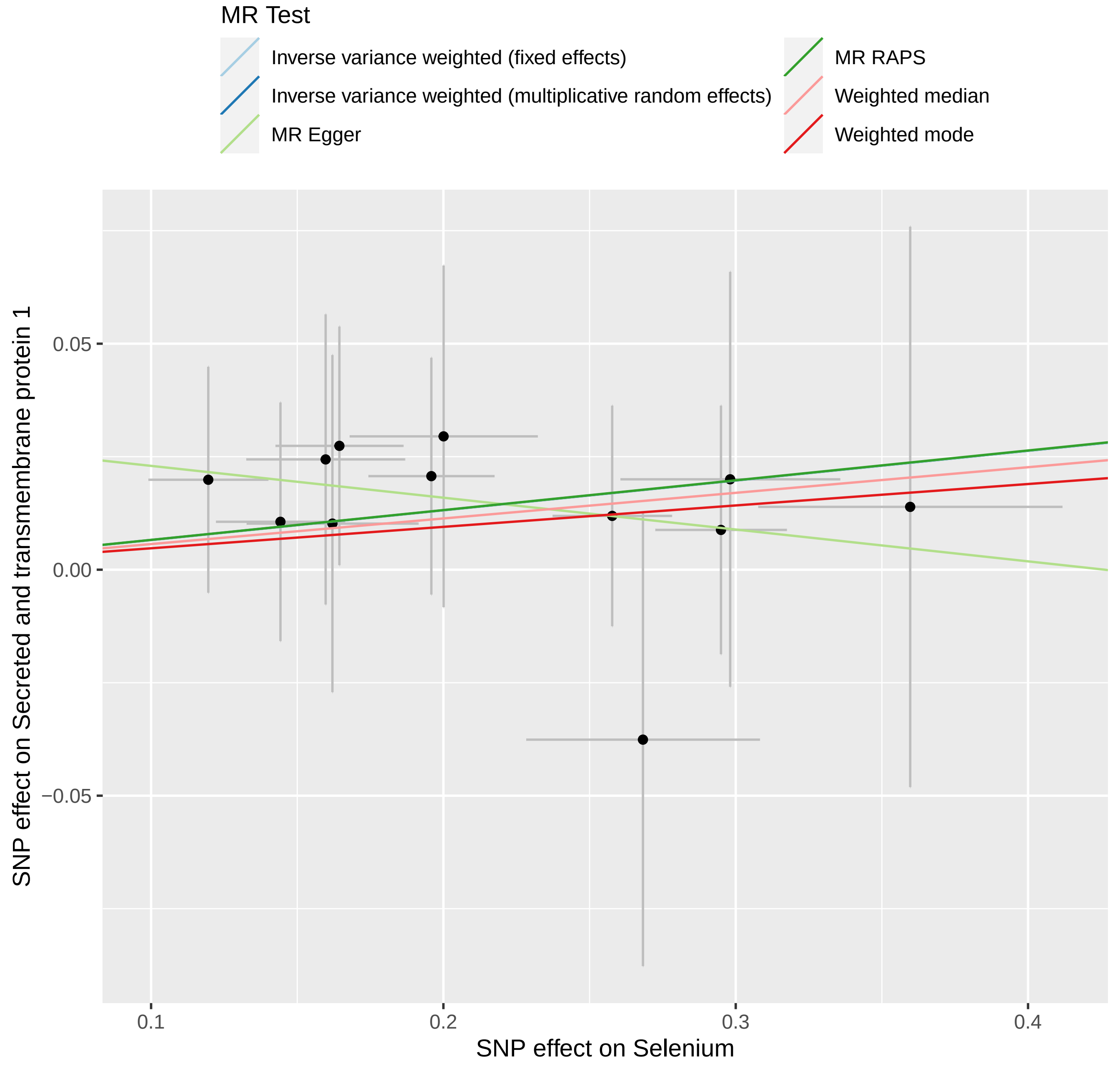

Batch 3−2664 : Selenium on Protein sel−1 homolog 2

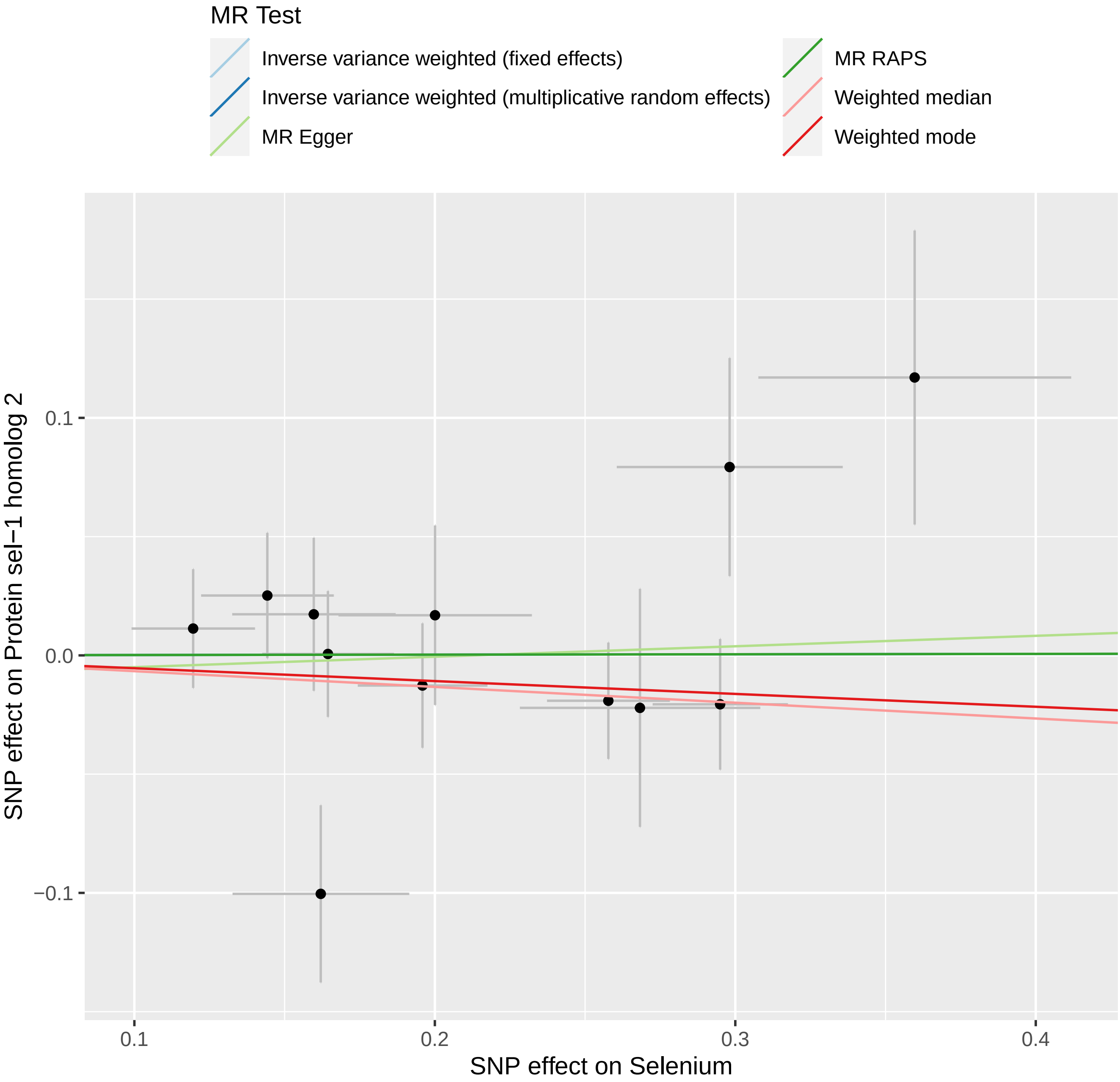

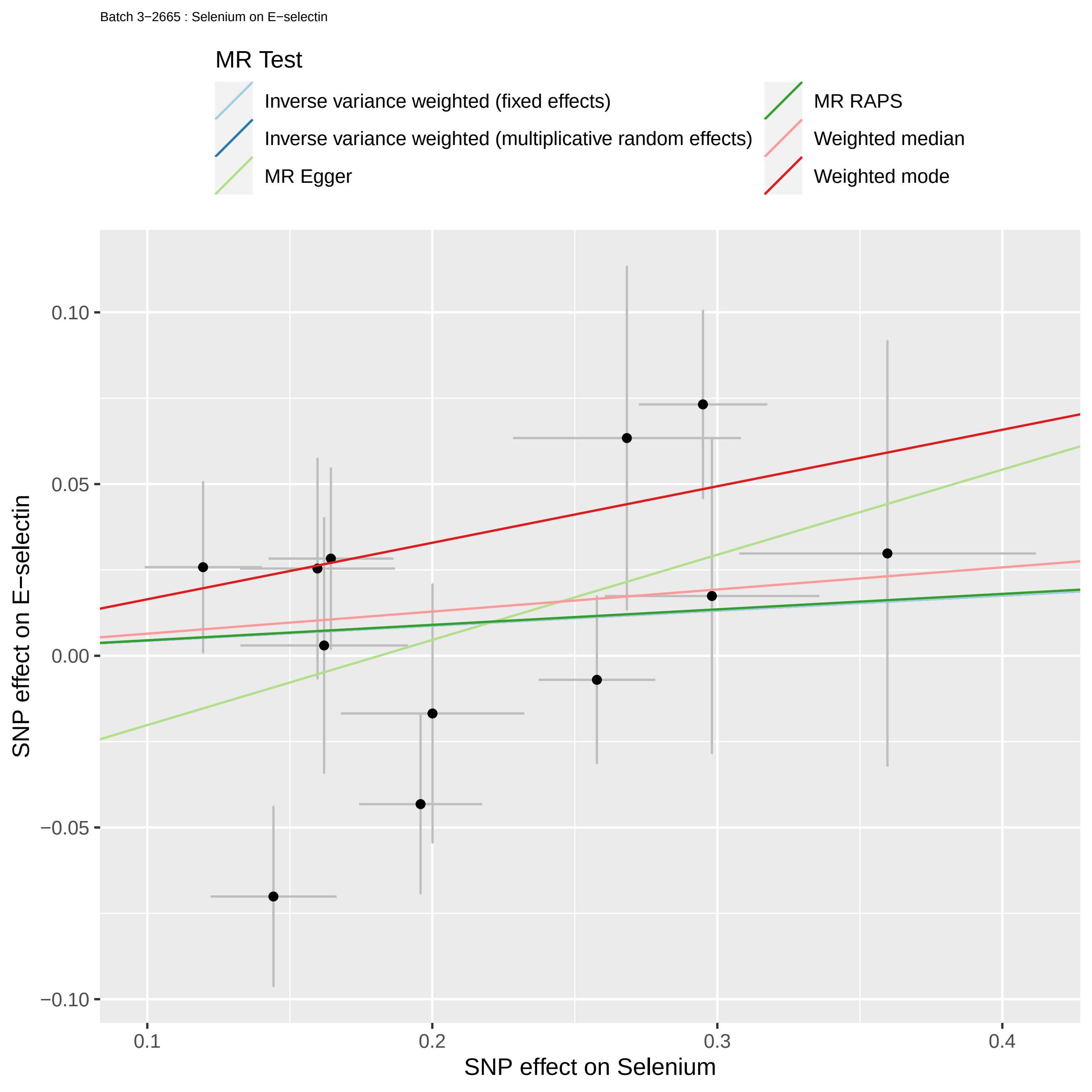

Batch 3−2665 : Selenium on E−selectin
MR Test
Inverse variance weighted (fixed effects)
Inverse variance weighted (multiplicative random effects)
MR Egger
MR RAPS
Weighted median
Weighted mode
0.10
0.05
0.00
−0.05
−0.10
0.1
0.2
0.3
0.4
SNP effect on Selenium
SNP effect on E−selectin

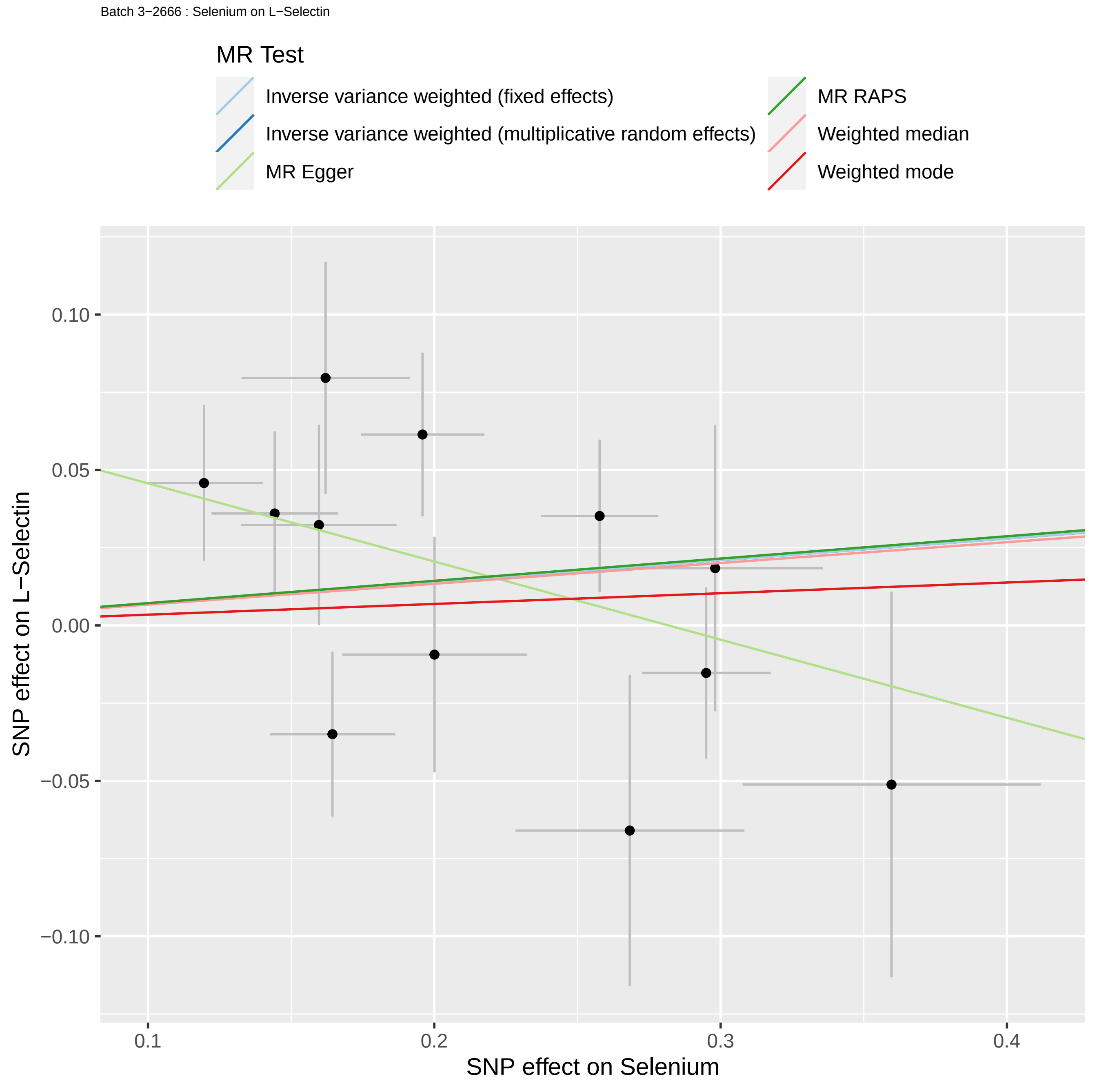
Batch 3−2666 : Selenium on L−Selectin
MR Test
Inverse variance weighted (fixed effects)
Inverse variance weighted (multiplicative random effects)
MR Egger
MR RAPS
Weighted median
Weighted mode
0.10
0.05
0.00
−0.05
−0.10
SNP effect on L−Selectin
0.1
0.2
0.3
0.4
SNP effect on Selenium

Batch 3−2667 : Selenium on P−selectin

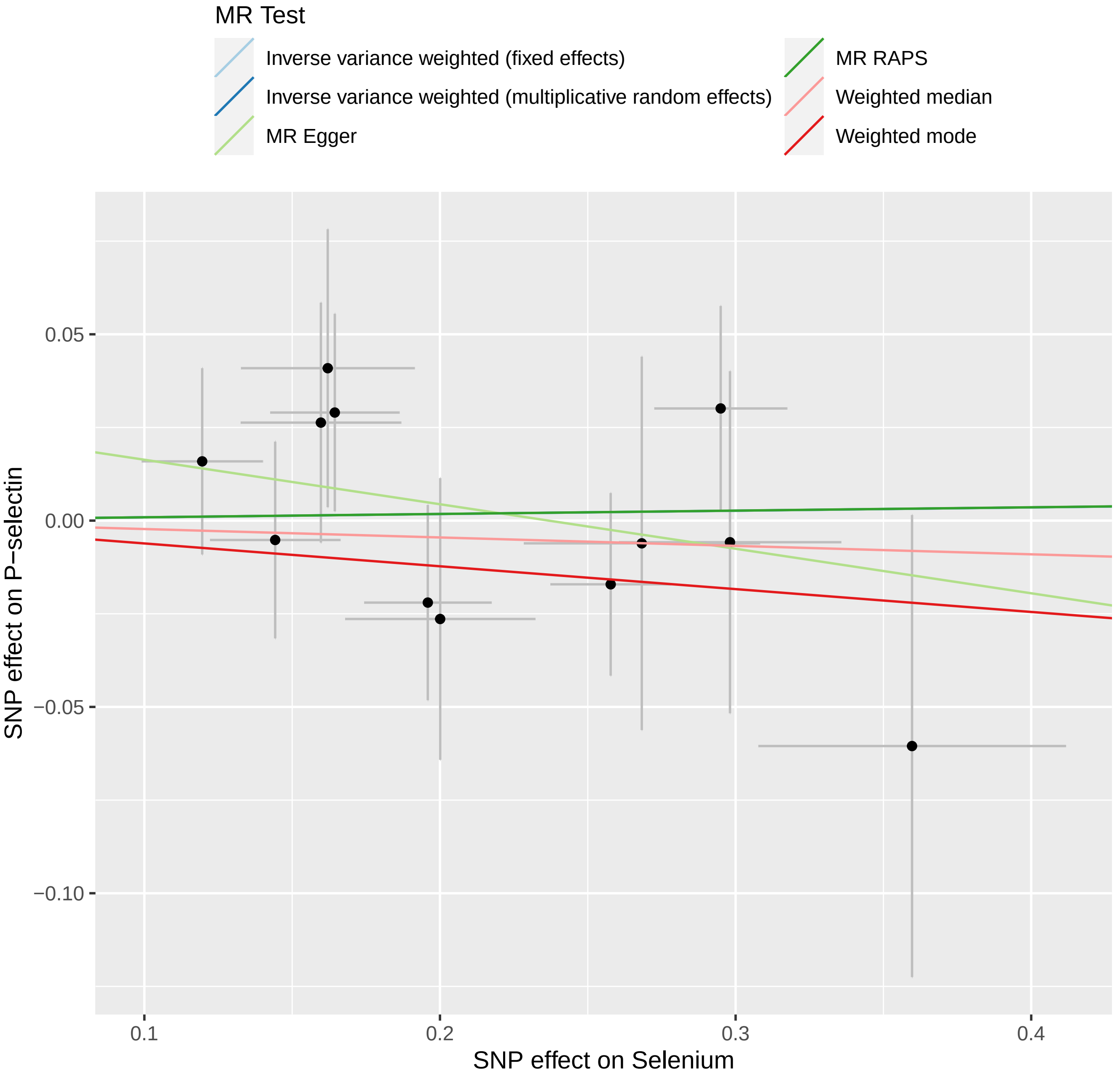

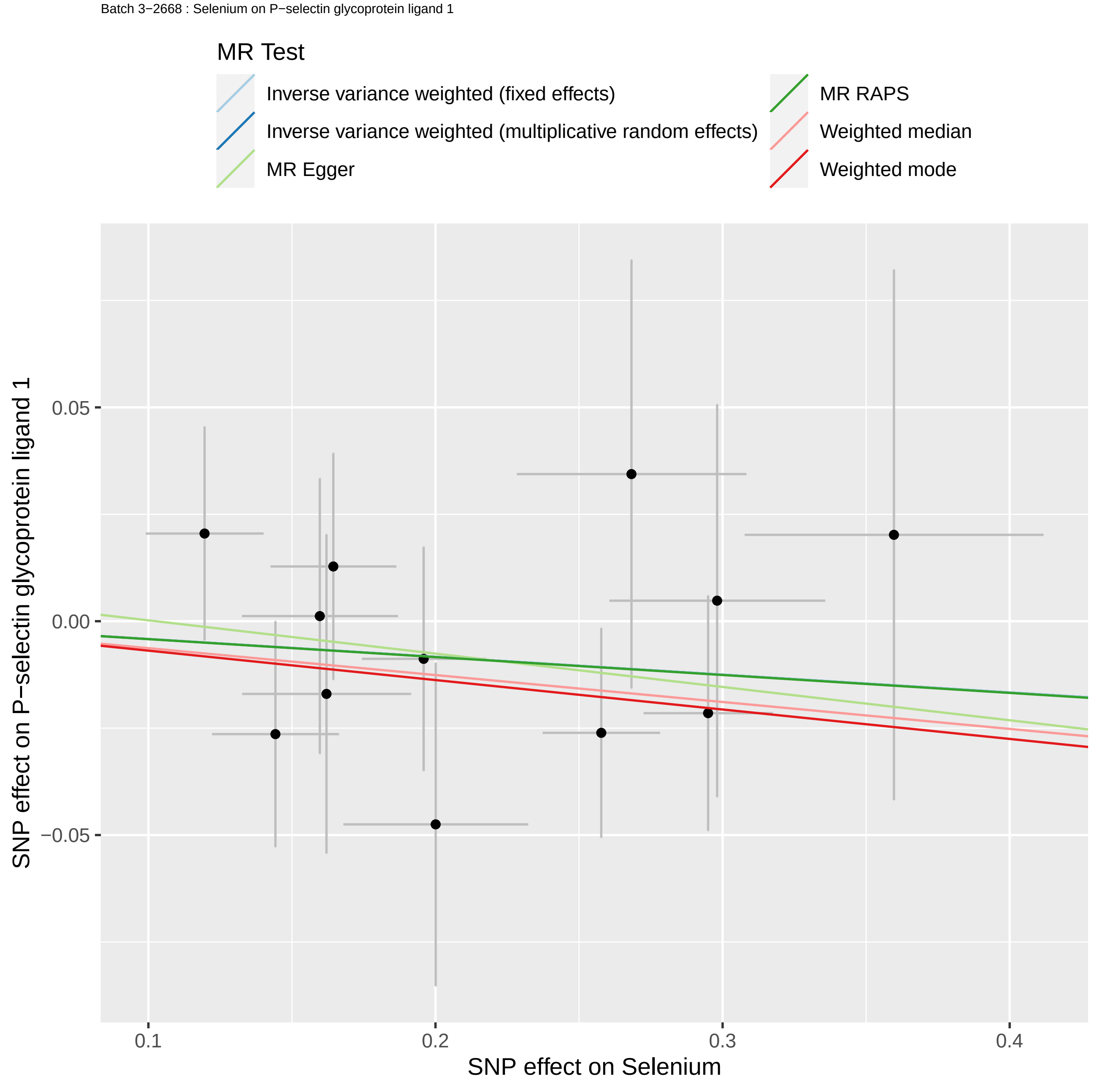

Batch 3−2668 : Selenium on P−selectin glycoprotein ligand 1
MR Test
Inverse variance weighted (fixed effects)
Inverse variance weighted (multiplicative random effects)
MR Egger
MR RAPS
Weighted median
Weighted mode
0.05
0.00
−0.05
0.1
0.2
0.3
0.4
SNP effect on P−selectin glycoprotein ligand 1
SNP effect on Selenium

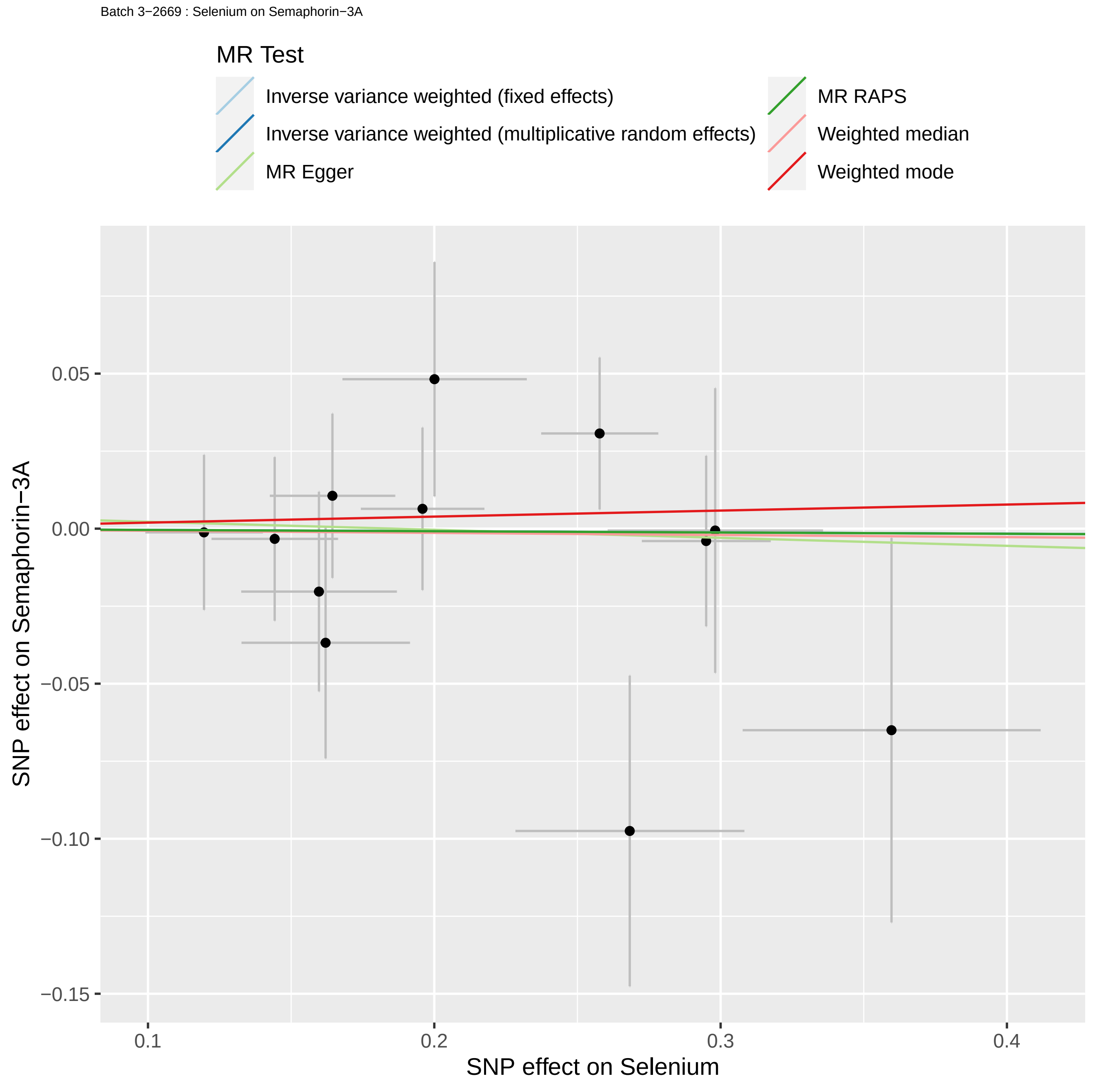

Batch 3−2669 : Selenium on Semaphorin−3A
MR Test
Inverse variance weighted (fixed effects)
Inverse variance weighted (multiplicative random effects)
MR Egger
MR RAPS
Weighted median
Weighted mode
SNP effect on Semaphorin−3A
0.05
0.00
−0.05
−0.10
−0.15
0.1
0.2
0.3
0.4
SNP effect on Selenium

Batch 3−2670 : Selenium on Semaphorin−3B

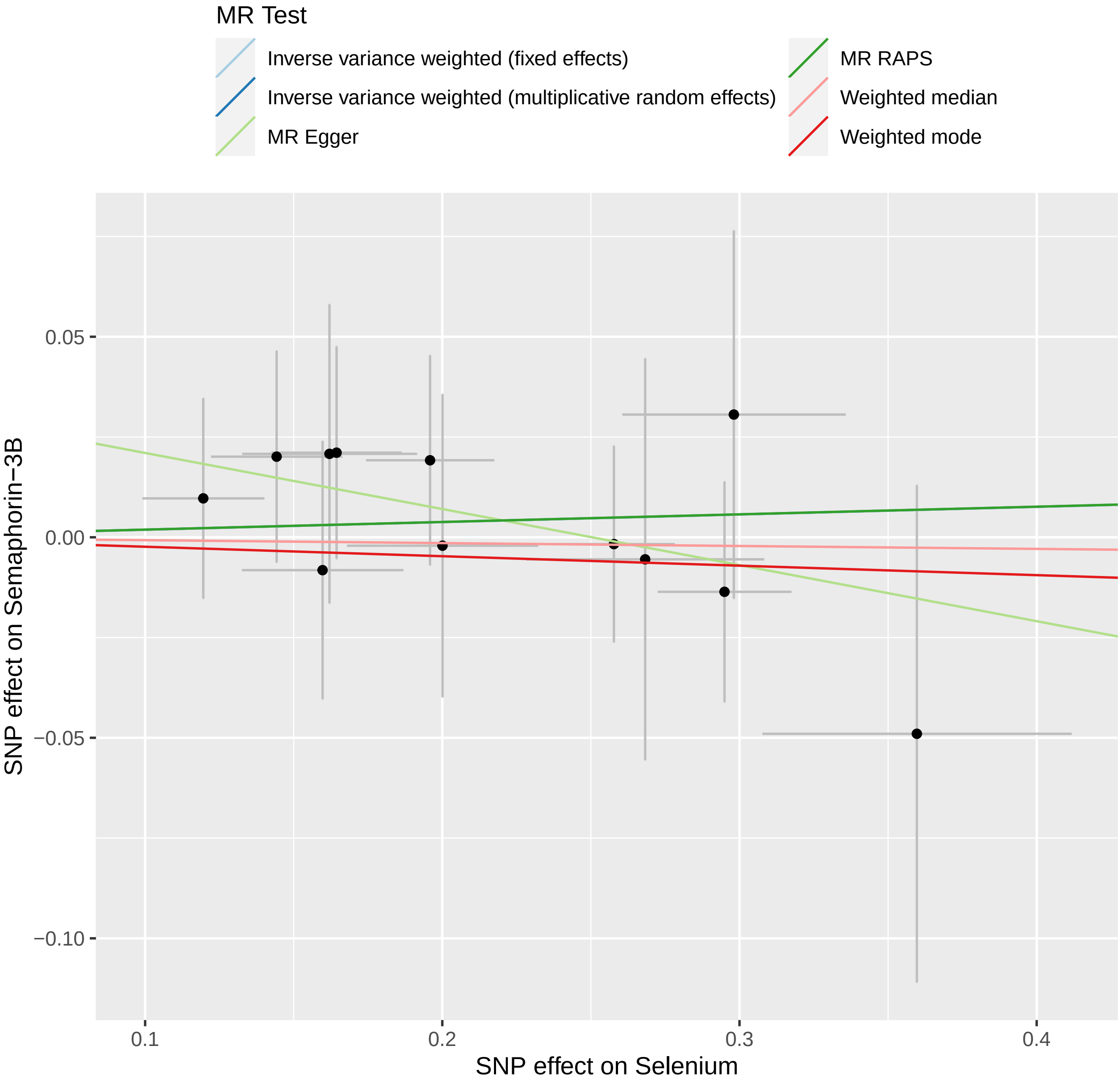

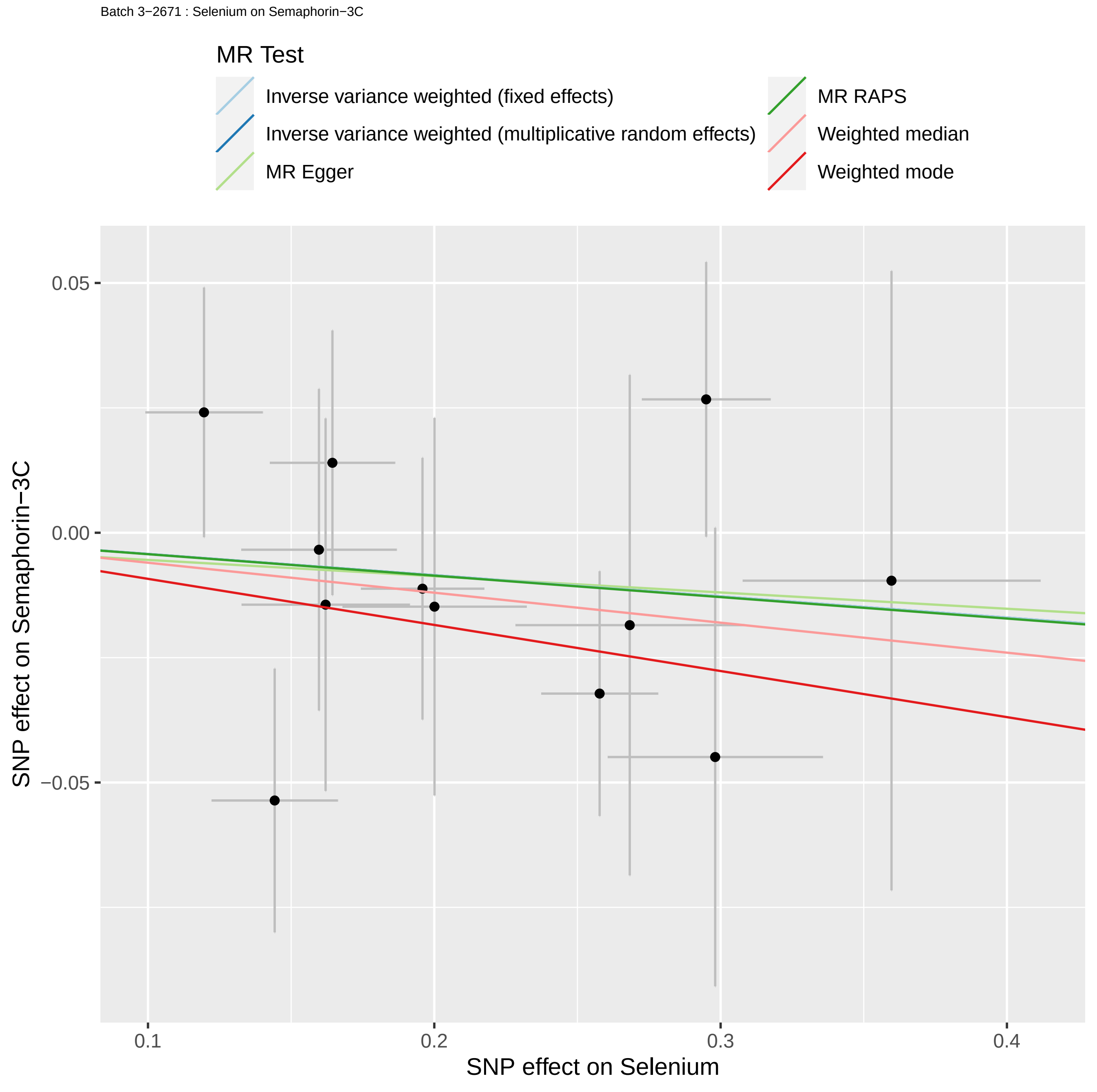

Batch 3−2671 : Selenium on Semaphorin−3C
MR Test
Inverse variance weighted (fixed effects)
Inverse variance weighted (multiplicative random effects)
MR Egger
MR RAPS
Weighted median
Weighted mode
0.05
0.00
−0.05
0.1
0.2
0.3
0.4
SNP effect on Semaphorin−3C
SNP effect on Selenium

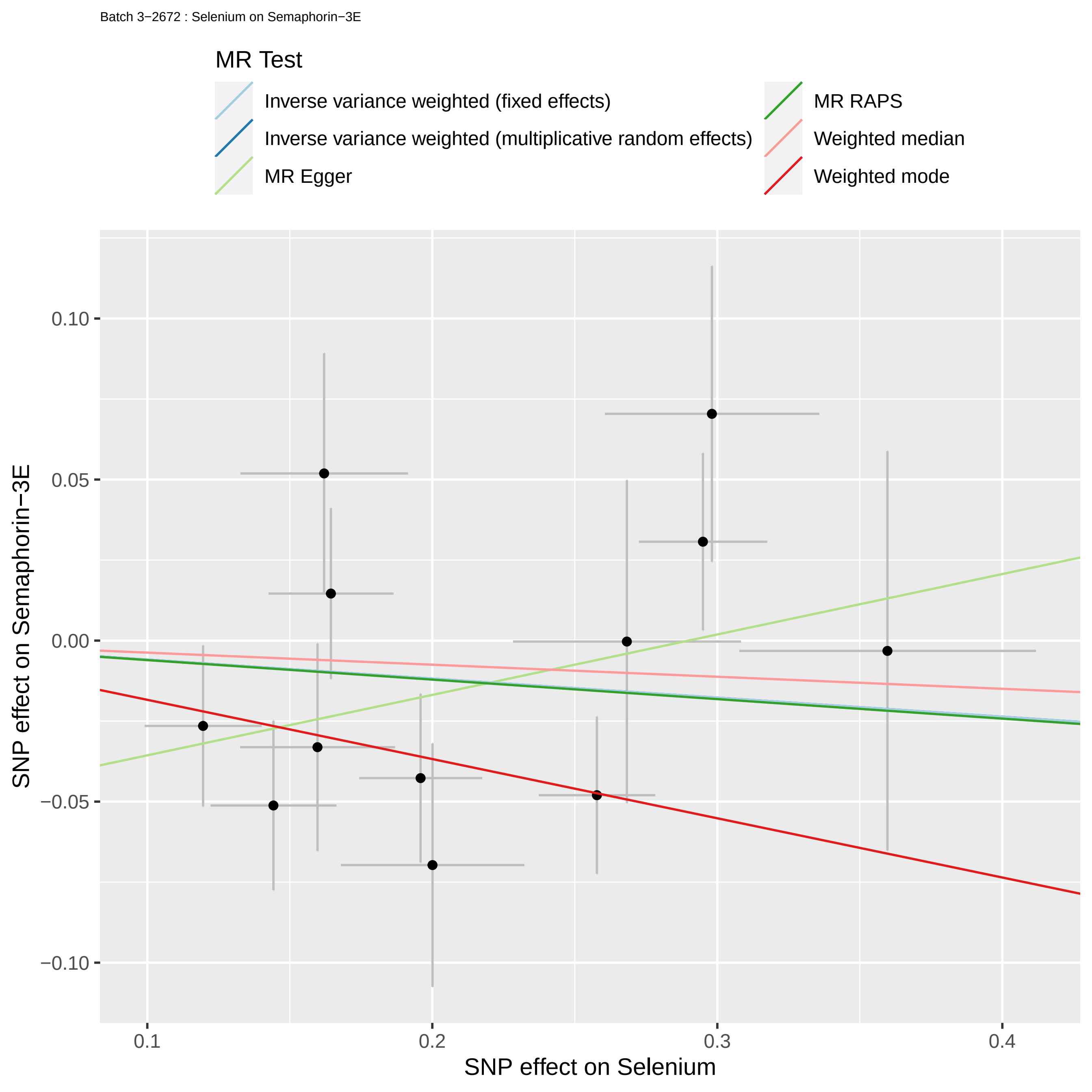
Batch 3−2672 : Selenium on Semaphorin−3E
MR Test
Inverse variance weighted (fixed effects)
Inverse variance weighted (multiplicative random effects)
MR Egger
MR RAPS
Weighted median
Weighted mode
0.10
0.05
0.00
−0.05
−0.10
0.1
0.2
0.3
0.4
SNP effect on Semaphorin−3E
SNP effect on Selenium

Batch 3−2673 : Selenium on Semaphorin−3G

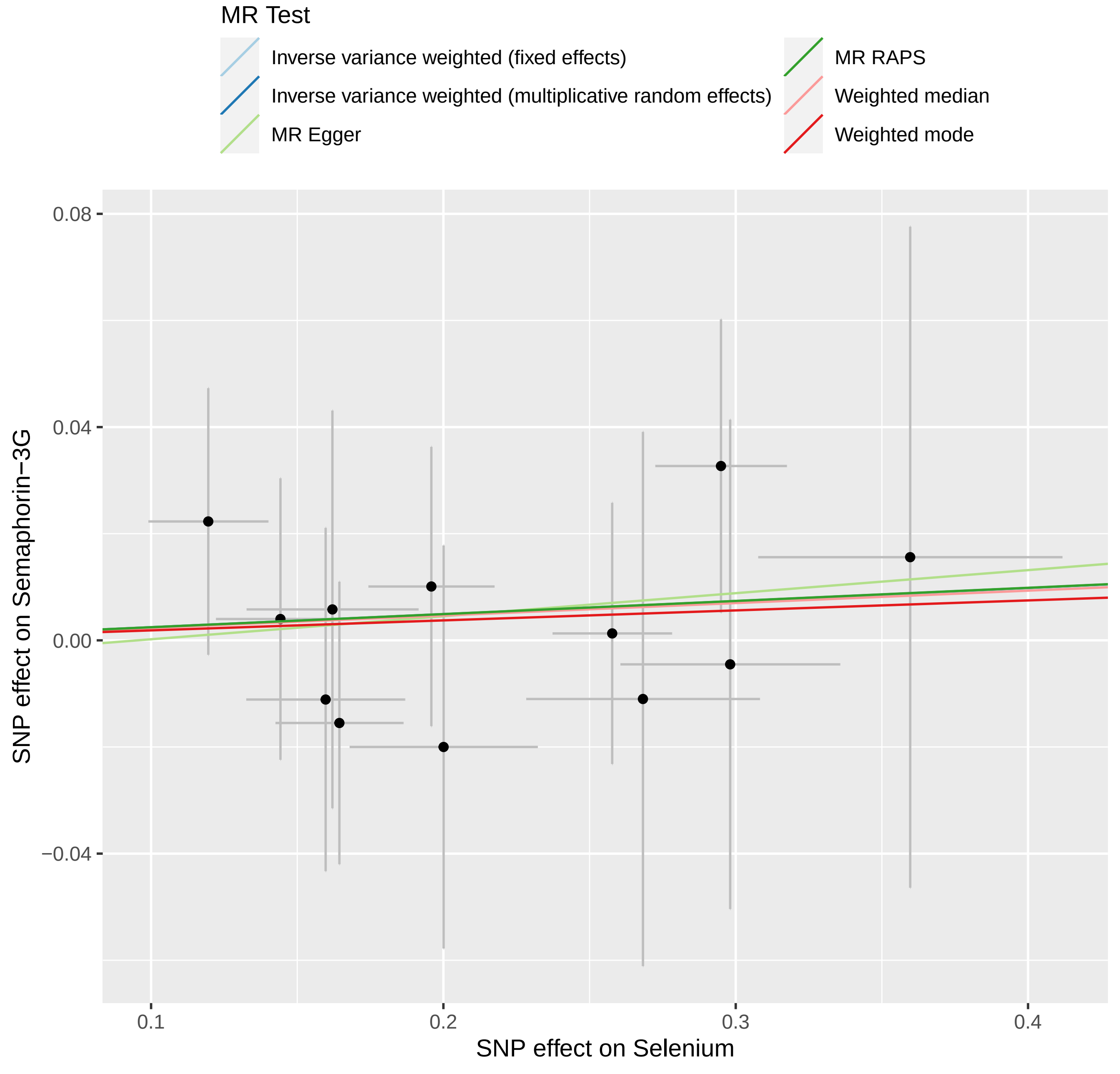

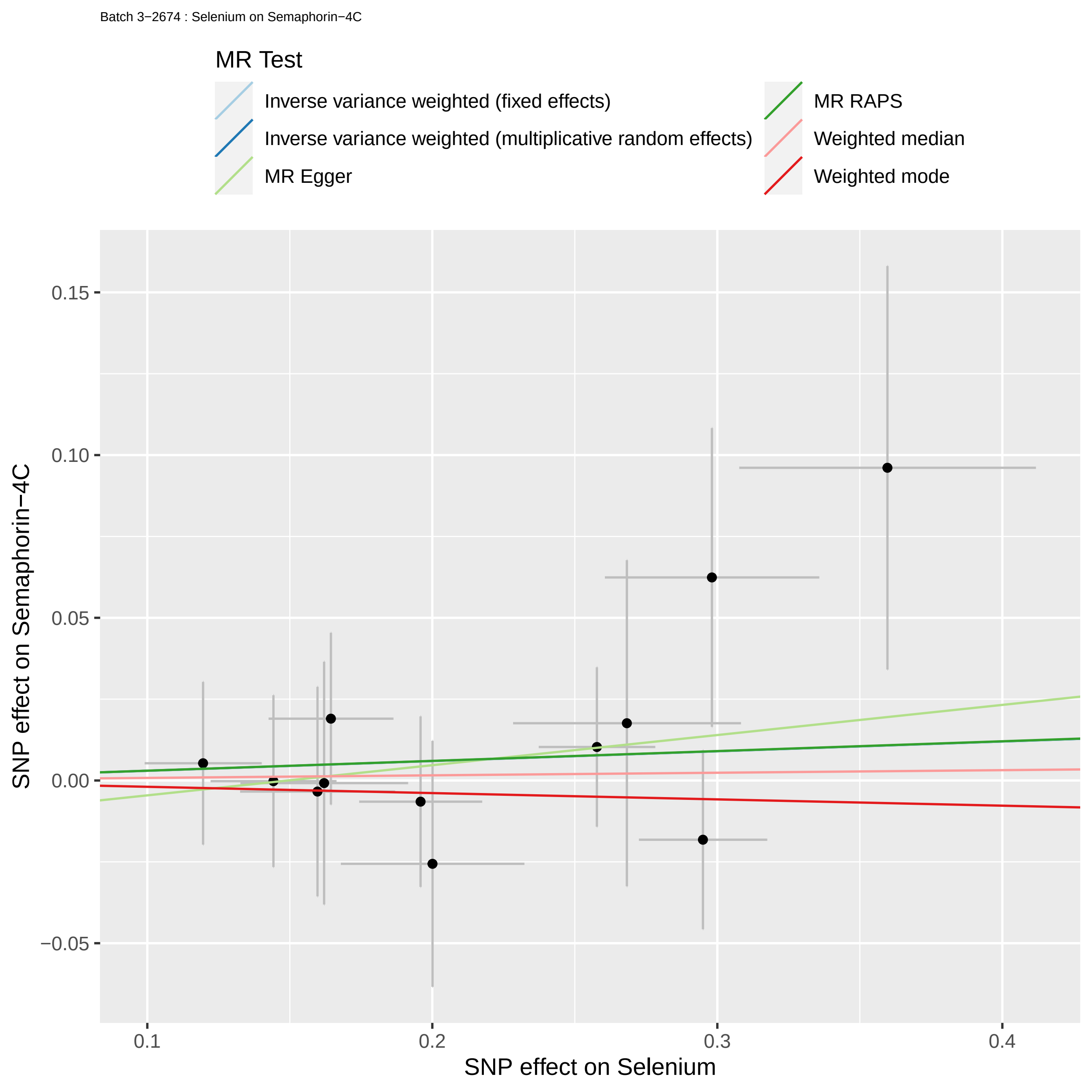
Batch 3−2674 : Selenium on Semaphorin−4C
MR Test
Inverse variance weighted (fixed effects)
Inverse variance weighted (multiplicative random effects)
MR Egger
MR RAPS
Weighted median
Weighted mode
SNP effect on Semaphorin−4C
0.15
0.10
0.05
0.00
−0.05
0.1
0.2
0.3
0.4
SNP effect on Selenium

Batch 3−2675 : Selenium on Semaphorin−4D

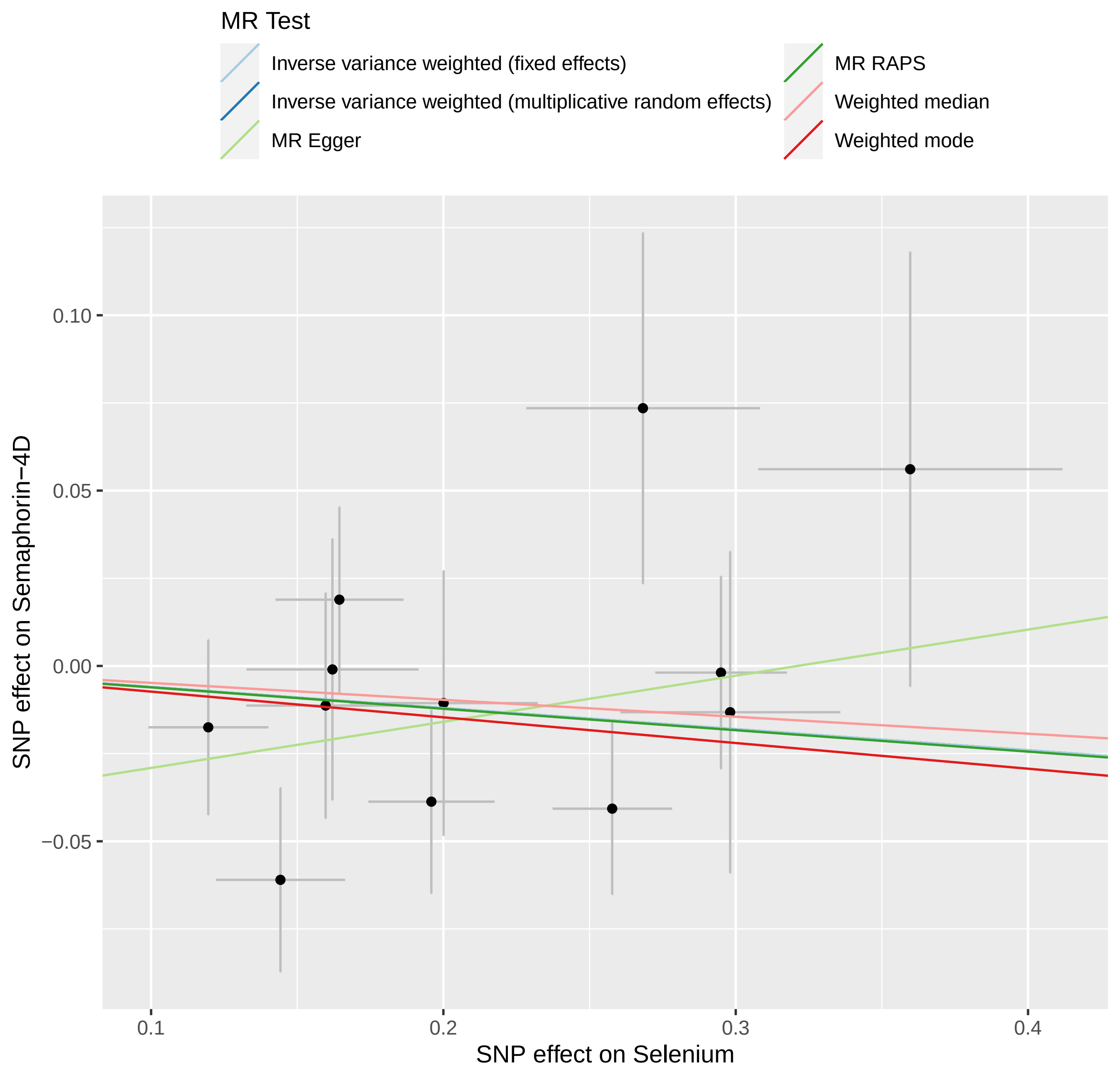

Batch 3−2676 : Selenium on Semaphorin−5A

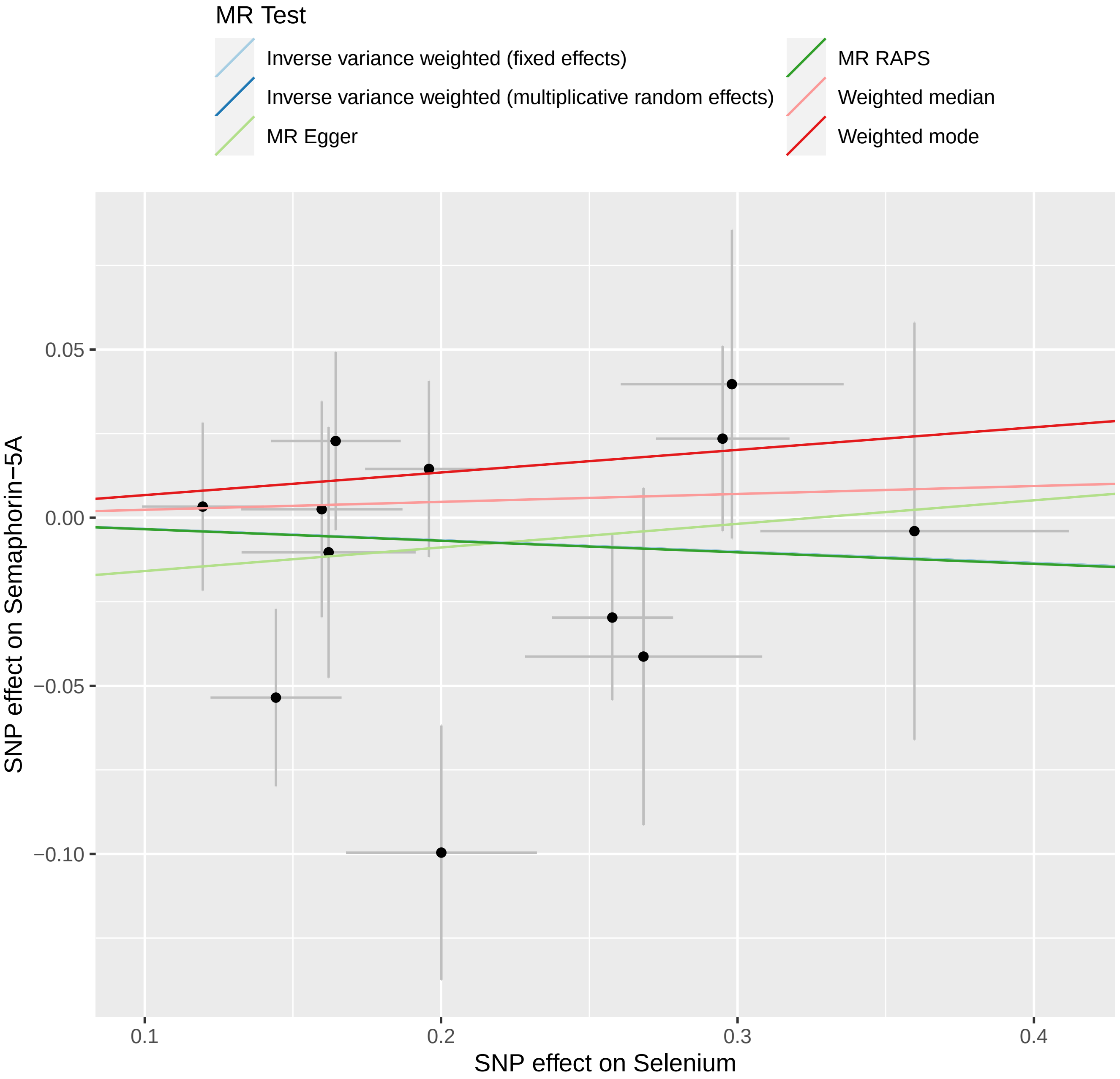

Batch 3−2677 : Selenium on Semaphorin−6A

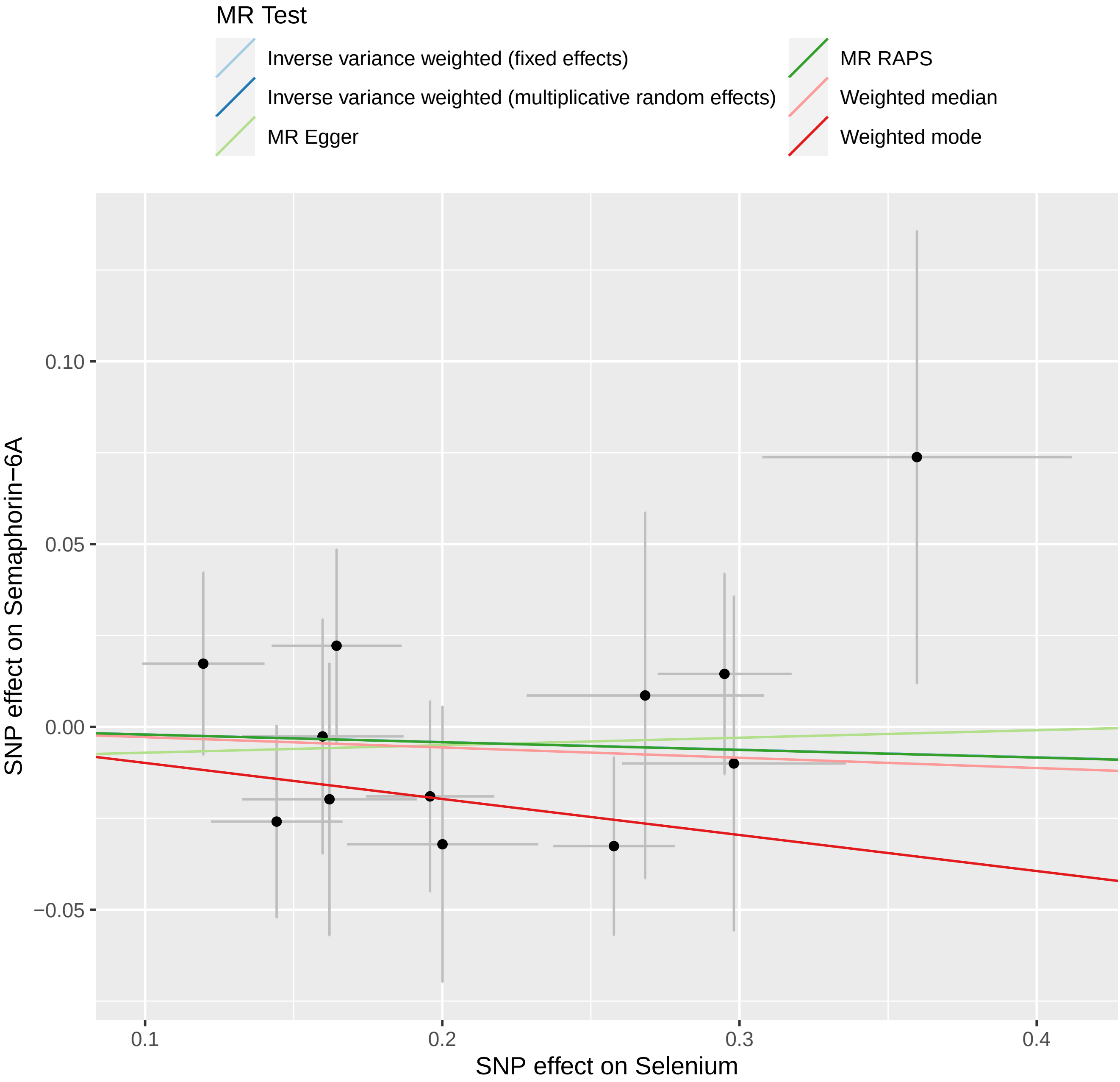

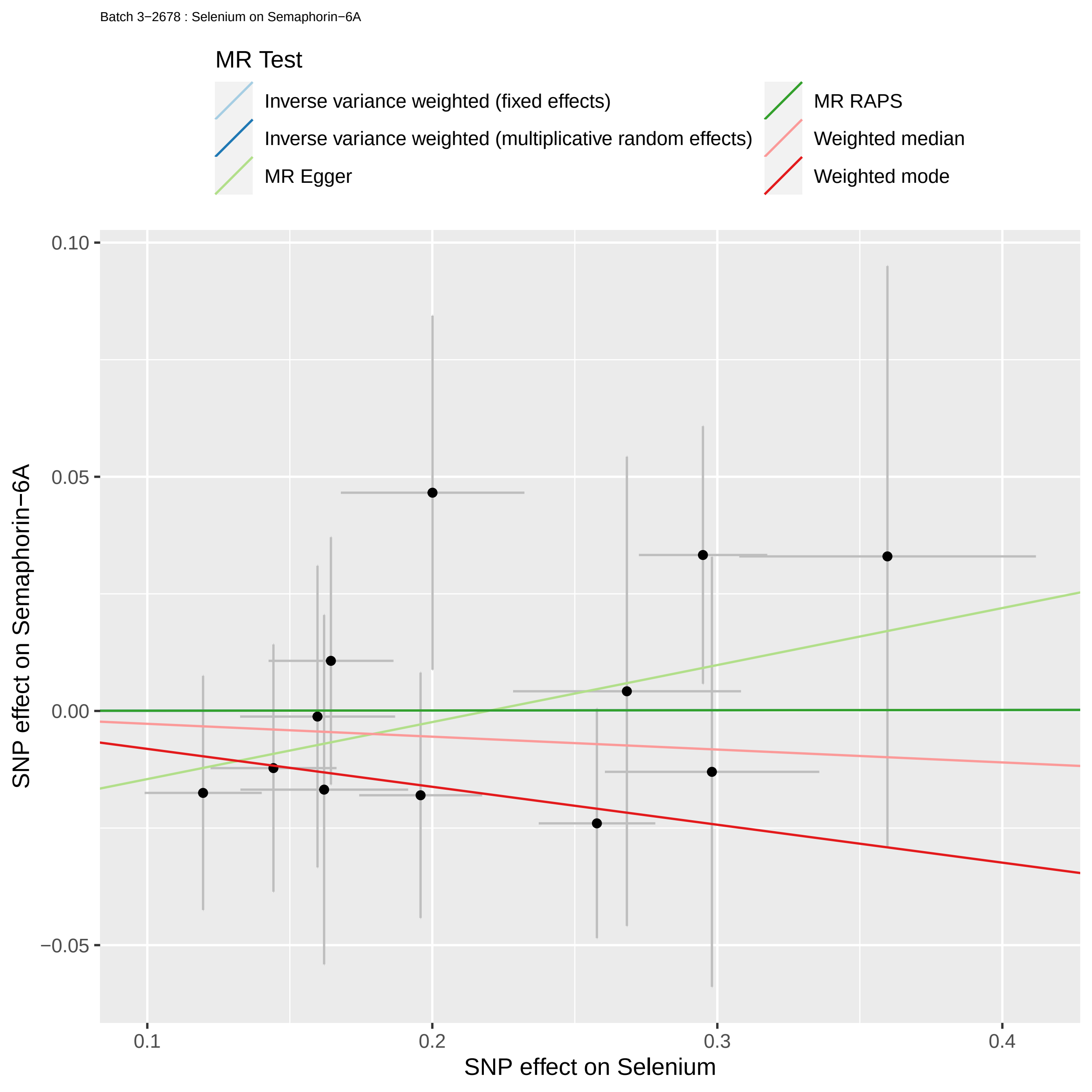
Batch 3−2678 : Selenium on Semaphorin−6A
MR Test
Inverse variance weighted (fixed effects)
Inverse variance weighted (multiplicative random effects)
MR Egger
MR RAPS
Weighted median
Weighted mode
0.10
0.05
0.00
−0.05
0.1
0.2
0.3
0.4
SNP effect on Semaphorin−6A
SNP effect on Selenium

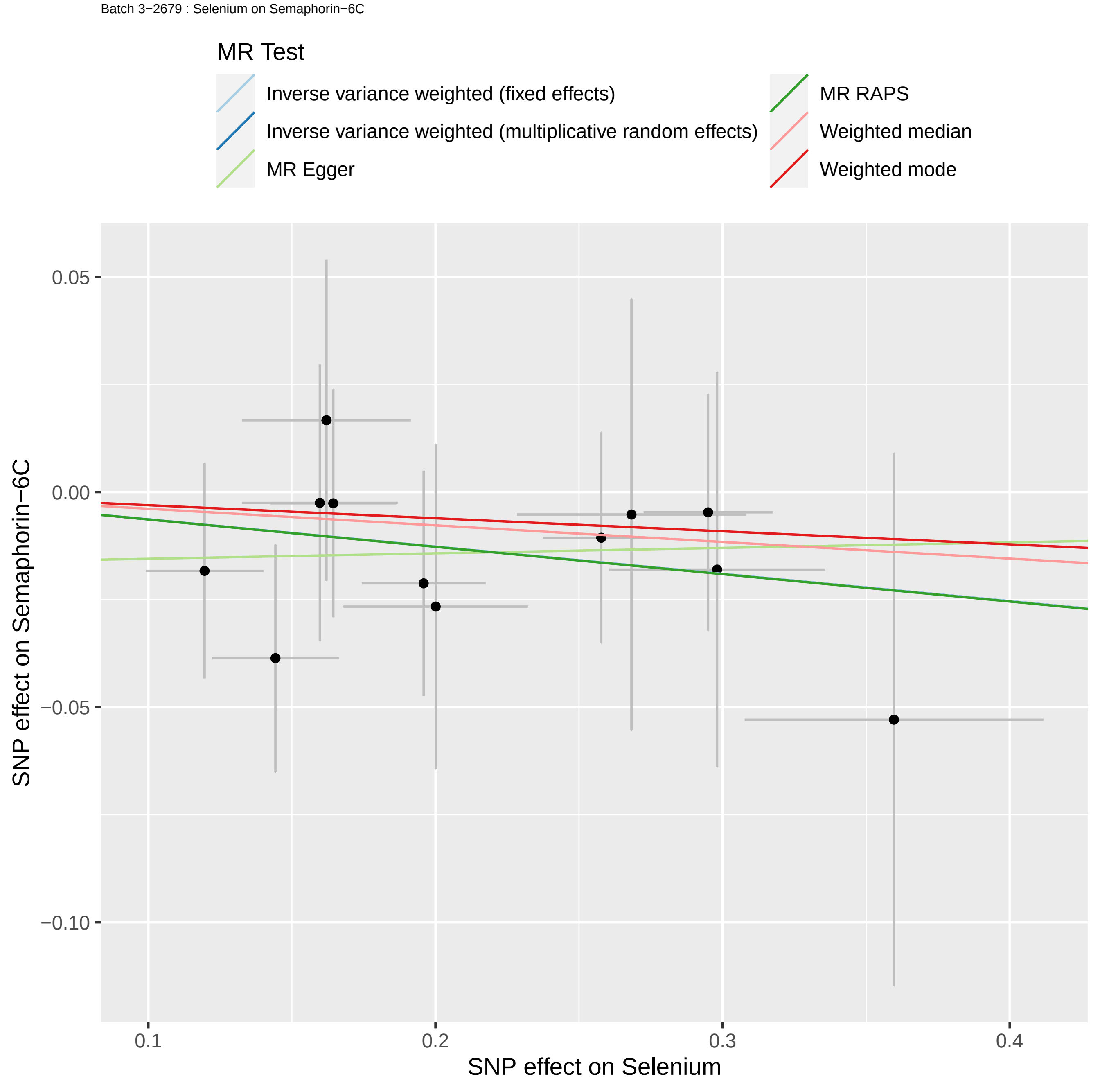

Batch 3−2679 : Selenium on Semaphorin−6C
MR Test
Inverse variance weighted (fixed effects)
Inverse variance weighted (multiplicative random effects)
MR Egger
MR RAPS
Weighted median
Weighted mode
0.05
0.00
−0.05
−0.10
0.1
0.2
0.3
0.4
SNP effect on Semaphorin−6C
SNP effect on Selenium

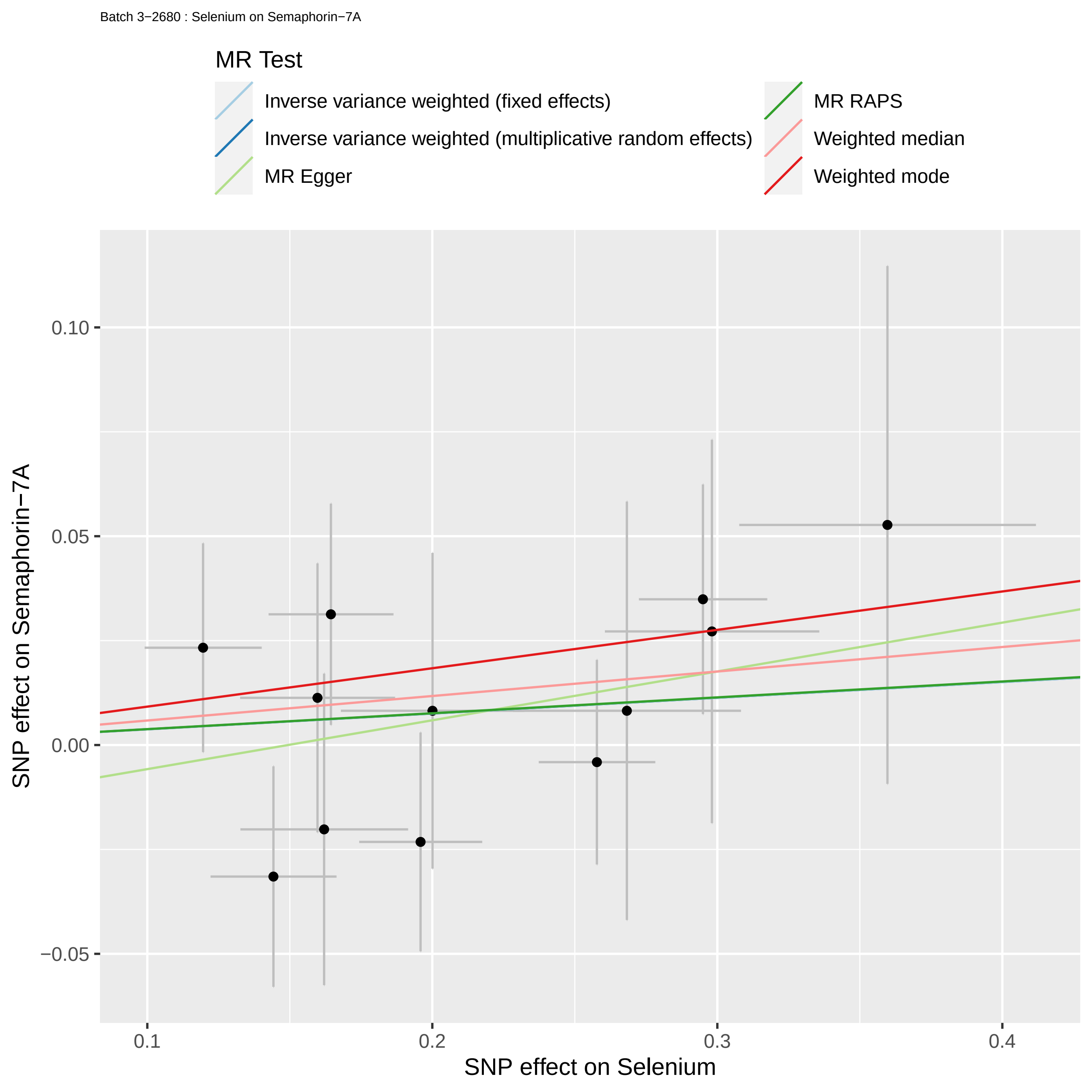

Batch 3−2680 : Selenium on Semaphorin−7A
MR Test
Inverse variance weighted (fixed effects)
Inverse variance weighted (multiplicative random effects)
MR Egger
MR RAPS
Weighted median
Weighted mode
SNP effect on Semaphorin−7A
0.10
0.05
0.00
−0.05
0.1
0.2
0.3
0.4
SNP effect on Selenium

Batch 3−2681 : Selenium on Semenogelin−1

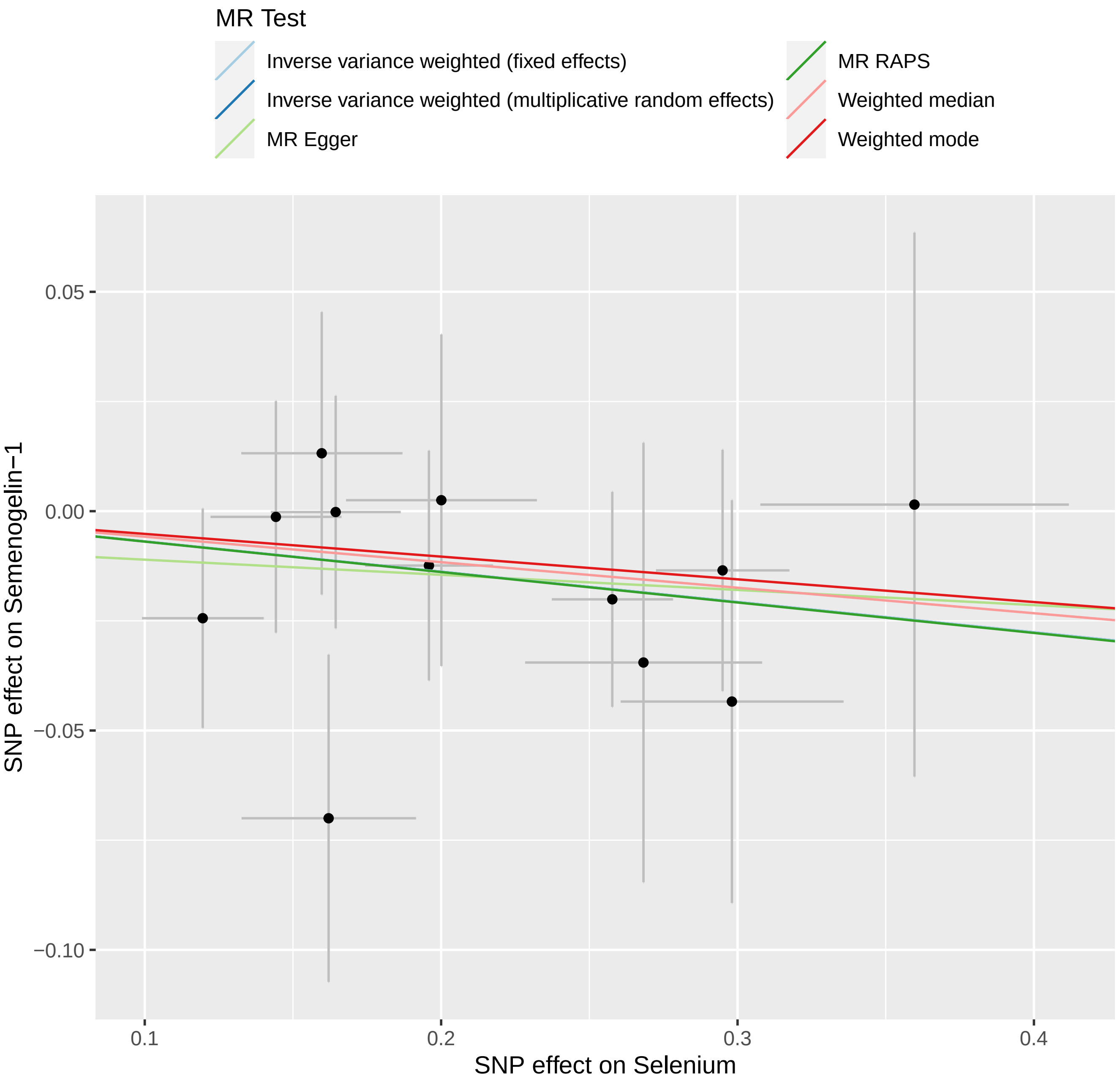

Batch 3−2682 : Selenium on Semenogelin−2

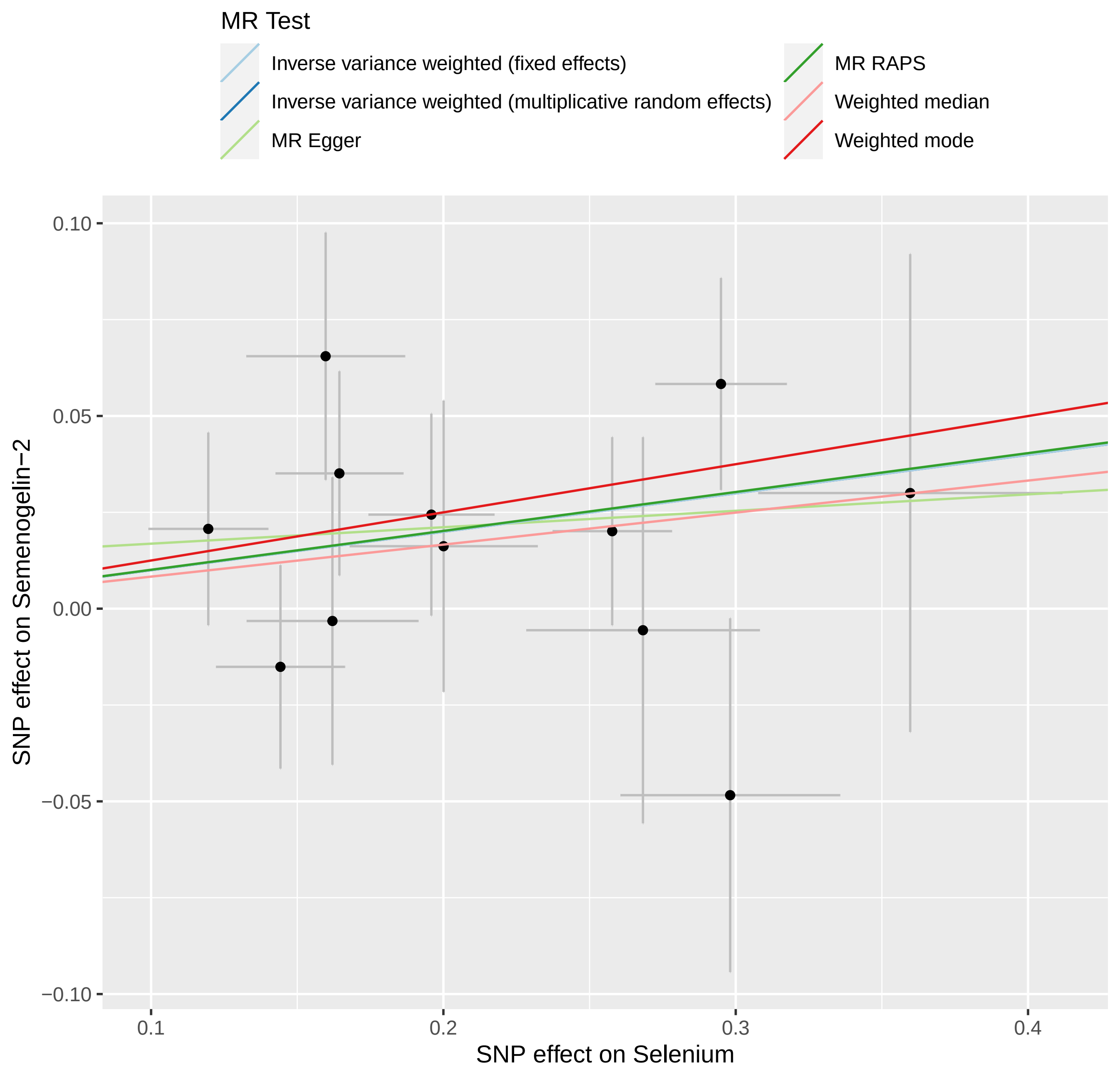

Batch 3−2683 : Selenium on Sentrin−specific protease 7

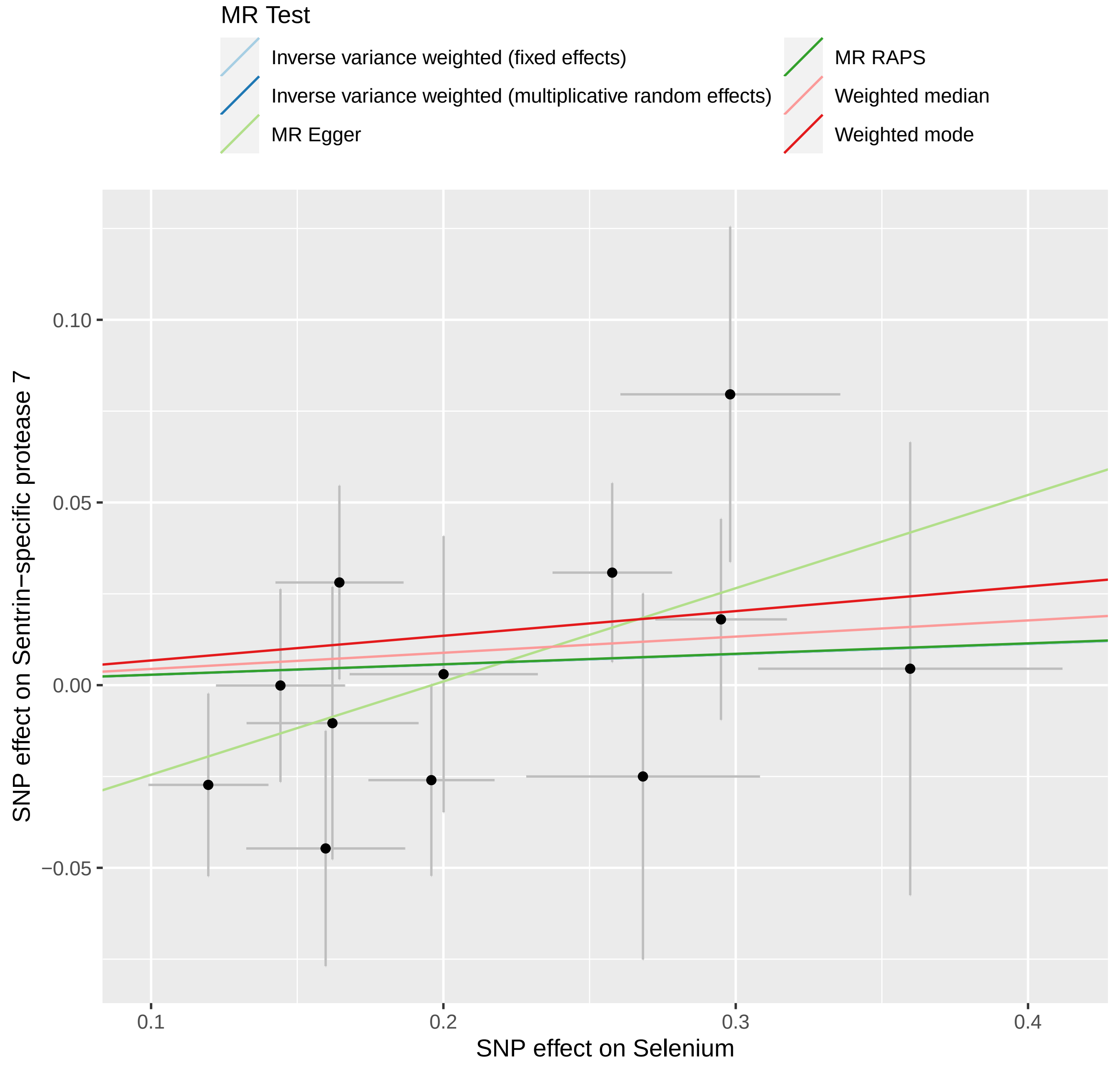

Batch 3−2684 : Selenium on Selenide, water dikinase 1

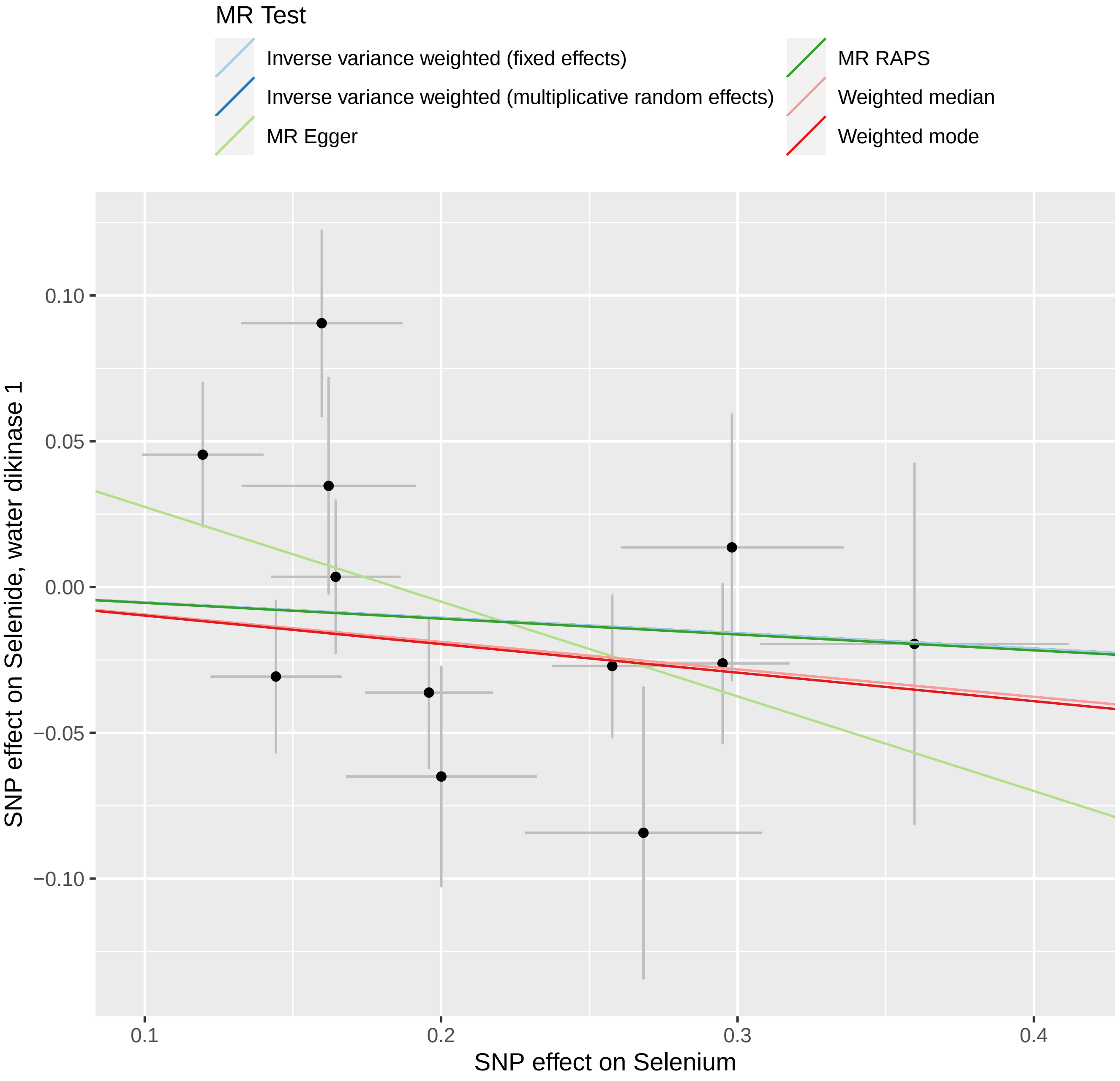

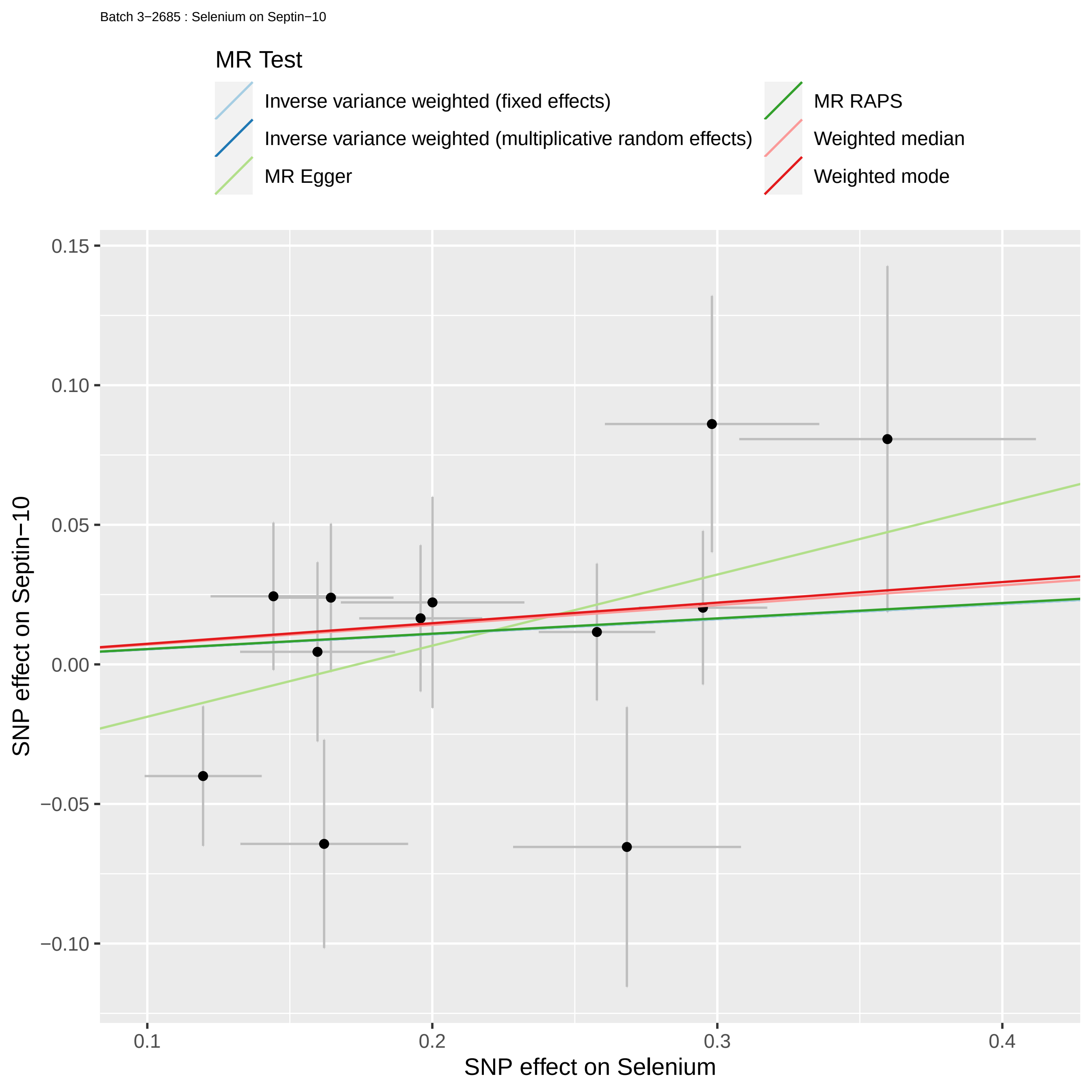
Batch 3−2685 : Selenium on Septin−10
MR Test
Inverse variance weighted (fixed effects)
Inverse variance weighted (multiplicative random effects)
MR Egger
MR RAPS
Weighted median
Weighted mode
0.15
0.10
0.05
0.00
−0.05
−0.10
0.1
0.2
0.3
0.4
SNP effect on Septin−10
SNP effect on Selenium

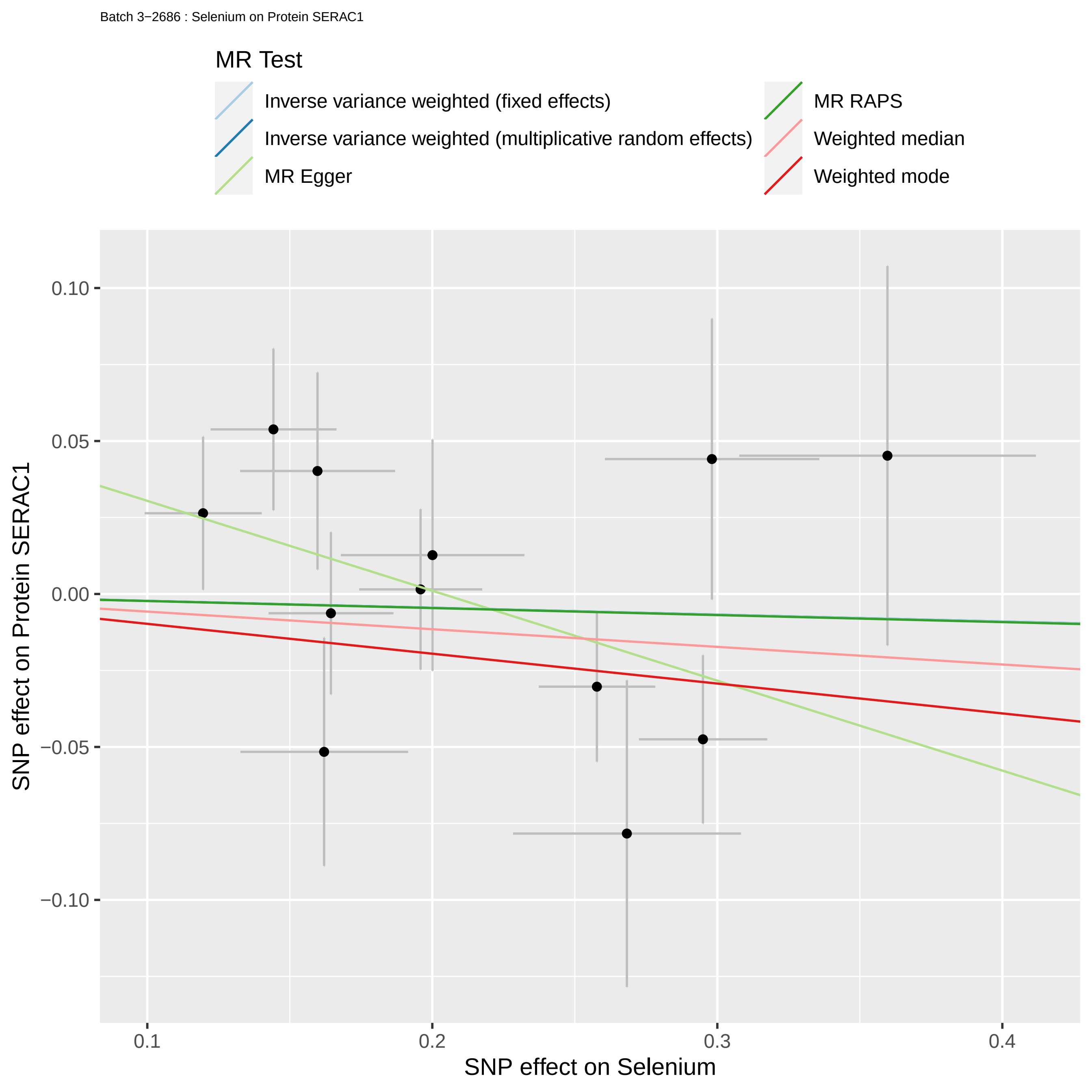

Batch 3−2686 : Selenium on Protein SERAC1
MR Test
Inverse variance weighted (fixed effects)
Inverse variance weighted (multiplicative random effects)
MR Egger
MR RAPS
Weighted median
Weighted mode
0.10
0.05
0.00
−0.05
−0.10
0.1
0.2
0.3
0.4
SNP effect on Protein SERAC1
SNP effect on Selenium

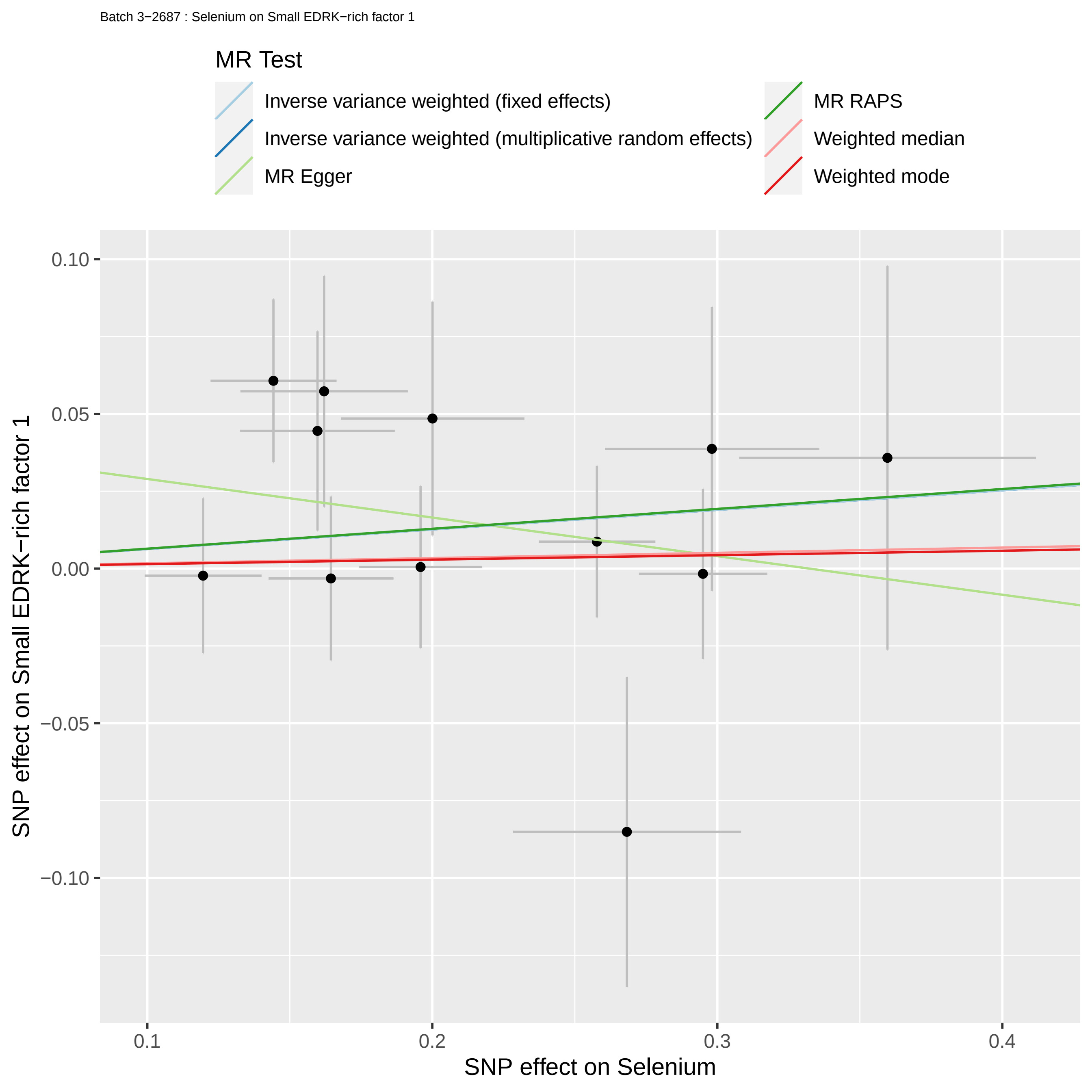
Batch 3−2687 : Selenium on Small EDRK−rich factor 1
MR Test
Inverse variance weighted (fixed effects)
Inverse variance weighted (multiplicative random effects)
MR Egger
MR RAPS
Weighted median
Weighted mode
0.10
0.05
0.00
−0.05
−0.10
0.1
0.2
0.3
0.4
SNP effect on Small EDRK−rich factor 1
SNP effect on Selenium

Batch 3−2688 : Selenium on Protein Z−dependent protease inhibitor

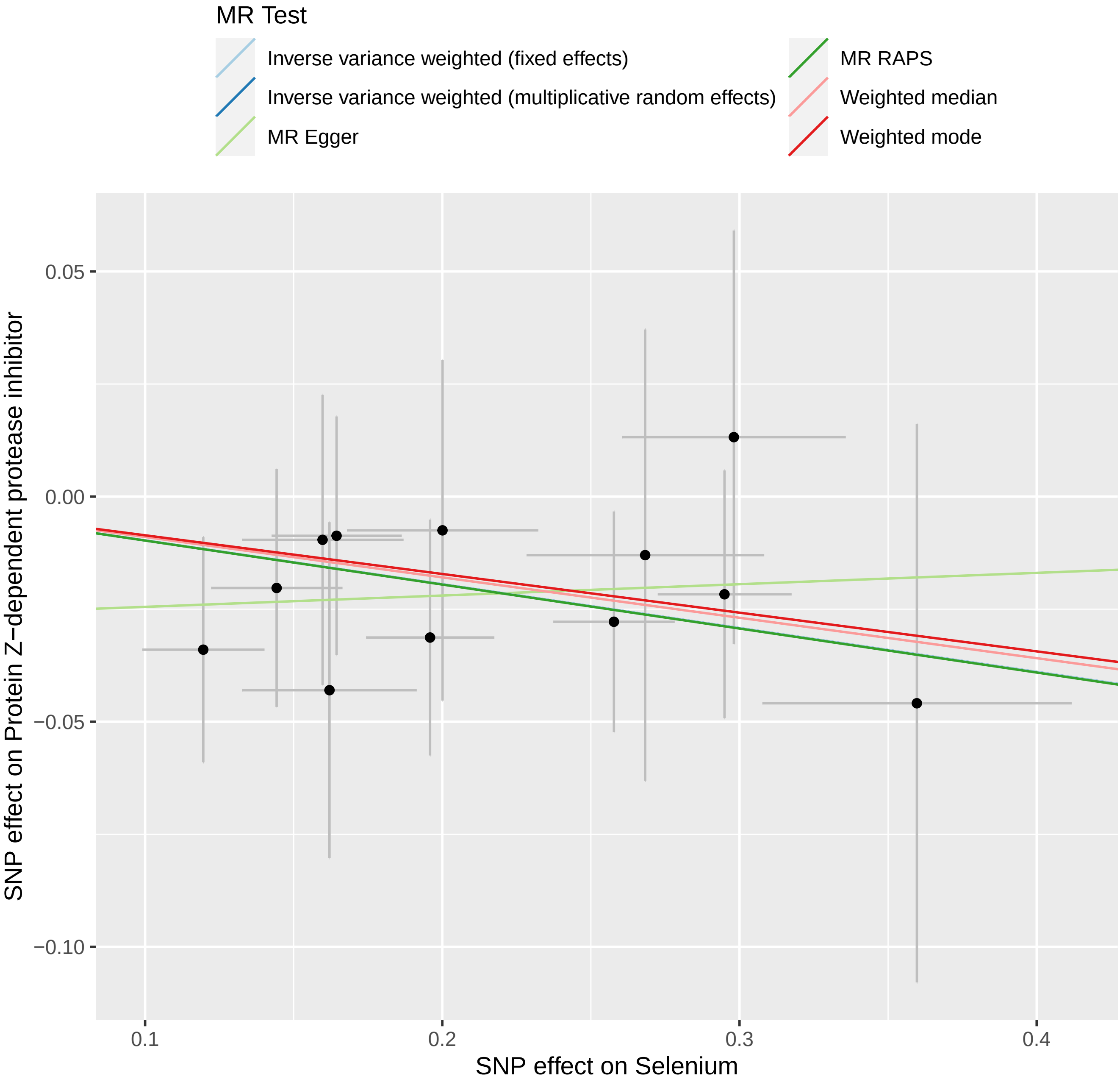

Batch 3−2689 : Selenium on Protein Z−dependent protease inhibitor

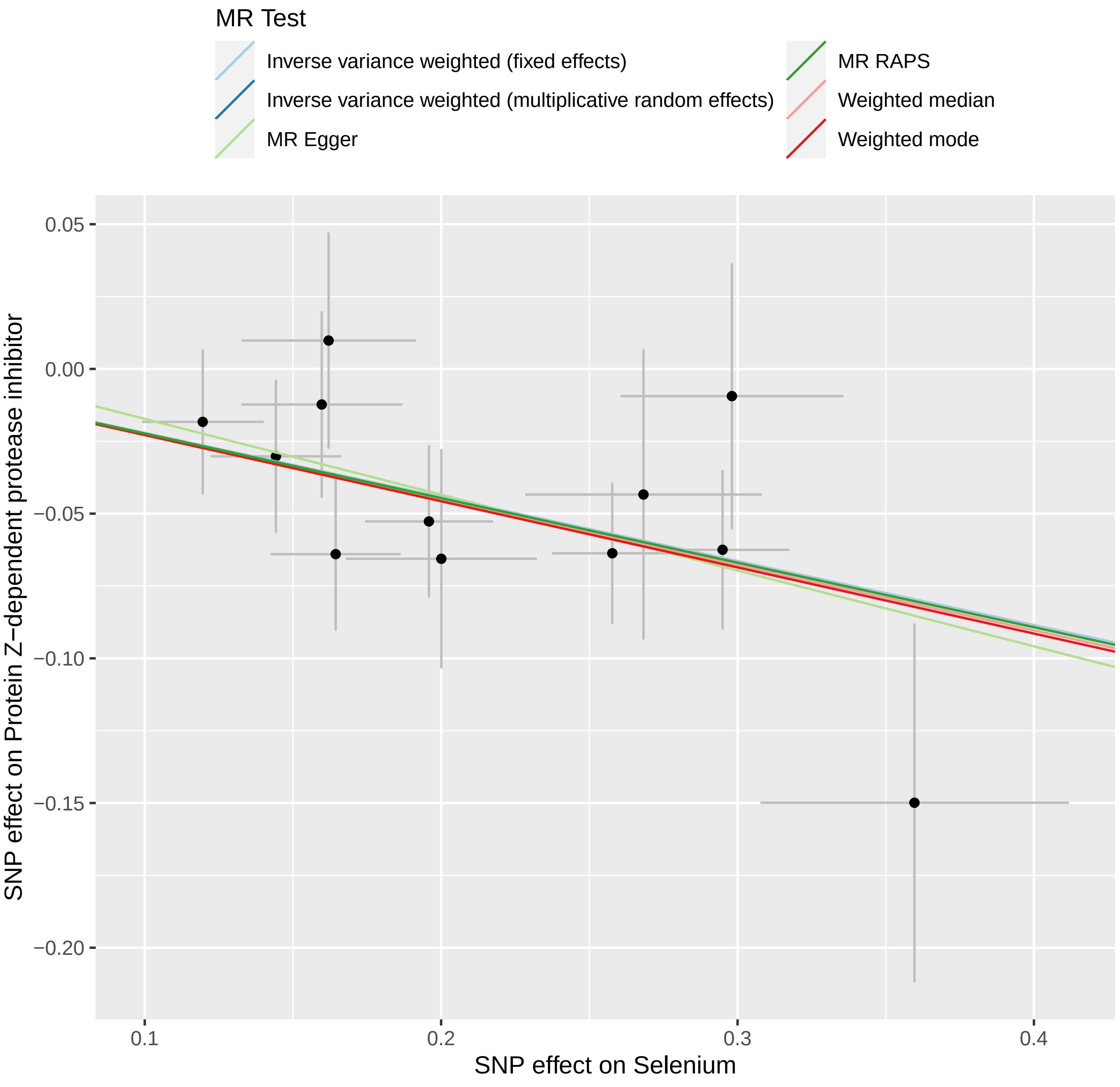

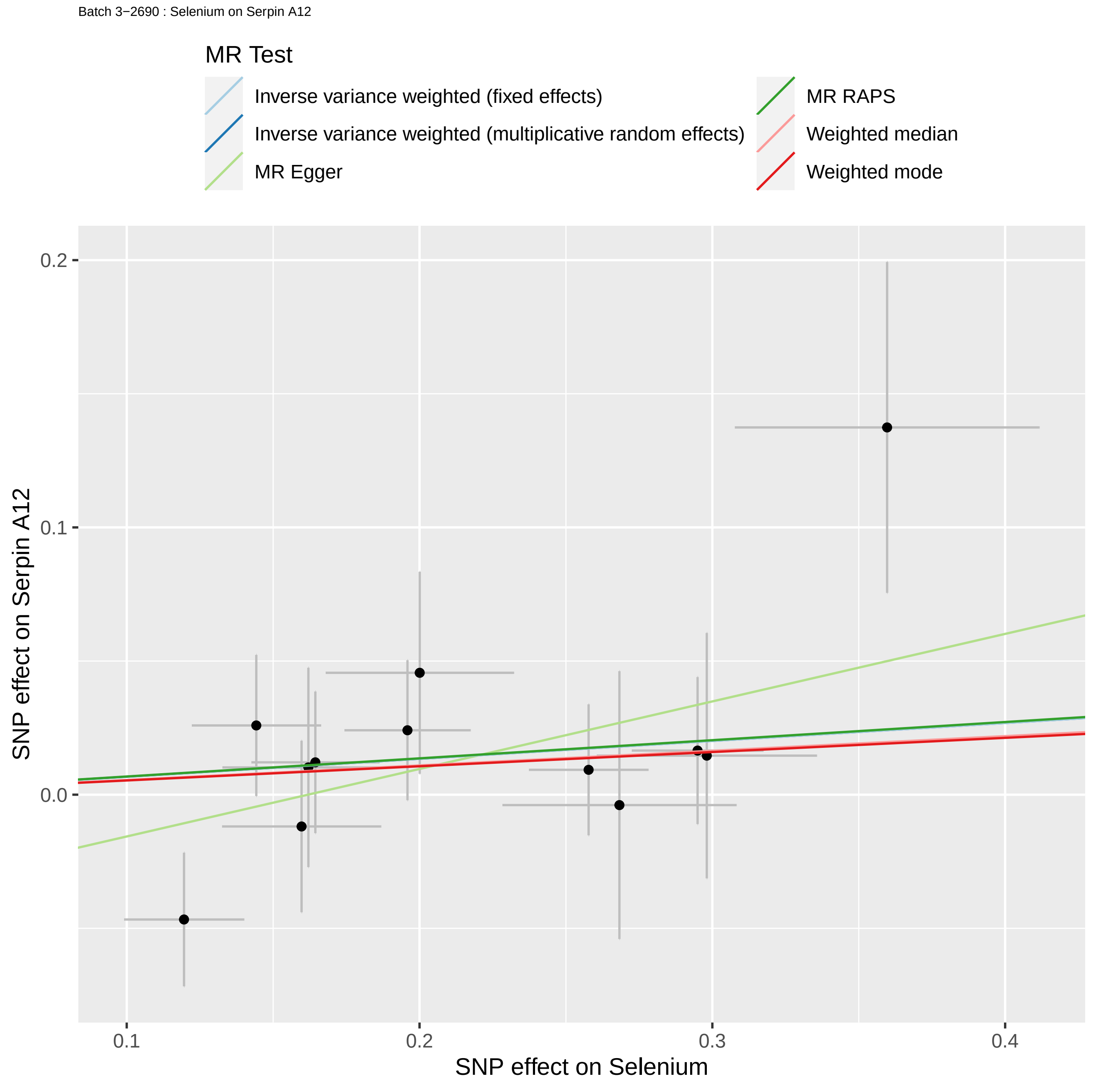

Batch 3−2690 : Selenium on Serpin A12
MR Test
Inverse variance weighted (fixed effects)
Inverse variance weighted (multiplicative random effects)
MR Egger
MR RAPS
Weighted median
Weighted mode
0.2
0.1
0.0
0.1
0.2
0.3
0.4
SNP effect on Serpin A12
SNP effect on Selenium

Batch 3−2691 : Selenium on Kallistatin

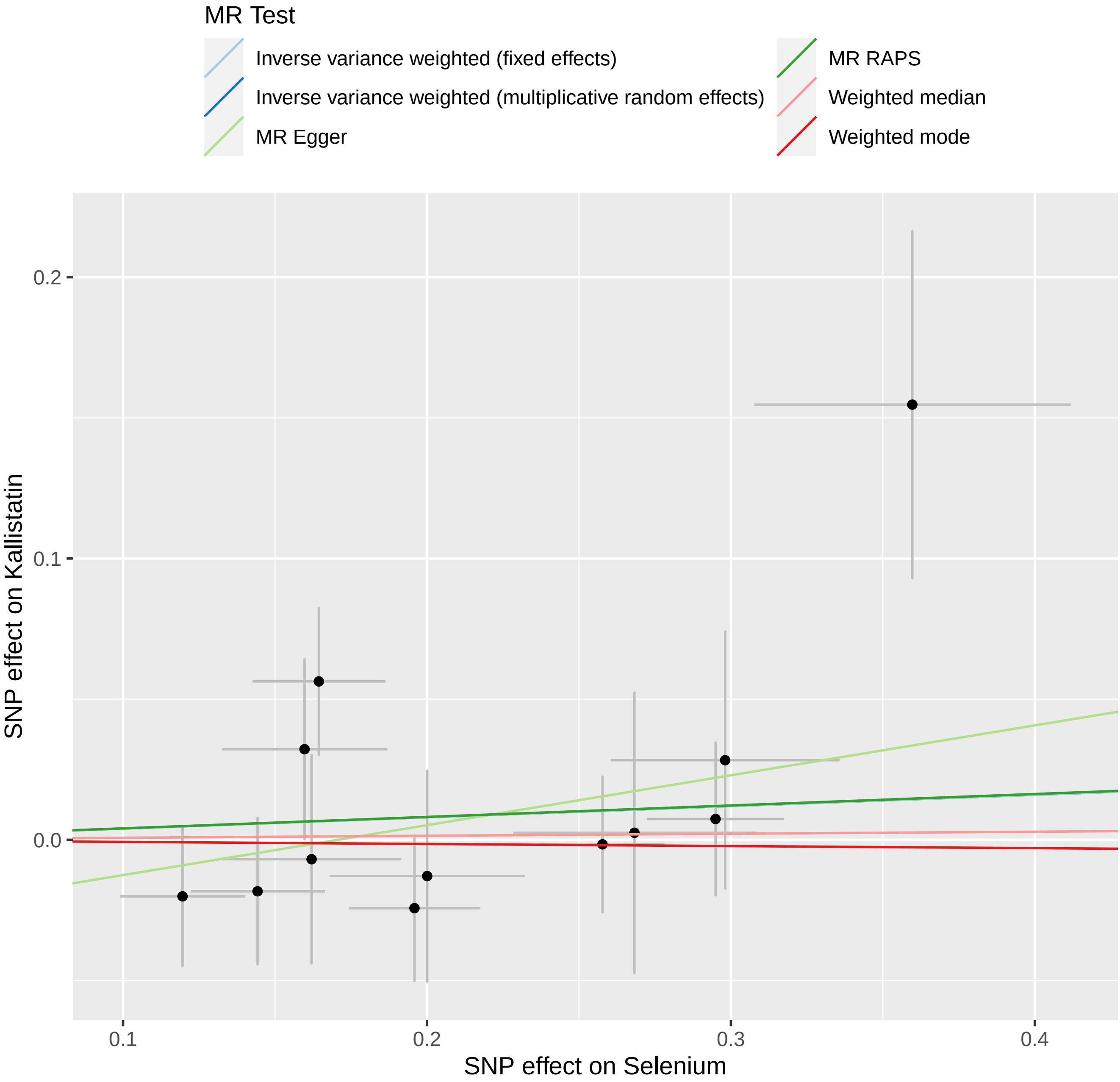

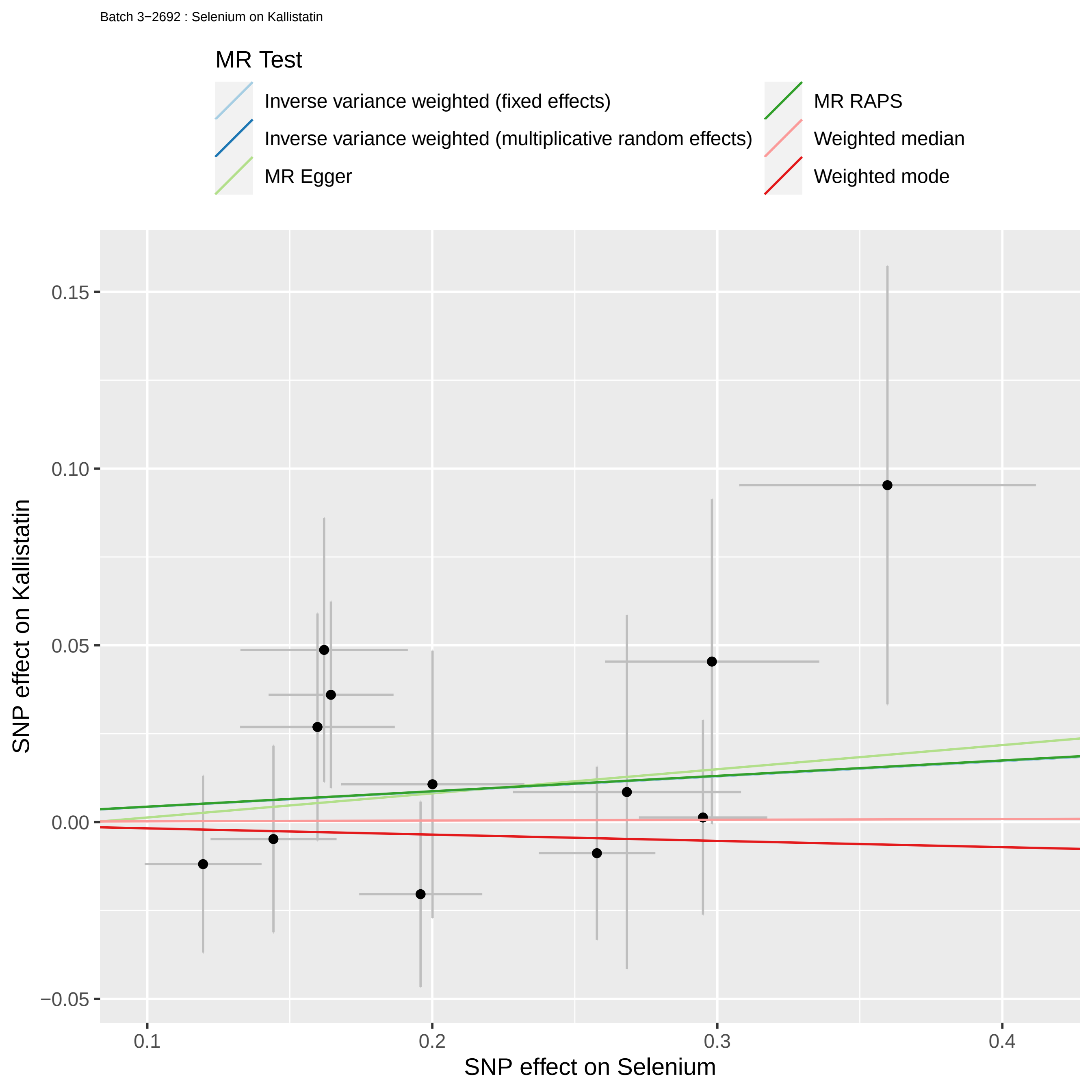

Batch 3−2692 : Selenium on Kallistatin
MR Test
Inverse variance weighted (fixed effects)
Inverse variance weighted (multiplicative random effects)
MR Egger
MR RAPS
Weighted median
Weighted mode
0.15
0.10
0.05
0.00
−0.05
0.1
0.2
0.3
0.4
SNP effect on Kallistatin
SNP effect on Selenium

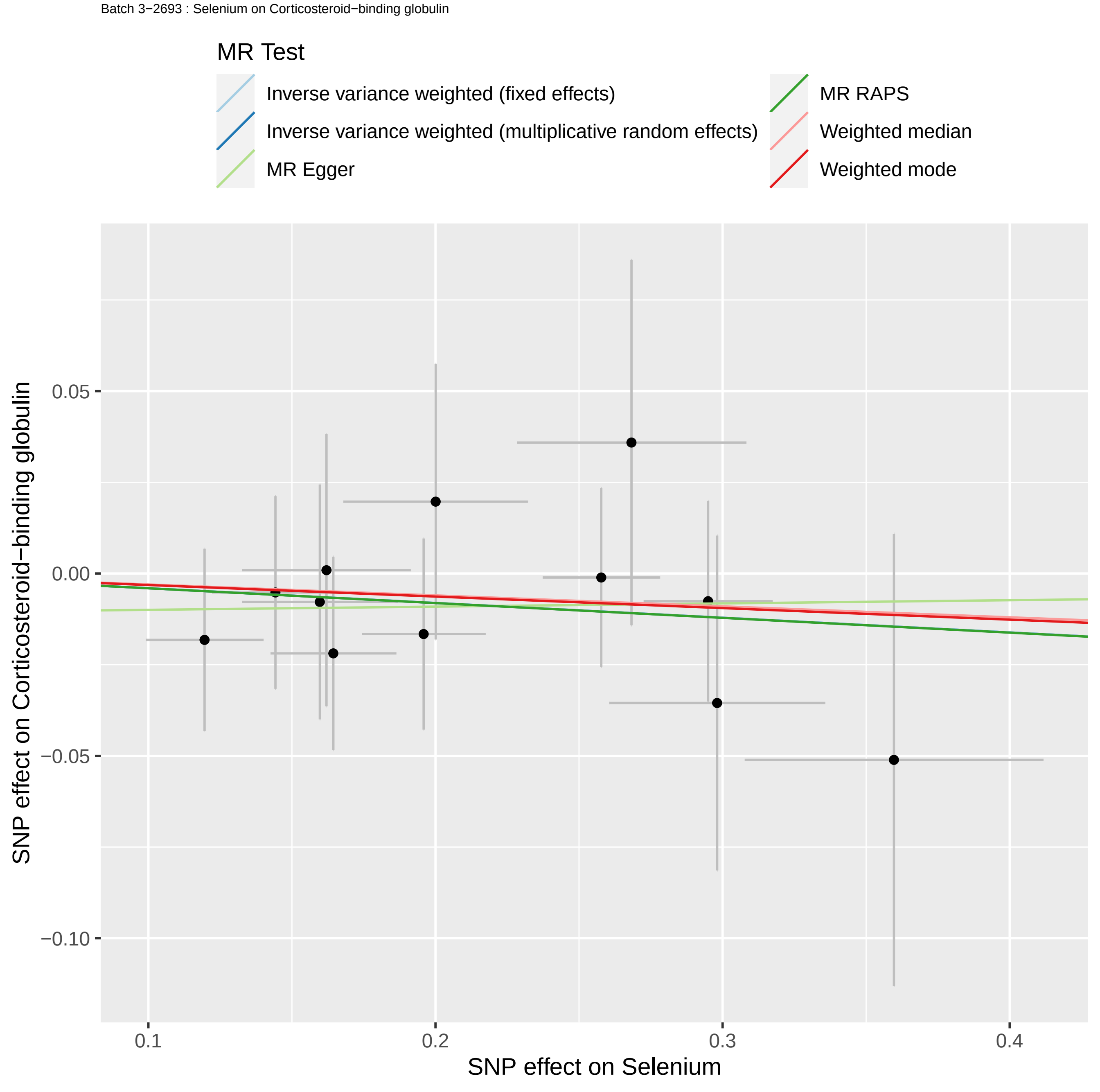

Batch 3−2693 : Selenium on Corticosteroid−binding globulin
MR Test
Inverse variance weighted (fixed effects)
Inverse variance weighted (multiplicative random effects)
MR Egger
MR RAPS
Weighted median
Weighted mode
0.05
0.00
−0.05
−0.10
0.1
0.2
0.3
0.4
SNP effect on Corticosteroid−binding globulin
SNP effect on Selenium

Batch 3−2694 : Selenium on Serpin A9

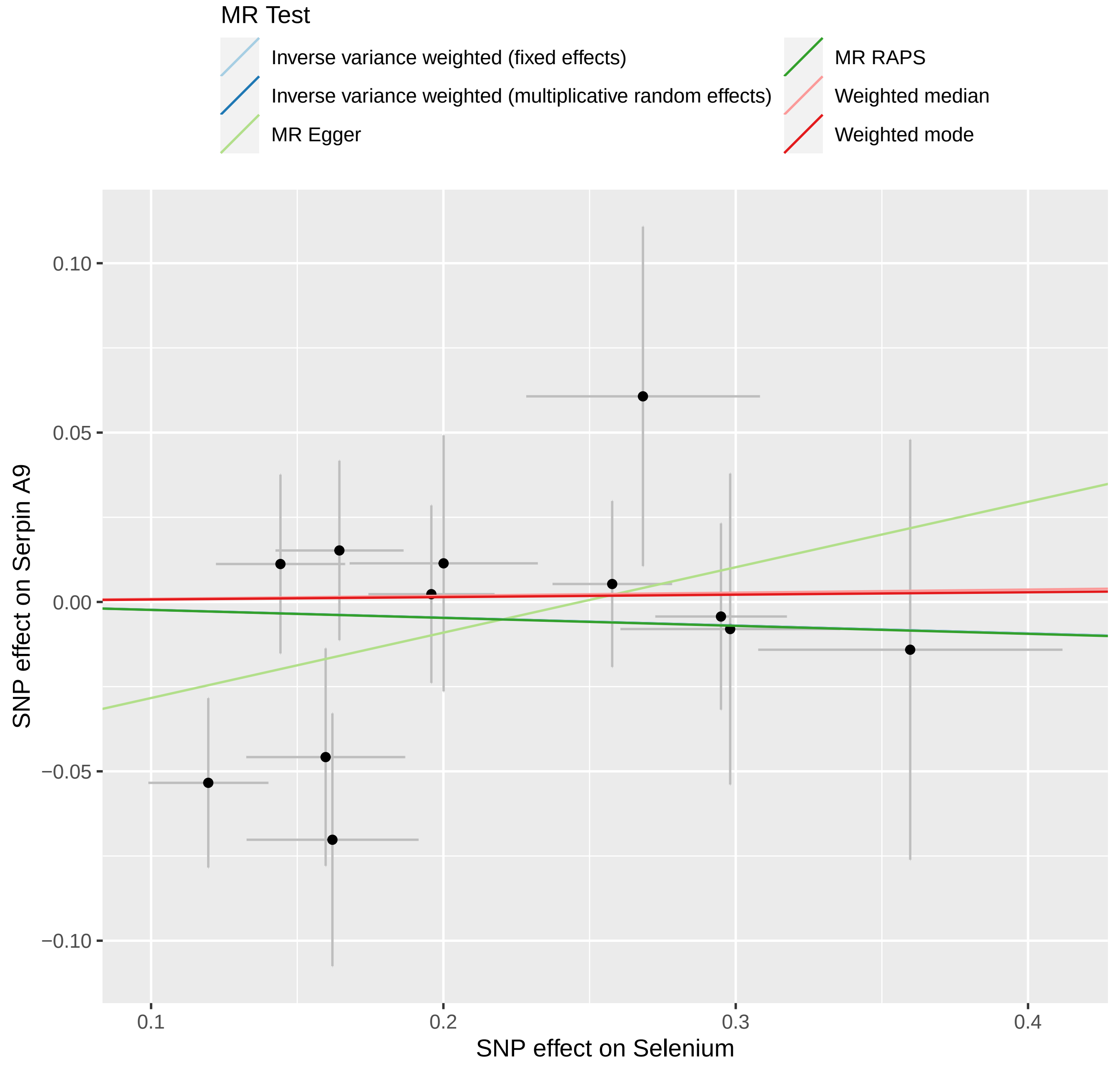

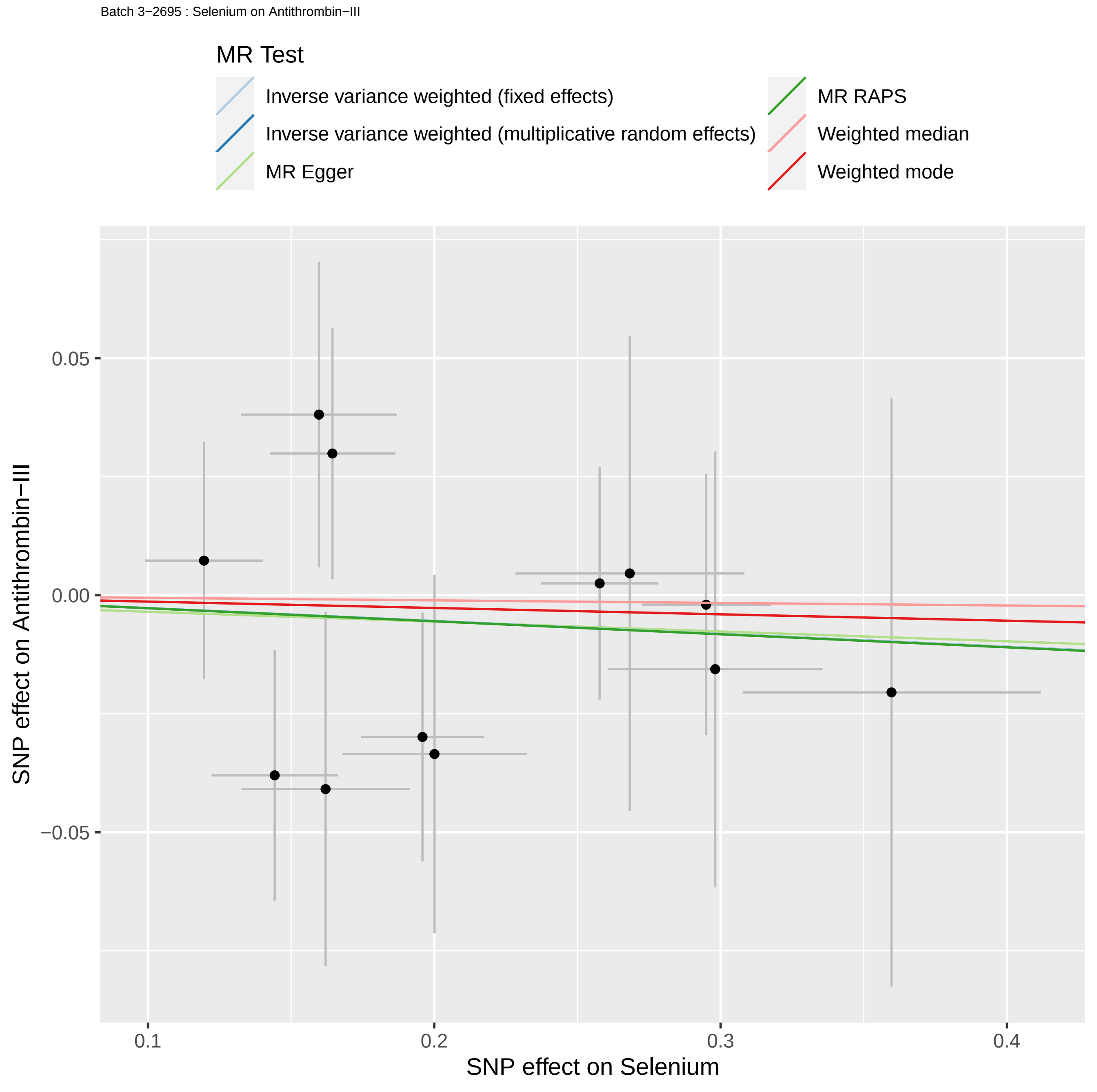
Batch 3−2695 : Selenium on Antithrombin−III
MR Test
Inverse variance weighted (fixed effects)
Inverse variance weighted (multiplicative random effects)
MR Egger
MR RAPS
Weighted median
Weighted mode
0.05
0.00
−0.05
0.1
0.2
0.3
0.4
SNP effect on Antithrombin−III
SNP effect on Selenium

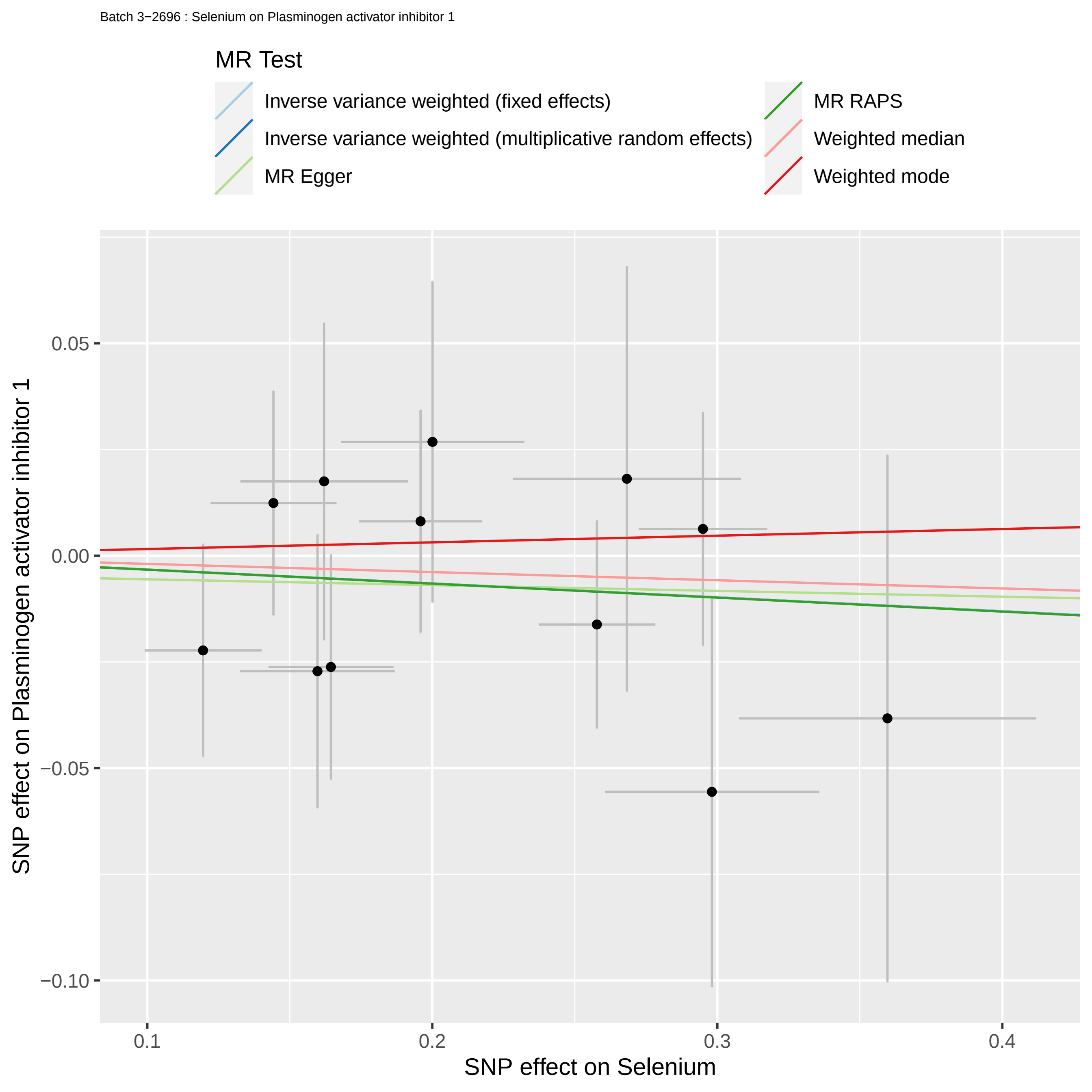
Batch 3−2696 : Selenium on Plasminogen activator inhibitor 1
MR Test
Inverse variance weighted (fixed effects)
Inverse variance weighted (multiplicative random effects)
MR Egger
MR RAPS
Weighted median
Weighted mode
0.05
0.00
−0.05
−0.10
0.1
0.2
0.3
0.4
SNP effect on Plasminogen activator inhibitor 1
SNP effect on Selenium

Batch 3−2697 : Selenium on Glia−derived nexin

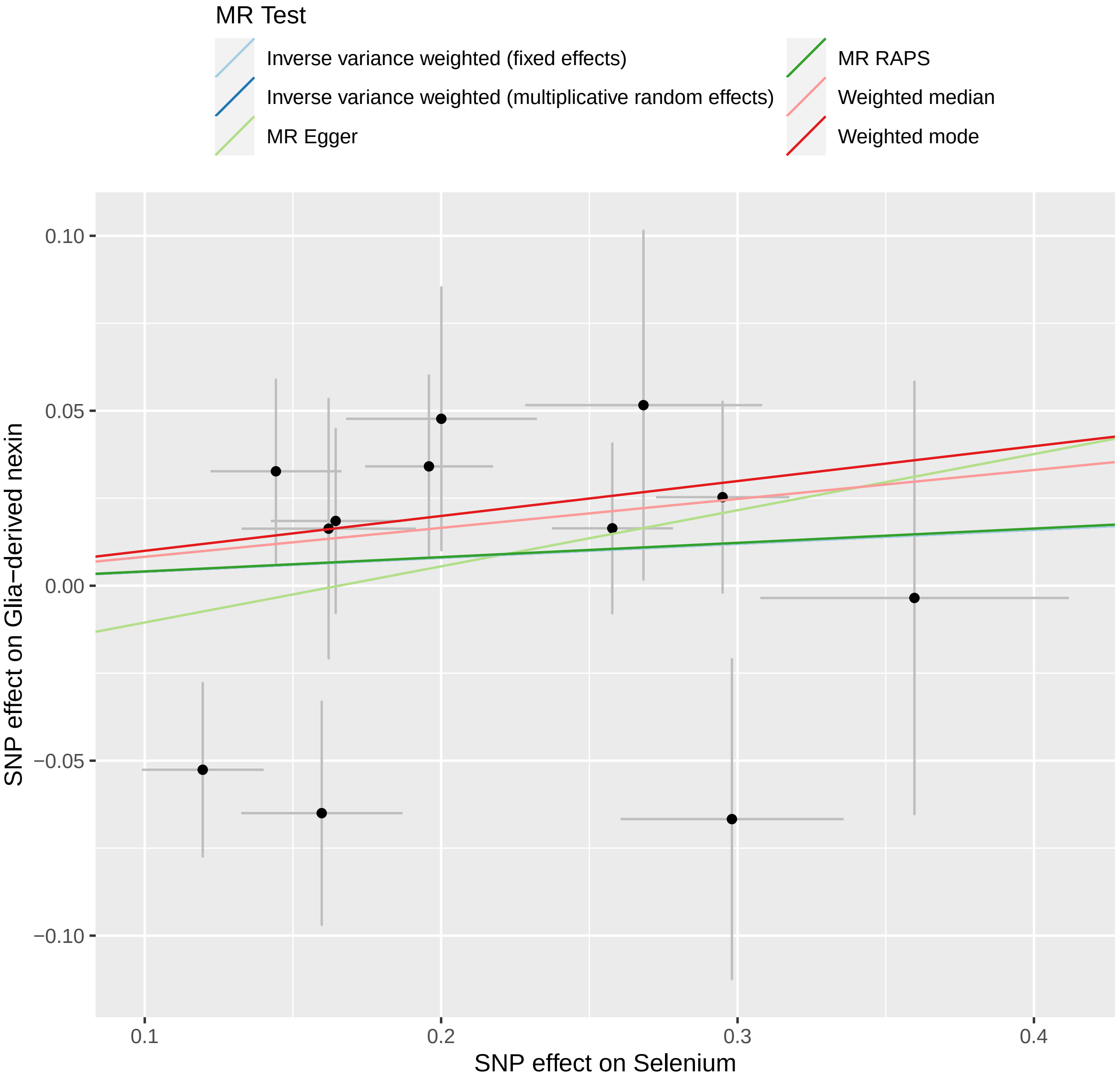

Batch 3−2698 : Selenium on Pigment epithelium−derived factor

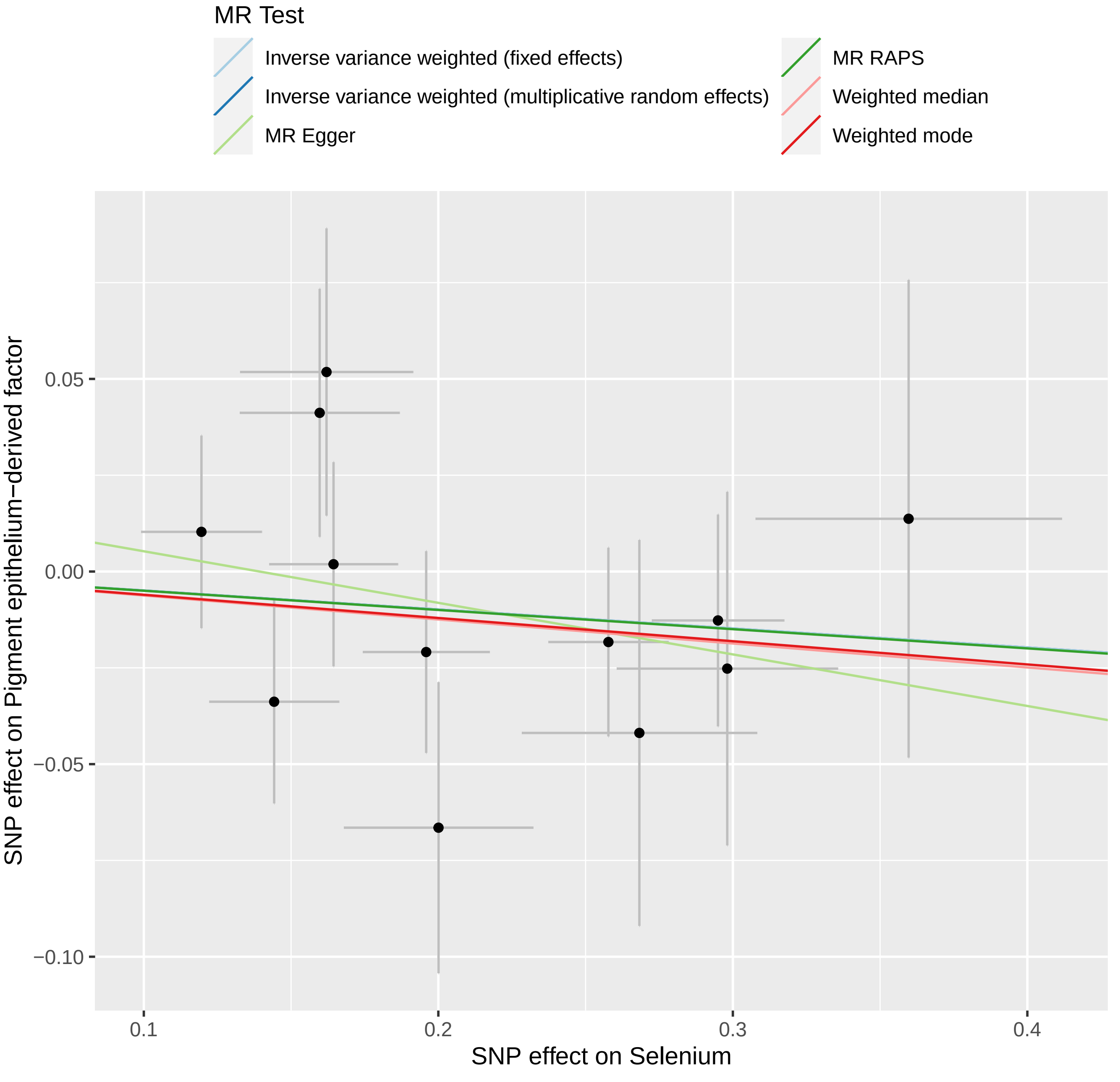

Batch 3−2699 : Selenium on Pigment epithelium−derived factor

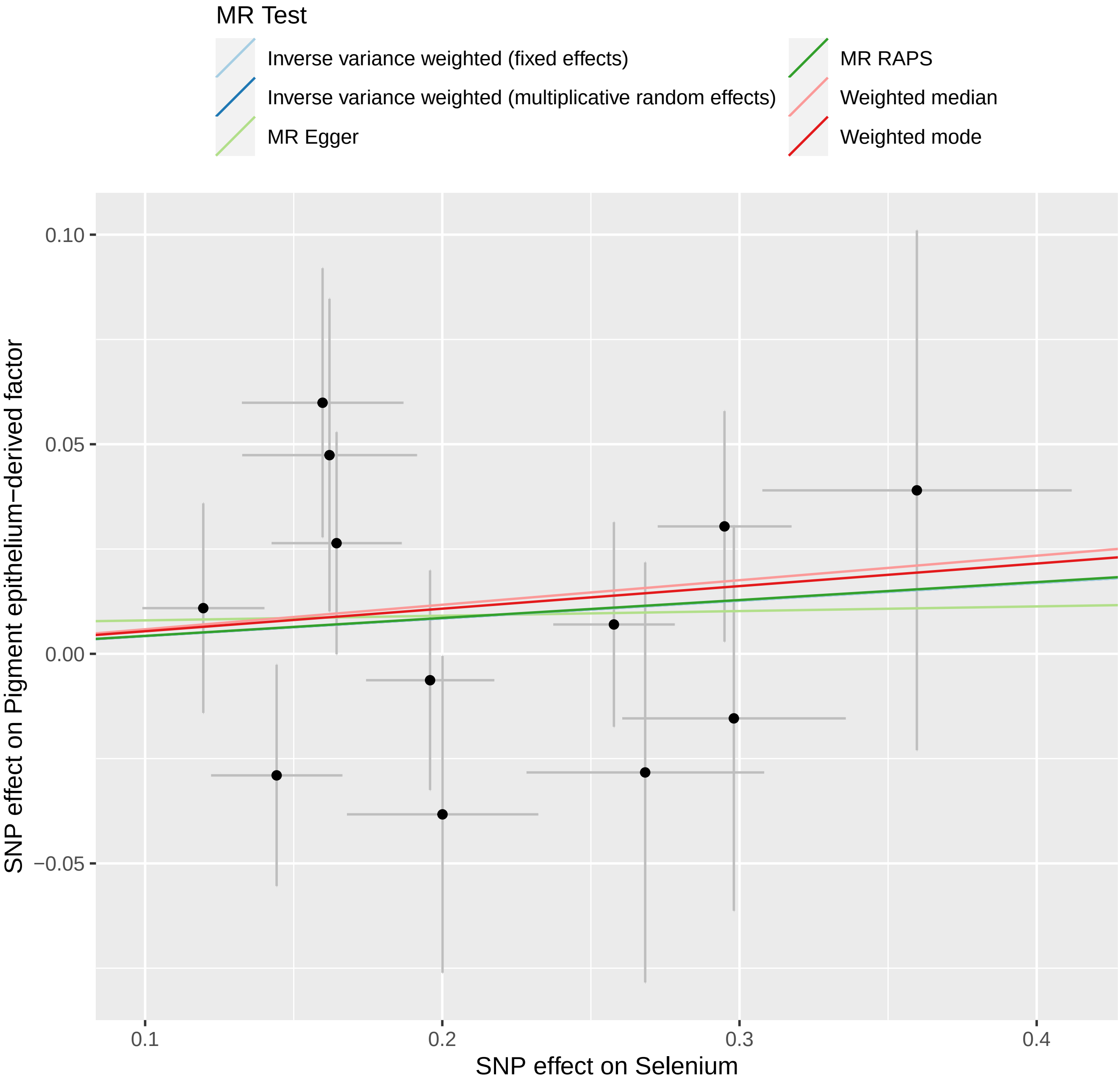

Batch 3−2700 : Selenium on Alpha−2−antiplasmin

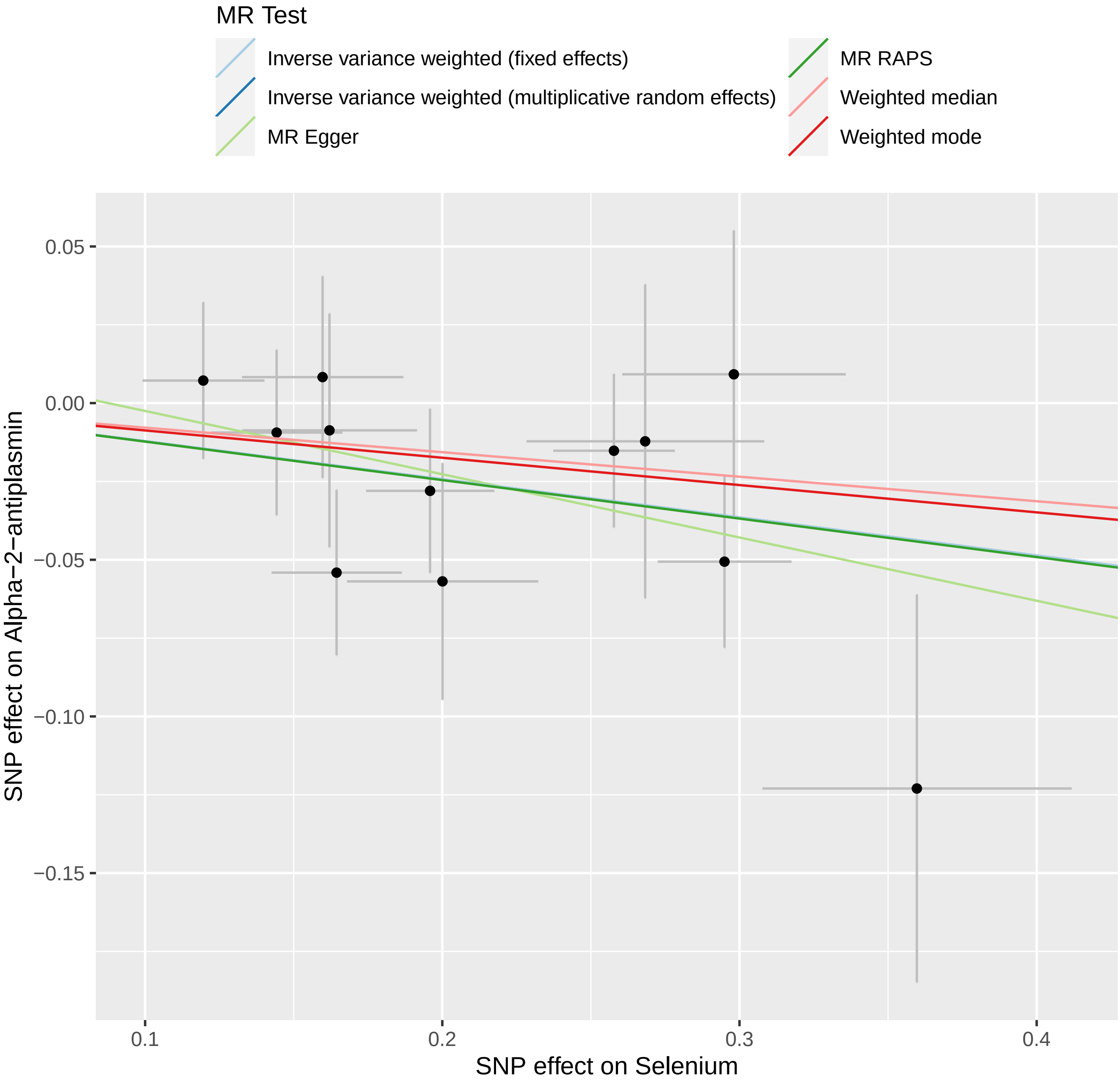

Batch 3−2701 : Selenium on Plasma protease C1 inhibitor

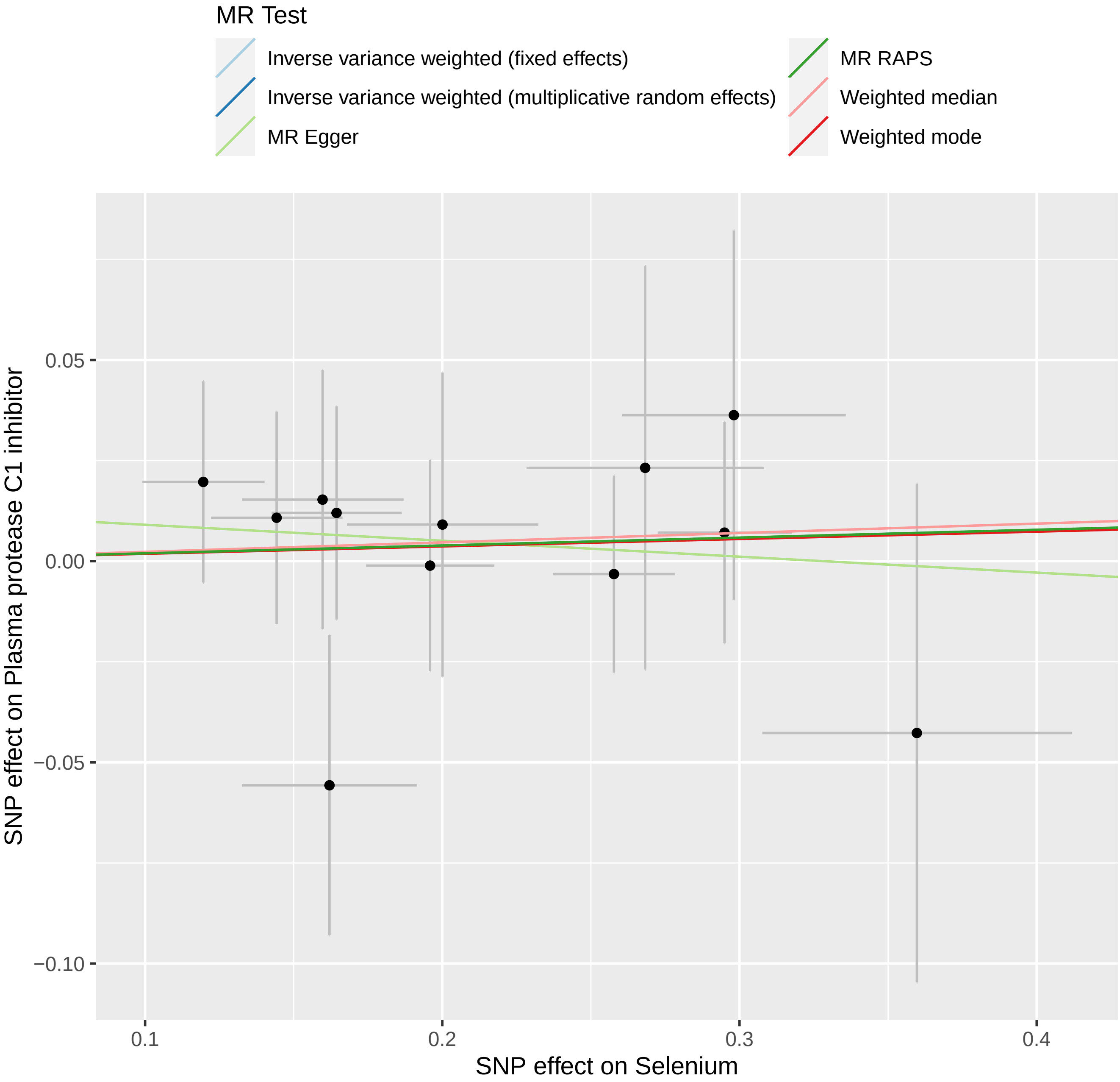

Batch 3−2702 : Selenium on Serpin I2

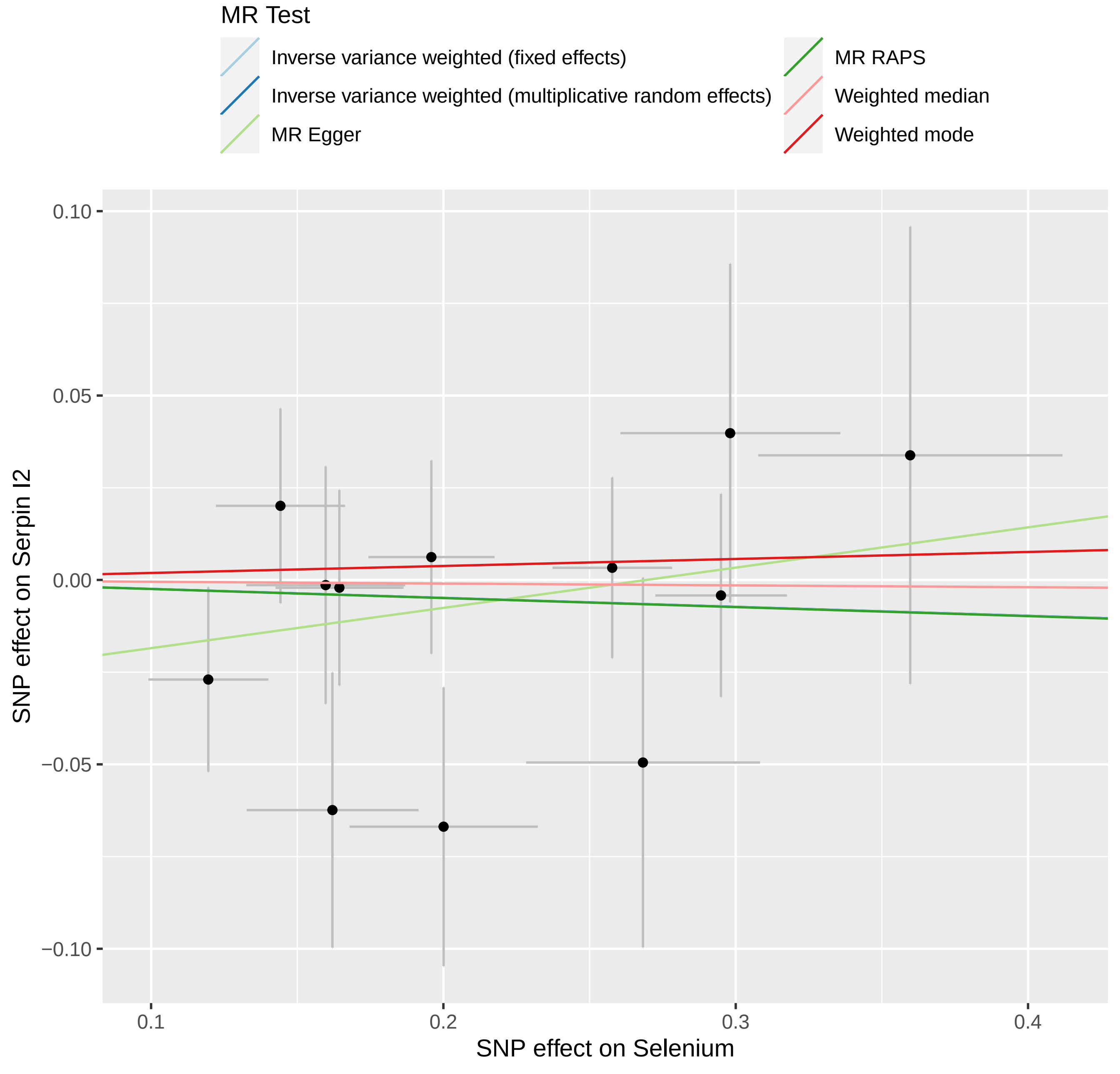

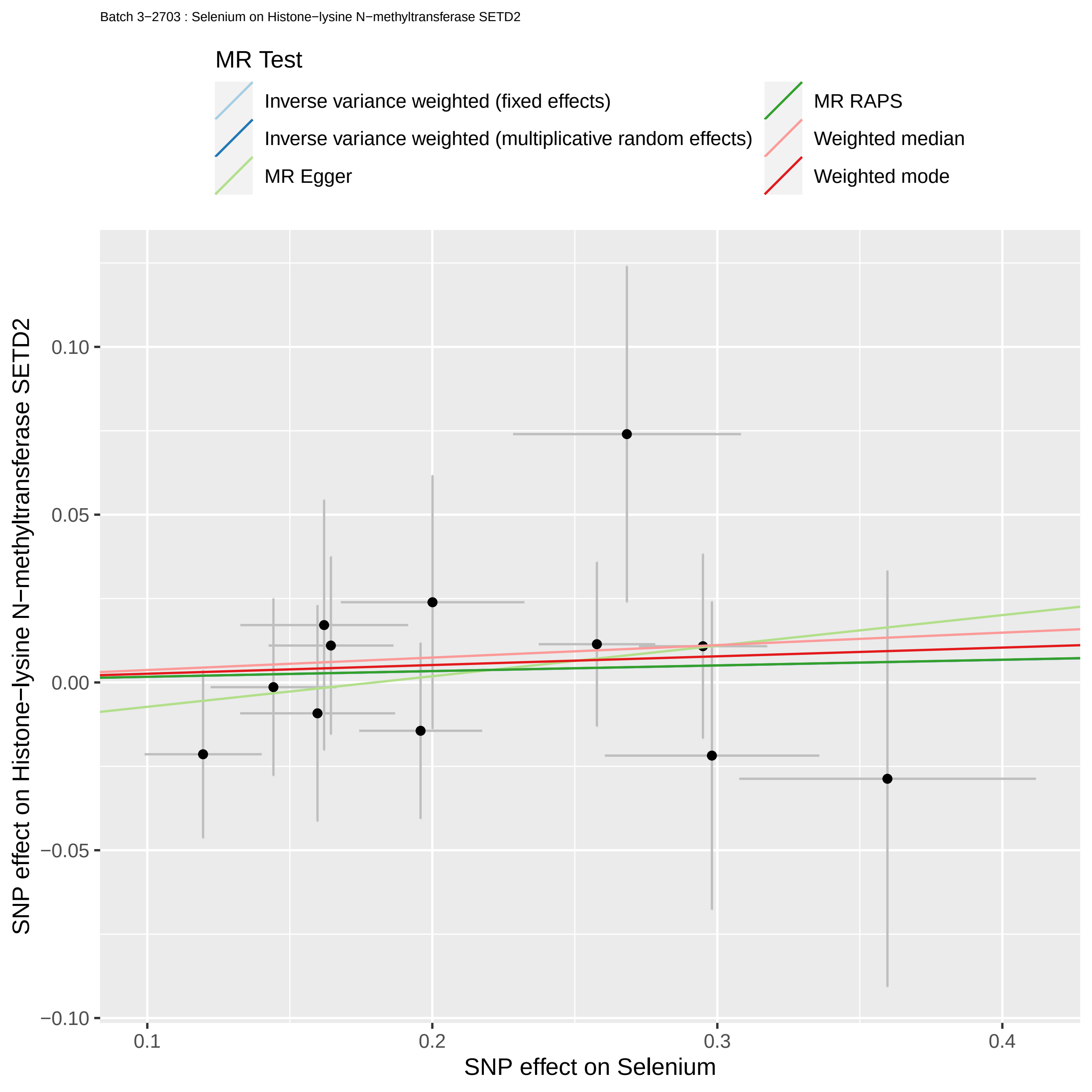

Batch 3−2703 : Selenium on Histone−lysine N−methyltransferase SETD2
MR Test
Inverse variance weighted (fixed effects)
Inverse variance weighted (multiplicative random effects)
MR Egger
MR RAPS
Weighted median
Weighted mode
SNP effect on Histone−lysine N−methyltransferase SETD2
0.10
0.05
0.00
−0.05
−0.10
0.1
0.2
0.3
0.4
SNP effect on Selenium

Batch 3−2704 : Selenium on Histone−lysine N−methyltransferase SETMAR

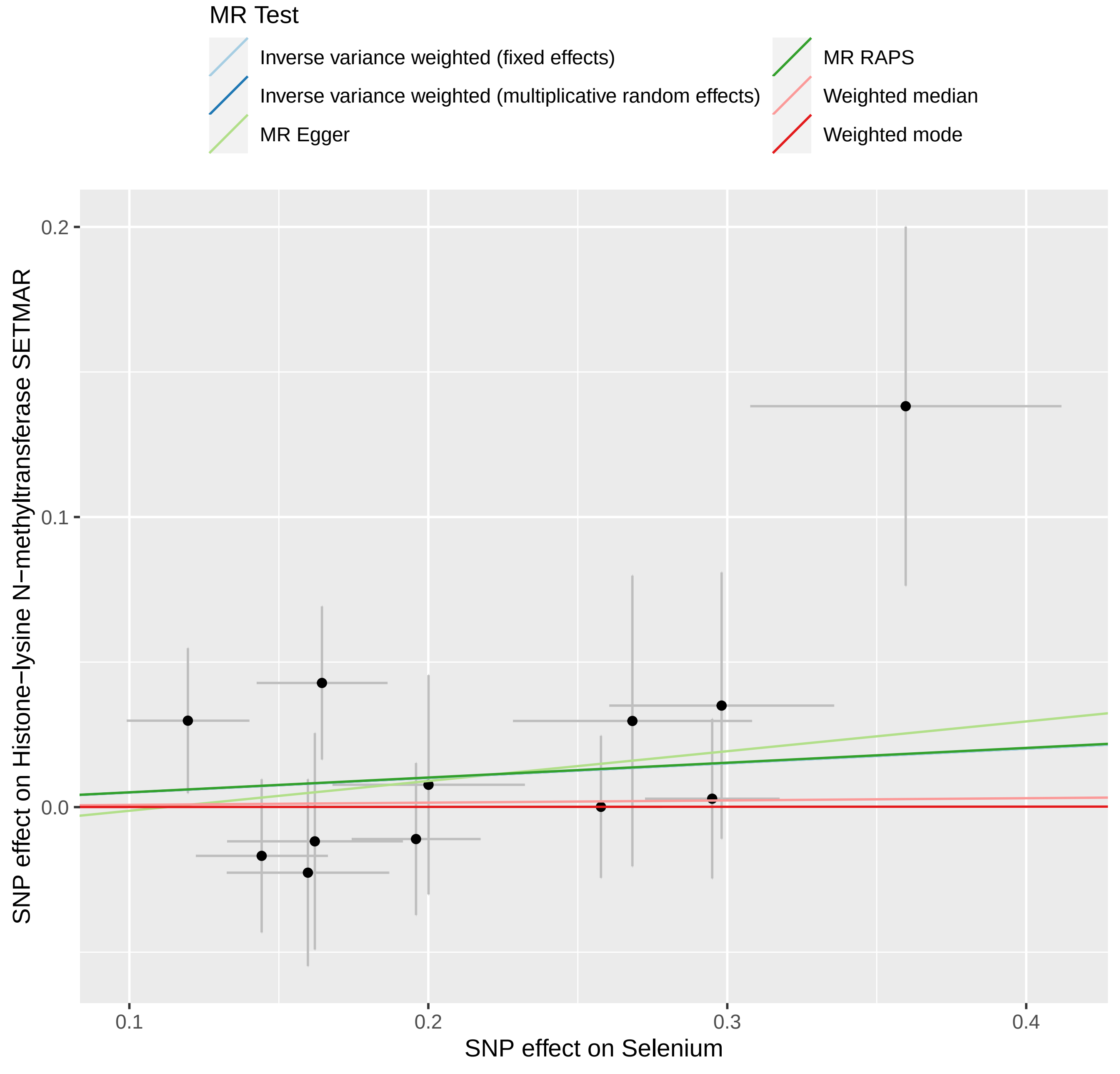

Batch 3−2705 : Selenium on Seizure 6−like protein 2

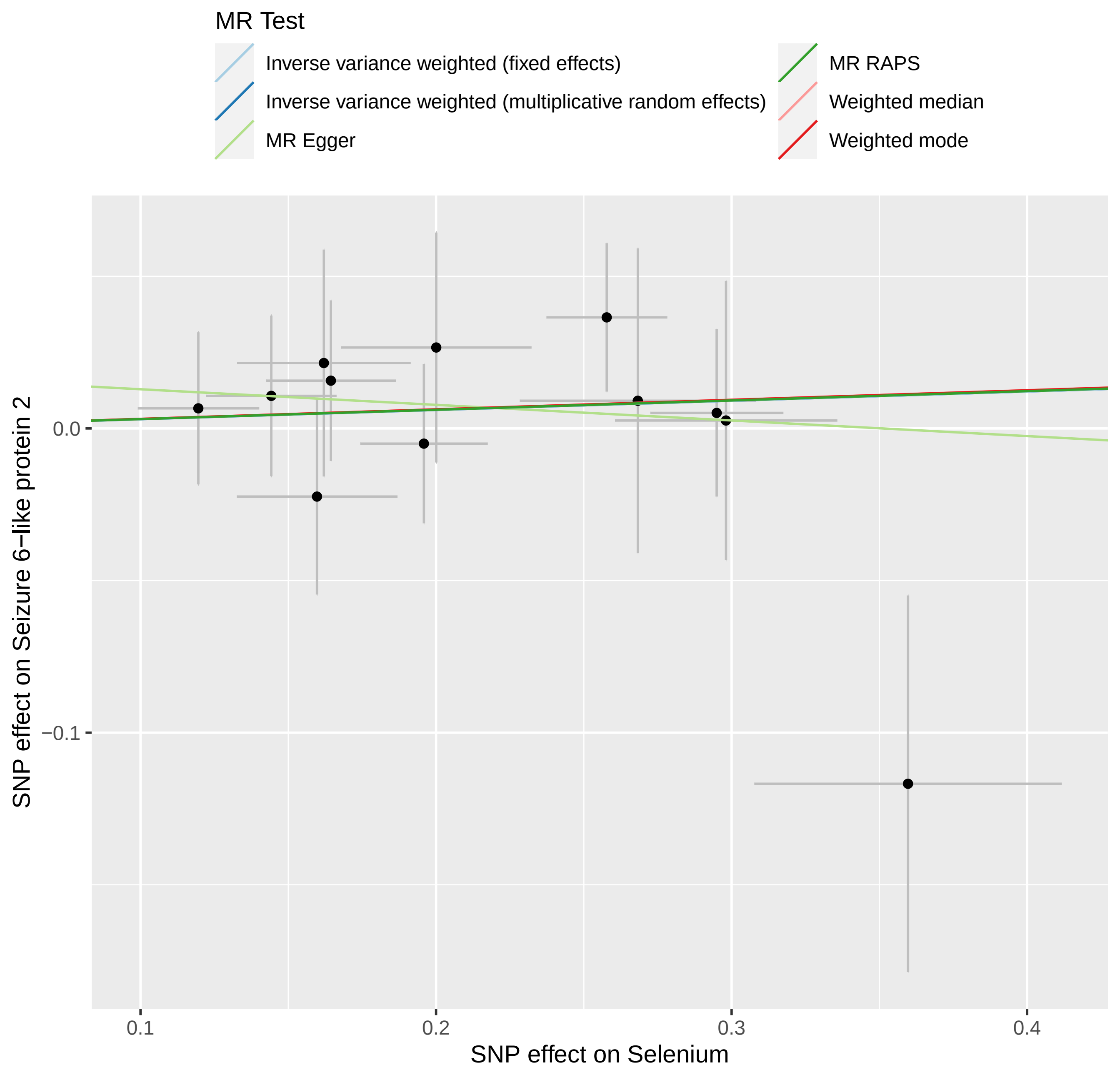

Batch 3−2706 : Selenium on Splicing factor 1

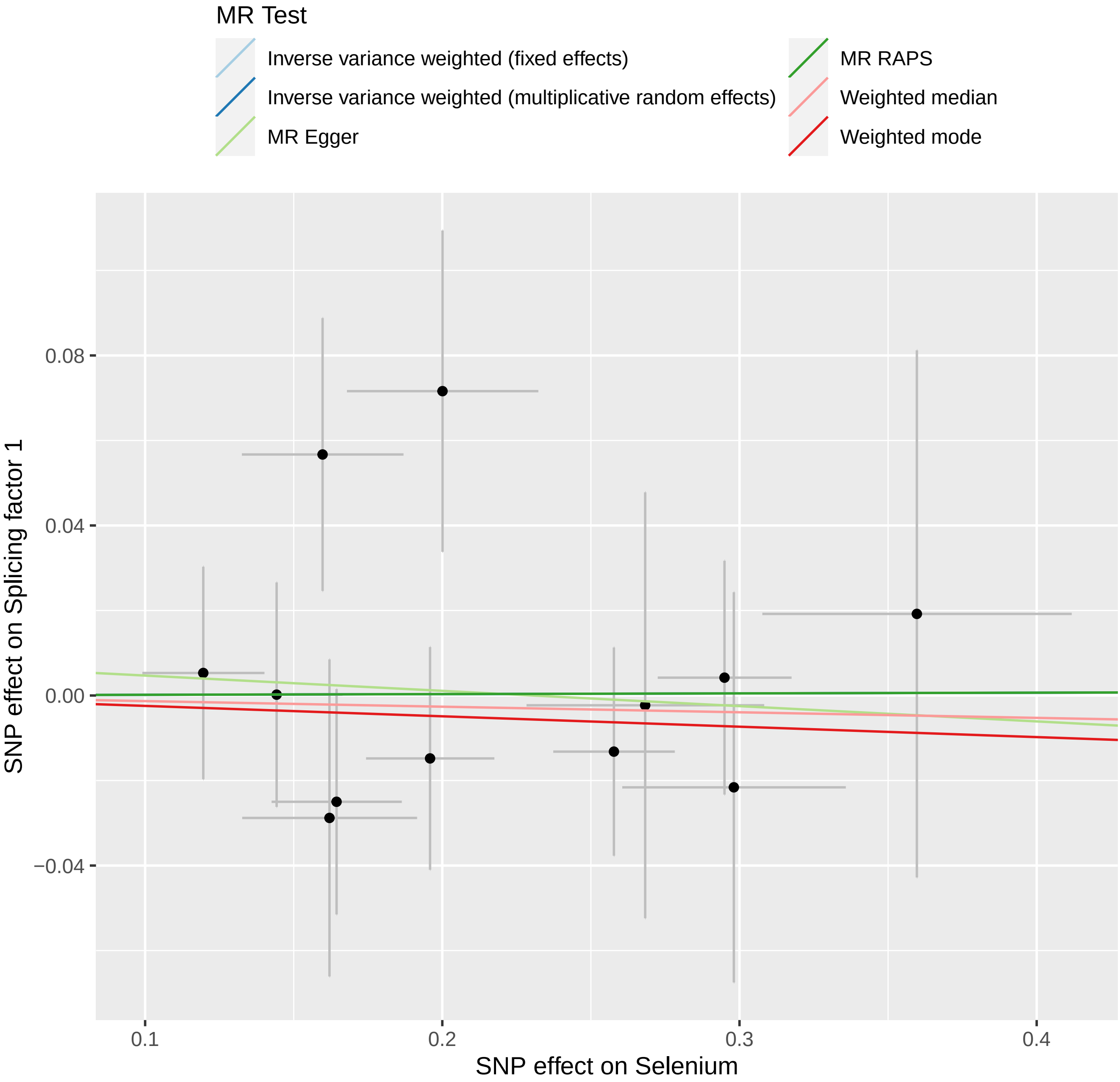

Batch 3−2707 : Selenium on Splicing factor 3B subunit 4

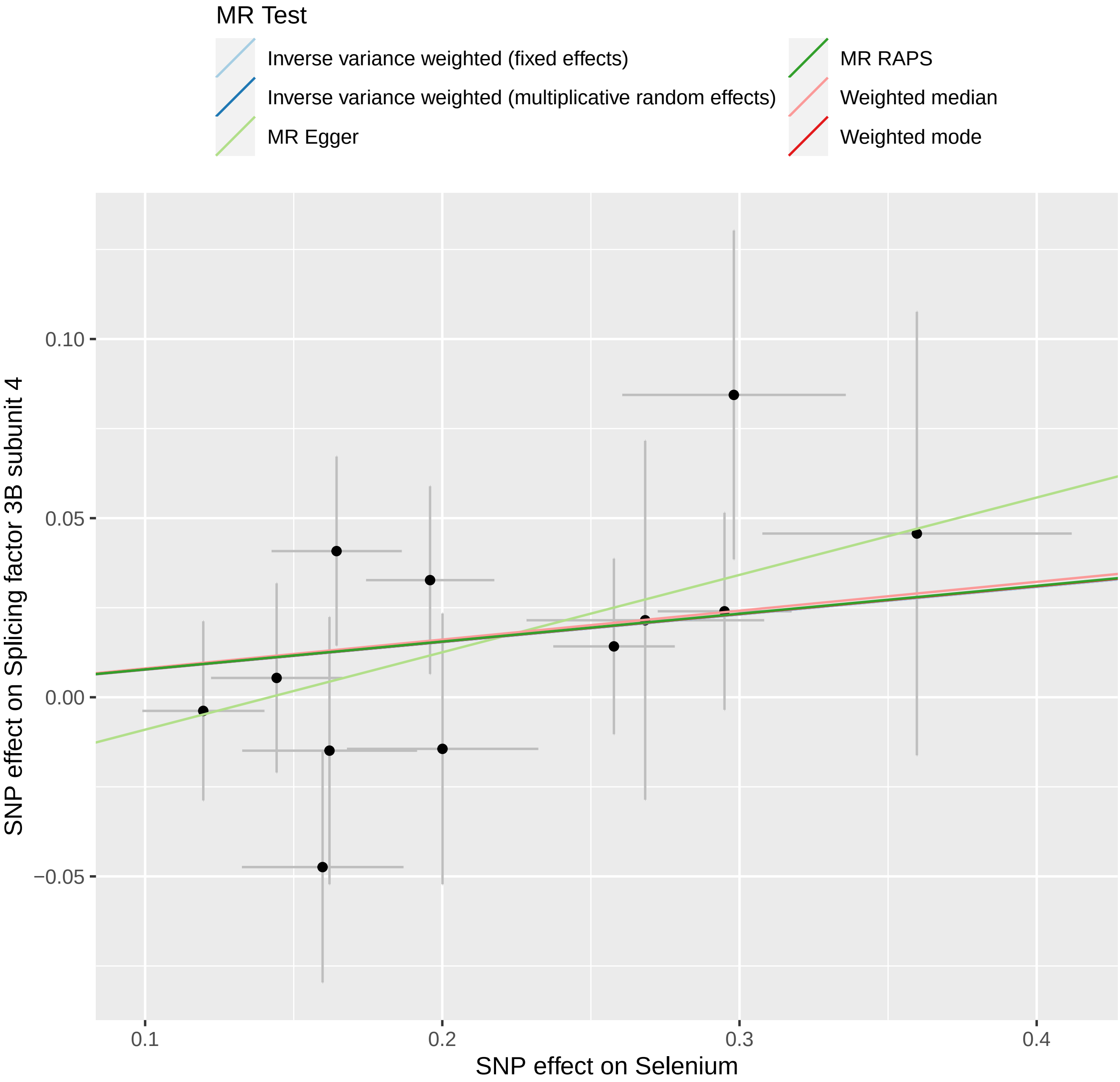

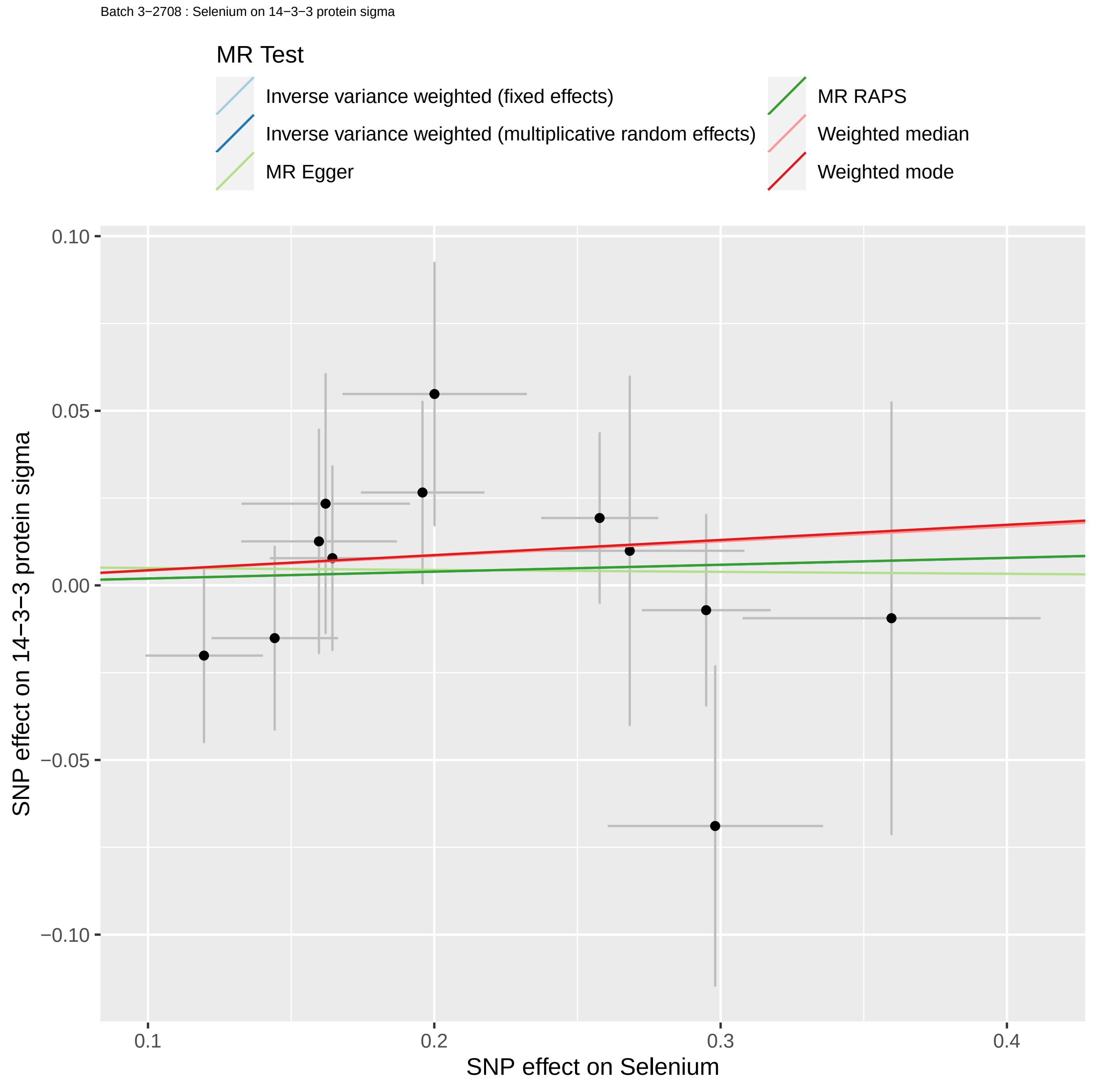

Batch 3−2708 : Selenium on 14−3−3 protein sigma
MR Test
Inverse variance weighted (fixed effects)
Inverse variance weighted (multiplicative random effects)
MR Egger
MR RAPS
Weighted median
Weighted mode
0.10
0.05
0.00
−0.05
−0.10
SNP effect on 14−3−3 protein sigma
0.1
0.2
0.3
0.4
SNP effect on Selenium

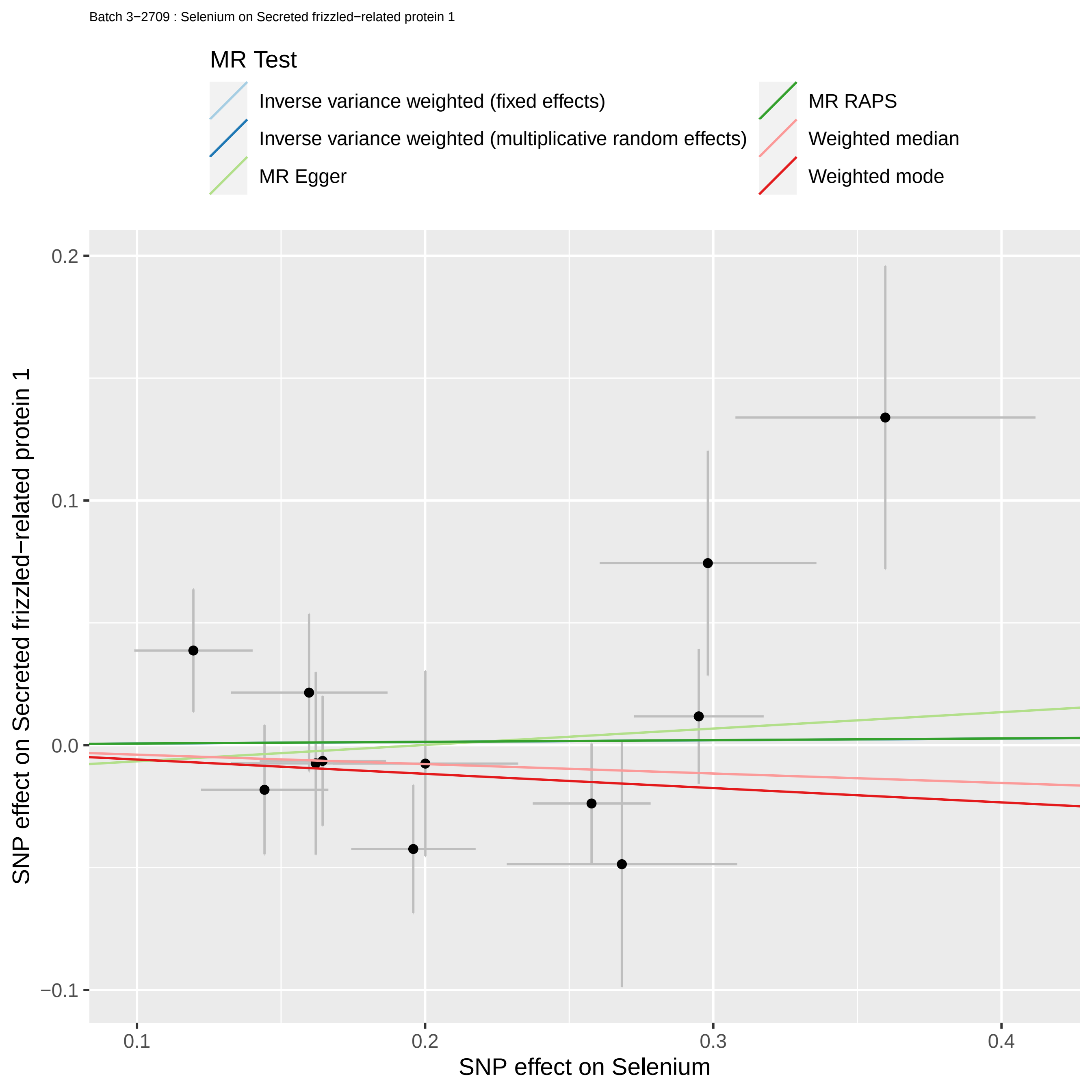

Batch 3−2709 : Selenium on Secreted frizzled−related protein 1
MR Test
Inverse variance weighted (fixed effects)
Inverse variance weighted (multiplicative random effects)
MR Egger
MR RAPS
Weighted median
Weighted mode
0.2
0.1
0.0
−0.1
0.1
0.2
0.3
0.4
SNP effect on Secreted frizzled−related protein 1
SNP effect on Selenium

Batch 3−2710 : Selenium on Secreted frizzled−related protein 2

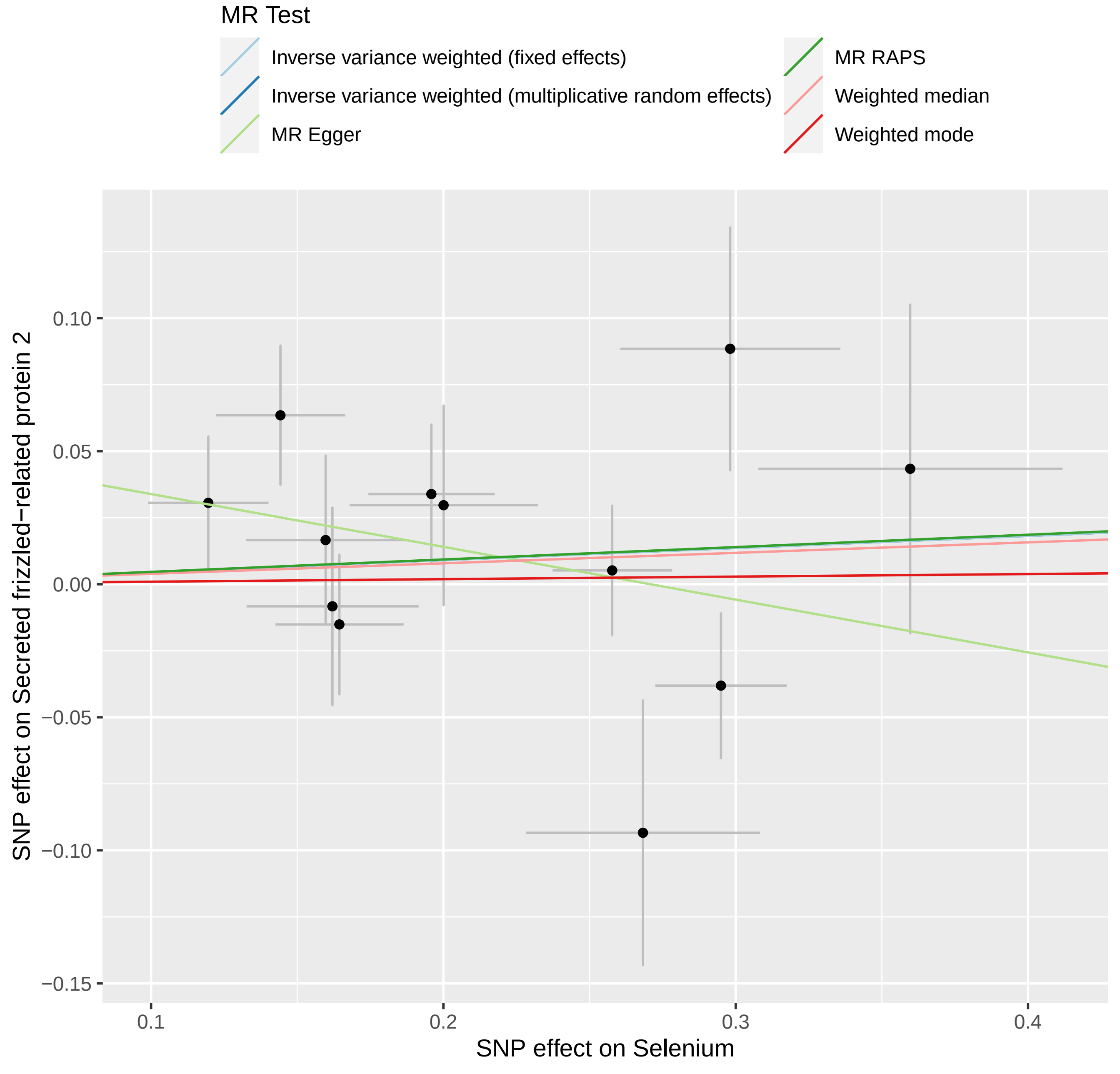

Batch 3−2711 : Selenium on Pulmonary surfactant−associated protein C

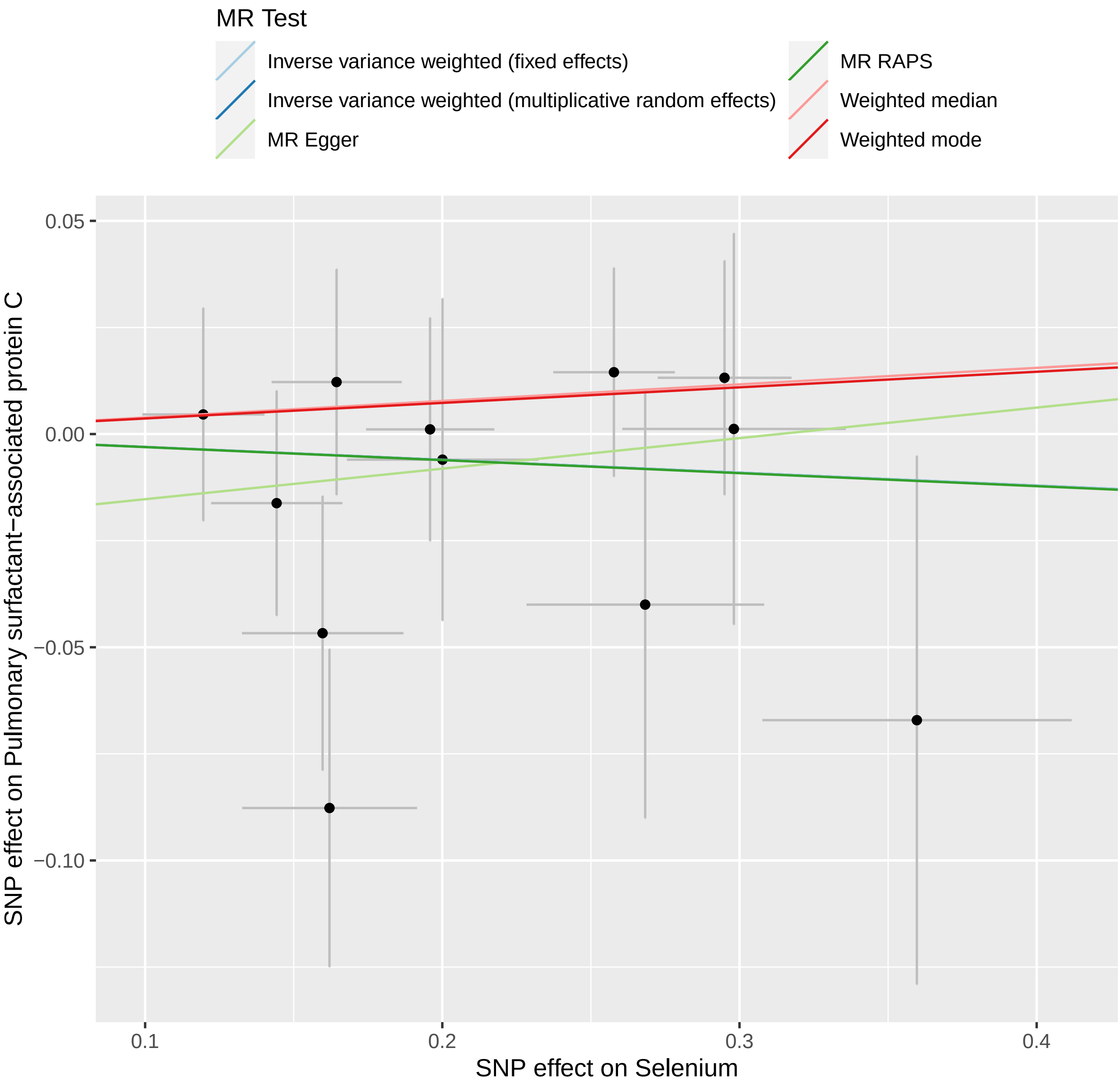

Batch 3−2712 : Selenium on Pulmonary surfactant−associated protein D

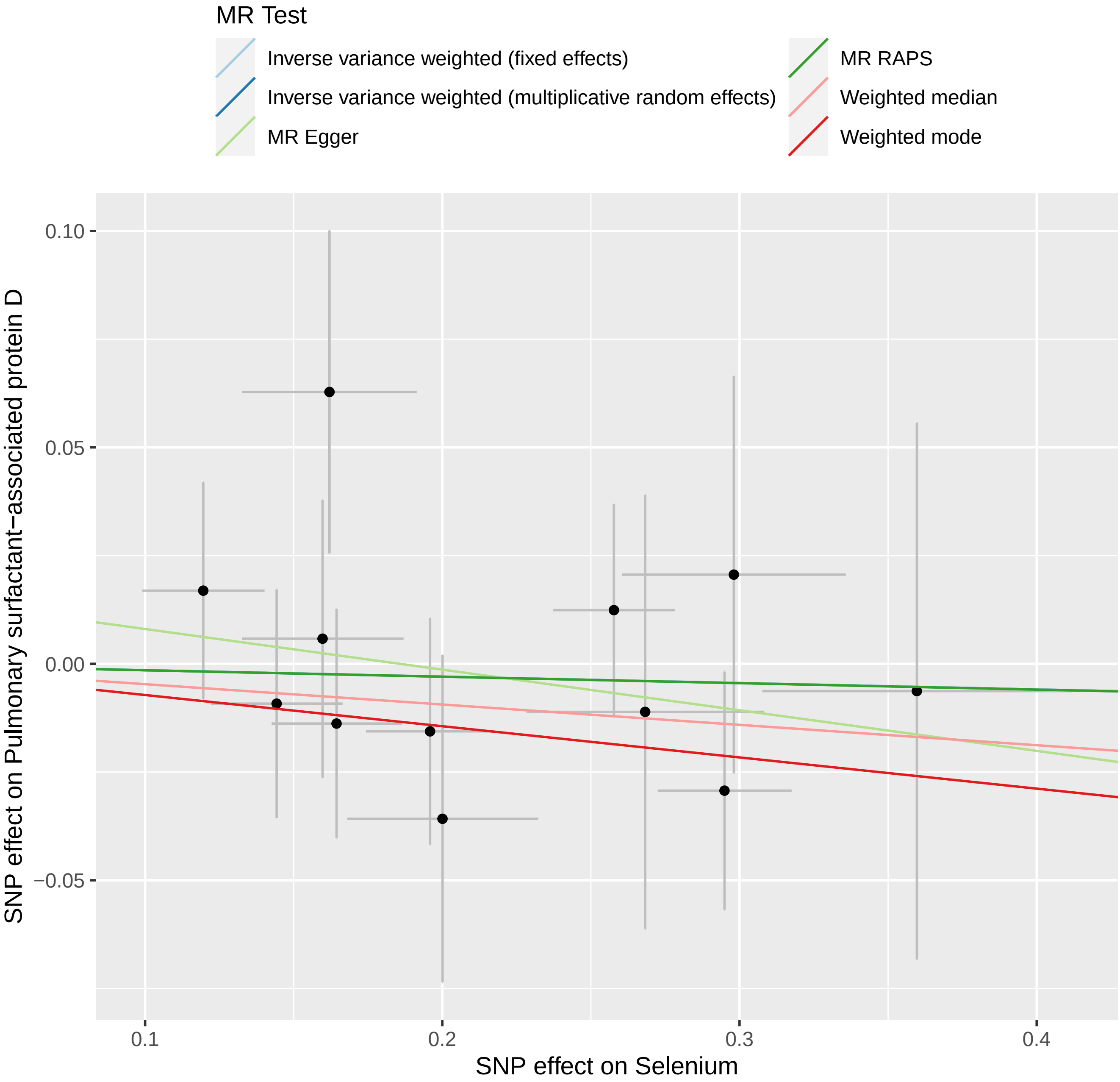

Batch 3−2713 : Selenium on Beta−sarcoglycan

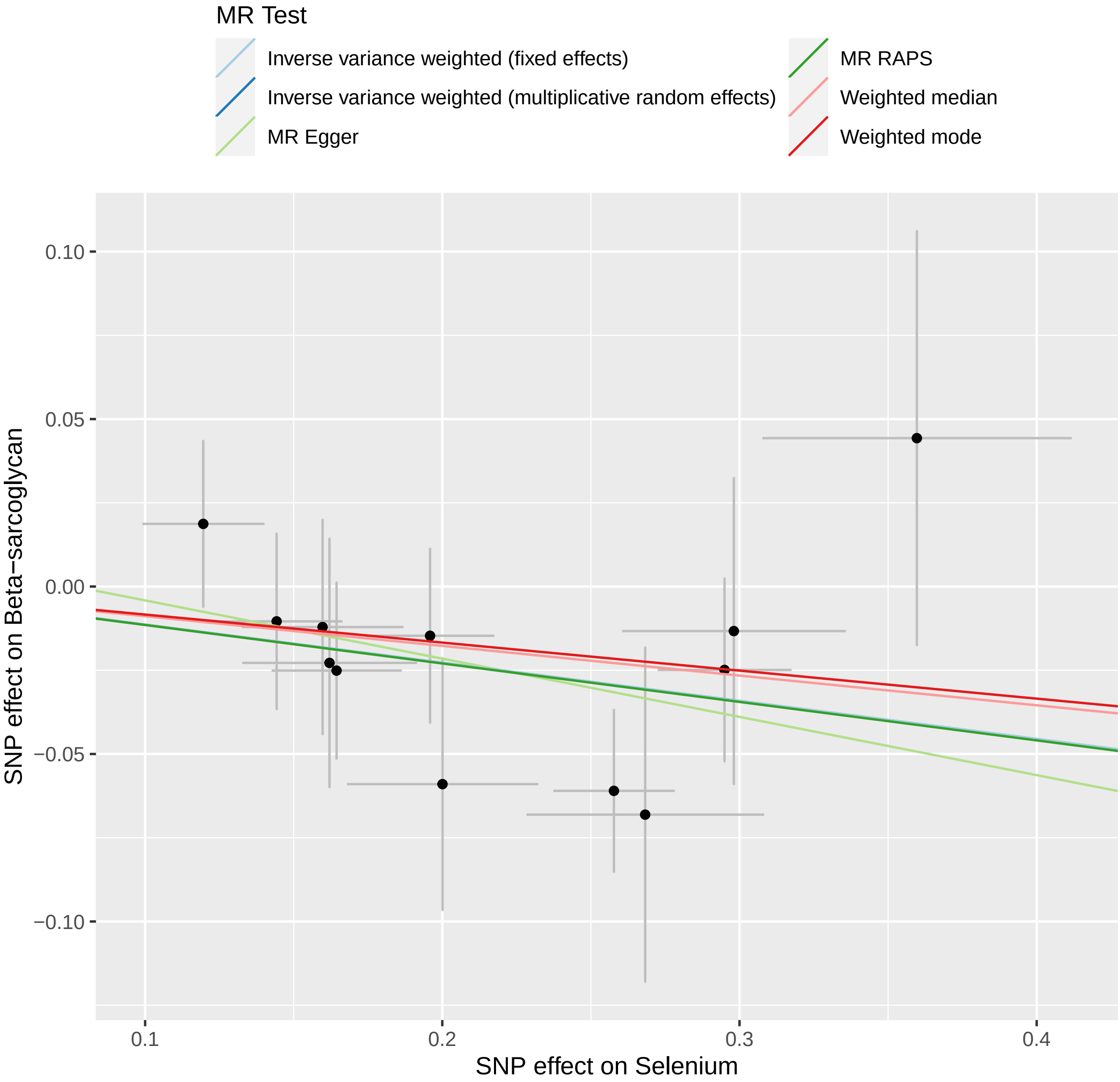

Batch 3−2714 : Selenium on Small glutamine−rich tetratricopeptide repeat−containing protein alpha

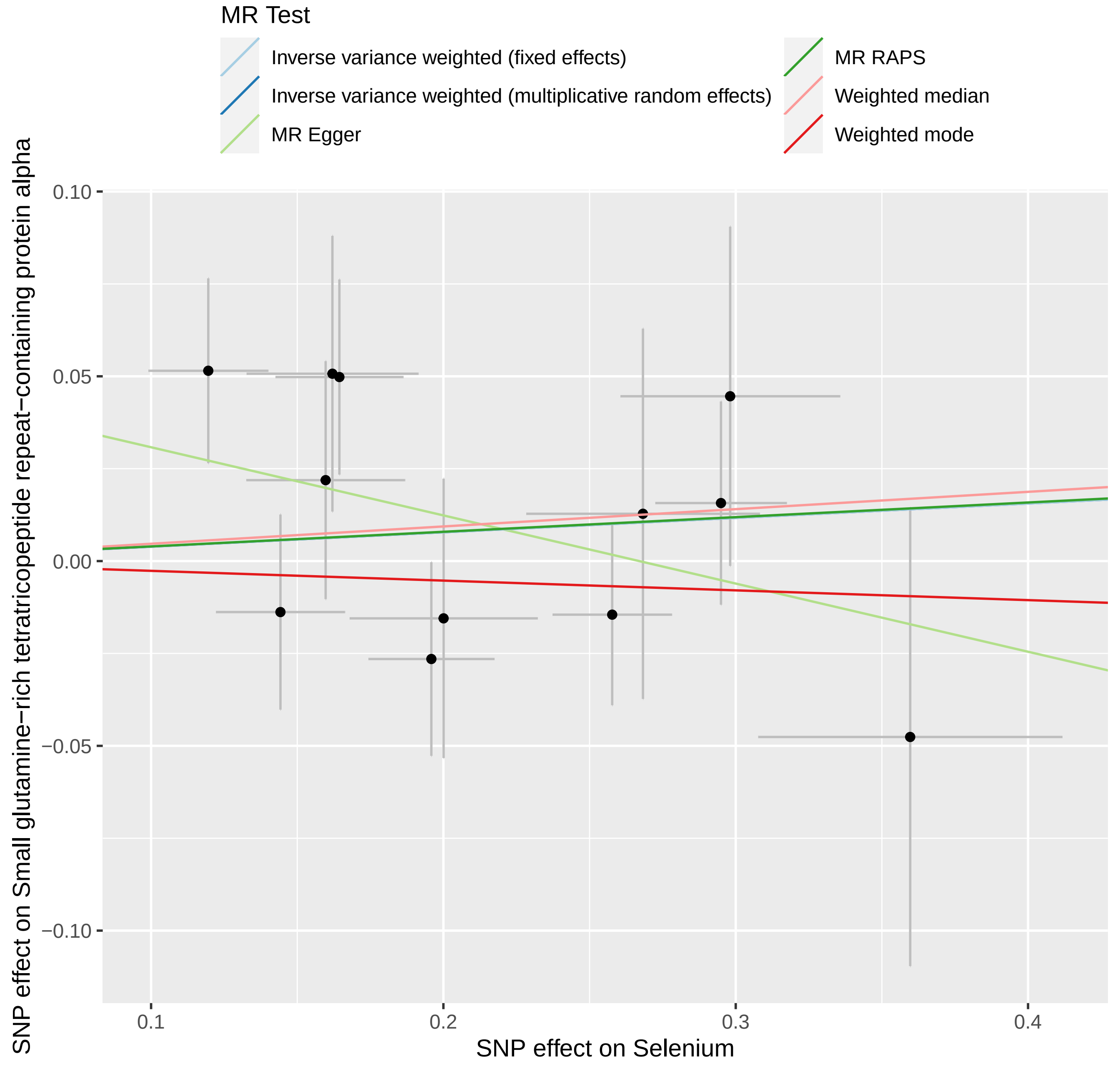

Batch 3−2715 : Selenium on SH2 domain−containing protein 1A

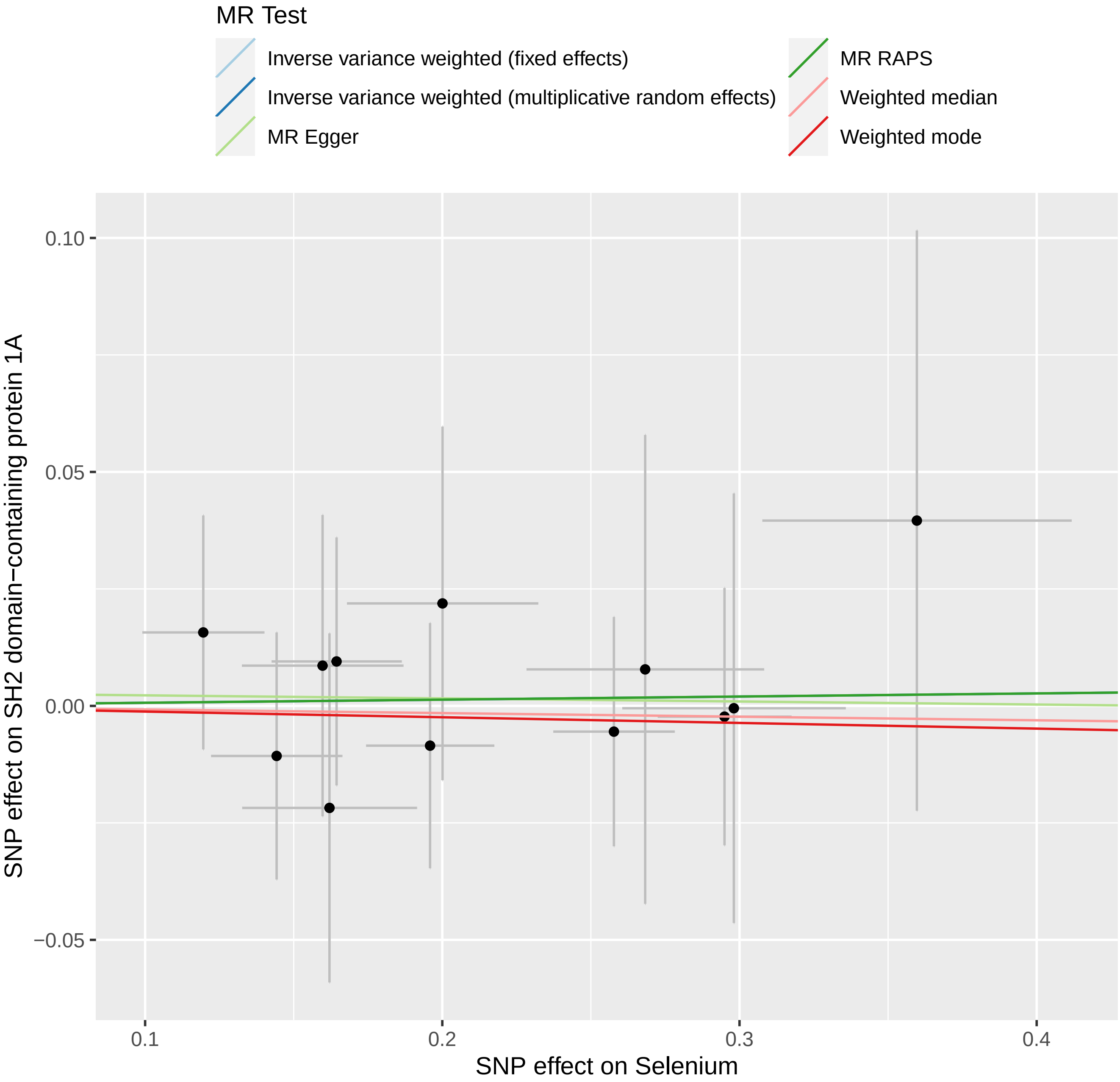

Batch 3−2716 : Selenium on SH3 domain−binding protein 2

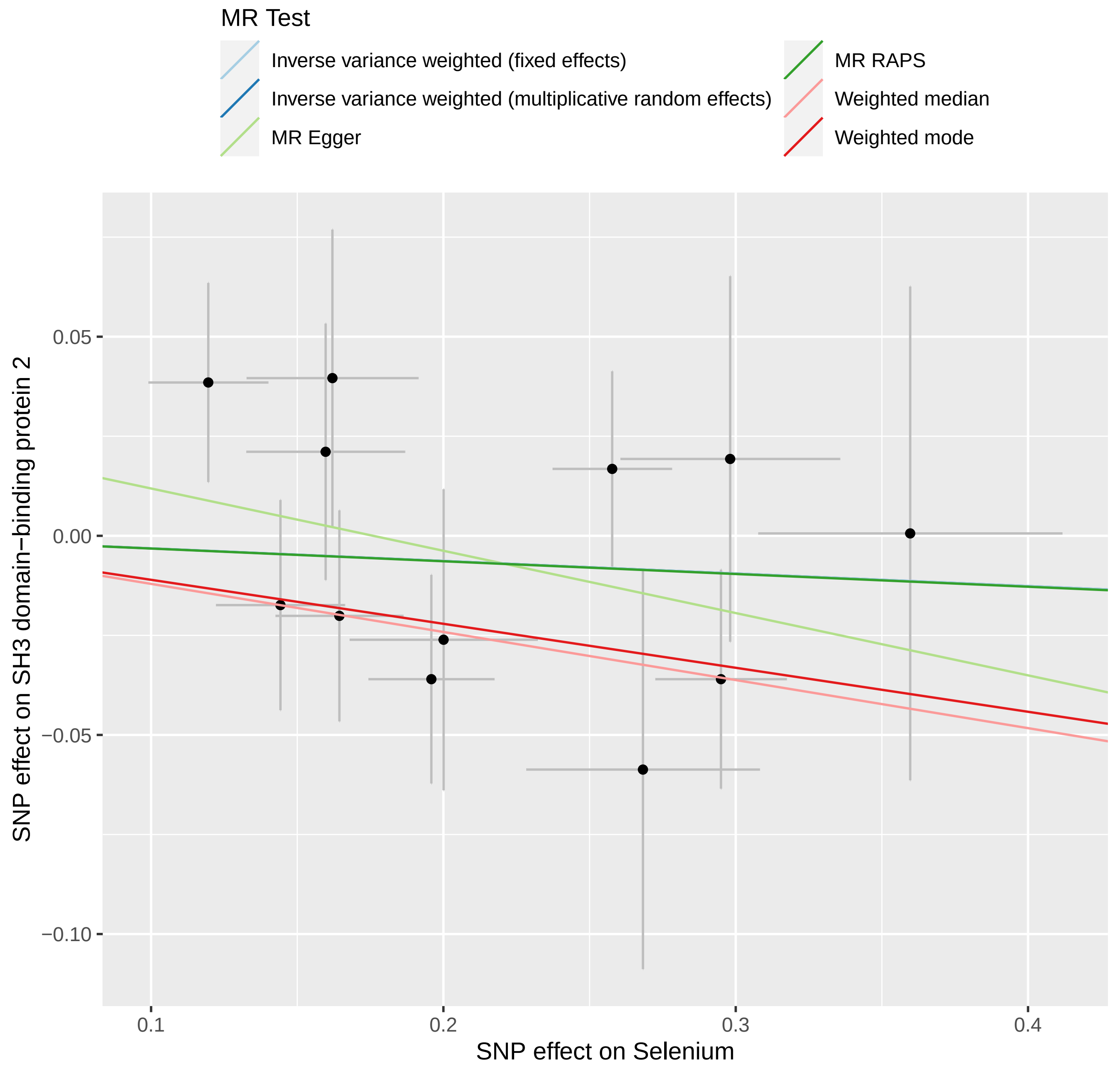

Batch 3−2717 : Selenium on SHC−transforming protein 1

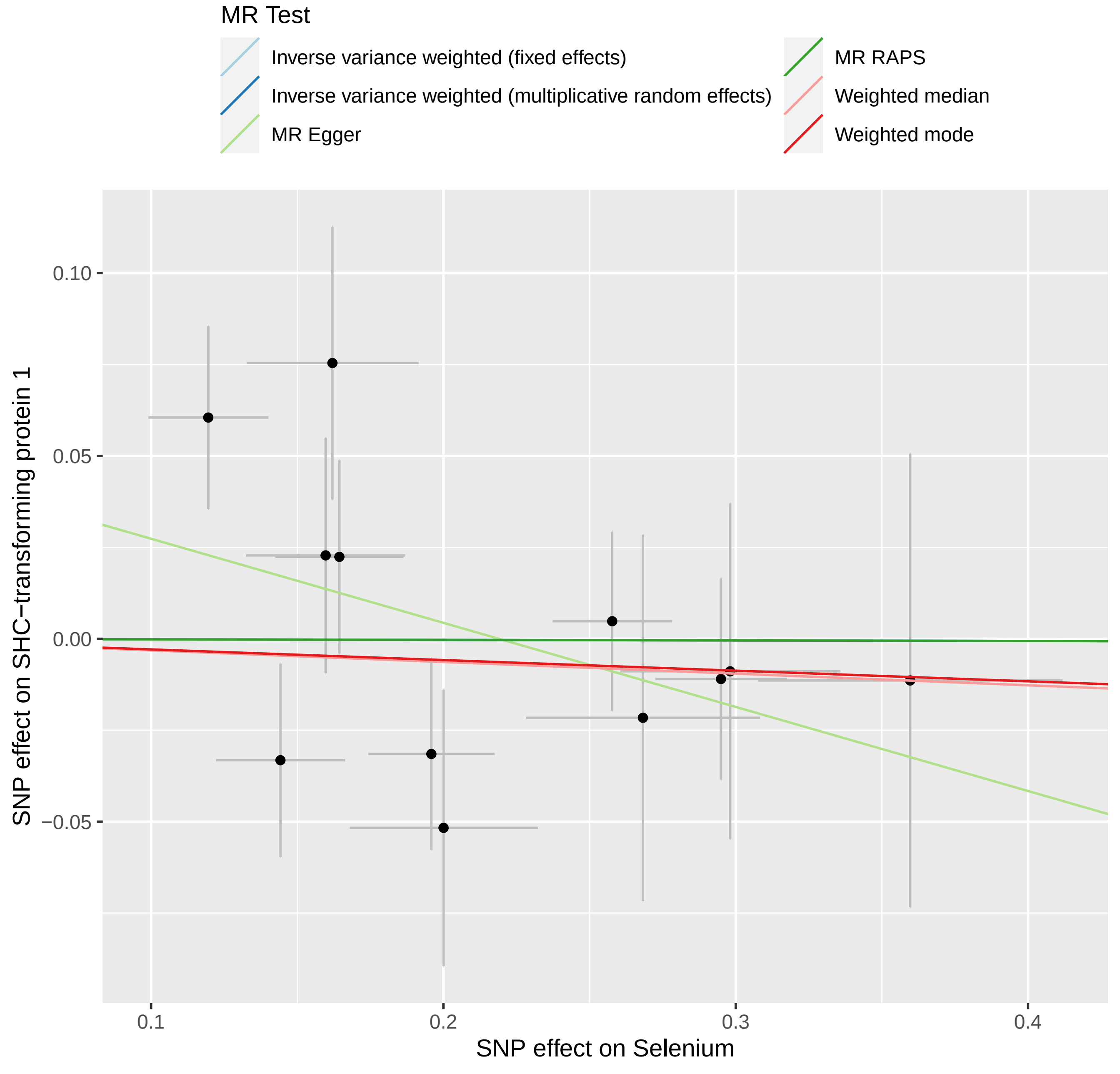

Batch 3−2718 : Selenium on SHC−transforming protein 2

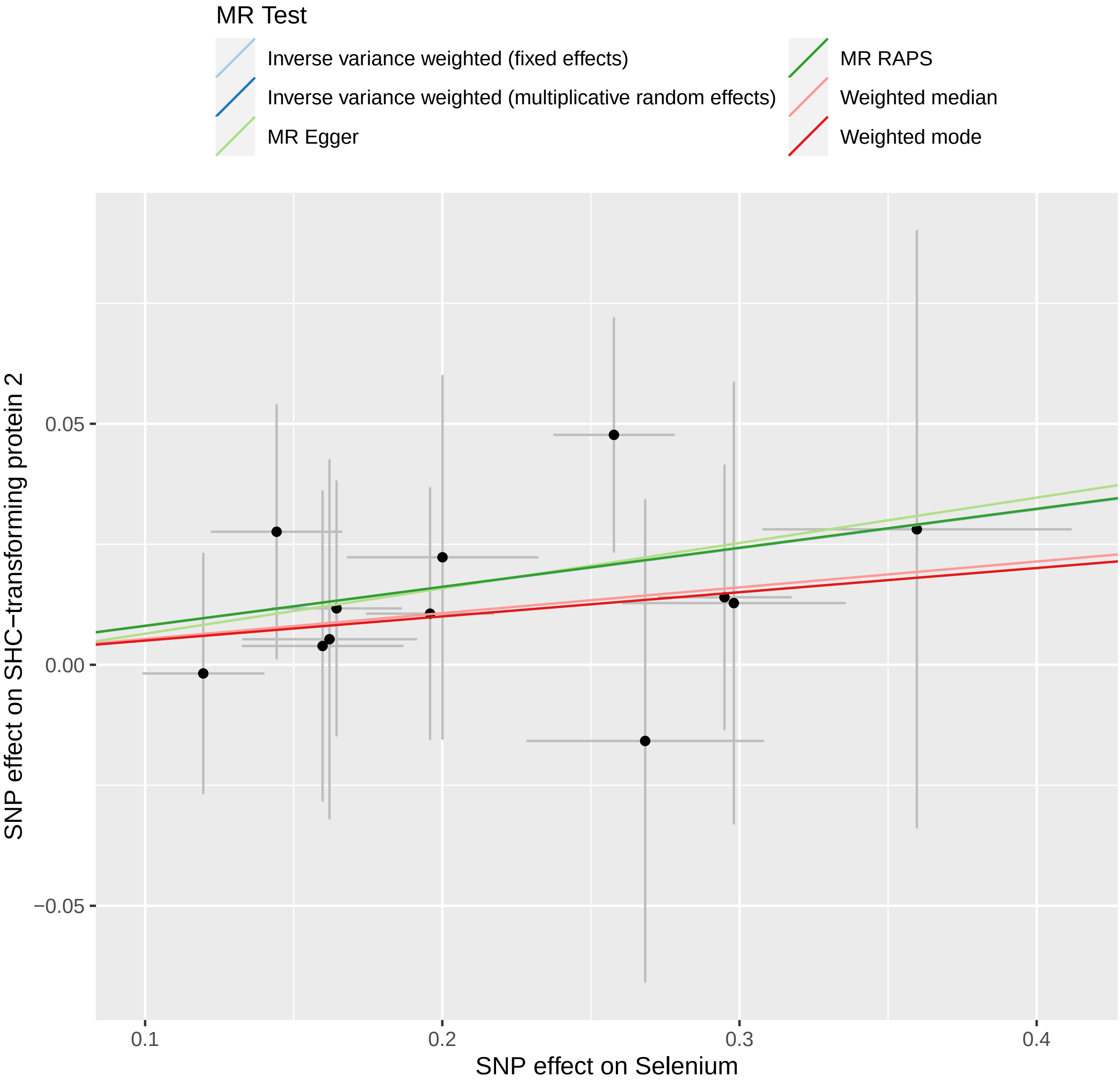

Batch 3−2719 : Selenium on SHC−transforming protein 4

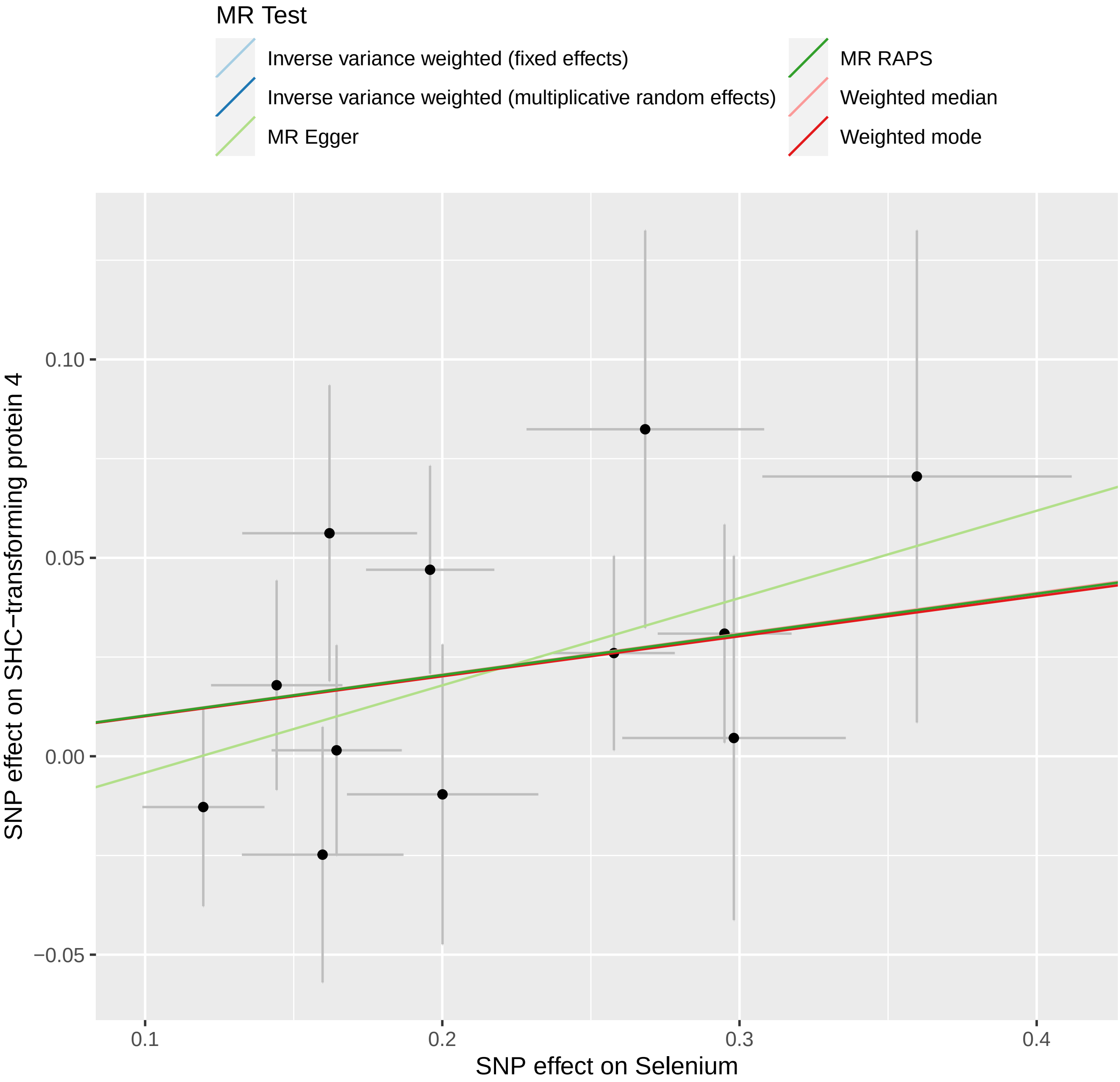

Batch 3−2720 : Selenium on Protein shisa−3 homolog

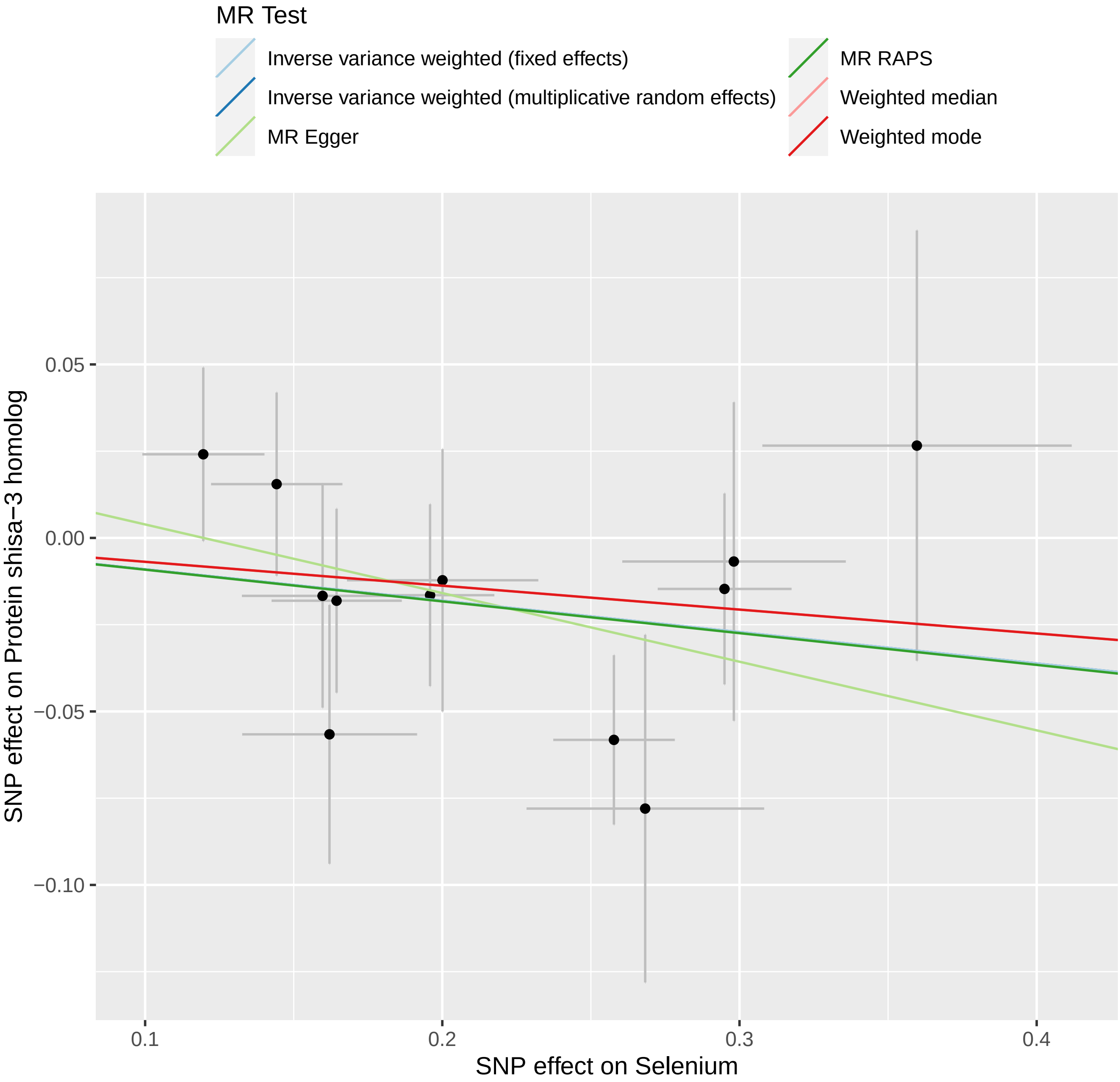

Batch 3−2721 : Selenium on Sialate O−acetylesterase

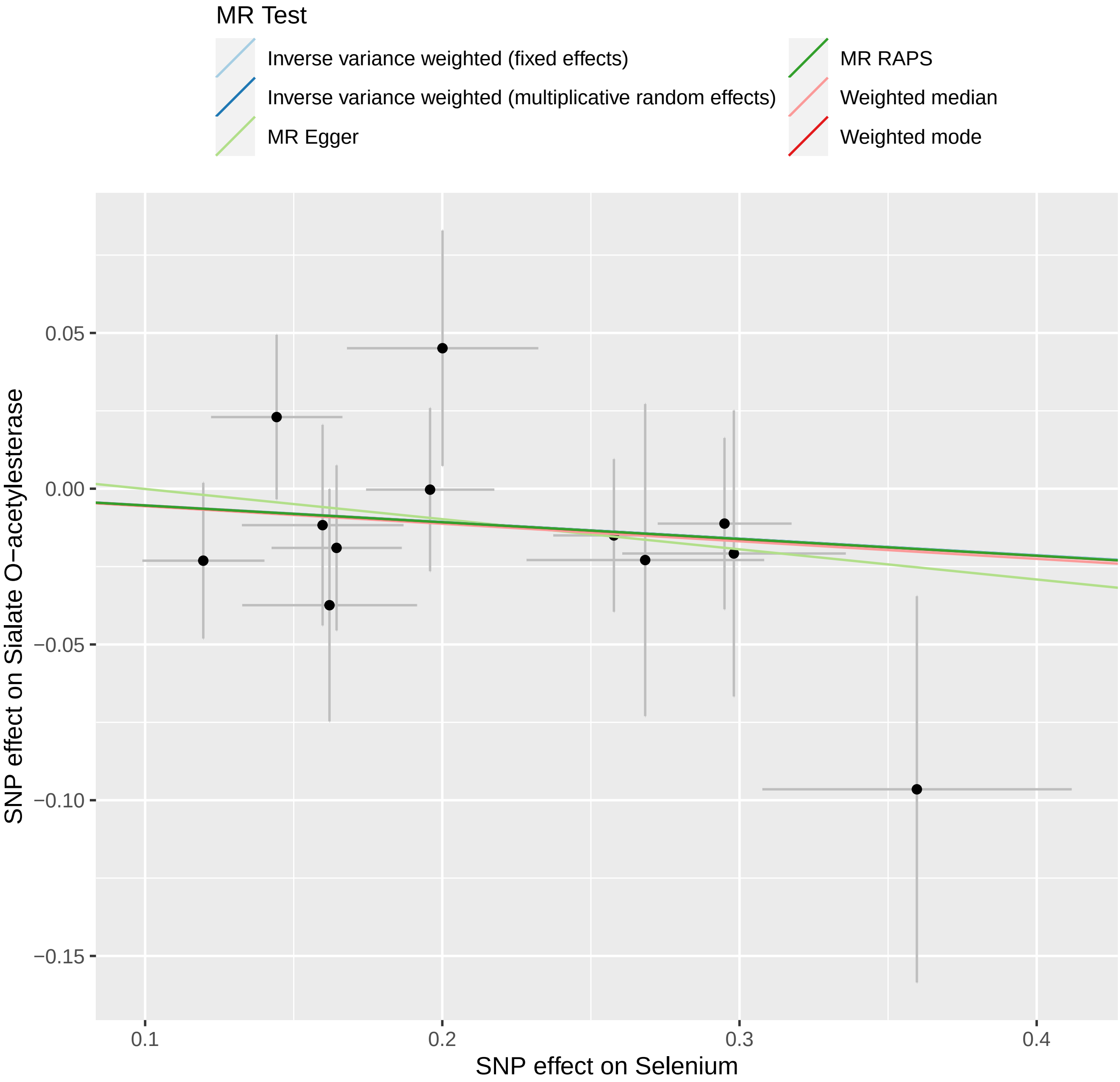

Batch 3−2722 : Selenium on Single Ig IL−1−related receptor

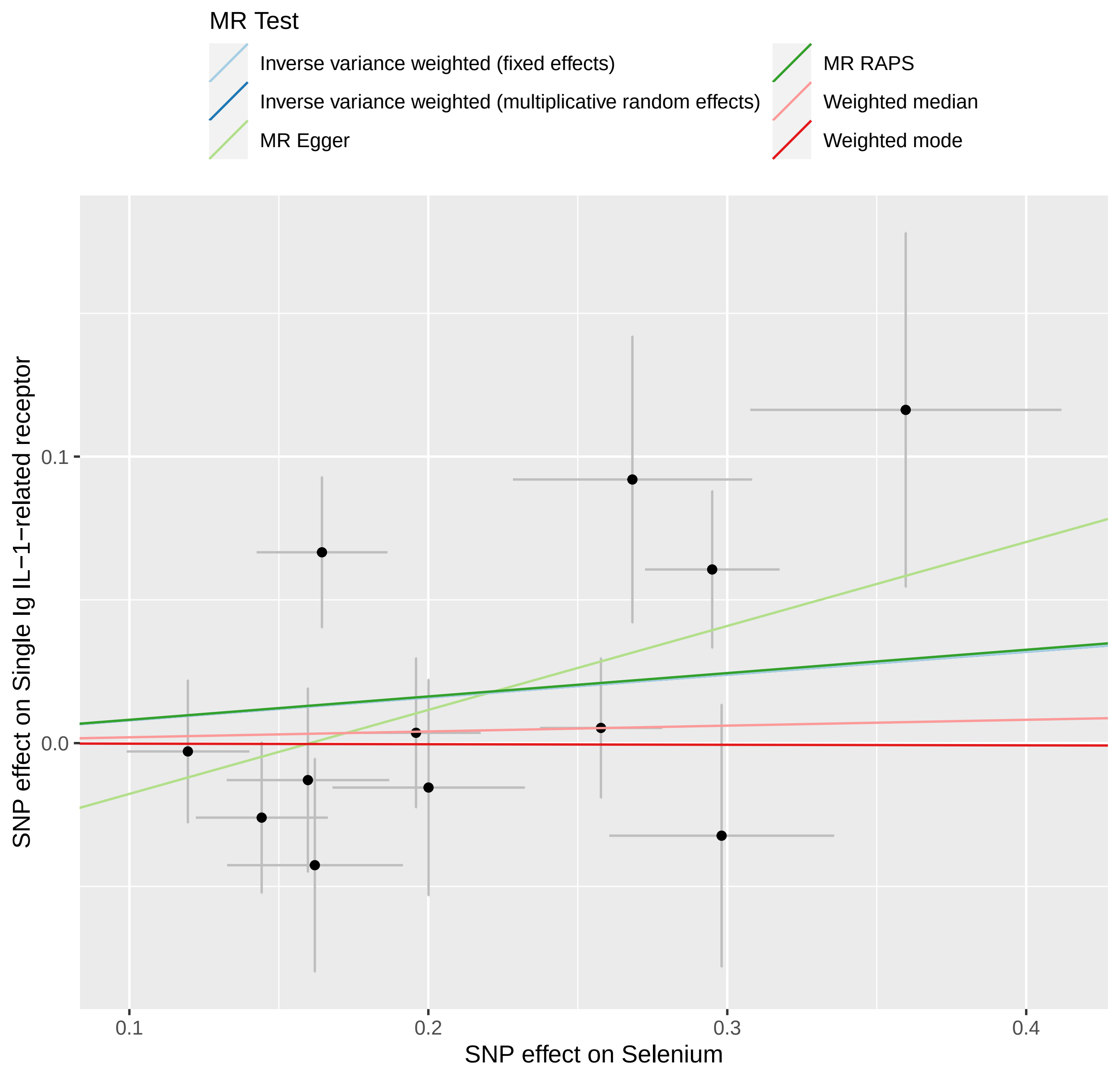

Batch 3−2723 : Selenium on Sialic acid−binding Ig−like lectin 10

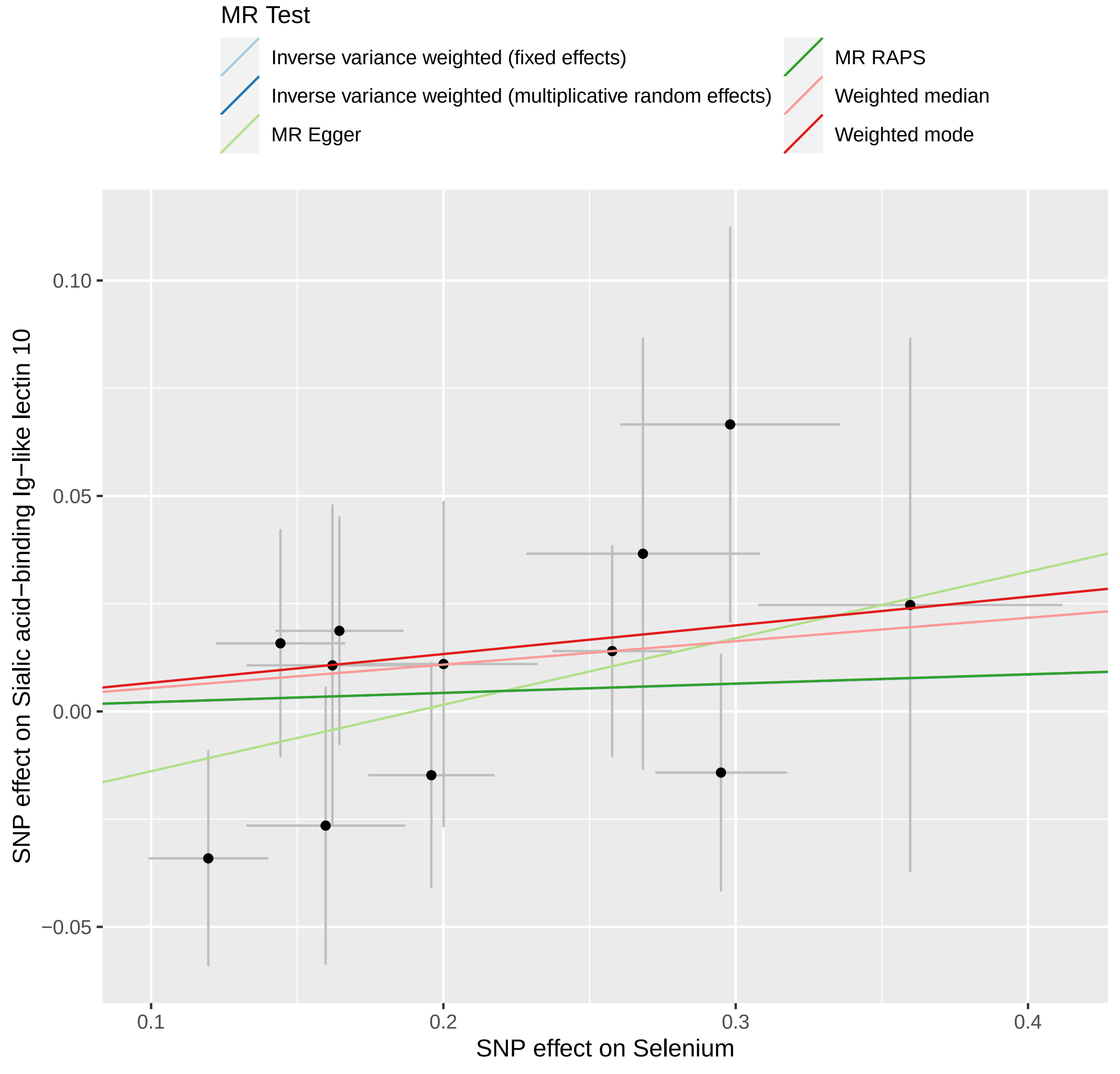

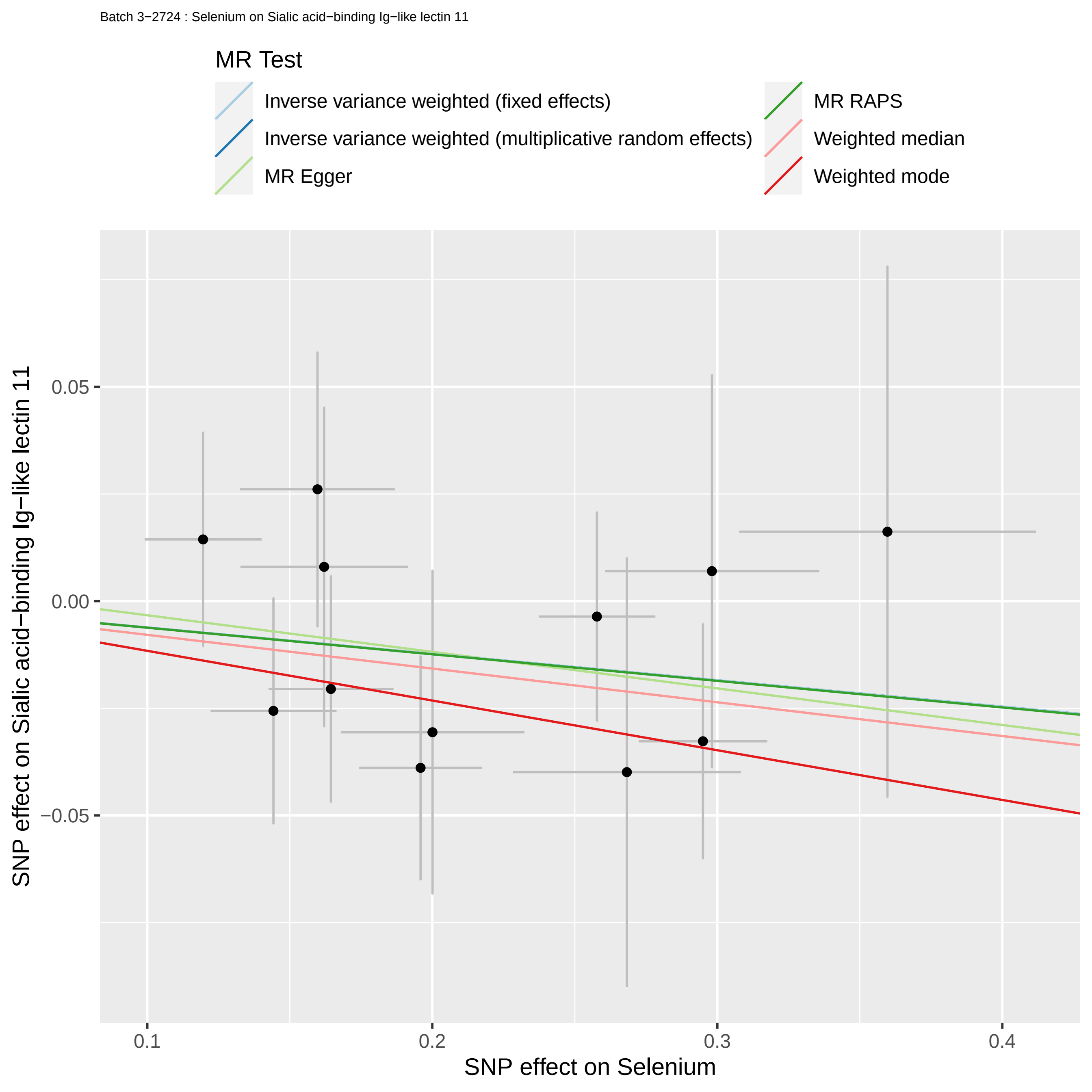
Batch 3−2724 : Selenium on Sialic acid−binding Ig−like lectin 11
MR Test
Inverse variance weighted (fixed effects)
Inverse variance weighted (multiplicative random effects)
MR Egger
MR RAPS
Weighted median
Weighted mode
0.05
0.00
−0.05
0.1
0.2
0.3
0.4
SNP effect on Sialic acid−binding Ig−like lectin 11
SNP effect on Selenium

Batch 3−2725 : Selenium on Sialic acid−binding Ig−like lectin 12

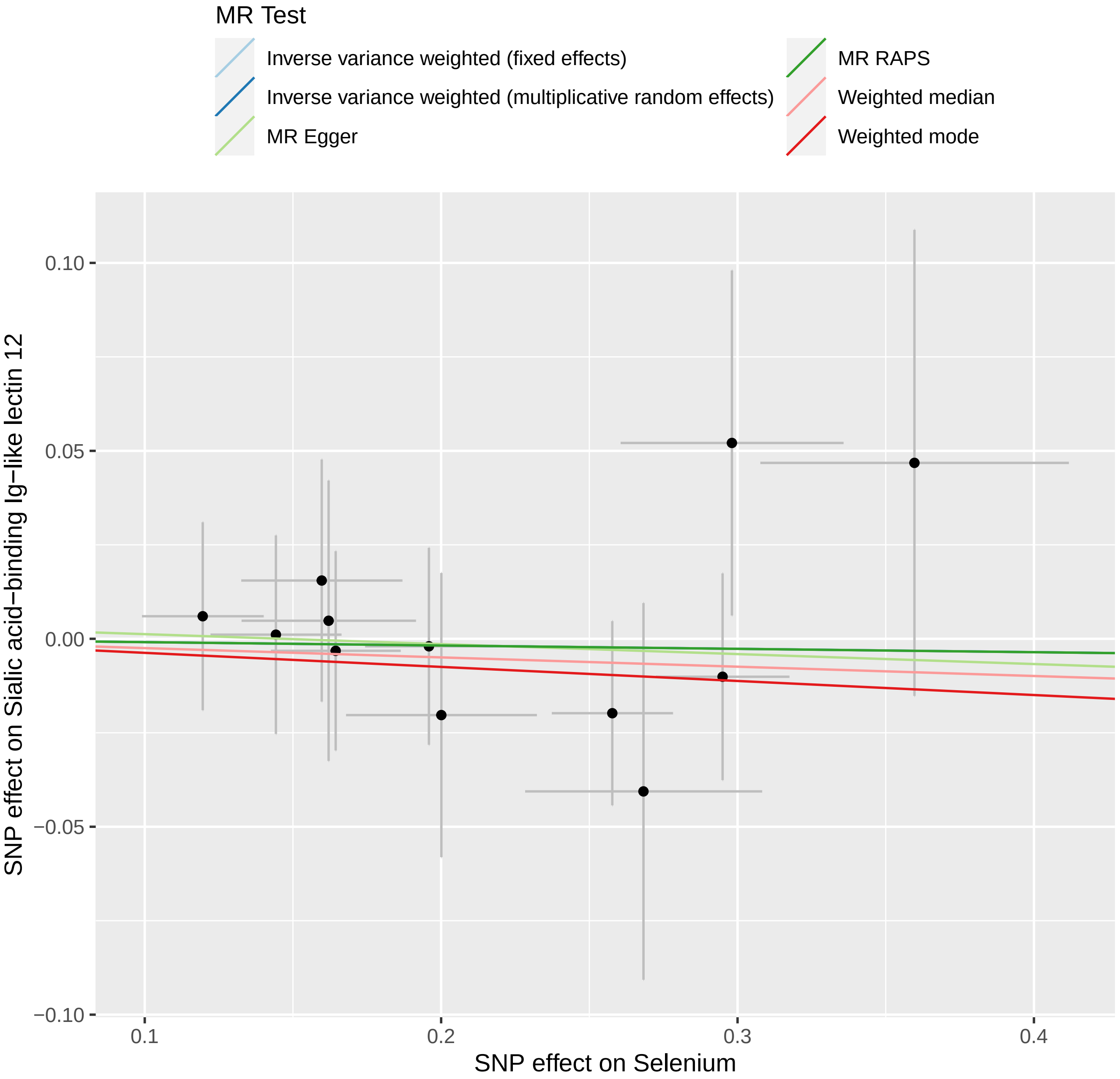

Batch 3−2726 : Selenium on Sialic acid−binding Ig−like lectin 12

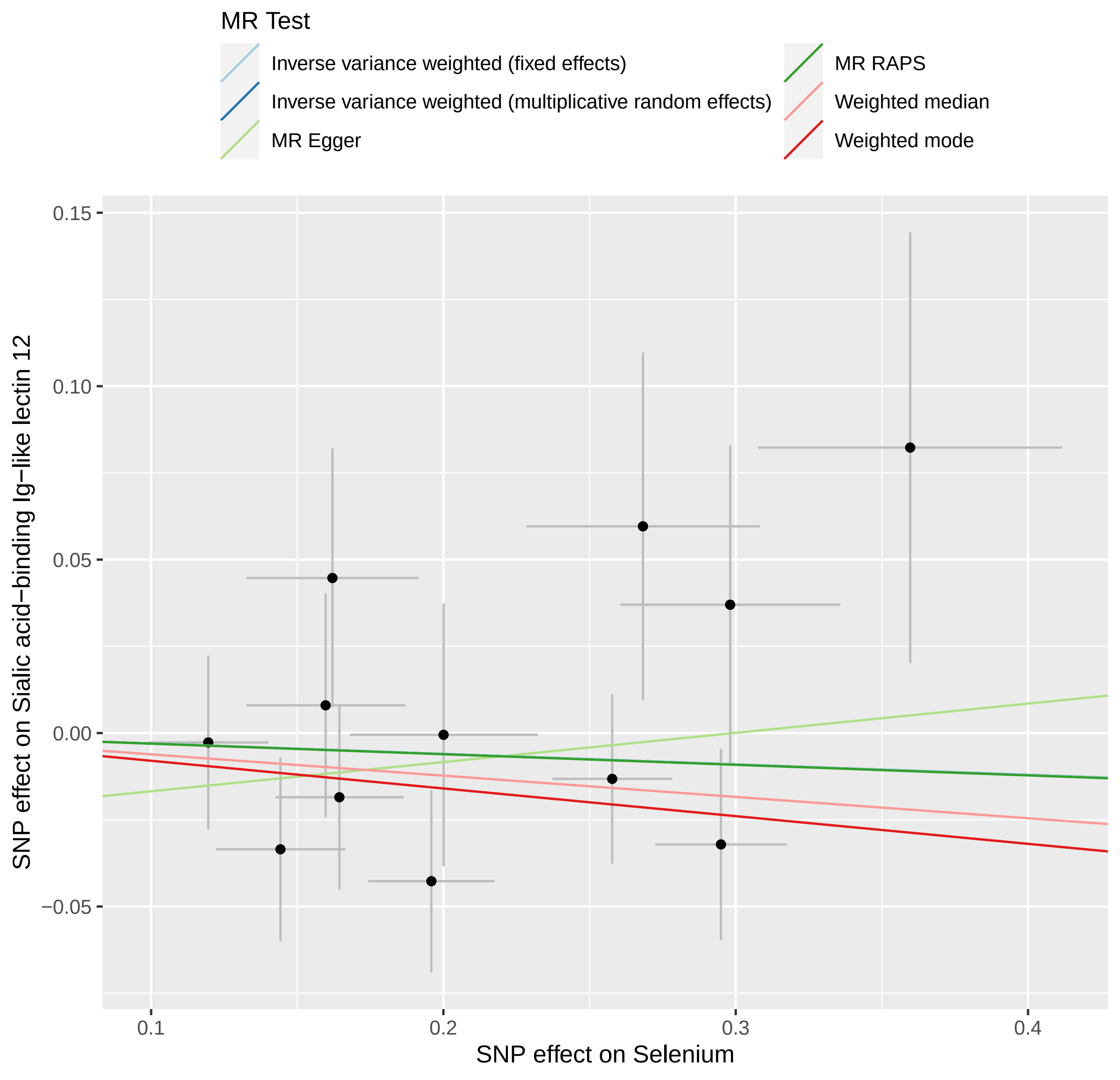

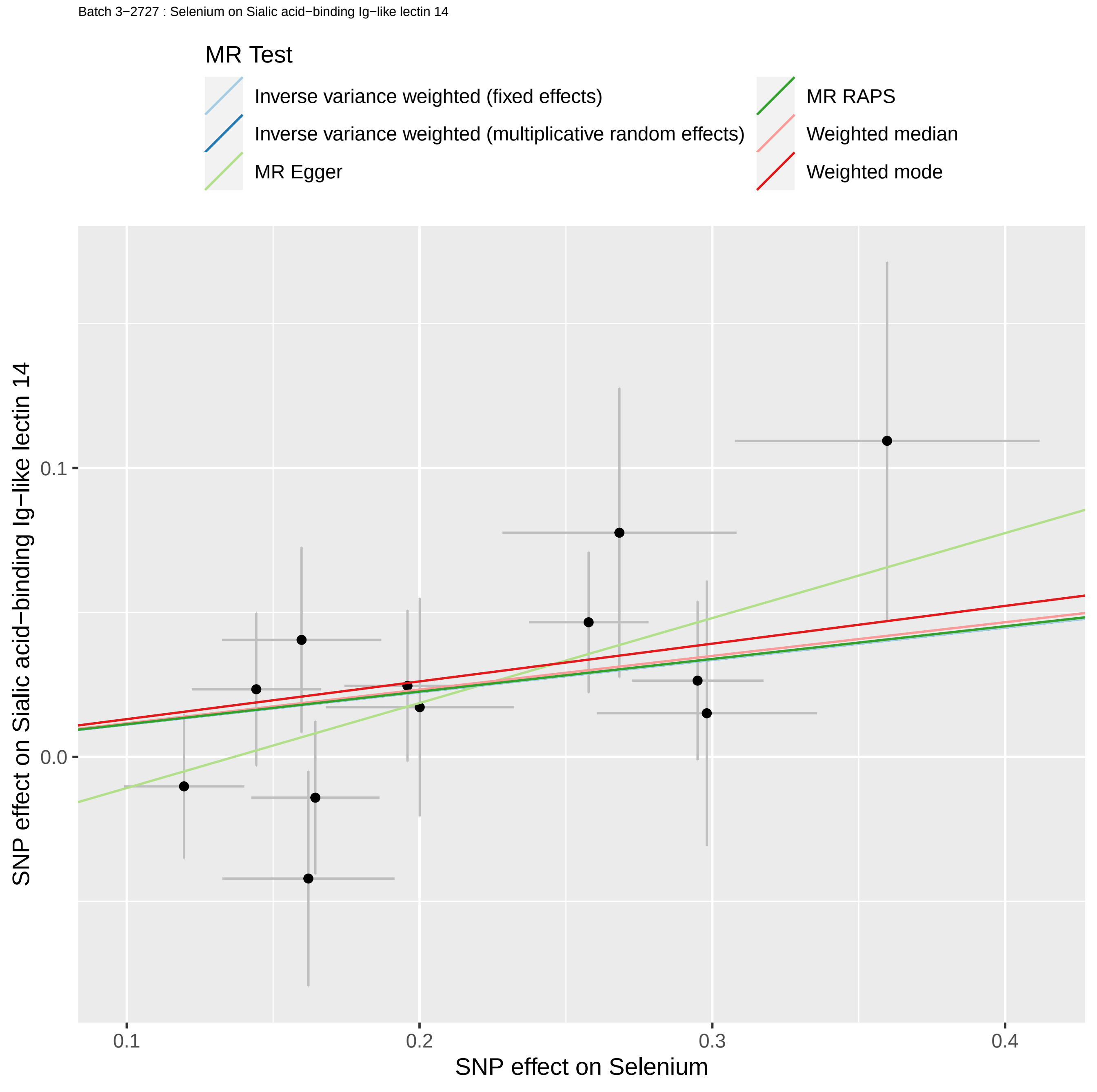

Batch 3−2727 : Selenium on Sialic acid−binding Ig−like lectin 14
MR Test
Inverse variance weighted (fixed effects)
Inverse variance weighted (multiplicative random effects)
MR Egger
MR RAPS
Weighted median
Weighted mode
SNP effect on Sialic acid−binding Ig−like lectin 14
0.1
0.0
0.1
0.2
0.3
0.4
SNP effect on Selenium

Batch 3−2728 : Selenium on Sialic acid−binding Ig−like lectin 14

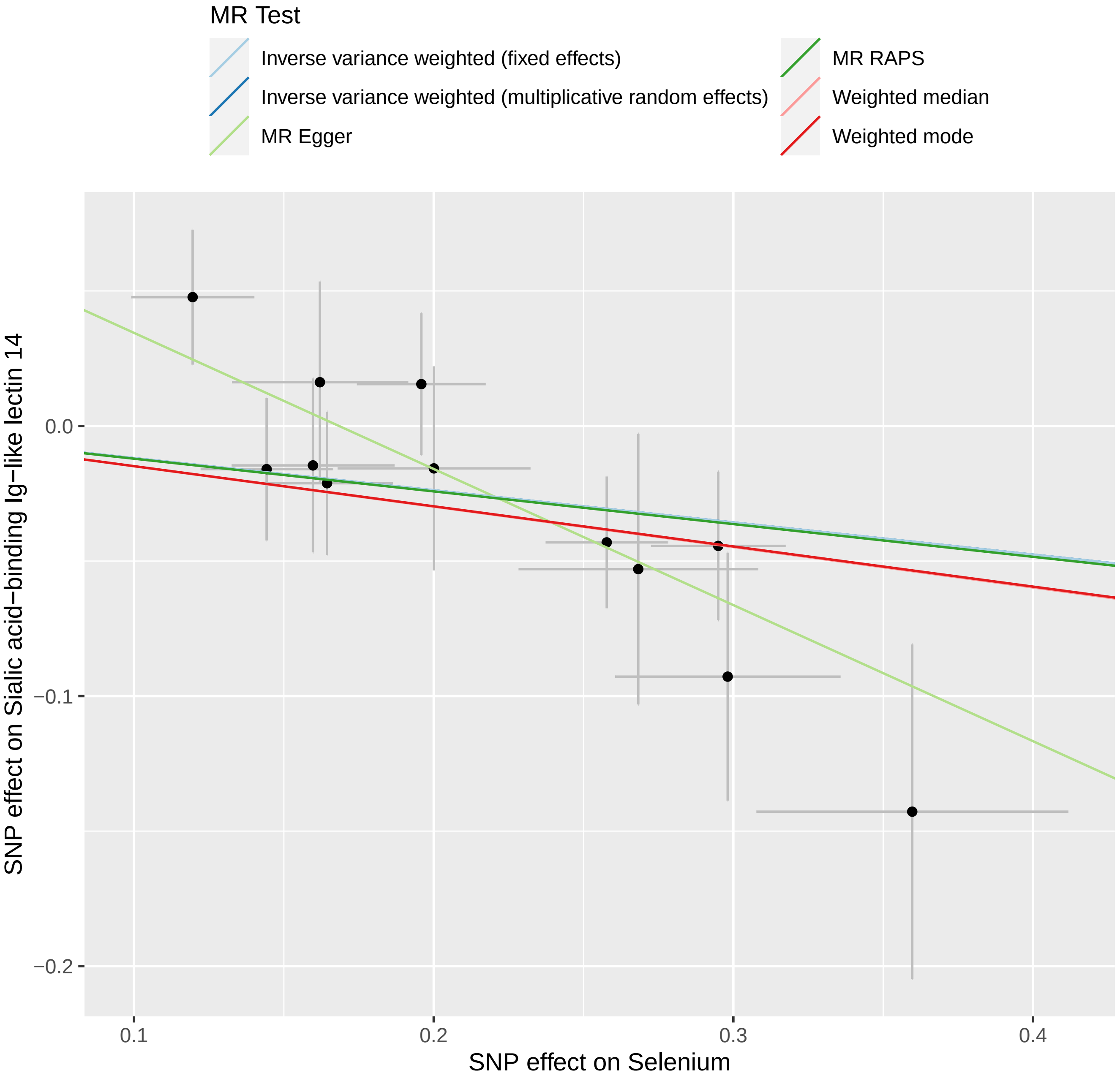

Batch 3−2729 : Selenium on Sialic acid−binding Ig−like lectin 7

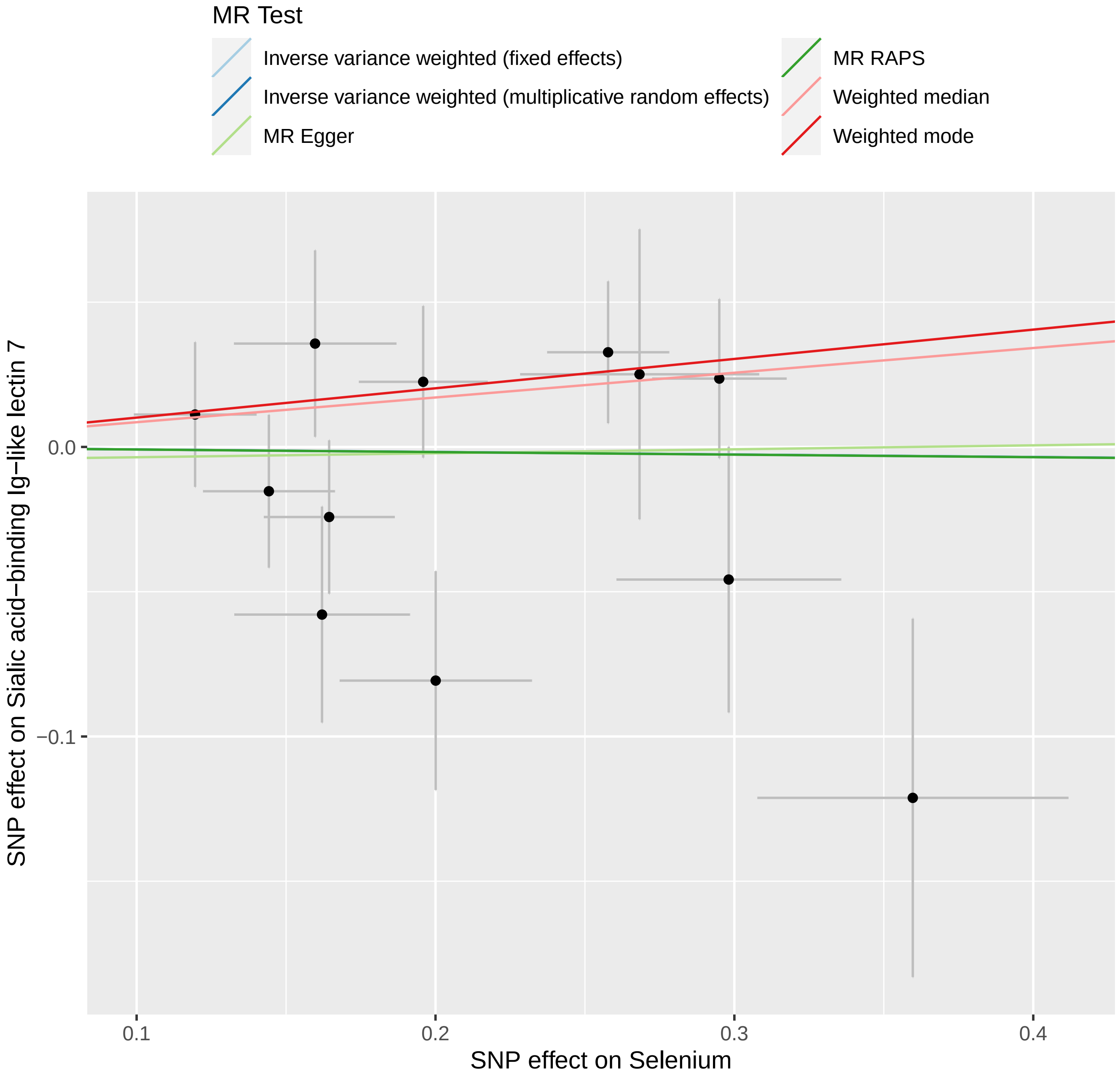

Batch 3−2730 : Selenium on Sialic acid−binding Ig−like lectin 8

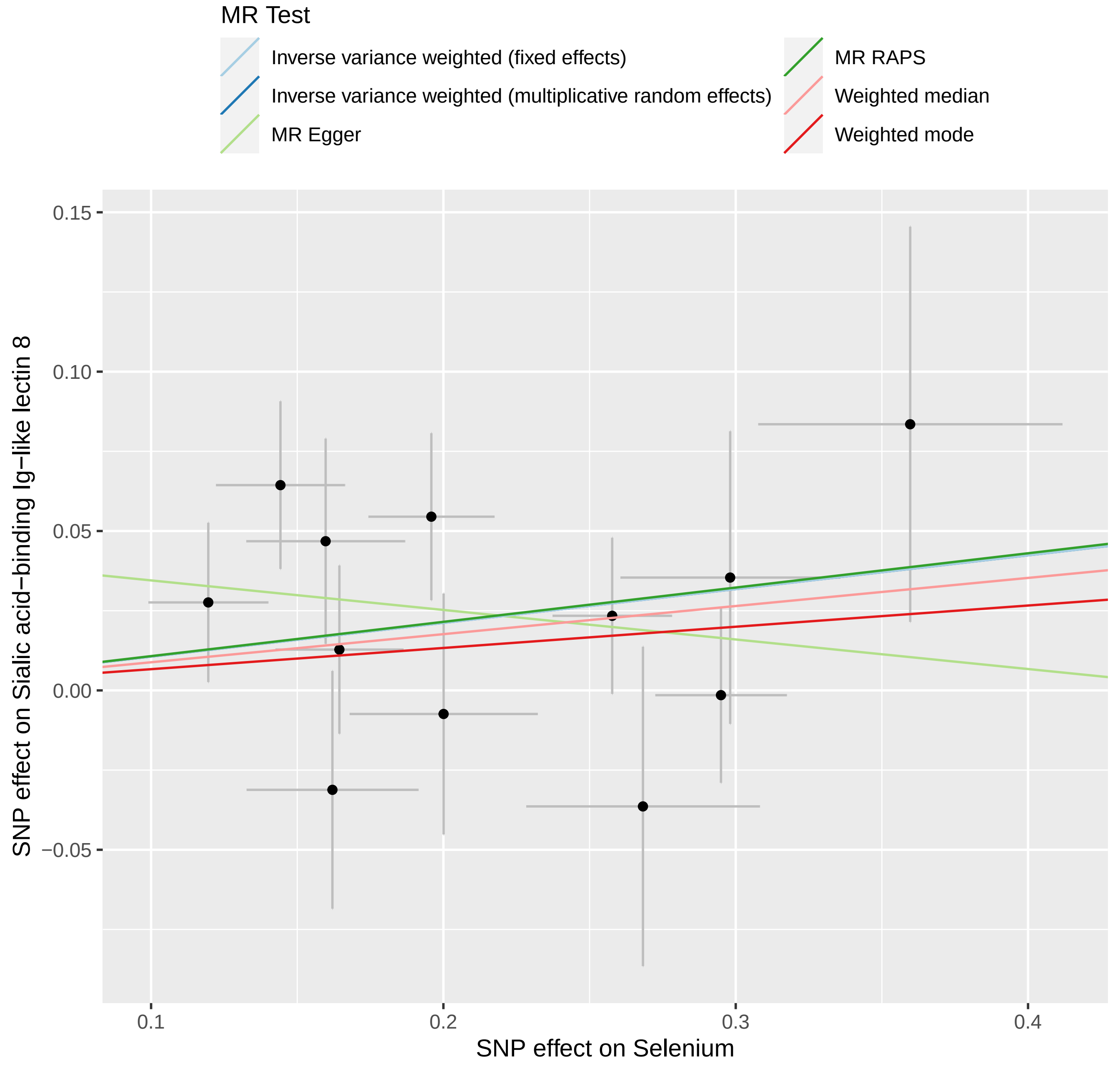

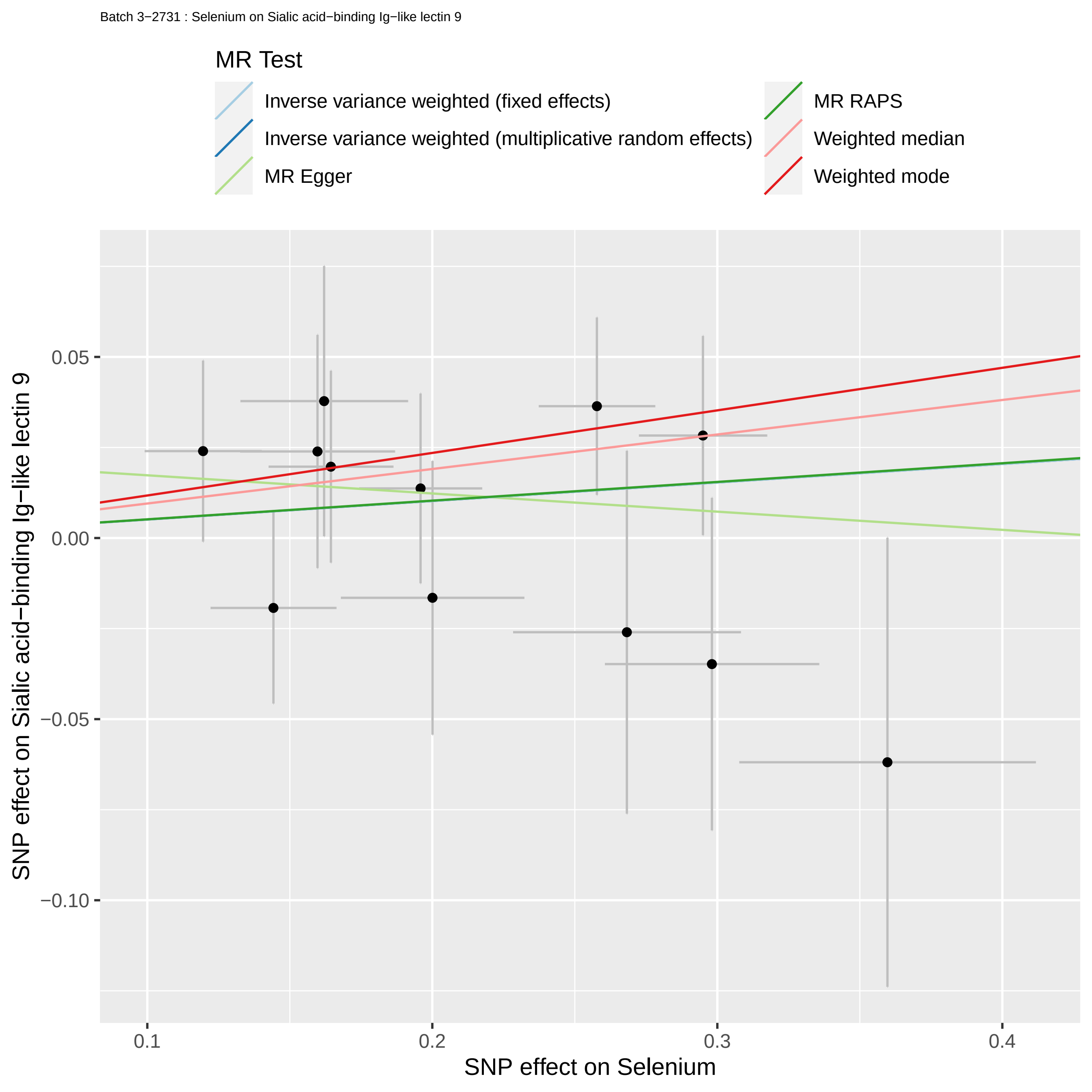

Batch 3−2731 : Selenium on Sialic acid−binding Ig−like lectin 9
MR Test
Inverse variance weighted (fixed effects)
Inverse variance weighted (multiplicative random effects)
MR Egger
MR RAPS
Weighted median
Weighted mode
SNP effect on Sialic acid−binding Ig−like lectin 9
0.05
0.00
−0.05
−0.10
0.1
0.2
0.3
0.4
SNP effect on Selenium

Batch 3−2732 : Selenium on Tyrosine−protein phosphatase non−receptor type substrate 1

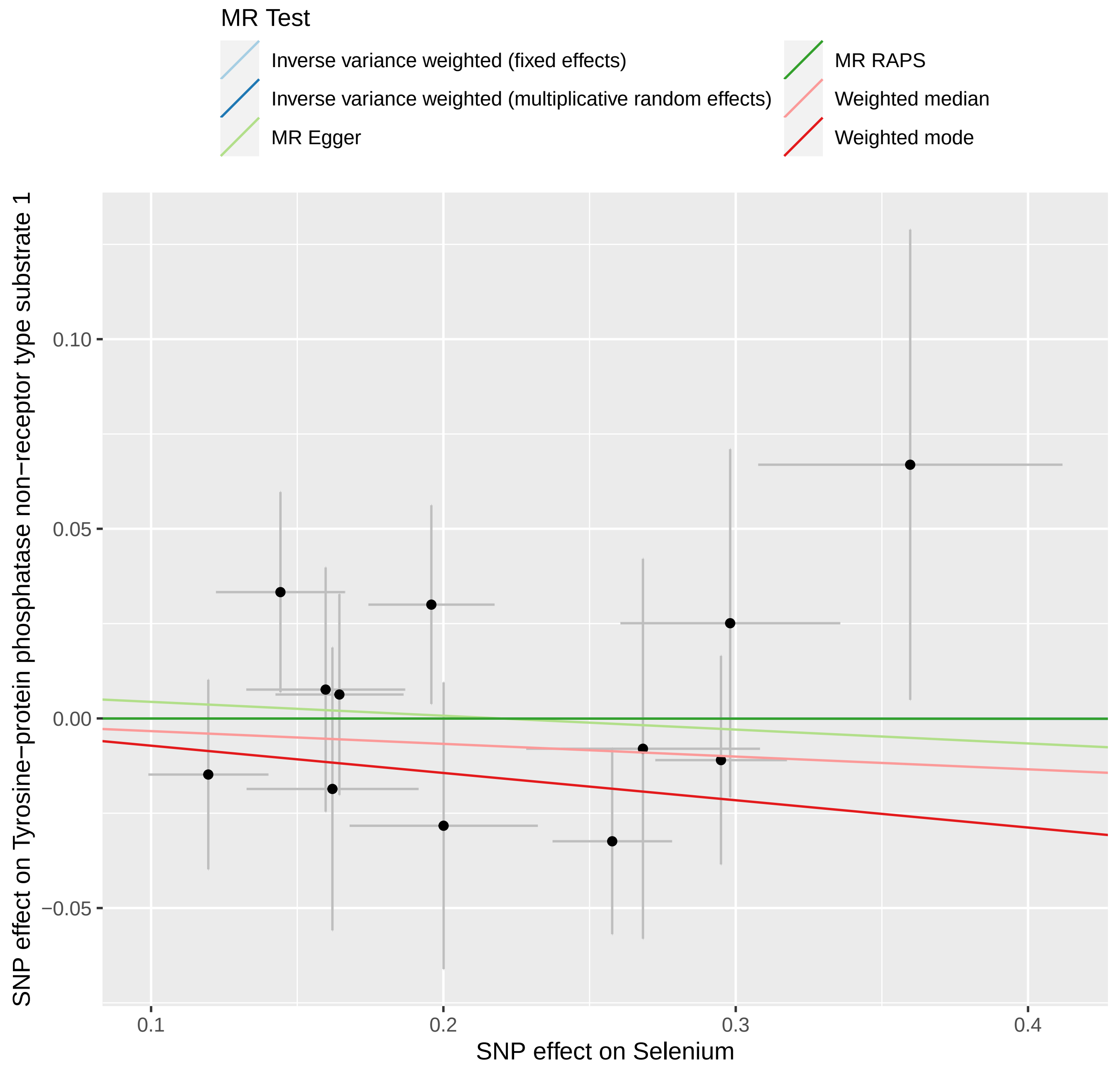

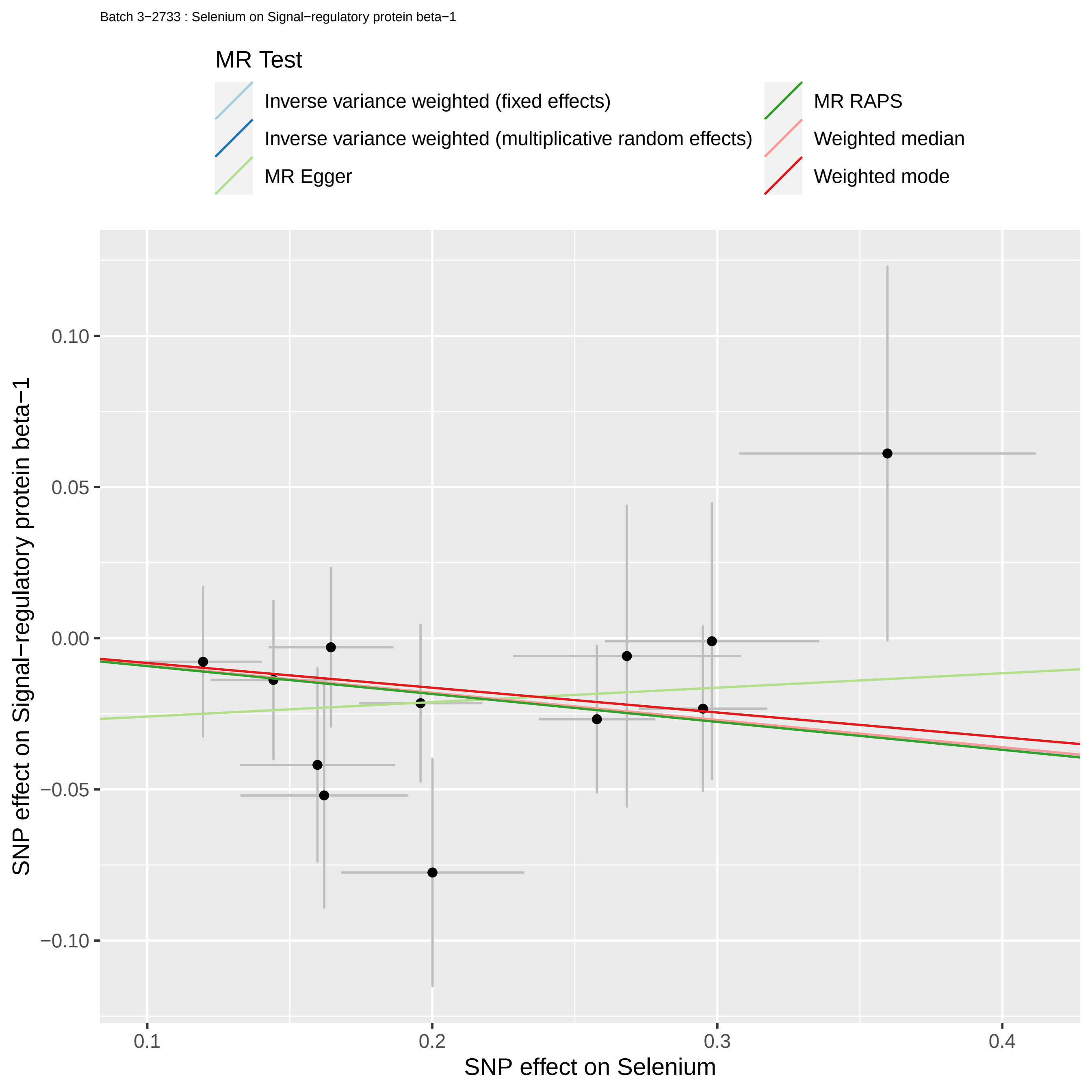

Batch 3−2733 : Selenium on Signal−regulatory protein beta−1
MR Test
Inverse variance weighted (fixed effects)
Inverse variance weighted (multiplicative random effects)
MR Egger
MR RAPS
Weighted median
Weighted mode
0.10
0.05
0.00
−0.05
−0.10
0.1
0.2
0.3
0.4
SNP effect on Selenium
SNP effect on Signal−regulatory protein beta−1

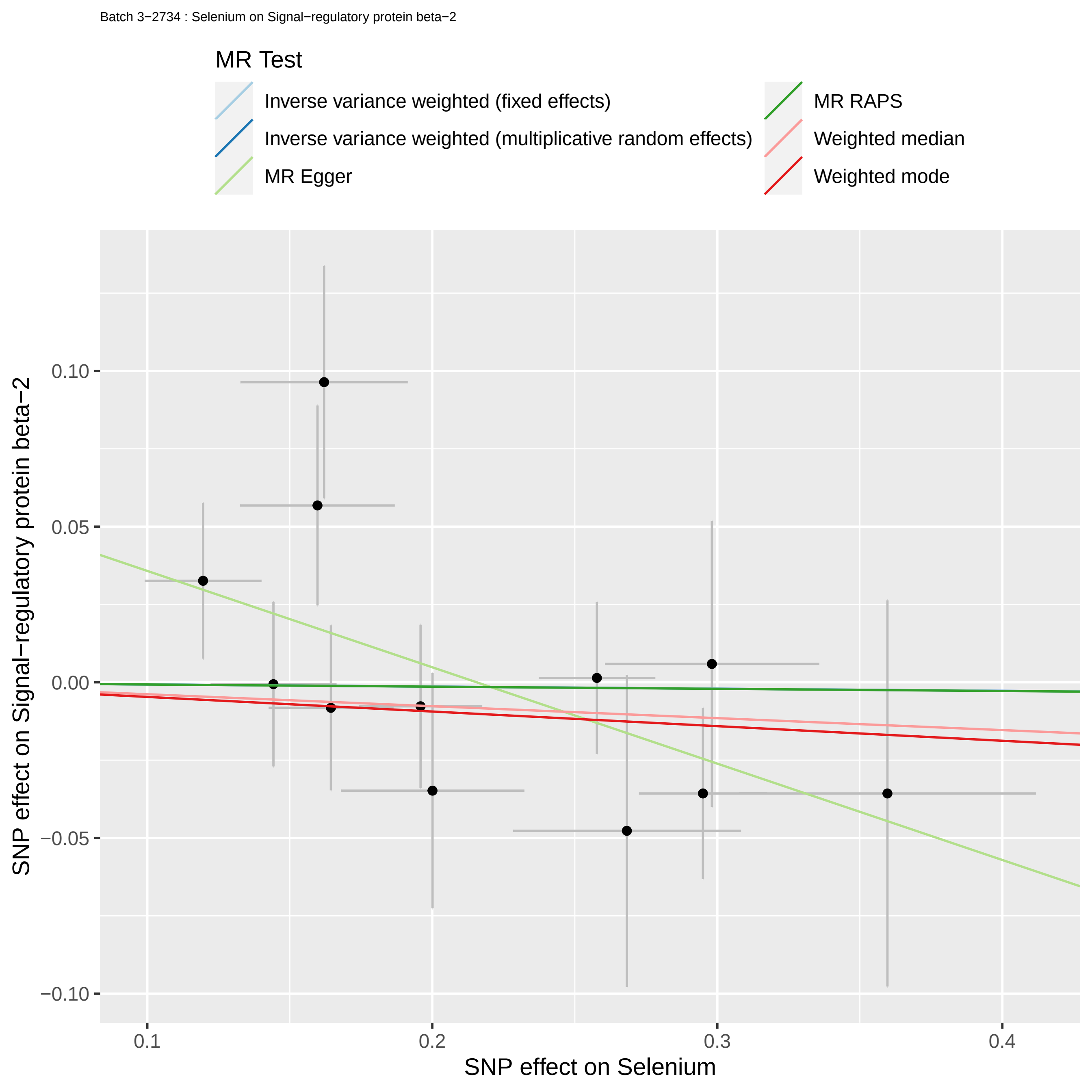

Batch 3−2734 : Selenium on Signal−regulatory protein beta−2
MR Test
Inverse variance weighted (fixed effects)
Inverse variance weighted (multiplicative random effects)
MR Egger
MR RAPS
Weighted median
Weighted mode
SNP effect on Signal−regulatory protein beta−2
0.10
0.05
0.00
−0.05
−0.10
0.1
0.2
0.3
0.4
SNP effect on Selenium

Batch 3−2735 : Selenium on Signal−regulatory protein gamma

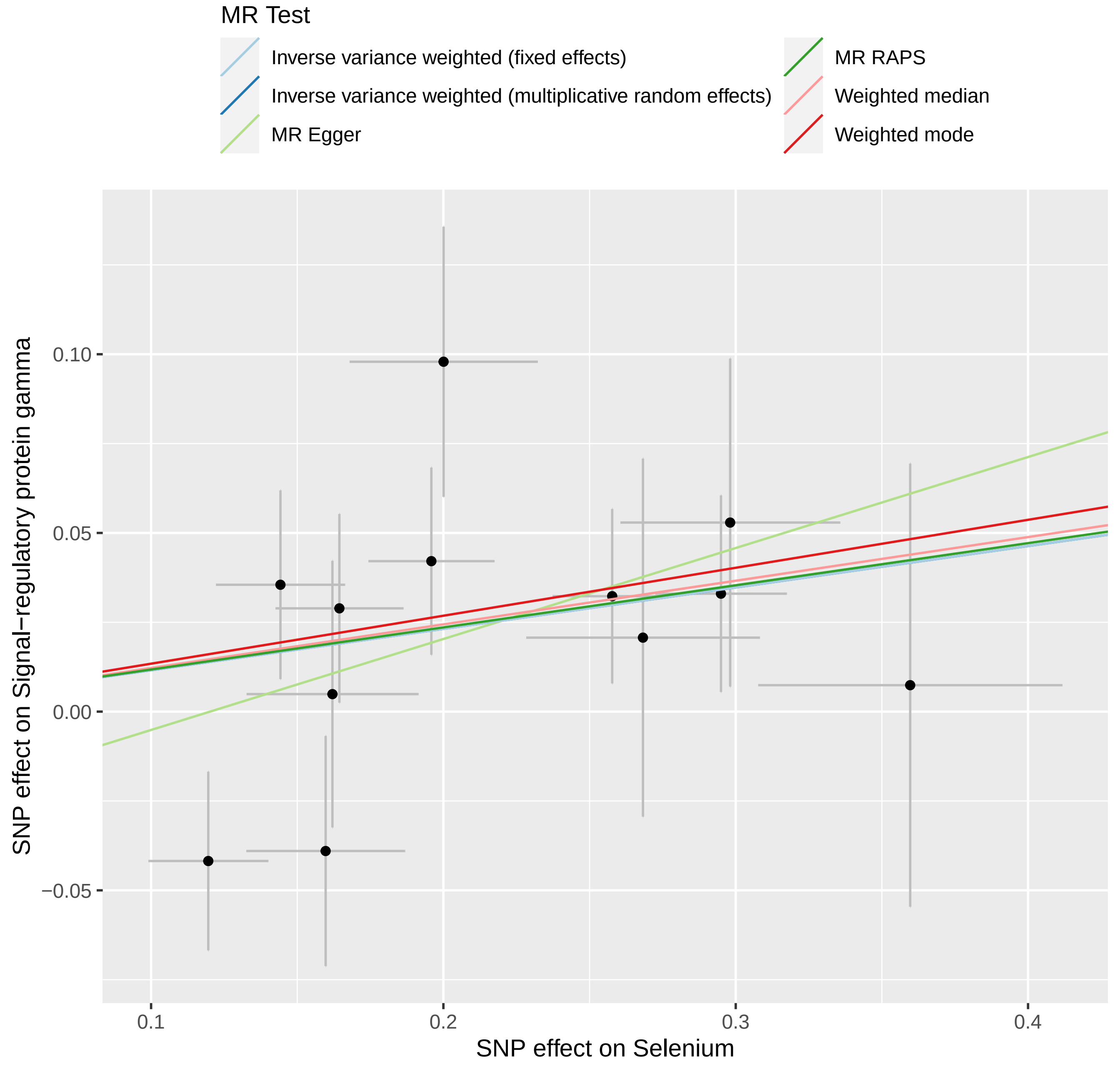

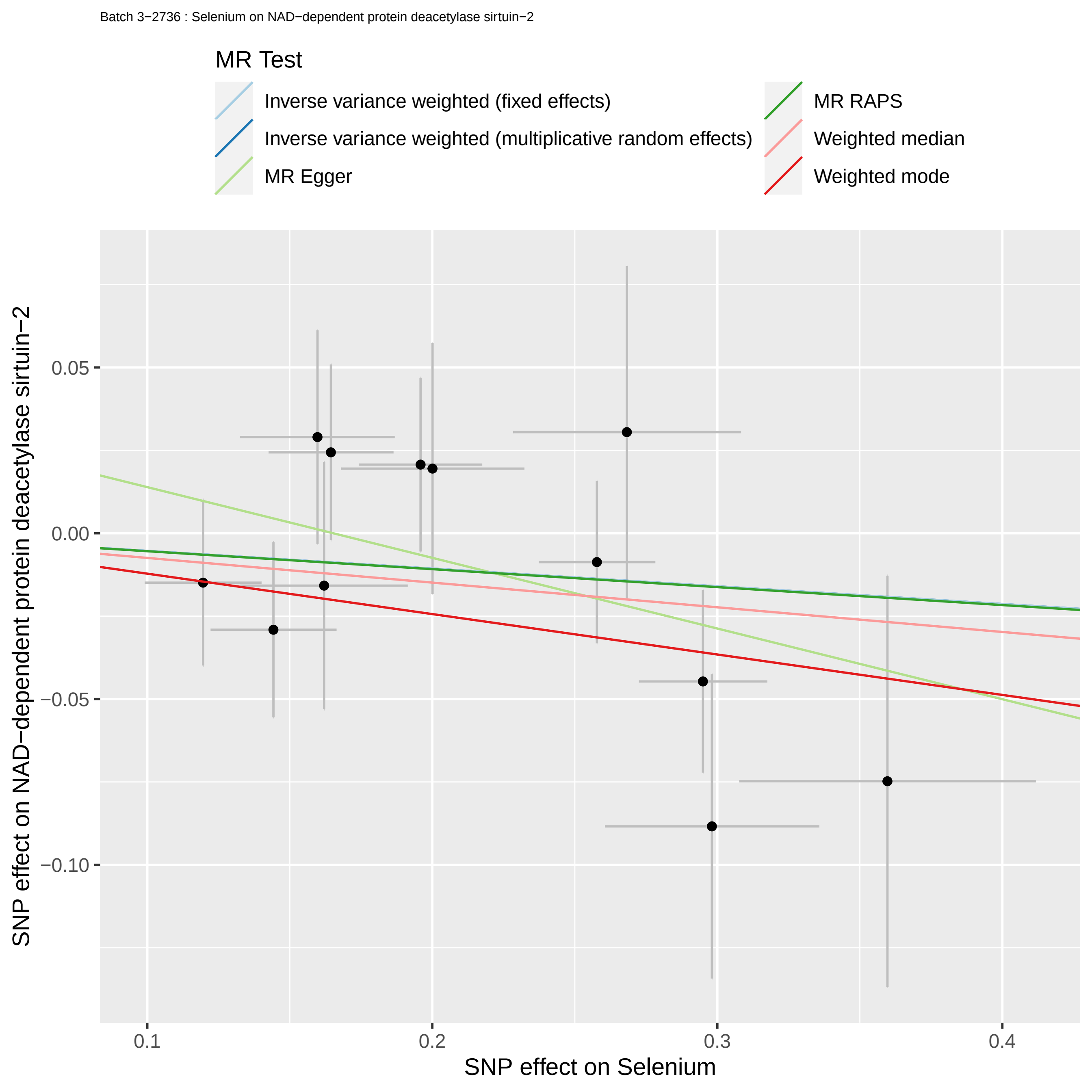

Batch 3−2736 : Selenium on NAD−dependent protein deacetylase sirtuin−2
MR Test
Inverse variance weighted (fixed effects)
Inverse variance weighted (multiplicative random effects)
MR Egger
MR RAPS
Weighted median
Weighted mode
SNP effect on NAD−dependent protein deacetylase sirtuin−2
0.05
0.00
−0.05
−0.10
0.1
0.2
0.3
0.4
SNP effect on Selenium

Batch 3−2737 : Selenium on NAD−dependent protein deacylase sirtuin−5, mitochondrial

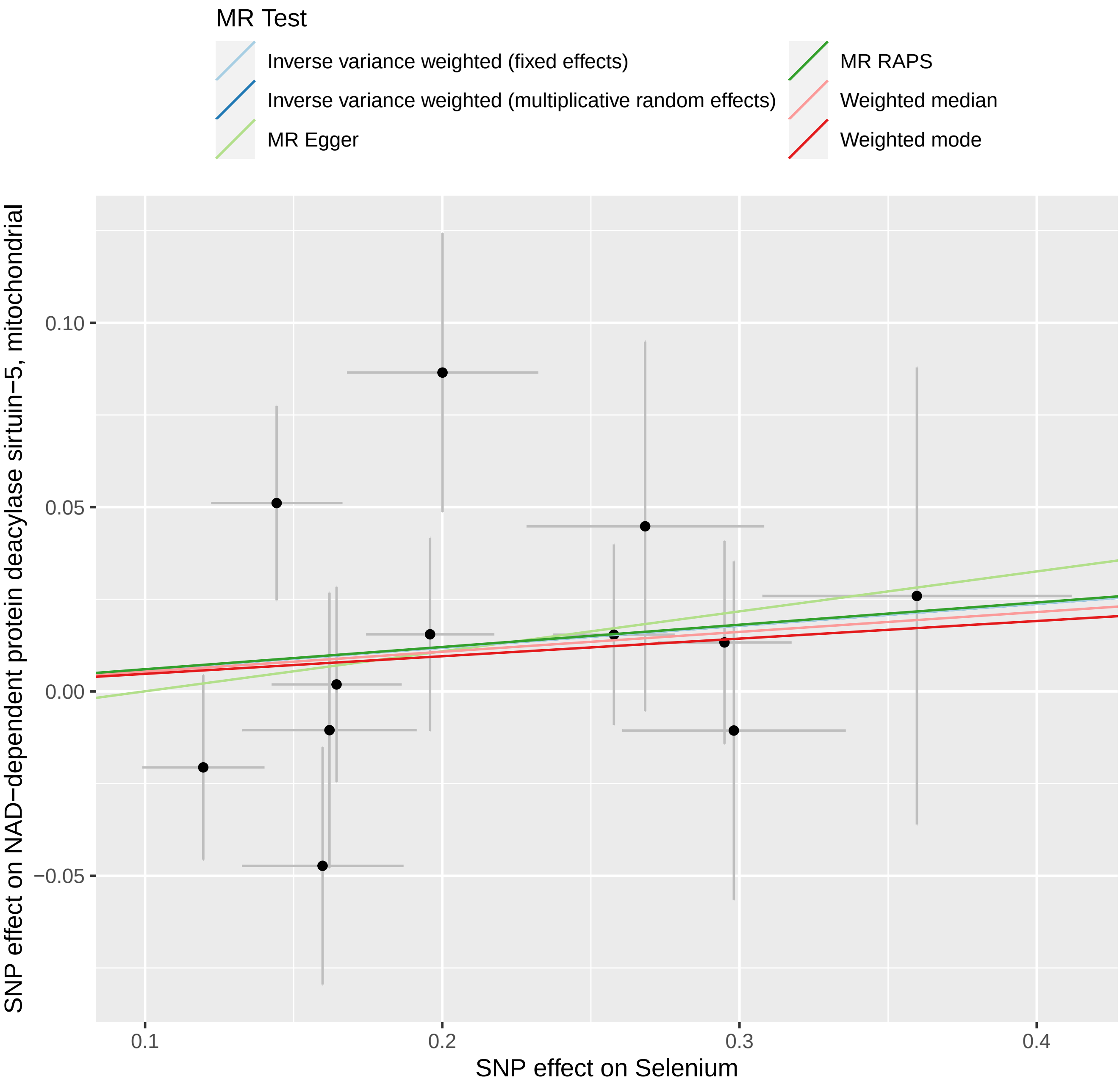

Batch 3−2738 : Selenium on Signaling threshold−regulating transmembrane adapter 1

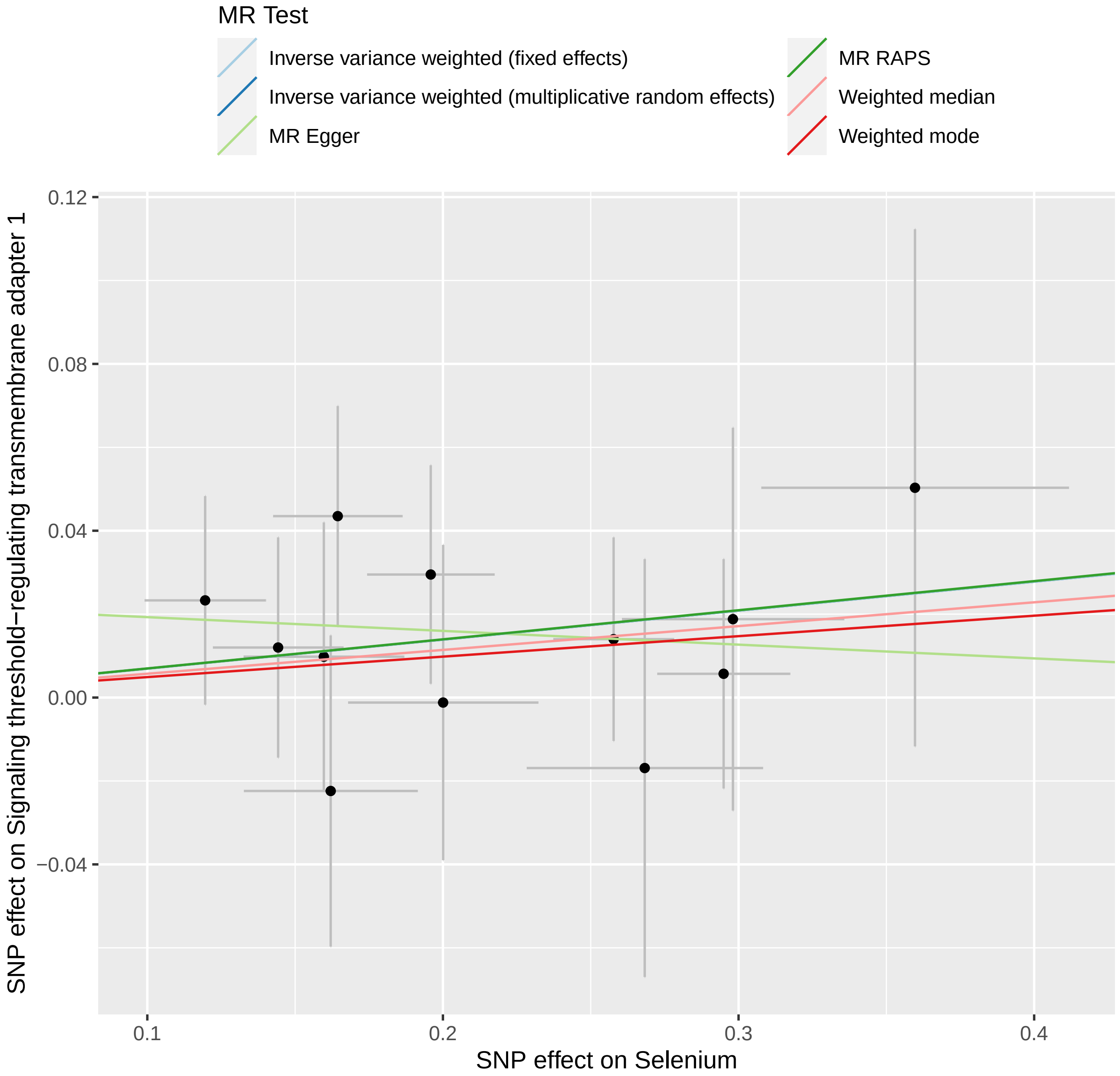

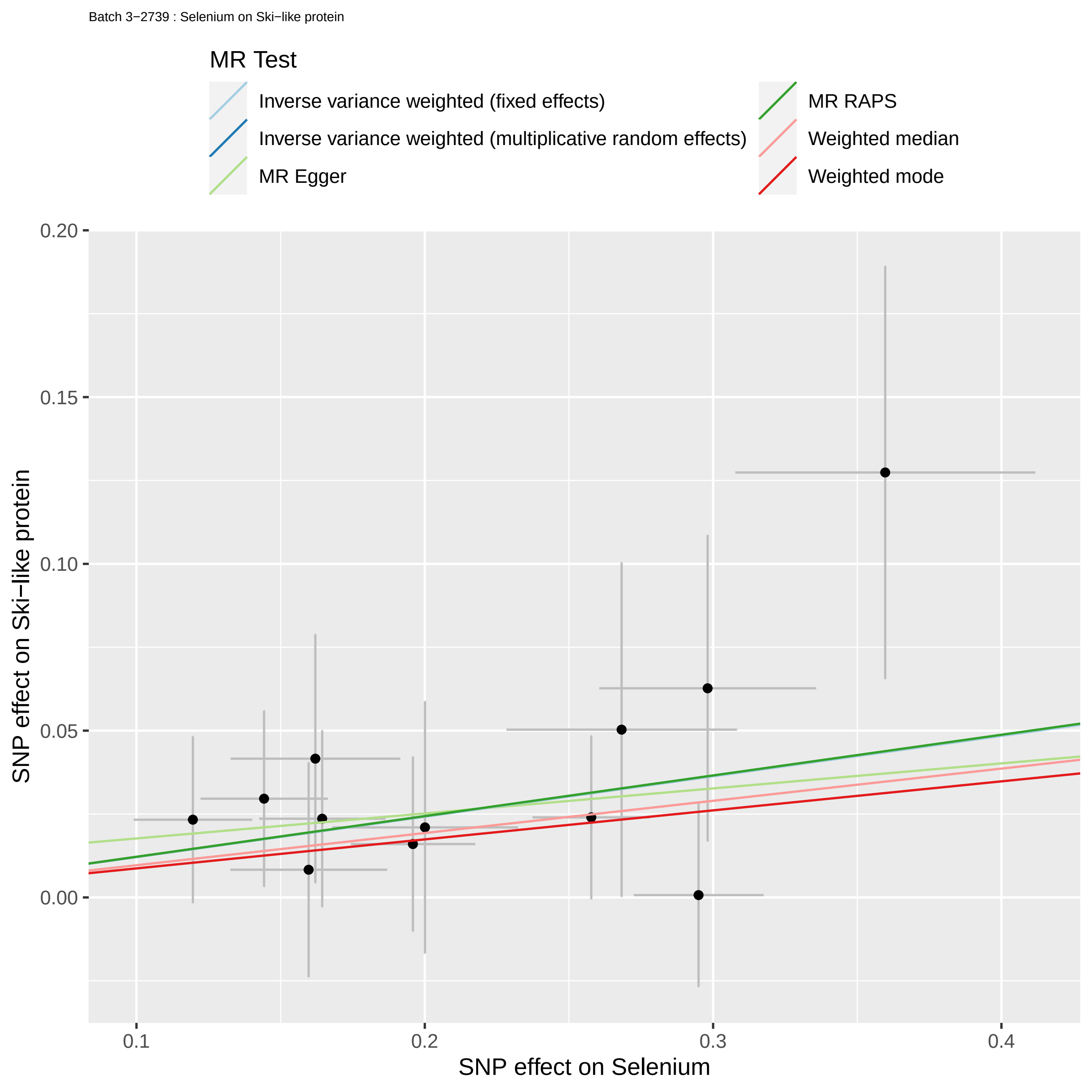

Batch 3−2739 : Selenium on Ski−like protein
MR Test
Inverse variance weighted (fixed effects)
Inverse variance weighted (multiplicative random effects)
MR Egger
MR RAPS
Weighted median
Weighted mode
0.20
0.15
0.10
0.05
0.00
0.1
0.2
0.3
0.4
SNP effect on Selenium
SNP effect on Ski−like protein

Batch 3−2740 : Selenium on S−phase kinase−associated protein 1

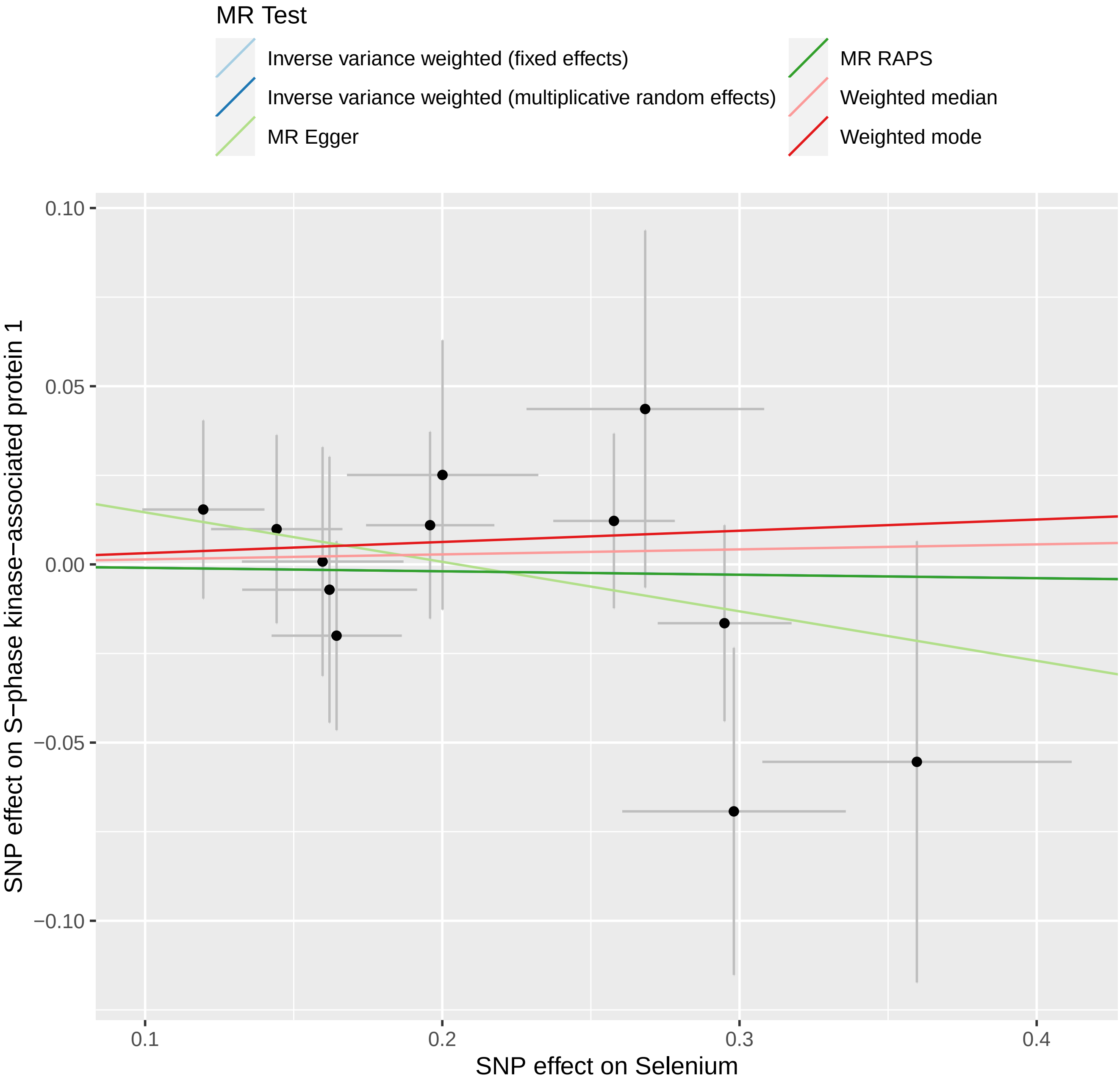

Batch 3−2741 : Selenium on Signaling lymphocytic activation molecule

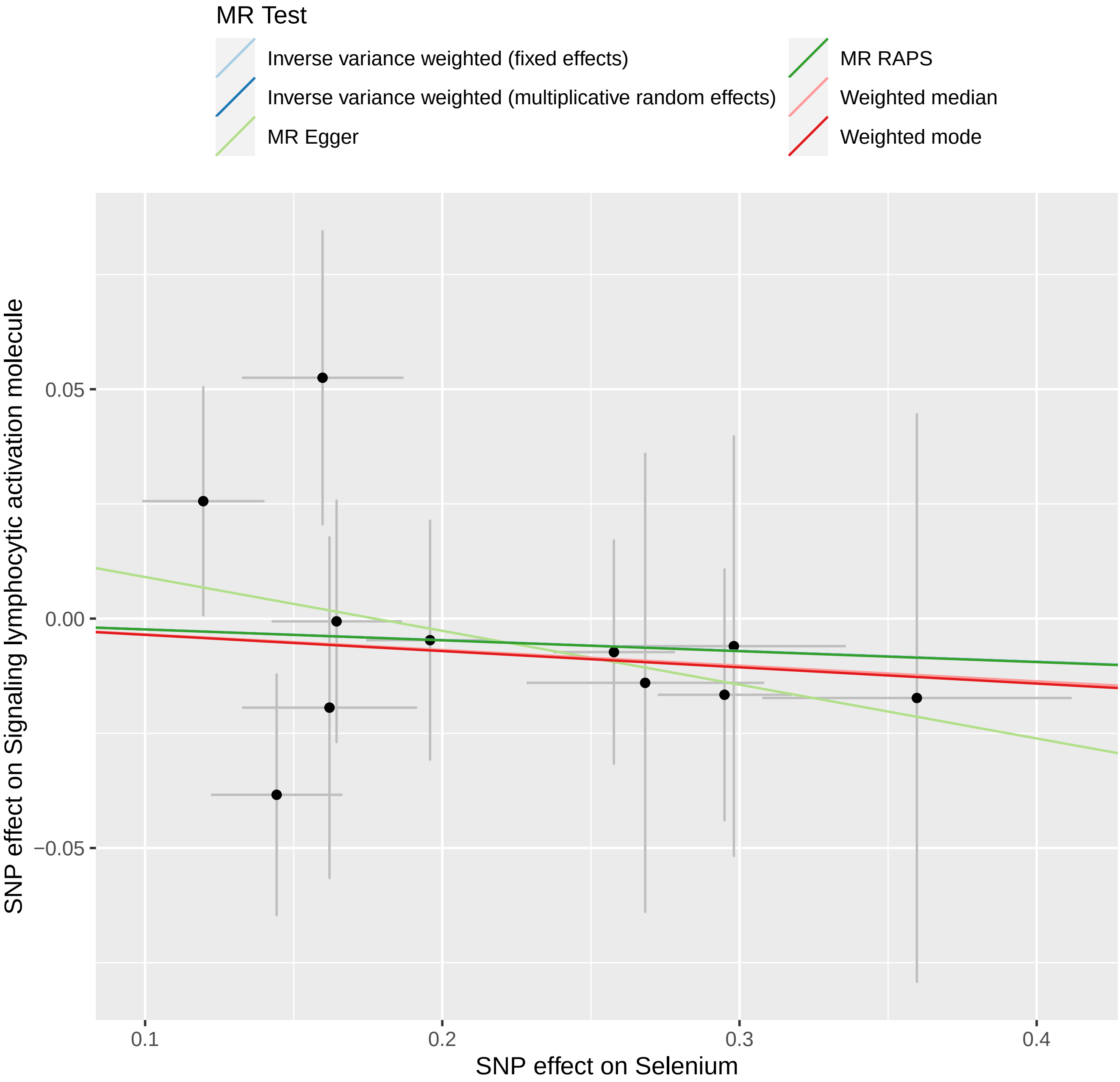

Batch 3−2742 : Selenium on SLAM family member 6

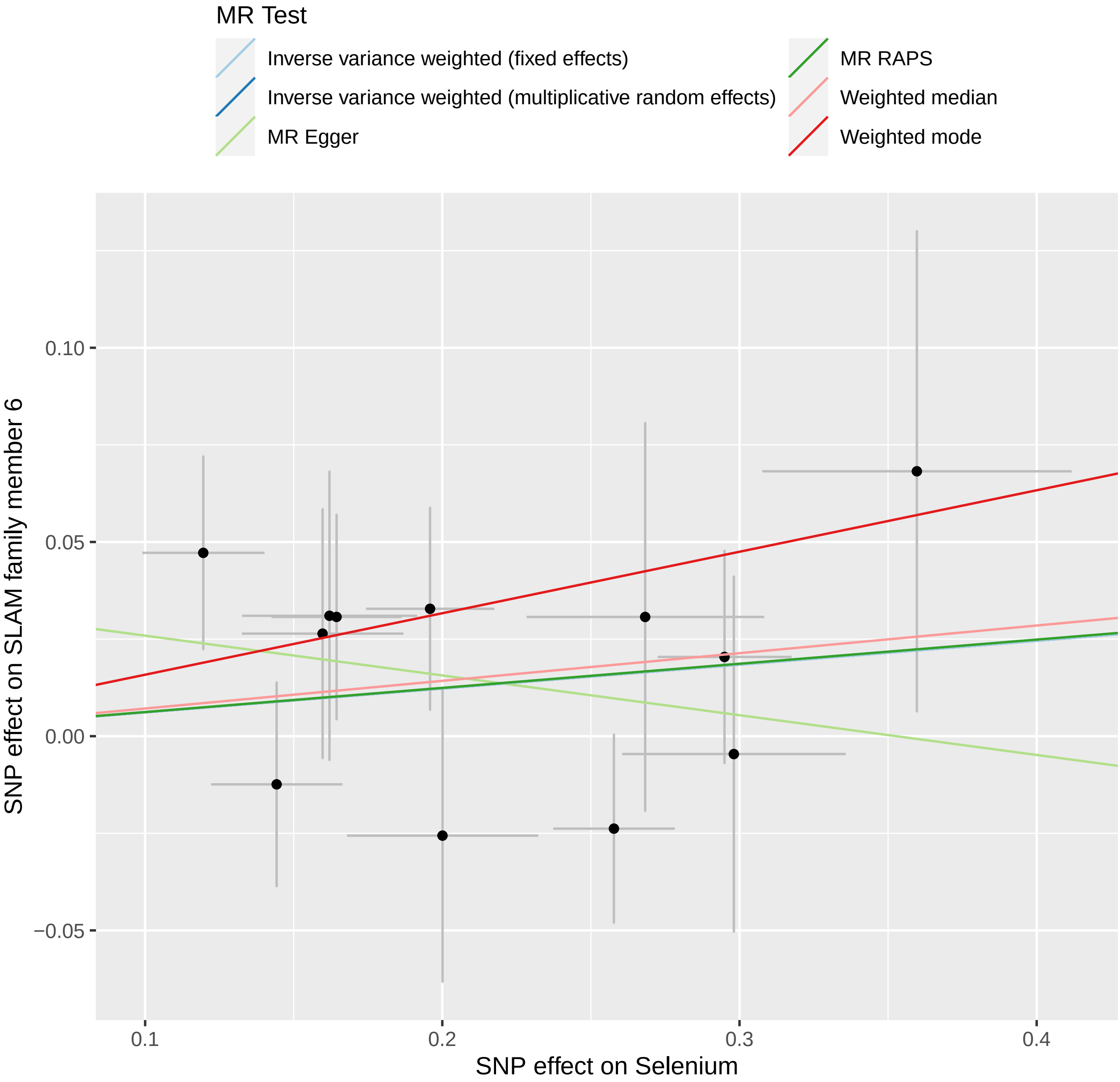

Batch 3−2743 : Selenium on SLAM family member 7

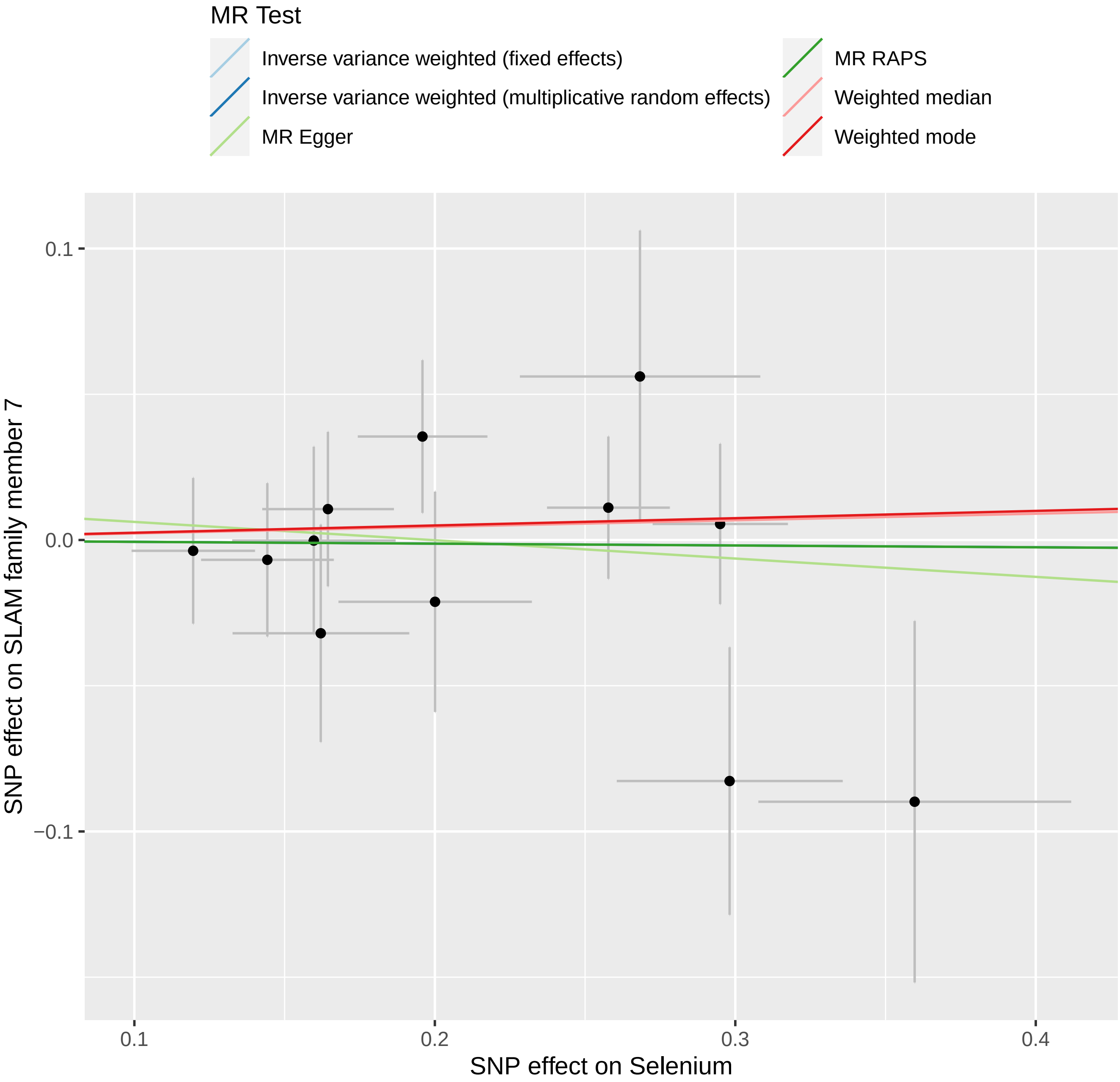

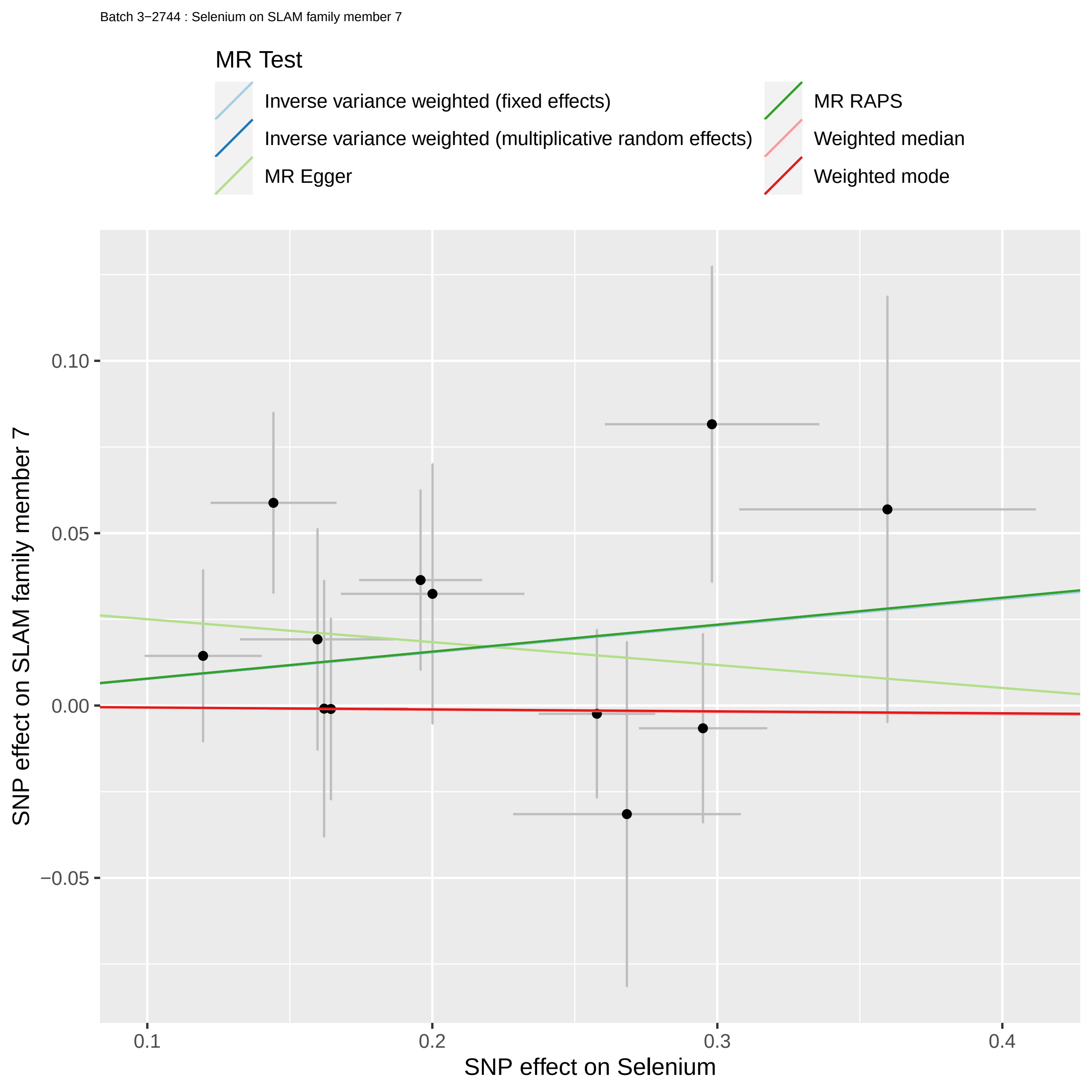

Batch 3−2744 : Selenium on SLAM family member 7
MR Test
Inverse variance weighted (fixed effects)
Inverse variance weighted (multiplicative random effects)
MR Egger
MR RAPS
Weighted median
Weighted mode
SNP effect on SLAM family member 7
0.10
0.05
0.00
−0.05
0.1
0.2
0.3
0.4
SNP effect on Selenium

Batch 3−2745 : Selenium on SLAM family member 8

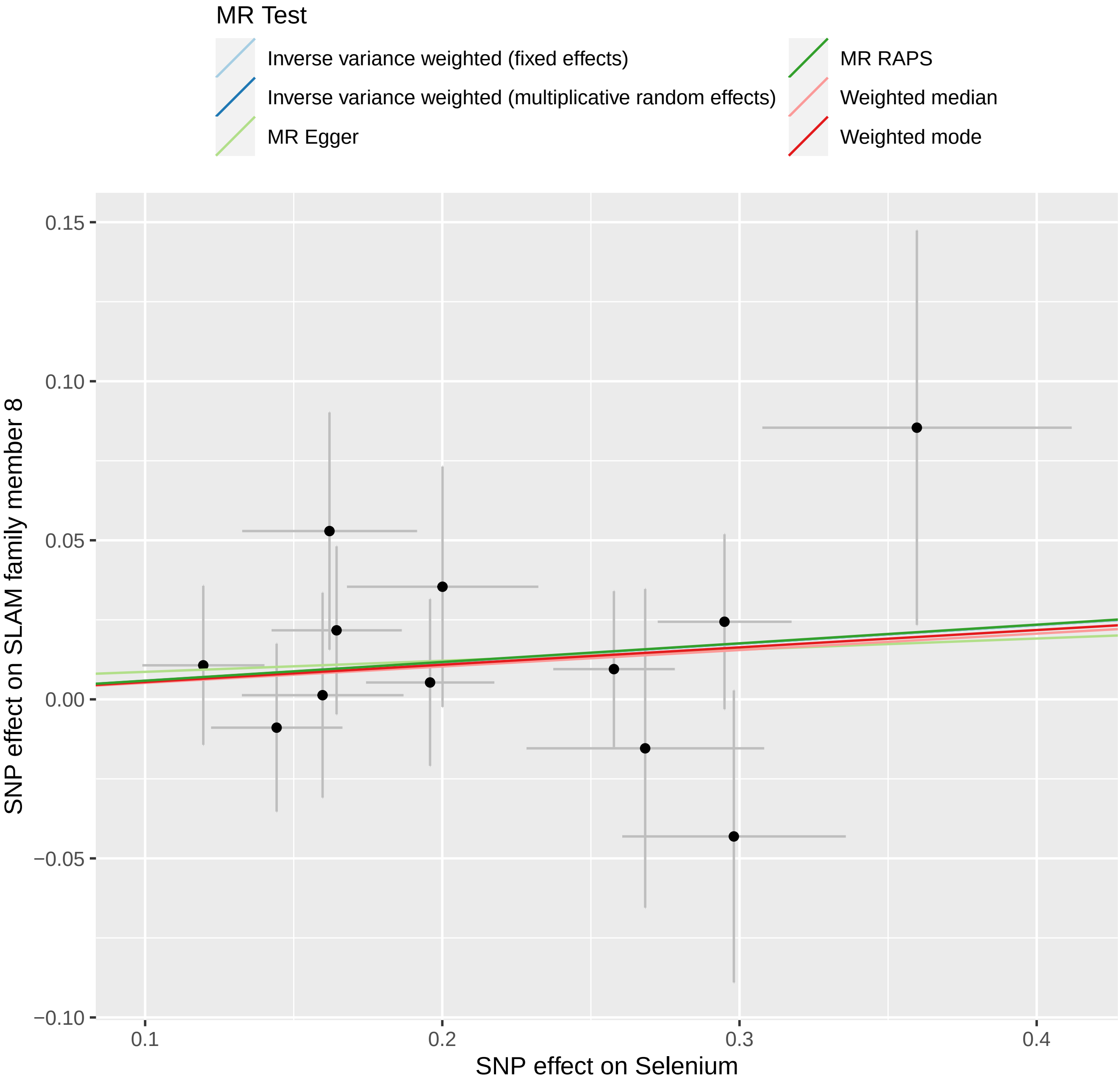

Batch 3−2746 : Selenium on Urea transporter 1

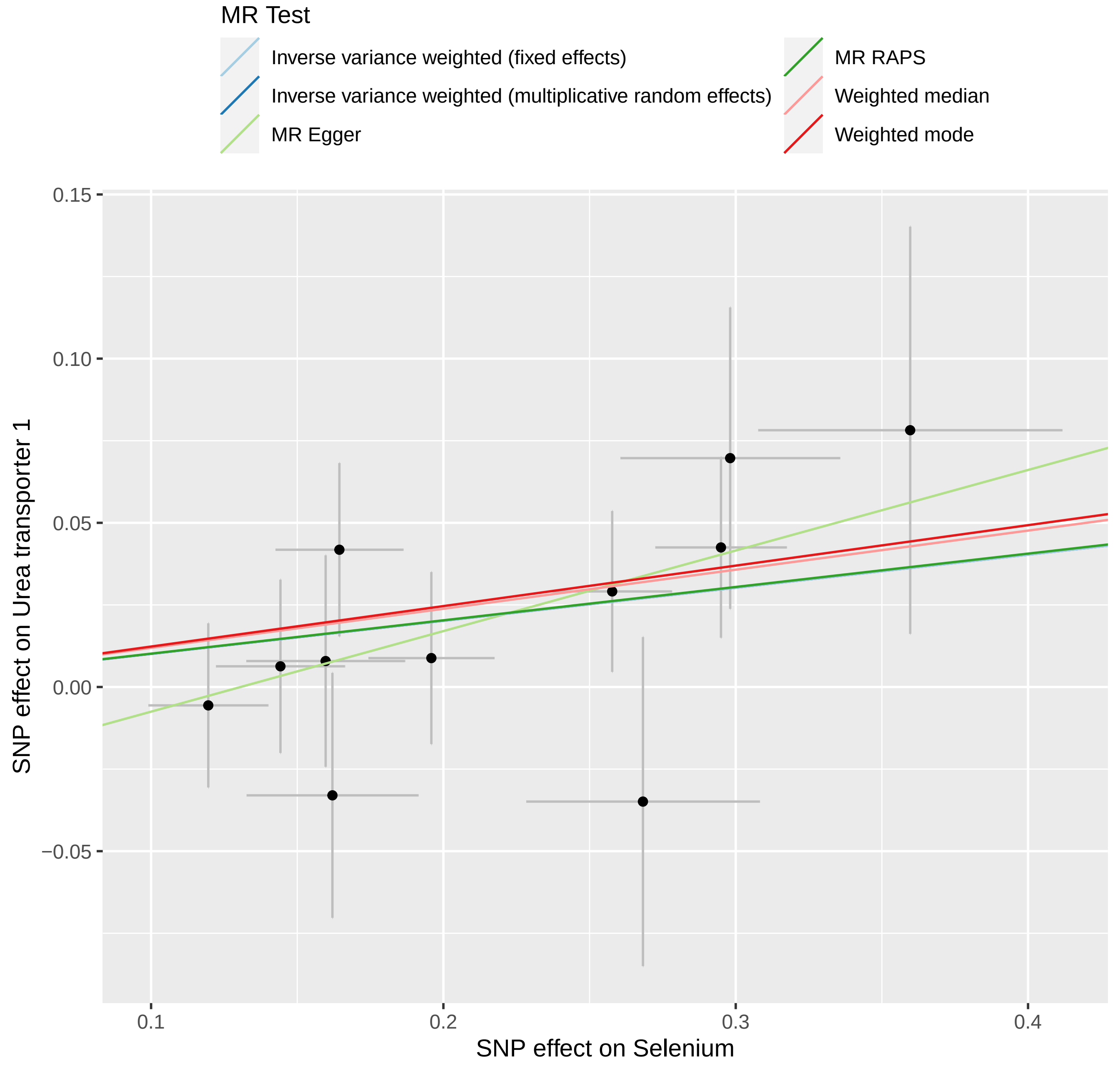

Batch 3−2747 : Selenium on Urea transporter 2

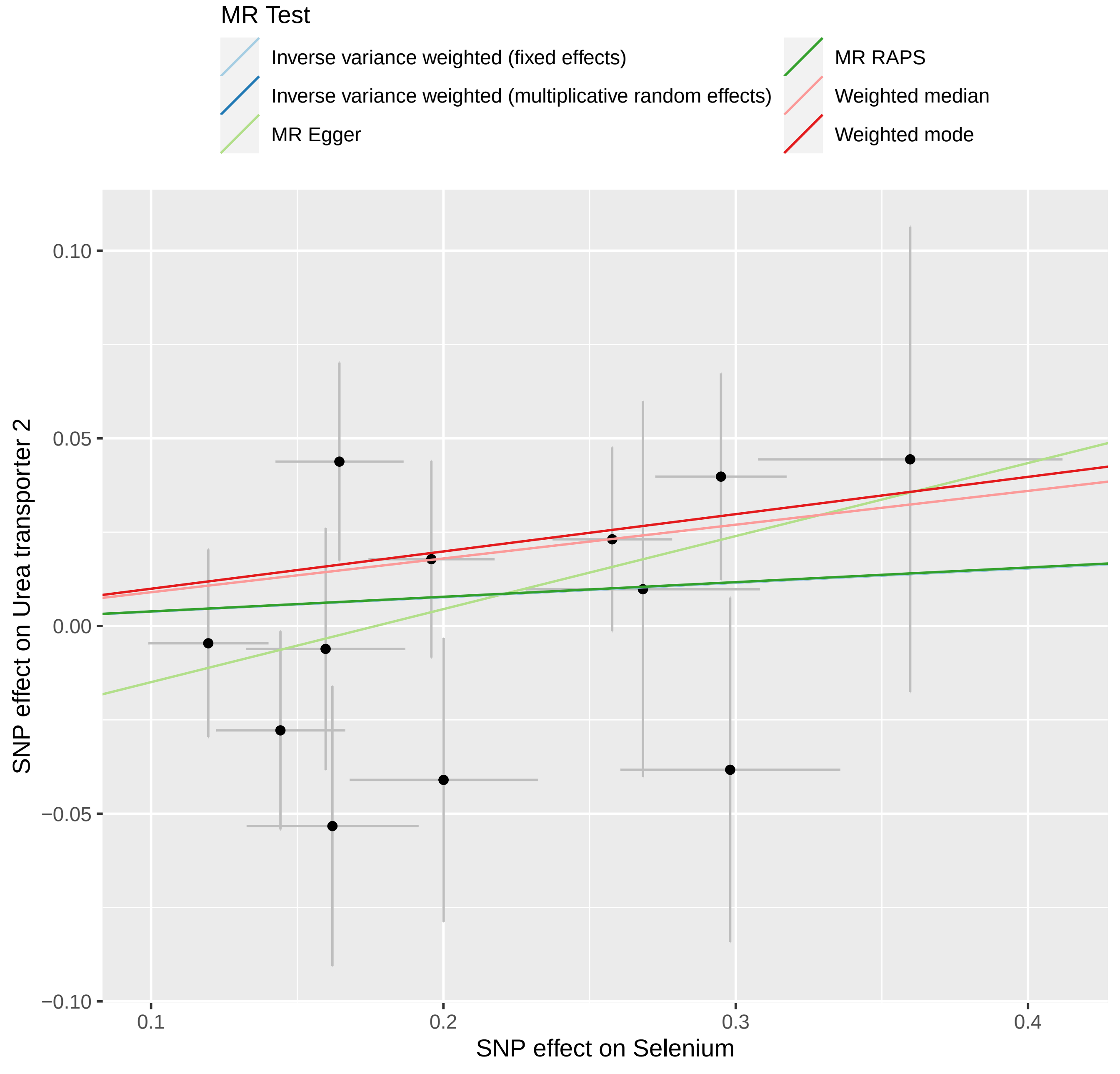

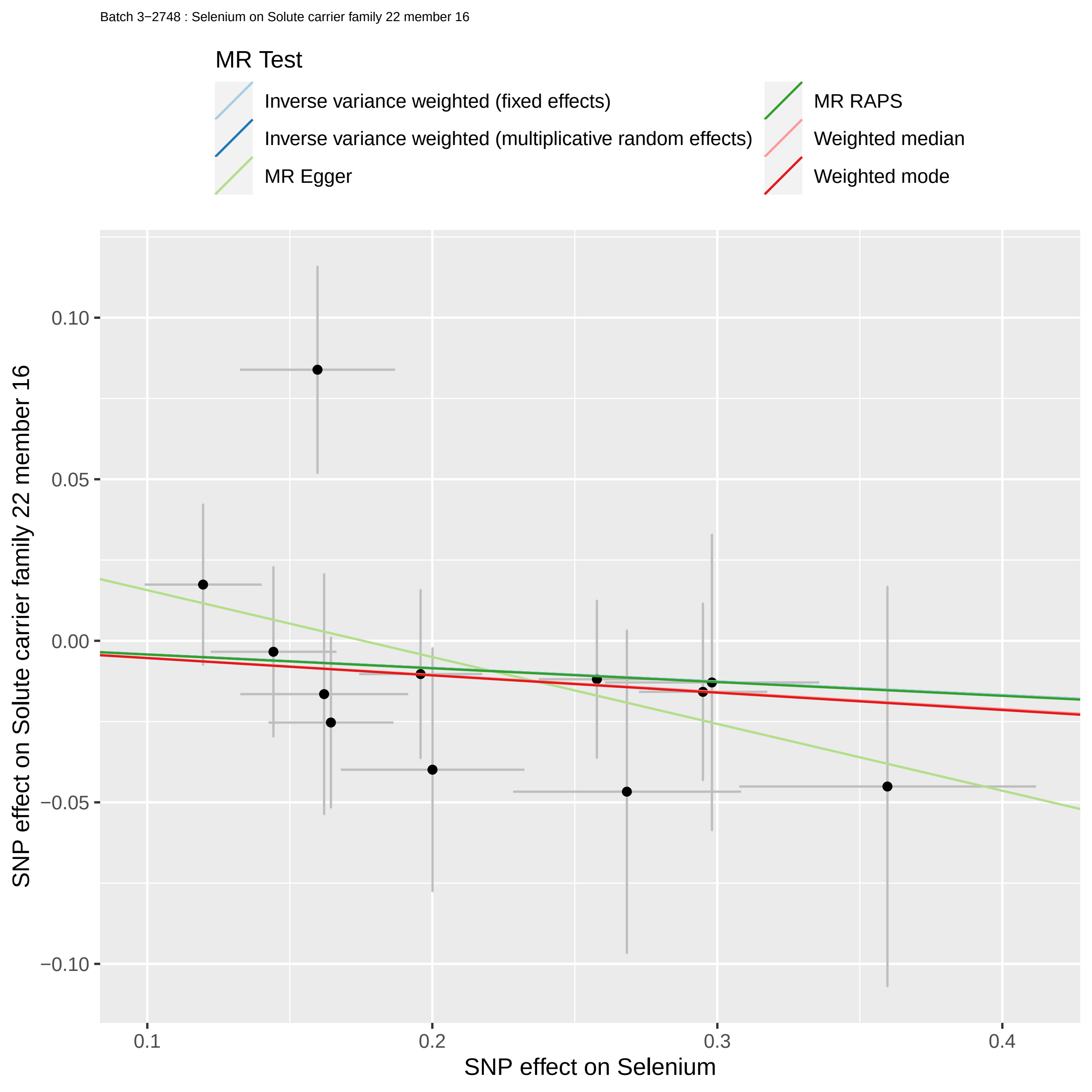
Batch 3−2748 : Selenium on Solute carrier family 22 member 16
MR Test
Inverse variance weighted (fixed effects)
Inverse variance weighted (multiplicative random effects)
MR Egger
MR RAPS
Weighted median
Weighted mode
0.10
0.05
0.00
−0.05
−0.10
SNP effect on Solute carrier family 22 member 16
0.1
0.2
0.3
0.4
SNP effect on Selenium

Batch 3−2749 : Selenium on Mitochondrial glutamate carrier 2

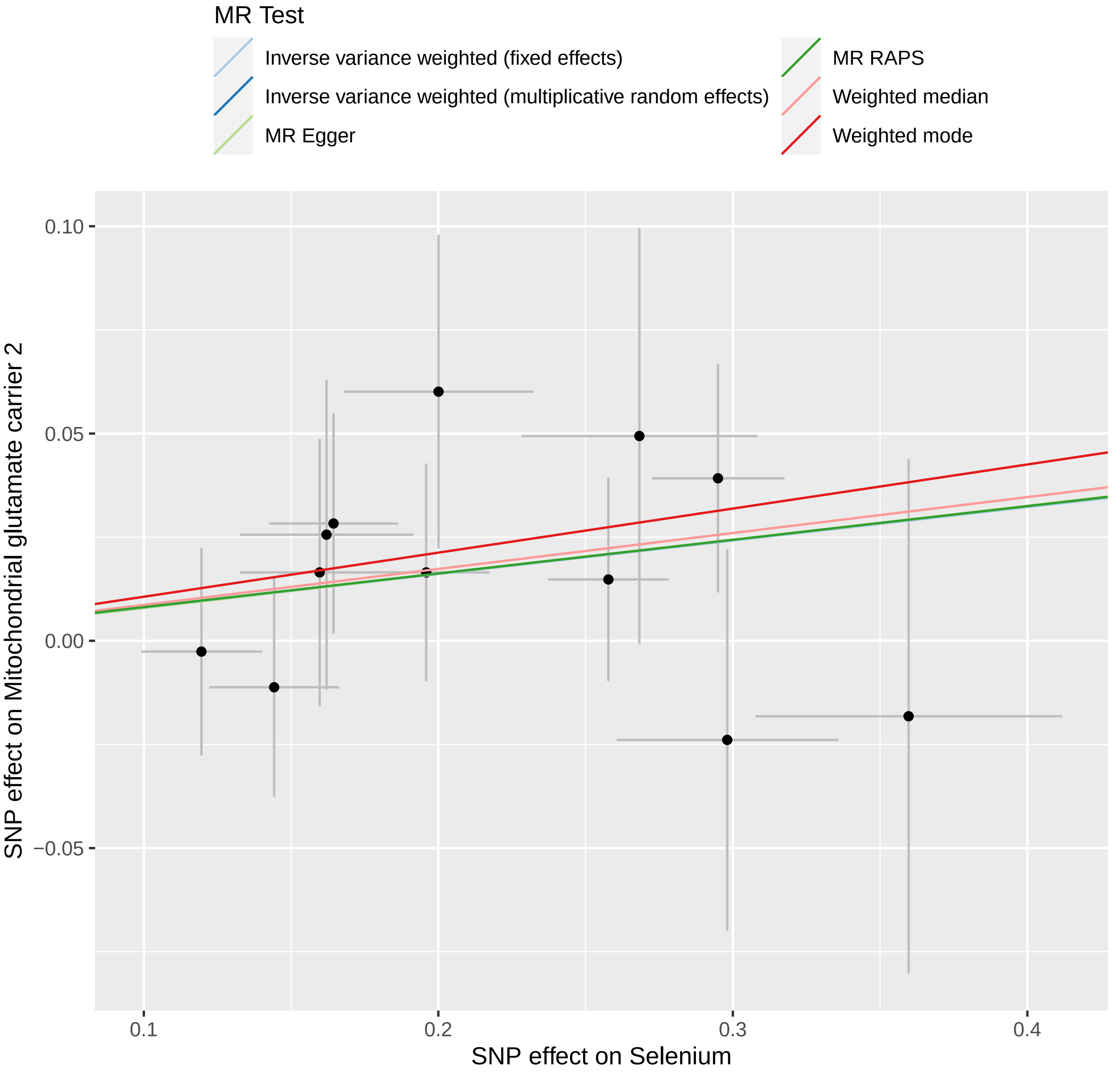

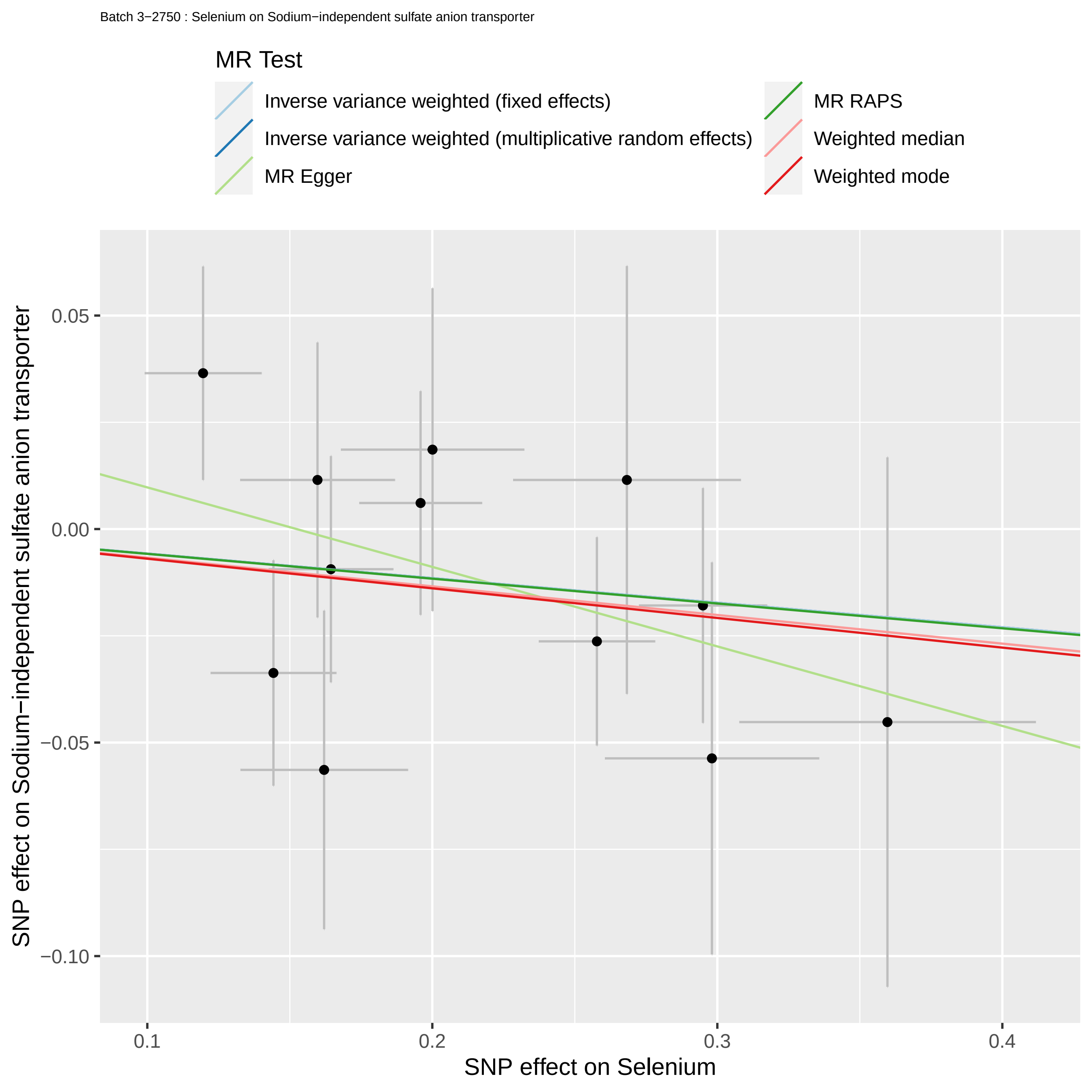

Batch 3−2750 : Selenium on Sodium−independent sulfate anion transporter
MR Test
Inverse variance weighted (fixed effects)
Inverse variance weighted (multiplicative random effects)
MR Egger
MR RAPS
Weighted median
Weighted mode
0.05
0.00
−0.05
−0.10
0.1
0.2
0.3
0.4
SNP effect on Sodium−independent sulfate anion transporter
SNP effect on Selenium

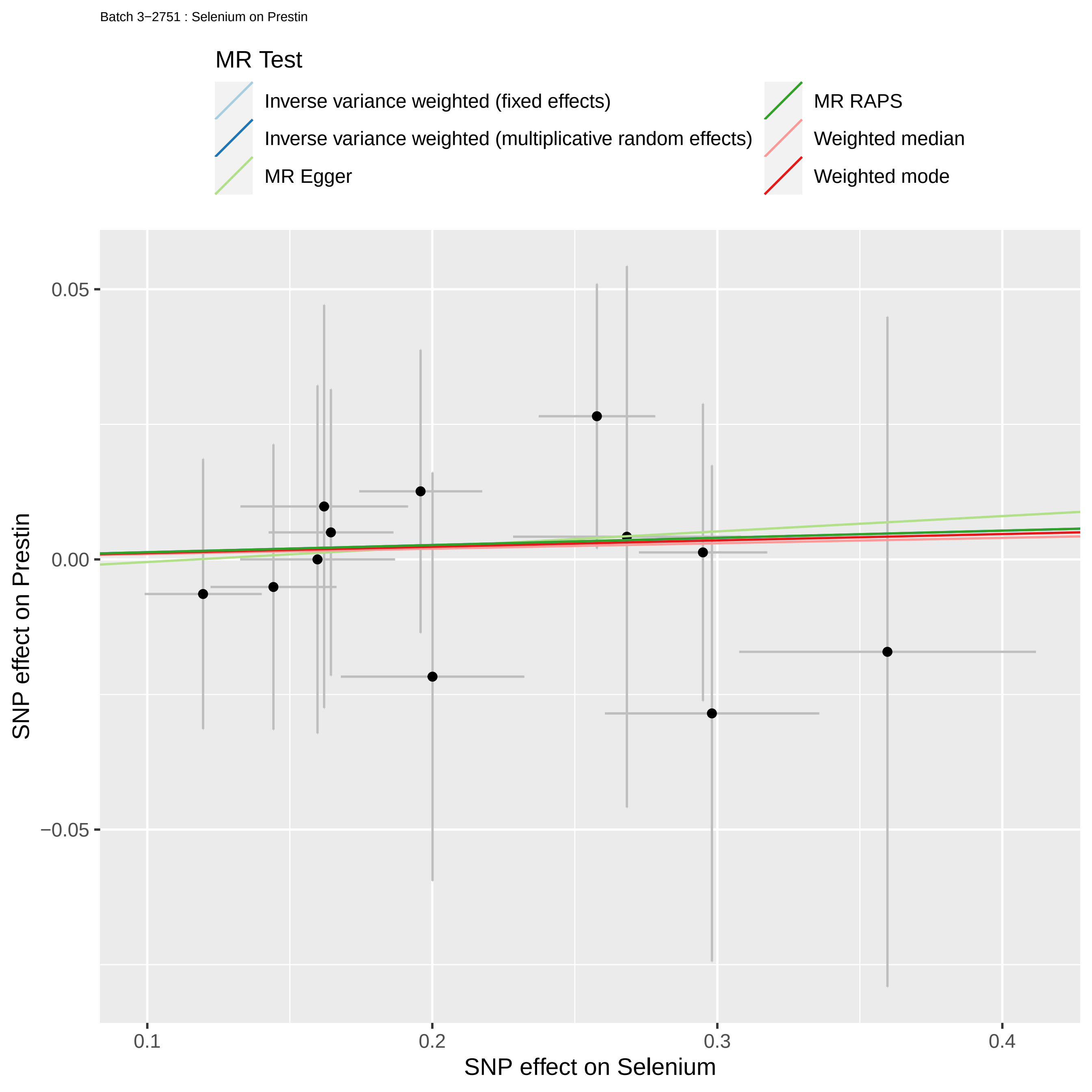
Batch 3−2751 : Selenium on Prestin
MR Test
Inverse variance weighted (fixed effects)
Inverse variance weighted (multiplicative random effects)
MR Egger
MR RAPS
Weighted median
Weighted mode
0.05
0.00
−0.05
0.1
0.2
0.3
0.4
SNP effect on Selenium
SNP effect on Prestin

Batch 3−2752 : Selenium on Very long−chain acyl−CoA synthetase

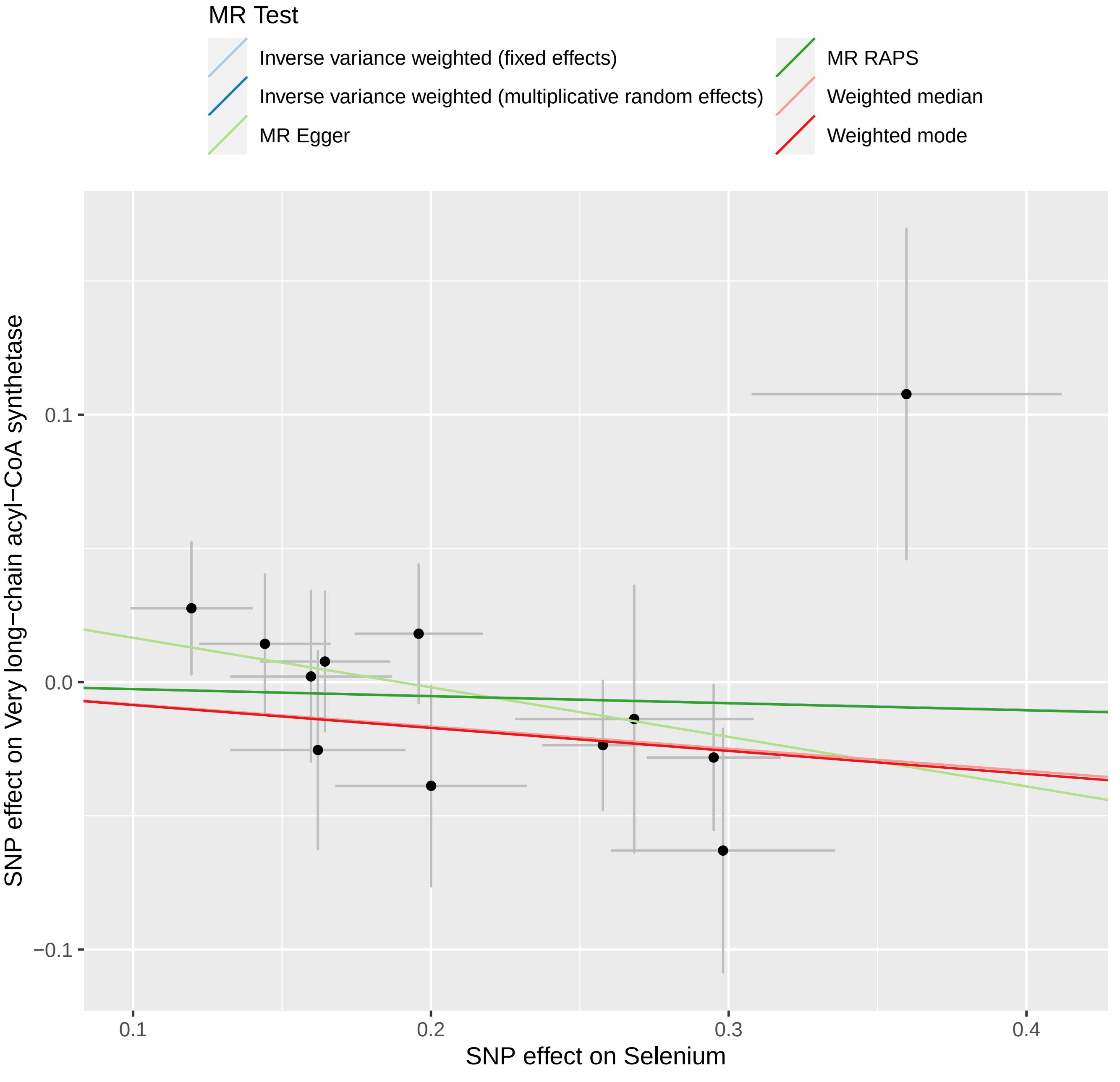

Batch 3−2753 : Selenium on Zinc transporter 3

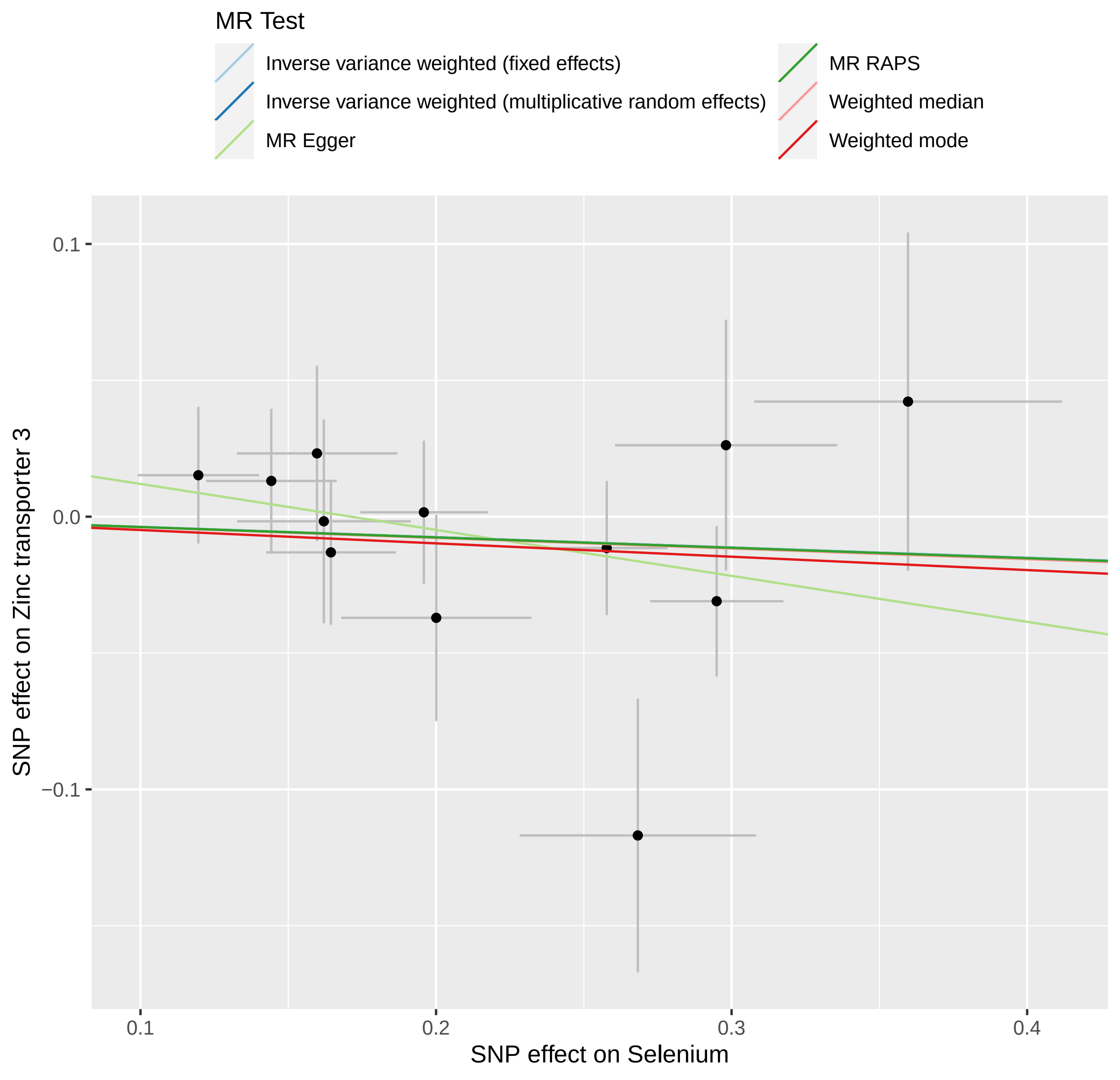

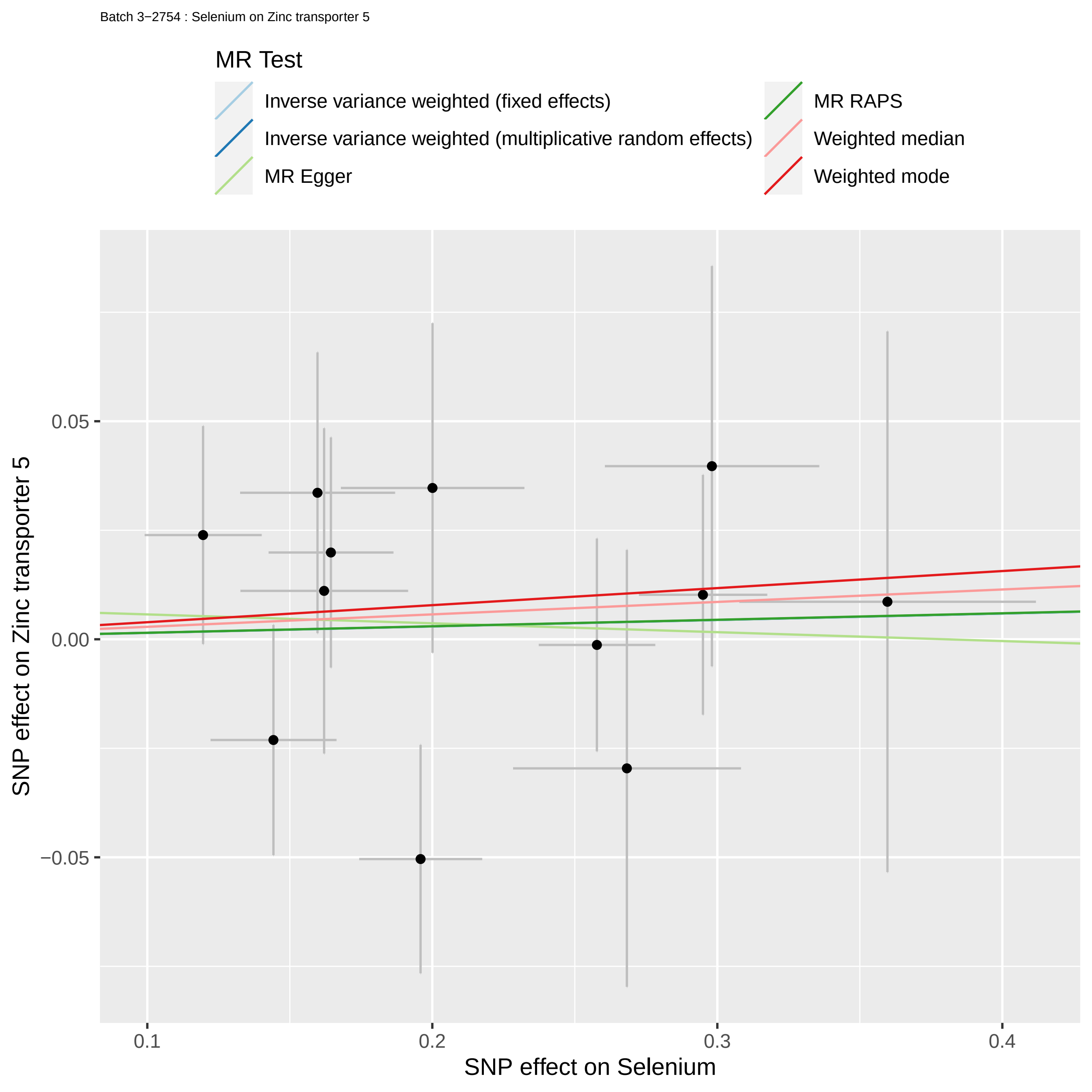
Batch 3−2754 : Selenium on Zinc transporter 5
MR Test
Inverse variance weighted (fixed effects)
Inverse variance weighted (multiplicative random effects)
MR Egger
MR RAPS
Weighted median
Weighted mode
0.05
0.00
−0.05
0.1
0.2
0.3
0.4
SNP effect on Zinc transporter 5
SNP effect on Selenium

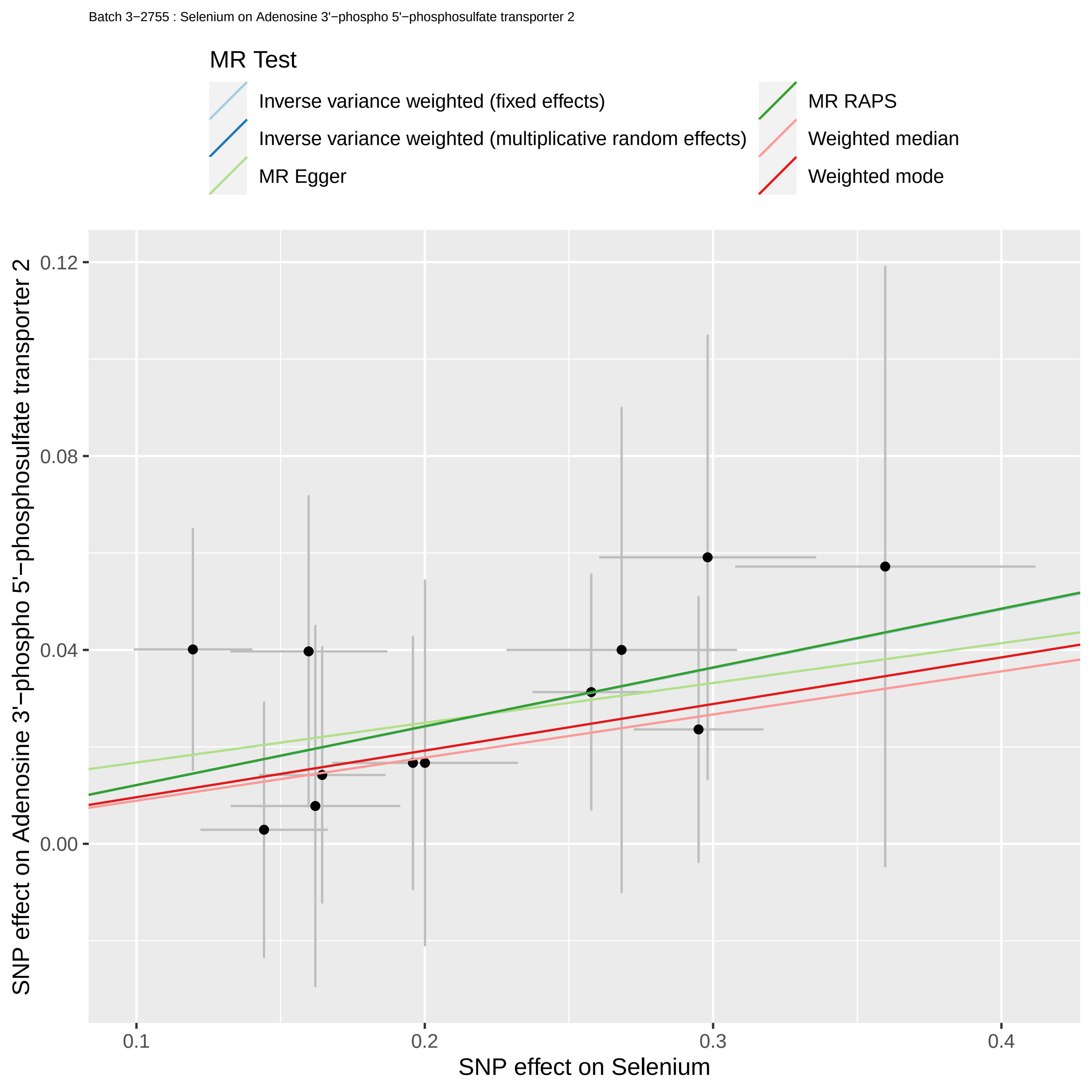
Batch 3−2755 : Selenium on Adenosine 3'−phospho 5'−phosphosulfate transporter 2
MR Test
Inverse variance weighted (fixed effects)
Inverse variance weighted (multiplicative random effects)
MR Egger
MR RAPS
Weighted median
Weighted mode
0.12
0.08
0.04
0.00
0.1
0.2
0.3
0.4
SNP effect on Selenium
SNP effect on Adenosine 3'−phospho 5'−phosphosulfate transporter 2

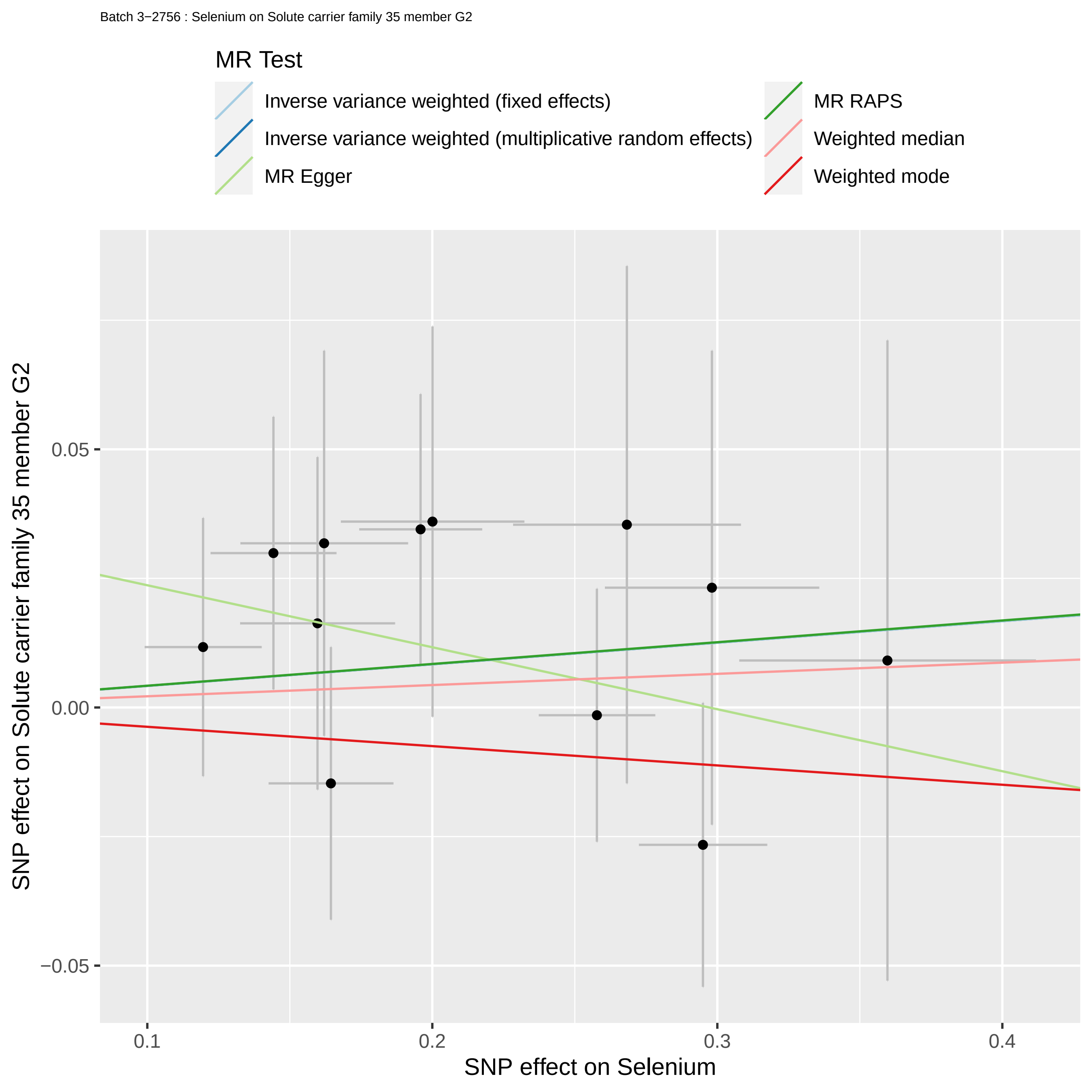

Batch 3−2756 : Selenium on Solute carrier family 35 member G2
MR Test
Inverse variance weighted (fixed effects)
Inverse variance weighted (multiplicative random effects)
MR Egger
MR RAPS
Weighted median
Weighted mode
SNP effect on Solute carrier family 35 member G2
0.05
0.00
−0.05
0.1
0.2
0.3
0.4
SNP effect on Selenium

Batch 3−2757 : Selenium on Solute carrier family 41 member 2

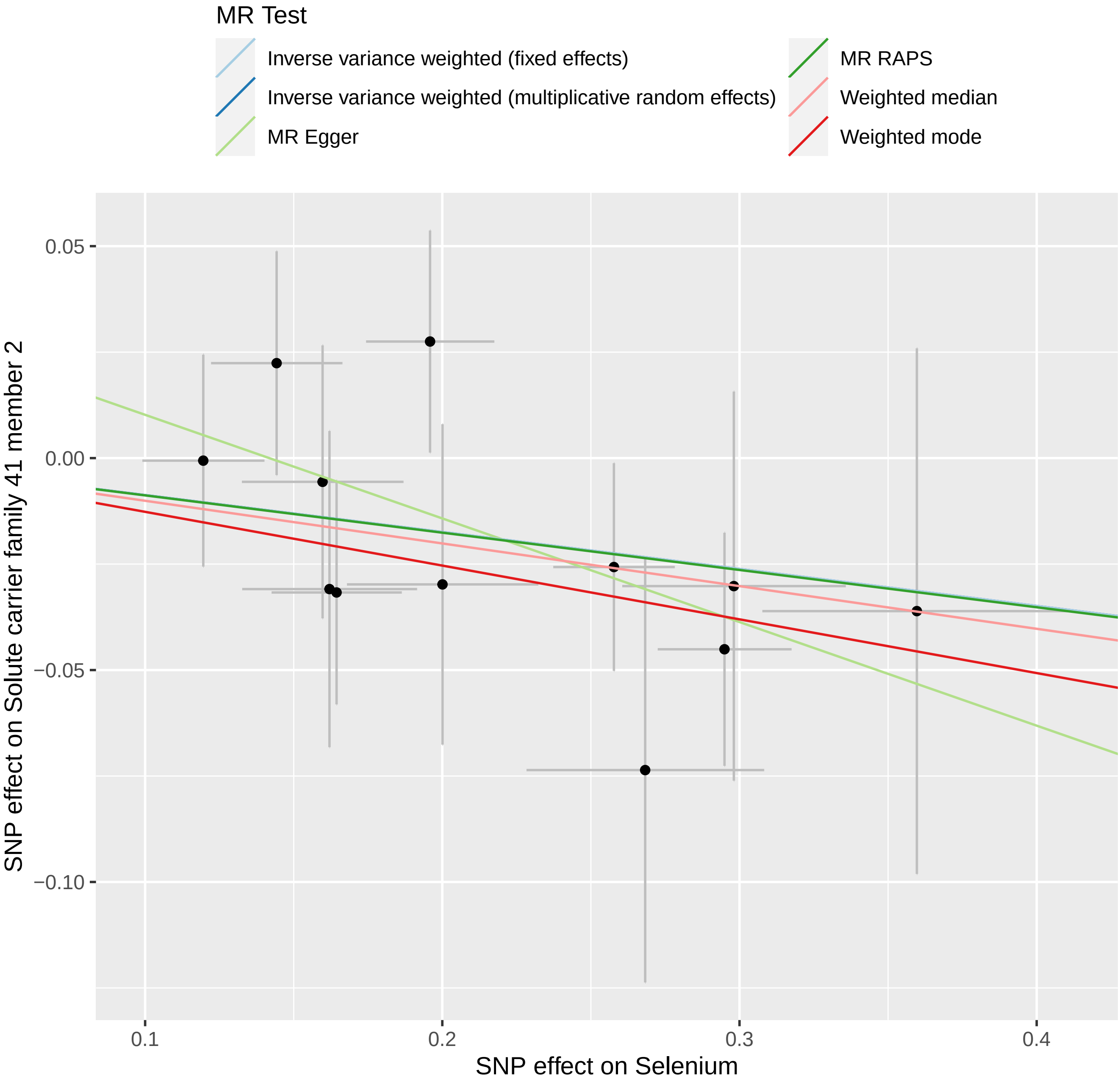

Batch 3−2758 : Selenium on Electroneutral sodium bicarbonate exchanger 1

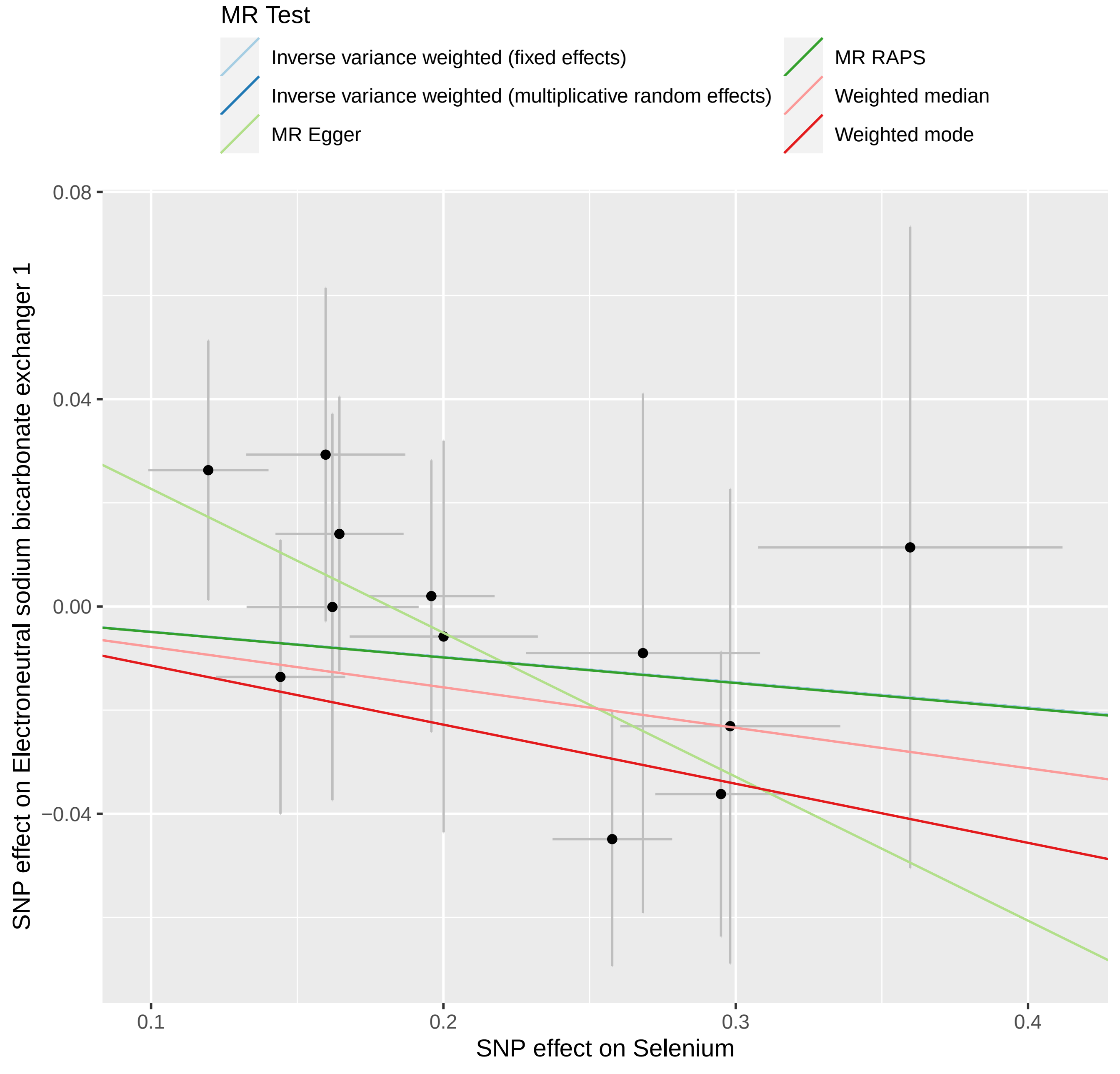

Batch 3−2759 : Selenium on Sodium/iodide cotransporter

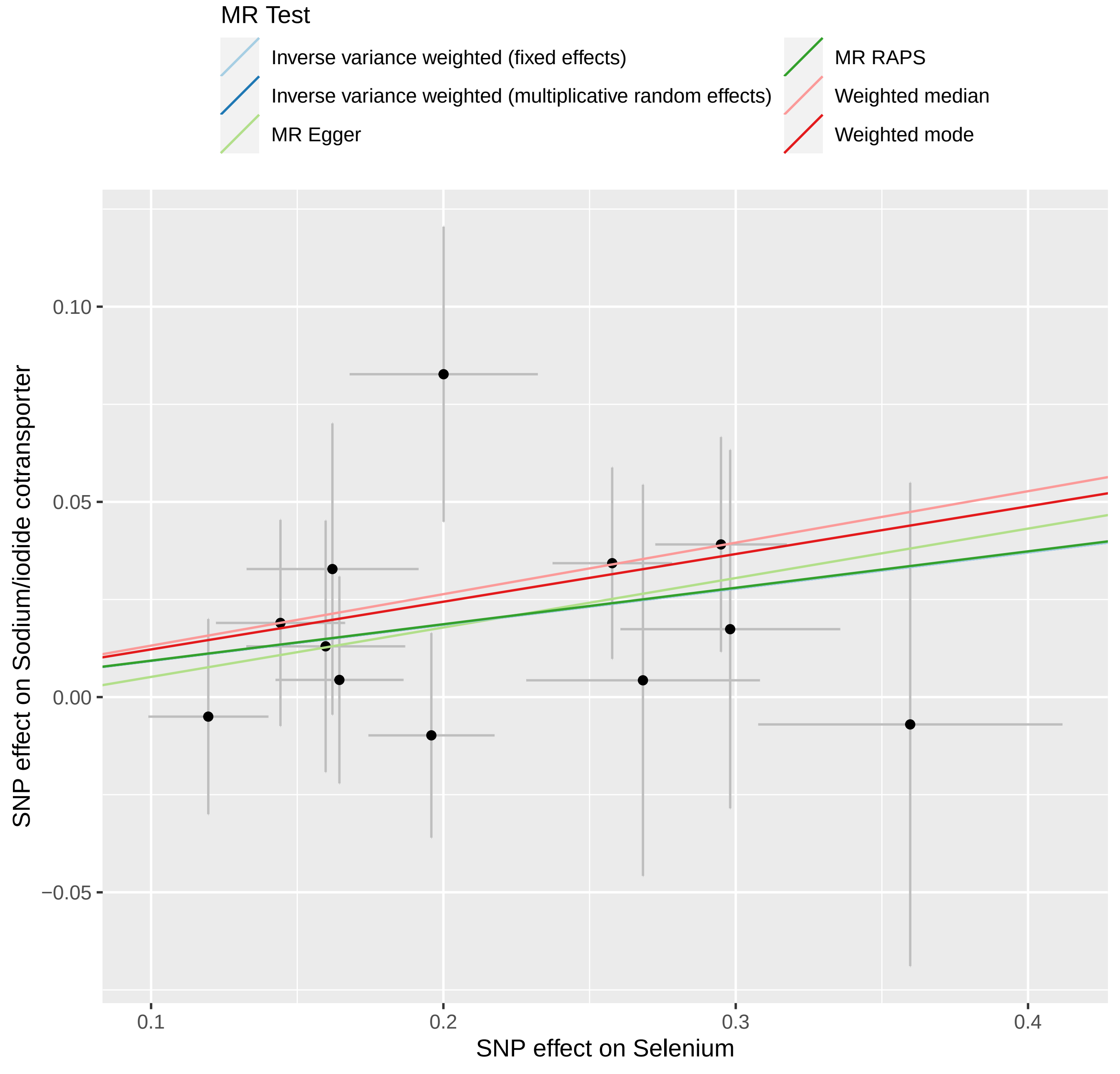

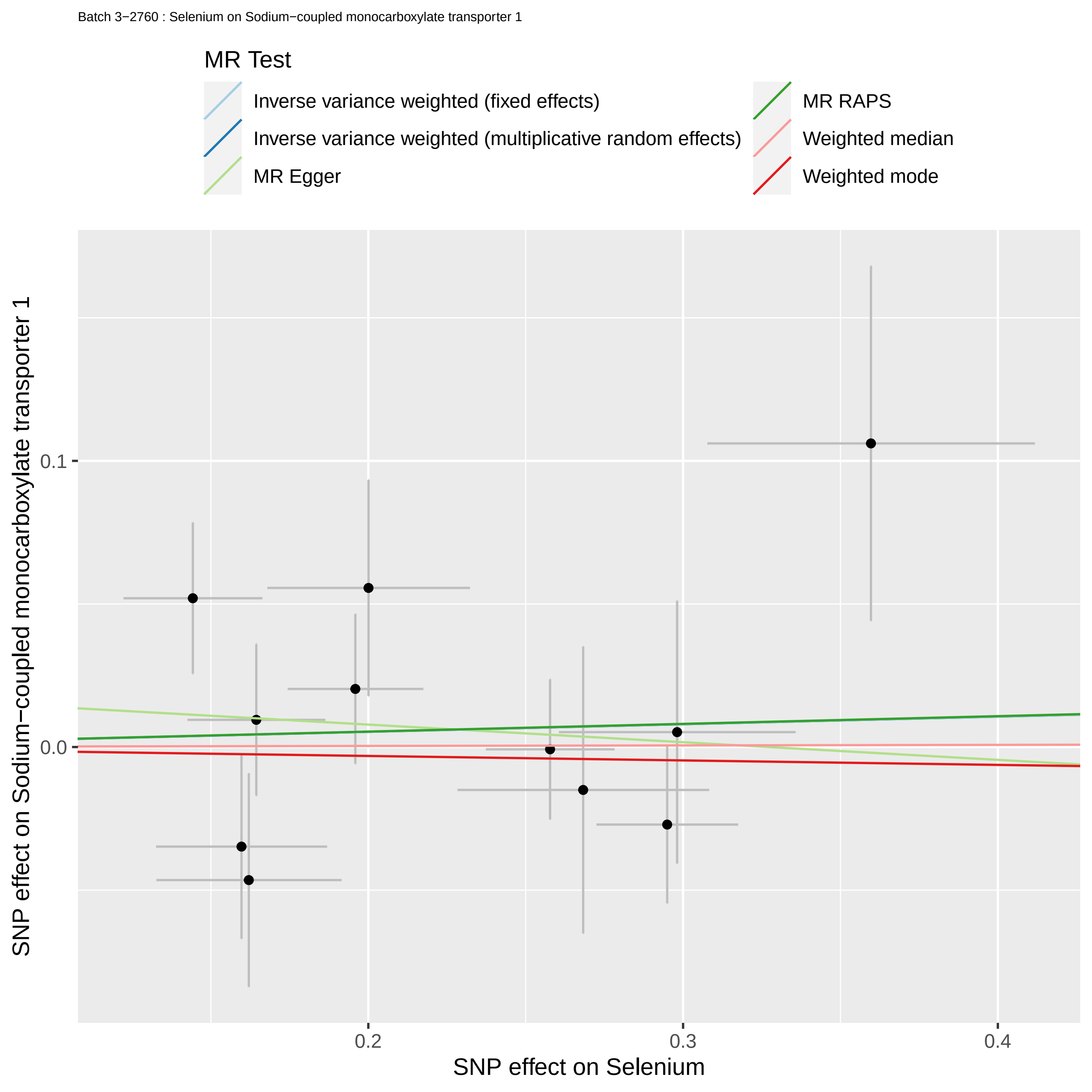

Batch 3−2760 : Selenium on Sodium−coupled monocarboxylate transporter 1
MR Test
Inverse variance weighted (fixed effects)
Inverse variance weighted (multiplicative random effects)
MR Egger
MR RAPS
Weighted median
Weighted mode
0.1
0.0
0.2
0.3
0.4
SNP effect on Sodium−coupled monocarboxylate transporter 1
SNP effect on Selenium

Batch 3−2761 : Selenium on Sodium− and chloride−dependent neutral and basic amino acid transporter B(0+)

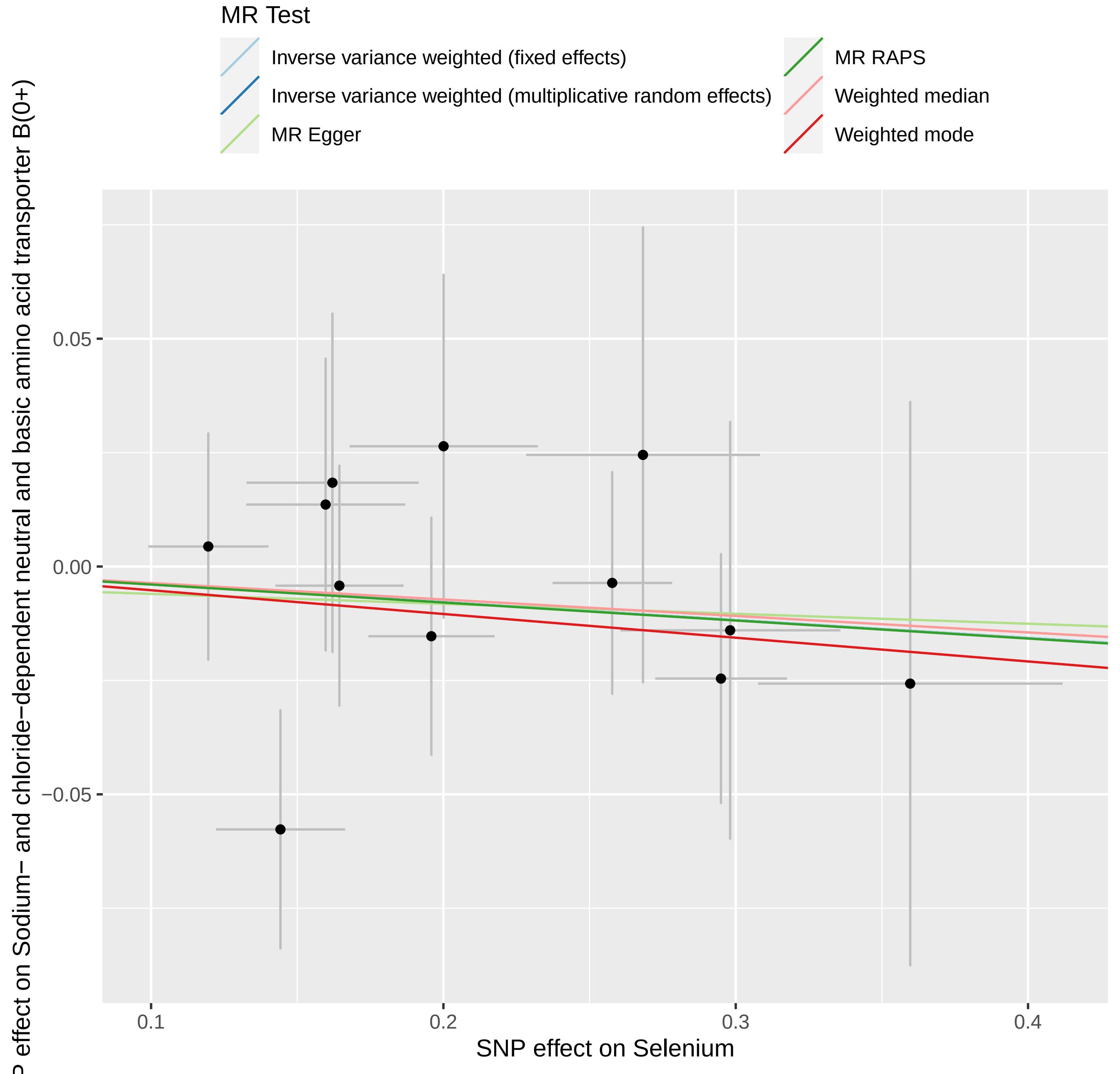

Batch 3−2762 : Selenium on Orphan sodium− and chloride−dependent neurotransmitter transporter NTT5

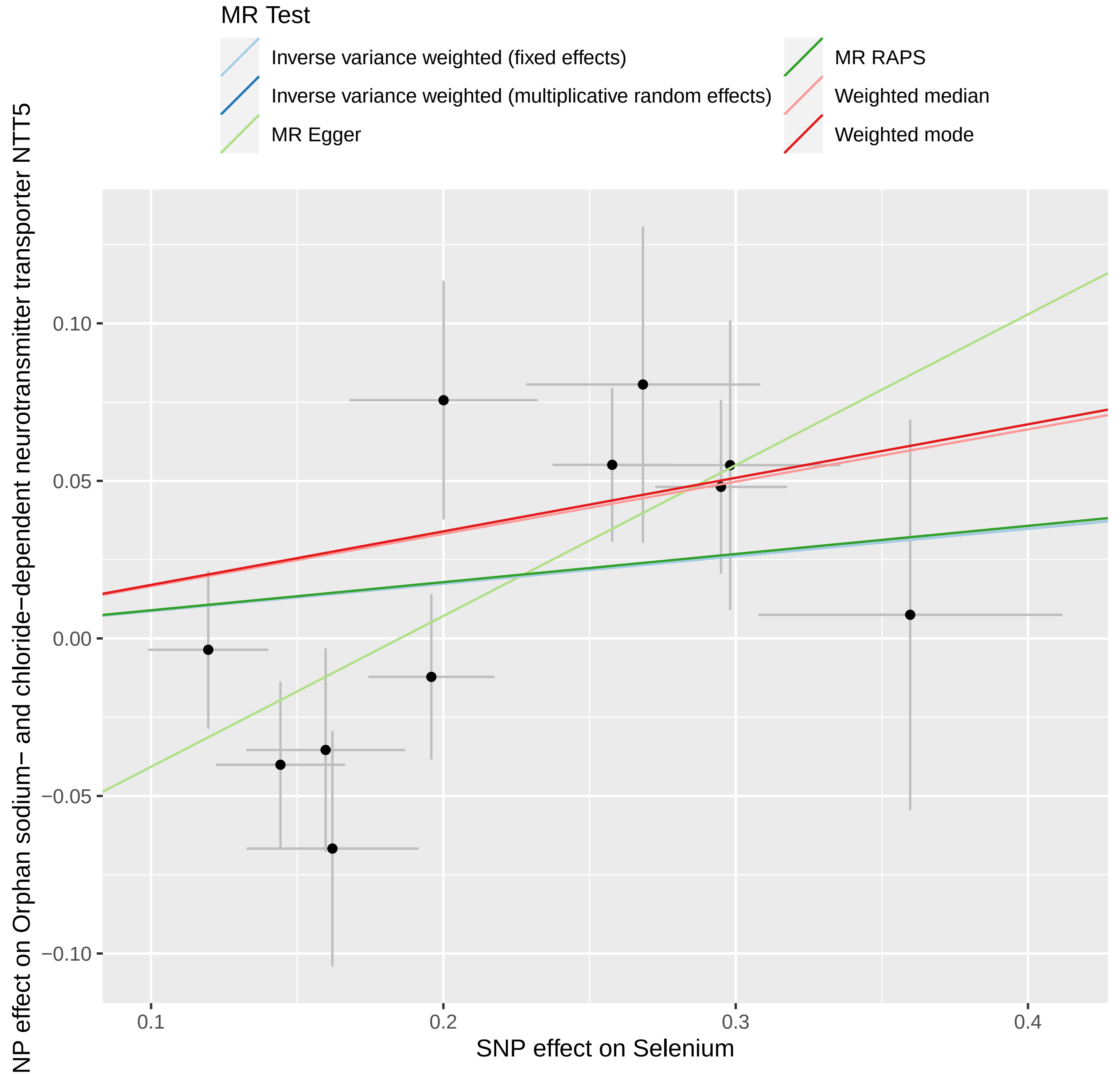

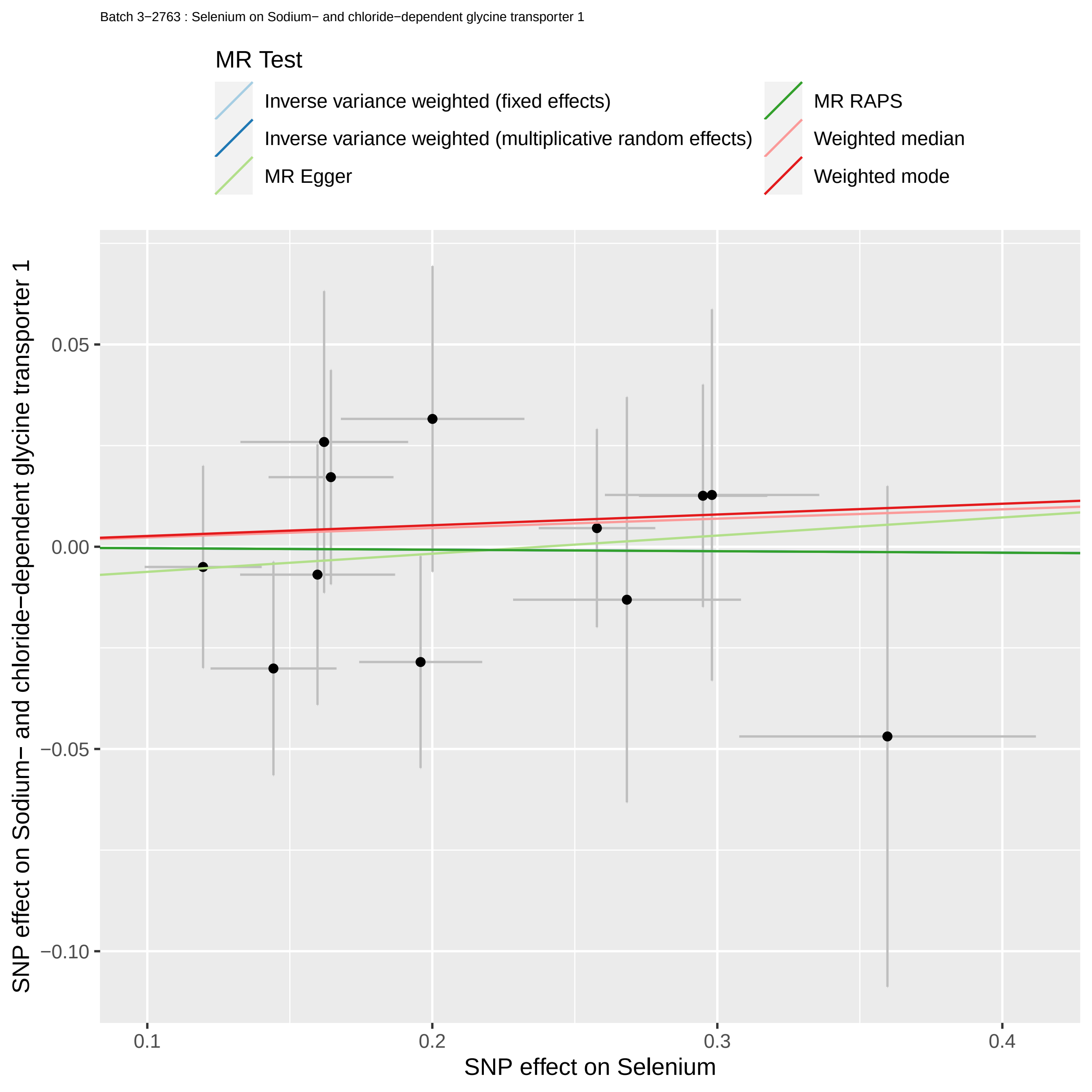

Batch 3−2763 : Selenium on Sodium− and chloride−dependent glycine transporter 1
MR Test
Inverse variance weighted (fixed effects)
Inverse variance weighted (multiplicative random effects)
MR Egger
MR RAPS
Weighted median
Weighted mode
SNP effect on Sodium− and chloride−dependent glycine transporter 1
0.05
0.00
−0.05
−0.10
0.1
0.2
0.3
0.4
SNP effect on Selenium

Batch 3−2764 : Selenium on Mitochondrial sodium/hydrogen exchanger 9B2

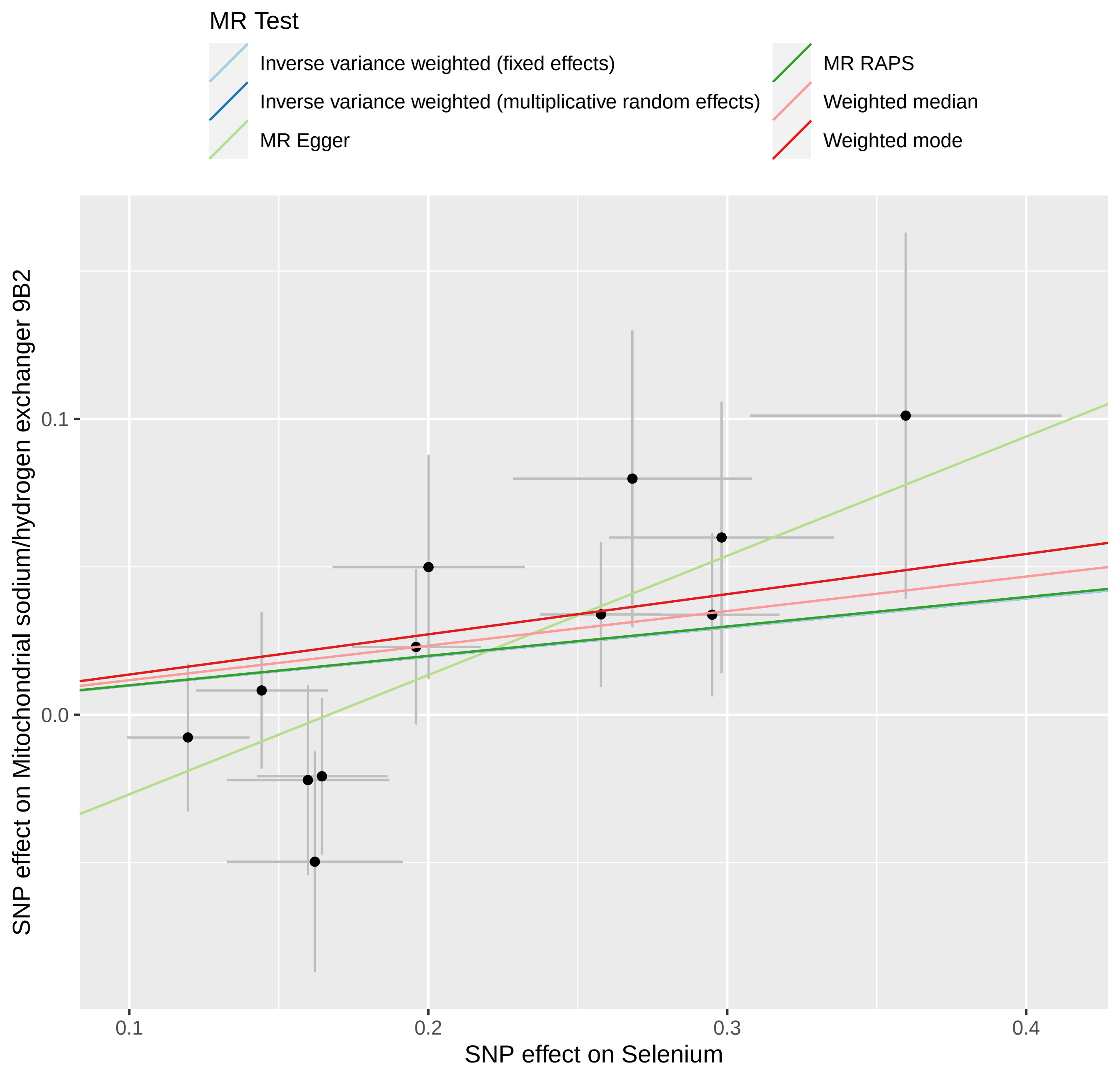

Batch 3−2765 : Selenium on Solute carrier organic anion transporter family member 5A1

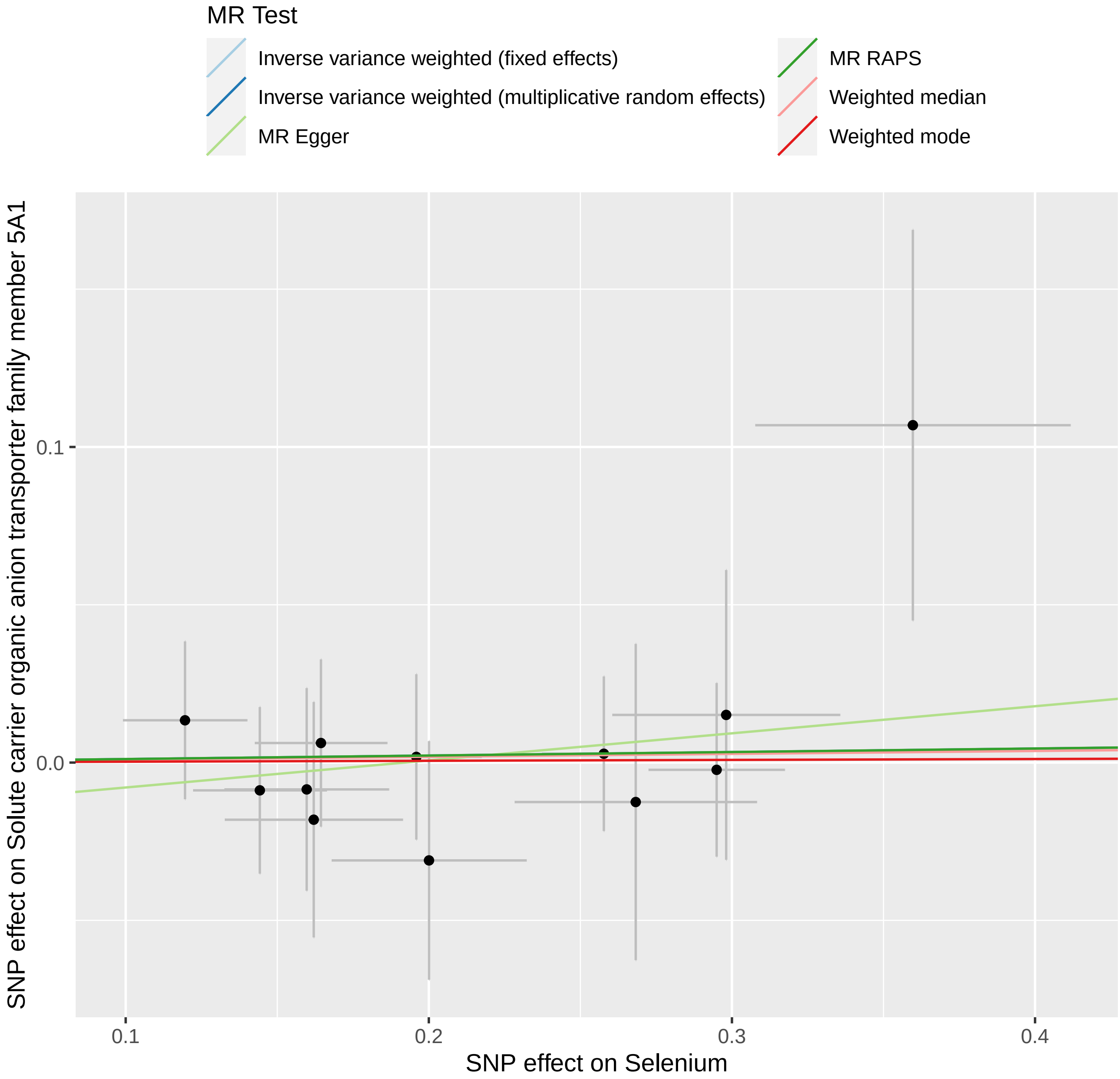

Batch 3−2766 : Selenium on SLIT and NTRK−like protein 1

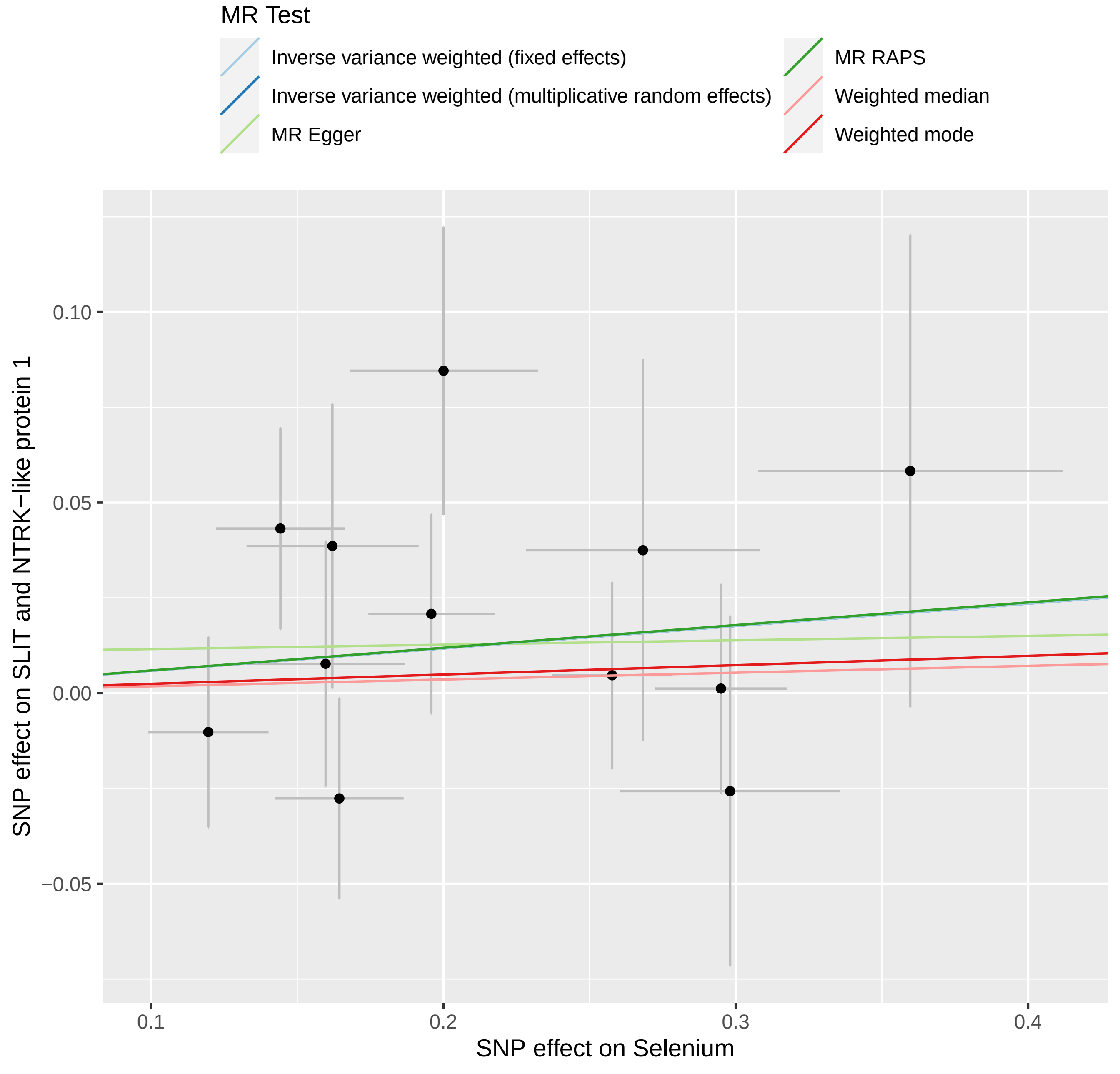

Batch 3−2767 : Selenium on SLIT and NTRK−like protein 3

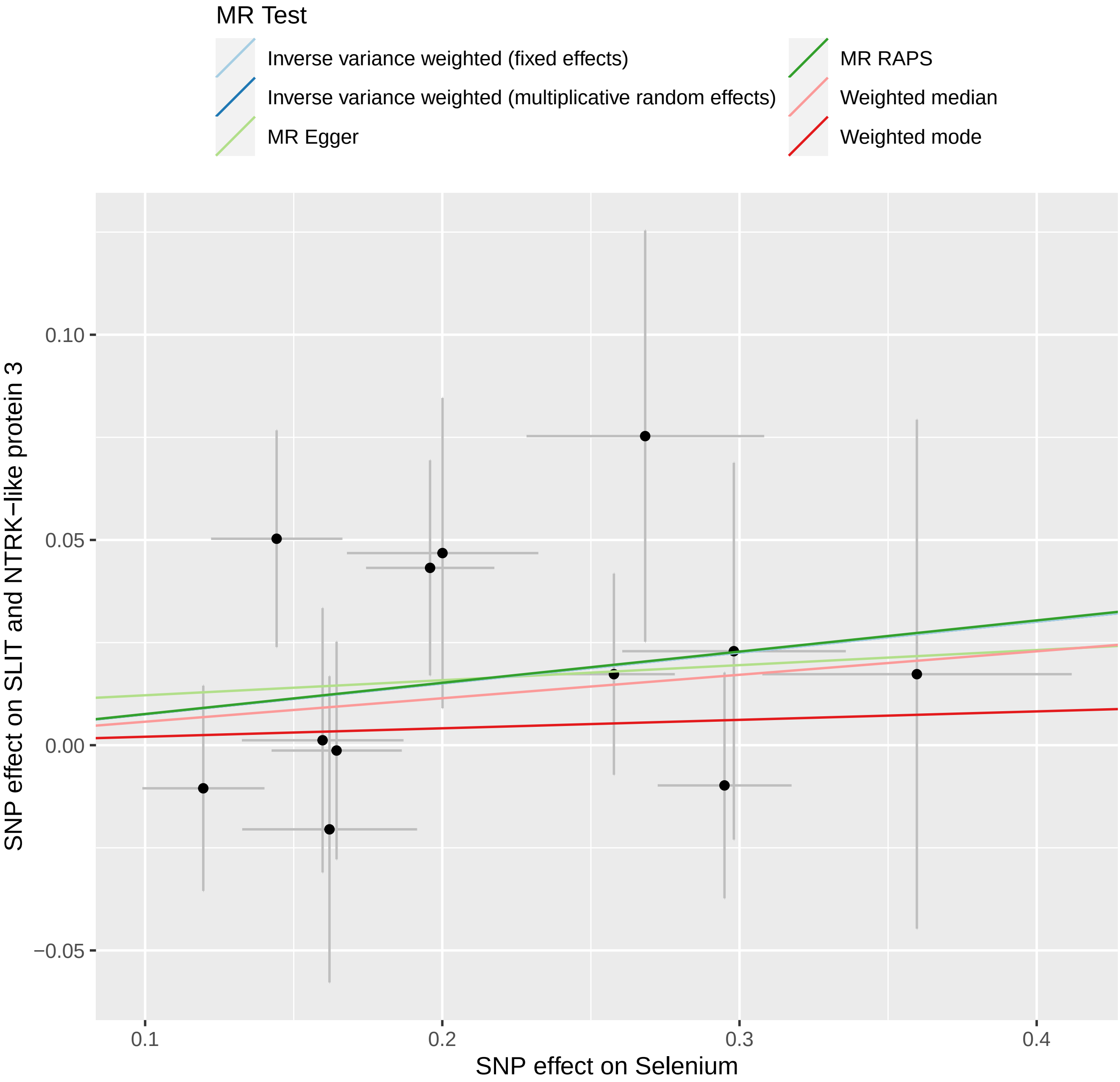

Batch 3−2768 : Selenium on SLIT and NTRK−like protein 4

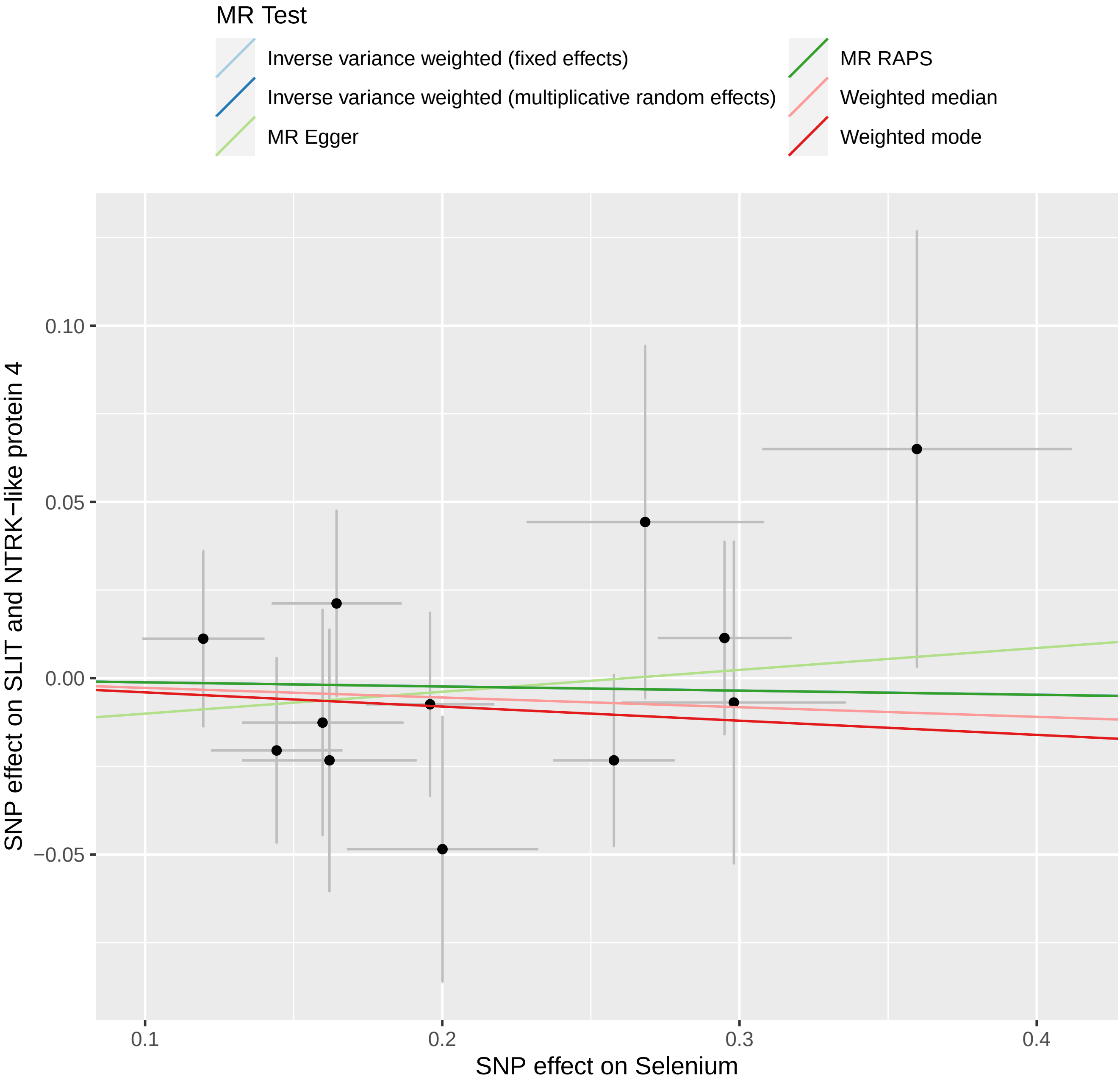

Batch 3−2769 : Selenium on SLIT and NTRK−like protein 5

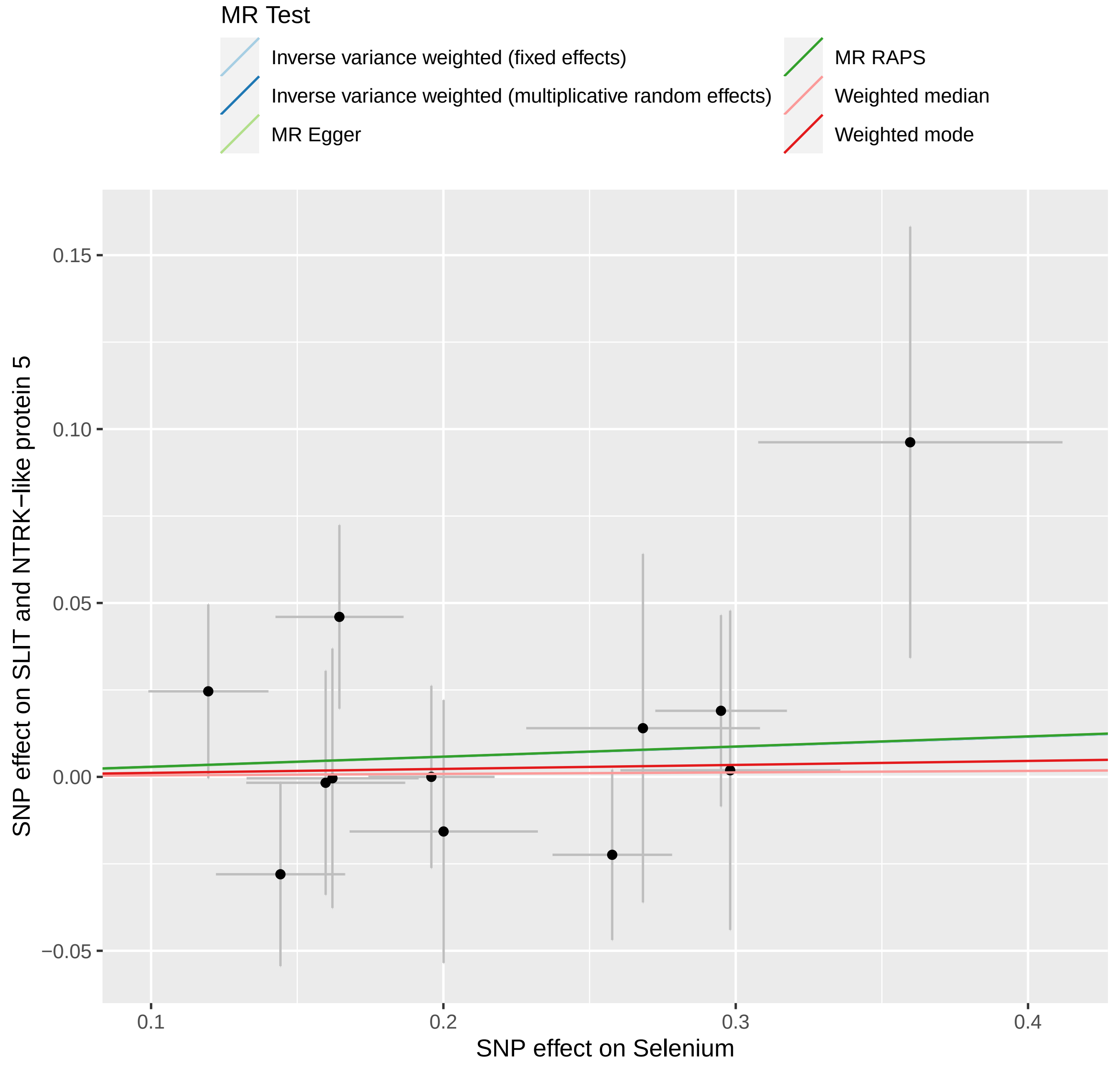

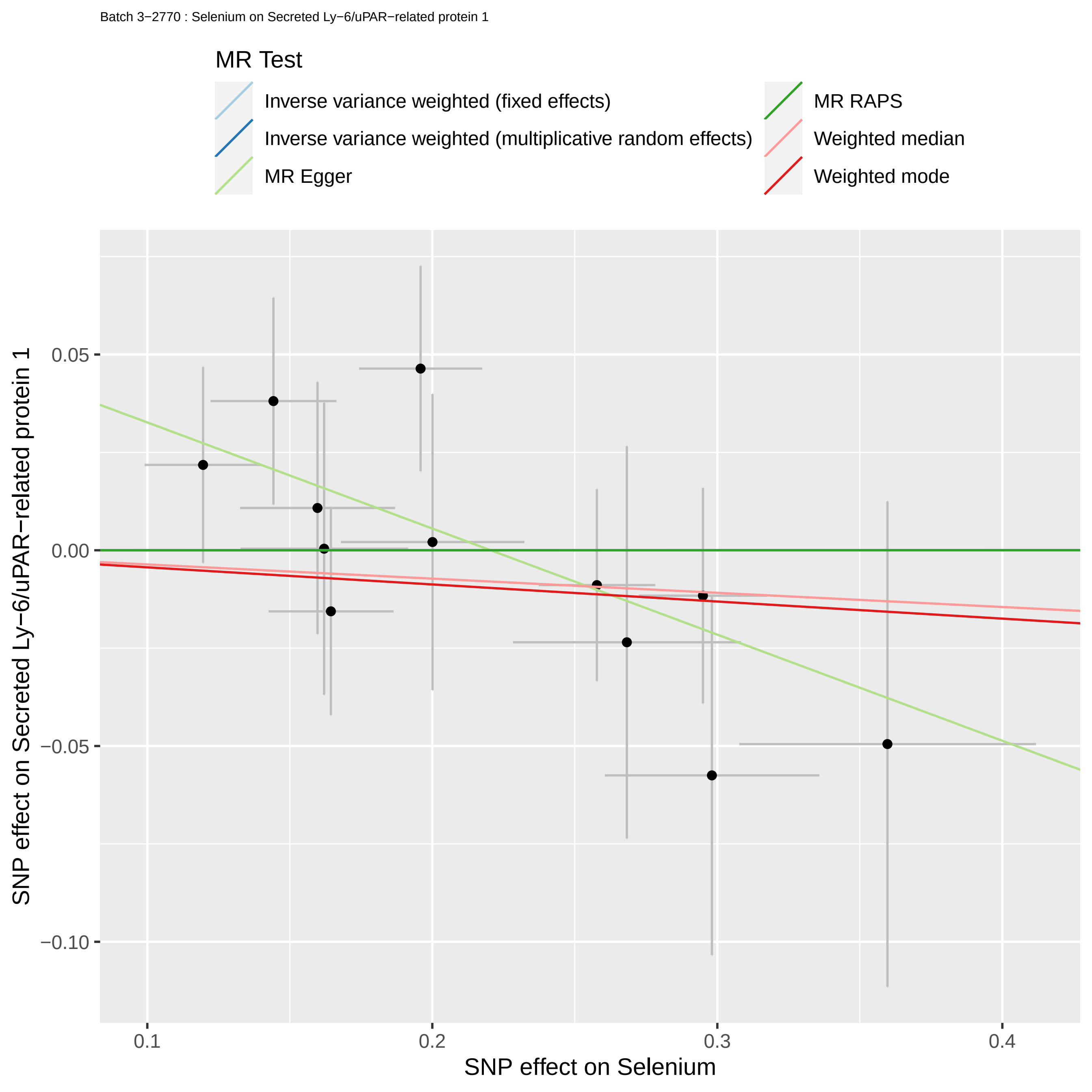

Batch 3−2770 : Selenium on Secreted Ly−6/uPAR−related protein 1
MR Test
Inverse variance weighted (fixed effects)
Inverse variance weighted (multiplicative random effects)
MR Egger
MR RAPS
Weighted median
Weighted mode
0.05
0.00
−0.05
−0.10
0.1
0.2
0.3
0.4
SNP effect on Selenium
SNP effect on Secreted Ly−6/uPAR−related protein 1

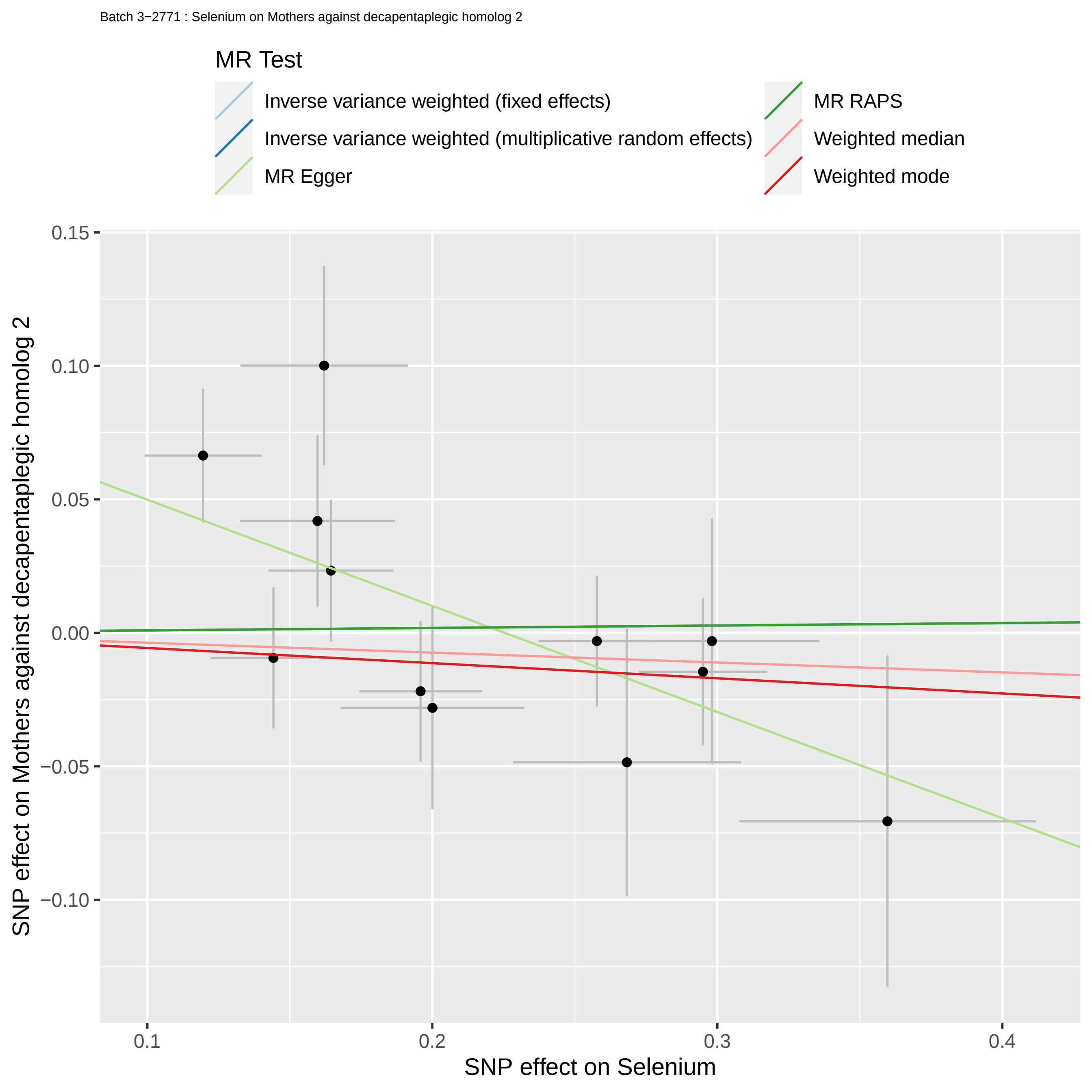

Batch 3−2771 : Selenium on Mothers against decapentaplegic homolog 2
MR Test
Inverse variance weighted (fixed effects)
Inverse variance weighted (multiplicative random effects)
MR Egger
MR RAPS
Weighted median
Weighted mode
0.15
0.10
0.05
0.00
−0.05
−0.10
0.1
0.2
0.3
0.4
SNP effect on Mothers against decapentaplegic homolog 2
SNP effect on Selenium

Batch 3−2772 : Selenium on Mothers against decapentaplegic homolog 4

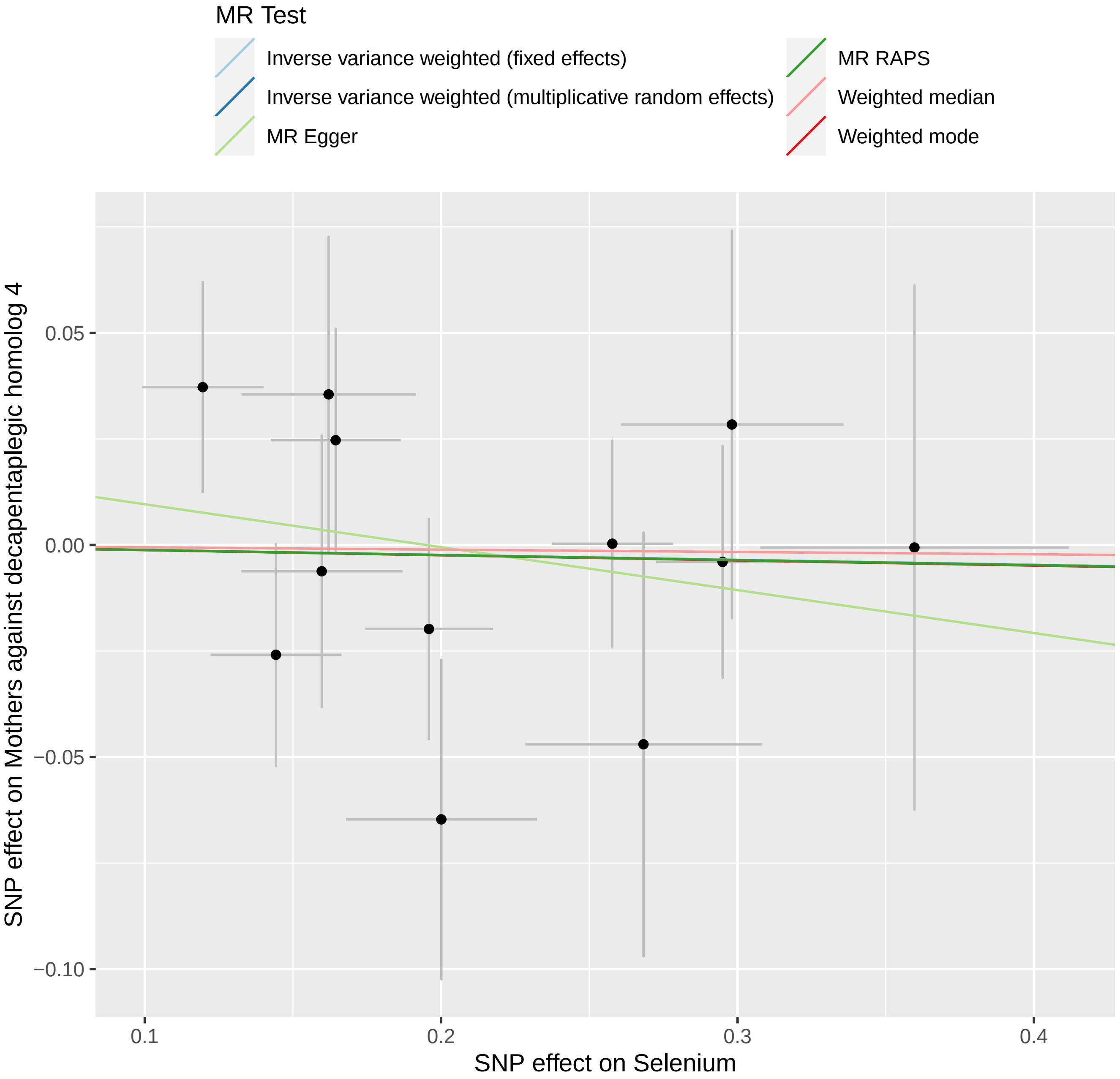

Batch 3−2773 : Selenium on Stromal membrane−associated protein 1

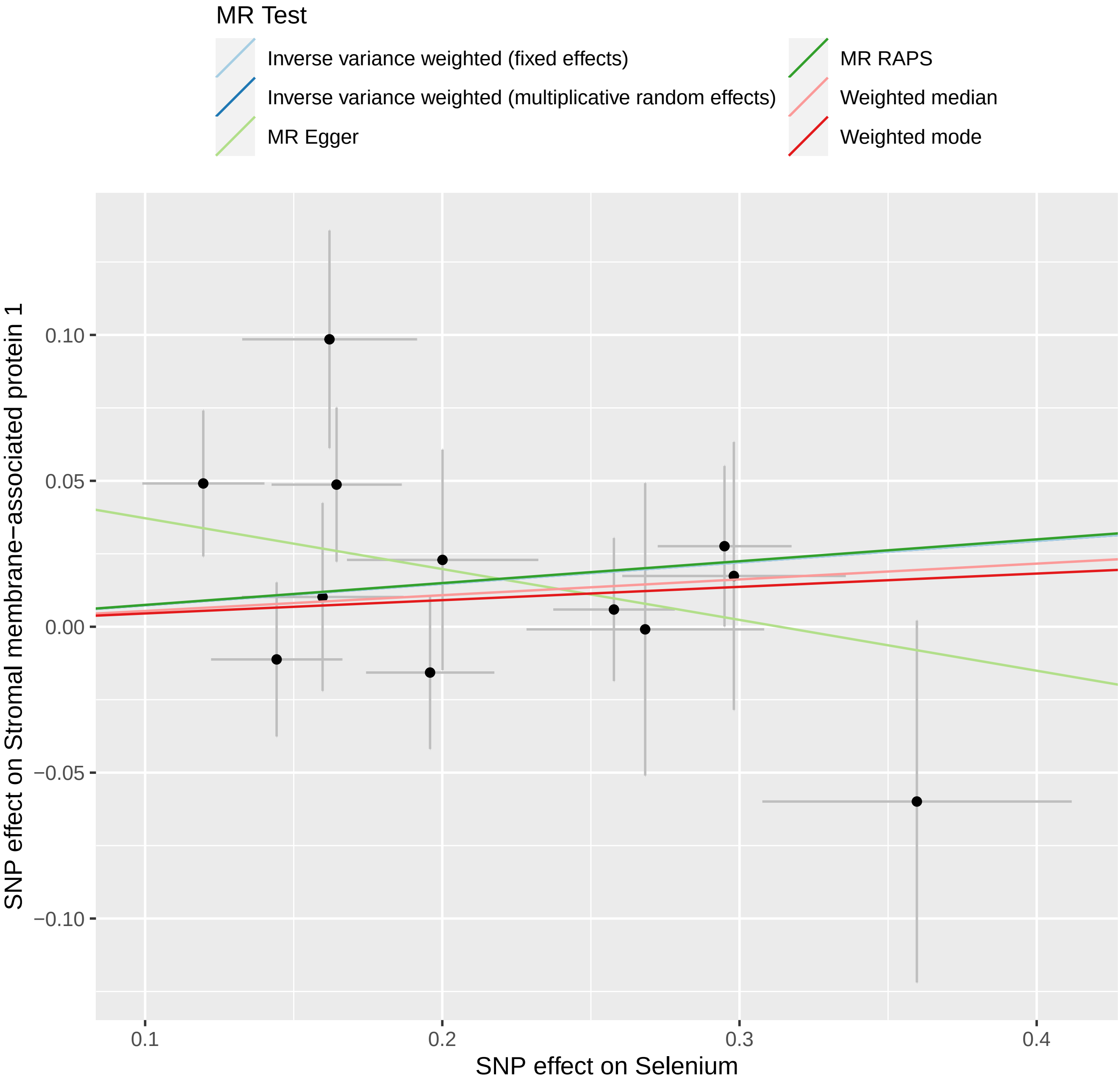

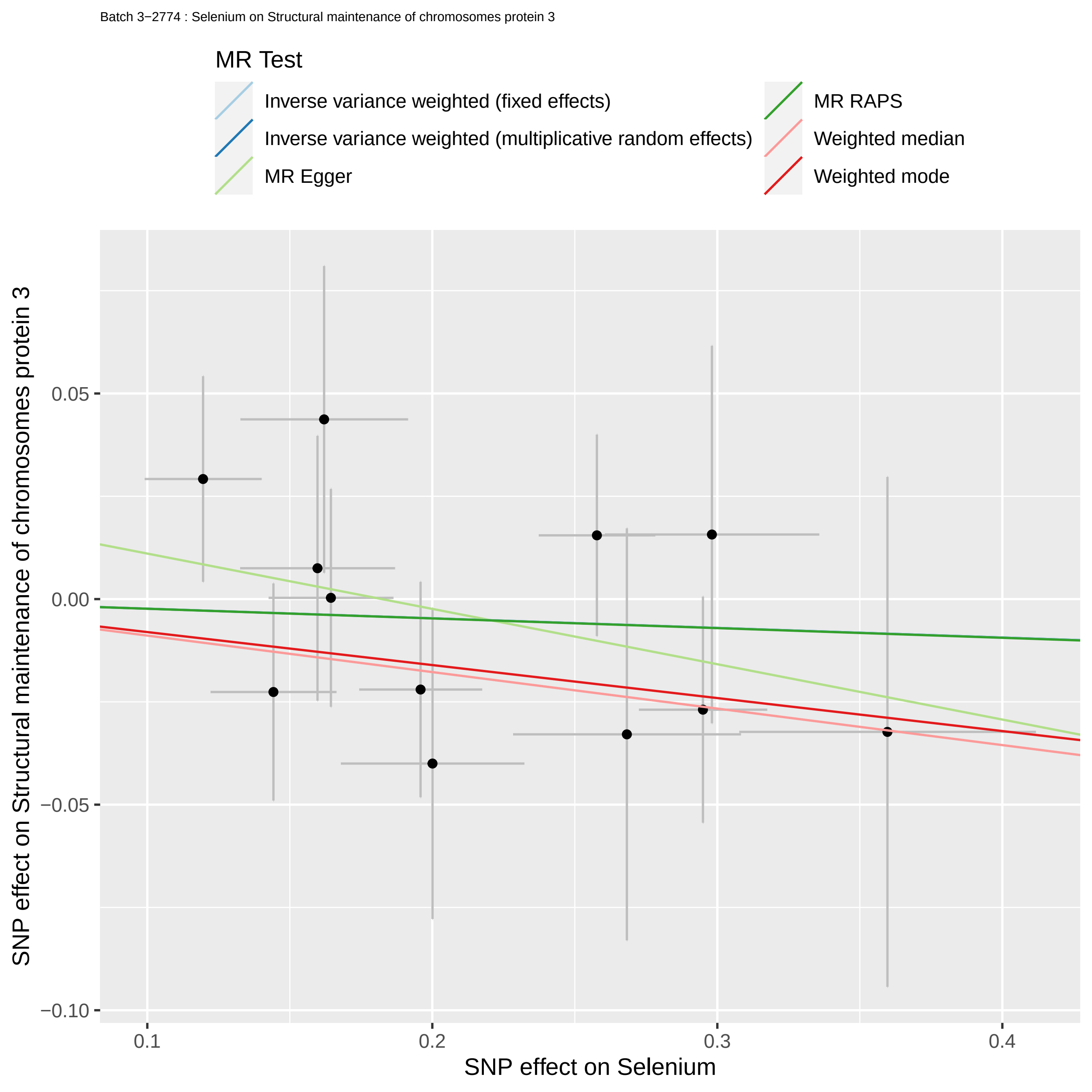
Batch 3−2774 : Selenium on Structural maintenance of chromosomes protein 3
MR Test
Inverse variance weighted (fixed effects)
Inverse variance weighted (multiplicative random effects)
MR Egger
MR RAPS
Weighted median
Weighted mode
0.05
0.00
−0.05
−0.10
0.1
0.2
0.3
0.4
SNP effect on Structural maintenance of chromosomes protein 3
SNP effect on Selenium

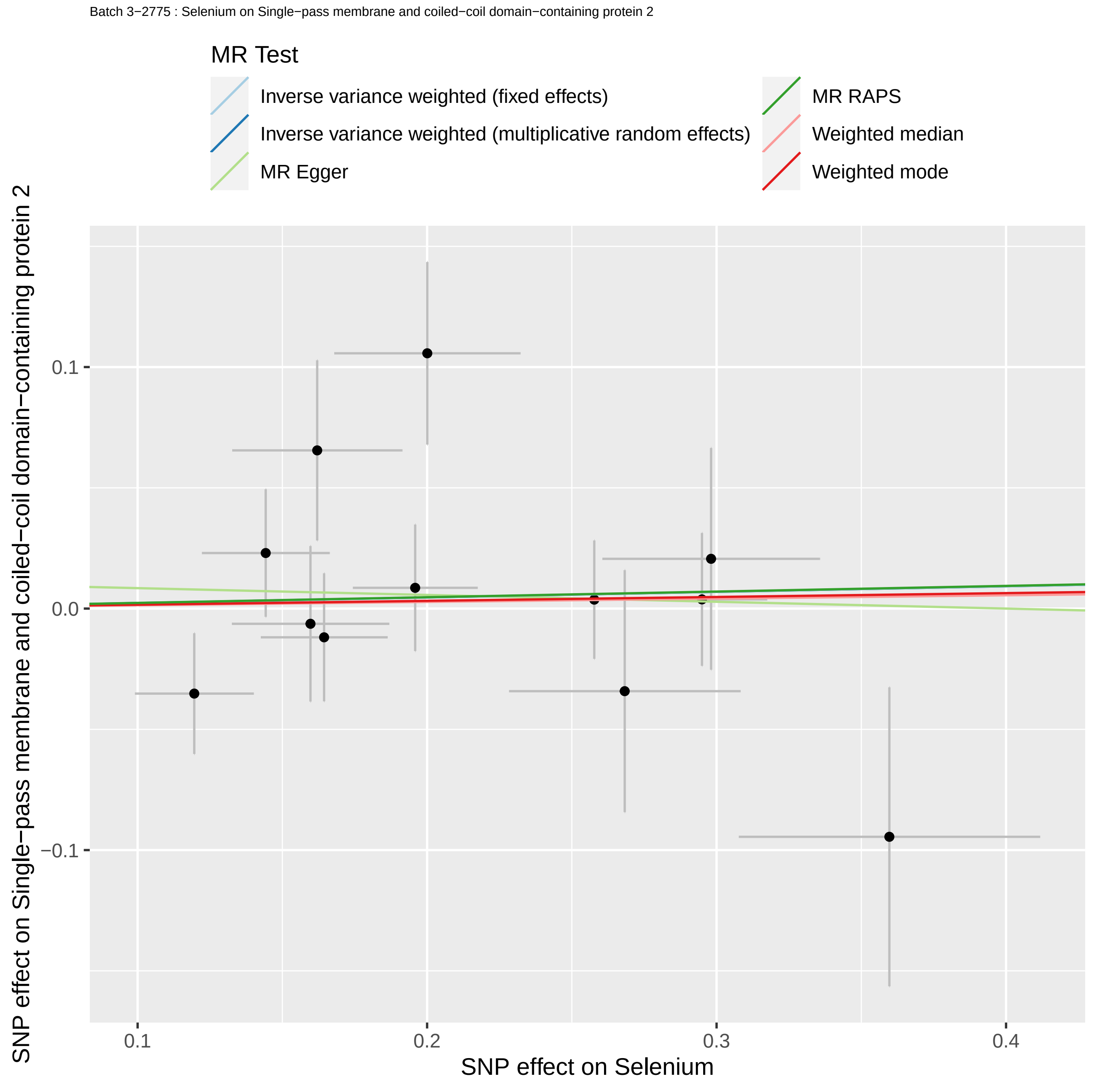

Batch 3−2775 : Selenium on Single−pass membrane and coiled−coil domain−containing protein 2
MR Test
Inverse variance weighted (fixed effects)
Inverse variance weighted (multiplicative random effects)
MR Egger
MR RAPS
Weighted median
Weighted mode
SNP effect on Single−pass membrane and coiled−coil domain−containing protein 2
0.1
0.0
−0.1
0.1
0.2
0.3
0.4
SNP effect on Selenium

Batch 3−2776 : Selenium on Essential MCU regulator, mitochondrial

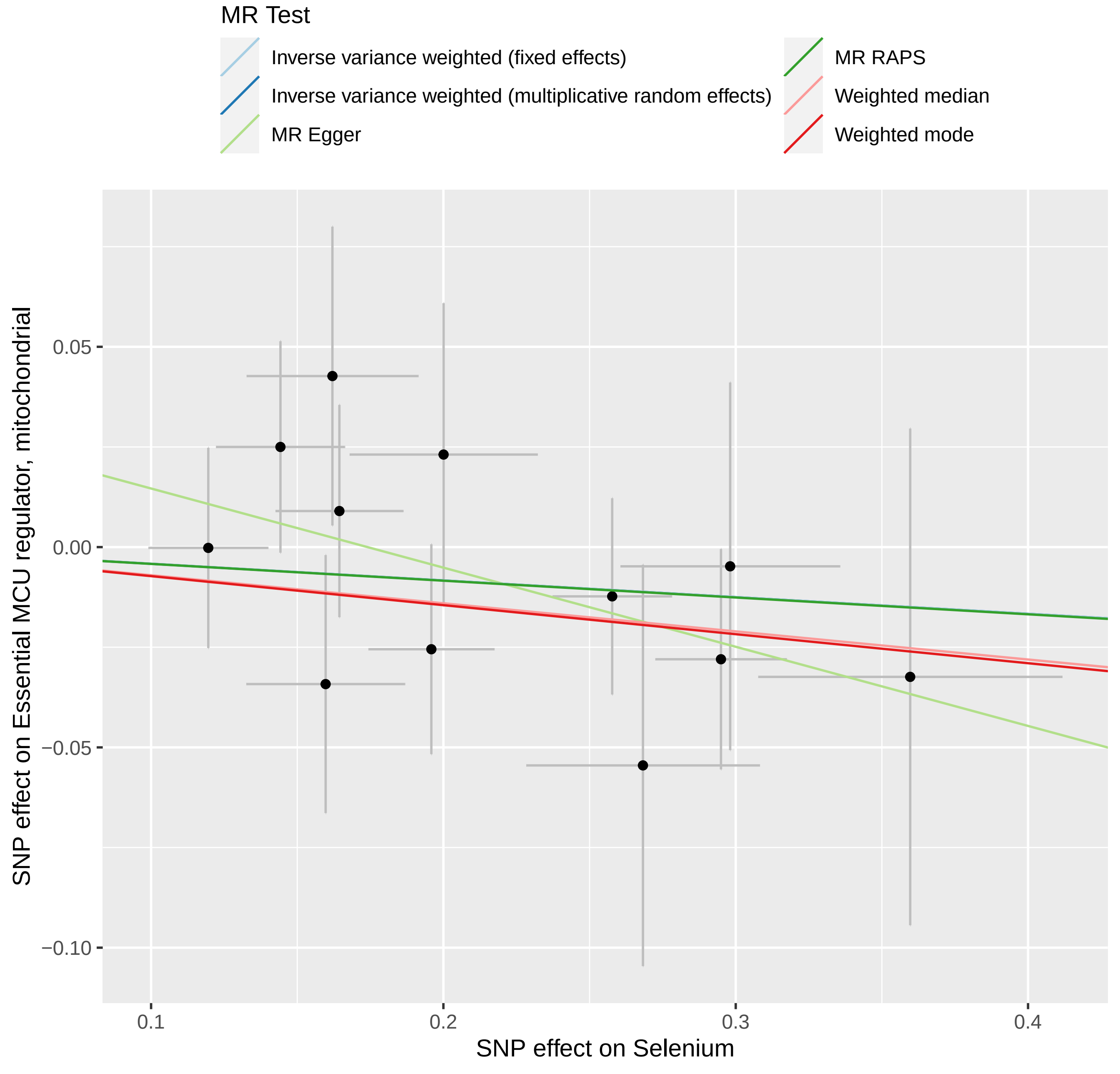

Batch 3−2777 : Selenium on Small integral membrane protein 10

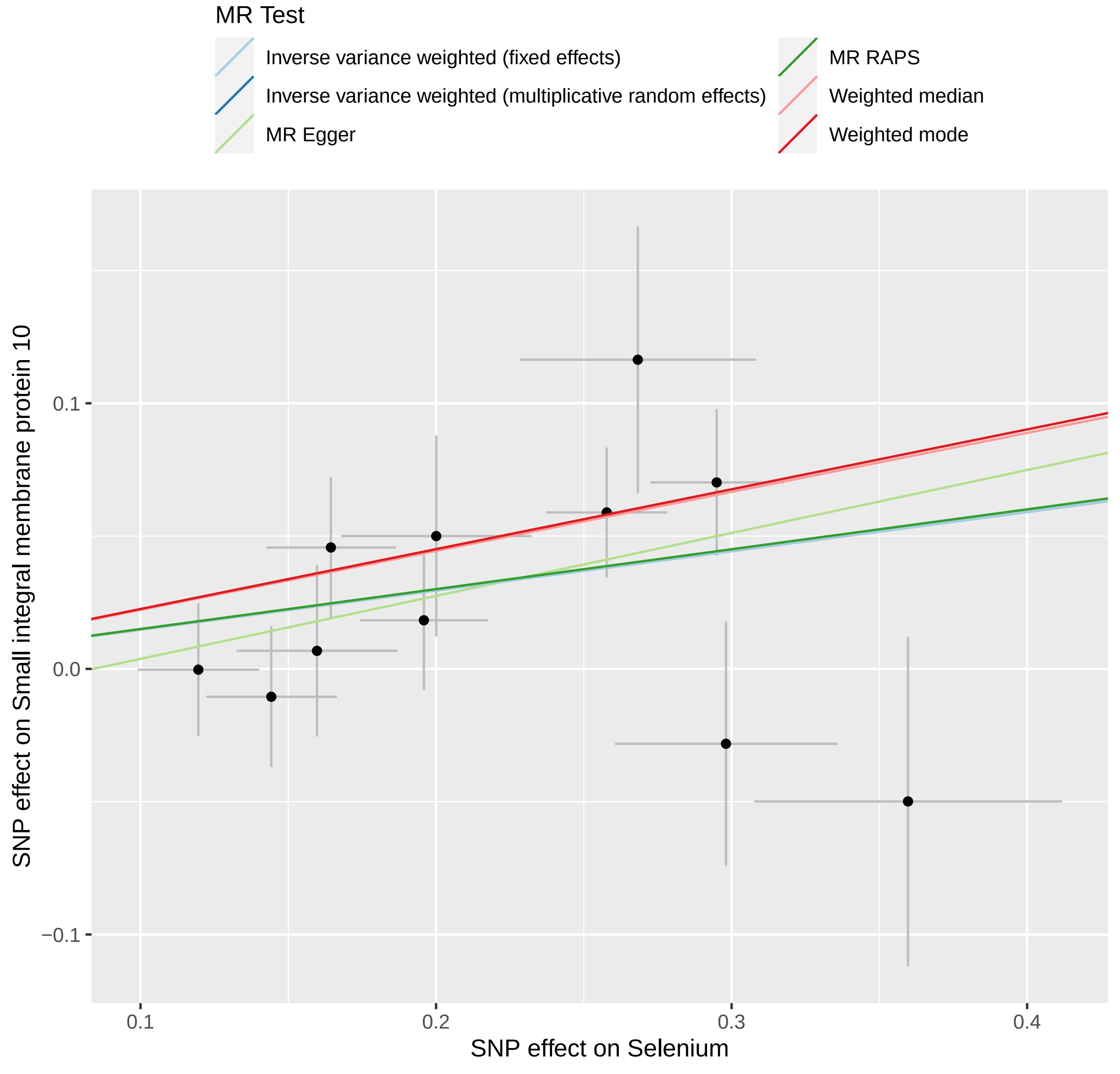

Batch 3−2778 : Selenium on Small integral membrane protein 9

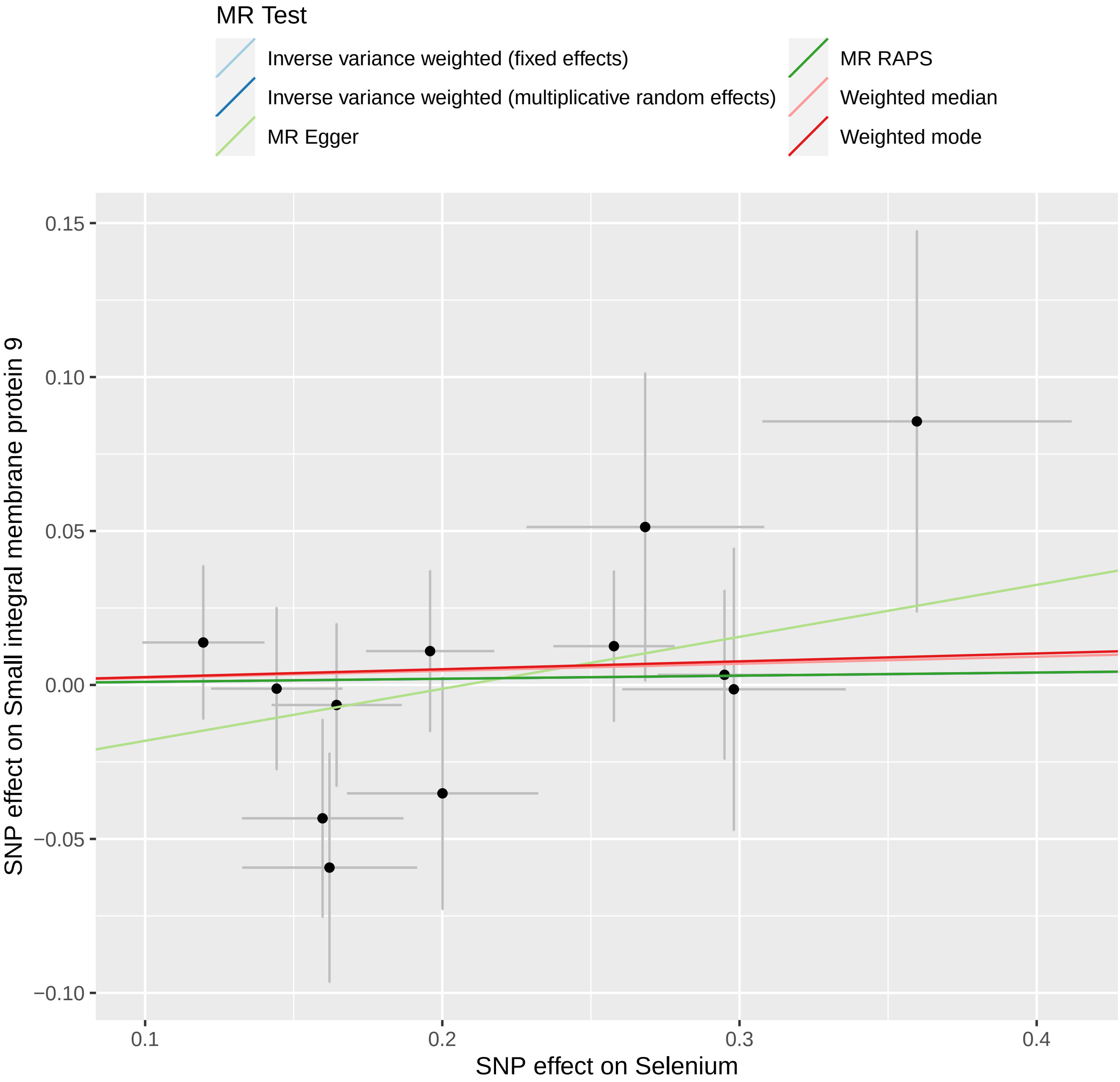

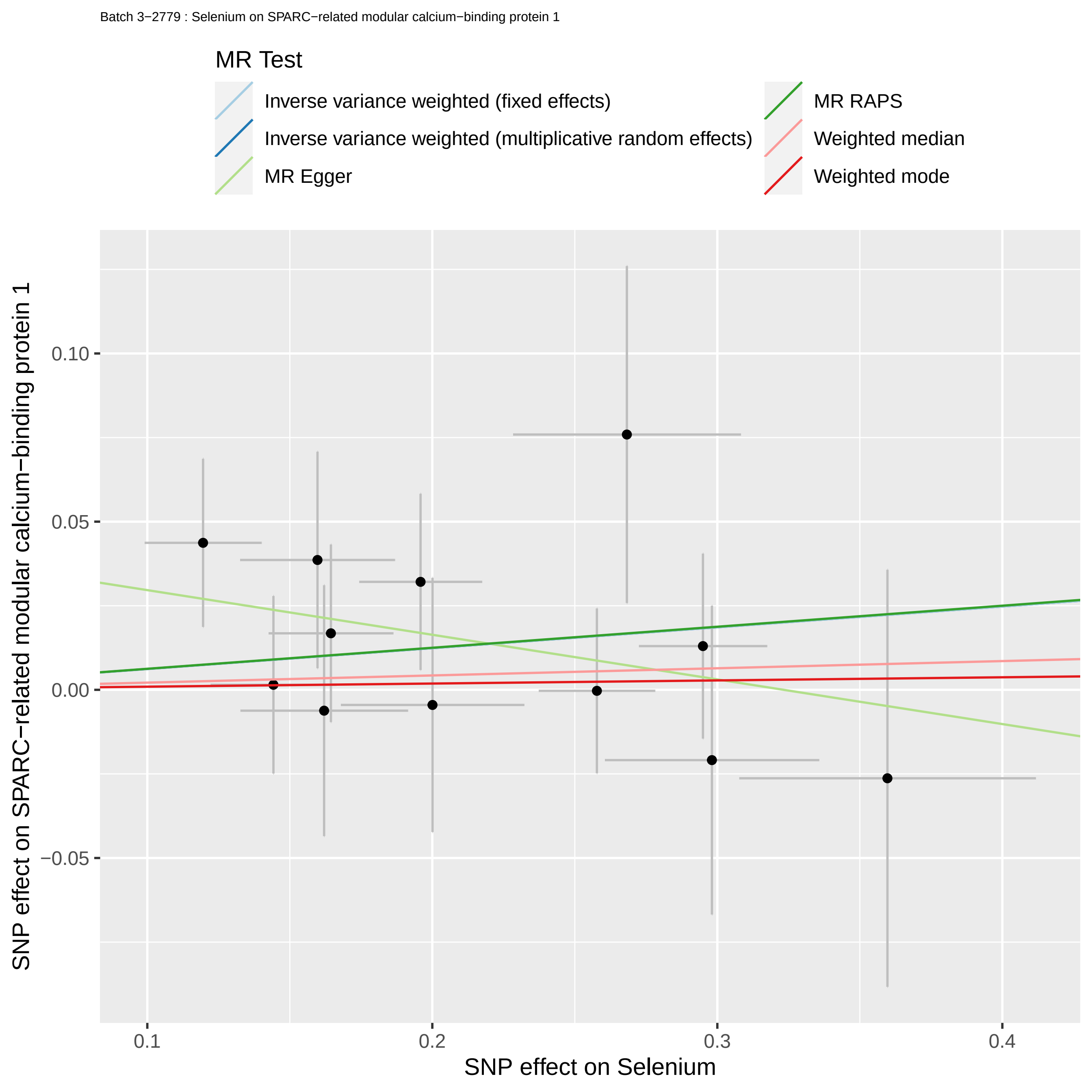
Batch 3−2779 : Selenium on SPARC−related modular calcium−binding protein 1
MR Test
Inverse variance weighted (fixed effects)
Inverse variance weighted (multiplicative random effects)
MR Egger
MR RAPS
Weighted median
Weighted mode
SNP effect on SPARC−related modular calcium−binding protein 1
0.10
0.05
0.00
−0.05
0.1
0.2
0.3
0.4
SNP effect on Selenium

Batch 3−2780 : Selenium on Acid sphingomyelinase−like phosphodiesterase 3a

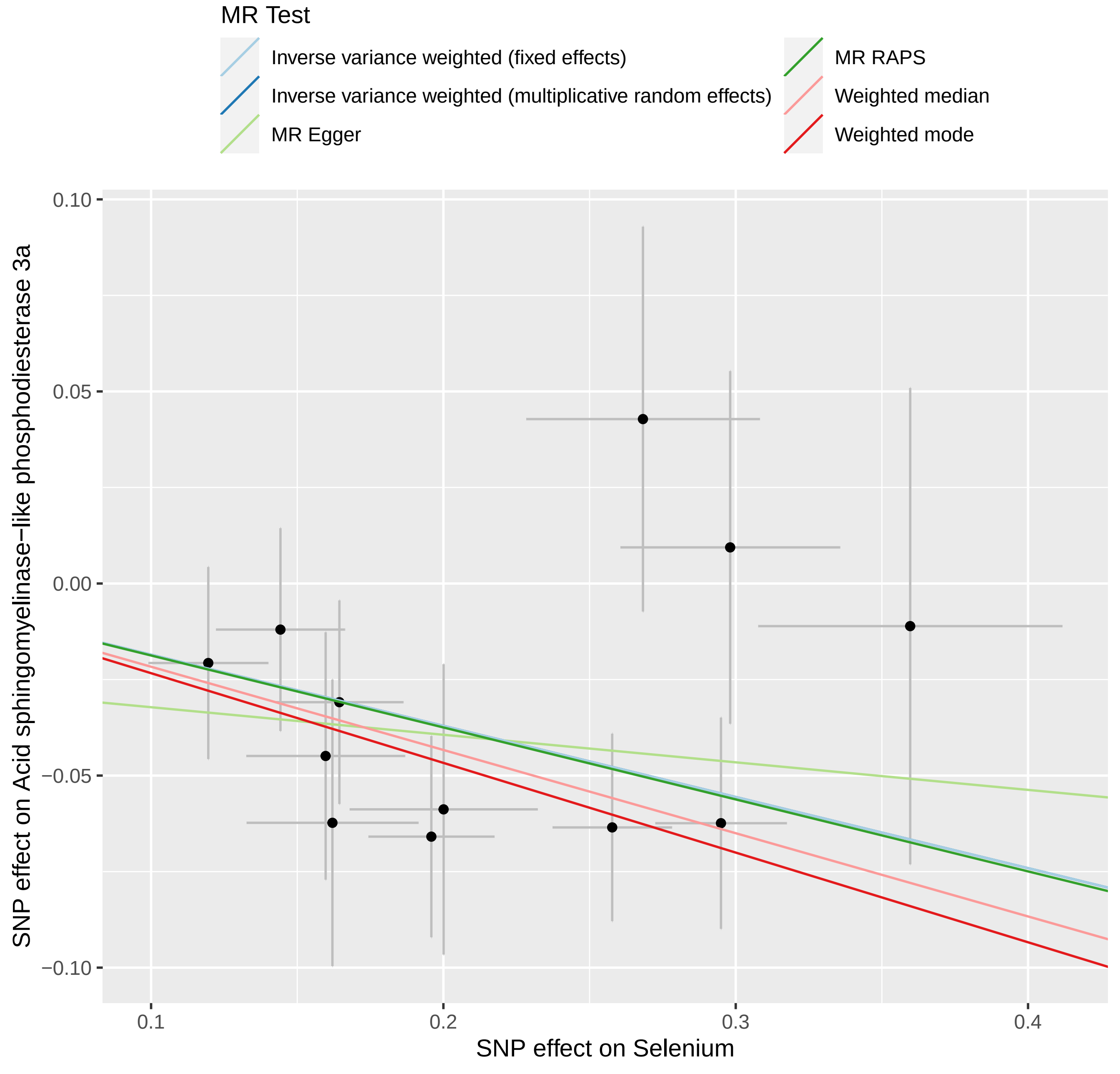

Batch 3−2781 : Selenium on Acid sphingomyelinase−like phosphodiesterase 3a

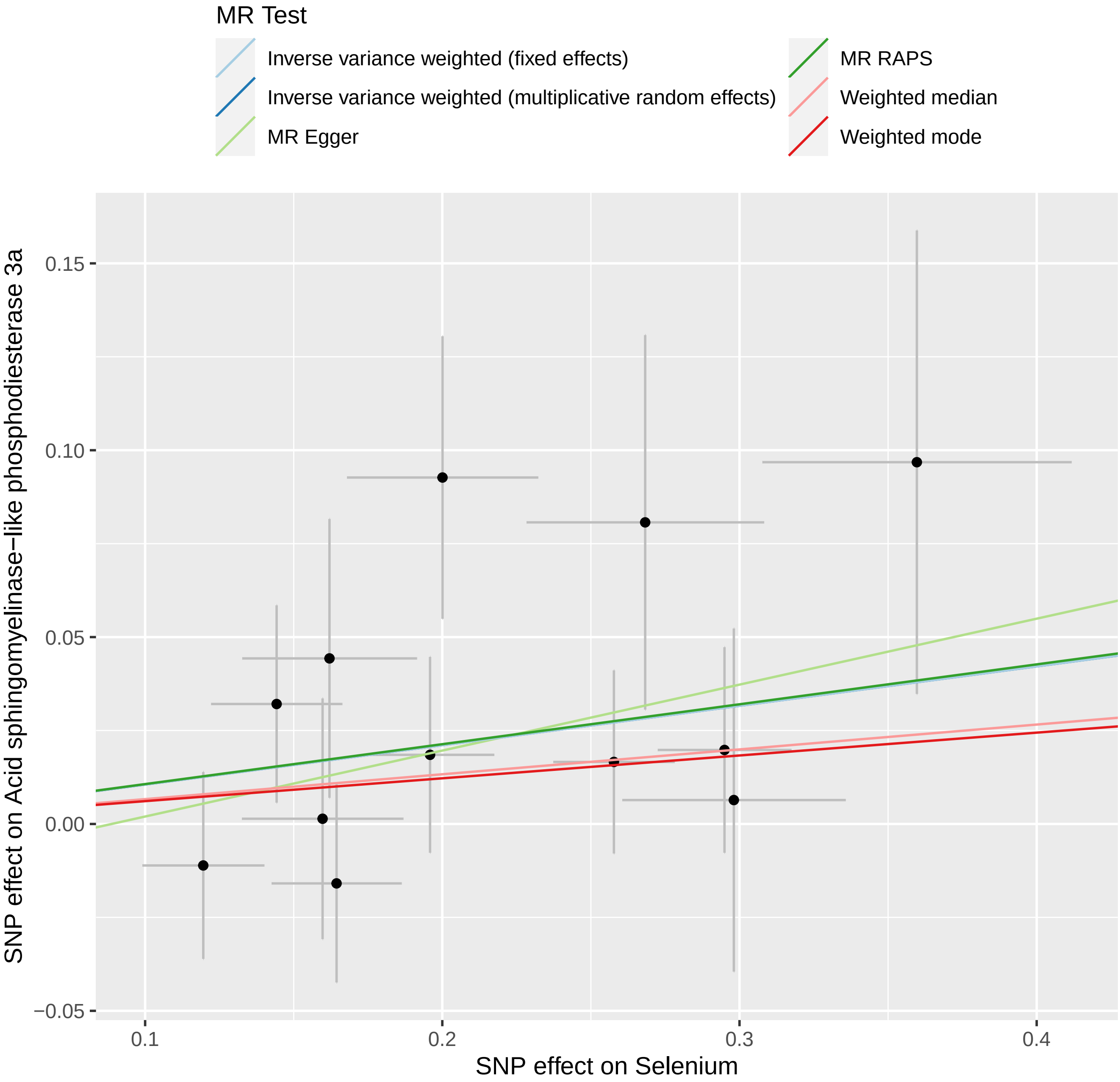

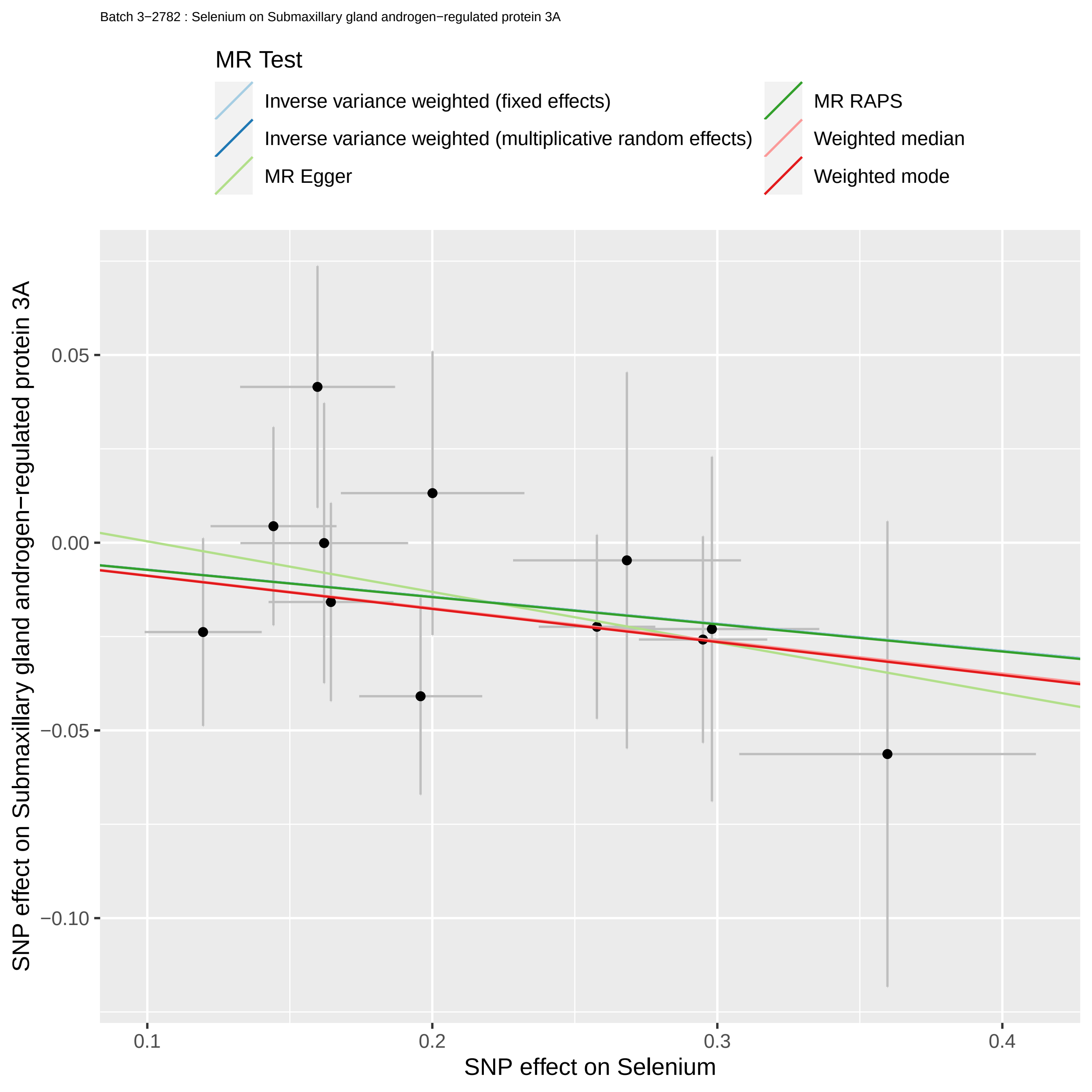
Batch 3−2782 : Selenium on Submaxillary gland androgen−regulated protein 3A
MR Test
Inverse variance weighted (fixed effects)
Inverse variance weighted (multiplicative random effects)
MR Egger
MR RAPS
Weighted median
Weighted mode
SNP effect on Submaxillary gland androgen−regulated protein 3A
0.05
0.00
−0.05
−0.10
0.1
0.2
0.3
0.4
SNP effect on Selenium

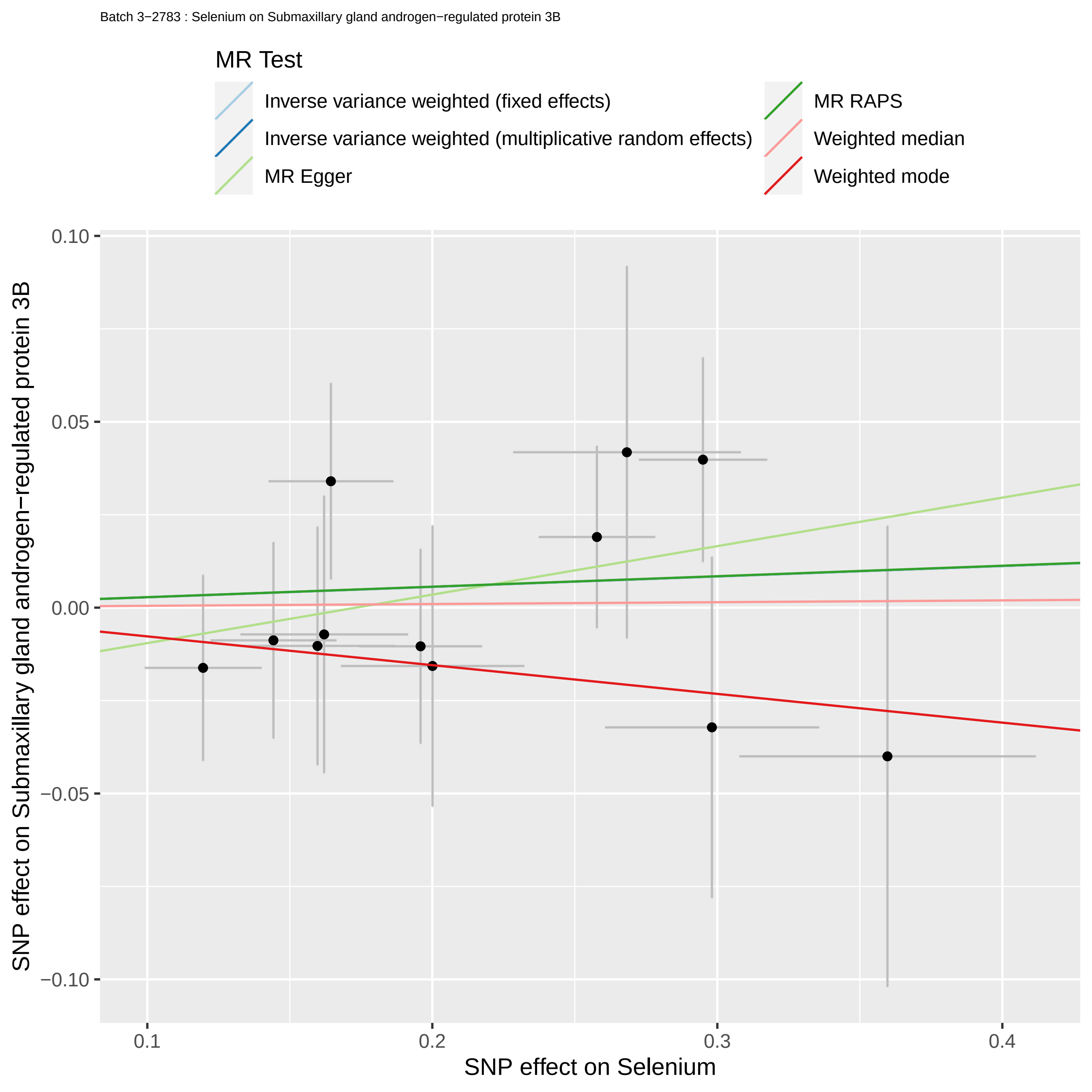

Batch 3−2783 : Selenium on Submaxillary gland androgen−regulated protein 3B
MR Test
Inverse variance weighted (fixed effects)
Inverse variance weighted (multiplicative random effects)
MR Egger
MR RAPS
Weighted median
Weighted mode
0.10
0.05
0.00
−0.05
−0.10
0.1
0.2
0.3
0.4
SNP effect on Submaxillary gland androgen−regulated protein 3B
SNP effect on Selenium

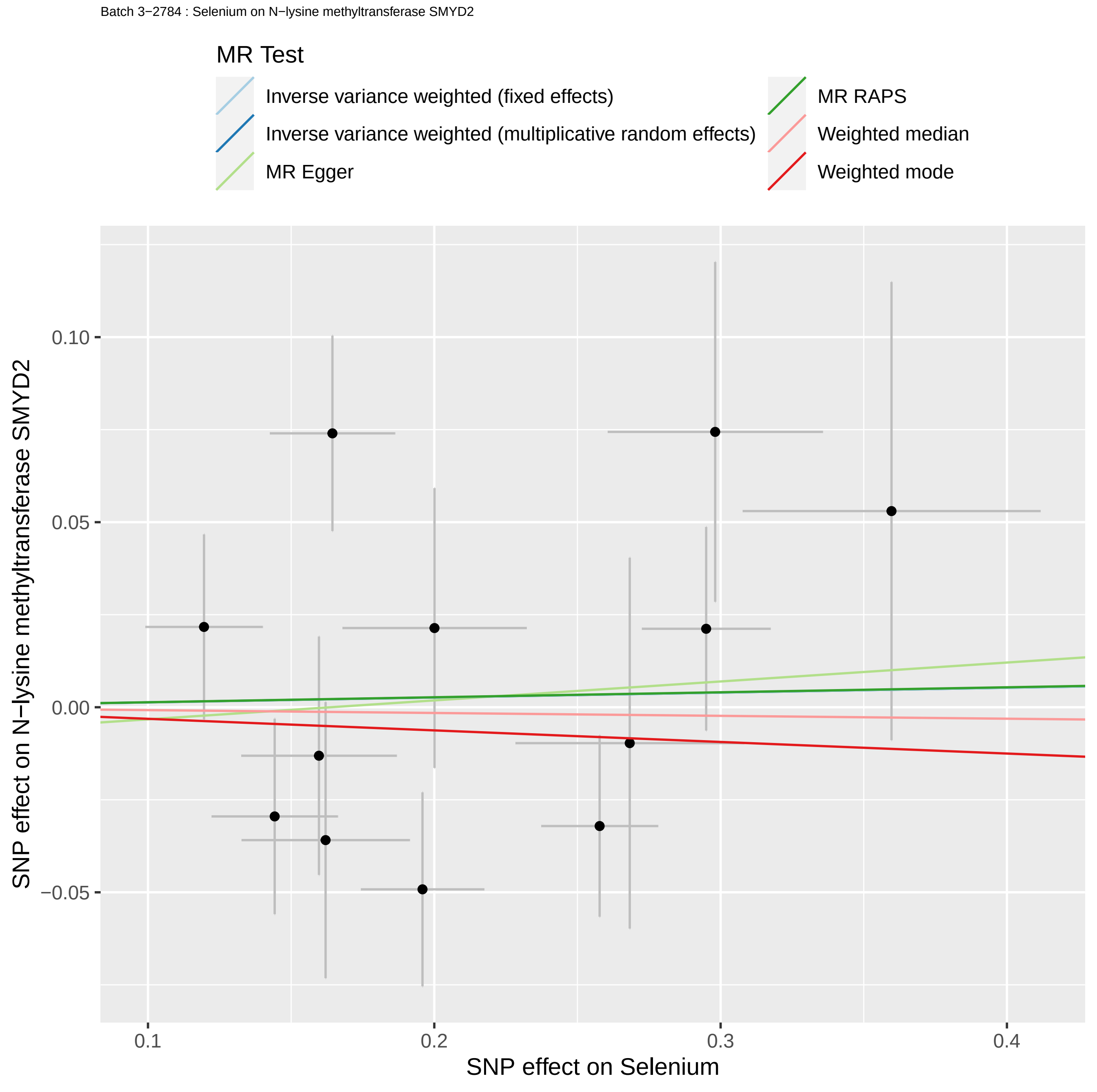

Batch 3−2784 : Selenium on N−lysine methyltransferase SMYD2
MR Test
Inverse variance weighted (fixed effects)
Inverse variance weighted (multiplicative random effects)
MR Egger
MR RAPS
Weighted median
Weighted mode
0.10
0.05
0.00
−0.05
0.1
0.2
0.3
0.4
SNP effect on Selenium
SNP effect on N−lysine methyltransferase SMYD2

Batch 3−2785 : Selenium on Zinc finger protein SNAI2

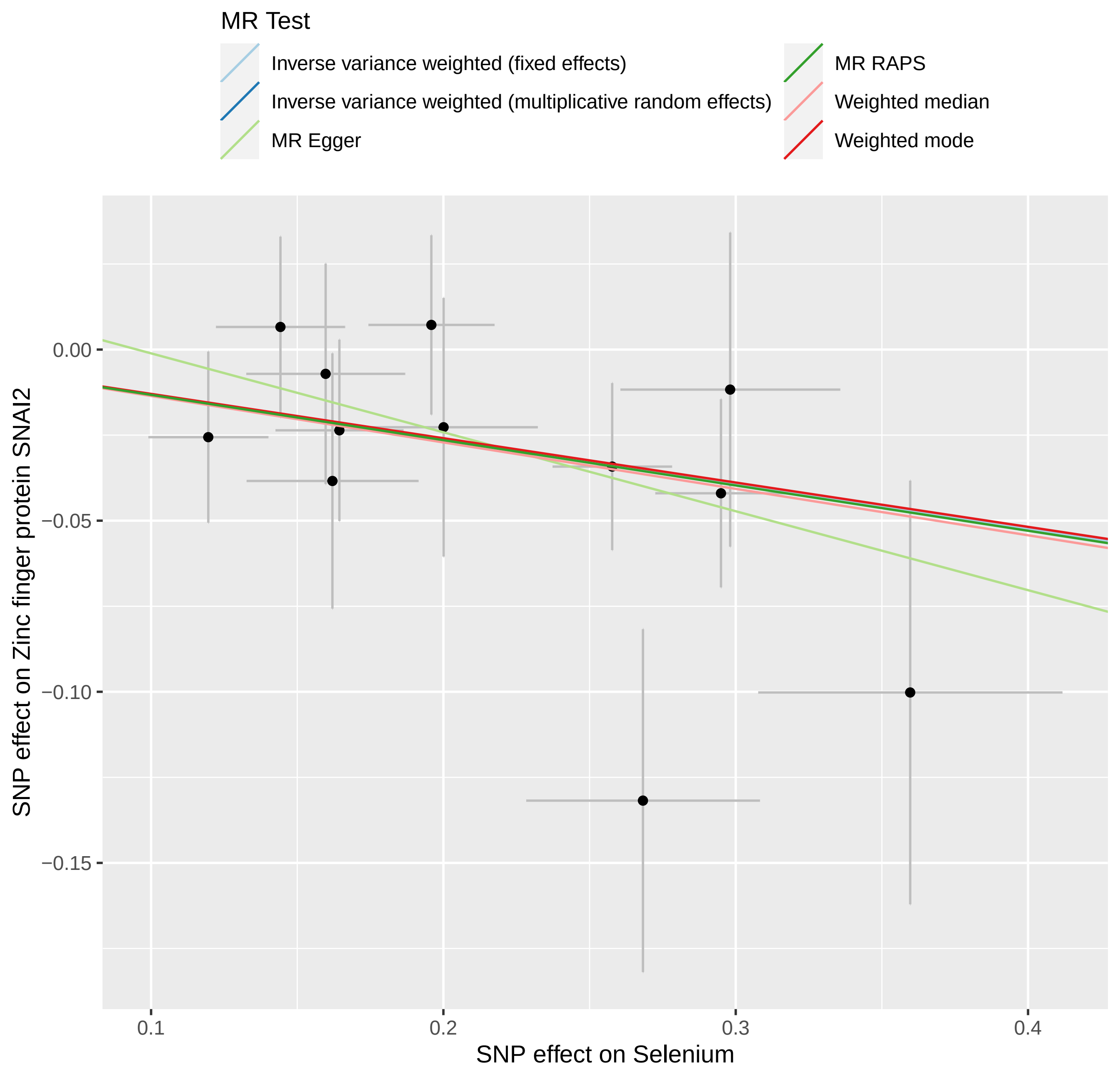

Batch 3−2786 : Selenium on Synaptosomal−associated protein 25

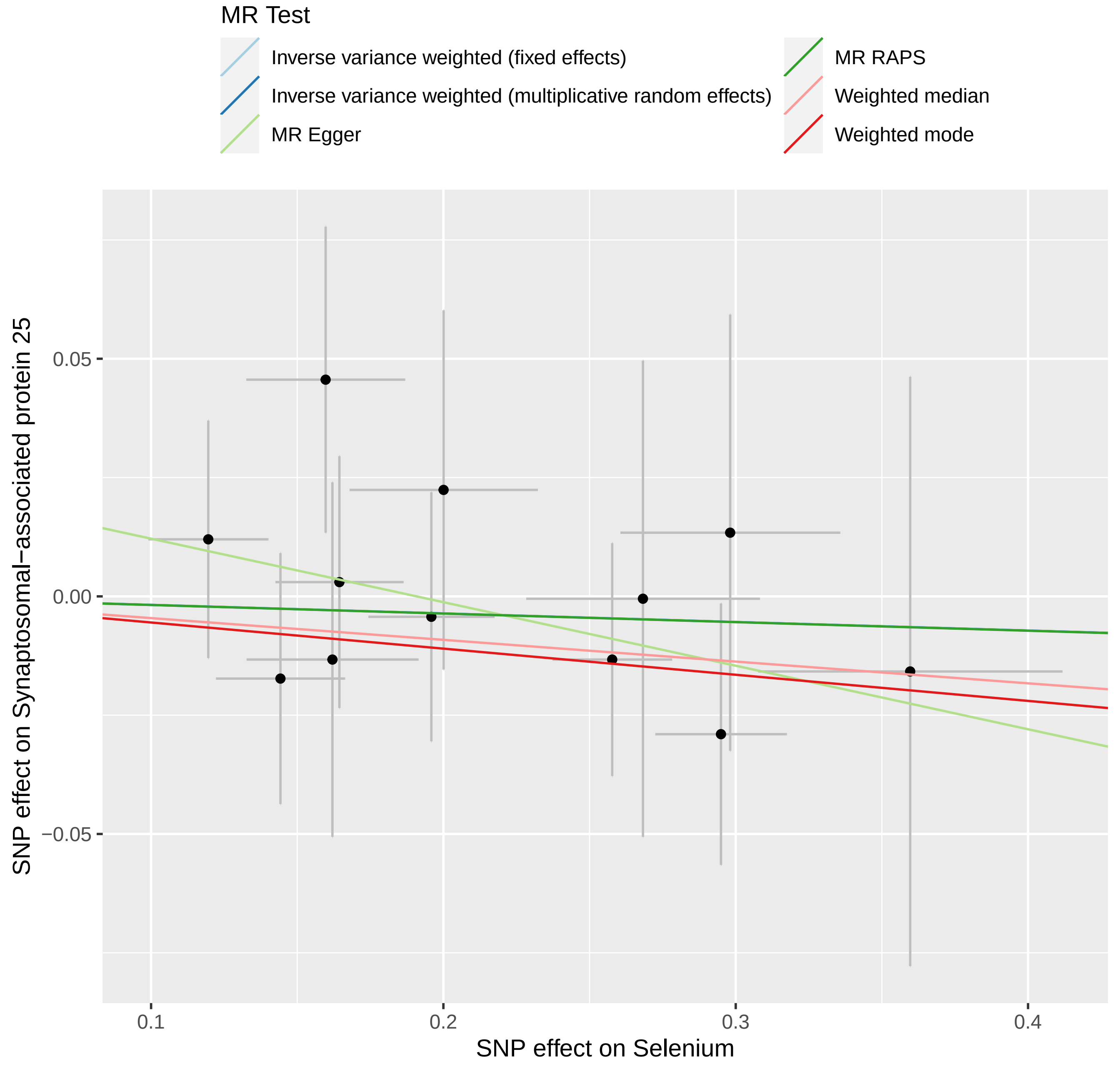

Batch 3−2787 : Selenium on Synaptosomal−associated protein 29

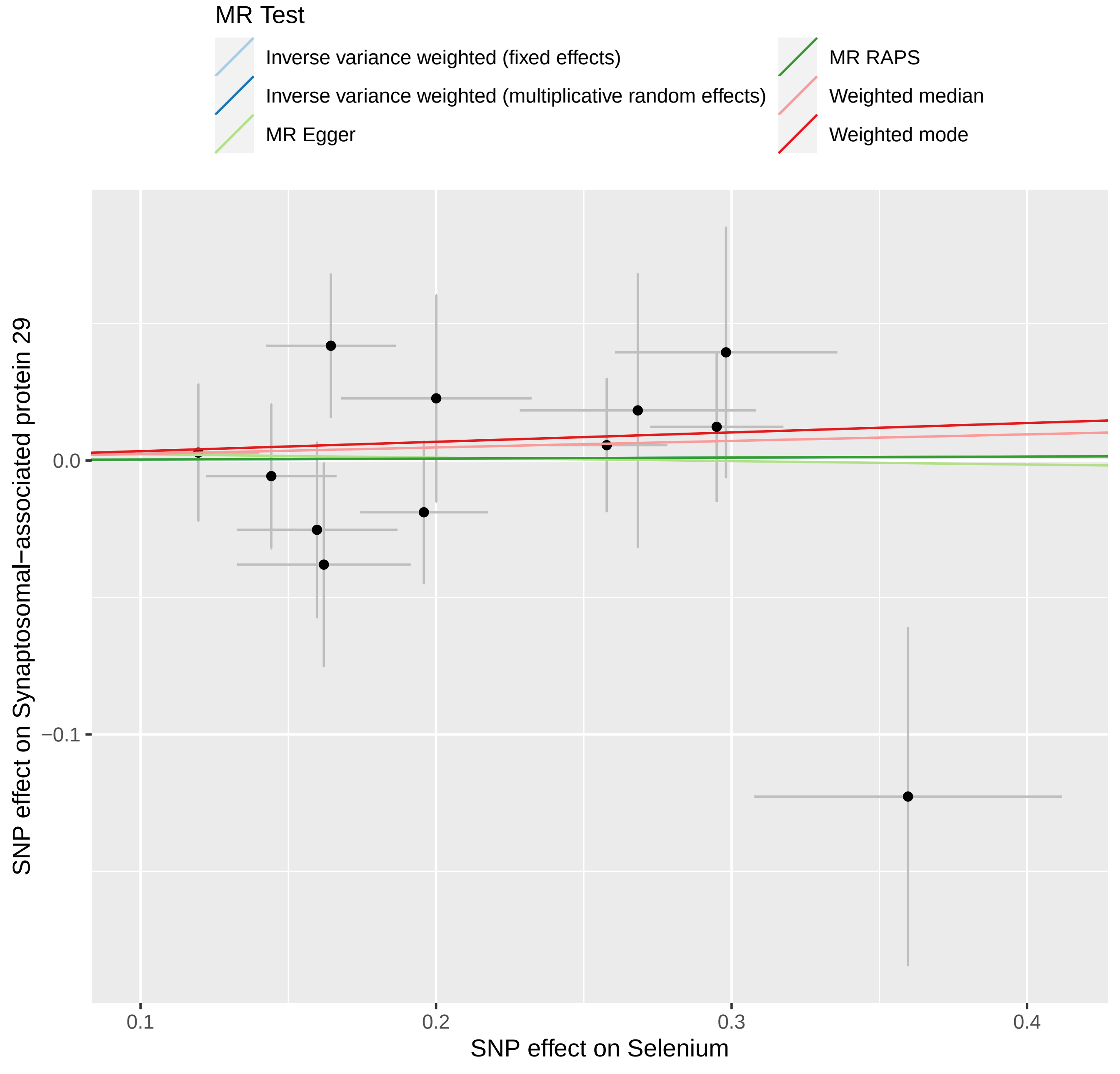

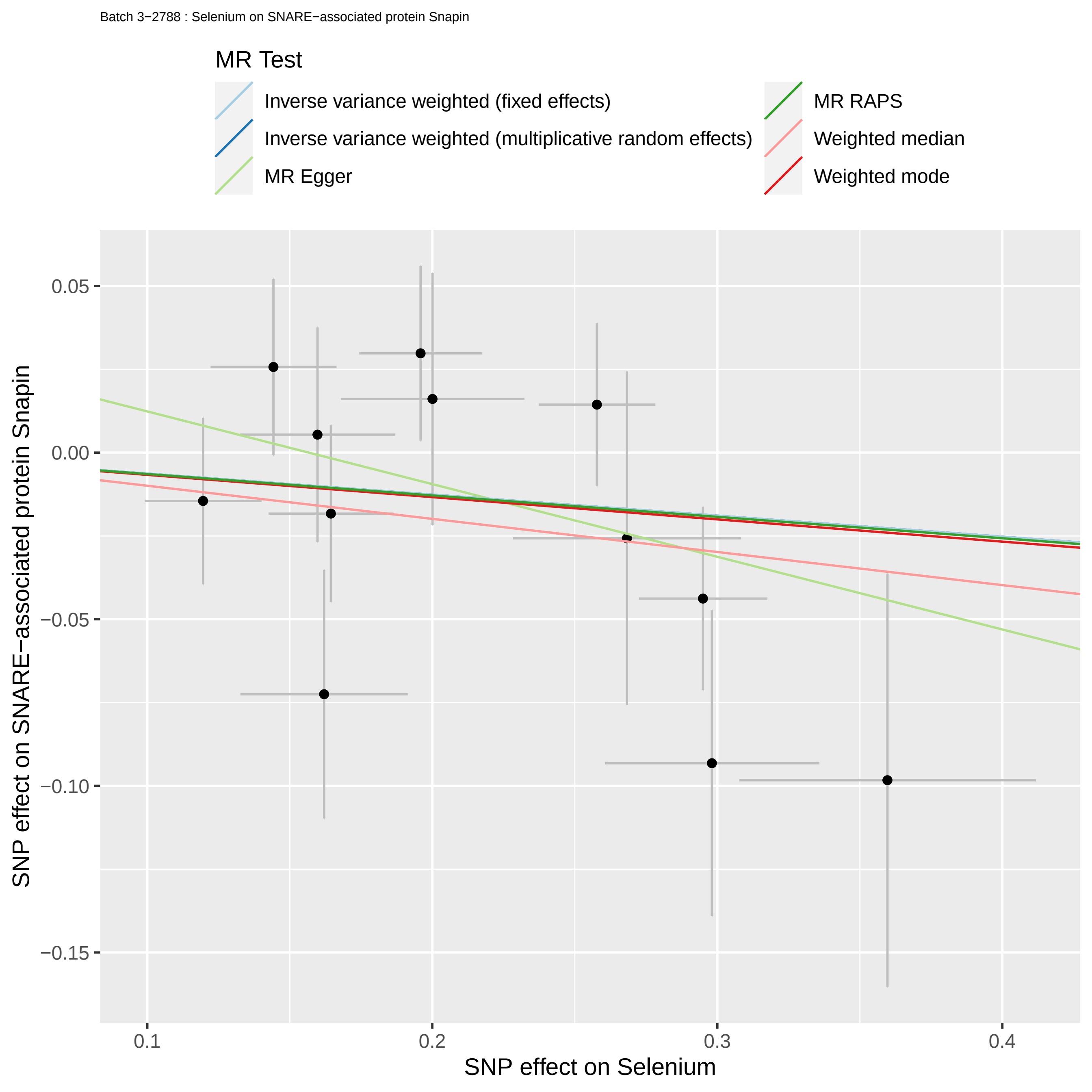

Batch 3−2788 : Selenium on SNARE−associated protein Snapin
MR Test
Inverse variance weighted (fixed effects)
Inverse variance weighted (multiplicative random effects)
MR Egger
MR RAPS
Weighted median
Weighted mode
0.05
0.00
−0.05
−0.10
−0.15
SNP effect on SNARE−associated protein Snapin
0.1
0.2
0.3
0.4
SNP effect on Selenium

Batch 3−2789 : Selenium on Alpha−synuclein

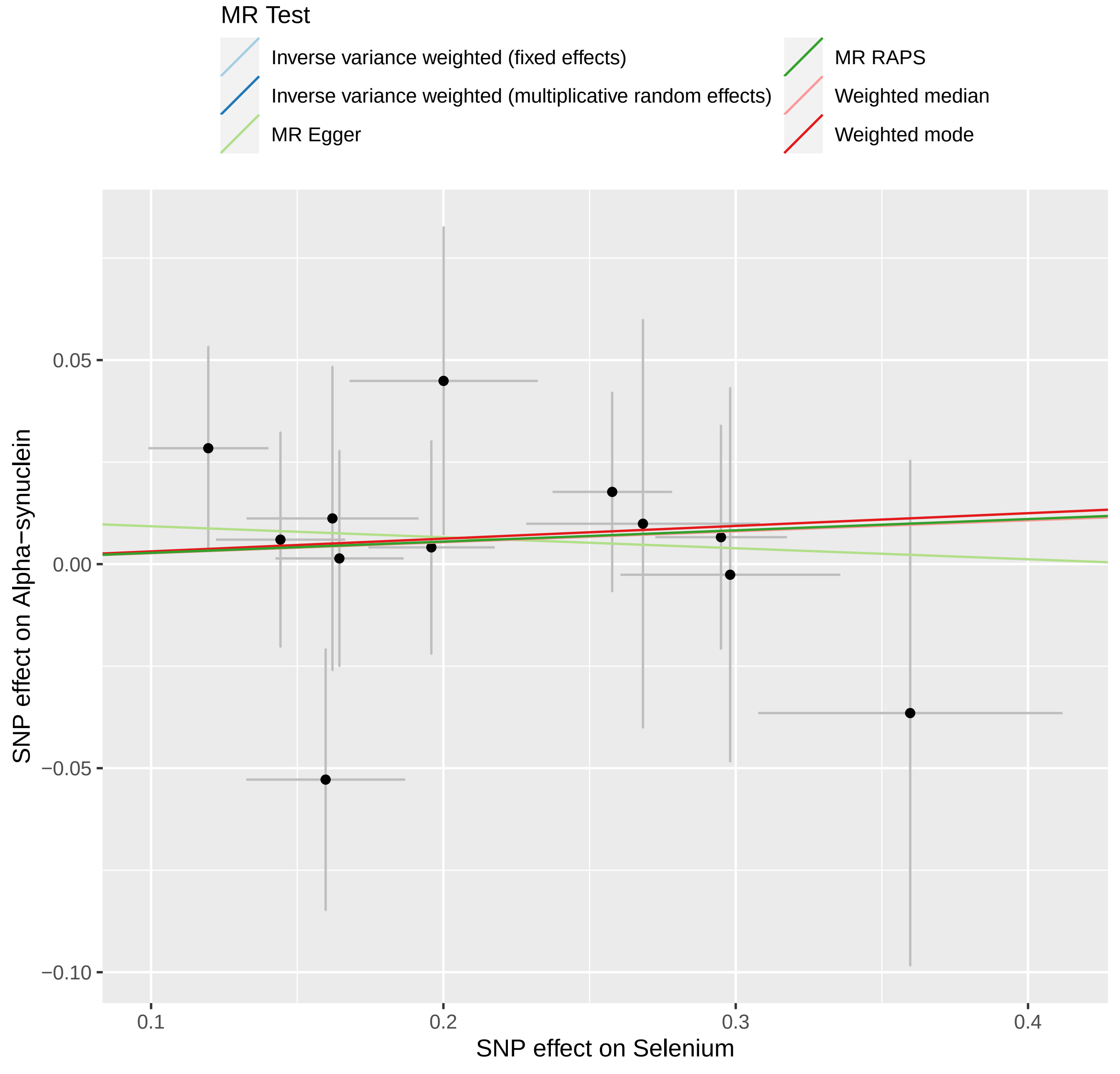

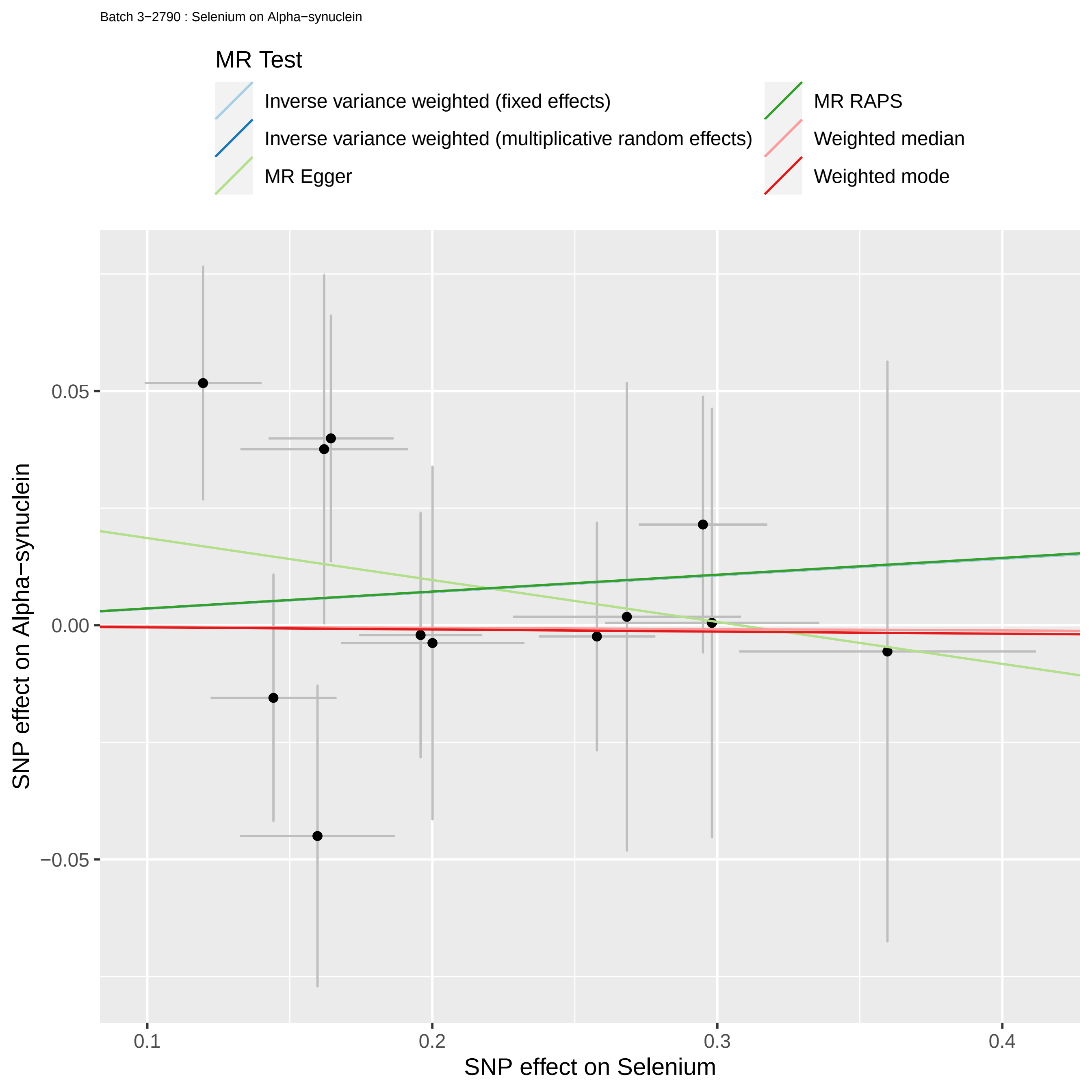
Batch 3−2790 : Selenium on Alpha−synuclein
MR Test
Inverse variance weighted (fixed effects)
Inverse variance weighted (multiplicative random effects)
MR Egger
MR RAPS
Weighted median
Weighted mode
0.05
0.00
−0.05
0.1
0.2
0.3
0.4
SNP effect on Alpha−synuclein
SNP effect on Selenium

Batch 3−2791 : Selenium on U1 small nuclear ribonucleoprotein A

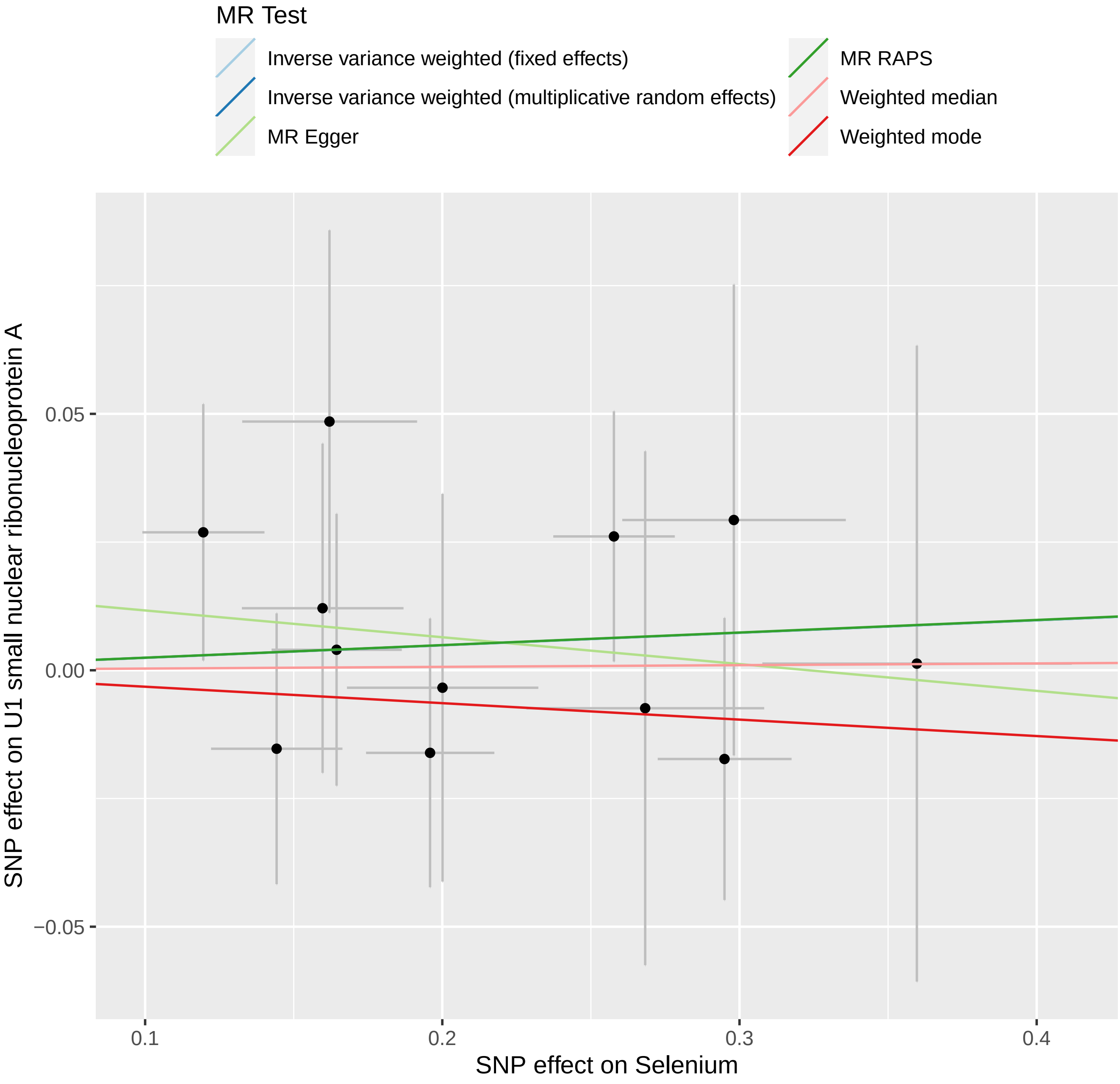

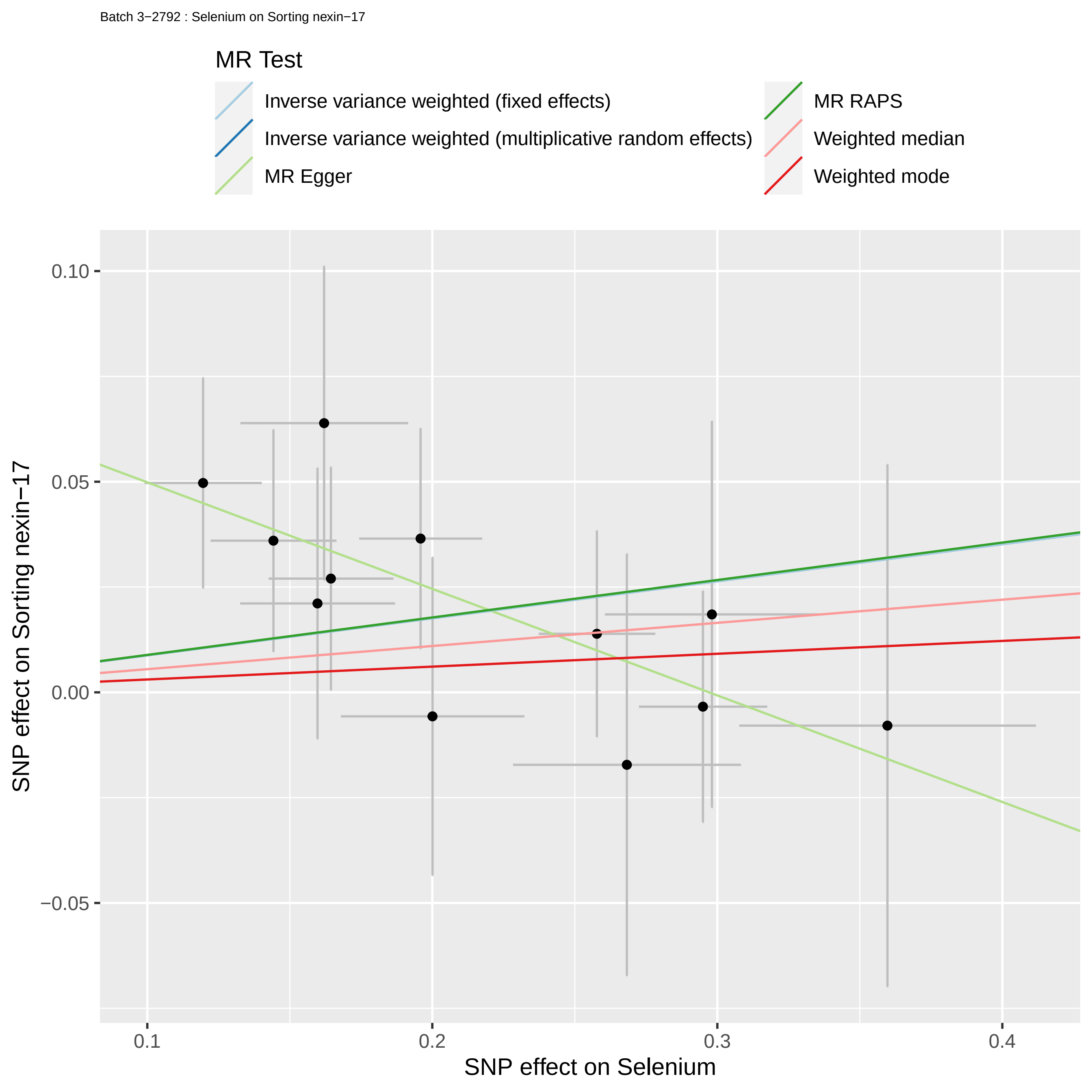
Batch 3−2792 : Selenium on Sorting nexin−17
MR Test
Inverse variance weighted (fixed effects)
Inverse variance weighted (multiplicative random effects)
MR Egger
MR RAPS
Weighted median
Weighted mode
0.10
0.05
0.00
−0.05
0.1
0.2
0.3
0.4
SNP effect on Sorting nexin−17
SNP effect on Selenium

Batch 3−2793 : Selenium on Sorting nexin−1

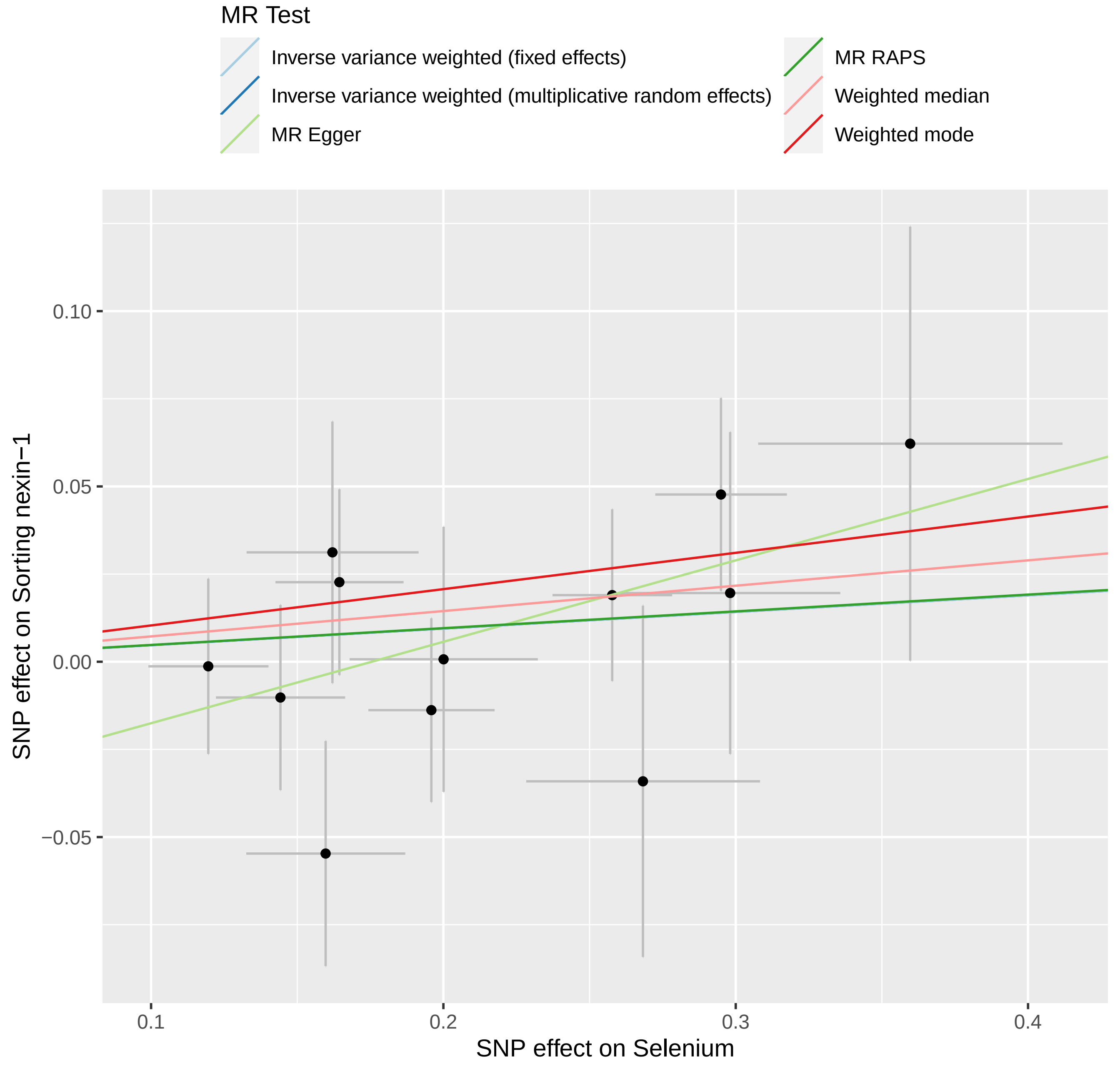

Batch 3−2794 : Selenium on Sorting nexin−4

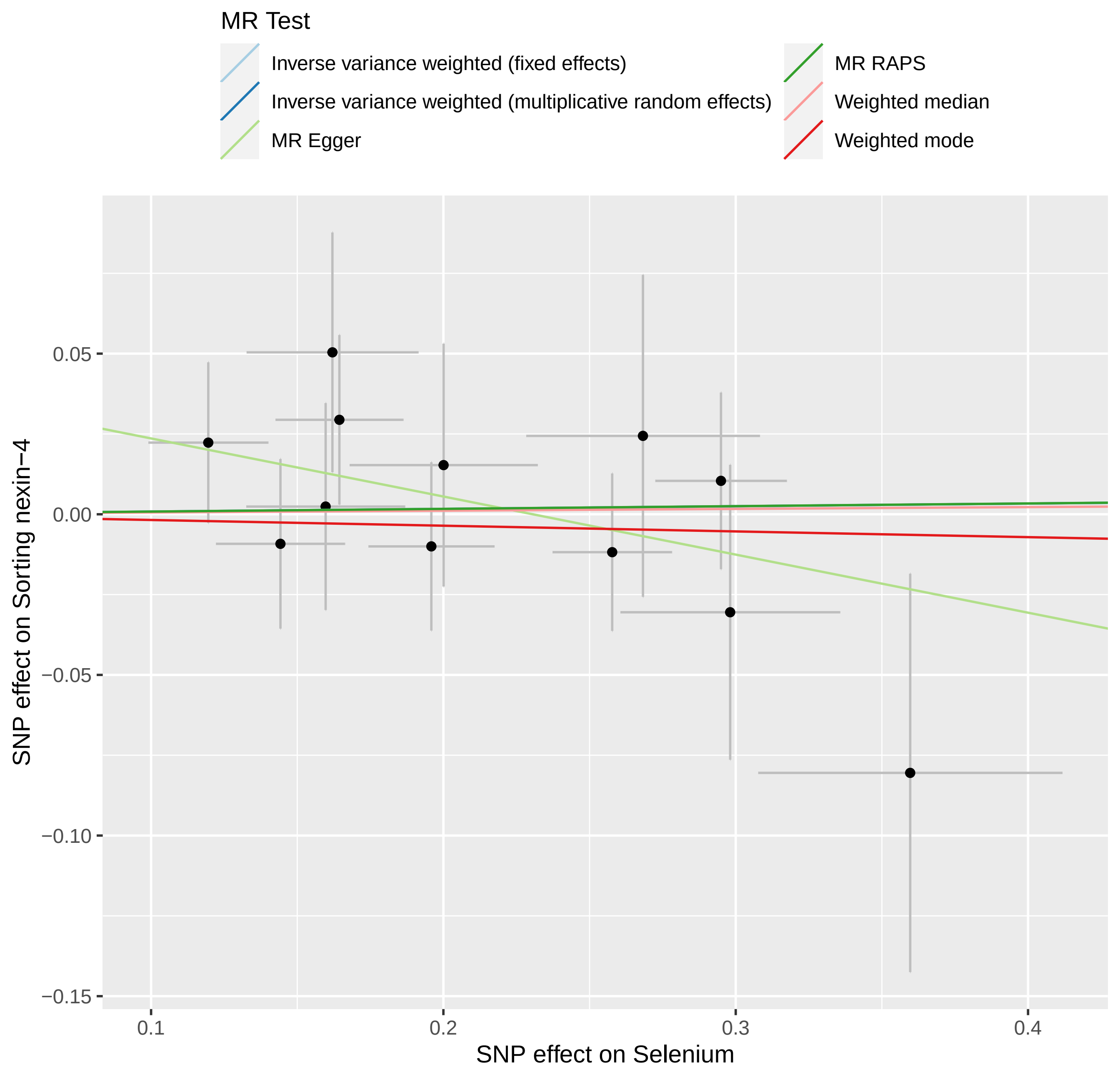

Batch 3−2795 : Selenium on Sorting nexin−7

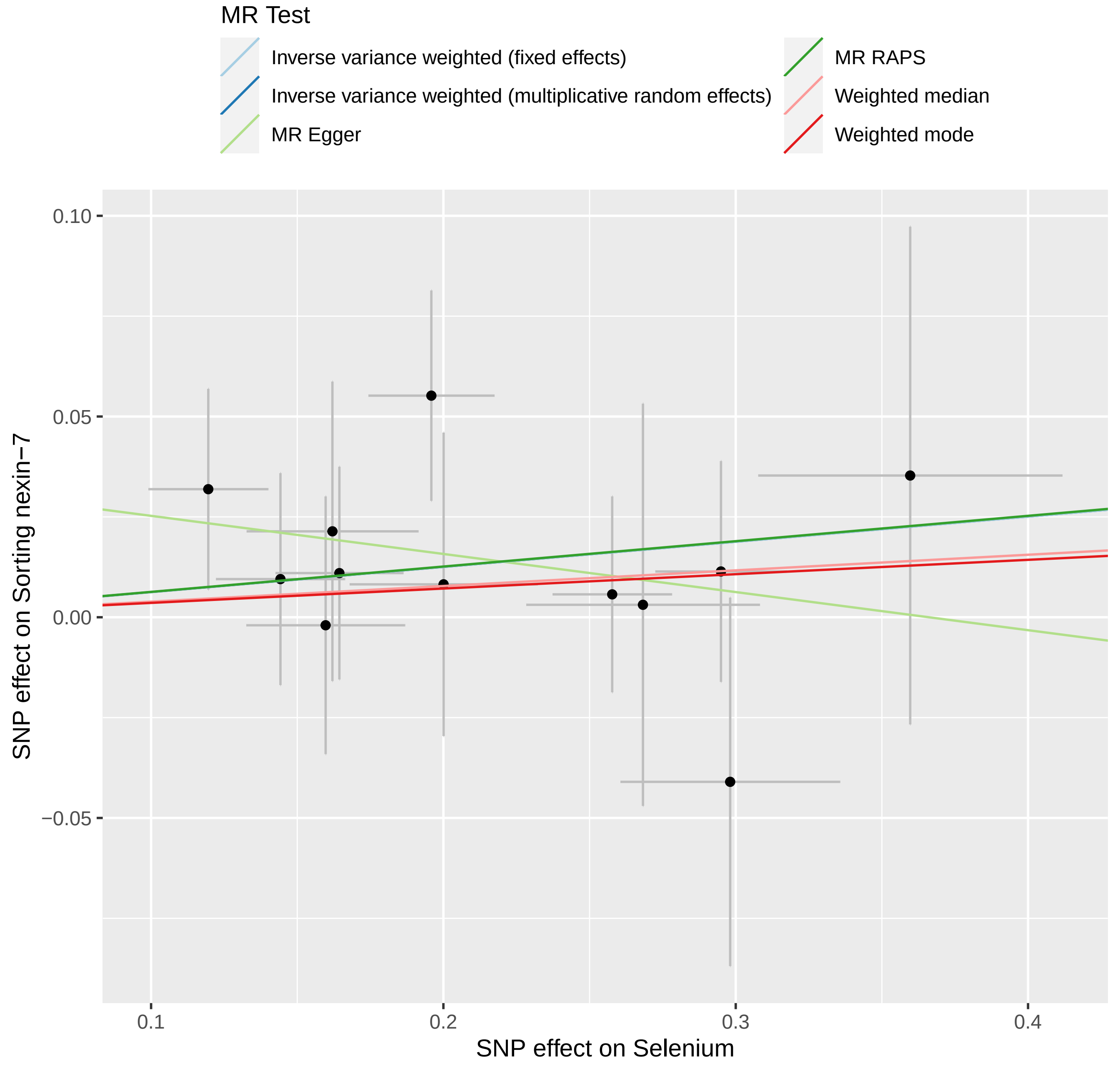

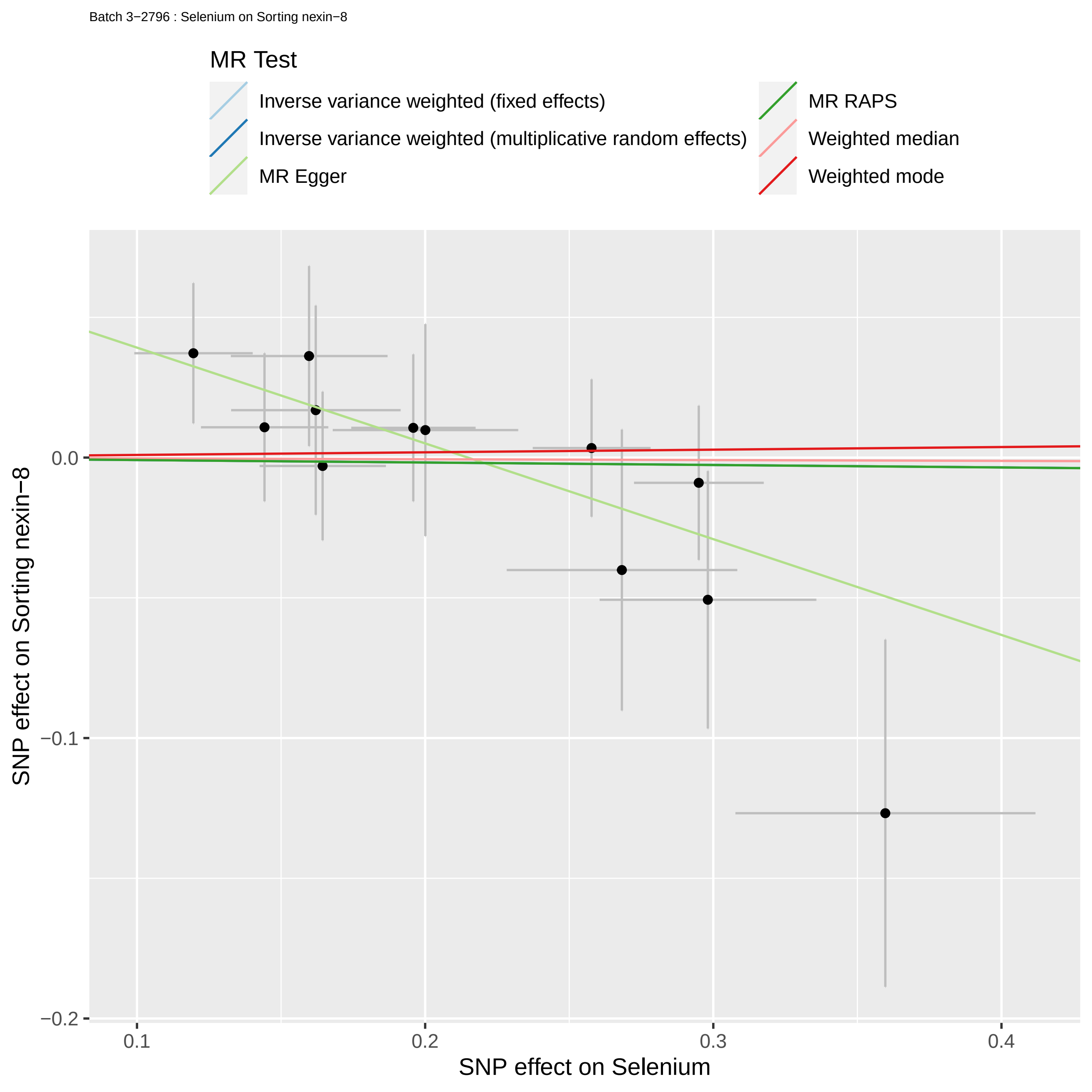
Batch 3−2796 : Selenium on Sorting nexin−8
MR Test
Inverse variance weighted (fixed effects)
Inverse variance weighted (multiplicative random effects)
MR Egger
MR RAPS
Weighted median
Weighted mode
SNP effect on Sorting nexin−8
0.0
−0.1
−0.2
0.1
0.2
0.3
0.4
SNP effect on Selenium

Batch 3−2797 : Selenium on Suppressor of cytokine signaling 3

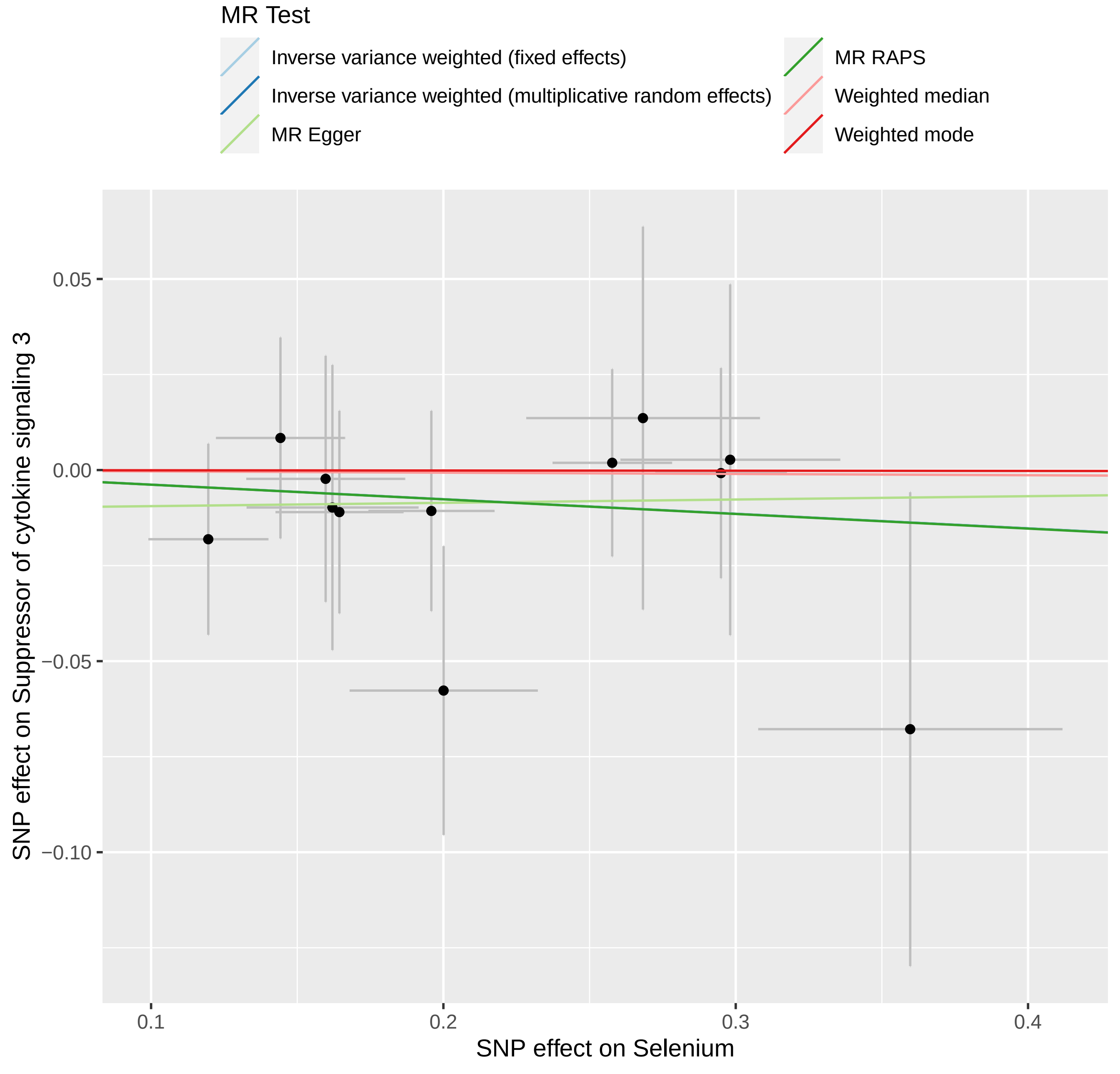

Batch 3−2798 : Selenium on Suppressor of cytokine signaling 7

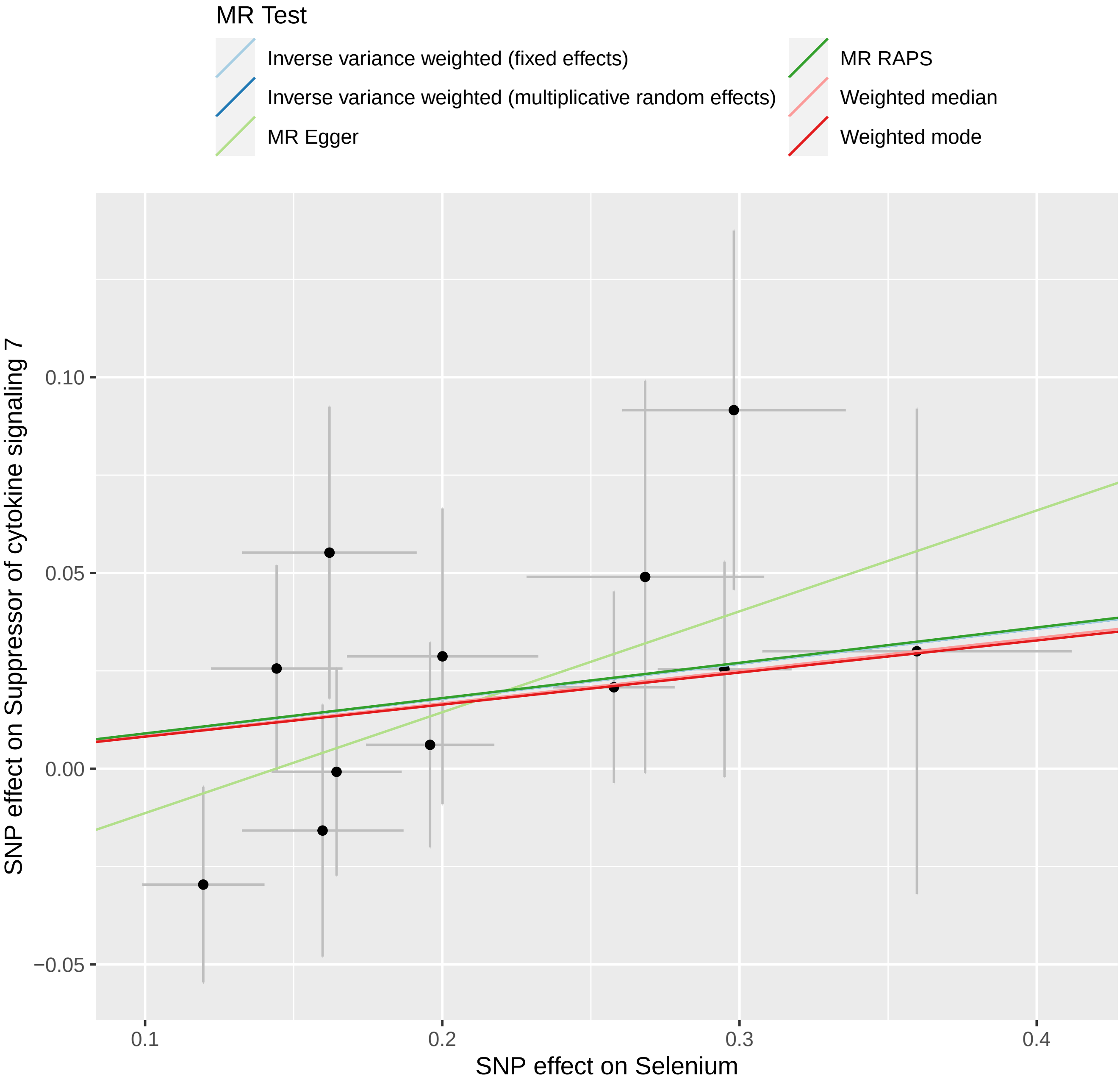

Batch 3−2799 : Selenium on Superoxide dismutase [Mn], mitochondrial

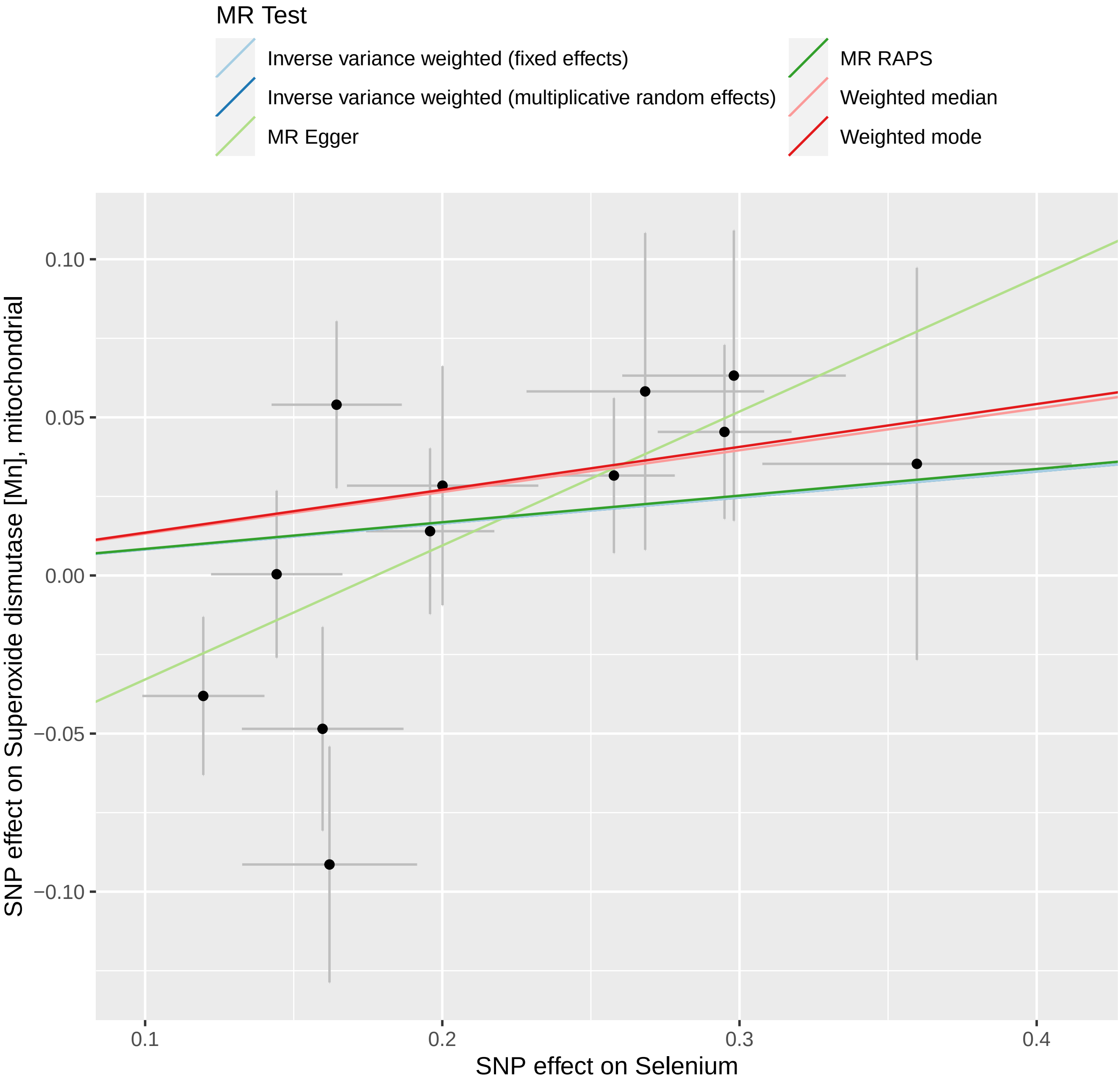

Batch 3−2800 : Selenium on Extracellular superoxide dismutase [Cu−Zn]

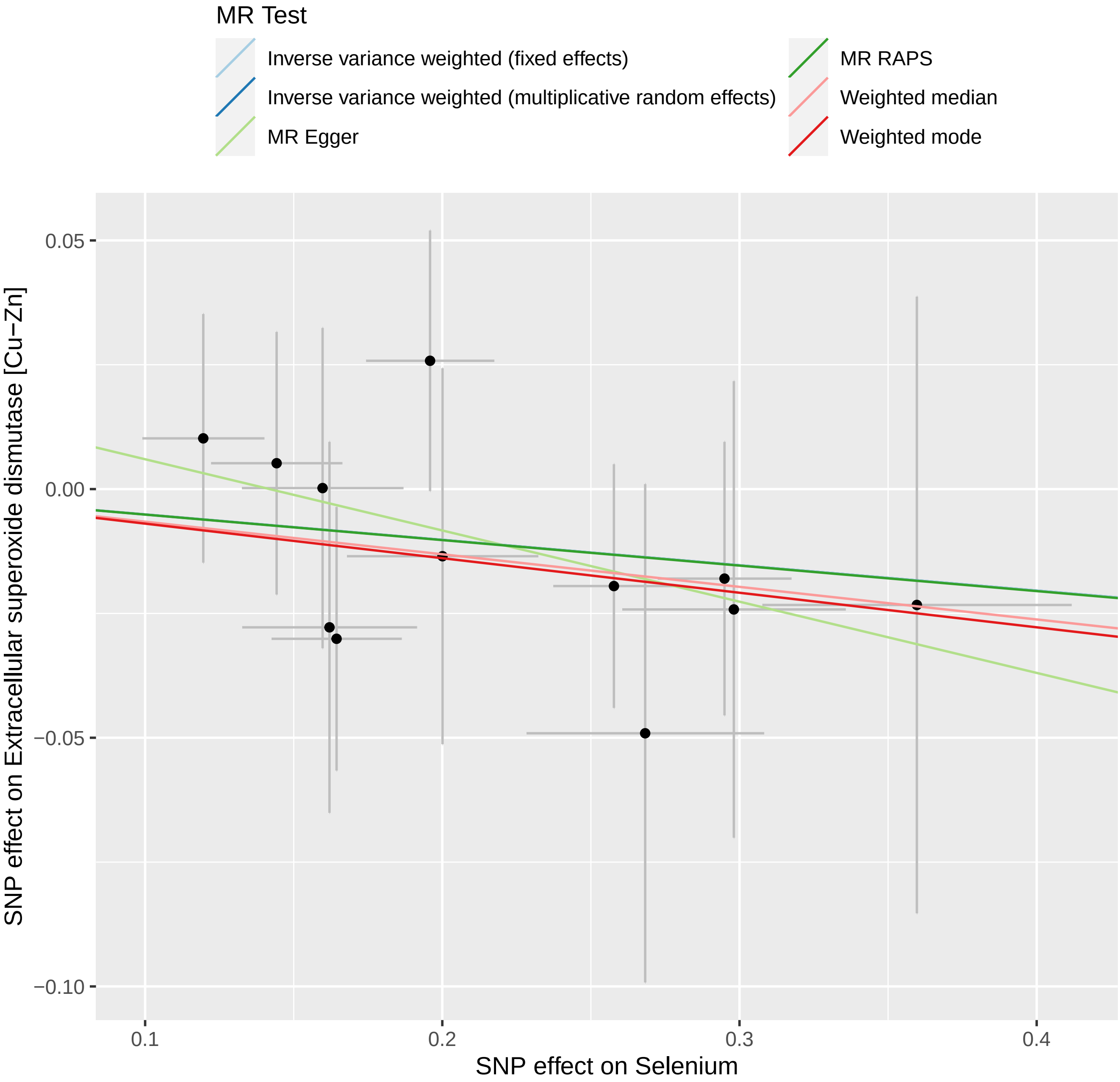

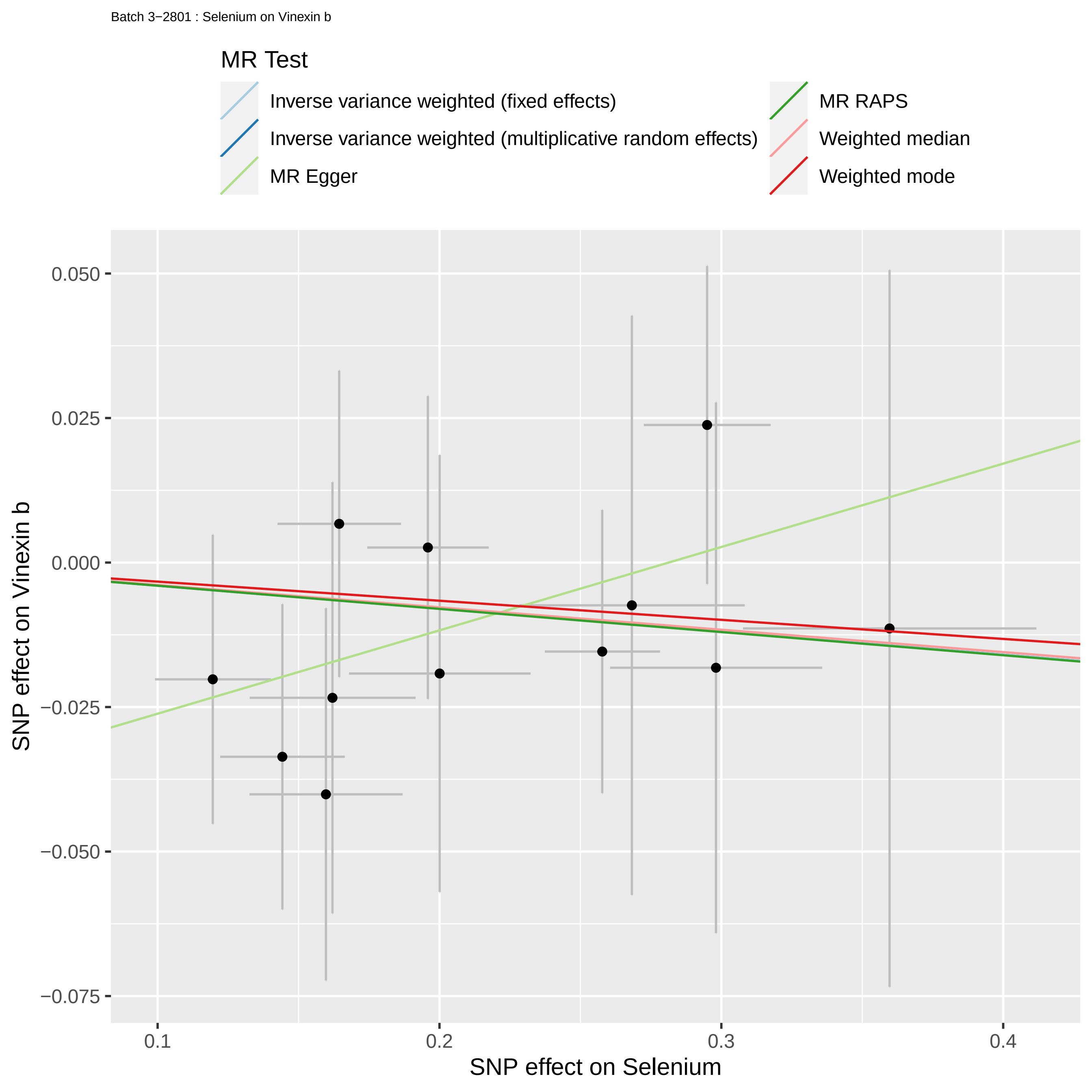
Batch 3−2801 : Selenium on Vinexin b
MR Test
Inverse variance weighted (fixed effects)
Inverse variance weighted (multiplicative random effects)
MR Egger
MR RAPS
Weighted median
Weighted mode
0.050
0.025
0.000
−0.025
−0.050
−0.075
0.1
0.2
0.3
0.4
SNP effect on Vinexin b
SNP effect on Selenium

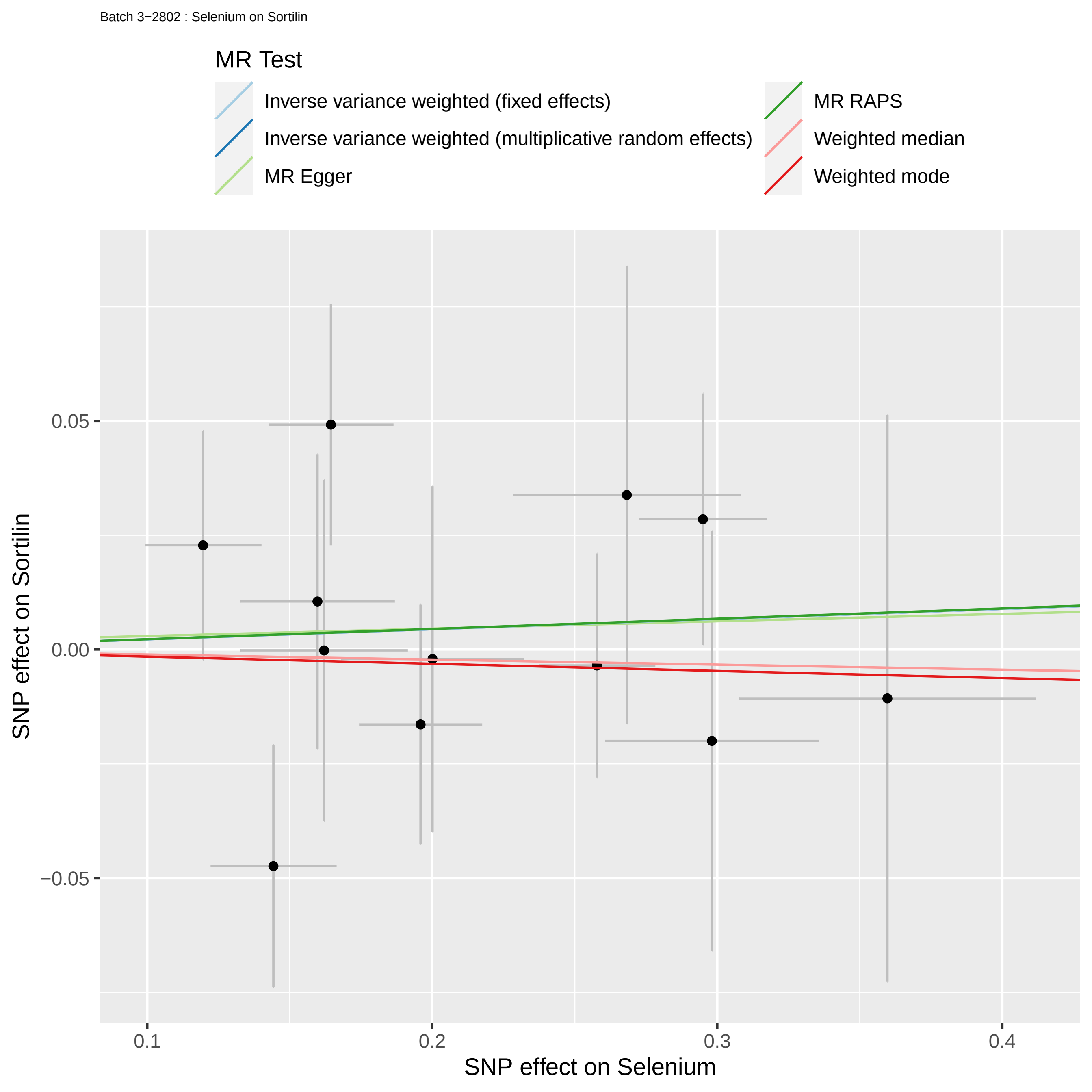
Batch 3−2802 : Selenium on Sortilin
MR Test
Inverse variance weighted (fixed effects)
Inverse variance weighted (multiplicative random effects)
MR Egger
MR RAPS
Weighted median
Weighted mode
0.05
0.00
−0.05
SNP effect on Sortilin
0.1
0.2
0.3
0.4
SNP effect on Selenium

Batch 3−2803 : Selenium on Sclerostin

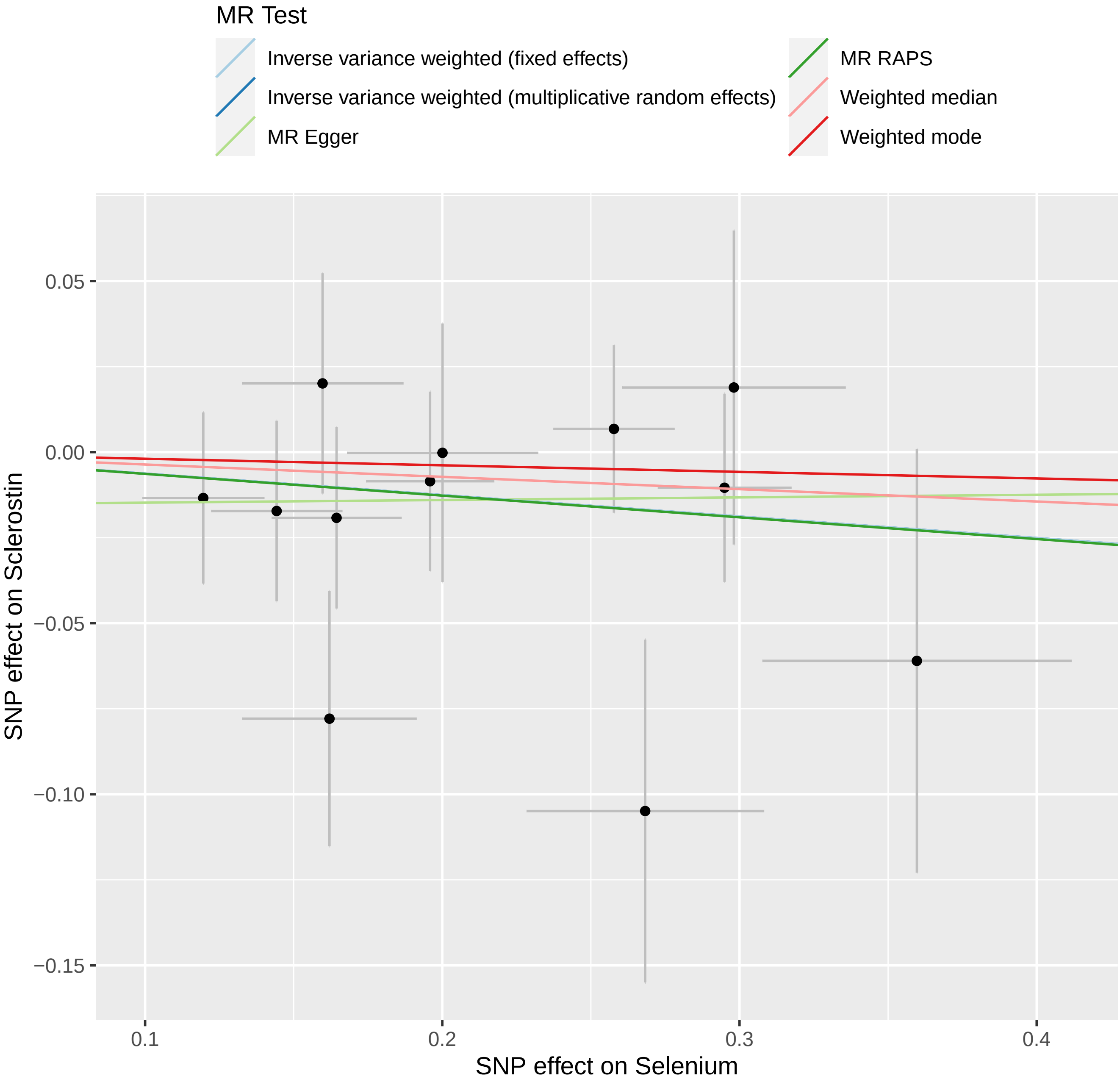

Batch 3−2804 : Selenium on Sperm acrosome membrane−associated protein 3

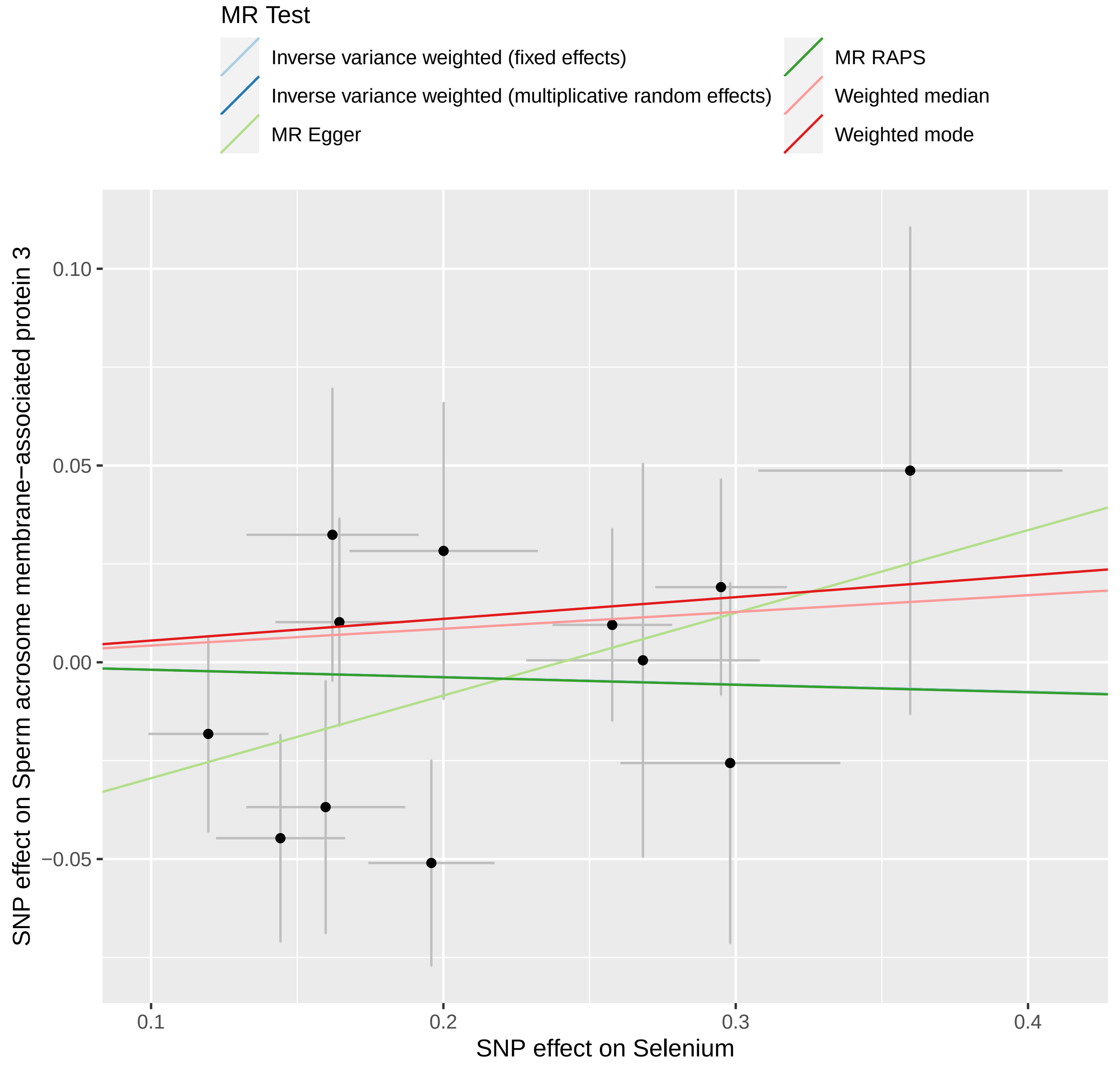

Batch 3−2805 : Selenium on Protein SPACA7

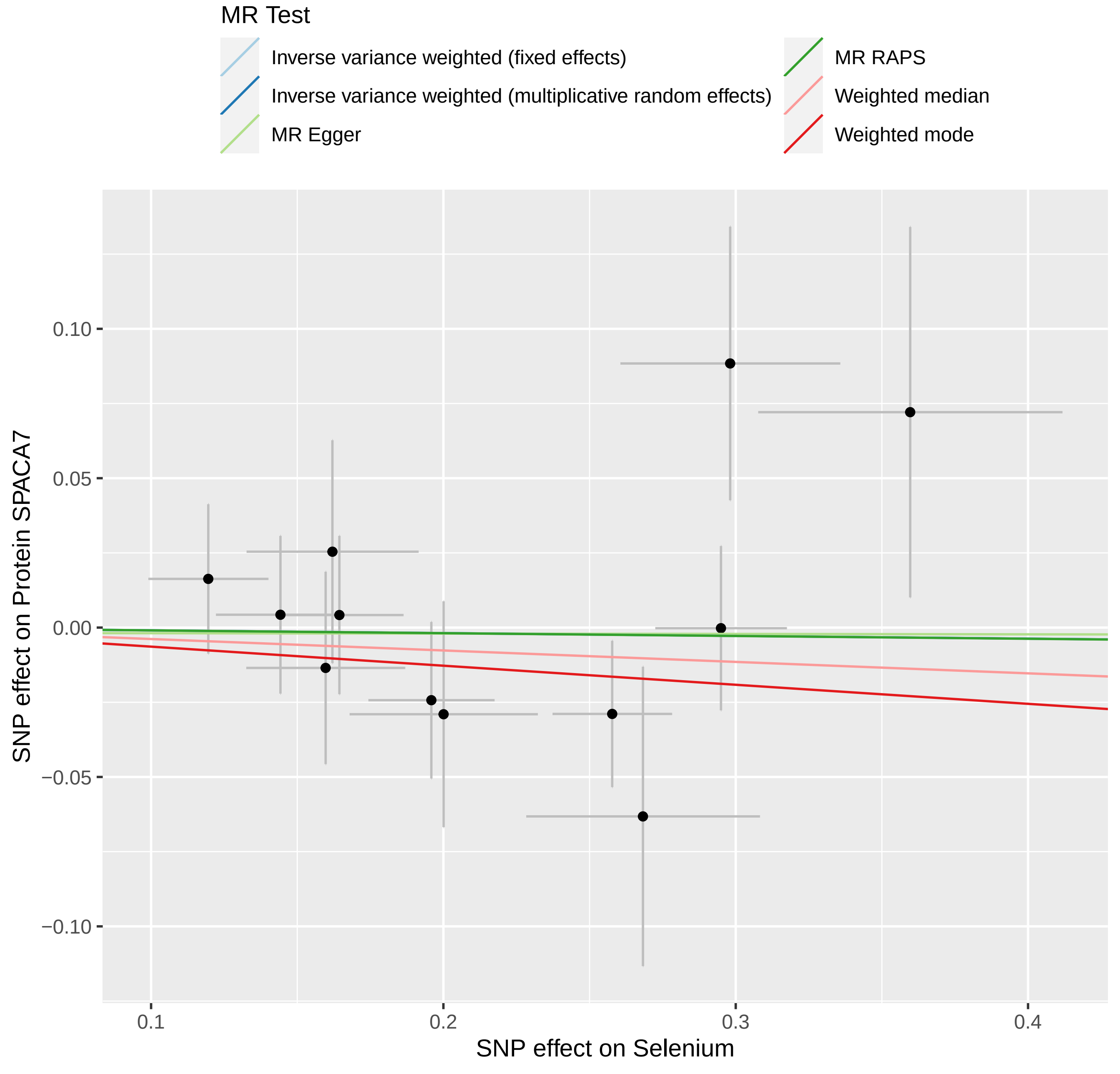

Batch 3−2806 : Selenium on Sperm−associated antigen 11A

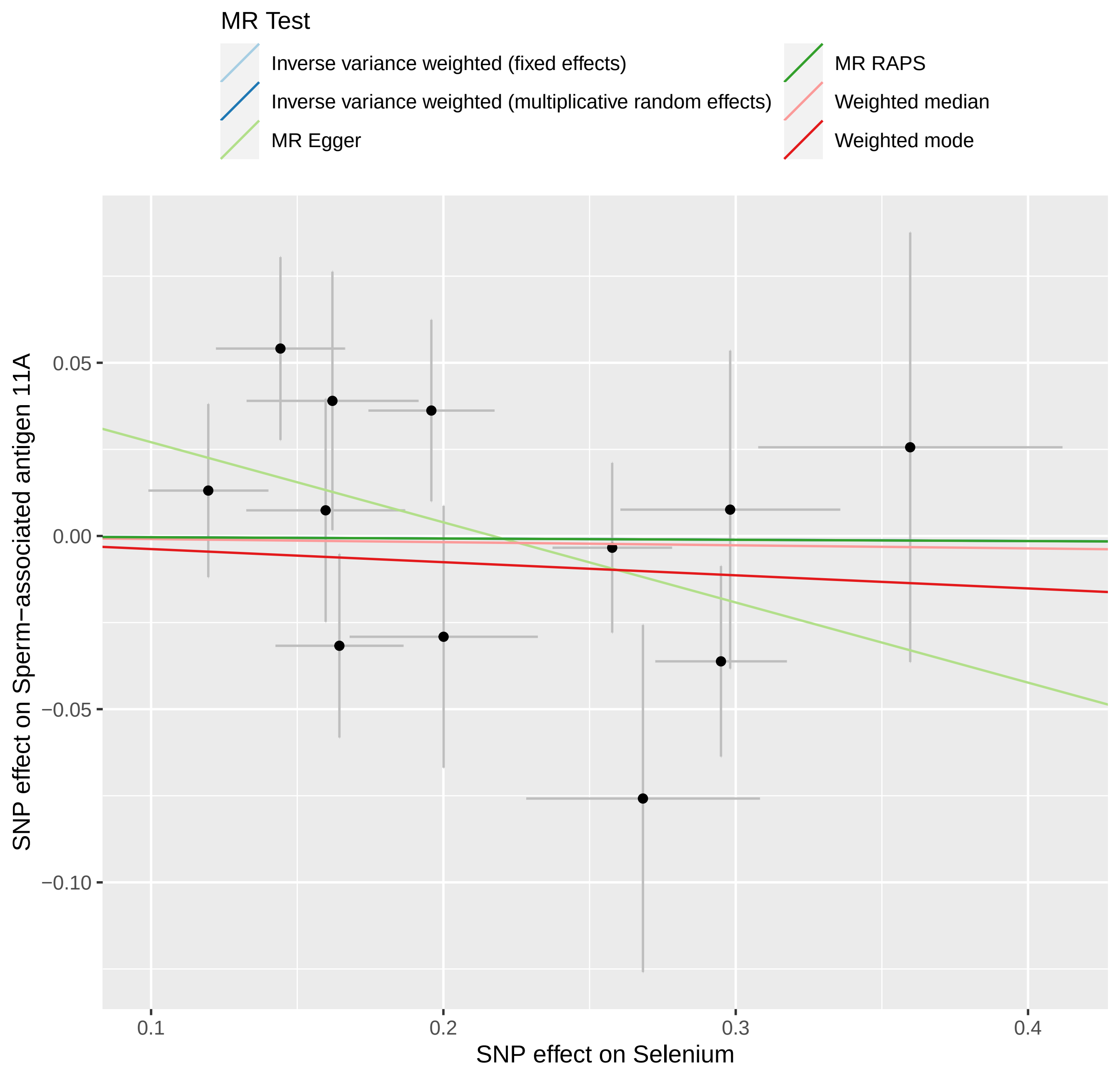

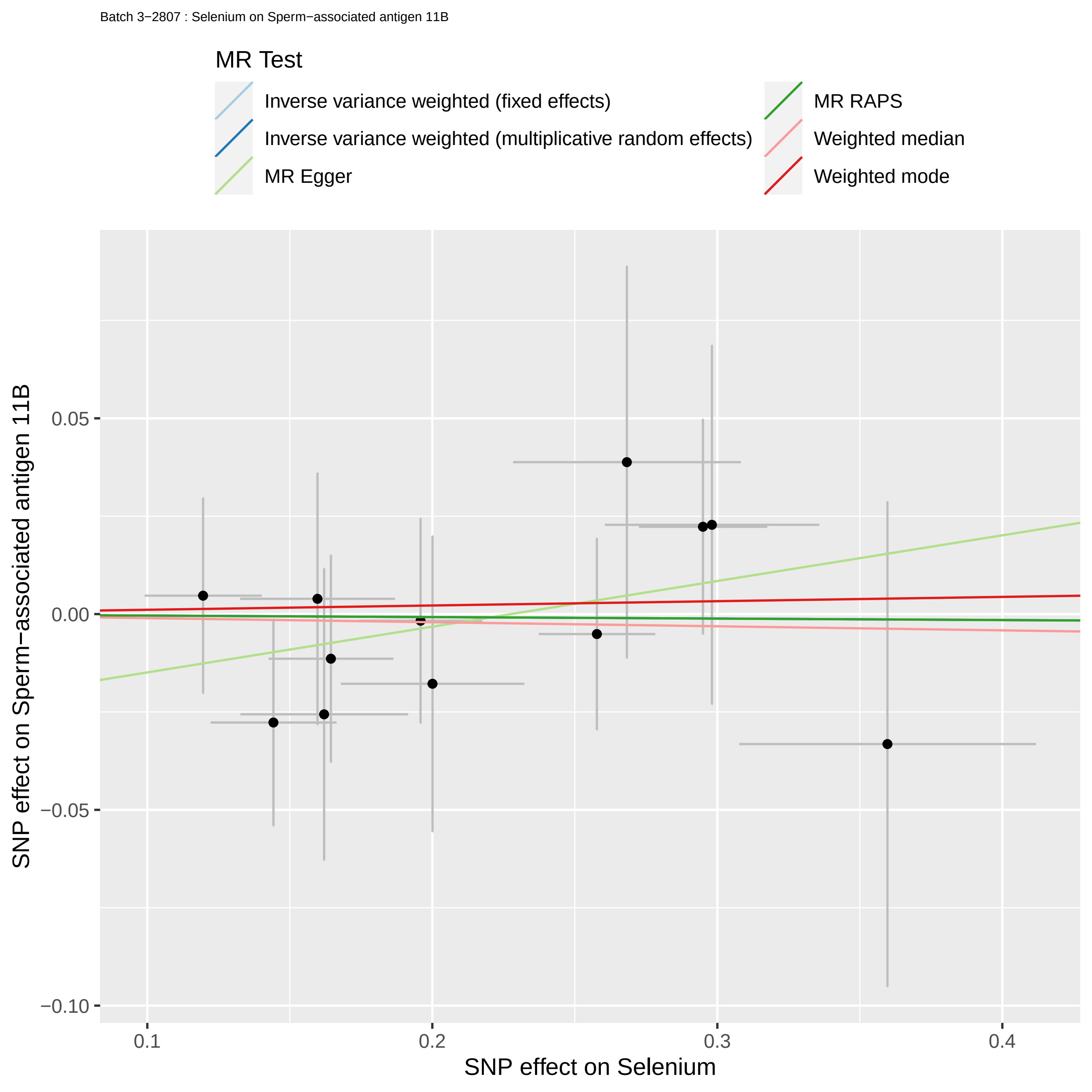
Batch 3−2807 : Selenium on Sperm−associated antigen 11B
MR Test
Inverse variance weighted (fixed effects)
Inverse variance weighted (multiplicative random effects)
MR Egger
MR RAPS
Weighted median
Weighted mode
0.05
0.00
−0.05
−0.10
0.1
0.2
0.3
0.4
SNP effect on Sperm−associated antigen 11B
SNP effect on Selenium

Batch 3−2808 : Selenium on SPARC

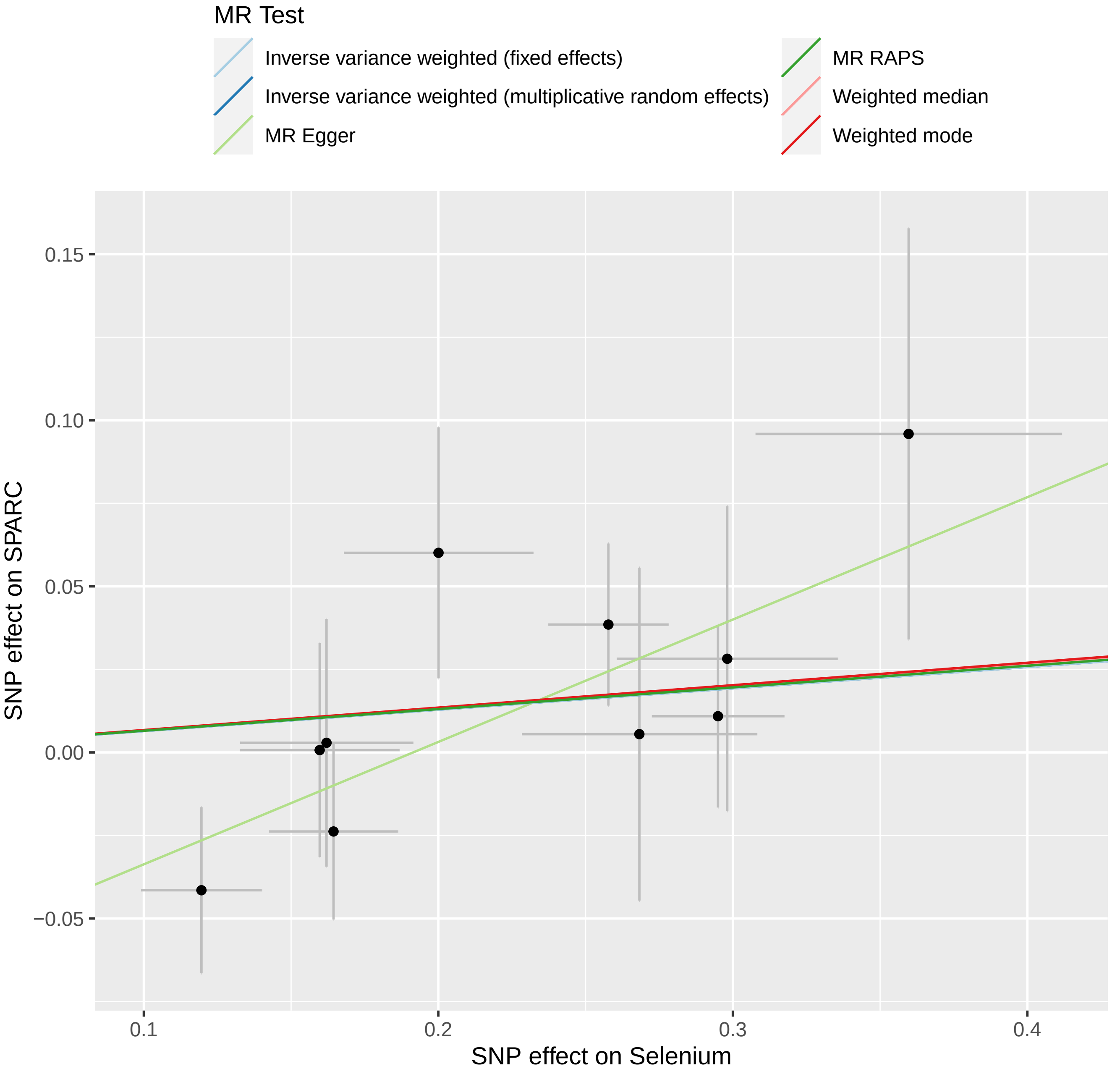

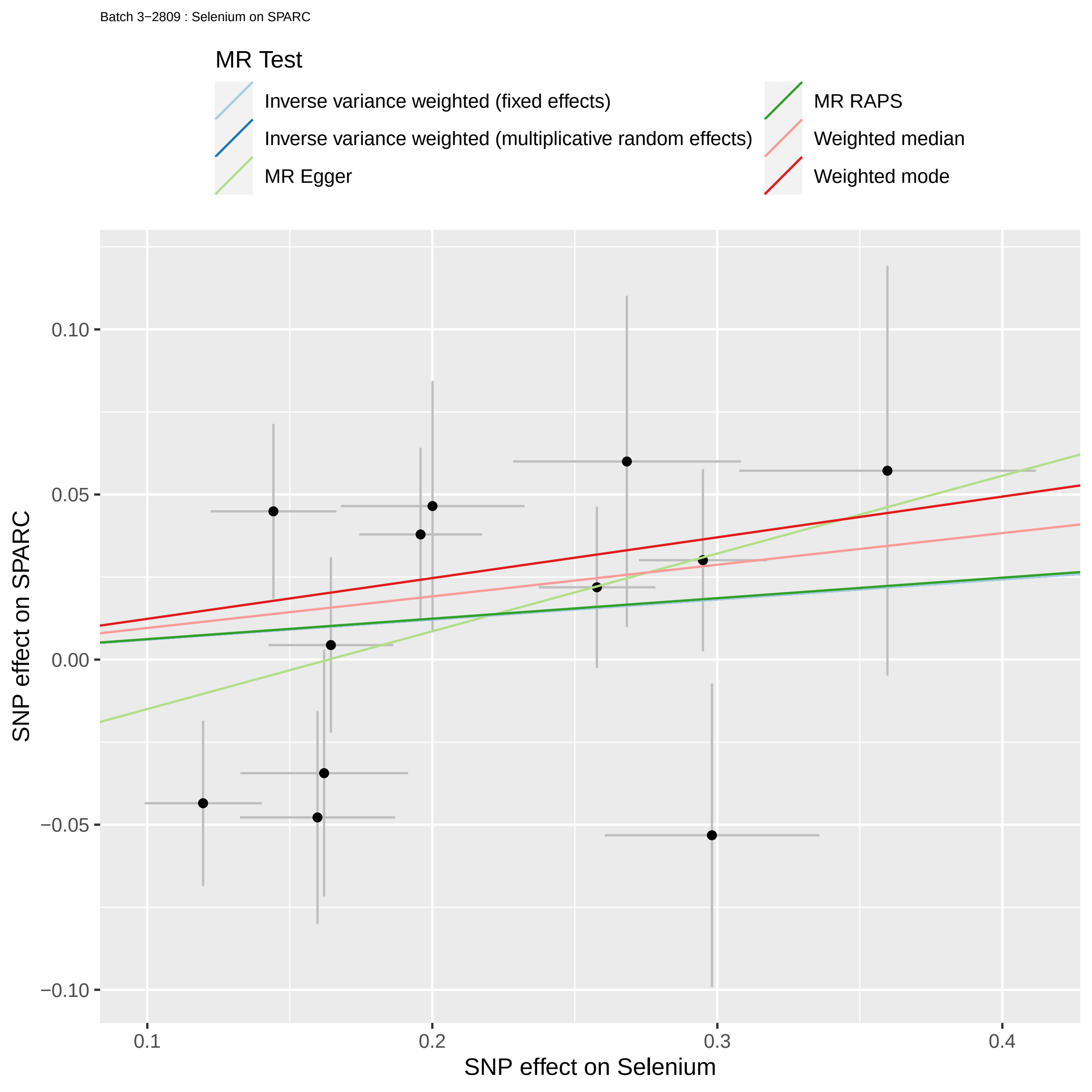

Batch 3−2809 : Selenium on SPARC
MR Test
Inverse variance weighted (fixed effects)
Inverse variance weighted (multiplicative random effects)
MR Egger
MR RAPS
Weighted median
Weighted mode
0.10
0.05
0.00
−0.05
−0.10
0.1
0.2
0.3
0.4
SNP effect on SPARC
SNP effect on Selenium

Batch 3−2810 : Selenium on SPARC−like protein 1

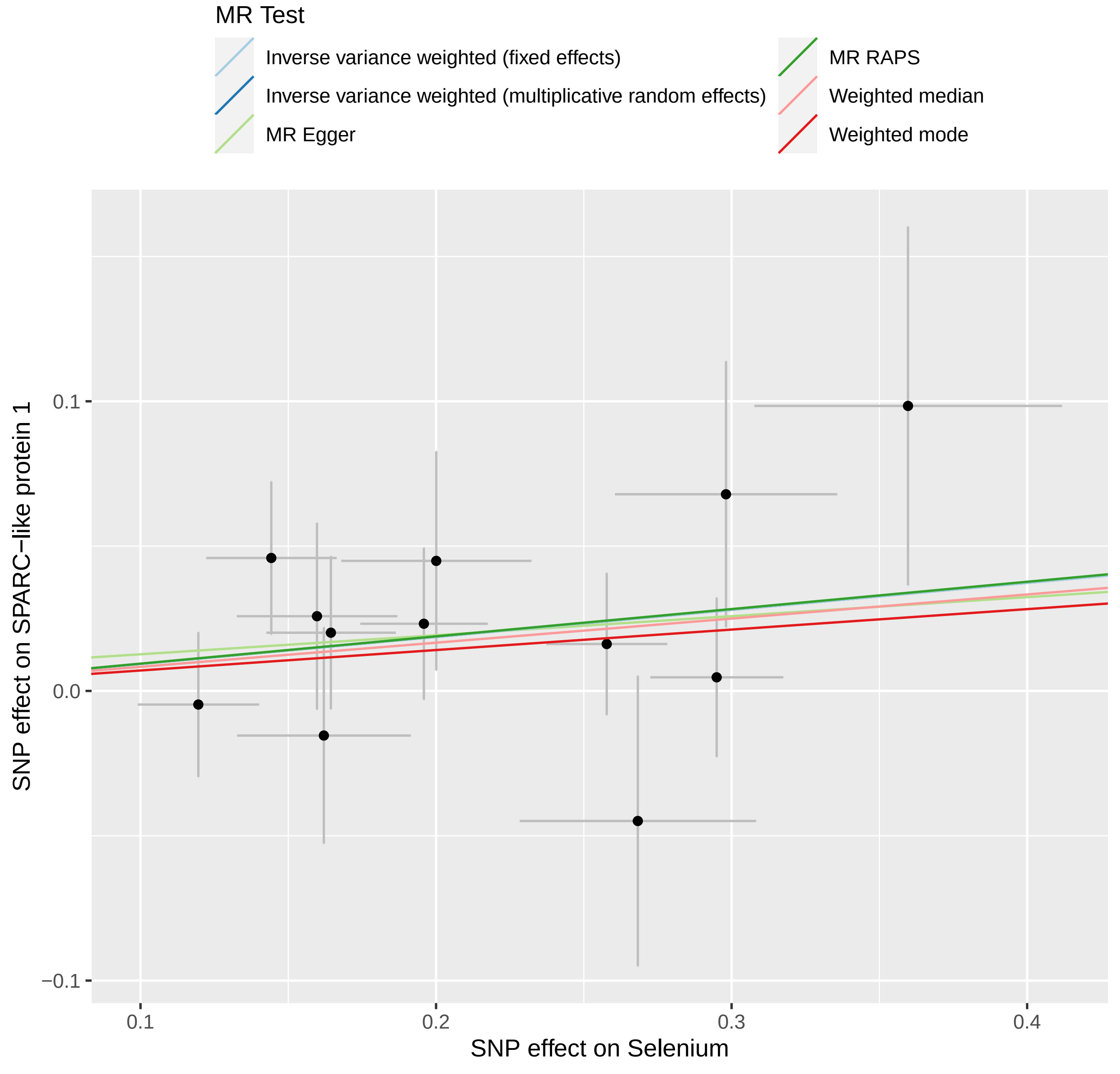

Batch 3−2811 : Selenium on SPARC−like protein 1

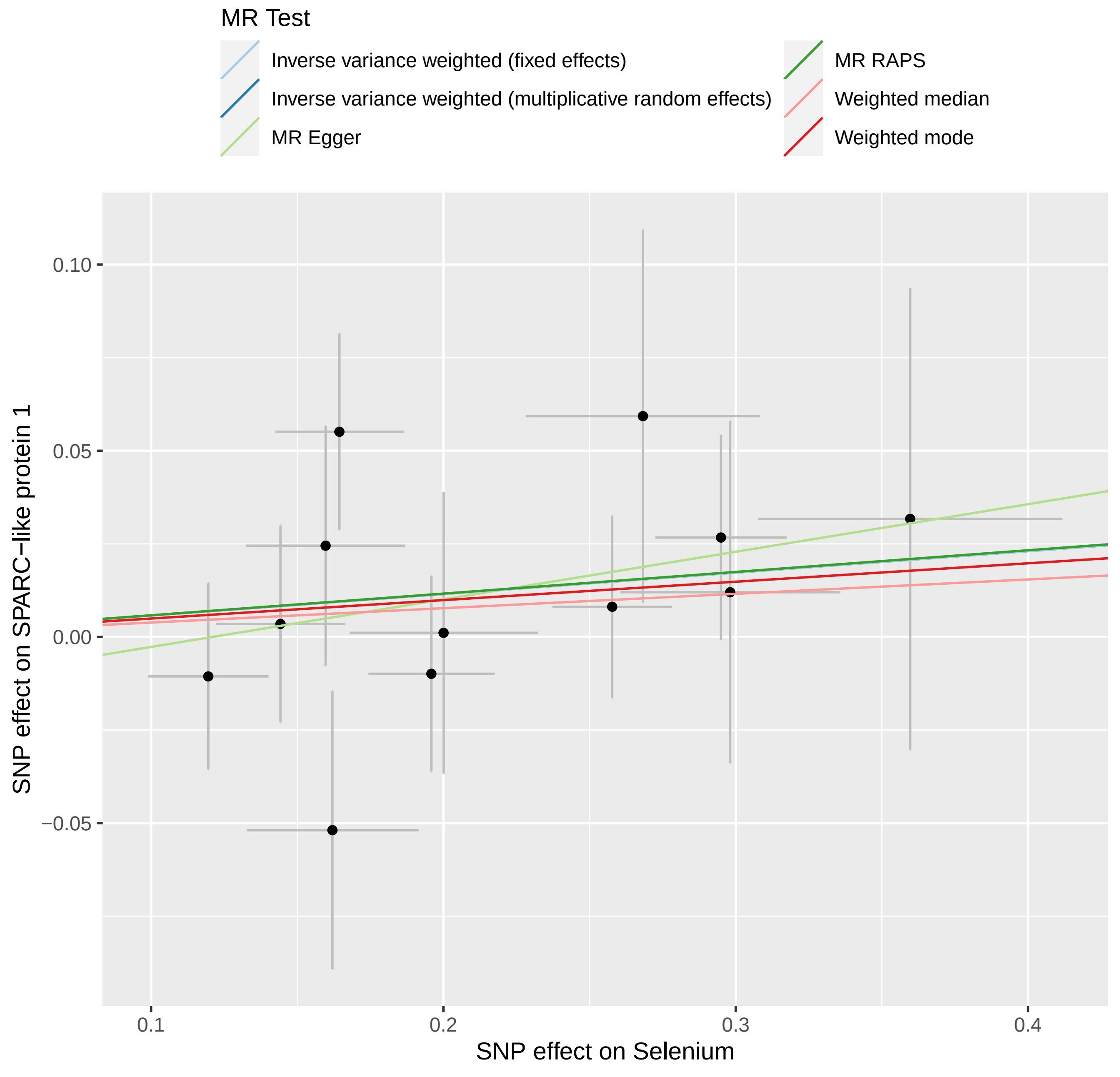

Batch 3−2812 : Selenium on Spastin

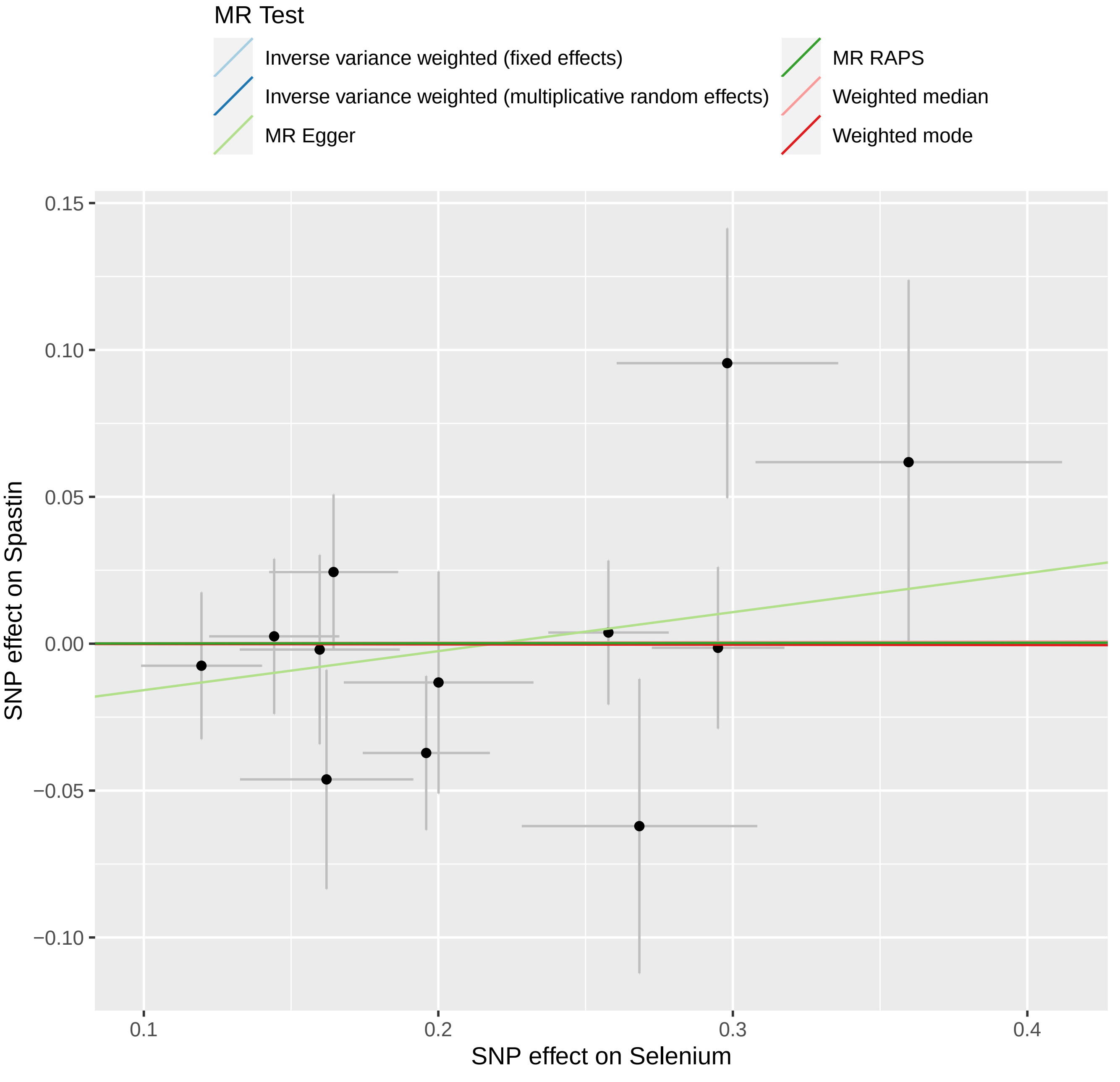

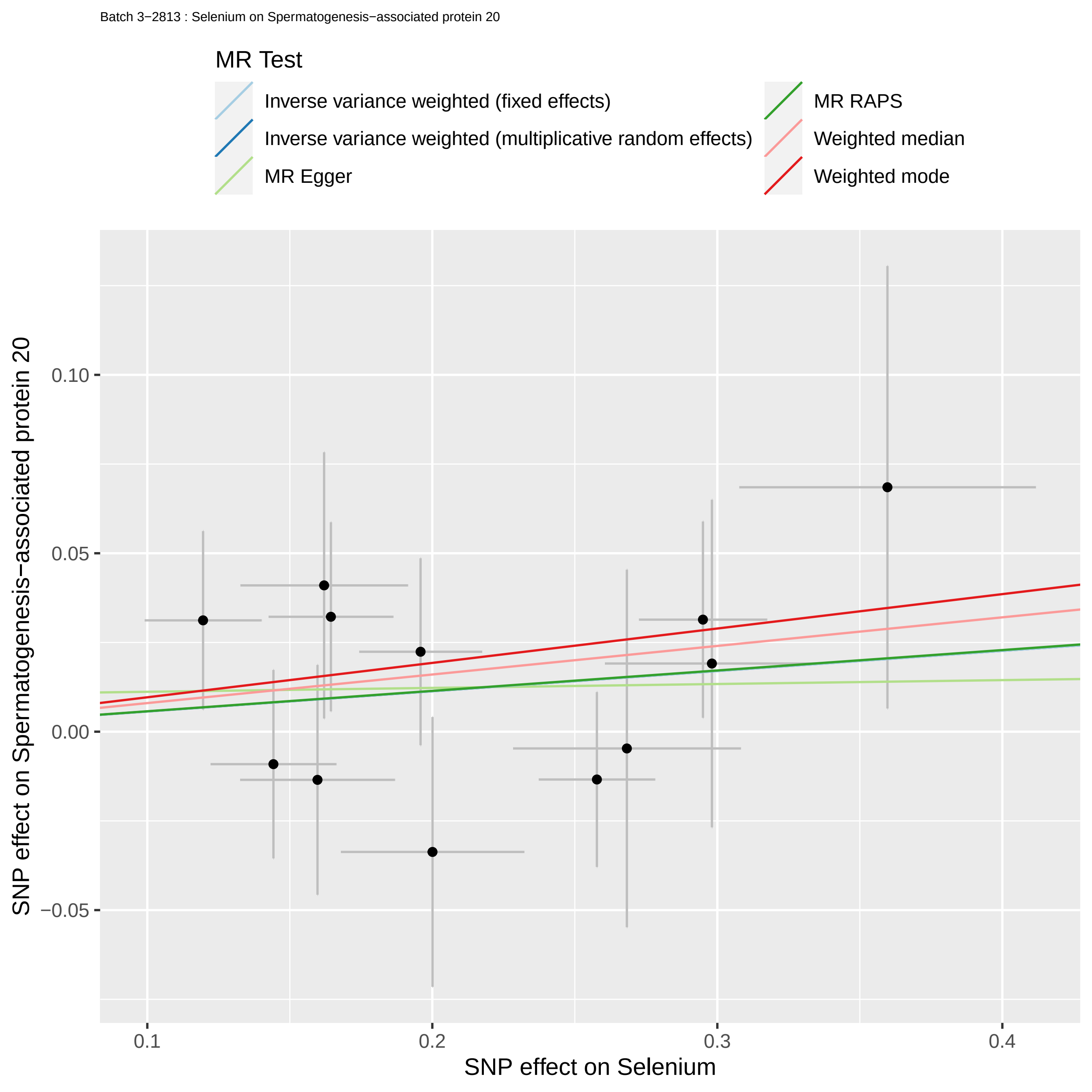

Batch 3−2813 : Selenium on Spermatogenesis−associated protein 20
MR Test
Inverse variance weighted (fixed effects)
Inverse variance weighted (multiplicative random effects)
MR Egger
MR RAPS
Weighted median
Weighted mode
0.10
0.05
0.00
−0.05
0.1
0.2
0.3
0.4
SNP effect on Spermatogenesis−associated protein 20
SNP effect on Selenium

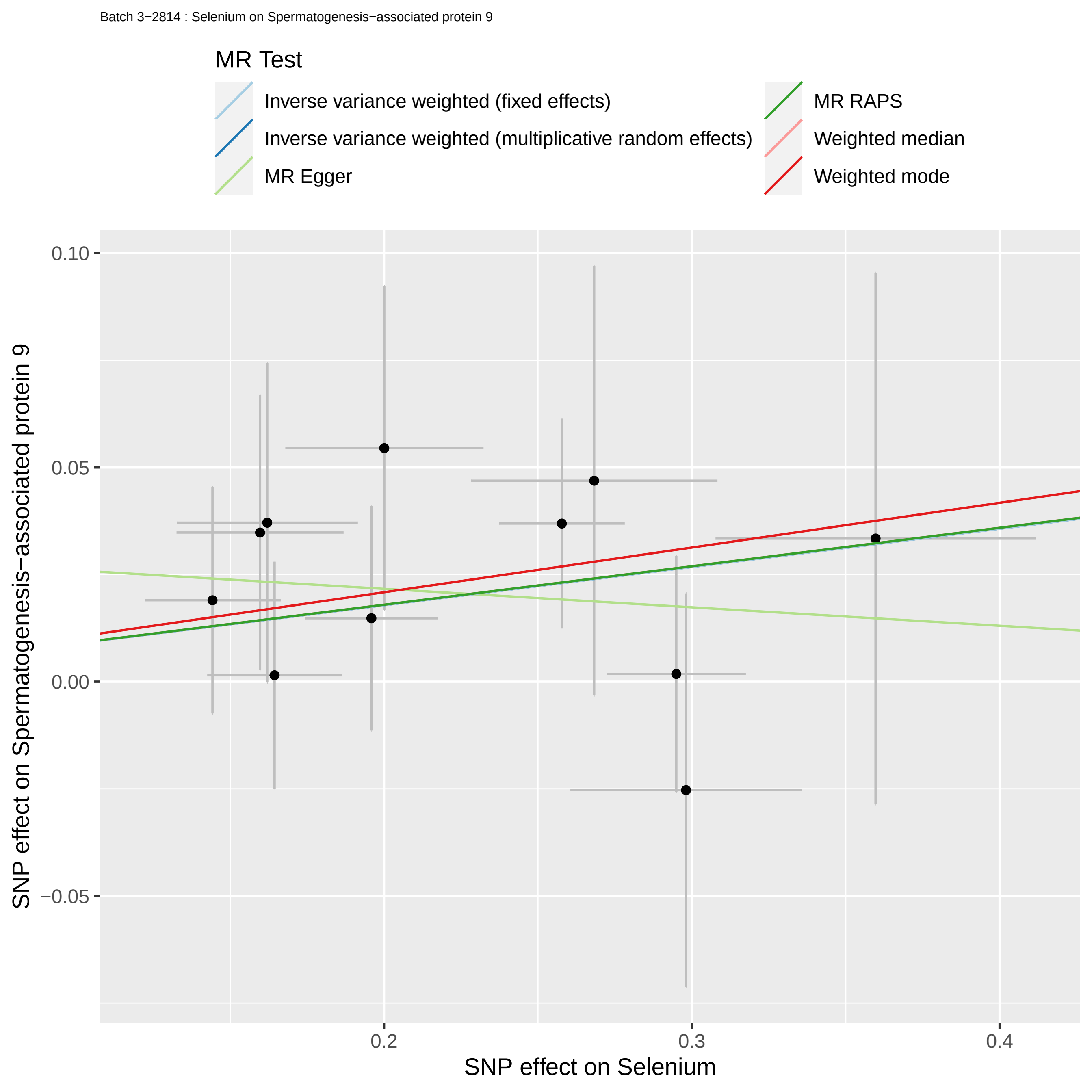

Batch 3−2814 : Selenium on Spermatogenesis−associated protein 9
MR Test
Inverse variance weighted (fixed effects)
Inverse variance weighted (multiplicative random effects)
MR Egger
MR RAPS
Weighted median
Weighted mode
0.10
0.05
0.00
−0.05
0.2
0.3
0.4
SNP effect on Spermatogenesis−associated protein 9
SNP effect on Selenium

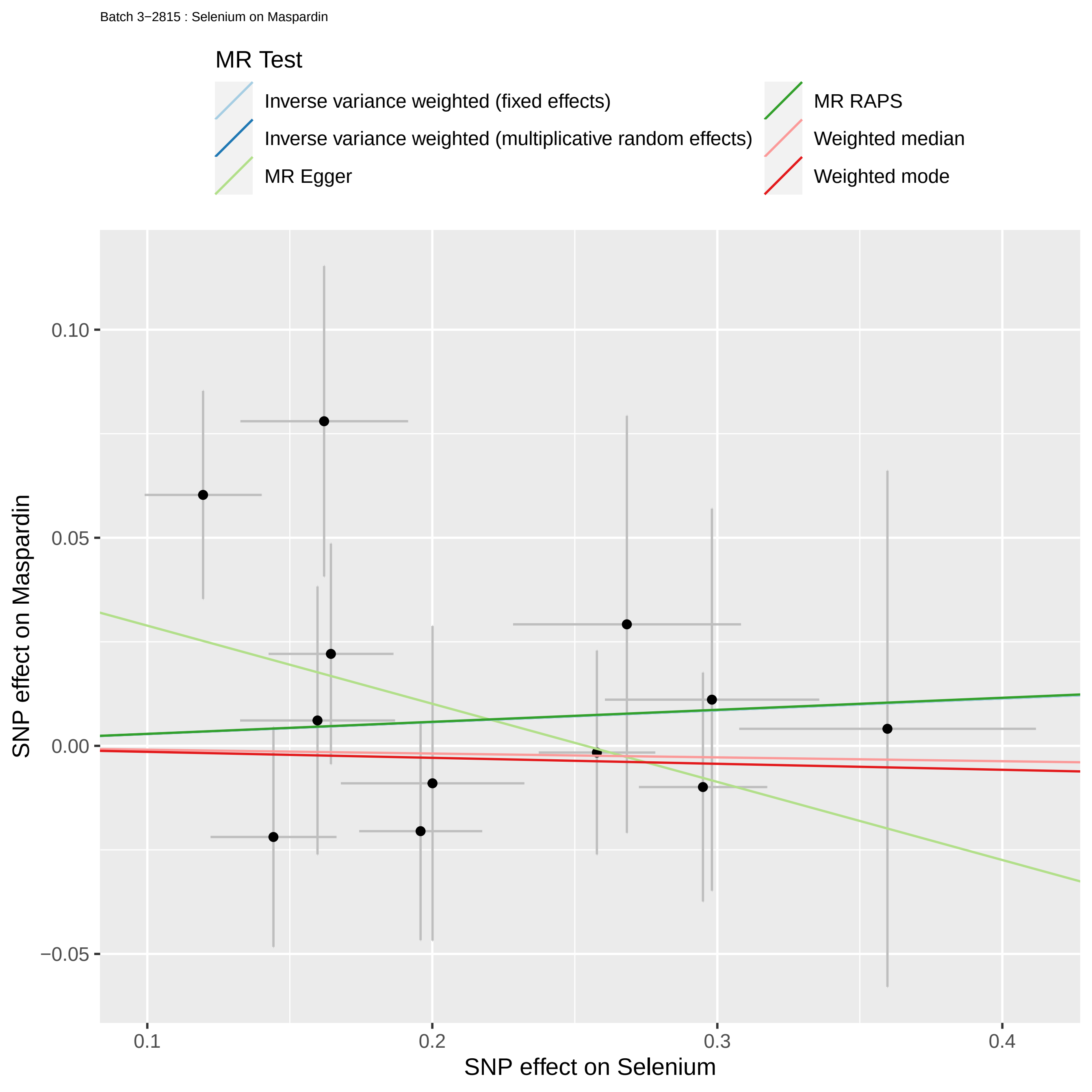
Batch 3−2815 : Selenium on Maspardin
MR Test
Inverse variance weighted (fixed effects)
Inverse variance weighted (multiplicative random effects)
MR Egger
MR RAPS
Weighted median
Weighted mode
0.10
0.05
0.00
−0.05
0.1
0.2
0.3
0.4
SNP effect on Selenium
SNP effect on Maspardin

Batch 3−2816 : Selenium on Sphingosine kinase 2

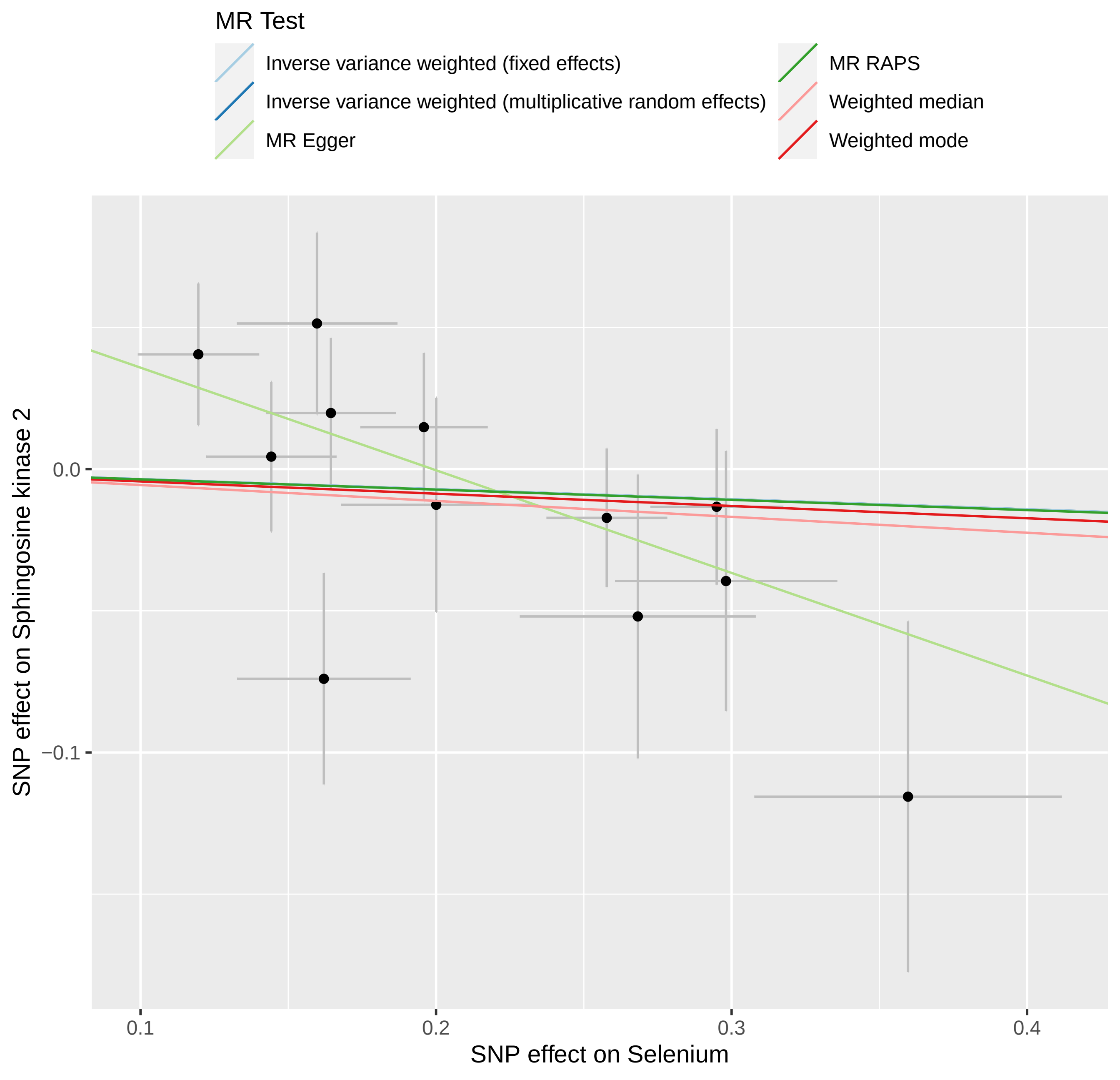

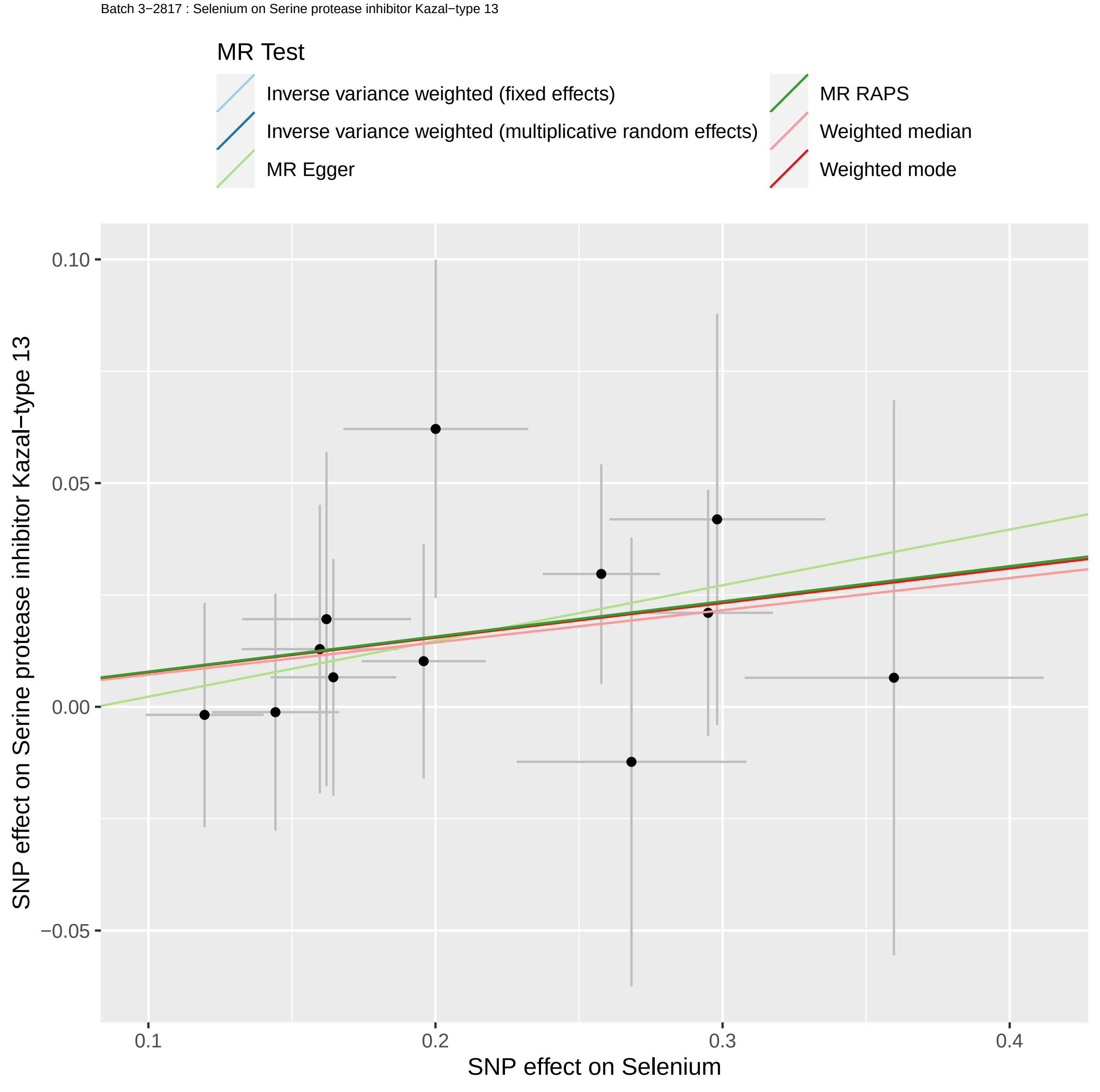

Batch 3−2817 : Selenium on Serine protease inhibitor Kazal−type 13
MR Test
Inverse variance weighted (fixed effects)
Inverse variance weighted (multiplicative random effects)
MR Egger
MR RAPS
Weighted median
Weighted mode
0.10
0.05
0.00
−0.05
0.1
0.2
0.3
0.4
SNP effect on Serine protease inhibitor Kazal−type 13
SNP effect on Selenium

Batch 3−2818 : Selenium on Serine protease inhibitor Kazal−type 2

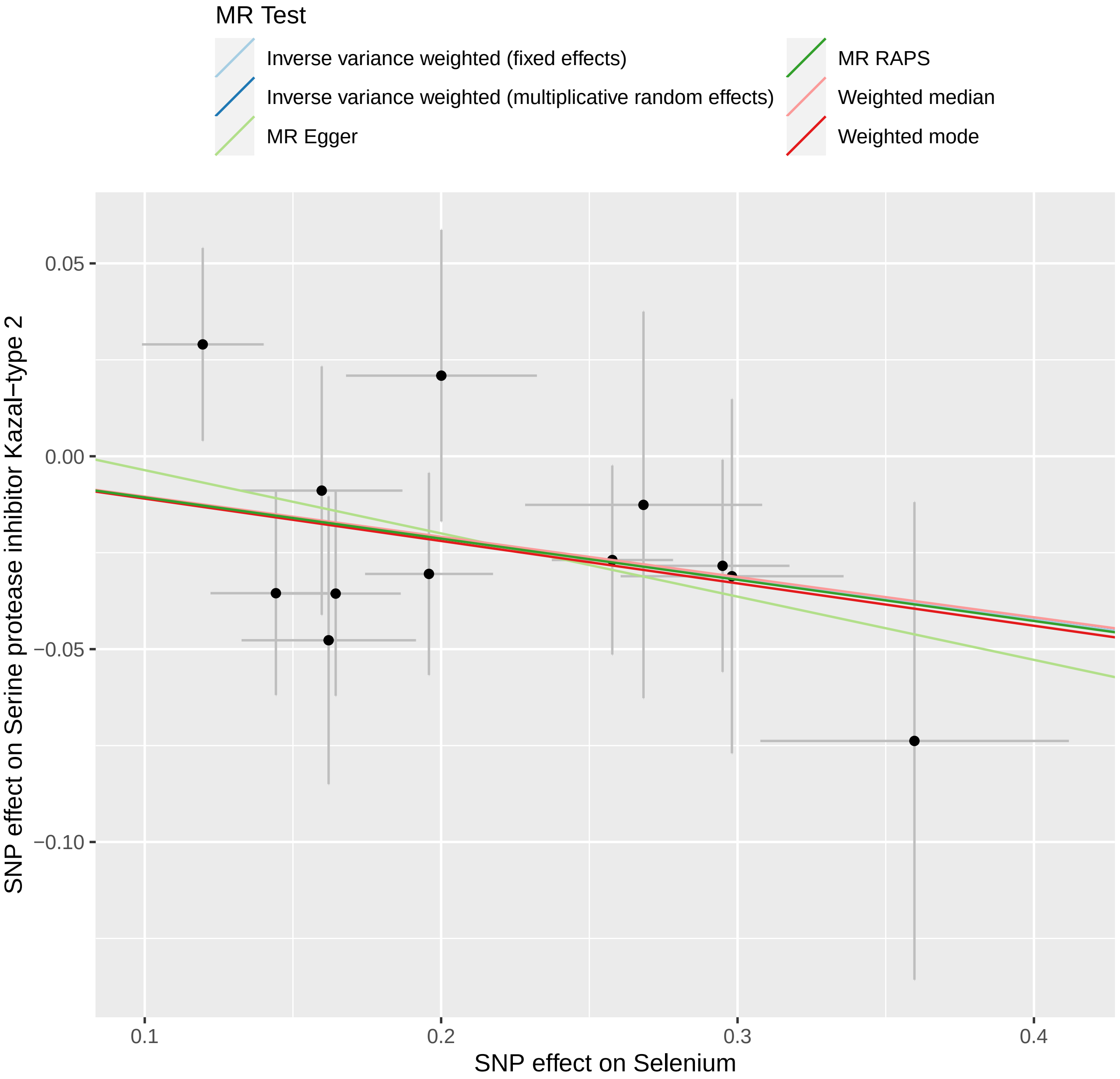

Batch 3−2819 : Selenium on Serine protease inhibitor Kazal−type 5

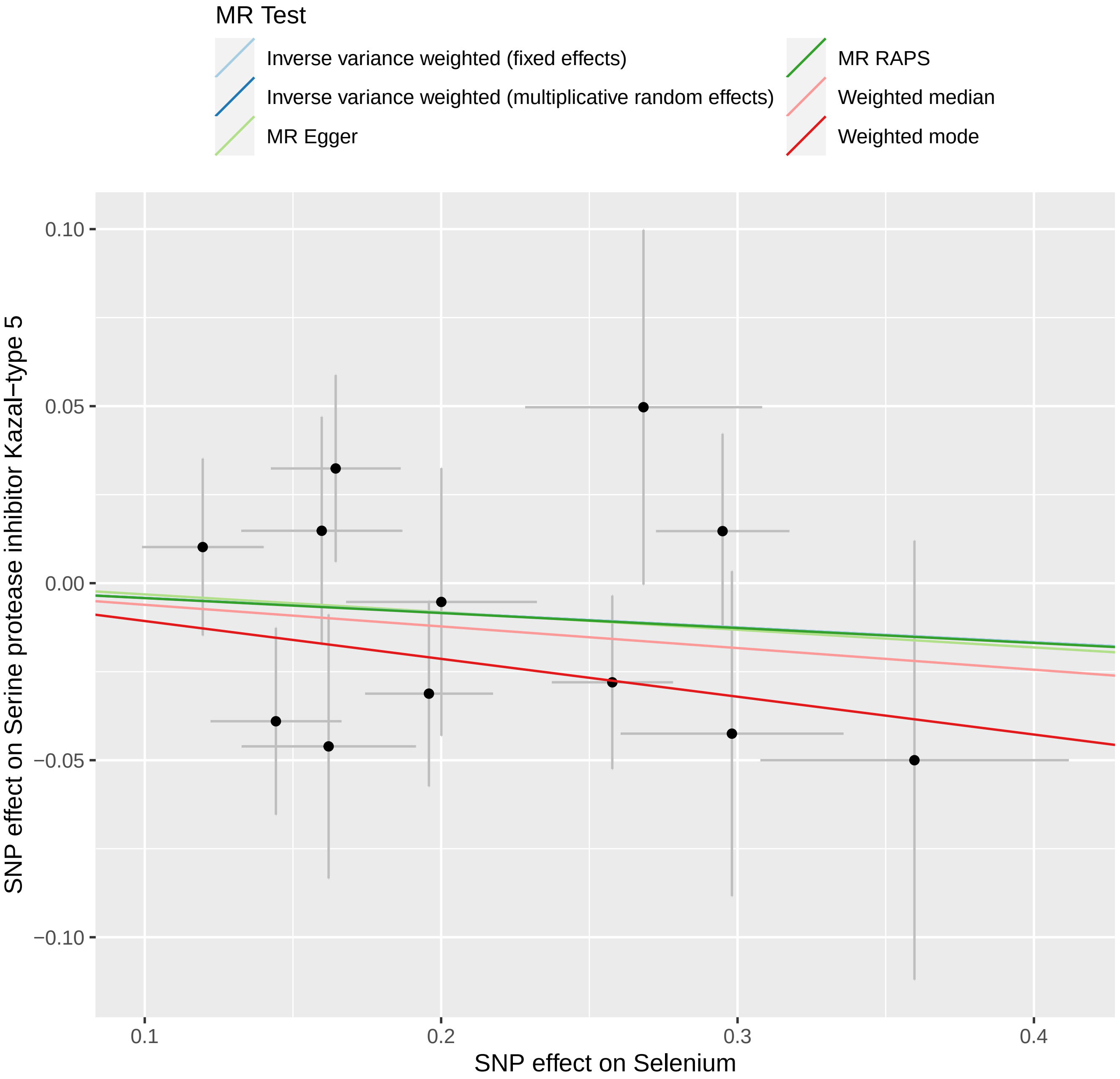

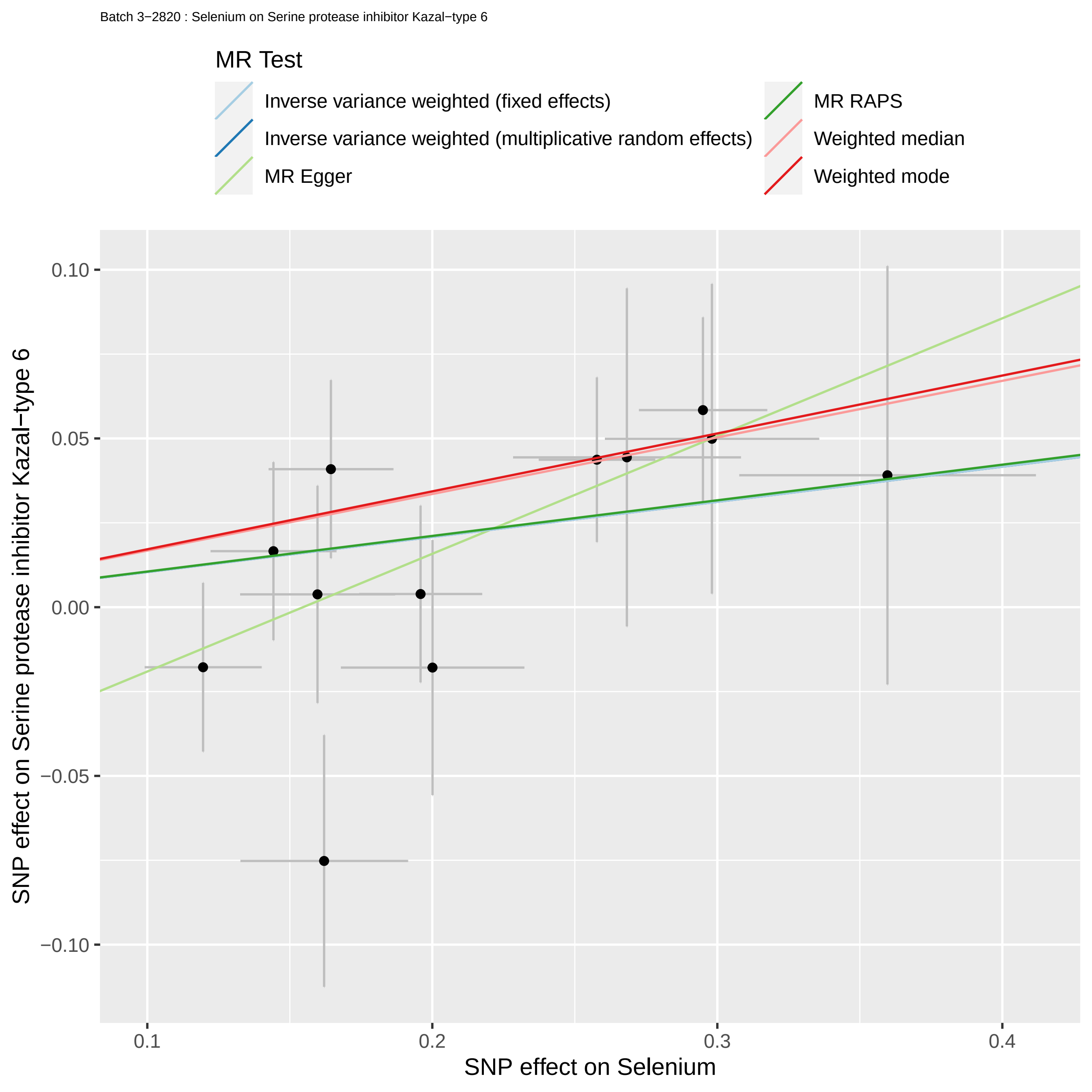

Batch 3−2820 : Selenium on Serine protease inhibitor Kazal−type 6
MR Test
Inverse variance weighted (fixed effects)
Inverse variance weighted (multiplicative random effects)
MR Egger
MR RAPS
Weighted median
Weighted mode
0.10
0.05
0.00
−0.05
−0.10
0.1
0.2
0.3
0.4
SNP effect on Selenium
SNP effect on Serine protease inhibitor Kazal−type 6

Batch 3−2821 : Selenium on Serine protease inhibitor Kazal−type 8

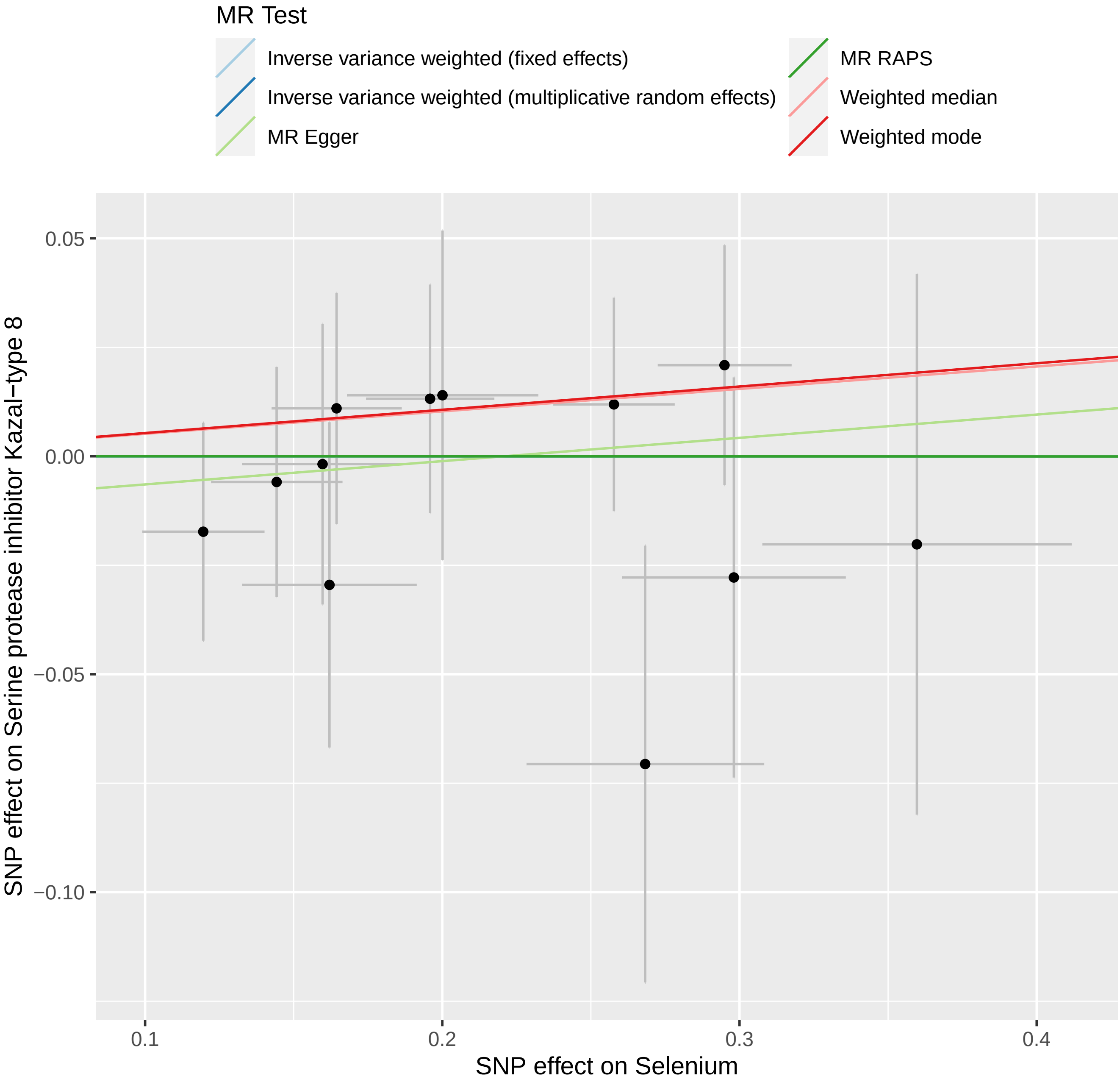

Batch 3−2822 : Selenium on Serine protease inhibitor Kazal−type 9

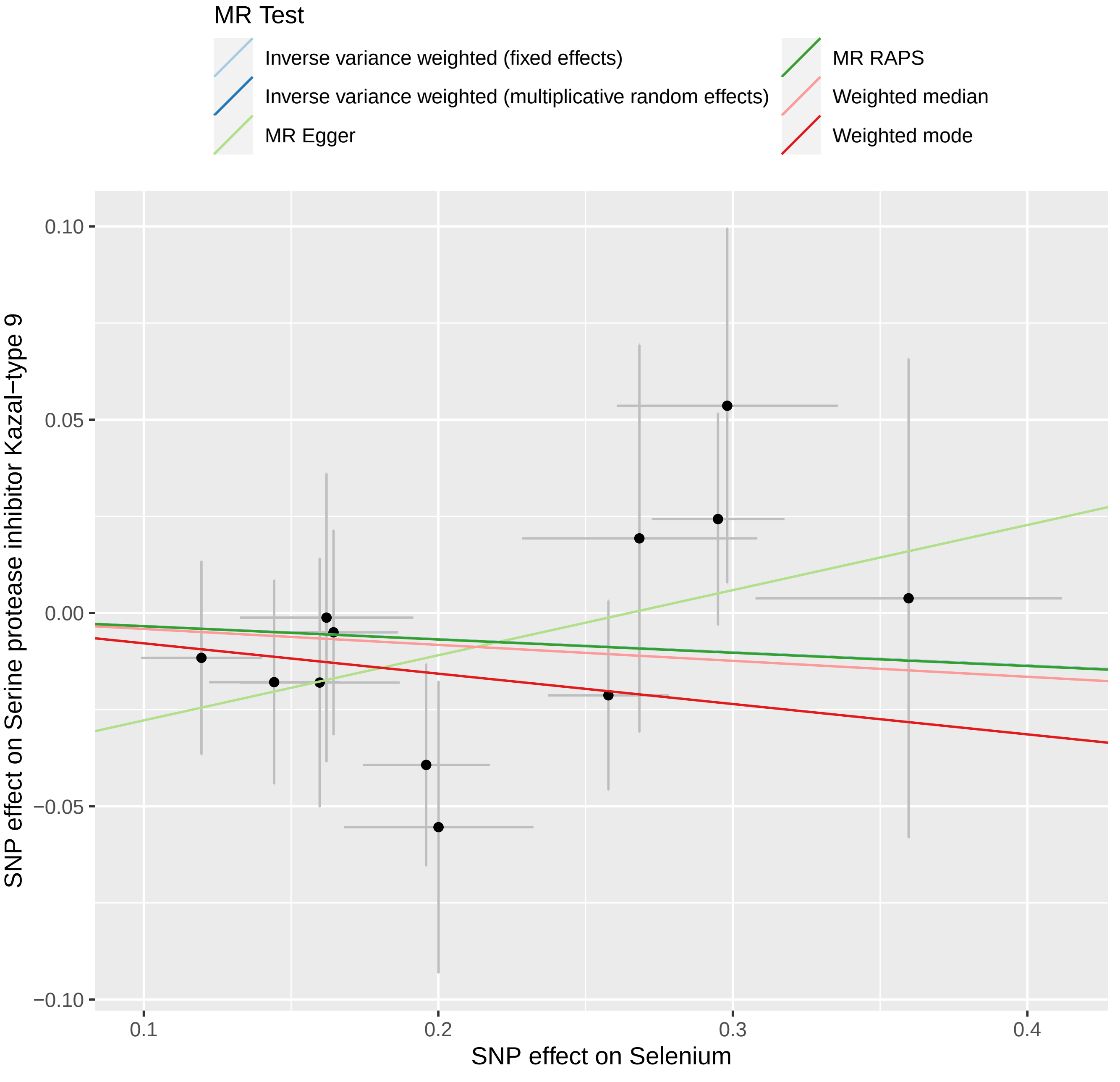

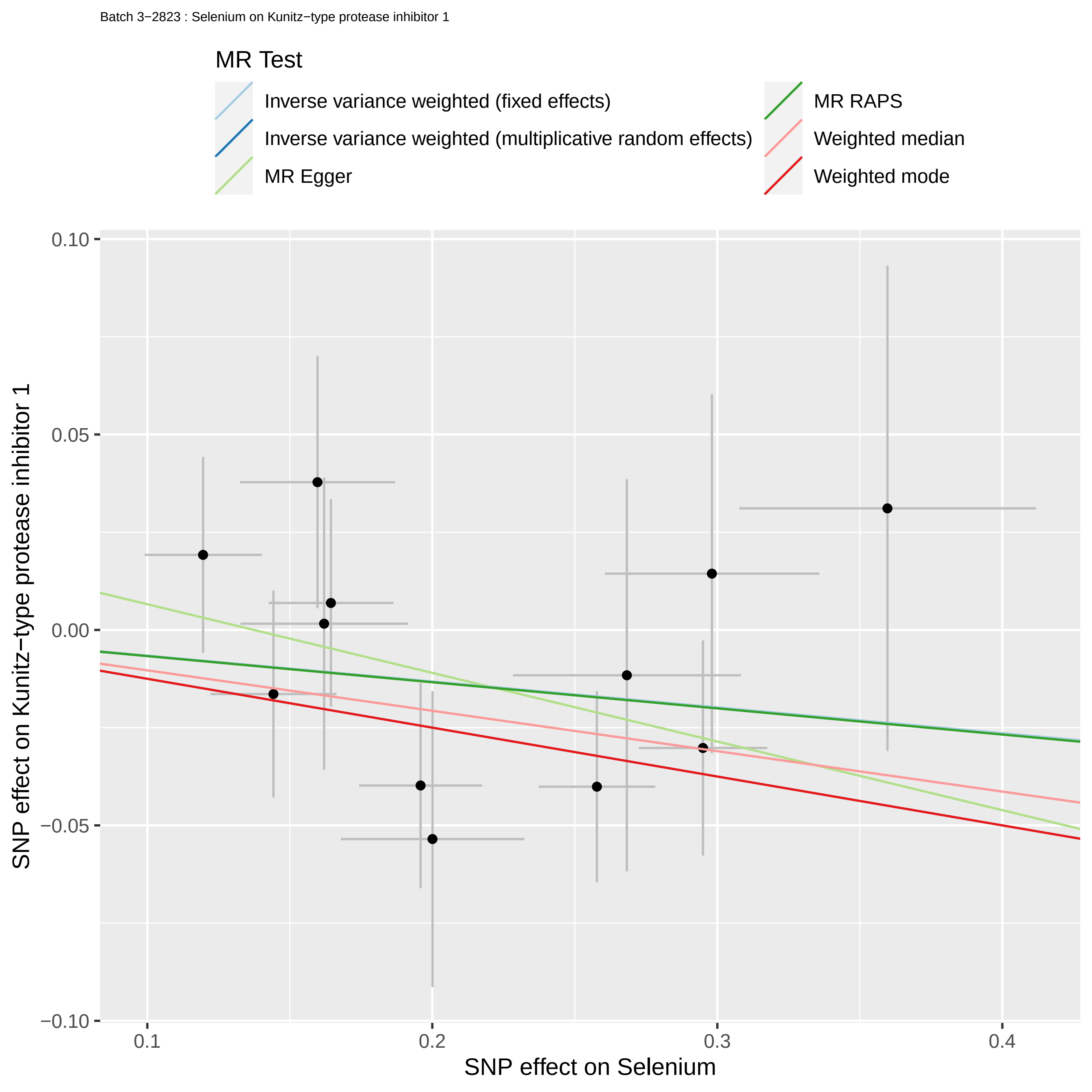

Batch 3−2823 : Selenium on Kunitz−type protease inhibitor 1
MR Test
Inverse variance weighted (fixed effects)
Inverse variance weighted (multiplicative random effects)
MR Egger
MR RAPS
Weighted median
Weighted mode
0.10
0.05
0.00
−0.05
−0.10
0.1
0.2
0.3
0.4
SNP effect on Kunitz−type protease inhibitor 1
SNP effect on Selenium

Batch 3−2824 : Selenium on Kunitz−type protease inhibitor 2

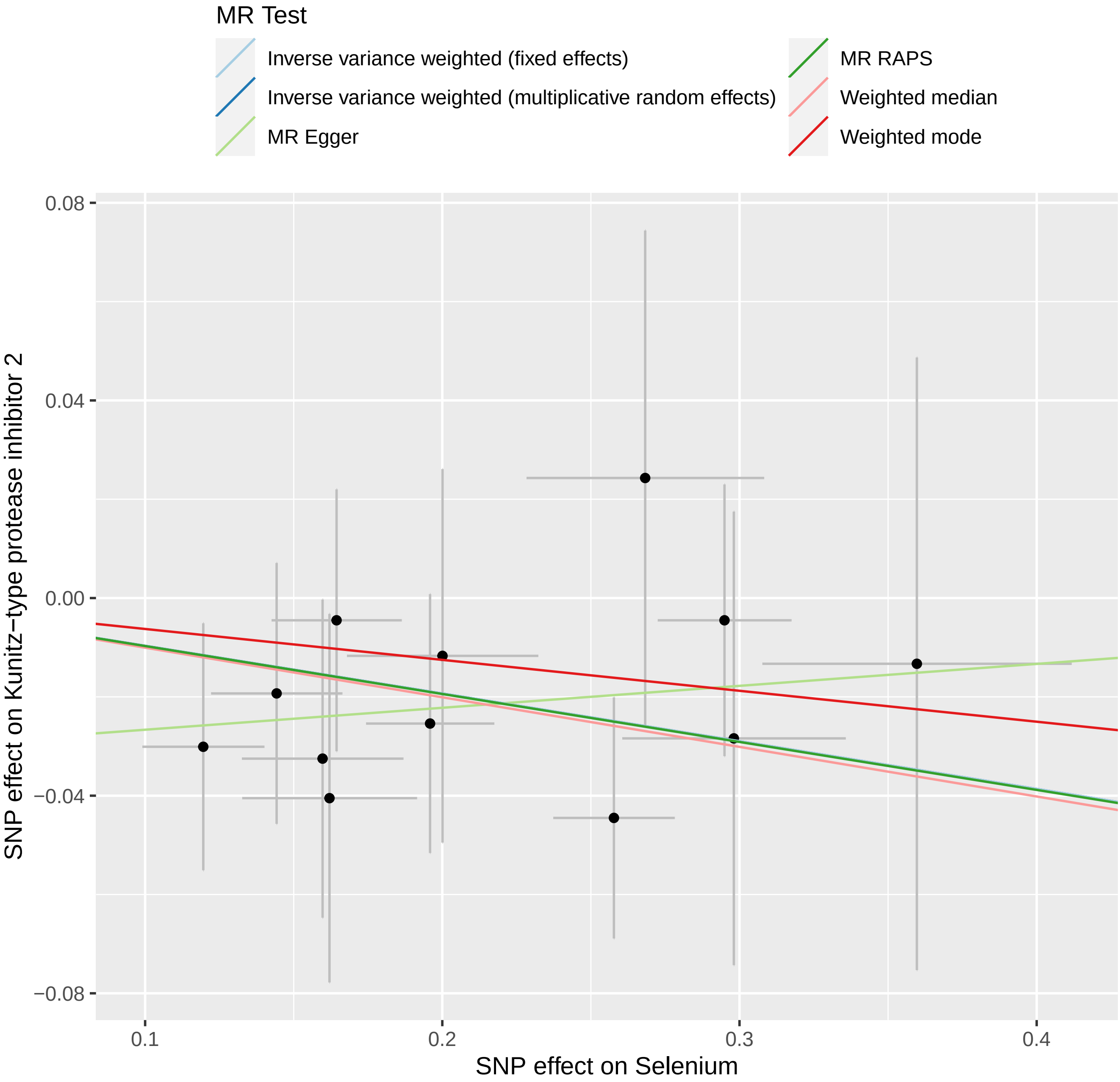

Batch 3−2825 : Selenium on Kunitz−type protease inhibitor 3

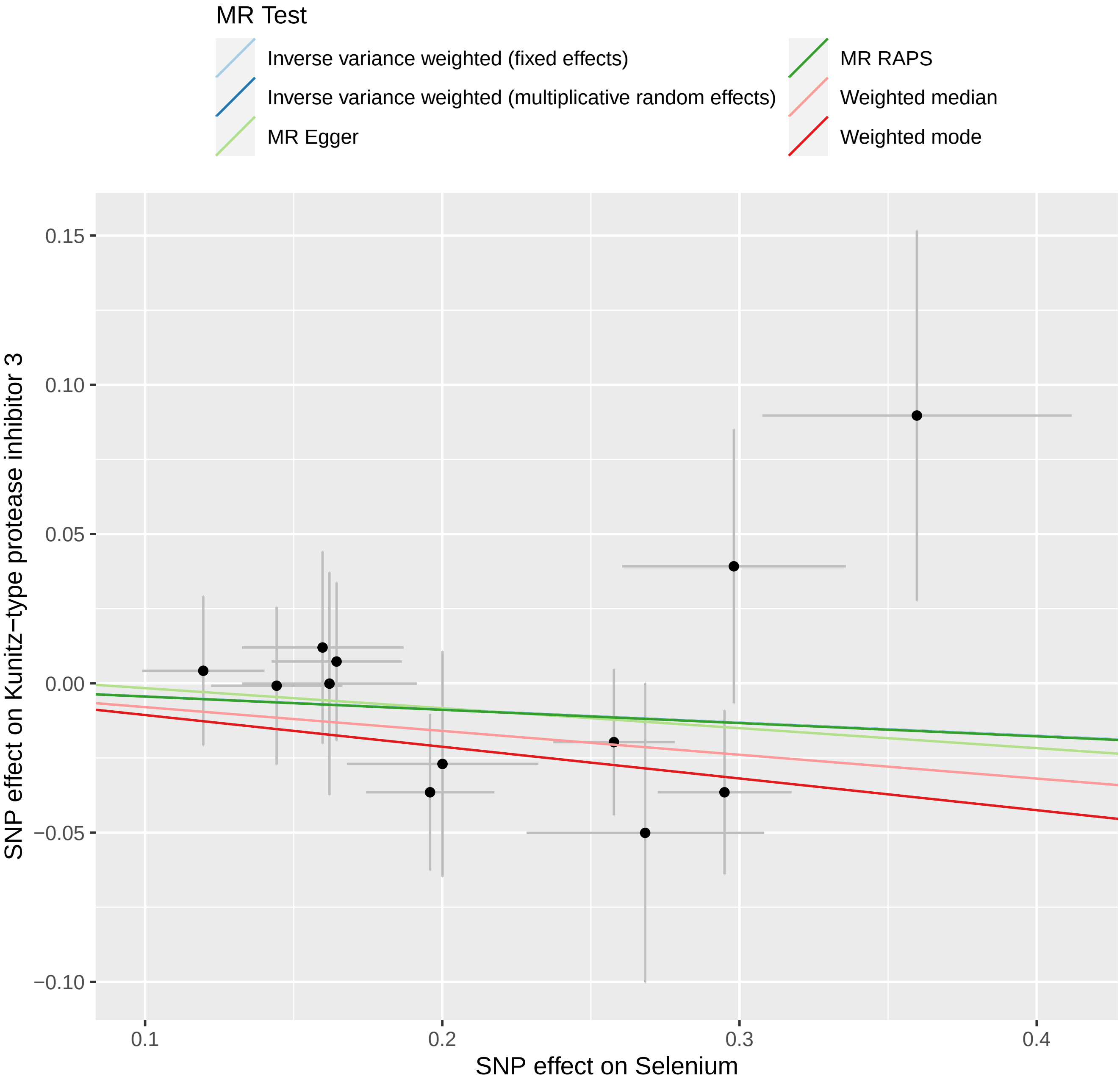

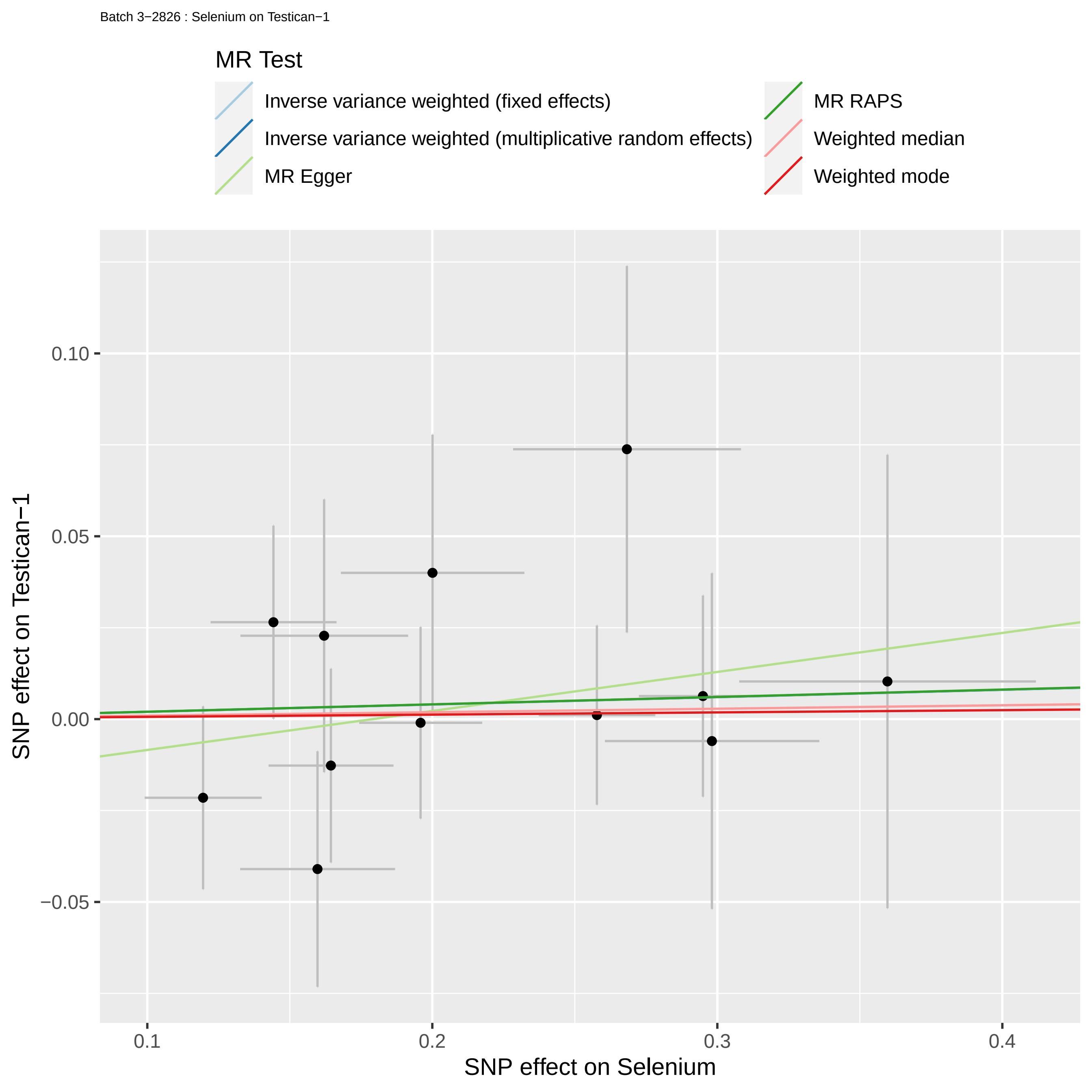

Batch 3−2826 : Selenium on Testican−1
MR Test
Inverse variance weighted (fixed effects)
Inverse variance weighted (multiplicative random effects)
MR Egger
MR RAPS
Weighted median
Weighted mode
0.10
0.05
0.00
−0.05
0.1
0.2
0.3
0.4
SNP effect on Testican−1
SNP effect on Selenium

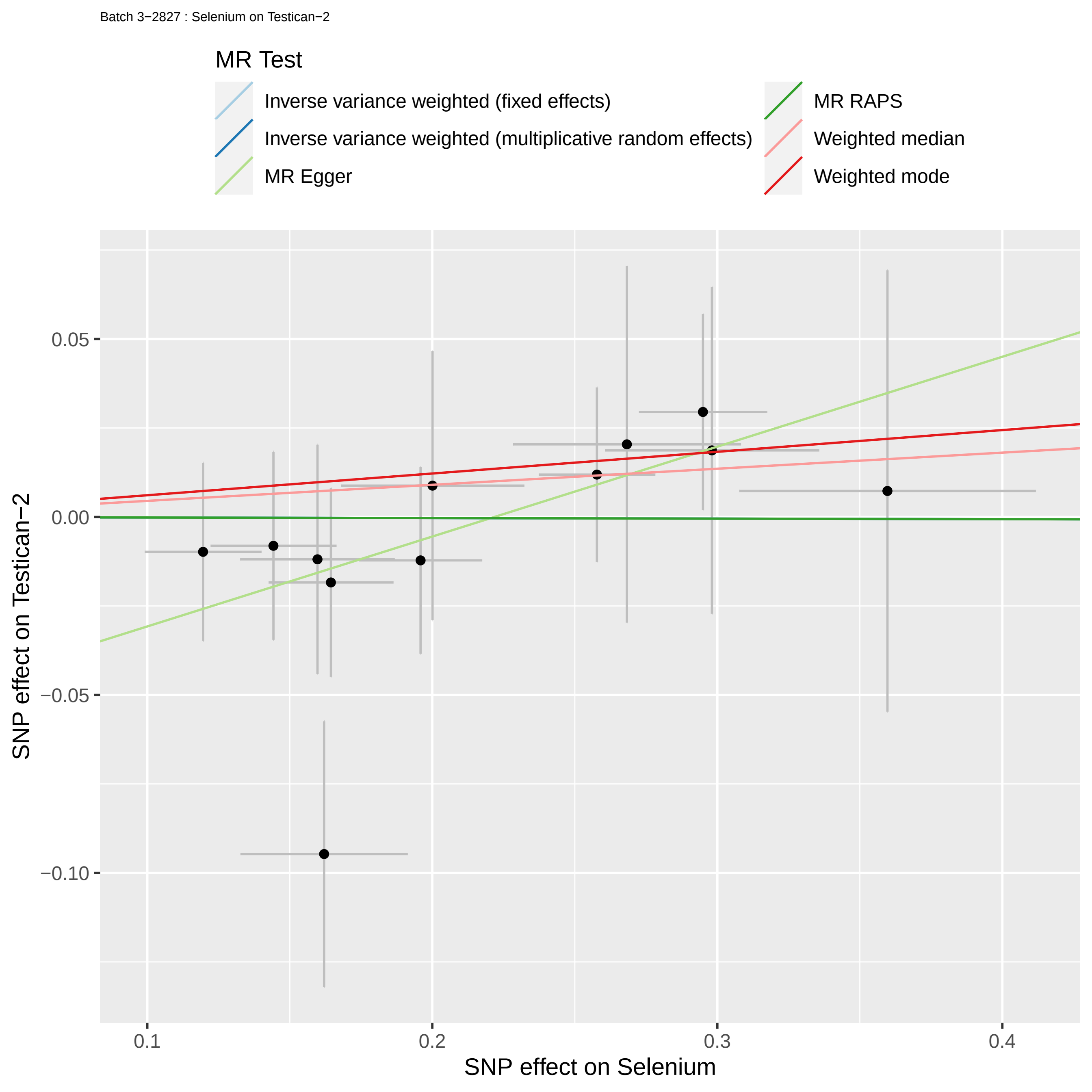

Batch 3−2827 : Selenium on Testican−2
MR Test
Inverse variance weighted (fixed effects)
Inverse variance weighted (multiplicative random effects)
MR Egger
MR RAPS
Weighted median
Weighted mode
0.05
0.00
−0.05
−0.10
0.1
0.2
0.3
0.4
SNP effect on Selenium
SNP effect on Testican−2

Batch 3−2828 : Selenium on Testican−3

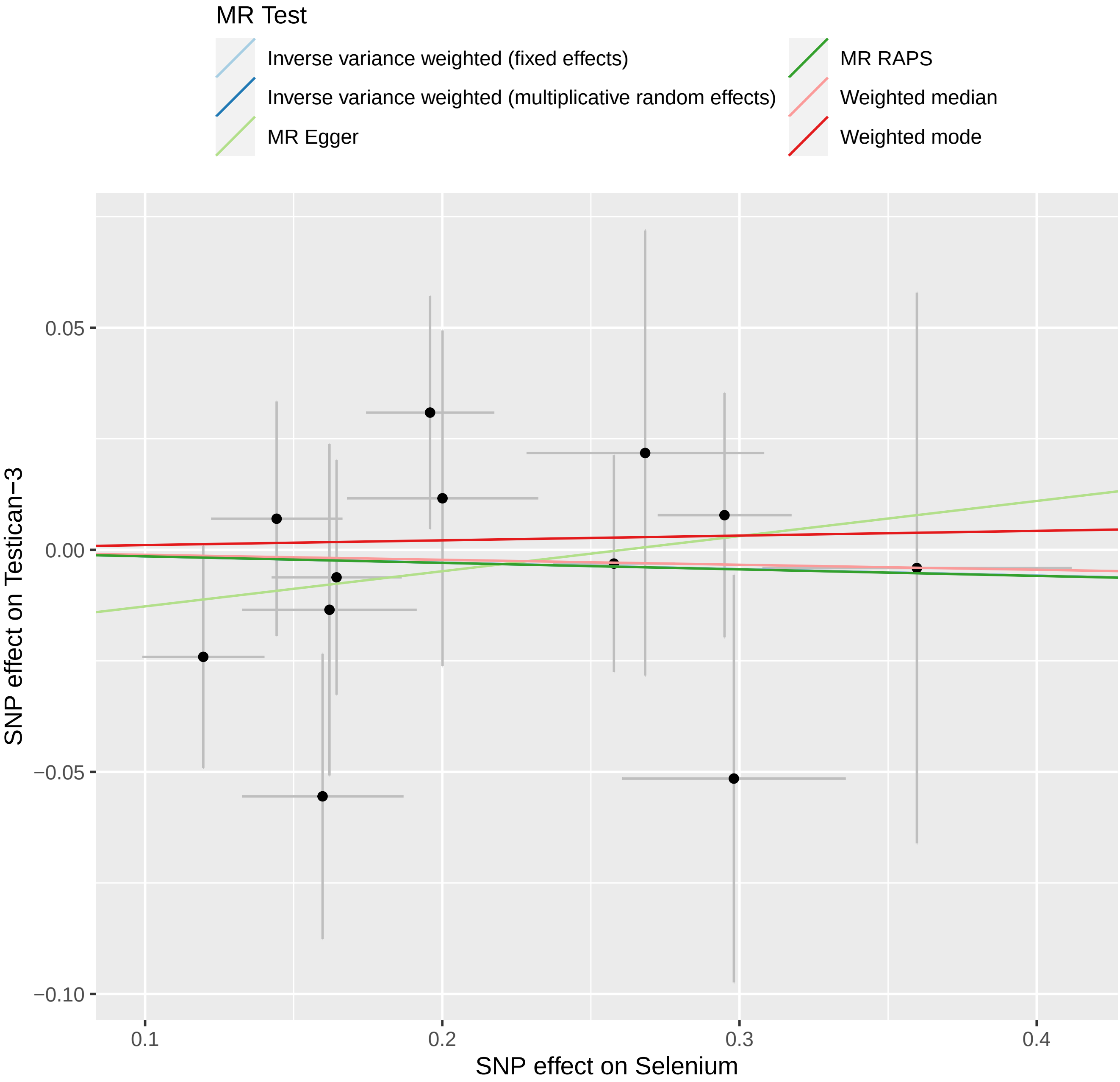

Batch 3−2829 : Selenium on Spondin−1

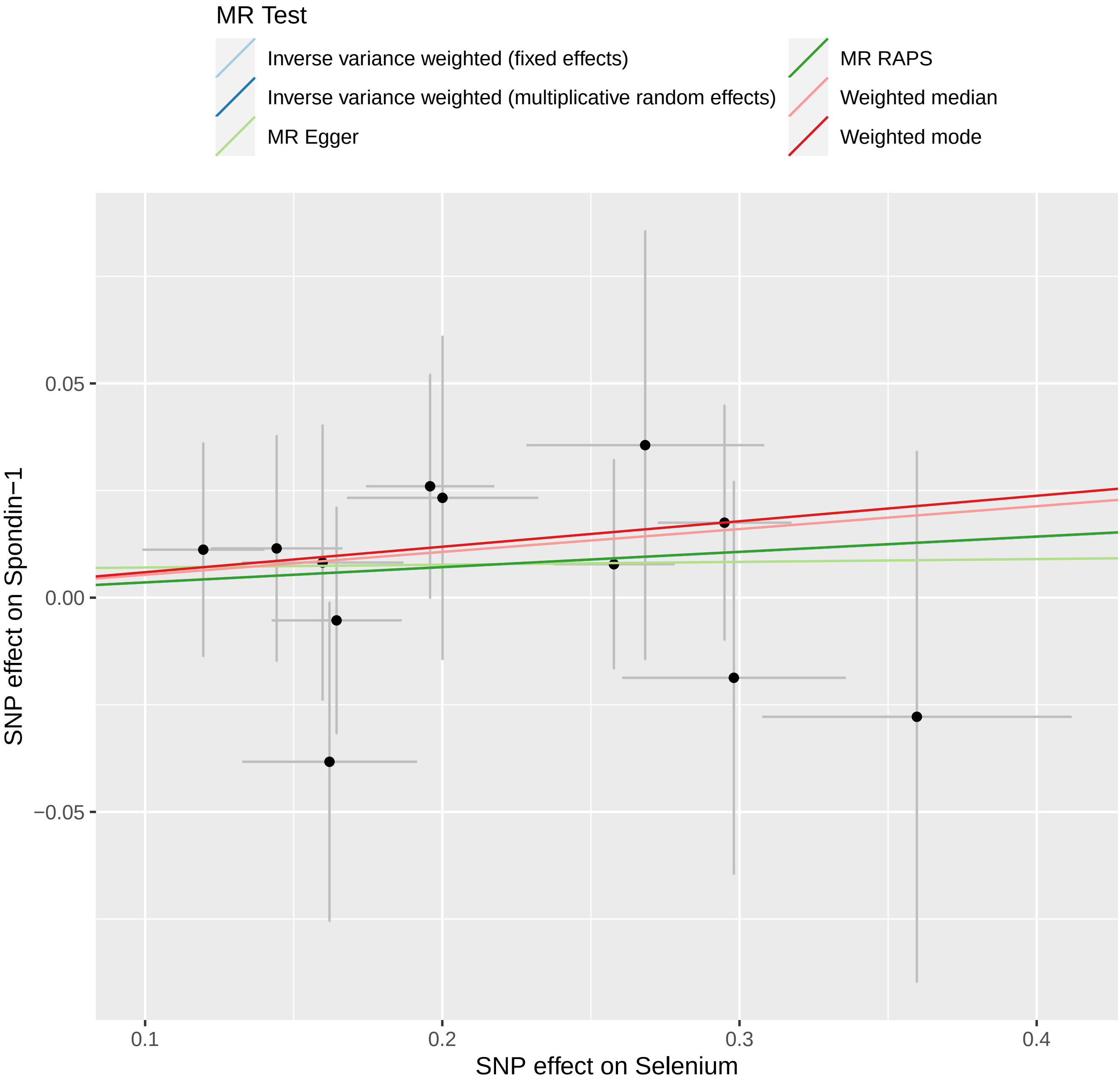

Batch 3−2830 : Selenium on Speckle−type POZ protein

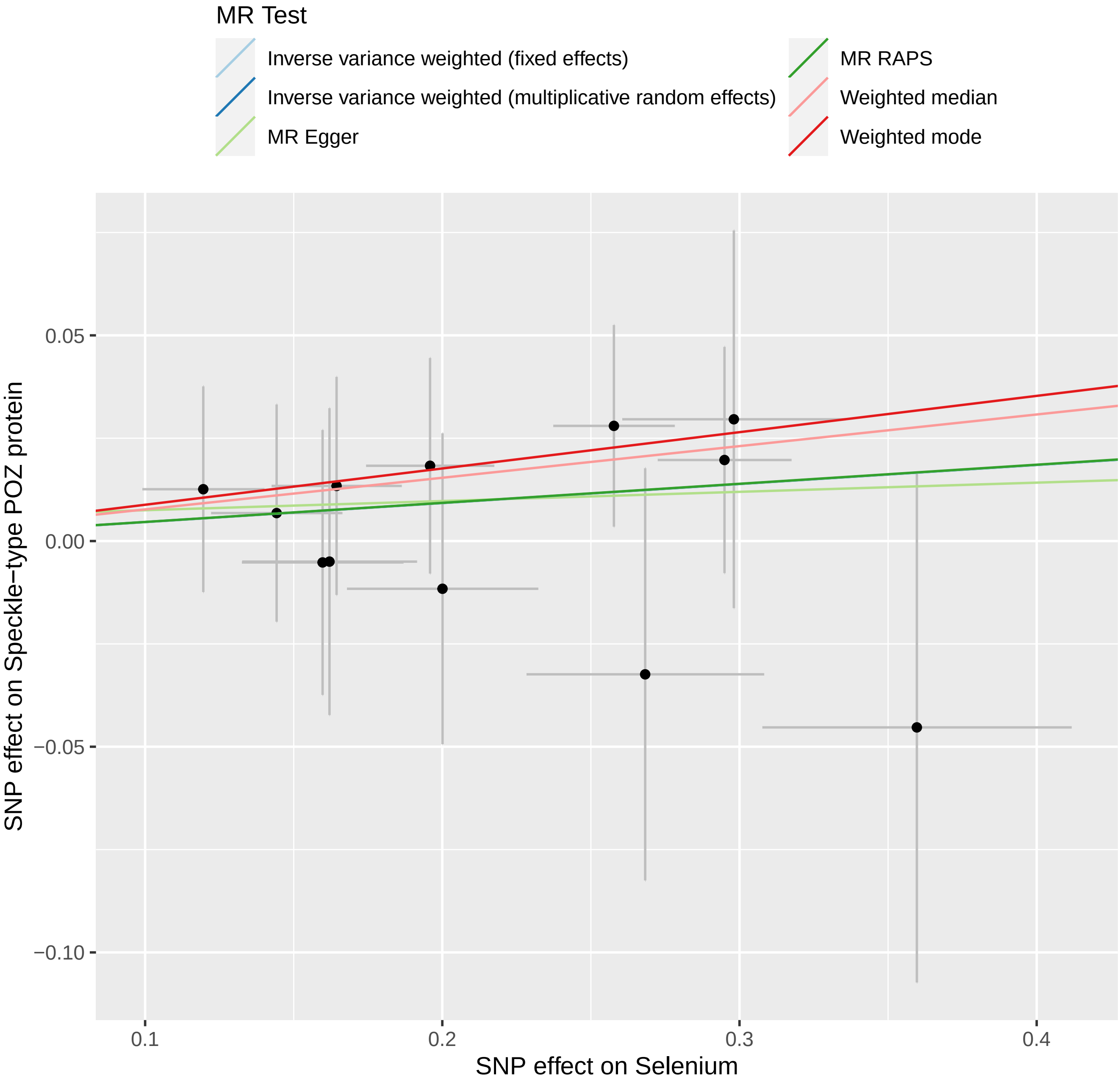

Batch 3−2831 : Selenium on Sepiapterin reductase

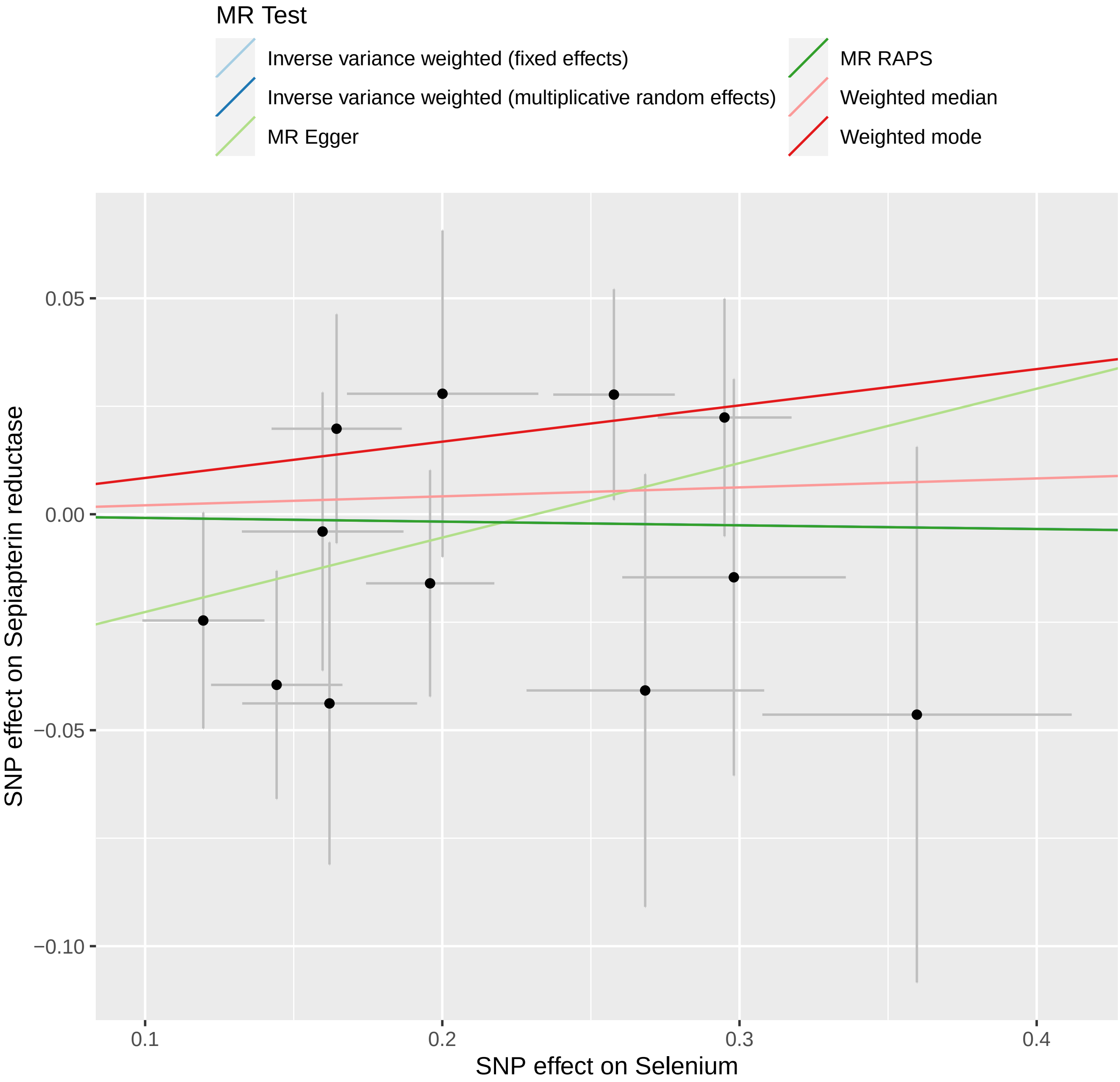

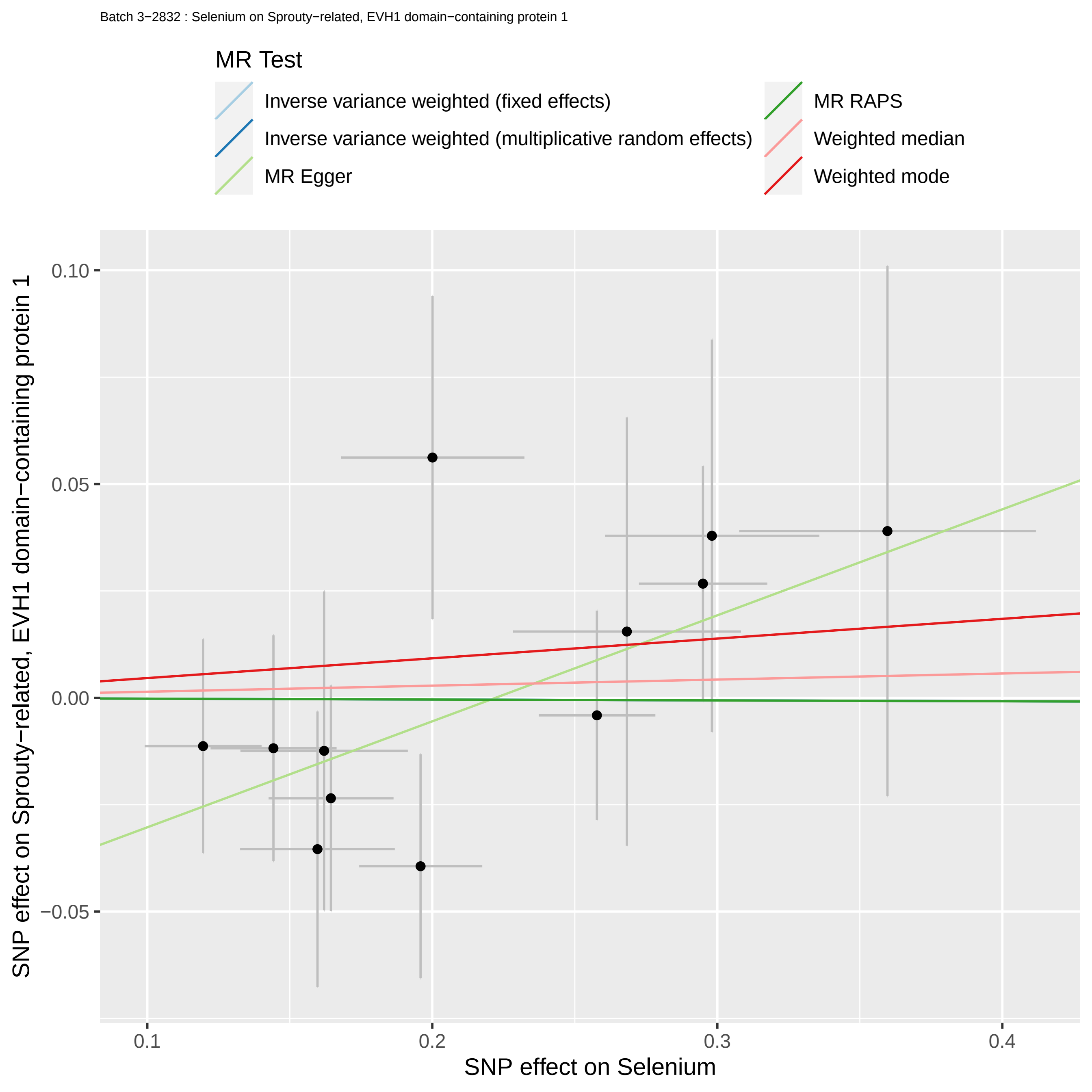

Batch 3−2832 : Selenium on Sprouty−related, EVH1 domain−containing protein 1
MR Test
Inverse variance weighted (fixed effects)
Inverse variance weighted (multiplicative random effects)
MR Egger
MR RAPS
Weighted median
Weighted mode
0.10
0.05
0.00
−0.05
0.1
0.2
0.3
0.4
SNP effect on Selenium
SNP effect on Sprouty−related, EVH1 domain−containing protein 1

Batch 3−2833 : Selenium on Protein sprouty homolog 1

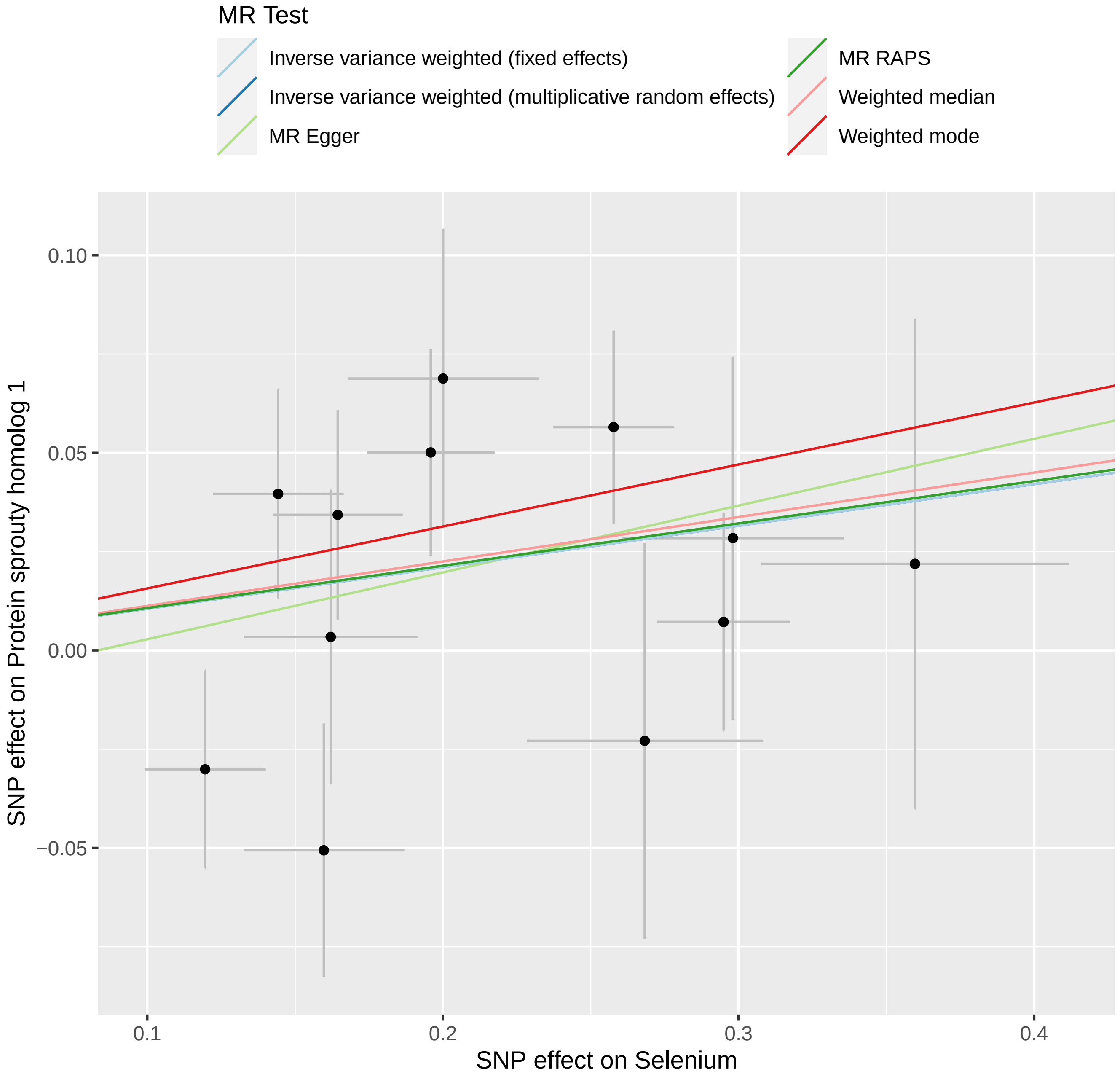

Batch 3−2834 : Selenium on SPRY domain−containing SOCS box protein 1

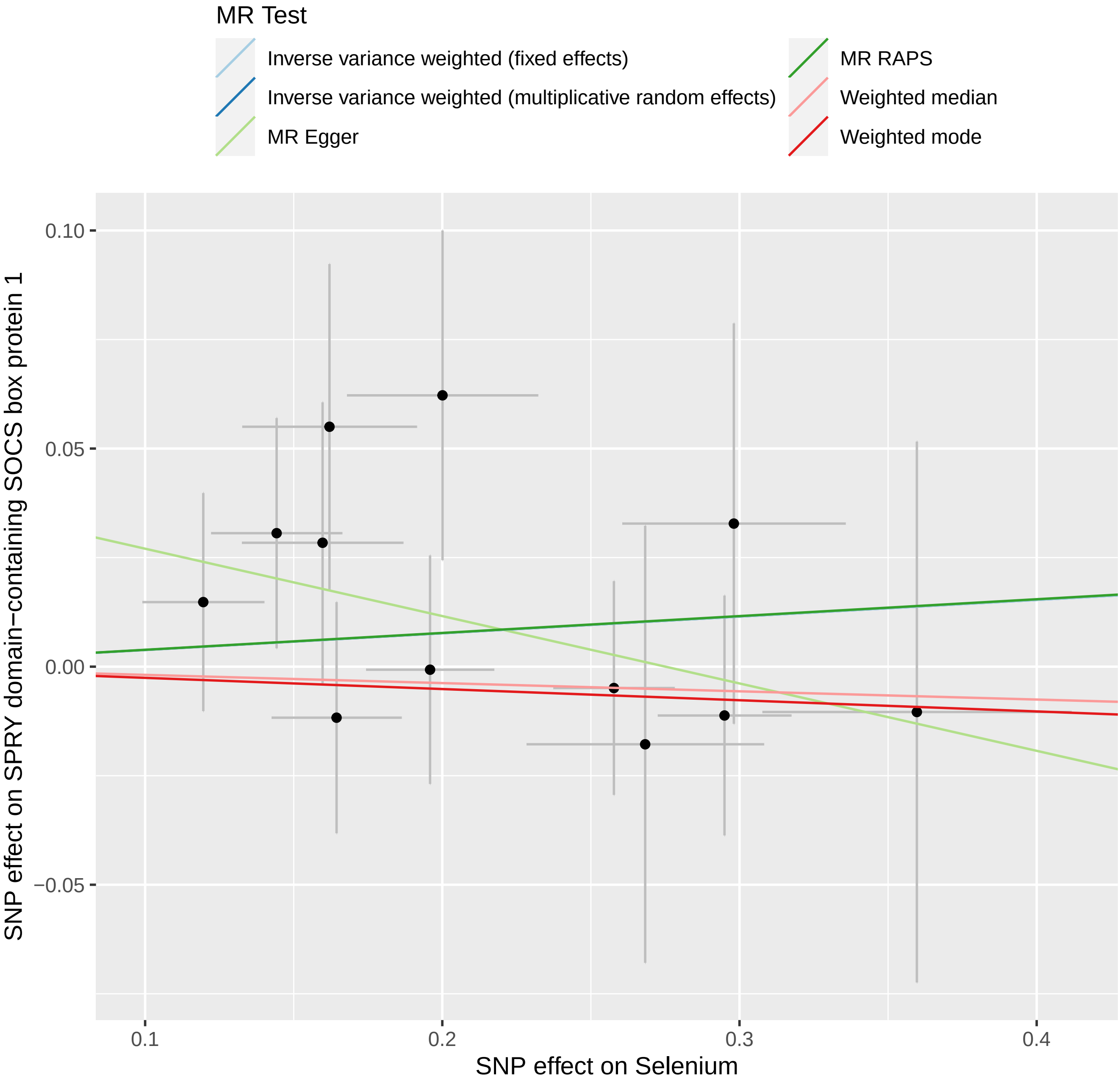

Batch 3−2835 : Selenium on Spectrin alpha chain, non−erythrocytic 1

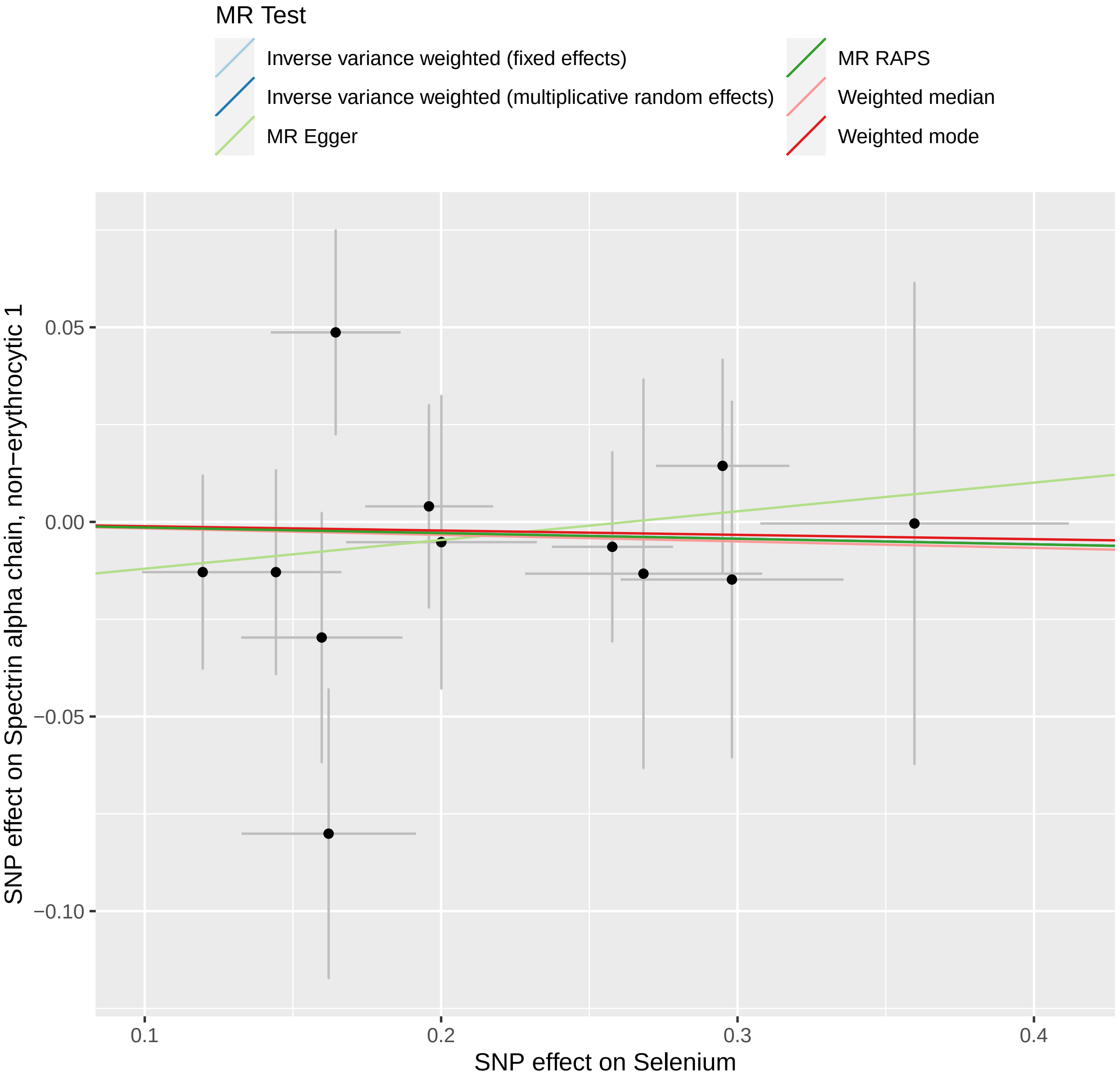

Batch 3−2836 : Selenium on Serine palmitoyltransferase 1

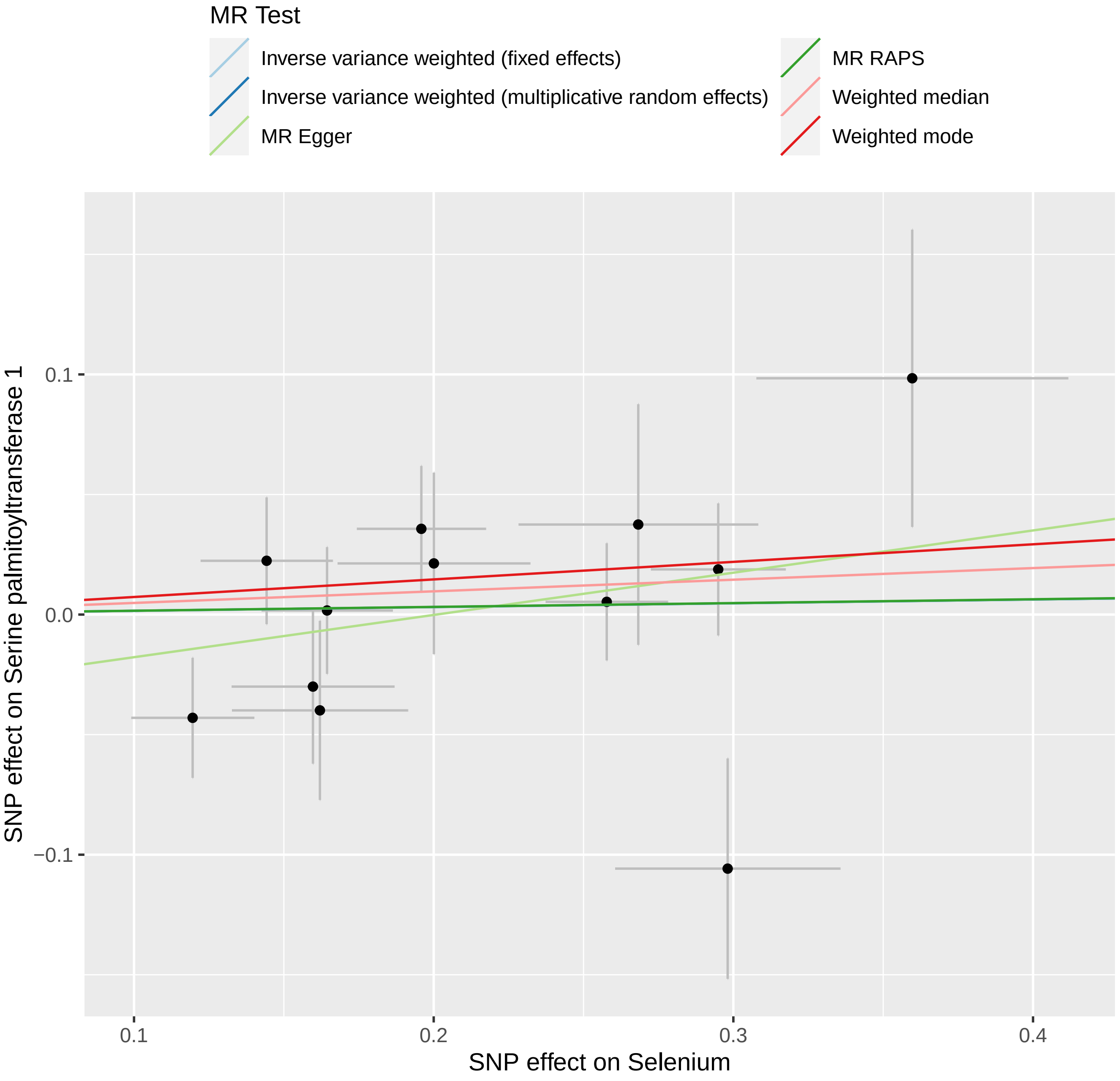

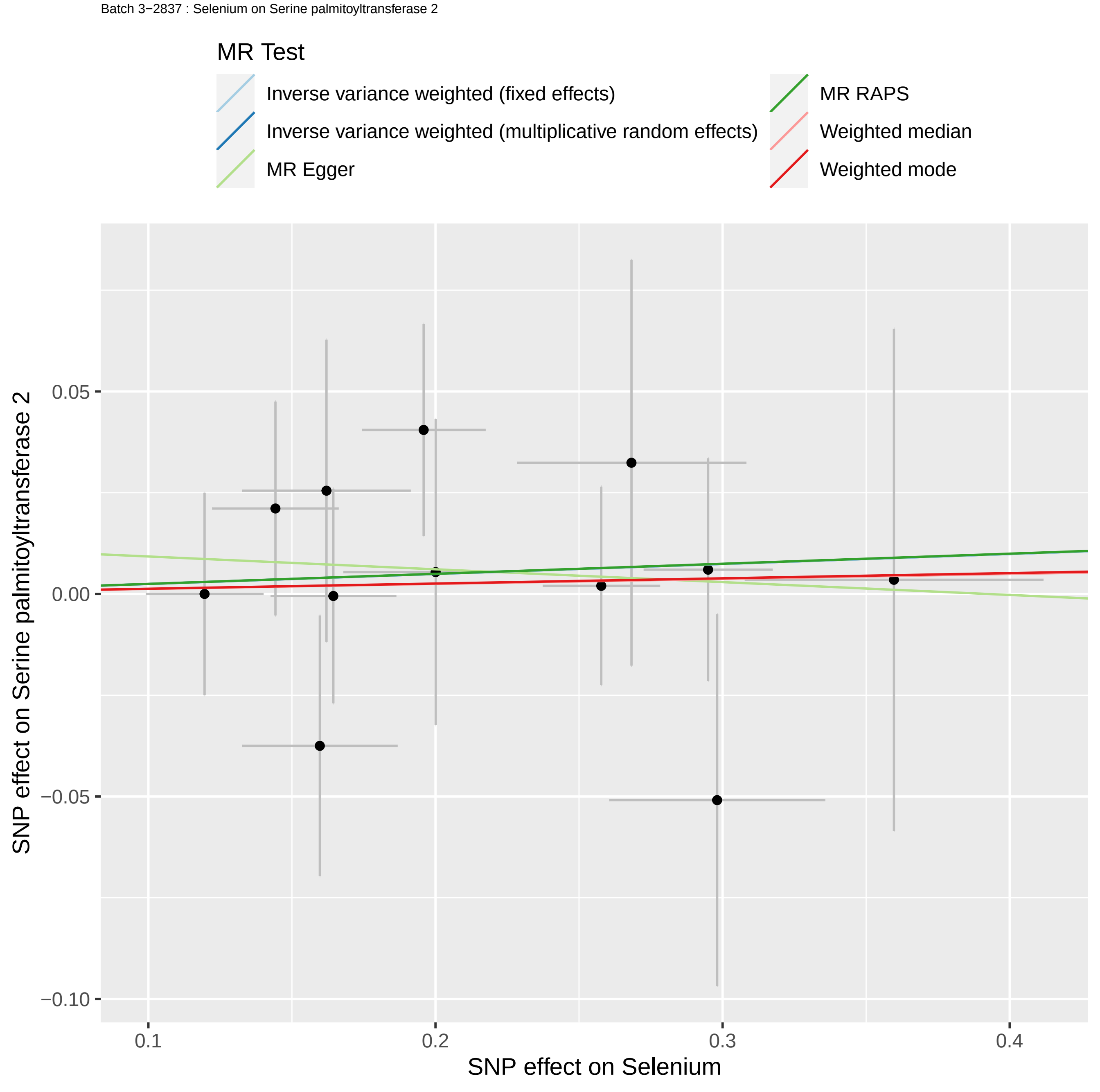

Batch 3−2837 : Selenium on Serine palmitoyltransferase 2
MR Test
Inverse variance weighted (fixed effects)
Inverse variance weighted (multiplicative random effects)
MR Egger
MR RAPS
Weighted median
Weighted mode
SNP effect on Serine palmitoyltransferase 2
0.05
0.00
−0.05
−0.10
0.1
0.2
0.3
0.4
SNP effect on Selenium

Batch 3−2838 : Selenium on Serine/arginine−rich splicing factor 6

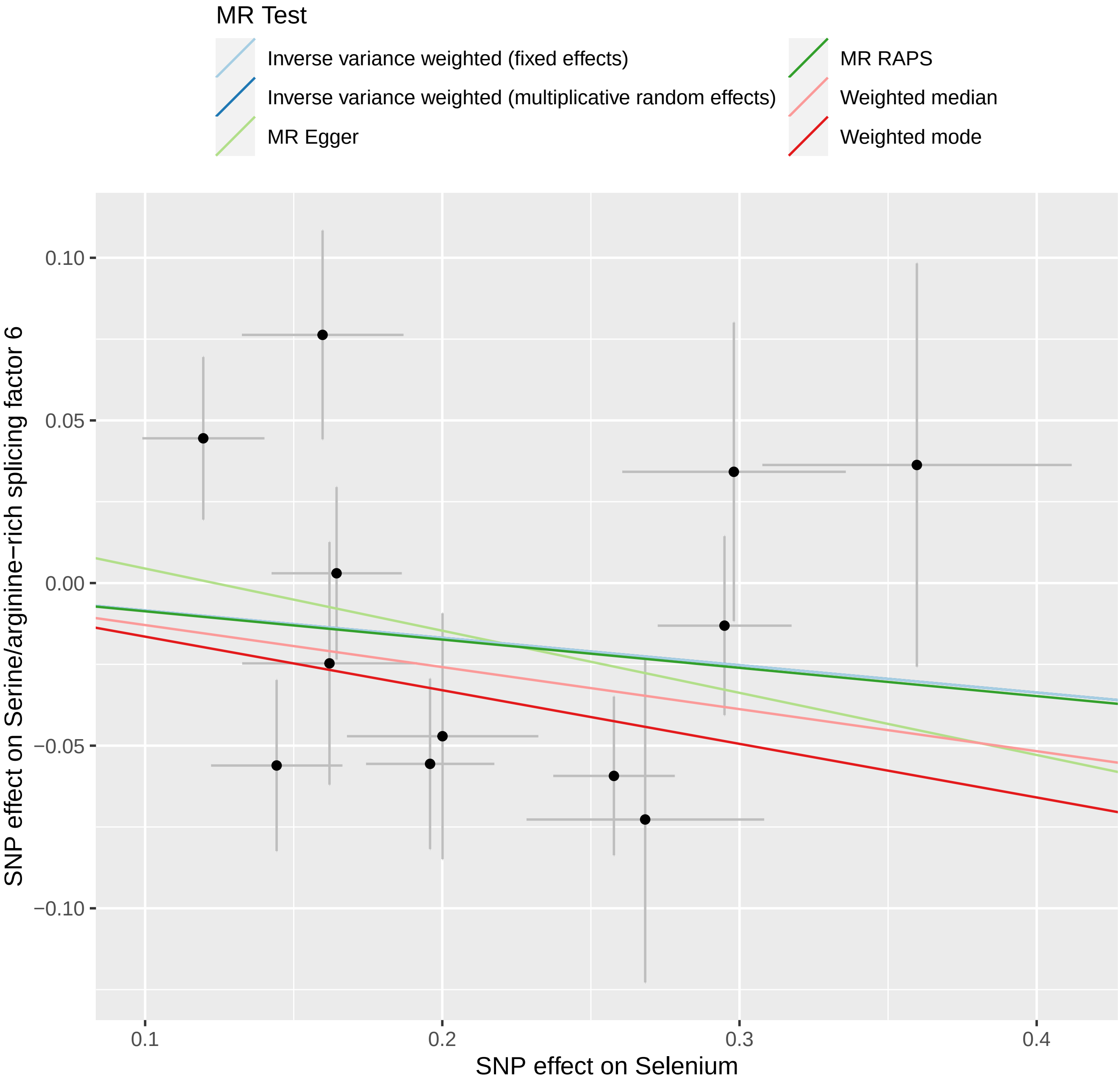

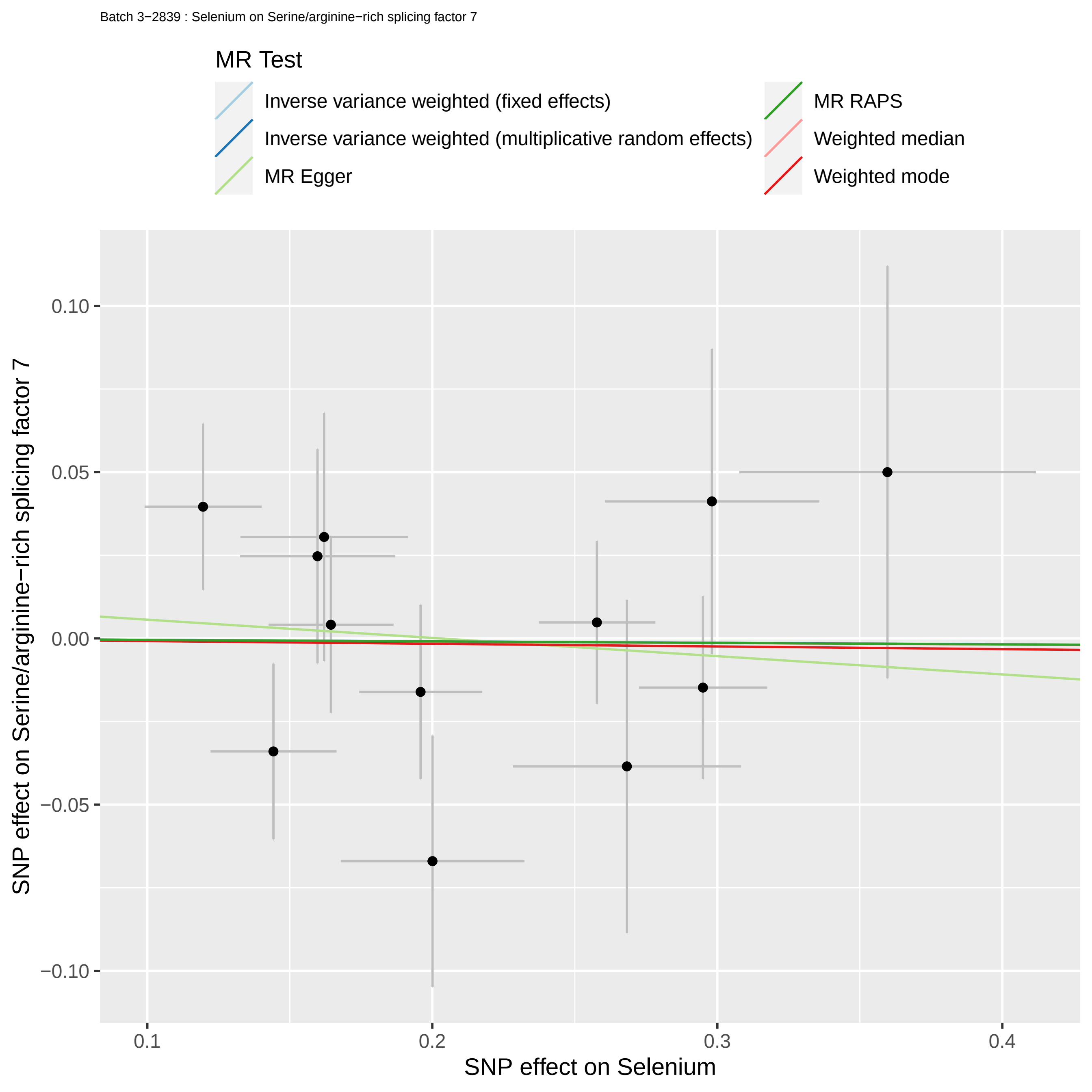

Batch 3−2839 : Selenium on Serine/arginine−rich splicing factor 7
MR Test
Inverse variance weighted (fixed effects)
Inverse variance weighted (multiplicative random effects)
MR Egger
MR RAPS
Weighted median
Weighted mode
0.10
0.05
0.00
−0.05
−0.10
0.1
0.2
0.3
0.4
SNP effect on Serine/arginine−rich splicing factor 7
SNP effect on Selenium

Batch 3−2840 : Selenium on Sulfiredoxin−1

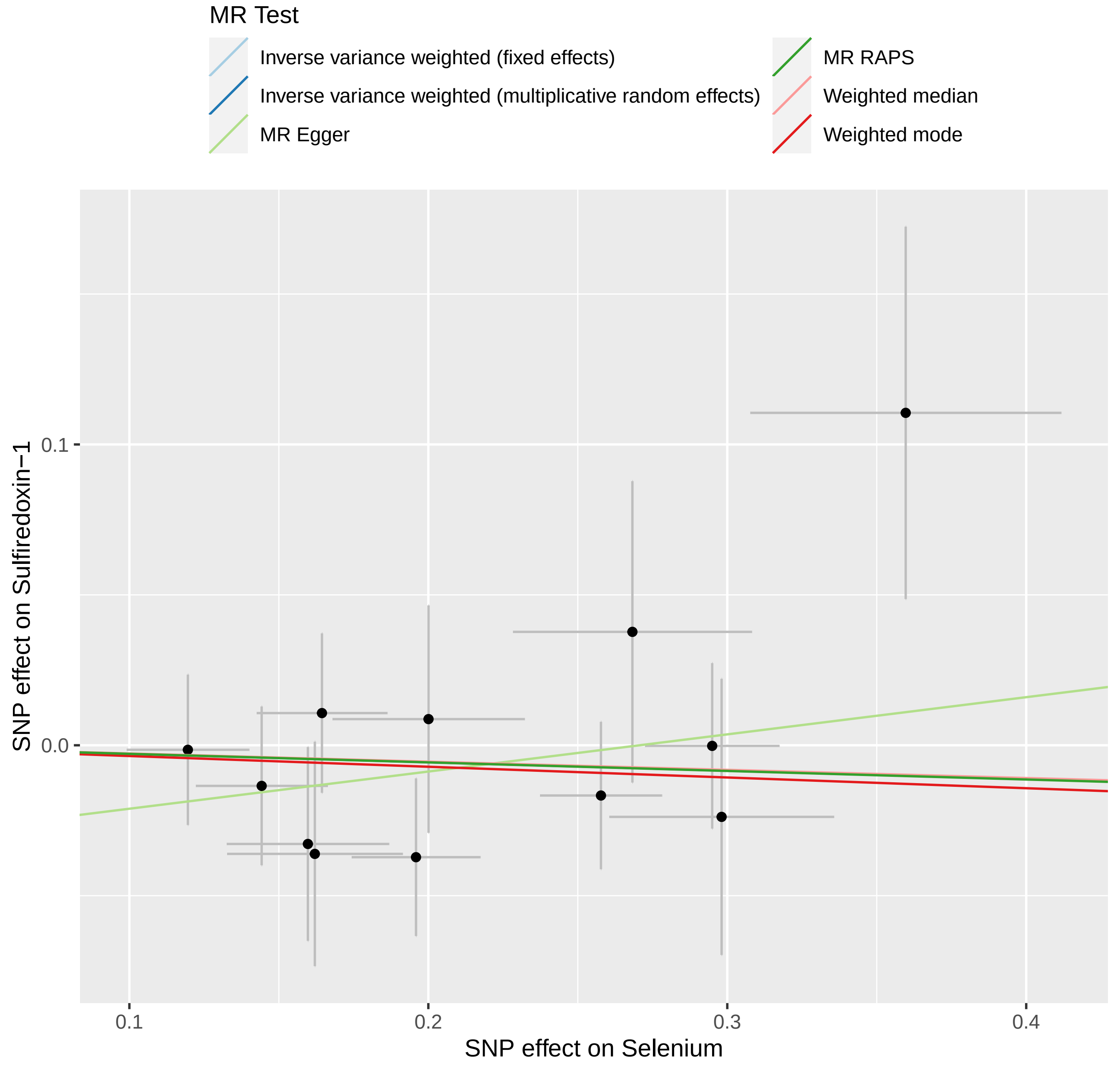

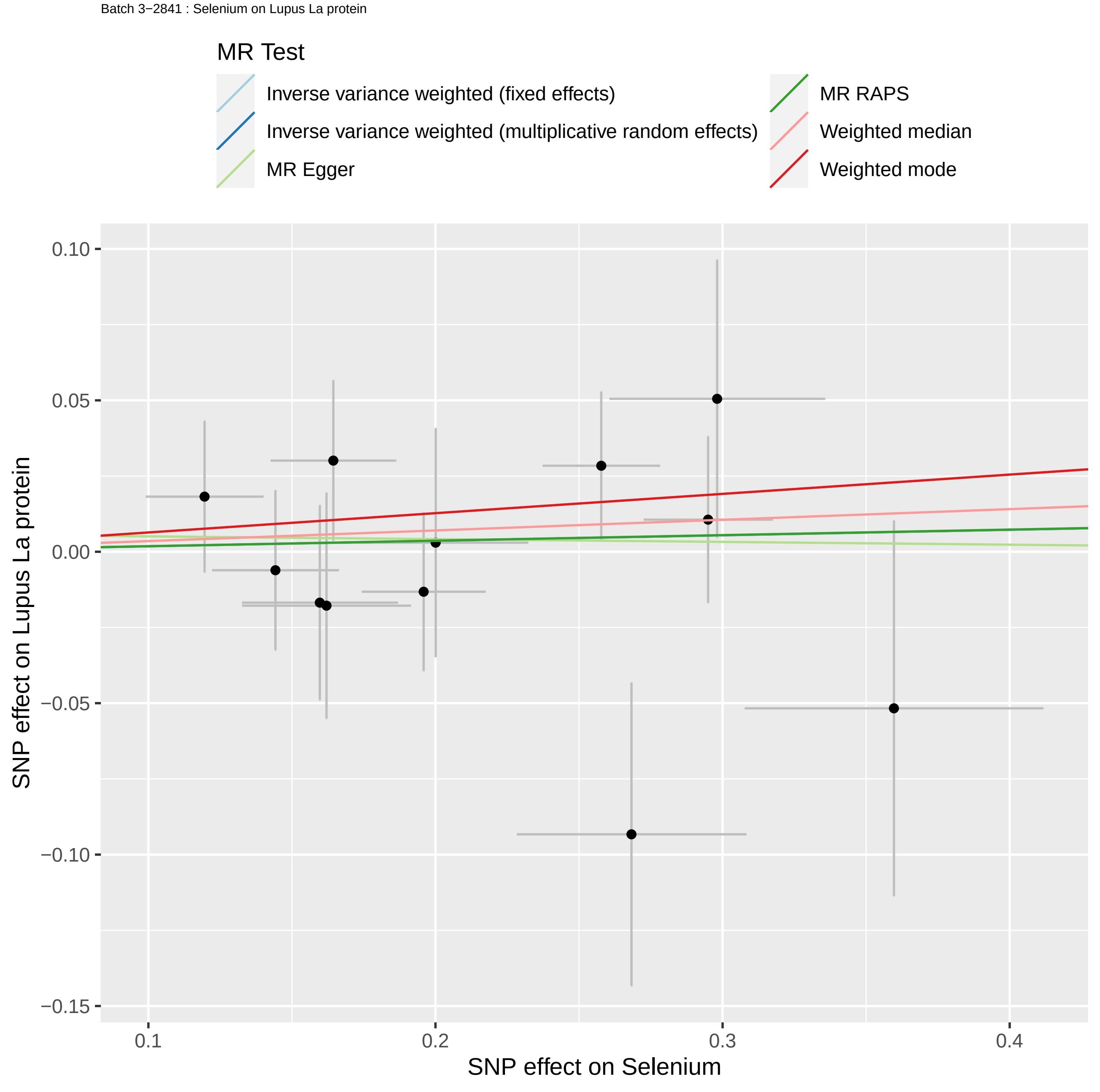

Batch 3−2841 : Selenium on Lupus La protein
MR Test
Inverse variance weighted (fixed effects)
Inverse variance weighted (multiplicative random effects)
MR Egger
MR RAPS
Weighted median
Weighted mode
0.10
0.05
0.00
−0.05
−0.10
−0.15
0.1
0.2
0.3
0.4
SNP effect on Lupus La protein
SNP effect on Selenium

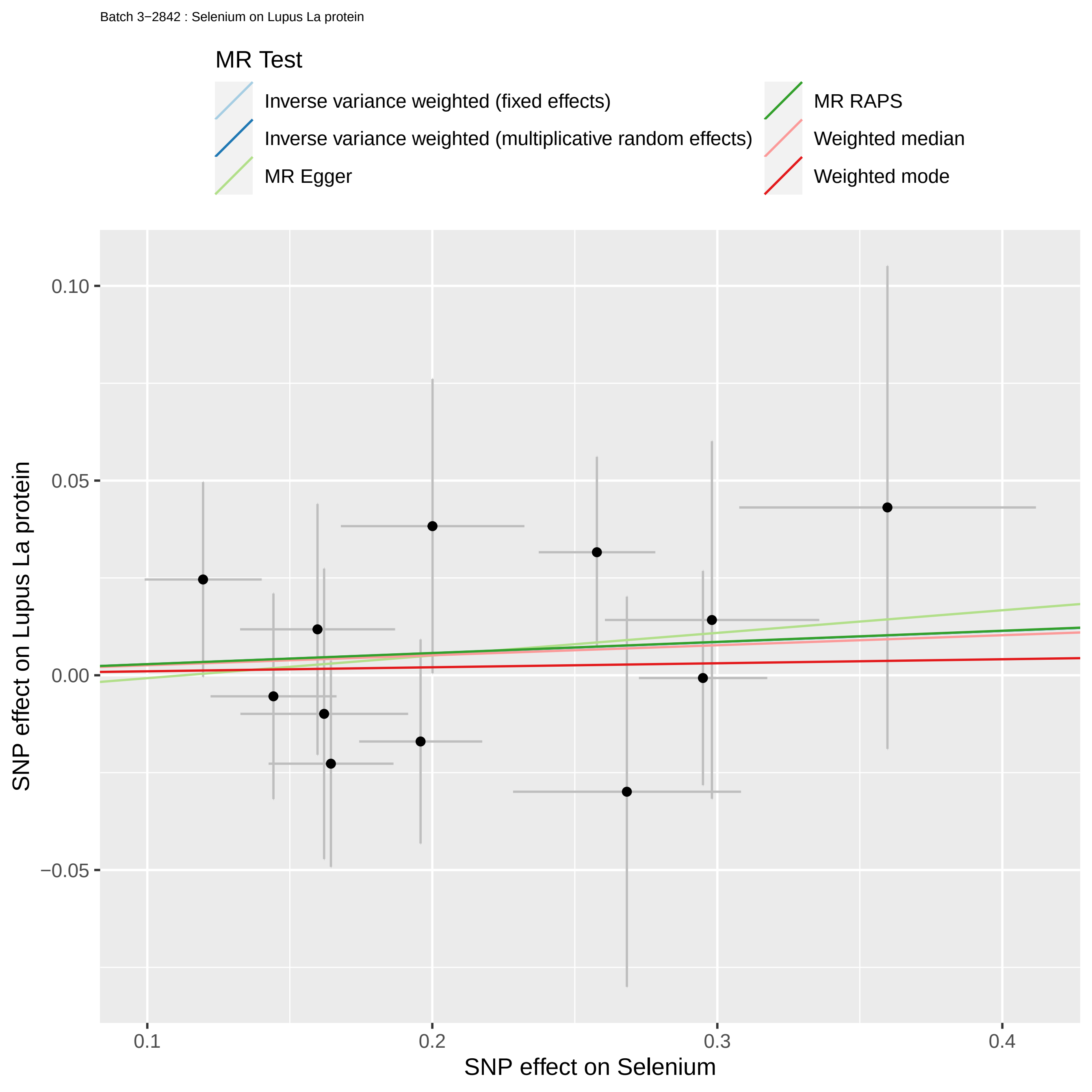
Batch 3−2842 : Selenium on Lupus La protein
MR Test
Inverse variance weighted (fixed effects)
Inverse variance weighted (multiplicative random effects)
MR Egger
MR RAPS
Weighted median
Weighted mode
0.10
0.05
0.00
−0.05
0.1
0.2
0.3
0.4
SNP effect on Lupus La protein
SNP effect on Selenium

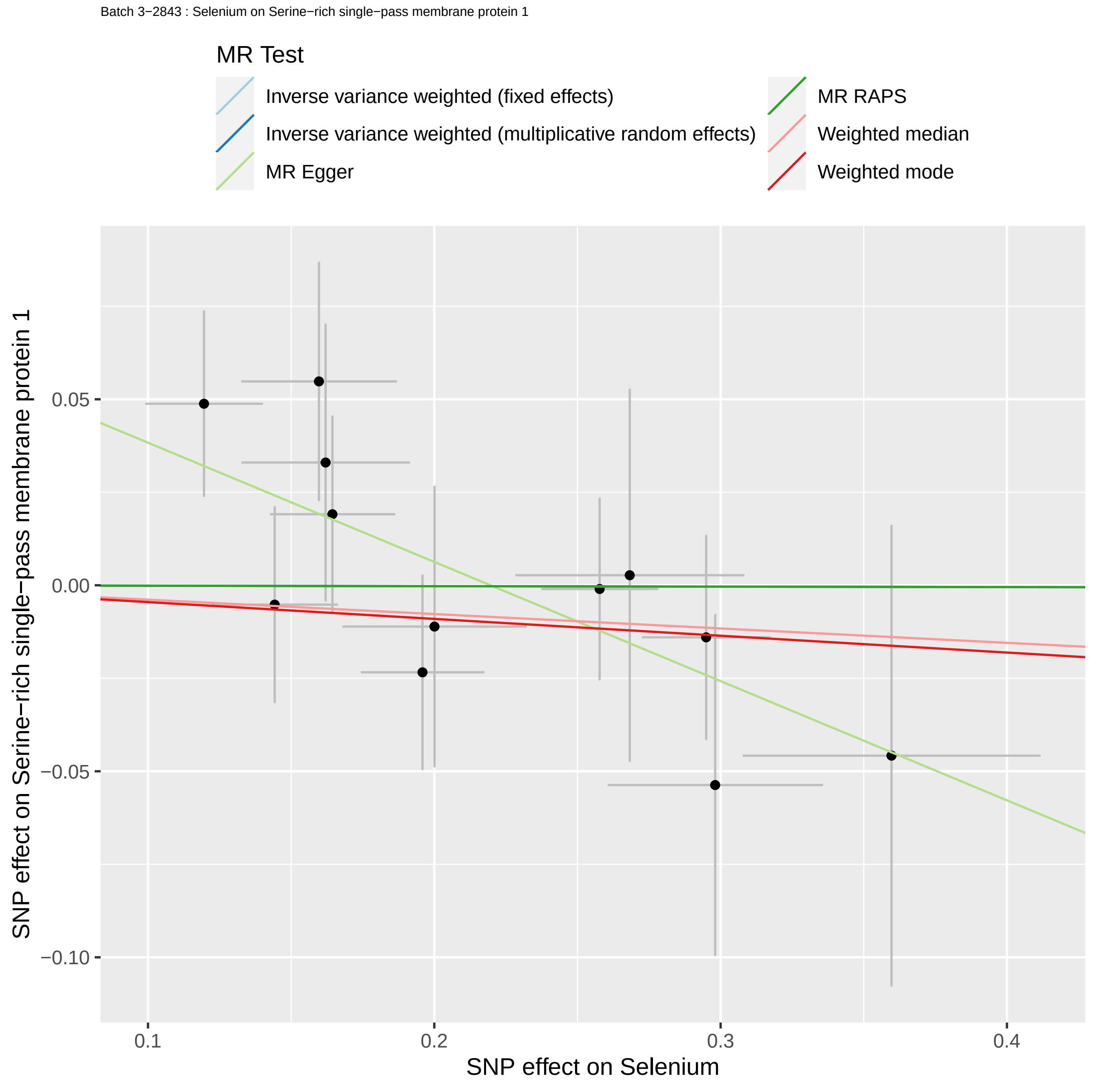
Batch 3−2843 : Selenium on Serine−rich single−pass membrane protein 1
MR Test
Inverse variance weighted (fixed effects)
Inverse variance weighted (multiplicative random effects)
MR Egger
MR RAPS
Weighted median
Weighted mode
SNP effect on Serine−rich single−pass membrane protein 1
0.05
0.00
−0.05
−0.10
0.1
0.2
0.3
0.4
SNP effect on Selenium

Batch 3−2844 : Selenium on Translocon−associated protein subunit alpha

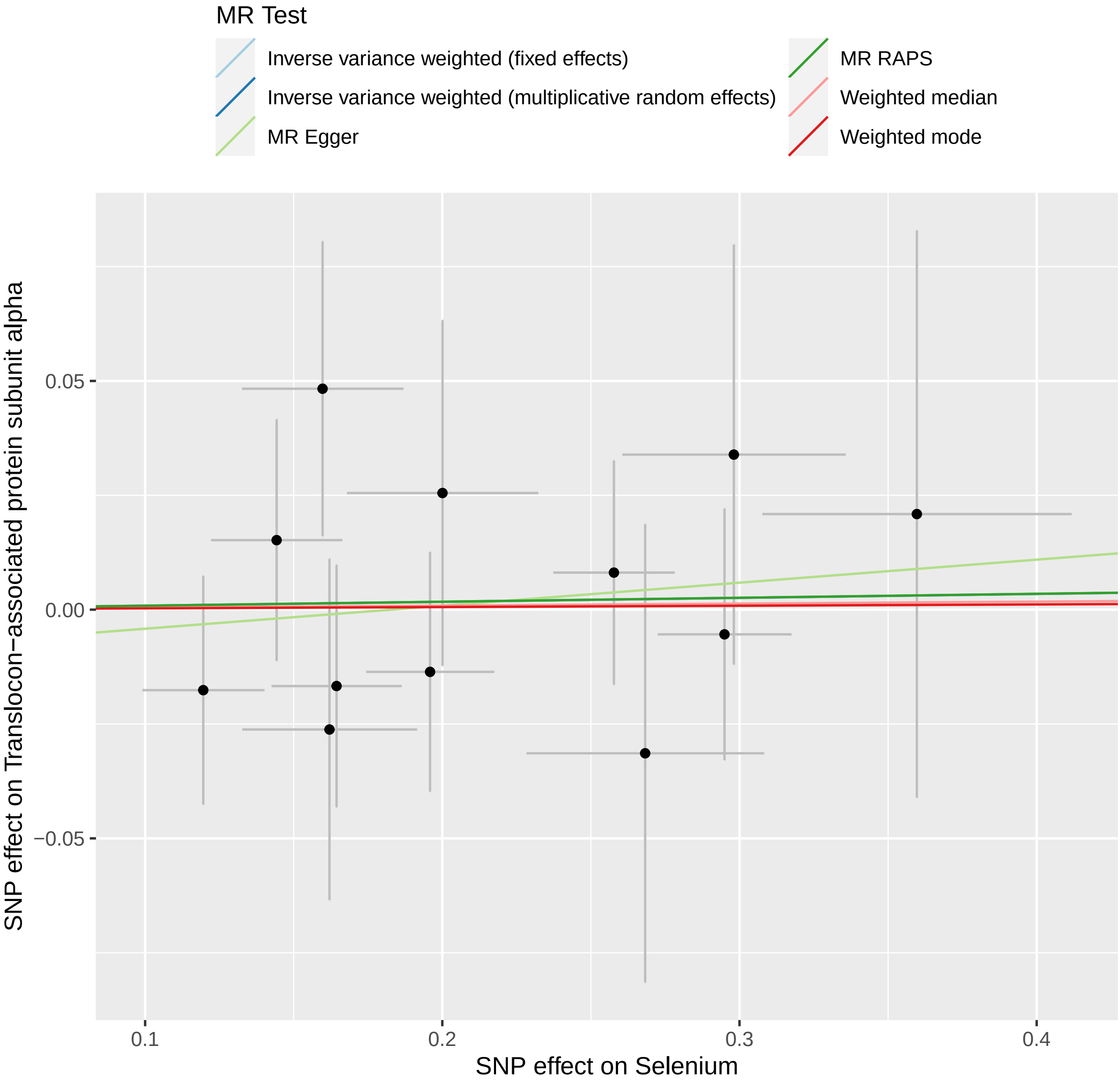

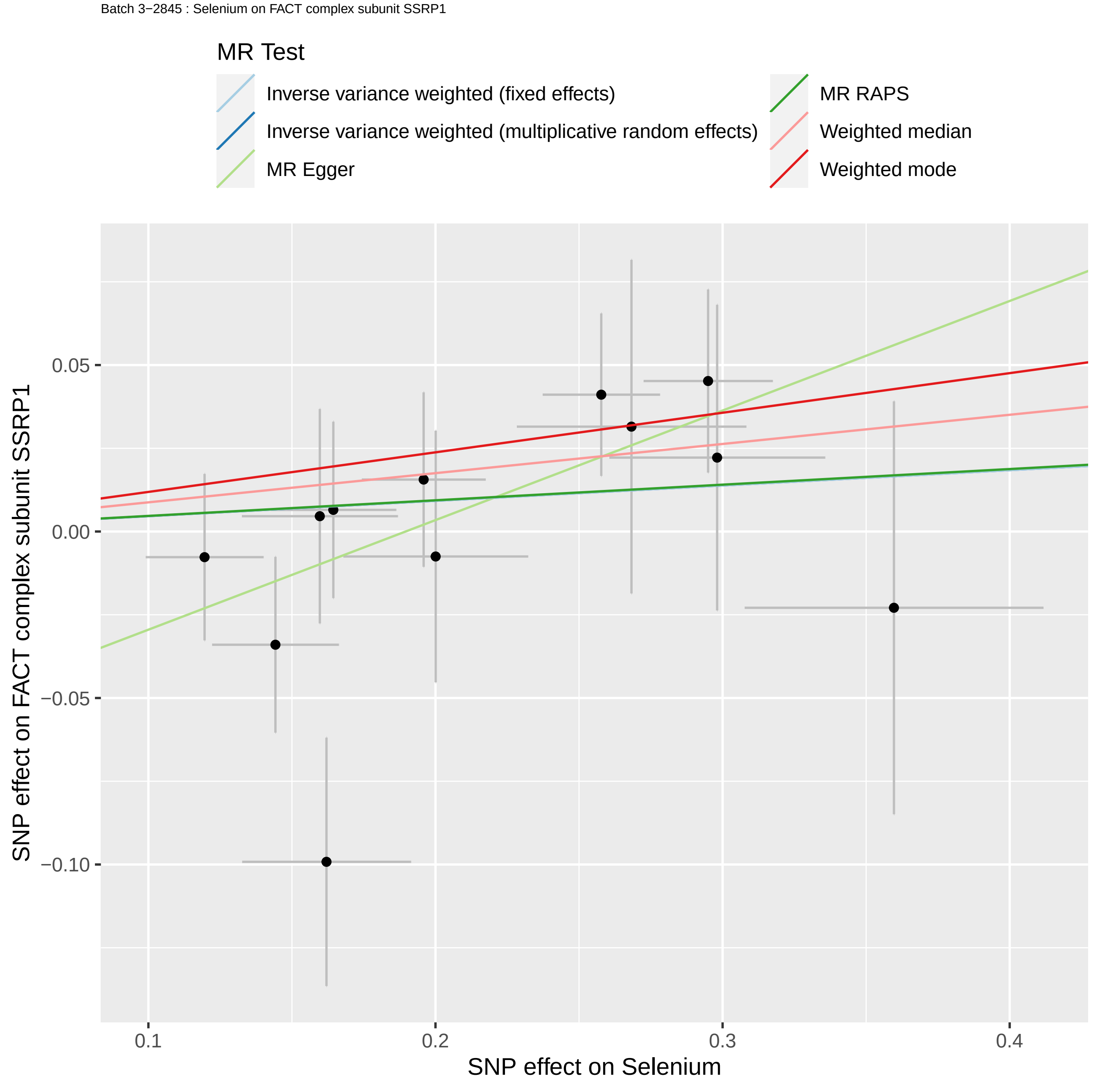

Batch 3−2845 : Selenium on FACT complex subunit SSRP1
MR Test
Inverse variance weighted (fixed effects)
Inverse variance weighted (multiplicative random effects)
MR Egger
MR RAPS
Weighted median
Weighted mode
0.05
0.00
−0.05
−0.10
SNP effect on FACT complex subunit SSRP1
0.1
0.2
0.3
0.4
SNP effect on Selenium

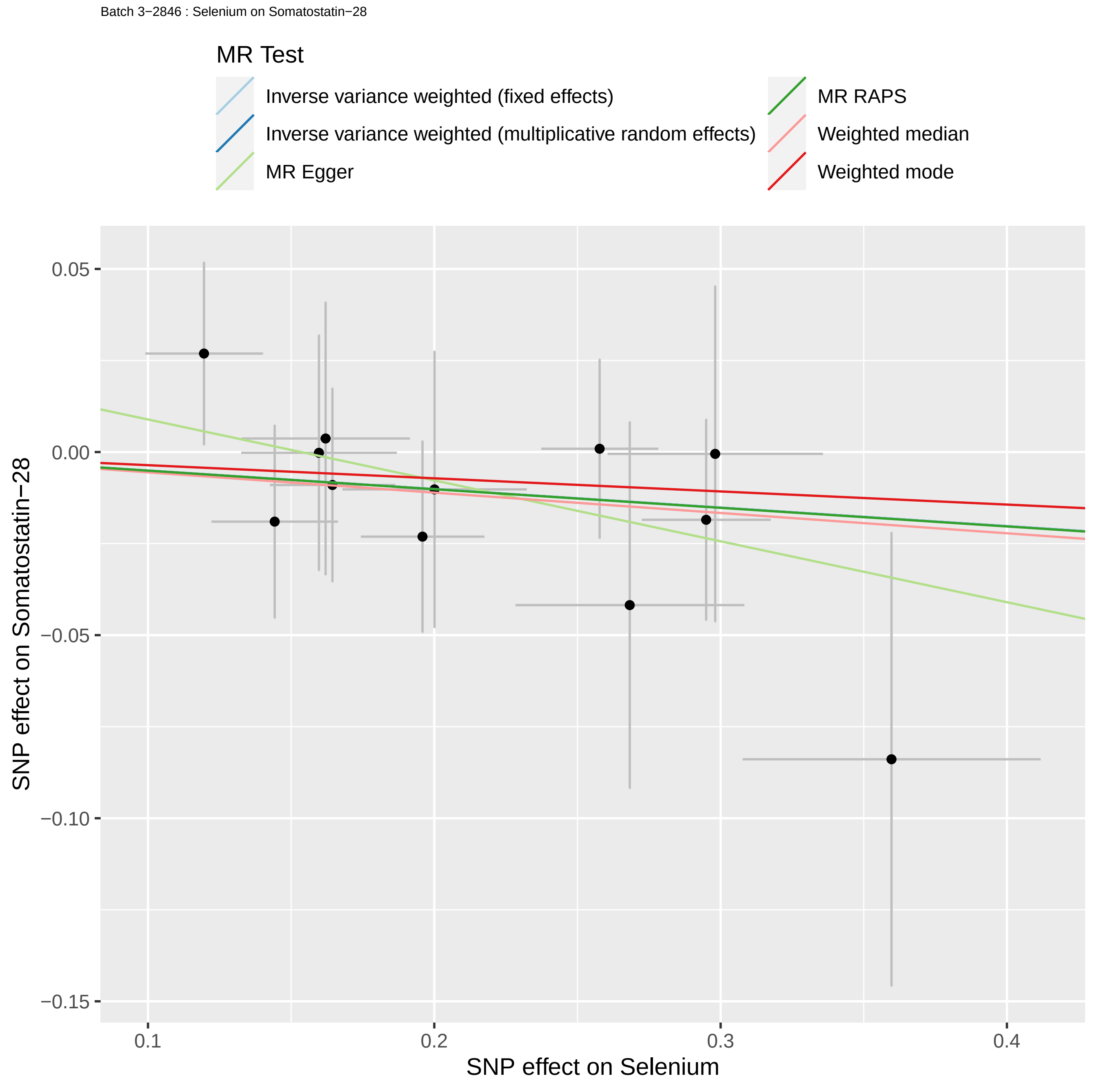

Batch 3−2846 : Selenium on Somatostatin−28
MR Test
Inverse variance weighted (fixed effects)
Inverse variance weighted (multiplicative random effects)
MR Egger
MR RAPS
Weighted median
Weighted mode
SNP effect on Somatostatin−28
0.05
0.00
−0.05
−0.10
−0.15
0.1
0.2
0.3
0.4
SNP effect on Selenium

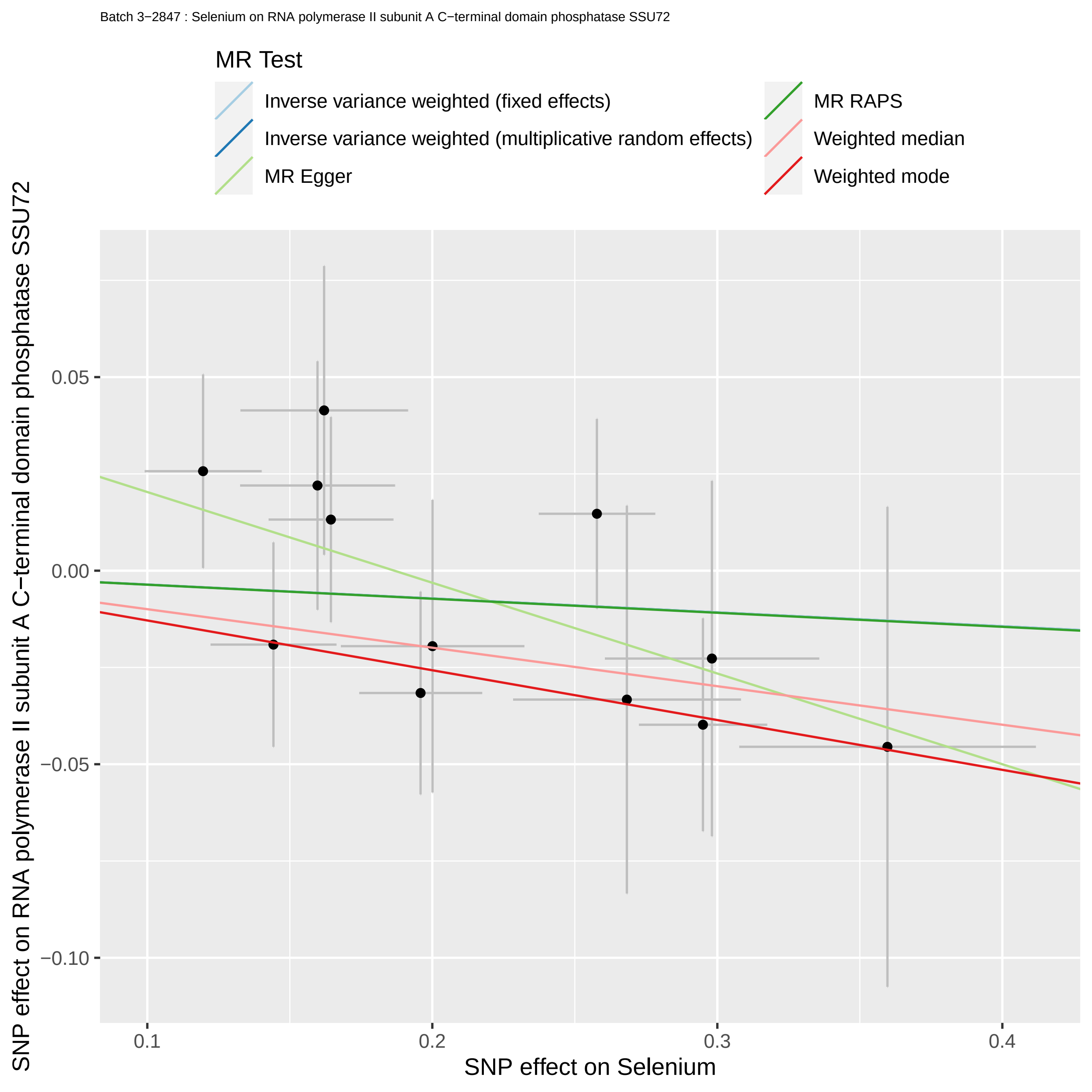

Batch 3−2847 : Selenium on RNA polymerase II subunit A C−terminal domain phosphatase SSU72
MR Test
Inverse variance weighted (fixed effects)
Inverse variance weighted (multiplicative random effects)
MR Egger
MR RAPS
Weighted median
Weighted mode
SNP effect on RNA polymerase II subunit A C−terminal domain phosphatase SSU72
0.05
0.00
−0.05
−0.10
0.1
0.2
0.3
0.4
SNP effect on Selenium

Batch 3−2848 : Selenium on CMP−N−acetylneuraminate−beta−galactosamide−alpha−2,3−sialyltransferase 1

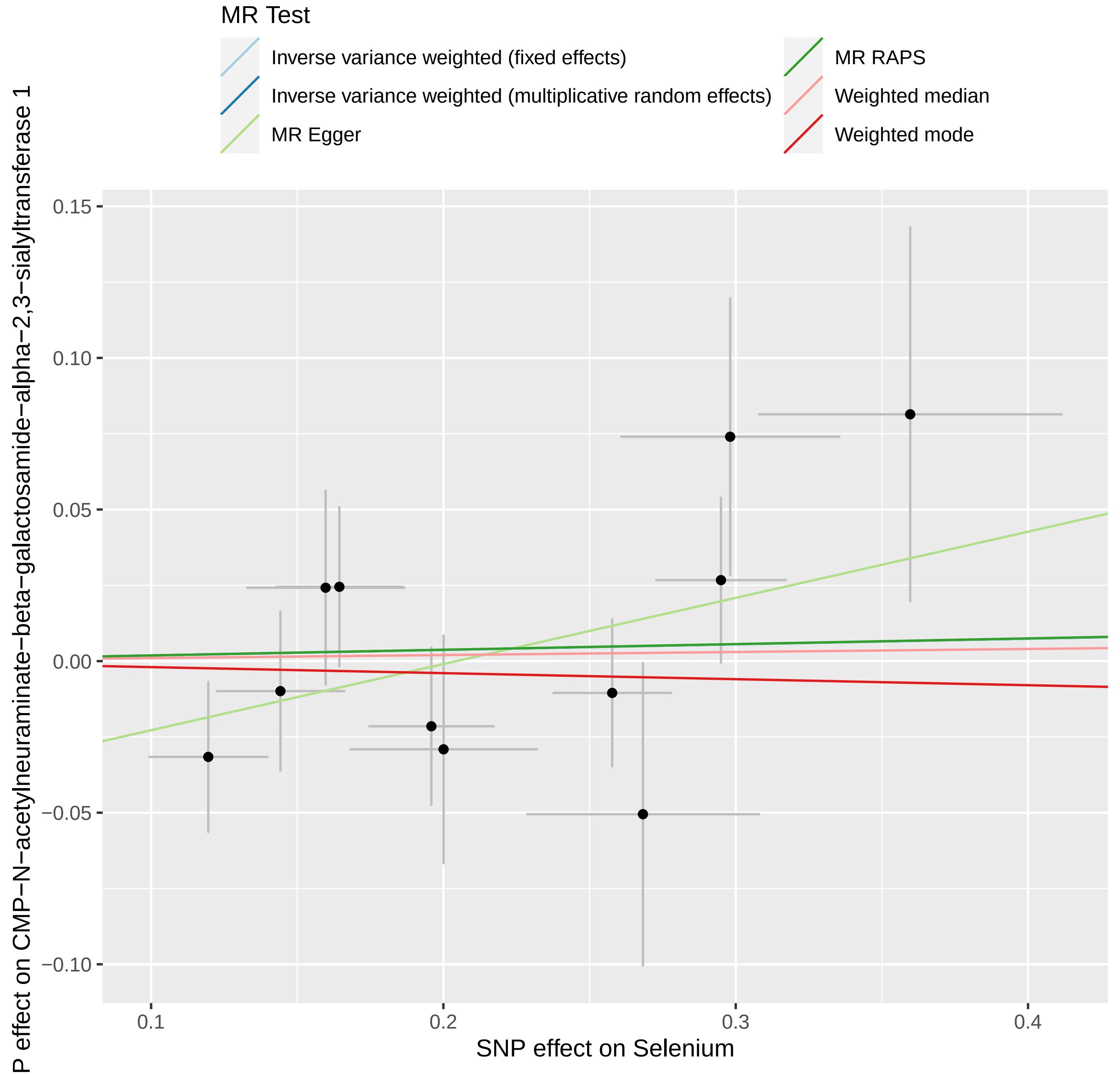

Batch 3−2849 : Selenium on CMP−N−acetylneuraminate−beta−galactosamide−alpha−2,3−sialyltransferase 2

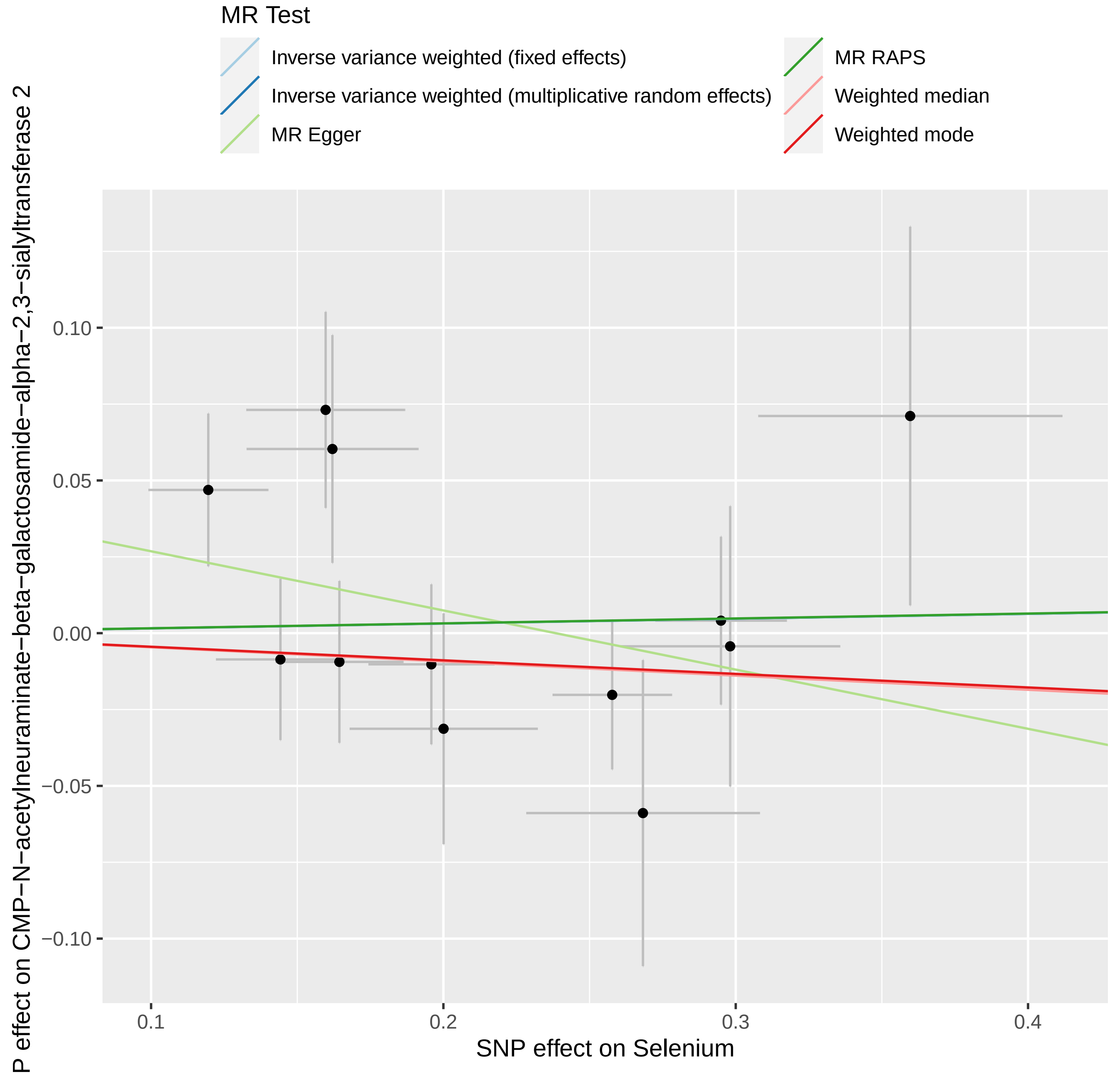

Batch 3−2850 : Selenium on Type 2 lactosamine alpha−2,3−sialyltransferase

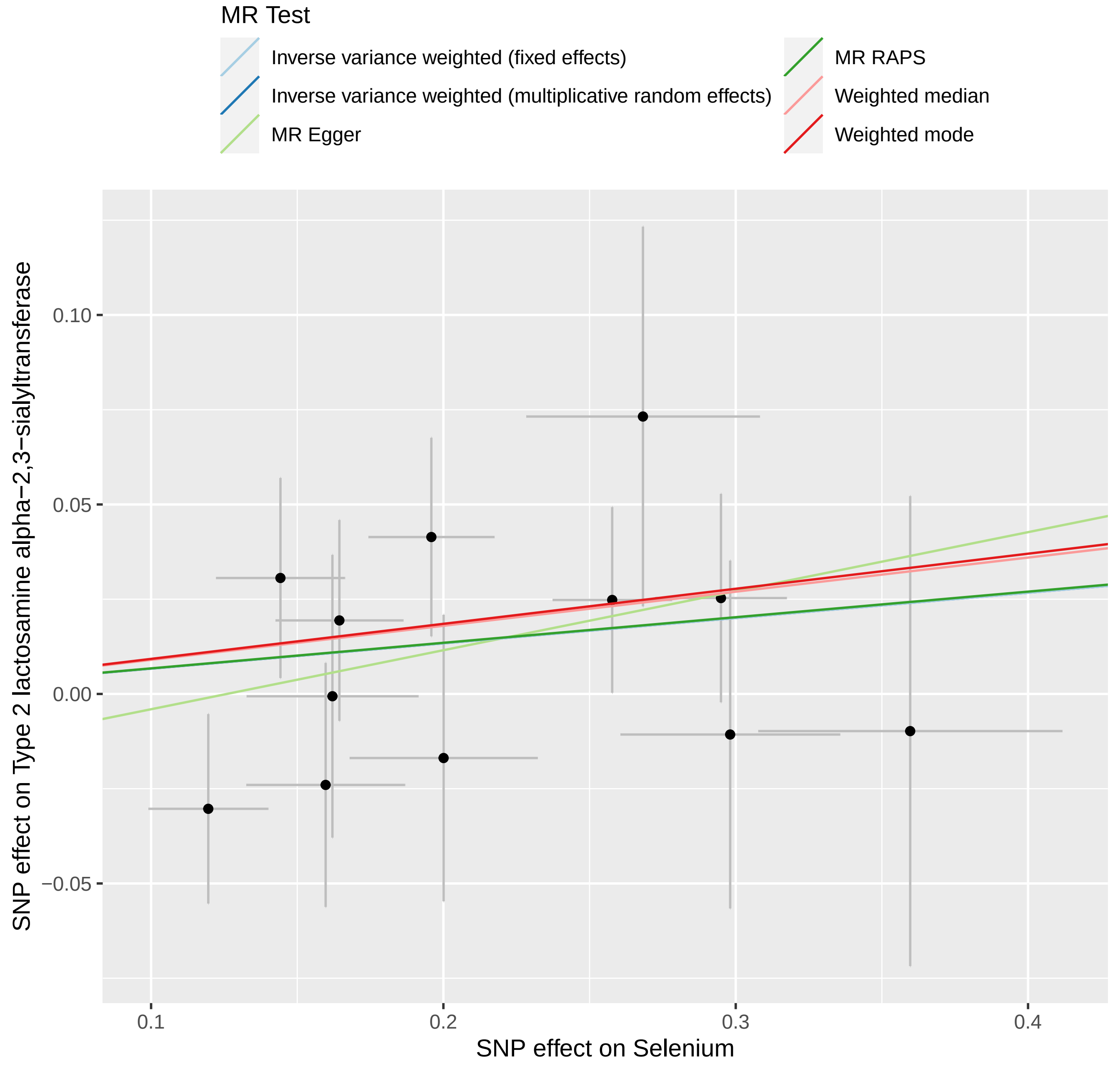

Batch 3−2851 : Selenium on Beta−galactoside alpha−2,6−sialyltransferase 1

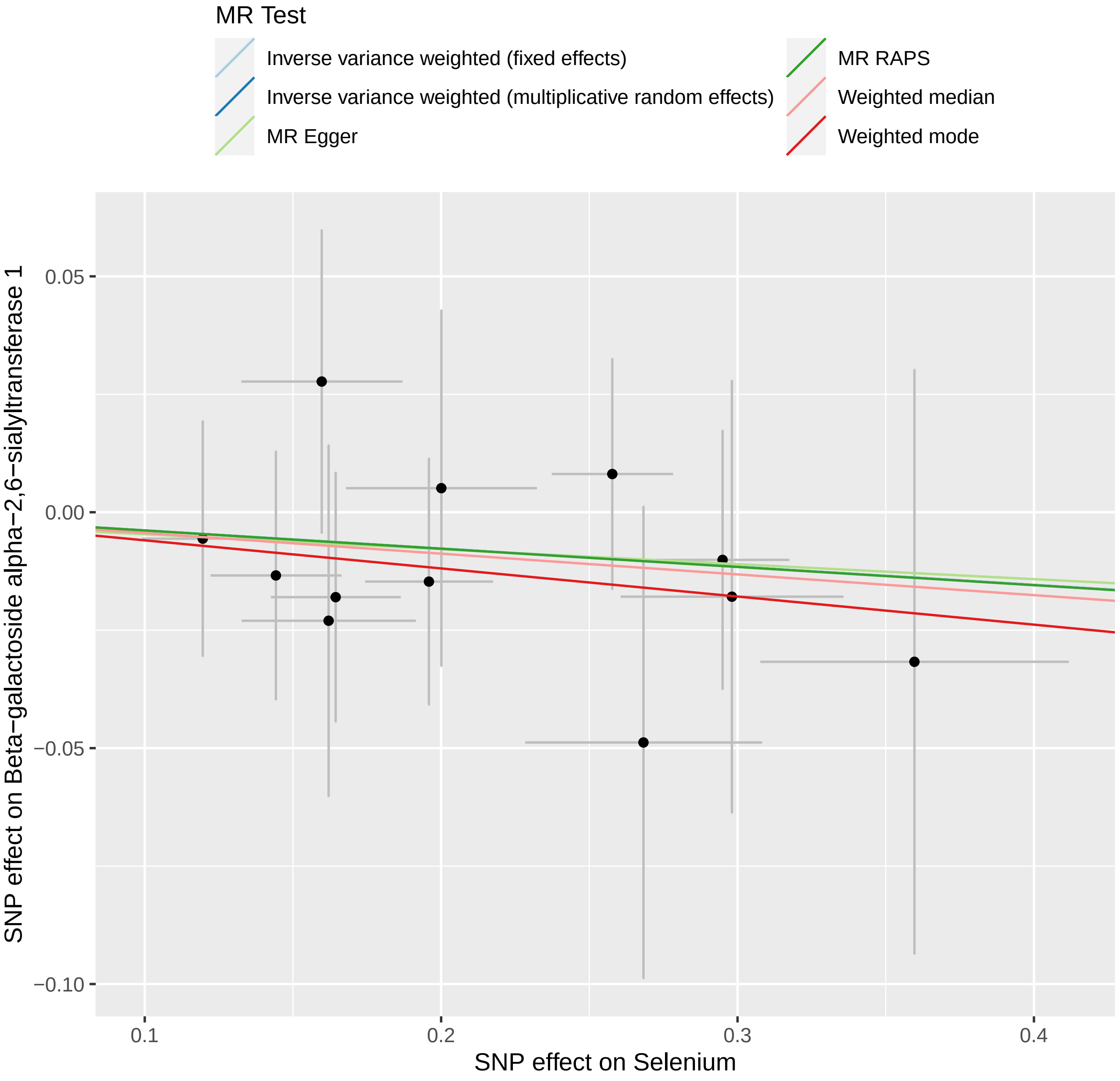

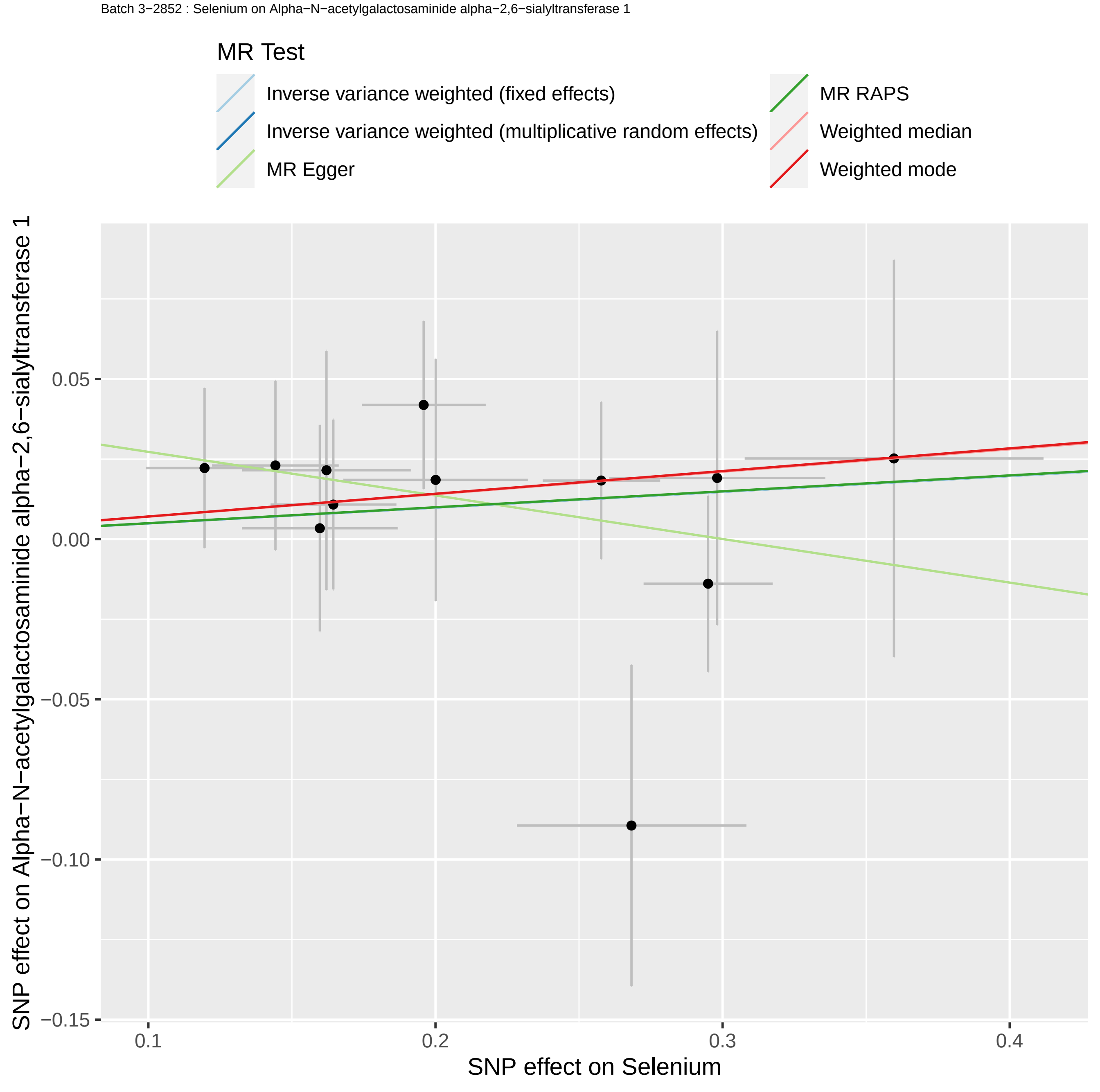

Batch 3−2852 : Selenium on Alpha−N−acetylgalactosaminide alpha−2,6−sialyltransferase 1
MR Test
Inverse variance weighted (fixed effects)
Inverse variance weighted (multiplicative random effects)
MR Egger
MR RAPS
Weighted median
Weighted mode
SNP effect on Alpha−N−acetylgalactosaminide alpha−2,6−sialyltransferase 1
0.05
0.00
−0.05
−0.10
−0.15
0.1
0.2
0.3
0.4
SNP effect on Selenium

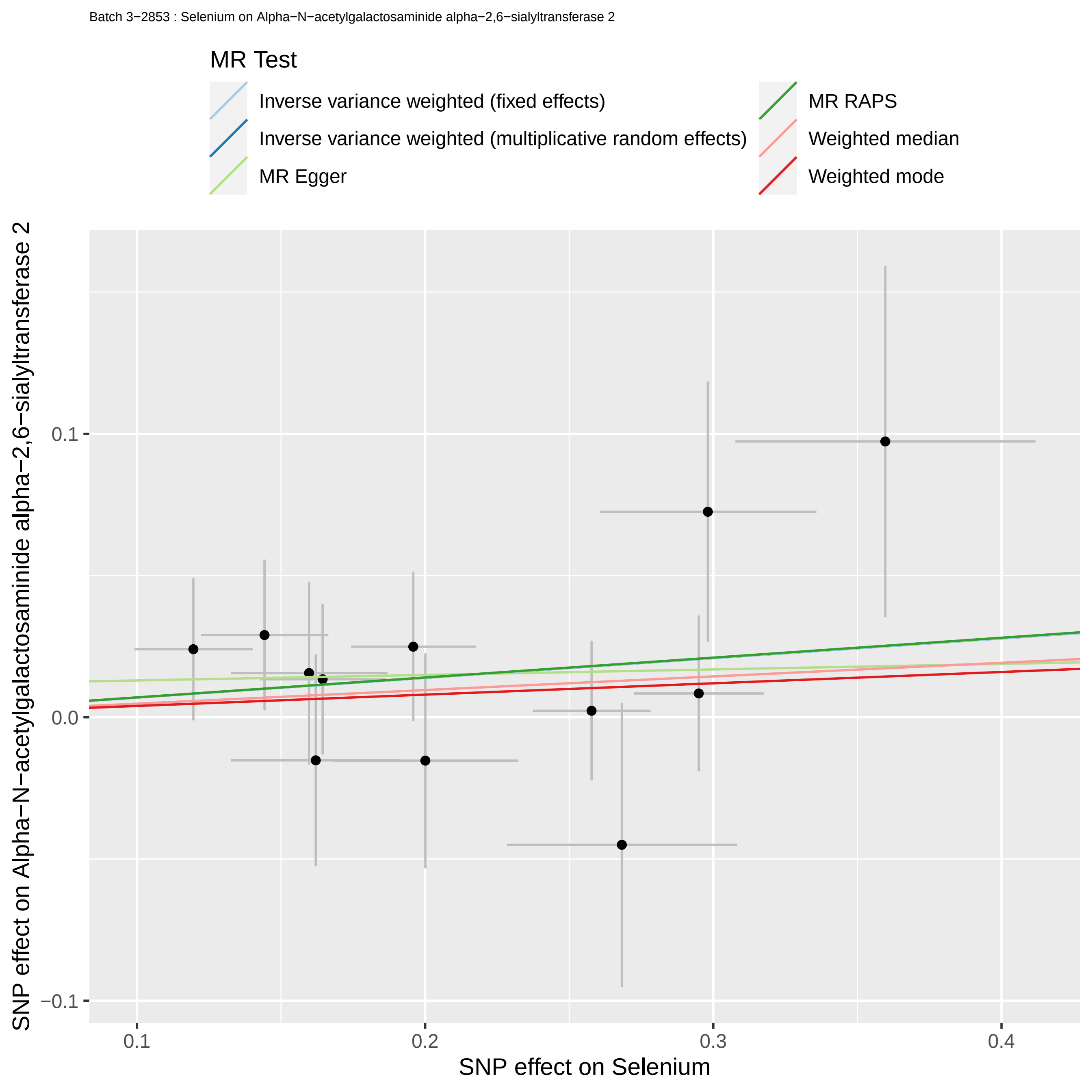

Batch 3−2853 : Selenium on Alpha−N−acetylgalactosaminide alpha−2,6−sialyltransferase 2
MR Test
Inverse variance weighted (fixed effects)
Inverse variance weighted (multiplicative random effects)
MR Egger
MR RAPS
Weighted median
Weighted mode
SNP effect on Alpha−N−acetylgalactosaminide alpha−2,6−sialyltransferase 2
0.1
0.0
−0.1
0.1
0.2
0.3
0.4
SNP effect on Selenium

Batch 3−2854 : Selenium on Alpha−N−acetylgalactosaminide alpha−2,6−sialyltransferase 3

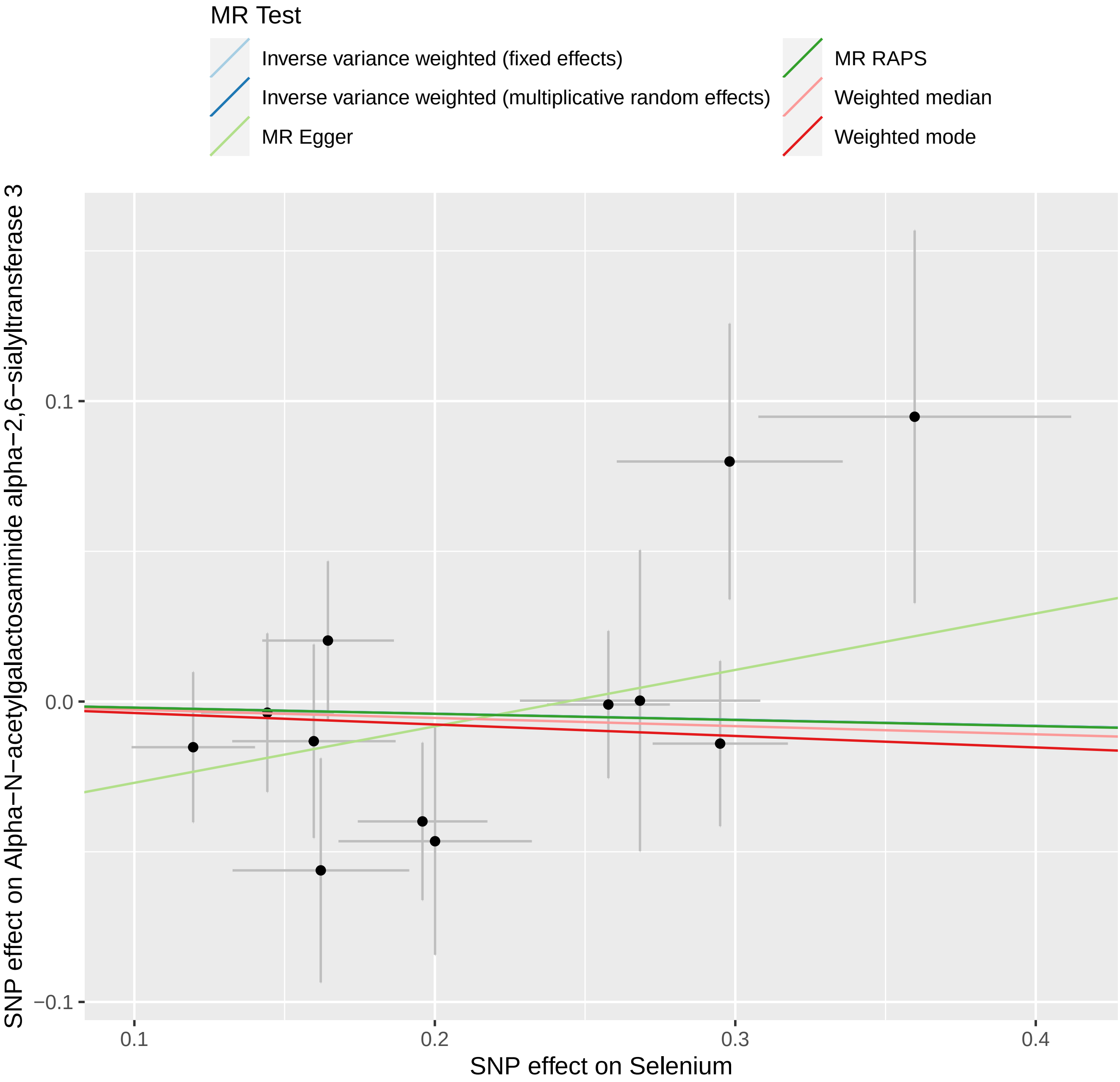

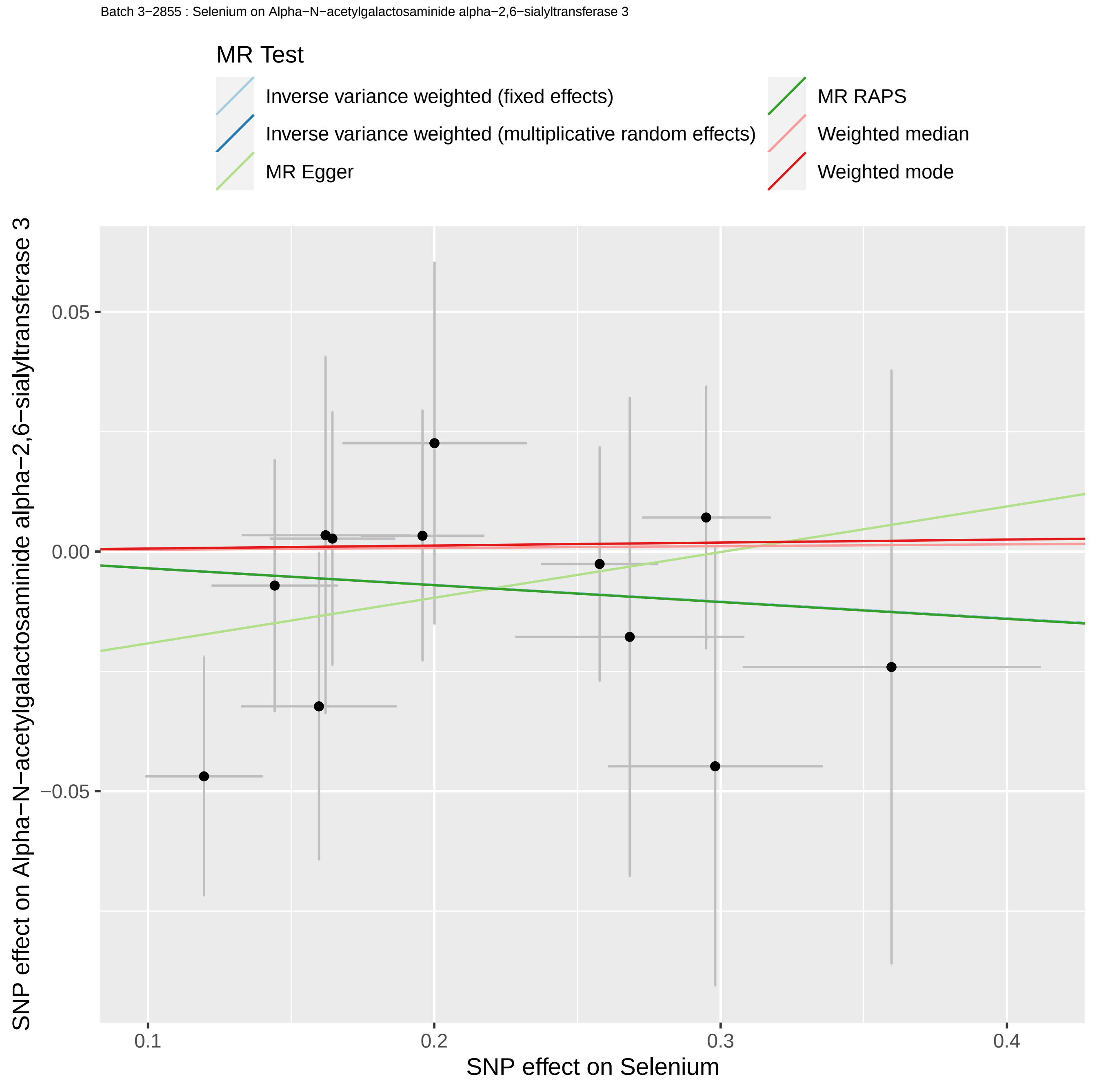
Batch 3−2855 : Selenium on Alpha−N−acetylgalactosaminide alpha−2,6−sialyltransferase 3
MR Test
Inverse variance weighted (fixed effects)
Inverse variance weighted (multiplicative random effects)
MR Egger
MR RAPS
Weighted median
Weighted mode
SNP effect on Alpha−N−acetylgalactosaminide alpha−2,6−sialyltransferase 3
0.05
0.00
−0.05
0.1
0.2
0.3
0.4
SNP effect on Selenium

Batch 3−2856 : Selenium on Alpha−N−acetylgalactosaminide alpha−2,6−sialyltransferase 5

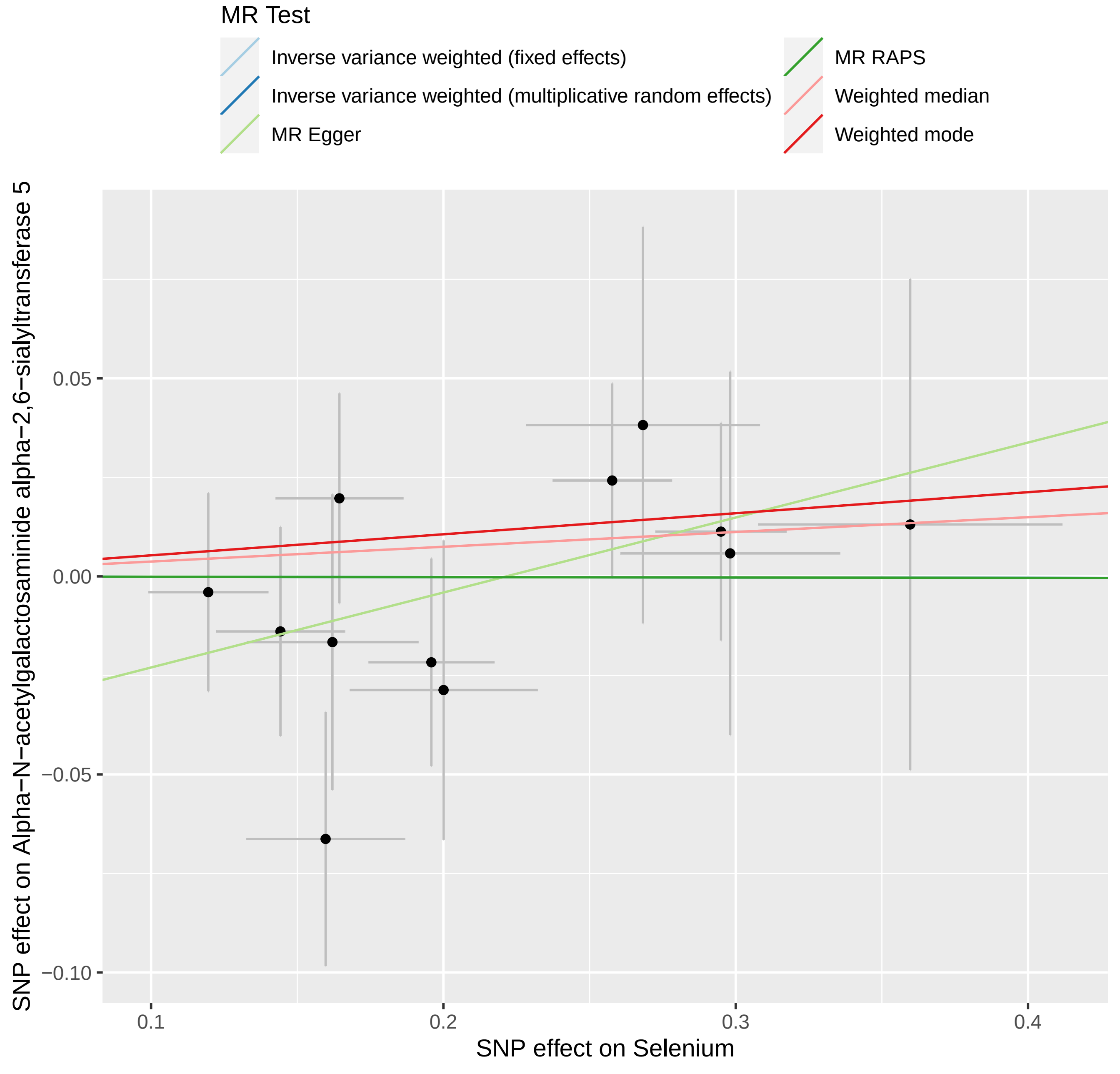

Batch 3−2857 : Selenium on Alpha−N−acetylgalactosaminide alpha−2,6−sialyltransferase 6

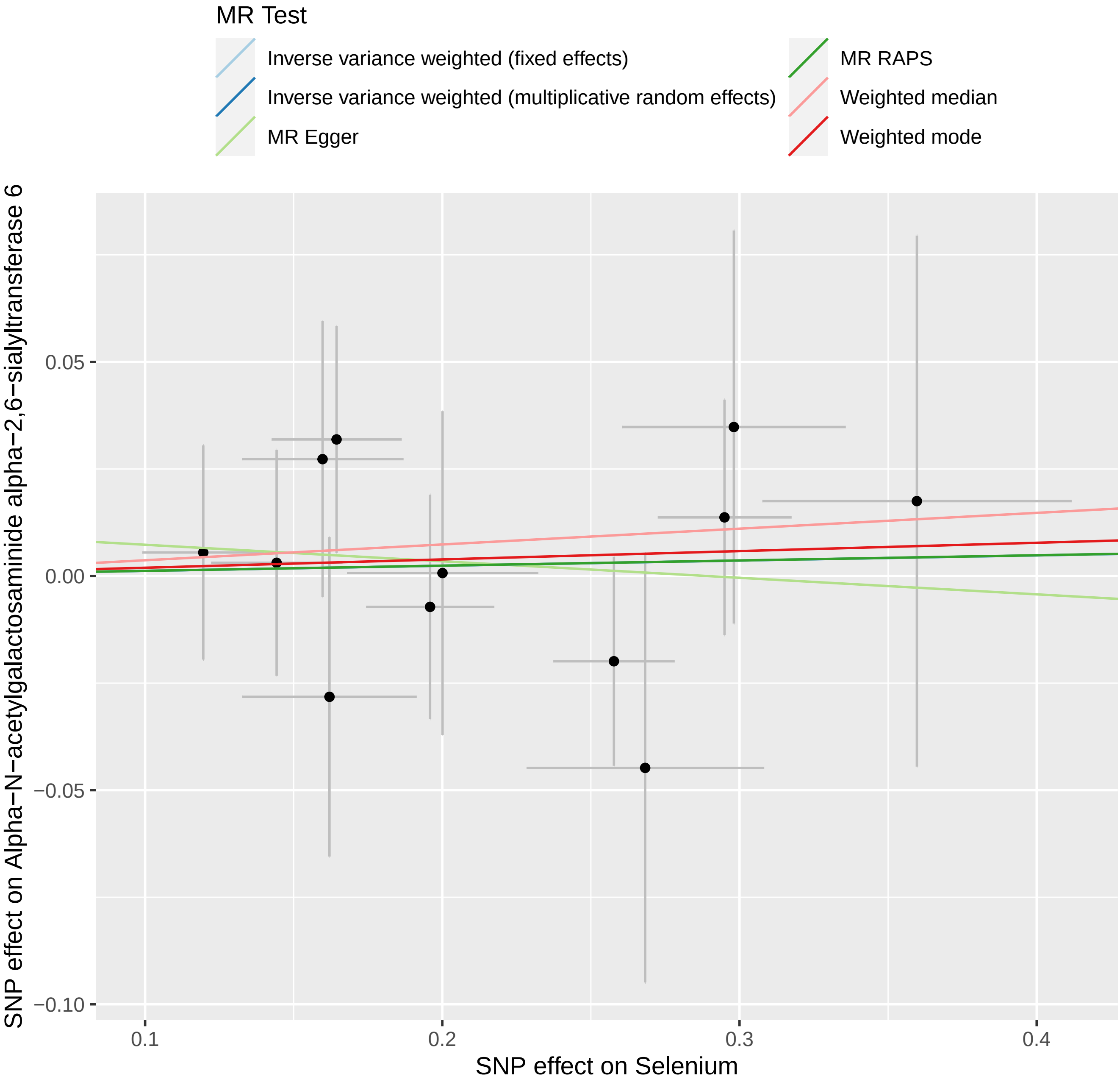

Batch 3−2858 : Selenium on Alpha−2,8−sialyltransferase 8B

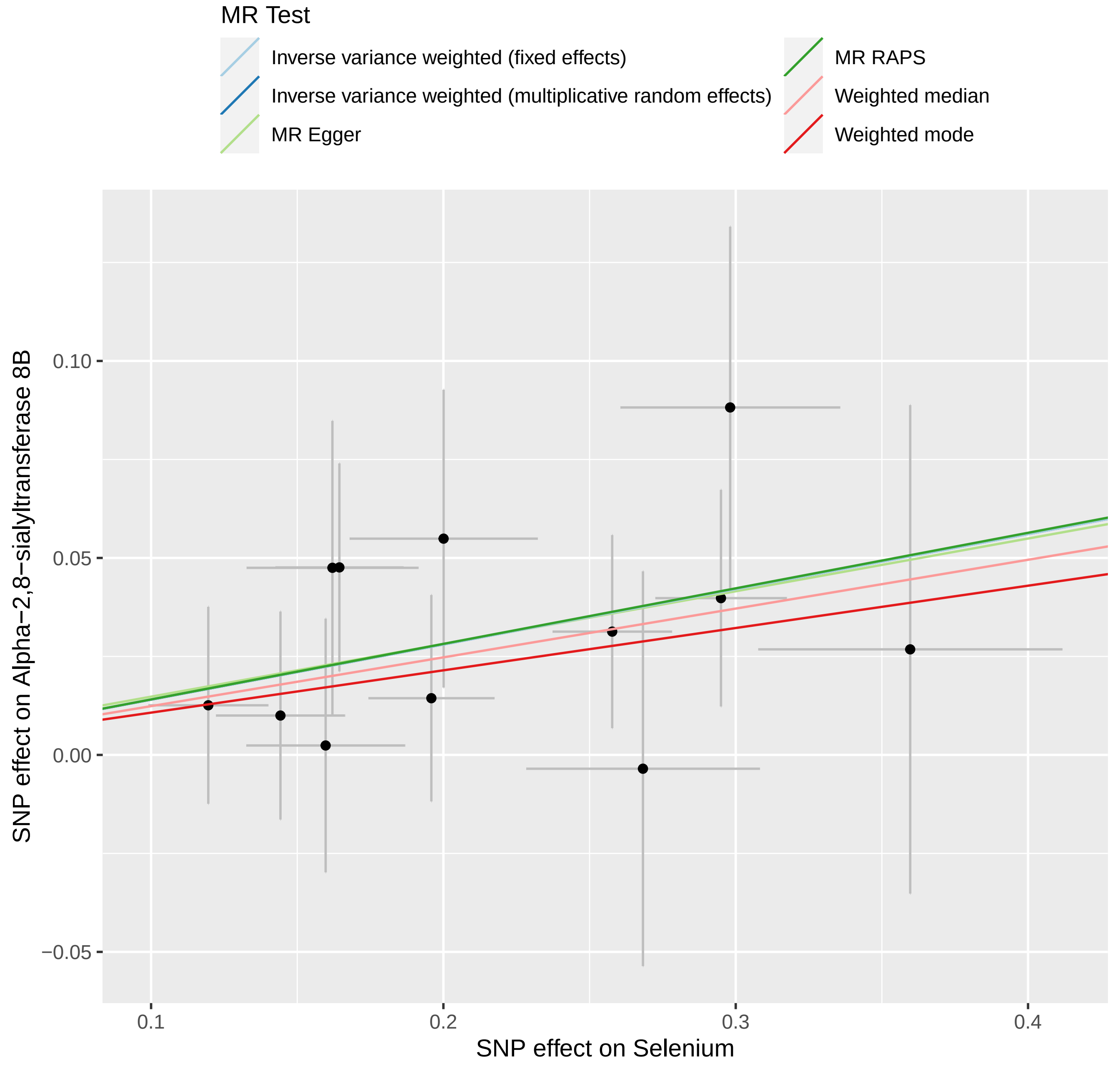

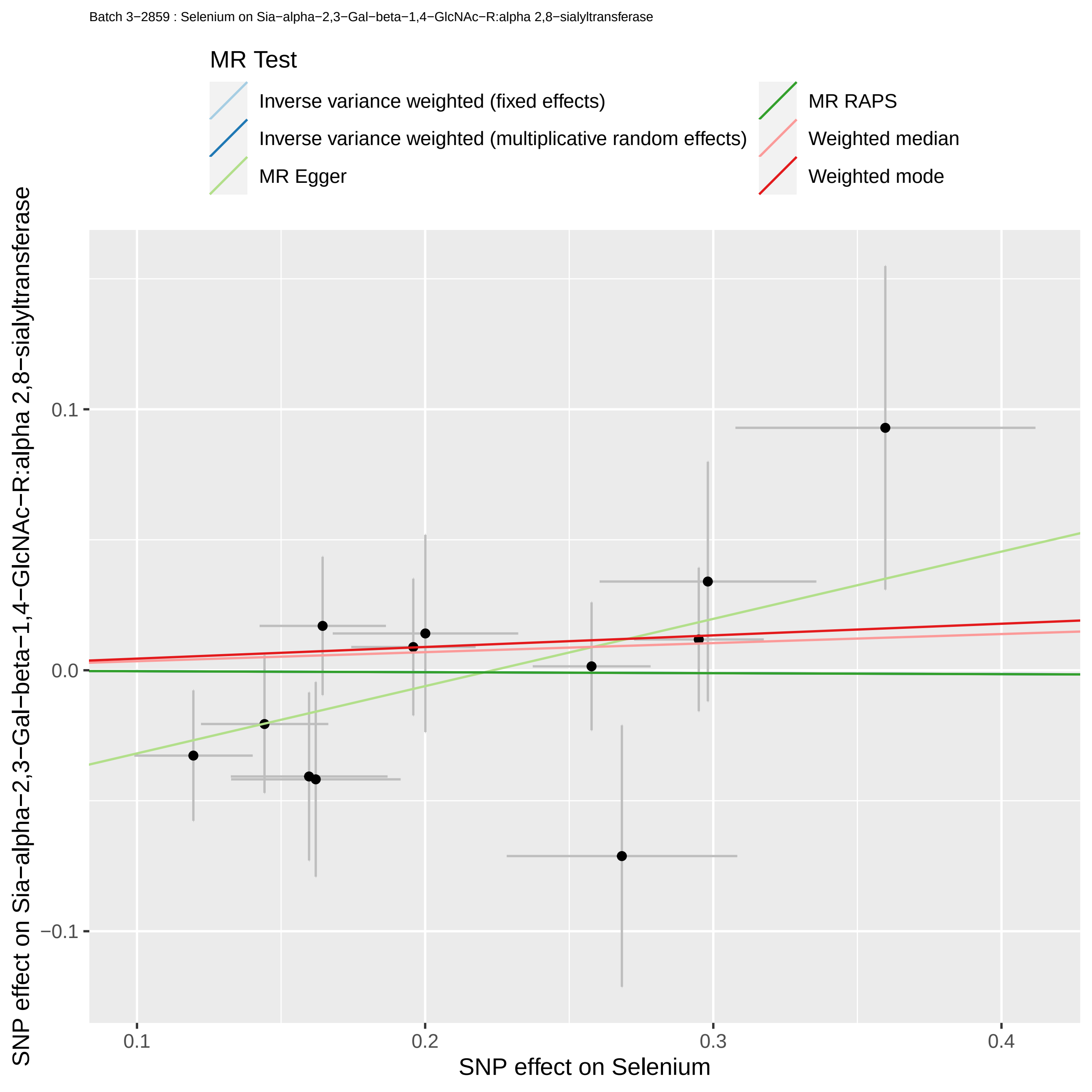
Batch 3−2859 : Selenium on Sia−alpha−2,3−Gal−beta−1,4−GlcNAc−R:alpha 2,8−sialyltransferase
MR Test
Inverse variance weighted (fixed effects)
Inverse variance weighted (multiplicative random effects)
MR Egger
MR RAPS
Weighted median
Weighted mode
0.1
0.0
−0.1
0.1
0.2
0.3
0.4
SNP effect on Sia−alpha−2,3−Gal−beta−1,4−GlcNAc−R:alpha 2,8−sialyltransferase
SNP effect on Selenium

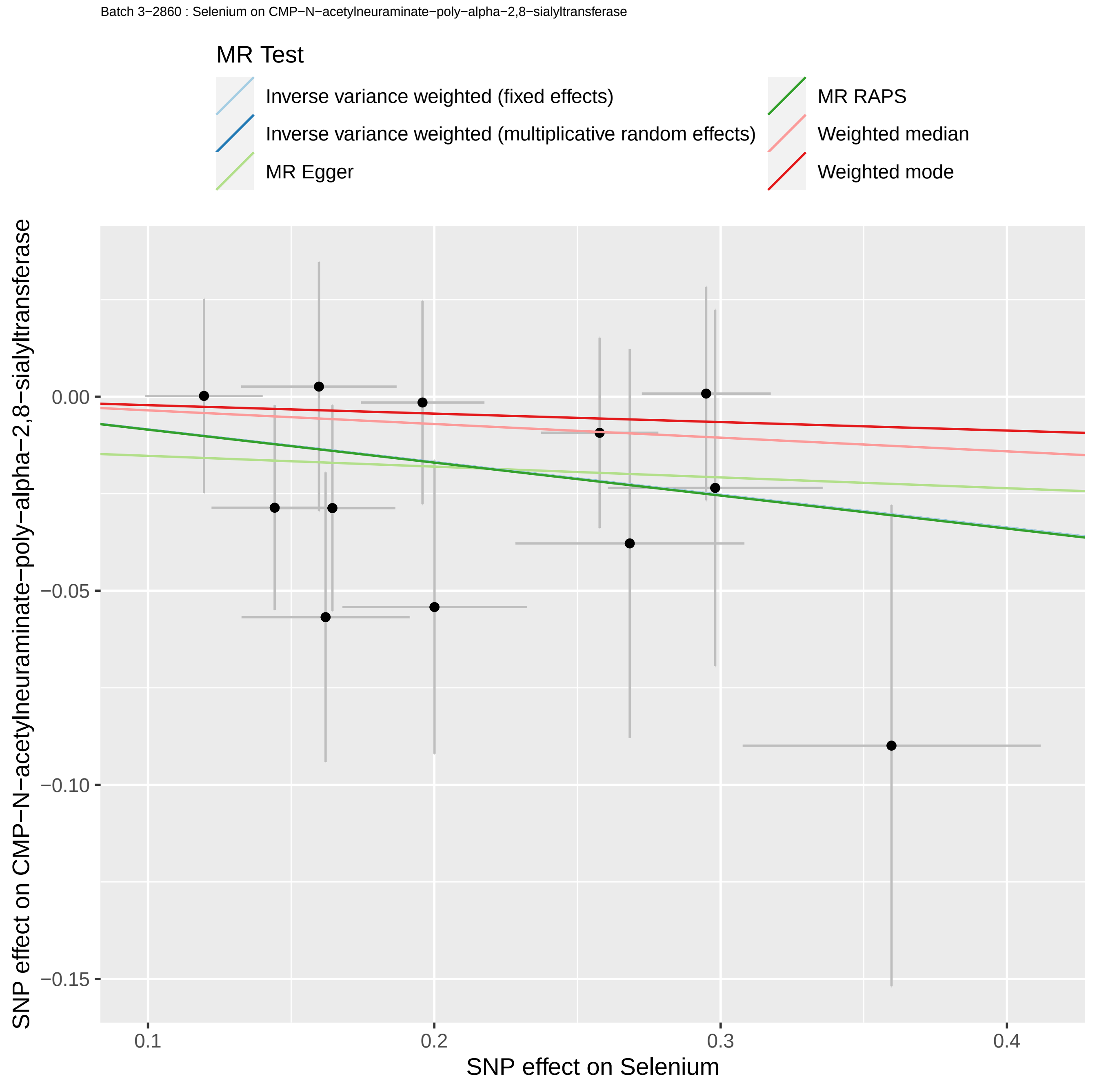

Batch 3−2860 : Selenium on CMP−N−acetylneuraminate−poly−alpha−2,8−sialyltransferase
MR Test
Inverse variance weighted (fixed effects)
Inverse variance weighted (multiplicative random effects)
MR Egger
MR RAPS
Weighted median
Weighted mode
SNP effect on CMP−N−acetylneuraminate−poly−alpha−2,8−sialyltransferase
0.00
−0.05
−0.10
−0.15
0.1
0.2
0.3
0.4
SNP effect on Selenium

Batch 3−2861 : Selenium on Alpha−2,8−sialyltransferase 8F

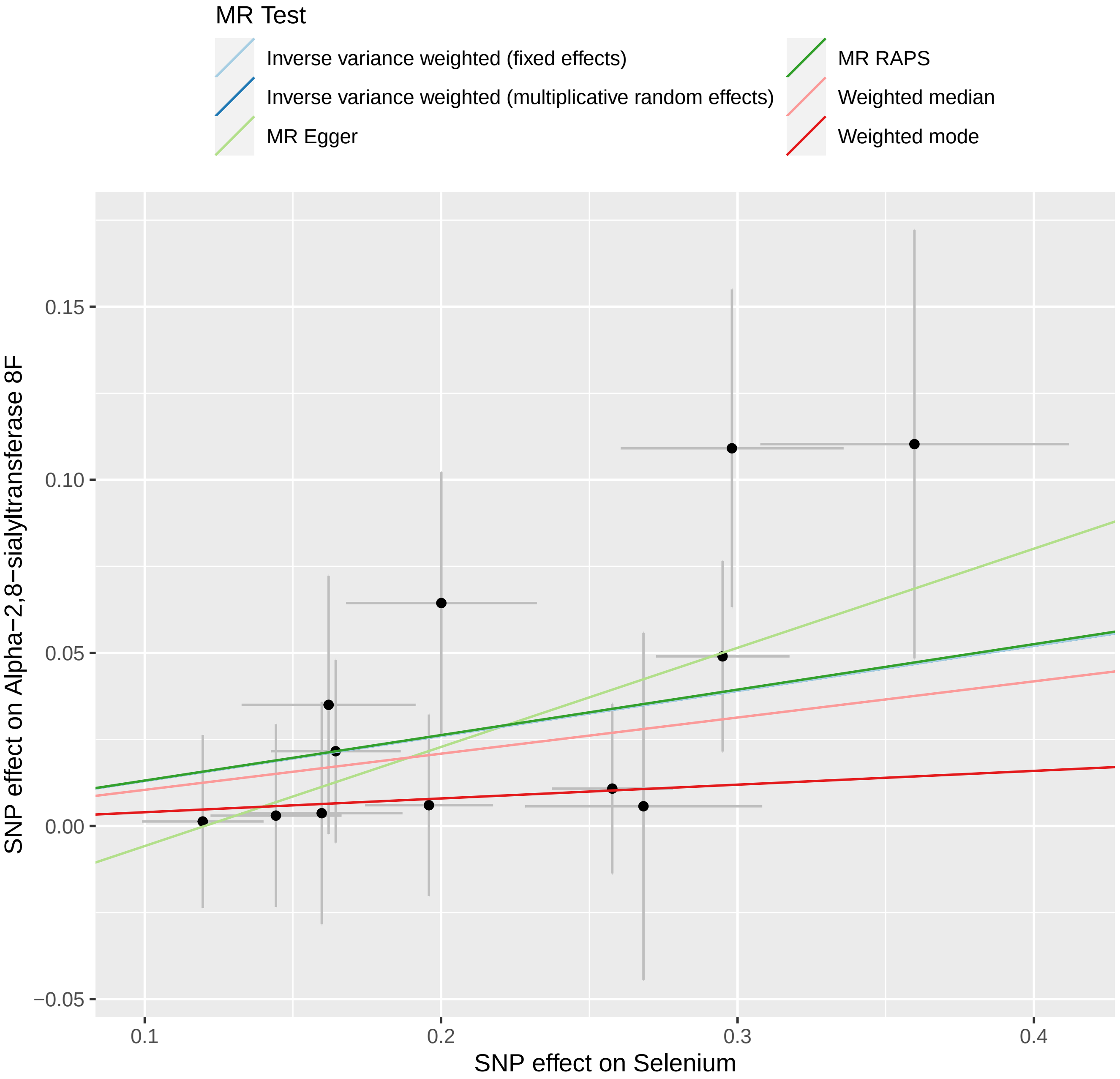

Batch 3−2862 : Selenium on Stabilin−1

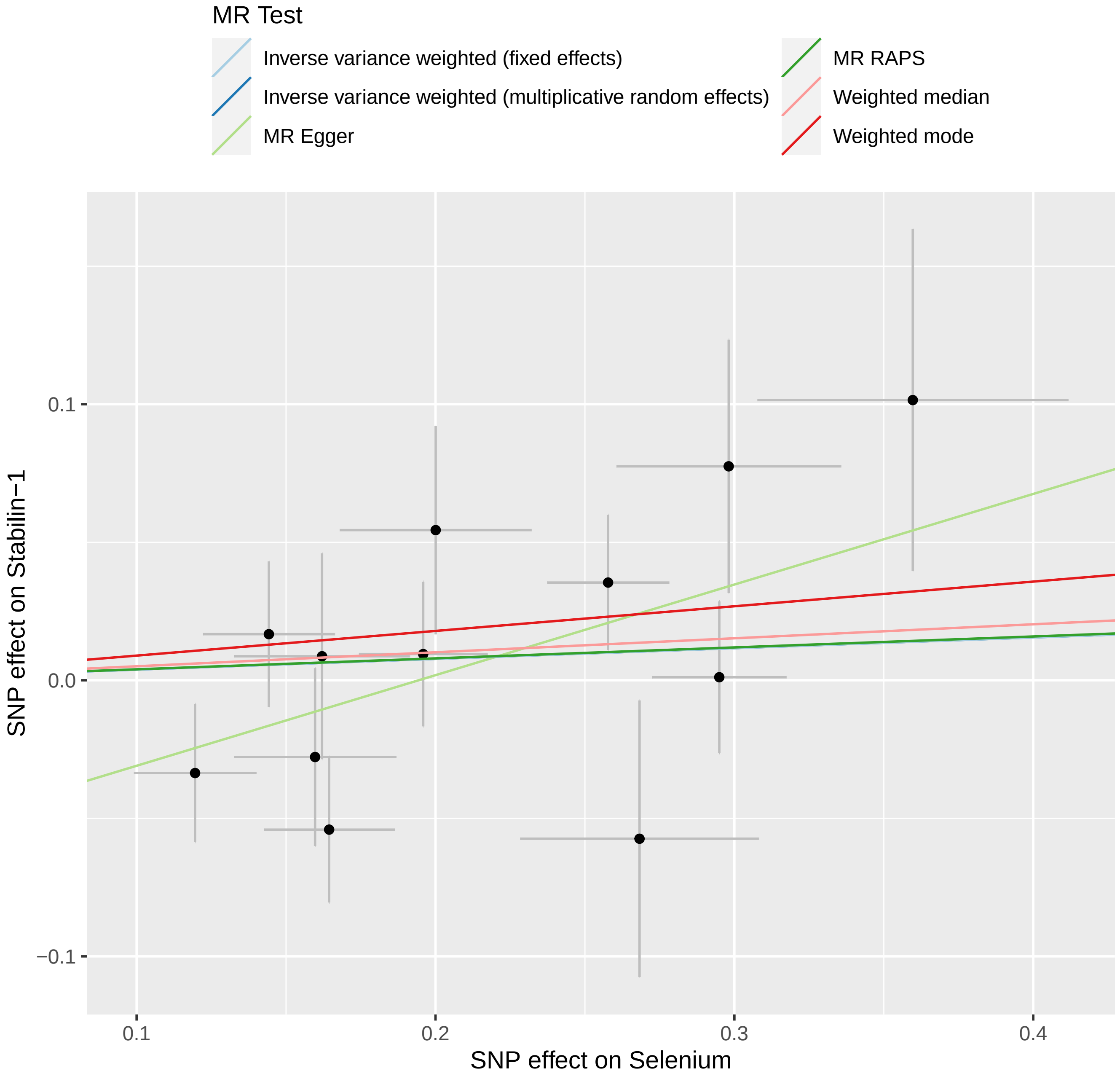

Batch 3−2863 : Selenium on Stabilin−2

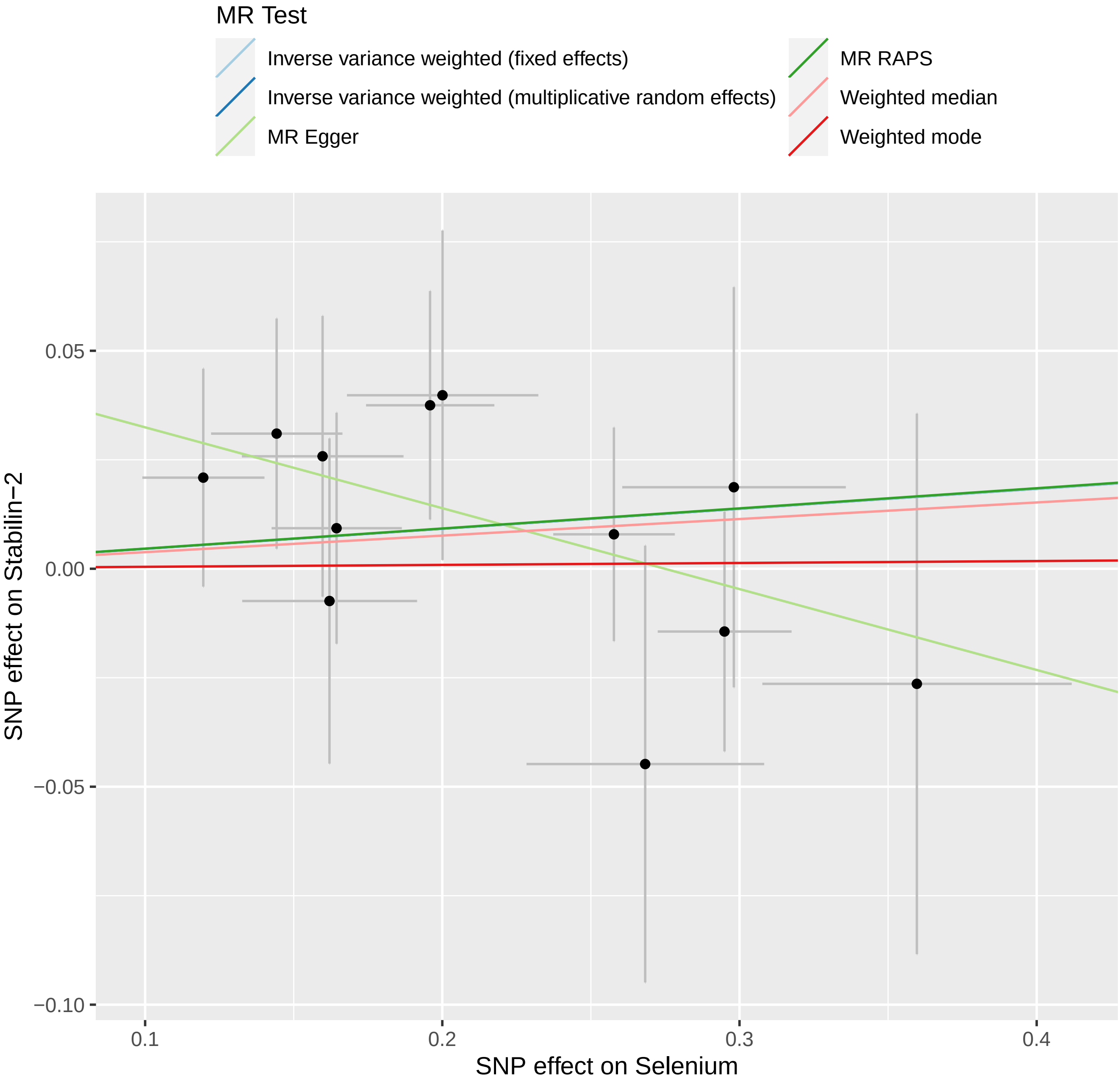

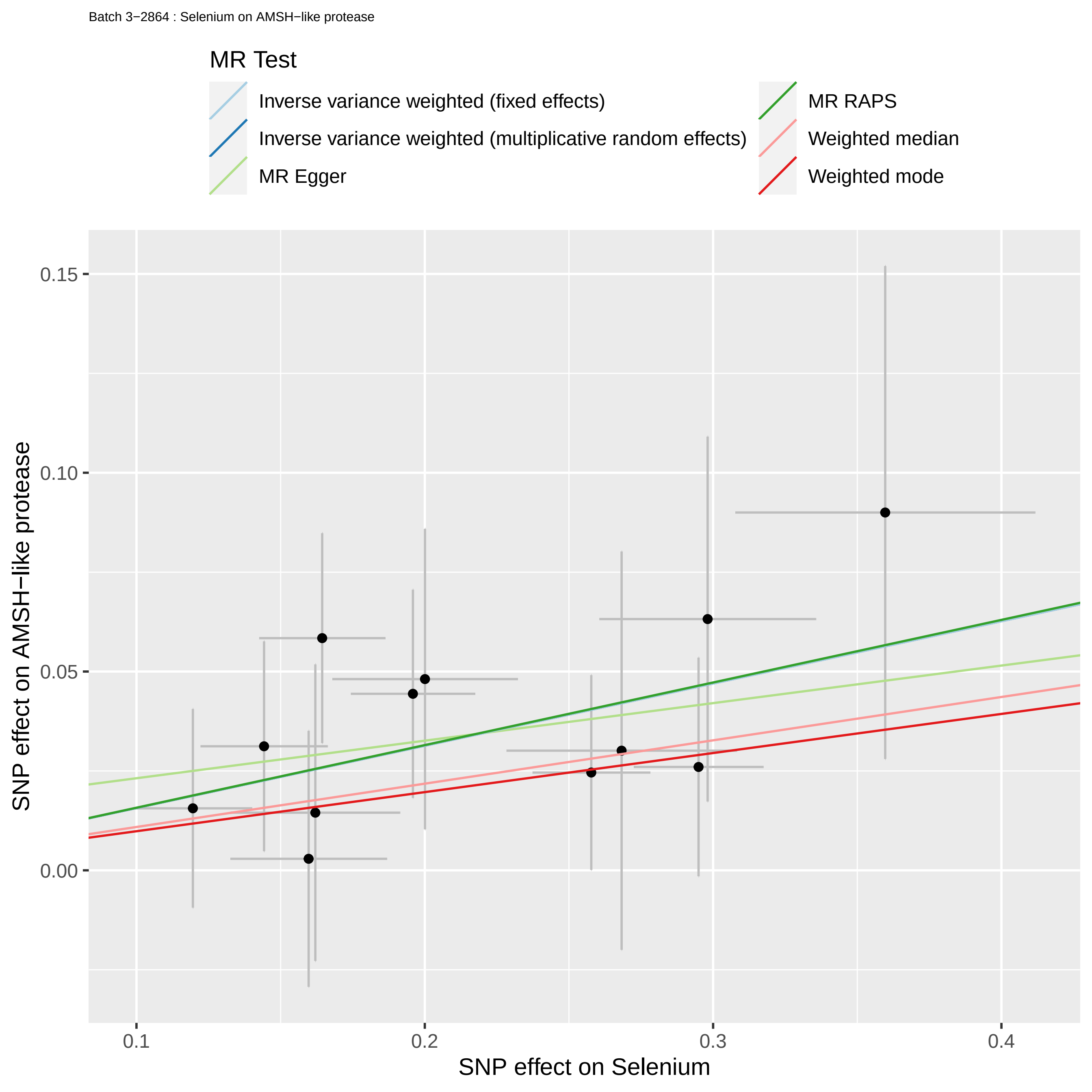

Batch 3−2864 : Selenium on AMSH−like protease
MR Test
Inverse variance weighted (fixed effects)
Inverse variance weighted (multiplicative random effects)
MR Egger
MR RAPS
Weighted median
Weighted mode
0.15
0.10
0.05
0.00
0.1
0.2
0.3
0.4
SNP effect on Selenium
SNP effect on AMSH−like protease

Batch 3−2865 : Selenium on Signal−transducing adaptor protein 1

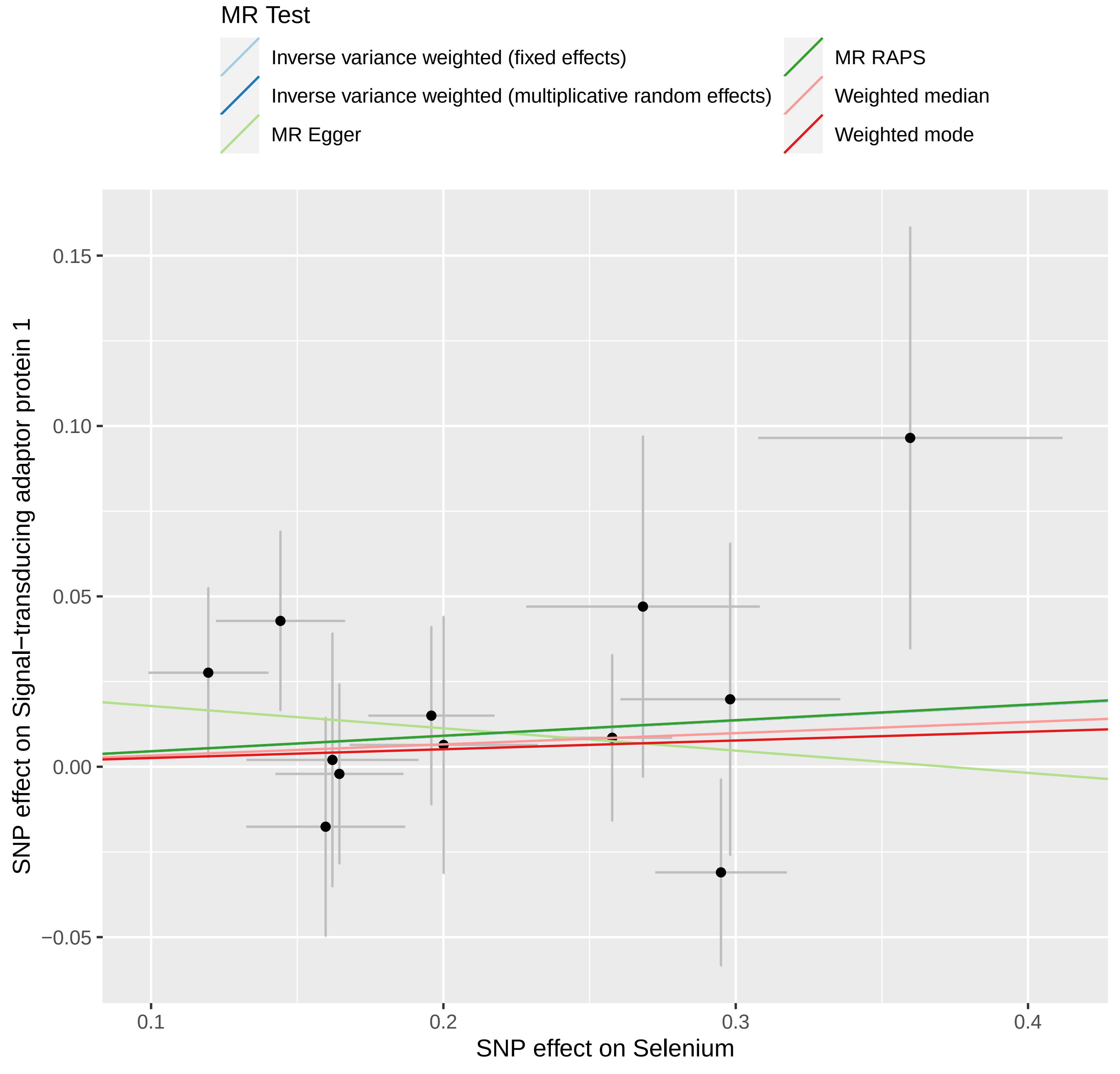

Batch 3−2866 : Selenium on Steroidogenic acute regulatory protein, mitochondrial

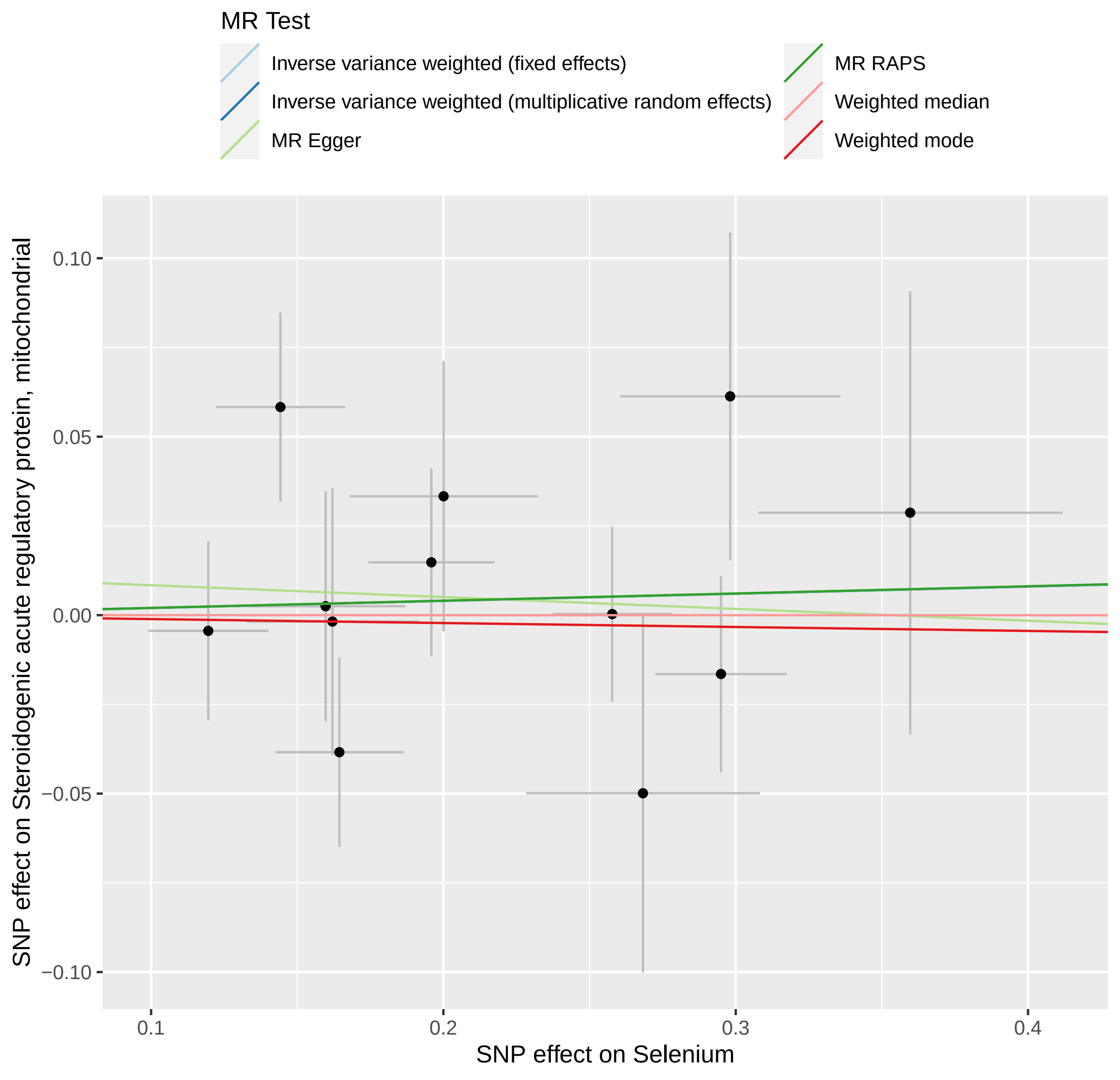

Batch 3−2867 : Selenium on Signal transducer and activator of transcription 1−alpha/beta

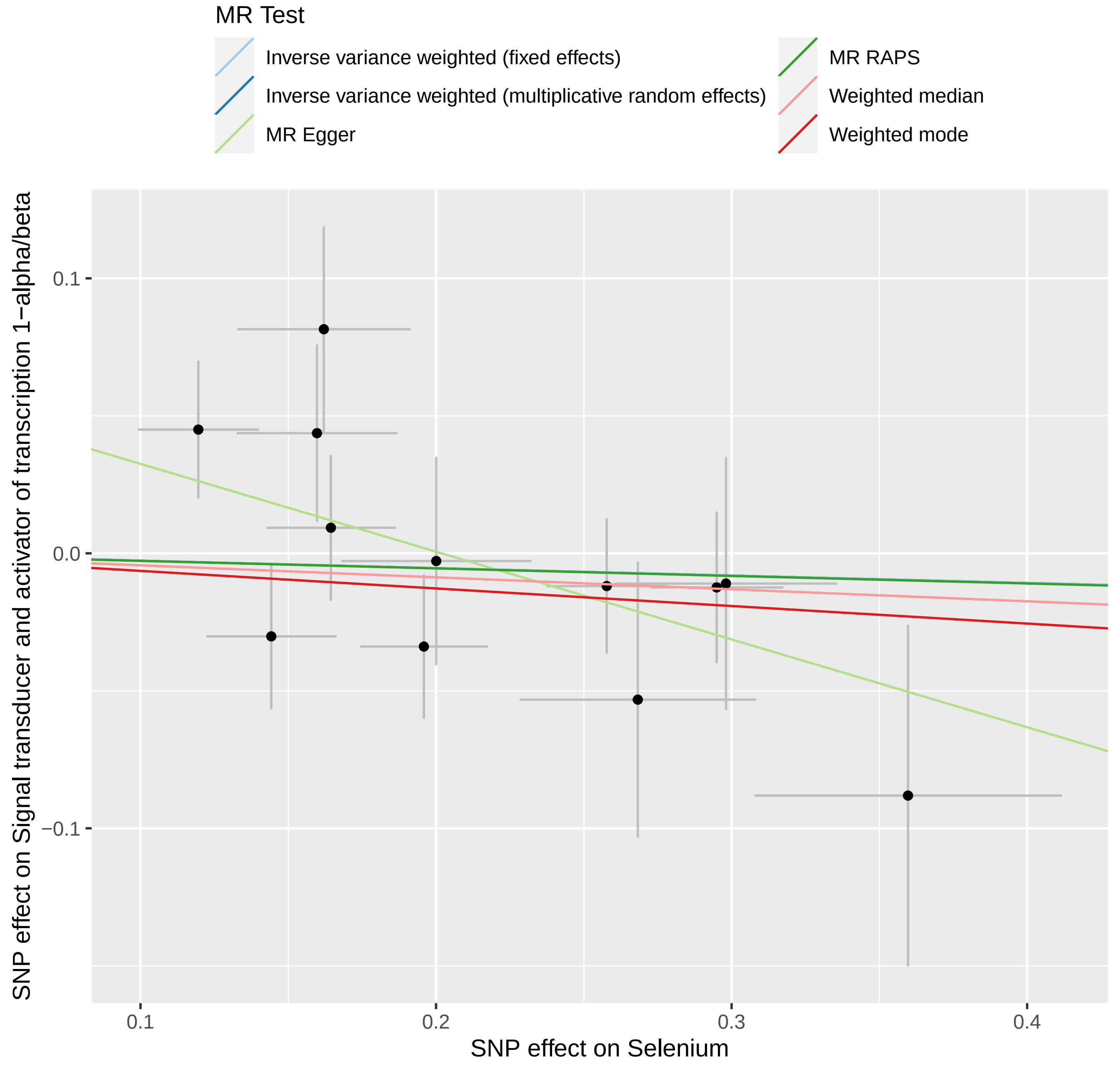

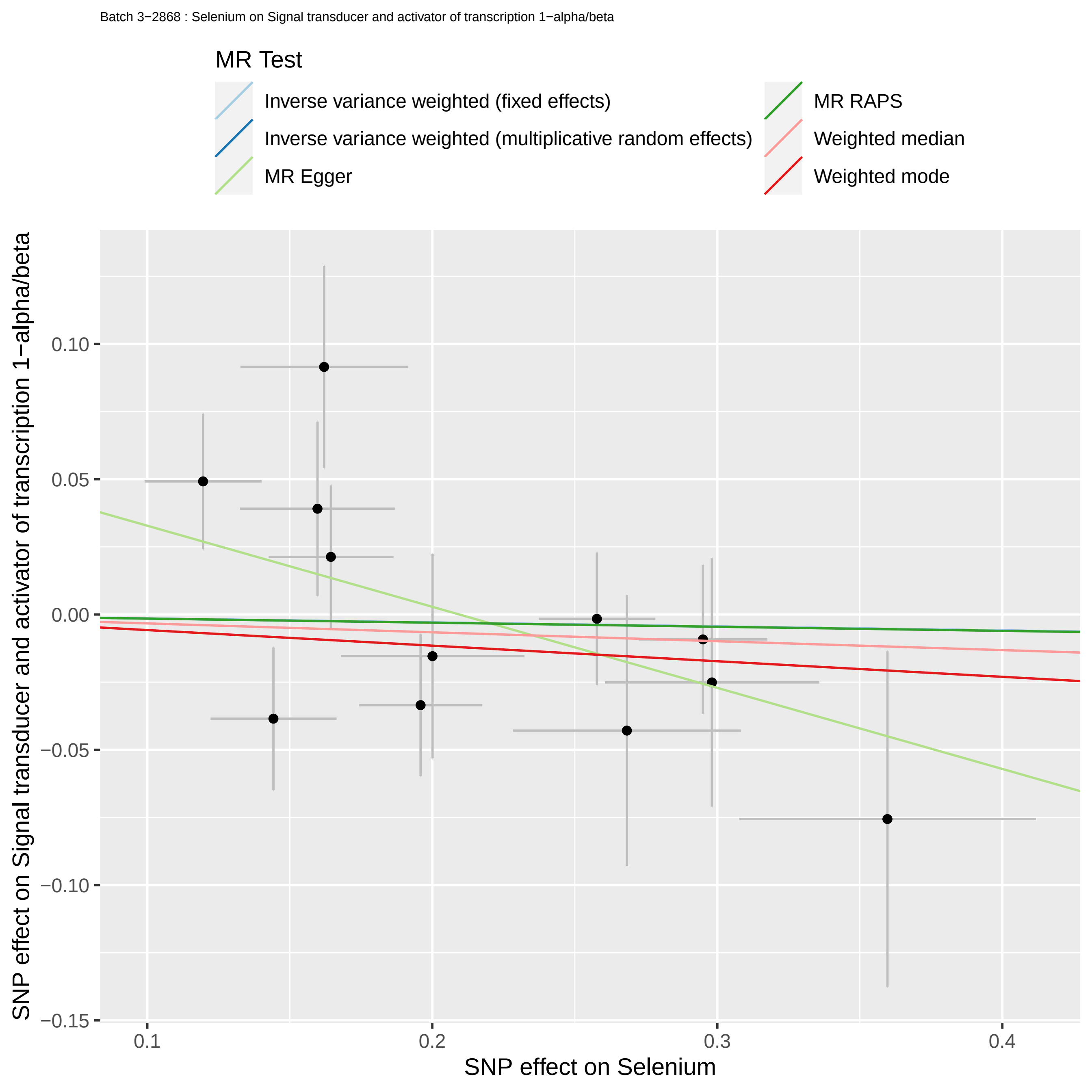
Batch 3−2868 : Selenium on Signal transducer and activator of transcription 1−alpha/beta
MR Test
Inverse variance weighted (fixed effects)
Inverse variance weighted (multiplicative random effects)
MR Egger
MR RAPS
Weighted median
Weighted mode
SNP effect on Signal transducer and activator of transcription 1−alpha/beta
0.10
0.05
0.00
−0.05
−0.10
−0.15
0.1
0.2
0.3
0.4
SNP effect on Selenium

Batch 3−2869 : Selenium on Signal transducer and activator of transcription 3

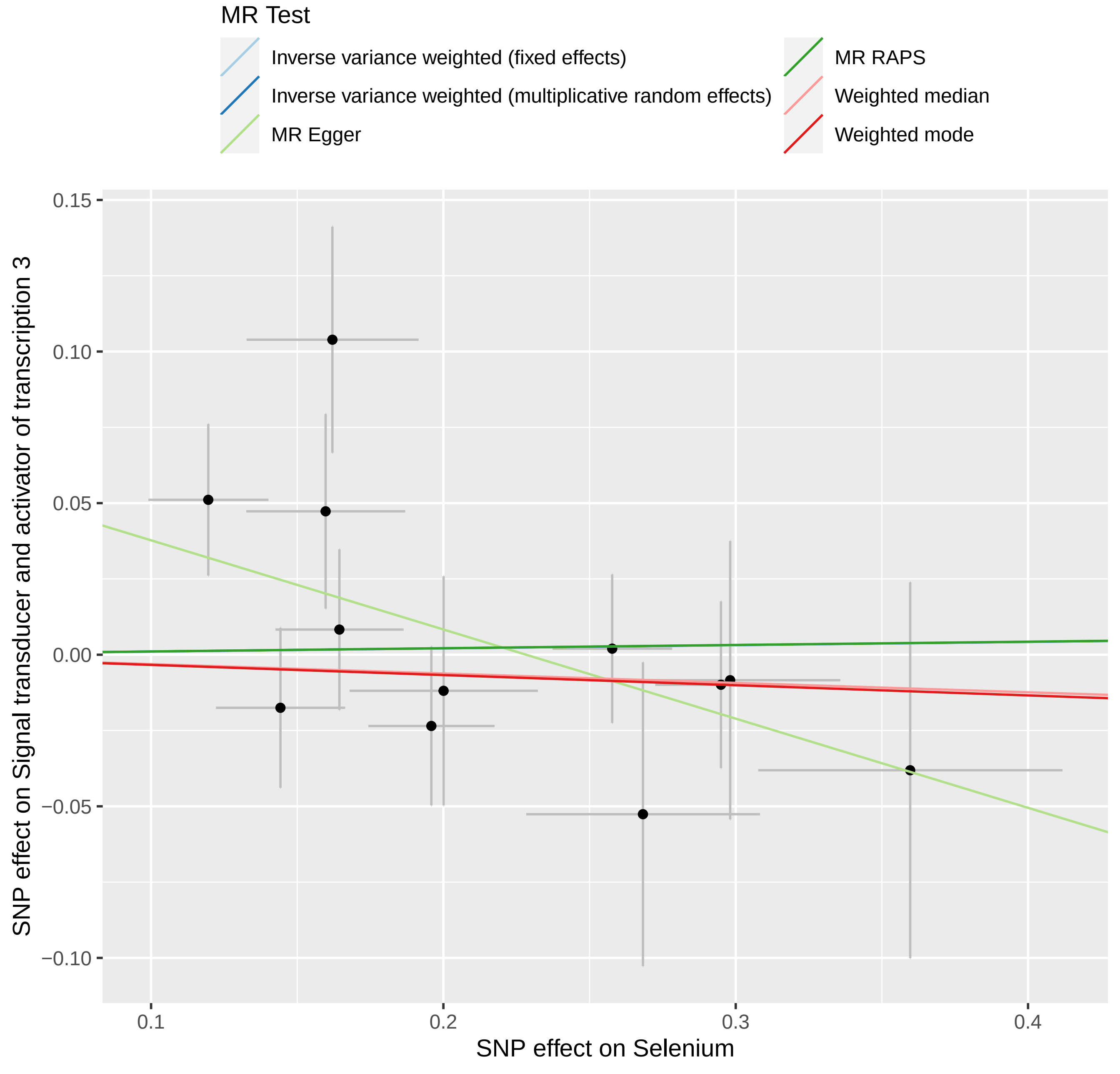

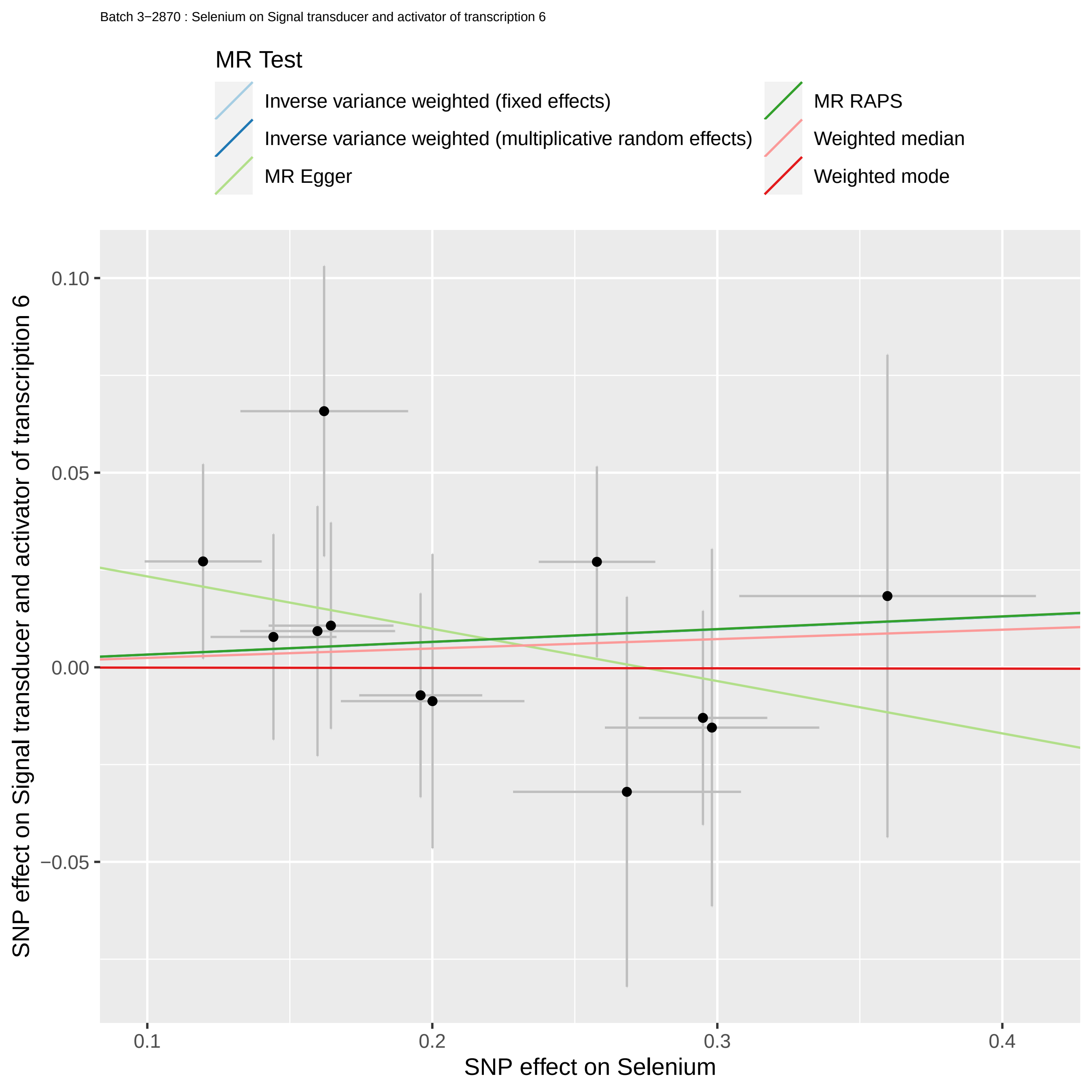

Batch 3−2870 : Selenium on Signal transducer and activator of transcription 6
MR Test
Inverse variance weighted (fixed effects)
Inverse variance weighted (multiplicative random effects)
MR Egger
MR RAPS
Weighted median
Weighted mode
0.10
0.05
0.00
−0.05
0.1
0.2
0.3
0.4
SNP effect on Signal transducer and activator of transcription 6
SNP effect on Selenium

Batch 3−2871 : Selenium on Double−stranded RNA−binding protein Staufen homolog 1

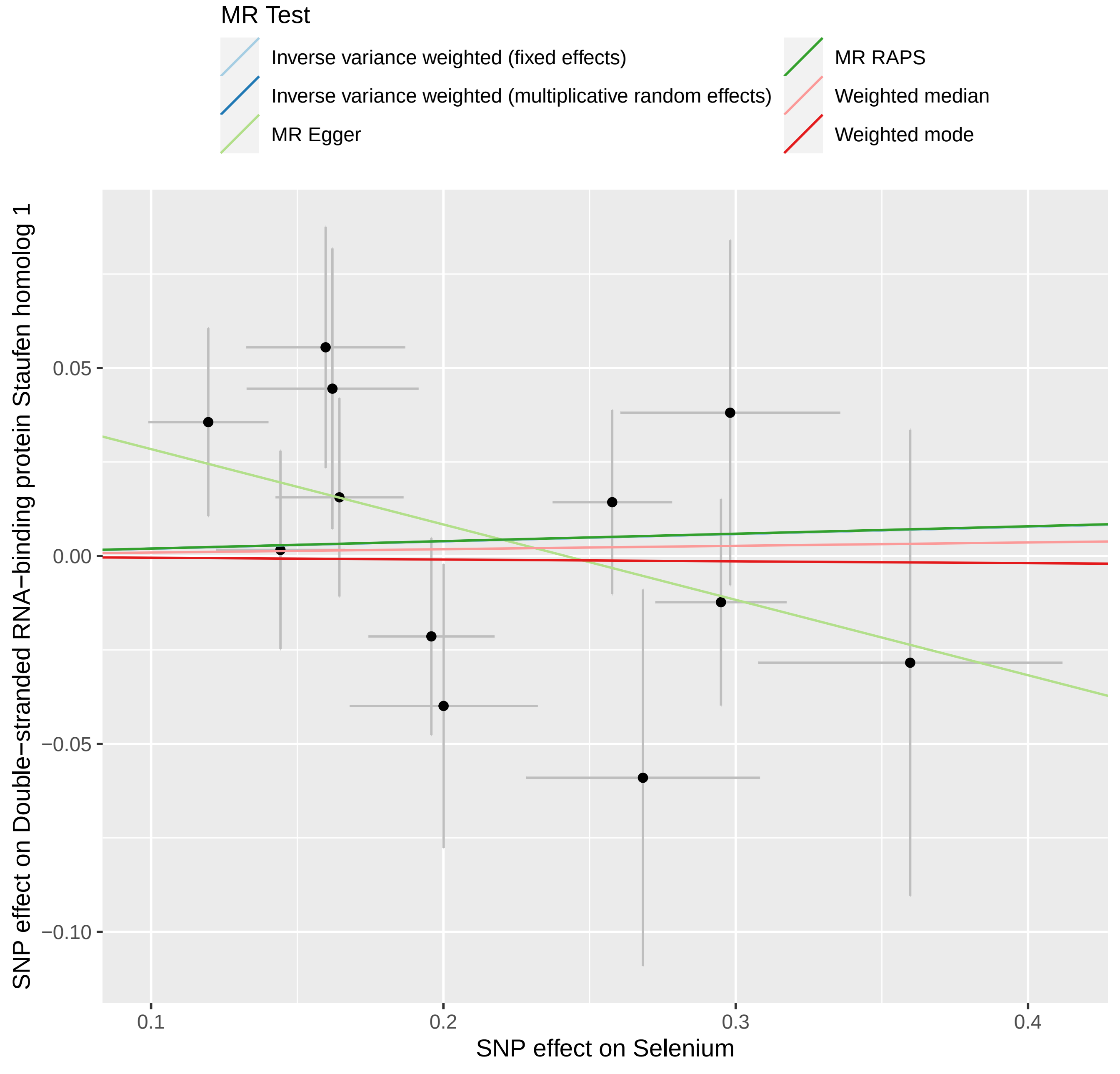

Batch 3−2872 : Selenium on Double−stranded RNA−binding protein Staufen homolog 2

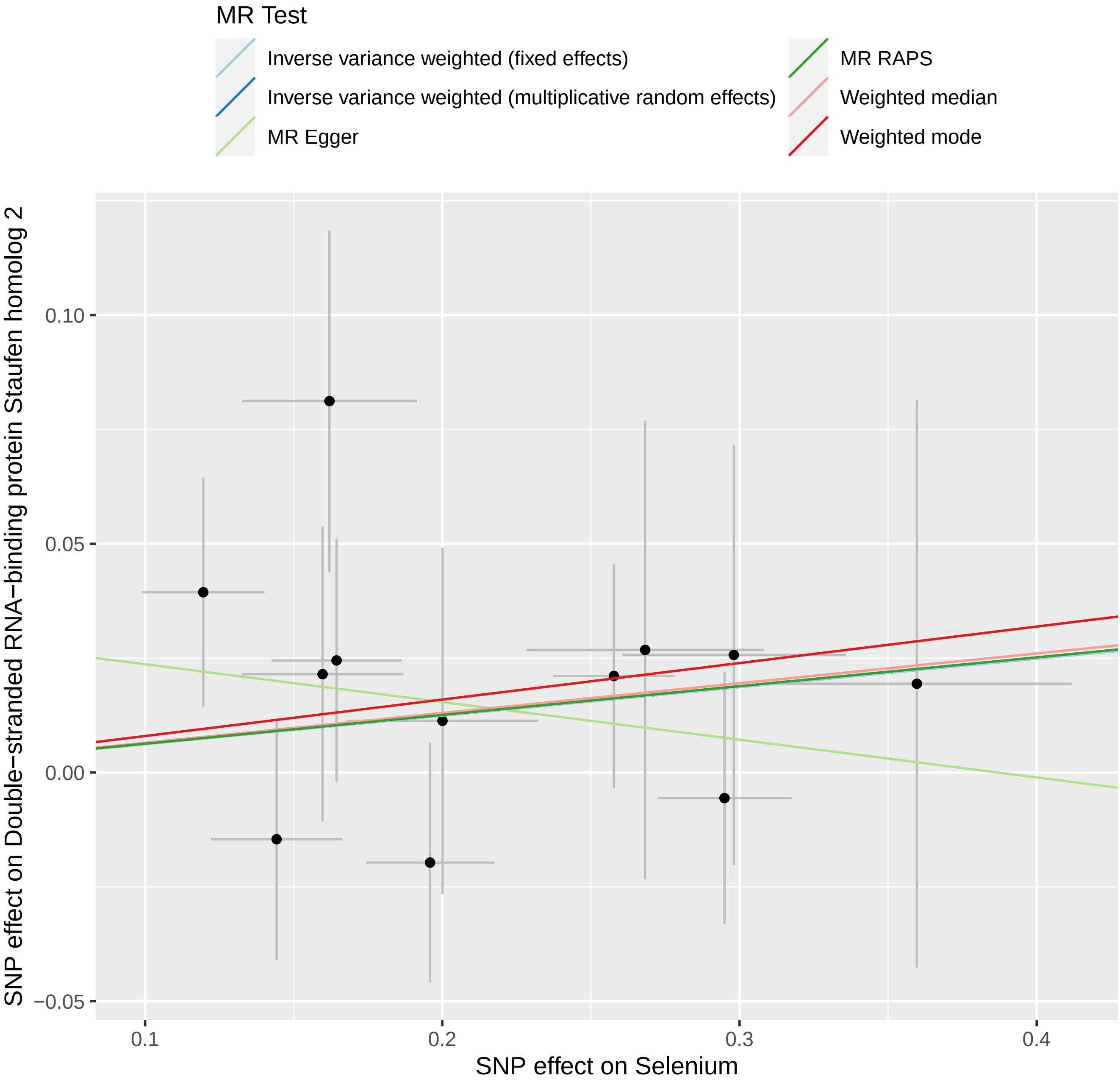

Batch 3−2873 : Selenium on Stanniocalcin−1

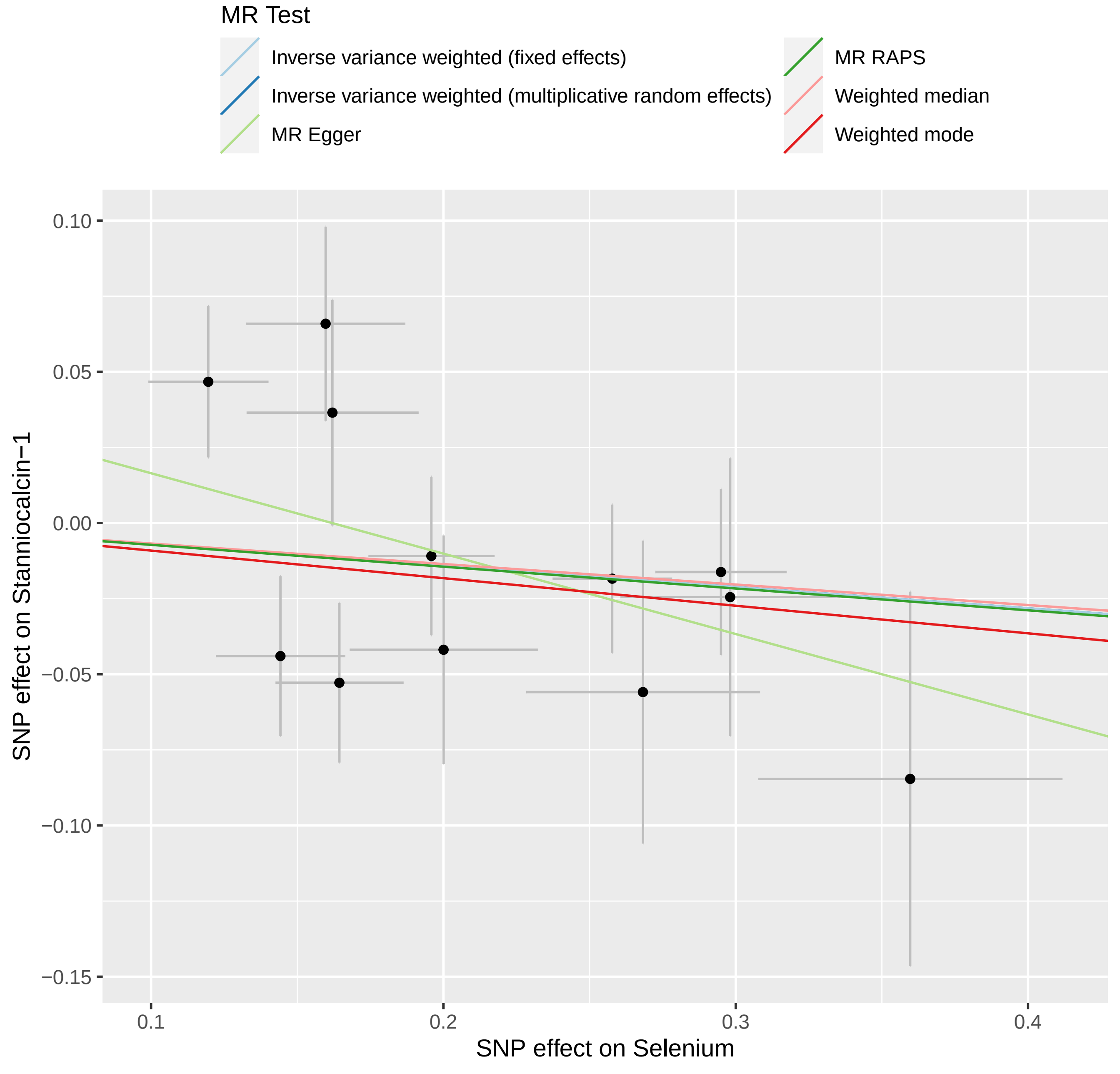

Batch 3−2874 : Selenium on Stanniocalcin−2

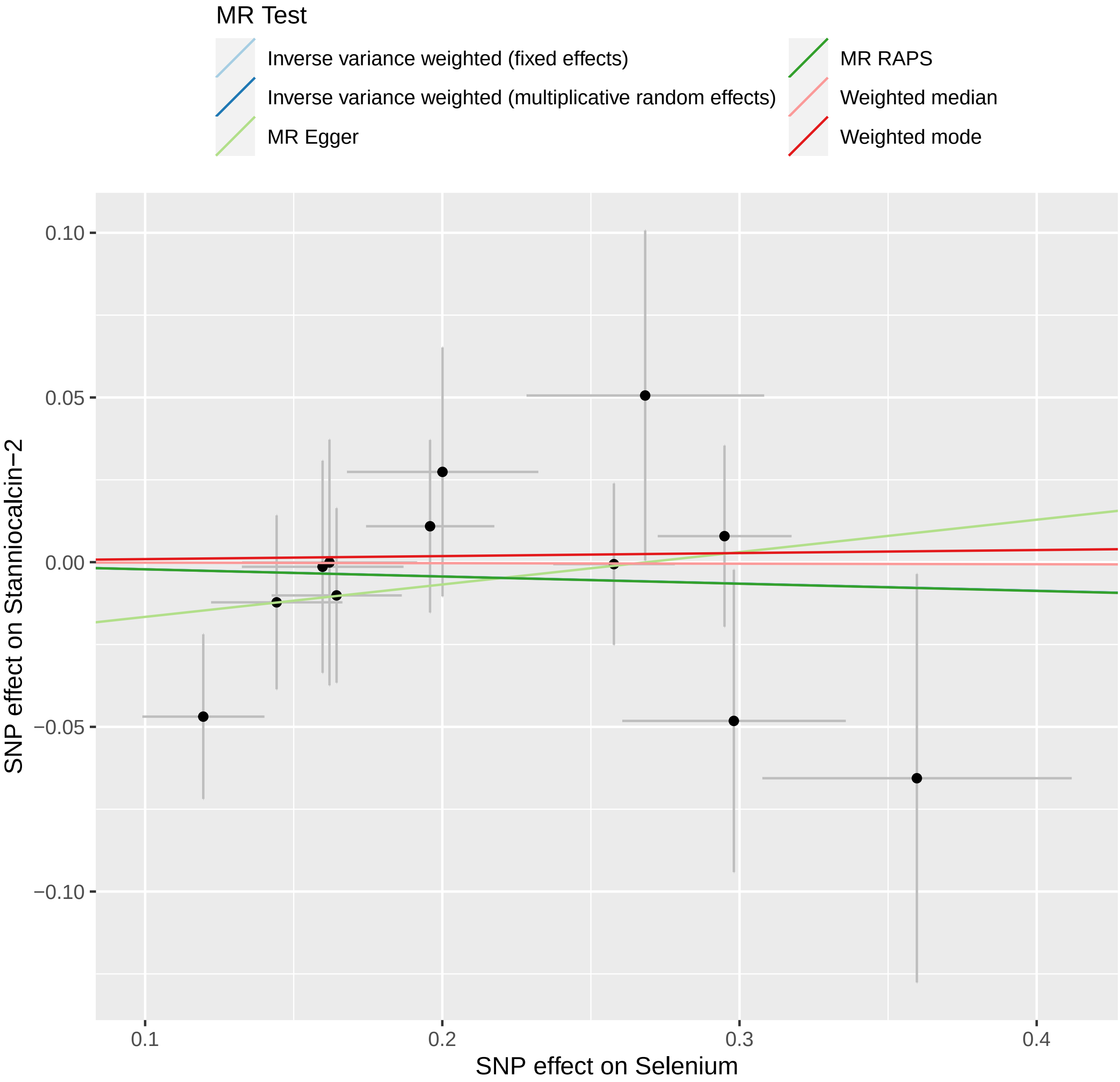

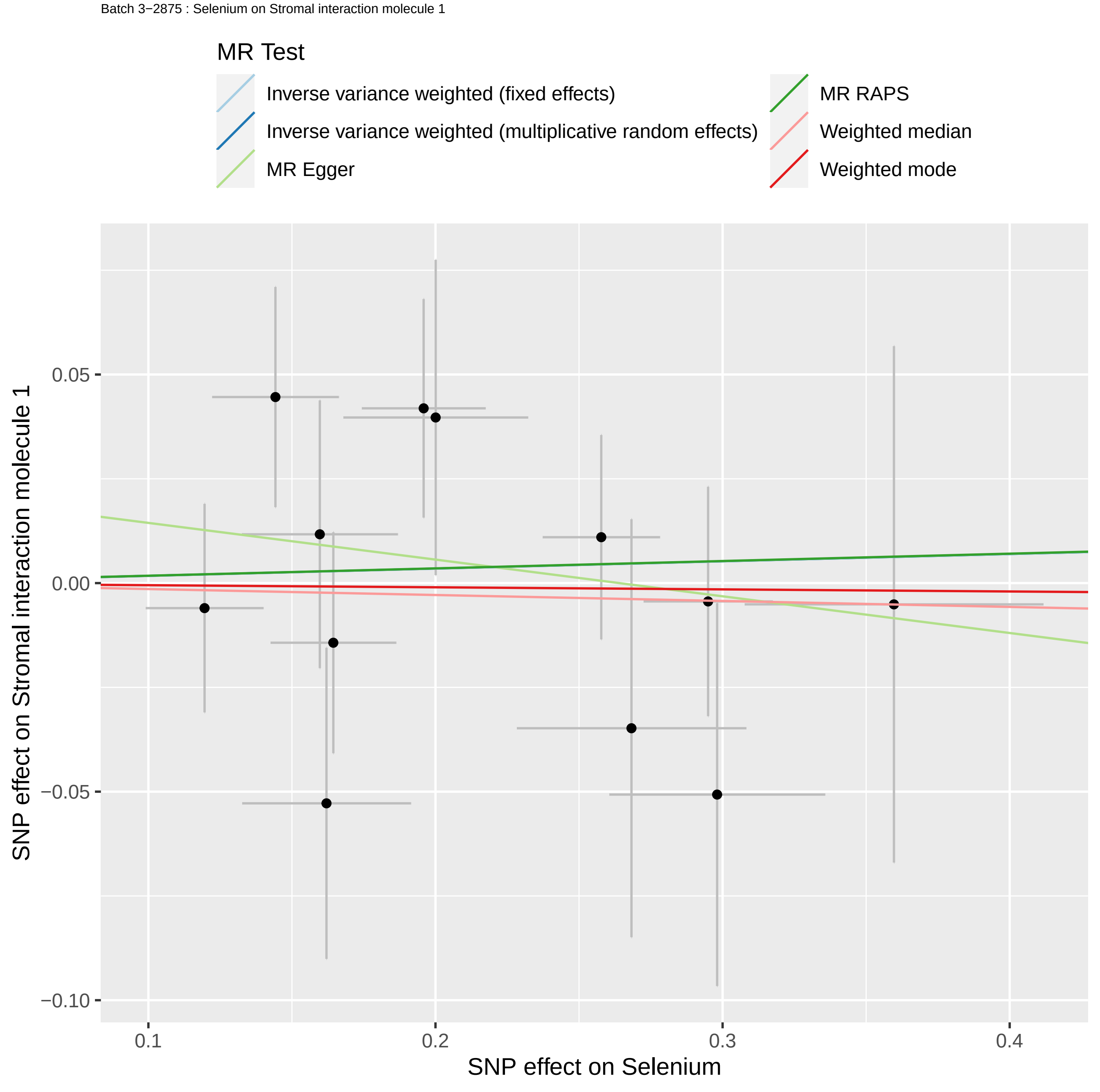

Batch 3−2875 : Selenium on Stromal interaction molecule 1
MR Test
Inverse variance weighted (fixed effects)
Inverse variance weighted (multiplicative random effects)
MR Egger
MR RAPS
Weighted median
Weighted mode
0.05
0.00
−0.05
−0.10
0.1
0.2
0.3
0.4
SNP effect on Stromal interaction molecule 1
SNP effect on Selenium

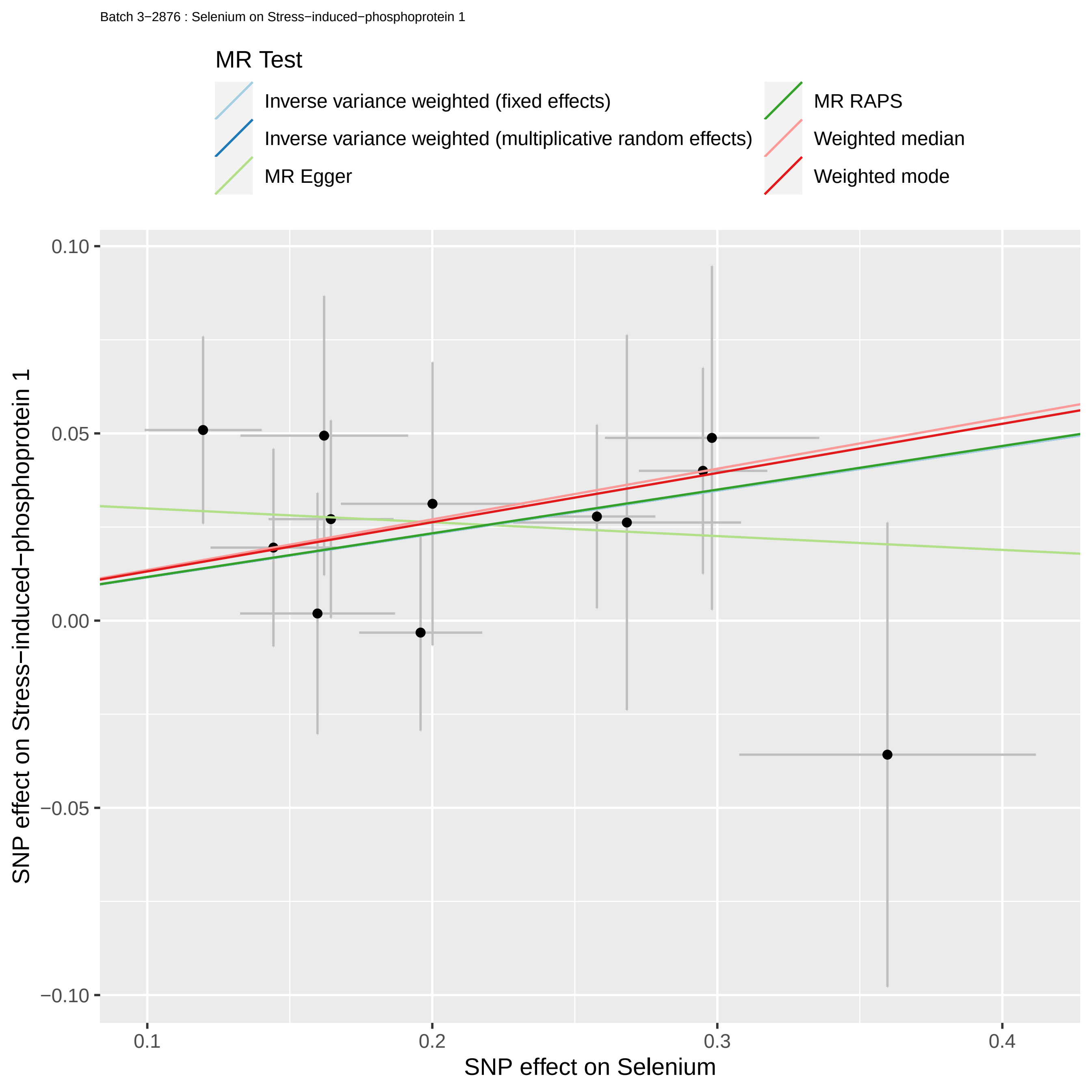

Batch 3−2876 : Selenium on Stress−induced−phosphoprotein 1
MR Test
Inverse variance weighted (fixed effects)
Inverse variance weighted (multiplicative random effects)
MR Egger
MR RAPS
Weighted median
Weighted mode
0.10
0.05
0.00
−0.05
−0.10
0.1
0.2
0.3
0.4
SNP effect on Selenium
SNP effect on Stress−induced−phosphoprotein 1

Batch 3−2877 : Selenium on Serine/threonine−protein kinase 16

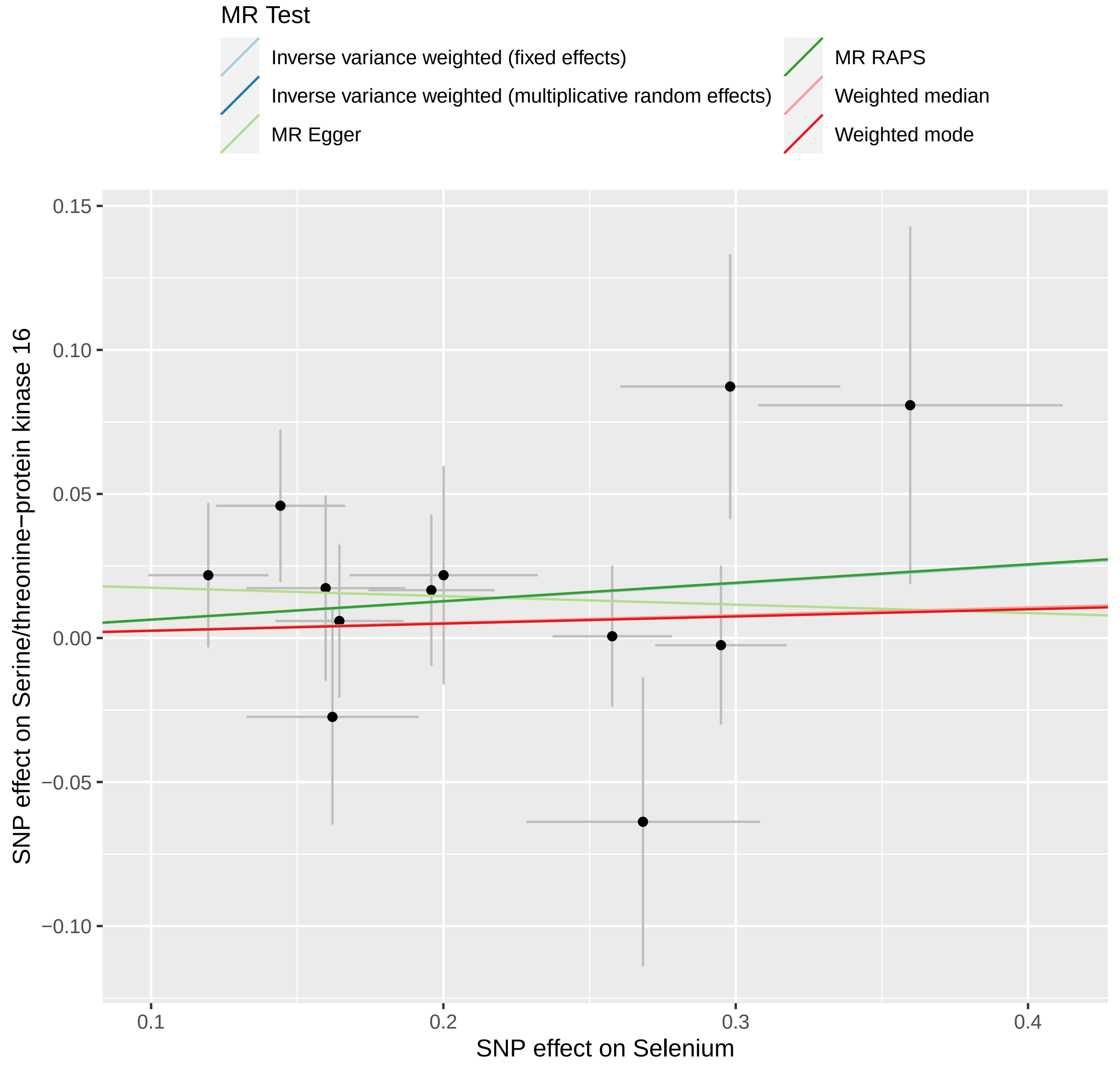

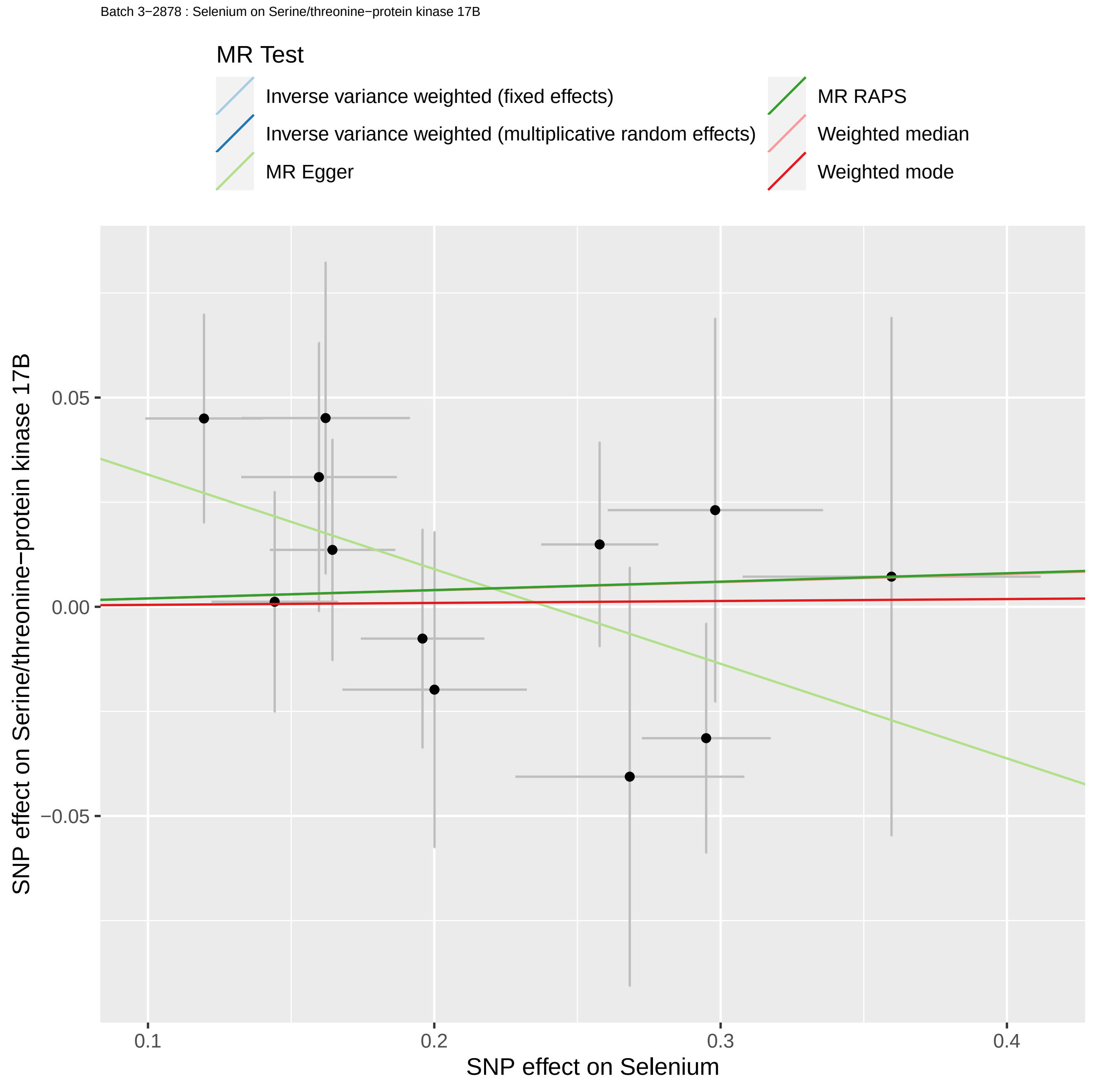

Batch 3−2878 : Selenium on Serine/threonine−protein kinase 17B
MR Test
Inverse variance weighted (fixed effects)
Inverse variance weighted (multiplicative random effects)
MR Egger
MR RAPS
Weighted median
Weighted mode
0.05
0.00
−0.05
0.1
0.2
0.3
0.4
SNP effect on Serine/threonine−protein kinase 17B
SNP effect on Selenium

Batch 3−2879 : Selenium on Stathmin−2

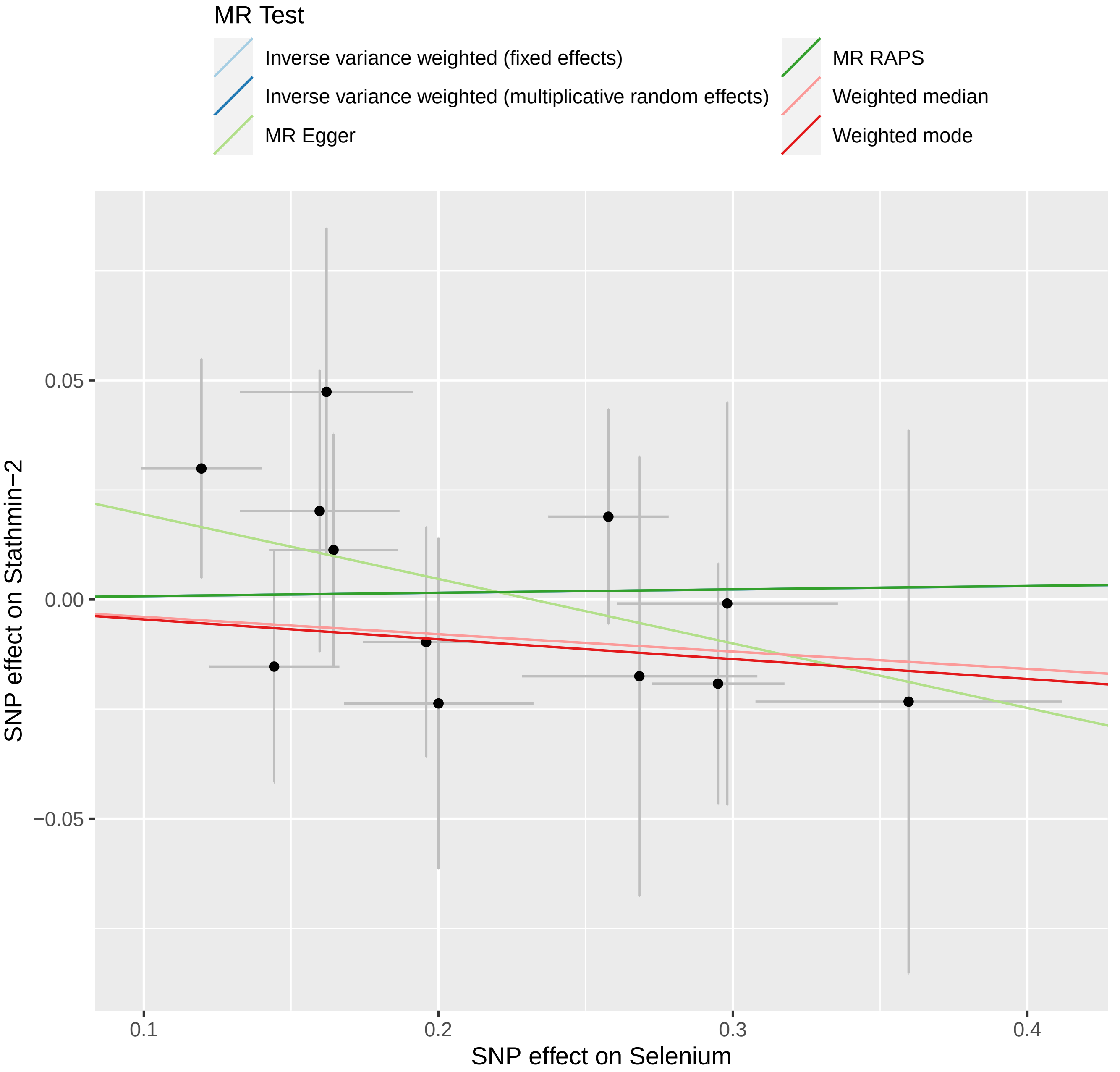

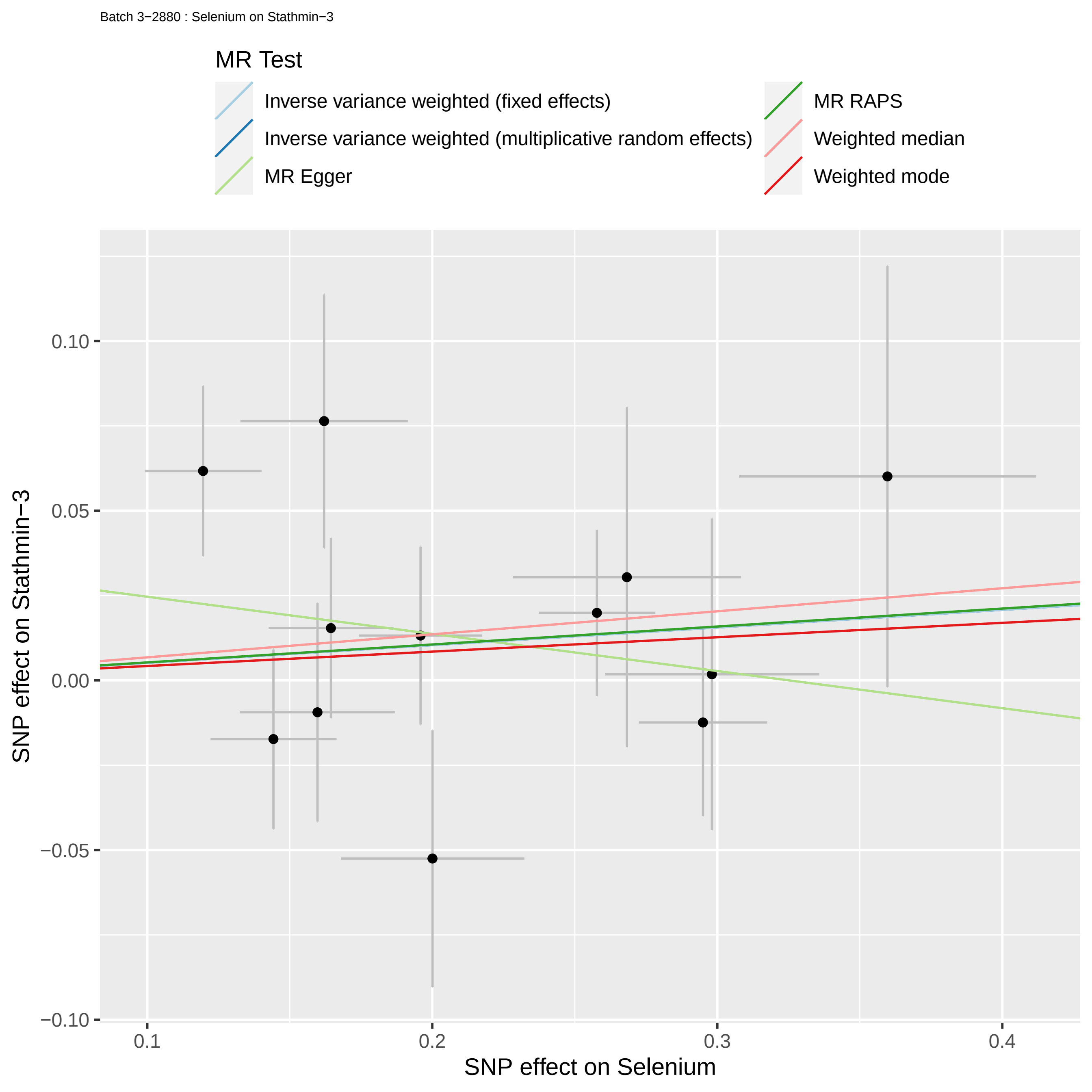
Batch 3−2880 : Selenium on Stathmin−3
MR Test
Inverse variance weighted (fixed effects)
Inverse variance weighted (multiplicative random effects)
MR Egger
MR RAPS
Weighted median
Weighted mode
0.10
0.05
0.00
−0.05
−0.10
0.1
0.2
0.3
0.4
SNP effect on Stathmin−3
SNP effect on Selenium

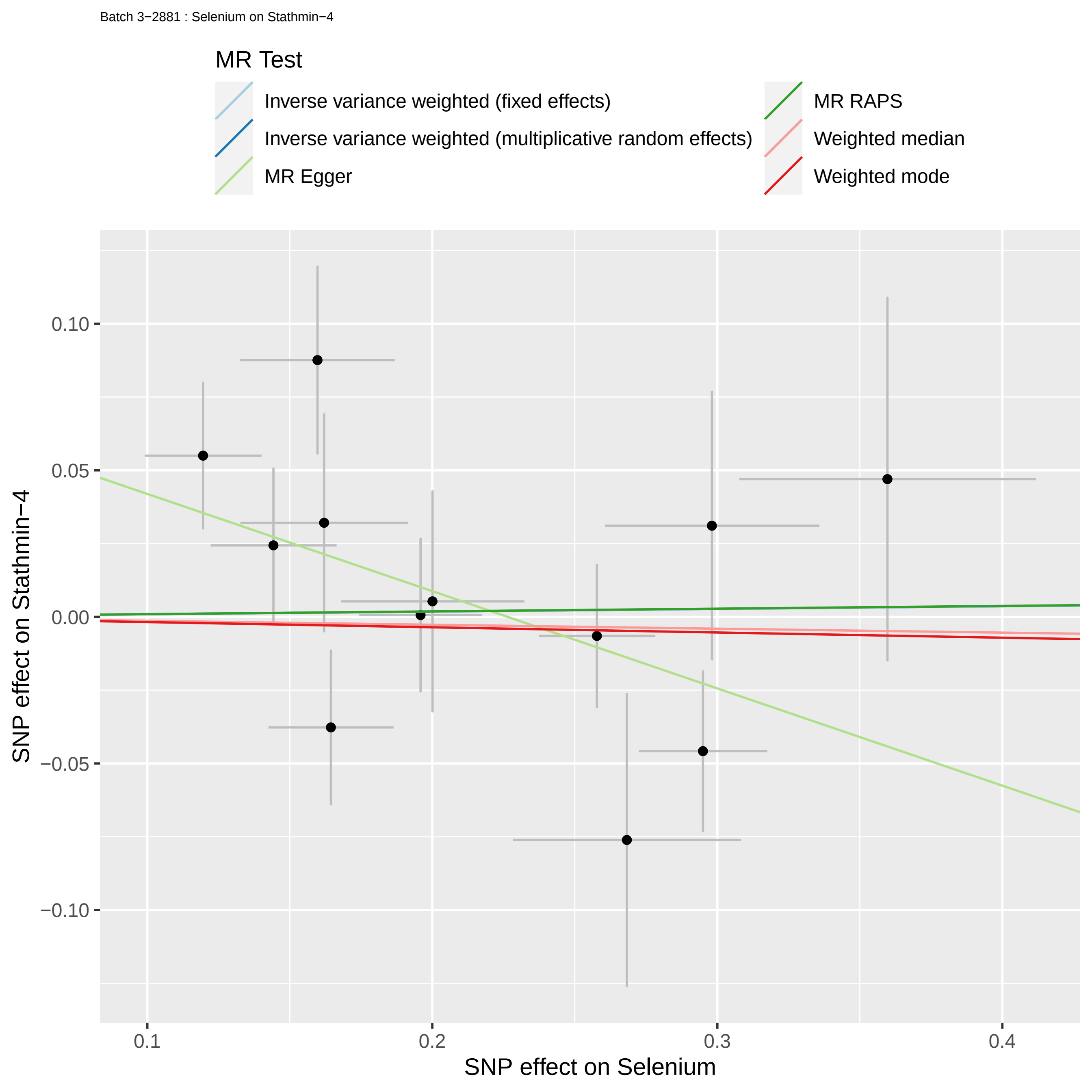

Batch 3−2881 : Selenium on Stathmin−4
MR Test
Inverse variance weighted (fixed effects)
Inverse variance weighted (multiplicative random effects)
MR Egger
MR RAPS
Weighted median
Weighted mode
0.10
0.05
0.00
−0.05
−0.10
0.1
0.2
0.3
0.4
SNP effect on Stathmin−4
SNP effect on Selenium

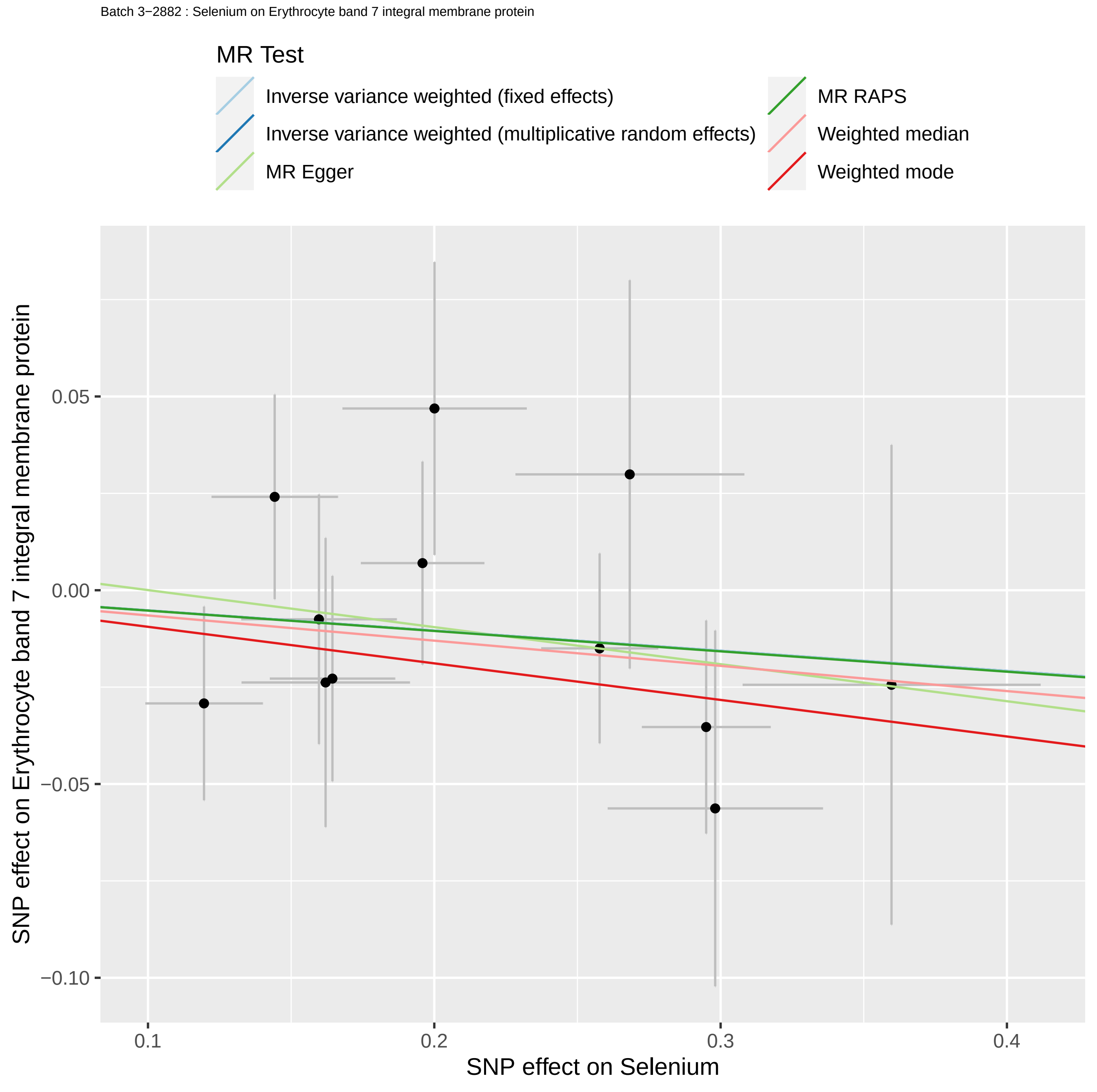
Batch 3−2882 : Selenium on Erythrocyte band 7 integral membrane protein
MR Test
Inverse variance weighted (fixed effects)
Inverse variance weighted (multiplicative random effects)
MR Egger
MR RAPS
Weighted median
Weighted mode
SNP effect on Erythrocyte band 7 integral membrane protein
0.05
0.00
−0.05
−0.10
0.1
0.2
0.3
0.4
SNP effect on Selenium

Batch 3−2883 : Selenium on Syntaxin−10

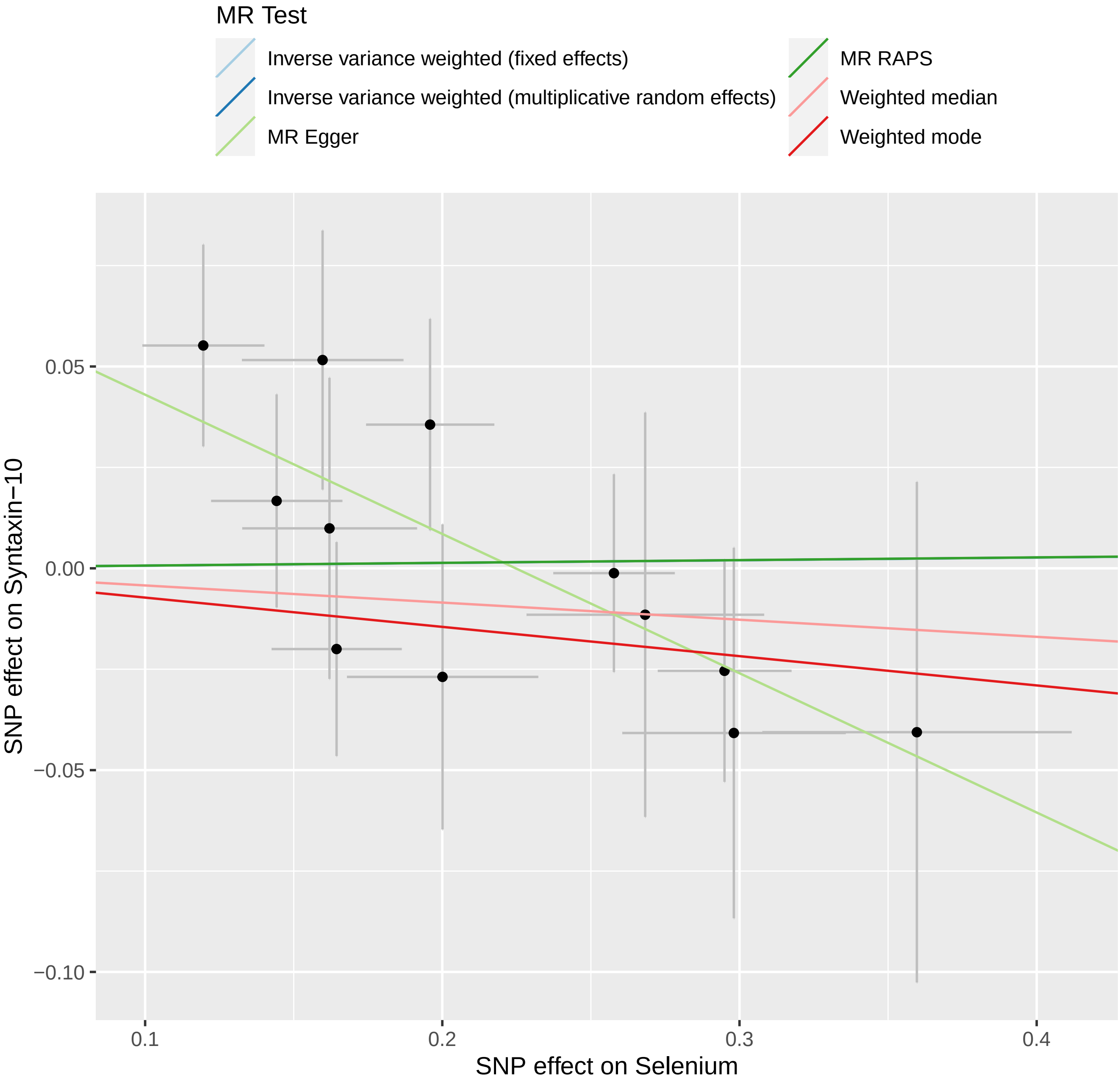

Batch 3−2884 : Selenium on Syntaxin−18

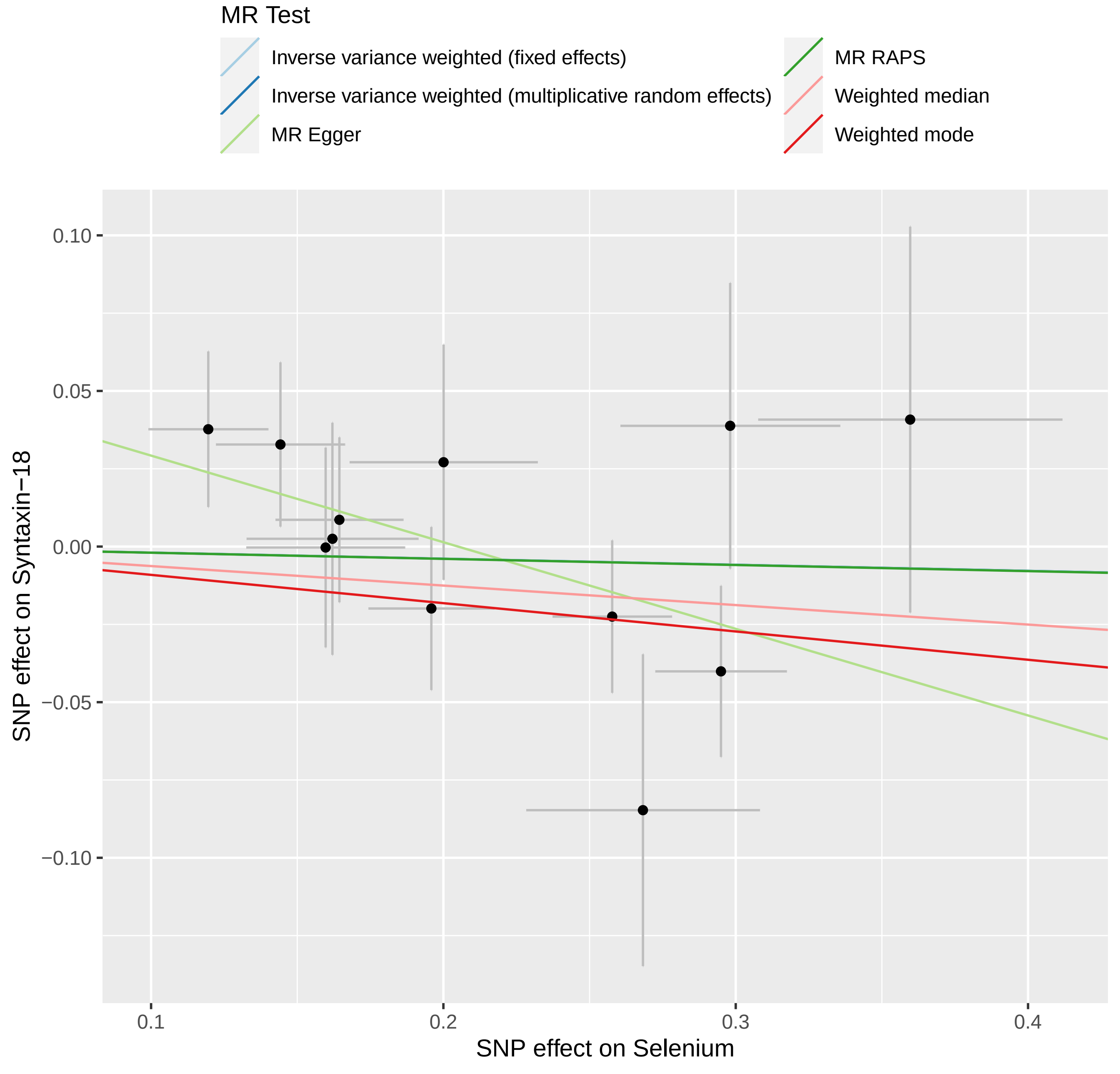

Batch 3−2885 : Selenium on Syntaxin−1A

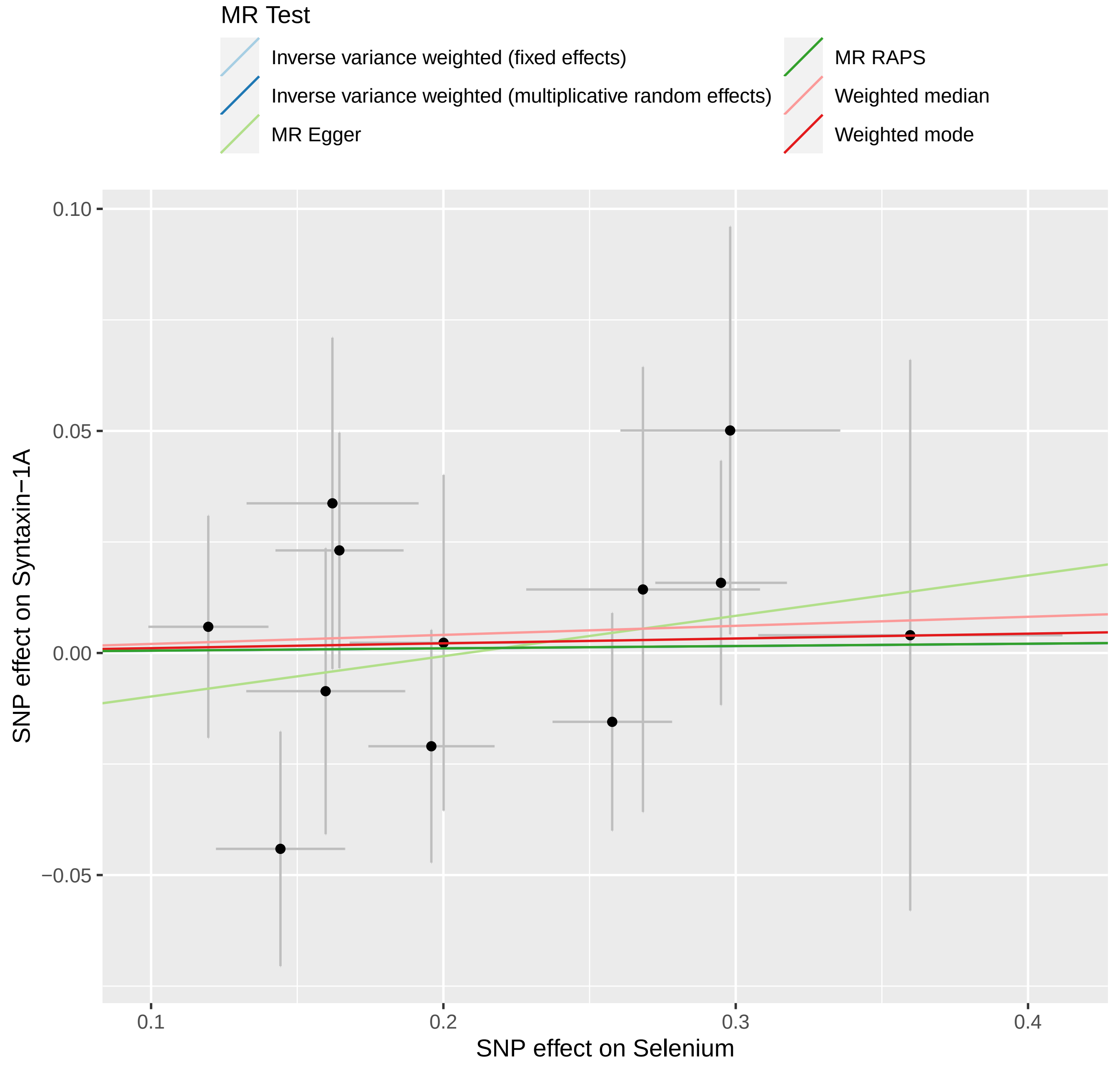

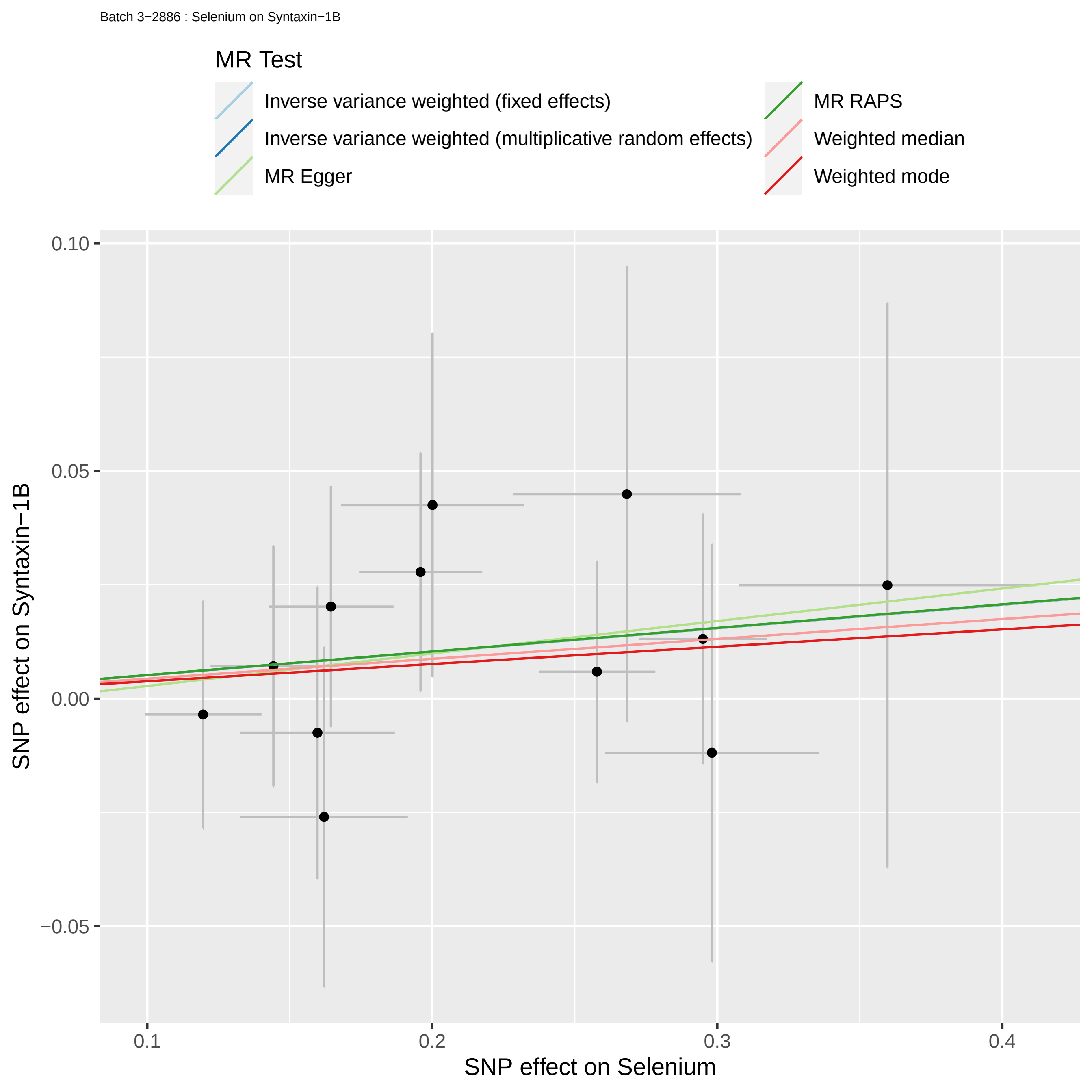
Batch 3−2886 : Selenium on Syntaxin−1B
MR Test
Inverse variance weighted (fixed effects)
Inverse variance weighted (multiplicative random effects)
MR Egger
MR RAPS
Weighted median
Weighted mode
0.10
0.05
0.00
−0.05
0.1
0.2
0.3
0.4
SNP effect on Syntaxin−1B
SNP effect on Selenium

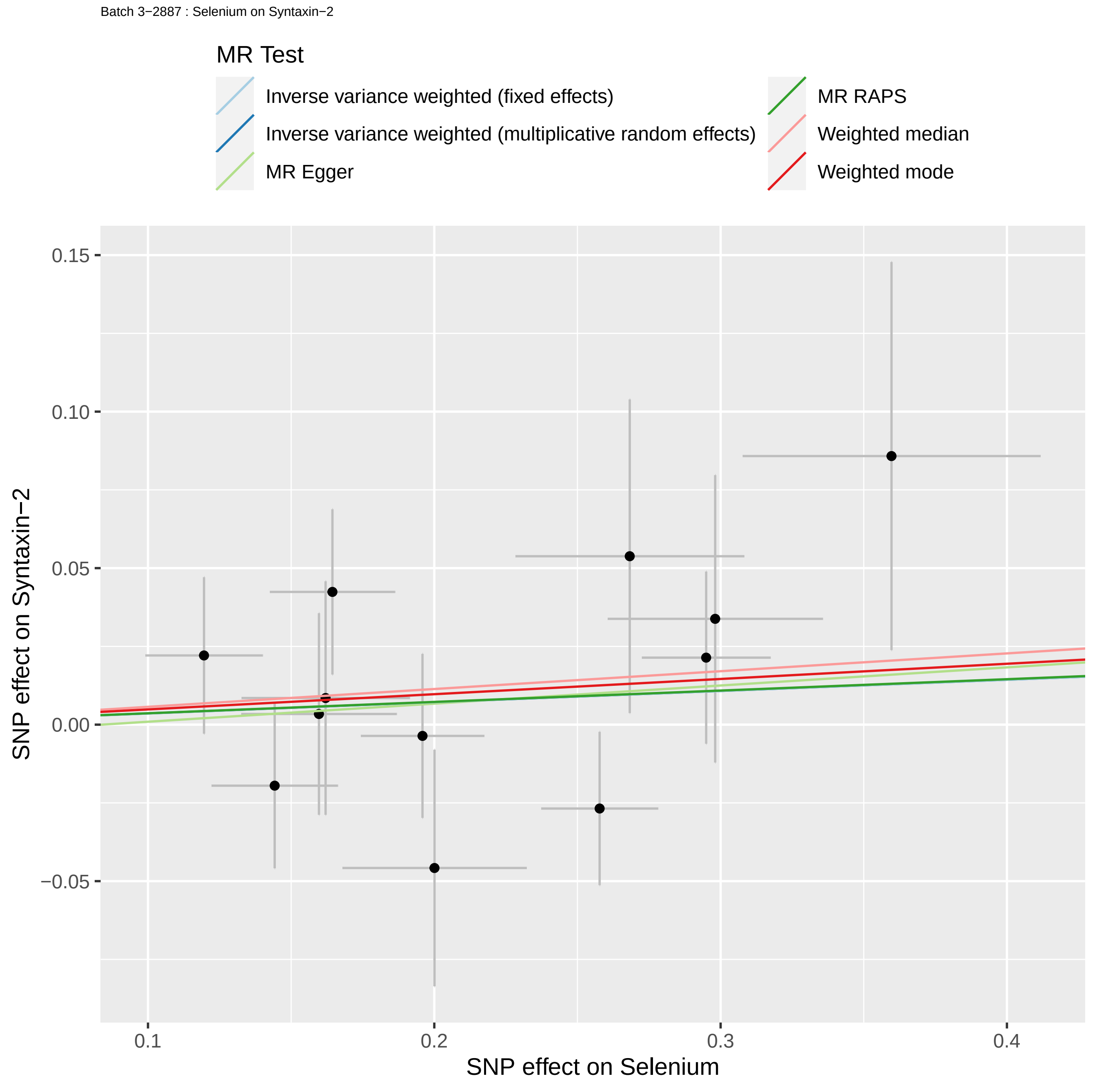
Batch 3−2887 : Selenium on Syntaxin−2
MR Test
Inverse variance weighted (fixed effects)
Inverse variance weighted (multiplicative random effects)
MR Egger
MR RAPS
Weighted median
Weighted mode
SNP effect on Syntaxin−2
0.15
0.10
0.05
0.00
−0.05
0.1
0.2
0.3
0.4
SNP effect on Selenium

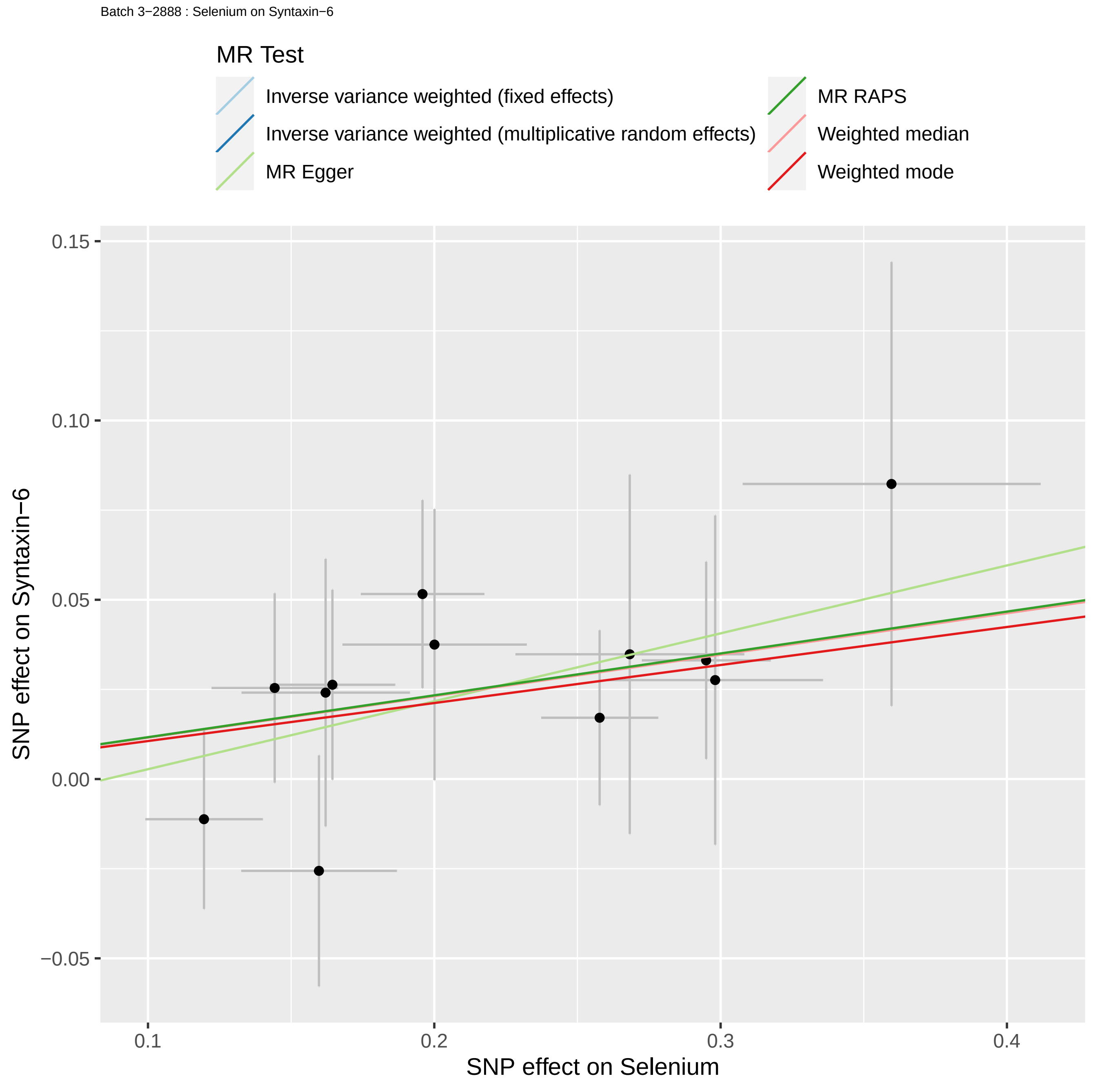
Batch 3−2888 : Selenium on Syntaxin−6
MR Test
Inverse variance weighted (fixed effects)
Inverse variance weighted (multiplicative random effects)
MR Egger
MR RAPS
Weighted median
Weighted mode
0.15
0.10
0.05
0.00
−0.05
0.1
0.2
0.3
0.4
SNP effect on Syntaxin−6
SNP effect on Selenium

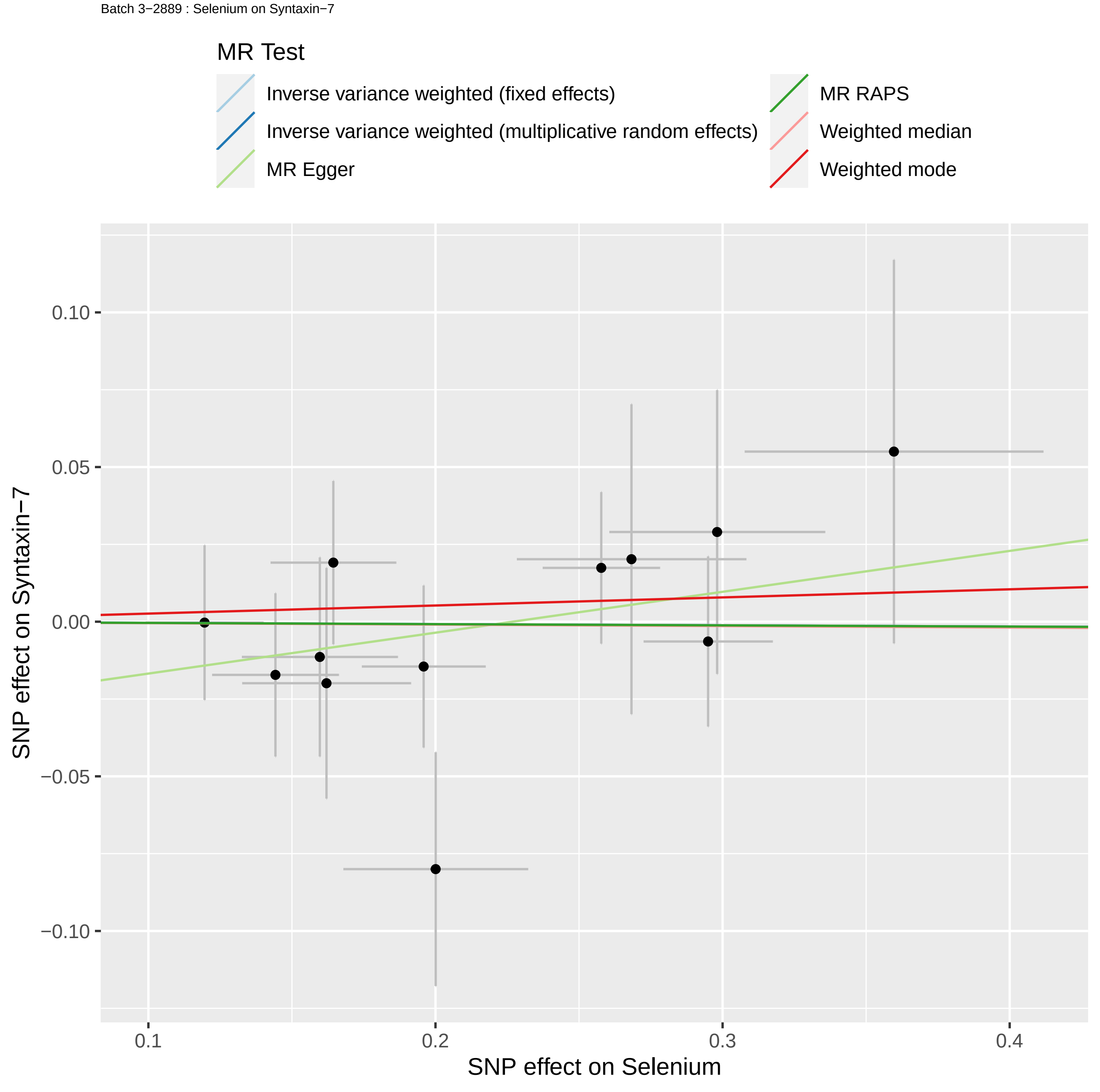
Batch 3−2889 : Selenium on Syntaxin−7
MR Test
Inverse variance weighted (fixed effects)
Inverse variance weighted (multiplicative random effects)
MR Egger
MR RAPS
Weighted median
Weighted mode
0.10
0.05
0.00
−0.05
−0.10
SNP effect on Syntaxin−7
0.1
0.2
0.3
0.4
SNP effect on Selenium

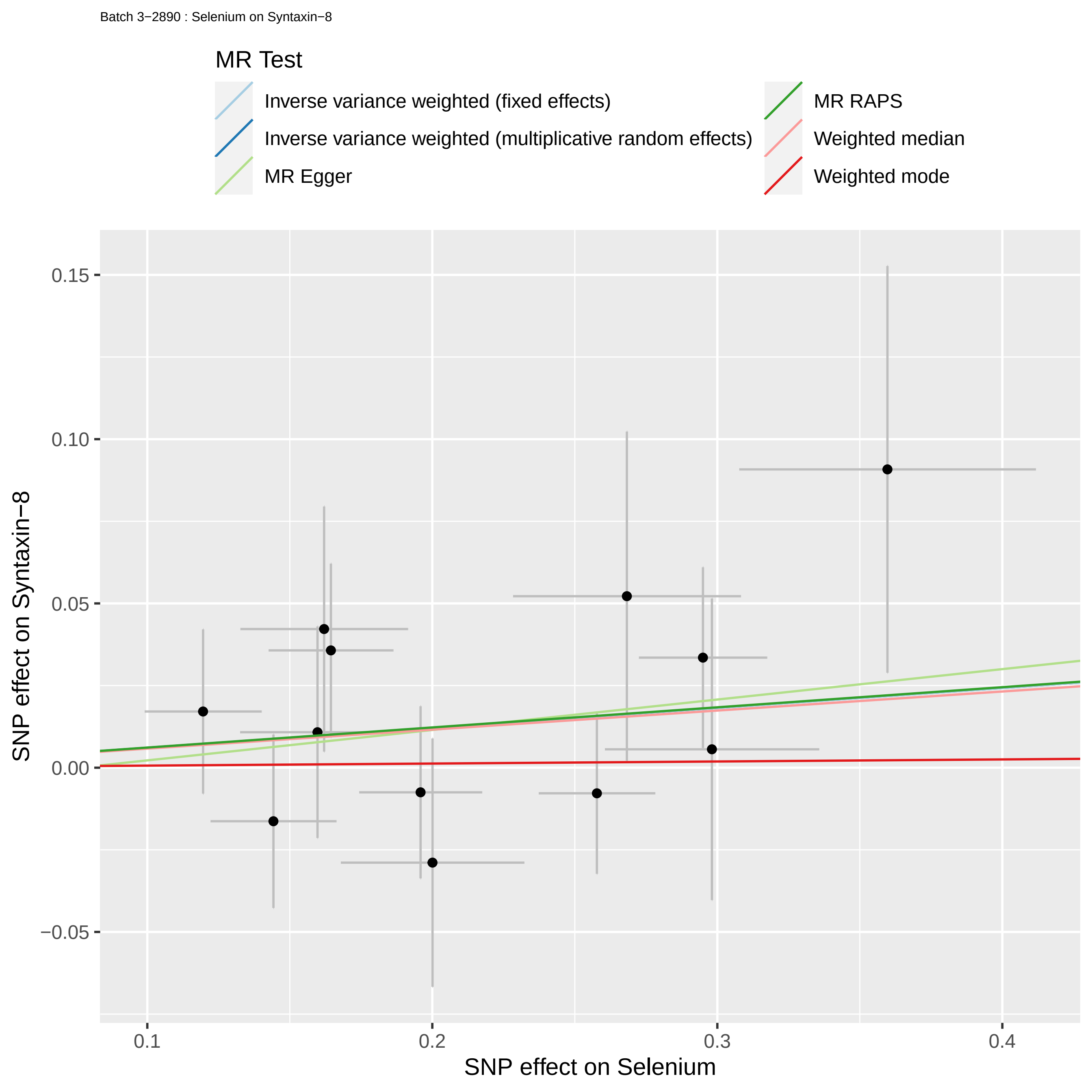
Batch 3−2890 : Selenium on Syntaxin−8
MR Test
Inverse variance weighted (fixed effects)
Inverse variance weighted (multiplicative random effects)
MR Egger
MR RAPS
Weighted median
Weighted mode
0.15
0.10
0.05
0.00
−0.05
0.1
0.2
0.3
0.4
SNP effect on Syntaxin−8
SNP effect on Selenium

Batch 3−2891 : Selenium on Extracellular sulfatase Sulf−2

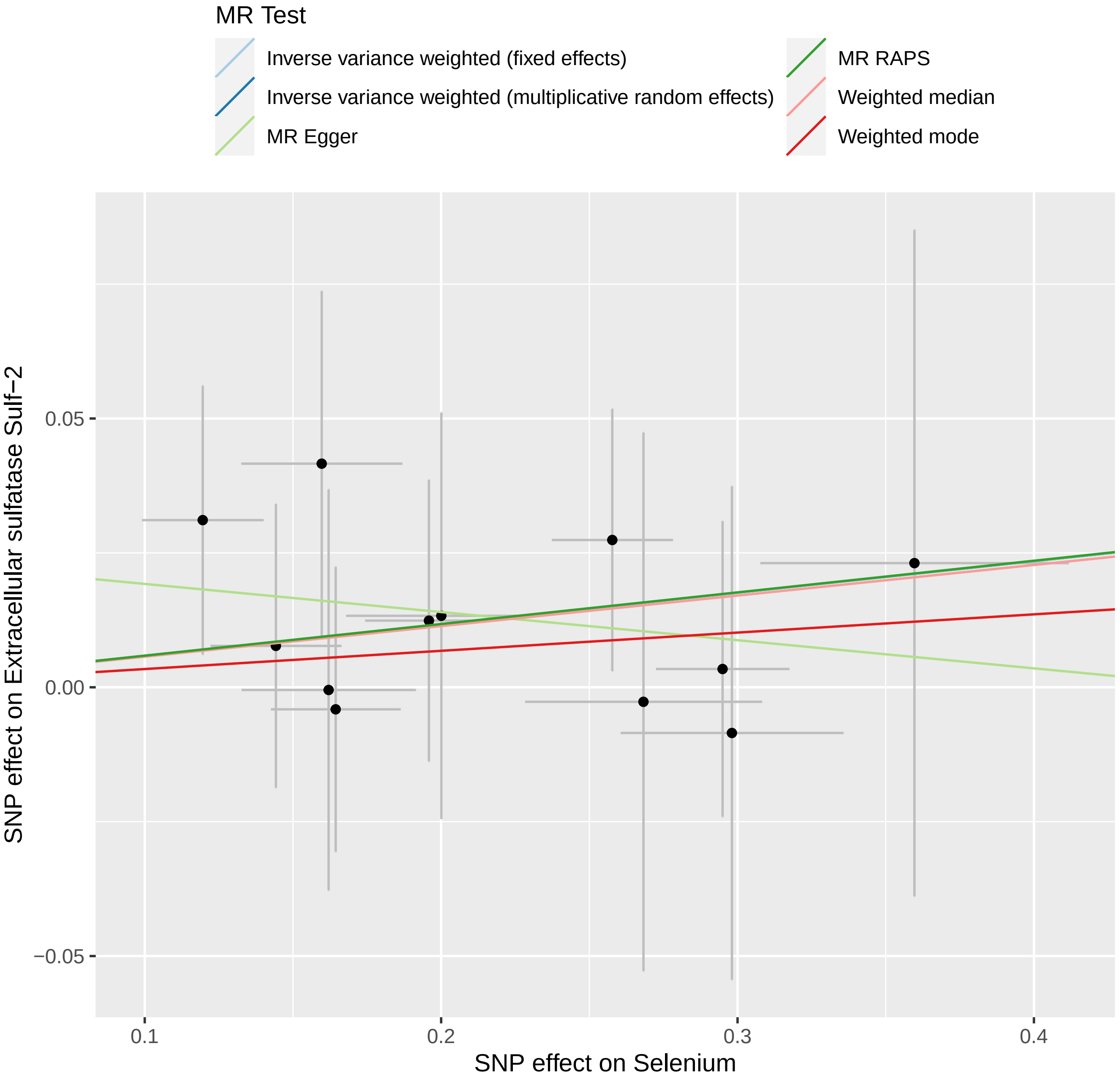

Batch 3−2892 : Selenium on Estrogen sulfotransferase

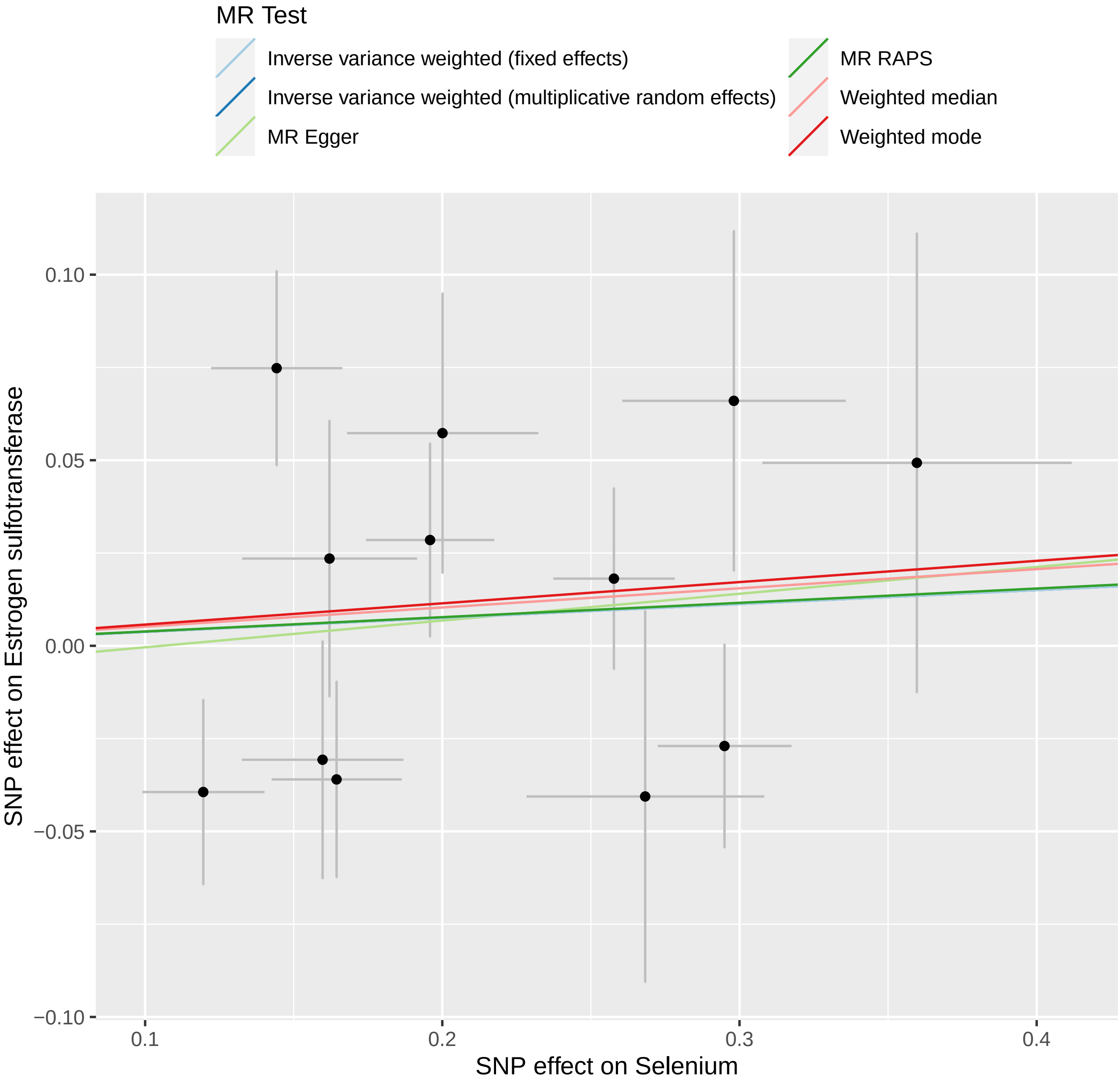

Batch 3−2893 : Selenium on Bile salt sulfotransferase

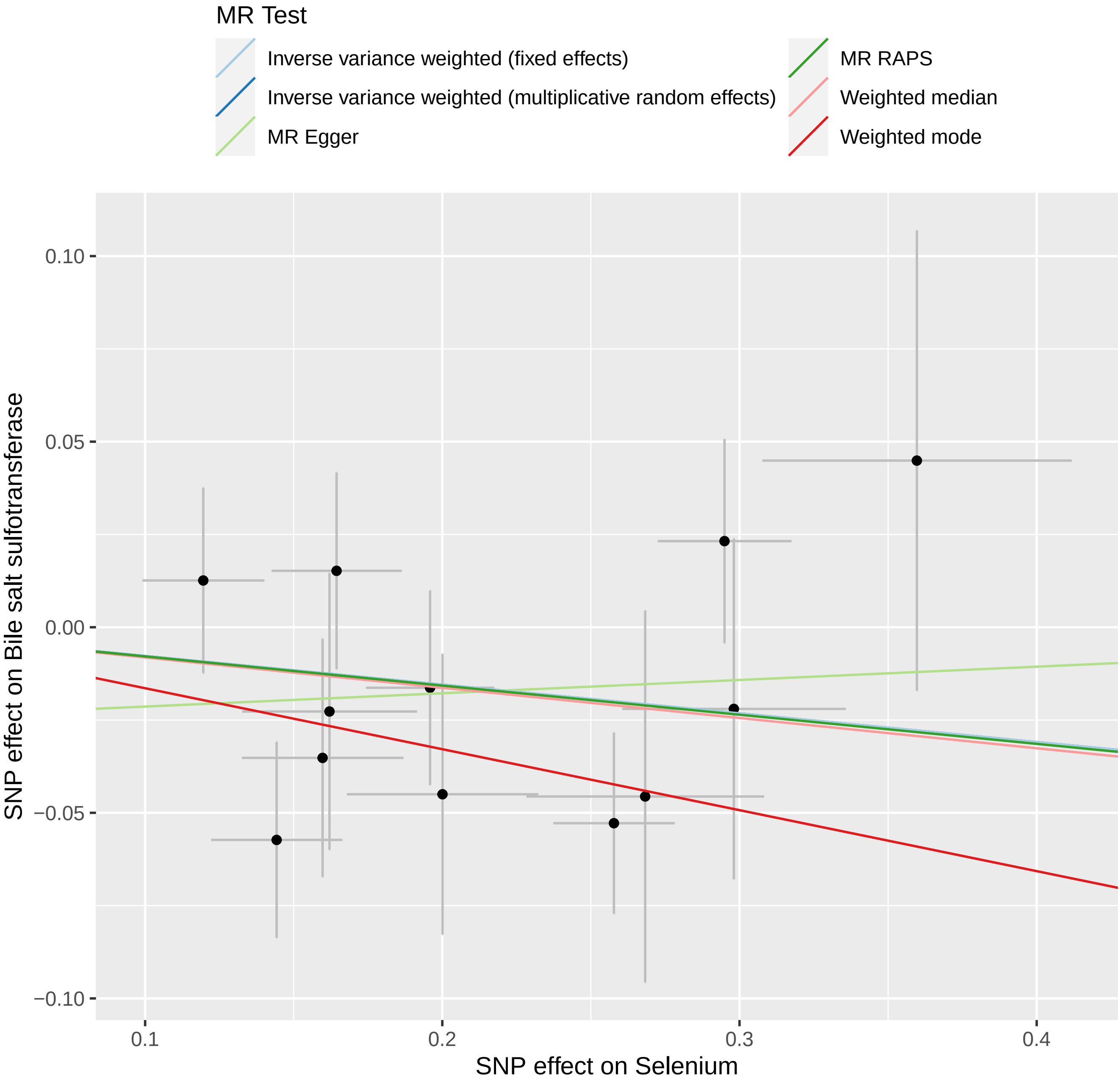

Batch 3−2894 : Selenium on Sulfotransferase 4A1

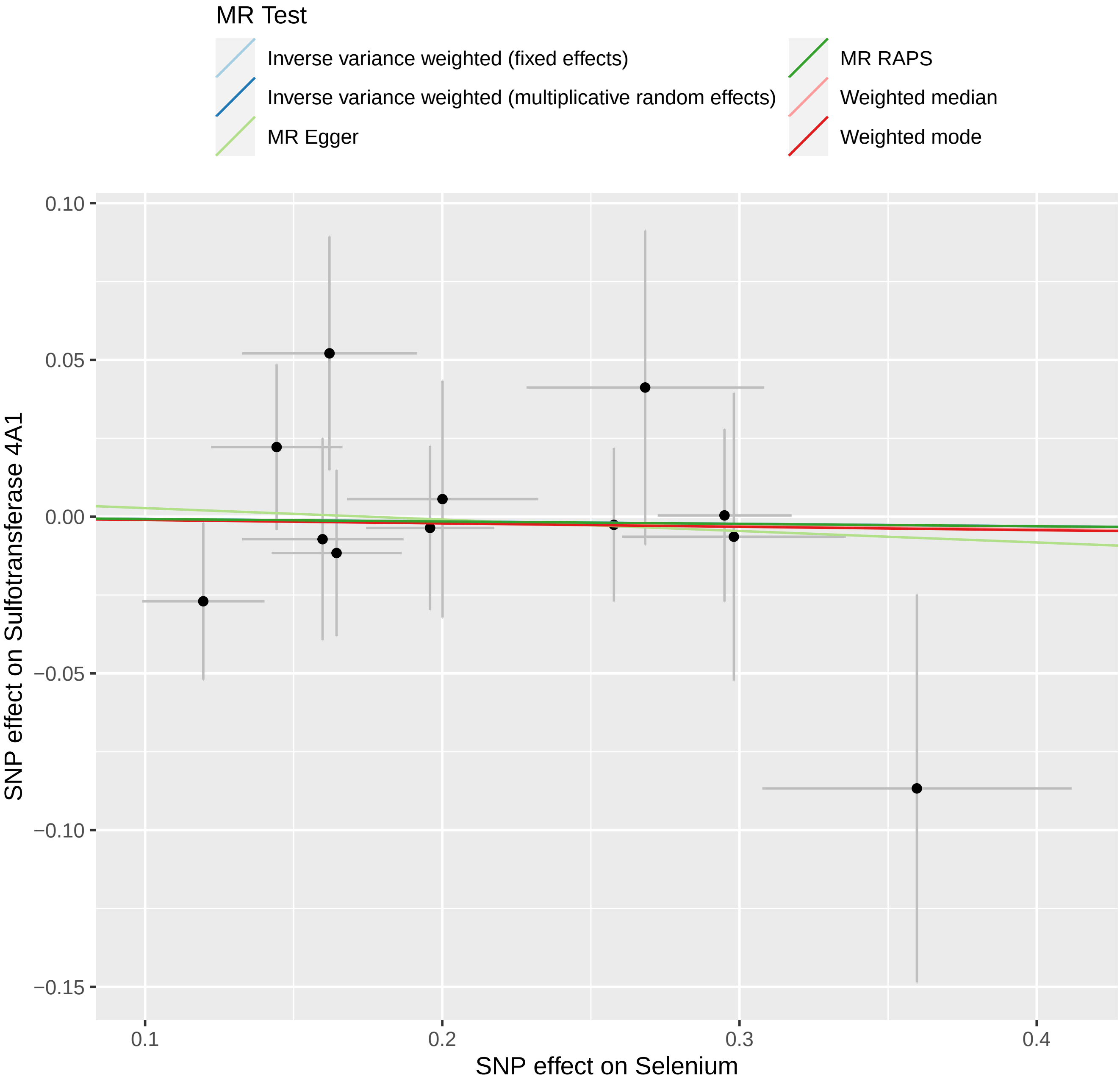

Batch 3−2895 : Selenium on Sulfatase−modifying factor 1

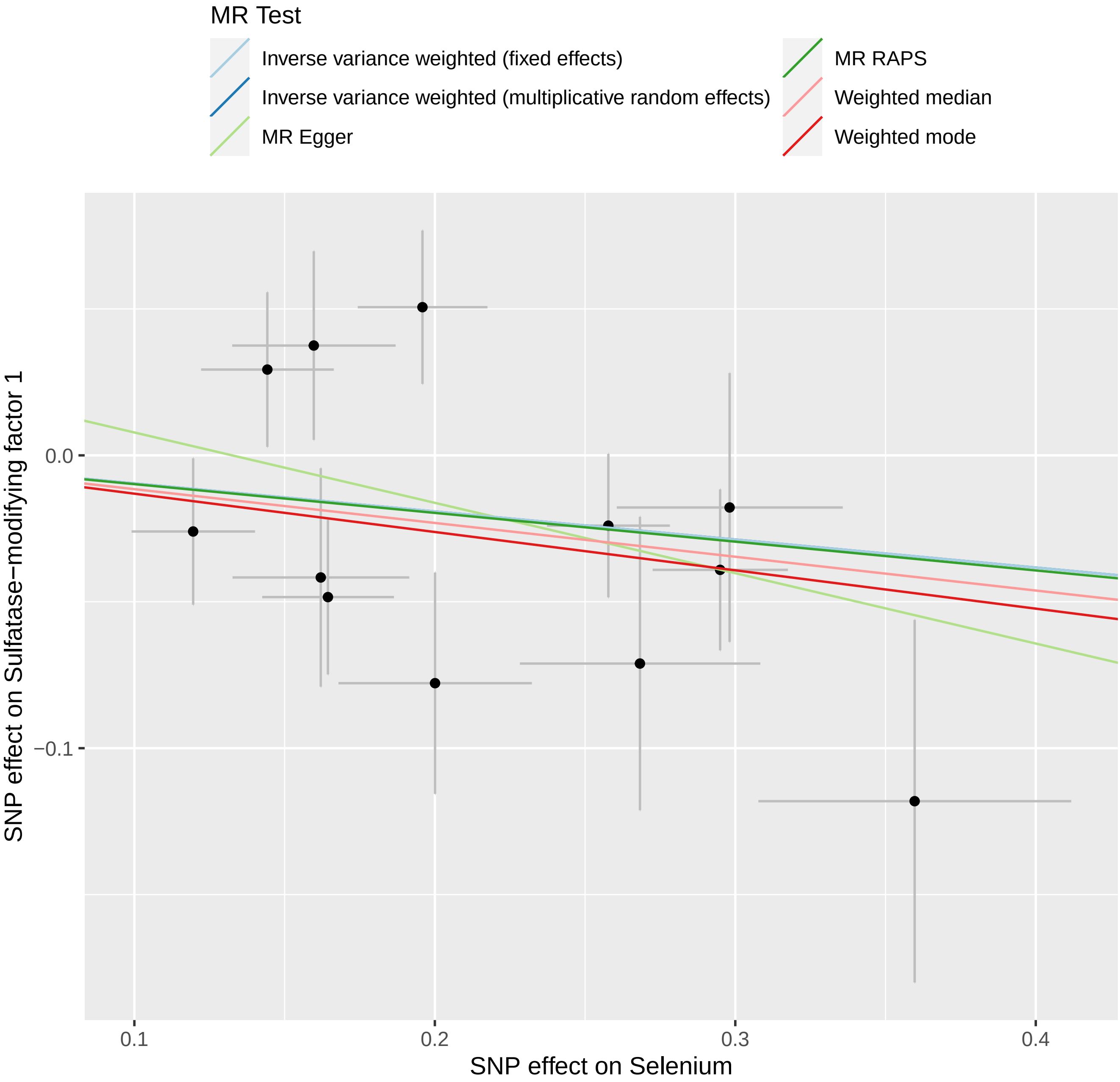

Batch 3−2896 : Selenium on Small ubiquitin−related modifier 3

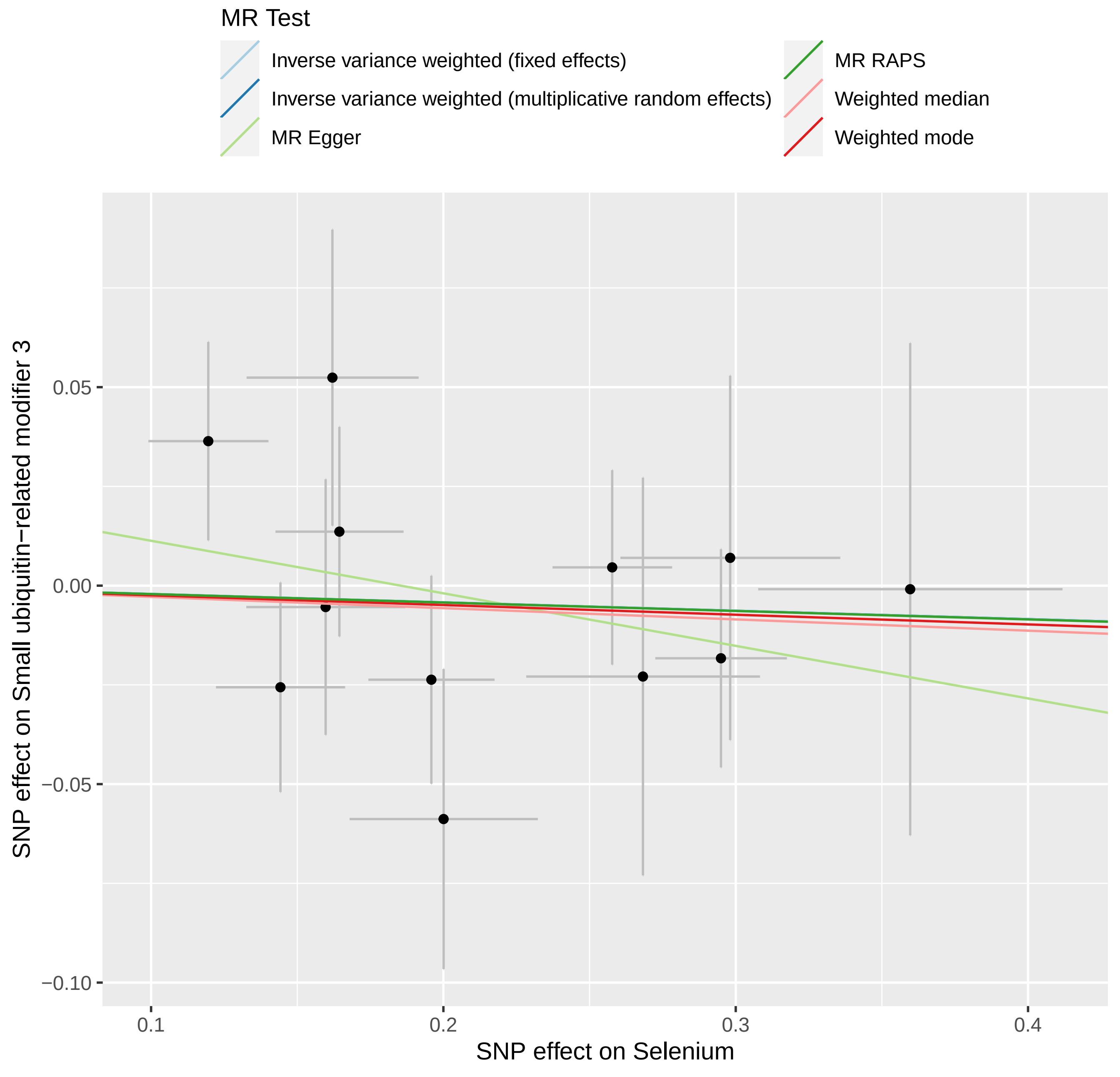

Batch 3−2897 : Selenium on Small ubiquitin−related modifier 3

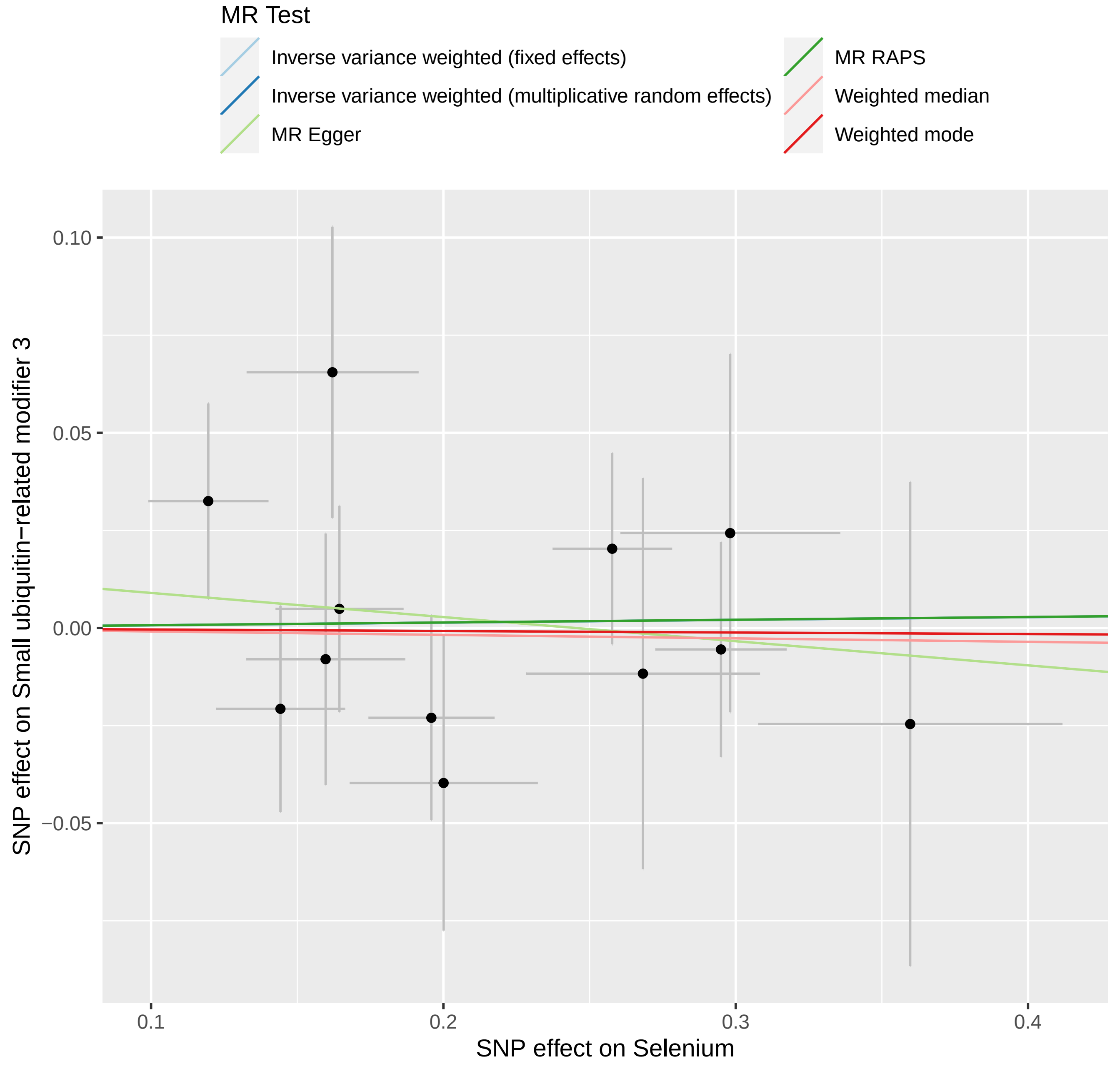

Batch 3−2898 : Selenium on SUN domain−containing protein 3

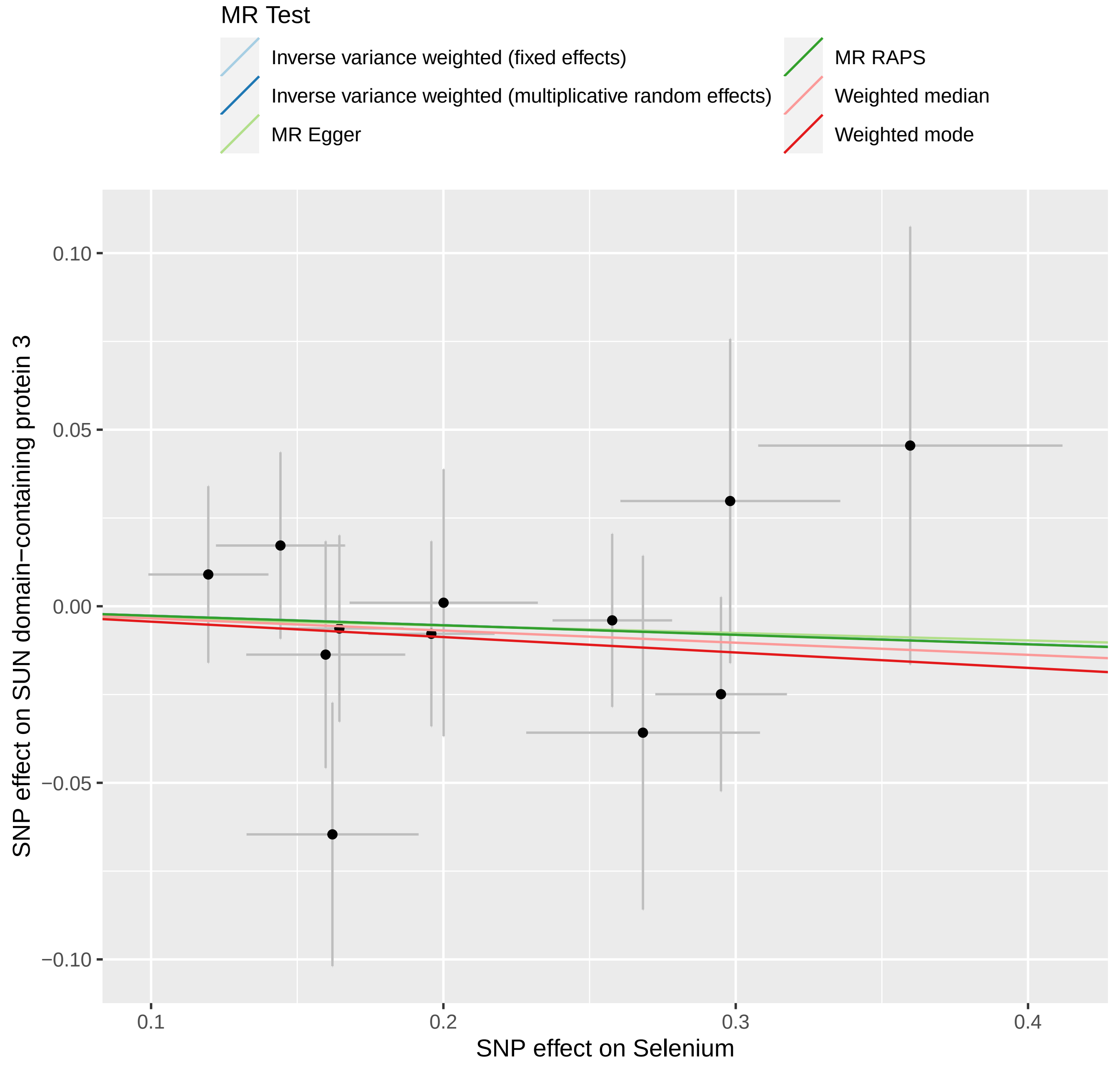

Batch 3−2899 : Selenium on SUN domain−containing protein 5

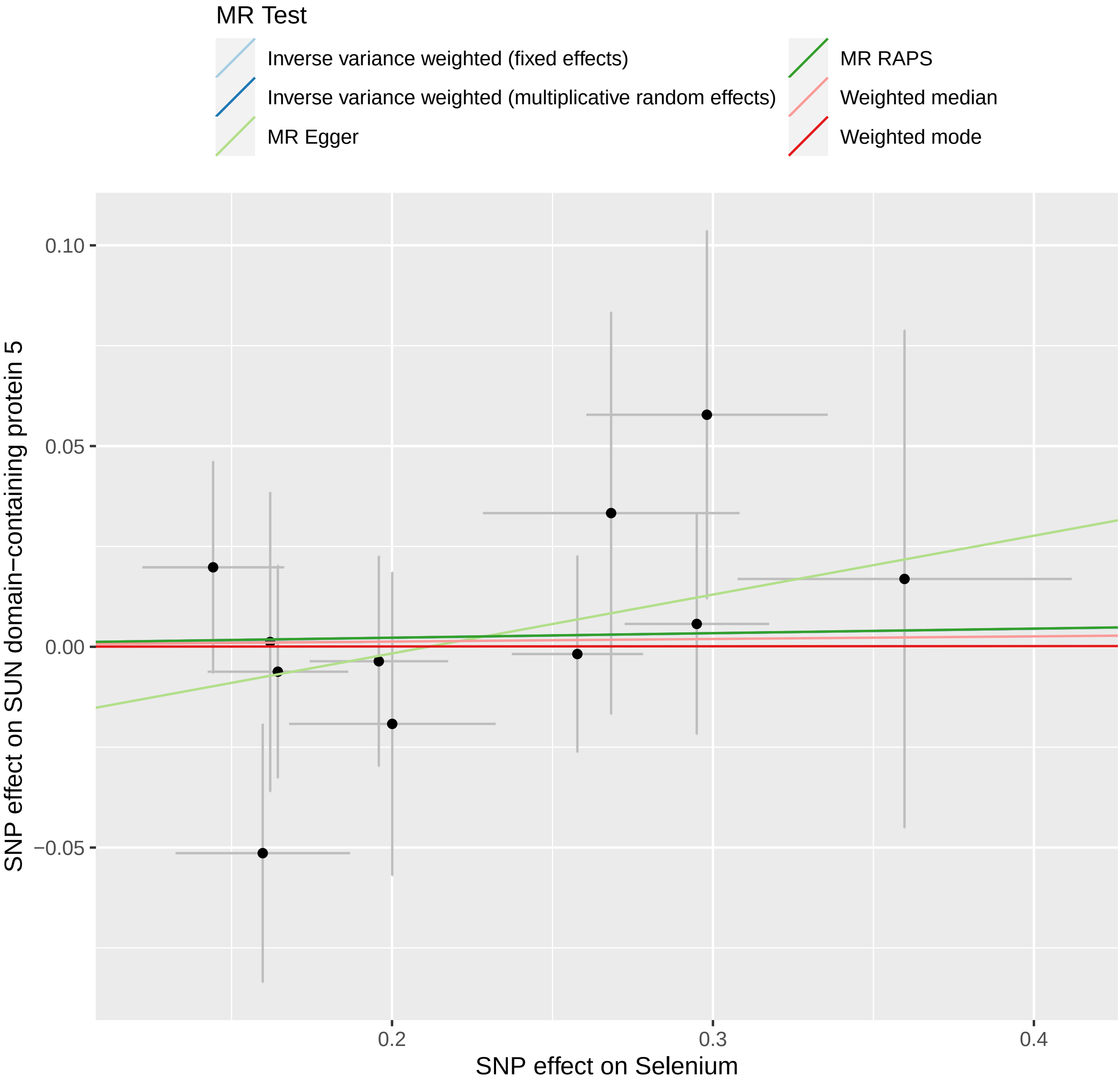

Batch 3−2900 : Selenium on Surfeit locus protein 1

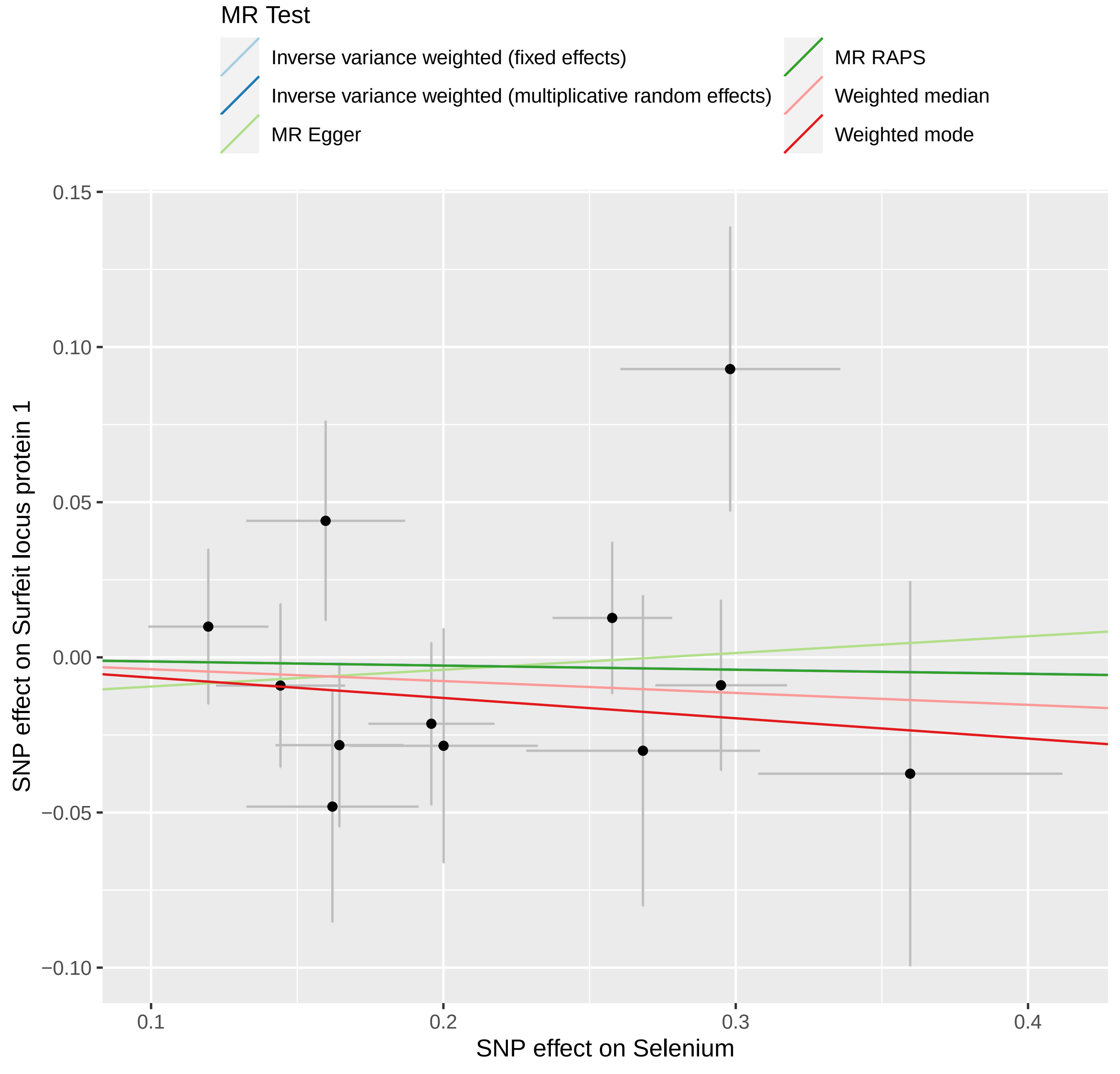

Batch 3−2901 : Selenium on Sushi domain−containing protein 1

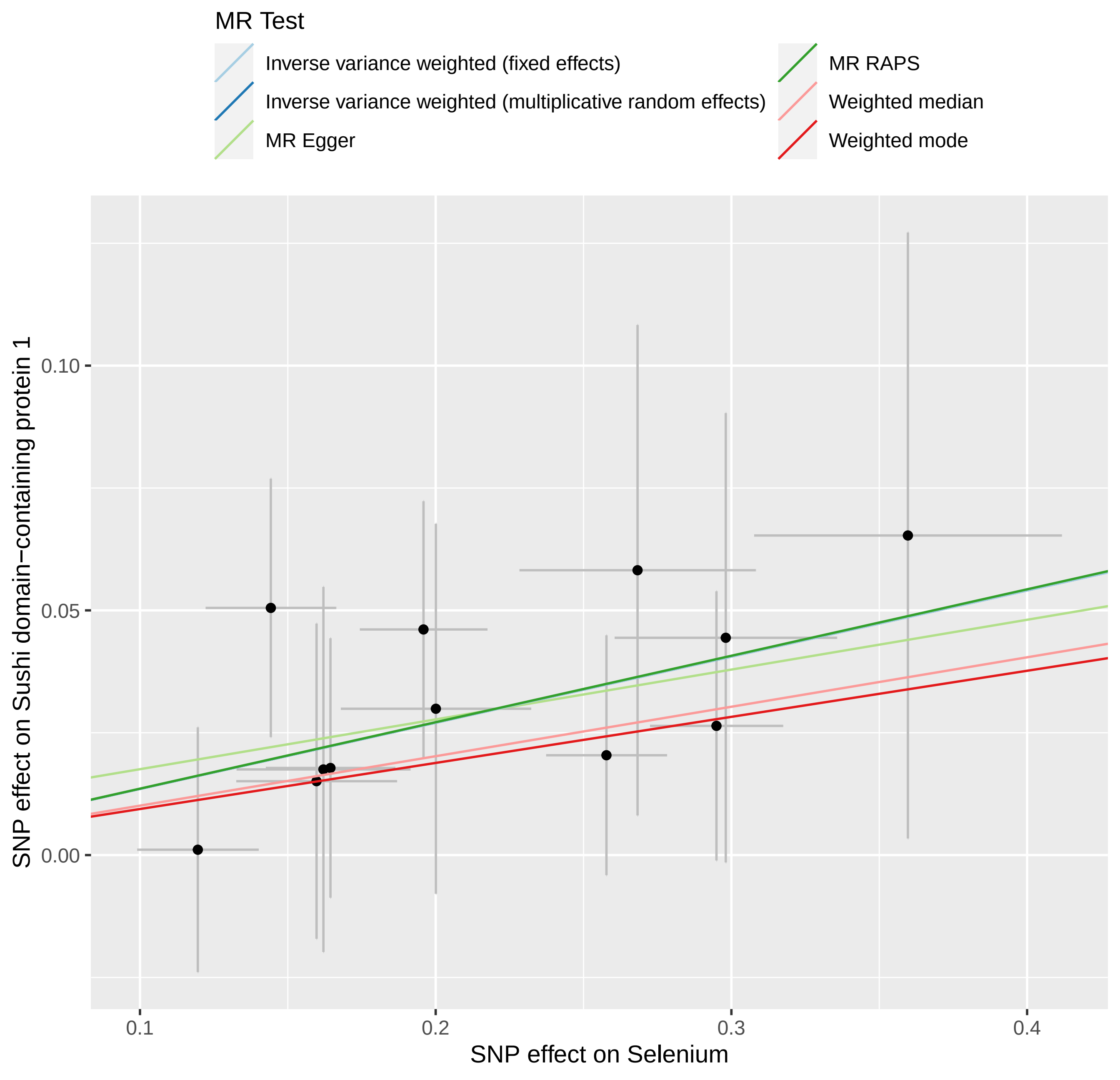

Batch 3−2902 : Selenium on Sushi domain−containing protein 2

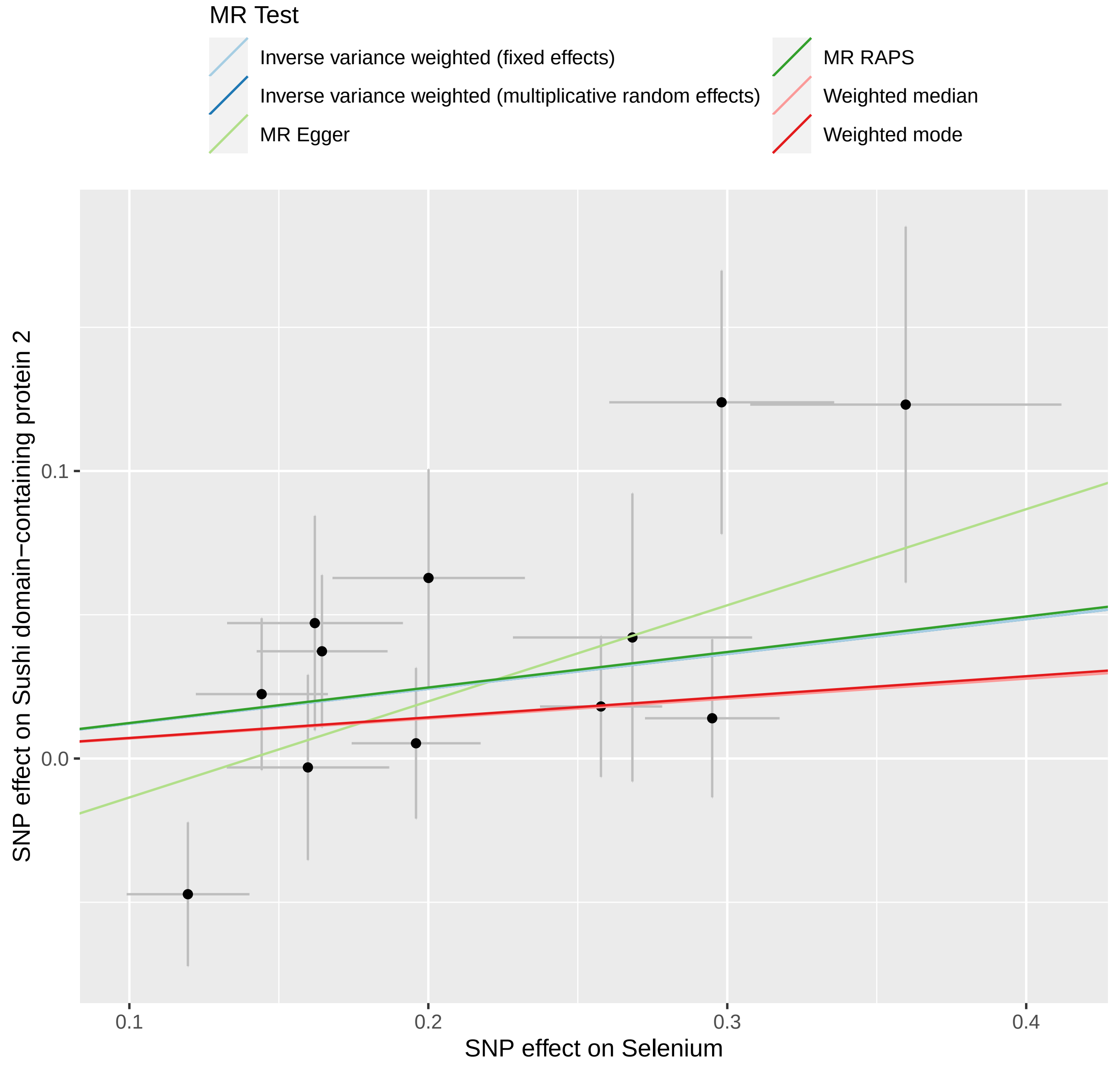

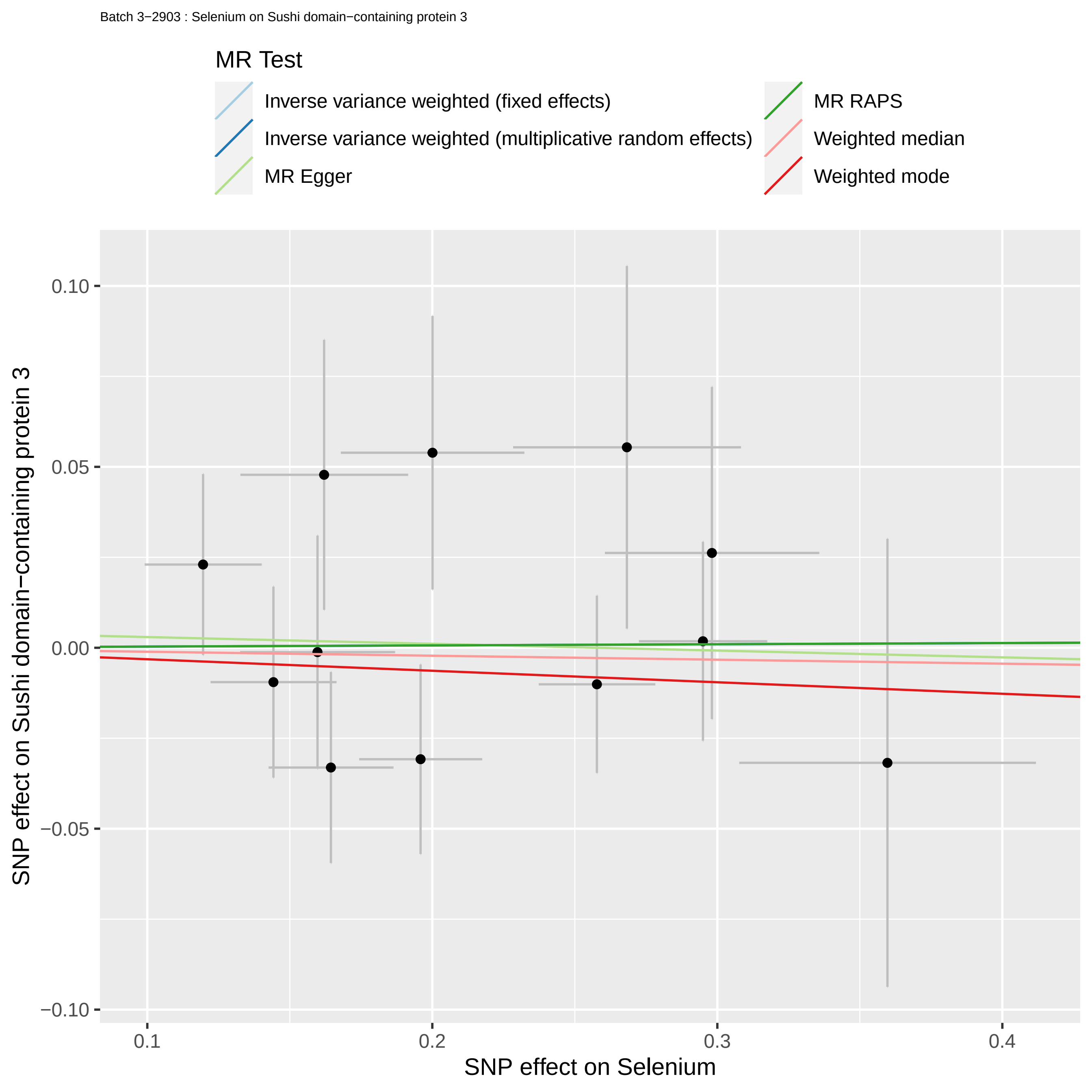
Batch 3−2903 : Selenium on Sushi domain−containing protein 3
MR Test
Inverse variance weighted (fixed effects)
Inverse variance weighted (multiplicative random effects)
MR Egger
MR RAPS
Weighted median
Weighted mode
SNP effect on Sushi domain−containing protein 3
0.10
0.05
0.00
−0.05
−0.10
SNP effect on Selenium
0.1
0.2
0.3
0.4

Batch 3−2904 : Selenium on Histone−lysine N−methyltransferase SUV420H2

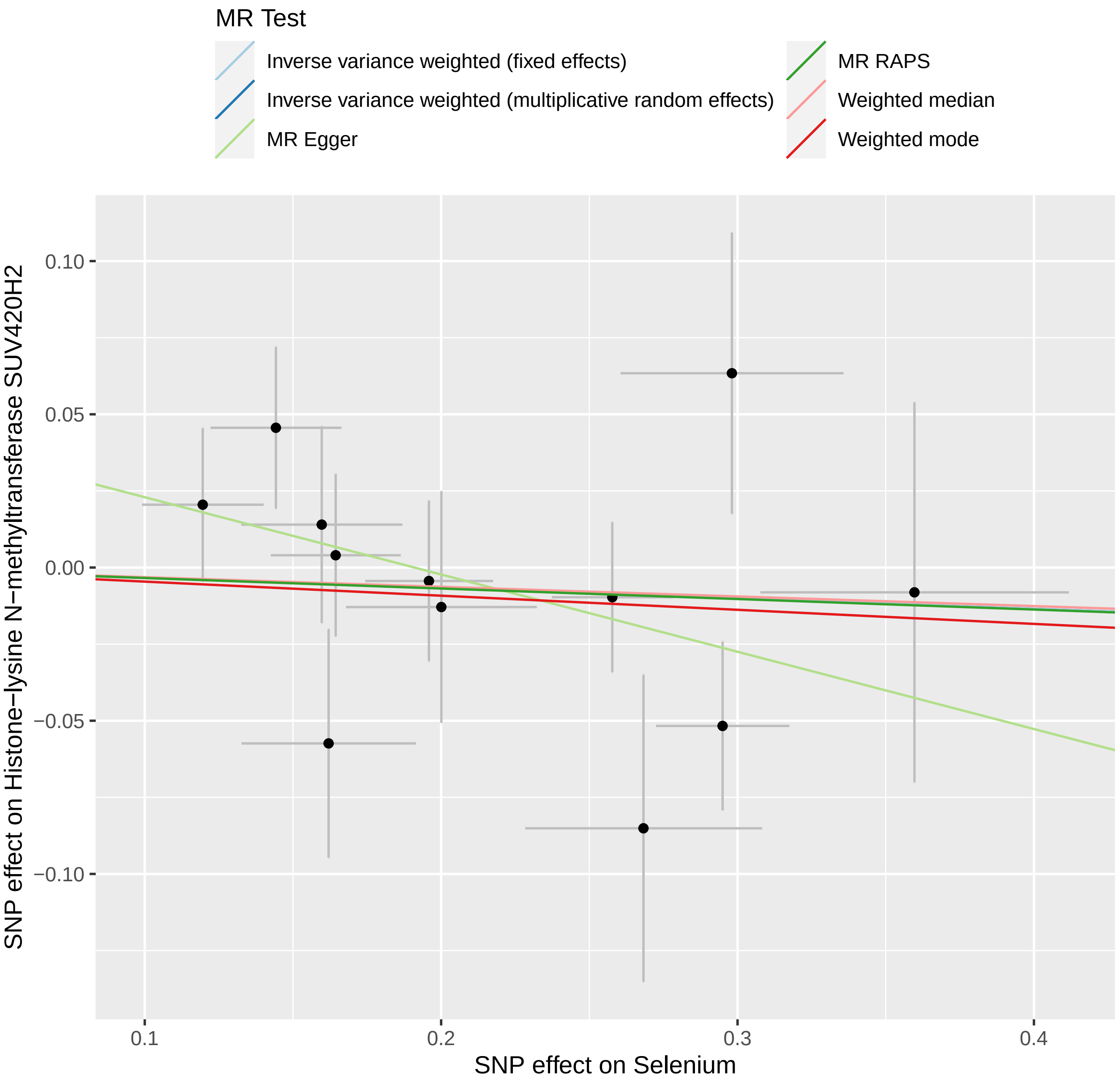

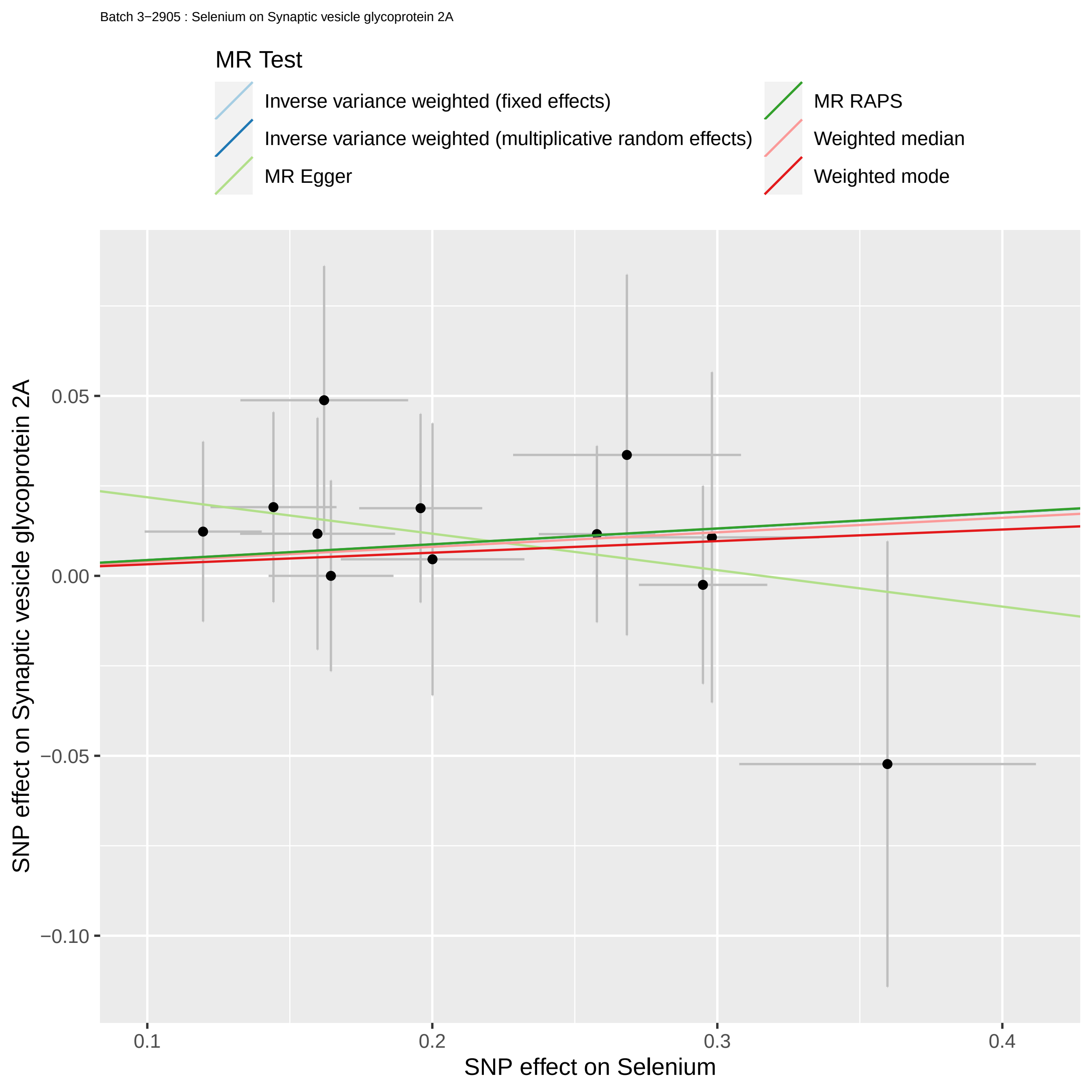

Batch 3−2905 : Selenium on Synaptic vesicle glycoprotein 2A
MR Test
Inverse variance weighted (fixed effects)
Inverse variance weighted (multiplicative random effects)
MR Egger
MR RAPS
Weighted median
Weighted mode
0.05
0.00
−0.05
−0.10
0.1
0.2
0.3
0.4
SNP effect on Selenium
SNP effect on Synaptic vesicle glycoprotein 2A

Batch 3−2906 : Selenium on Sushi, von Willebrand factor type A, EGF and pentraxin domain−containing protein 1

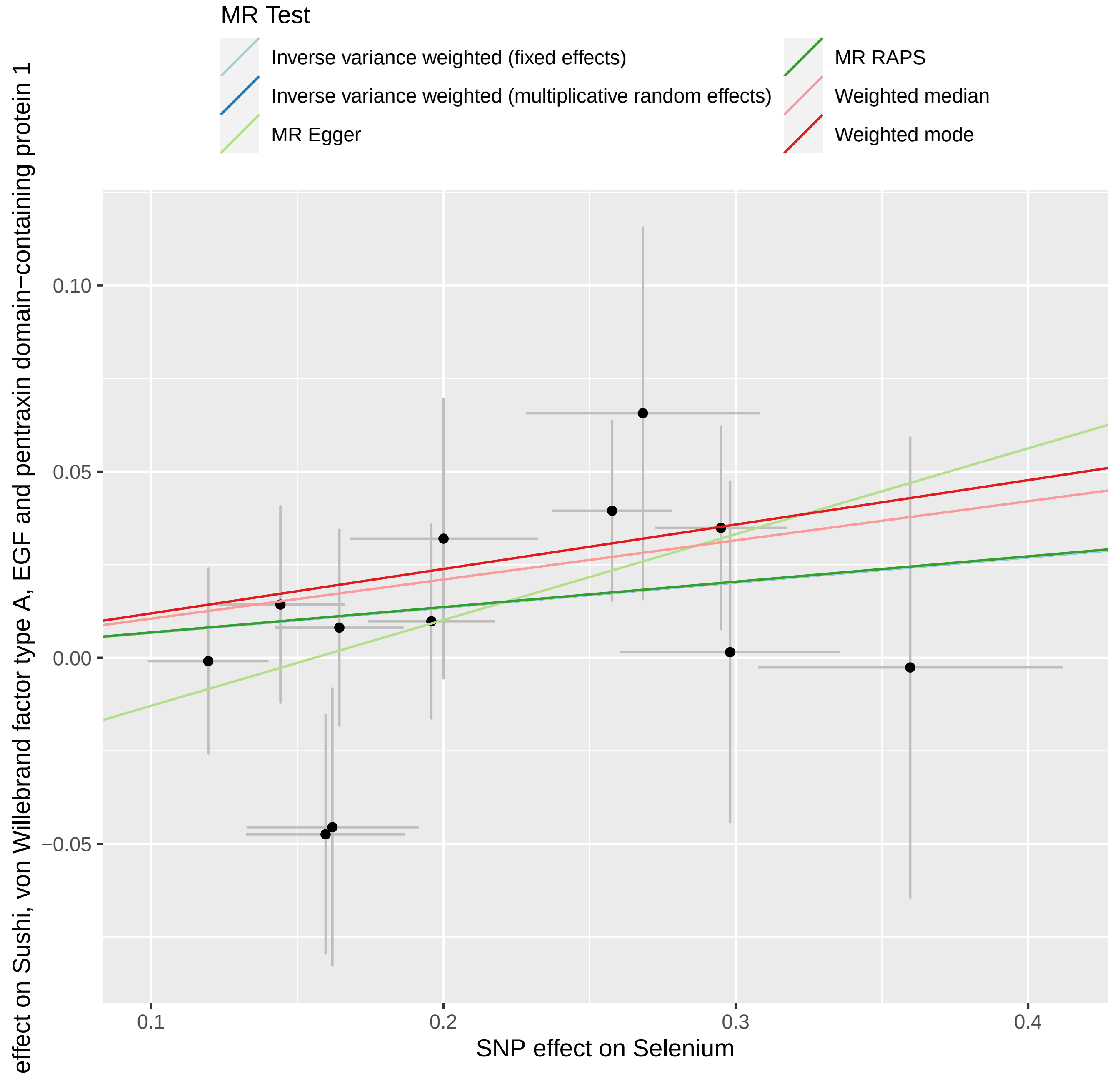

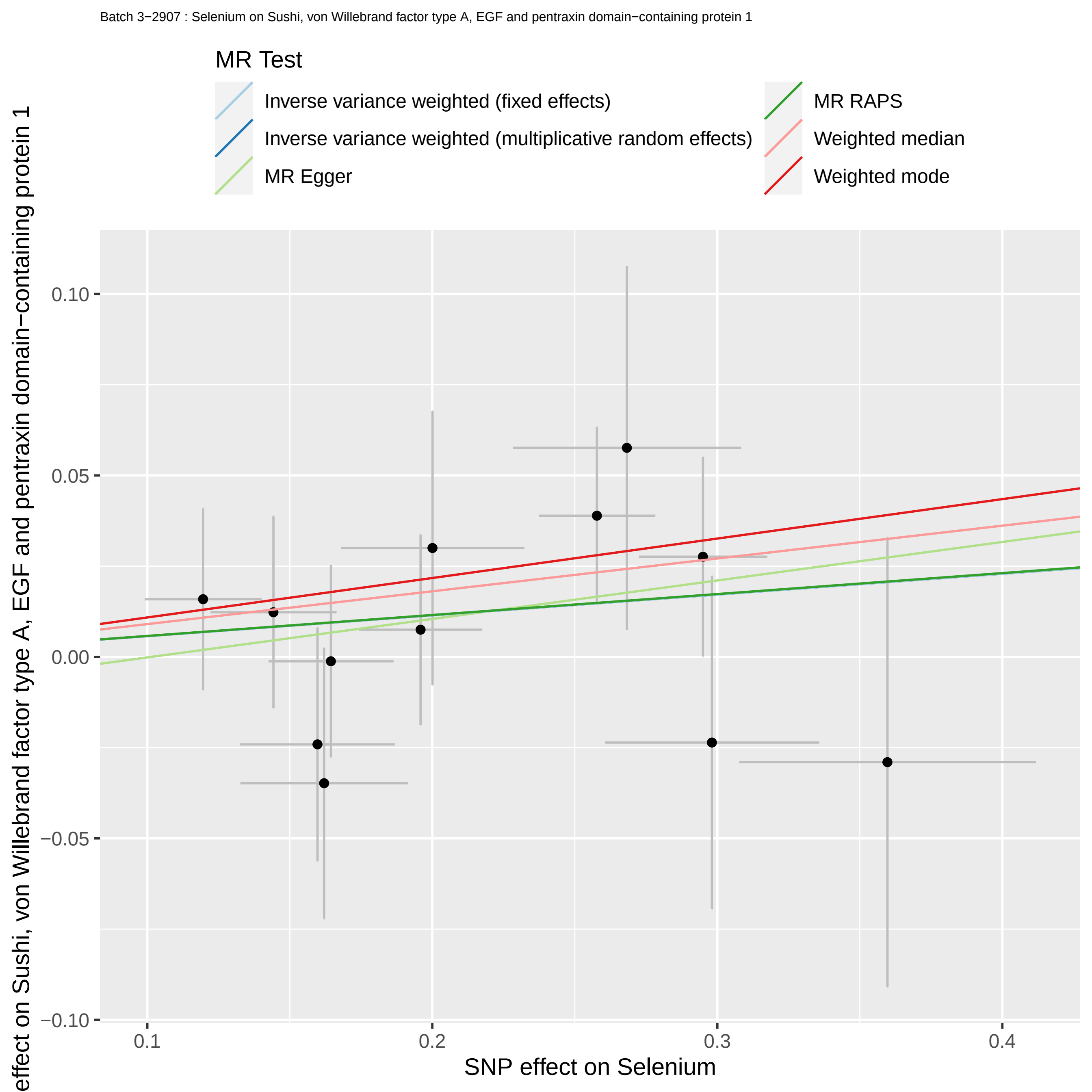
Batch 3−2907 : Selenium on Sushi, von Willebrand factor type A, EGF and pentraxin domain−containing protein 1
MR Test
Inverse variance weighted (fixed effects)
Inverse variance weighted (multiplicative random effects)
MR Egger
MR RAPS
Weighted median
Weighted mode
0.10
0.05
0.00
−0.05
−0.10
0.1
0.2
0.3
0.4
SNP effect on Selenium
effect on Sushi, von Willebrand factor type A, EGF and pentraxin domain−containing protein 1

Batch 3−2908 : Selenium on Switch−associated protein 70

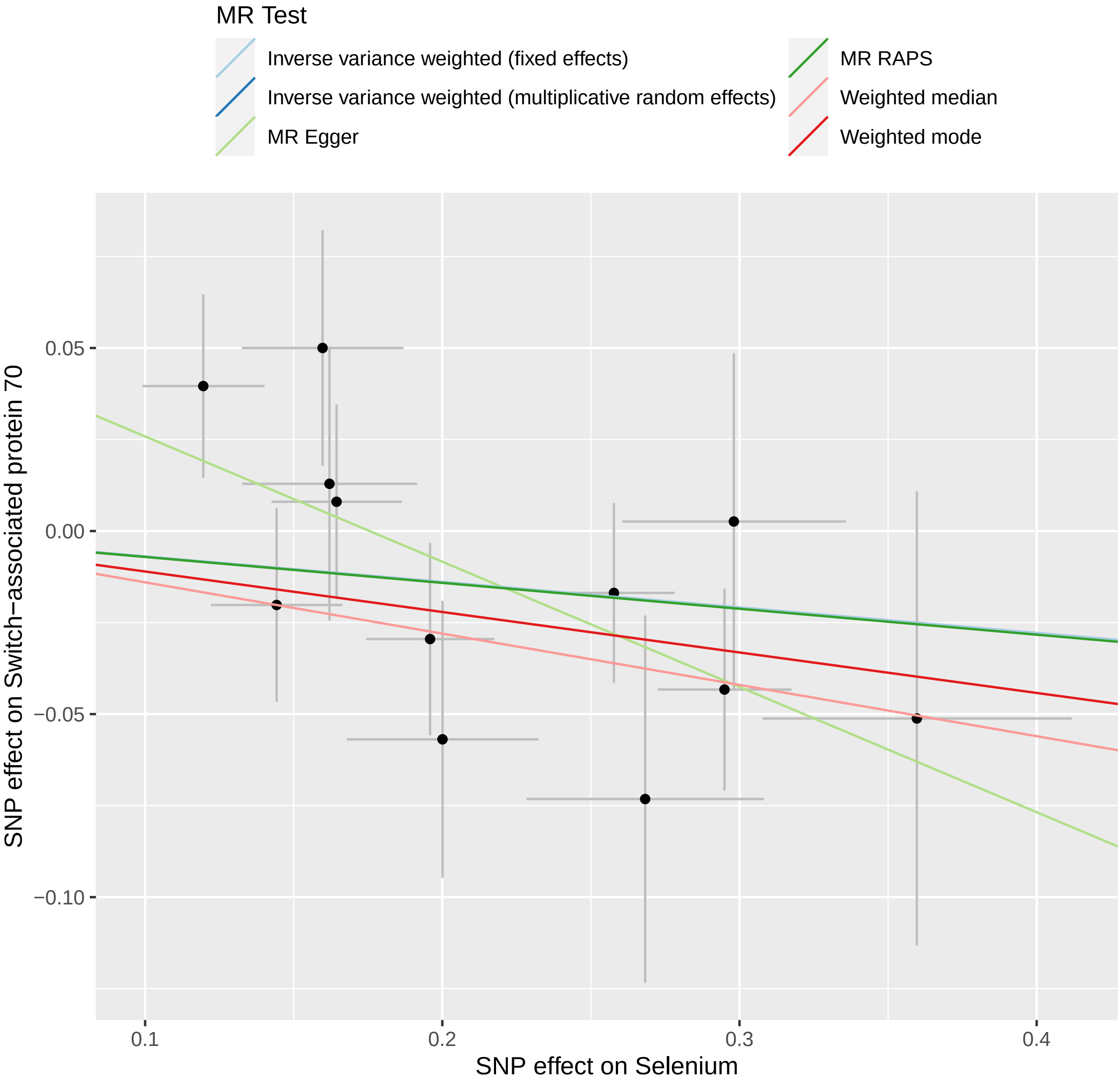

Batch 3−2909 : Selenium on Heterogeneous nuclear ribonucleoprotein Q

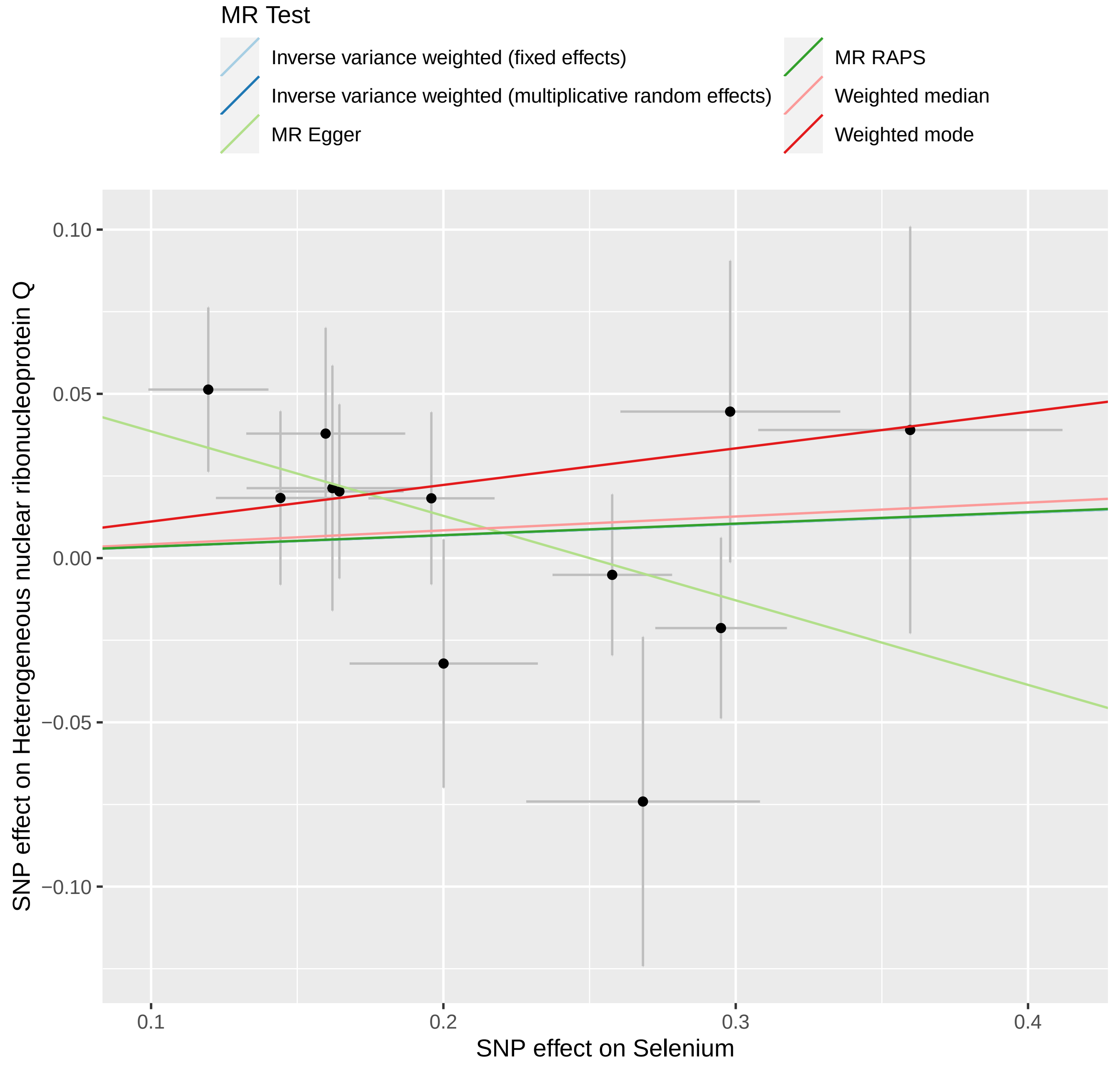

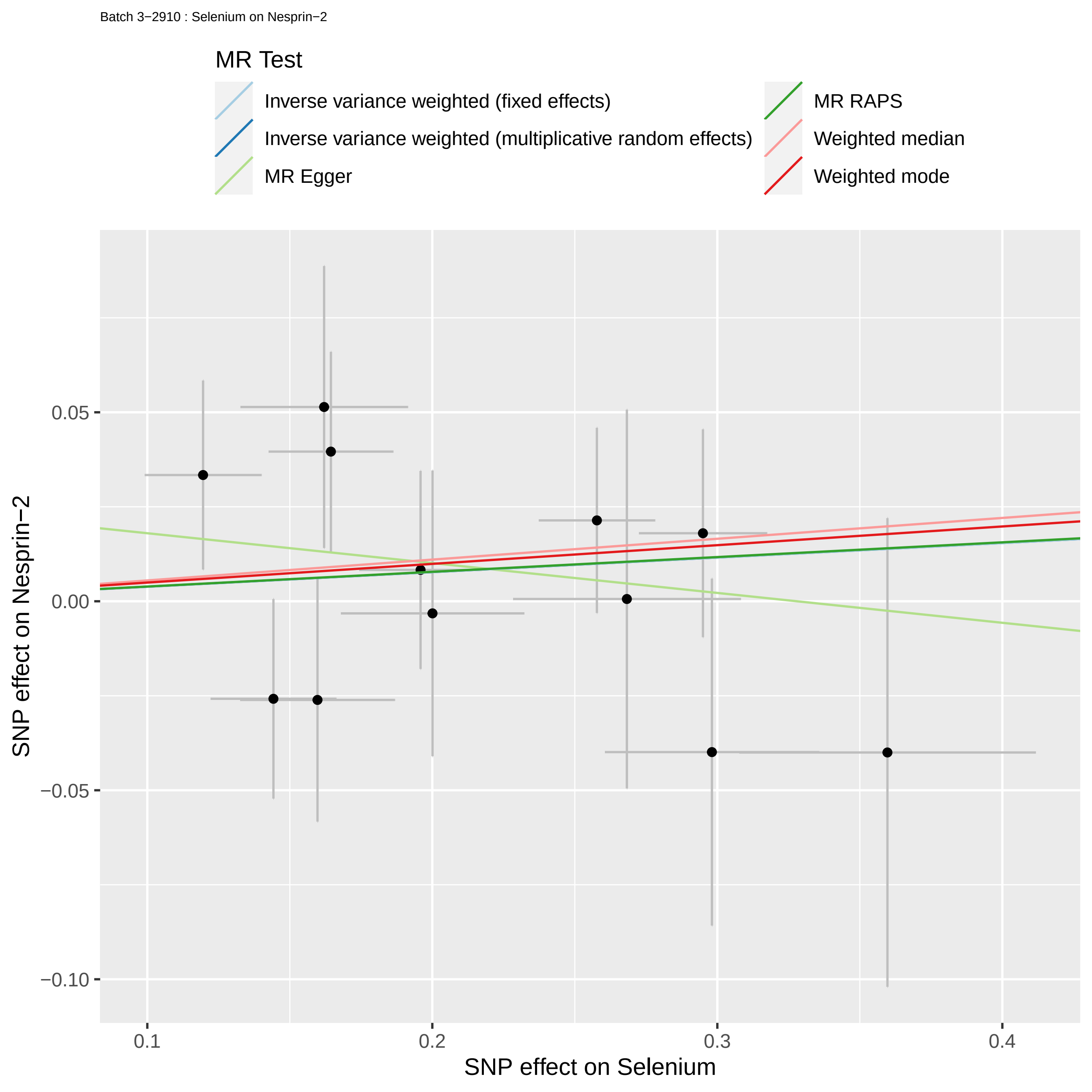
Batch 3−2910 : Selenium on Nesprin−2
MR Test
Inverse variance weighted (fixed effects)
Inverse variance weighted (multiplicative random effects)
MR Egger
MR RAPS
Weighted median
Weighted mode
SNP effect on Nesprin−2
0.05
0.00
−0.05
−0.10
0.1
0.2
0.3
0.4
SNP effect on Selenium

Batch 3−2911 : Selenium on Synaptotagmin−11

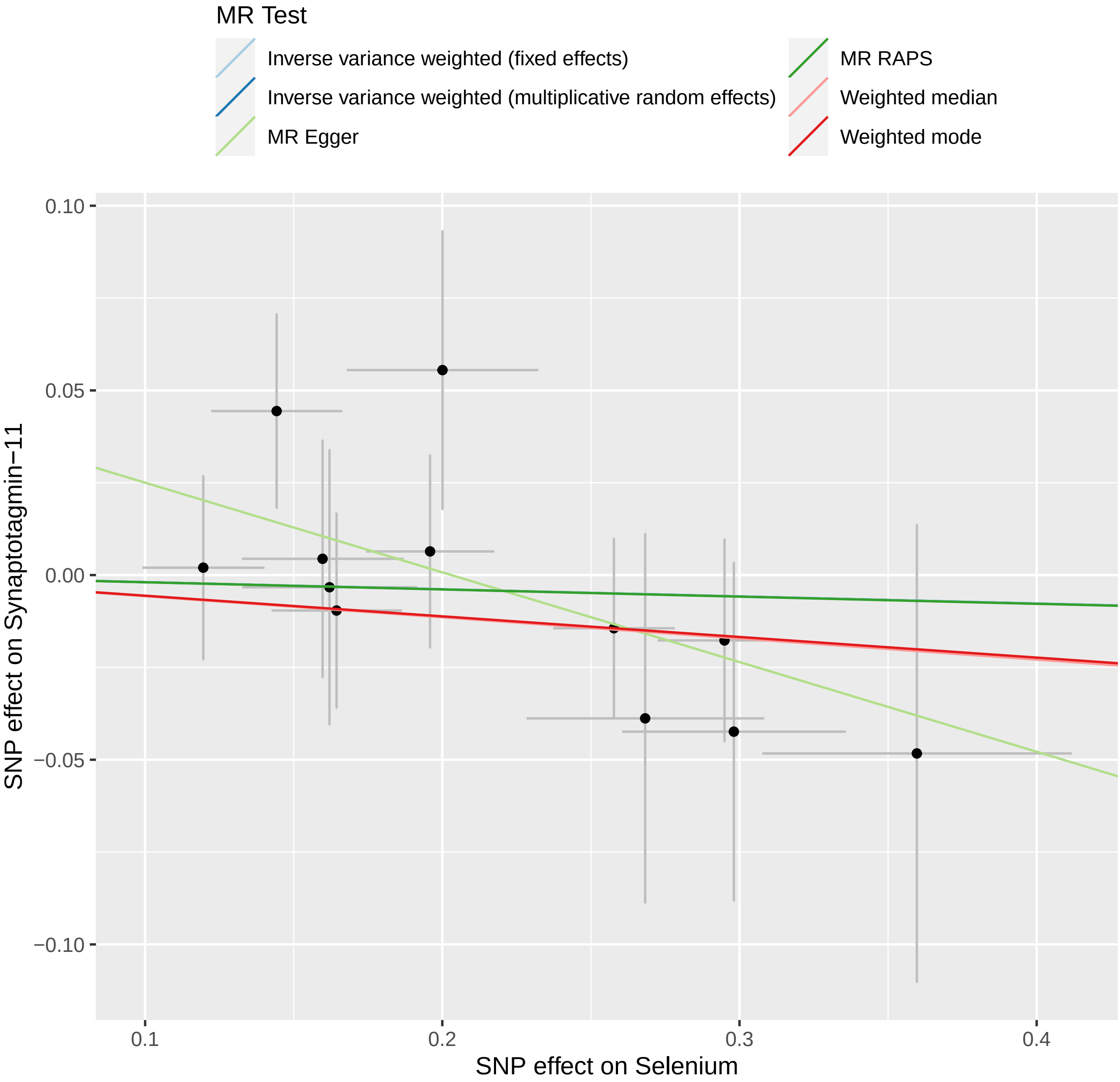

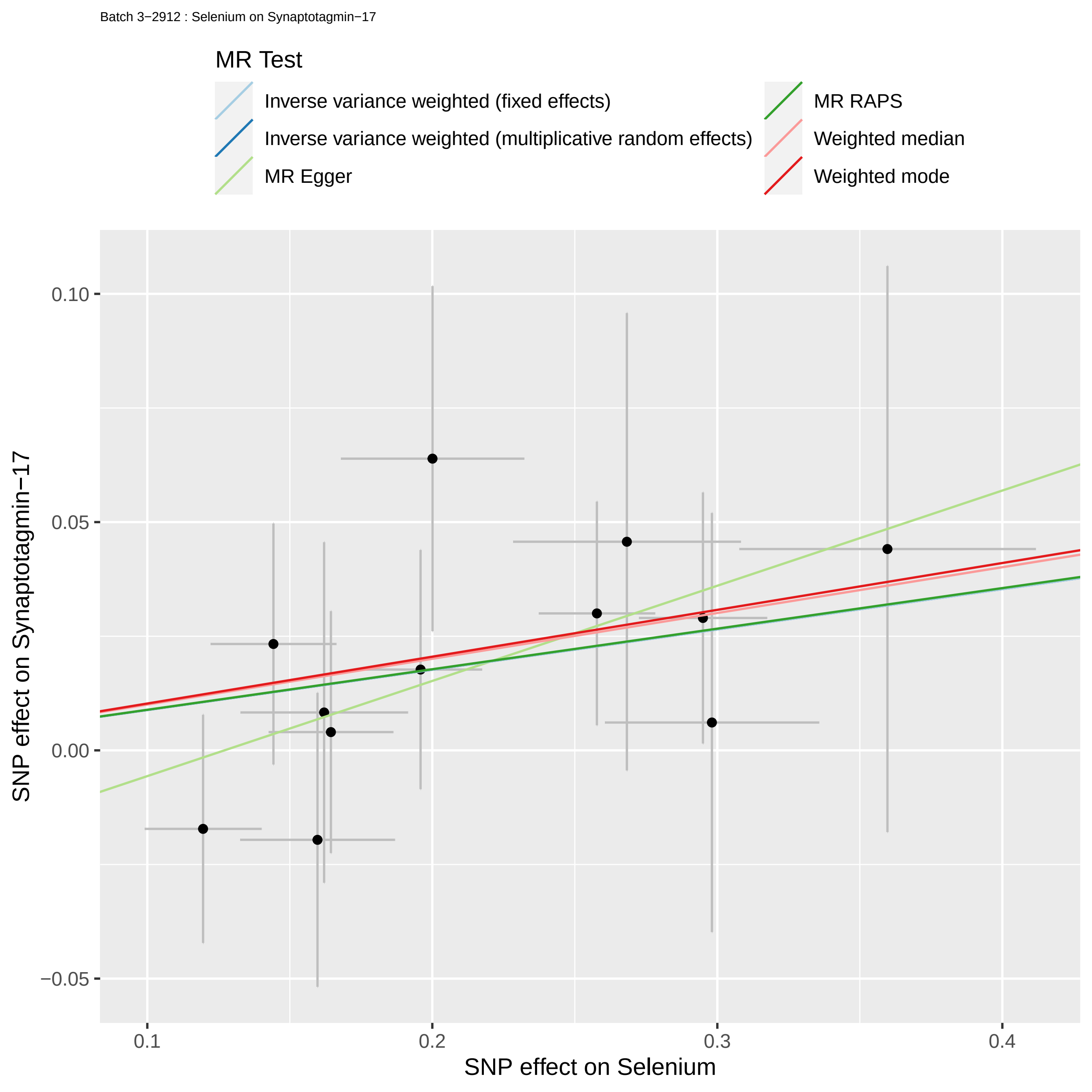
Batch 3−2912 : Selenium on Synaptotagmin−17
MR Test
Inverse variance weighted (fixed effects)
Inverse variance weighted (multiplicative random effects)
MR Egger
MR RAPS
Weighted median
Weighted mode
0.10
0.05
0.00
−0.05
0.1
0.2
0.3
0.4
SNP effect on Synaptotagmin−17
SNP effect on Selenium

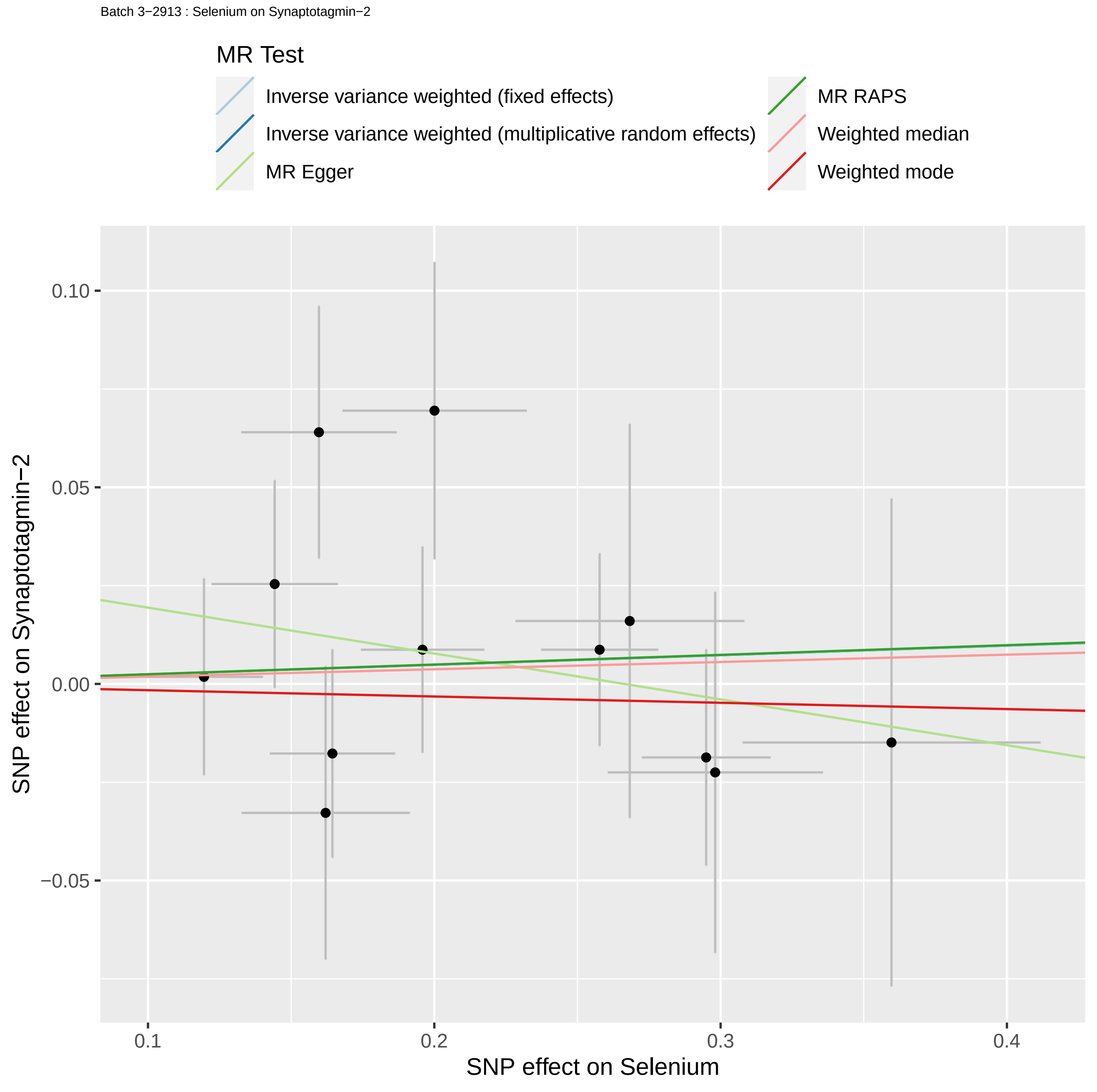
Batch 3−2913 : Selenium on Synaptotagmin−2
MR Test
Inverse variance weighted (fixed effects)
Inverse variance weighted (multiplicative random effects)
MR Egger
MR RAPS
Weighted median
Weighted mode
0.10
0.05
0.00
−0.05
0.1
0.2
0.3
0.4
SNP effect on Selenium
SNP effect on Synaptotagmin−2

Batch 3−2914 : Selenium on Synaptotagmin−3

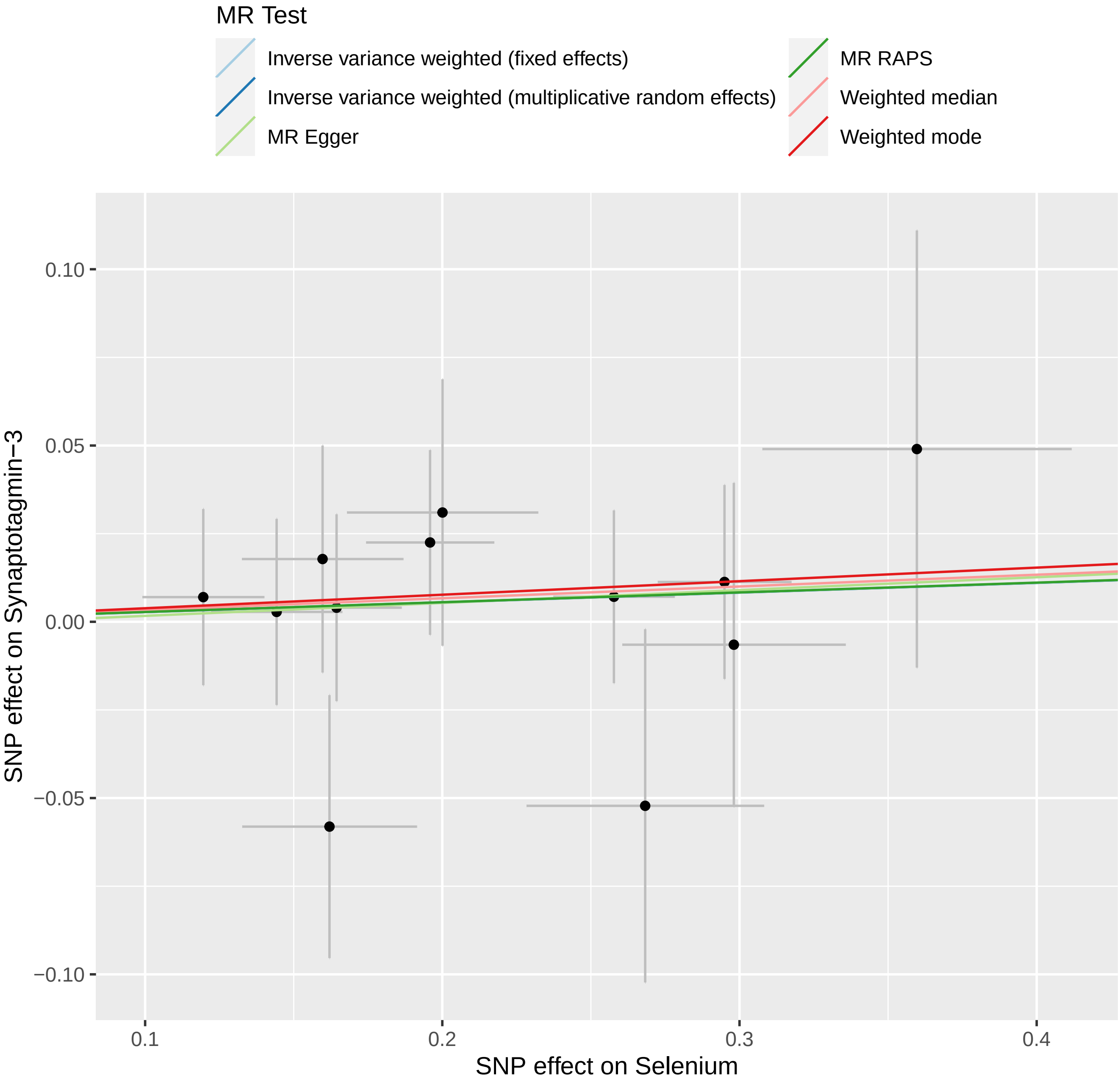

Batch 3−2915 : Selenium on Synaptotagmin−5

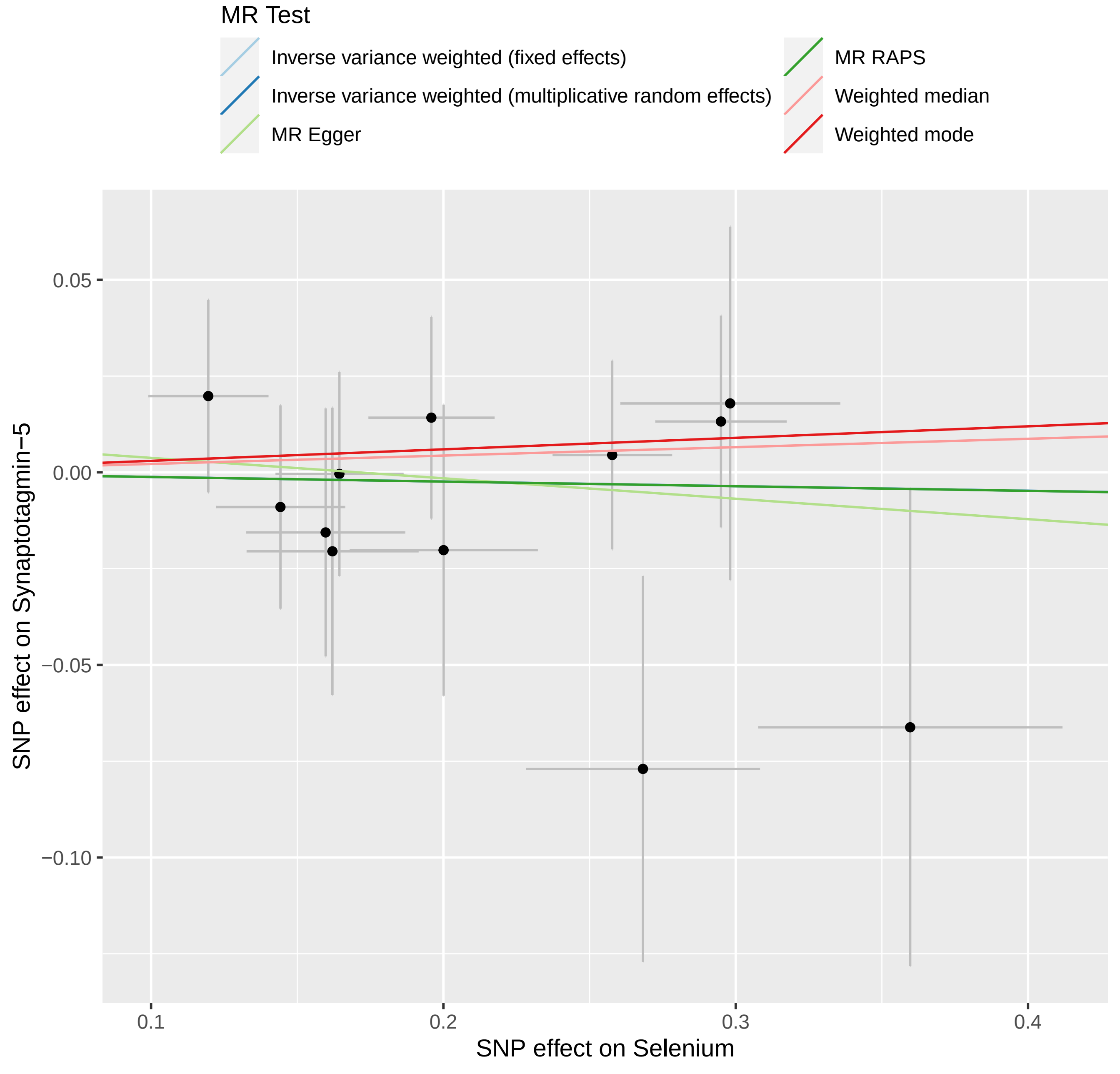

Batch 3−2916 : Selenium on Synaptotagmin−7

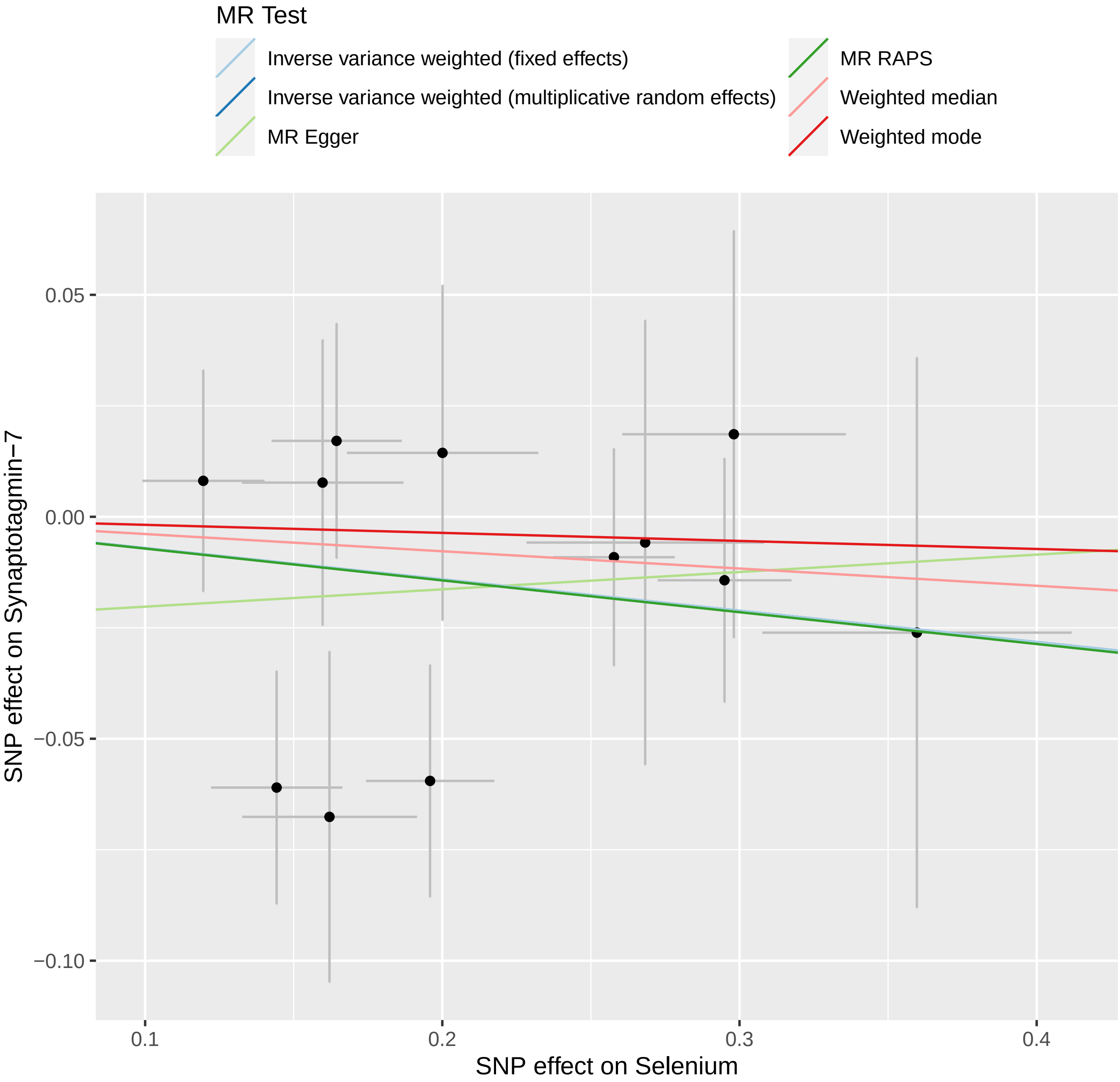

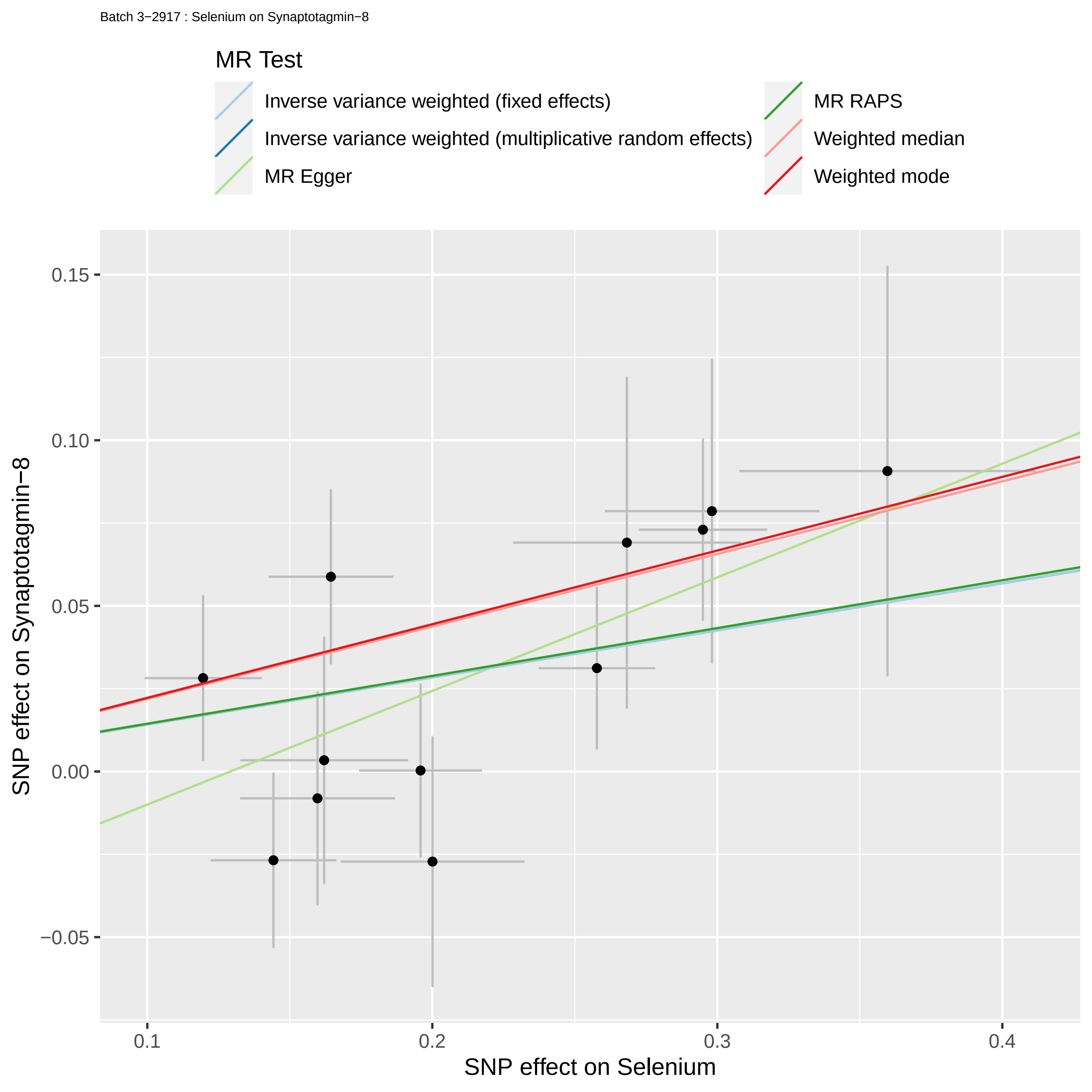
Batch 3−2917 : Selenium on Synaptotagmin−8
MR Test
Inverse variance weighted (fixed effects)
Inverse variance weighted (multiplicative random effects)
MR Egger
MR RAPS
Weighted median
Weighted mode
0.15
0.10
0.05
0.00
−0.05
0.1
0.2
0.3
0.4
SNP effect on Synaptotagmin−8
SNP effect on Selenium

Batch 3−2918 : Selenium on Synaptotagmin−like protein 1

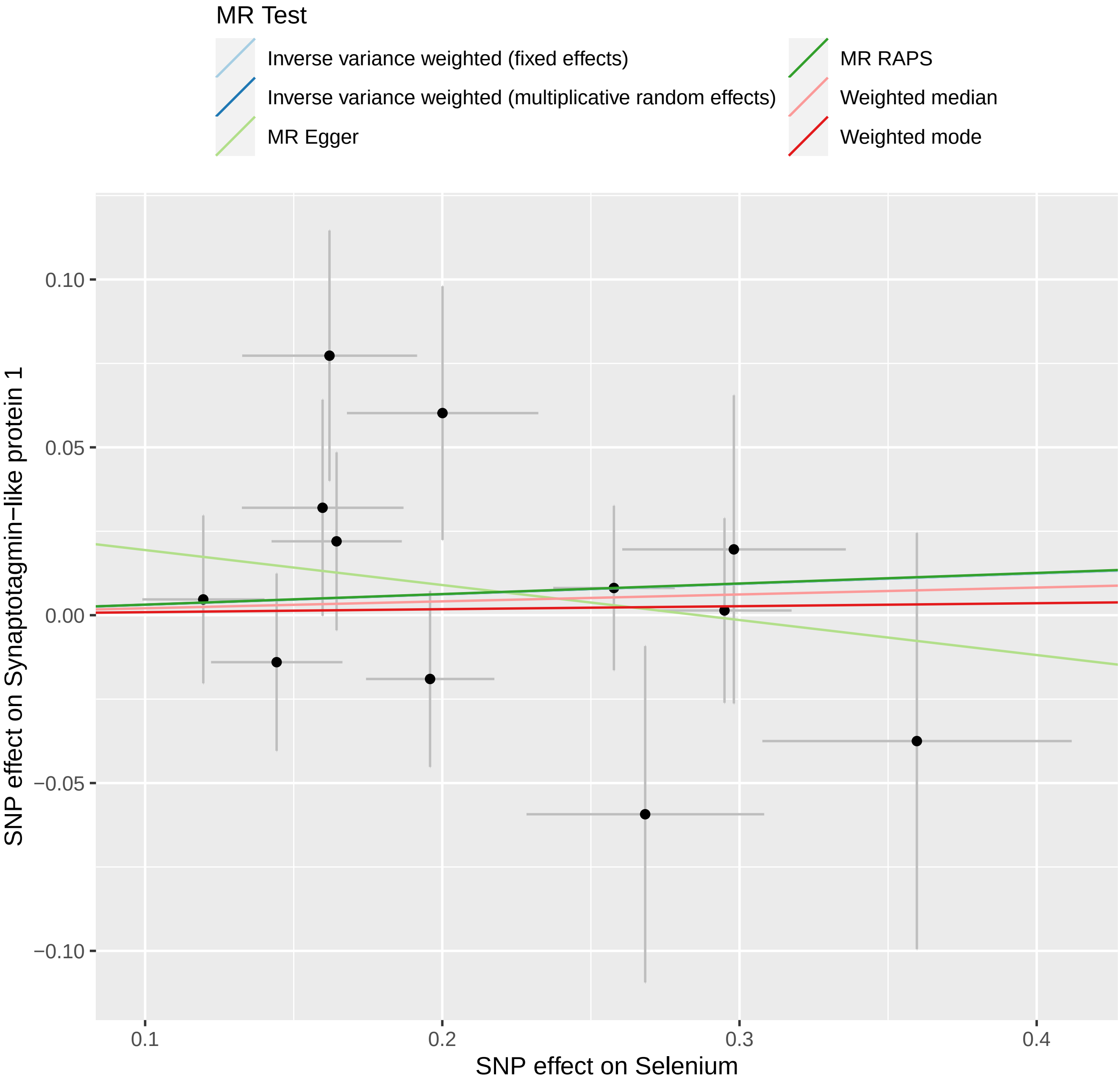

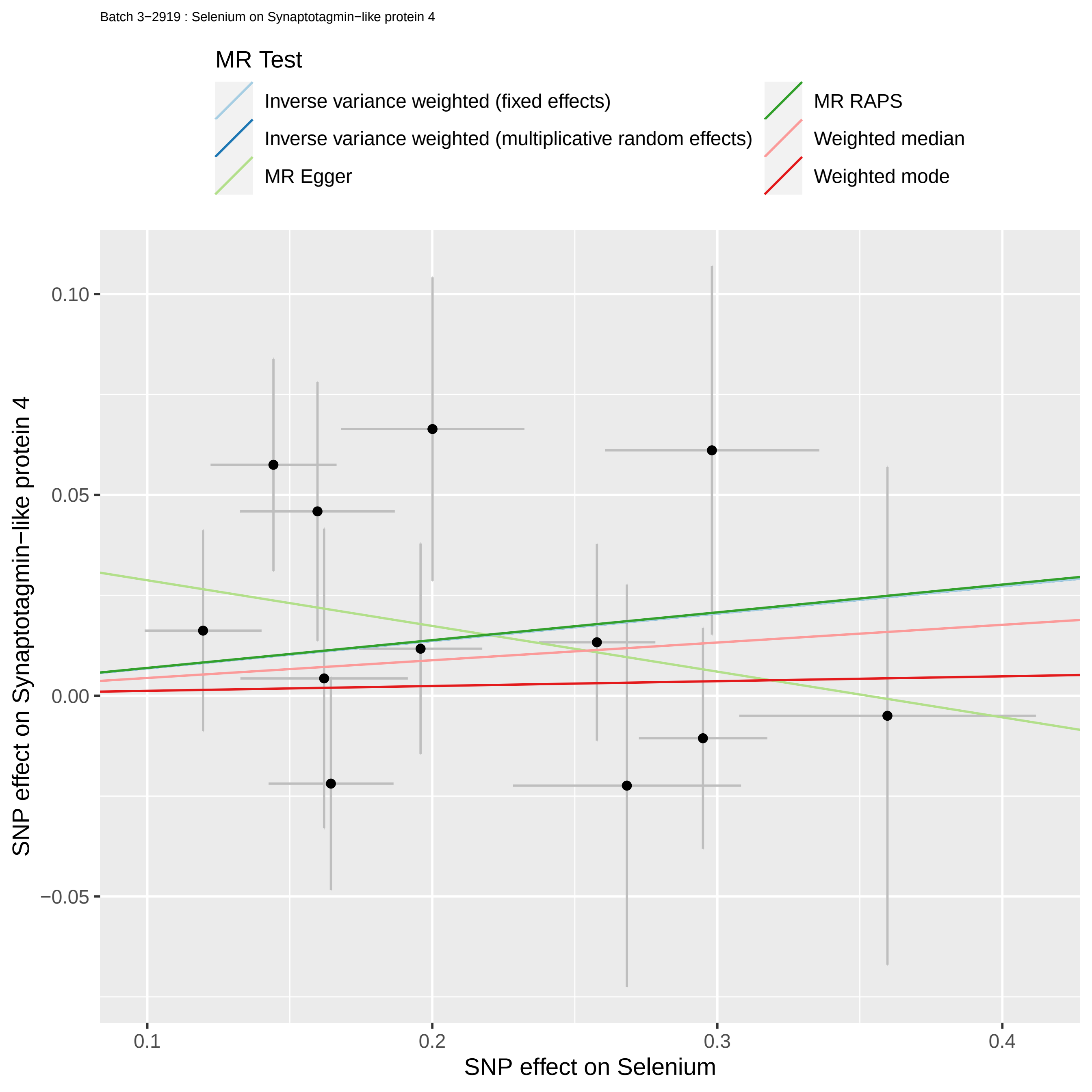
Batch 3−2919 : Selenium on Synaptotagmin−like protein 4
MR Test
Inverse variance weighted (fixed effects)
Inverse variance weighted (multiplicative random effects)
MR Egger
MR RAPS
Weighted median
Weighted mode
0.10
0.05
0.00
−0.05
0.1
0.2
0.3
0.4
SNP effect on Synaptotagmin−like protein 4
SNP effect on Selenium

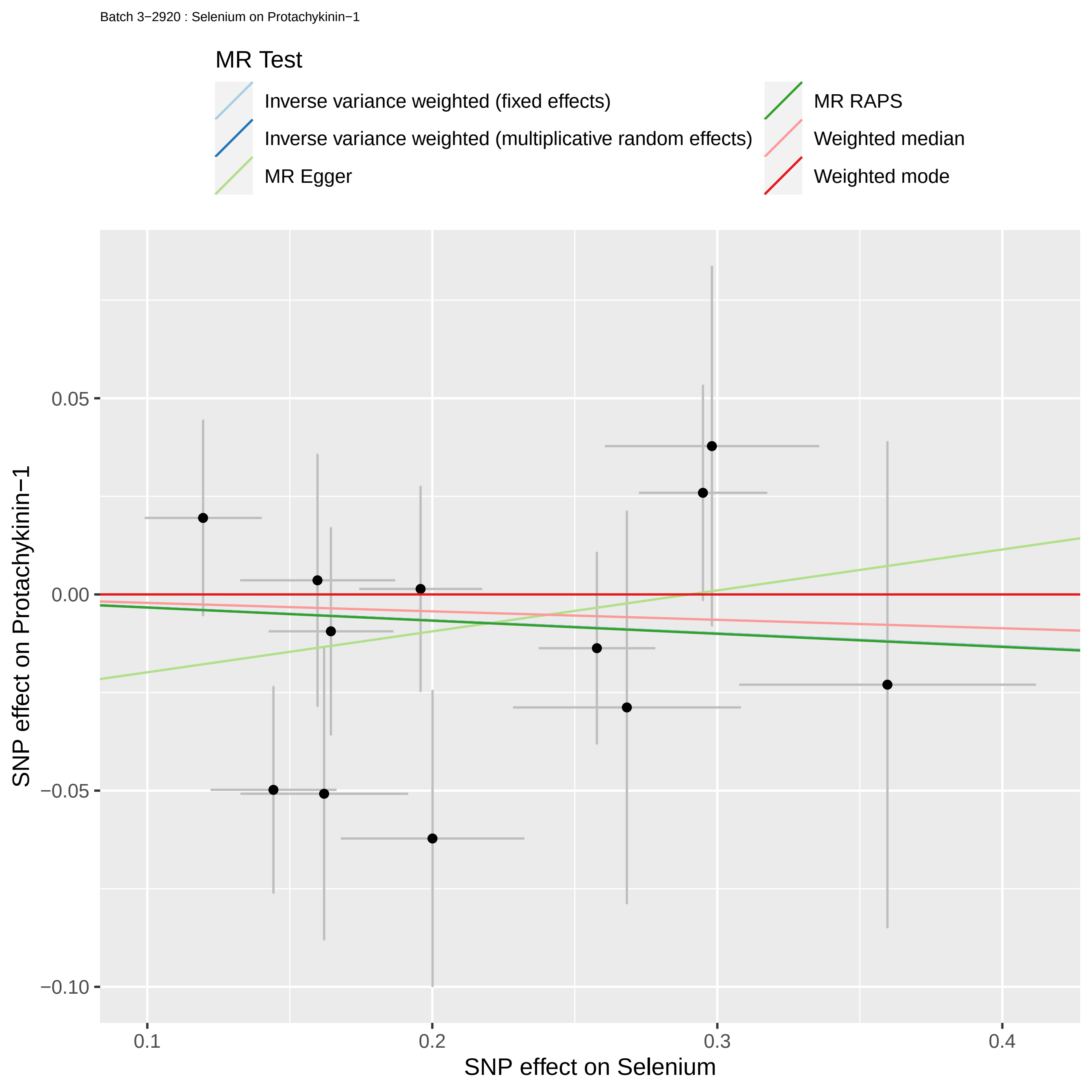

Batch 3−2920 : Selenium on Protachykinin−1
MR Test
Inverse variance weighted (fixed effects)
Inverse variance weighted (multiplicative random effects)
MR Egger
MR RAPS
Weighted median
Weighted mode
0.05
0.00
−0.05
−0.10
0.1
0.2
0.3
0.4
SNP effect on Protachykinin−1
SNP effect on Selenium

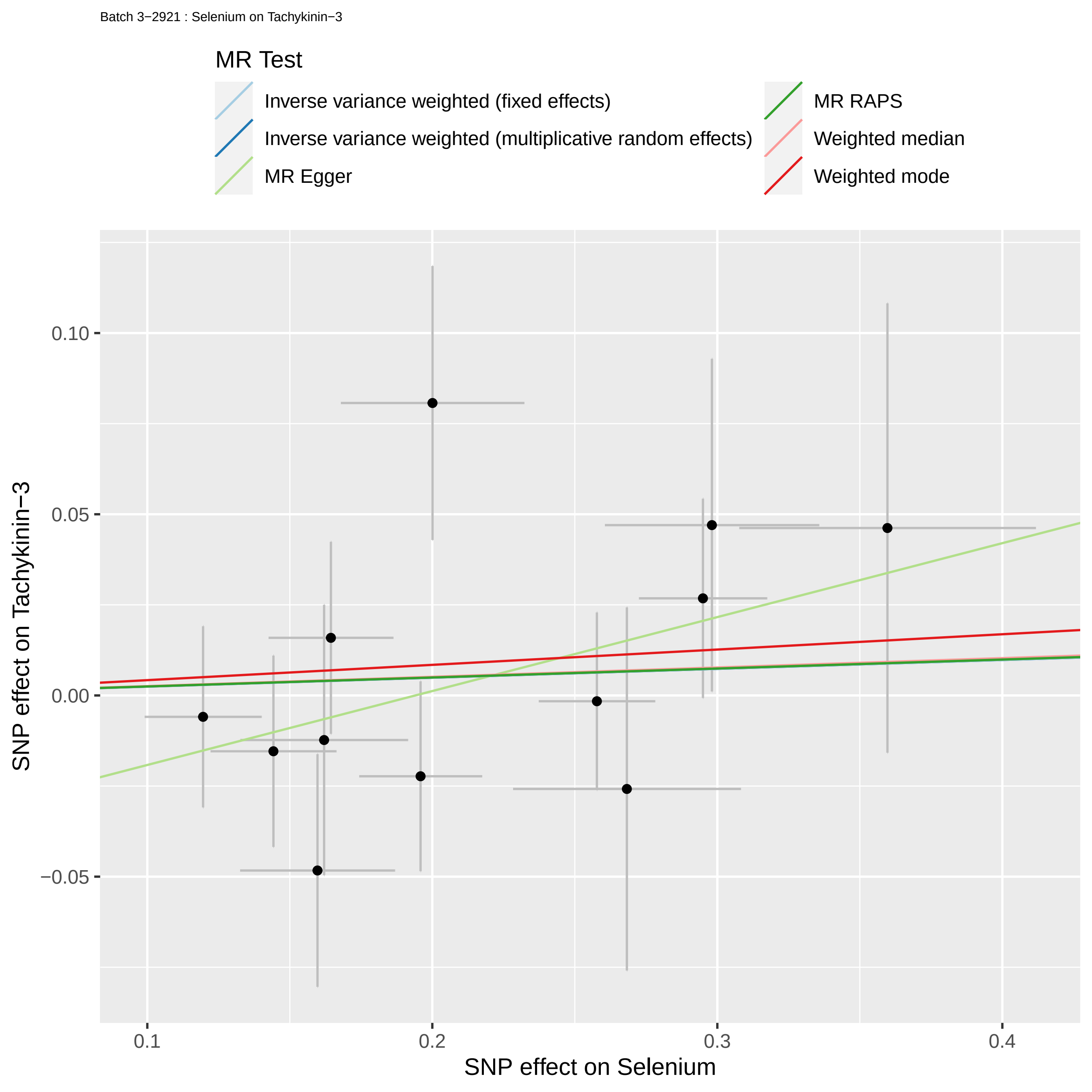

Batch 3−2921 : Selenium on Tachykinin−3
MR Test
Inverse variance weighted (fixed effects)
Inverse variance weighted (multiplicative random effects)
MR Egger
MR RAPS
Weighted median
Weighted mode
0.10
0.05
0.00
−0.05
0.1
0.2
0.3
0.4
SNP effect on Tachykinin−3
SNP effect on Selenium

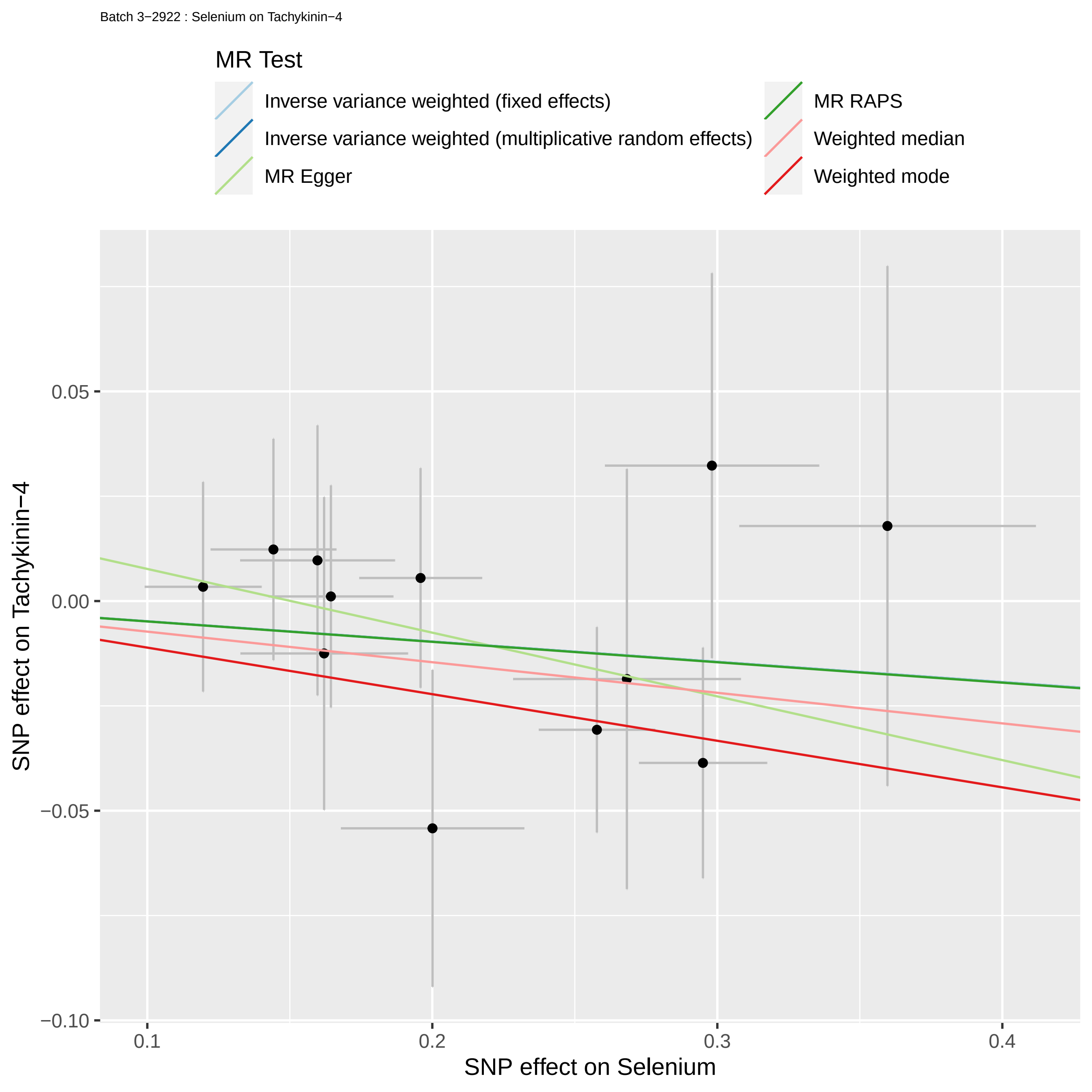
Batch 3−2922 : Selenium on Tachykinin−4
MR Test
Inverse variance weighted (fixed effects)
Inverse variance weighted (multiplicative random effects)
MR Egger
MR RAPS
Weighted median
Weighted mode
0.05
0.00
−0.05
−0.10
0.1
0.2
0.3
0.4
SNP effect on Tachykinin−4
SNP effect on Selenium

Batch 3−2923 : Selenium on Tumor−associated calcium signal transducer 2

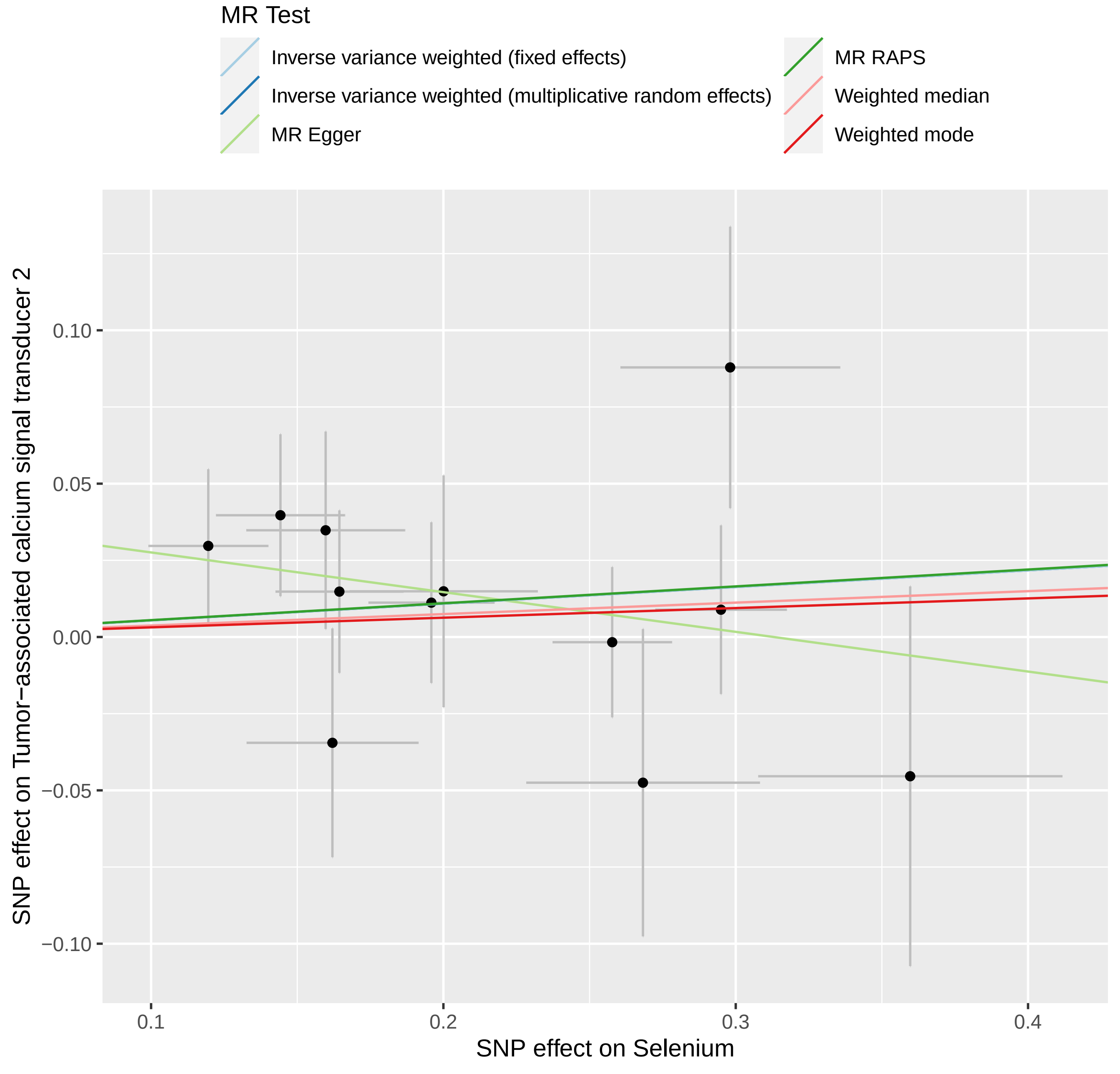

Batch 3−2924 : Selenium on Tumor−associated calcium signal transducer 2

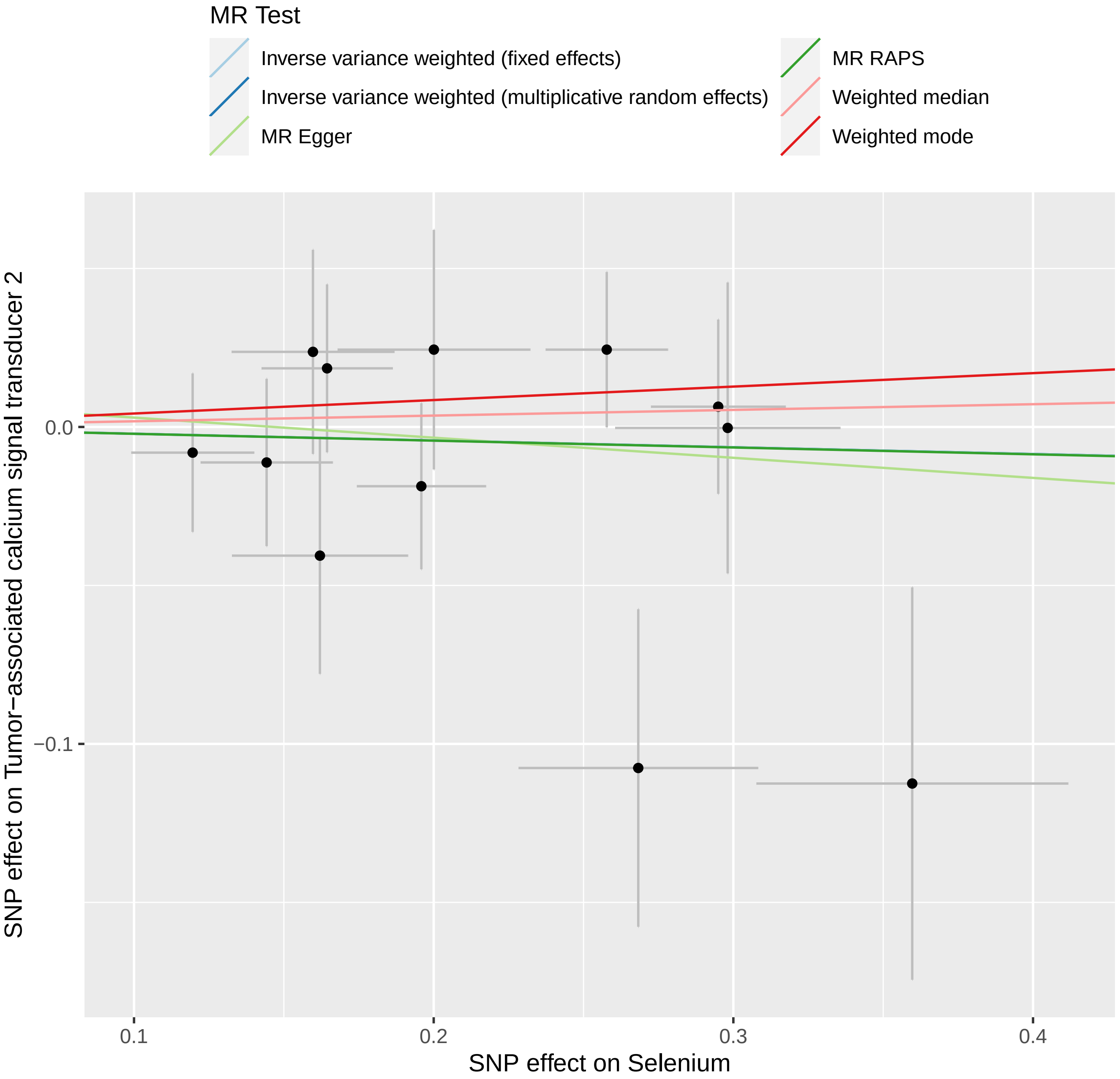

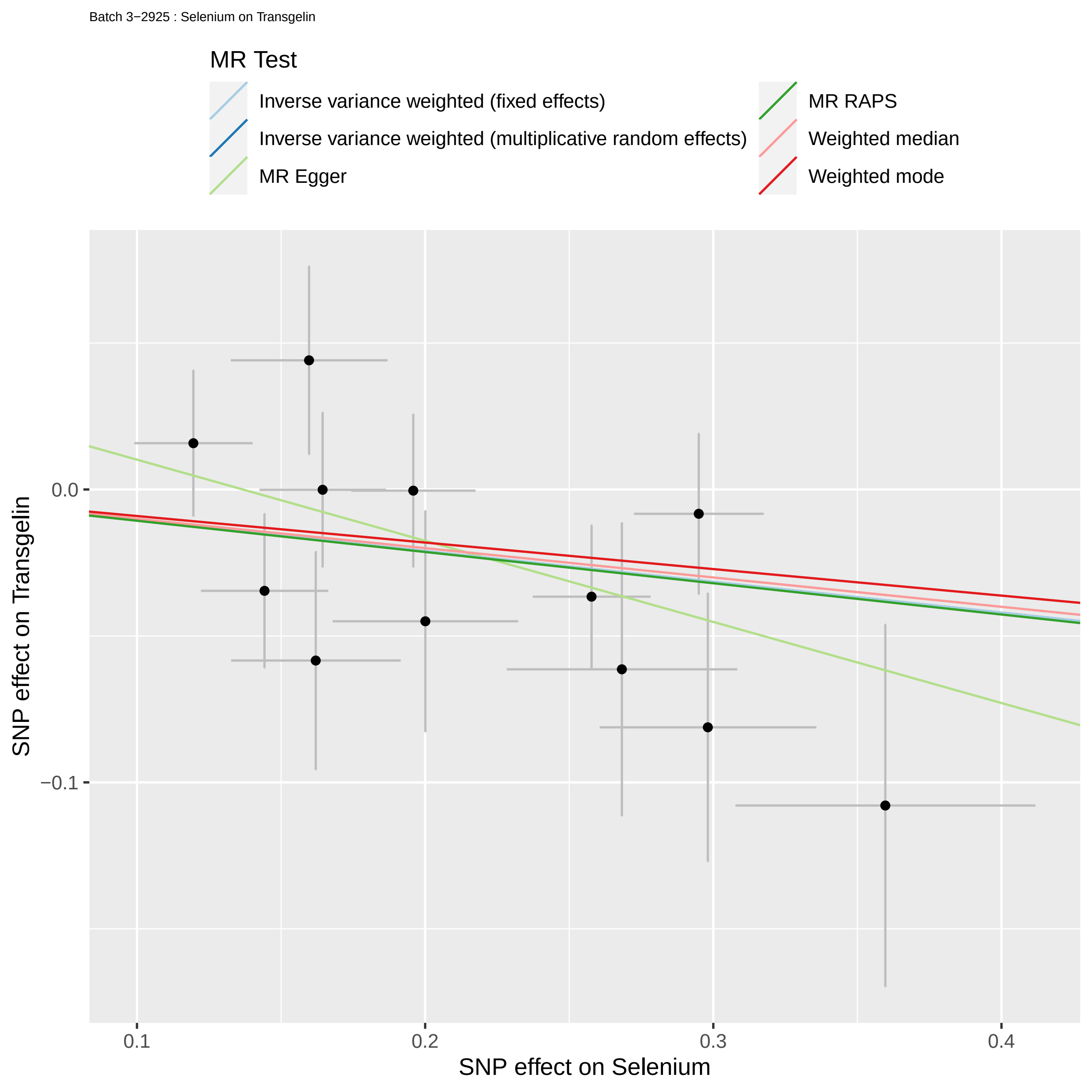
Batch 3−2925 : Selenium on Transgelin
MR Test
Inverse variance weighted (fixed effects)
Inverse variance weighted (multiplicative random effects)
MR Egger
MR RAPS
Weighted median
Weighted mode
0.0
−0.1
0.1
0.2
0.3
0.4
SNP effect on Transgelin
SNP effect on Selenium

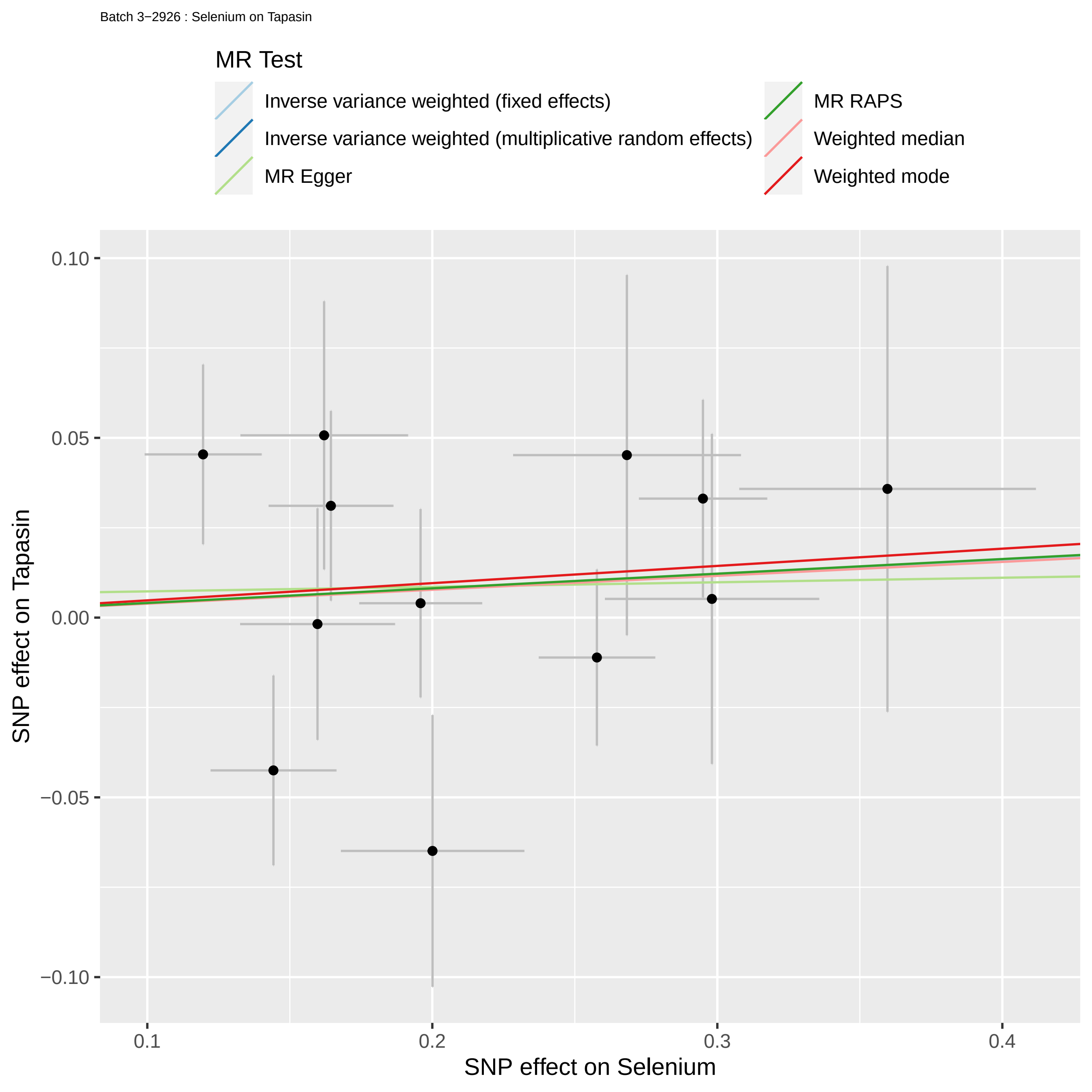
Batch 3−2926 : Selenium on Tapasin
MR Test
Inverse variance weighted (fixed effects)
Inverse variance weighted (multiplicative random effects)
MR Egger
MR RAPS
Weighted median
Weighted mode
0.10
0.05
0.00
−0.05
−0.10
SNP effect on Tapasin
0.1
0.2
0.3
0.4
SNP effect on Selenium

Batch 3−2927 : Selenium on Tapasin−related protein

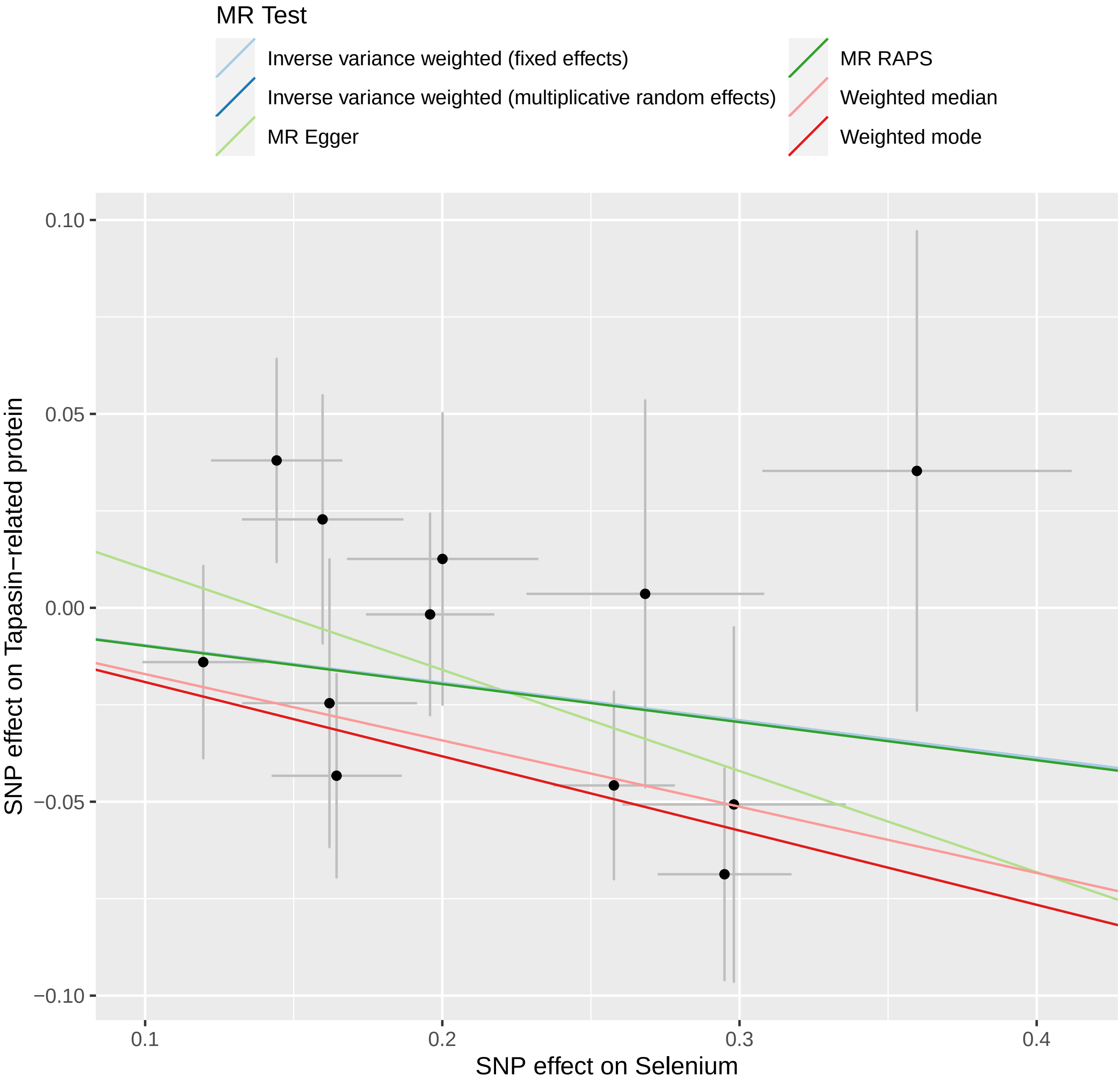

Batch 3−2928 : Selenium on Tax1−binding protein 1

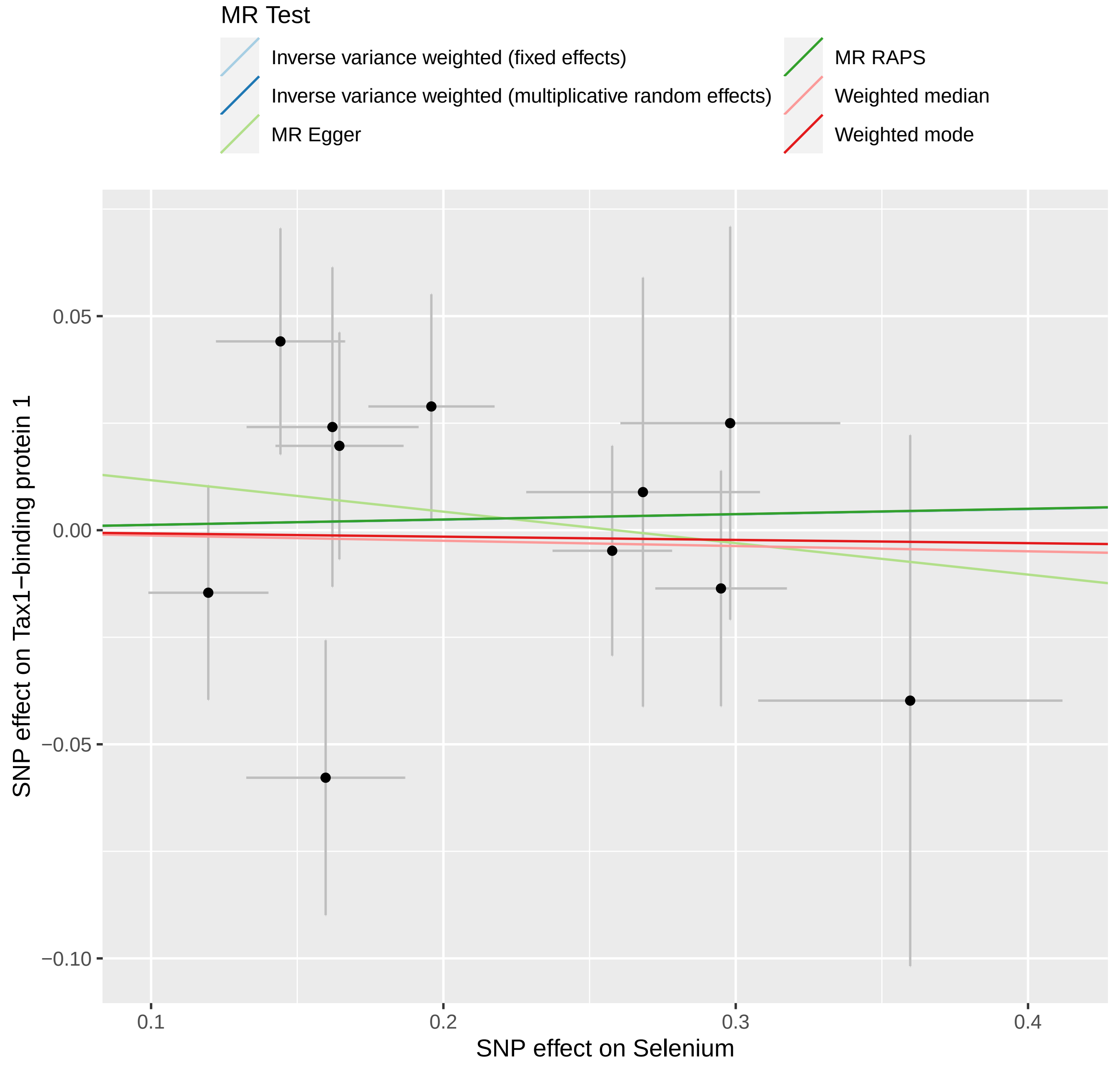

Batch 3−2929 : Selenium on Tax1−binding protein 3

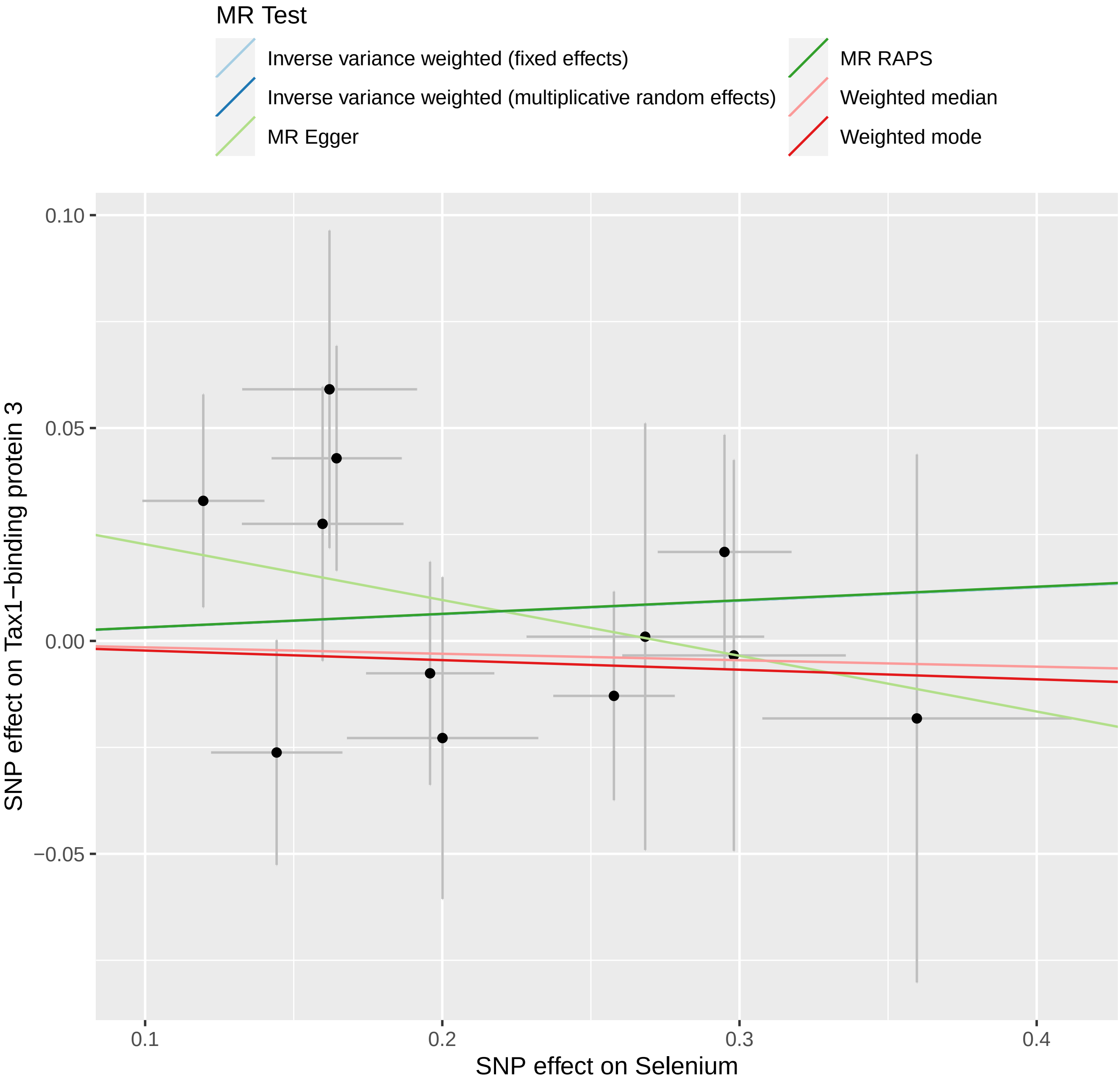

Batch 3−2930 : Selenium on Tubulin−specific chaperone A

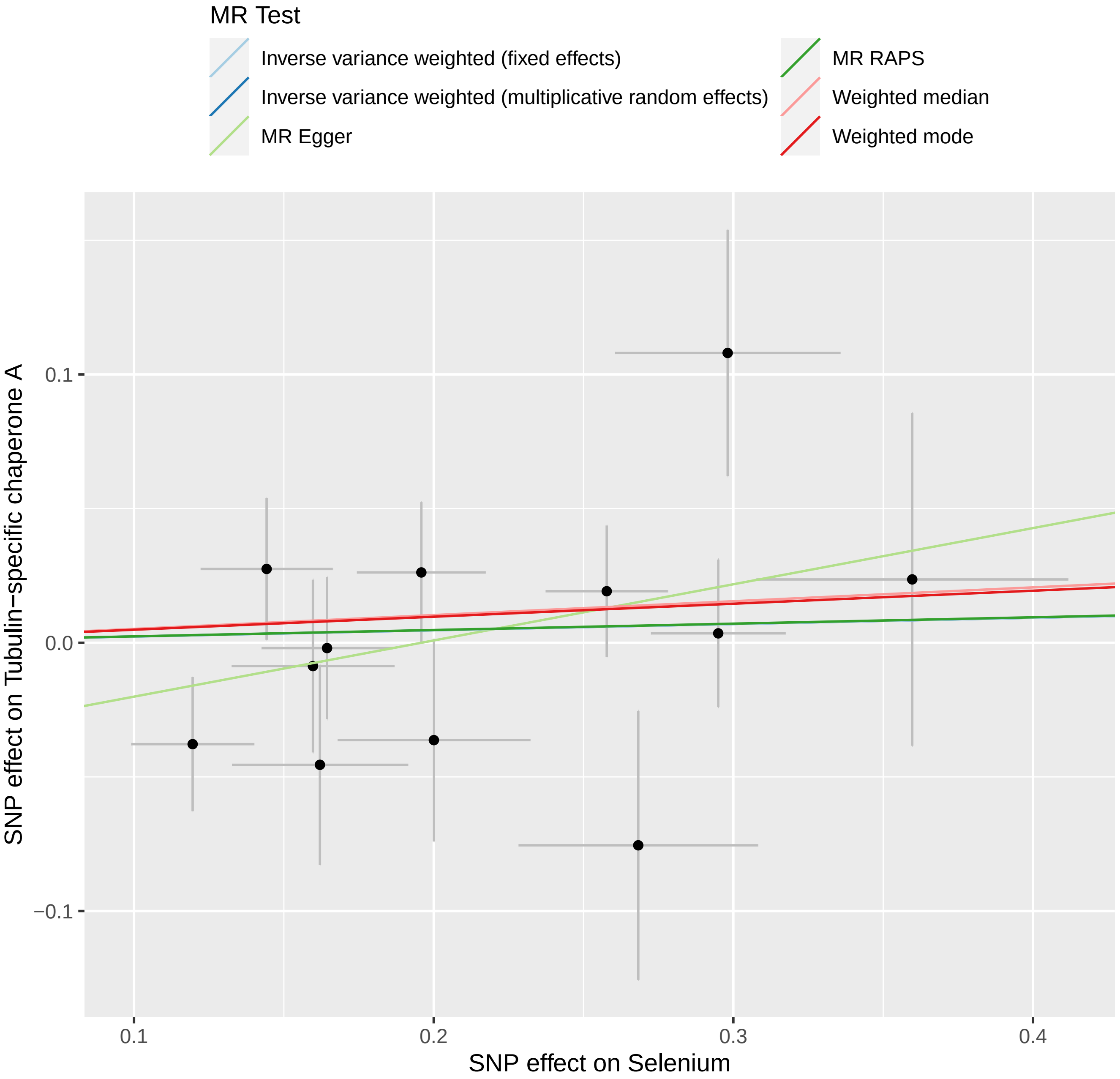

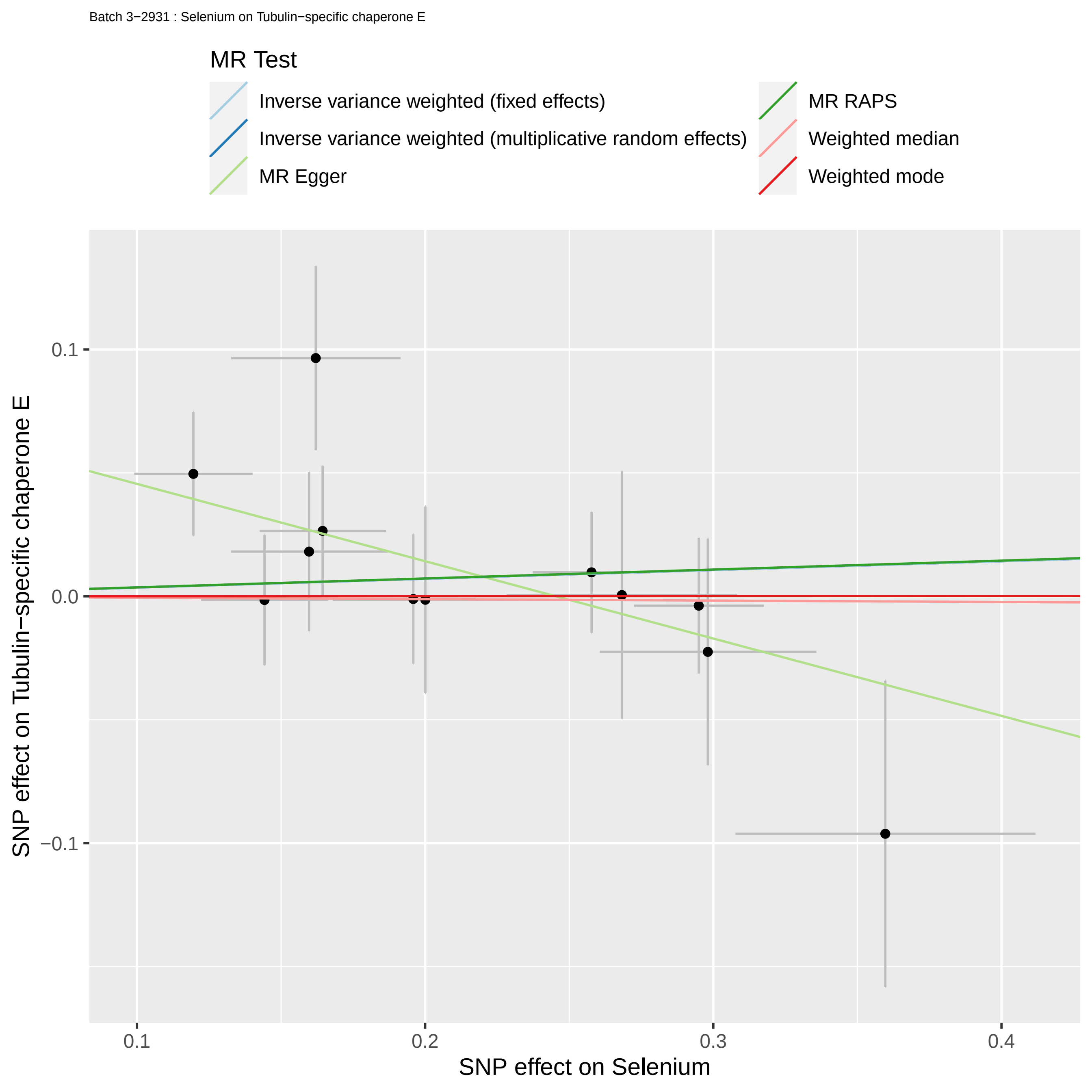

Batch 3−2931 : Selenium on Tubulin−specific chaperone E
MR Test
Inverse variance weighted (fixed effects)
Inverse variance weighted (multiplicative random effects)
MR Egger
MR RAPS
Weighted median
Weighted mode
0.1
0.0
−0.1
0.1
0.2
0.3
0.4
SNP effect on Tubulin−specific chaperone E
SNP effect on Selenium

Batch 3−2932 : Selenium on Serine/threonine−protein kinase TBK1

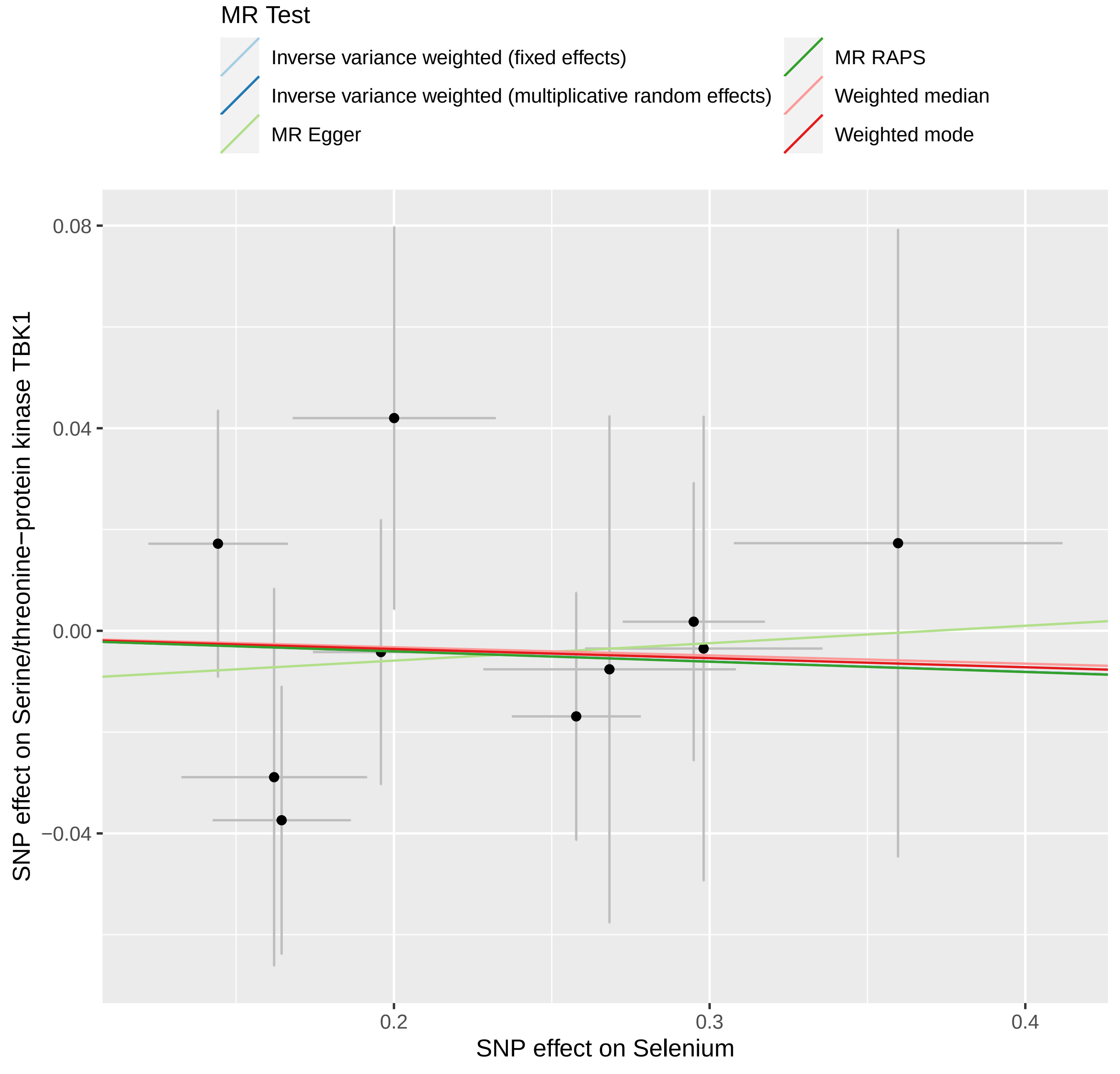

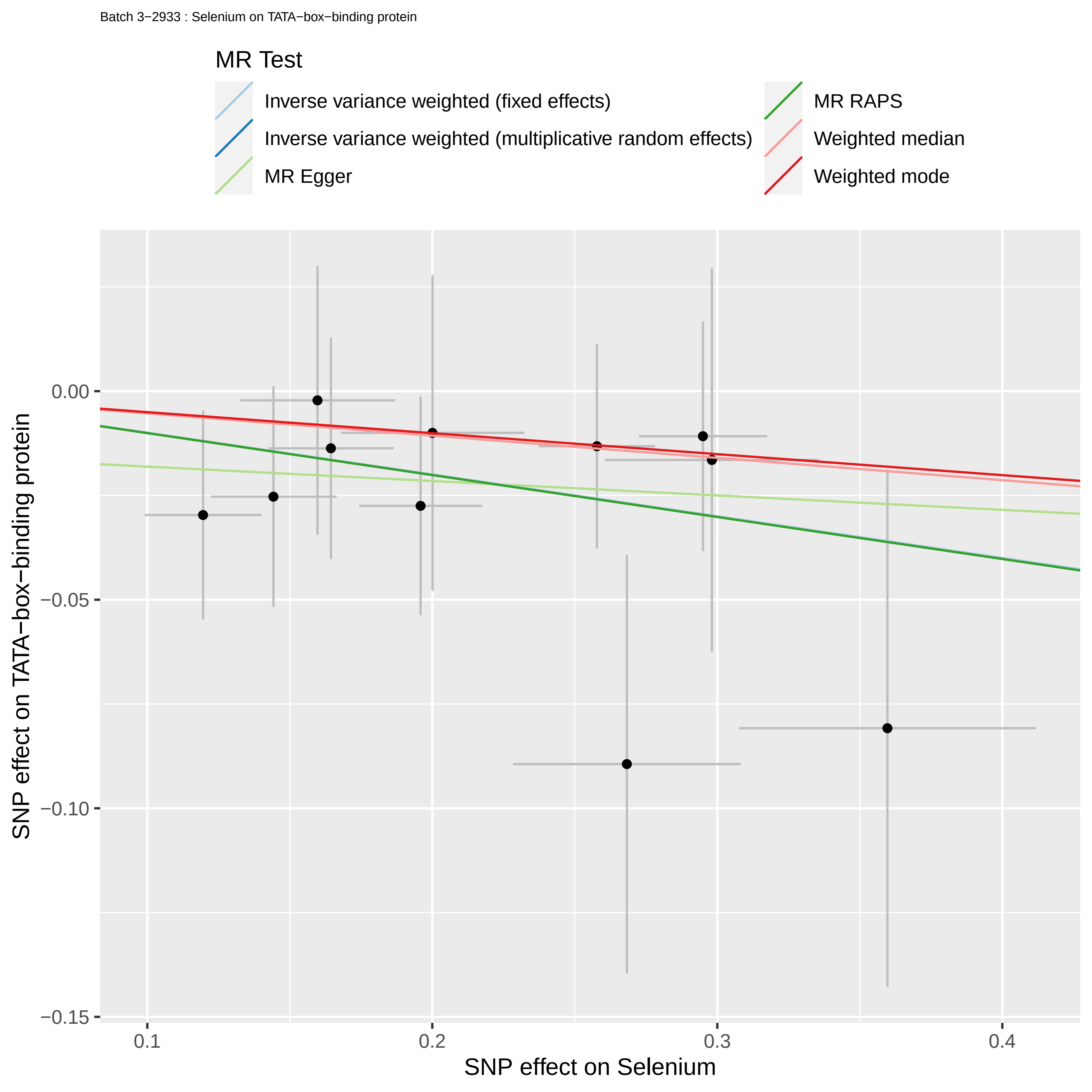
Batch 3−2933 : Selenium on TATA−box−binding protein
MR Test
Inverse variance weighted (fixed effects)
Inverse variance weighted (multiplicative random effects)
MR Egger
MR RAPS
Weighted median
Weighted mode
SNP effect on TATA−box−binding protein
0.00
−0.05
−0.10
−0.15
0.1
0.2
0.3
0.4
SNP effect on Selenium

Batch 3−2934 : Selenium on T−box transcription factor TBX22

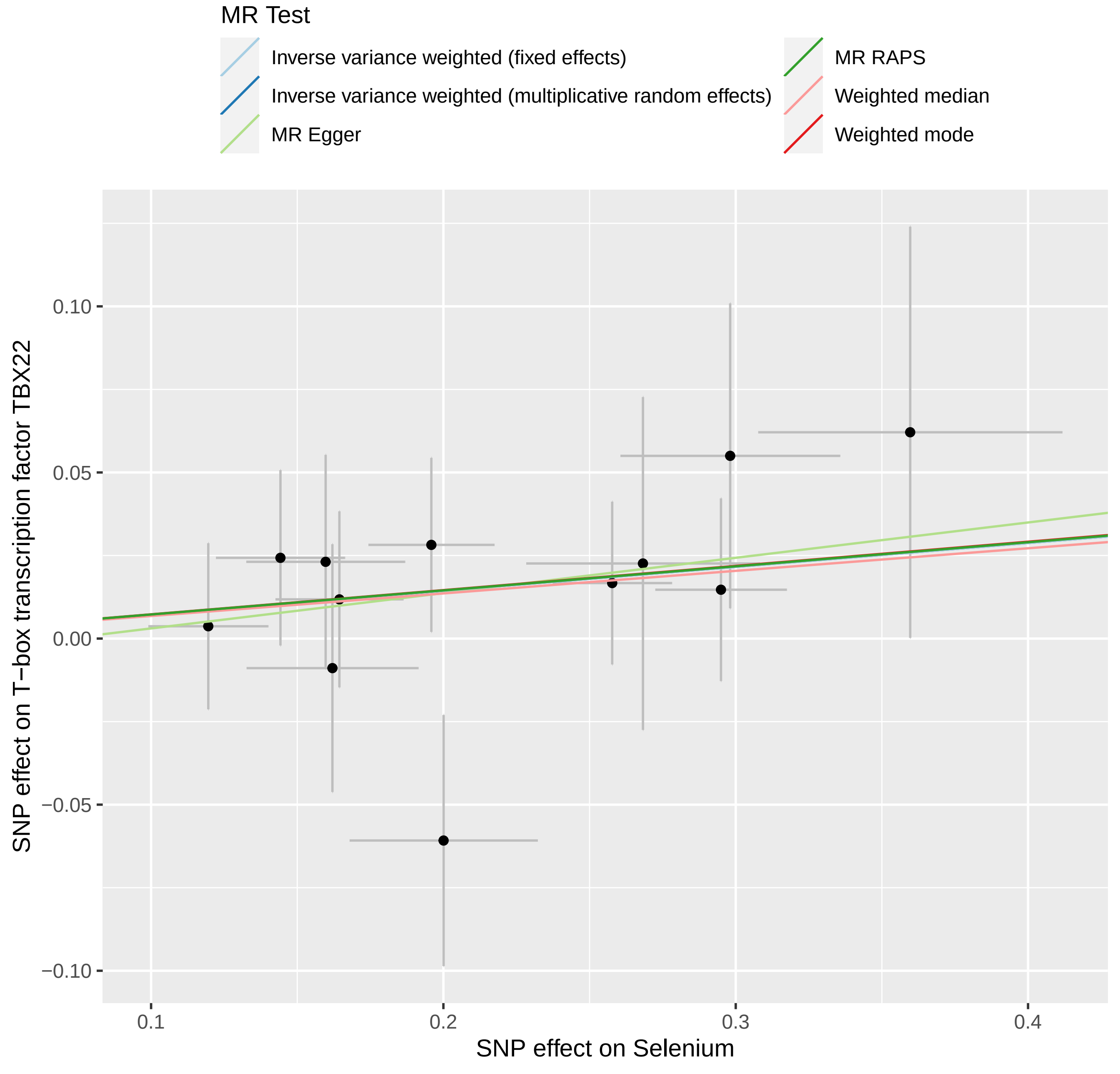

Batch 3−2935 : Selenium on T−box transcription factor TBX3

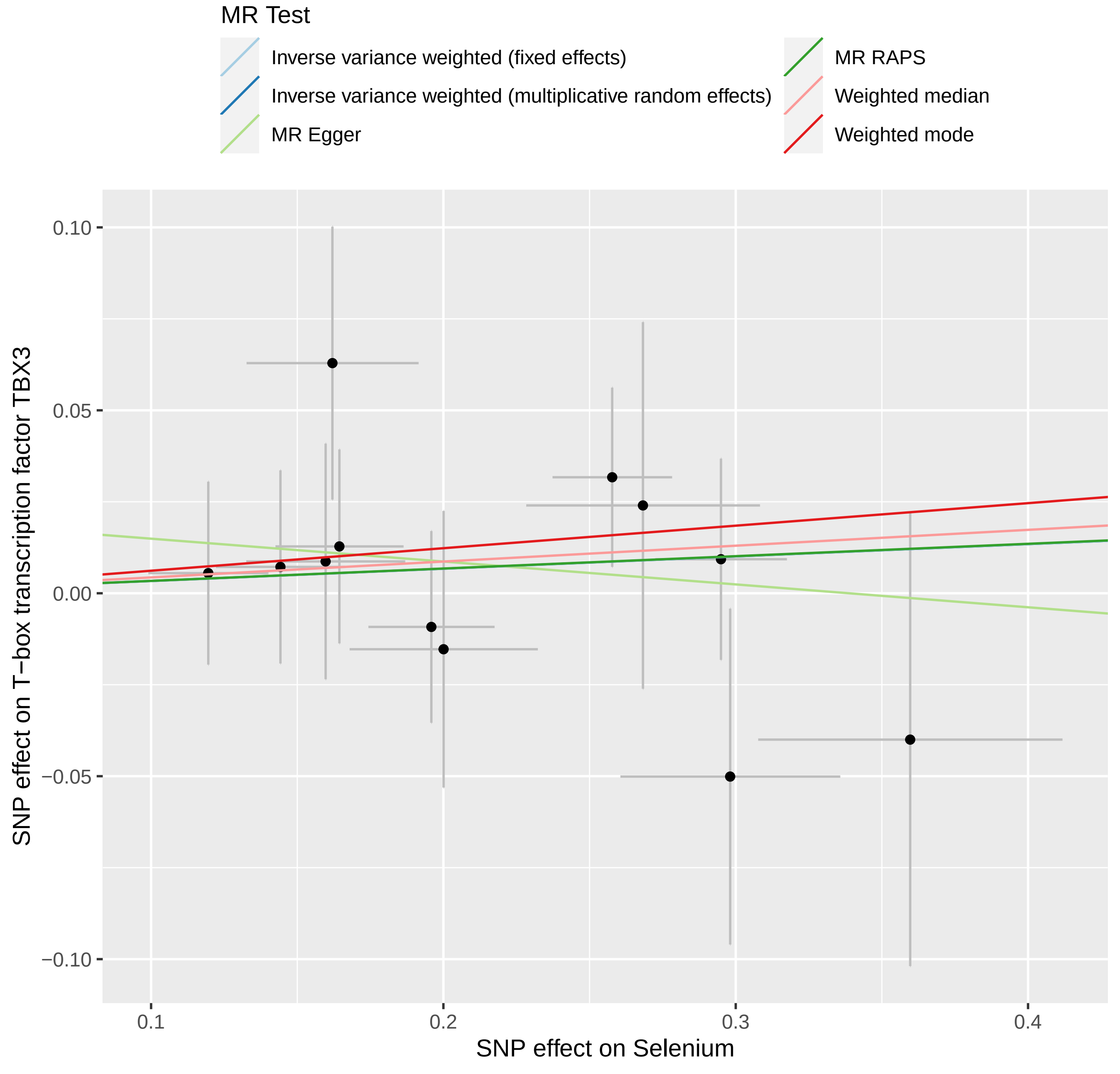

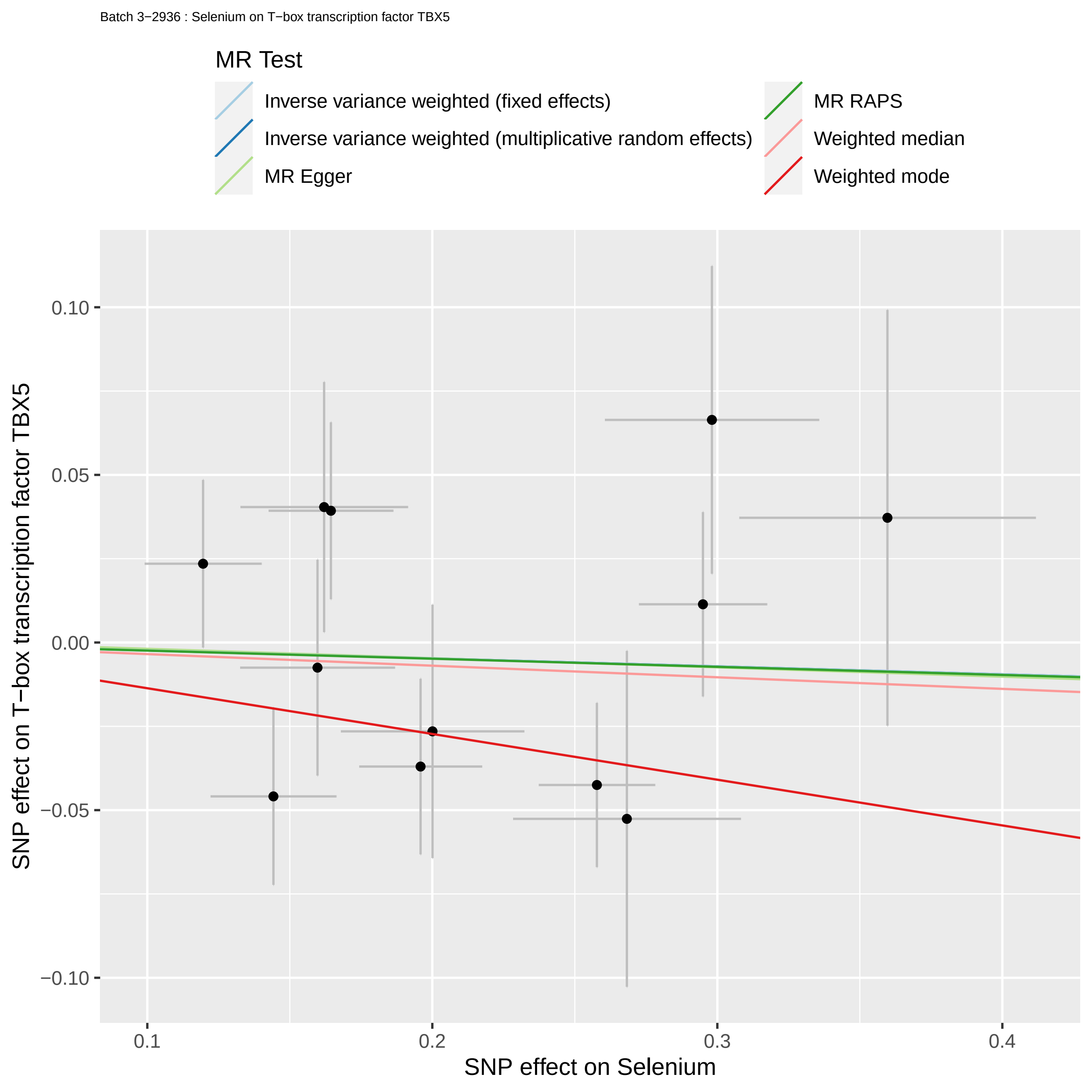
Batch 3−2936 : Selenium on T−box transcription factor TBX5
MR Test
Inverse variance weighted (fixed effects)
Inverse variance weighted (multiplicative random effects)
MR Egger
MR RAPS
Weighted median
Weighted mode
0.10
0.05
0.00
−0.05
−0.10
0.1
0.2
0.3
0.4
SNP effect on Selenium
SNP effect on T−box transcription factor TBX5

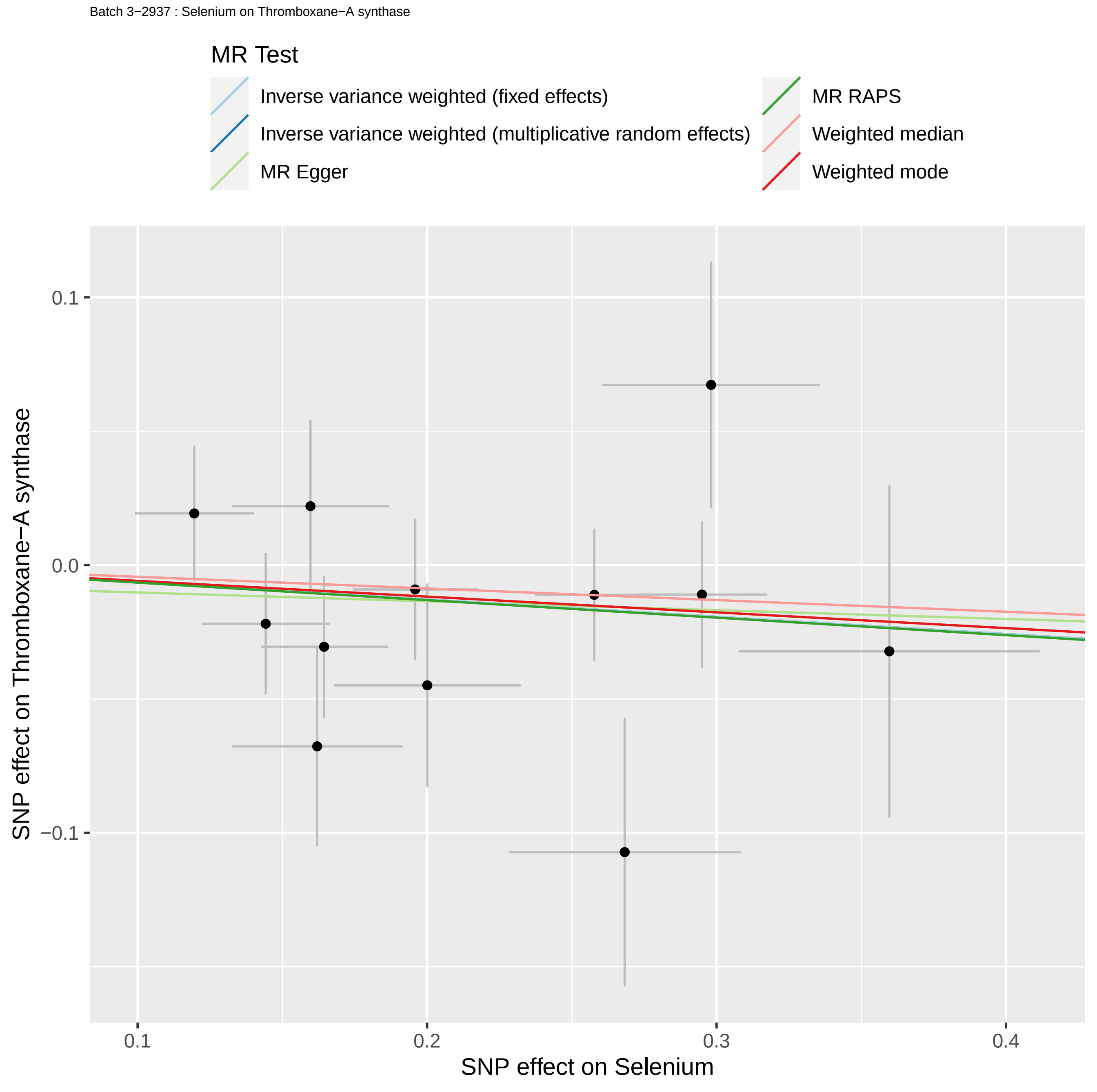

Batch 3−2937 : Selenium on Thromboxane−A synthase
MR Test
Inverse variance weighted (fixed effects)
Inverse variance weighted (multiplicative random effects)
MR Egger
MR RAPS
Weighted median
Weighted mode
0.1
0.0
−0.1
0.1
0.2
0.3
0.4
SNP effect on Thromboxane−A synthase
SNP effect on Selenium

Batch 3−2938 : Selenium on Transcobalamin−1

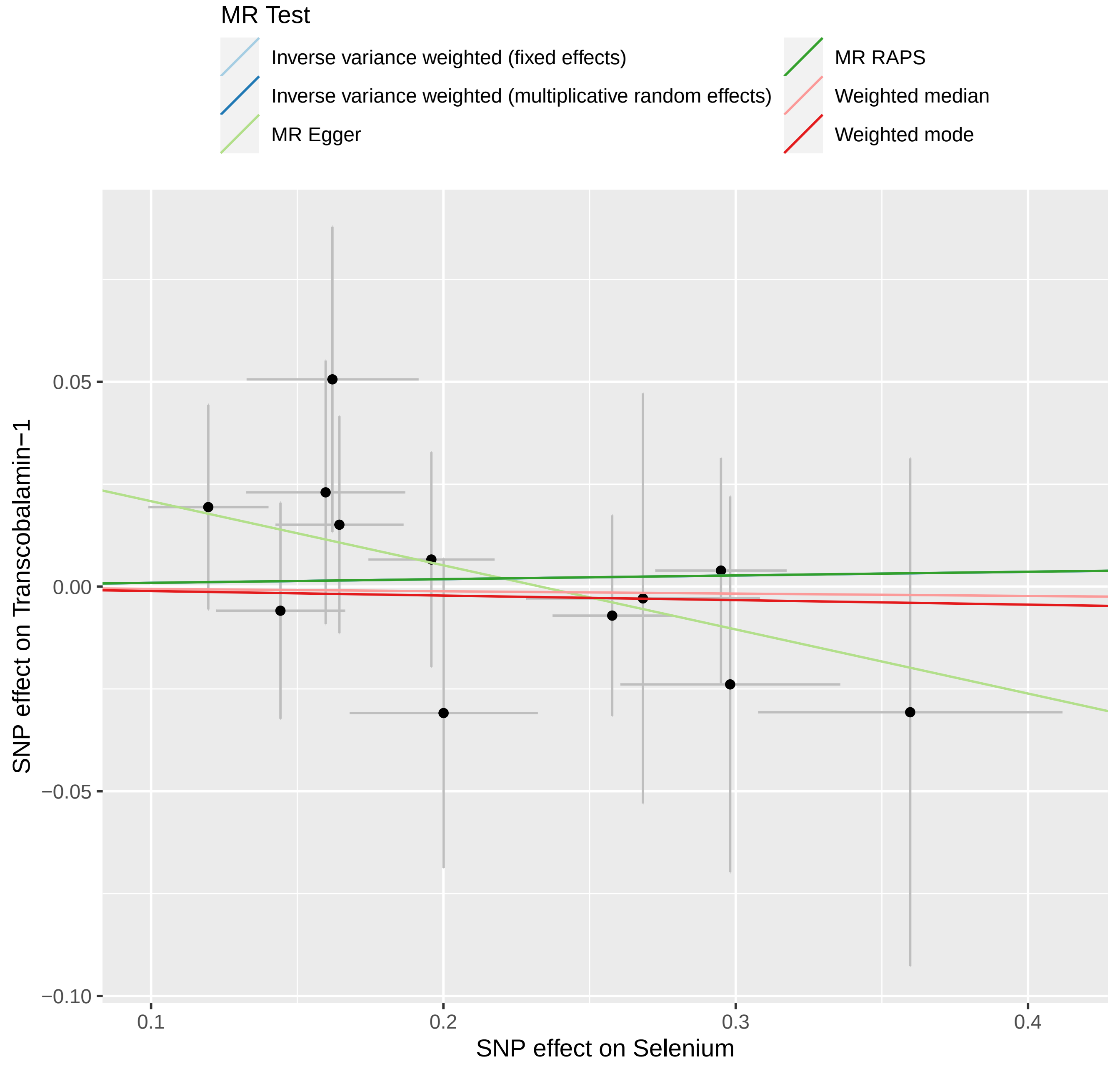

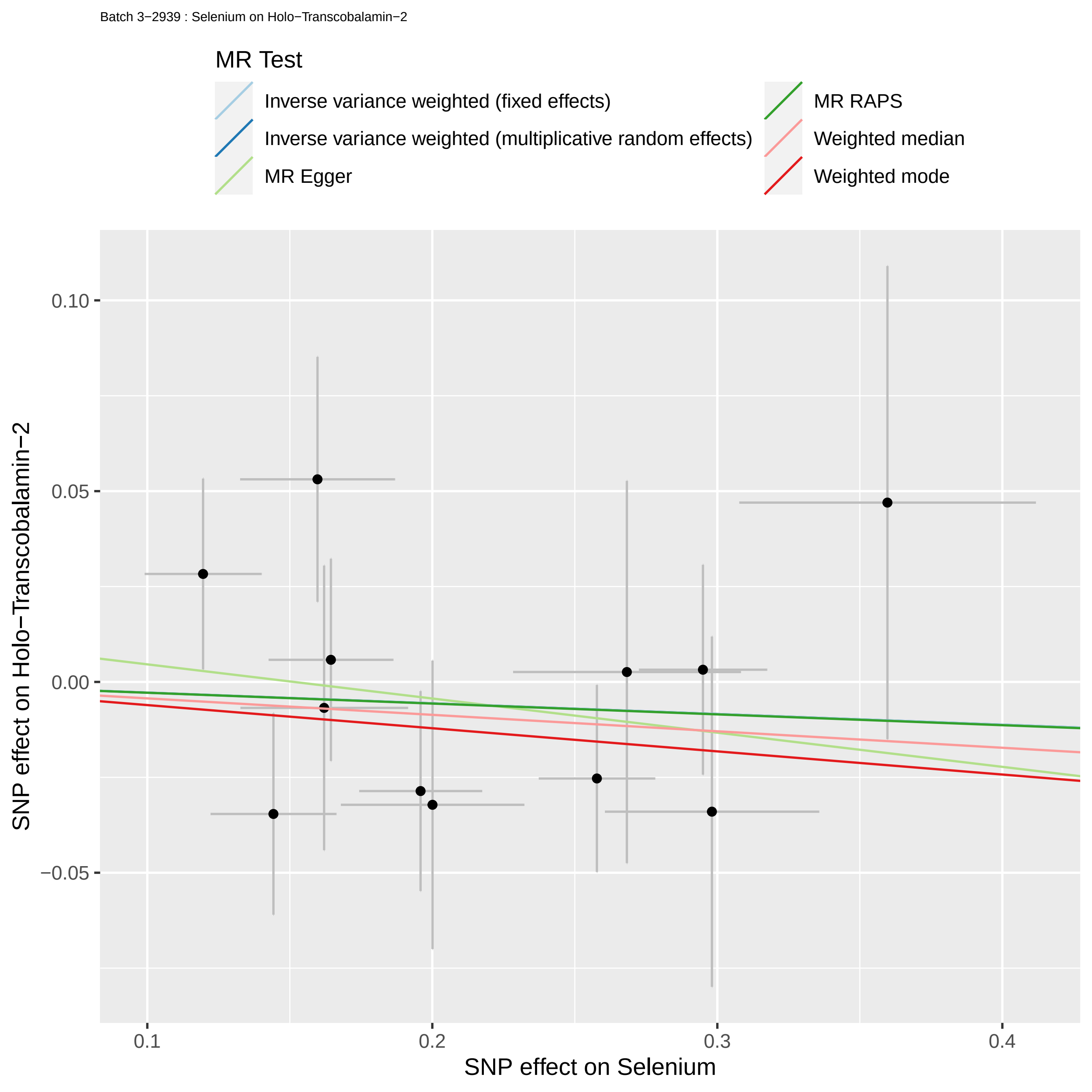
Batch 3−2939 : Selenium on Holo−Transcobalamin−2
MR Test
Inverse variance weighted (fixed effects)
Inverse variance weighted (multiplicative random effects)
MR Egger
MR RAPS
Weighted median
Weighted mode
0.10
0.05
0.00
−0.05
0.1
0.2
0.3
0.4
SNP effect on Holo−Transcobalamin−2
SNP effect on Selenium

Batch 3−2940 : Selenium on Teratocarcinoma−derived growth factor 1

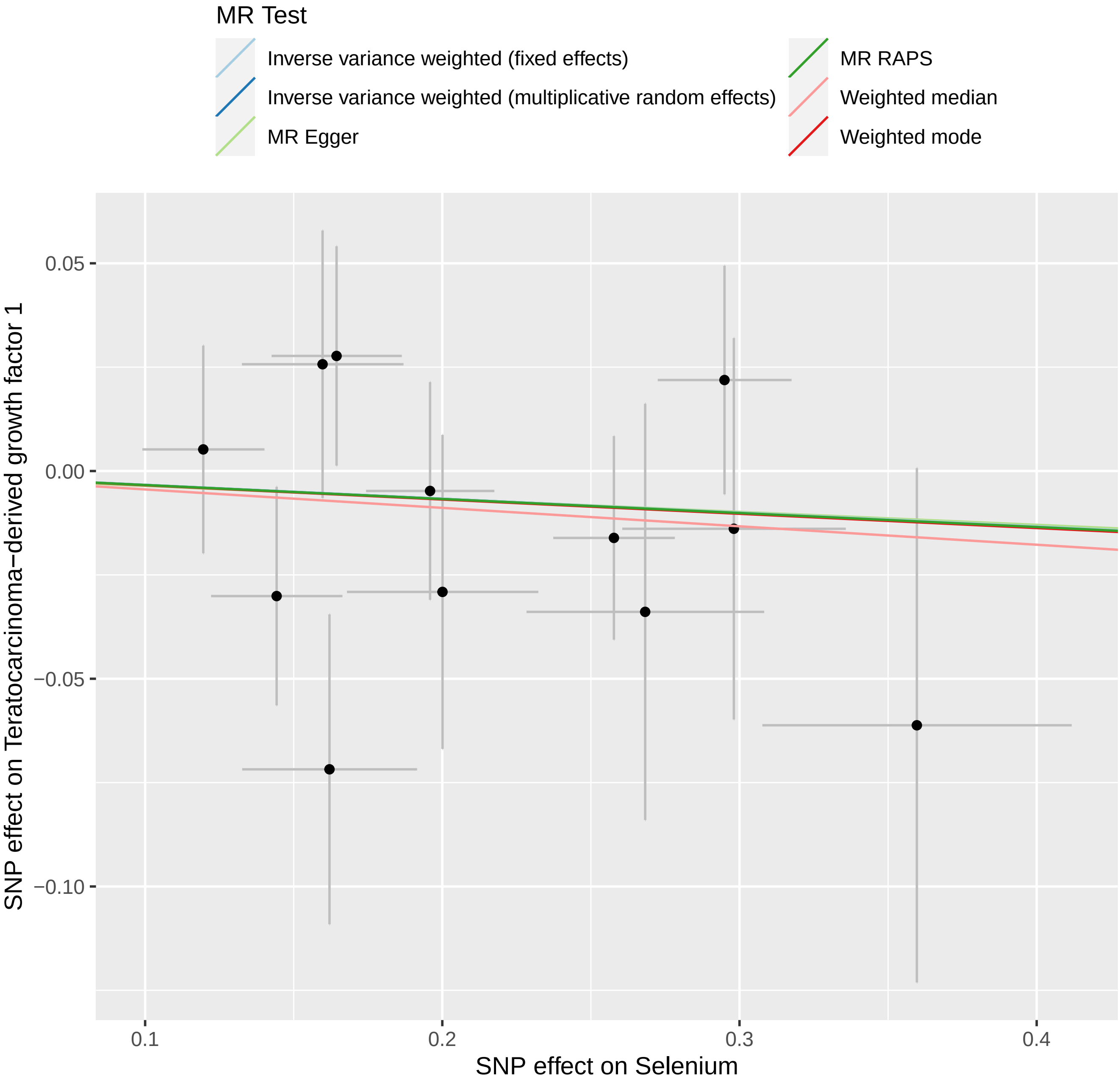

Batch 3−2941 : Selenium on Tyrosyl−DNA phosphodiesterase 1

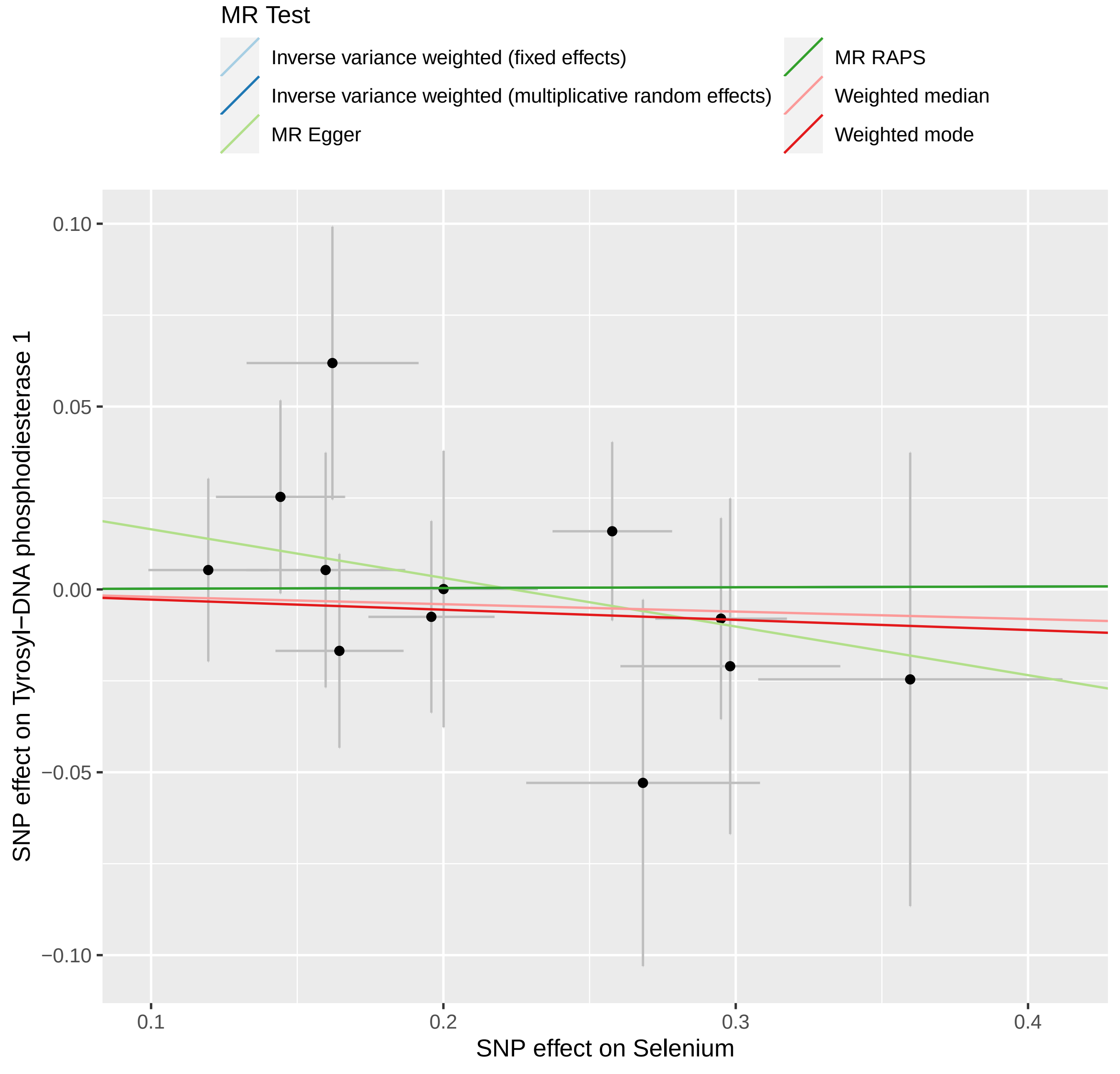

Batch 3−2942 : Selenium on Transcriptional enhancer factor TEF−5

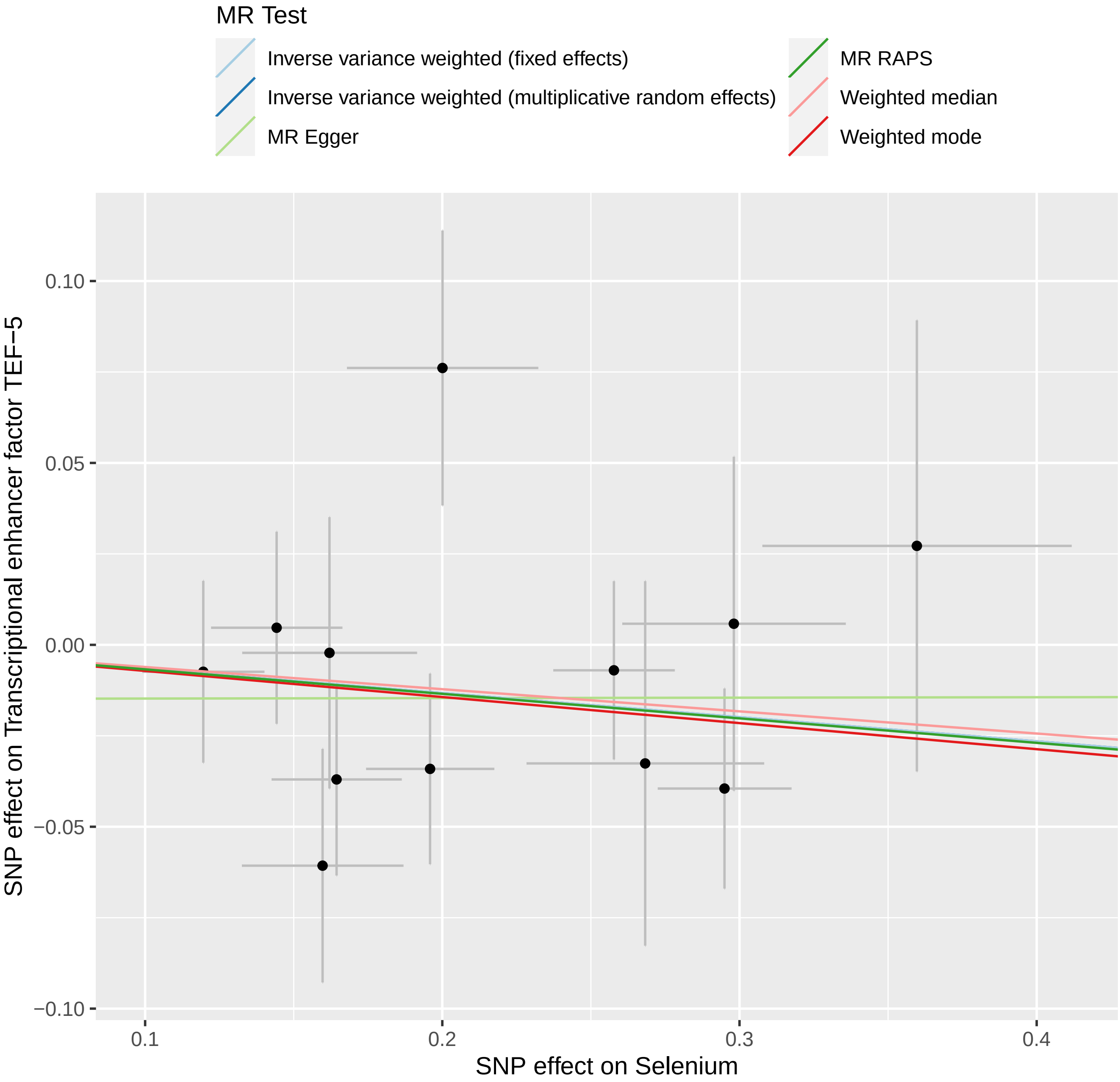

Batch 3−2943 : Selenium on Transcriptional enhancer factor TEF−3

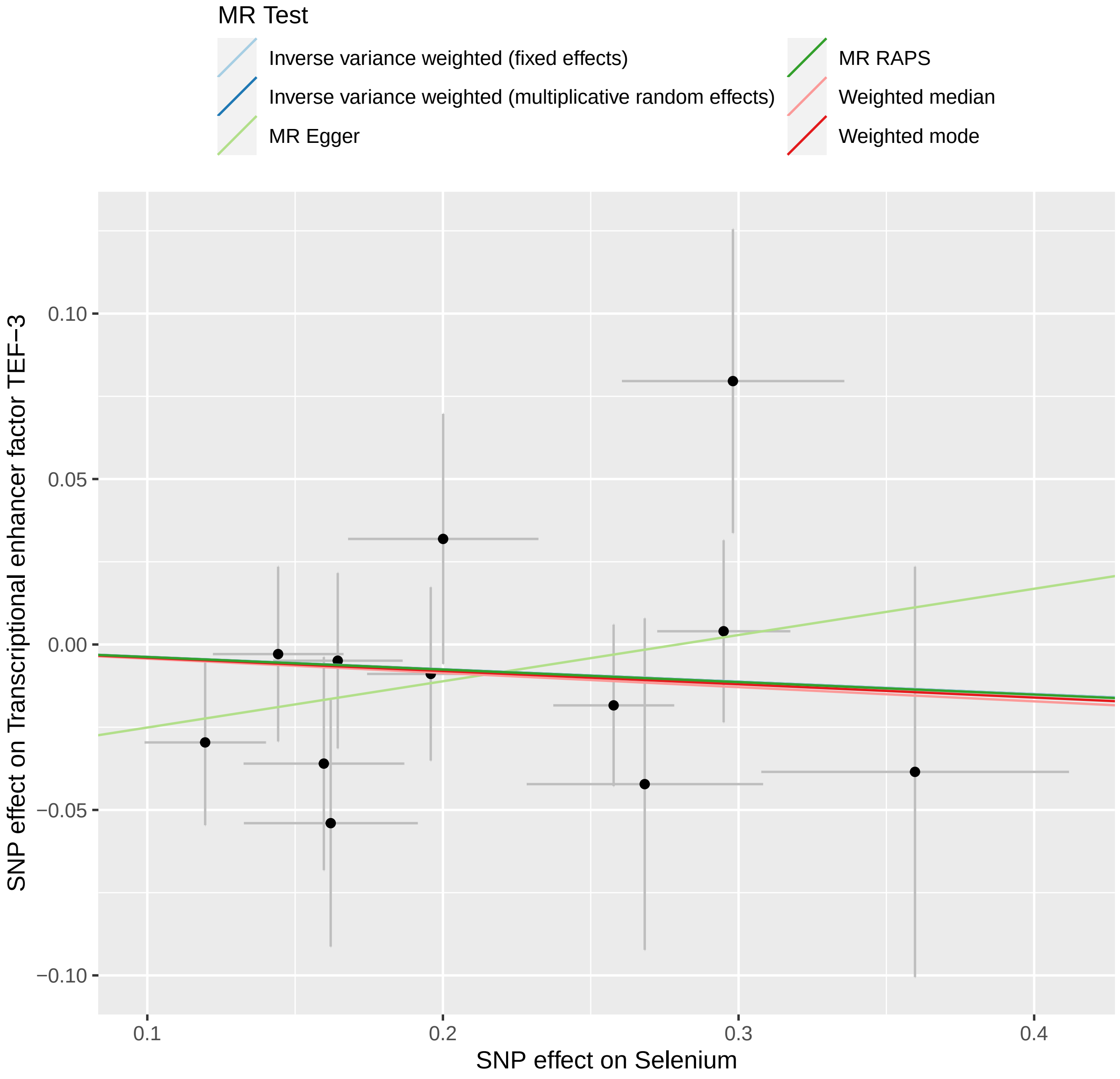

Batch 3−2944 : Selenium on Angiopoietin−1 receptor, soluble

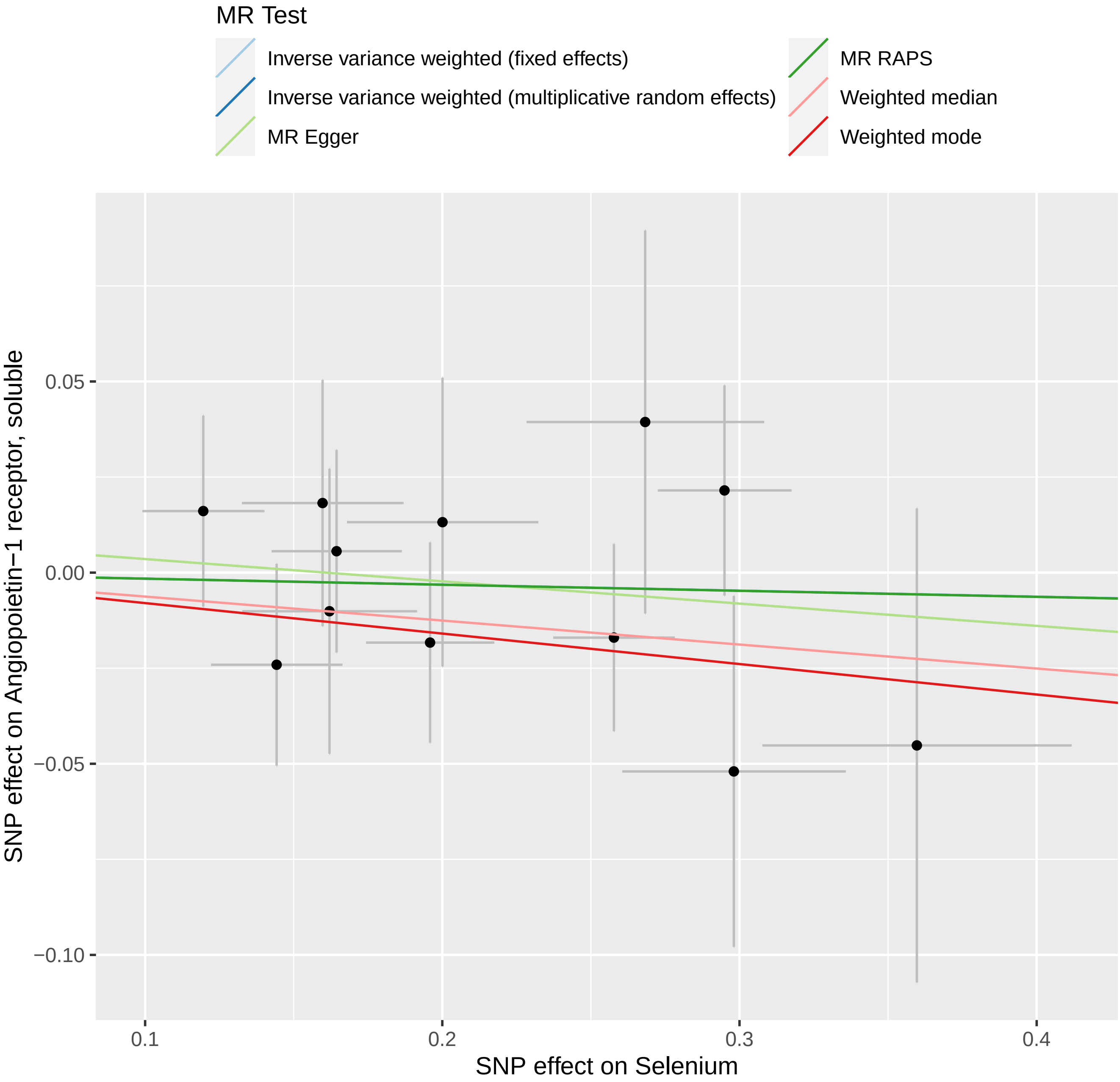

Batch 3−2945 : Selenium on Teneurin−3

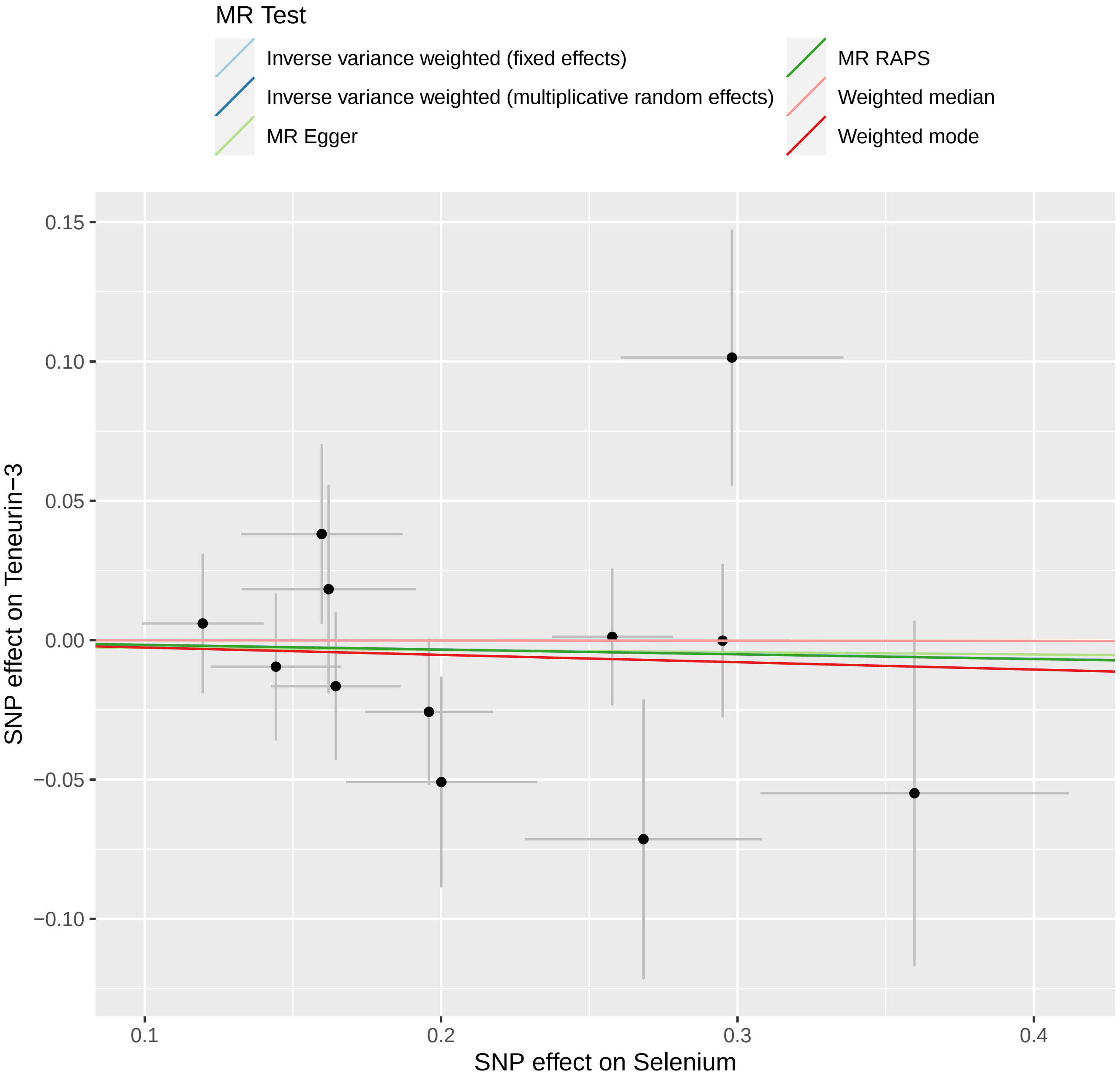

Batch 3−2946 : Selenium on Teneurin−4

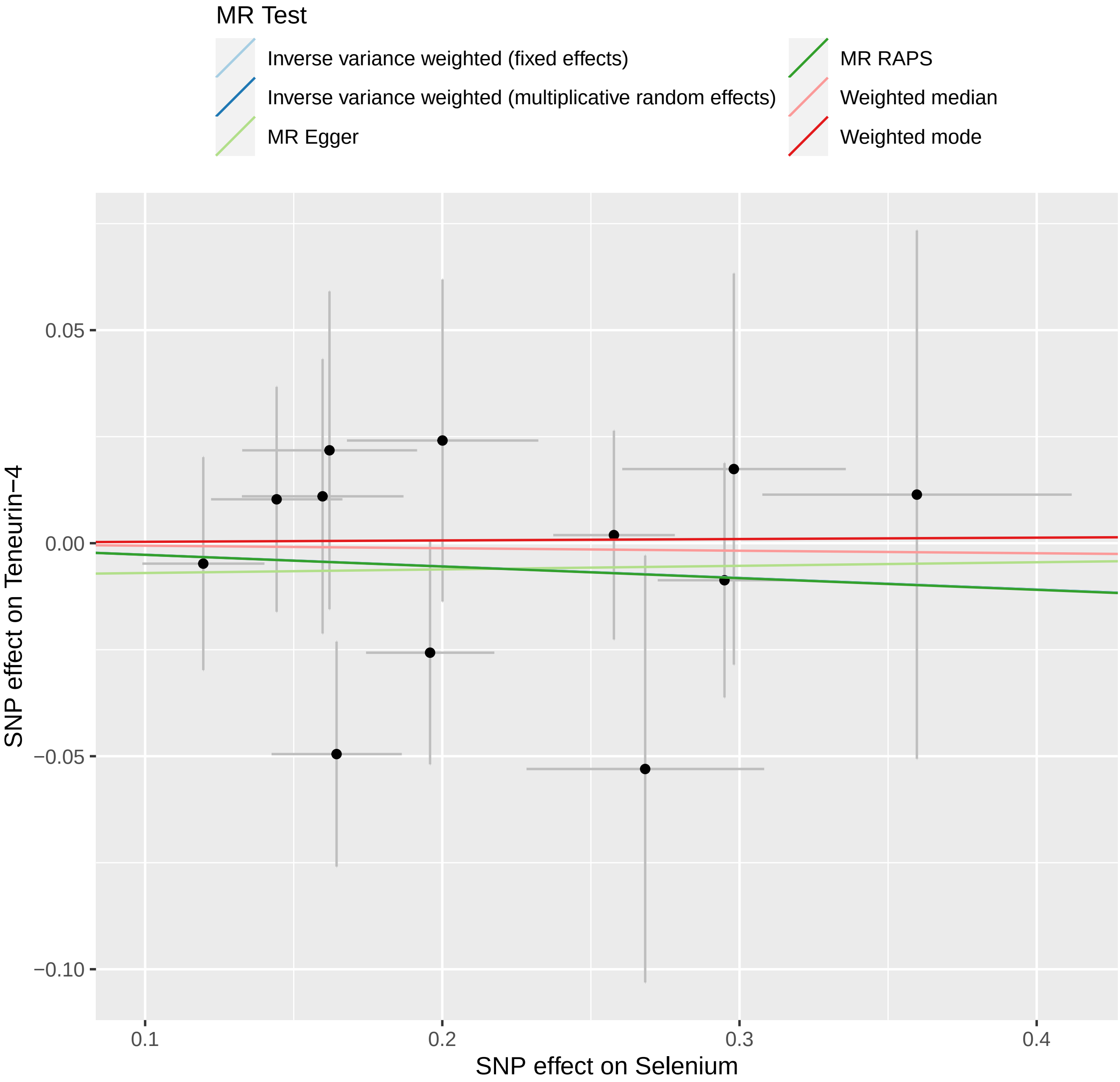

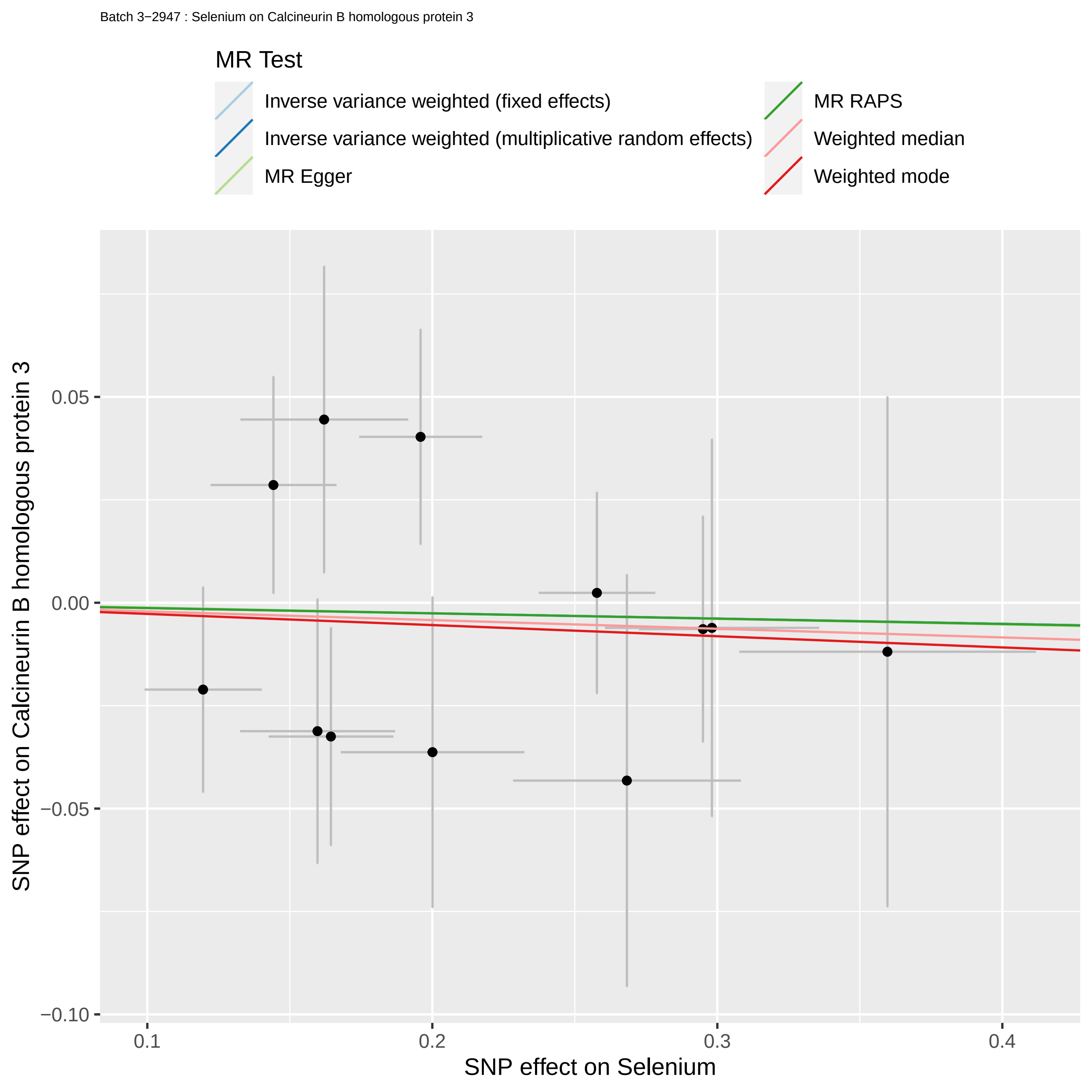

Batch 3−2947 : Selenium on Calcineurin B homologous protein 3
MR Test
Inverse variance weighted (fixed effects)
Inverse variance weighted (multiplicative random effects)
MR Egger
MR RAPS
Weighted median
Weighted mode
SNP effect on Calcineurin B homologous protein 3
0.05
0.00
−0.05
−0.10
0.1
0.2
0.3
0.4
SNP effect on Selenium

Batch 3−2948 : Selenium on Testis−expressed sequence 29 protein

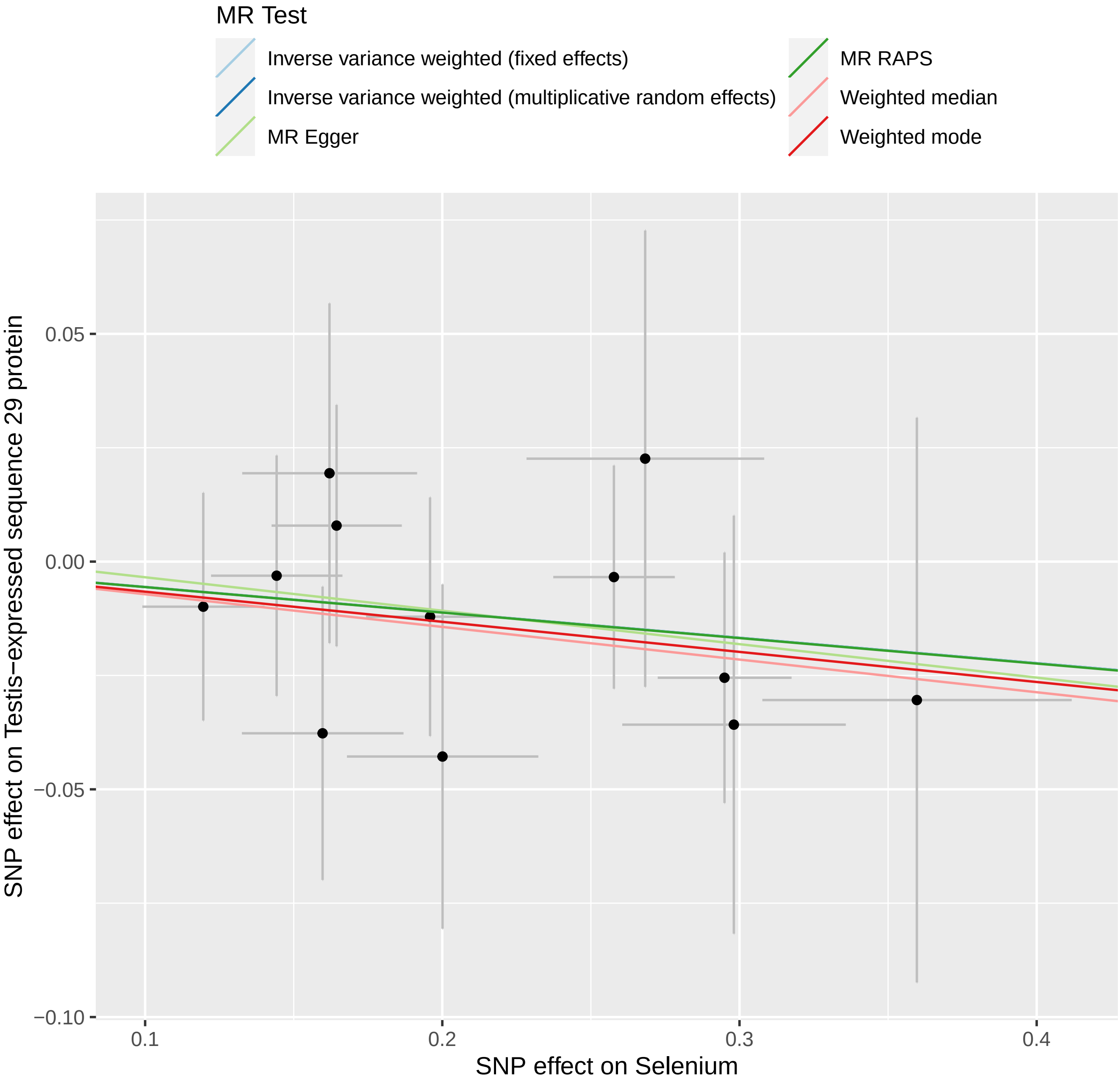

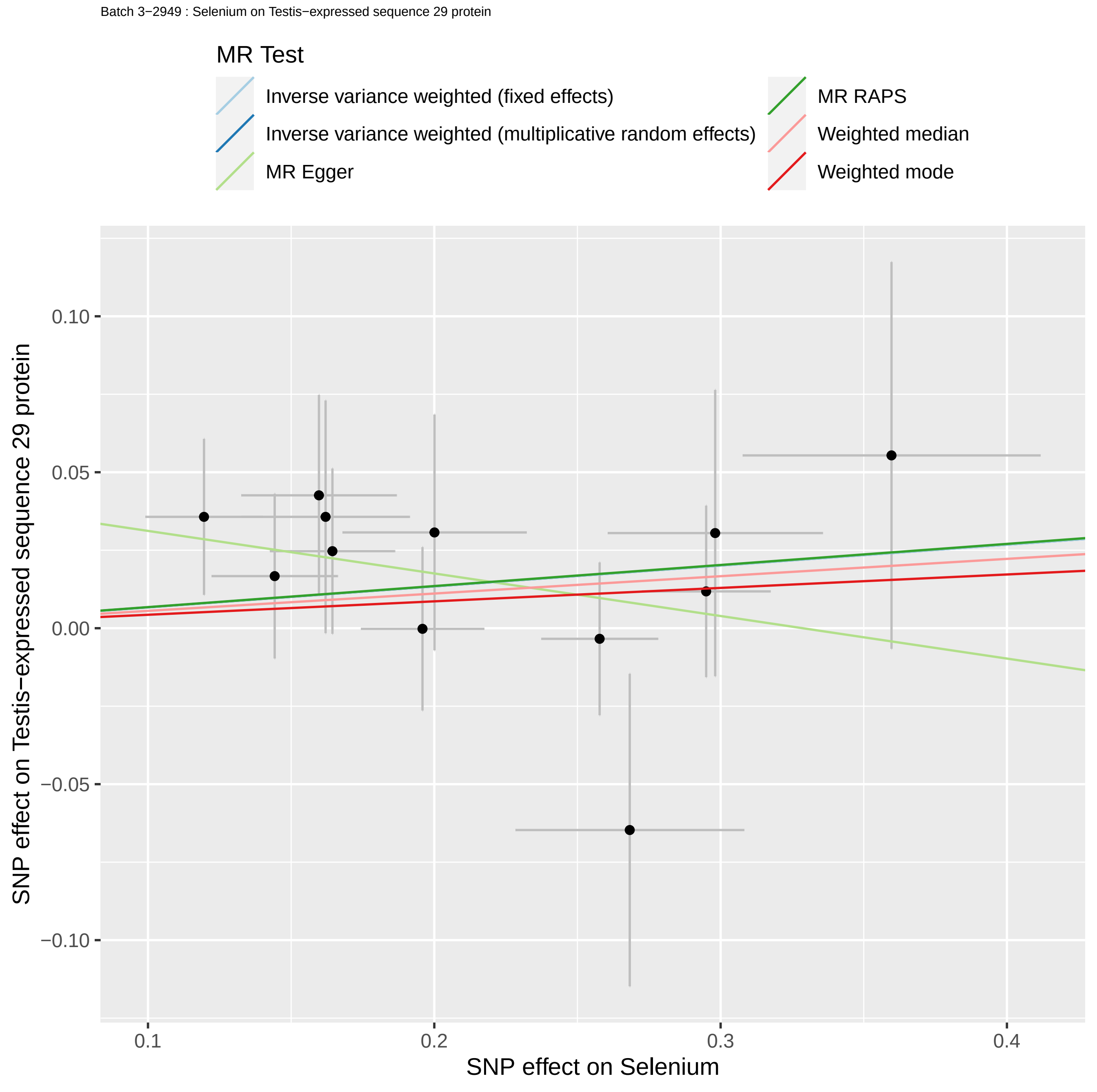
Batch 3−2949 : Selenium on Testis−expressed sequence 29 protein
MR Test
Inverse variance weighted (fixed effects)
Inverse variance weighted (multiplicative random effects)
MR Egger
MR RAPS
Weighted median
Weighted mode
0.10
0.05
0.00
−0.05
−0.10
0.1
0.2
0.3
0.4
SNP effect on Testis−expressed sequence 29 protein
SNP effect on Selenium

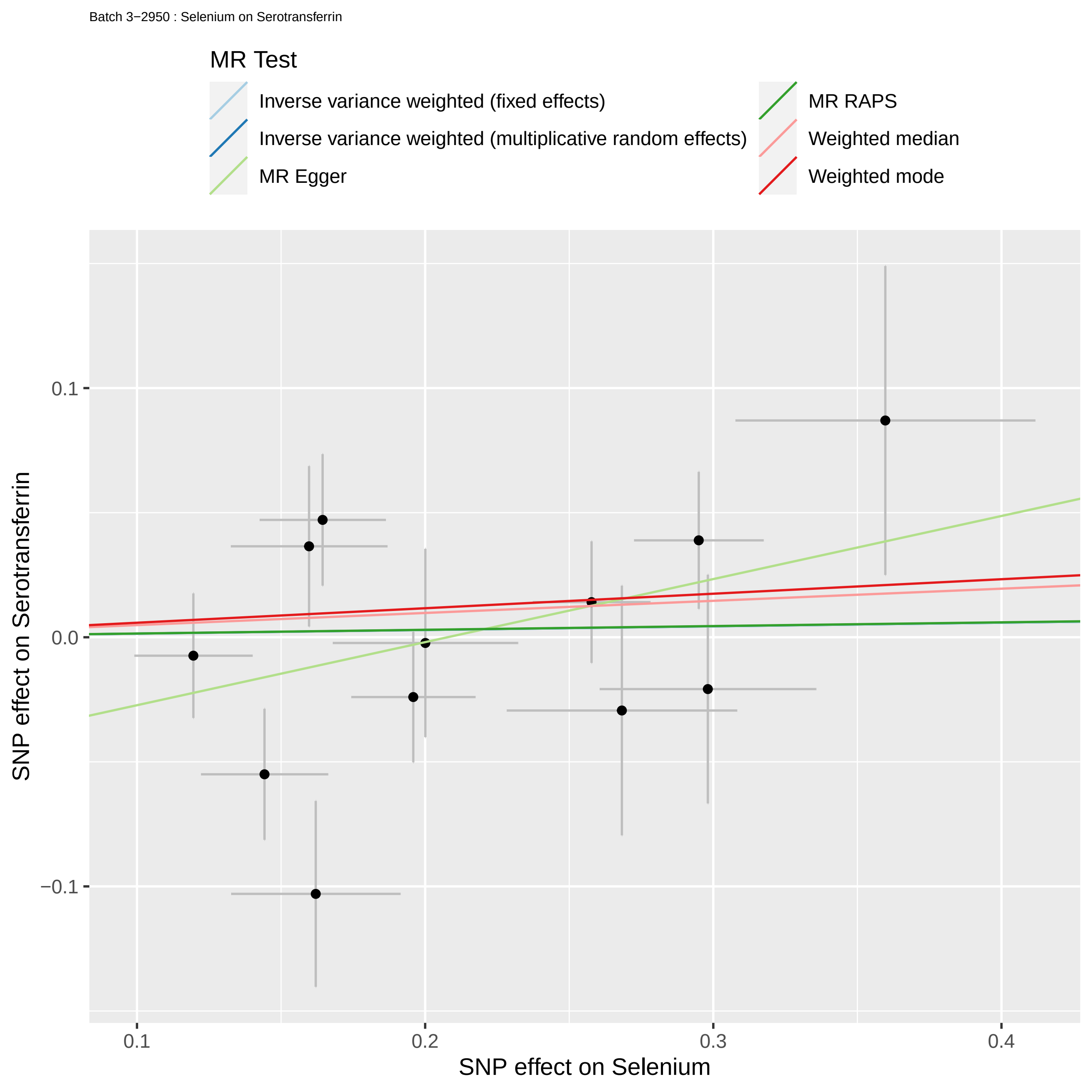

Batch 3−2950 : Selenium on Serotransferrin
MR Test
Inverse variance weighted (fixed effects)
Inverse variance weighted (multiplicative random effects)
MR Egger
MR RAPS
Weighted median
Weighted mode
0.1
0.0
−0.1
0.1
0.2
0.3
0.4
SNP effect on Serotransferrin
SNP effect on Selenium

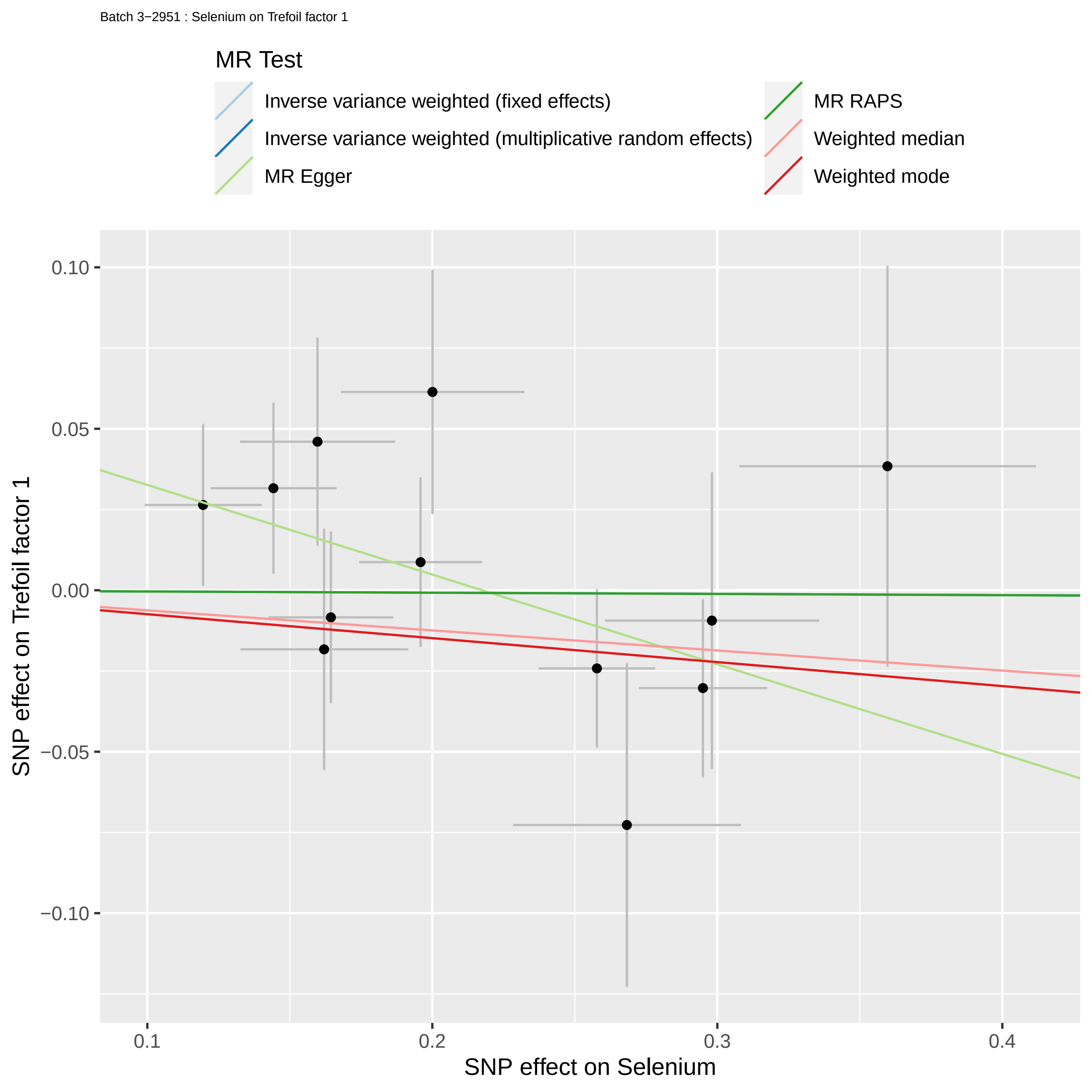

Batch 3−2951 : Selenium on Trefoil factor 1
MR Test
Inverse variance weighted (fixed effects)
Inverse variance weighted (multiplicative random effects)
MR Egger
MR RAPS
Weighted median
Weighted mode
SNP effect on Trefoil factor 1
0.10
0.05
0.00
−0.05
−0.10
0.1
0.2
0.3
0.4
SNP effect on Selenium

Batch 3−2952 : Selenium on Trefoil factor 2

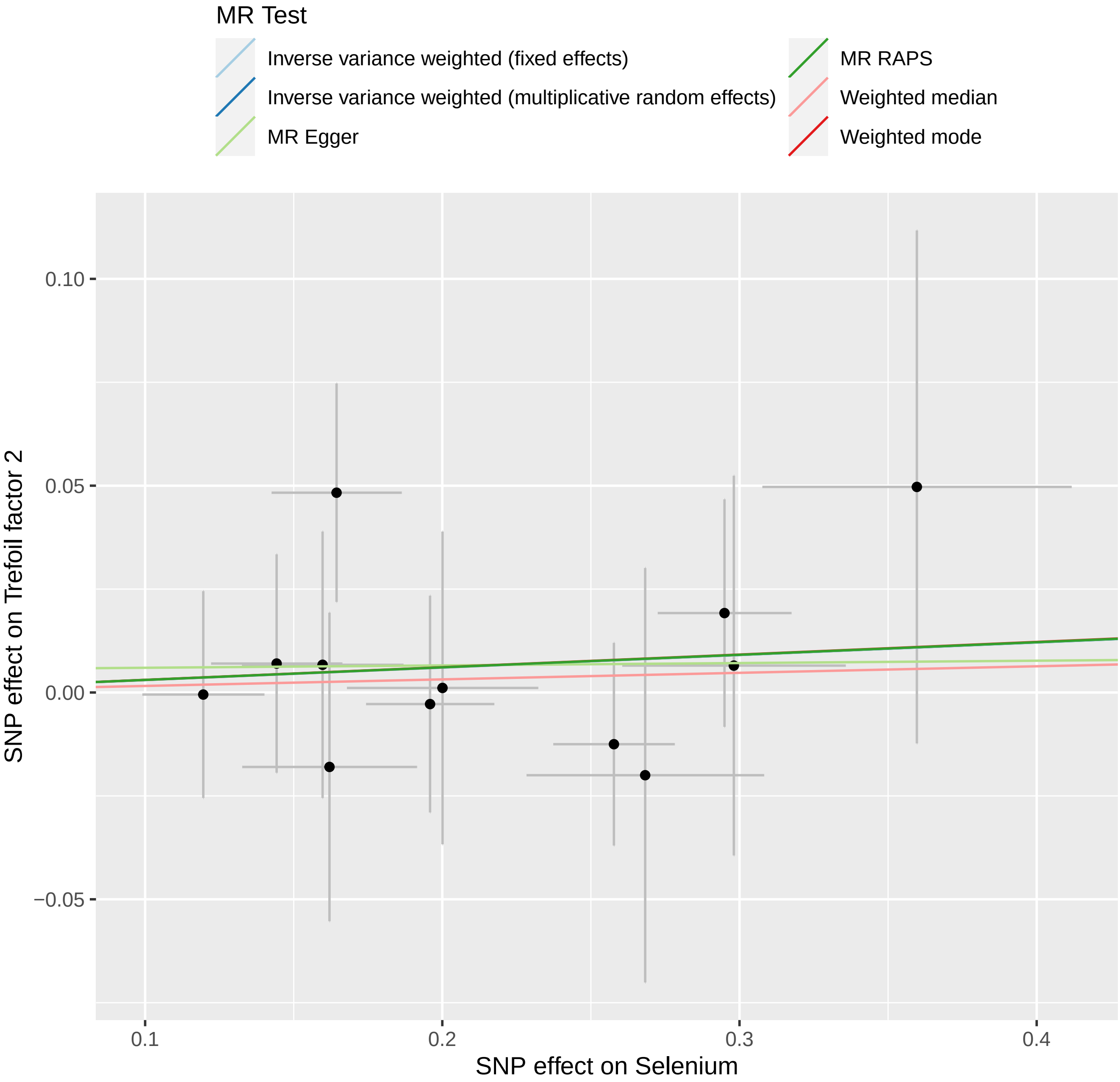

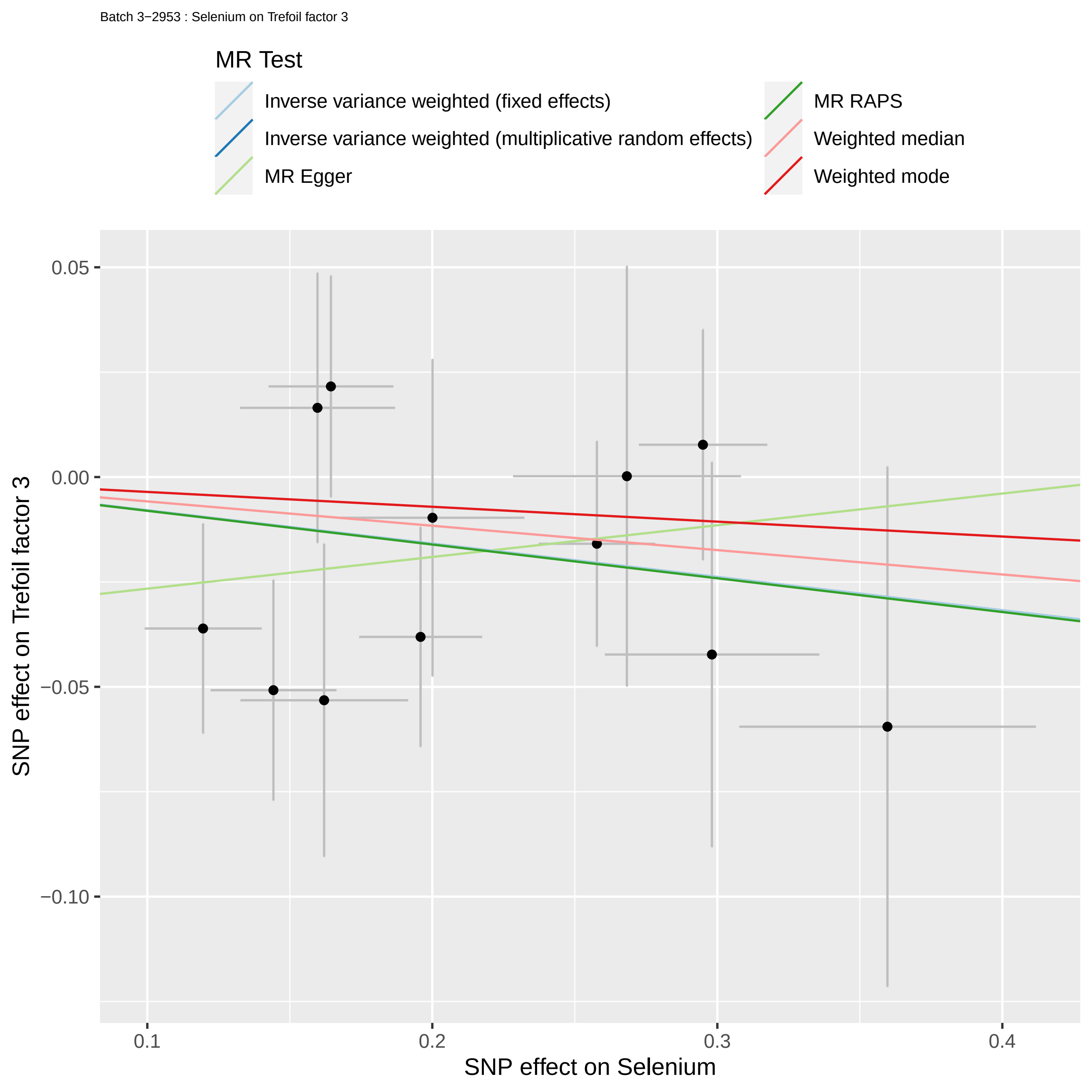

Batch 3−2953 : Selenium on Trefoil factor 3
MR Test
Inverse variance weighted (fixed effects)
Inverse variance weighted (multiplicative random effects)
MR Egger
MR RAPS
Weighted median
Weighted mode
0.05
0.00
−0.05
−0.10
0.1
0.2
0.3
0.4
SNP effect on Trefoil factor 3
SNP effect on Selenium

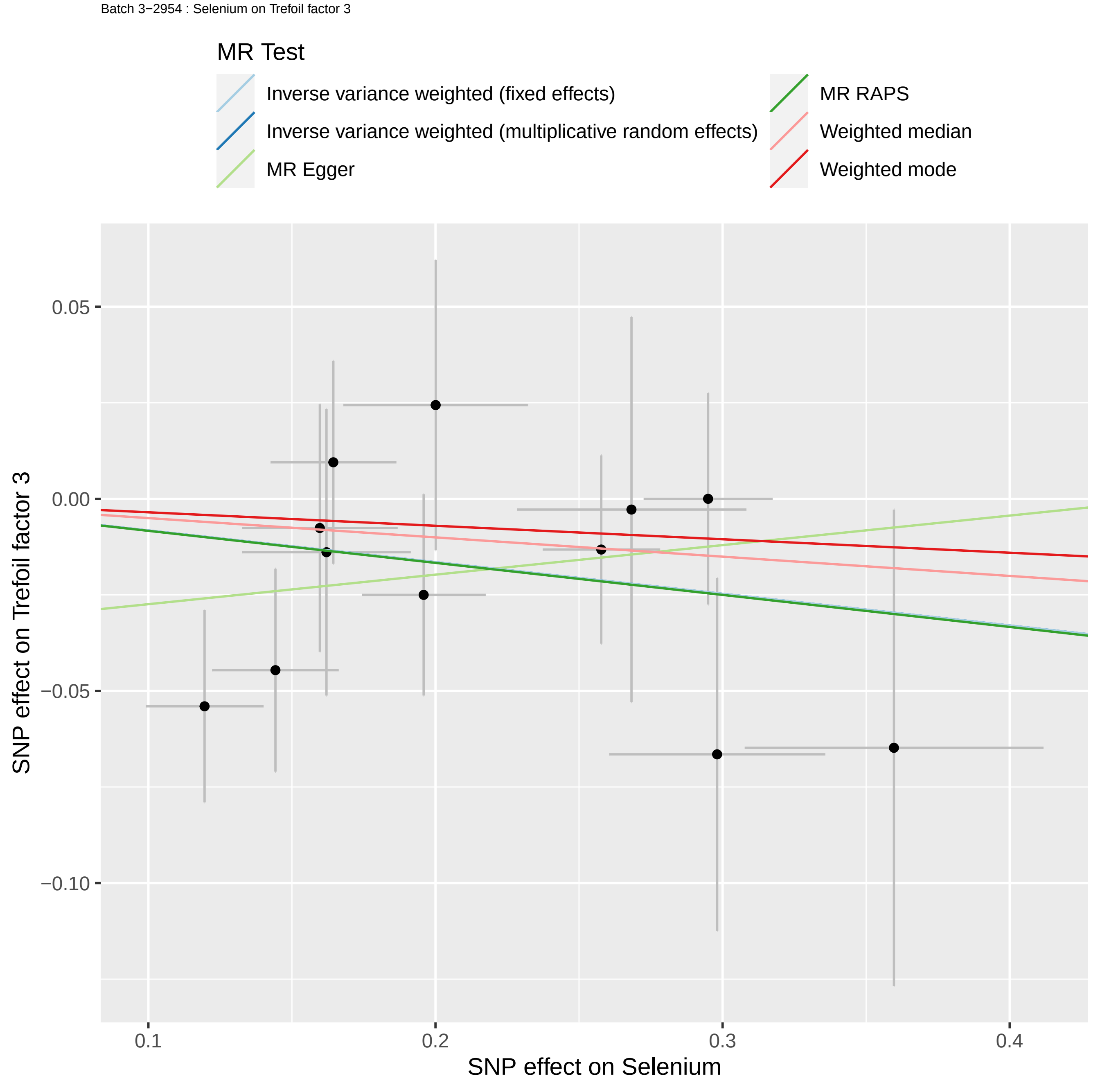
Batch 3−2954 : Selenium on Trefoil factor 3
MR Test
Inverse variance weighted (fixed effects)
Inverse variance weighted (multiplicative random effects)
MR Egger
MR RAPS
Weighted median
Weighted mode
0.05
0.00
−0.05
−0.10
0.1
0.2
0.3
0.4
SNP effect on Trefoil factor 3
SNP effect on Selenium

Batch 3−2955 : Selenium on Tuftelin−interacting protein 11

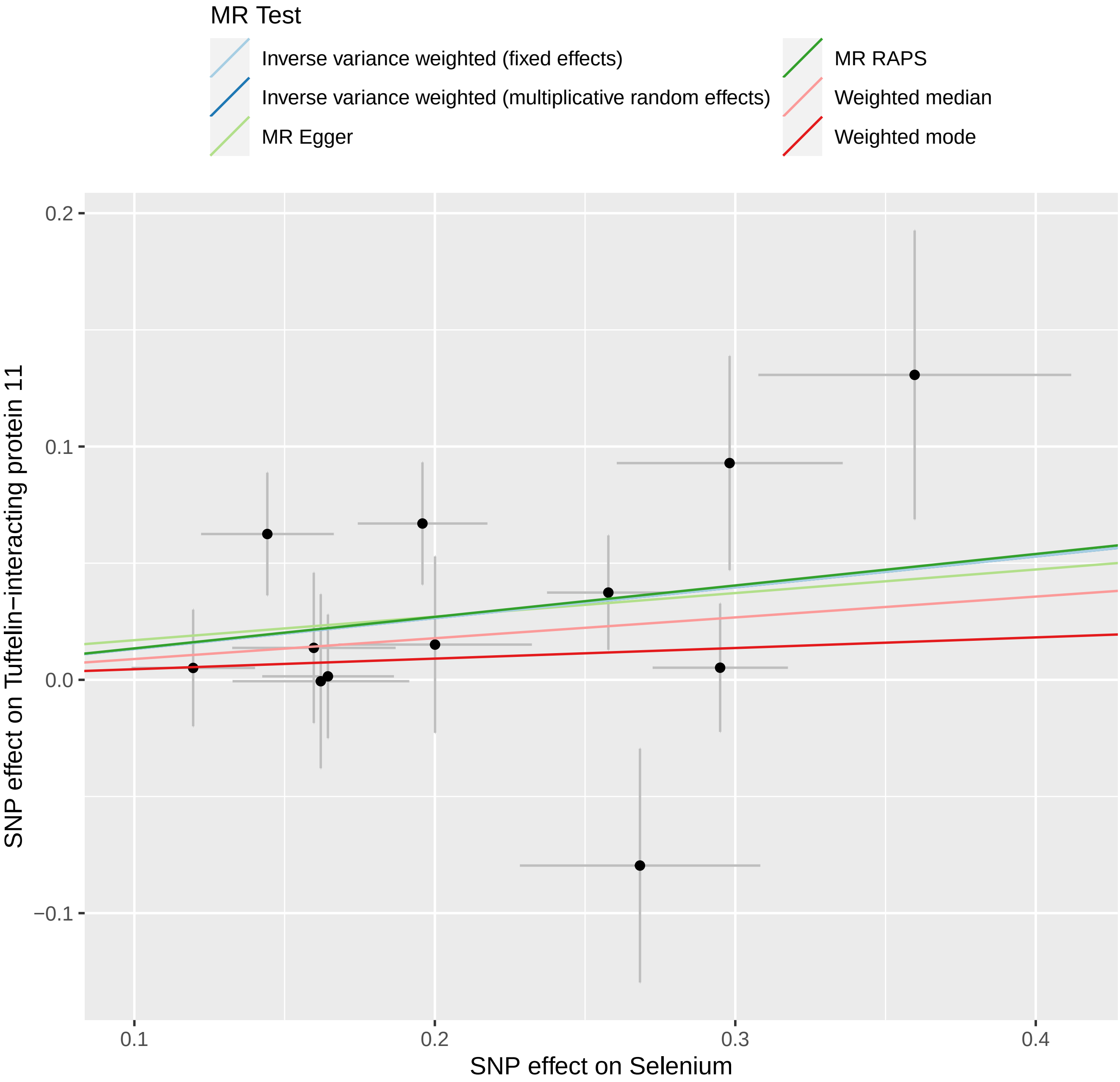

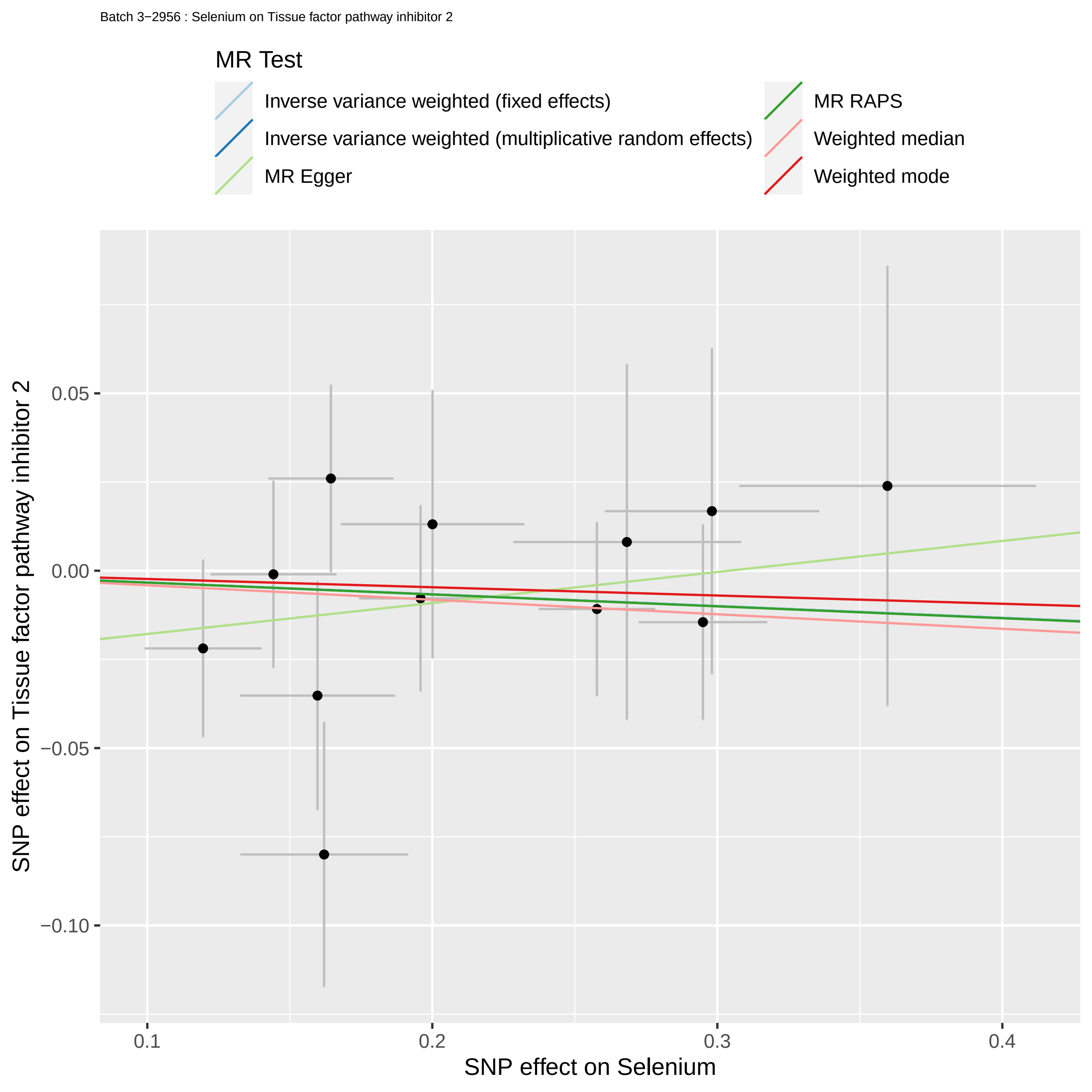
Batch 3−2956 : Selenium on Tissue factor pathway inhibitor 2
MR Test
Inverse variance weighted (fixed effects)
Inverse variance weighted (multiplicative random effects)
MR Egger
MR RAPS
Weighted median
Weighted mode
SNP effect on Tissue factor pathway inhibitor 2
0.05
0.00
−0.05
−0.10
0.1
0.2
0.3
0.4
SNP effect on Selenium

Batch 3−2957 : Selenium on Tissue factor pathway inhibitor

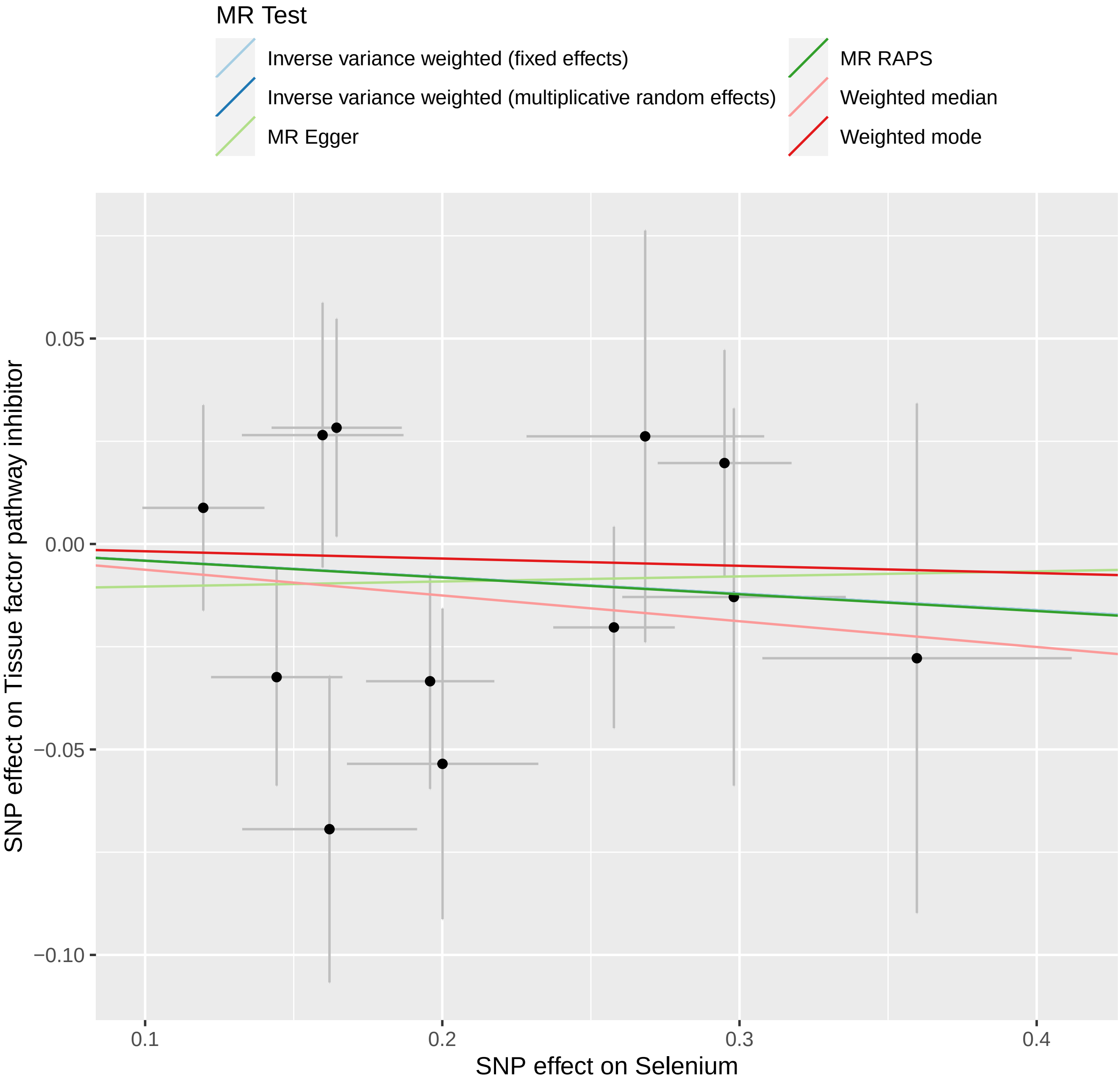

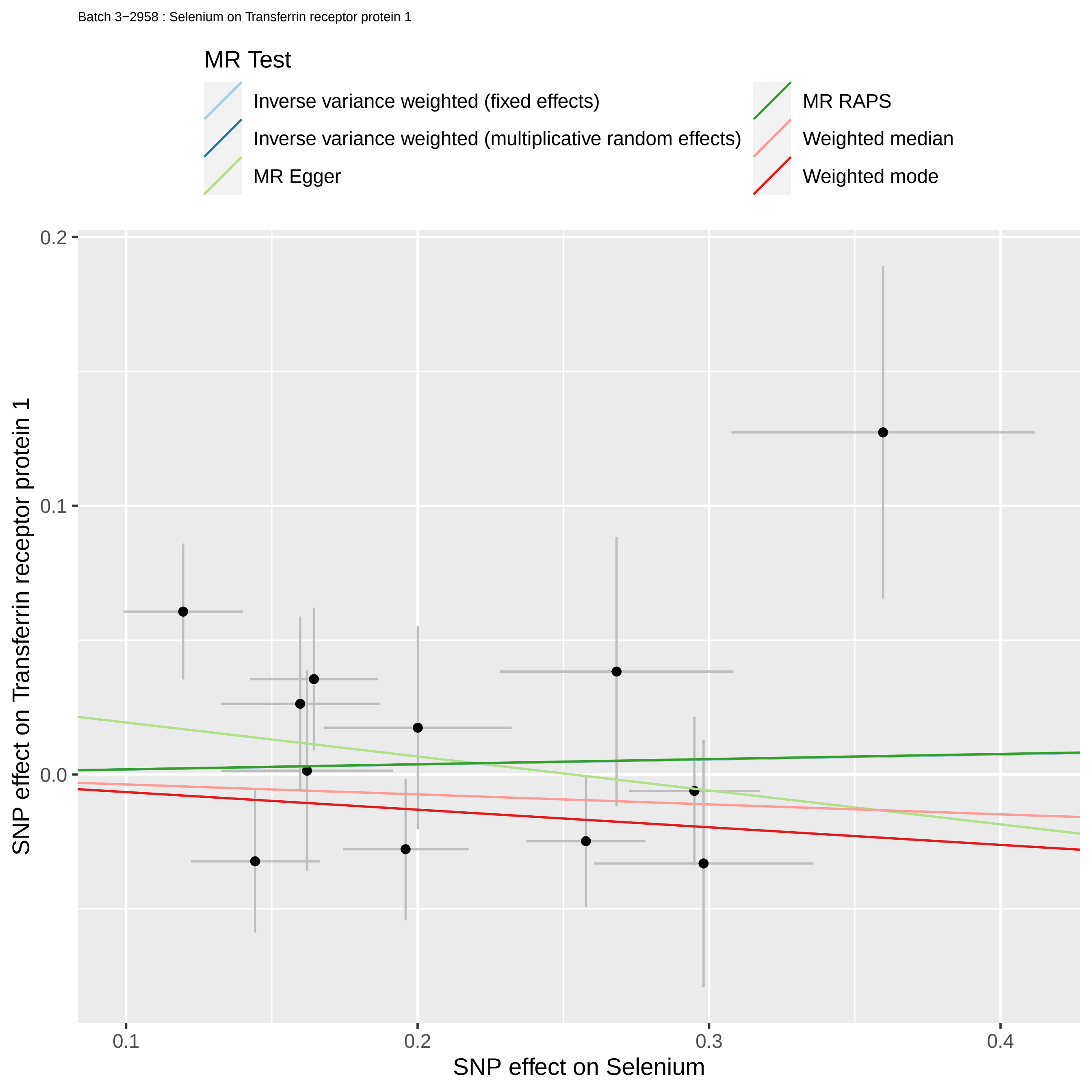

Batch 3−2958 : Selenium on Transferrin receptor protein 1
MR Test
Inverse variance weighted (fixed effects)
Inverse variance weighted (multiplicative random effects)
MR Egger
MR RAPS
Weighted median
Weighted mode
0.2
0.1
0.0
0.1
0.2
0.3
0.4
SNP effect on Transferrin receptor protein 1
SNP effect on Selenium

Batch 3−2959 : Selenium on Transferrin receptor protein 1

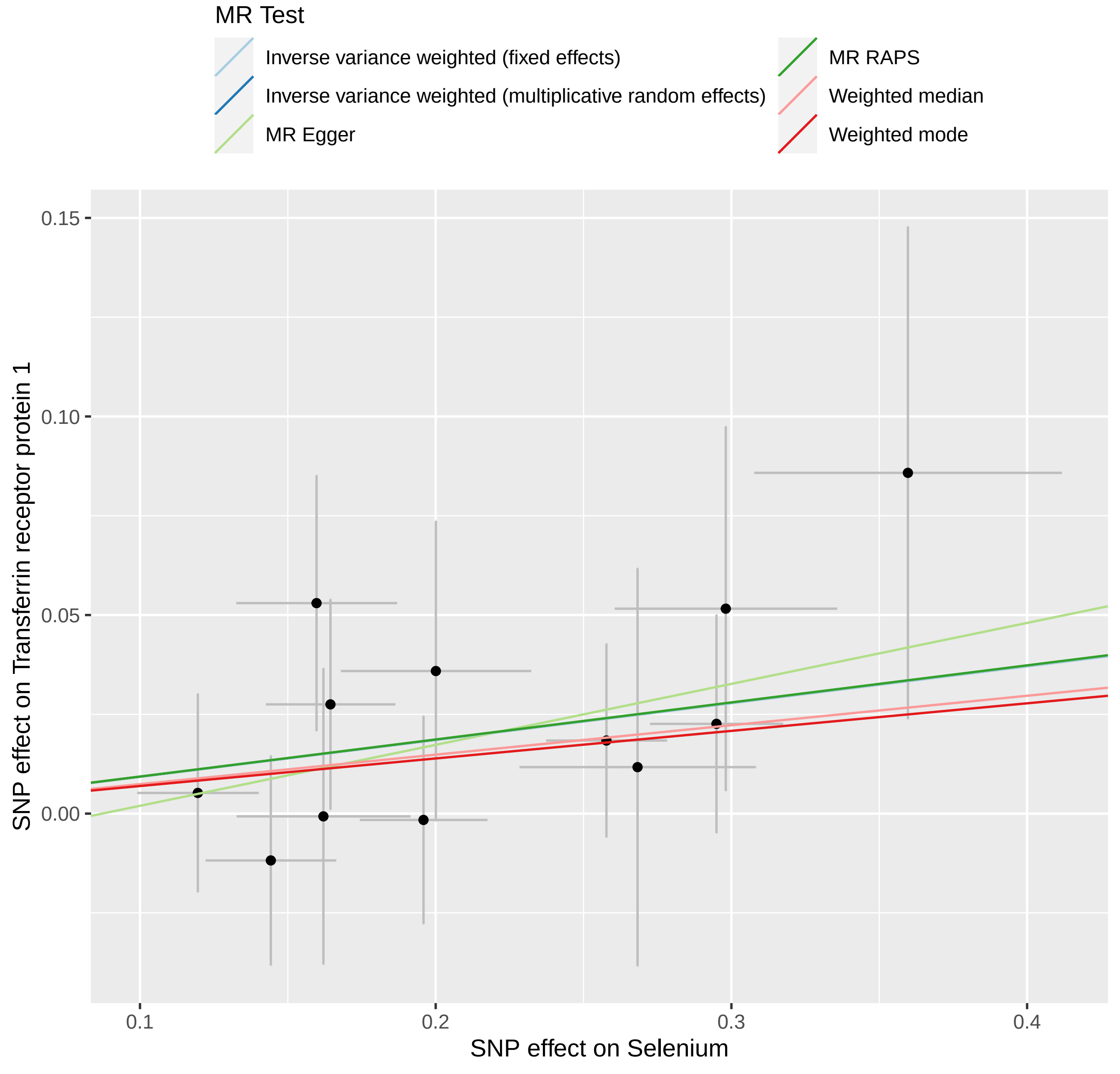

Batch 3−2960 : Selenium on Thyroglobulin

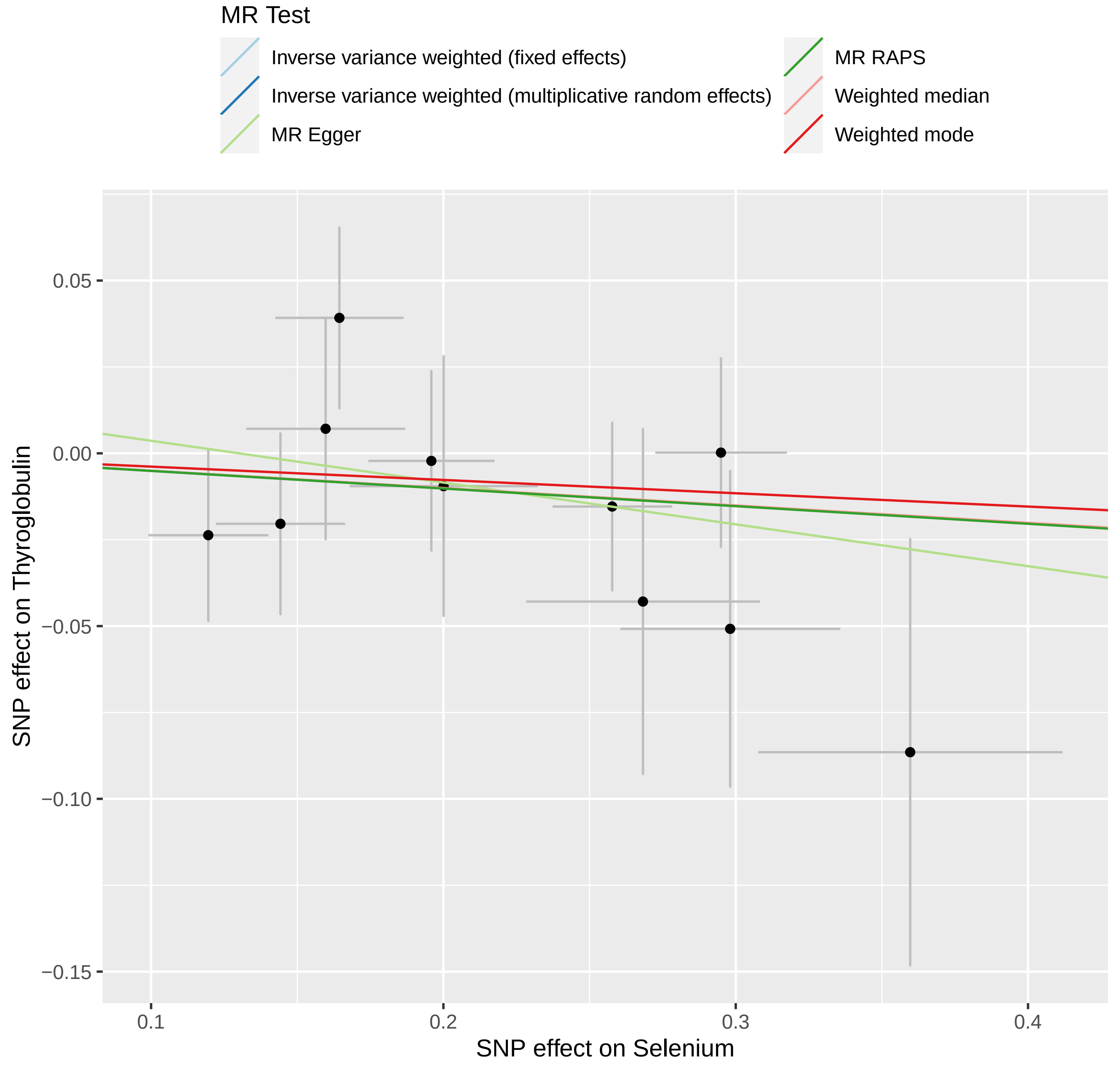

Batch 3−2961 : Selenium on Transforming growth factor alpha

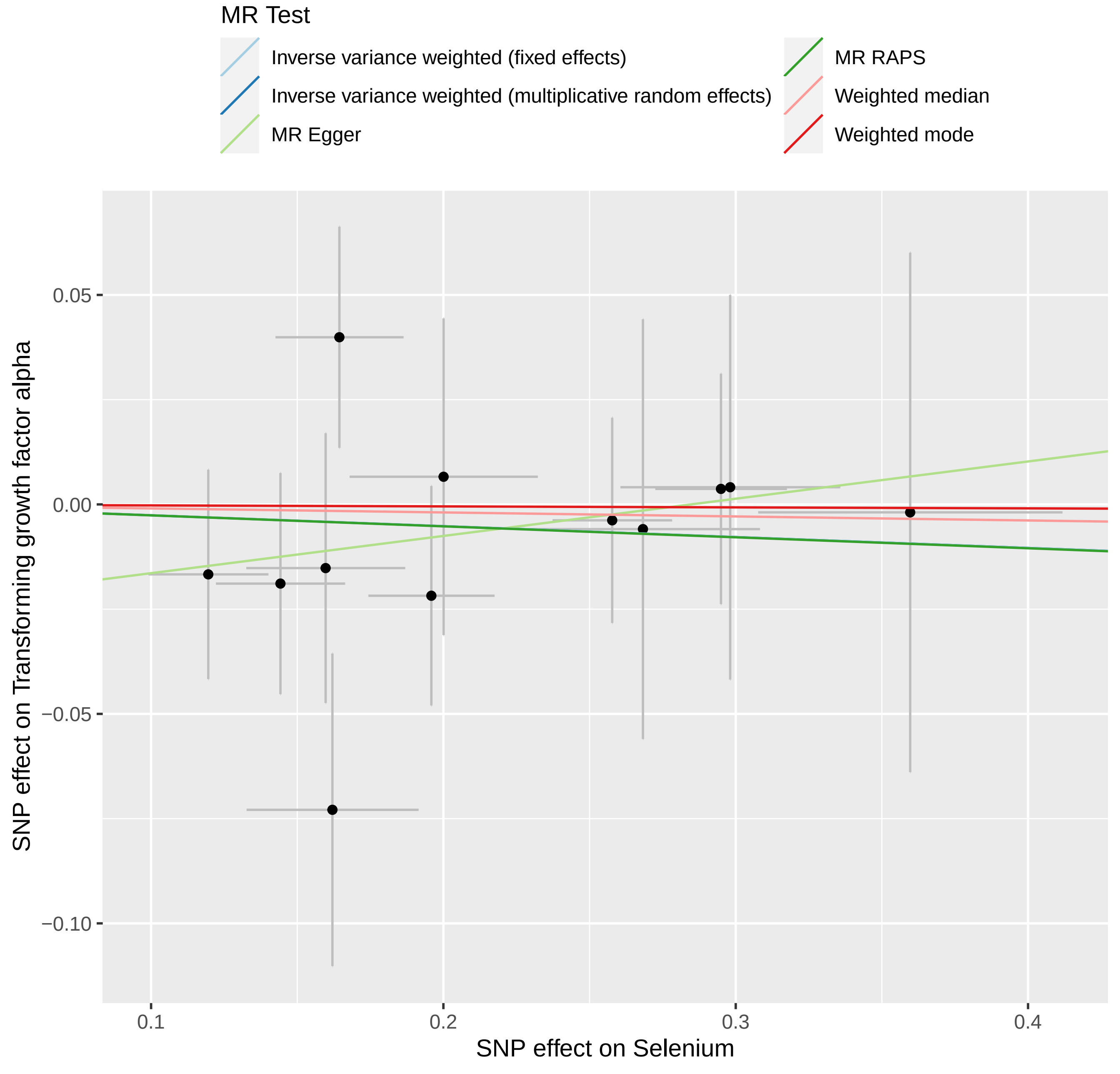

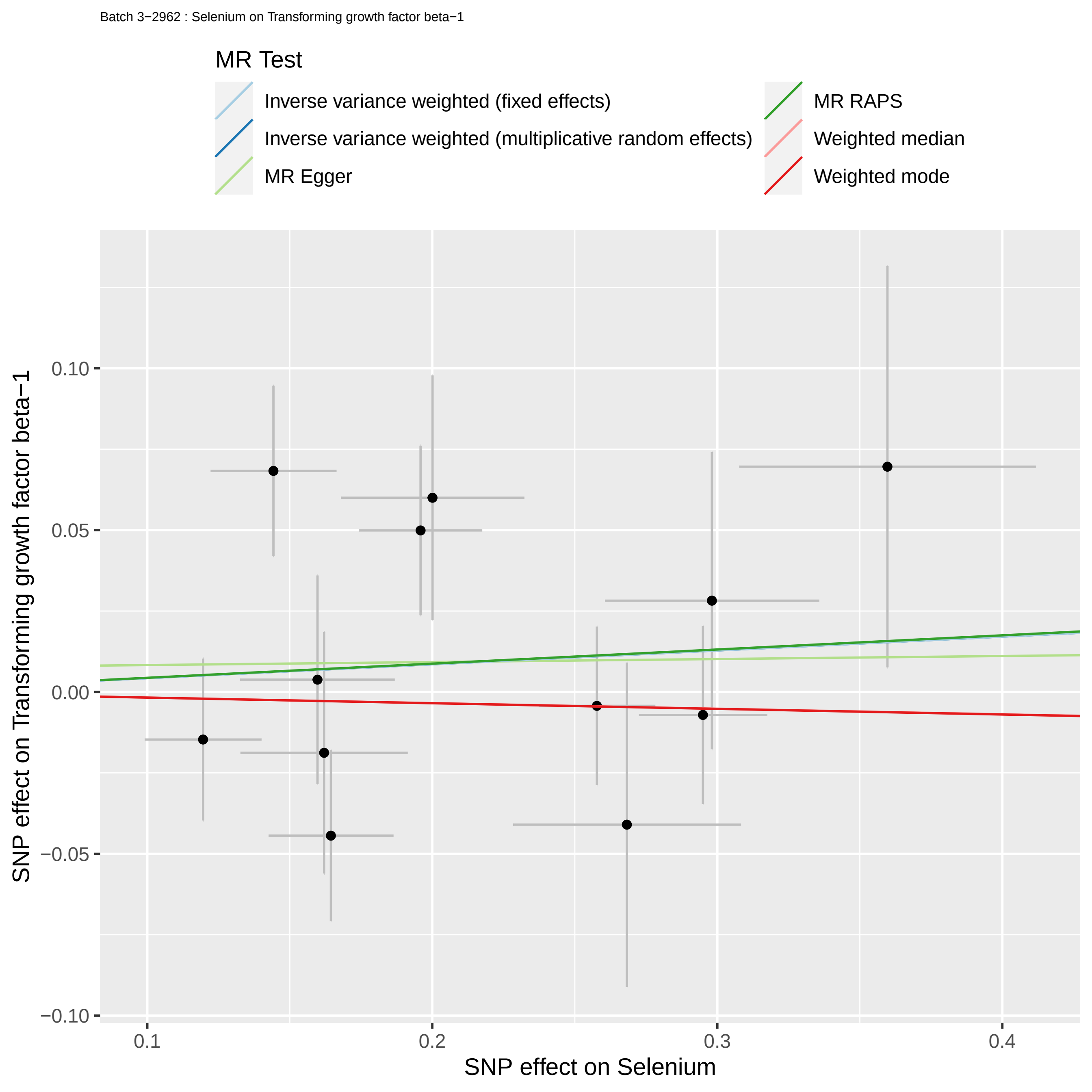
Batch 3−2962 : Selenium on Transforming growth factor beta−1
MR Test
Inverse variance weighted (fixed effects)
Inverse variance weighted (multiplicative random effects)
MR Egger
MR RAPS
Weighted median
Weighted mode
0.10
0.05
0.00
−0.05
−0.10
0.1
0.2
0.3
0.4
SNP effect on Transforming growth factor beta−1
SNP effect on Selenium

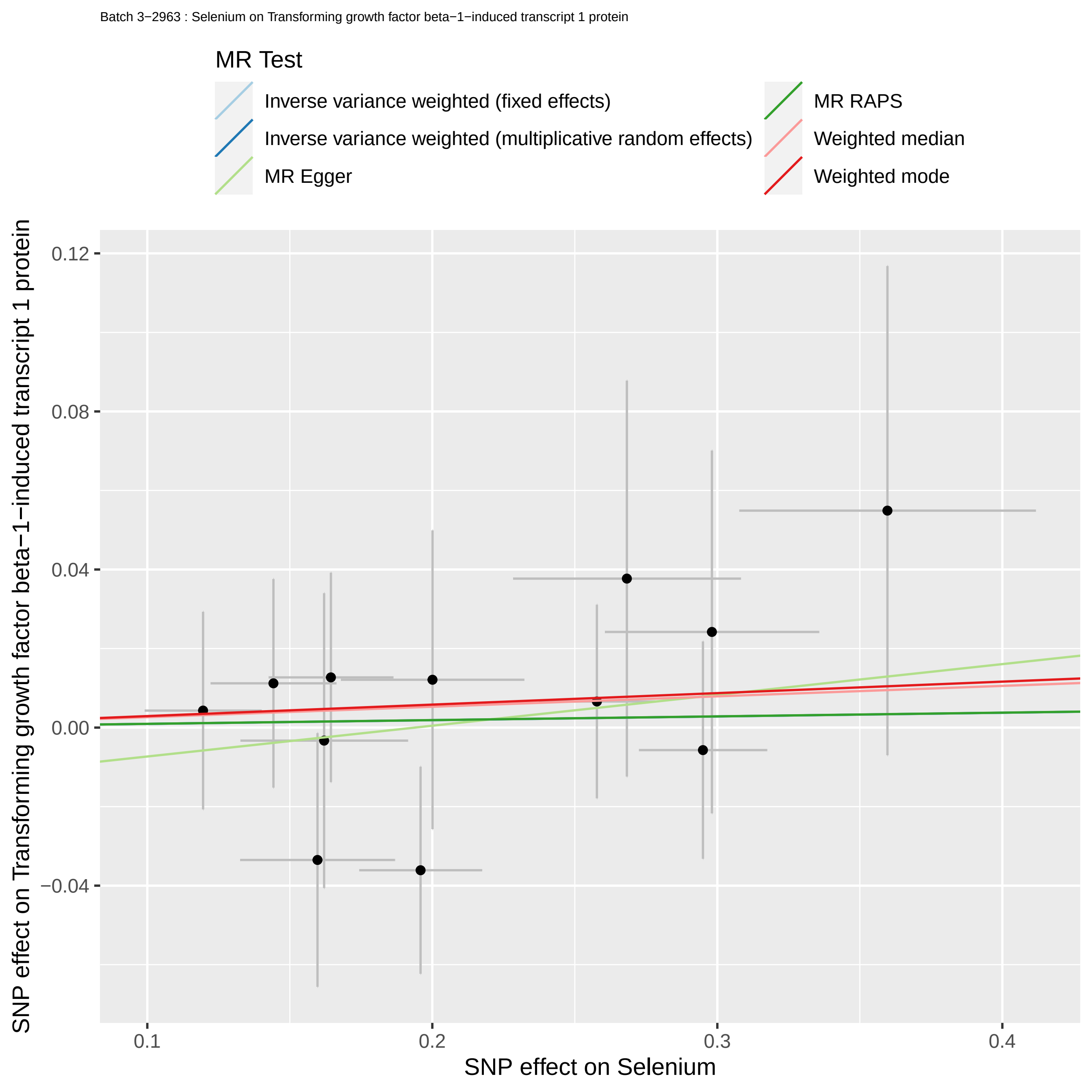

Batch 3−2963 : Selenium on Transforming growth factor beta−1−induced transcript 1 protein
MR Test
Inverse variance weighted (fixed effects)
Inverse variance weighted (multiplicative random effects)
MR Egger
MR RAPS
Weighted median
Weighted mode
0.12
0.08
0.04
0.00
−0.04
0.1
0.2
0.3
0.4
SNP effect on Transforming growth factor beta−1−induced transcript 1 protein
SNP effect on Selenium

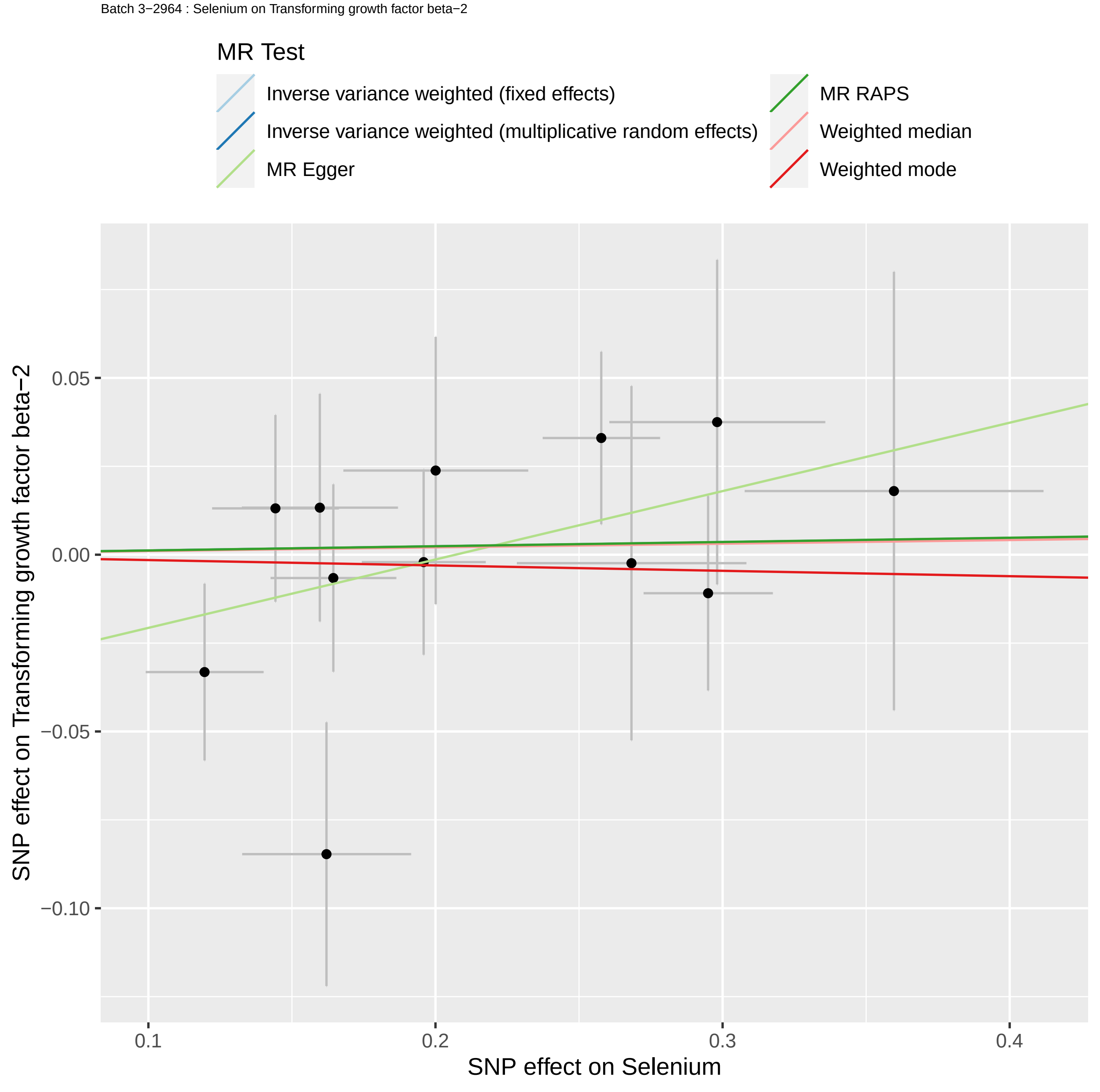
Batch 3−2964 : Selenium on Transforming growth factor beta−2
MR Test
Inverse variance weighted (fixed effects)
Inverse variance weighted (multiplicative random effects)
MR Egger
MR RAPS
Weighted median
Weighted mode
0.05
0.00
−0.05
−0.10
0.1
0.2
0.3
0.4
SNP effect on Selenium
SNP effect on Transforming growth factor beta−2

Batch 3−2965 : Selenium on Transforming growth factor beta−3

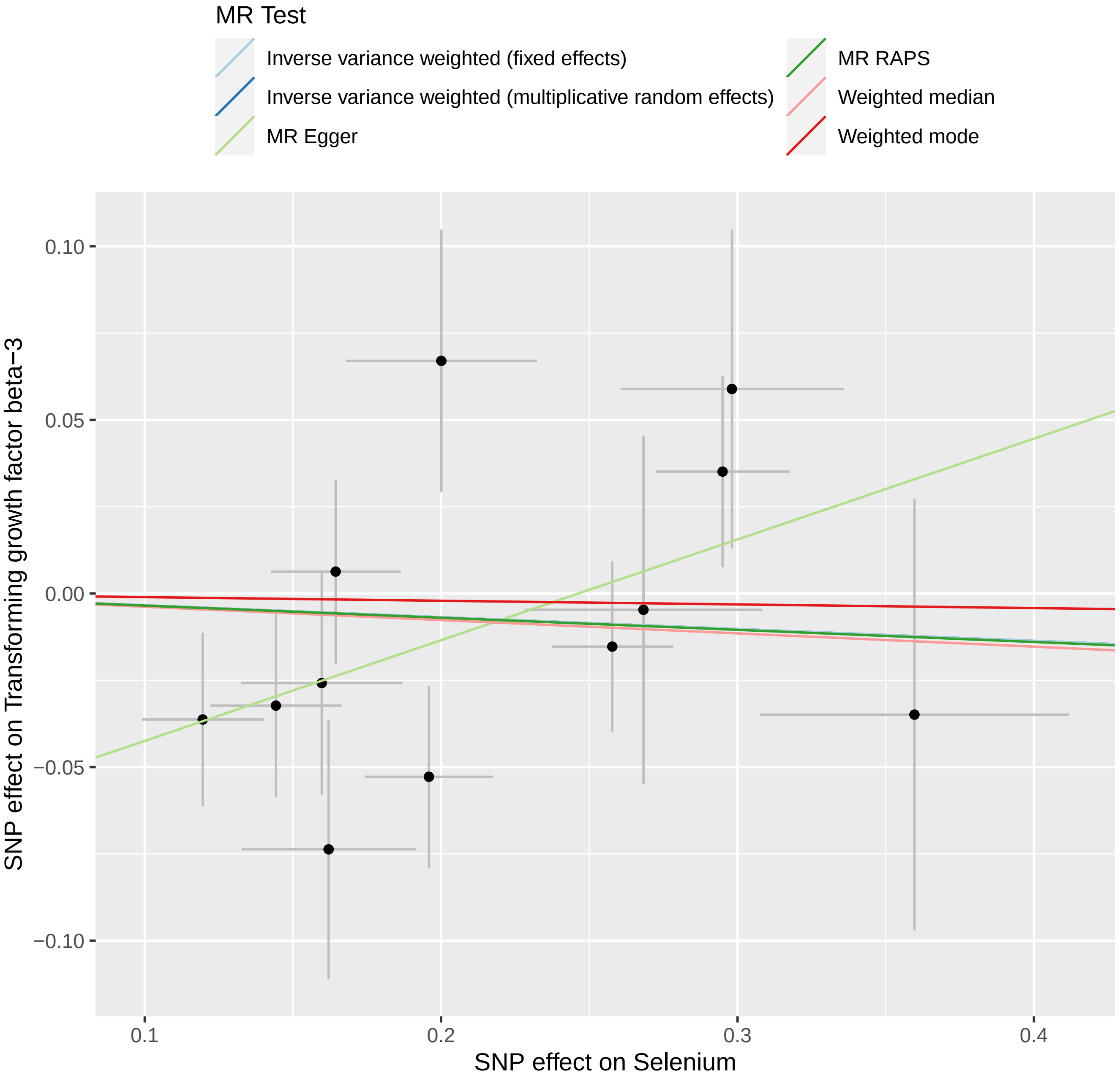

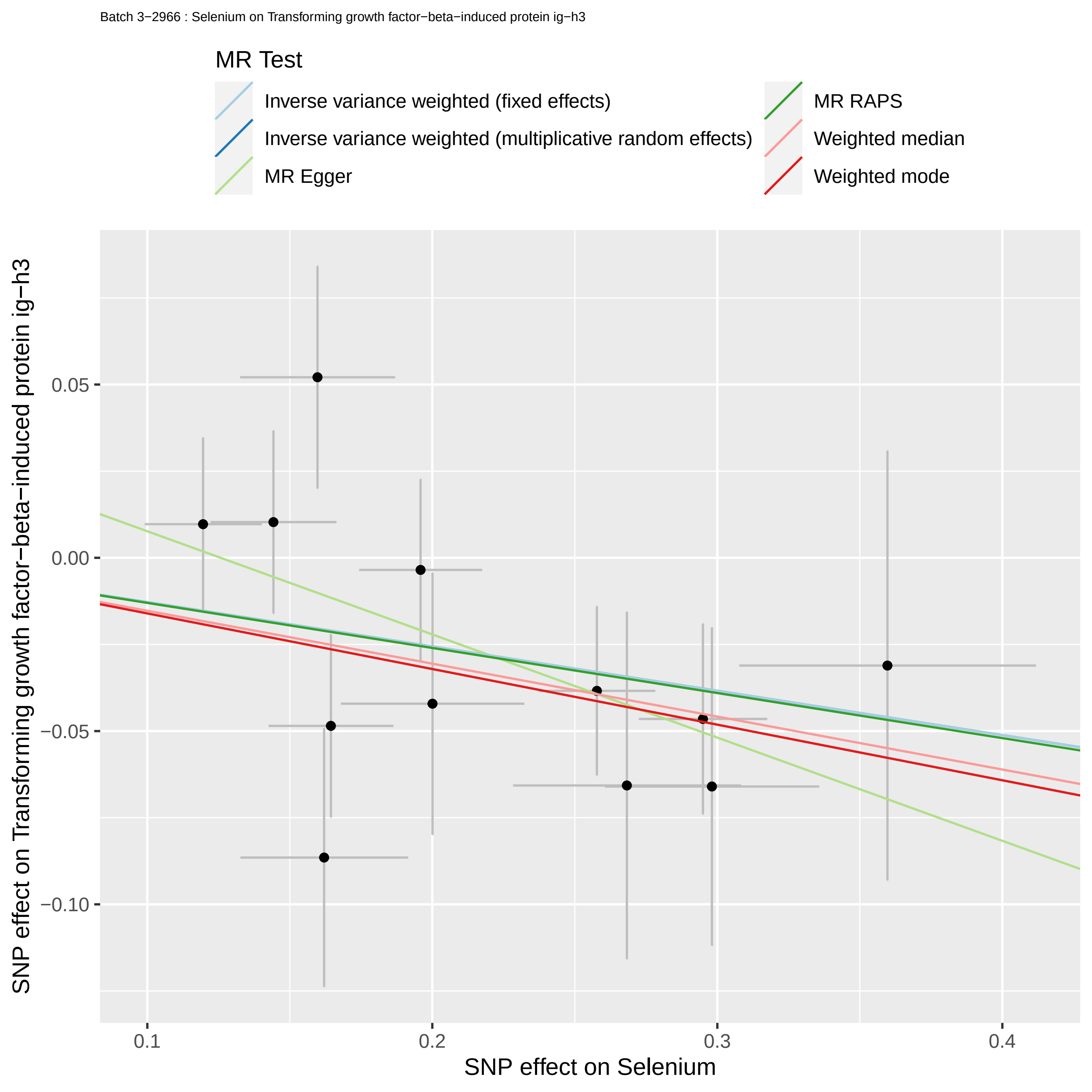

Batch 3−2966 : Selenium on Transforming growth factor−beta−induced protein ig−h3
MR Test
Inverse variance weighted (fixed effects)
Inverse variance weighted (multiplicative random effects)
MR Egger
MR RAPS
Weighted median
Weighted mode
0.05
0.00
−0.05
−0.10
0.1
0.2
0.3
0.4
SNP effect on Selenium
SNP effect on Transforming growth factor−beta−induced protein ig−h3

Batch 3−2967 : Selenium on TGF−beta receptor type−2

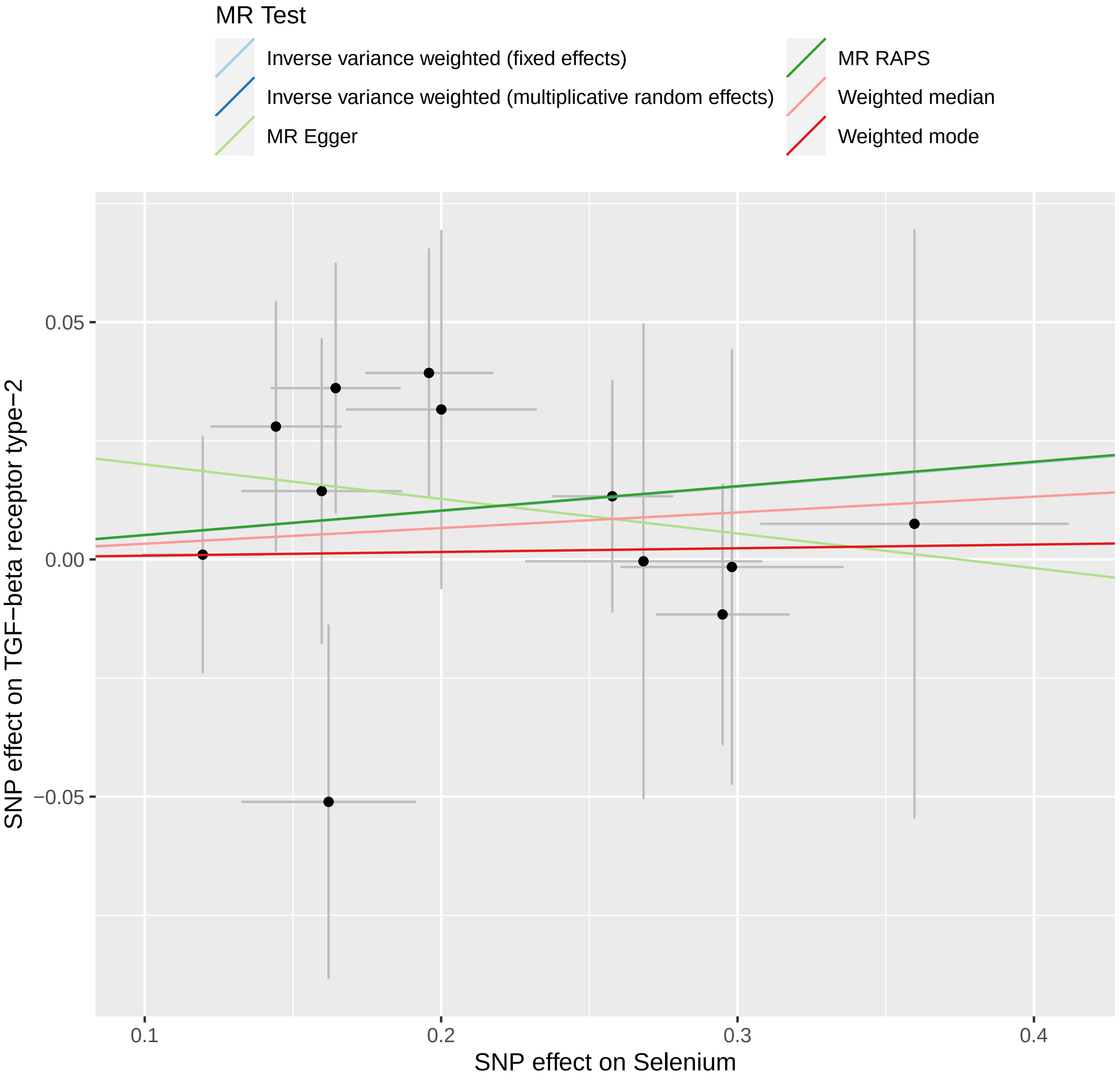

Batch 3−2968 : Selenium on Transforming growth factor beta receptor type 3

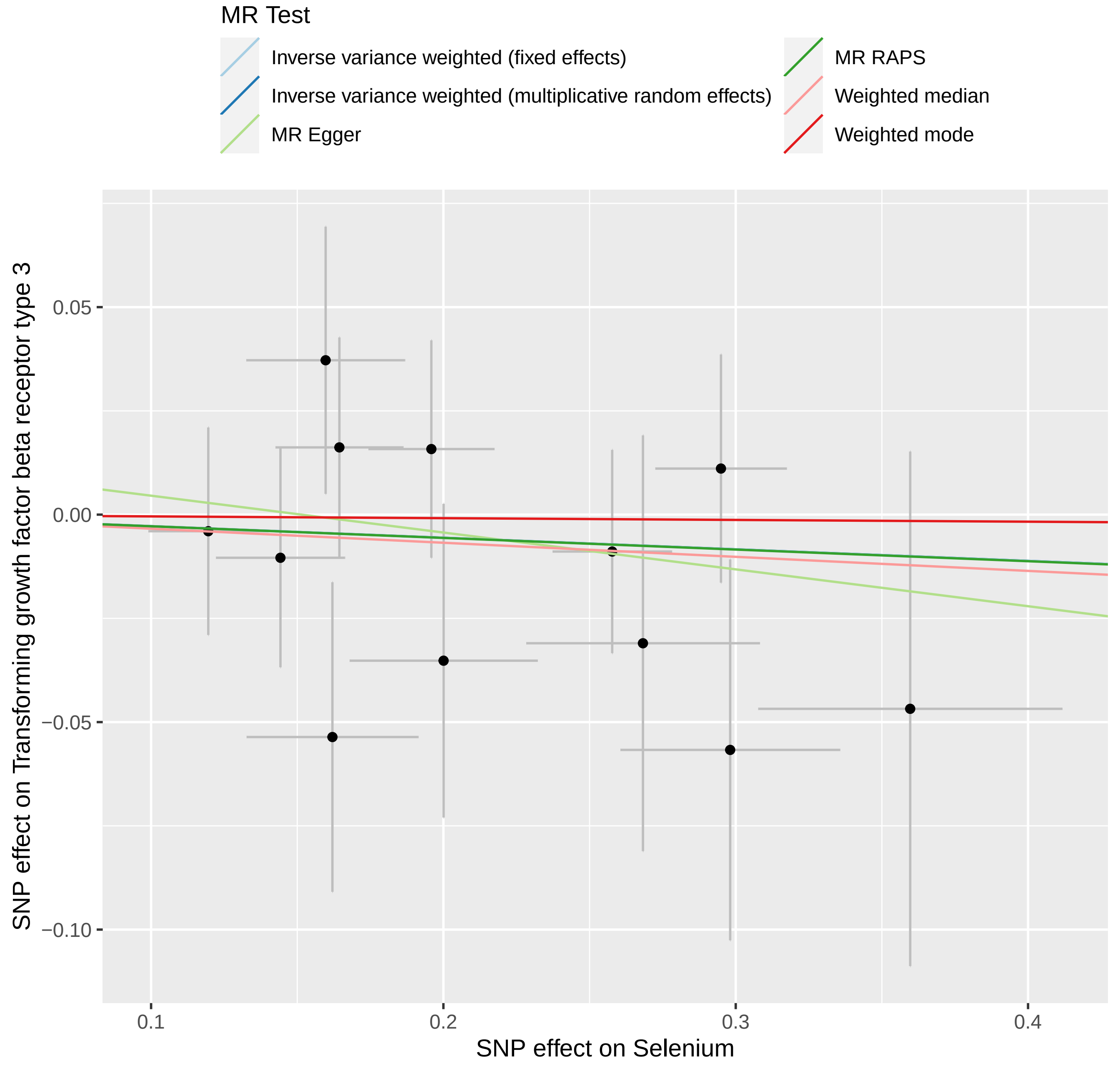

Batch 3−2969 : Selenium on Homeobox protein TGIF2

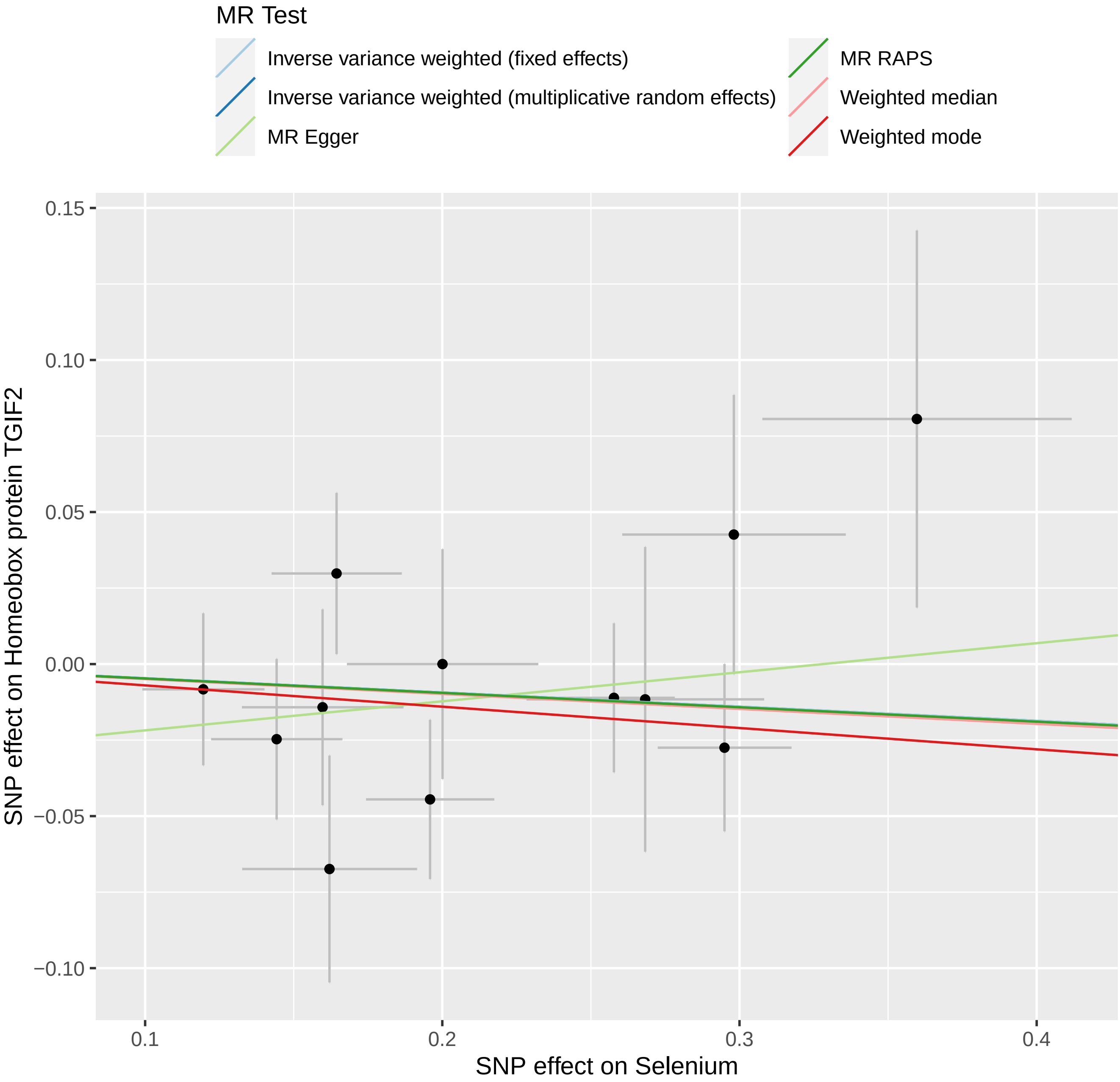

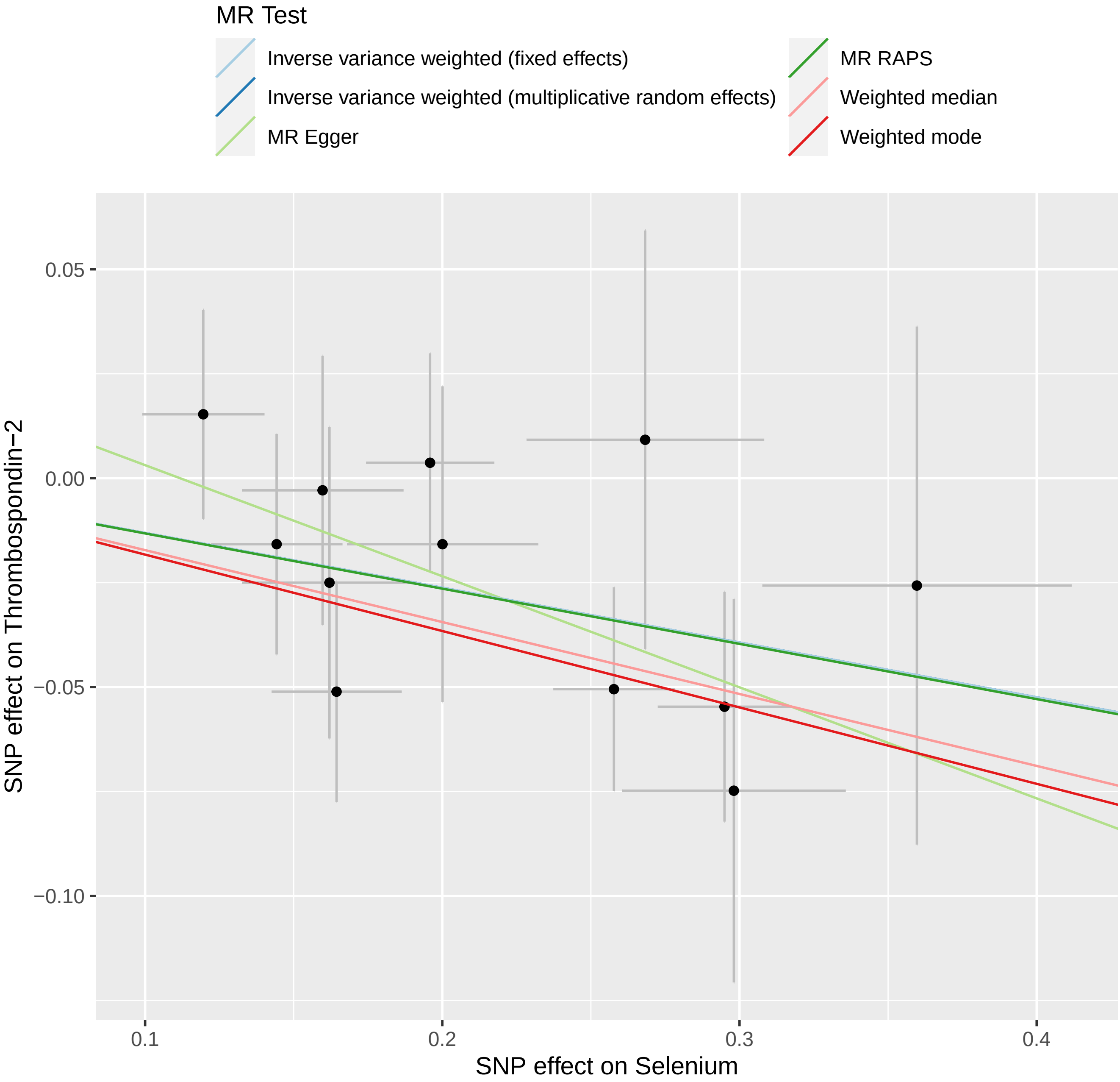
MR Test
Inverse variance weighted (fixed effects)
Inverse variance weighted (multiplicative random effects)
MR Egger
MR RAPS
Weighted median
Weighted mode
0.05
0.00
−0.05
−0.10
0.1
0.2
0.3
0.4
SNP effect on Thrombospondin−2
SNP effect on Selenium

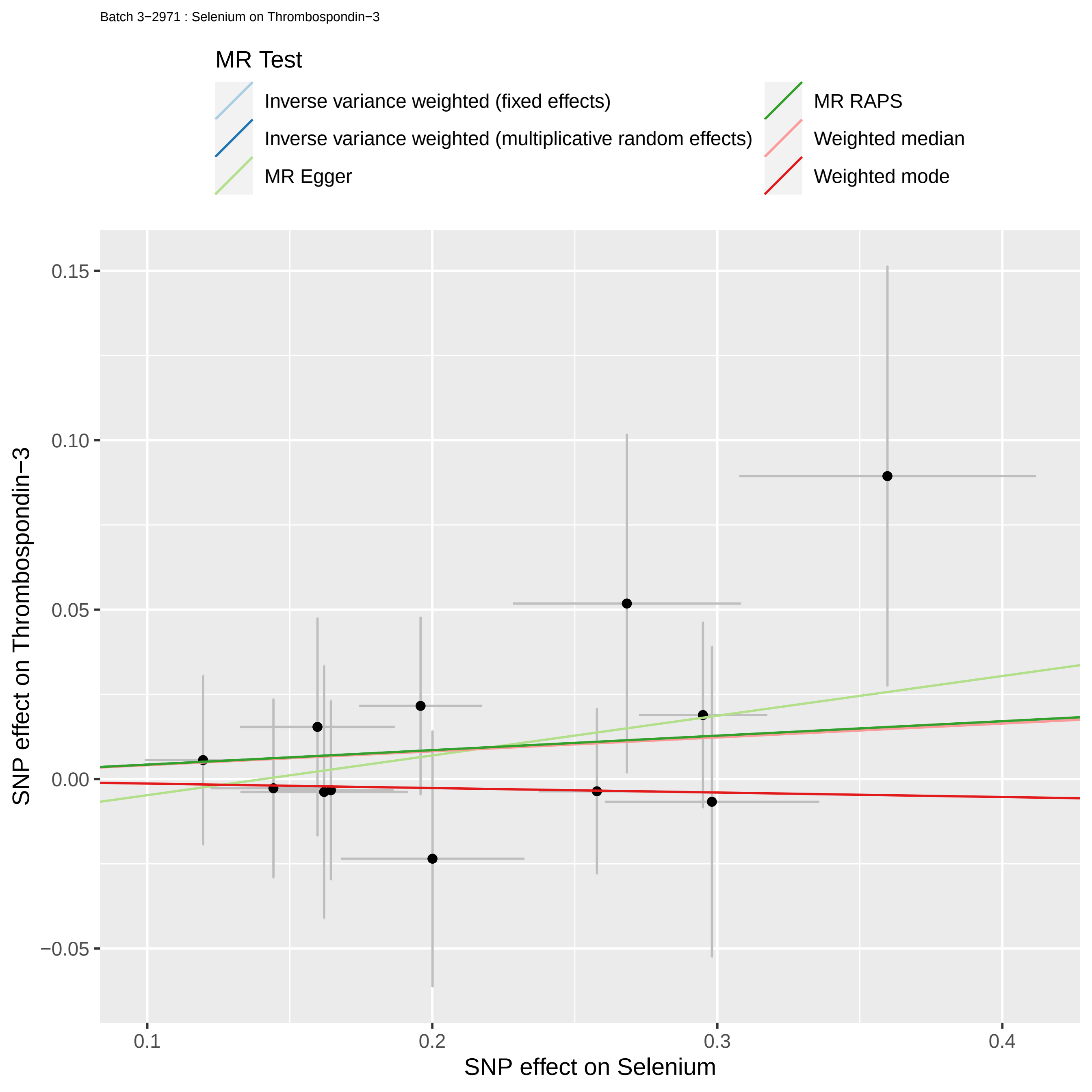
Batch 3−2971 : Selenium on Thrombospondin−3
MR Test
Inverse variance weighted (fixed effects)
Inverse variance weighted (multiplicative random effects)
MR Egger
MR RAPS
Weighted median
Weighted mode
0.15
0.10
0.05
0.00
−0.05
0.1
0.2
0.3
0.4
SNP effect on Thrombospondin−3
SNP effect on Selenium

Batch 3−2972 : Selenium on Thrombopoietin

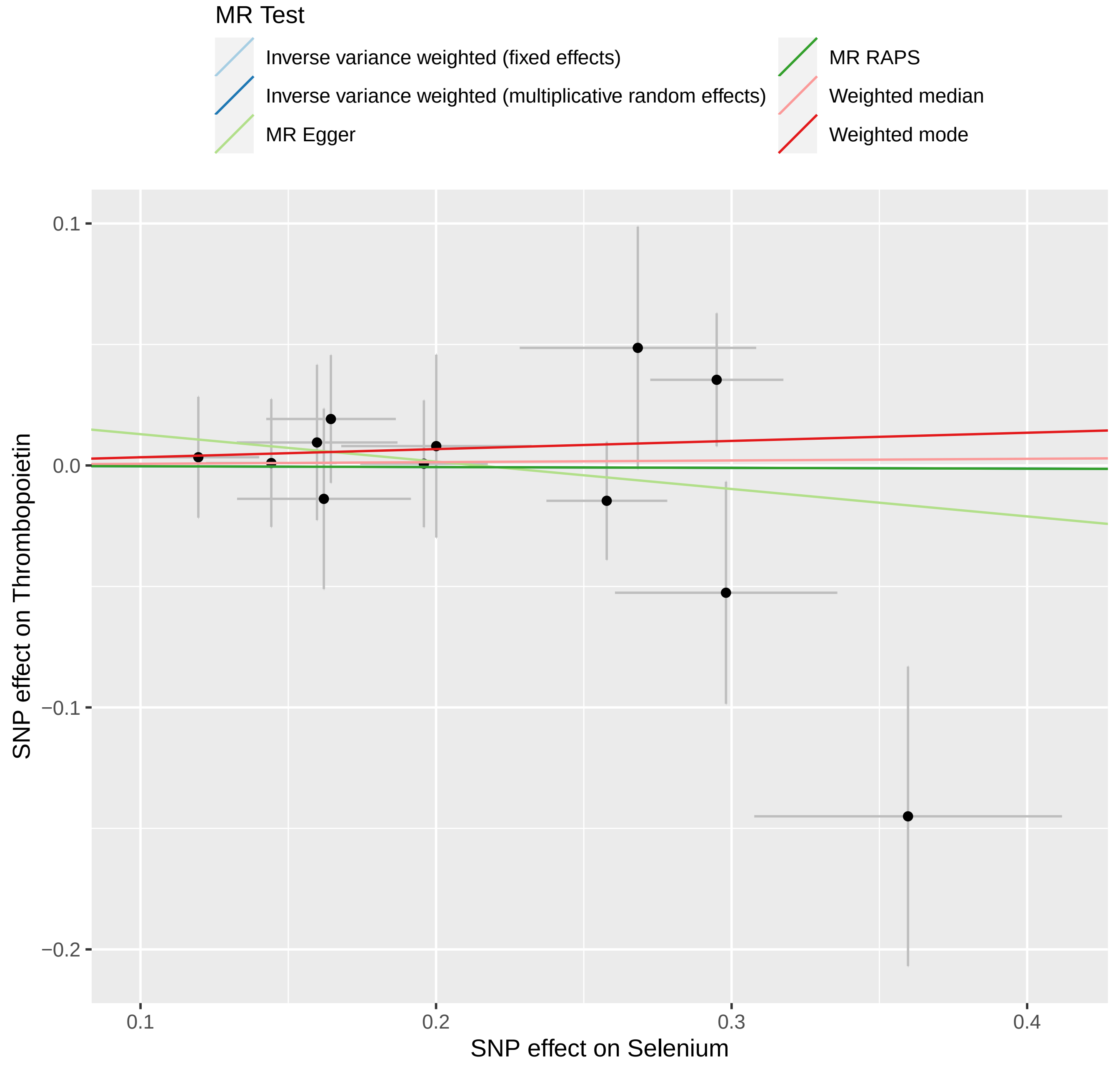

Batch 3−2973 : Selenium on Thrombopoietin

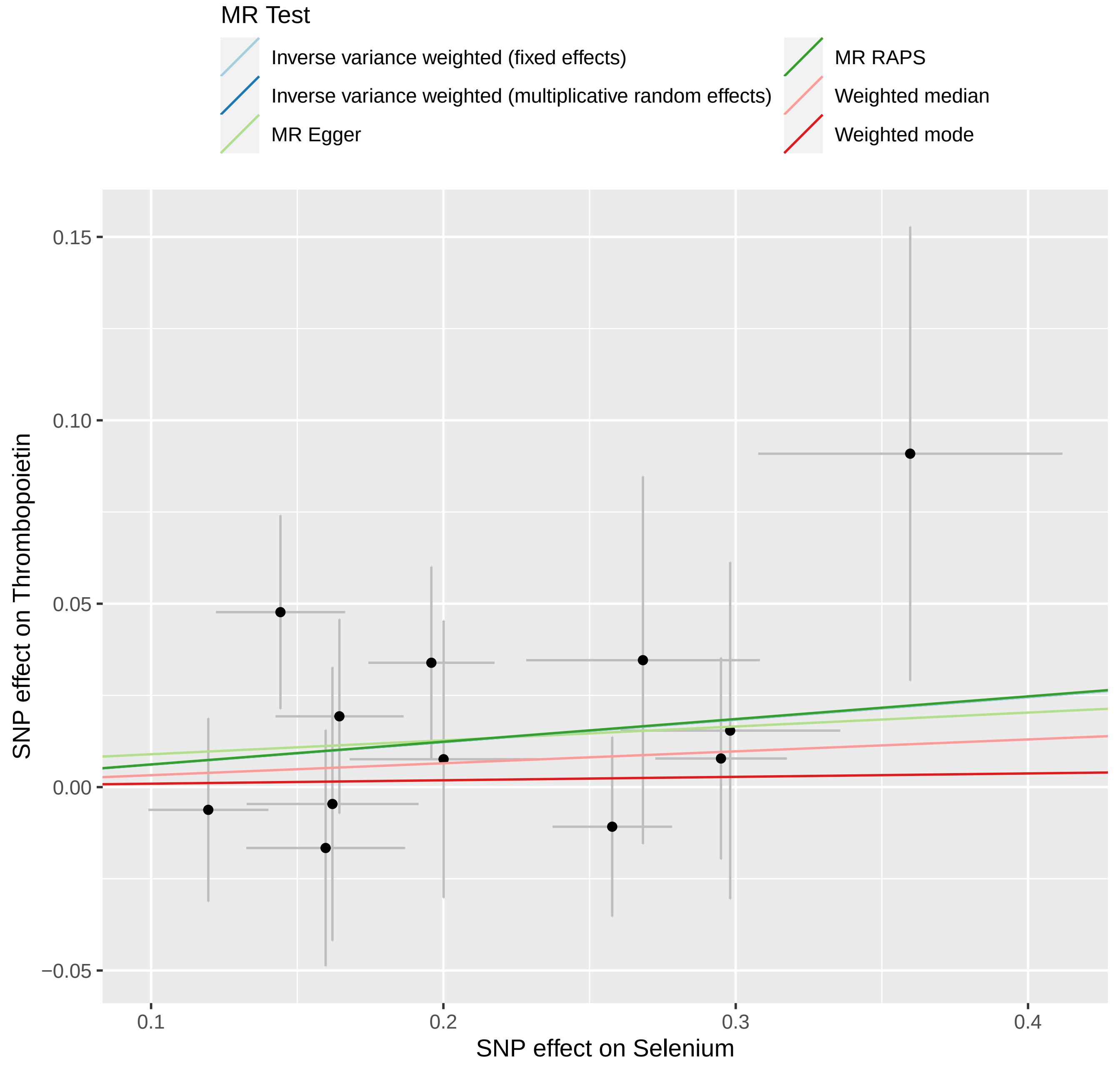

Batch 3−2974 : Selenium on Thyroid hormone receptor alpha

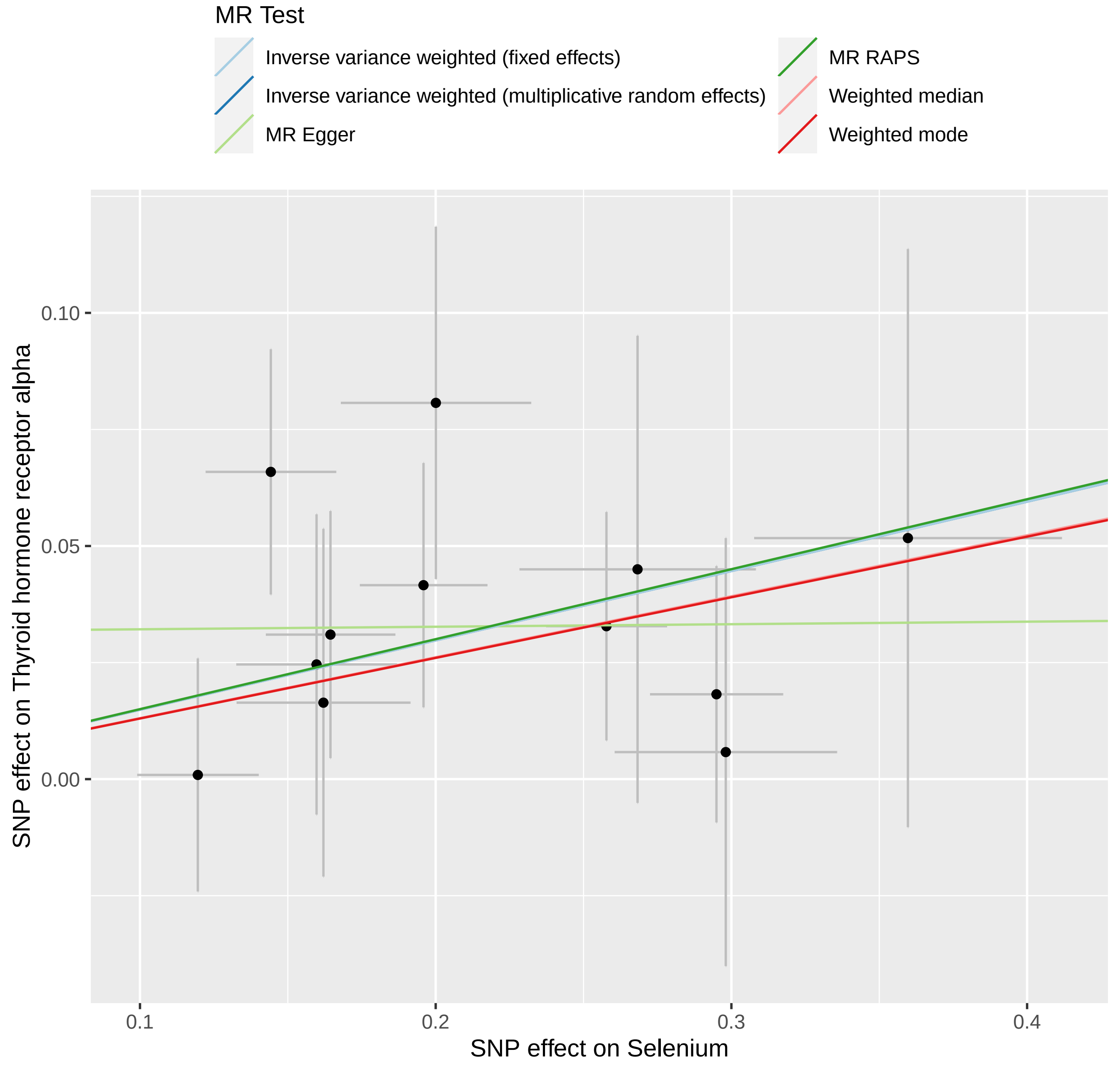

Batch 3−2975 : Selenium on Thrombospondin type−1 domain−containing protein 1

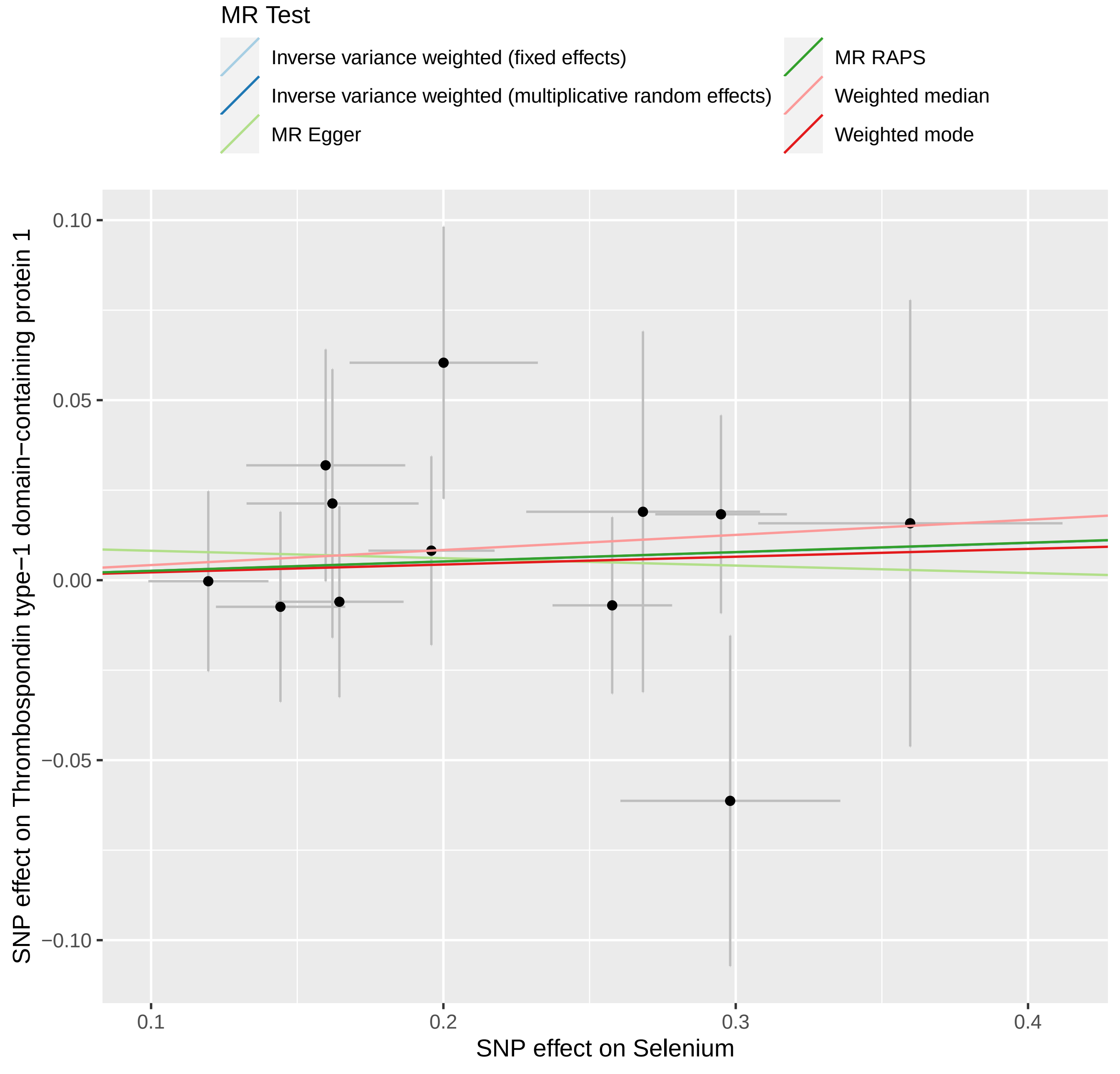

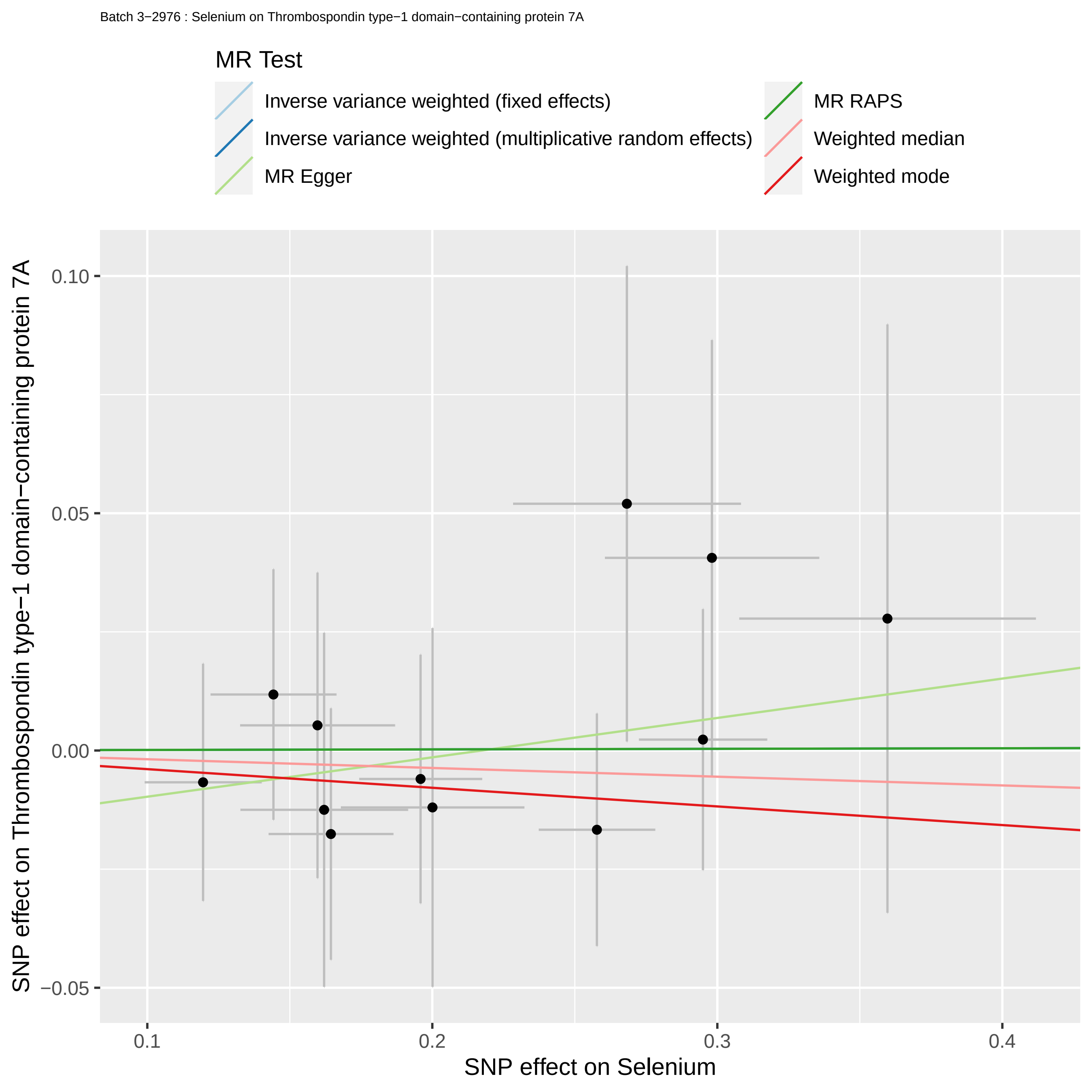

Batch 3−2976 : Selenium on Thrombospondin type−1 domain−containing protein 7A
MR Test
Inverse variance weighted (fixed effects)
Inverse variance weighted (multiplicative random effects)
MR Egger
MR RAPS
Weighted median
Weighted mode
SNP effect on Thrombospondin type−1 domain−containing protein 7A
0.10
0.05
0.00
−0.05
0.1
0.2
0.3
0.4
SNP effect on Selenium

Batch 3−2977 : Selenium on Thymocyte nuclear protein 1

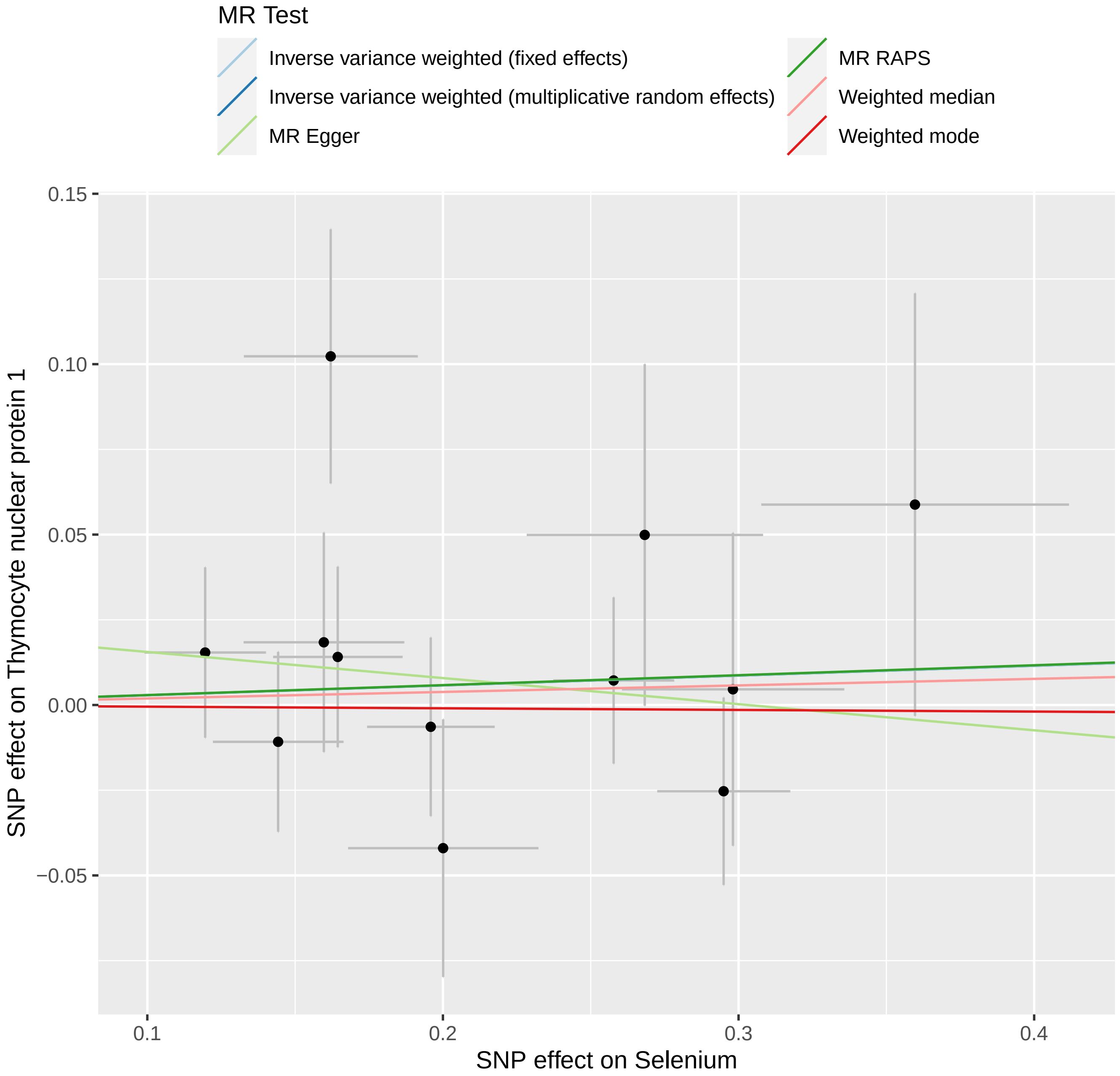

Batch 3−2978 : Selenium on Tyrosine−protein kinase receptor Tie−1, soluble

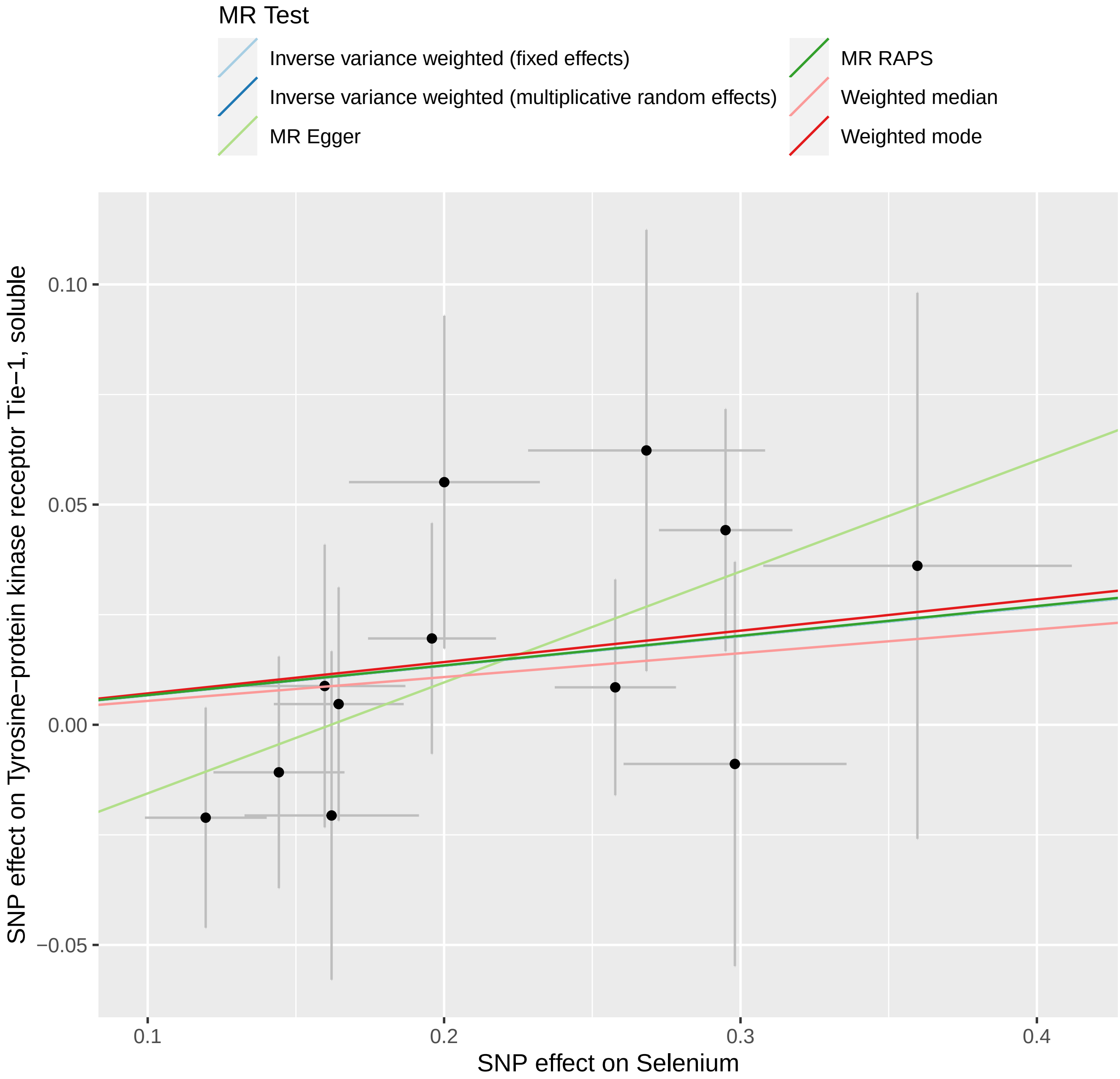

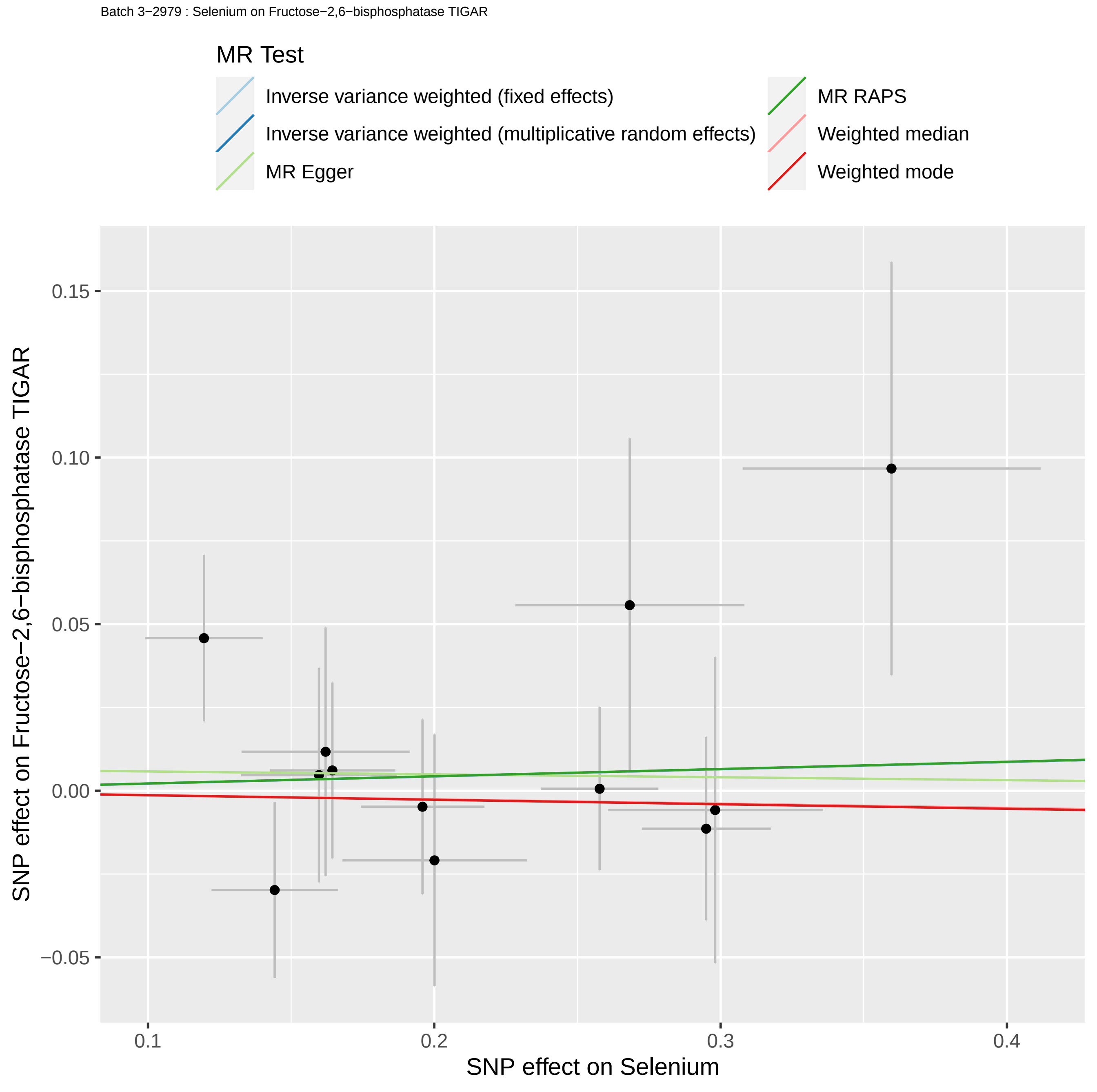

Batch 3−2979 : Selenium on Fructose−2,6−bisphosphatase TIGAR
MR Test
Inverse variance weighted (fixed effects)
Inverse variance weighted (multiplicative random effects)
MR Egger
MR RAPS
Weighted median
Weighted mode
0.15
0.10
0.05
0.00
−0.05
0.1
0.2
0.3
0.4
SNP effect on Fructose−2,6−bisphosphatase TIGAR
SNP effect on Selenium

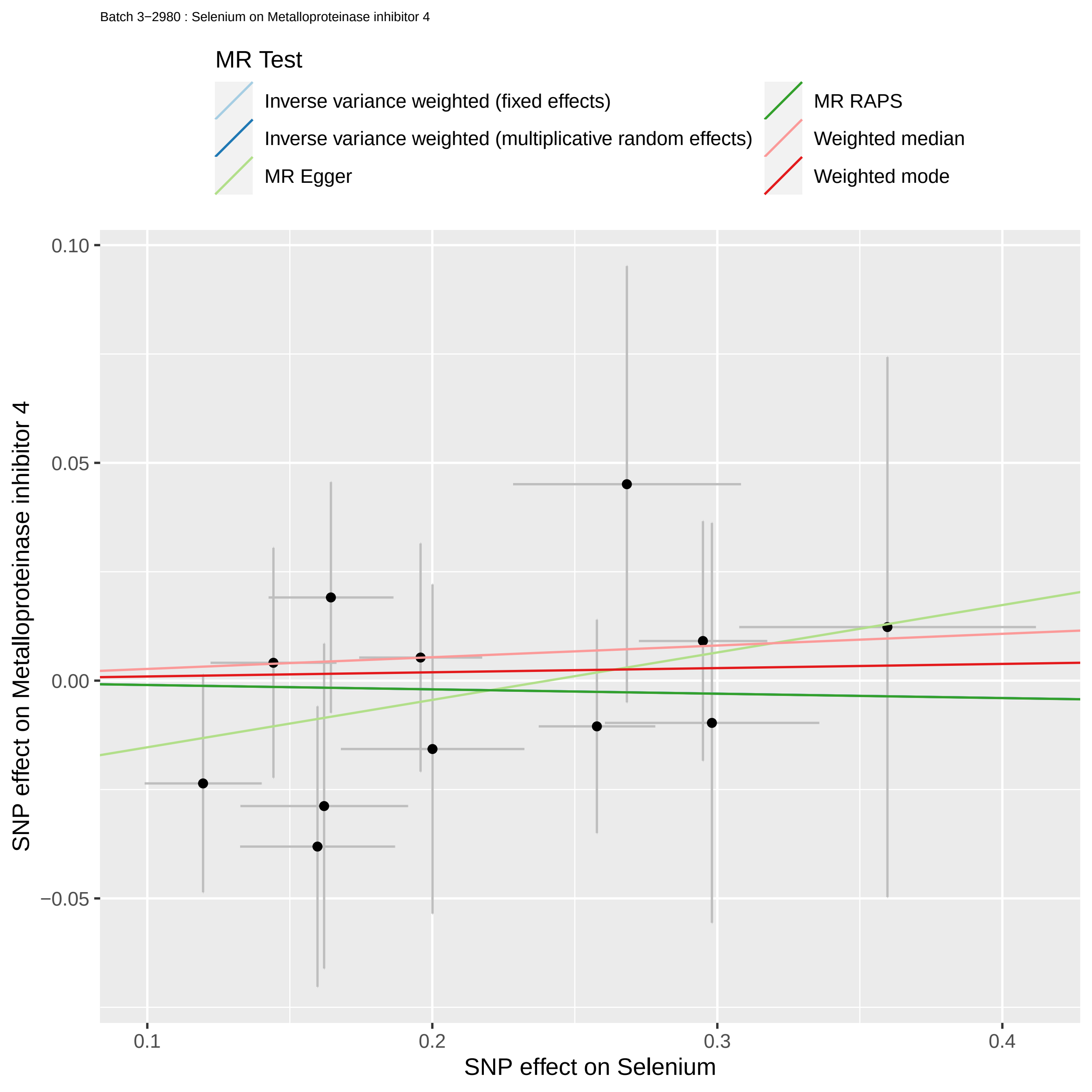
Batch 3−2980 : Selenium on Metalloproteinase inhibitor 4
MR Test
Inverse variance weighted (fixed effects)
Inverse variance weighted (multiplicative random effects)
MR Egger
MR RAPS
Weighted median
Weighted mode
0.10
0.05
0.00
−0.05
0.1
0.2
0.3
0.4
SNP effect on Metalloproteinase inhibitor 4
SNP effect on Selenium

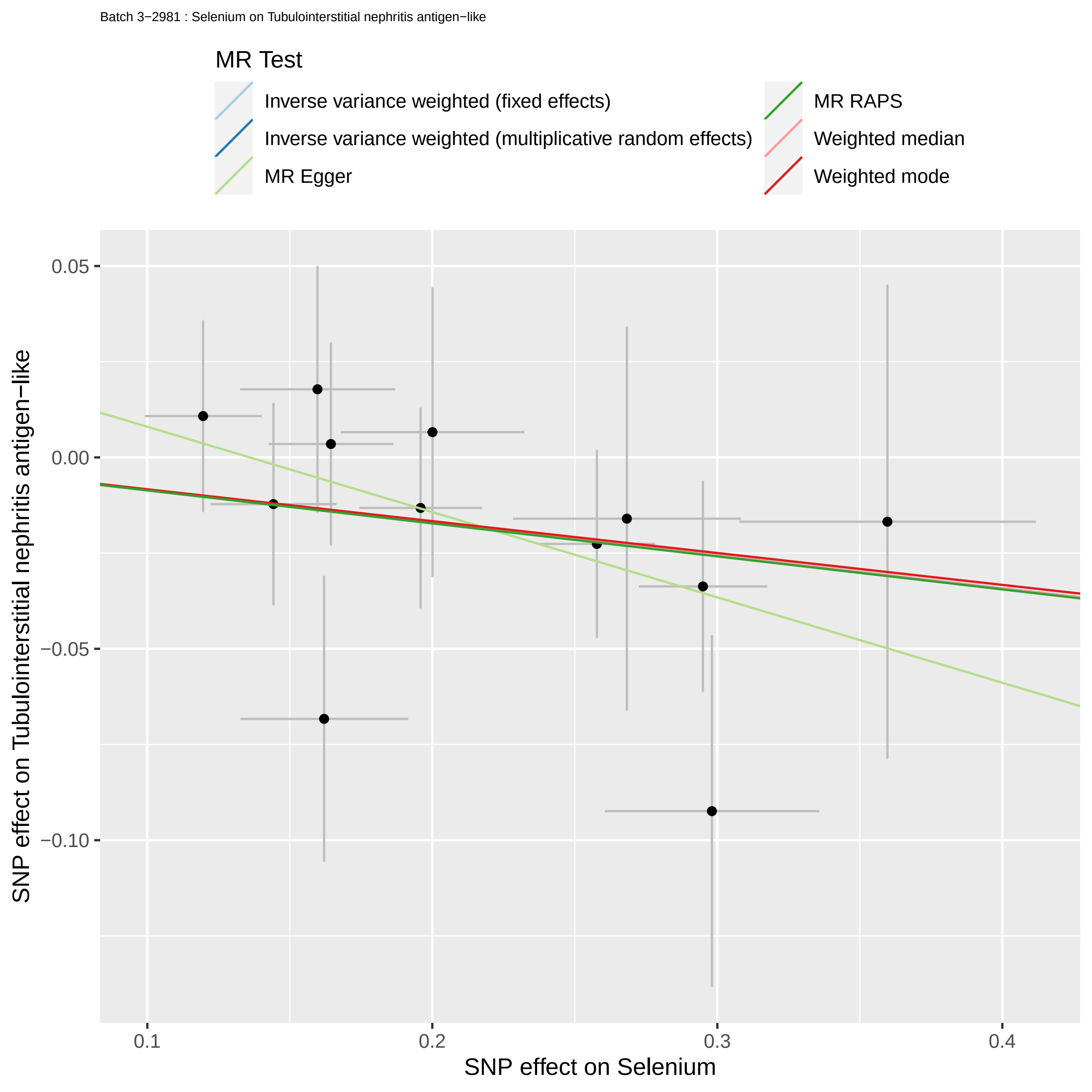

Batch 3−2981 : Selenium on Tubulointerstitial nephritis antigen−like
MR Test
Inverse variance weighted (fixed effects)
Inverse variance weighted (multiplicative random effects)
MR Egger
MR RAPS
Weighted median
Weighted mode
0.05
0.00
−0.05
−0.10
0.1
0.2
0.3
0.4
SNP effect on Tubulointerstitial nephritis antigen−like
SNP effect on Selenium

Batch 3−2982 : Selenium on Toll/interleukin−1 receptor domain−containing adapter protein

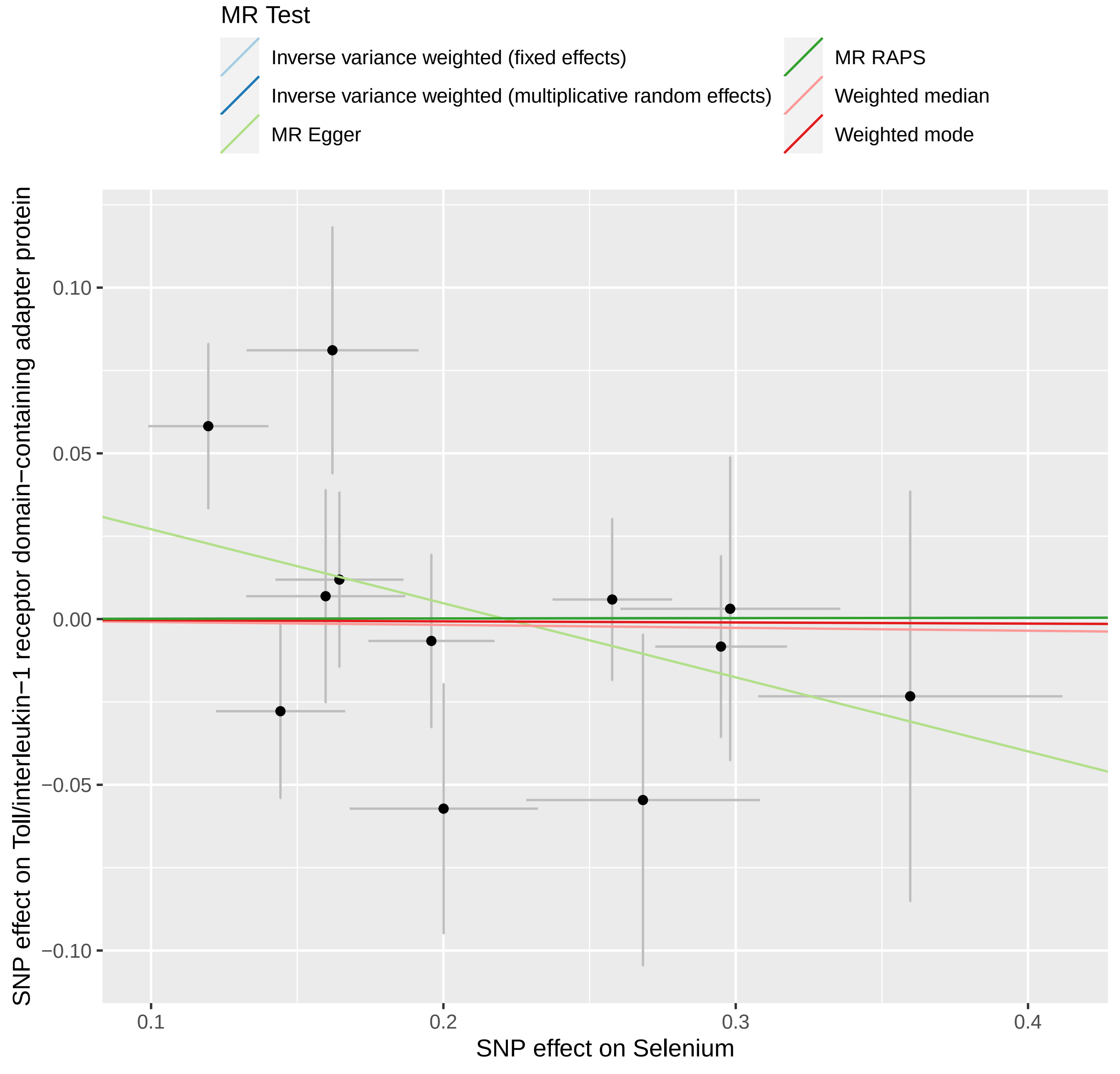

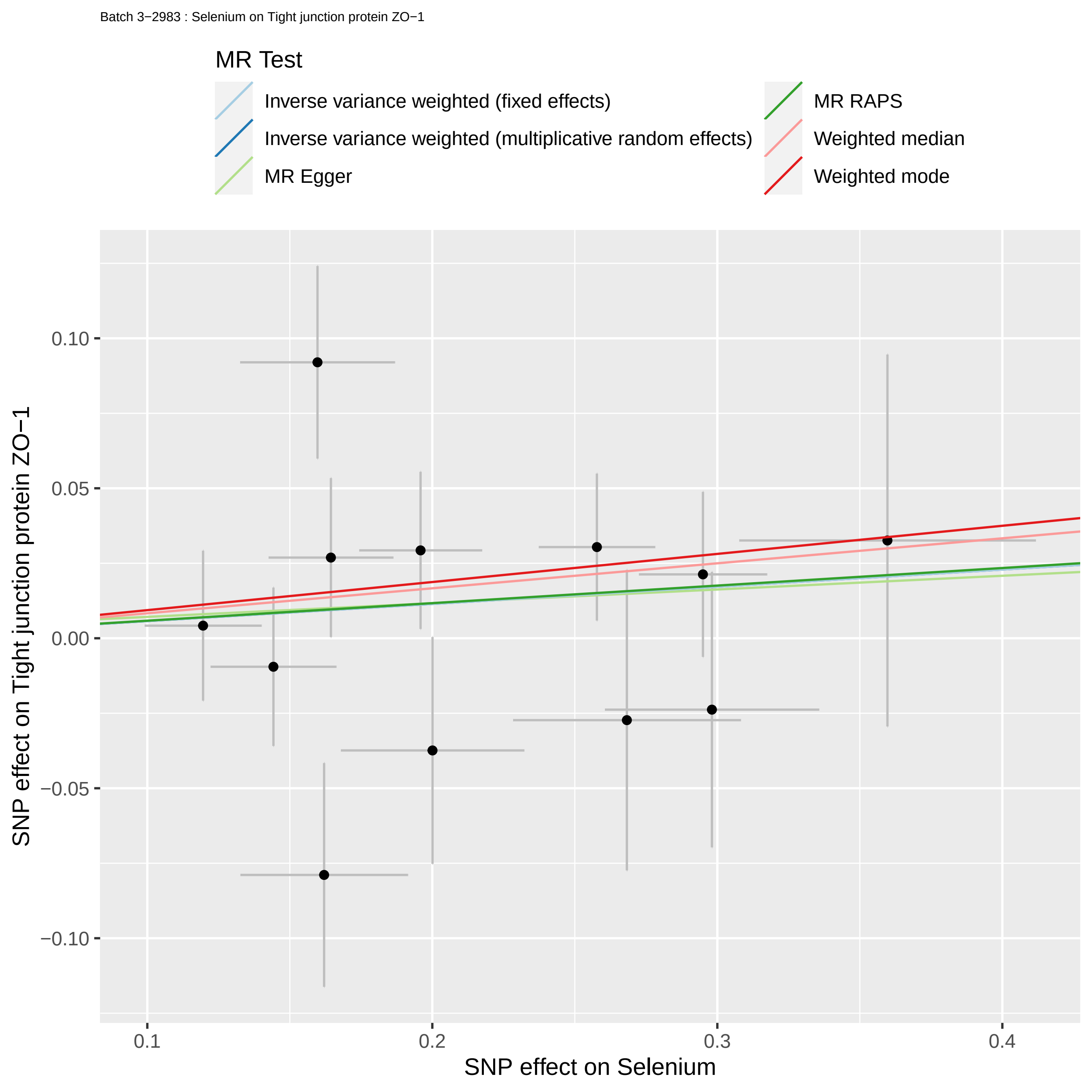

Batch 3−2983 : Selenium on Tight junction protein ZO−1
MR Test
Inverse variance weighted (fixed effects)
Inverse variance weighted (multiplicative random effects)
MR Egger
MR RAPS
Weighted median
Weighted mode
SNP effect on Tight junction protein ZO−1
0.10
0.05
0.00
−0.05
−0.10
0.1
0.2
0.3
0.4
SNP effect on Selenium

Batch 3−2984 : Selenium on Thymidine kinase, cytosolic

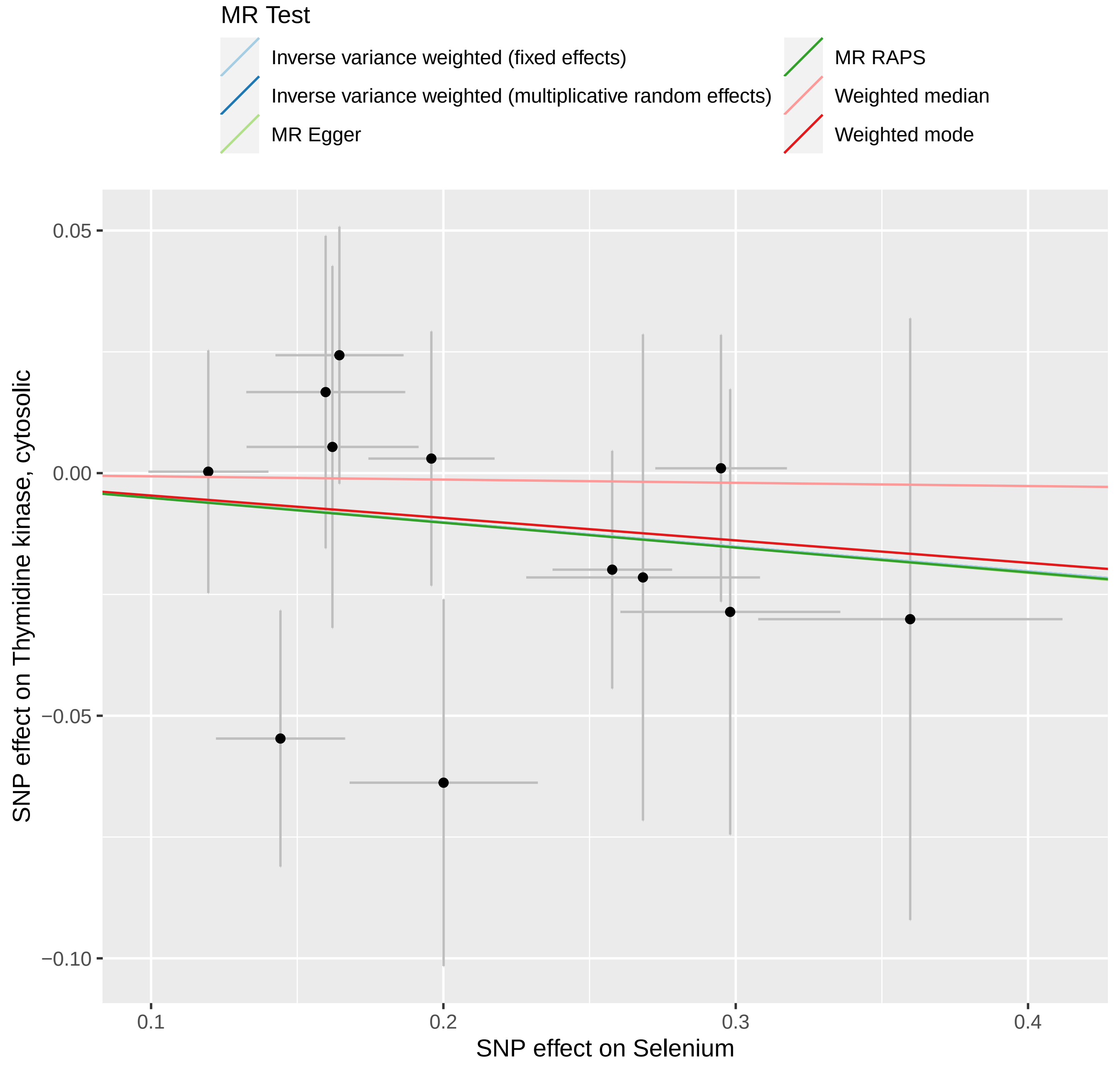

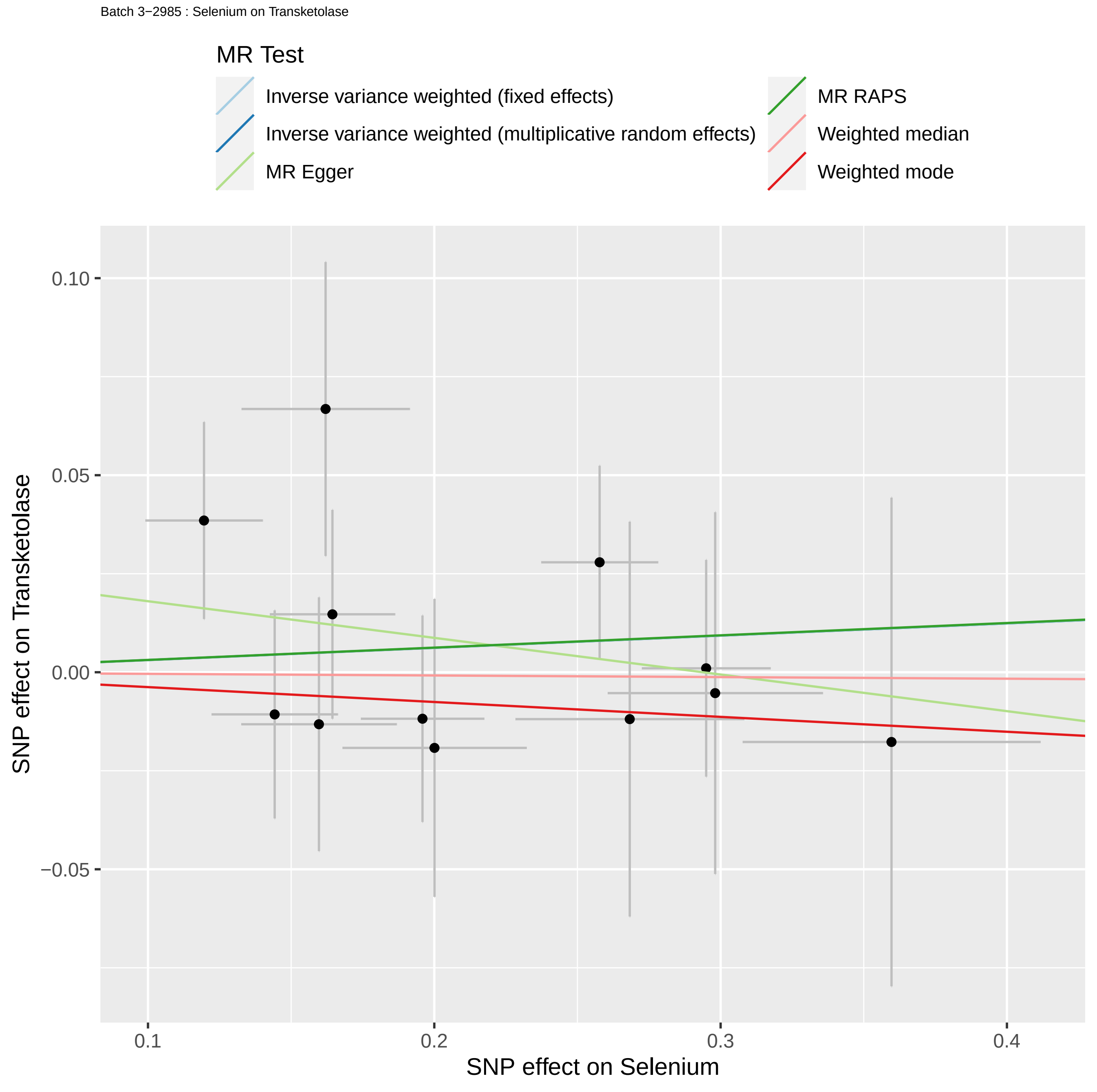
Batch 3−2985 : Selenium on Transketolase
MR Test
Inverse variance weighted (fixed effects)
Inverse variance weighted (multiplicative random effects)
MR Egger
MR RAPS
Weighted median
Weighted mode
0.10
0.05
0.00
−0.05
0.1
0.2
0.3
0.4
SNP effect on Transketolase
SNP effect on Selenium

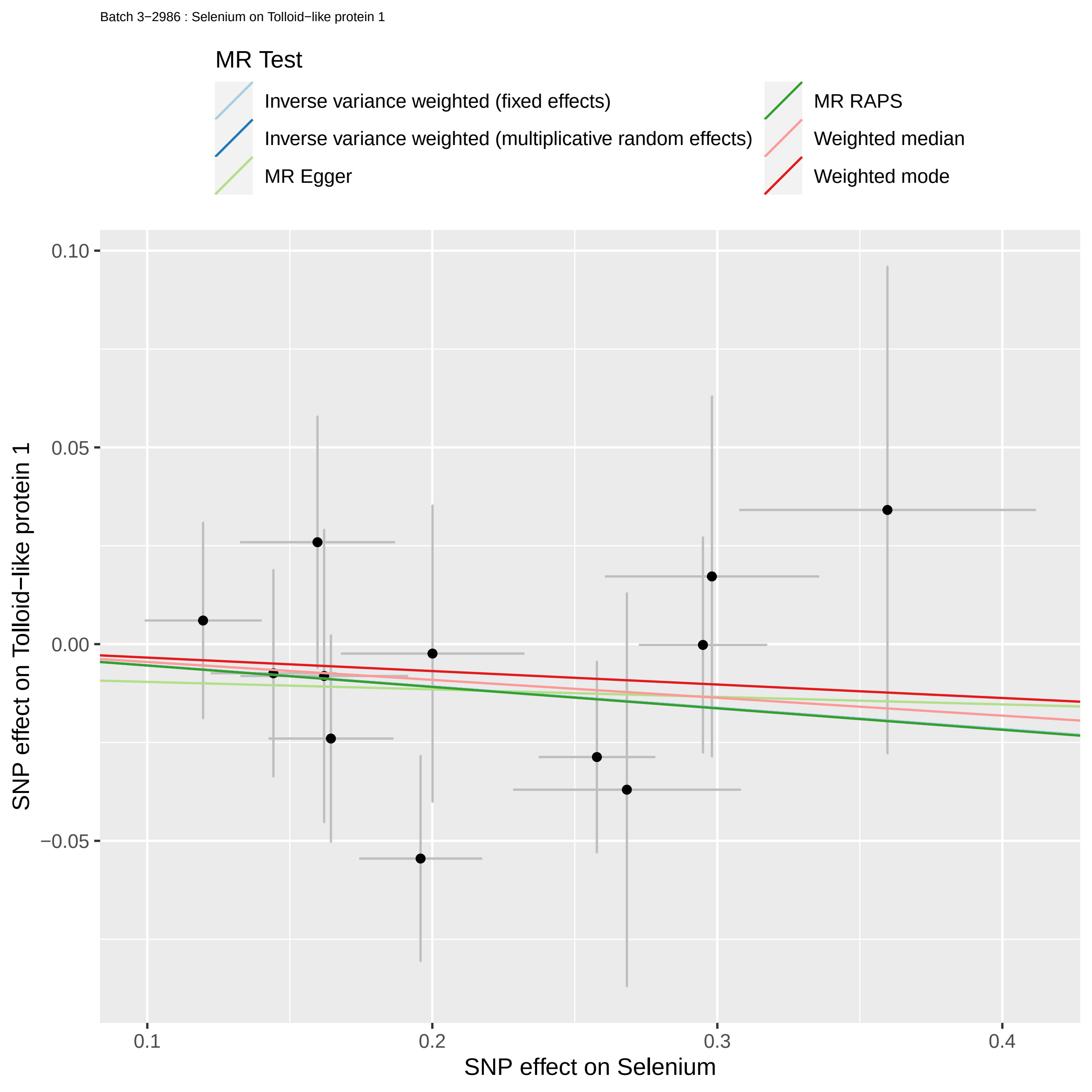
Batch 3−2986 : Selenium on Tolloid−like protein 1
MR Test
Inverse variance weighted (fixed effects)
Inverse variance weighted (multiplicative random effects)
MR Egger
MR RAPS
Weighted median
Weighted mode
0.10
0.05
0.00
−0.05
0.1
0.2
0.3
0.4
SNP effect on Tolloid−like protein 1
SNP effect on Selenium

Batch 3−2987 : Selenium on Talin−2

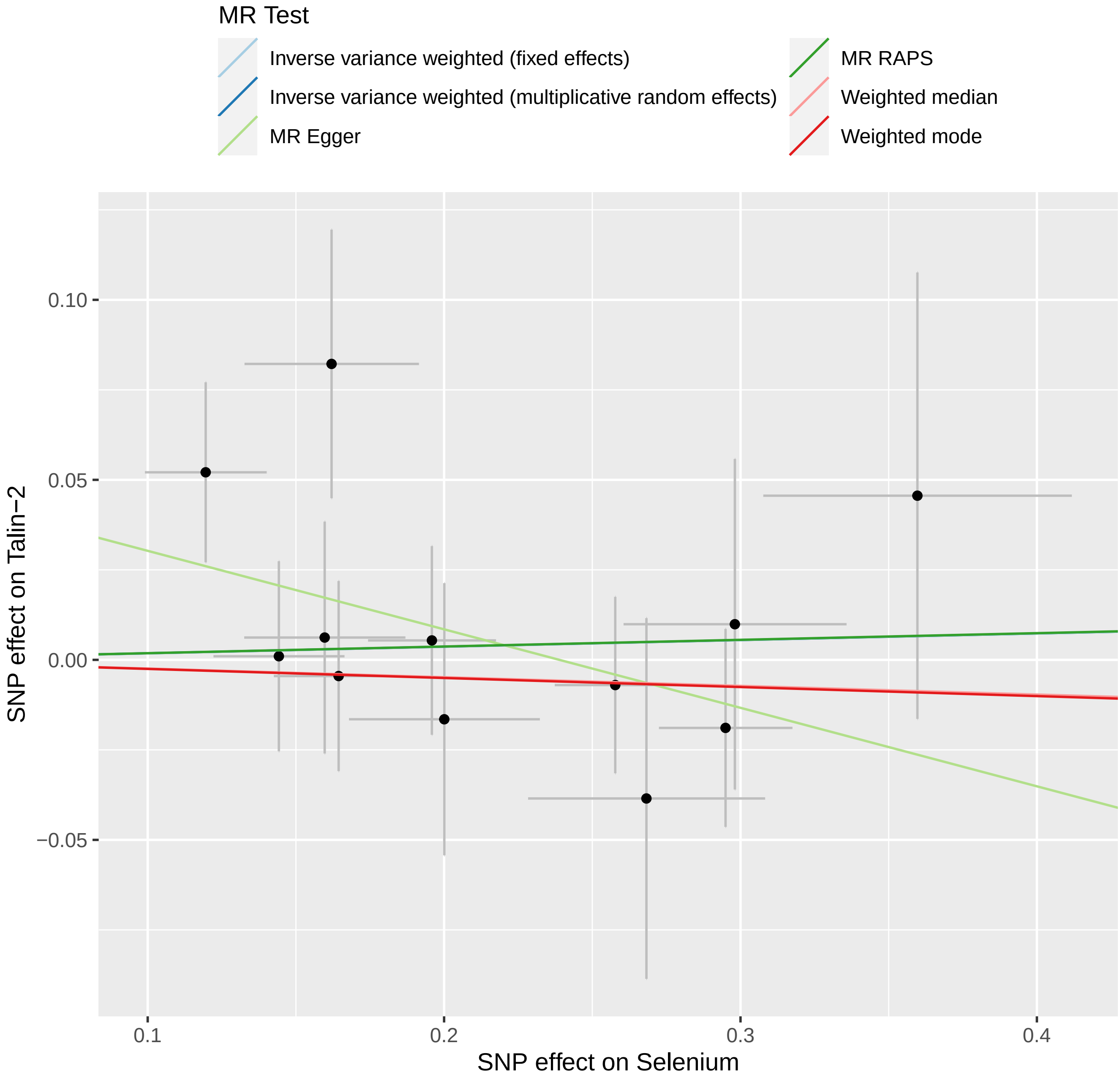

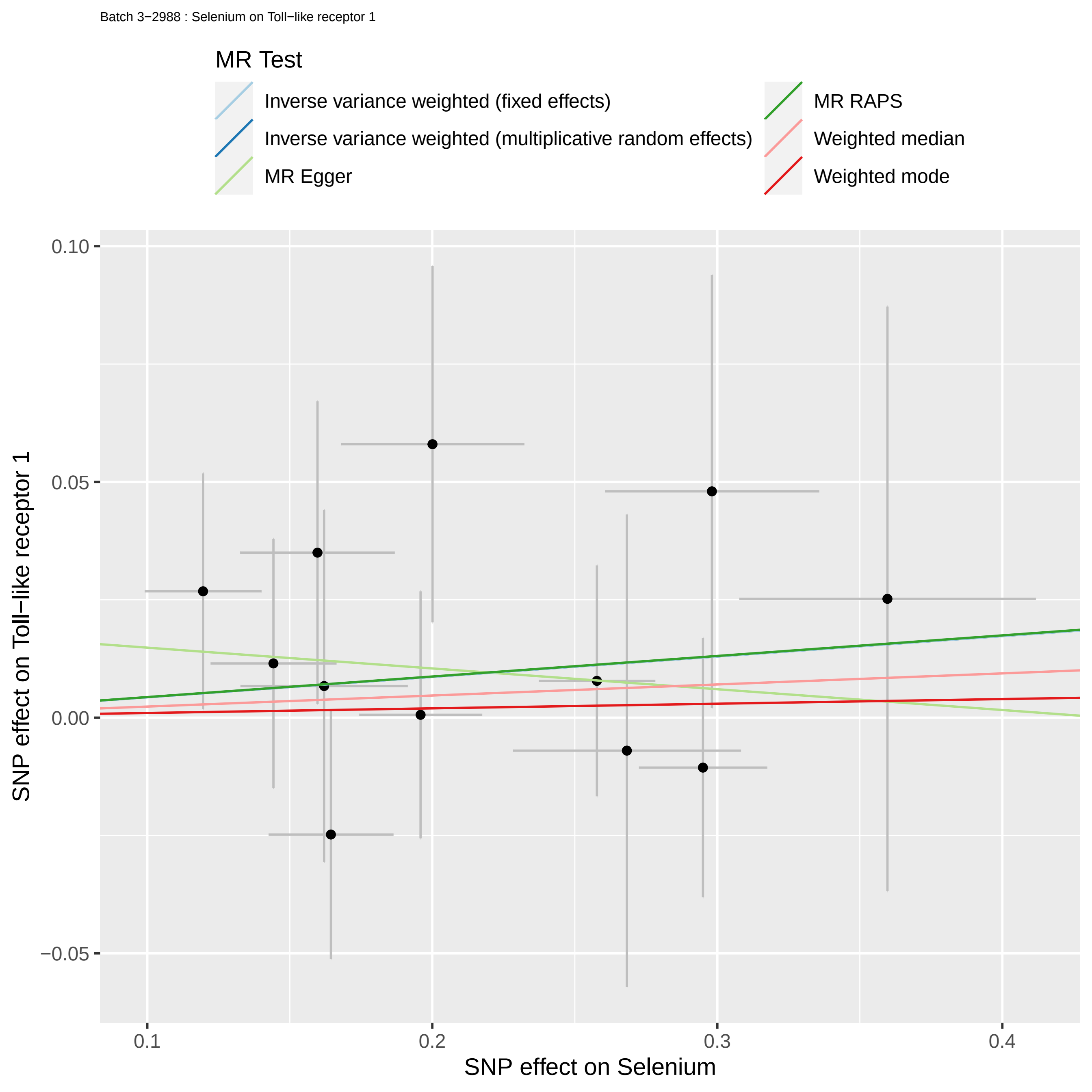

Batch 3−2988 : Selenium on Toll−like receptor 1
MR Test
Inverse variance weighted (fixed effects)
Inverse variance weighted (multiplicative random effects)
MR Egger
MR RAPS
Weighted median
Weighted mode
0.10
0.05
0.00
−0.05
0.1
0.2
0.3
0.4
SNP effect on Toll−like receptor 1
SNP effect on Selenium

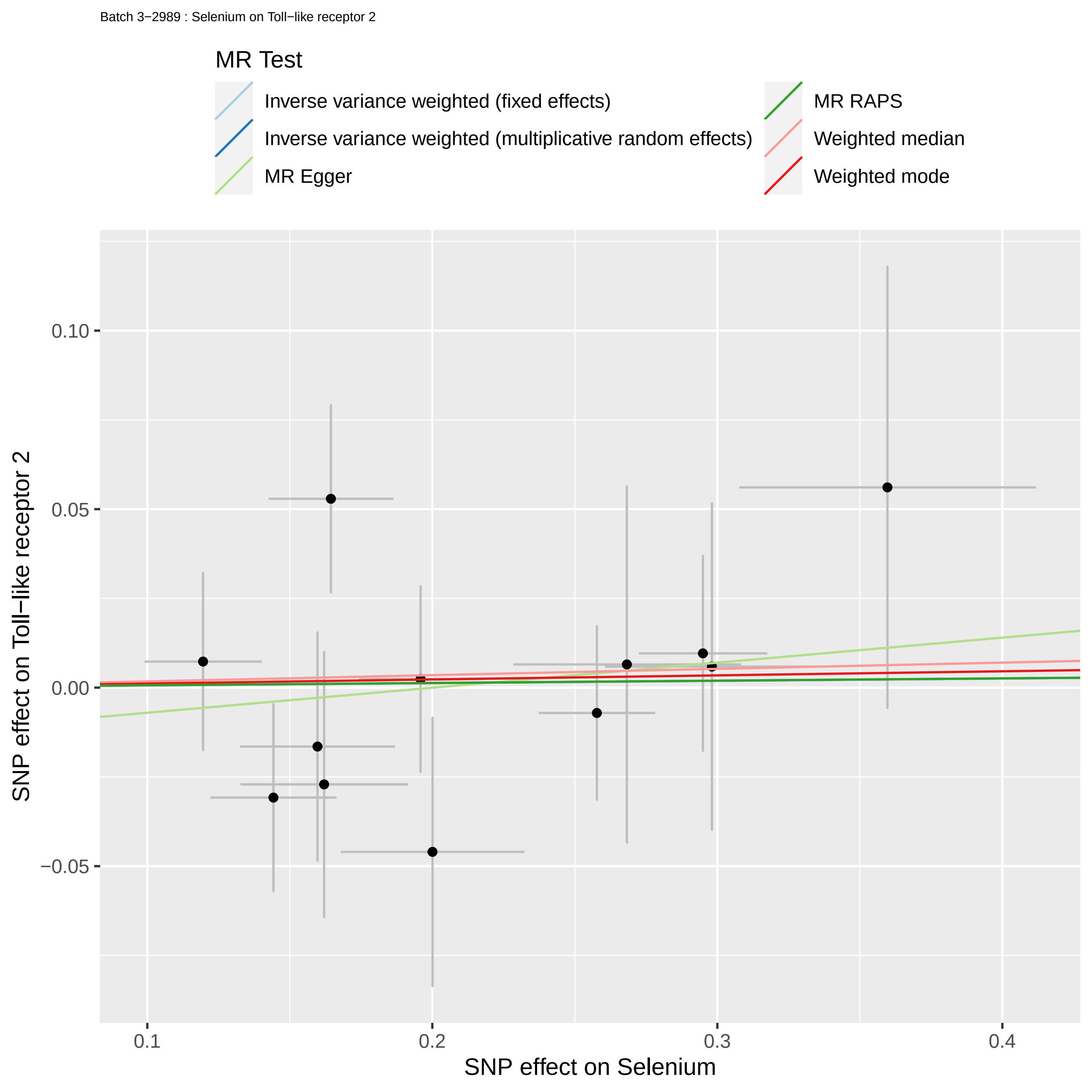
Batch 3−2989 : Selenium on Toll−like receptor 2
MR Test
Inverse variance weighted (fixed effects)
Inverse variance weighted (multiplicative random effects)
MR Egger
MR RAPS
Weighted median
Weighted mode
SNP effect on Toll−like receptor 2
0.10
0.05
0.00
−0.05
0.1
0.2
0.3
0.4
SNP effect on Selenium

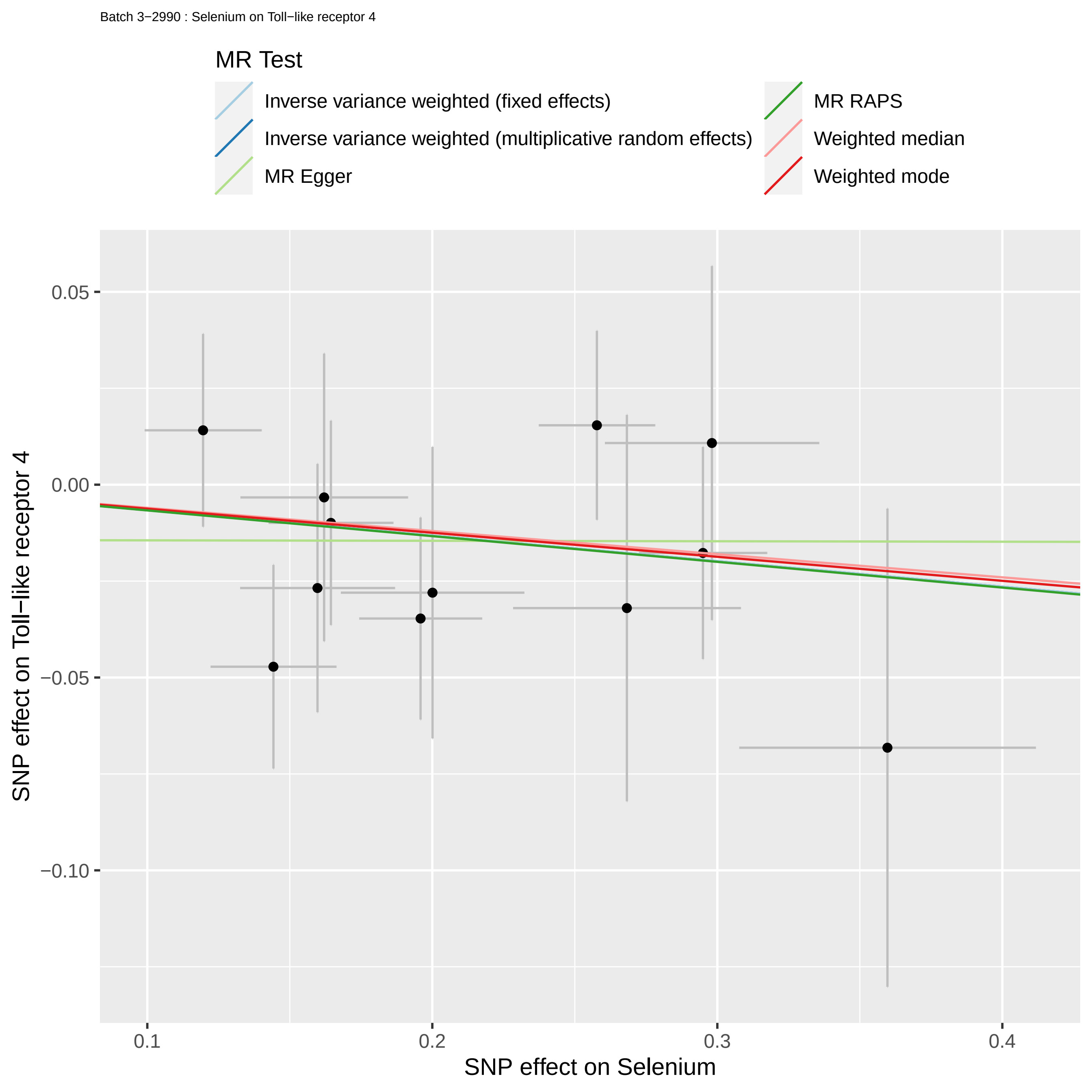

Batch 3−2990 : Selenium on Toll−like receptor 4
MR Test
Inverse variance weighted (fixed effects)
Inverse variance weighted (multiplicative random effects)
MR Egger
MR RAPS
Weighted median
Weighted mode
0.05
0.00
−0.05
−0.10
SNP effect on Toll−like receptor 4
0.1
0.2
0.3
0.4
SNP effect on Selenium

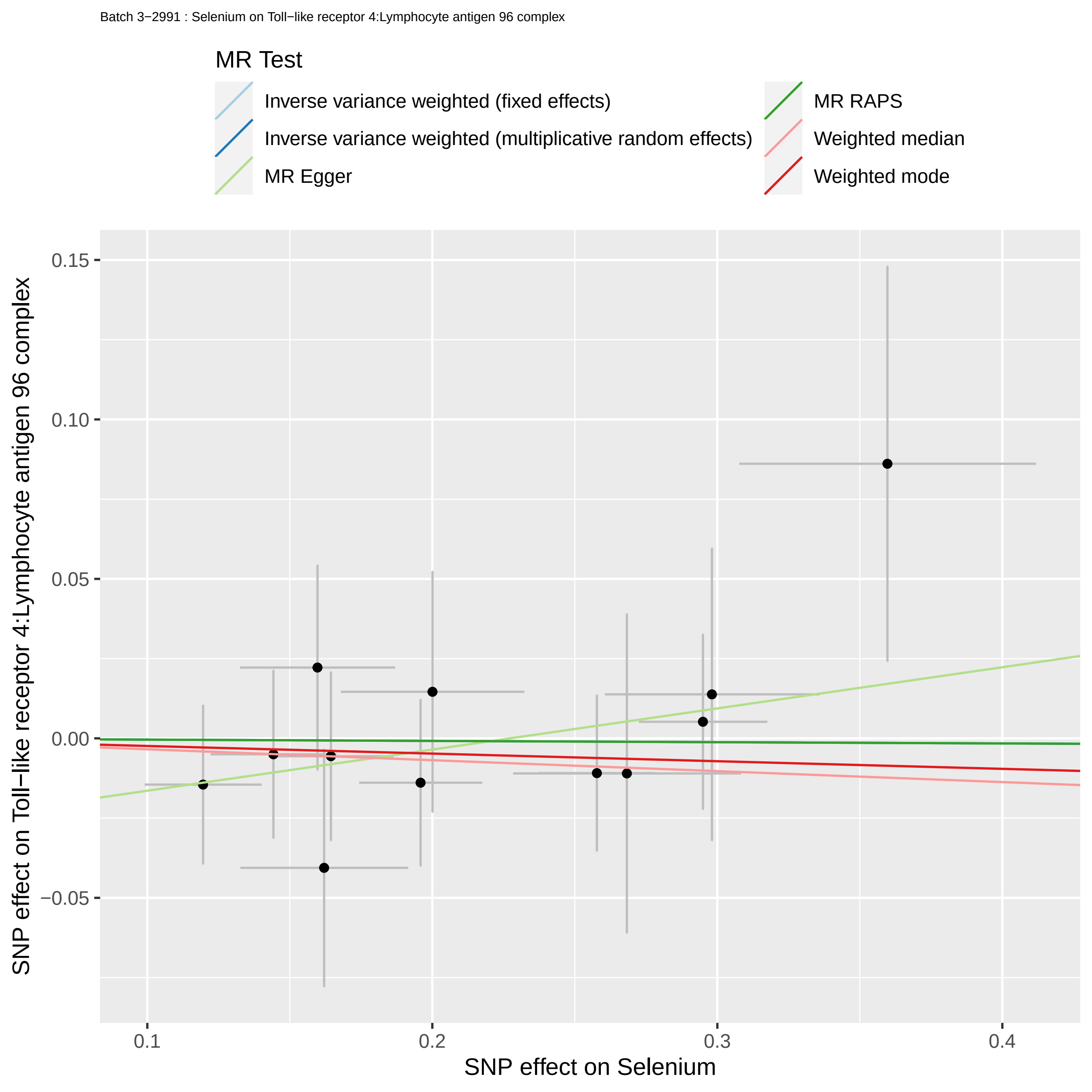

Batch 3−2991 : Selenium on Toll−like receptor 4:Lymphocyte antigen 96 complex
MR Test
Inverse variance weighted (fixed effects)
Inverse variance weighted (multiplicative random effects)
MR Egger
MR RAPS
Weighted median
Weighted mode
0.15
0.10
0.05
0.00
−0.05
0.1
0.2
0.3
0.4
SNP effect on Toll−like receptor 4:Lymphocyte antigen 96 complex
SNP effect on Selenium

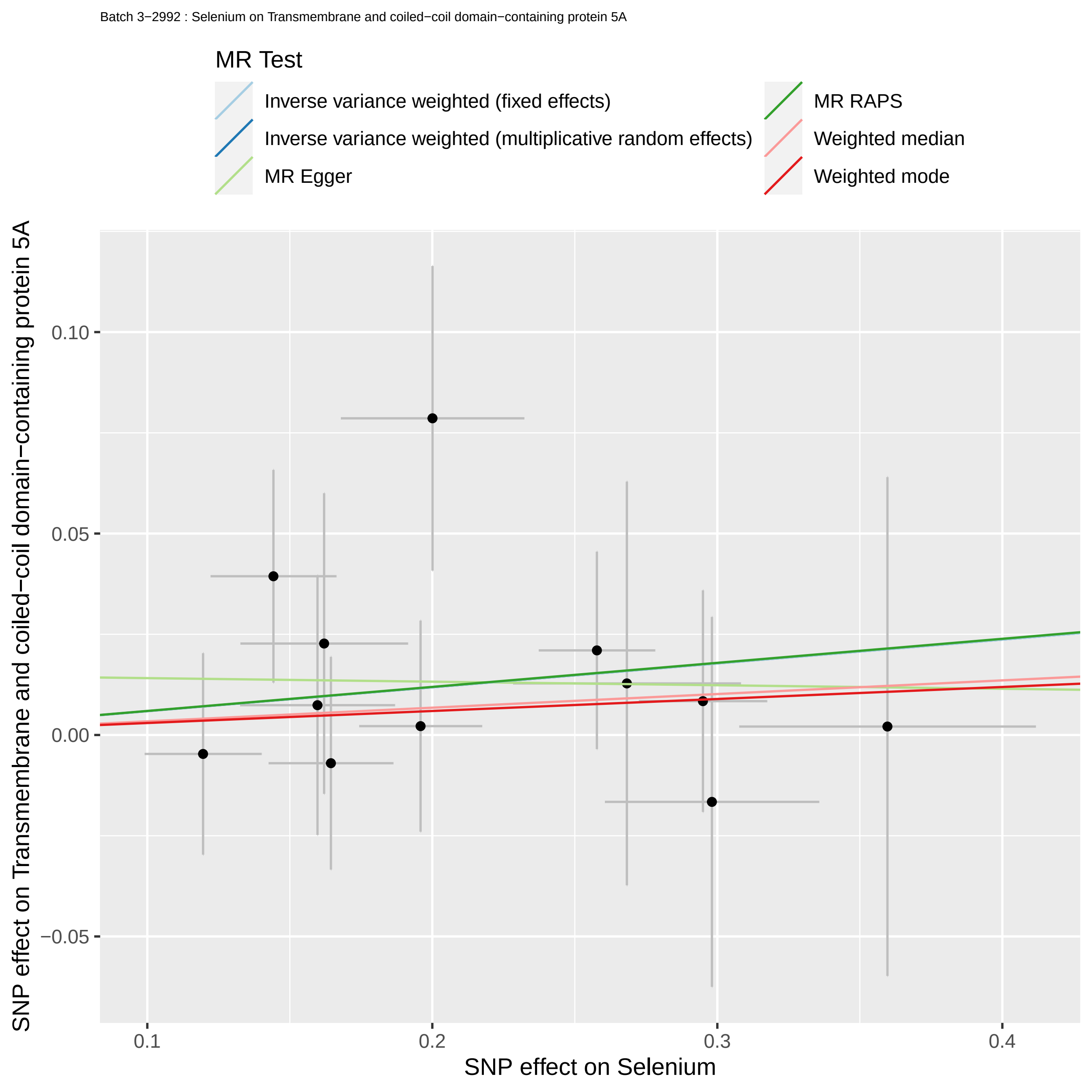
Batch 3−2992 : Selenium on Transmembrane and coiled−coil domain−containing protein 5A
MR Test
Inverse variance weighted (fixed effects)
Inverse variance weighted (multiplicative random effects)
MR Egger
MR RAPS
Weighted median
Weighted mode
SNP effect on Transmembrane and coiled−coil domain−containing protein 5A
0.10
0.05
0.00
−0.05
0.1
0.2
0.3
0.4
SNP effect on Selenium

Batch 3−2993 : Selenium on Transmembrane emp24 domain−containing protein 10

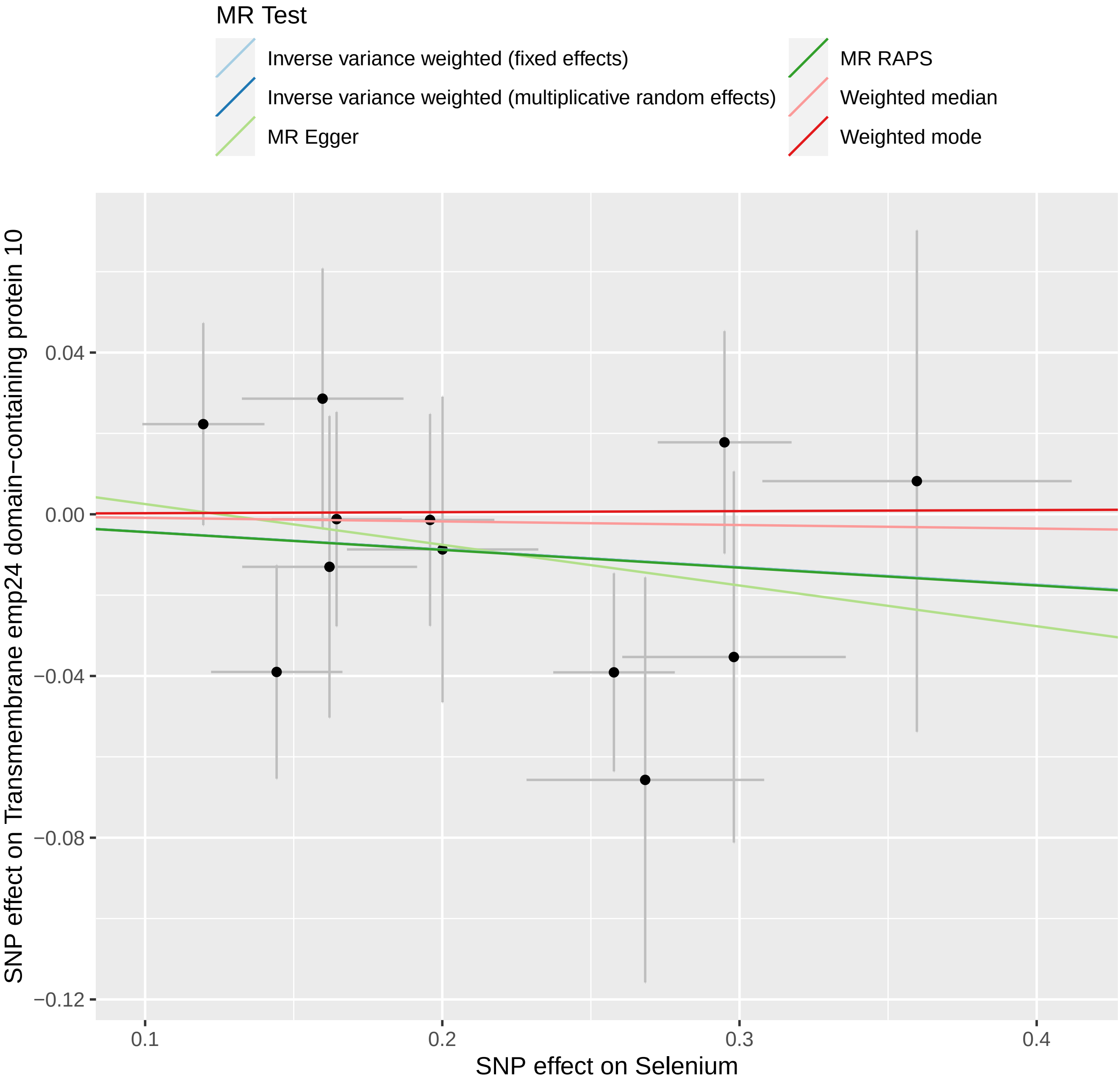

Batch 3−2994 : Selenium on Transmembrane emp24 domain−containing protein 2

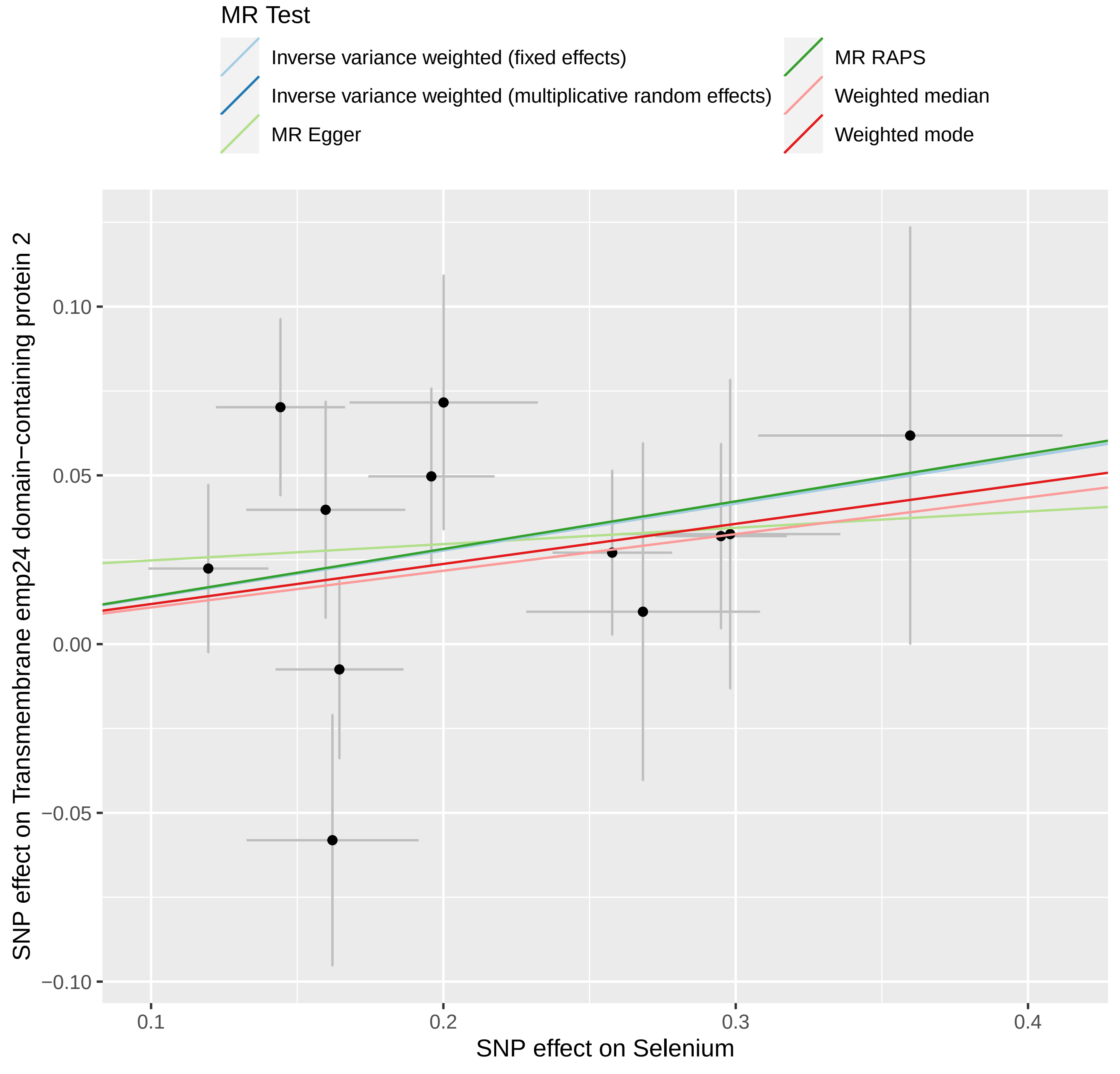

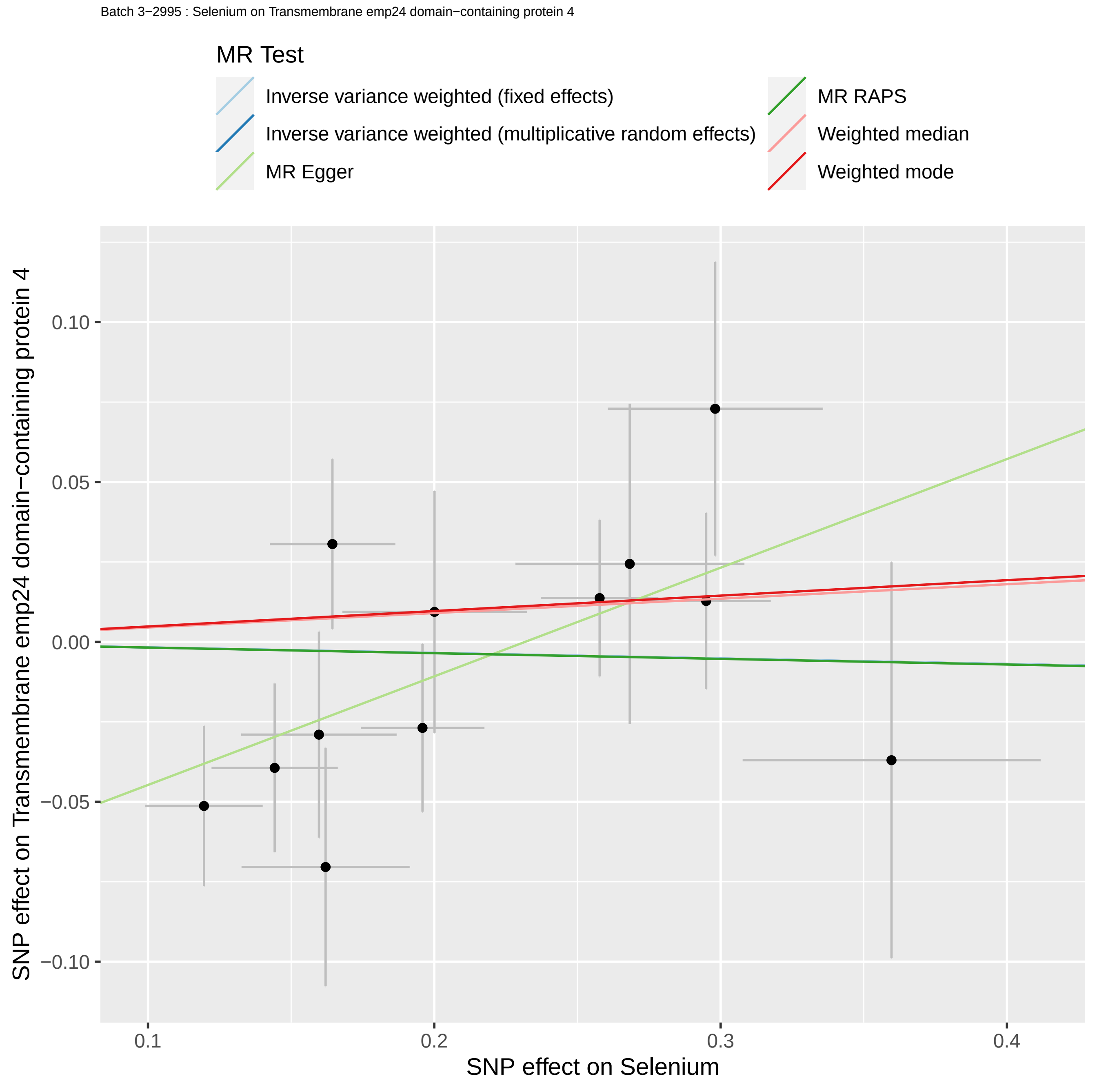
Batch 3−2995 : Selenium on Transmembrane emp24 domain−containing protein 4
MR Test
Inverse variance weighted (fixed effects)
Inverse variance weighted (multiplicative random effects)
MR Egger
MR RAPS
Weighted median
Weighted mode
SNP effect on Transmembrane emp24 domain−containing protein 4
0.10
0.05
0.00
−0.05
−0.10
0.1
0.2
0.3
0.4
SNP effect on Selenium

Batch 3−2996 : Selenium on Transmembrane protein 119

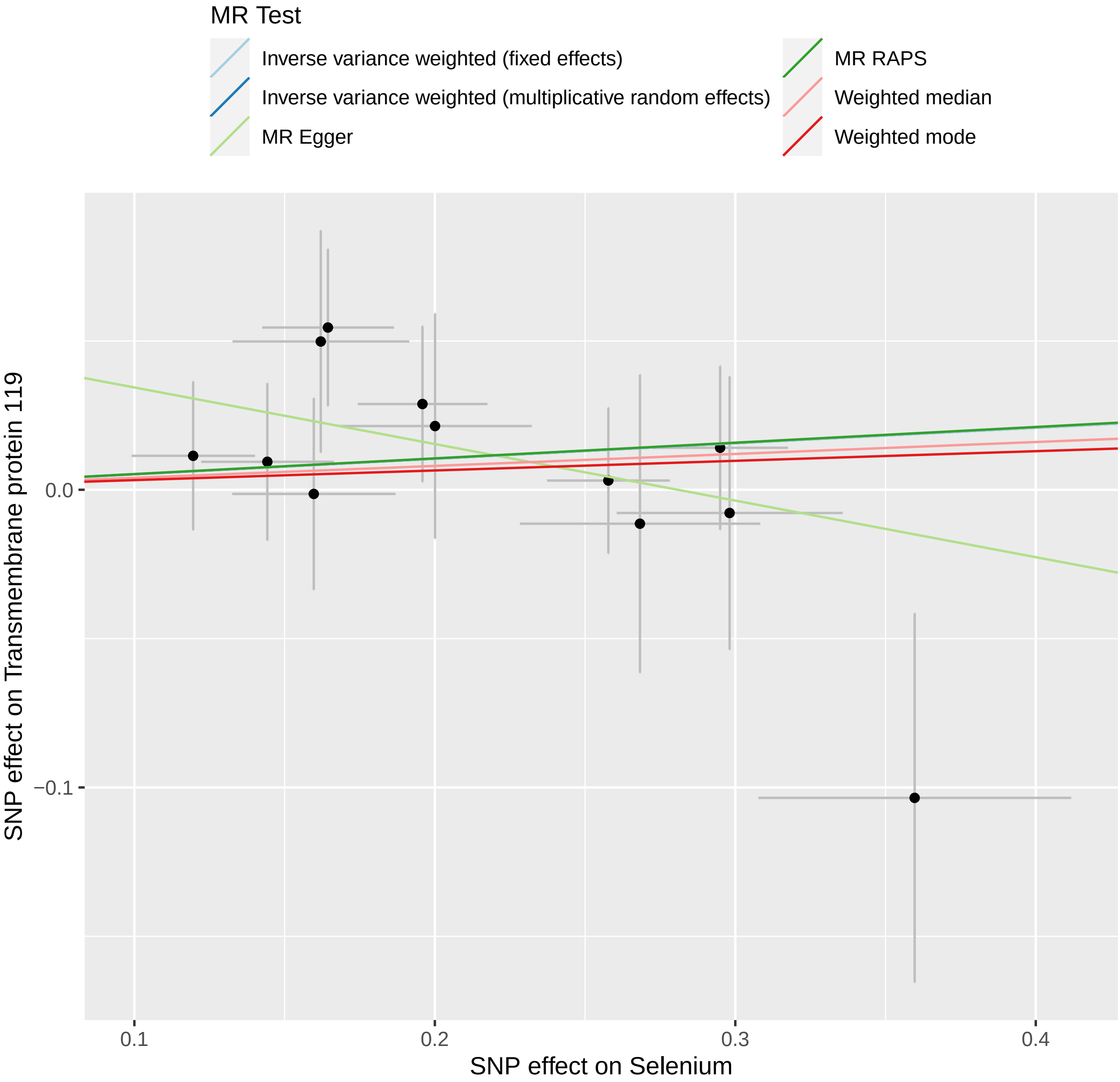

Batch 3−2997 : Selenium on Transmembrane protein 132A

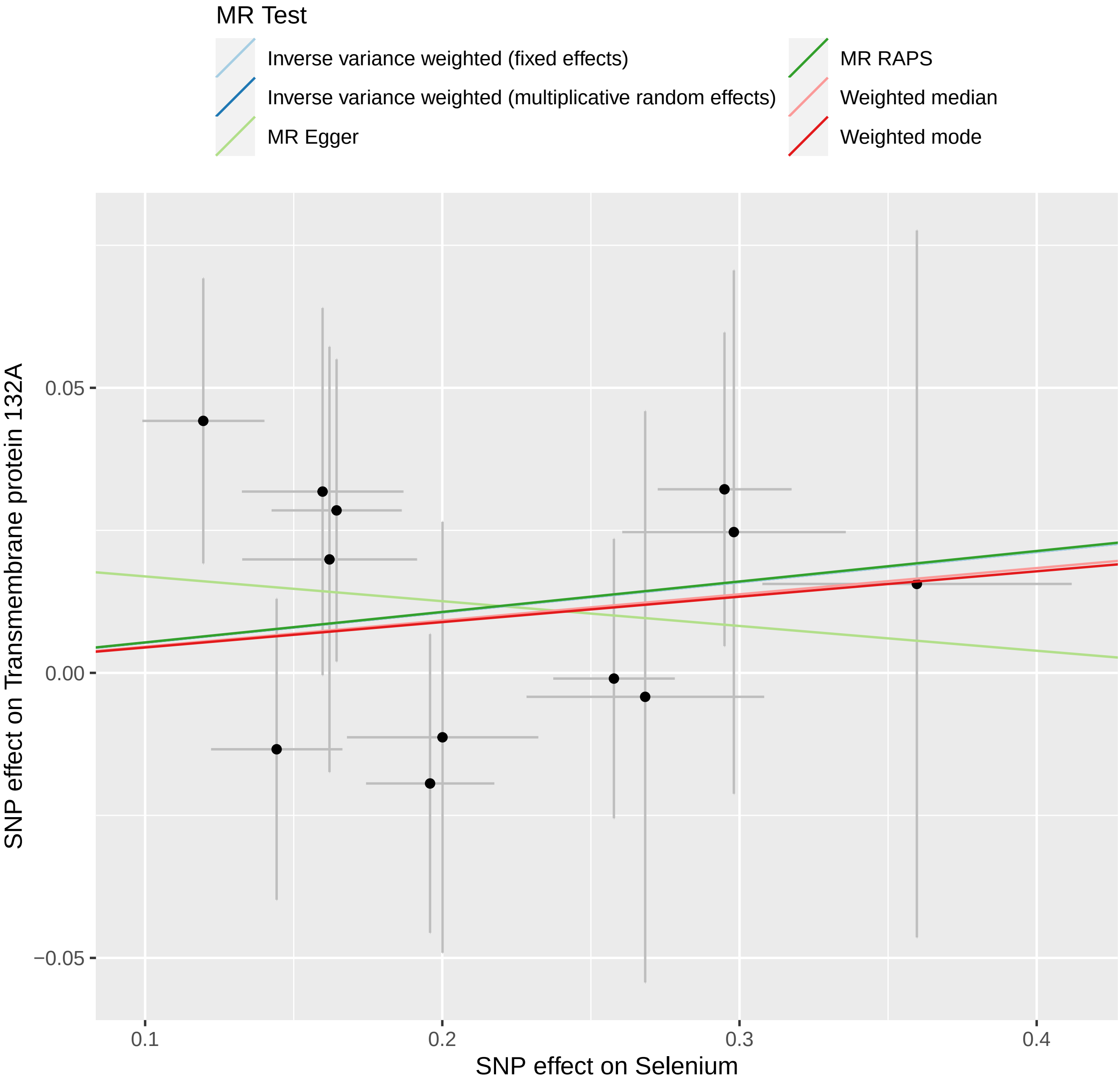

Batch 3−2998 : Selenium on Transmembrane protein 132B

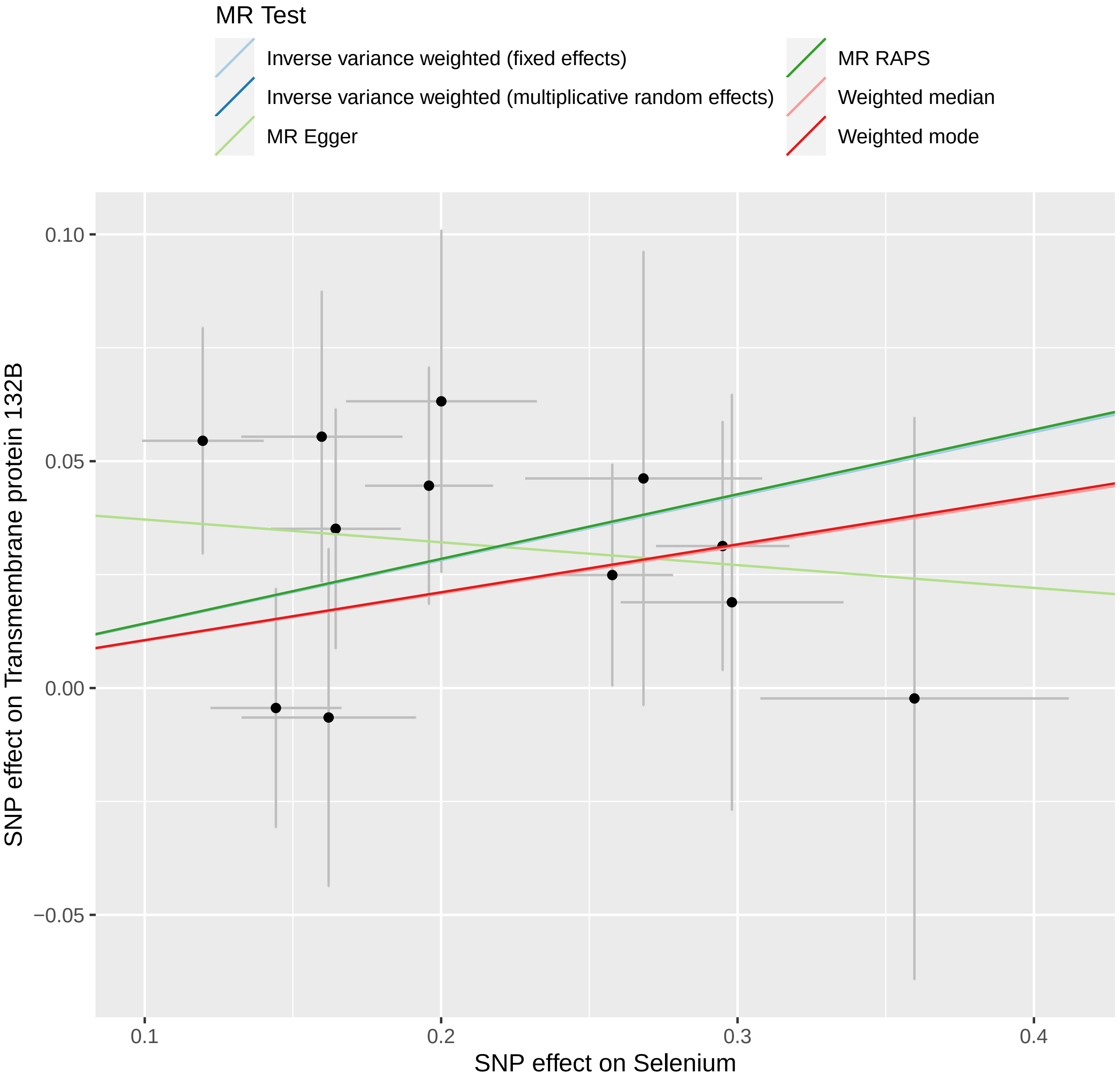

Batch 3−2999 : Selenium on Transmembrane protein 132C

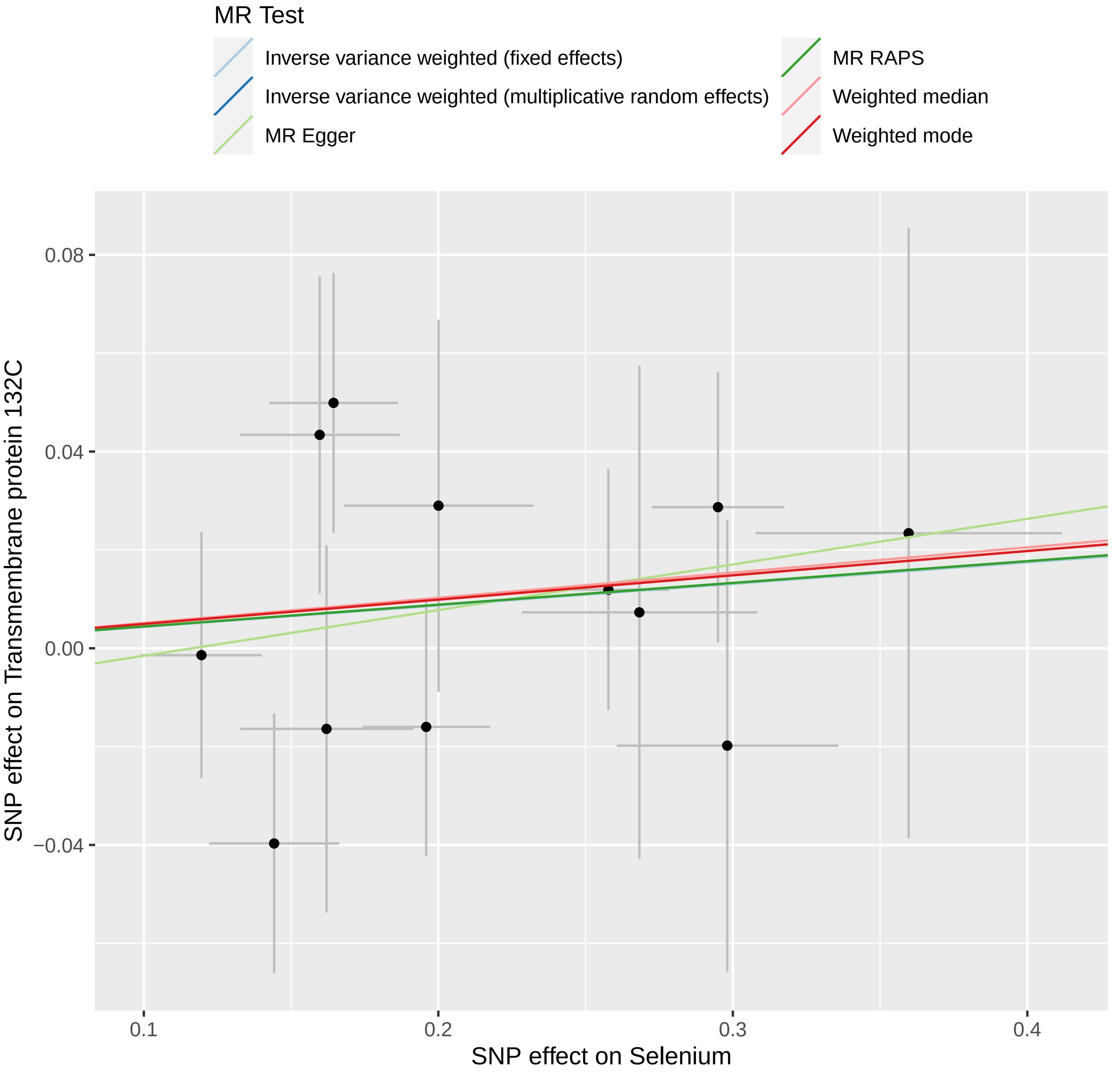

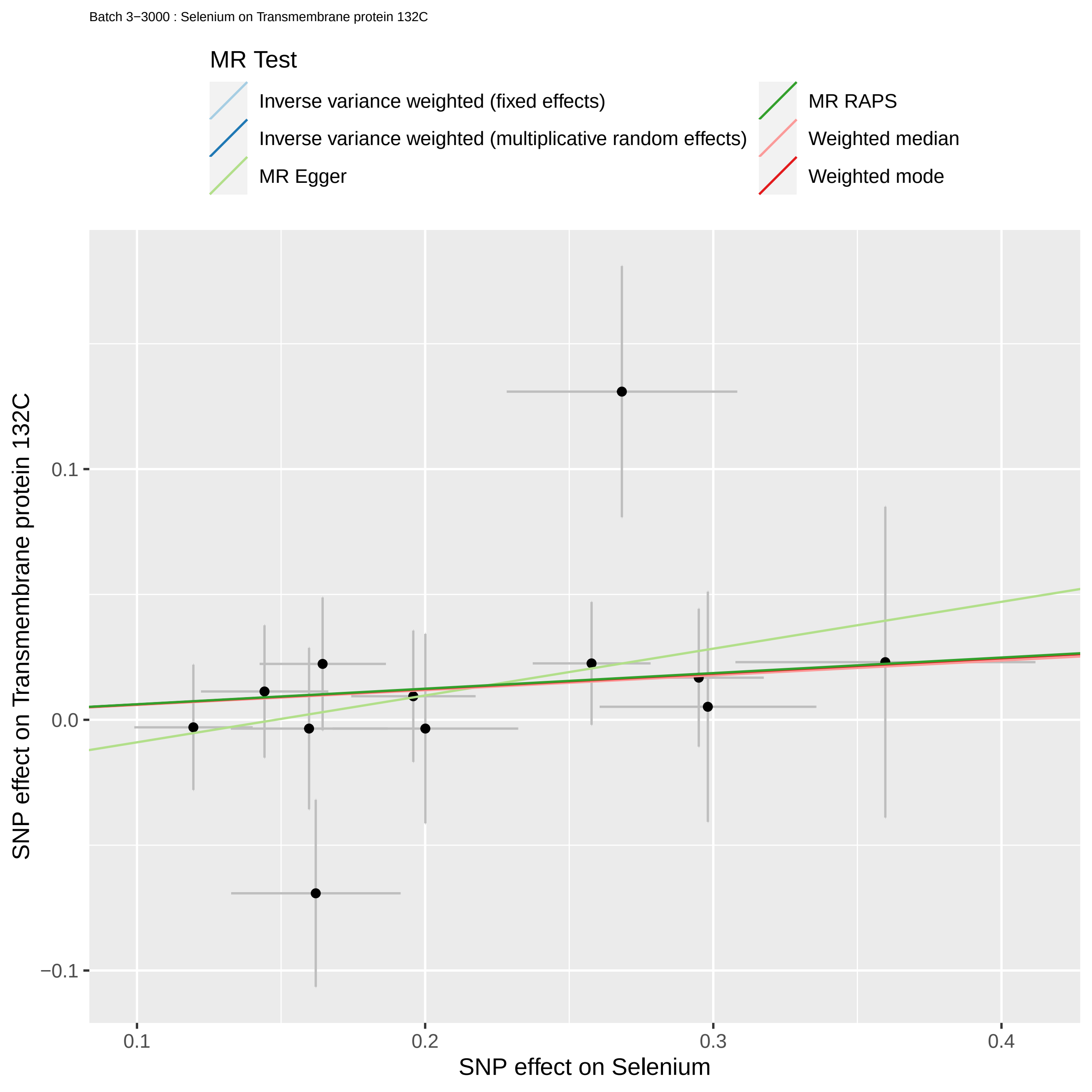
Batch 3−3000 : Selenium on Transmembrane protein 132C
MR Test
Inverse variance weighted (fixed effects)
Inverse variance weighted (multiplicative random effects)
MR Egger
MR RAPS
Weighted median
Weighted mode
SNP effect on Transmembrane protein 132C
0.1
0.0
−0.1
0.1
0.2
0.3
0.4
SNP effect on Selenium

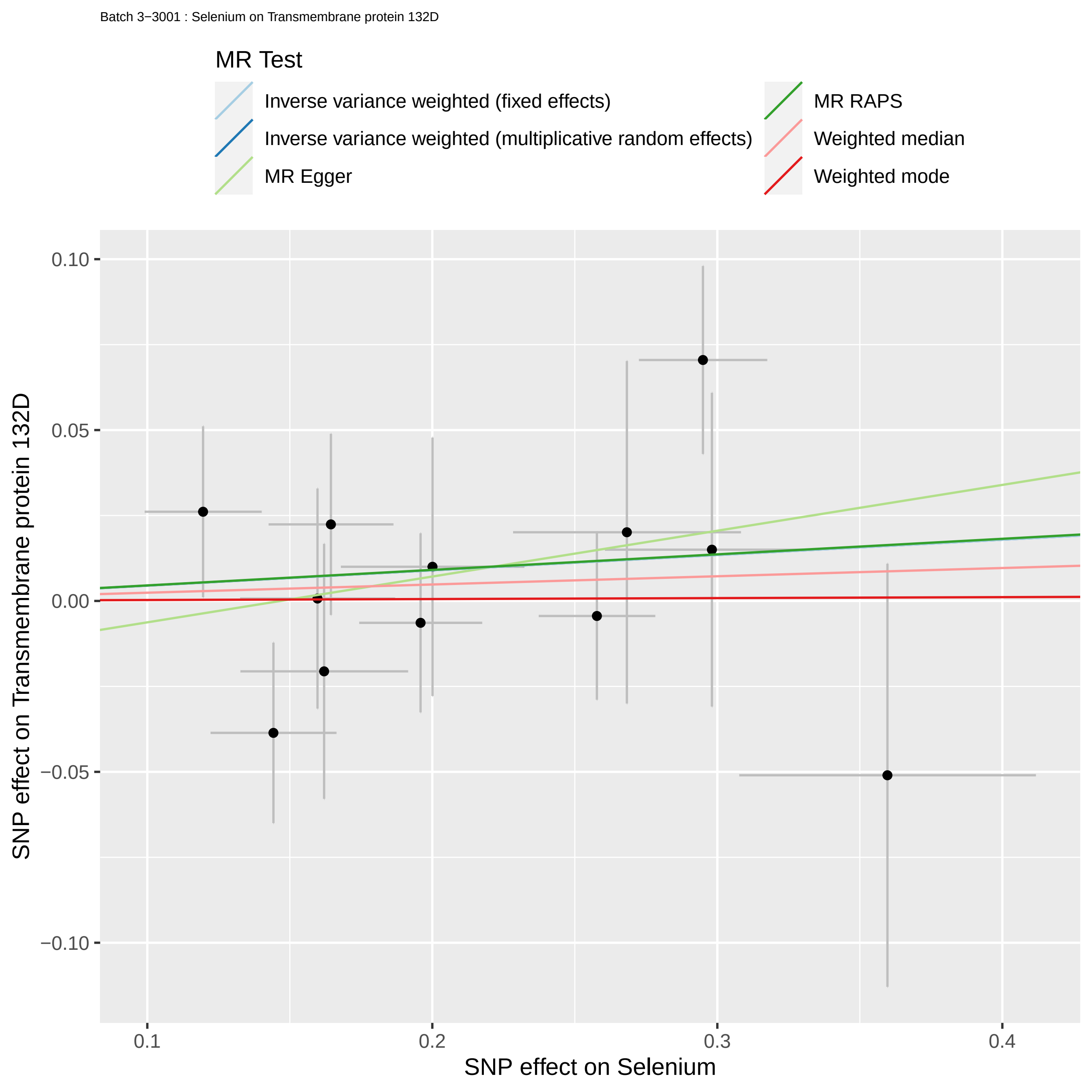
Batch 3−3001 : Selenium on Transmembrane protein 132D
MR Test
Inverse variance weighted (fixed effects)
Inverse variance weighted (multiplicative random effects)
MR Egger
MR RAPS
Weighted median
Weighted mode
0.10
0.05
0.00
−0.05
−0.10
0.1
0.2
0.3
0.4
SNP effect on Transmembrane protein 132D
SNP effect on Selenium

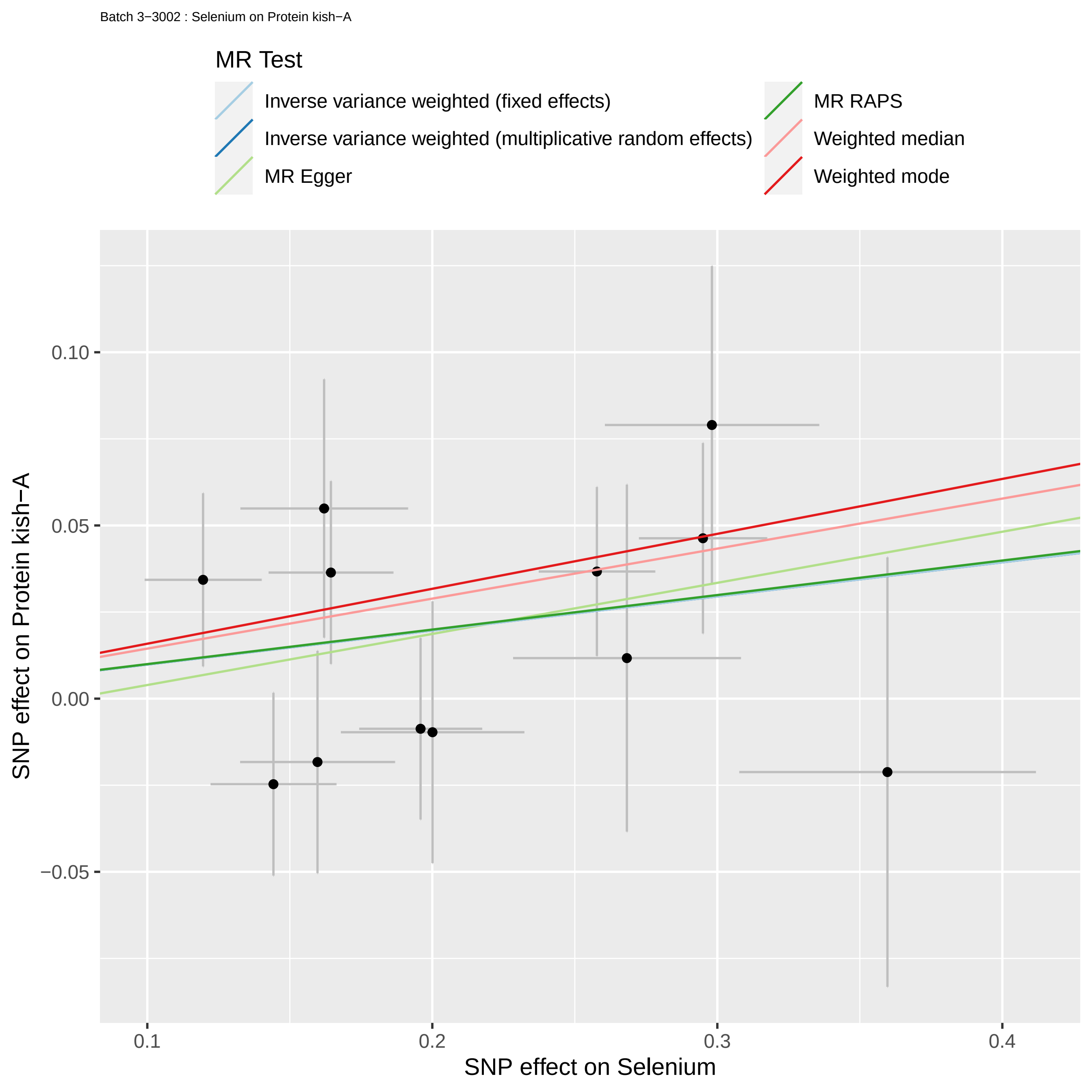

Batch 3−3002 : Selenium on Protein kish−A
MR Test
Inverse variance weighted (fixed effects)
Inverse variance weighted (multiplicative random effects)
MR Egger
MR RAPS
Weighted median
Weighted mode
0.10
0.05
0.00
−0.05
0.1
0.2
0.3
0.4
SNP effect on Protein kish−A
SNP effect on Selenium

Batch 3−3003 : Selenium on Protein kish−B

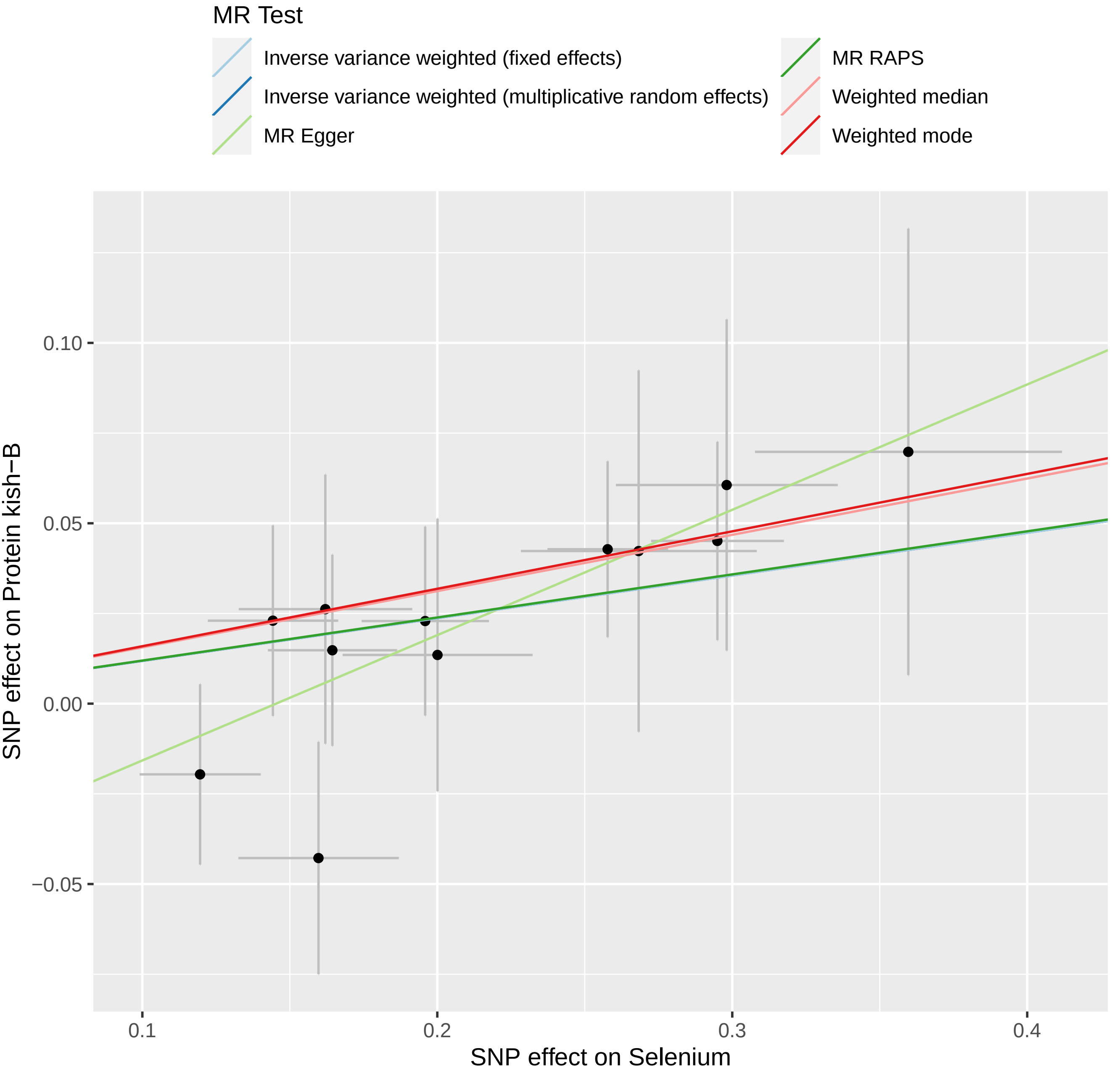

Batch 3−3004 : Selenium on Transmembrane protein 190

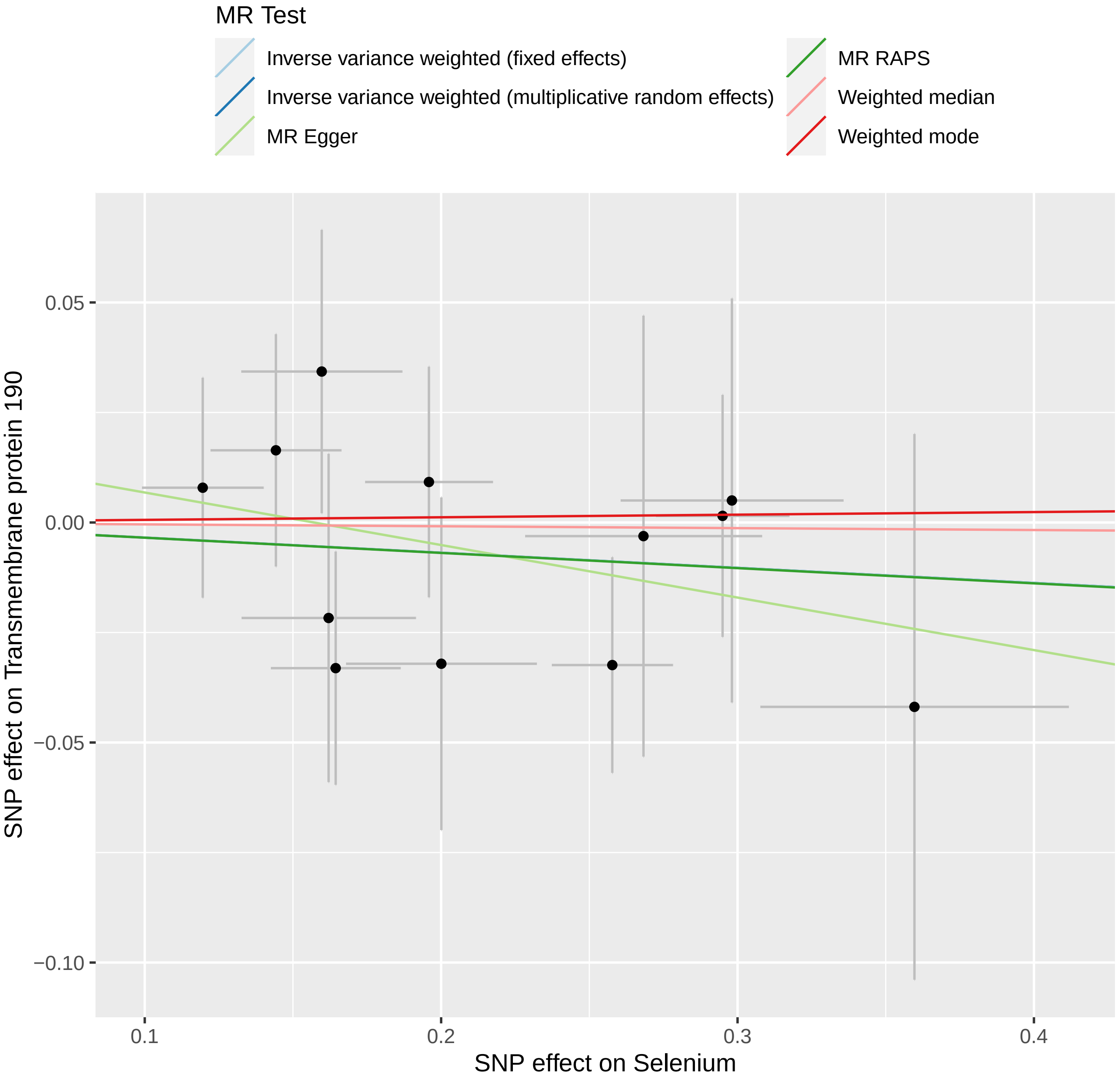

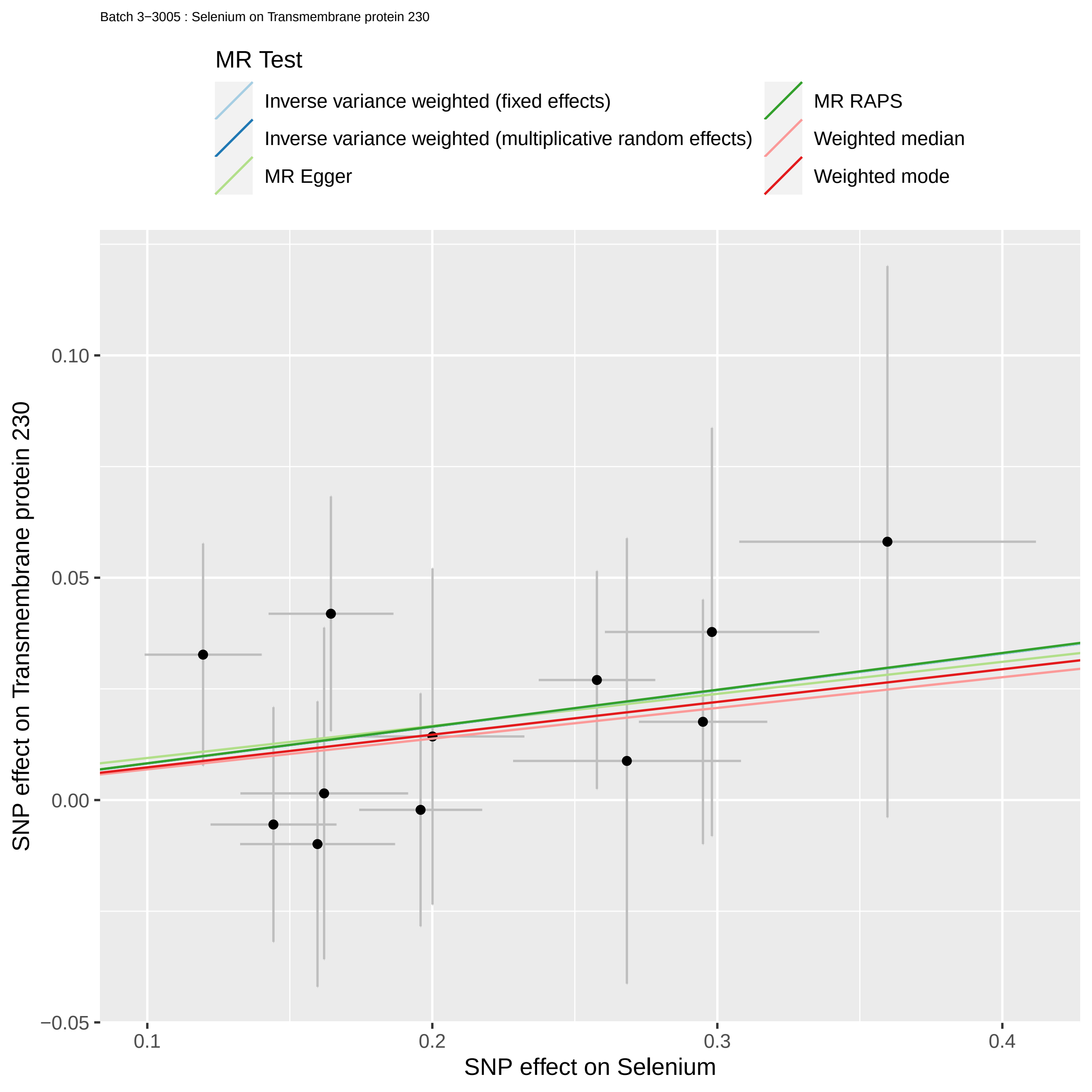

Batch 3−3005 : Selenium on Transmembrane protein 230
MR Test
Inverse variance weighted (fixed effects)
Inverse variance weighted (multiplicative random effects)
MR Egger
MR RAPS
Weighted median
Weighted mode
0.10
0.05
0.00
−0.05
0.1
0.2
0.3
0.4
SNP effect on Selenium
SNP effect on Transmembrane protein 230

Batch 3−3006 : Selenium on Transmembrane protein 234

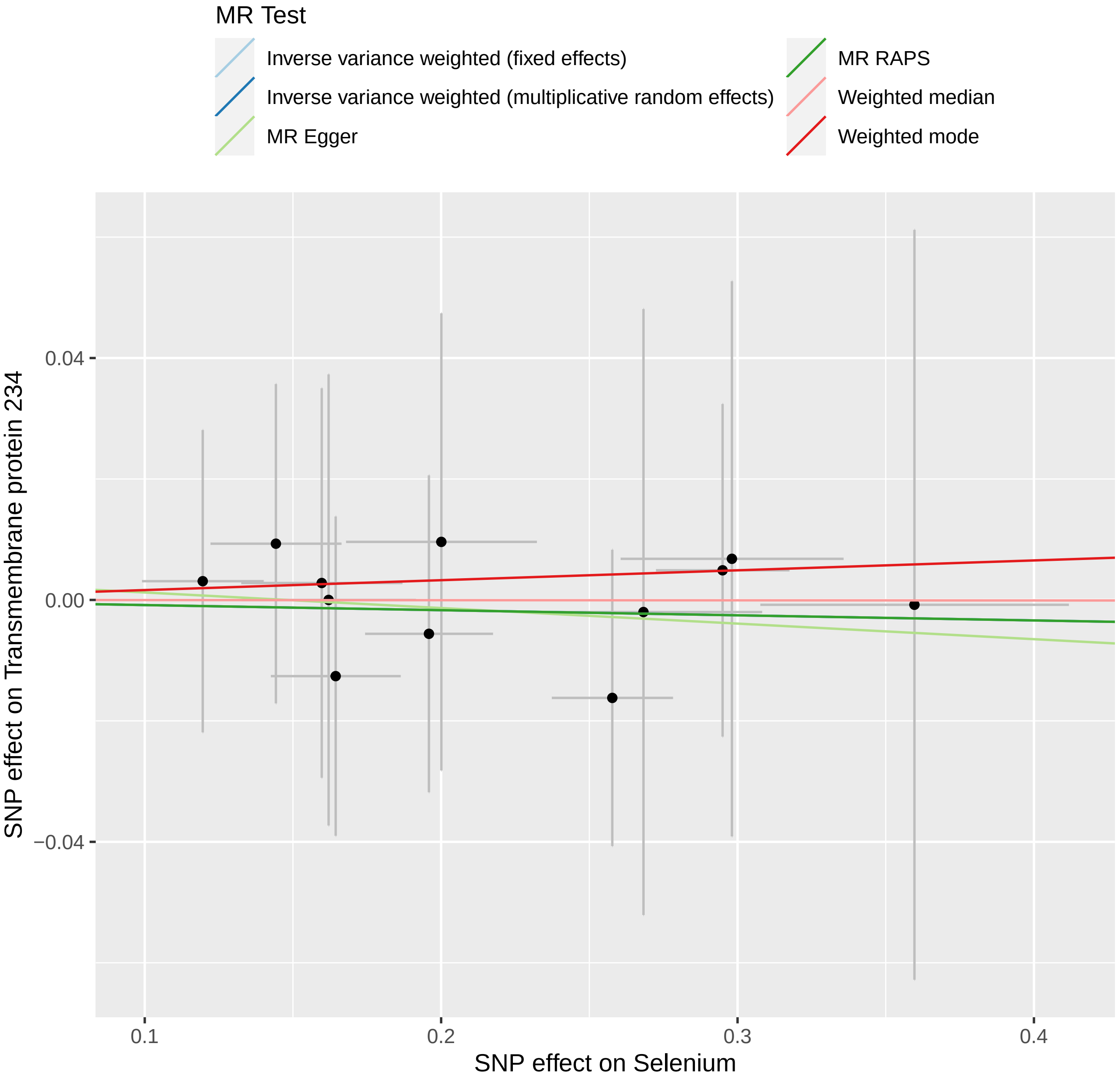

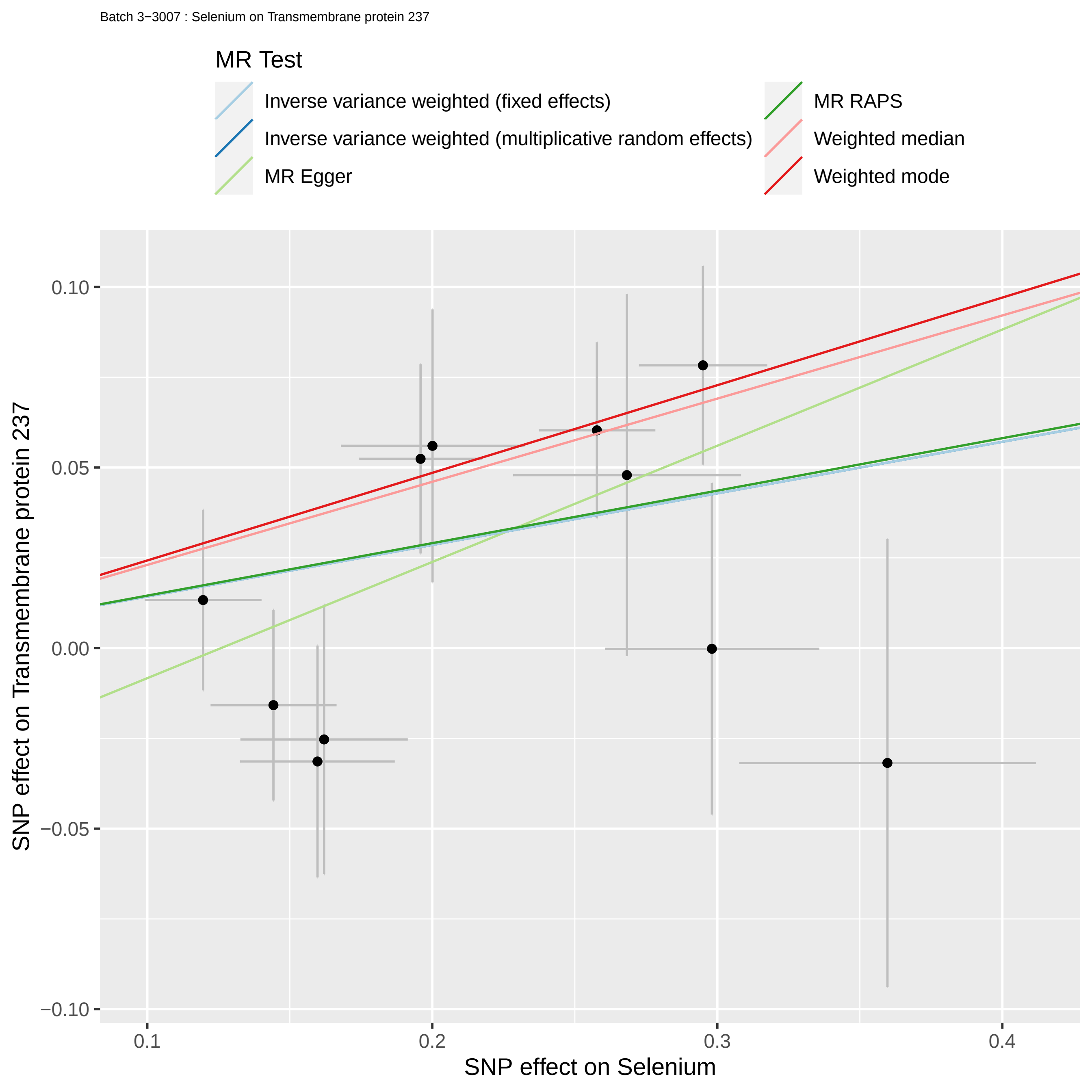

Batch 3−3007 : Selenium on Transmembrane protein 237
MR Test
Inverse variance weighted (fixed effects)
Inverse variance weighted (multiplicative random effects)
MR Egger
MR RAPS
Weighted median
Weighted mode
0.10
0.05
0.00
−0.05
−0.10
0.1
0.2
0.3
0.4
SNP effect on Transmembrane protein 237
SNP effect on Selenium

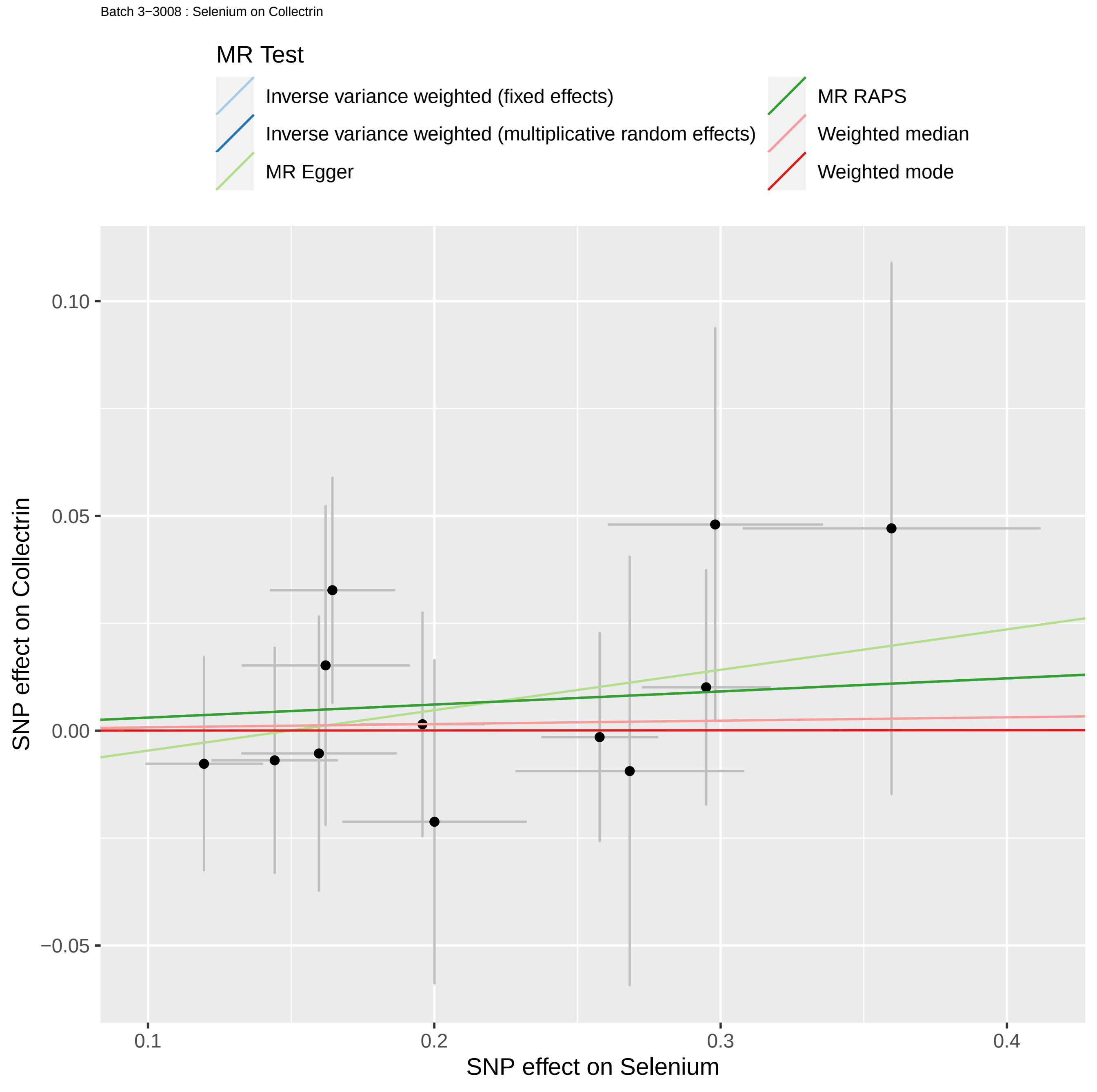

Batch 3−3008 : Selenium on Collectrin
MR Test
Inverse variance weighted (fixed effects)
Inverse variance weighted (multiplicative random effects)
MR Egger
MR RAPS
Weighted median
Weighted mode
0.10
0.05
0.00
−0.05
0.1
0.2
0.3
0.4
SNP effect on Collectrin
SNP effect on Selenium

Batch 3−3009 : Selenium on Transmembrane protein 2

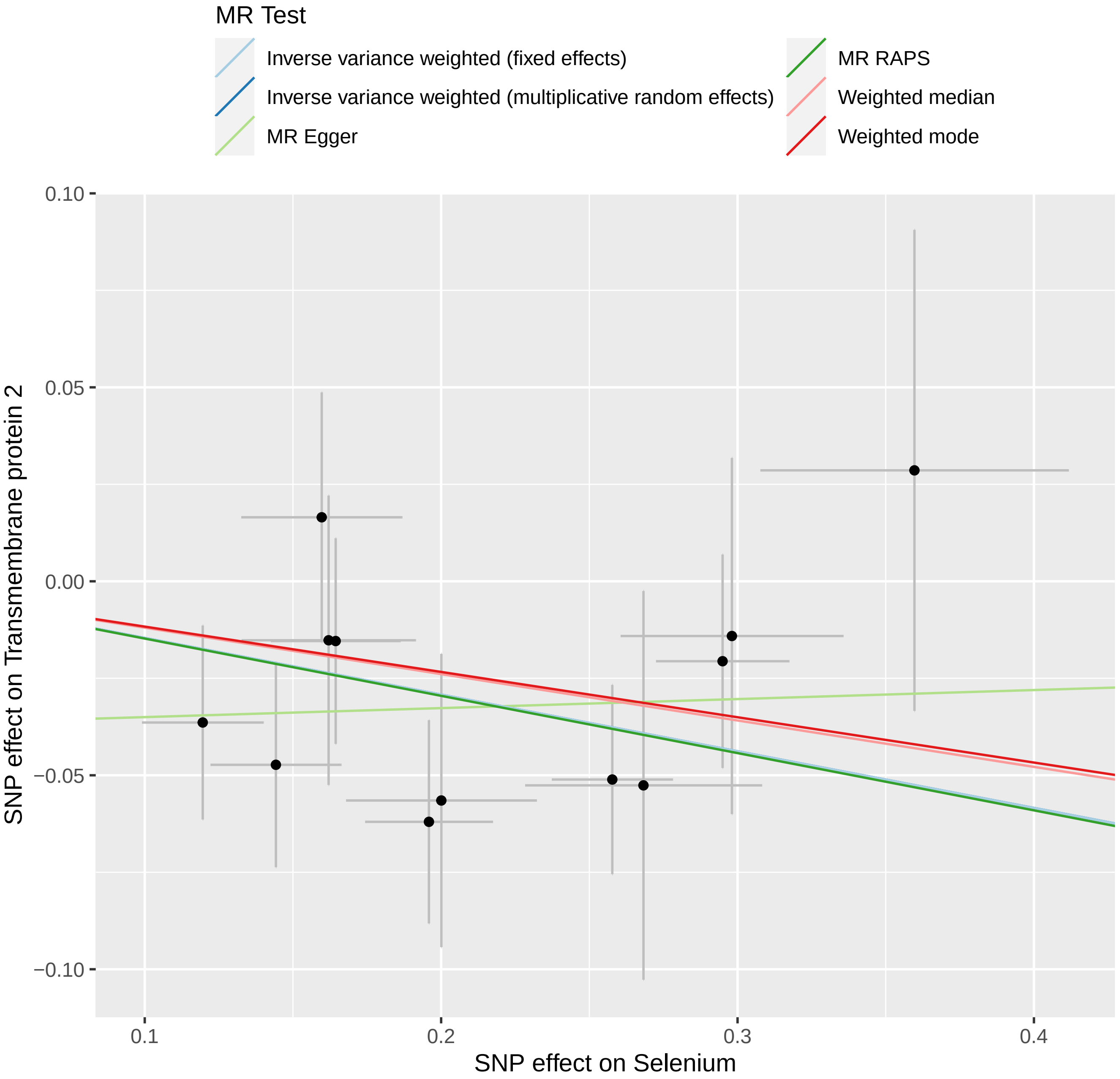

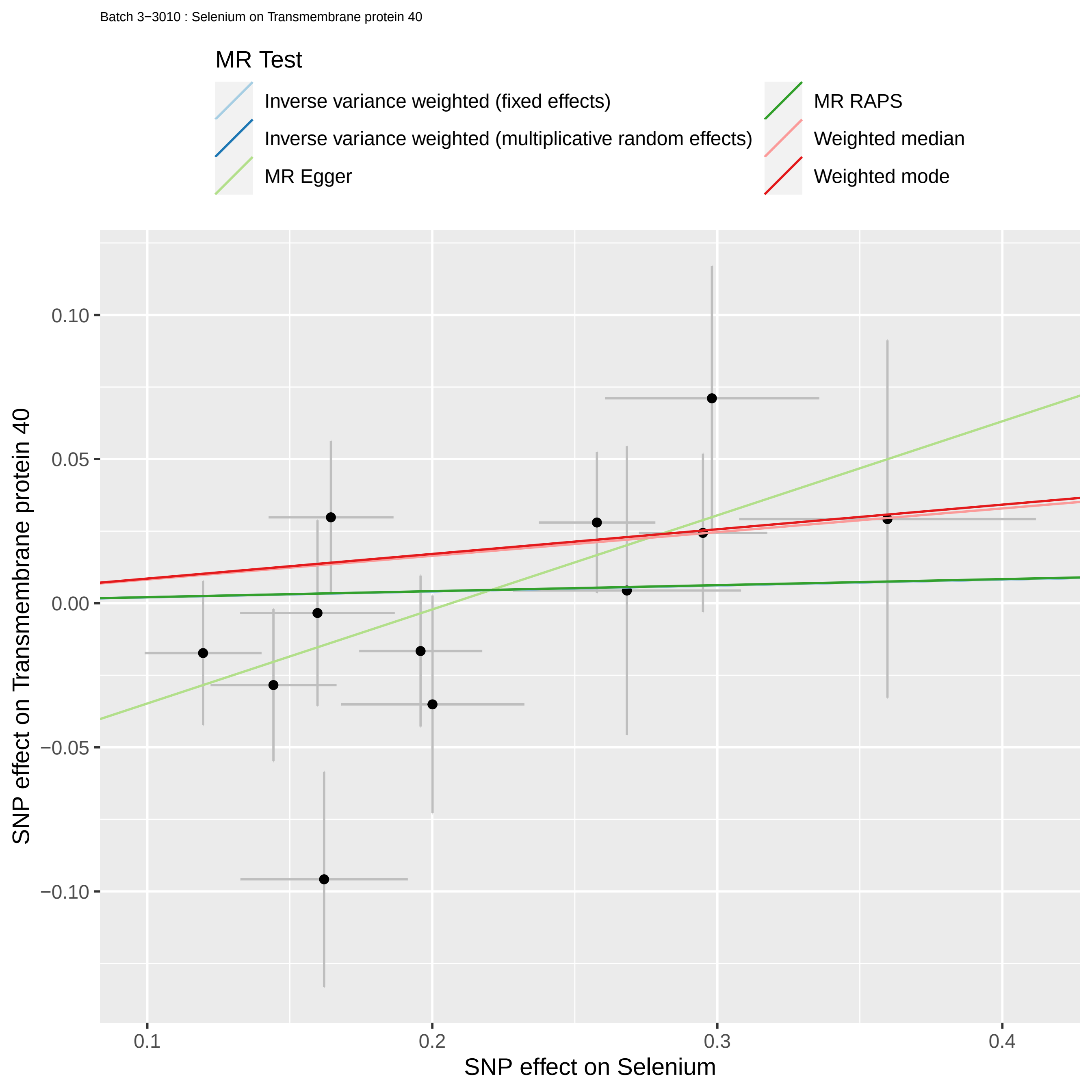

Batch 3−3010 : Selenium on Transmembrane protein 40
MR Test
Inverse variance weighted (fixed effects)
Inverse variance weighted (multiplicative random effects)
MR Egger
MR RAPS
Weighted median
Weighted mode
0.10
0.05
0.00
−0.05
−0.10
0.1
0.2
0.3
0.4
SNP effect on Transmembrane protein 40
SNP effect on Selenium

Batch 3−3011 : Selenium on Transmembrane protein 41B

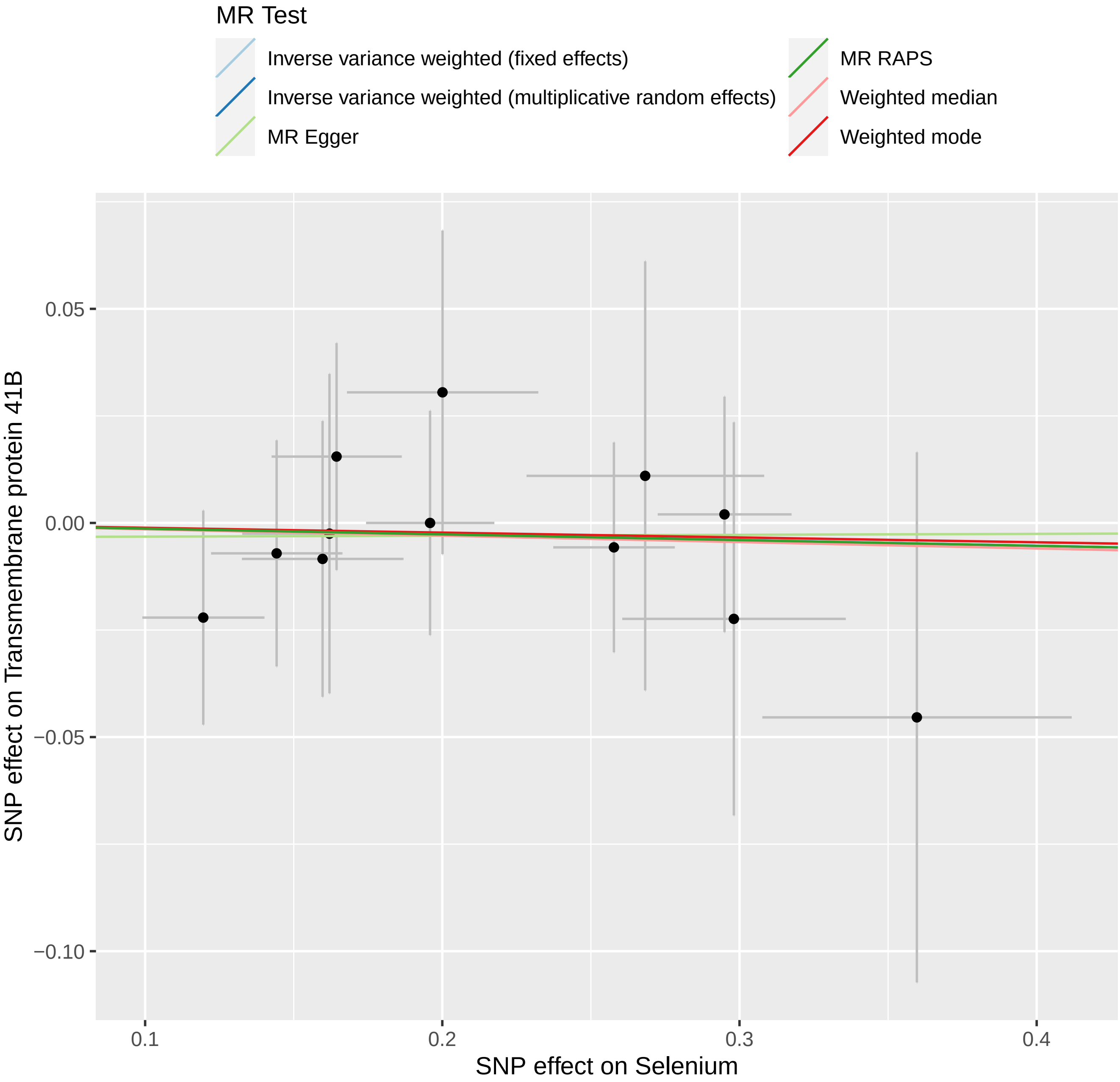

Batch 3−3012 : Selenium on Transmembrane protein 52

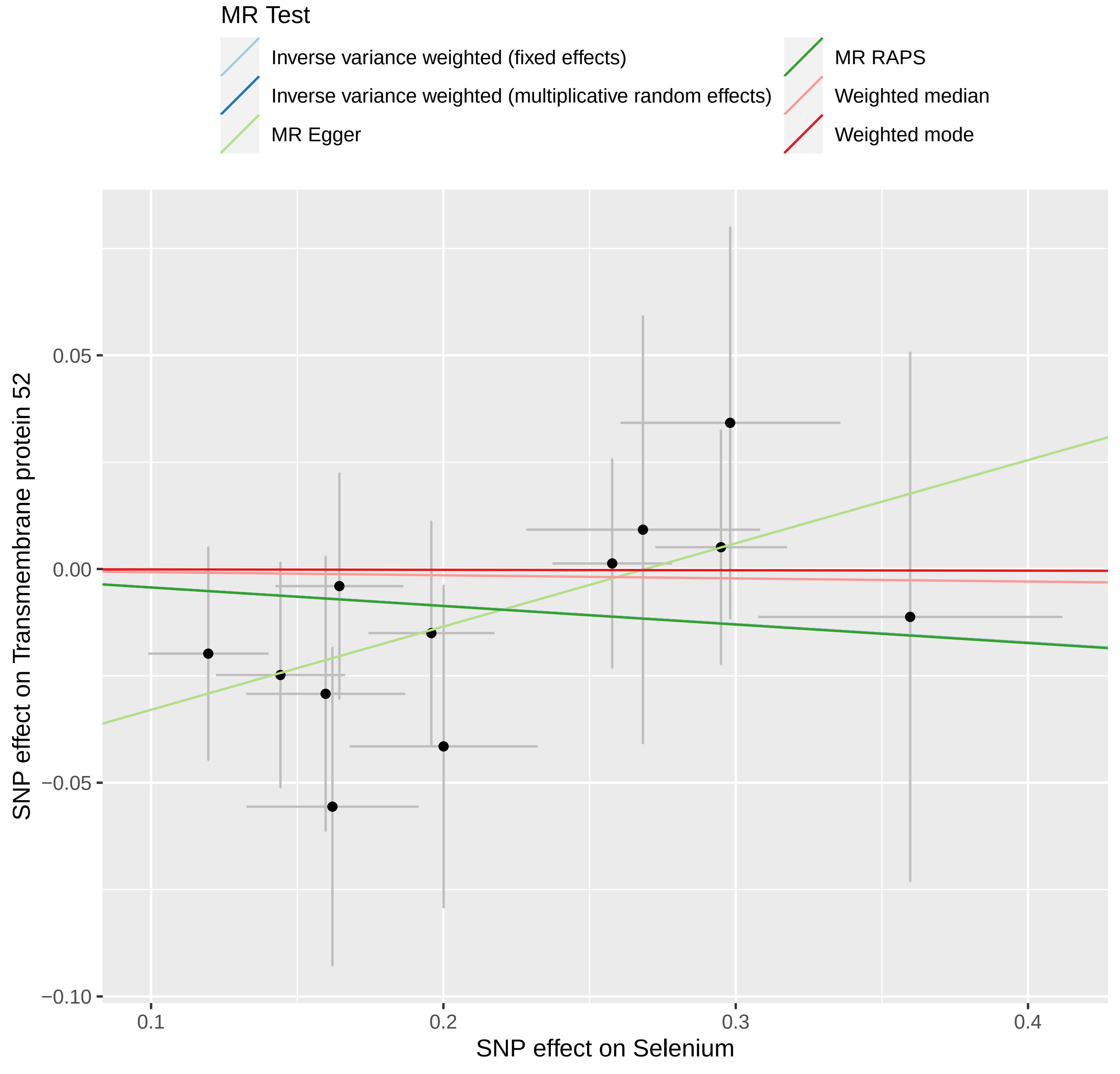

Batch 3−3013 : Selenium on Macoilin

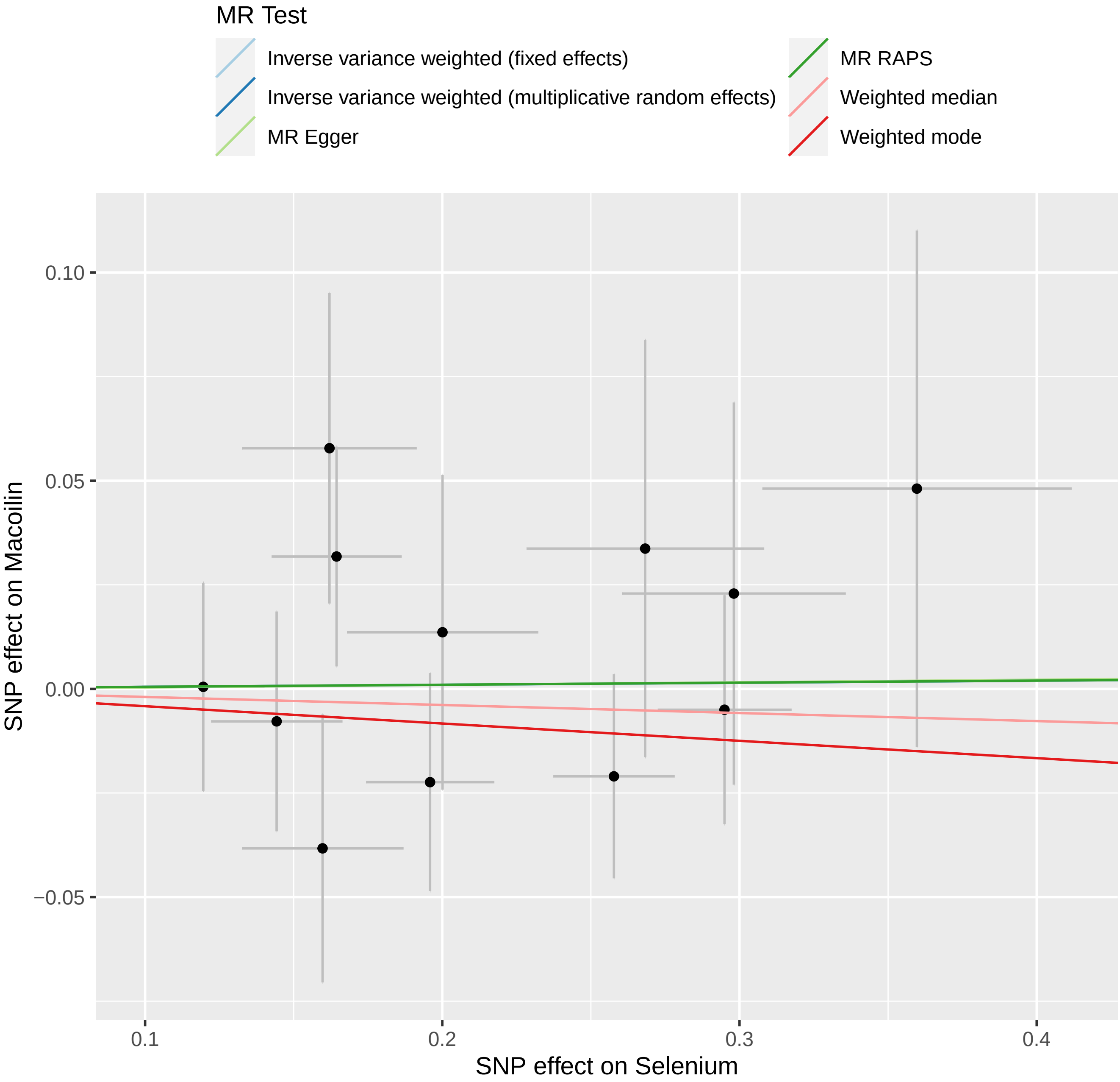

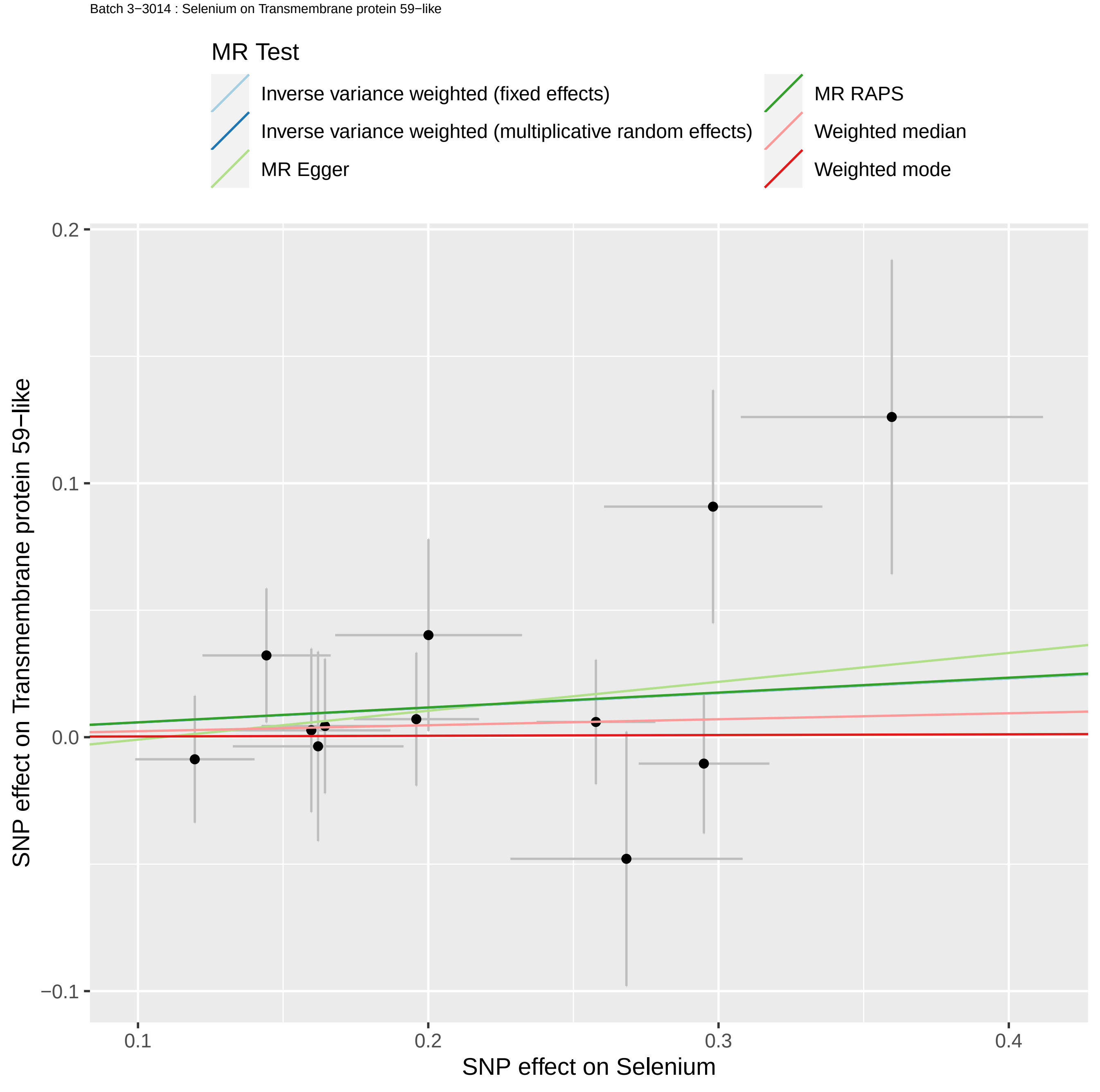
Batch 3−3014 : Selenium on Transmembrane protein 59−like
MR Test
Inverse variance weighted (fixed effects)
Inverse variance weighted (multiplicative random effects)
MR Egger
MR RAPS
Weighted median
Weighted mode
0.2
0.1
0.0
−0.1
0.1
0.2
0.3
0.4
SNP effect on Transmembrane protein 59−like
SNP effect on Selenium

Batch 3−3015 : Selenium on Transmembrane protein 70, mitochondrial

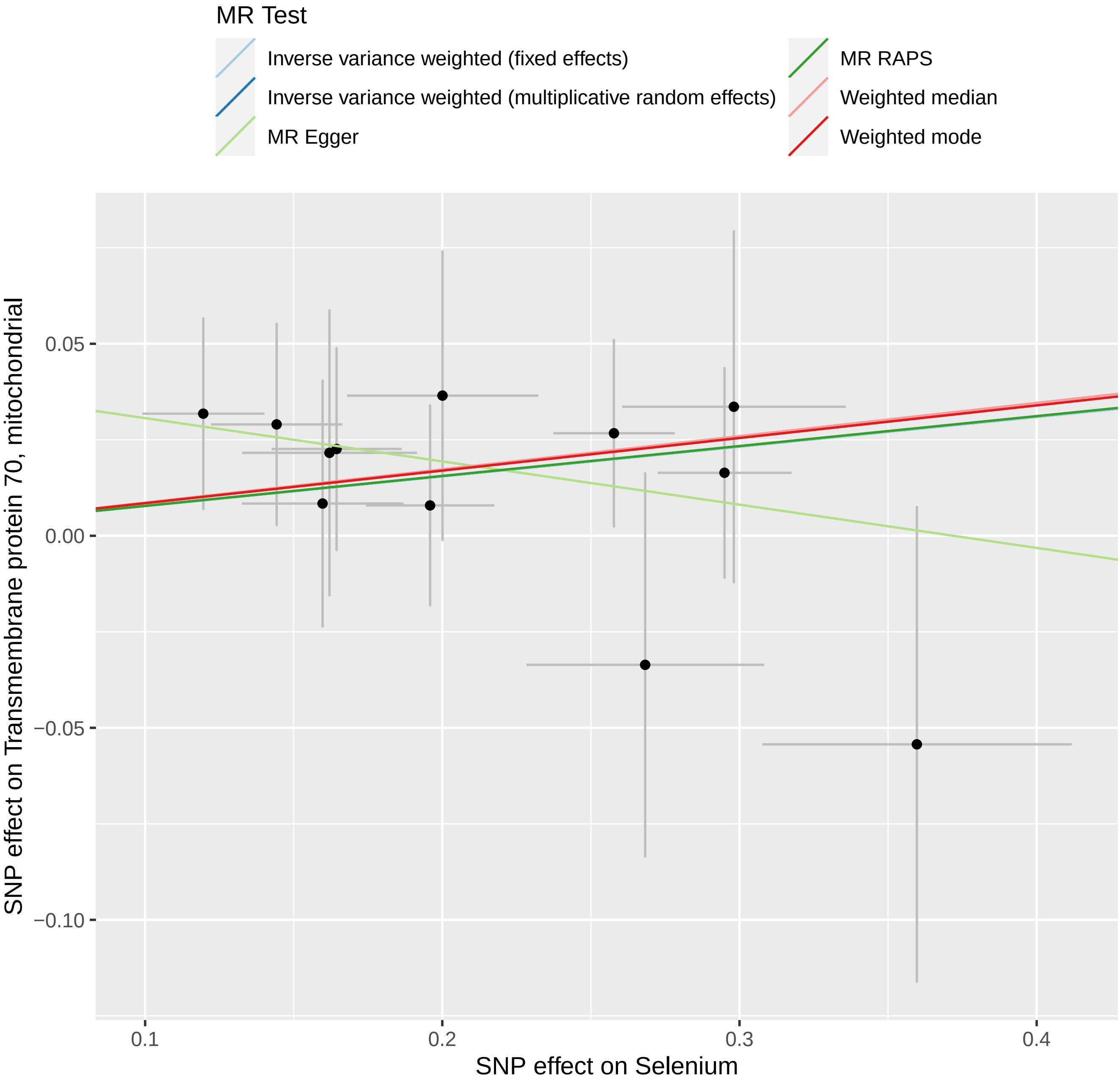

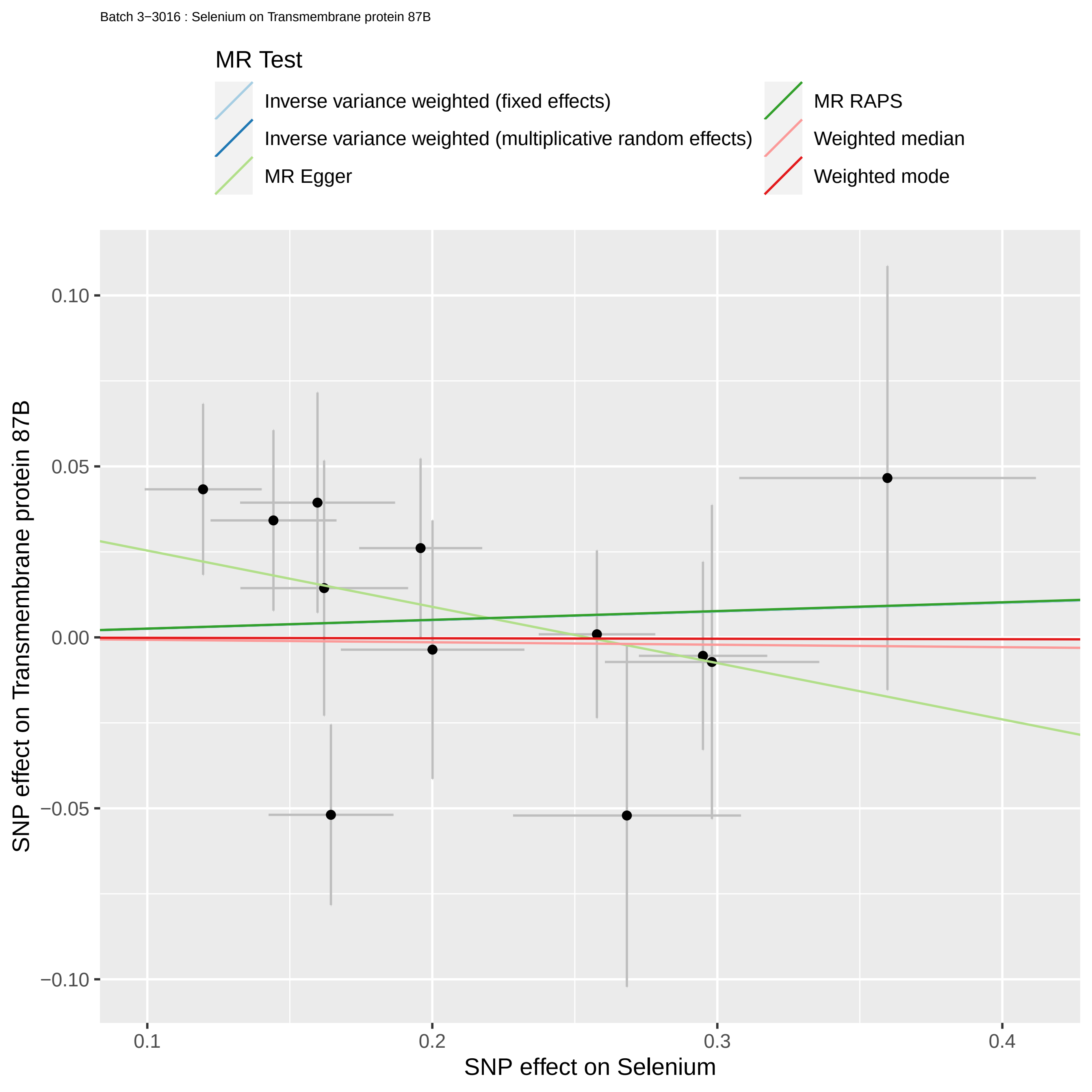

Batch 3−3016 : Selenium on Transmembrane protein 87B
MR Test
Inverse variance weighted (fixed effects)
Inverse variance weighted (multiplicative random effects)
MR Egger
MR RAPS
Weighted median
Weighted mode
0.10
0.05
0.00
−0.05
−0.10
0.1
0.2
0.3
0.4
SNP effect on Selenium
SNP effect on Transmembrane protein 87B

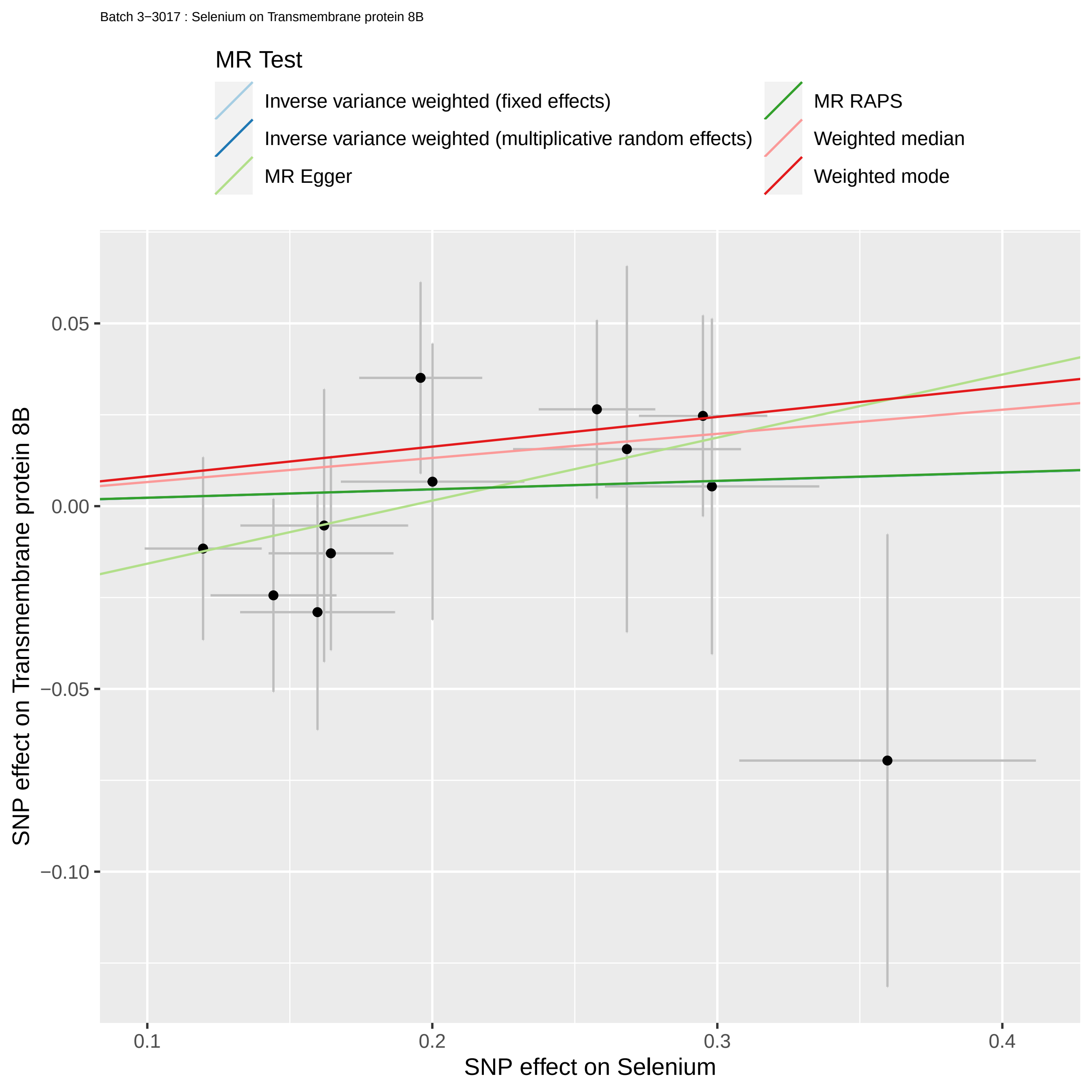
Batch 3−3017 : Selenium on Transmembrane protein 8B
MR Test
Inverse variance weighted (fixed effects)
Inverse variance weighted (multiplicative random effects)
MR Egger
MR RAPS
Weighted median
Weighted mode
0.05
0.00
−0.05
−0.10
0.1
0.2
0.3
0.4
SNP effect on Selenium
SNP effect on Transmembrane protein 8B

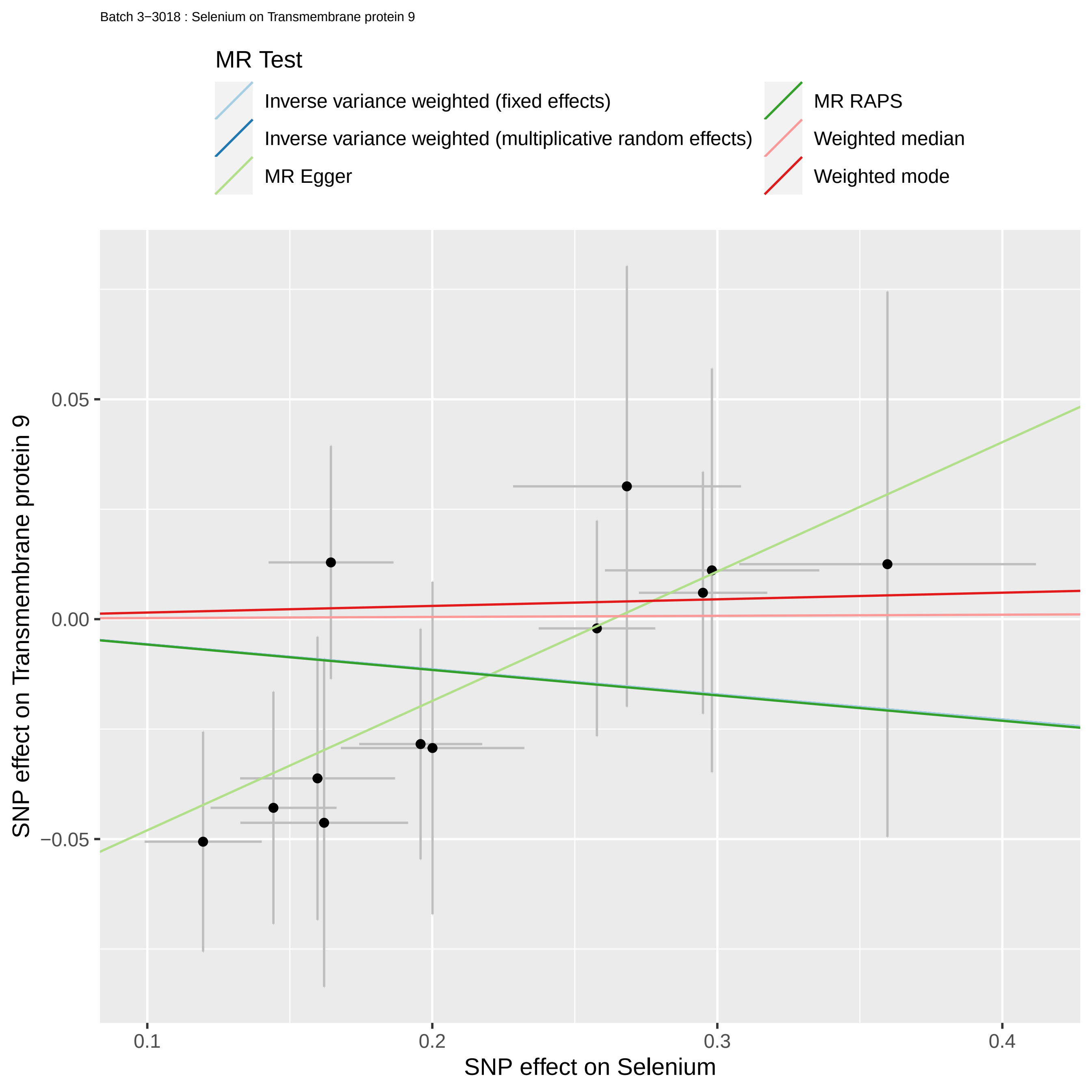
Batch 3−3018 : Selenium on Transmembrane protein 9
MR Test
Inverse variance weighted (fixed effects)
Inverse variance weighted (multiplicative random effects)
MR Egger
MR RAPS
Weighted median
Weighted mode
0.05
0.00
−0.05
0.1
0.2
0.3
0.4
SNP effect on Transmembrane protein 9
SNP effect on Selenium

Batch 3−3019 : Selenium on Transmembrane inner ear expressed protein

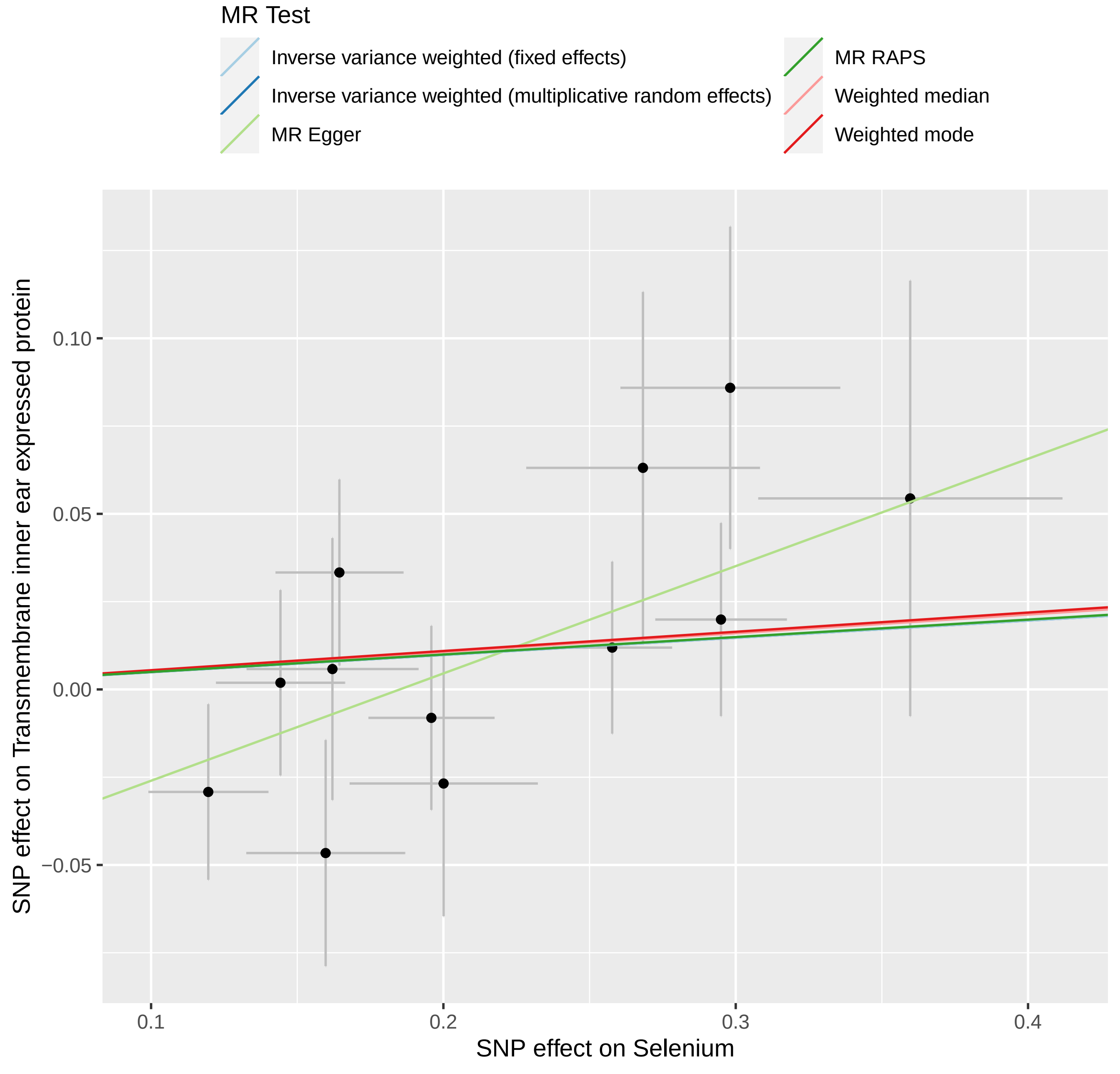

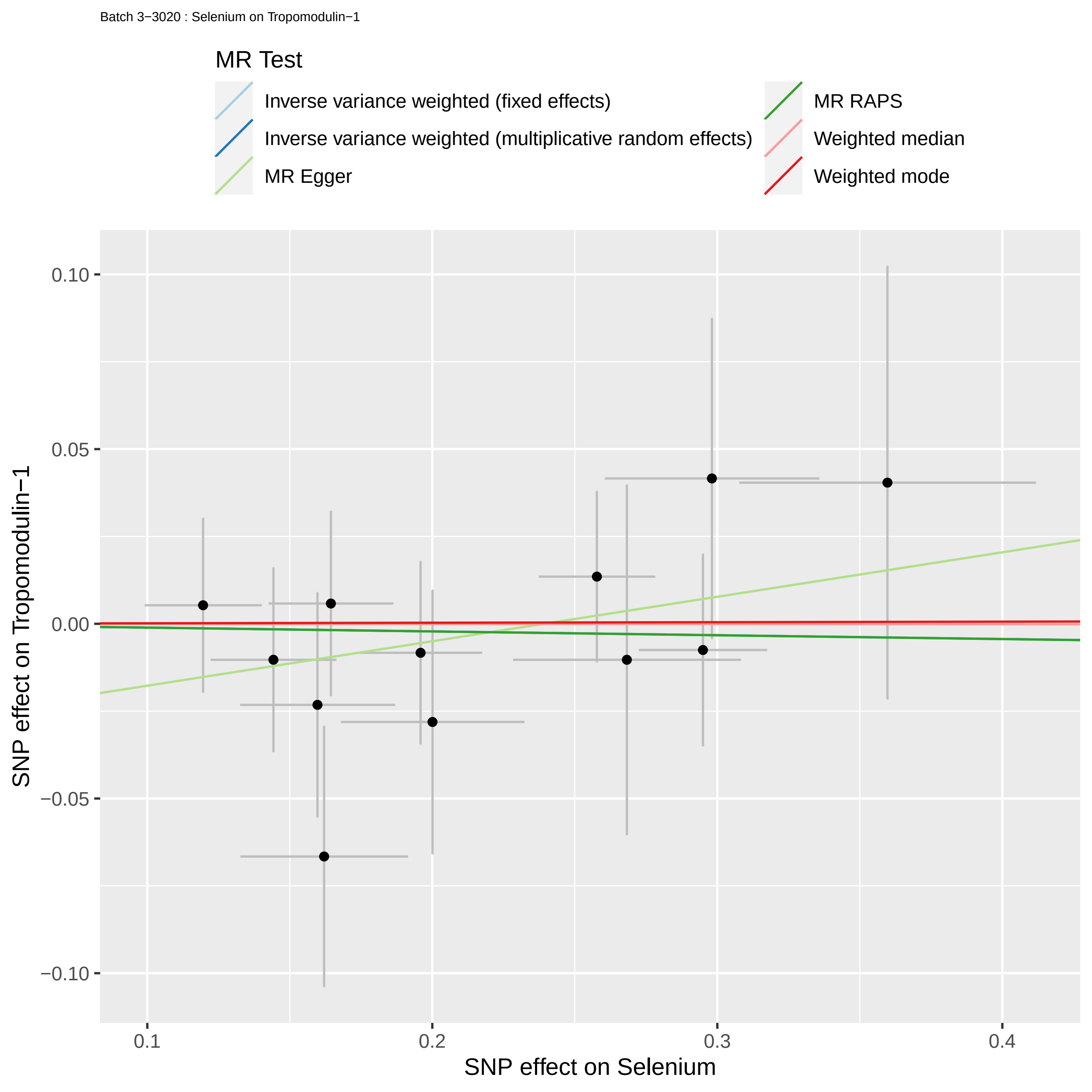
Batch 3−3020 : Selenium on Tropomodulin−1
MR Test
Inverse variance weighted (fixed effects)
Inverse variance weighted (multiplicative random effects)
MR Egger
MR RAPS
Weighted median
Weighted mode
0.10
0.05
0.00
−0.05
−0.10
0.1
0.2
0.3
0.4
SNP effect on Tropomodulin−1
SNP effect on Selenium

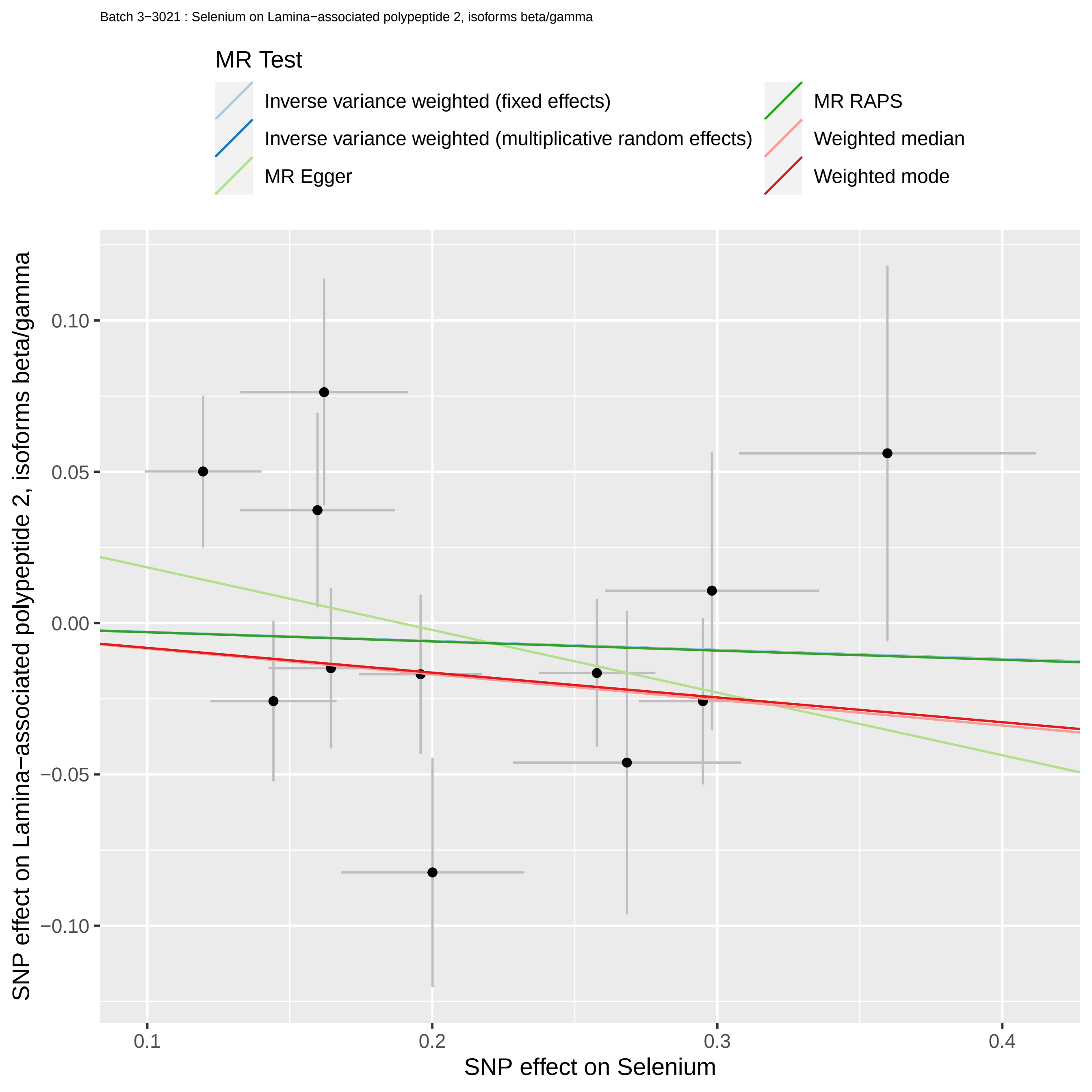

Batch 3−3021 : Selenium on Lamina−associated polypeptide 2, isoforms beta/gamma
MR Test
Inverse variance weighted (fixed effects)
Inverse variance weighted (multiplicative random effects)
MR Egger
MR RAPS
Weighted median
Weighted mode
SNP effect on Lamina−associated polypeptide 2, isoforms beta/gamma
0.10
0.05
0.00
−0.05
−0.10
0.1
0.2
0.3
0.4
SNP effect on Selenium

Batch 3−3022 : Selenium on Transmembrane protease serine 11A

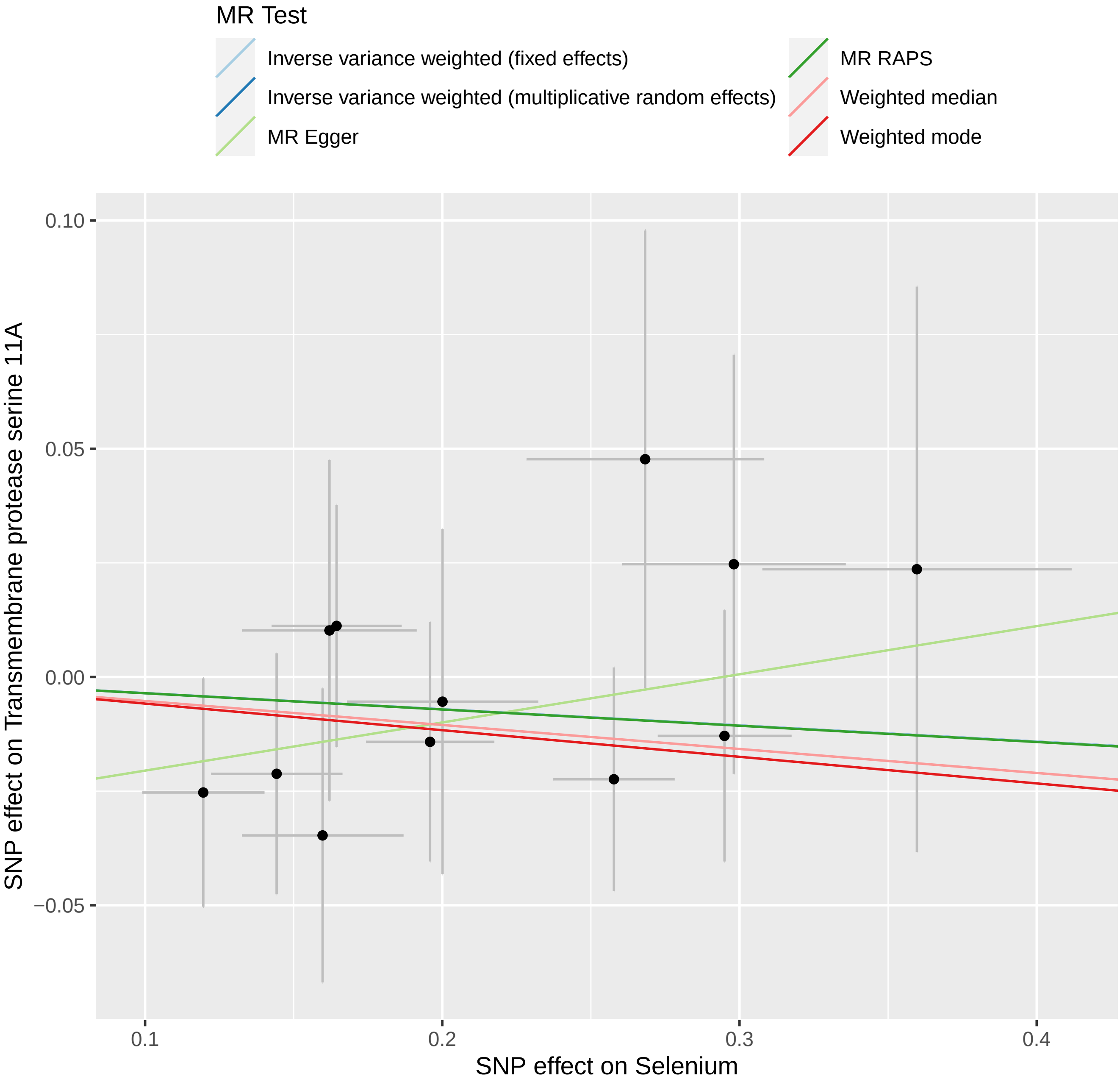

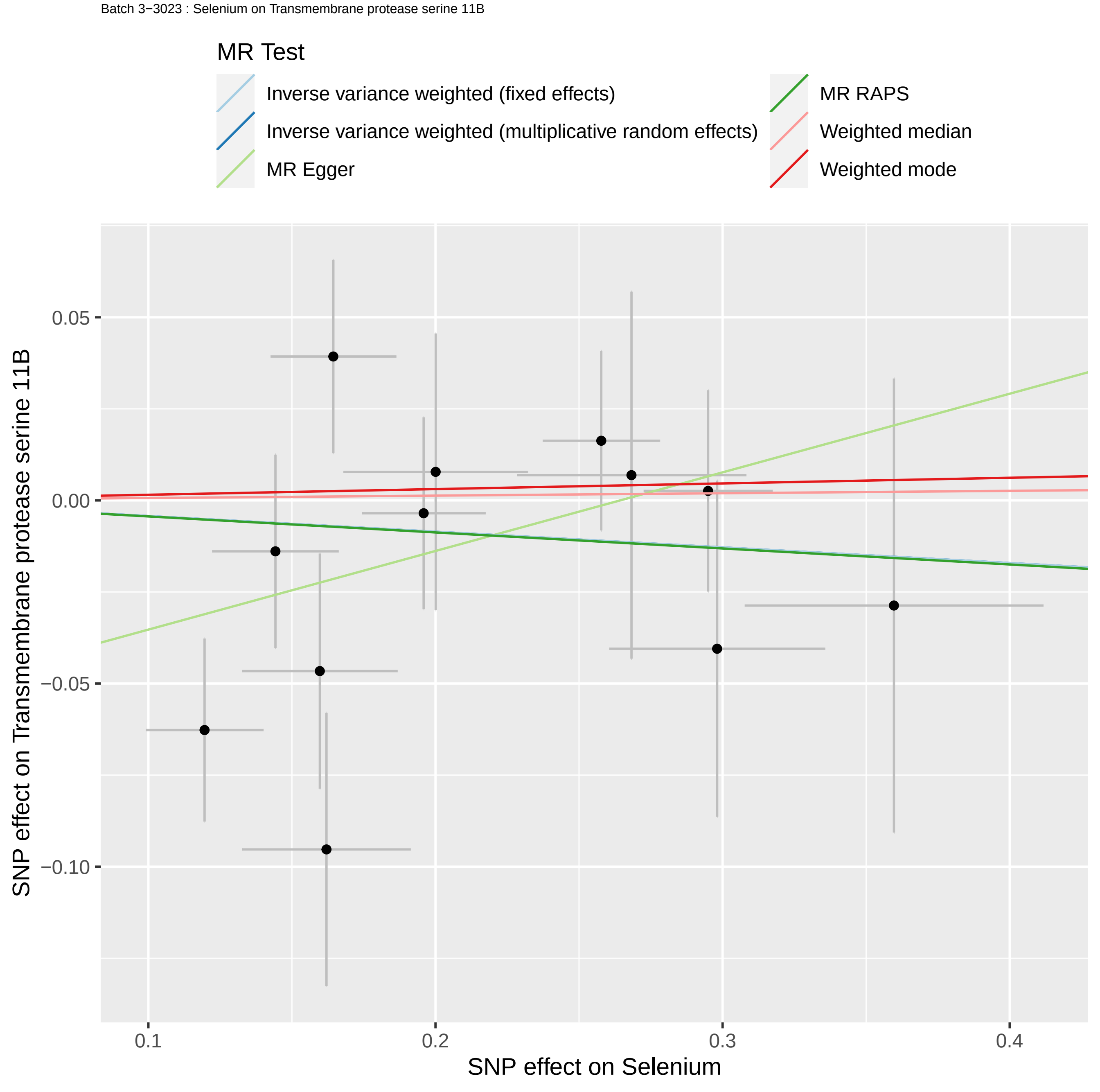

Batch 3−3023 : Selenium on Transmembrane protease serine 11B
MR Test
Inverse variance weighted (fixed effects)
Inverse variance weighted (multiplicative random effects)
MR Egger
MR RAPS
Weighted median
Weighted mode
0.05
0.00
−0.05
−0.10
0.1
0.2
0.3
0.4
SNP effect on Transmembrane protease serine 11B
SNP effect on Selenium

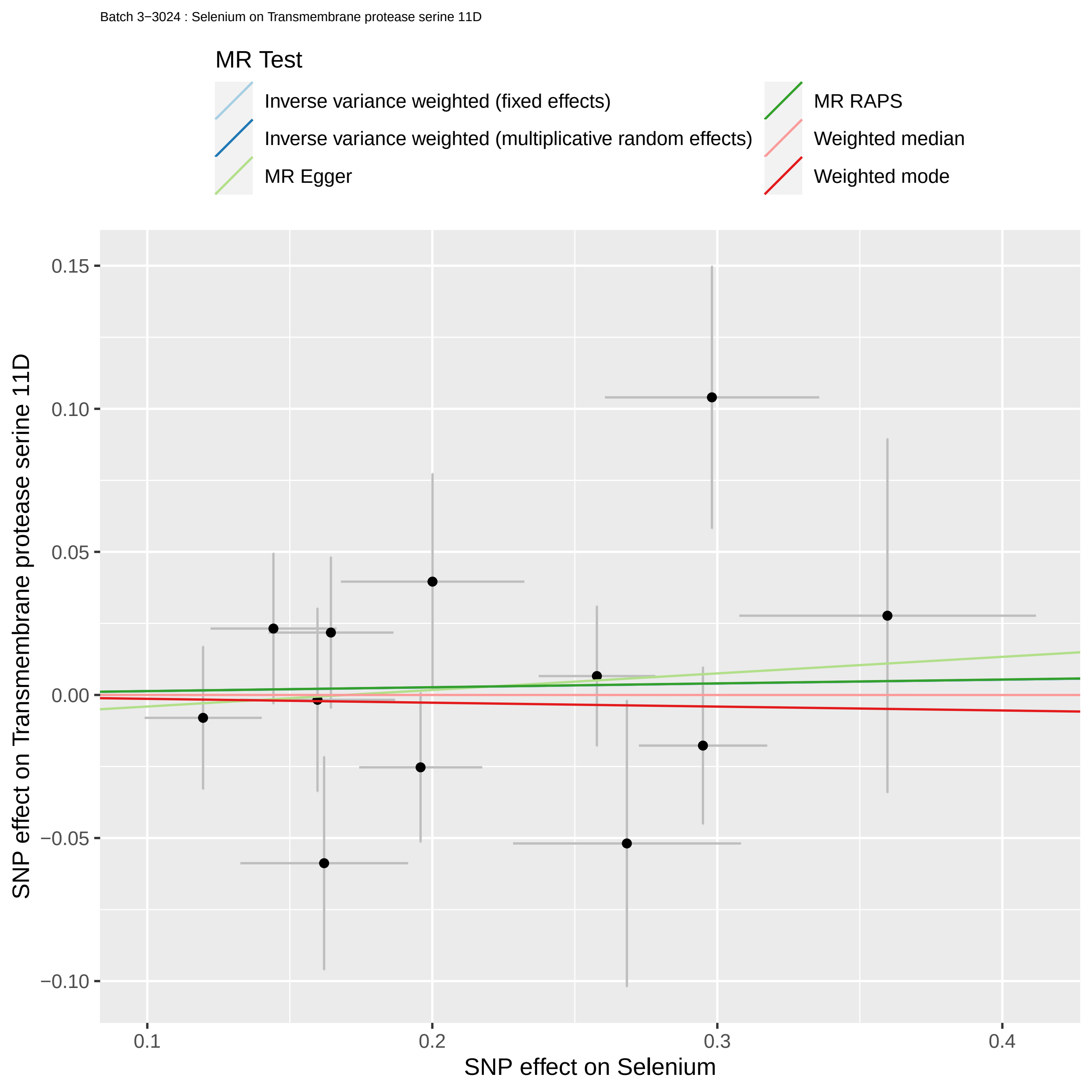

Batch 3−3024 : Selenium on Transmembrane protease serine 11D
MR Test
Inverse variance weighted (fixed effects)
Inverse variance weighted (multiplicative random effects)
MR Egger
MR RAPS
Weighted median
Weighted mode
SNP effect on Transmembrane protease serine 11D
0.15
0.10
0.05
0.00
−0.05
−0.10
0.1
0.2
0.3
0.4
SNP effect on Selenium

Batch 3−3025 : Selenium on Enteropeptidase

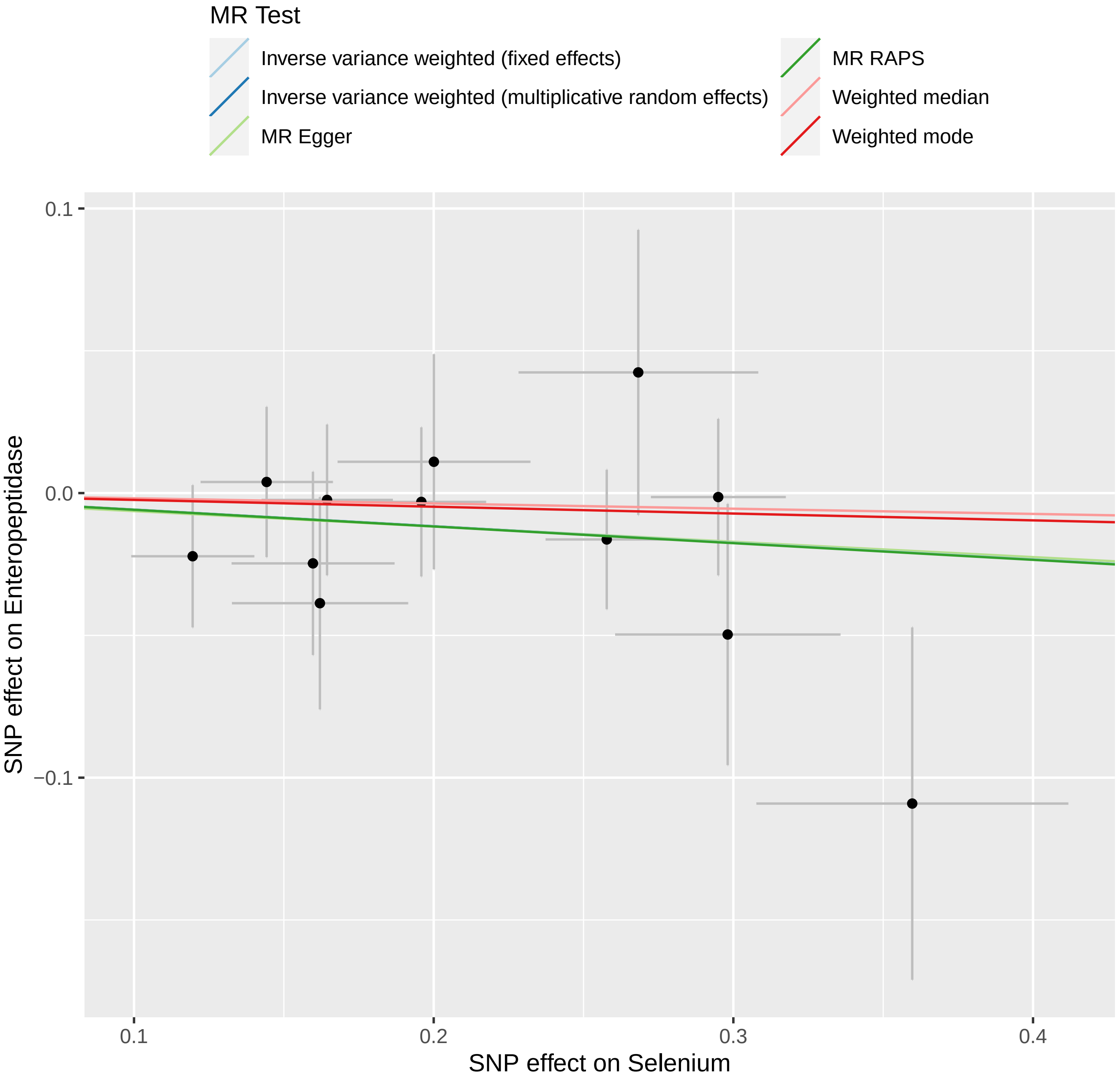

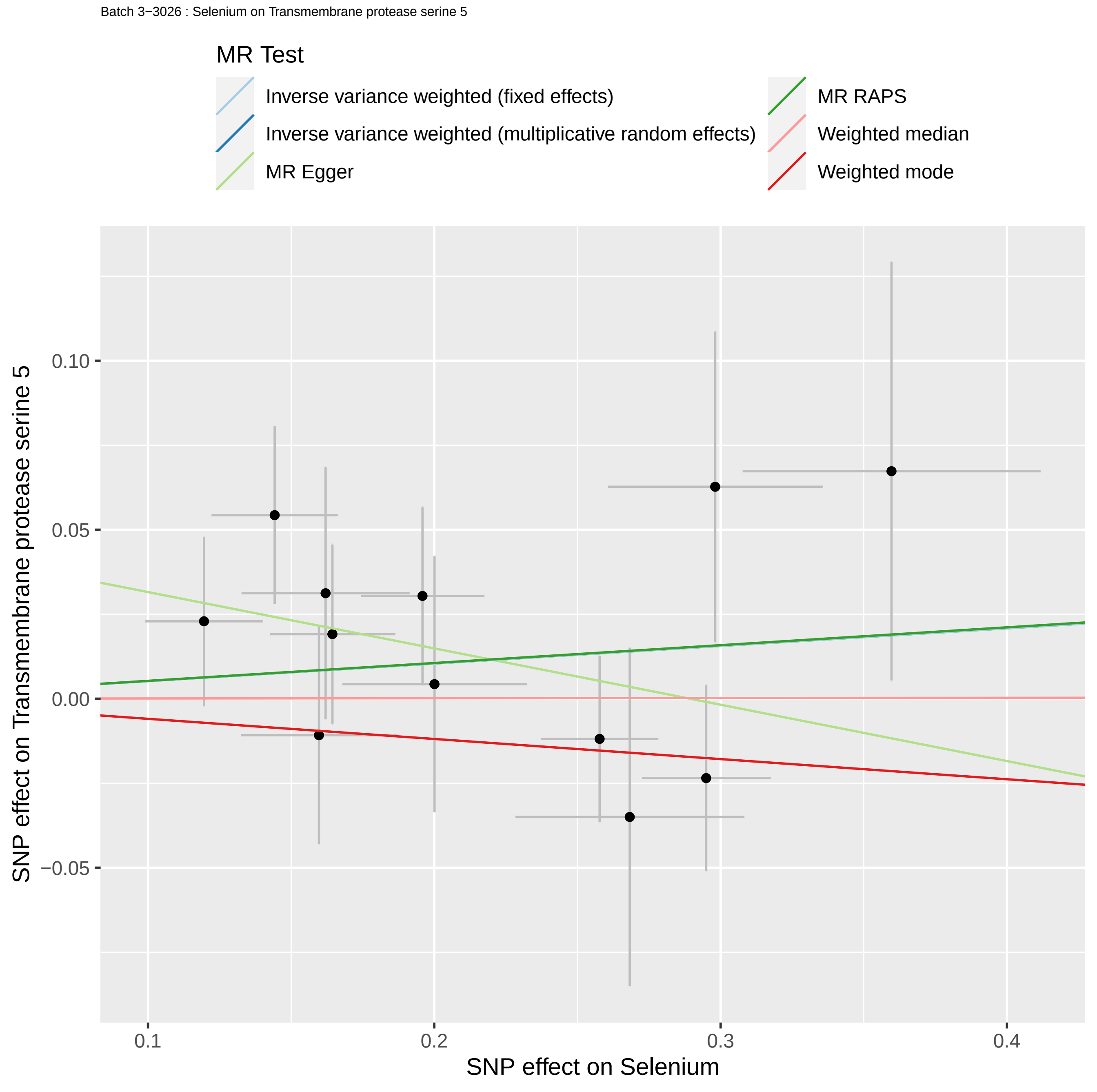

Batch 3−3026 : Selenium on Transmembrane protease serine 5
MR Test
Inverse variance weighted (fixed effects)
Inverse variance weighted (multiplicative random effects)
MR Egger
MR RAPS
Weighted median
Weighted mode
SNP effect on Transmembrane protease serine 5
0.10
0.05
0.00
−0.05
0.1
0.2
0.3
0.4
SNP effect on Selenium

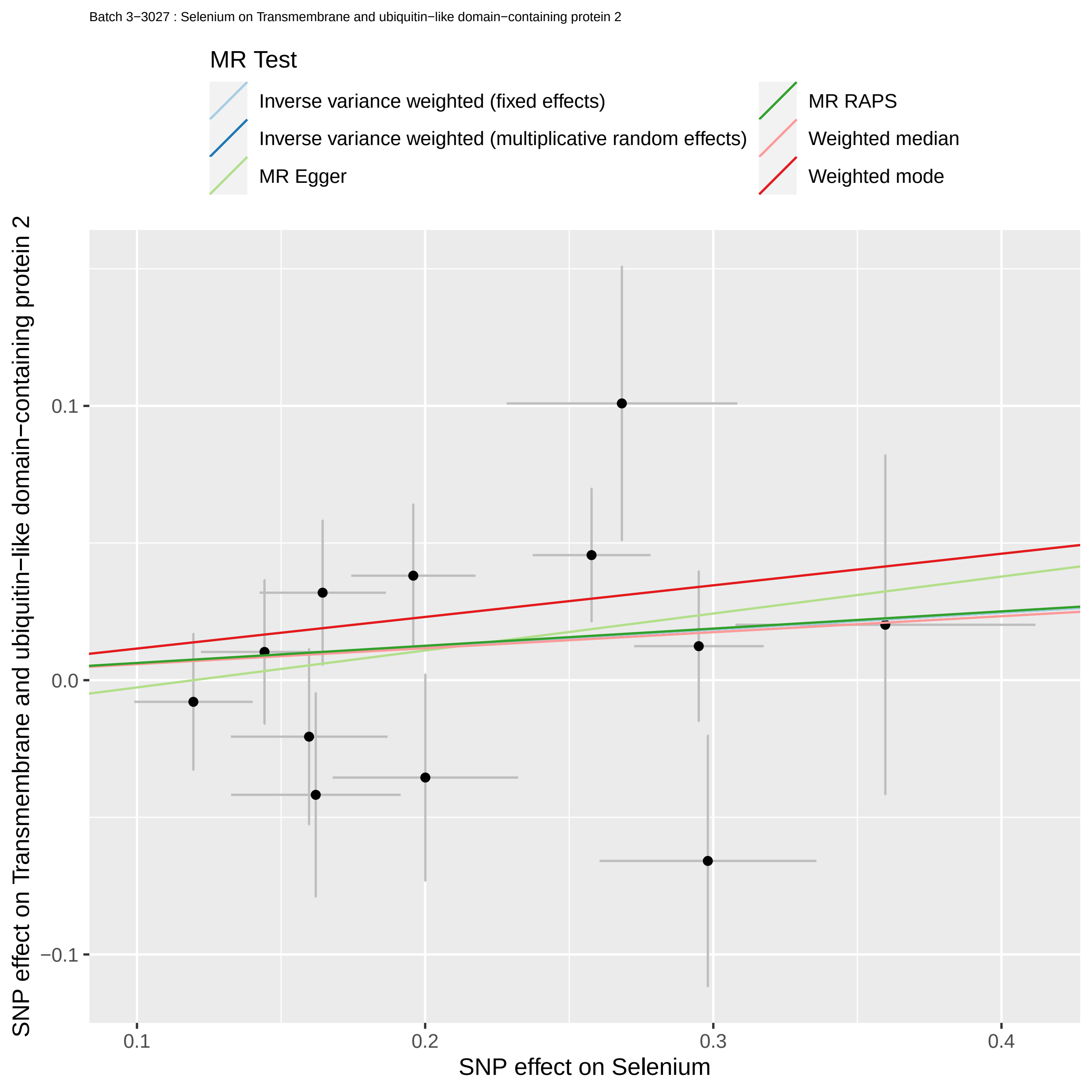
Batch 3−3027 : Selenium on Transmembrane and ubiquitin−like domain−containing protein 2
MR Test
Inverse variance weighted (fixed effects)
Inverse variance weighted (multiplicative random effects)
MR Egger
MR RAPS
Weighted median
Weighted mode
SNP effect on Transmembrane and ubiquitin−like domain−containing protein 2
0.1
0.0
−0.1
0.1
0.2
0.3
0.4
SNP effect on Selenium

Batch 3−3028 : Selenium on Tenascin

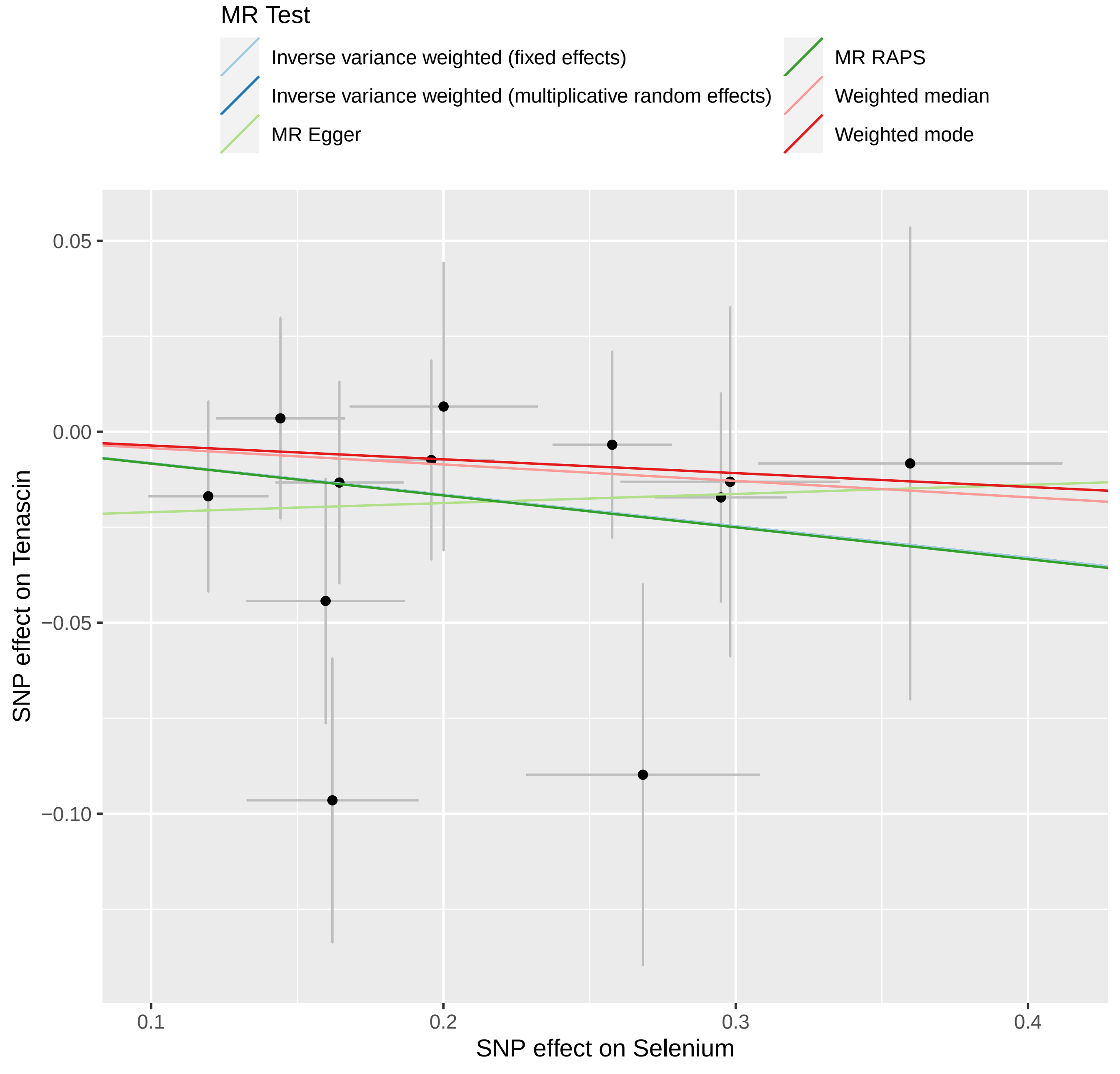

Batch 3−3029 : Selenium on Tumor necrosis factor

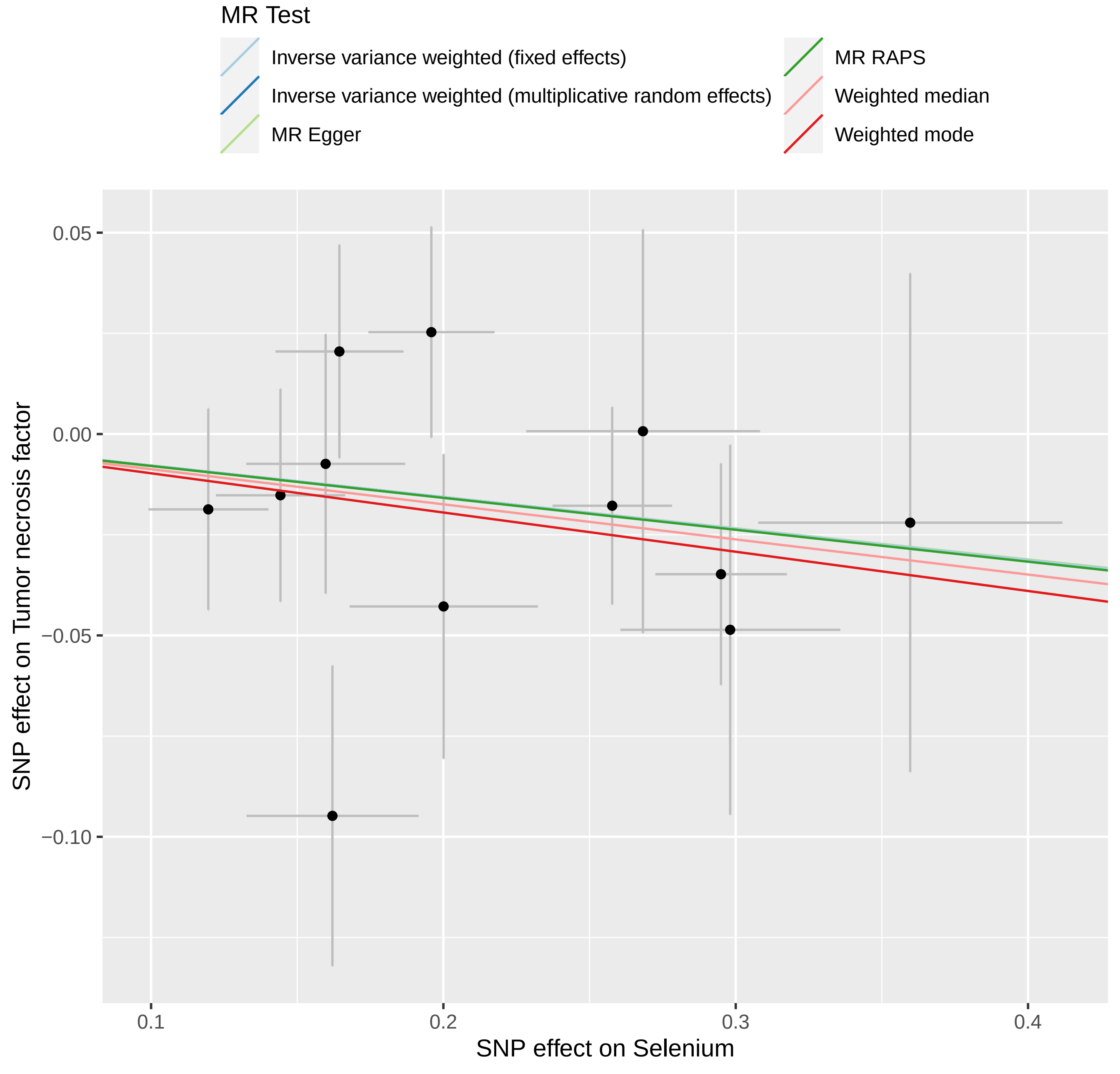

Batch 3−3030 : Selenium on Tumor necrosis factor alpha−induced protein 3

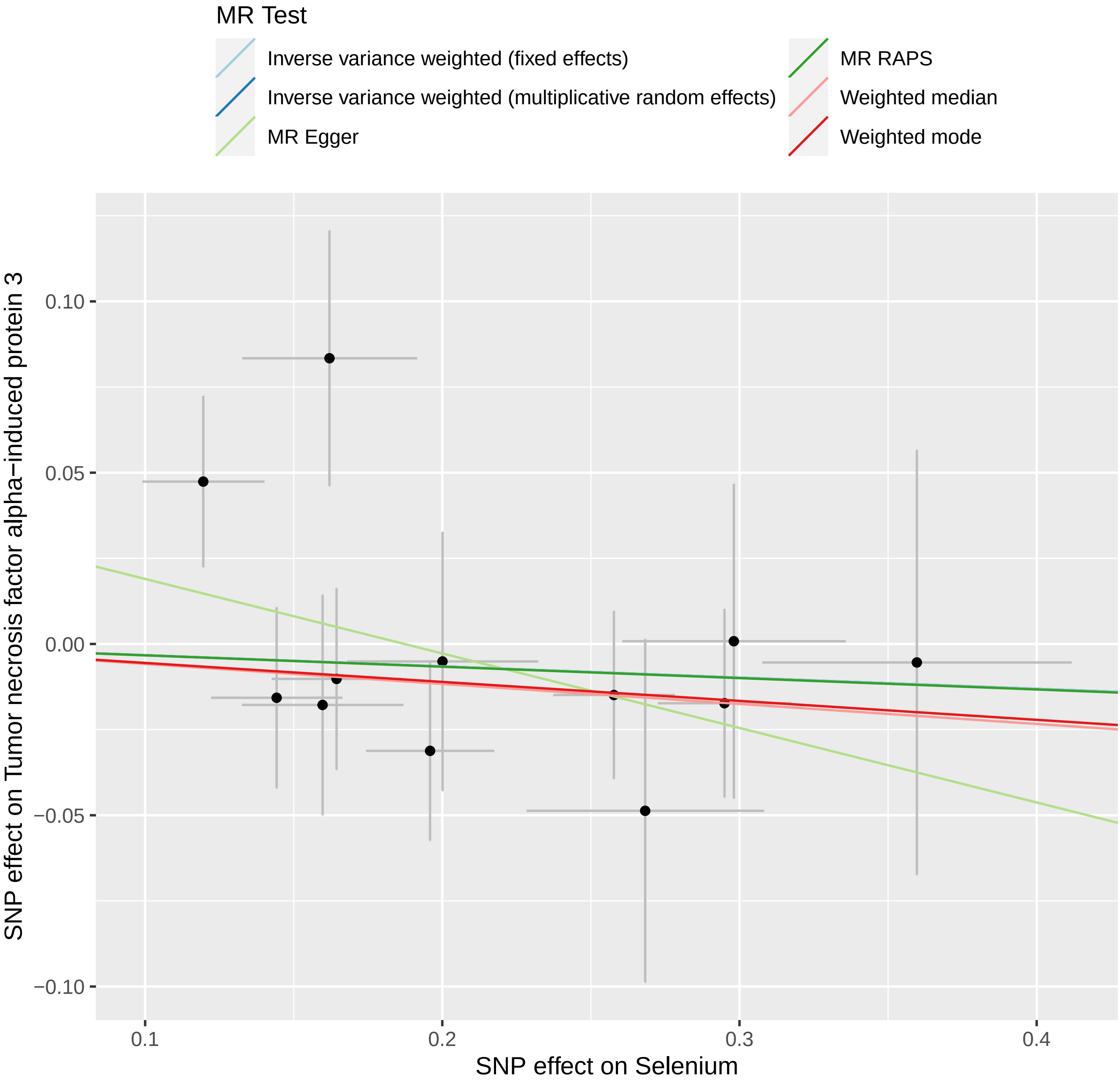

Batch 3−3031 : Selenium on Tumor necrosis factor−inducible gene 6 protein

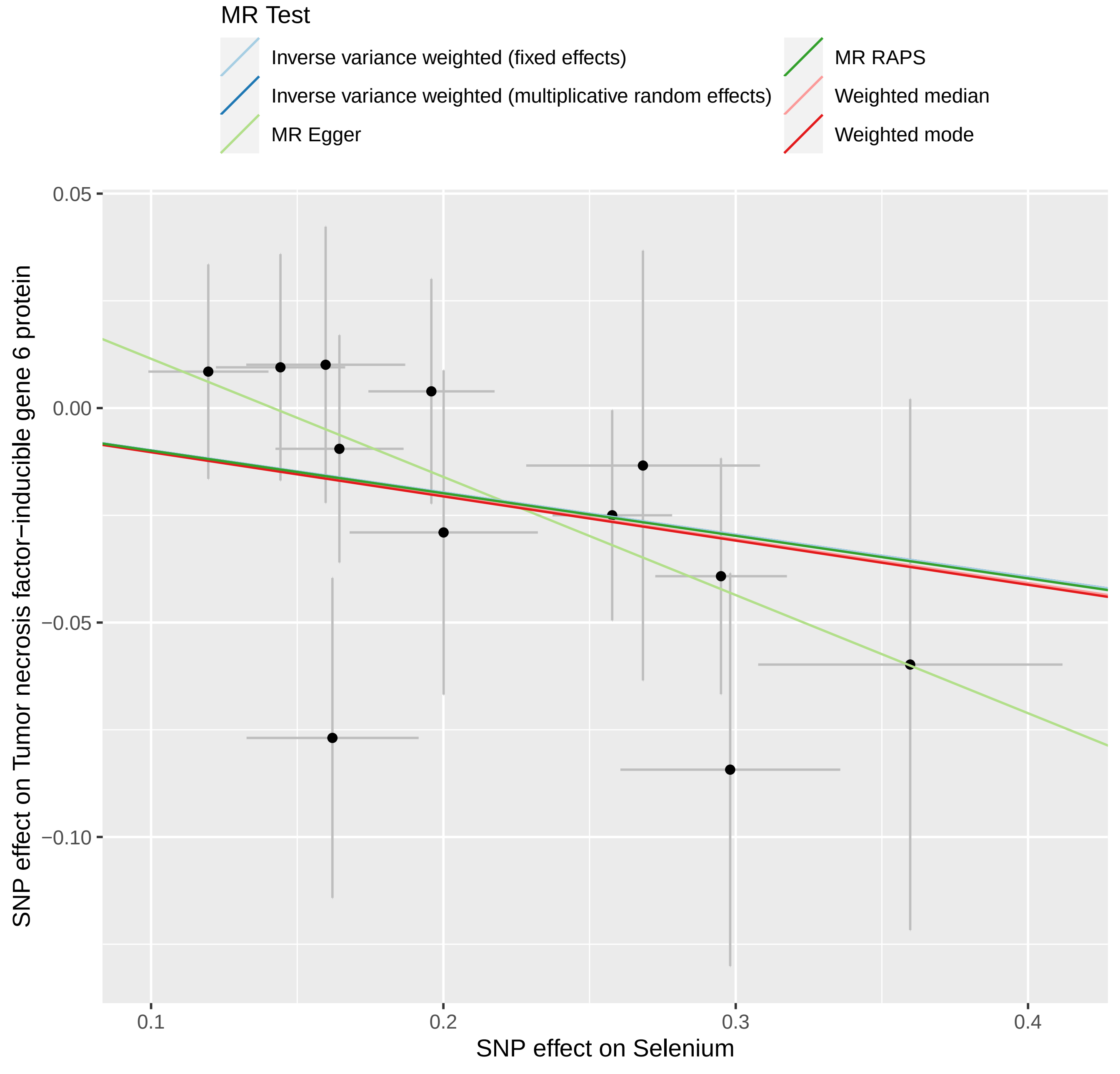

Batch 3−3032 : Selenium on Tumor necrosis factor receptor superfamily member 10A

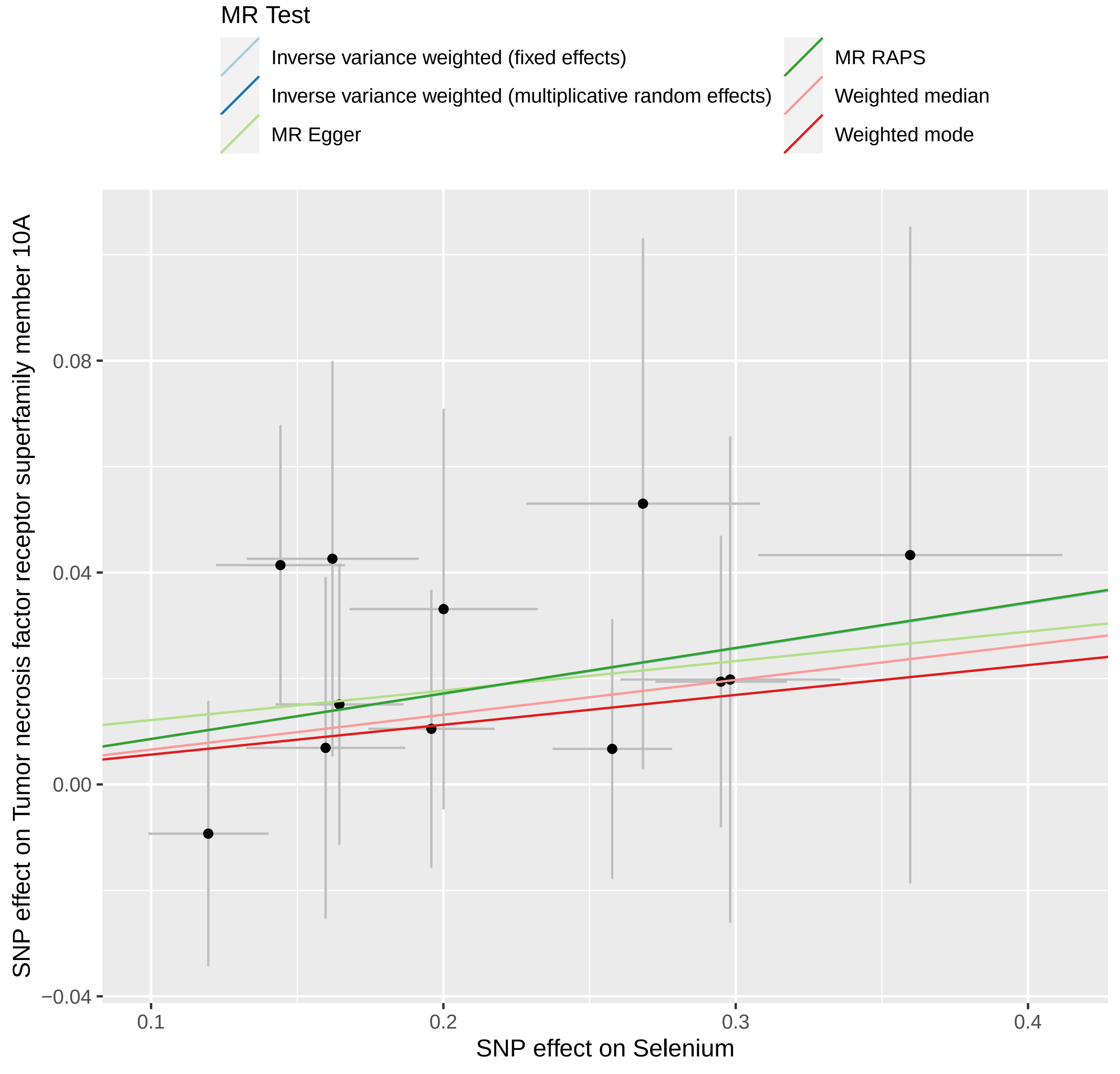

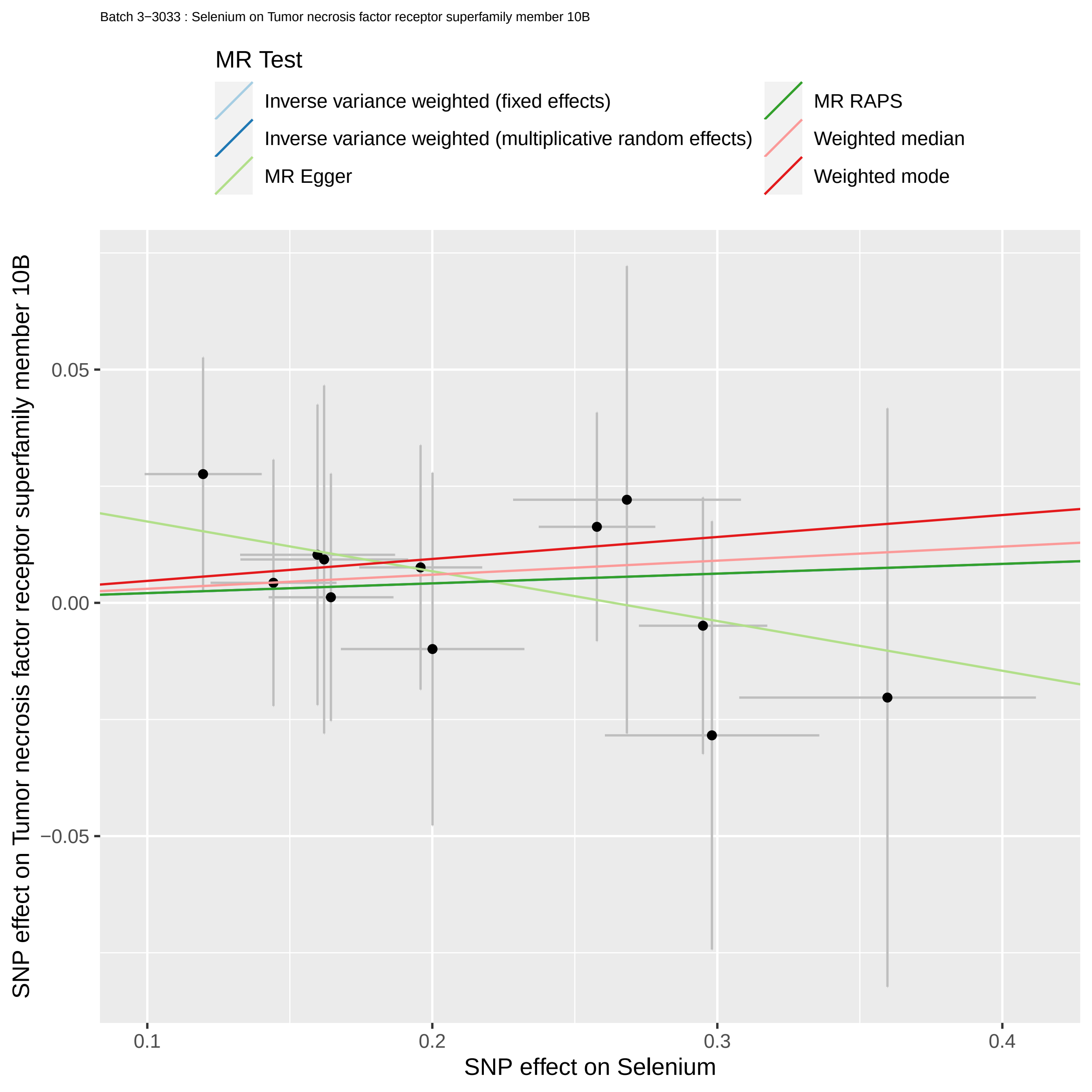
Batch 3−3033 : Selenium on Tumor necrosis factor receptor superfamily member 10B
MR Test
Inverse variance weighted (fixed effects)
Inverse variance weighted (multiplicative random effects)
MR Egger
MR RAPS
Weighted median
Weighted mode
0.05
0.00
−0.05
0.1
0.2
0.3
0.4
SNP effect on Selenium
SNP effect on Tumor necrosis factor receptor superfamily member 10B

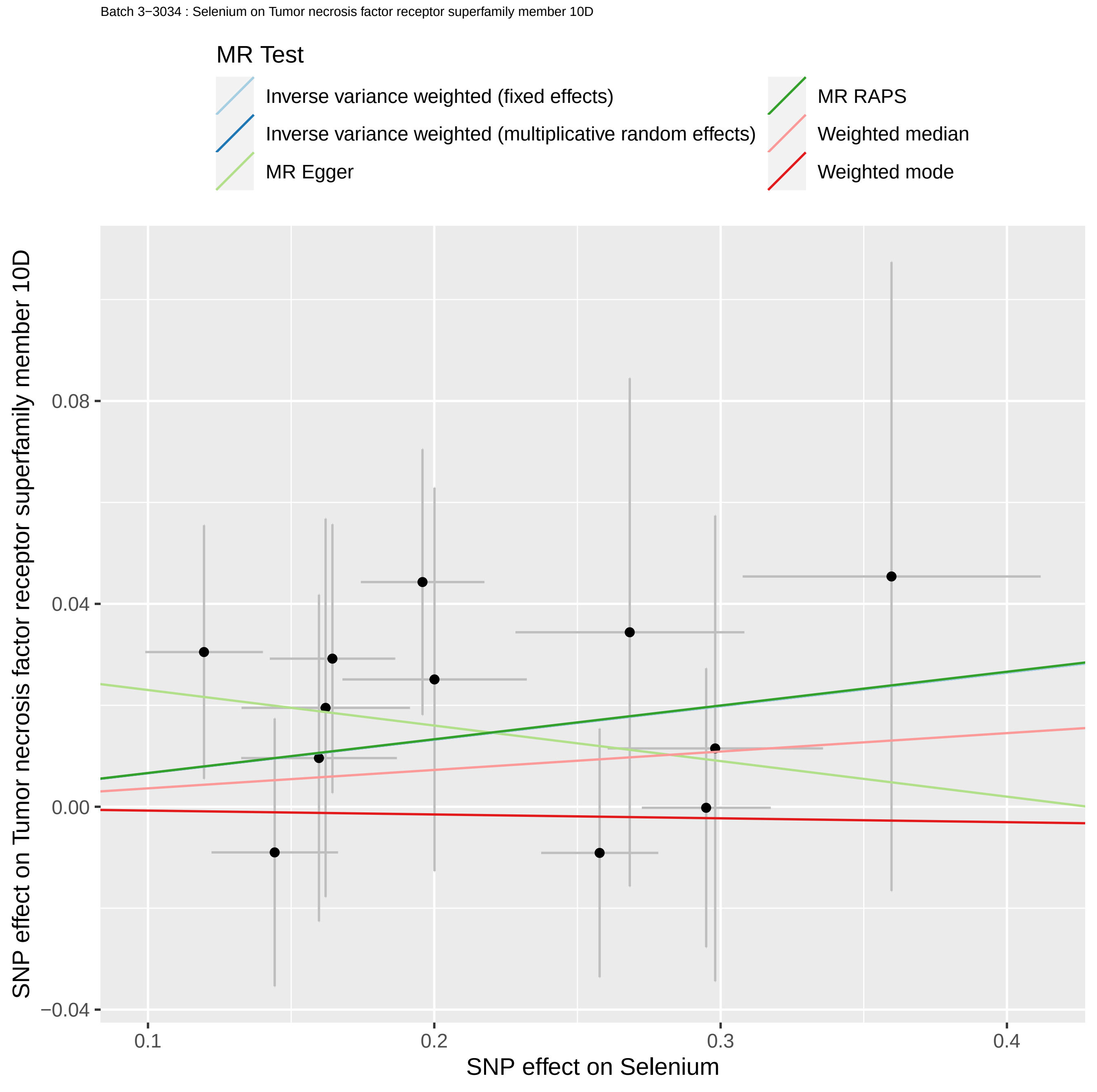

Batch 3−3034 : Selenium on Tumor necrosis factor receptor superfamily member 10D
MR Test
Inverse variance weighted (fixed effects)
Inverse variance weighted (multiplicative random effects)
MR Egger
MR RAPS
Weighted median
Weighted mode
SNP effect on Tumor necrosis factor receptor superfamily member 10D
0.08
0.04
0.00
−0.04
0.1
0.2
0.3
0.4
SNP effect on Selenium

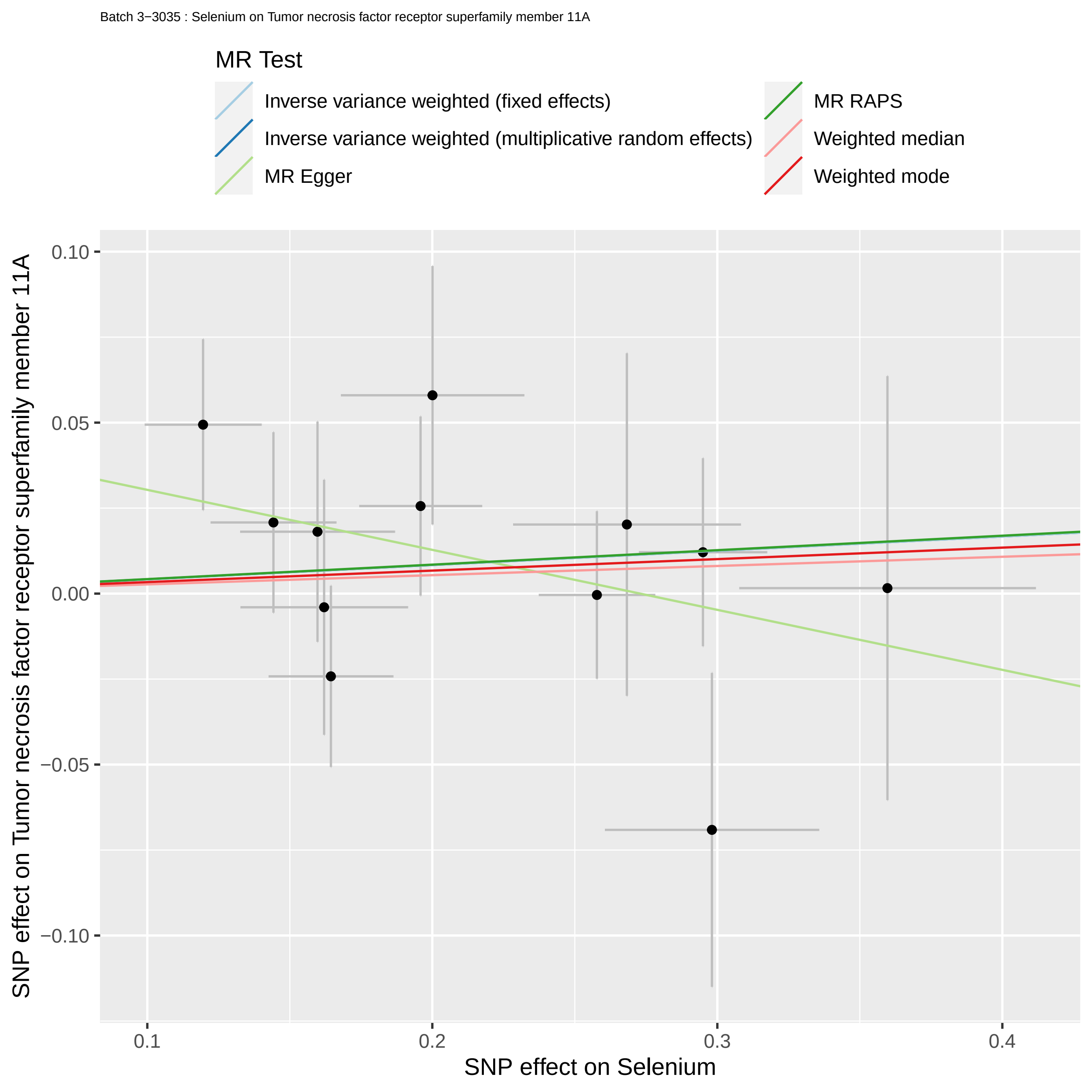
Batch 3−3035 : Selenium on Tumor necrosis factor receptor superfamily member 11A
MR Test
Inverse variance weighted (fixed effects)
Inverse variance weighted (multiplicative random effects)
MR Egger
MR RAPS
Weighted median
Weighted mode
0.10
0.05
0.00
−0.05
−0.10
0.1
0.2
0.3
0.4
SNP effect on Selenium
SNP effect on Tumor necrosis factor receptor superfamily member 11A

Batch 3−3036 : Selenium on Tumor necrosis factor receptor superfamily member 11A

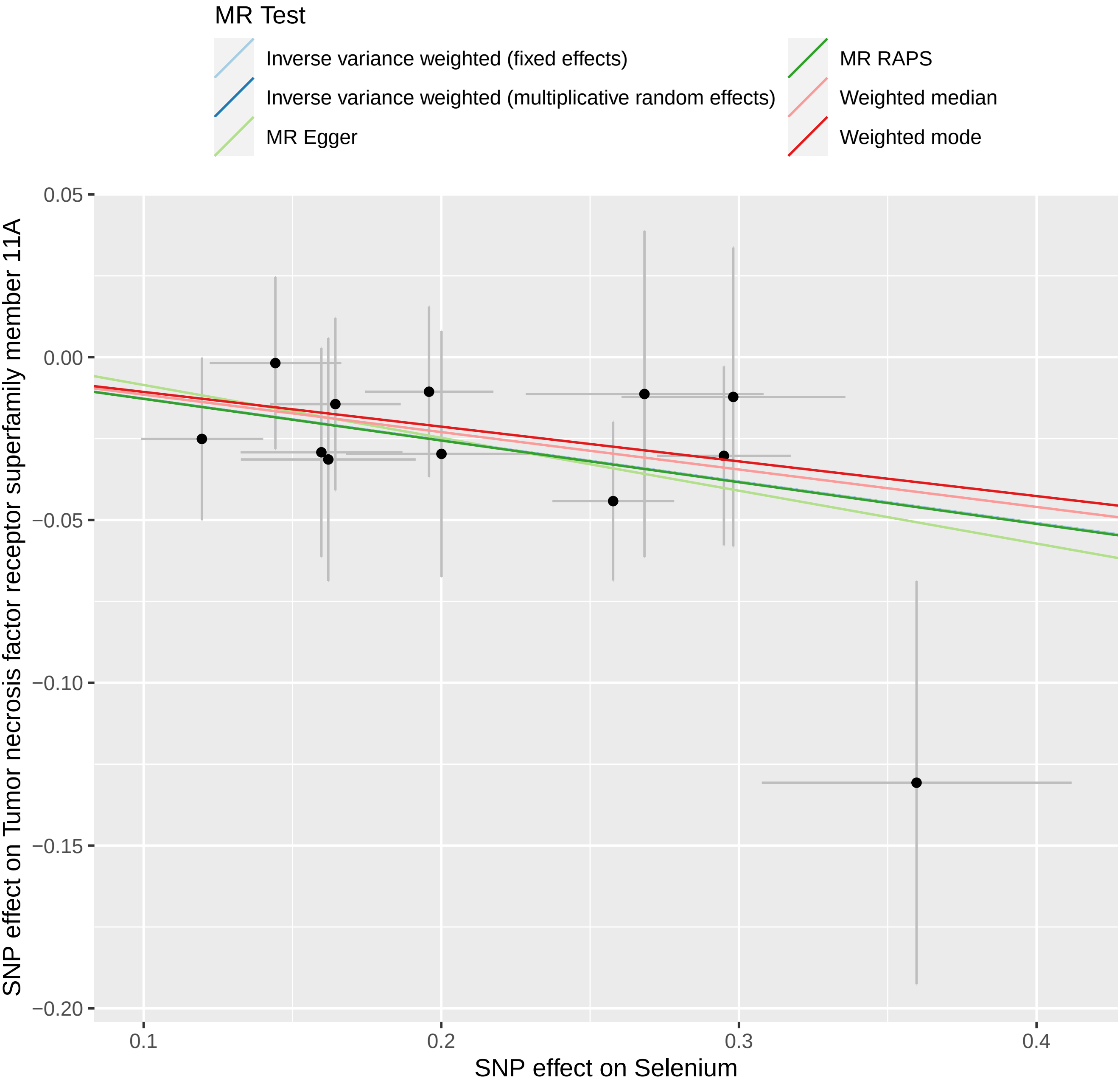

Batch 3−3037 : Selenium on Tumor necrosis factor receptor superfamily member 11A

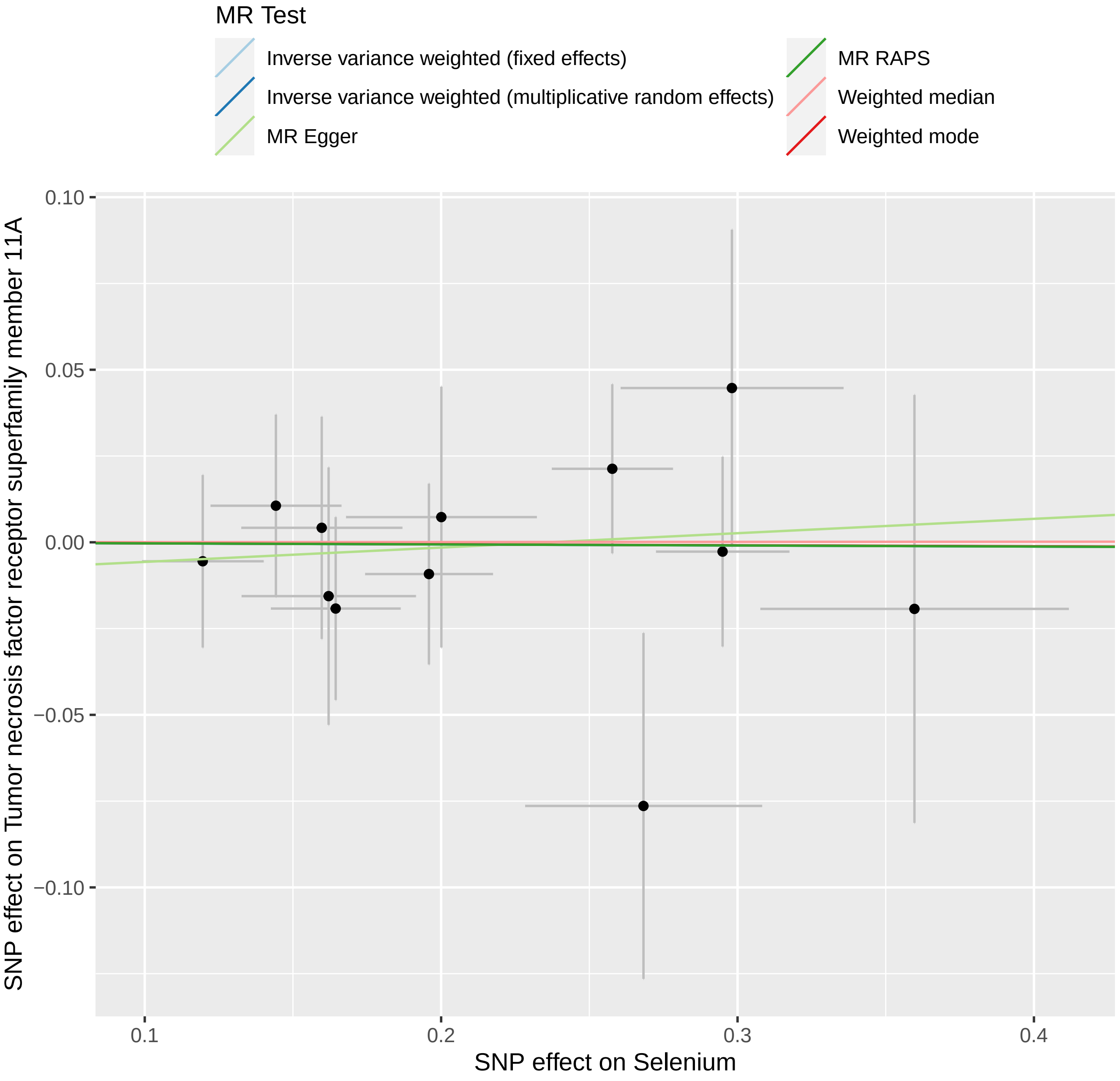

Batch 3−3038 : Selenium on Tumor necrosis factor receptor superfamily member 11B

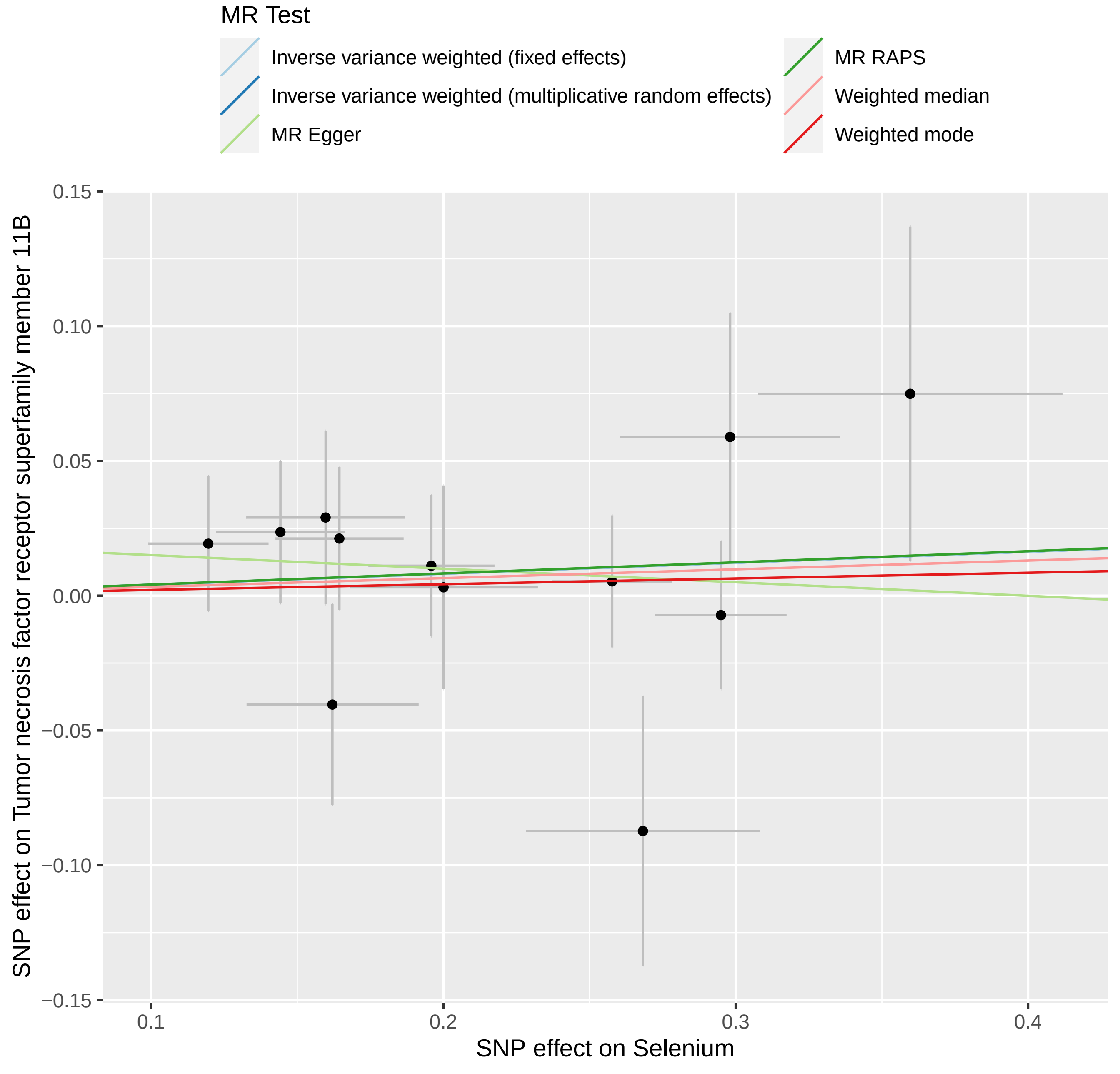

Batch 3−3039 : Selenium on Tumor necrosis factor receptor superfamily member 11B

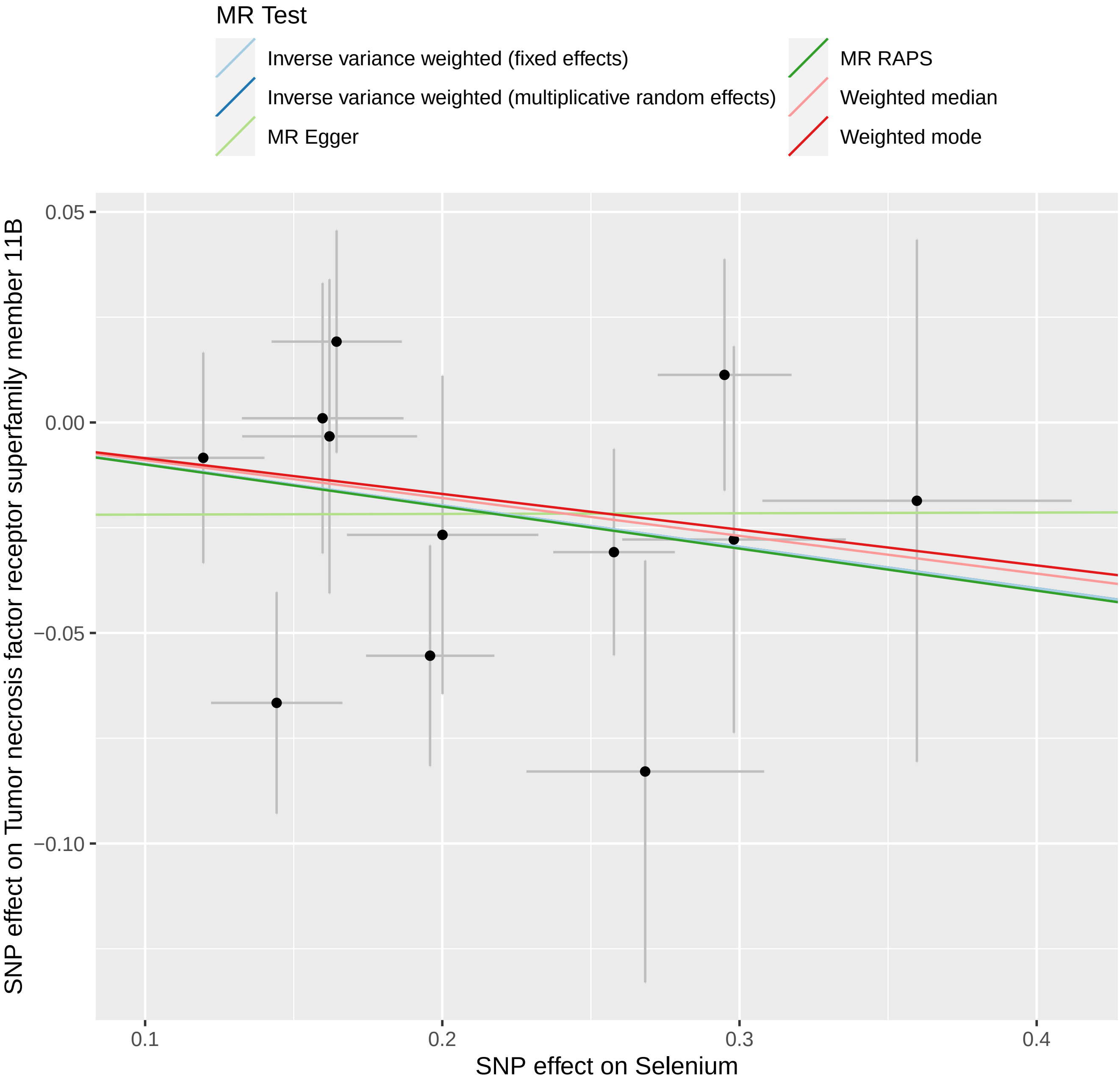

Batch 3−3040 : Selenium on Tumor necrosis factor receptor superfamily member 12A

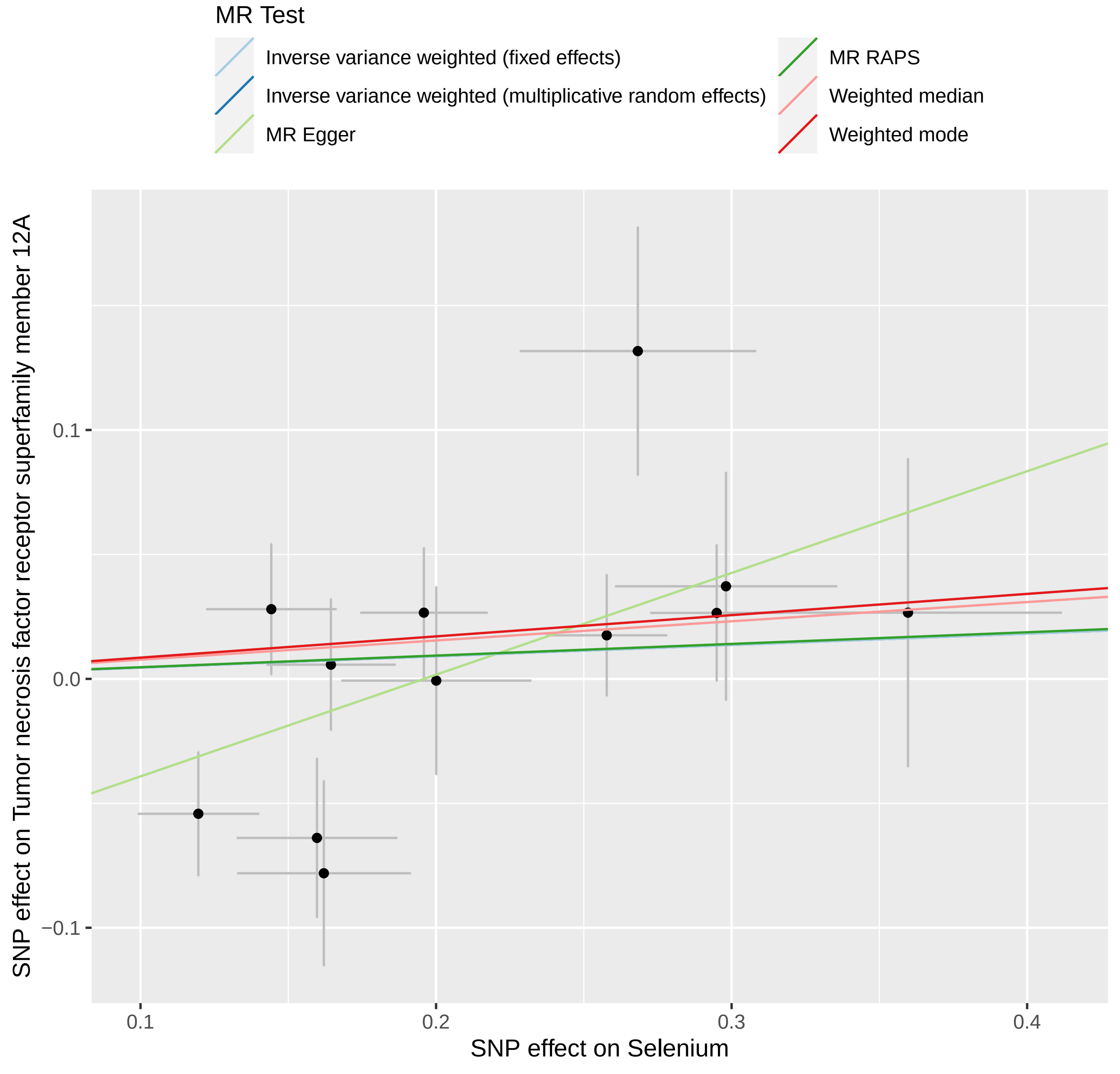

Batch 3−3041 : Selenium on Tumor necrosis factor receptor superfamily member 13B

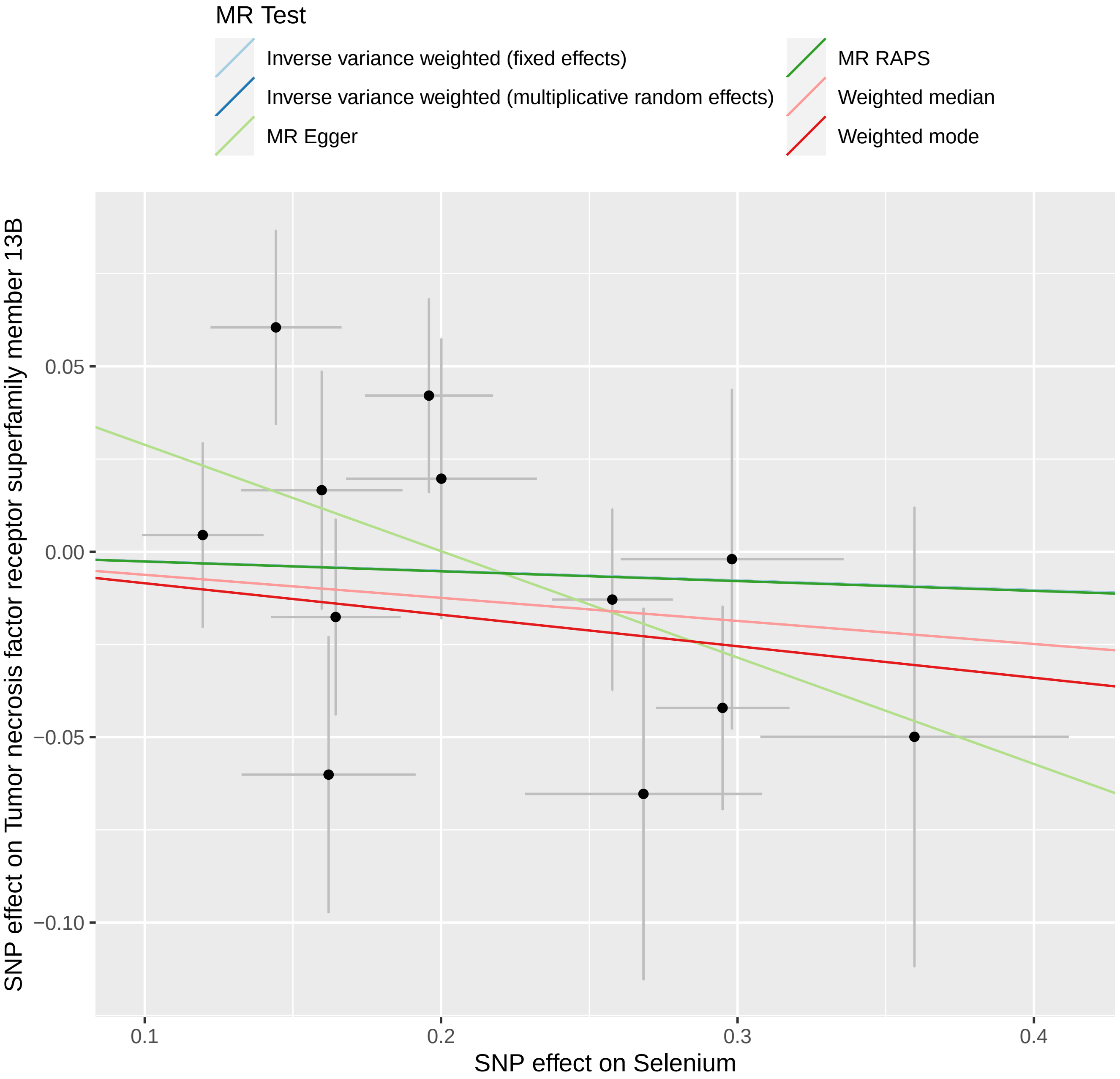

Batch 3−3042 : Selenium on Tumor necrosis factor receptor superfamily member 14

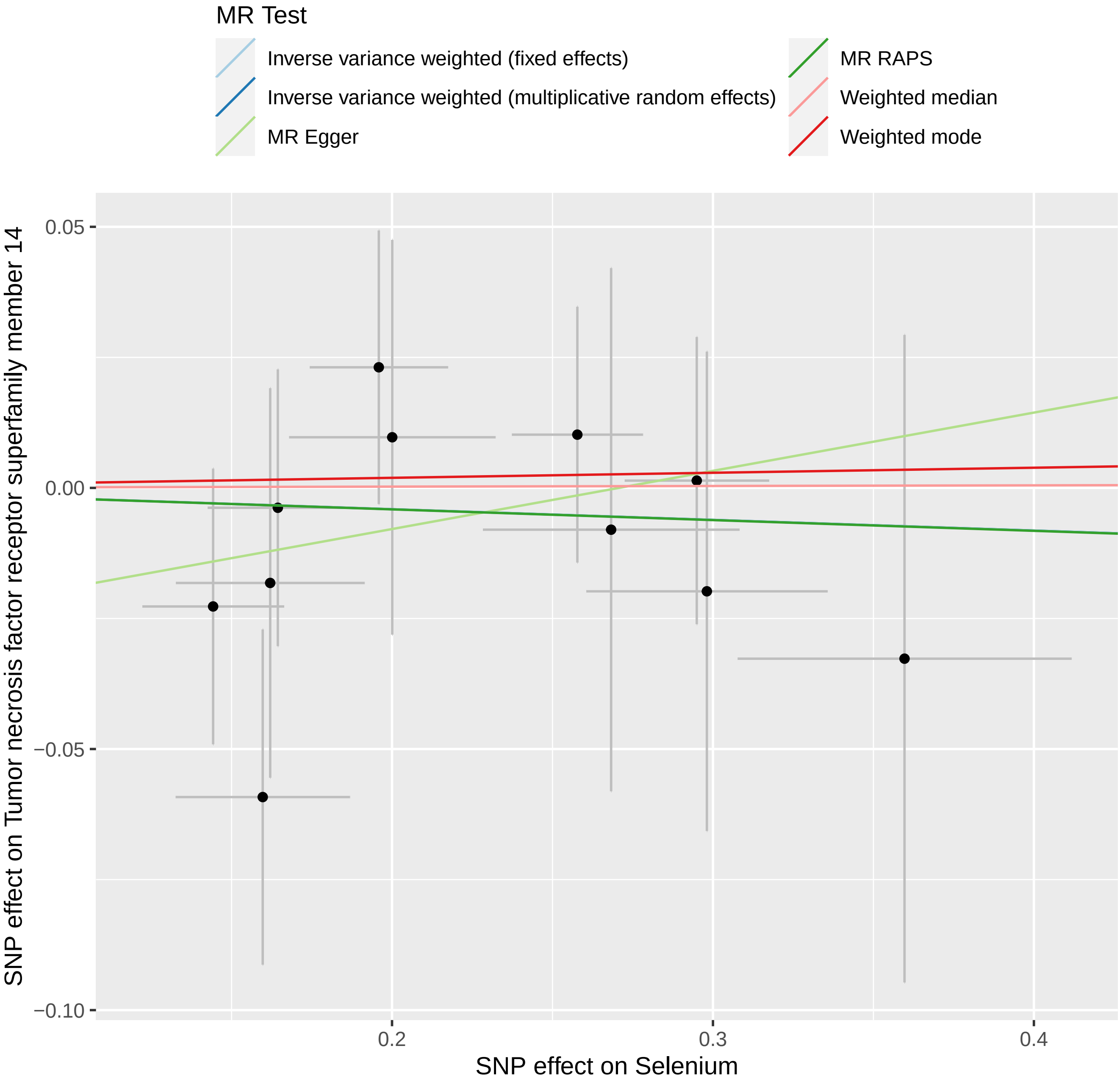

Batch 3−3043 : Selenium on Tumor necrosis factor receptor superfamily member 17

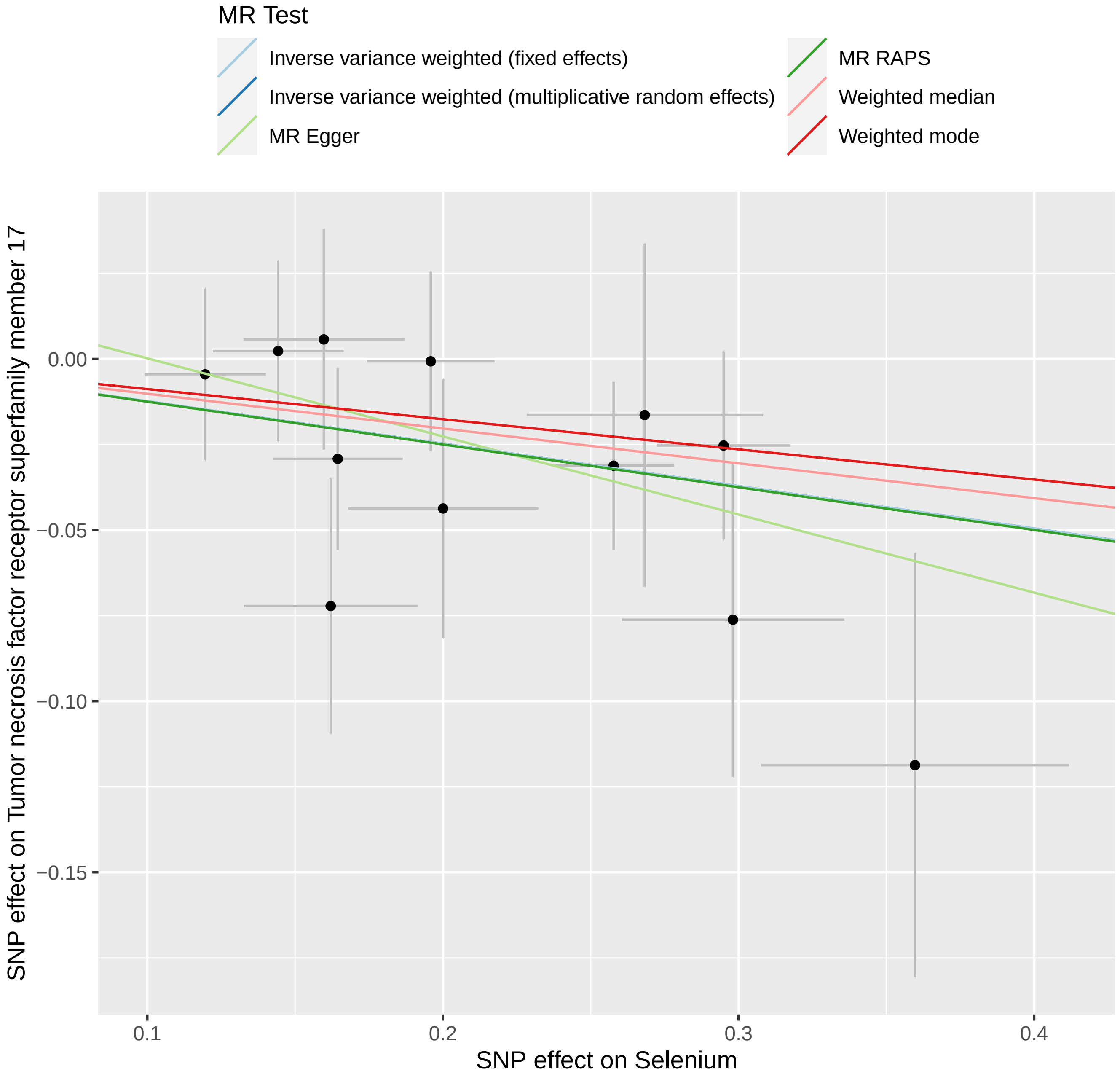

Batch 3−3044 : Selenium on Tumor necrosis factor receptor superfamily member 18

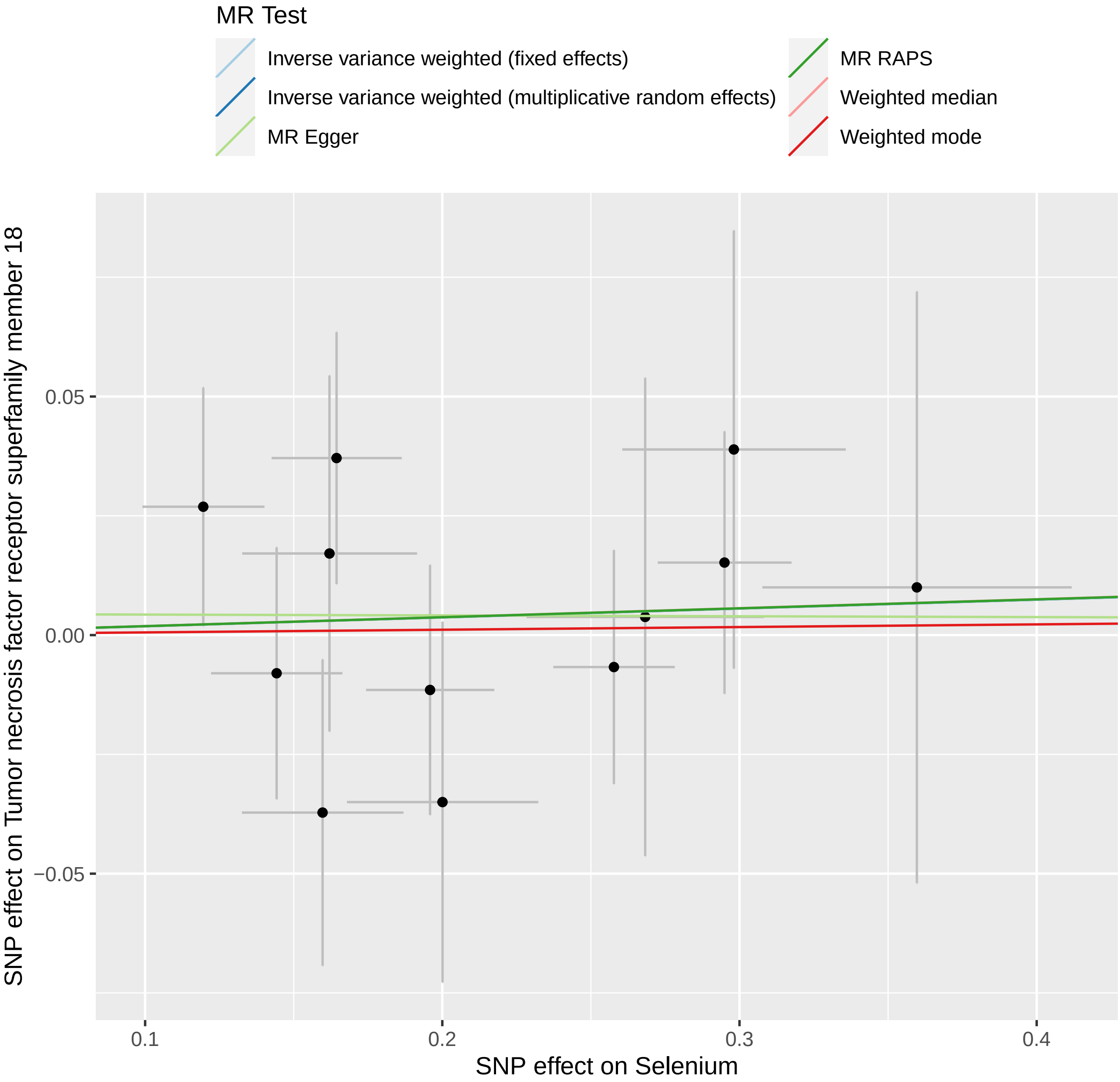

Batch 3−3045 : Selenium on Tumor necrosis factor receptor superfamily member 19

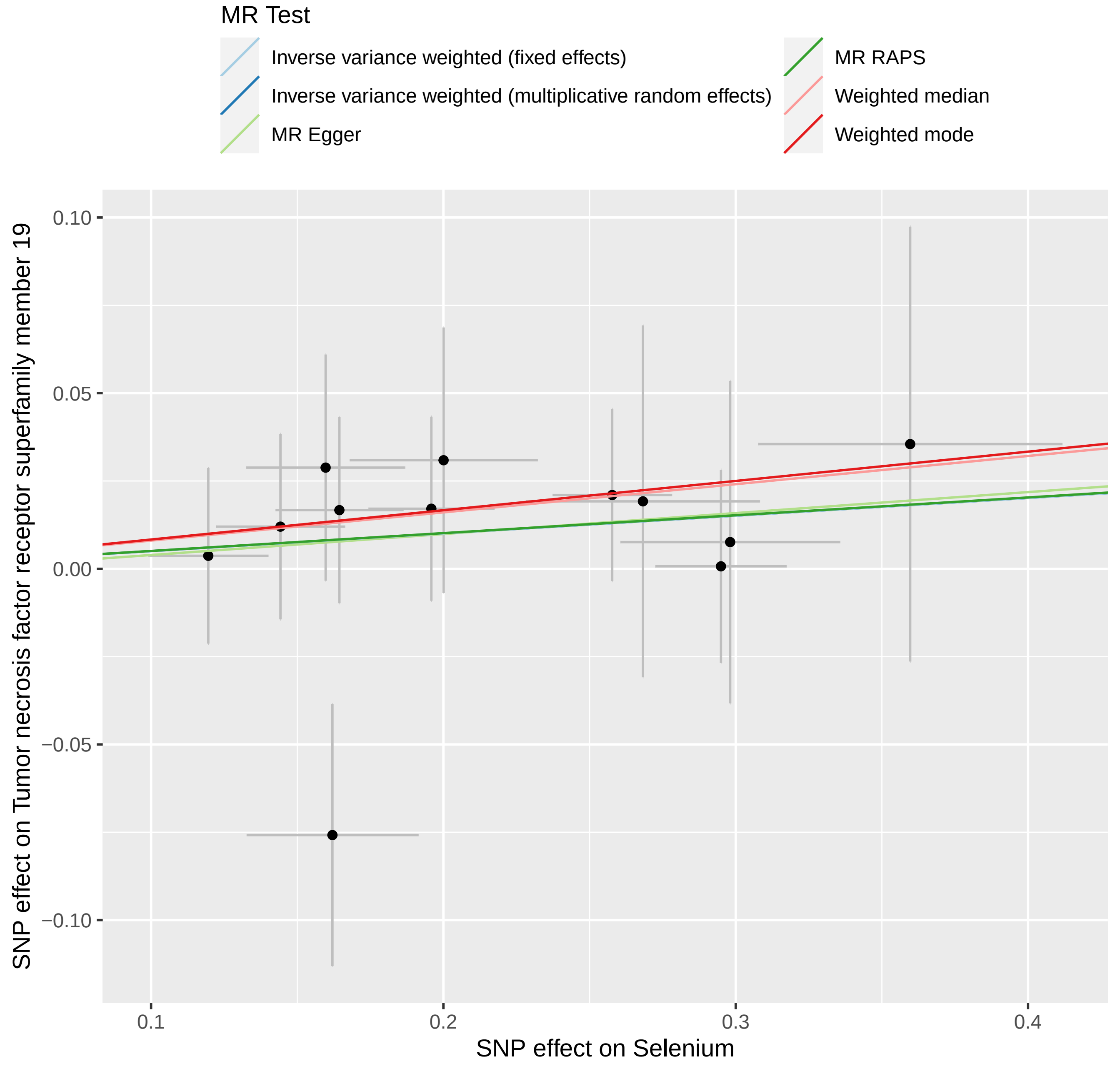

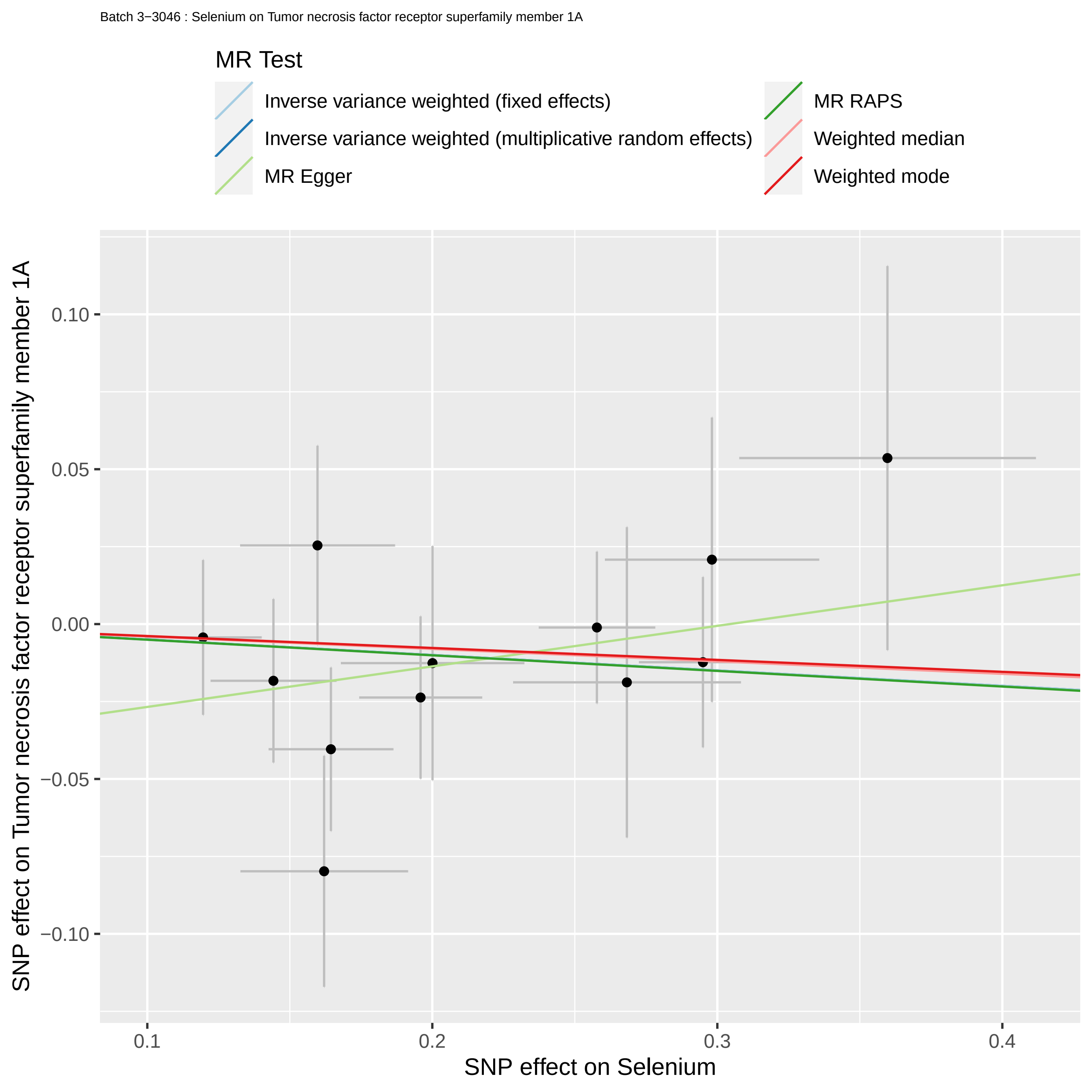

Batch 3−3046 : Selenium on Tumor necrosis factor receptor superfamily member 1A
MR Test
Inverse variance weighted (fixed effects)
Inverse variance weighted (multiplicative random effects)
MR Egger
MR RAPS
Weighted median
Weighted mode
SNP effect on Tumor necrosis factor receptor superfamily member 1A
0.10
0.05
0.00
−0.05
−0.10
0.1
0.2
0.3
0.4
SNP effect on Selenium

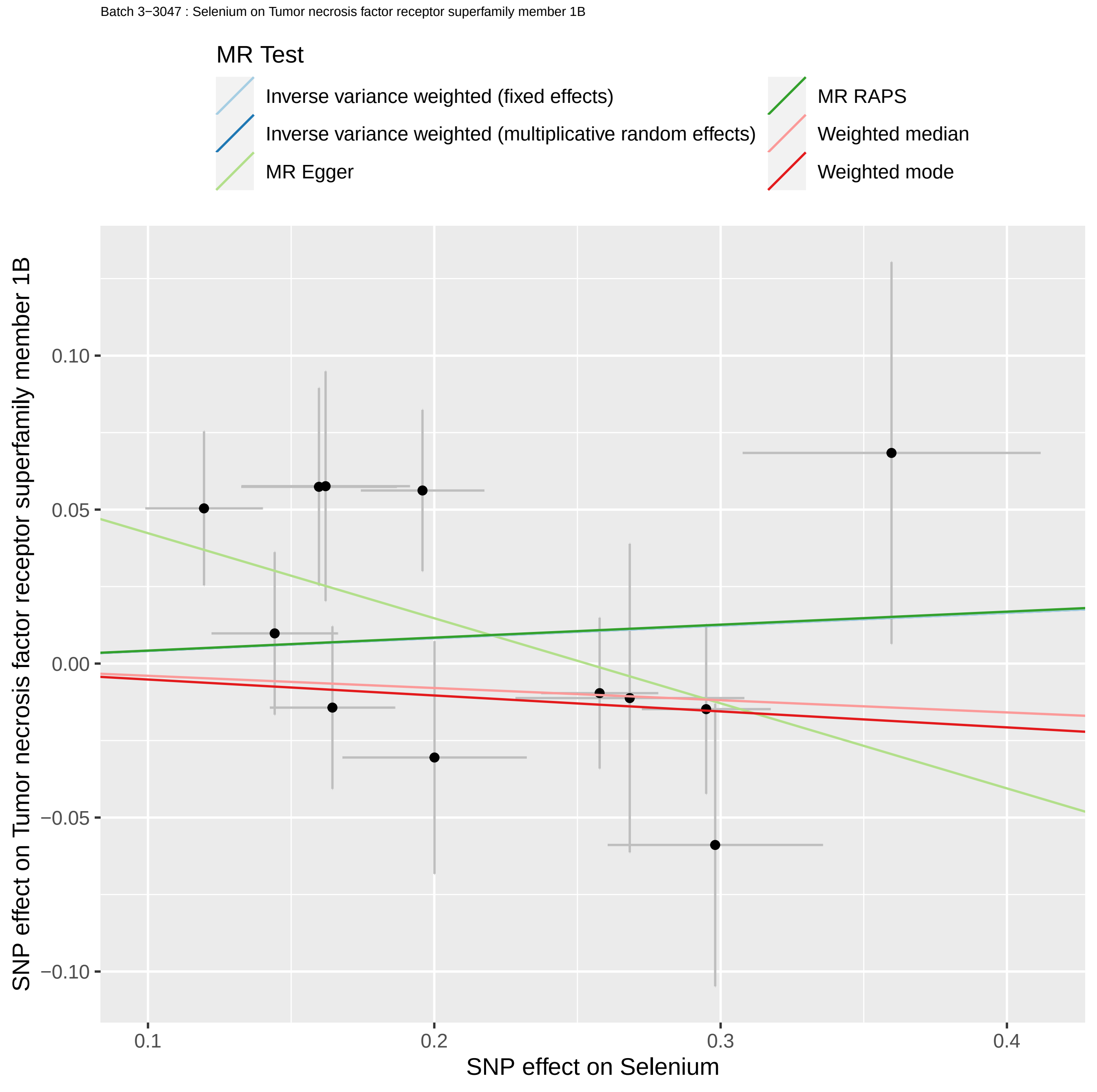

Batch 3−3047 : Selenium on Tumor necrosis factor receptor superfamily member 1B
MR Test
Inverse variance weighted (fixed effects)
Inverse variance weighted (multiplicative random effects)
MR Egger
MR RAPS
Weighted median
Weighted mode
0.10
0.05
0.00
−0.05
−0.10
0.1
0.2
0.3
0.4
SNP effect on Selenium
SNP effect on Tumor necrosis factor receptor superfamily member 1B

Batch 3−3048 : Selenium on Tumor necrosis factor receptor superfamily member 1B

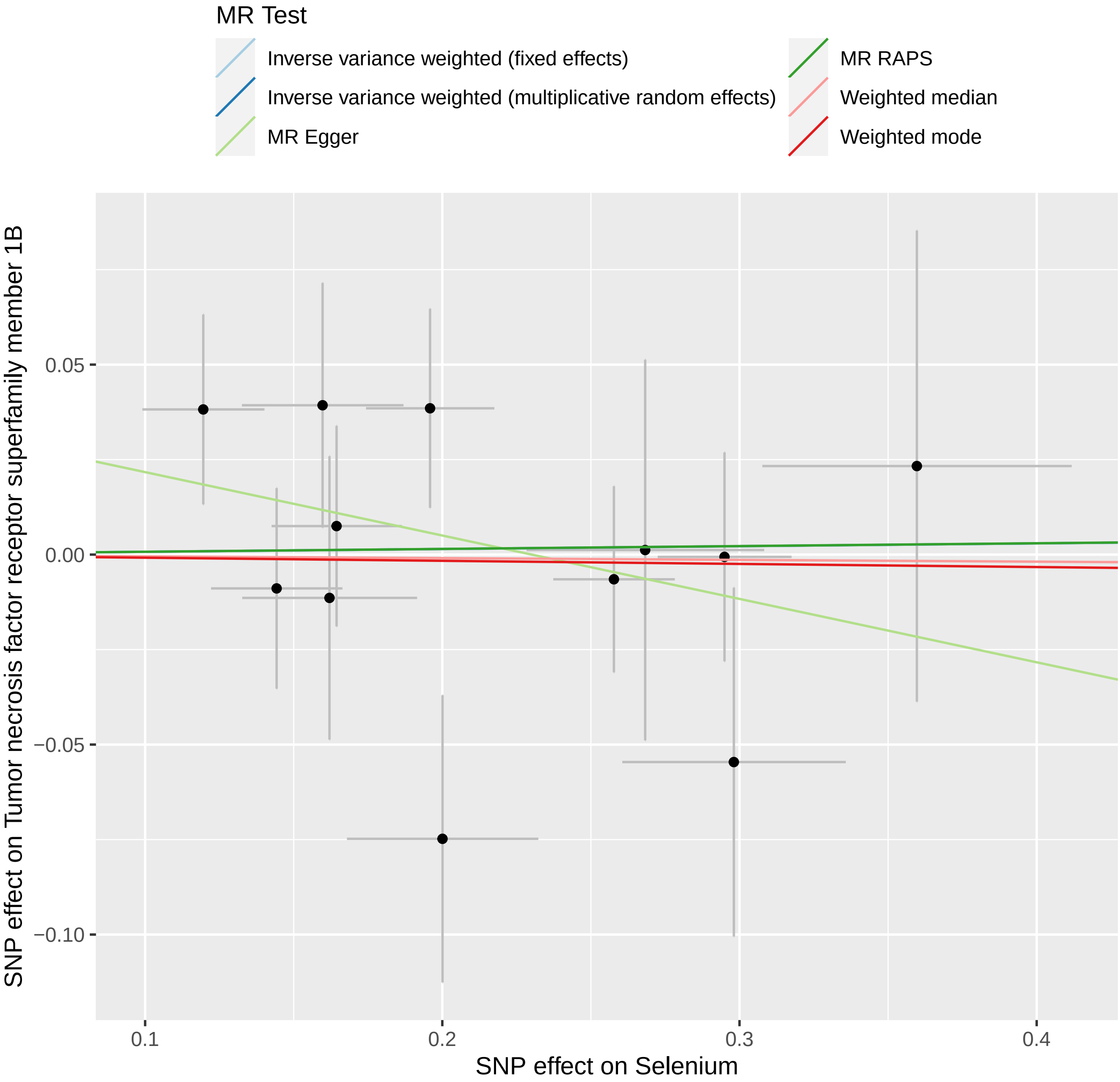

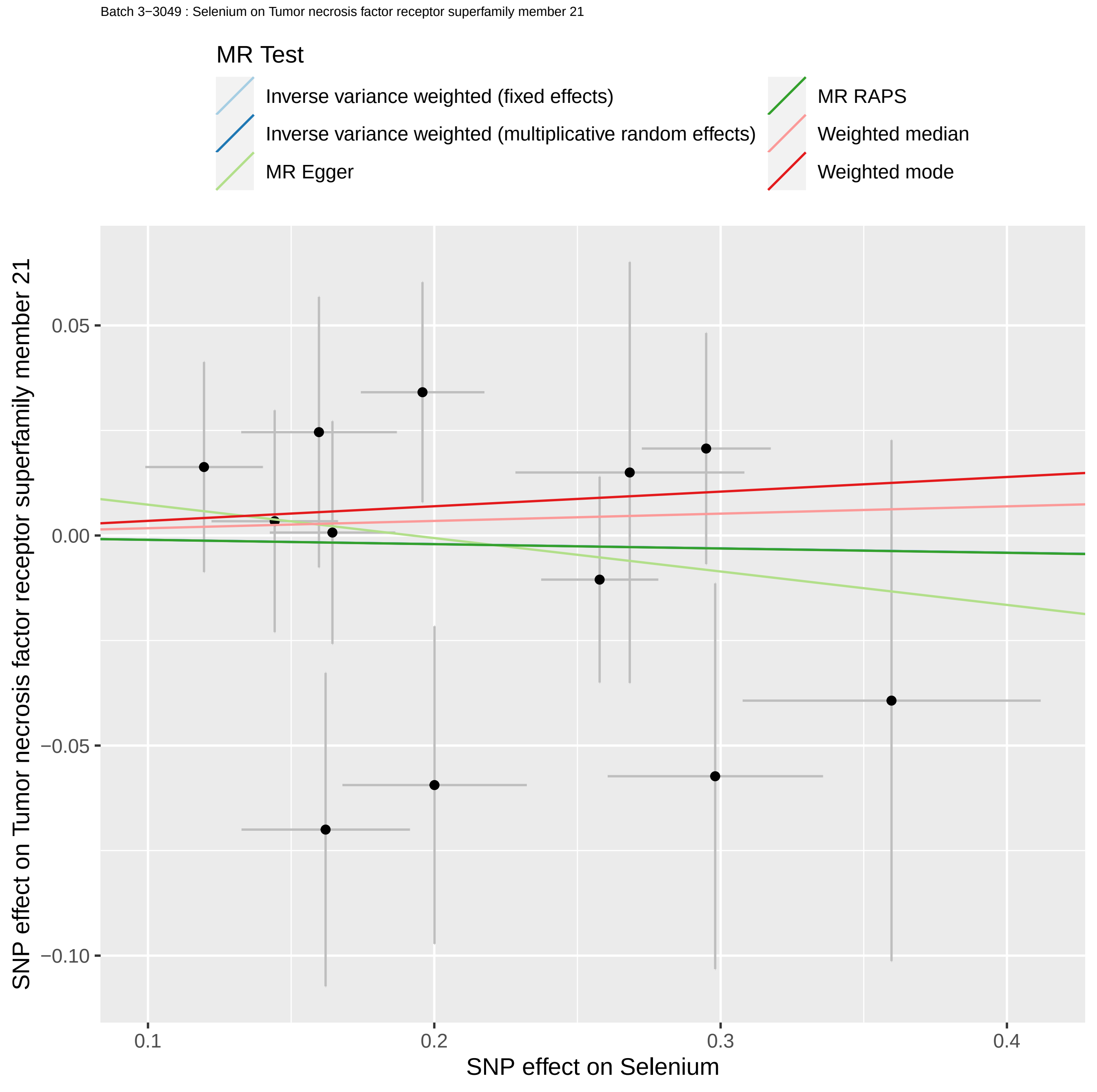

Batch 3−3049 : Selenium on Tumor necrosis factor receptor superfamily member 21
MR Test
Inverse variance weighted (fixed effects)
Inverse variance weighted (multiplicative random effects)
MR Egger
MR RAPS
Weighted median
Weighted mode
SNP effect on Tumor necrosis factor receptor superfamily member 21
0.05
0.00
−0.05
−0.10
0.1
0.2
0.3
0.4
SNP effect on Selenium

Batch 3−3050 : Selenium on Tumor necrosis factor receptor superfamily member 21

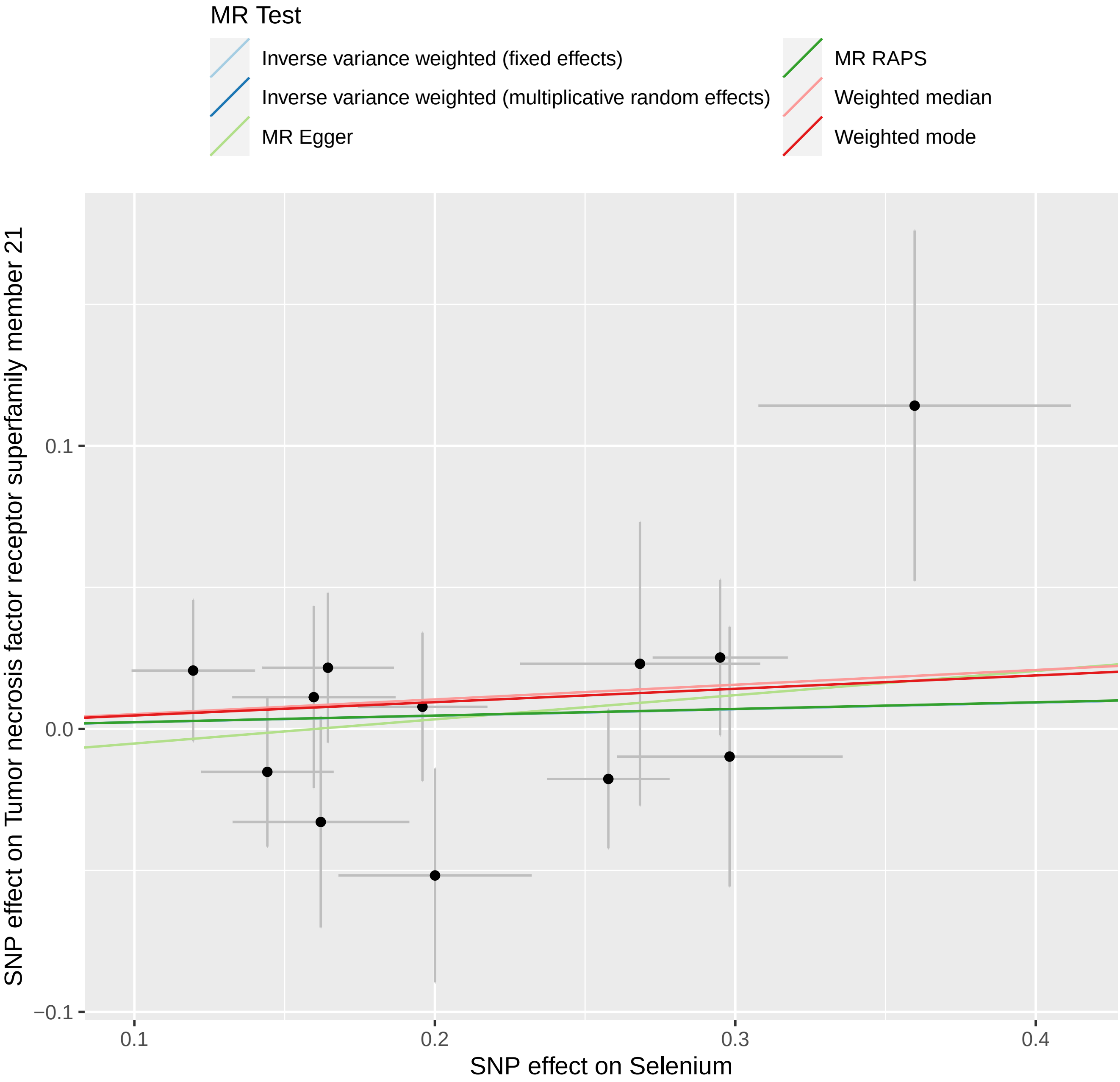

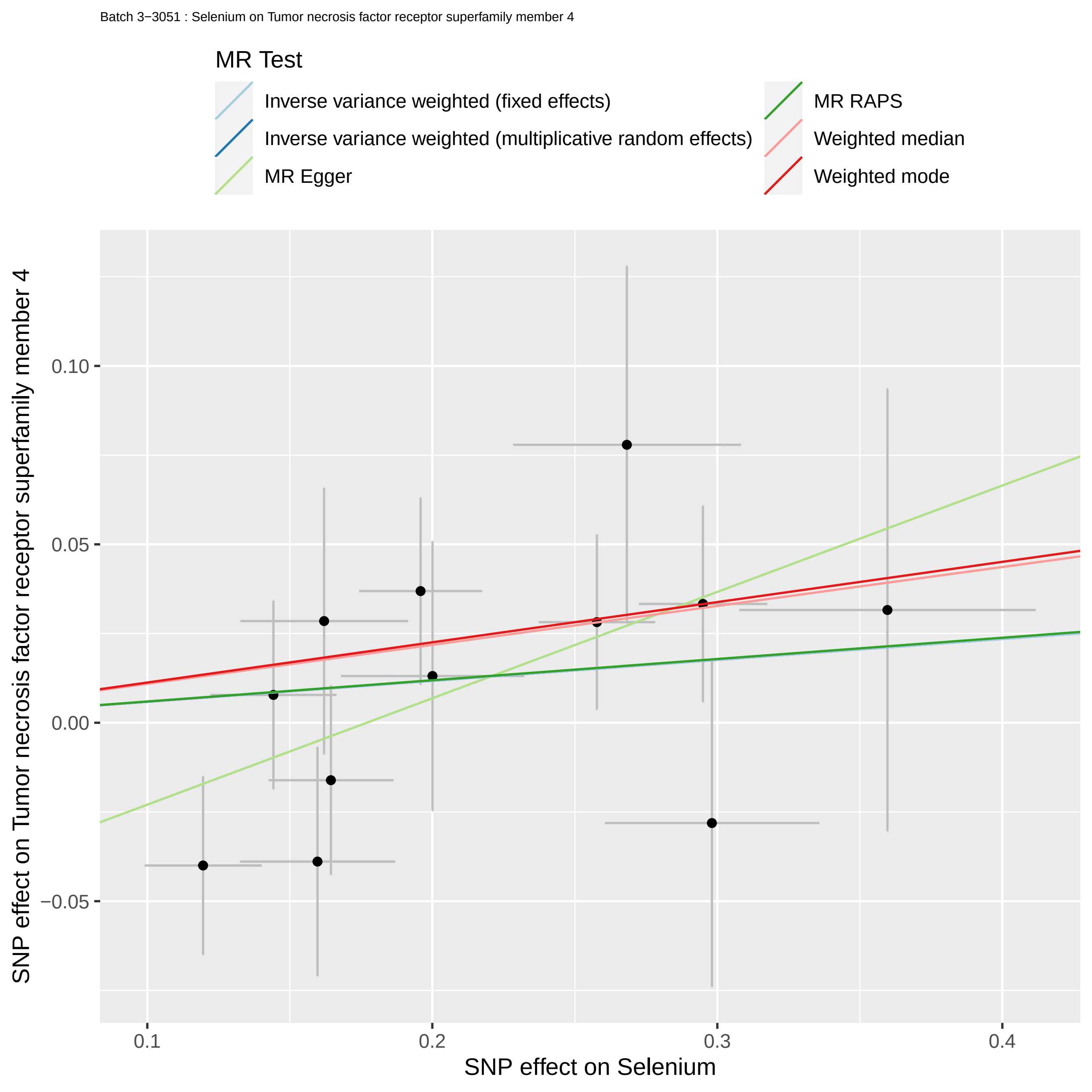
Batch 3−3051 : Selenium on Tumor necrosis factor receptor superfamily member 4
MR Test
Inverse variance weighted (fixed effects)
Inverse variance weighted (multiplicative random effects)
MR Egger
MR RAPS
Weighted median
Weighted mode
0.10
0.05
0.00
−0.05
0.1
0.2
0.3
0.4
SNP effect on Selenium
SNP effect on Tumor necrosis factor receptor superfamily member 4

Batch 3−3052 : Selenium on Tumor necrosis factor receptor superfamily member 6B

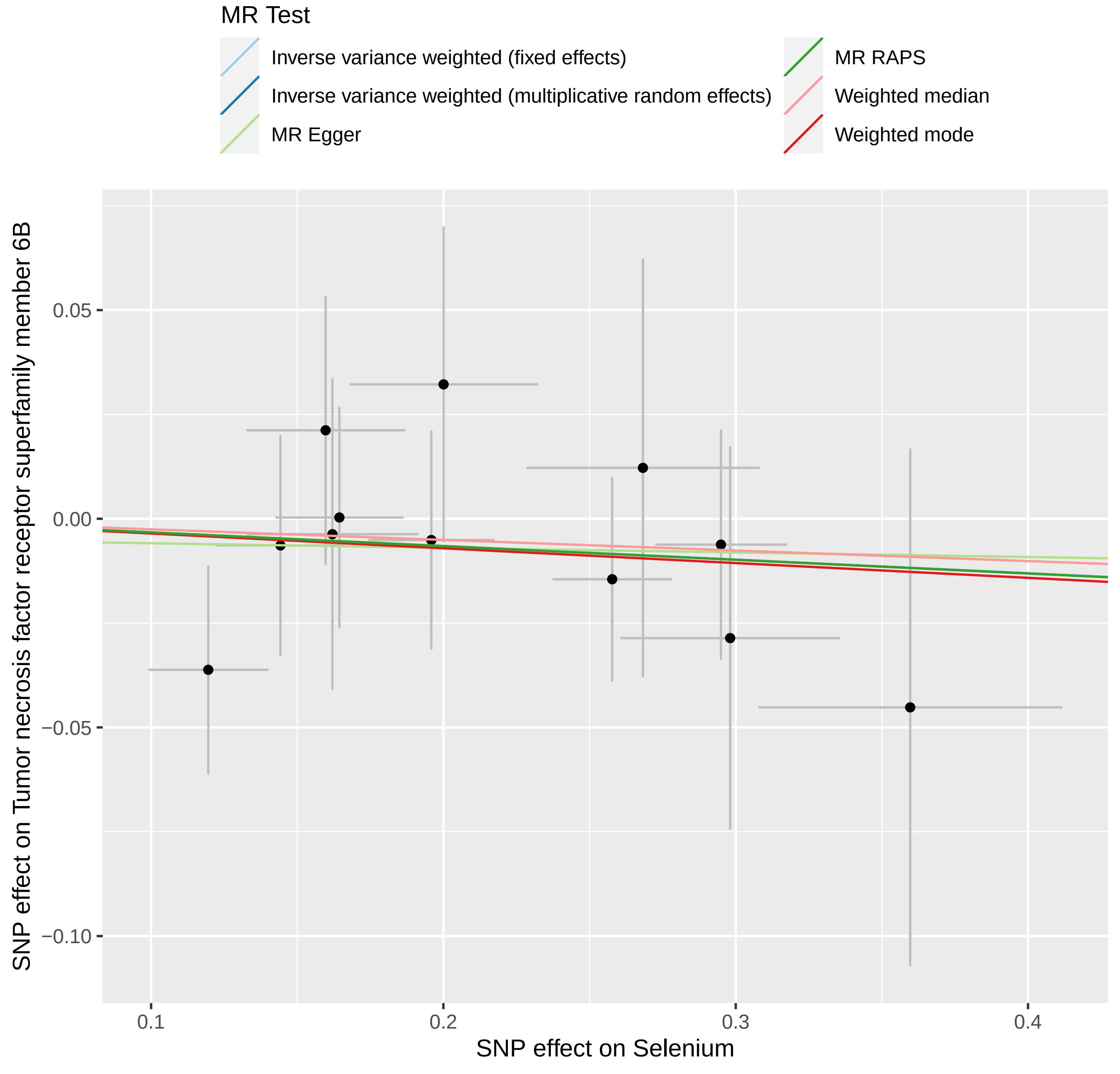

Batch 3−3053 : Selenium on Tumor necrosis factor receptor superfamily member 8

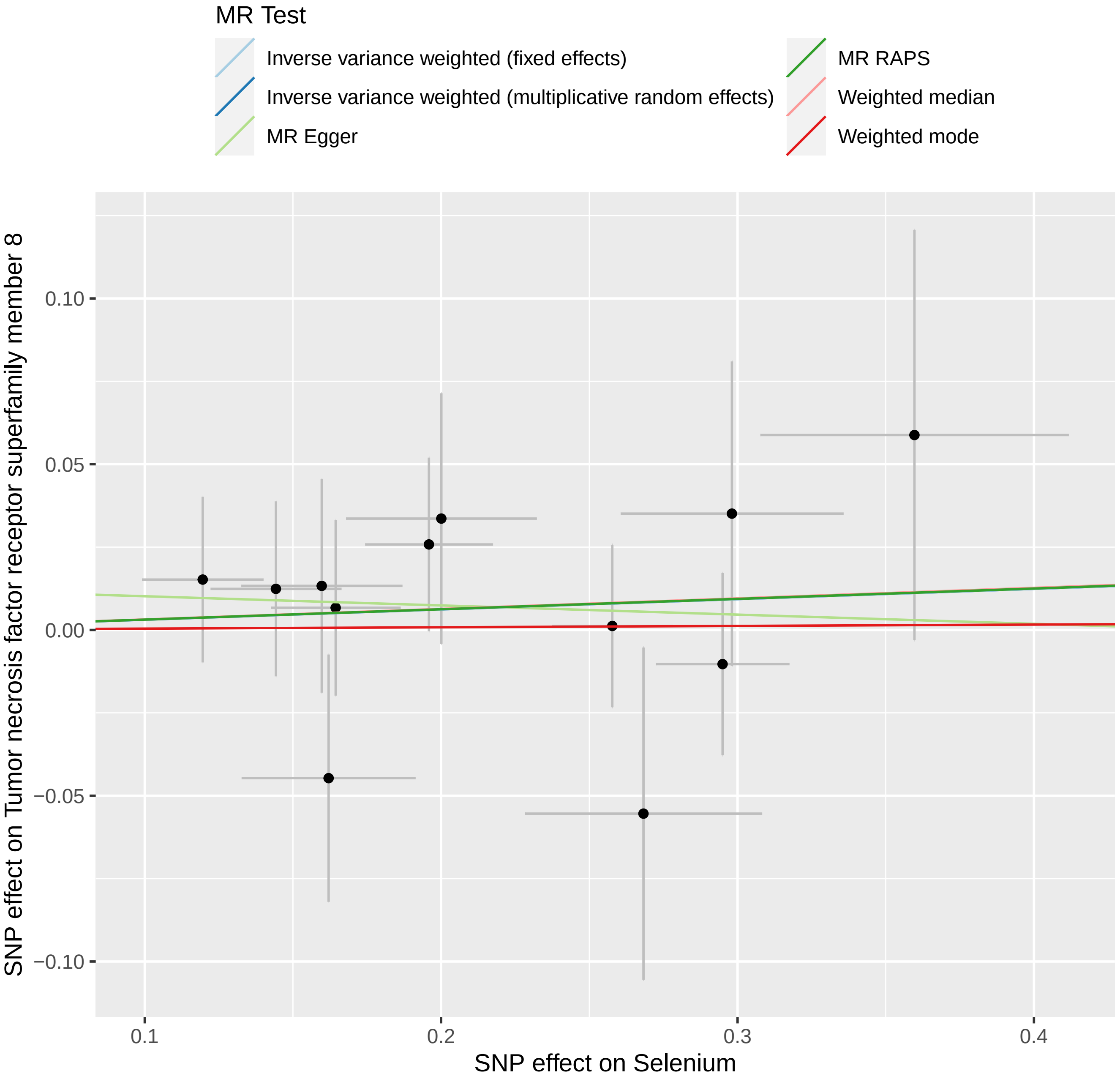

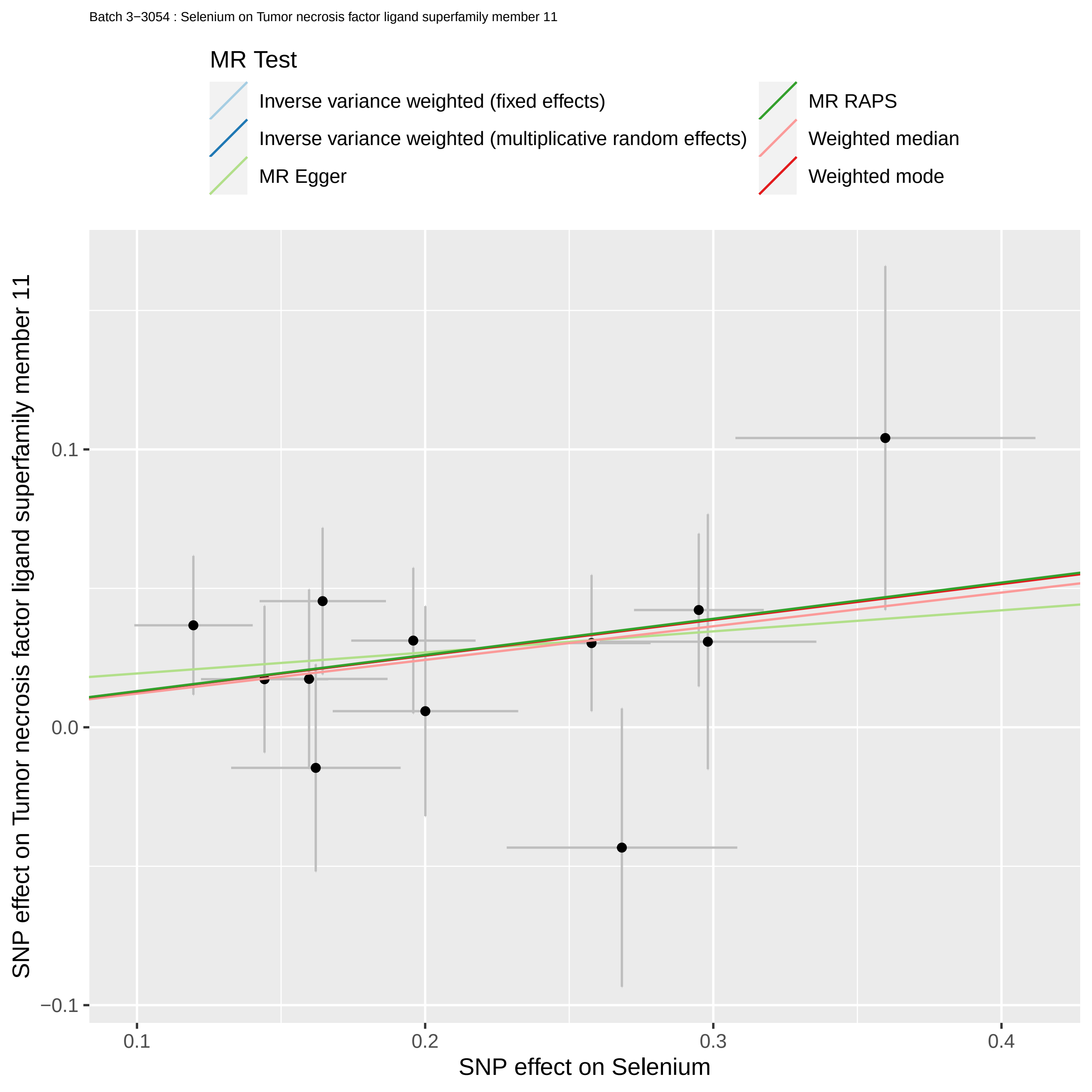

Batch 3−3054 : Selenium on Tumor necrosis factor ligand superfamily member 11
MR Test
Inverse variance weighted (fixed effects)
Inverse variance weighted (multiplicative random effects)
MR Egger
MR RAPS
Weighted median
Weighted mode
SNP effect on Tumor necrosis factor ligand superfamily member 11
0.1
0.0
−0.1
0.1
0.2
0.3
0.4
SNP effect on Selenium

Batch 3−3055 : Selenium on Tumor necrosis factor ligand superfamily member 12

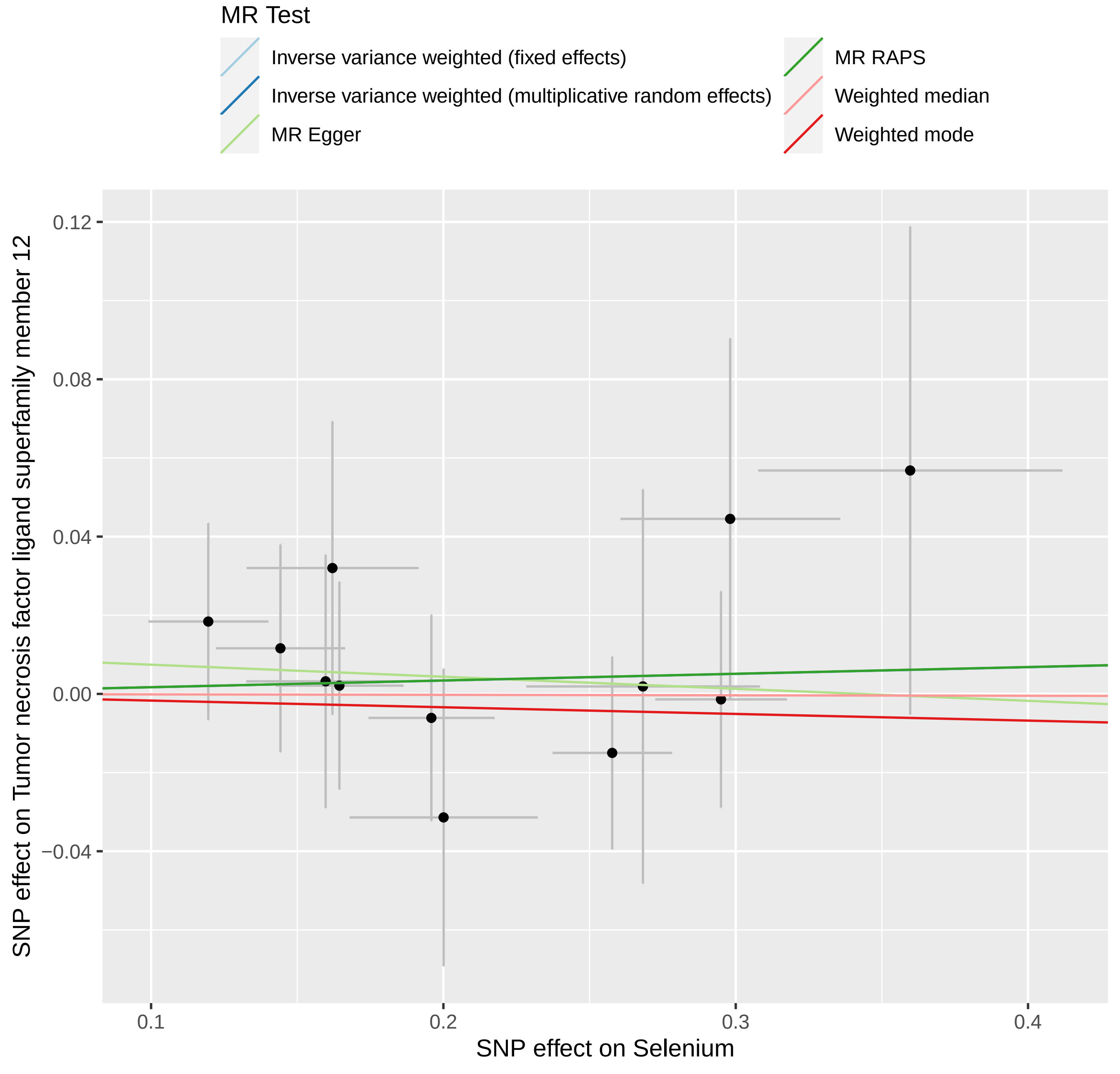

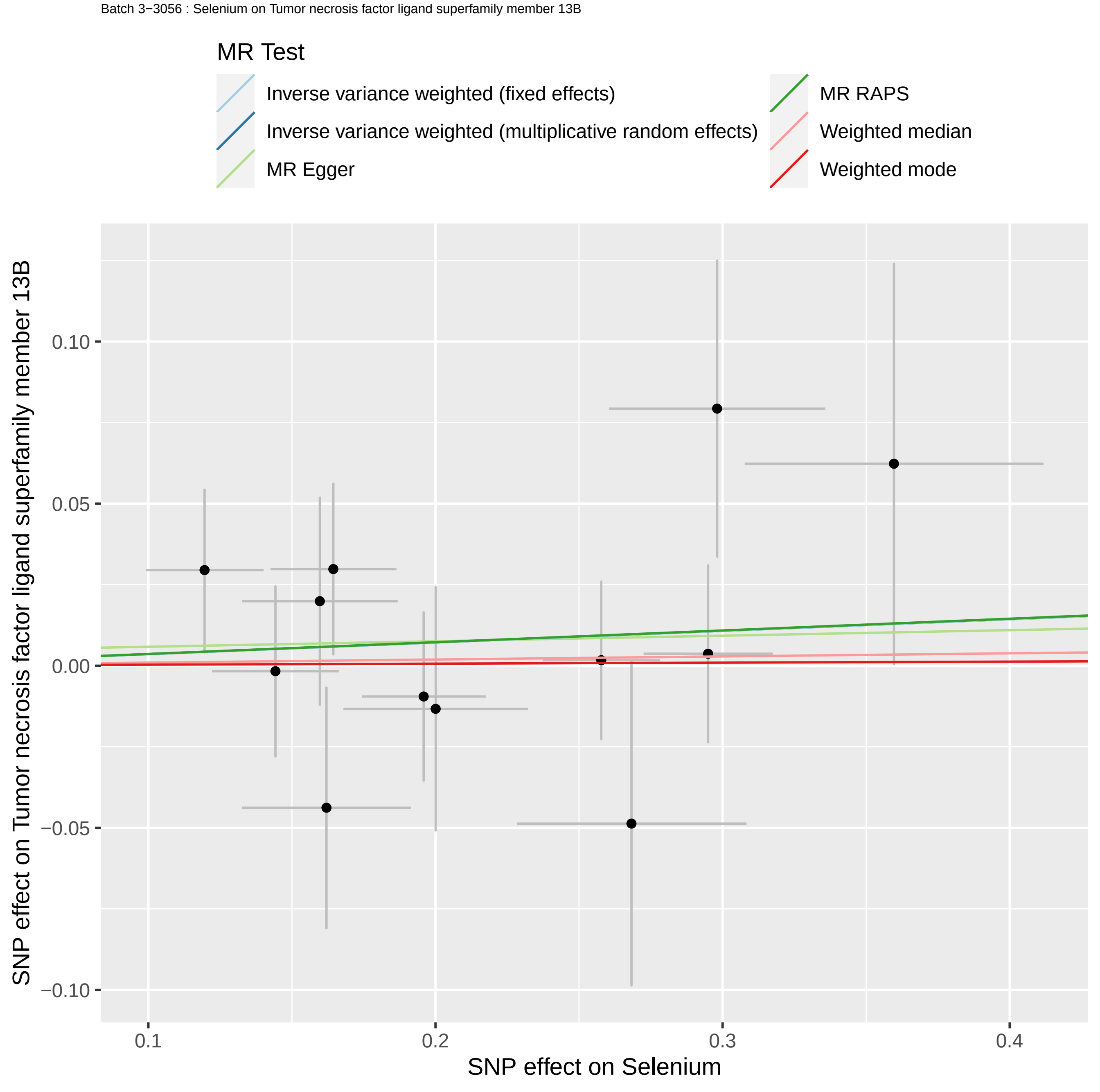

Batch 3−3056 : Selenium on Tumor necrosis factor ligand superfamily member 13B
MR Test
Inverse variance weighted (fixed effects)
Inverse variance weighted (multiplicative random effects)
MR Egger
MR RAPS
Weighted median
Weighted mode
SNP effect on Tumor necrosis factor ligand superfamily member 13B
0.10
0.05
0.00
−0.05
−0.10
0.1
0.2
0.3
0.4
SNP effect on Selenium

Batch 3−3057 : Selenium on Tumor necrosis factor ligand superfamily member 14

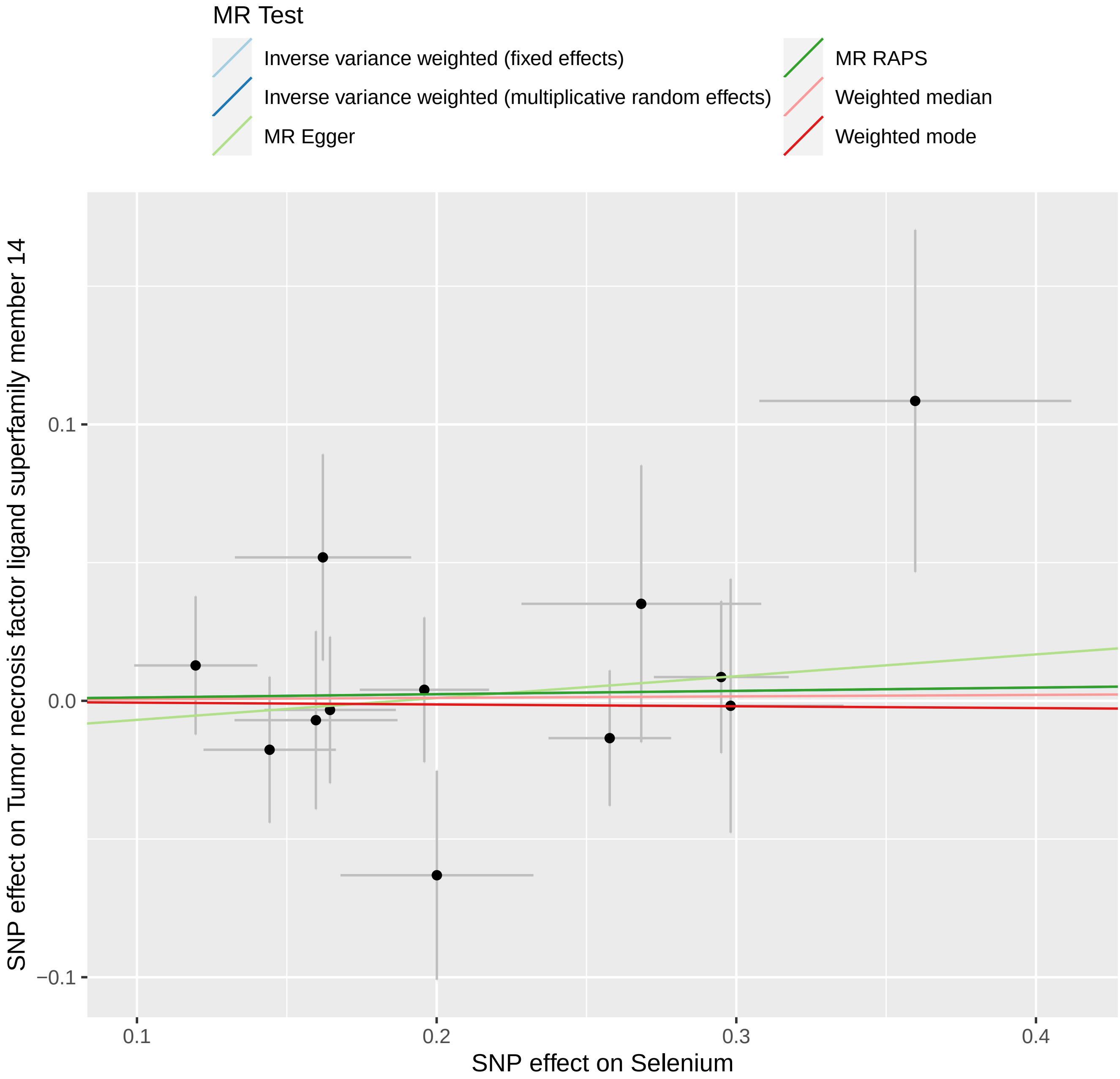

Batch 3−3058 : Selenium on Tumor necrosis factor ligand superfamily member 15

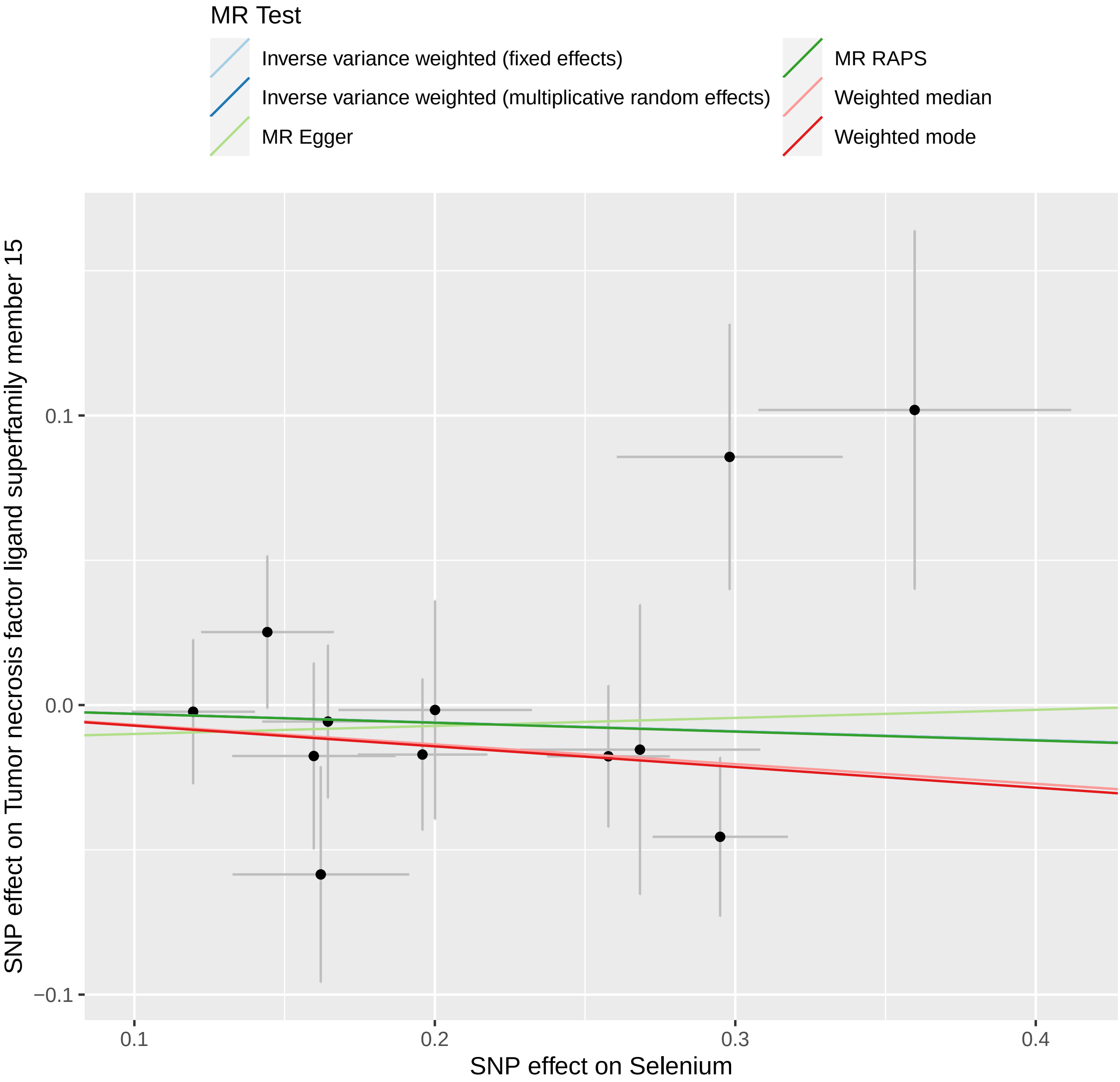

Batch 3−3059 : Selenium on Tumor necrosis factor ligand superfamily member 15

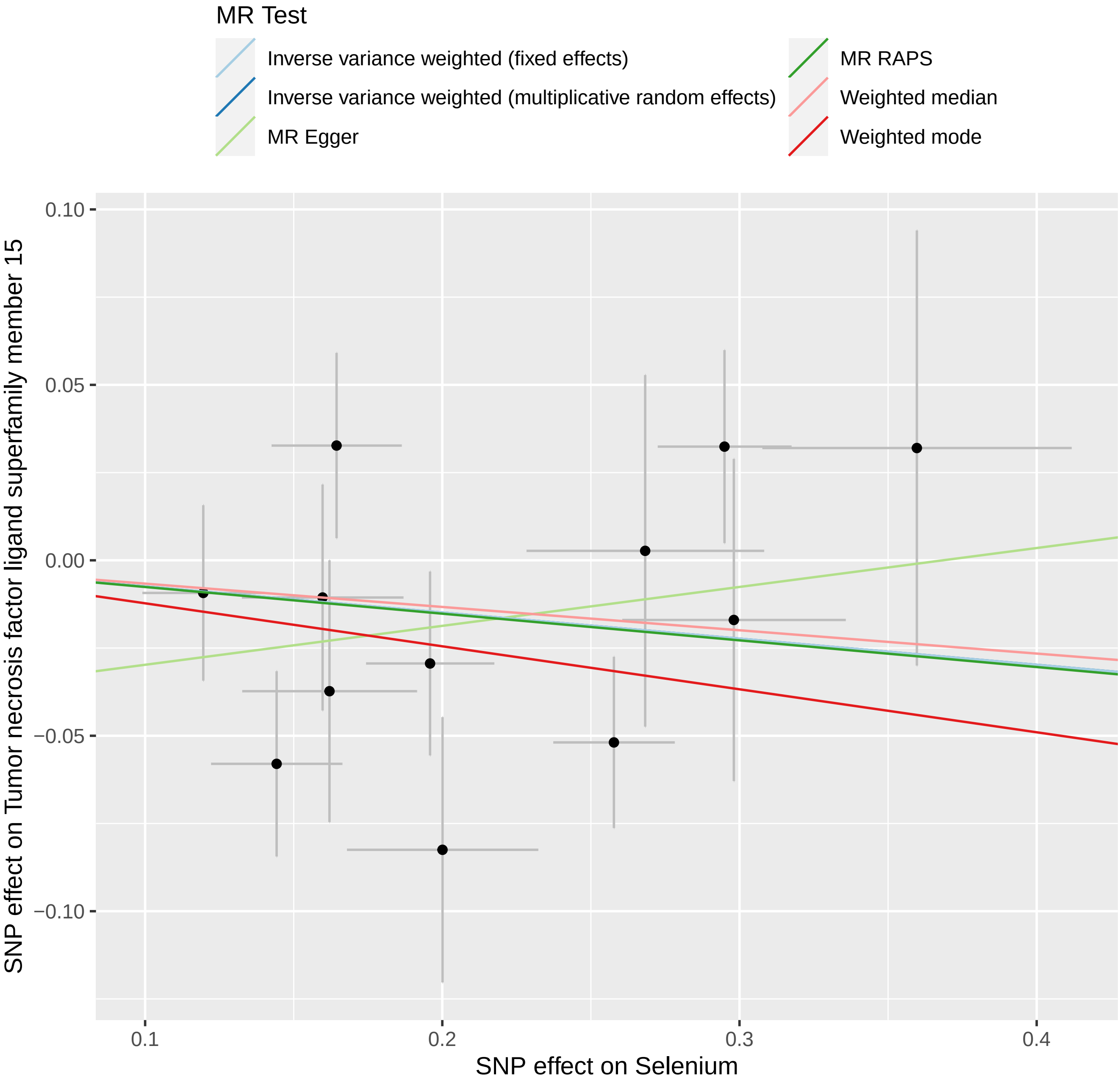

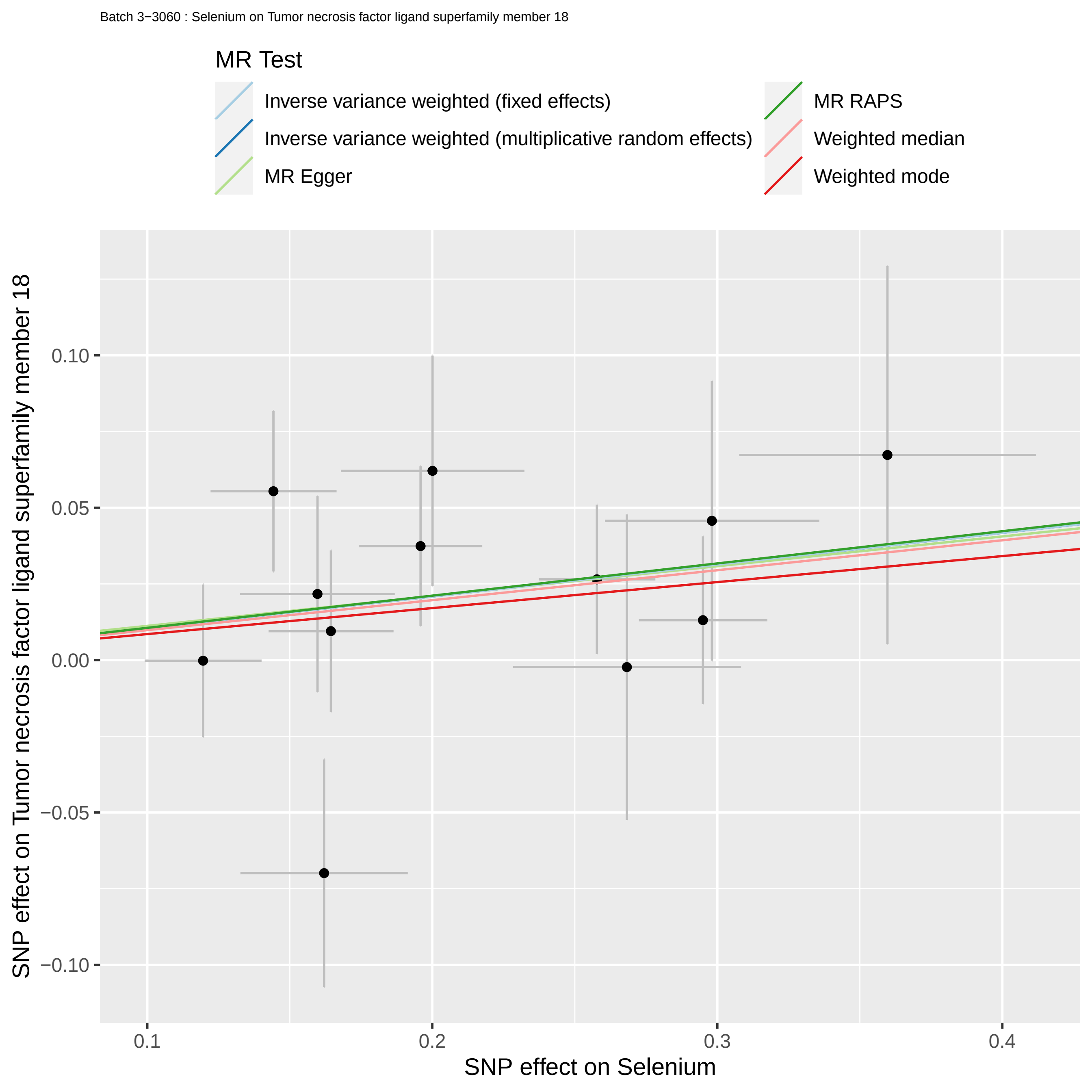

Batch 3−3060 : Selenium on Tumor necrosis factor ligand superfamily member 18
MR Test
Inverse variance weighted (fixed effects)
Inverse variance weighted (multiplicative random effects)
MR Egger
MR RAPS
Weighted median
Weighted mode
SNP effect on Tumor necrosis factor ligand superfamily member 18
0.10
0.05
0.00
−0.05
−0.10
0.1
0.2
0.3
0.4
SNP effect on Selenium

Batch 3−3061 : Selenium on Tumor necrosis factor ligand superfamily member 4

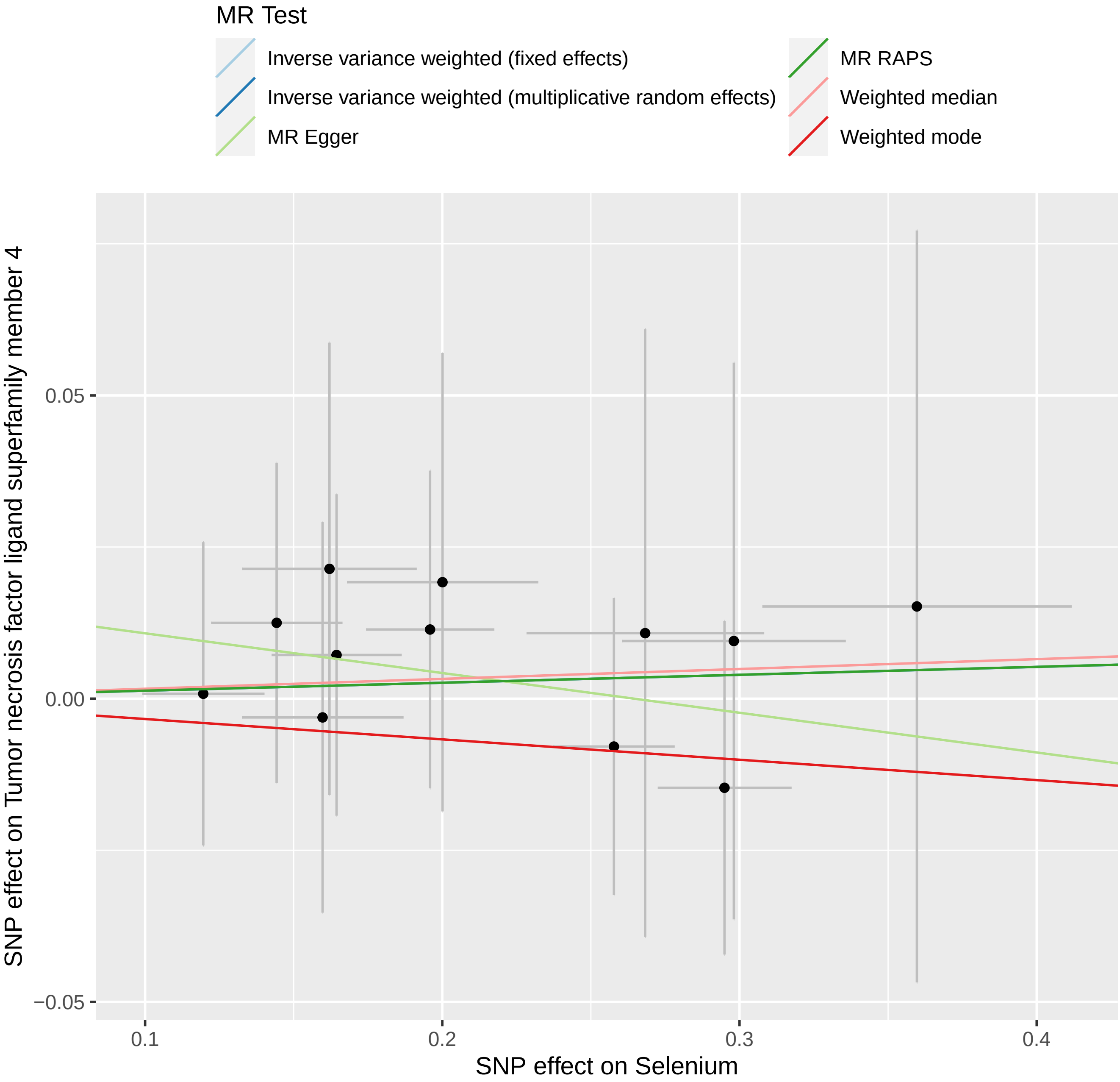

Batch 3−3062 : Selenium on Tumor necrosis factor ligand superfamily member 4

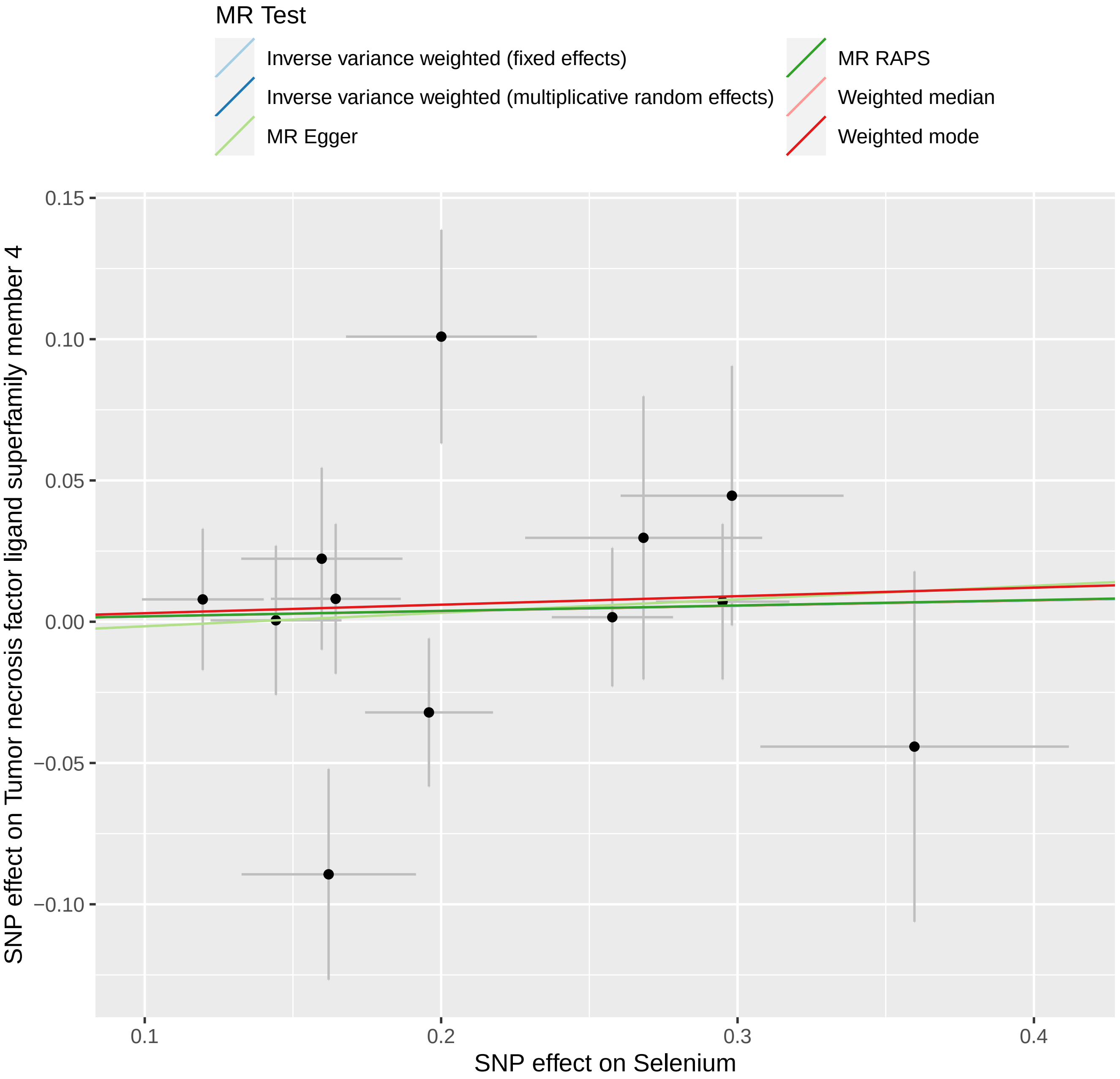

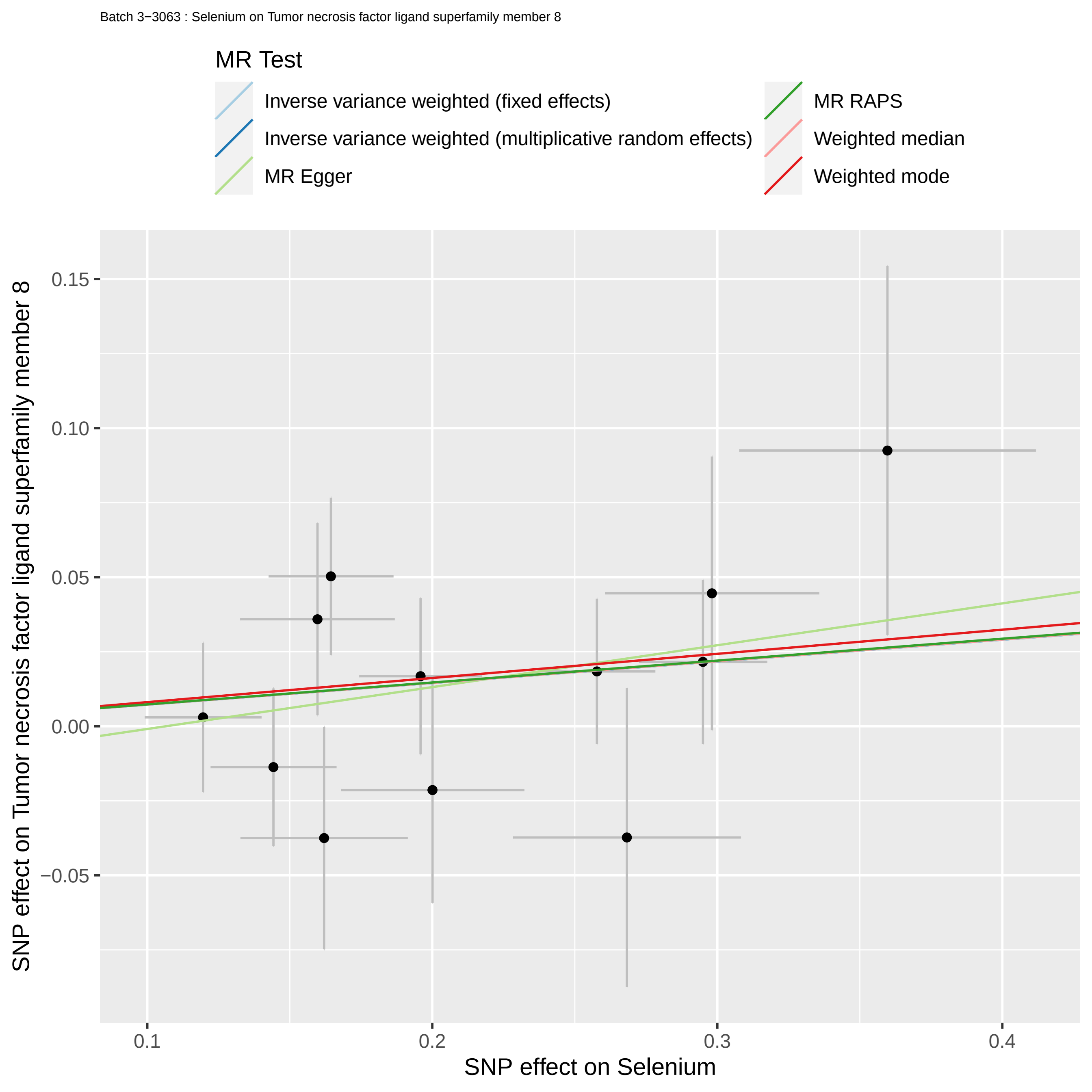
Batch 3−3063 : Selenium on Tumor necrosis factor ligand superfamily member 8
MR Test
Inverse variance weighted (fixed effects)
Inverse variance weighted (multiplicative random effects)
MR Egger
MR RAPS
Weighted median
Weighted mode
SNP effect on Tumor necrosis factor ligand superfamily member 8
0.15
0.10
0.05
0.00
−0.05
0.1
0.2
0.3
0.4
SNP effect on Selenium

Batch 3−3064 : Selenium on Tumor necrosis factor ligand superfamily member 9

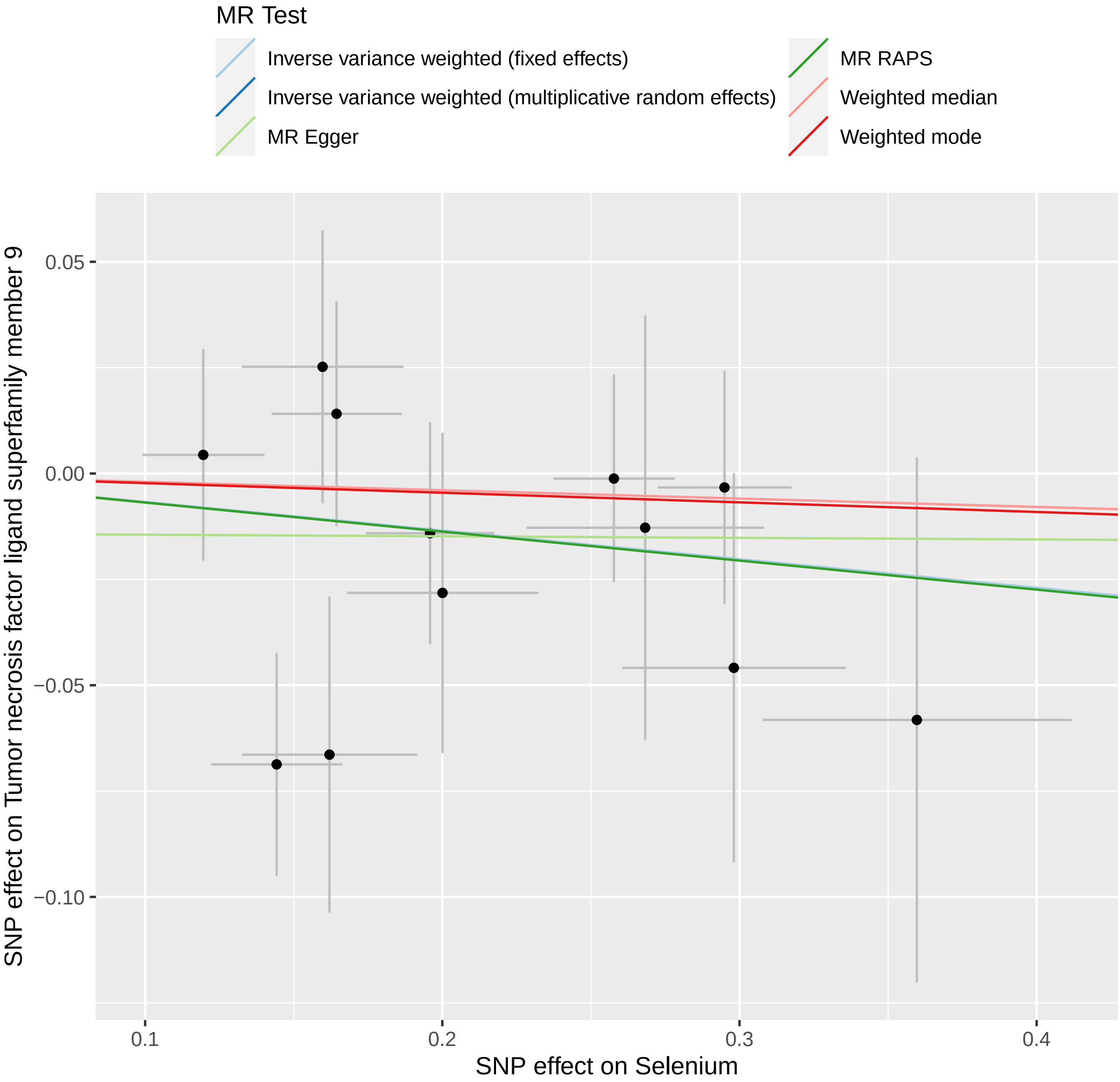

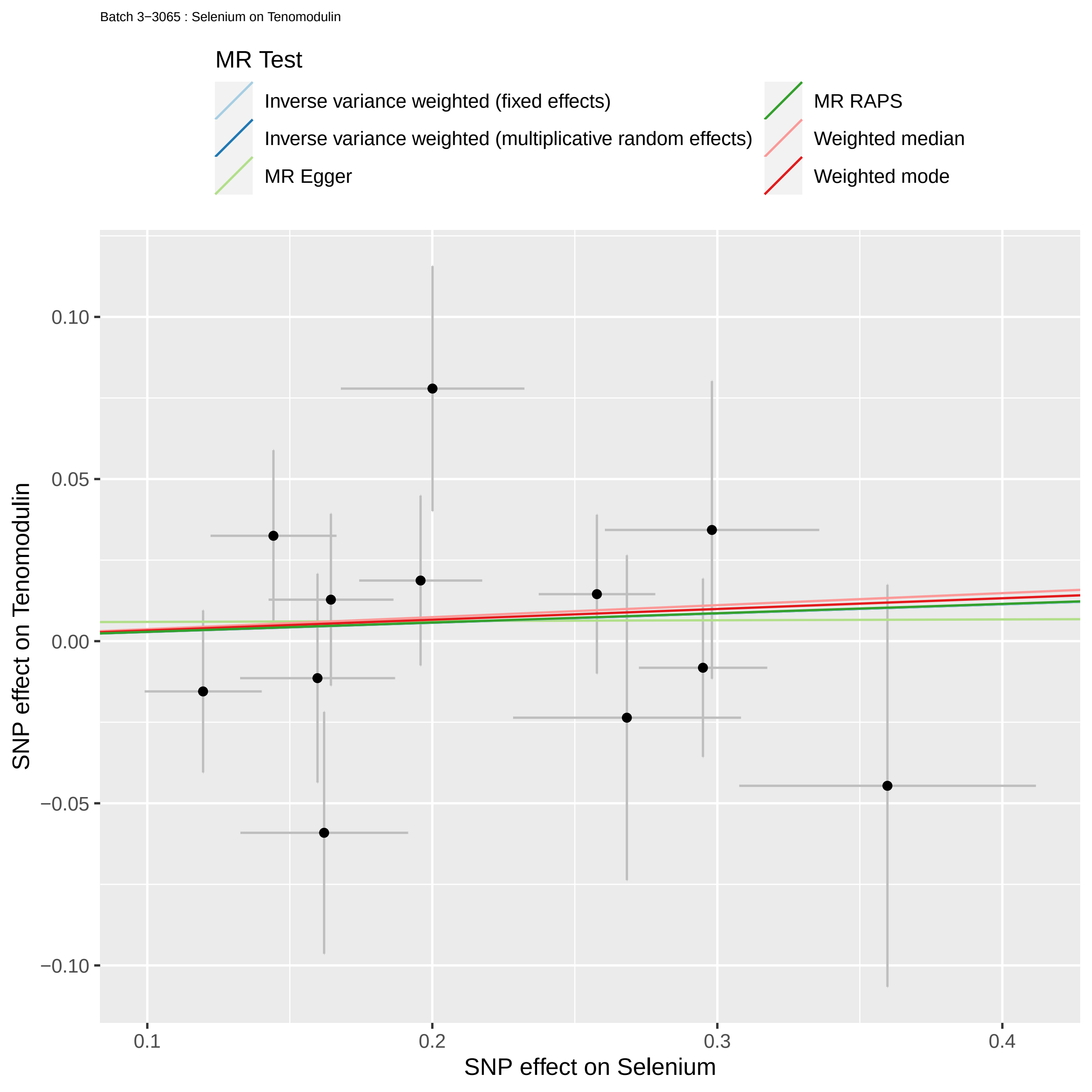

Batch 3−3065 : Selenium on Tenomodulin
MR Test
Inverse variance weighted (fixed effects)
Inverse variance weighted (multiplicative random effects)
MR Egger
MR RAPS
Weighted median
Weighted mode
0.10
0.05
0.00
−0.05
−0.10
SNP effect on Tenomodulin
0.1
0.2
0.3
0.4
SNP effect on Selenium

Batch 3−3066 : Selenium on Troponin I, cardiac muscle

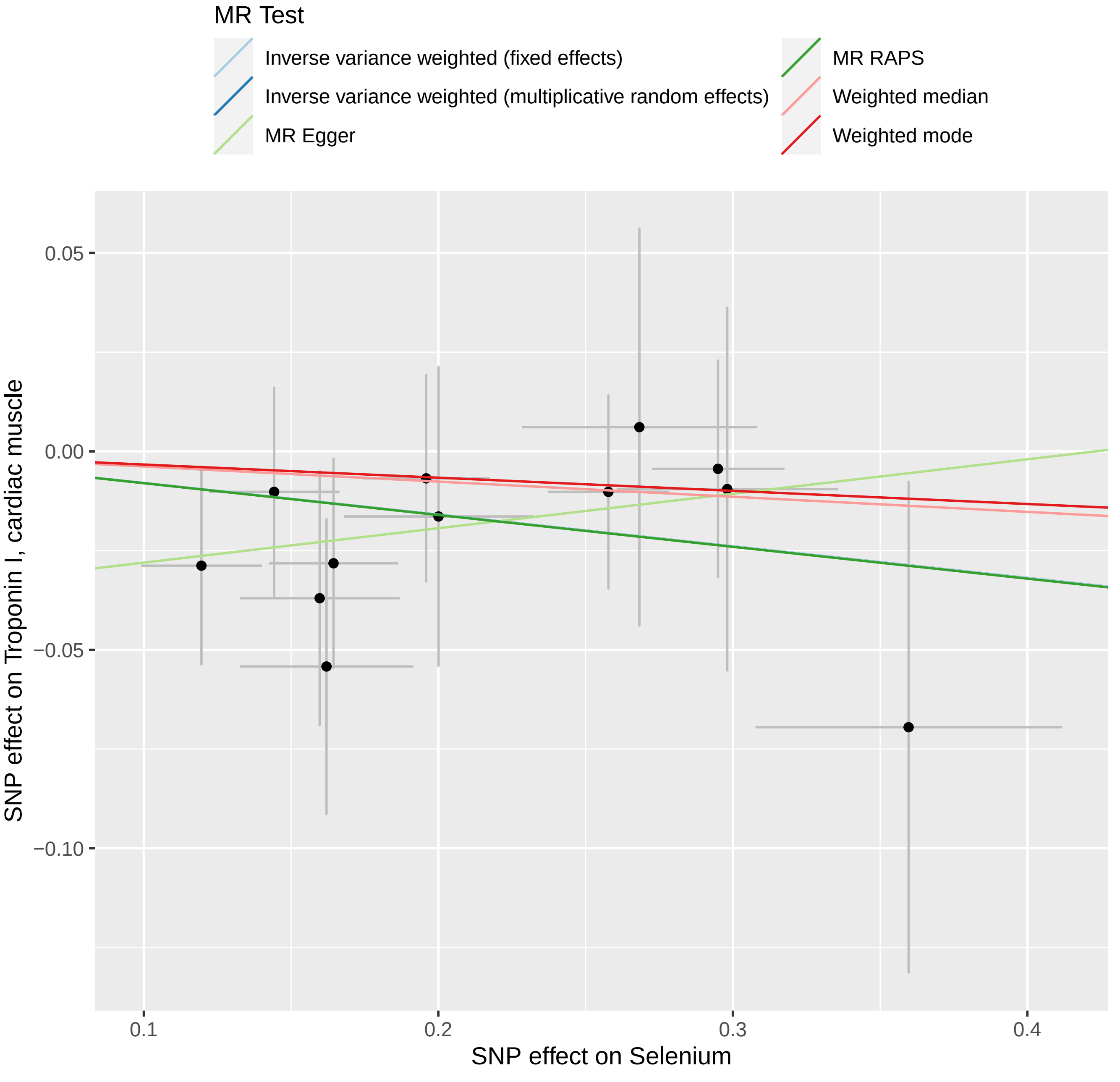

Batch 3−3067 : Selenium on Troponin I, cardiac muscle

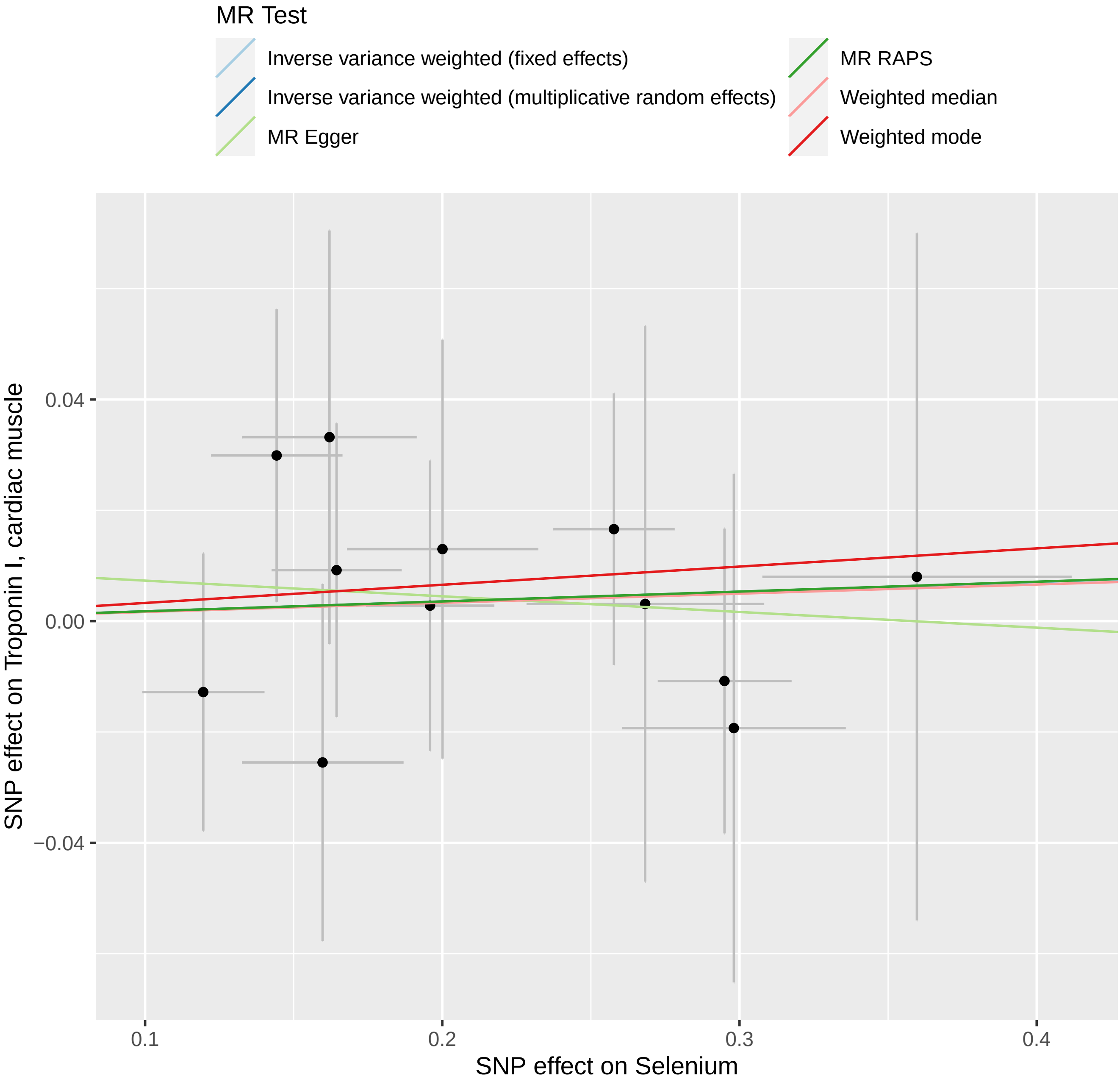

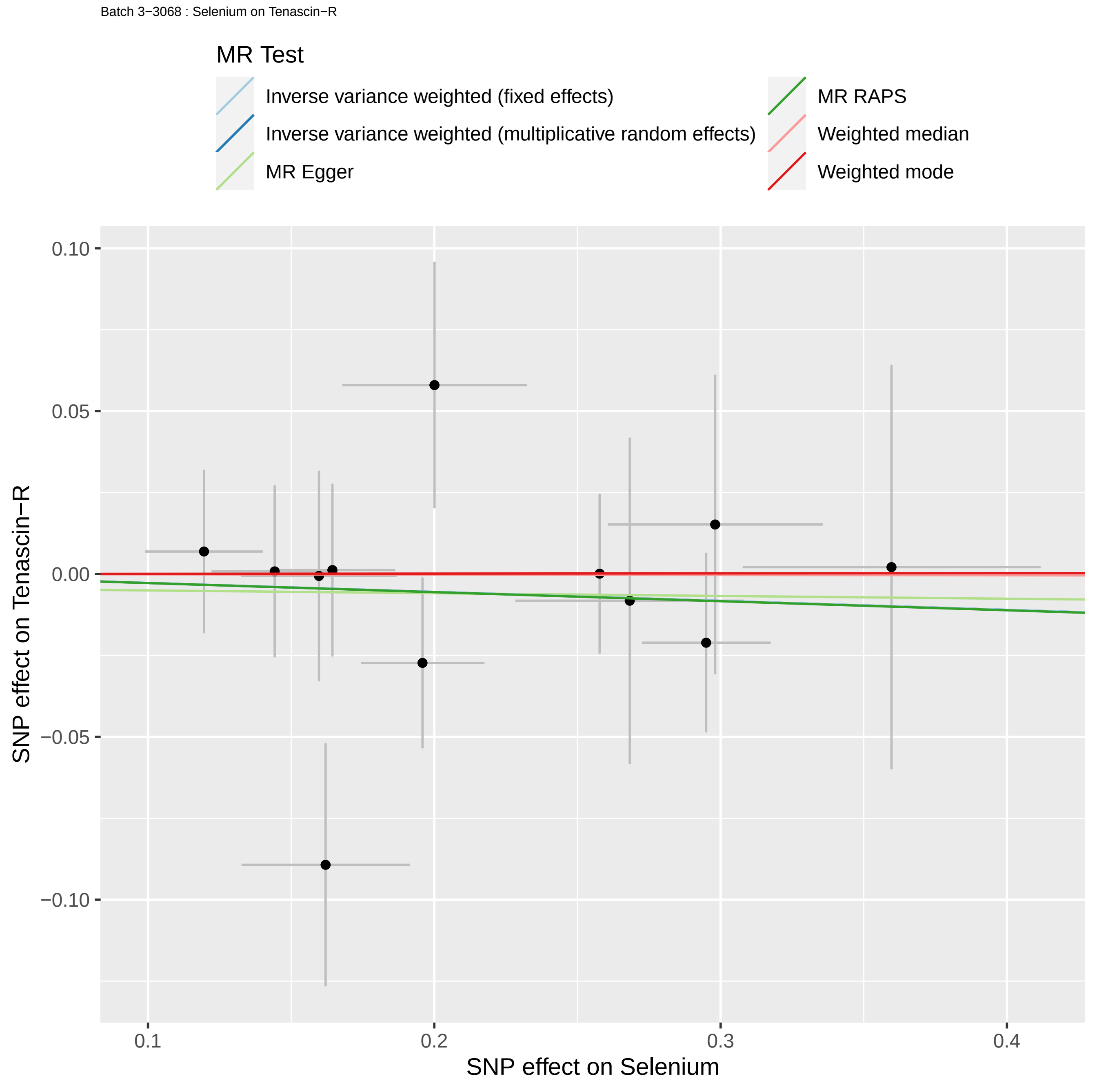
Batch 3−3068 : Selenium on Tenascin−R
MR Test
Inverse variance weighted (fixed effects)
Inverse variance weighted (multiplicative random effects)
MR Egger
MR RAPS
Weighted median
Weighted mode
0.10
0.05
0.00
−0.05
−0.10
0.1
0.2
0.3
0.4
SNP effect on Tenascin−R
SNP effect on Selenium

Batch 3−3069 : Selenium on Tensin−2

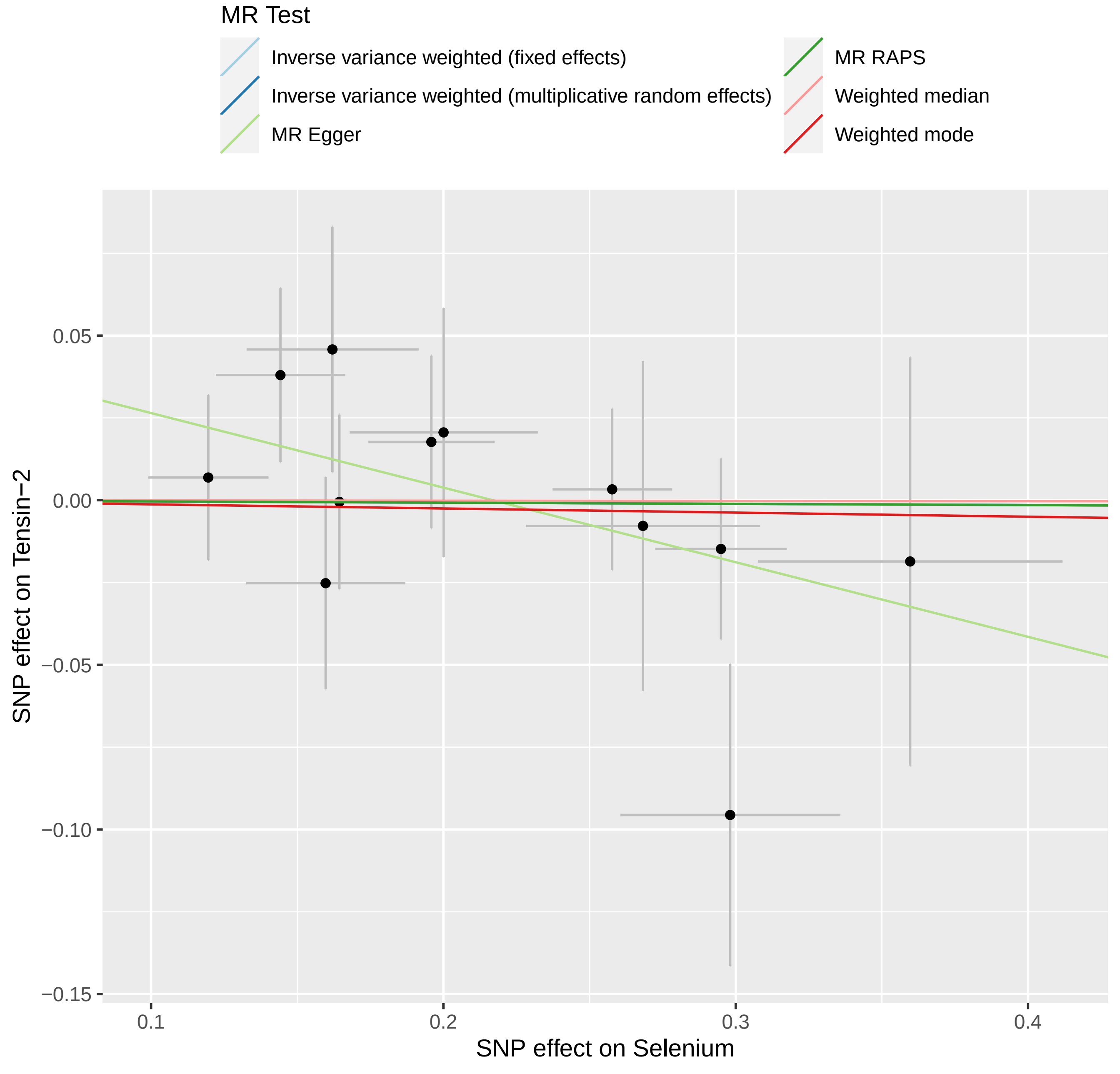

Batch 3−3070 : Selenium on Tensin−4

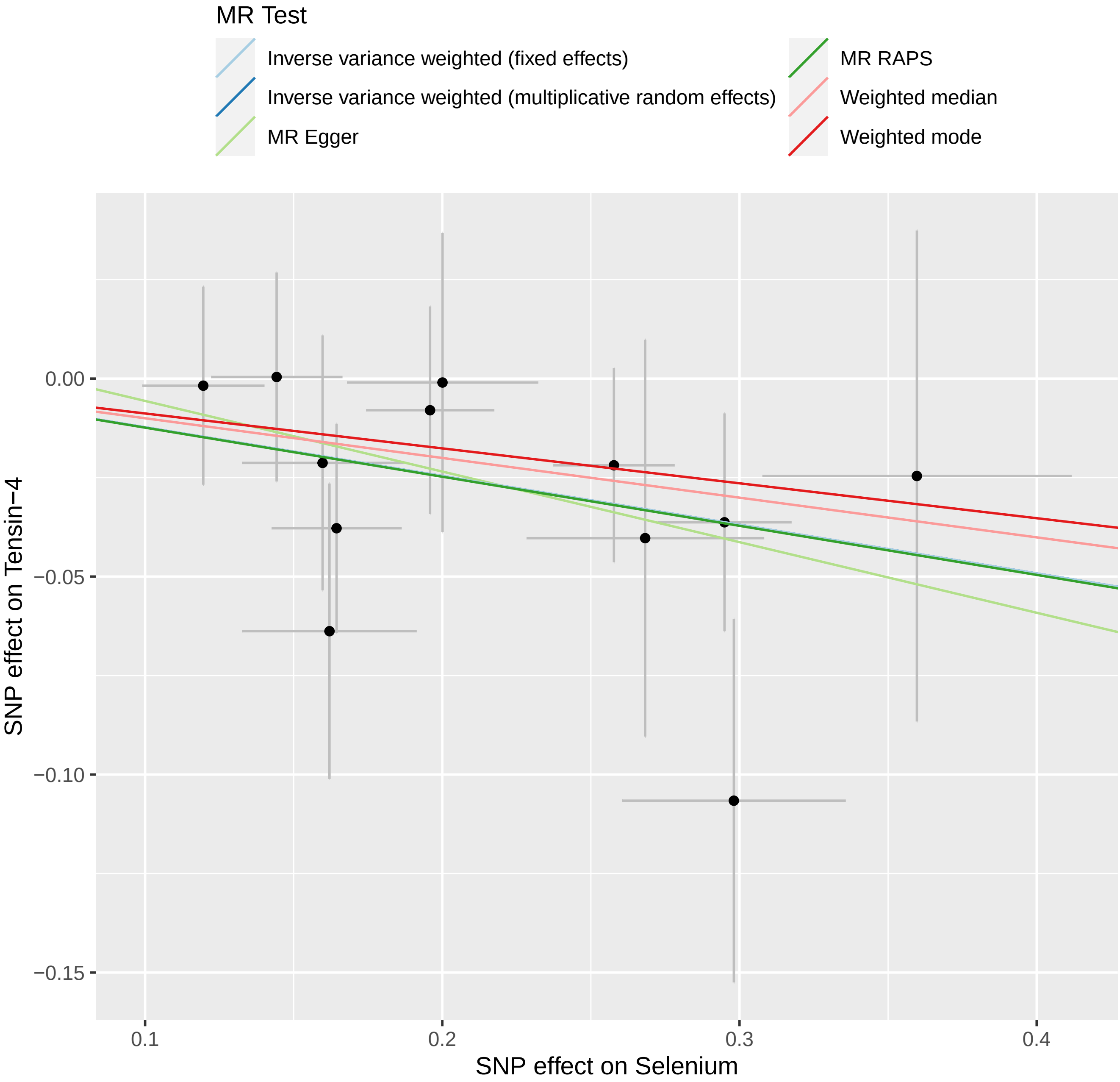

Batch 3−3071 : Selenium on Tenascin−X

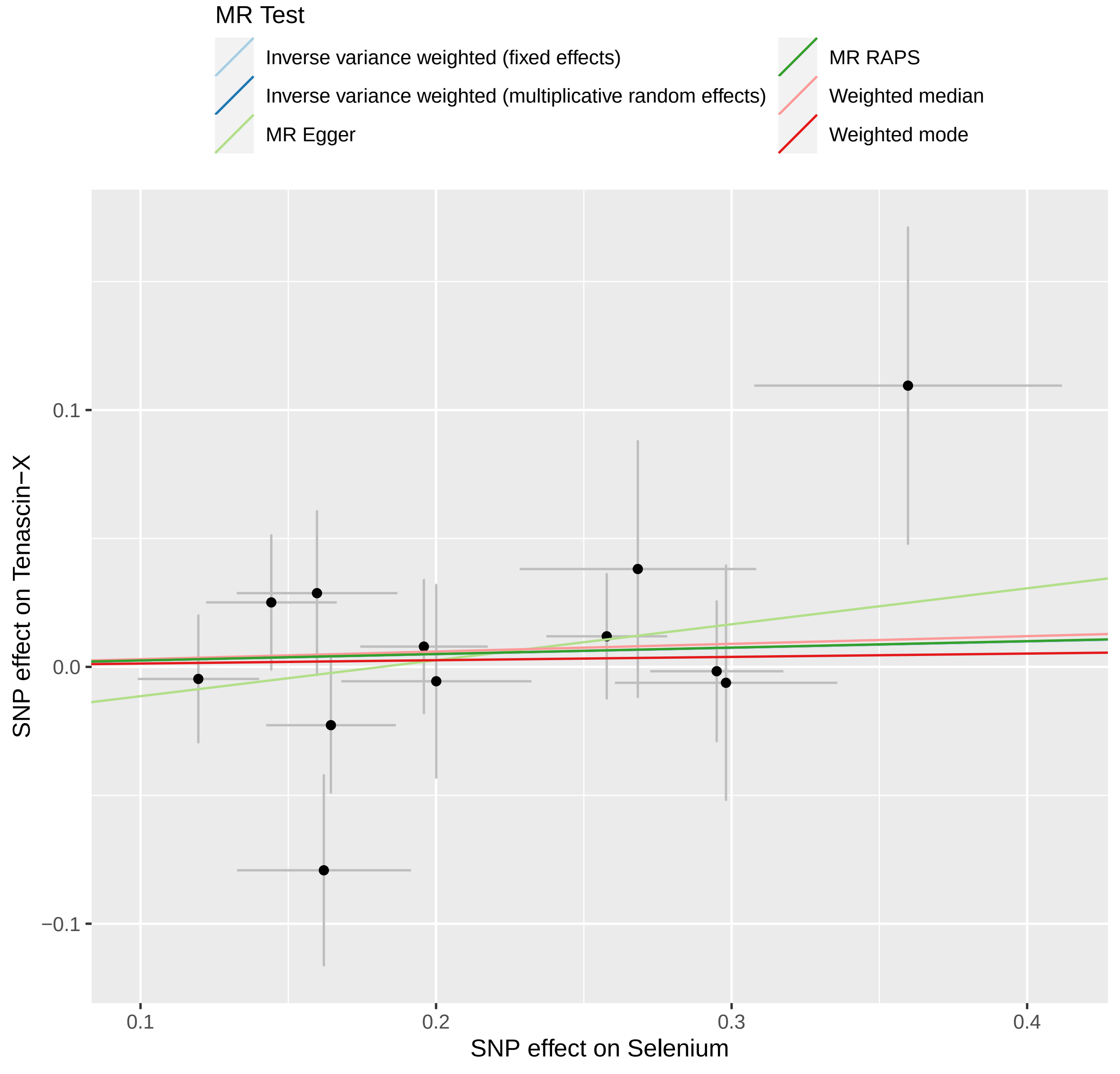

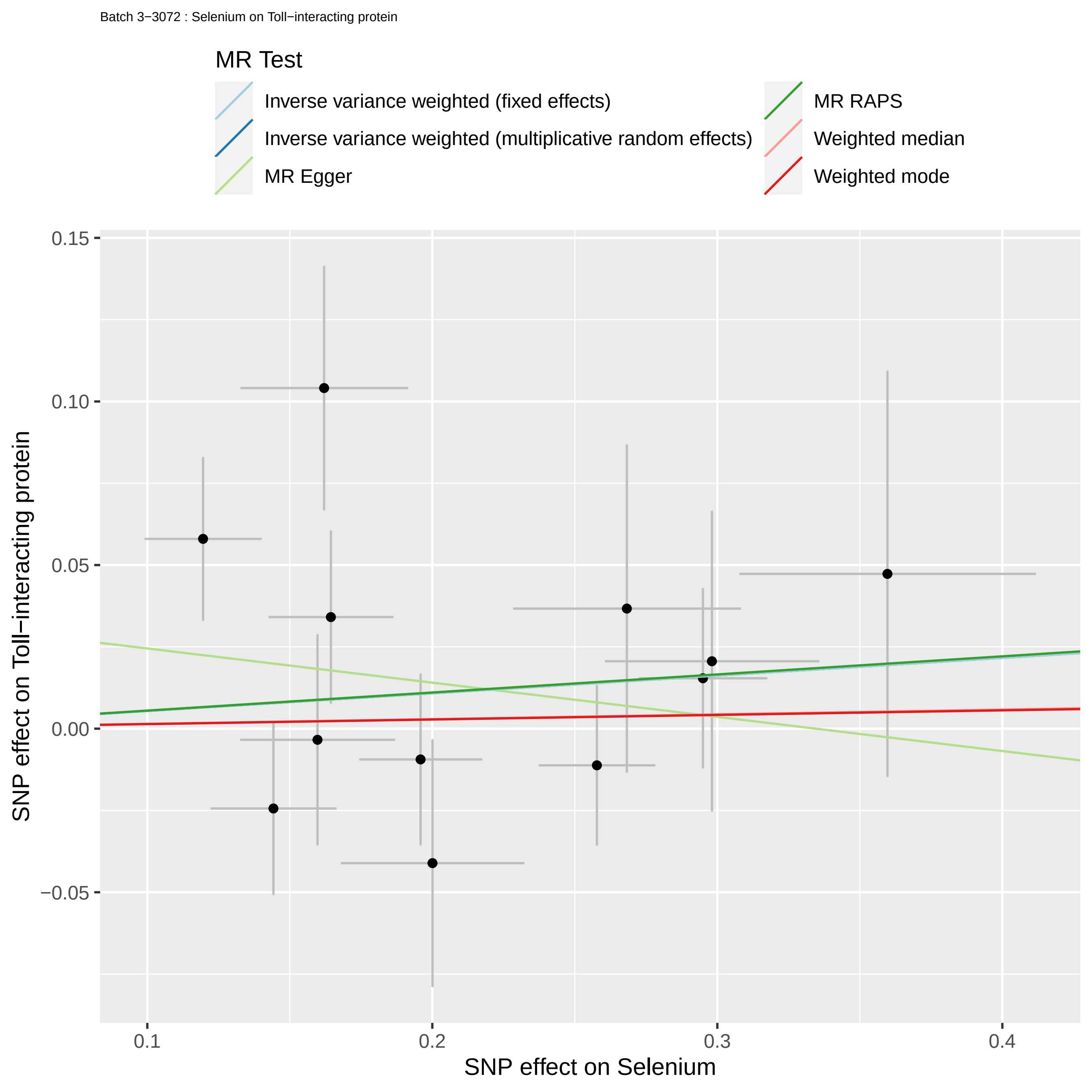
Batch 3−3072 : Selenium on Toll−interacting protein
MR Test
Inverse variance weighted (fixed effects)
Inverse variance weighted (multiplicative random effects)
MR Egger
MR RAPS
Weighted median
Weighted mode
0.15
0.10
0.05
0.00
−0.05
0.1
0.2
0.3
0.4
SNP effect on Toll−interacting protein
SNP effect on Selenium

Batch 3−3073 : Selenium on TOM1−like protein 1

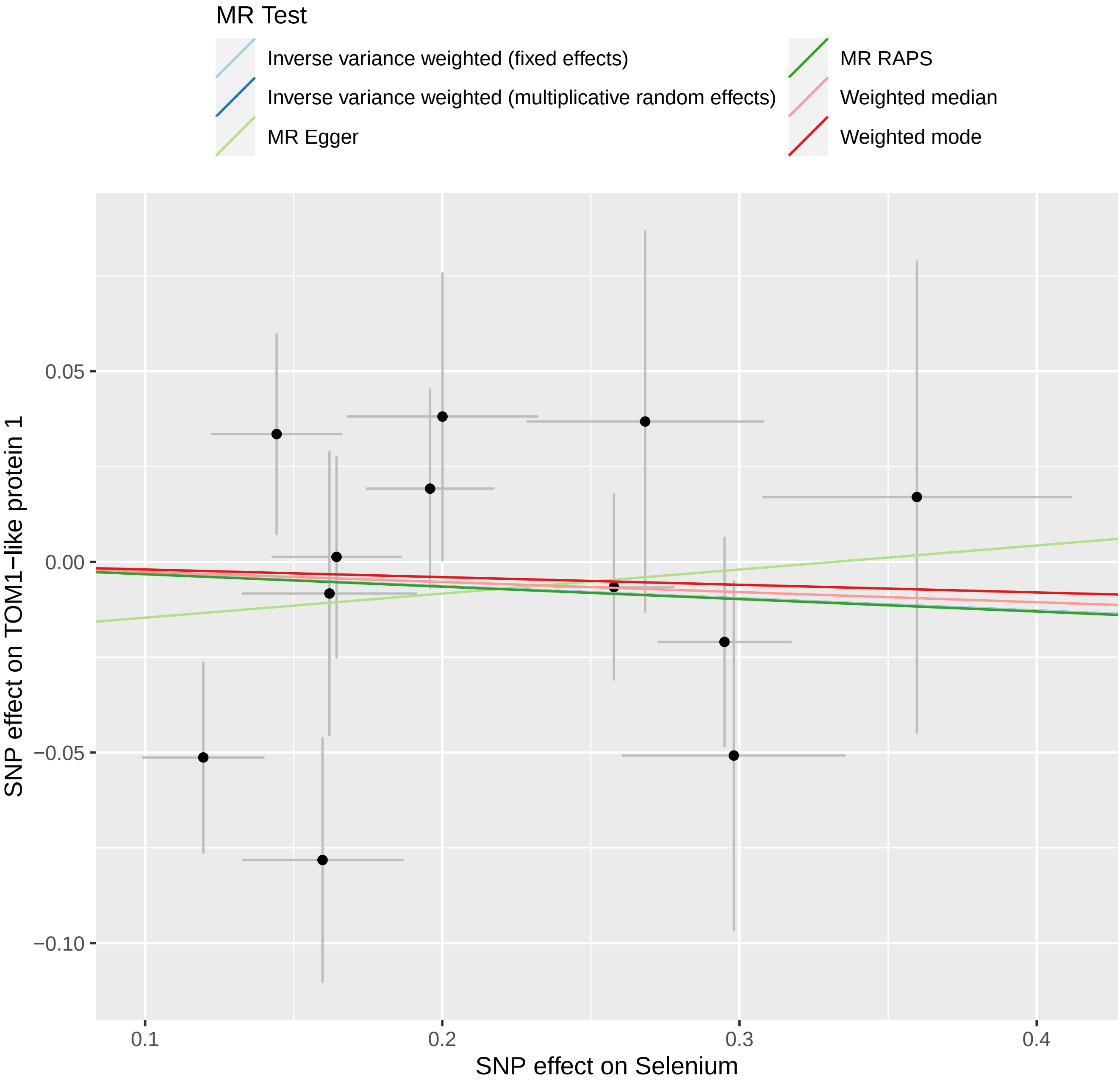

Batch 3−3074 : Selenium on DNA topoisomerase 1

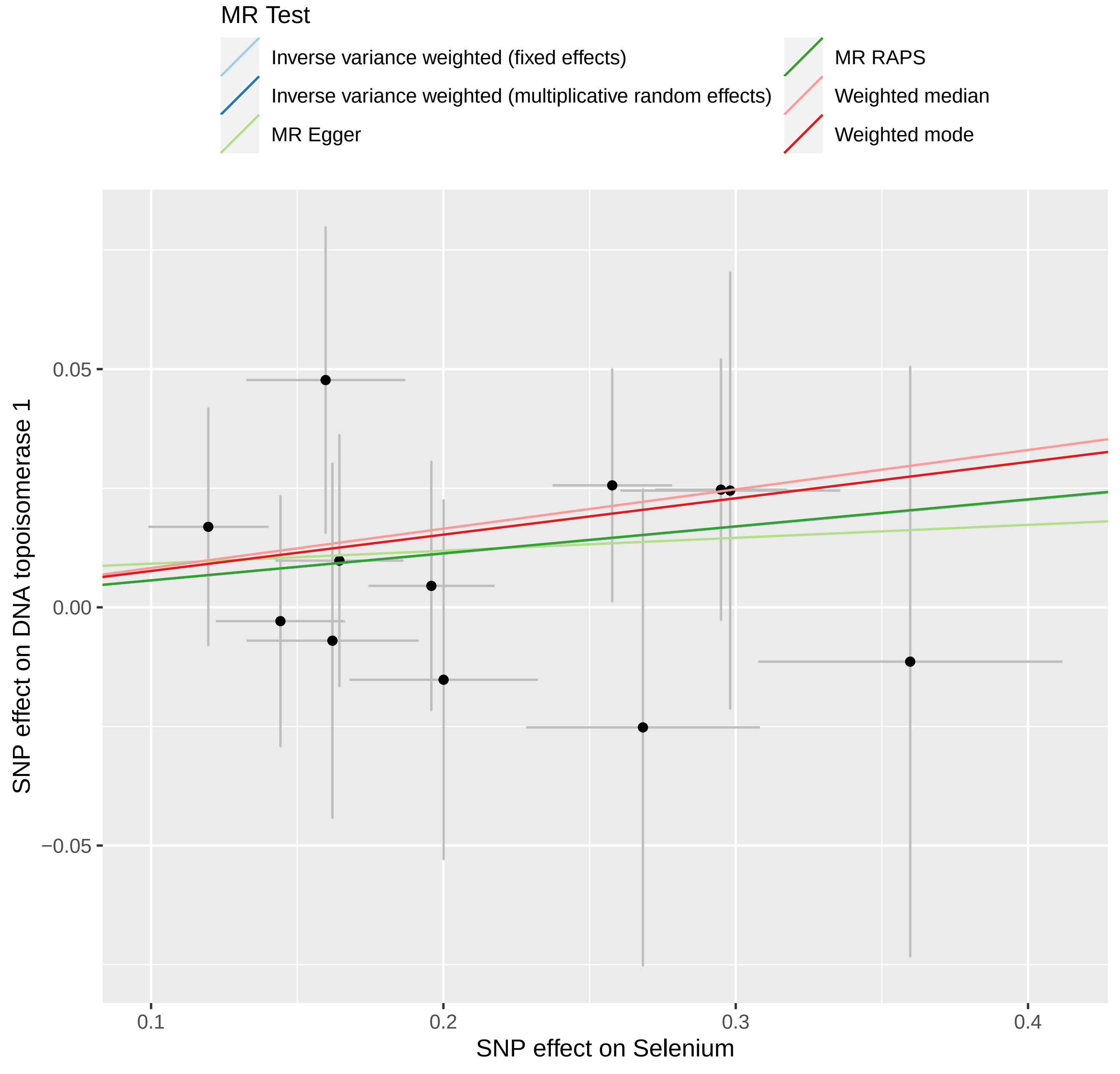

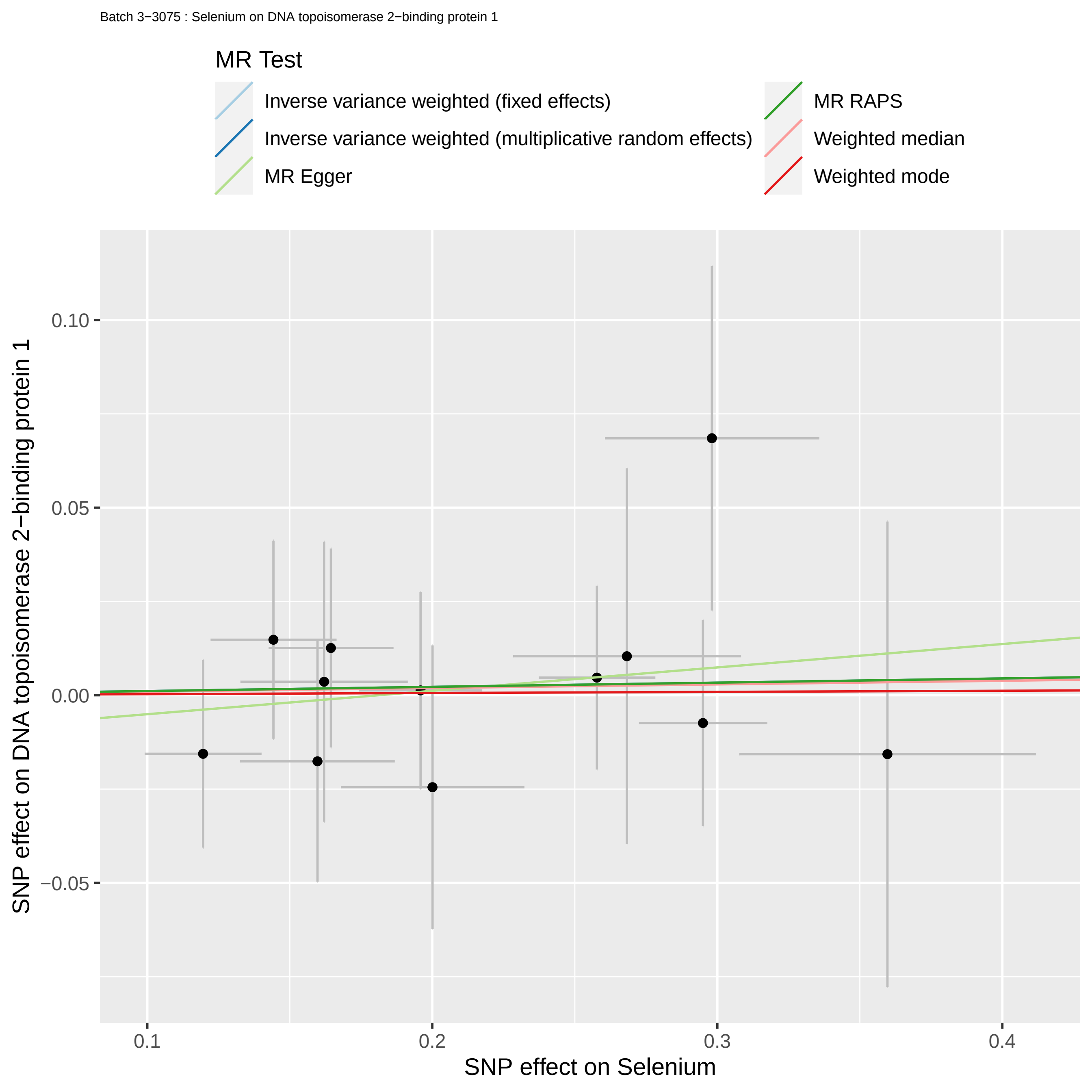

Batch 3−3075 : Selenium on DNA topoisomerase 2−binding protein 1
MR Test
Inverse variance weighted (fixed effects)
Inverse variance weighted (multiplicative random effects)
MR Egger
MR RAPS
Weighted median
Weighted mode
0.10
0.05
0.00
−0.05
0.1
0.2
0.3
0.4
SNP effect on Selenium
SNP effect on DNA topoisomerase 2−binding protein 1

Batch 3−3076 : Selenium on Torsin−1A−interacting protein 1

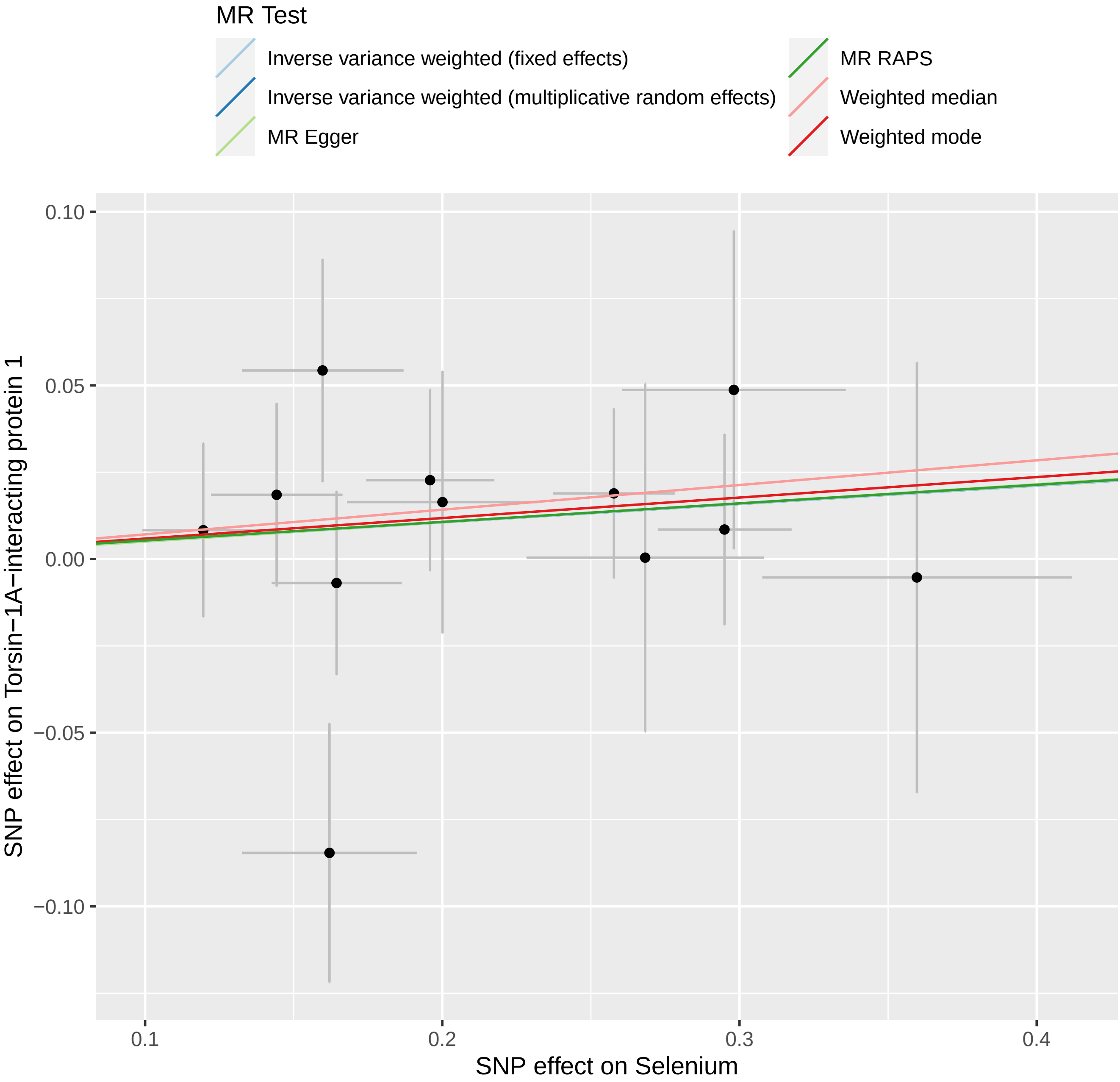

Batch 3−3077 : Selenium on Torsin−1A−interacting protein 2

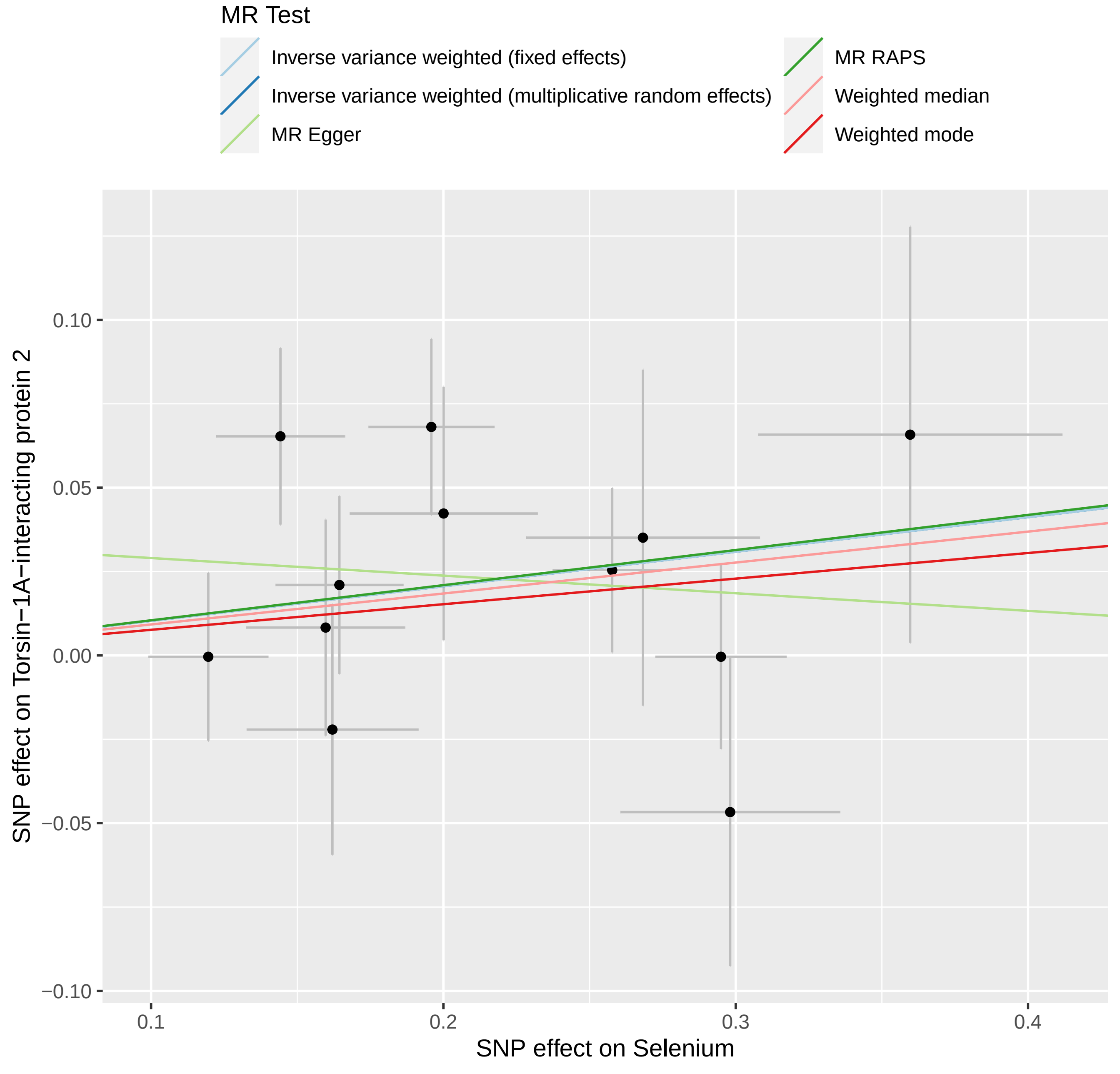

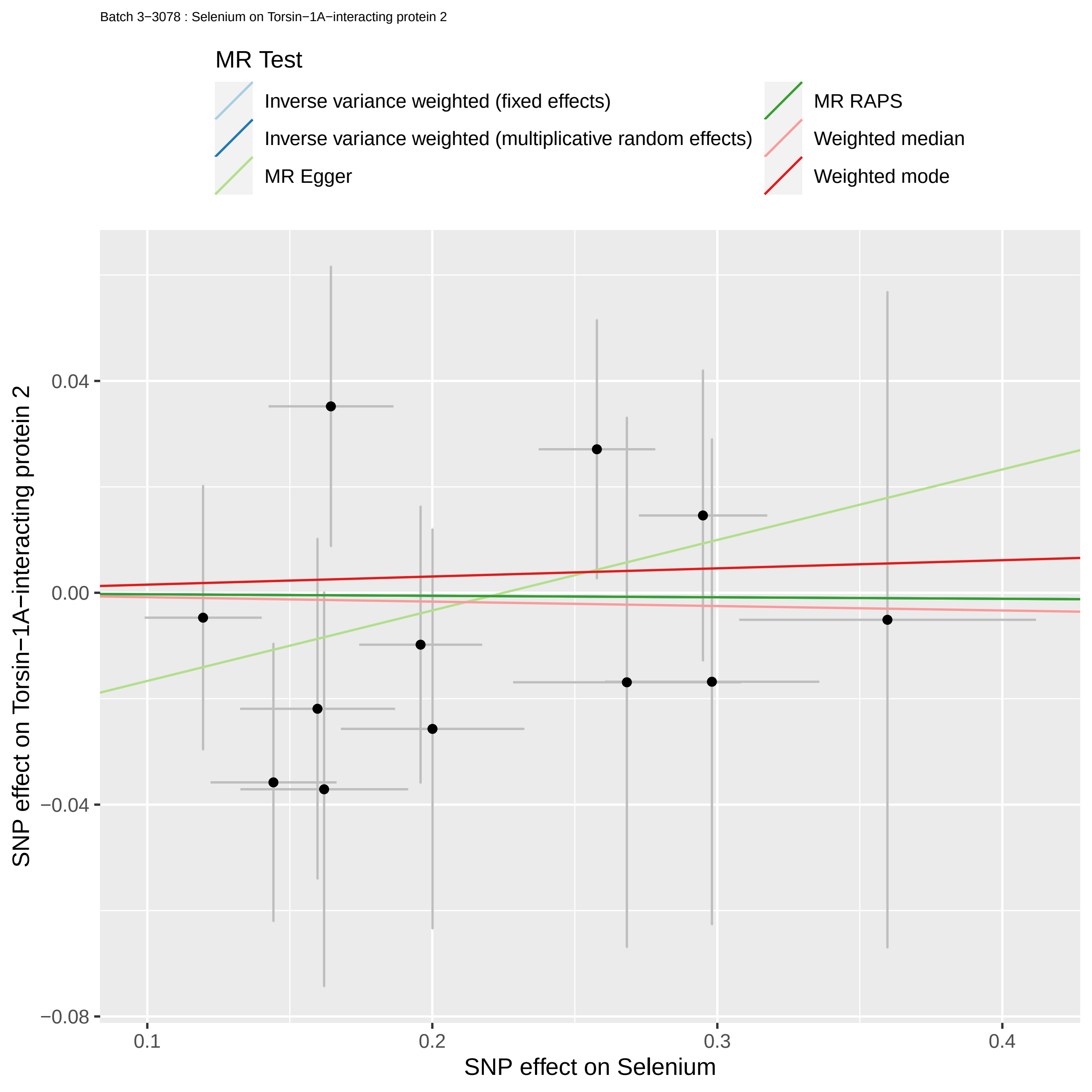

Batch 3−3078 : Selenium on Torsin−1A−interacting protein 2
MR Test
Inverse variance weighted (fixed effects)
Inverse variance weighted (multiplicative random effects)
MR Egger
MR RAPS
Weighted median
Weighted mode
SNP effect on Torsin−1A−interacting protein 2
0.04
0.00
−0.04
−0.08
0.1
0.2
0.3
0.4
SNP effect on Selenium

Batch 3−3079 : Selenium on TOX high mobility group box family member 3

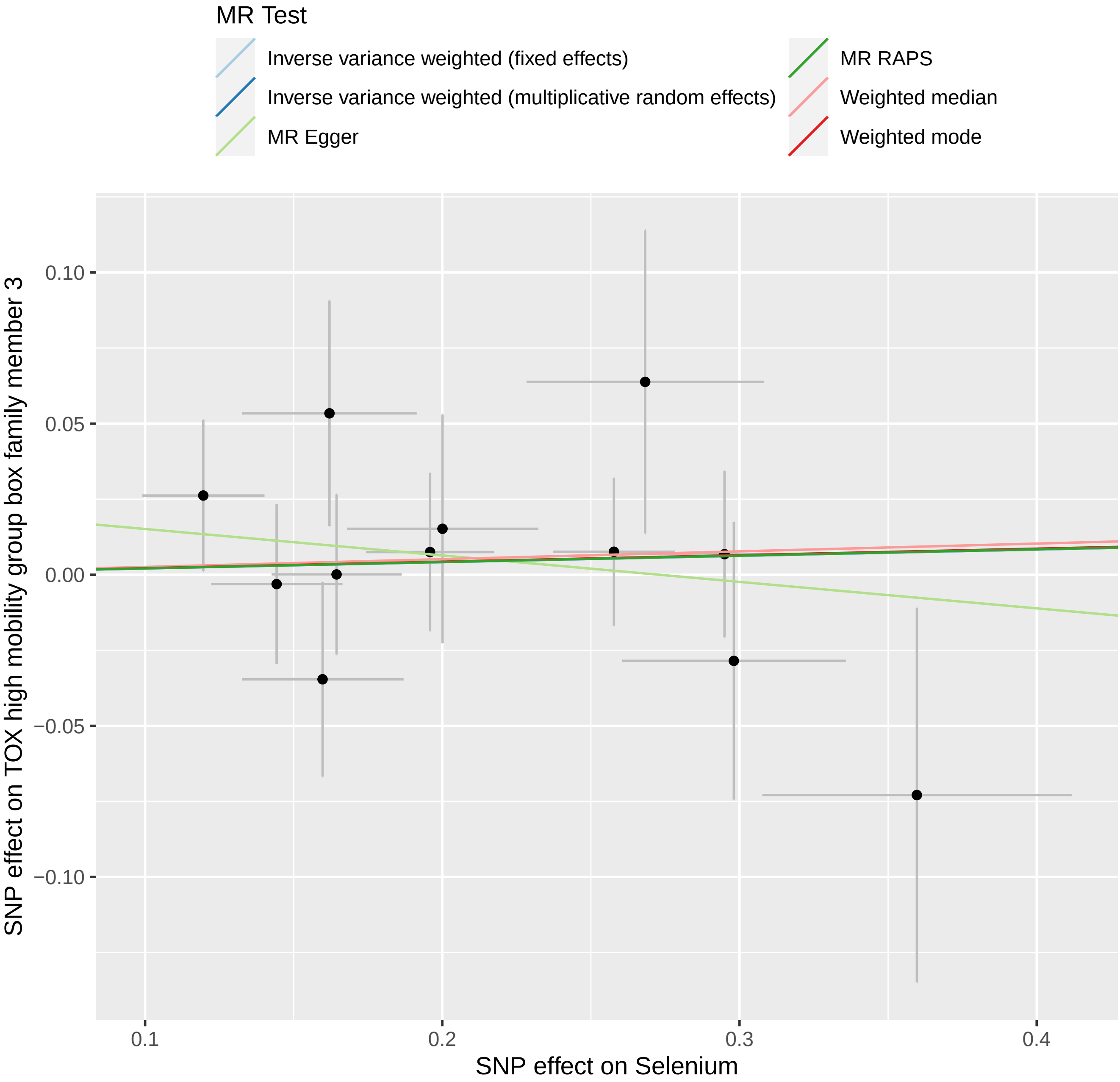

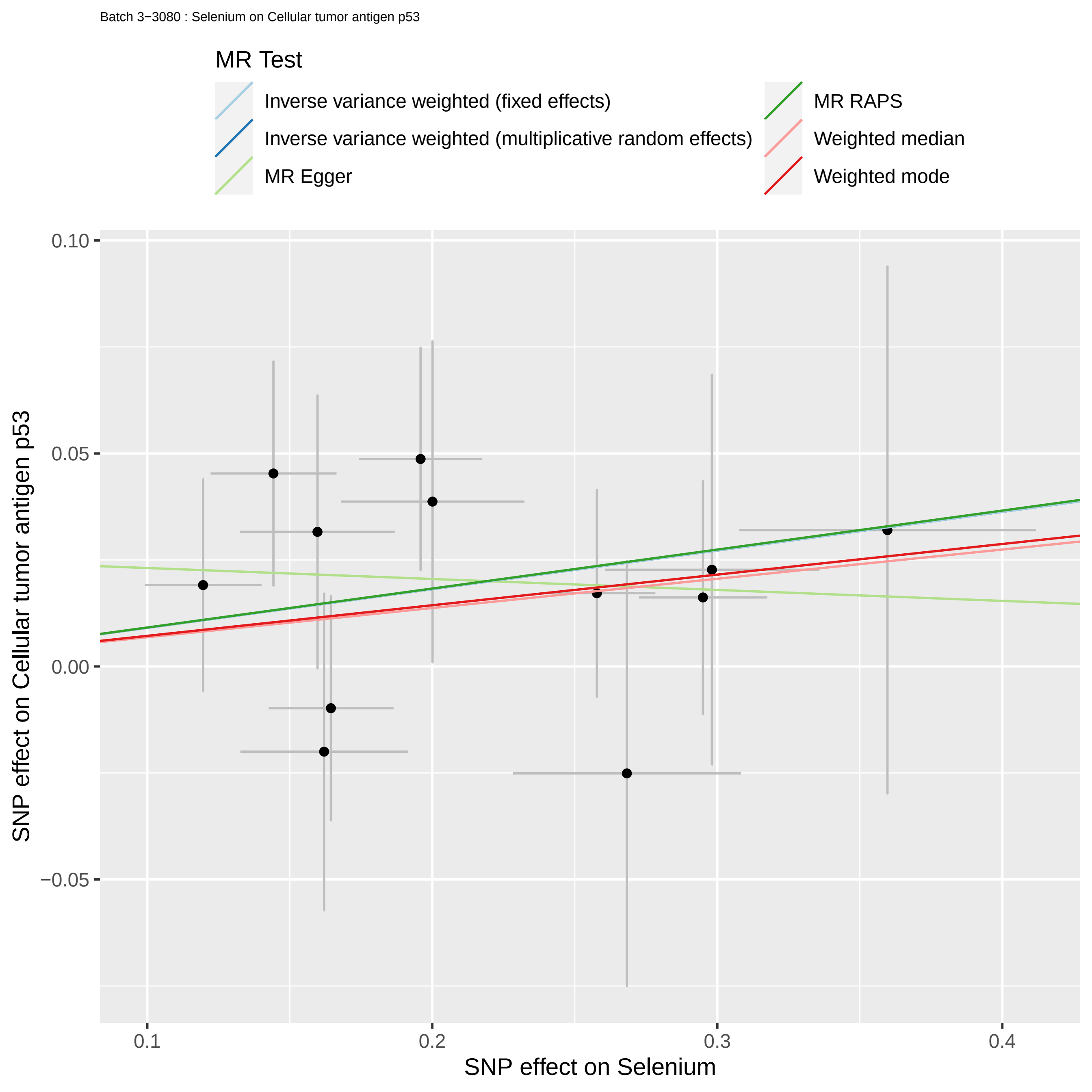

Batch 3−3080 : Selenium on Cellular tumor antigen p53
MR Test
Inverse variance weighted (fixed effects)
Inverse variance weighted (multiplicative random effects)
MR Egger
MR RAPS
Weighted median
Weighted mode
0.10
0.05
0.00
−0.05
0.1
0.2
0.3
0.4
SNP effect on Cellular tumor antigen p53
SNP effect on Selenium

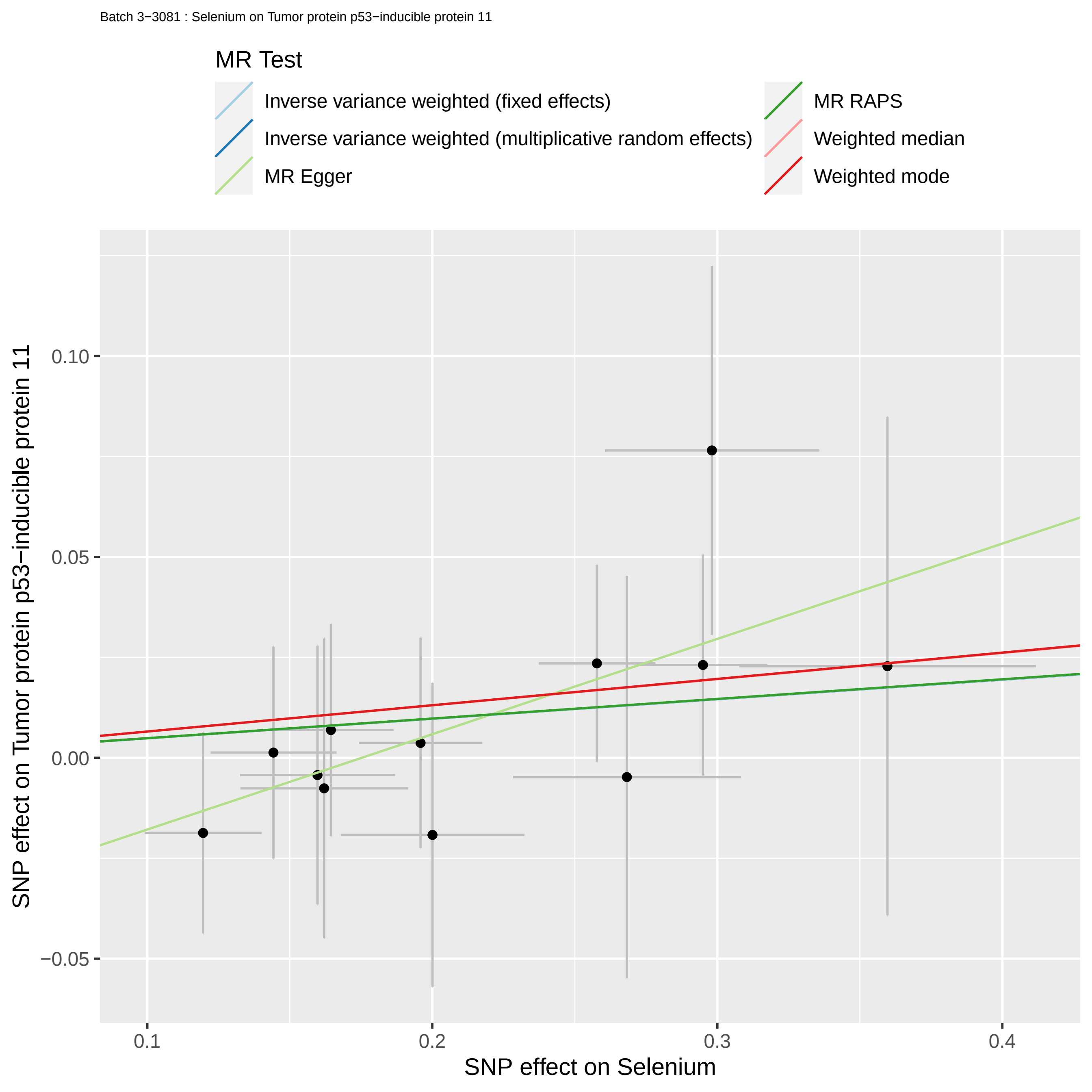

Batch 3−3081 : Selenium on Tumor protein p53−inducible protein 11
MR Test
Inverse variance weighted (fixed effects)
Inverse variance weighted (multiplicative random effects)
MR Egger
MR RAPS
Weighted median
Weighted mode
SNP effect on Tumor protein p53−inducible protein 11
0.10
0.05
0.00
−0.05
0.1
0.2
0.3
0.4
SNP effect on Selenium

Batch 3−3082 : Selenium on Tumor protein 63

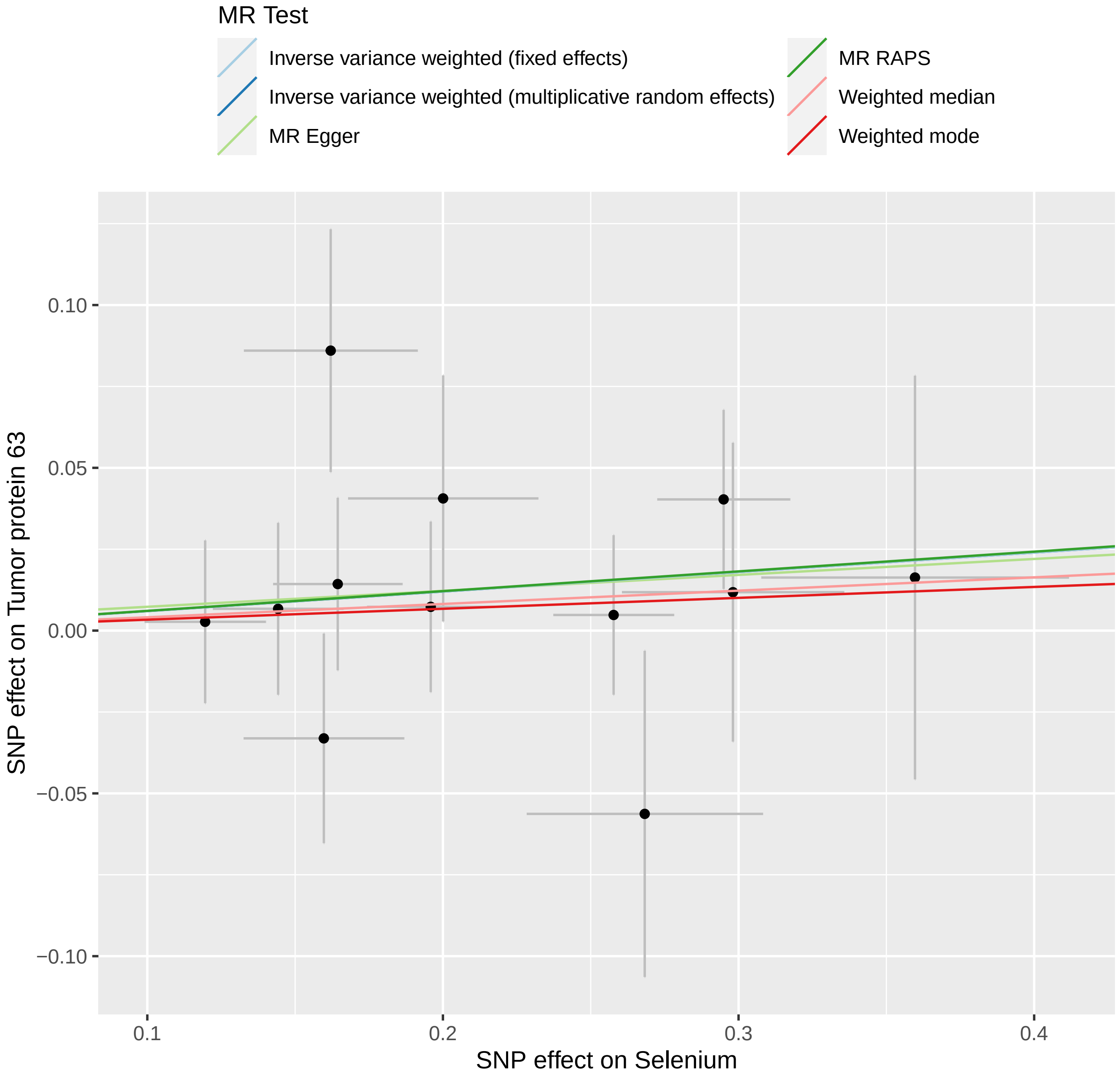

Batch 3−3083 : Selenium on Triosephosphate isomerase

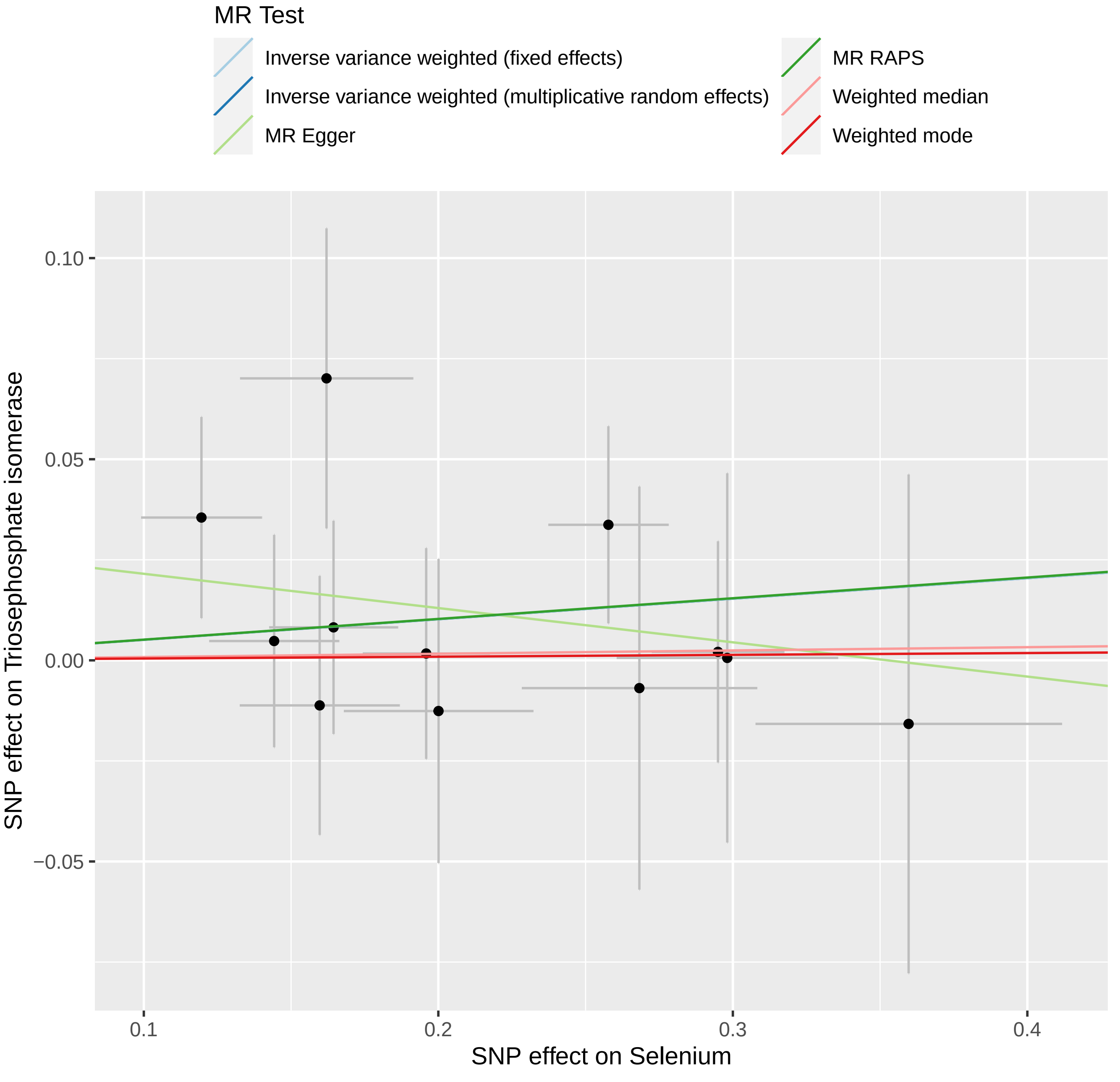

Batch 3−3084 : Selenium on Thiamin pyrophosphokinase 1

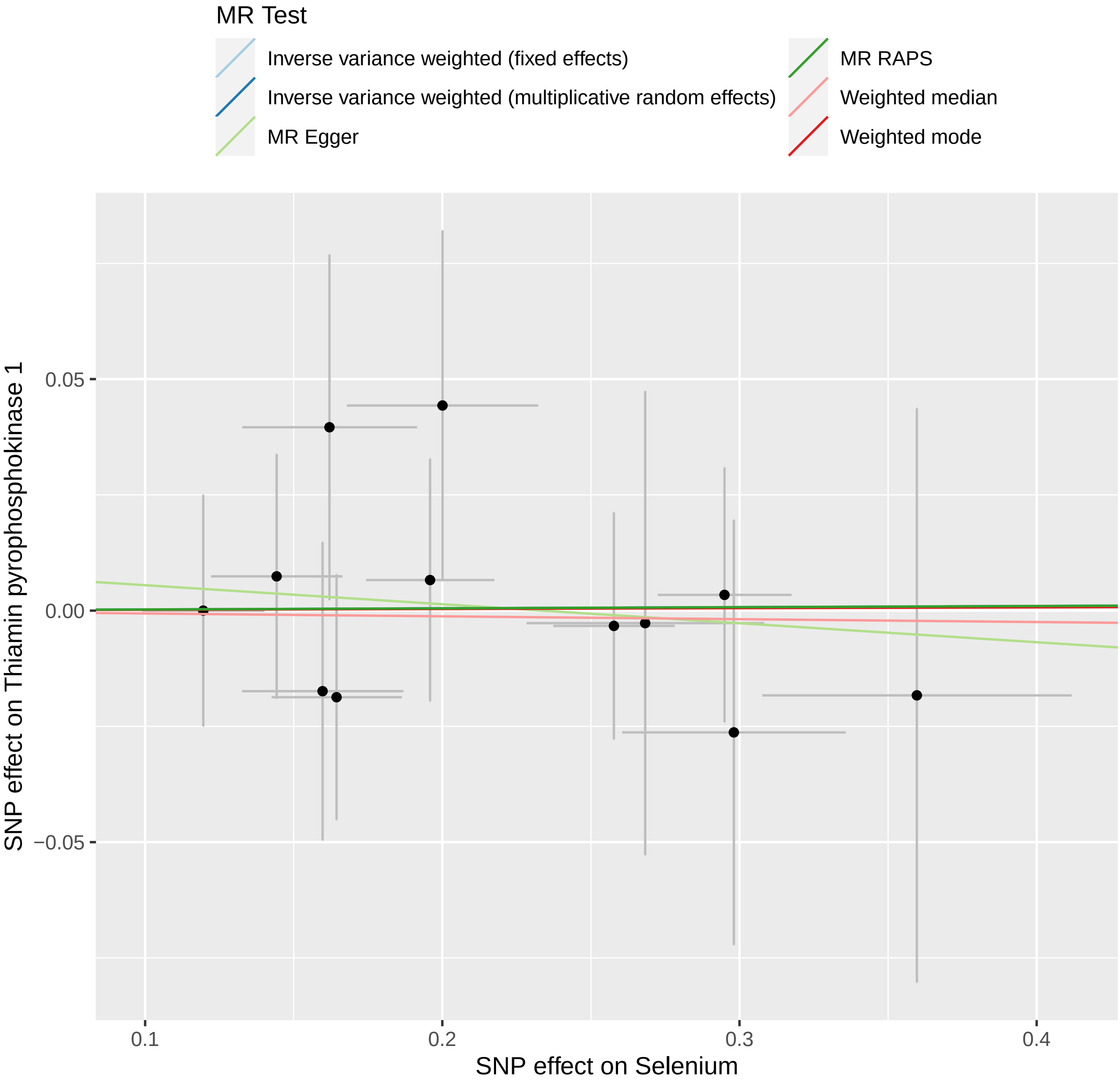

Batch 3−3085 : Selenium on Tropomyosin alpha−1 chain

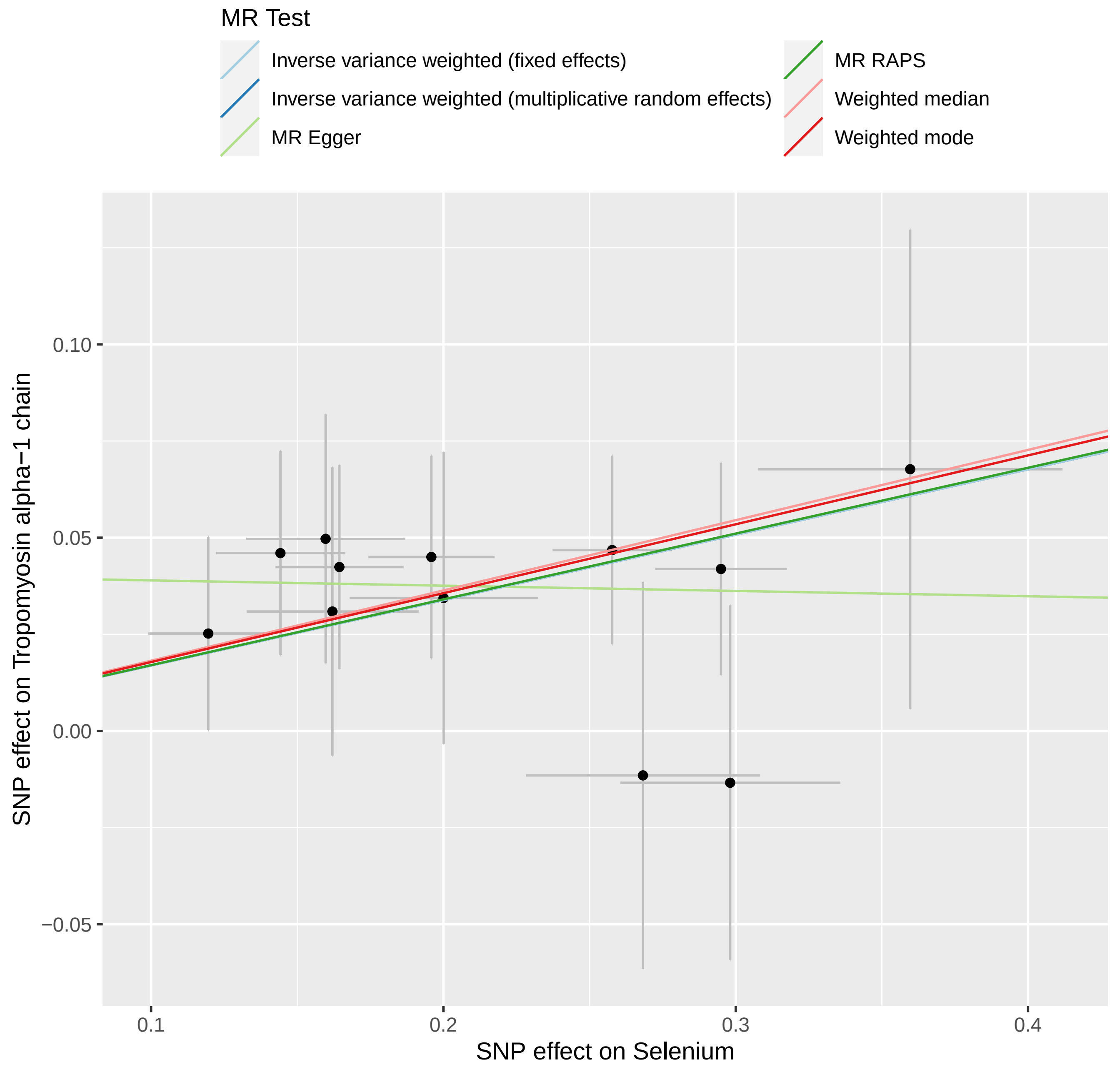

Batch 3−3086 : Selenium on Tropomyosin beta chain

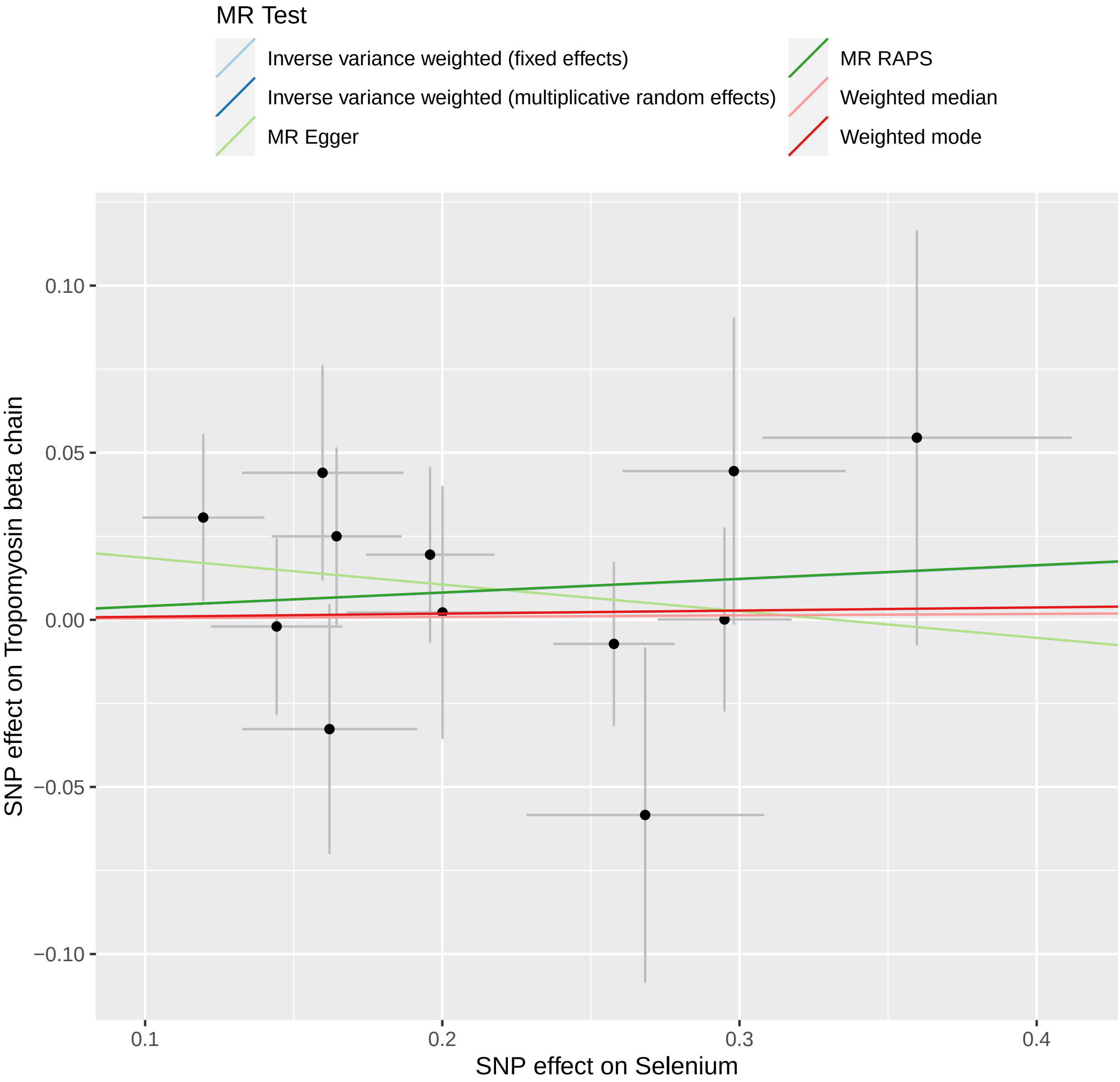

Batch 3−3087 : Selenium on Tropomyosin alpha−3 chain

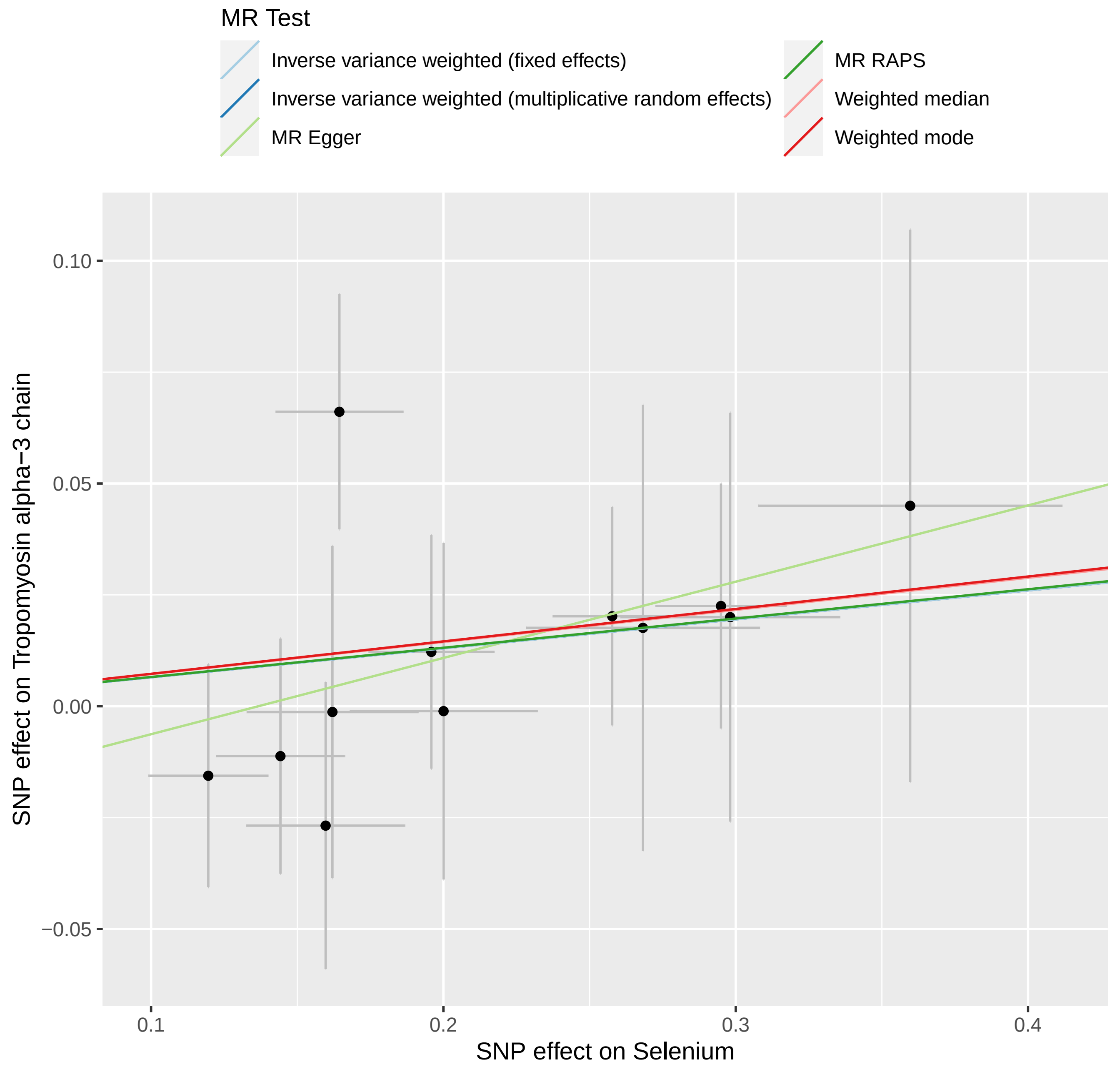

Batch 3−3088 : Selenium on Thyroid peroxidase

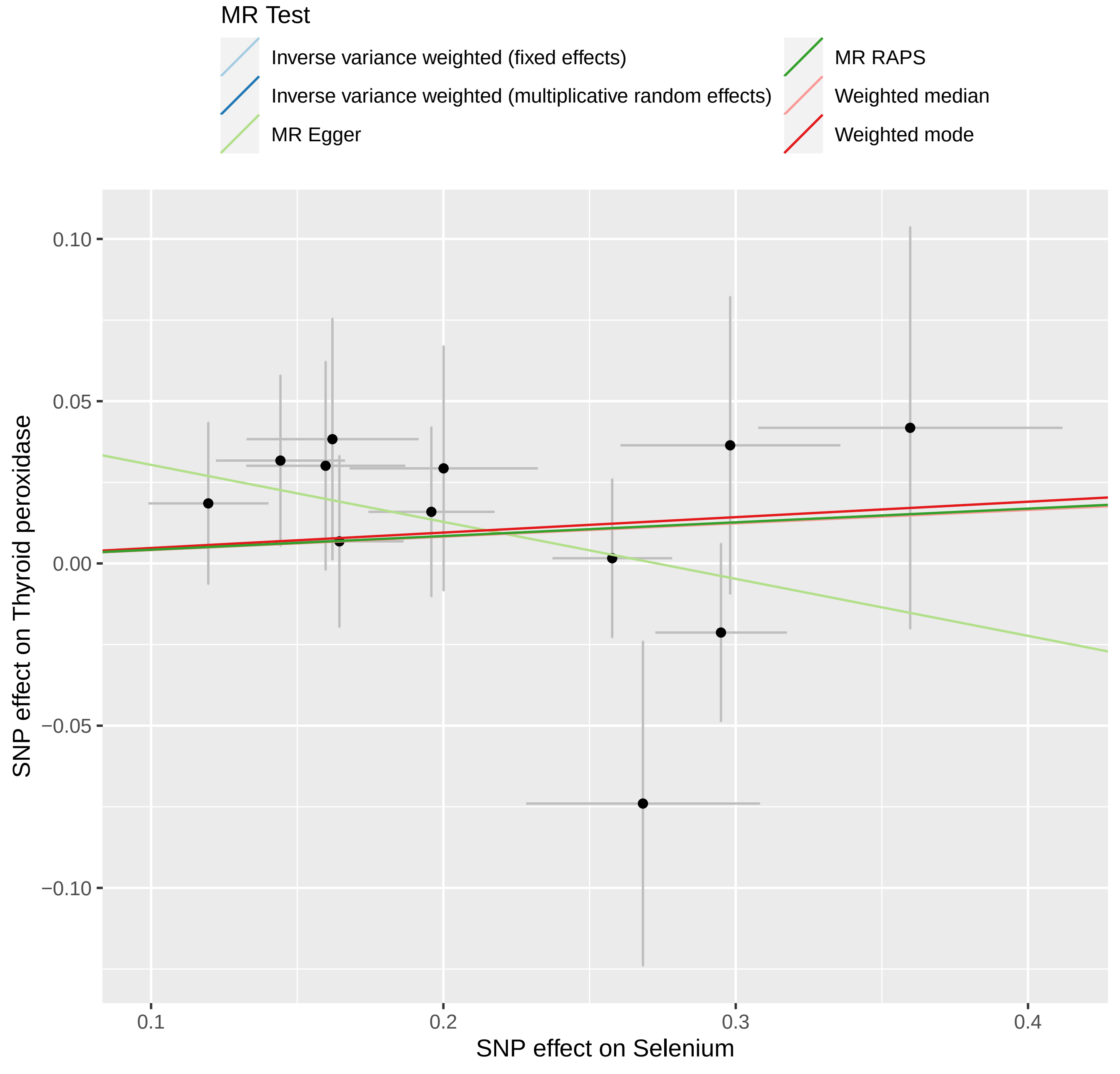

Batch 3−3089 : Selenium on Tubulin polymerization−promoting protein family member 2

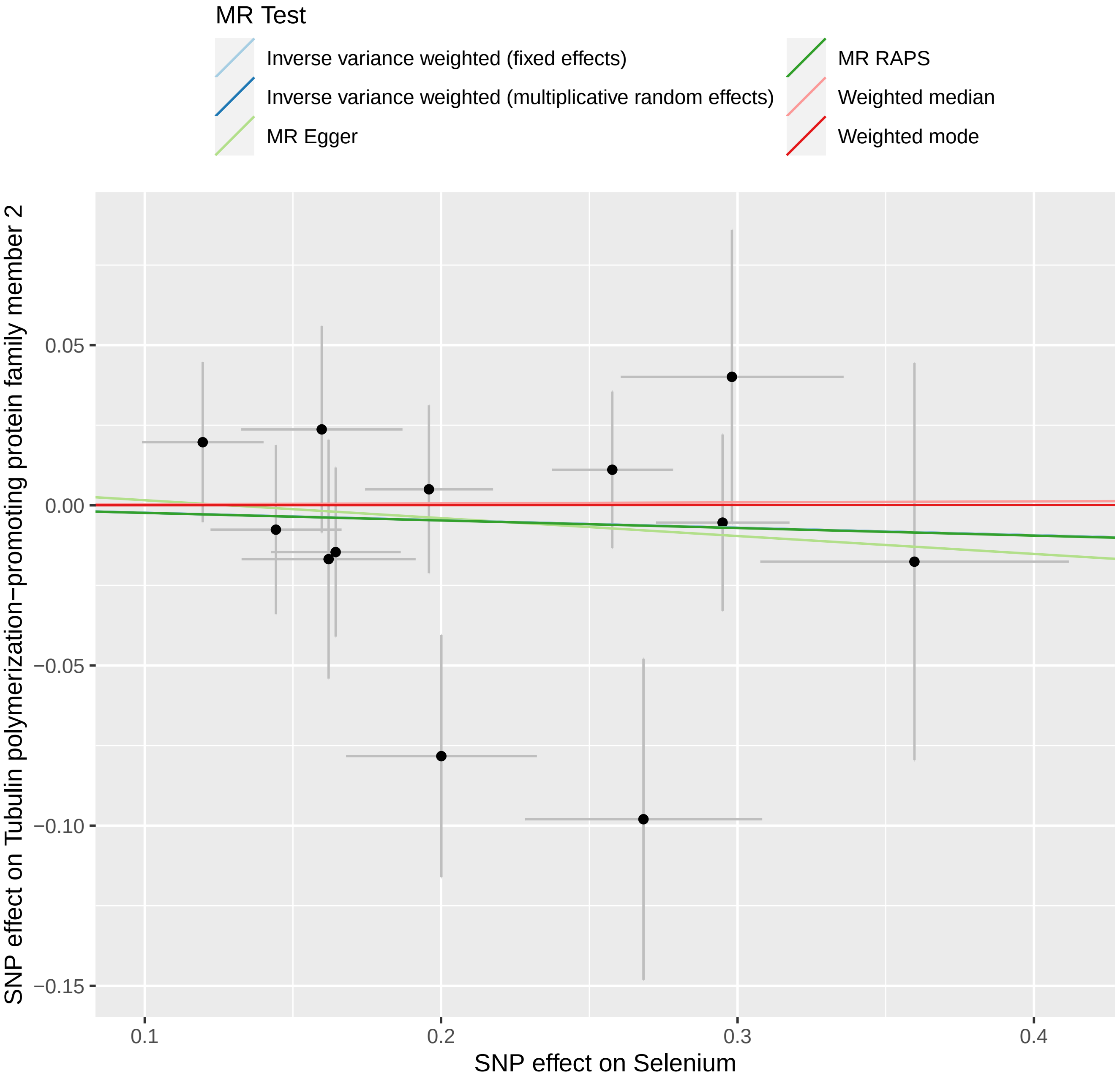

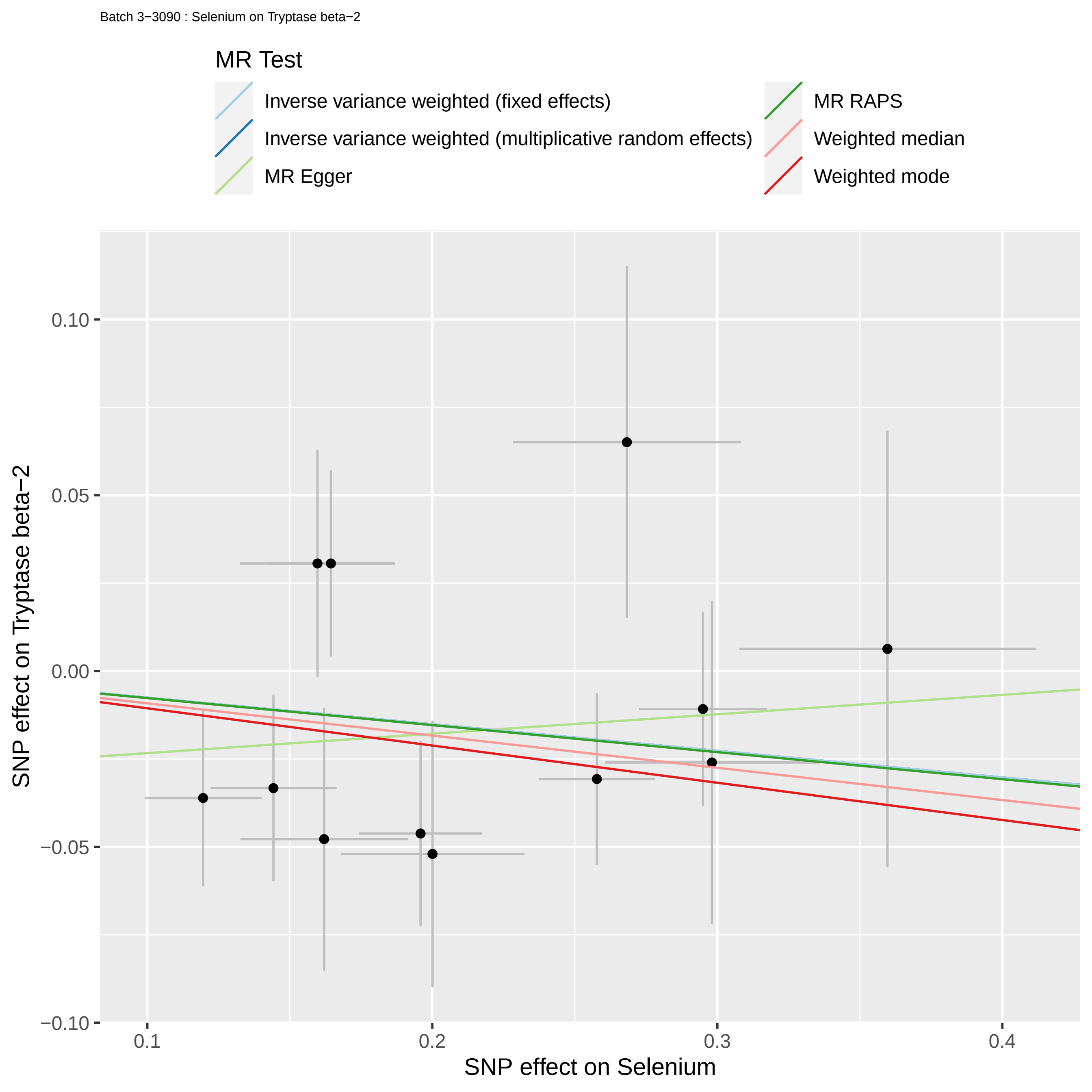

Batch 3−3090 : Selenium on Tryptase beta−2
MR Test
Inverse variance weighted (fixed effects)
Inverse variance weighted (multiplicative random effects)
MR Egger
MR RAPS
Weighted median
Weighted mode
0.10
0.05
0.00
−0.05
−0.10
0.1
0.2
0.3
0.4
SNP effect on Tryptase beta−2
SNP effect on Selenium

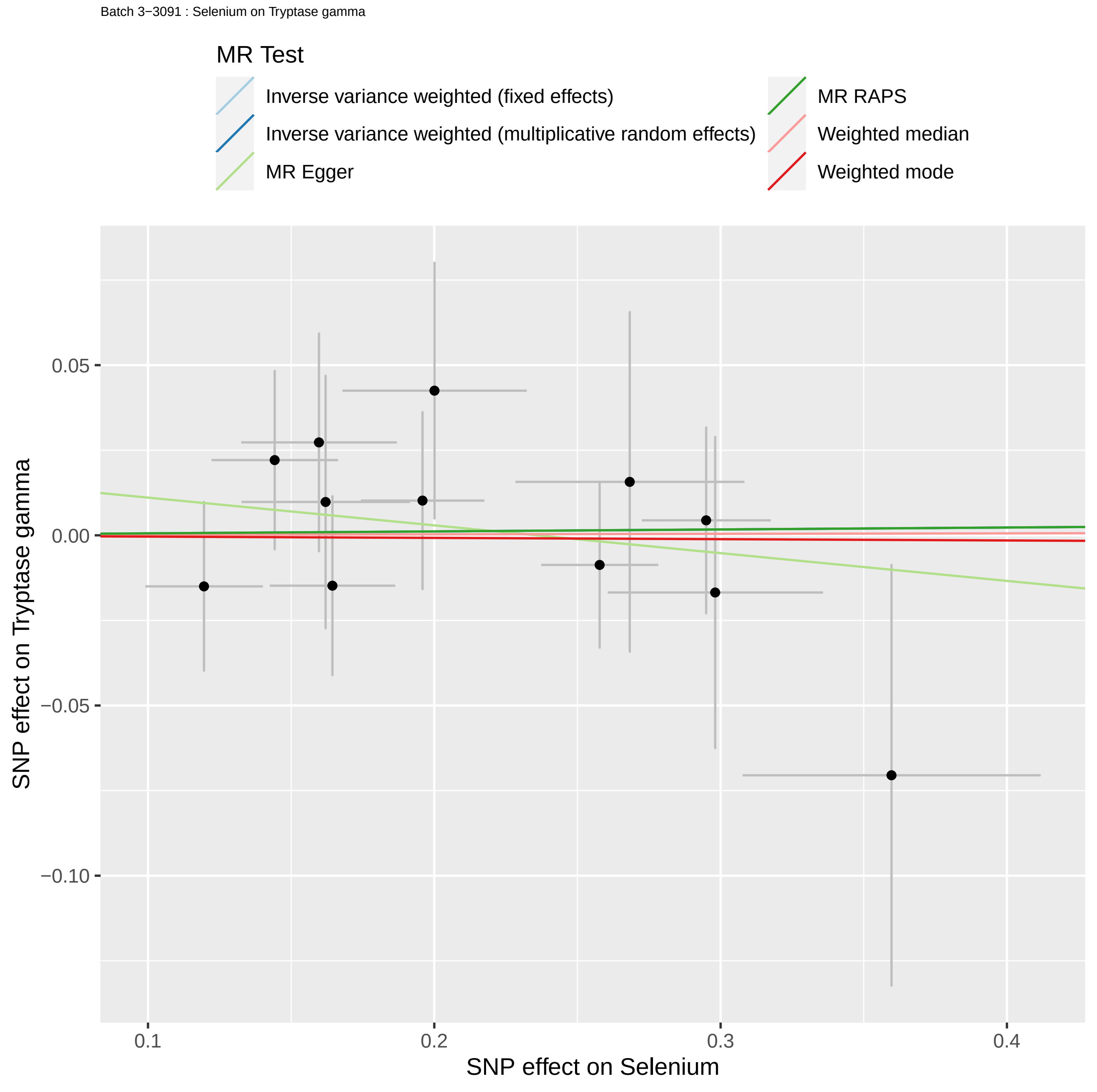

Batch 3−3091 : Selenium on Tryptase gamma
MR Test
Inverse variance weighted (fixed effects)
Inverse variance weighted (multiplicative random effects)
MR Egger
MR RAPS
Weighted median
Weighted mode
0.05
0.00
−0.05
−0.10
0.1
0.2
0.3
0.4
SNP effect on Tryptase gamma
SNP effect on Selenium

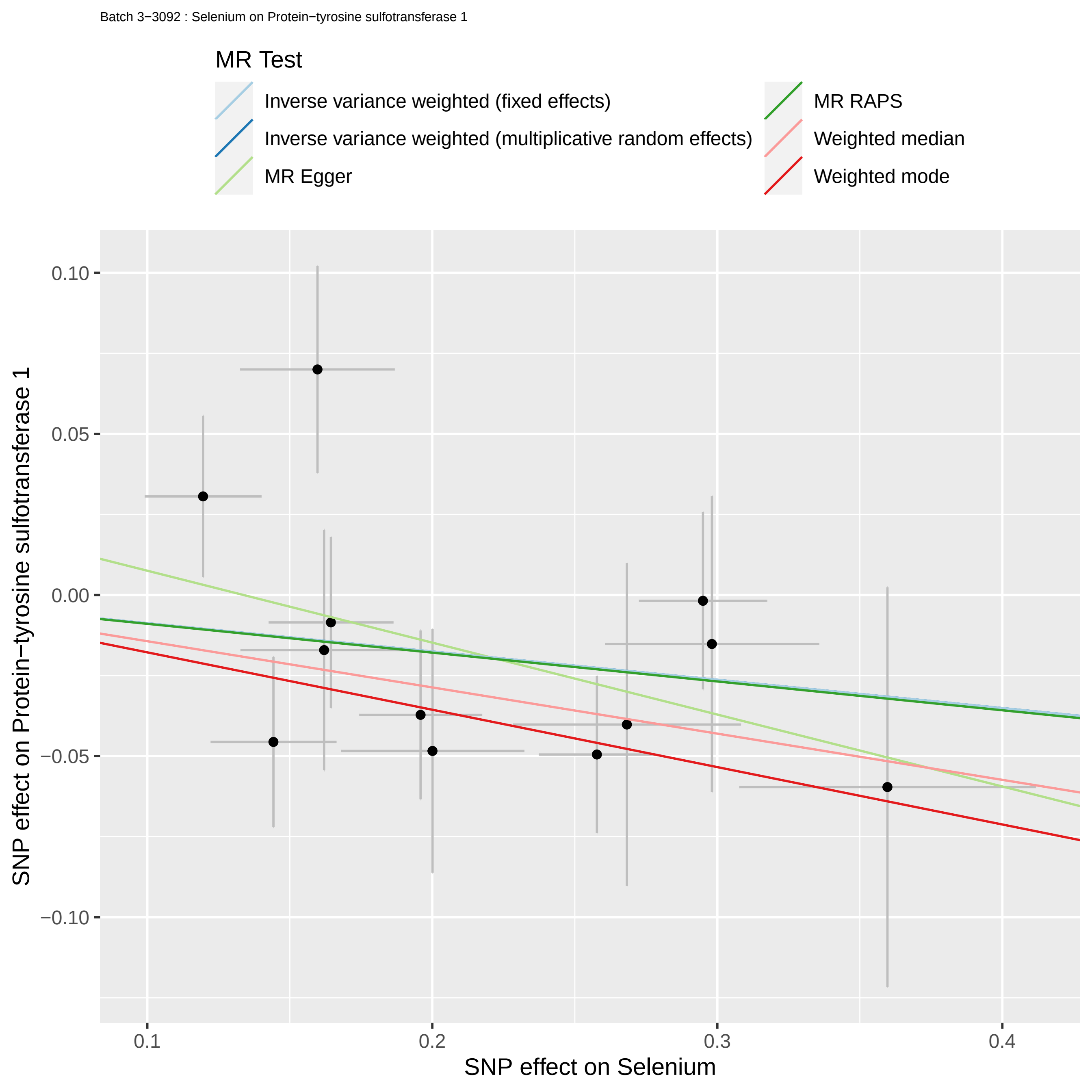
Batch 3−3092 : Selenium on Protein−tyrosine sulfotransferase 1
MR Test
Inverse variance weighted (fixed effects)
Inverse variance weighted (multiplicative random effects)
MR Egger
MR RAPS
Weighted median
Weighted mode
0.10
0.05
0.00
−0.05
−0.10
0.1
0.2
0.3
0.4
SNP effect on Protein−tyrosine sulfotransferase 1
SNP effect on Selenium

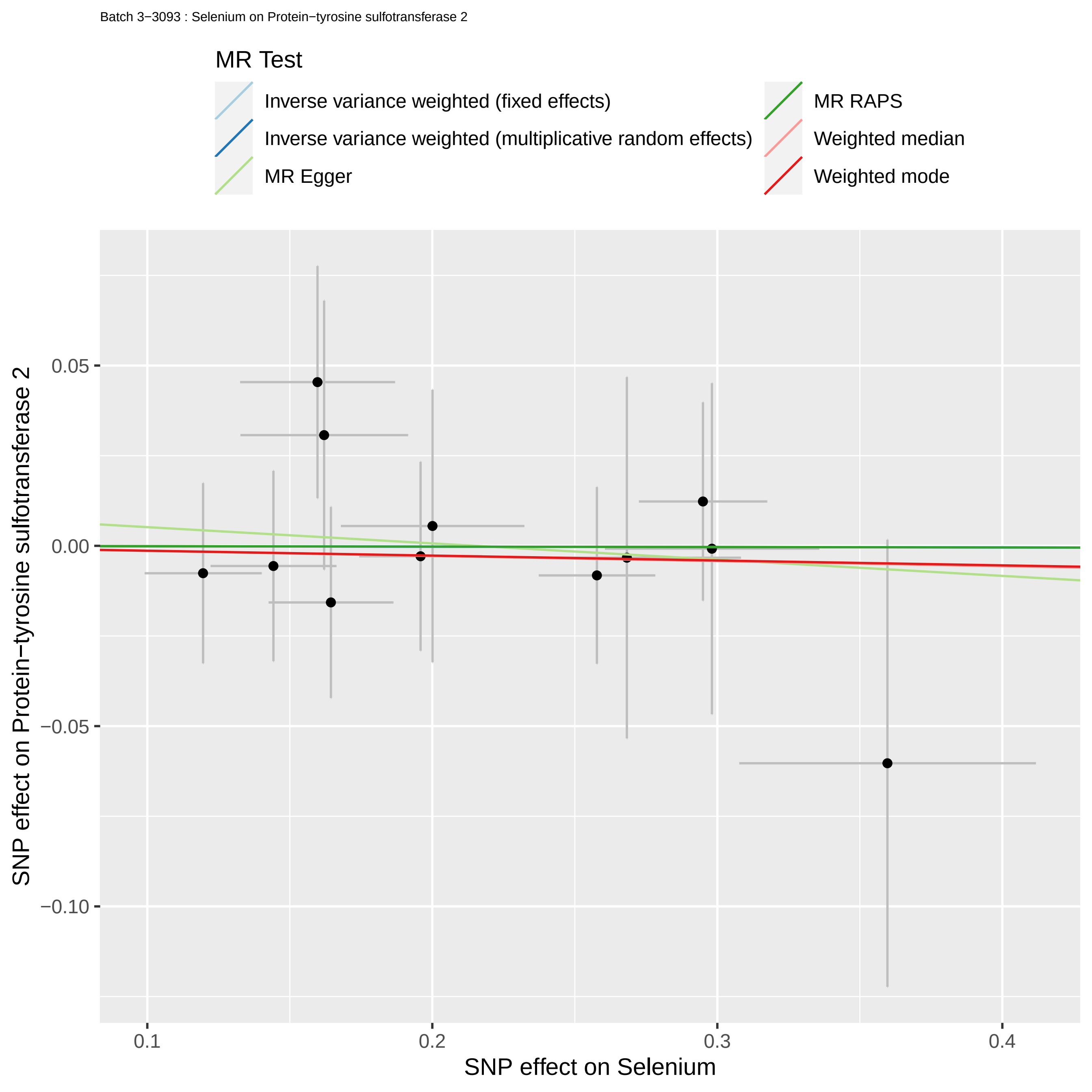

Batch 3−3093 : Selenium on Protein−tyrosine sulfotransferase 2
MR Test
Inverse variance weighted (fixed effects)
Inverse variance weighted (multiplicative random effects)
MR Egger
MR RAPS
Weighted median
Weighted mode
0.05
0.00
−0.05
−0.10
0.1
0.2
0.3
0.4
SNP effect on Protein−tyrosine sulfotransferase 2
SNP effect on Selenium

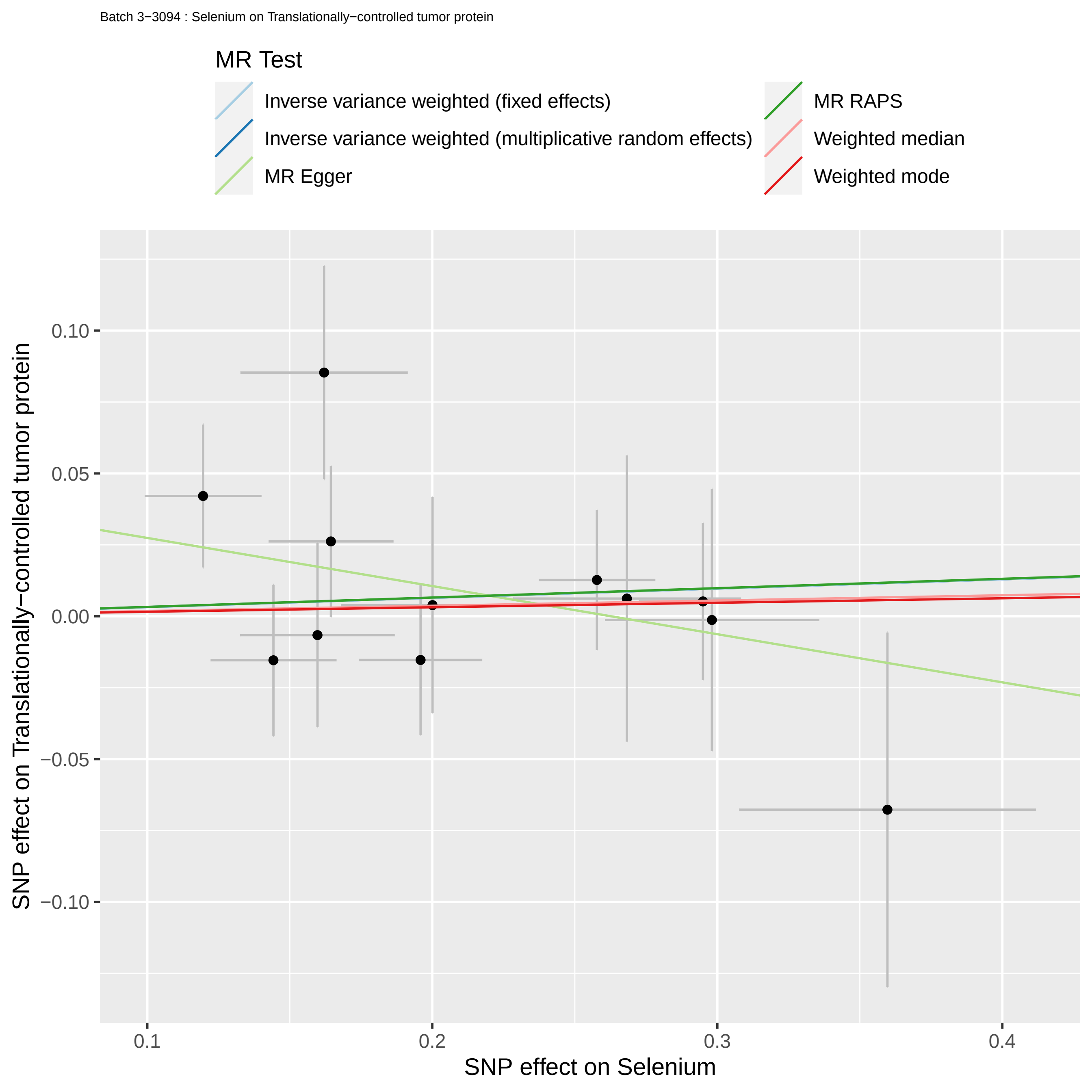

Batch 3−3094 : Selenium on Translationally−controlled tumor protein
MR Test
Inverse variance weighted (fixed effects)
Inverse variance weighted (multiplicative random effects)
MR Egger
MR RAPS
Weighted median
Weighted mode
SNP effect on Translationally−controlled tumor protein
0.10
0.05
0.00
−0.05
−0.10
0.1
0.2
0.3
0.4
SNP effect on Selenium

Batch 3−3095 : Selenium on Transformer−2 protein homolog beta

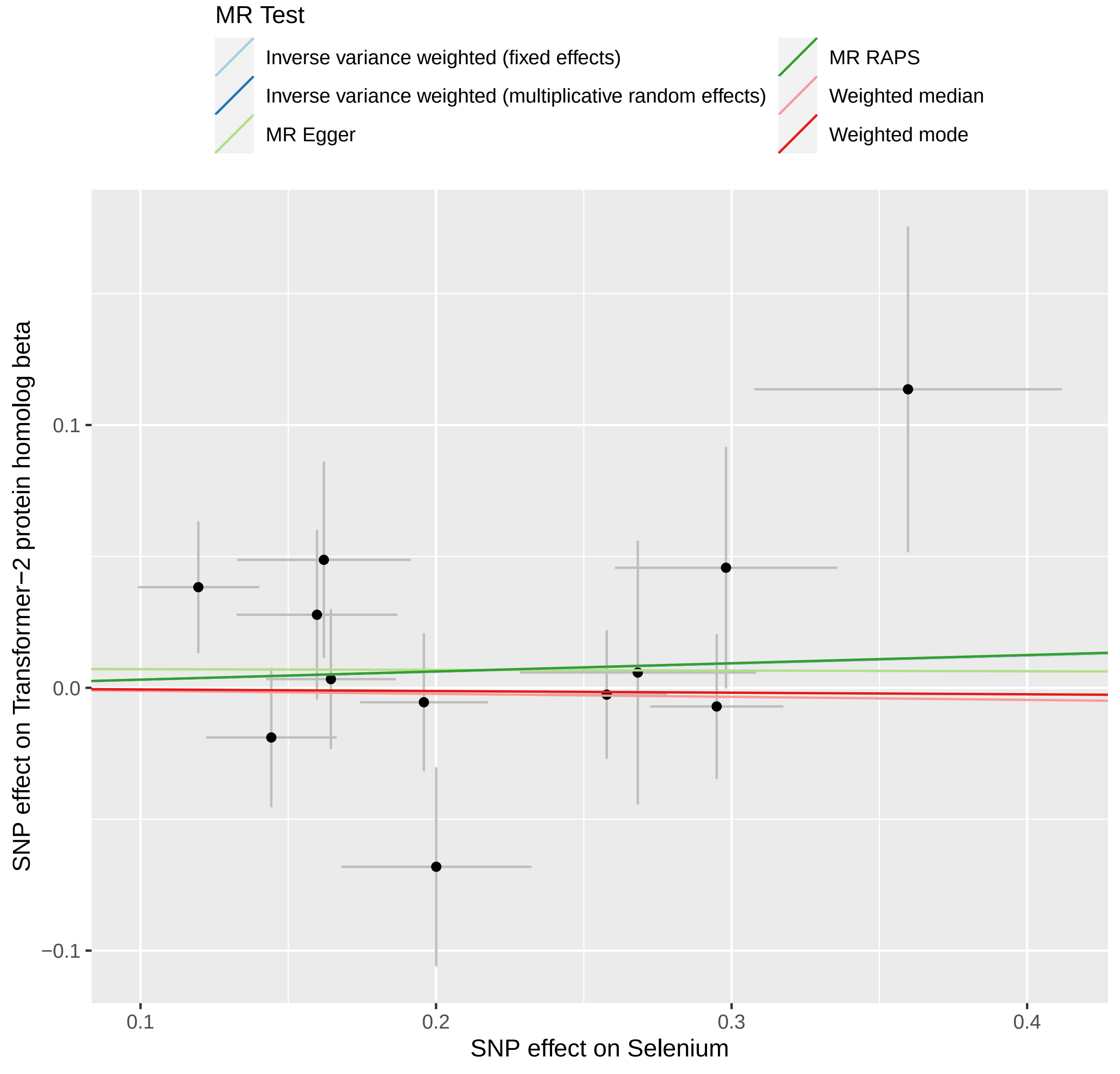

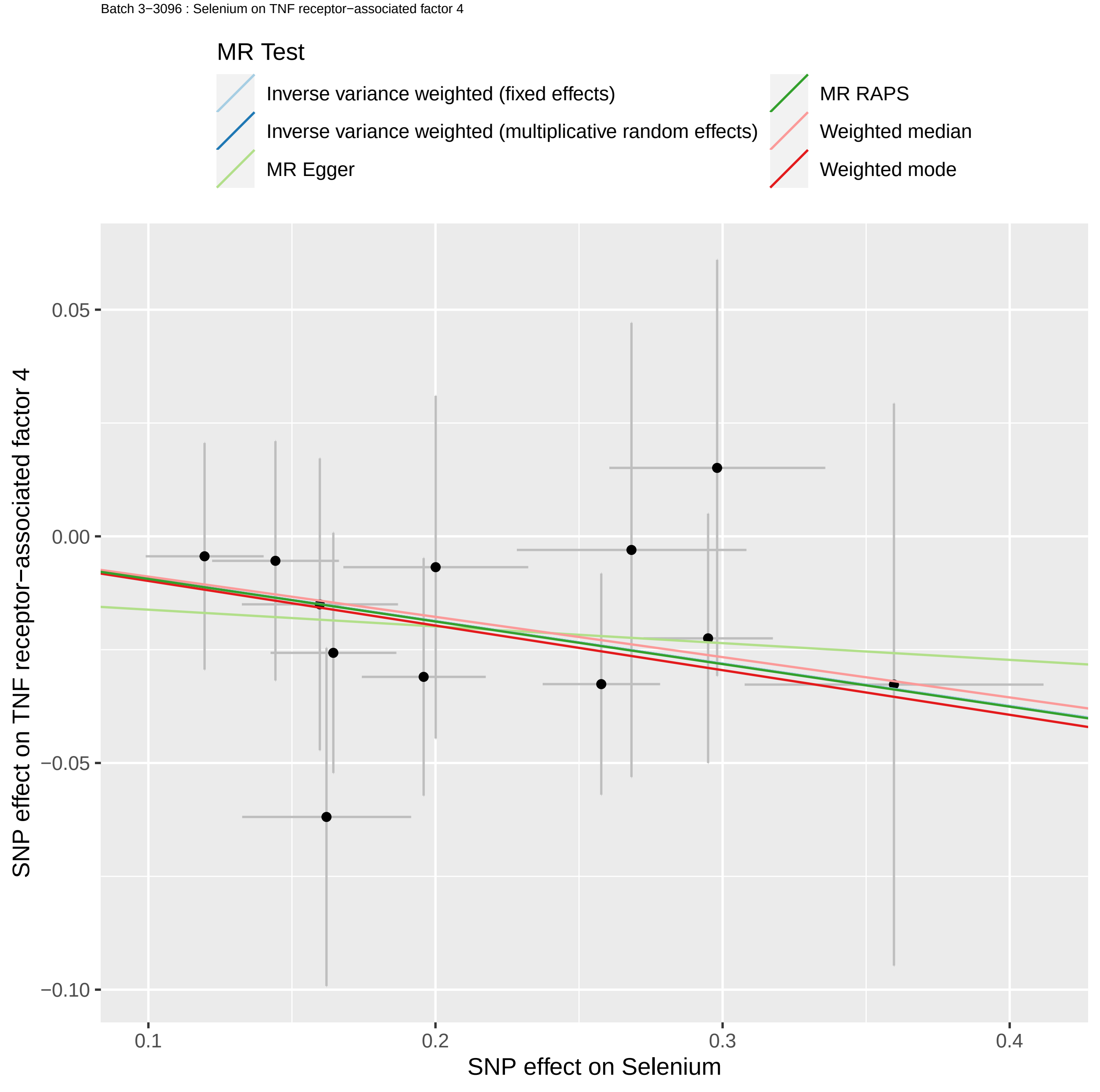
Batch 3−3096 : Selenium on TNF receptor−associated factor 4
MR Test
Inverse variance weighted (fixed effects)
Inverse variance weighted (multiplicative random effects)
MR Egger
MR RAPS
Weighted median
Weighted mode
0.05
0.00
−0.05
−0.10
0.1
0.2
0.3
0.4
SNP effect on TNF receptor−associated factor 4
SNP effect on Selenium

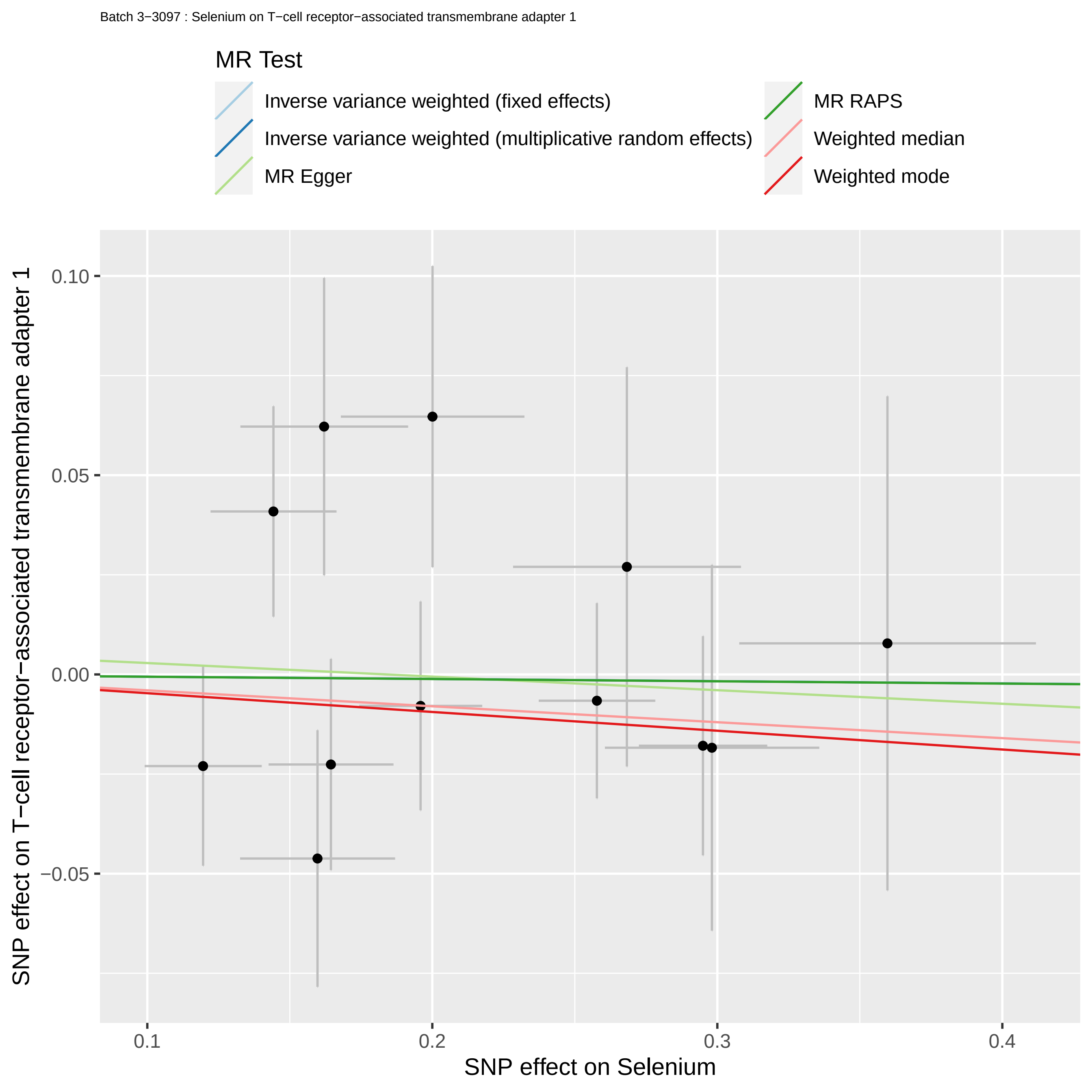
Batch 3−3097 : Selenium on T−cell receptor−associated transmembrane adapter 1
MR Test
Inverse variance weighted (fixed effects)
Inverse variance weighted (multiplicative random effects)
MR Egger
MR RAPS
Weighted median
Weighted mode
SNP effect on T−cell receptor−associated transmembrane adapter 1
0.10
0.05
0.00
−0.05
0.1
0.2
0.3
0.4
SNP effect on Selenium

Batch 3−3098 : Selenium on Triggering receptor expressed on myeloid cells 1

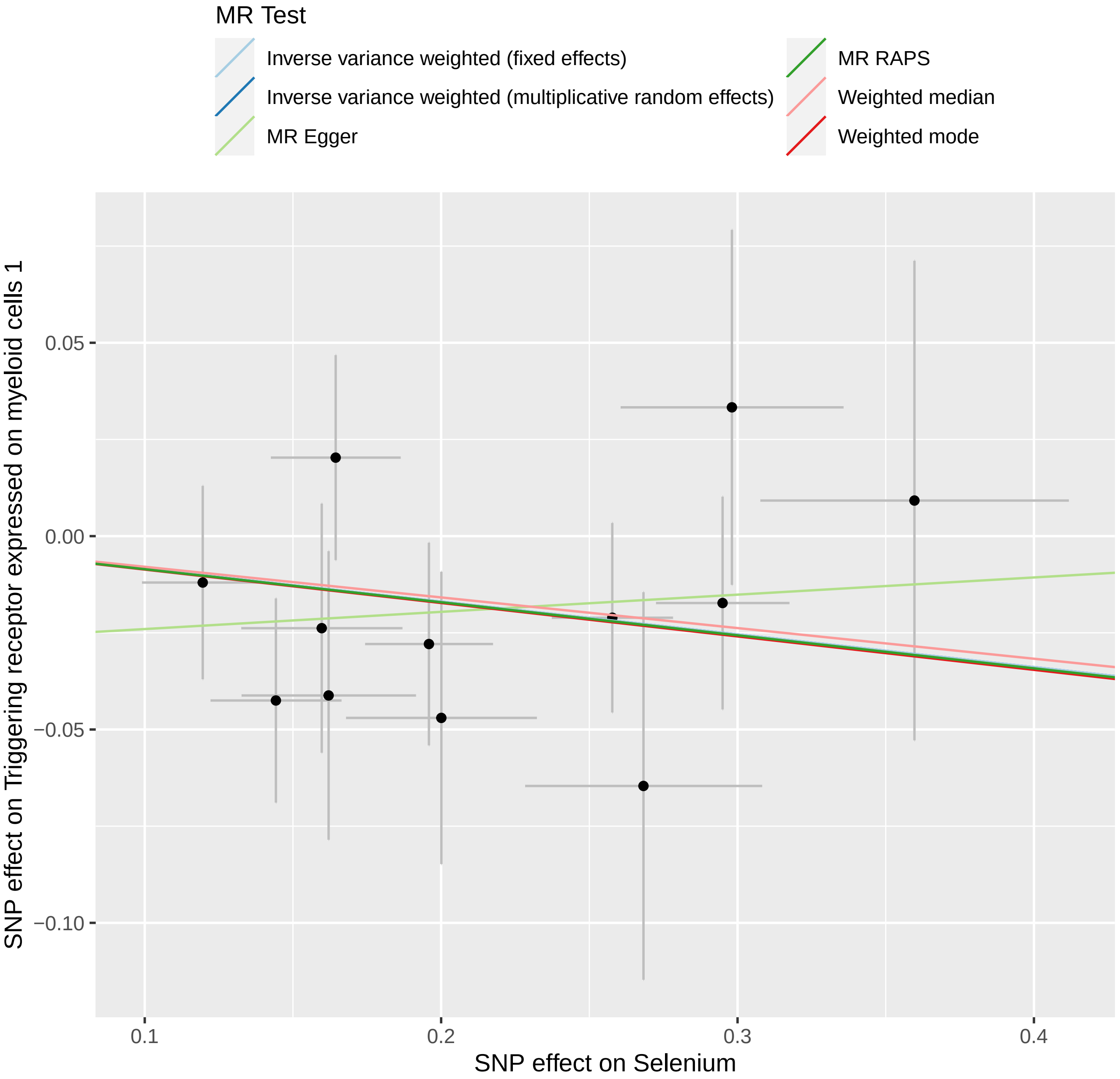

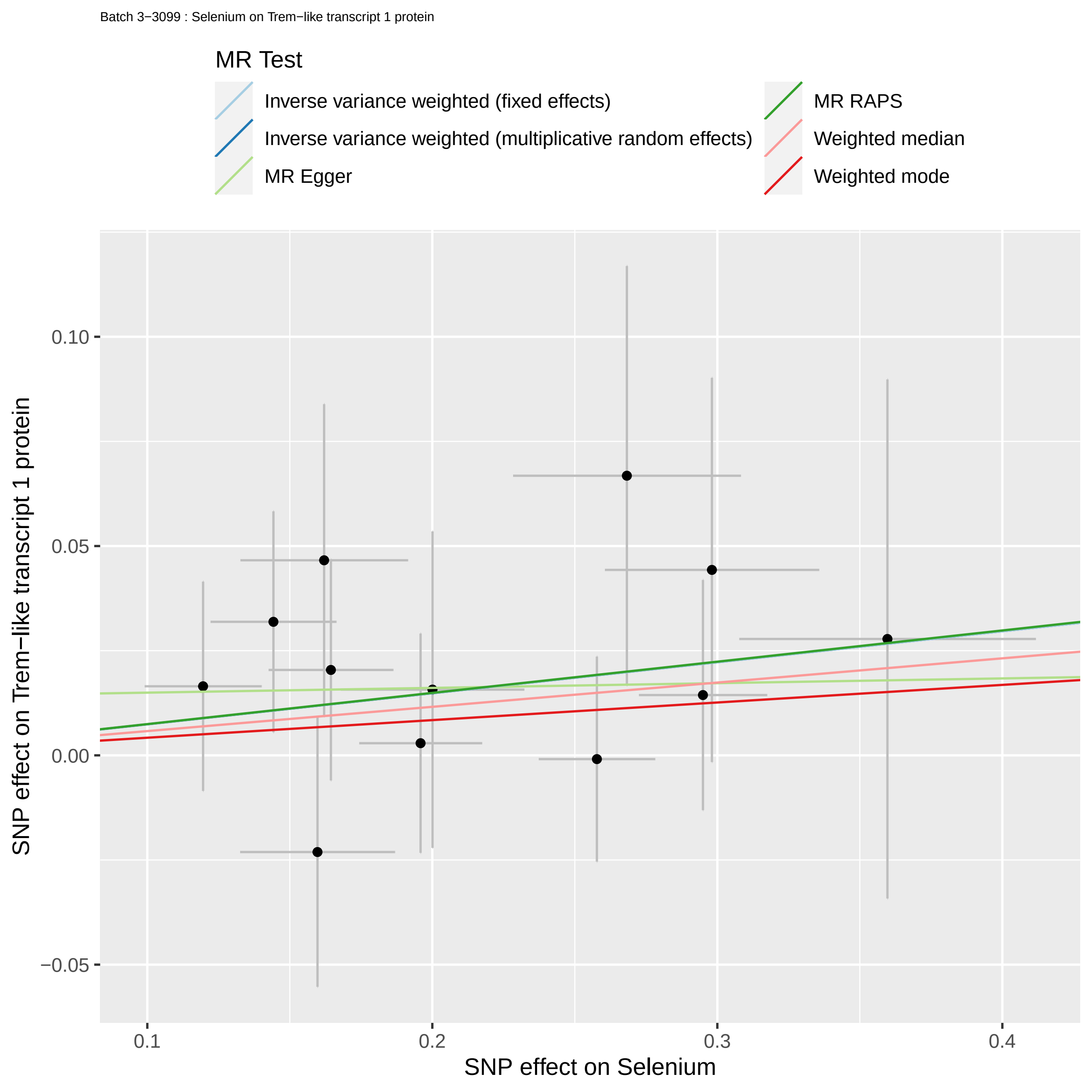
Batch 3−3099 : Selenium on Trem−like transcript 1 protein
MR Test
Inverse variance weighted (fixed effects)
Inverse variance weighted (multiplicative random effects)
MR Egger
MR RAPS
Weighted median
Weighted mode
0.10
0.05
0.00
−0.05
0.1
0.2
0.3
0.4
SNP effect on Trem−like transcript 1 protein
SNP effect on Selenium

Batch 3−3100 : Selenium on Trem−like transcript 2 protein

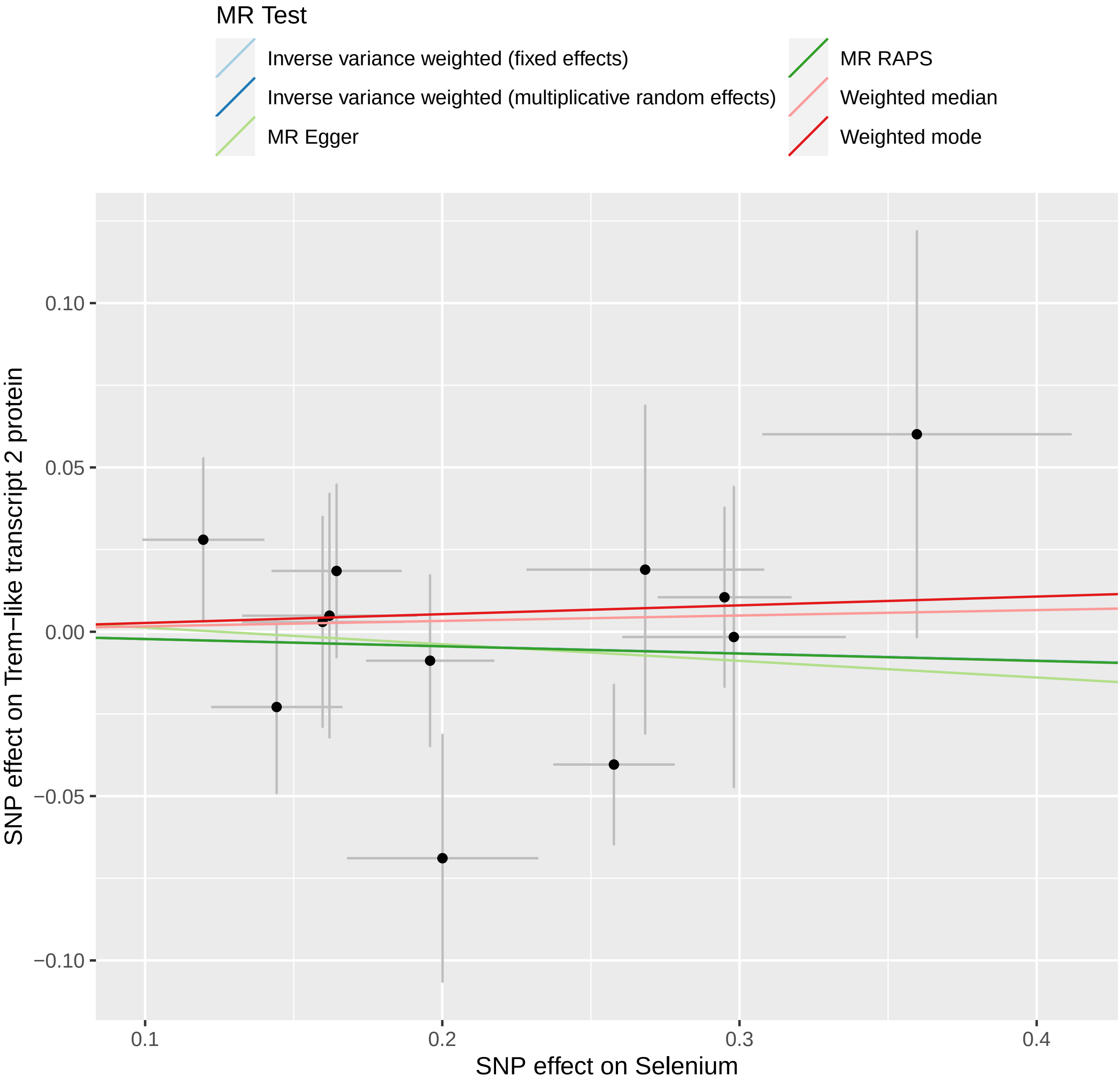

Batch 3−3101 : Selenium on Trem−like transcript 4 protein

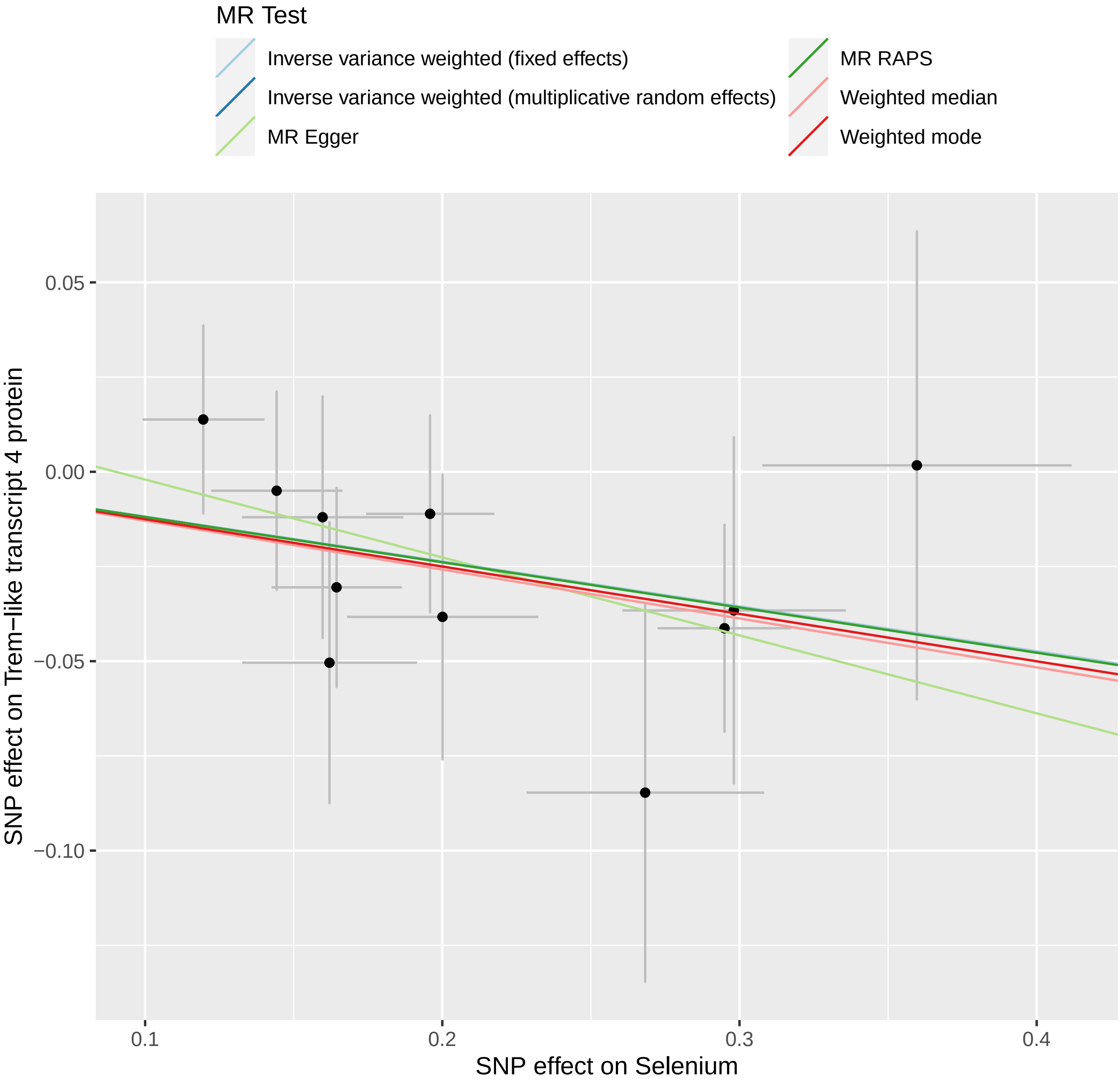

Batch 3−3102 : Selenium on Thyrotropin−releasing hormone

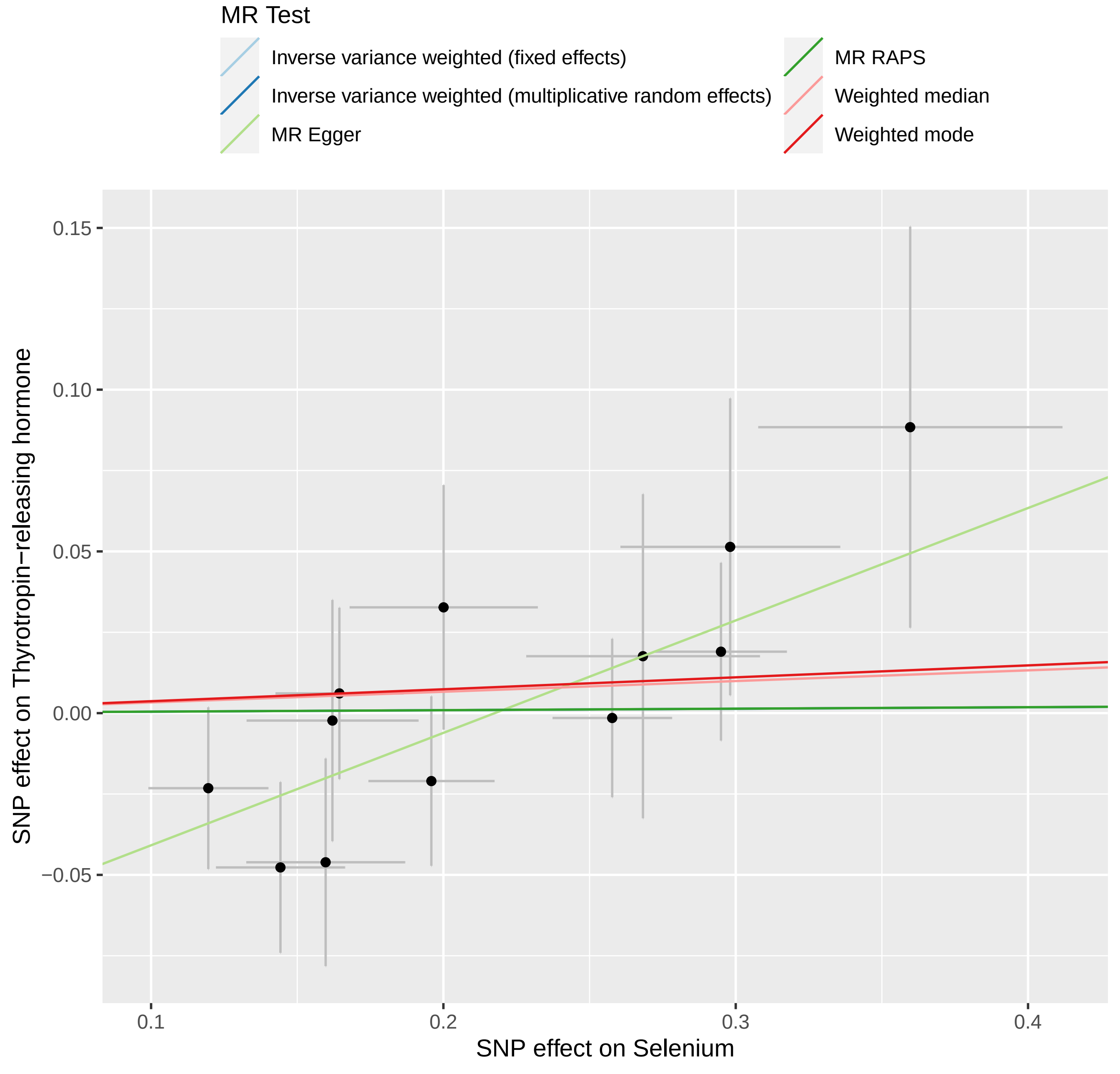

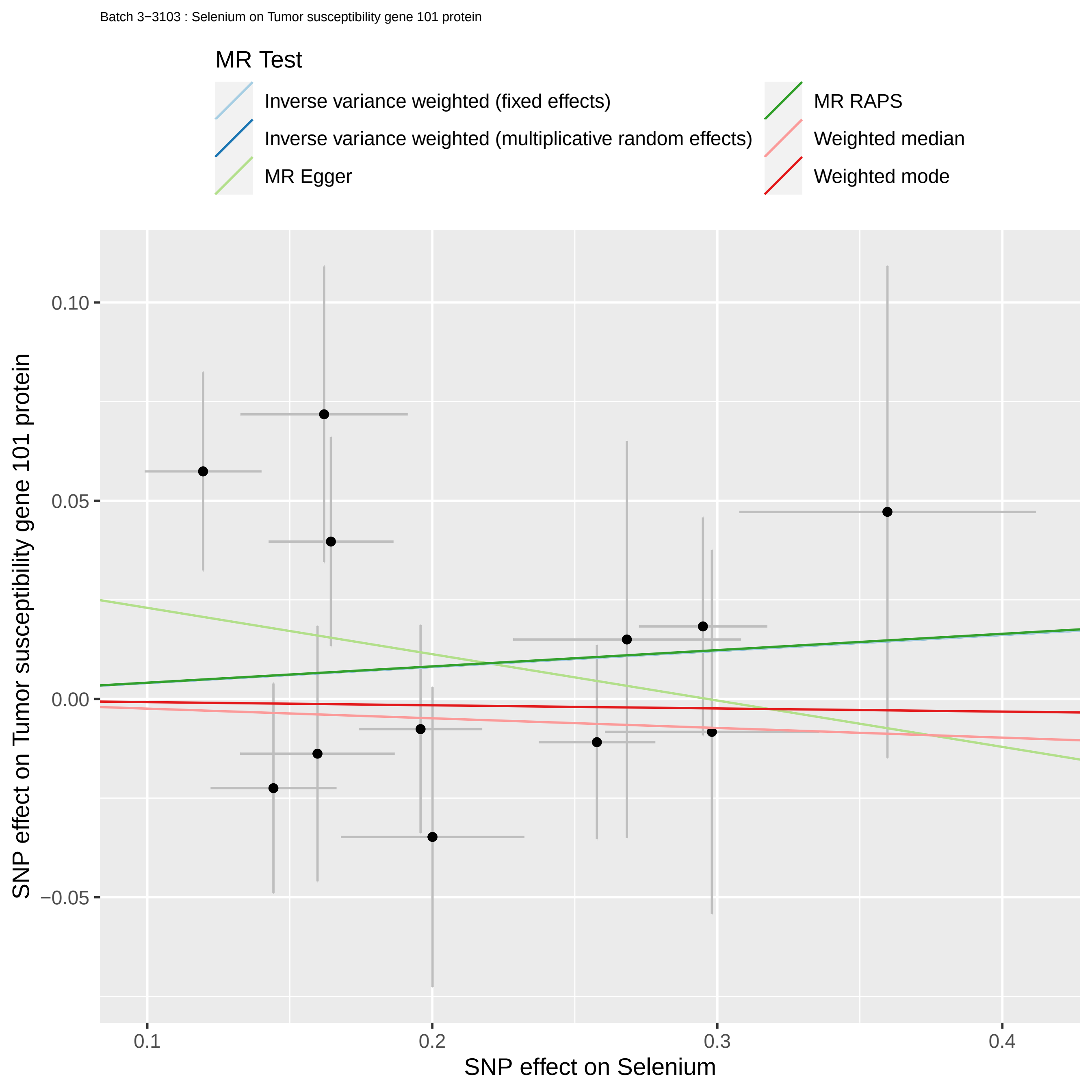

Batch 3−3103 : Selenium on Tumor susceptibility gene 101 protein
MR Test
Inverse variance weighted (fixed effects)
Inverse variance weighted (multiplicative random effects)
MR Egger
MR RAPS
Weighted median
Weighted mode
0.10
0.05
0.00
−0.05
0.1
0.2
0.3
0.4
SNP effect on Tumor susceptibility gene 101 protein
SNP effect on Selenium

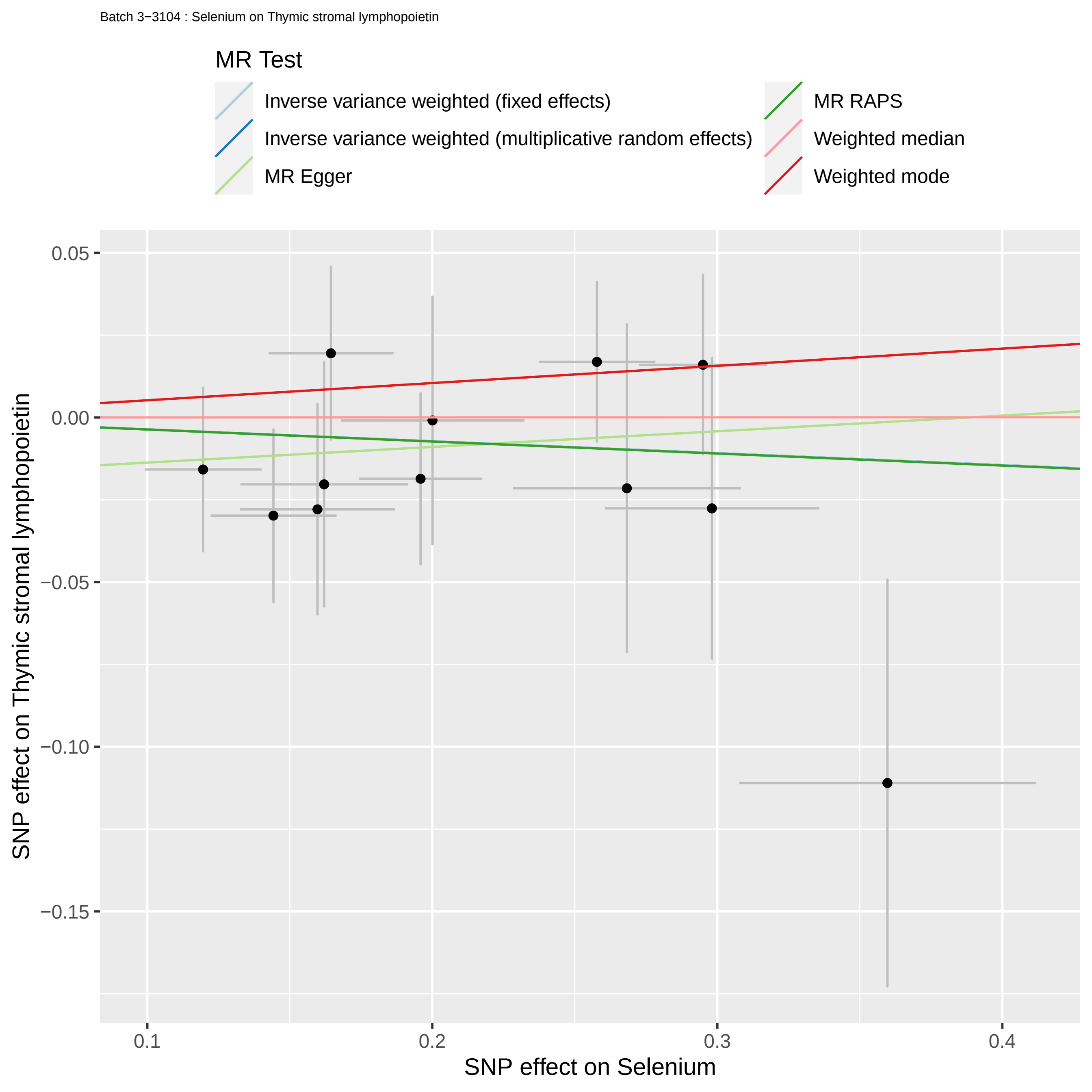

Batch 3−3104 : Selenium on Thymic stromal lymphopoietin
MR Test
Inverse variance weighted (fixed effects)
Inverse variance weighted (multiplicative random effects)
MR Egger
MR RAPS
Weighted median
Weighted mode
0.05
0.00
−0.05
−0.10
−0.15
0.1
0.2
0.3
0.4
SNP effect on Thymic stromal lymphopoietin
SNP effect on Selenium

Batch 3−3105 : Selenium on Translin

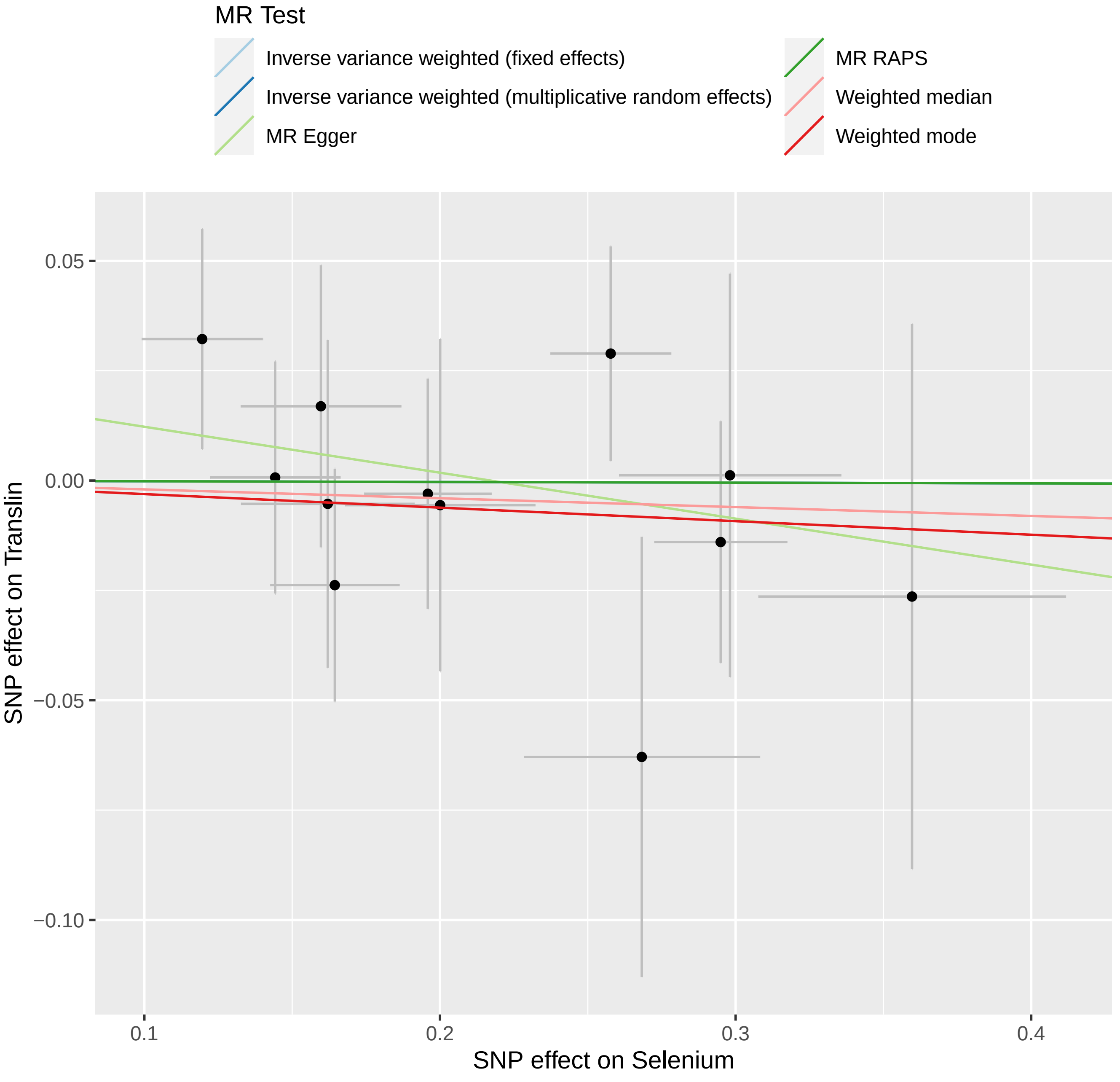

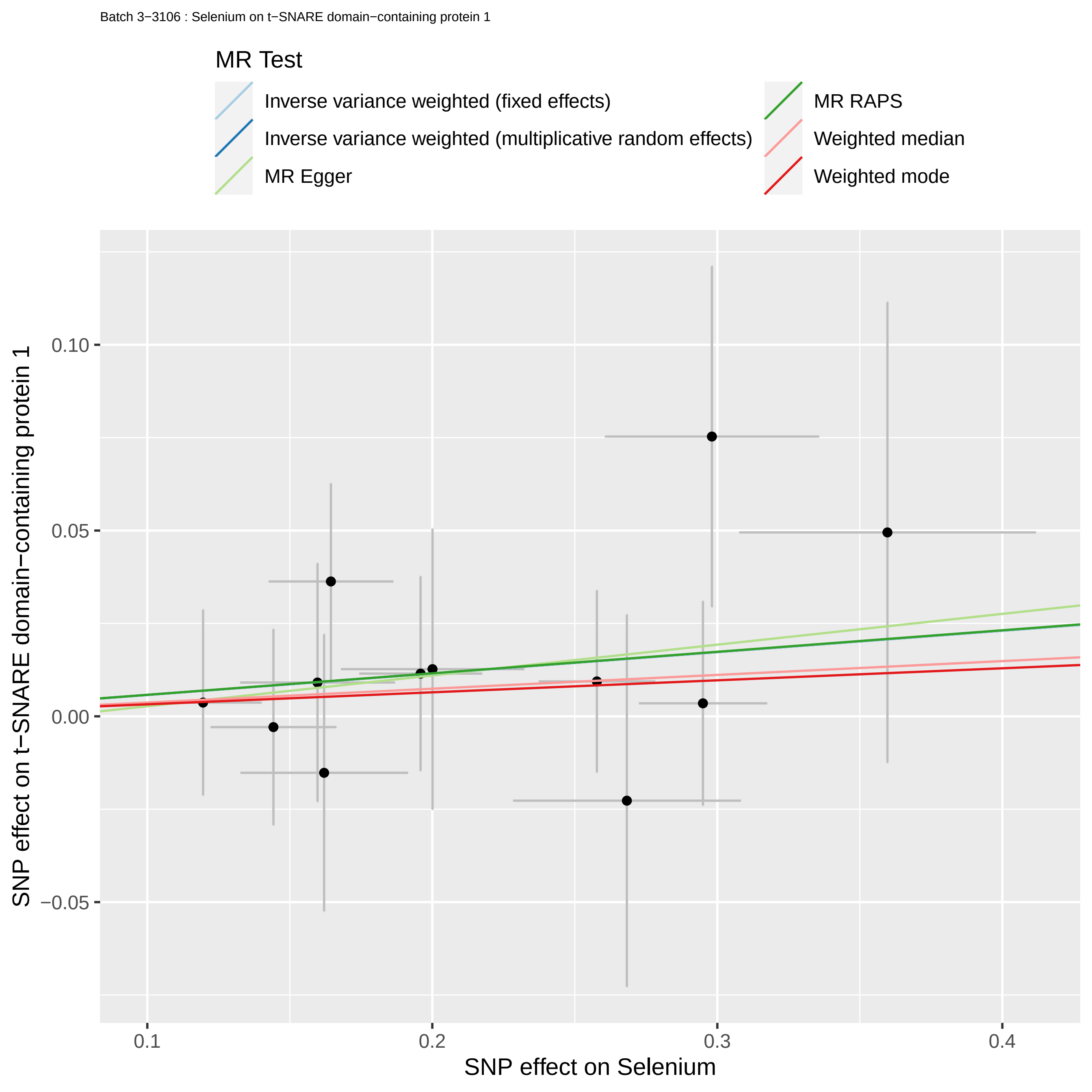

Batch 3−3106 : Selenium on t−SNARE domain−containing protein 1
MR Test
Inverse variance weighted (fixed effects)
Inverse variance weighted (multiplicative random effects)
MR Egger
MR RAPS
Weighted median
Weighted mode
0.10
0.05
0.00
−0.05
0.1
0.2
0.3
0.4
SNP effect on t−SNARE domain−containing protein 1
SNP effect on Selenium

Batch 3−3107 : Selenium on Ribosome biogenesis protein TSR3 homolog

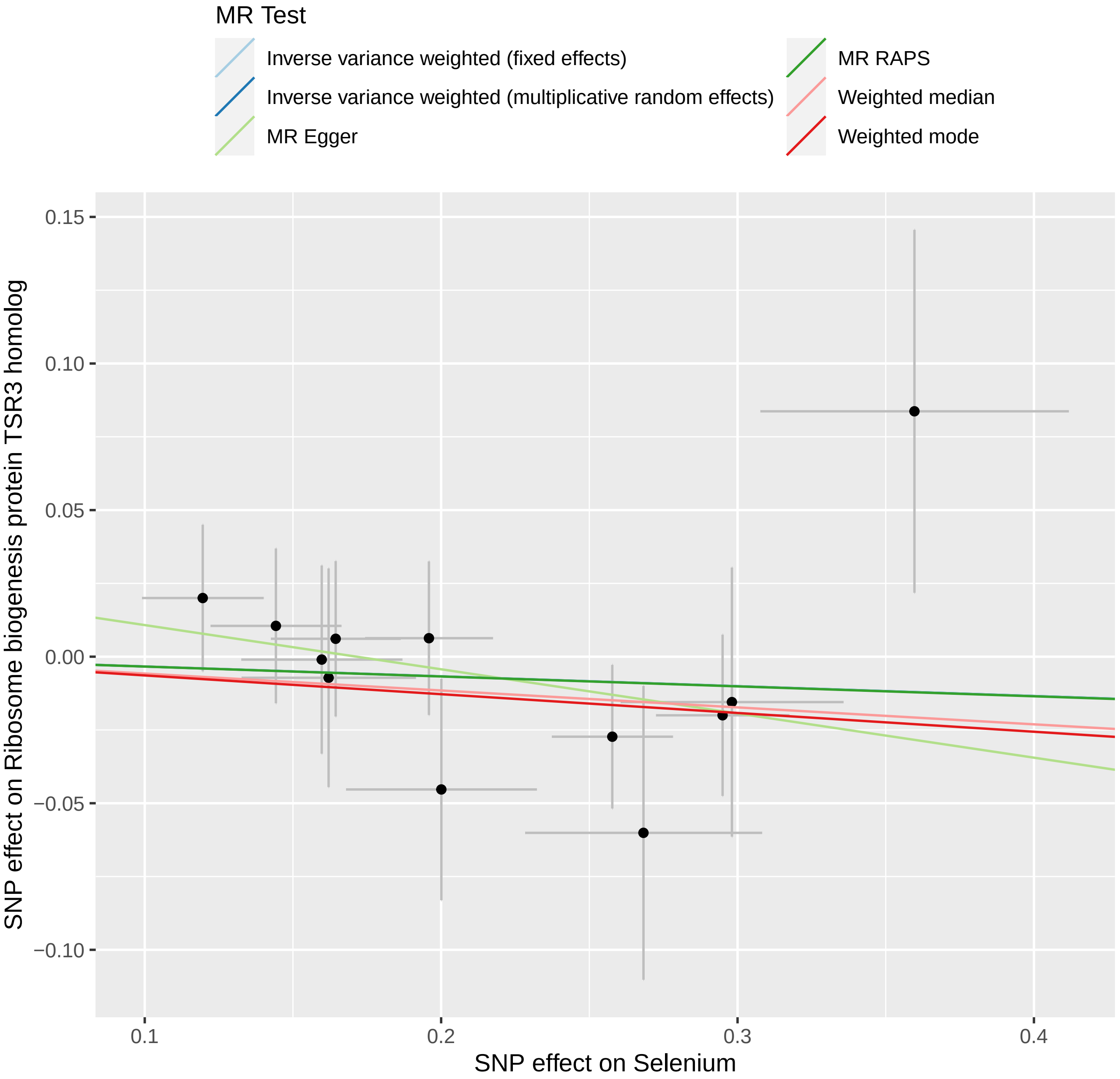

Batch 3−3108 : Selenium on Testis−specific serine/threonine−protein kinase 2

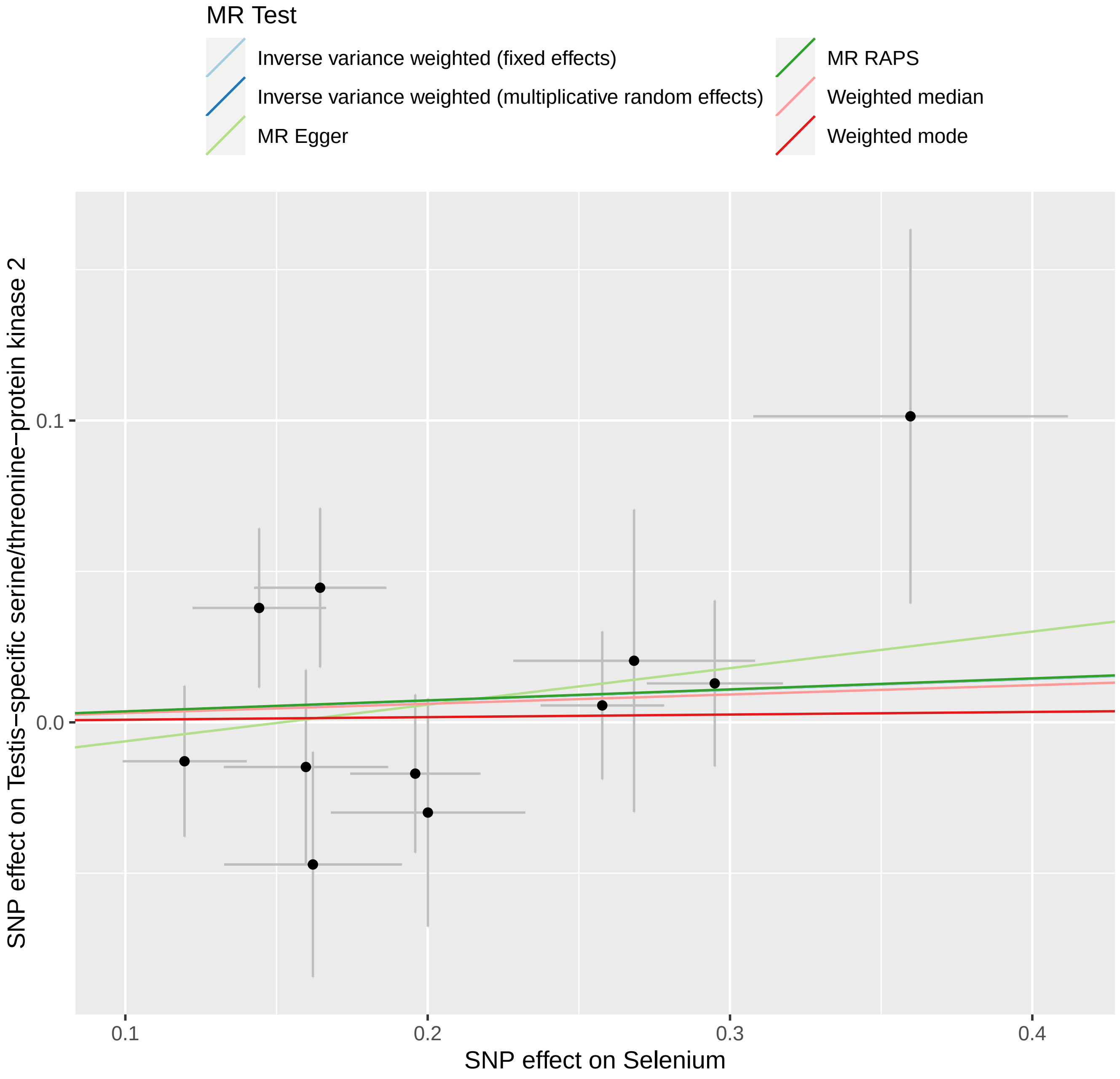

Batch 3−3109 : Selenium on Thiosulfate sulfurtransferase

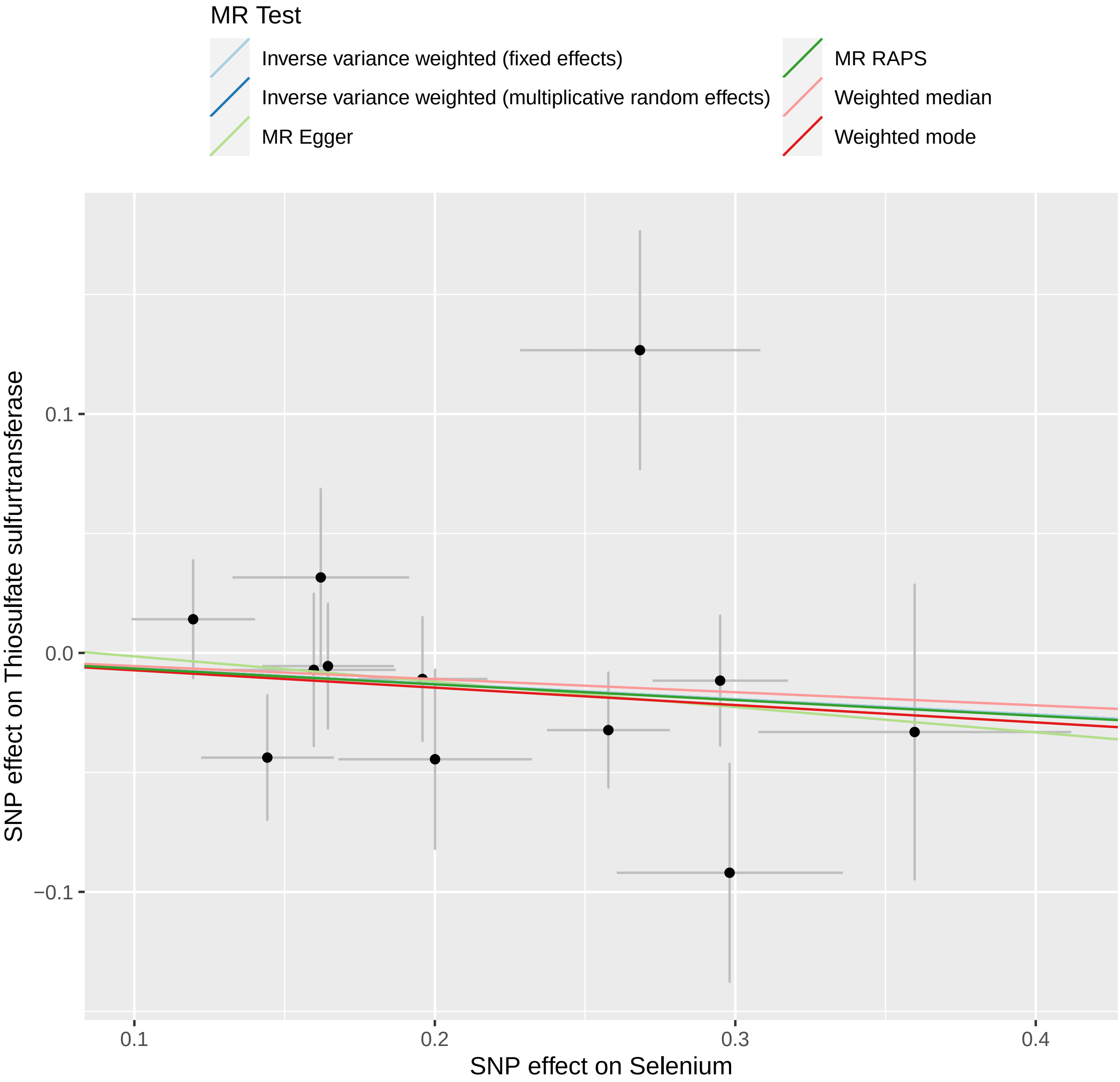

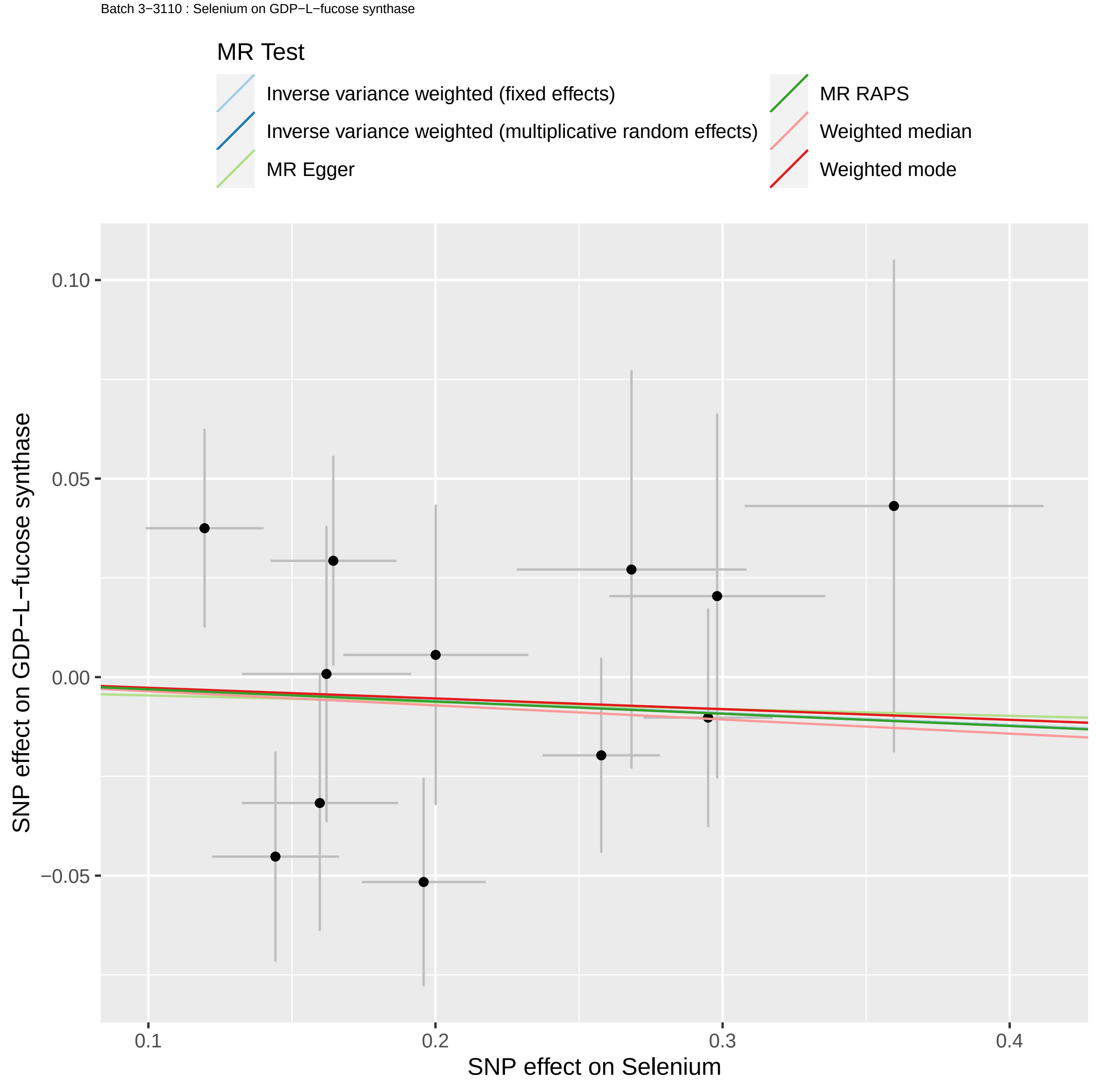
Batch 3−3110 : Selenium on GDP−L−fucose synthase
MR Test
Inverse variance weighted (fixed effects)
Inverse variance weighted (multiplicative random effects)
MR Egger
MR RAPS
Weighted median
Weighted mode
0.10
0.05
0.00
−0.05
0.1
0.2
0.3
0.4
SNP effect on GDP−L−fucose synthase
SNP effect on Selenium

Batch 3−3111 : Selenium on Tetratricopeptide repeat protein 17

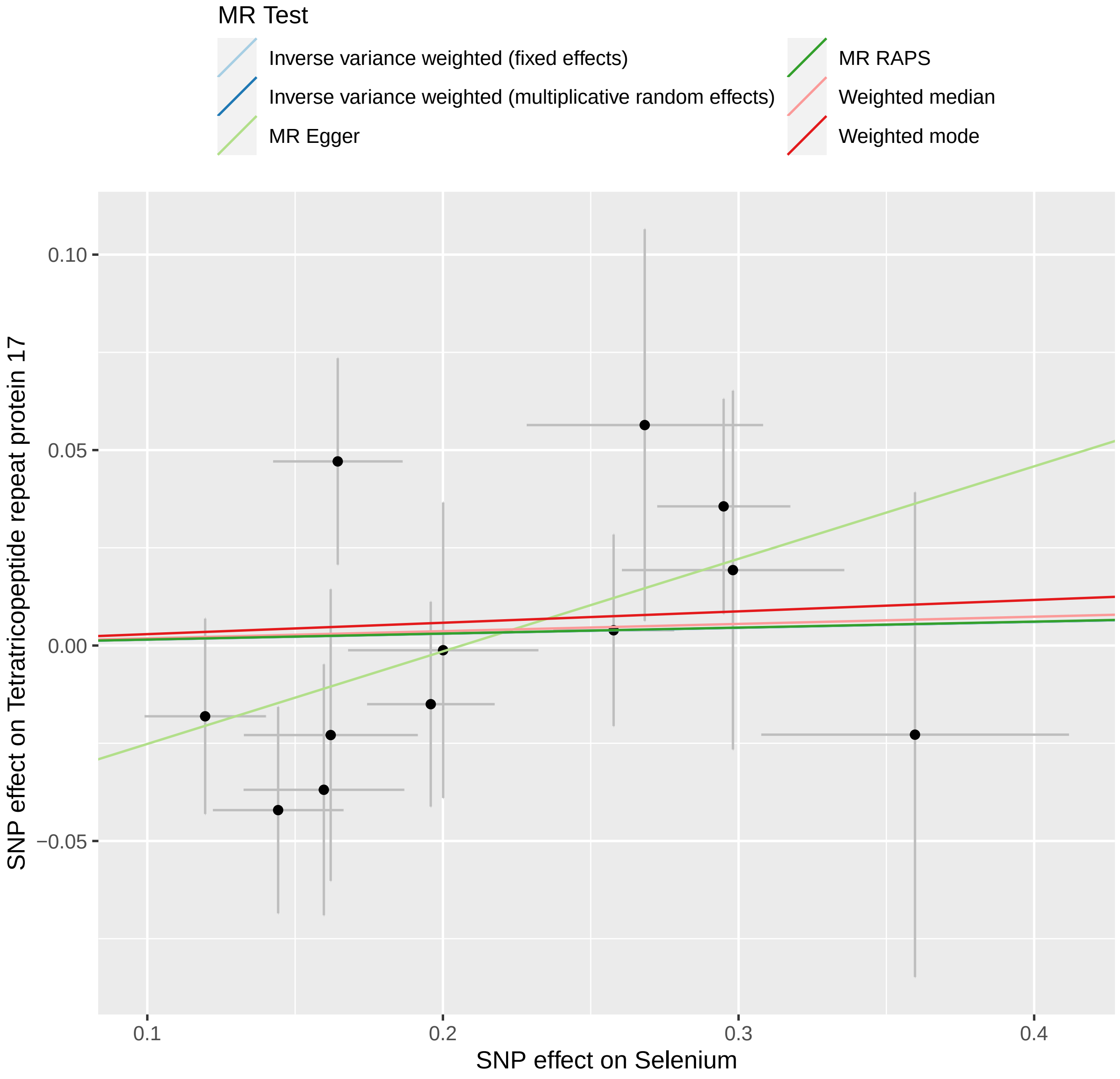

Batch 3−3112 : Selenium on Tetratricopeptide repeat protein 17

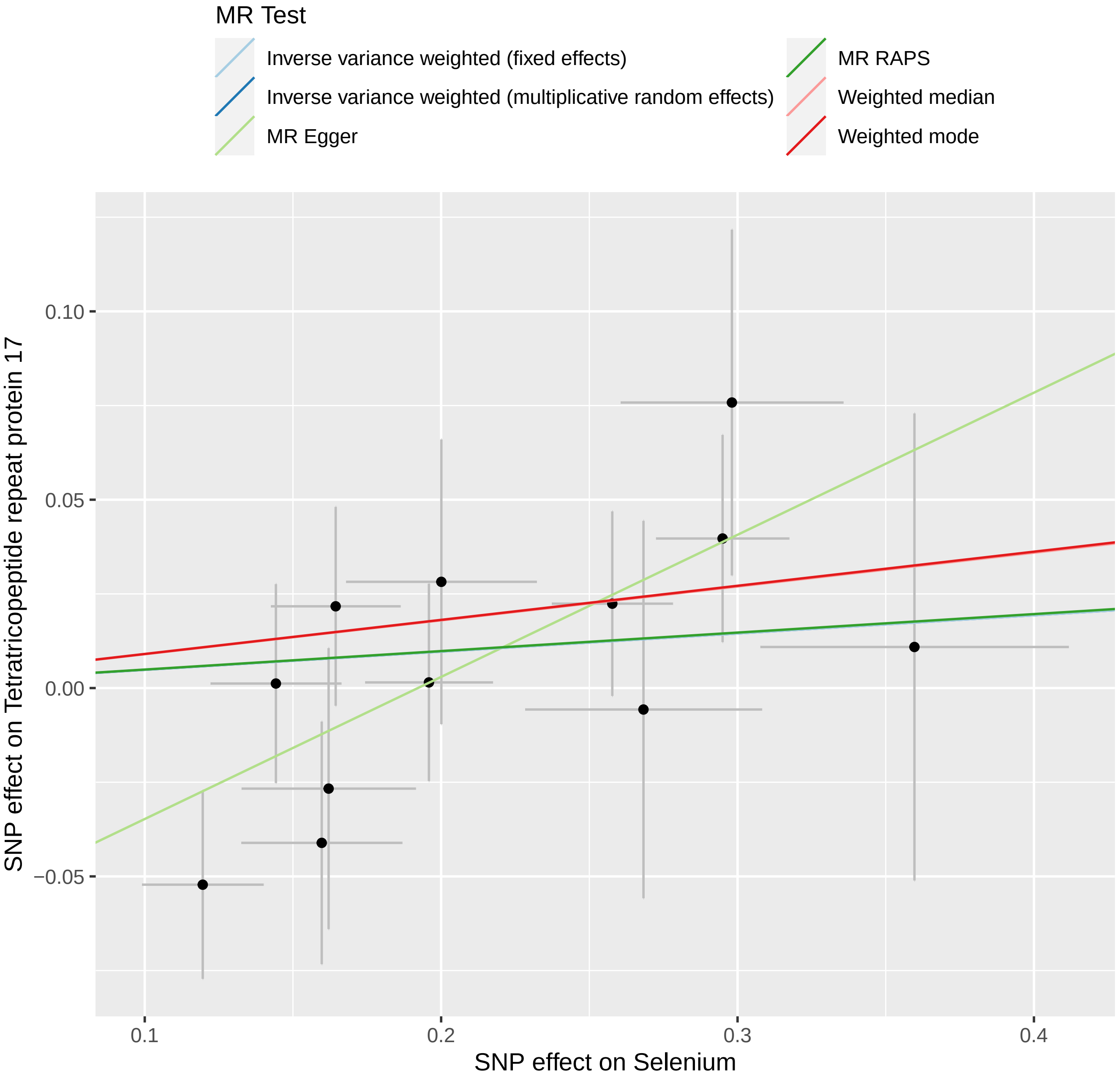

Batch 3−3113 : Selenium on Tetratricopeptide repeat protein 9B

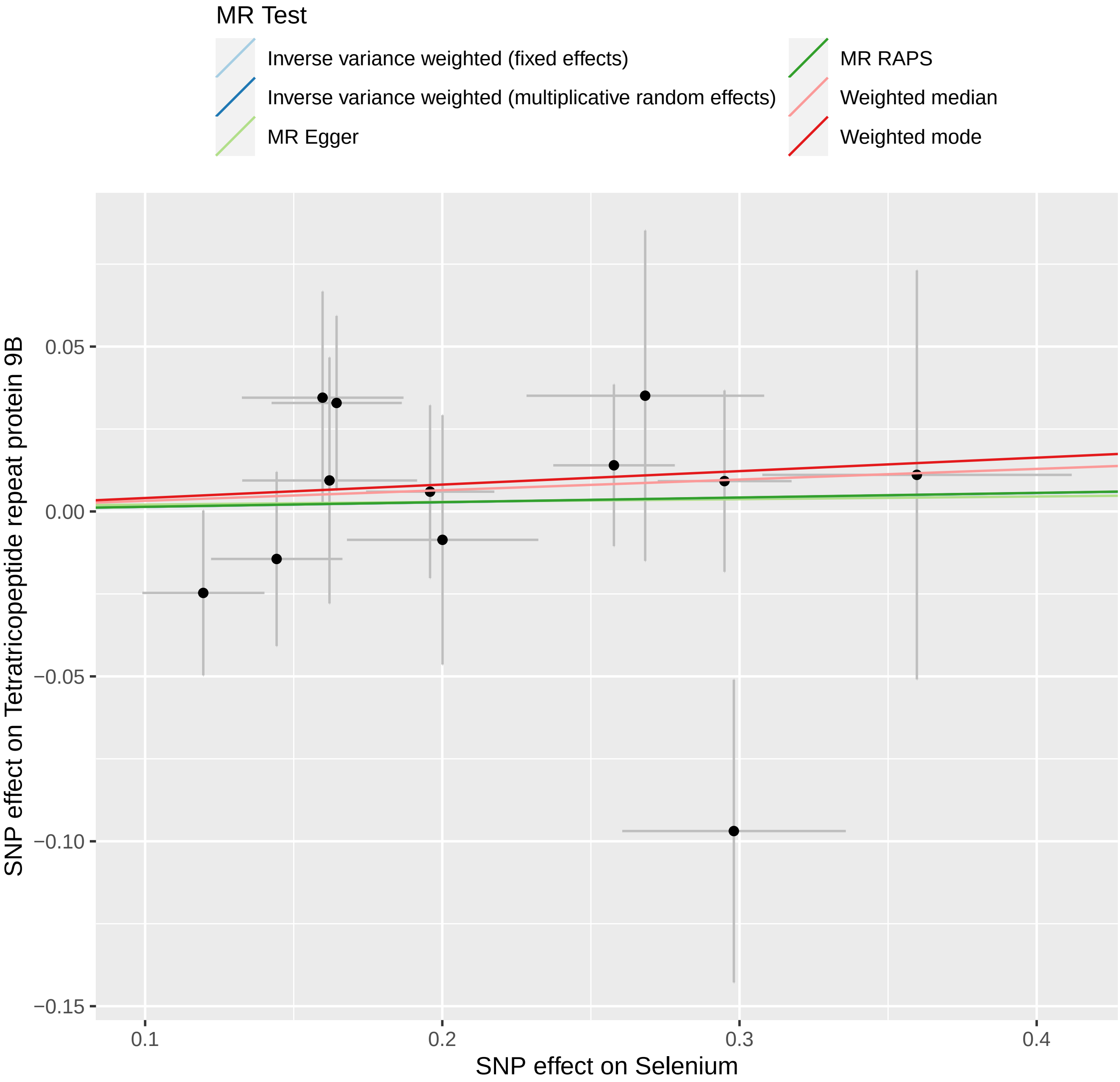

Batch 3−3114 : Selenium on Tubulin−−tyrosine ligase

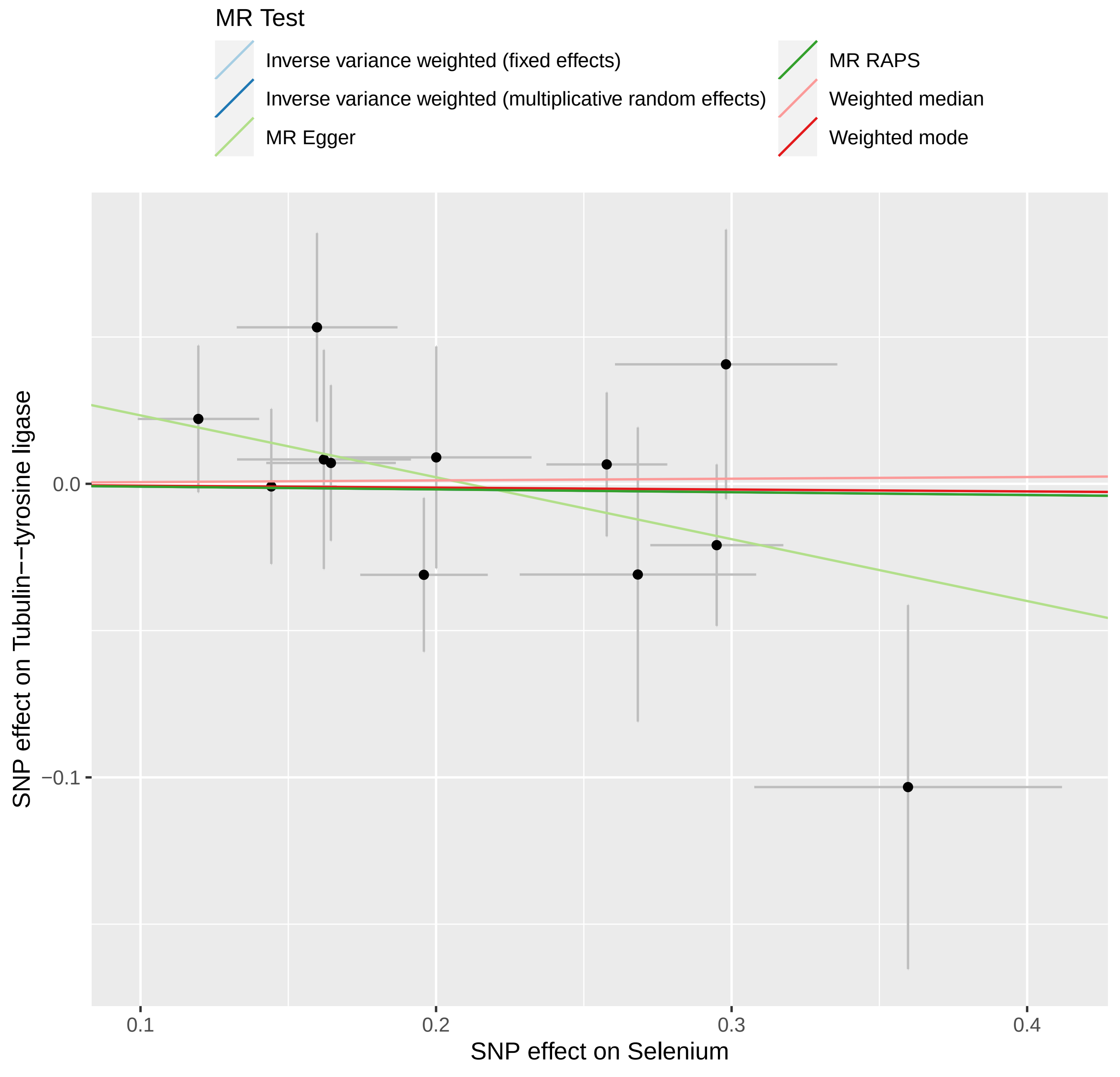

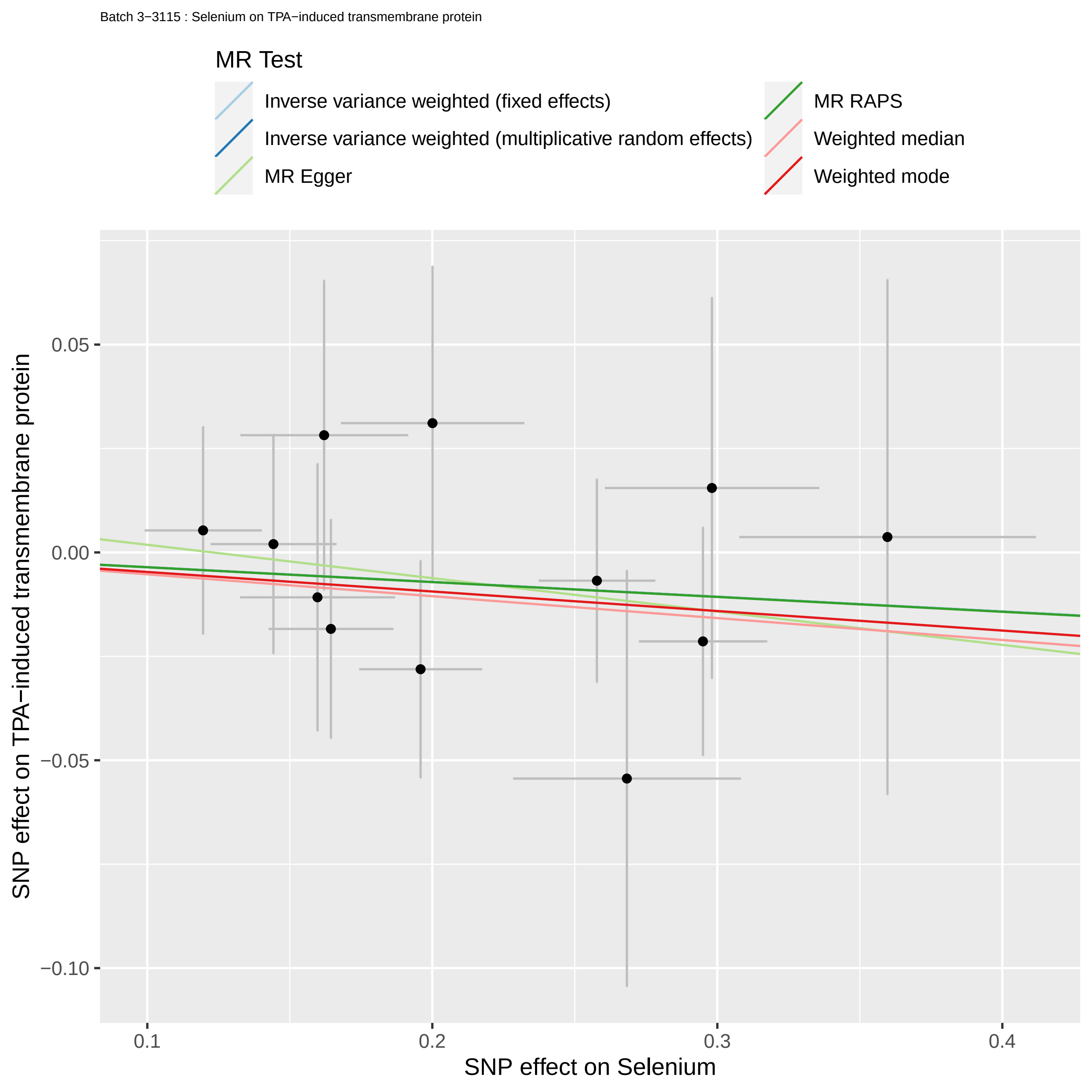
Batch 3−3115 : Selenium on TPA−induced transmembrane protein
MR Test
Inverse variance weighted (fixed effects)
Inverse variance weighted (multiplicative random effects)
MR Egger
MR RAPS
Weighted median
Weighted mode
SNP effect on TPA−induced transmembrane protein
0.05
0.00
−0.05
−0.10
0.1
0.2
0.3
0.4
SNP effect on Selenium

Batch 3−3116 : Selenium on Titin

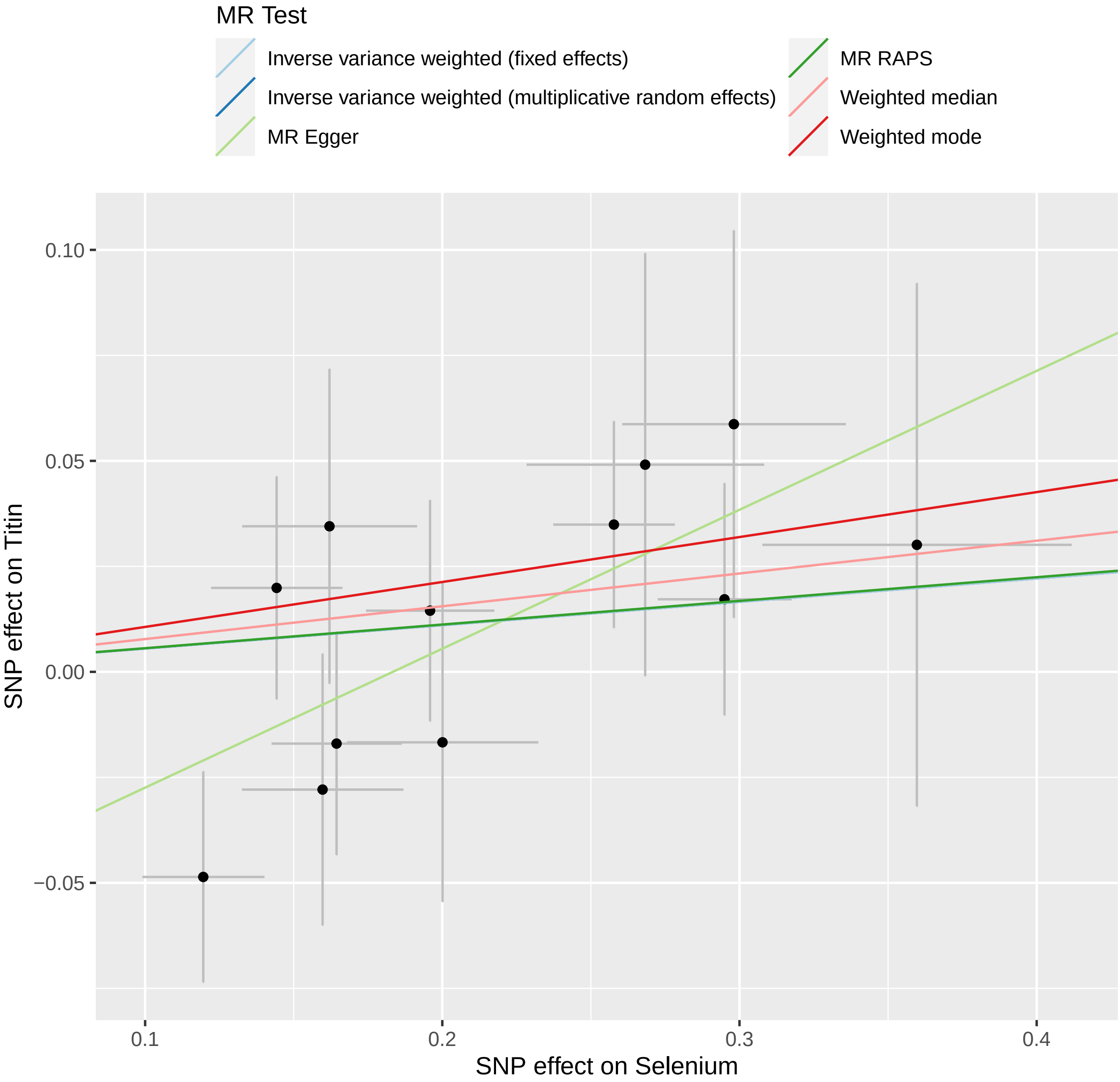

Batch 3−3117 : Selenium on Tuftelin

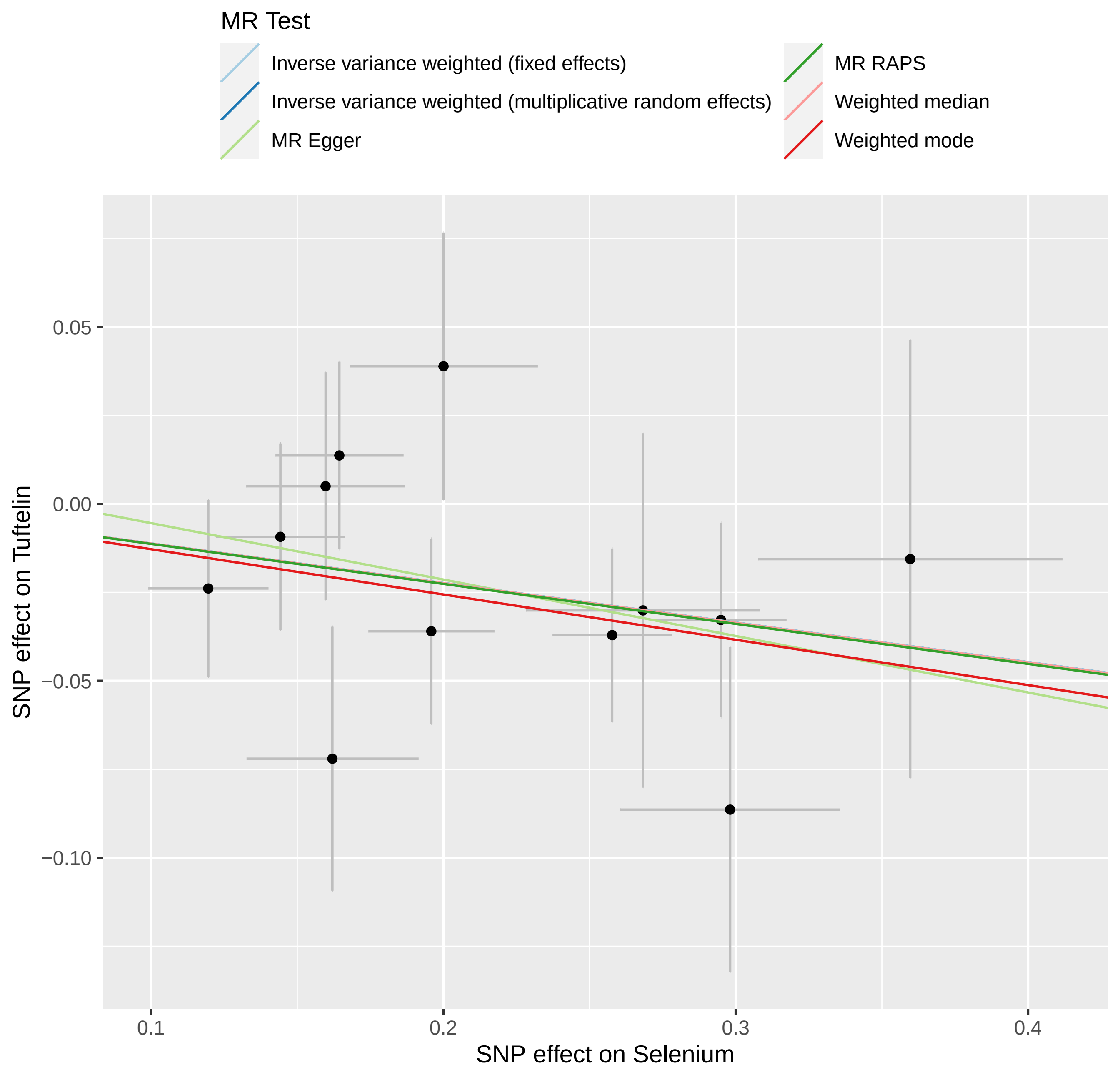

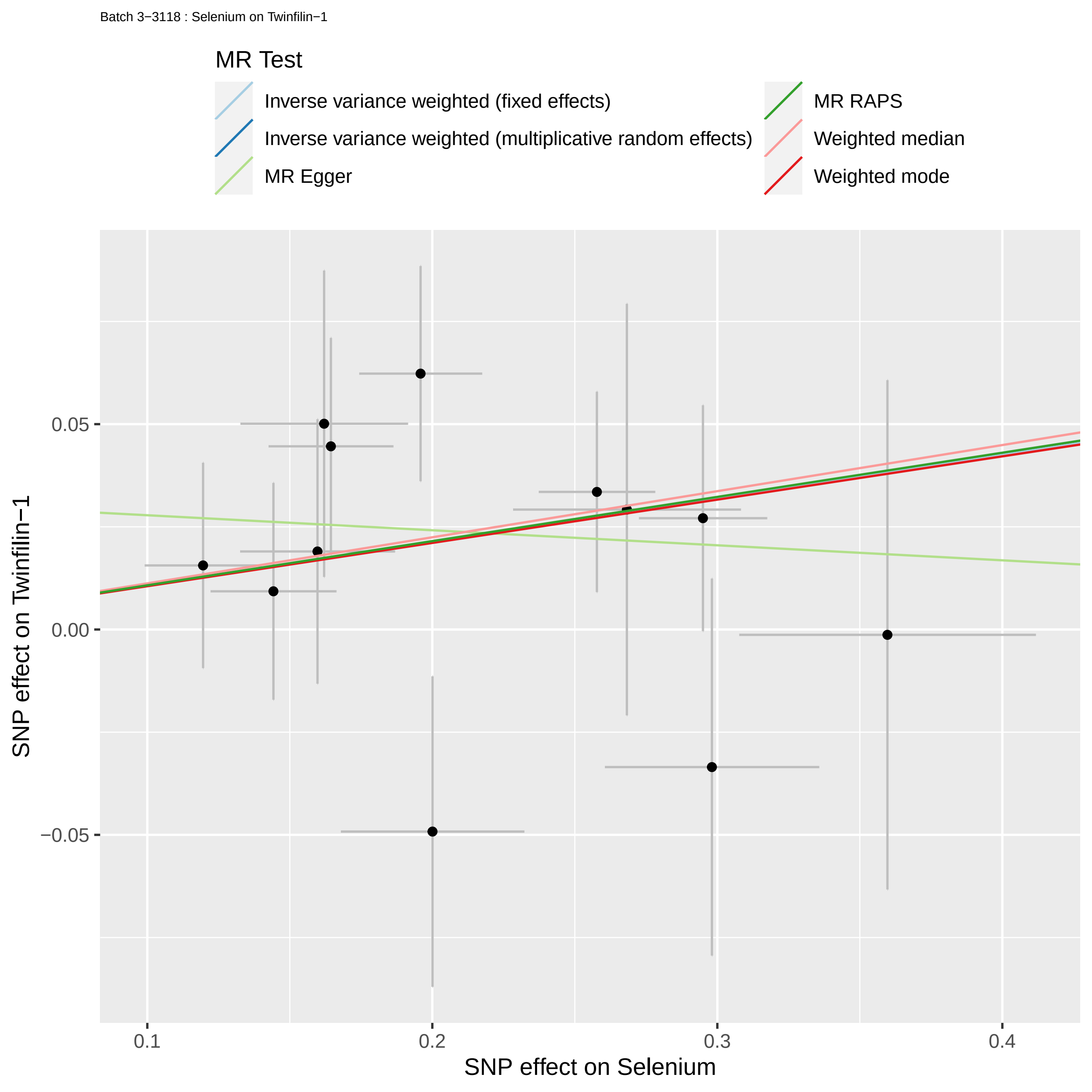
Batch 3−3118 : Selenium on Twinfilin−1
MR Test
Inverse variance weighted (fixed effects)
Inverse variance weighted (multiplicative random effects)
MR Egger
MR RAPS
Weighted median
Weighted mode
0.05
0.00
−0.05
0.1
0.2
0.3
0.4
SNP effect on Twinfilin−1
SNP effect on Selenium

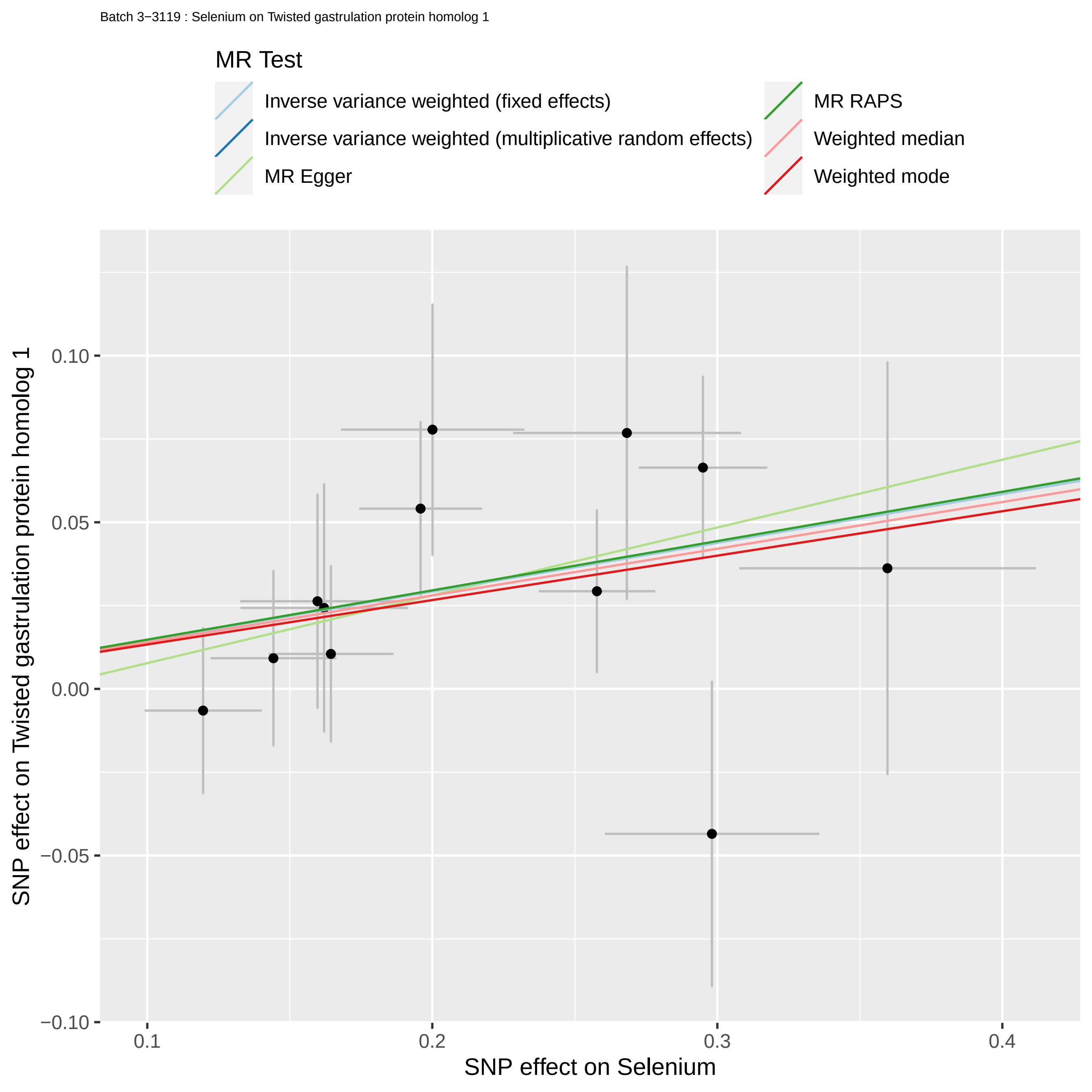

Batch 3−3119 : Selenium on Twisted gastrulation protein homolog 1
MR Test
Inverse variance weighted (fixed effects)
Inverse variance weighted (multiplicative random effects)
MR Egger
MR RAPS
Weighted median
Weighted mode
SNP effect on Twisted gastrulation protein homolog 1
0.10
0.05
0.00
−0.05
−0.10
0.1
0.2
0.3
0.4
SNP effect on Selenium

Batch 3−3120 : Selenium on Alpha−taxilin

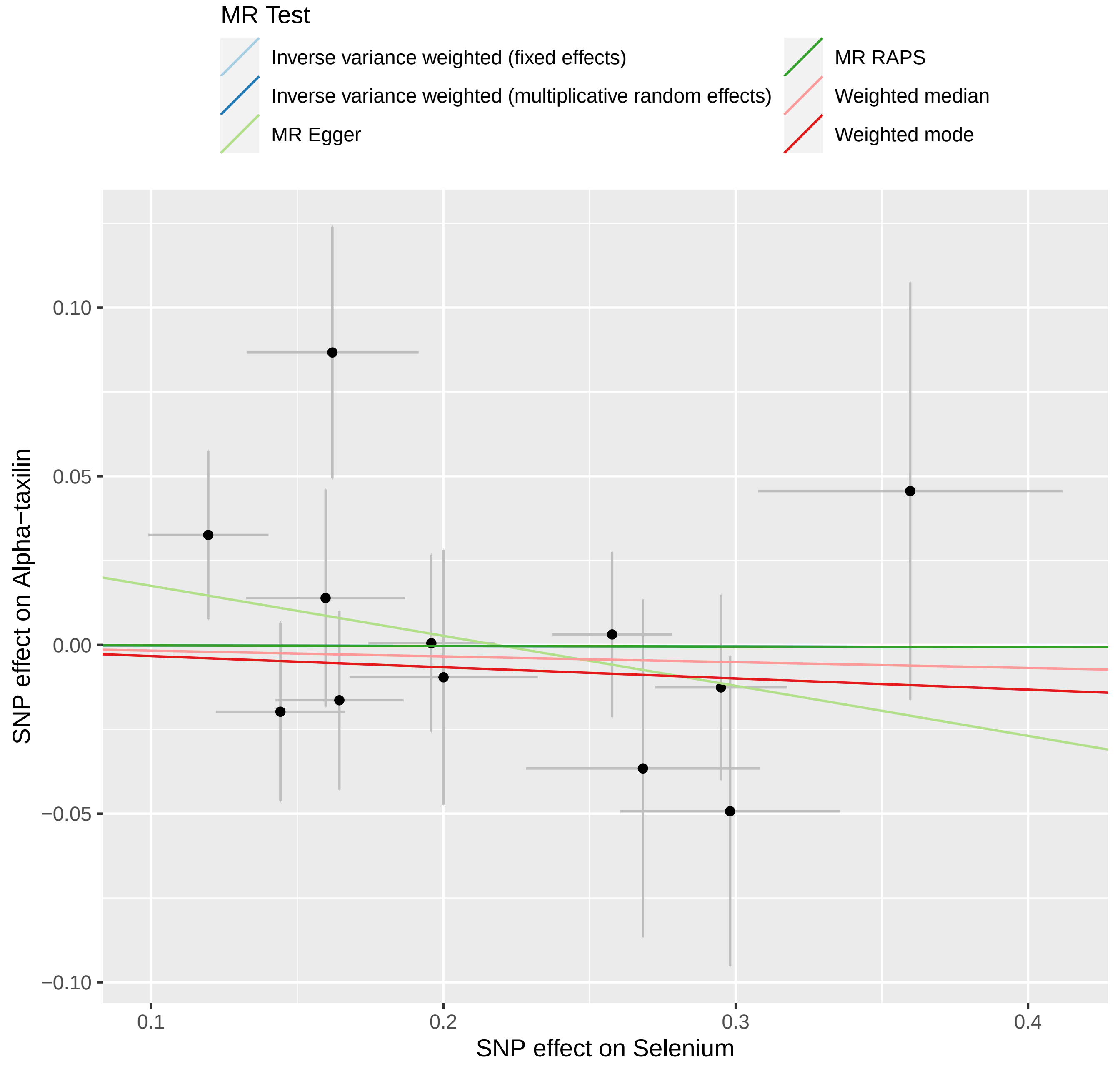

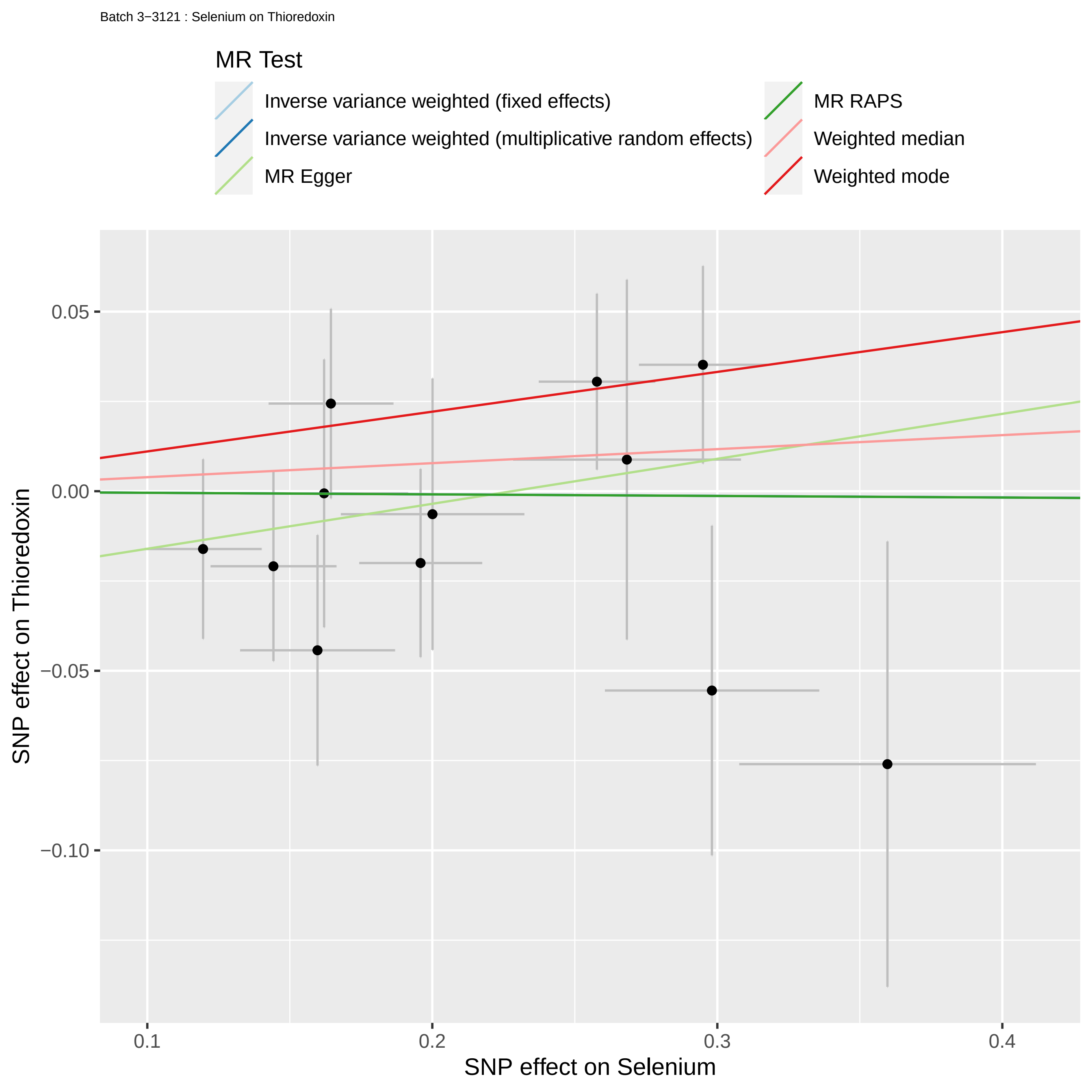
Batch 3−3121 : Selenium on Thioredoxin
MR Test
Inverse variance weighted (fixed effects)
Inverse variance weighted (multiplicative random effects)
MR Egger
MR RAPS
Weighted median
Weighted mode
0.05
0.00
−0.05
−0.10
0.1
0.2
0.3
0.4
SNP effect on Thioredoxin
SNP effect on Selenium

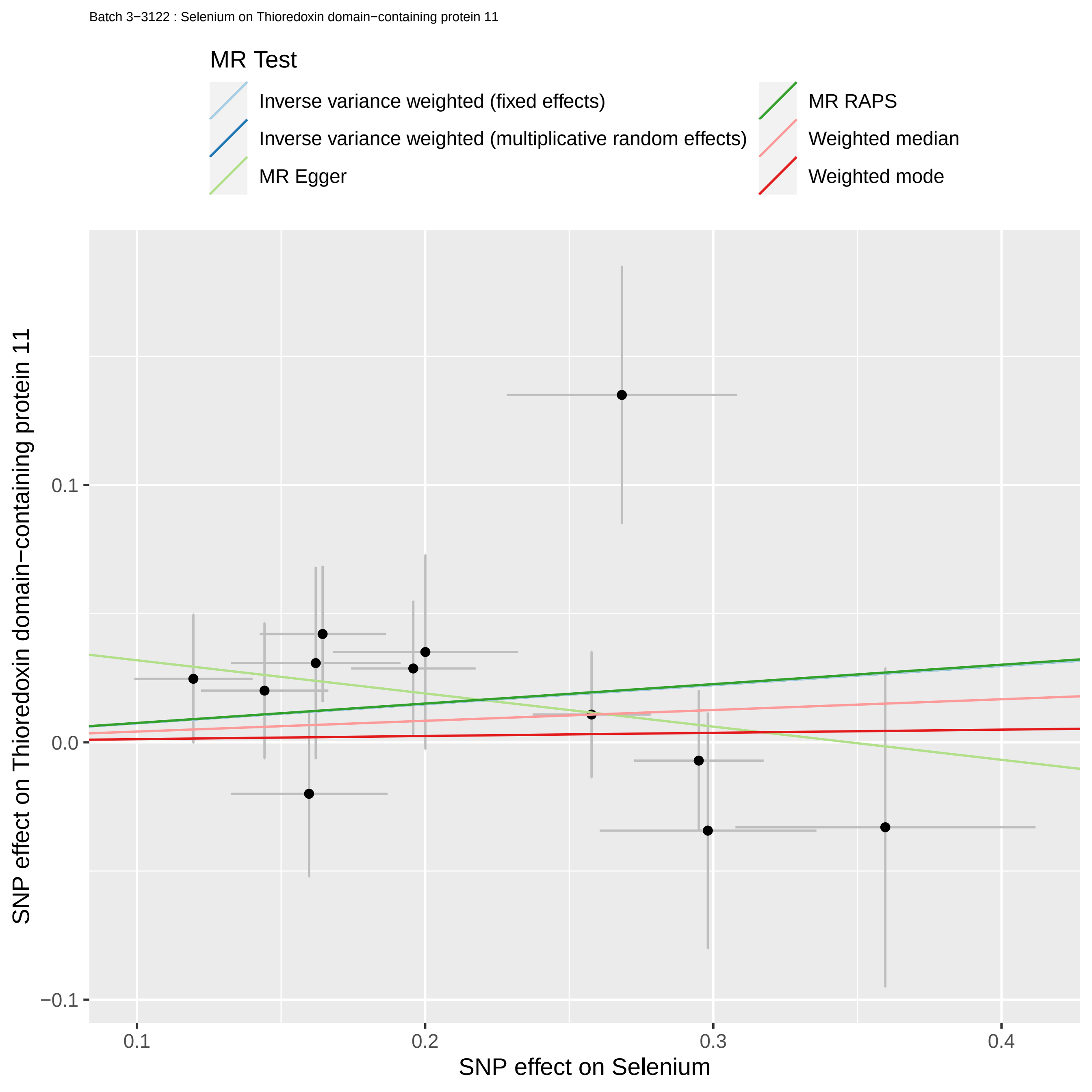

Batch 3−3122 : Selenium on Thioredoxin domain−containing protein 11
MR Test
Inverse variance weighted (fixed effects)
Inverse variance weighted (multiplicative random effects)
MR Egger
MR RAPS
Weighted median
Weighted mode
SNP effect on Thioredoxin domain−containing protein 11
0.1
0.0
−0.1
0.1
0.2
0.3
0.4
SNP effect on Selenium

Batch 3−3123 : Selenium on Thioredoxin domain−containing protein 12

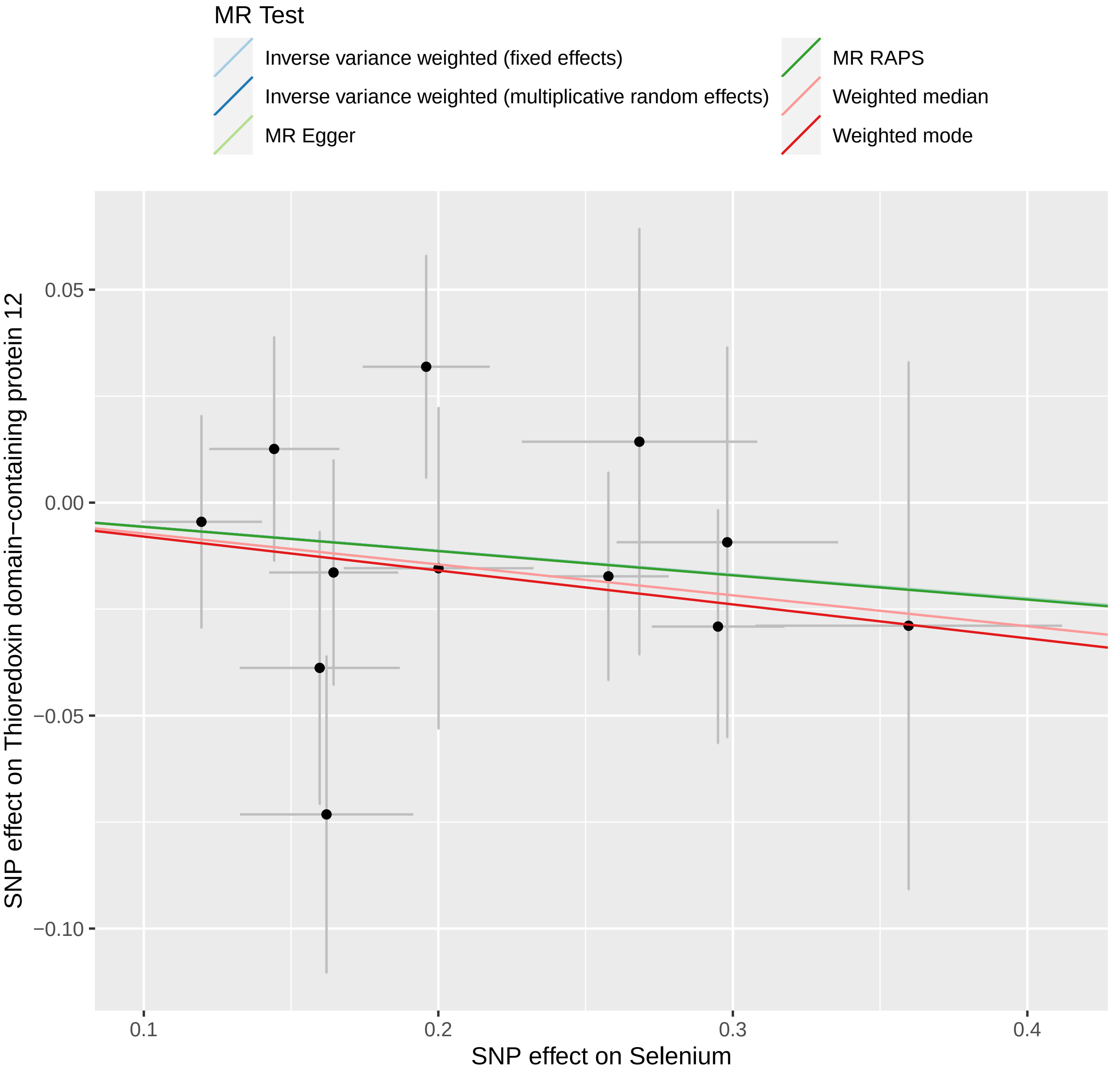

Batch 3−3124 : Selenium on Thioredoxin domain−containing protein 5

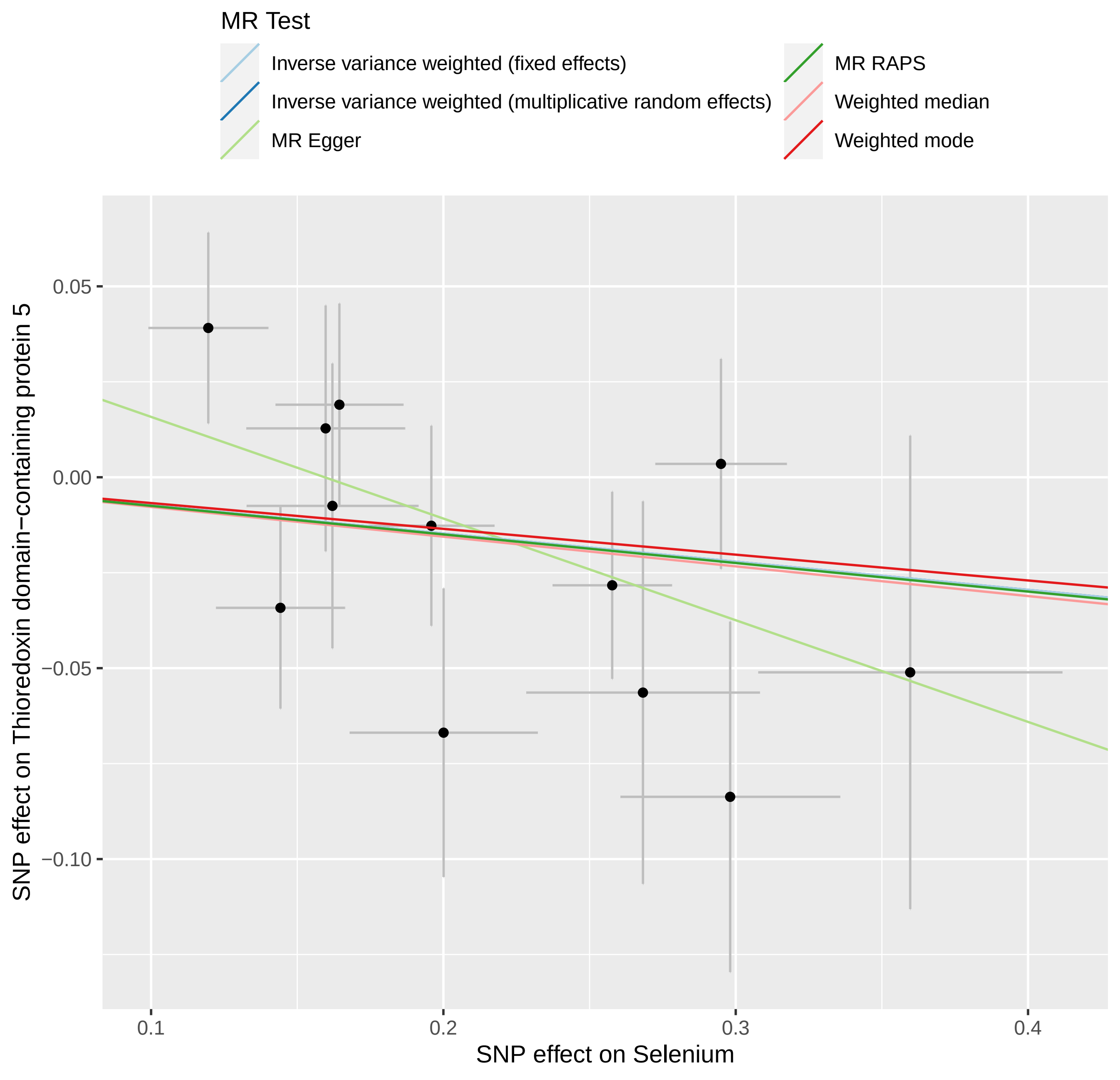

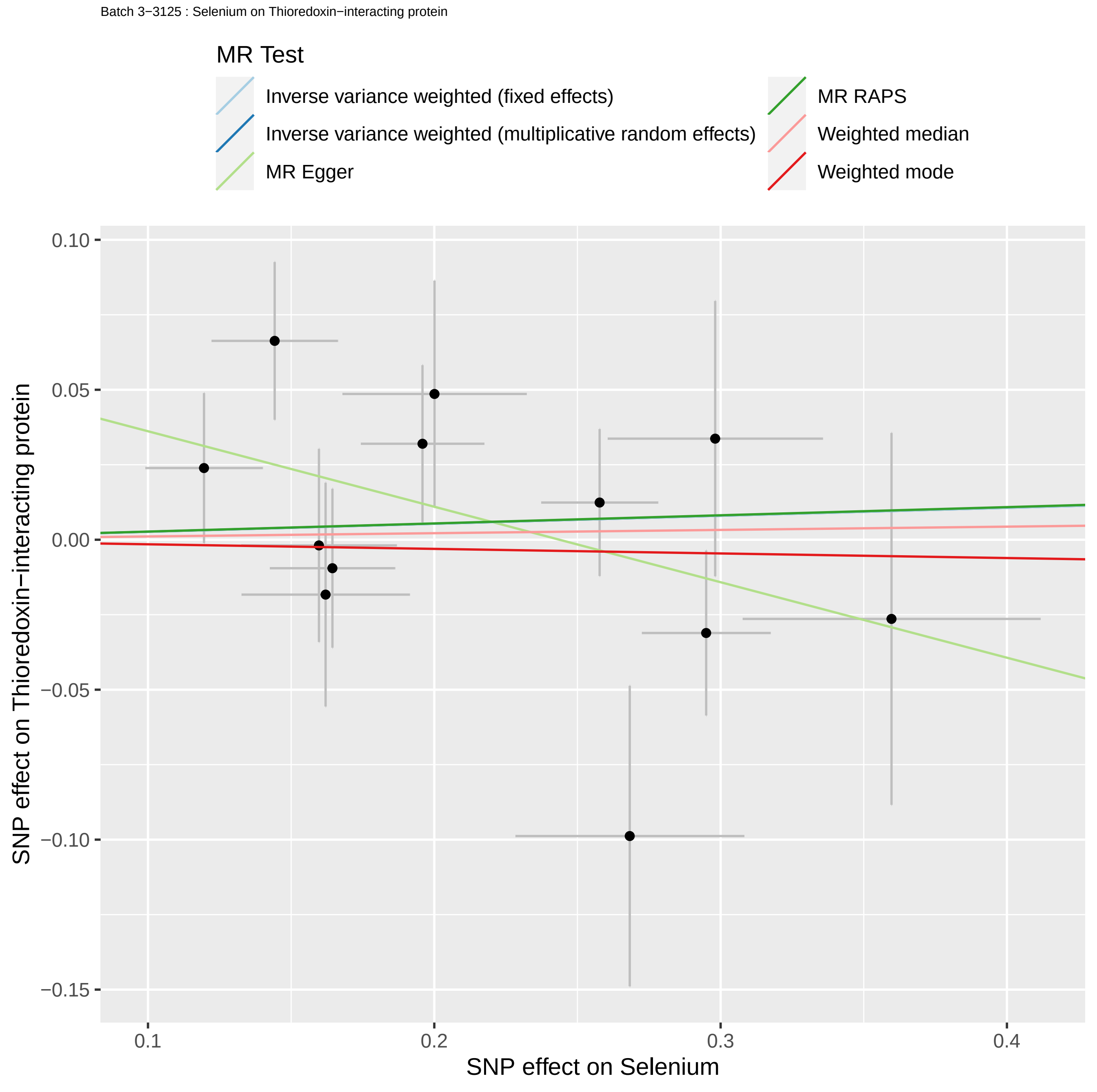

Batch 3−3125 : Selenium on Thioredoxin−interacting protein
MR Test
Inverse variance weighted (fixed effects)
Inverse variance weighted (multiplicative random effects)
MR Egger
MR RAPS
Weighted median
Weighted mode
0.10
0.05
0.00
−0.05
−0.10
−0.15
0.1
0.2
0.3
0.4
SNP effect on Thioredoxin−interacting protein
SNP effect on Selenium

Batch 3−3126 : Selenium on Thioredoxin reductase 1, cytoplasmic

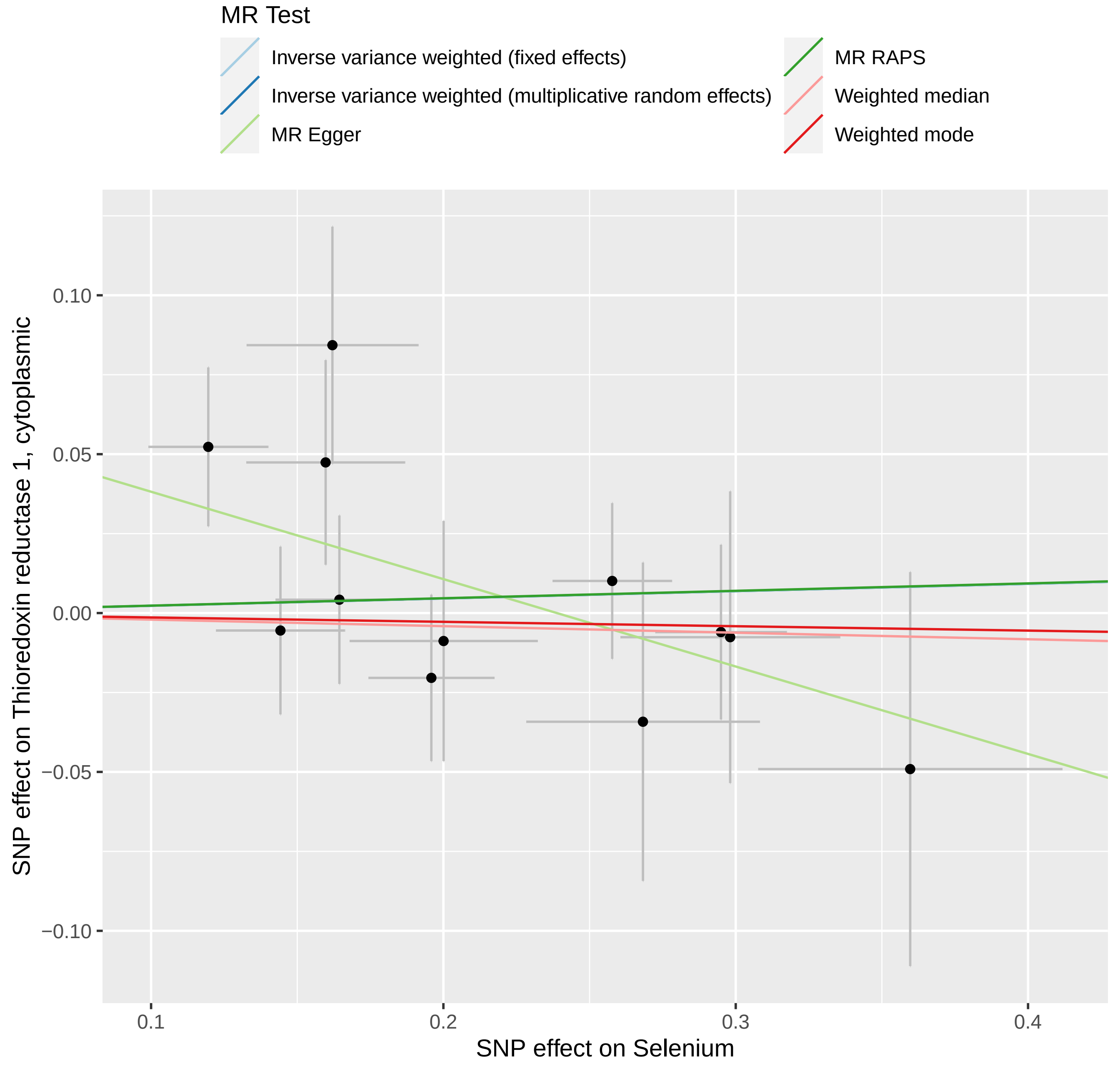

Batch 3−3127 : Selenium on Non−receptor tyrosine−protein kinase TYK2

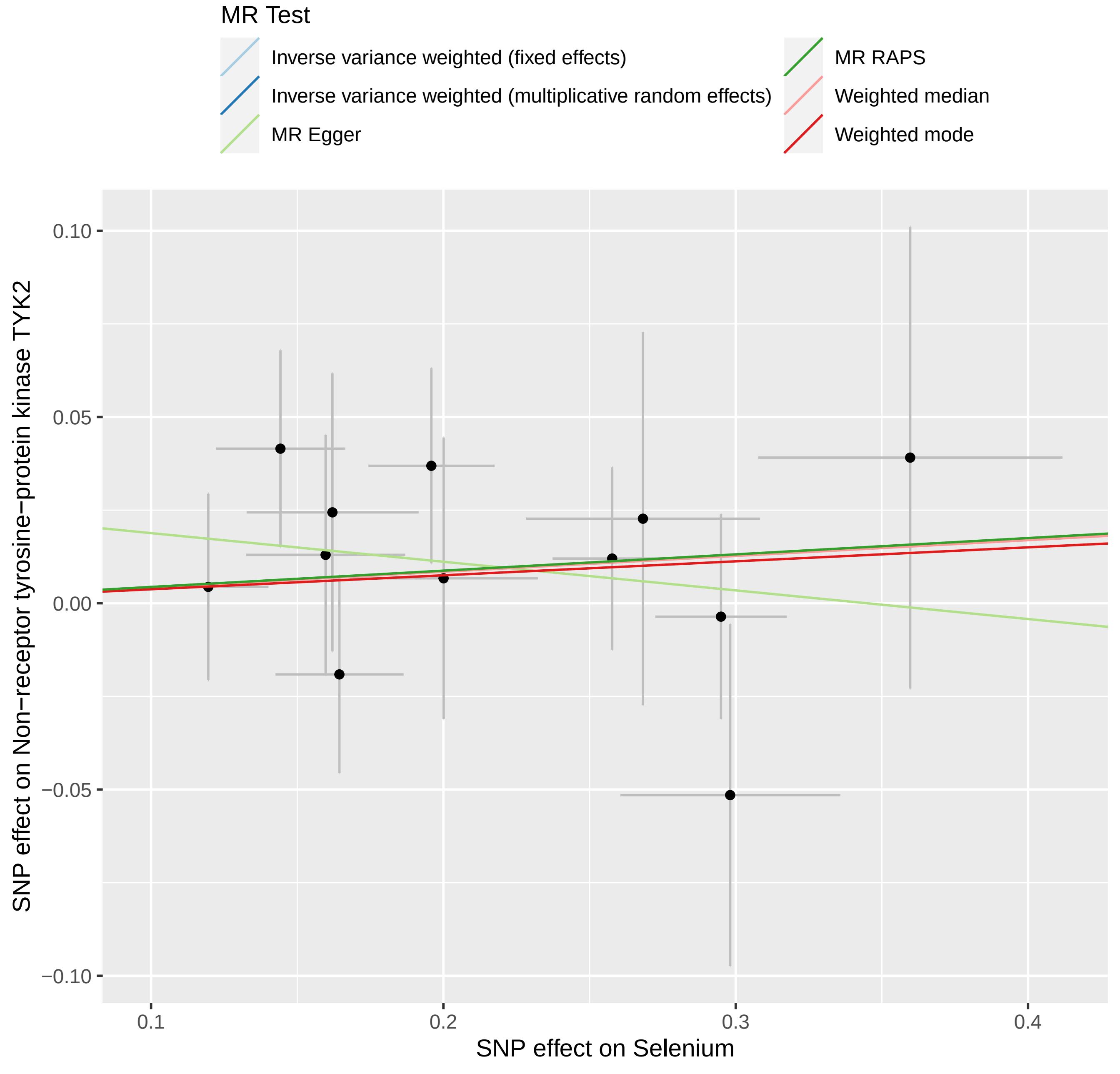

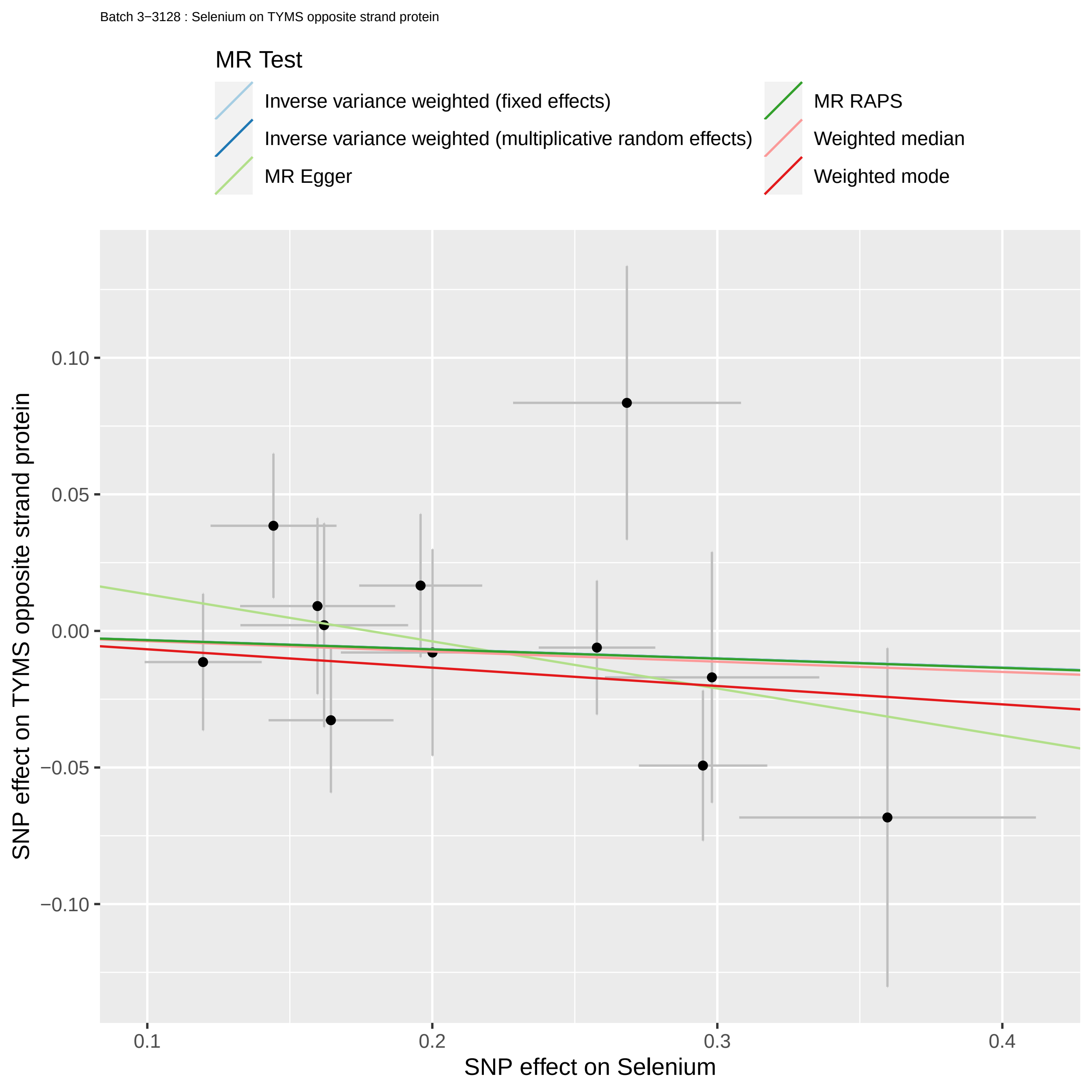
Batch 3−3128 : Selenium on TYMS opposite strand protein
MR Test
Inverse variance weighted (fixed effects)
Inverse variance weighted (multiplicative random effects)
MR Egger
MR RAPS
Weighted median
Weighted mode
SNP effect on TYMS opposite strand protein
0.10
0.05
0.00
−0.05
−0.10
0.1
0.2
0.3
0.4
SNP effect on Selenium

Batch 3−3129 : Selenium on Tyrosine−protein kinase receptor TYRO3

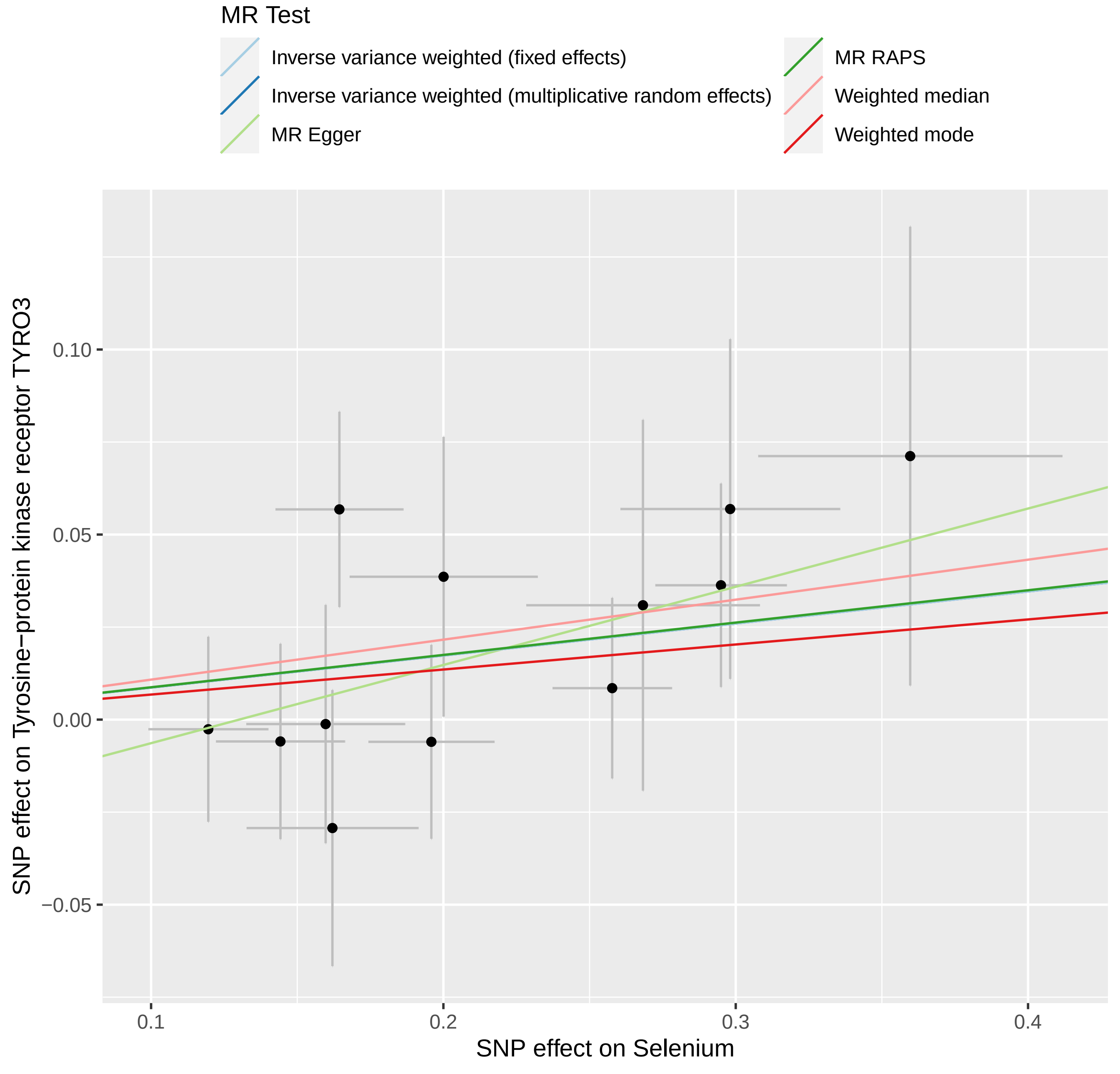

Batch 3−3130 : Selenium on Splicing factor U2AF 65 kDa subunit

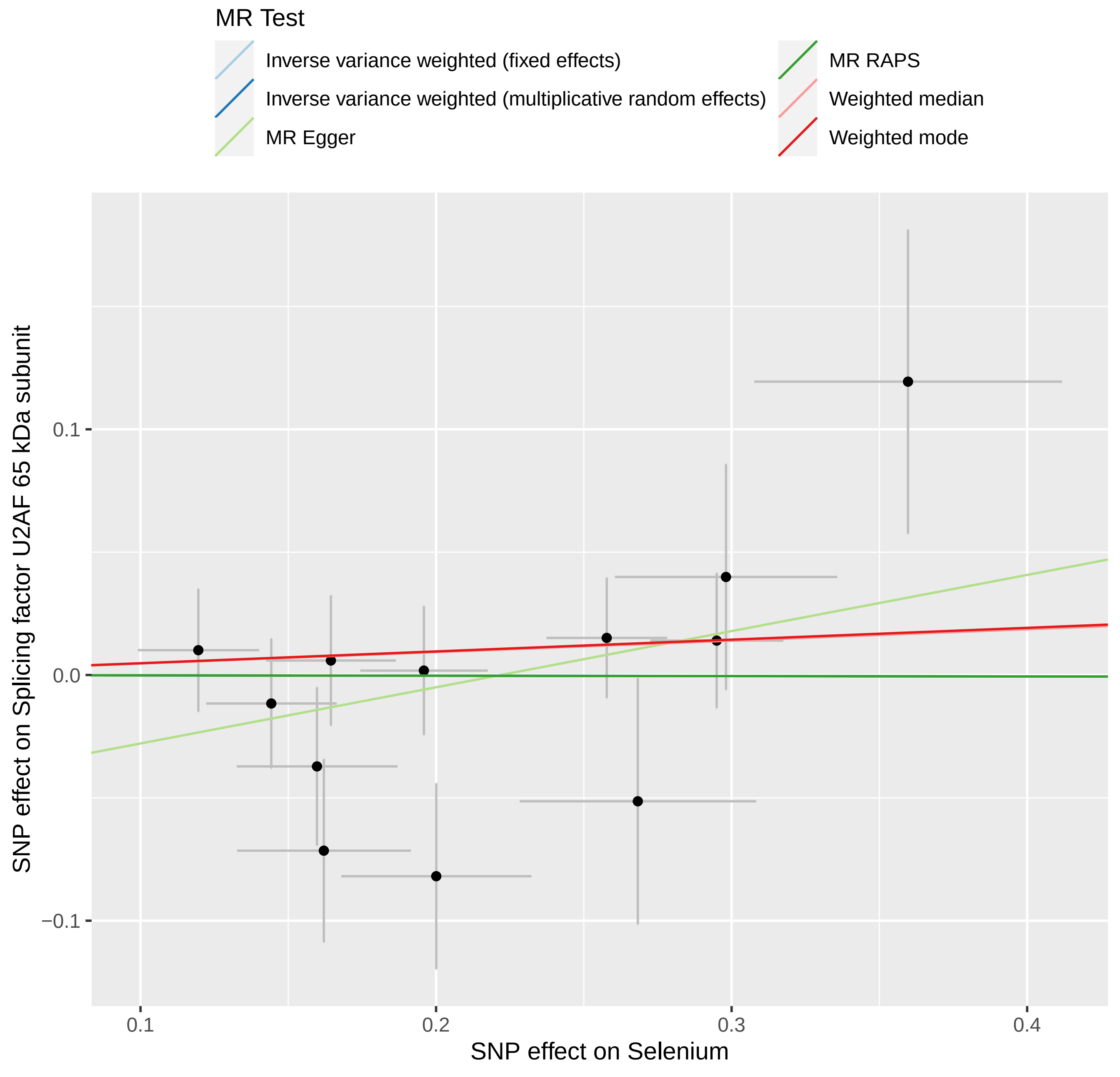

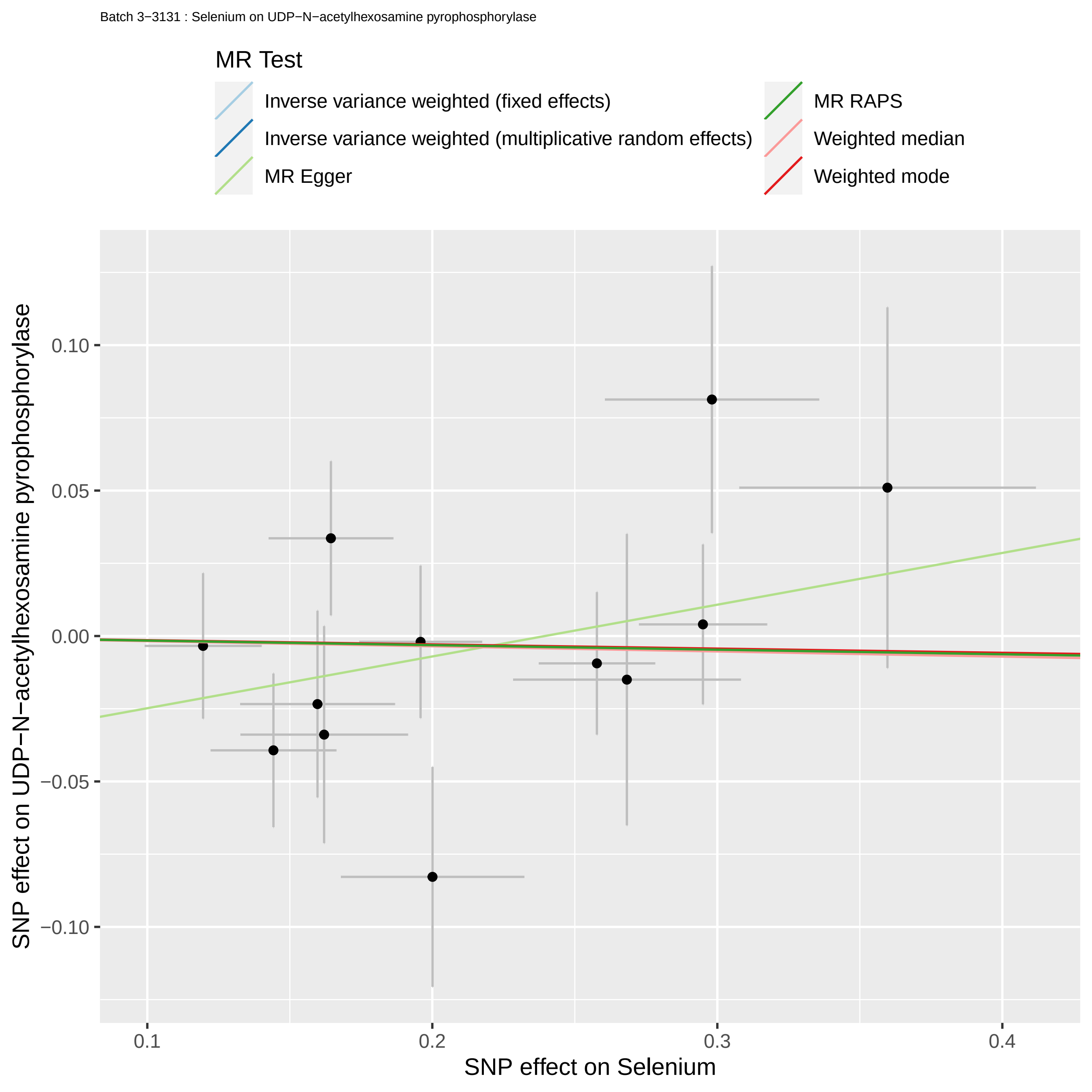

Batch 3−3131 : Selenium on UDP−N−acetylhexosamine pyrophosphorylase
MR Test
Inverse variance weighted (fixed effects)
Inverse variance weighted (multiplicative random effects)
MR Egger
MR RAPS
Weighted median
Weighted mode
0.10
0.05
0.00
−0.05
−0.10
0.1
0.2
0.3
0.4
SNP effect on Selenium
SNP effect on UDP−N−acetylhexosamine pyrophosphorylase

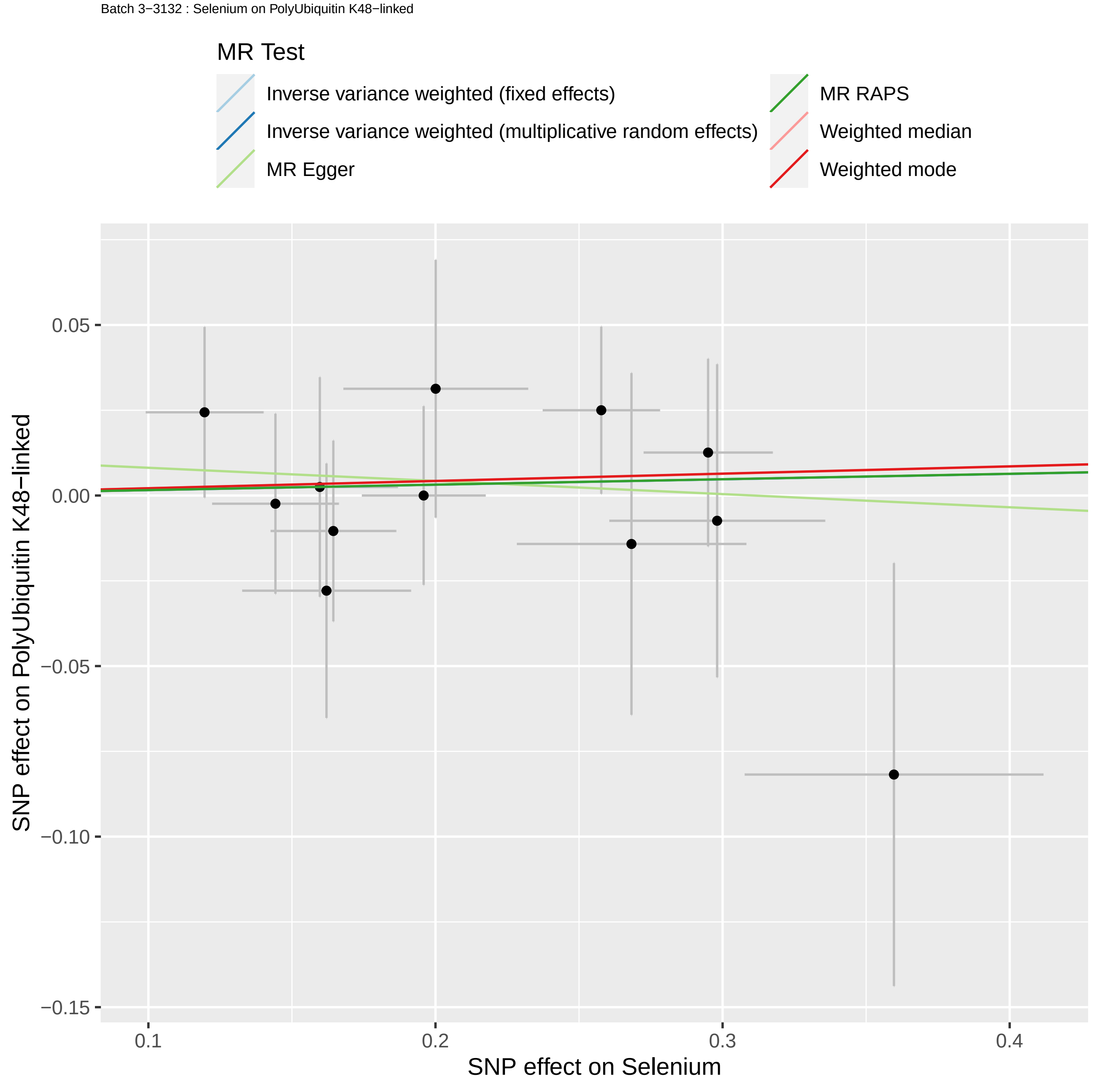
Batch 3−3132 : Selenium on PolyUbiquitin K48−linked
MR Test
Inverse variance weighted (fixed effects)
Inverse variance weighted (multiplicative random effects)
MR Egger
MR RAPS
Weighted median
Weighted mode
0.05
0.00
−0.05
−0.10
−0.15
0.1
0.2
0.3
0.4
SNP effect on PolyUbiquitin K48−linked
SNP effect on Selenium

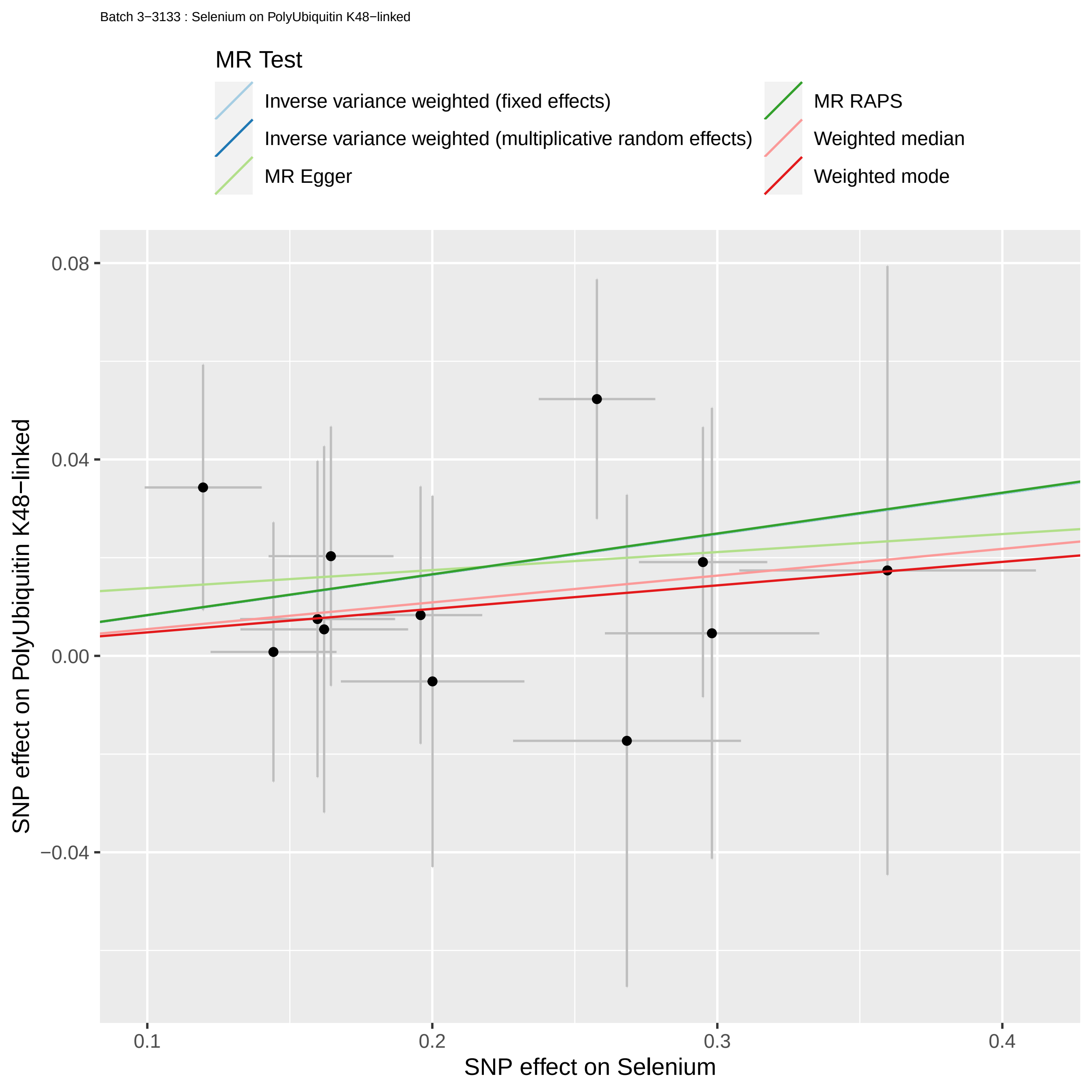
Batch 3−3133 : Selenium on PolyUbiquitin K48−linked
MR Test
Inverse variance weighted (fixed effects)
Inverse variance weighted (multiplicative random effects)
MR Egger
MR RAPS
Weighted median
Weighted mode
0.08
0.04
0.00
−0.04
0.1
0.2
0.3
0.4
SNP effect on PolyUbiquitin K48−linked
SNP effect on Selenium

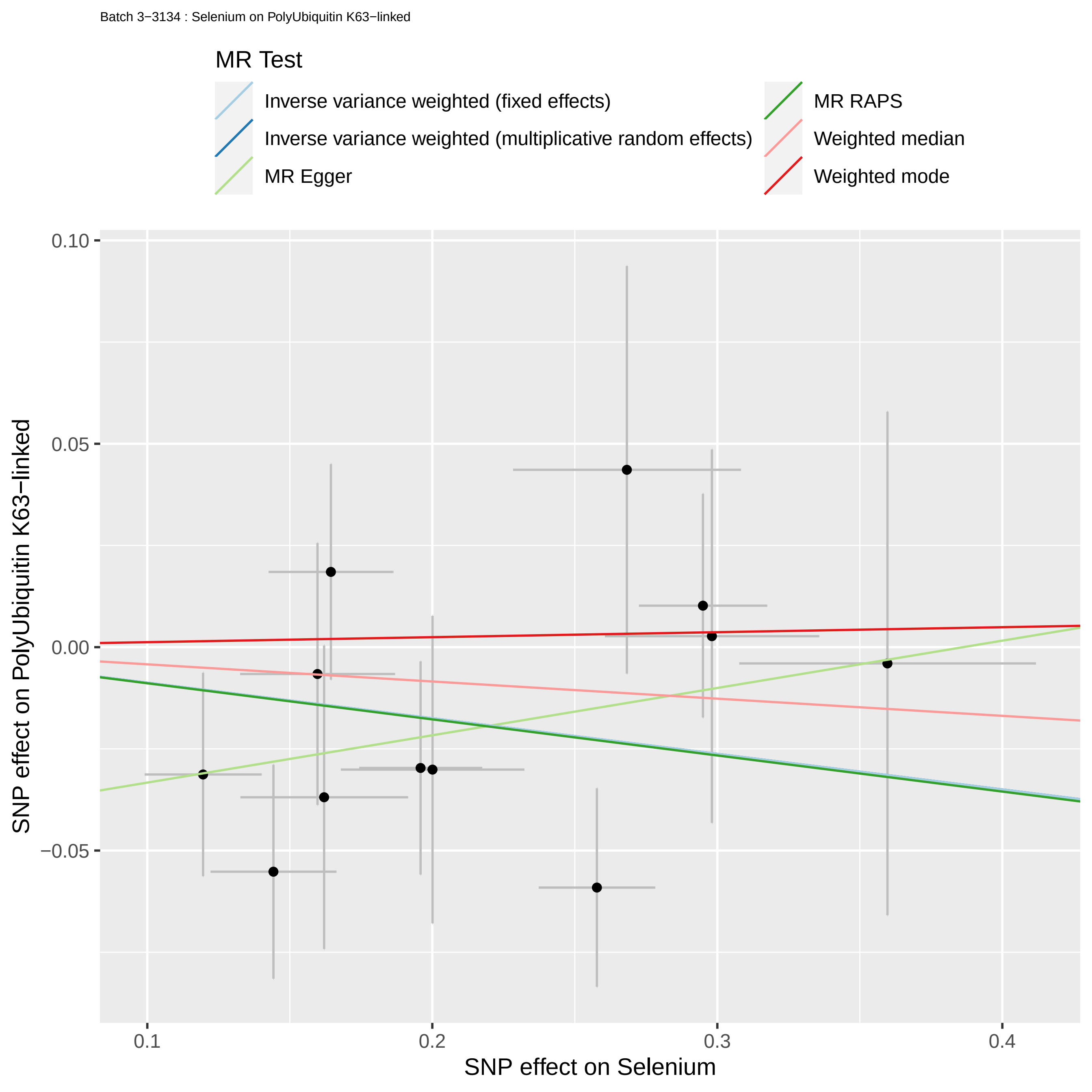
Batch 3−3134 : Selenium on PolyUbiquitin K63−linked
MR Test
Inverse variance weighted (fixed effects)
Inverse variance weighted (multiplicative random effects)
MR Egger
MR RAPS
Weighted median
Weighted mode
0.10
0.05
0.00
−0.05
0.1
0.2
0.3
0.4
SNP effect on Selenium
SNP effect on PolyUbiquitin K63−linked

Batch 3−3135 : Selenium on Ubiquitin−conjugating enzyme E2 B

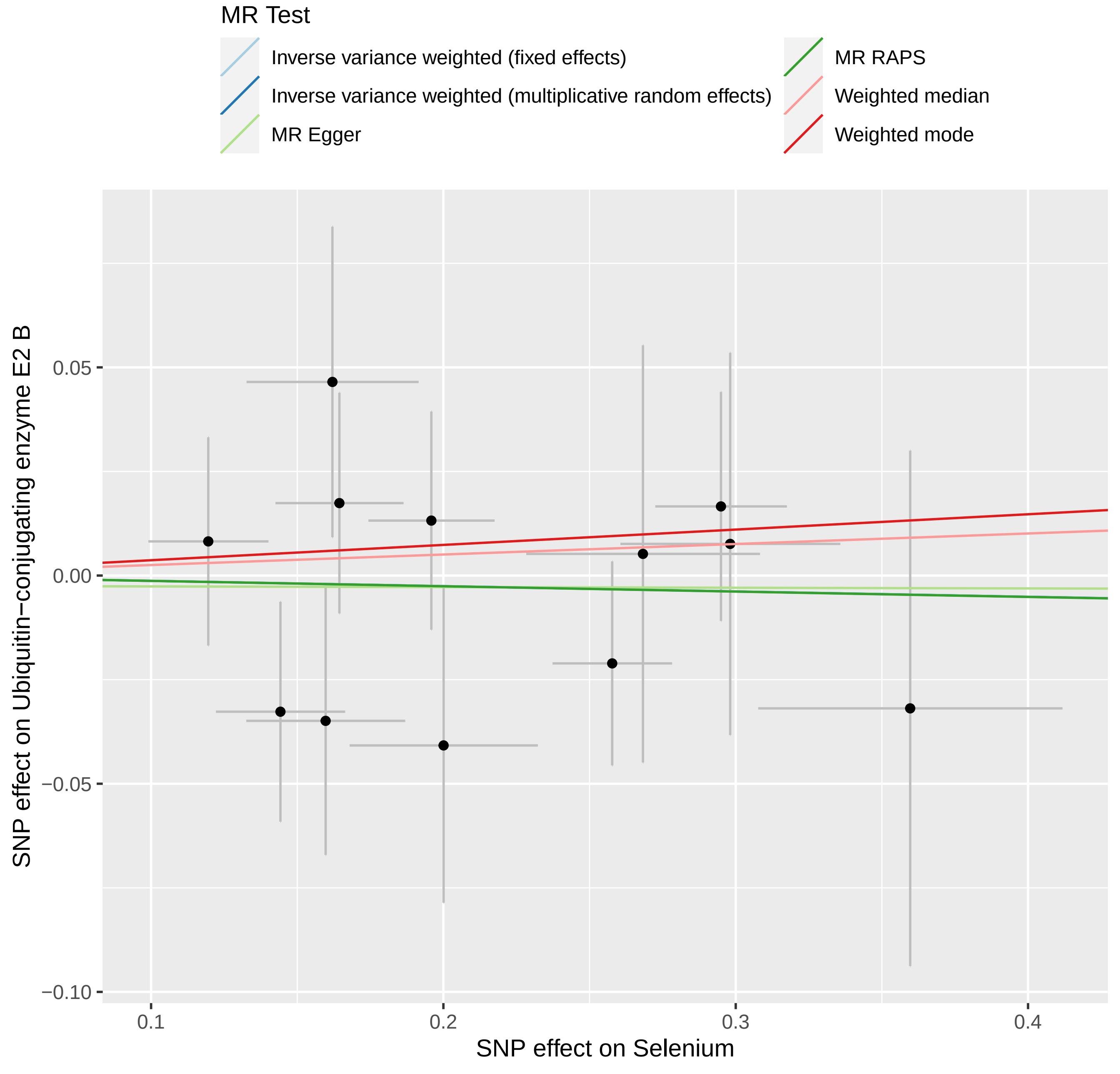

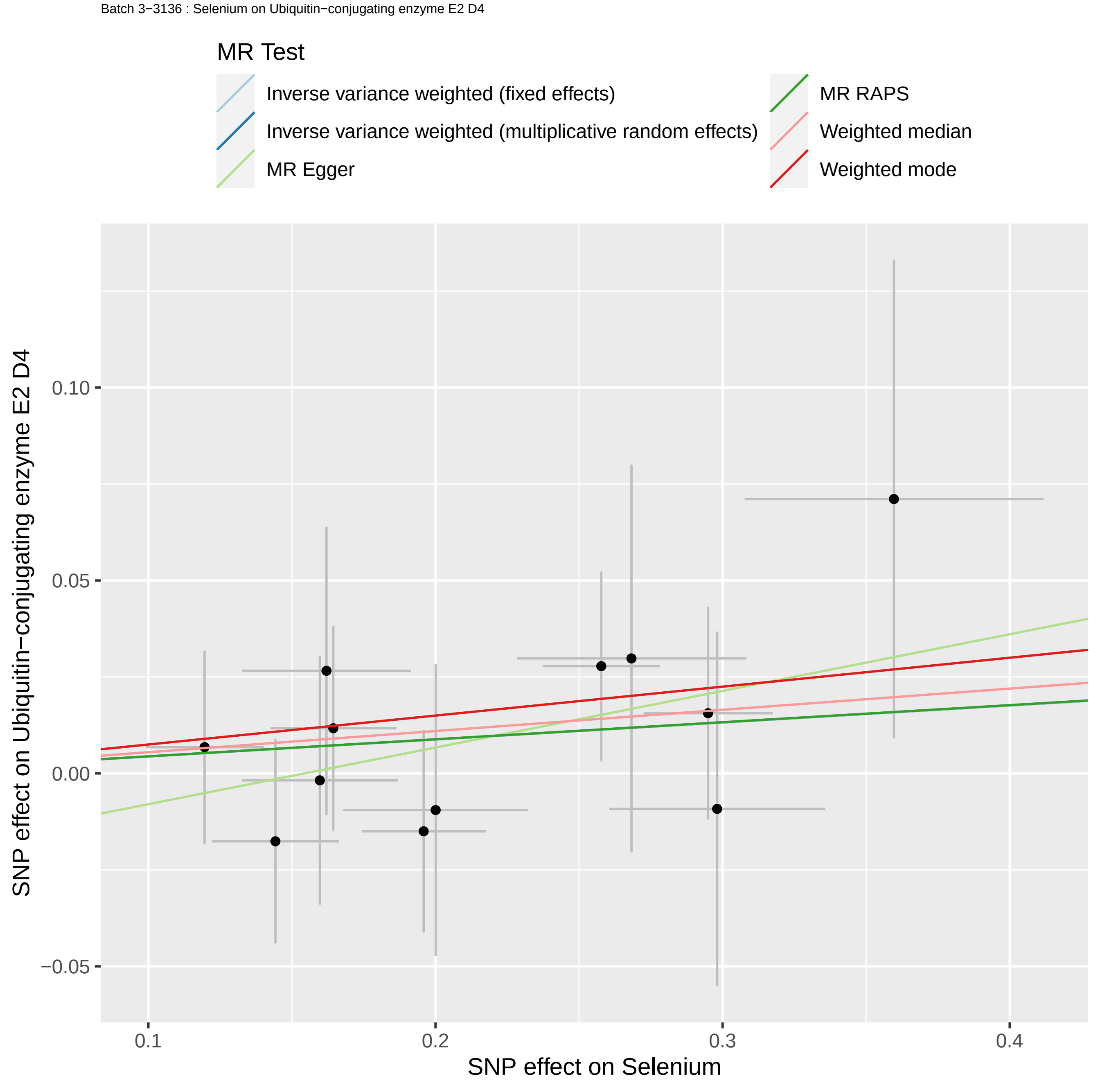

Batch 3−3136 : Selenium on Ubiquitin−conjugating enzyme E2 D4
MR Test
Inverse variance weighted (fixed effects)
Inverse variance weighted (multiplicative random effects)
MR Egger
MR RAPS
Weighted median
Weighted mode
0.10
0.05
0.00
−0.05
0.1
0.2
0.3
0.4
SNP effect on Selenium
SNP effect on Ubiquitin−conjugating enzyme E2 D4

Batch 3−3137 : Selenium on Ubiquitin−conjugating enzyme E2 G2

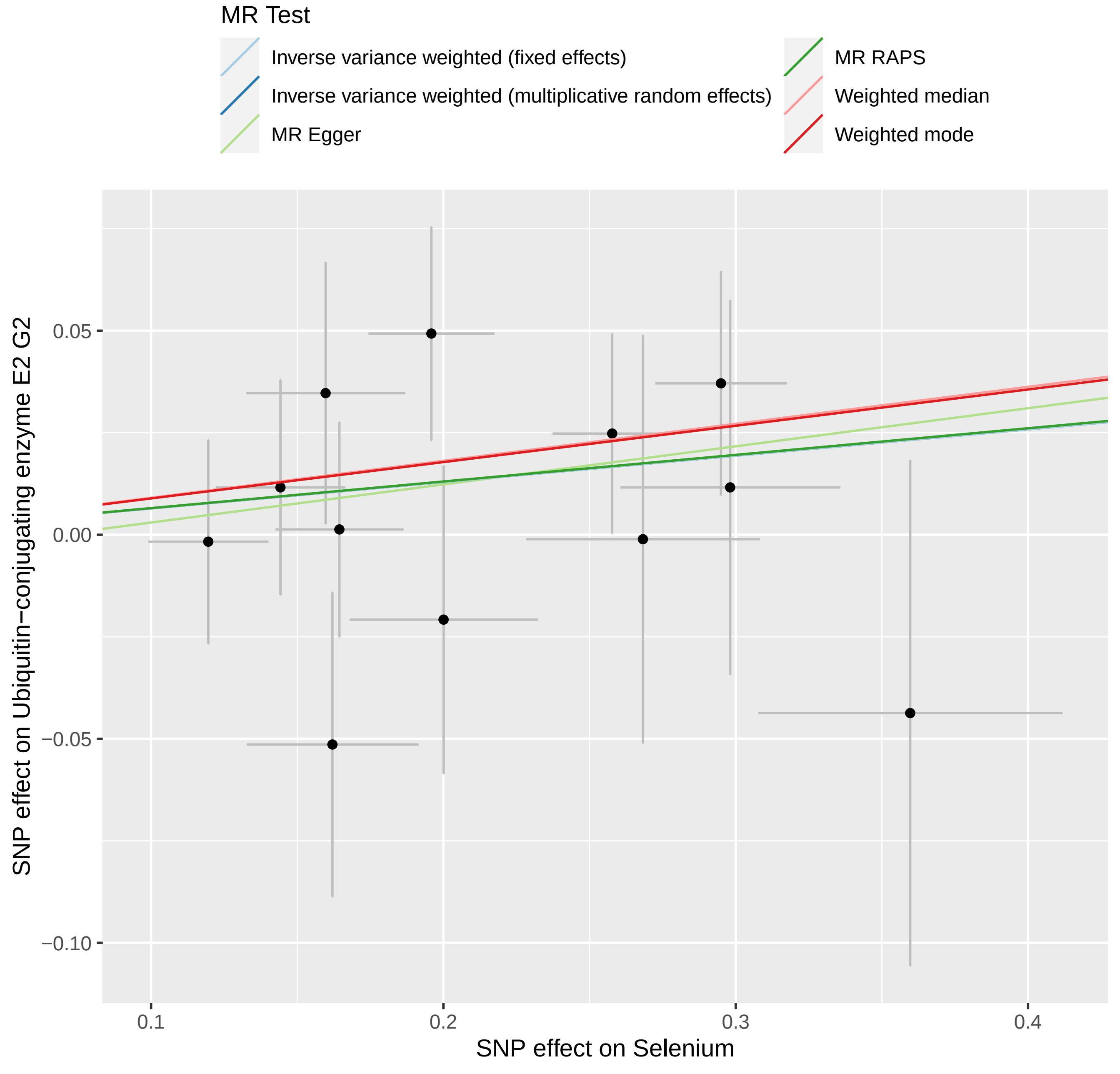

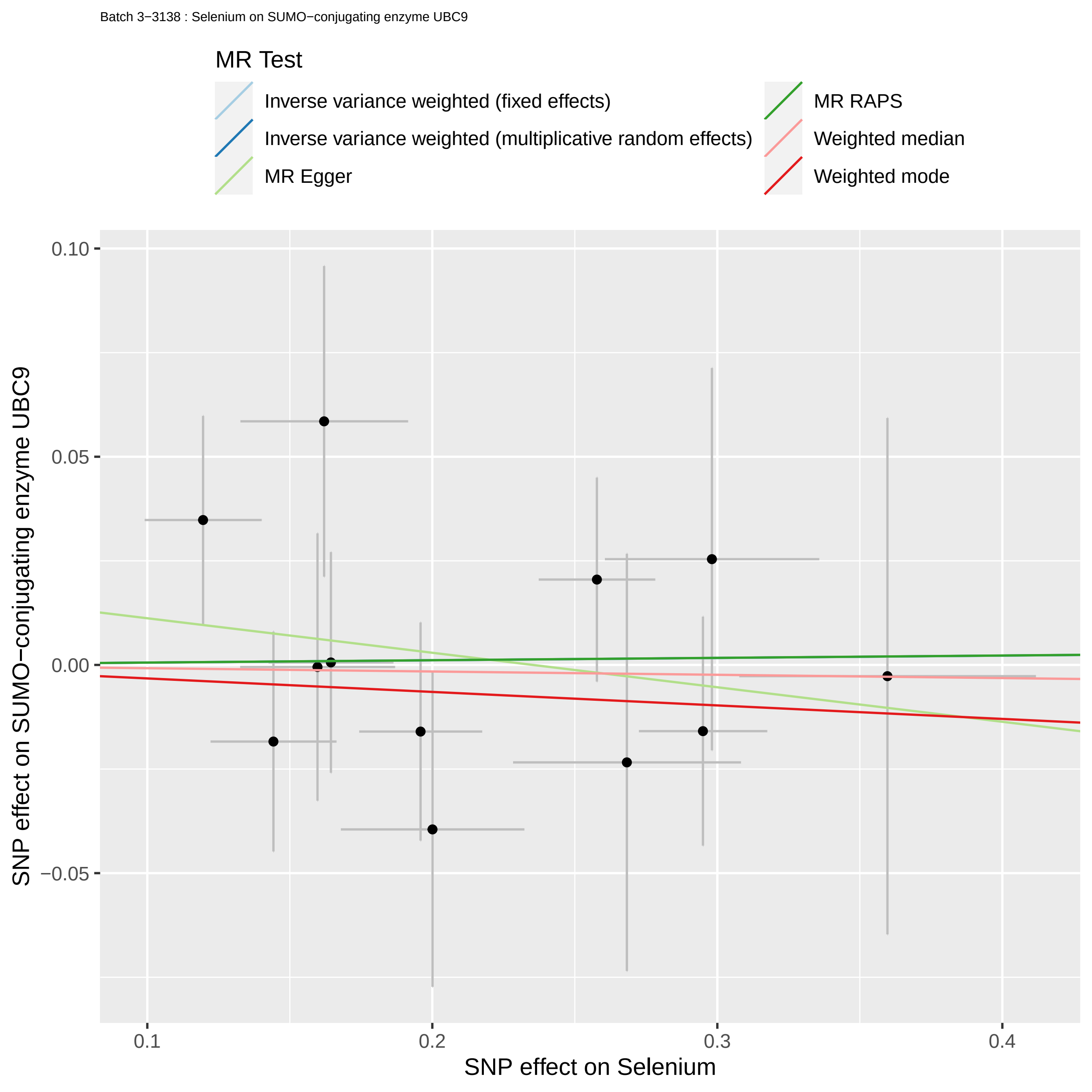
Batch 3−3138 : Selenium on SUMO−conjugating enzyme UBC9
MR Test
Inverse variance weighted (fixed effects)
Inverse variance weighted (multiplicative random effects)
MR Egger
MR RAPS
Weighted median
Weighted mode
0.10
0.05
0.00
−0.05
0.1
0.2
0.3
0.4
SNP effect on SUMO−conjugating enzyme UBC9
SNP effect on Selenium

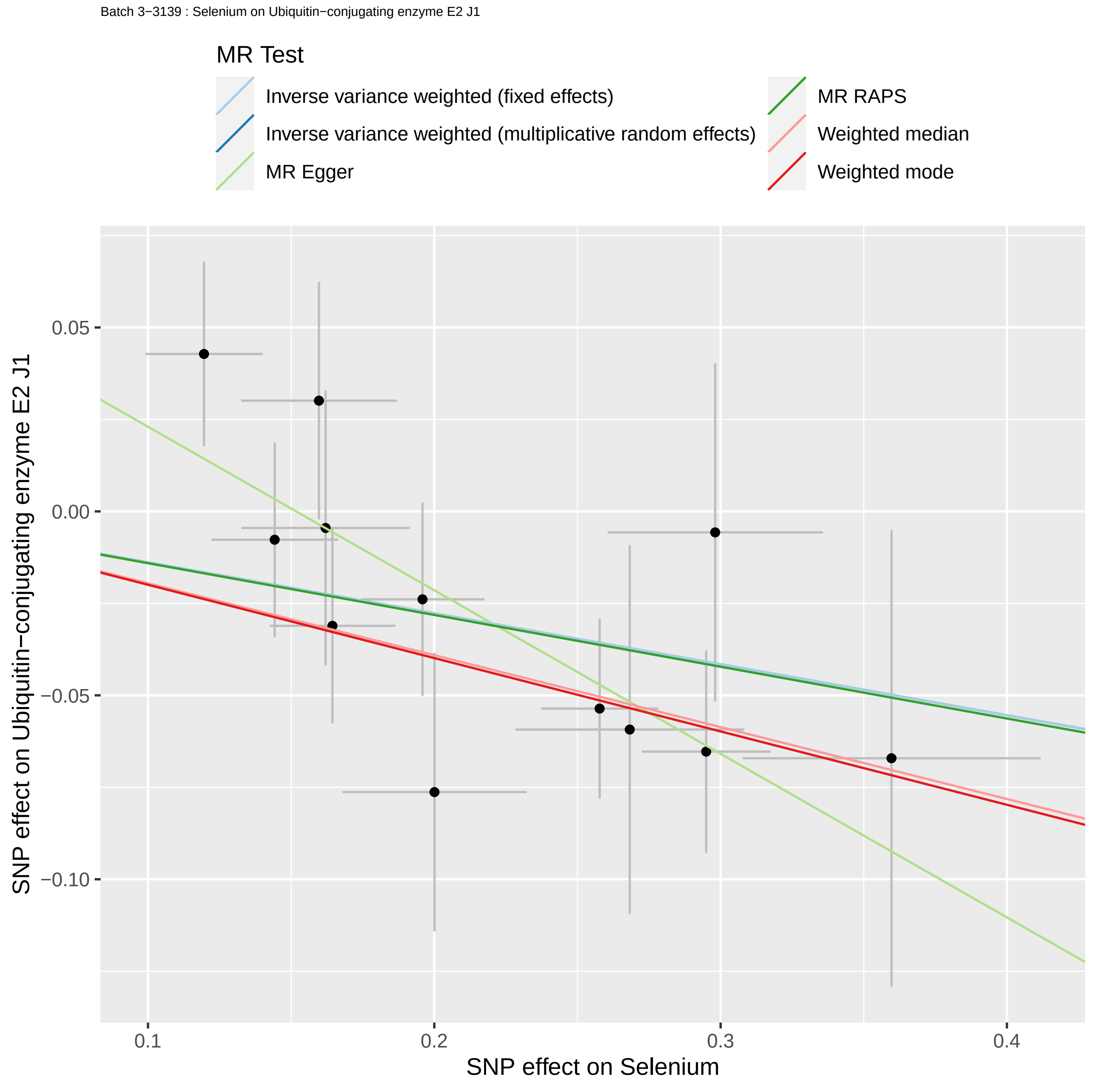

Batch 3−3139 : Selenium on Ubiquitin−conjugating enzyme E2 J1
MR Test
Inverse variance weighted (fixed effects)
Inverse variance weighted (multiplicative random effects)
MR Egger
MR RAPS
Weighted median
Weighted mode
SNP effect on Ubiquitin−conjugating enzyme E2 J1
0.05
0.00
−0.05
−0.10
0.1
0.2
0.3
0.4
SNP effect on Selenium

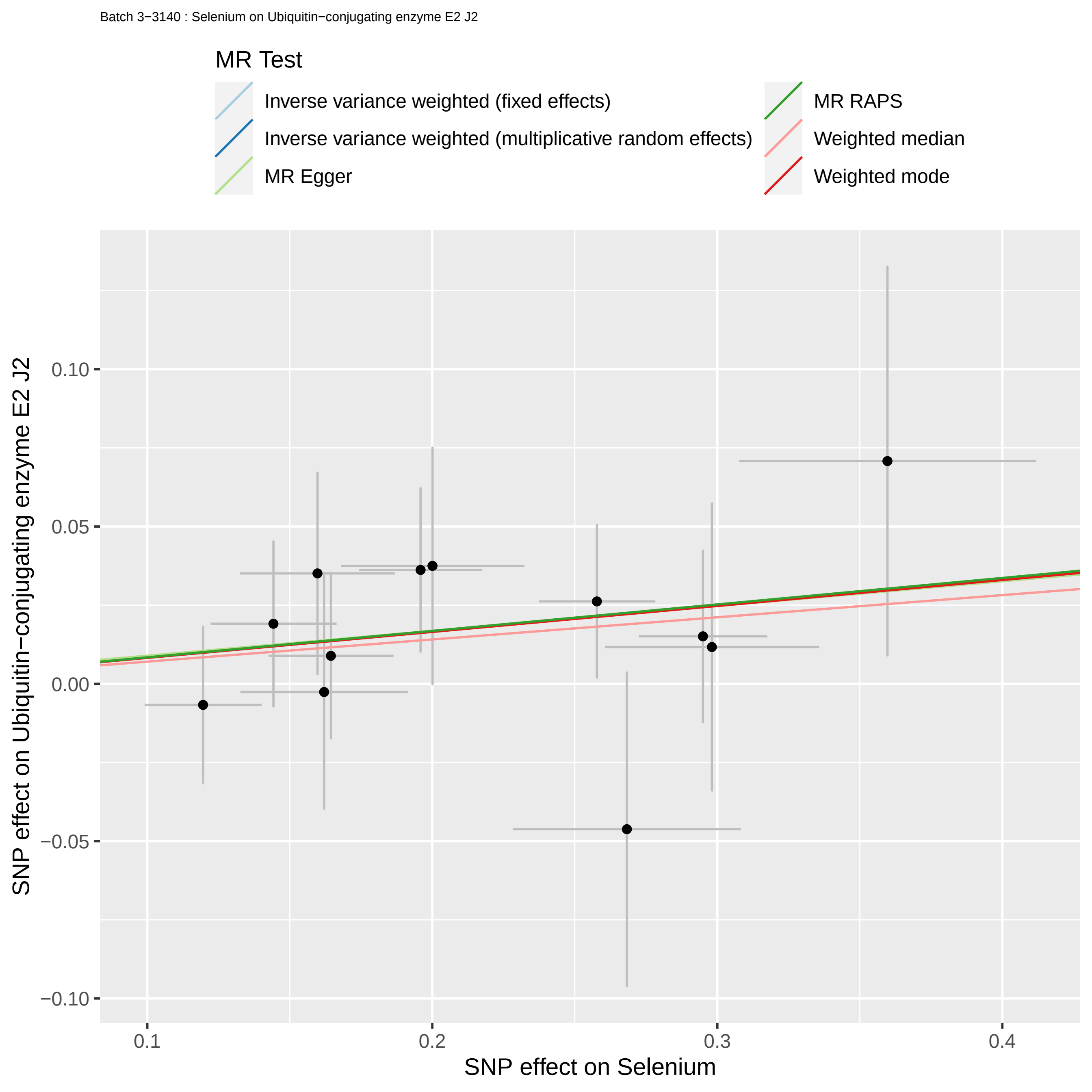
Batch 3−3140 : Selenium on Ubiquitin−conjugating enzyme E2 J2
MR Test
Inverse variance weighted (fixed effects)
Inverse variance weighted (multiplicative random effects)
MR Egger
MR RAPS
Weighted median
Weighted mode
SNP effect on Ubiquitin−conjugating enzyme E2 J2
0.10
0.05
0.00
−0.05
−0.10
0.1
0.2
0.3
0.4
SNP effect on Selenium

Batch 3−3141 : Selenium on Ubiquitin−conjugating enzyme E2 J2

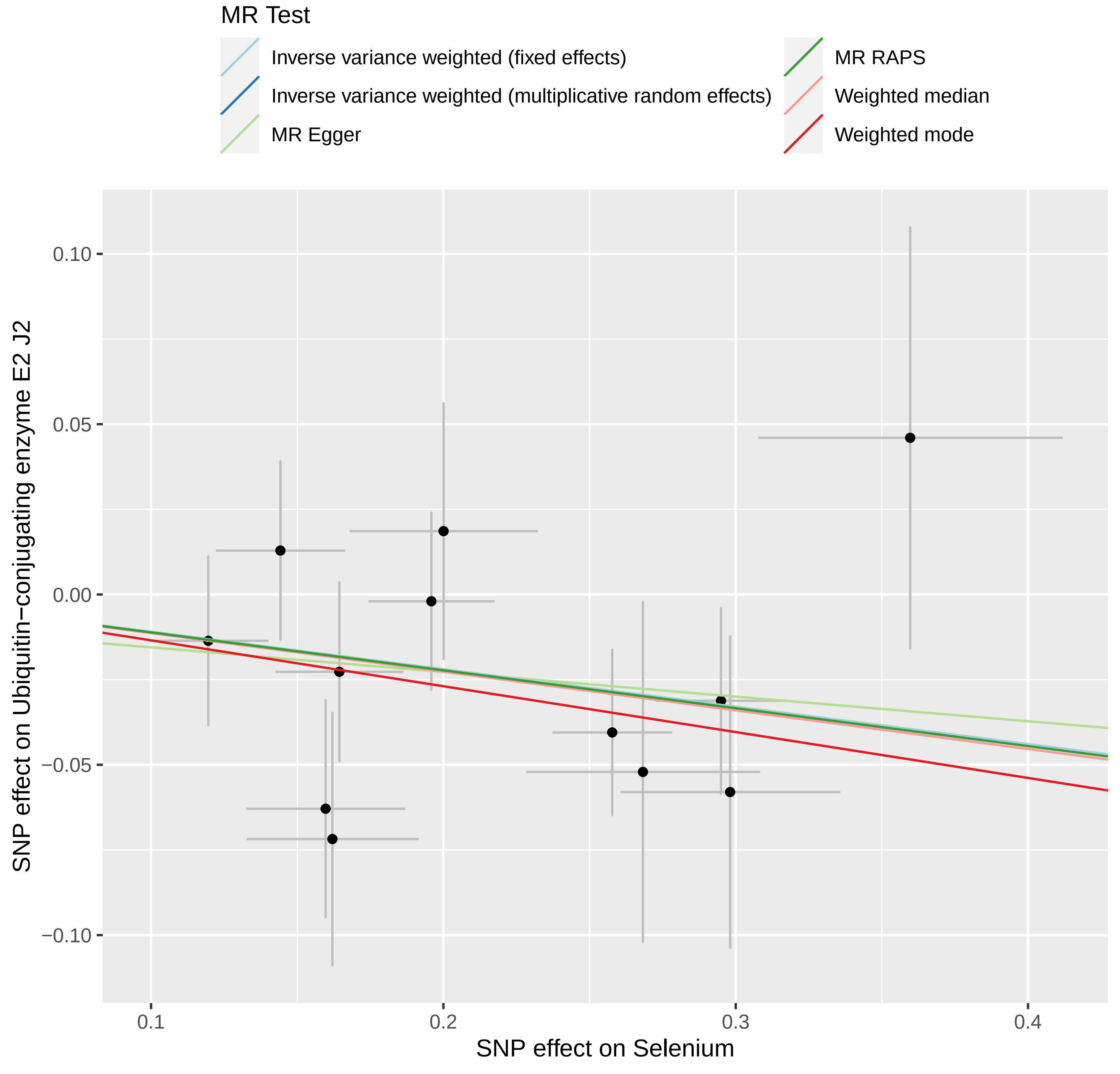

Batch 3−3142 : Selenium on Ubiquitin−conjugating enzyme E2 L3

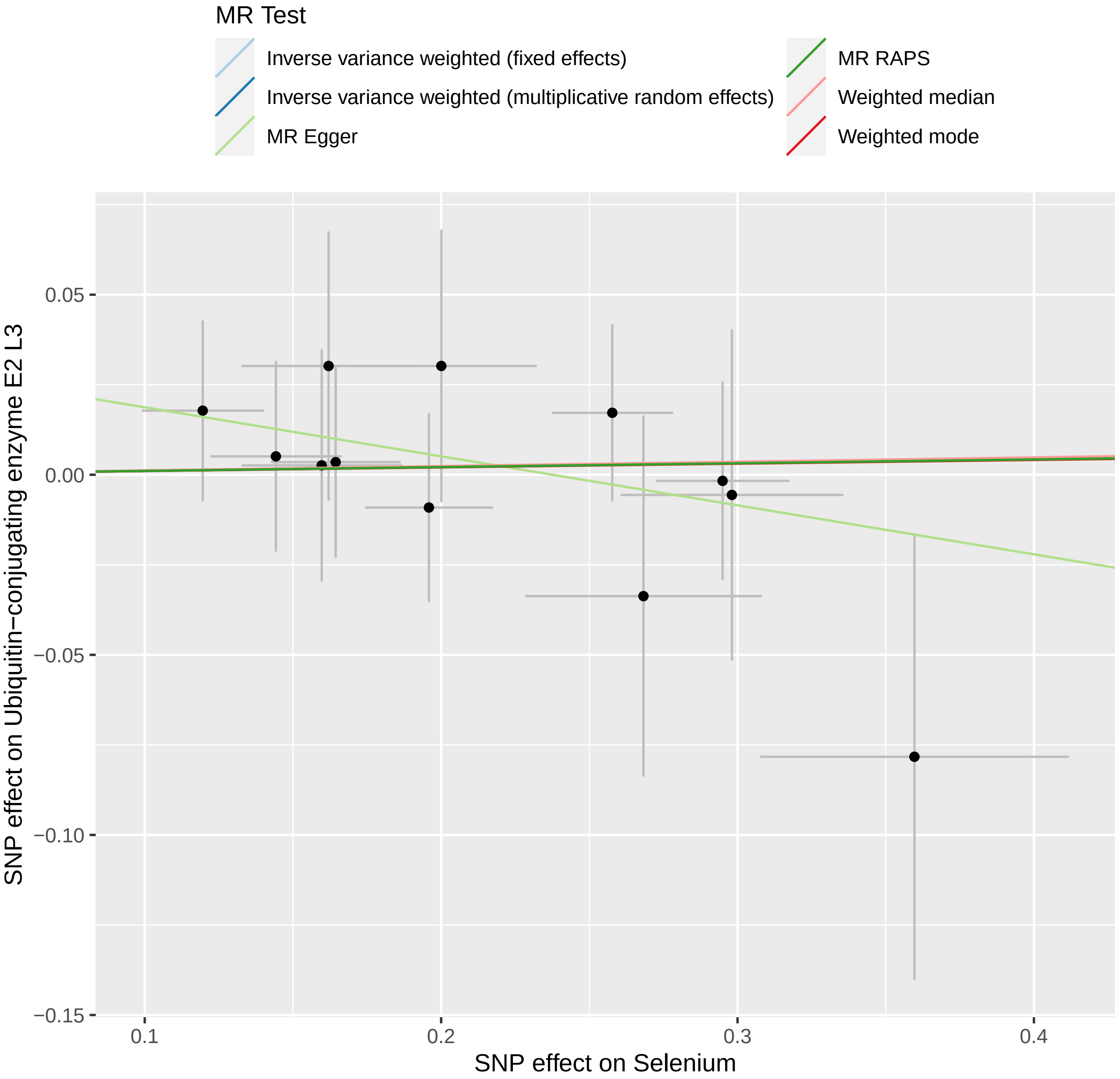

Batch 3−3143 : Selenium on Ubiquitin−conjugating enzyme E2 N

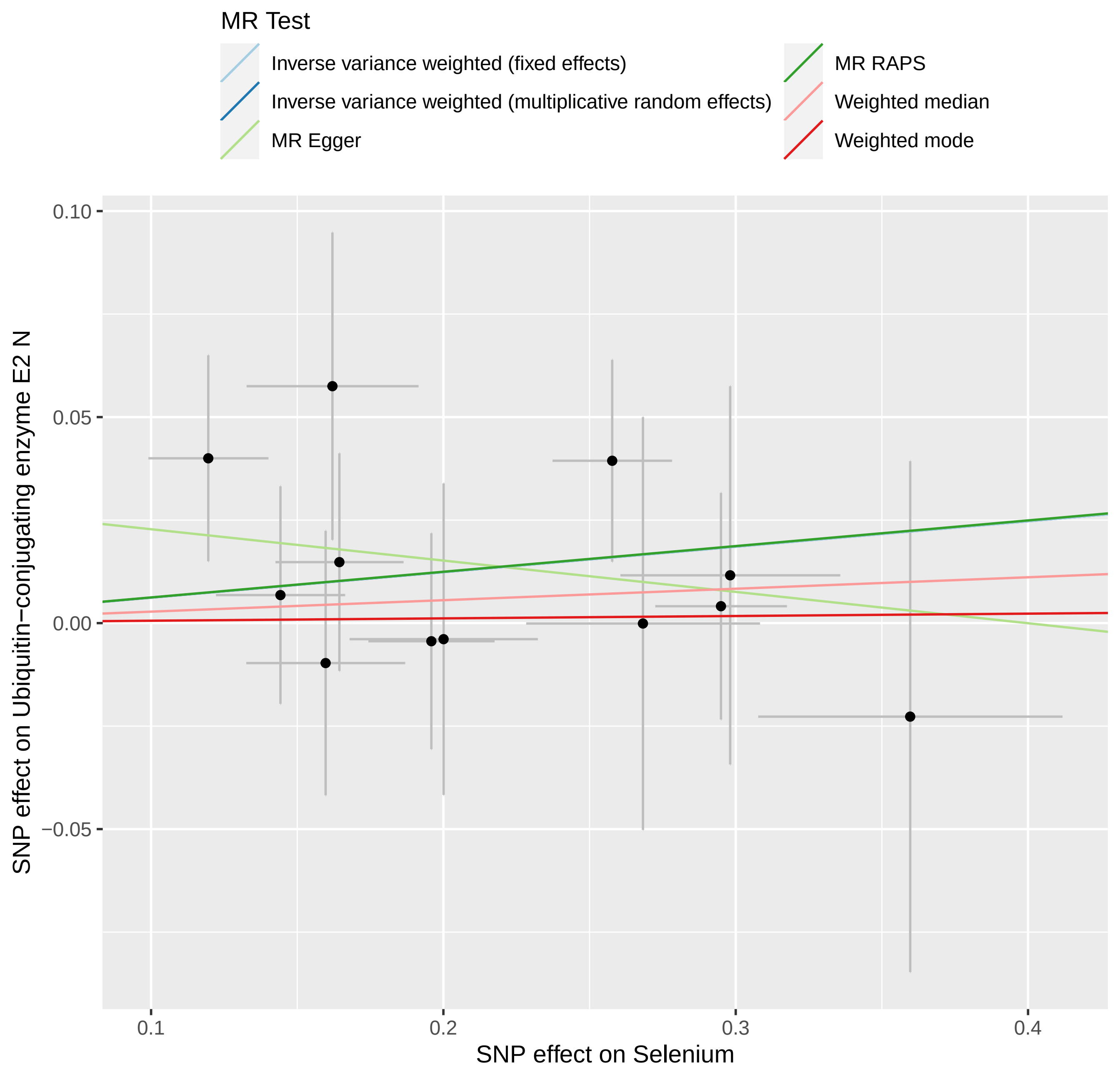

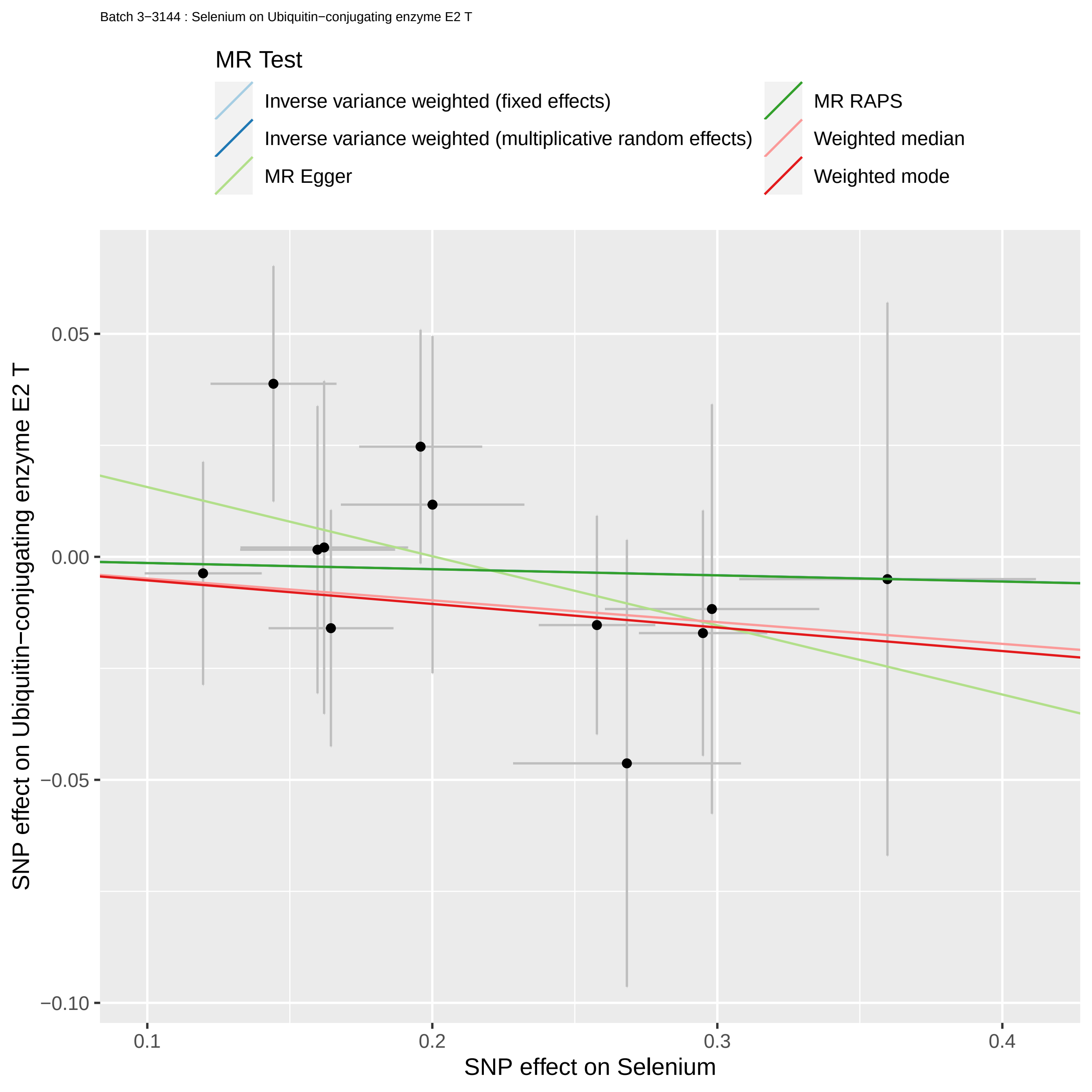
Batch 3−3144 : Selenium on Ubiquitin−conjugating enzyme E2 T
MR Test
Inverse variance weighted (fixed effects)
Inverse variance weighted (multiplicative random effects)
MR Egger
MR RAPS
Weighted median
Weighted mode
0.05
0.00
−0.05
−0.10
0.1
0.2
0.3
0.4
SNP effect on Ubiquitin−conjugating enzyme E2 T
SNP effect on Selenium

Batch 3−3145 : Selenium on Ubiquitin−conjugating enzyme E2 variant 1

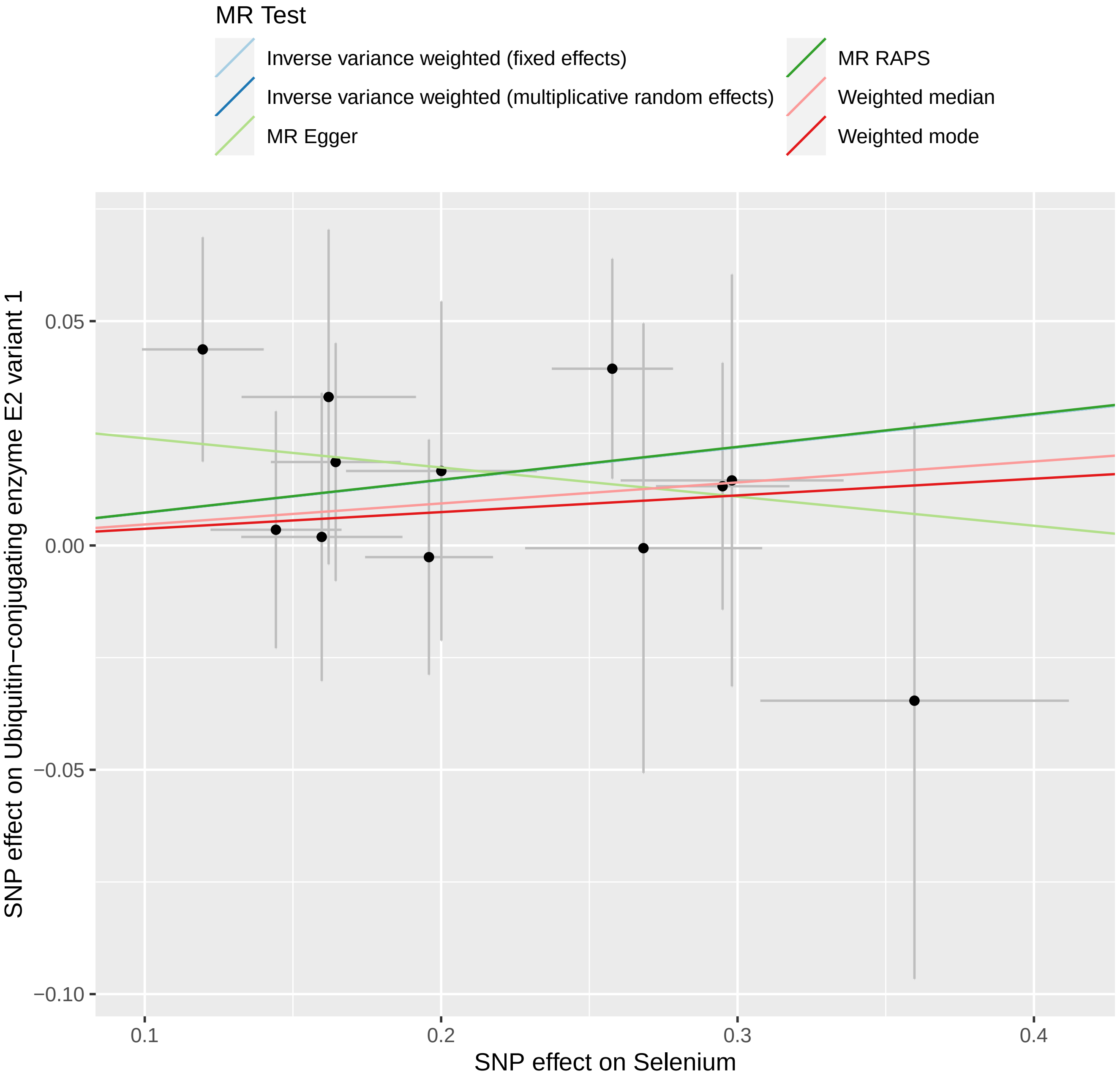

Batch 3−3146 : Selenium on Ubiquitin−like protein 4A

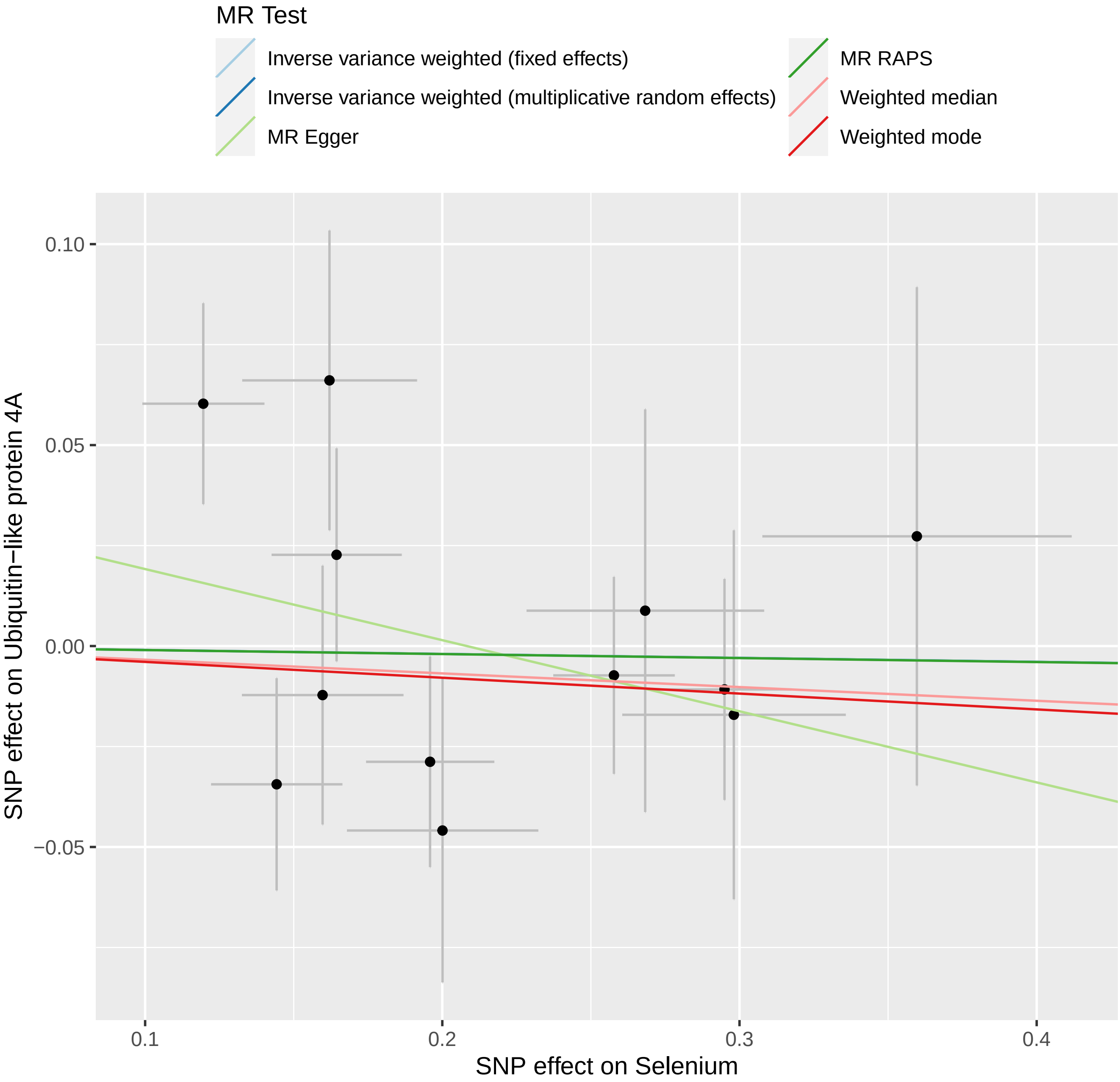

Batch 3−3147 : Selenium on Ubiquilin−4

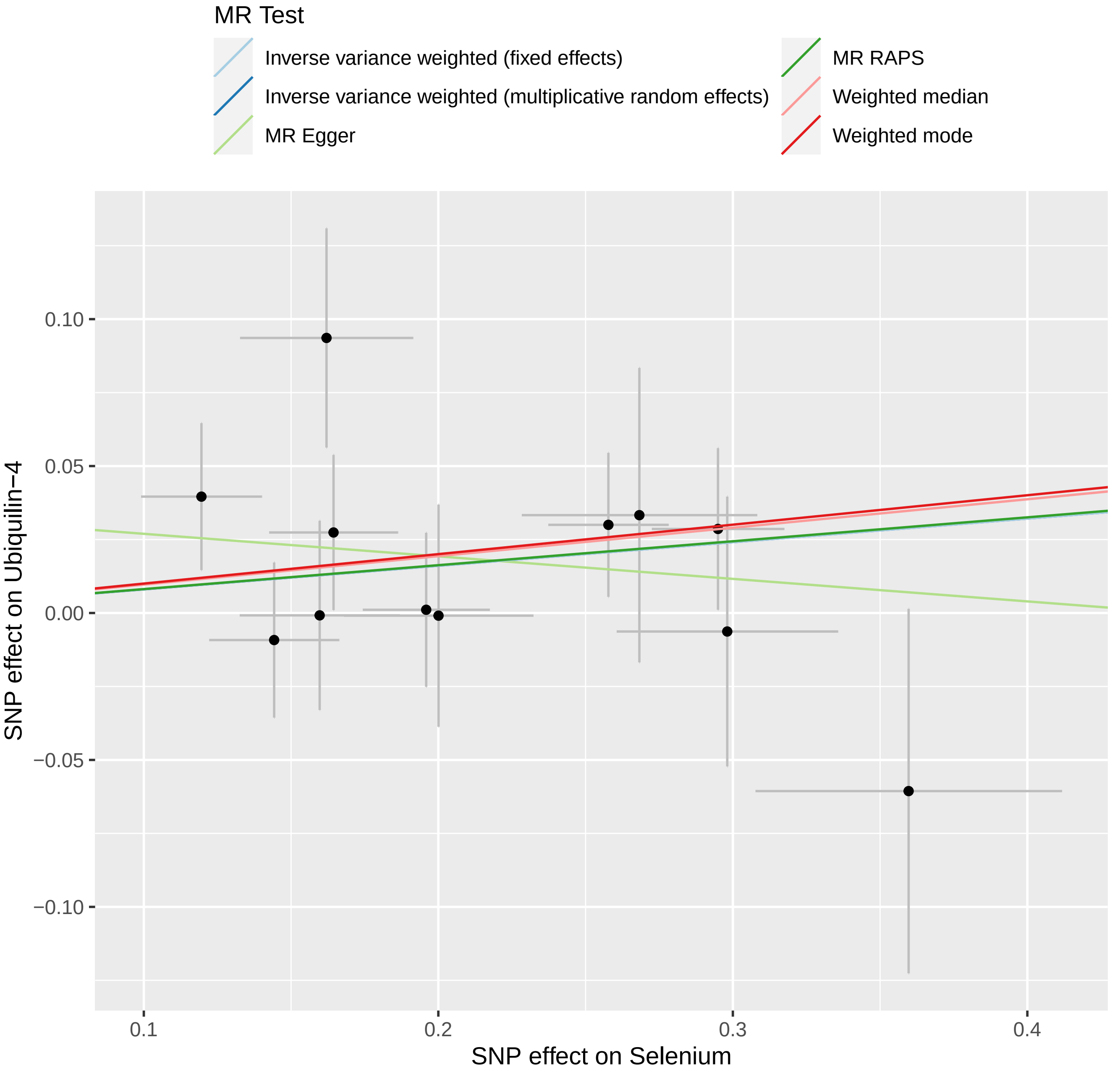

Batch 3−3148 : Selenium on Ubiquitin domain−containing protein 2

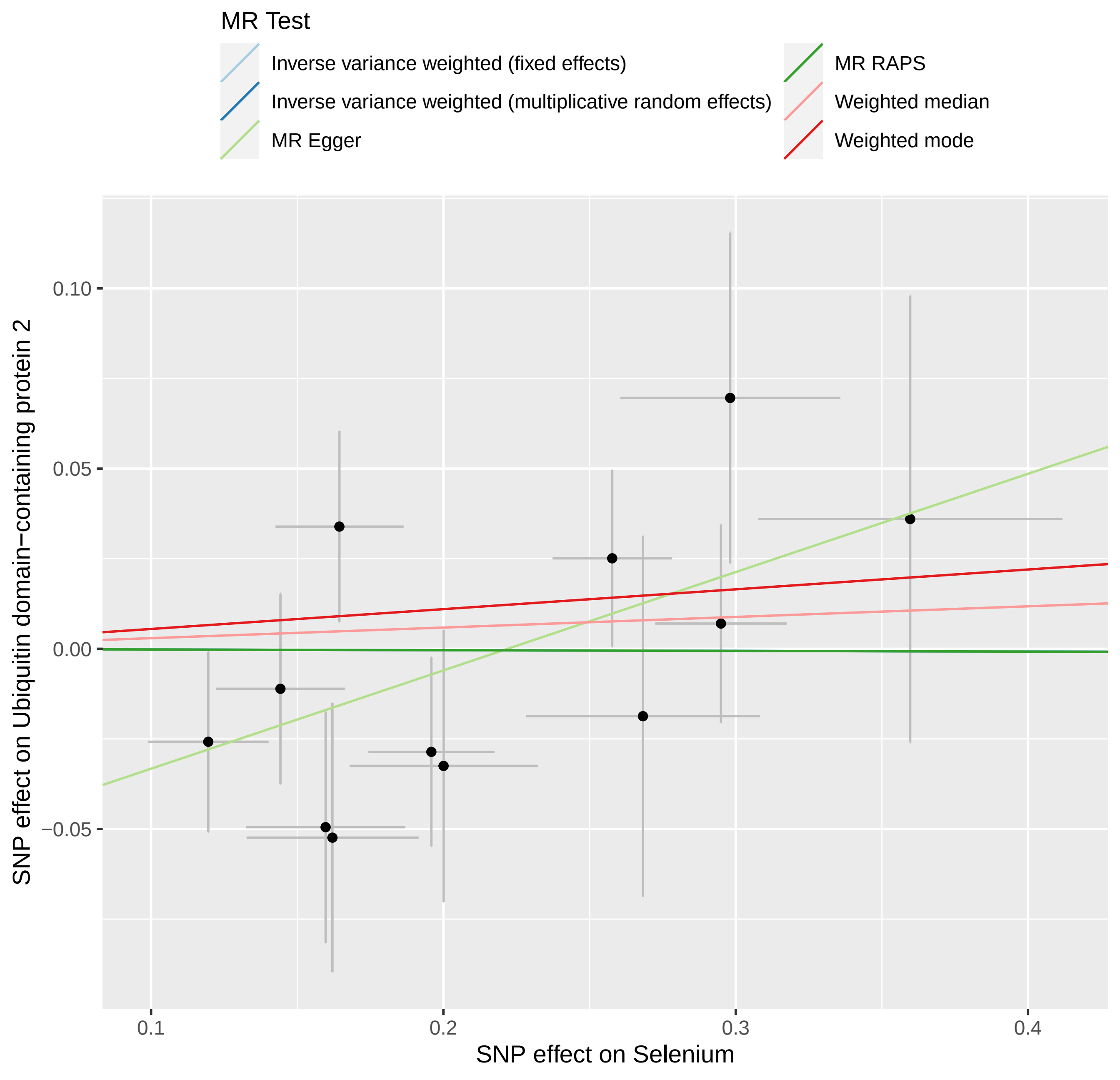

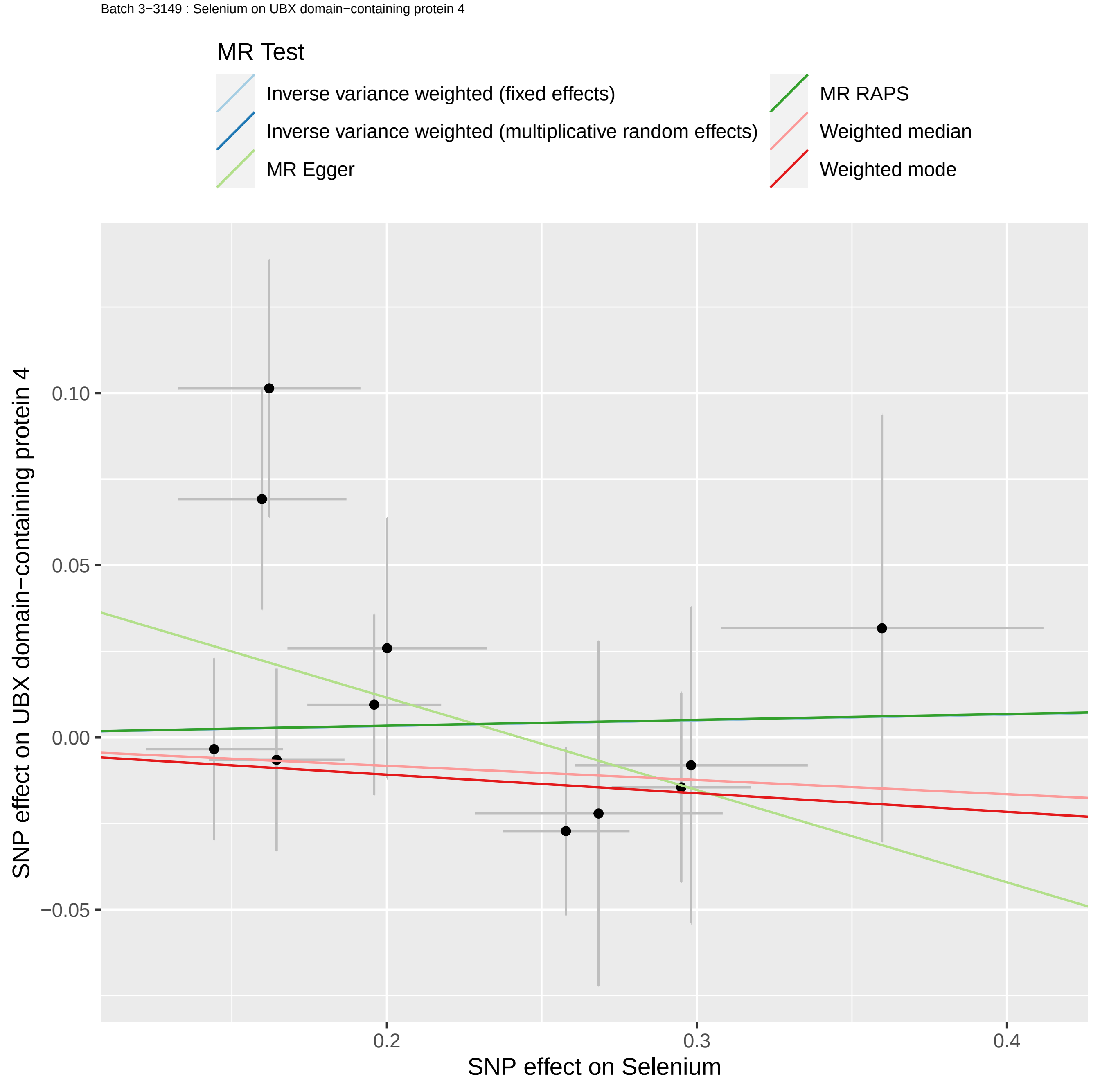

Batch 3−3149 : Selenium on UBX domain−containing protein 4
MR Test
Inverse variance weighted (fixed effects)
Inverse variance weighted (multiplicative random effects)
MR Egger
MR RAPS
Weighted median
Weighted mode
SNP effect on UBX domain−containing protein 4
0.10
0.05
0.00
−0.05
0.2
0.3
0.4
SNP effect on Selenium

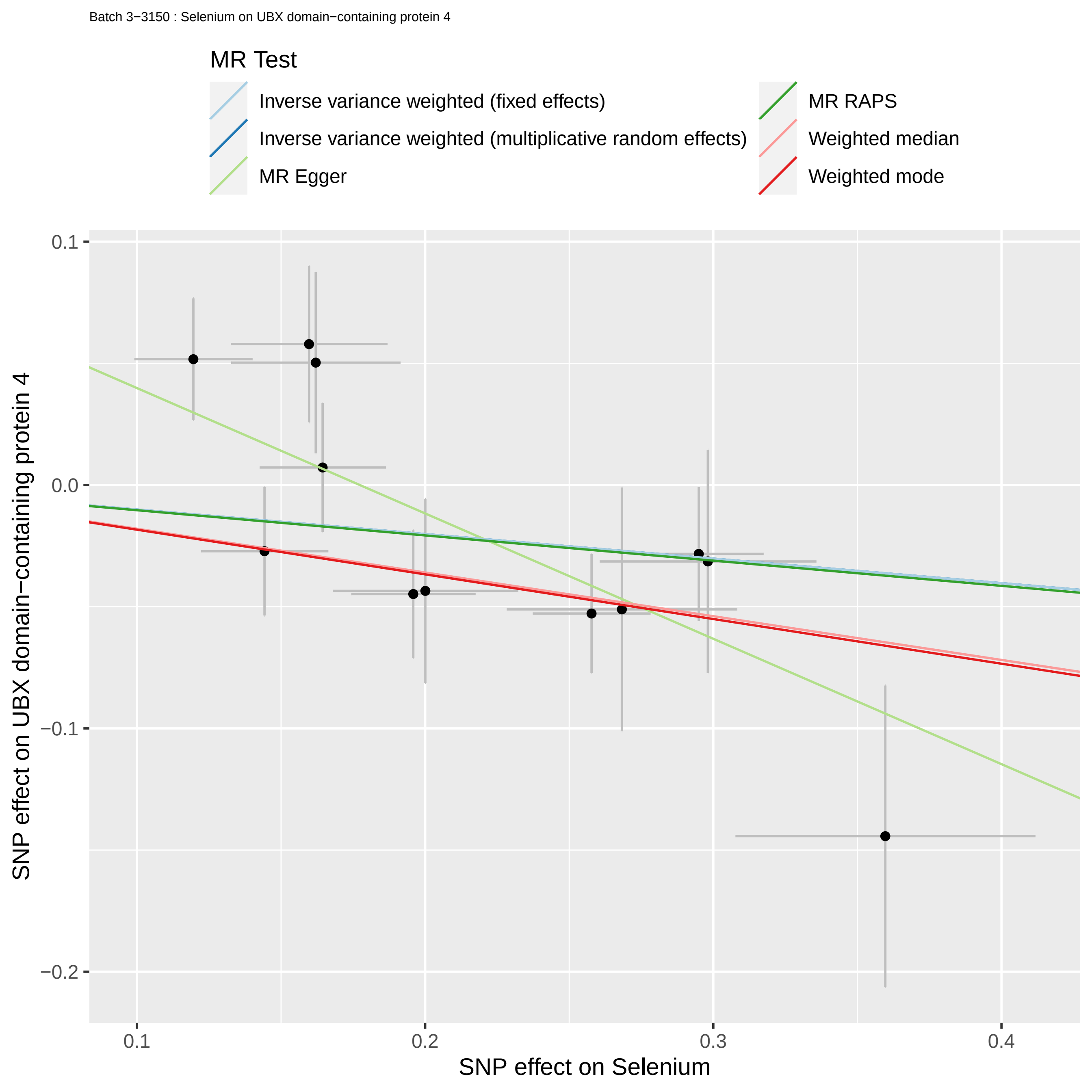

Batch 3−3150 : Selenium on UBX domain−containing protein 4
MR Test
Inverse variance weighted (fixed effects)
Inverse variance weighted (multiplicative random effects)
MR Egger
MR RAPS
Weighted median
Weighted mode
0.1
0.0
−0.1
−0.2
0.1
0.2
0.3
0.4
SNP effect on UBX domain−containing protein 4
SNP effect on Selenium

Batch 3−3151 : Selenium on Uridine−cytidine kinase 2

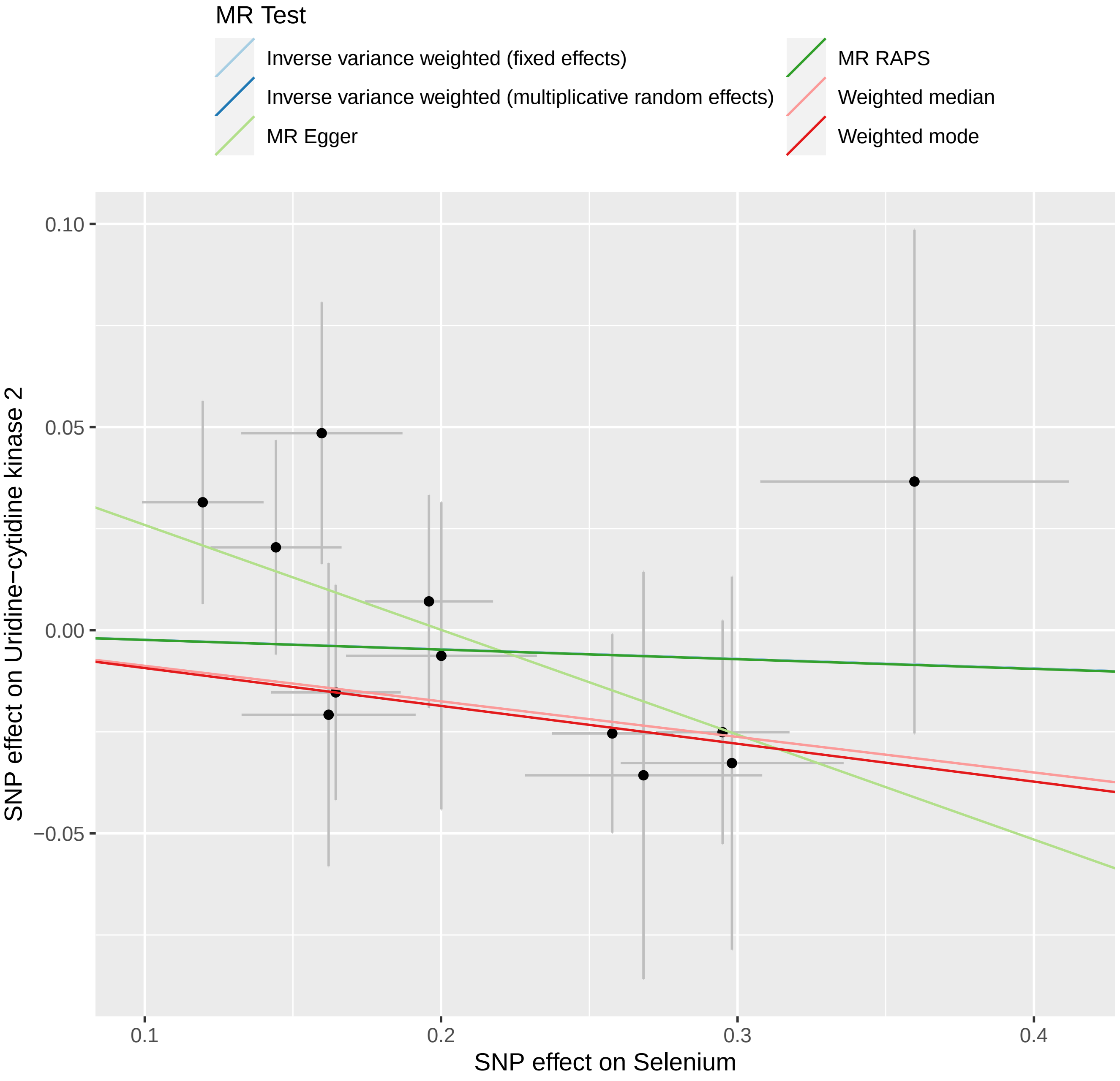

Batch 3−3152 : Selenium on Unique cartilage matrix−associated protein

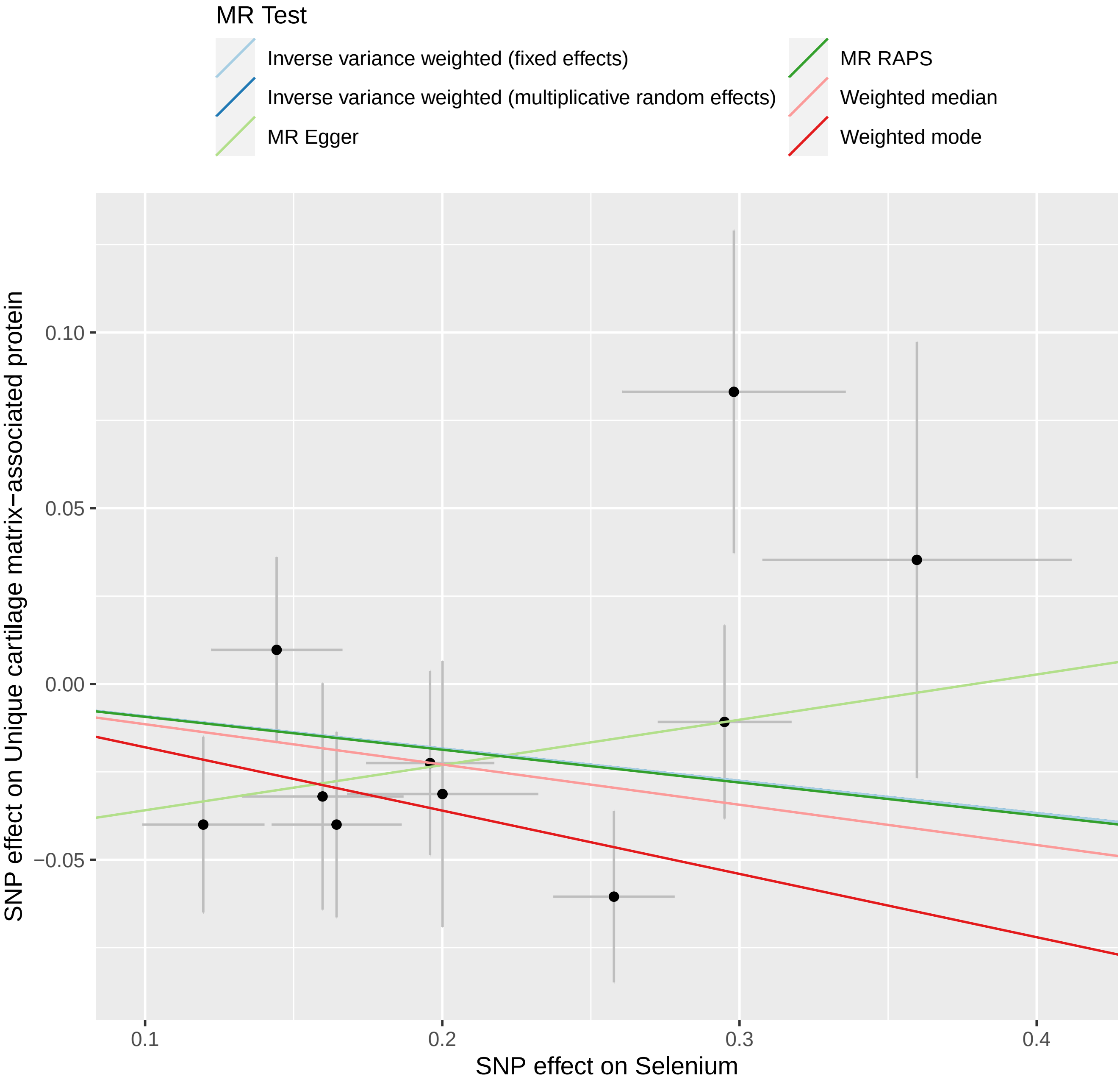

Batch 3−3153 : Selenium on Ubiquitin−fold modifier−conjugating enzyme 1

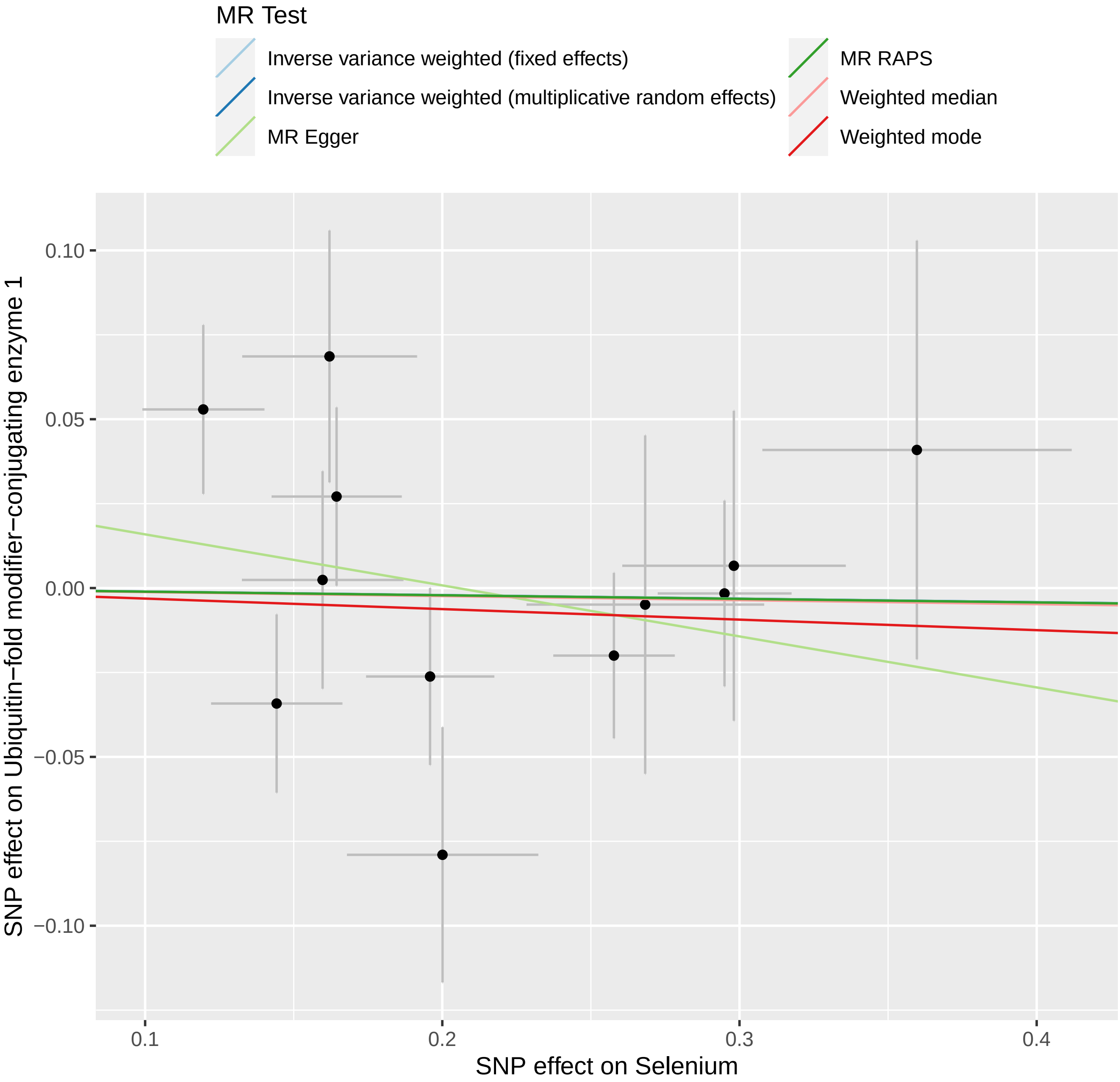

Batch 3−3154 : Selenium on Ubiquitin−fold modifier 1

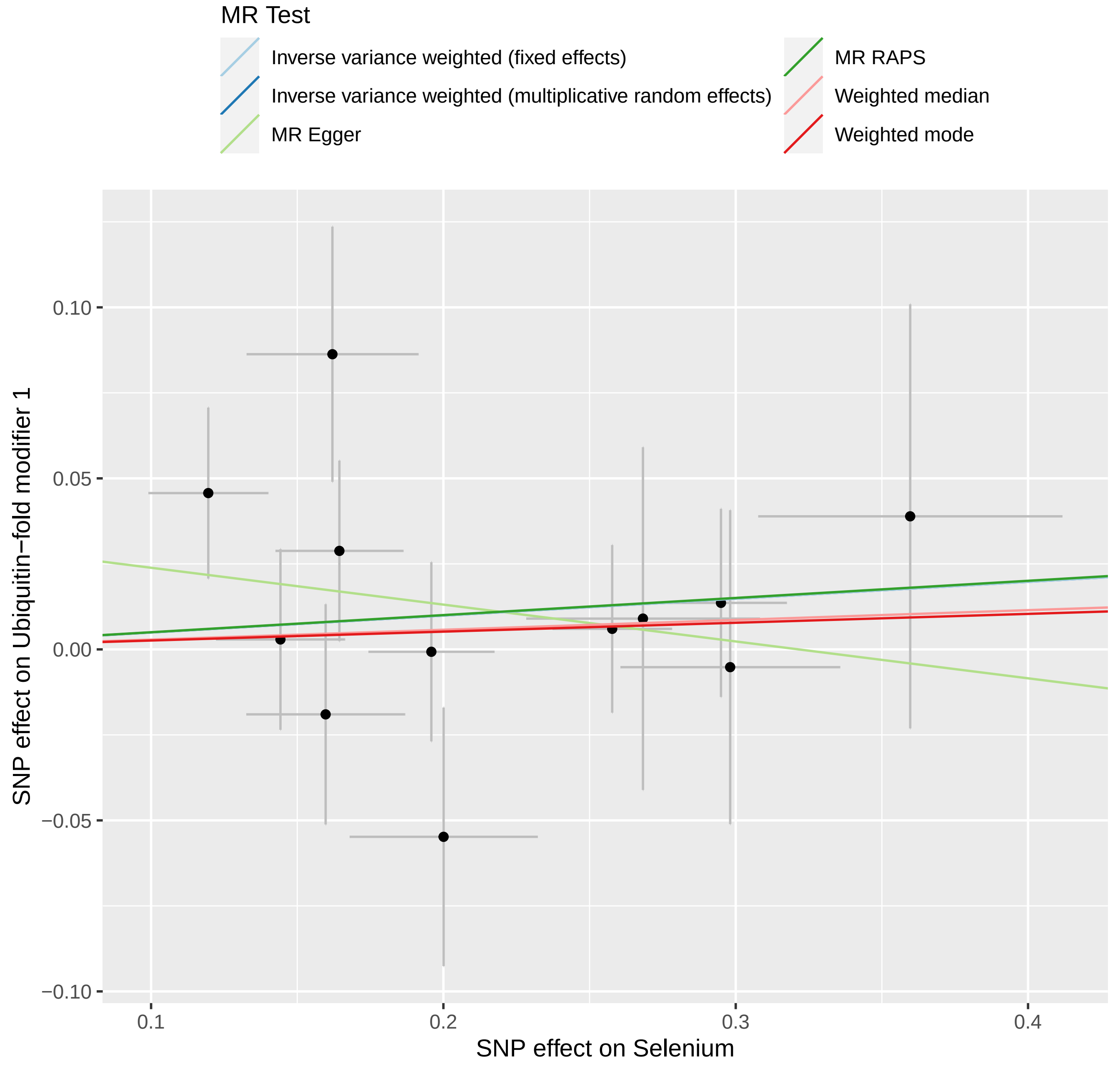

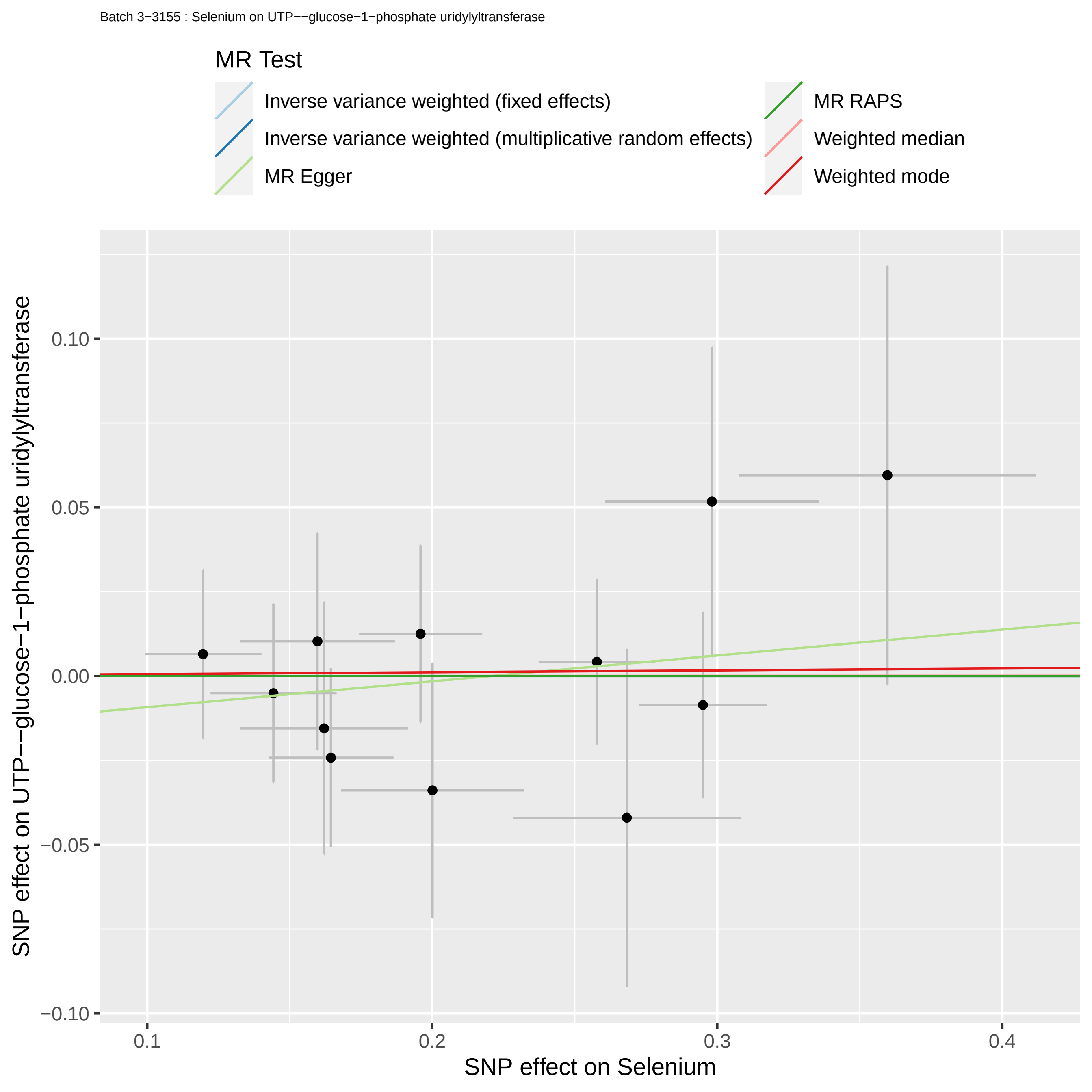

Batch 3−3155 : Selenium on UTP−−glucose−1−phosphate uridylyltransferase
MR Test
Inverse variance weighted (fixed effects)
Inverse variance weighted (multiplicative random effects)
MR Egger
MR RAPS
Weighted median
Weighted mode
SNP effect on UTP−−glucose−1−phosphate uridylyltransferase
0.10
0.05
0.00
−0.05
−0.10
0.1
0.2
0.3
0.4
SNP effect on Selenium

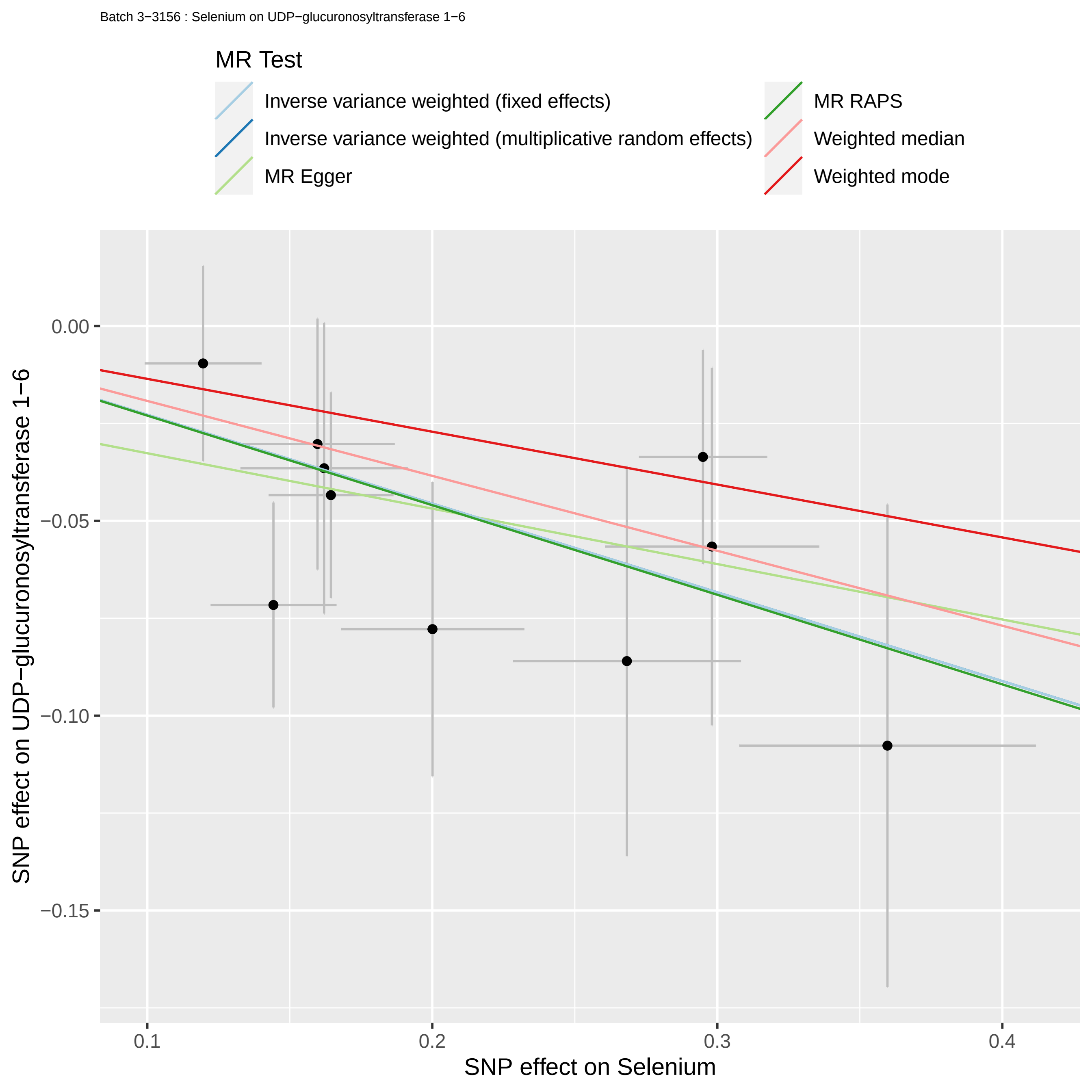
Batch 3−3156 : Selenium on UDP−glucuronosyltransferase 1−6
MR Test
Inverse variance weighted (fixed effects)
Inverse variance weighted (multiplicative random effects)
MR Egger
MR RAPS
Weighted median
Weighted mode
0.00
−0.05
−0.10
−0.15
0.1
0.2
0.3
0.4
SNP effect on Selenium
SNP effect on UDP−glucuronosyltransferase 1−6

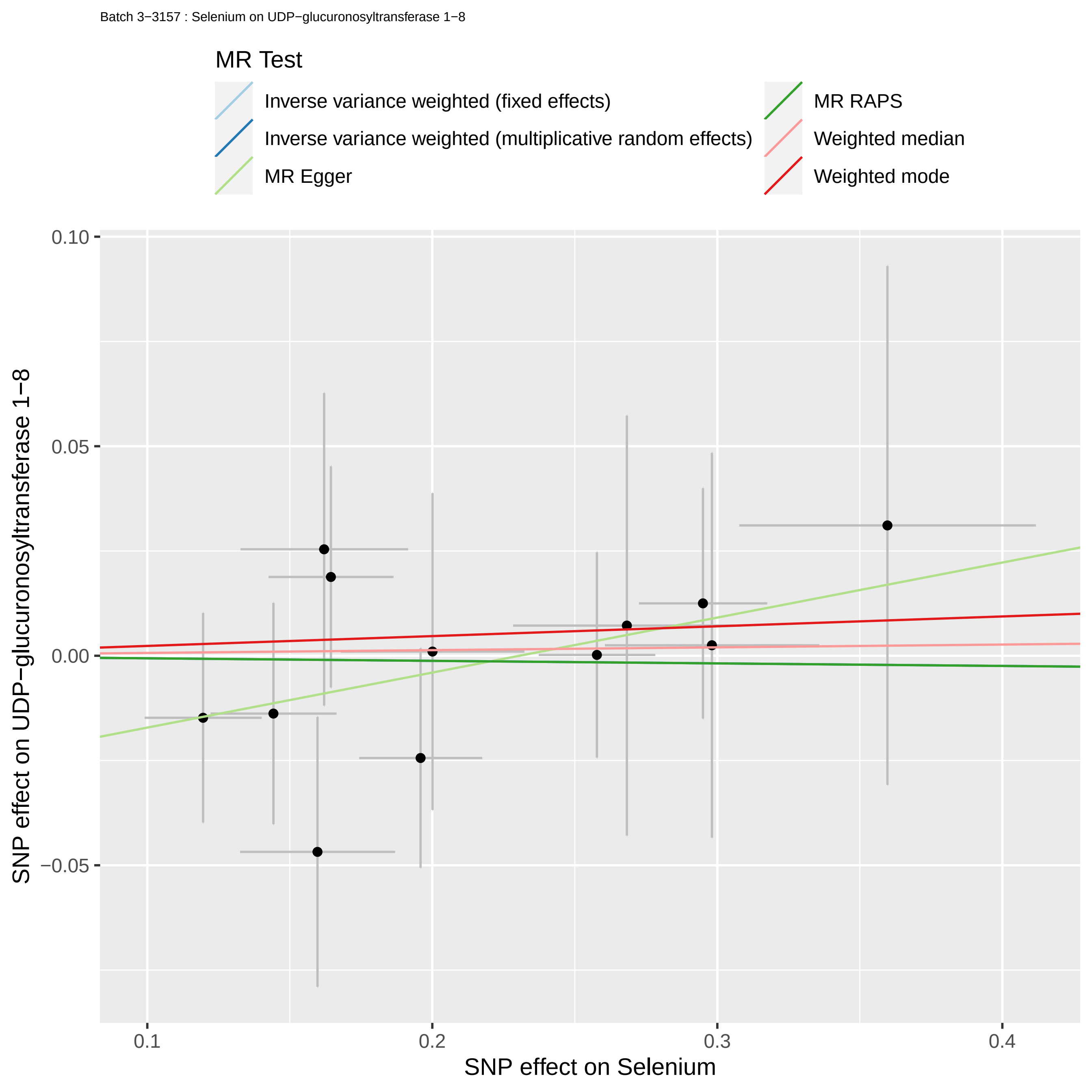

Batch 3−3157 : Selenium on UDP−glucuronosyltransferase 1−8
MR Test
Inverse variance weighted (fixed effects)
Inverse variance weighted (multiplicative random effects)
MR Egger
MR RAPS
Weighted median
Weighted mode
0.10
0.05
0.00
−0.05
0.1
0.2
0.3
0.4
SNP effect on UDP−glucuronosyltransferase 1−8
SNP effect on Selenium

Batch 3−3158 : Selenium on UDP−glucuronosyltransferase 2A1

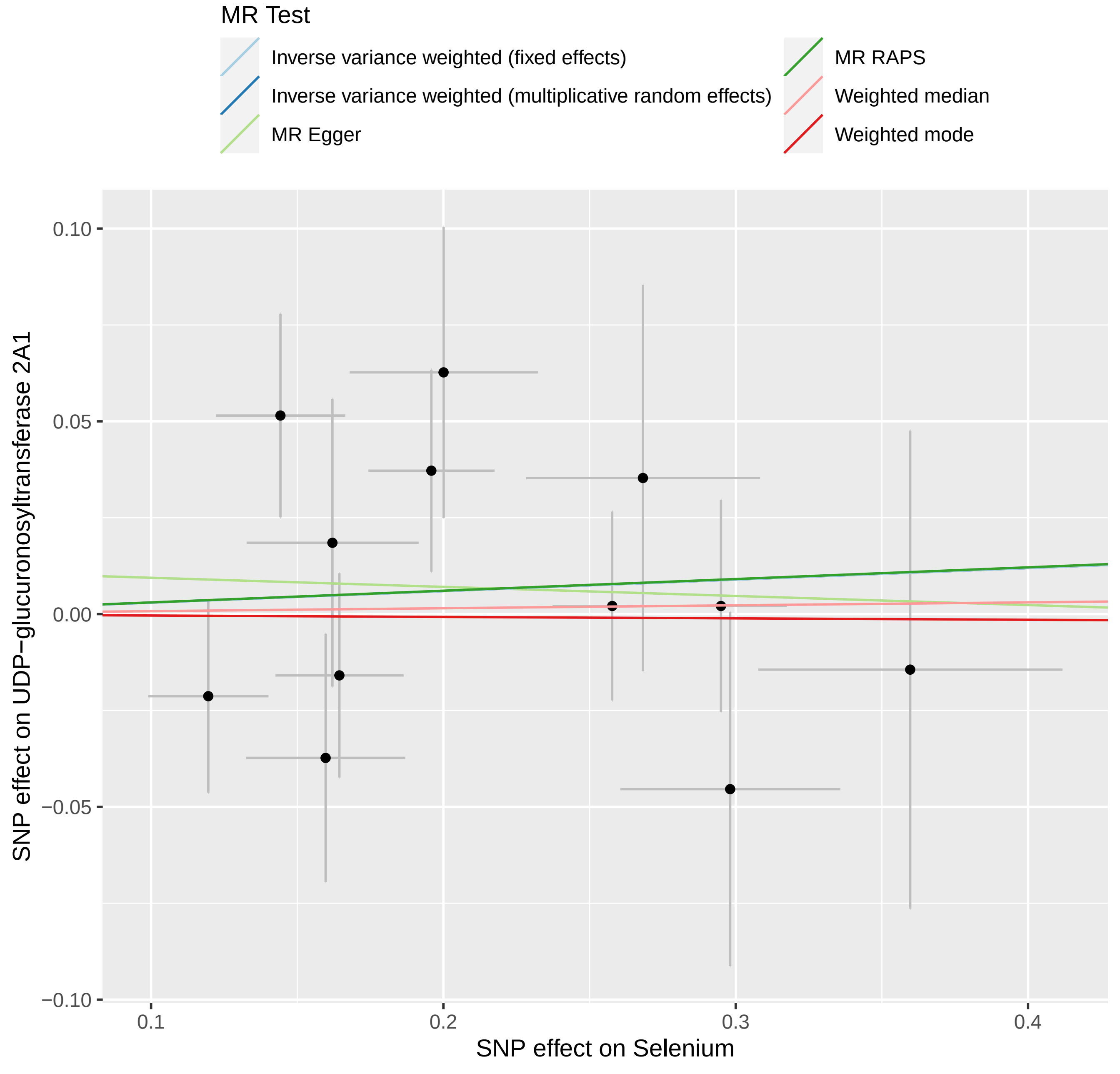

Batch 3−3159 : Selenium on NKG2D ligand 1

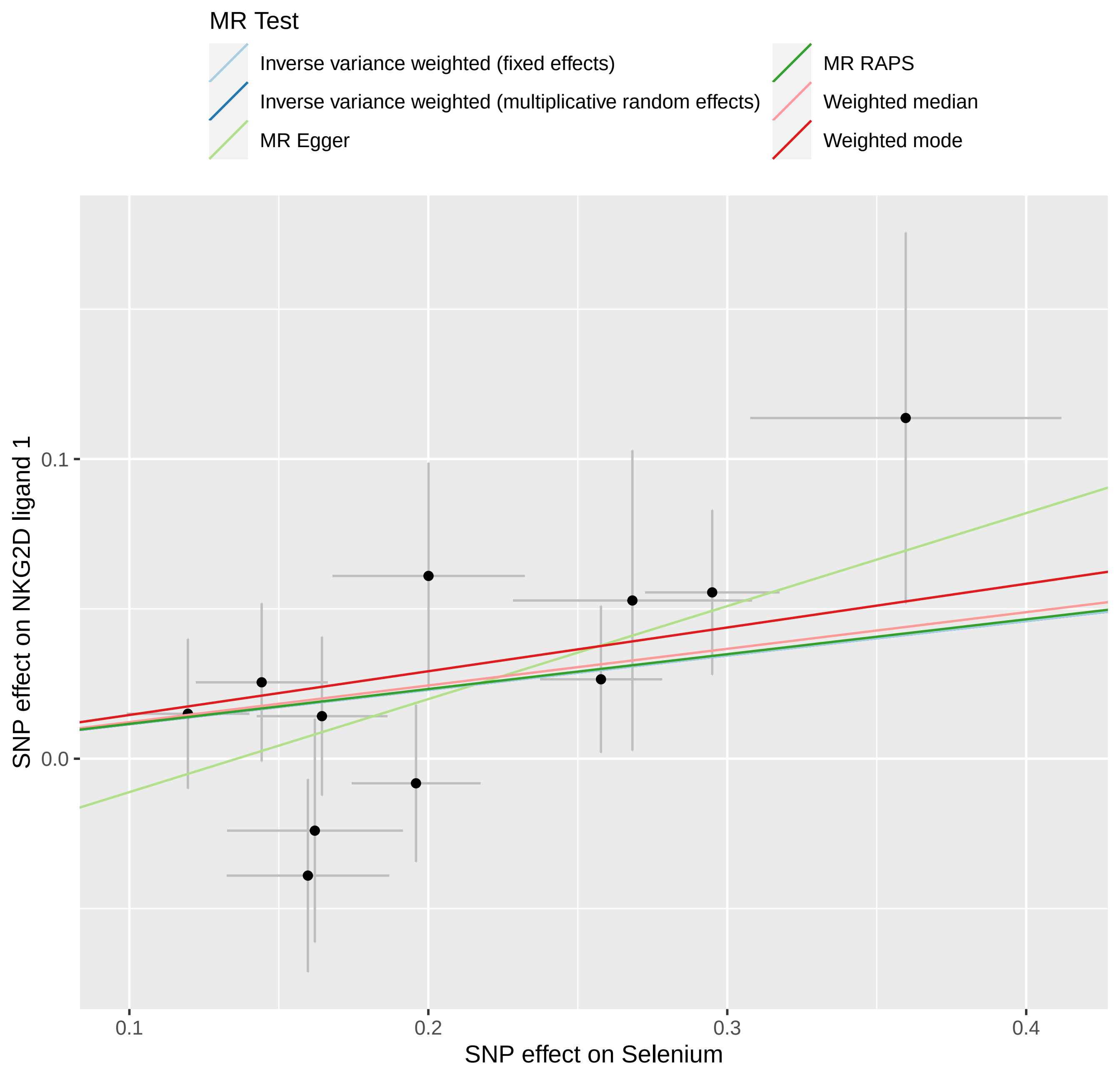

Batch 3−3160 : Selenium on NKG2D ligand 1

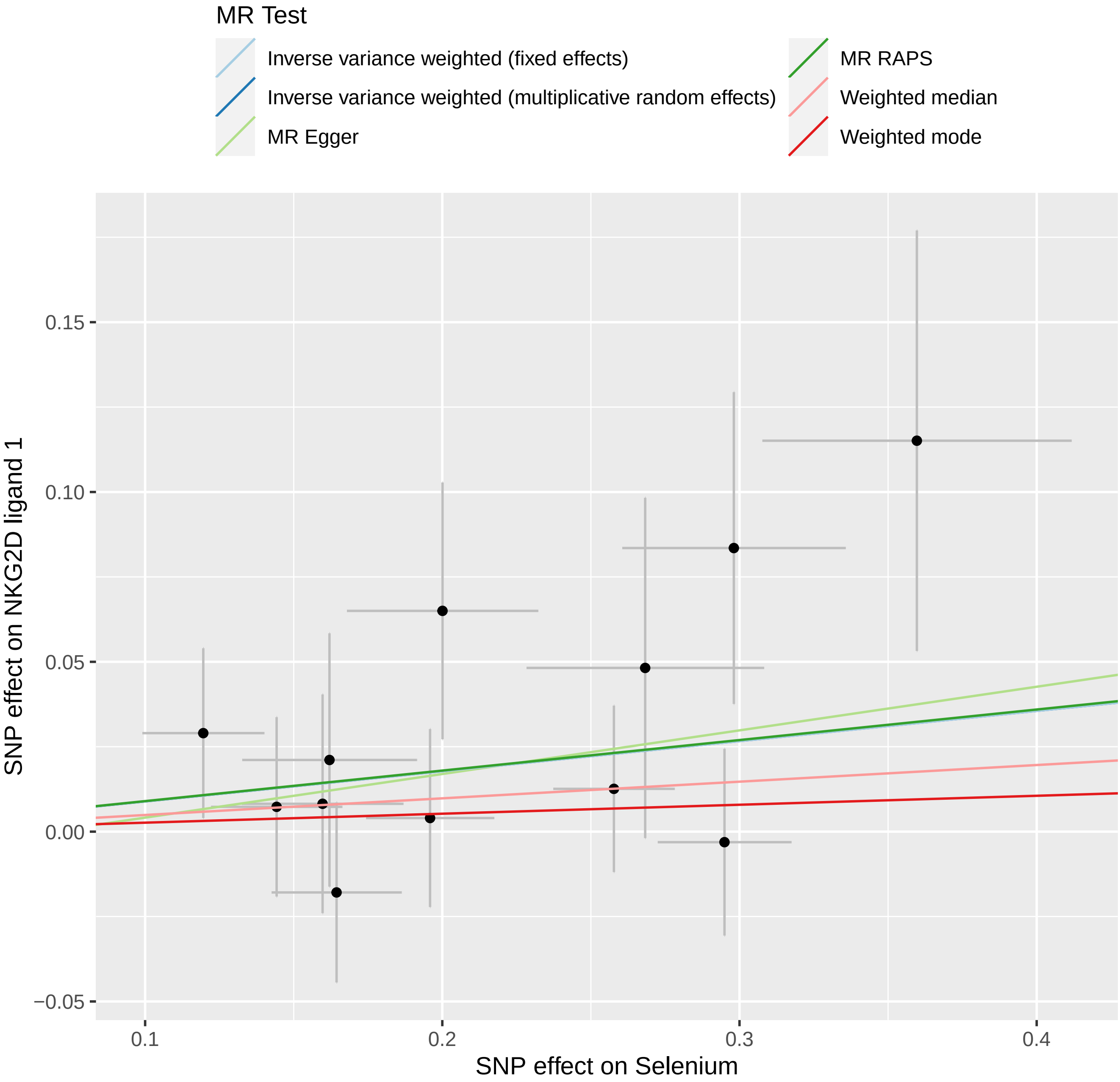

Batch 3−3161 : Selenium on NKG2D ligand 2

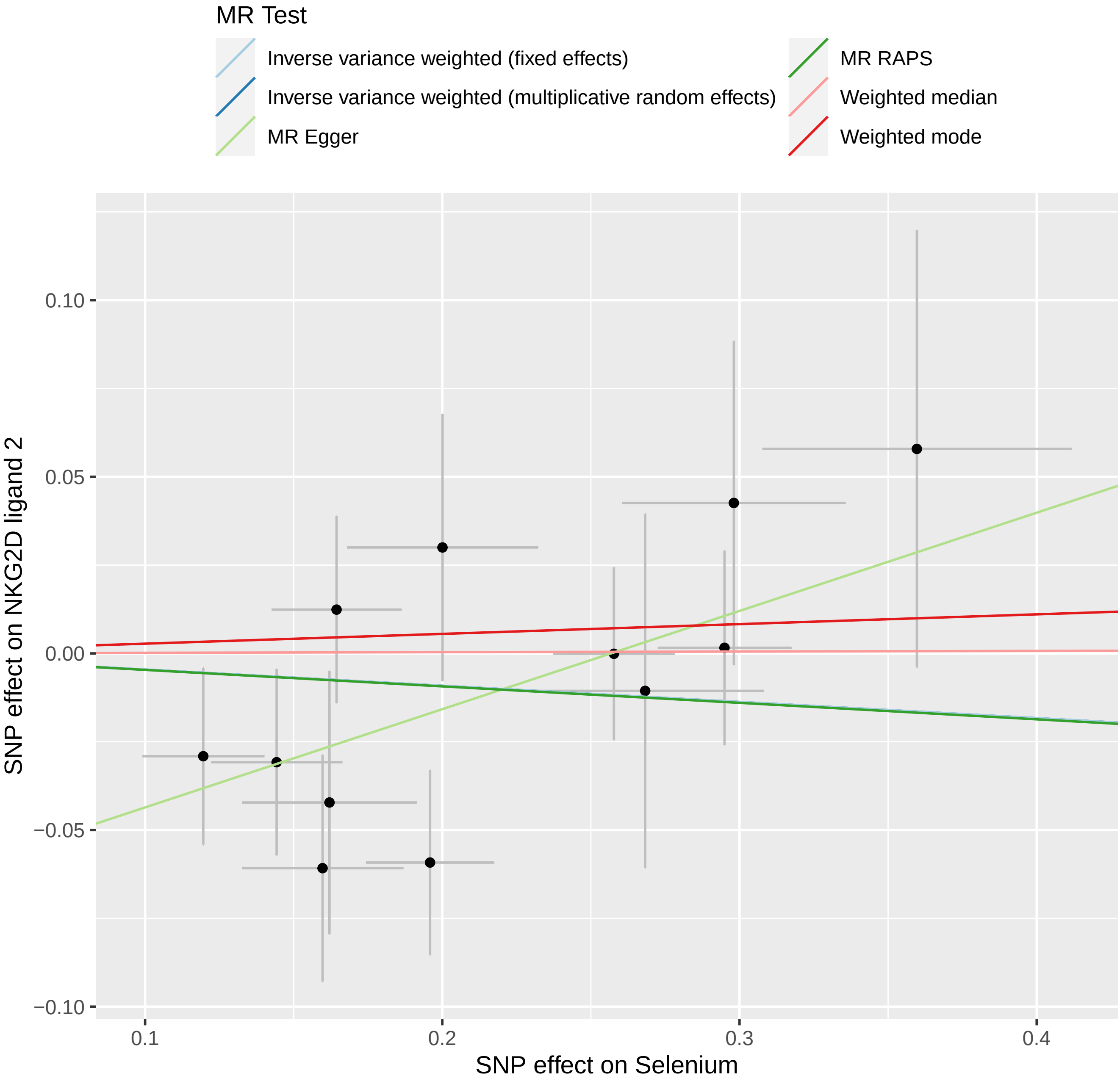

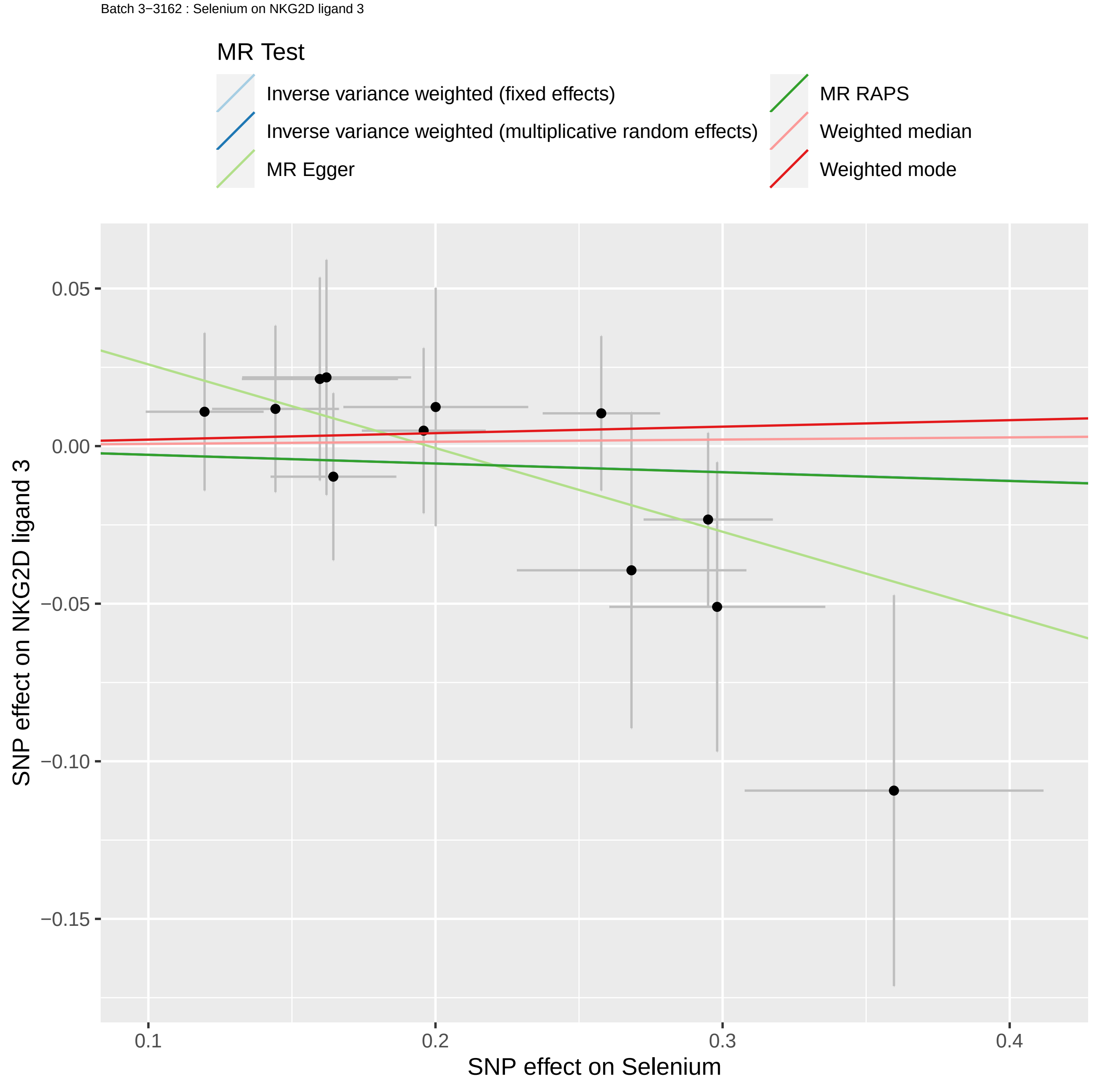

Batch 3−3162 : Selenium on NKG2D ligand 3
MR Test
Inverse variance weighted (fixed effects)
Inverse variance weighted (multiplicative random effects)
MR Egger
MR RAPS
Weighted median
Weighted mode
0.05
0.00
−0.05
−0.10
−0.15
0.1
0.2
0.3
0.4
SNP effect on NKG2D ligand 3
SNP effect on Selenium

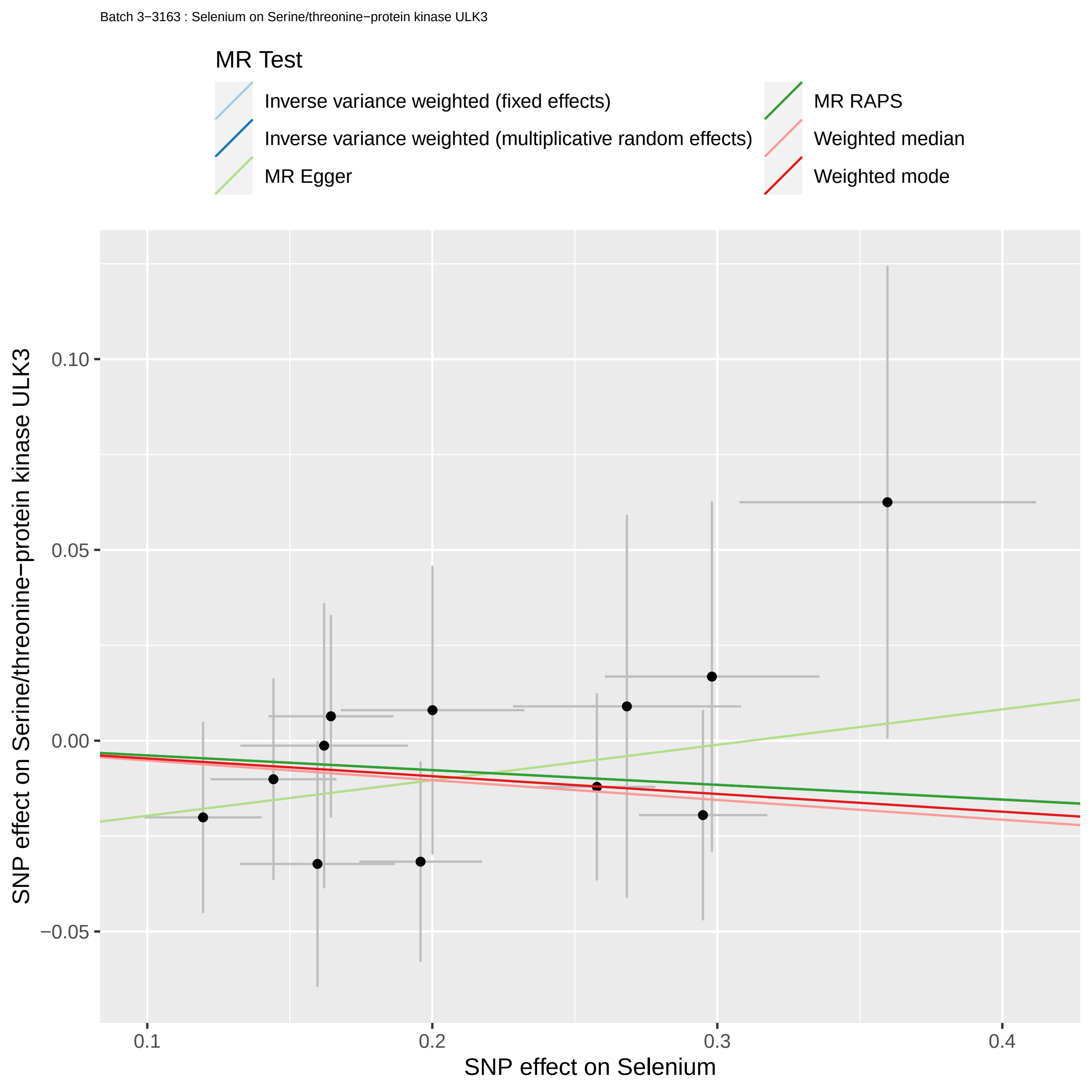

Batch 3−3163 : Selenium on Serine/threonine−protein kinase ULK3
MR Test
Inverse variance weighted (fixed effects)
Inverse variance weighted (multiplicative random effects)
MR Egger
MR RAPS
Weighted median
Weighted mode
0.10
0.05
0.00
−0.05
0.1
0.2
0.3
0.4
SNP effect on Serine/threonine−protein kinase ULK3
SNP effect on Selenium

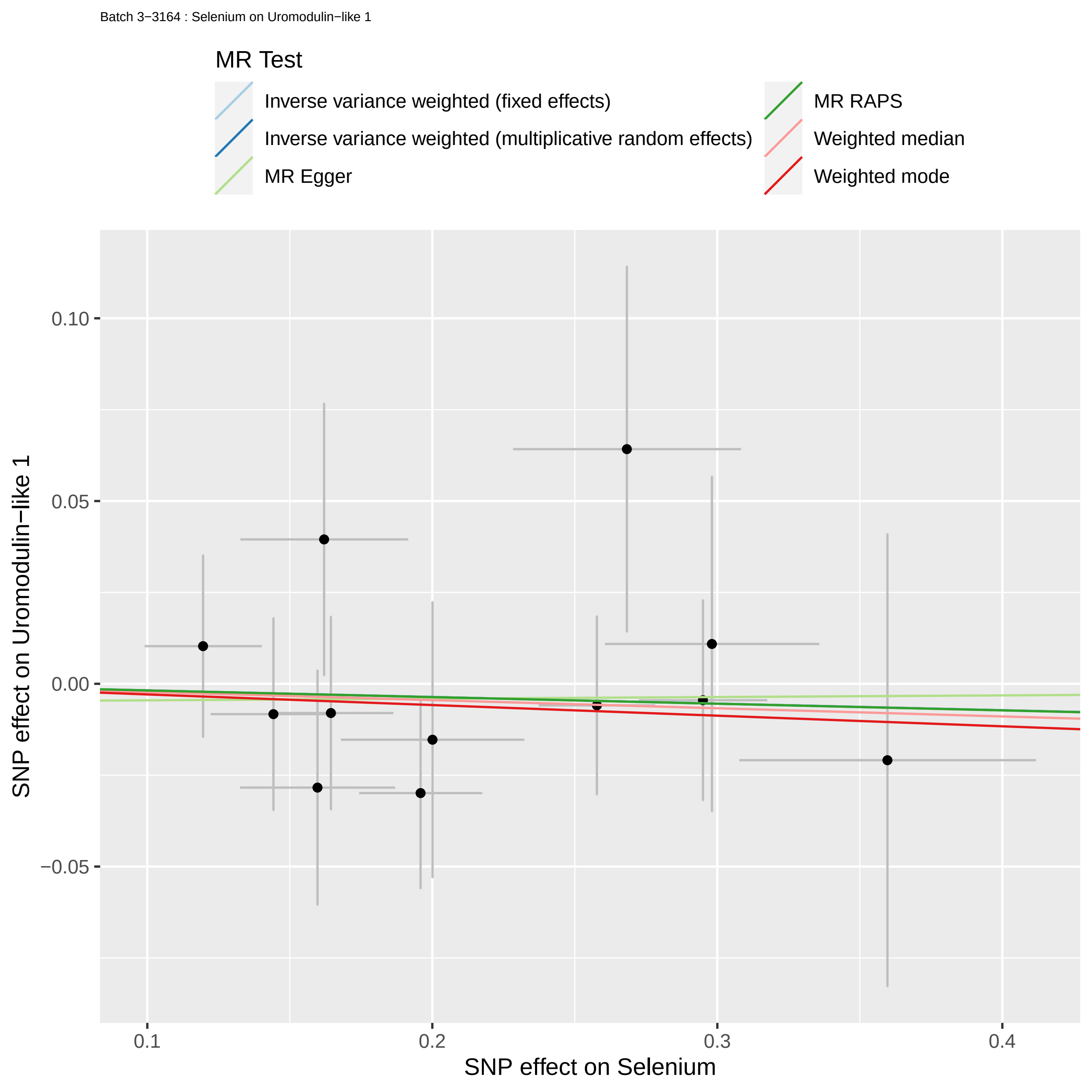

Batch 3−3164 : Selenium on Uromodulin−like 1
MR Test
Inverse variance weighted (fixed effects)
Inverse variance weighted (multiplicative random effects)
MR Egger
MR RAPS
Weighted median
Weighted mode
0.10
0.05
0.00
−0.05
0.1
0.2
0.3
0.4
SNP effect on Selenium
SNP effect on Uromodulin−like 1

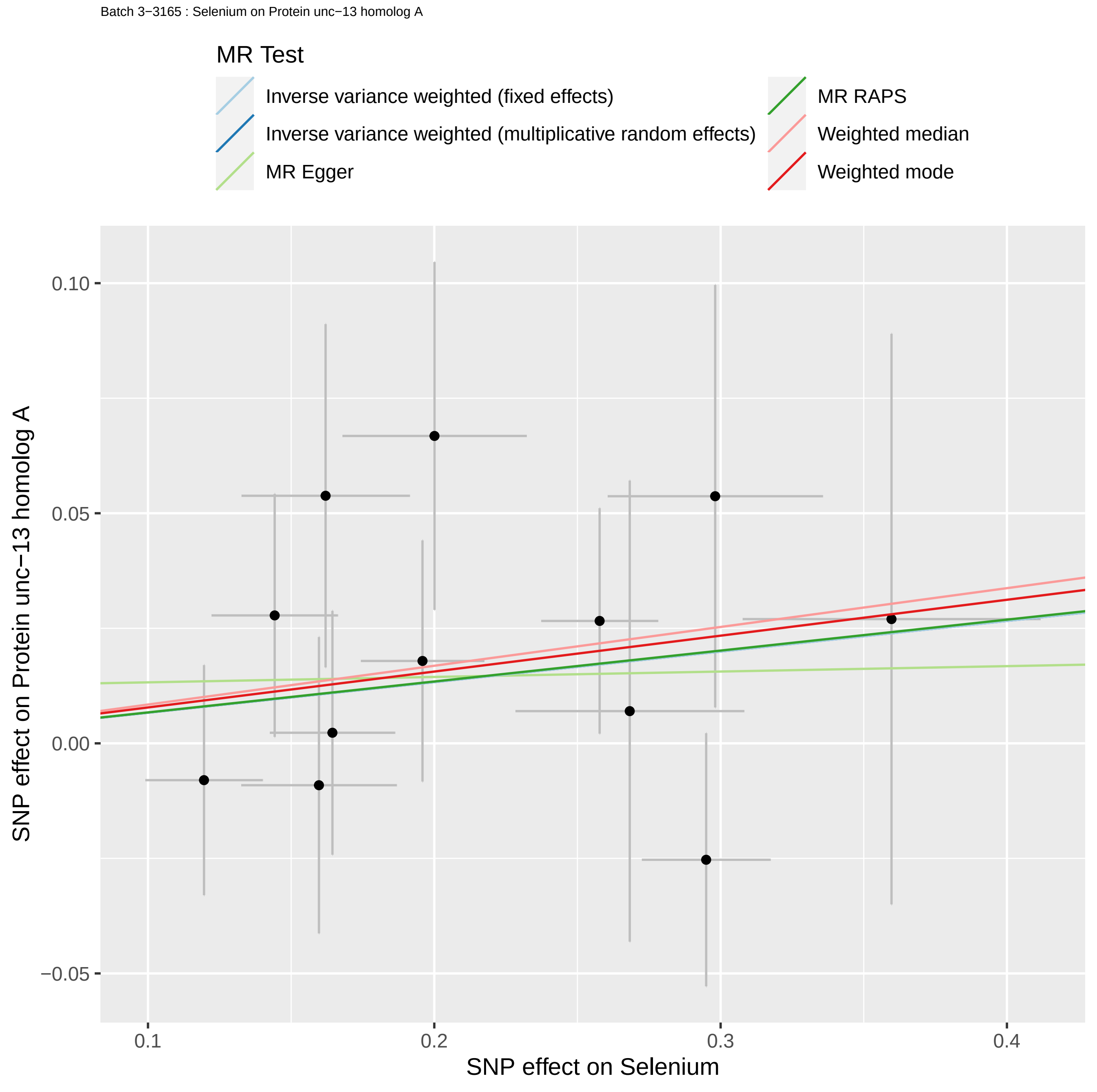

Batch 3−3165 : Selenium on Protein unc−13 homolog A
MR Test
Inverse variance weighted (fixed effects)
Inverse variance weighted (multiplicative random effects)
MR Egger
MR RAPS
Weighted median
Weighted mode
0.10
0.05
0.00
−0.05
0.1
0.2
0.3
0.4
SNP effect on Protein unc−13 homolog A
SNP effect on Selenium

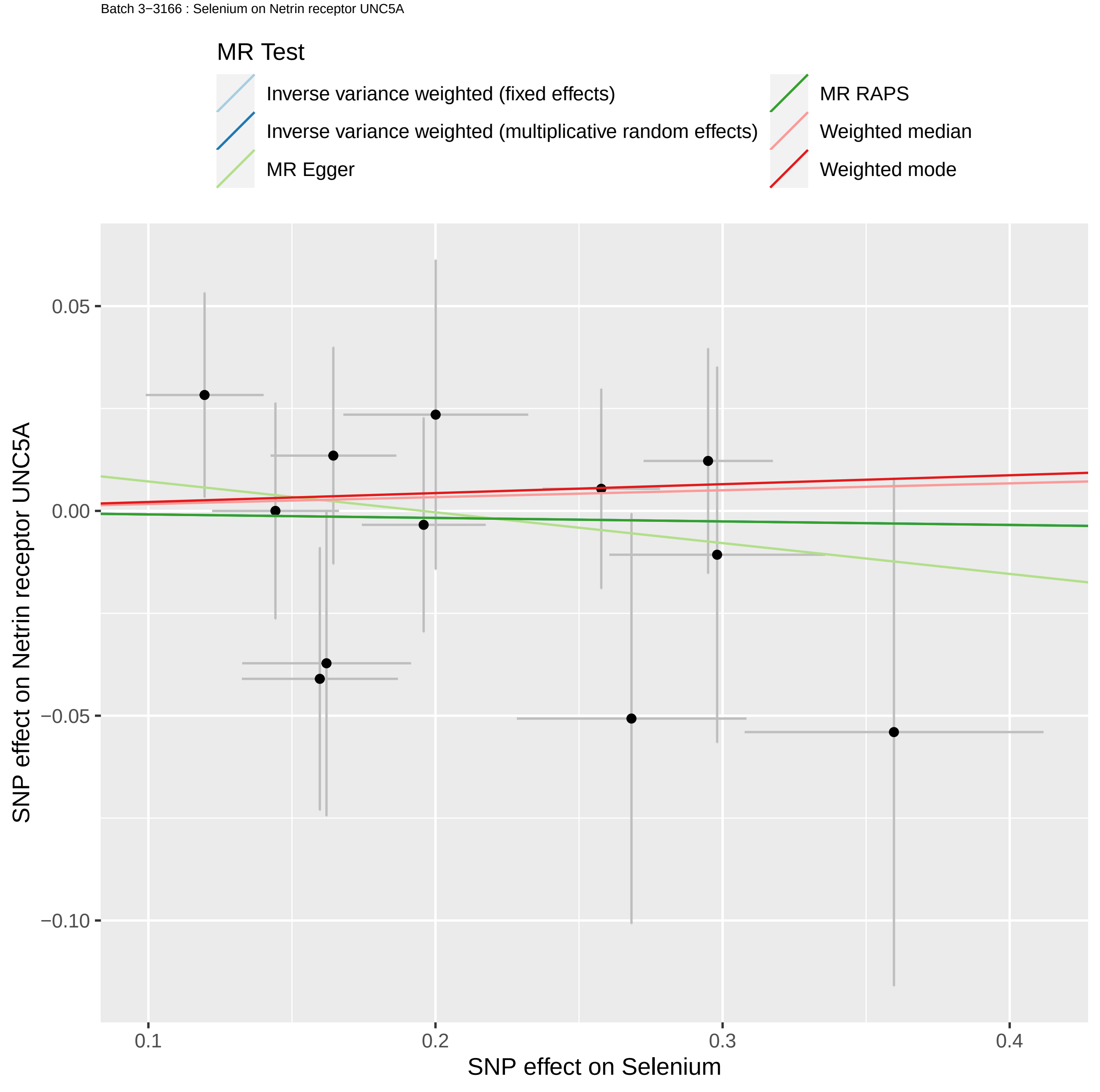
Batch 3−3166 : Selenium on Netrin receptor UNC5A
MR Test
Inverse variance weighted (fixed effects)
Inverse variance weighted (multiplicative random effects)
MR Egger
MR RAPS
Weighted median
Weighted mode
SNP effect on Netrin receptor UNC5A
0.05
0.00
−0.05
−0.10
0.1
0.2
0.3
0.4
SNP effect on Selenium

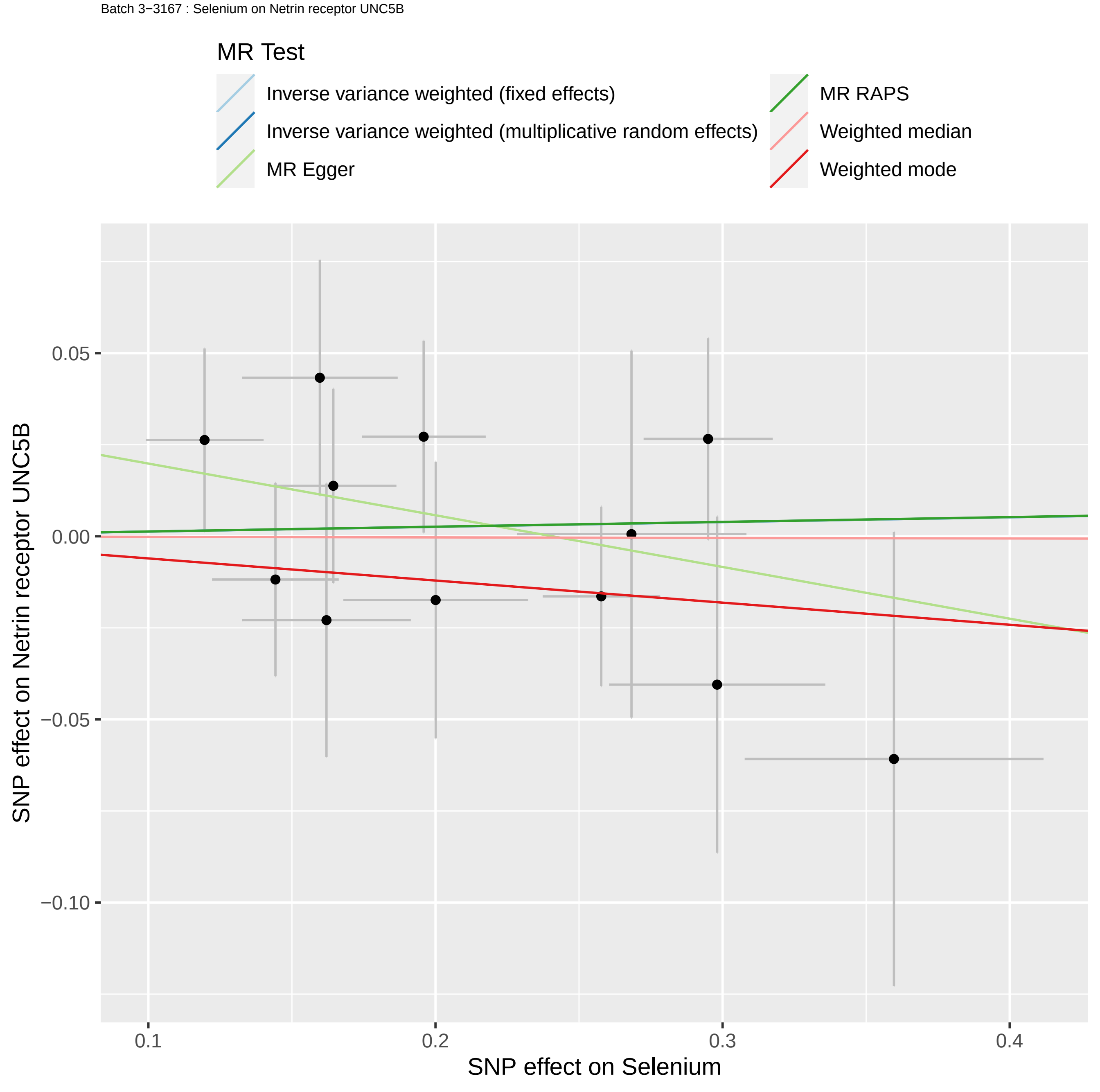
Batch 3−3167 : Selenium on Netrin receptor UNC5B
MR Test
Inverse variance weighted (fixed effects)
Inverse variance weighted (multiplicative random effects)
MR Egger
MR RAPS
Weighted median
Weighted mode
0.05
0.00
−0.05
−0.10
0.1
0.2
0.3
0.4
SNP effect on Netrin receptor UNC5B
SNP effect on Selenium

Batch 3−3168 : Selenium on Netrin receptor UNC5C

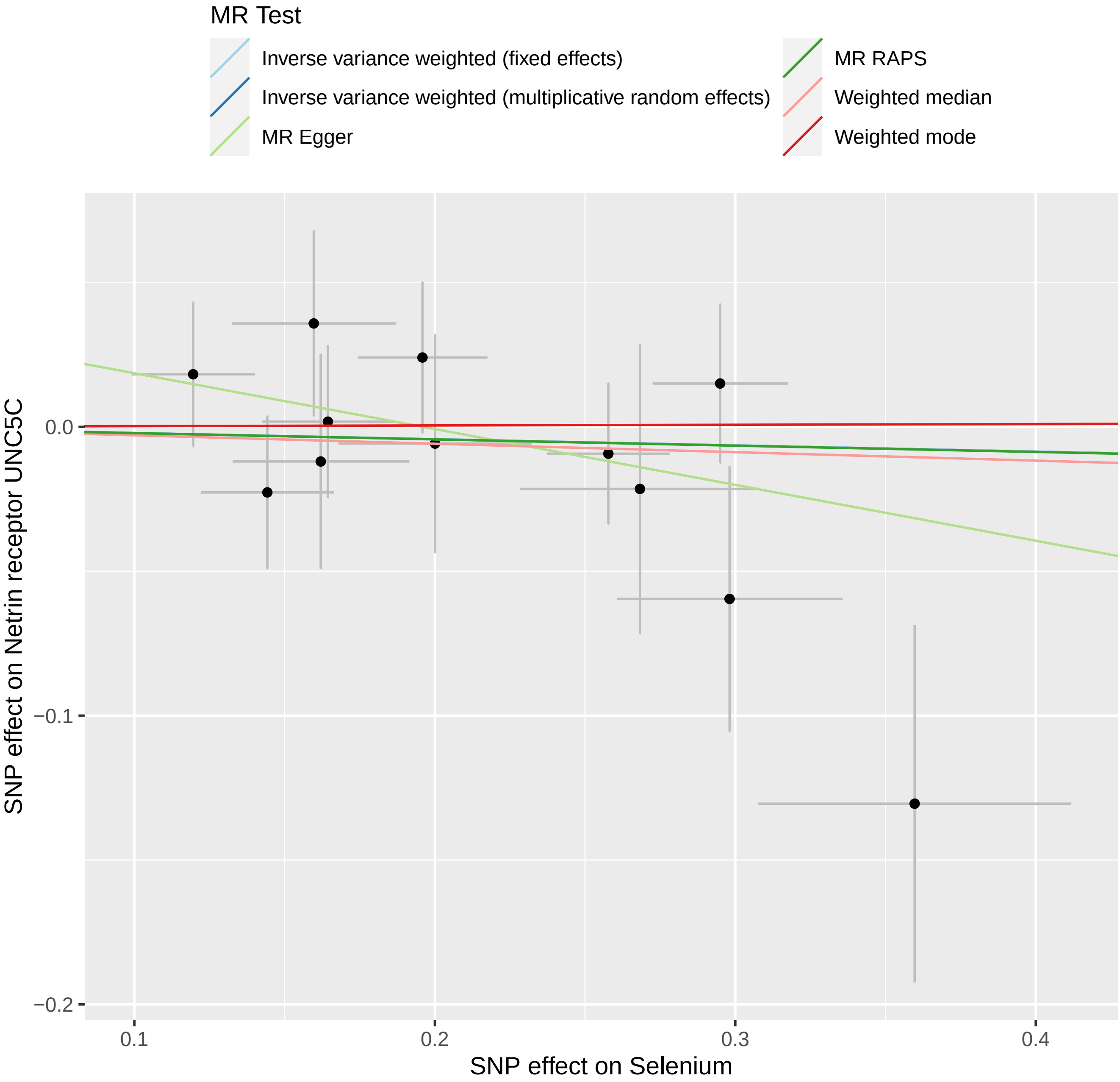

Batch 3−3169 : Selenium on Netrin receptor UNC5D

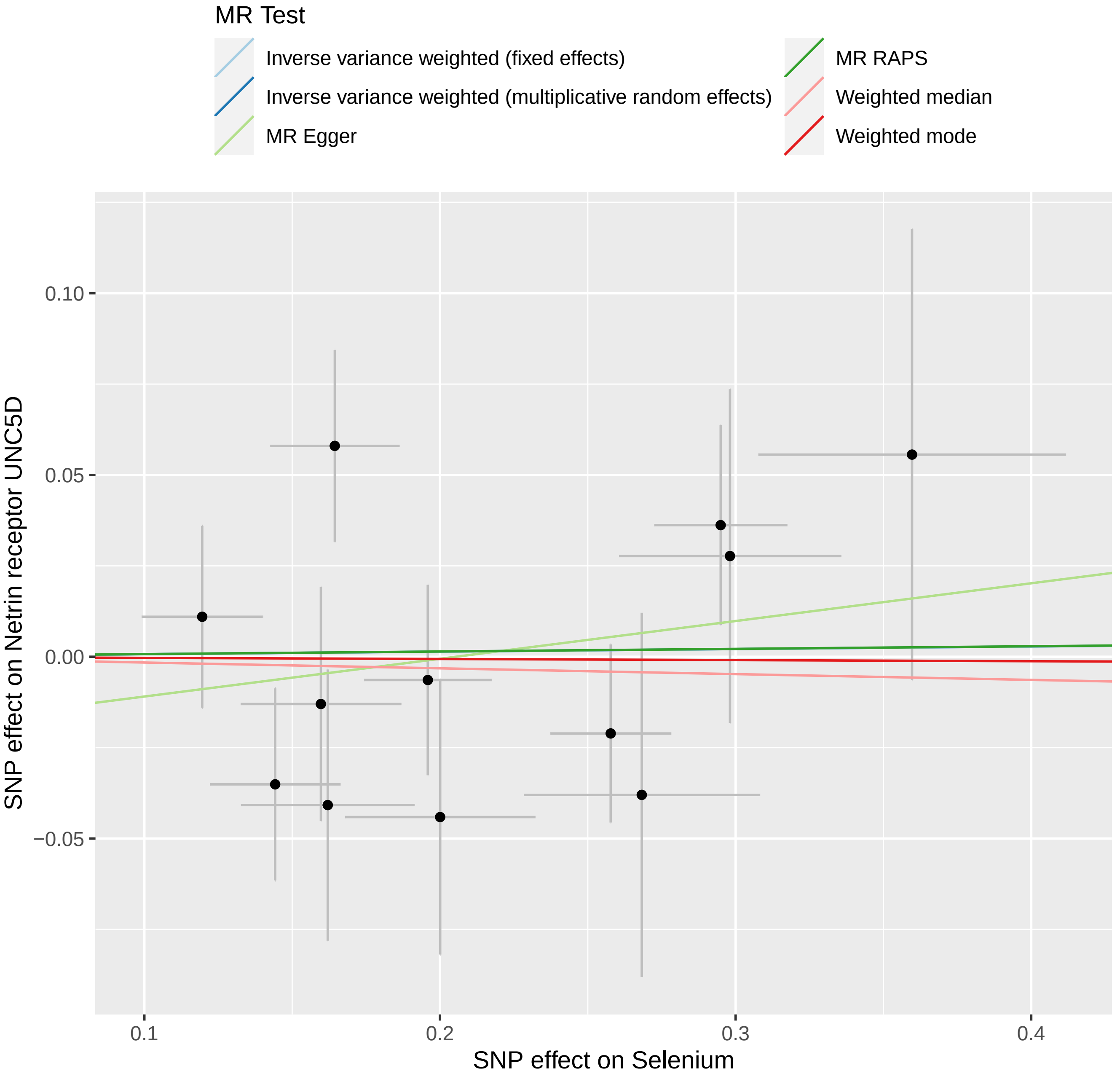

Batch 3−3170 : Selenium on Netrin receptor UNC5D

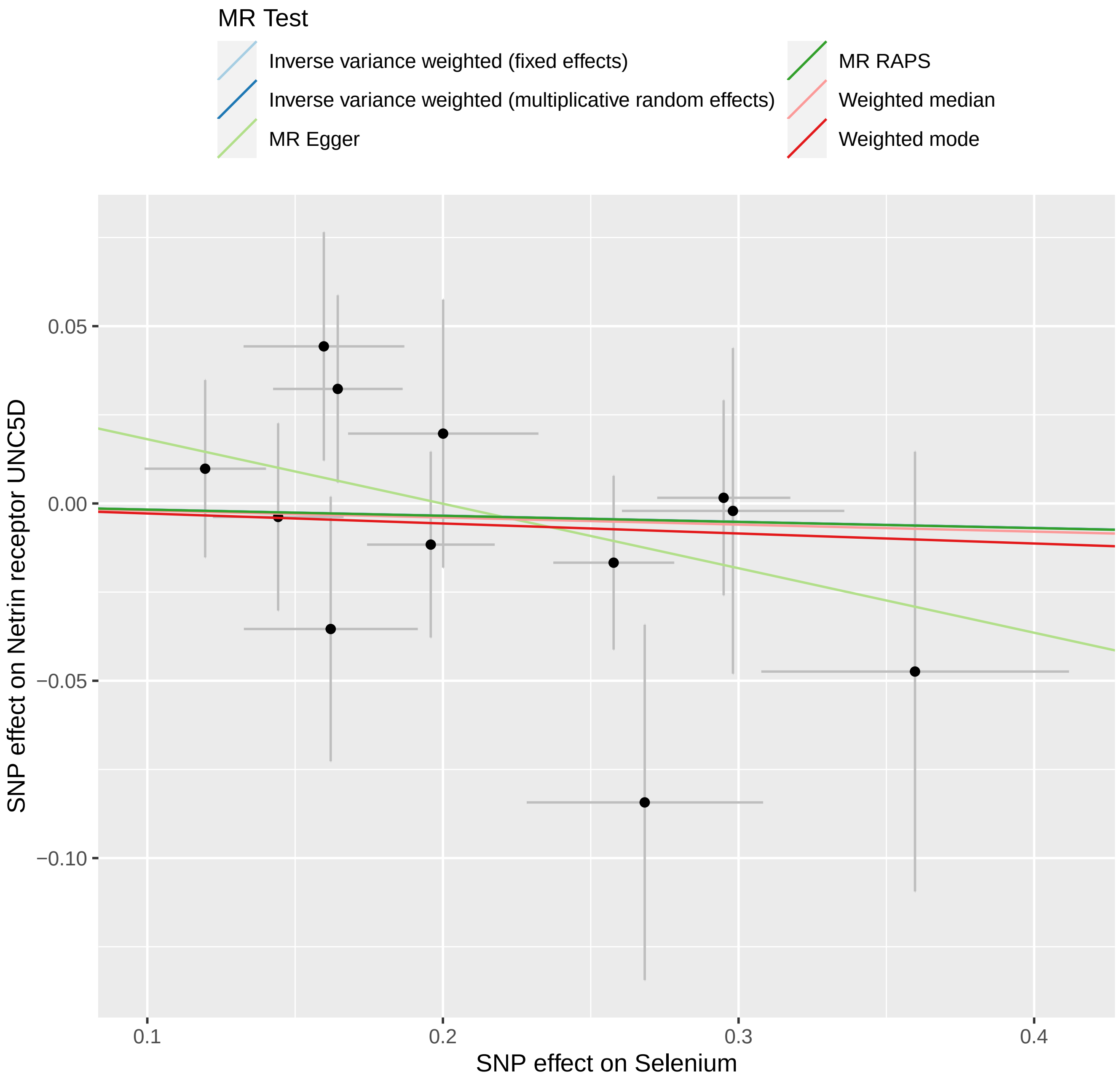

Batch 3−3171 : Selenium on Protein unc−93 homolog B1

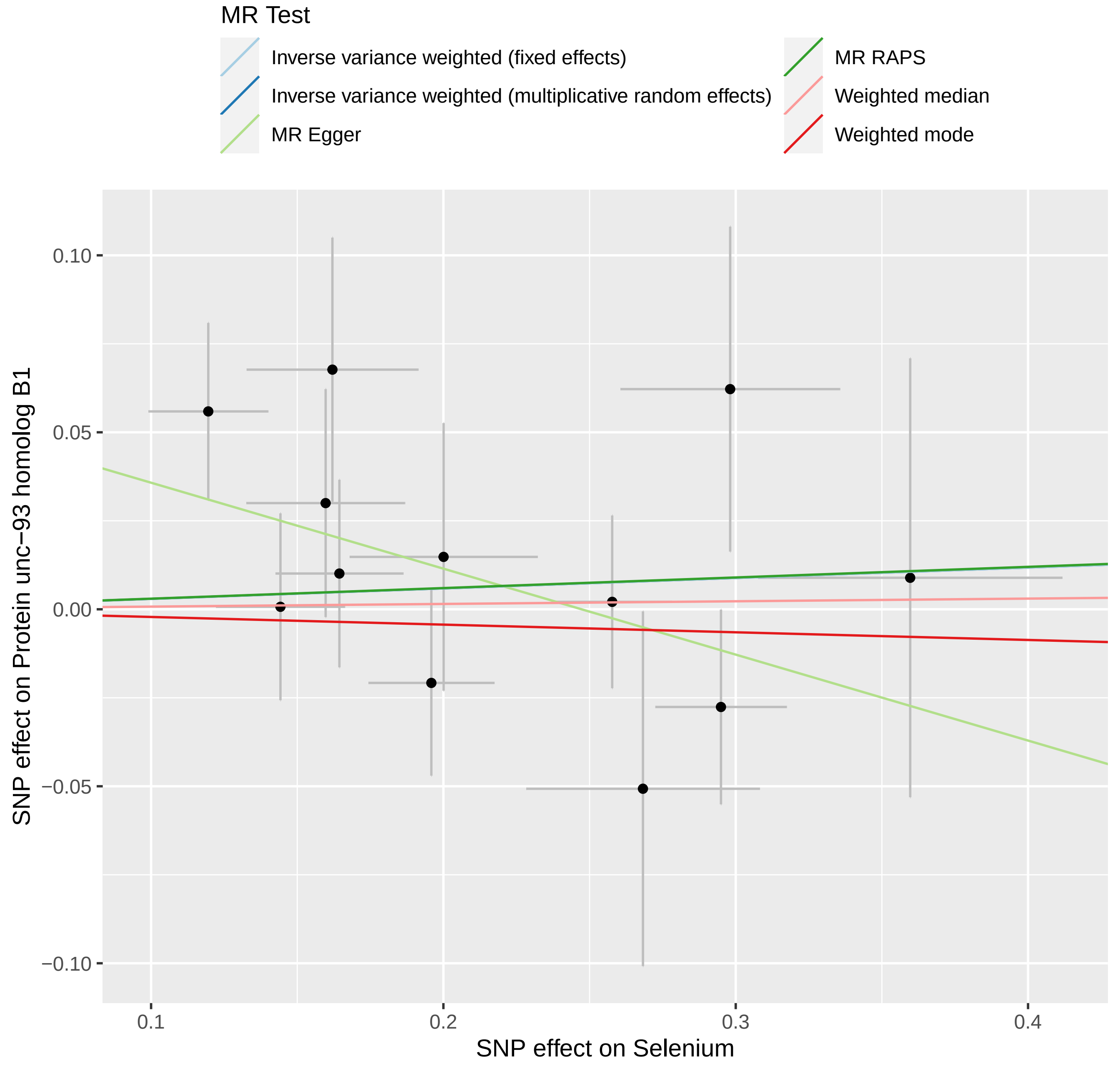

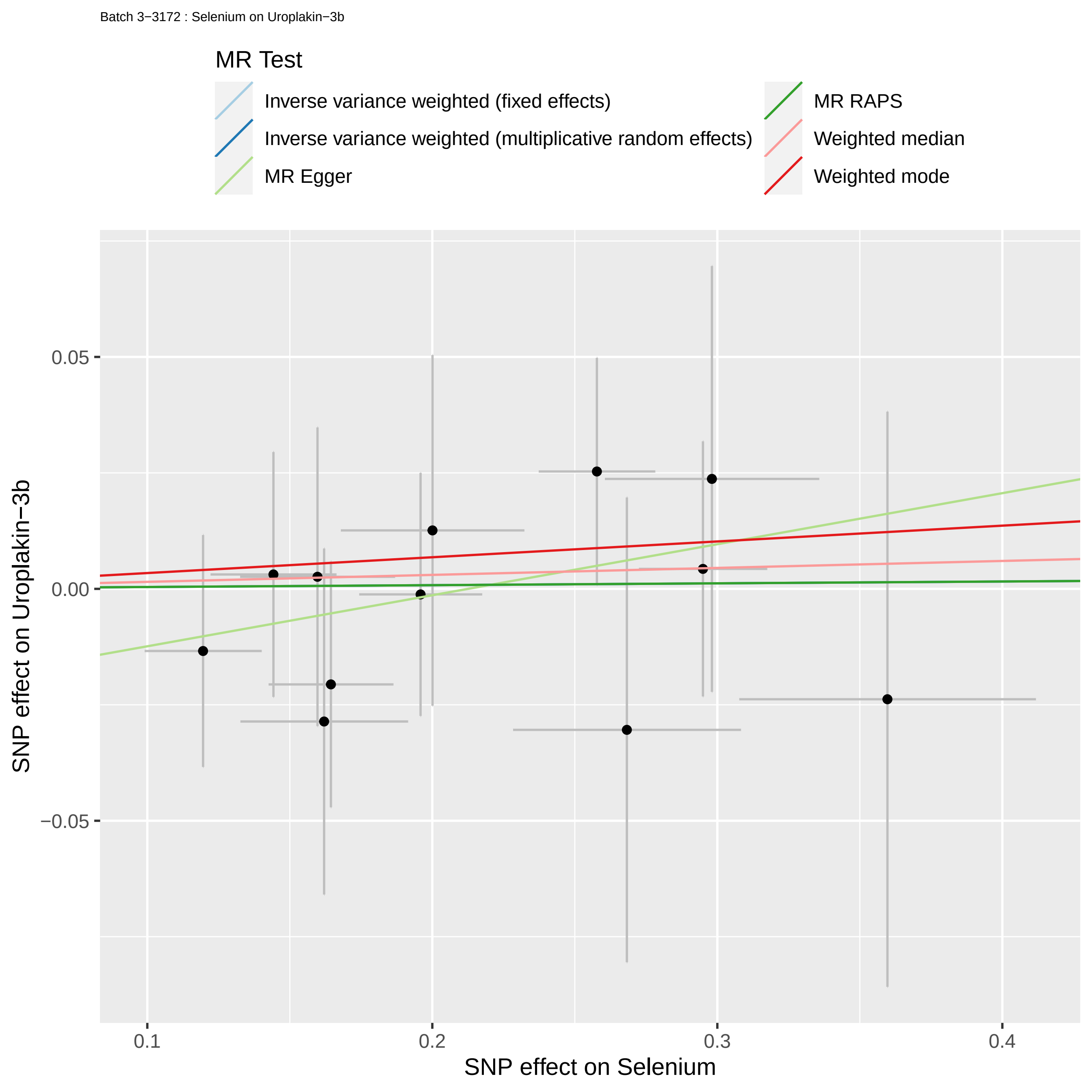

Batch 3−3172 : Selenium on Uroplakin−3b
MR Test
Inverse variance weighted (fixed effects)
Inverse variance weighted (multiplicative random effects)
MR Egger
MR RAPS
Weighted median
Weighted mode
0.05
0.00
−0.05
0.1
0.2
0.3
0.4
SNP effect on Uroplakin−3b
SNP effect on Selenium

Batch 3−3173 : Selenium on Uroplakin−3b−like protein

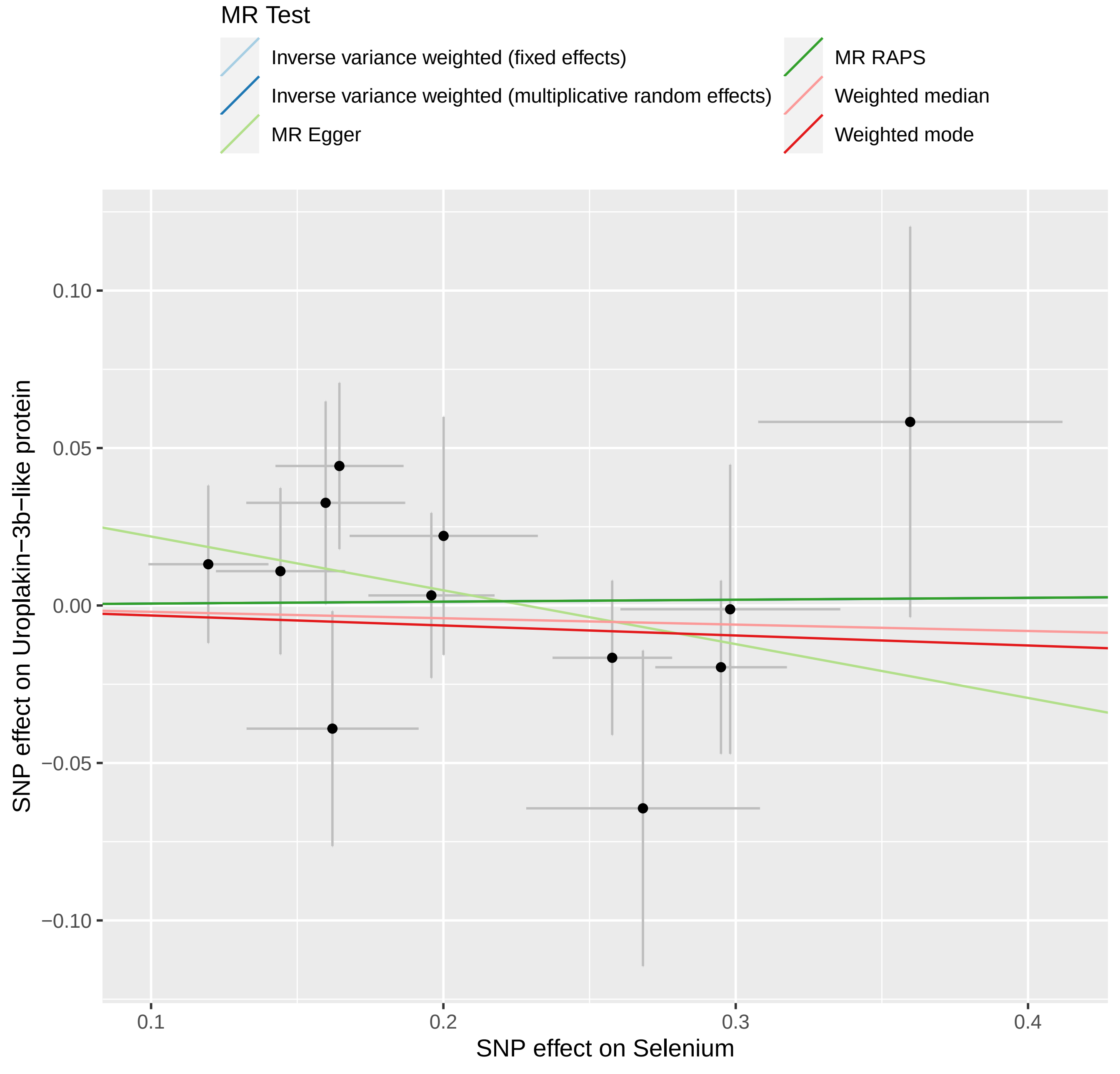

Batch 3−3174 : Selenium on Ubiquinol−cytochrome−c reductase complex assembly factor 3

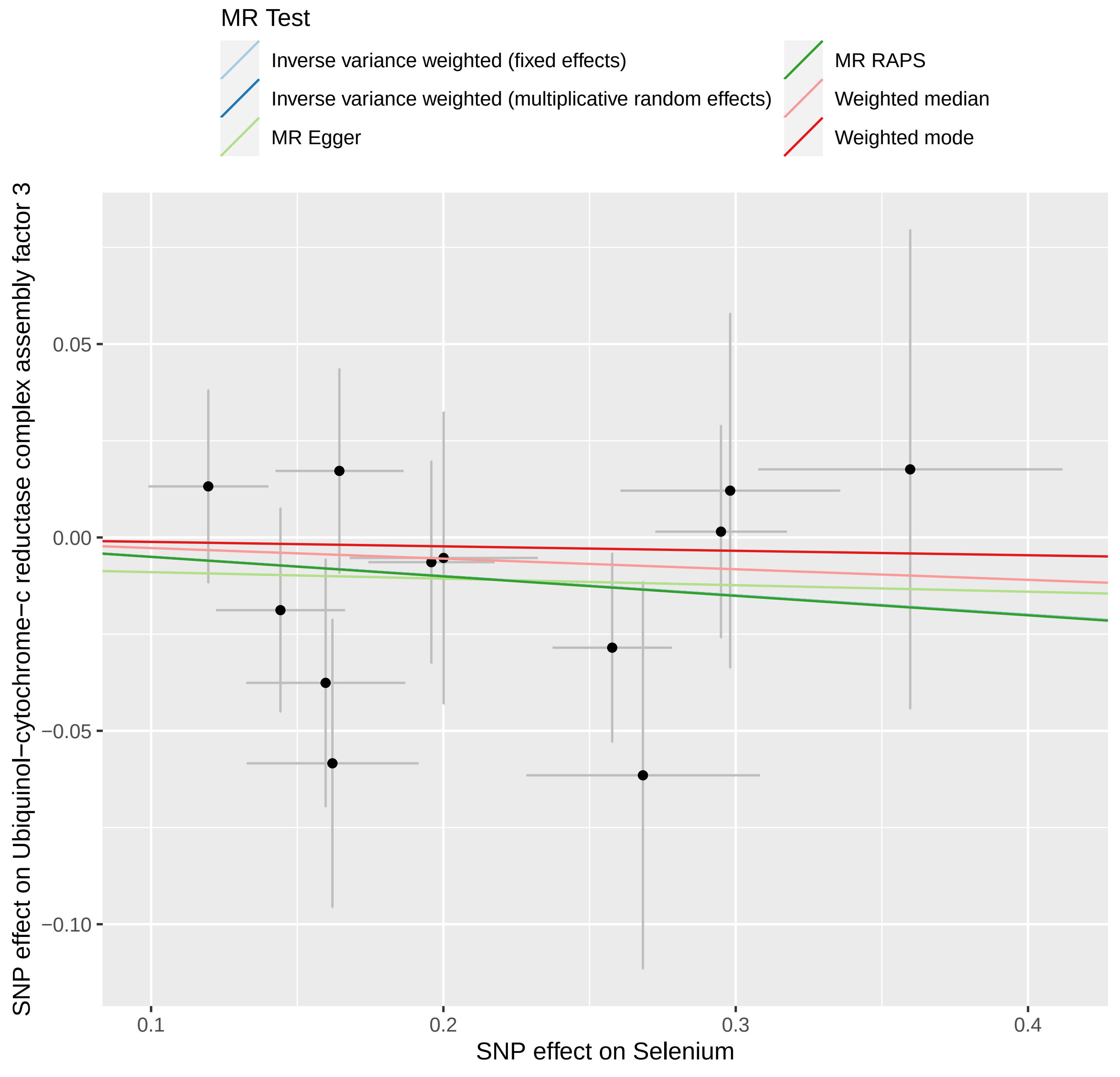

Batch 3−3175 : Selenium on Cytochrome b−c1 complex subunit 7

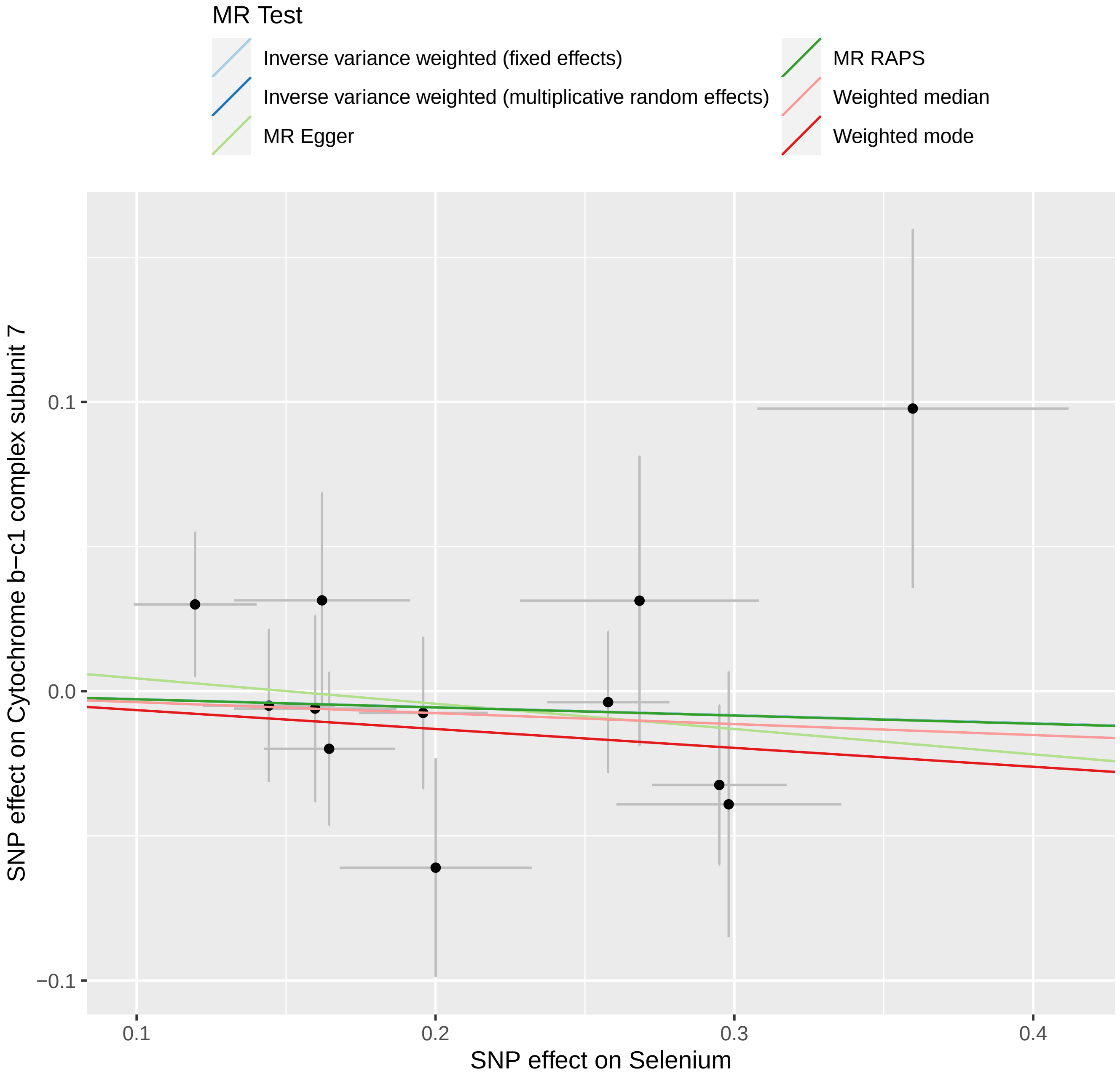

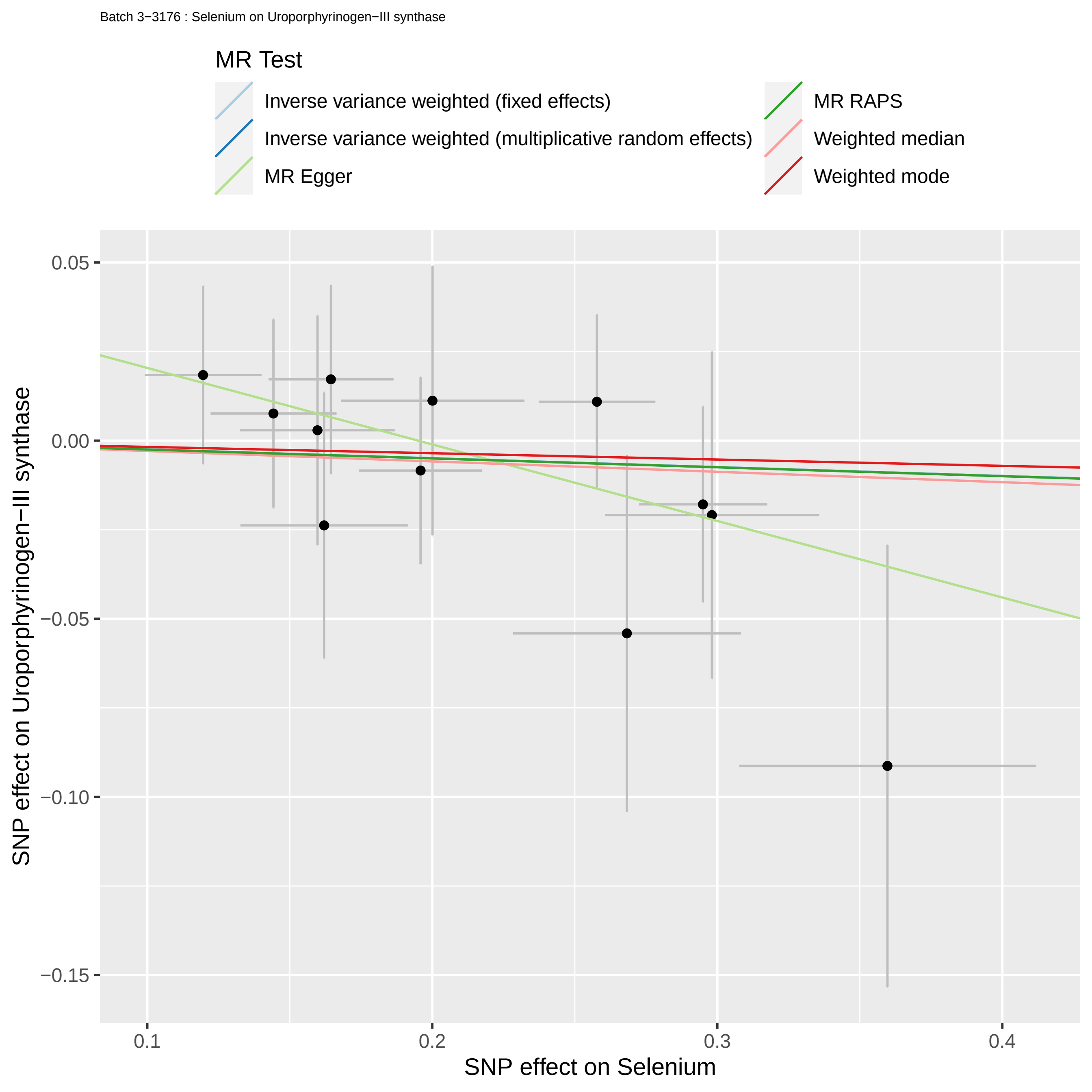

Batch 3−3176 : Selenium on Uroporphyrinogen−III synthase
MR Test
Inverse variance weighted (fixed effects)
Inverse variance weighted (multiplicative random effects)
MR Egger
MR RAPS
Weighted median
Weighted mode
0.05
0.00
−0.05
−0.10
−0.15
0.1
0.2
0.3
0.4
SNP effect on Uroporphyrinogen−III synthase
SNP effect on Selenium

Batch 3−3177 : Selenium on U6 snRNA phosphodiesterase

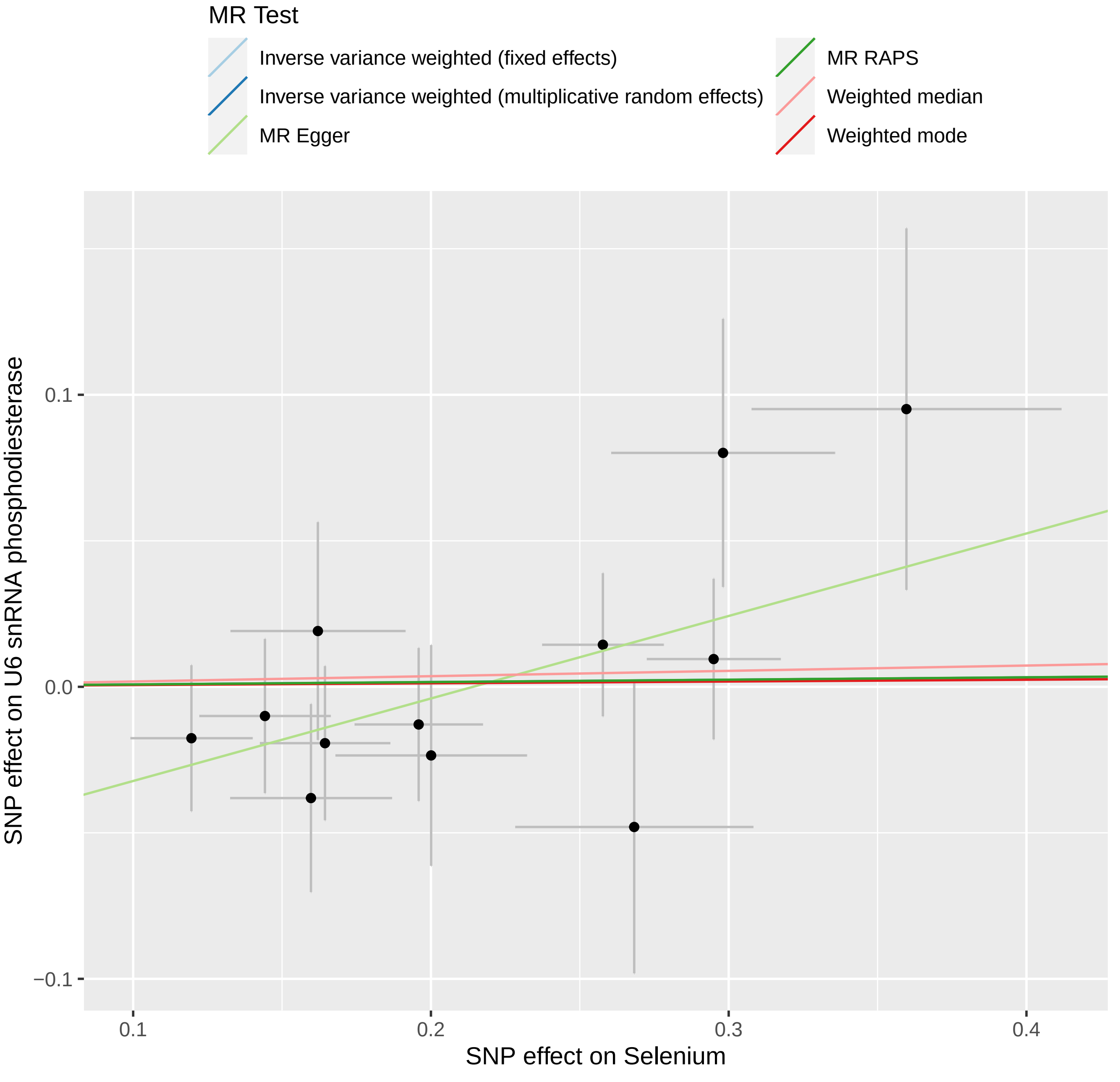

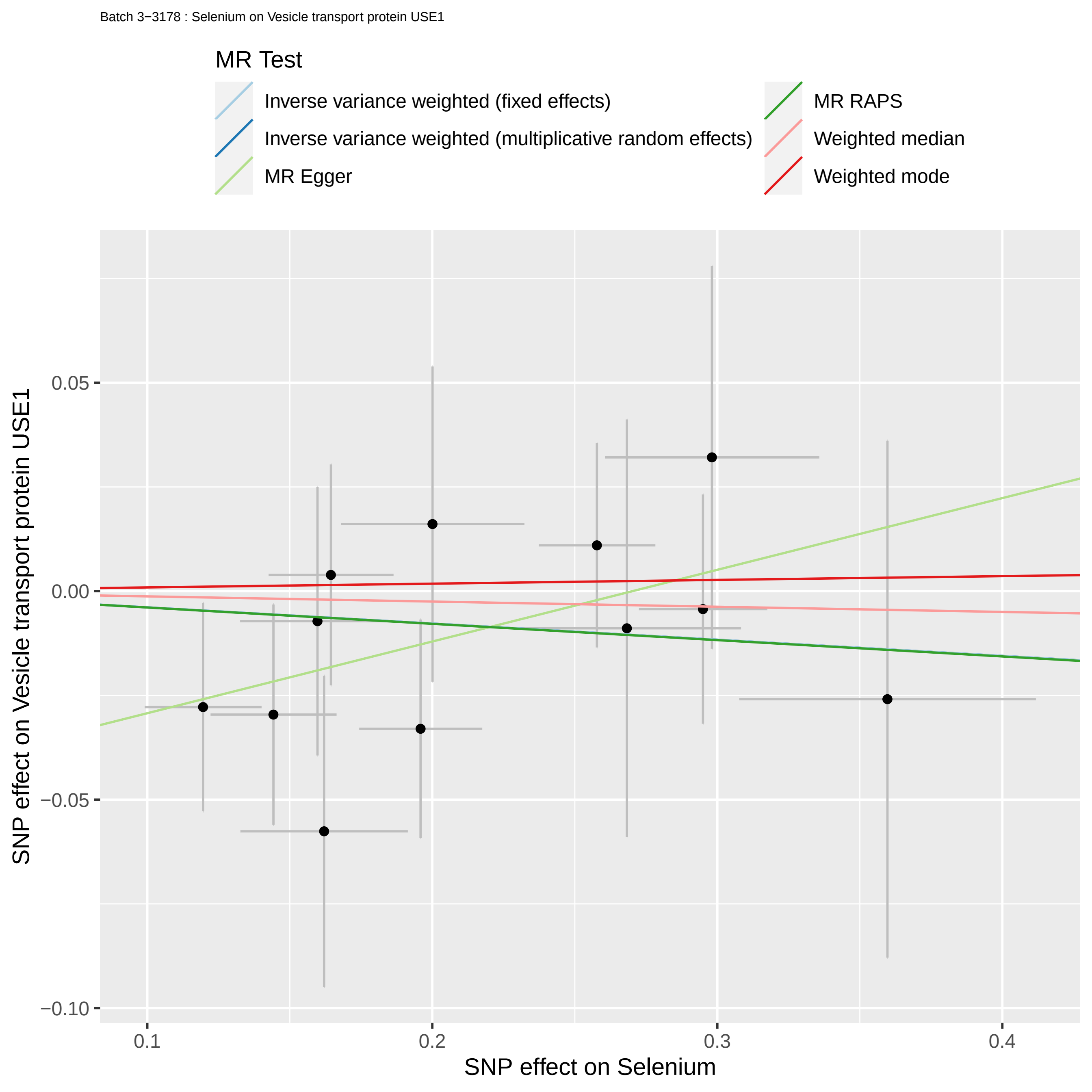

Batch 3−3178 : Selenium on Vesicle transport protein USE1
MR Test
Inverse variance weighted (fixed effects)
Inverse variance weighted (multiplicative random effects)
MR Egger
MR RAPS
Weighted median
Weighted mode
0.05
0.00
−0.05
−0.10
0.1
0.2
0.3
0.4
SNP effect on Vesicle transport protein USE1
SNP effect on Selenium

Batch 3−3179 : Selenium on Ubiquitin carboxyl−terminal hydrolase 21

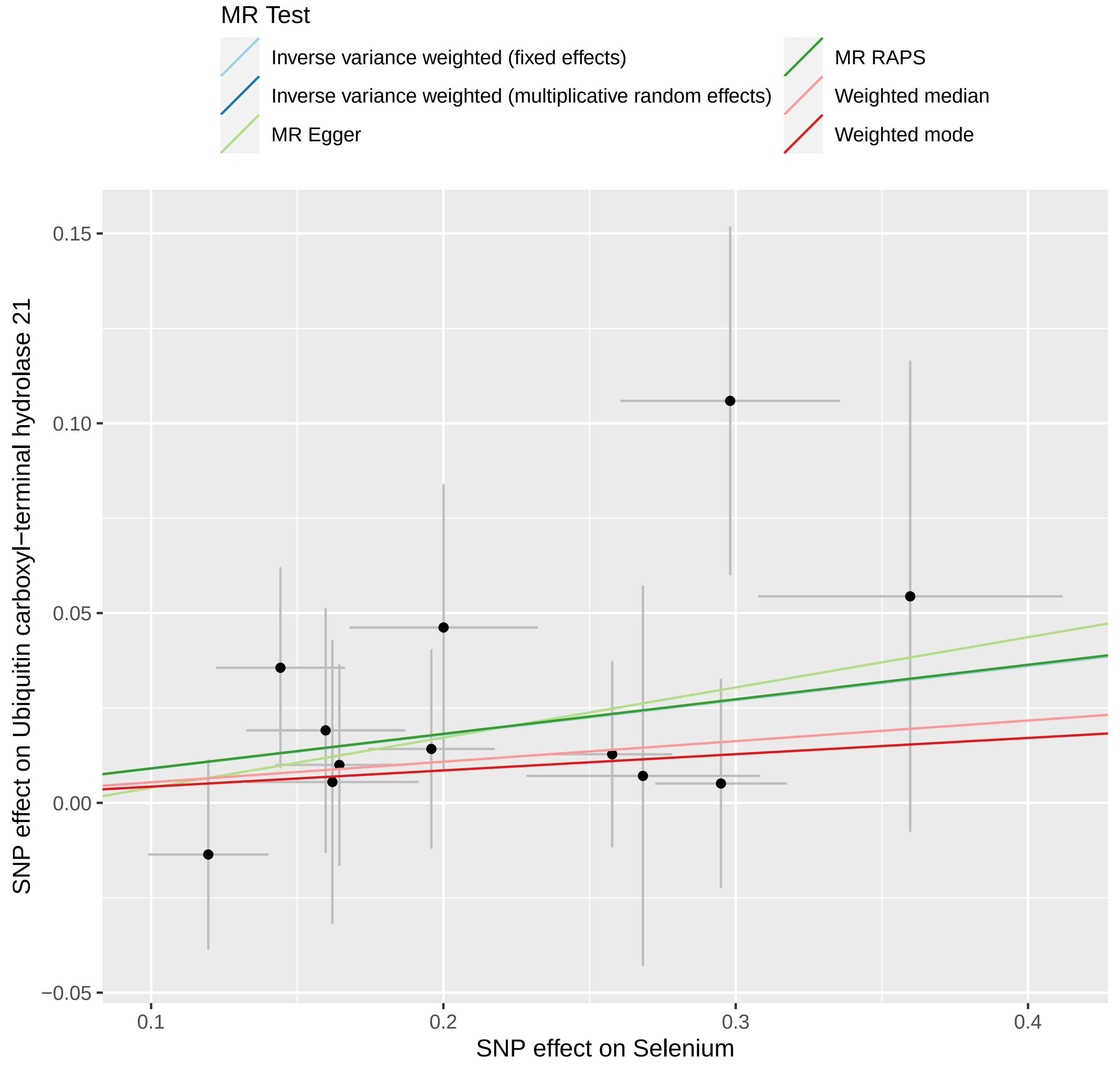

Batch 3−3180 : Selenium on Ubiquitin carboxyl−terminal hydrolase 25

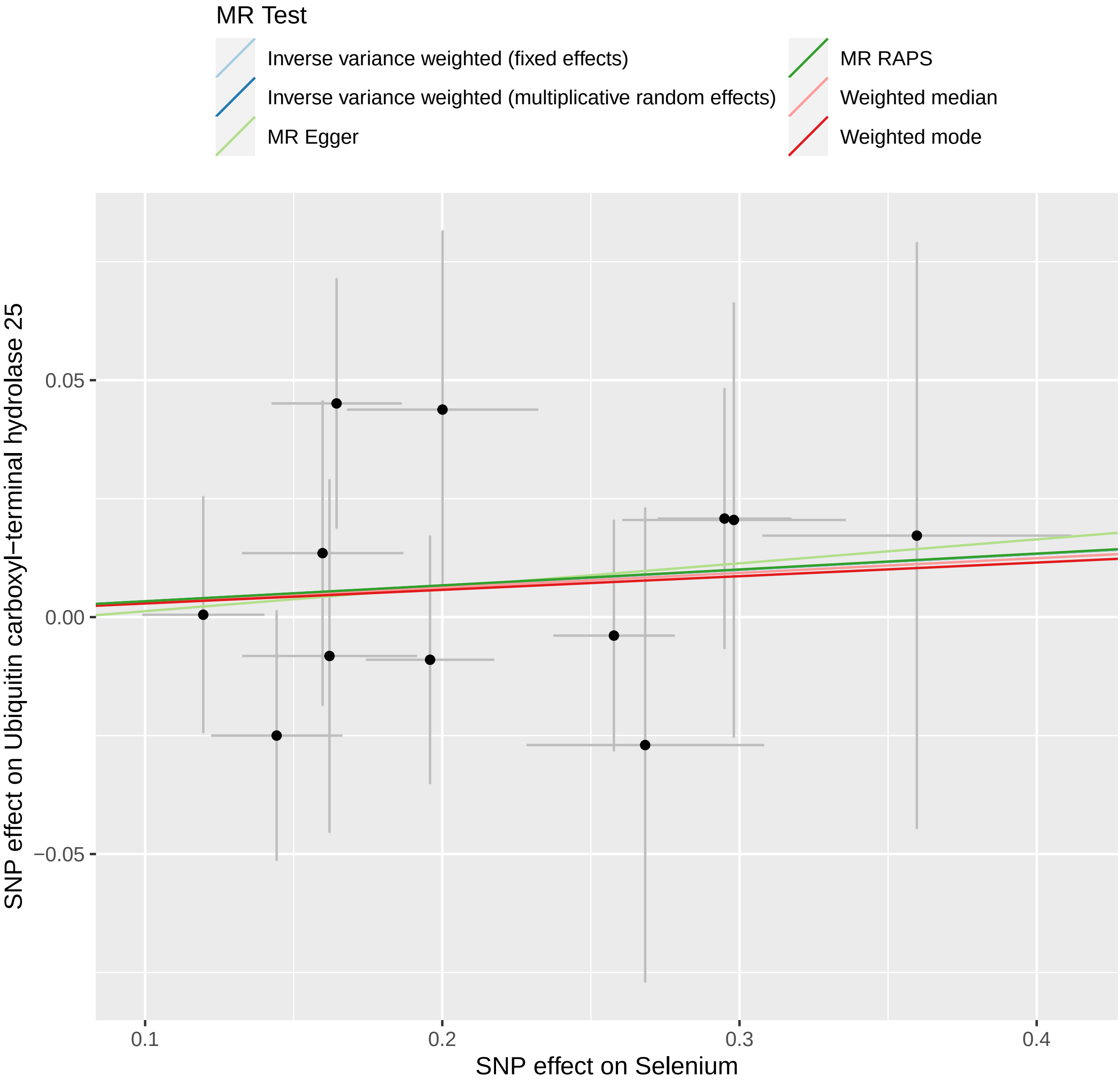

Batch 3−3181 : Selenium on Ubiquitin carboxyl−terminal hydrolase 8

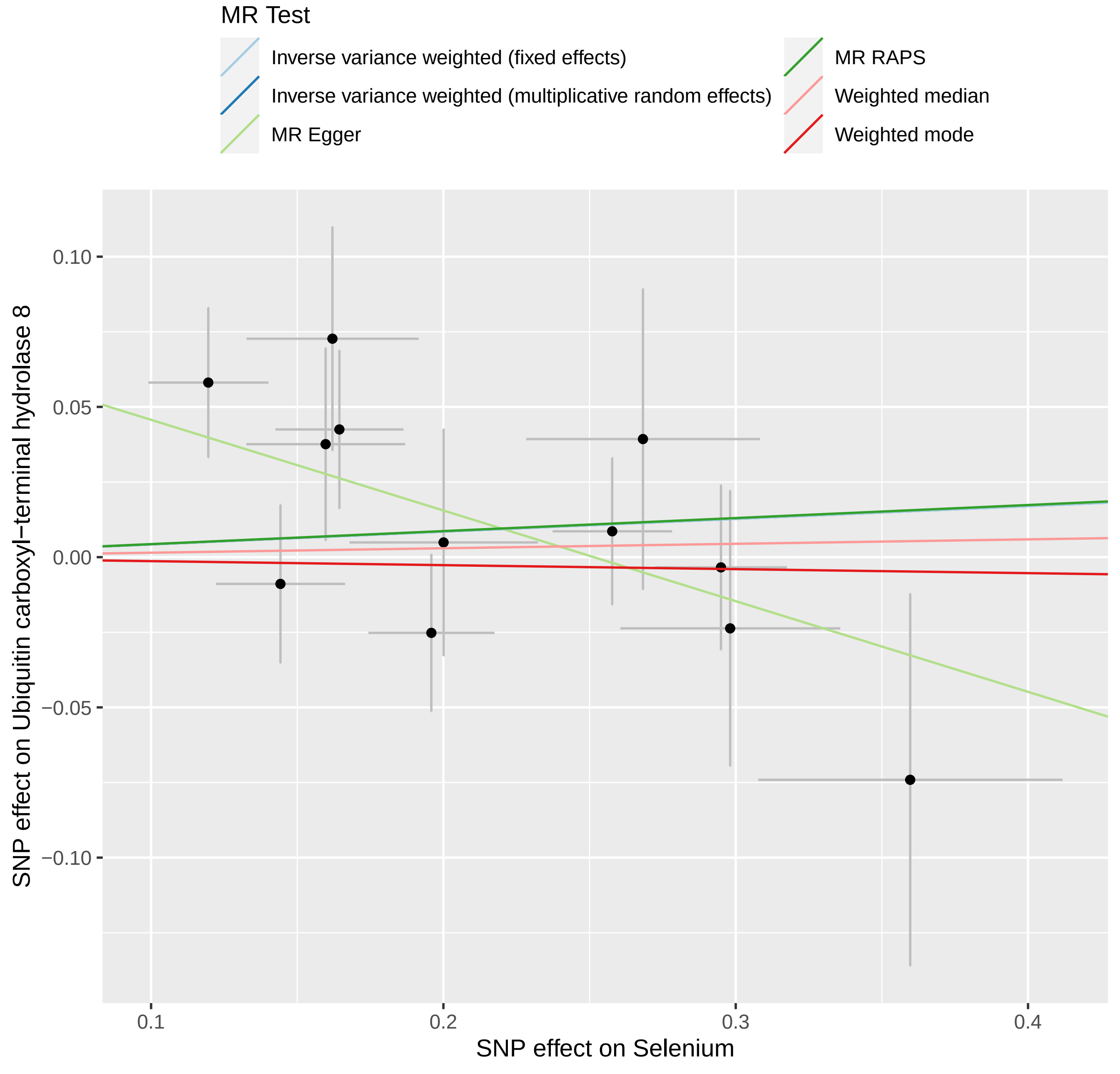

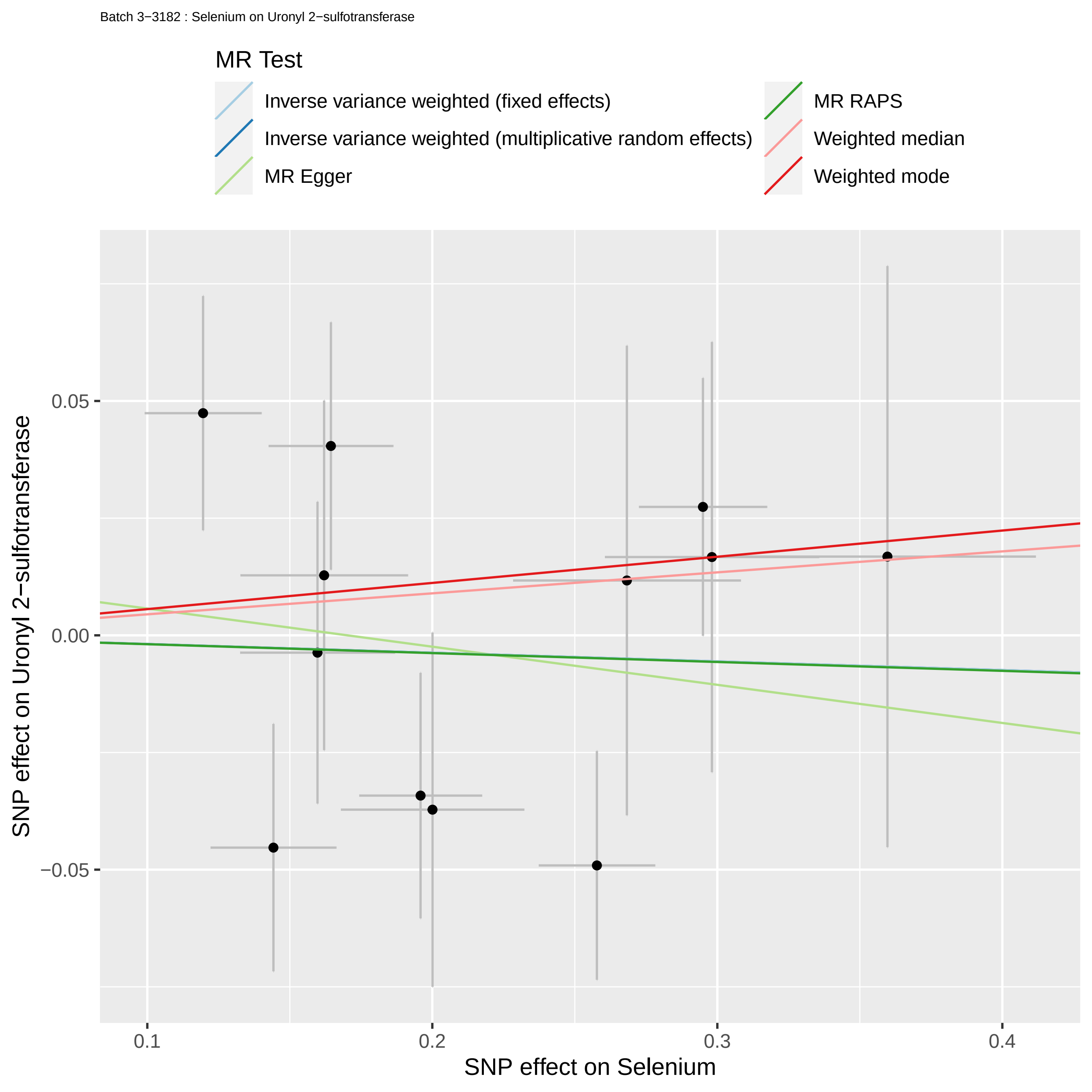
Batch 3−3182 : Selenium on Uronyl 2−sulfotransferase
MR Test
Inverse variance weighted (fixed effects)
Inverse variance weighted (multiplicative random effects)
MR Egger
MR RAPS
Weighted median
Weighted mode
0.05
0.00
−0.05
0.1
0.2
0.3
0.4
SNP effect on Selenium
SNP effect on Uronyl 2−sulfotransferase

Batch 3−3183 : Selenium on Urotensin−2 receptor

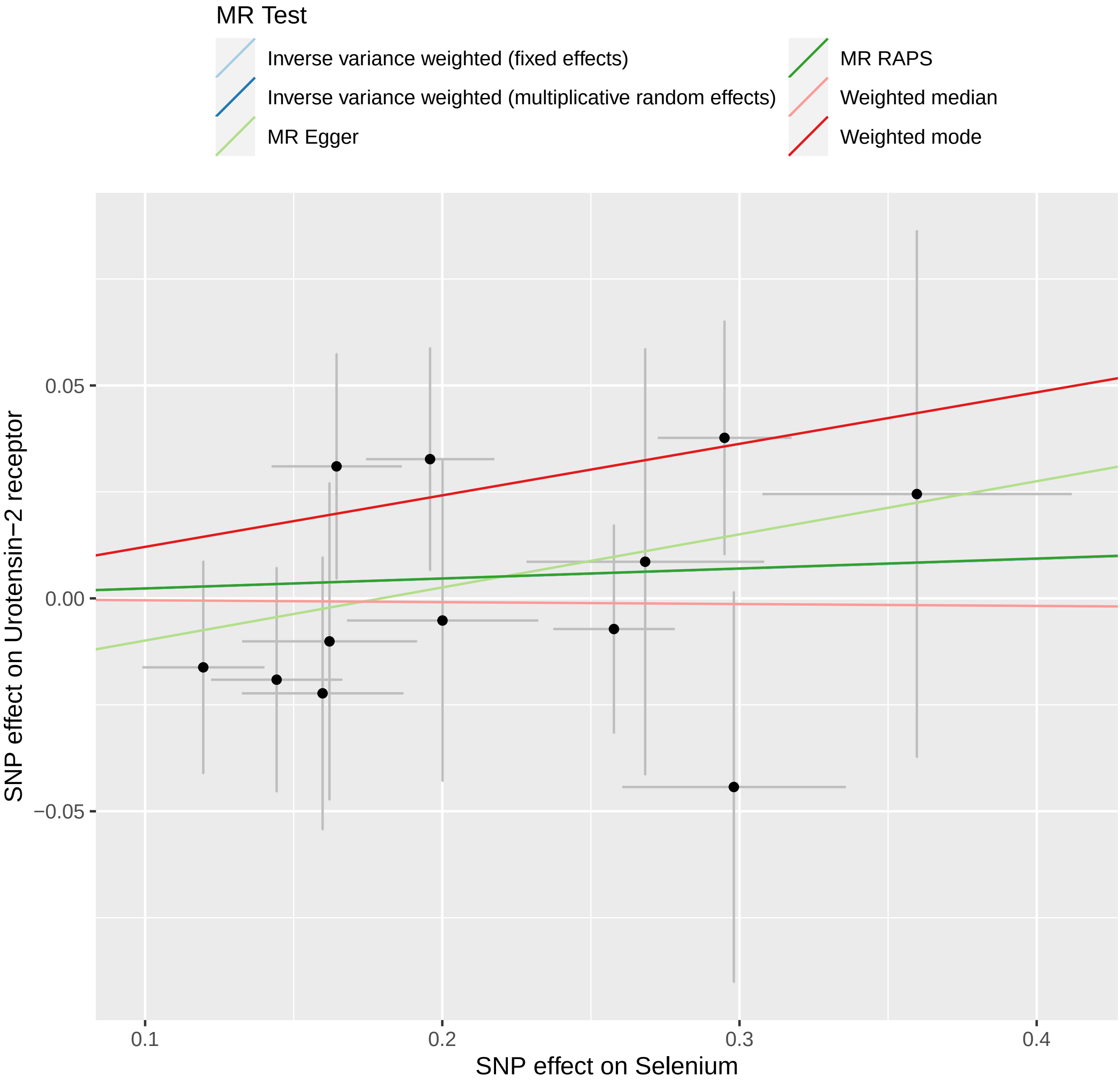

Batch 3−3184 : Selenium on UDP−glucuronic acid decarboxylase 1

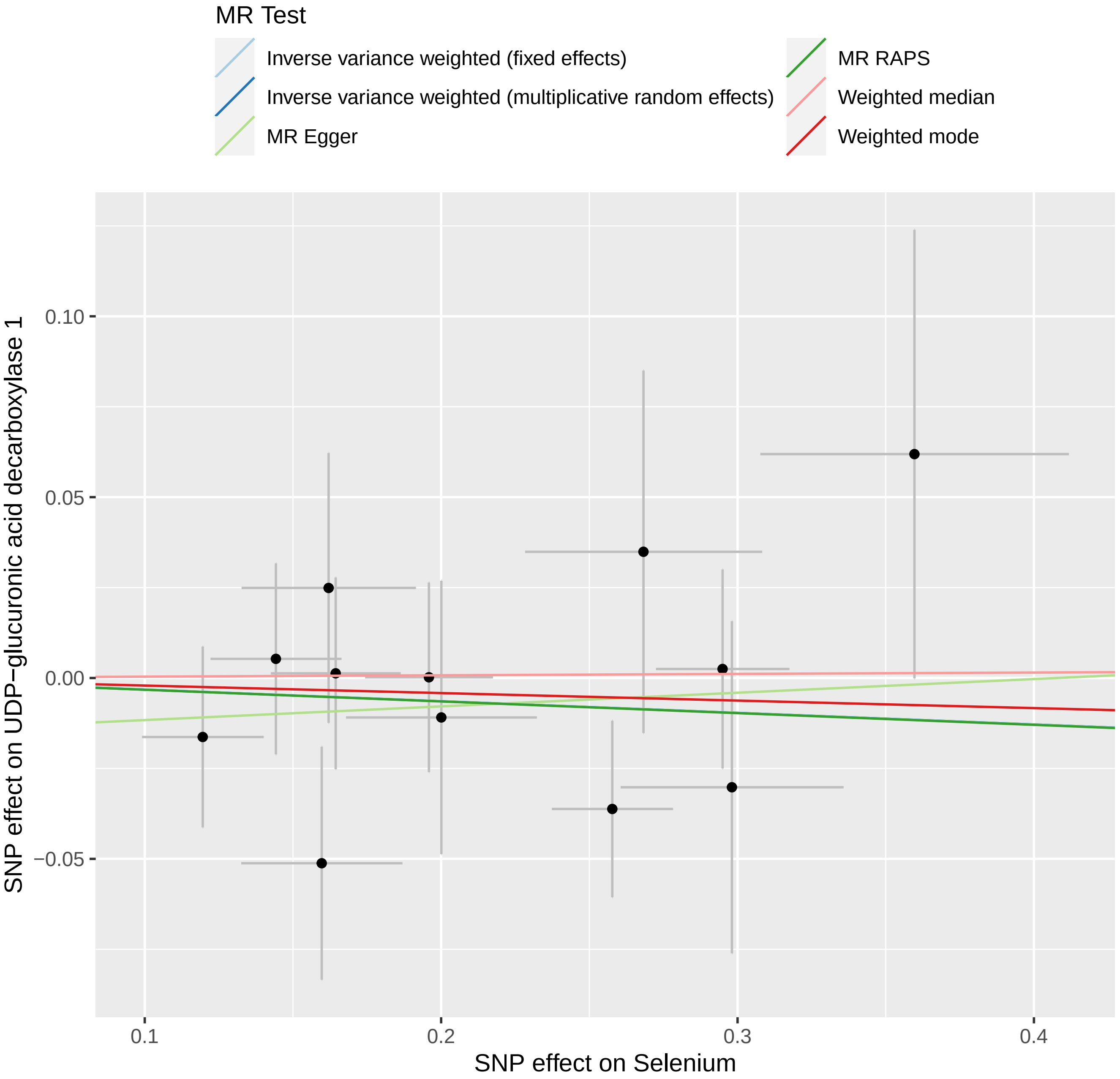

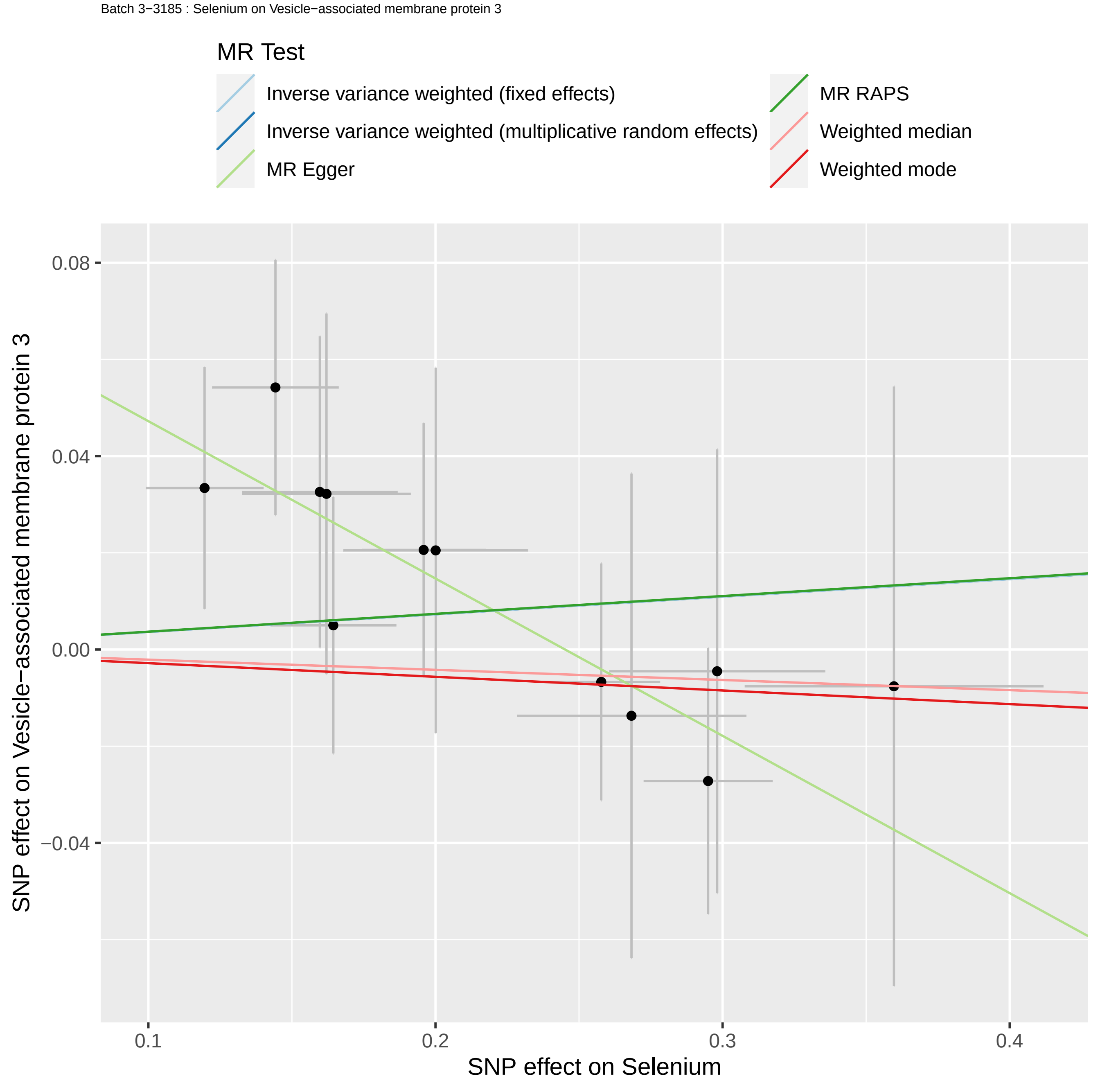
Batch 3−3185 : Selenium on Vesicle−associated membrane protein 3
MR Test
Inverse variance weighted (fixed effects)
Inverse variance weighted (multiplicative random effects)
MR Egger
MR RAPS
Weighted median
Weighted mode
0.08
0.04
0.00
−0.04
0.1
0.2
0.3
0.4
SNP effect on Vesicle−associated membrane protein 3
SNP effect on Selenium

Batch 3−3186 : Selenium on Vesicle−associated membrane protein 4

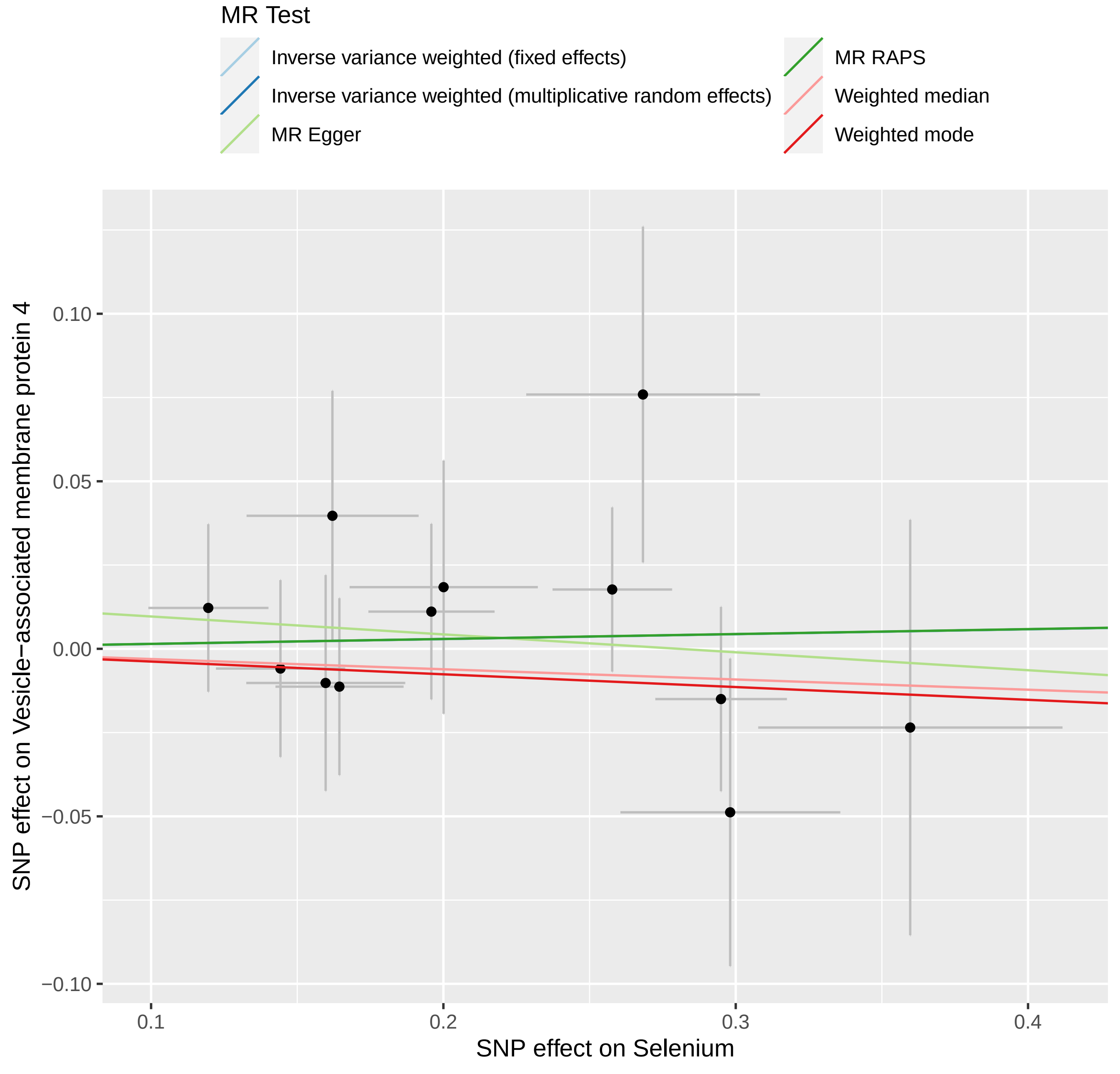

Batch 3−3187 : Selenium on Vesicle−associated membrane protein 5

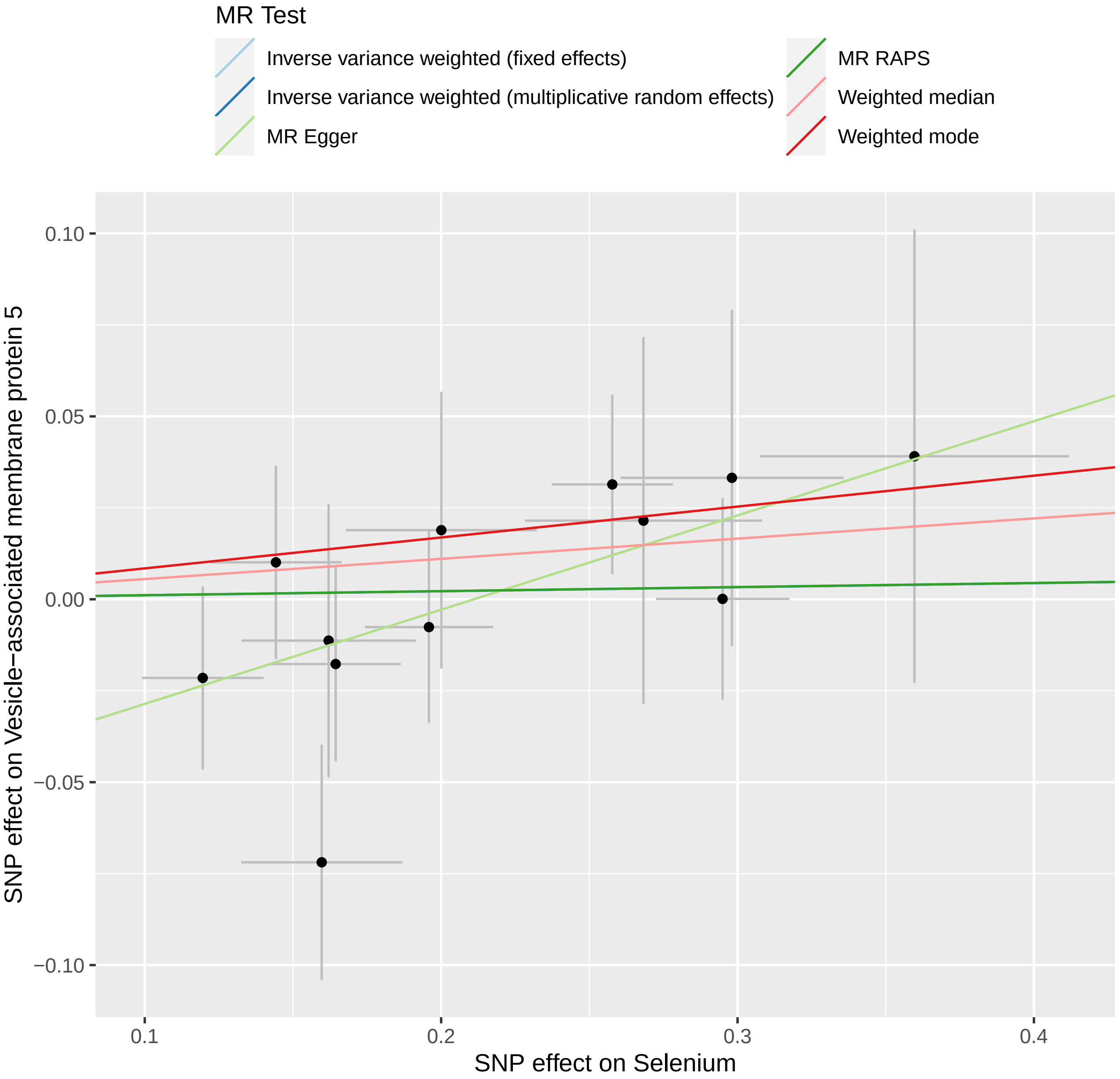

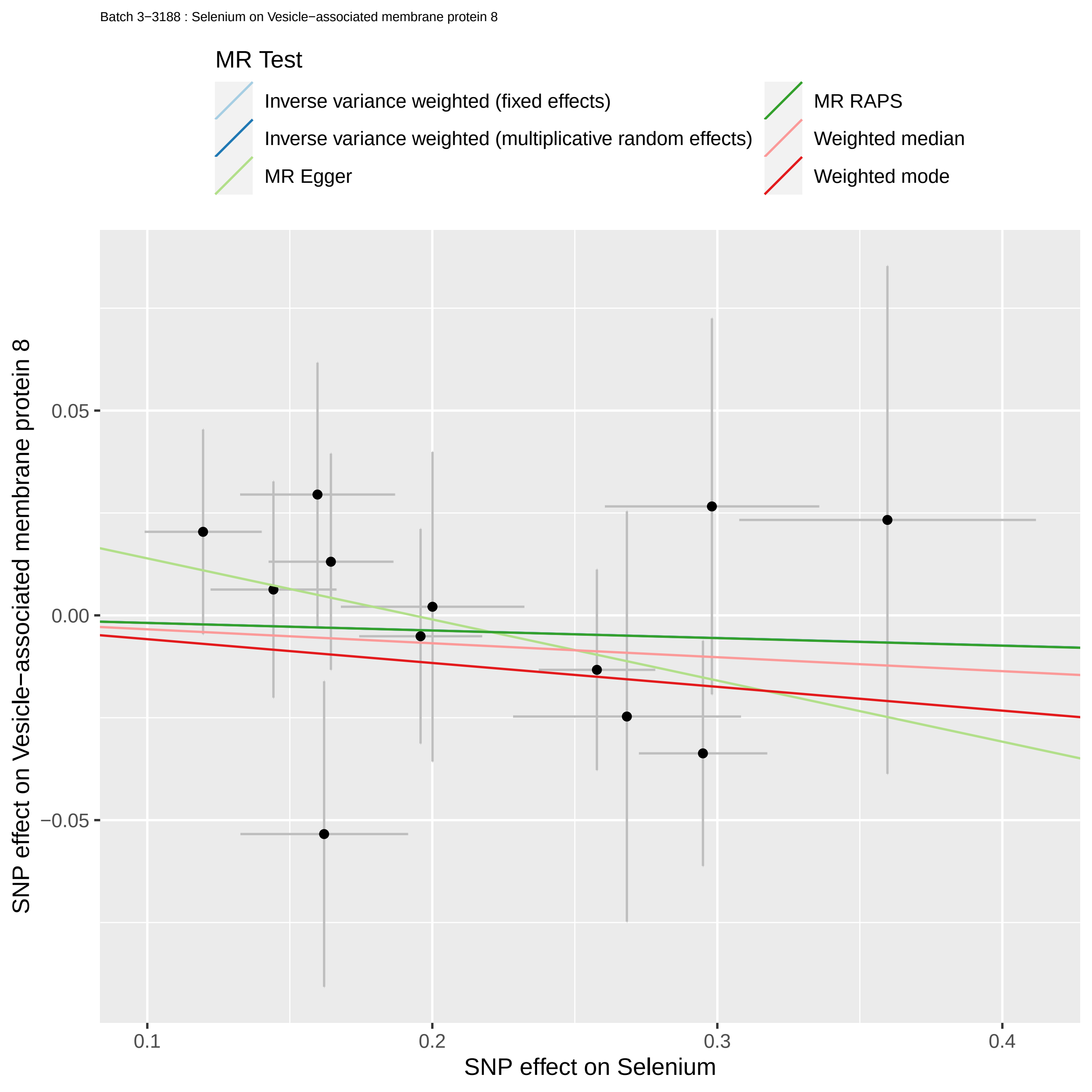

Batch 3−3188 : Selenium on Vesicle−associated membrane protein 8
MR Test
Inverse variance weighted (fixed effects)
Inverse variance weighted (multiplicative random effects)
MR Egger
MR RAPS
Weighted median
Weighted mode
SNP effect on Vesicle−associated membrane protein 8
0.05
0.00
−0.05
0.1
0.2
0.3
0.4
SNP effect on Selenium

Batch 3−3189 : Selenium on Vesicle−associated membrane protein−associated protein A

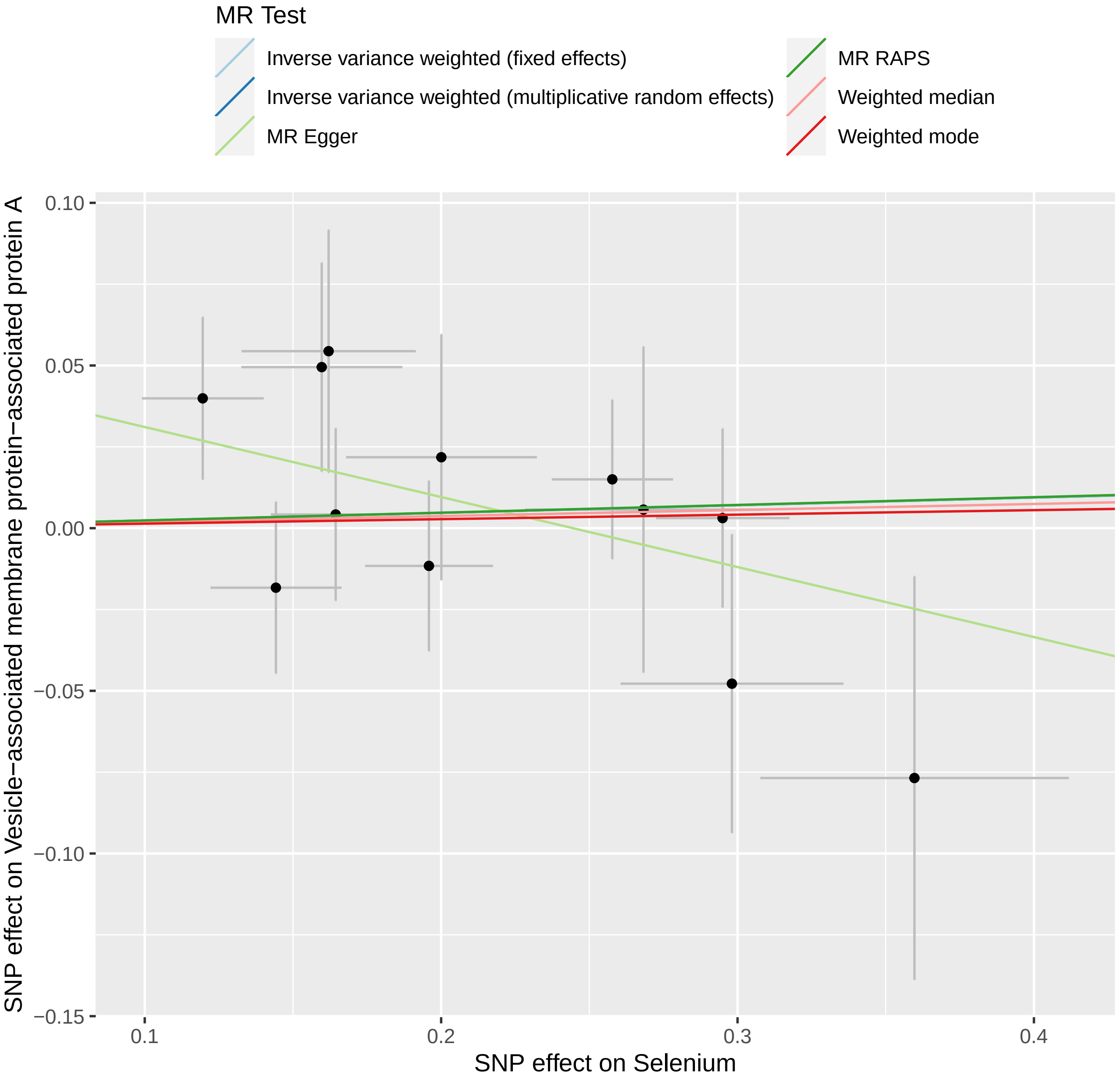

Batch 3−3190 : Selenium on Vesicle−associated membrane protein−associated protein B/C

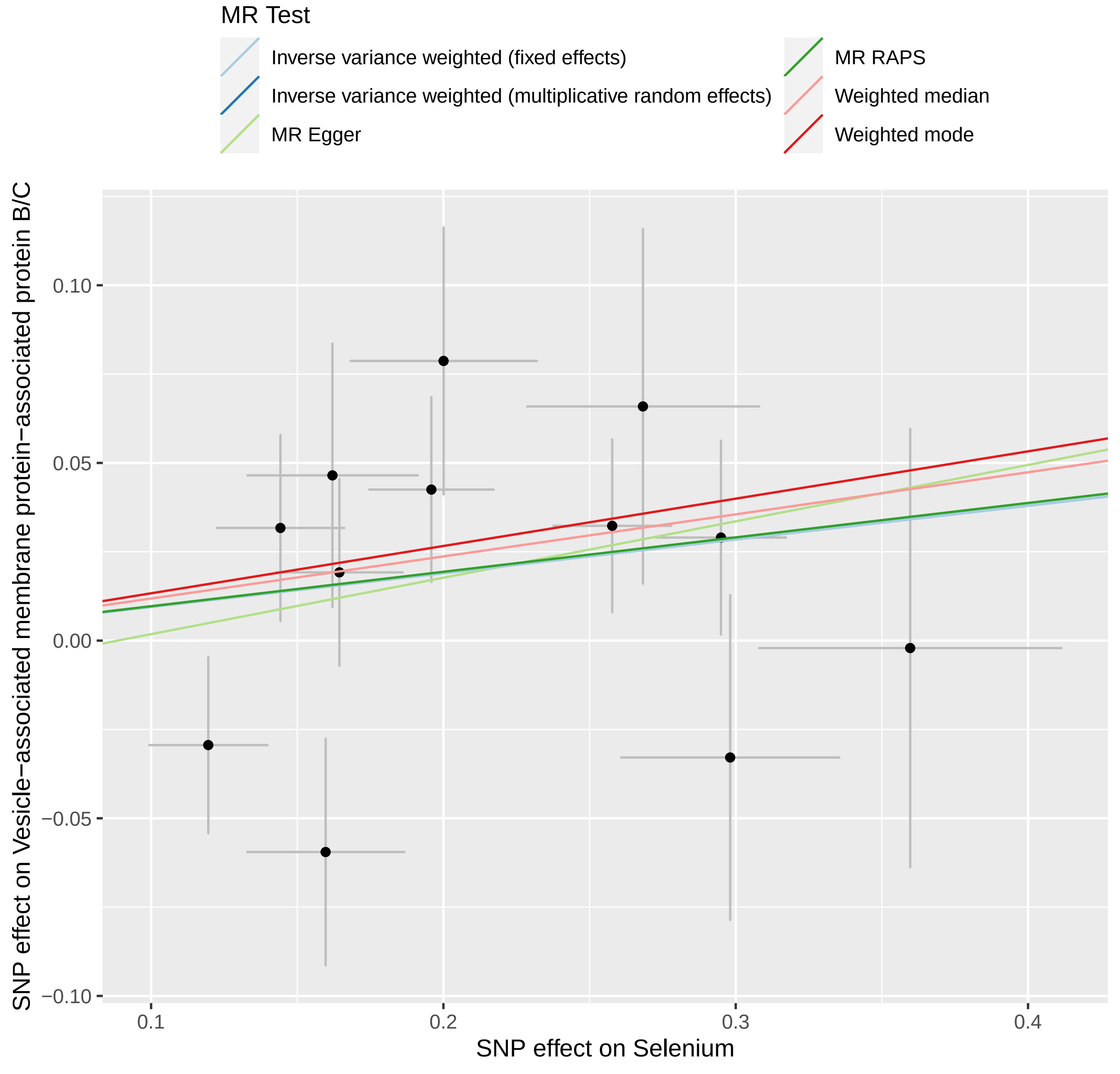

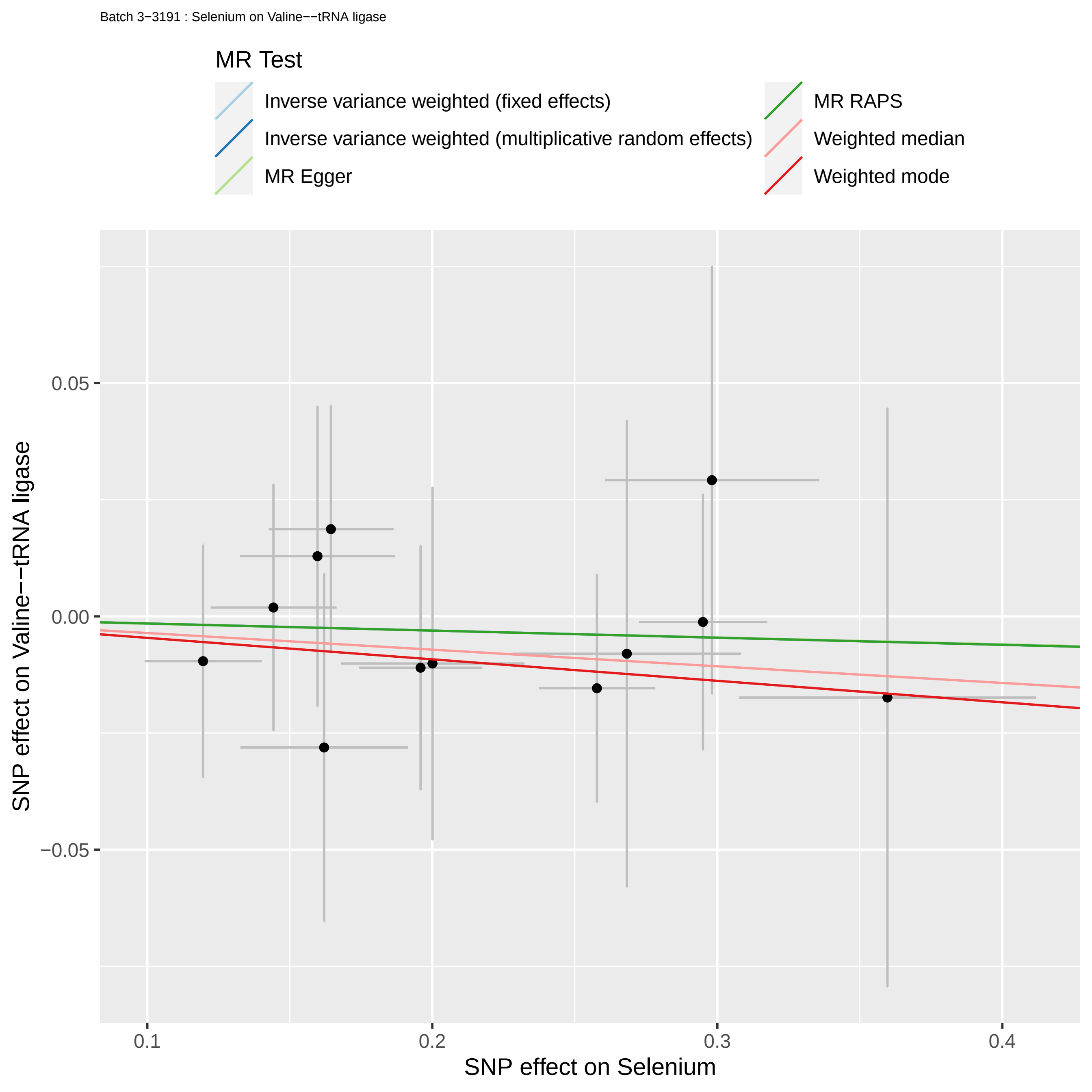

Batch 3−3191 : Selenium on Valine−−tRNA ligase
MR Test
Inverse variance weighted (fixed effects)
Inverse variance weighted (multiplicative random effects)
MR Egger
MR RAPS
Weighted median
Weighted mode
0.05
0.00
−0.05
0.1
0.2
0.3
0.4
SNP effect on Valine−−tRNA ligase
SNP effect on Selenium

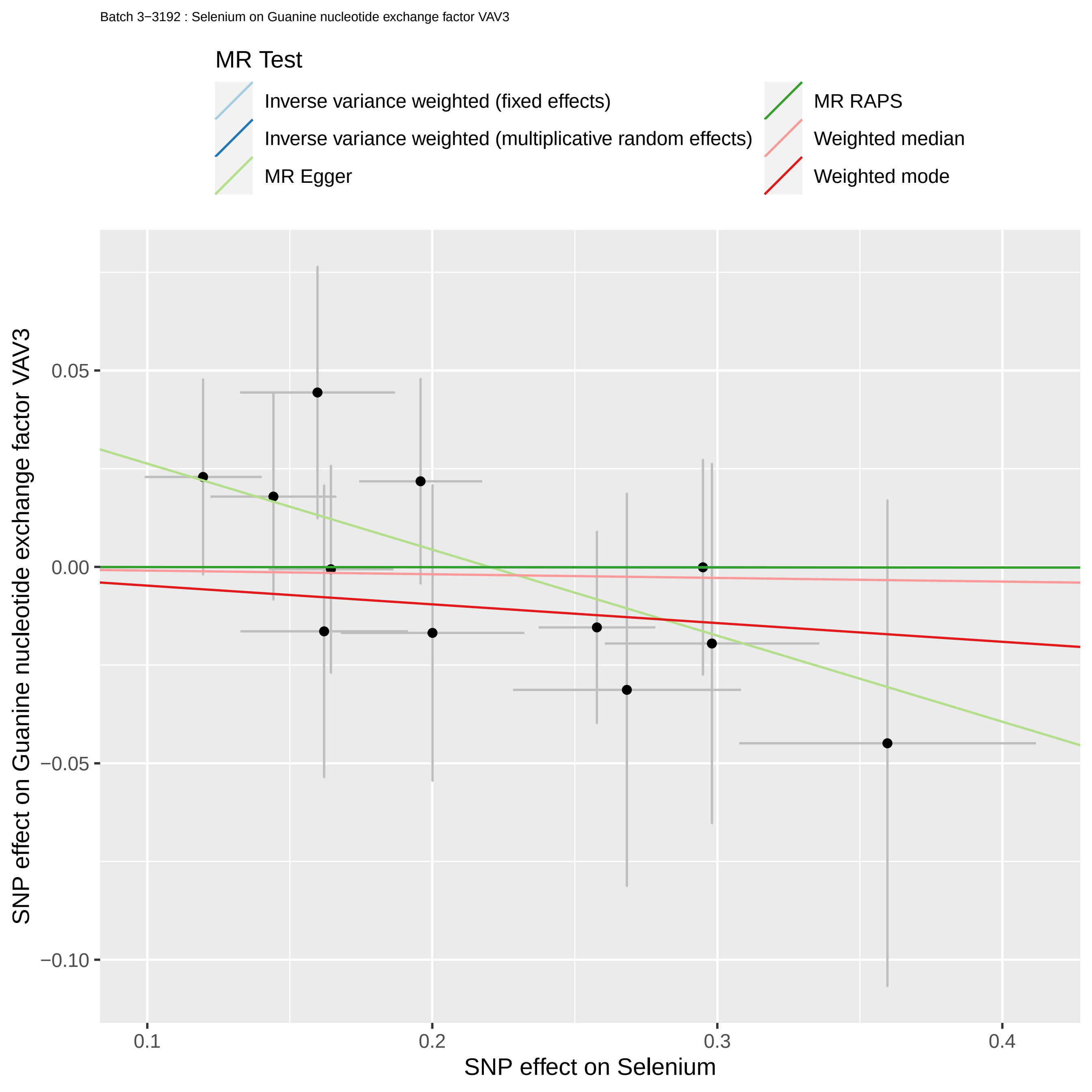
Batch 3−3192 : Selenium on Guanine nucleotide exchange factor VAV3
MR Test
Inverse variance weighted (fixed effects)
Inverse variance weighted (multiplicative random effects)
MR Egger
MR RAPS
Weighted median
Weighted mode
0.05
0.00
−0.05
−0.10
0.1
0.2
0.3
0.4
SNP effect on Guanine nucleotide exchange factor VAV3
SNP effect on Selenium

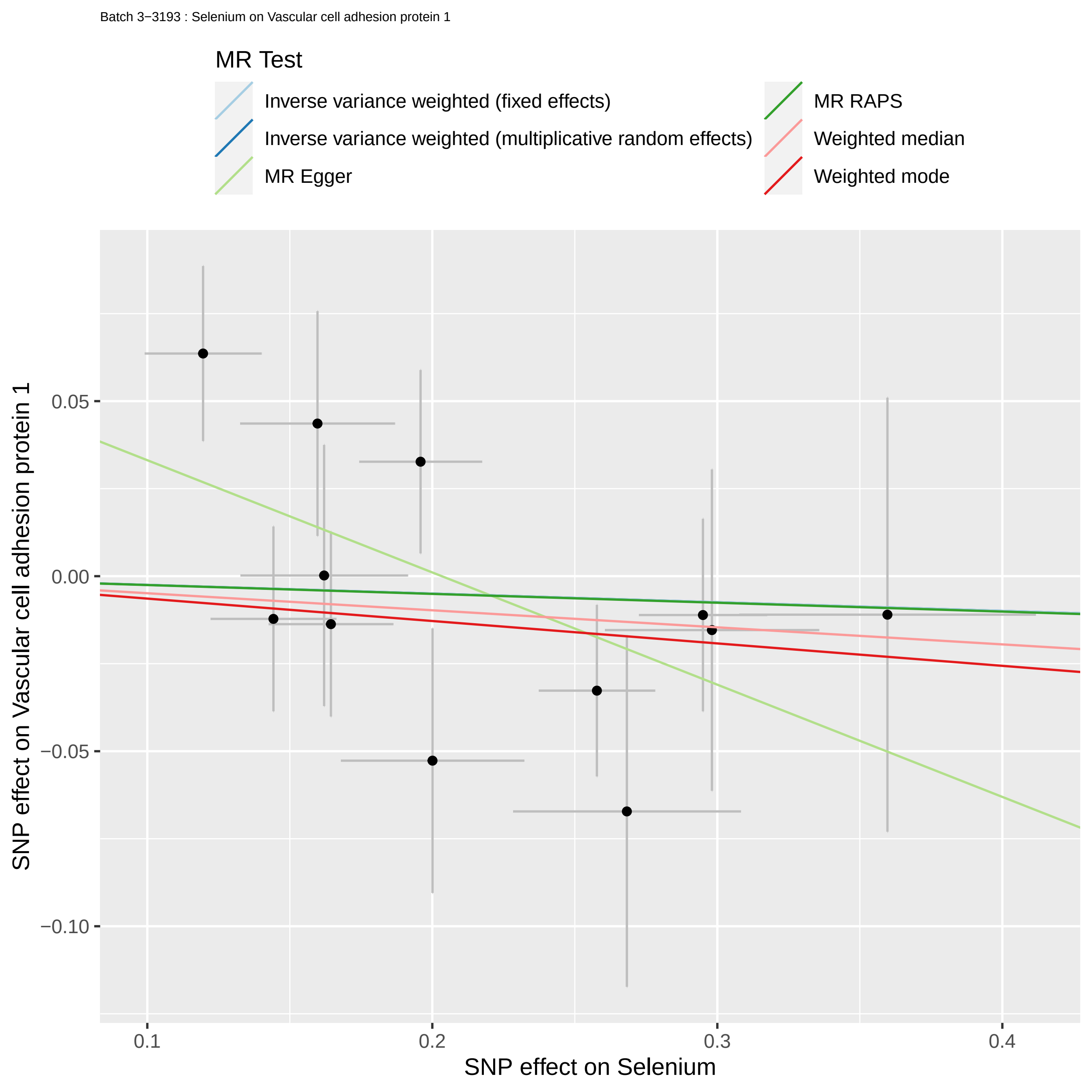
Batch 3−3193 : Selenium on Vascular cell adhesion protein 1
MR Test
Inverse variance weighted (fixed effects)
Inverse variance weighted (multiplicative random effects)
MR Egger
MR RAPS
Weighted median
Weighted mode
SNP effect on Vascular cell adhesion protein 1
0.05
0.00
−0.05
−0.10
0.1
0.2
0.3
0.4
SNP effect on Selenium

Batch 3−3194 : Selenium on Versican core protein

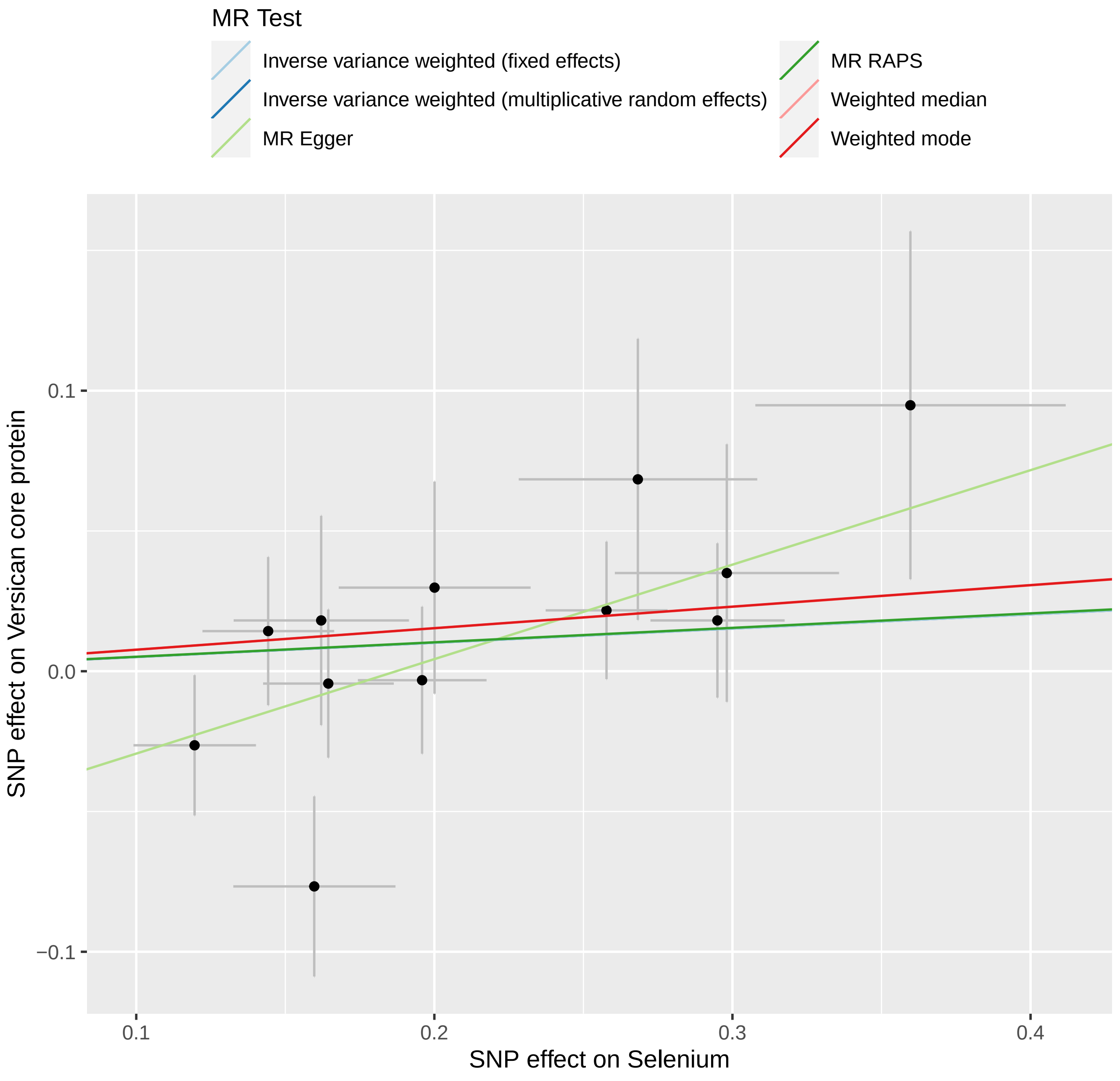

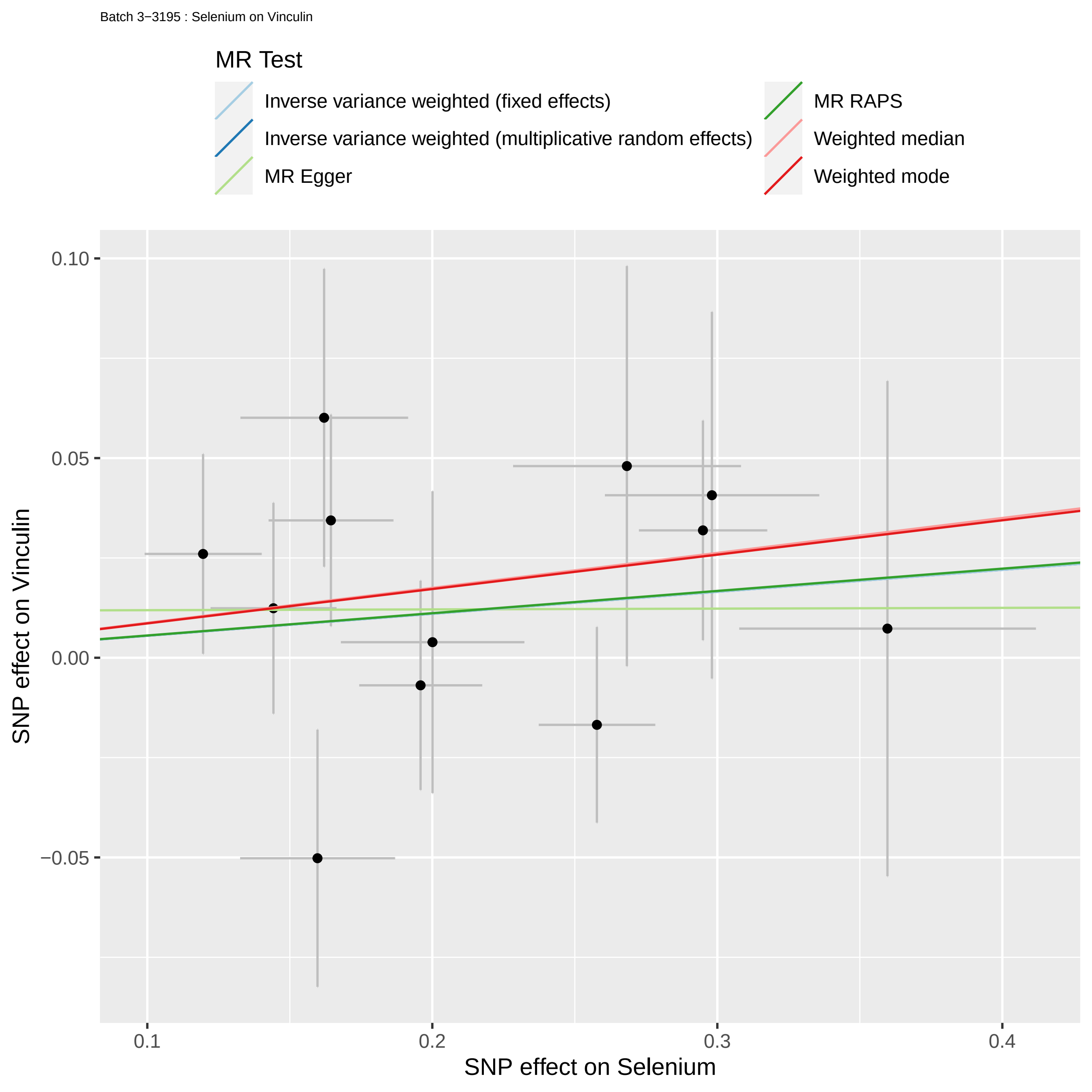

Batch 3−3195 : Selenium on Vinculin
MR Test
Inverse variance weighted (fixed effects)
Inverse variance weighted (multiplicative random effects)
MR Egger
MR RAPS
Weighted median
Weighted mode
0.10
0.05
0.00
−0.05
0.1
0.2
0.3
0.4
SNP effect on Vinculin
SNP effect on Selenium

Batch 3−3196 : Selenium on Testis−specific basic protein Y 1

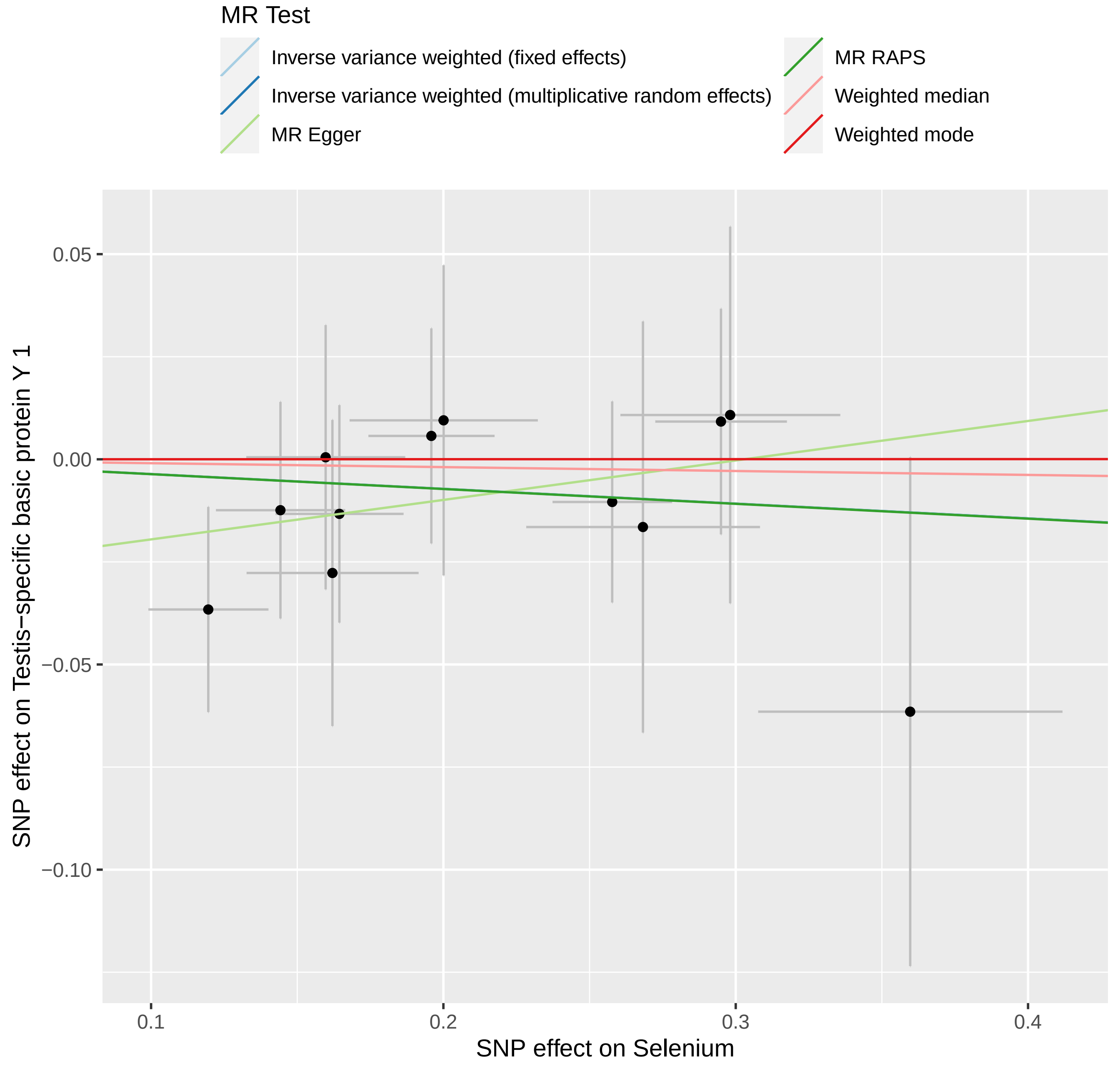

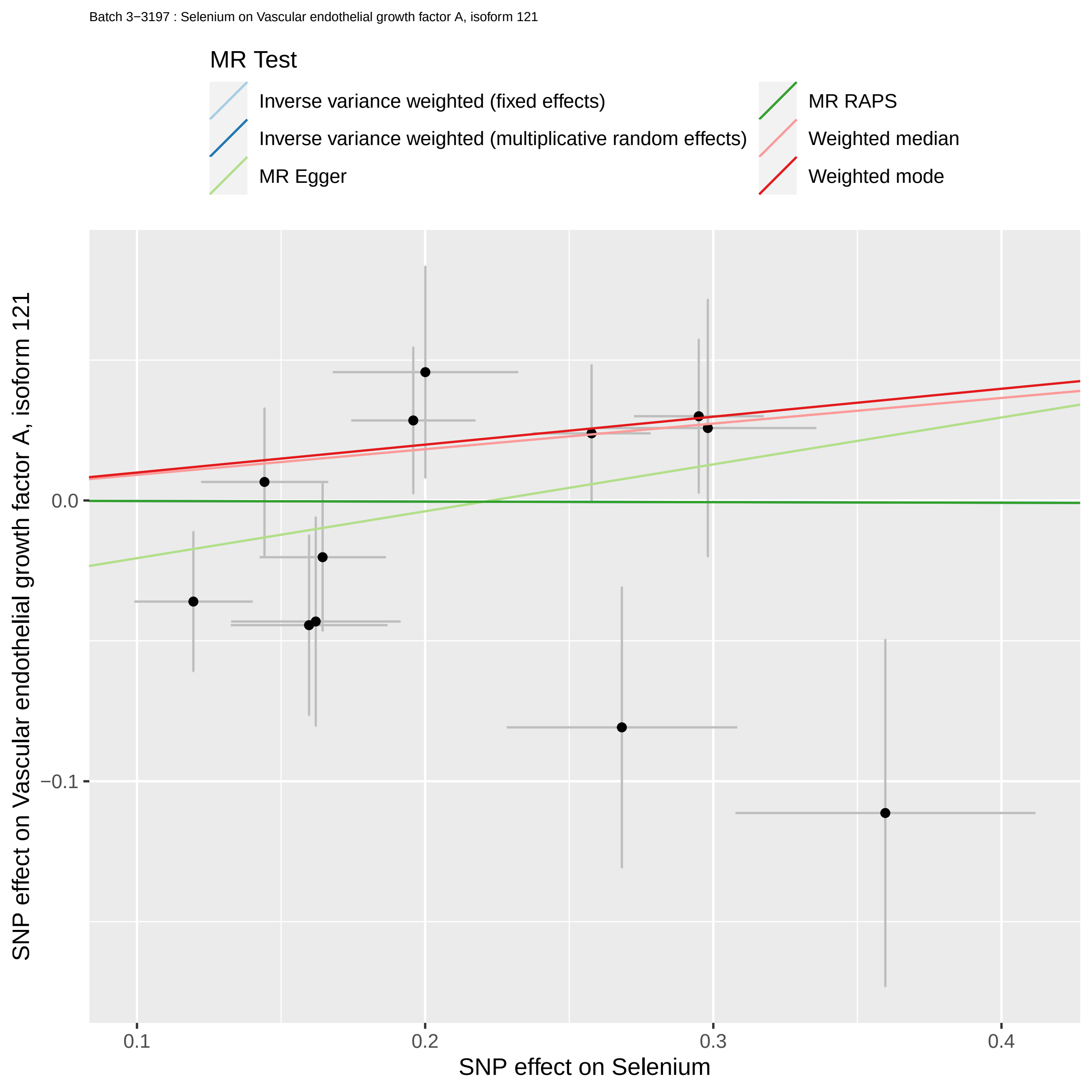

Batch 3−3197 : Selenium on Vascular endothelial growth factor A, isoform 121
MR Test
Inverse variance weighted (fixed effects)
Inverse variance weighted (multiplicative random effects)
MR Egger
MR RAPS
Weighted median
Weighted mode
SNP effect on Vascular endothelial growth factor A, isoform 121
0.0
−0.1
0.1
0.2
0.3
0.4
SNP effect on Selenium

Batch 3−3198 : Selenium on Vascular endothelial growth factor A

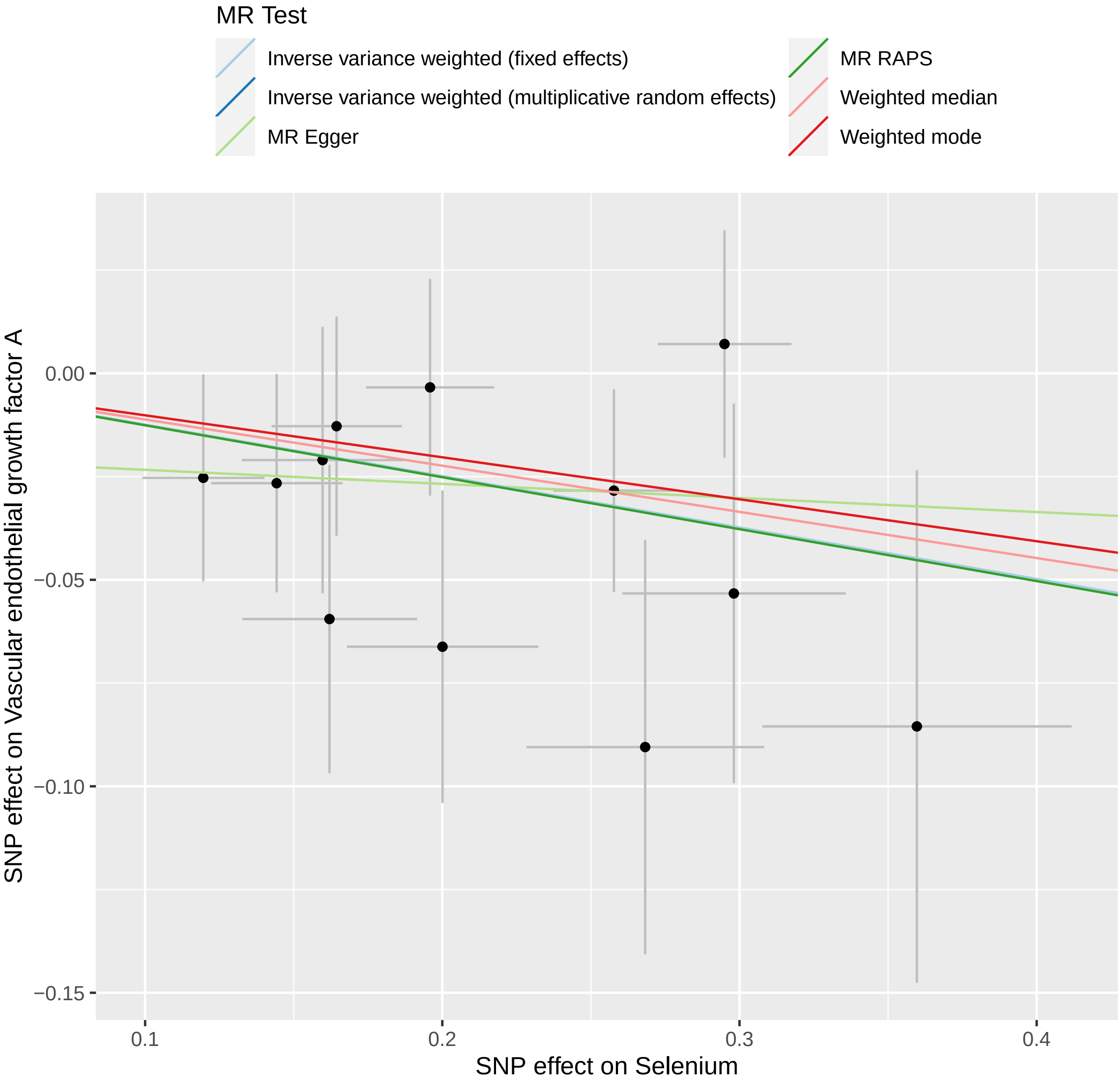

Batch 3−3199 : Selenium on Vascular endothelial growth factor C

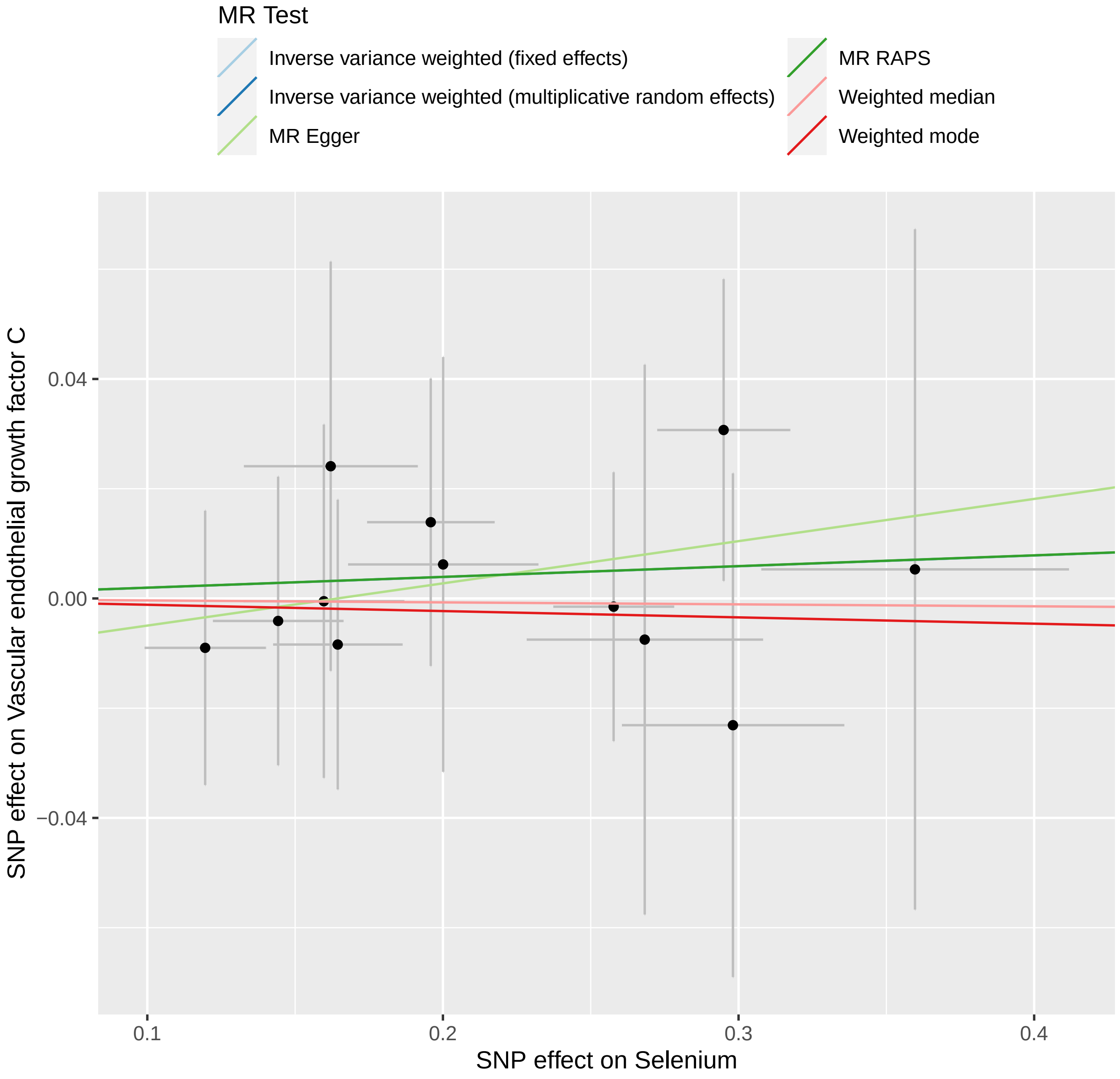

Batch 3−3200 : Selenium on Selenoprotein S

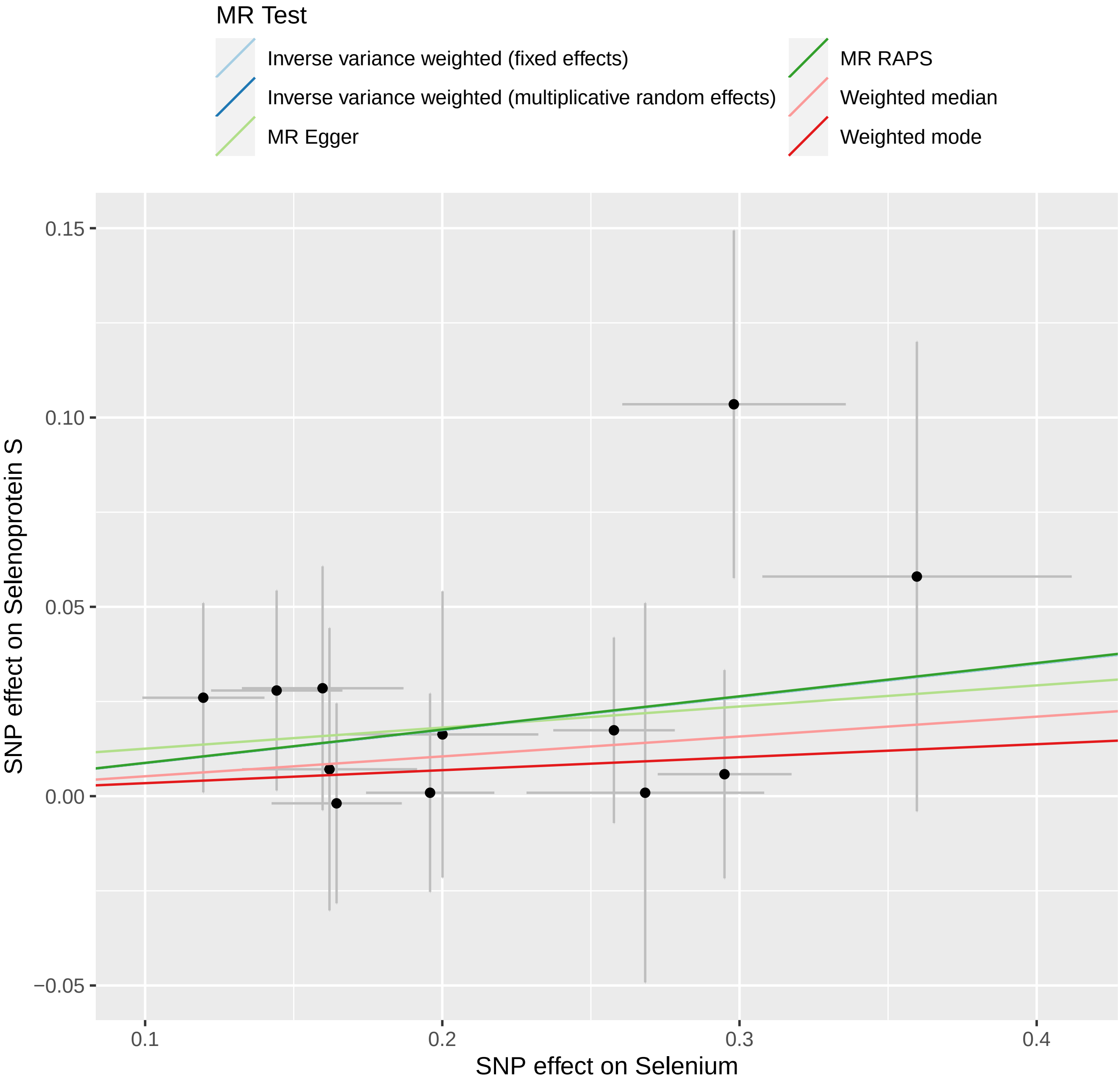

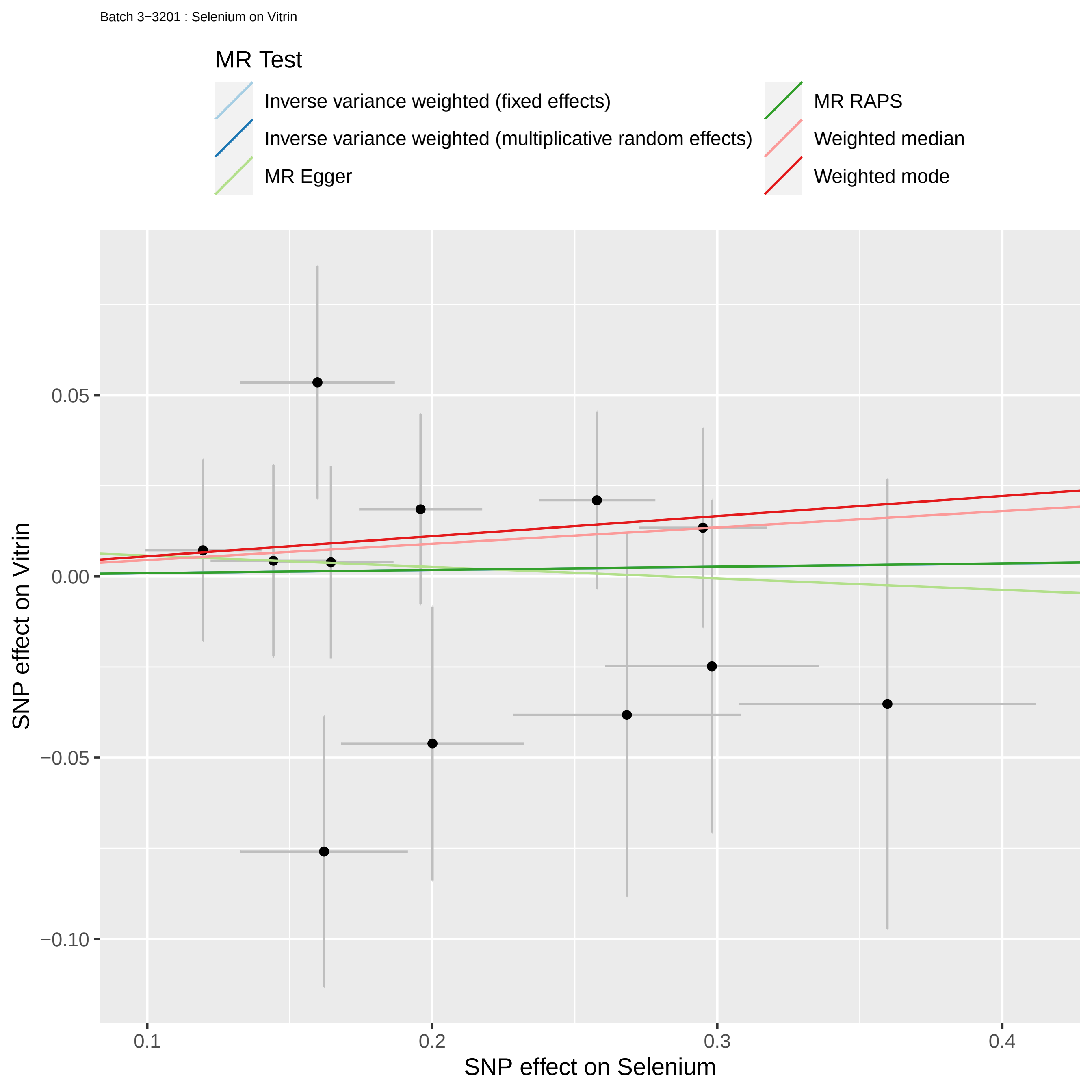
Batch 3−3201 : Selenium on Vitrin
MR Test
Inverse variance weighted (fixed effects)
Inverse variance weighted (multiplicative random effects)
MR Egger
MR RAPS
Weighted median
Weighted mode
0.05
0.00
−0.05
−0.10
SNP effect on Vitrin
0.1
0.2
0.3
0.4
SNP effect on Selenium

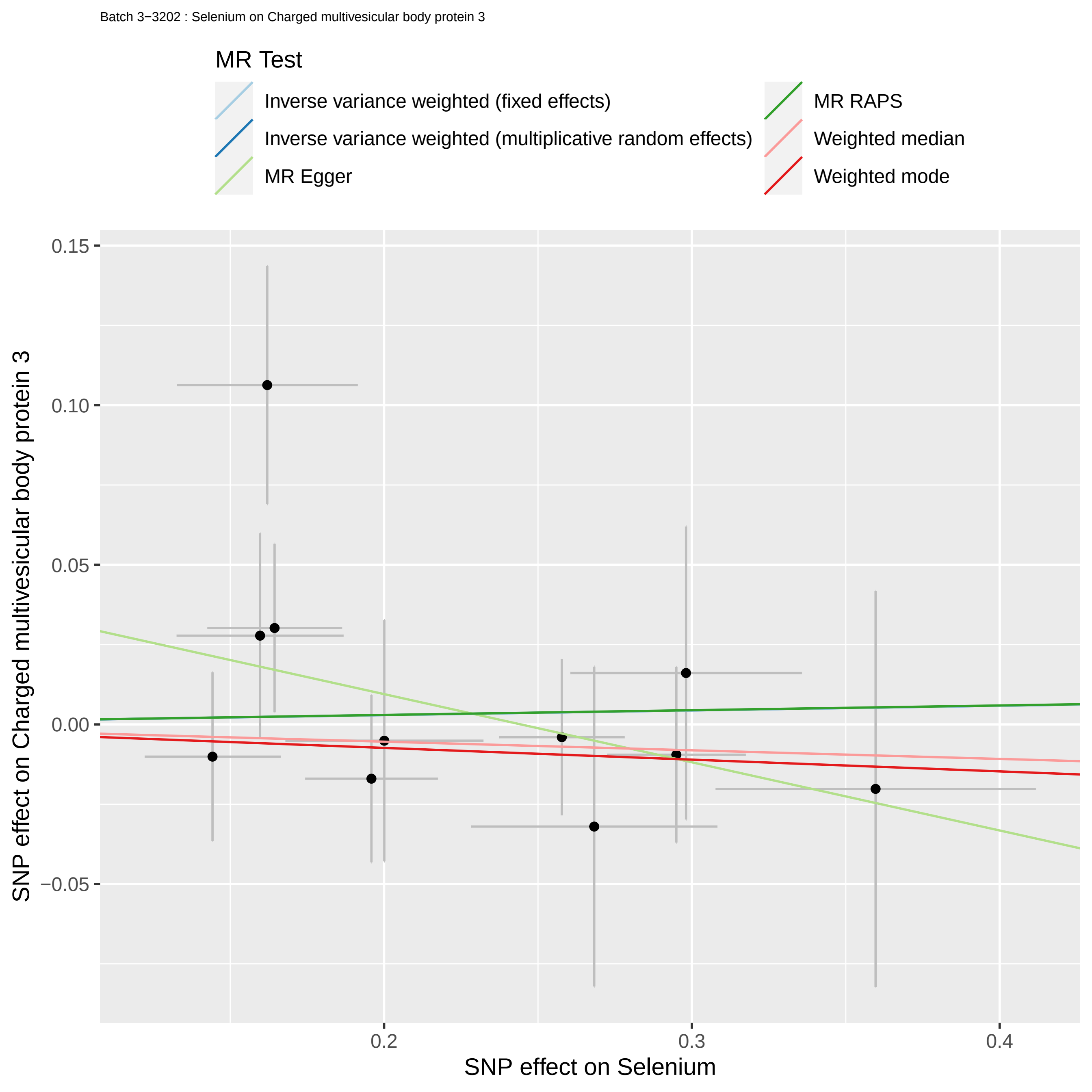
Batch 3−3202 : Selenium on Charged multivesicular body protein 3
MR Test
Inverse variance weighted (fixed effects)
Inverse variance weighted (multiplicative random effects)
MR Egger
MR RAPS
Weighted median
Weighted mode
0.15
0.10
0.05
0.00
−0.05
0.2
0.3
0.4
SNP effect on Selenium
SNP effect on Charged multivesicular body protein 3

Batch 3−3203 : Selenium on Vacuolar protein sorting−associated protein 29

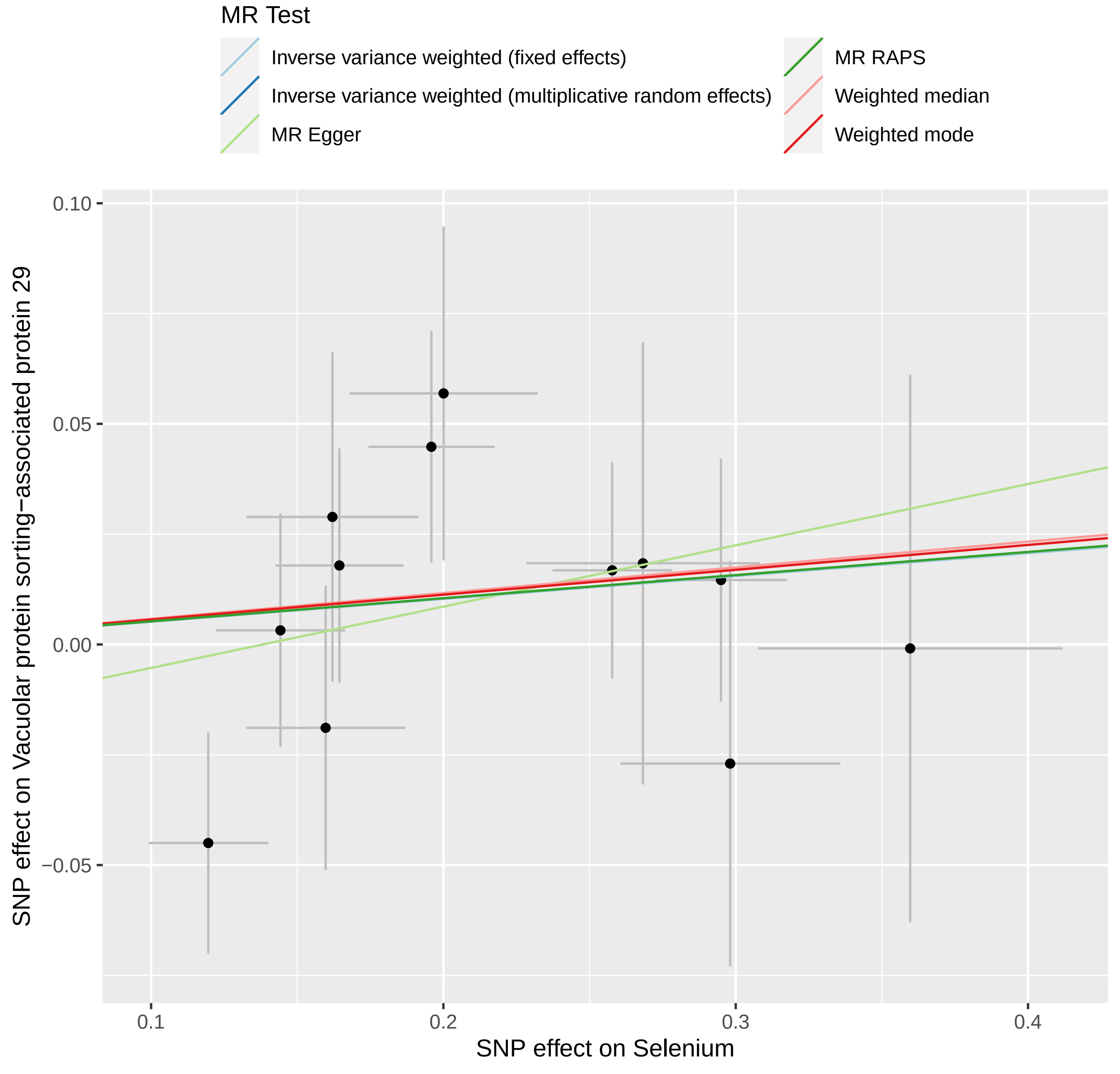

Batch 3−3204 : Selenium on Vacuolar protein sorting−associated protein 4A

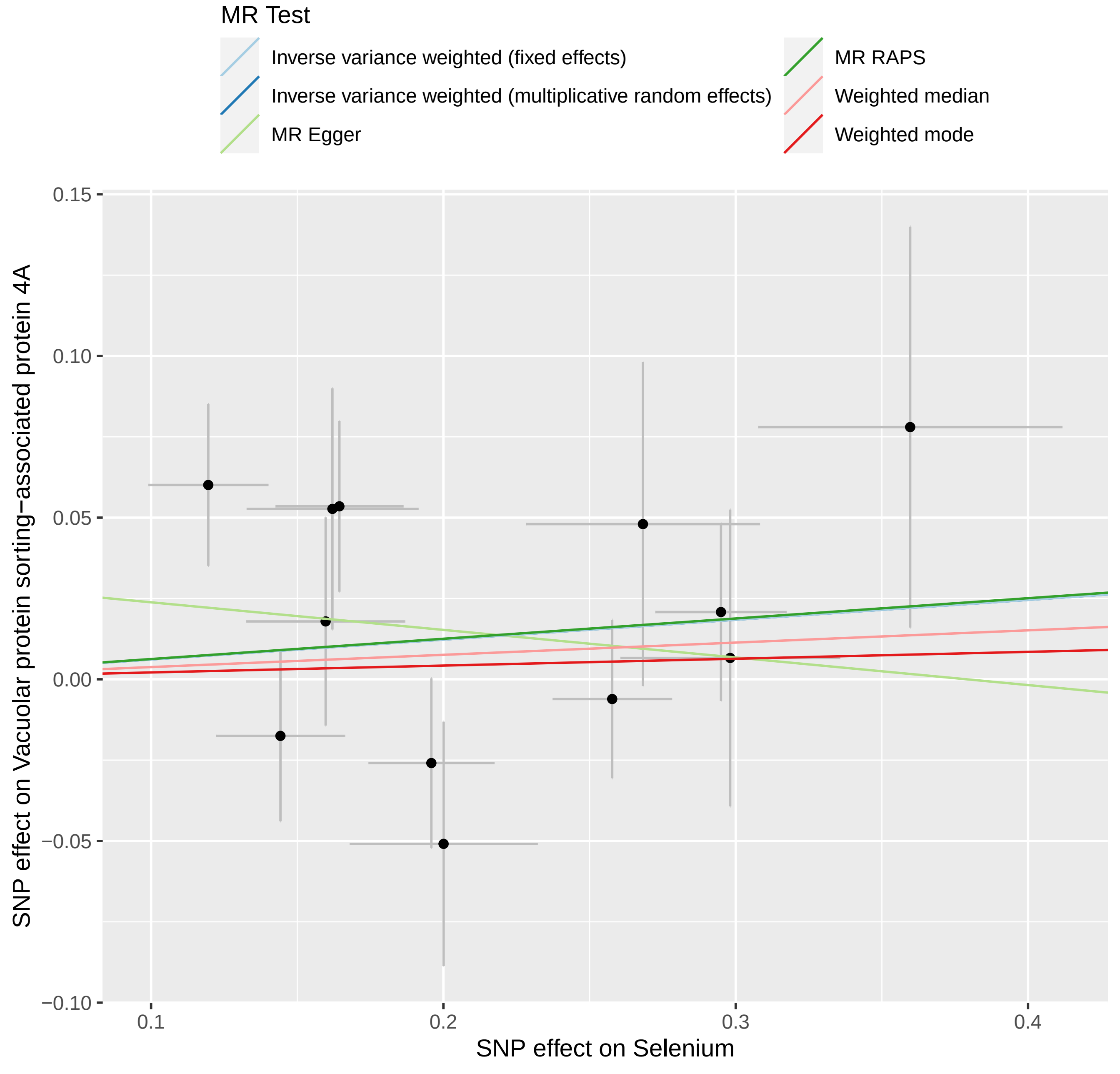

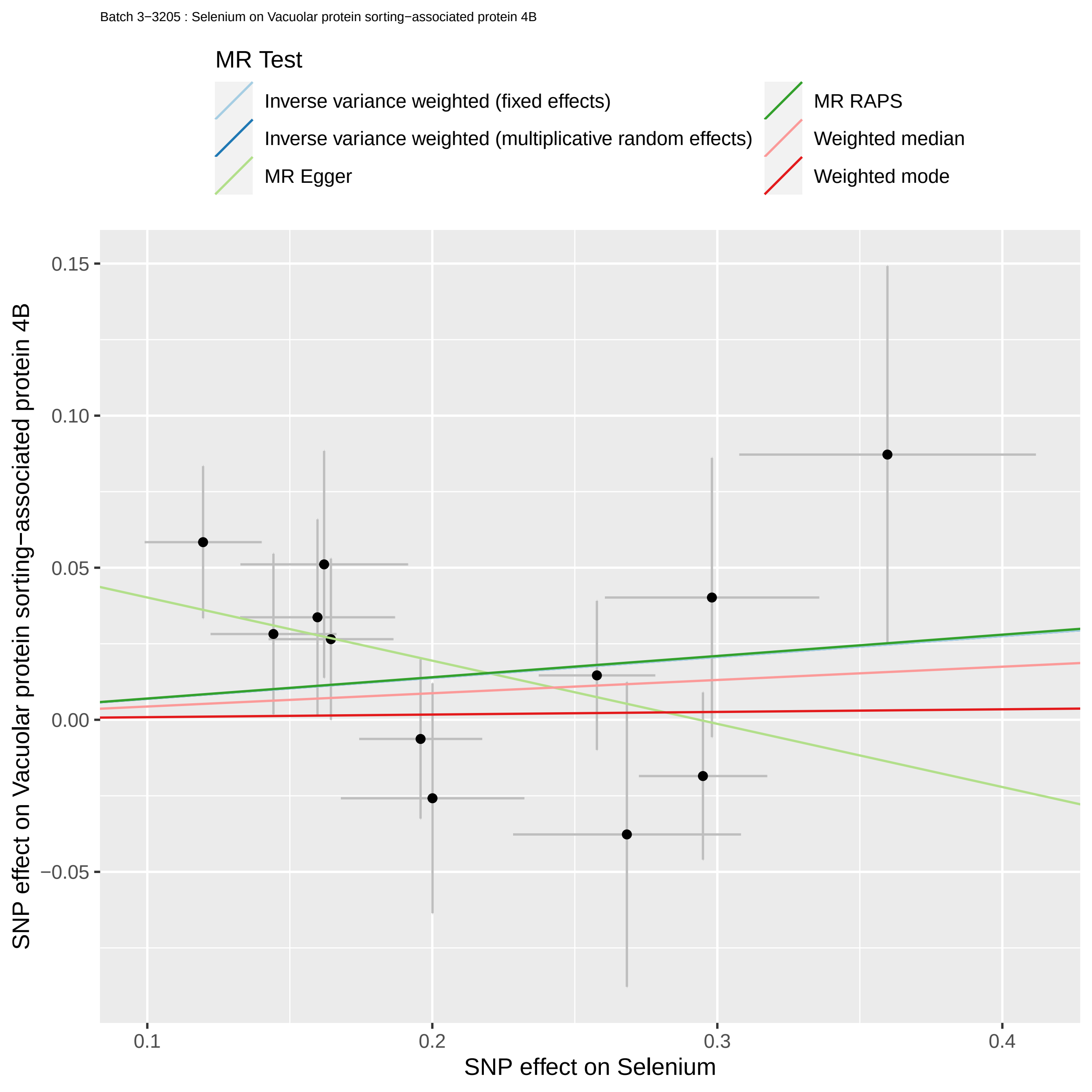
Batch 3−3205 : Selenium on Vacuolar protein sorting−associated protein 4B
MR Test
Inverse variance weighted (fixed effects)
Inverse variance weighted (multiplicative random effects)
MR Egger
MR RAPS
Weighted median
Weighted mode
SNP effect on Vacuolar protein sorting−associated protein 4B
0.15
0.10
0.05
0.00
−0.05
0.1
0.2
0.3
0.4
SNP effect on Selenium

Batch 3−3206 : Selenium on Serine/threonine−protein kinase VRK1

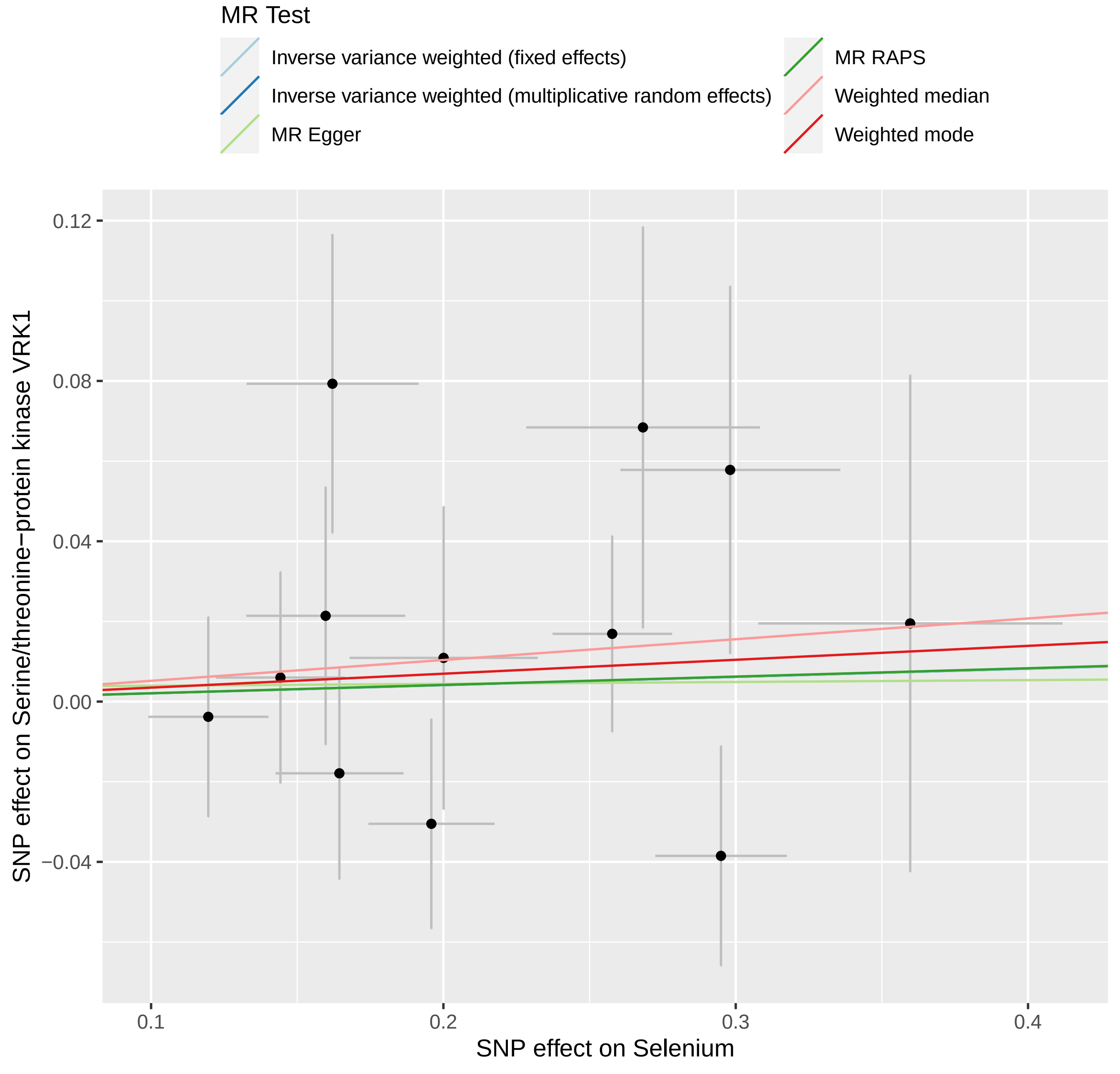

Batch 3−3207 : Selenium on V−set and immunoglobulin domain−containing protein 1

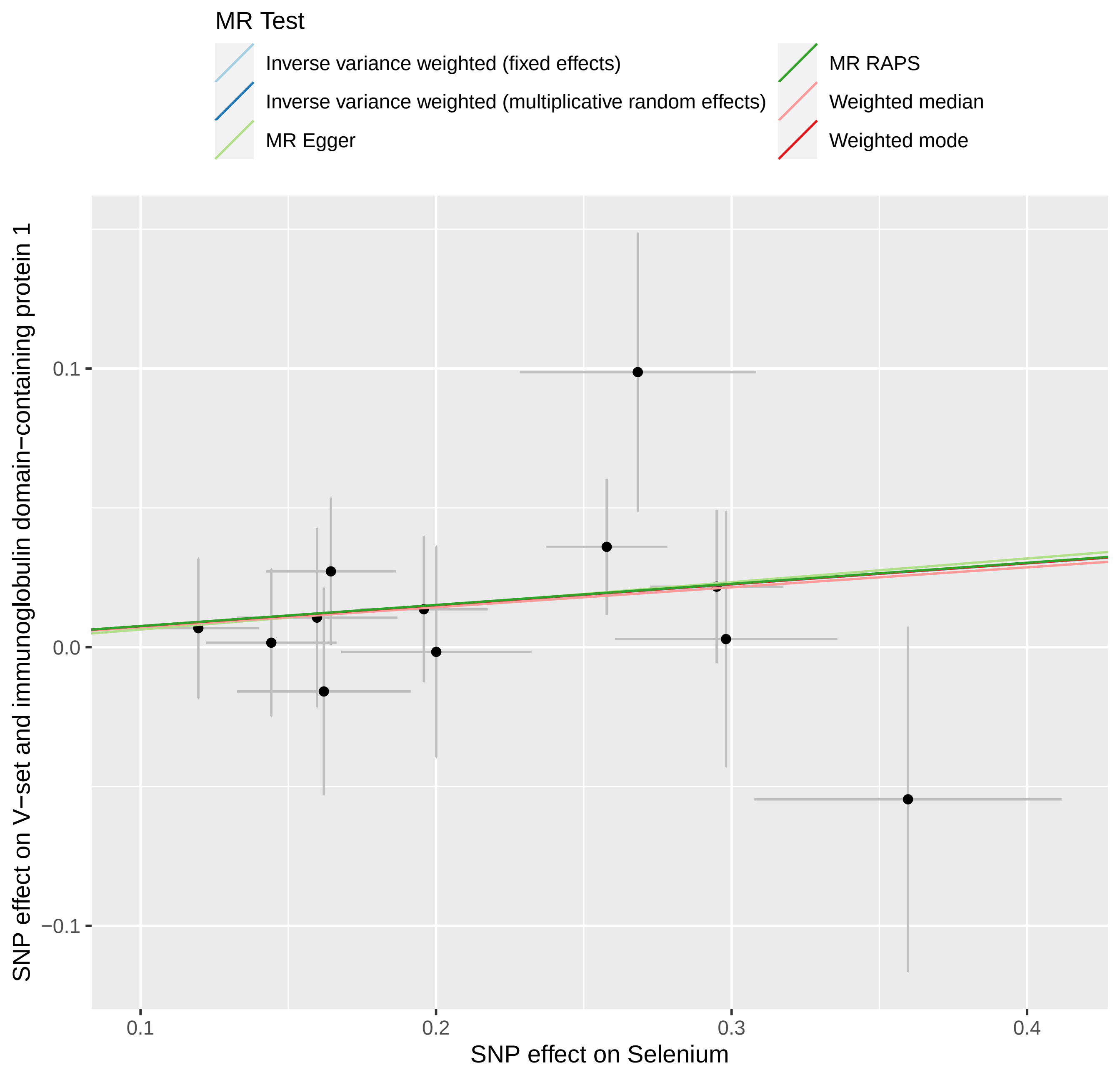

Batch 3−3208 : Selenium on V−set and immunoglobulin domain−containing protein 2

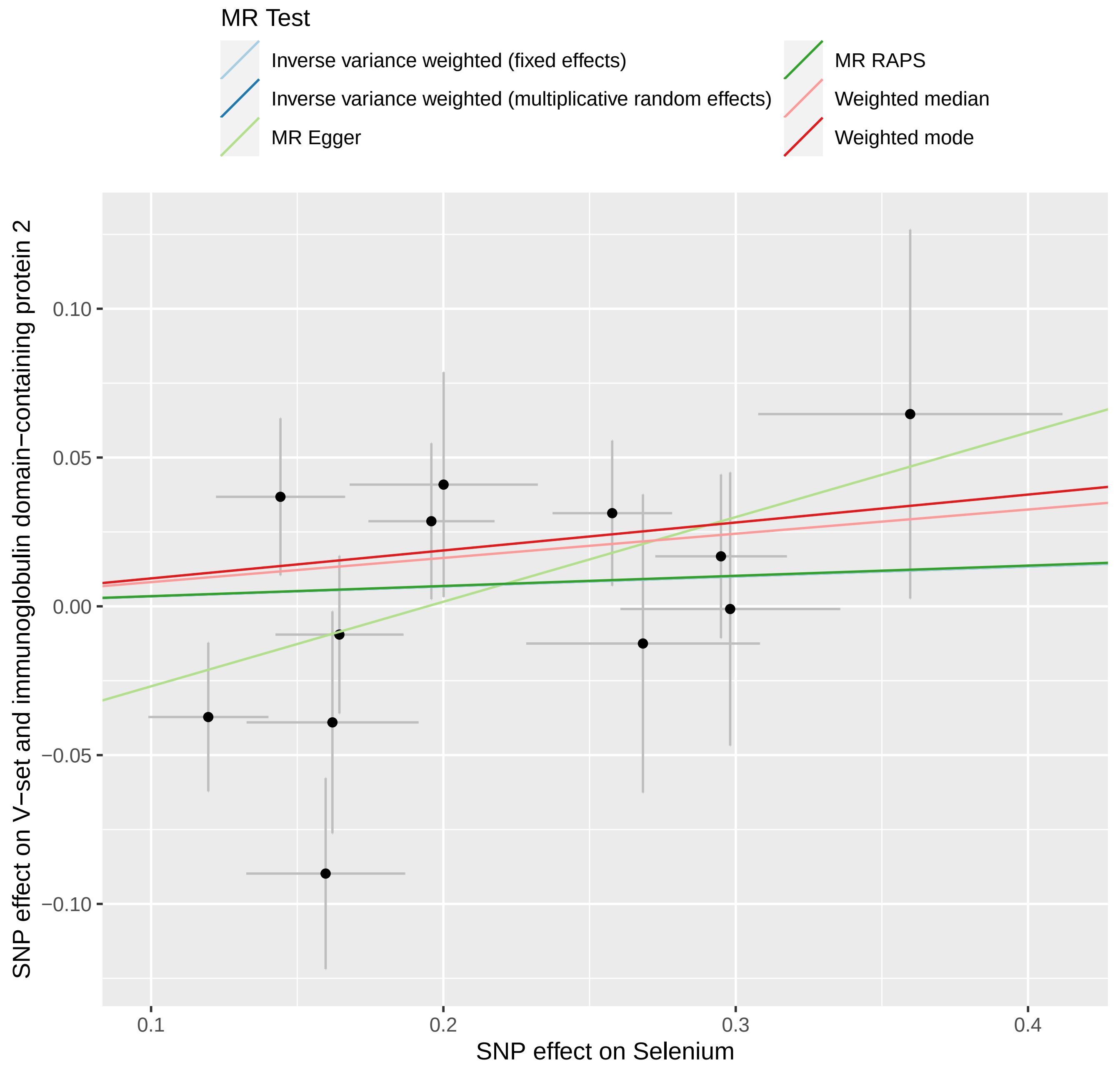

Batch 3−3209 : Selenium on V−set and transmembrane domain−containing protein 2−like protein

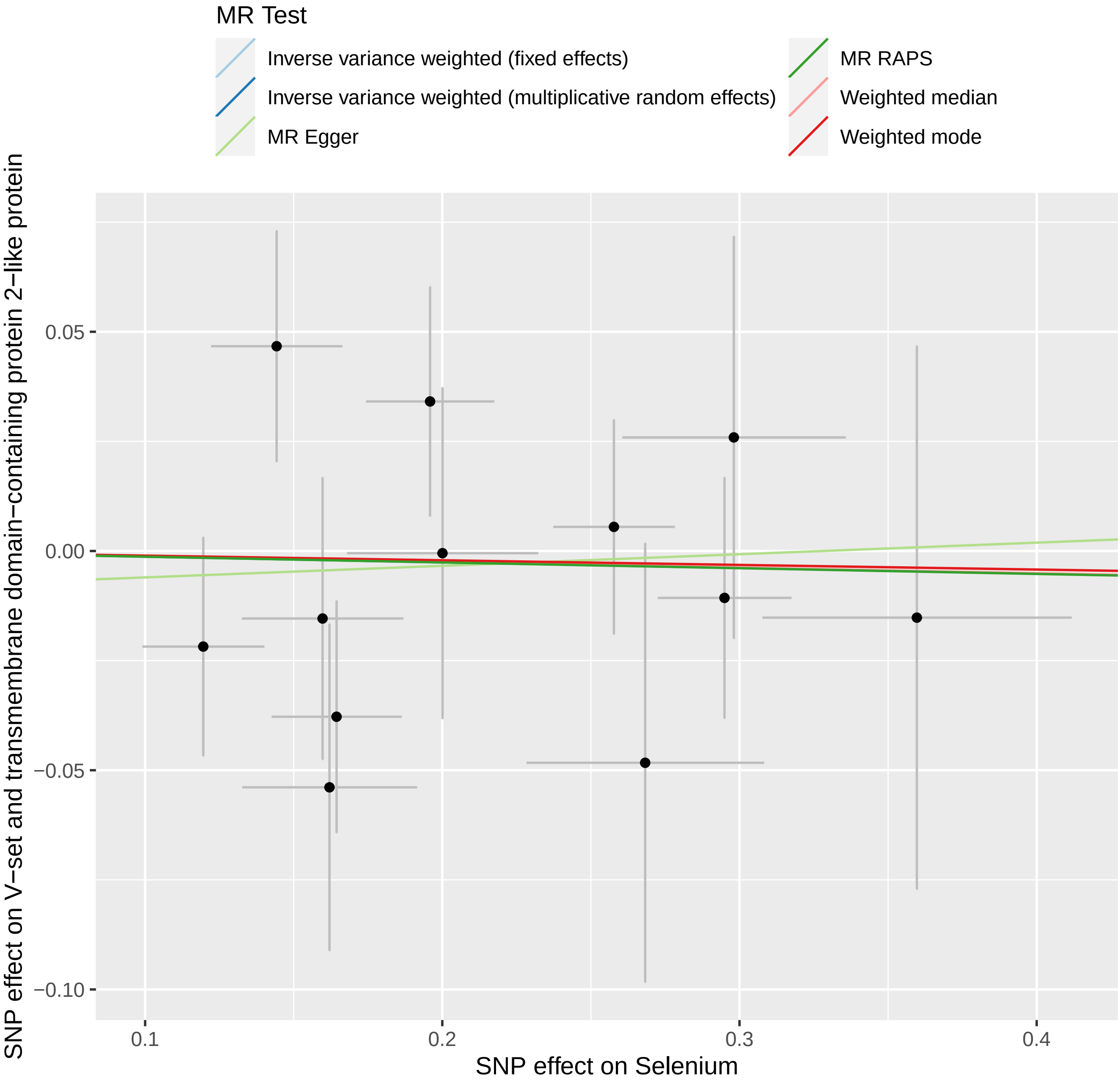

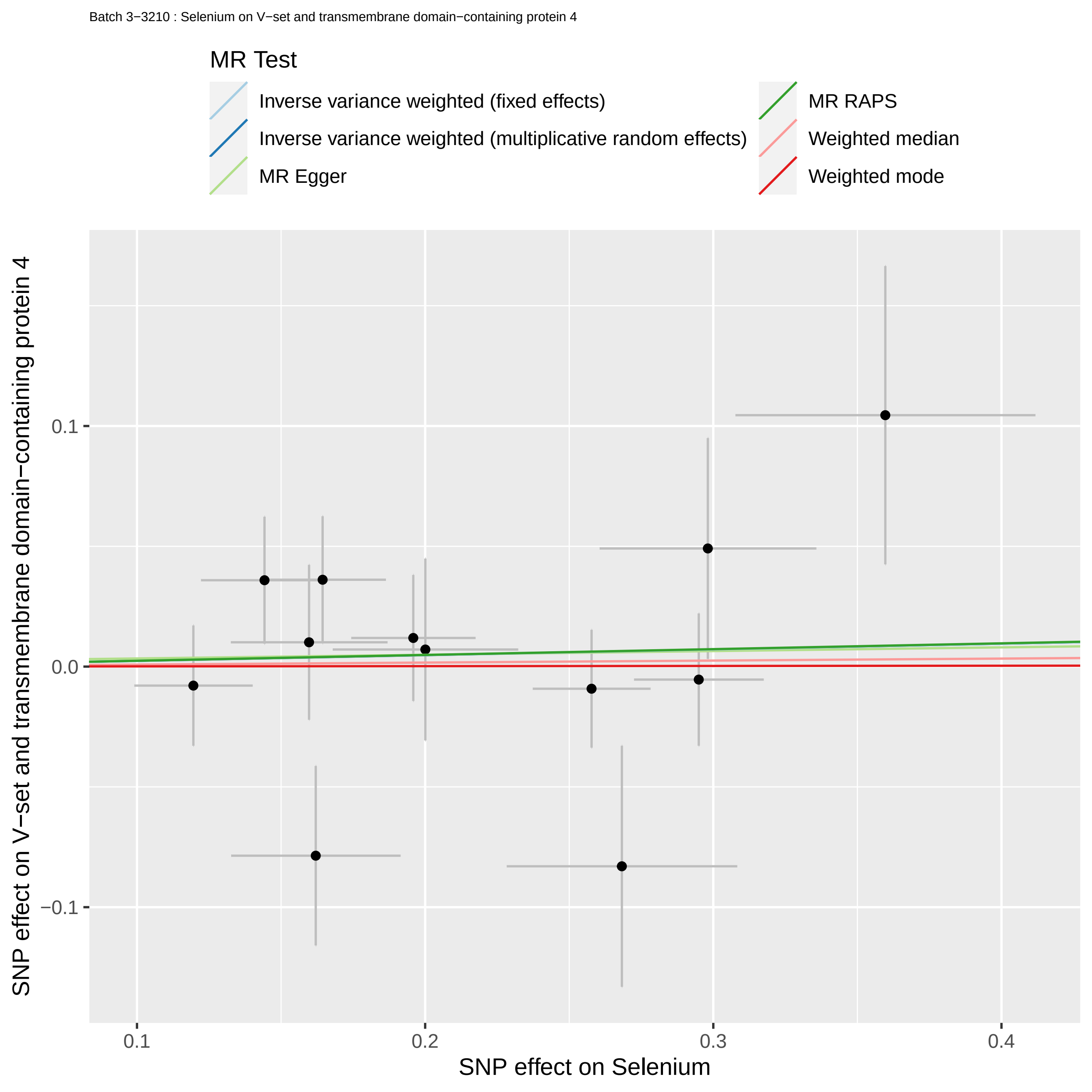

Batch 3−3210 : Selenium on V−set and transmembrane domain−containing protein 4
MR Test
Inverse variance weighted (fixed effects)
Inverse variance weighted (multiplicative random effects)
MR Egger
MR RAPS
Weighted median
Weighted mode
SNP effect on V−set and transmembrane domain−containing protein 4
0.1
0.0
−0.1
0.1
0.2
0.3
0.4
SNP effect on Selenium

Batch 3−3211 : Selenium on Vacuolar protein sorting−associated protein VTA1 homolog

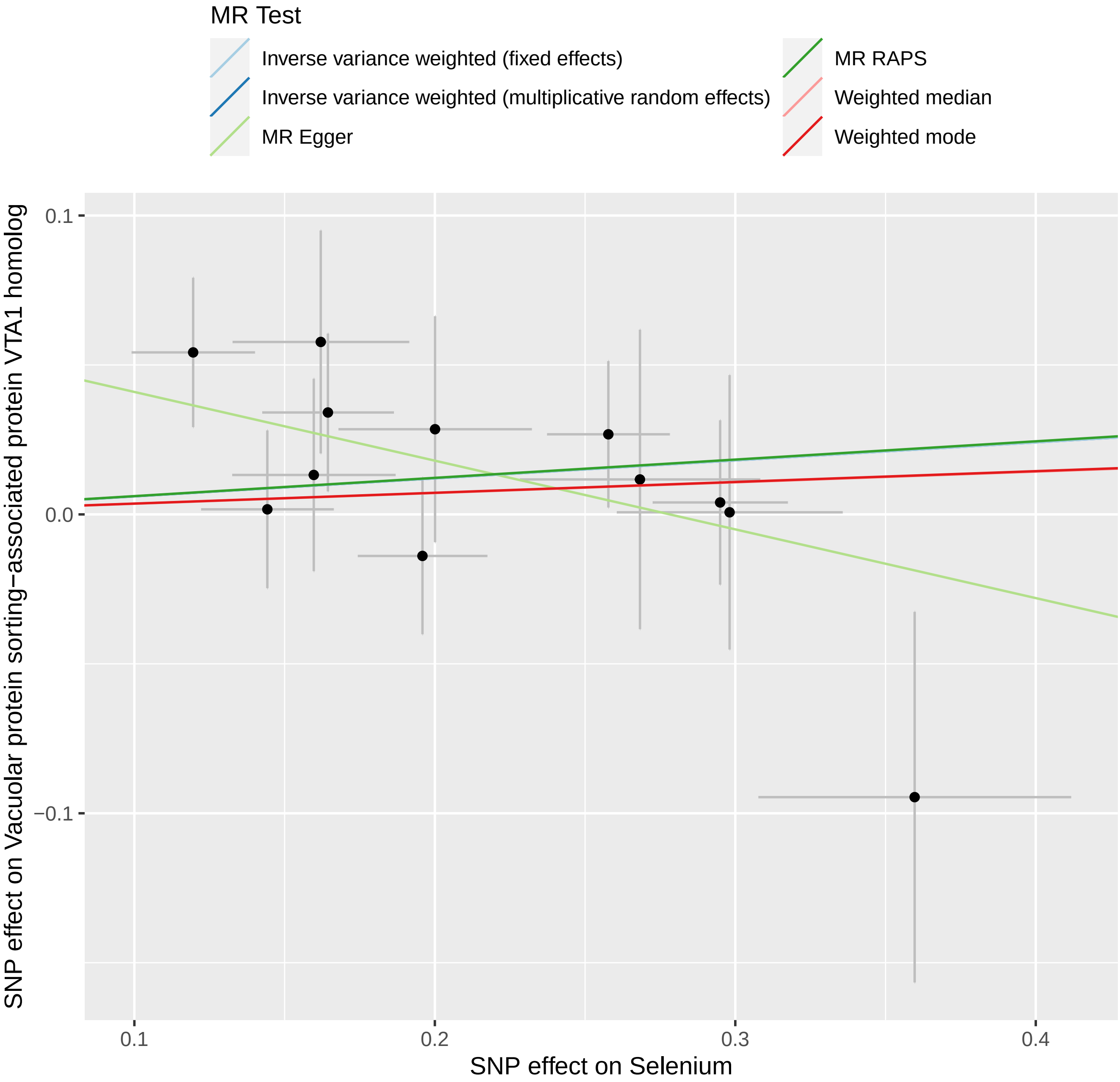

Batch 3−3212 : Selenium on Vesicle transport through interaction with t−SNAREs homolog 1A

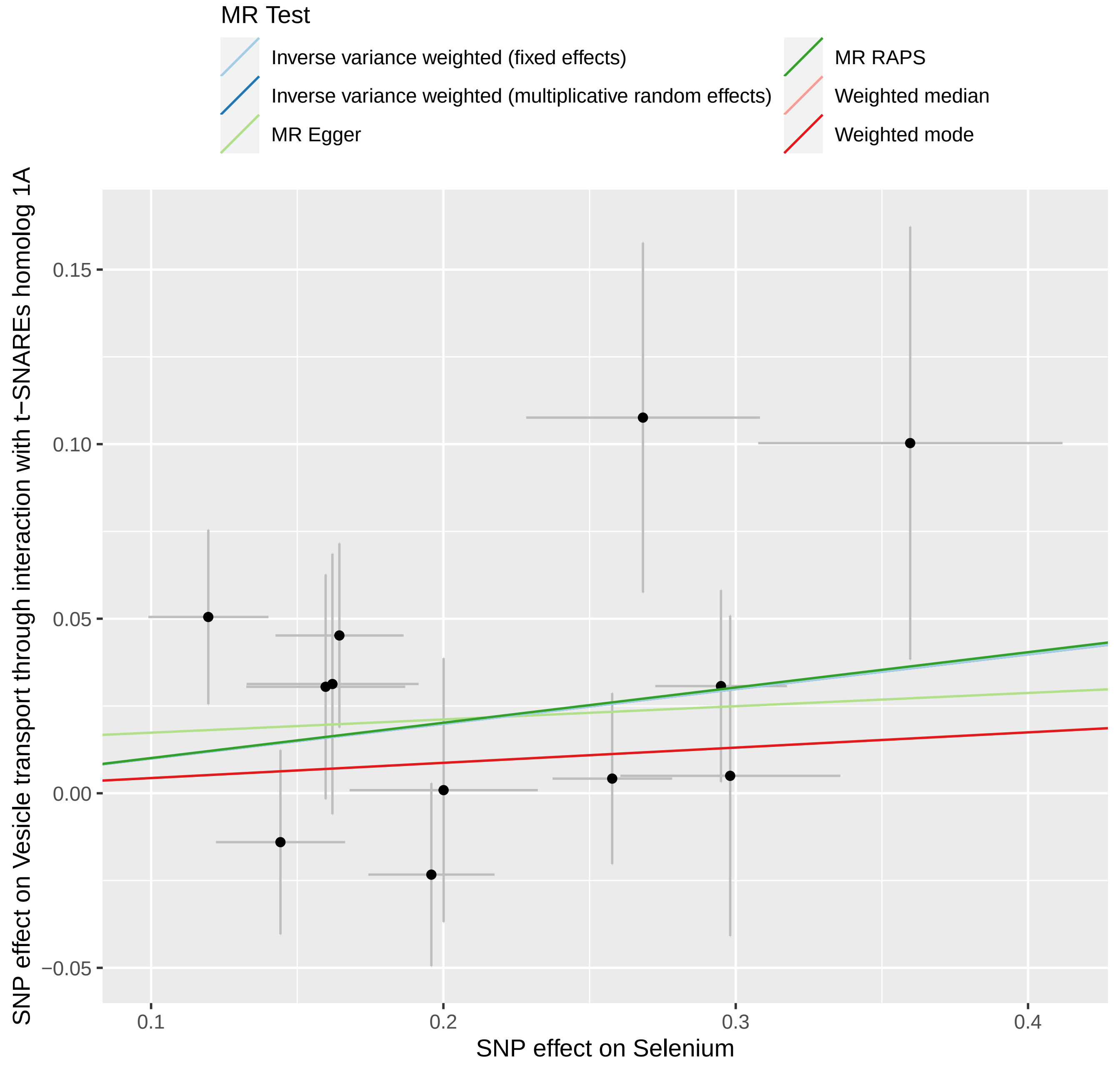

Batch 3−3213 : Selenium on Vesicle transport through interaction with t−SNAREs homolog 1B

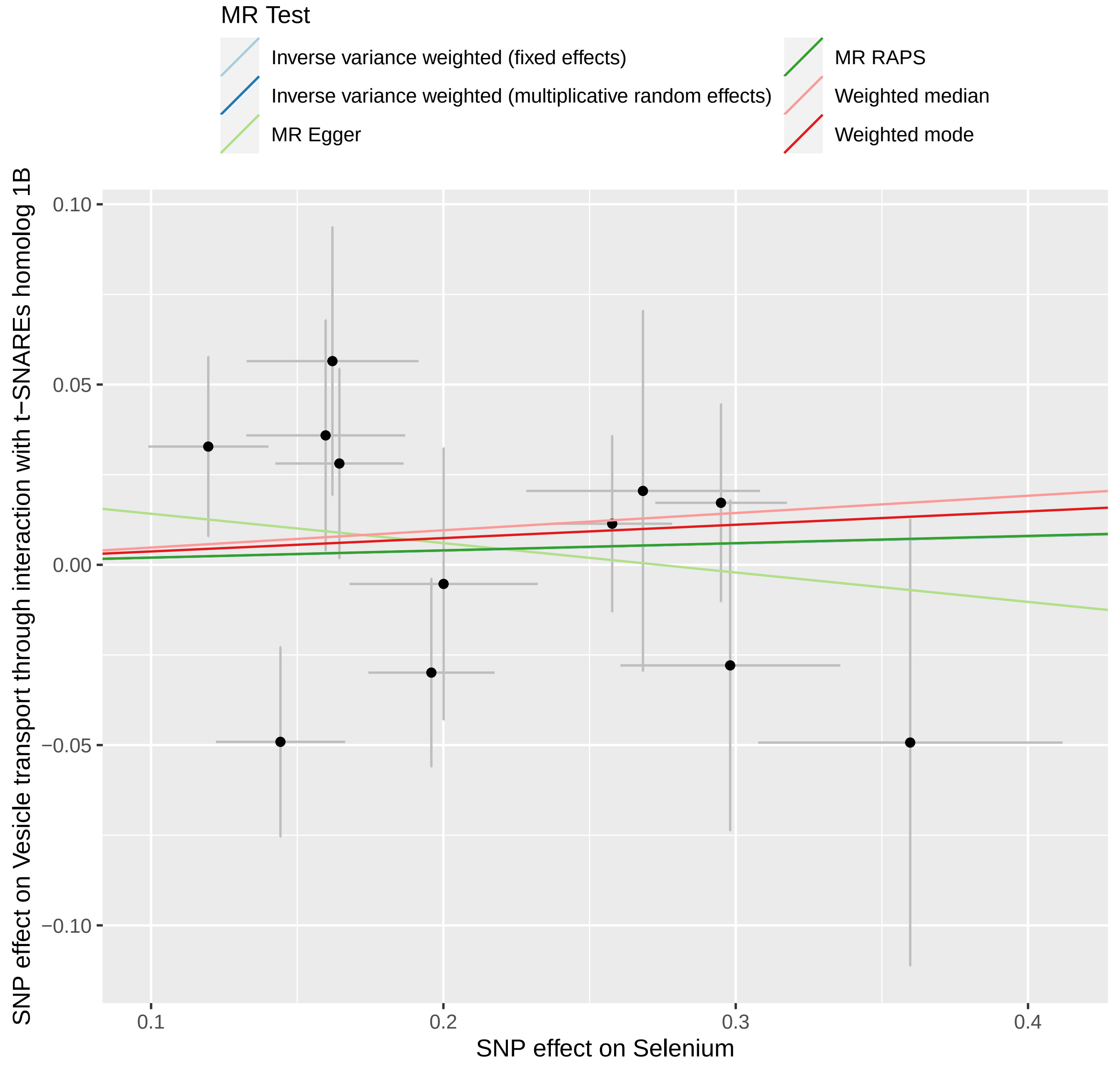

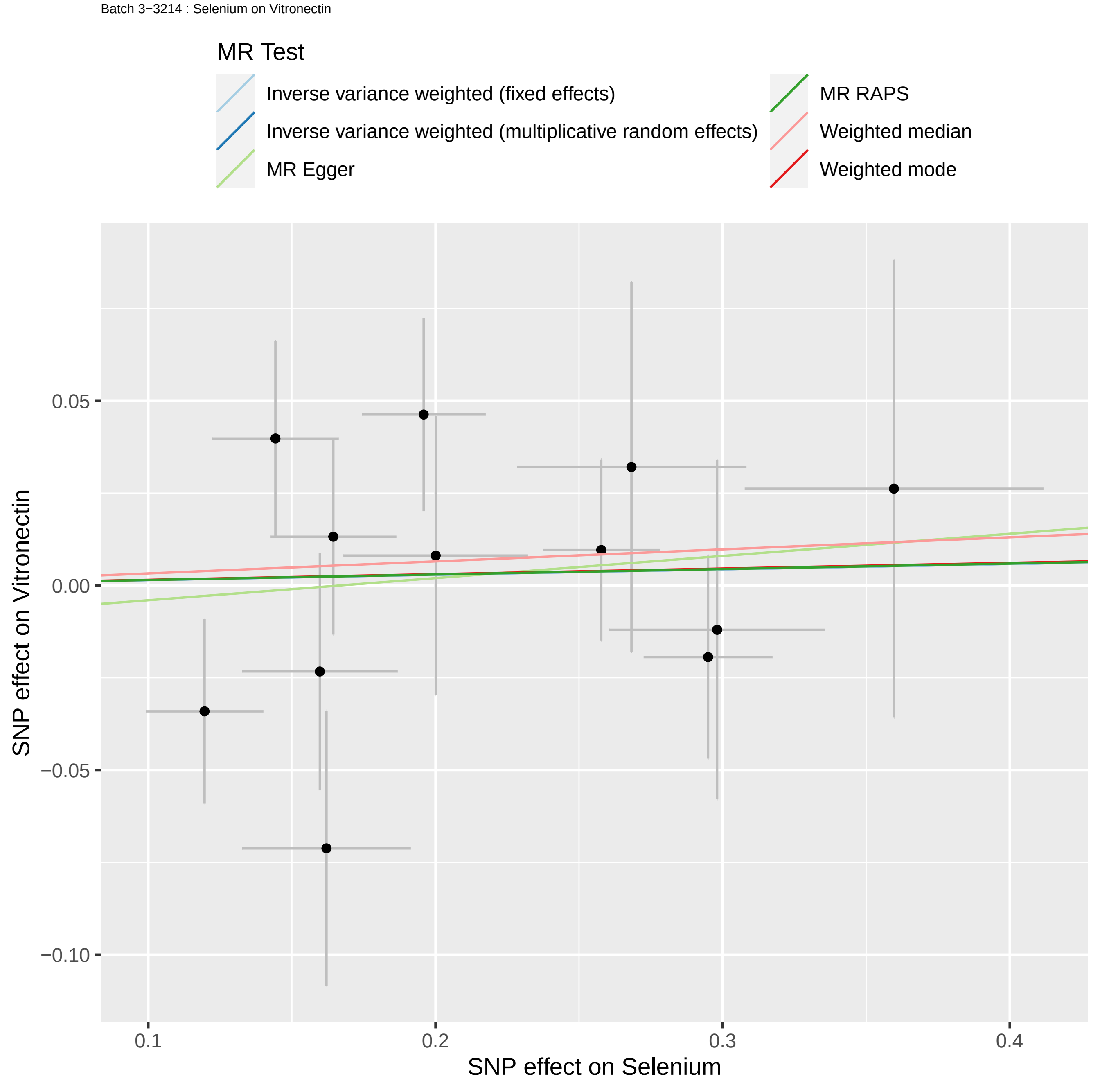

Batch 3−3214 : Selenium on Vitronectin
MR Test
Inverse variance weighted (fixed effects)
Inverse variance weighted (multiplicative random effects)
MR Egger
MR RAPS
Weighted median
Weighted mode
0.05
0.00
−0.05
−0.10
0.1
0.2
0.3
0.4
SNP effect on Vitronectin
SNP effect on Selenium

Batch 3−3215 : Selenium on Vitronectin

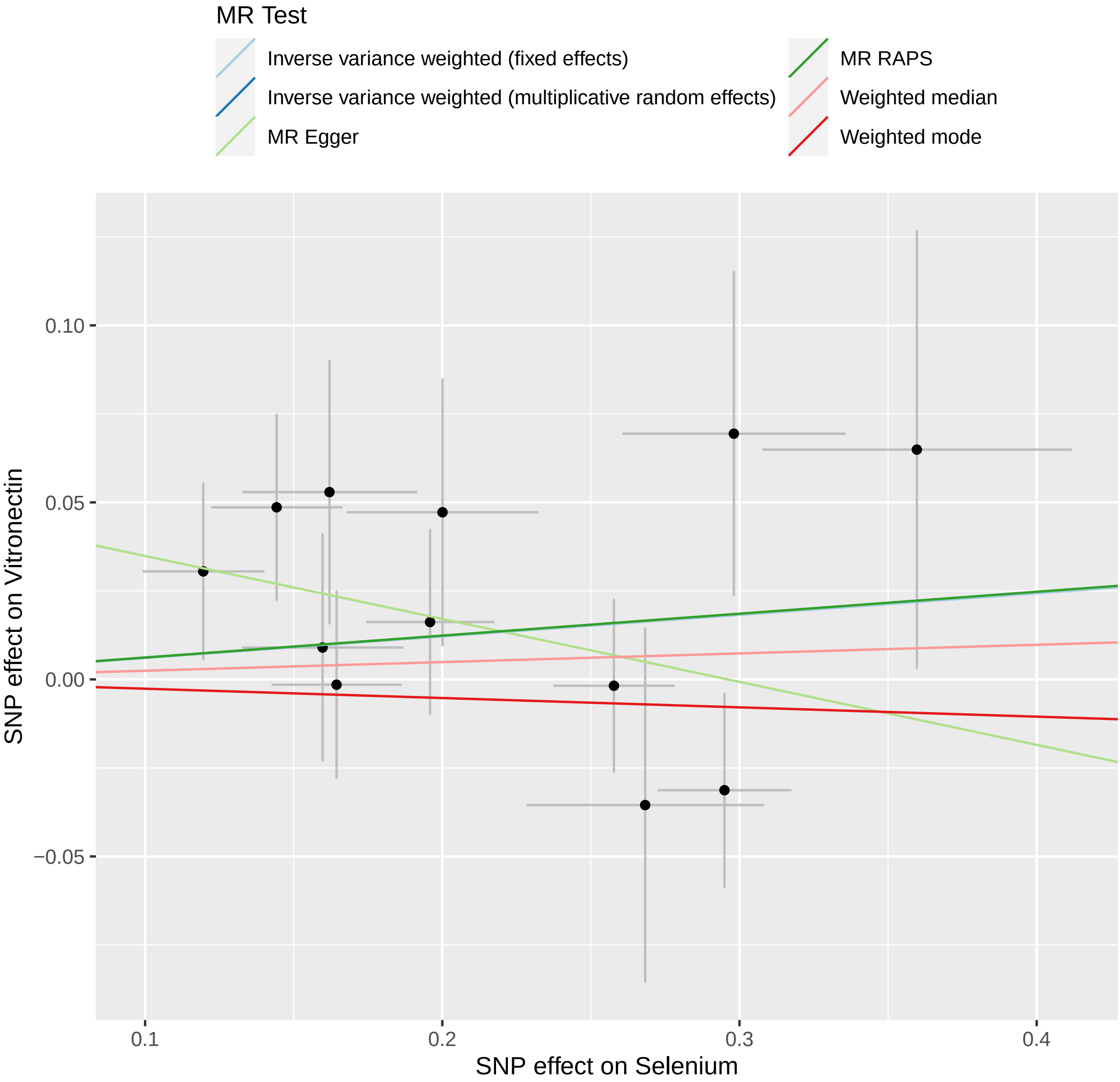

Batch 3−3216 : Selenium on Brorin

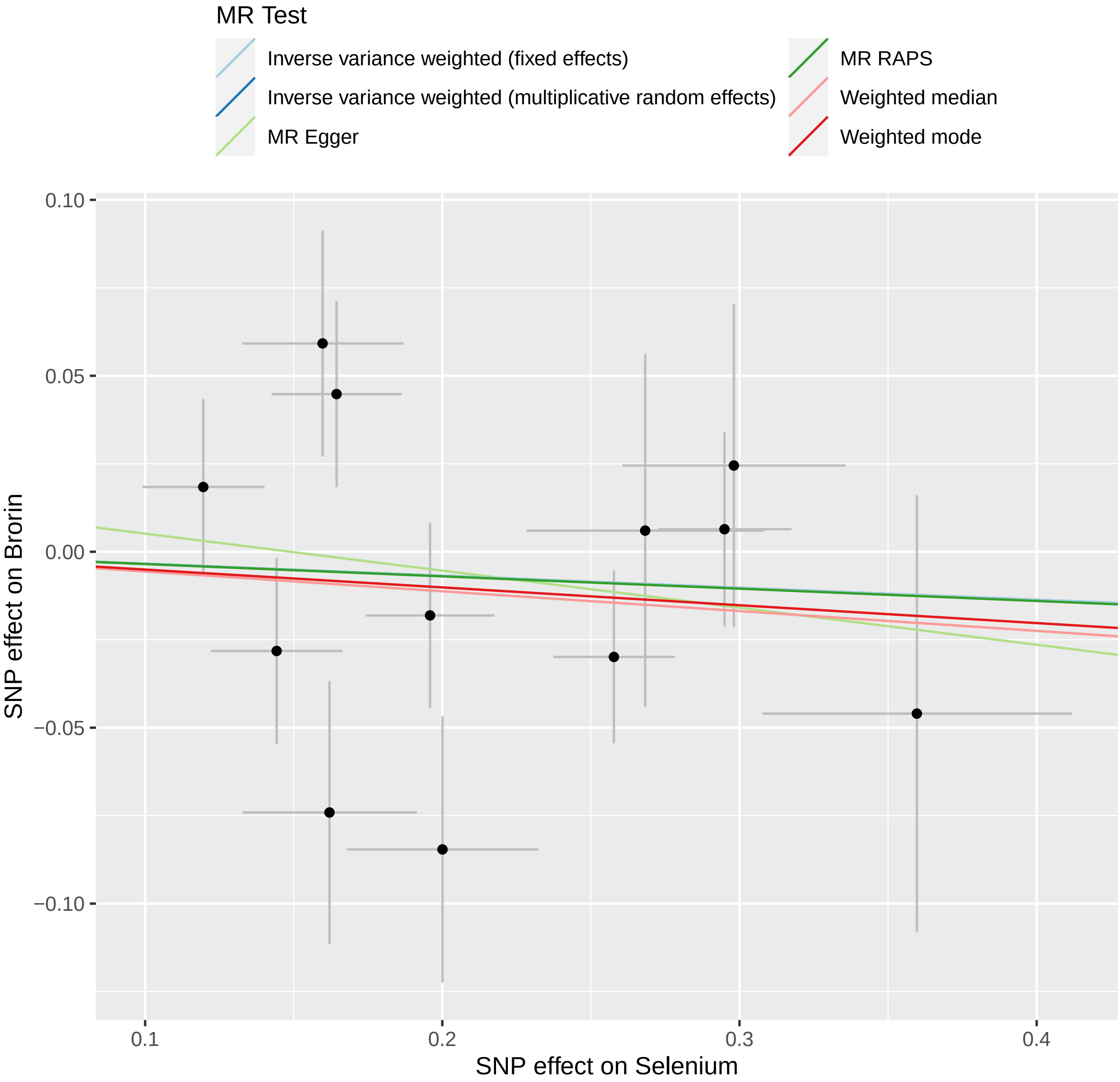

Batch 3−3217 : Selenium on von Willebrand factor C domain−containing protein 2−like

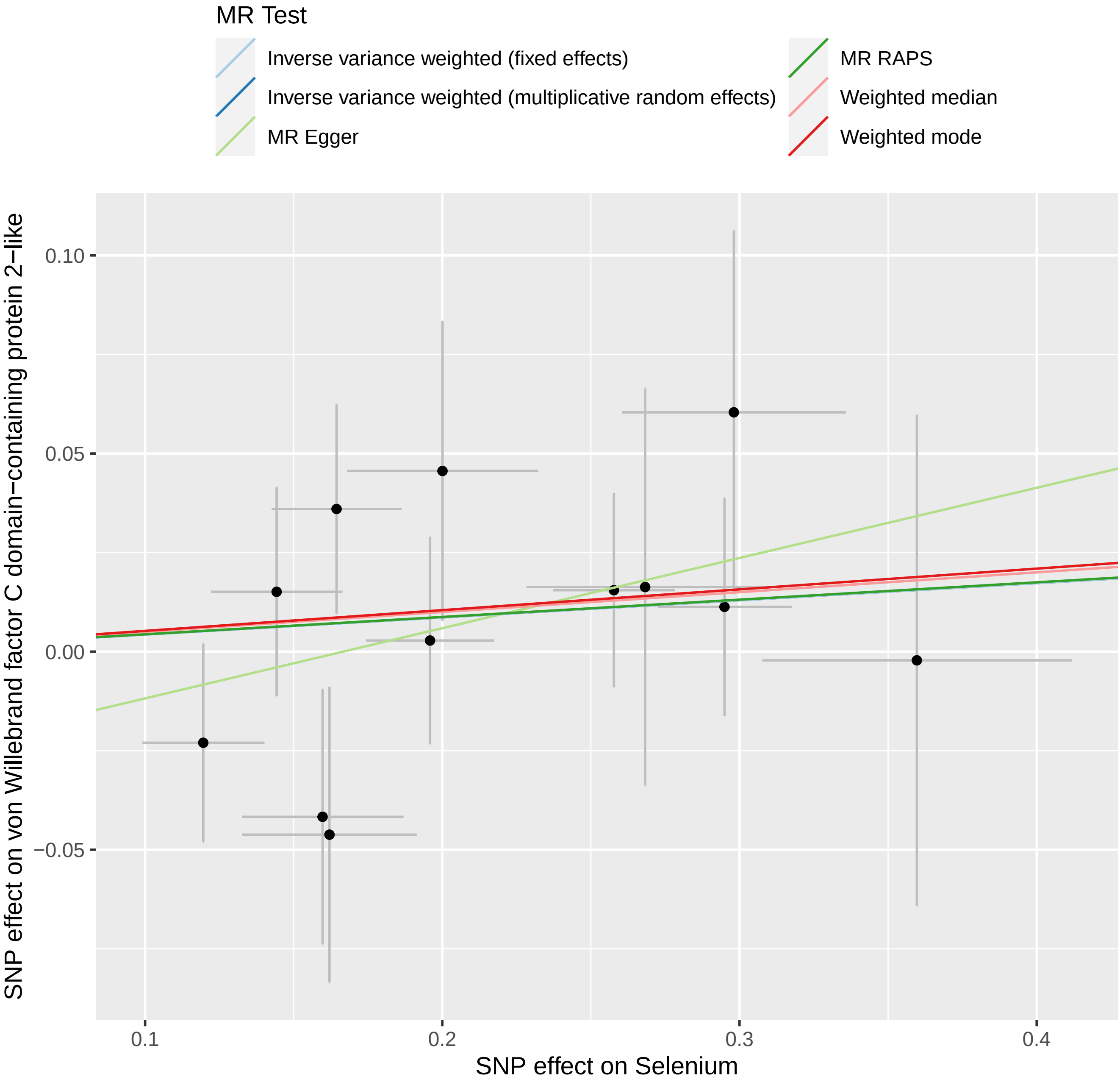

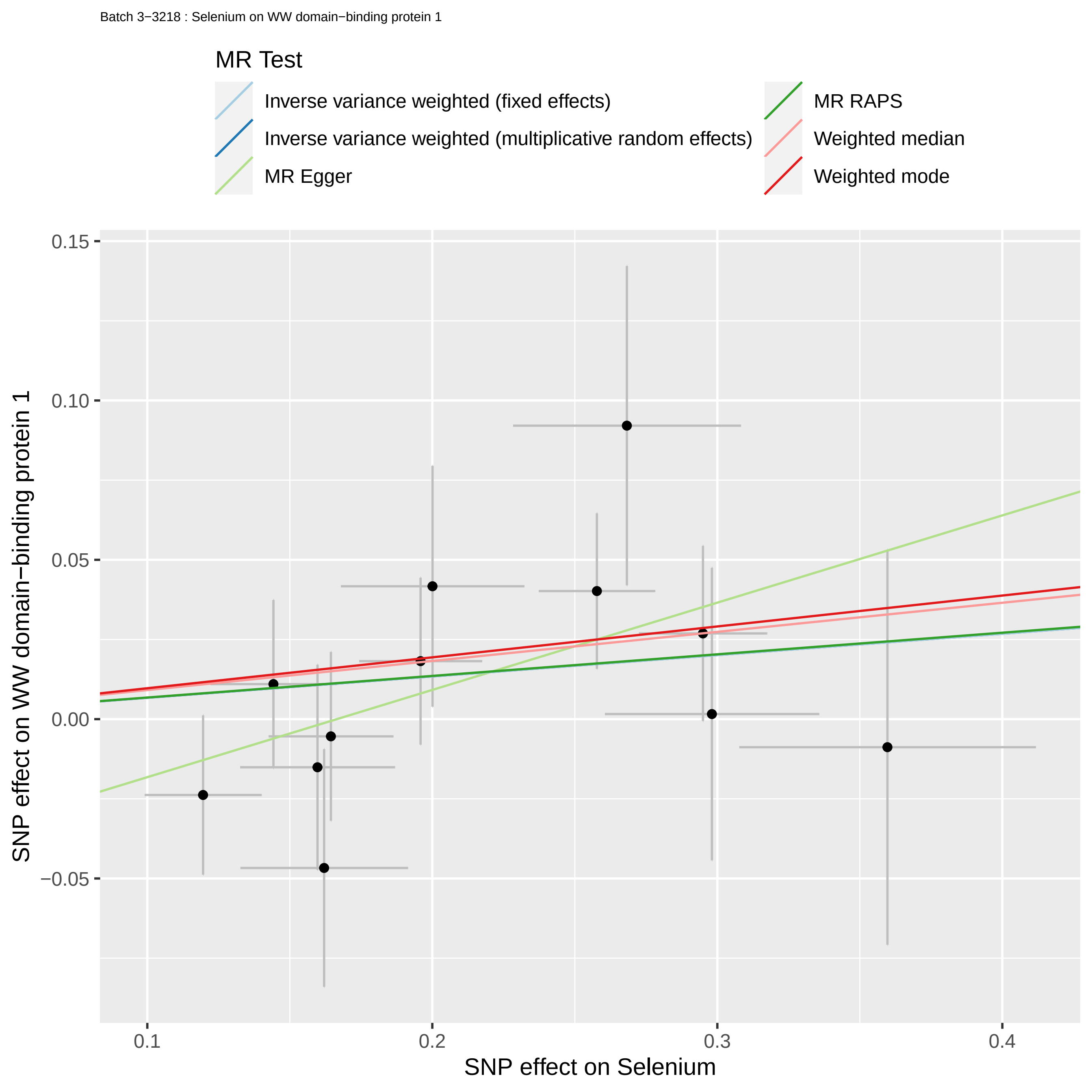

Batch 3−3218 : Selenium on WW domain−binding protein 1
MR Test
Inverse variance weighted (fixed effects)
Inverse variance weighted (multiplicative random effects)
MR Egger
MR RAPS
Weighted median
Weighted mode
0.15
0.10
0.05
0.00
−0.05
0.1
0.2
0.3
0.4
SNP effect on WW domain−binding protein 1
SNP effect on Selenium

Batch 3−3219 : Selenium on WW domain binding protein 1−like

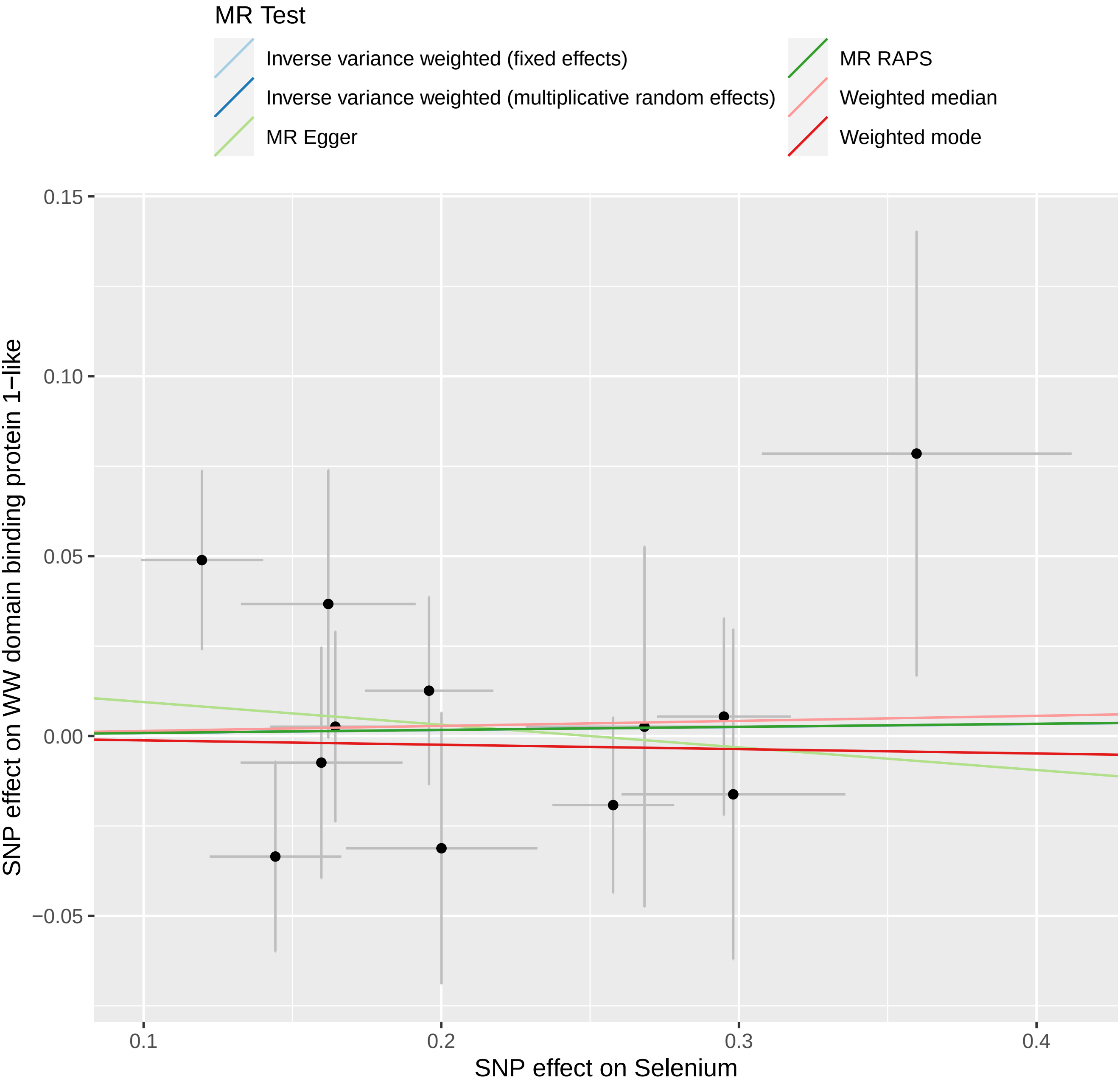

Batch 3−3220 : Selenium on WD repeat−containing protein 1

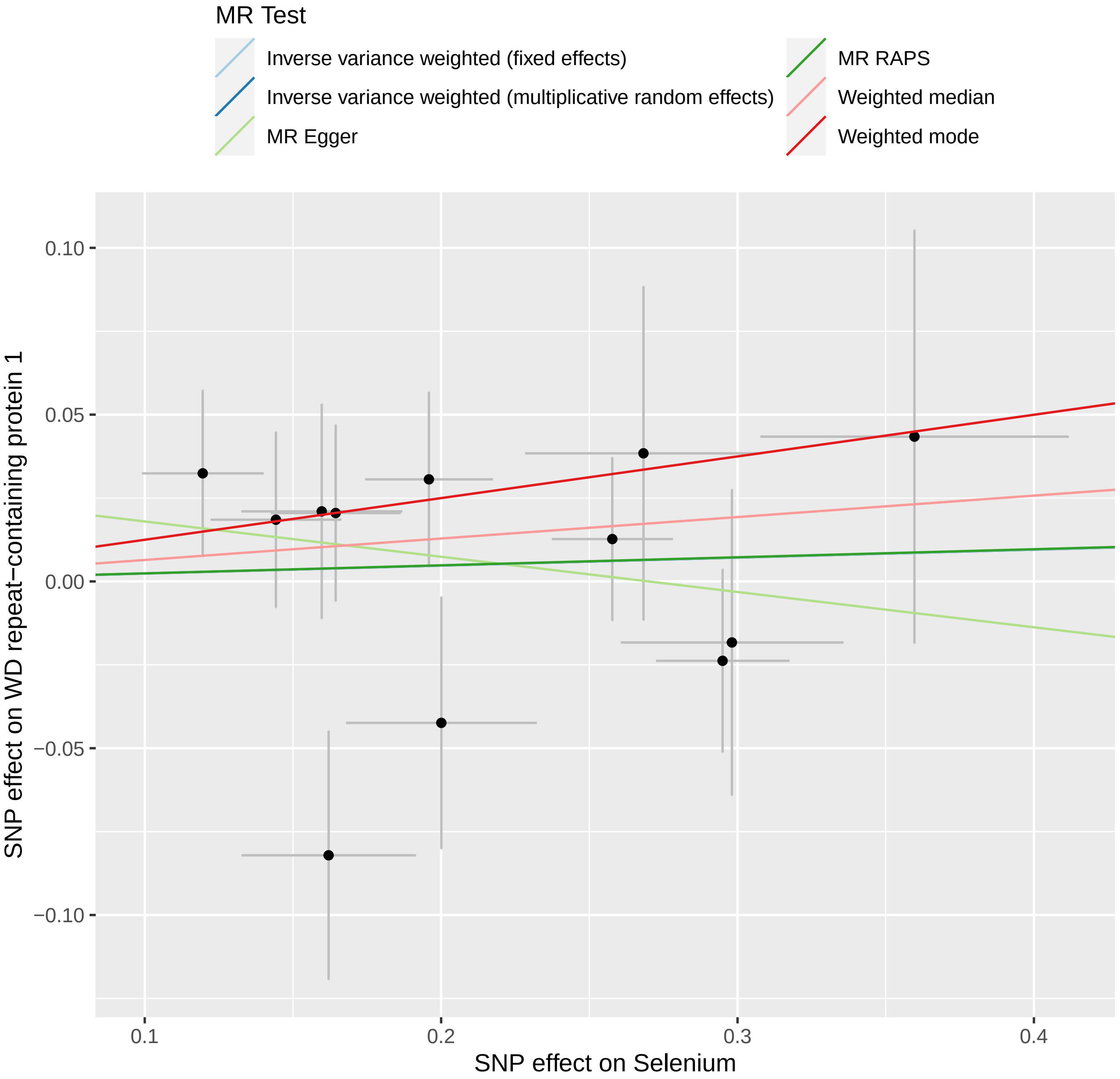

Batch 3−3221 : Selenium on WAP four−disulfide core domain protein 10A

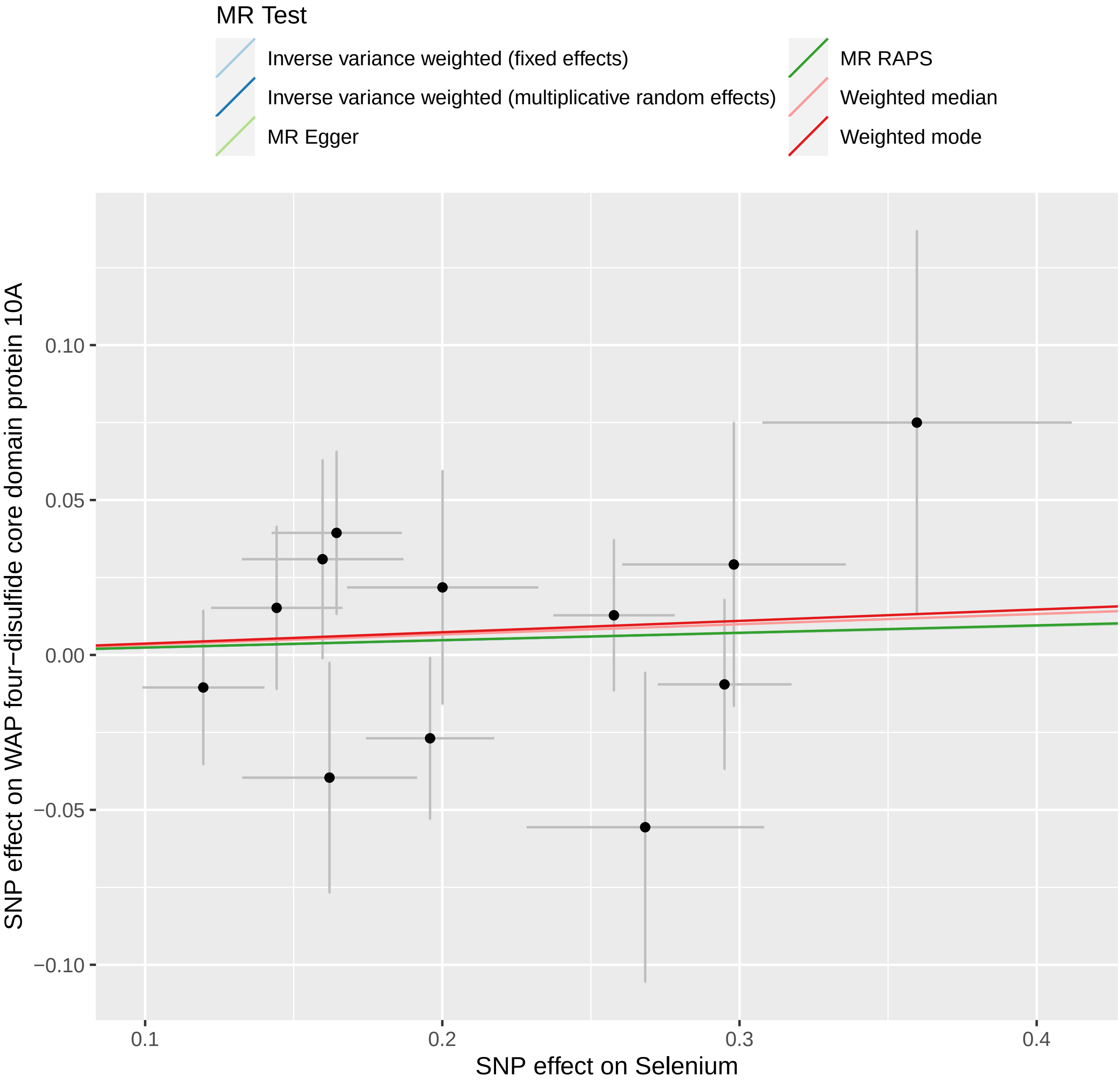

Batch 3−3222 : Selenium on WAP four−disulfide core domain protein 12

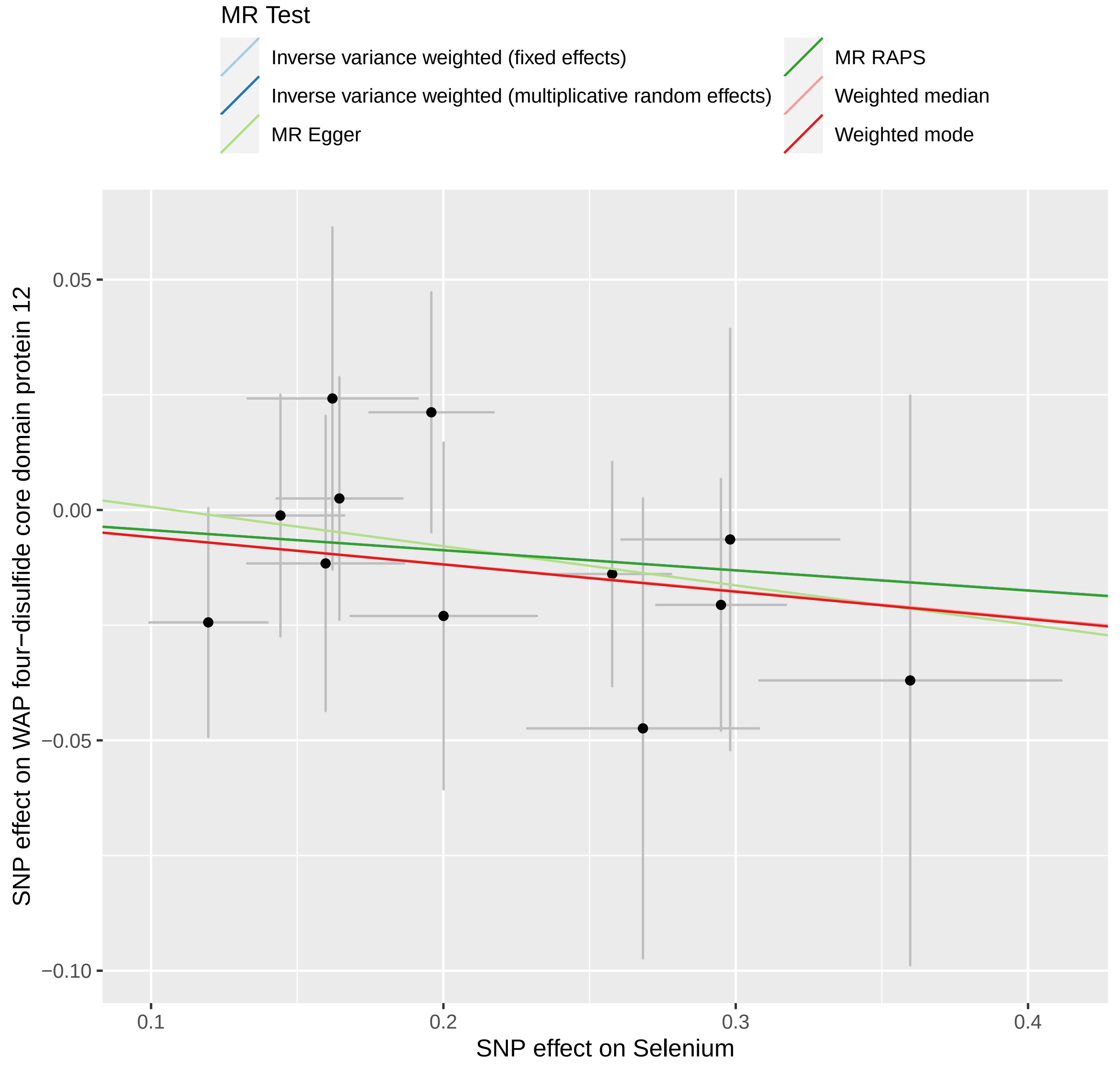

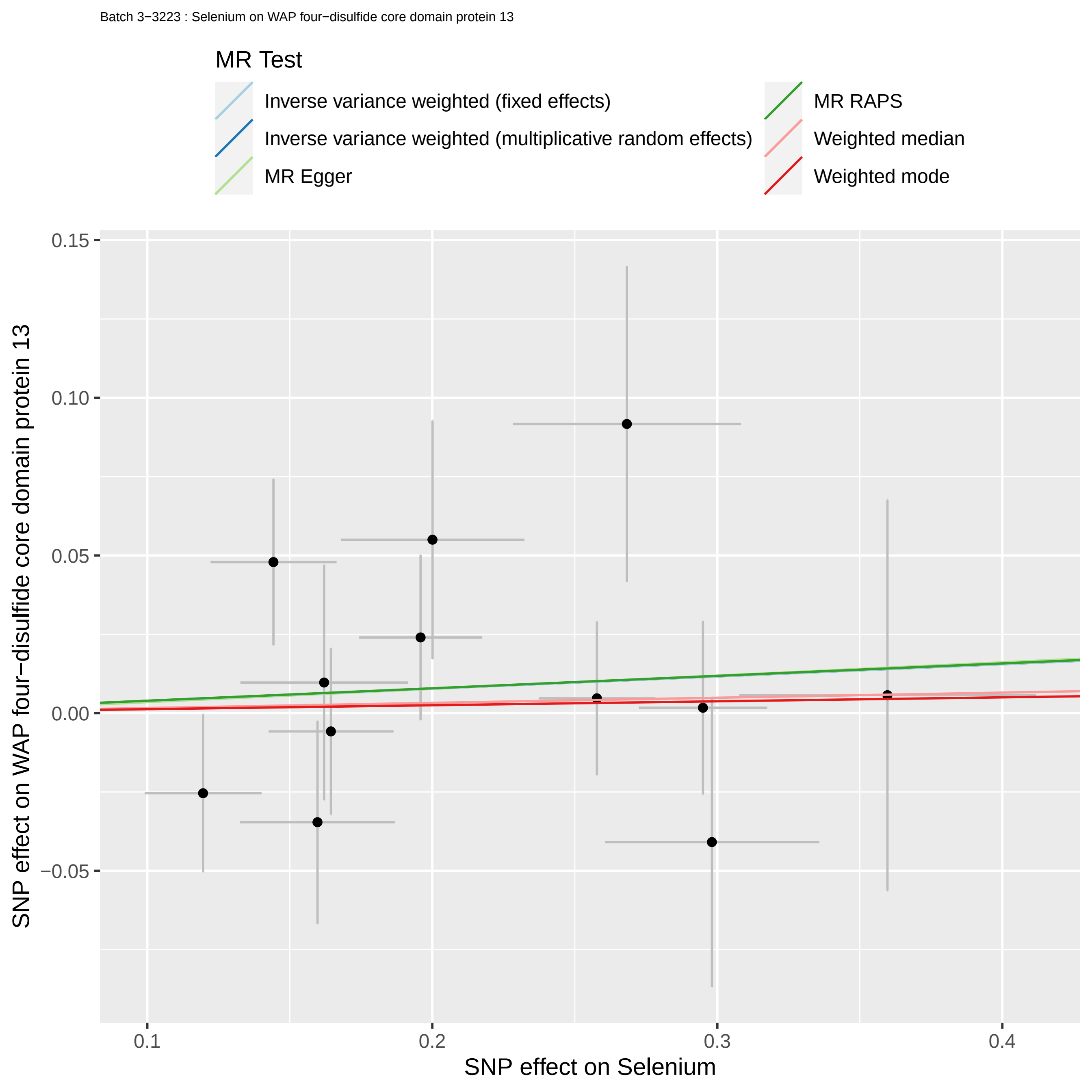

Batch 3−3223 : Selenium on WAP four−disulfide core domain protein 13
MR Test
Inverse variance weighted (fixed effects)
Inverse variance weighted (multiplicative random effects)
MR Egger
MR RAPS
Weighted median
Weighted mode
0.15
0.10
0.05
0.00
−0.05
0.1
0.2
0.3
0.4
SNP effect on WAP four−disulfide core domain protein 13
SNP effect on Selenium

Batch 3−3224 : Selenium on WAP four−disulfide core domain protein 2

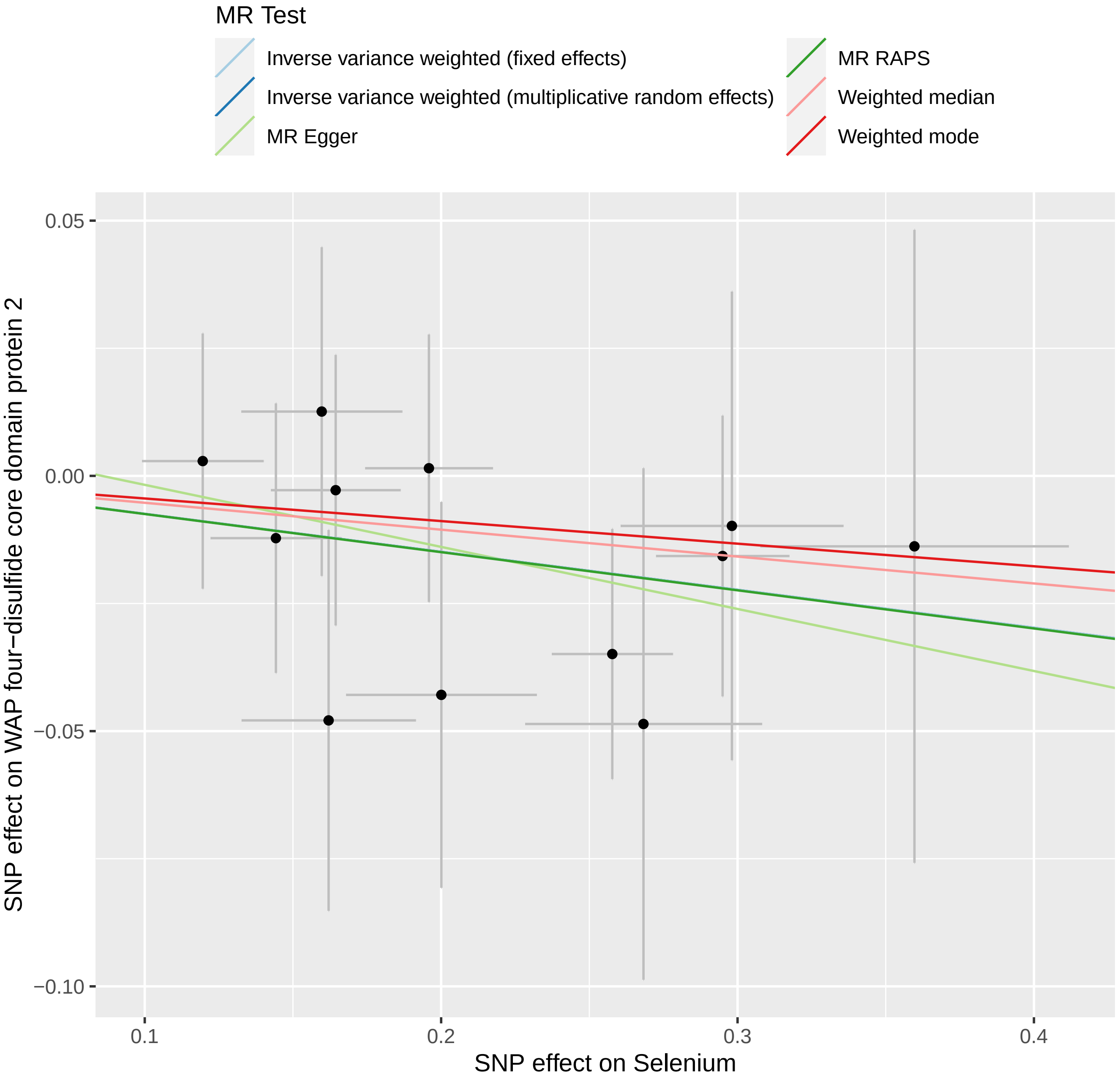

Batch 3−3225 : Selenium on WAP four−disulfide core domain protein 3

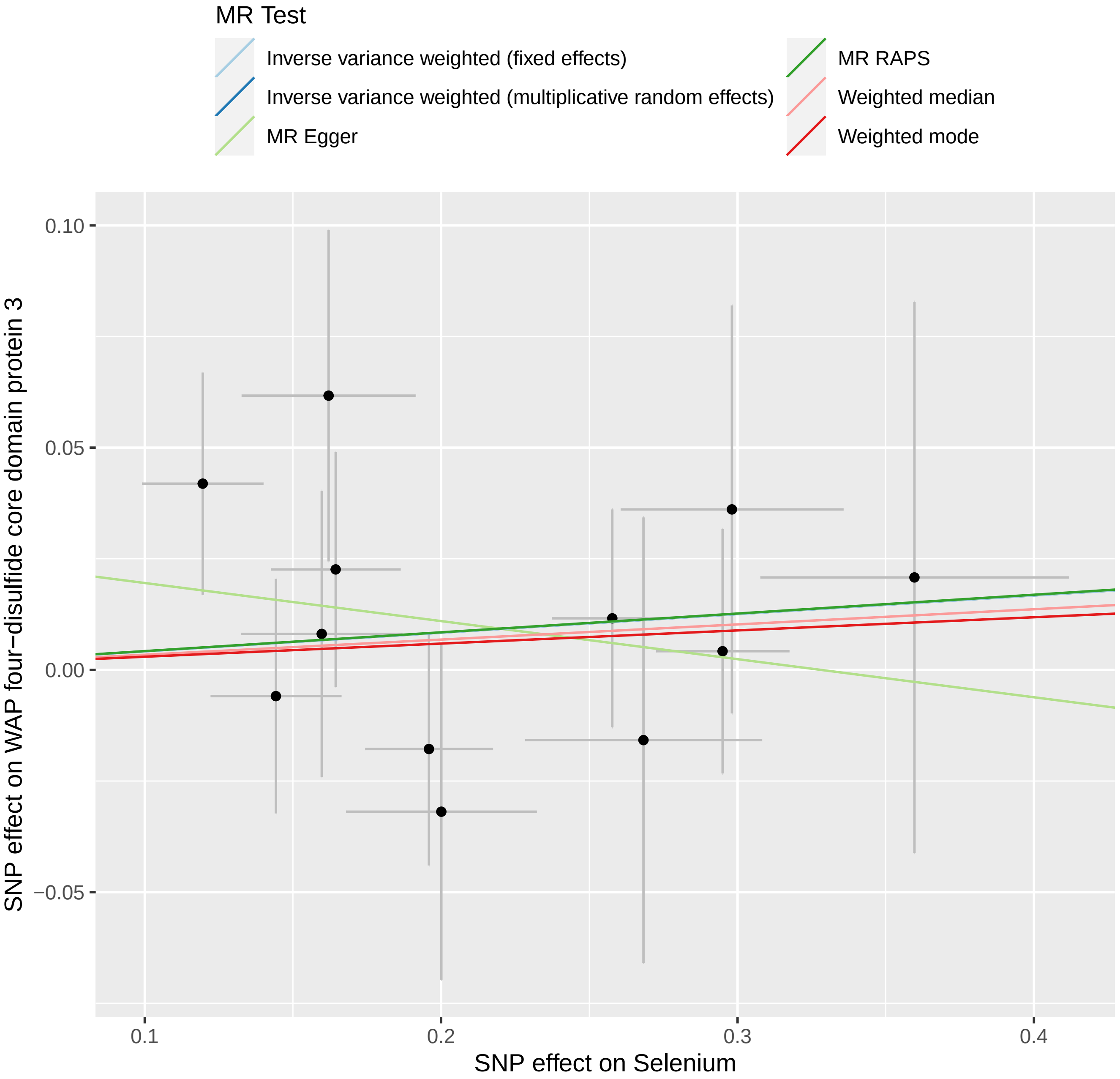

Batch 3−3226 : Selenium on WAP four−disulfide core domain protein 5

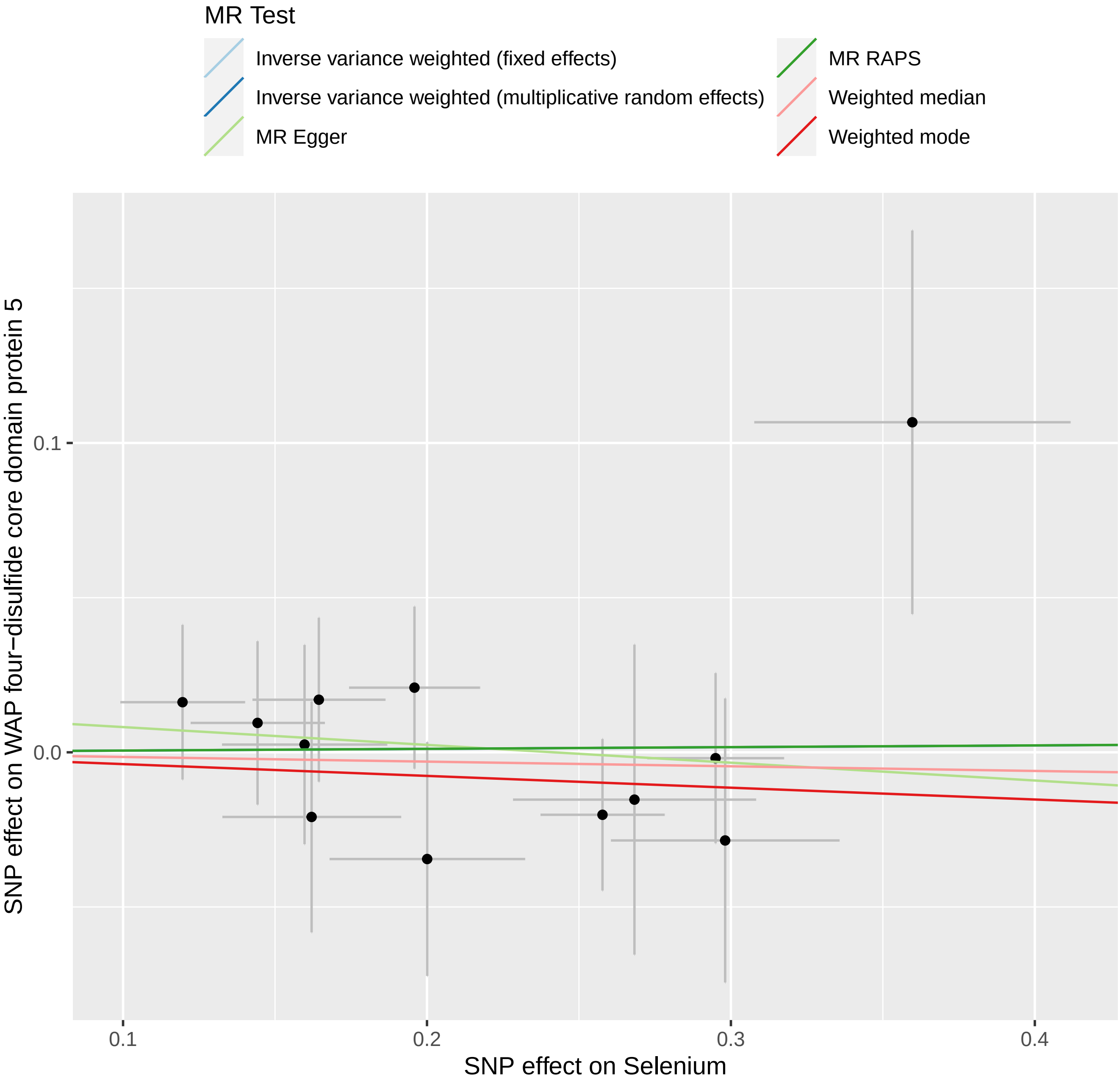

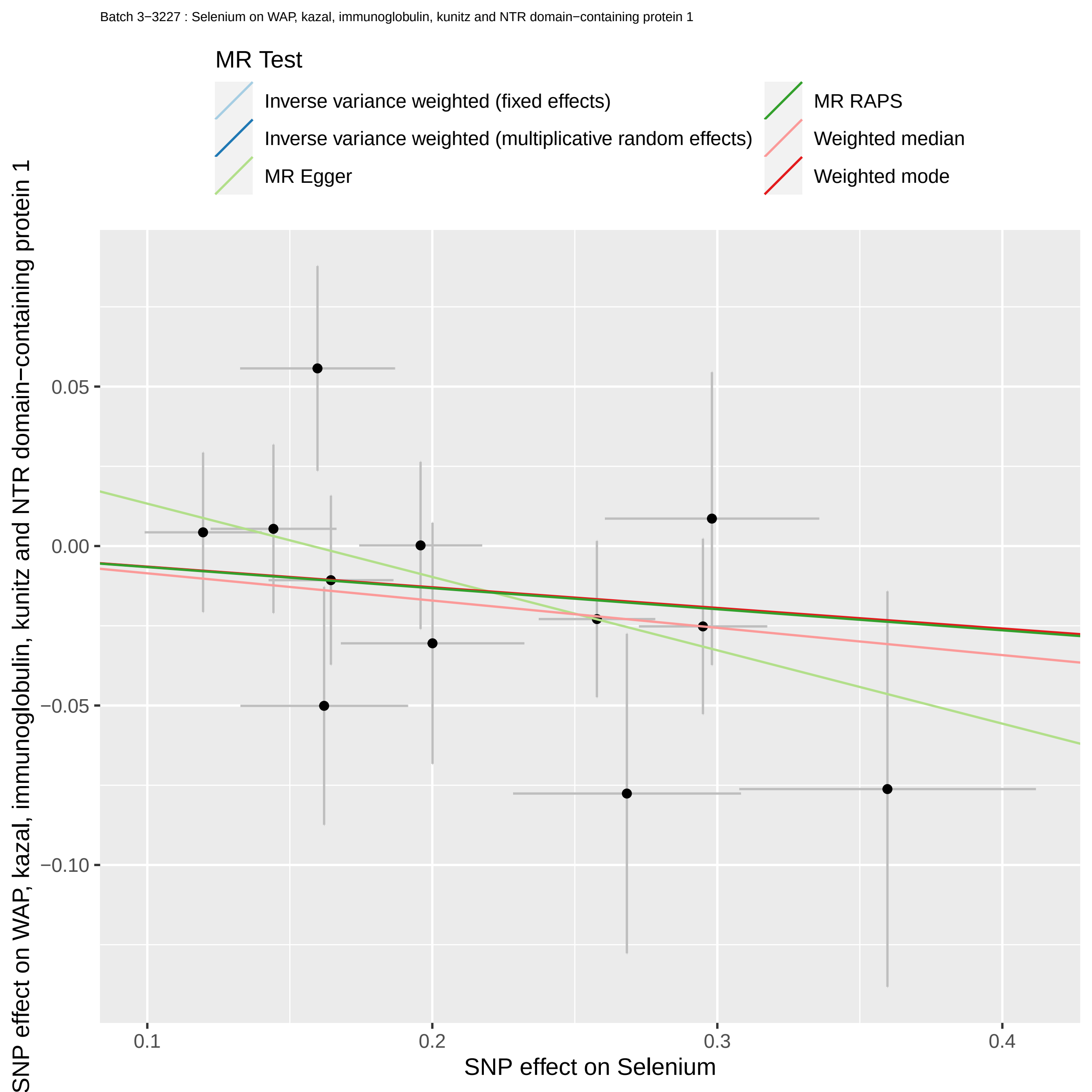

Batch 3−3227 : Selenium on WAP, kazal, immunoglobulin, kunitz and NTR domain−containing protein 1
MR Test
Inverse variance weighted (fixed effects)
Inverse variance weighted (multiplicative random effects)
MR Egger
MR RAPS
Weighted median
Weighted mode
0.05
0.00
−0.05
−0.10
0.1
0.2
0.3
0.4
SNP effect on Selenium
SNP effect on WAP, kazal, immunoglobulin, kunitz and NTR domain−containing protein 1

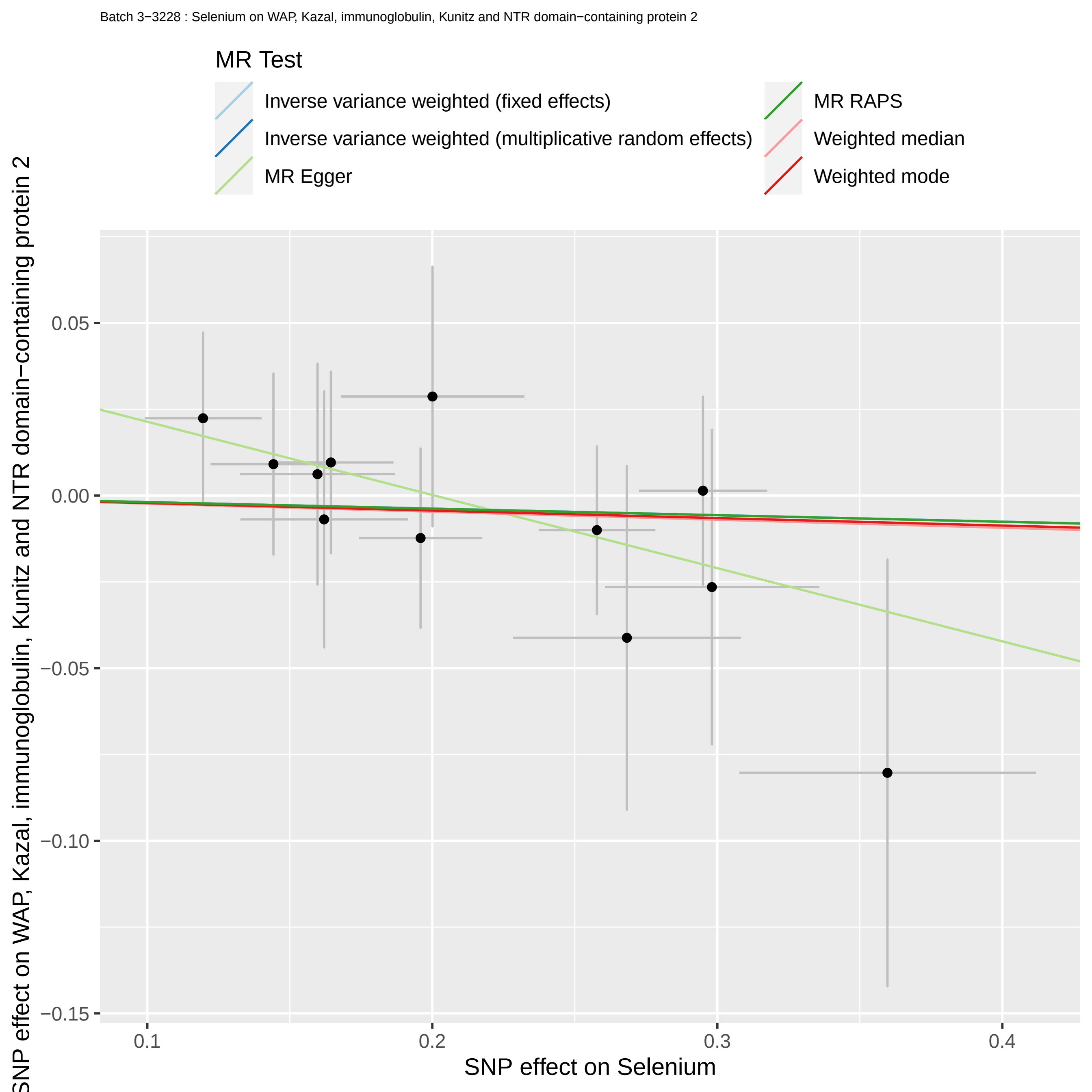

Batch 3−3228 : Selenium on WAP, Kazal, immunoglobulin, Kunitz and NTR domain−containing protein 2
MR Test
Inverse variance weighted (fixed effects)
Inverse variance weighted (multiplicative random effects)
MR Egger
MR RAPS
Weighted median
Weighted mode
0.05
0.00
−0.05
−0.10
−0.15
0.1
0.2
0.3
0.4
SNP effect on Selenium
SNP effect on WAP, Kazal, immunoglobulin, Kunitz and NTR domain−containing protein 2

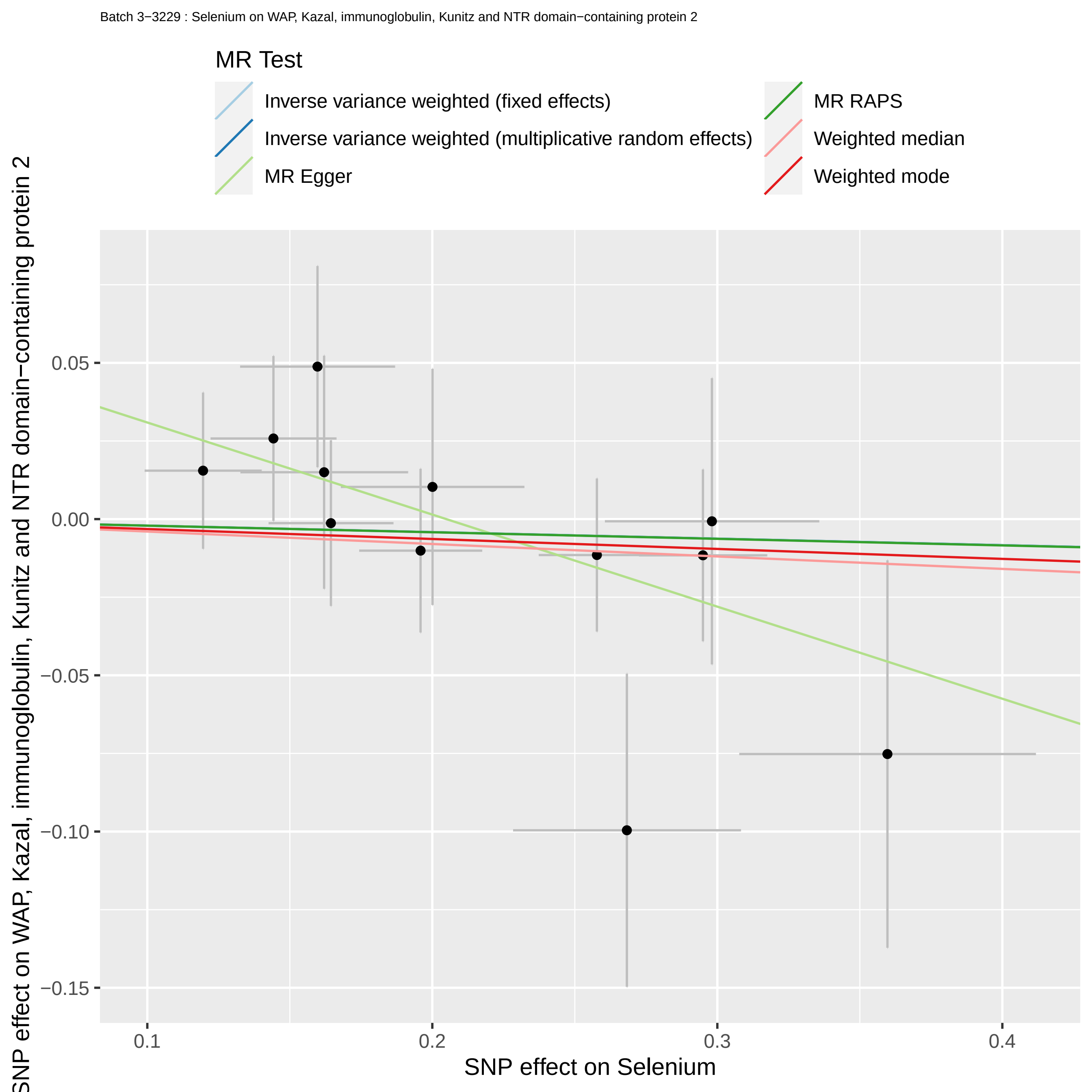
Batch 3−3229 : Selenium on WAP, Kazal, immunoglobulin, Kunitz and NTR domain−containing protein 2
MR Test
Inverse variance weighted (fixed effects)
Inverse variance weighted (multiplicative random effects)
MR Egger
MR RAPS
Weighted median
Weighted mode
0.05
0.00
−0.05
−0.10
−0.15
0.1
0.2
0.3
0.4
SNP effect on Selenium
SNP effect on WAP, Kazal, immunoglobulin, Kunitz and NTR domain−containing protein 2

Batch 3−3230 : Selenium on Wnt inhibitory factor 1

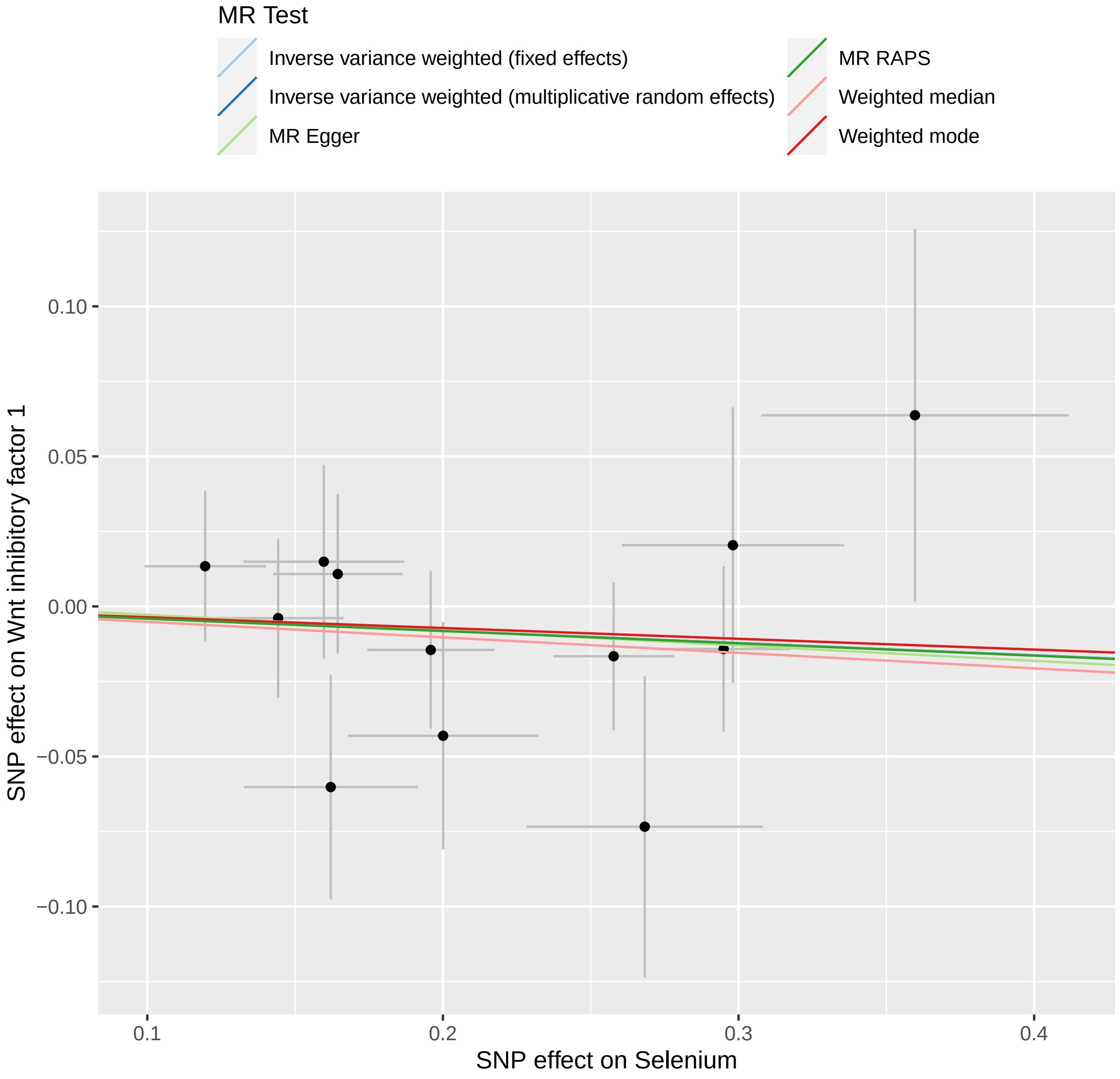

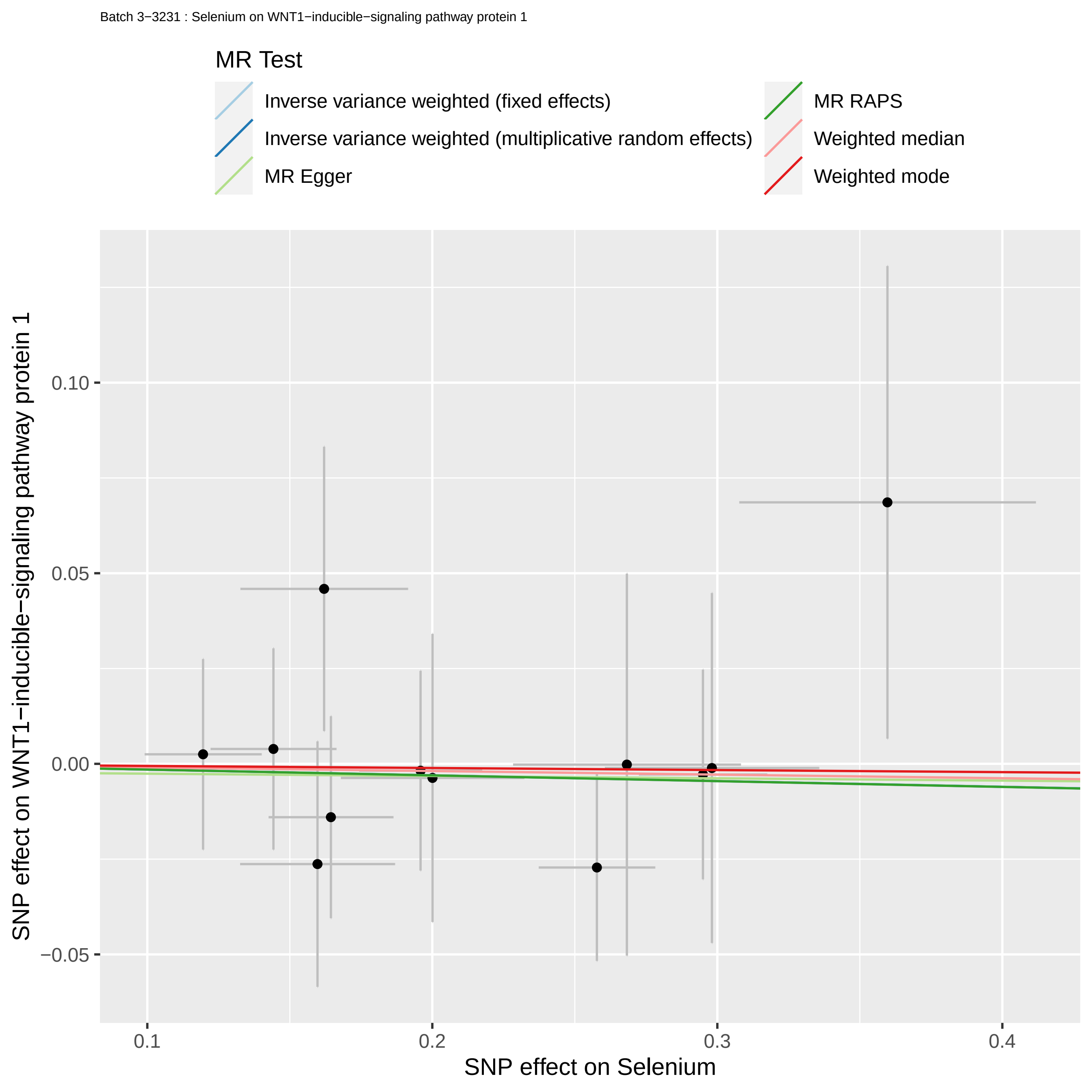
Batch 3−3231 : Selenium on WNT1−inducible−signaling pathway protein 1
MR Test
Inverse variance weighted (fixed effects)
Inverse variance weighted (multiplicative random effects)
MR Egger
MR RAPS
Weighted median
Weighted mode
SNP effect on WNT1−inducible−signaling pathway protein 1
0.10
0.05
0.00
−0.05
0.1
0.2
0.3
0.4
SNP effect on Selenium

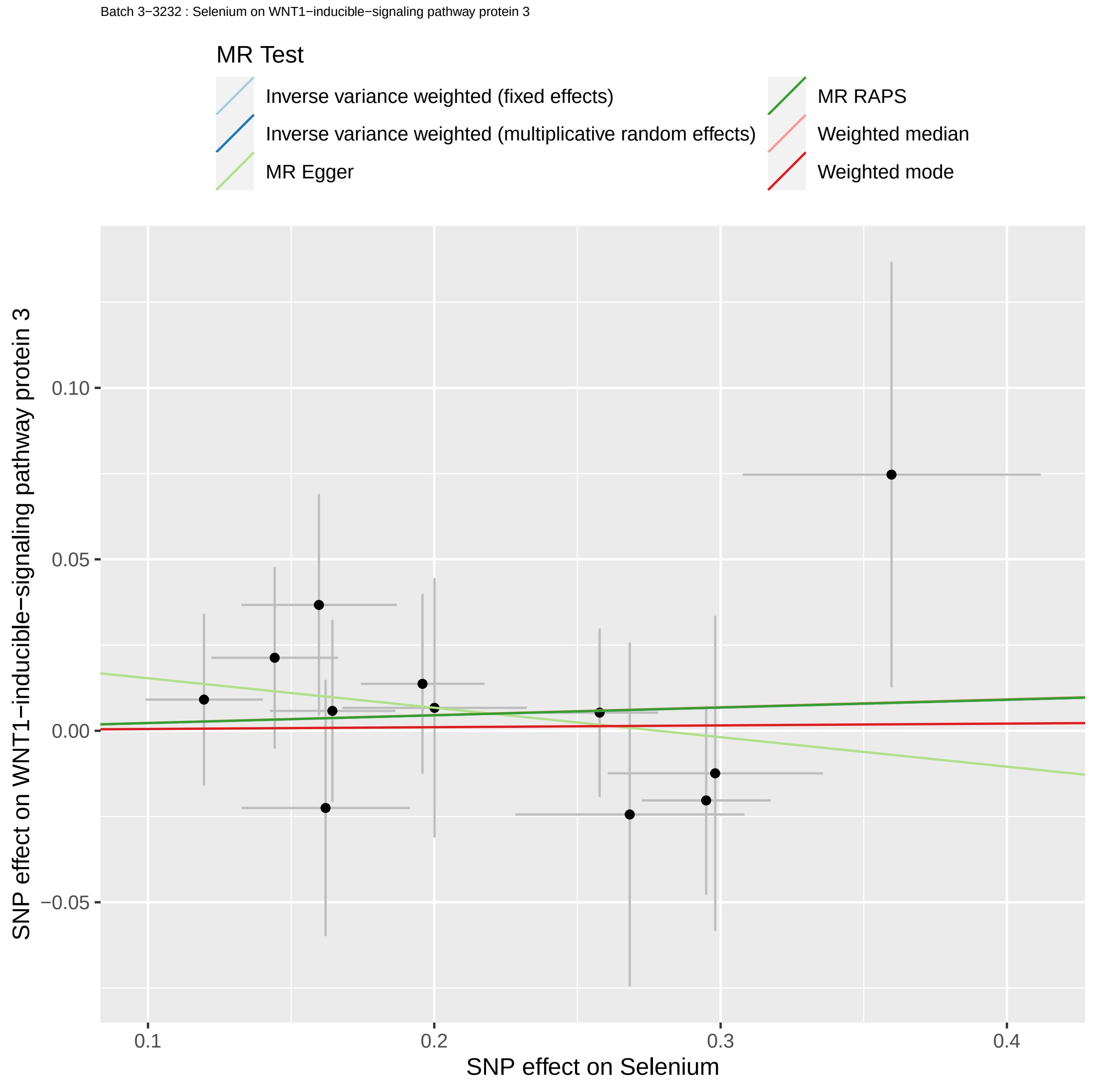

Batch 3−3232 : Selenium on WNT1−inducible−signaling pathway protein 3
MR Test
Inverse variance weighted (fixed effects)
Inverse variance weighted (multiplicative random effects)
MR Egger
MR RAPS
Weighted median
Weighted mode
SNP effect on WNT1−inducible−signaling pathway protein 3
0.10
0.05
0.00
−0.05
0.1
0.2
0.3
0.4
SNP effect on Selenium

Batch 3−3233 : Selenium on Serine/threonine−protein kinase WNK3

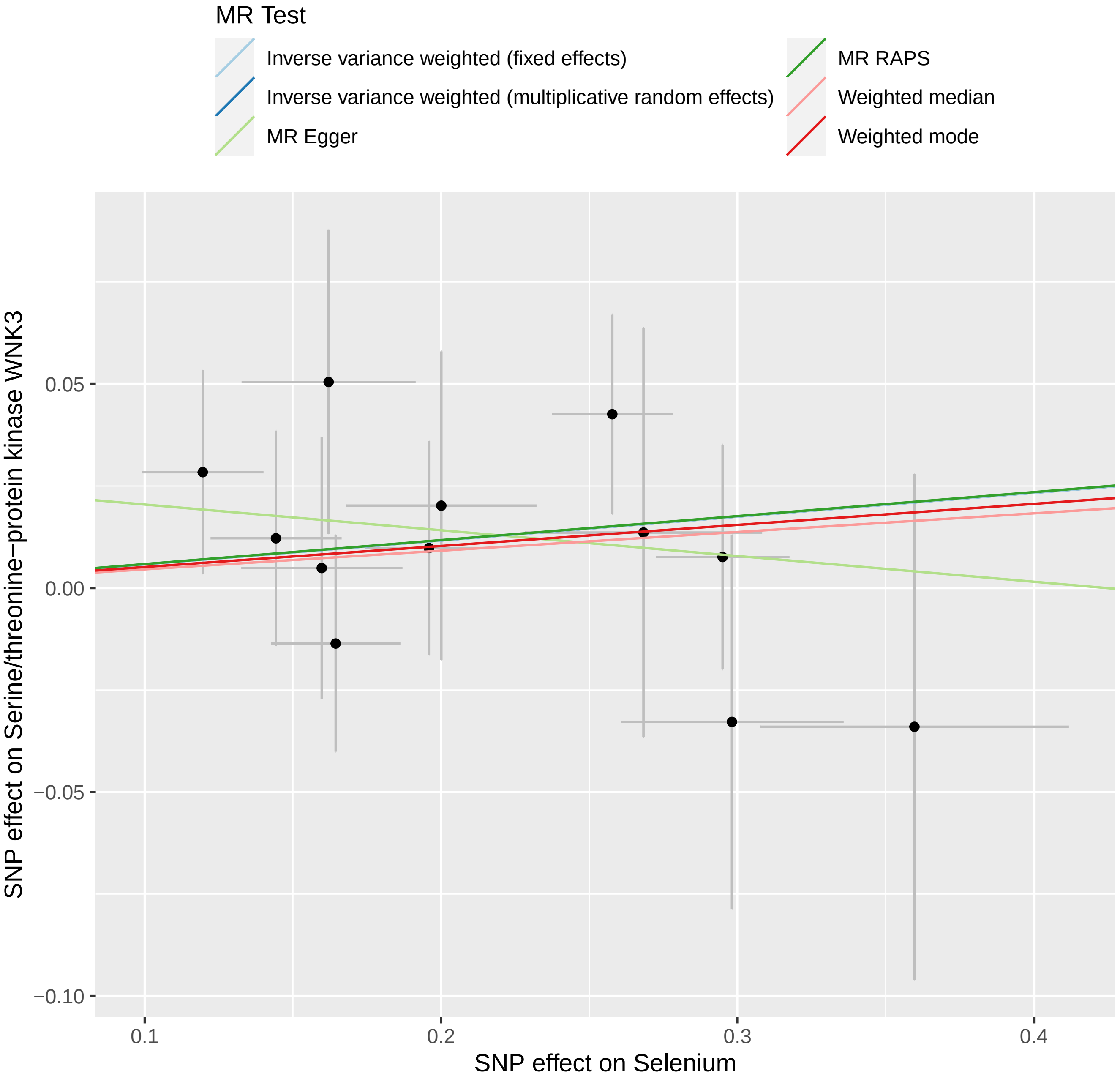

Batch 3−3234 : Selenium on Protein Wnt−7a

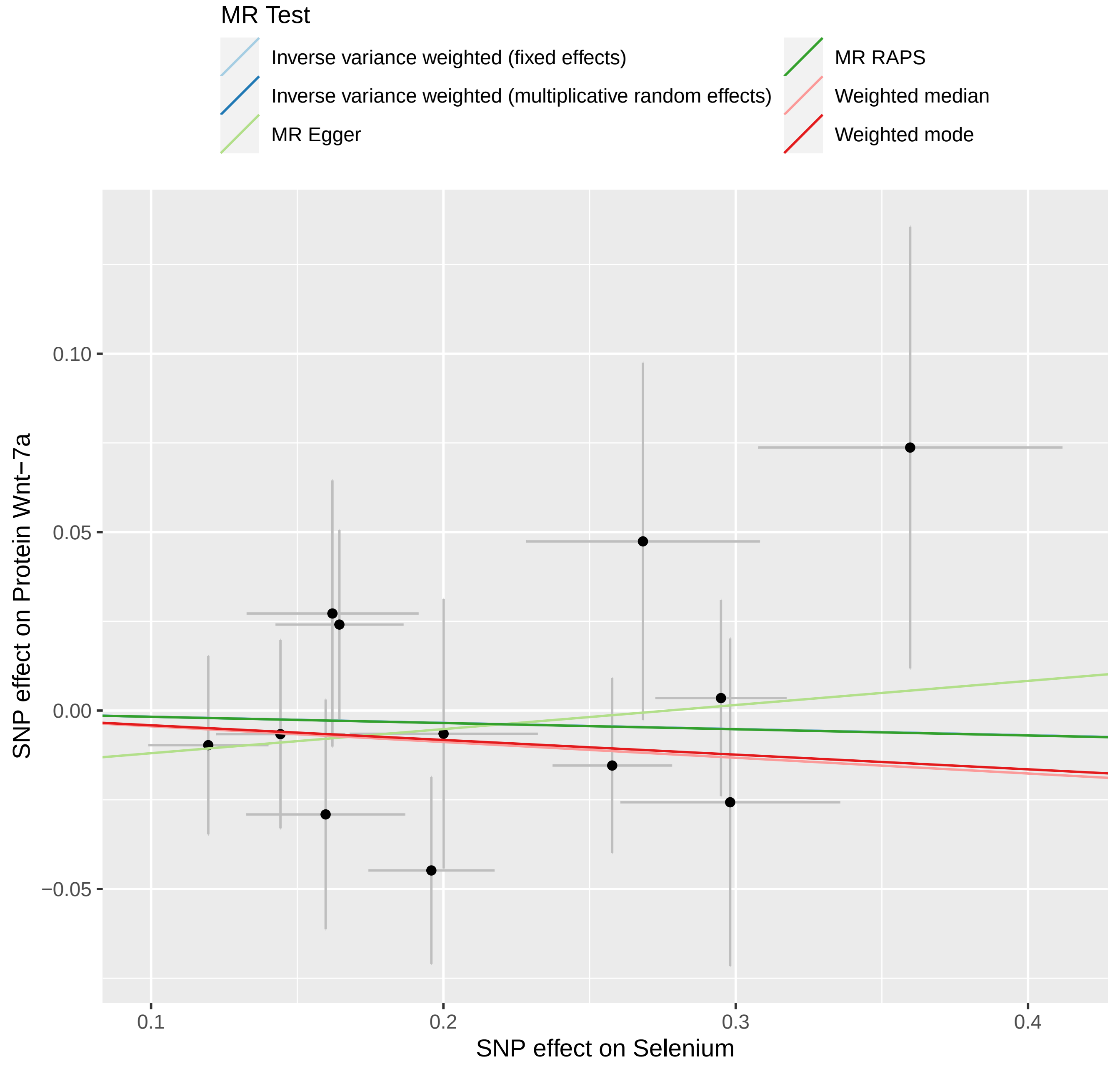

Batch 3−3235 : Selenium on WSC domain−containing protein 2

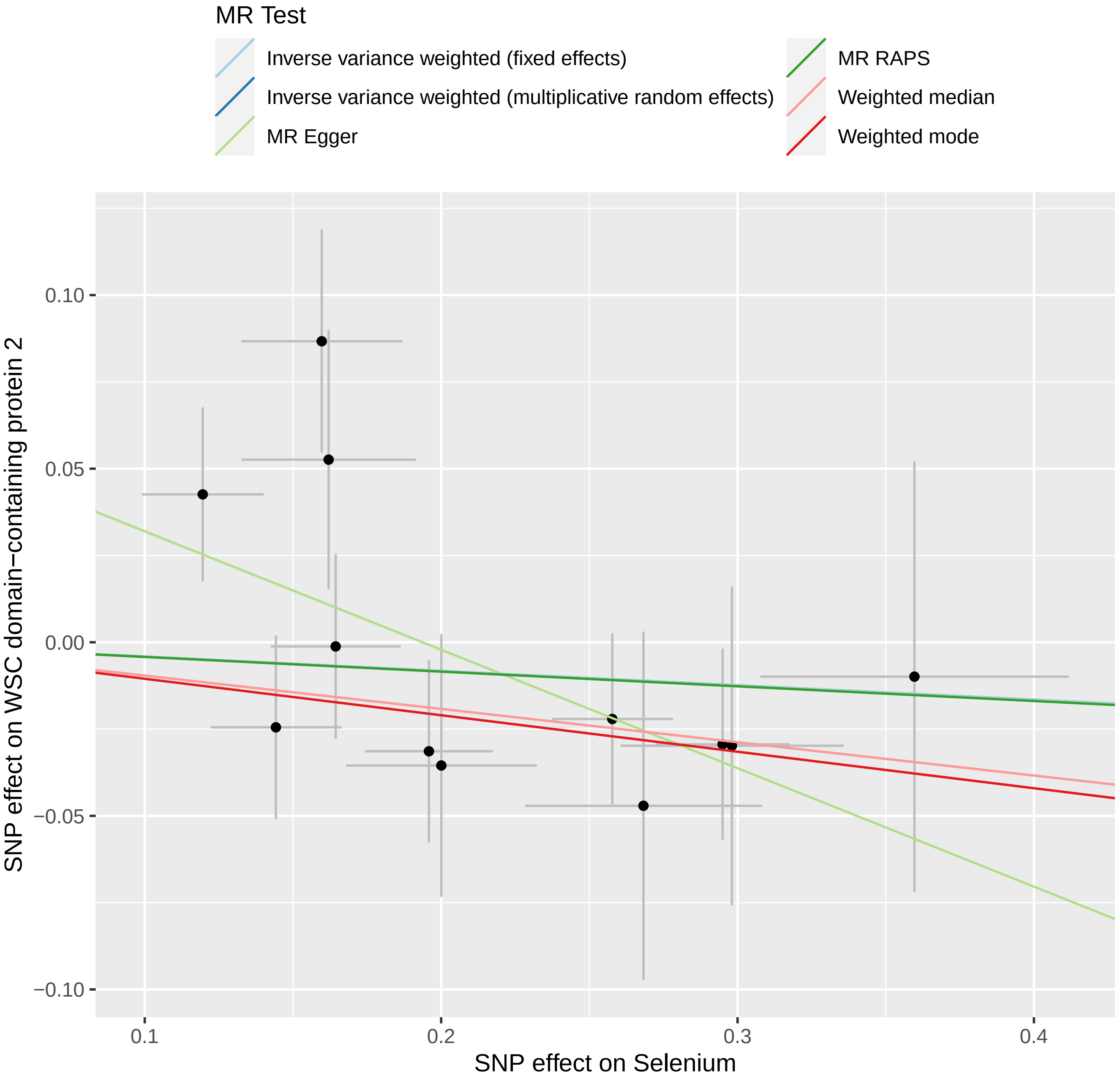

Batch 3−3236 : Selenium on Lymphotactin

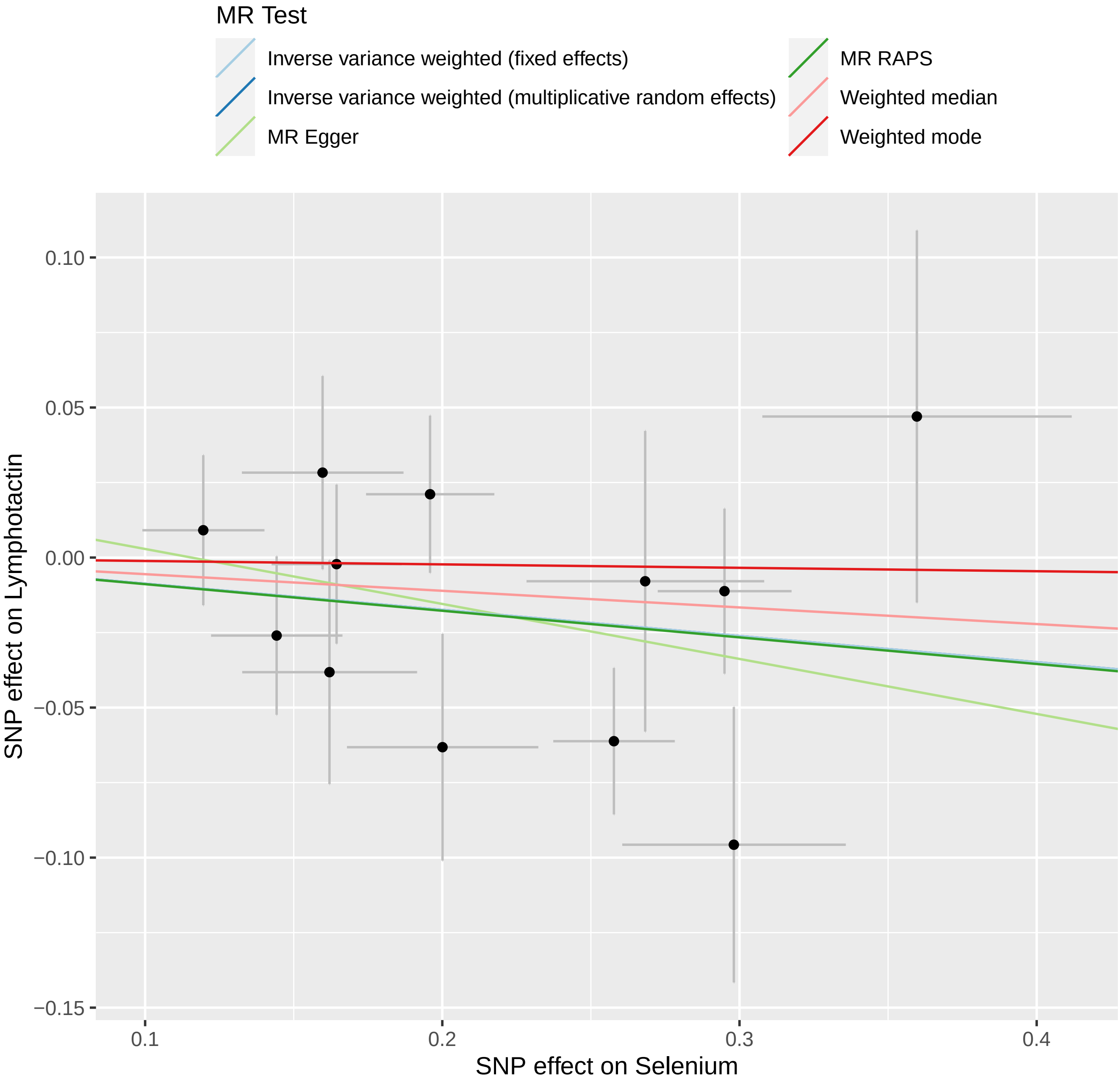

Batch 3−3237 : Selenium on Lymphotactin

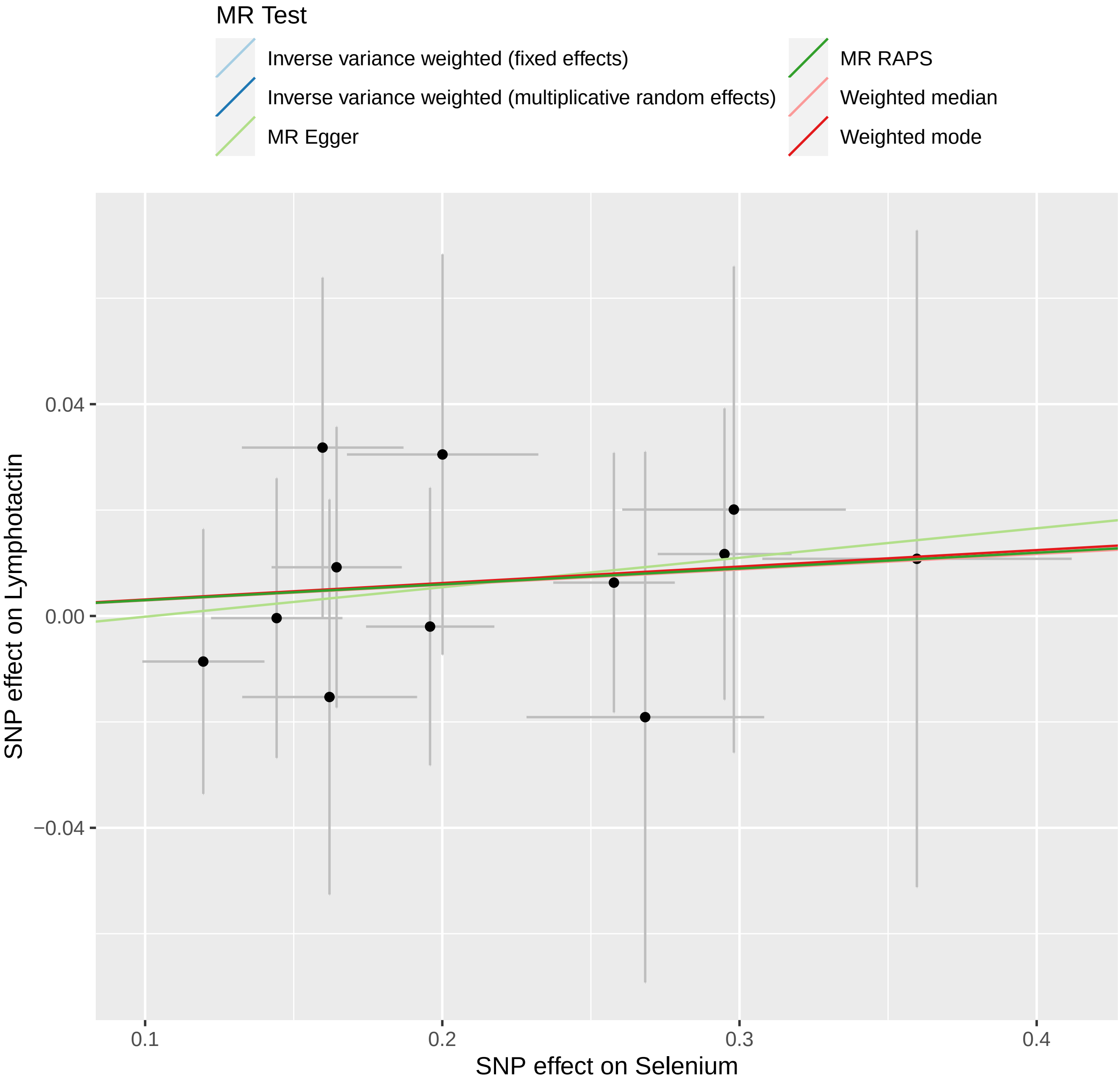

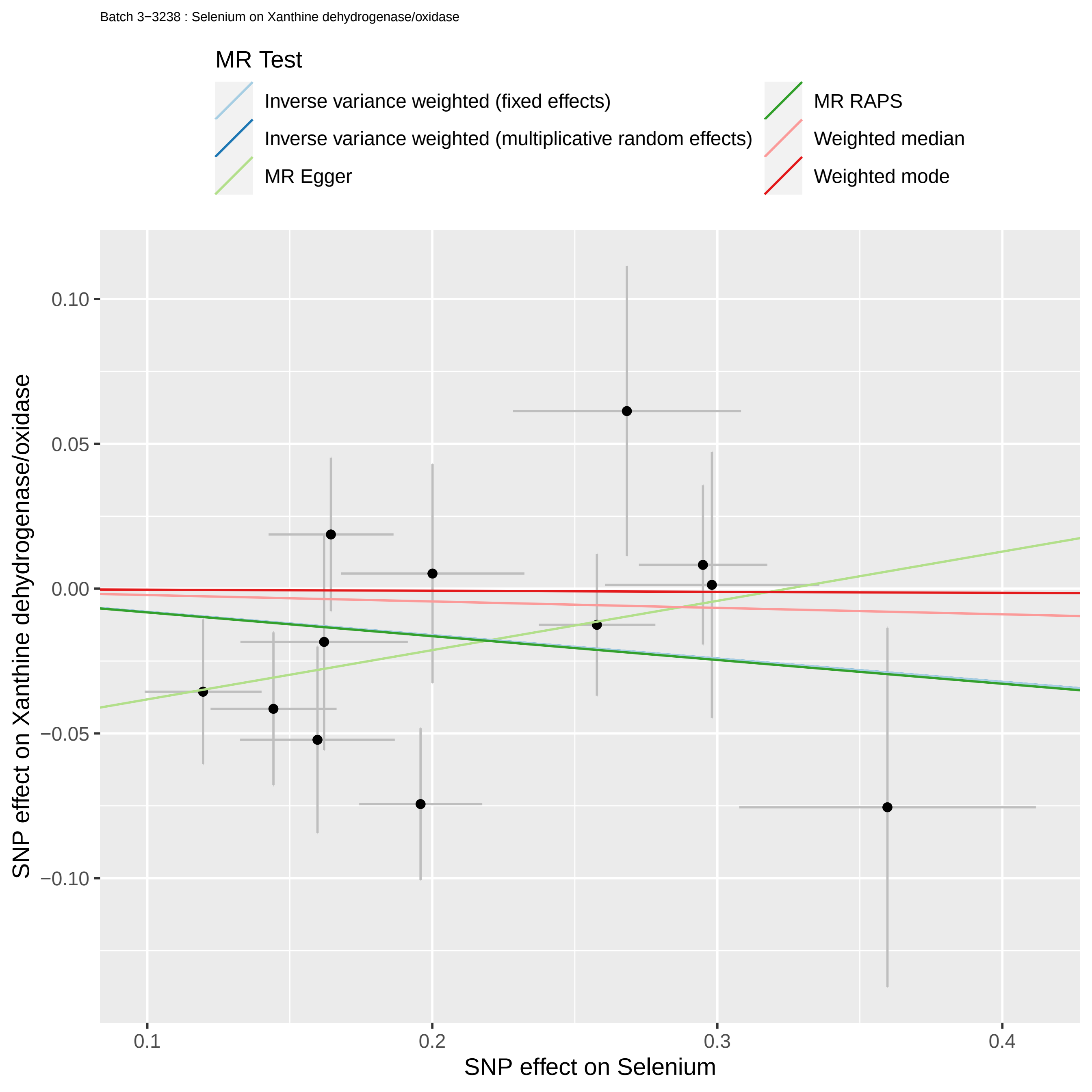

Batch 3−3238 : Selenium on Xanthine dehydrogenase/oxidase
MR Test
Inverse variance weighted (fixed effects)
Inverse variance weighted (multiplicative random effects)
MR Egger
MR RAPS
Weighted median
Weighted mode
0.10
0.05
0.00
−0.05
−0.10
SNP effect on Xanthine dehydrogenase/oxidase
0.1
0.2
0.3
0.4
SNP effect on Selenium

Batch 3−3239 : Selenium on Glycoprotein Xg

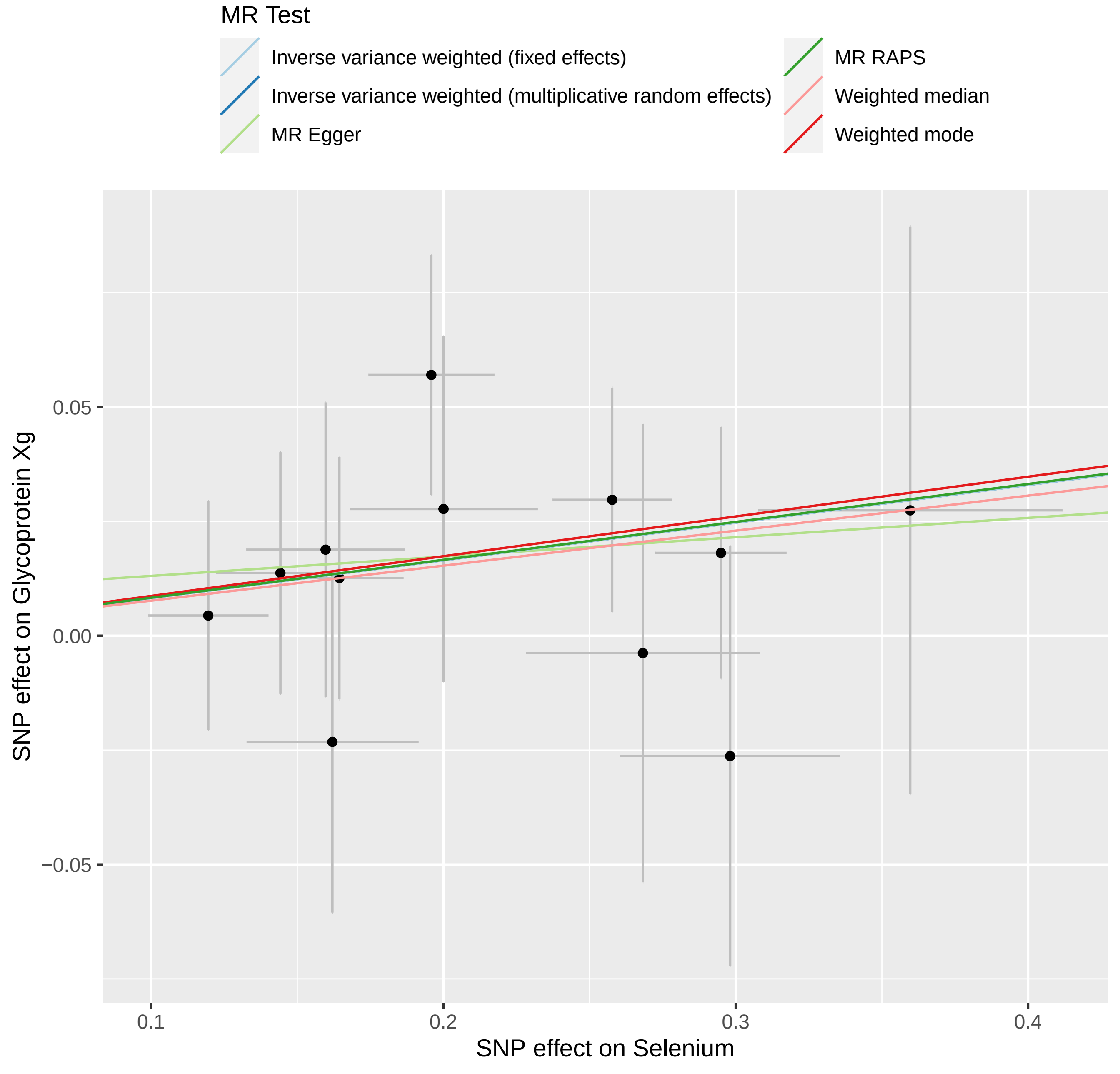

Batch 3−3240 : Selenium on Xaa−Pro aminopeptidase 1

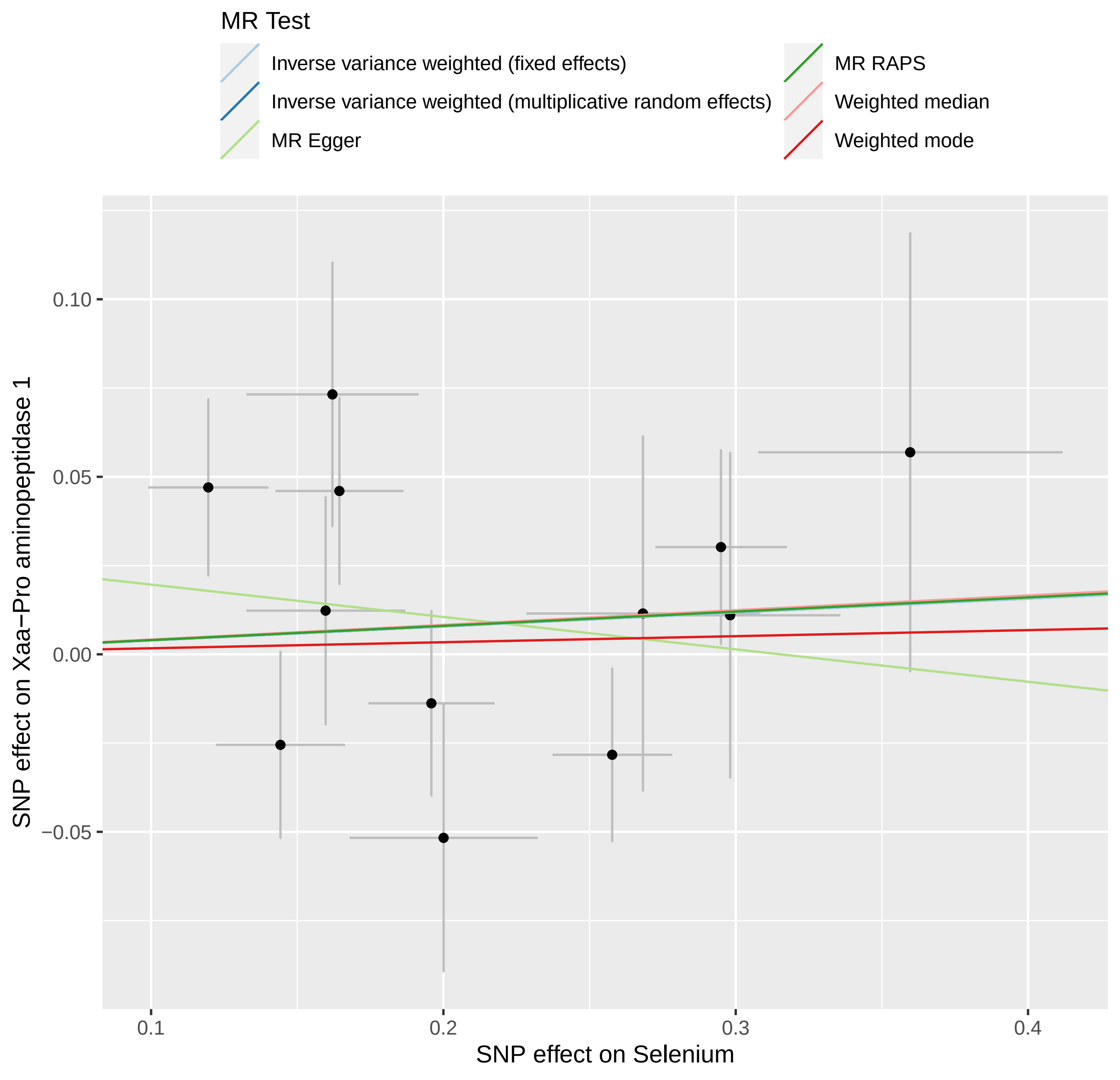

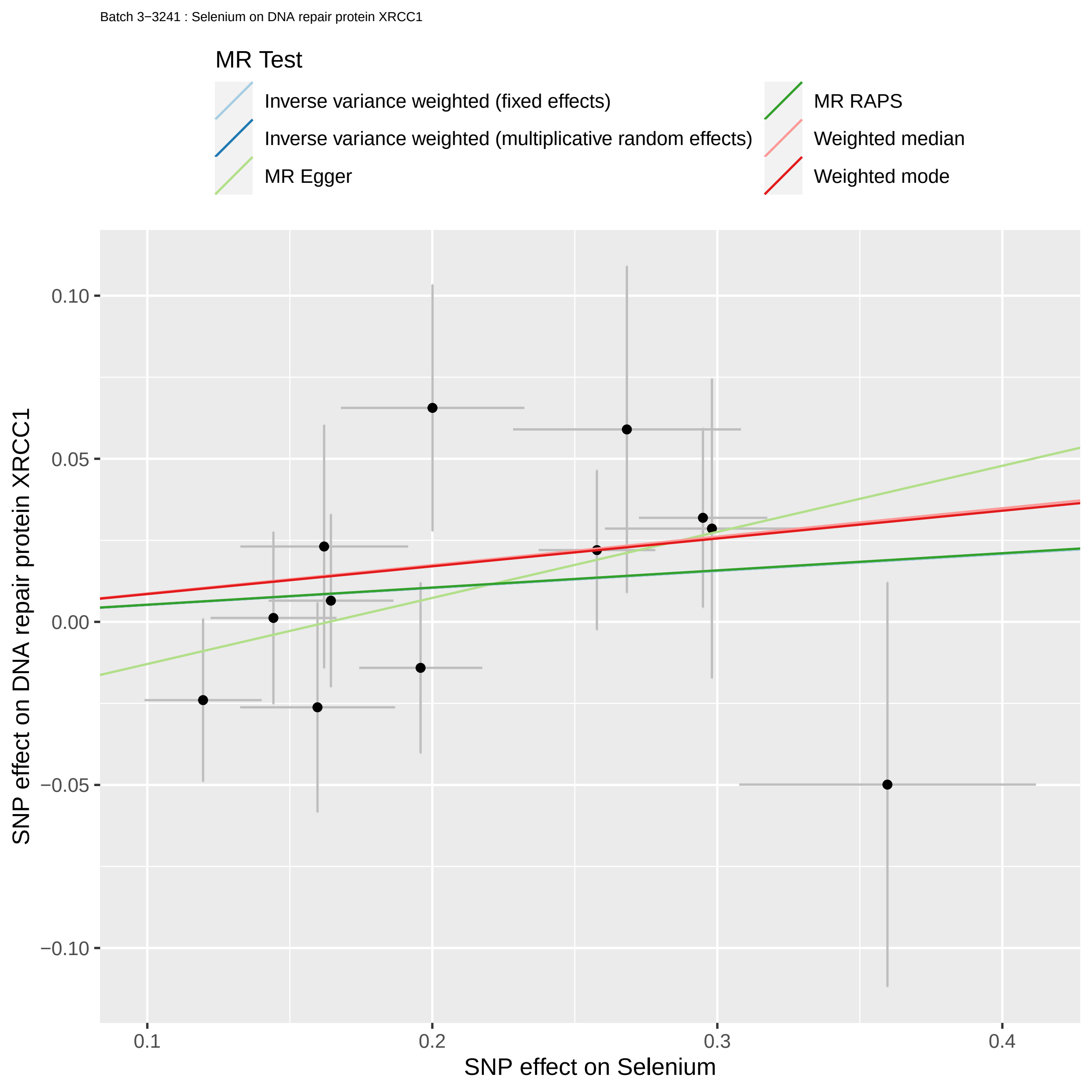

Batch 3−3241 : Selenium on DNA repair protein XRCC1
MR Test
Inverse variance weighted (fixed effects)
Inverse variance weighted (multiplicative random effects)
MR Egger
MR RAPS
Weighted median
Weighted mode
0.10
0.05
0.00
−0.05
−0.10
0.1
0.2
0.3
0.4
SNP effect on DNA repair protein XRCC1
SNP effect on Selenium

Batch 3−3242 : Selenium on DNA repair protein XRCC4

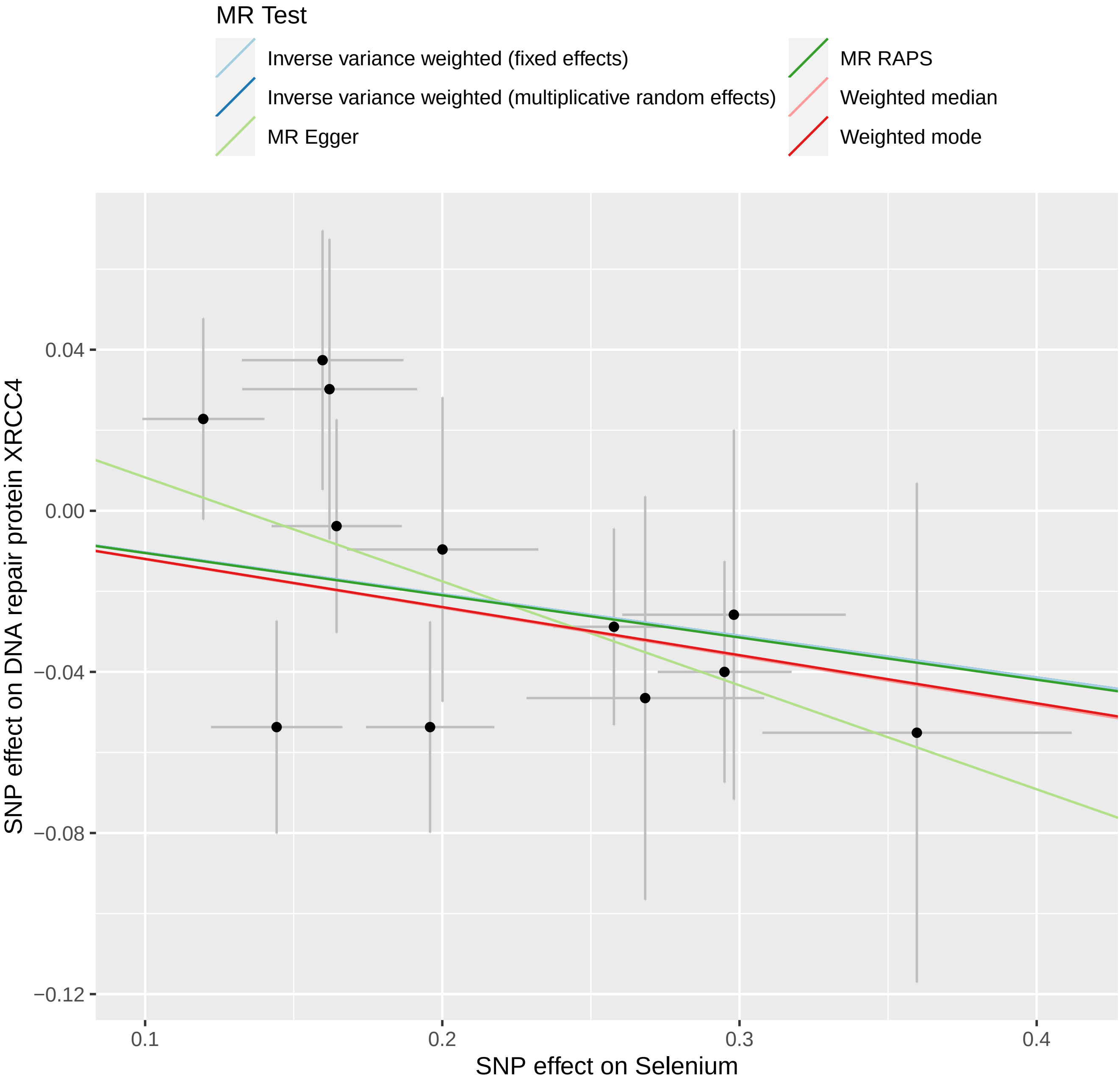

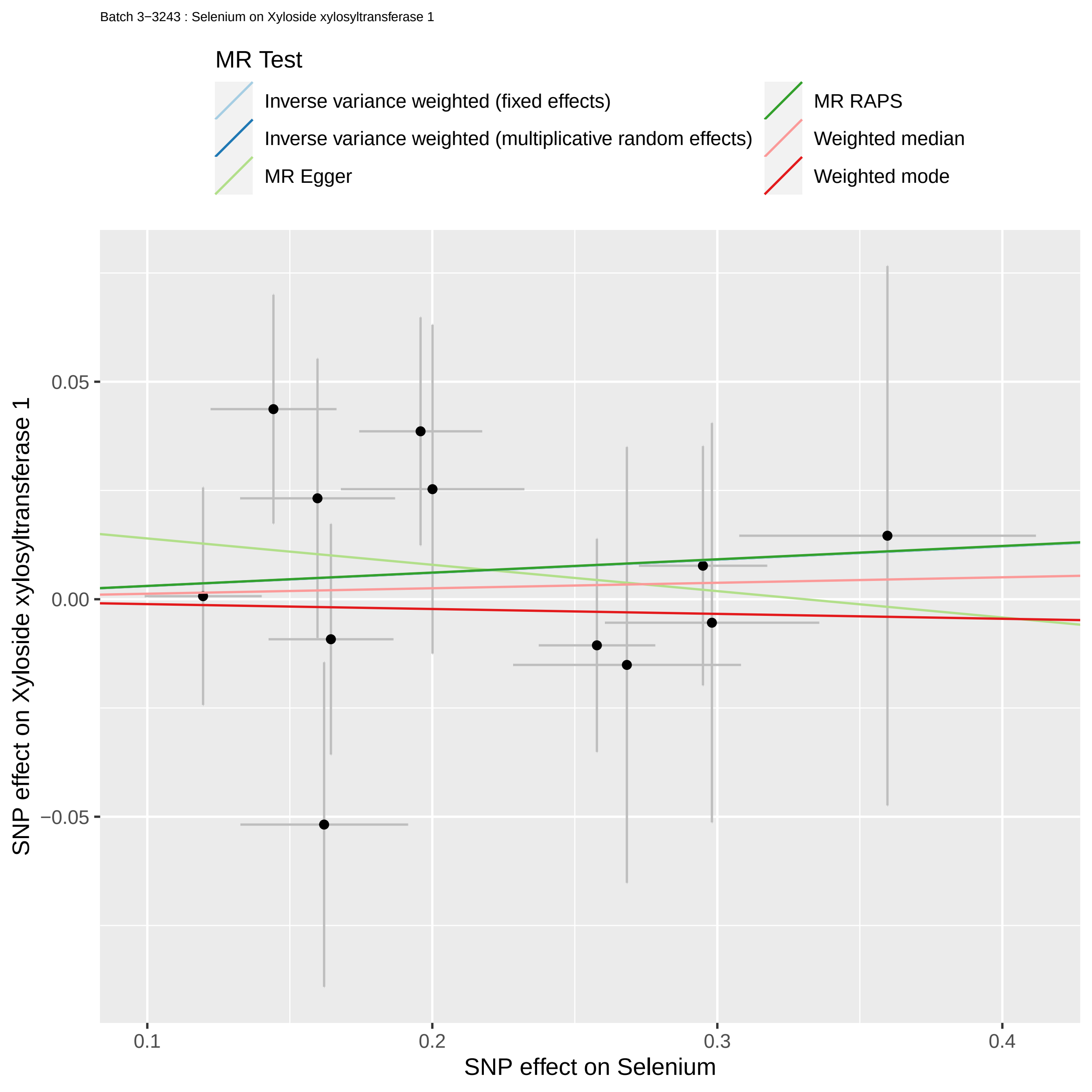
Batch 3−3243 : Selenium on Xyloside xylosyltransferase 1
MR Test
Inverse variance weighted (fixed effects)
Inverse variance weighted (multiplicative random effects)
MR Egger
MR RAPS
Weighted median
Weighted mode
0.05
0.00
−0.05
0.1
0.2
0.3
0.4
SNP effect on Xyloside xylosyltransferase 1
SNP effect on Selenium

Batch 3−3244 : Selenium on Y−box−binding protein 2

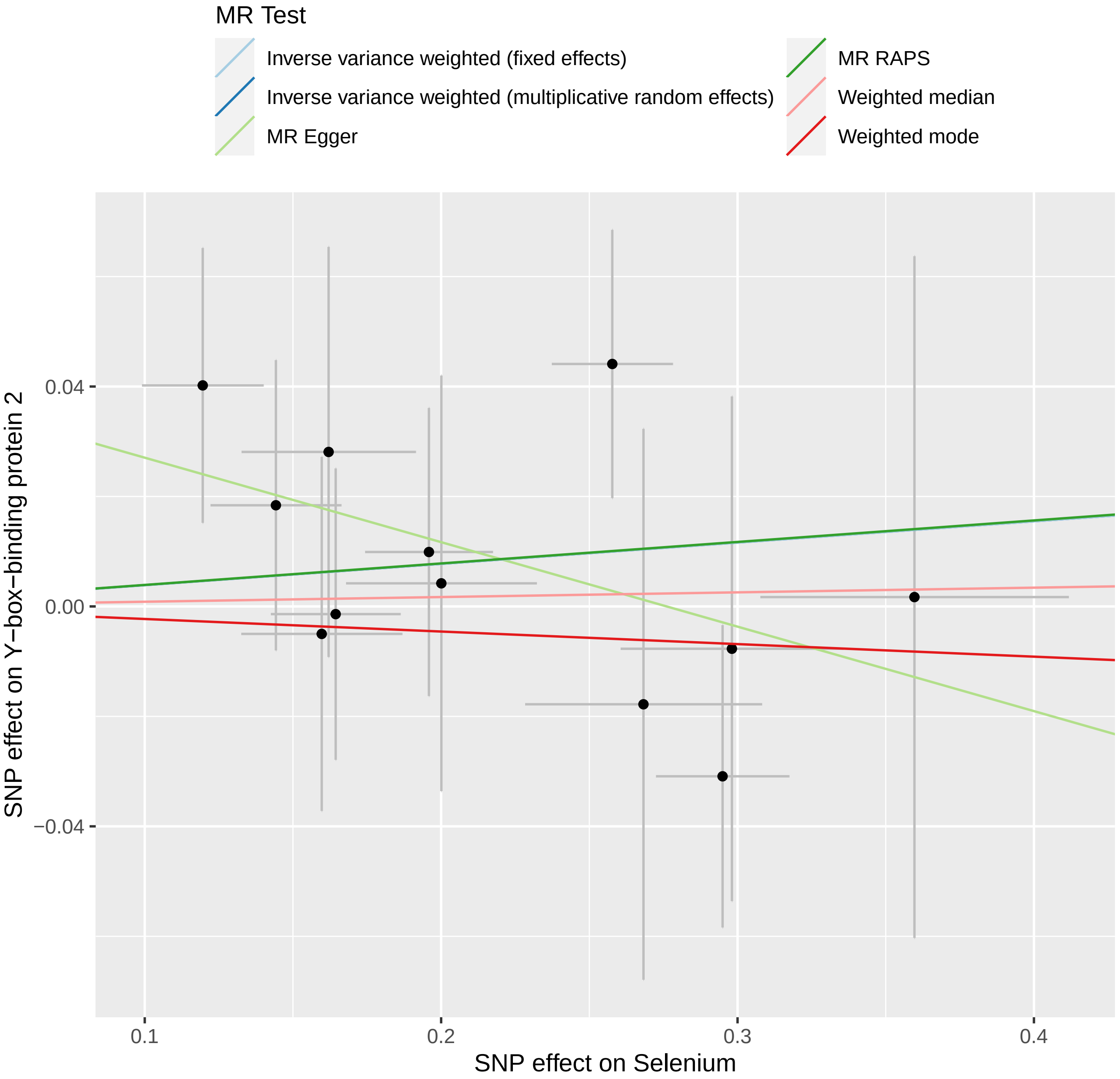

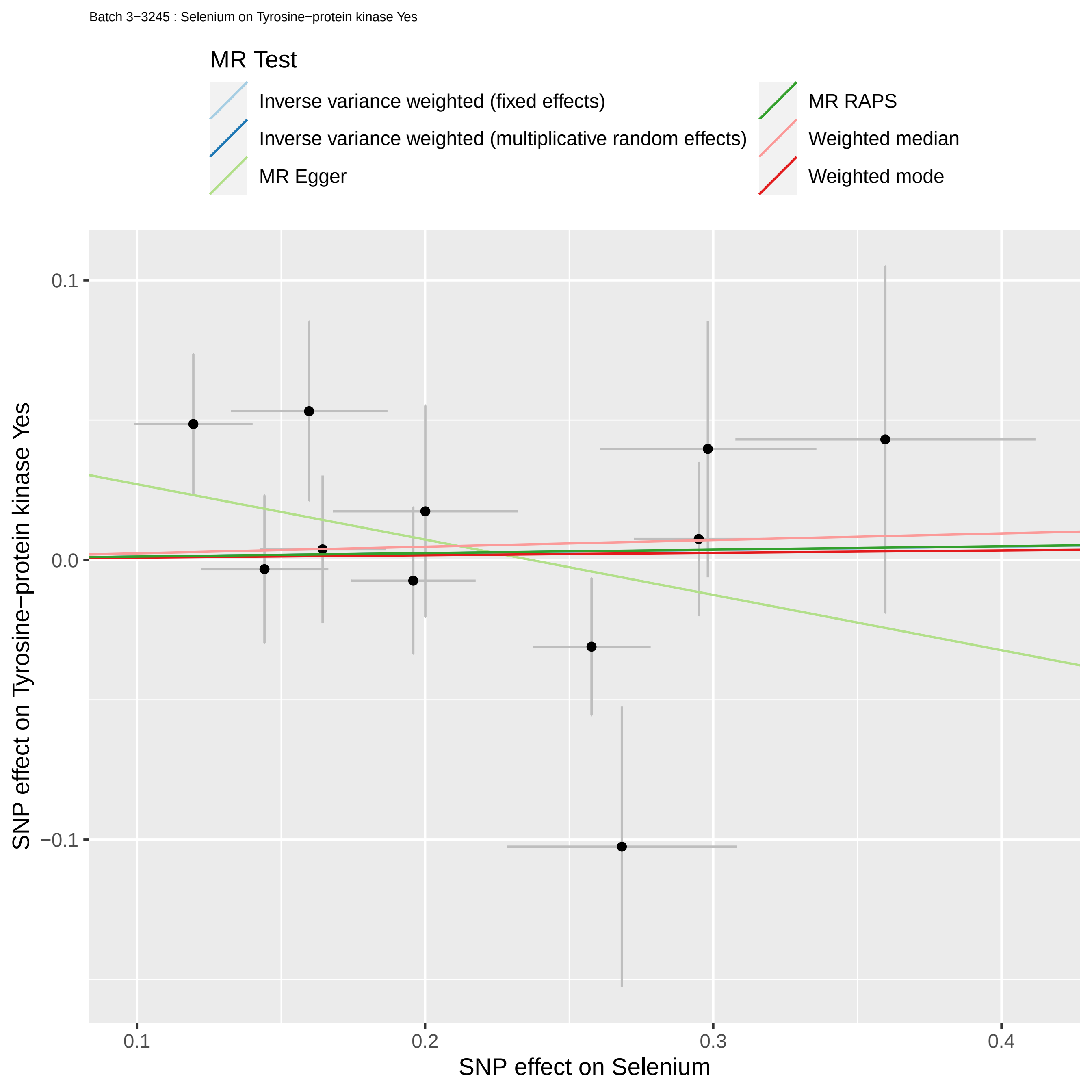

Batch 3−3245 : Selenium on Tyrosine−protein kinase Yes
MR Test
Inverse variance weighted (fixed effects)
Inverse variance weighted (multiplicative random effects)
MR Egger
MR RAPS
Weighted median
Weighted mode
SNP effect on Tyrosine−protein kinase Yes
0.1
0.0
−0.1
0.1
0.2
0.3
0.4
SNP effect on Selenium

Batch 3−3246 : Selenium on Protein YIPF6

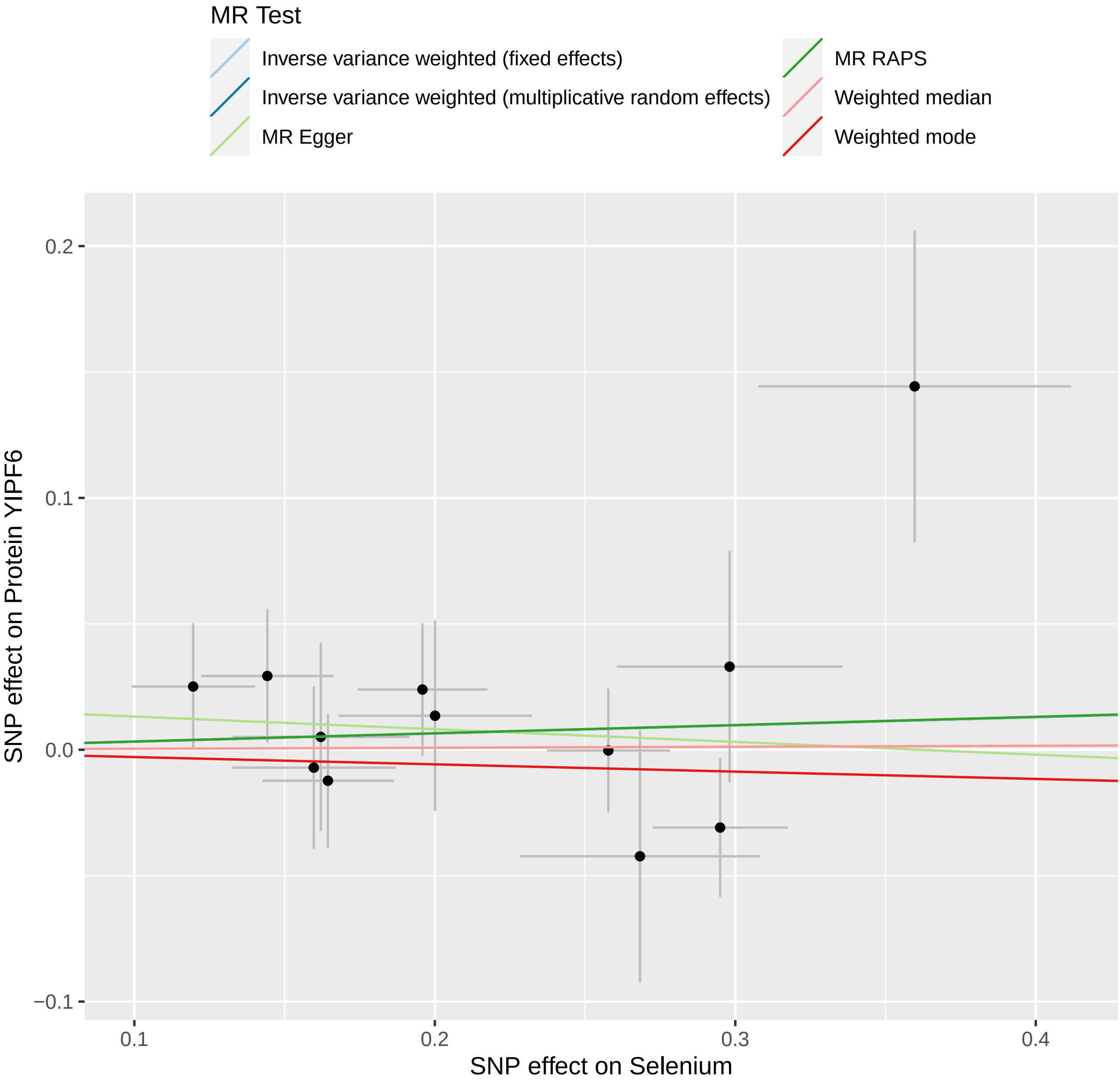

Batch 3−3247 : Selenium on YTH domain−containing protein 1

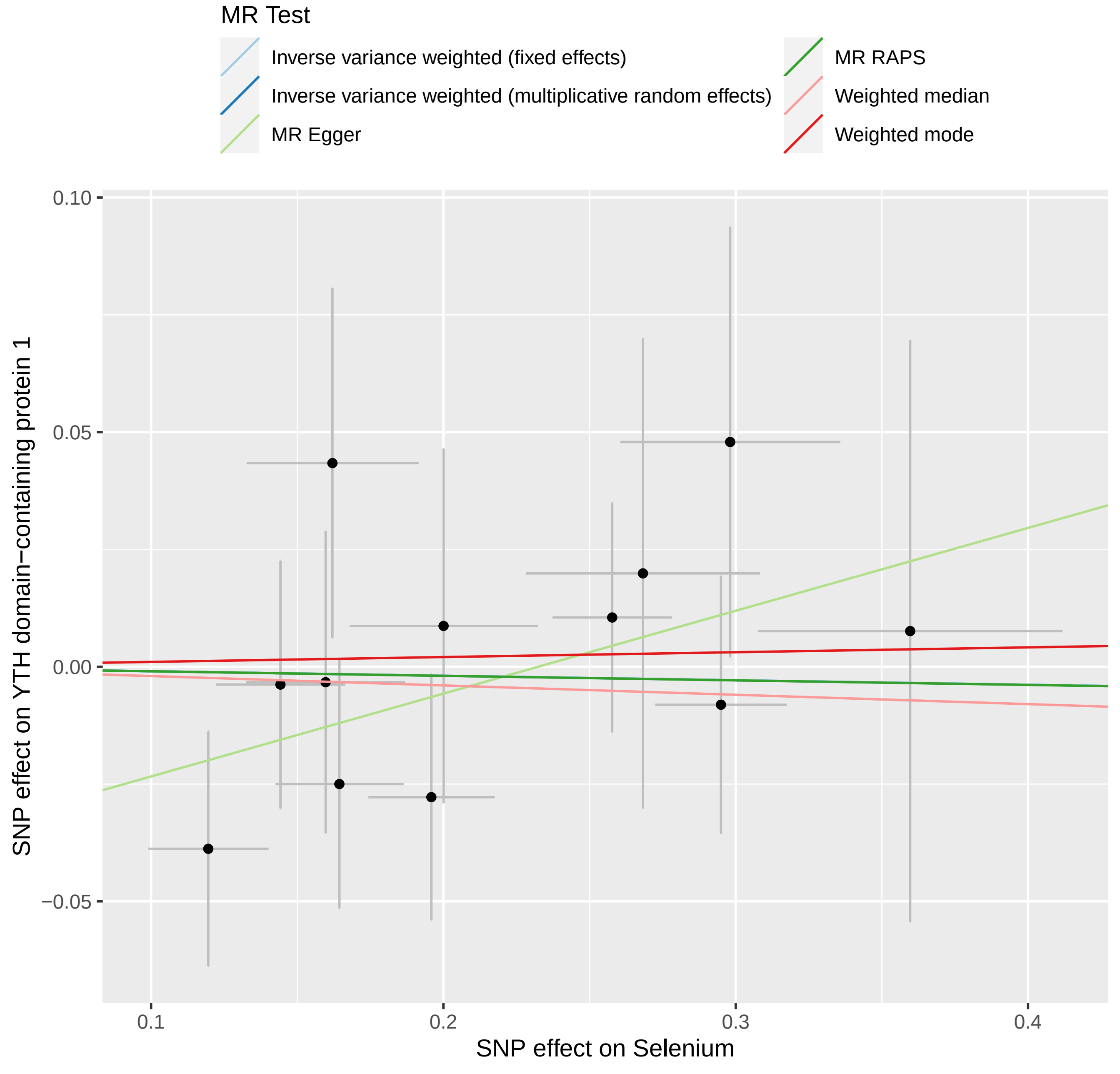

Batch 3−3248 : Selenium on 14−3−3 protein family

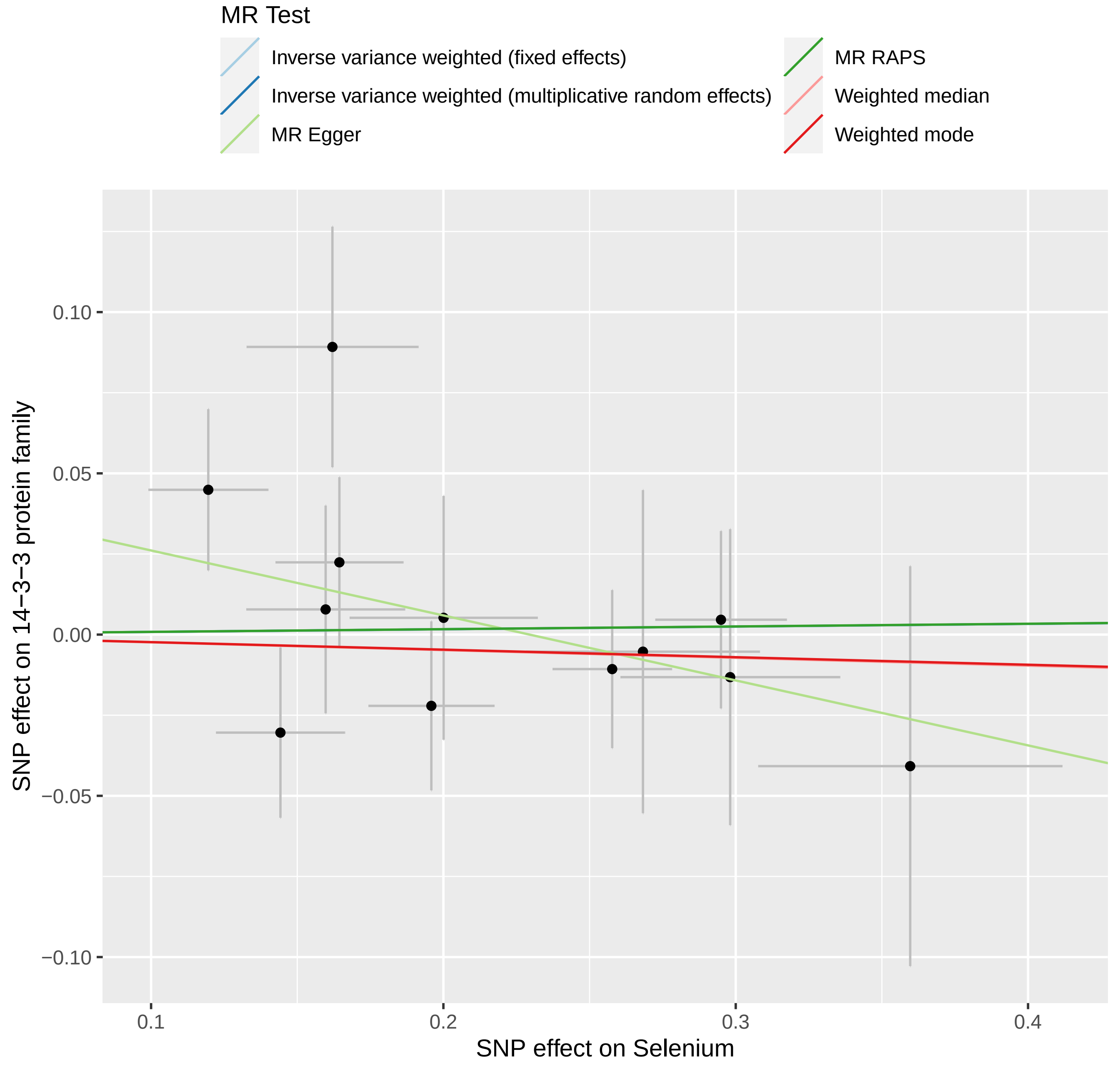

Batch 3−3249 : Selenium on 14−3−3 protein epsilon

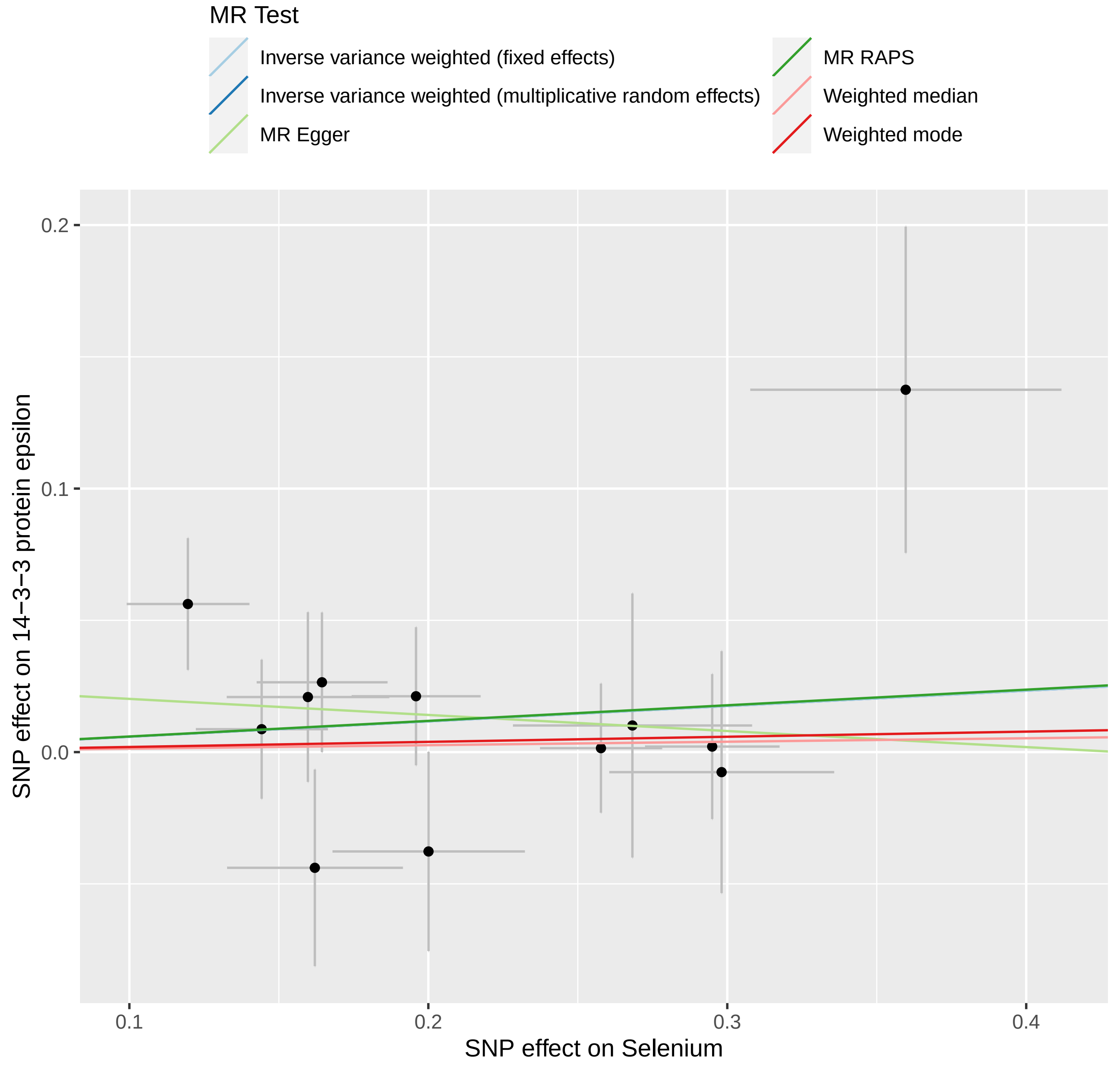

Batch 3−3250 : Selenium on 14−3−3 protein theta

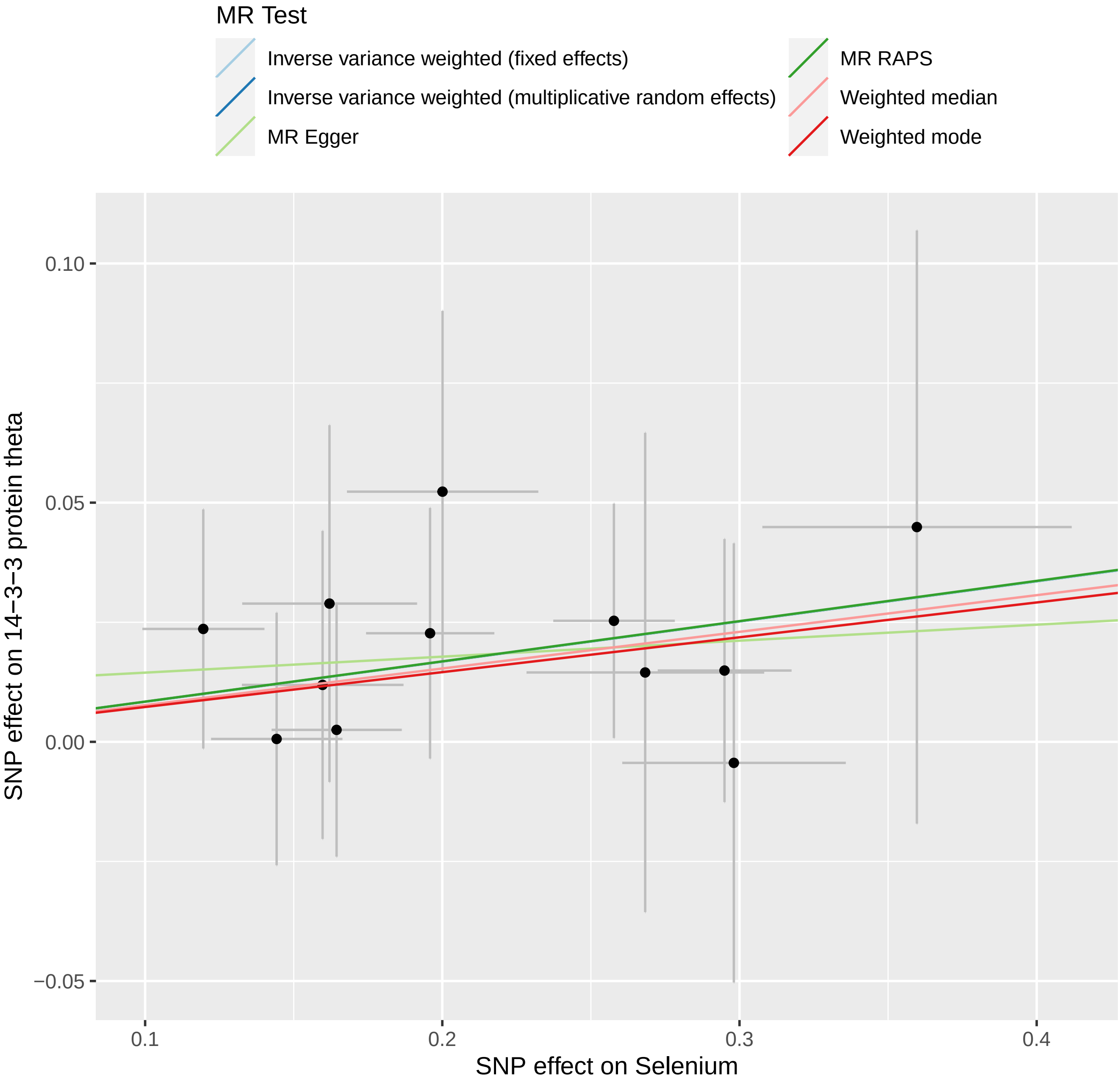

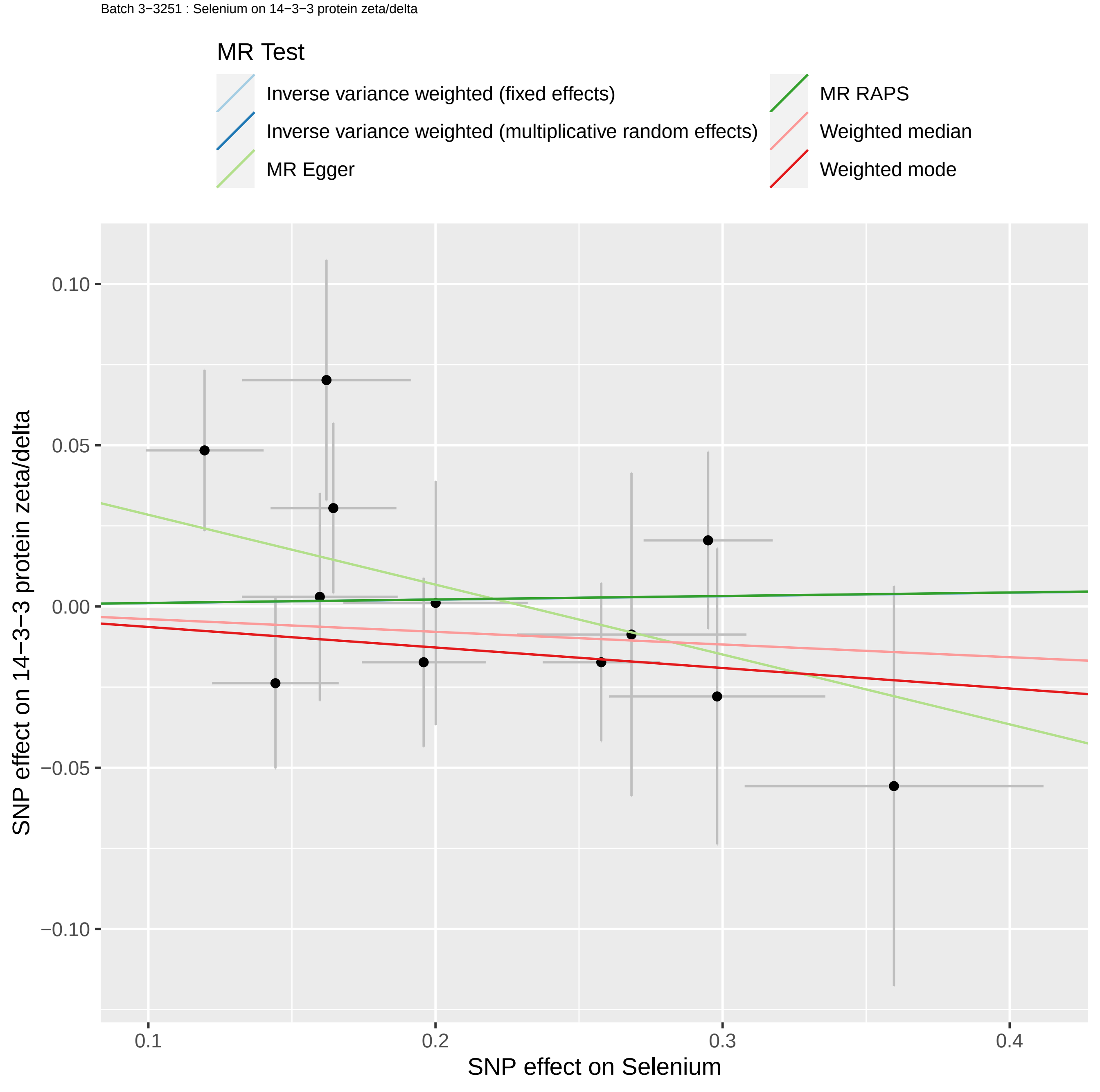
Batch 3−3251 : Selenium on 14−3−3 protein zeta/delta
MR Test
Inverse variance weighted (fixed effects)
Inverse variance weighted (multiplicative random effects)
MR Egger
MR RAPS
Weighted median
Weighted mode
0.10
0.05
0.00
−0.05
−0.10
0.1
0.2
0.3
0.4
SNP effect on 14−3−3 protein zeta/delta
SNP effect on Selenium

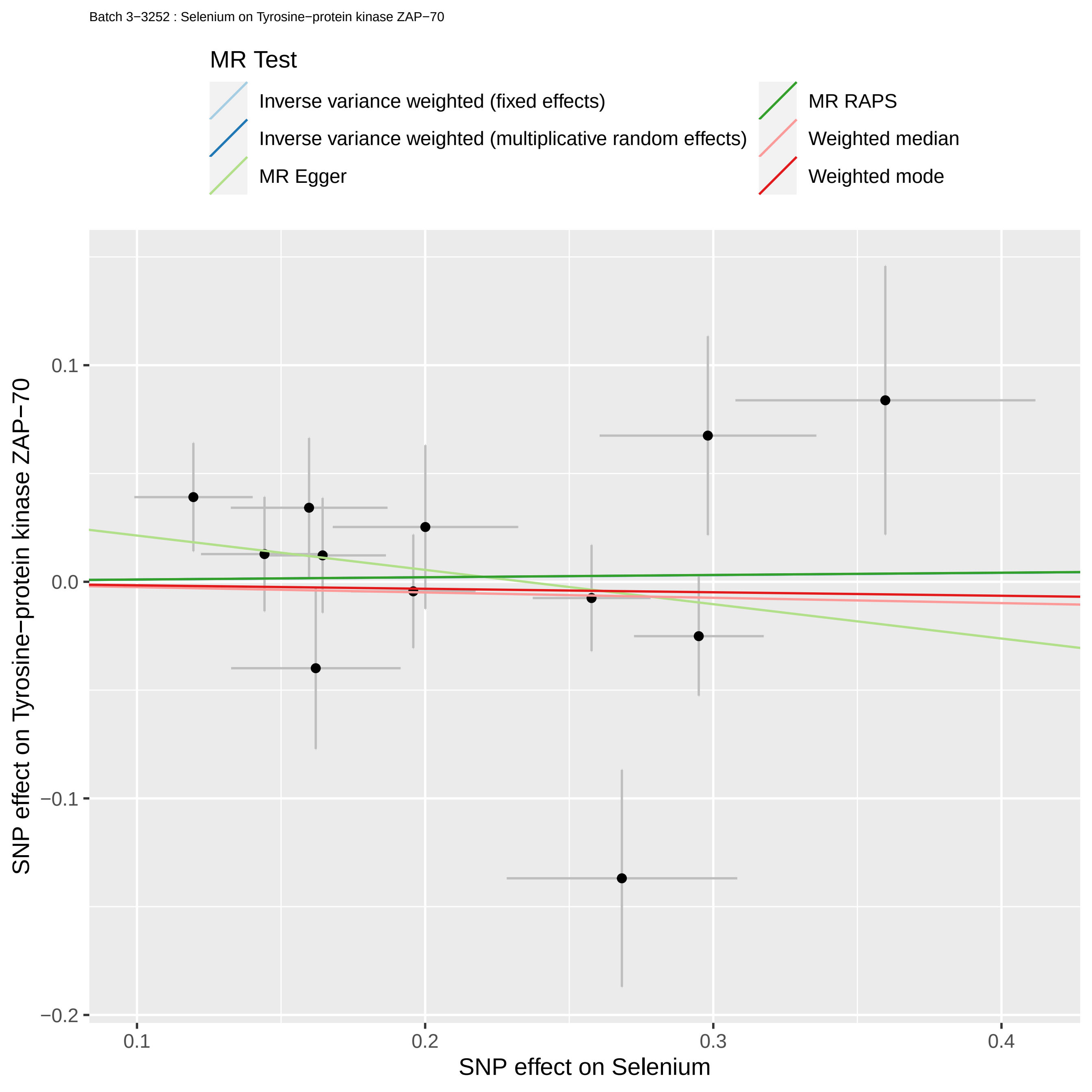

Batch 3−3252 : Selenium on Tyrosine−protein kinase ZAP−70
MR Test
Inverse variance weighted (fixed effects)
Inverse variance weighted (multiplicative random effects)
MR Egger
MR RAPS
Weighted median
Weighted mode
0.1
0.0
−0.1
−0.2
0.1
0.2
0.3
0.4
SNP effect on Tyrosine−protein kinase ZAP−70
SNP effect on Selenium

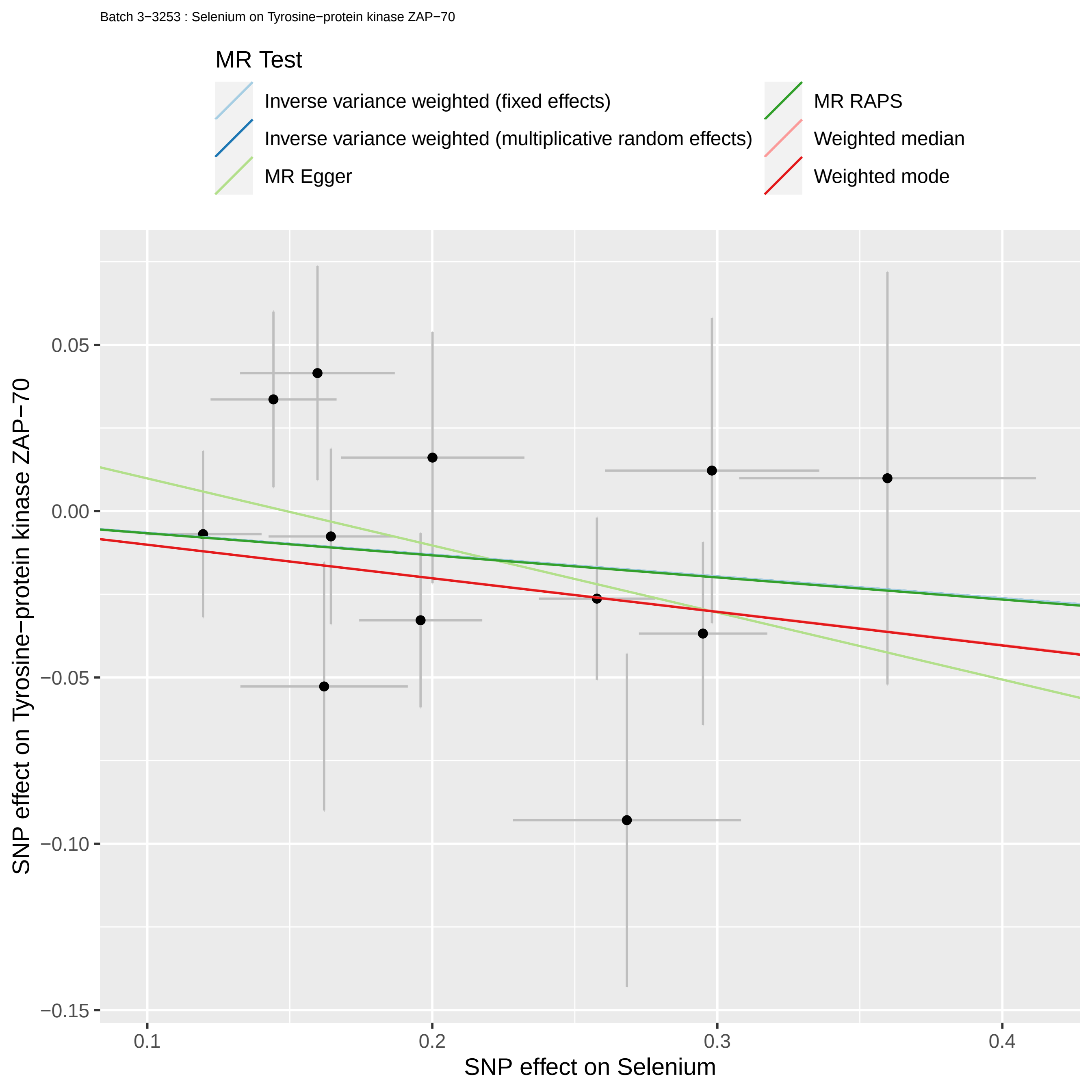

Batch 3−3253 : Selenium on Tyrosine−protein kinase ZAP−70
MR Test
Inverse variance weighted (fixed effects)
Inverse variance weighted (multiplicative random effects)
MR Egger
MR RAPS
Weighted median
Weighted mode
SNP effect on Tyrosine−protein kinase ZAP−70
0.05
0.00
−0.05
−0.10
−0.15
0.1
0.2
0.3
0.4
SNP effect on Selenium

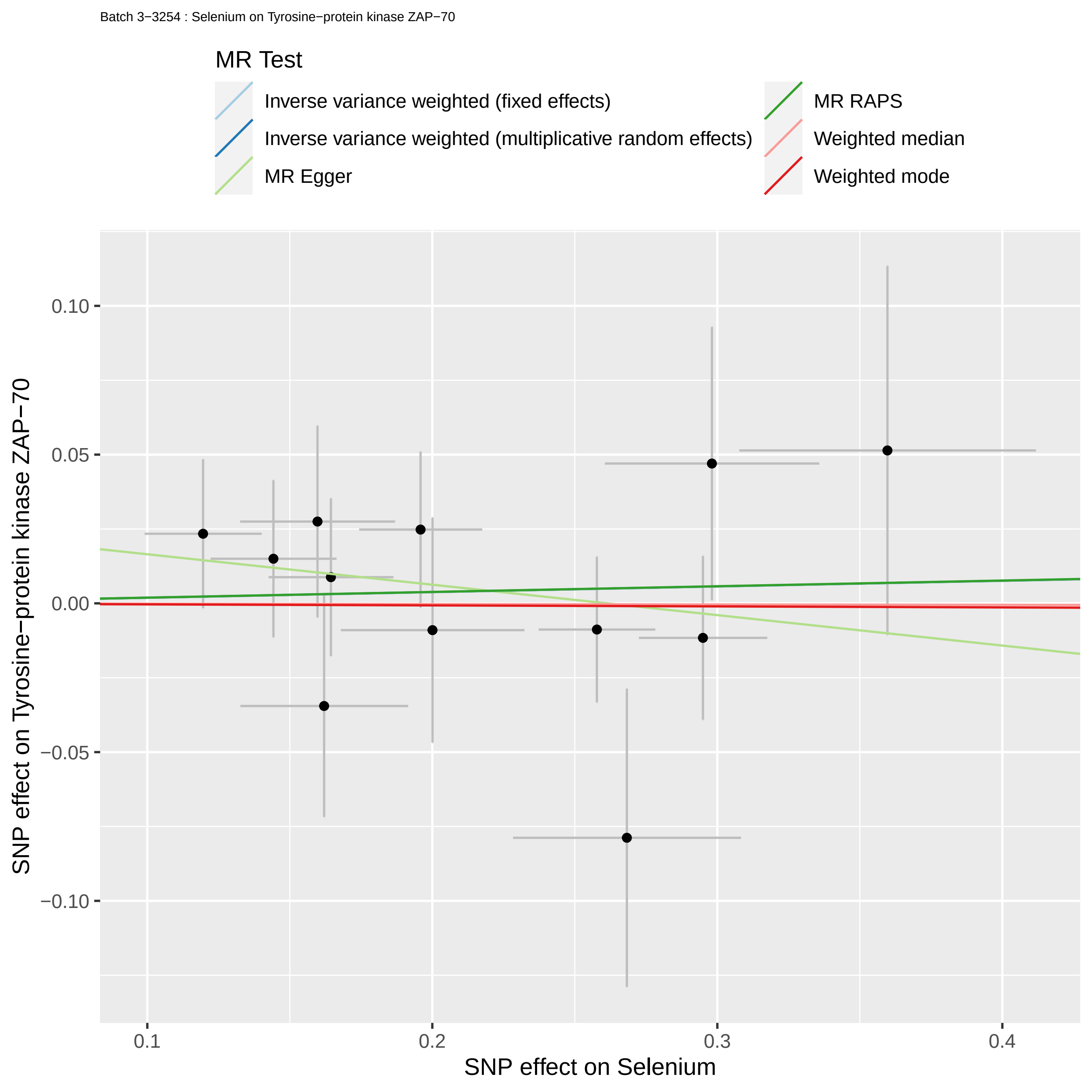
Batch 3−3254 : Selenium on Tyrosine−protein kinase ZAP−70
MR Test
Inverse variance weighted (fixed effects)
Inverse variance weighted (multiplicative random effects)
MR Egger
MR RAPS
Weighted median
Weighted mode
0.10
0.05
0.00
−0.05
−0.10
0.1
0.2
0.3
0.4
SNP effect on Selenium
SNP effect on Tyrosine−protein kinase ZAP−70

Batch 3−3255 : Selenium on Transcriptional regulator Kaiso

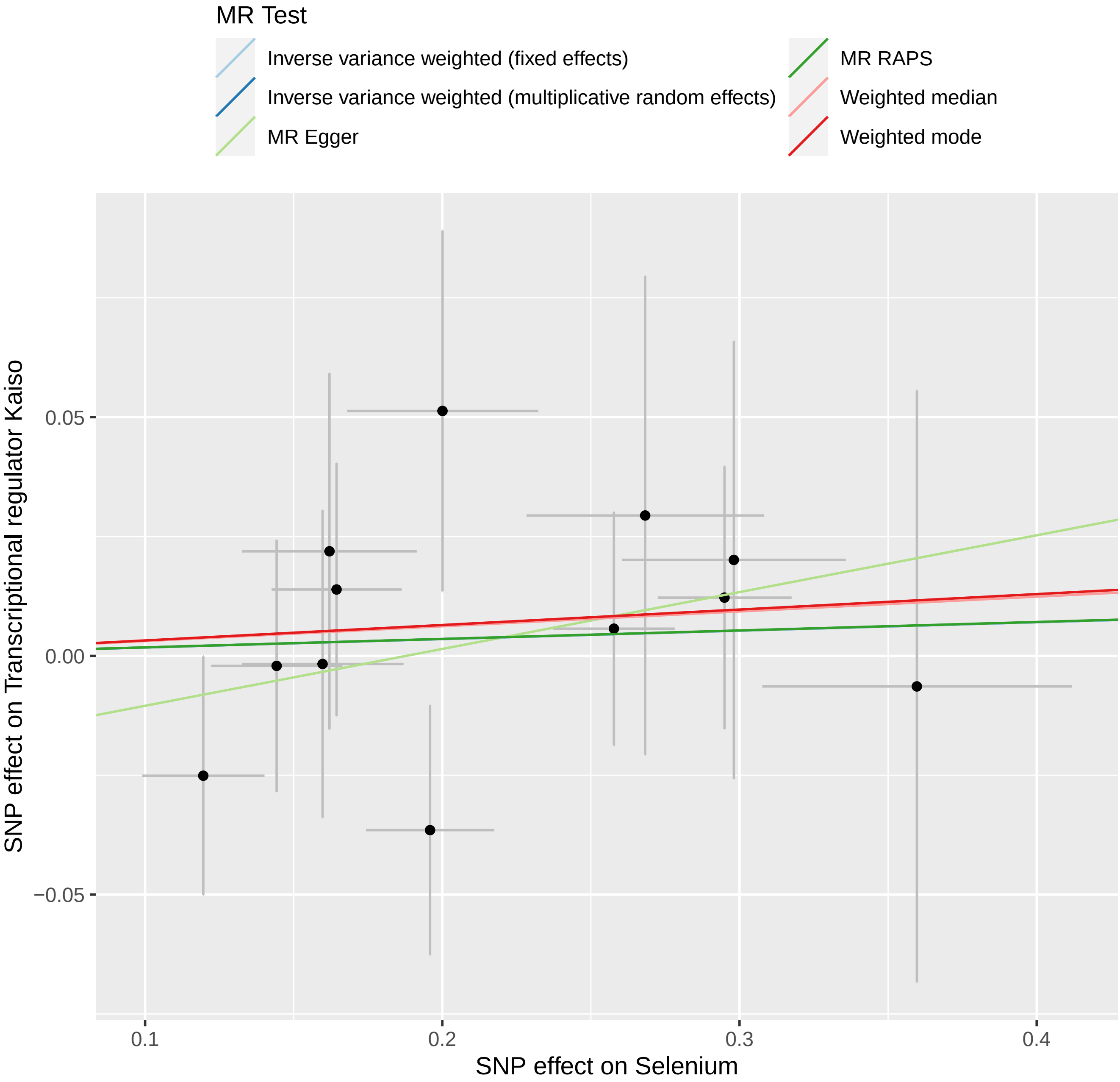

Batch 3−3256 : Selenium on E3 ubiquitin−protein ligase ZFP91

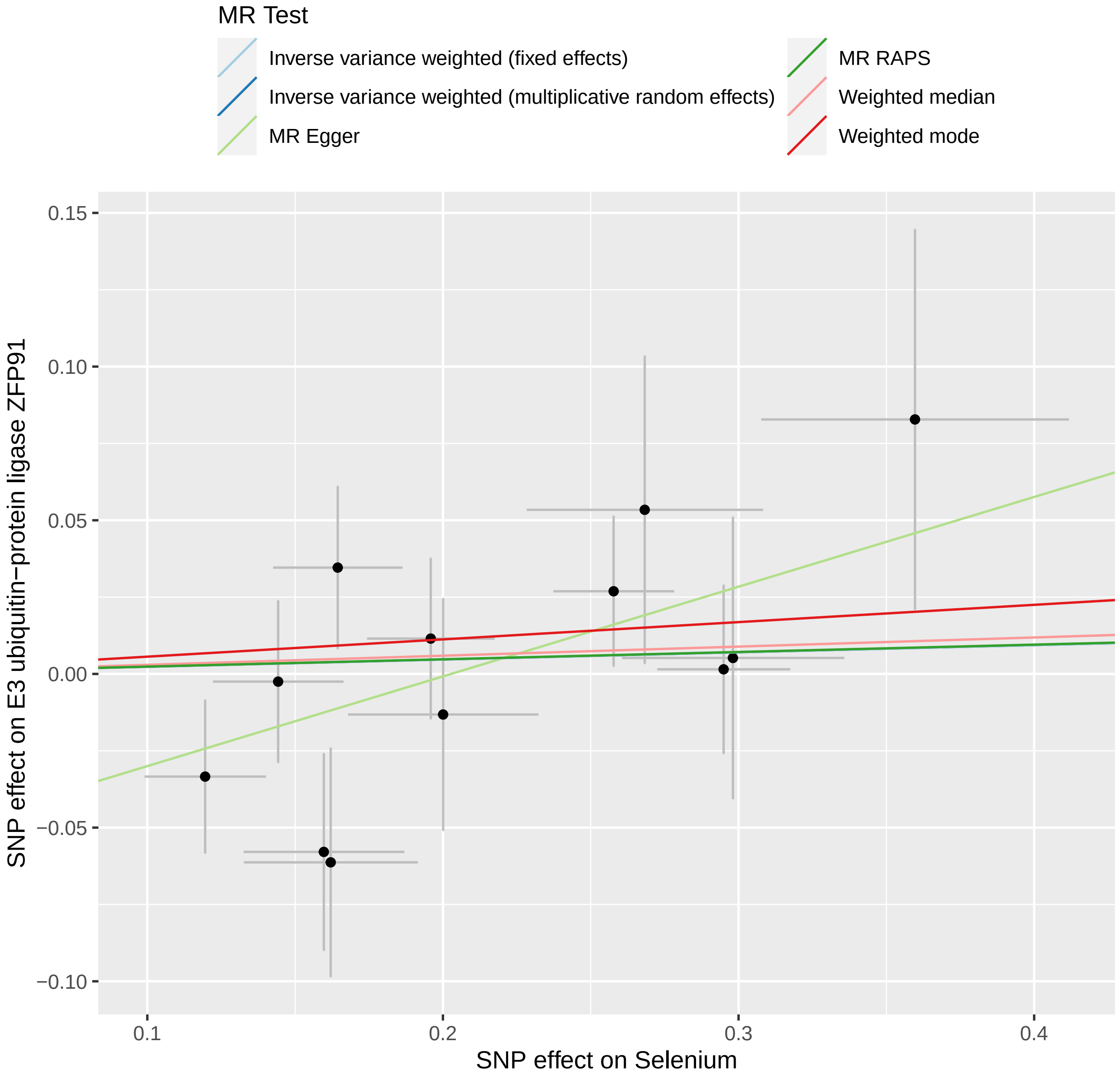

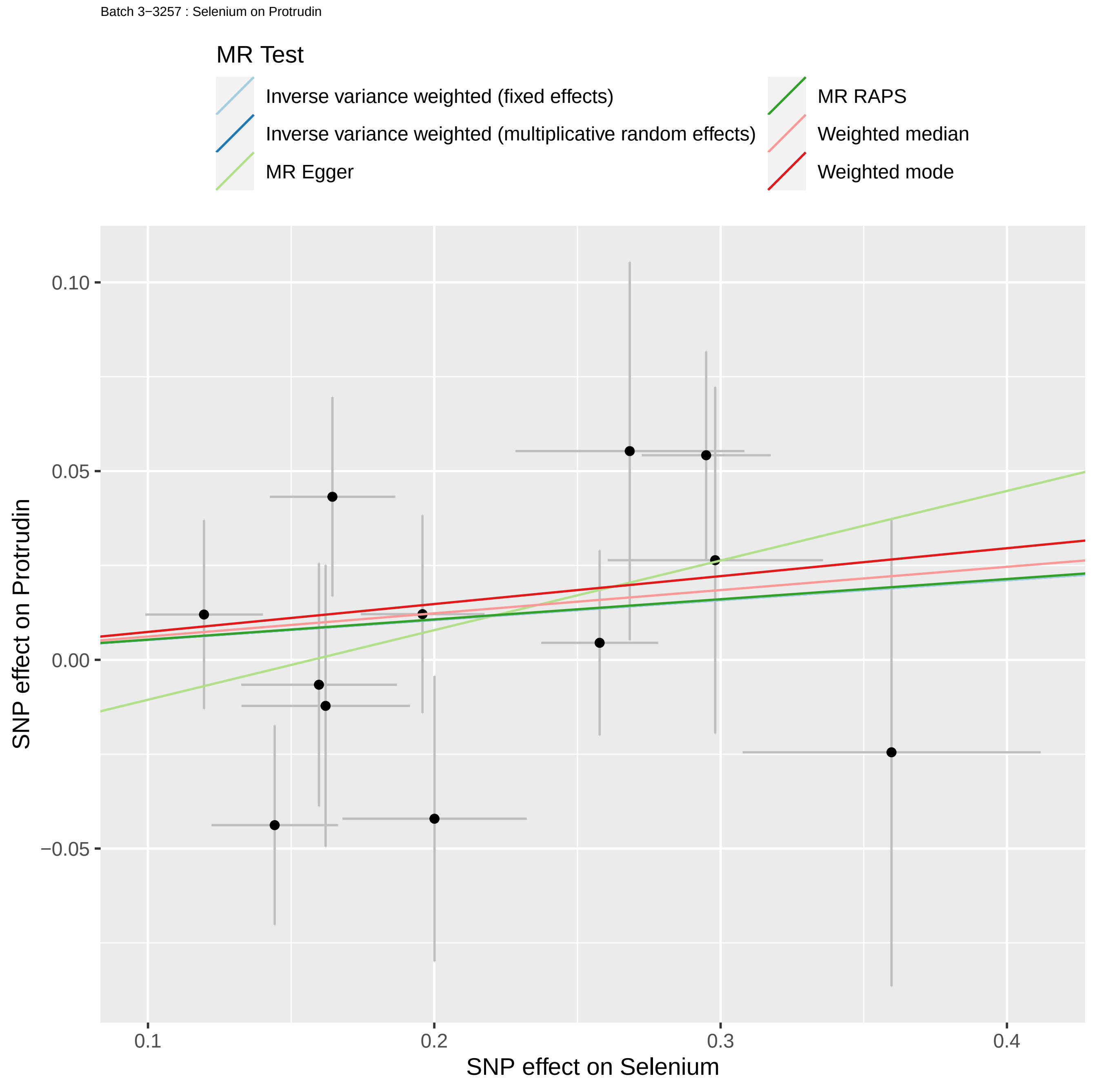
Batch 3−3257 : Selenium on Protrudin
MR Test
Inverse variance weighted (fixed effects)
Inverse variance weighted (multiplicative random effects)
MR Egger
MR RAPS
Weighted median
Weighted mode
0.10
0.05
0.00
−0.05
SNP effect on Protrudin
0.1
0.2
0.3
0.4
SNP effect on Selenium

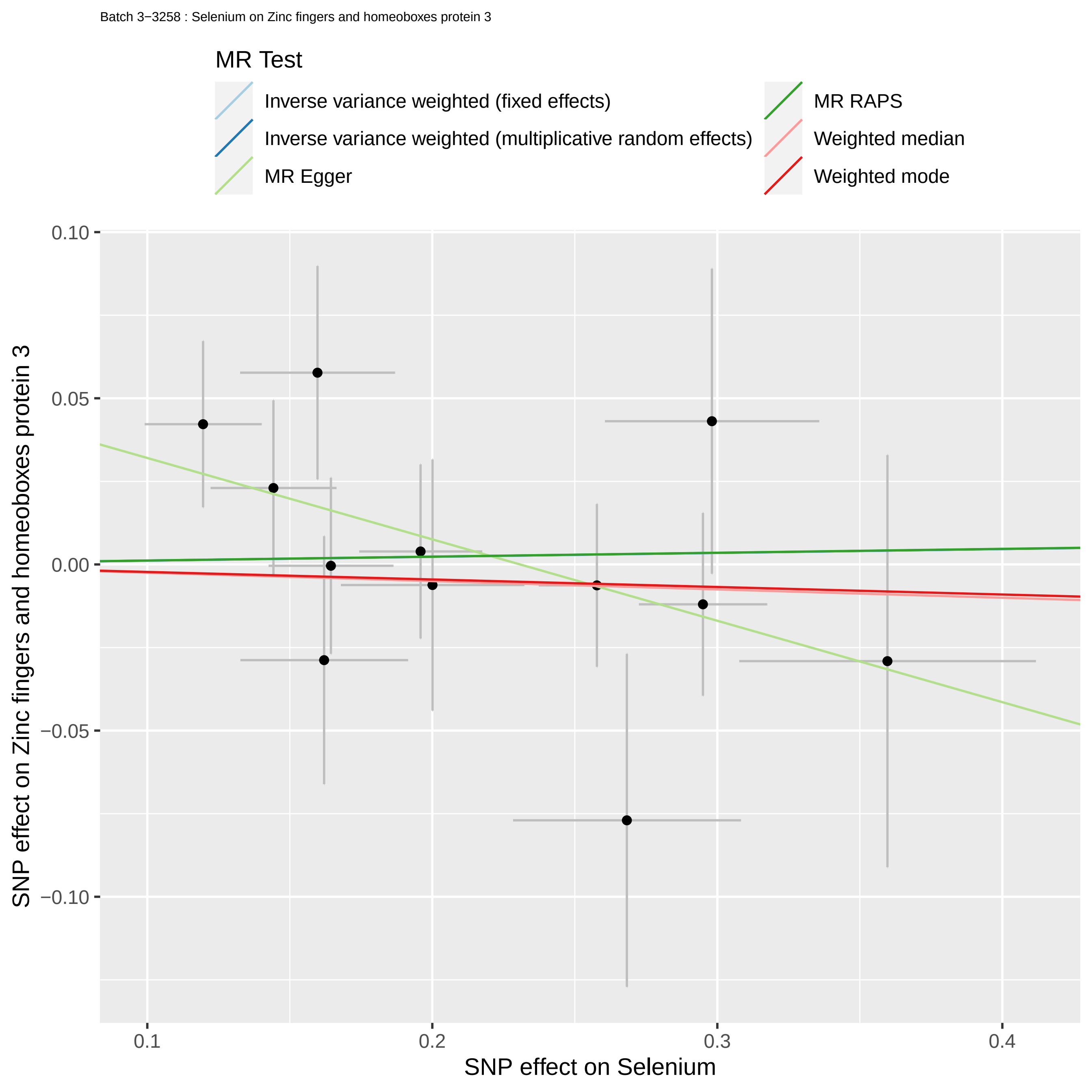

Batch 3−3258 : Selenium on Zinc fingers and homeoboxes protein 3
MR Test
Inverse variance weighted (fixed effects)
Inverse variance weighted (multiplicative random effects)
MR Egger
MR RAPS
Weighted median
Weighted mode
0.10
0.05
0.00
−0.05
−0.10
0.1
0.2
0.3
0.4
SNP effect on Zinc fingers and homeoboxes protein 3
SNP effect on Selenium

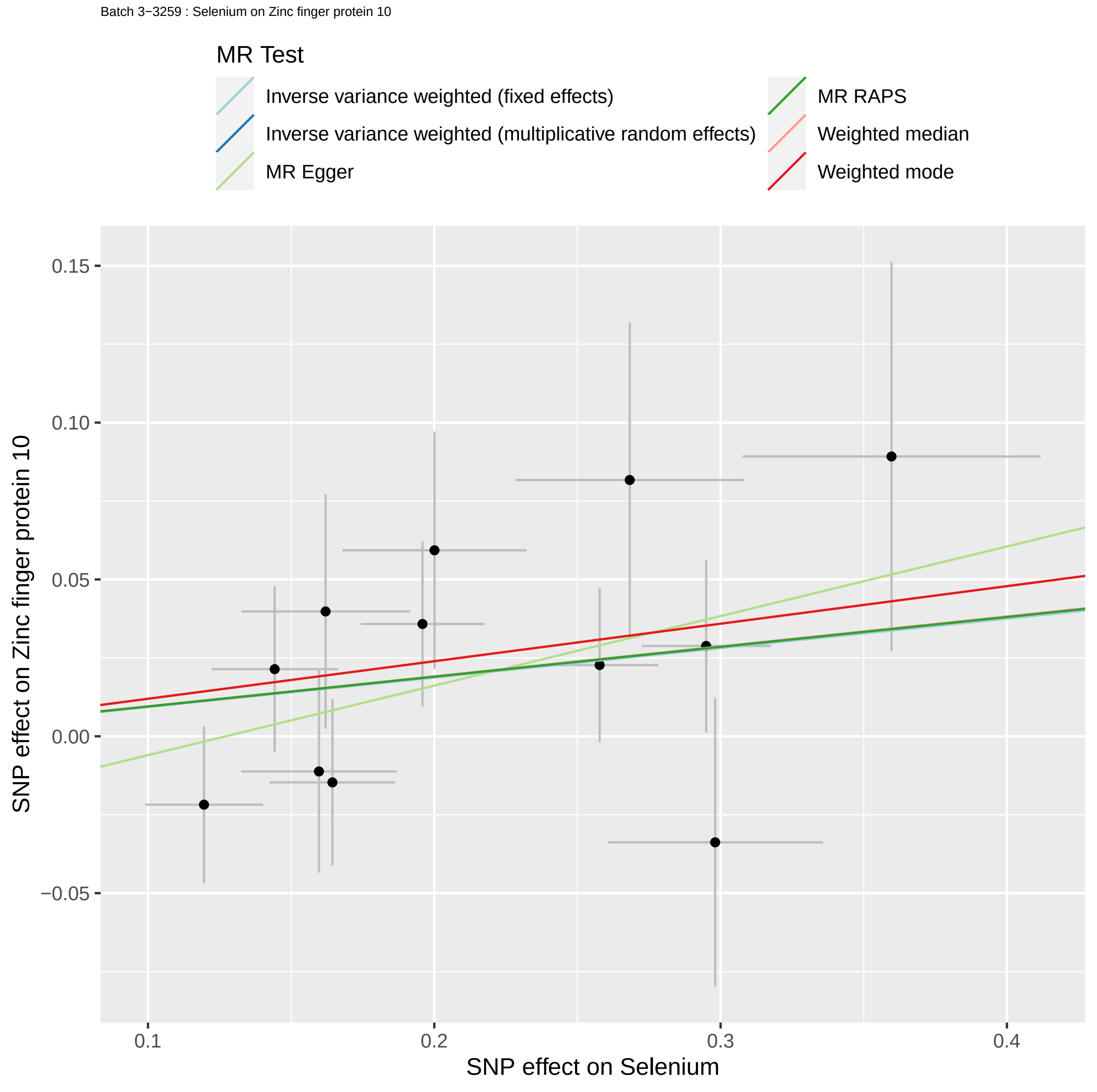

Batch 3−3259 : Selenium on Zinc finger protein 10
MR Test
Inverse variance weighted (fixed effects)
Inverse variance weighted (multiplicative random effects)
MR Egger
MR RAPS
Weighted median
Weighted mode
0.15
0.10
0.05
0.00
−0.05
0.1
0.2
0.3
0.4
SNP effect on Zinc finger protein 10
SNP effect on Selenium

Batch 3−3260 : Selenium on Zinc finger protein 134

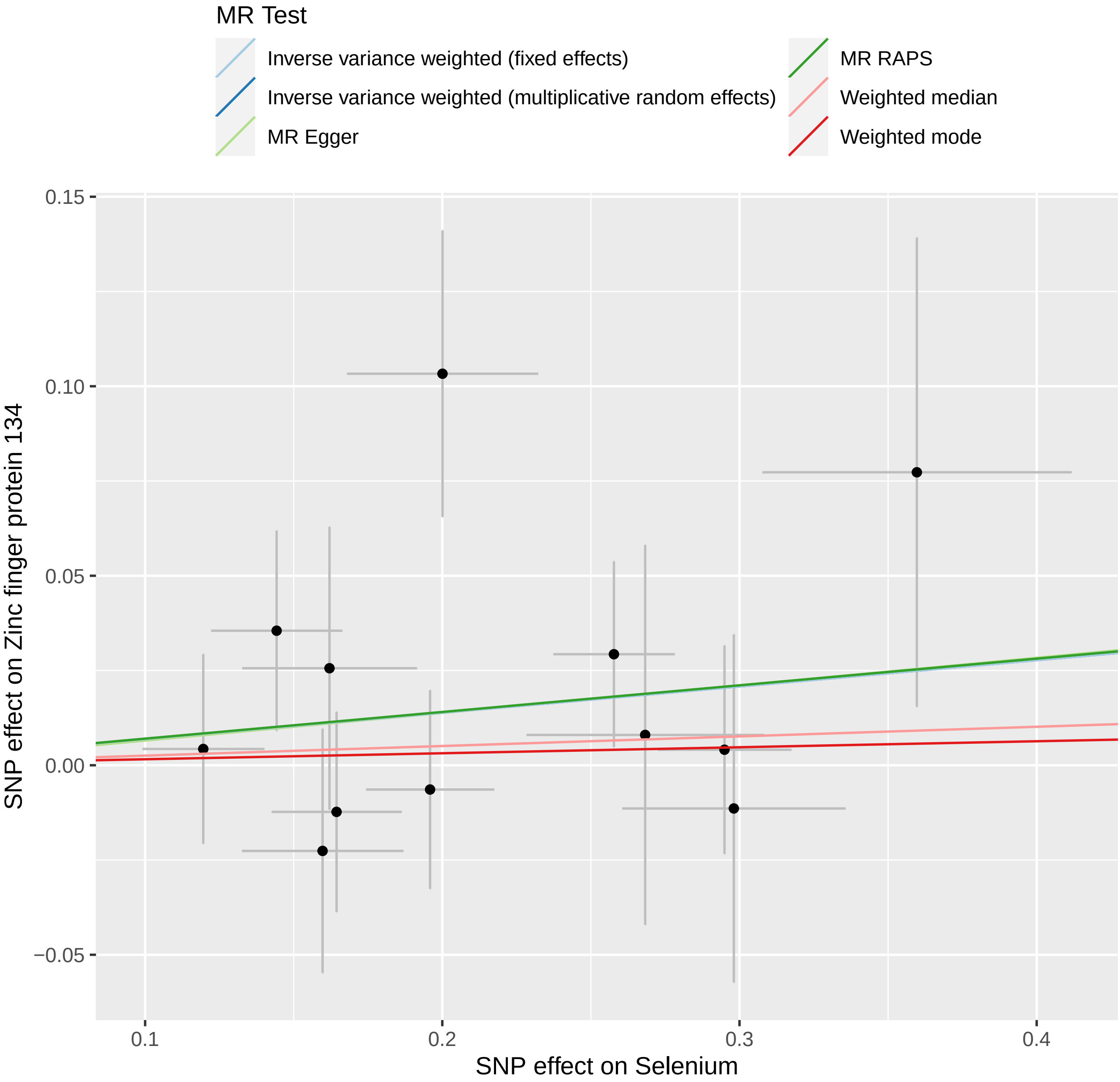

Batch 3−3261 : Selenium on Zinc finger protein 174

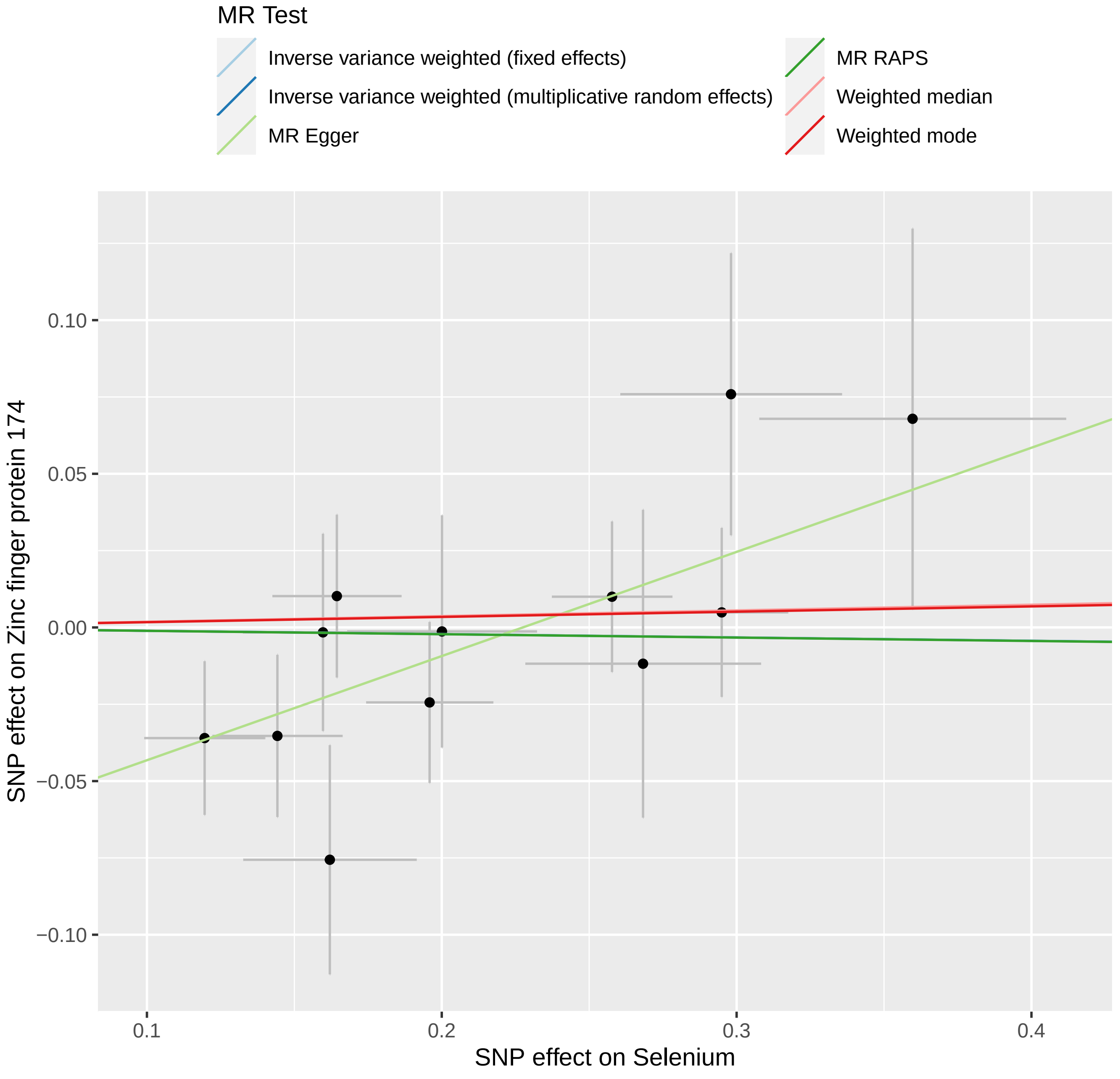

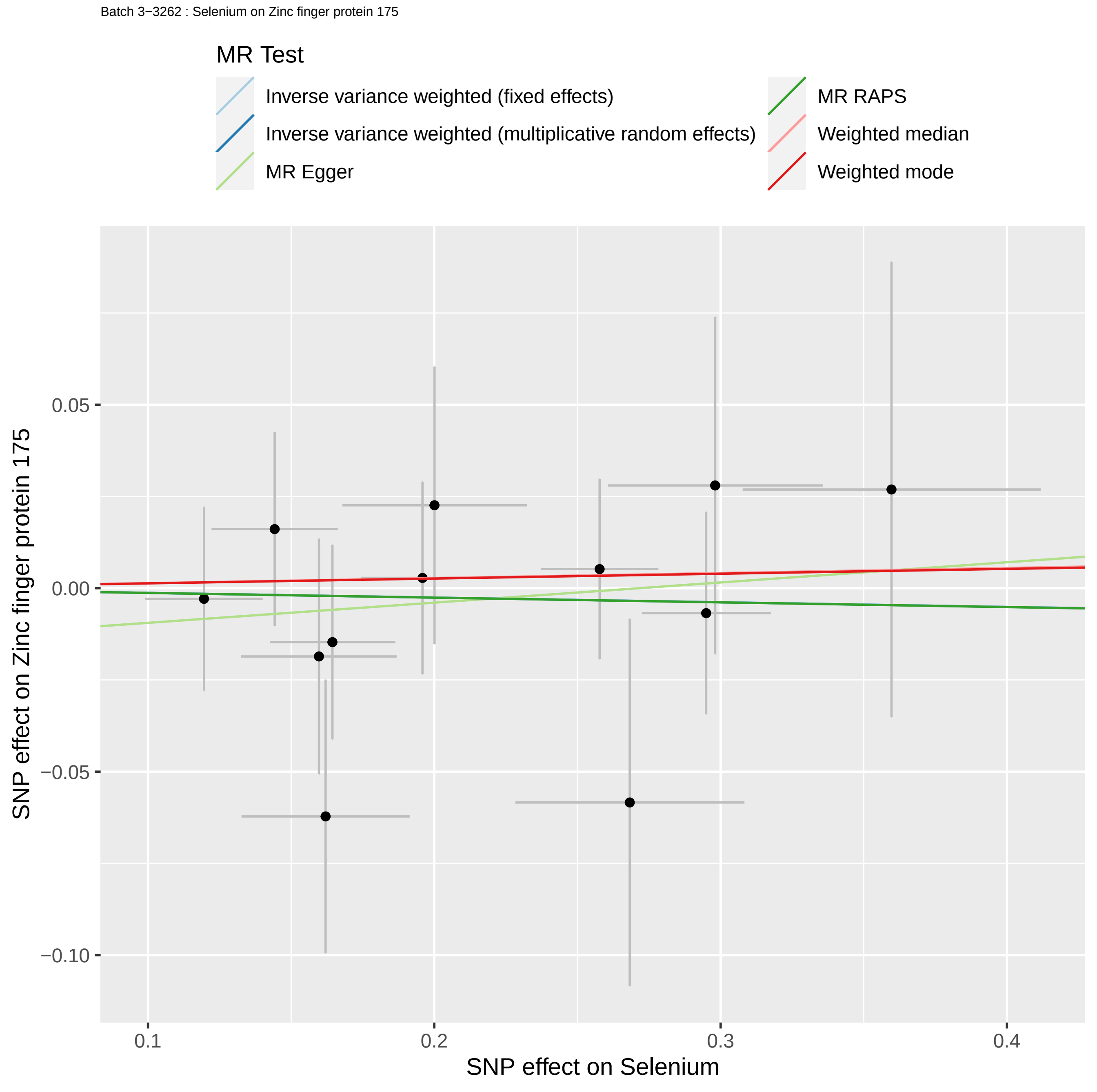
Batch 3−3262 : Selenium on Zinc finger protein 175
MR Test
Inverse variance weighted (fixed effects)
Inverse variance weighted (multiplicative random effects)
MR Egger
MR RAPS
Weighted median
Weighted mode
0.05
0.00
−0.05
−0.10
0.1
0.2
0.3
0.4
SNP effect on Zinc finger protein 175
SNP effect on Selenium

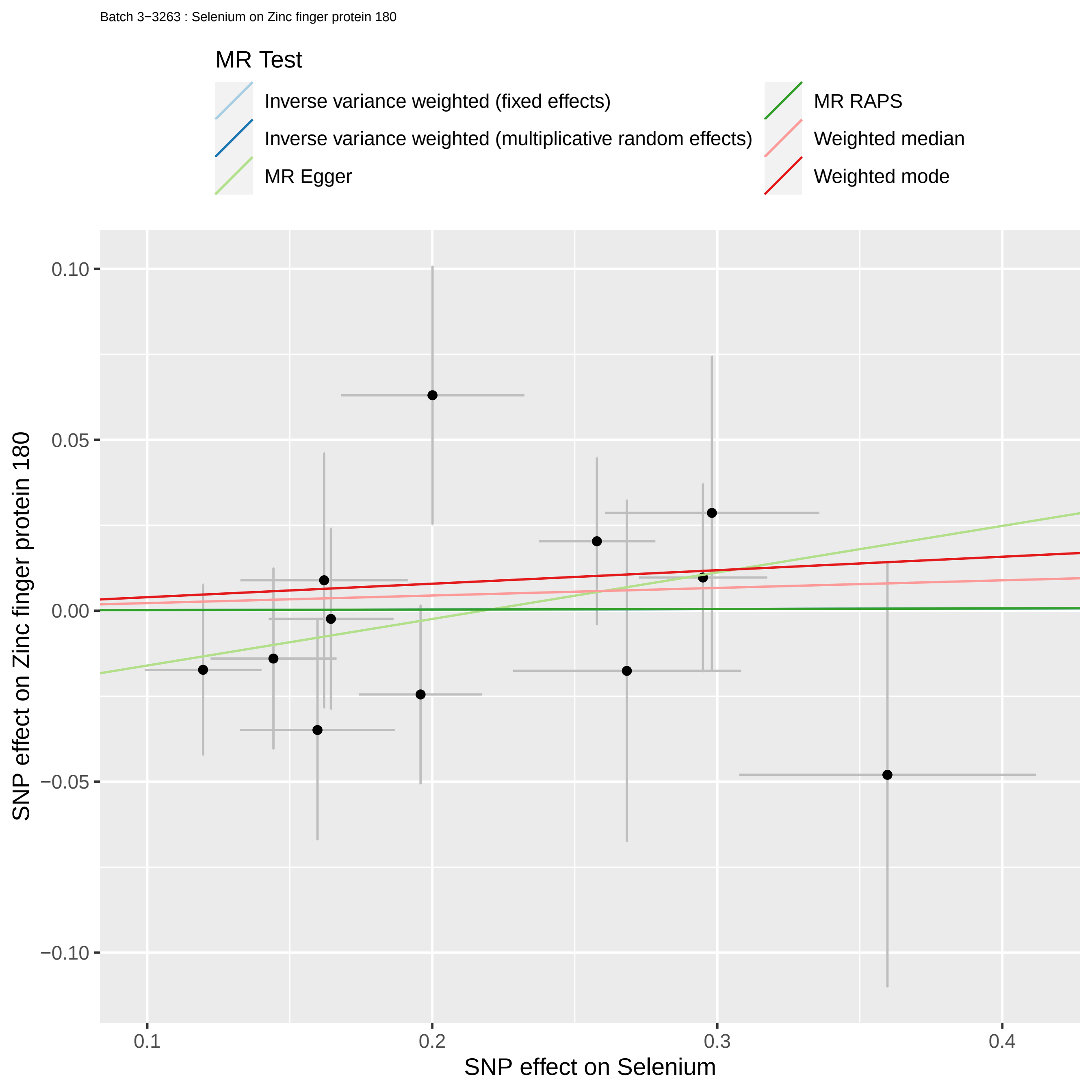
Batch 3−3263 : Selenium on Zinc finger protein 180
MR Test
Inverse variance weighted (fixed effects)
Inverse variance weighted (multiplicative random effects)
MR Egger
MR RAPS
Weighted median
Weighted mode
0.10
0.05
0.00
−0.05
−0.10
0.1
0.2
0.3
0.4
SNP effect on Zinc finger protein 180
SNP effect on Selenium

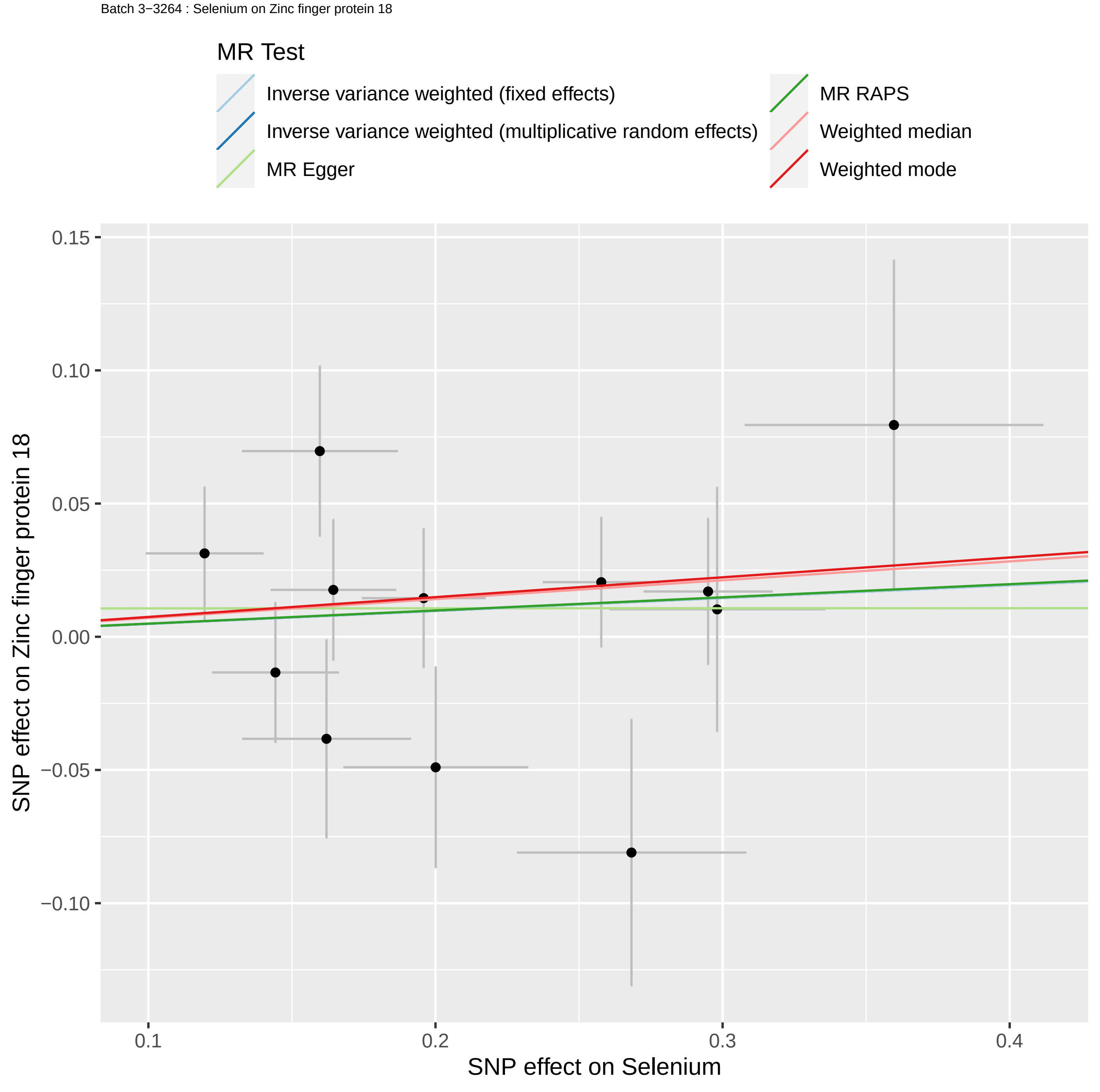

Batch 3−3264 : Selenium on Zinc finger protein 18
MR Test
Inverse variance weighted (fixed effects)
Inverse variance weighted (multiplicative random effects)
MR Egger
MR RAPS
Weighted median
Weighted mode
0.15
0.10
0.05
0.00
−0.05
−0.10
0.1
0.2
0.3
0.4
SNP effect on Selenium
SNP effect on Zinc finger protein 18

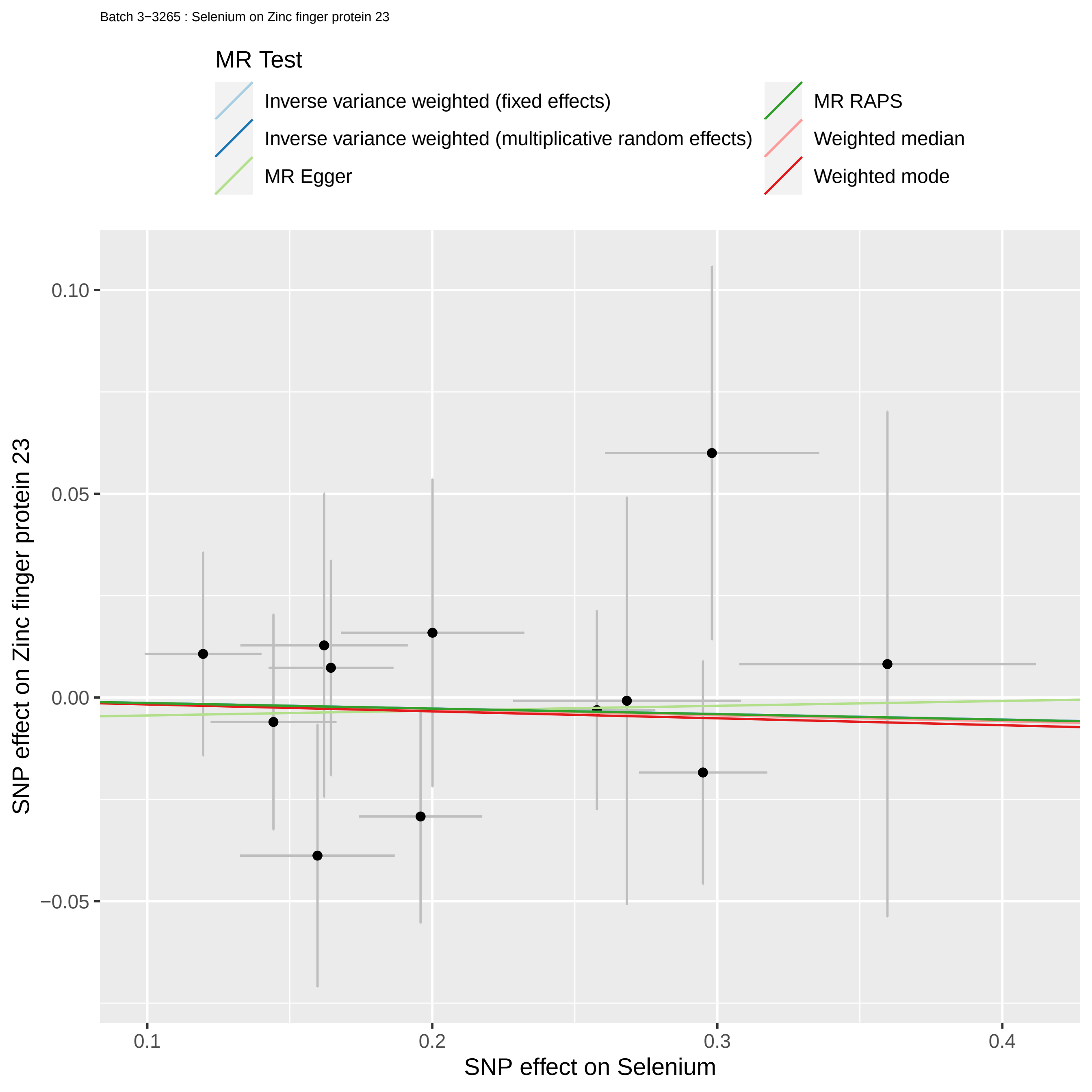

Batch 3−3265 : Selenium on Zinc finger protein 23
MR Test
Inverse variance weighted (fixed effects)
Inverse variance weighted (multiplicative random effects)
MR Egger
MR RAPS
Weighted median
Weighted mode
0.10
0.05
0.00
−0.05
0.1
0.2
0.3
0.4
SNP effect on Zinc finger protein 23
SNP effect on Selenium

Batch 3−3266 : Selenium on Zinc finger protein 264

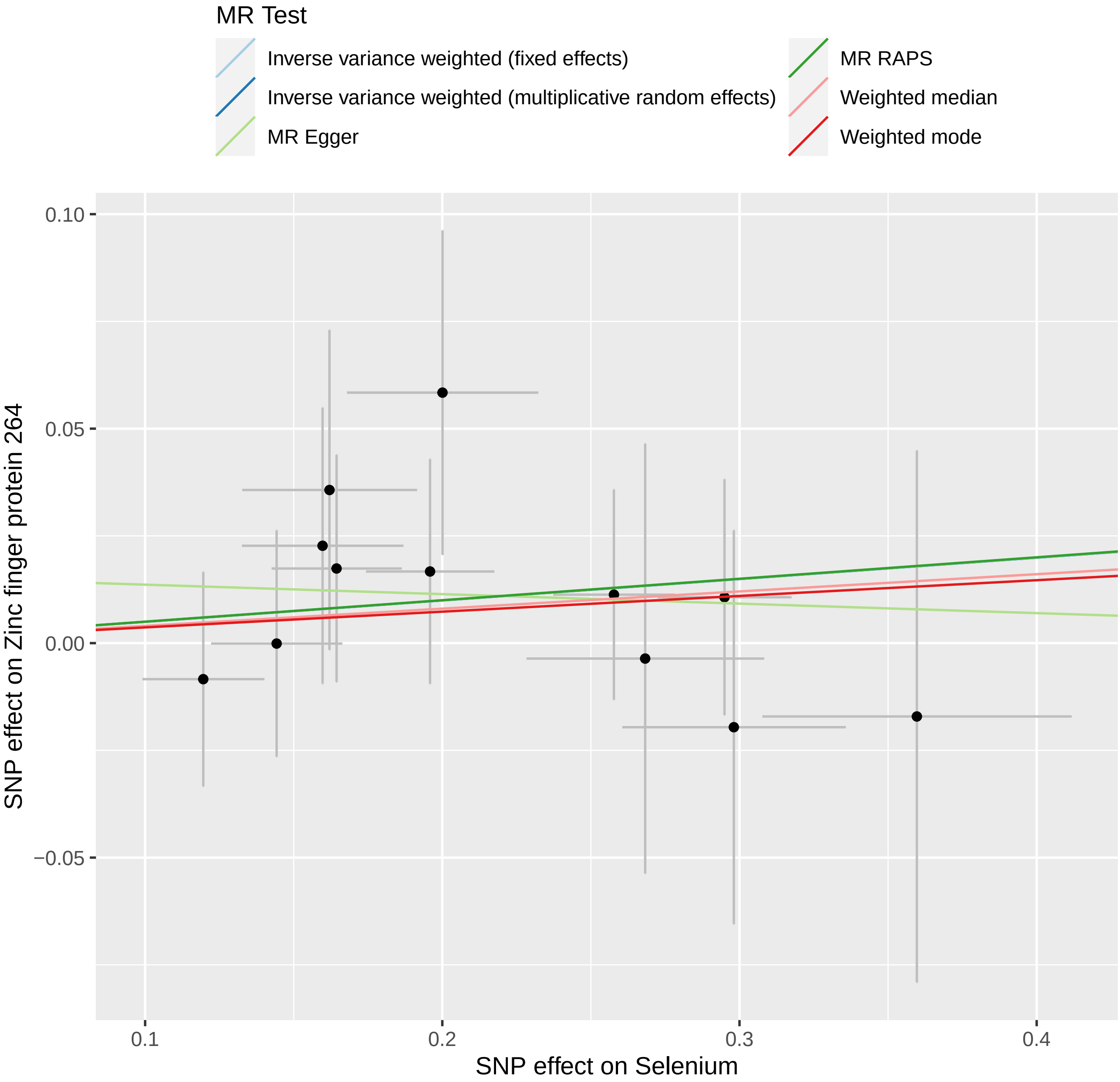

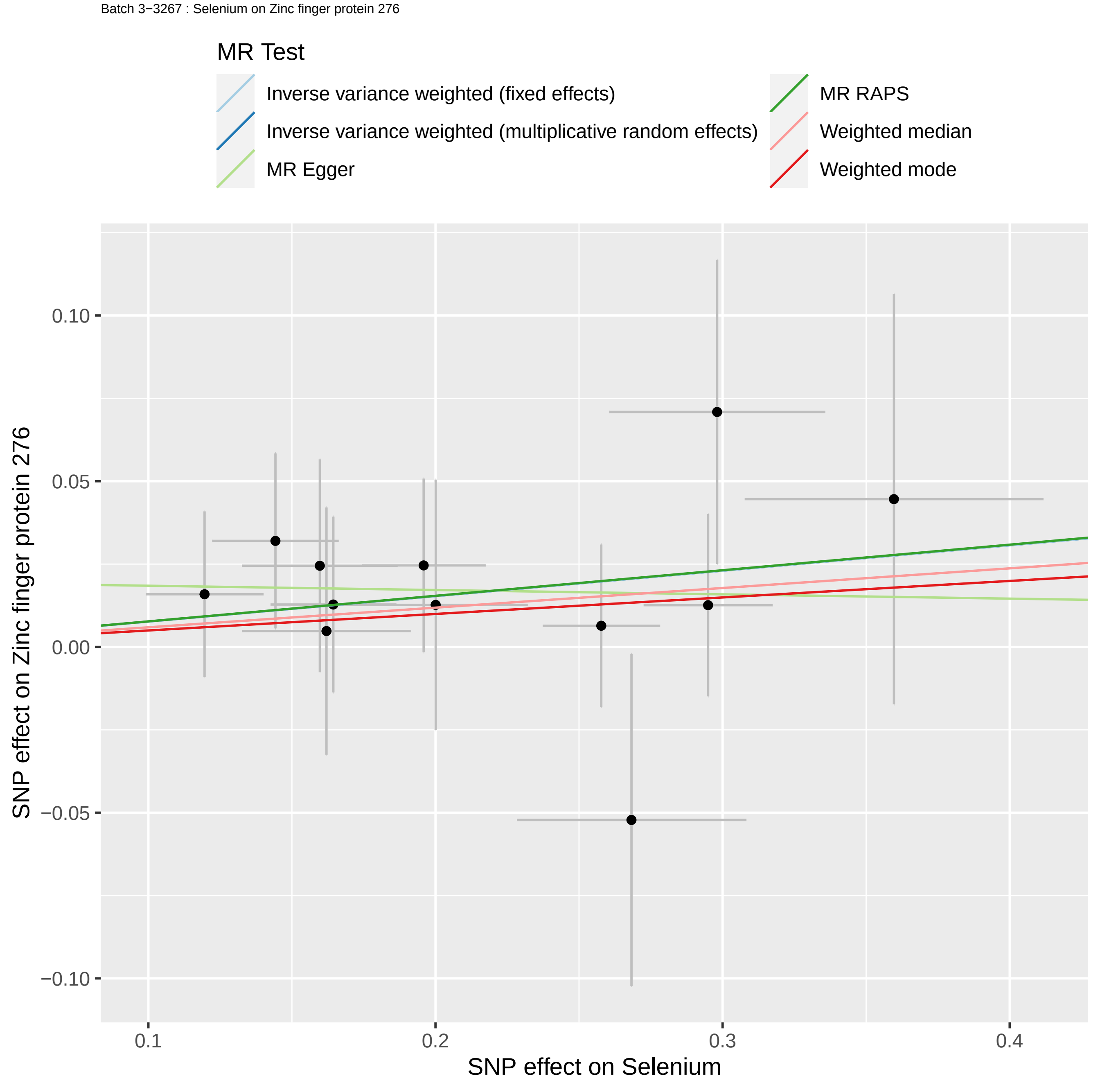

Batch 3−3267 : Selenium on Zinc finger protein 276
MR Test
Inverse variance weighted (fixed effects)
Inverse variance weighted (multiplicative random effects)
MR Egger
MR RAPS
Weighted median
Weighted mode
SNP effect on Zinc finger protein 276
0.10
0.05
0.00
−0.05
−0.10
0.1
0.2
0.3
0.4
SNP effect on Selenium

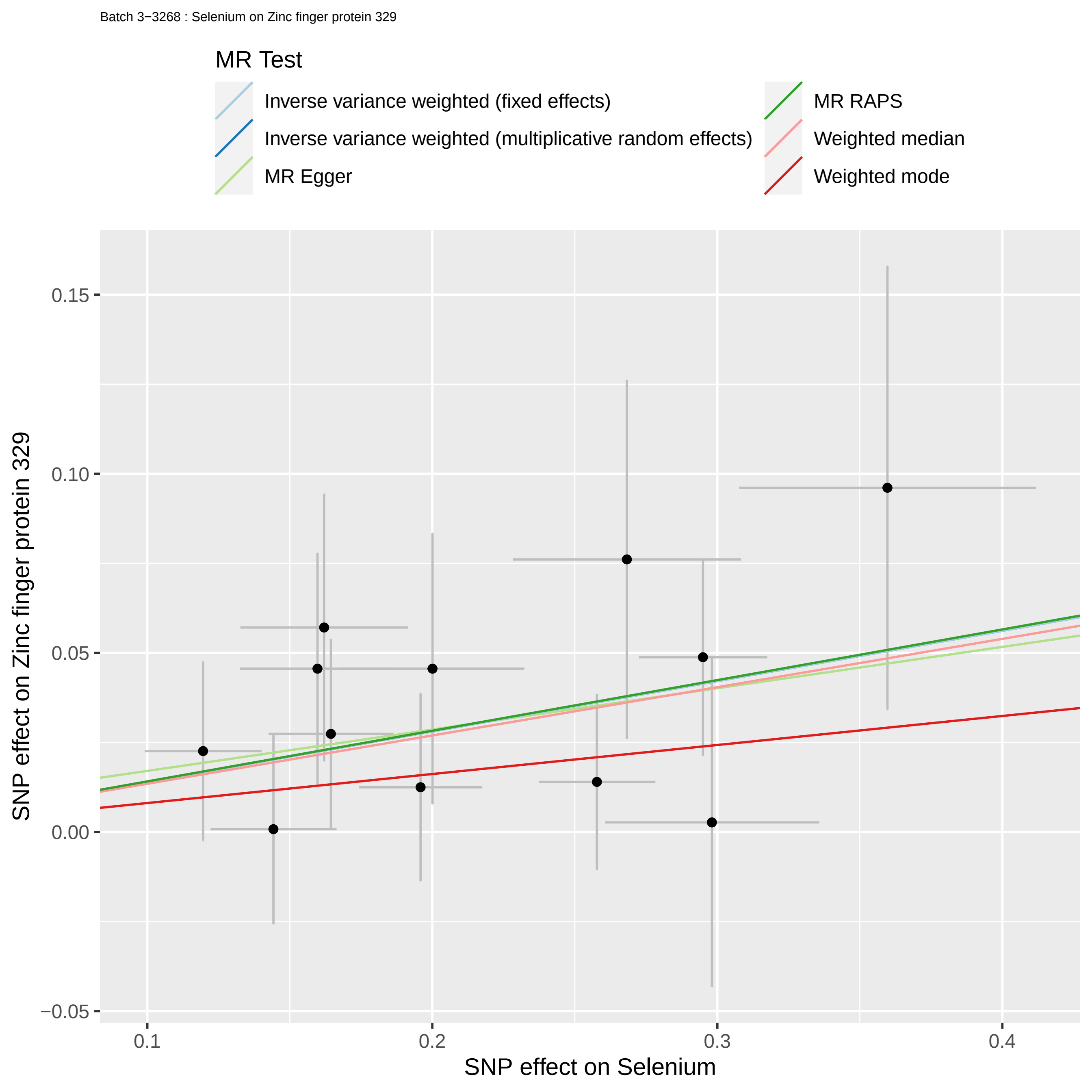
Batch 3−3268 : Selenium on Zinc finger protein 329
MR Test
Inverse variance weighted (fixed effects)
Inverse variance weighted (multiplicative random effects)
MR Egger
MR RAPS
Weighted median
Weighted mode
0.15
0.10
0.05
0.00
−0.05
0.1
0.2
0.3
0.4
SNP effect on Zinc finger protein 329
SNP effect on Selenium

Batch 3−3269 : Selenium on Zinc finger protein 334

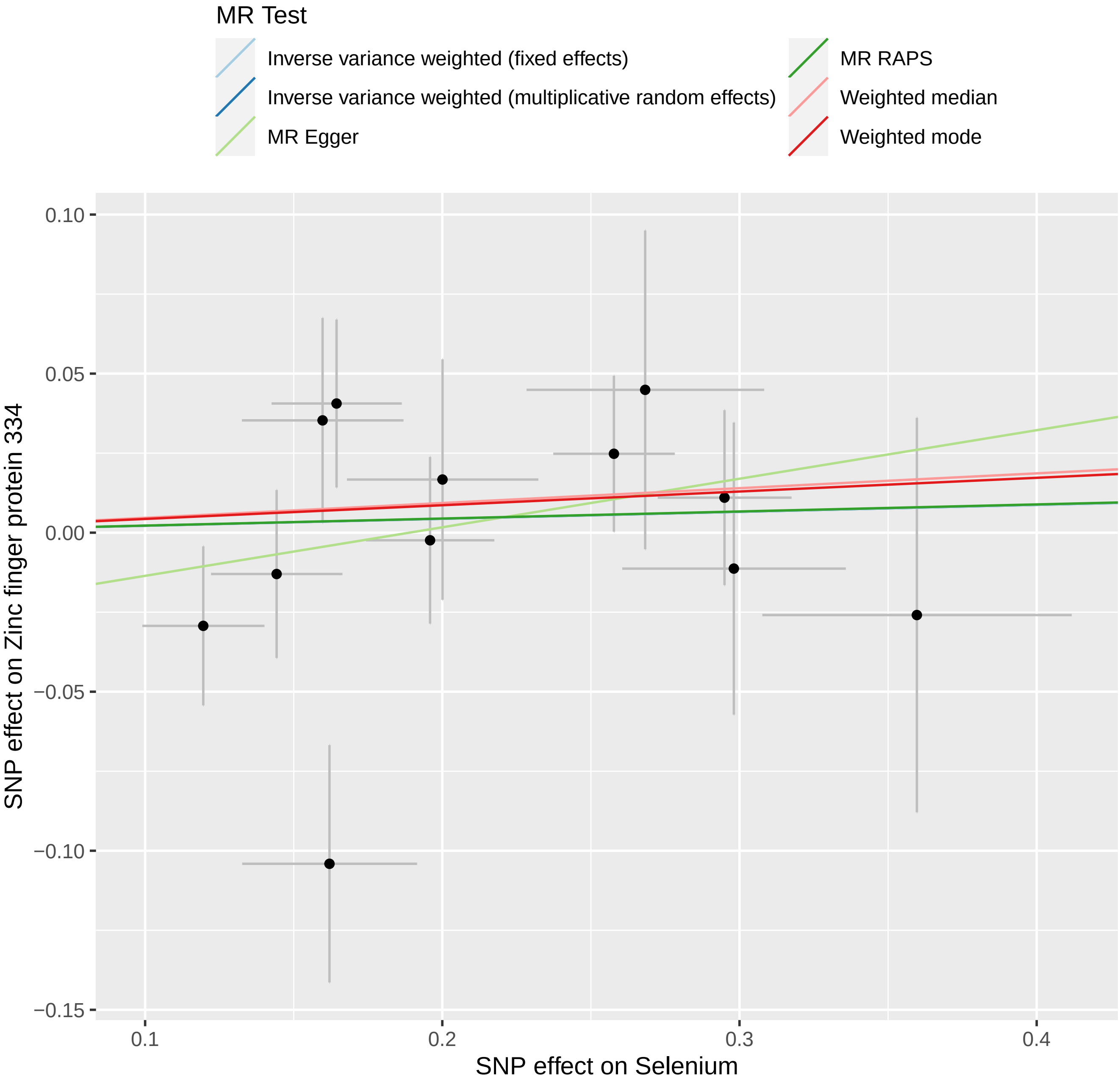

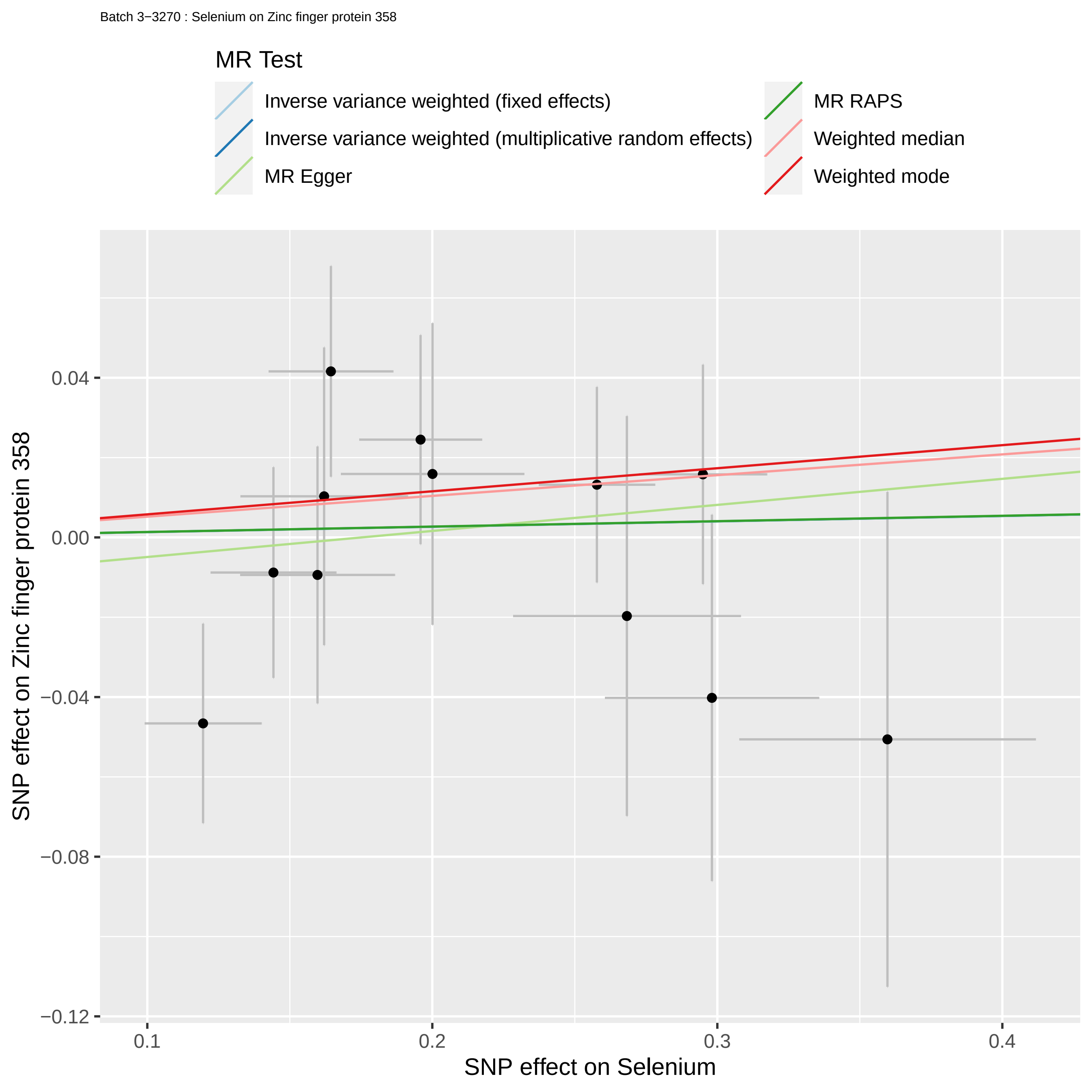
Batch 3−3270 : Selenium on Zinc finger protein 358
MR Test
Inverse variance weighted (fixed effects)
Inverse variance weighted (multiplicative random effects)
MR Egger
MR RAPS
Weighted median
Weighted mode
SNP effect on Zinc finger protein 358
0.04
0.00
−0.04
−0.08
−0.12
0.1
0.2
0.3
0.4
SNP effect on Selenium

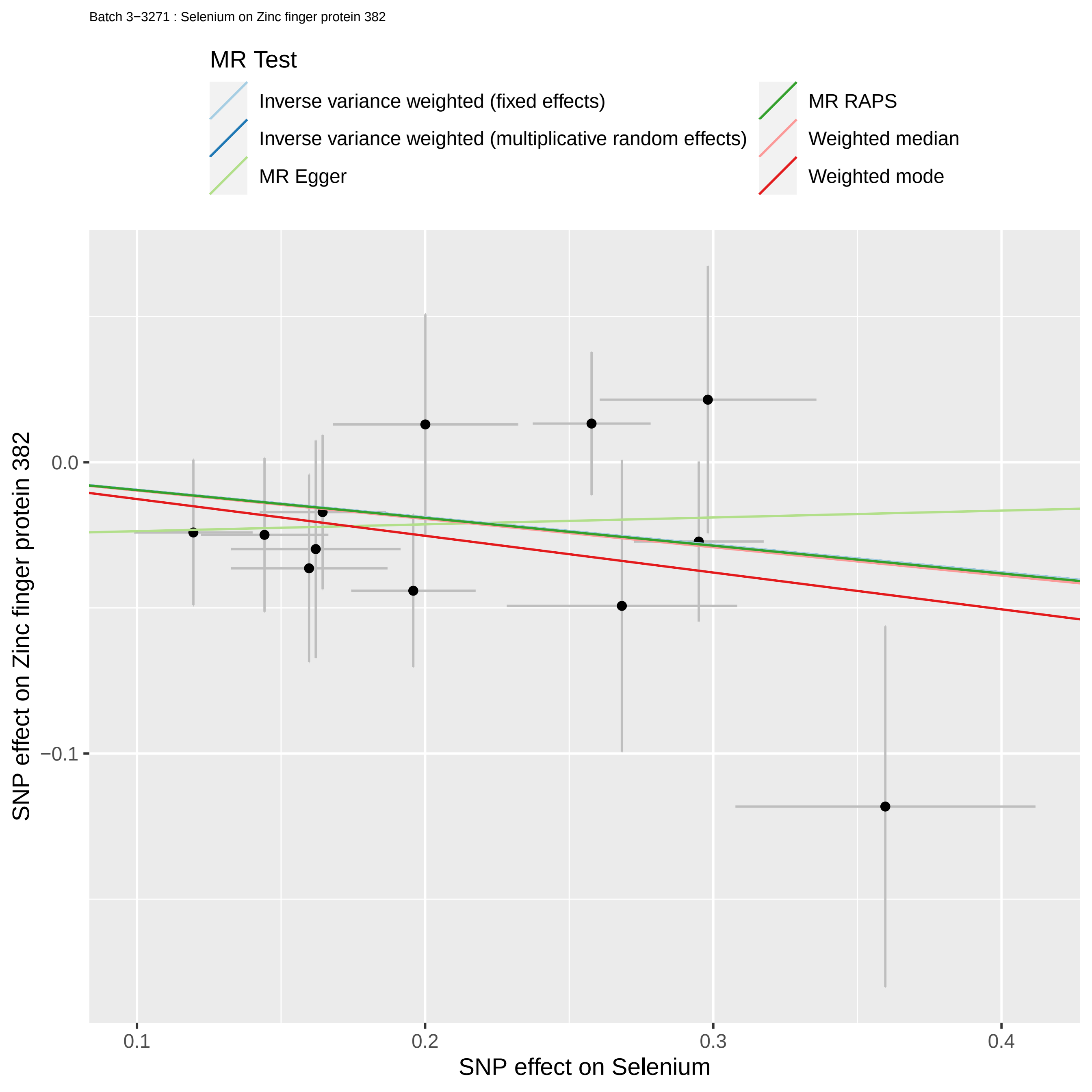
Batch 3−3271 : Selenium on Zinc finger protein 382
MR Test
Inverse variance weighted (fixed effects)
Inverse variance weighted (multiplicative random effects)
MR Egger
MR RAPS
Weighted median
Weighted mode
0.0
−0.1
0.1
0.2
0.3
0.4
SNP effect on Selenium
SNP effect on Zinc finger protein 382

Batch 3−3272 : Selenium on Zinc finger protein 410

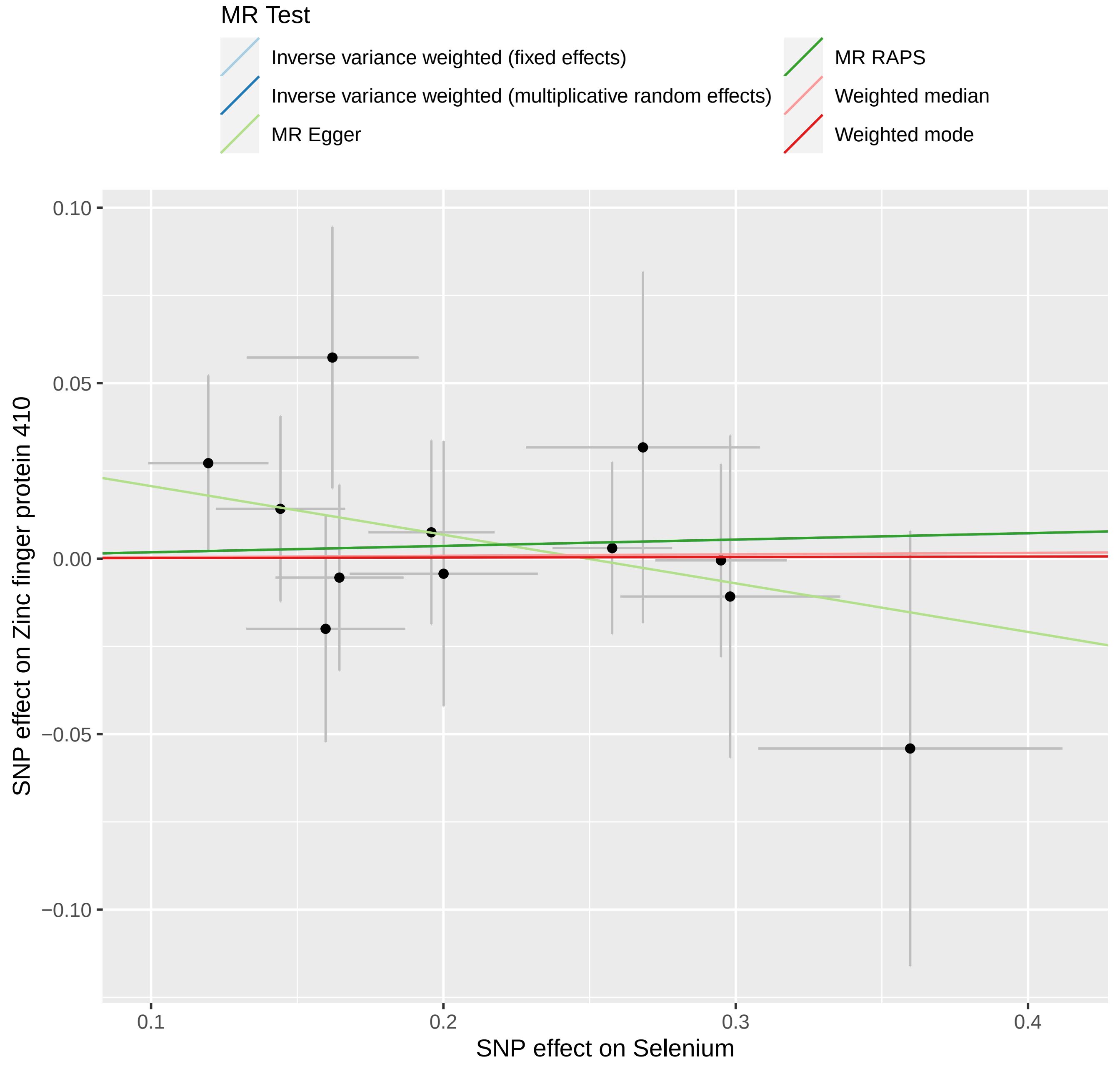

Batch 3−3273 : Selenium on Zinc finger protein 415

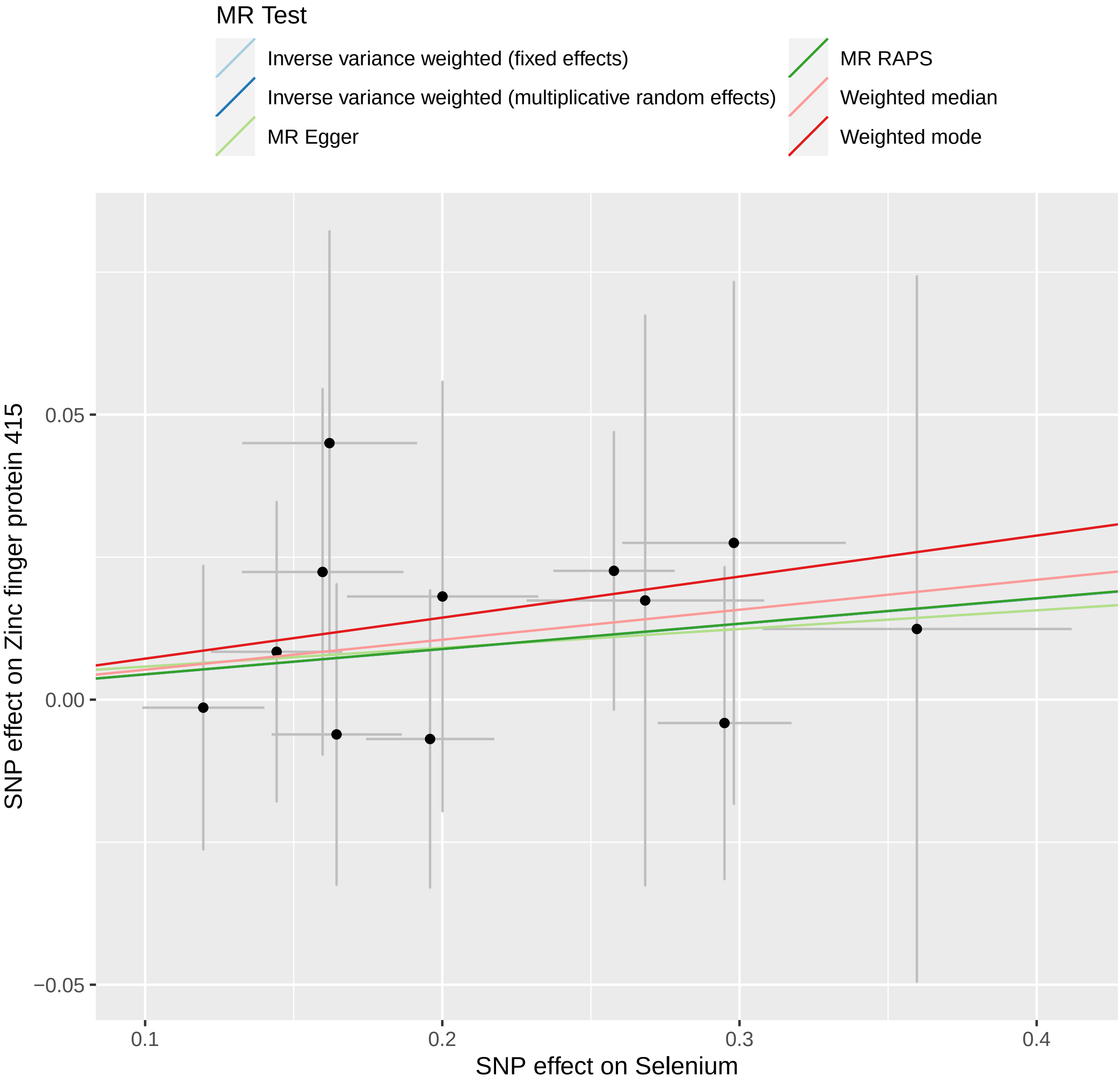

Batch 3−3274 : Selenium on Zinc finger protein 566

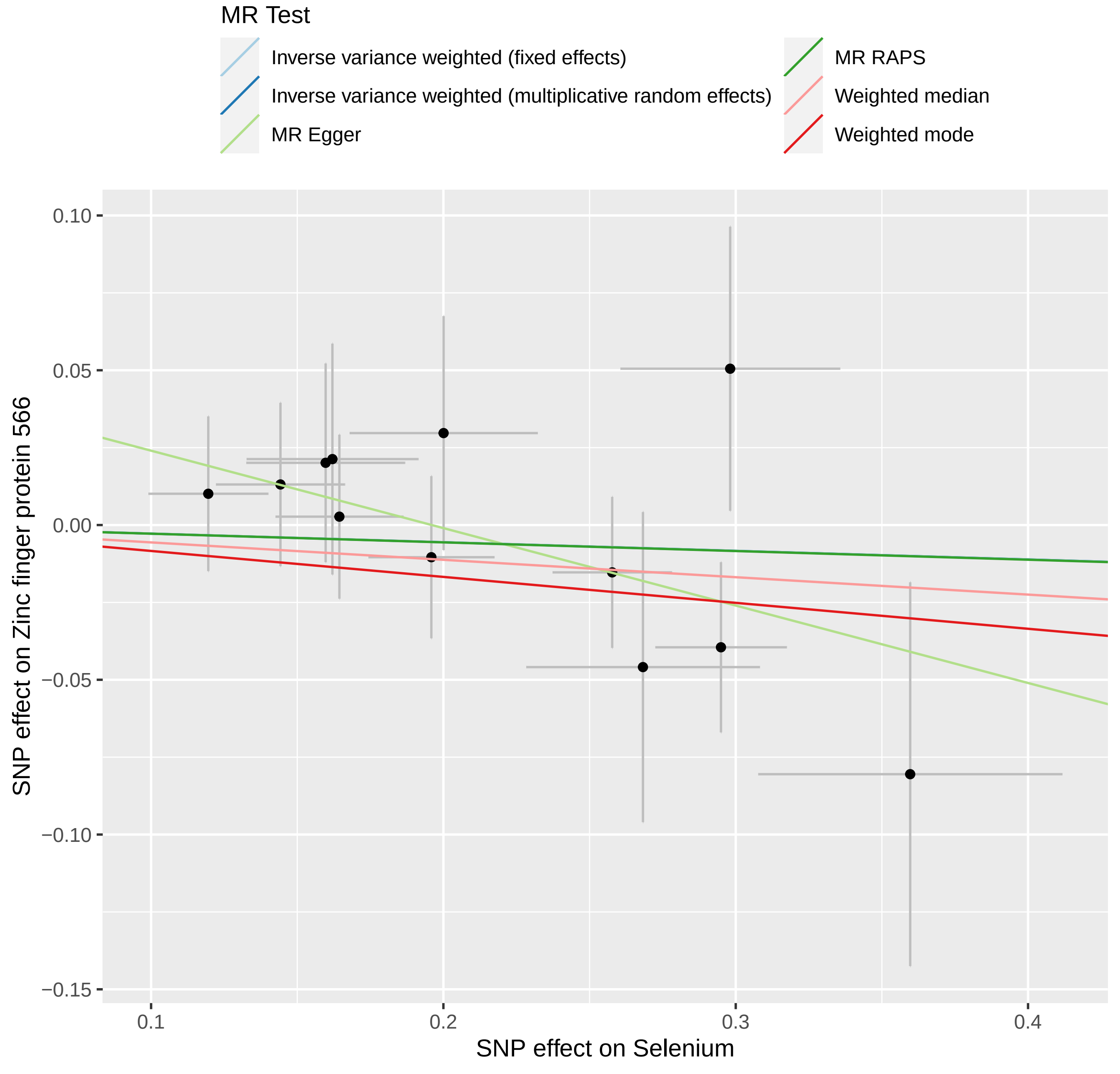

Batch 3−3275 : Selenium on Zinc finger protein 75D

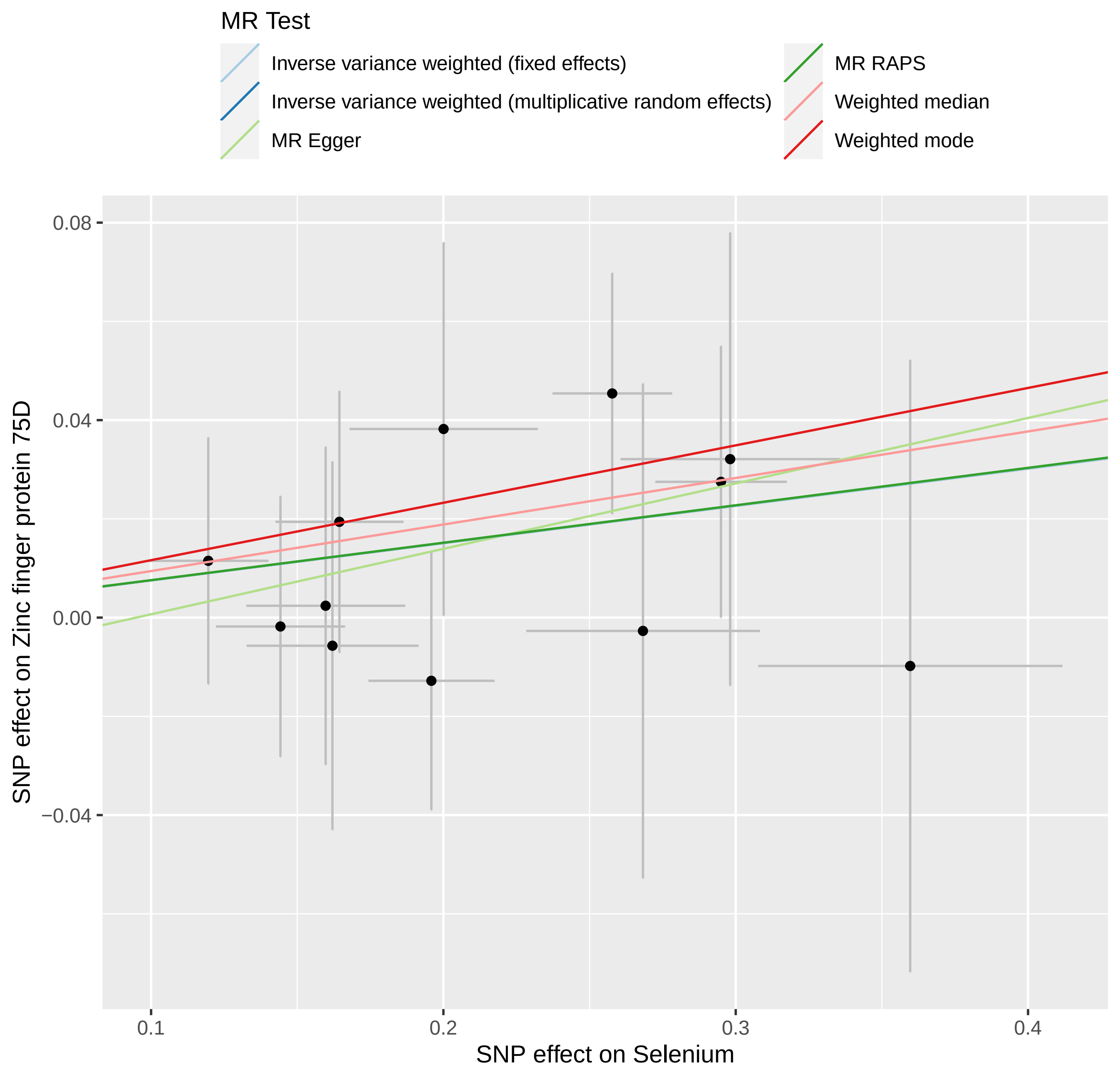

Batch 3−3276 : Selenium on Zinc finger protein 774

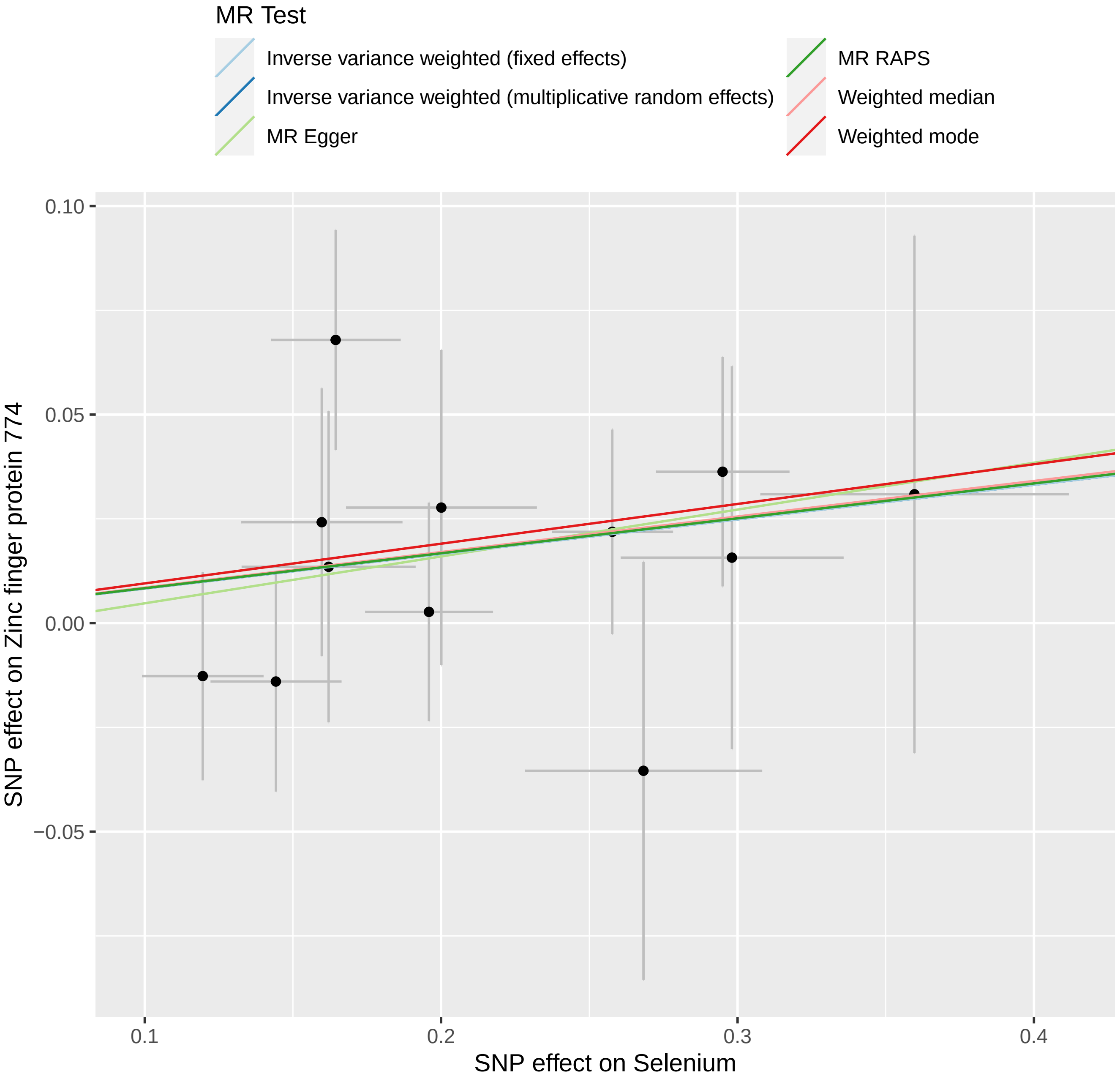

Batch 3−3277 : Selenium on Zinc finger protein 843

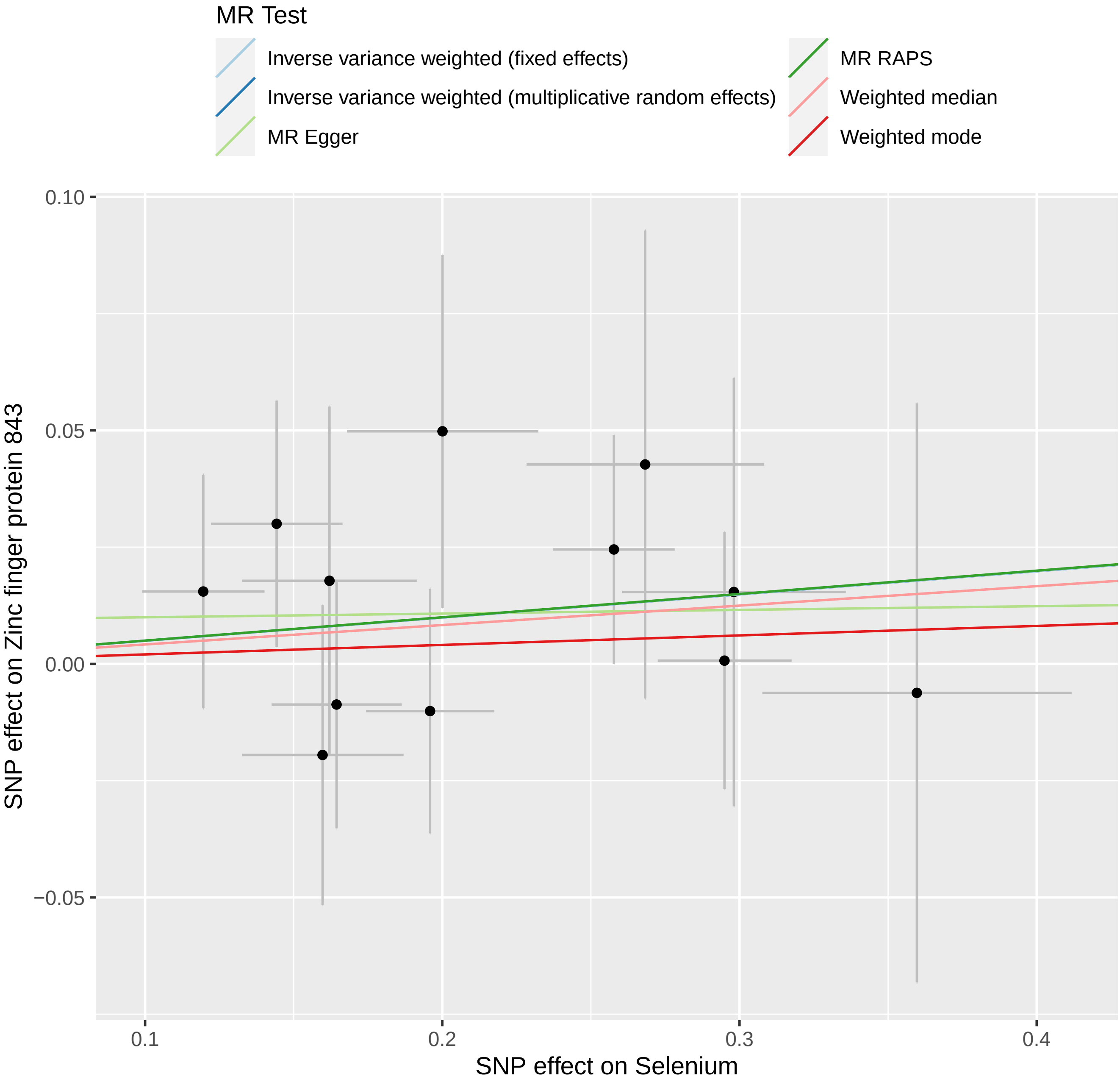

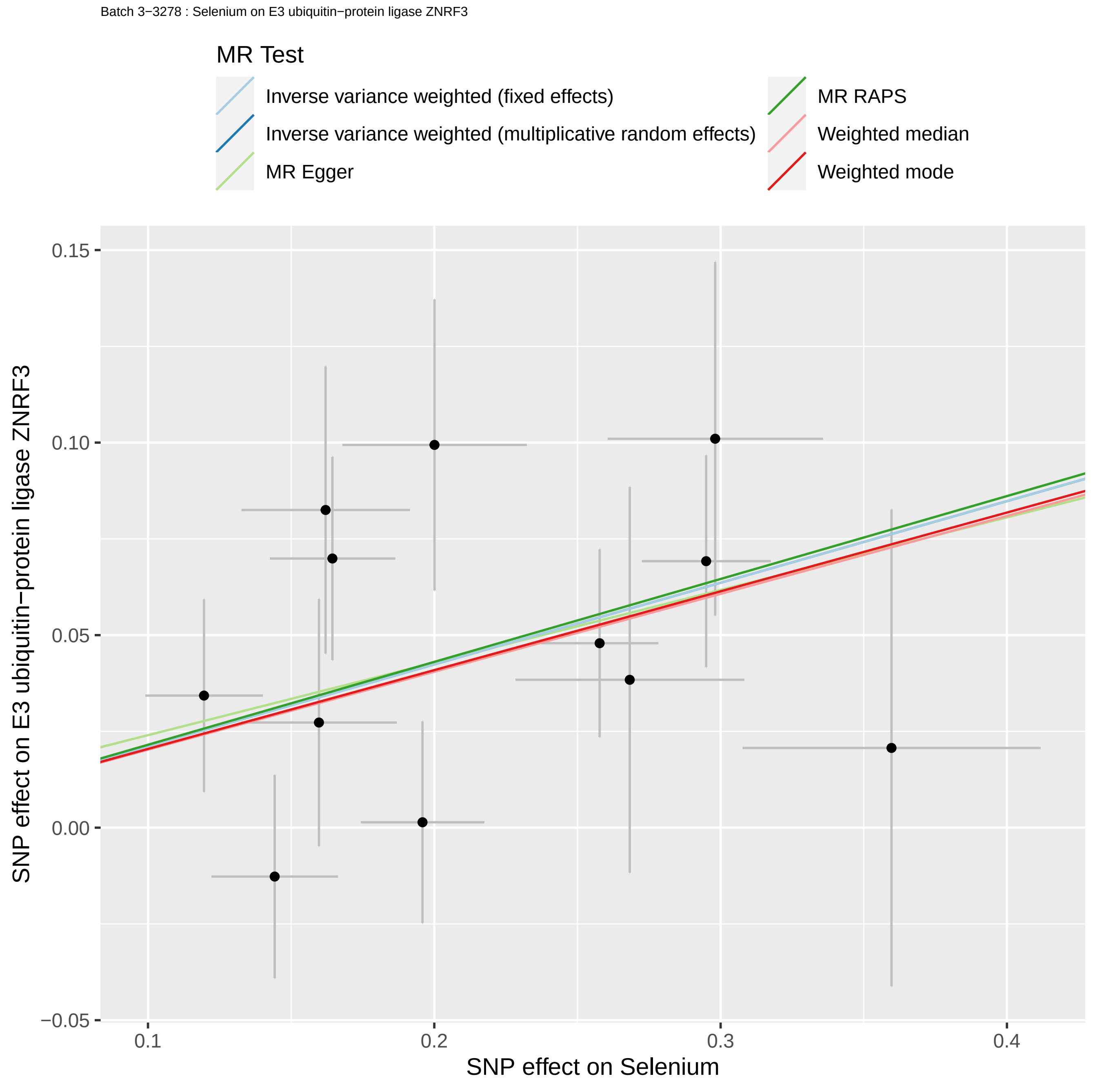

Batch 3−3278 : Selenium on E3 ubiquitin−protein ligase ZNRF3
MR Test
Inverse variance weighted (fixed effects)
Inverse variance weighted (multiplicative random effects)
MR Egger
MR RAPS
Weighted median
Weighted mode
0.15
0.10
0.05
0.00
−0.05
0.1
0.2
0.3
0.4
SNP effect on E3 ubiquitin−protein ligase ZNRF3
SNP effect on Selenium

Batch 3−3279 : Selenium on E3 ubiquitin−protein ligase ZNRF3

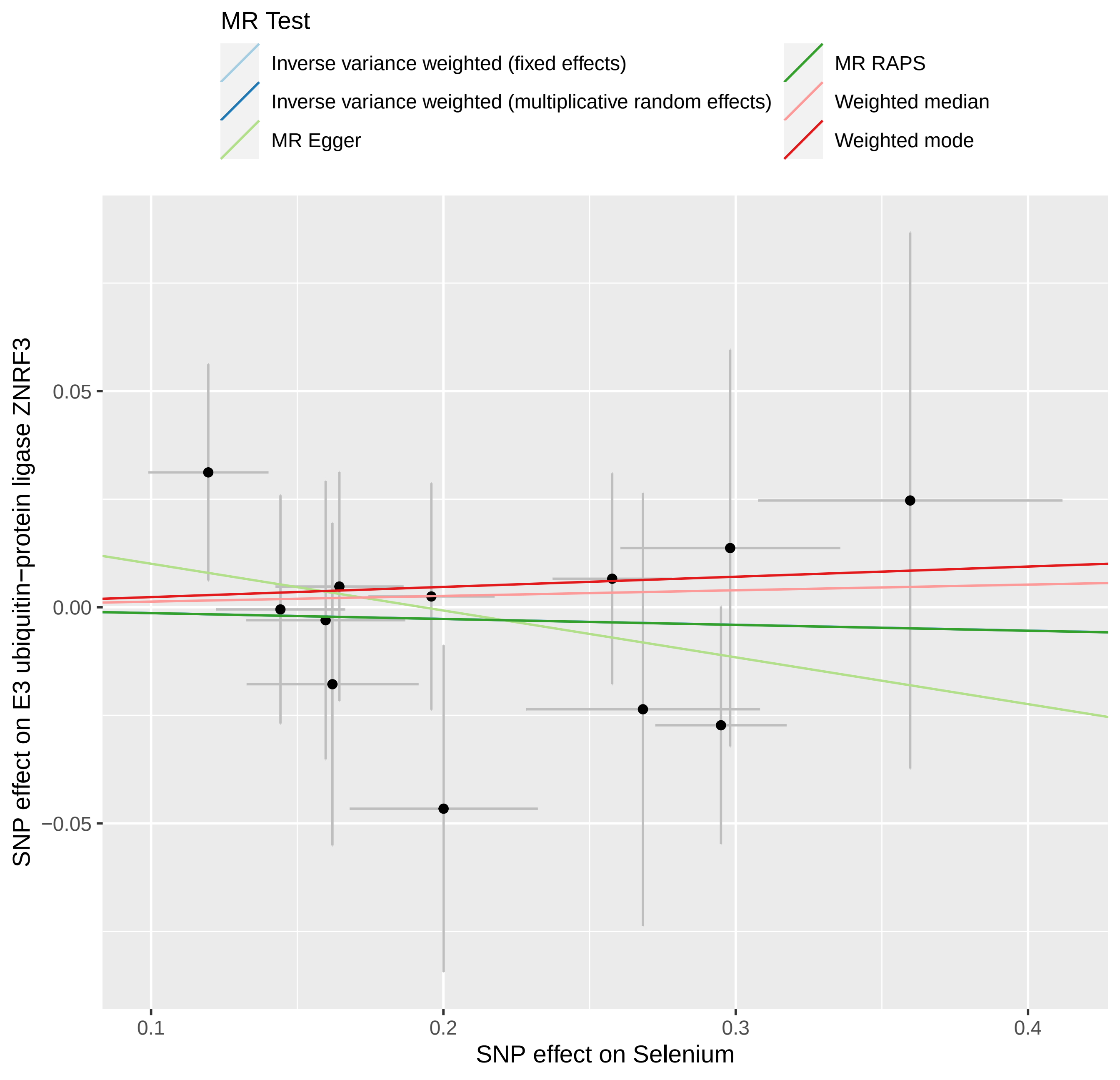

Batch 3−3280 : Selenium on Zinc/RING finger protein 4

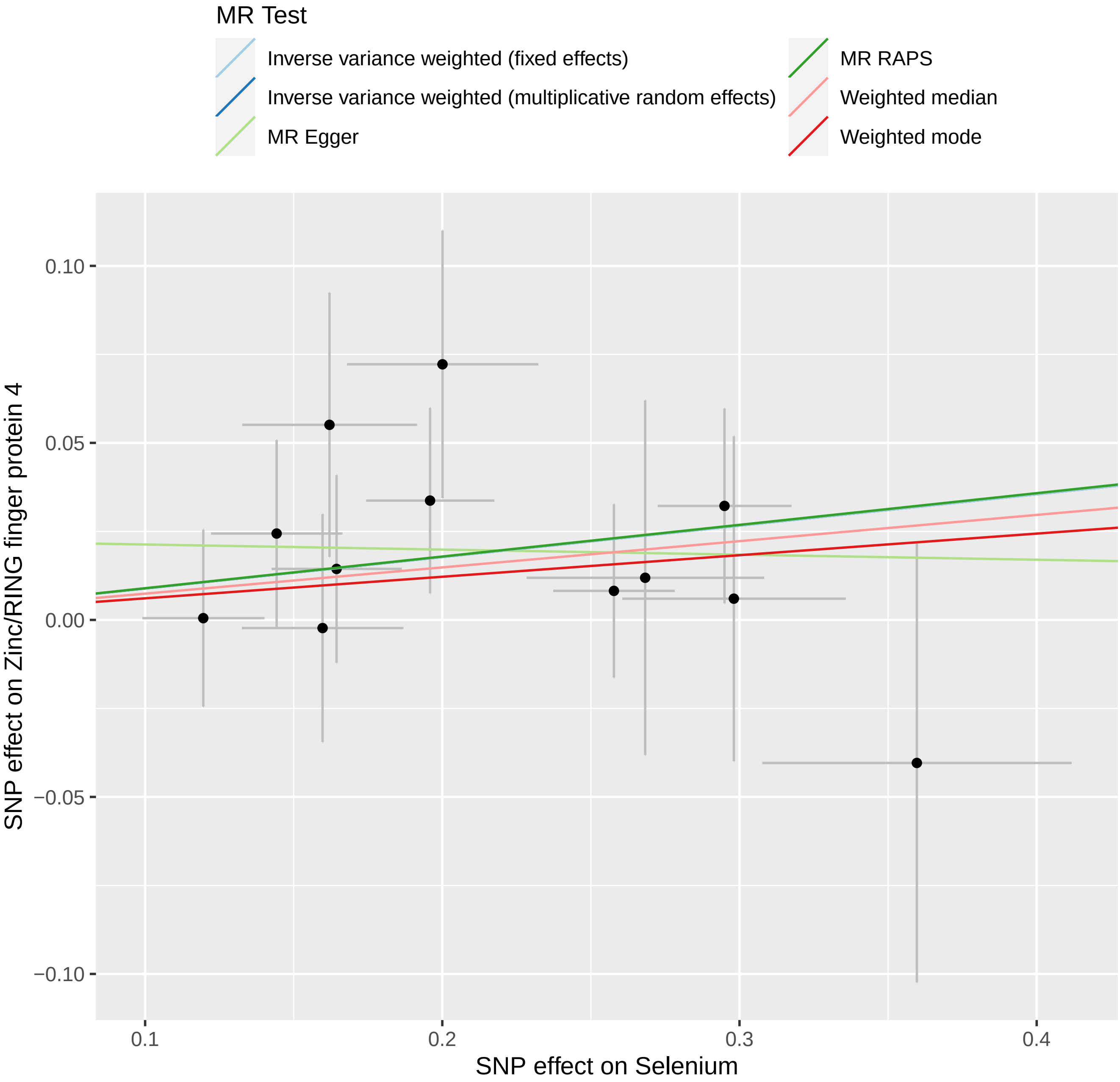

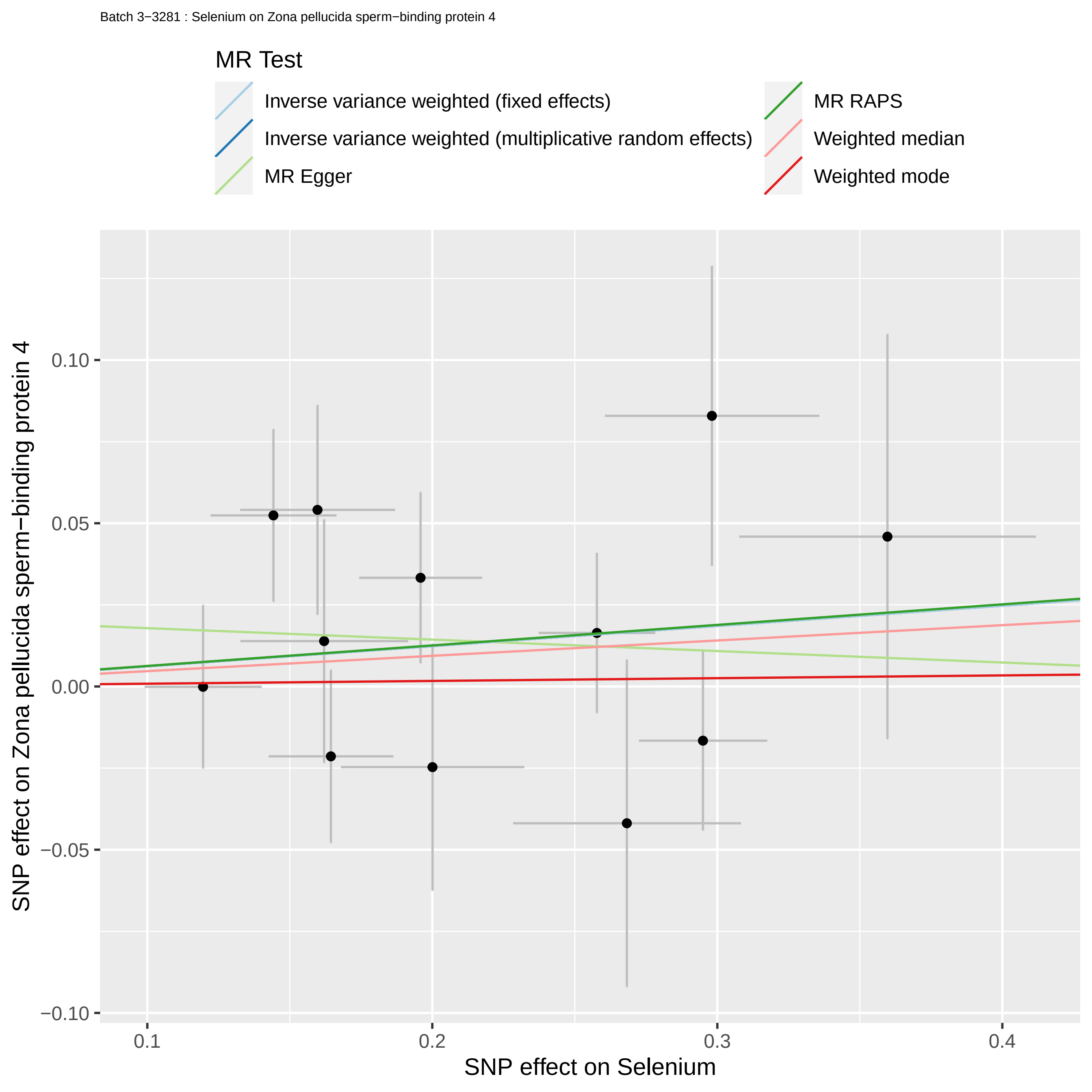
Batch 3−3281 : Selenium on Zona pellucida sperm−binding protein 4
MR Test
Inverse variance weighted (fixed effects)
Inverse variance weighted (multiplicative random effects)
MR Egger
MR RAPS
Weighted median
Weighted mode
SNP effect on Zona pellucida sperm−binding protein 4
0.10
0.05
0.00
−0.05
−0.10
0.1
0.2
0.3
0.4
SNP effect on Selenium

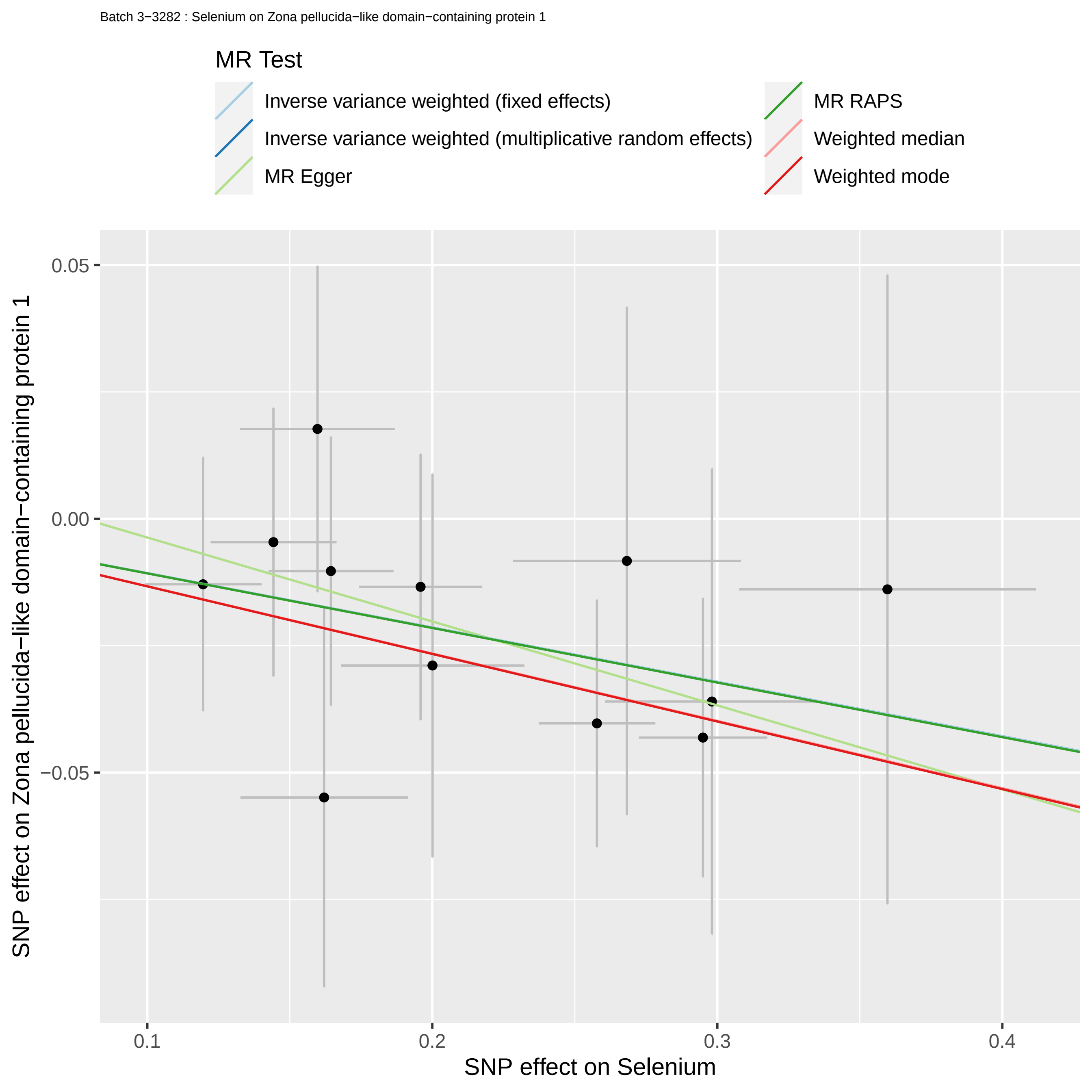
Batch 3−3282 : Selenium on Zona pellucida−like domain−containing protein 1
MR Test
Inverse variance weighted (fixed effects)
Inverse variance weighted (multiplicative random effects)
MR Egger
MR RAPS
Weighted median
Weighted mode
0.05
0.00
−0.05
0.1
0.2
0.3
0.4
SNP effect on Zona pellucida−like domain−containing protein 1
SNP effect on Selenium

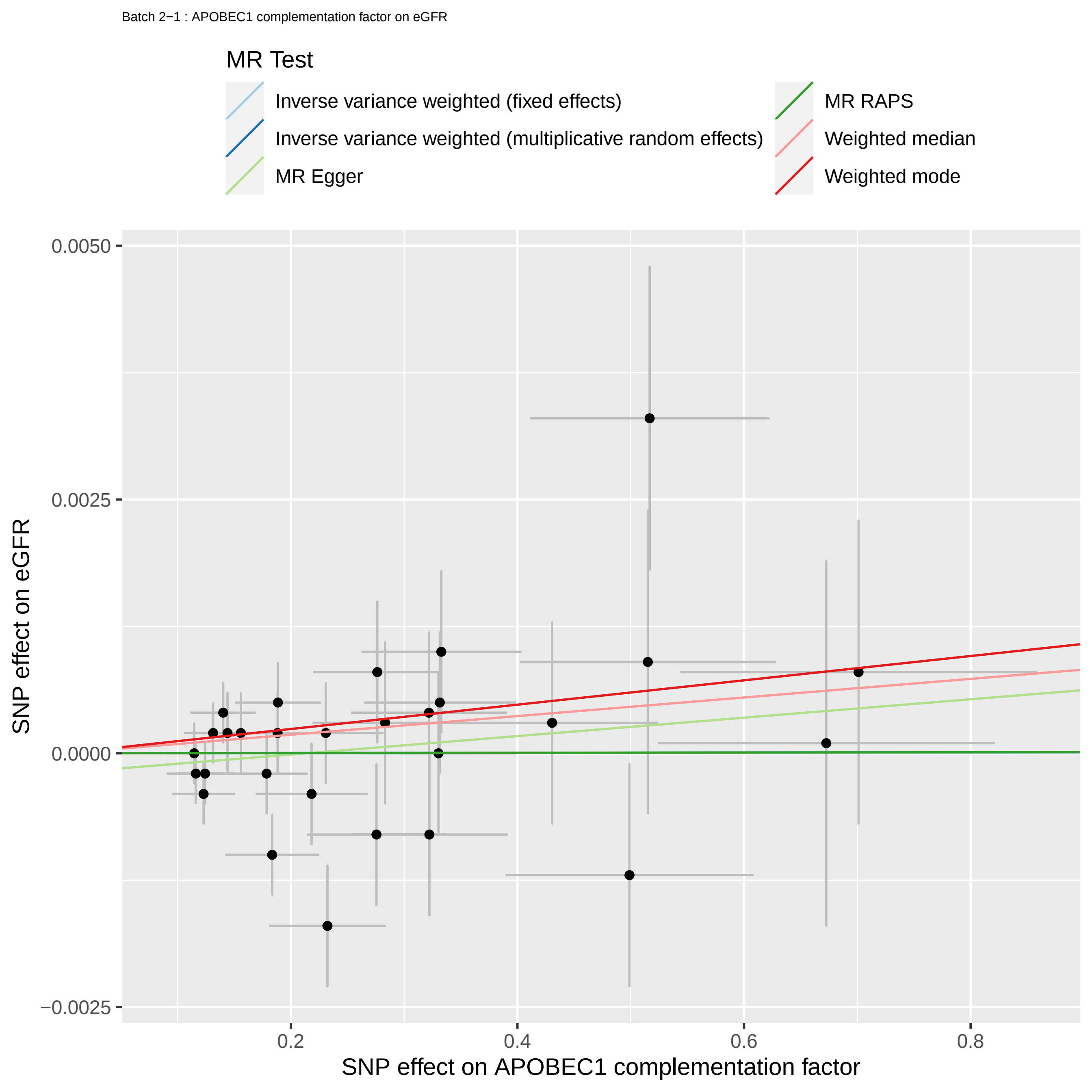
Batch 2−1 : APOBEC1 complementation factor on eGFR
MR Test
Inverse variance weighted (fixed effects)
Inverse variance weighted (multiplicative random effects)
MR Egger
MR RAPS
Weighted median
Weighted mode
0.0050
0.0025
0.0000
−0.0025
0.2
0.4
0.6
0.8
SNP effect on eGFR
SNP effect on APOBEC1 complementation factor

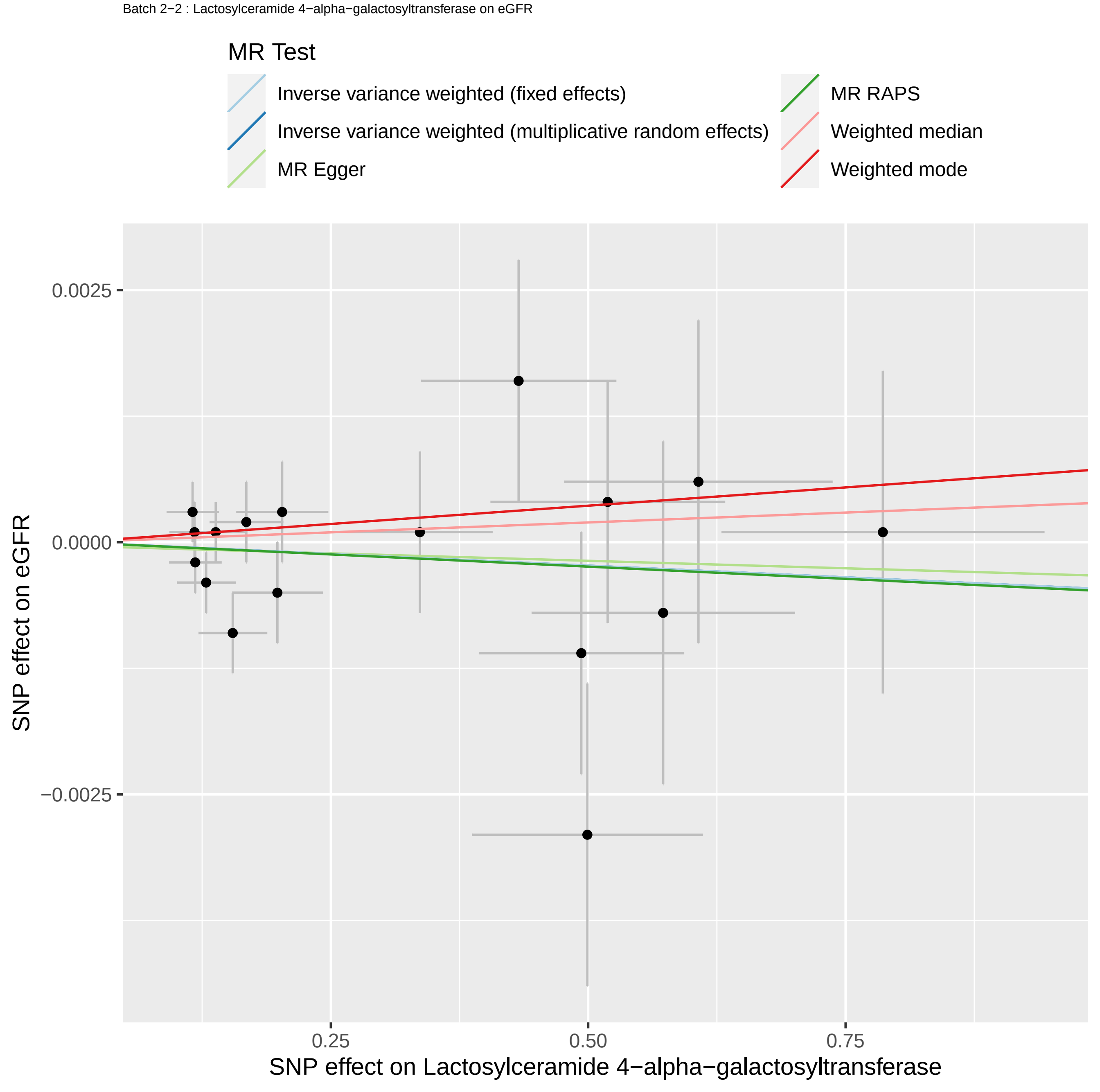

Batch 2−2 : Lactosylceramide 4−alpha−galactosyltransferase on eGFR
MR Test
Inverse variance weighted (fixed effects)
Inverse variance weighted (multiplicative random effects)
MR Egger
MR RAPS
Weighted median
Weighted mode
0.0025
0.0000
−0.0025
SNP effect on eGFR
0.25
0.50
0.75
SNP effect on Lactosylceramide 4−alpha−galactosyltransferase

Batch 2−3 : Alanine−−tRNA ligase, cytoplasmic on eGFR

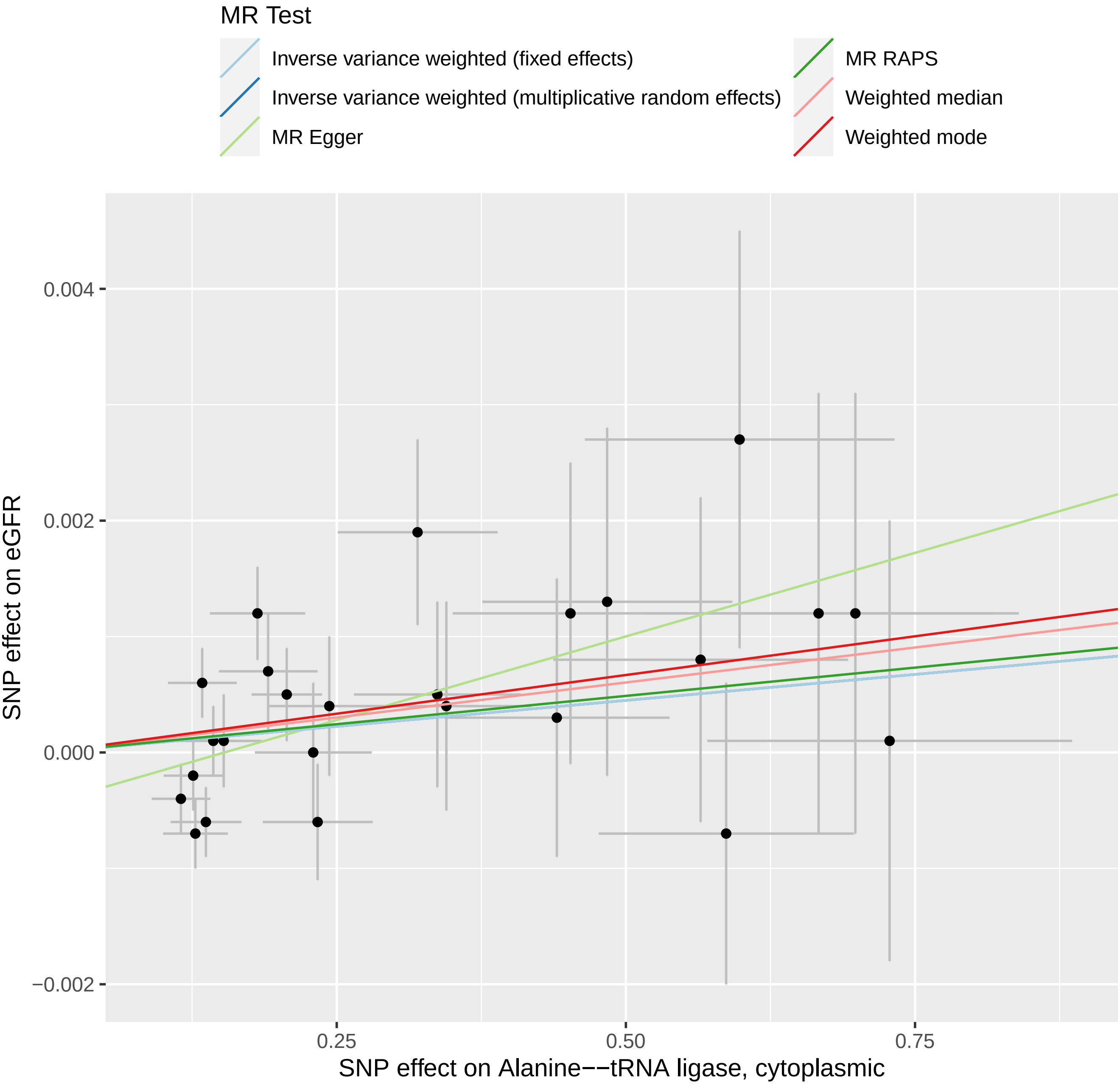

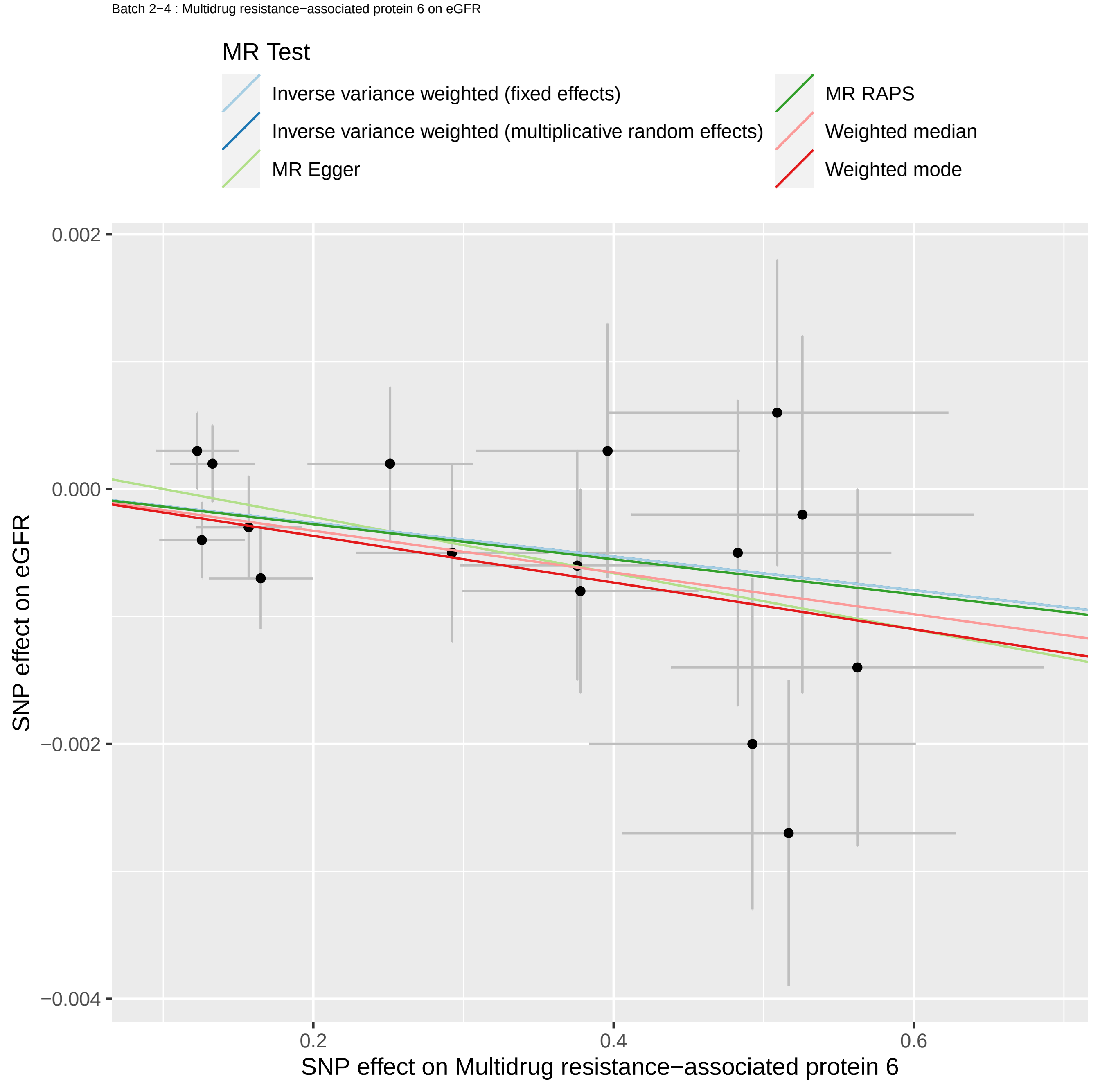

Batch 2−4 : Multidrug resistance−associated protein 6 on eGFR
MR Test
Inverse variance weighted (fixed effects)
Inverse variance weighted (multiplicative random effects)
MR Egger
MR RAPS
Weighted median
Weighted mode
0.002
0.000
−0.002
−0.004
0.2
0.4
0.6
SNP effect on eGFR
SNP effect on Multidrug resistance−associated protein 6

Batch 2−5 : Monoacylglycerol lipase ABHD12 on eGFR

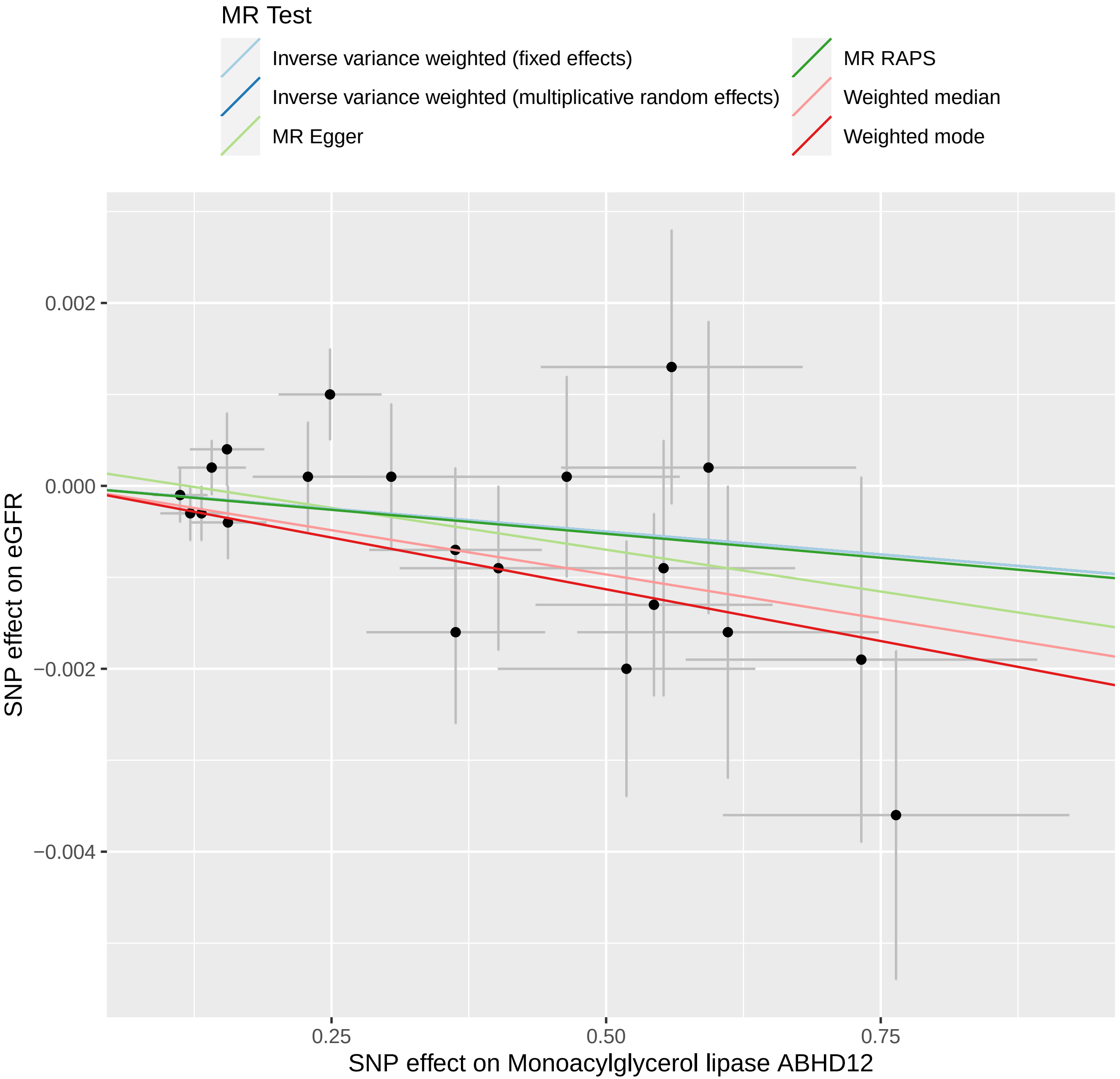

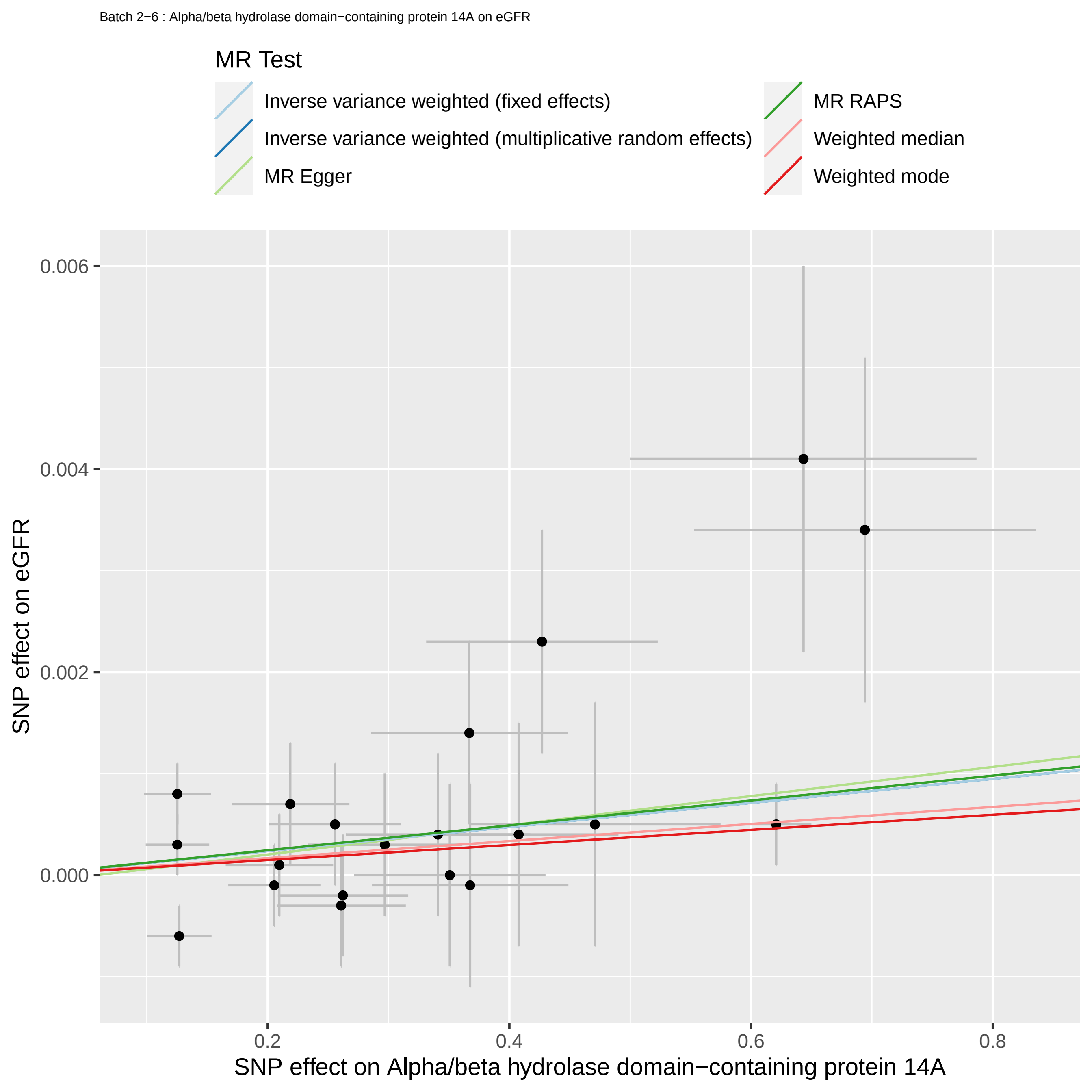
Batch 2−6 : Alpha/beta hydrolase domain−containing protein 14A on eGFR
MR Test
Inverse variance weighted (fixed effects)
Inverse variance weighted (multiplicative random effects)
MR Egger
MR RAPS
Weighted median
Weighted mode
0.006
0.004
0.002
0.000
SNP effect on eGFR
0.2
0.4
0.6
0.8
SNP effect on Alpha/beta hydrolase domain−containing protein 14A

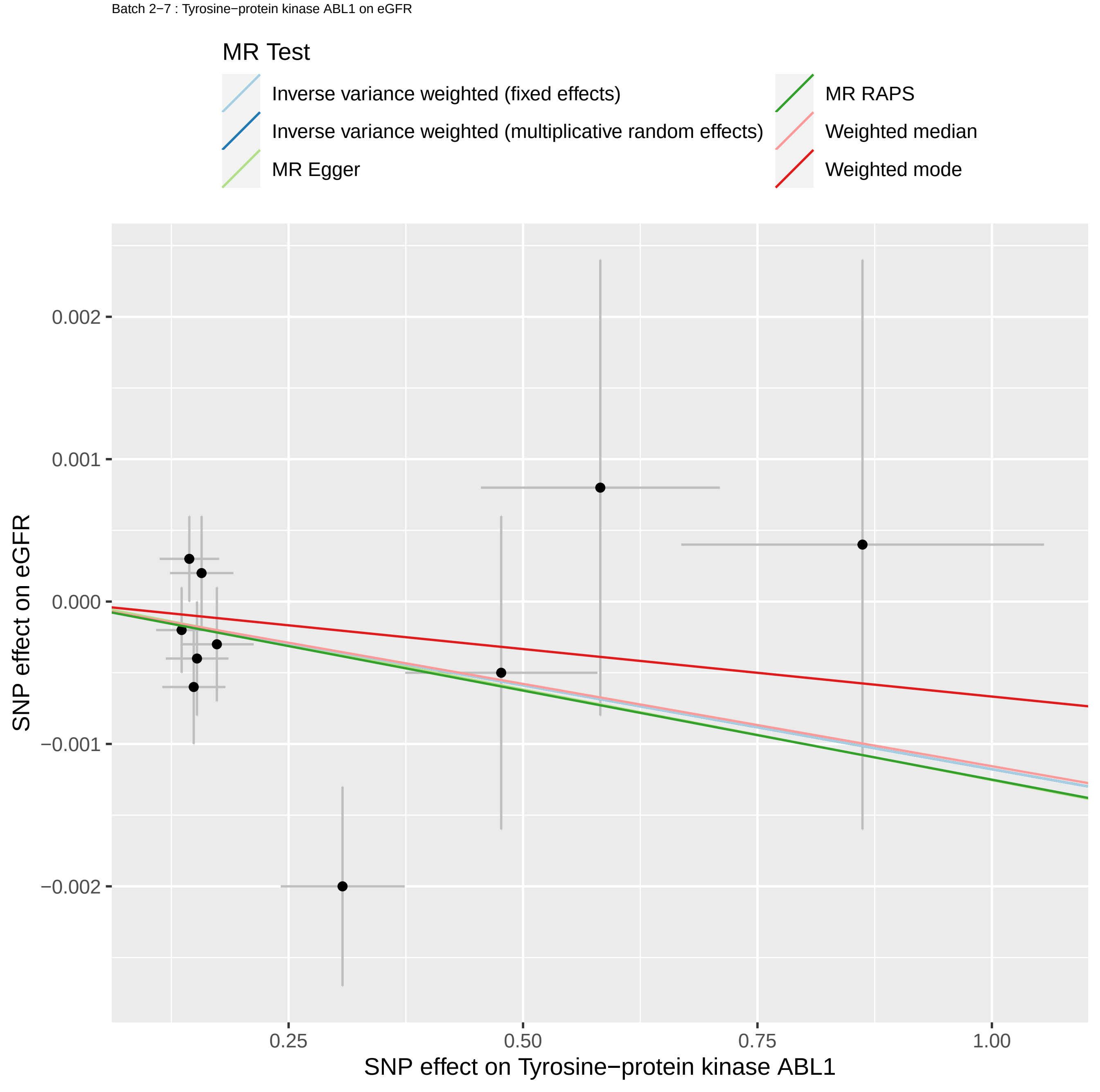

Batch 2−7 : Tyrosine−protein kinase ABL1 on eGFR
MR Test
Inverse variance weighted (fixed effects)
Inverse variance weighted (multiplicative random effects)
MR Egger
MR RAPS
Weighted median
Weighted mode
0.002
0.001
0.000
−0.001
−0.002
0.25
0.50
0.75
1.00
SNP effect on eGFR
SNP effect on Tyrosine−protein kinase ABL1

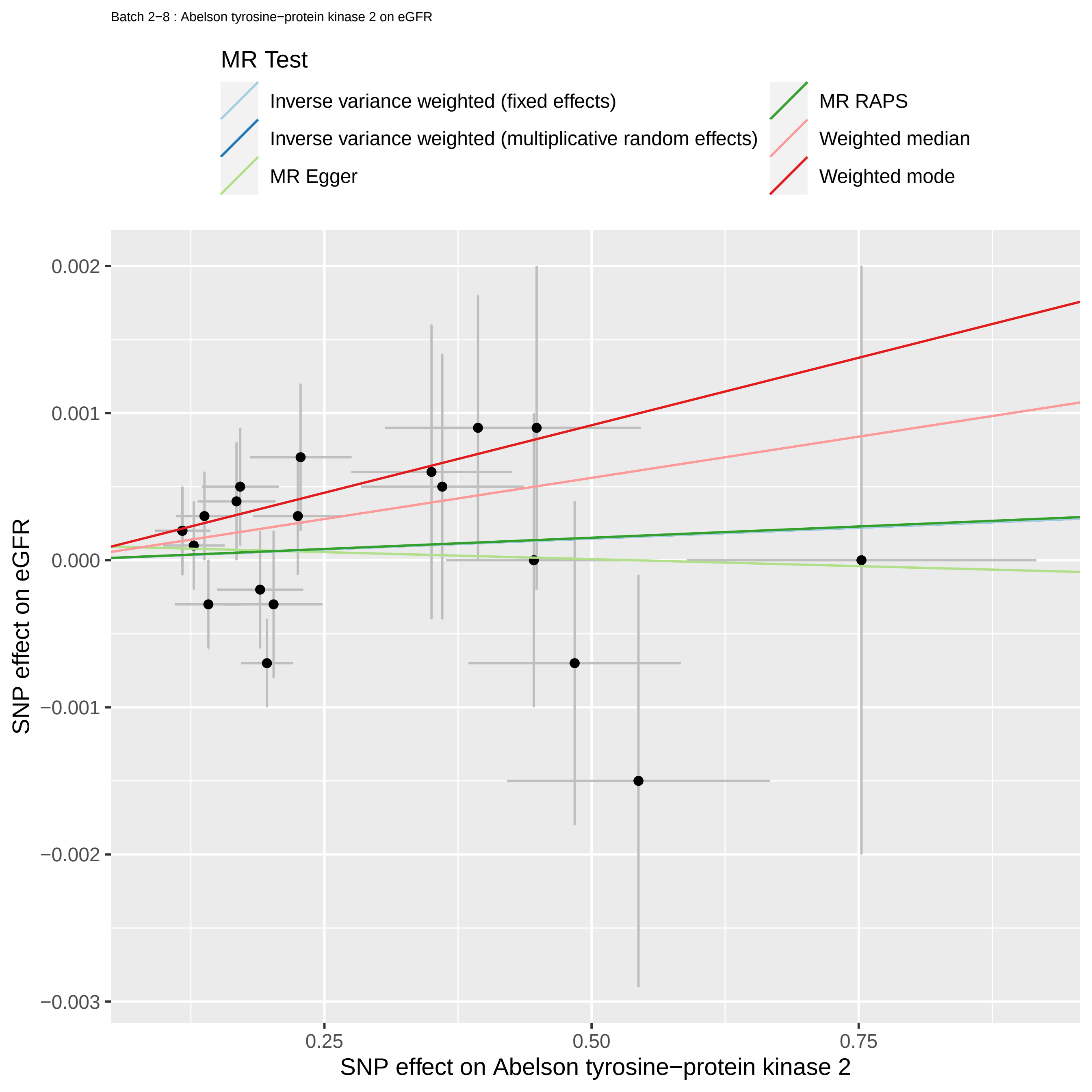

Batch 2−8 : Abelson tyrosine−protein kinase 2 on eGFR
MR Test
Inverse variance weighted (fixed effects)
Inverse variance weighted (multiplicative random effects)
MR Egger
MR RAPS
Weighted median
Weighted mode
0.002
0.001
0.000
−0.001
−0.002
−0.003
SNP effect on eGFR
0.25
0.50
0.75
SNP effect on Abelson tyrosine−protein kinase 2

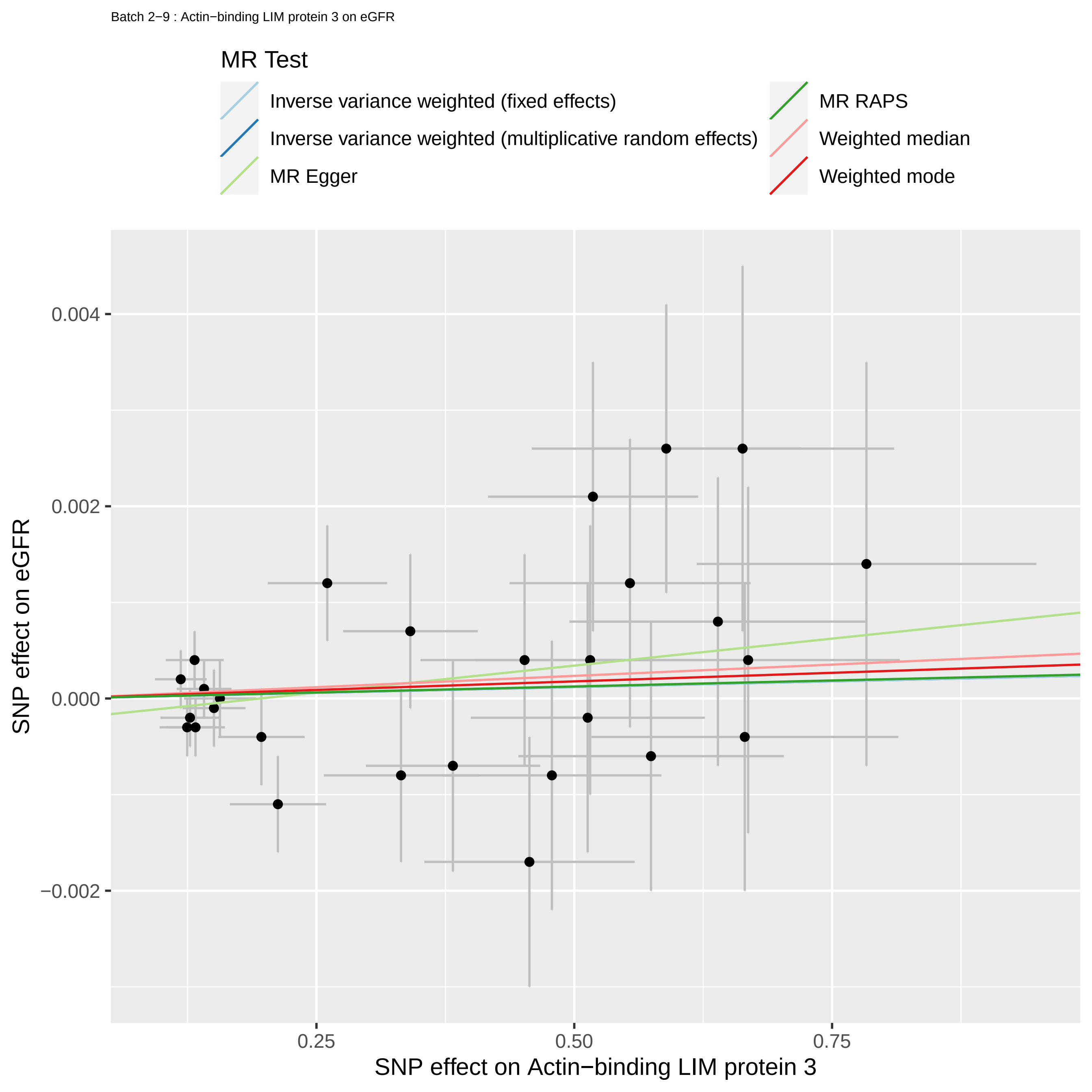

Batch 2−9 : Actin−binding LIM protein 3 on eGFR
MR Test
Inverse variance weighted (fixed effects)
Inverse variance weighted (multiplicative random effects)
MR Egger
MR RAPS
Weighted median
Weighted mode
SNP effect on eGFR
0.004
0.002
0.000
−0.002
0.25
0.50
0.75
SNP effect on Actin−binding LIM protein 3

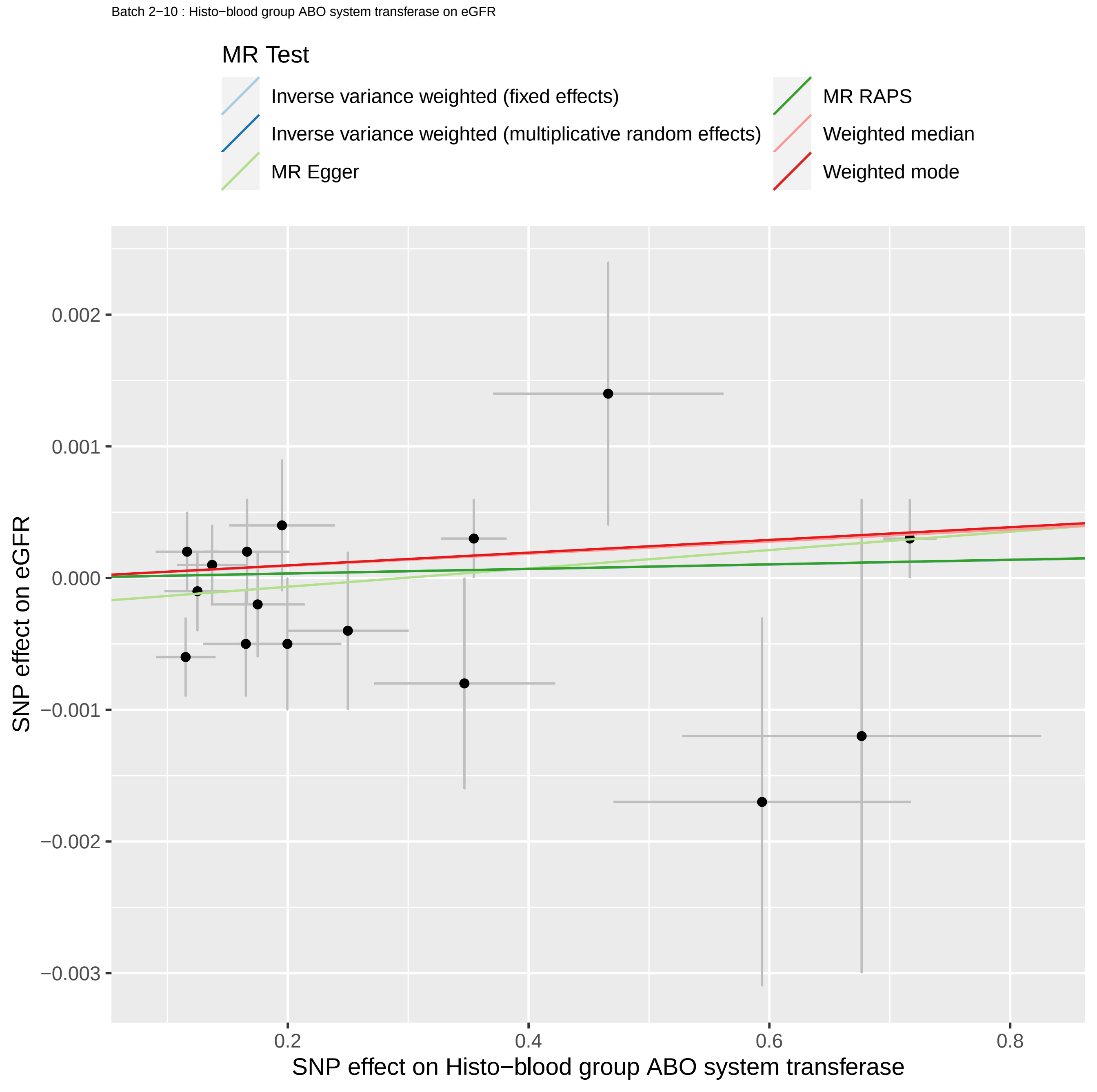
Batch 2−10 : Histo−blood group ABO system transferase on eGFR
MR Test
Inverse variance weighted (fixed effects)
Inverse variance weighted (multiplicative random effects)
MR Egger
MR RAPS
Weighted median
Weighted mode
0.002
0.001
0.000
−0.001
−0.002
−0.003
SNP effect on eGFR
0.2
0.4
0.6
0.8
SNP effect on Histo−blood group ABO system transferase

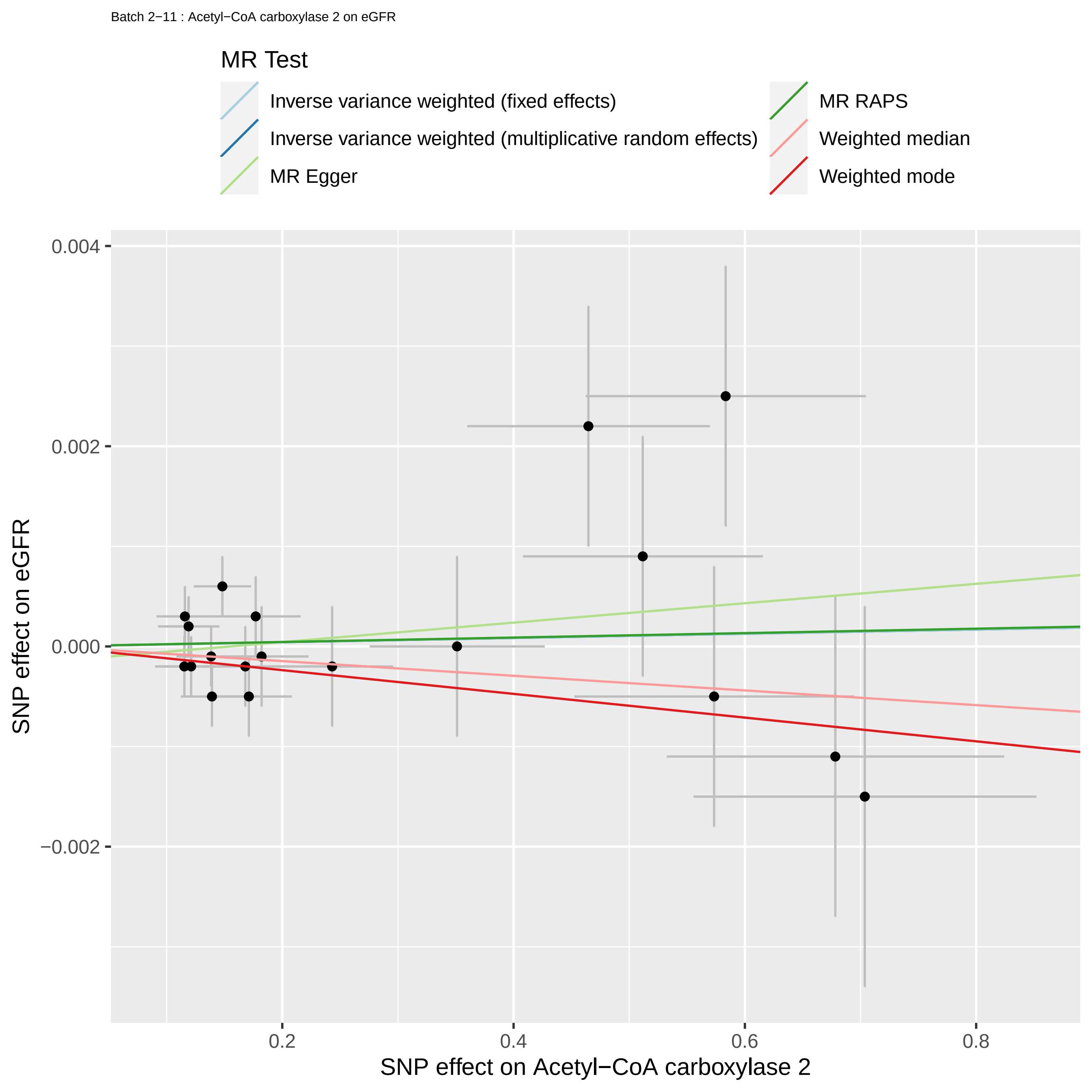

Batch 2−11 : Acetyl−CoA carboxylase 2 on eGFR
MR Test
Inverse variance weighted (fixed effects)
Inverse variance weighted (multiplicative random effects)
MR Egger
MR RAPS
Weighted median
Weighted mode
0.004
0.002
0.000
−0.002
0.2
0.4
0.6
0.8
SNP effect on eGFR
SNP effect on Acetyl−CoA carboxylase 2

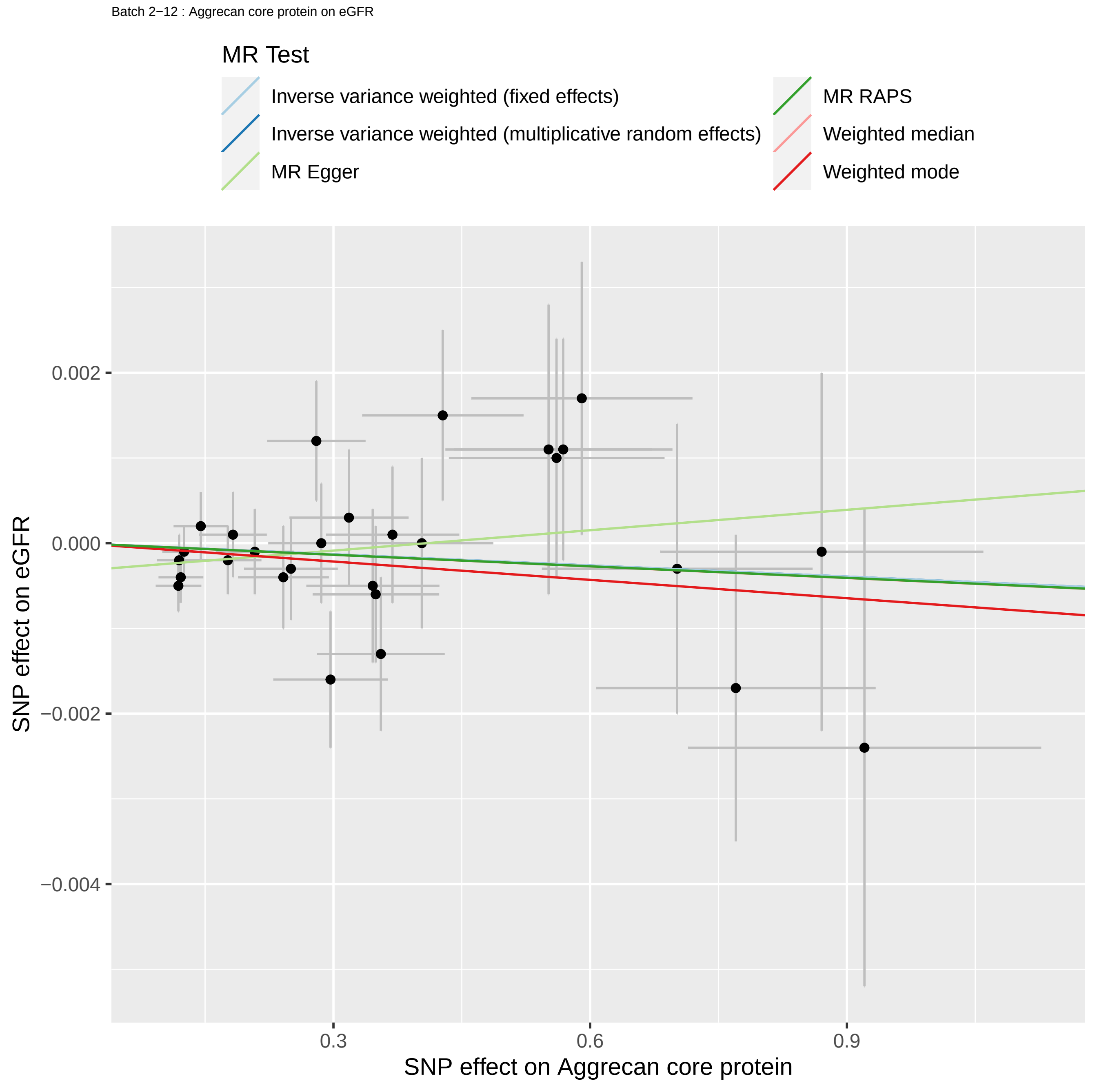

Batch 2−12 : Aggrecan core protein on eGFR
MR Test
Inverse variance weighted (fixed effects)
Inverse variance weighted (multiplicative random effects)
MR Egger
MR RAPS
Weighted median
Weighted mode
0.002
0.000
−0.002
−0.004
0.3
0.6
0.9
SNP effect on eGFR
SNP effect on Aggrecan core protein

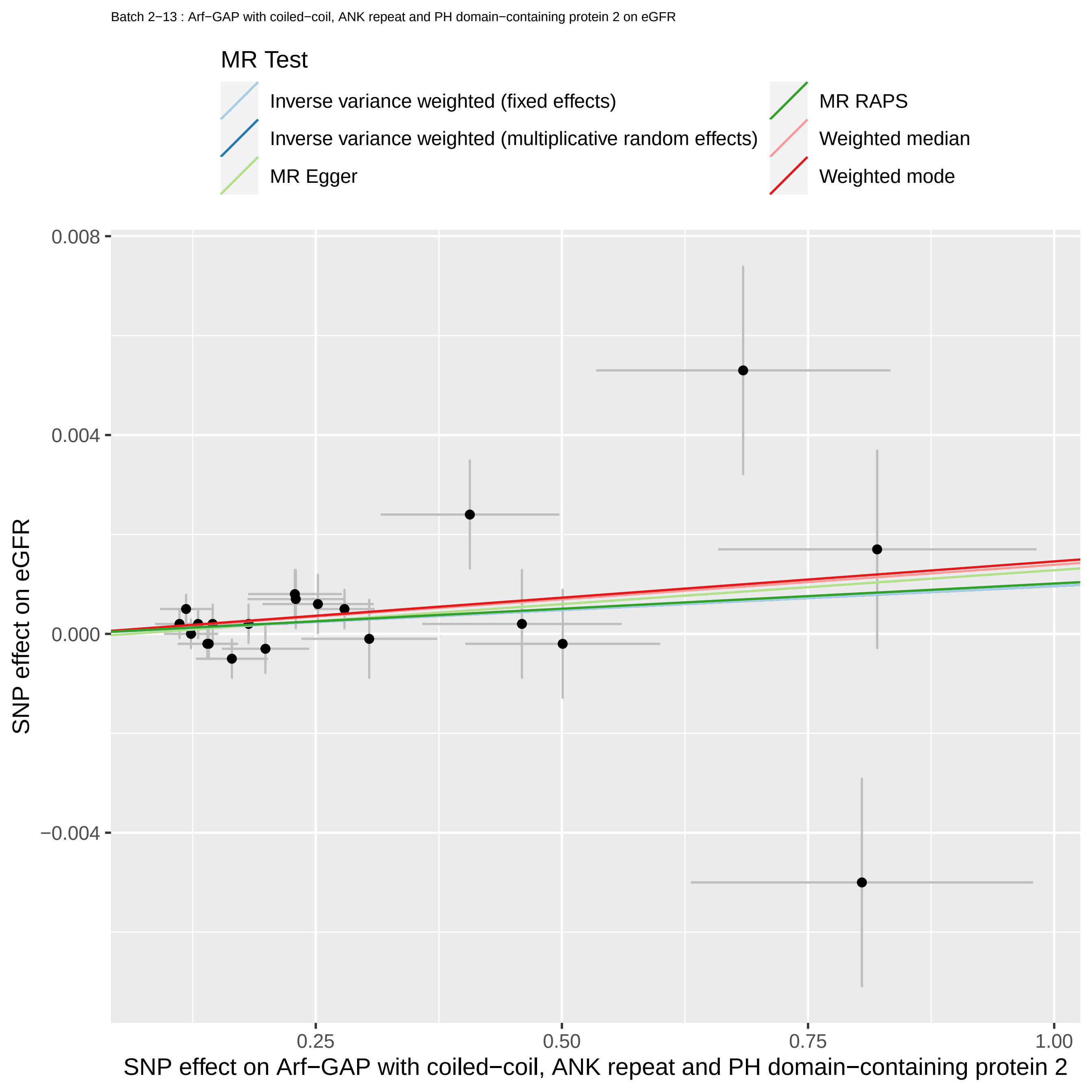

Batch 2−13 : Arf−GAP with coiled−coil, ANK repeat and PH domain−containing protein 2 on eGFR
MR Test
Inverse variance weighted (fixed effects)
Inverse variance weighted (multiplicative random effects)
MR Egger
MR RAPS
Weighted median
Weighted mode
0.008
0.004
0.000
−0.004
0.25
0.50
0.75
1.00
SNP effect on eGFR
SNP effect on Arf−GAP with coiled−coil, ANK repeat and PH domain−containing protein 2

Batch 2−14 : Acyl−CoA−binding domain−containing protein 6 on eGFR

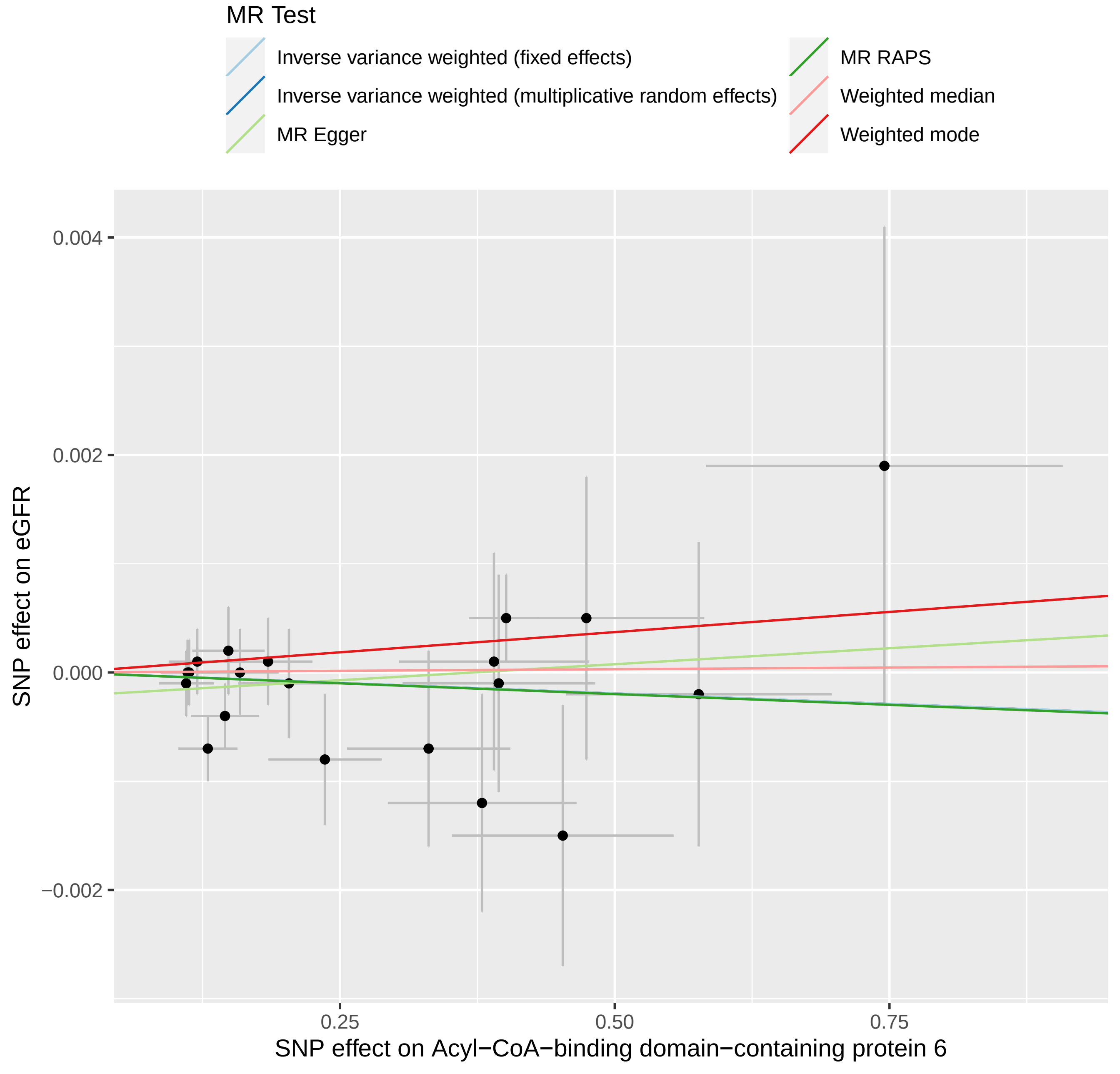

Batch 2−15 : Acyl−CoA−binding domain−containing protein 7 on eGFR

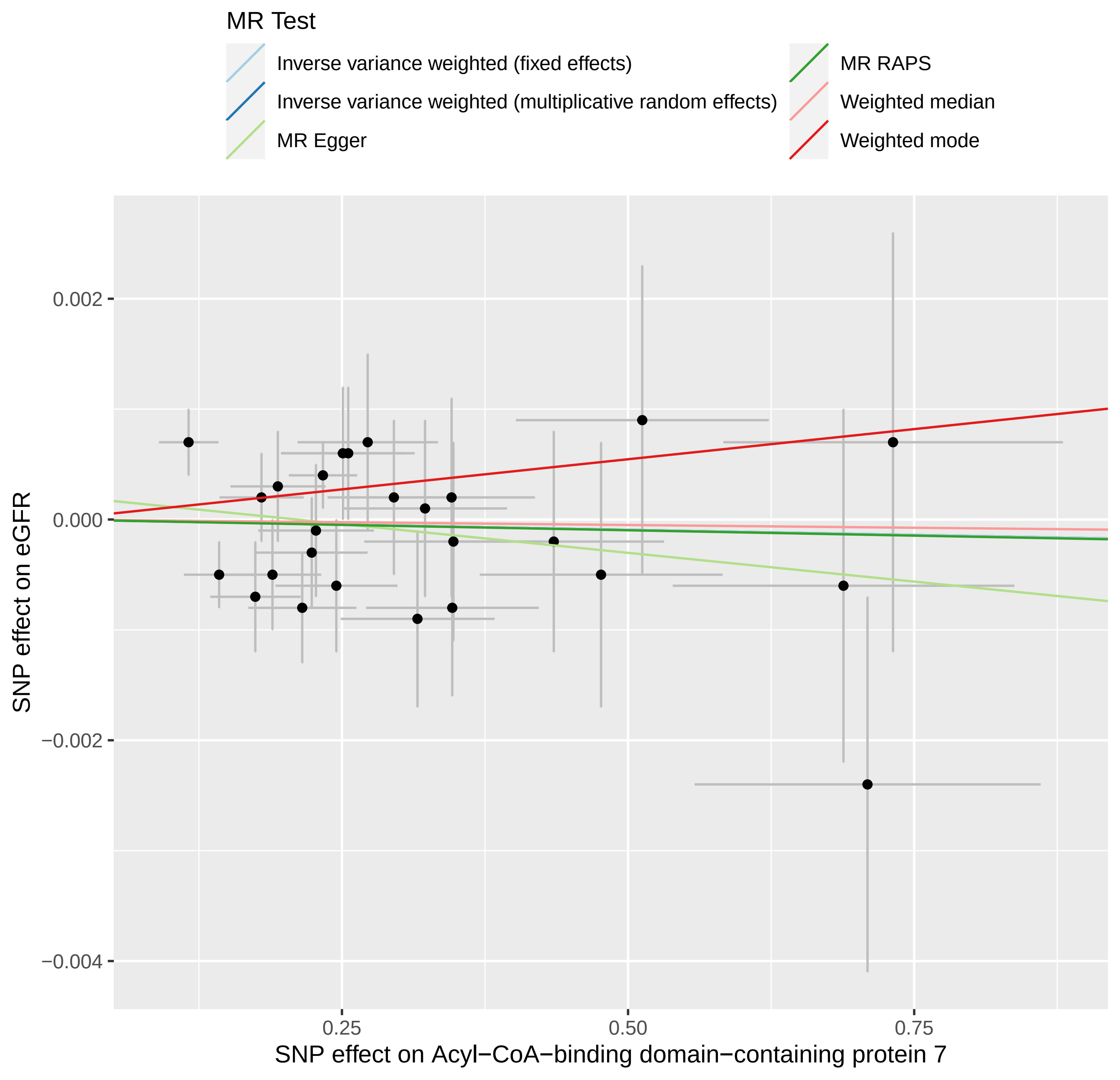

Batch 2−16 : Angiotensin−converting enzyme 2 on eGFR

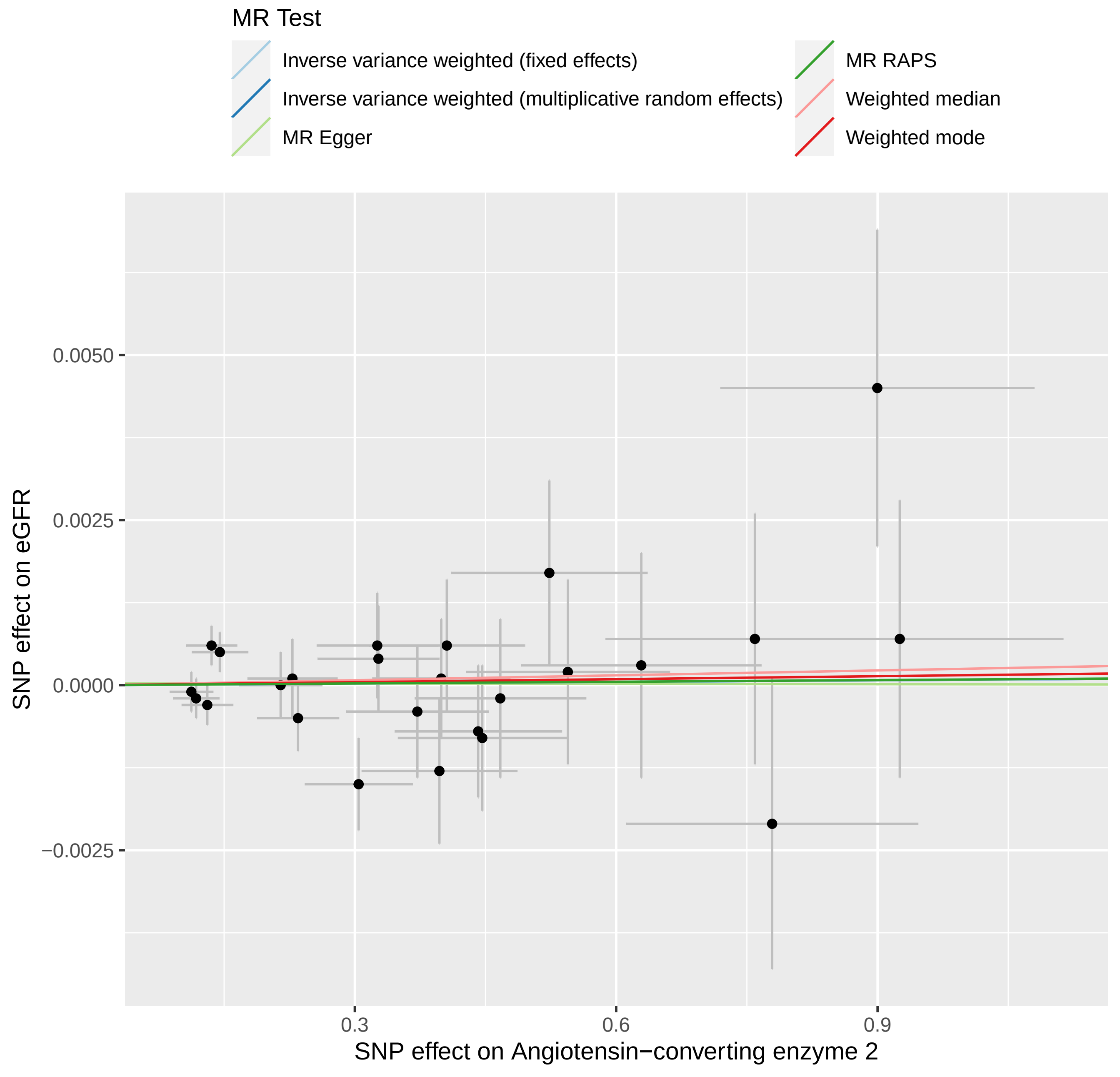

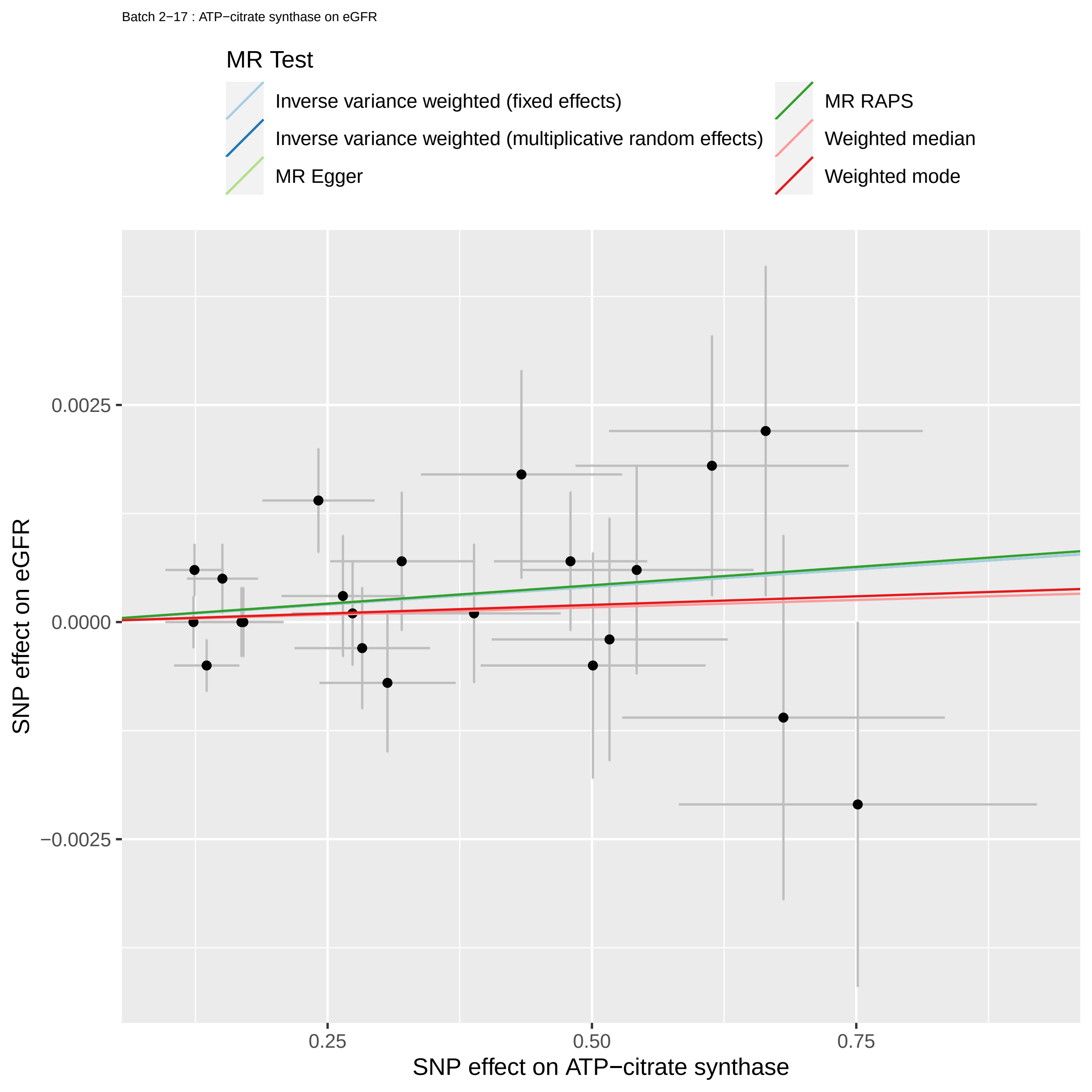

Batch 2−17 : ATP−citrate synthase on eGFR
MR Test
Inverse variance weighted (fixed effects)
Inverse variance weighted (multiplicative random effects)
MR Egger
MR RAPS
Weighted median
Weighted mode
0.0025
0.0000
−0.0025
0.25
0.50
0.75
SNP effect on eGFR
SNP effect on ATP−citrate synthase

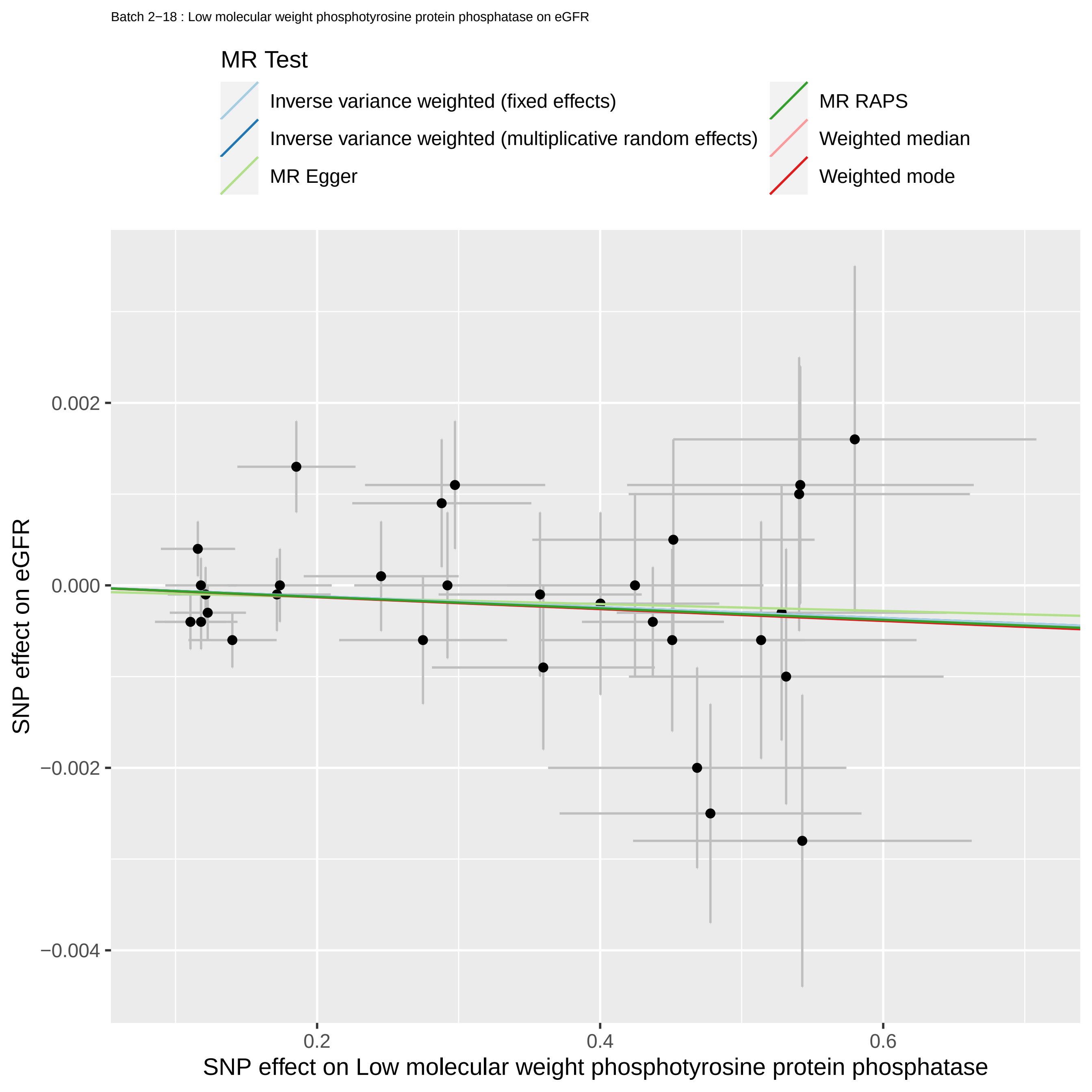
Batch 2−18 : Low molecular weight phosphotyrosine protein phosphatase on eGFR
MR Test
Inverse variance weighted (fixed effects)
Inverse variance weighted (multiplicative random effects)
MR Egger
MR RAPS
Weighted median
Weighted mode
0.002
0.000
−0.002
−0.004
0.2
0.4
0.6
SNP effect on eGFR
SNP effect on Low molecular weight phosphotyrosine protein phosphatase

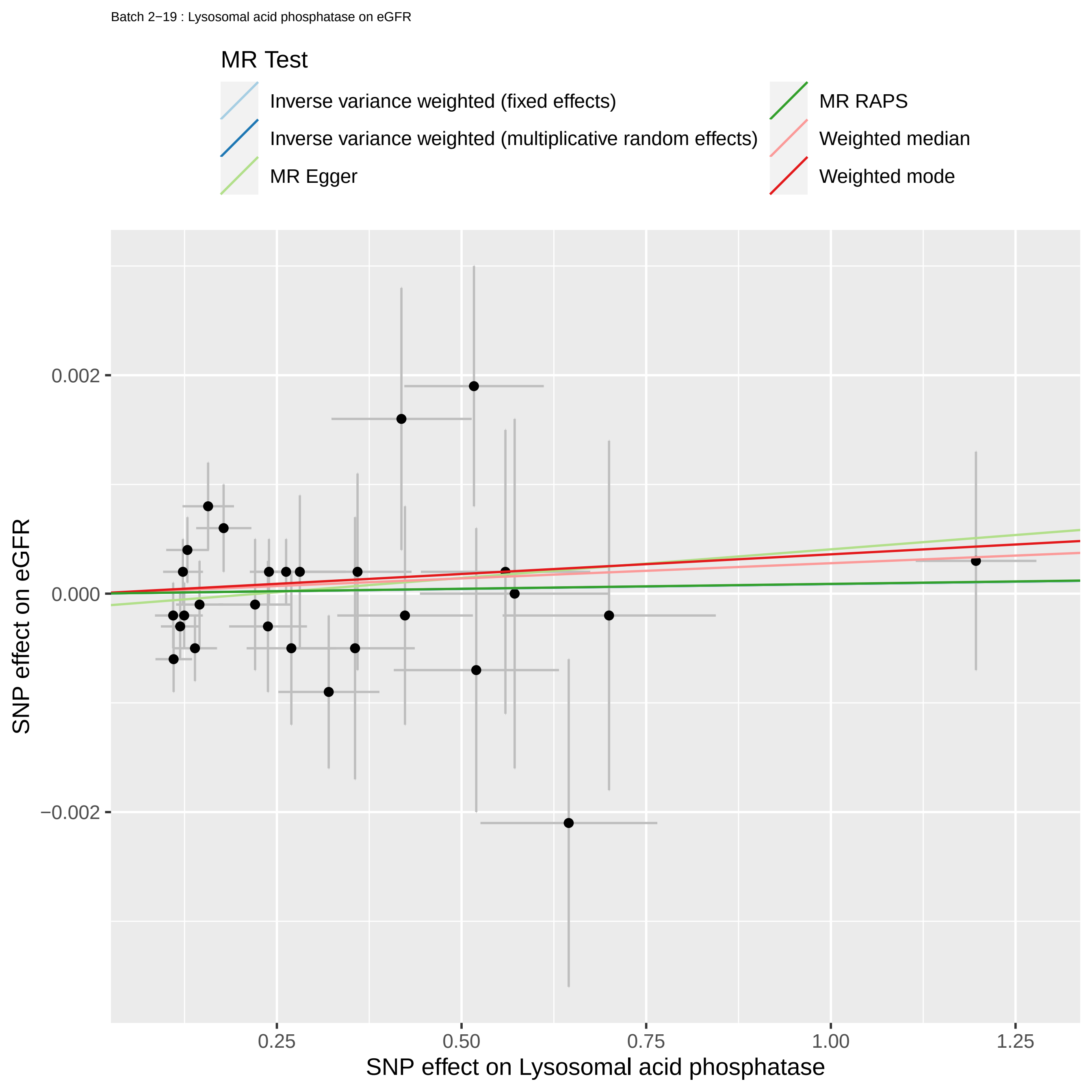

Batch 2−19 : Lysosomal acid phosphatase on eGFR
MR Test
Inverse variance weighted (fixed effects)
Inverse variance weighted (multiplicative random effects)
MR Egger
MR RAPS
Weighted median
Weighted mode
0.002
0.000
−0.002
0.25
0.50
0.75
1.00
1.25
SNP effect on eGFR
SNP effect on Lysosomal acid phosphatase

Batch 2−20 : Tartrate−resistant acid phosphatase type 5 on eGFR

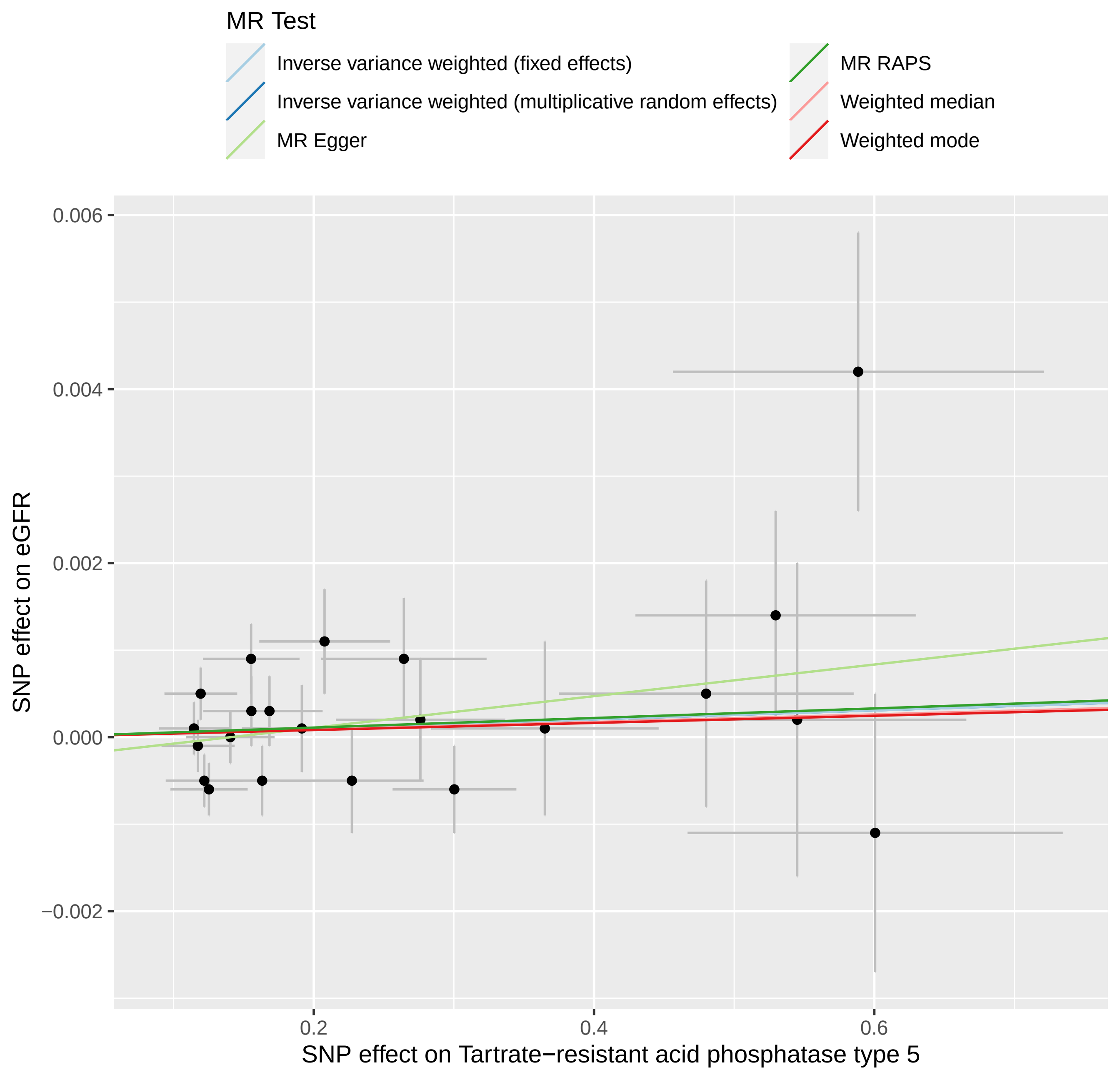

Batch 2−21 : 2−phosphoxylose phosphatase 1 on eGFR

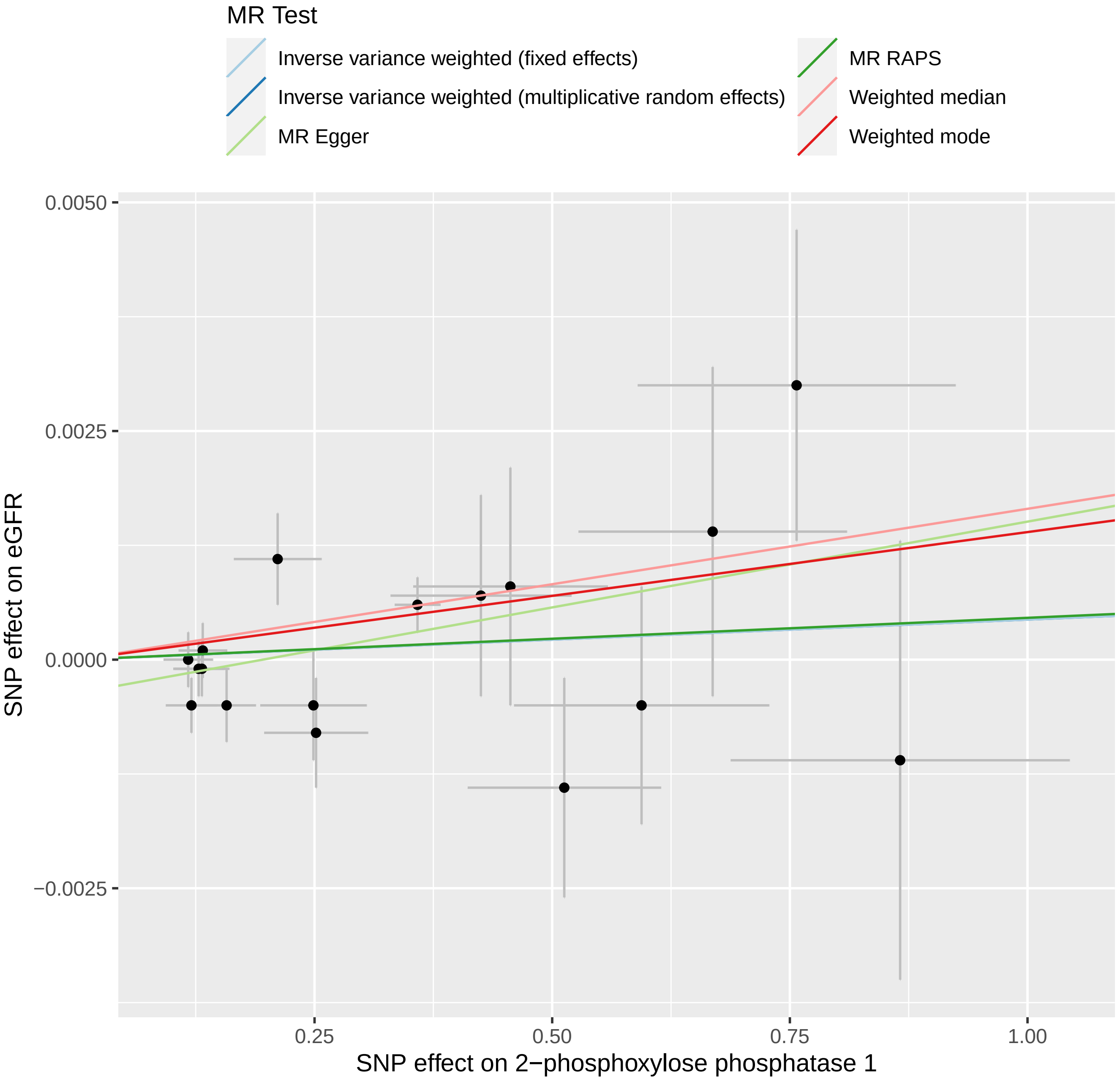

Batch 2−22 : Activin receptor type−1B on eGFR

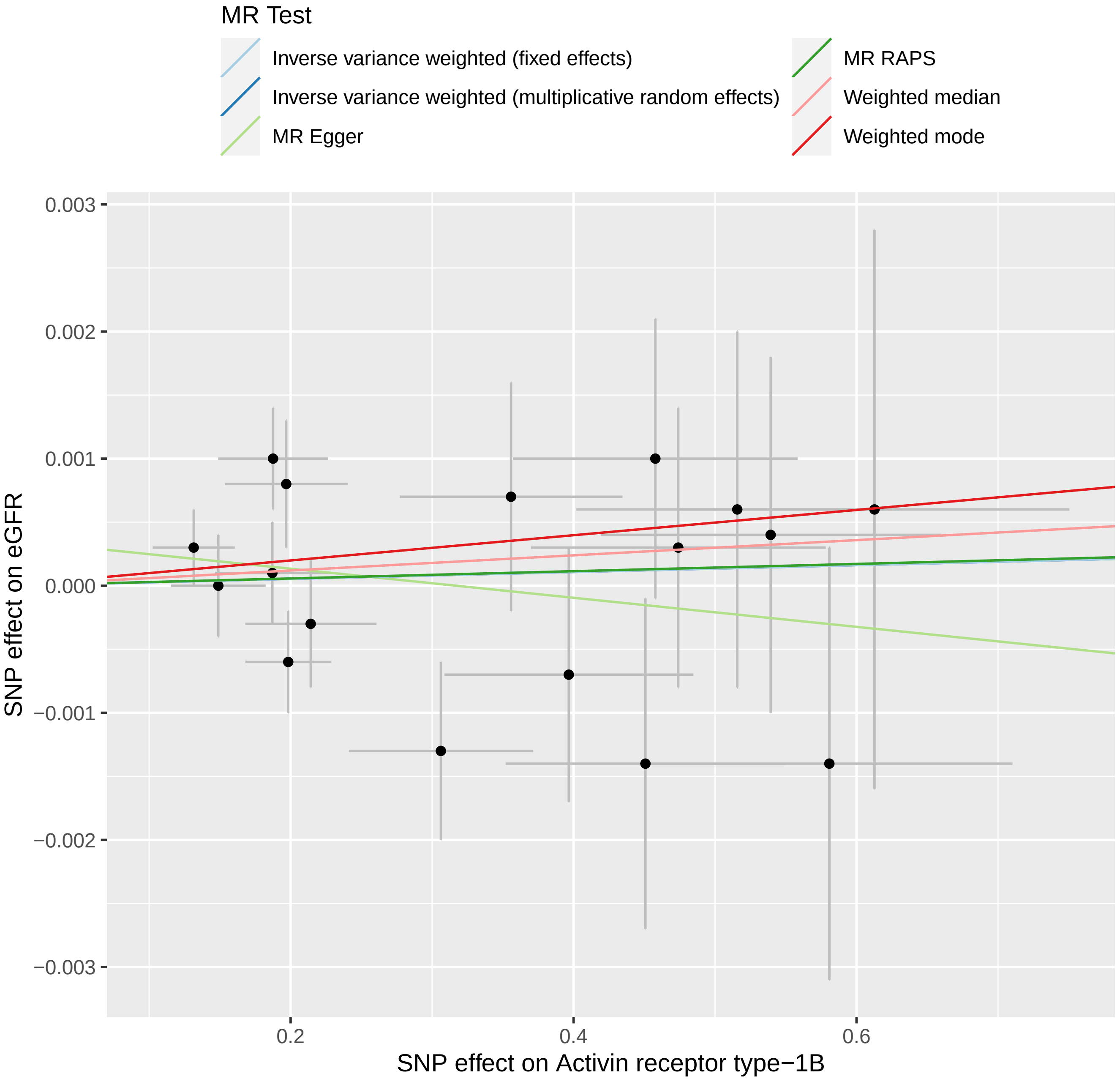

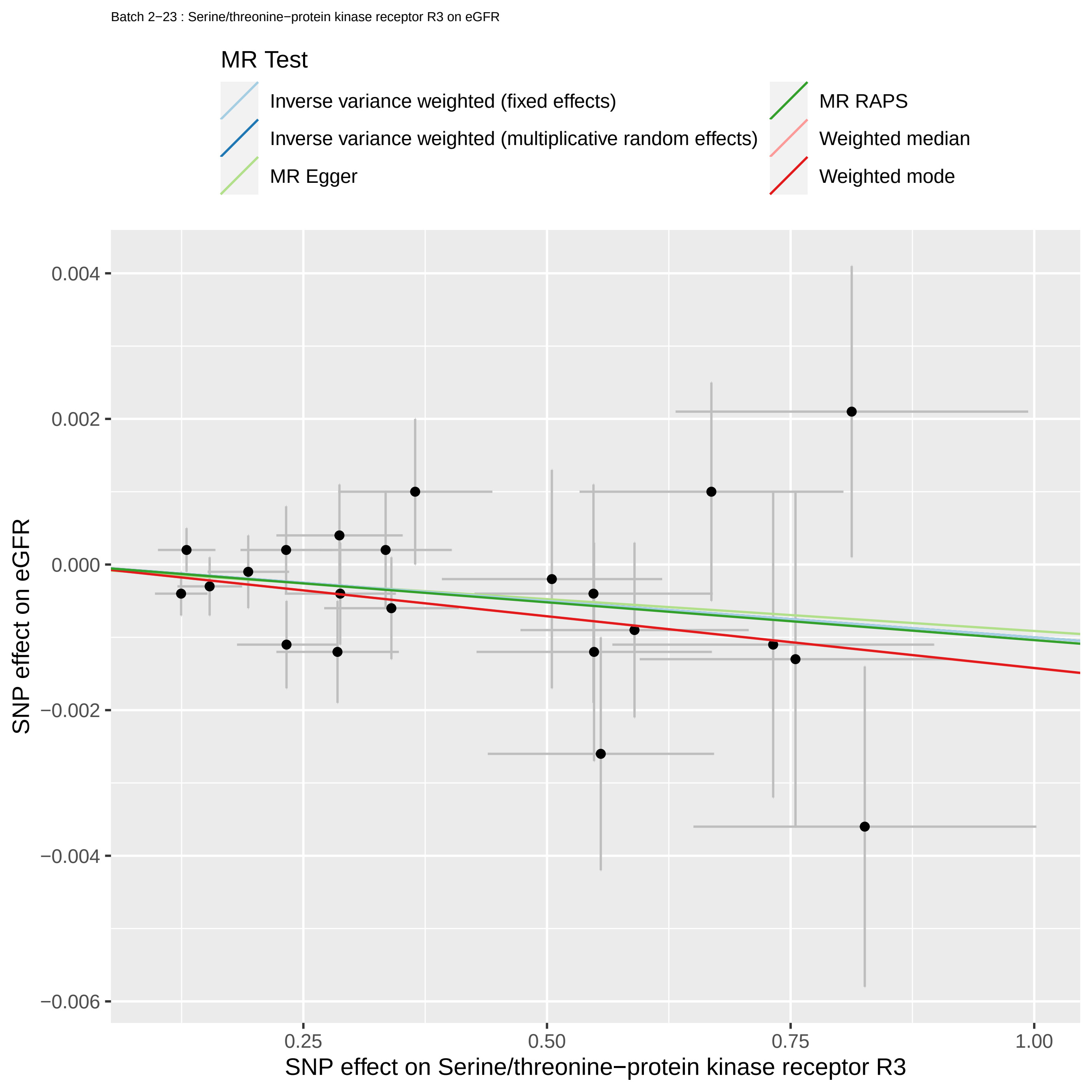
Batch 2−23 : Serine/threonine−protein kinase receptor R3 on eGFR
MR Test
Inverse variance weighted (fixed effects)
Inverse variance weighted (multiplicative random effects)
MR Egger
MR RAPS
Weighted median
Weighted mode
0.004
0.002
0.000
−0.002
−0.004
−0.006
0.25
0.50
0.75
1.00
SNP effect on eGFR
SNP effect on Serine/threonine−protein kinase receptor R3

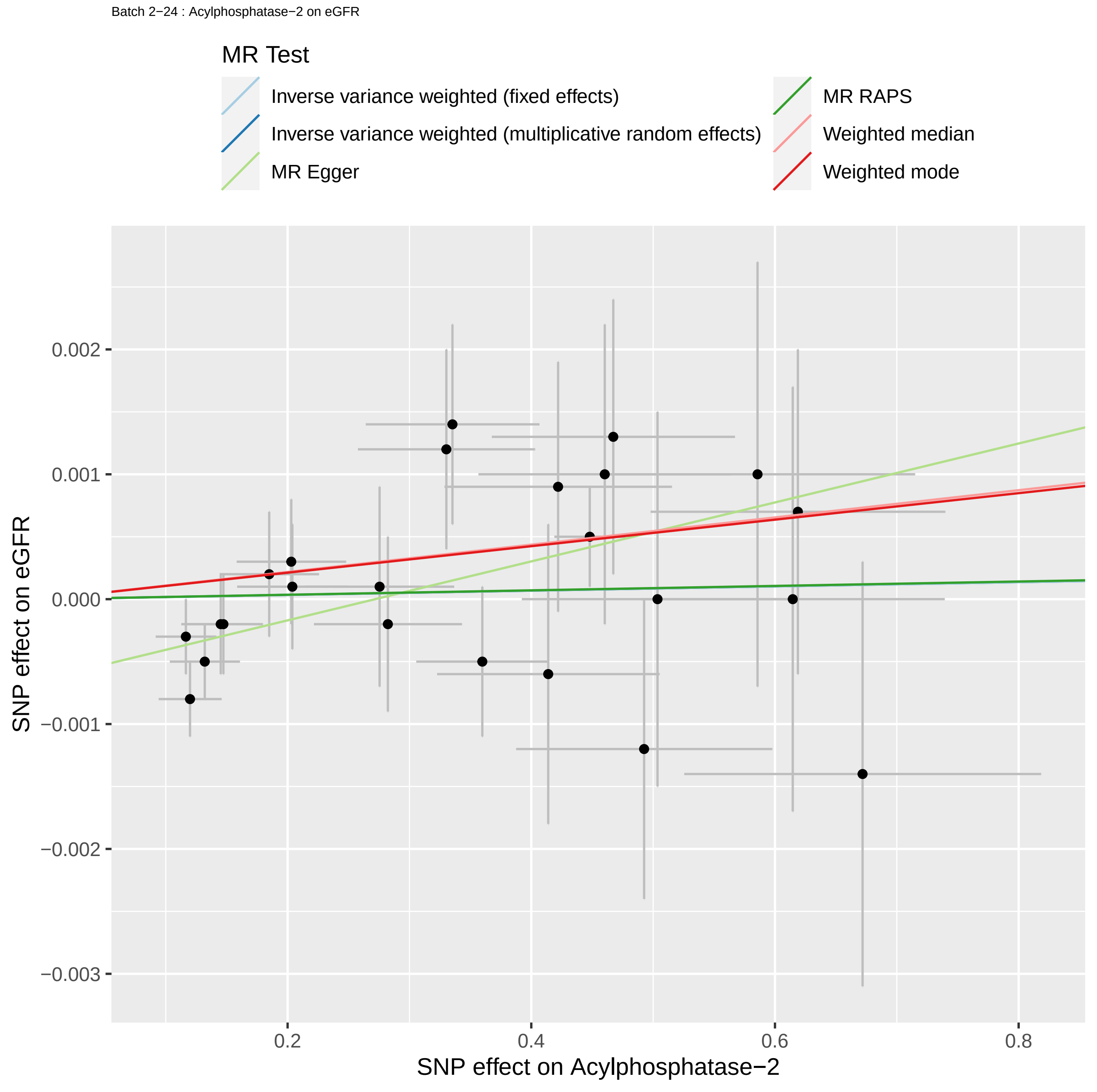

Batch 2−24 : Acylphosphatase−2 on eGFR
MR Test
Inverse variance weighted (fixed effects)
Inverse variance weighted (multiplicative random effects)
MR Egger
MR RAPS
Weighted median
Weighted mode
0.002
0.001
0.000
−0.001
−0.002
−0.003
0.2
0.4
0.6
0.8
SNP effect on eGFR
SNP effect on Acylphosphatase−2

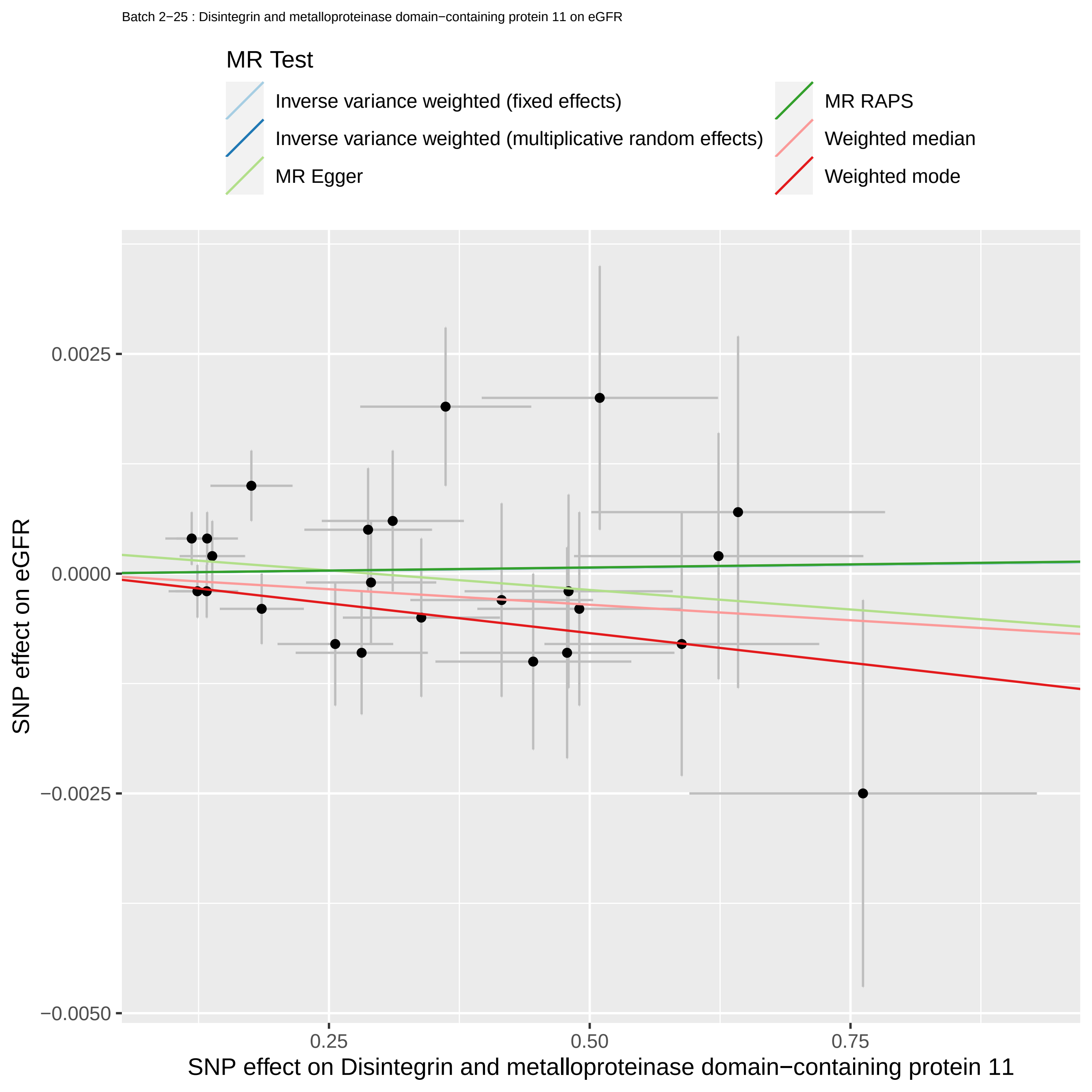

Batch 2−25 : Disintegrin and metalloproteinase domain−containing protein 11 on eGFR
MR Test
Inverse variance weighted (fixed effects)
Inverse variance weighted (multiplicative random effects)
MR Egger
MR RAPS
Weighted median
Weighted mode
0.0025
0.0000
−0.0025
−0.0050
0.25
0.50
0.75
SNP effect on eGFR
SNP effect on Disintegrin and metalloproteinase domain−containing protein 11

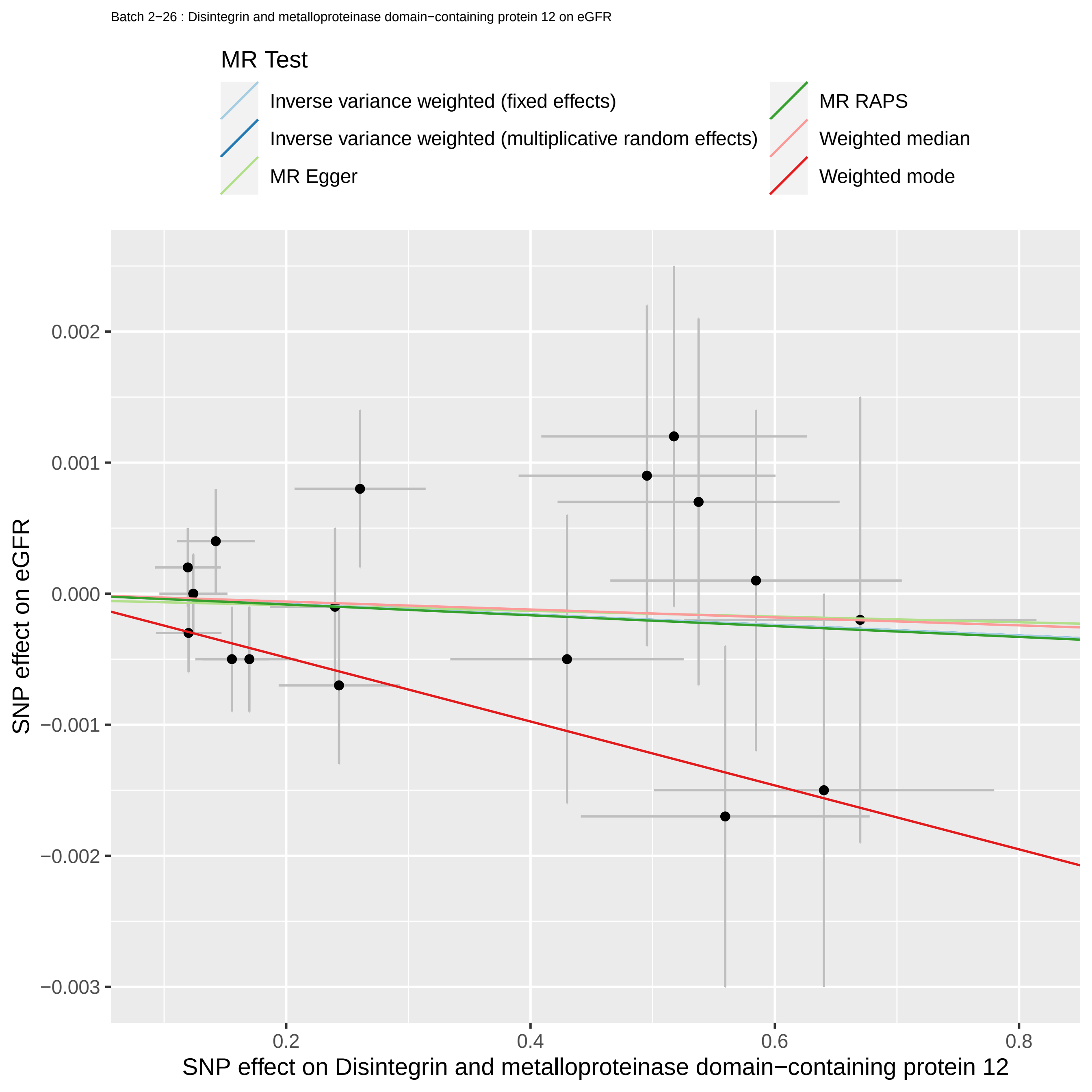

Batch 2−26 : Disintegrin and metalloproteinase domain−containing protein 12 on eGFR
MR Test
Inverse variance weighted (fixed effects)
Inverse variance weighted (multiplicative random effects)
MR Egger
MR RAPS
Weighted median
Weighted mode
0.002
0.001
0.000
−0.001
−0.002
−0.003
0.2
0.4
0.6
0.8
SNP effect on eGFR
SNP effect on Disintegrin and metalloproteinase domain−containing protein 12

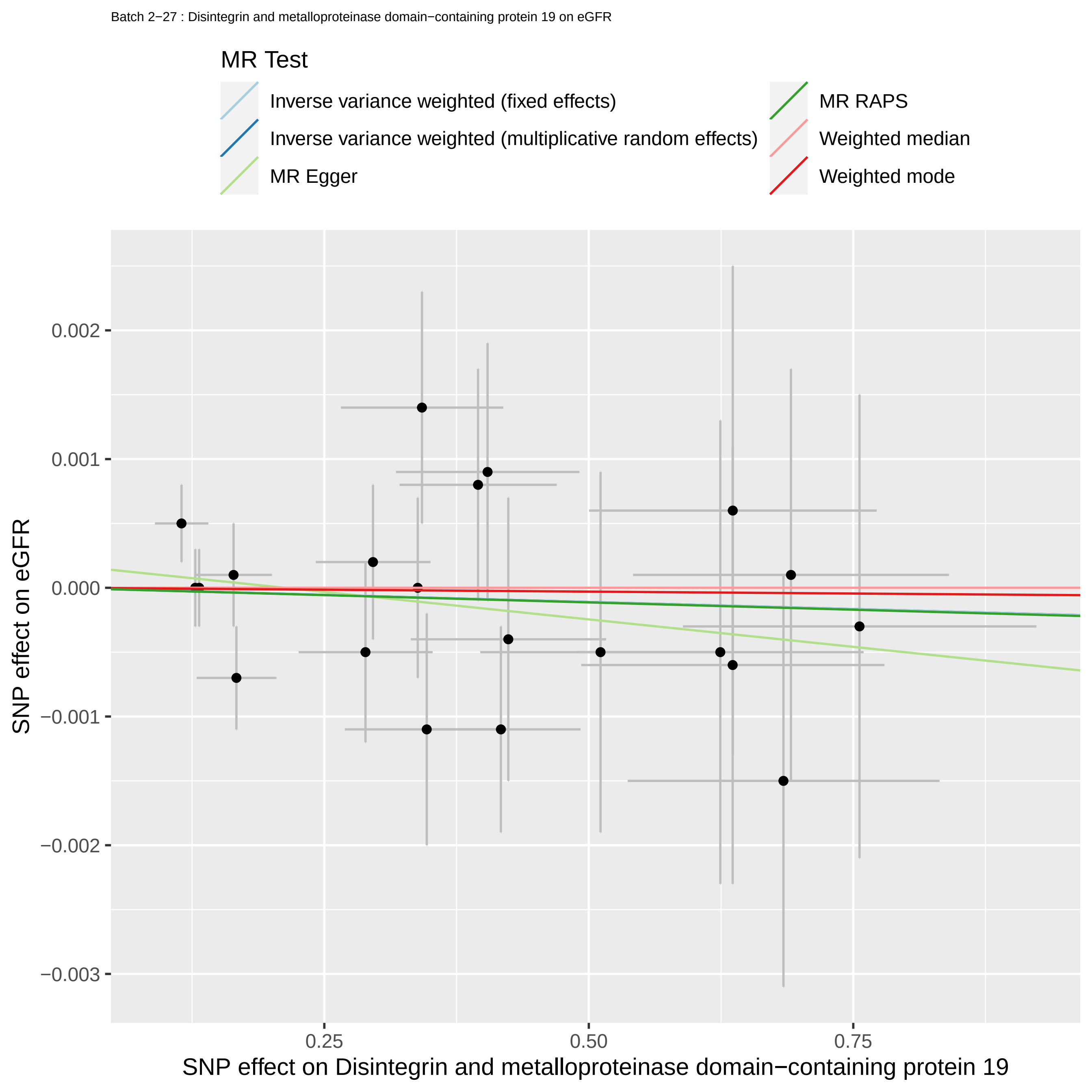
Batch 2−27 : Disintegrin and metalloproteinase domain−containing protein 19 on eGFR
MR Test
Inverse variance weighted (fixed effects)
Inverse variance weighted (multiplicative random effects)
MR Egger
MR RAPS
Weighted median
Weighted mode
0.002
0.001
0.000
−0.001
−0.002
−0.003
0.25
0.50
0.75
SNP effect on eGFR
SNP effect on Disintegrin and metalloproteinase domain−containing protein 19

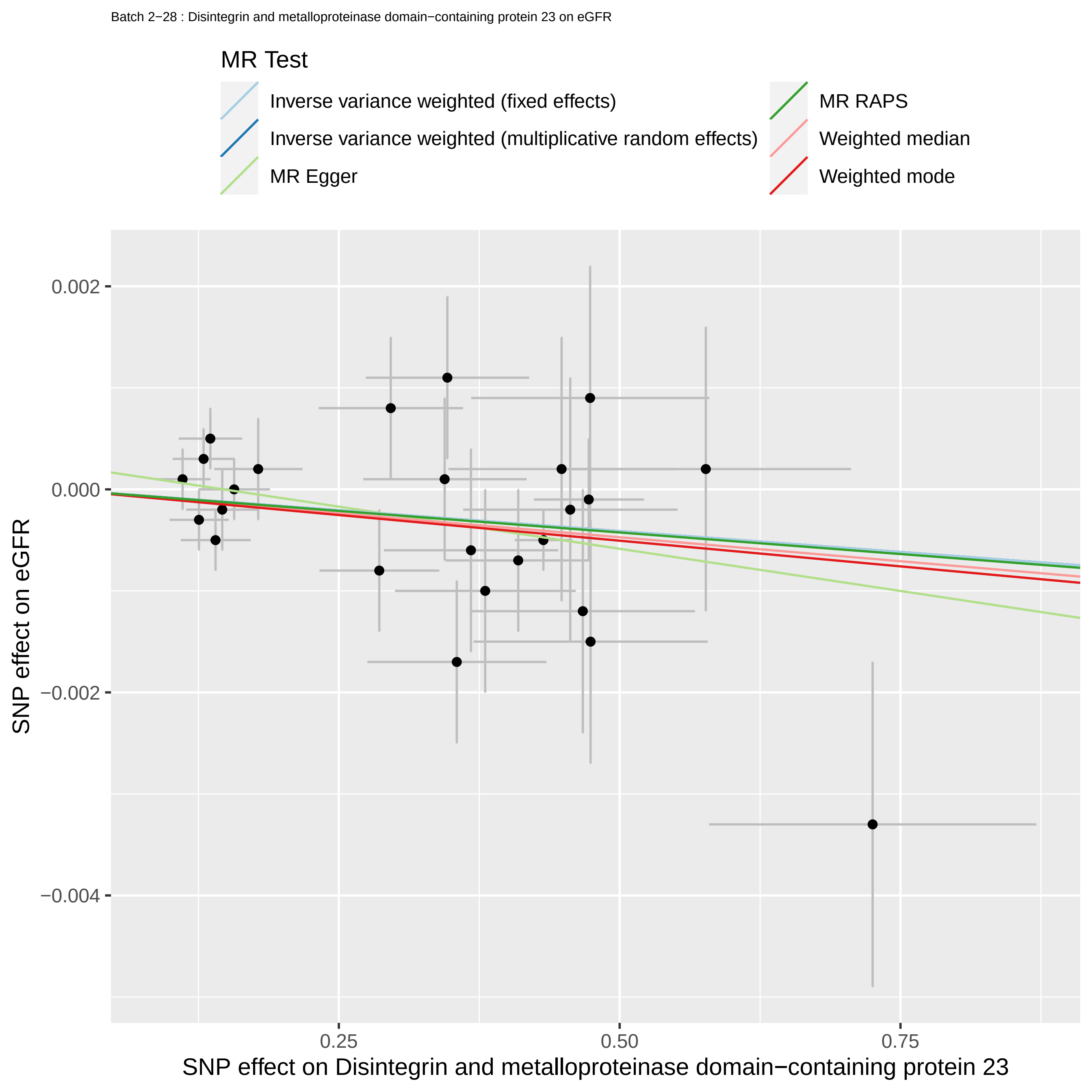

Batch 2−28 : Disintegrin and metalloproteinase domain−containing protein 23 on eGFR
MR Test
Inverse variance weighted (fixed effects)
Inverse variance weighted (multiplicative random effects)
MR Egger
MR RAPS
Weighted median
Weighted mode
0.002
0.000
−0.002
−0.004
0.25
0.50
0.75
SNP effect on eGFR
SNP effect on Disintegrin and metalloproteinase domain−containing protein 23

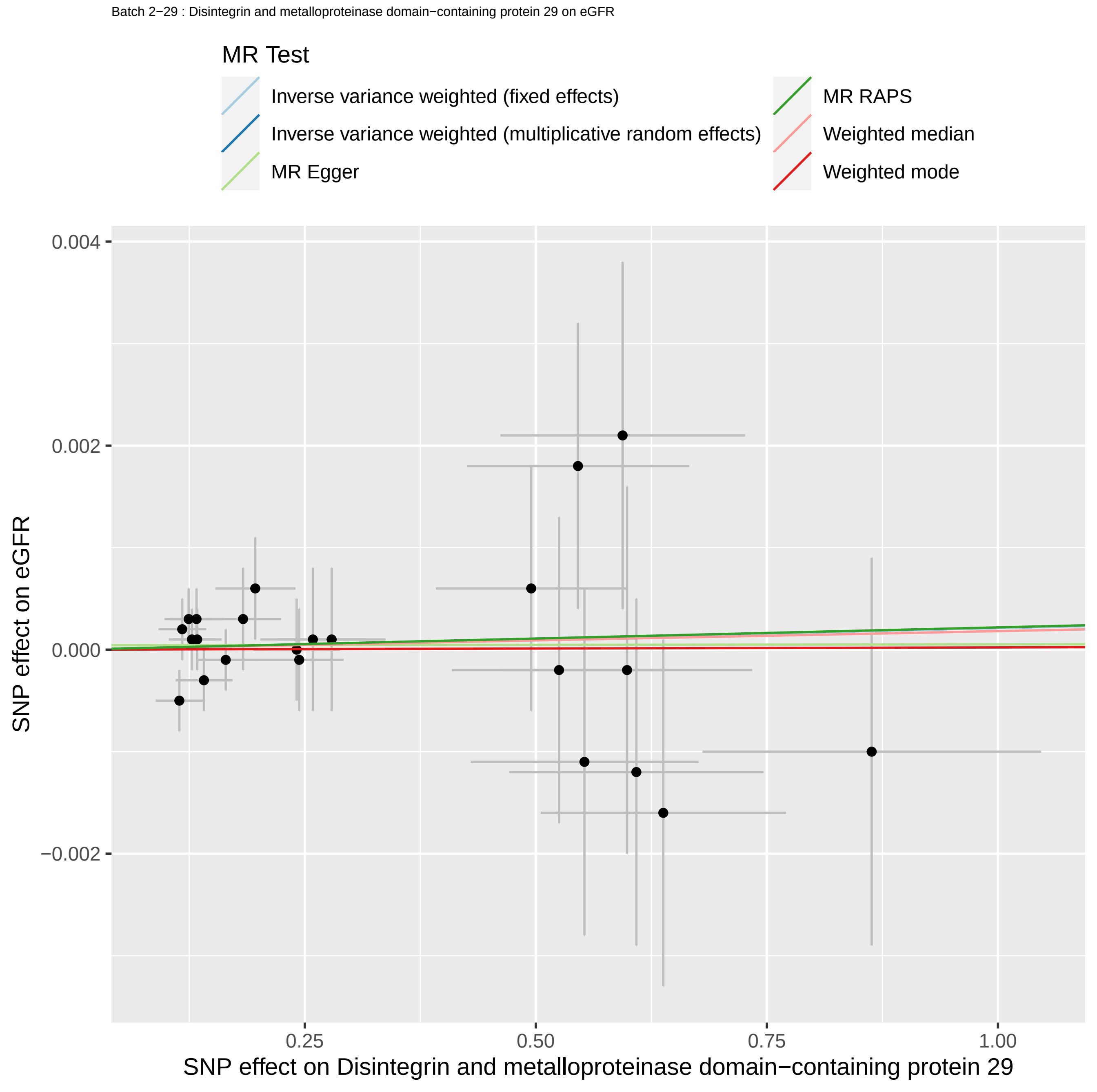
Batch 2−29 : Disintegrin and metalloproteinase domain−containing protein 29 on eGFR
MR Test
Inverse variance weighted (fixed effects)
Inverse variance weighted (multiplicative random effects)
MR Egger
MR RAPS
Weighted median
Weighted mode
0.004
0.002
0.000
−0.002
0.25
0.50
0.75
1.00
SNP effect on eGFR
SNP effect on Disintegrin and metalloproteinase domain−containing protein 29

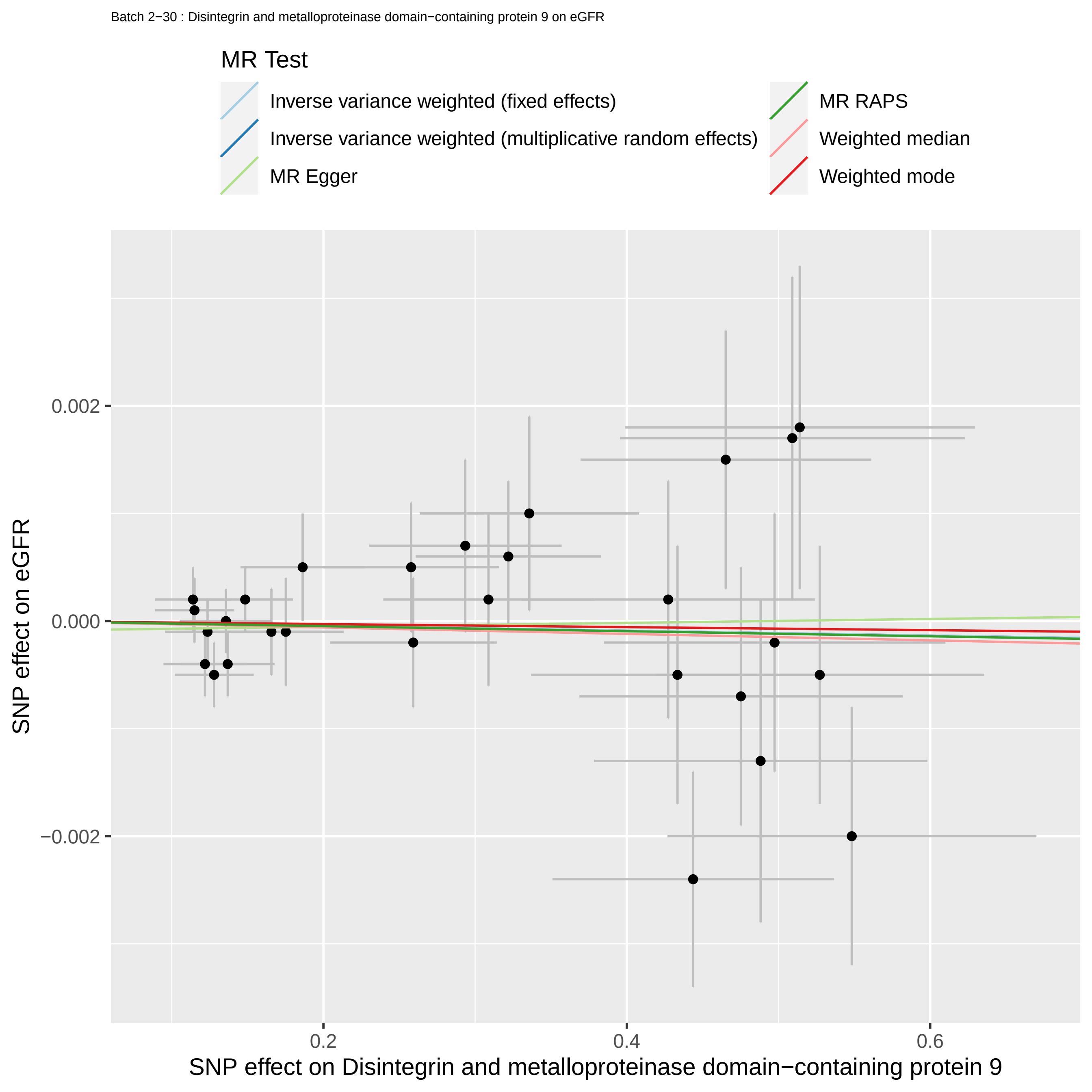

Batch 2−30 : Disintegrin and metalloproteinase domain−containing protein 9 on eGFR
MR Test
Inverse variance weighted (fixed effects)
Inverse variance weighted (multiplicative random effects)
MR Egger
MR RAPS
Weighted median
Weighted mode
0.002
0.000
−0.002
0.2
0.4
0.6
SNP effect on eGFR
SNP effect on Disintegrin and metalloproteinase domain−containing protein 9

Batch 2−31 : A disintegrin and metalloproteinase with thrombospondin motifs 1 on eGFR

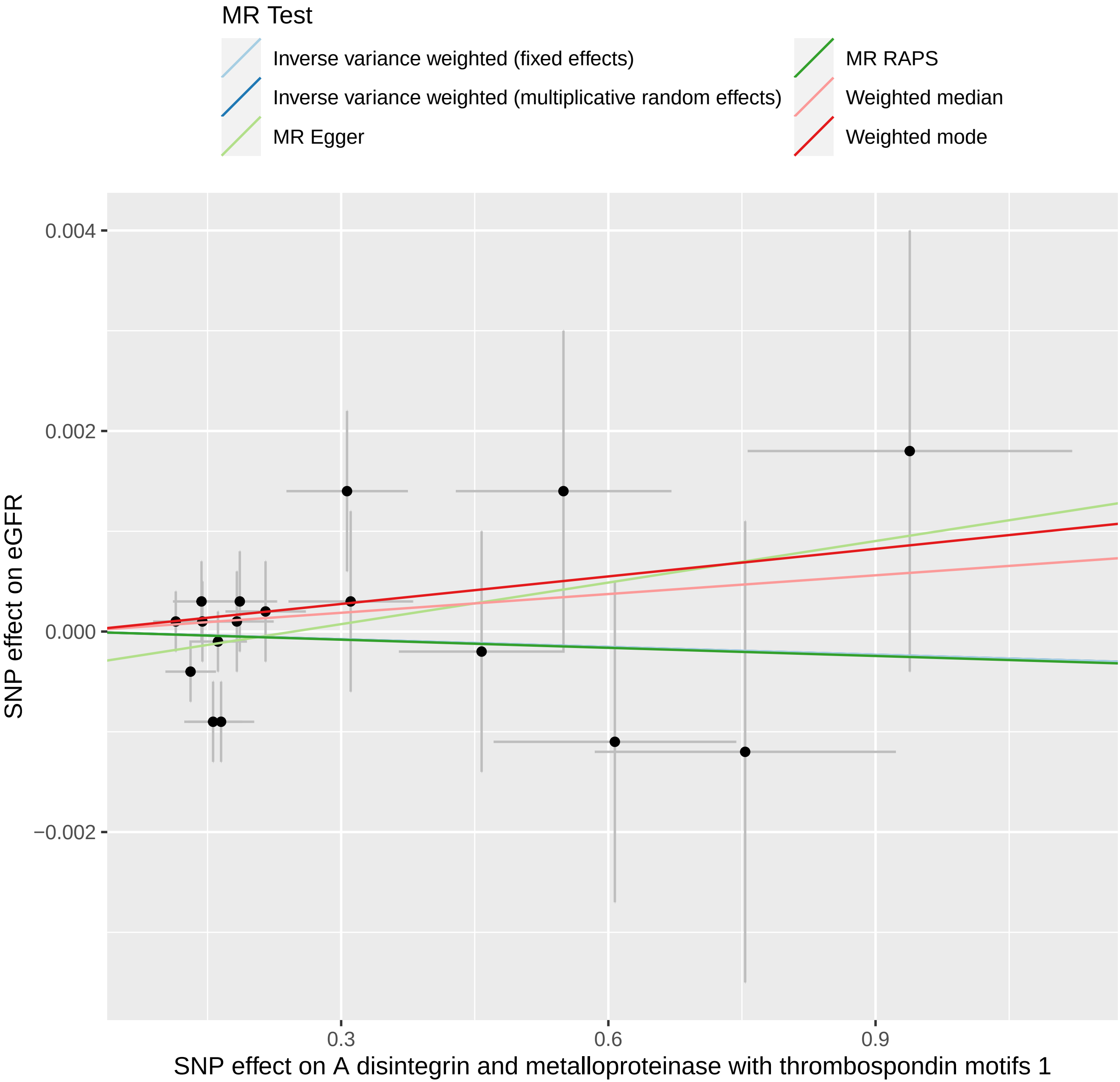

Batch 2−32 : A disintegrin and metalloproteinase with thrombospondin motifs 13 on eGFR

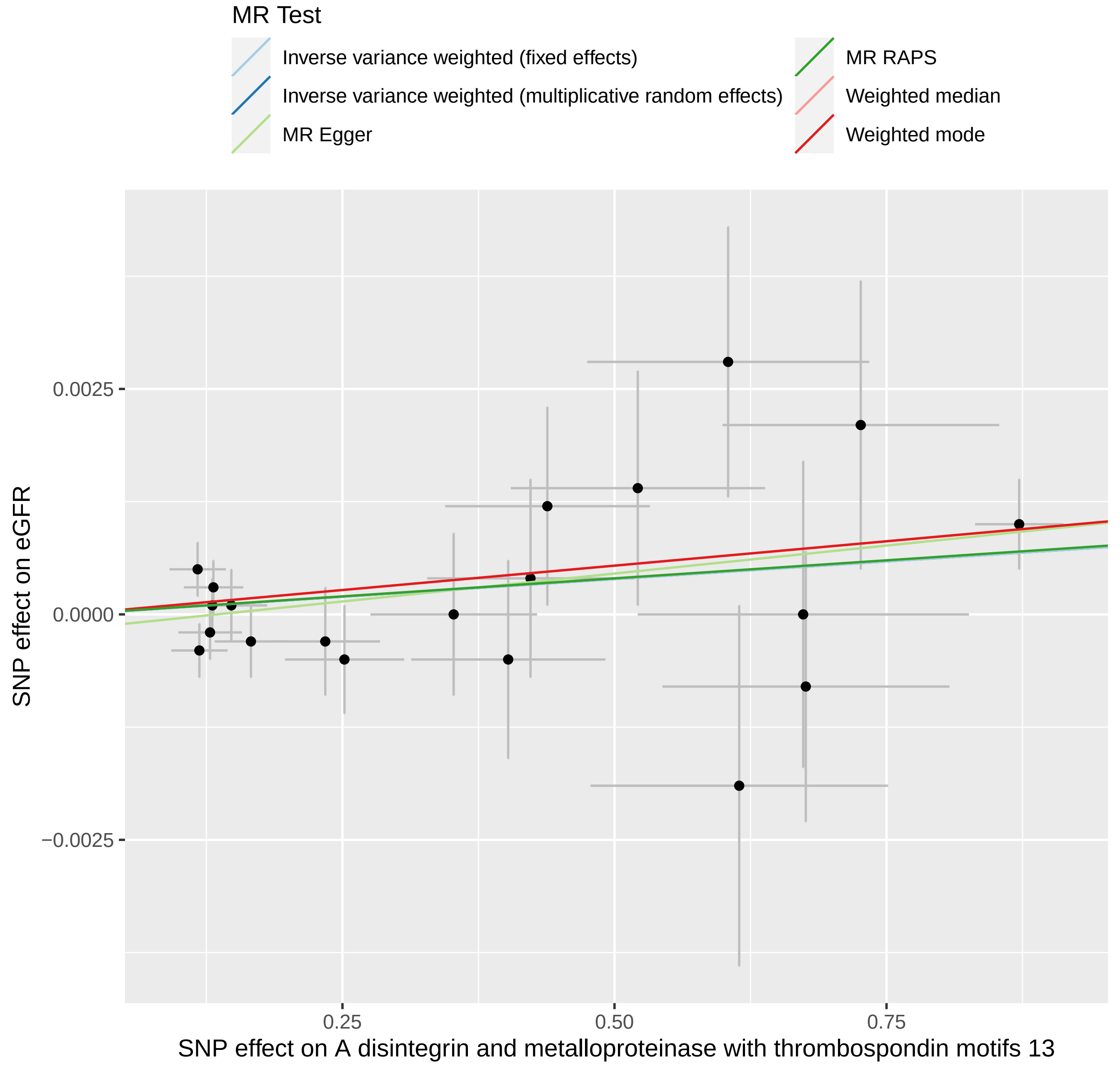

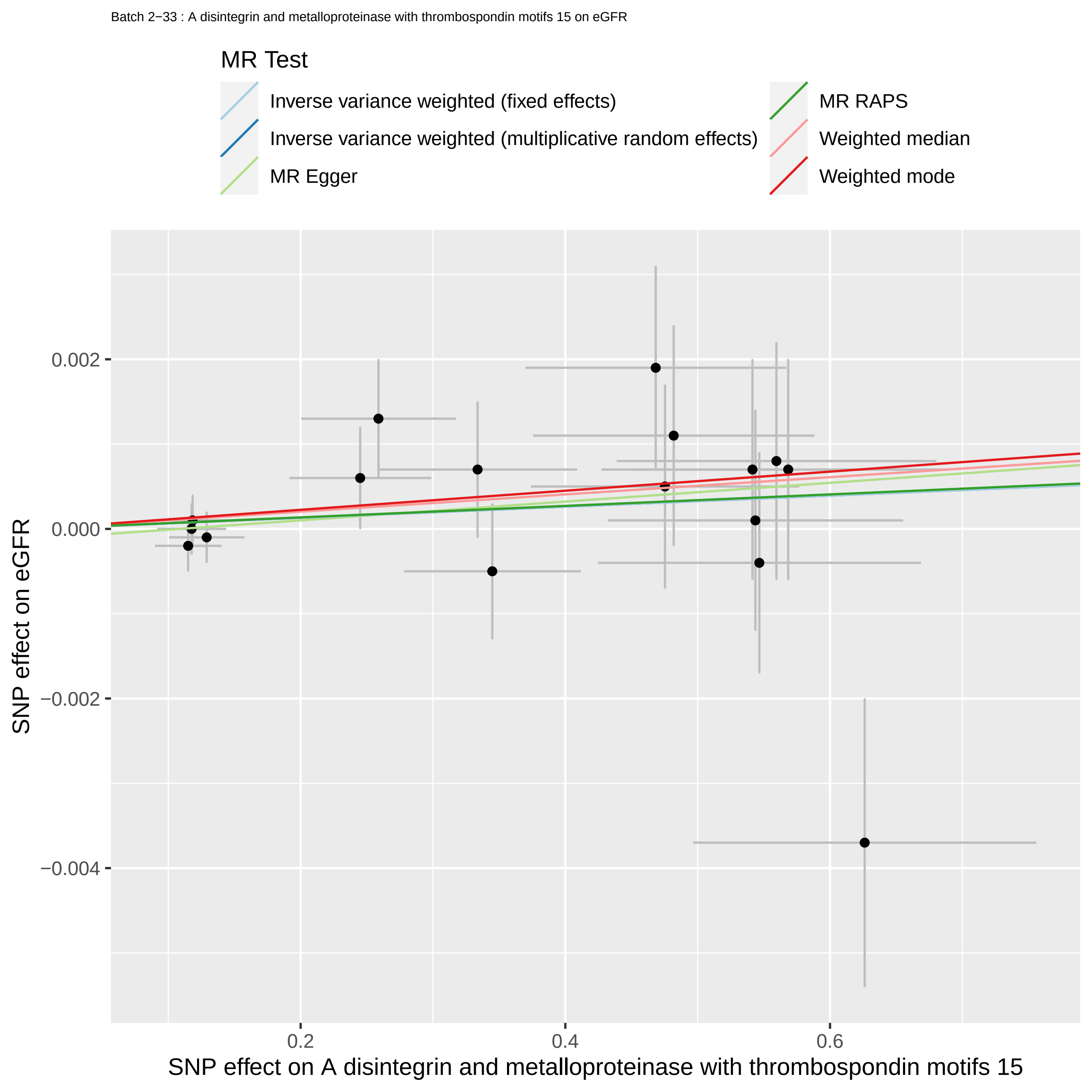

Batch 2−33 : A disintegrin and metalloproteinase with thrombospondin motifs 15 on eGFR
MR Test
Inverse variance weighted (fixed effects)
Inverse variance weighted (multiplicative random effects)
MR Egger
MR RAPS
Weighted median
Weighted mode
0.002
0.000
−0.002
−0.004
0.2
0.4
0.6
SNP effect on eGFR
SNP effect on A disintegrin and metalloproteinase with thrombospondin motifs 15

Batch 2−34 : A disintegrin and metalloproteinase with thrombospondin motifs 3 on eGFR

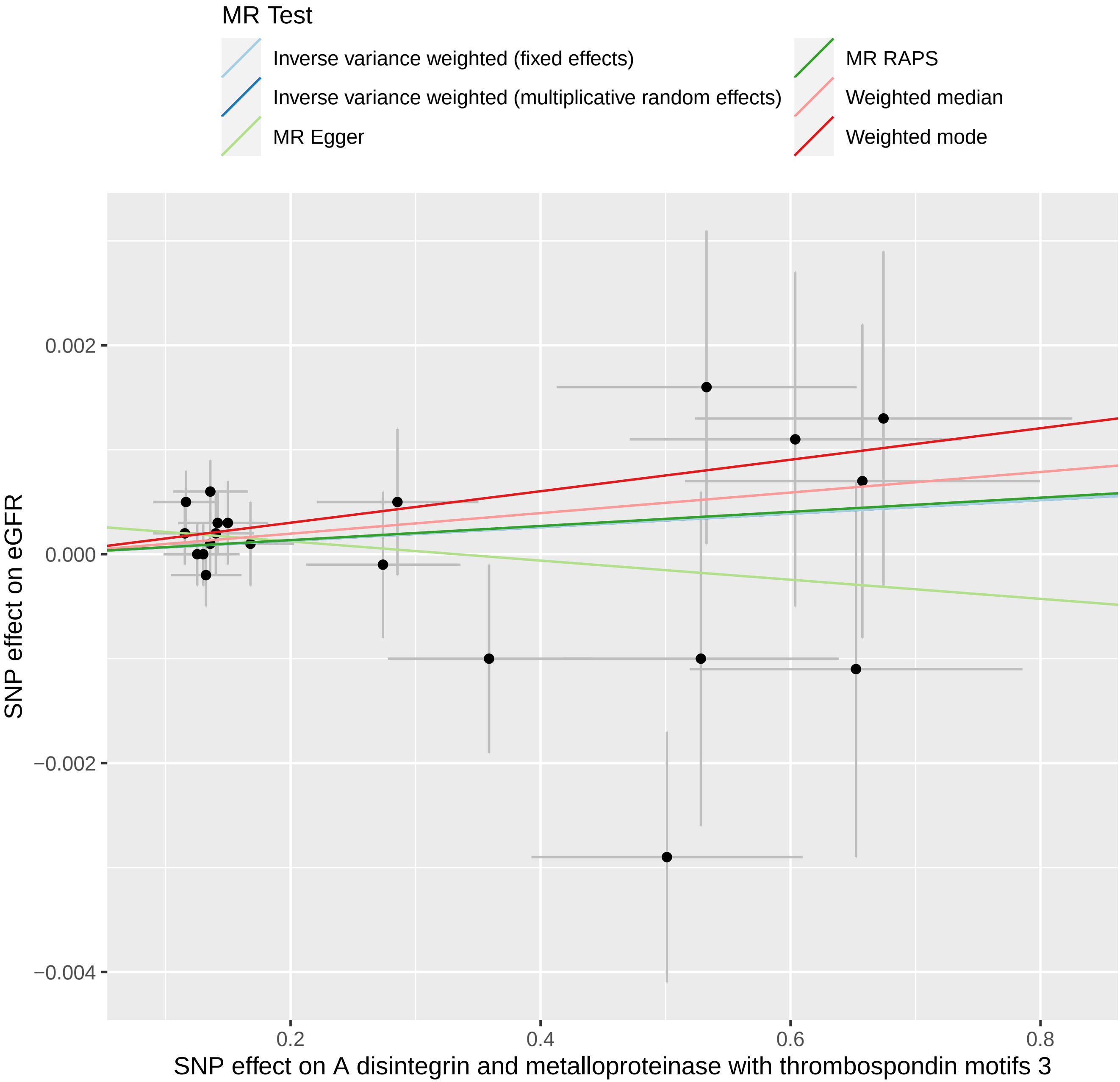

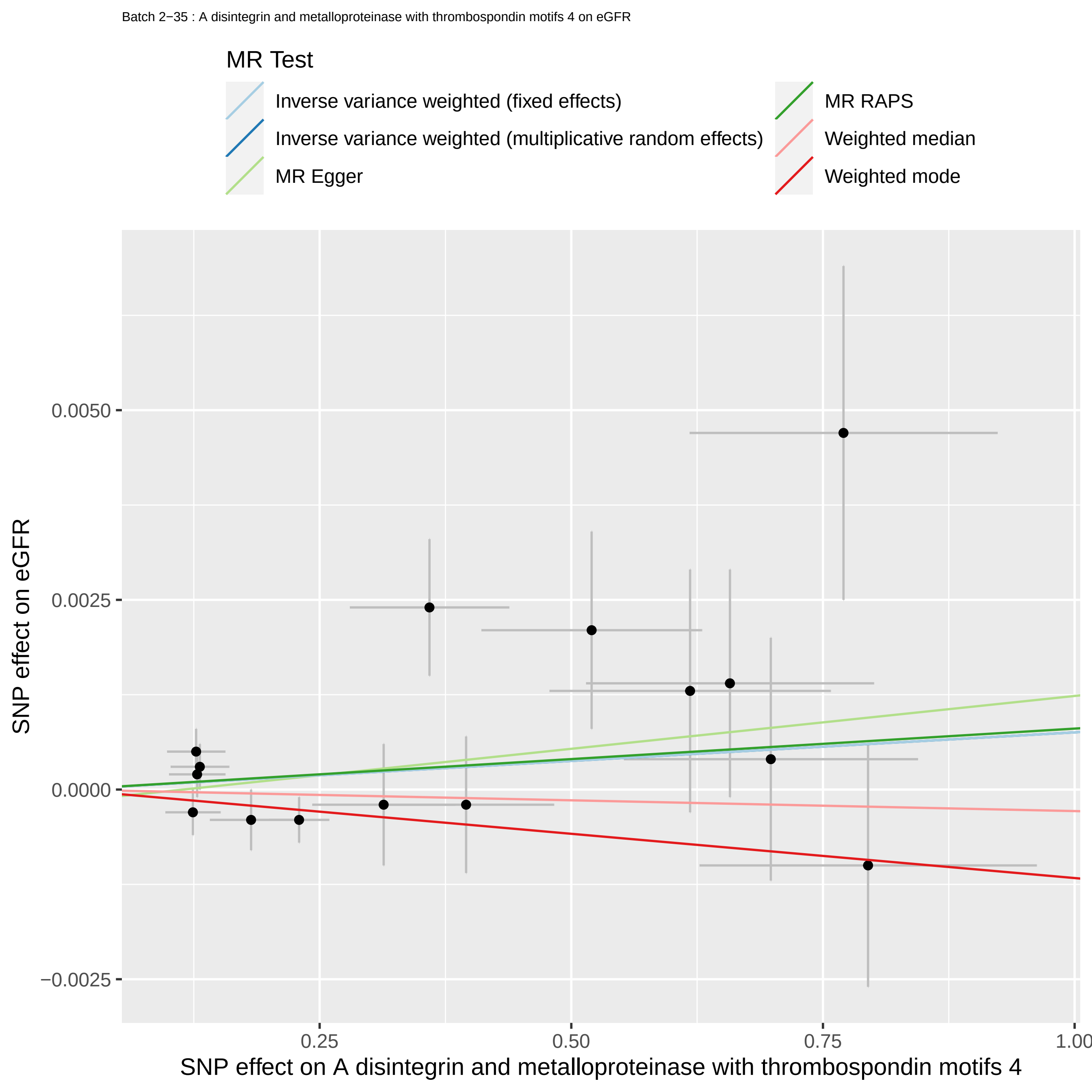
Batch 2−35 : A disintegrin and metalloproteinase with thrombospondin motifs 4 on eGFR
MR Test
Inverse variance weighted (fixed effects)
Inverse variance weighted (multiplicative random effects)
MR Egger
MR RAPS
Weighted median
Weighted mode
0.0050
0.0025
0.0000
−0.0025
0.25
0.50
0.75
1.00
SNP effect on eGFR
SNP effect on A disintegrin and metalloproteinase with thrombospondin motifs 4

Batch 2−36 : A disintegrin and metalloproteinase with thrombospondin motifs 5 on eGFR

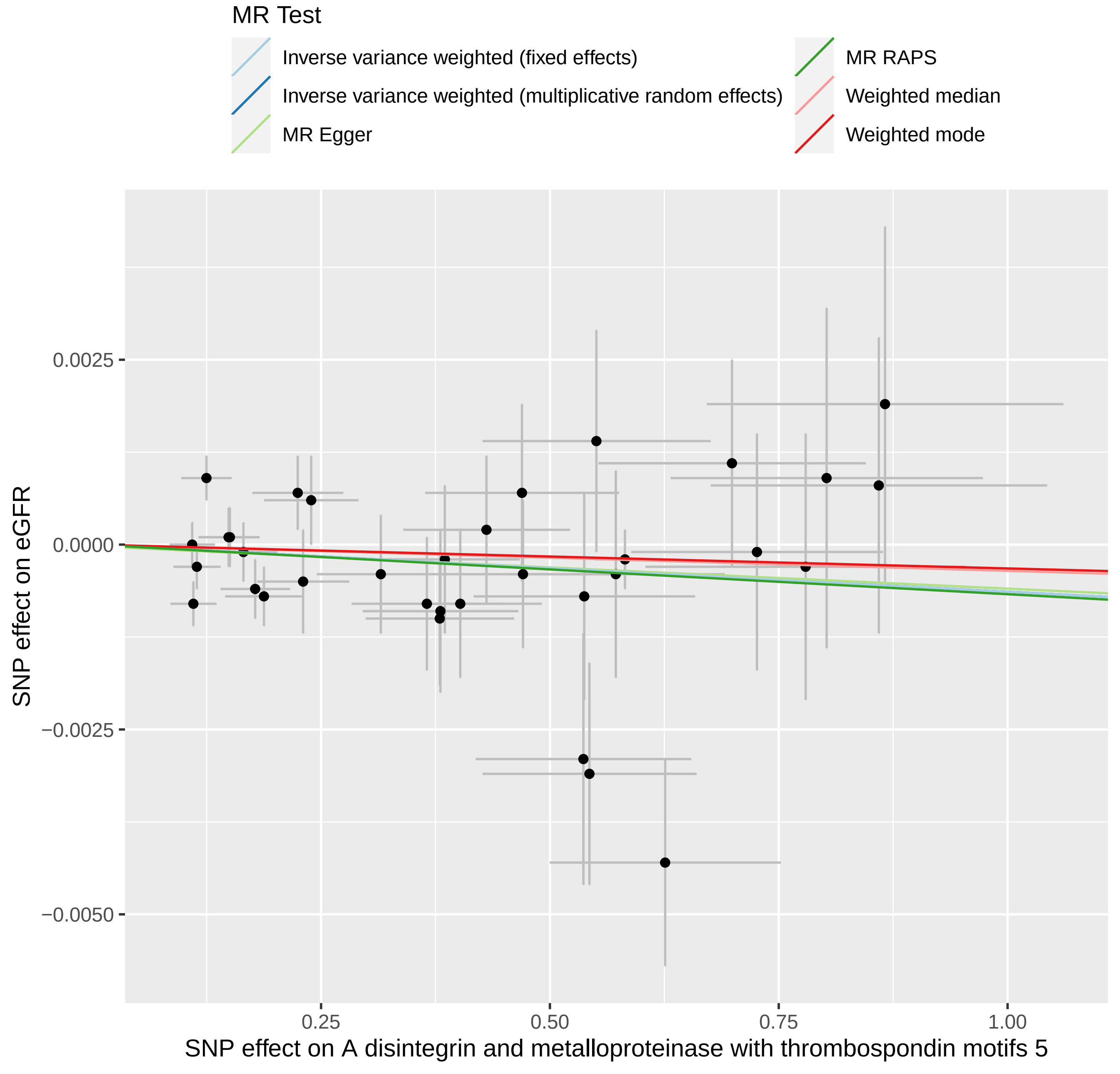

Batch 2−37 : A disintegrin and metalloproteinase with thrombospondin motifs 6 on eGFR

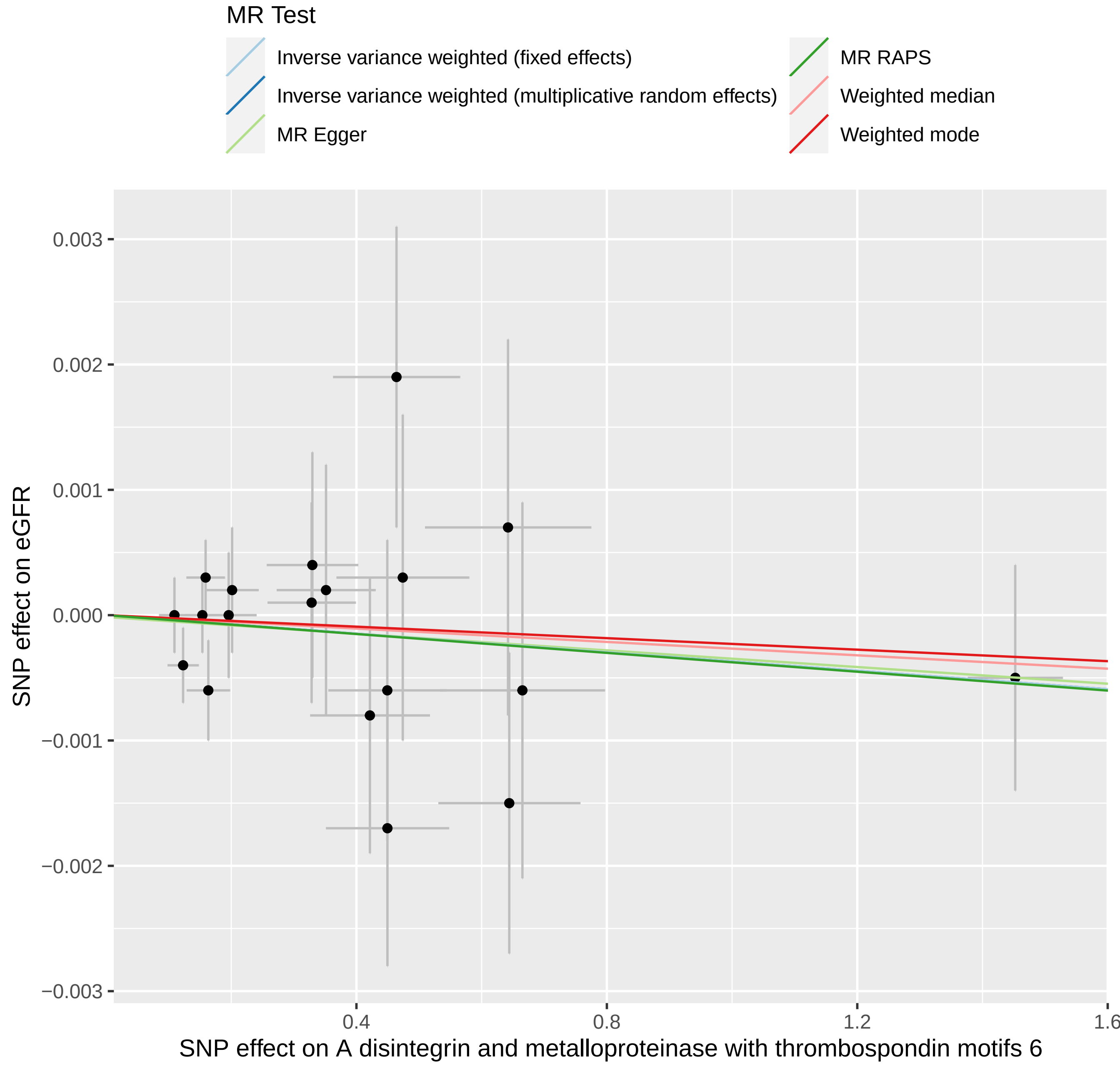

Batch 2−38 : ADAMTS−like protein 1 on eGFR

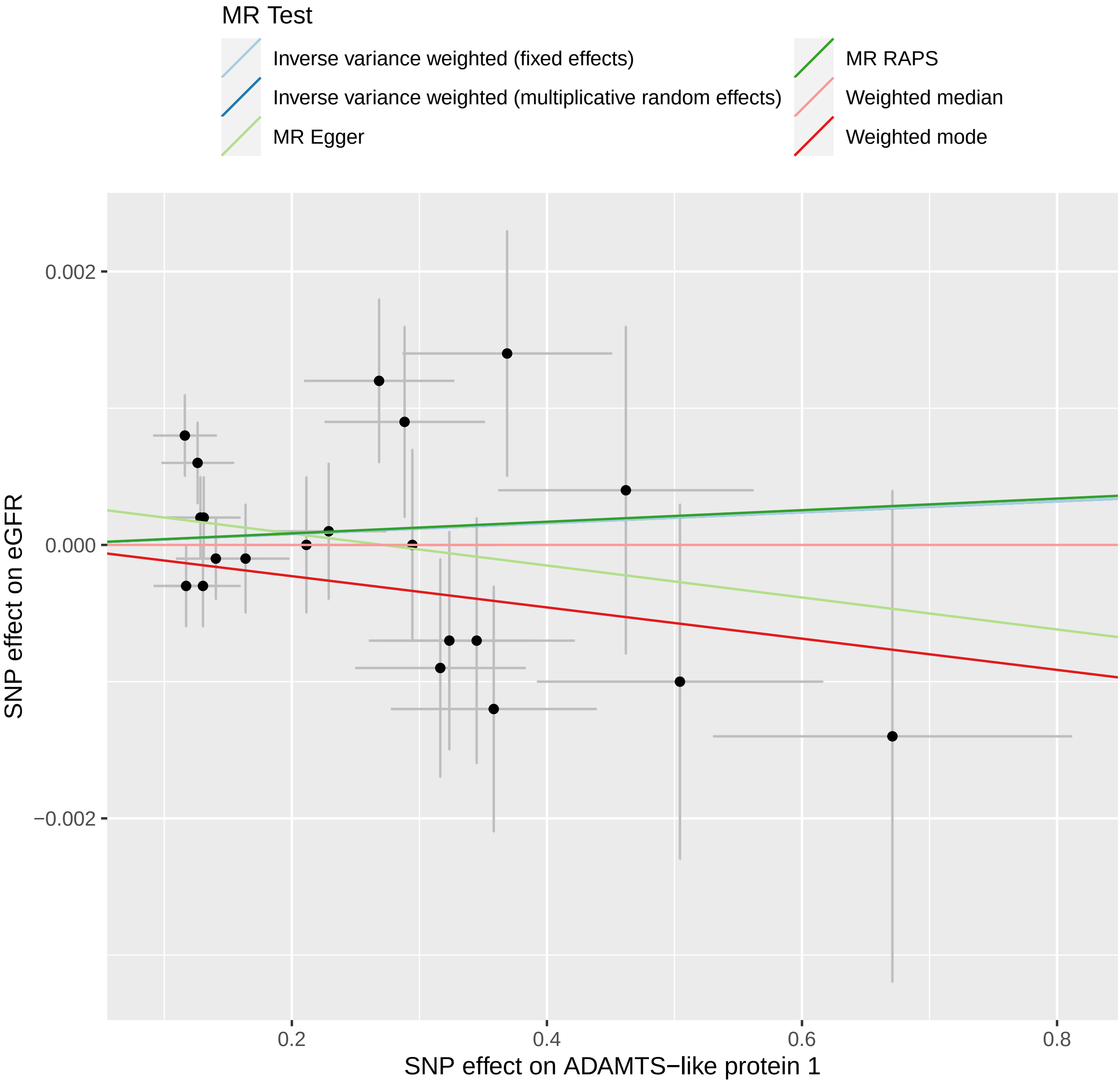

Batch 2−39 : AarF domain−containing protein kinase 4 on eGFR

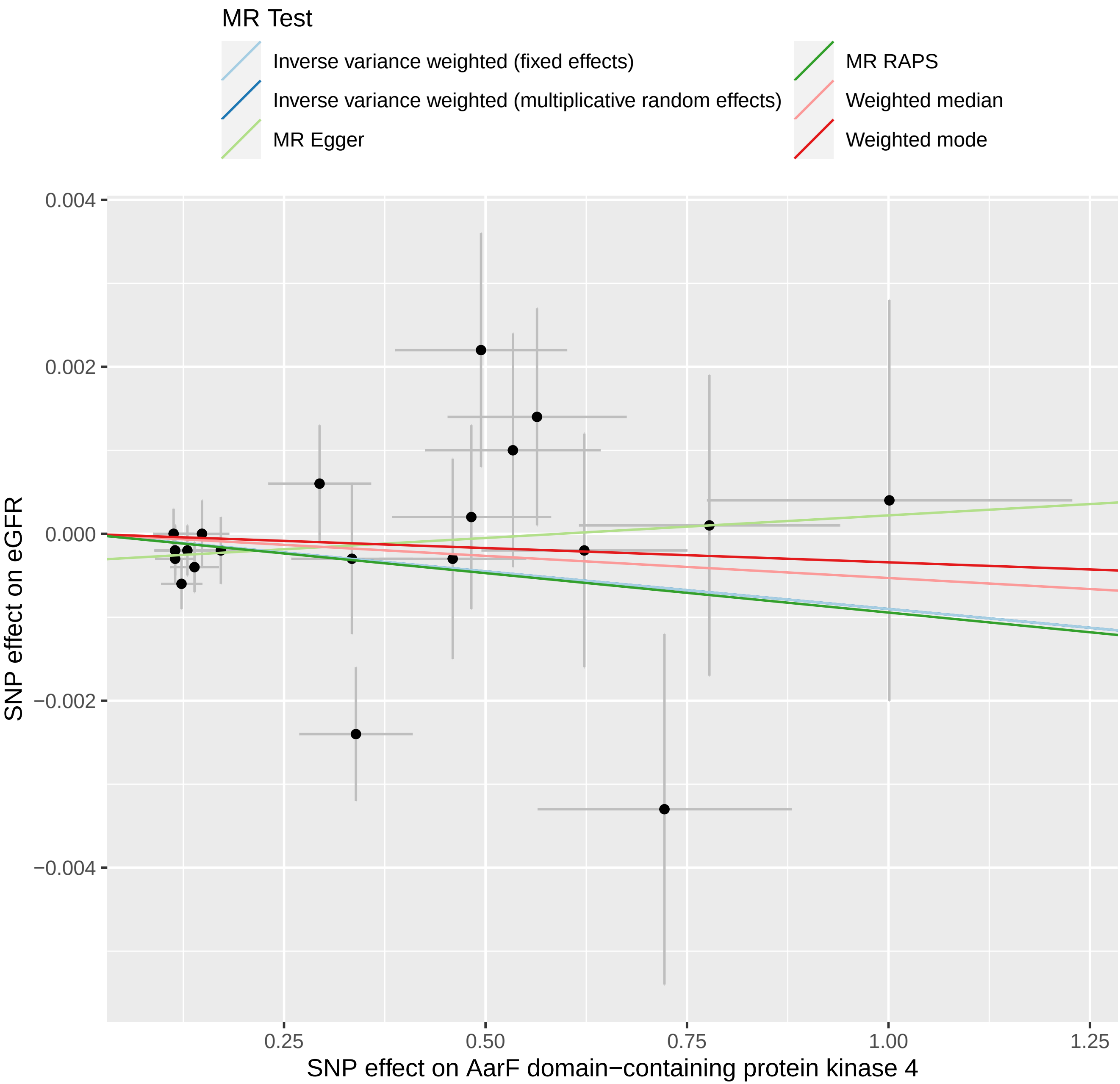

Batch 2−40 : Pituitary adenylate cyclase−activating polypeptide 27 on eGFR

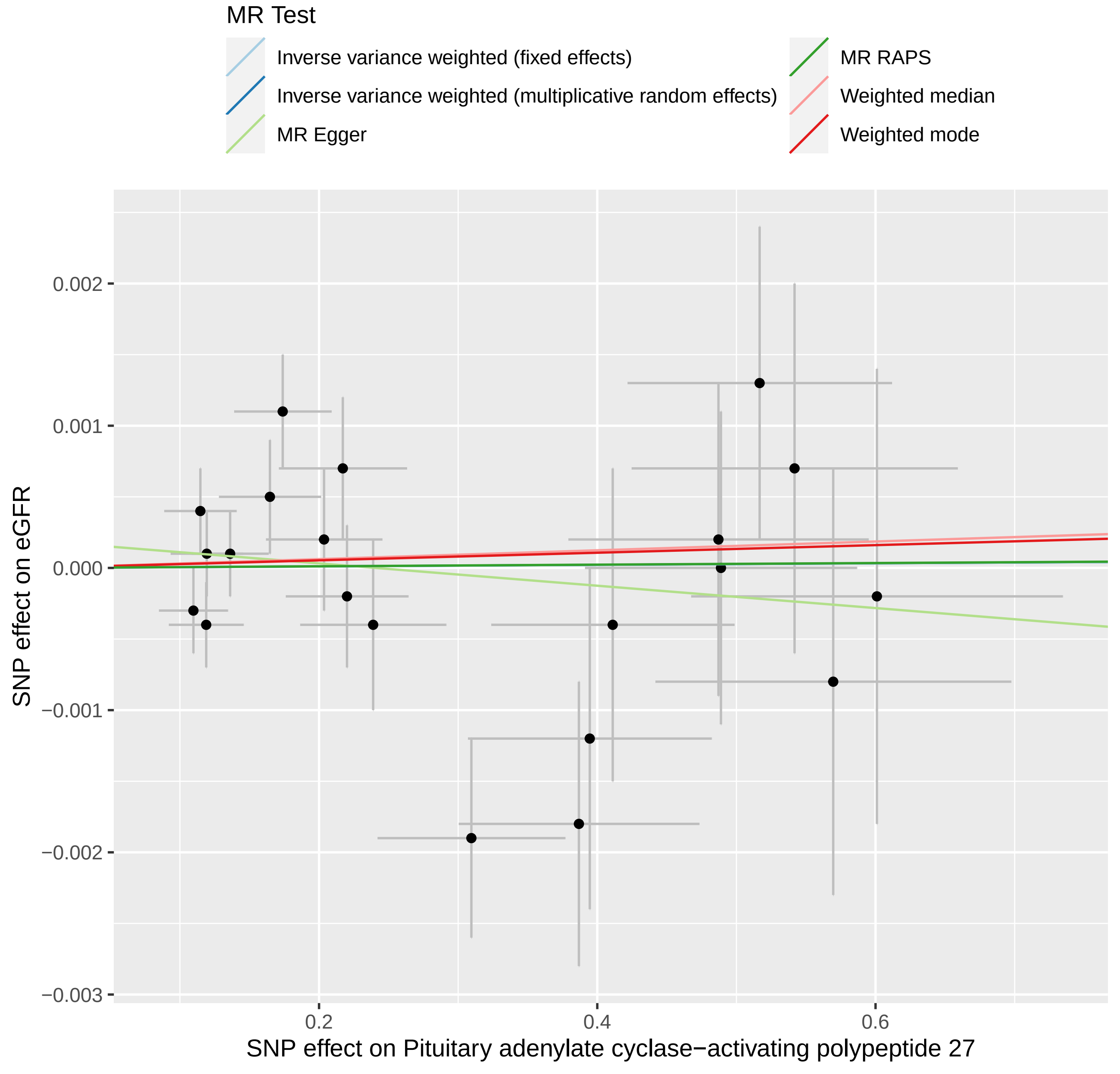

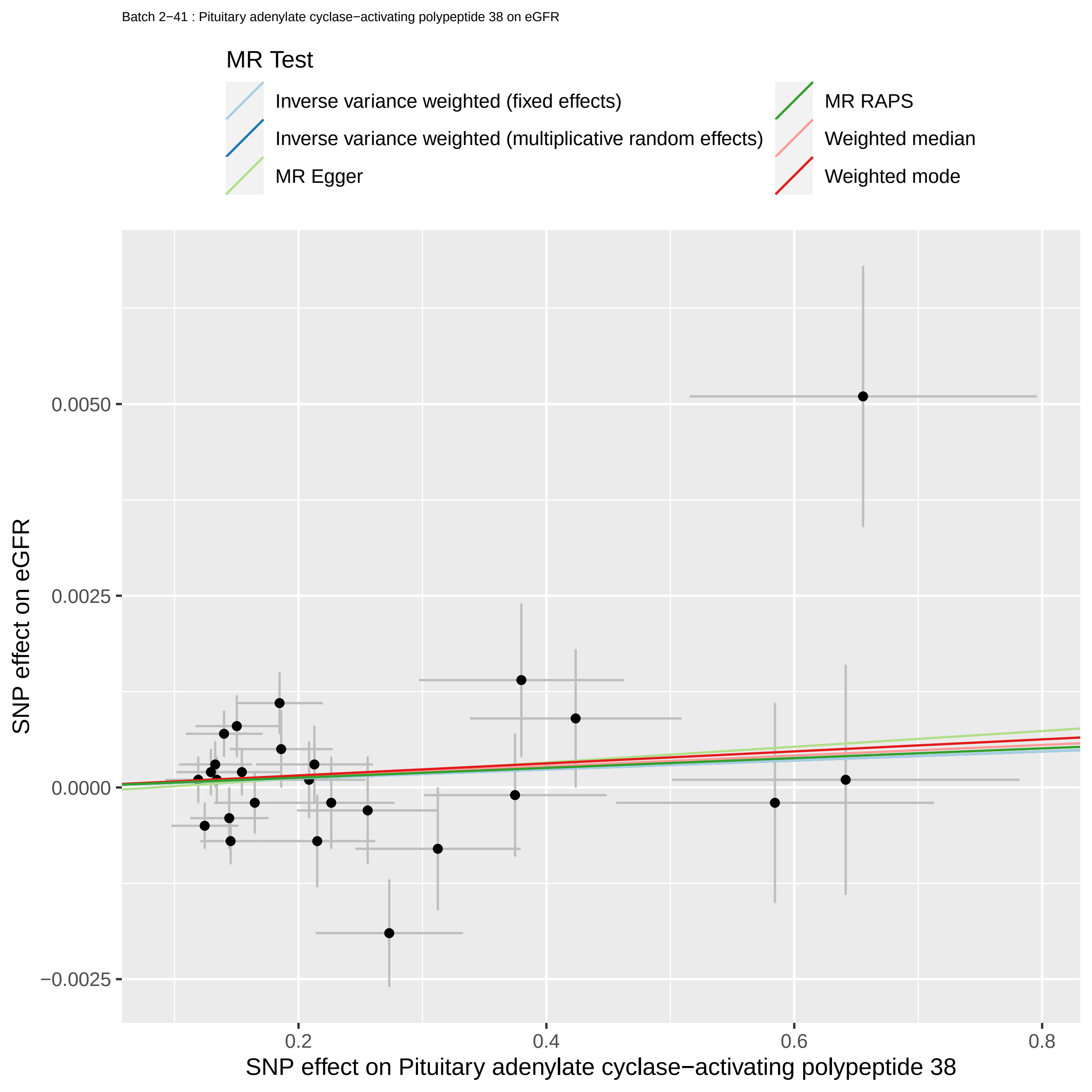
Batch 2−41 : Pituitary adenylate cyclase−activating polypeptide 38 on eGFR
MR Test
Inverse variance weighted (fixed effects)
Inverse variance weighted (multiplicative random effects)
MR Egger
MR RAPS
Weighted median
Weighted mode
0.0050
0.0025
0.0000
−0.0025
SNP effect on eGFR
0.2
0.4
0.6
0.8
SNP effect on Pituitary adenylate cyclase−activating polypeptide 38

Batch 2−42 : Pituitary adenylate cyclase−activating polypeptide on eGFR

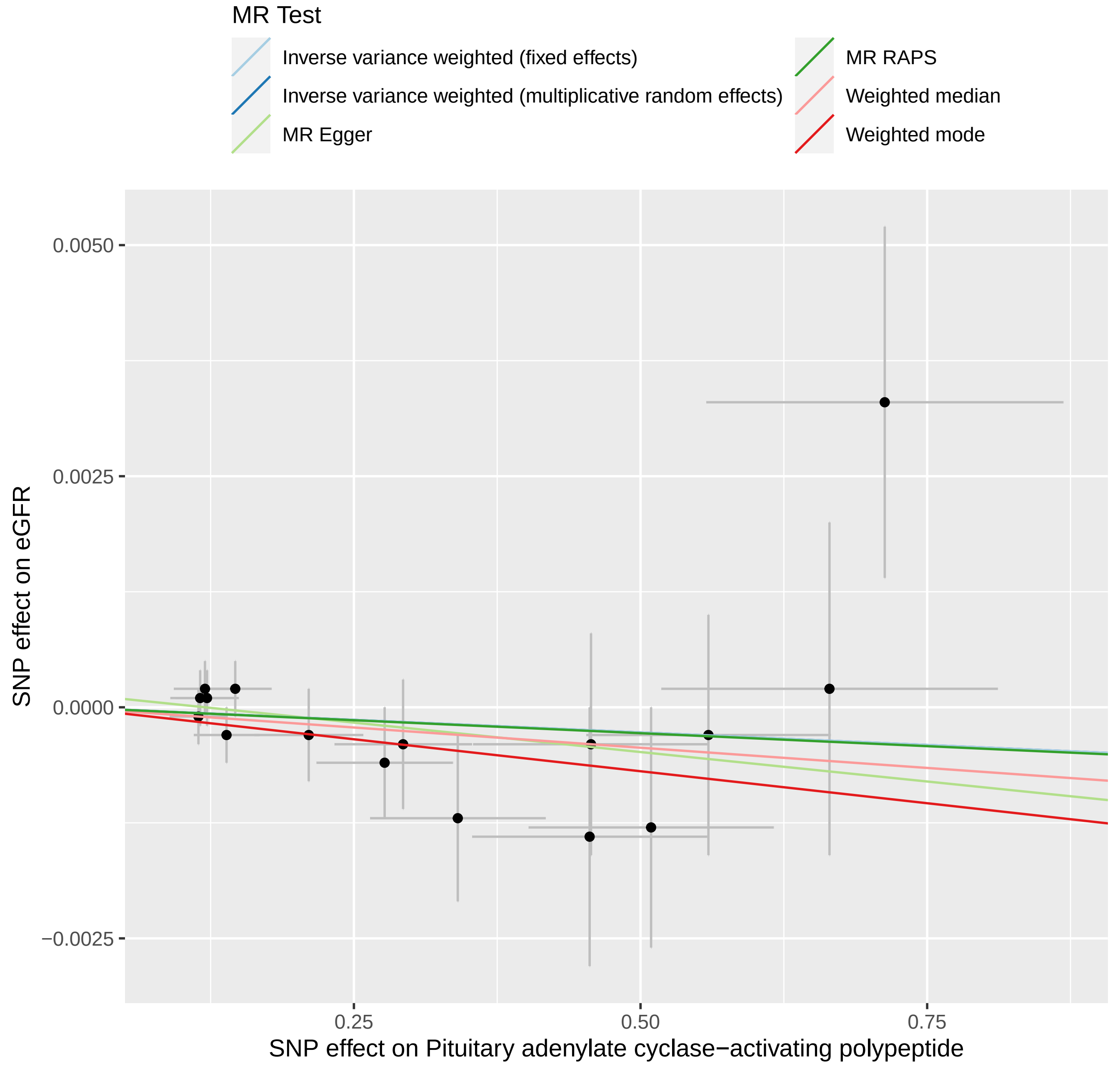

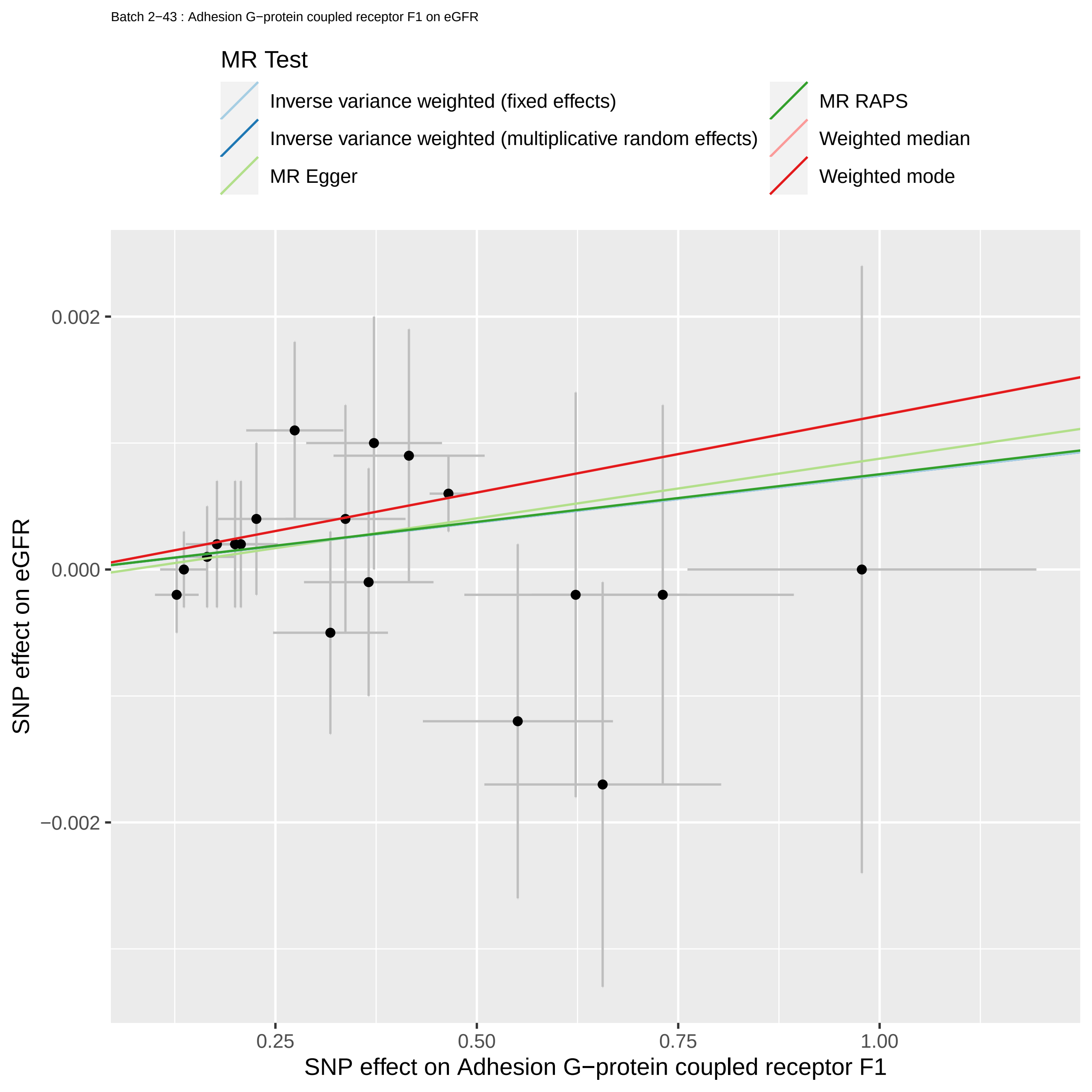
Batch 2−43 : Adhesion G−protein coupled receptor F1 on eGFR
MR Test
Inverse variance weighted (fixed effects)
Inverse variance weighted (multiplicative random effects)
MR Egger
MR RAPS
Weighted median
Weighted mode
0.002
0.000
−0.002
0.25
0.50
0.75
1.00
SNP effect on eGFR
SNP effect on Adhesion G−protein coupled receptor F1

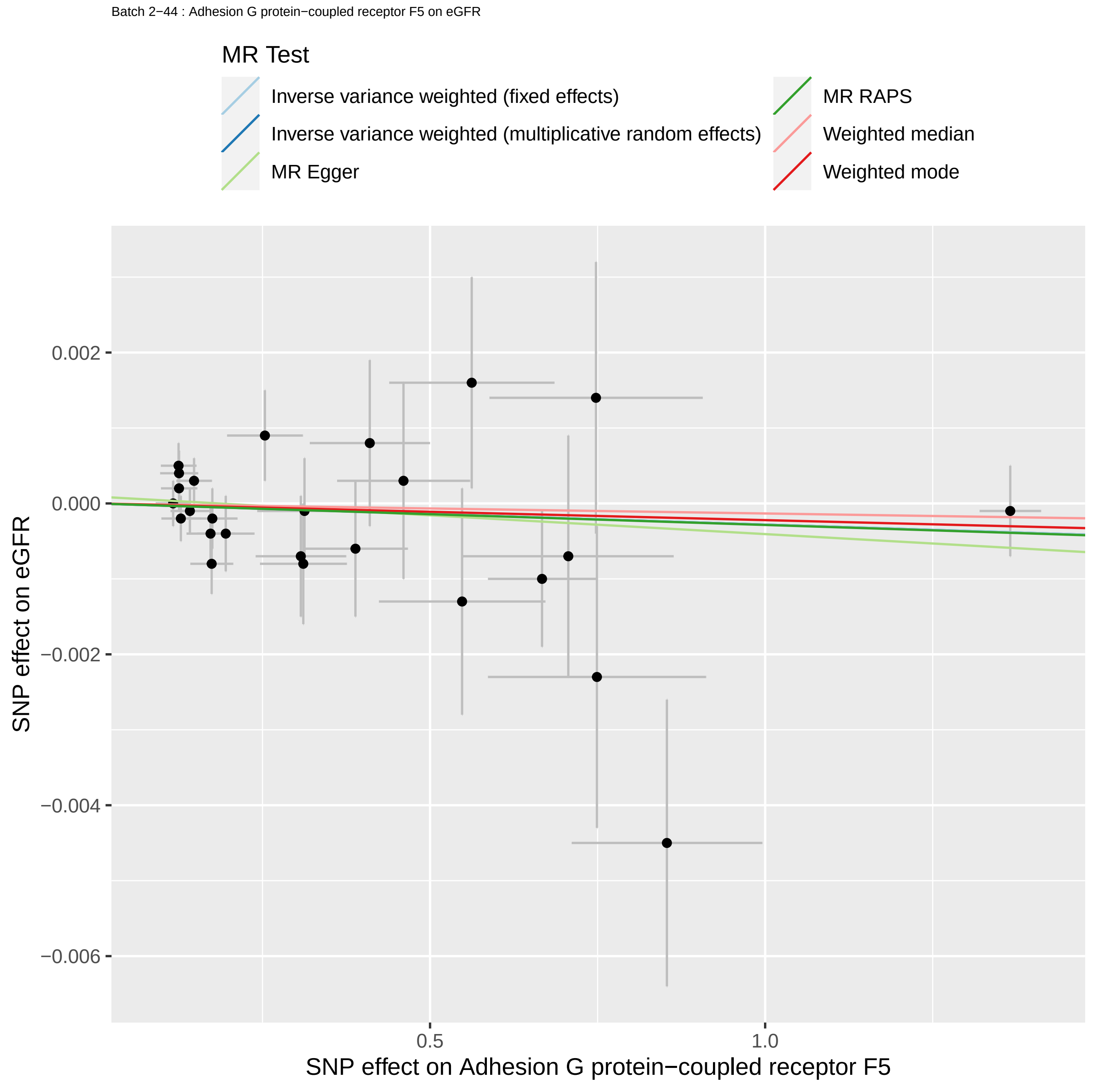

Batch 2−44 : Adhesion G protein−coupled receptor F5 on eGFR
MR Test
Inverse variance weighted (fixed effects)
Inverse variance weighted (multiplicative random effects)
MR Egger
MR RAPS
Weighted median
Weighted mode
0.002
0.000
−0.002
−0.004
−0.006
0.5
1.0
SNP effect on eGFR
SNP effect on Adhesion G protein−coupled receptor F5

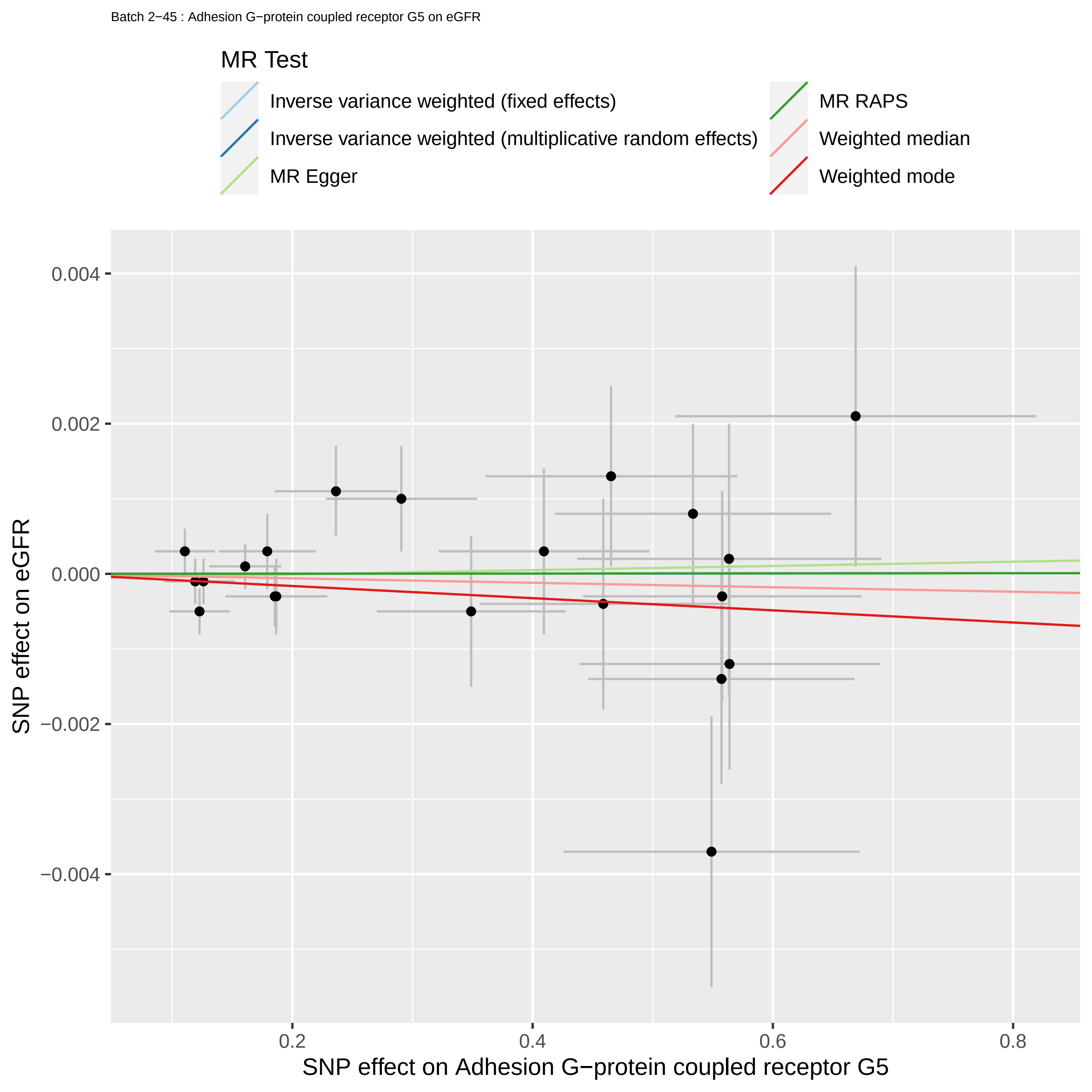

Batch 2−45 : Adhesion G−protein coupled receptor G5 on eGFR
MR Test
Inverse variance weighted (fixed effects)
Inverse variance weighted (multiplicative random effects)
MR Egger
MR RAPS
Weighted median
Weighted mode
0.004
0.002
0.000
−0.002
−0.004
0.2
0.4
0.6
0.8
SNP effect on eGFR
SNP effect on Adhesion G−protein coupled receptor G5

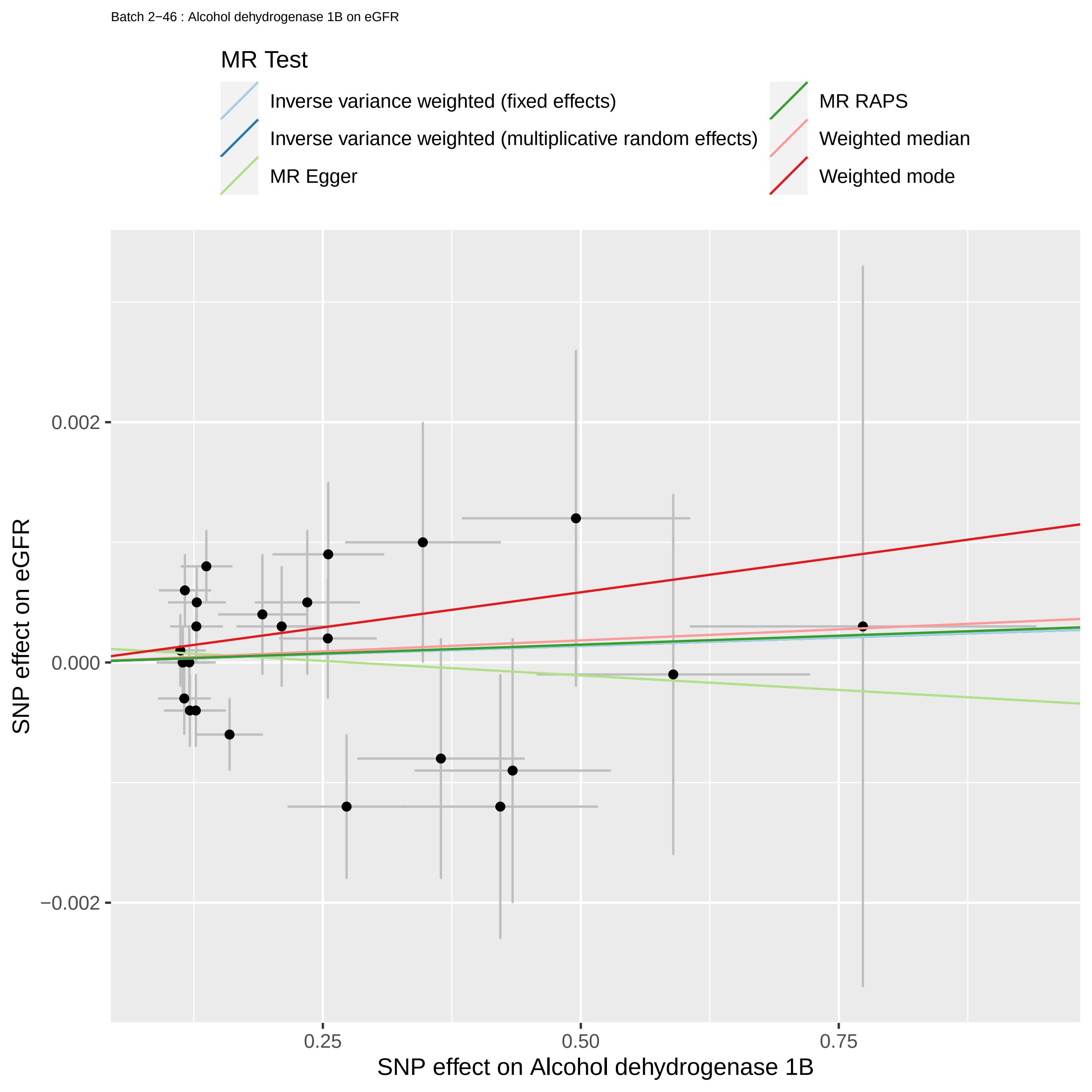

Batch 2−46 : Alcohol dehydrogenase 1B on eGFR
MR Test
Inverse variance weighted (fixed effects)
Inverse variance weighted (multiplicative random effects)
MR Egger
MR RAPS
Weighted median
Weighted mode
0.002
0.000
−0.002
0.25
0.50
0.75
SNP effect on eGFR
SNP effect on Alcohol dehydrogenase 1B

Batch 2−47 : Alcohol dehydrogenase 4 on eGFR

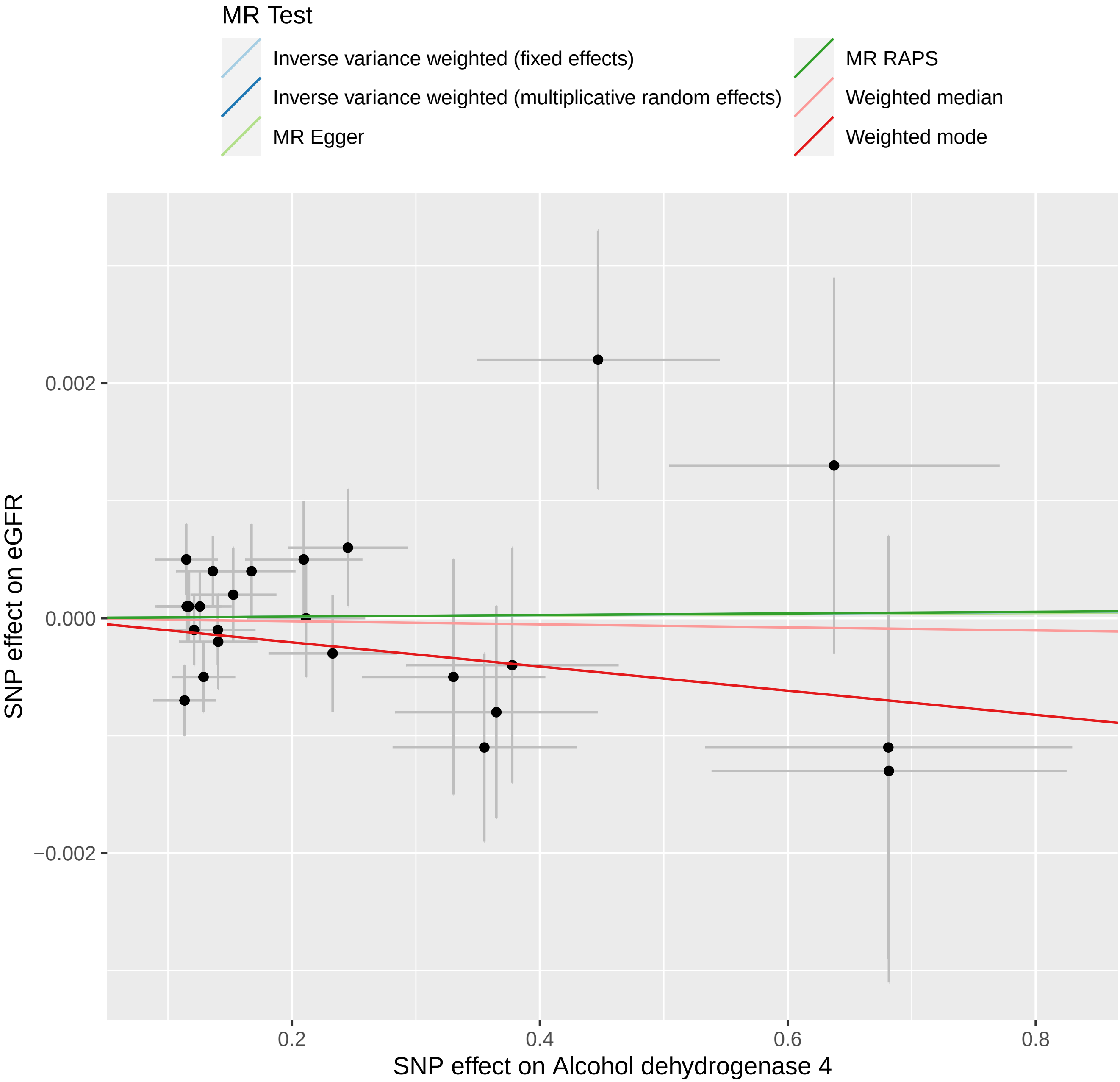

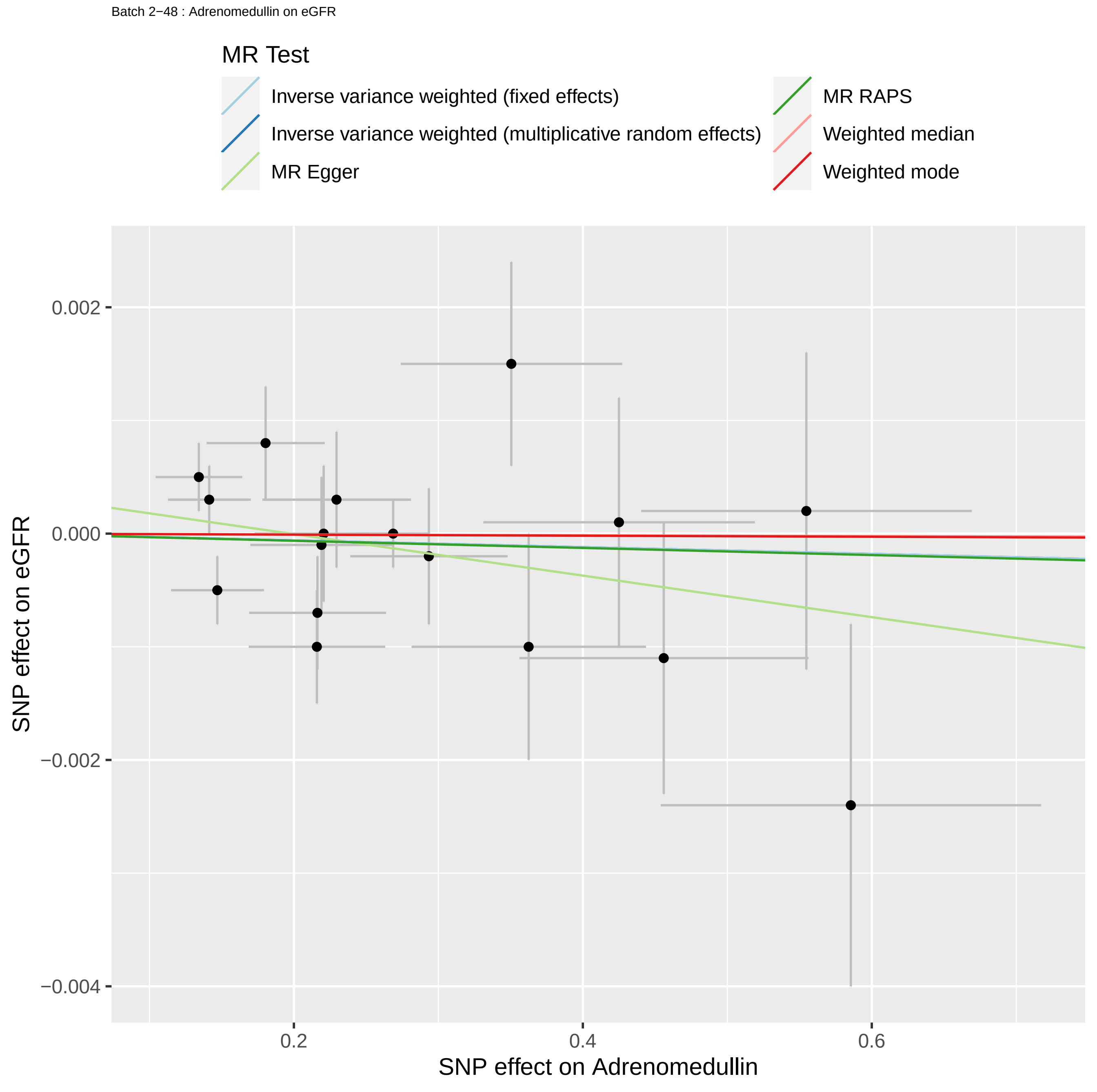
Batch 2−48 : Adrenomedullin on eGFR
MR Test
Inverse variance weighted (fixed effects)
Inverse variance weighted (multiplicative random effects)
MR Egger
MR RAPS
Weighted median
Weighted mode
0.002
0.000
−0.002
−0.004
SNP effect on eGFR
0.2
0.4
0.6
SNP effect on Adrenomedullin

Batch 2−49 : ADP−dependent glucokinase on eGFR

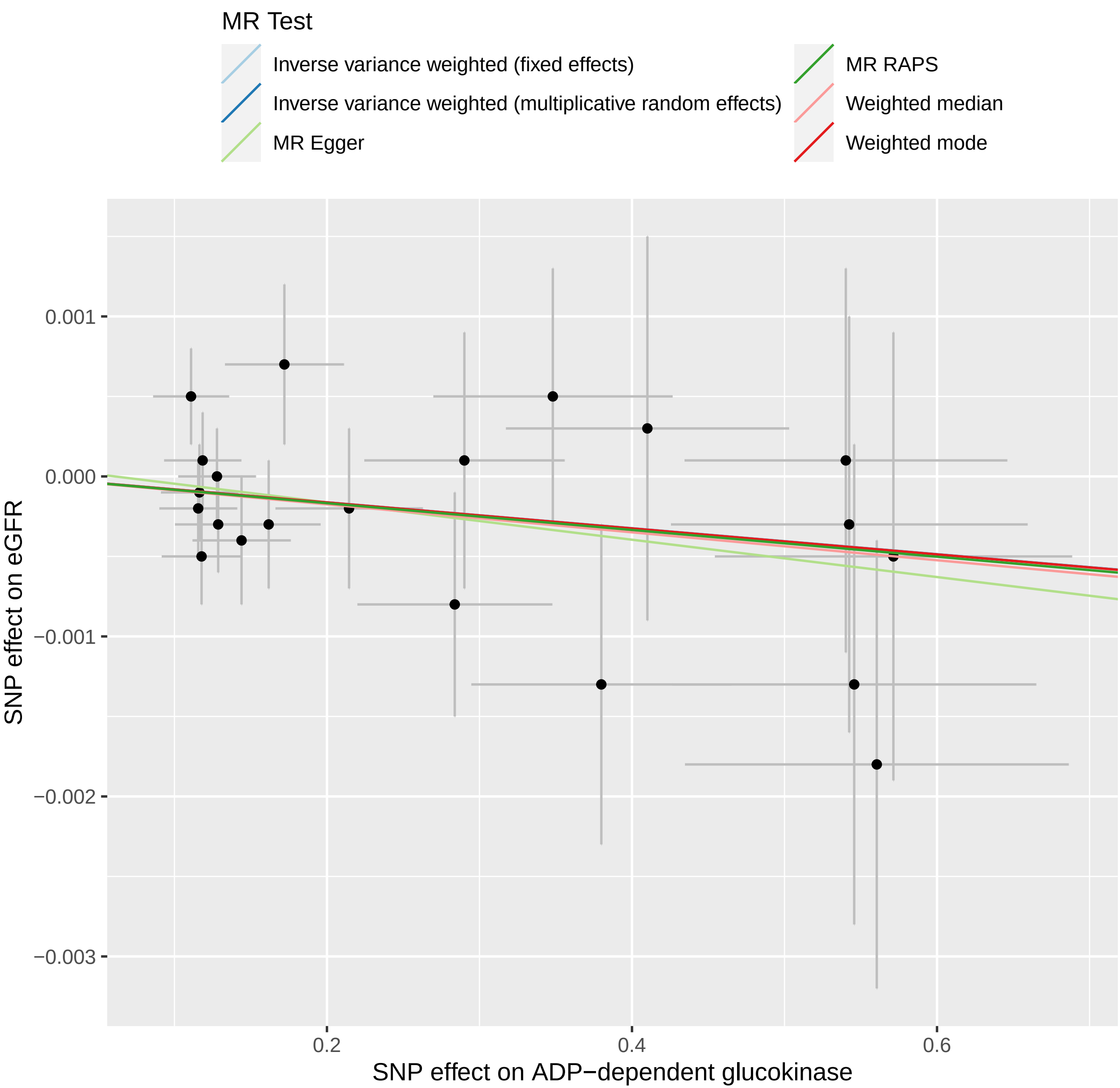

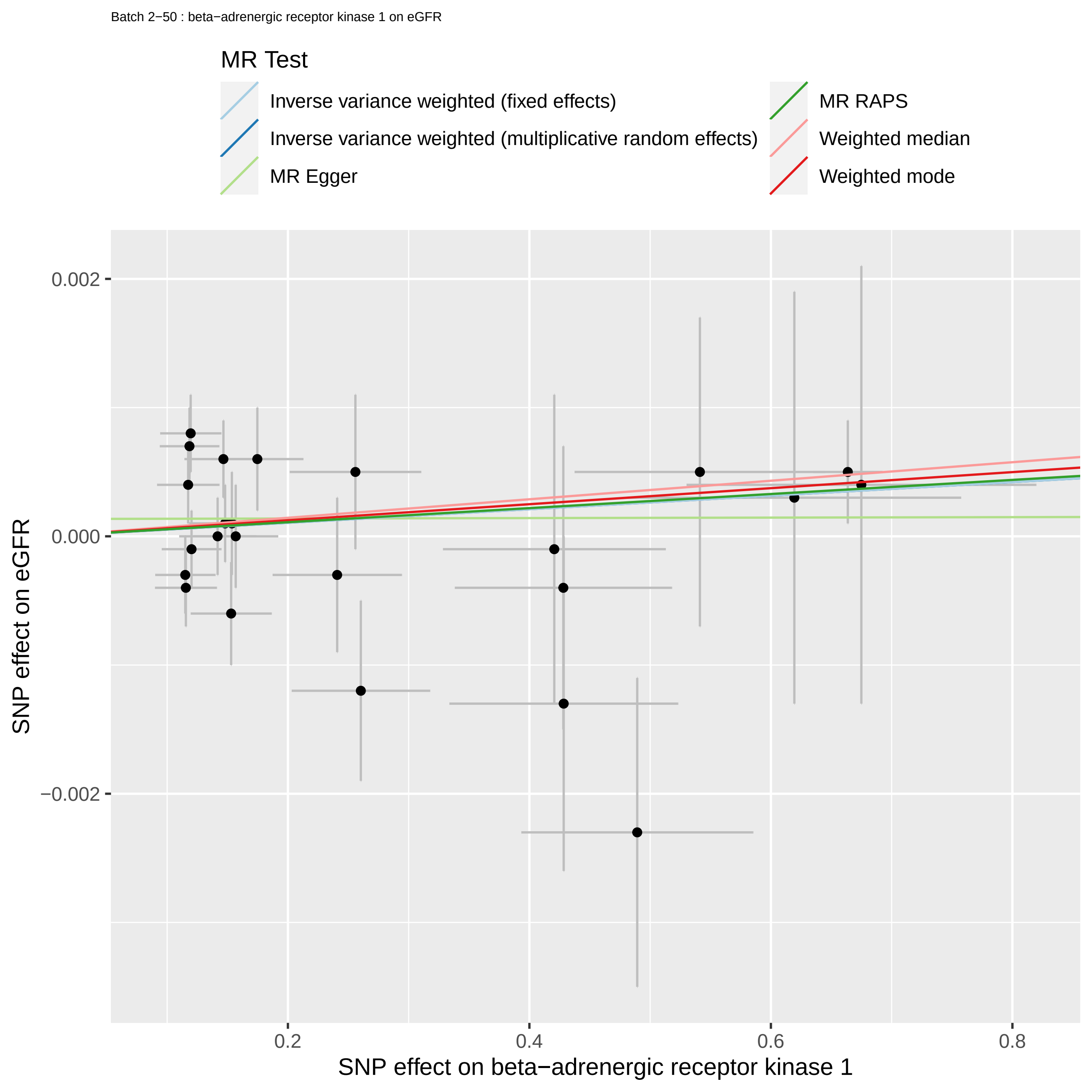

Batch 2−50 : beta−adrenergic receptor kinase 1 on eGFR
MR Test
Inverse variance weighted (fixed effects)
Inverse variance weighted (multiplicative random effects)
MR Egger
MR RAPS
Weighted median
Weighted mode
0.002
0.000
−0.002
0.2
0.4
0.6
0.8
SNP effect on eGFR
SNP effect on beta−adrenergic receptor kinase 1

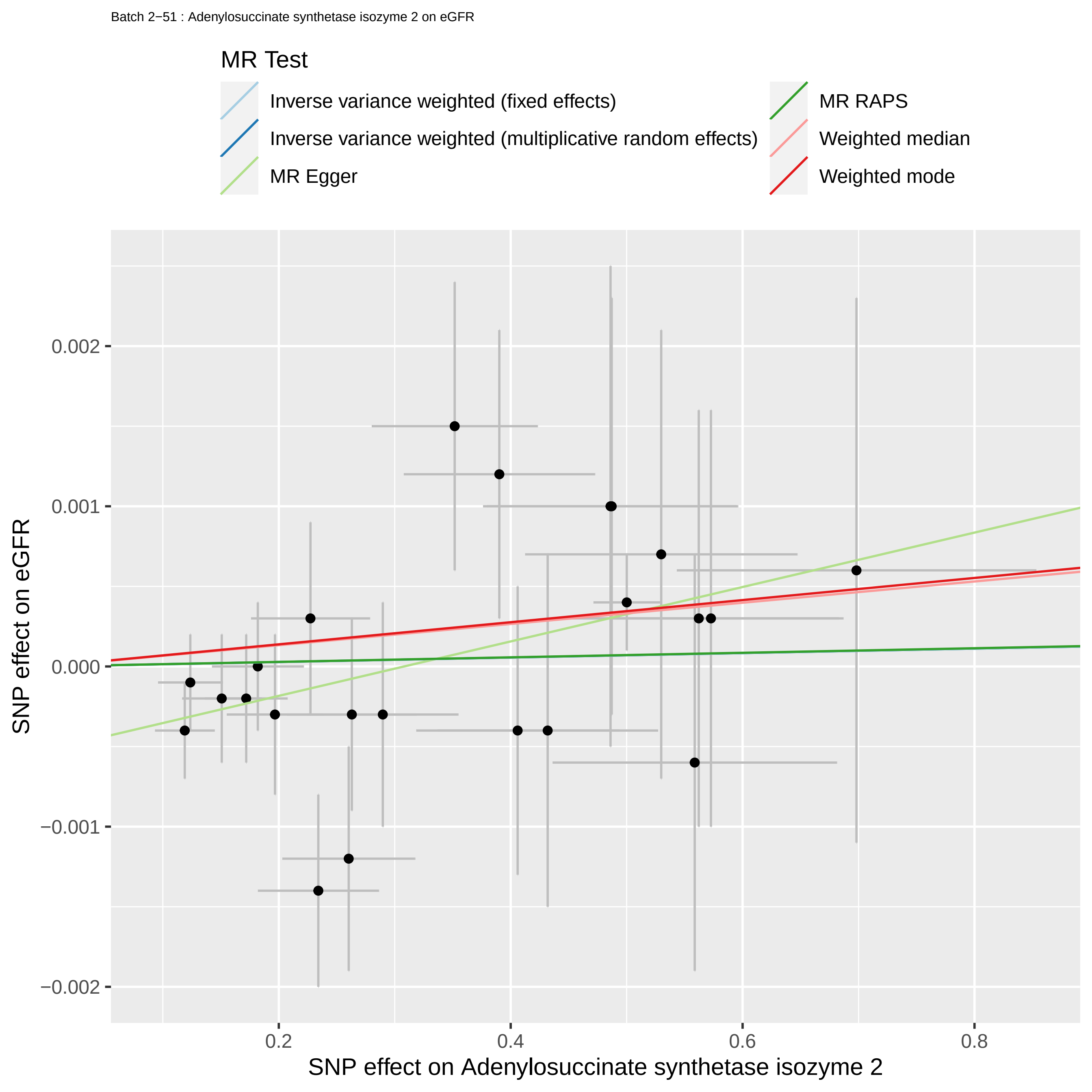

Batch 2−51 : Adenylosuccinate synthetase isozyme 2 on eGFR
MR Test
Inverse variance weighted (fixed effects)
Inverse variance weighted (multiplicative random effects)
MR Egger
MR RAPS
Weighted median
Weighted mode
0.002
0.001
0.000
−0.001
−0.002
0.2
0.4
0.6
0.8
SNP effect on eGFR
SNP effect on Adenylosuccinate synthetase isozyme 2

Batch 2−52 : Adenylosuccinate synthetase isozyme 1 on eGFR

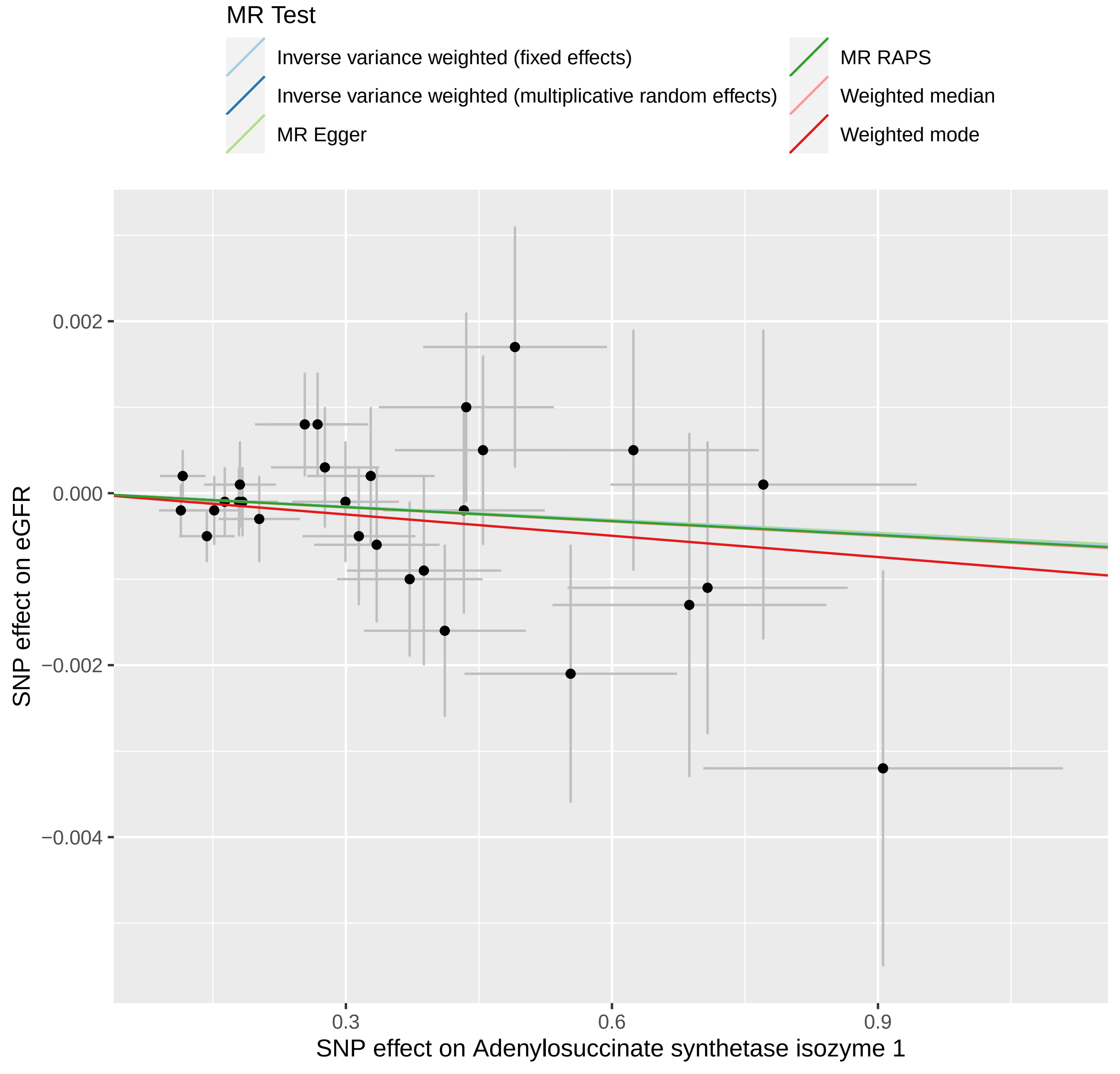

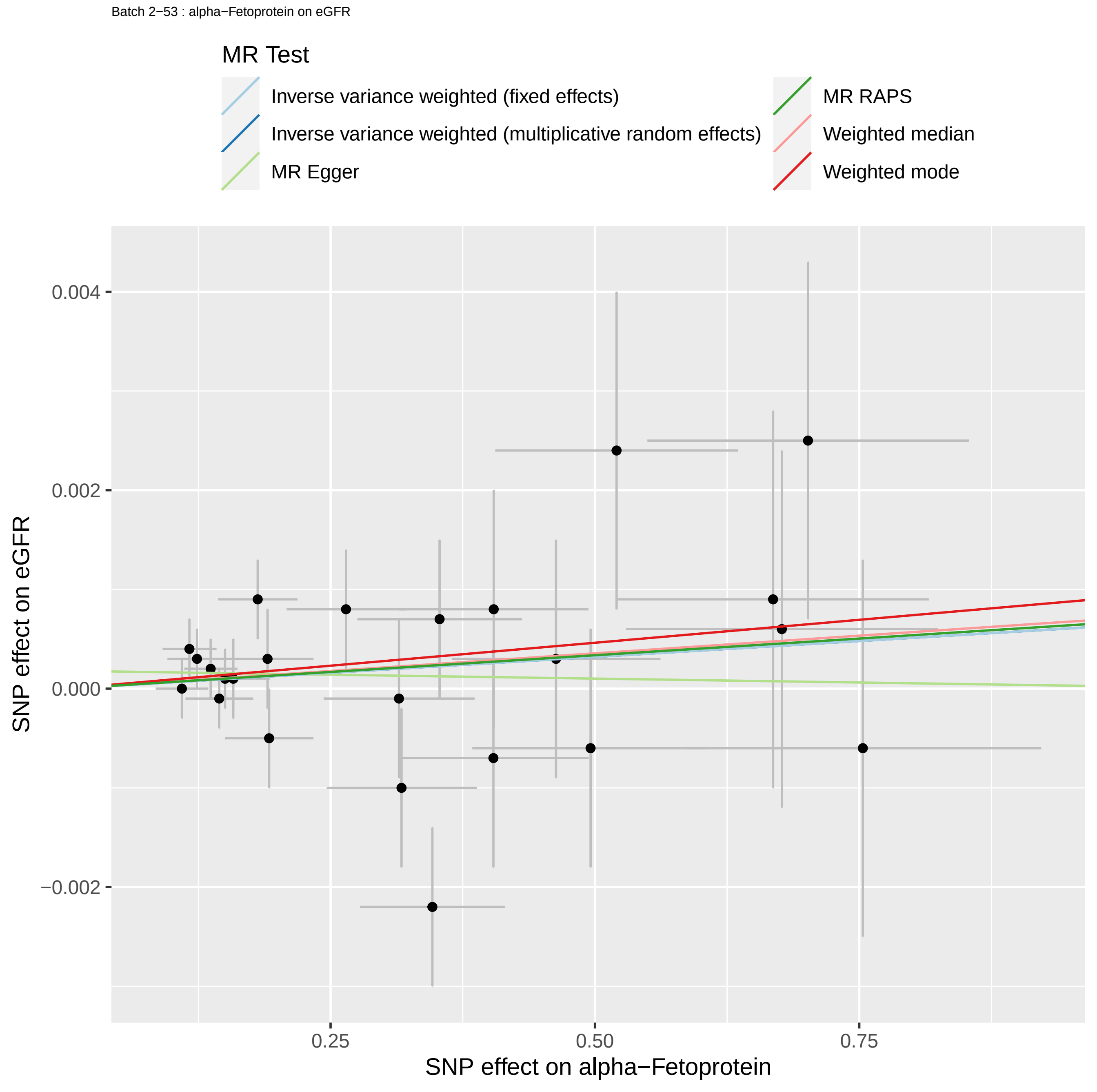
Batch 2−53 : alpha−Fetoprotein on eGFR
MR Test
Inverse variance weighted (fixed effects)
Inverse variance weighted (multiplicative random effects)
MR Egger
MR RAPS
Weighted median
Weighted mode
0.004
0.002
0.000
−0.002
SNP effect on eGFR
0.25
0.50
0.75
SNP effect on alpha−Fetoprotein

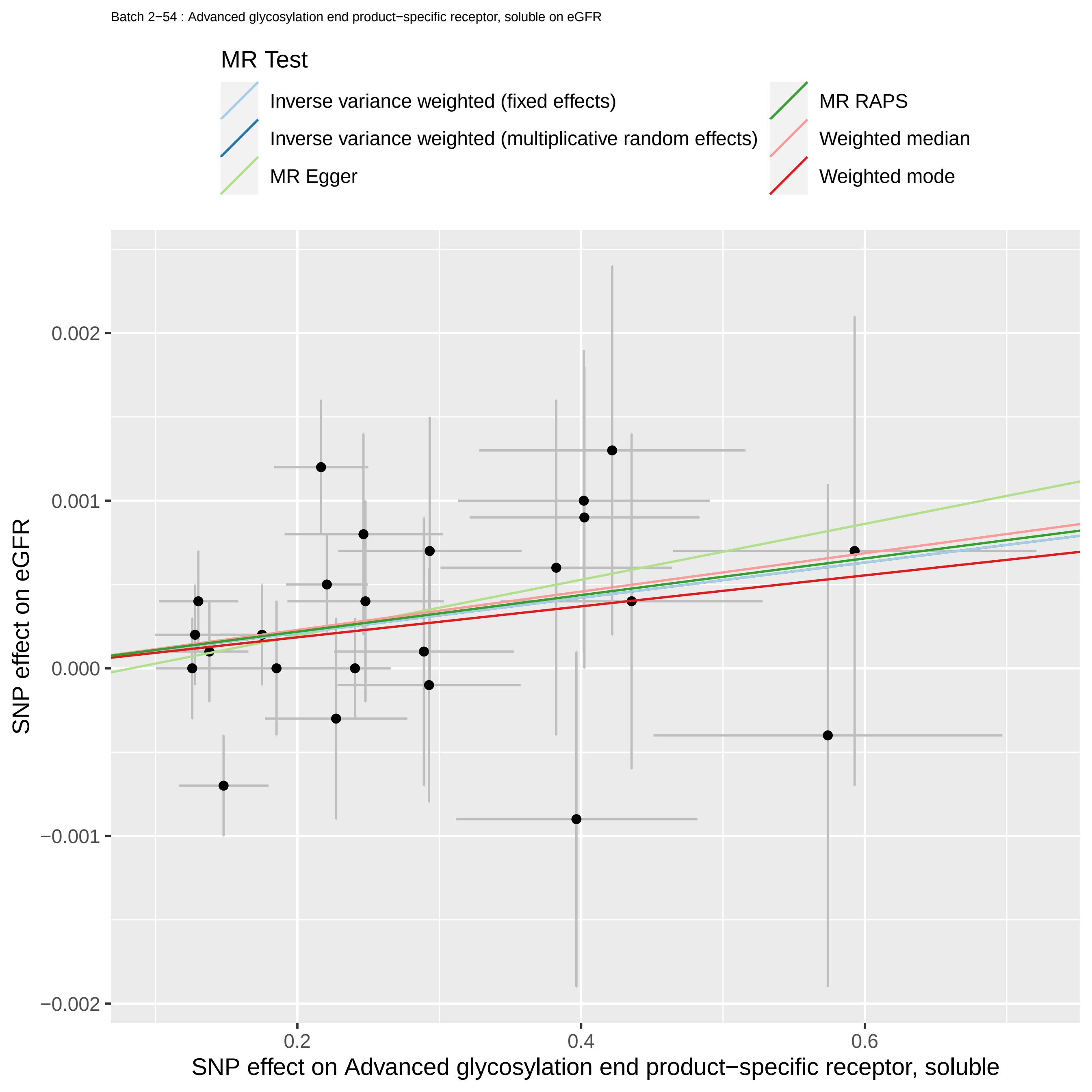
Batch 2−54 : Advanced glycosylation end product−specific receptor, soluble on eGFR
MR Test
Inverse variance weighted (fixed effects)
Inverse variance weighted (multiplicative random effects)
MR Egger
MR RAPS
Weighted median
Weighted mode
0.002
0.001
0.000
−0.001
−0.002
SNP effect on eGFR
0.2
0.4
0.6
SNP effect on Advanced glycosylation end product−specific receptor, soluble

Batch 2−55 : Arf−GAP domain and FG repeat−containing protein 1 on eGFR

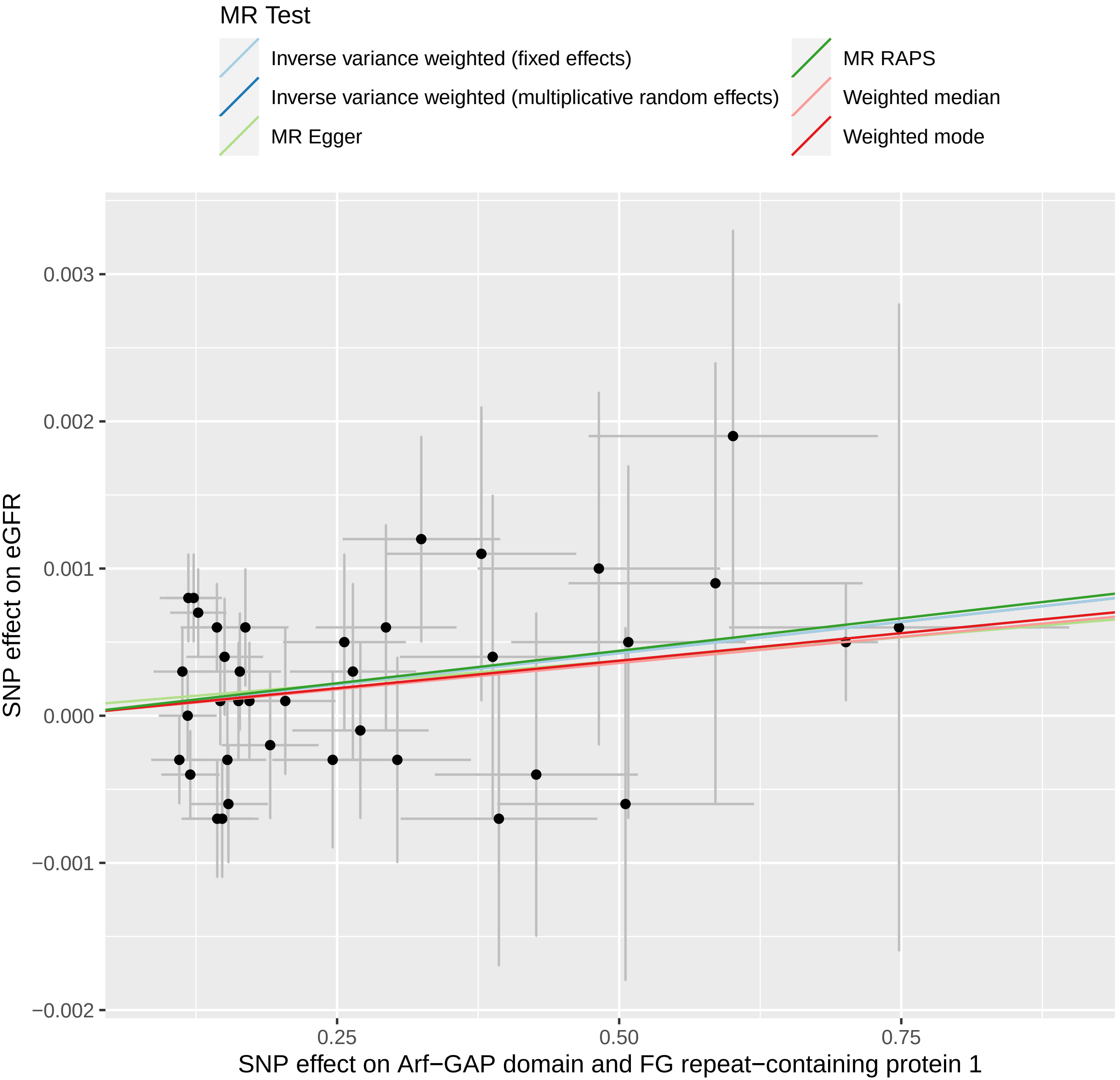

Batch 2−56 : Angiogenic factor with G patch and FHA domains 1 on eGFR

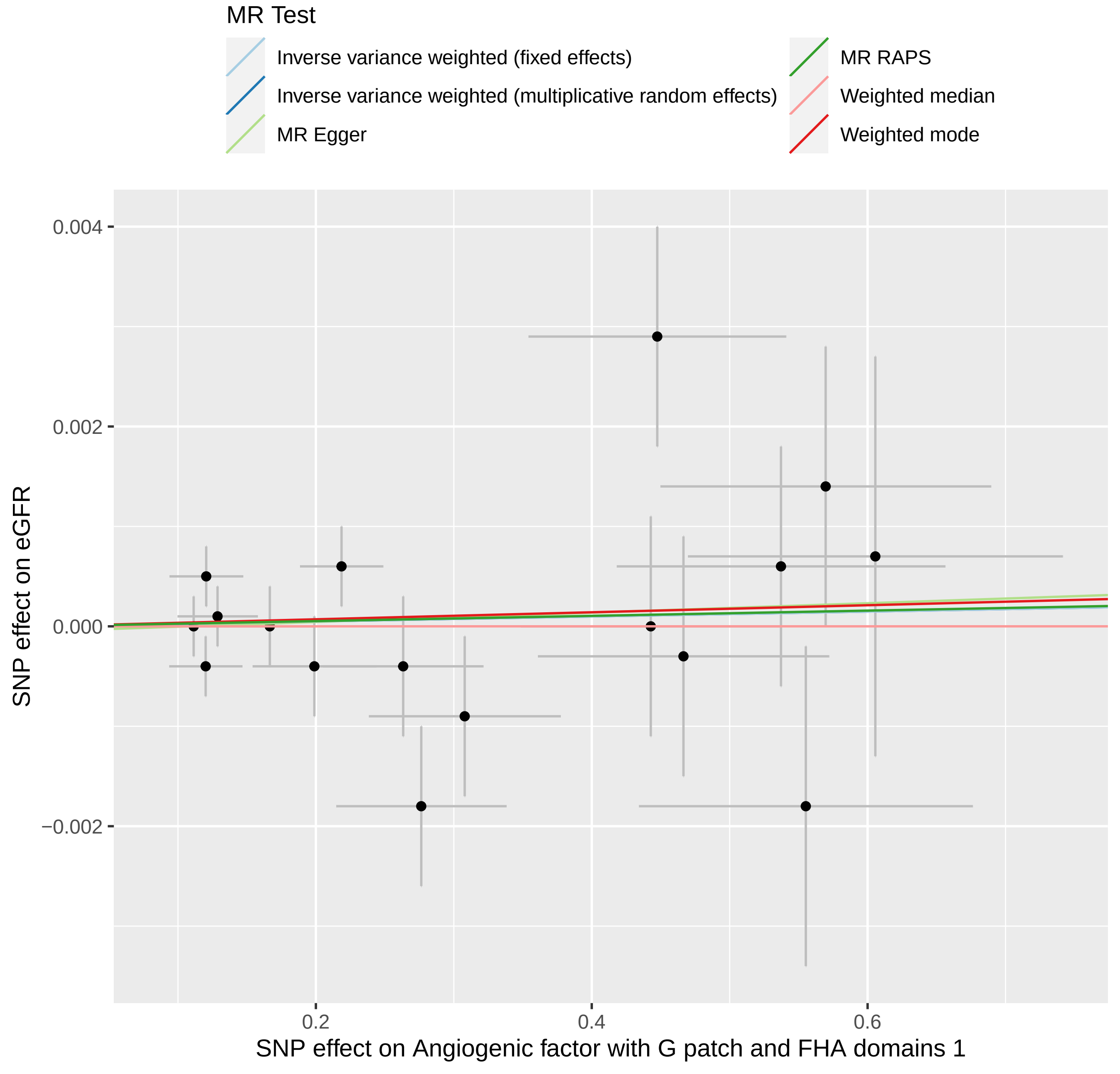

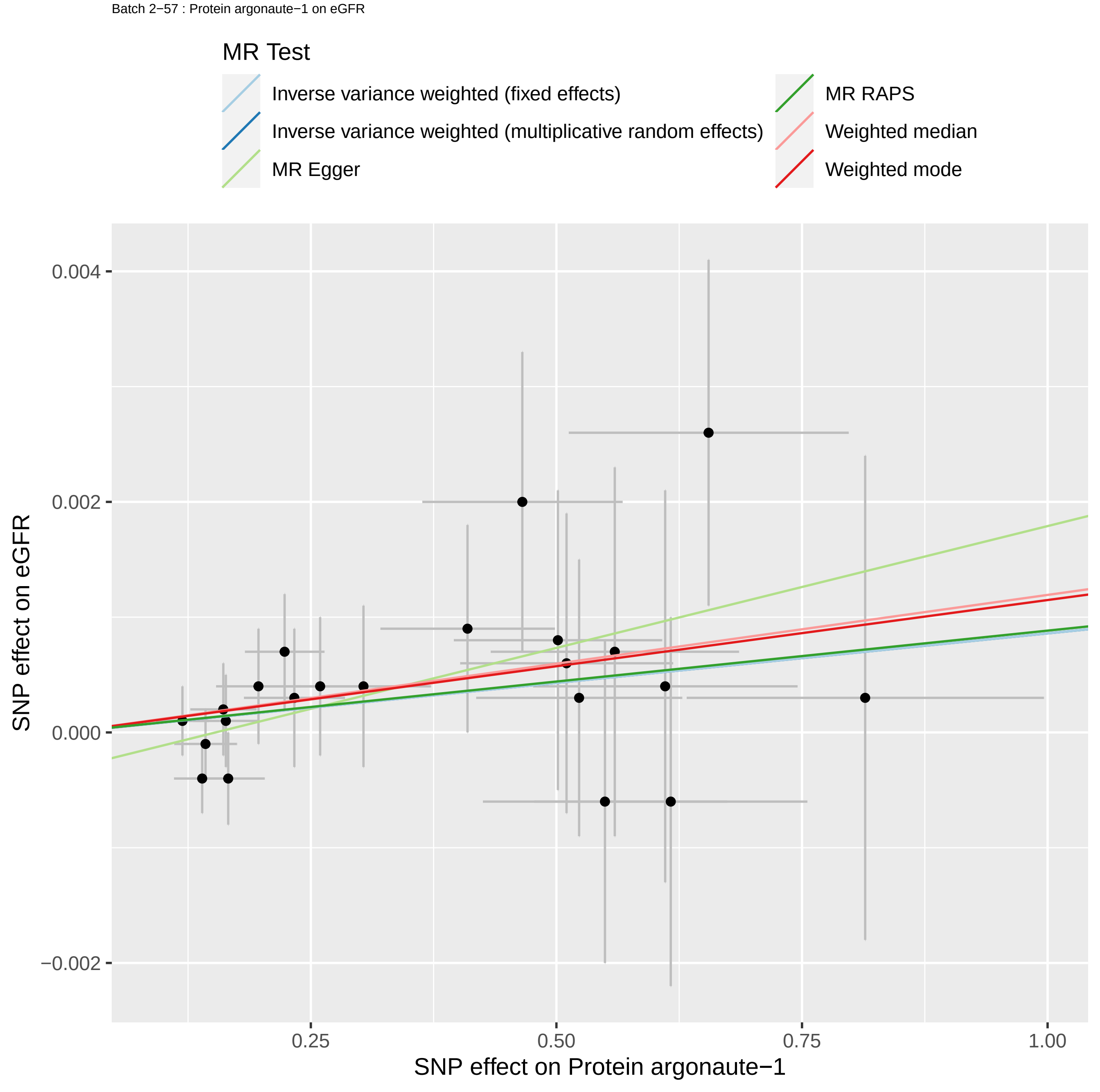
Batch 2−57 : Protein argonaute−1 on eGFR
MR Test
Inverse variance weighted (fixed effects)
Inverse variance weighted (multiplicative random effects)
MR Egger
MR RAPS
Weighted median
Weighted mode
0.004
0.002
0.000
−0.002
SNP effect on eGFR
0.25
0.50
0.75
1.00
SNP effect on Protein argonaute−1

Batch 2−58 : Anterior gradient protein 2 homolog on eGFR

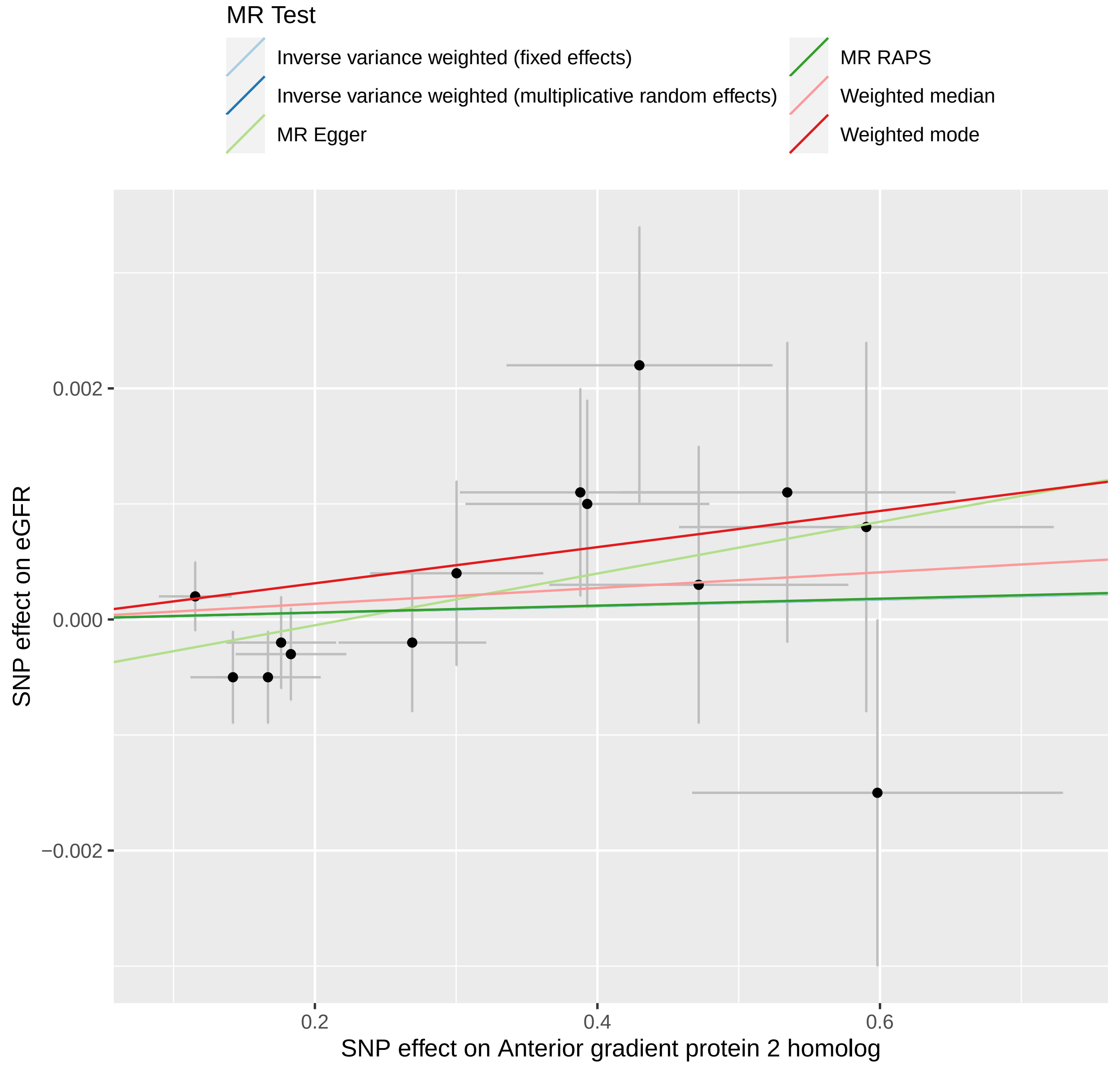

Batch 2−59 : Agouti−related protein on eGFR

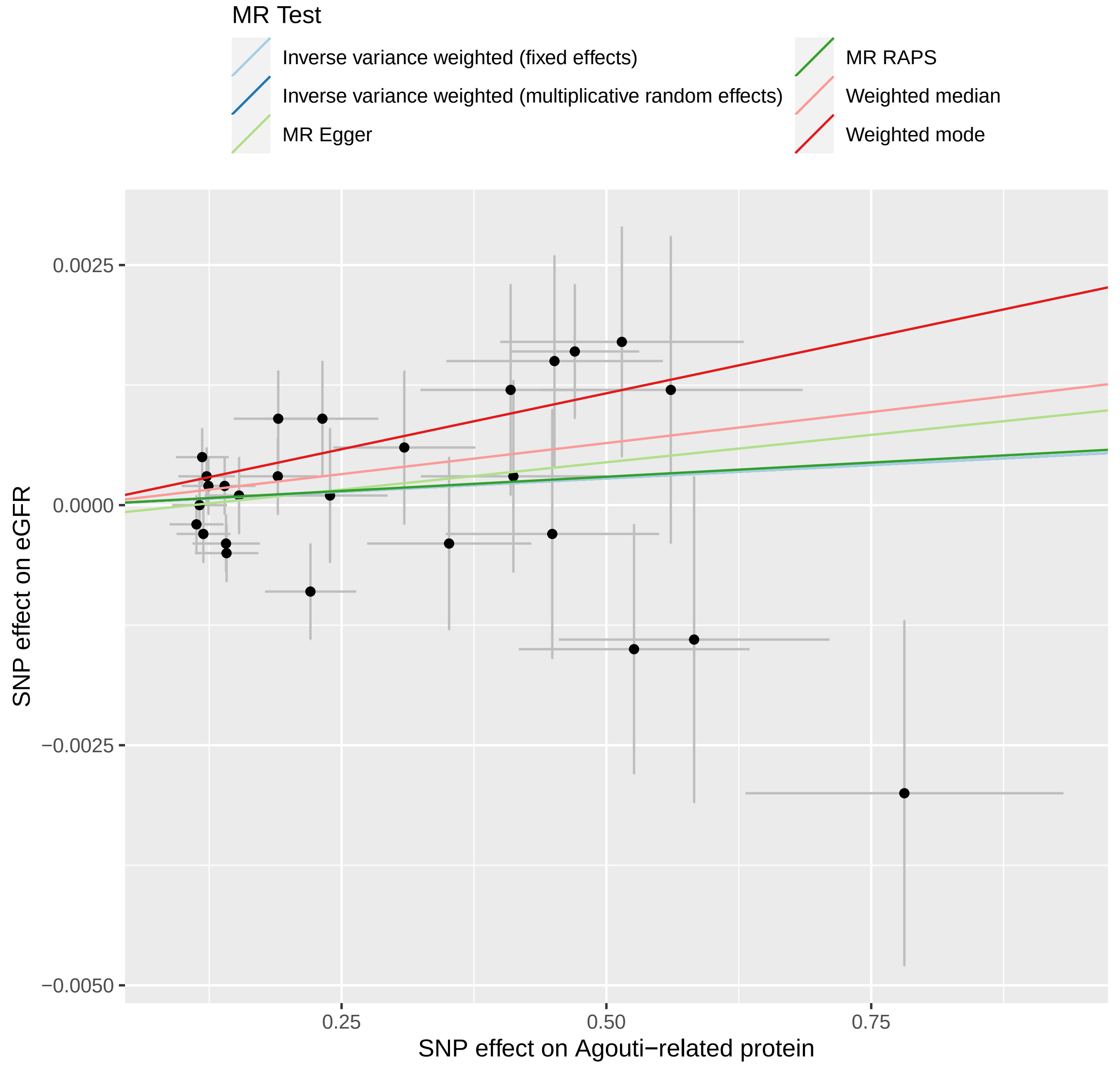

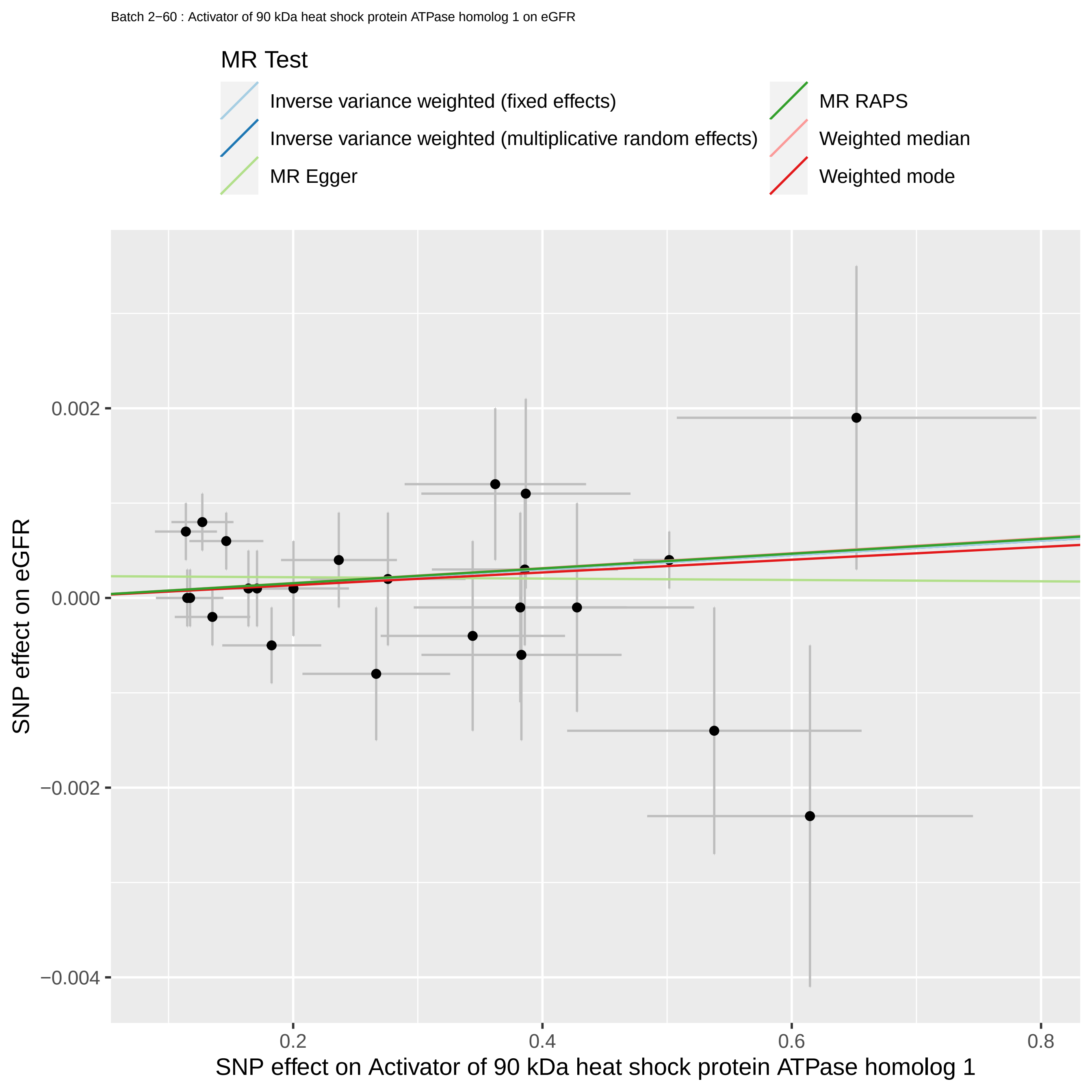
Batch 2−60 : Activator of 90 kDa heat shock protein ATPase homolog 1 on eGFR
MR Test
Inverse variance weighted (fixed effects)
Inverse variance weighted (multiplicative random effects)
MR Egger
MR RAPS
Weighted median
Weighted mode
0.002
0.000
−0.002
−0.004
0.2
0.4
0.6
0.8
SNP effect on eGFR
SNP effect on Activator of 90 kDa heat shock protein ATPase homolog 1

Batch 2−61 : Alpha−2−HS−glycoprotein on eGFR

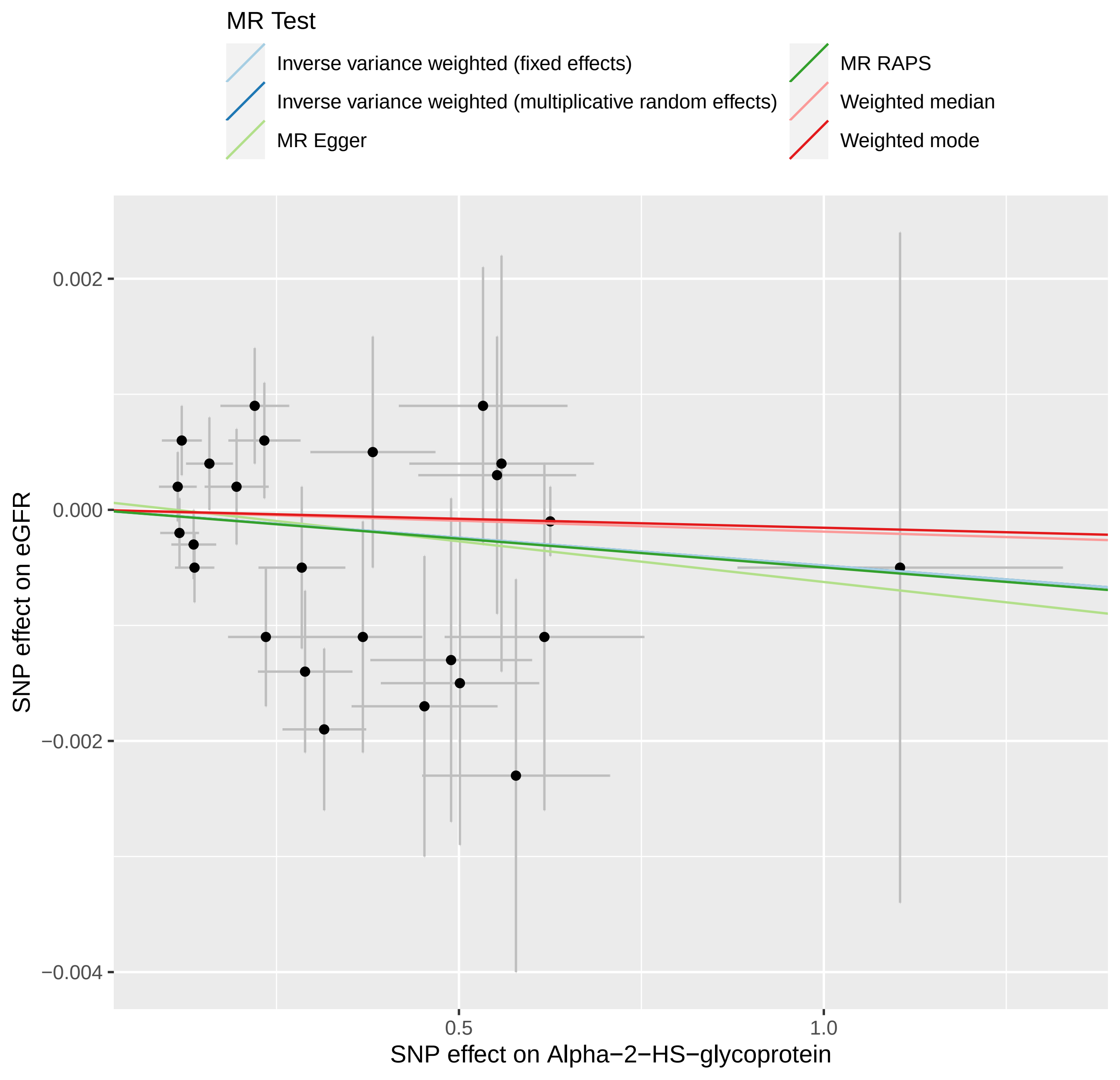

Batch 2−62 : Allograft inflammatory factor 1 on eGFR

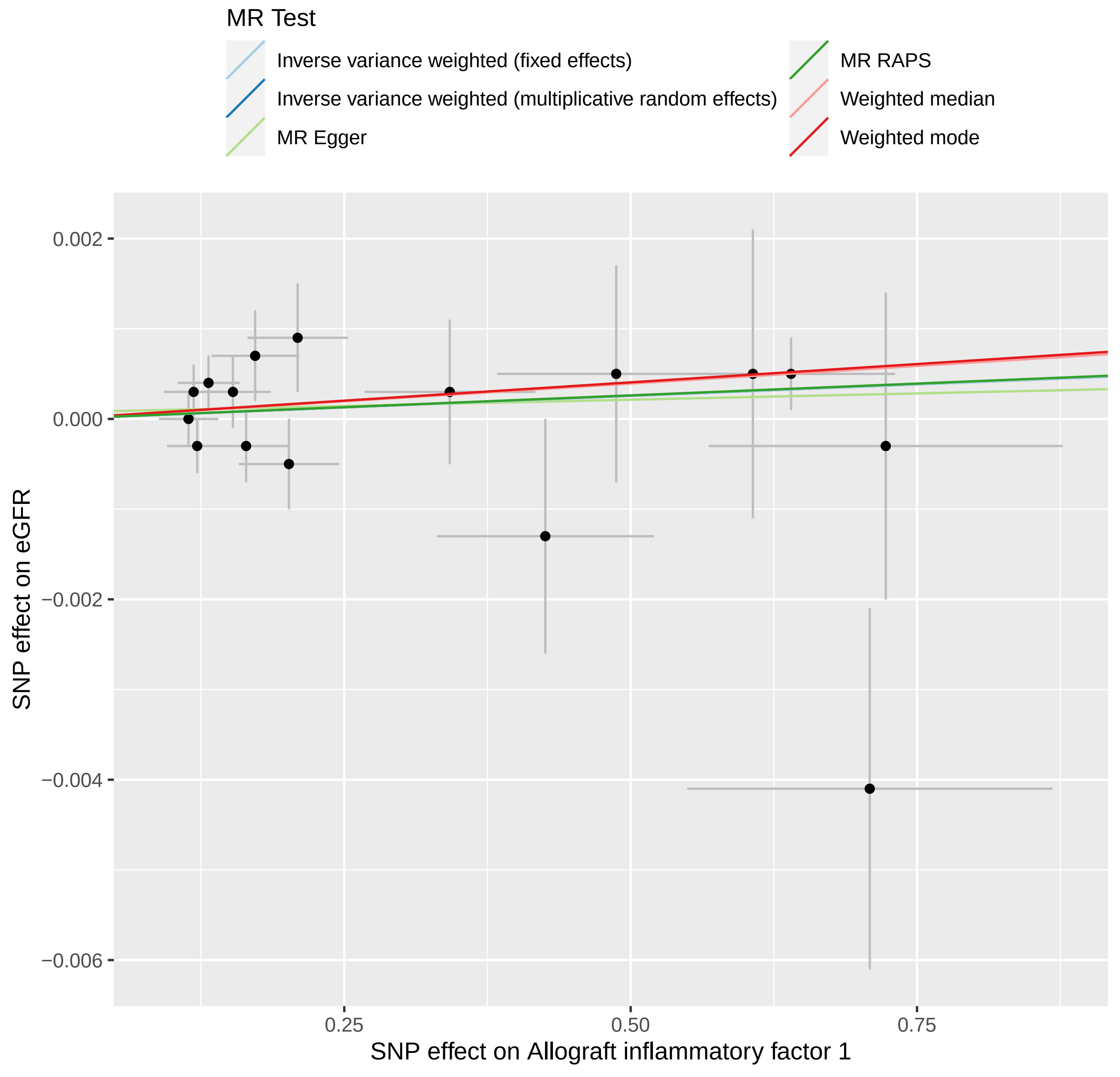

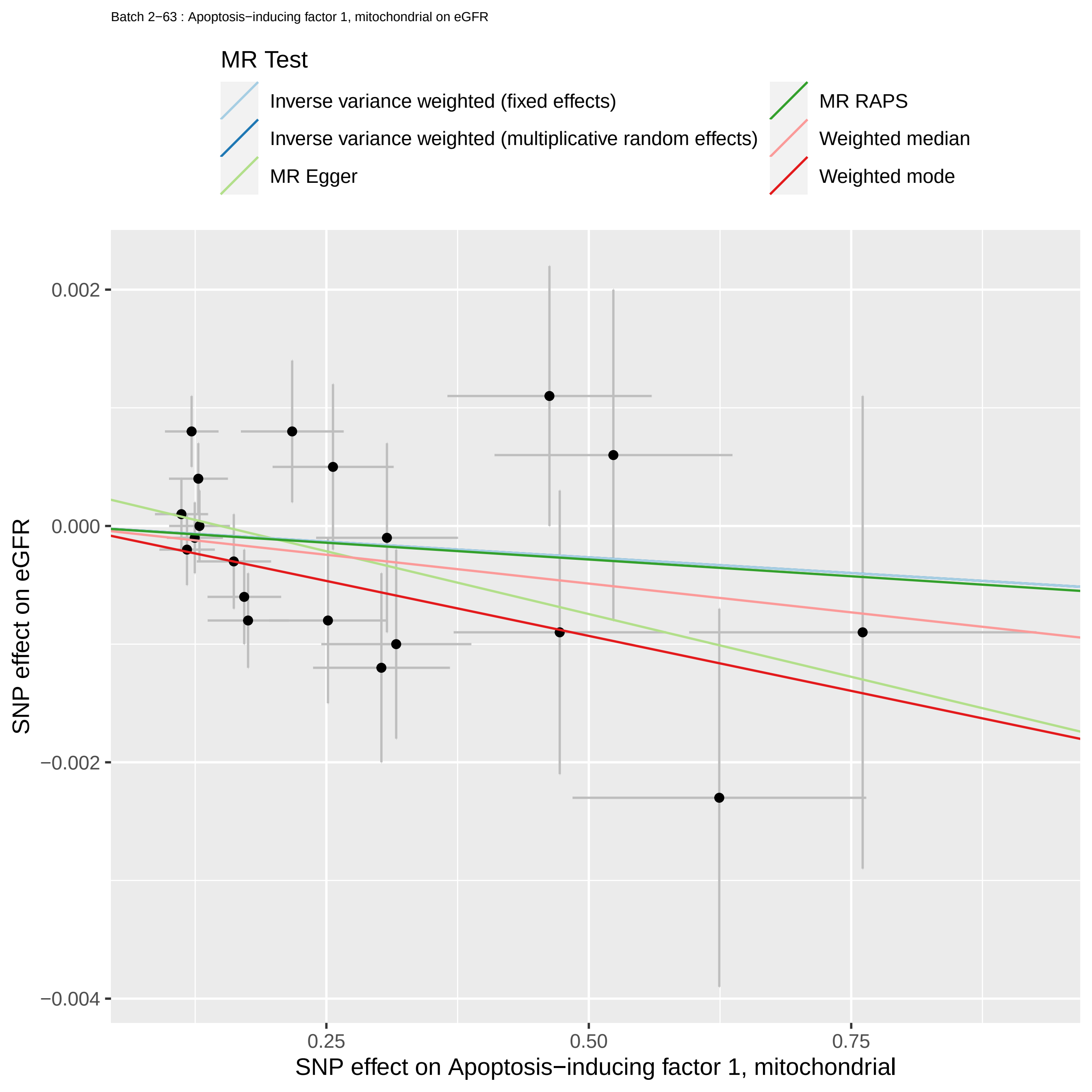
Batch 2−63 : Apoptosis−inducing factor 1, mitochondrial on eGFR
MR Test
Inverse variance weighted (fixed effects)
Inverse variance weighted (multiplicative random effects)
MR Egger
MR RAPS
Weighted median
Weighted mode
0.002
0.000
−0.002
−0.004
0.25
0.50
0.75
SNP effect on eGFR
SNP effect on Apoptosis−inducing factor 1, mitochondrial

Batch 2−64 : Apoptosis−inducing factor 1, mitochondrial on eGFR

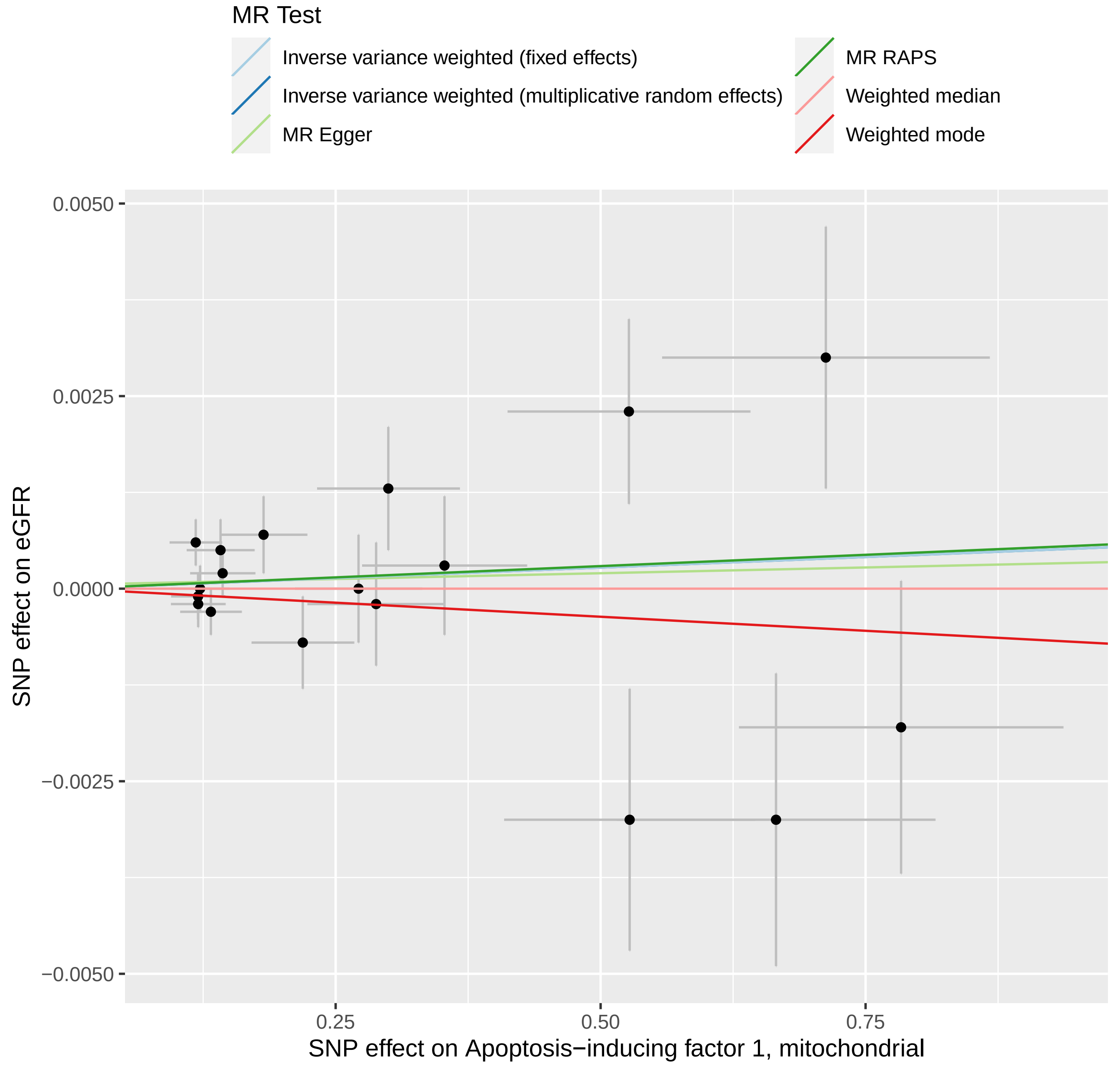

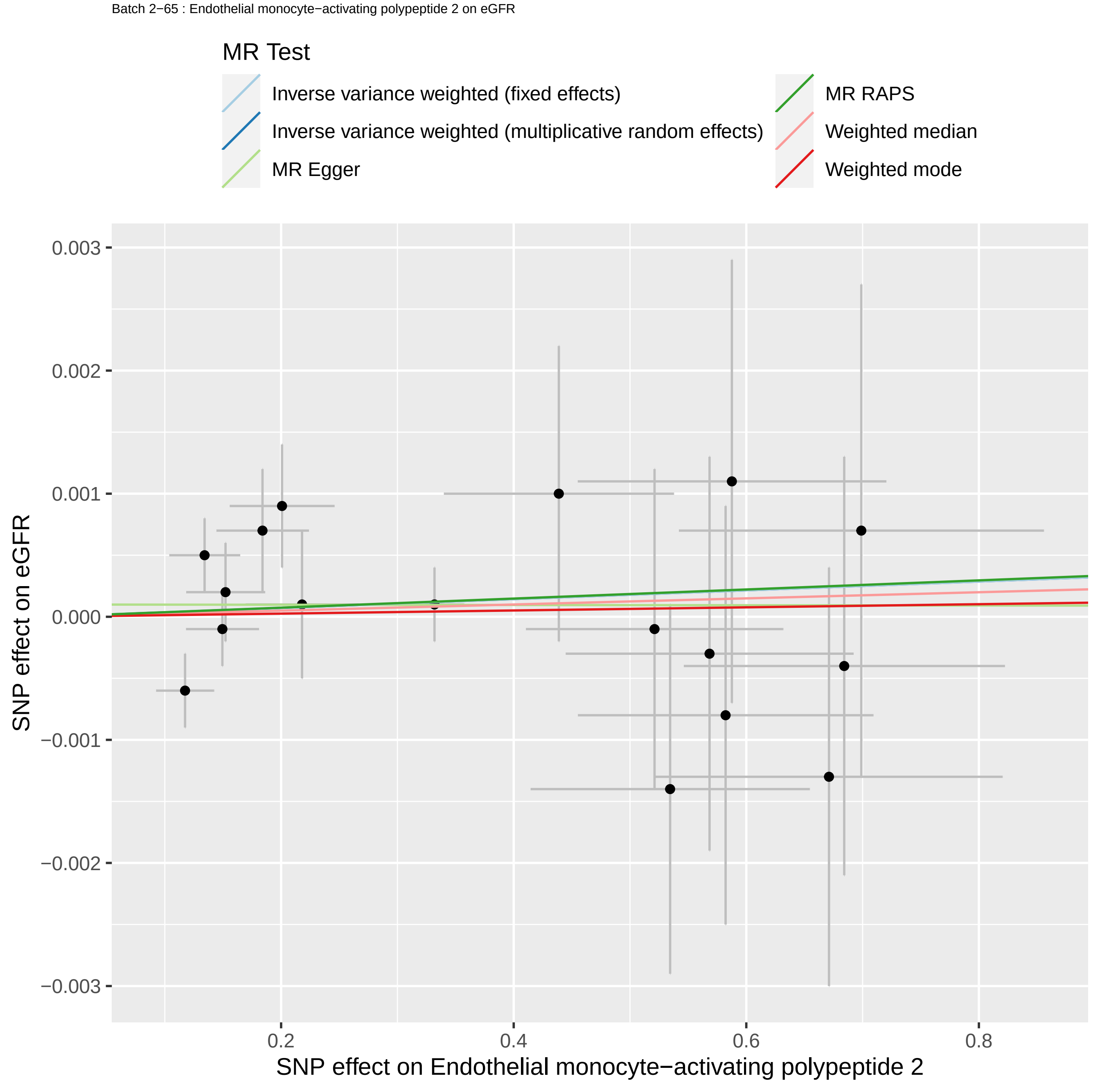
Batch 2−65 : Endothelial monocyte−activating polypeptide 2 on eGFR
MR Test
Inverse variance weighted (fixed effects)
Inverse variance weighted (multiplicative random effects)
MR Egger
MR RAPS
Weighted median
Weighted mode
0.003
0.002
0.001
0.000
−0.001
−0.002
−0.003
0.2
0.4
0.6
0.8
SNP effect on eGFR
SNP effect on Endothelial monocyte−activating polypeptide 2

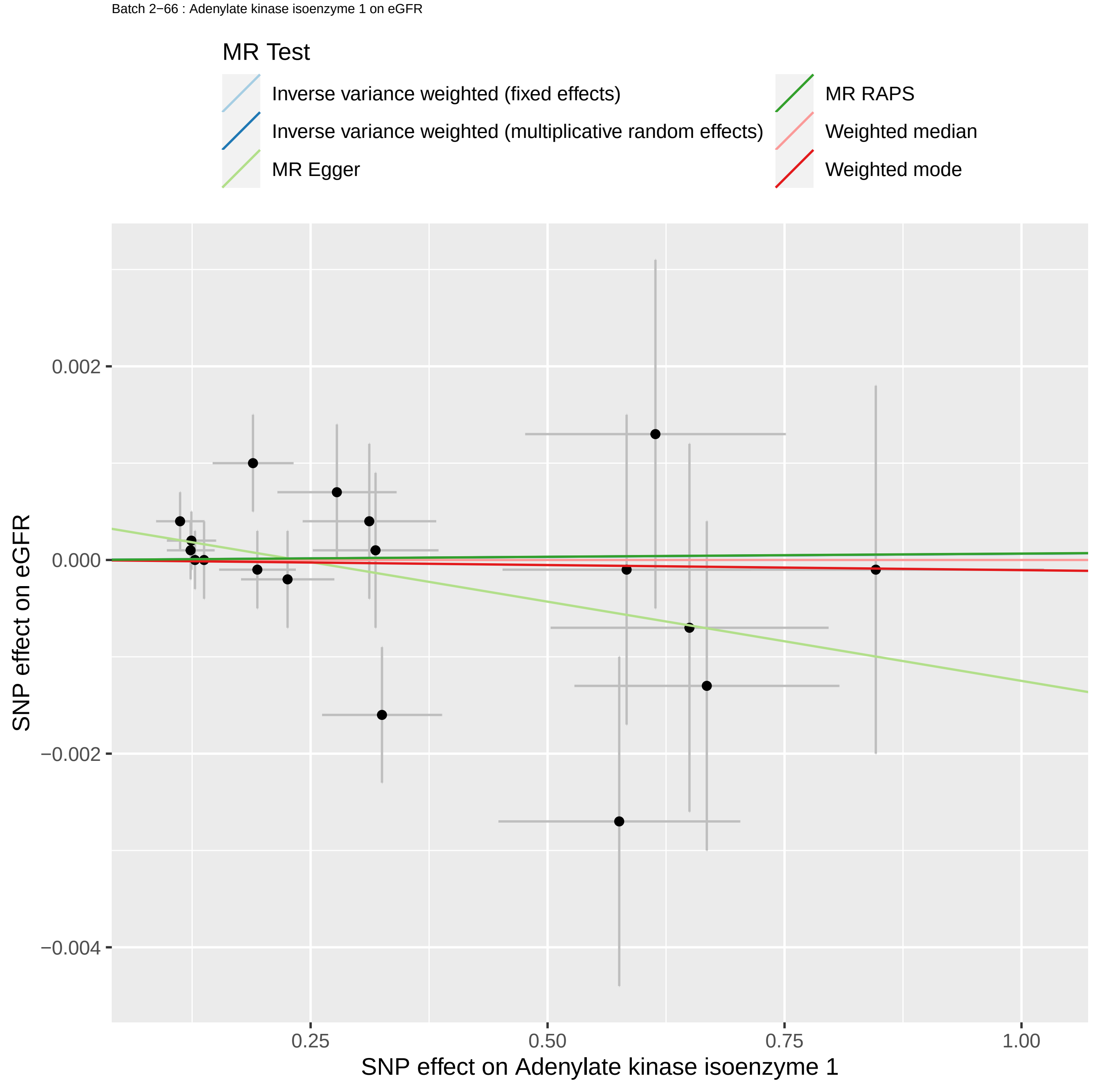

Batch 2−66 : Adenylate kinase isoenzyme 1 on eGFR
MR Test
Inverse variance weighted (fixed effects)
Inverse variance weighted (multiplicative random effects)
MR Egger
MR RAPS
Weighted median
Weighted mode
0.002
0.000
−0.002
−0.004
0.25
0.50
0.75
1.00
SNP effect on eGFR
SNP effect on Adenylate kinase isoenzyme 1

Batch 2−67 : Alcohol dehydrogenase [NADP(+)] on eGFR

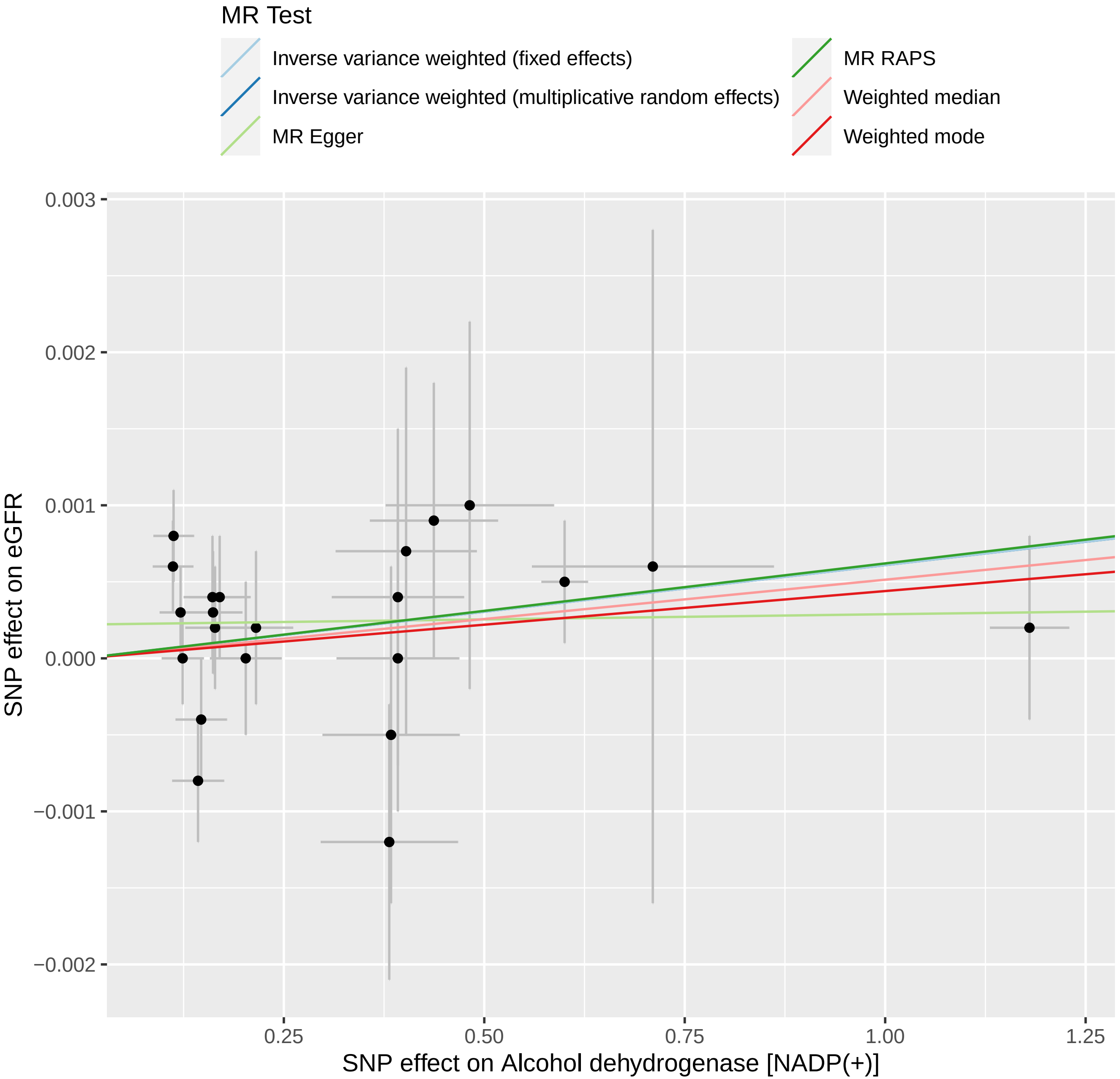

Batch 2−68 : Aldose reductase on eGFR

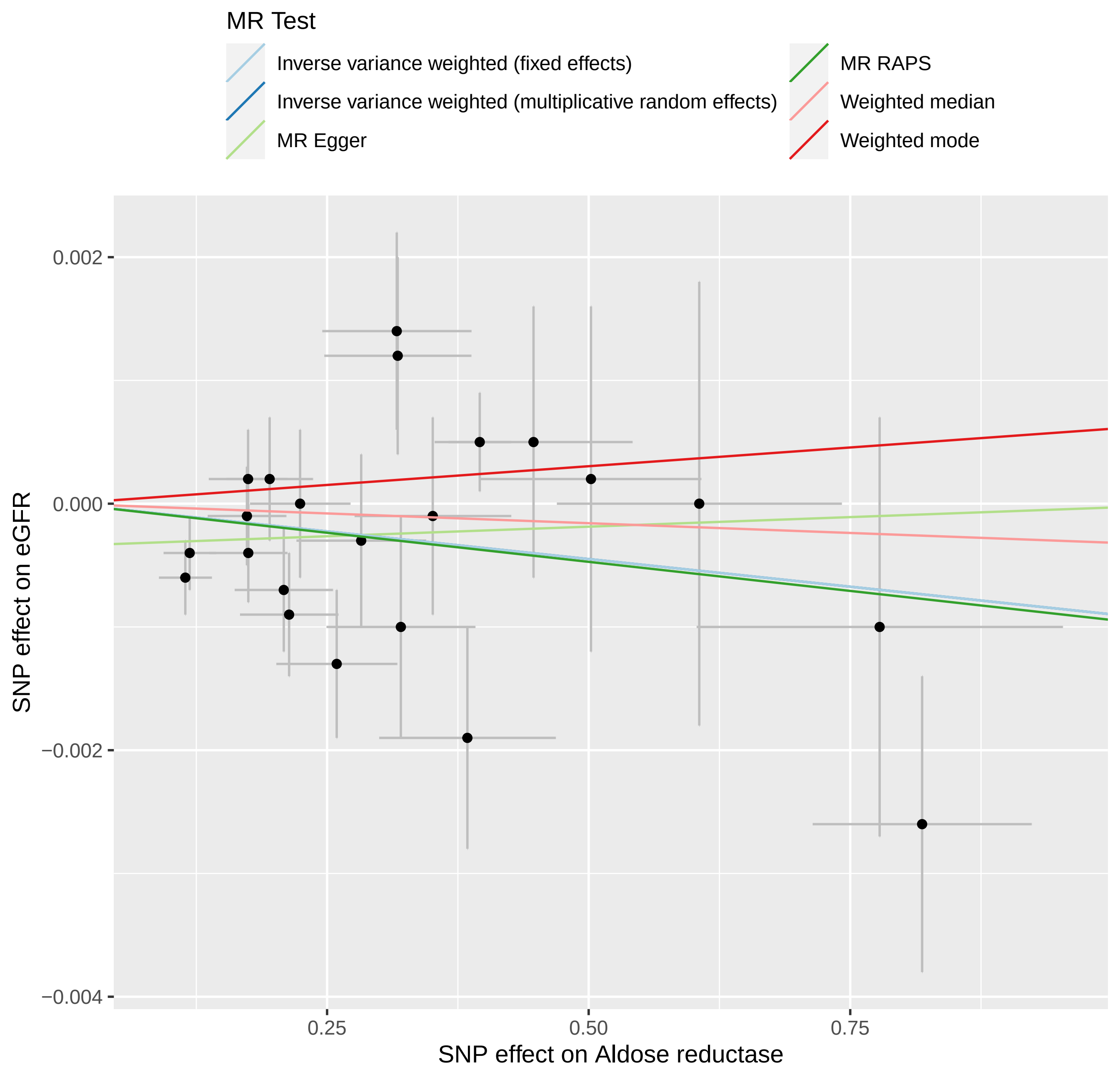

Batch 2−69 : Aldo−keto reductase family 1 member C1 on eGFR

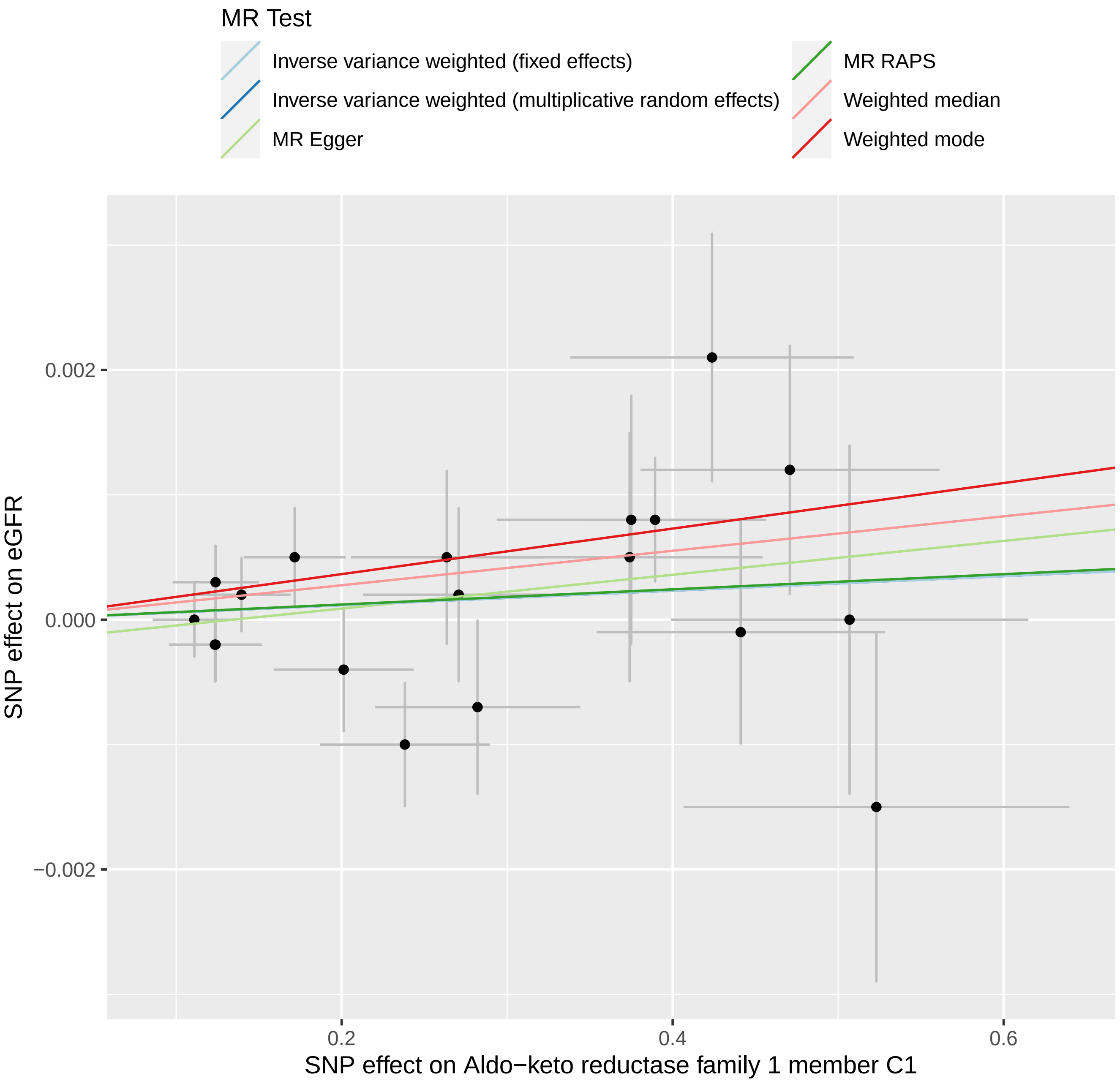

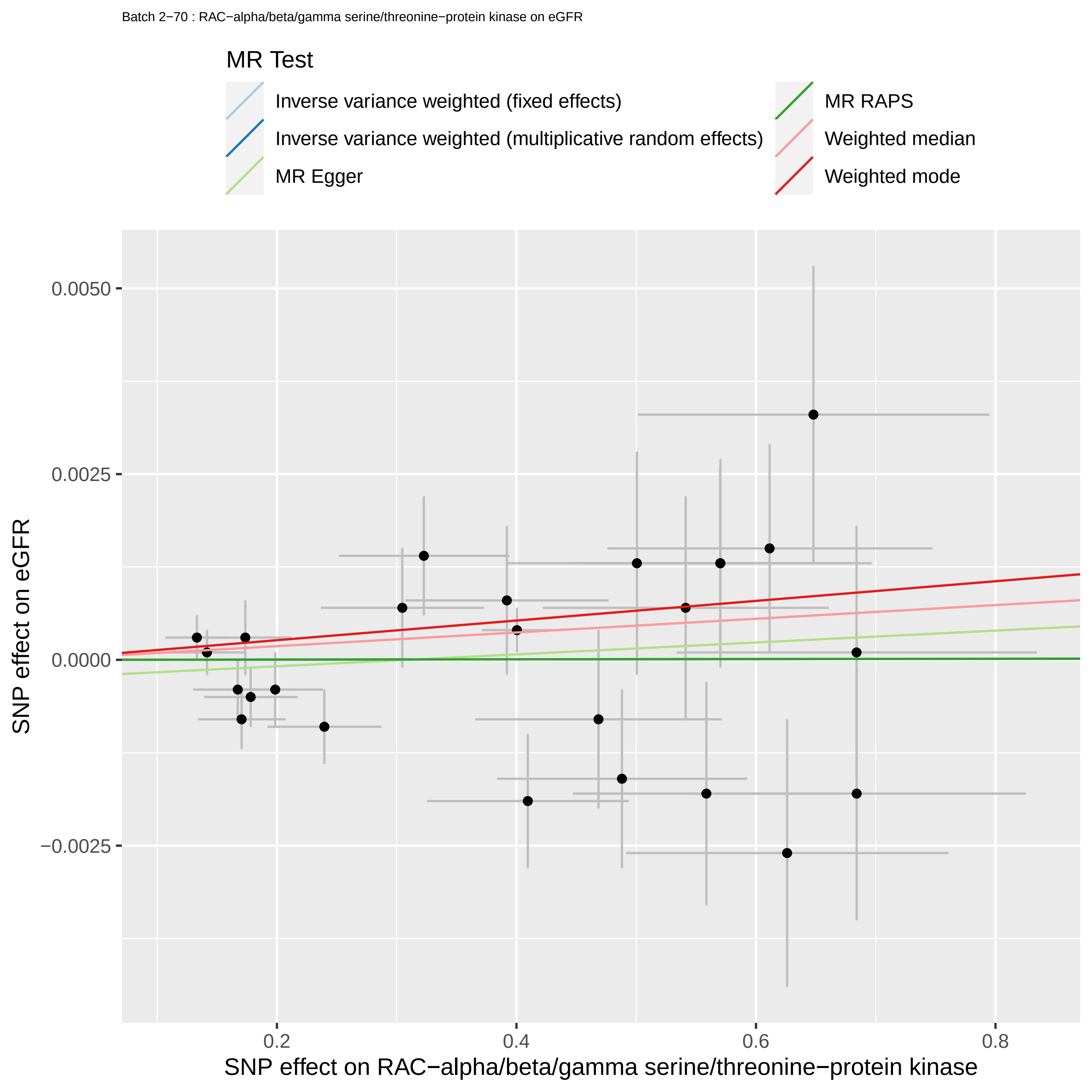
Batch 2−70 : RAC−alpha/beta/gamma serine/threonine−protein kinase on eGFR
MR Test
Inverse variance weighted (fixed effects)
Inverse variance weighted (multiplicative random effects)
MR Egger
MR RAPS
Weighted median
Weighted mode
0.0050
0.0025
0.0000
−0.0025
0.2
0.4
0.6
0.8
SNP effect on eGFR
SNP effect on RAC−alpha/beta/gamma serine/threonine−protein kinase

Batch 2−71 : RAC−alpha/beta/gamma serine/threonine−protein kinase on eGFR

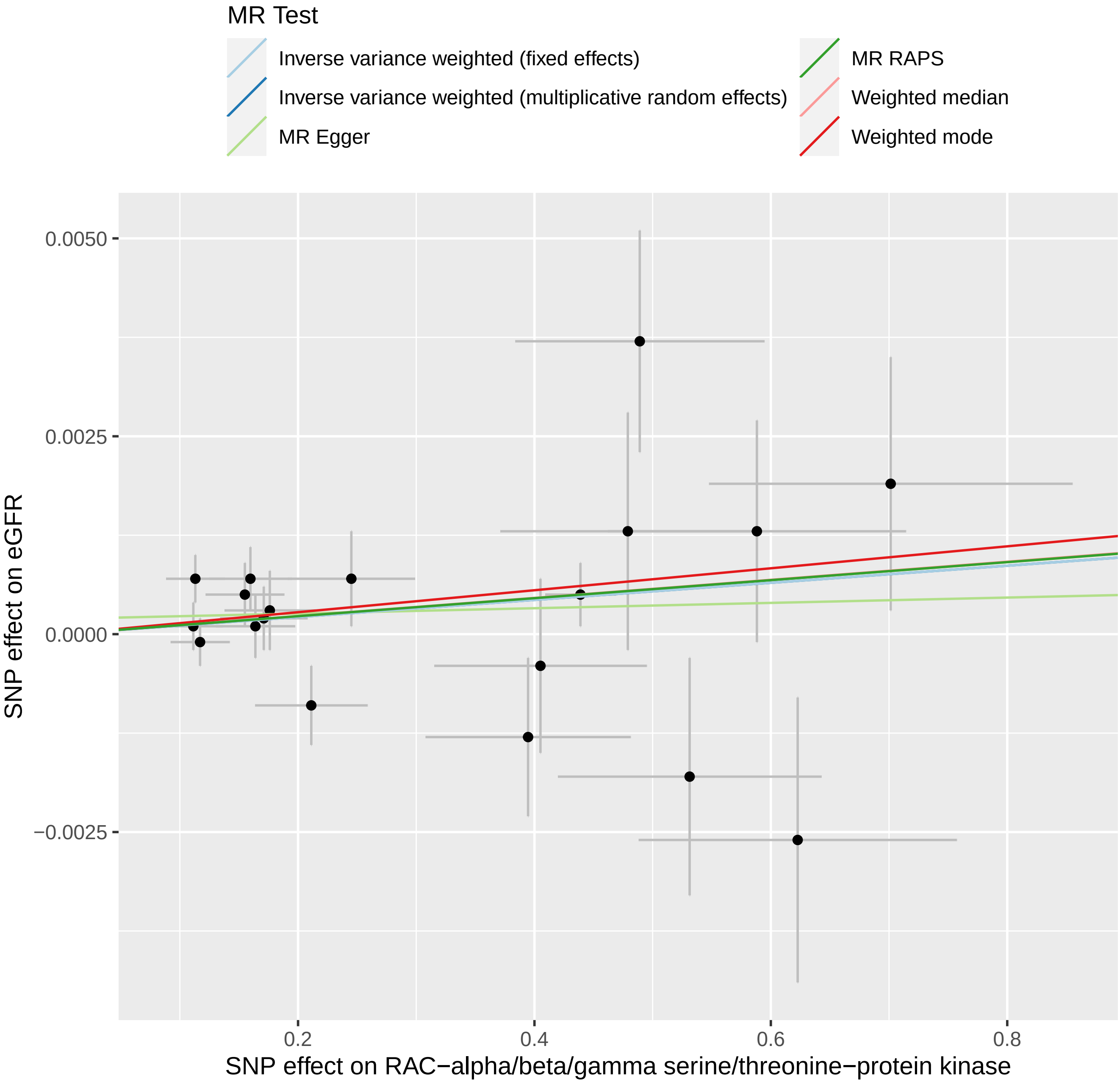

Batch 2−72 : RAC−beta serine/threonine−protein kinase on eGFR

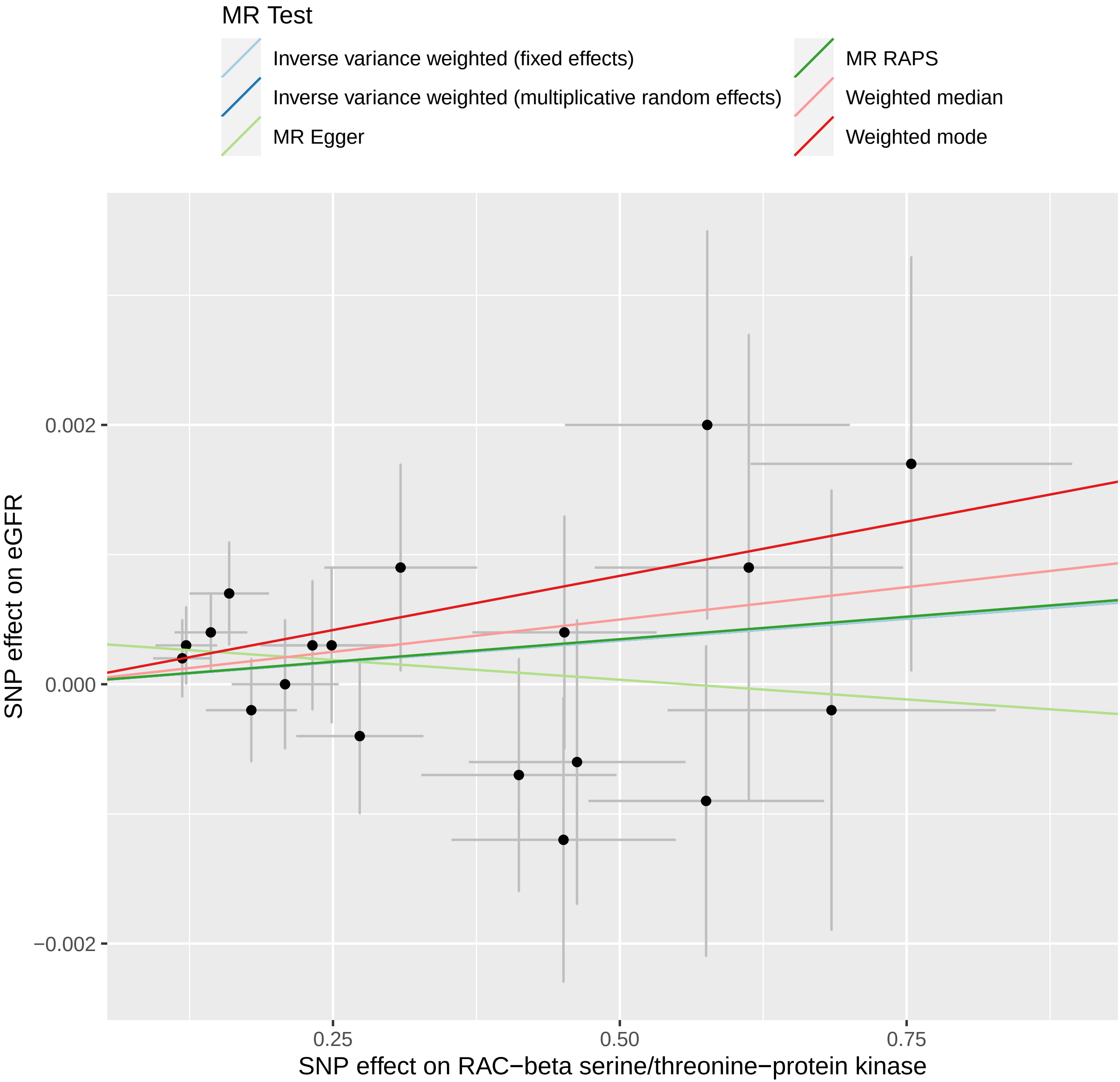

Batch 2−73 : RAC−beta serine/threonine−protein kinase on eGFR

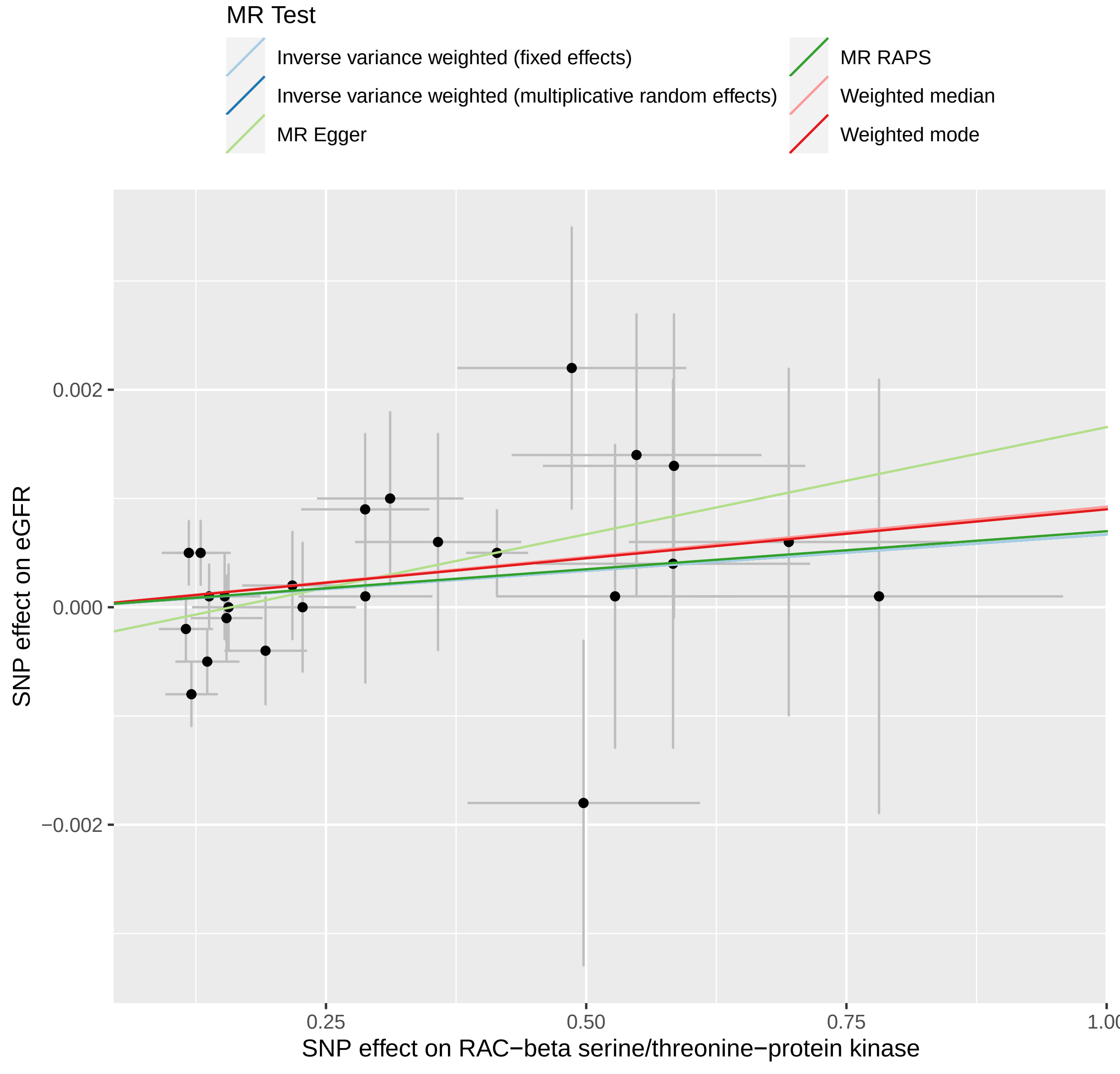

Batch 2−74 : CD166 antigen on eGFR

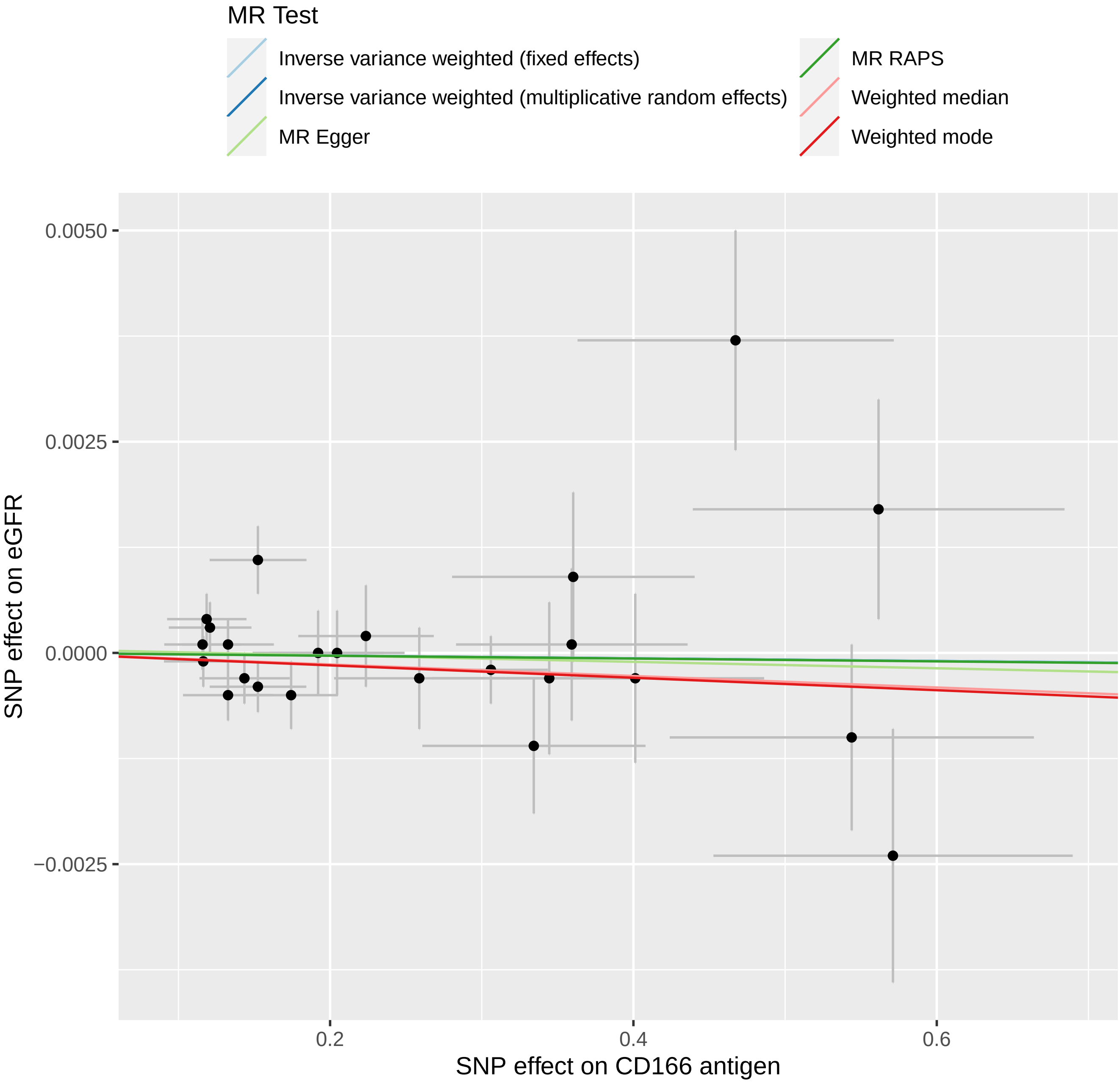

Batch 2−75 : Aldehyde dehydrogenase, dimeric NADP−preferring on eGFR

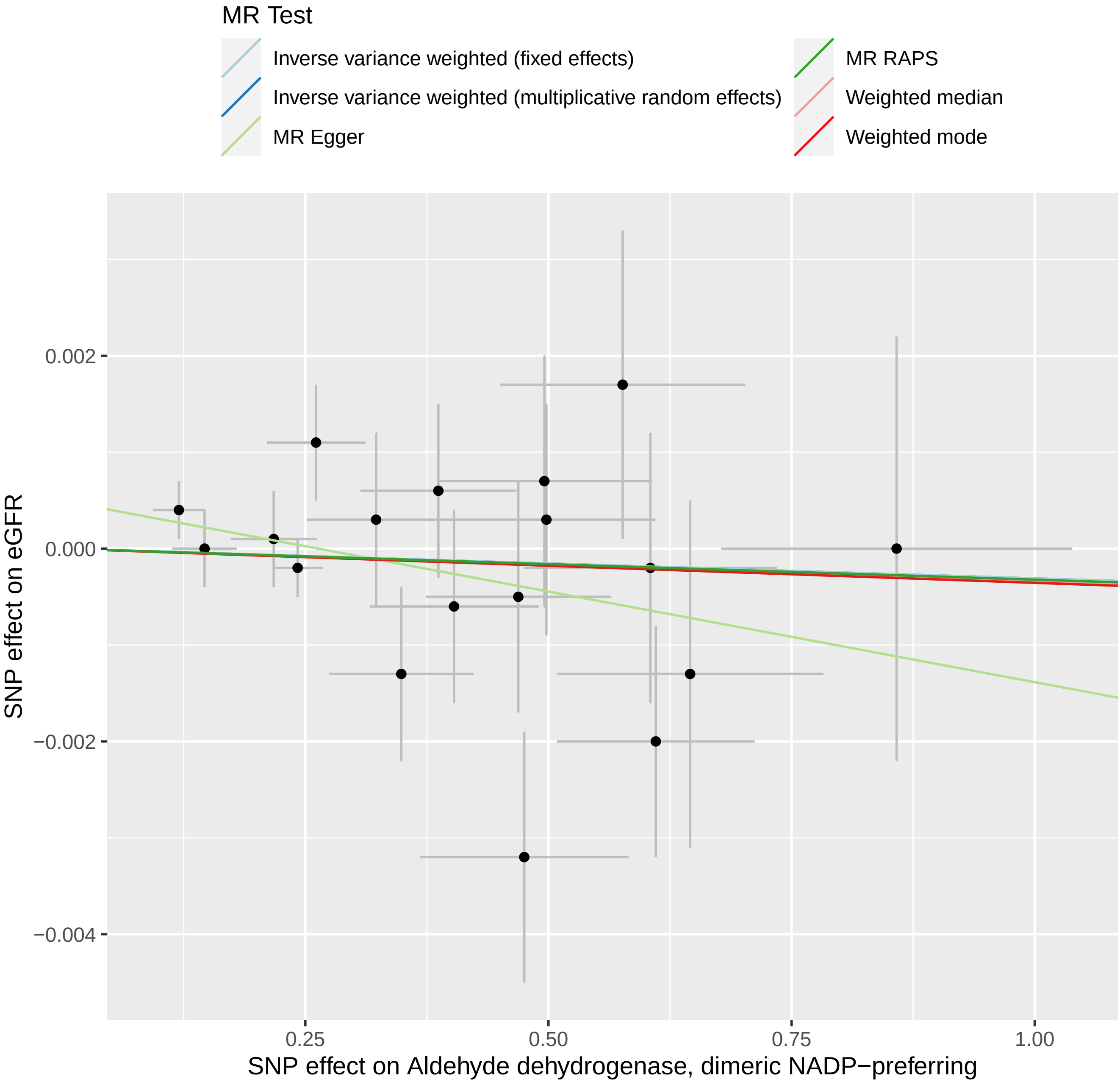

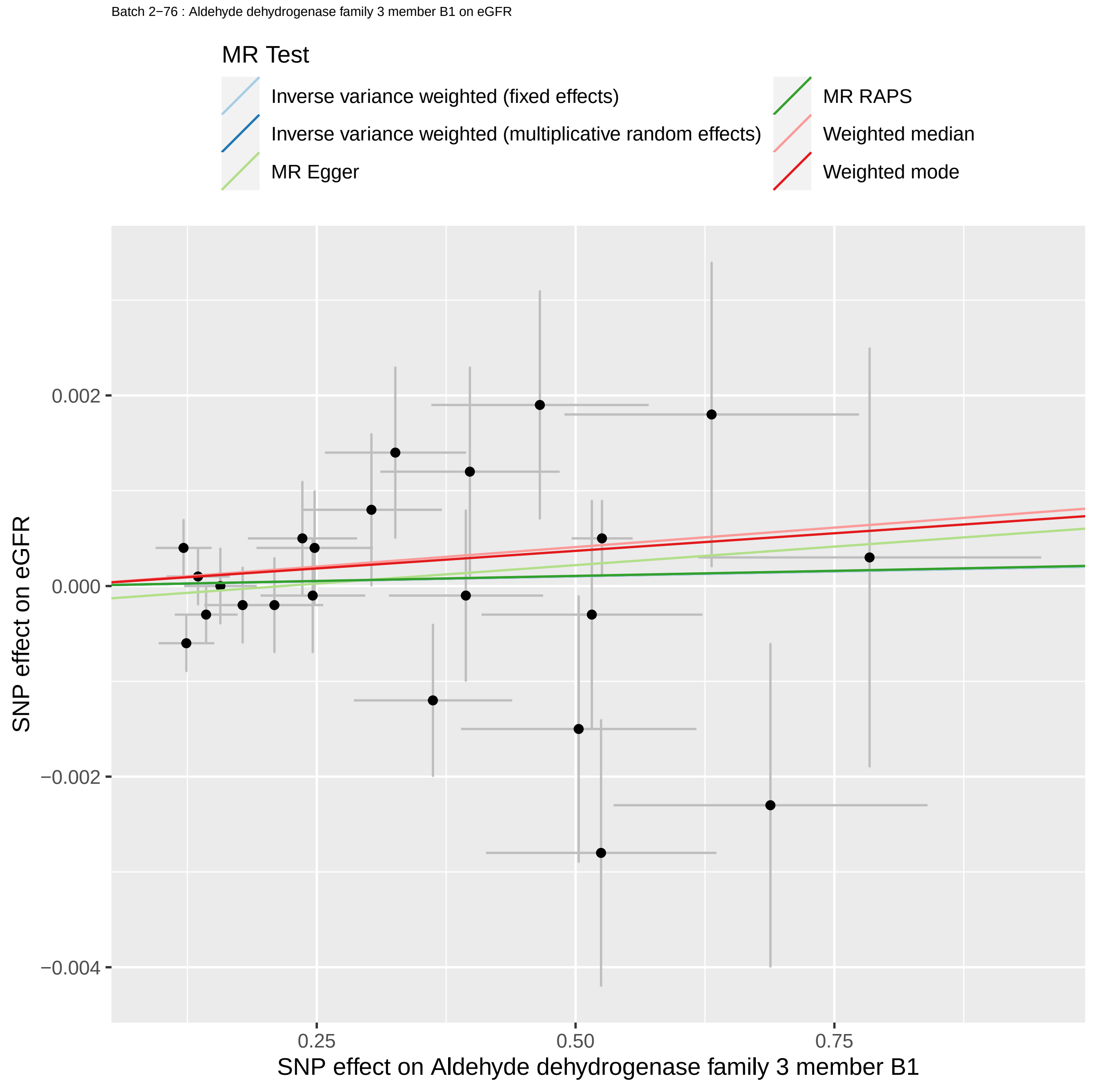
Batch 2−76 : Aldehyde dehydrogenase family 3 member B1 on eGFR
MR Test
Inverse variance weighted (fixed effects)
Inverse variance weighted (multiplicative random effects)
MR Egger
MR RAPS
Weighted median
Weighted mode
0.002
0.000
−0.002
−0.004
0.25
0.50
0.75
SNP effect on eGFR
SNP effect on Aldehyde dehydrogenase family 3 member B1

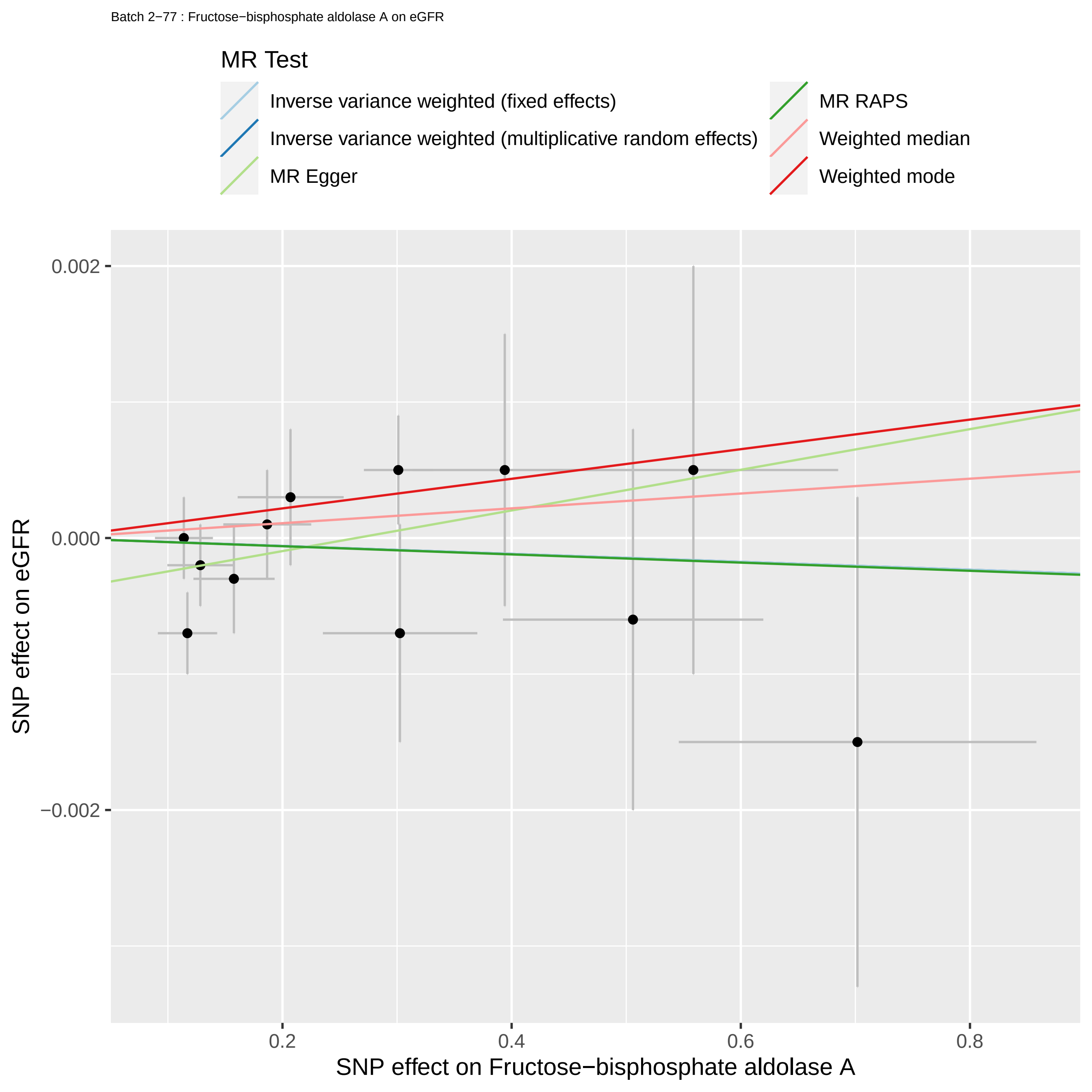

Batch 2−77 : Fructose−bisphosphate aldolase A on eGFR
MR Test
Inverse variance weighted (fixed effects)
Inverse variance weighted (multiplicative random effects)
MR Egger
MR RAPS
Weighted median
Weighted mode
0.002
0.000
−0.002
0.2
0.4
0.6
0.8
SNP effect on eGFR
SNP effect on Fructose−bisphosphate aldolase A

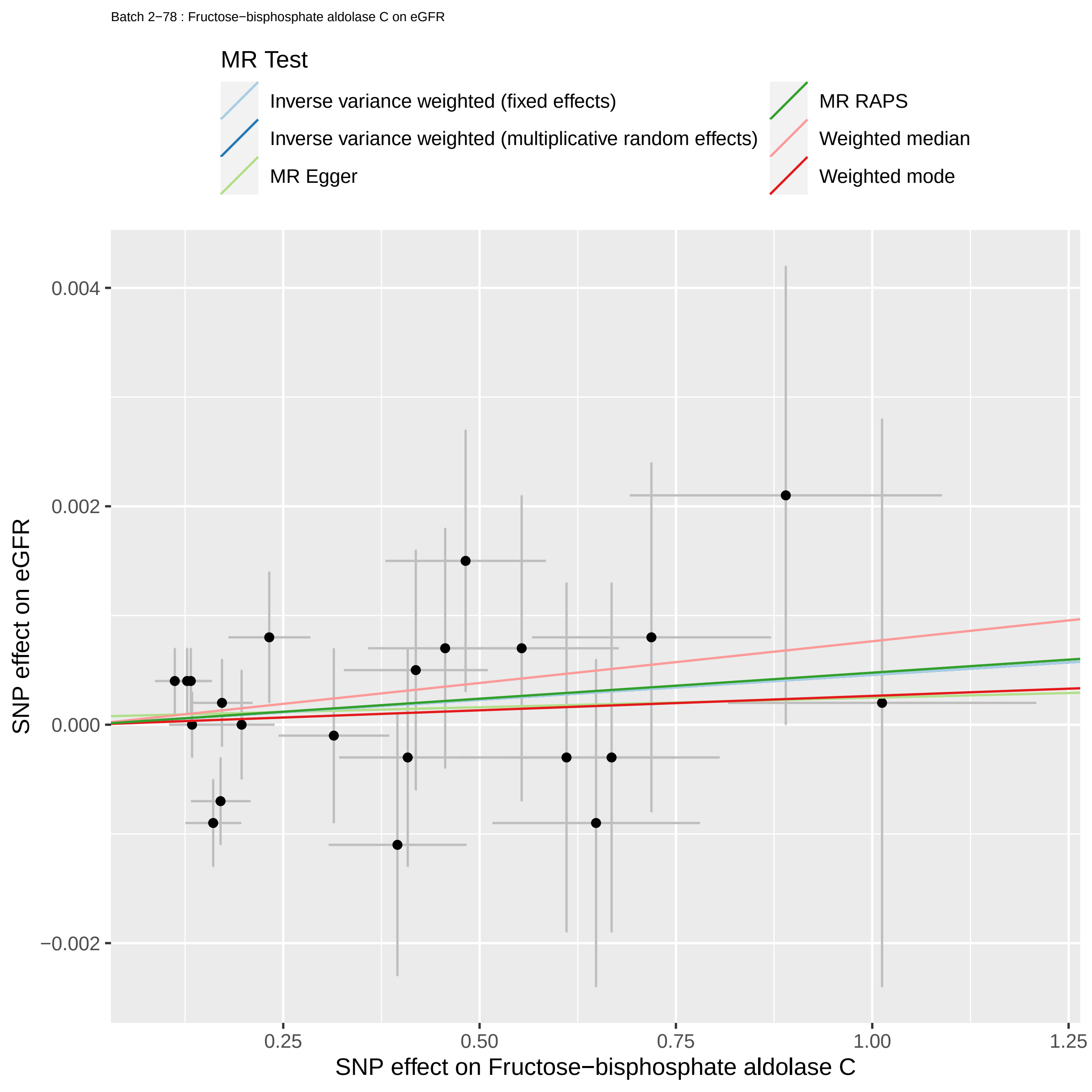

Batch 2−78 : Fructose−bisphosphate aldolase C on eGFR
MR Test
Inverse variance weighted (fixed effects)
Inverse variance weighted (multiplicative random effects)
MR Egger
MR RAPS
Weighted median
Weighted mode
0.004
0.002
0.000
−0.002
SNP effect on eGFR
0.25
0.50
0.75
1.00
1.25
SNP effect on Fructose−bisphosphate aldolase C

Batch 2−79 : RNA demethylase ALKBH5 on eGFR

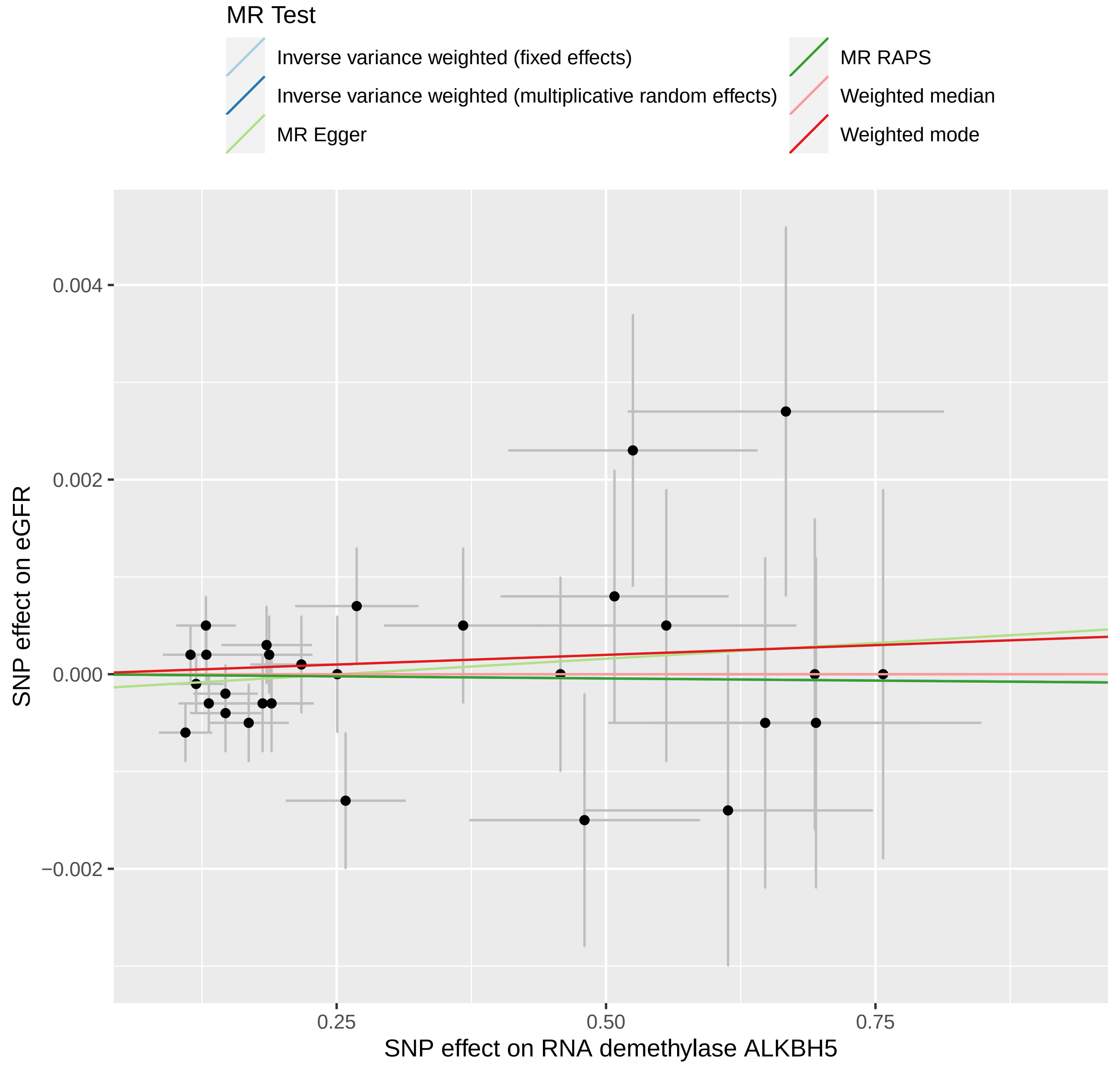

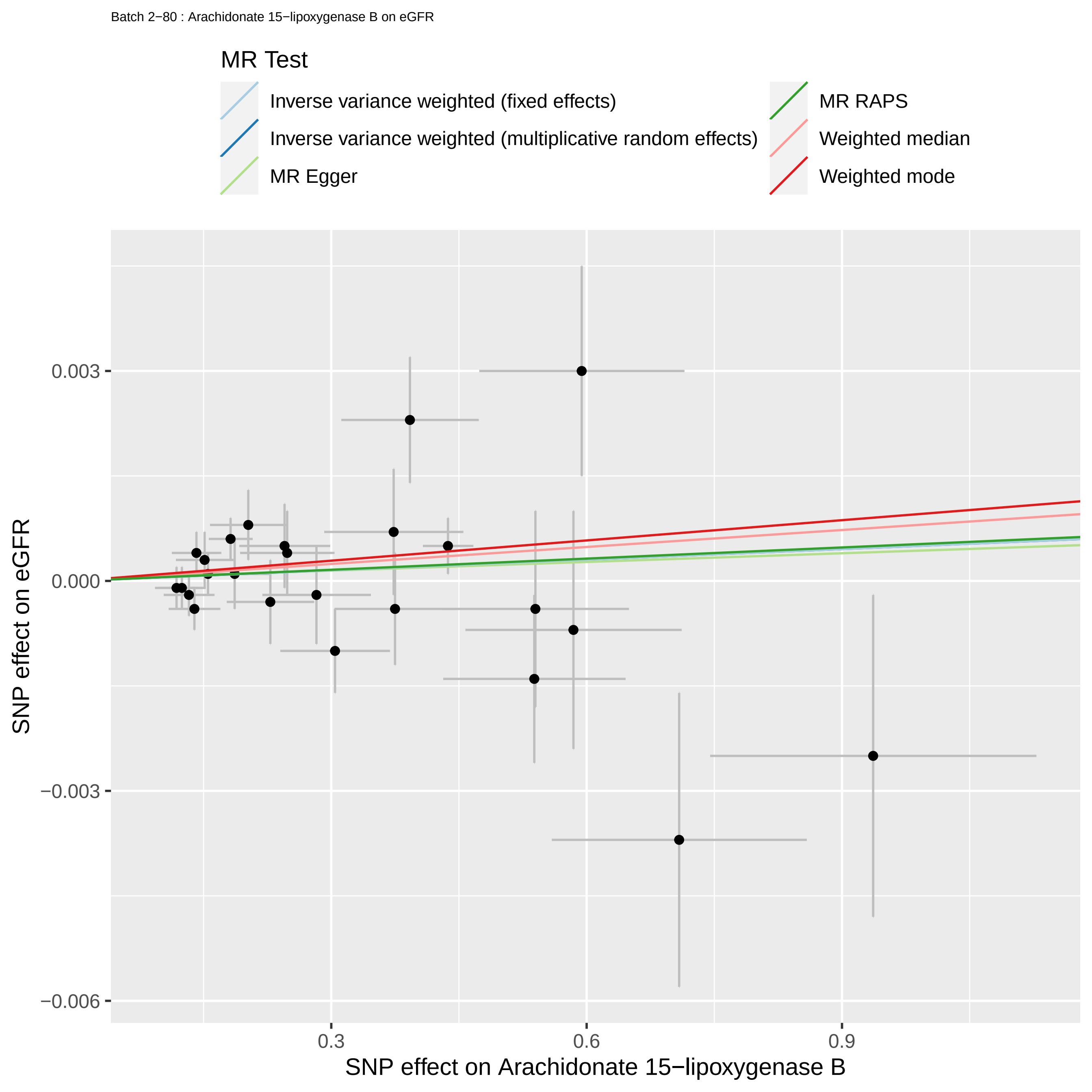
Batch 2−80 : Arachidonate 15−lipoxygenase B on eGFR
MR Test
Inverse variance weighted (fixed effects)
Inverse variance weighted (multiplicative random effects)
MR Egger
MR RAPS
Weighted median
Weighted mode
0.003
0.000
−0.003
−0.006
0.3
0.6
0.9
SNP effect on eGFR
SNP effect on Arachidonate 15−lipoxygenase B

Batch 2−81 : Intestinal−type alkaline phosphatase on eGFR

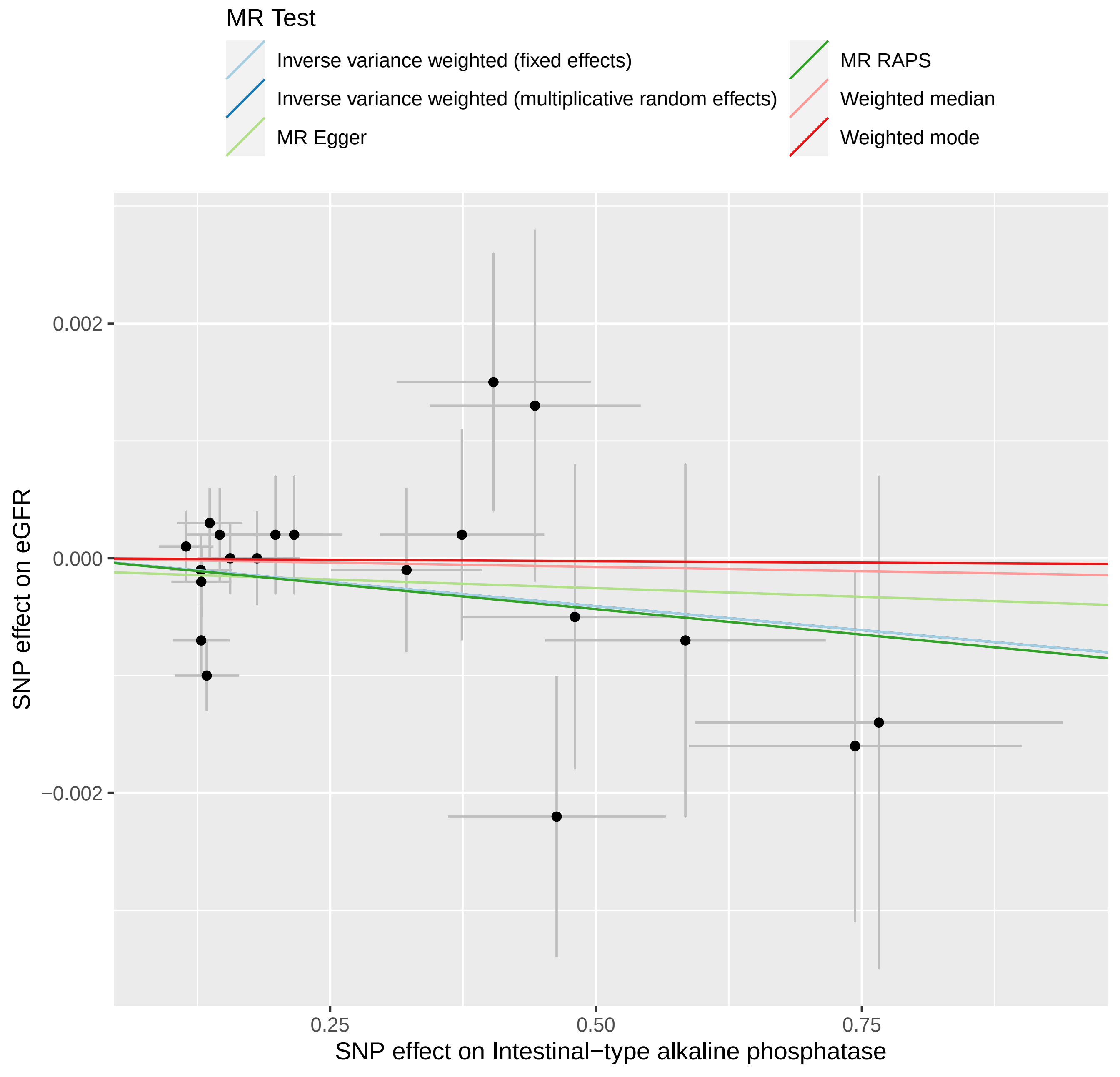

Batch 2−82 : Ameloblastin on eGFR

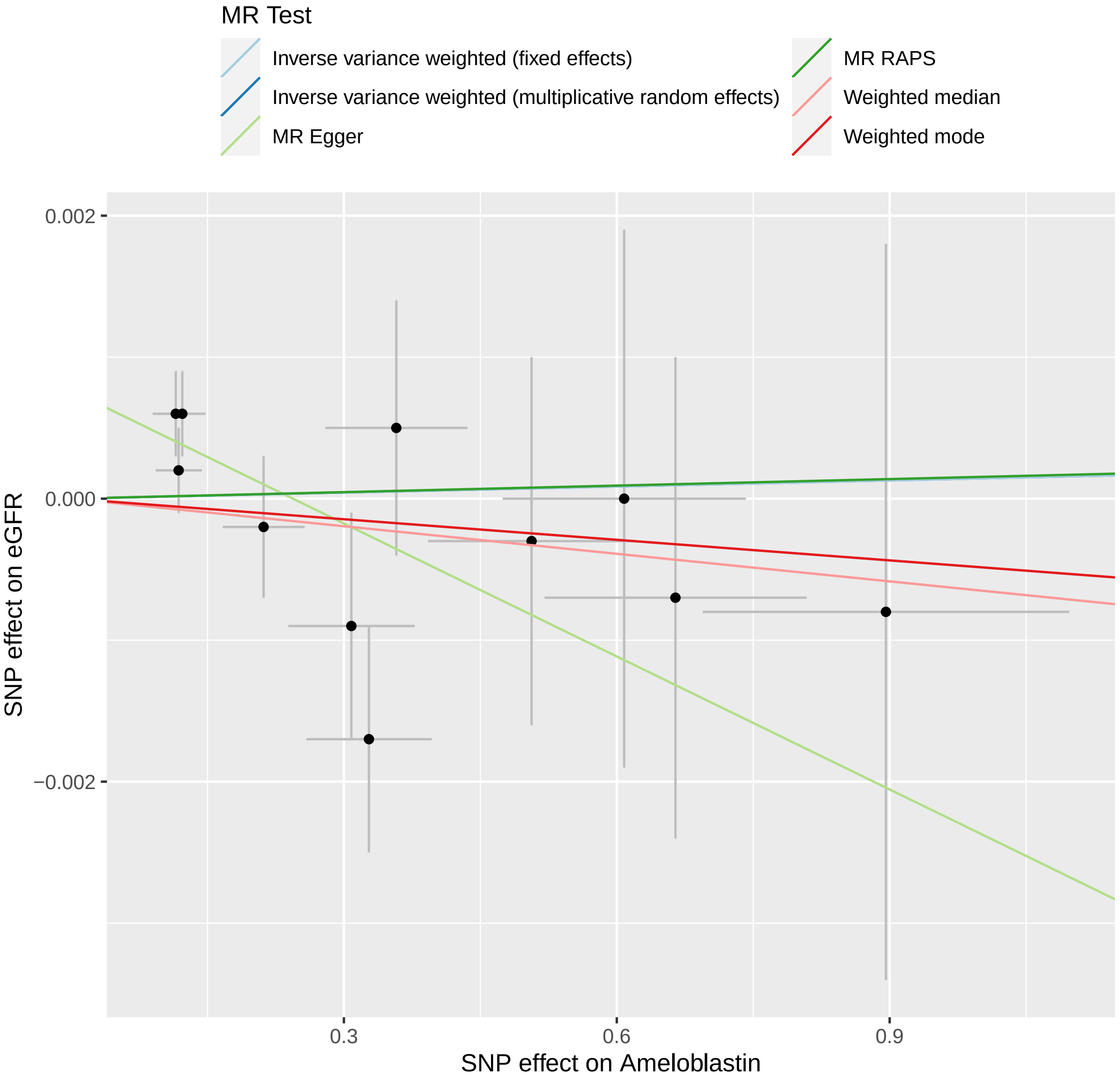

Batch 2−83 : Amelogenin, X isoform on eGFR

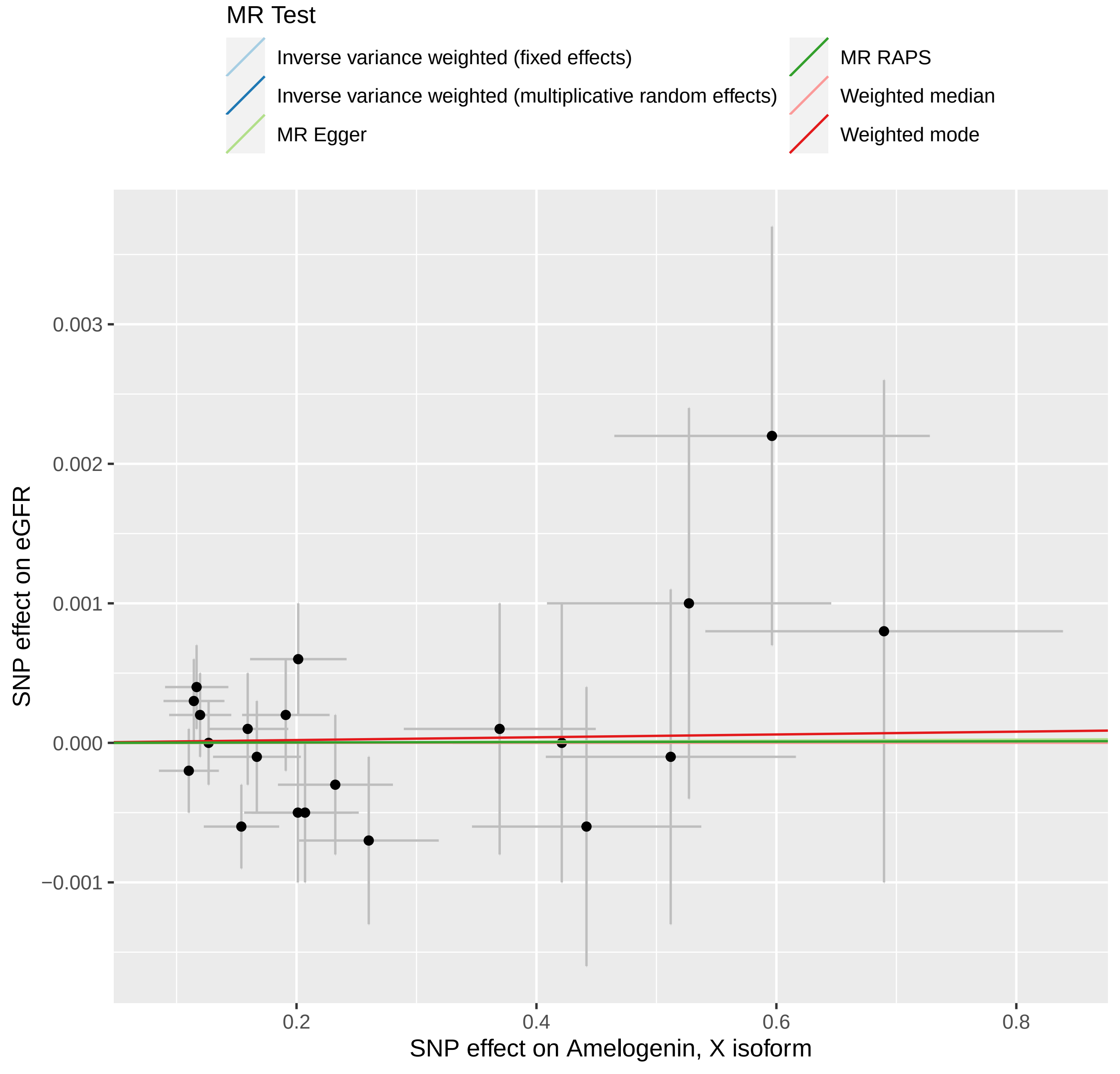

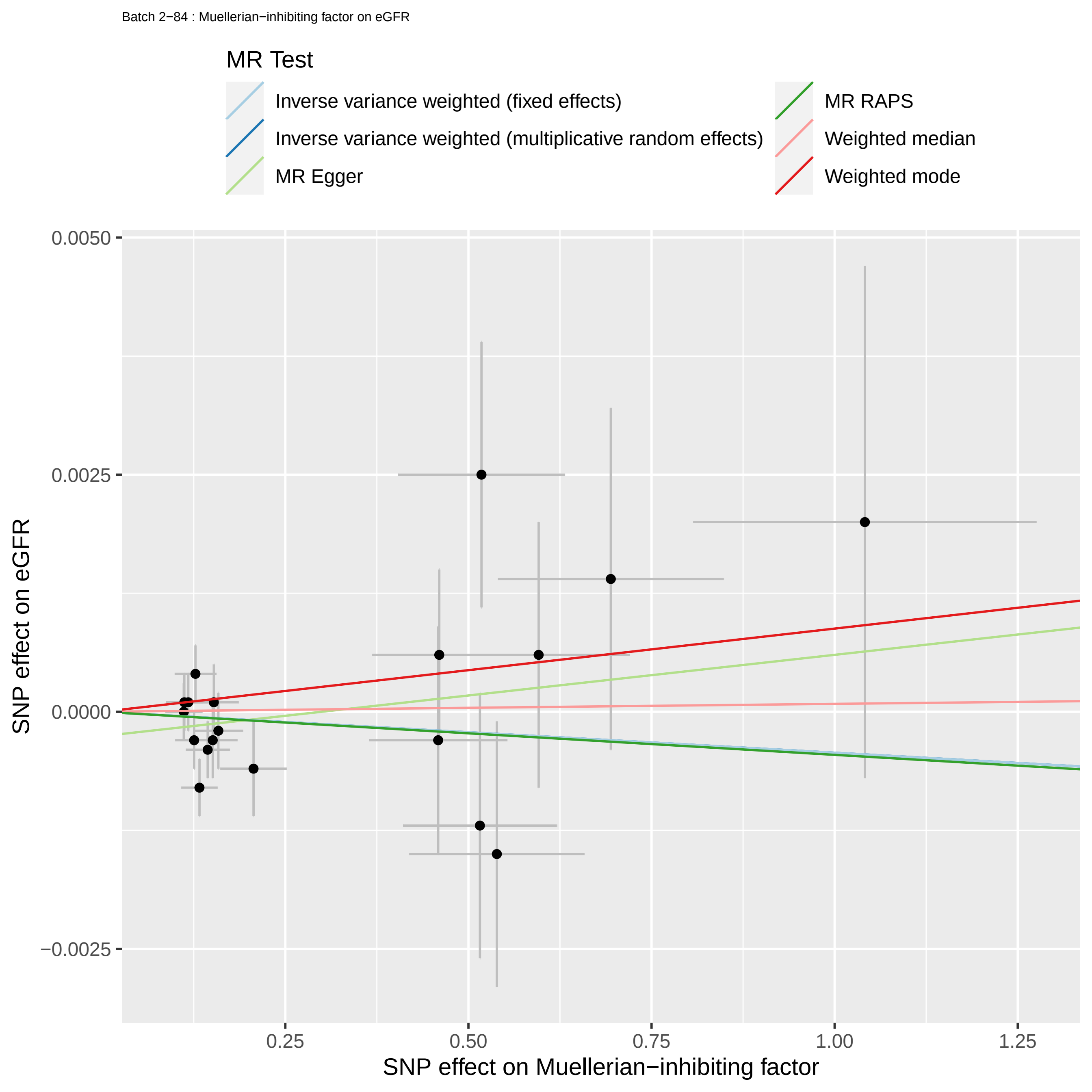
Batch 2−84 : Muellerian−inhibiting factor on eGFR
MR Test
Inverse variance weighted (fixed effects)
Inverse variance weighted (multiplicative random effects)
MR Egger
MR RAPS
Weighted median
Weighted mode
0.0050
0.0025
0.0000
−0.0025
0.25
0.50
0.75
1.00
1.25
SNP effect on eGFR
SNP effect on Muellerian−inhibiting factor

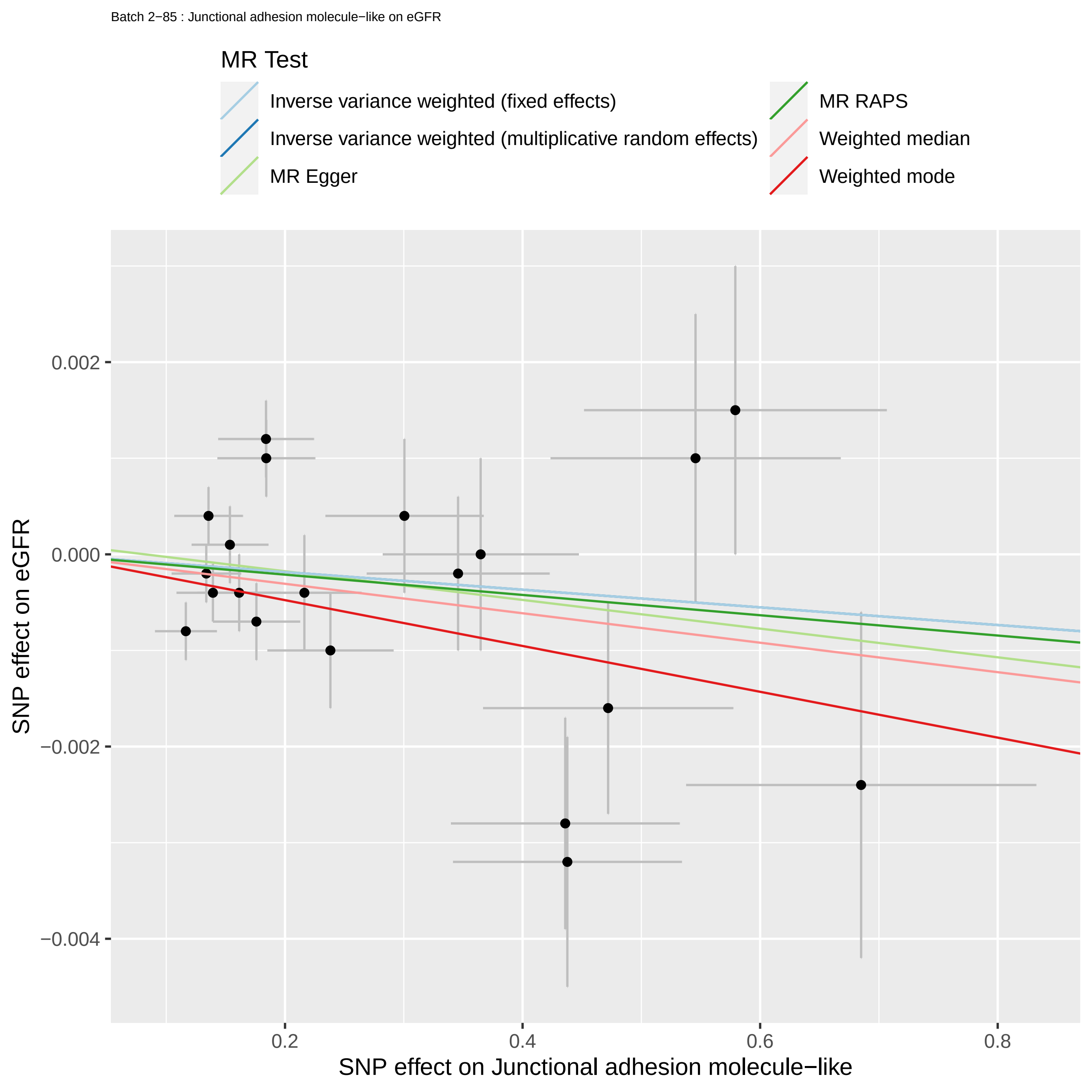

Batch 2−85 : Junctional adhesion molecule−like on eGFR
MR Test
Inverse variance weighted (fixed effects)
Inverse variance weighted (multiplicative random effects)
MR Egger
MR RAPS
Weighted median
Weighted mode
0.002
0.000
−0.002
−0.004
0.2
0.4
0.6
0.8
SNP effect on eGFR
SNP effect on Junctional adhesion molecule−like

Batch 2−86 : Junctional adhesion molecule−like on eGFR

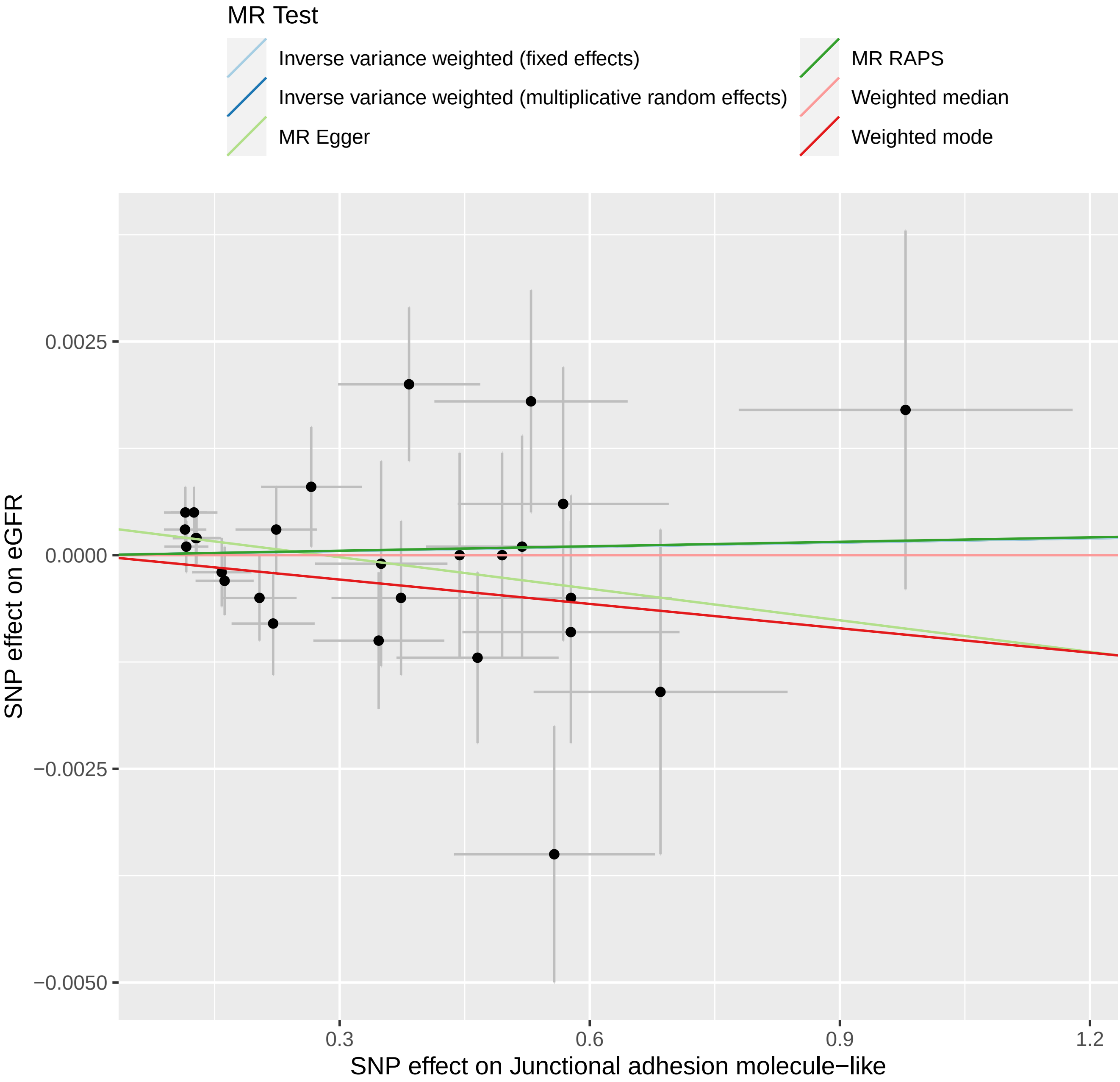

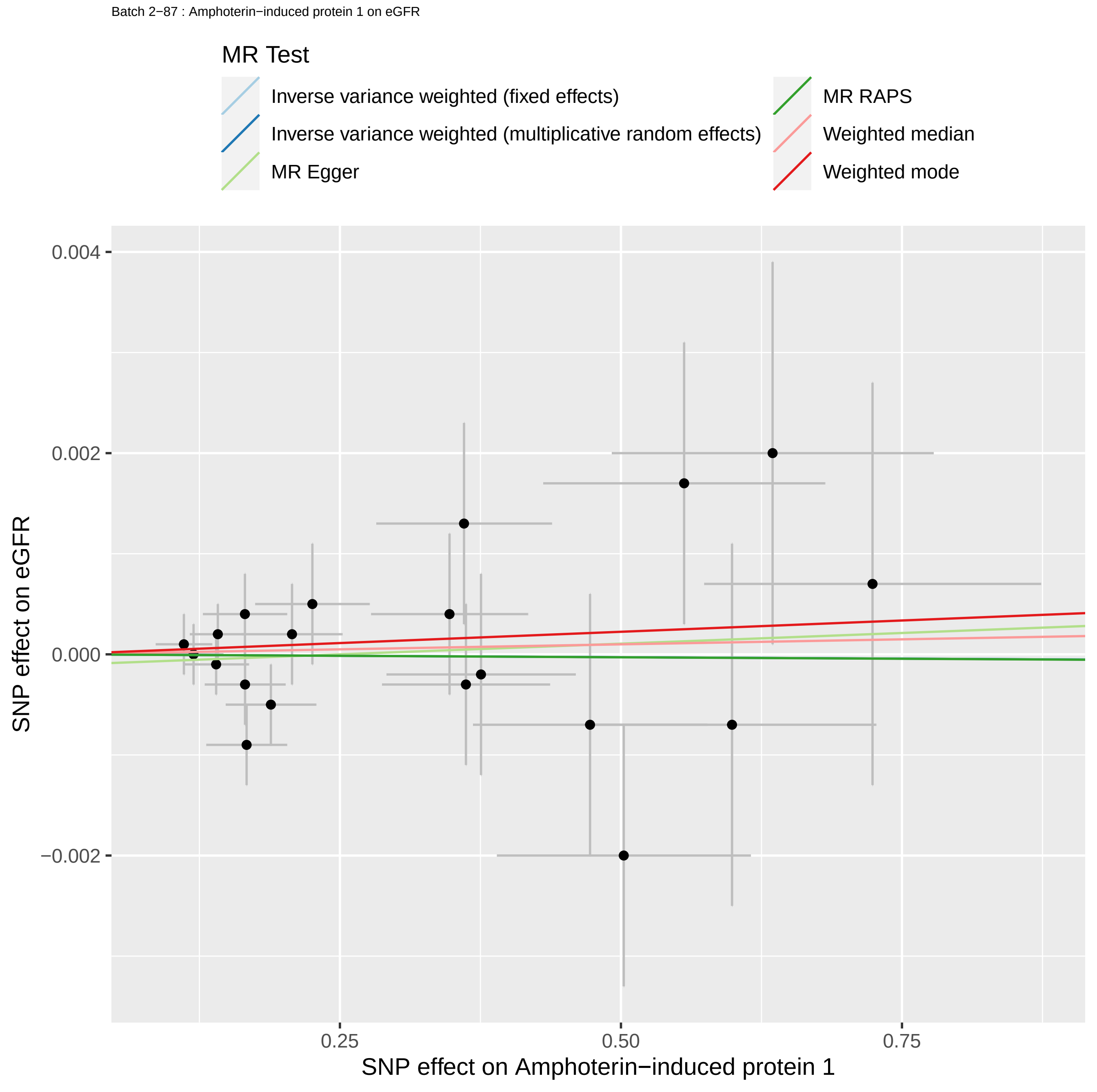

Batch 2−87 : Amphoterin−induced protein 1 on eGFR
MR Test
Inverse variance weighted (fixed effects)
Inverse variance weighted (multiplicative random effects)
MR Egger
MR RAPS
Weighted median
Weighted mode
0.004
0.002
0.000
−0.002
0.25
0.50
0.75
SNP effect on eGFR
SNP effect on Amphoterin−induced protein 1

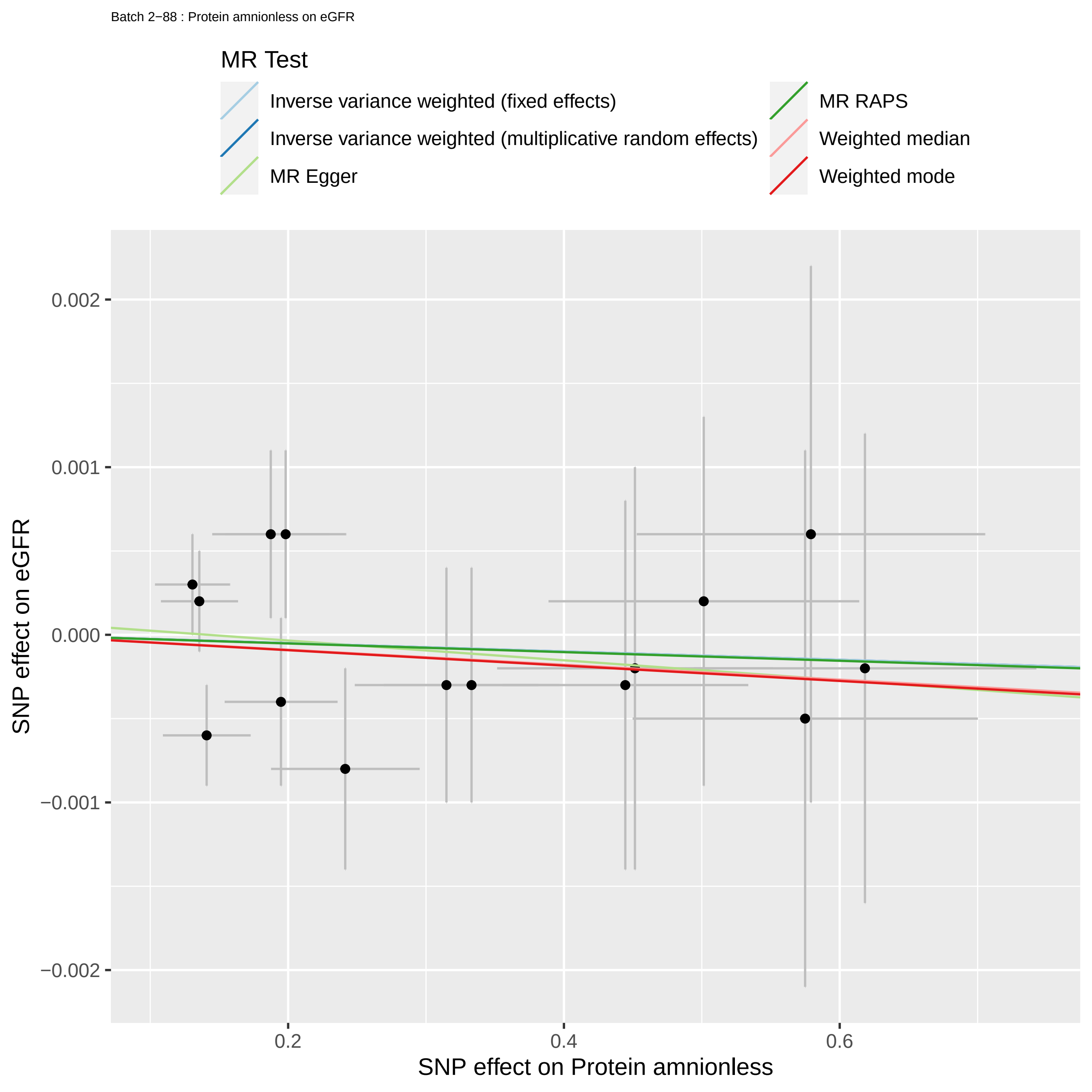
Batch 2−88 : Protein amnionless on eGFR
MR Test
Inverse variance weighted (fixed effects)
Inverse variance weighted (multiplicative random effects)
MR Egger
MR RAPS
Weighted median
Weighted mode
0.002
0.001
0.000
−0.001
−0.002
0.2
0.4
0.6
SNP effect on eGFR
SNP effect on Protein amnionless

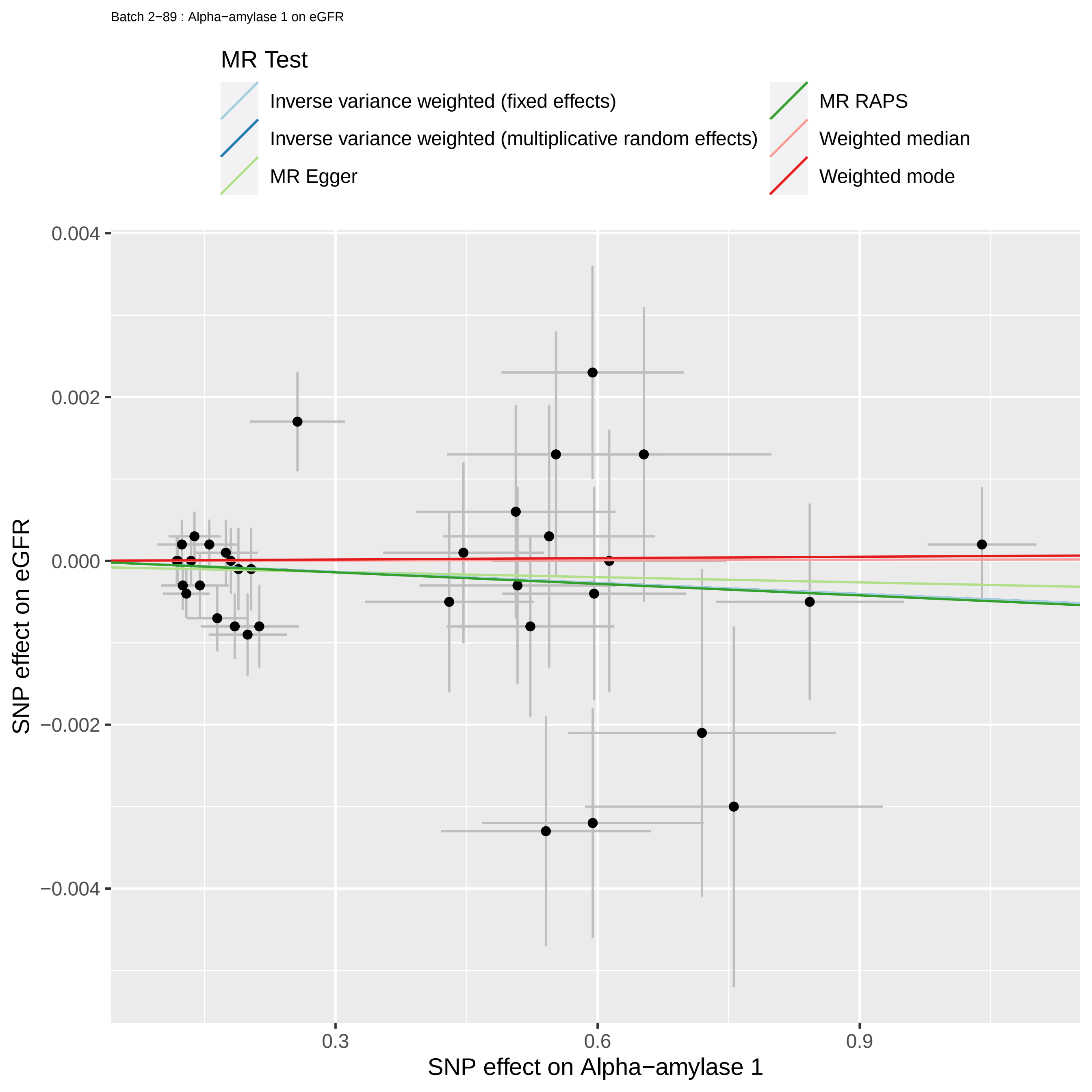
Batch 2−89 : Alpha−amylase 1 on eGFR
MR Test
Inverse variance weighted (fixed effects)
Inverse variance weighted (multiplicative random effects)
MR Egger
MR RAPS
Weighted median
Weighted mode
0.004
0.002
0.000
−0.002
−0.004
SNP effect on eGFR
0.3
0.6
0.9
SNP effect on Alpha−amylase 1

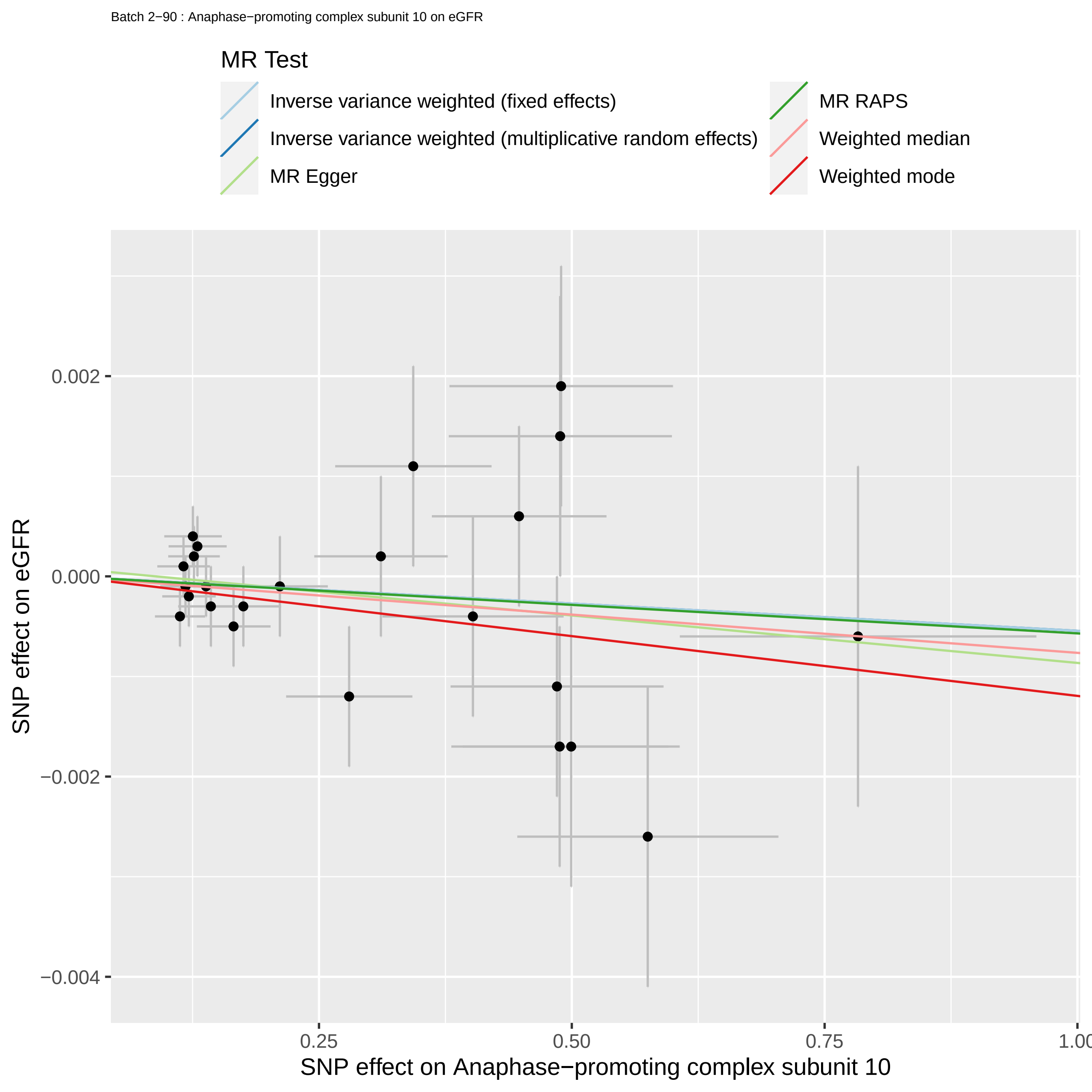

Batch 2−90 : Anaphase−promoting complex subunit 10 on eGFR
MR Test
Inverse variance weighted (fixed effects)
Inverse variance weighted (multiplicative random effects)
MR Egger
MR RAPS
Weighted median
Weighted mode
0.002
0.000
−0.002
−0.004
0.25
0.50
0.75
1.00
SNP effect on eGFR
SNP effect on Anaphase−promoting complex subunit 10

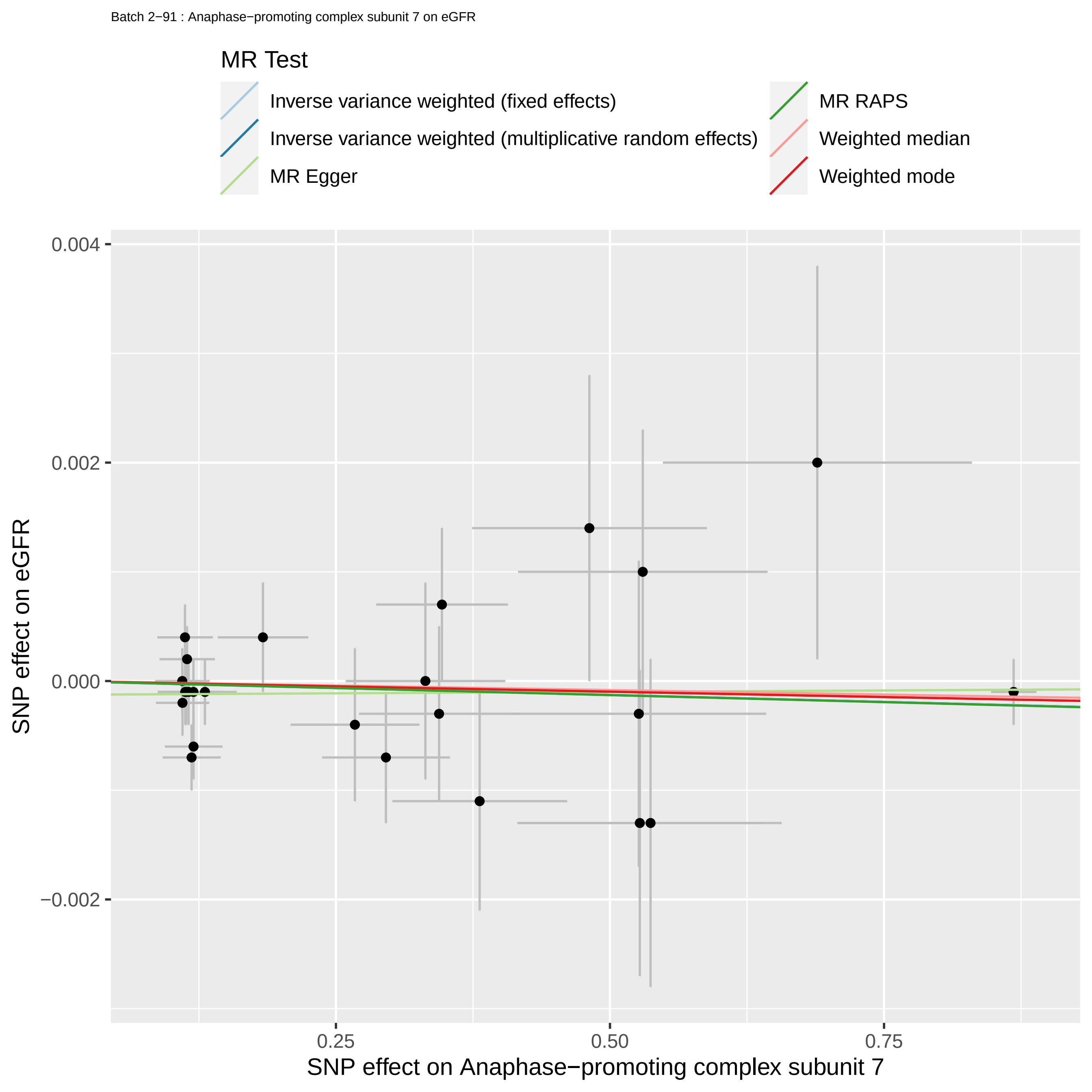
Batch 2−91 : Anaphase−promoting complex subunit 7 on eGFR
MR Test
Inverse variance weighted (fixed effects)
Inverse variance weighted (multiplicative random effects)
MR Egger
MR RAPS
Weighted median
Weighted mode
0.004
0.002
0.000
−0.002
0.25
0.50
0.75
SNP effect on eGFR
SNP effect on Anaphase−promoting complex subunit 7

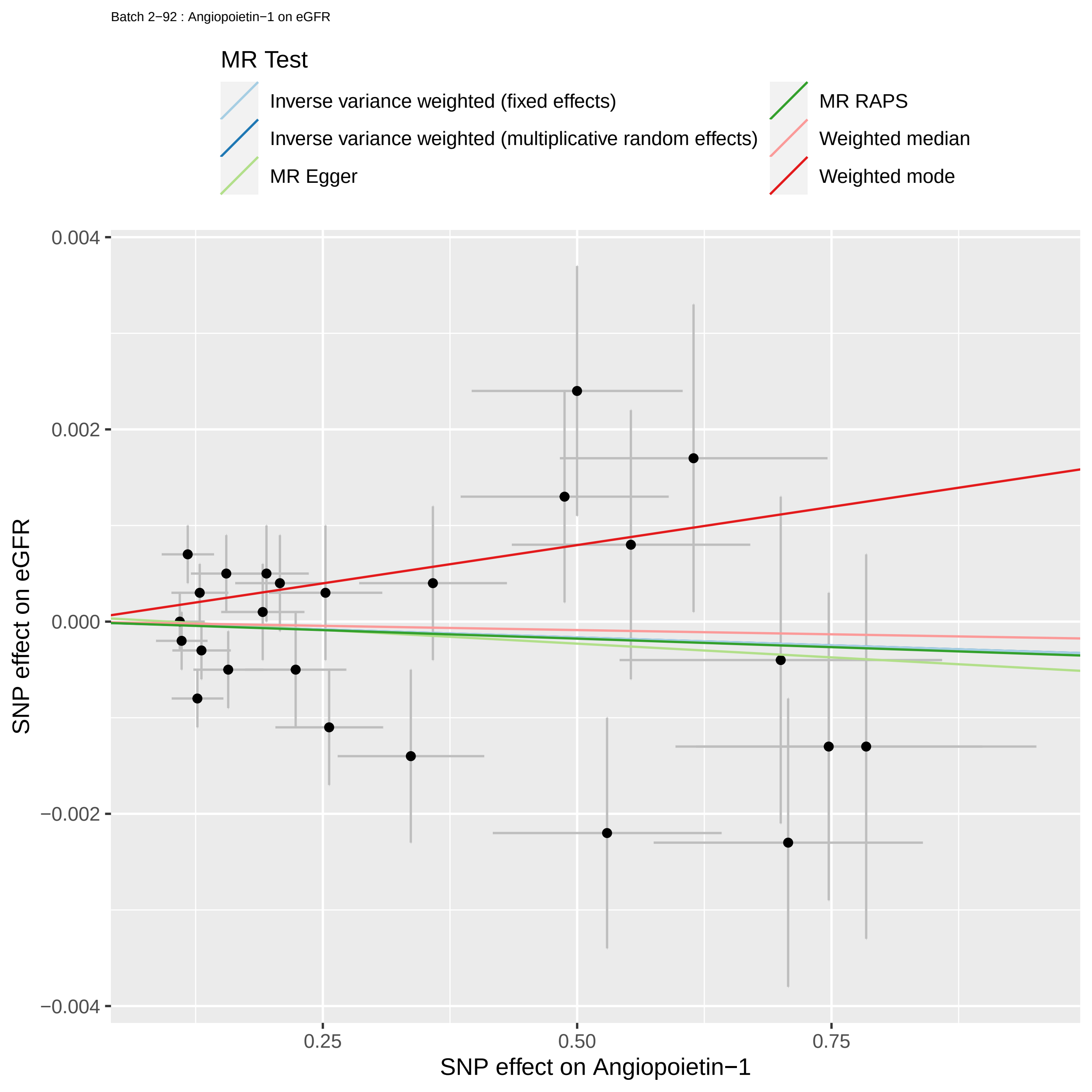

Batch 2−92 : Angiopoietin−1 on eGFR
MR Test
Inverse variance weighted (fixed effects)
Inverse variance weighted (multiplicative random effects)
MR Egger
MR RAPS
Weighted median
Weighted mode
0.004
0.002
0.000
−0.002
−0.004
SNP effect on eGFR
0.25
0.50
0.75
SNP effect on Angiopoietin−1

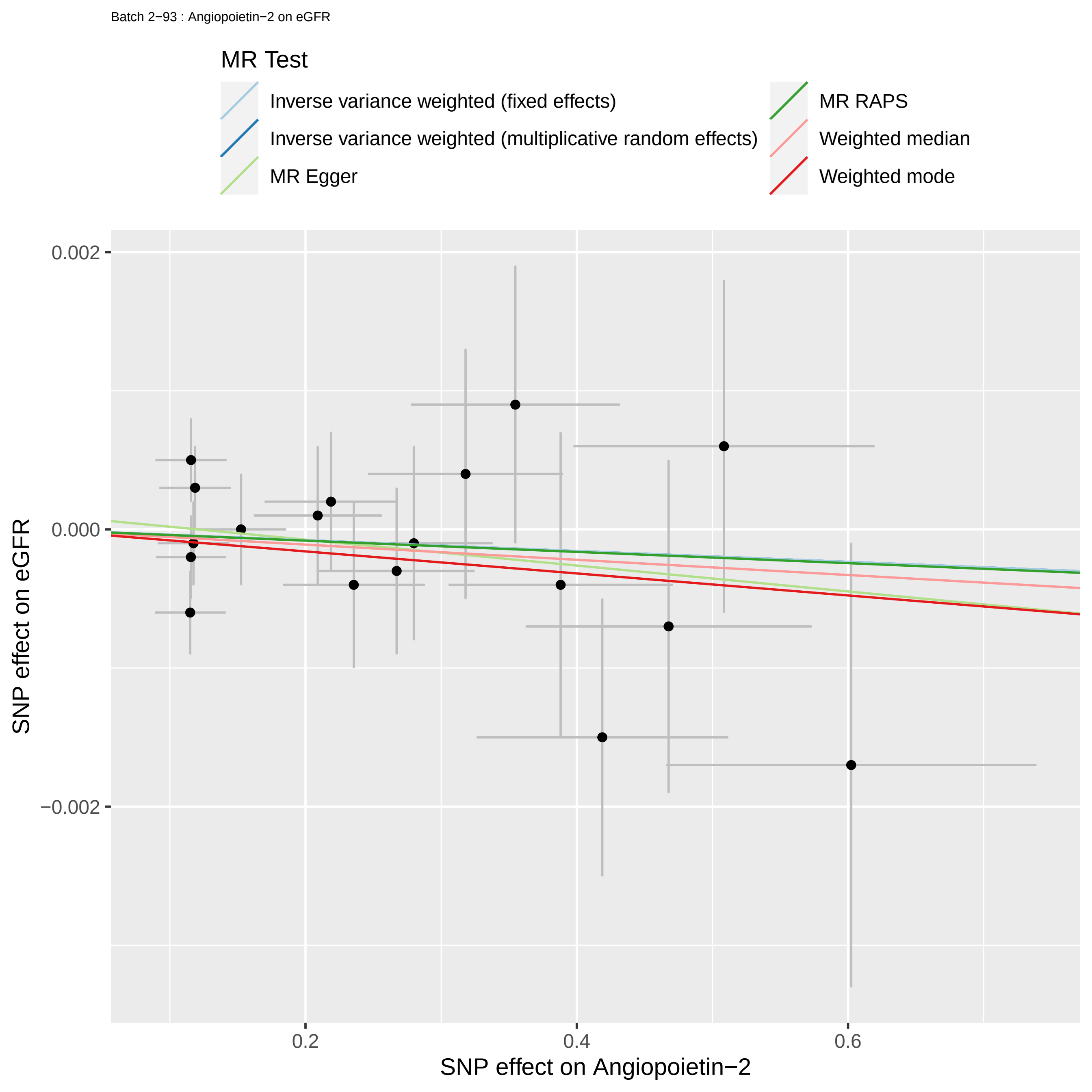
Batch 2−93 : Angiopoietin−2 on eGFR
MR Test
Inverse variance weighted (fixed effects)
Inverse variance weighted (multiplicative random effects)
MR Egger
MR RAPS
Weighted median
Weighted mode
0.002
0.000
−0.002
0.2
0.4
0.6
SNP effect on eGFR
SNP effect on Angiopoietin−2

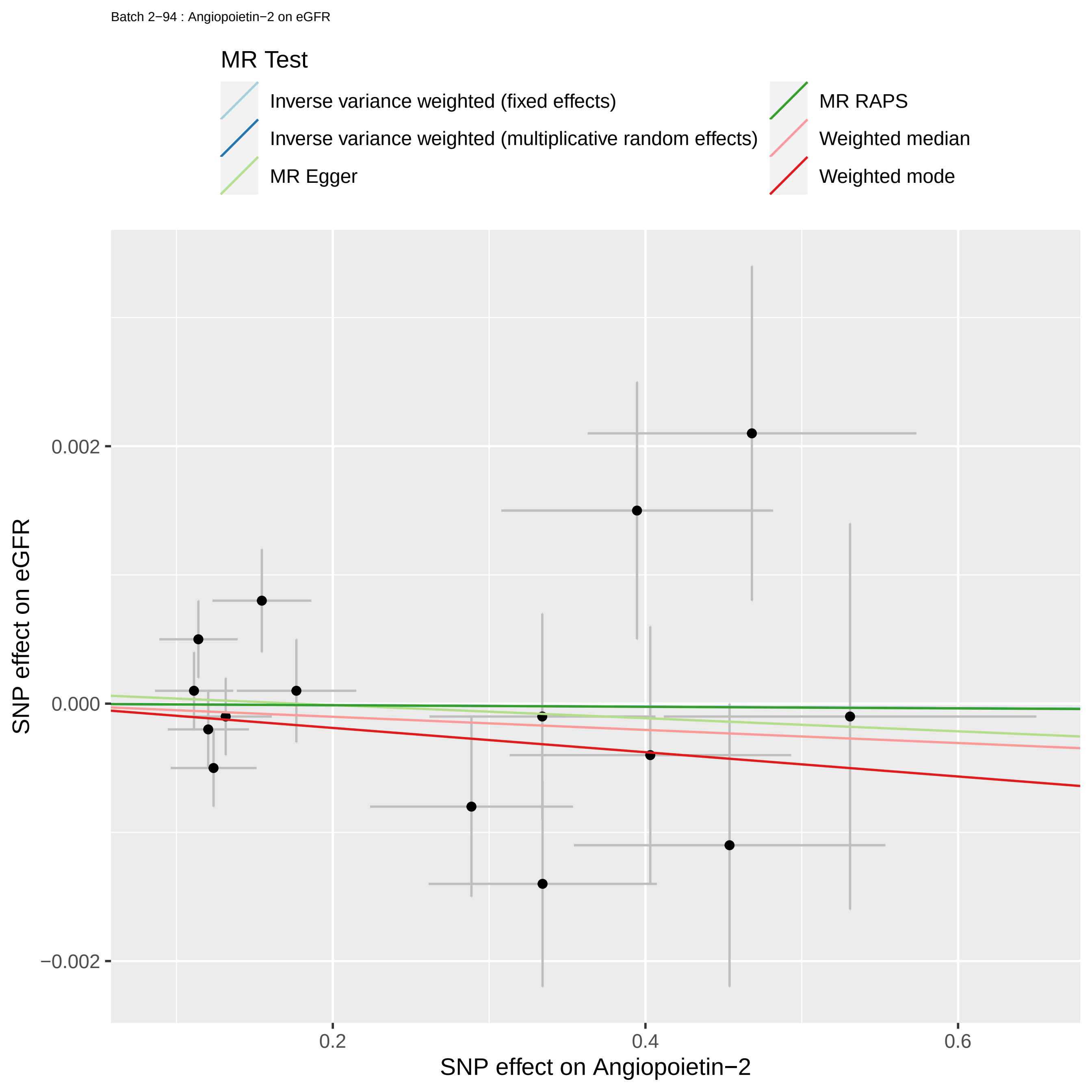

Batch 2−94 : Angiopoietin−2 on eGFR
MR Test
Inverse variance weighted (fixed effects)
Inverse variance weighted (multiplicative random effects)
MR Egger
MR RAPS
Weighted median
Weighted mode
0.002
0.000
−0.002
SNP effect on eGFR
0.2
0.4
0.6
SNP effect on Angiopoietin−2

Batch 2−95 : Angiopoietin−4 on eGFR

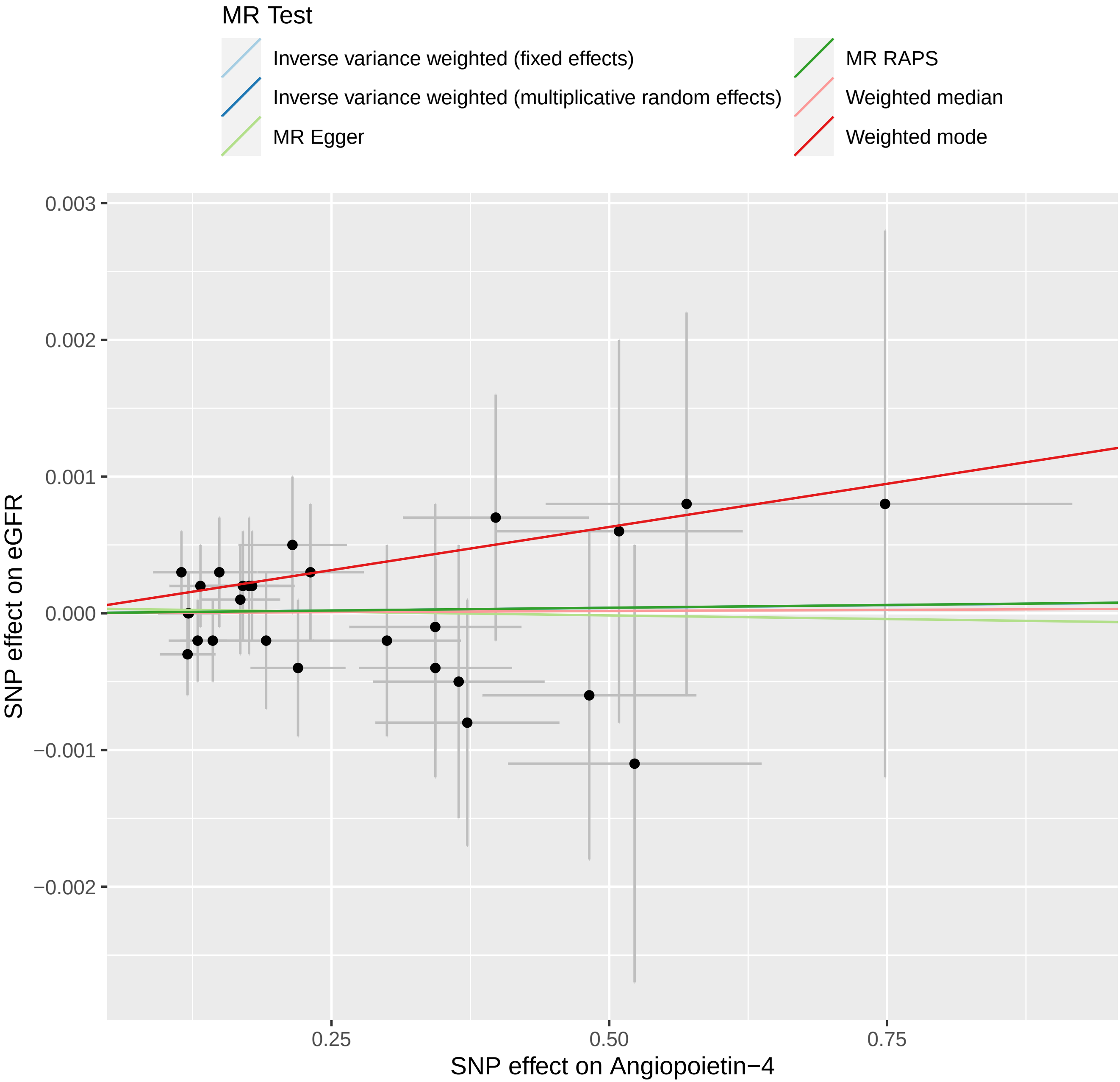

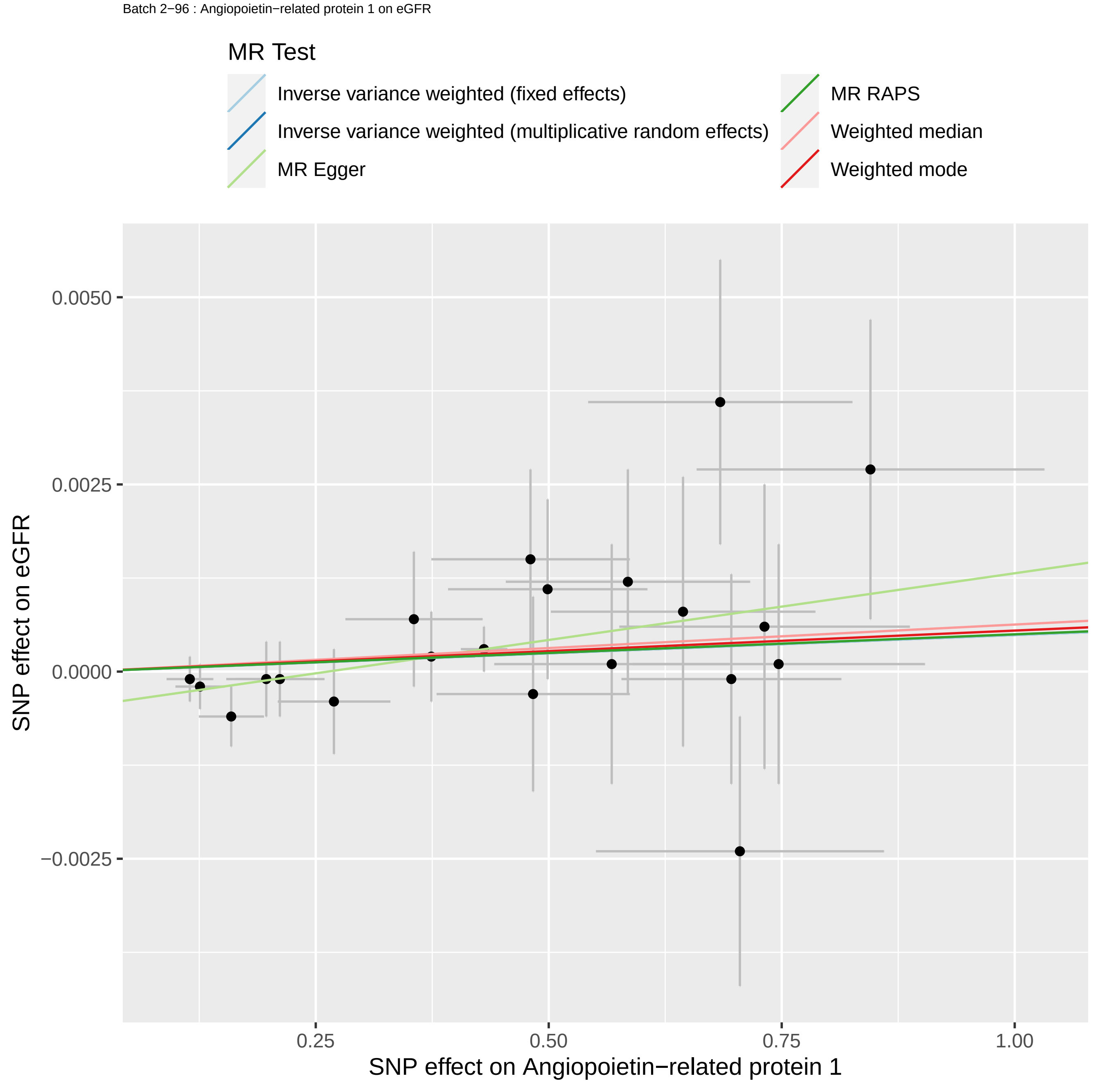

Batch 2−96 : Angiopoietin−related protein 1 on eGFR
MR Test
Inverse variance weighted (fixed effects)
Inverse variance weighted (multiplicative random effects)
MR Egger
MR RAPS
Weighted median
Weighted mode
0.0050
0.0025
0.0000
−0.0025
SNP effect on eGFR
0.25
0.50
0.75
1.00
SNP effect on Angiopoietin−related protein 1

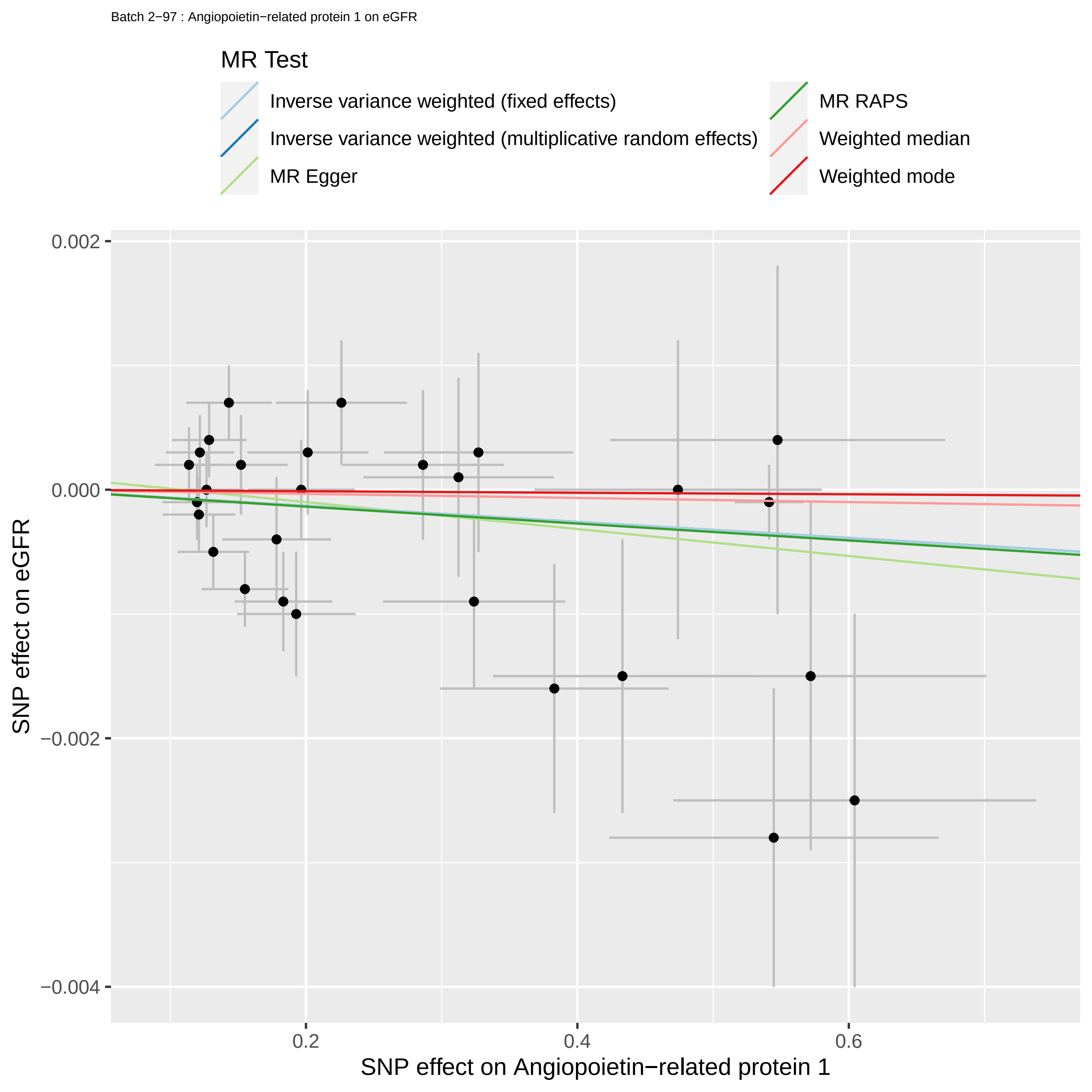
Batch 2−97 : Angiopoietin−related protein 1 on eGFR
MR Test
Inverse variance weighted (fixed effects)
Inverse variance weighted (multiplicative random effects)
MR Egger
MR RAPS
Weighted median
Weighted mode
0.002
0.000
−0.002
−0.004
0.2
0.4
0.6
SNP effect on eGFR
SNP effect on Angiopoietin−related protein 1

Batch 2−98 : Angiopoietin−related protein 3 on eGFR

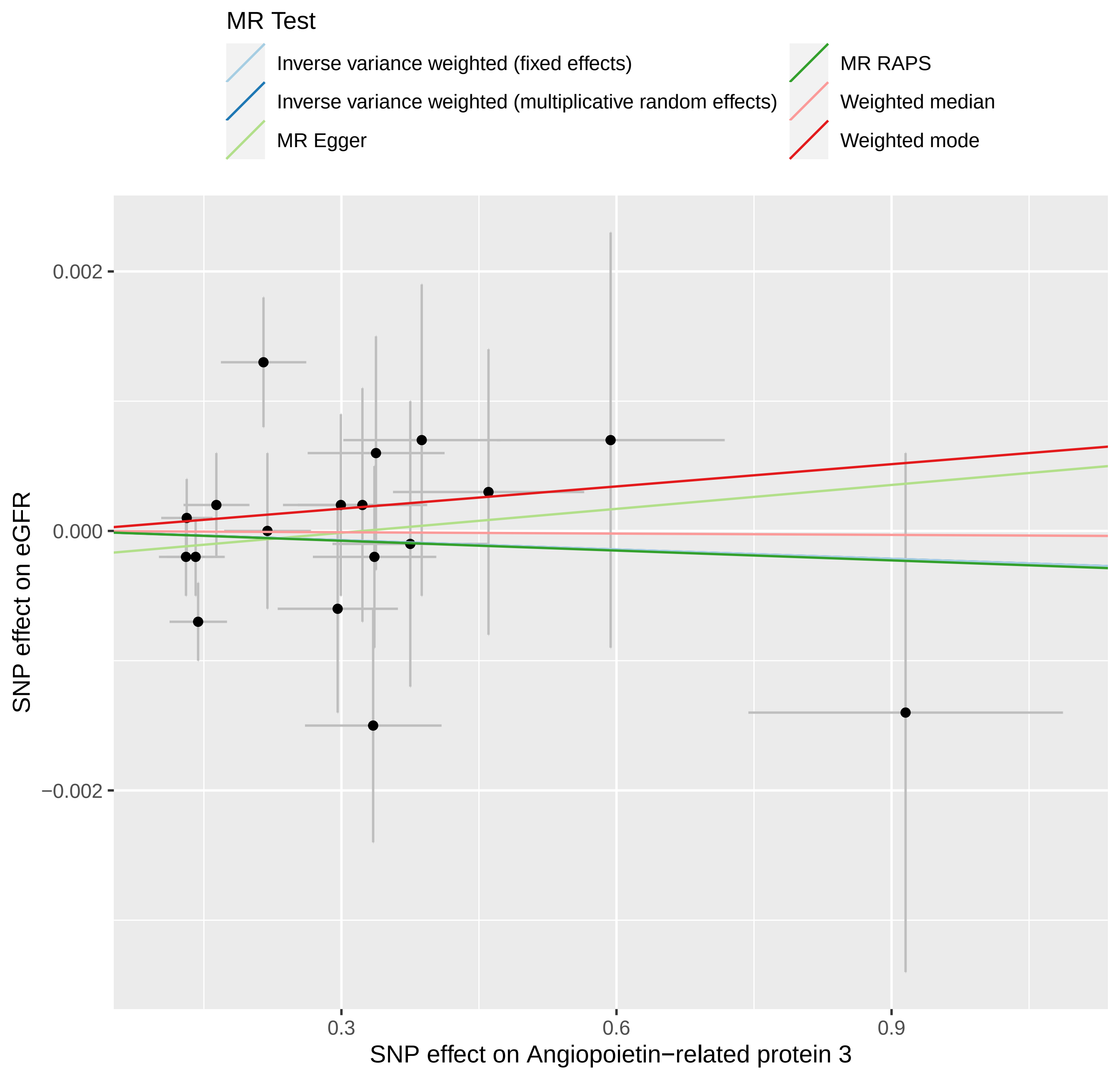

Batch 2−99 : Angiopoietin−related protein 4 on eGFR

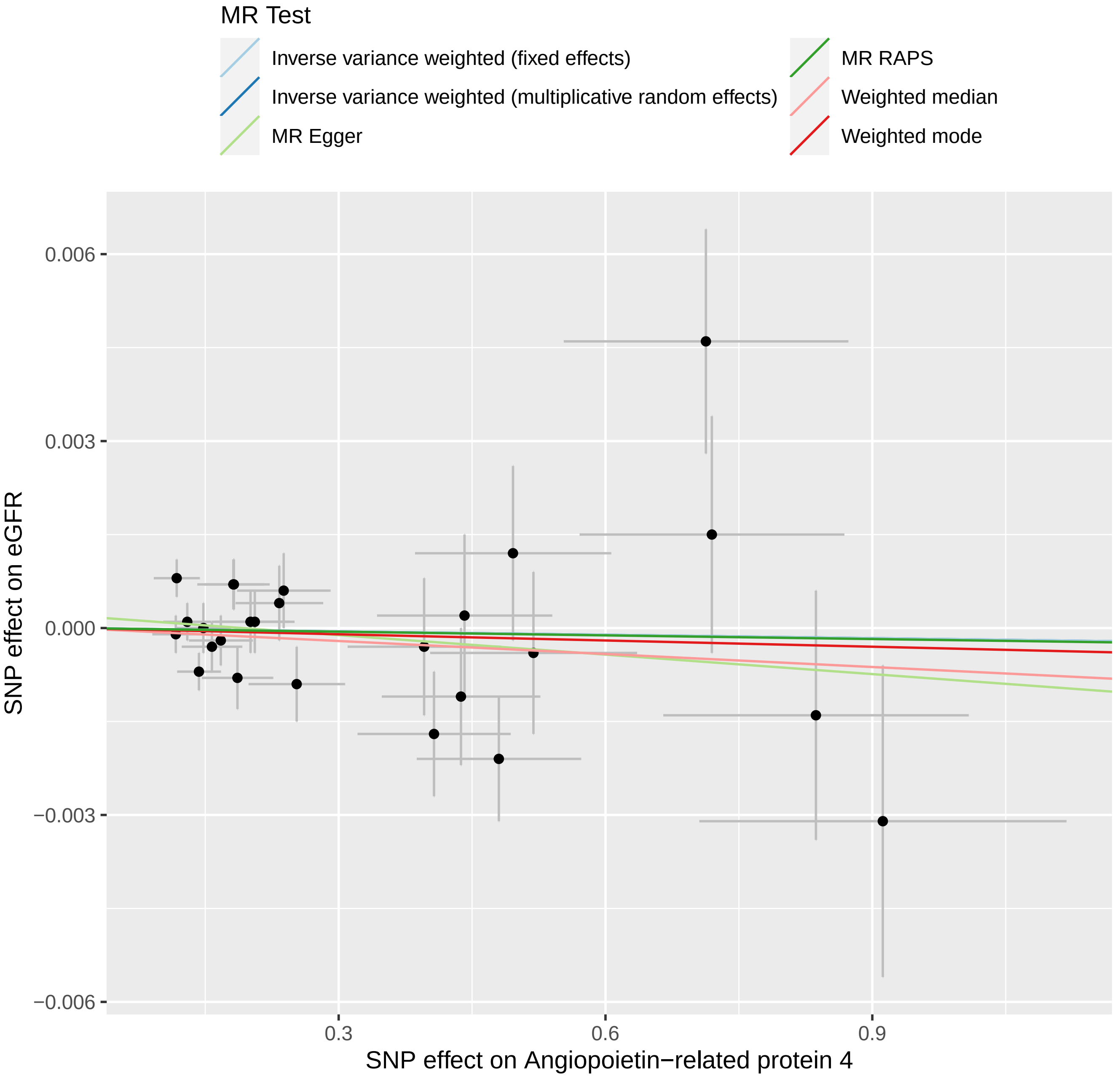

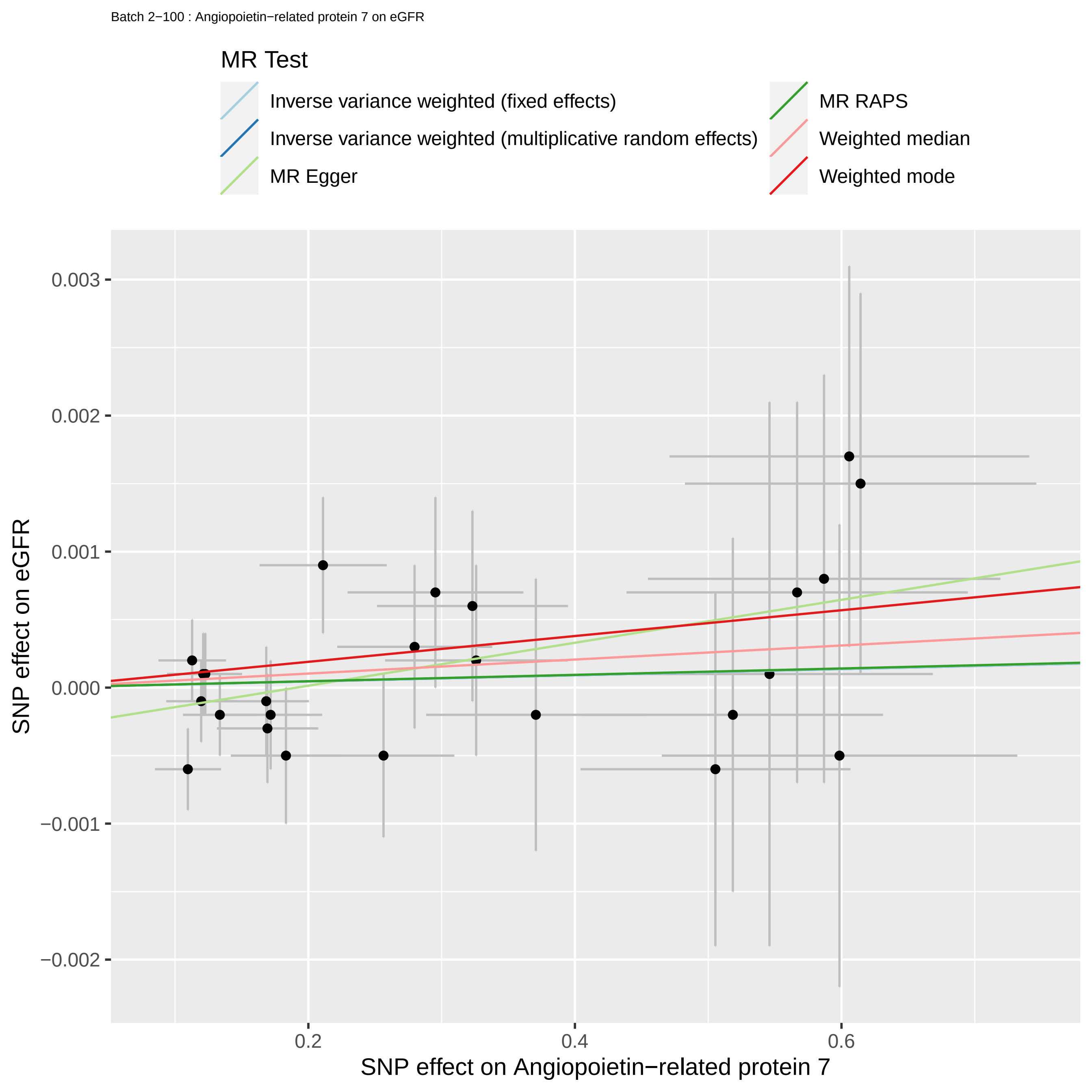
Batch 2−100 : Angiopoietin−related protein 7 on eGFR
MR Test
Inverse variance weighted (fixed effects)
Inverse variance weighted (multiplicative random effects)
MR Egger
MR RAPS
Weighted median
Weighted mode
0.003
0.002
0.001
0.000
−0.001
−0.002
SNP effect on eGFR
0.2
0.4
0.6
SNP effect on Angiopoietin−related protein 7

Batch 2−101 : Ankyrin−2 on eGFR

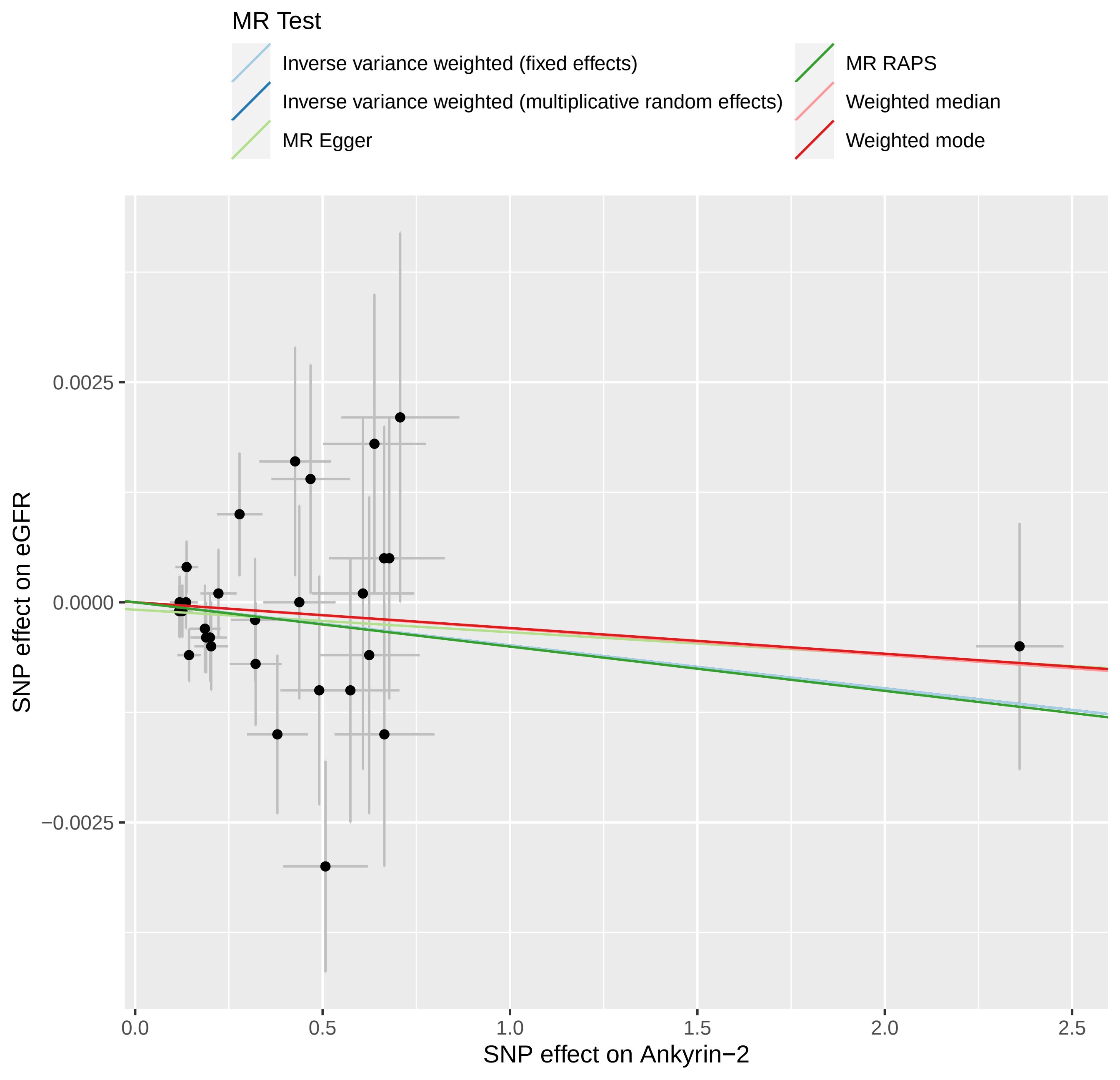

Batch 2−102 : Ankyrin repeat domain−containing protein 27 on eGFR

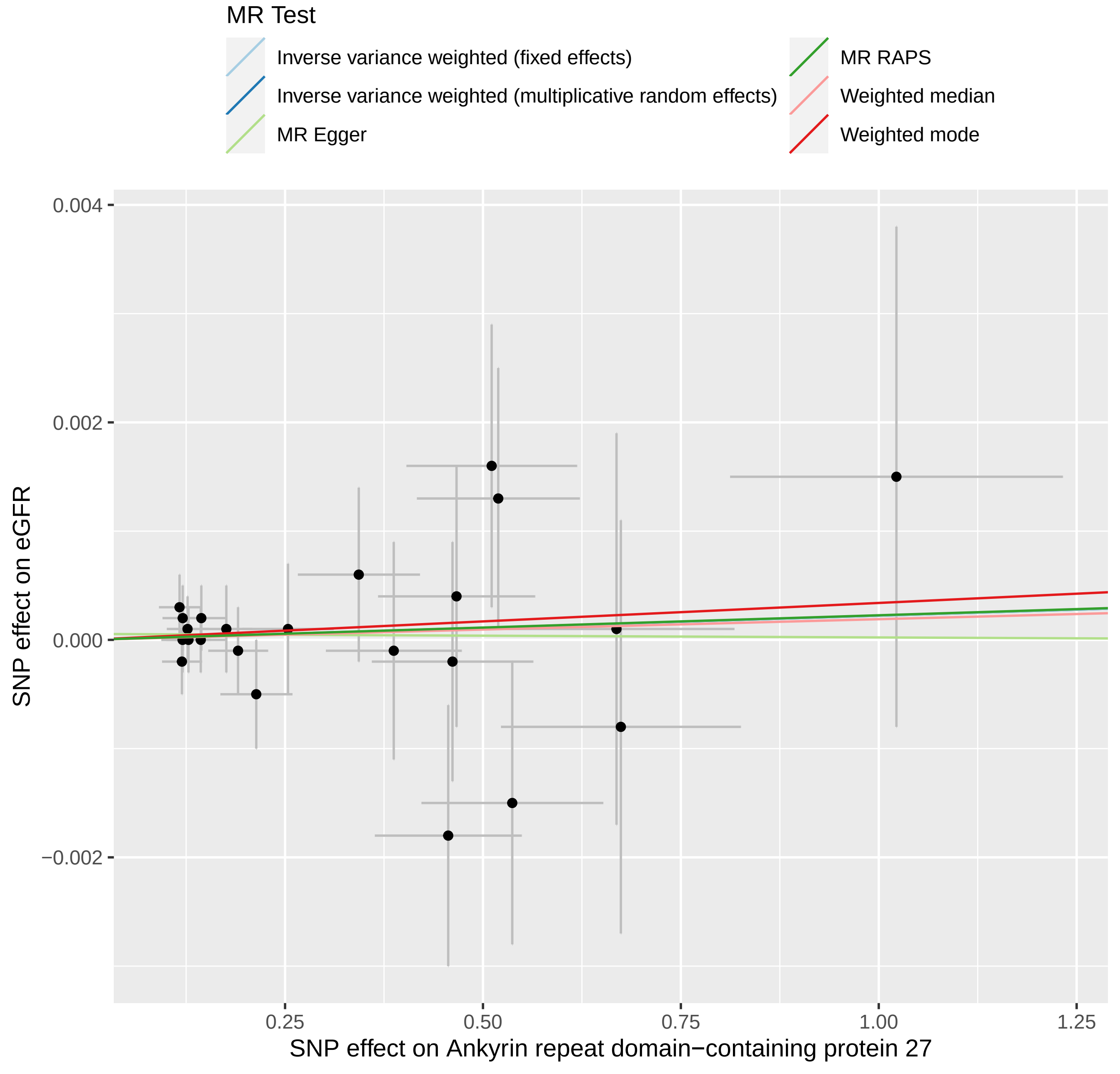

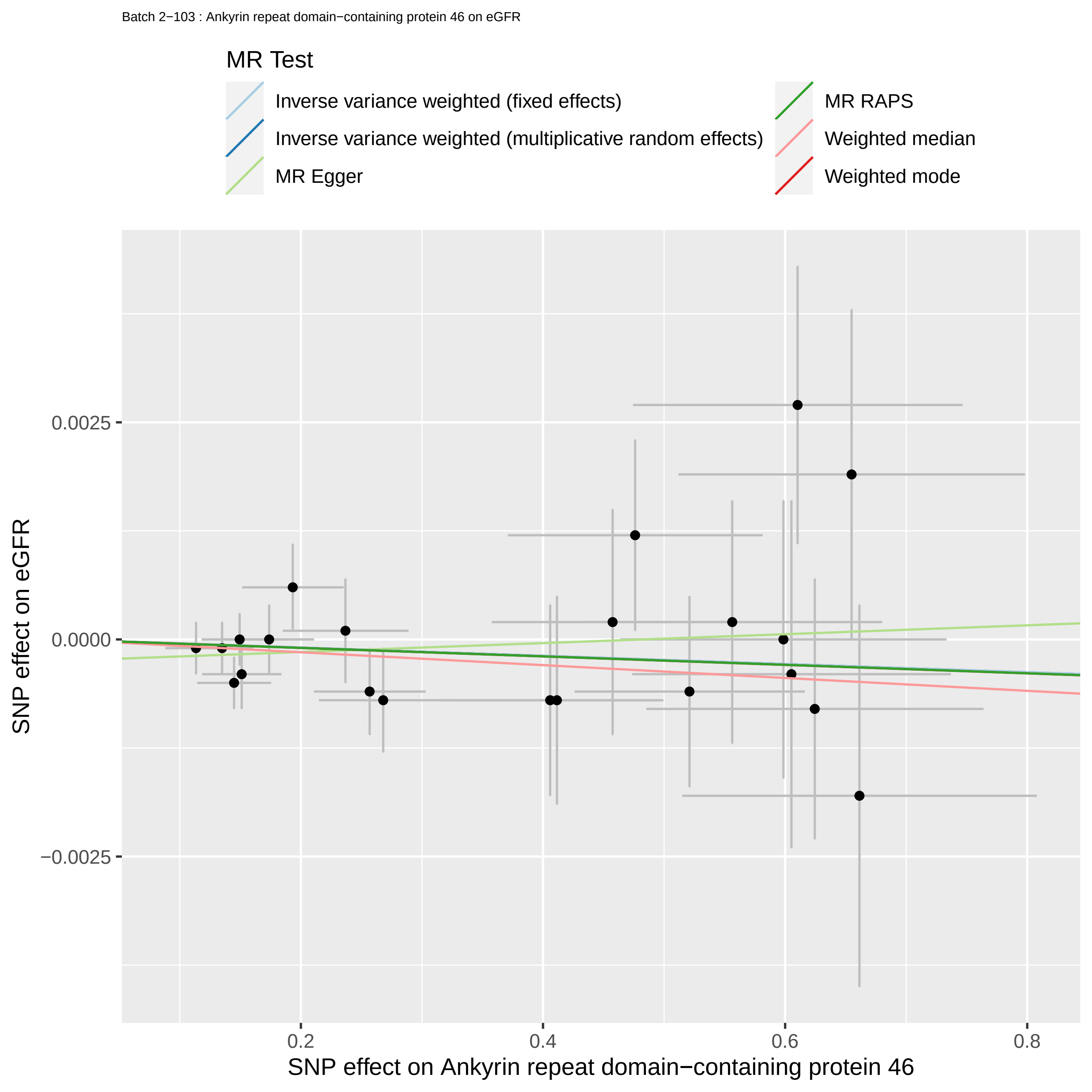

Batch 2−103 : Ankyrin repeat domain−containing protein 46 on eGFR
MR Test
Inverse variance weighted (fixed effects)
Inverse variance weighted (multiplicative random effects)
MR Egger
MR RAPS
Weighted median
Weighted mode
0.0025
0.0000
−0.0025
0.2
0.4
0.6
0.8
SNP effect on eGFR
SNP effect on Ankyrin repeat domain−containing protein 46

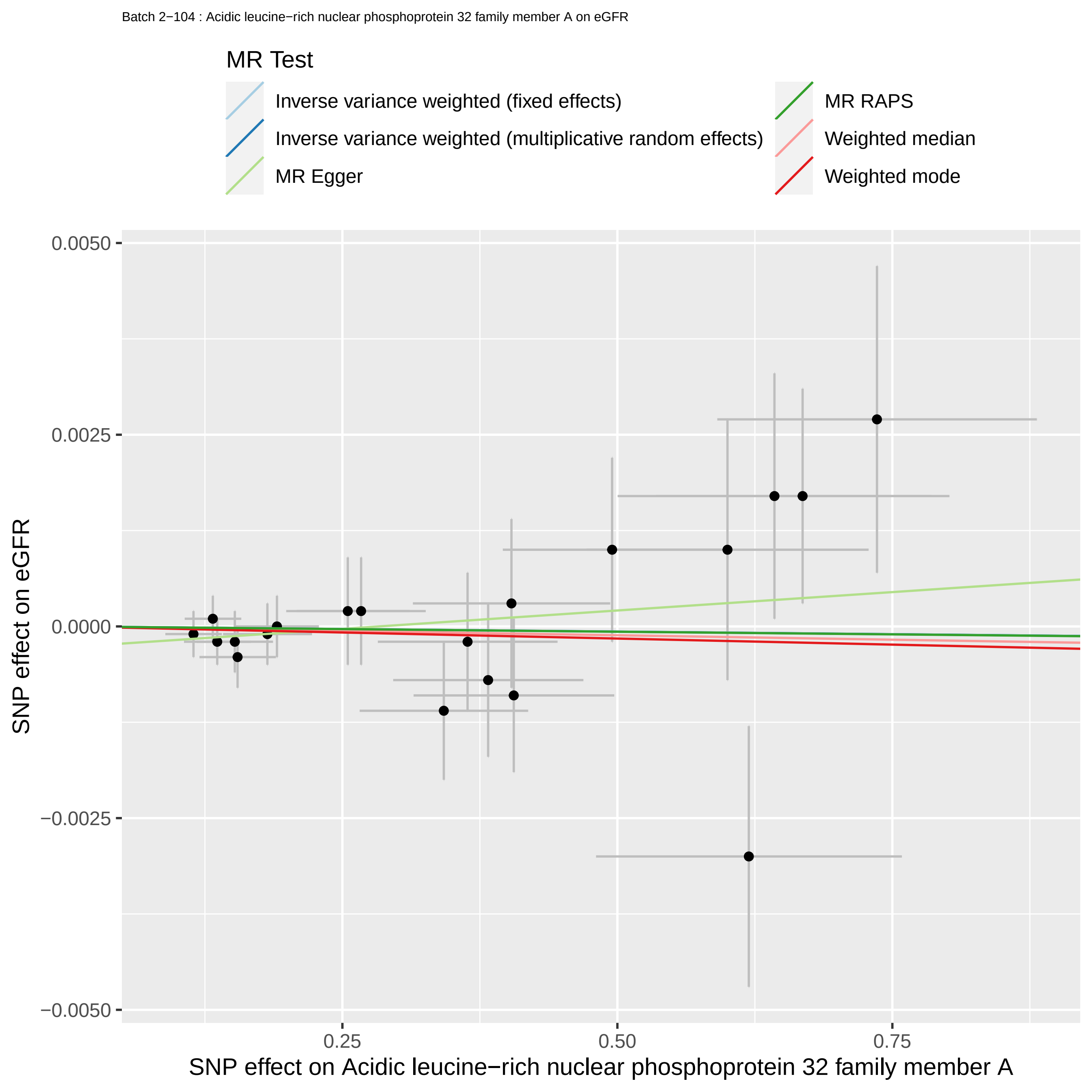
Batch 2−104 : Acidic leucine−rich nuclear phosphoprotein 32 family member A on eGFR
MR Test
Inverse variance weighted (fixed effects)
Inverse variance weighted (multiplicative random effects)
MR Egger
MR RAPS
Weighted median
Weighted mode
0.0050
0.0025
0.0000
−0.0025
−0.0050
0.25
0.50
0.75
SNP effect on eGFR
SNP effect on Acidic leucine−rich nuclear phosphoprotein 32 family member A

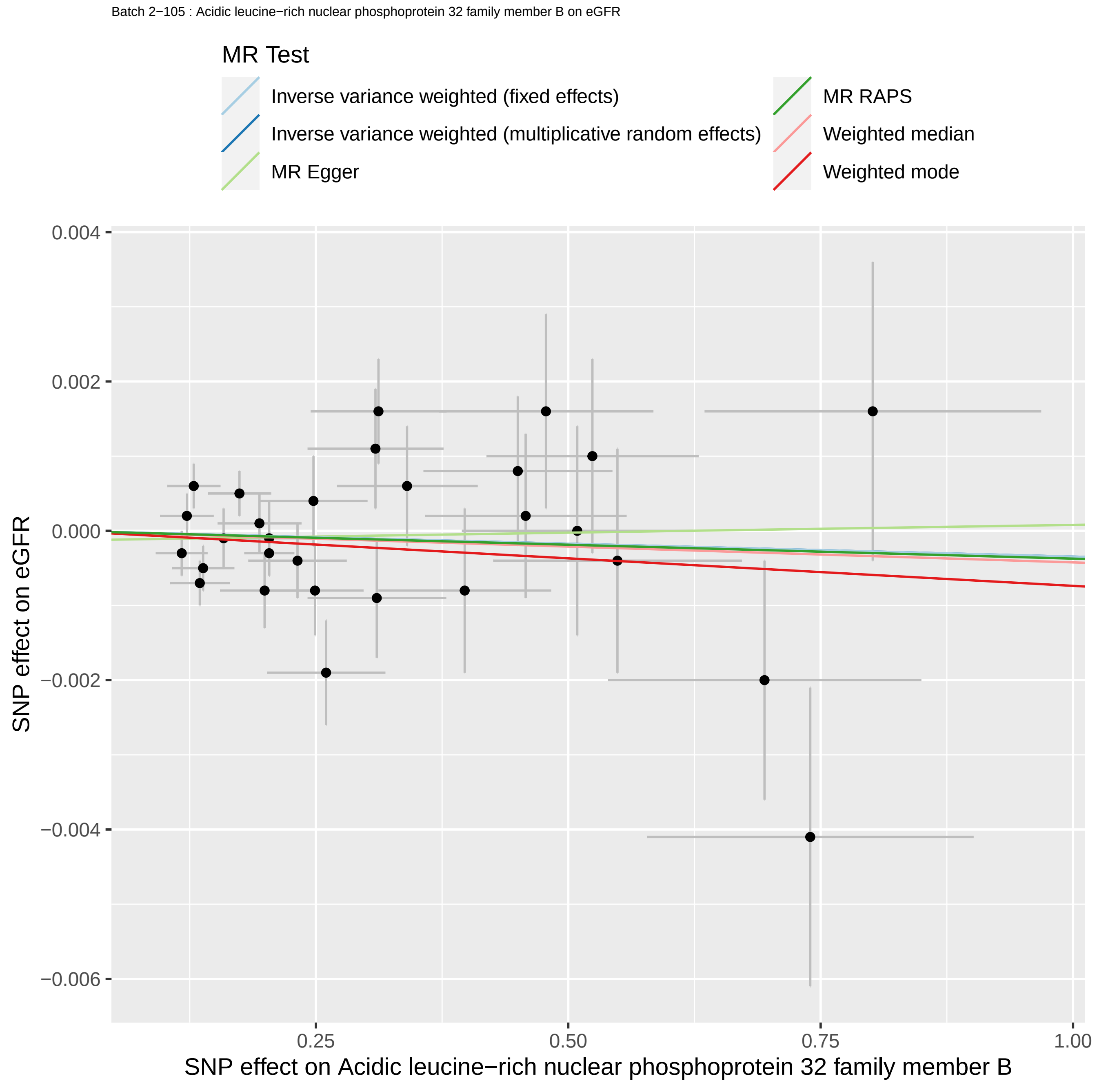
Batch 2−105 : Acidic leucine−rich nuclear phosphoprotein 32 family member B on eGFR
MR Test
Inverse variance weighted (fixed effects)
Inverse variance weighted (multiplicative random effects)
MR Egger
MR RAPS
Weighted median
Weighted mode
0.004
0.002
0.000
−0.002
−0.004
−0.006
0.25
0.50
0.75
1.00
SNP effect on eGFR
SNP effect on Acidic leucine−rich nuclear phosphoprotein 32 family member B

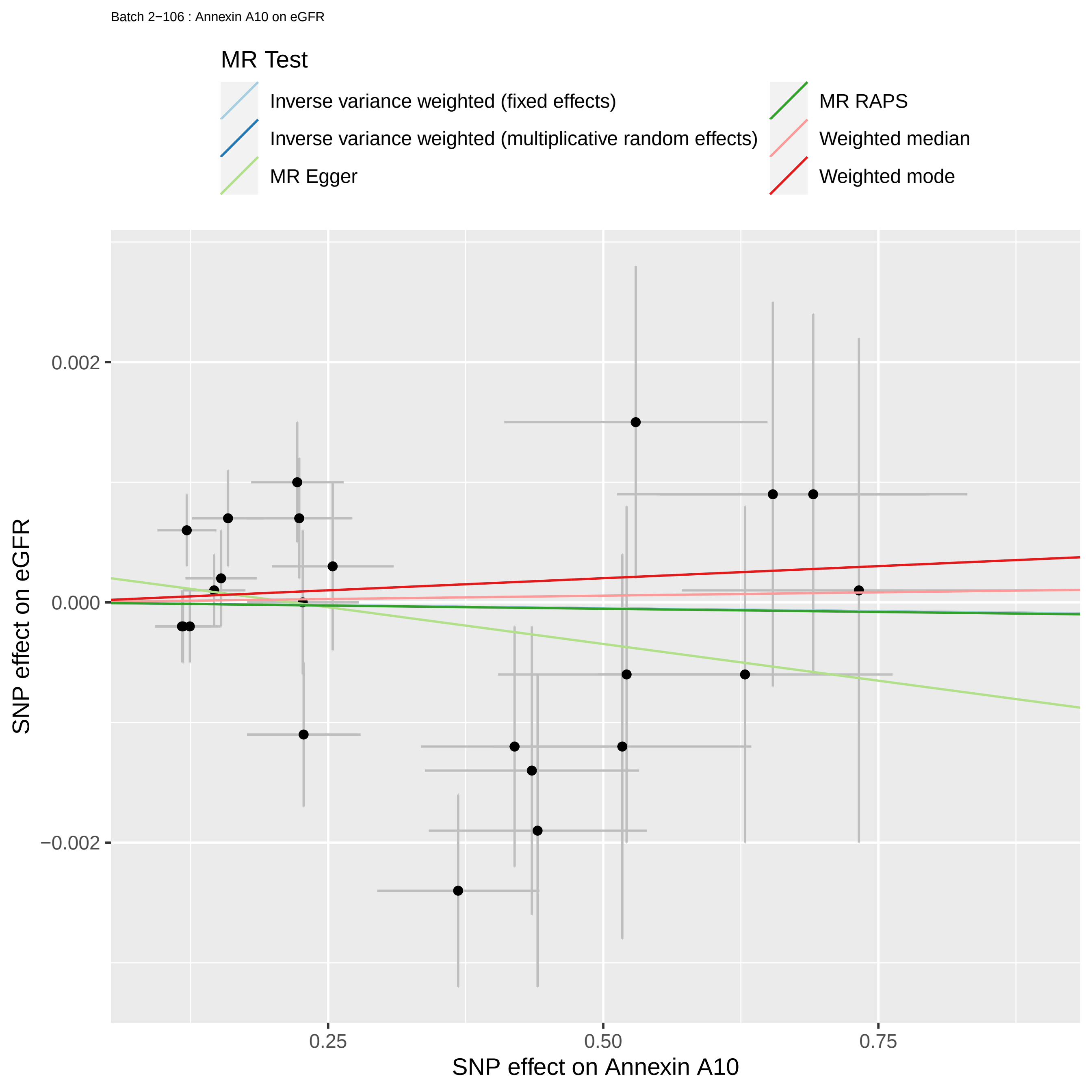

Batch 2−106 : Annexin A10 on eGFR
MR Test
Inverse variance weighted (fixed effects)
Inverse variance weighted (multiplicative random effects)
MR Egger
MR RAPS
Weighted median
Weighted mode
0.002
0.000
−0.002
0.25
0.50
0.75
SNP effect on Annexin A10
SNP effect on eGFR

Batch 2−107 : Annexin A1 on eGFR

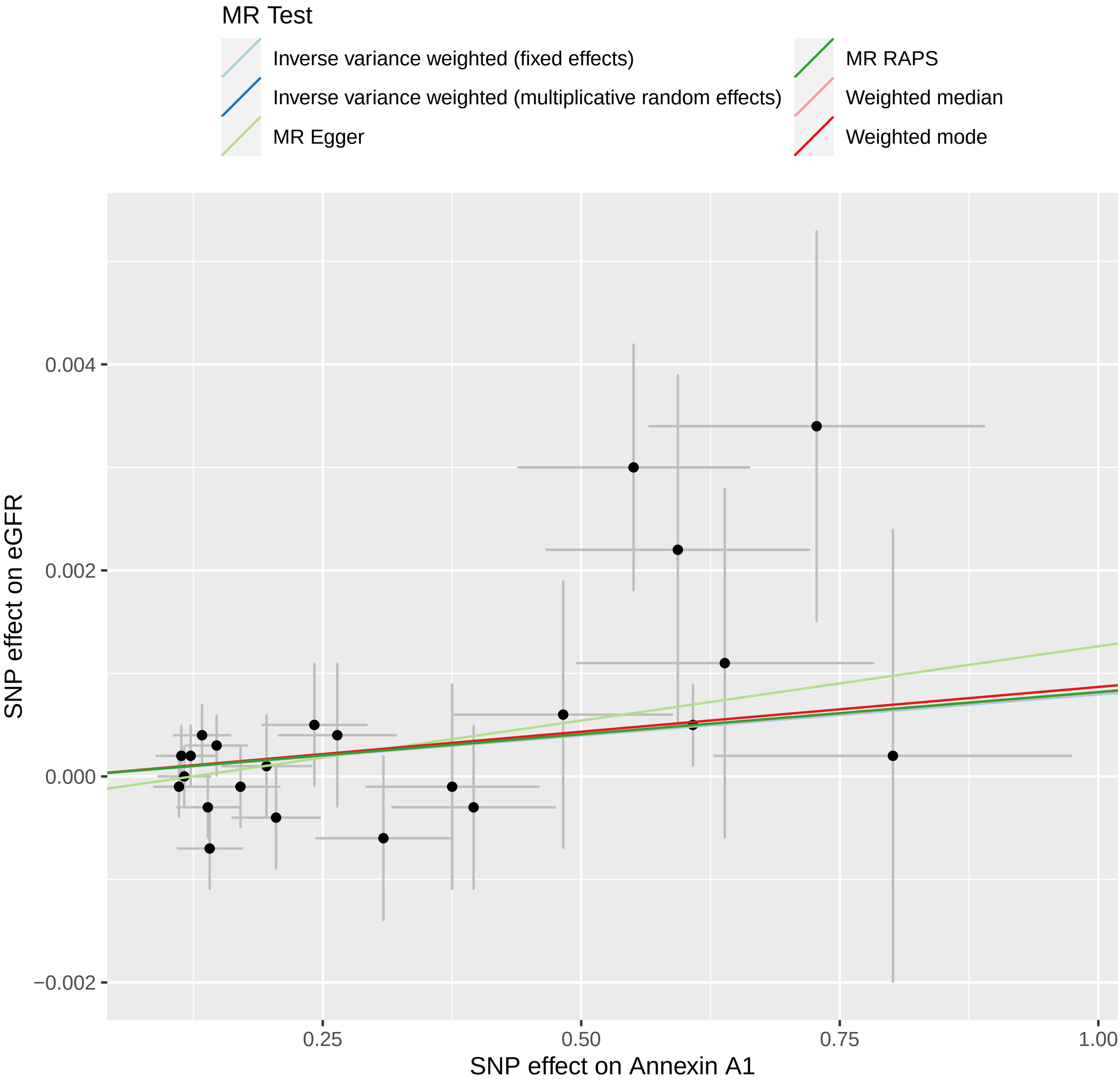

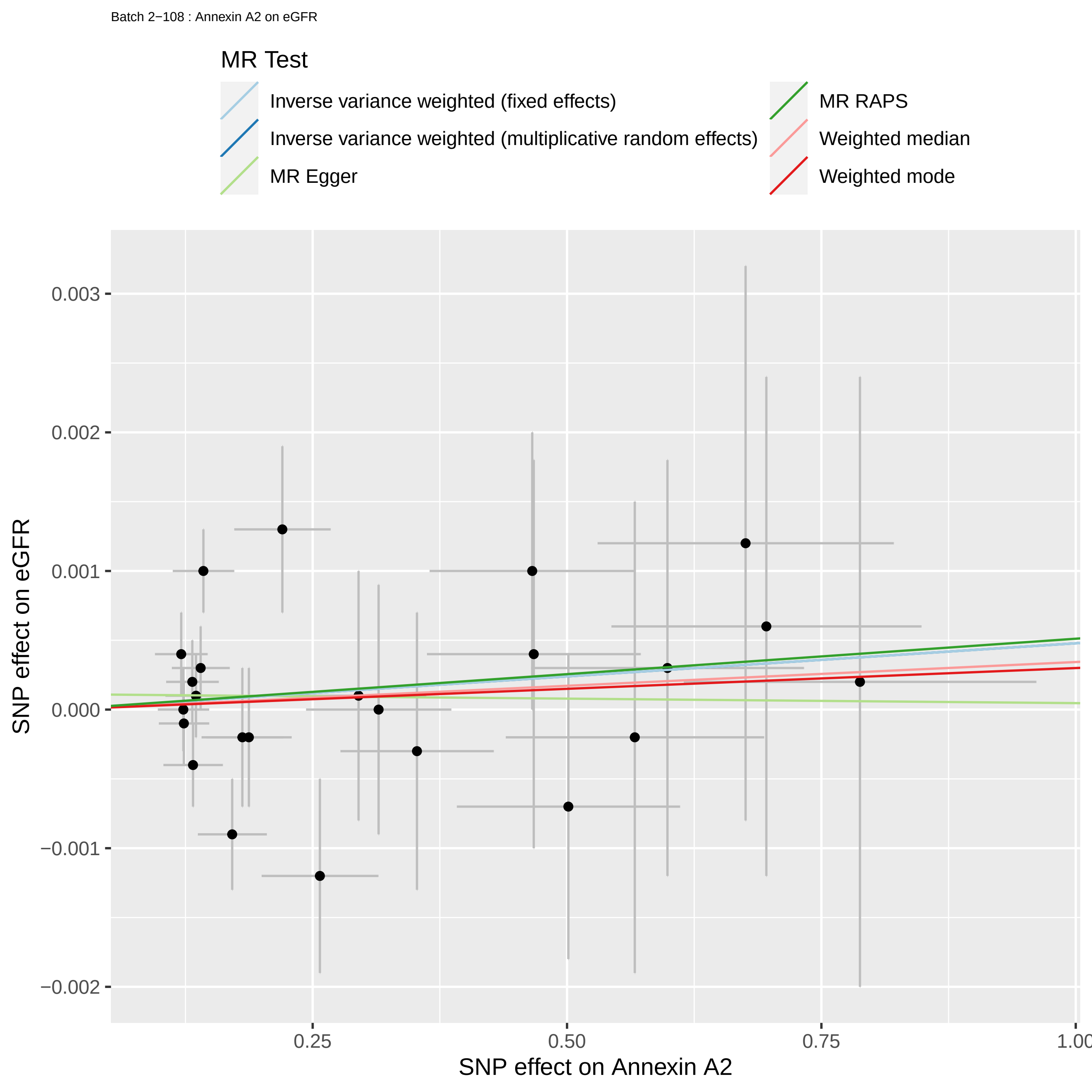
Batch 2−108 : Annexin A2 on eGFR
MR Test
Inverse variance weighted (fixed effects)
Inverse variance weighted (multiplicative random effects)
MR Egger
MR RAPS
Weighted median
Weighted mode
0.003
0.002
0.001
0.000
−0.001
−0.002
0.25
0.50
0.75
1.00
SNP effect on Annexin A2
SNP effect on eGFR

Batch 2−109 : Annexin A2 on eGFR

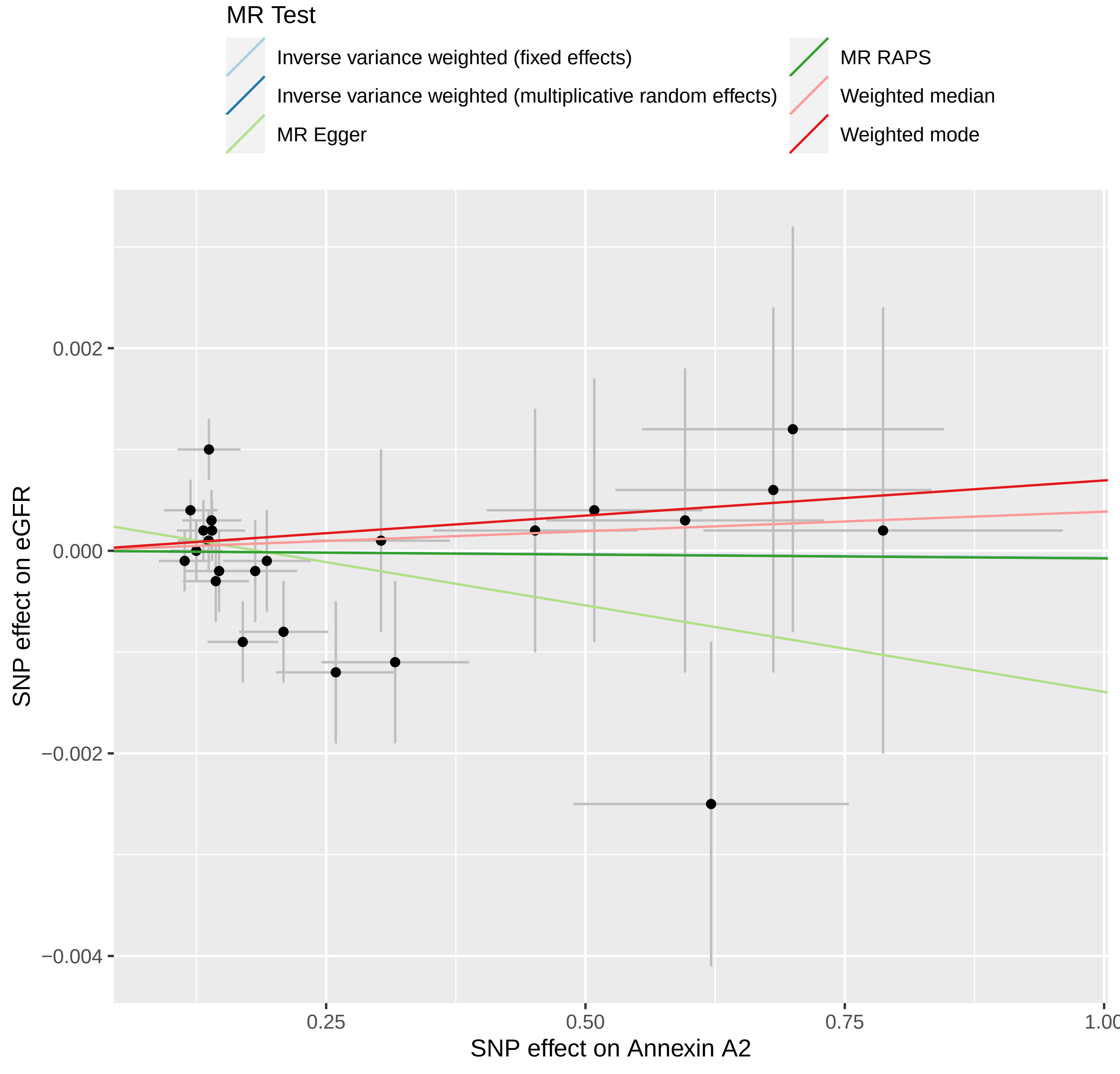

Batch 2−110 : Annexin A9 on eGFR

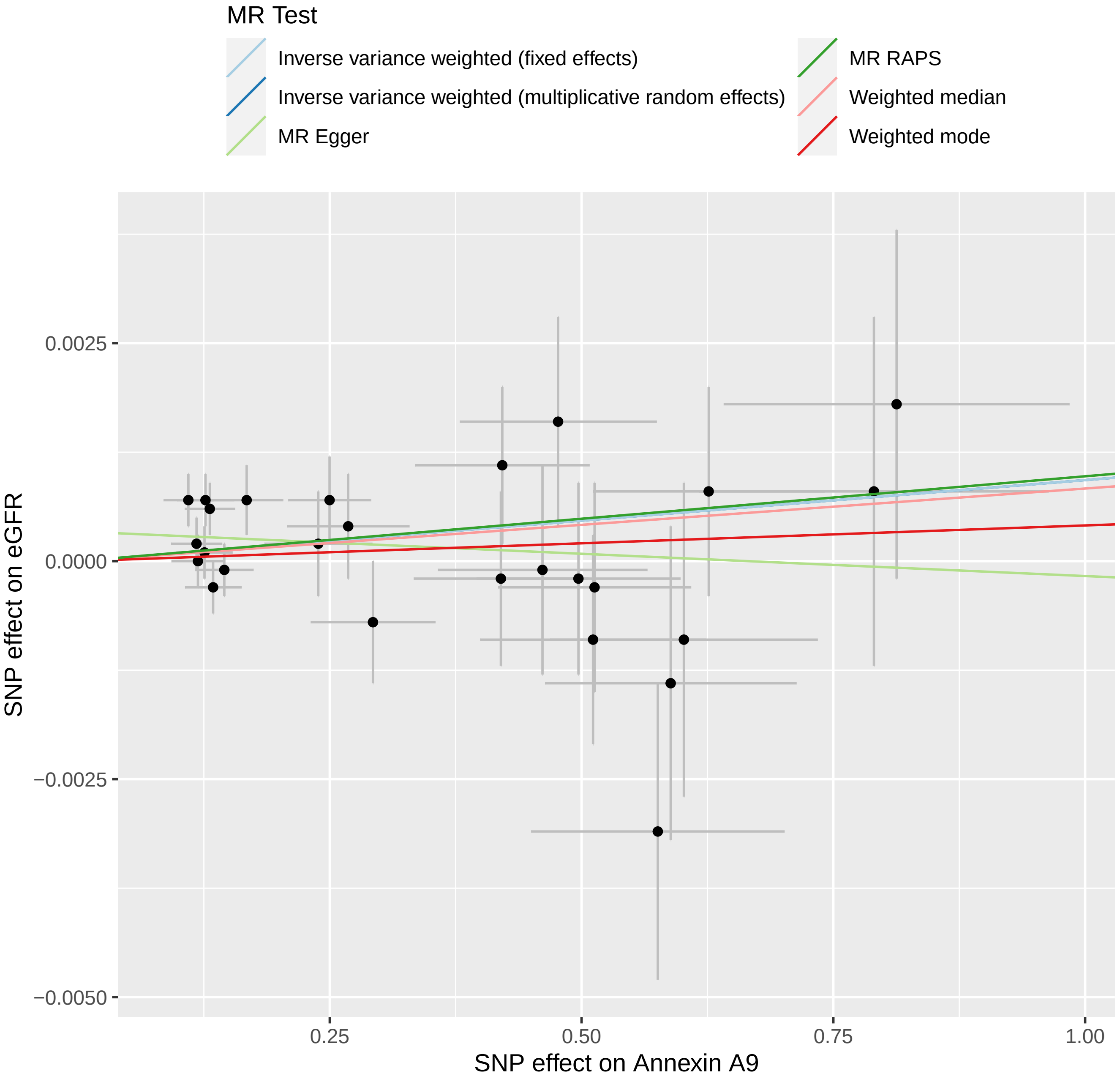

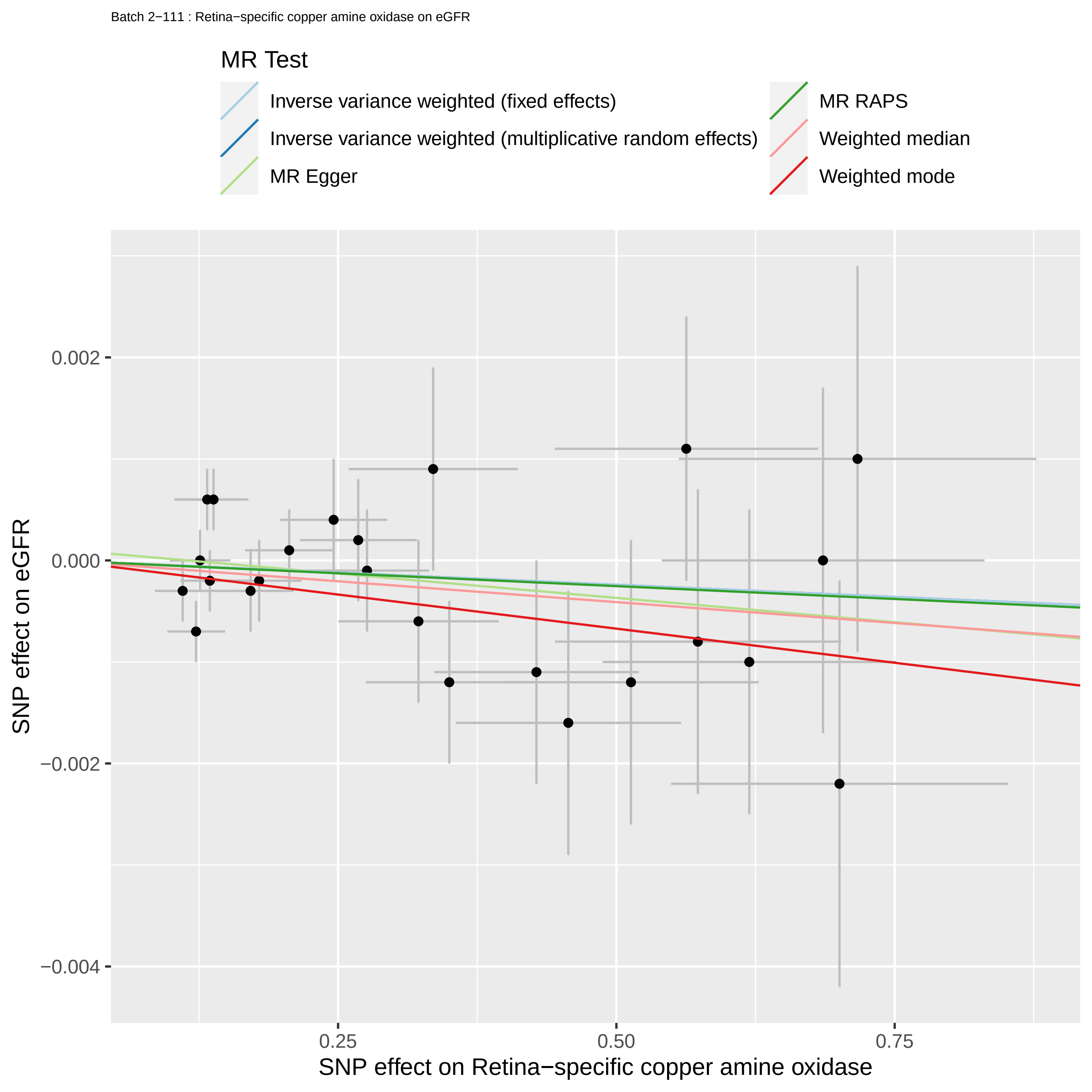
Batch 2−111 : Retina−specific copper amine oxidase on eGFR
MR Test
Inverse variance weighted (fixed effects)
Inverse variance weighted (multiplicative random effects)
MR Egger
MR RAPS
Weighted median
Weighted mode
0.002
0.000
−0.002
−0.004
0.25
0.50
0.75
SNP effect on eGFR
SNP effect on Retina−specific copper amine oxidase

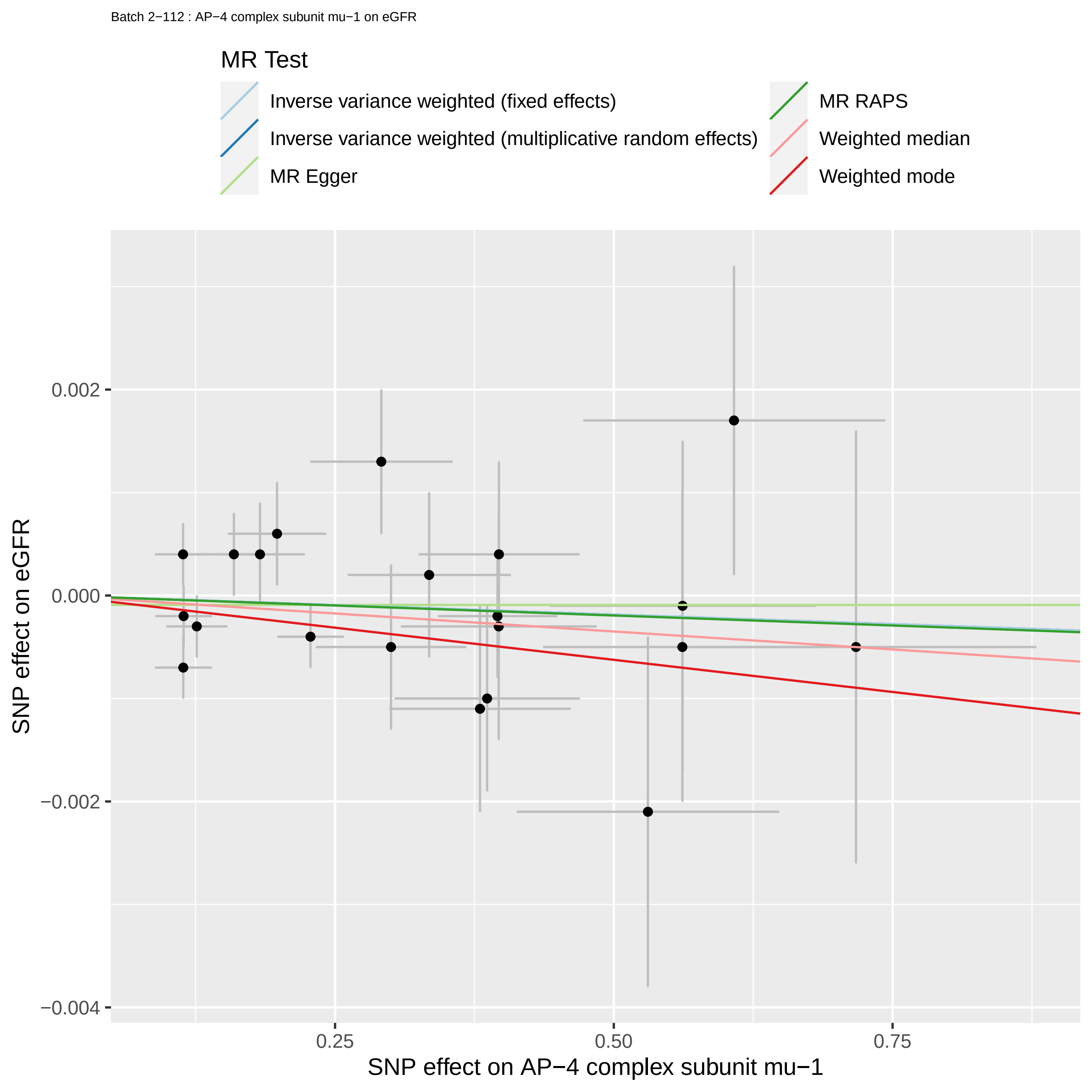
Batch 2−112 : AP−4 complex subunit mu−1 on eGFR
MR Test
Inverse variance weighted (fixed effects)
Inverse variance weighted (multiplicative random effects)
MR Egger
MR RAPS
Weighted median
Weighted mode
0.002
0.000
−0.002
−0.004
0.25
0.50
0.75
SNP effect on eGFR
SNP effect on AP−4 complex subunit mu−1

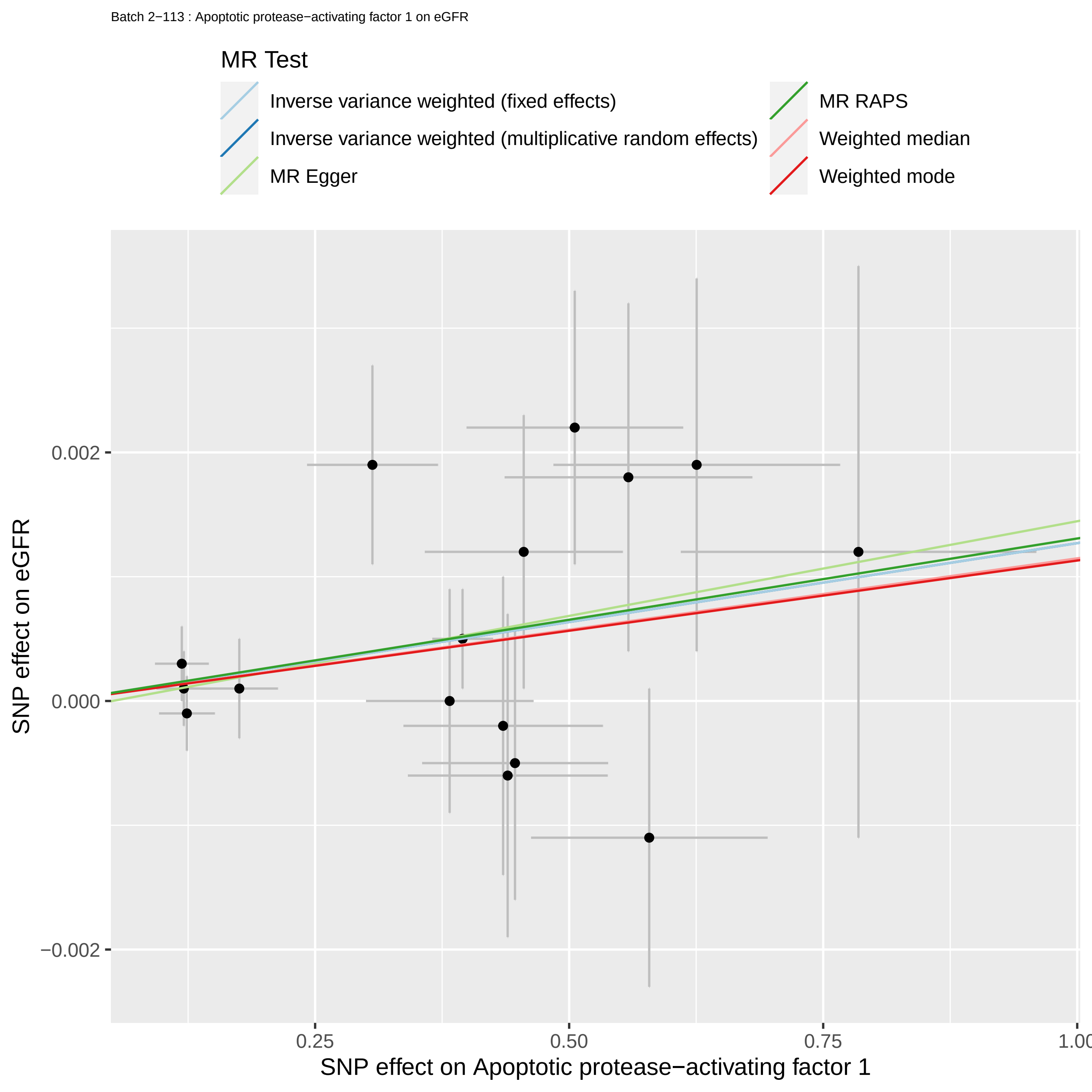

Batch 2−113 : Apoptotic protease−activating factor 1 on eGFR
MR Test
Inverse variance weighted (fixed effects)
Inverse variance weighted (multiplicative random effects)
MR Egger
MR RAPS
Weighted median
Weighted mode
0.002
0.000
−0.002
0.25
0.50
0.75
1.00
SNP effect on eGFR
SNP effect on Apoptotic protease−activating factor 1

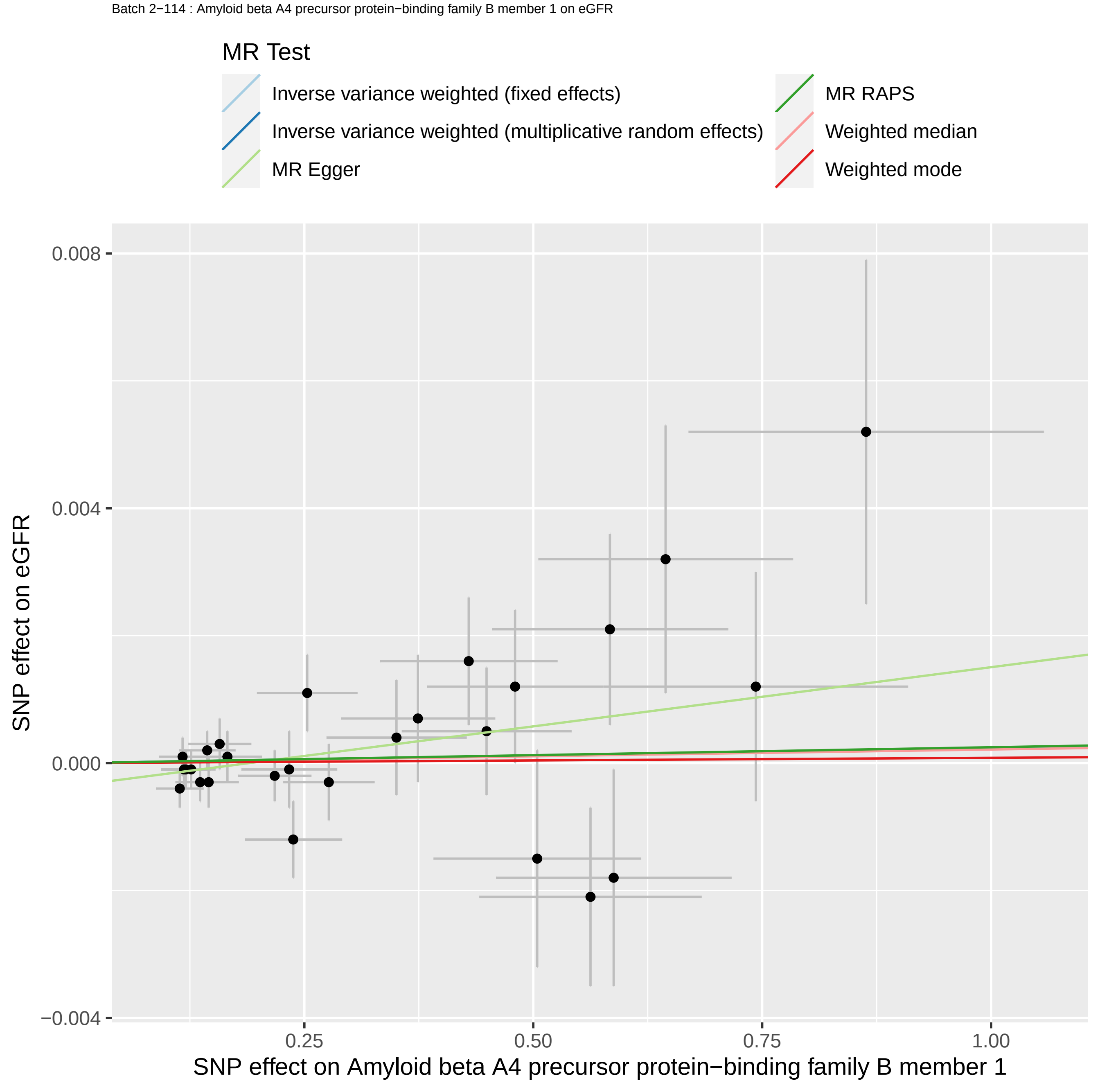

Batch 2−114 : Amyloid beta A4 precursor protein−binding family B member 1 on eGFR
MR Test
Inverse variance weighted (fixed effects)
Inverse variance weighted (multiplicative random effects)
MR Egger
MR RAPS
Weighted median
Weighted mode
0.008
0.004
0.000
−0.004
0.25
0.50
0.75
1.00
SNP effect on eGFR
SNP effect on Amyloid beta A4 precursor protein−binding family B member 1

Batch 2−115 : Amyloid beta A4 precursor protein−binding family B member 1 on eGFR

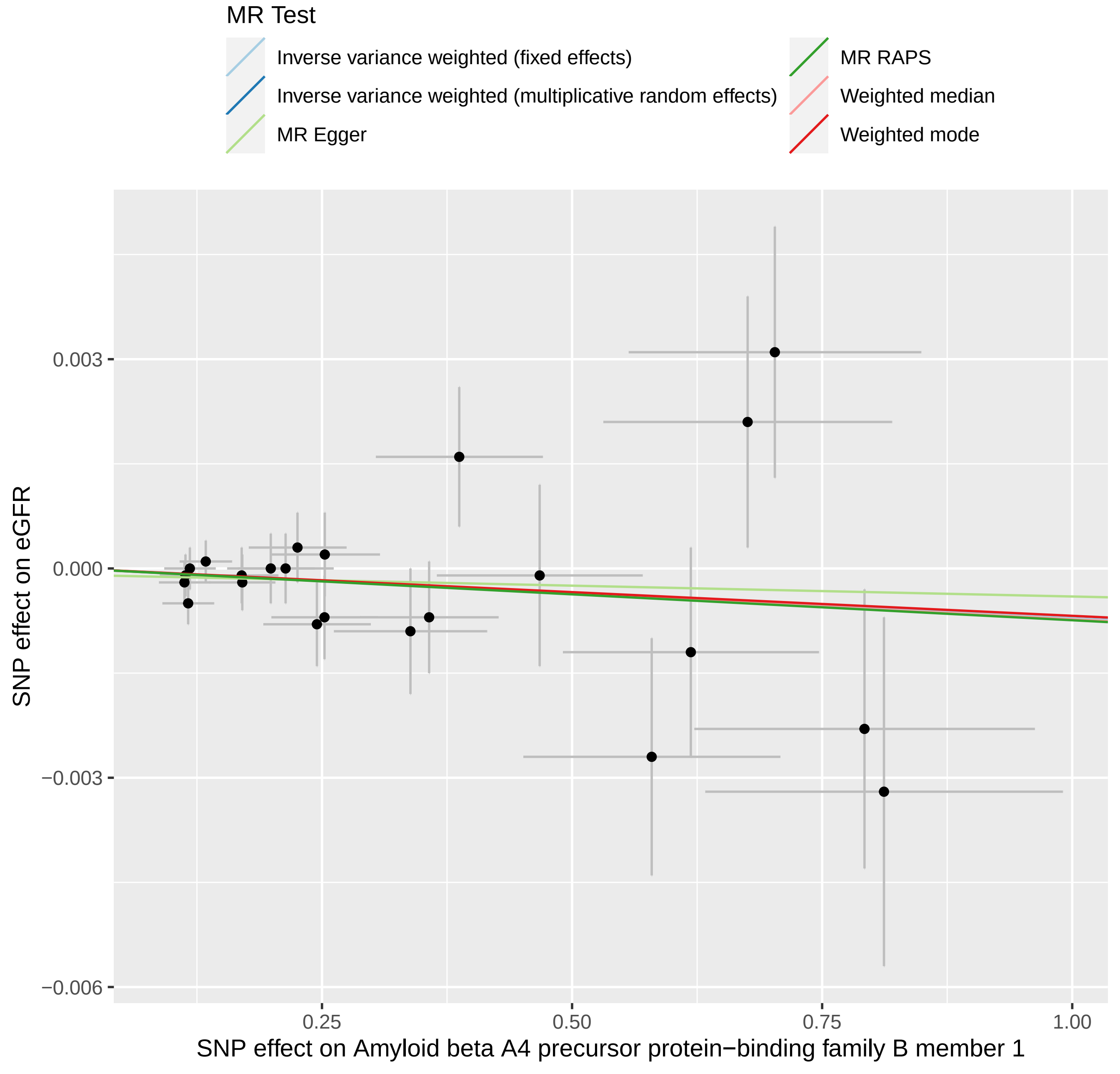

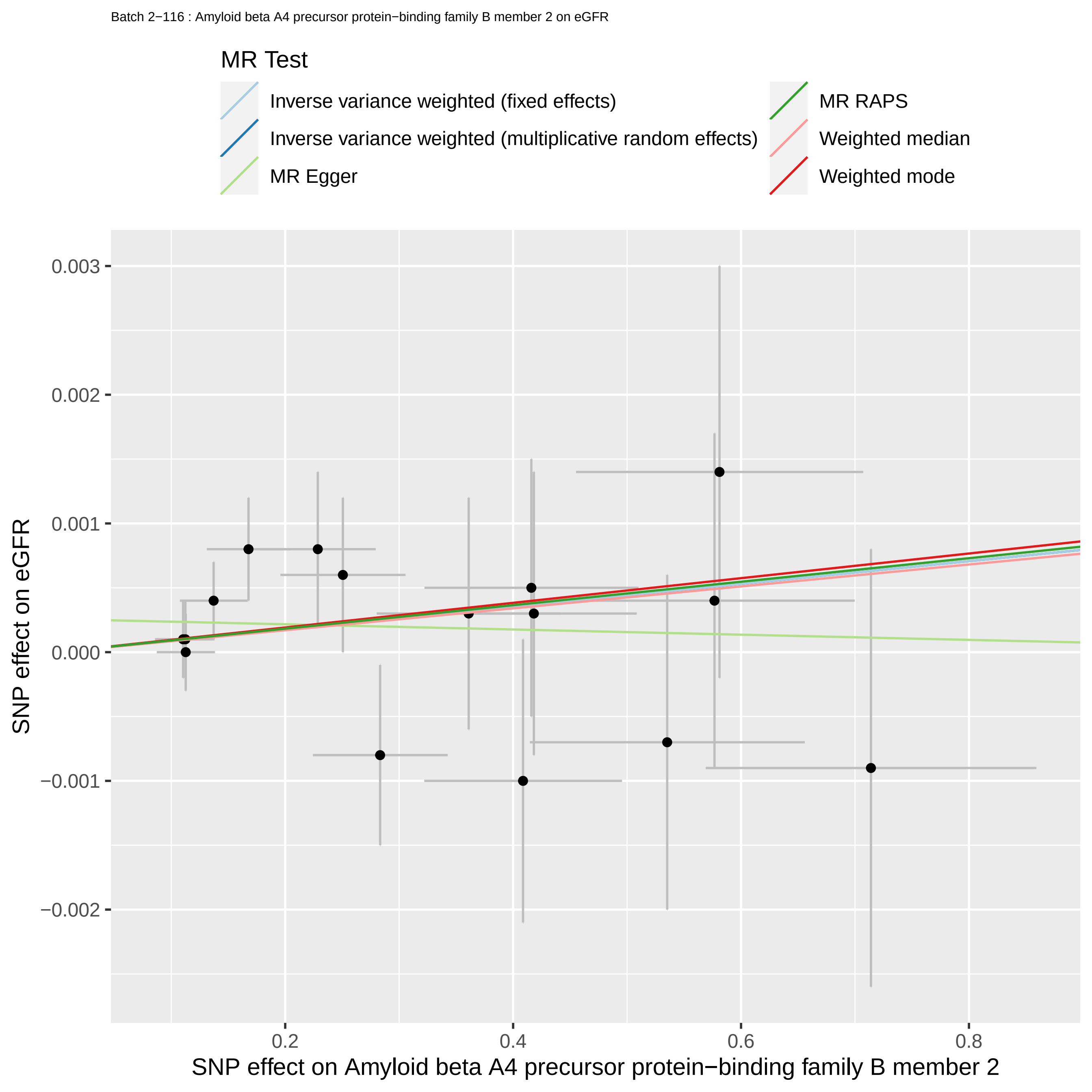

Batch 2−116 : Amyloid beta A4 precursor protein−binding family B member 2 on eGFR
MR Test
Inverse variance weighted (fixed effects)
Inverse variance weighted (multiplicative random effects)
MR Egger
MR RAPS
Weighted median
Weighted mode
0.003
0.002
0.001
0.000
−0.001
−0.002
0.2
0.4
0.6
0.8
SNP effect on eGFR
SNP effect on Amyloid beta A4 precursor protein−binding family B member 2

Batch 2−117 : Amyloid beta A4 precursor protein−binding family B member 2 on eGFR

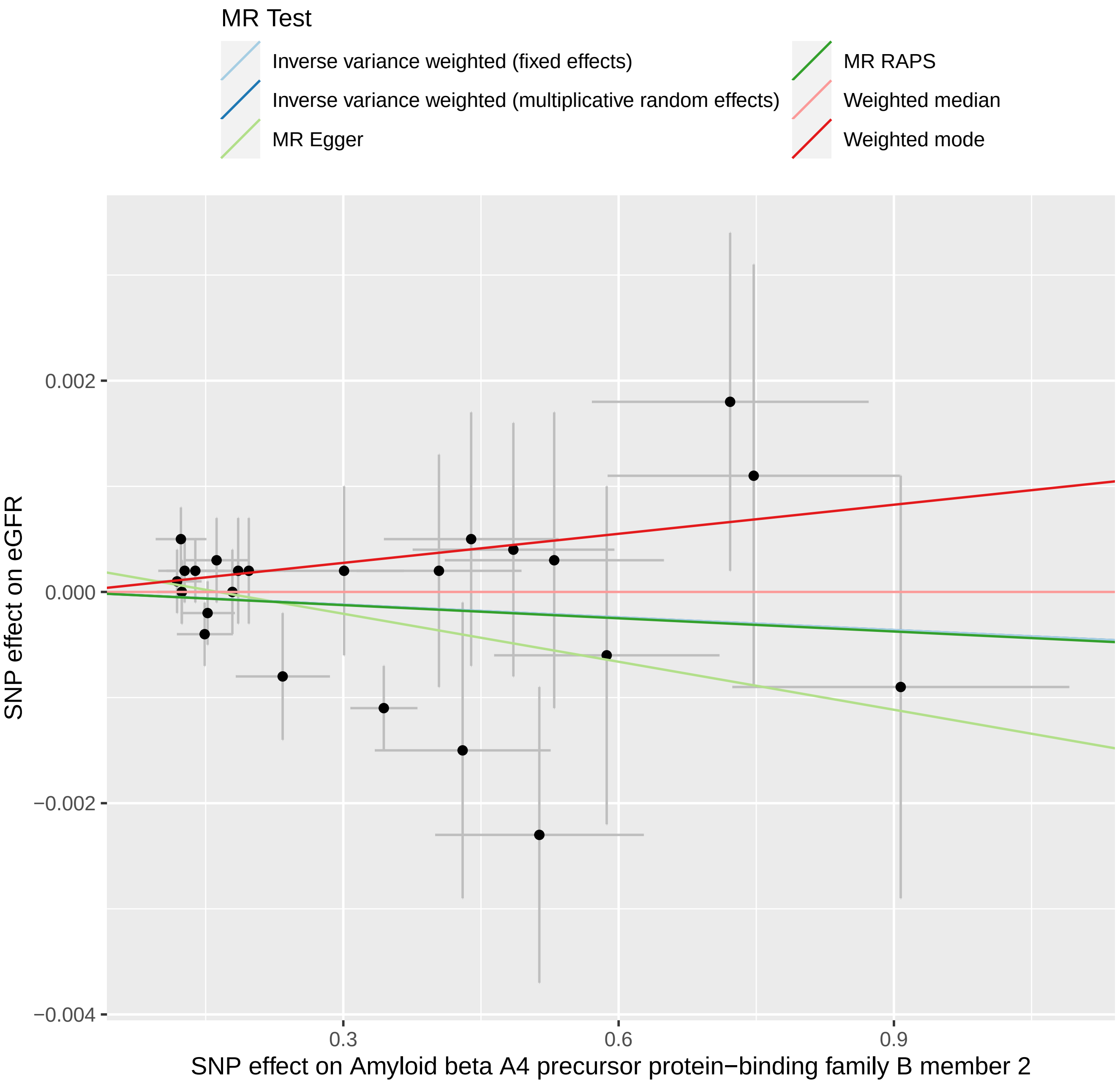

Batch 2−118 : Amyloid beta A4 precursor protein−binding family B member 3 on eGFR

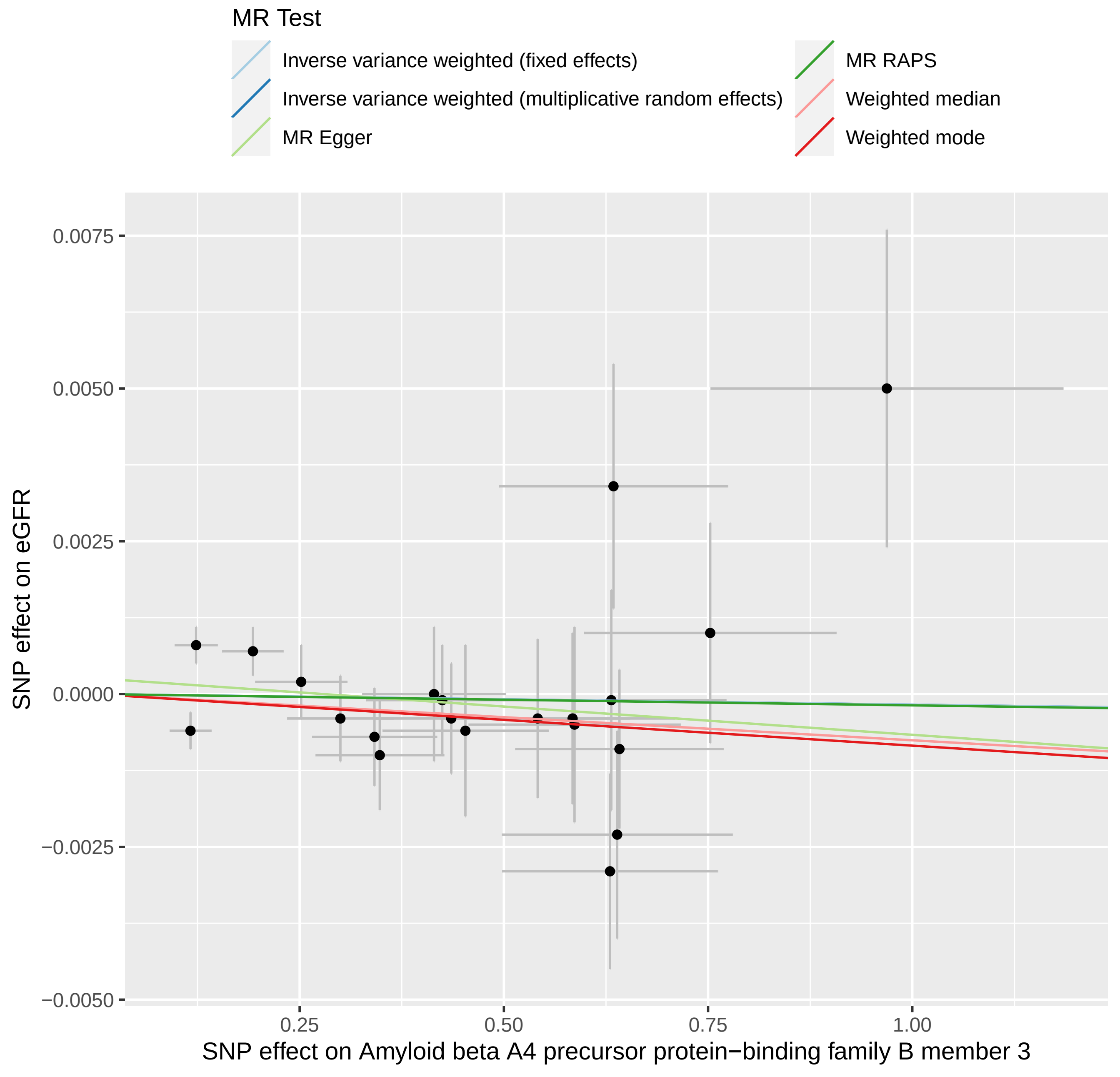

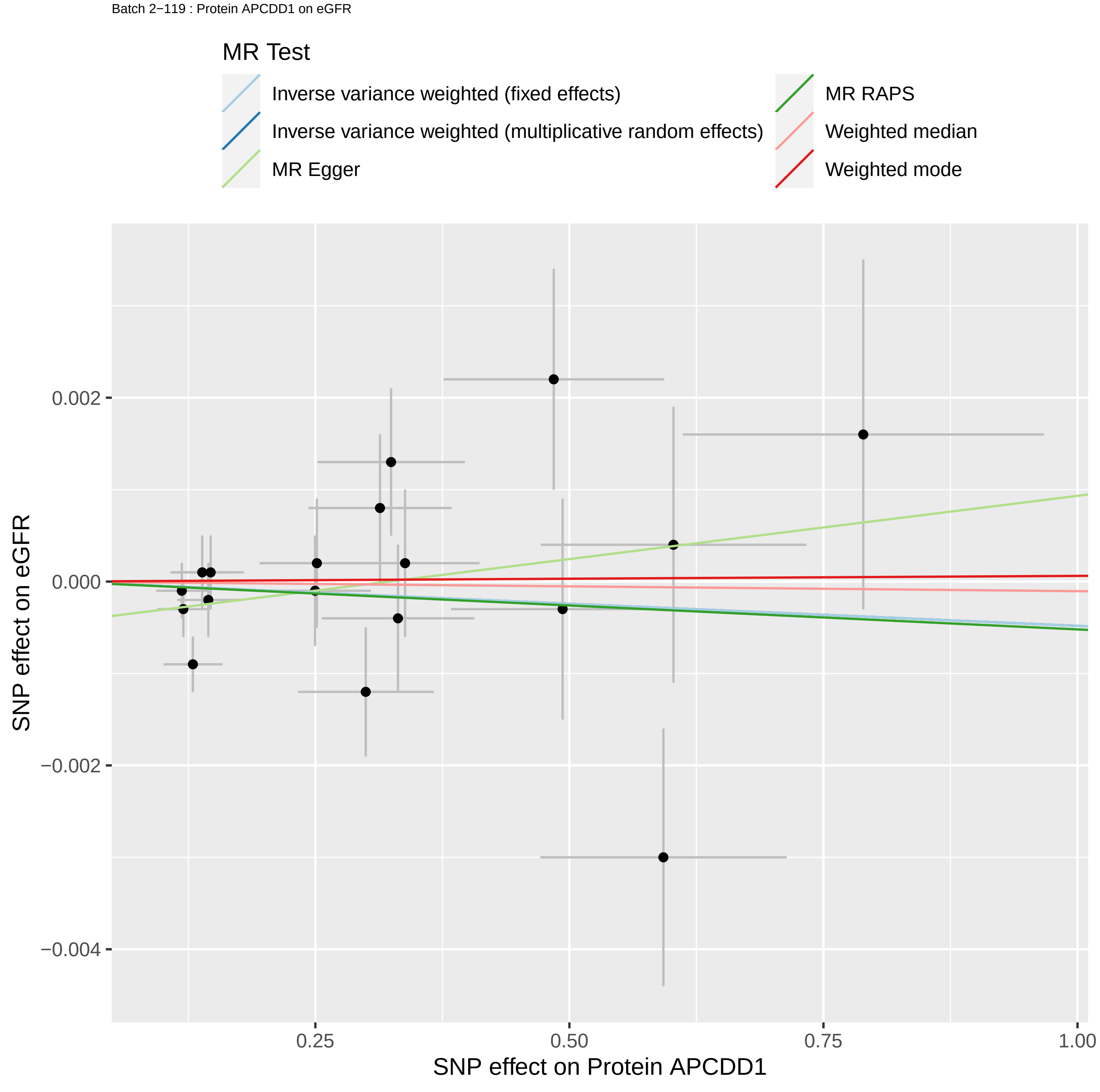
Batch 2−119 : Protein APCDD1 on eGFR
MR Test
Inverse variance weighted (fixed effects)
Inverse variance weighted (multiplicative random effects)
MR Egger
MR RAPS
Weighted median
Weighted mode
0.002
0.000
−0.002
−0.004
SNP effect on eGFR
0.25
0.50
0.75
1.00
SNP effect on Protein APCDD1

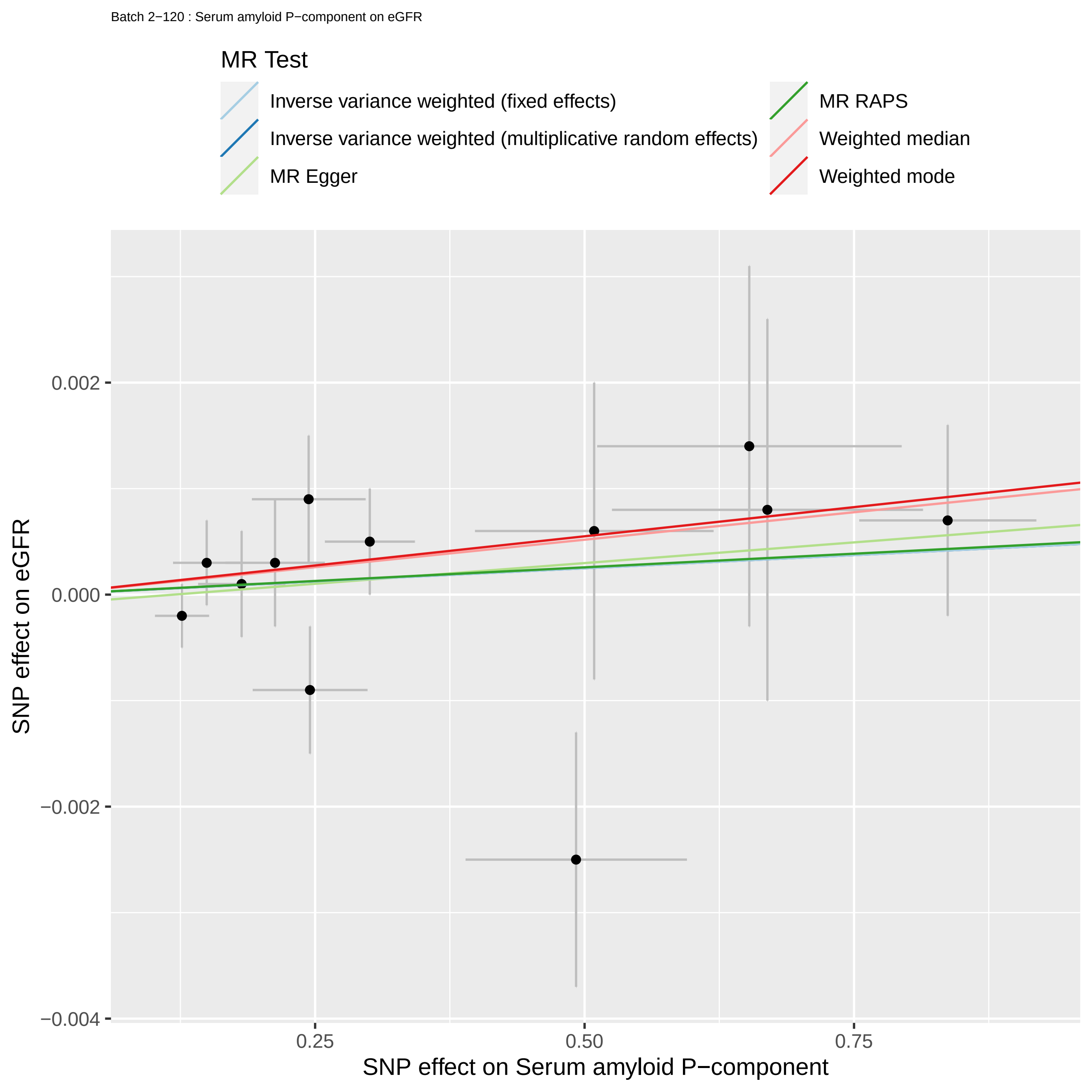
Batch 2−120 : Serum amyloid P−component on eGFR
MR Test
Inverse variance weighted (fixed effects)
Inverse variance weighted (multiplicative random effects)
MR Egger
MR RAPS
Weighted median
Weighted mode
0.002
0.000
−0.002
−0.004
SNP effect on eGFR
0.25
0.50
0.75
SNP effect on Serum amyloid P−component

Batch 2−121 : DNA−(apurinic or apyrimidinic site) lyase on eGFR

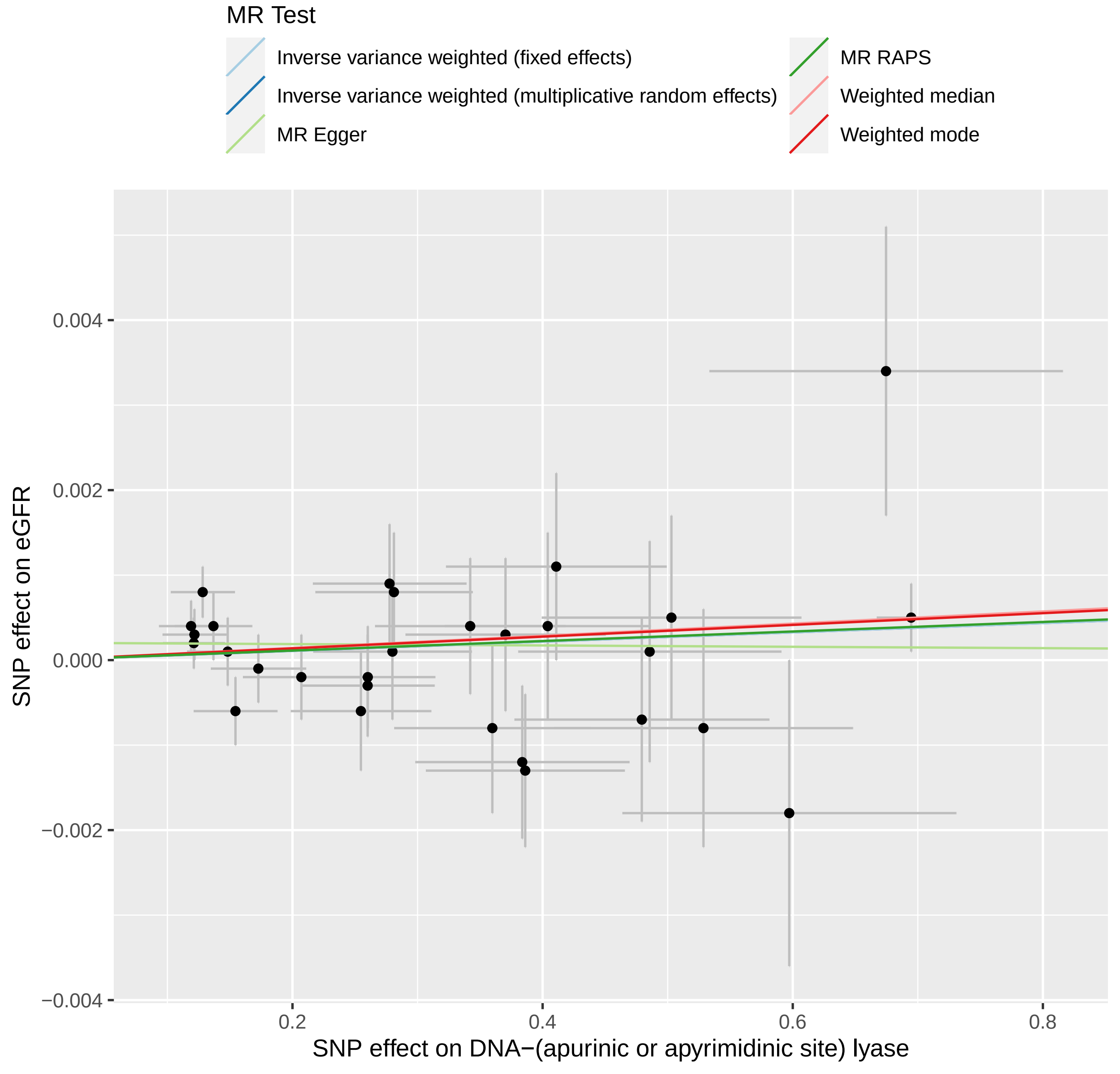

Batch 2−122 : Amyloid−like protein 2 on eGFR

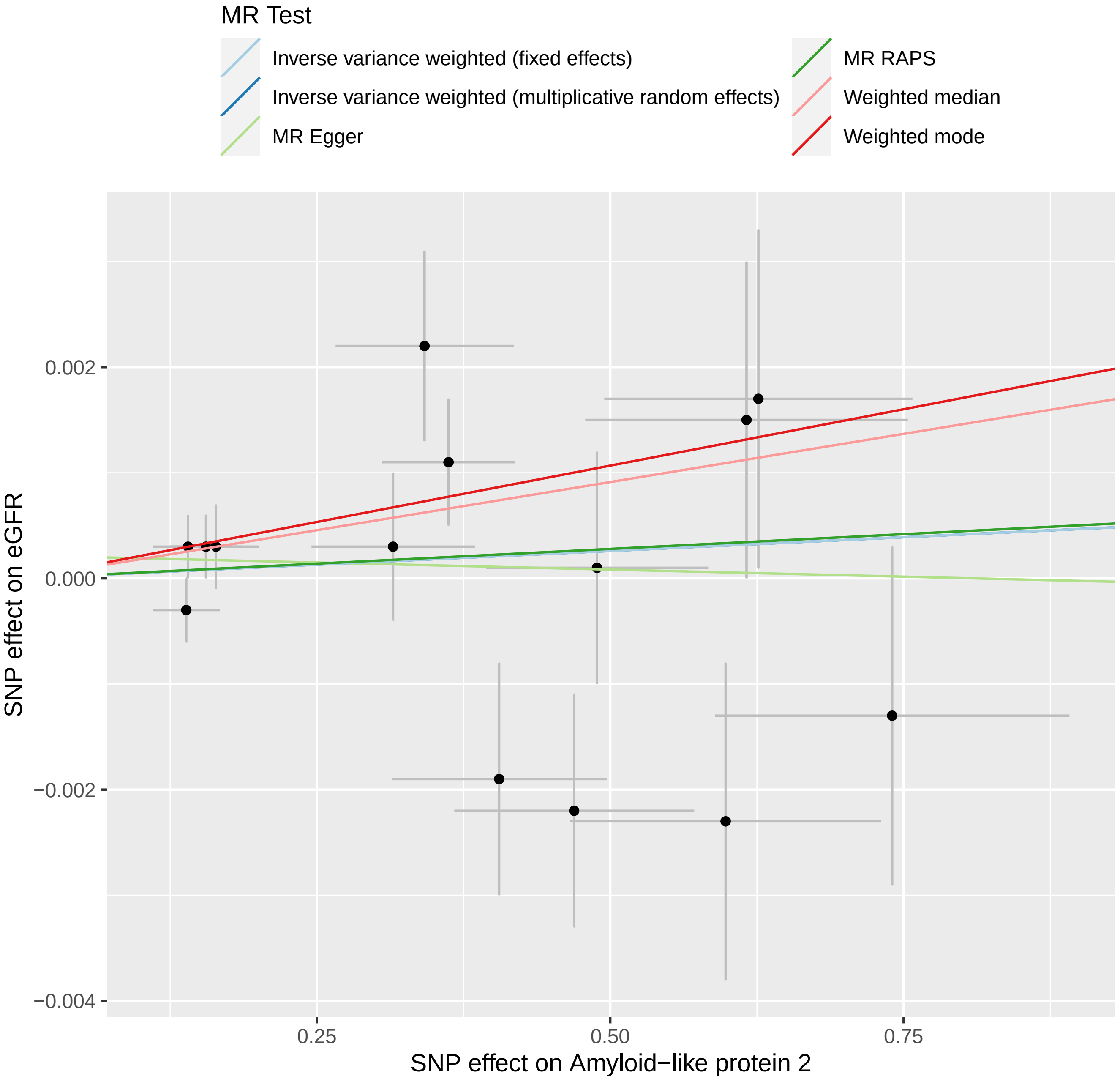

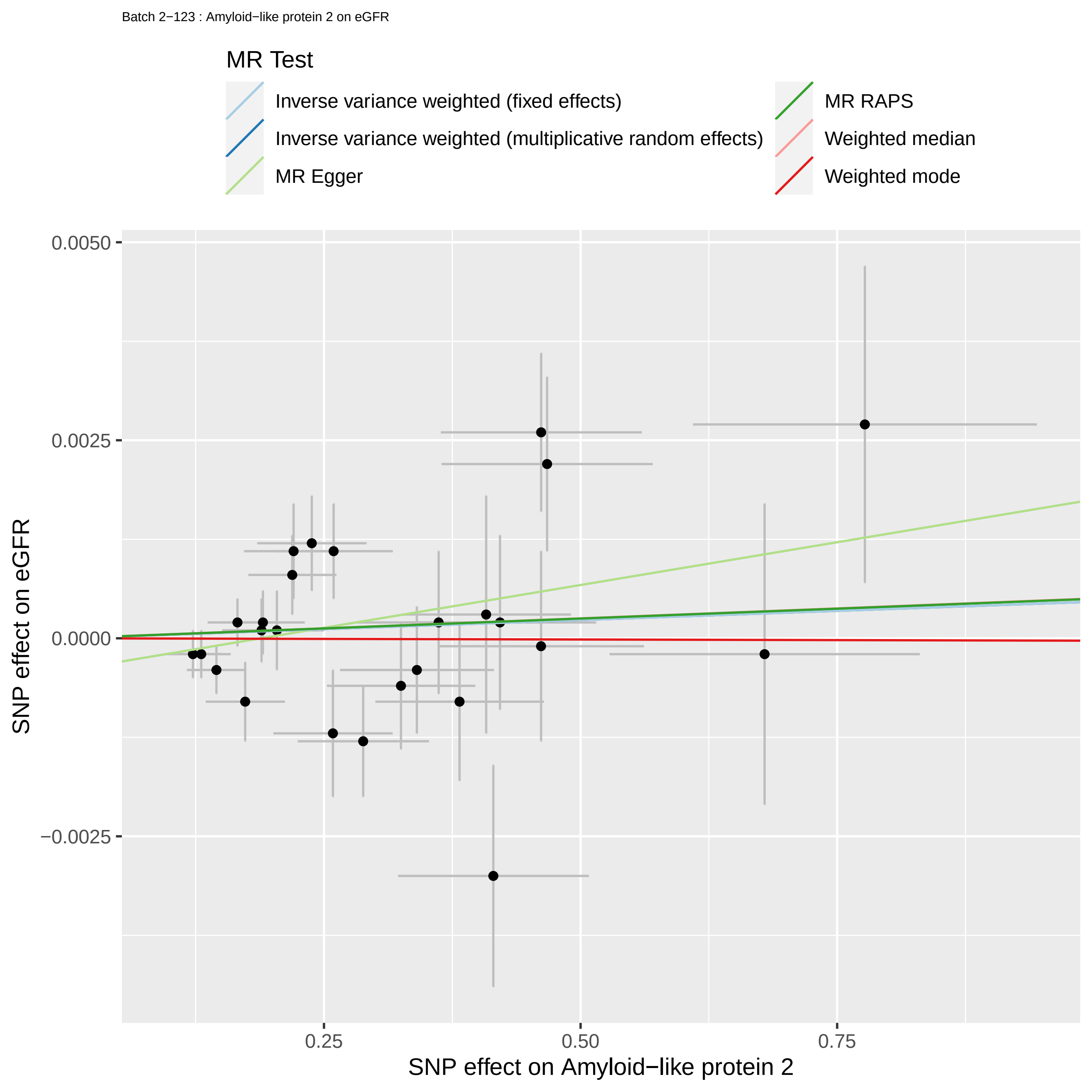

Batch 2−123 : Amyloid−like protein 2 on eGFR
MR Test
Inverse variance weighted (fixed effects)
Inverse variance weighted (multiplicative random effects)
MR Egger
MR RAPS
Weighted median
Weighted mode
0.0050
0.0025
0.0000
−0.0025
0.25
0.50
0.75
SNP effect on eGFR
SNP effect on Amyloid−like protein 2

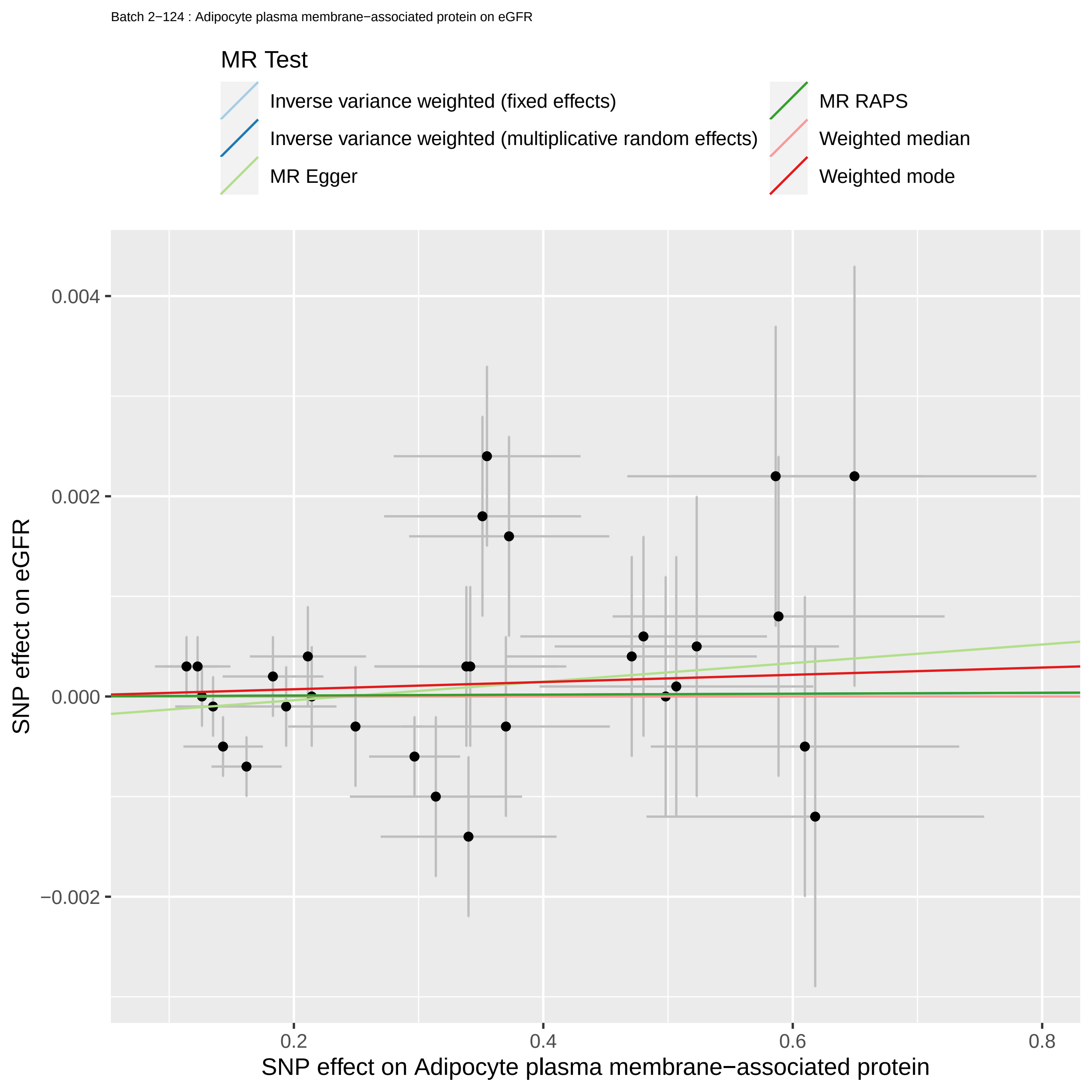
Batch 2−124 : Adipocyte plasma membrane−associated protein on eGFR
MR Test
Inverse variance weighted (fixed effects)
Inverse variance weighted (multiplicative random effects)
MR Egger
MR RAPS
Weighted median
Weighted mode
0.004
0.002
0.000
−0.002
0.2
0.4
0.6
0.8
SNP effect on eGFR
SNP effect on Adipocyte plasma membrane−associated protein

Batch 2−125 : Apolipoprotein A−V on eGFR

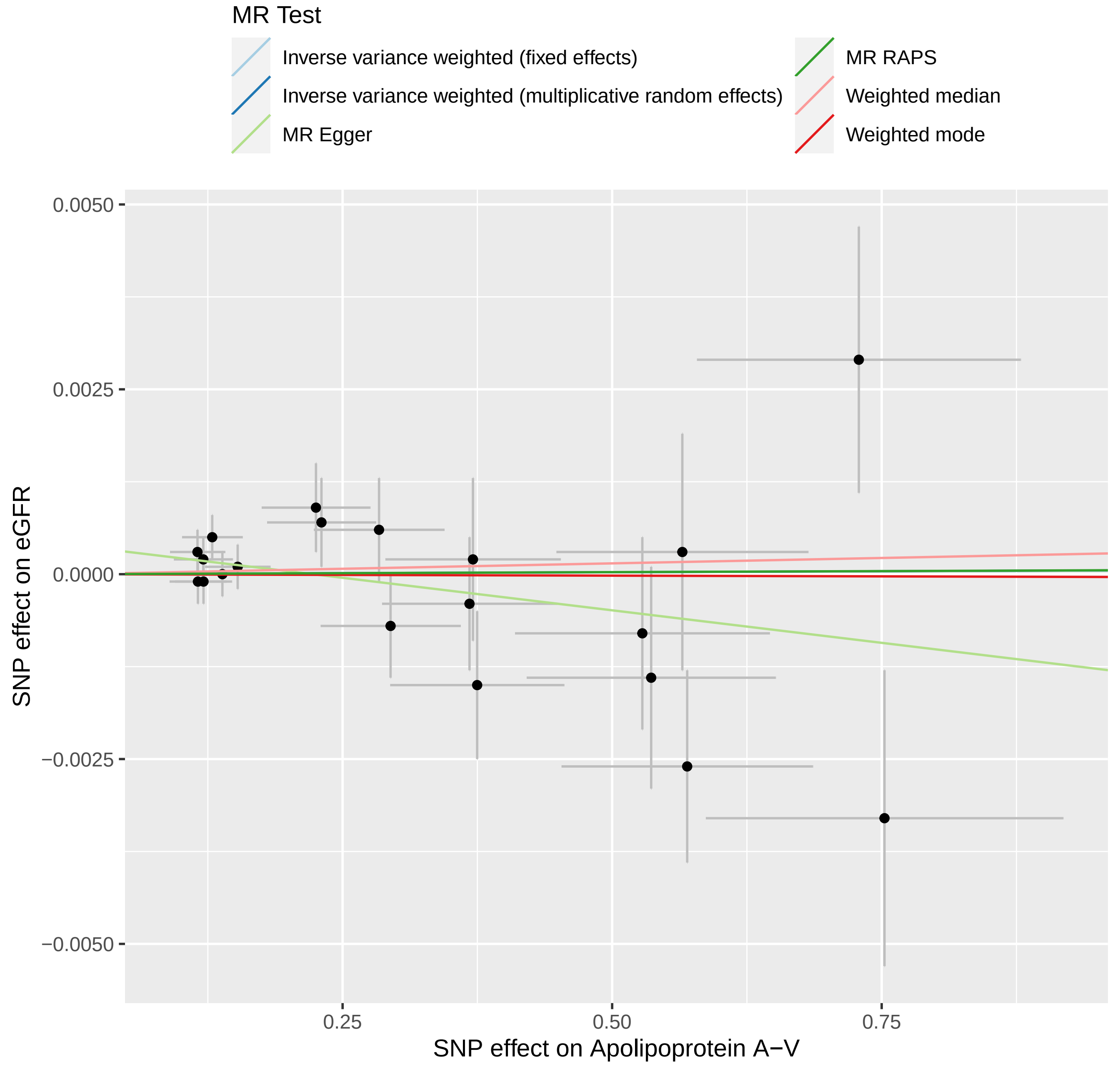

Batch 2−126 : Apolipoprotein A−V on eGFR

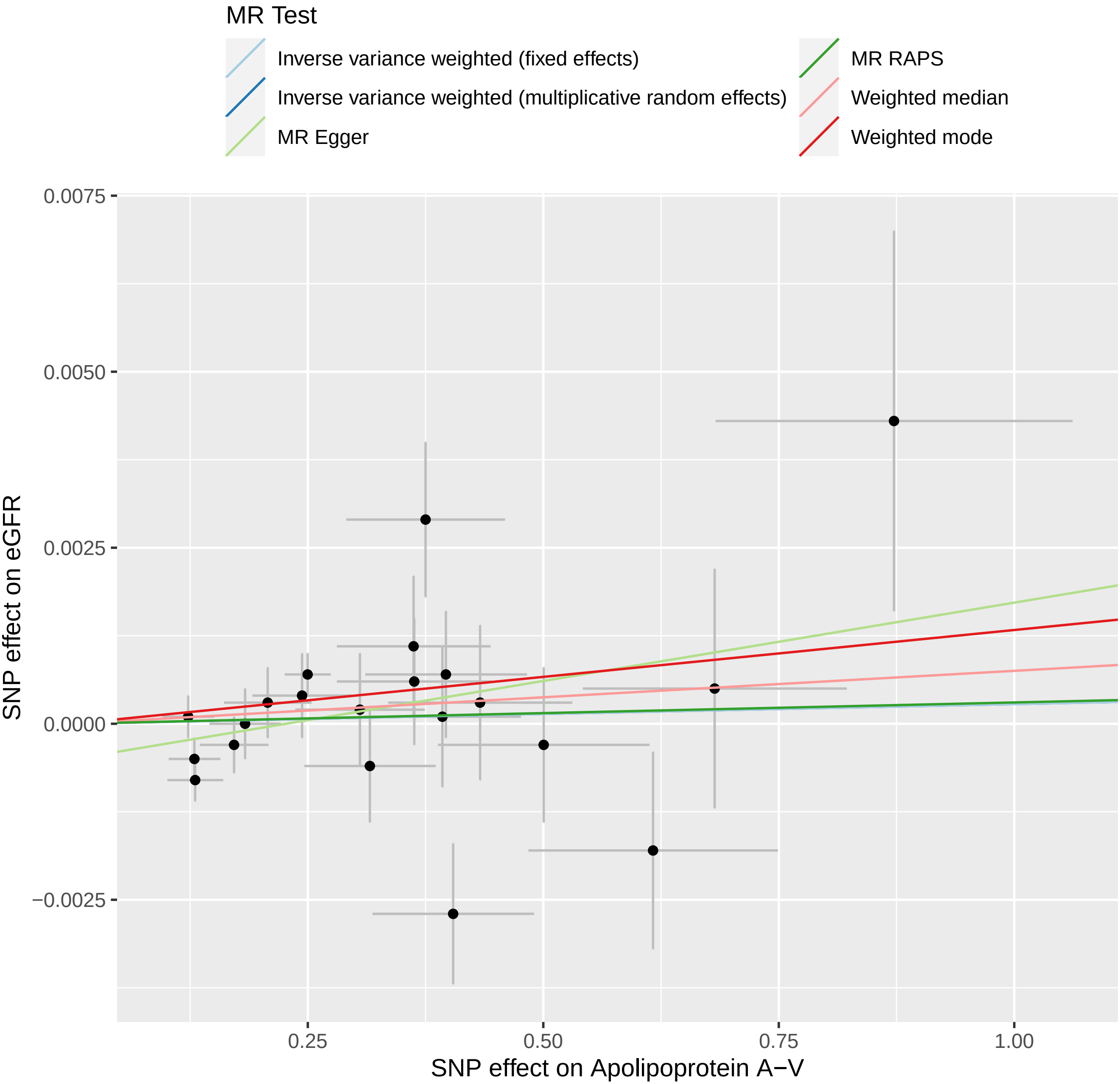

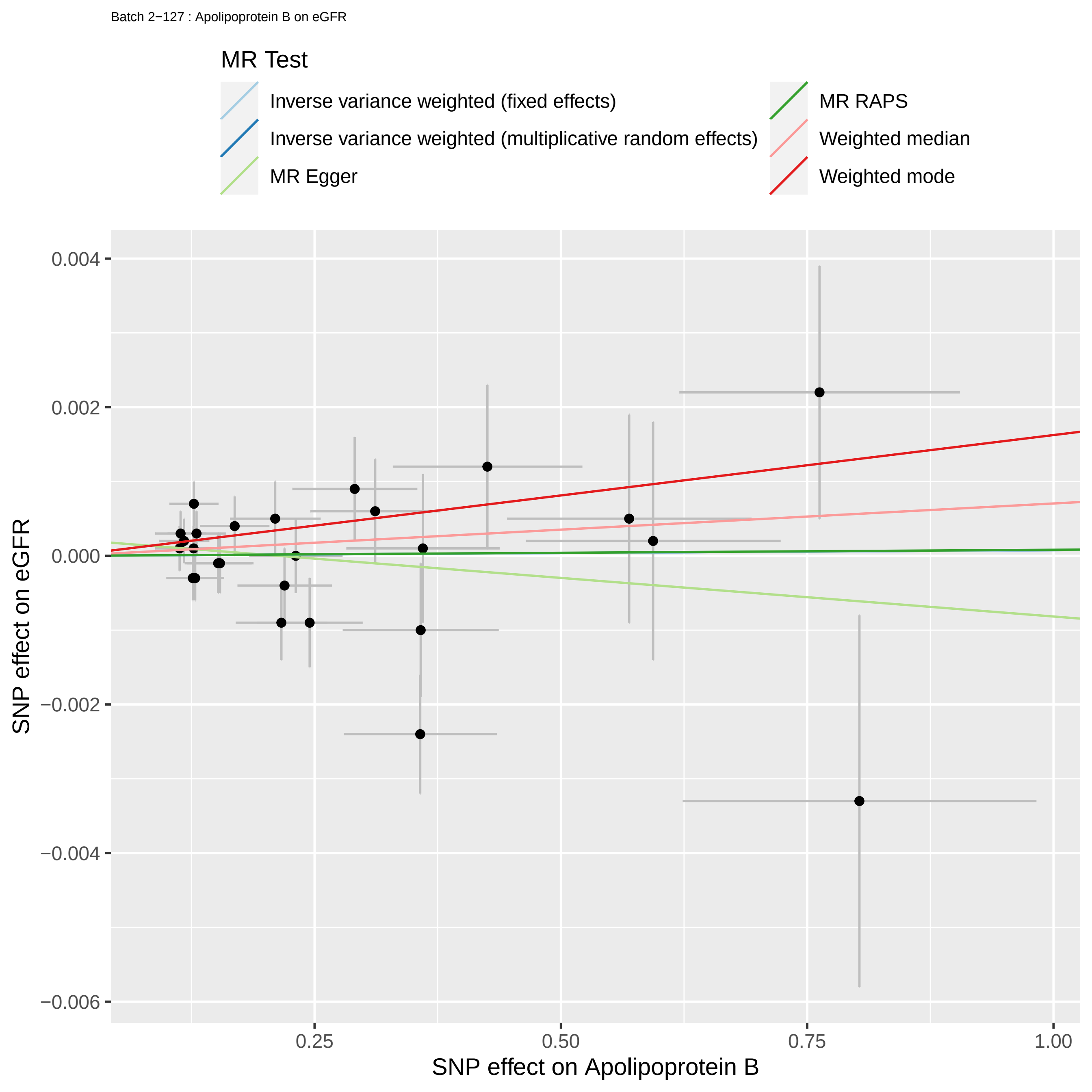

Batch 2−127 : Apolipoprotein B on eGFR
MR Test
Inverse variance weighted (fixed effects)
Inverse variance weighted (multiplicative random effects)
MR Egger
MR RAPS
Weighted median
Weighted mode
0.004
0.002
0.000
−0.002
−0.004
−0.006
SNP effect on eGFR
0.25
0.50
0.75
1.00
SNP effect on Apolipoprotein B

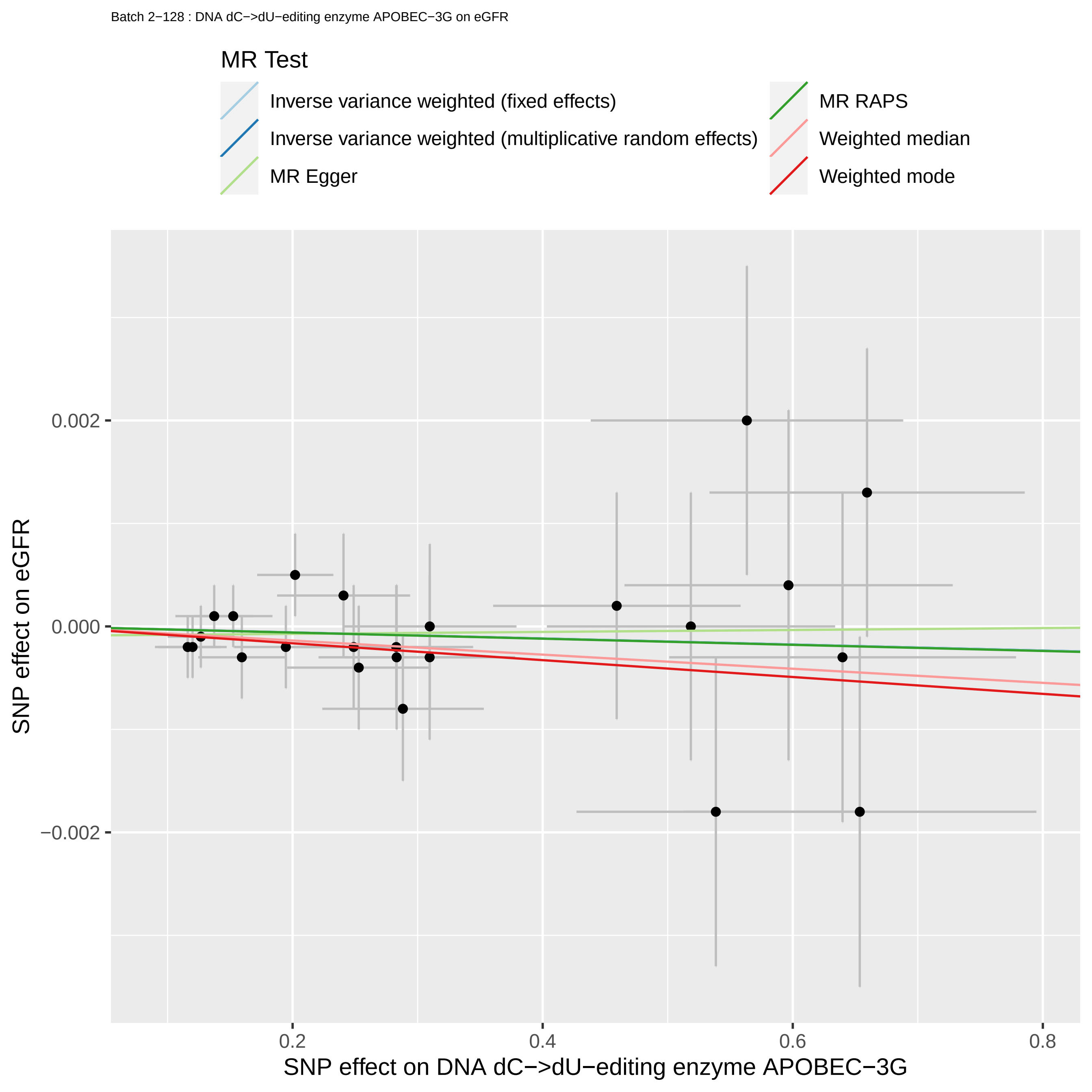

Batch 2−128 : DNA dC−>dU−editing enzyme APOBEC−3G on eGFR
MR Test
Inverse variance weighted (fixed effects)
Inverse variance weighted (multiplicative random effects)
MR Egger
MR RAPS
Weighted median
Weighted mode
SNP effect on eGFR
0.002
0.000
−0.002
0.2
0.4
0.6
0.8
SNP effect on DNA dC−>dU−editing enzyme APOBEC−3G

Batch 2−129 : Apolipoprotein D on eGFR

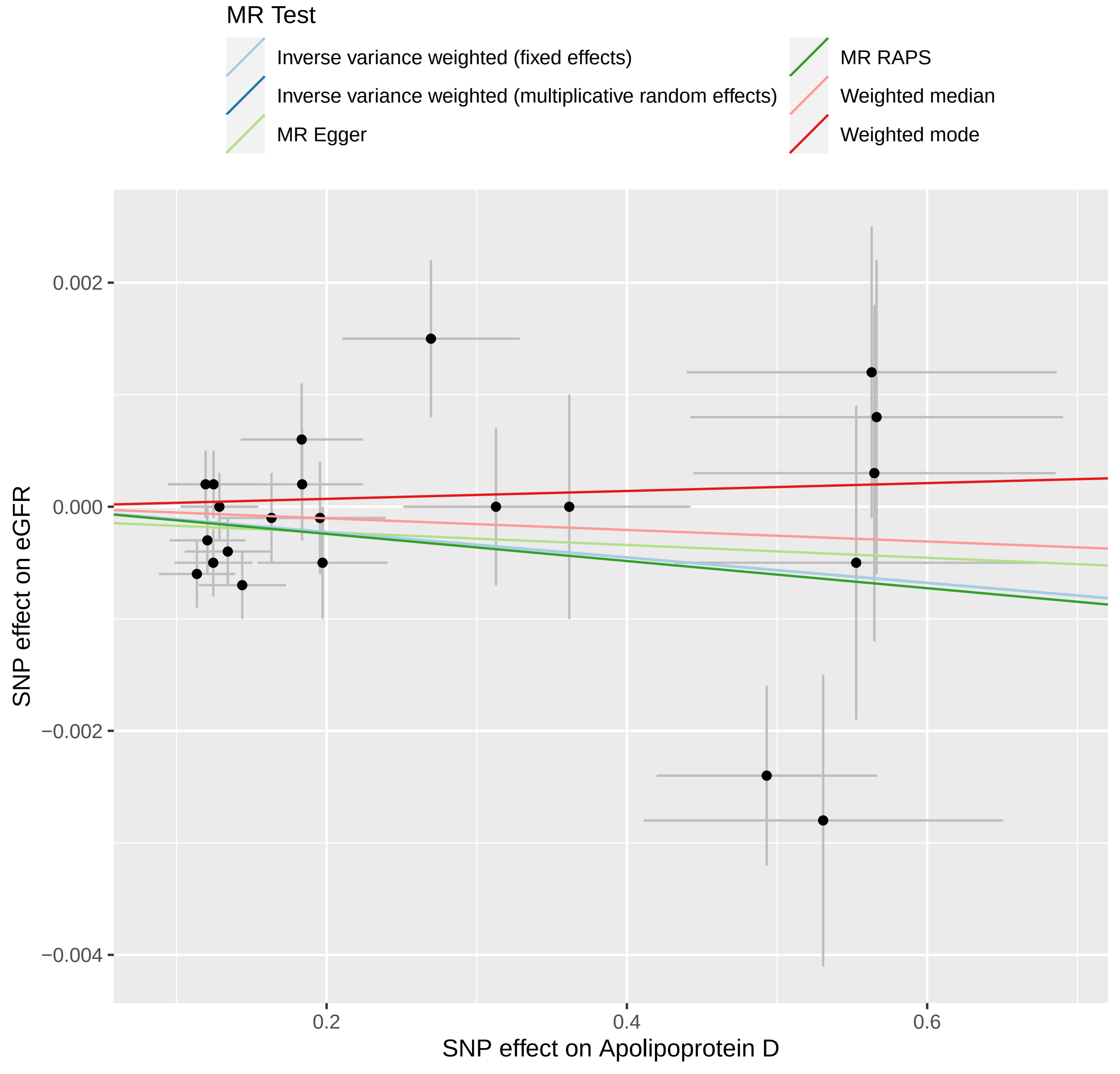

Batch 2−130 : Apolipoprotein D on eGFR

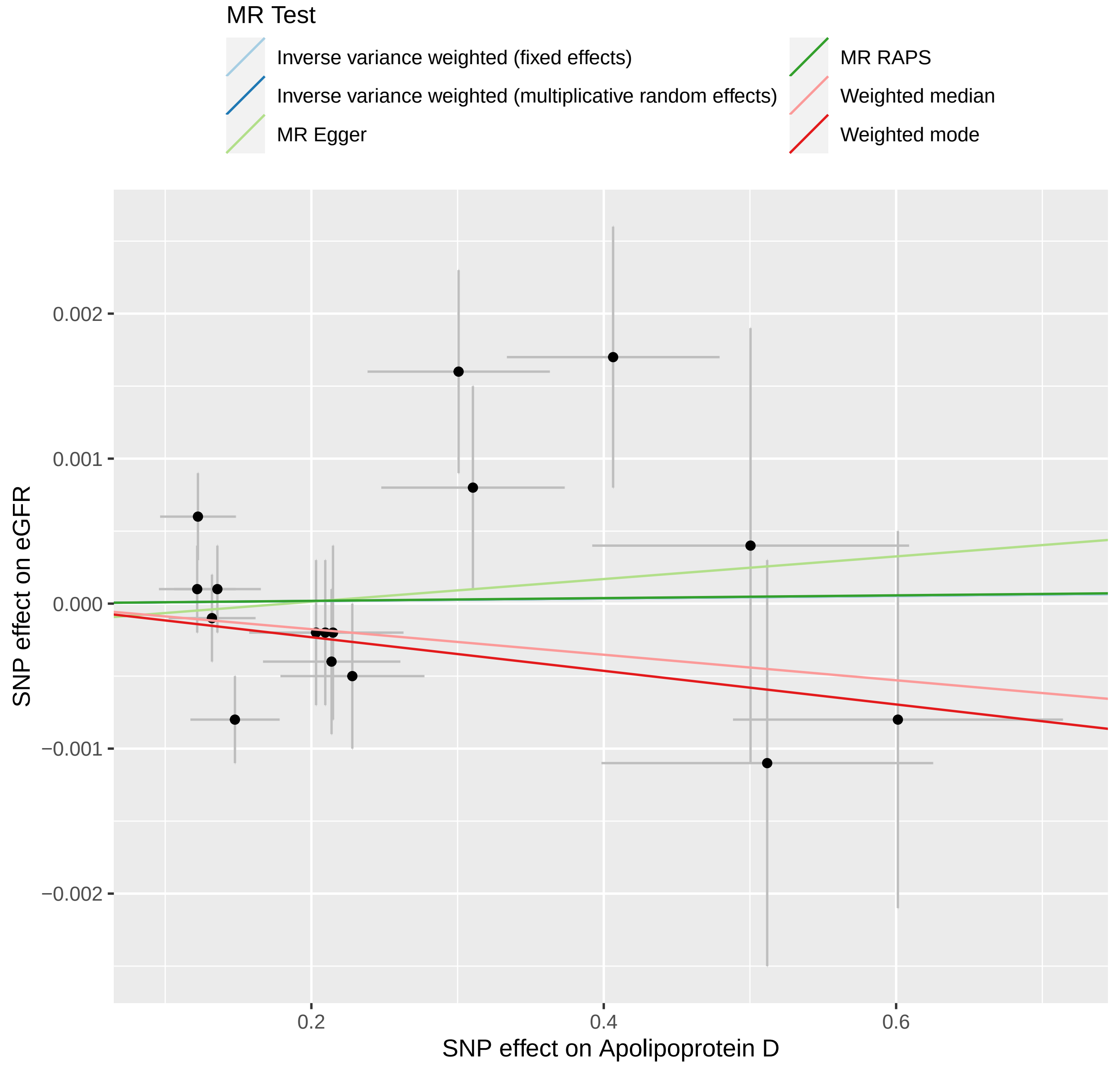

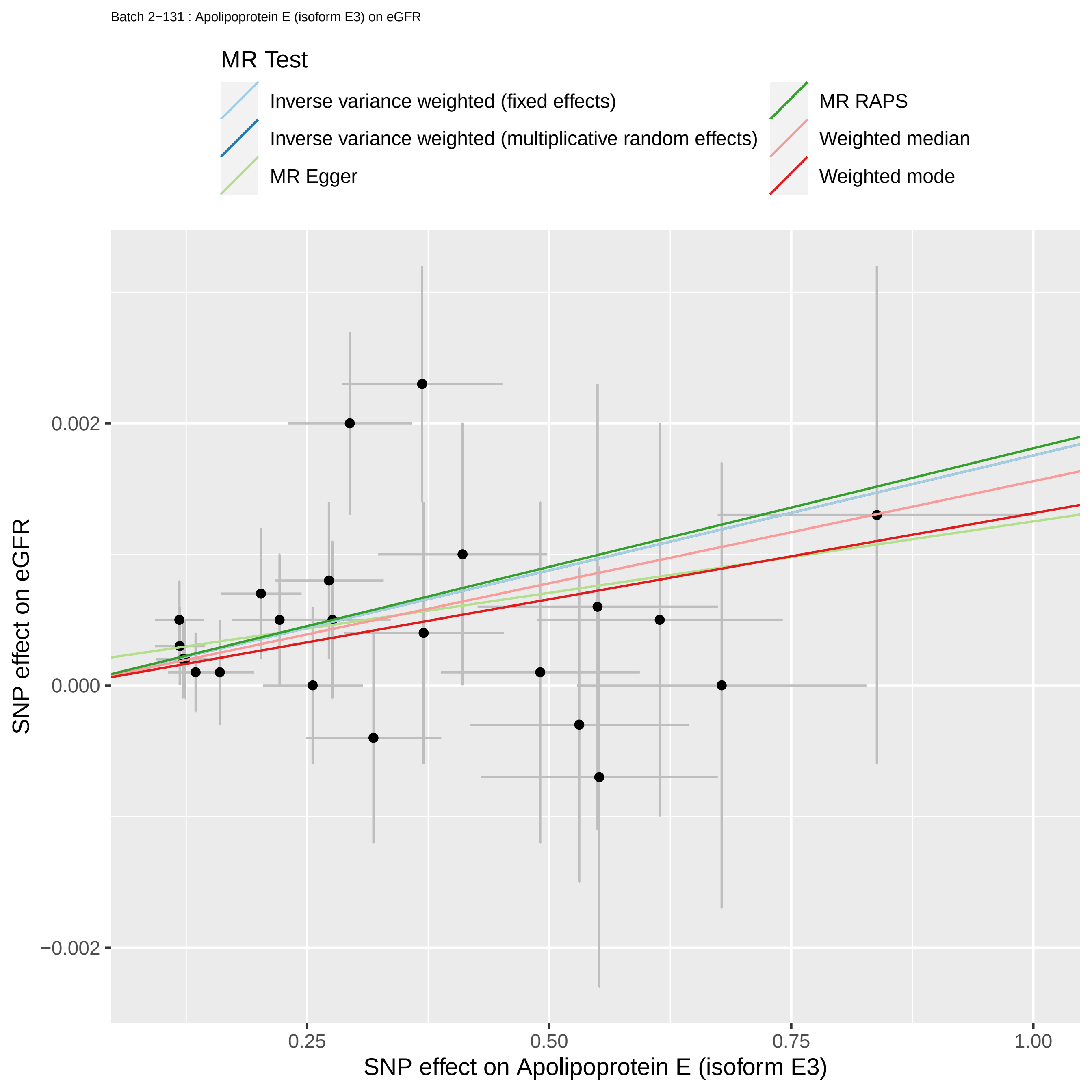

Batch 2−131 : Apolipoprotein E (isoform E3) on eGFR
MR Test
Inverse variance weighted (fixed effects)
Inverse variance weighted (multiplicative random effects)
MR Egger
MR RAPS
Weighted median
Weighted mode
0.002
0.000
−0.002
0.25
0.50
0.75
1.00
SNP effect on eGFR
SNP effect on Apolipoprotein E (isoform E3)

Batch 2−132 : Apolipoprotein E (isoform E2) on eGFR

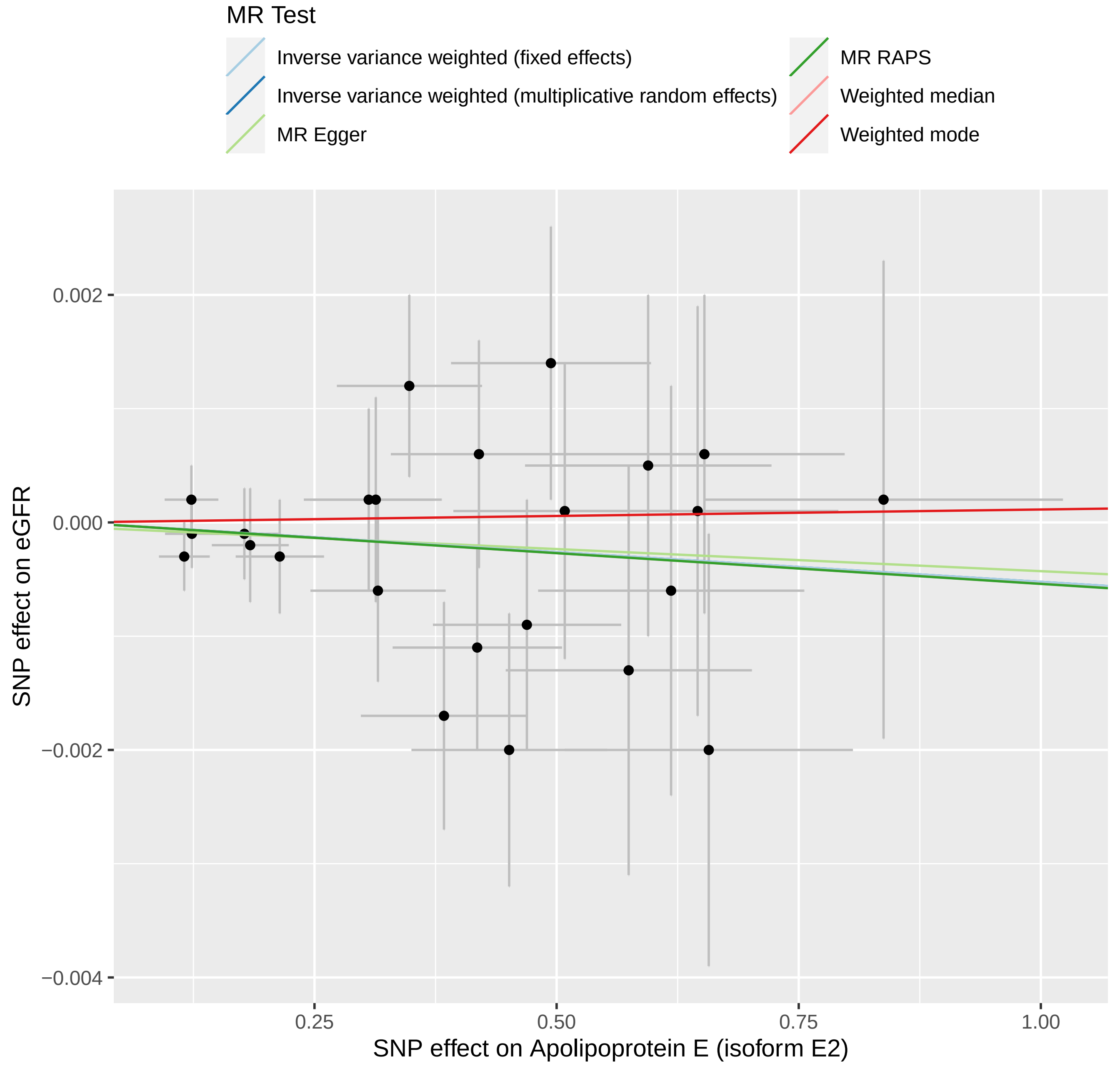

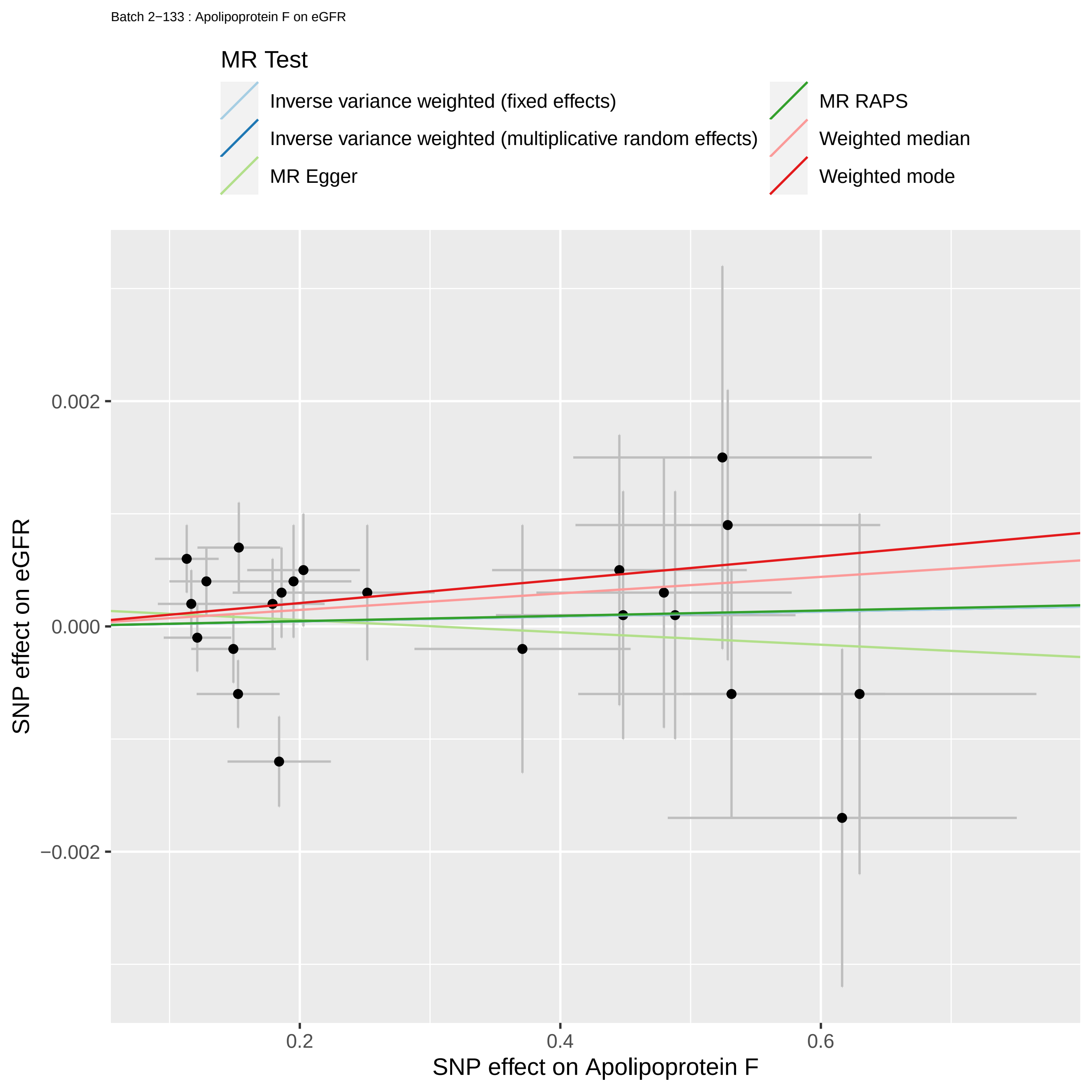
Batch 2−133 : Apolipoprotein F on eGFR
MR Test
Inverse variance weighted (fixed effects)
Inverse variance weighted (multiplicative random effects)
MR Egger
MR RAPS
Weighted median
Weighted mode
0.002
0.000
−0.002
SNP effect on eGFR
0.2
0.4
0.6
SNP effect on Apolipoprotein F

Batch 2−134 : Apolipoprotein L1 on eGFR

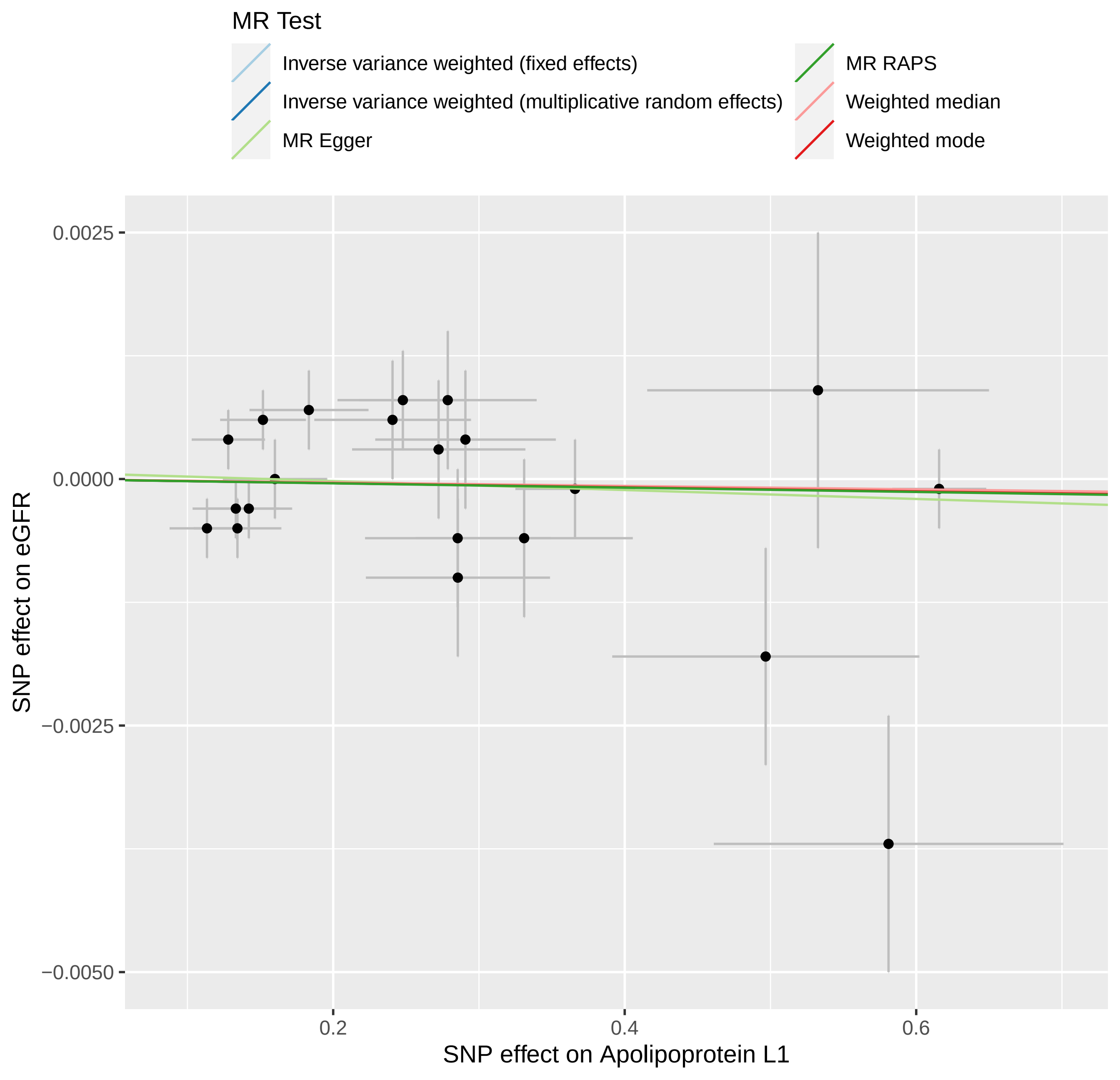

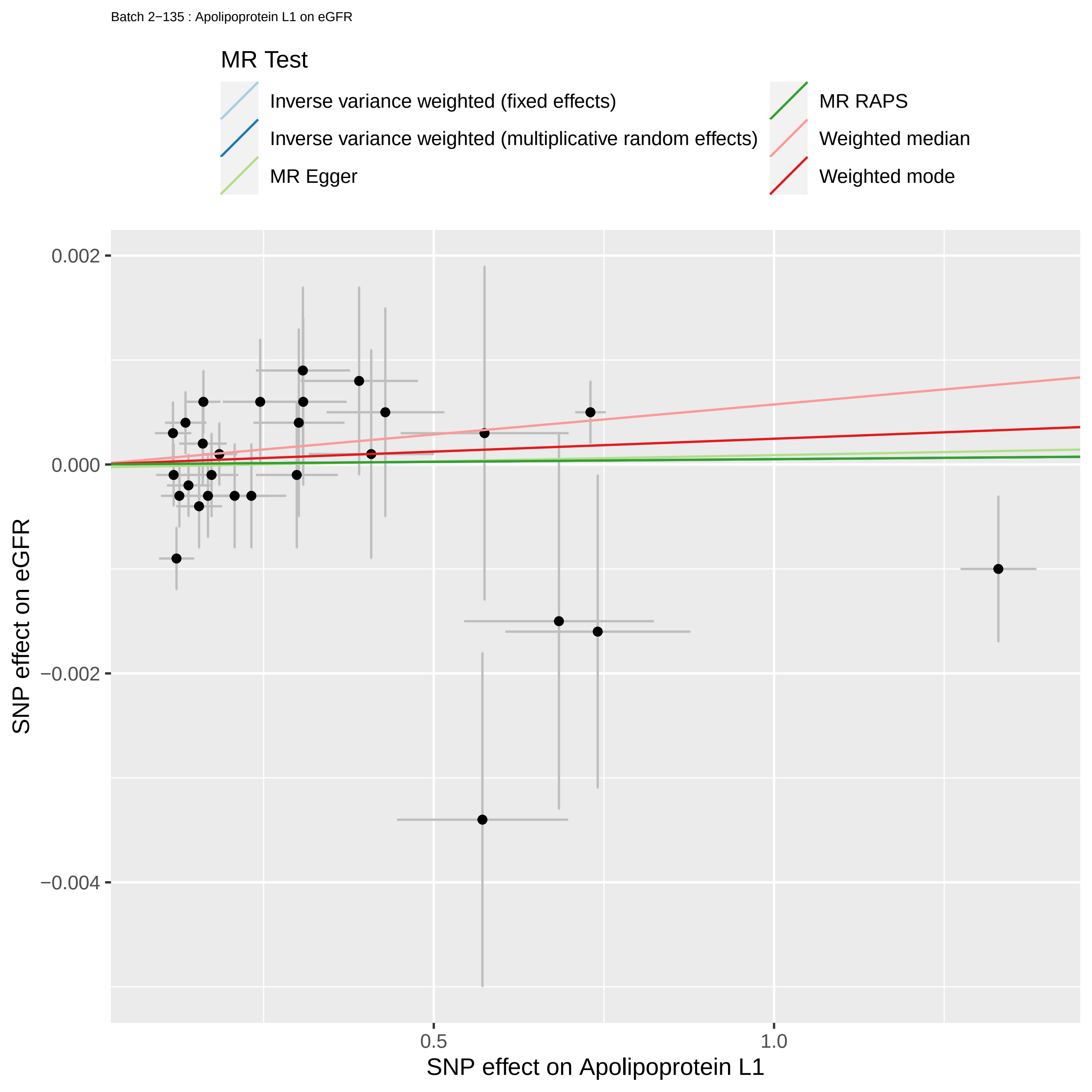
Batch 2−135 : Apolipoprotein L1 on eGFR
MR Test
Inverse variance weighted (fixed effects)
Inverse variance weighted (multiplicative random effects)
MR Egger
MR RAPS
Weighted median
Weighted mode
0.002
0.000
−0.002
−0.004
SNP effect on eGFR
0.5
1.0
SNP effect on Apolipoprotein L1

Batch 2−136 : Apolipoprotein M on eGFR

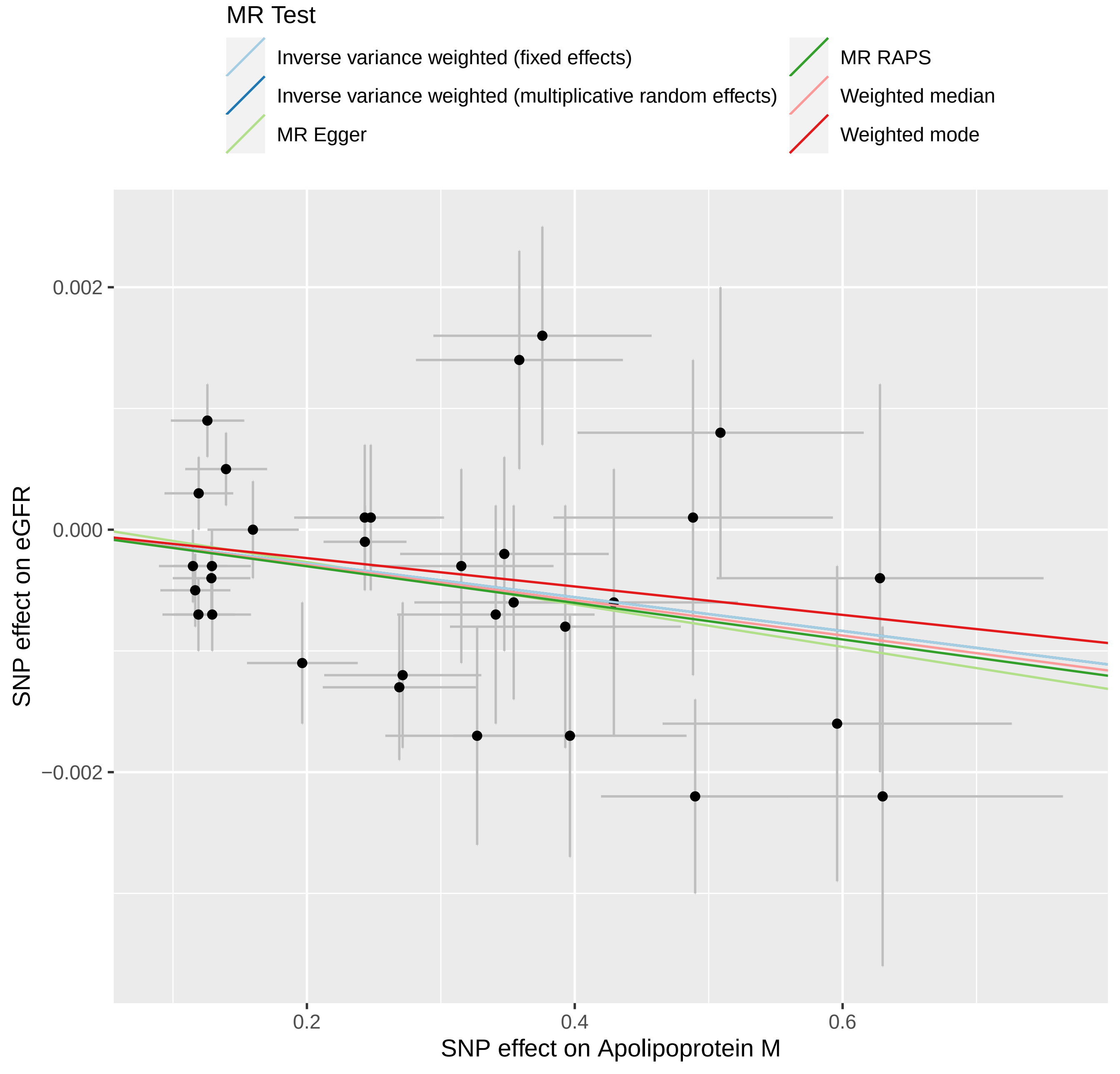

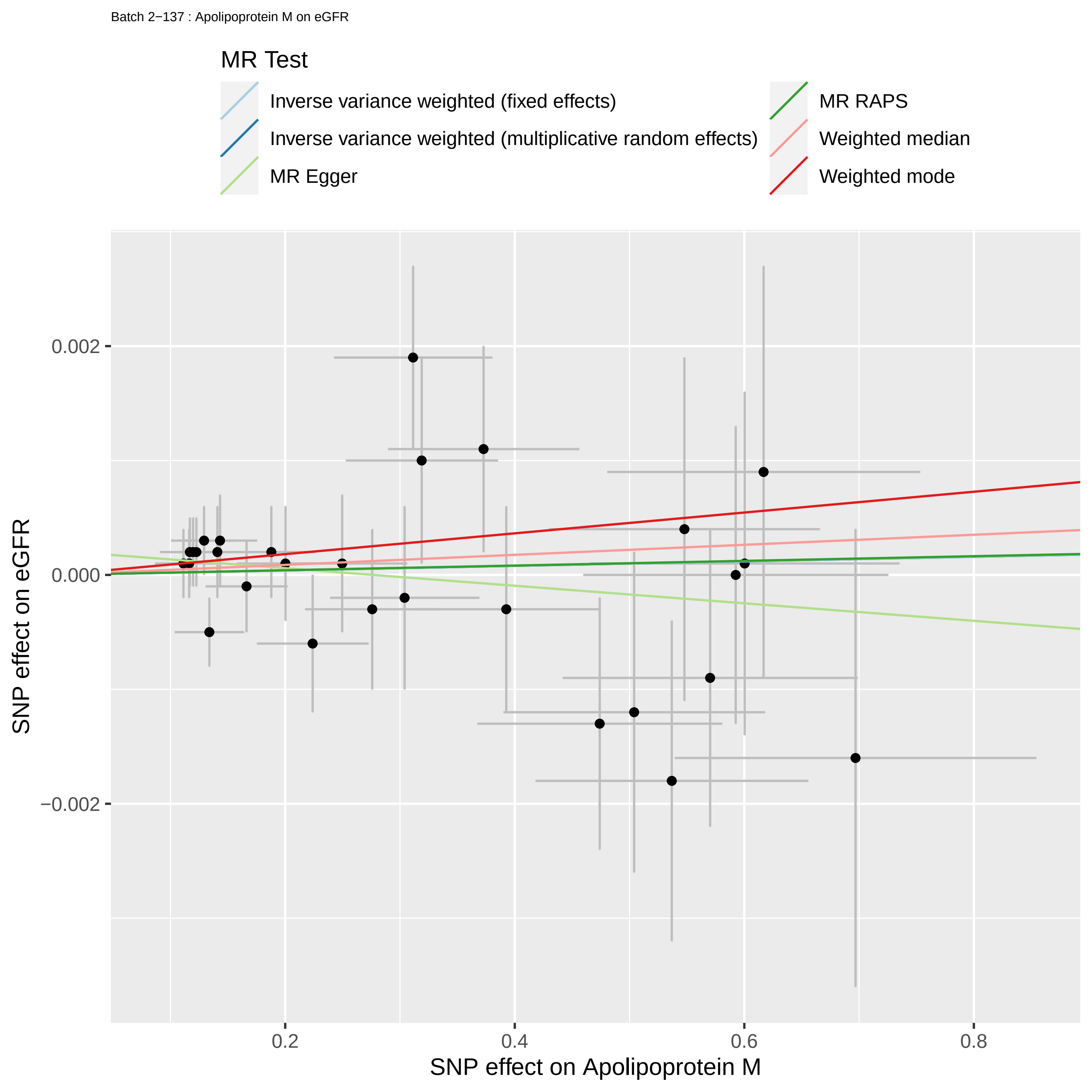

Batch 2−137 : Apolipoprotein M on eGFR
MR Test
Inverse variance weighted (fixed effects)
Inverse variance weighted (multiplicative random effects)
MR Egger
MR RAPS
Weighted median
Weighted mode
0.002
0.000
−0.002
0.2
0.4
0.6
0.8
SNP effect on Apolipoprotein M
SNP effect on eGFR

Batch 2−138 : MICOS complex subunit MIC26 on eGFR

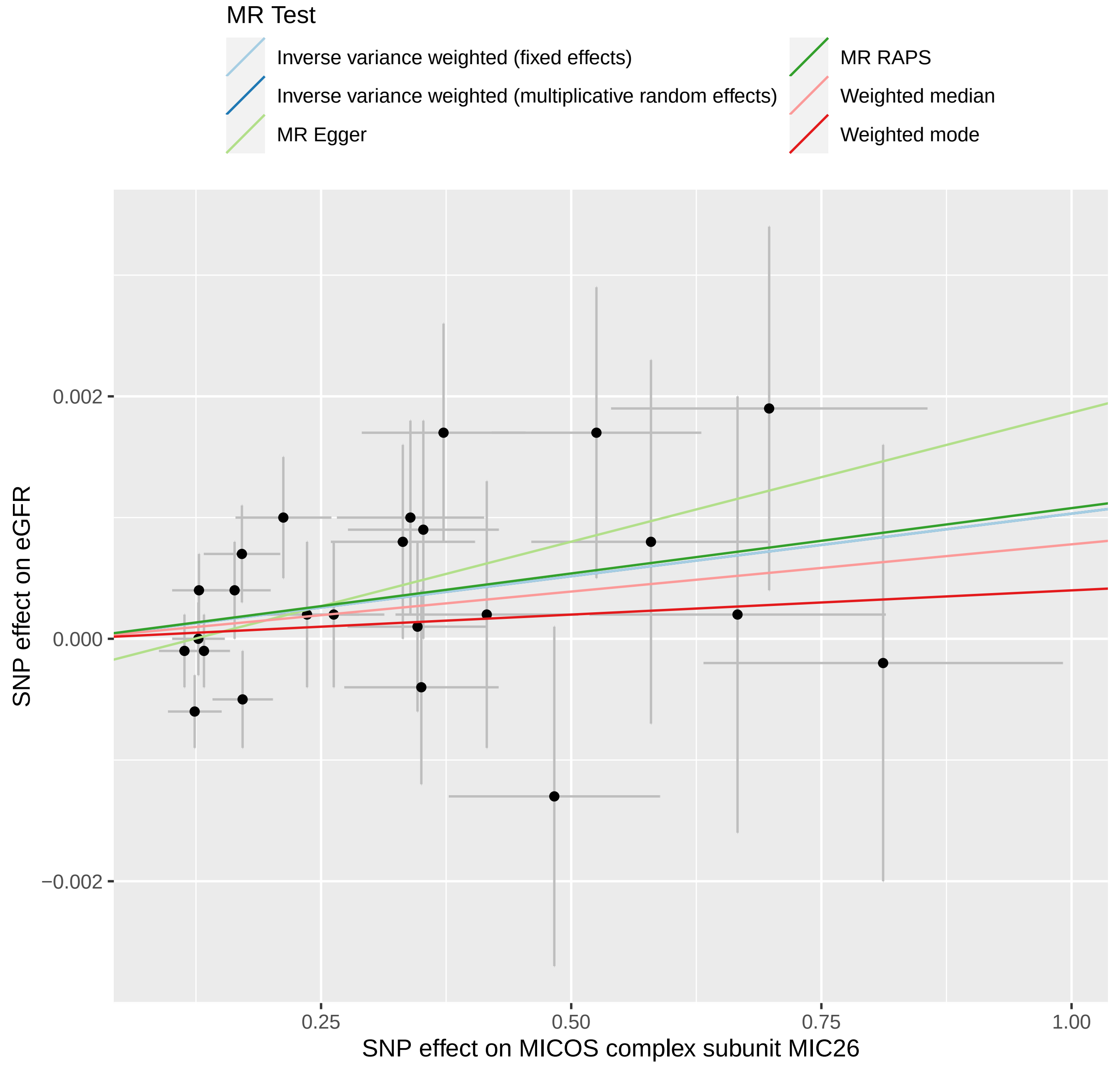

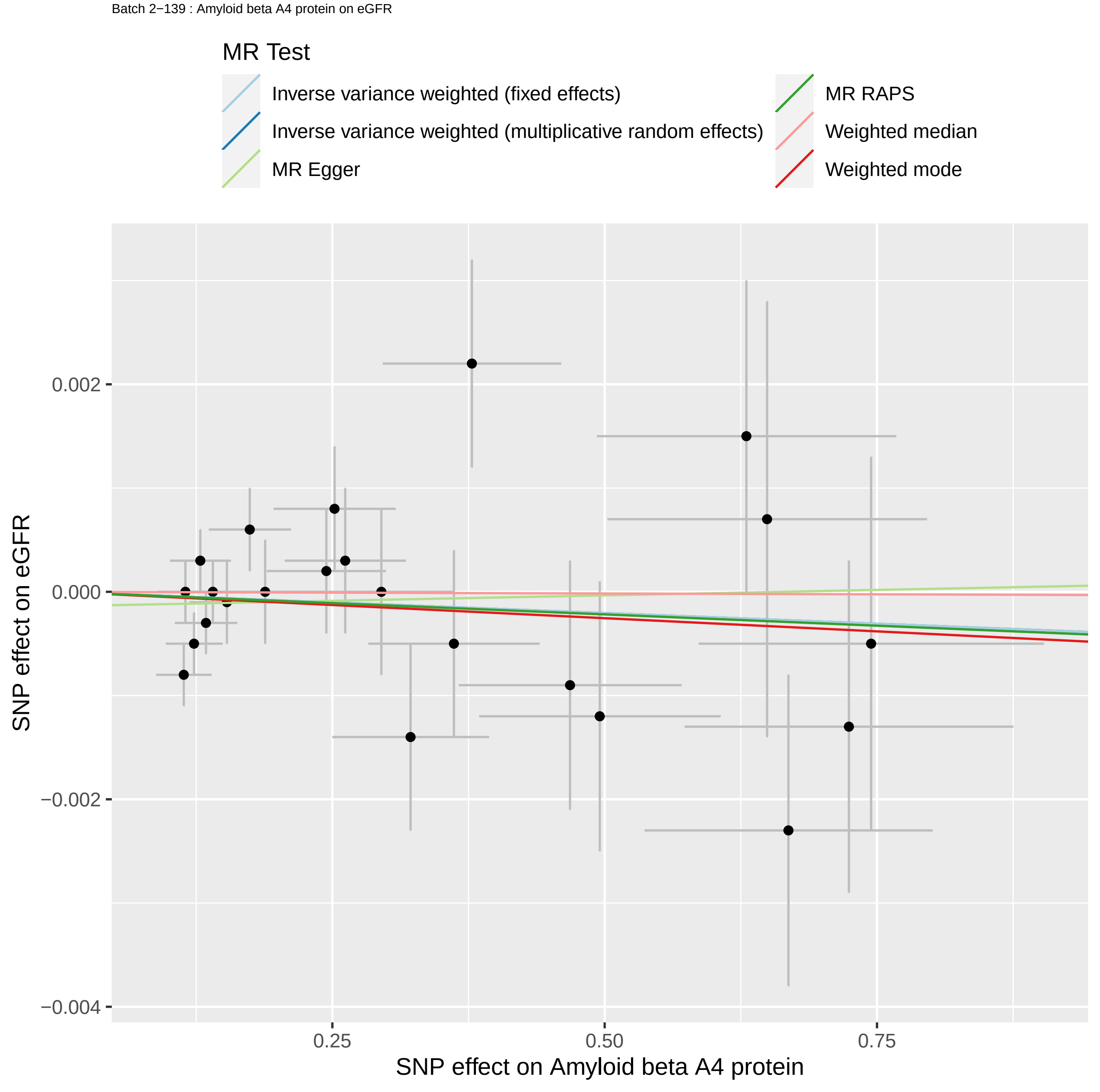

Batch 2−139 : Amyloid beta A4 protein on eGFR
MR Test
Inverse variance weighted (fixed effects)
Inverse variance weighted (multiplicative random effects)
MR Egger
MR RAPS
Weighted median
Weighted mode
0.002
0.000
−0.002
−0.004
SNP effect on eGFR
0.25
0.50
0.75
SNP effect on Amyloid beta A4 protein

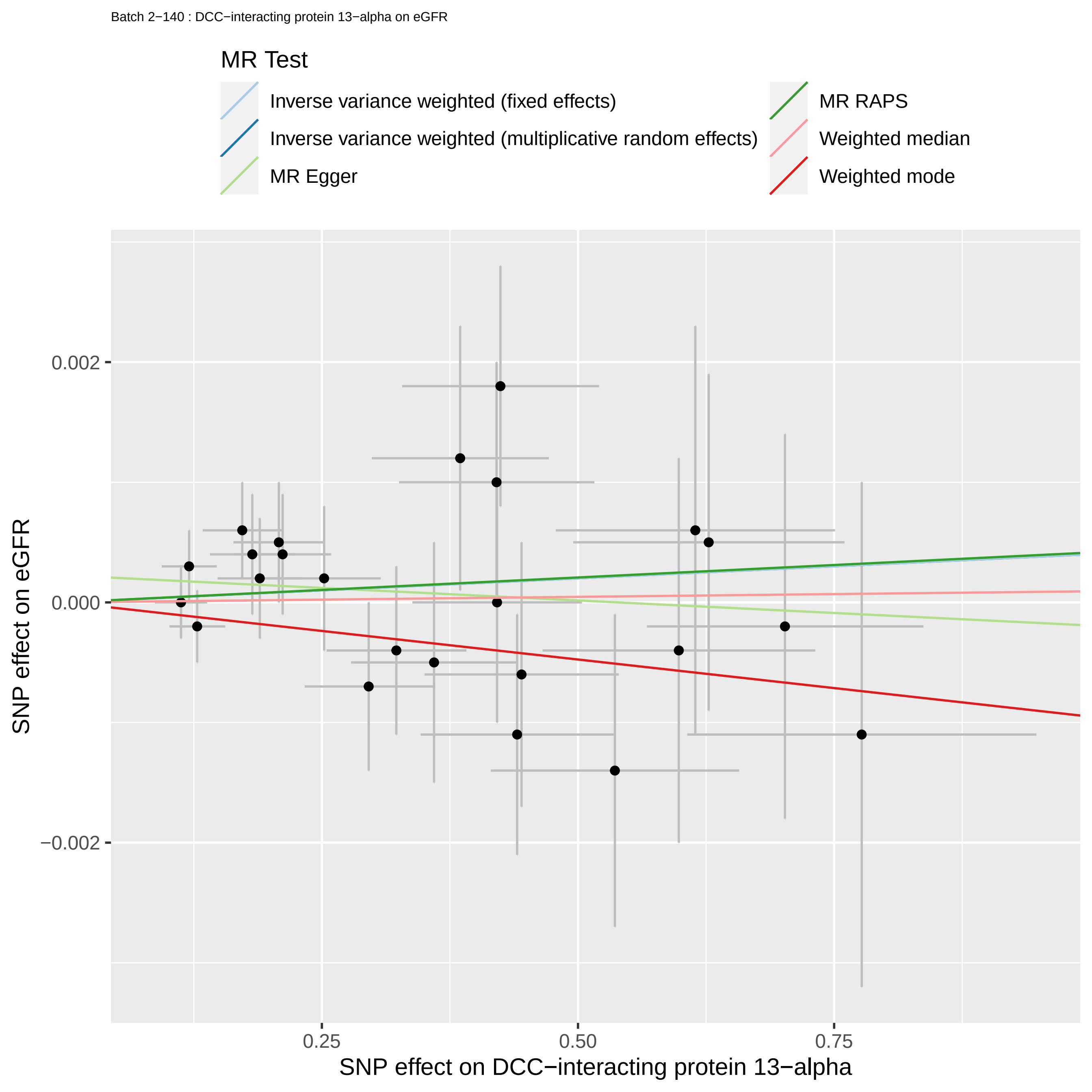

Batch 2−140 : DCC−interacting protein 13−alpha on eGFR
MR Test
Inverse variance weighted (fixed effects)
Inverse variance weighted (multiplicative random effects)
MR Egger
MR RAPS
Weighted median
Weighted mode
0.002
0.000
−0.002
0.25
0.50
0.75
SNP effect on eGFR
SNP effect on DCC−interacting protein 13−alpha

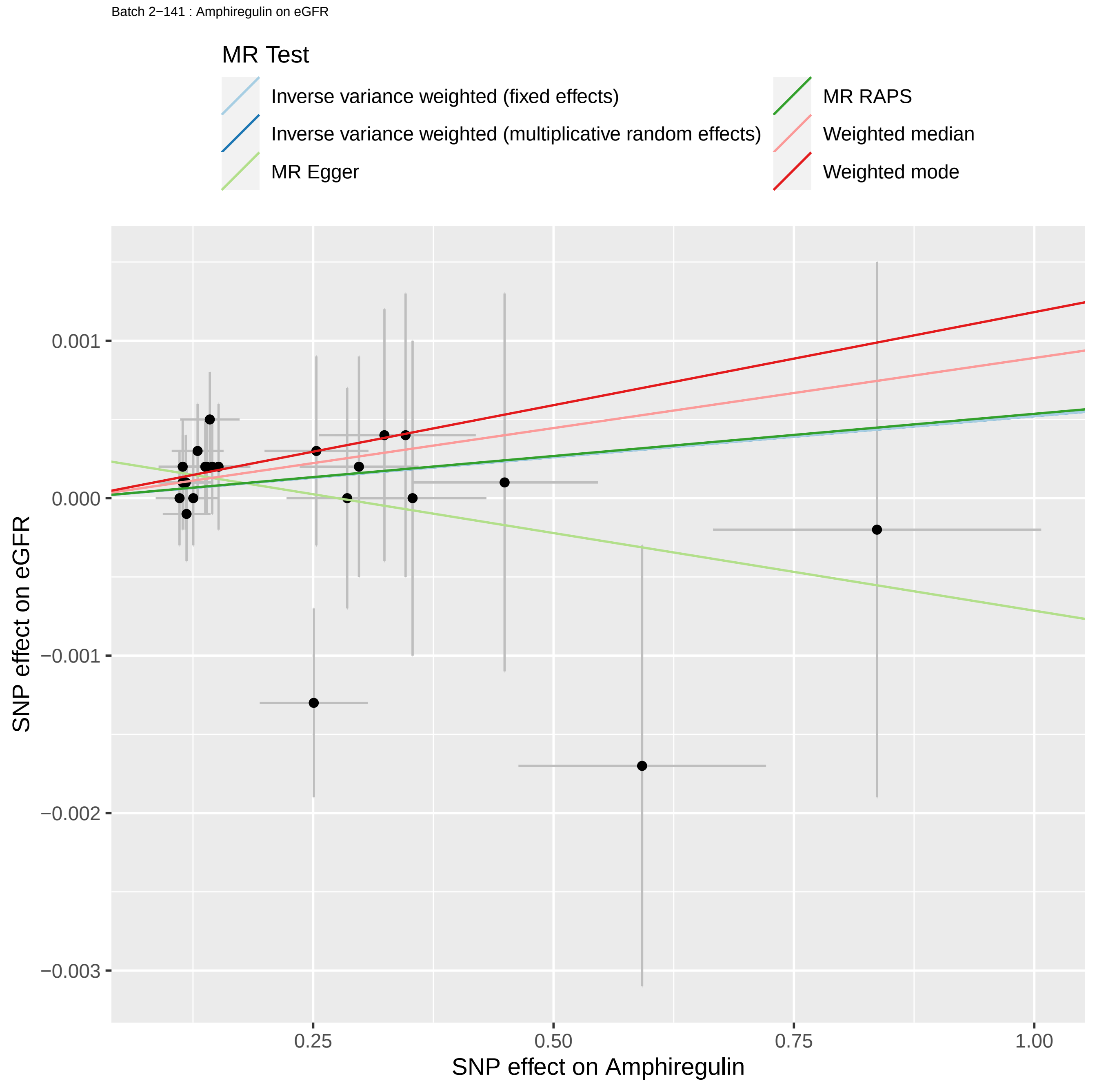
Batch 2−141 : Amphiregulin on eGFR
MR Test
Inverse variance weighted (fixed effects)
Inverse variance weighted (multiplicative random effects)
MR Egger
MR RAPS
Weighted median
Weighted mode
0.001
0.000
−0.001
−0.002
−0.003
0.25
0.50
0.75
1.00
SNP effect on eGFR
SNP effect on Amphiregulin

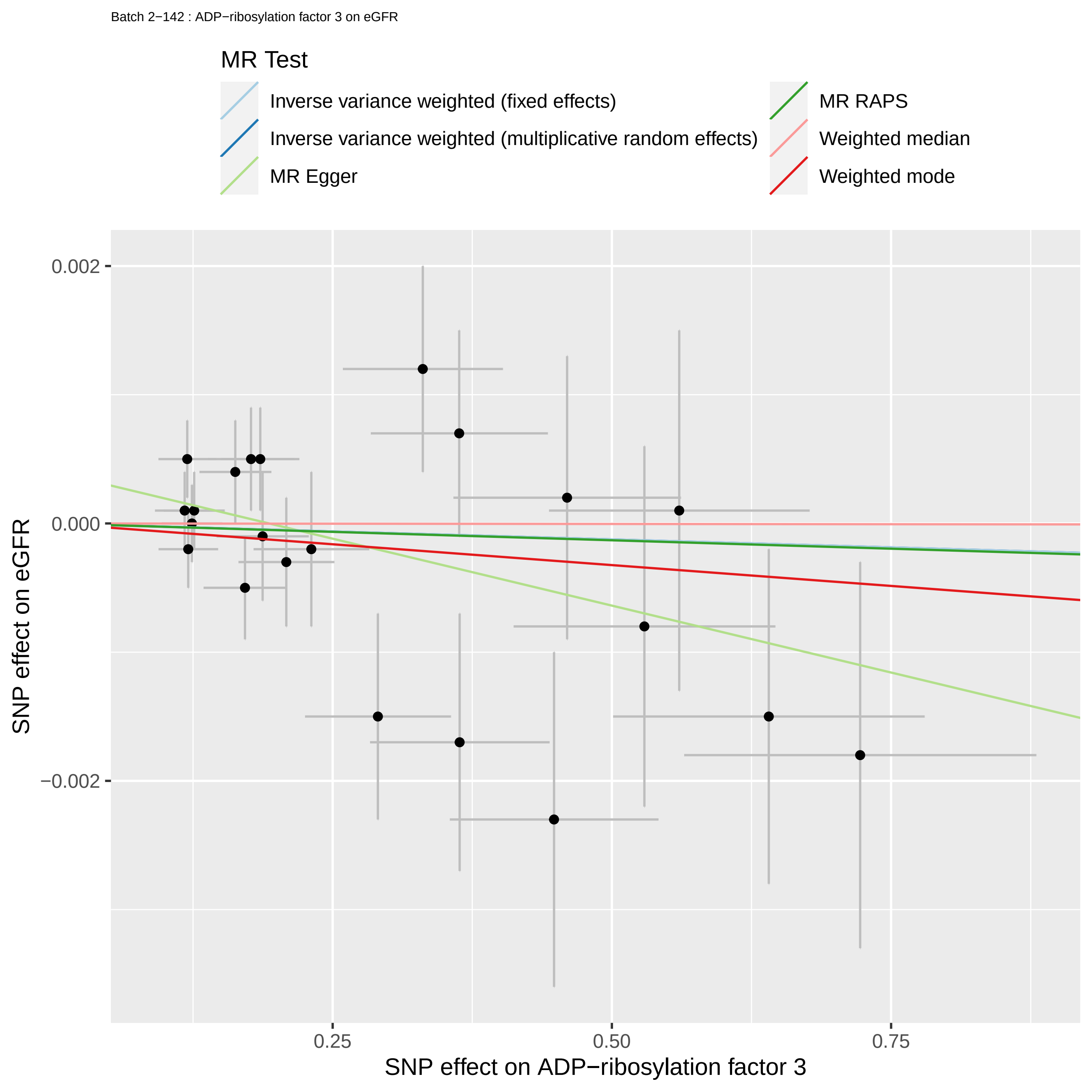
Batch 2−142 : ADP−ribosylation factor 3 on eGFR
MR Test
Inverse variance weighted (fixed effects)
Inverse variance weighted (multiplicative random effects)
MR Egger
MR RAPS
Weighted median
Weighted mode
0.002
0.000
−0.002
0.25
0.50
0.75
SNP effect on eGFR
SNP effect on ADP−ribosylation factor 3

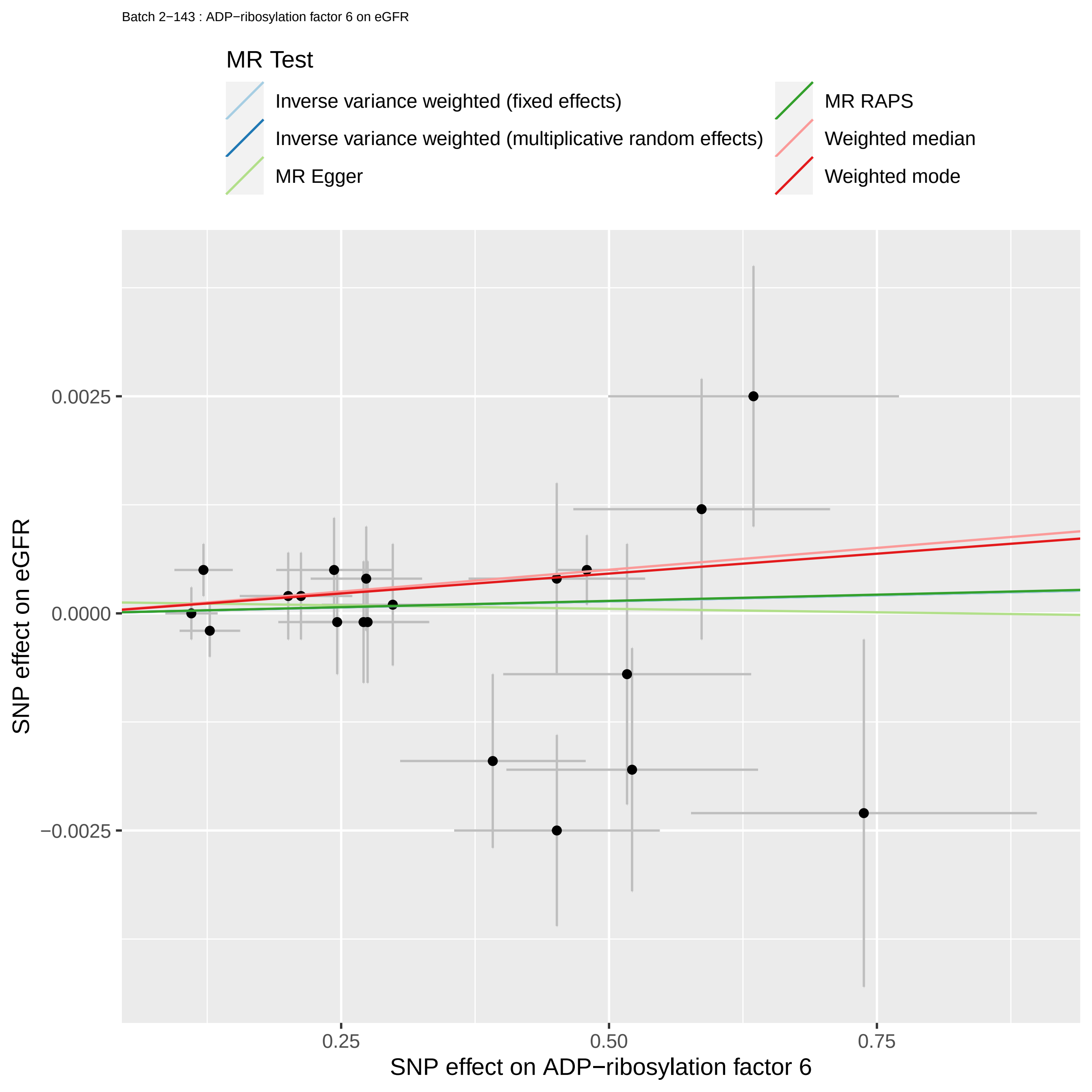
Batch 2−143 : ADP−ribosylation factor 6 on eGFR
MR Test
Inverse variance weighted (fixed effects)
Inverse variance weighted (multiplicative random effects)
MR Egger
MR RAPS
Weighted median
Weighted mode
0.0025
0.0000
−0.0025
SNP effect on eGFR
0.25
0.50
0.75
SNP effect on ADP−ribosylation factor 6

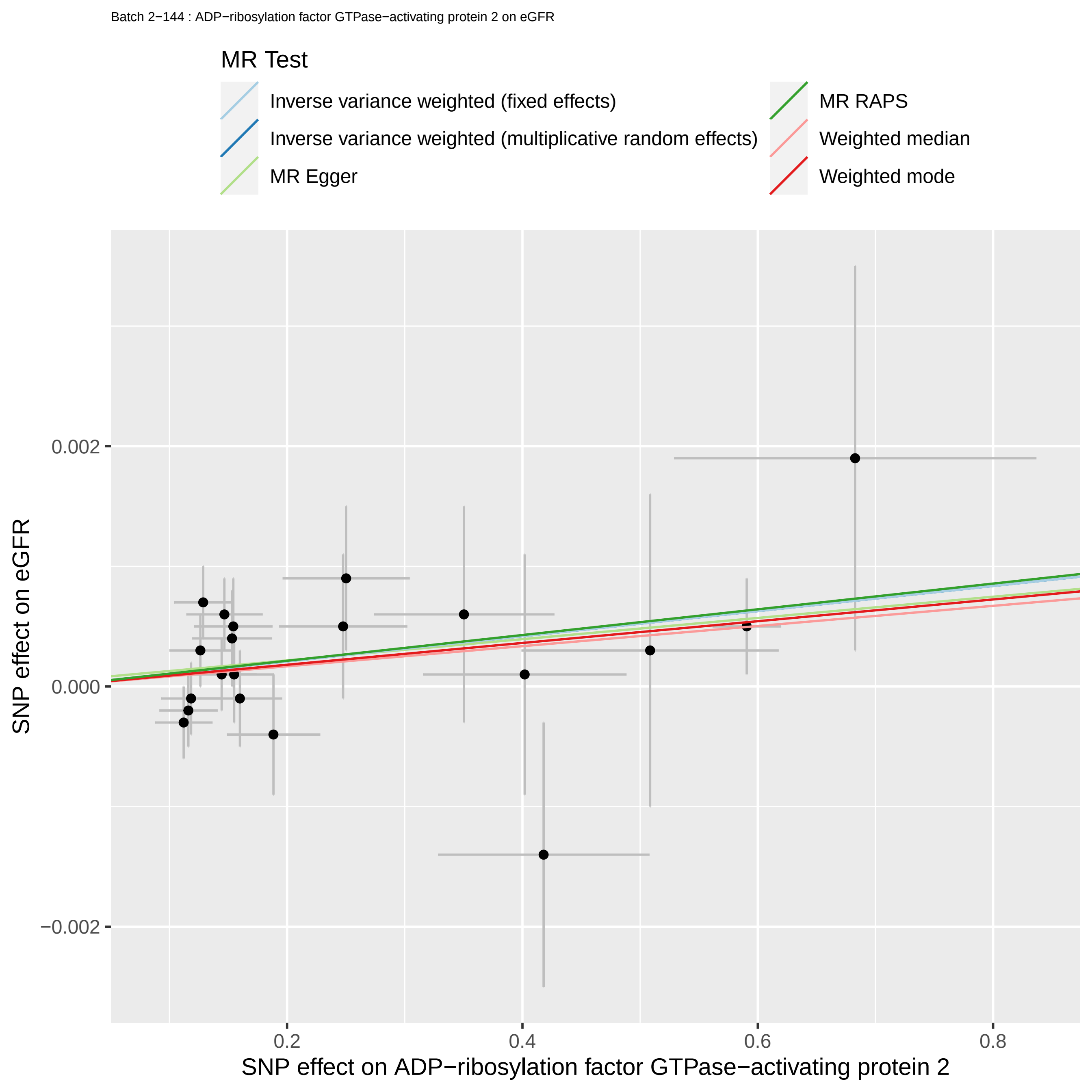

Batch 2−144 : ADP−ribosylation factor GTPase−activating protein 2 on eGFR
MR Test
Inverse variance weighted (fixed effects)
Inverse variance weighted (multiplicative random effects)
MR Egger
MR RAPS
Weighted median
Weighted mode
0.002
0.000
−0.002
0.2
0.4
0.6
0.8
SNP effect on eGFR
SNP effect on ADP−ribosylation factor GTPase−activating protein 2

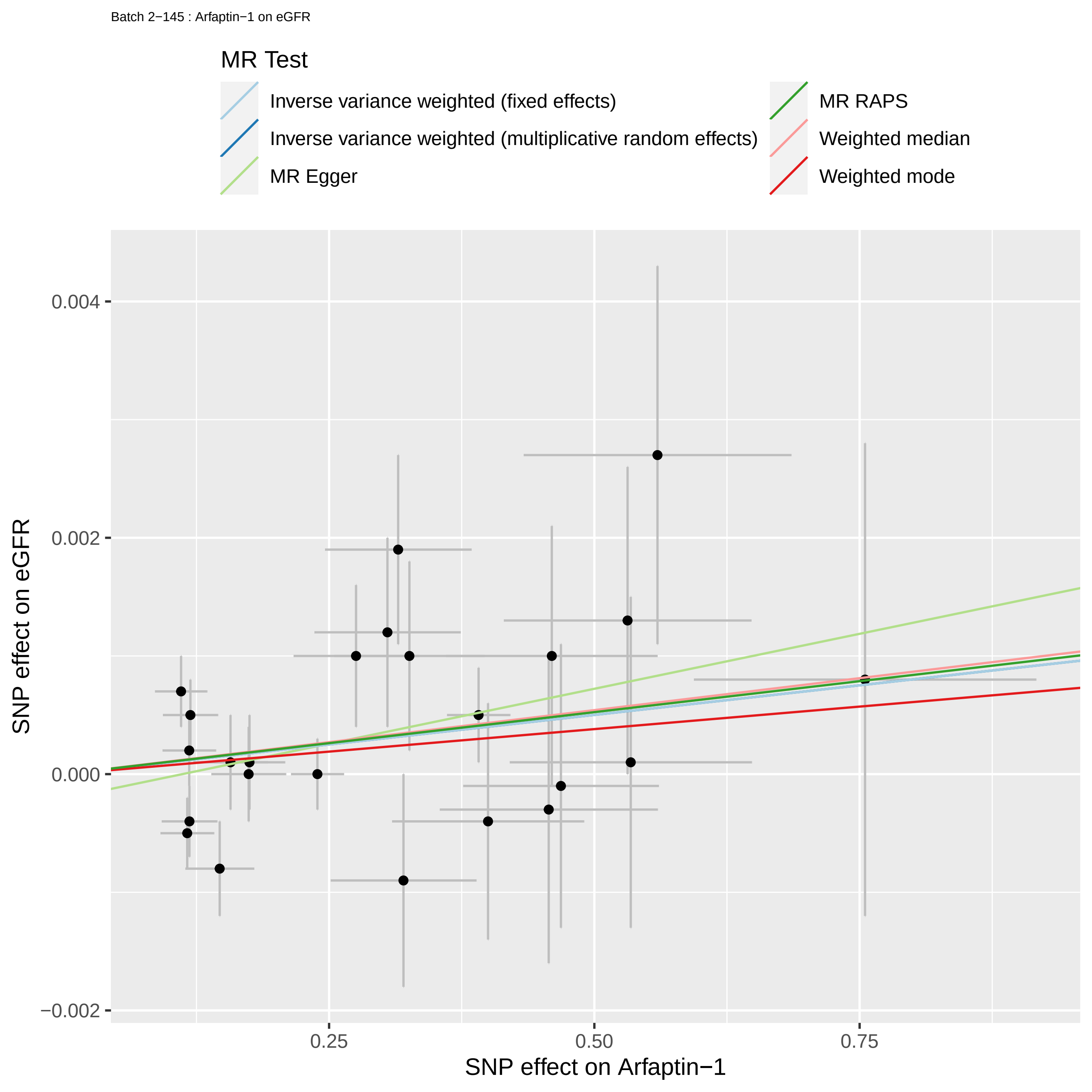

Batch 2−145 : Arfaptin−1 on eGFR
MR Test
Inverse variance weighted (fixed effects)
Inverse variance weighted (multiplicative random effects)
MR Egger
MR RAPS
Weighted median
Weighted mode
0.004
0.002
0.000
−0.002
0.25
0.50
0.75
SNP effect on eGFR
SNP effect on Arfaptin−1

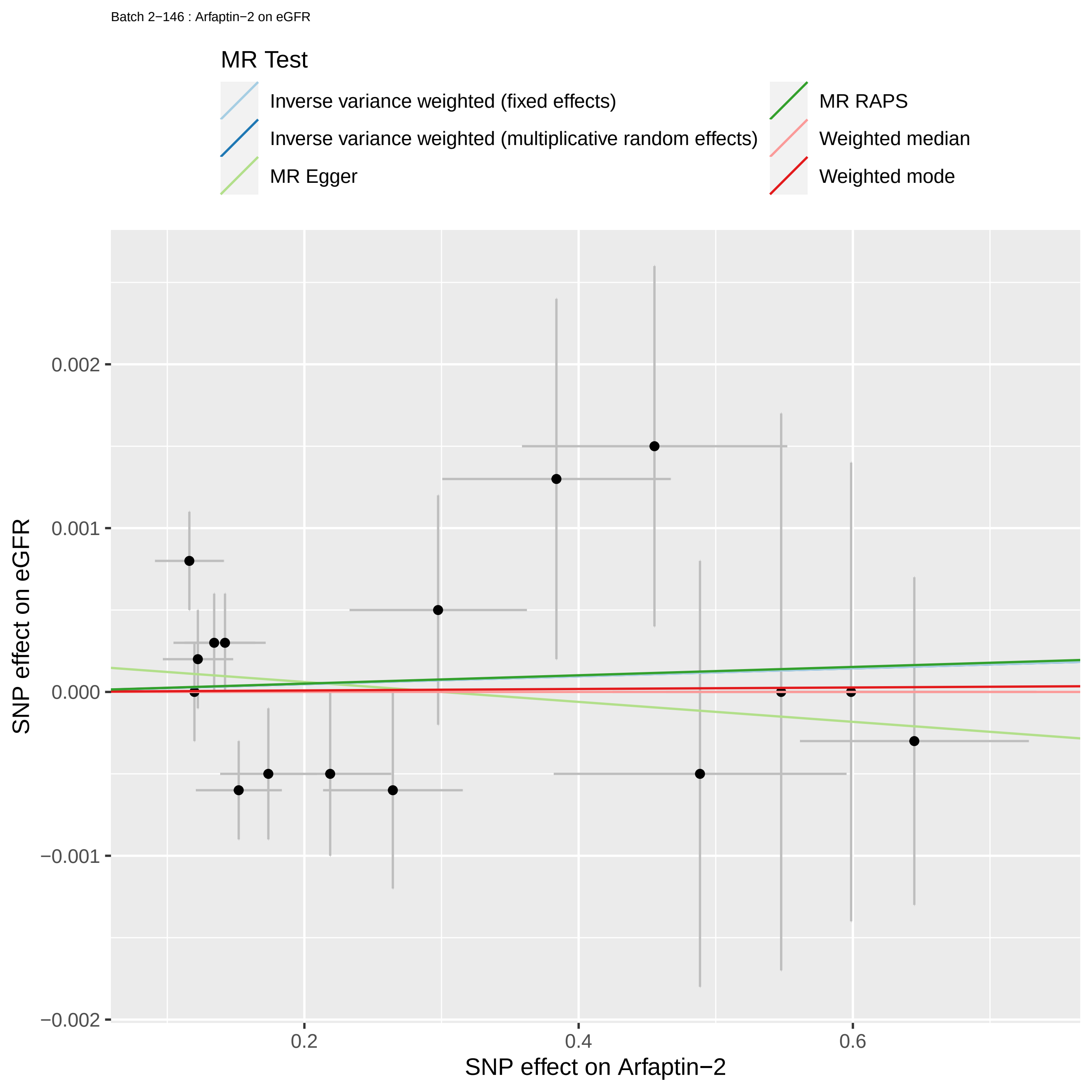

Batch 2−146 : Arfaptin−2 on eGFR
MR Test
Inverse variance weighted (fixed effects)
Inverse variance weighted (multiplicative random effects)
MR Egger
MR RAPS
Weighted median
Weighted mode
0.002
0.001
0.000
−0.001
−0.002
SNP effect on eGFR
0.2
0.4
0.6
SNP effect on Arfaptin−2

Batch 2−147 : Rho GTPase−activating protein 1 on eGFR

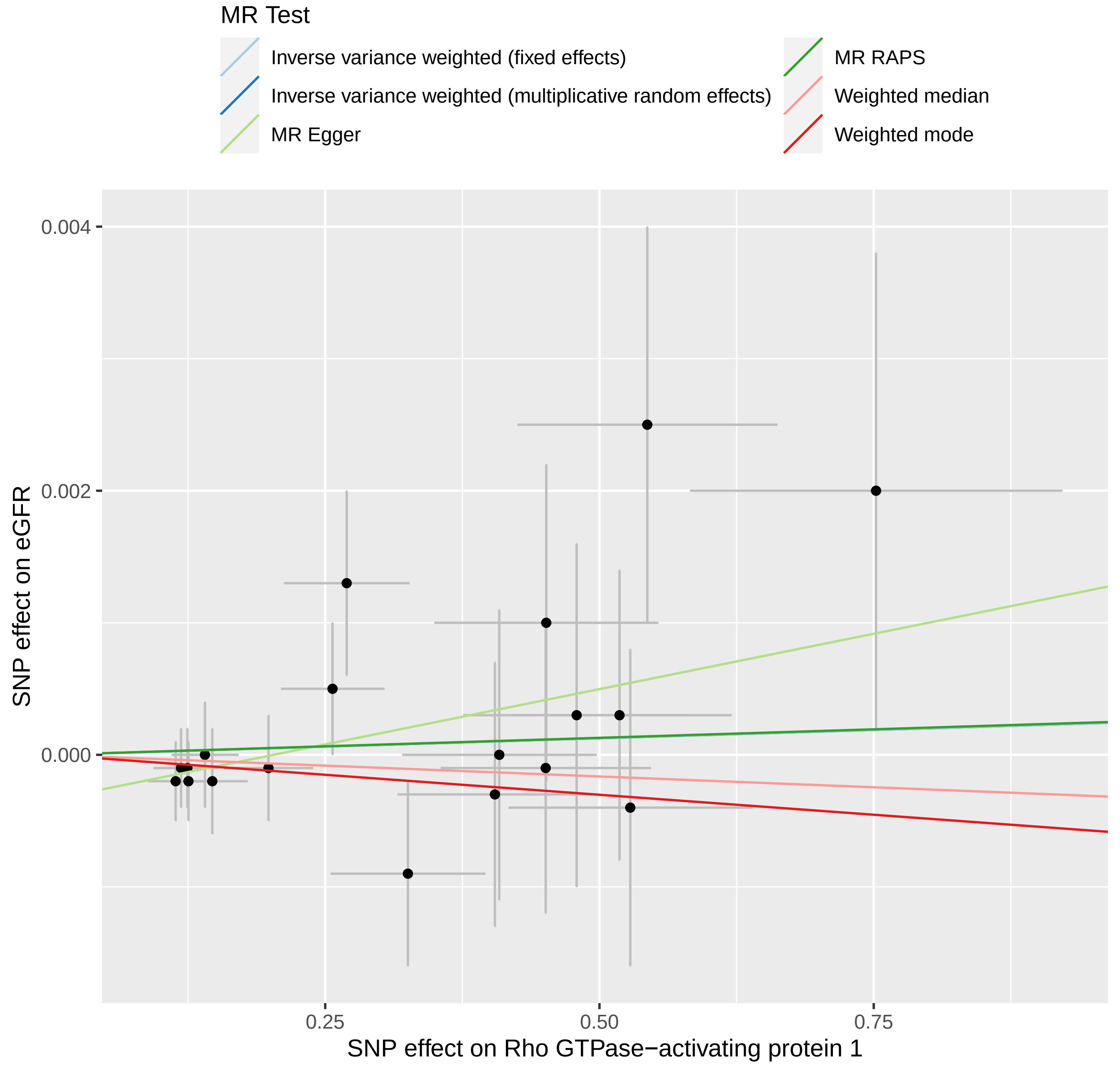

Batch 2−148 : Rho GTPase−activating protein 25 on eGFR

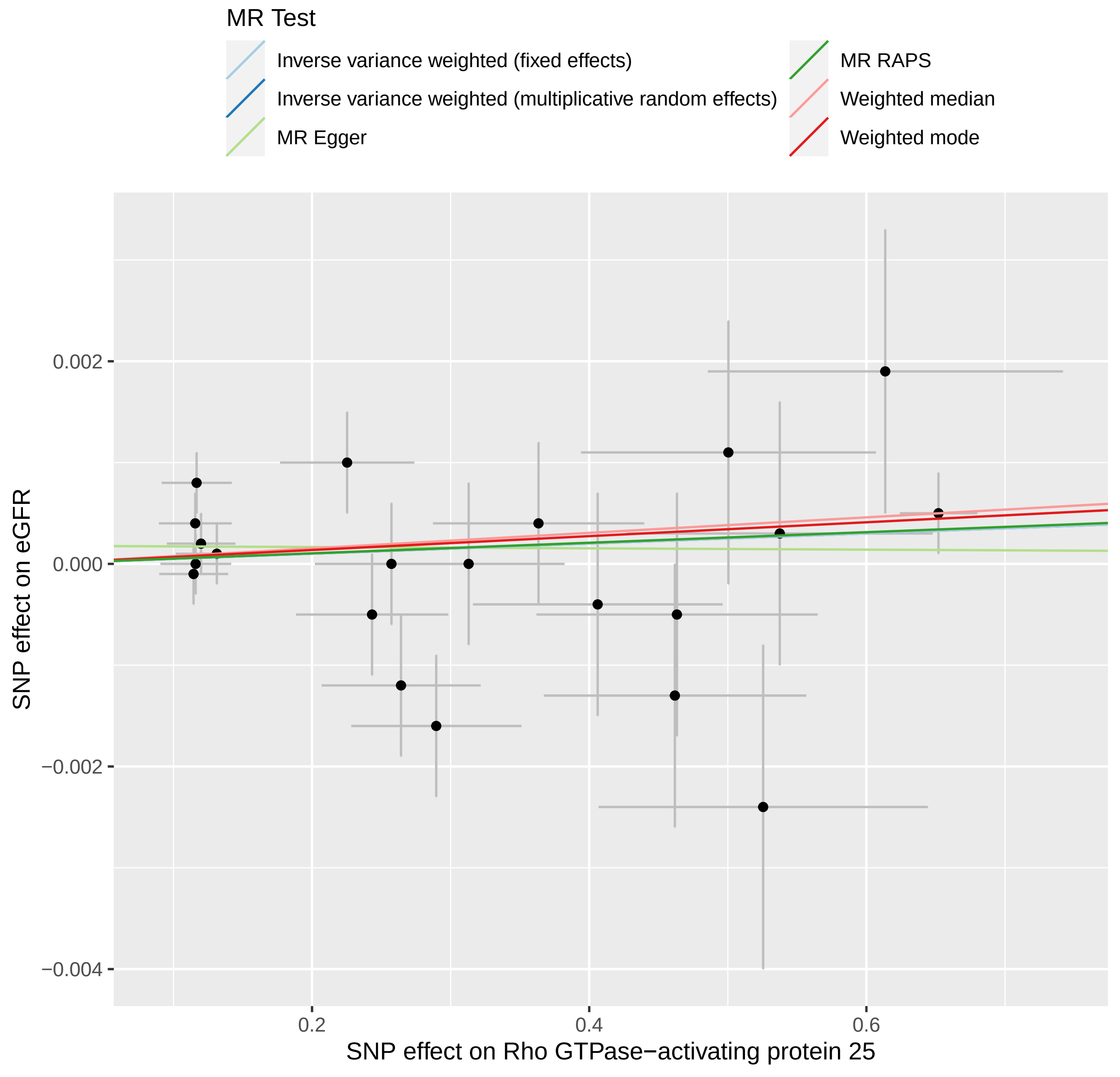

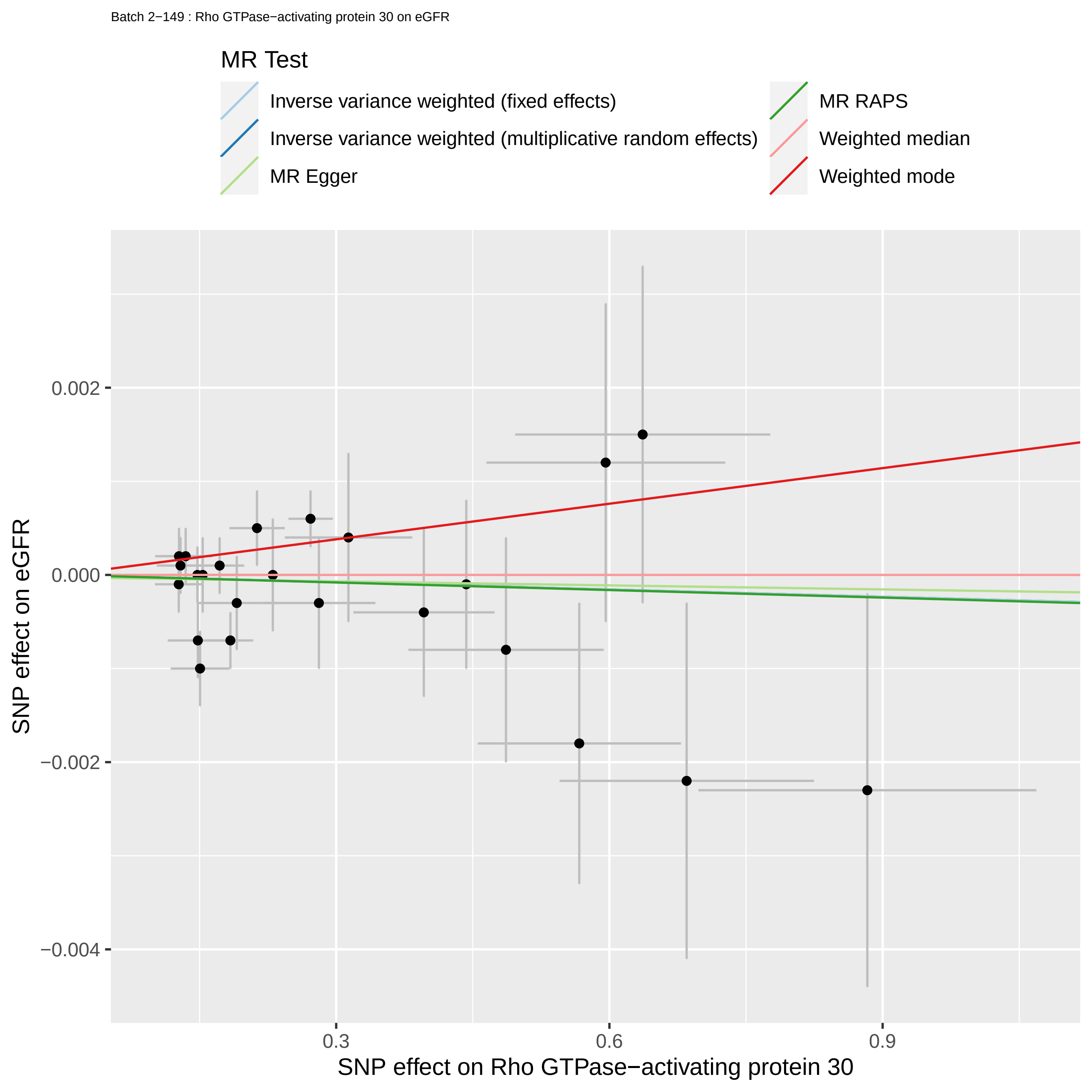

Batch 2−149 : Rho GTPase−activating protein 30 on eGFR
MR Test
Inverse variance weighted (fixed effects)
Inverse variance weighted (multiplicative random effects)
MR Egger
MR RAPS
Weighted median
Weighted mode
0.002
0.000
−0.002
−0.004
0.3
0.6
0.9
SNP effect on eGFR
SNP effect on Rho GTPase−activating protein 30

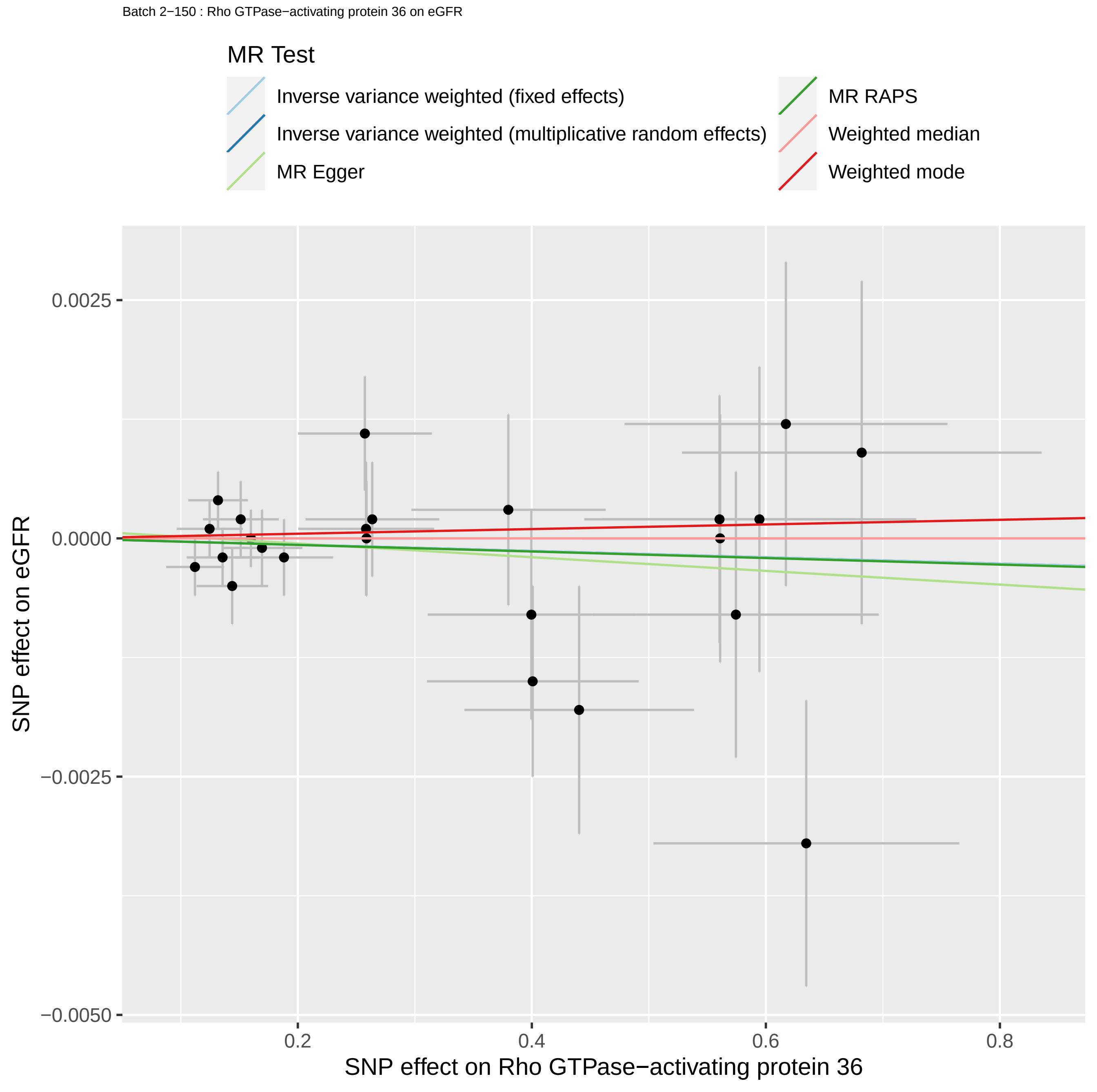

Batch 2−150 : Rho GTPase−activating protein 36 on eGFR
MR Test
Inverse variance weighted (fixed effects)
Inverse variance weighted (multiplicative random effects)
MR Egger
MR RAPS
Weighted median
Weighted mode
0.0025
0.0000
−0.0025
−0.0050
0.2
0.4
0.6
0.8
SNP effect on eGFR
SNP effect on Rho GTPase−activating protein 36

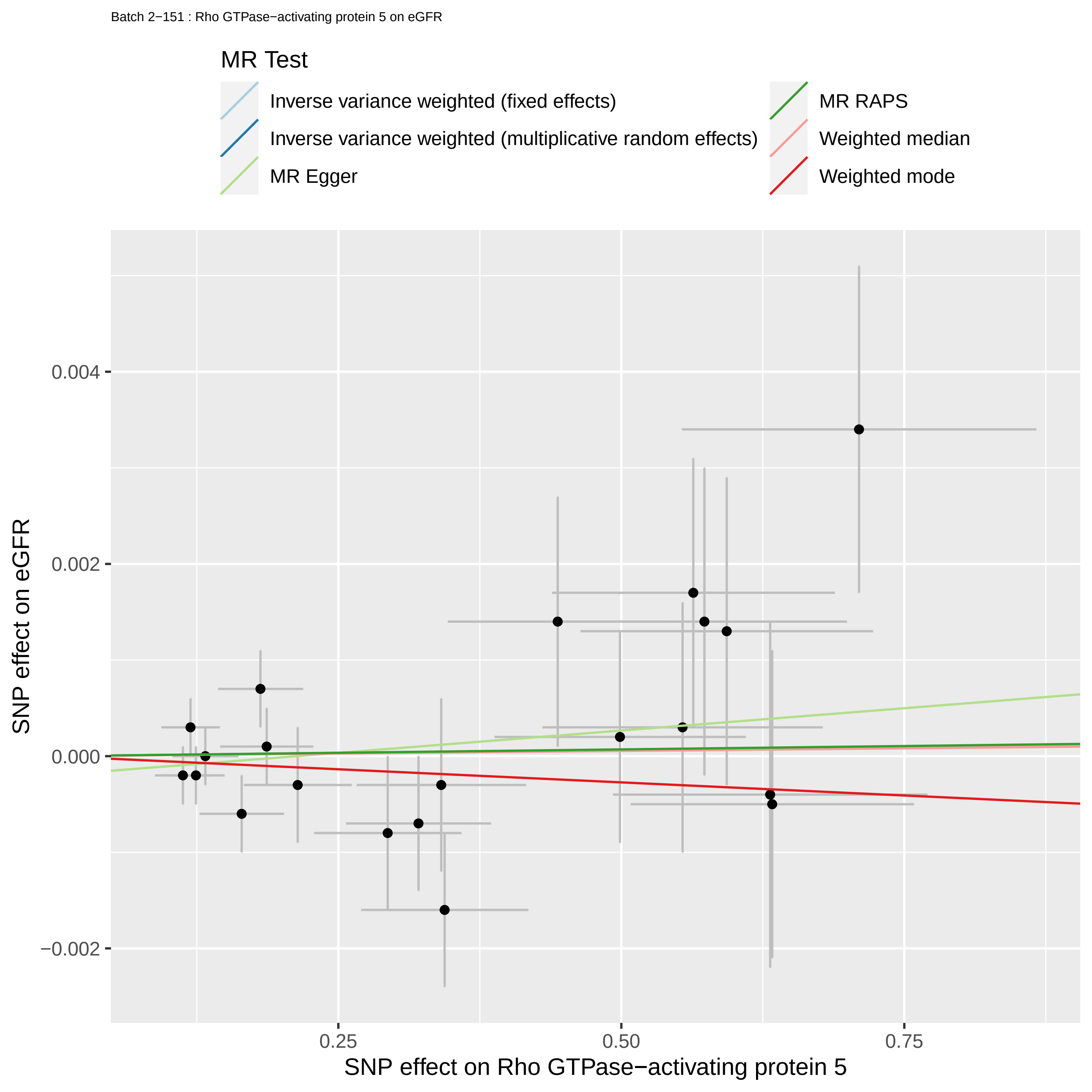

Batch 2−151 : Rho GTPase−activating protein 5 on eGFR
MR Test
Inverse variance weighted (fixed effects)
Inverse variance weighted (multiplicative random effects)
MR Egger
MR RAPS
Weighted median
Weighted mode
0.004
0.002
0.000
−0.002
0.25
0.50
0.75
SNP effect on eGFR
SNP effect on Rho GTPase−activating protein 5

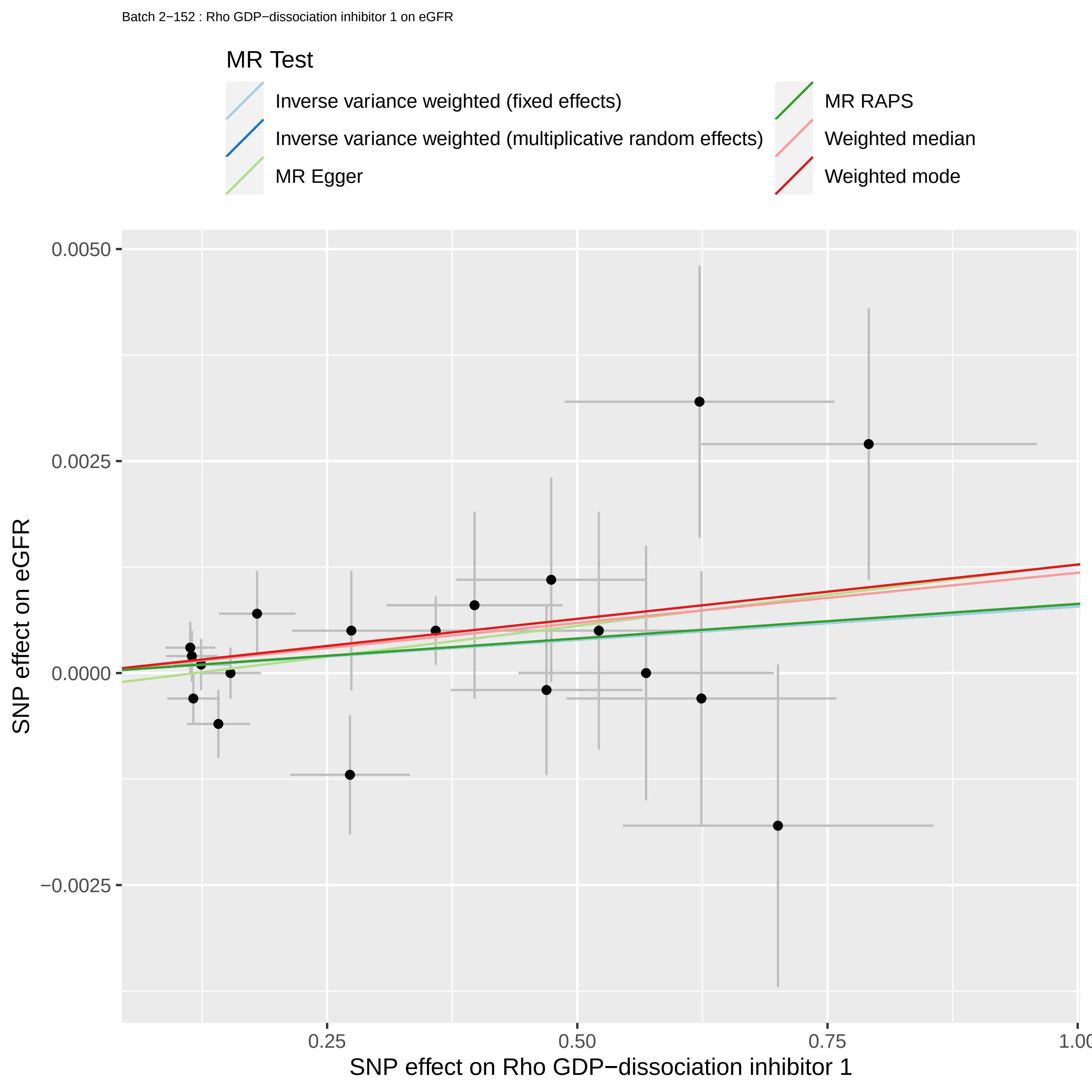
Batch 2−152 : Rho GDP−dissociation inhibitor 1 on eGFR
MR Test
Inverse variance weighted (fixed effects)
Inverse variance weighted (multiplicative random effects)
MR Egger
MR RAPS
Weighted median
Weighted mode
0.0050
0.0025
0.0000
−0.0025
0.25
0.50
0.75
1.00
SNP effect on eGFR
SNP effect on Rho GDP−dissociation inhibitor 1

Batch 2−153 : Rho GDP−dissociation inhibitor 2 on eGFR

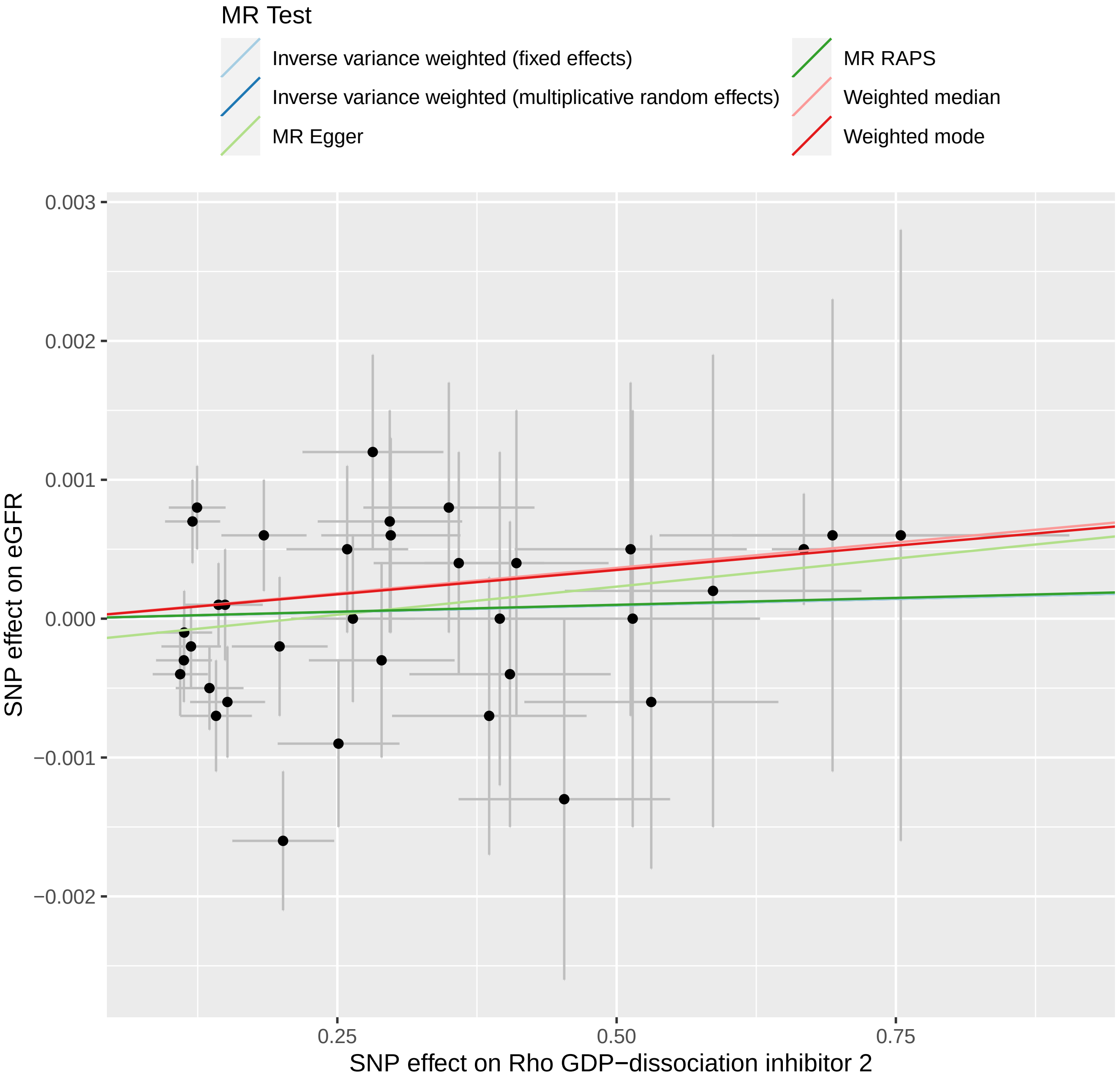

Batch 2−154 : Rho guanine nucleotide exchange factor 10 on eGFR

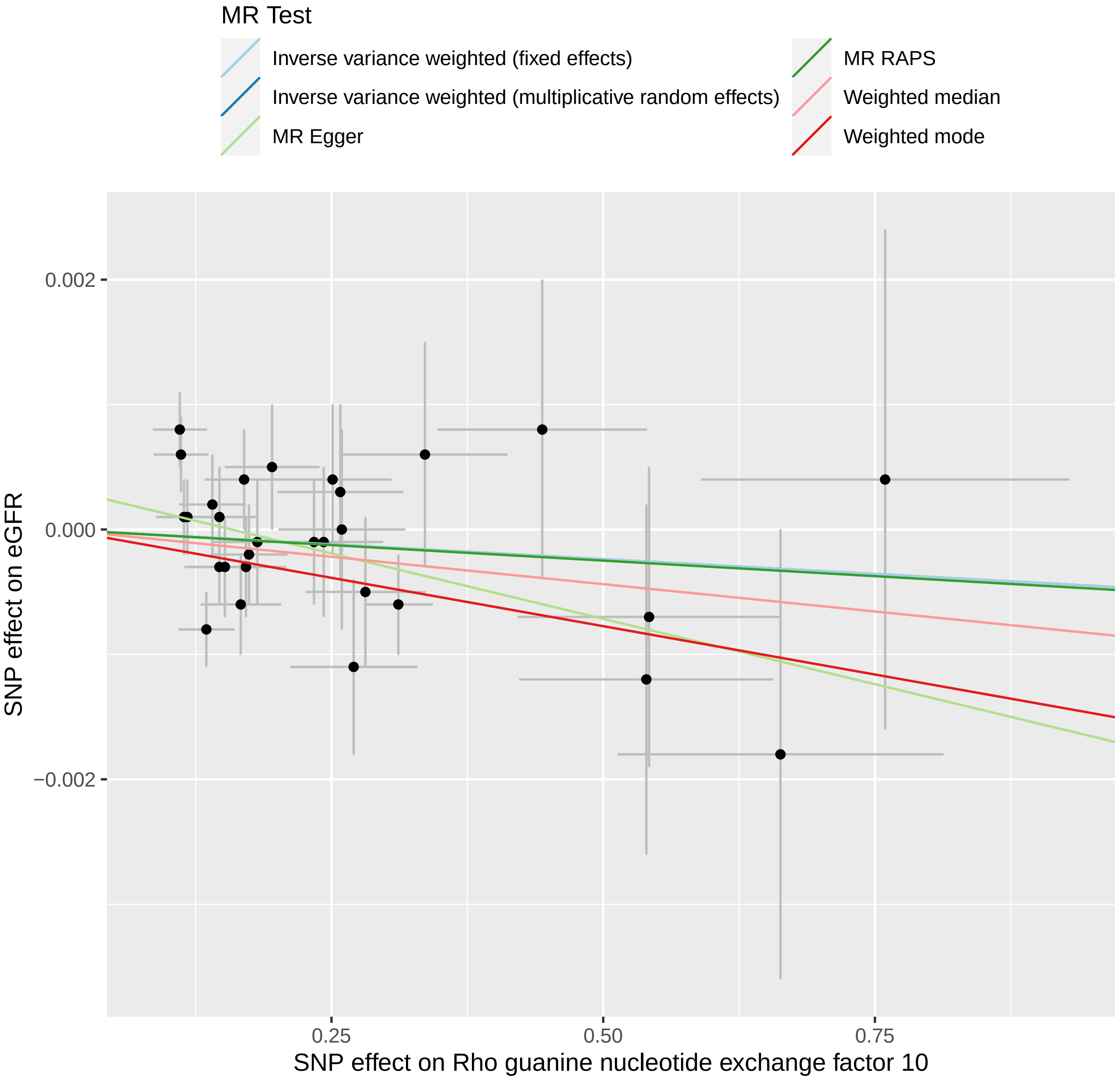

Batch 2−155 : Rho guanine nucleotide exchange factor 1 on eGFR

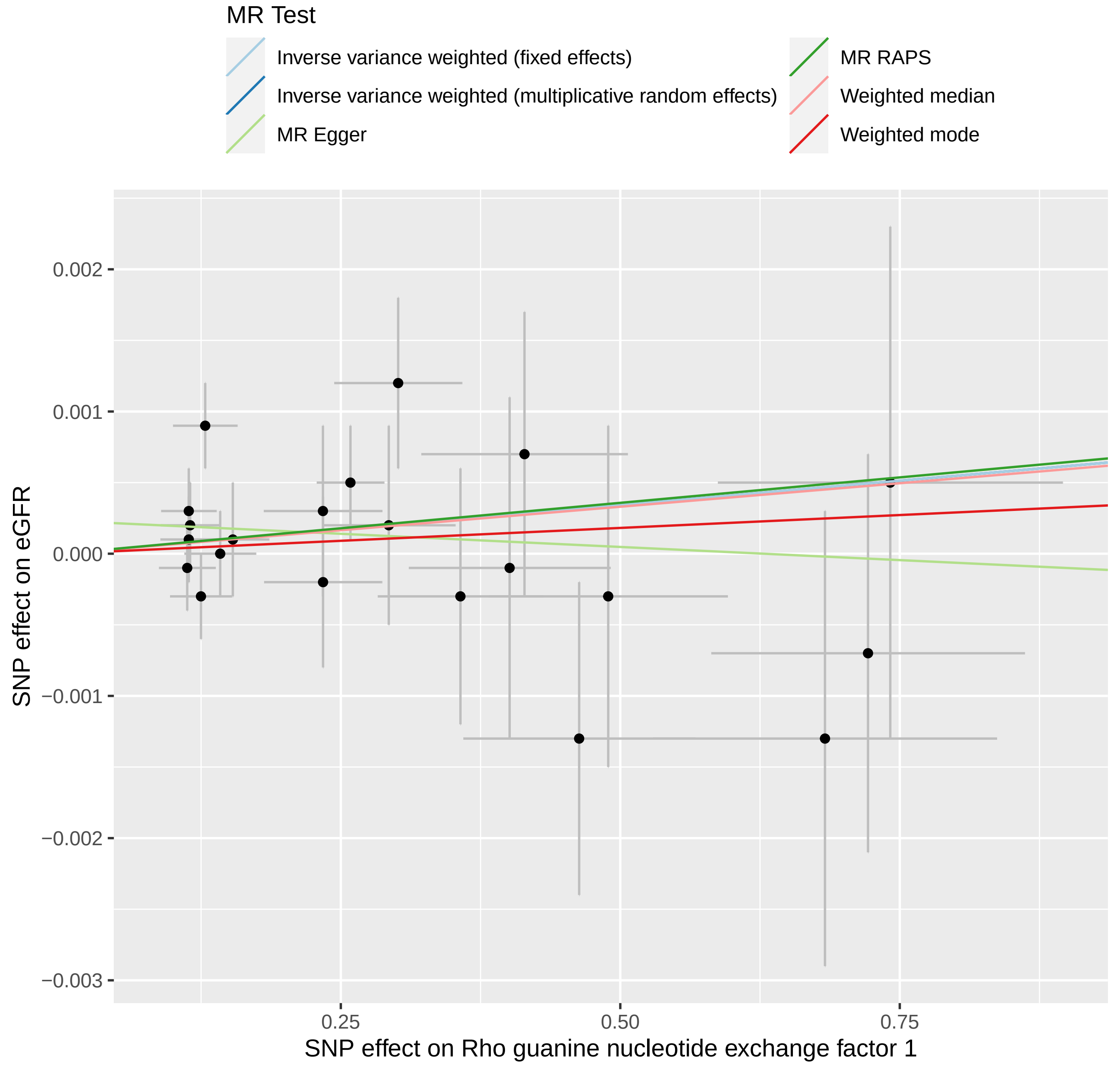

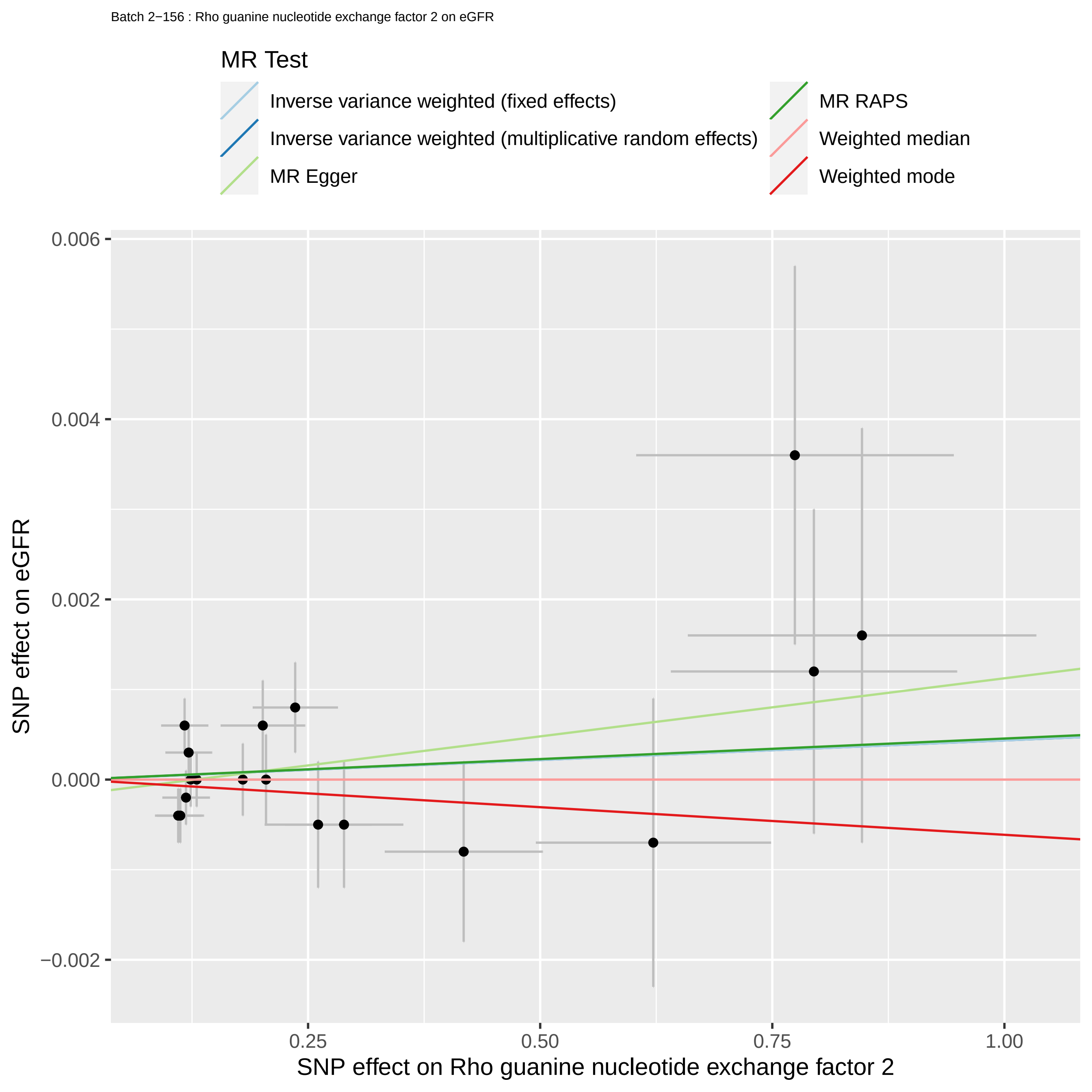

Batch 2−156 : Rho guanine nucleotide exchange factor 2 on eGFR
MR Test
Inverse variance weighted (fixed effects)
Inverse variance weighted (multiplicative random effects)
MR Egger
MR RAPS
Weighted median
Weighted mode
0.006
0.004
0.002
0.000
−0.002
0.25
0.50
0.75
1.00
SNP effect on eGFR
SNP effect on Rho guanine nucleotide exchange factor 2

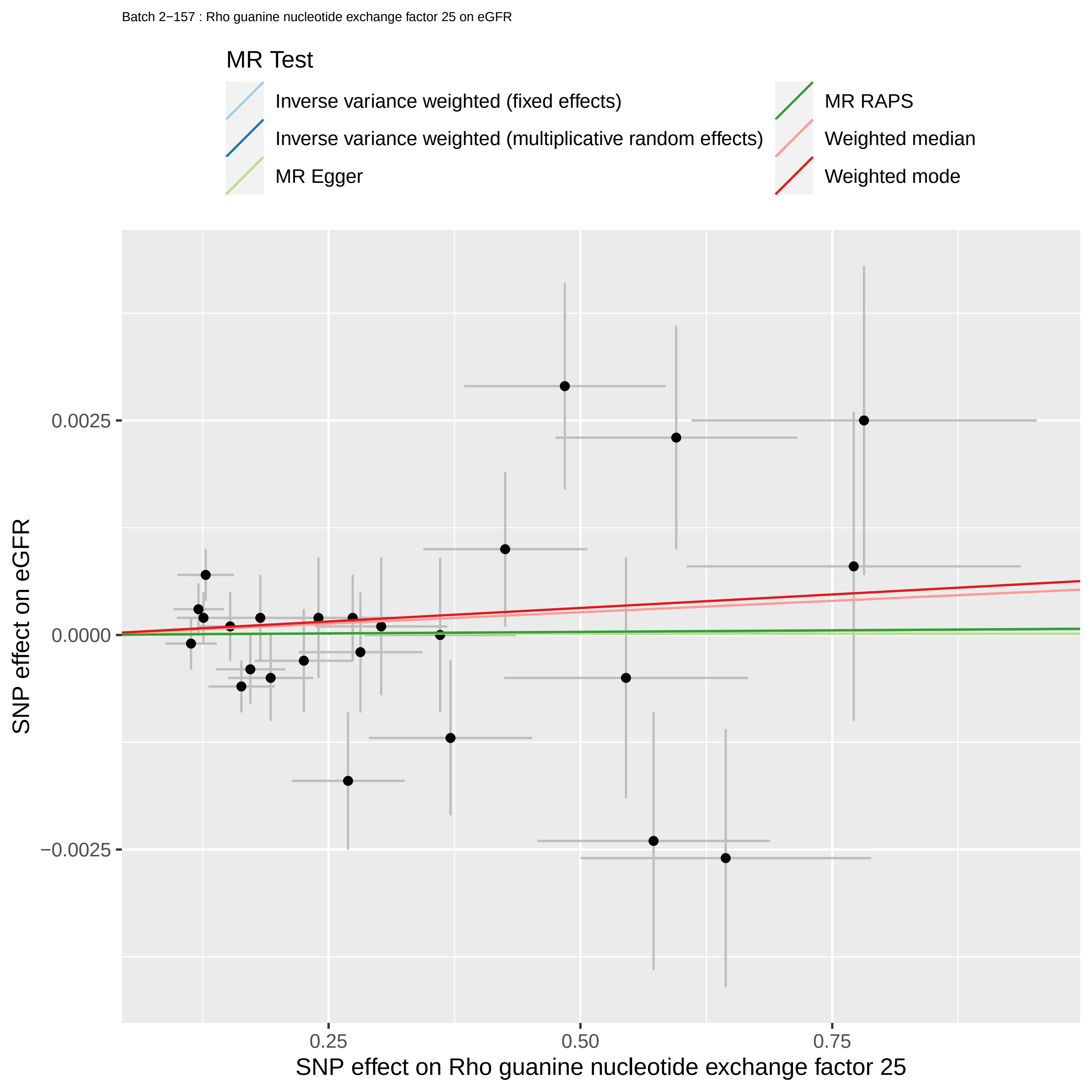

Batch 2−157 : Rho guanine nucleotide exchange factor 25 on eGFR
MR Test
Inverse variance weighted (fixed effects)
Inverse variance weighted (multiplicative random effects)
MR Egger
MR RAPS
Weighted median
Weighted mode
0.0025
0.0000
−0.0025
SNP effect on eGFR
0.25
0.50
0.75
SNP effect on Rho guanine nucleotide exchange factor 25

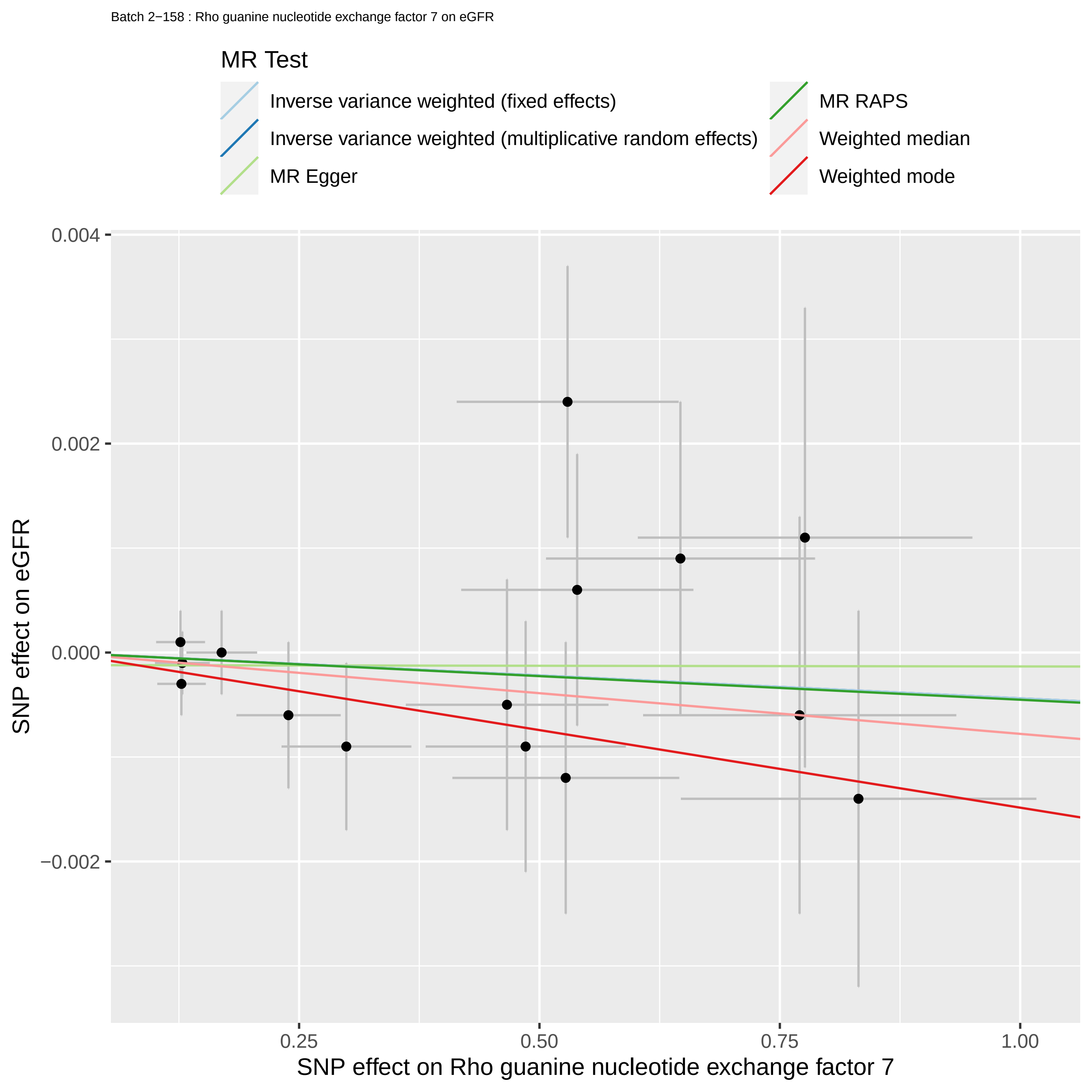
Batch 2−158 : Rho guanine nucleotide exchange factor 7 on eGFR
MR Test
Inverse variance weighted (fixed effects)
Inverse variance weighted (multiplicative random effects)
MR Egger
MR RAPS
Weighted median
Weighted mode
0.004
0.002
0.000
−0.002
SNP effect on eGFR
0.25
0.50
0.75
1.00
SNP effect on Rho guanine nucleotide exchange factor 7

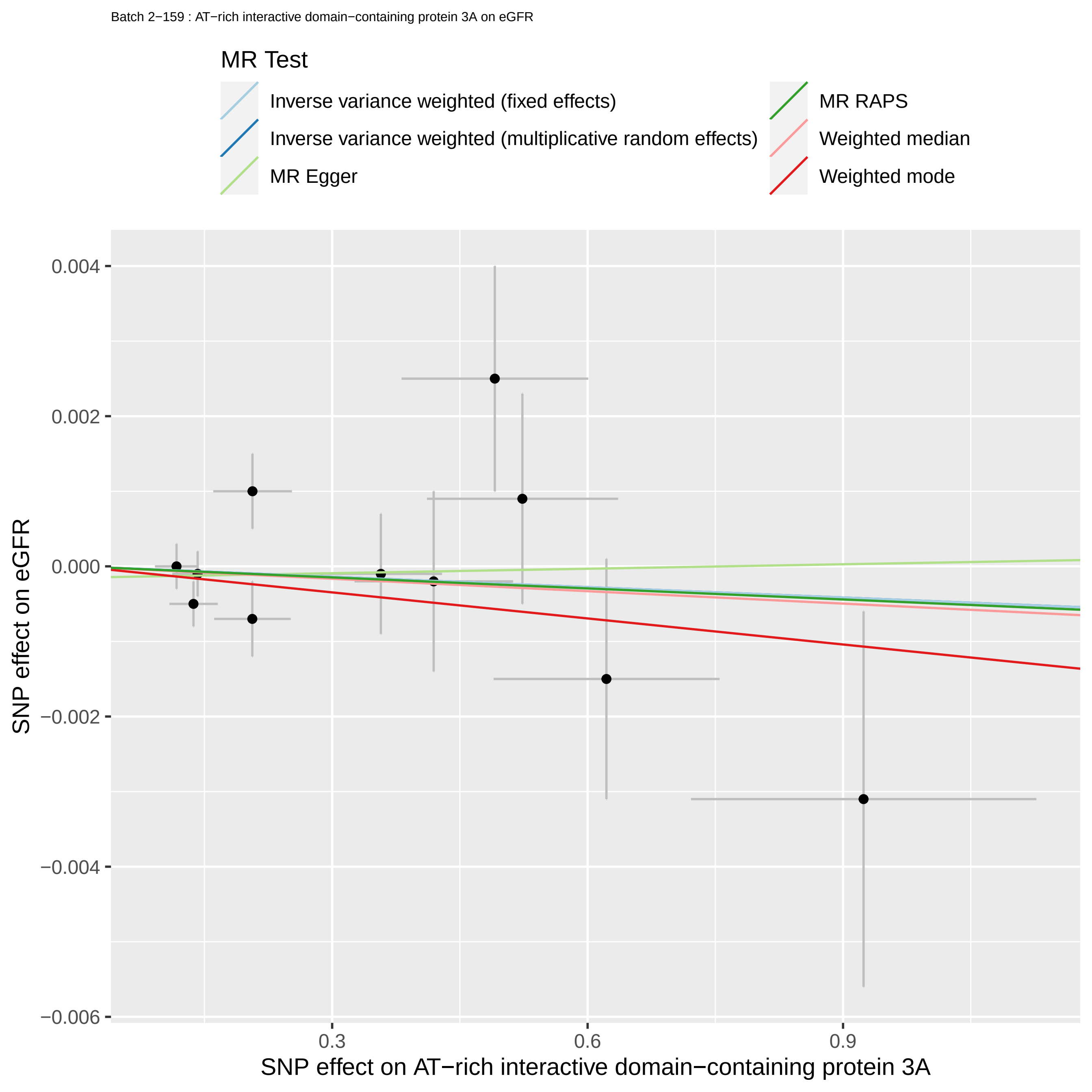

Batch 2−159 : AT−rich interactive domain−containing protein 3A on eGFR
MR Test
Inverse variance weighted (fixed effects)
Inverse variance weighted (multiplicative random effects)
MR Egger
MR RAPS
Weighted median
Weighted mode
0.004
0.002
0.000
−0.002
−0.004
−0.006
0.3
0.6
0.9
SNP effect on eGFR
SNP effect on AT−rich interactive domain−containing protein 3A

Batch 2−160 : ADP−ribosylation factor−like protein 11 on eGFR

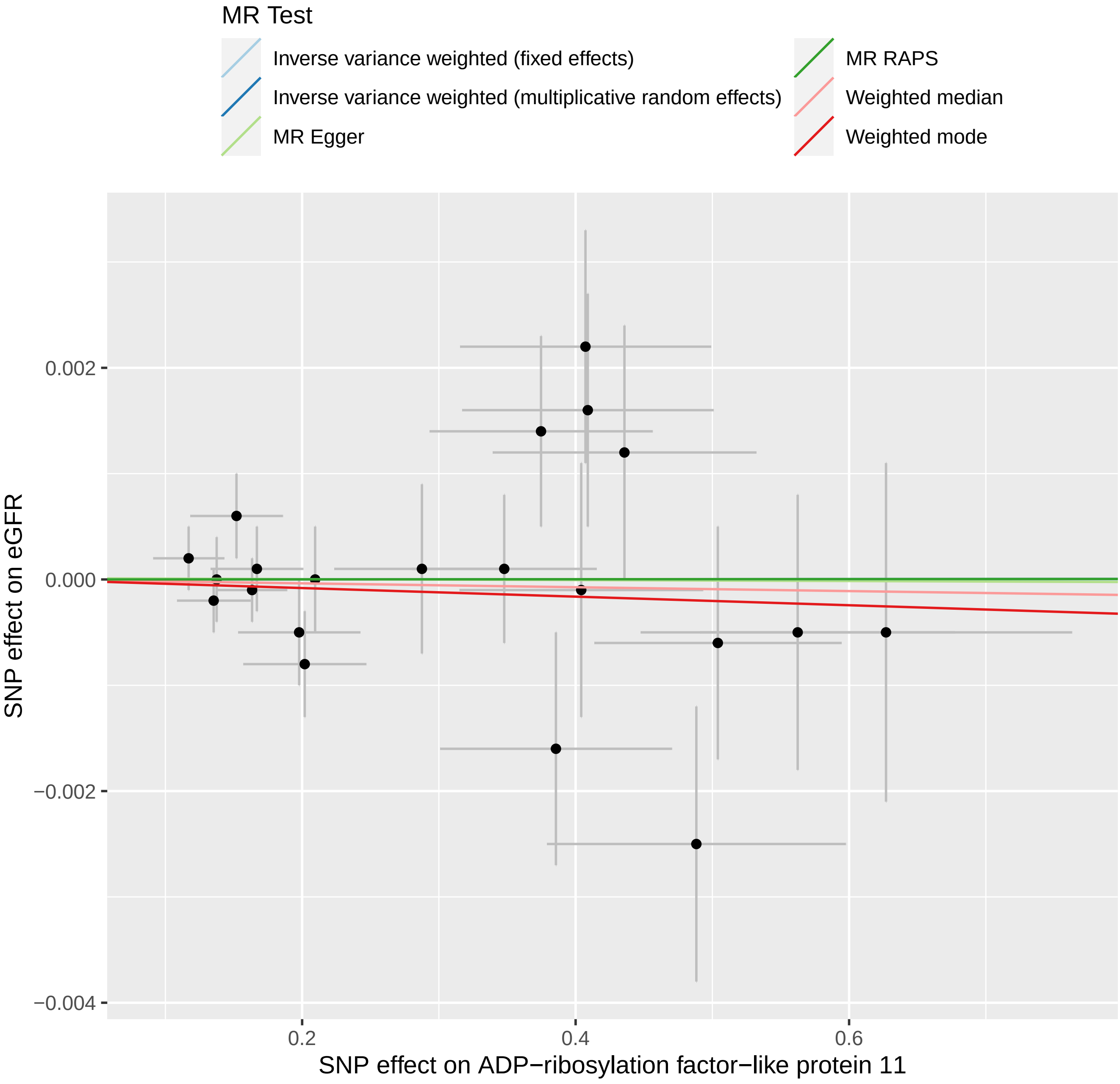

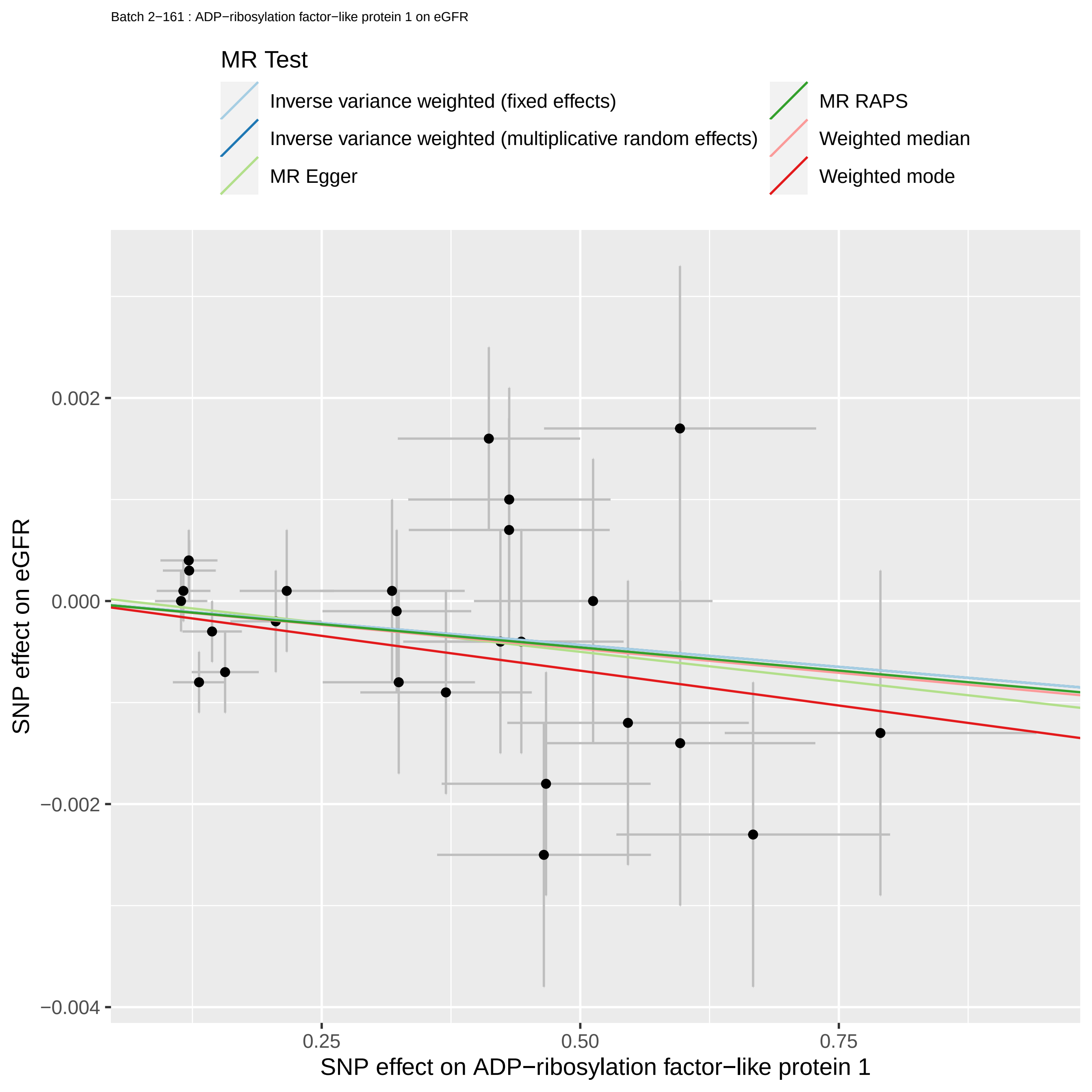

Batch 2−161 : ADP−ribosylation factor−like protein 1 on eGFR
MR Test
Inverse variance weighted (fixed effects)
Inverse variance weighted (multiplicative random effects)
MR Egger
MR RAPS
Weighted median
Weighted mode
0.002
0.000
−0.002
−0.004
SNP effect on eGFR
0.25
0.50
0.75
SNP effect on ADP−ribosylation factor−like protein 1

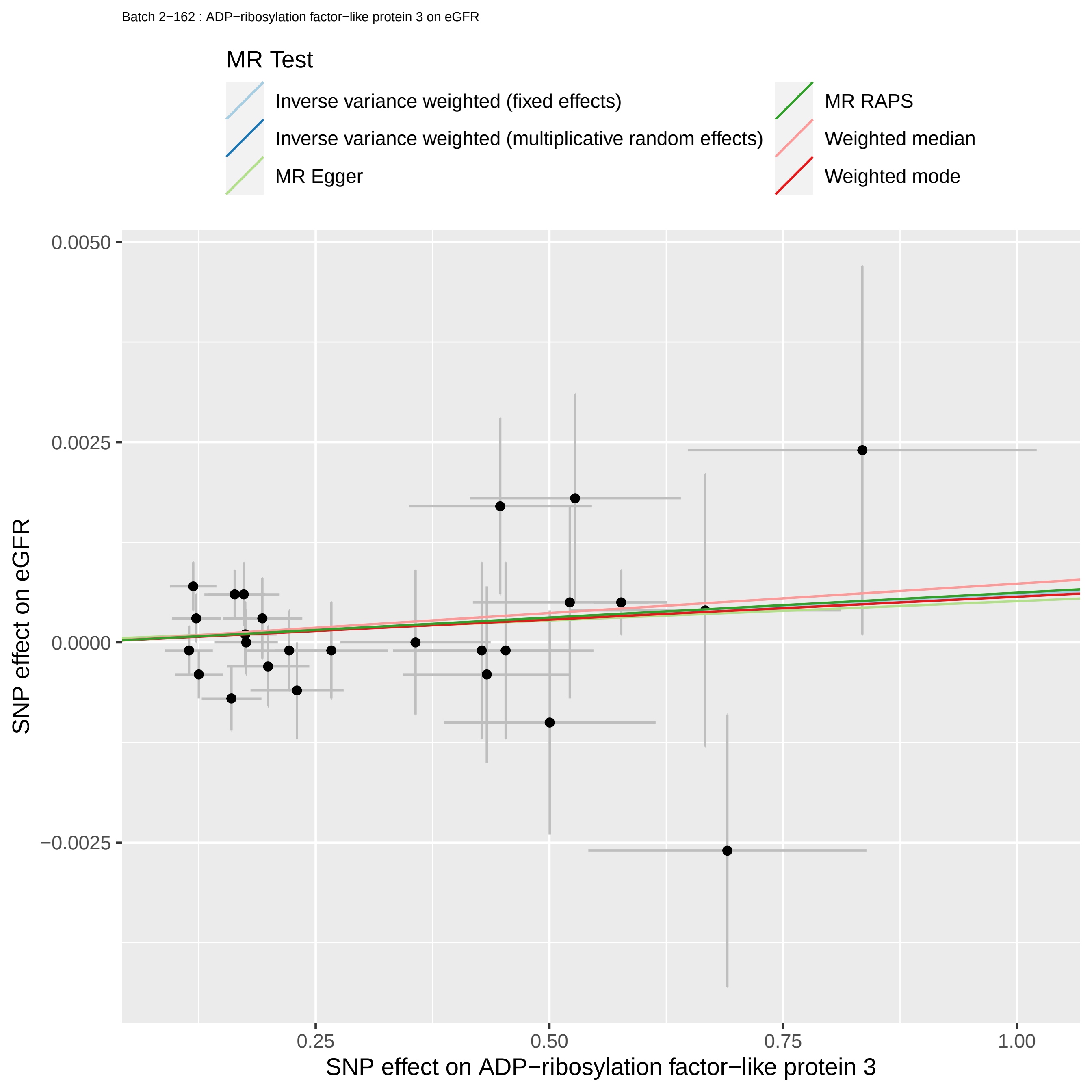
Batch 2−162 : ADP−ribosylation factor−like protein 3 on eGFR
MR Test
Inverse variance weighted (fixed effects)
Inverse variance weighted (multiplicative random effects)
MR Egger
MR RAPS
Weighted median
Weighted mode
0.0050
0.0025
0.0000
−0.0025
0.25
0.50
0.75
1.00
SNP effect on eGFR
SNP effect on ADP−ribosylation factor−like protein 3

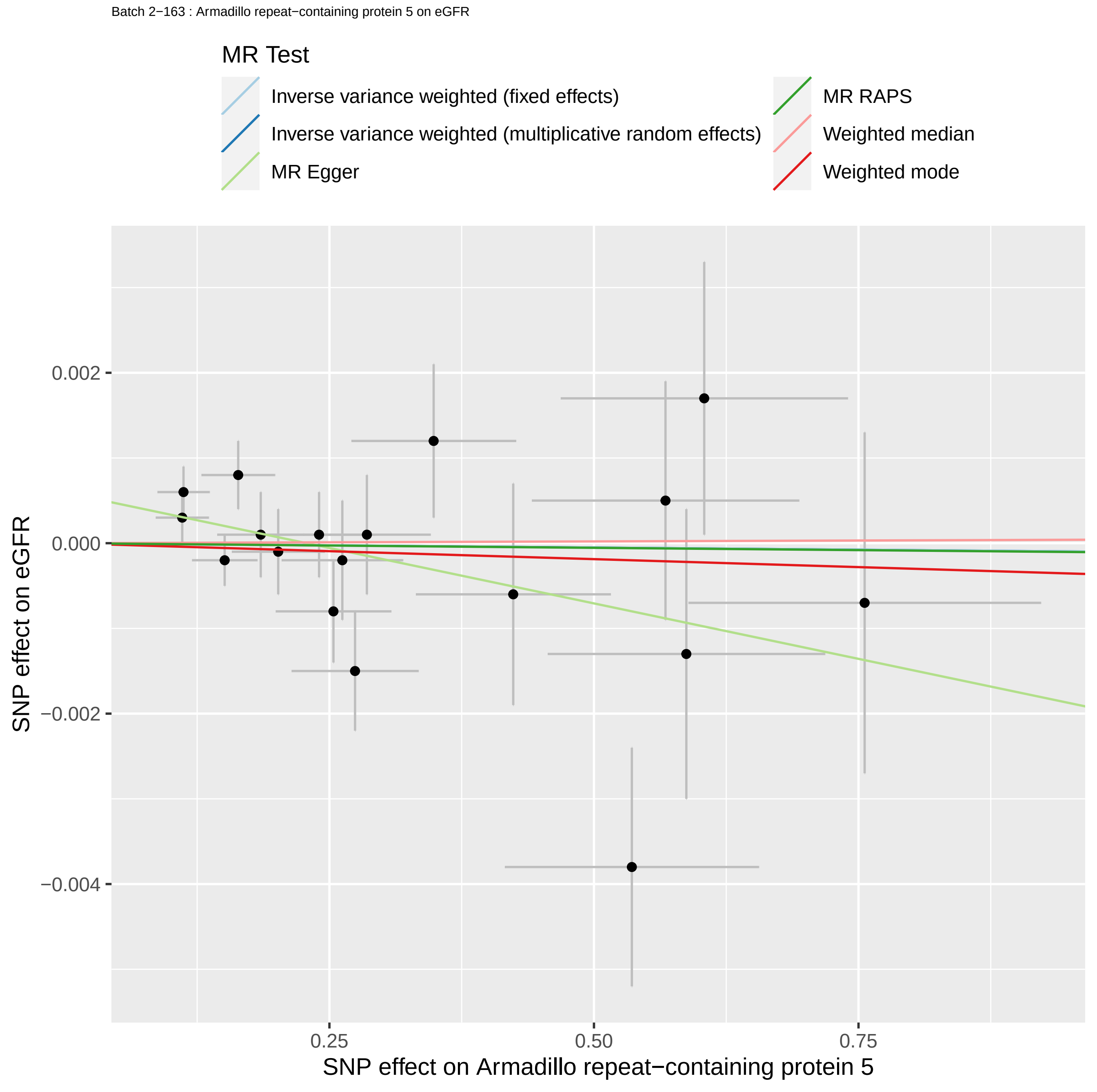
Batch 2−163 : Armadillo repeat−containing protein 5 on eGFR
MR Test
Inverse variance weighted (fixed effects)
Inverse variance weighted (multiplicative random effects)
MR Egger
MR RAPS
Weighted median
Weighted mode
0.002
0.000
−0.002
−0.004
0.25
0.50
0.75
SNP effect on eGFR
SNP effect on Armadillo repeat−containing protein 5

Batch 2−164 : Armadillo repeat−containing protein 5 on eGFR

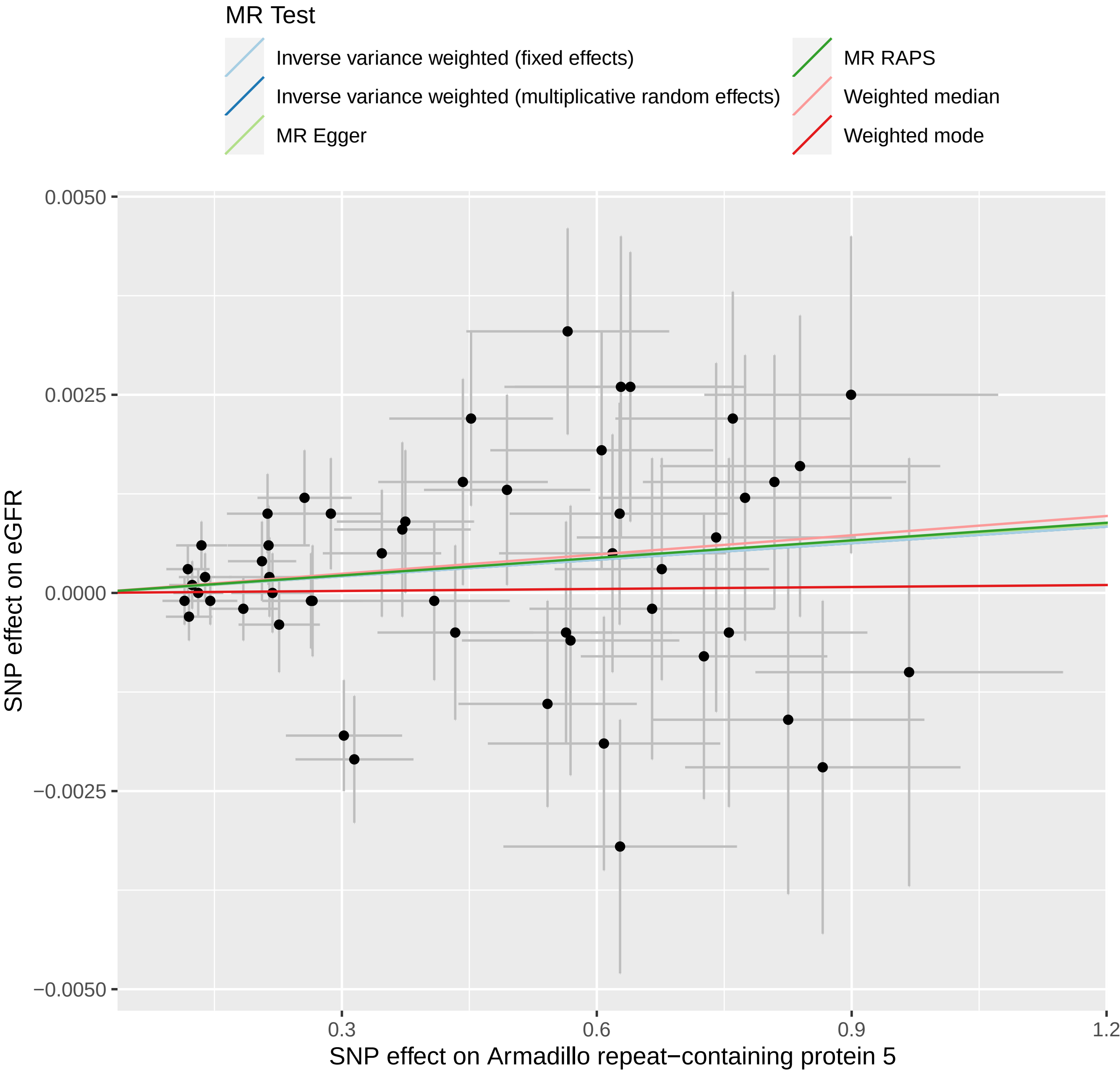

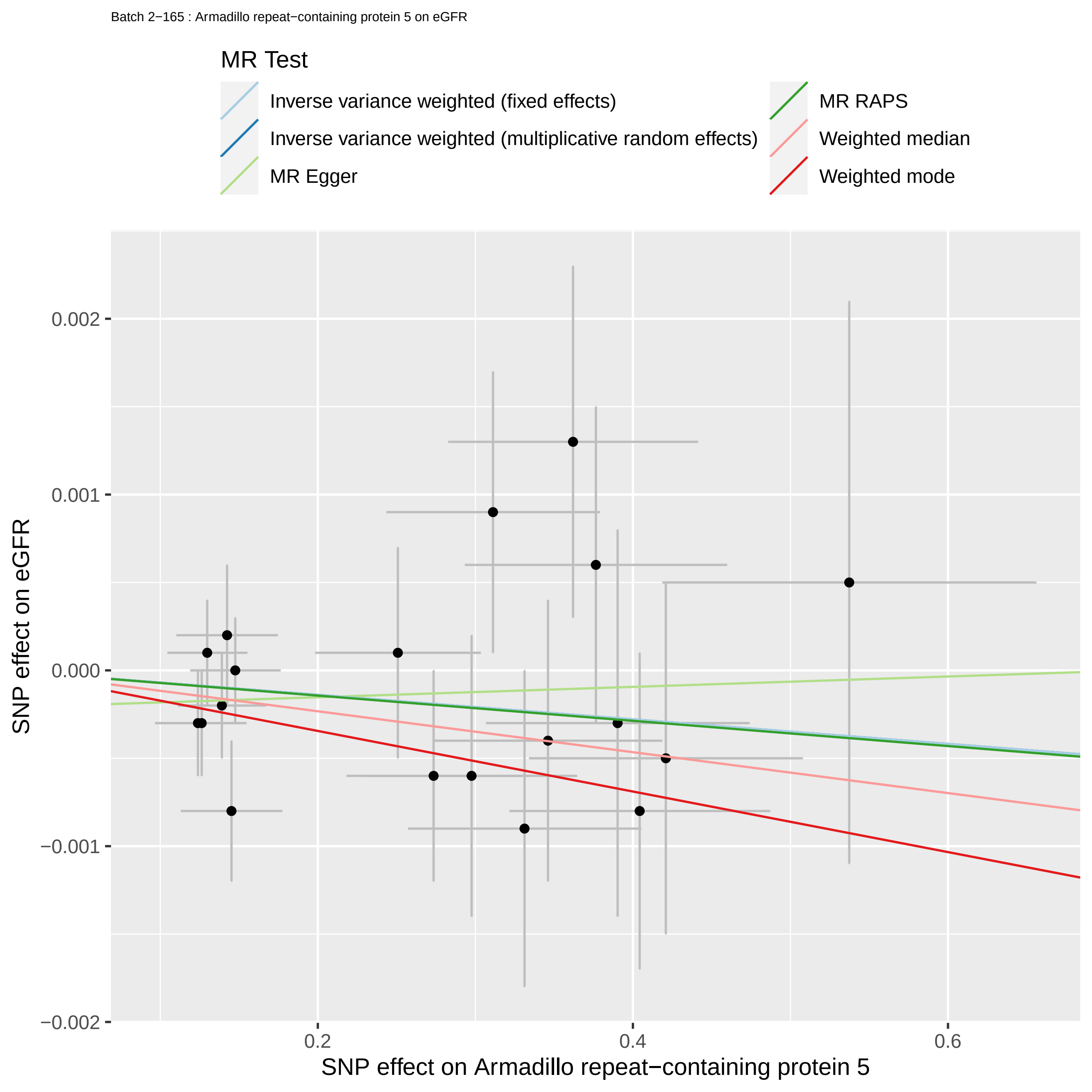

Batch 2−165 : Armadillo repeat−containing protein 5 on eGFR
MR Test
Inverse variance weighted (fixed effects)
Inverse variance weighted (multiplicative random effects)
MR Egger
MR RAPS
Weighted median
Weighted mode
0.002
0.001
0.000
−0.001
−0.002
0.2
0.4
0.6
SNP effect on eGFR
SNP effect on Armadillo repeat−containing protein 5

Batch 2−166 : Actin−related protein 2/3 complex subunit 3 on eGFR

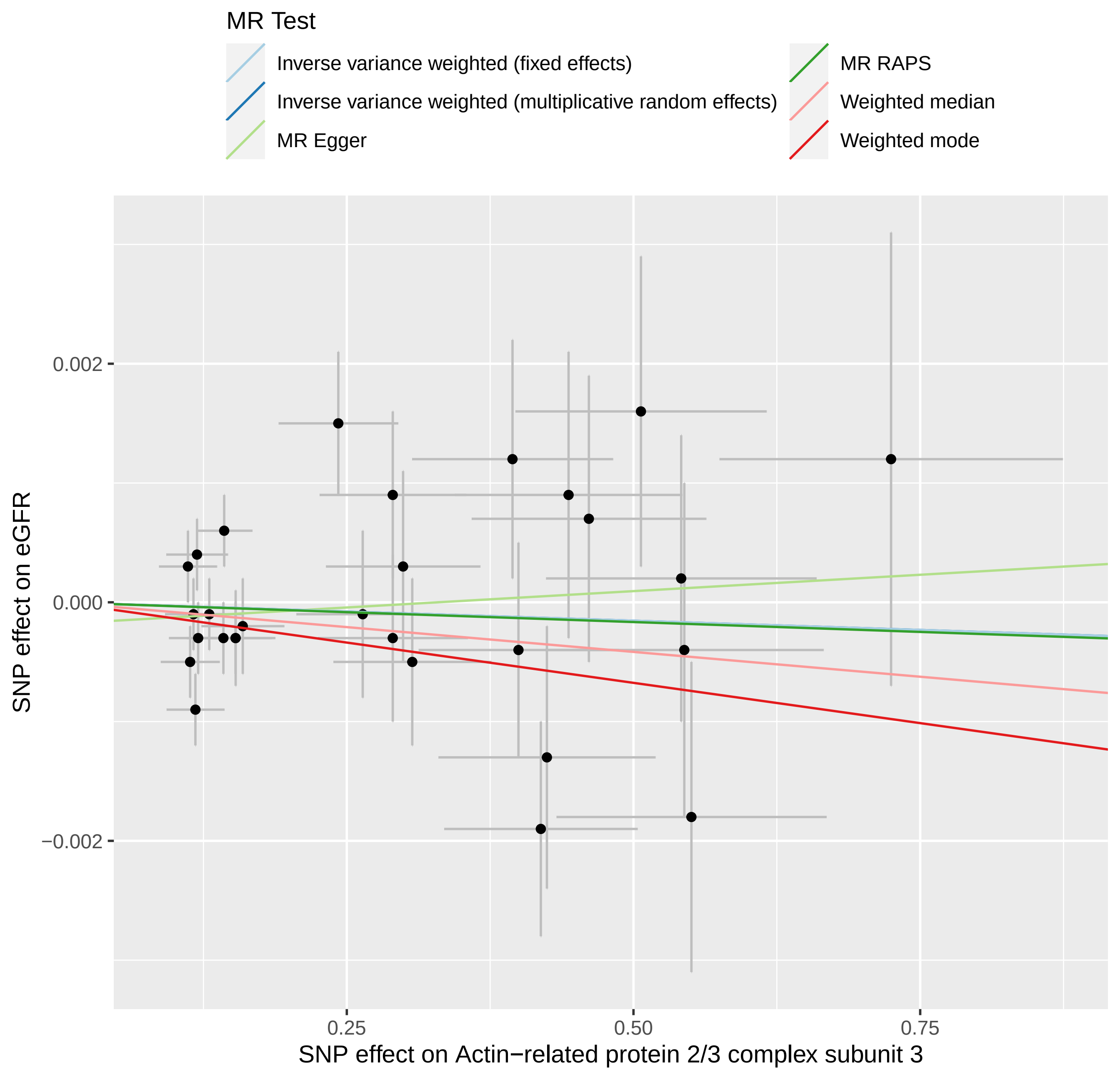

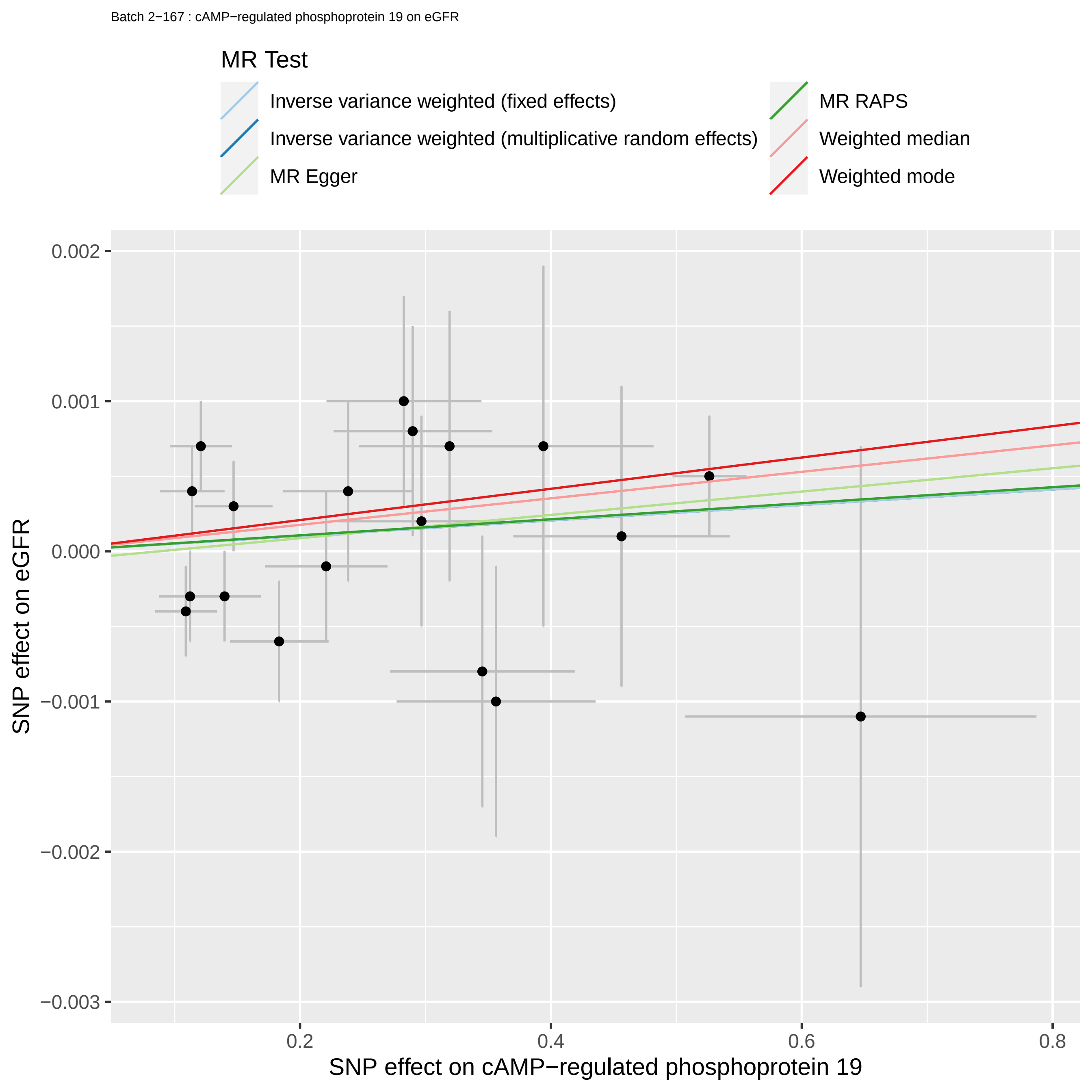
Batch 2−167 : cAMP−regulated phosphoprotein 19 on eGFR
MR Test
Inverse variance weighted (fixed effects)
Inverse variance weighted (multiplicative random effects)
MR Egger
MR RAPS
Weighted median
Weighted mode
0.002
0.001
0.000
−0.001
−0.002
−0.003
SNP effect on eGFR
0.2
0.4
0.6
0.8
SNP effect on cAMP−regulated phosphoprotein 19

Batch 2−168 : cAMP−regulated phosphoprotein 21 on eGFR

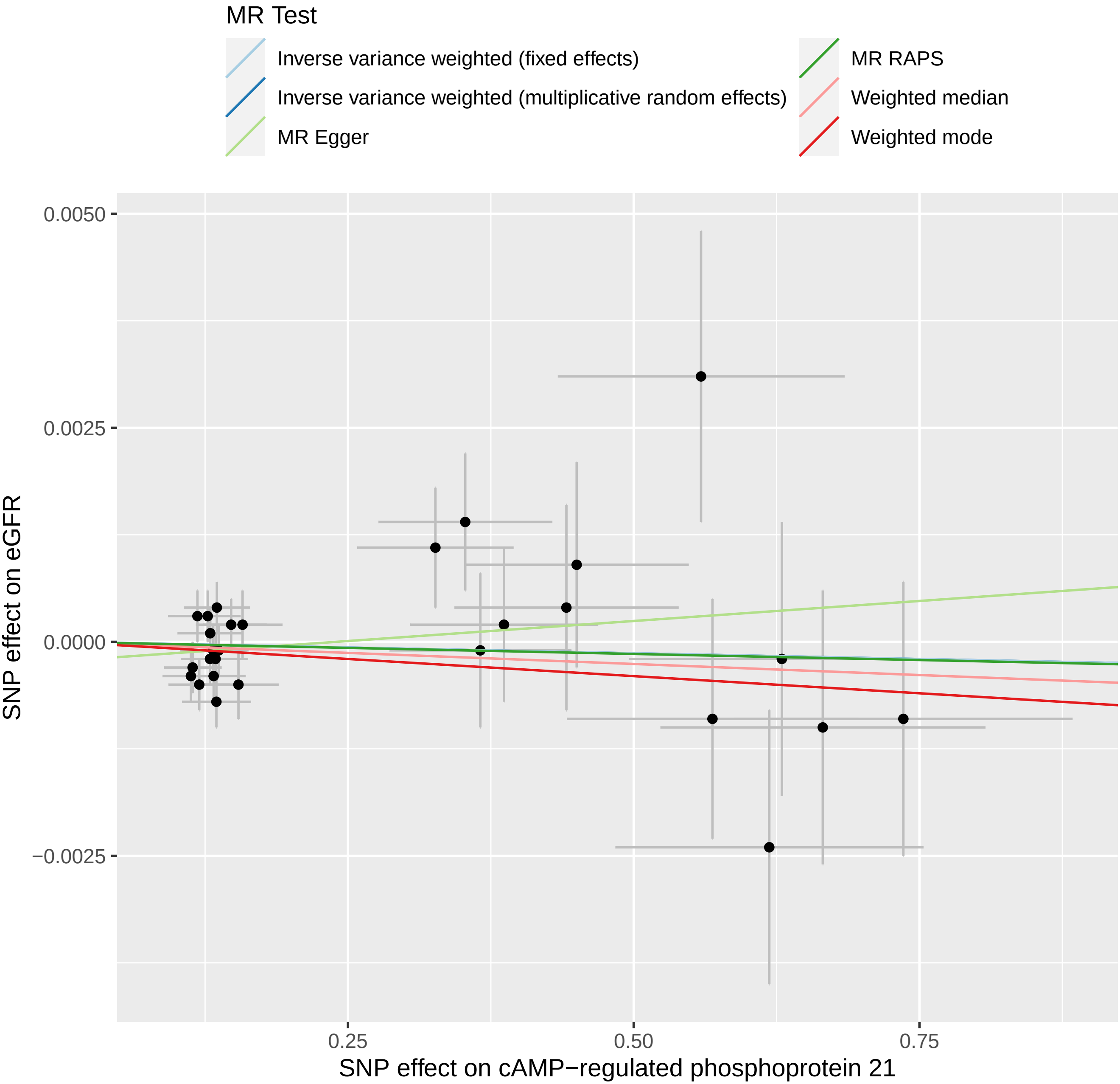

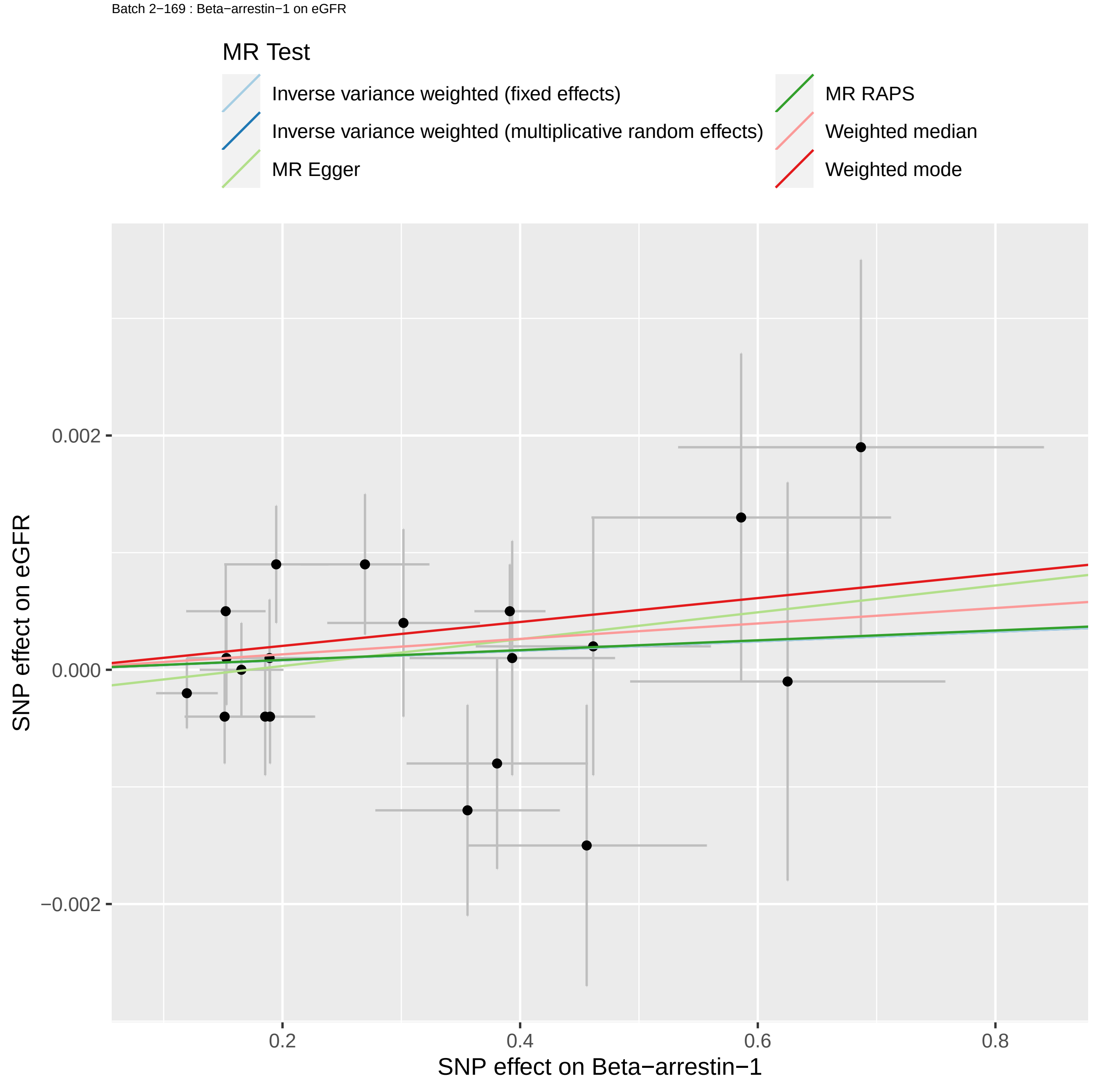

Batch 2−169 : Beta−arrestin−1 on eGFR
MR Test
Inverse variance weighted (fixed effects)
Inverse variance weighted (multiplicative random effects)
MR Egger
MR RAPS
Weighted median
Weighted mode
0.002
0.000
−0.002
0.2
0.4
0.6
0.8
SNP effect on eGFR
SNP effect on Beta−arrestin−1

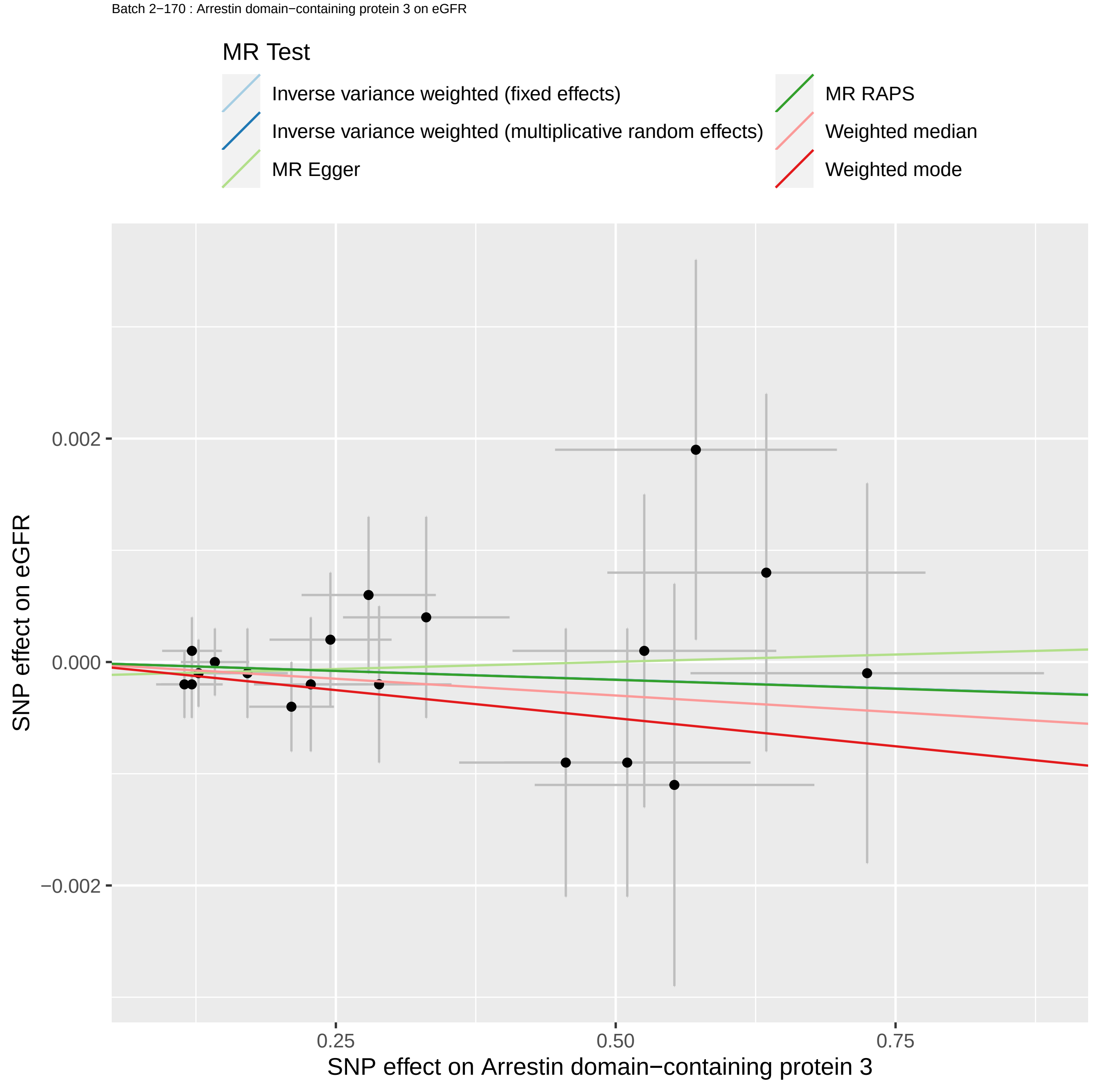
Batch 2−170 : Arrestin domain−containing protein 3 on eGFR
MR Test
Inverse variance weighted (fixed effects)
Inverse variance weighted (multiplicative random effects)
MR Egger
MR RAPS
Weighted median
Weighted mode
0.002
0.000
−0.002
0.25
0.50
0.75
SNP effect on eGFR
SNP effect on Arrestin domain−containing protein 3

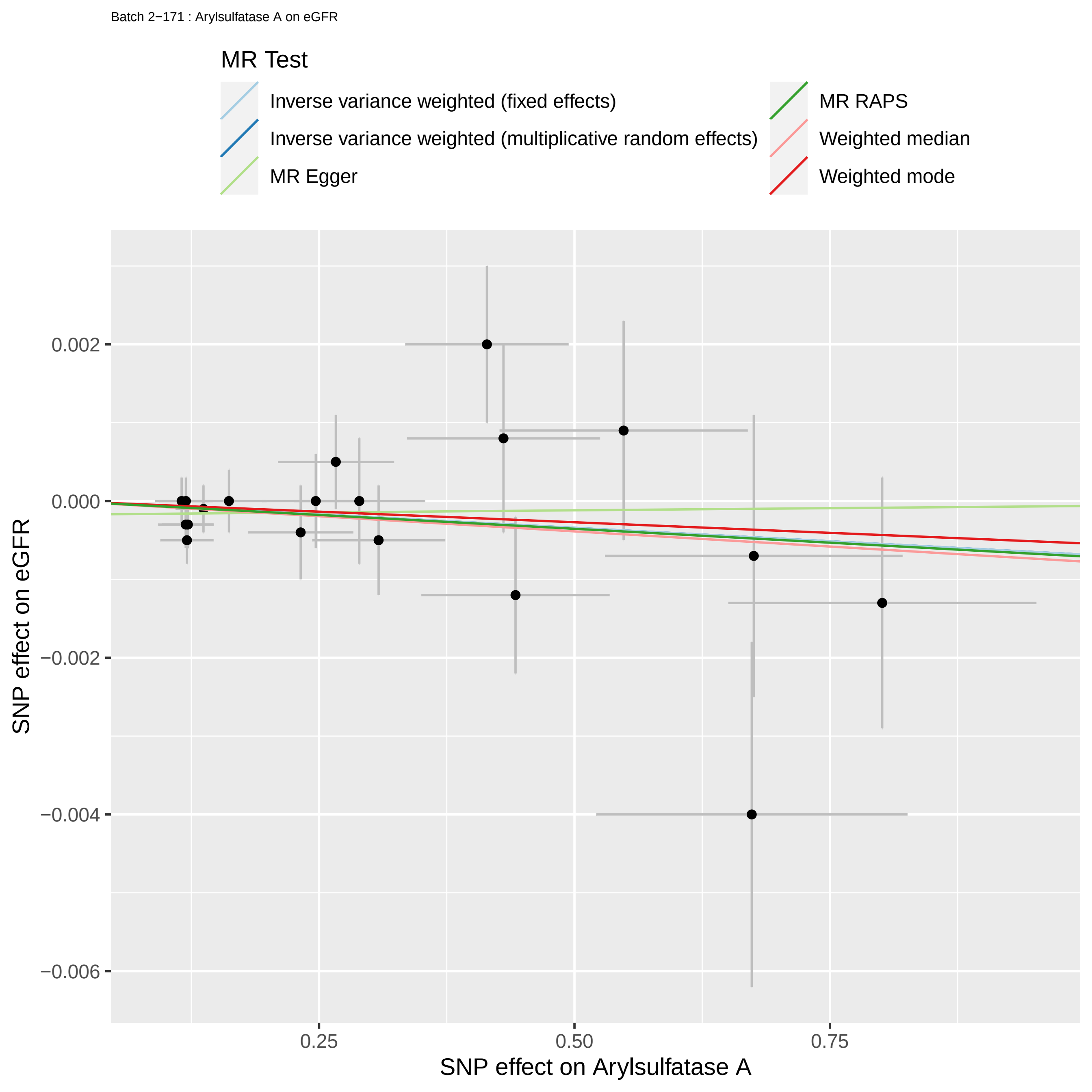

Batch 2−171 : Arylsulfatase A on eGFR
MR Test
Inverse variance weighted (fixed effects)
Inverse variance weighted (multiplicative random effects)
MR Egger
MR RAPS
Weighted median
Weighted mode
0.002
0.000
−0.002
−0.004
−0.006
SNP effect on eGFR
0.25
0.50
0.75
SNP effect on Arylsulfatase A

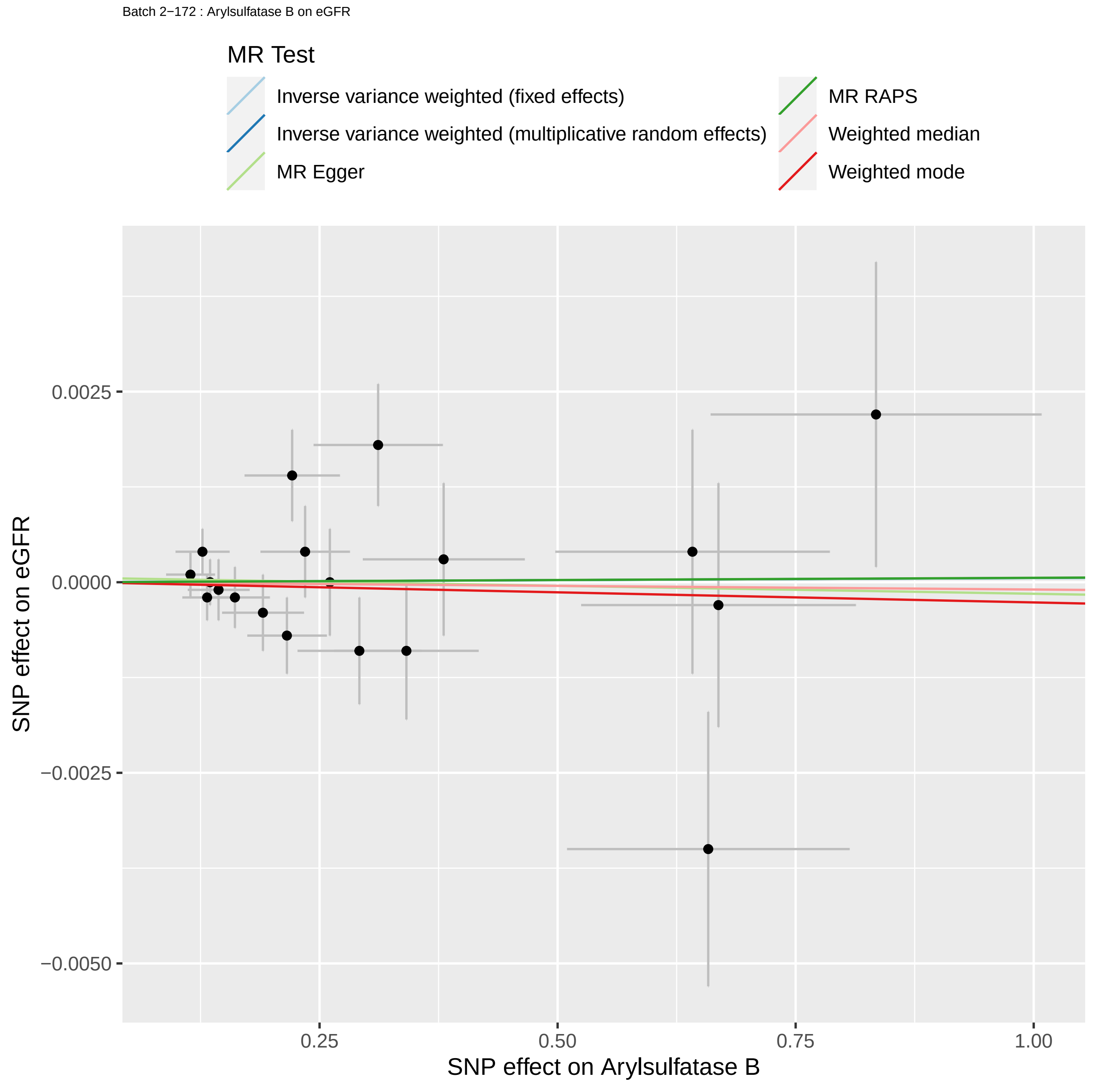

Batch 2−172 : Arylsulfatase B on eGFR
MR Test
Inverse variance weighted (fixed effects)
Inverse variance weighted (multiplicative random effects)
MR Egger
MR RAPS
Weighted median
Weighted mode
0.0025
0.0000
−0.0025
−0.0050
0.25
0.50
0.75
1.00
SNP effect on eGFR
SNP effect on Arylsulfatase B

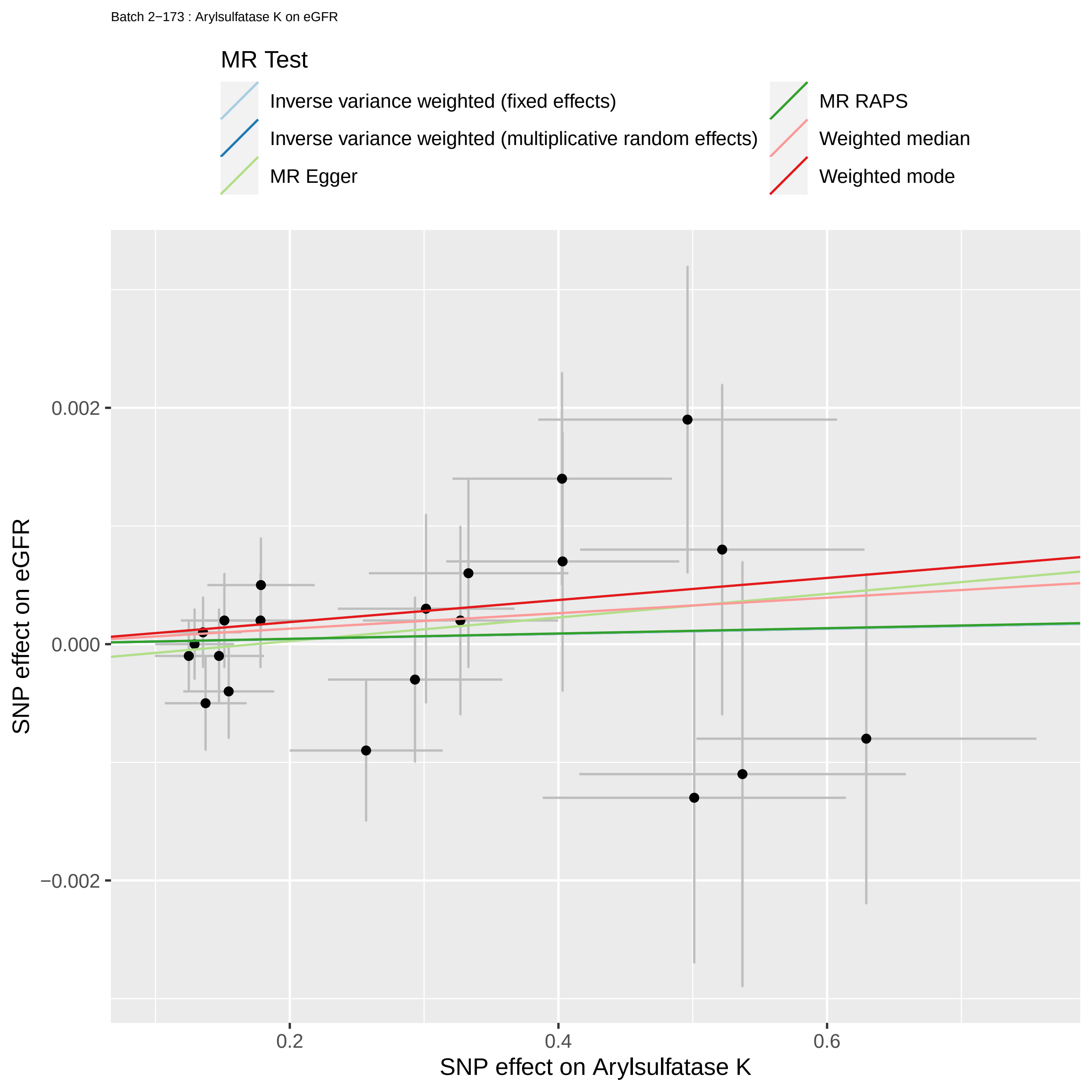

Batch 2−173 : Arylsulfatase K on eGFR
MR Test
Inverse variance weighted (fixed effects)
Inverse variance weighted (multiplicative random effects)
MR Egger
MR RAPS
Weighted median
Weighted mode
0.002
0.000
−0.002
SNP effect on eGFR
0.2
0.4
0.6
SNP effect on Arylsulfatase K

Batch 2−174 : Ecto−ADP−ribosyltransferase 3 on eGFR

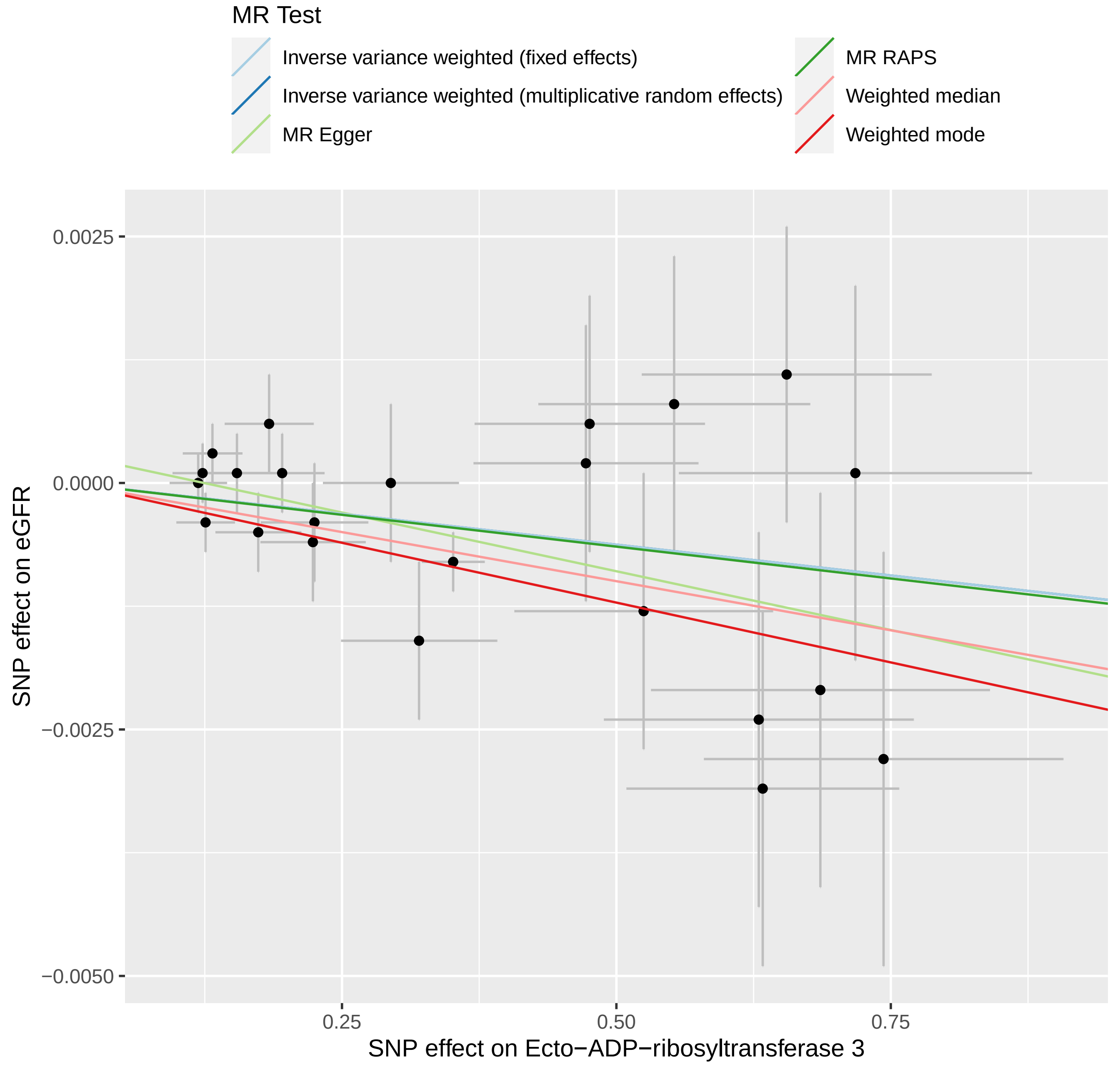

Batch 2−175 : Ecto−ADP−ribosyltransferase 3 on eGFR

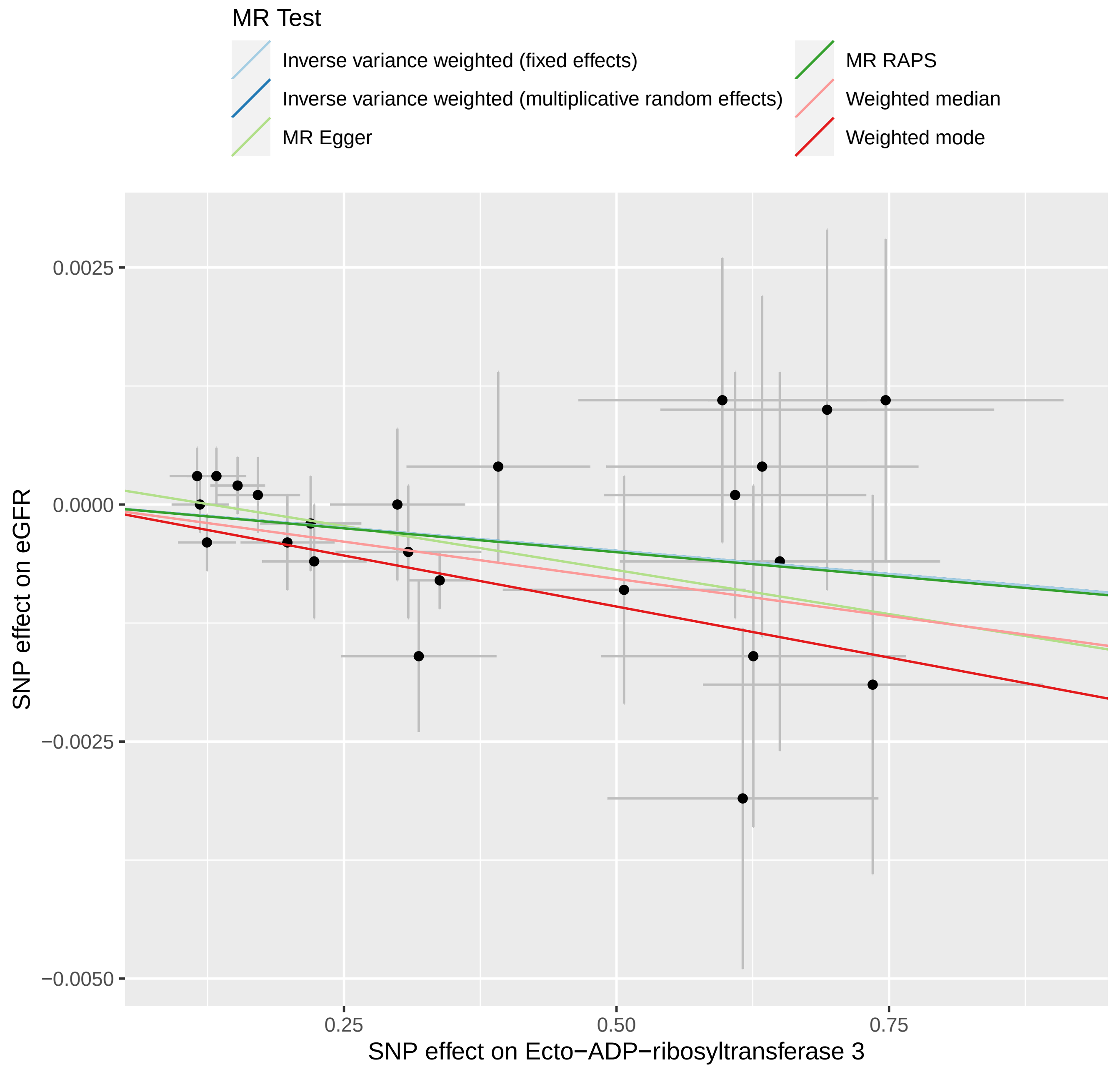

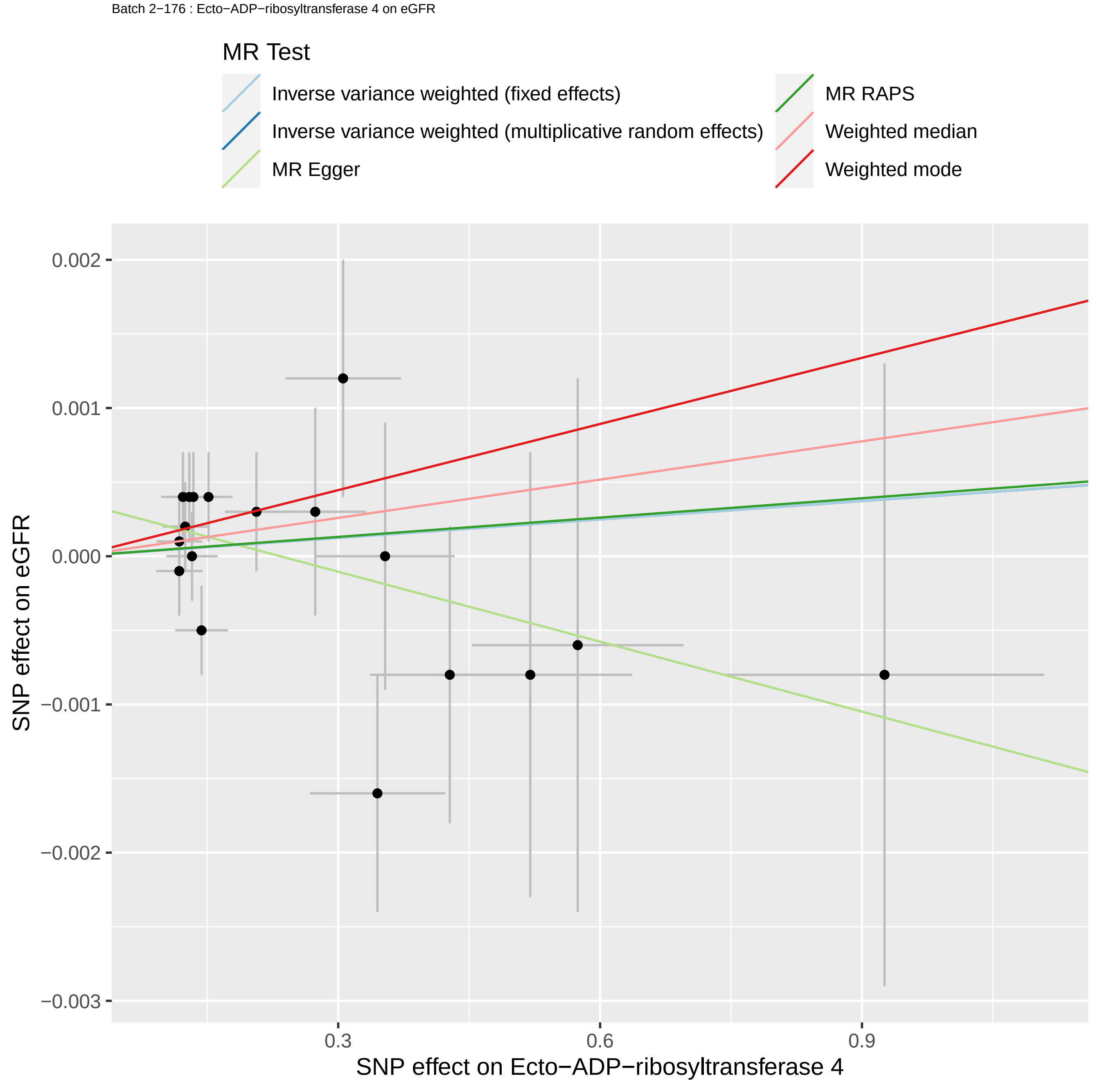

Batch 2−176 : Ecto−ADP−ribosyltransferase 4 on eGFR
MR Test
Inverse variance weighted (fixed effects)
Inverse variance weighted (multiplicative random effects)
MR Egger
MR RAPS
Weighted median
Weighted mode
0.002
0.001
0.000
−0.001
−0.002
−0.003
0.3
0.6
0.9
SNP effect on eGFR
SNP effect on Ecto−ADP−ribosyltransferase 4

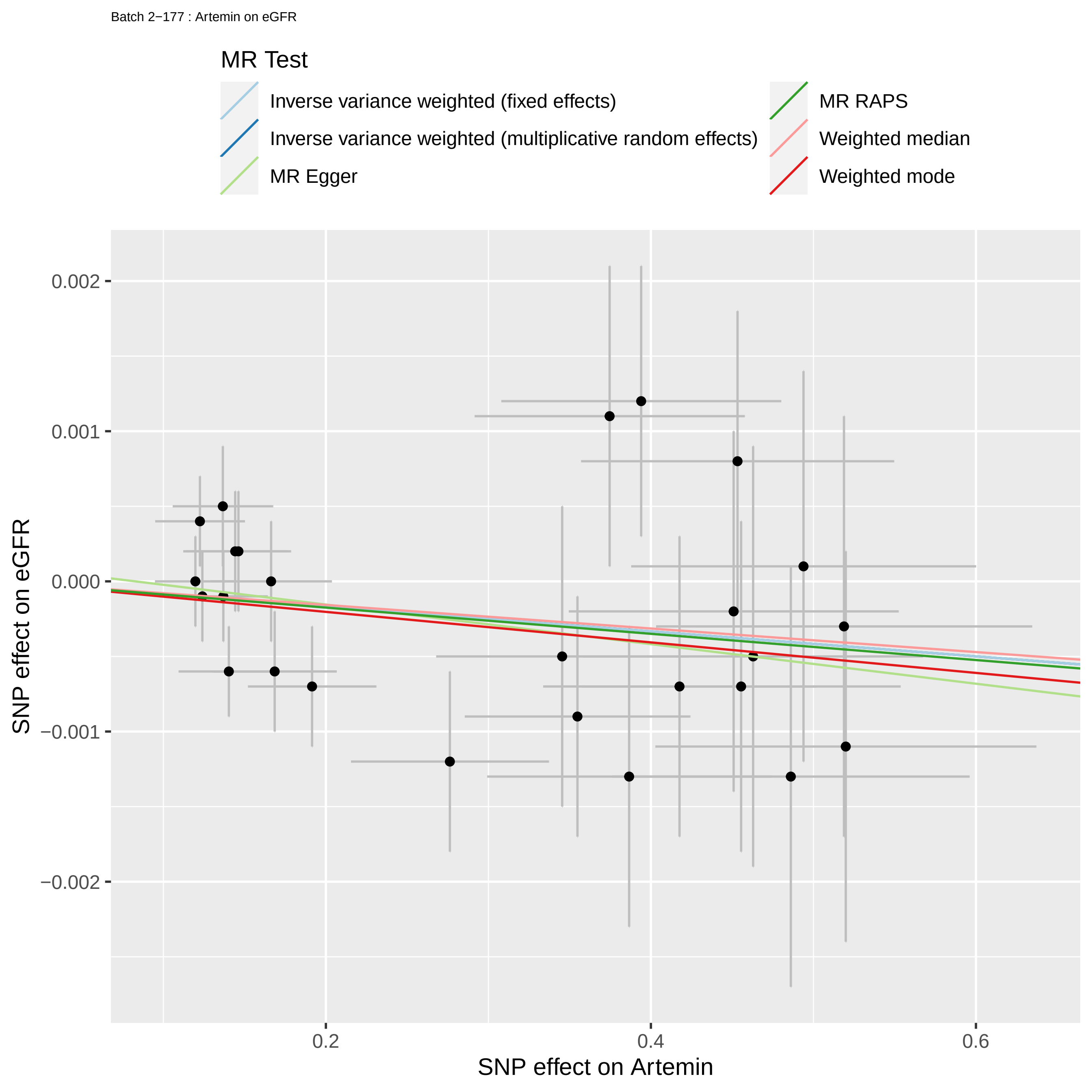
Batch 2−177 : Artemin on eGFR
MR Test
Inverse variance weighted (fixed effects)
Inverse variance weighted (multiplicative random effects)
MR Egger
MR RAPS
Weighted median
Weighted mode
0.002
0.001
0.000
−0.001
−0.002
0.2
0.4
0.6
SNP effect on Artemin
SNP effect on eGFR

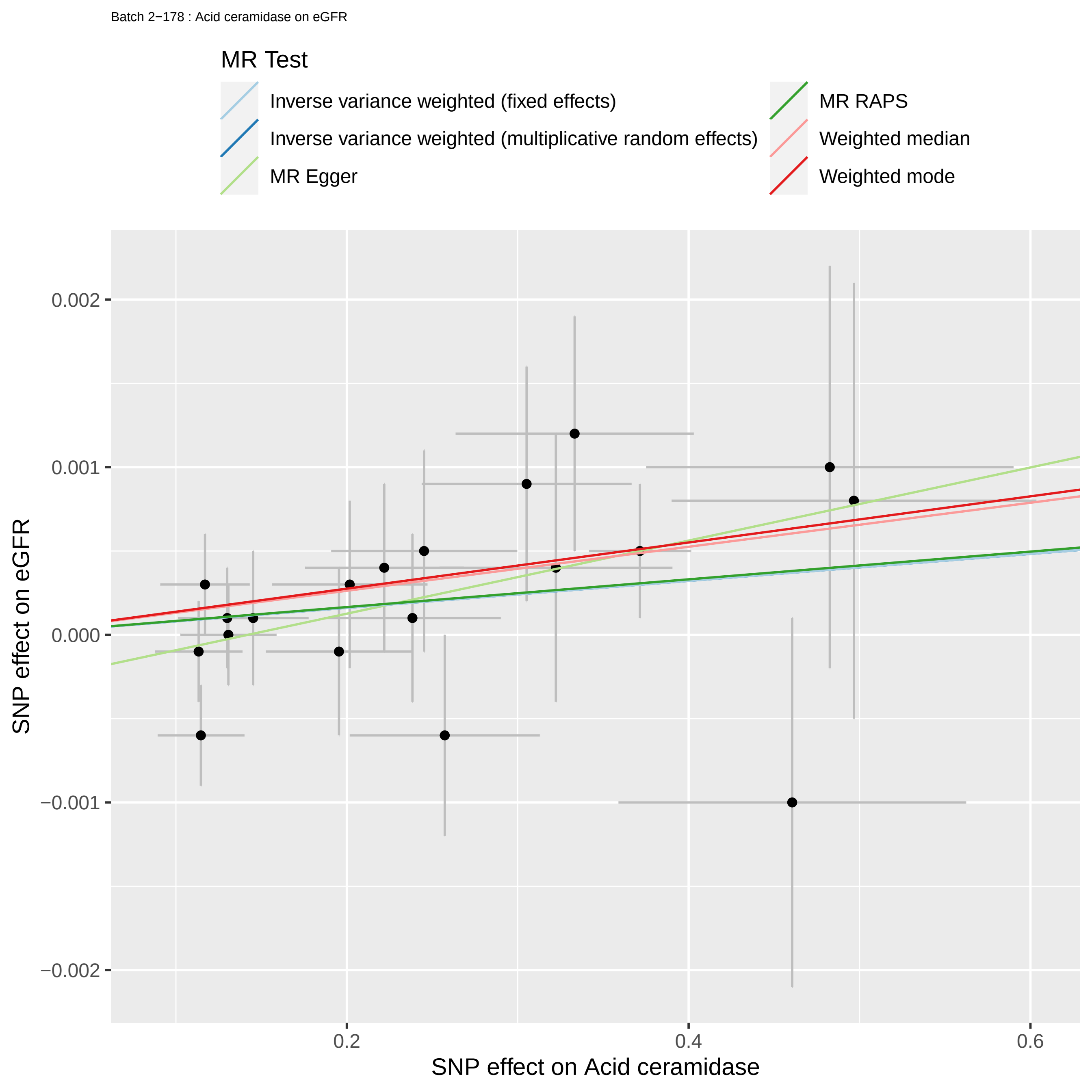

Batch 2−178 : Acid ceramidase on eGFR
MR Test
Inverse variance weighted (fixed effects)
Inverse variance weighted (multiplicative random effects)
MR Egger
MR RAPS
Weighted median
Weighted mode
0.002
0.001
0.000
−0.001
−0.002
SNP effect on eGFR
0.2
0.4
0.6
SNP effect on Acid ceramidase

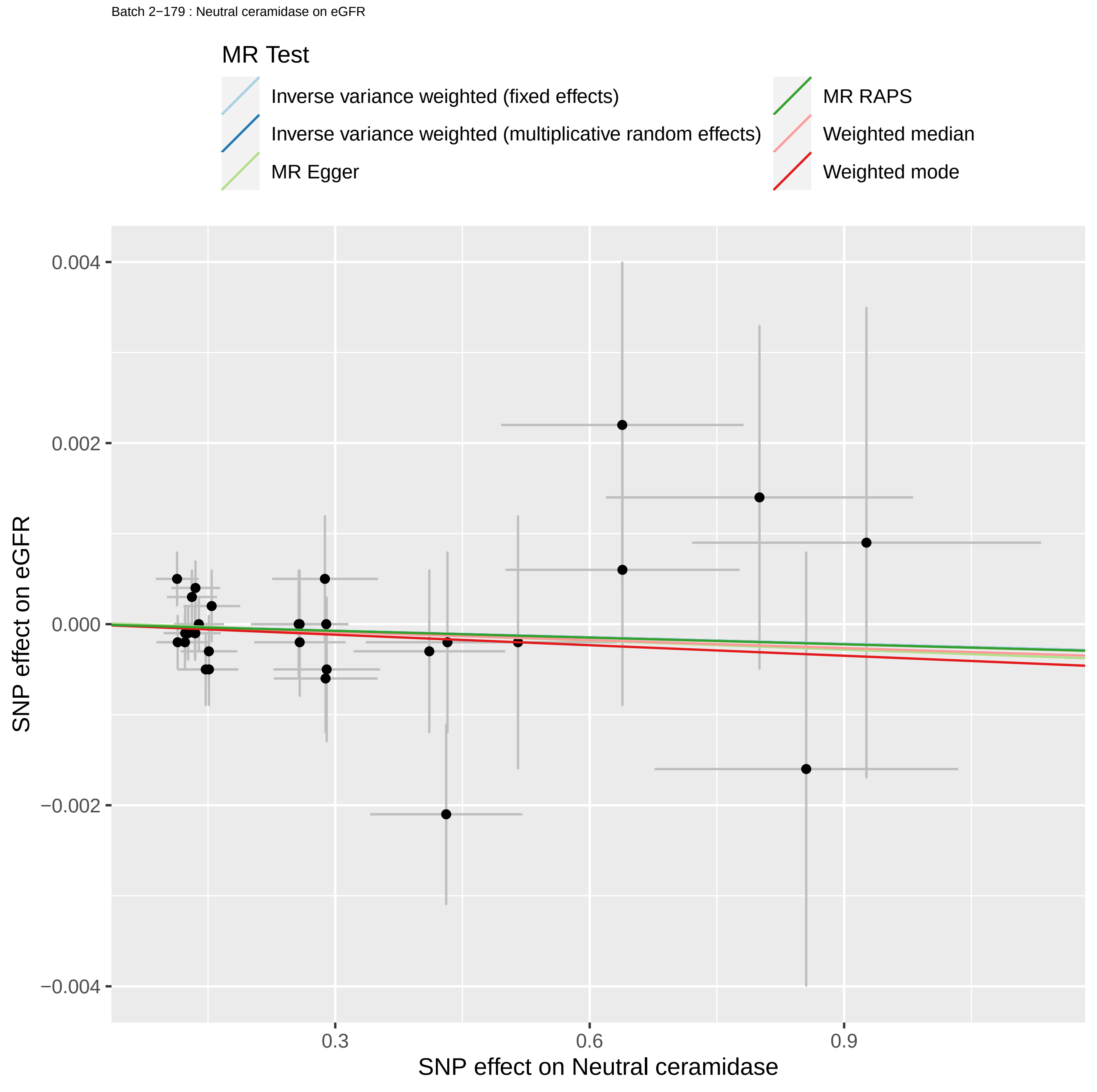
Batch 2−179 : Neutral ceramidase on eGFR
MR Test
Inverse variance weighted (fixed effects)
Inverse variance weighted (multiplicative random effects)
MR Egger
MR RAPS
Weighted median
Weighted mode
0.004
0.002
0.000
−0.002
−0.004
SNP effect on eGFR
0.3
0.6
0.9
SNP effect on Neutral ceramidase

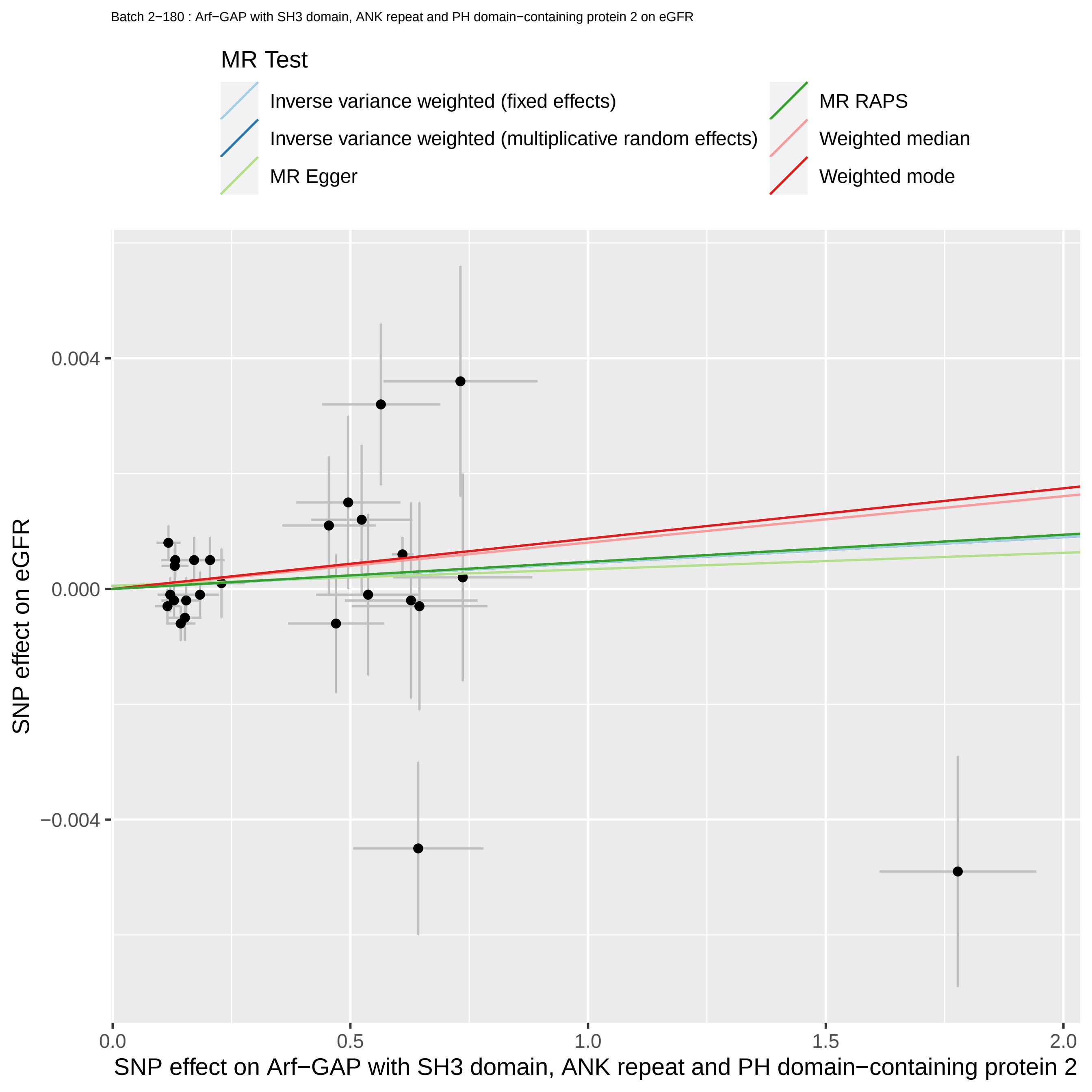
Batch 2−180 : Arf−GAP with SH3 domain, ANK repeat and PH domain−containing protein 2 on eGFR
MR Test
Inverse variance weighted (fixed effects)
Inverse variance weighted (multiplicative random effects)
MR Egger
MR RAPS
Weighted median
Weighted mode
0.004
0.000
−0.004
0.0
0.5
1.0
1.5
2.0
SNP effect on eGFR
SNP effect on Arf−GAP with SH3 domain, ANK repeat and PH domain−containing protein 2

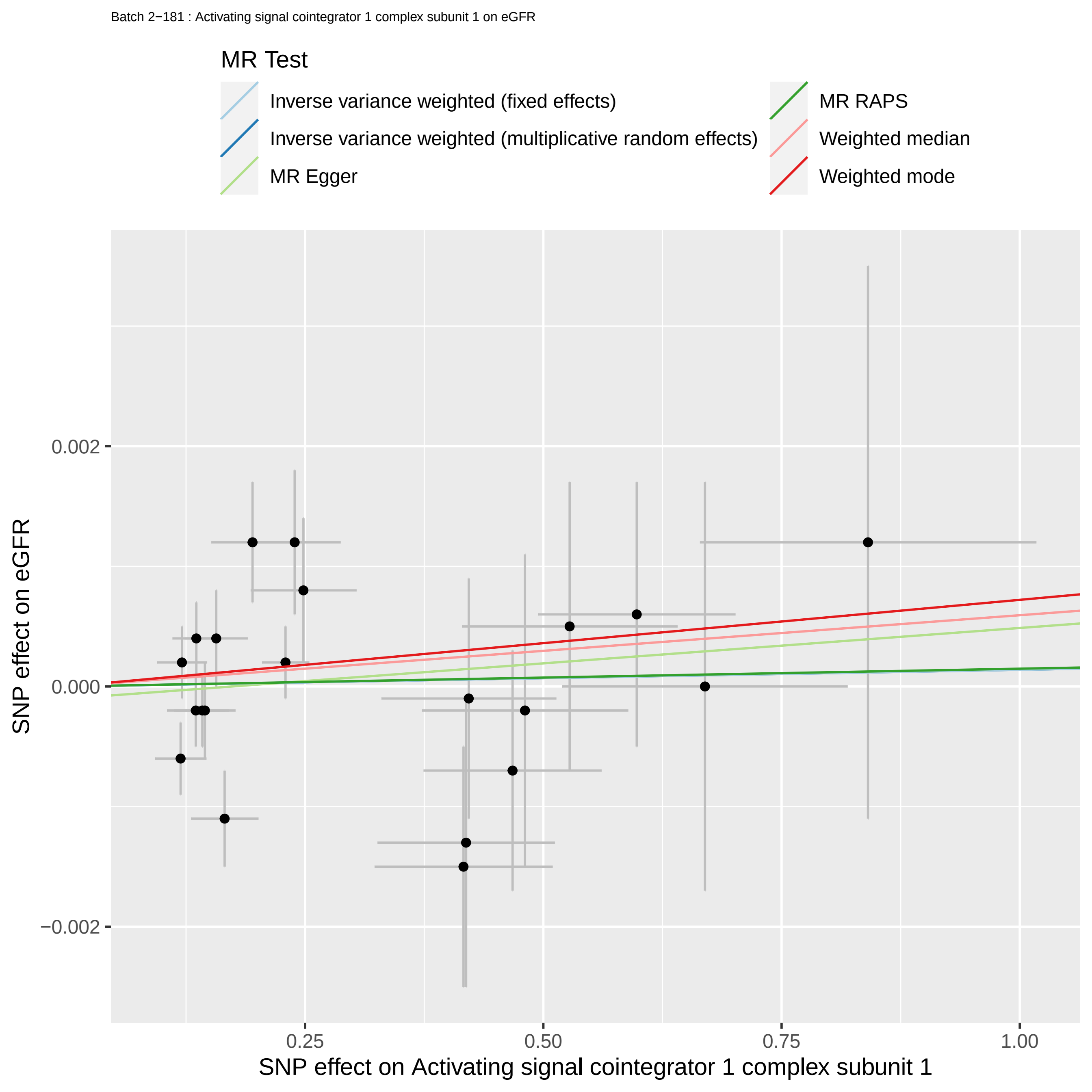

Batch 2−181 : Activating signal cointegrator 1 complex subunit 1 on eGFR
MR Test
Inverse variance weighted (fixed effects)
Inverse variance weighted (multiplicative random effects)
MR Egger
MR RAPS
Weighted median
Weighted mode
0.002
0.000
−0.002
SNP effect on eGFR
0.25
0.50
0.75
1.00
SNP effect on Activating signal cointegrator 1 complex subunit 1

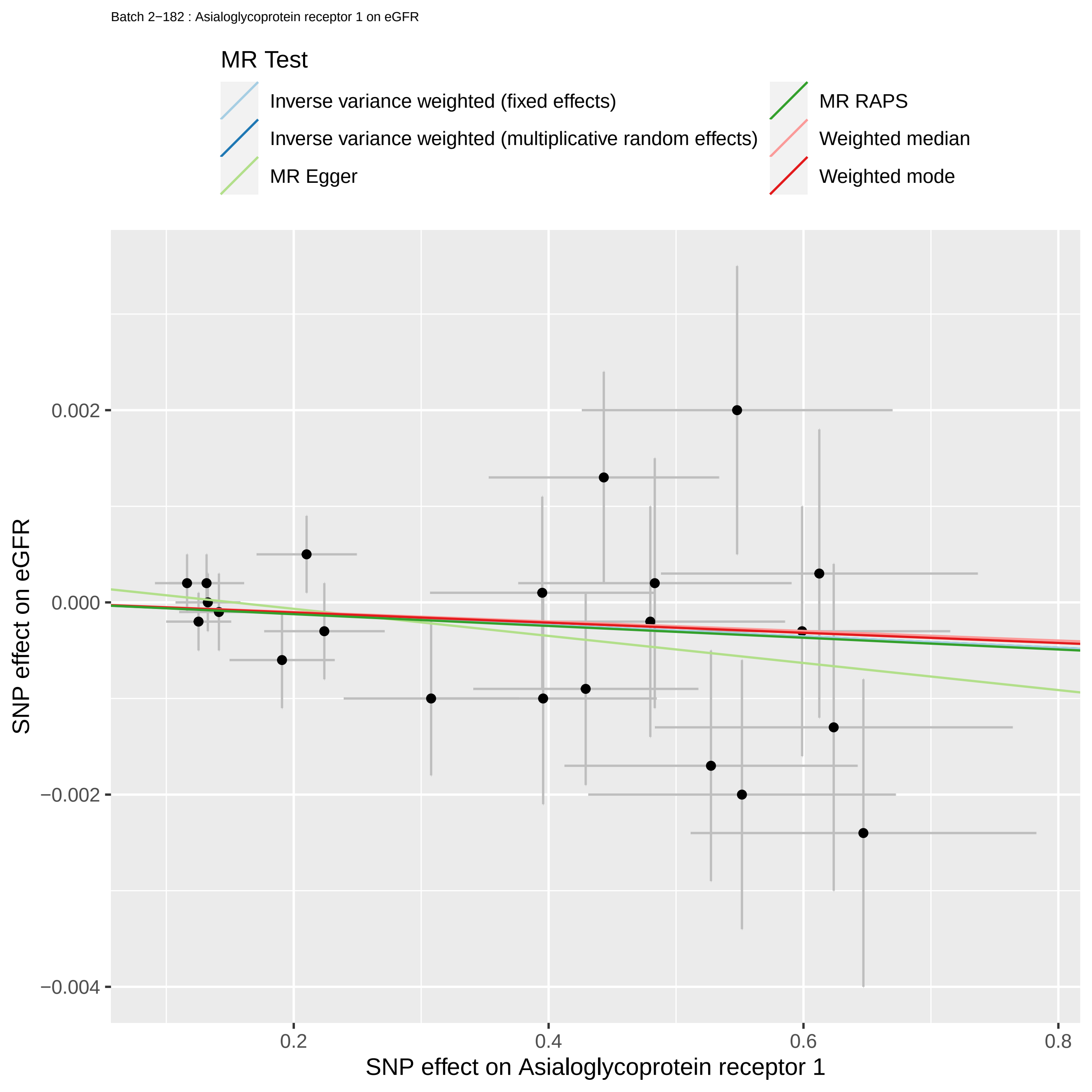

Batch 2−182 : Asialoglycoprotein receptor 1 on eGFR
MR Test
Inverse variance weighted (fixed effects)
Inverse variance weighted (multiplicative random effects)
MR Egger
MR RAPS
Weighted median
Weighted mode
0.002
0.000
−0.002
−0.004
0.2
0.4
0.6
0.8
SNP effect on eGFR
SNP effect on Asialoglycoprotein receptor 1

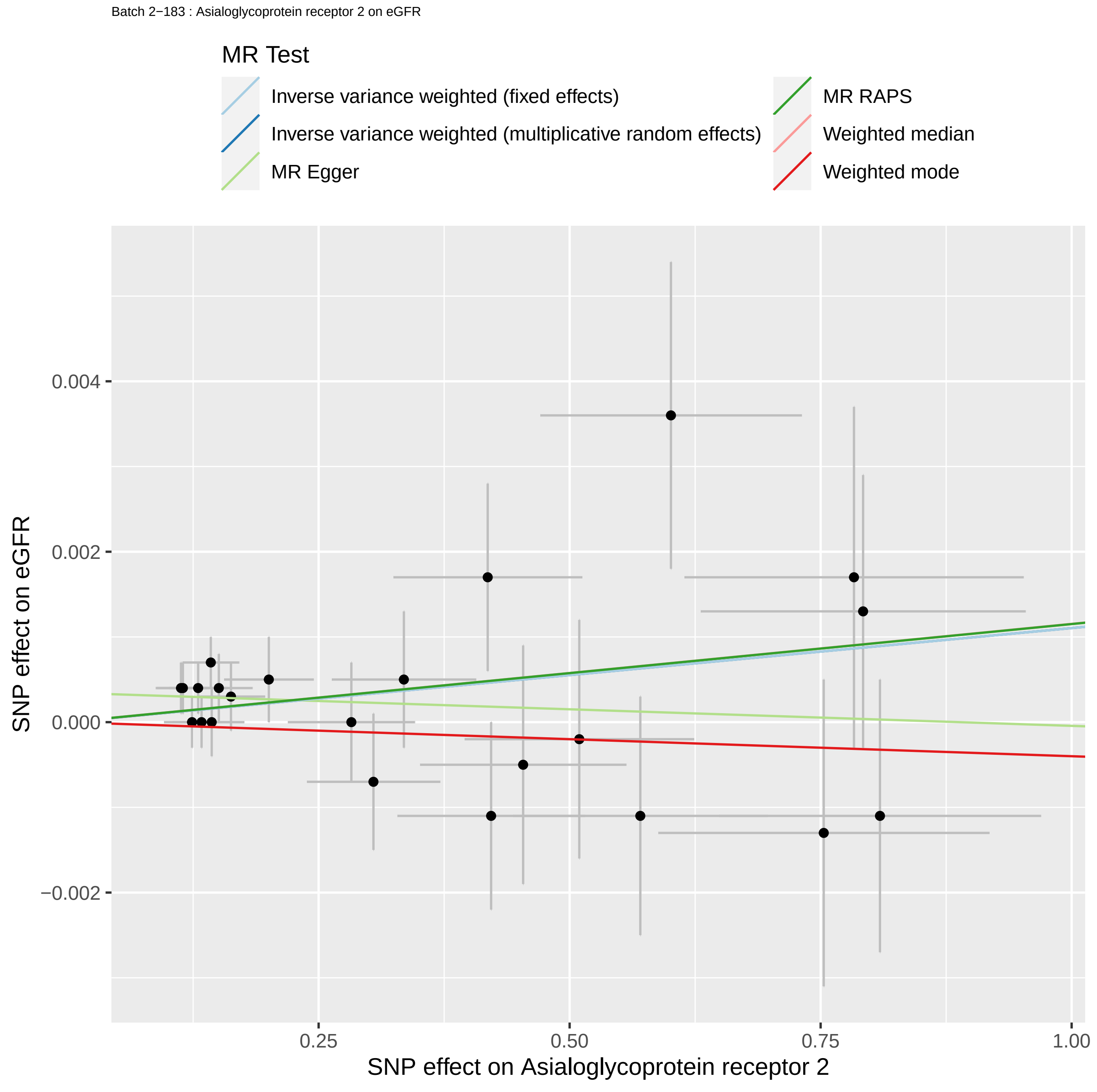

Batch 2−183 : Asialoglycoprotein receptor 2 on eGFR
MR Test
Inverse variance weighted (fixed effects)
Inverse variance weighted (multiplicative random effects)
MR Egger
MR RAPS
Weighted median
Weighted mode
0.004
0.002
0.000
−0.002
0.25
0.50
0.75
1.00
SNP effect on eGFR
SNP effect on Asialoglycoprotein receptor 2

Batch 2−184 : Histone−lysine N−methyltransferase ASH1L on eGFR

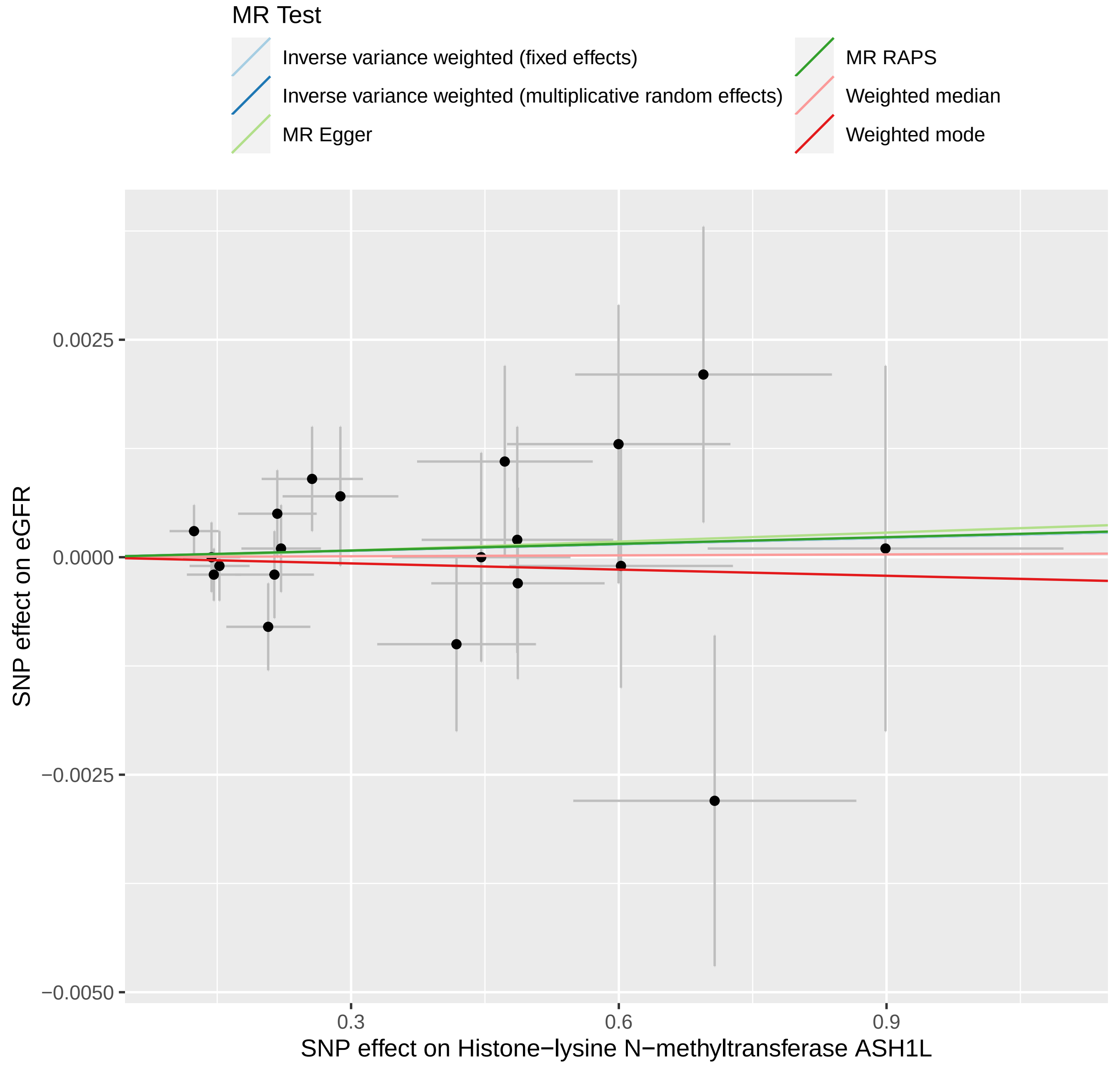

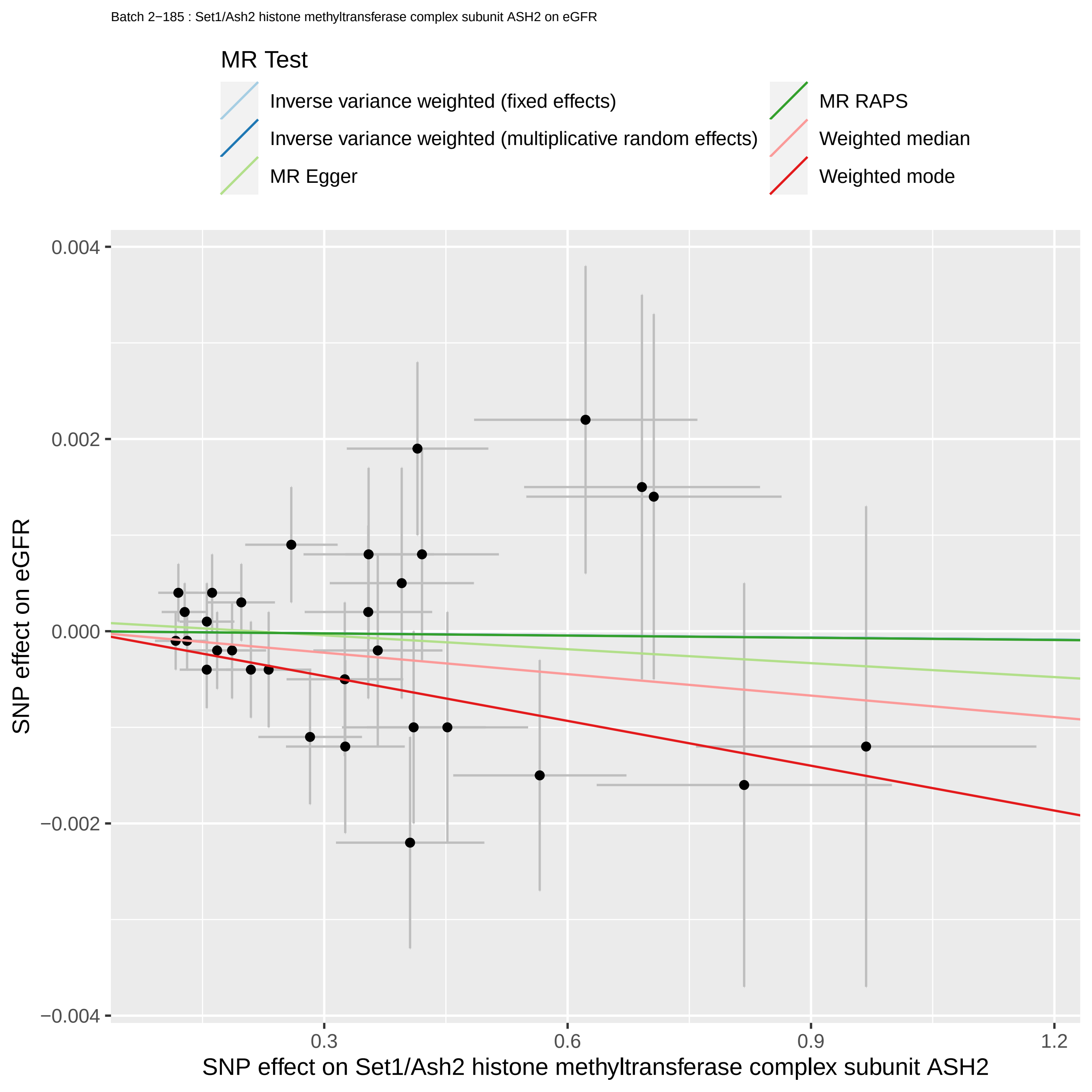

Batch 2−185 : Set1/Ash2 histone methyltransferase complex subunit ASH2 on eGFR
MR Test
Inverse variance weighted (fixed effects)
Inverse variance weighted (multiplicative random effects)
MR Egger
MR RAPS
Weighted median
Weighted mode
SNP effect on eGFR
0.004
0.002
0.000
−0.002
−0.004
0.3
0.6
0.9
1.2
SNP effect on Set1/Ash2 histone methyltransferase complex subunit ASH2

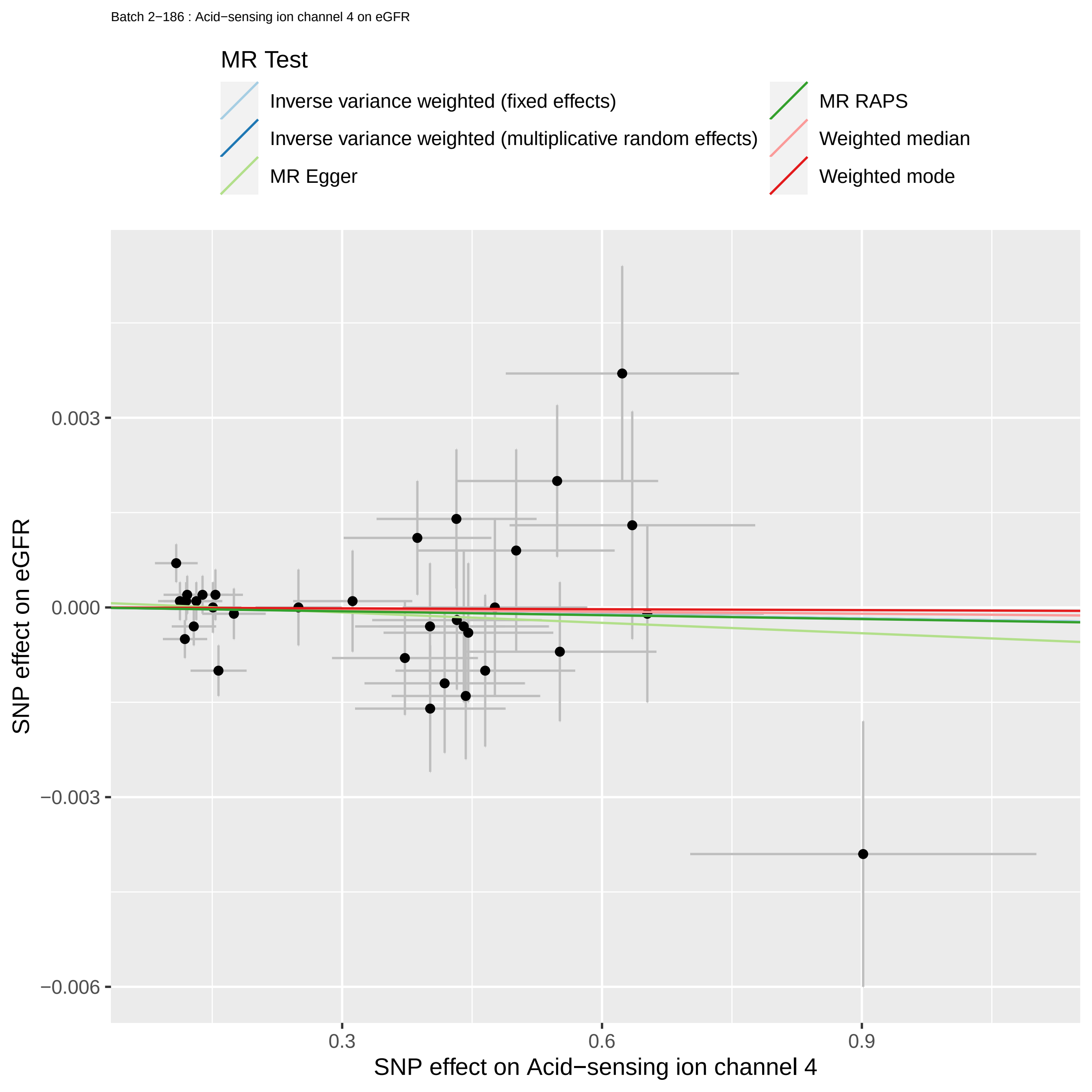
Batch 2−186 : Acid−sensing ion channel 4 on eGFR
MR Test
Inverse variance weighted (fixed effects)
Inverse variance weighted (multiplicative random effects)
MR Egger
MR RAPS
Weighted median
Weighted mode
0.003
0.000
−0.003
−0.006
0.3
0.6
0.9
SNP effect on eGFR
SNP effect on Acid−sensing ion channel 4

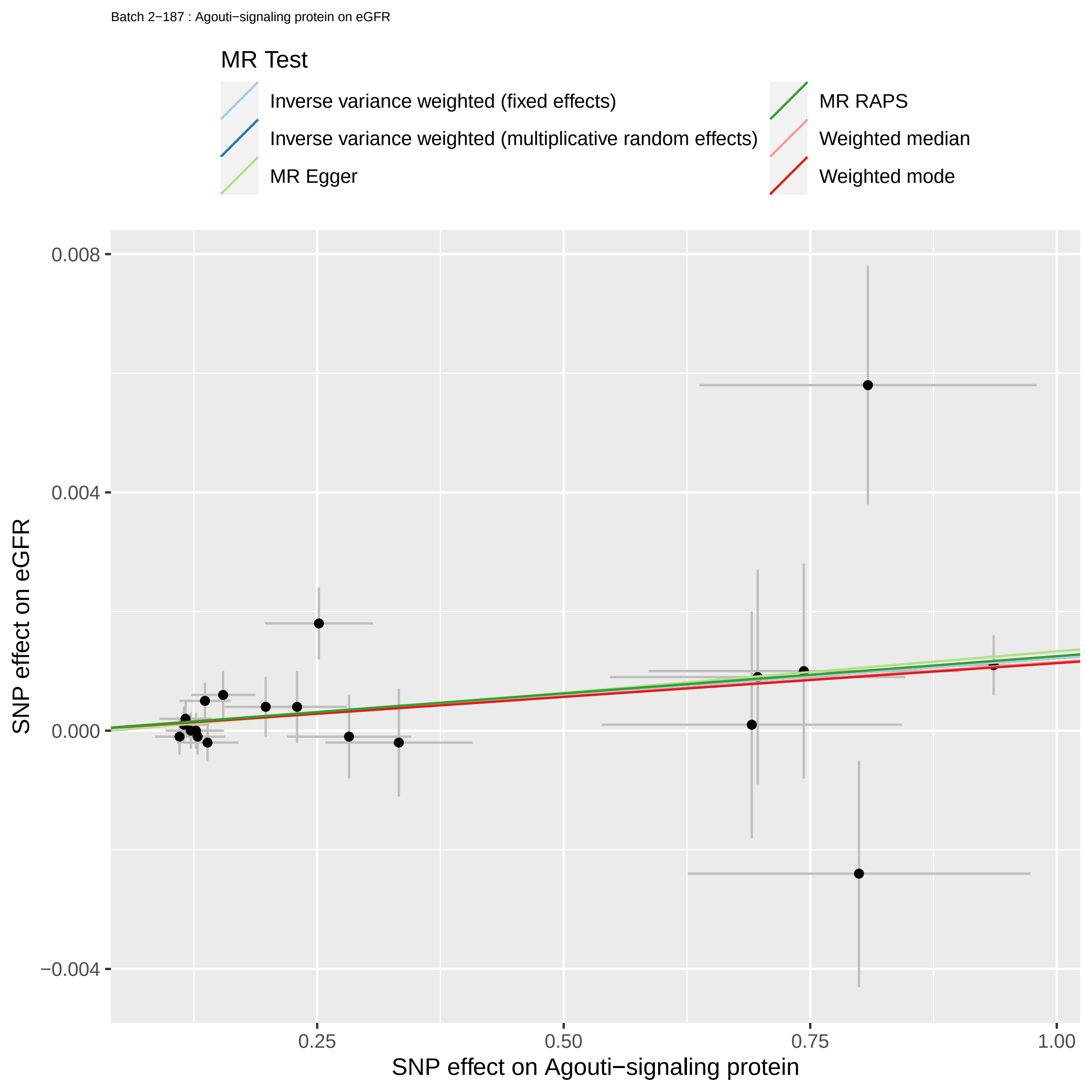
Batch 2−187 : Agouti−signaling protein on eGFR
MR Test
Inverse variance weighted (fixed effects)
Inverse variance weighted (multiplicative random effects)
MR Egger
MR RAPS
Weighted median
Weighted mode
0.008
0.004
0.000
−0.004
0.25
0.50
0.75
1.00
SNP effect on eGFR
SNP effect on Agouti−signaling protein

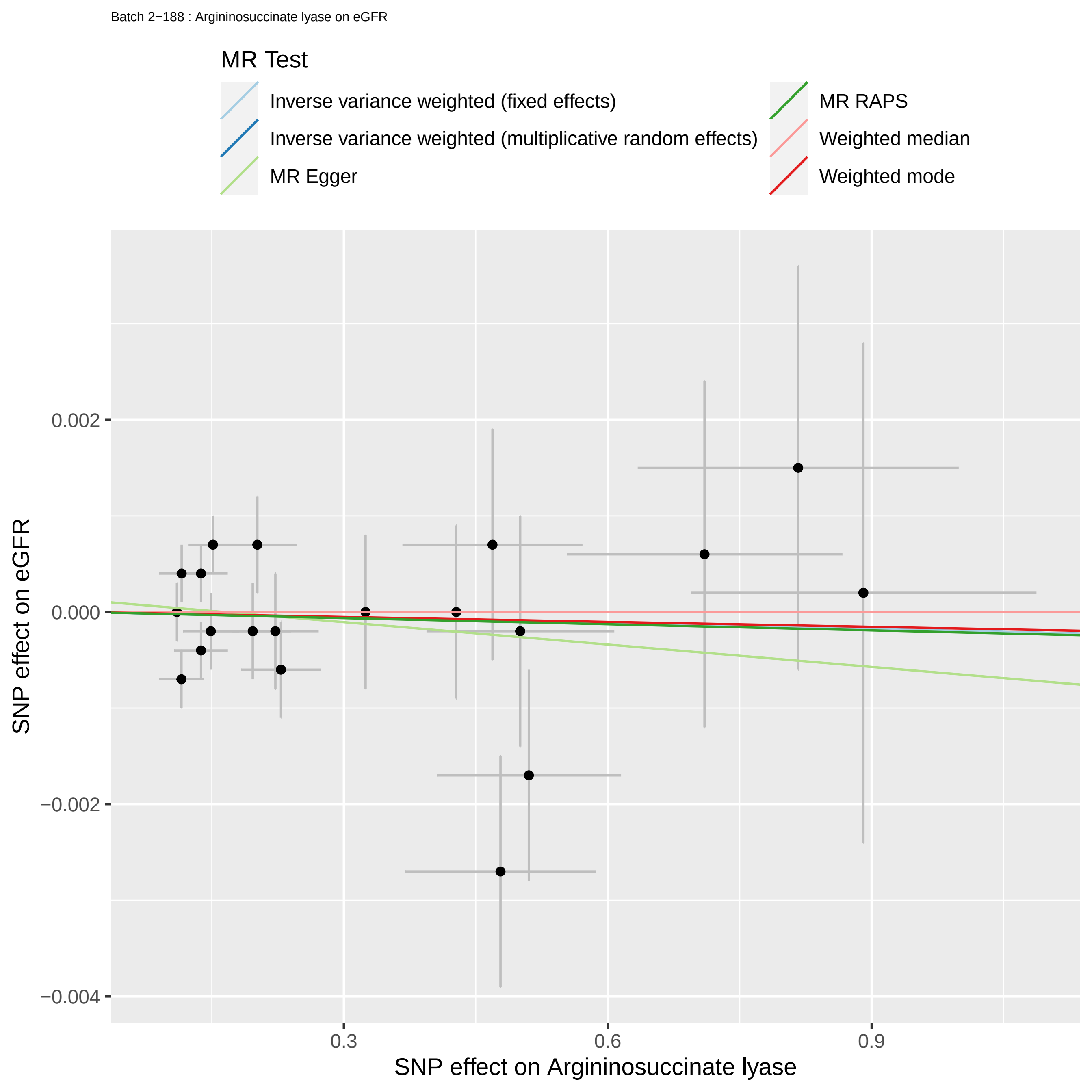

Batch 2−188 : Argininosuccinate lyase on eGFR
MR Test
Inverse variance weighted (fixed effects)
Inverse variance weighted (multiplicative random effects)
MR Egger
MR RAPS
Weighted median
Weighted mode
0.002
0.000
−0.002
−0.004
0.3
0.6
0.9
SNP effect on eGFR
SNP effect on Argininosuccinate lyase

Batch 2−189 : N−acetylserotonin O−methyltransferase−like protein on eGFR

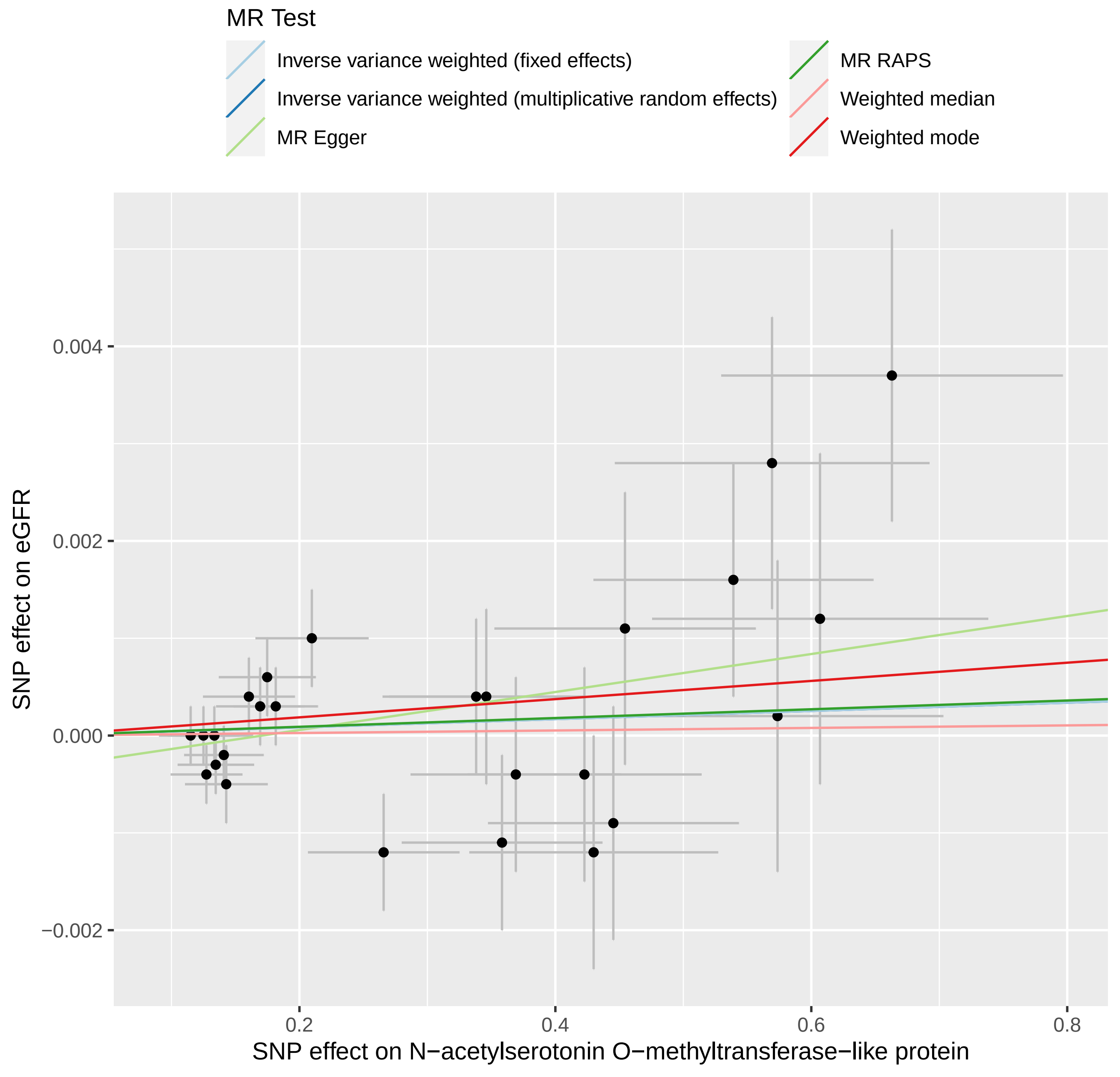

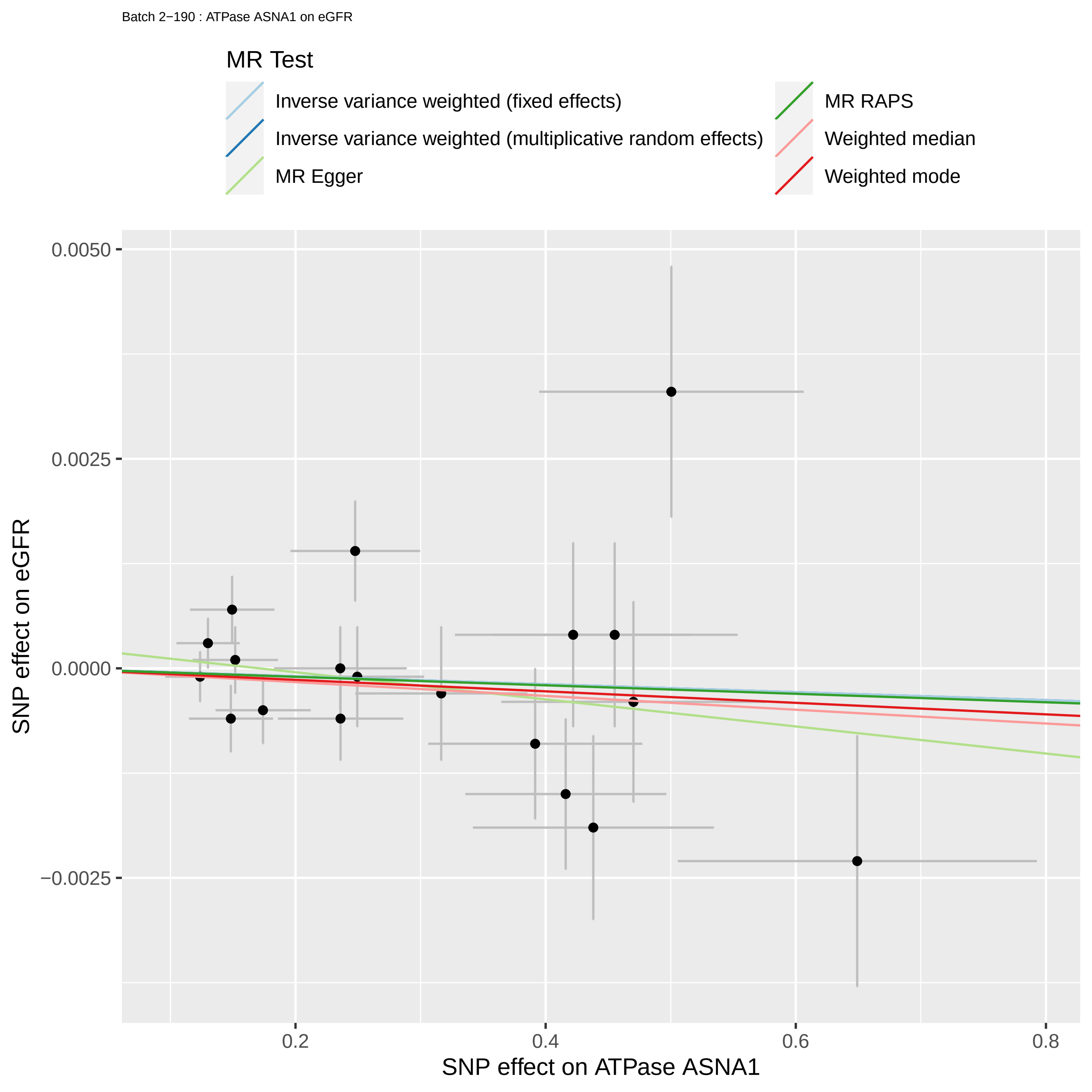

Batch 2−190 : ATPase ASNA1 on eGFR
MR Test
Inverse variance weighted (fixed effects)
Inverse variance weighted (multiplicative random effects)
MR Egger
MR RAPS
Weighted median
Weighted mode
0.0050
0.0025
0.0000
−0.0025
SNP effect on eGFR
0.2
0.4
0.6
0.8
SNP effect on ATPase ASNA1

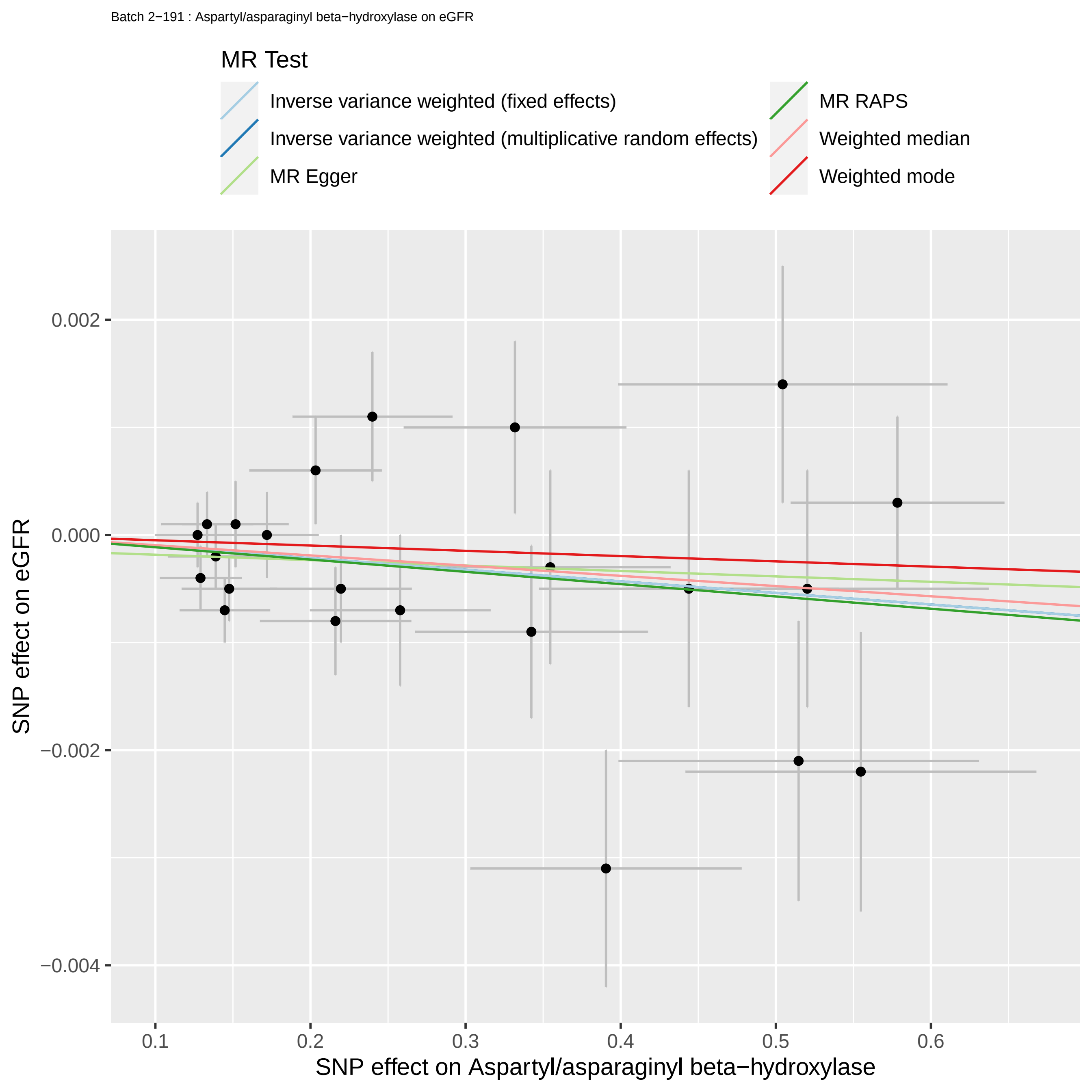

Batch 2−191 : Aspartyl/asparaginyl beta−hydroxylase on eGFR
MR Test
Inverse variance weighted (fixed effects)
Inverse variance weighted (multiplicative random effects)
MR Egger
MR RAPS
Weighted median
Weighted mode
0.002
0.000
−0.002
−0.004
SNP effect on eGFR
0.1
0.2
0.3
0.4
0.5
0.6
SNP effect on Aspartyl/asparaginyl beta−hydroxylase

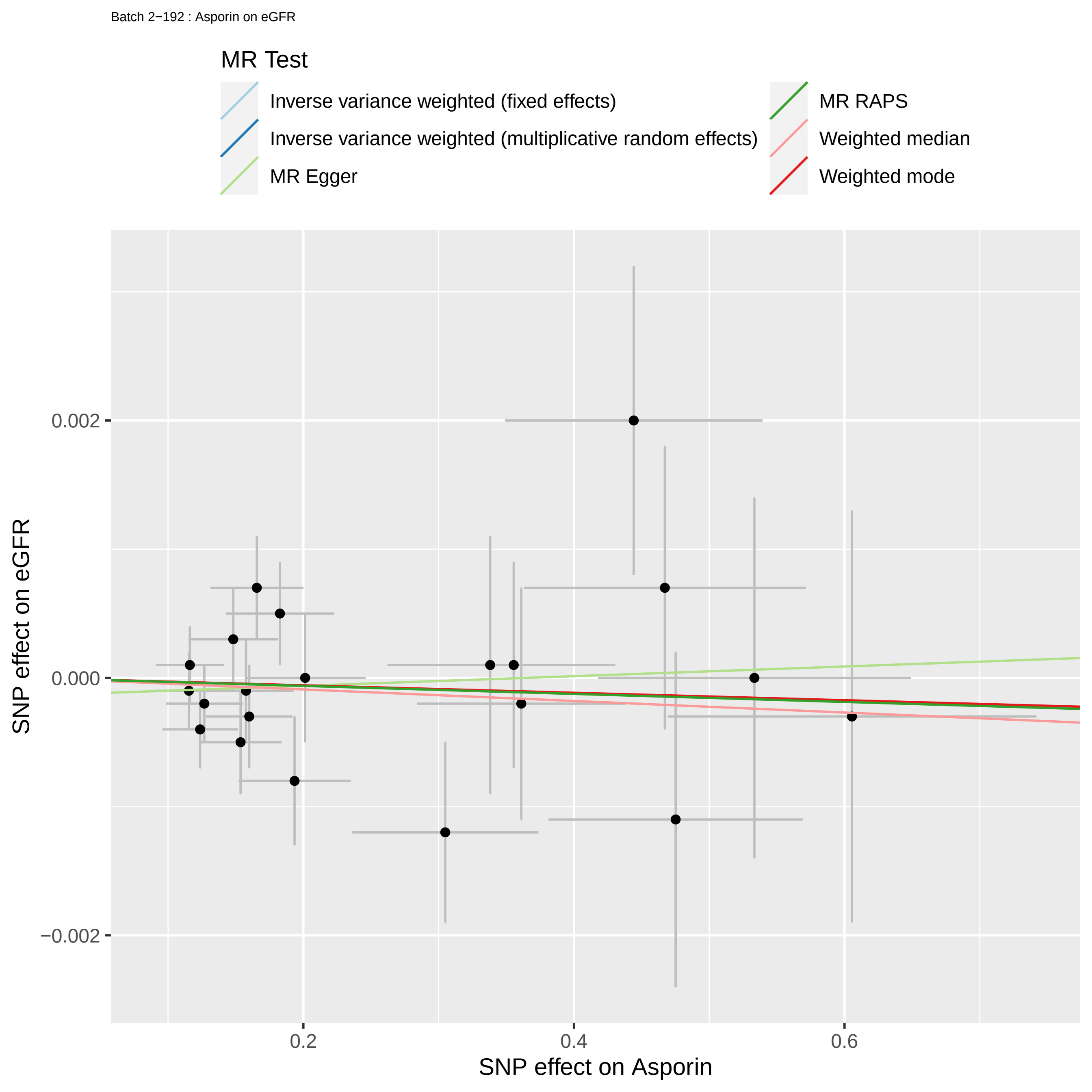

Batch 2−192 : Asporin on eGFR
MR Test
Inverse variance weighted (fixed effects)
Inverse variance weighted (multiplicative random effects)
MR Egger
MR RAPS
Weighted median
Weighted mode
0.002
0.000
−0.002
0.2
0.4
0.6
SNP effect on eGFR
SNP effect on Asporin

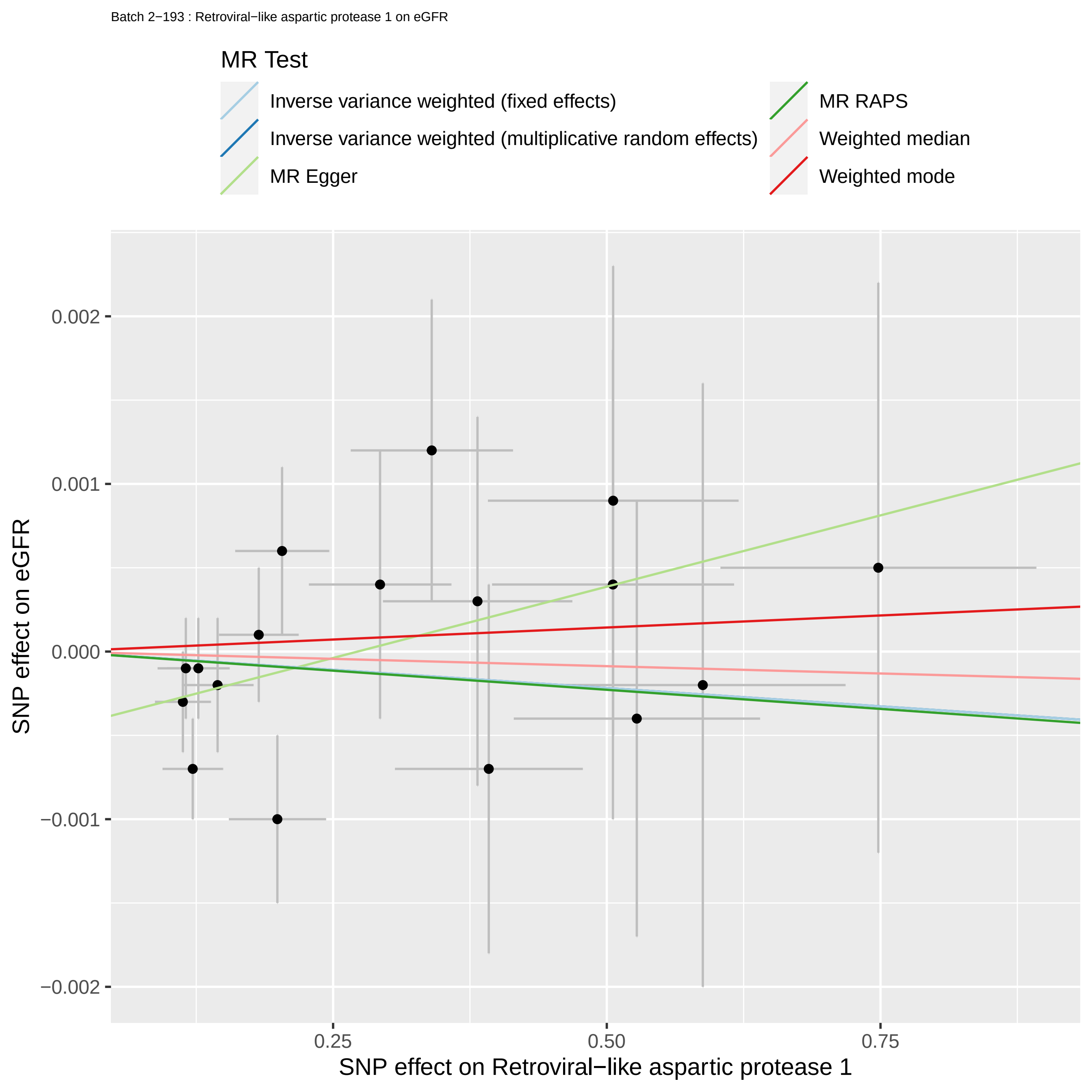

Batch 2−193 : Retroviral−like aspartic protease 1 on eGFR
MR Test
Inverse variance weighted (fixed effects)
Inverse variance weighted (multiplicative random effects)
MR Egger
MR RAPS
Weighted median
Weighted mode
0.002
0.001
0.000
−0.001
−0.002
0.25
0.50
0.75
SNP effect on eGFR
SNP effect on Retroviral−like aspartic protease 1

Batch 2−194 : Astacin−like metalloendopeptidase on eGFR

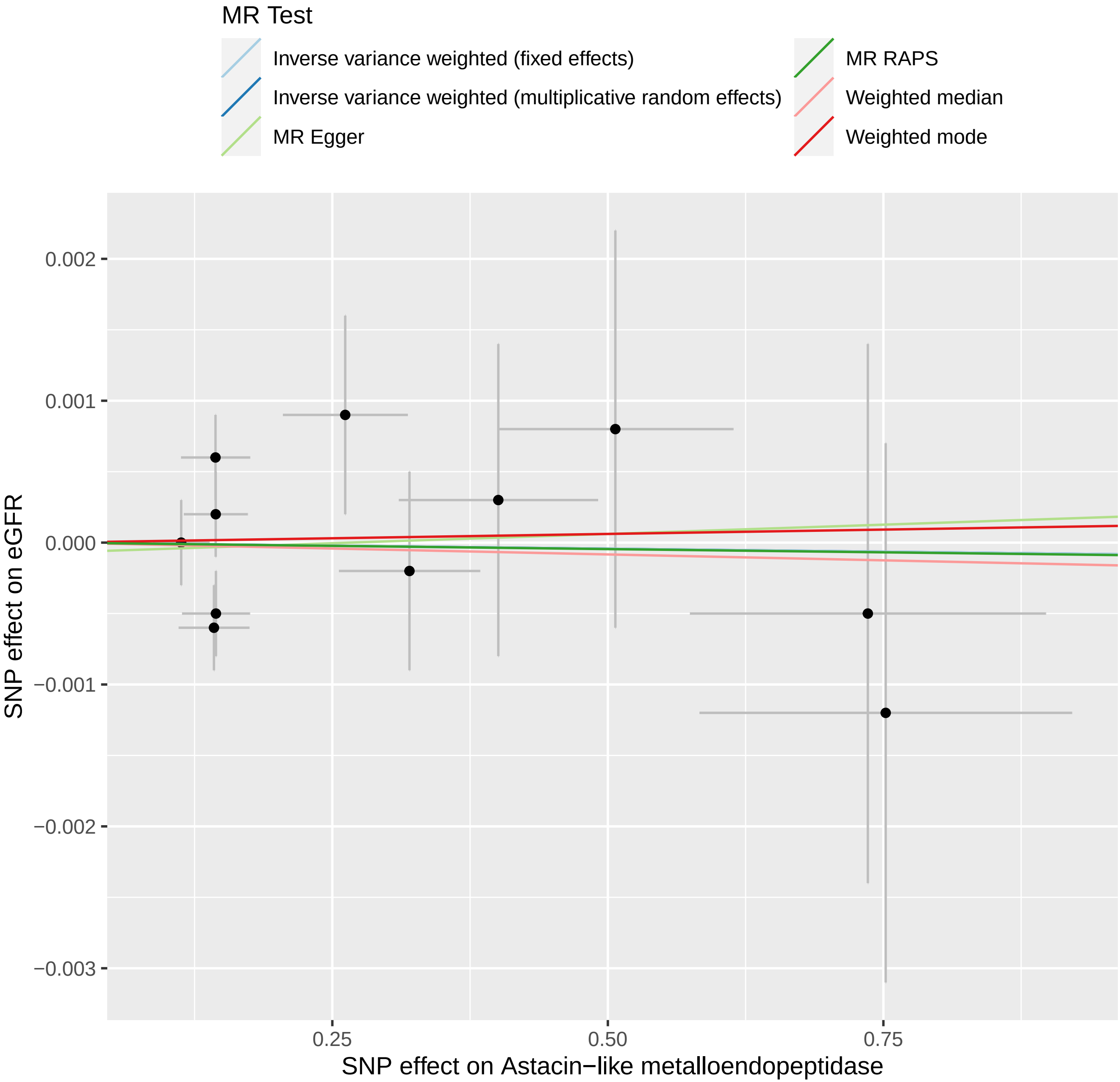

Batch 2−195 : ATPase family AAA domain−containing protein 1 on eGFR

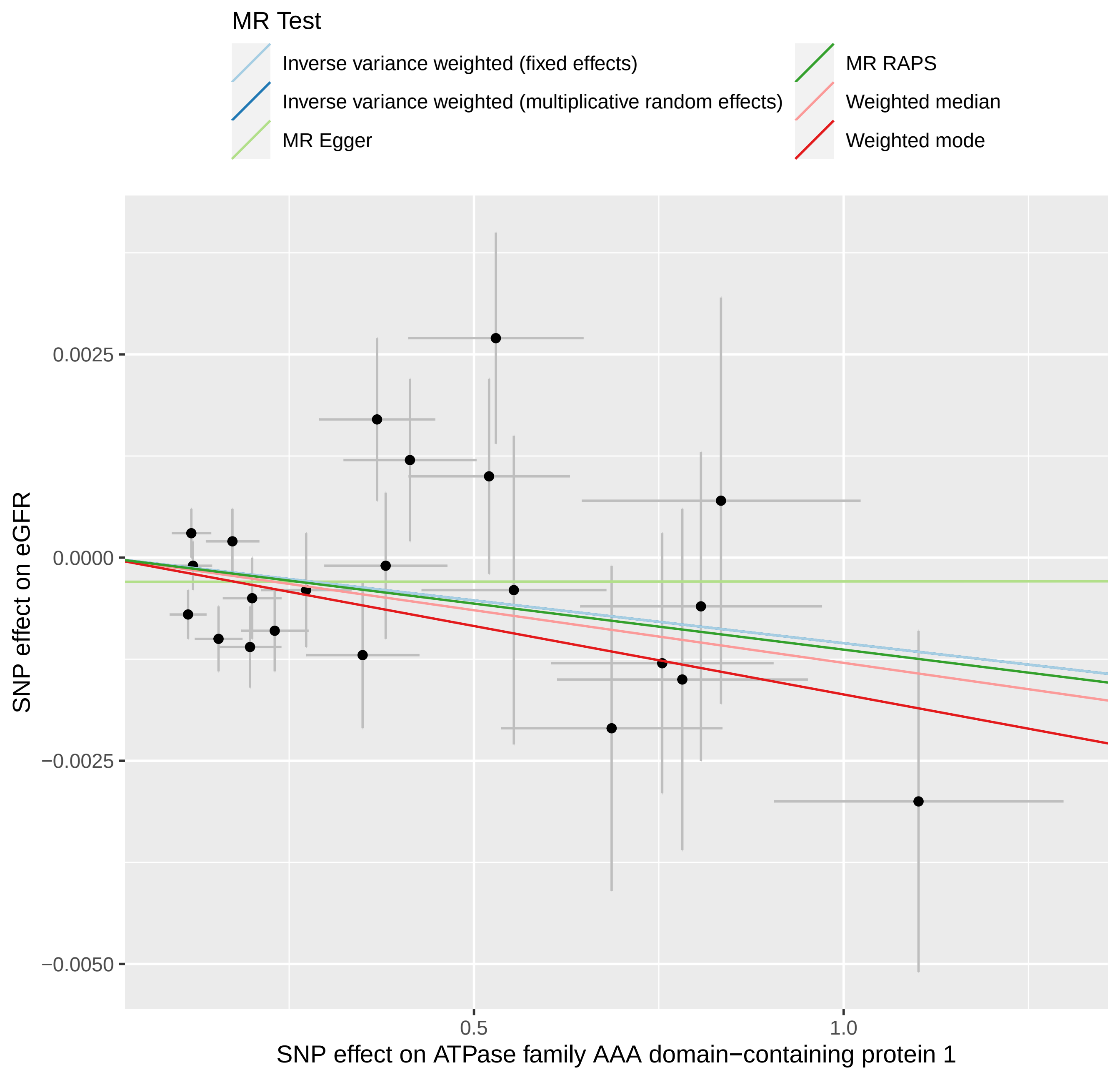

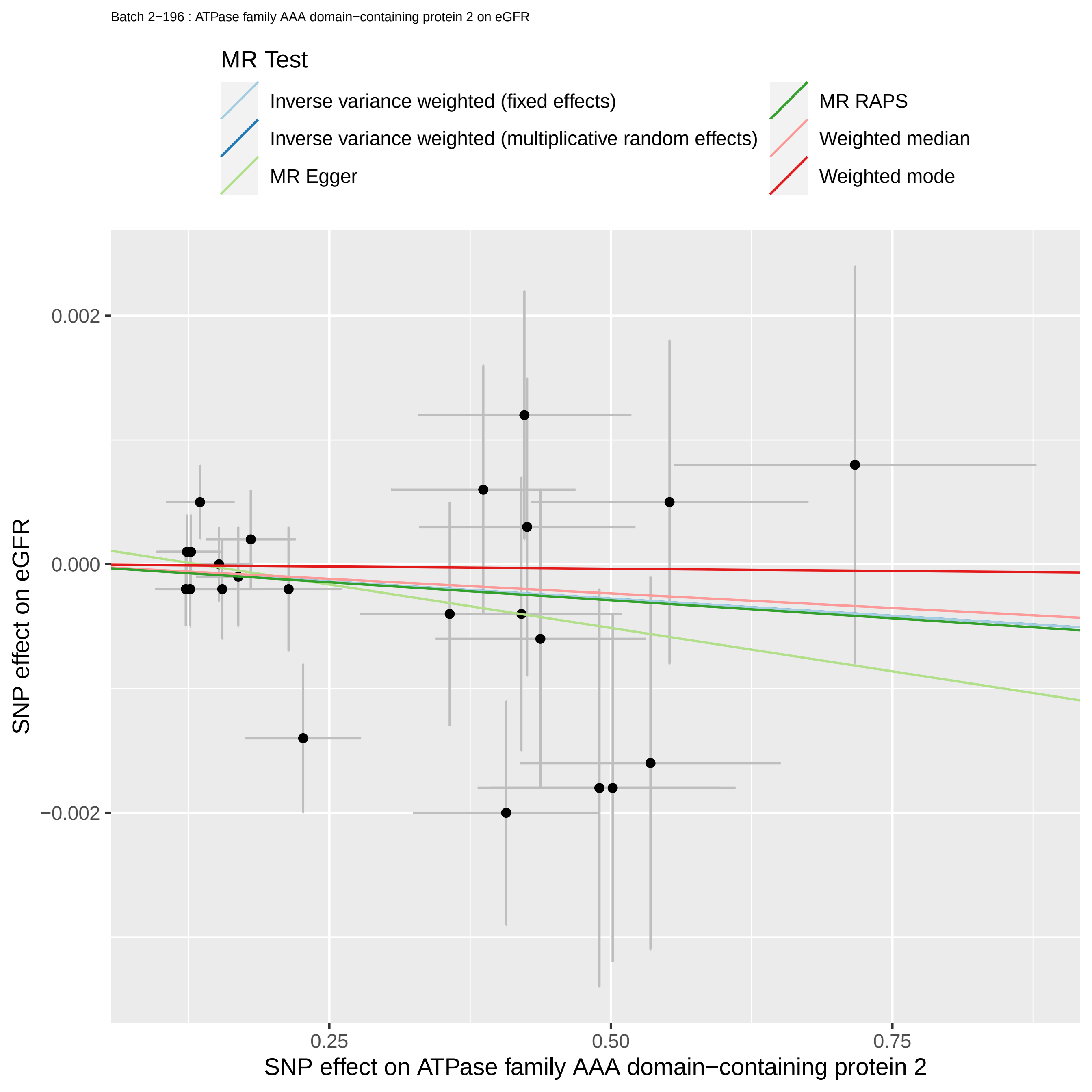
Batch 2−196 : ATPase family AAA domain−containing protein 2 on eGFR
MR Test
Inverse variance weighted (fixed effects)
Inverse variance weighted (multiplicative random effects)
MR Egger
MR RAPS
Weighted median
Weighted mode
0.002
0.000
−0.002
0.25
0.50
0.75
SNP effect on eGFR
SNP effect on ATPase family AAA domain−containing protein 2

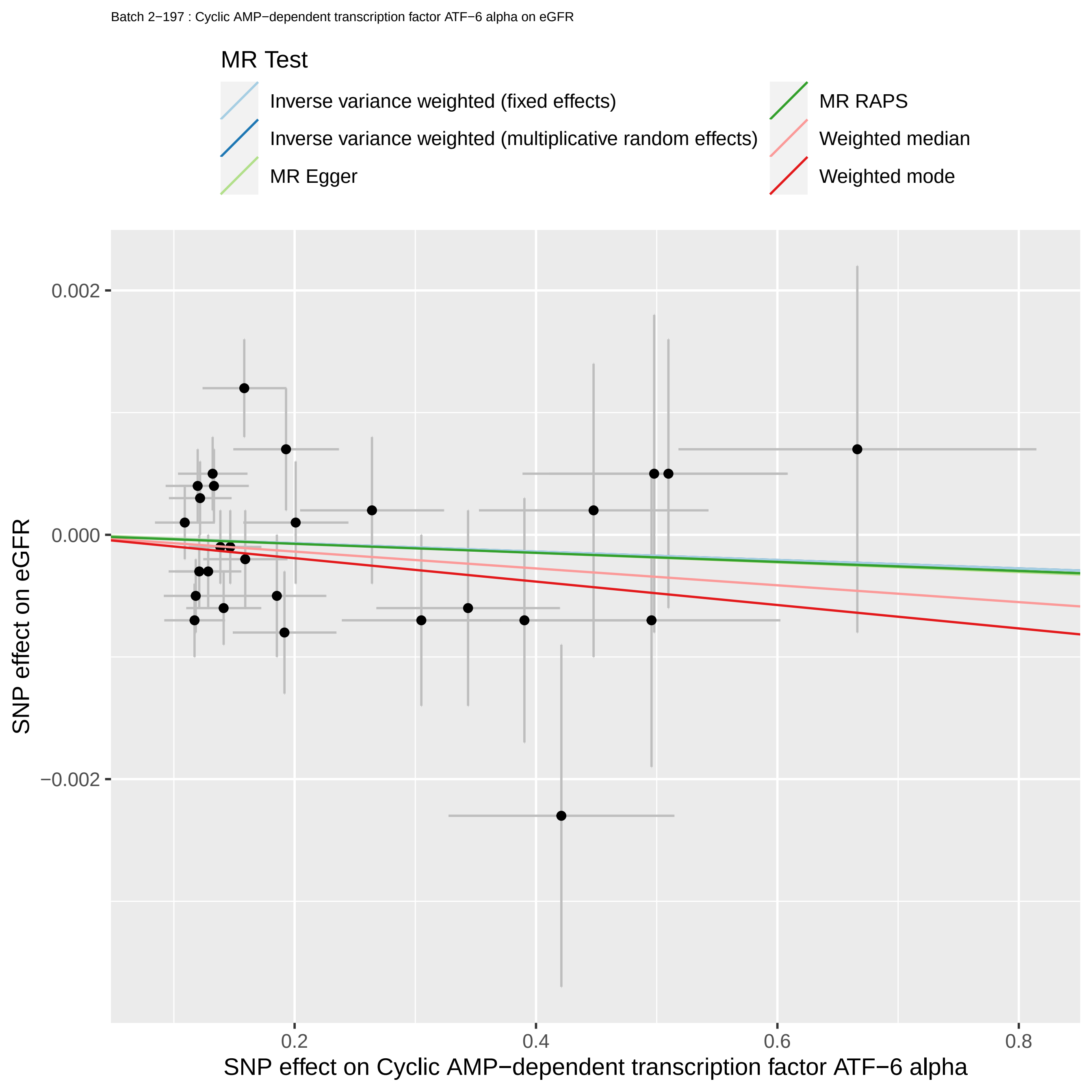
Batch 2−197 : Cyclic AMP−dependent transcription factor ATF−6 alpha on eGFR
MR Test
Inverse variance weighted (fixed effects)
Inverse variance weighted (multiplicative random effects)
MR Egger
MR RAPS
Weighted median
Weighted mode
0.002
0.000
−0.002
SNP effect on eGFR
0.2
0.4
0.6
0.8
SNP effect on Cyclic AMP−dependent transcription factor ATF−6 alpha

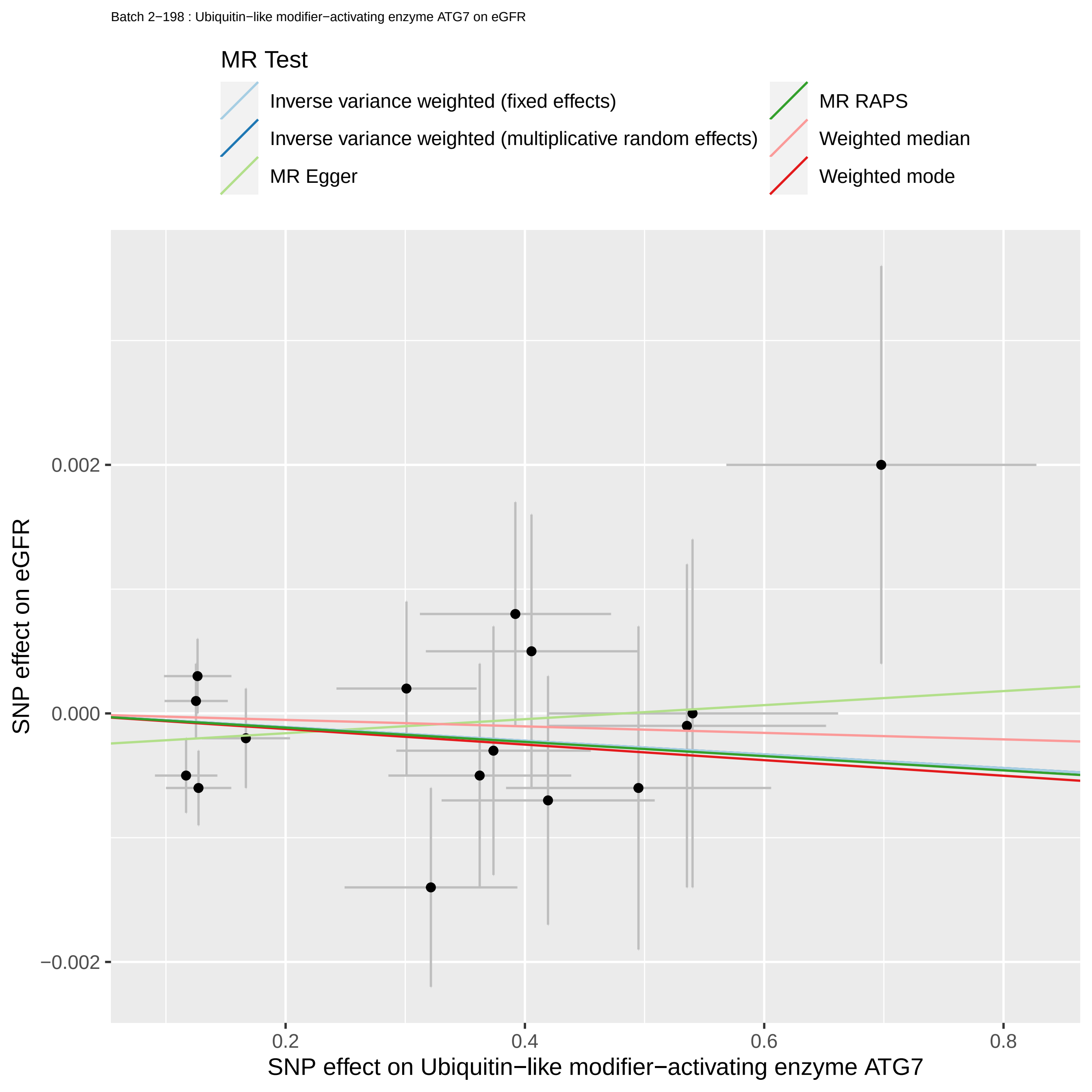
Batch 2−198 : Ubiquitin−like modifier−activating enzyme ATG7 on eGFR
MR Test
Inverse variance weighted (fixed effects)
Inverse variance weighted (multiplicative random effects)
MR Egger
MR RAPS
Weighted median
Weighted mode
0.002
0.000
−0.002
0.2
0.4
0.6
0.8
SNP effect on eGFR
SNP effect on Ubiquitin−like modifier−activating enzyme ATG7

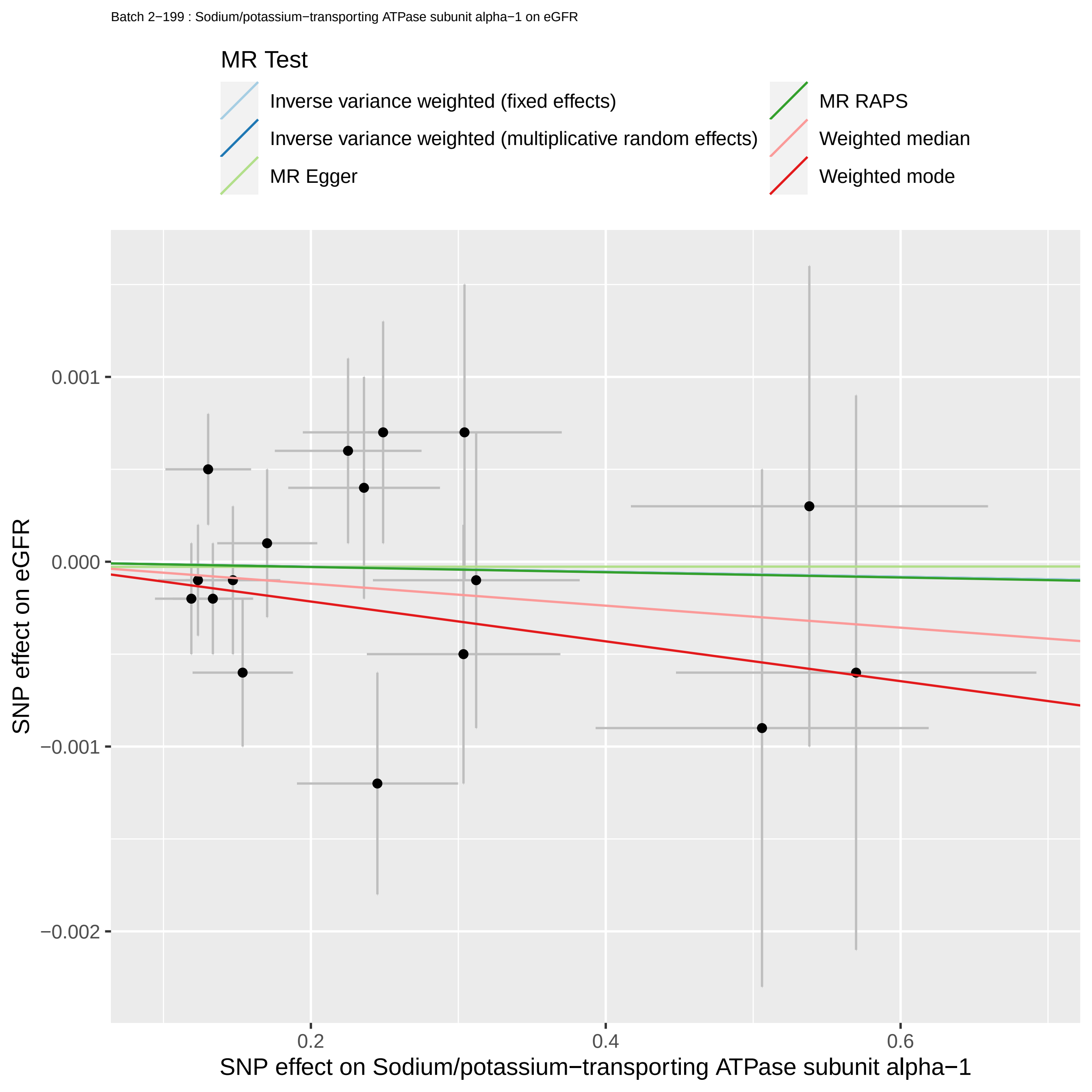
Batch 2−199 : Sodium/potassium−transporting ATPase subunit alpha−1 on eGFR
MR Test
Inverse variance weighted (fixed effects)
Inverse variance weighted (multiplicative random effects)
MR Egger
MR RAPS
Weighted median
Weighted mode
0.001
0.000
−0.001
−0.002
0.2
0.4
0.6
SNP effect on eGFR
SNP effect on Sodium/potassium−transporting ATPase subunit alpha−1

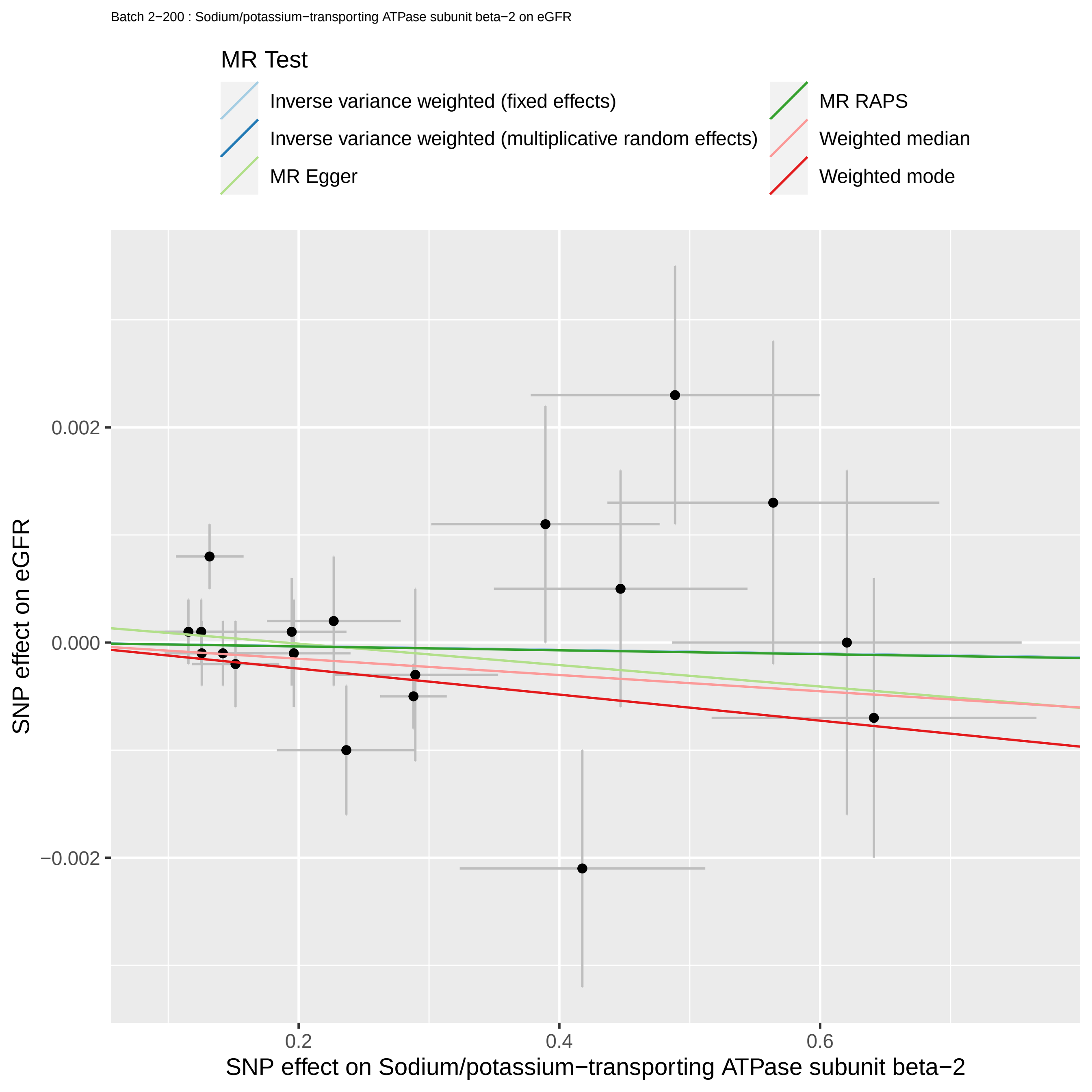
Batch 2−200 : Sodium/potassium−transporting ATPase subunit beta−2 on eGFR
MR Test
Inverse variance weighted (fixed effects)
Inverse variance weighted (multiplicative random effects)
MR Egger
MR RAPS
Weighted median
Weighted mode
0.002
0.000
−0.002
0.2
0.4
0.6
SNP effect on eGFR
SNP effect on Sodium/potassium−transporting ATPase subunit beta−2

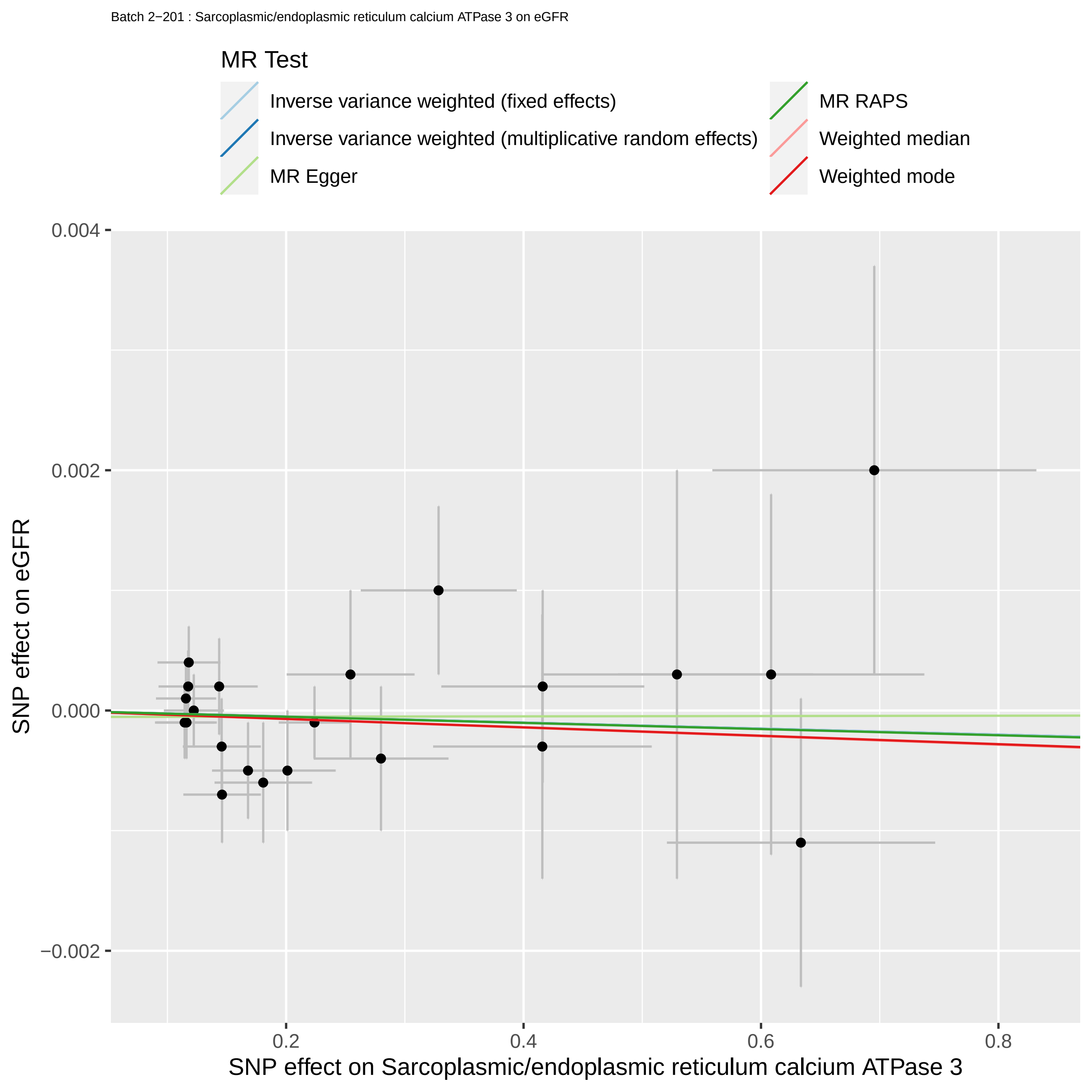

Batch 2−201 : Sarcoplasmic/endoplasmic reticulum calcium ATPase 3 on eGFR
MR Test
Inverse variance weighted (fixed effects)
Inverse variance weighted (multiplicative random effects)
MR Egger
MR RAPS
Weighted median
Weighted mode
0.004
0.002
0.000
−0.002
0.2
0.4
0.6
0.8
SNP effect on eGFR
SNP effect on Sarcoplasmic/endoplasmic reticulum calcium ATPase 3

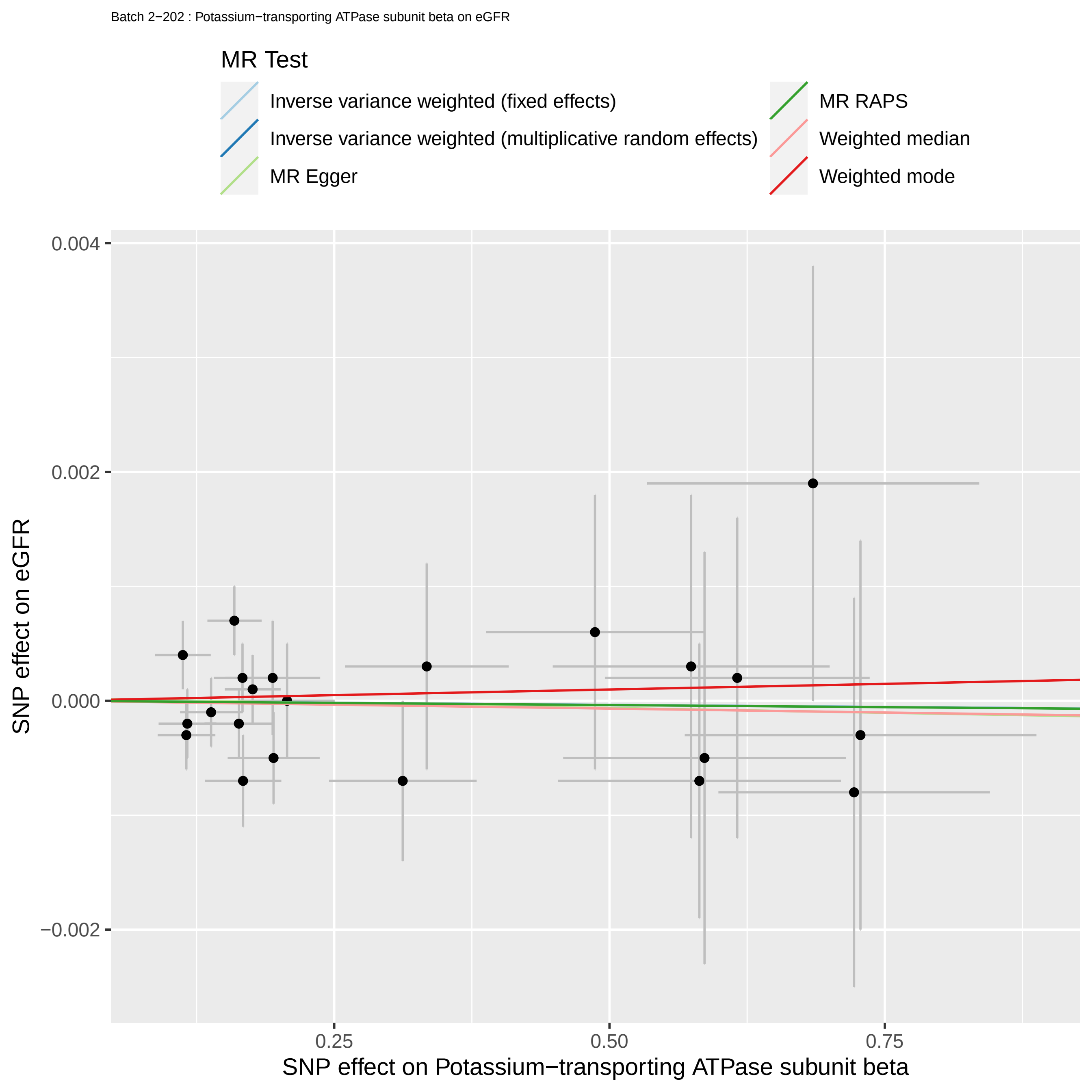

Batch 2−202 : Potassium−transporting ATPase subunit beta on eGFR
MR Test
Inverse variance weighted (fixed effects)
Inverse variance weighted (multiplicative random effects)
MR Egger
MR RAPS
Weighted median
Weighted mode
0.004
0.002
0.000
−0.002
0.25
0.50
0.75
SNP effect on eGFR
SNP effect on Potassium−transporting ATPase subunit beta

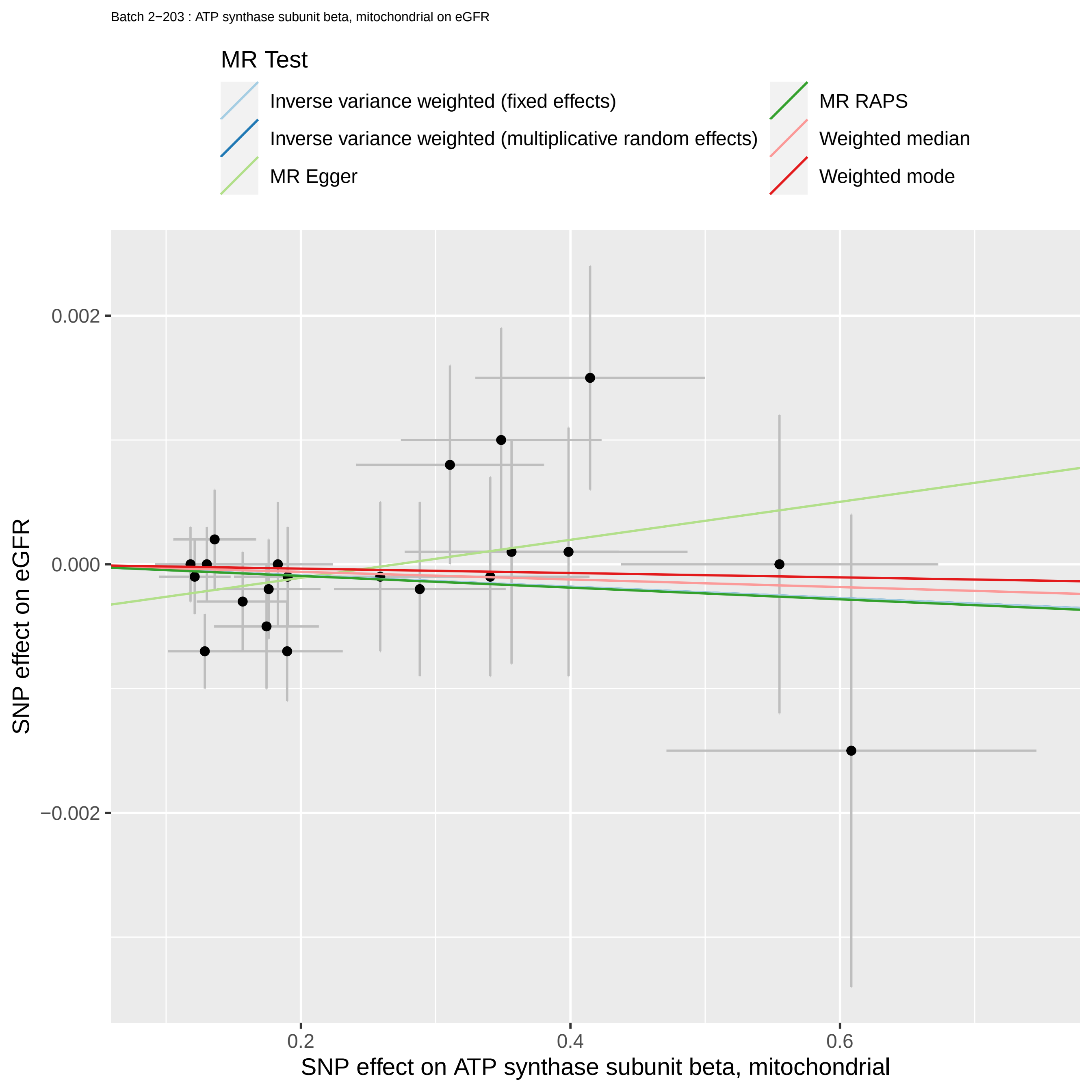

Batch 2−203 : ATP synthase subunit beta, mitochondrial on eGFR
MR Test
Inverse variance weighted (fixed effects)
Inverse variance weighted (multiplicative random effects)
MR Egger
MR RAPS
Weighted median
Weighted mode
0.002
0.000
−0.002
0.2
0.4
0.6
SNP effect on eGFR
SNP effect on ATP synthase subunit beta, mitochondrial

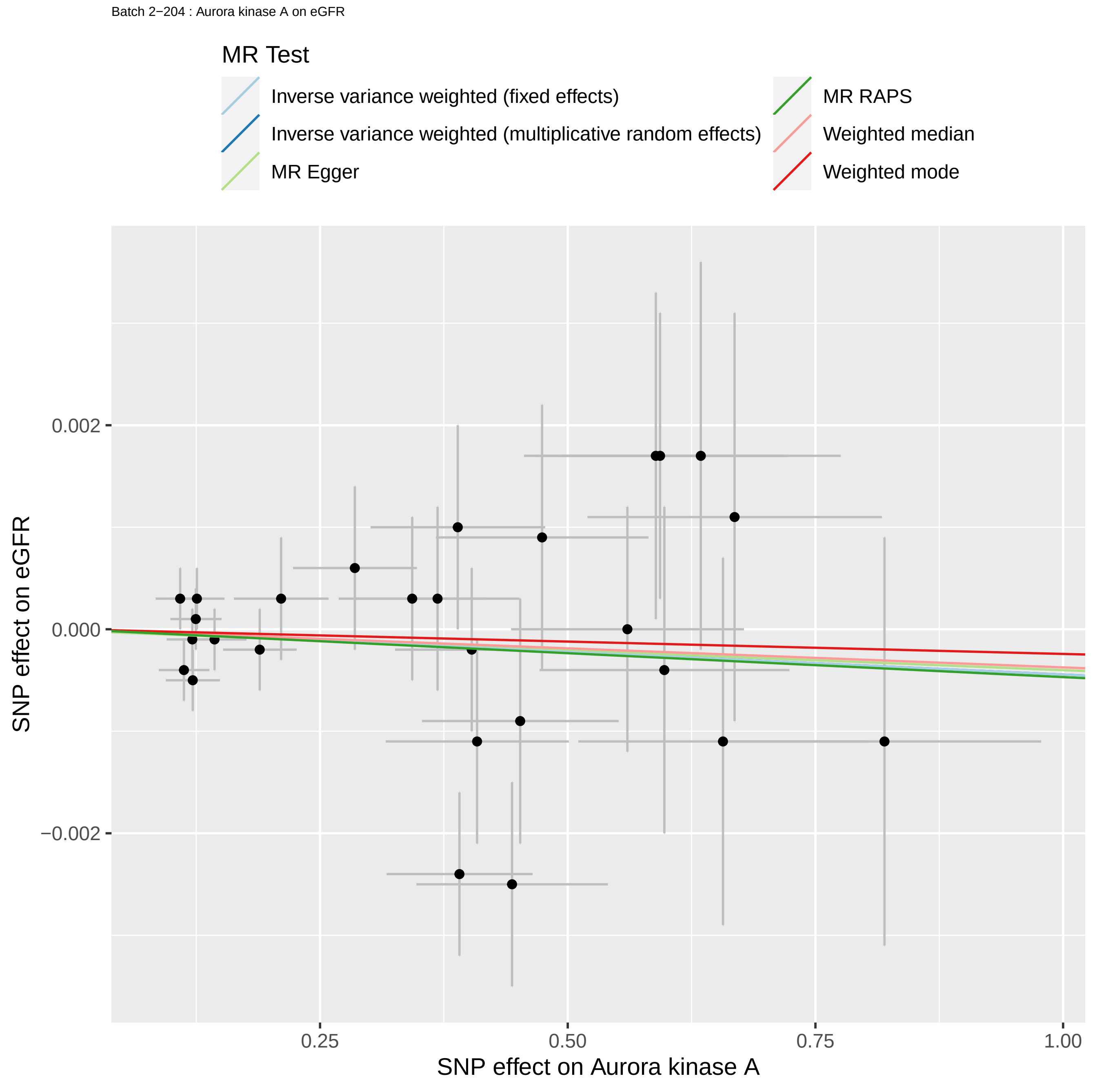
Batch 2−204 : Aurora kinase A on eGFR
MR Test
Inverse variance weighted (fixed effects)
Inverse variance weighted (multiplicative random effects)
MR Egger
MR RAPS
Weighted median
Weighted mode
0.002
0.000
−0.002
0.25
0.50
0.75
1.00
SNP effect on eGFR
SNP effect on Aurora kinase A

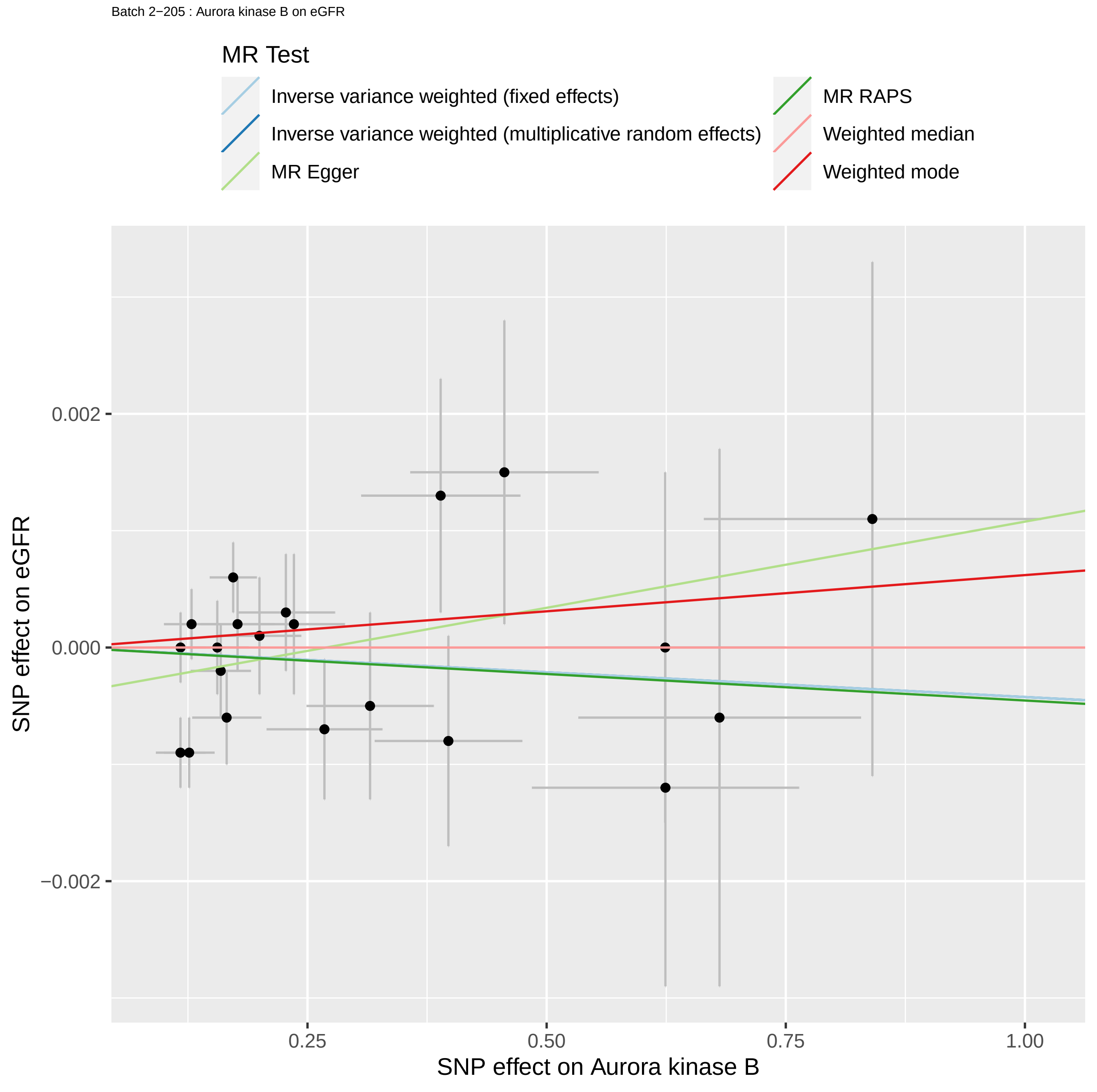
Batch 2−205 : Aurora kinase B on eGFR
MR Test
Inverse variance weighted (fixed effects)
Inverse variance weighted (multiplicative random effects)
MR Egger
MR RAPS
Weighted median
Weighted mode
0.002
0.000
−0.002
0.25
0.50
0.75
1.00
SNP effect on eGFR
SNP effect on Aurora kinase B

Batch 2−206 : Axin−2 on eGFR

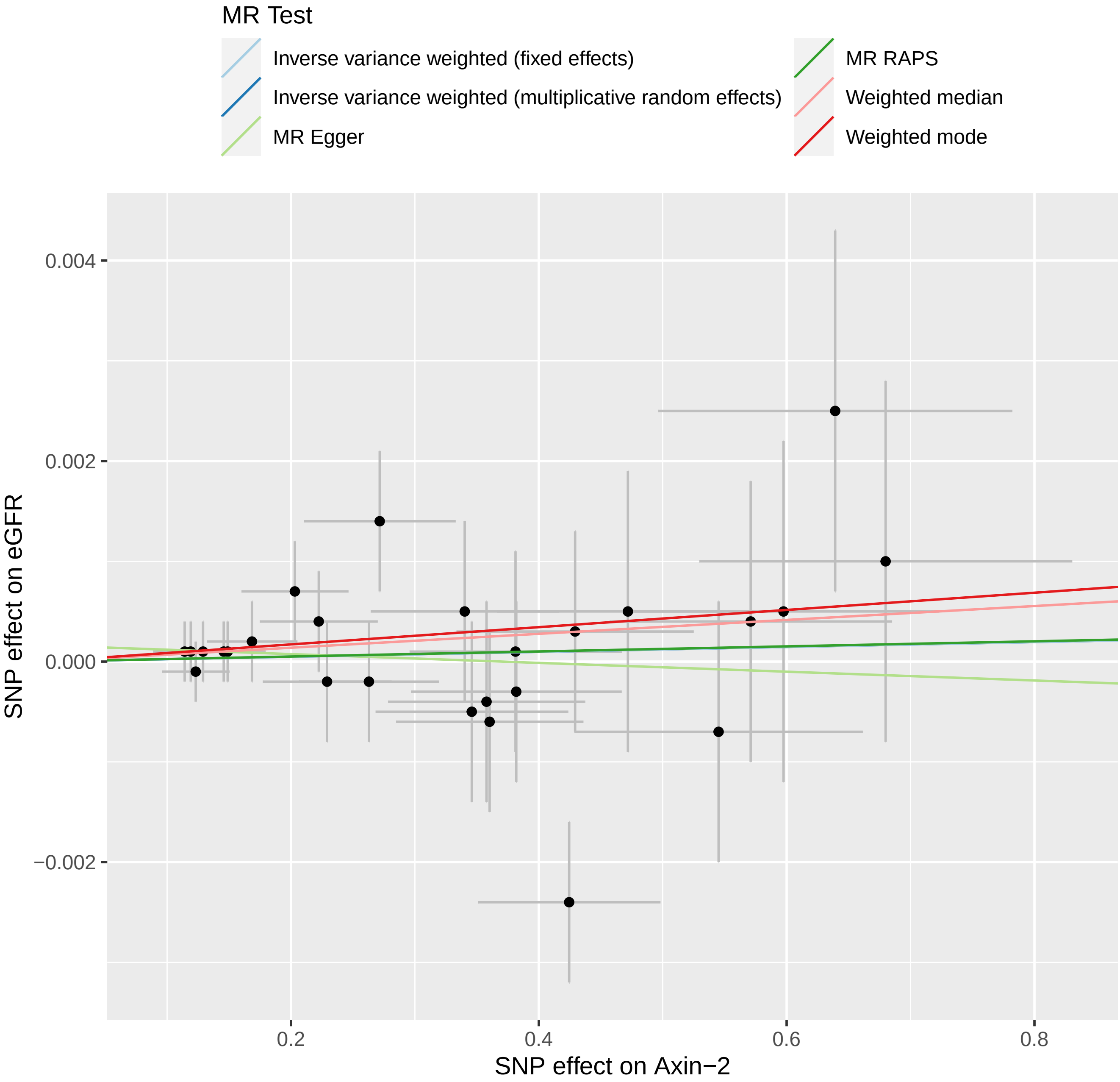

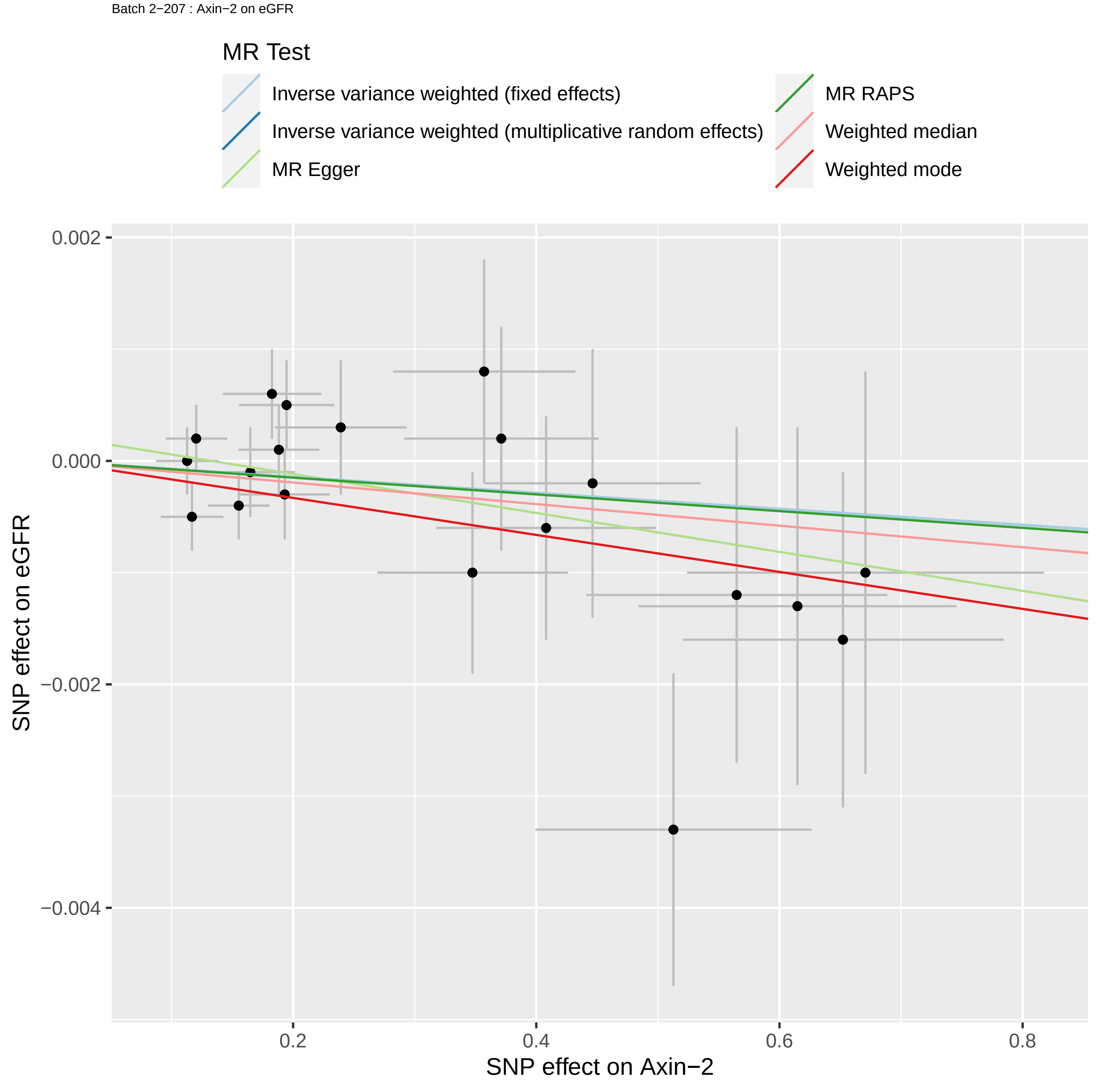

Batch 2−207 : Axin−2 on eGFR
MR Test
Inverse variance weighted (fixed effects)
Inverse variance weighted (multiplicative random effects)
MR Egger
MR RAPS
Weighted median
Weighted mode
0.002
0.000
−0.002
−0.004
SNP effect on eGFR
0.2
0.4
0.6
0.8
SNP effect on Axin−2

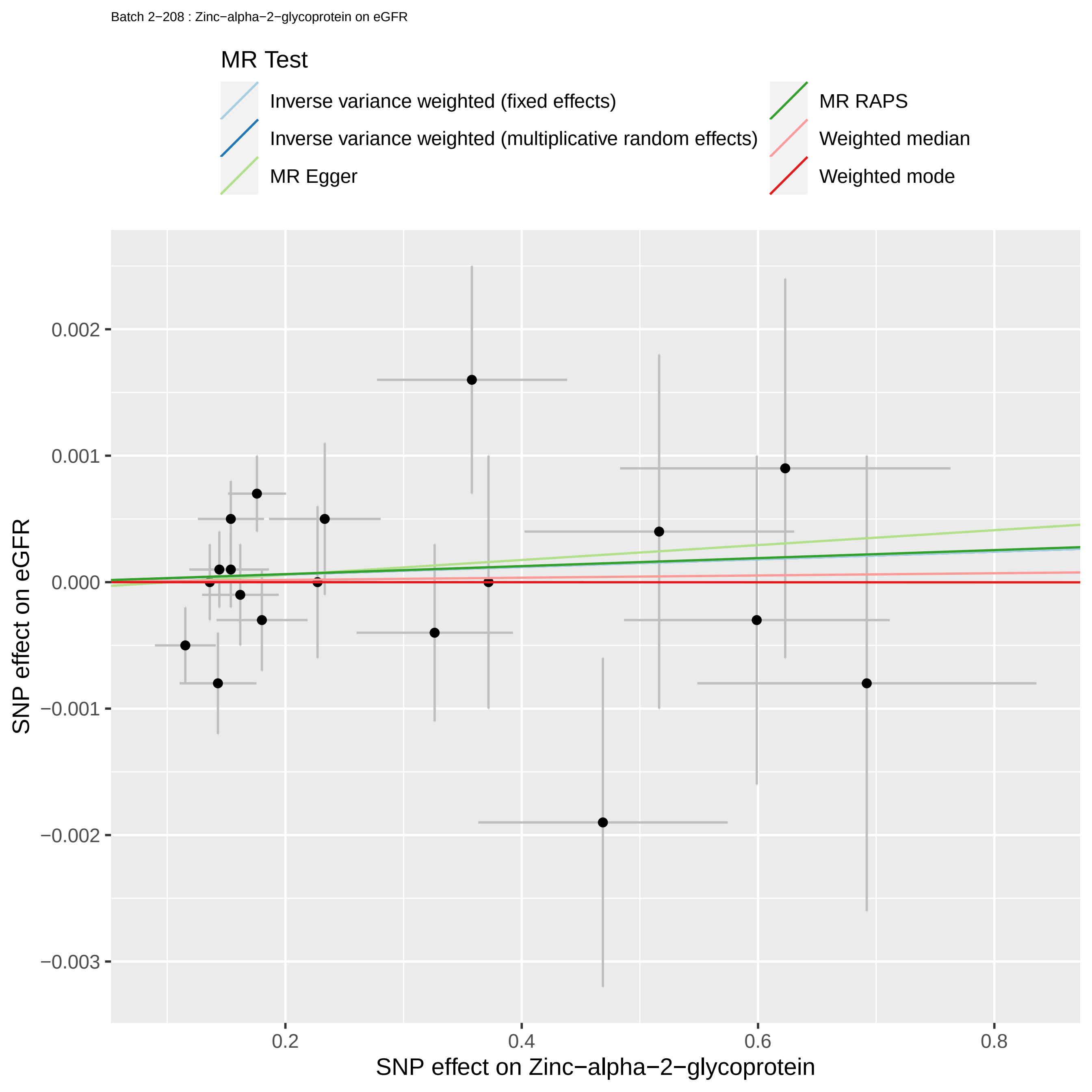

Batch 2−208 : Zinc−alpha−2−glycoprotein on eGFR
MR Test
Inverse variance weighted (fixed effects)
Inverse variance weighted (multiplicative random effects)
MR Egger
MR RAPS
Weighted median
Weighted mode
SNP effect on eGFR
0.002
0.001
0.000
−0.001
−0.002
−0.003
0.2
0.4
0.6
0.8
SNP effect on Zinc−alpha−2−glycoprotein

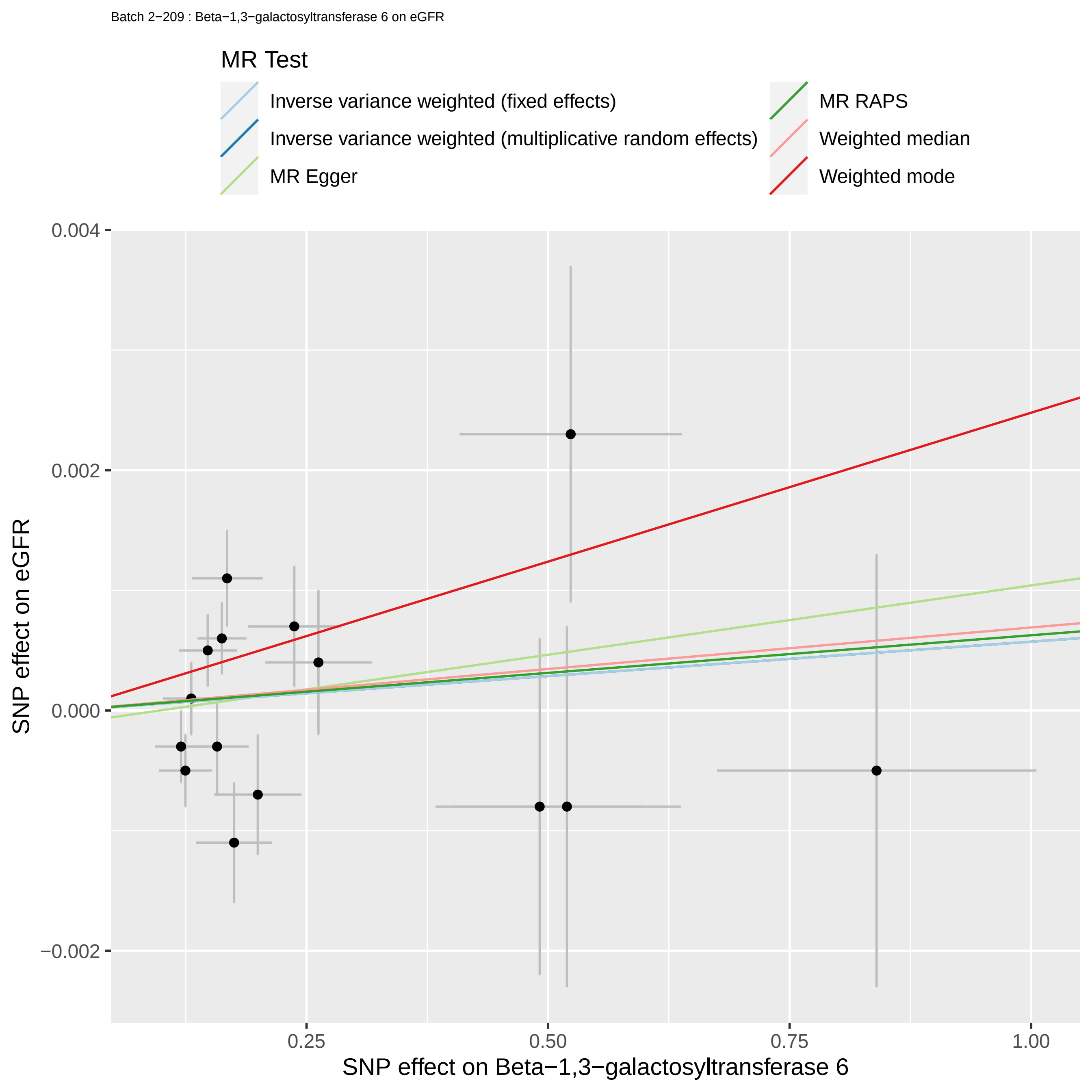

Batch 2−209 : Beta−1,3−galactosyltransferase 6 on eGFR
MR Test
Inverse variance weighted (fixed effects)
Inverse variance weighted (multiplicative random effects)
MR Egger
MR RAPS
Weighted median
Weighted mode
0.004
0.002
0.000
−0.002
0.25
0.50
0.75
1.00
SNP effect on eGFR
SNP effect on Beta−1,3−galactosyltransferase 6

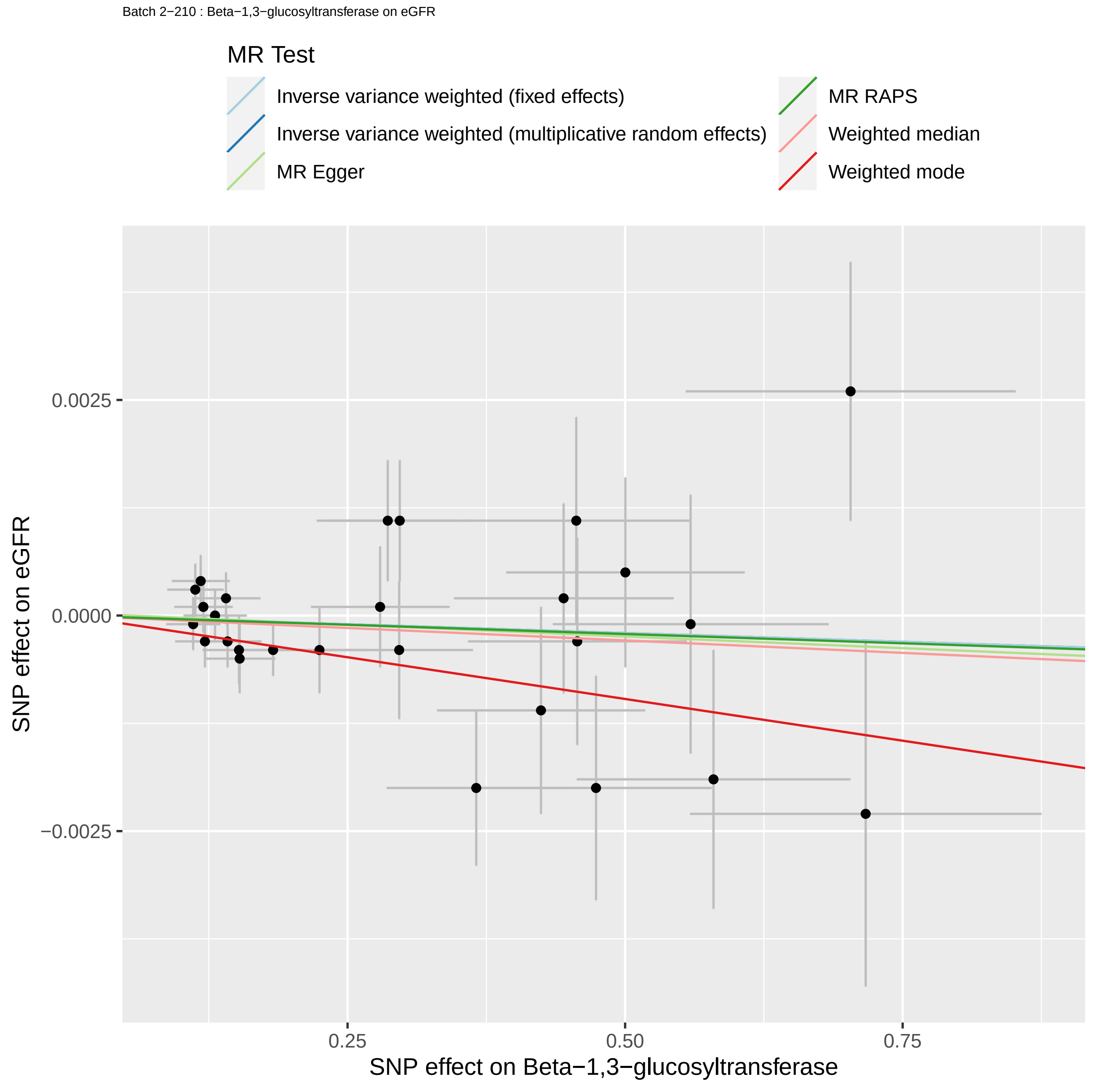

Batch 2−210 : Beta−1,3−glucosyltransferase on eGFR
MR Test
Inverse variance weighted (fixed effects)
Inverse variance weighted (multiplicative random effects)
MR Egger
MR RAPS
Weighted median
Weighted mode
0.0025
0.0000
−0.0025
0.25
0.50
0.75
SNP effect on eGFR
SNP effect on Beta−1,3−glucosyltransferase

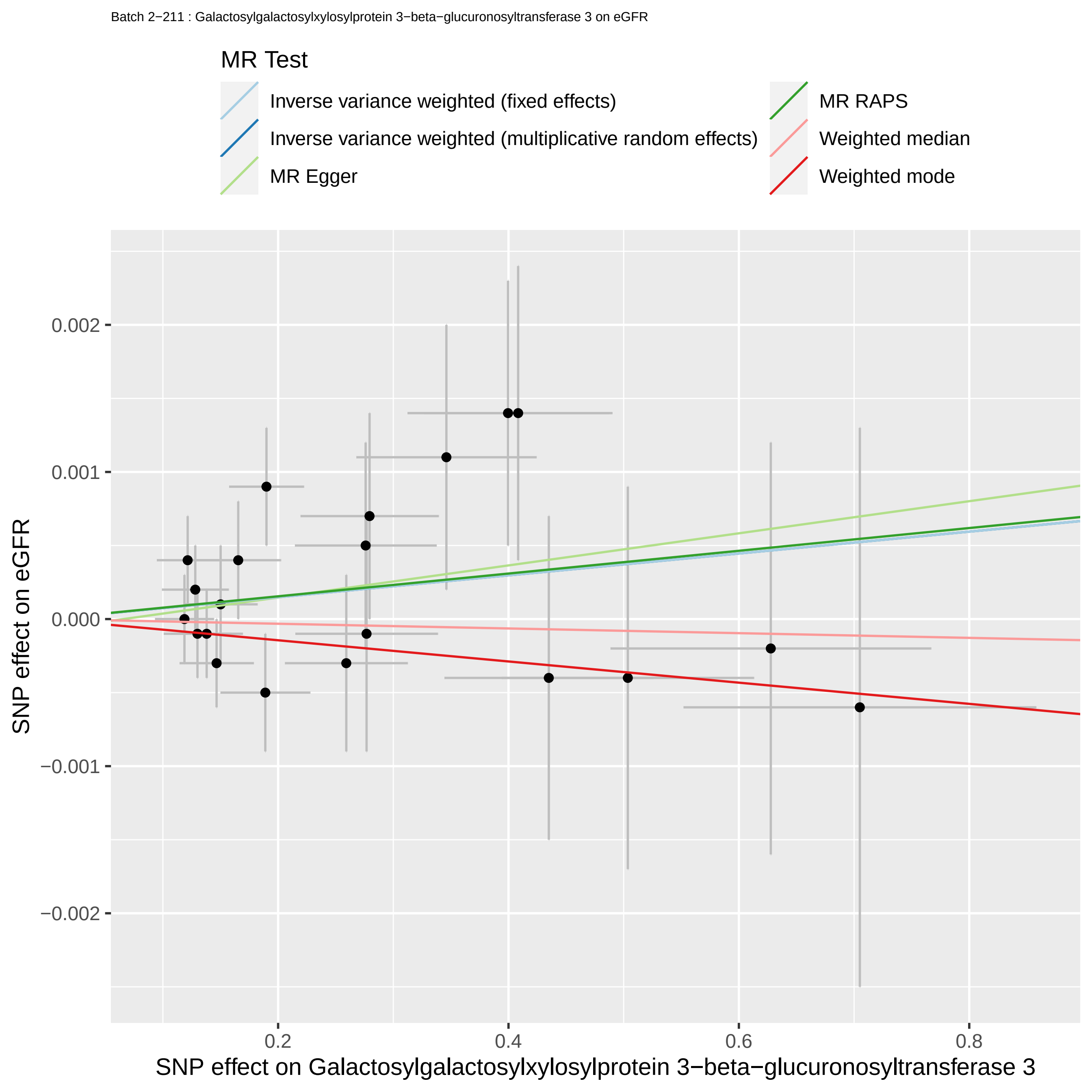

Batch 2−211 : Galactosylgalactosylxylosylprotein 3−beta−glucuronosyltransferase 3 on eGFR
MR Test
Inverse variance weighted (fixed effects)
Inverse variance weighted (multiplicative random effects)
MR Egger
MR RAPS
Weighted median
Weighted mode
0.002
0.001
0.000
−0.001
−0.002
0.2
0.4
0.6
0.8
SNP effect on eGFR
SNP effect on Galactosylgalactosylxylosylprotein 3−beta−glucuronosyltransferase 3

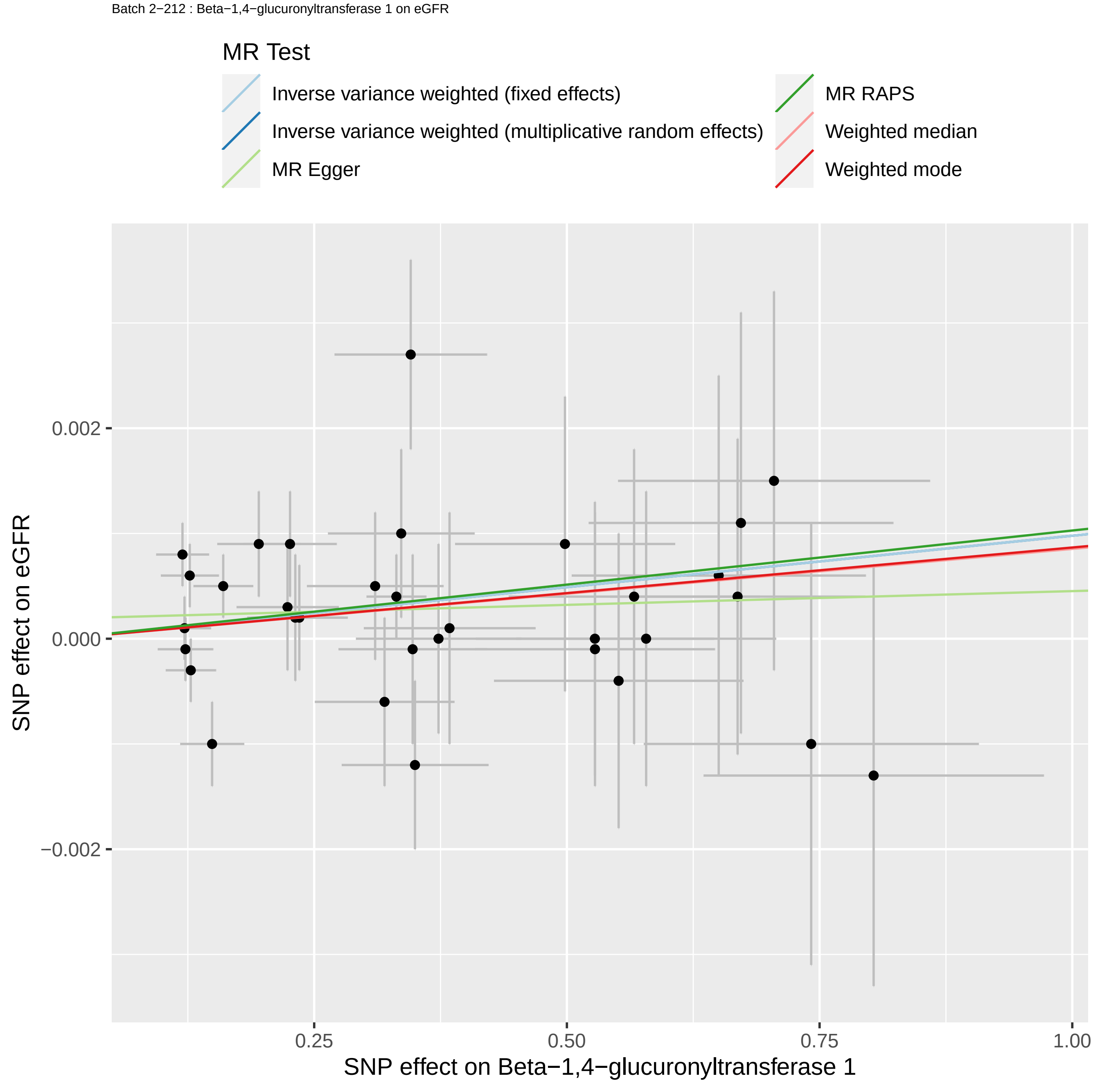
Batch 2−212 : Beta−1,4−glucuronyltransferase 1 on eGFR
MR Test
Inverse variance weighted (fixed effects)
Inverse variance weighted (multiplicative random effects)
MR Egger
MR RAPS
Weighted median
Weighted mode
0.002
0.000
−0.002
0.25
0.50
0.75
1.00
SNP effect on eGFR
SNP effect on Beta−1,4−glucuronyltransferase 1

Batch 2−213 : N−acetyllactosaminide beta−1,3−N−acetylglucosaminyltransferase 2 on eGFR

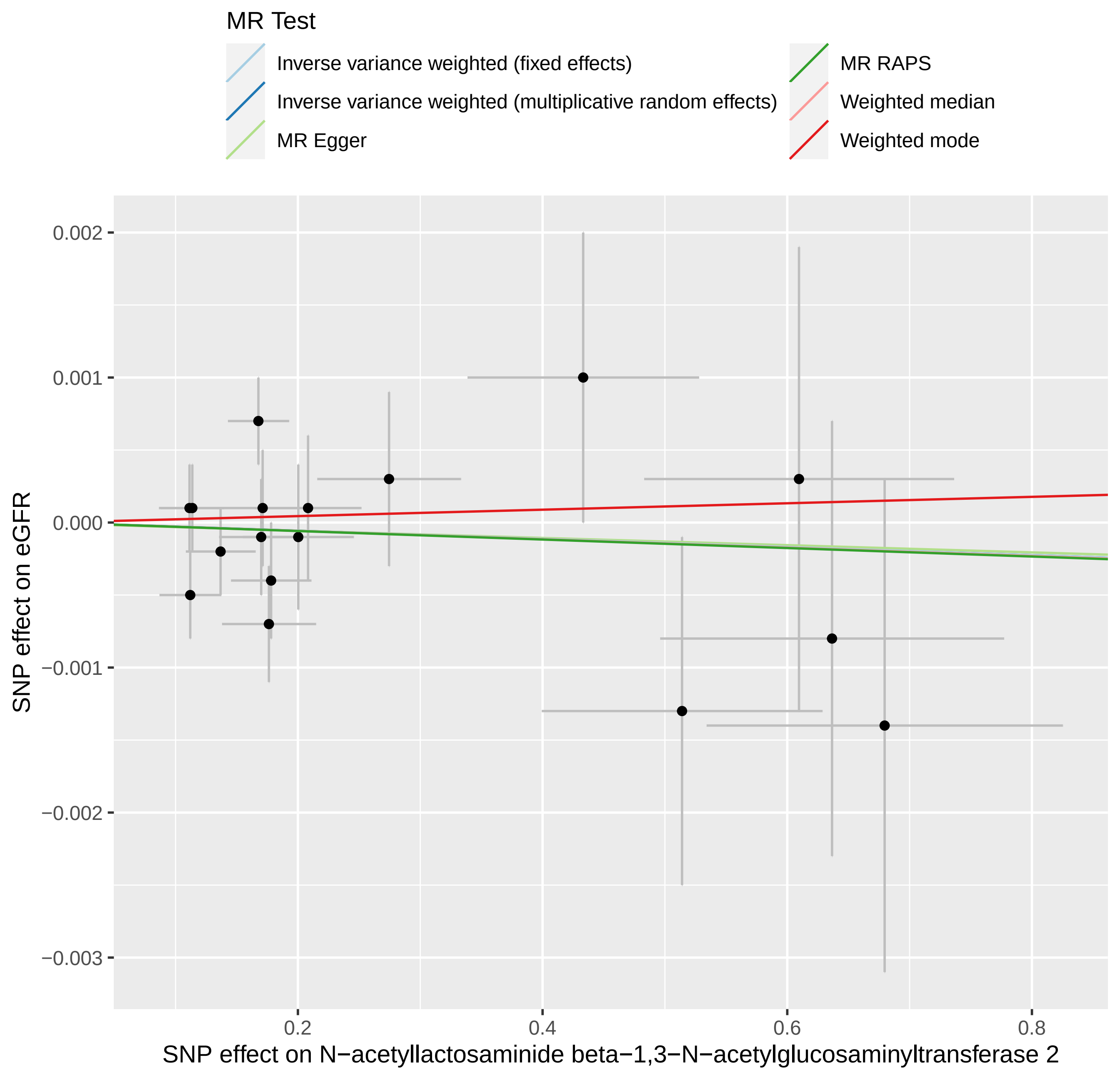

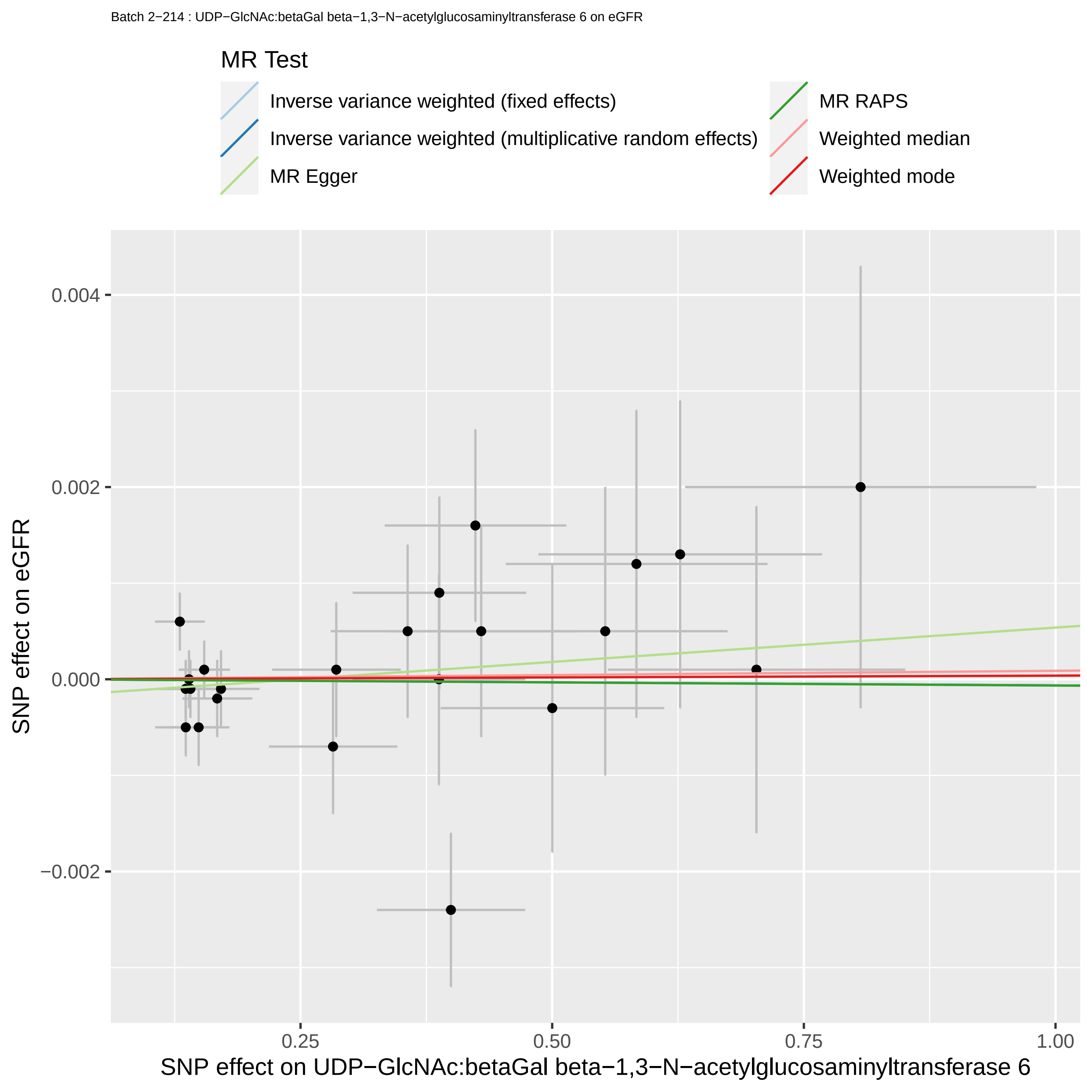
Batch 2−214 : UDP−GlcNAc:betaGal beta−1,3−N−acetylglucosaminyltransferase 6 on eGFR
MR Test
Inverse variance weighted (fixed effects)
Inverse variance weighted (multiplicative random effects)
MR Egger
MR RAPS
Weighted median
Weighted mode
0.004
0.002
0.000
−0.002
0.25
0.50
0.75
1.00
SNP effect on eGFR
SNP effect on UDP−GlcNAc:betaGal beta−1,3−N−acetylglucosaminyltransferase 6

Batch 2−215 : UDP−GlcNAc:betaGal beta−1,3−N−acetylglucosaminyltransferase 8 on eGFR

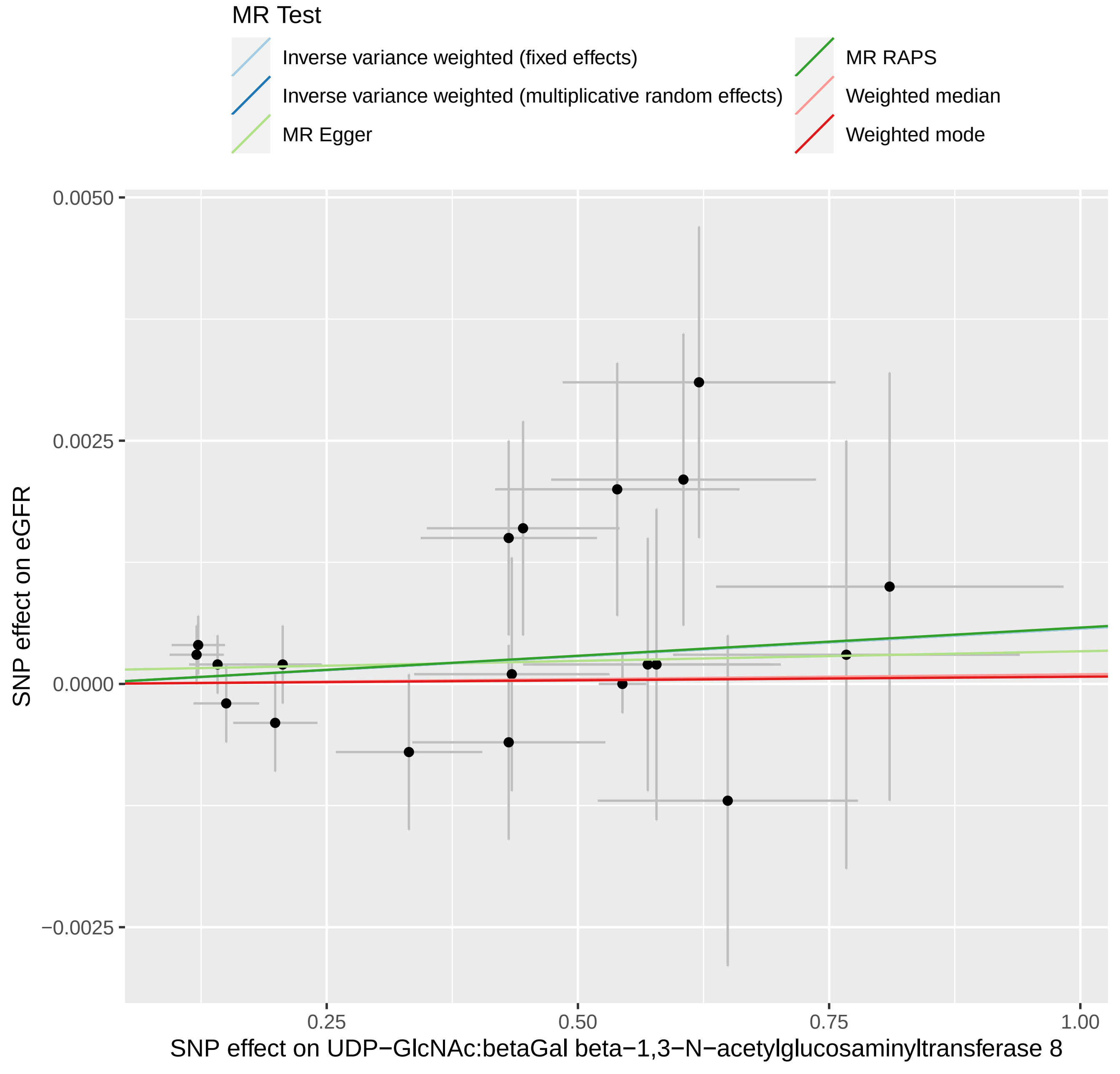

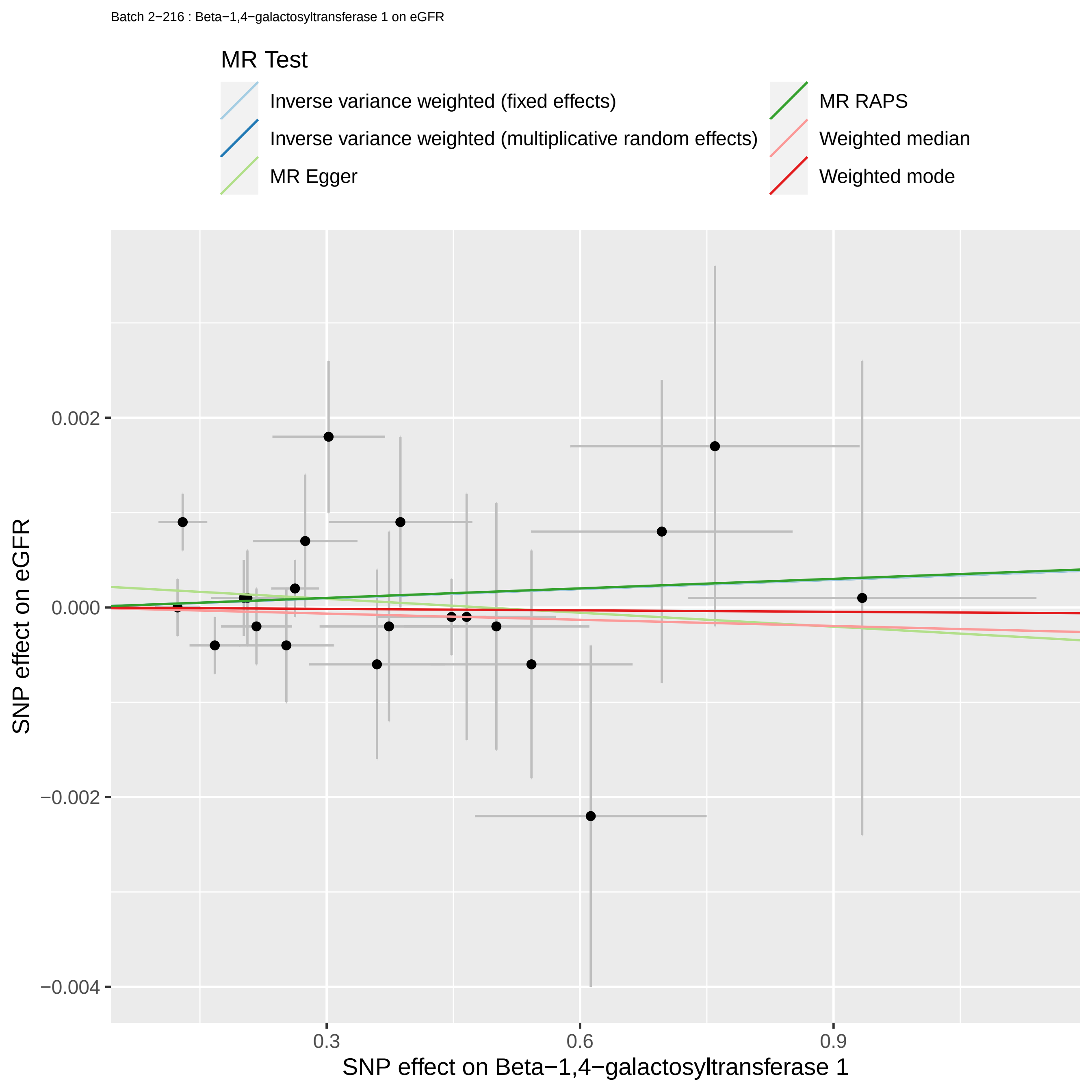
Batch 2−216 : Beta−1,4−galactosyltransferase 1 on eGFR
MR Test
Inverse variance weighted (fixed effects)
Inverse variance weighted (multiplicative random effects)
MR Egger
MR RAPS
Weighted median
Weighted mode
0.002
0.000
−0.002
−0.004
SNP effect on eGFR
0.3
0.6
0.9
SNP effect on Beta−1,4−galactosyltransferase 1

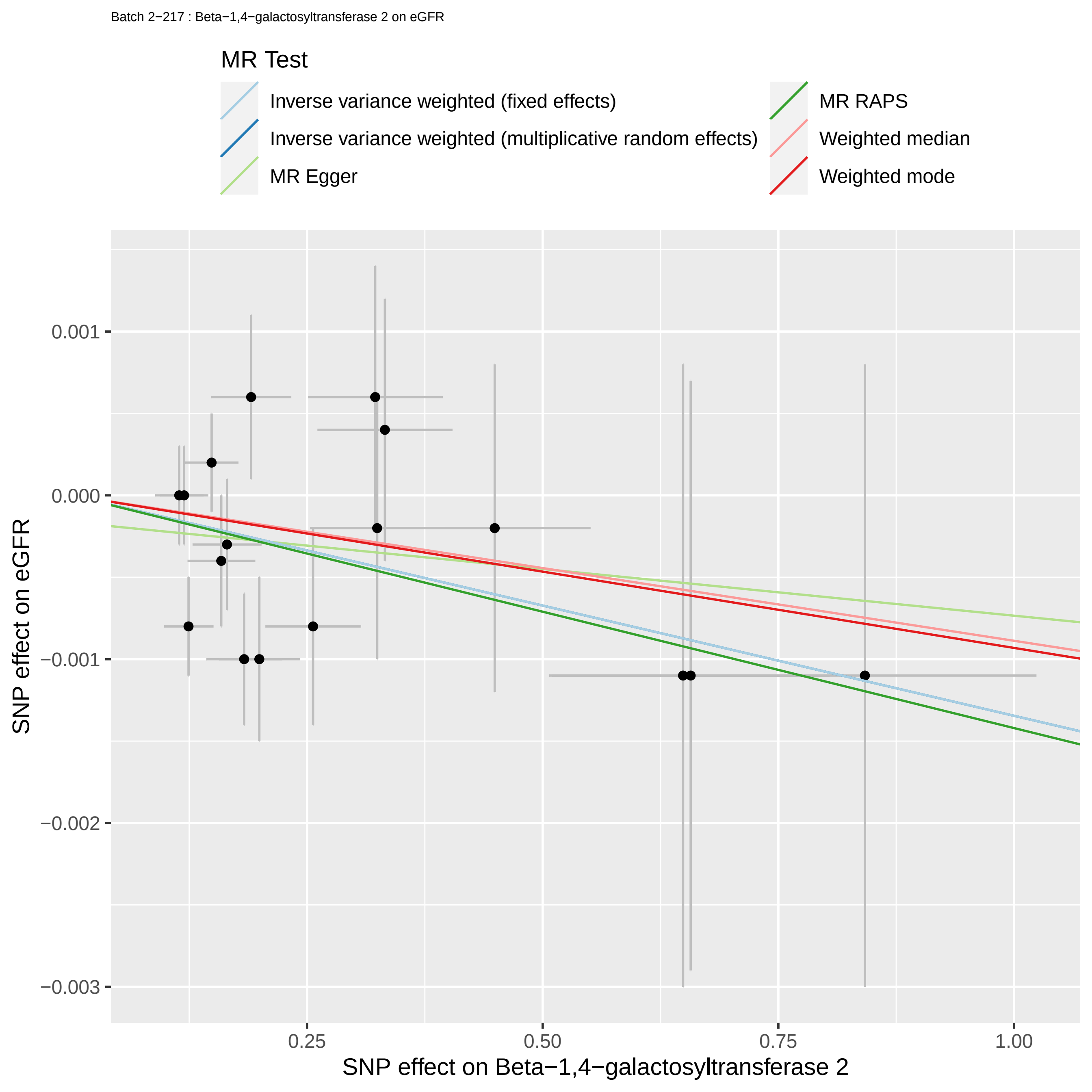
Batch 2−217 : Beta−1,4−galactosyltransferase 2 on eGFR
MR Test
Inverse variance weighted (fixed effects)
Inverse variance weighted (multiplicative random effects)
MR Egger
MR RAPS
Weighted median
Weighted mode
0.001
0.000
−0.001
−0.002
−0.003
0.25
0.50
0.75
1.00
SNP effect on eGFR
SNP effect on Beta−1,4−galactosyltransferase 2

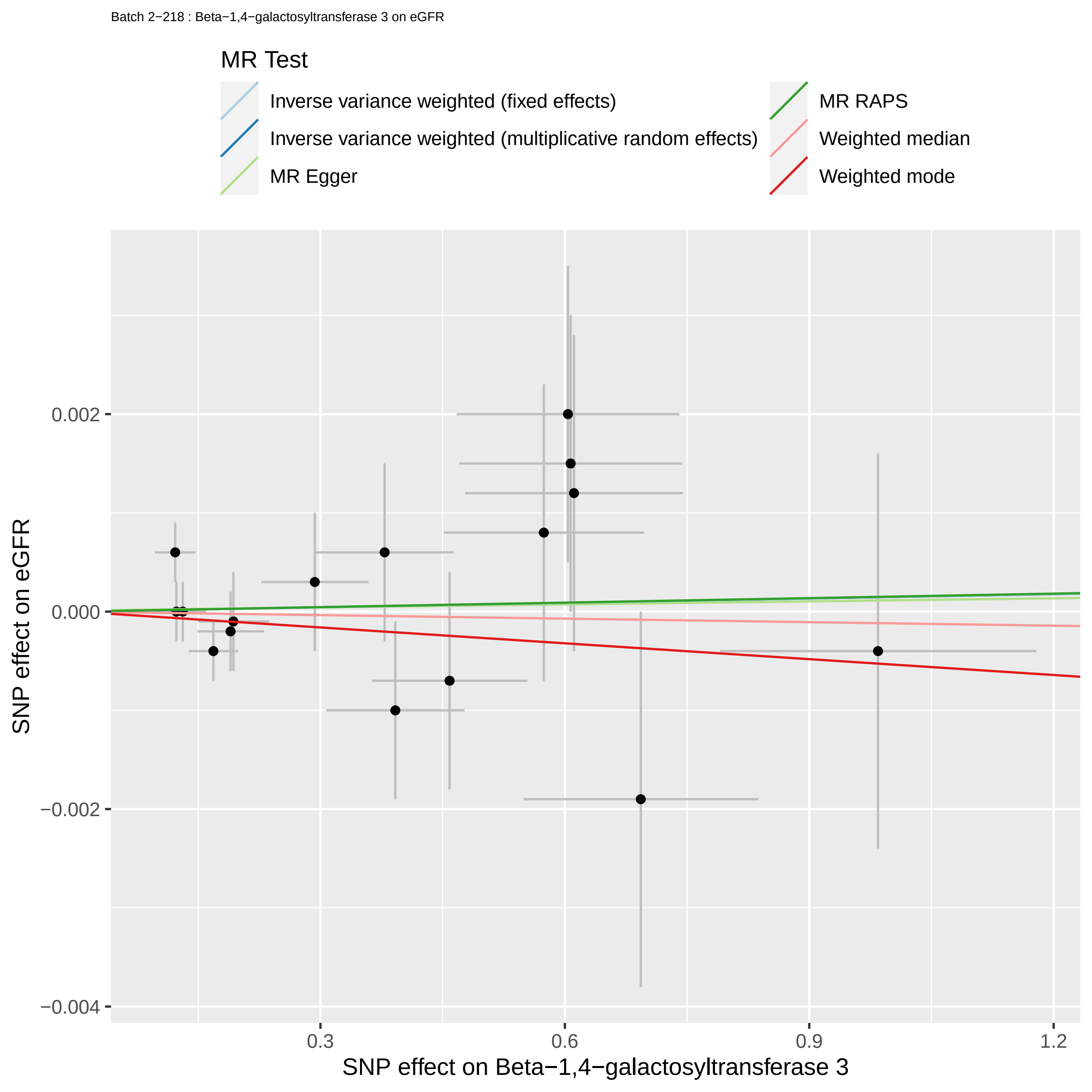
Batch 2−218 : Beta−1,4−galactosyltransferase 3 on eGFR
MR Test
Inverse variance weighted (fixed effects)
Inverse variance weighted (multiplicative random effects)
MR Egger
MR RAPS
Weighted median
Weighted mode
0.002
0.000
−0.002
−0.004
0.3
0.6
0.9
1.2
SNP effect on eGFR
SNP effect on Beta−1,4−galactosyltransferase 3

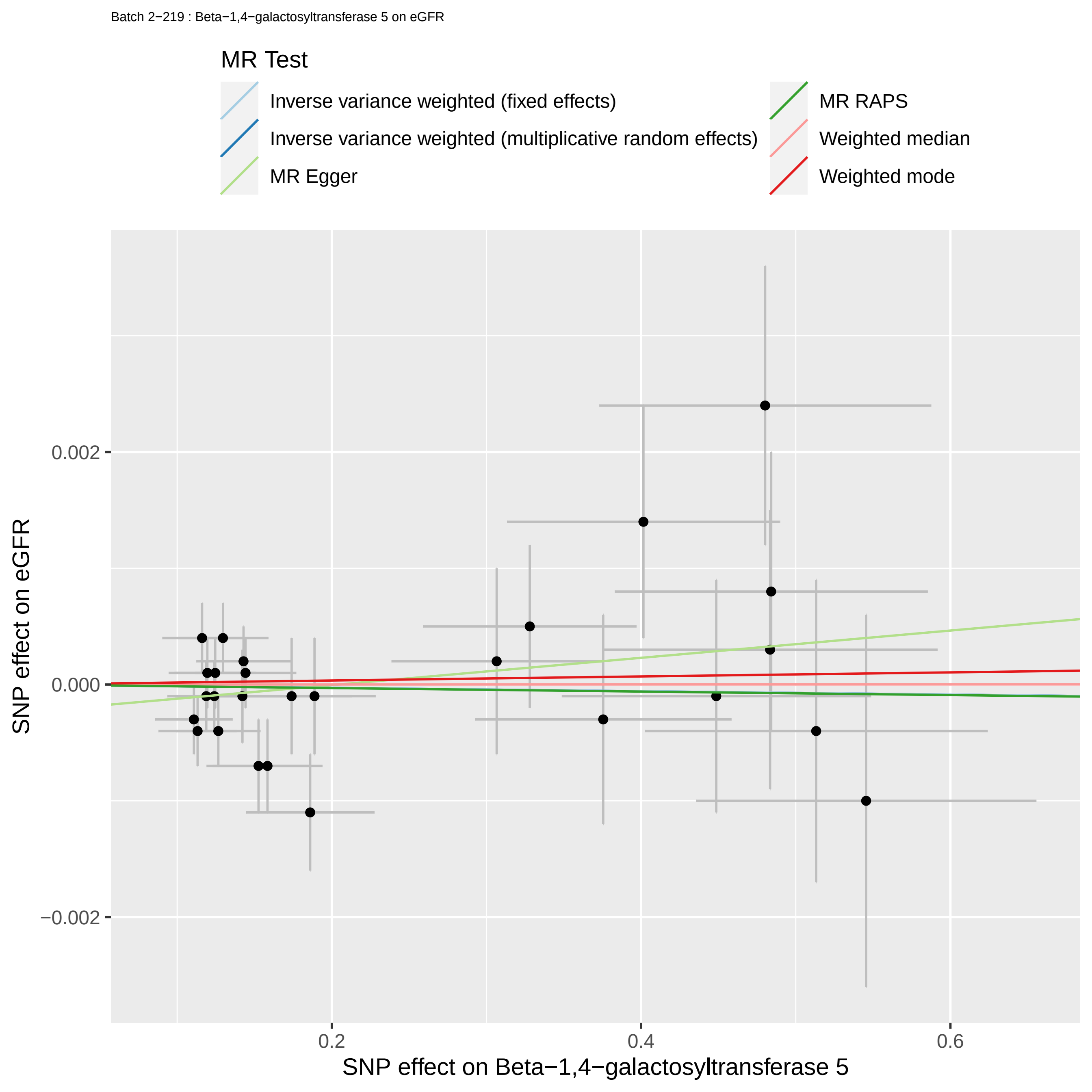

Batch 2−219 : Beta−1,4−galactosyltransferase 5 on eGFR
MR Test
Inverse variance weighted (fixed effects)
Inverse variance weighted (multiplicative random effects)
MR Egger
MR RAPS
Weighted median
Weighted mode
SNP effect on eGFR
0.002
0.000
−0.002
0.2
0.4
0.6
SNP effect on Beta−1,4−galactosyltransferase 5

Batch 2−220 : Beta−1,4−galactosyltransferase 6 on eGFR

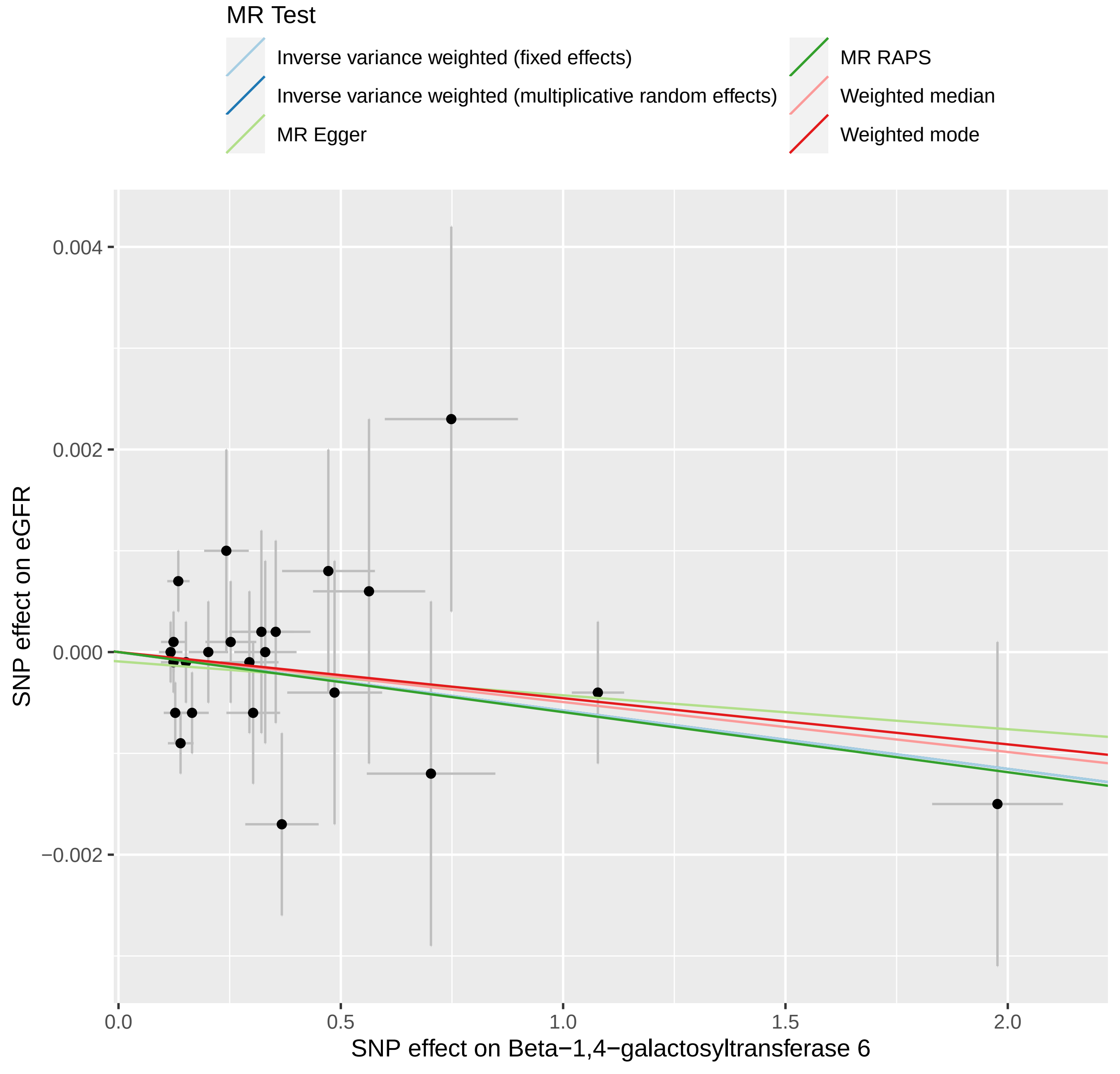

Batch 2−221 : Beta−1,4−galactosyltransferase 7 on eGFR

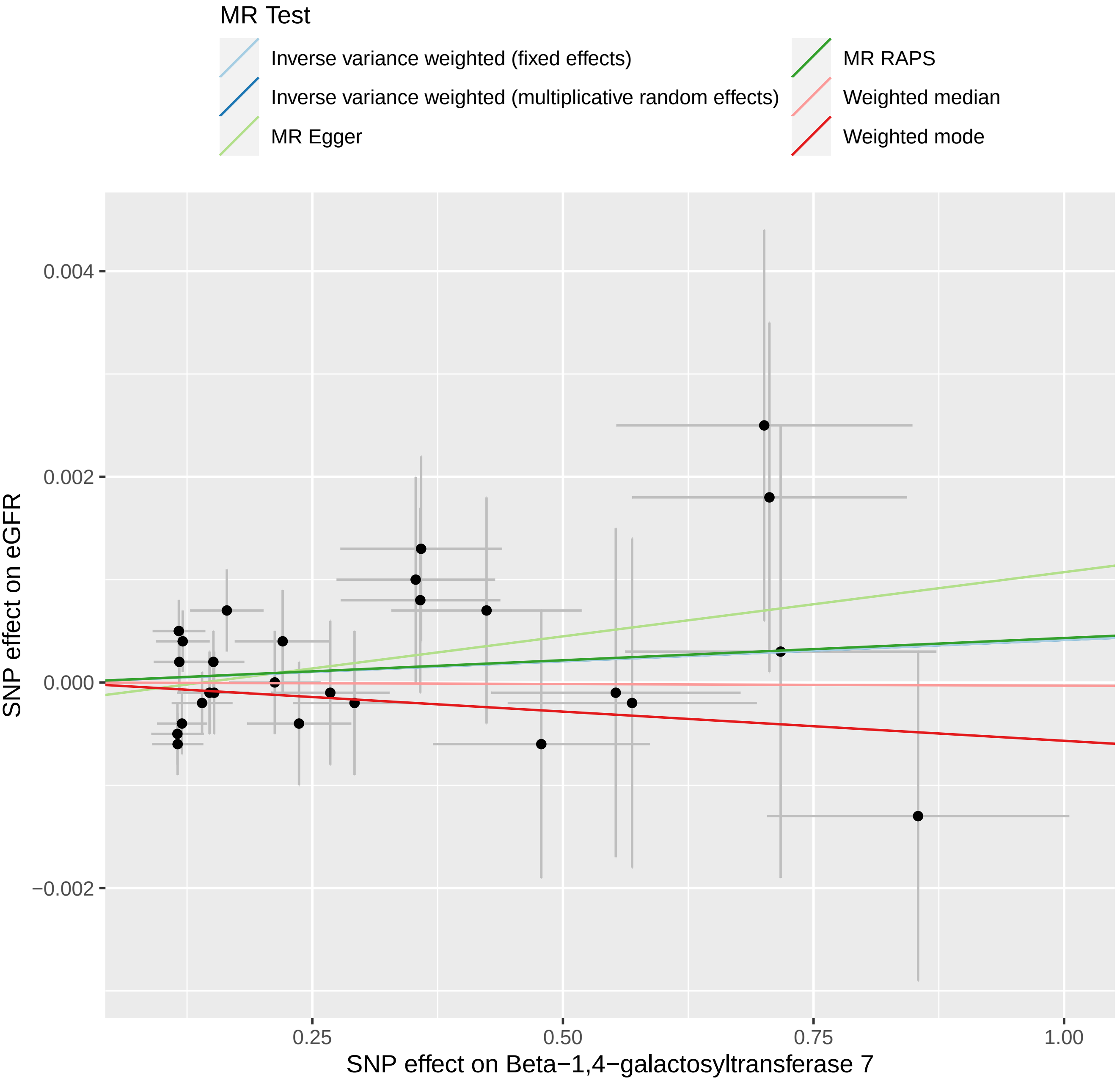

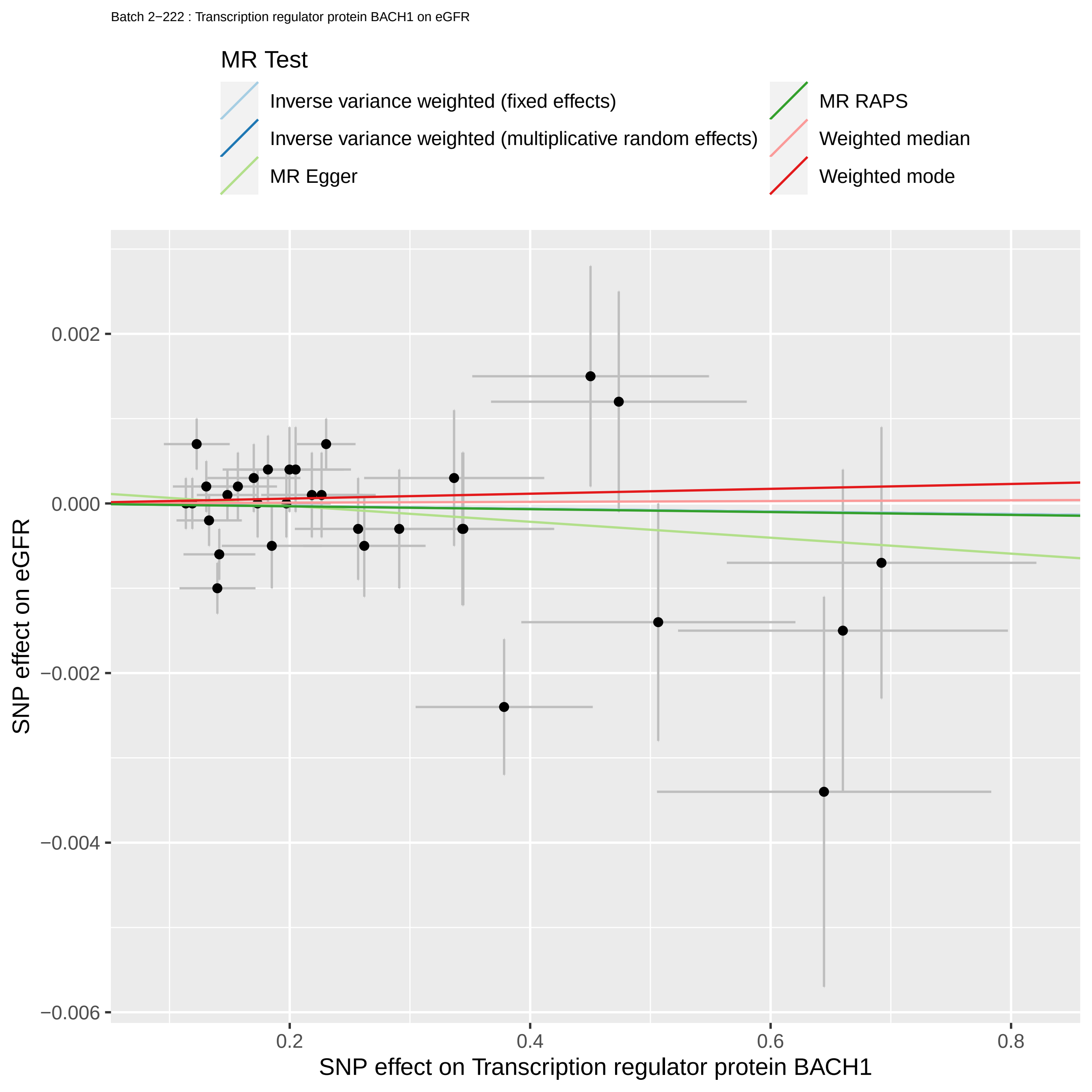

Batch 2−222 : Transcription regulator protein BACH1 on eGFR
MR Test
Inverse variance weighted (fixed effects)
Inverse variance weighted (multiplicative random effects)
MR Egger
MR RAPS
Weighted median
Weighted mode
0.002
0.000
−0.002
−0.004
−0.006
0.2
0.4
0.6
0.8
SNP effect on eGFR
SNP effect on Transcription regulator protein BACH1

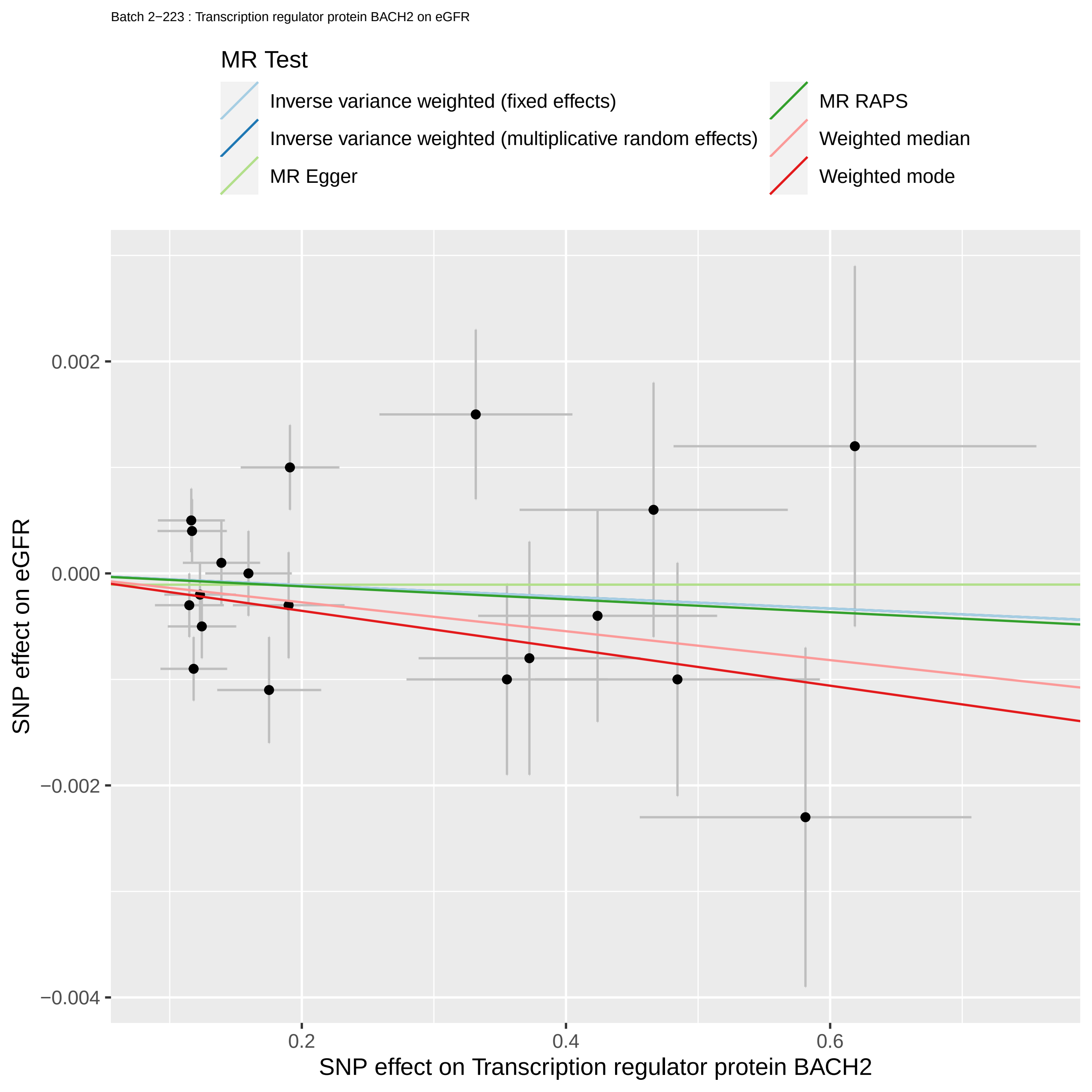

Batch 2−223 : Transcription regulator protein BACH2 on eGFR
MR Test
Inverse variance weighted (fixed effects)
Inverse variance weighted (multiplicative random effects)
MR Egger
MR RAPS
Weighted median
Weighted mode
0.002
0.000
−0.002
−0.004
0.2
0.4
0.6
SNP effect on eGFR
SNP effect on Transcription regulator protein BACH2

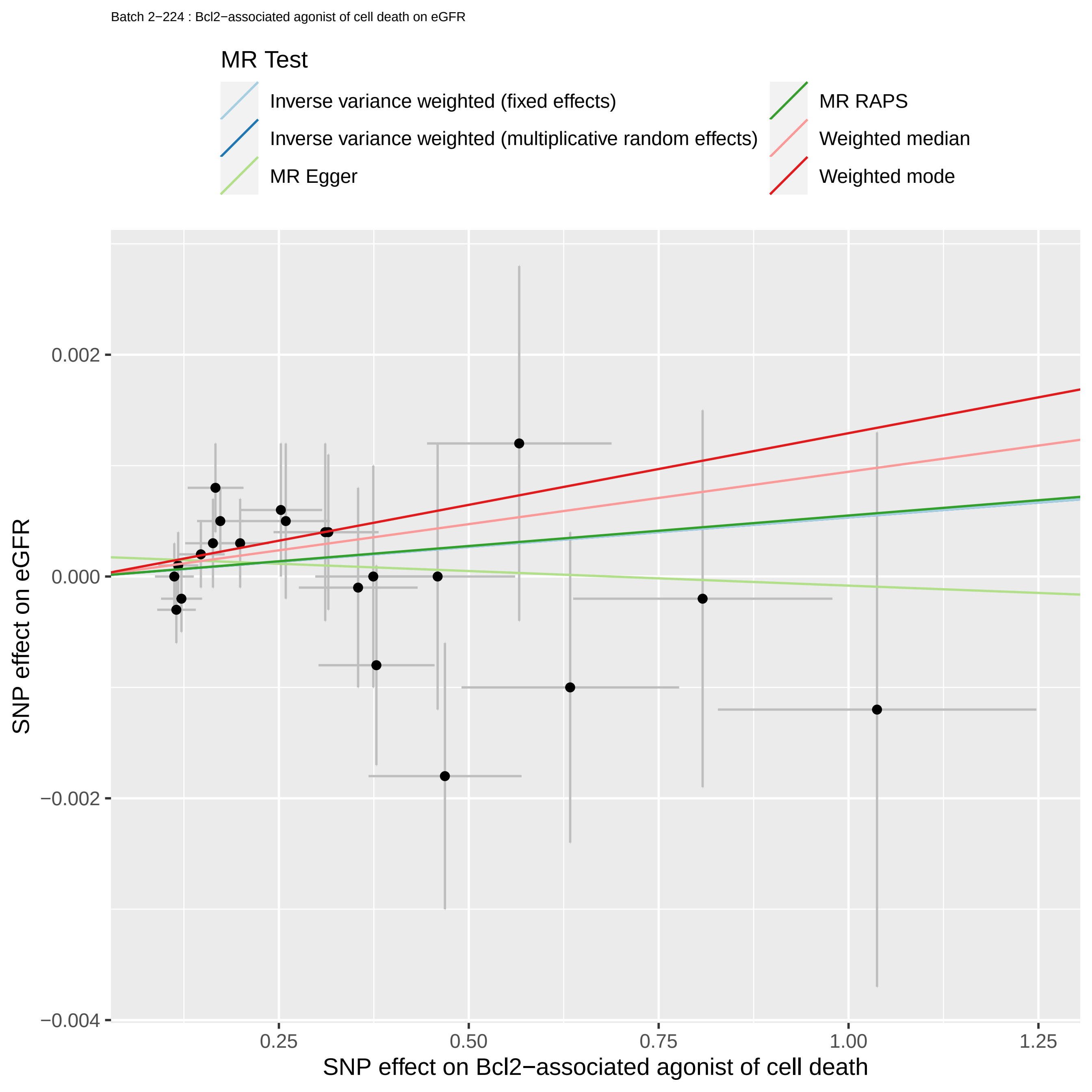
Batch 2−224 : Bcl2−associated agonist of cell death on eGFR
MR Test
Inverse variance weighted (fixed effects)
Inverse variance weighted (multiplicative random effects)
MR Egger
MR RAPS
Weighted median
Weighted mode
0.002
0.000
−0.002
−0.004
0.25
0.50
0.75
1.00
1.25
SNP effect on eGFR
SNP effect on Bcl2−associated agonist of cell death

Batch 2−225 : BAG family molecular chaperone regulator 3 on eGFR

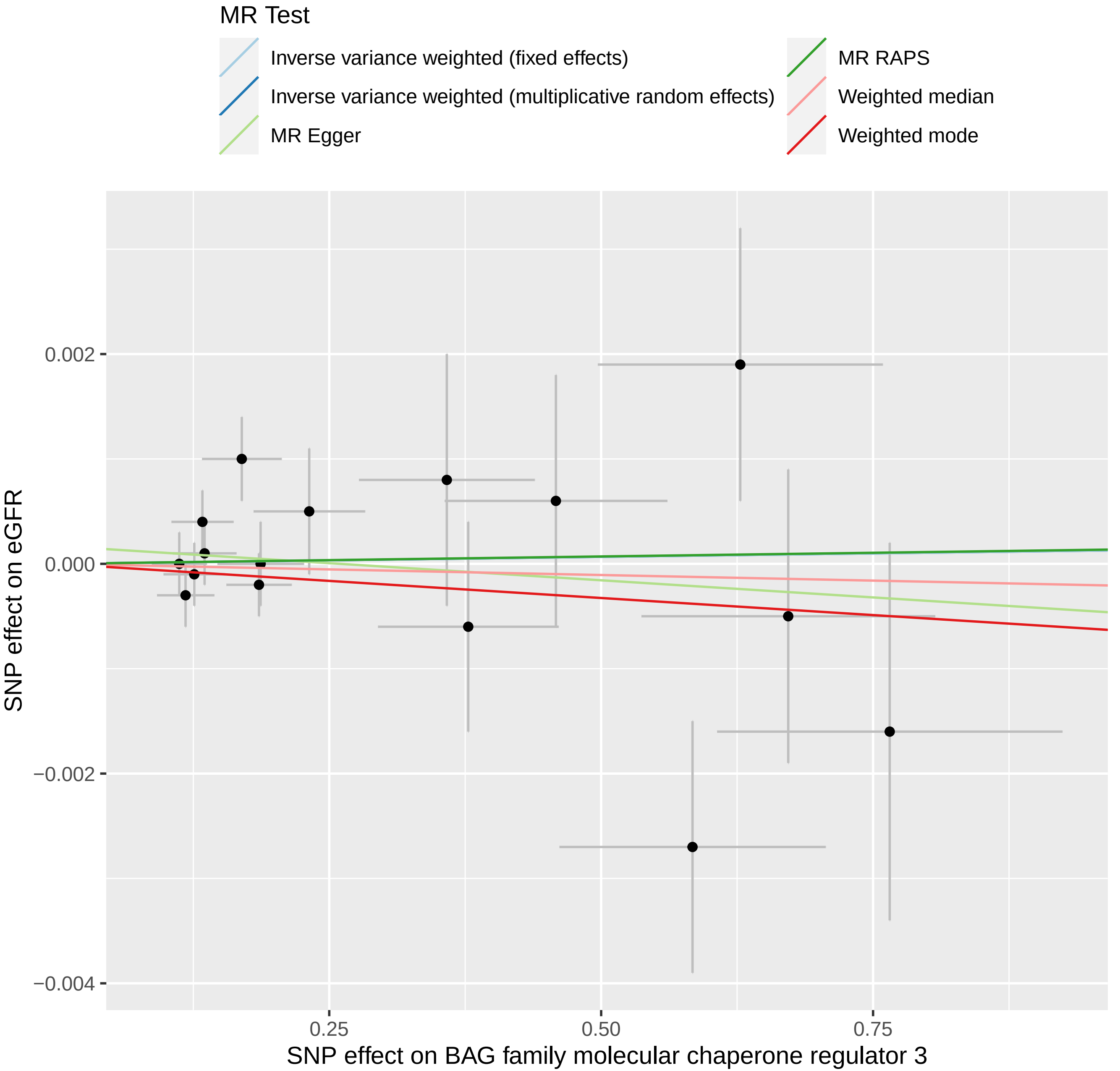

Batch 2−226 : BAG family molecular chaperone regulator 4 on eGFR

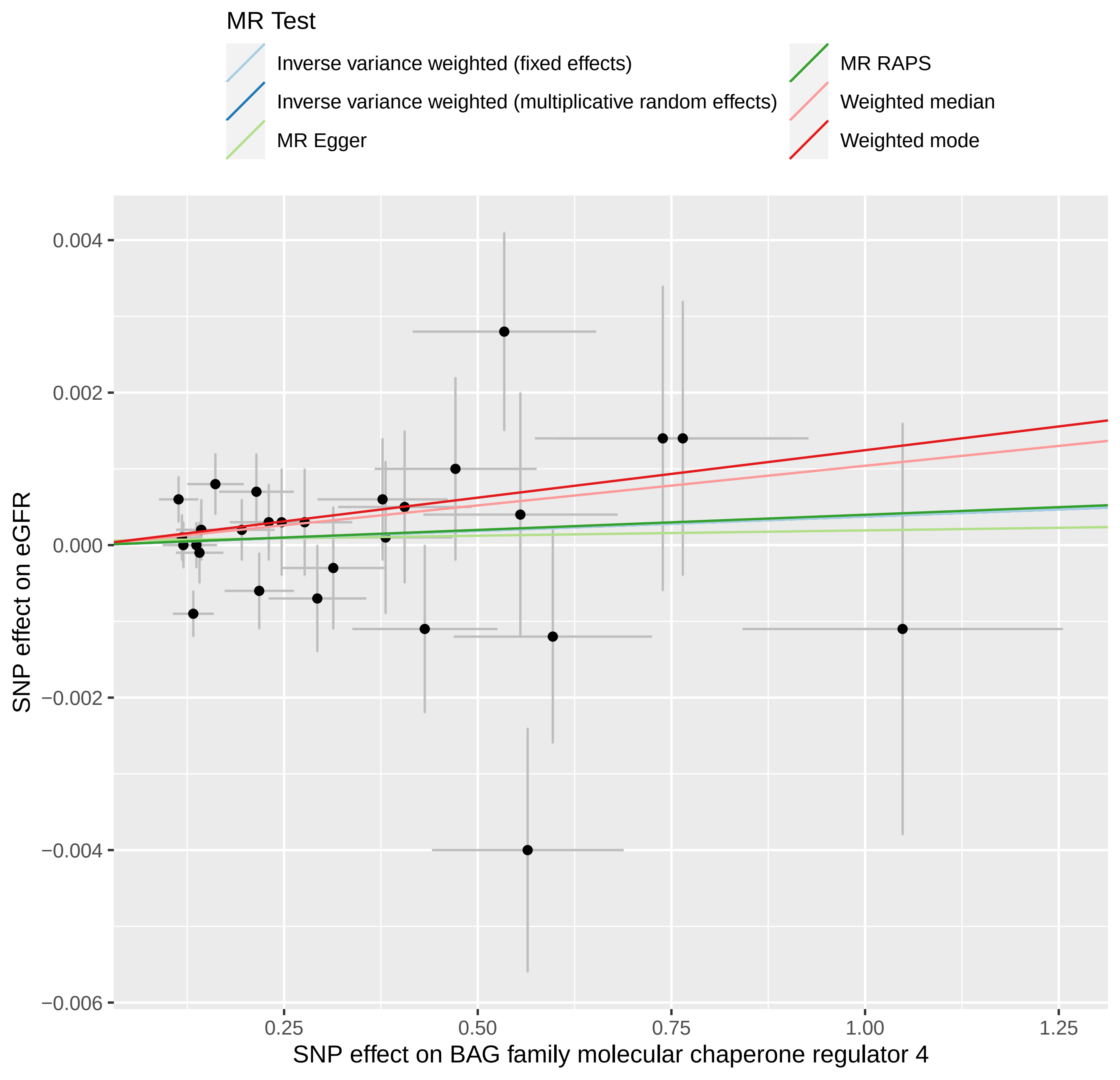

Batch 2−227 : B melanoma antigen 2 on eGFR

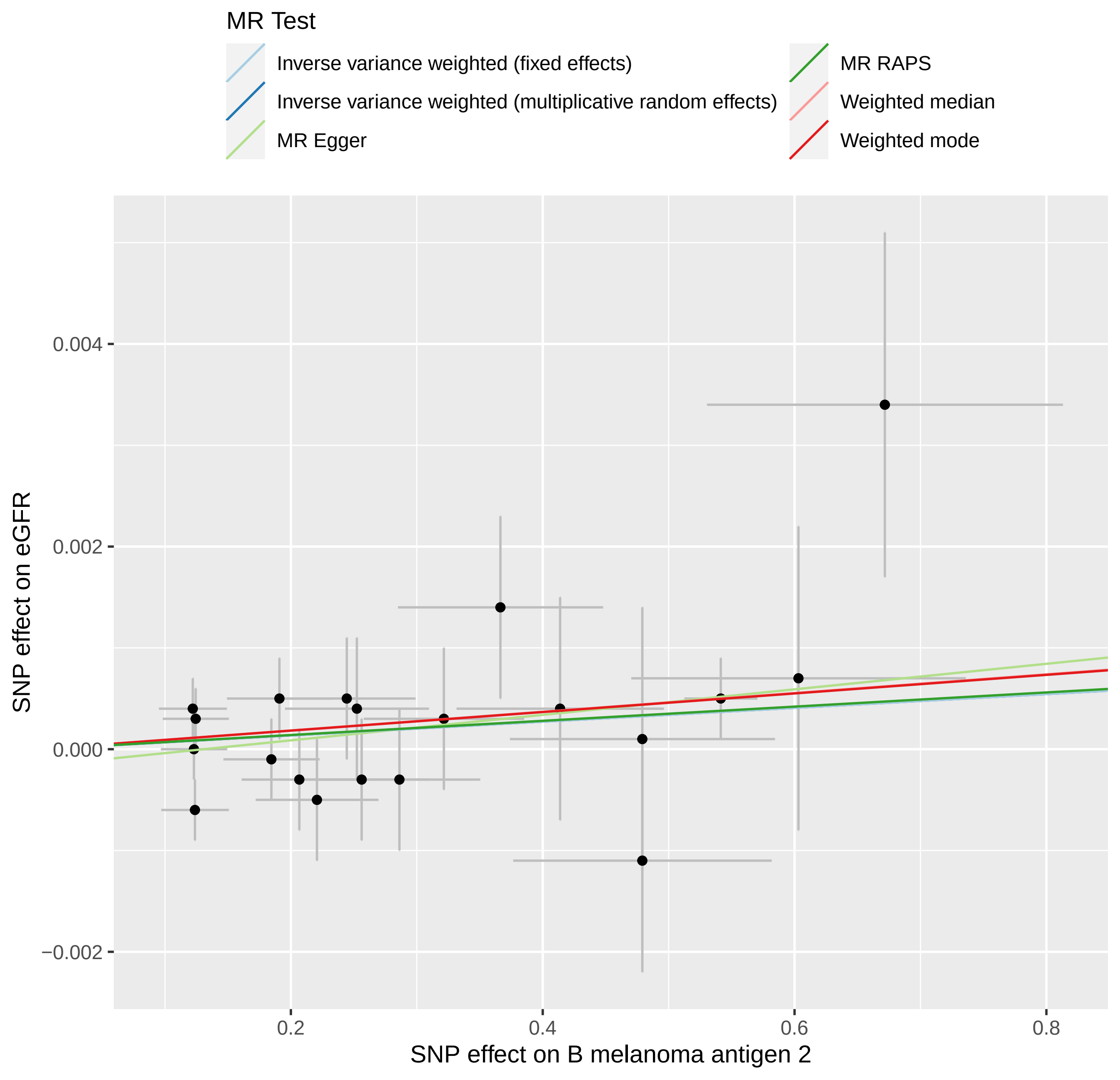

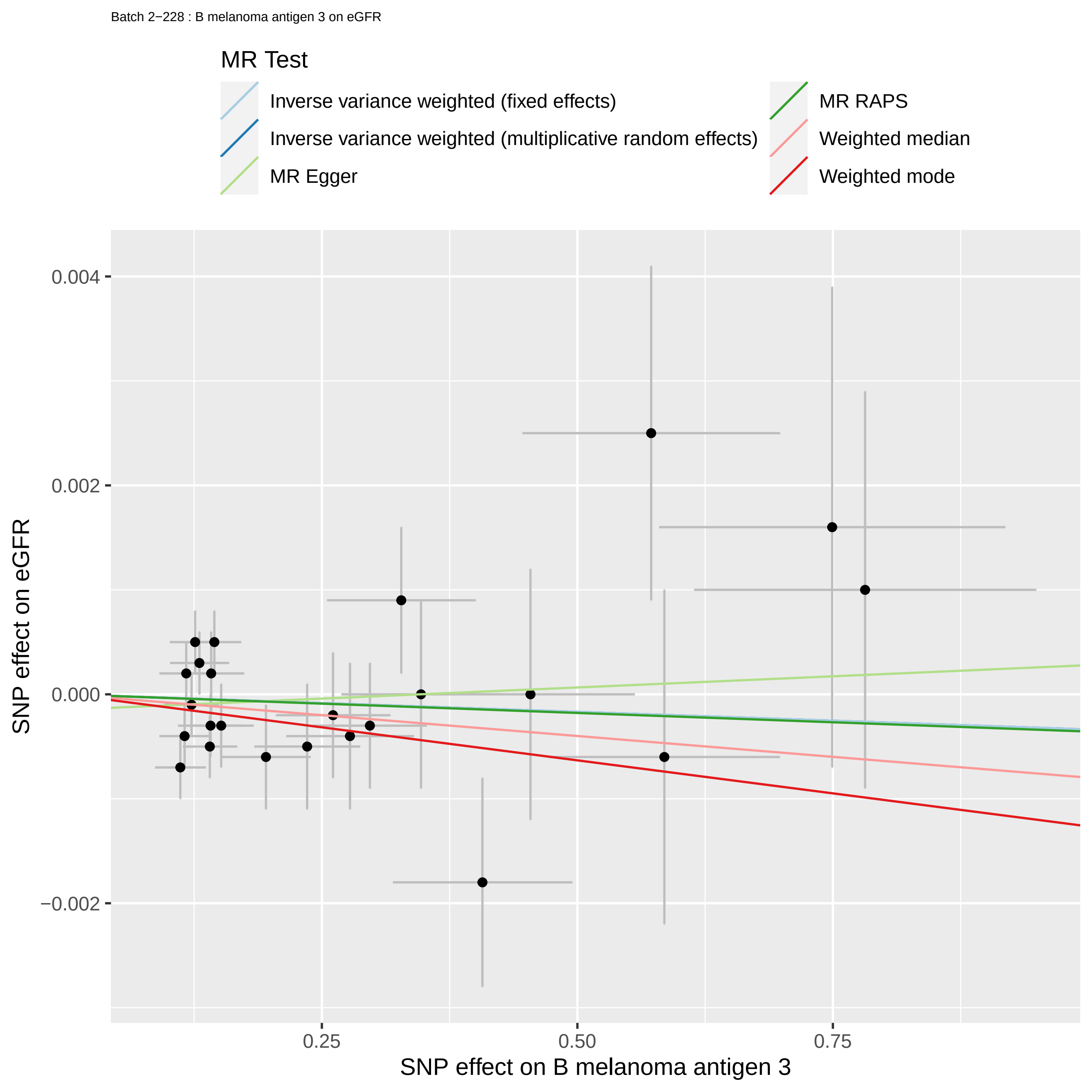
Batch 2−228 : B melanoma antigen 3 on eGFR
MR Test
Inverse variance weighted (fixed effects)
Inverse variance weighted (multiplicative random effects)
MR Egger
MR RAPS
Weighted median
Weighted mode
0.004
0.002
0.000
−0.002
0.25
0.50
0.75
SNP effect on eGFR
SNP effect on B melanoma antigen 3

Batch 2−229 : BRCA1−associated RING domain protein 1 on eGFR

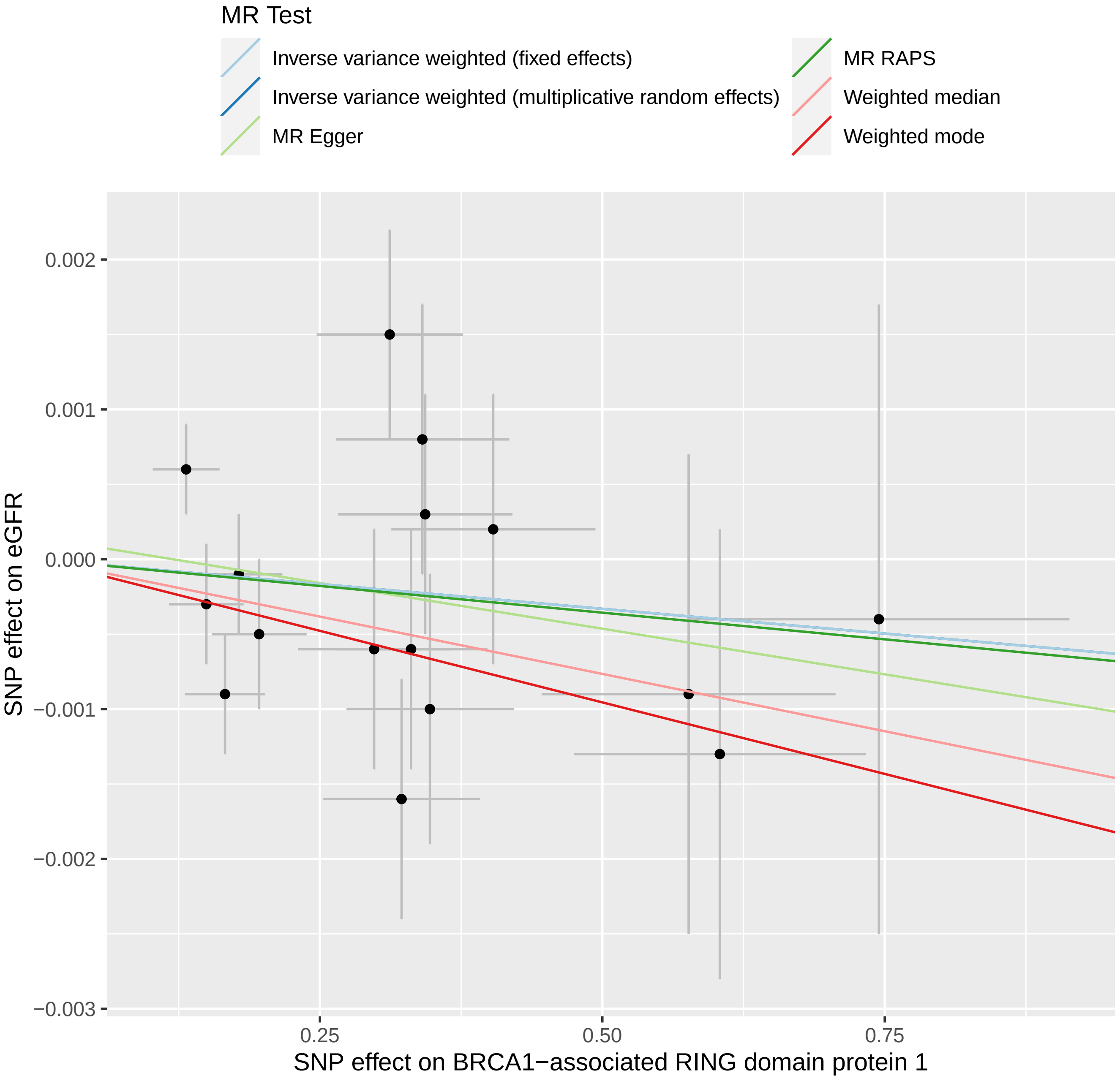

Batch 2−230 : Basic leucine zipper transcriptional factor ATF−like 3 on eGFR

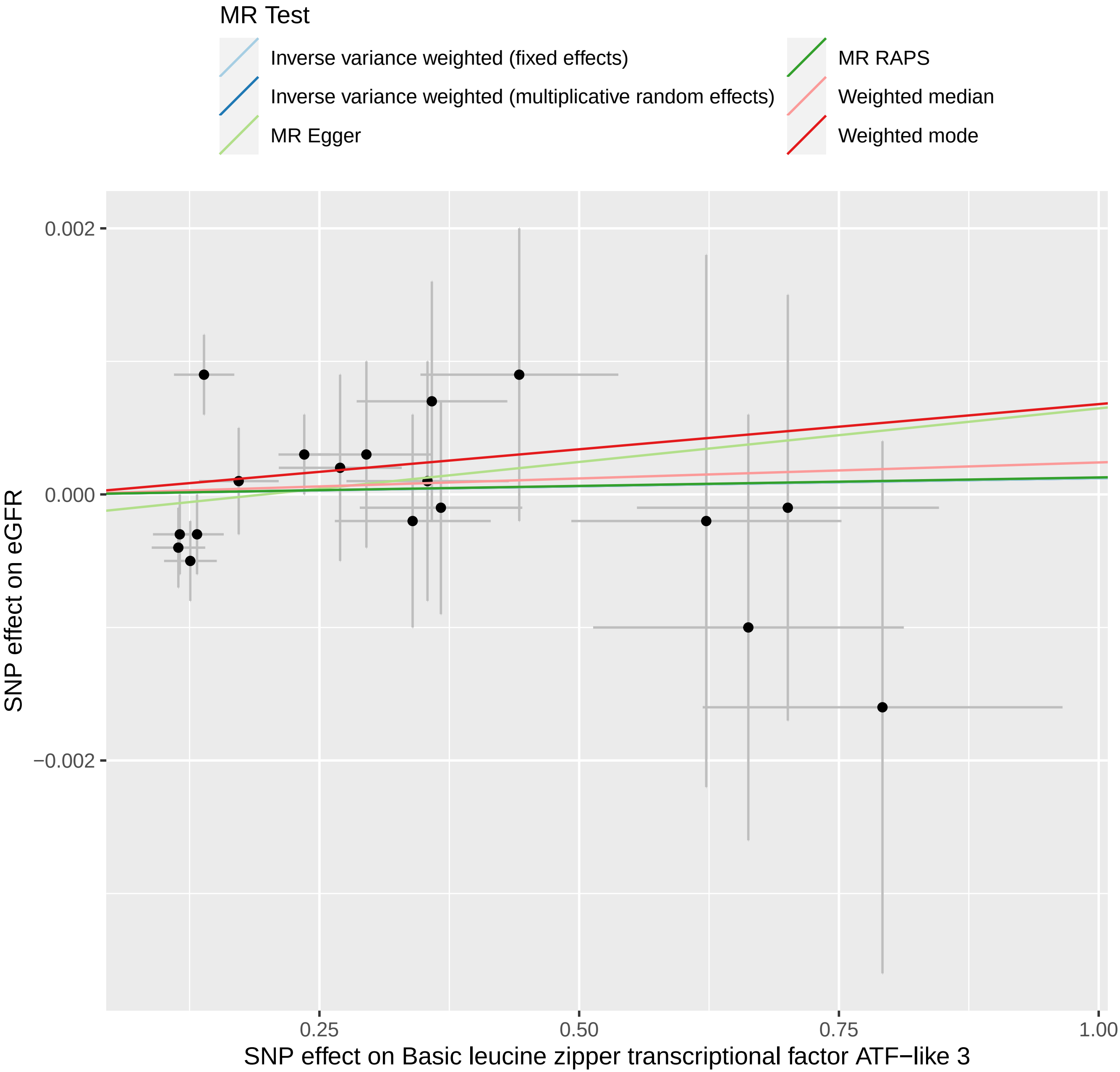

Batch 2−231 : Basal Cell Adhesion Molecule on eGFR

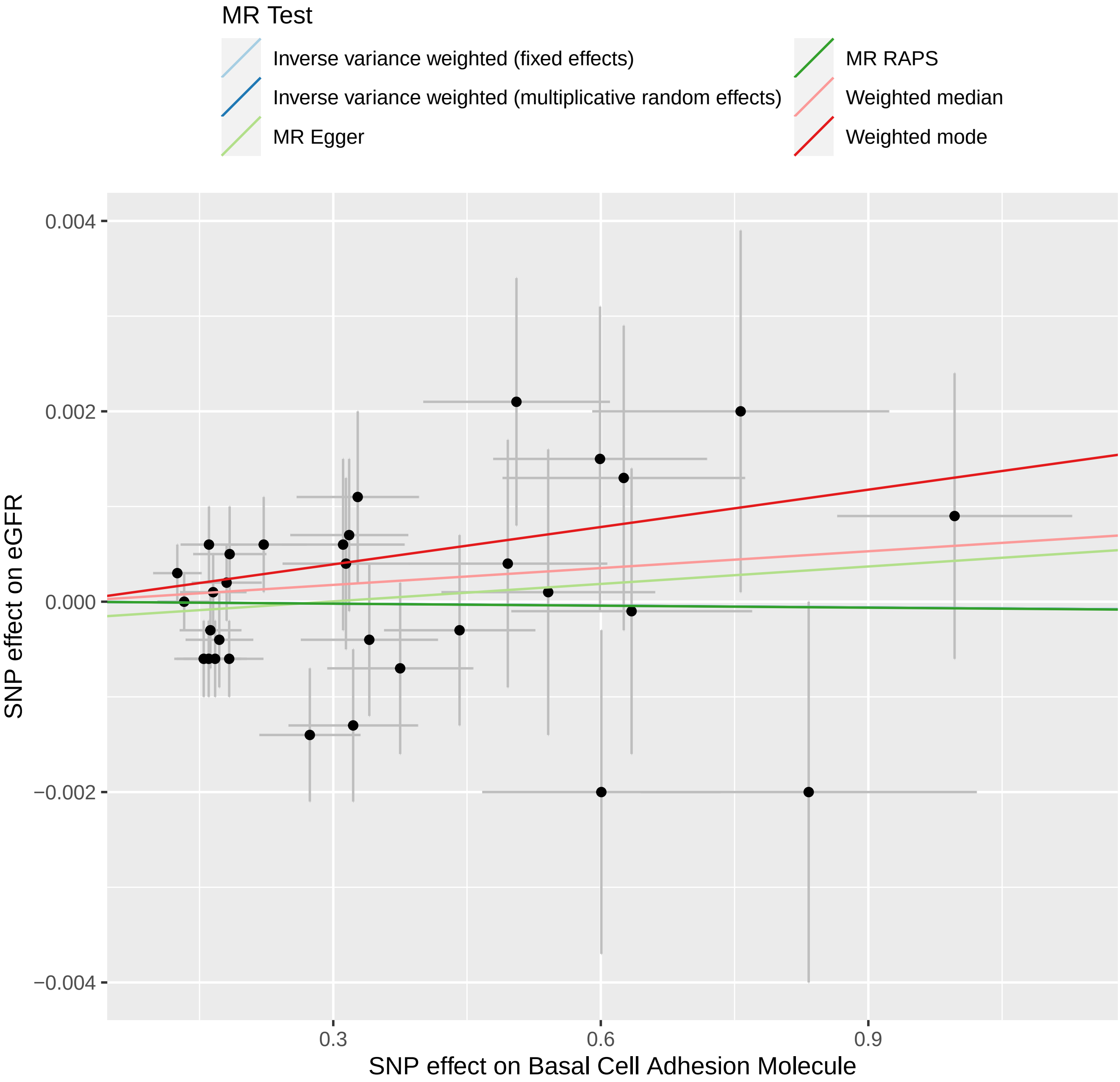

Batch 2−232 : Brevican core protein on eGFR

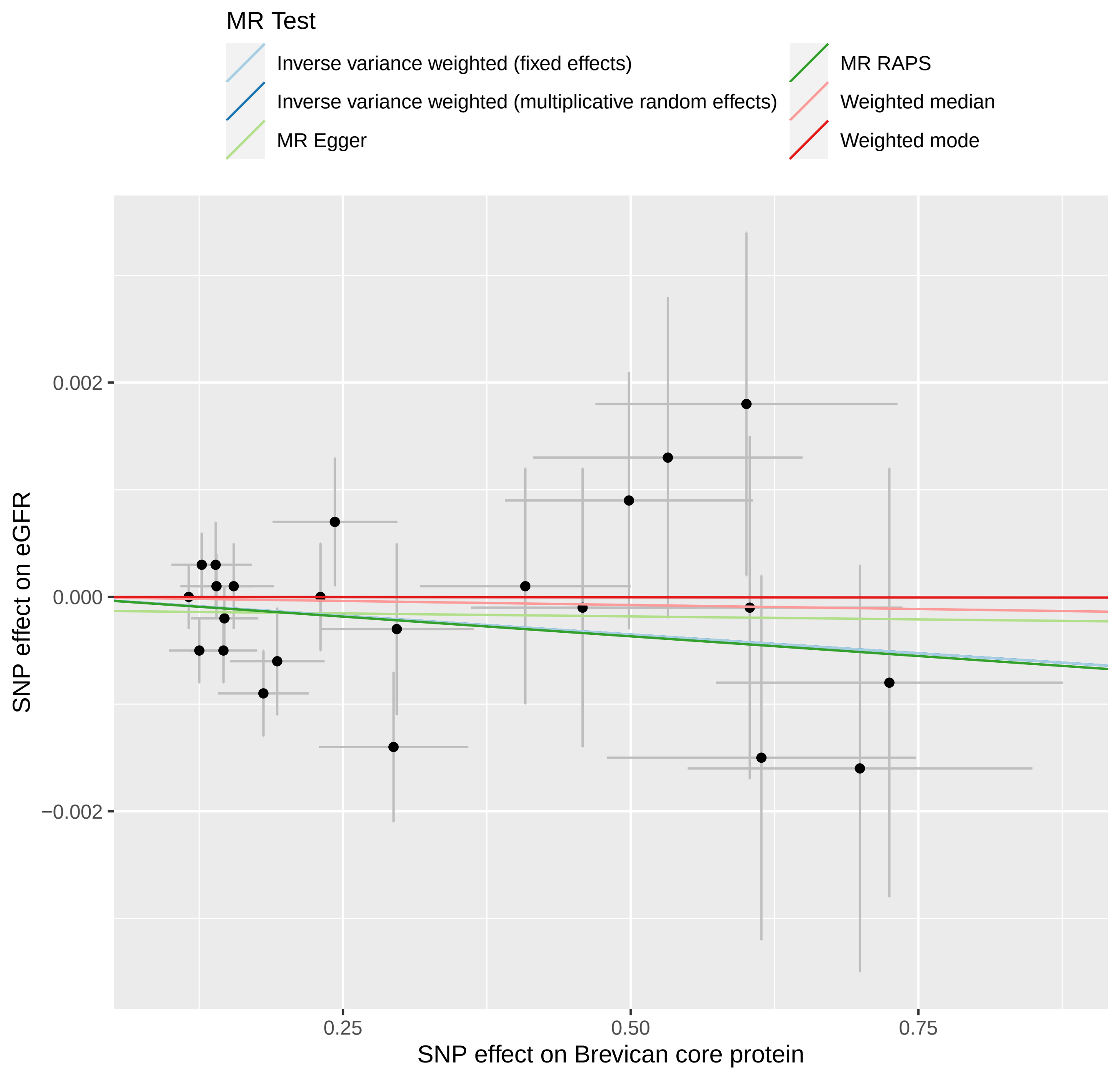

Batch 2−233 : B−cell receptor−associated protein 29 on eGFR

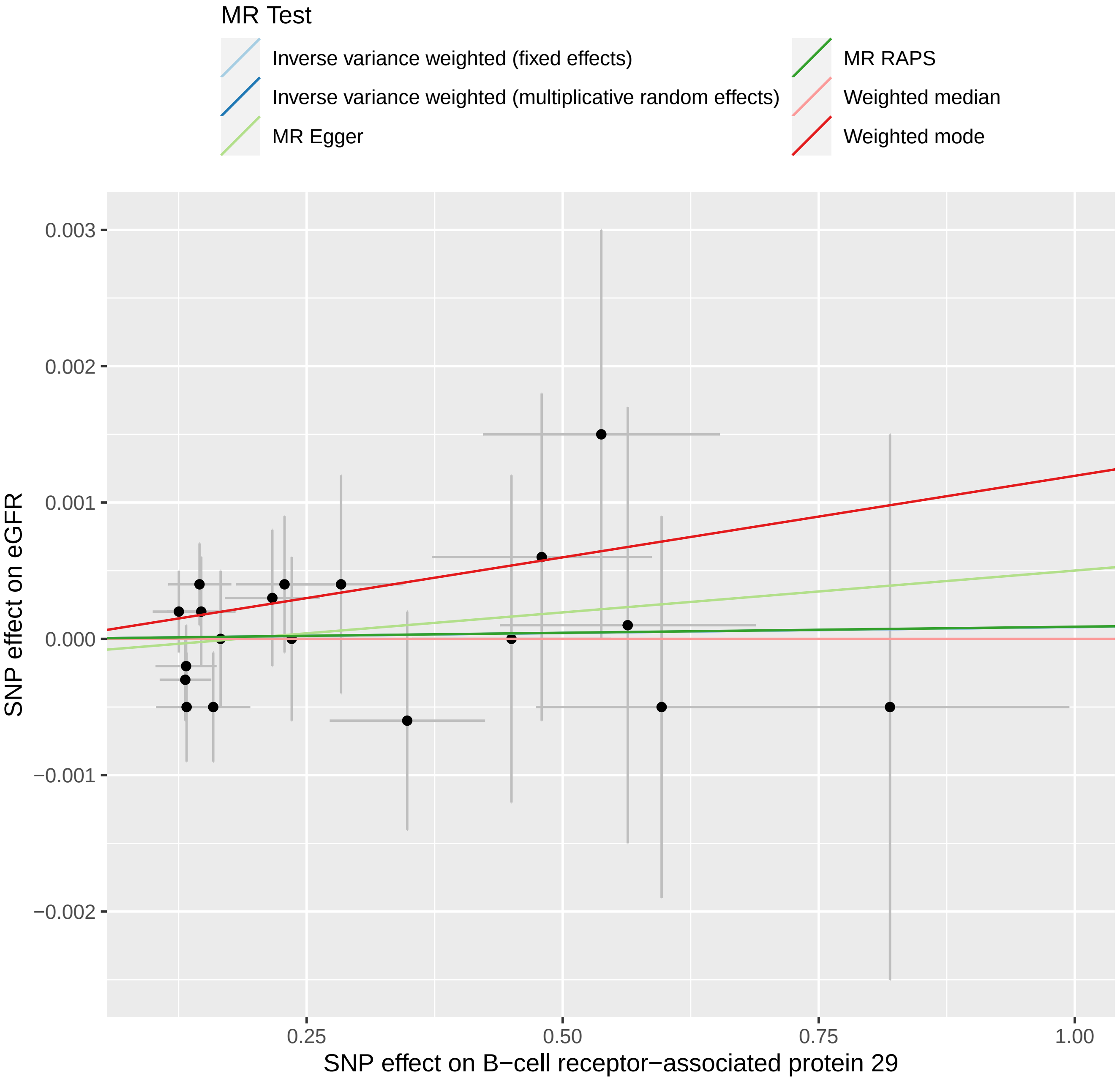

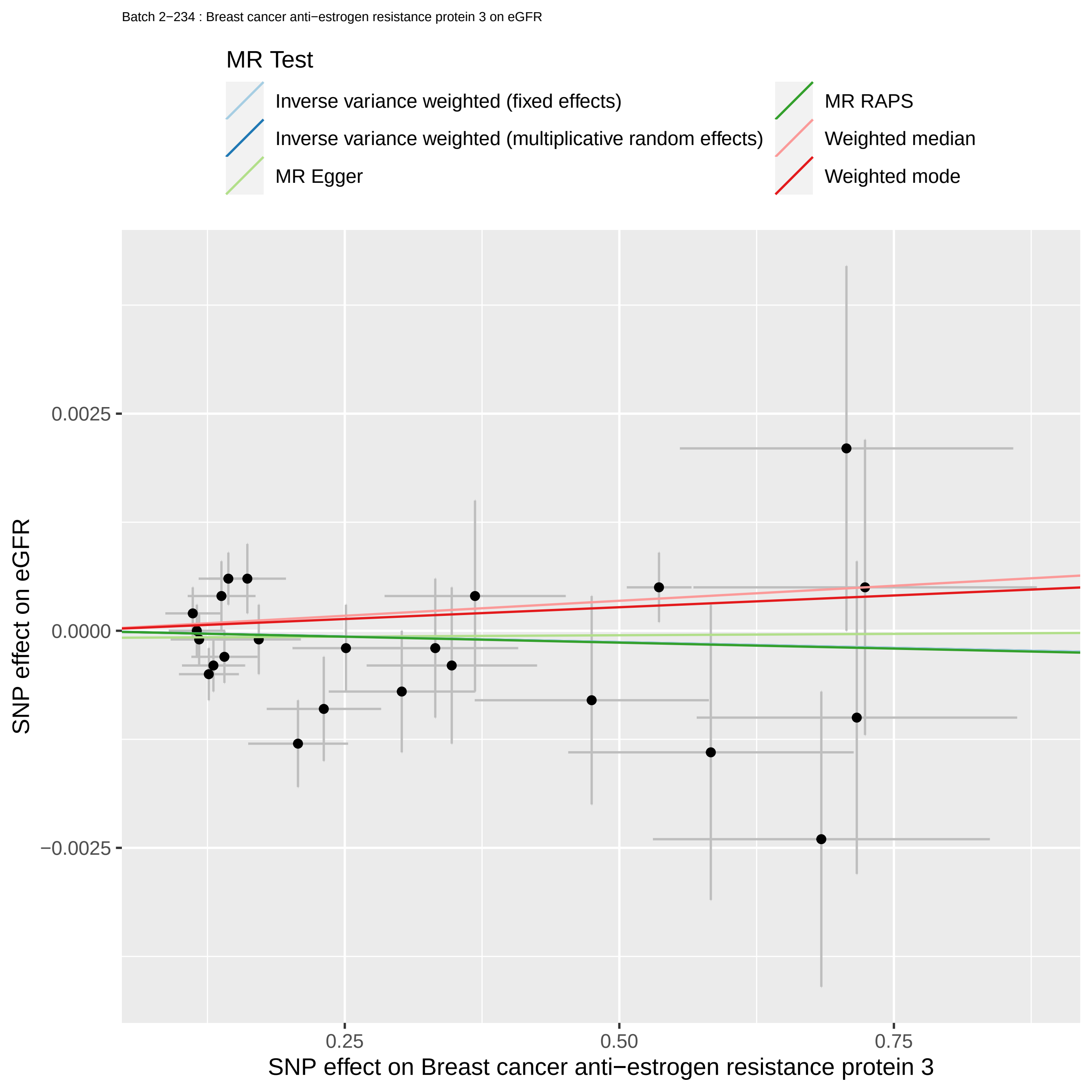

Batch 2−234 : Breast cancer anti−estrogen resistance protein 3 on eGFR
MR Test
Inverse variance weighted (fixed effects)
Inverse variance weighted (multiplicative random effects)
MR Egger
MR RAPS
Weighted median
Weighted mode
0.0025
0.0000
−0.0025
0.25
0.50
0.75
SNP effect on eGFR
SNP effect on Breast cancer anti−estrogen resistance protein 3

Batch 2−235 : B−cell lymphoma/leukemia 10 on eGFR

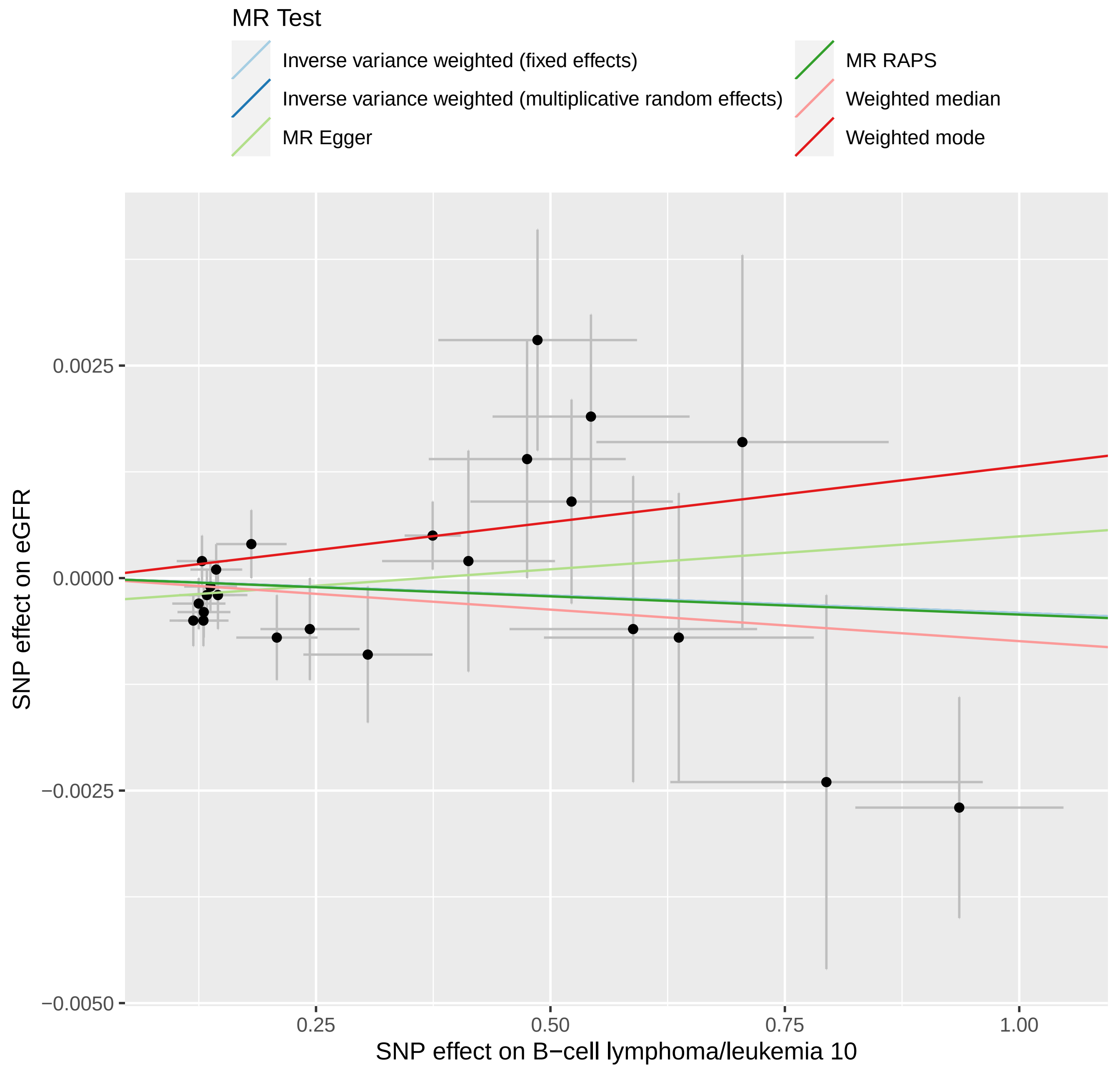

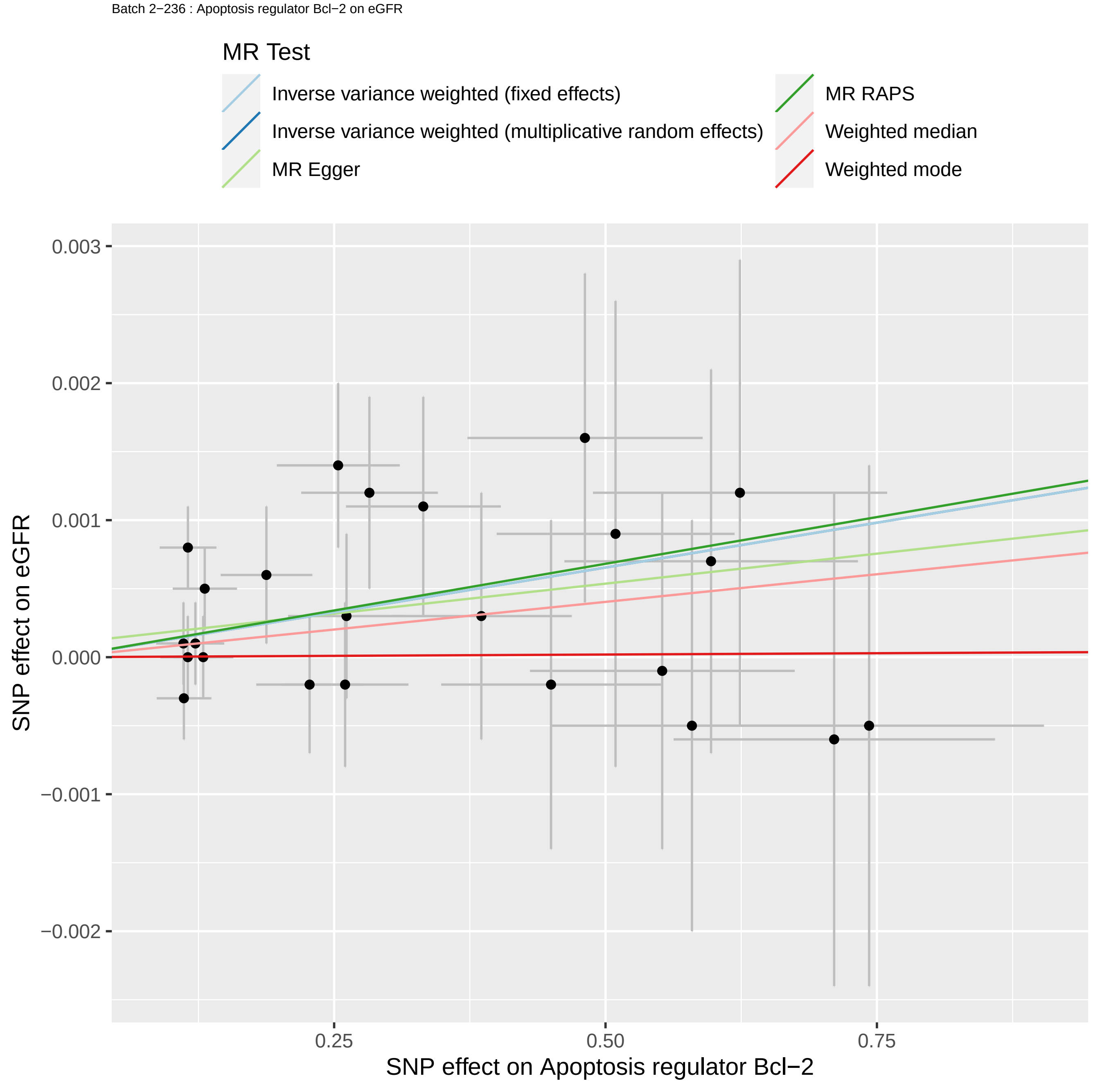

Batch 2−236 : Apoptosis regulator Bcl−2 on eGFR
MR Test
Inverse variance weighted (fixed effects)
Inverse variance weighted (multiplicative random effects)
MR Egger
MR RAPS
Weighted median
Weighted mode
0.003
0.002
0.001
0.000
−0.001
−0.002
SNP effect on eGFR
0.25
0.50
0.75
SNP effect on Apoptosis regulator Bcl−2

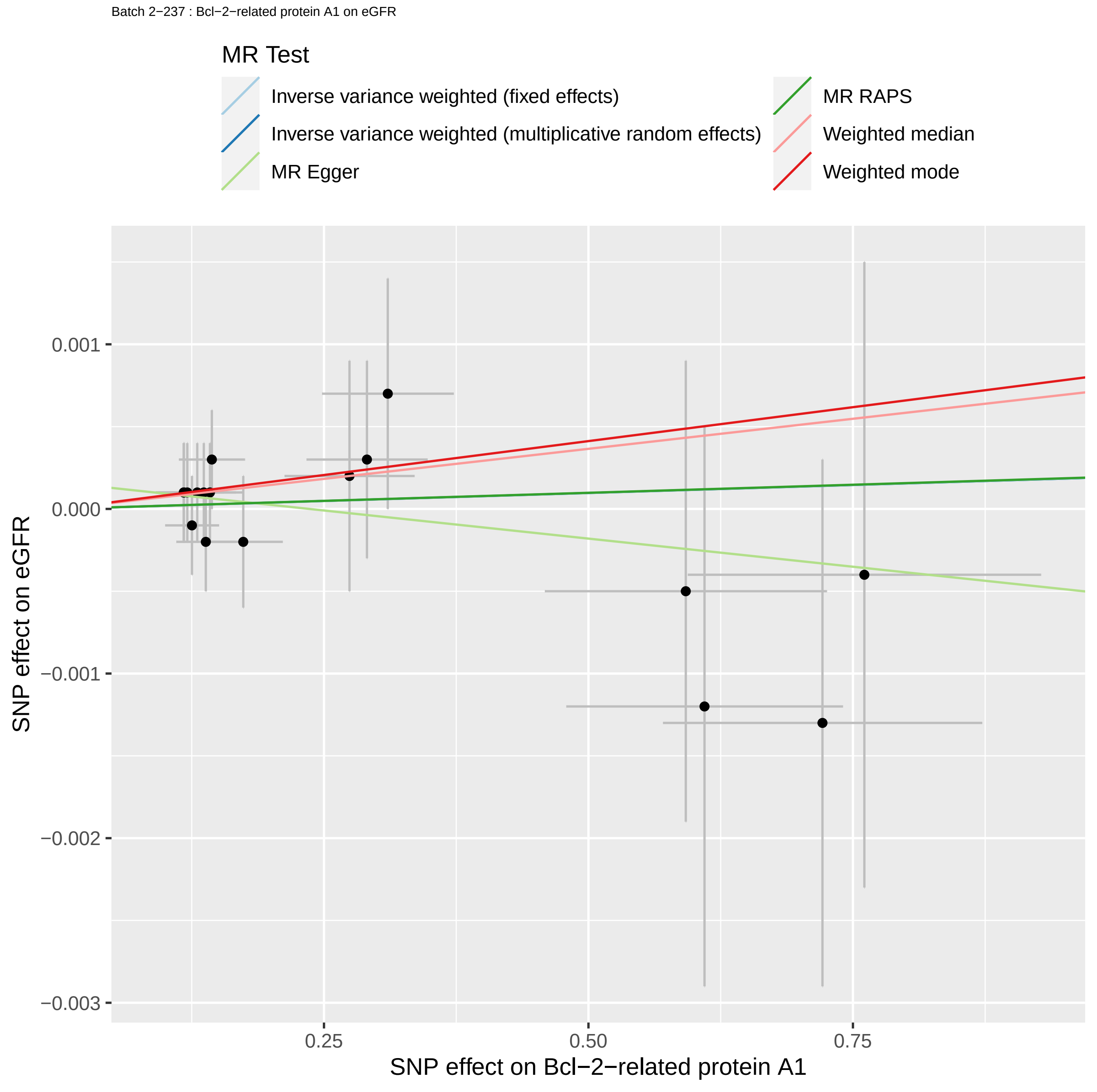

Batch 2−237 : Bcl−2−related protein A1 on eGFR
MR Test
Inverse variance weighted (fixed effects)
Inverse variance weighted (multiplicative random effects)
MR Egger
MR RAPS
Weighted median
Weighted mode
0.001
0.000
−0.001
−0.002
−0.003
0.25
0.50
0.75
SNP effect on eGFR
SNP effect on Bcl−2−related protein A1

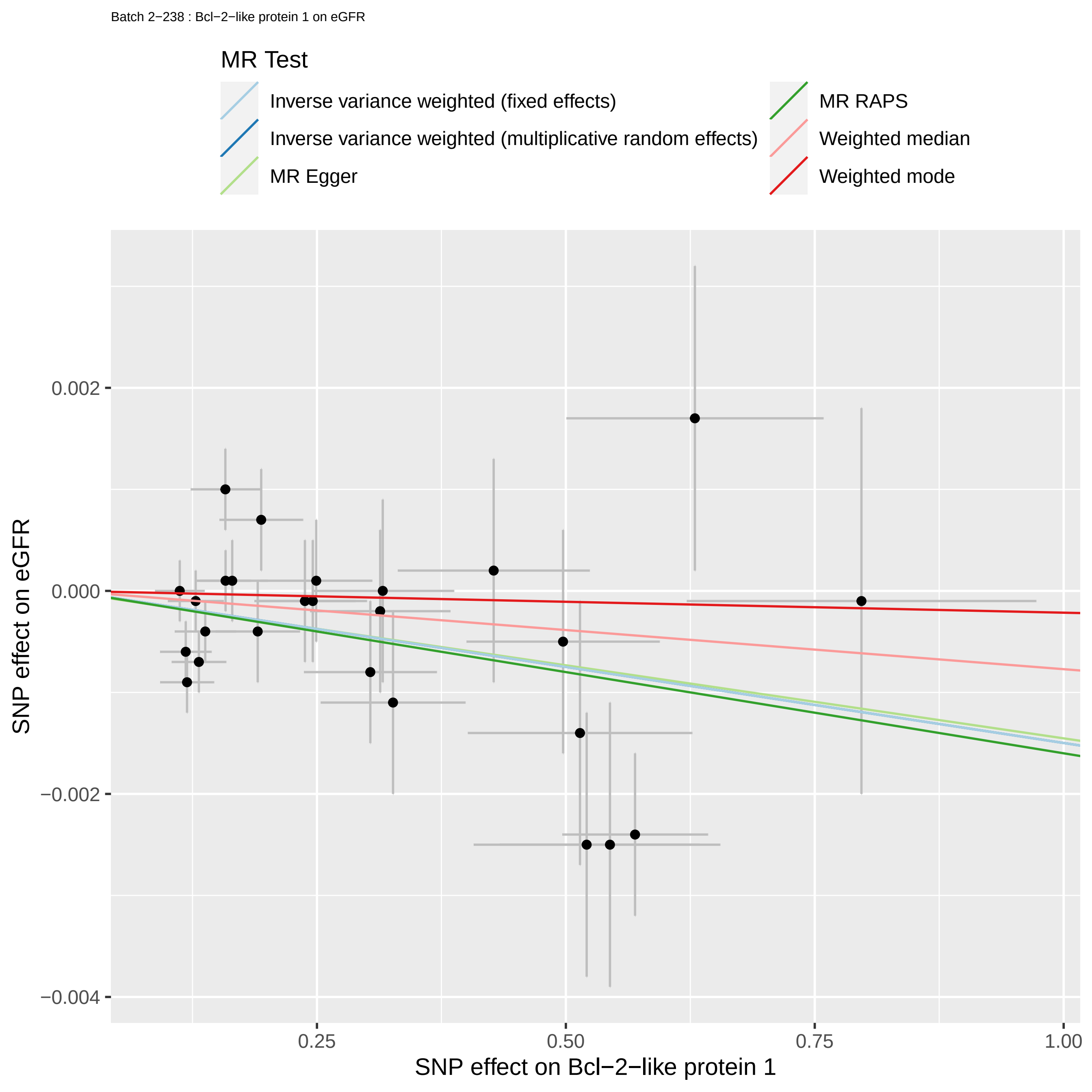

Batch 2−238 : Bcl−2−like protein 1 on eGFR
MR Test
Inverse variance weighted (fixed effects)
Inverse variance weighted (multiplicative random effects)
MR Egger
MR RAPS
Weighted median
Weighted mode
0.002
0.000
−0.002
−0.004
0.25
0.50
0.75
1.00
SNP effect on eGFR
SNP effect on Bcl−2−like protein 1

Batch 2−239 : Bcl−2−like protein 2 on eGFR

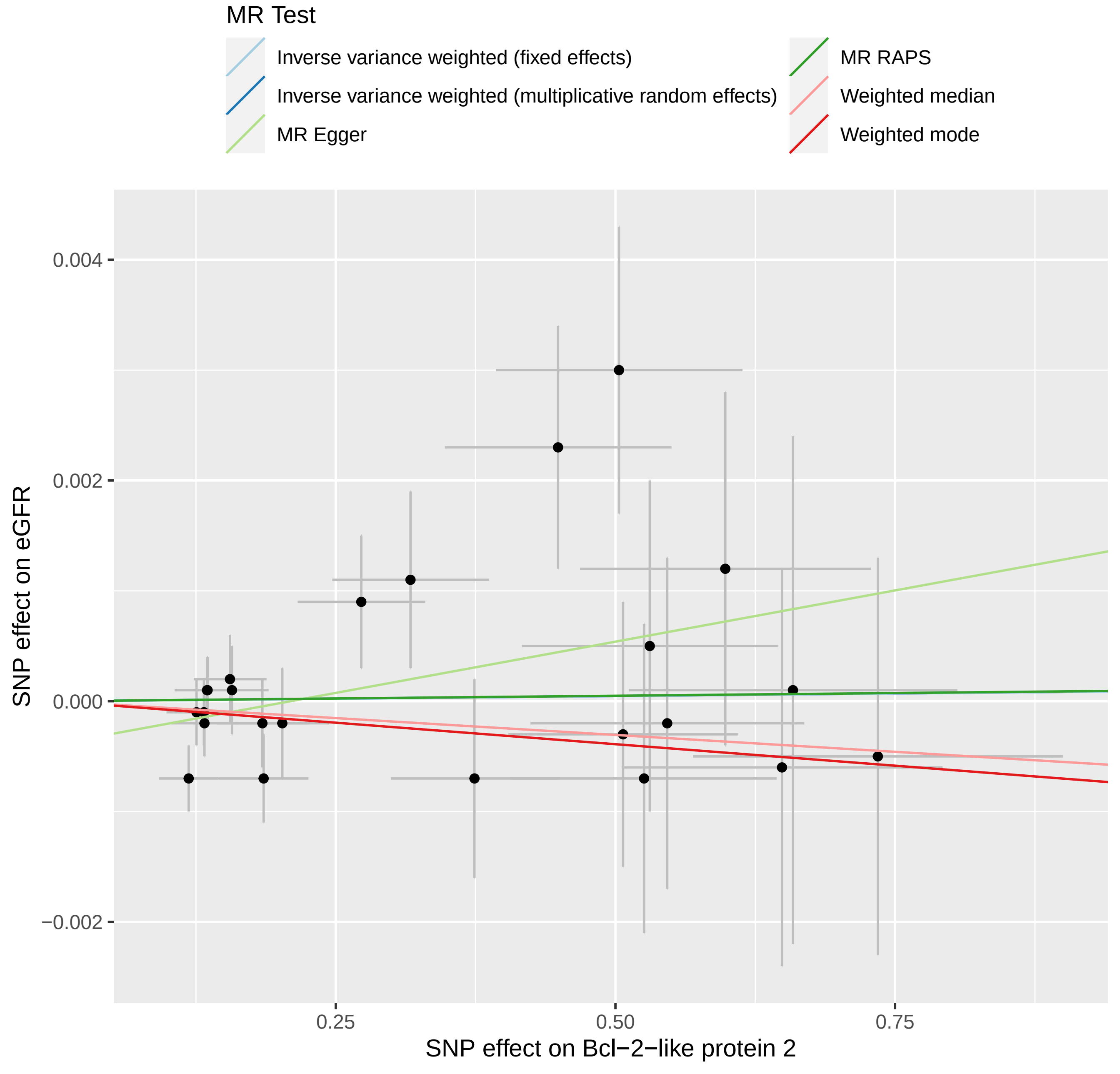

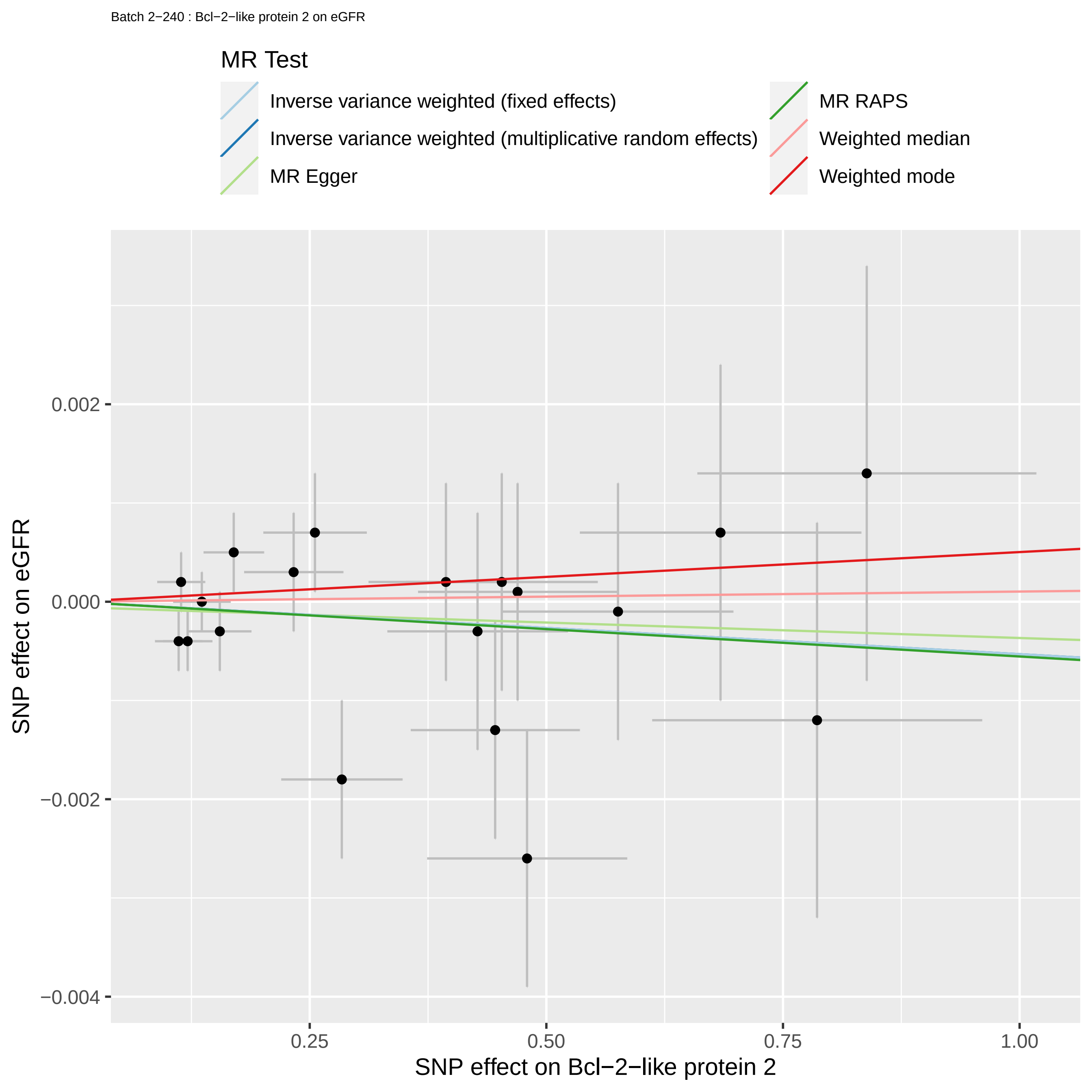

Batch 2−240 : Bcl−2−like protein 2 on eGFR
MR Test
Inverse variance weighted (fixed effects)
Inverse variance weighted (multiplicative random effects)
MR Egger
MR RAPS
Weighted median
Weighted mode
0.002
0.000
−0.002
−0.004
0.25
0.50
0.75
1.00
SNP effect on eGFR
SNP effect on Bcl−2−like protein 2

Batch 2−241 : B−cell lymphoma 6 protein on eGFR

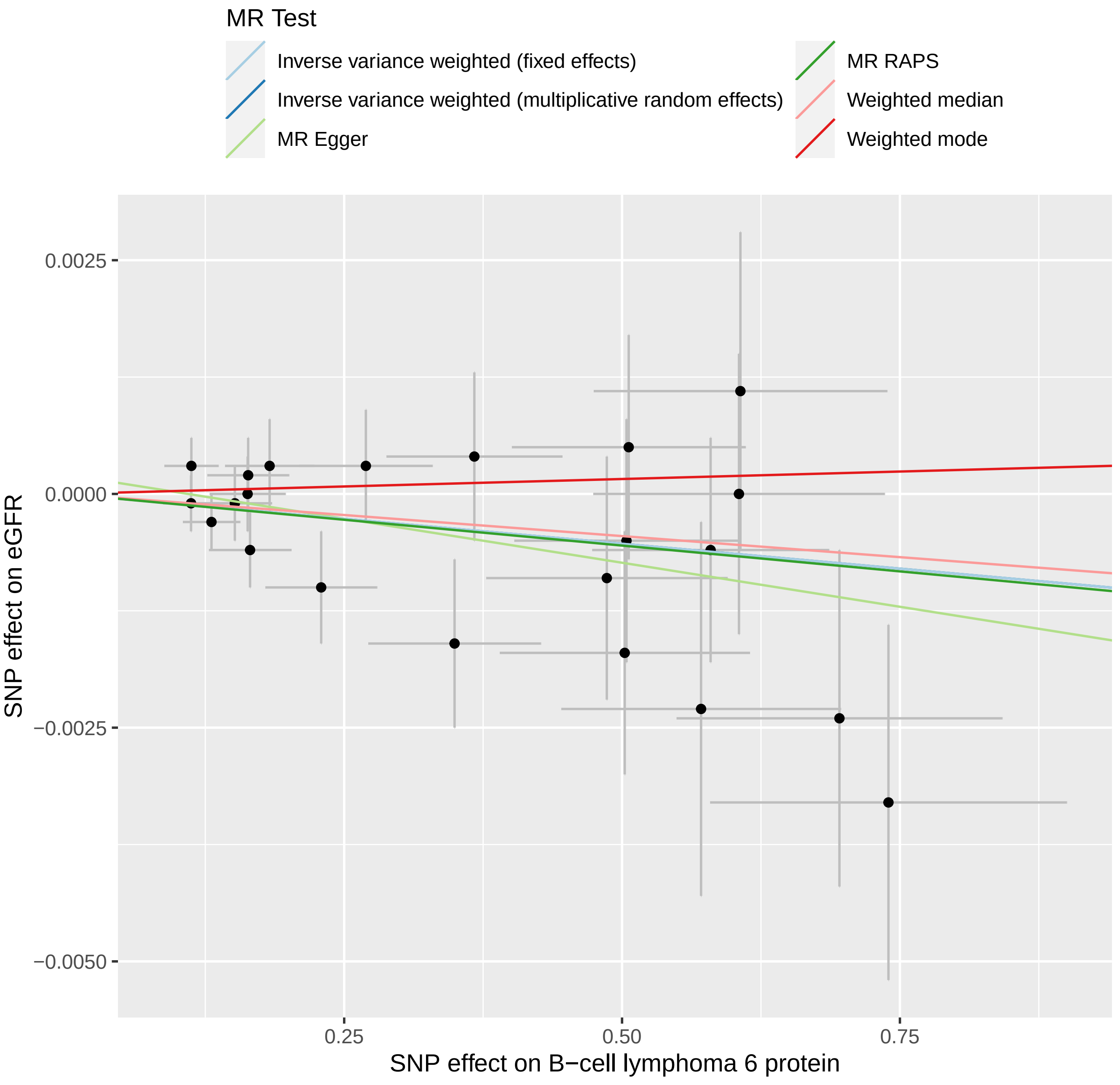

Batch 2−242 : Brain−derived neurotrophic factor on eGFR

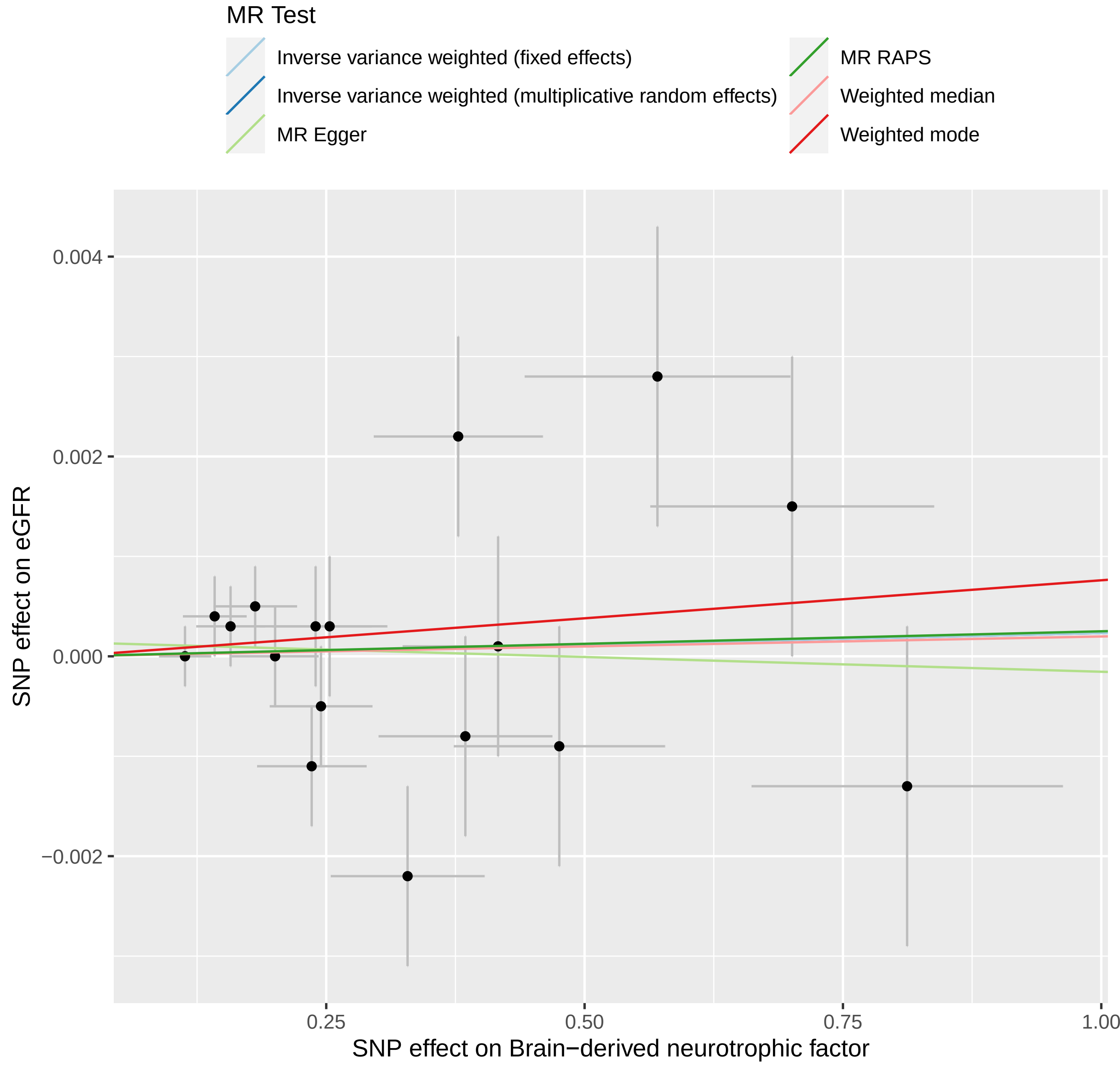

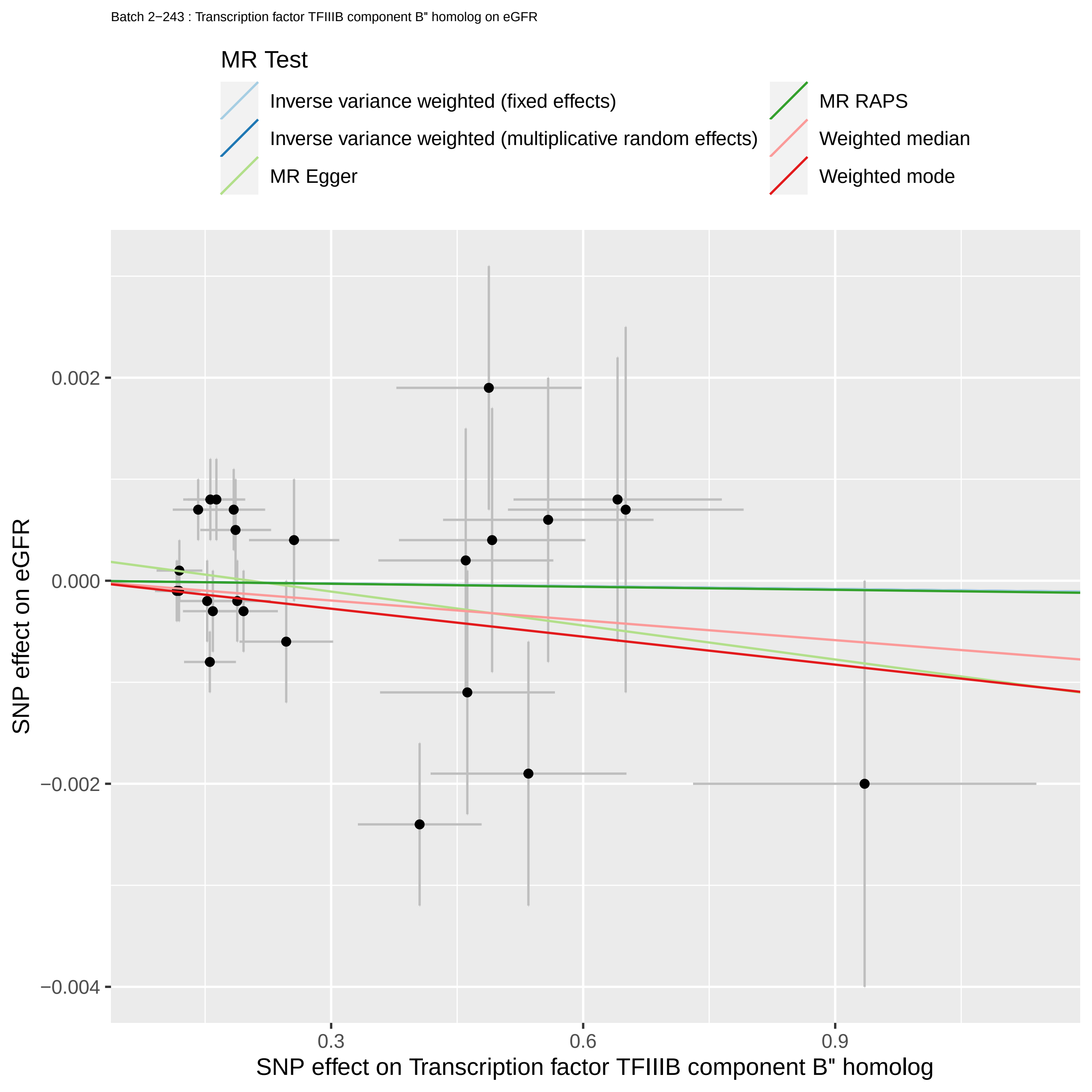
Batch 2−243 : Transcription factor TFIIIB component B" homolog on eGFR
MR Test
Inverse variance weighted (fixed effects)
Inverse variance weighted (multiplicative random effects)
MR Egger
MR RAPS
Weighted median
Weighted mode
0.002
0.000
−0.002
−0.004
0.3
0.6
0.9
SNP effect on eGFR
SNP effect on Transcription factor TFIIIB component B" homolog

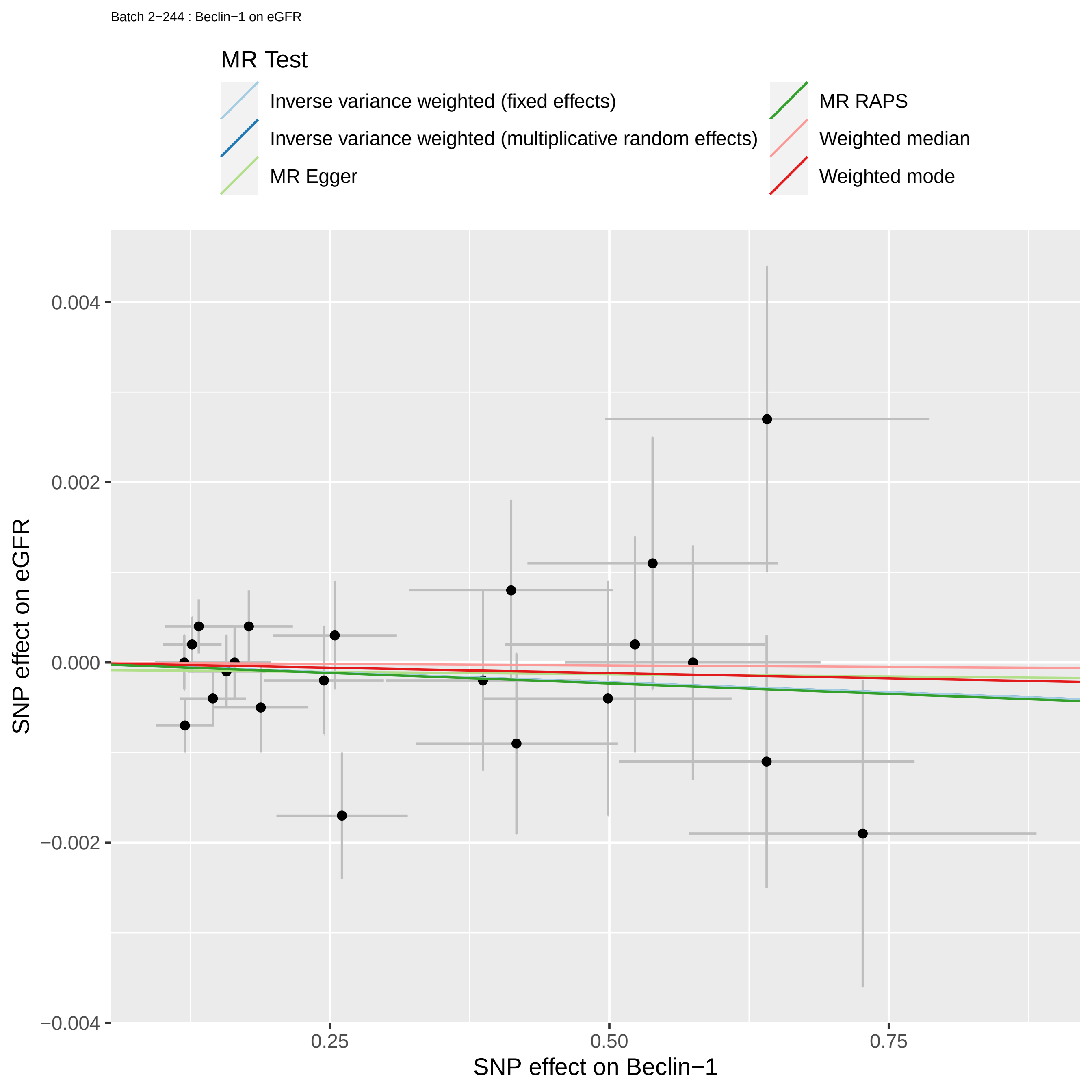

Batch 2−244 : Beclin−1 on eGFR
MR Test
Inverse variance weighted (fixed effects)
Inverse variance weighted (multiplicative random effects)
MR Egger
MR RAPS
Weighted median
Weighted mode
0.004
0.002
0.000
−0.002
−0.004
0.25
0.50
0.75
SNP effect on eGFR
SNP effect on Beclin−1

Batch 2−245 : BET1−like protein on eGFR

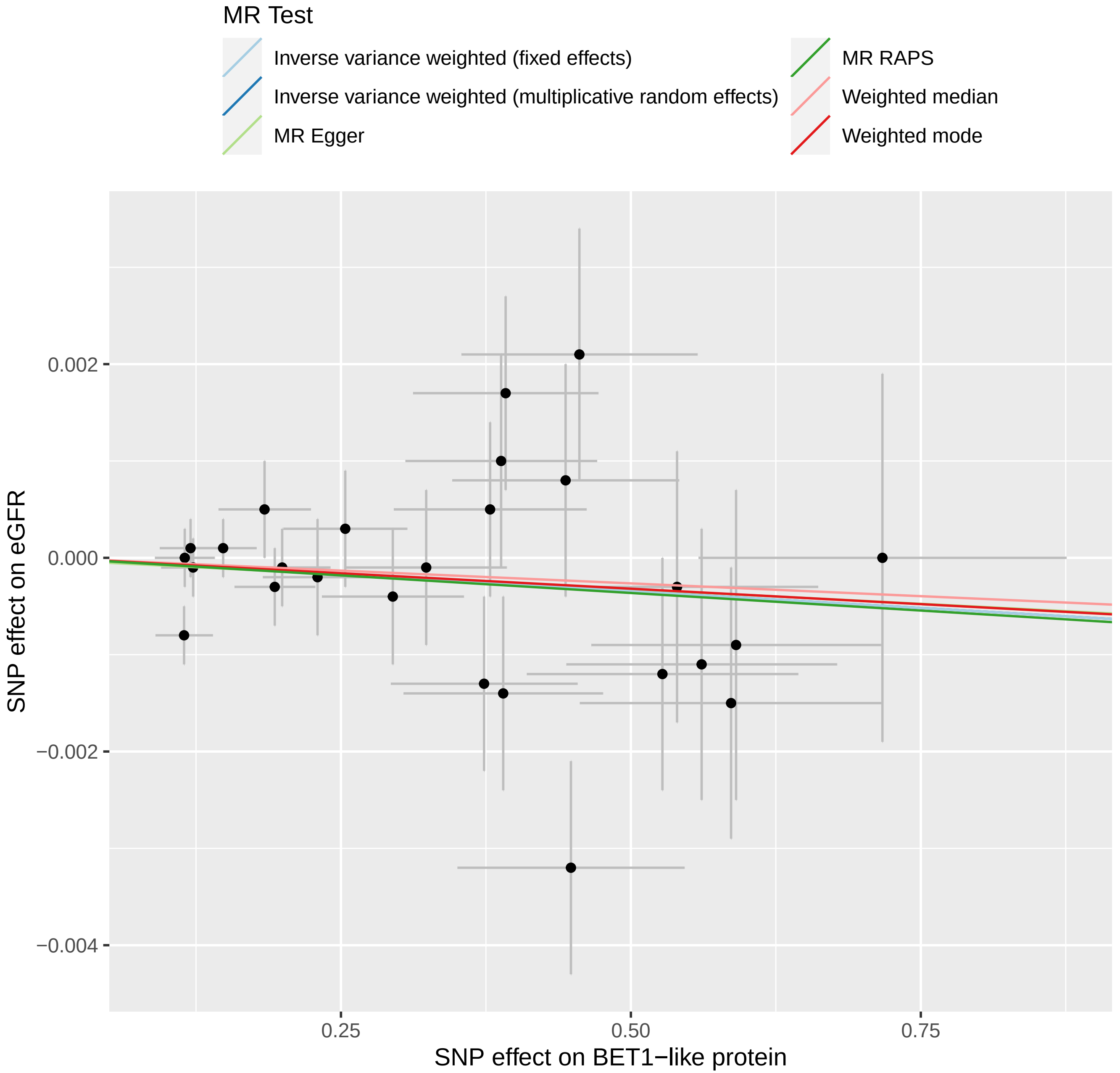

Batch 2−246 : Osteocalcin on eGFR

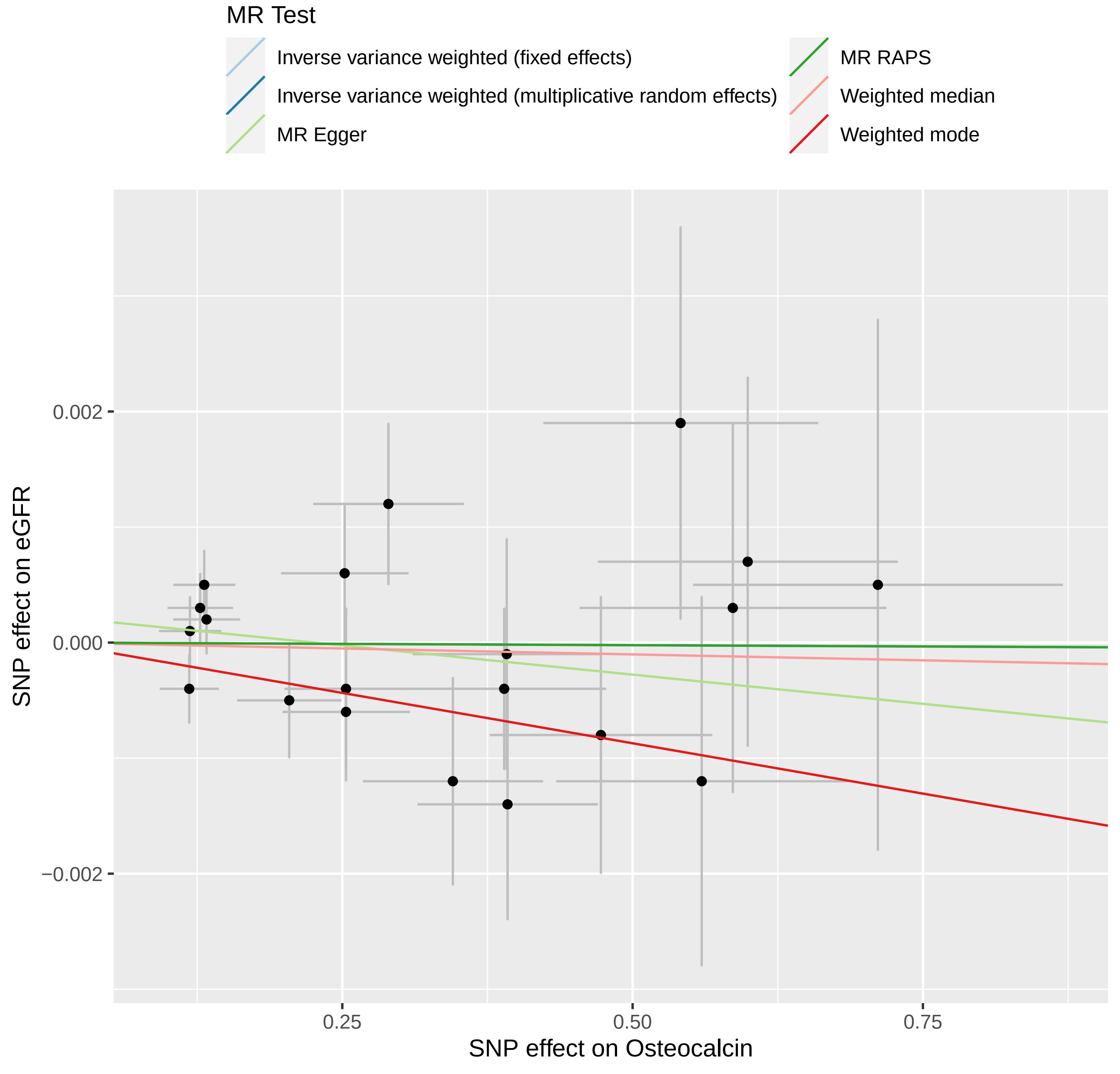

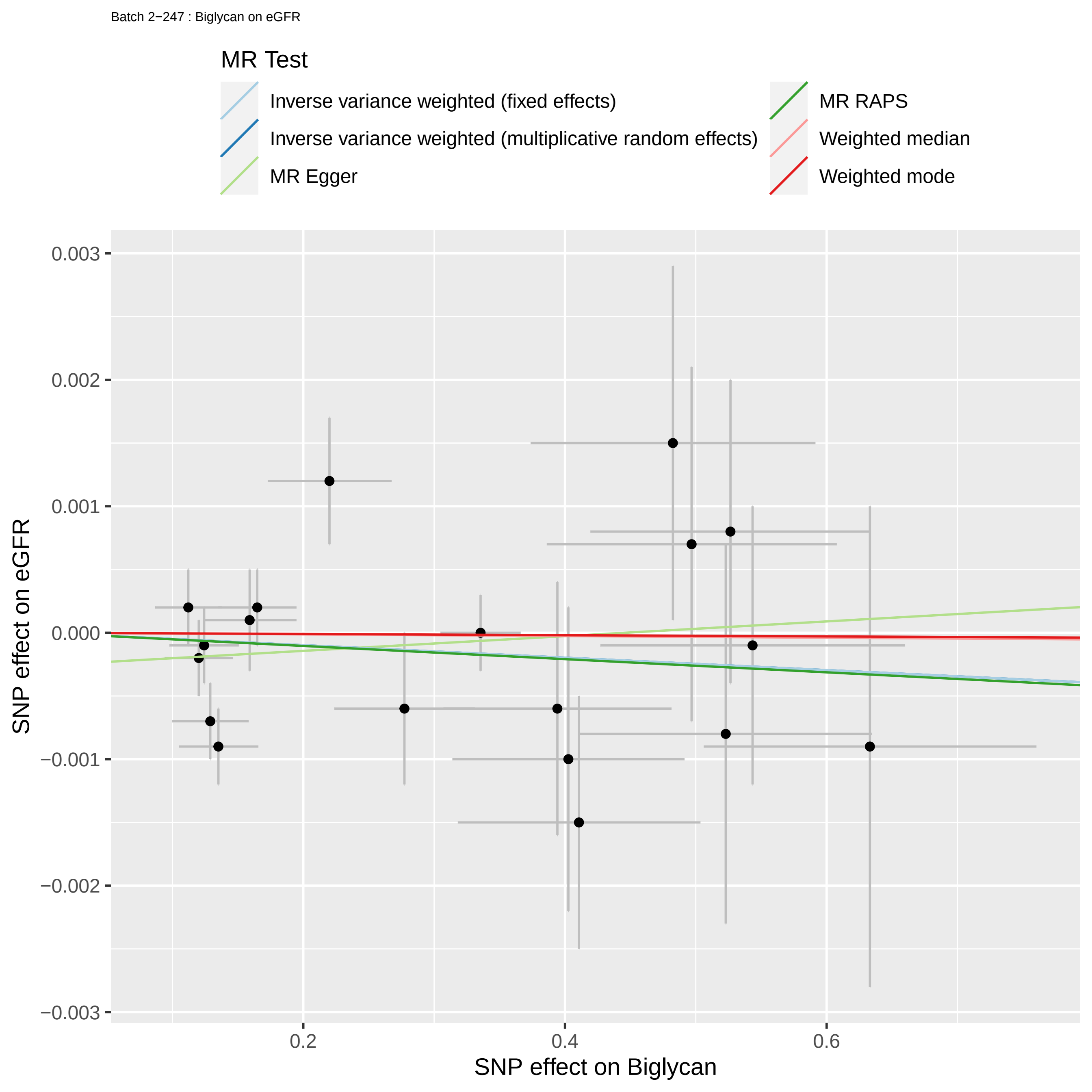

Batch 2−247 : Biglycan on eGFR
MR Test
Inverse variance weighted (fixed effects)
Inverse variance weighted (multiplicative random effects)
MR Egger
MR RAPS
Weighted median
Weighted mode
0.003
0.002
0.001
0.000
−0.001
−0.002
−0.003
SNP effect on eGFR
0.2
0.4
0.6
SNP effect on Biglycan

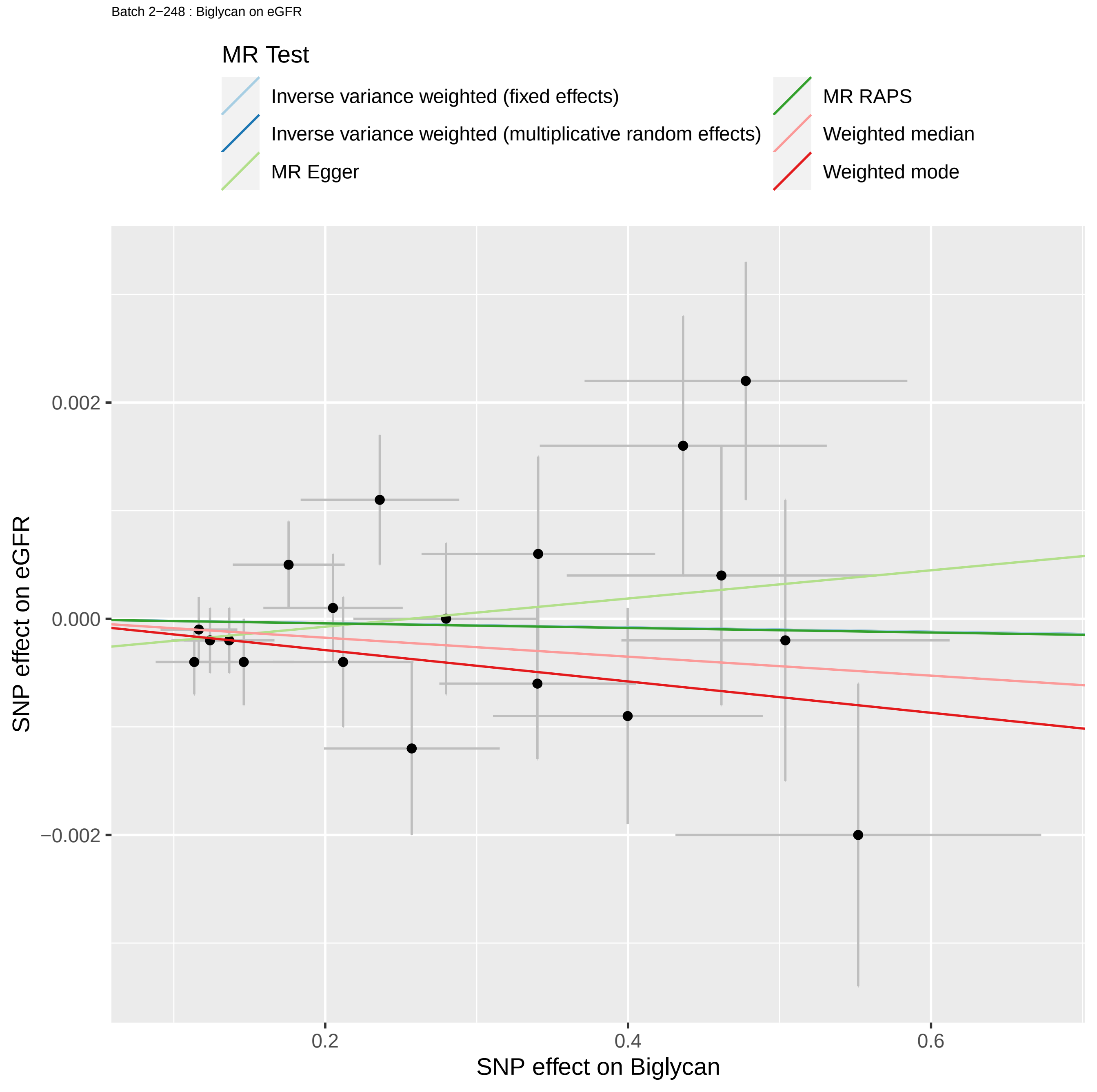

Batch 2−248 : Biglycan on eGFR
MR Test
Inverse variance weighted (fixed effects)
Inverse variance weighted (multiplicative random effects)
MR Egger
MR RAPS
Weighted median
Weighted mode
0.002
0.000
−0.002
0.2
0.4
0.6
SNP effect on eGFR
SNP effect on Biglycan

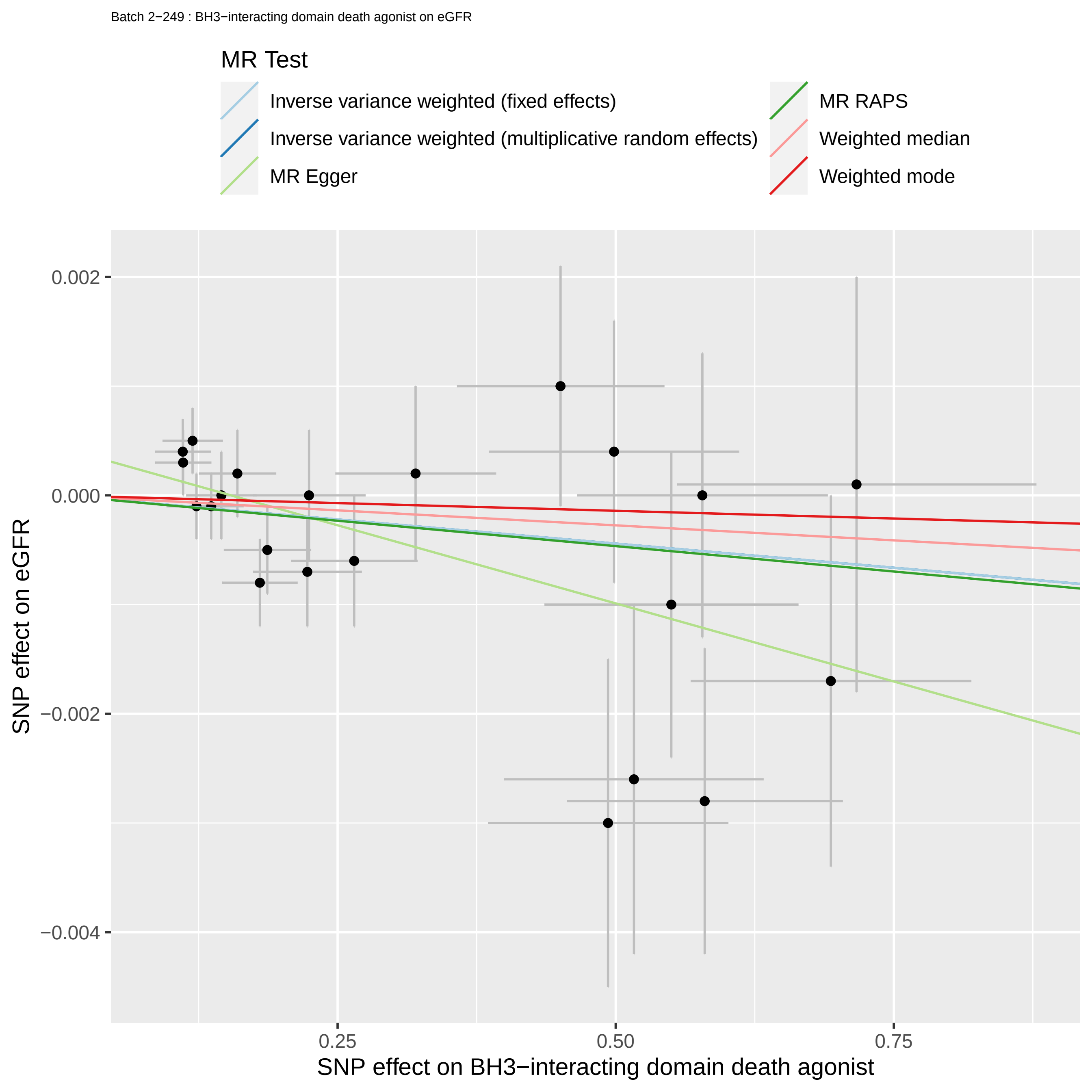

Batch 2−249 : BH3−interacting domain death agonist on eGFR
MR Test
Inverse variance weighted (fixed effects)
Inverse variance weighted (multiplicative random effects)
MR Egger
MR RAPS
Weighted median
Weighted mode
0.002
0.000
−0.002
−0.004
0.25
0.50
0.75
SNP effect on eGFR
SNP effect on BH3−interacting domain death agonist

Batch 2−250 : Myc box−dependent−interacting protein 1 on eGFR

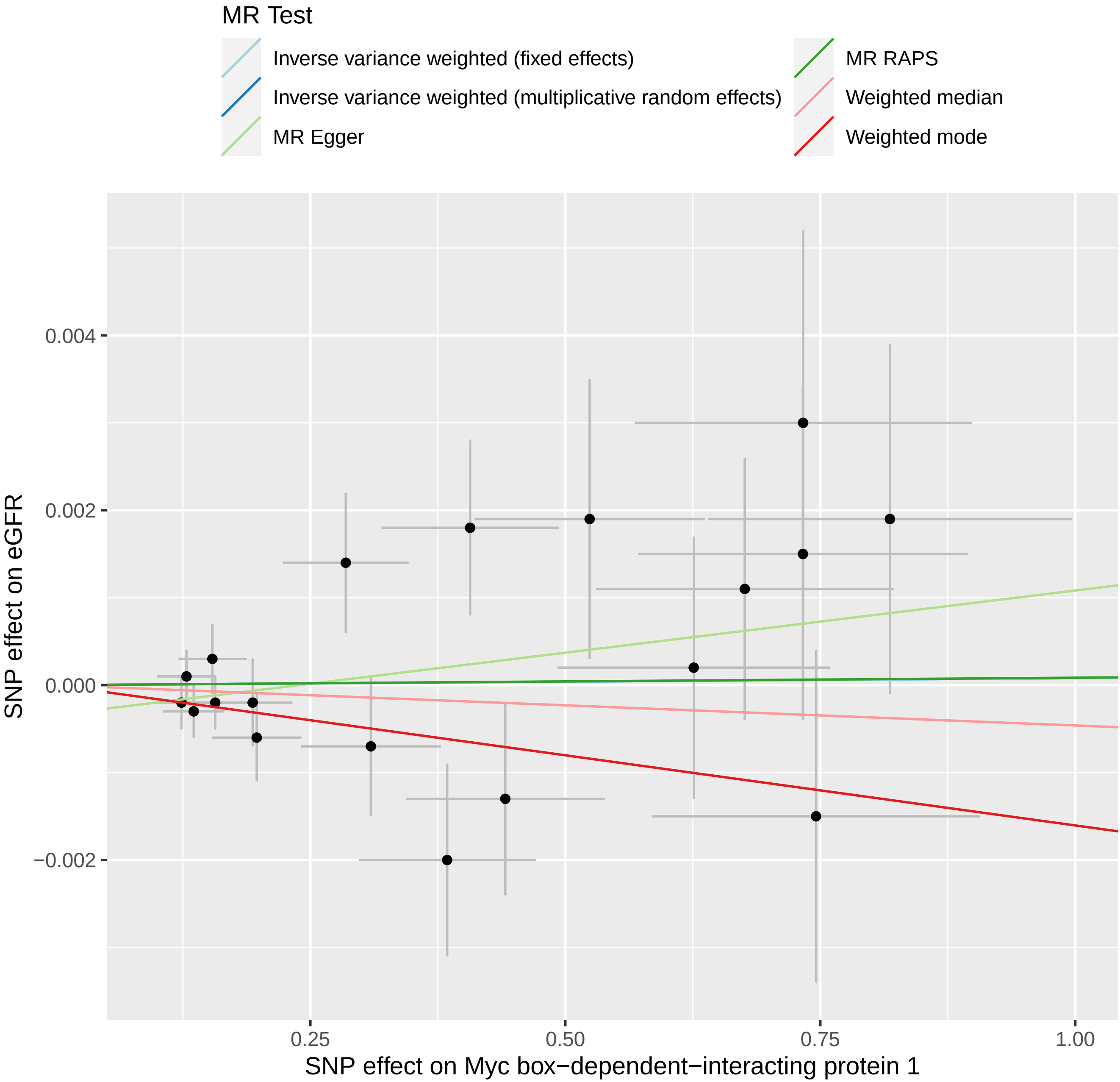

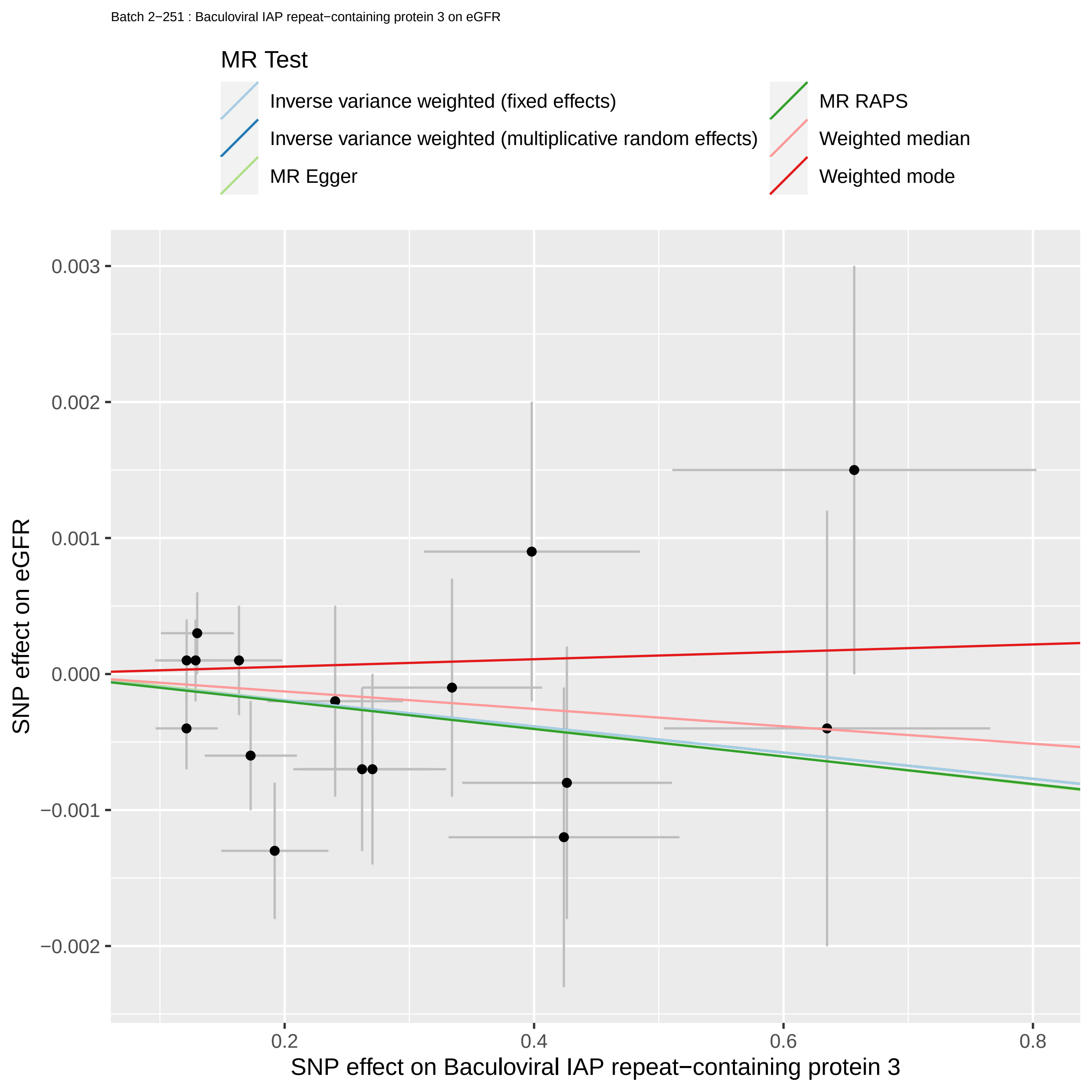
Batch 2−251 : Baculoviral IAP repeat−containing protein 3 on eGFR
MR Test
Inverse variance weighted (fixed effects)
Inverse variance weighted (multiplicative random effects)
MR Egger
MR RAPS
Weighted median
Weighted mode
0.003
0.002
0.001
0.000
−0.001
−0.002
0.2
0.4
0.6
0.8
SNP effect on eGFR
SNP effect on Baculoviral IAP repeat−containing protein 3

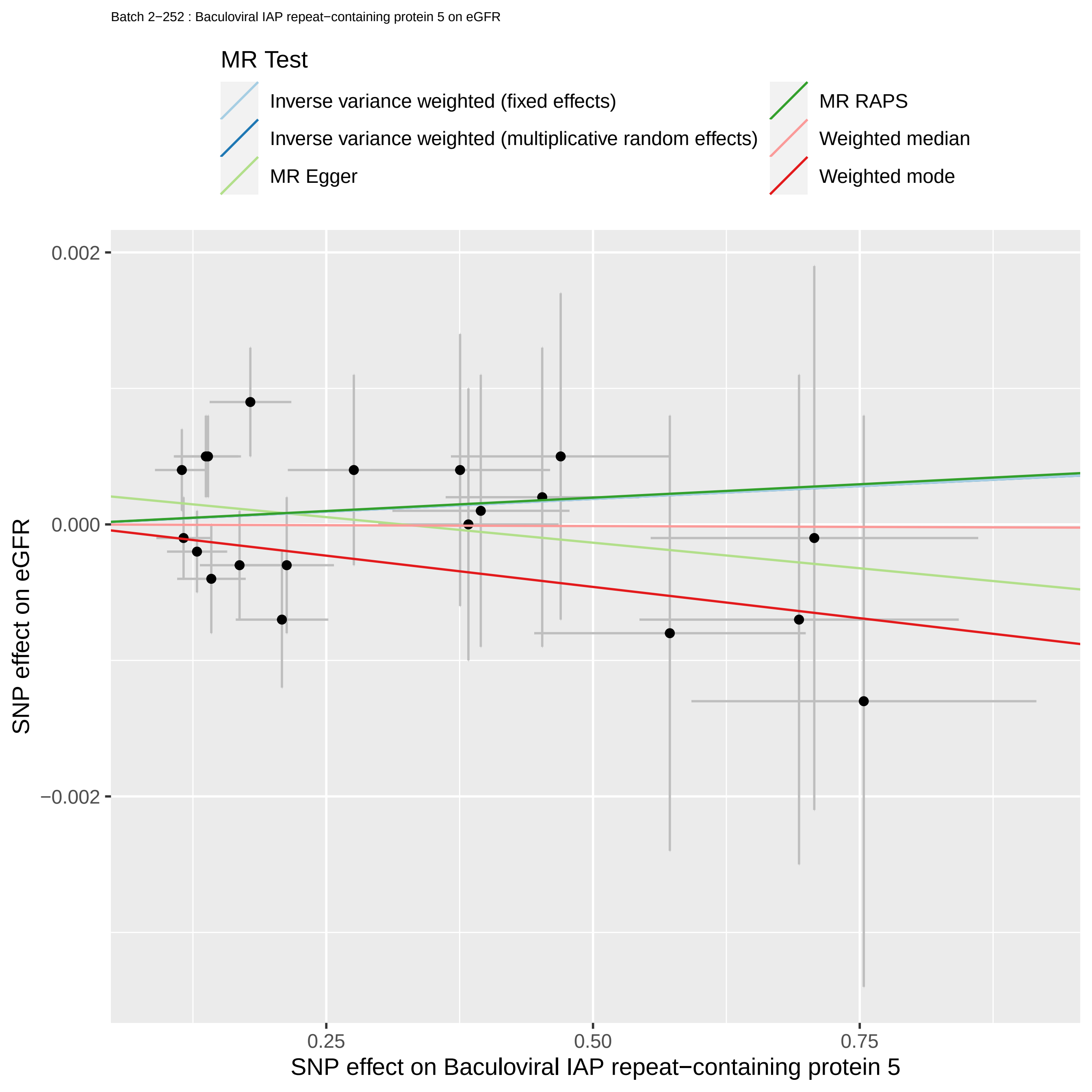

Batch 2−252 : Baculoviral IAP repeat−containing protein 5 on eGFR
MR Test
Inverse variance weighted (fixed effects)
Inverse variance weighted (multiplicative random effects)
MR Egger
MR RAPS
Weighted median
Weighted mode
0.002
0.000
−0.002
0.25
0.50
0.75
SNP effect on eGFR
SNP effect on Baculoviral IAP repeat−containing protein 5

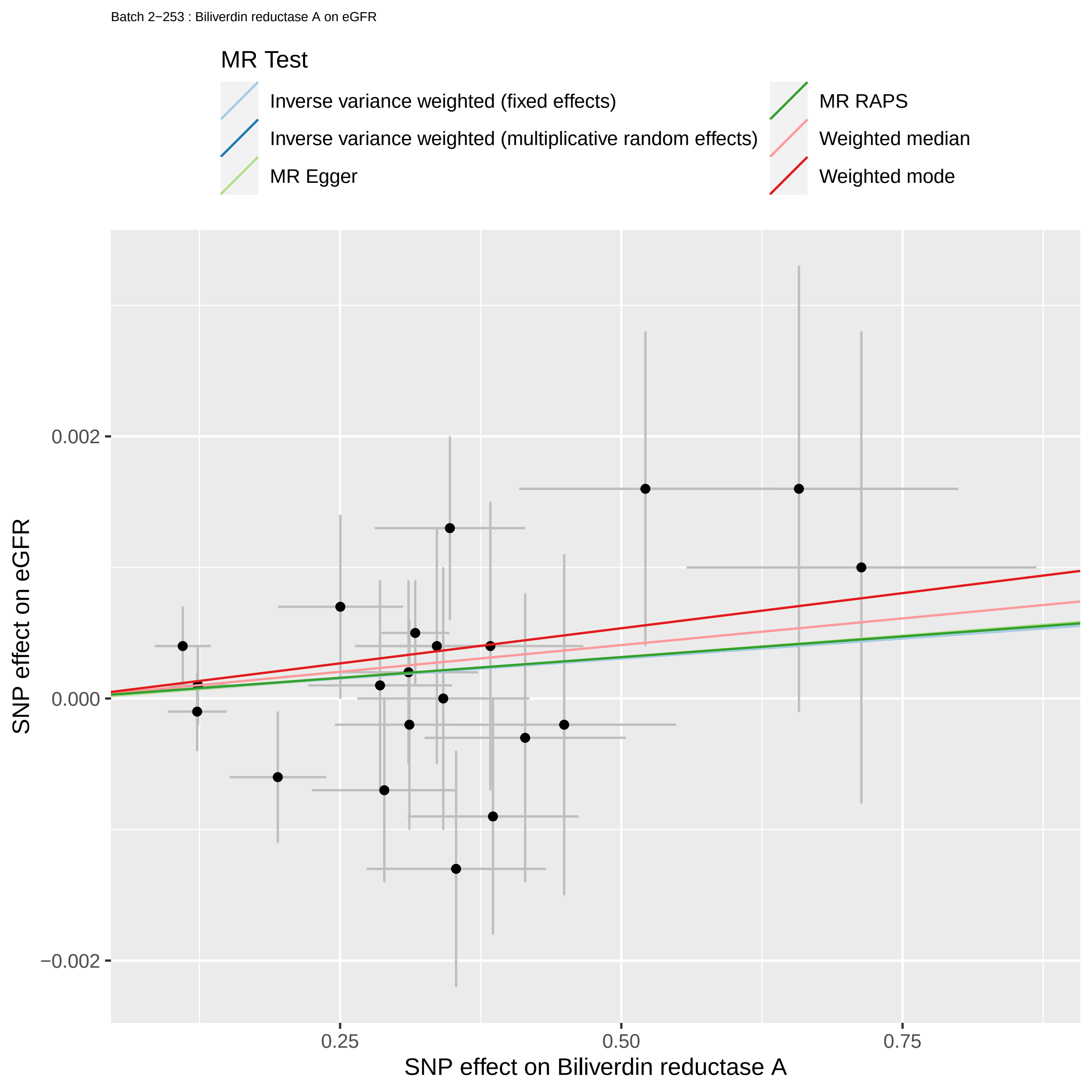

Batch 2−253 : Biliverdin reductase A on eGFR
MR Test
Inverse variance weighted (fixed effects)
Inverse variance weighted (multiplicative random effects)
MR Egger
MR RAPS
Weighted median
Weighted mode
0.002
0.000
−0.002
0.25
0.50
0.75
SNP effect on eGFR
SNP effect on Biliverdin reductase A

Batch 2−254 : Bone morphogenetic protein 10 on eGFR

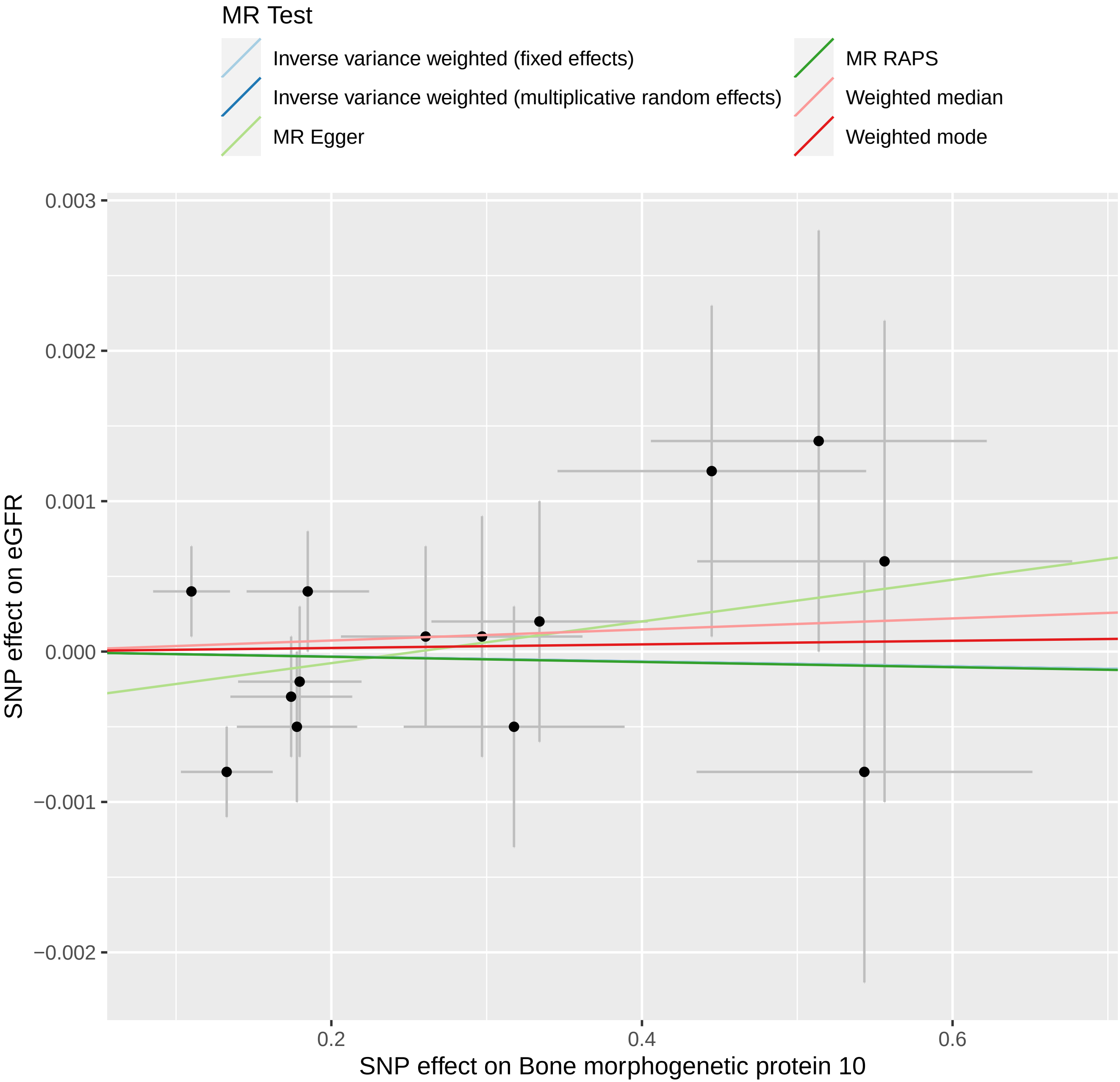

Batch 2−255 : Bone morphogenetic protein 6 on eGFR

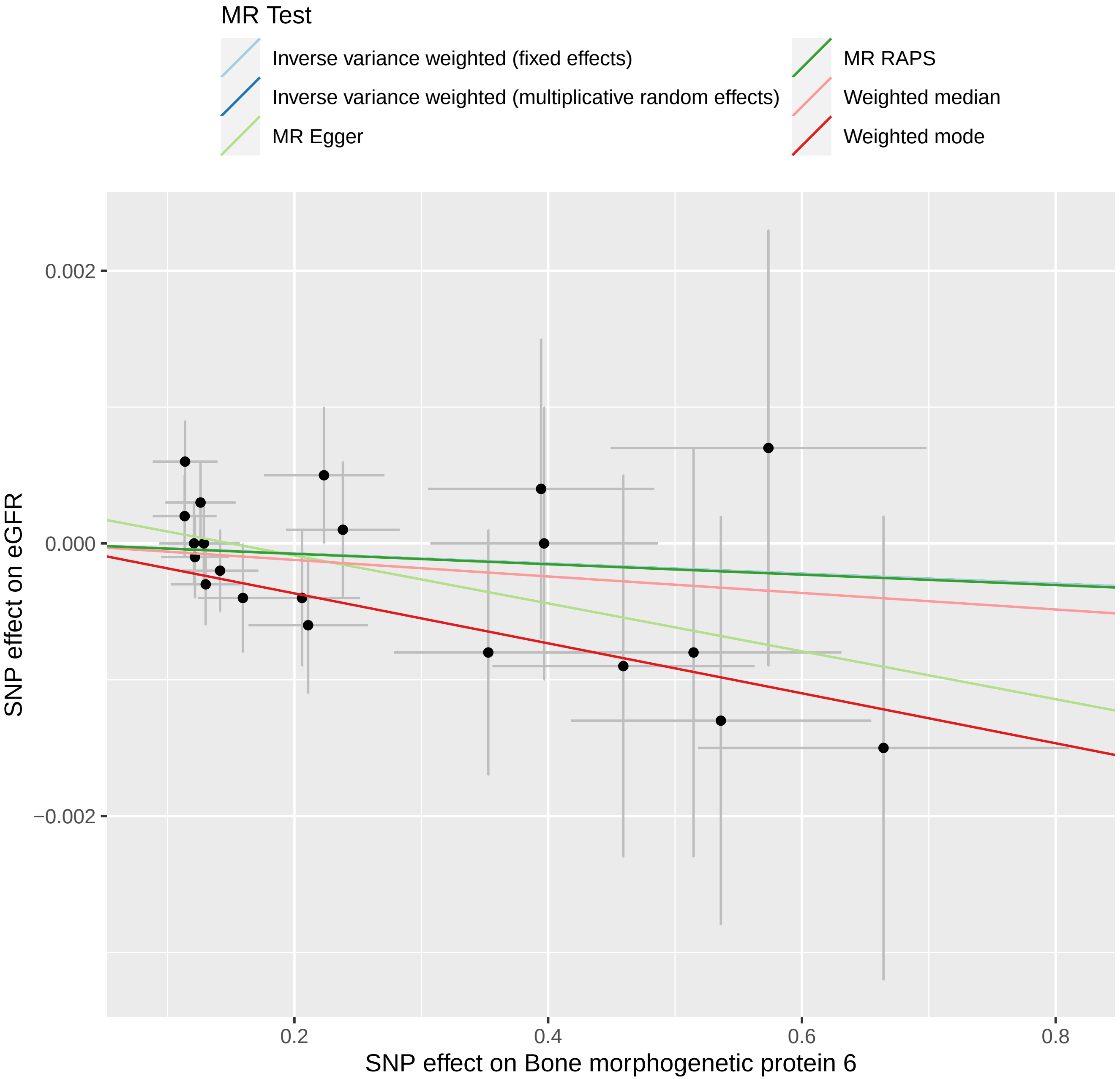

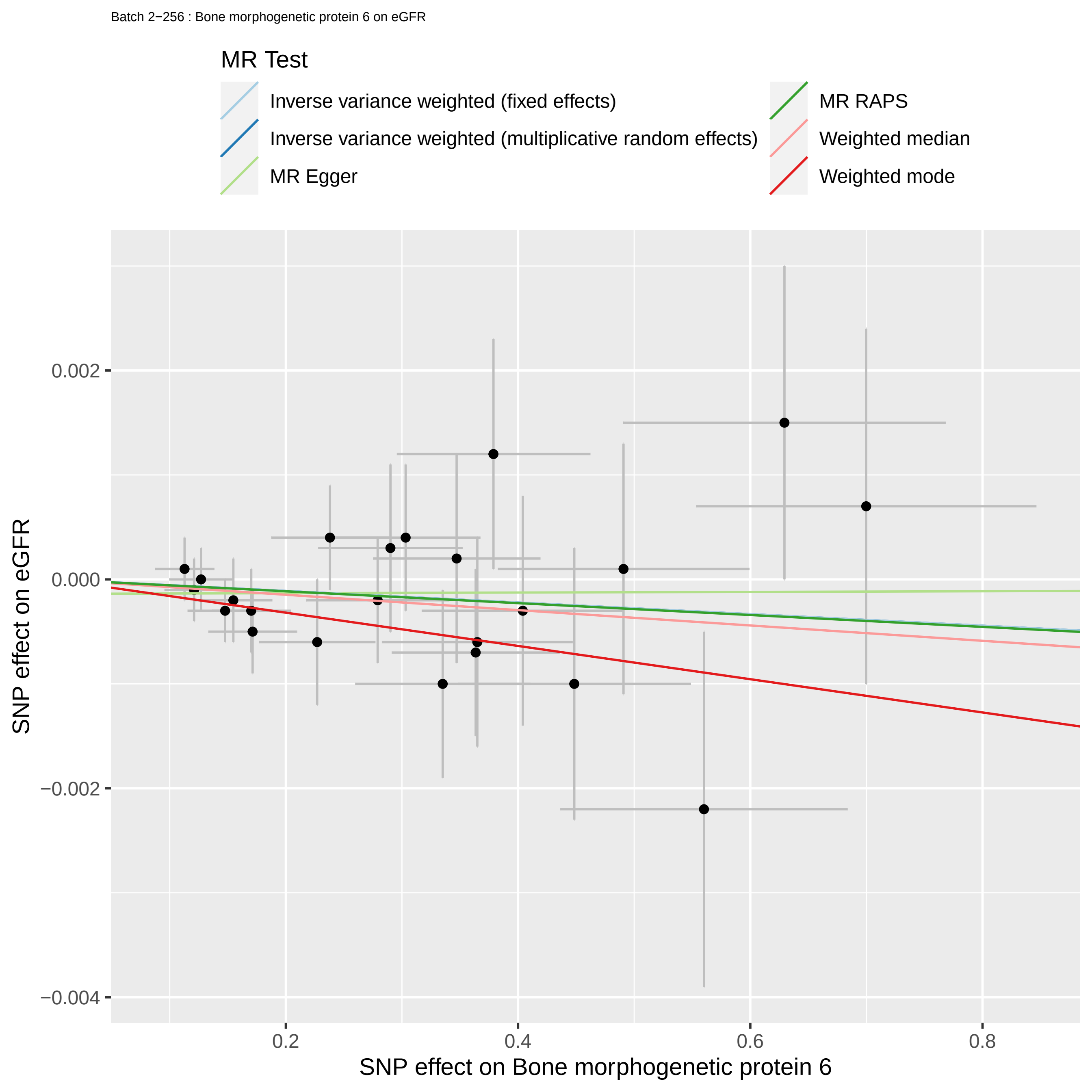
Batch 2−256 : Bone morphogenetic protein 6 on eGFR
MR Test
Inverse variance weighted (fixed effects)
Inverse variance weighted (multiplicative random effects)
MR Egger
MR RAPS
Weighted median
Weighted mode
0.002
0.000
−0.002
−0.004
0.2
0.4
0.6
0.8
SNP effect on eGFR
SNP effect on Bone morphogenetic protein 6

Batch 2−257 : Bone morphogenetic protein 7 on eGFR

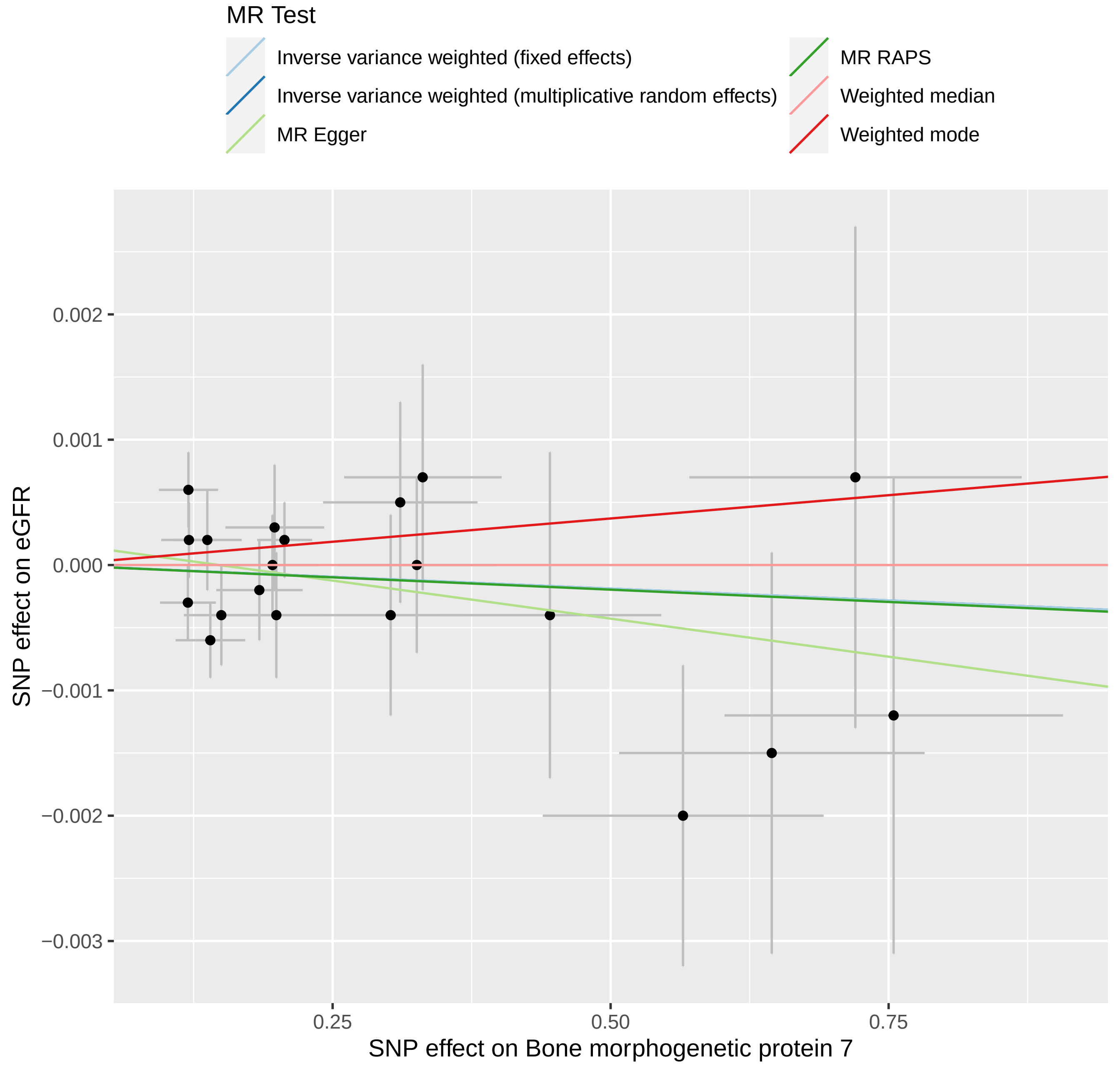

Batch 2−258 : BMP−binding endothelial regulator protein on eGFR

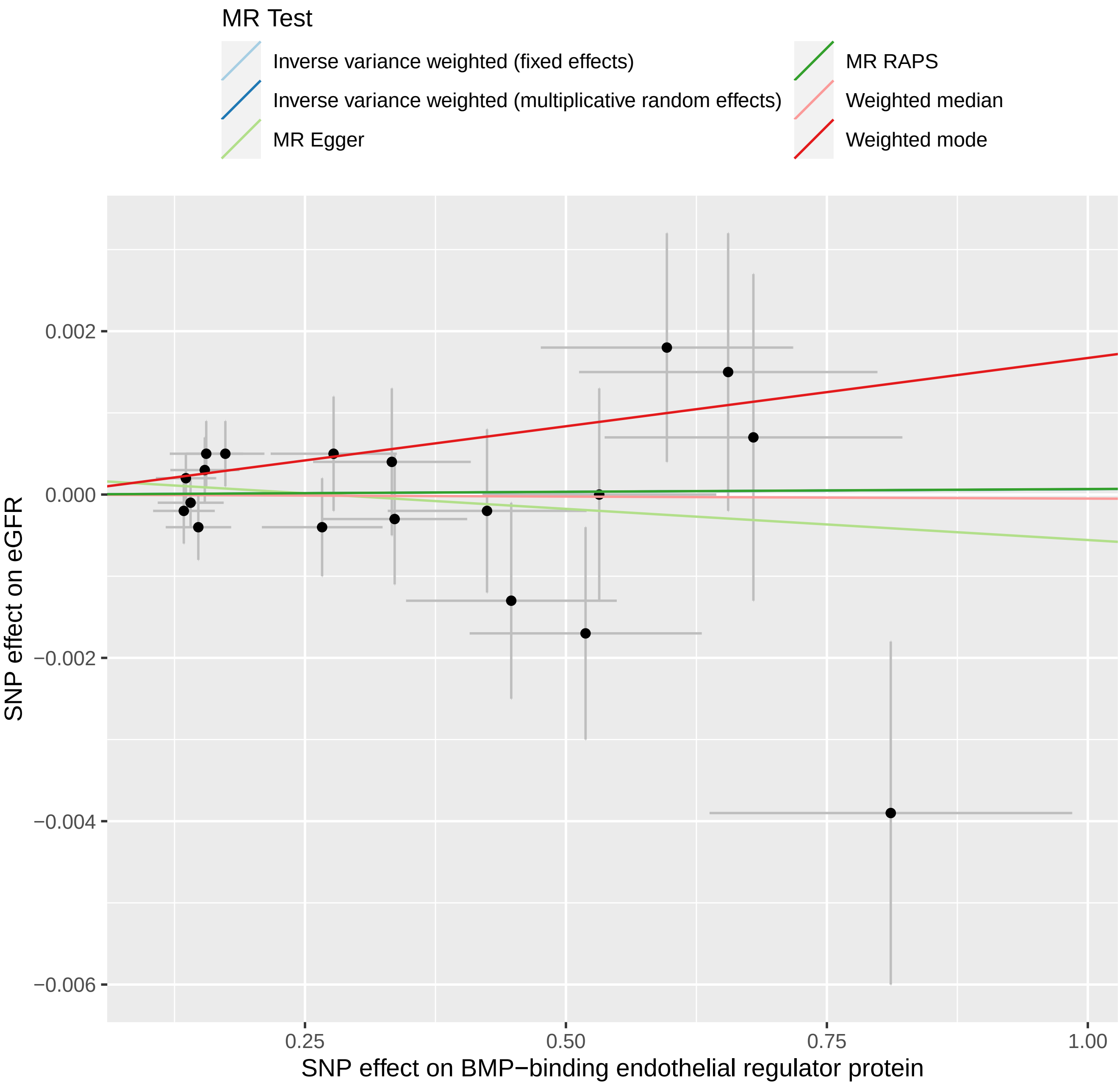

Batch 2−259 : Bone morphogenetic protein receptor type−1A on eGFR

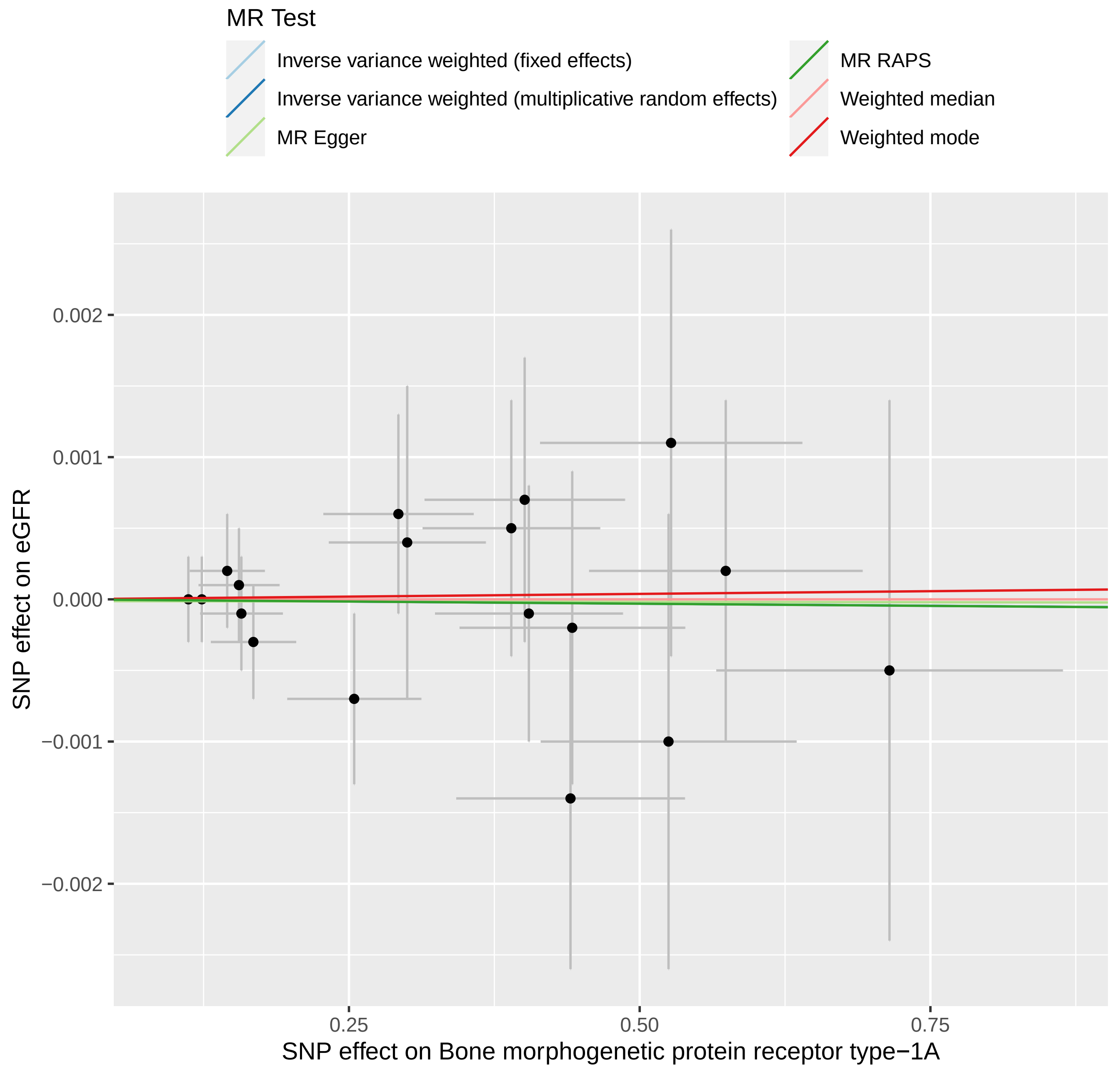

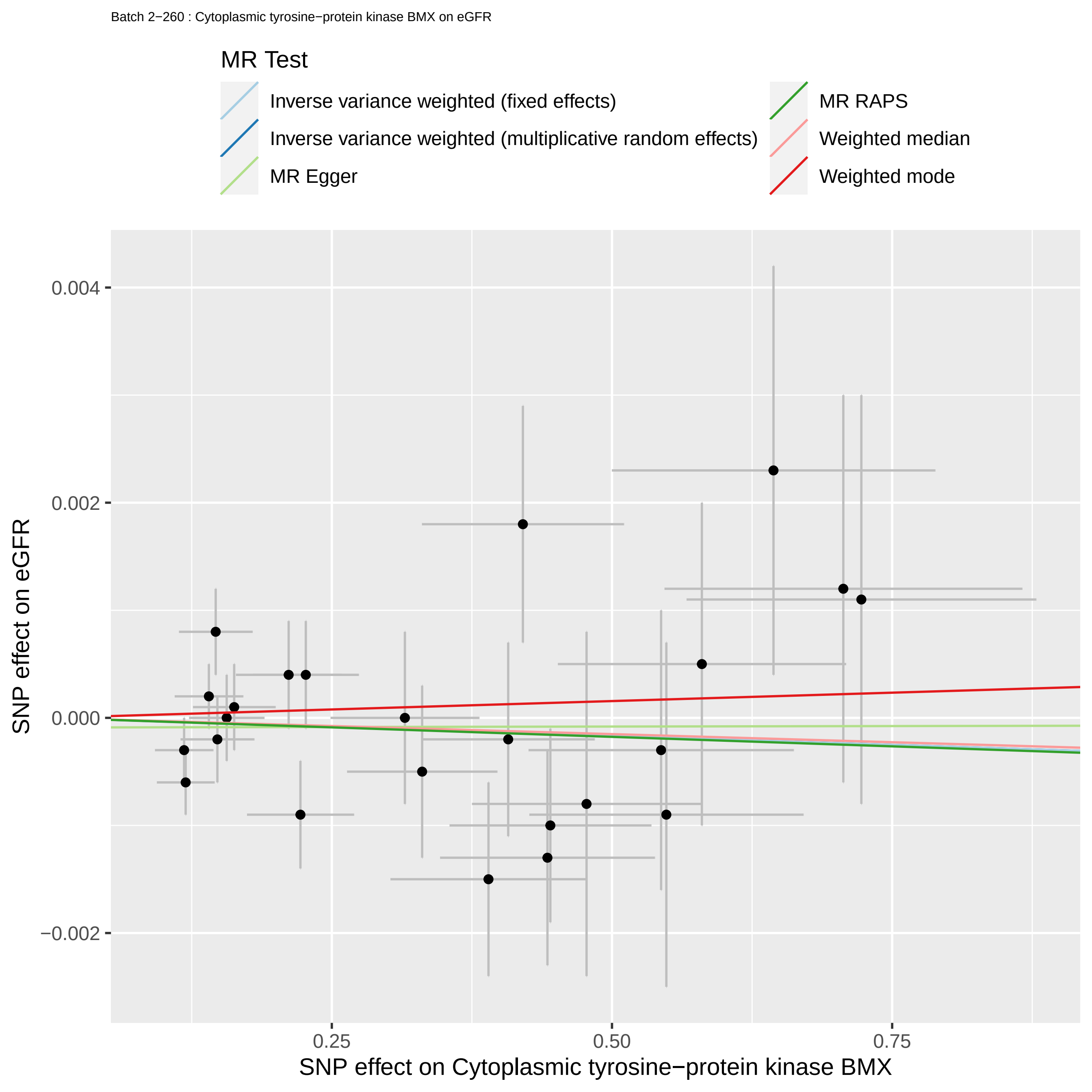

Batch 2−260 : Cytoplasmic tyrosine−protein kinase BMX on eGFR
MR Test
Inverse variance weighted (fixed effects)
Inverse variance weighted (multiplicative random effects)
MR Egger
MR RAPS
Weighted median
Weighted mode
0.004
0.002
0.000
−0.002
0.25
0.50
0.75
SNP effect on eGFR
SNP effect on Cytoplasmic tyrosine−protein kinase BMX

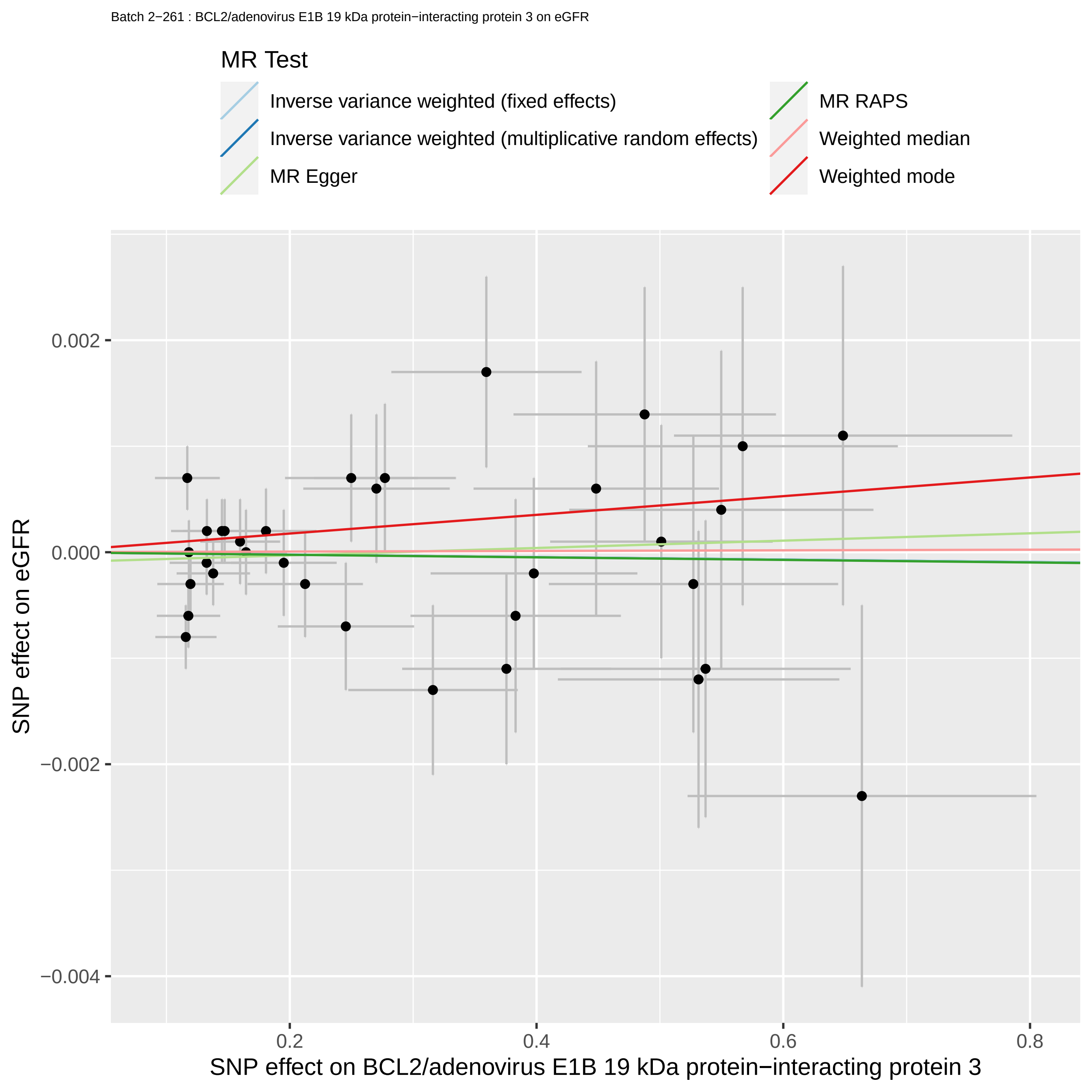

Batch 2−261 : BCL2/adenovirus E1B 19 kDa protein−interacting protein 3 on eGFR
MR Test
Inverse variance weighted (fixed effects)
Inverse variance weighted (multiplicative random effects)
MR Egger
MR RAPS
Weighted median
Weighted mode
0.002
0.000
−0.002
−0.004
0.2
0.4
0.6
0.8
SNP effect on eGFR
SNP effect on BCL2/adenovirus E1B 19 kDa protein−interacting protein 3

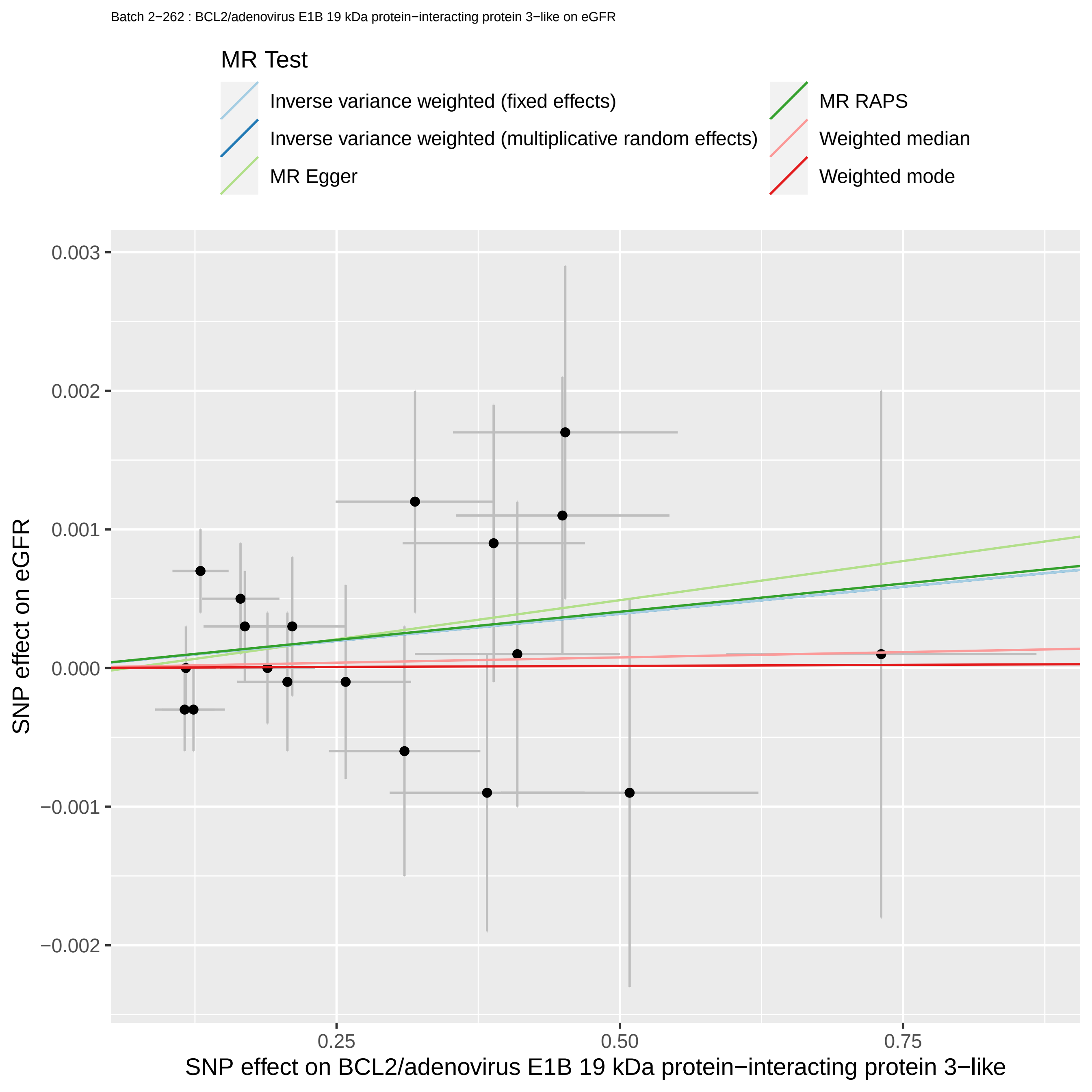

Batch 2−262 : BCL2/adenovirus E1B 19 kDa protein−interacting protein 3−like on eGFR
MR Test
Inverse variance weighted (fixed effects)
Inverse variance weighted (multiplicative random effects)
MR Egger
MR RAPS
Weighted median
Weighted mode
0.003
0.002
0.001
0.000
−0.001
−0.002
SNP effect on eGFR
0.25
0.50
0.75
SNP effect on BCL2/adenovirus E1B 19 kDa protein−interacting protein 3−like

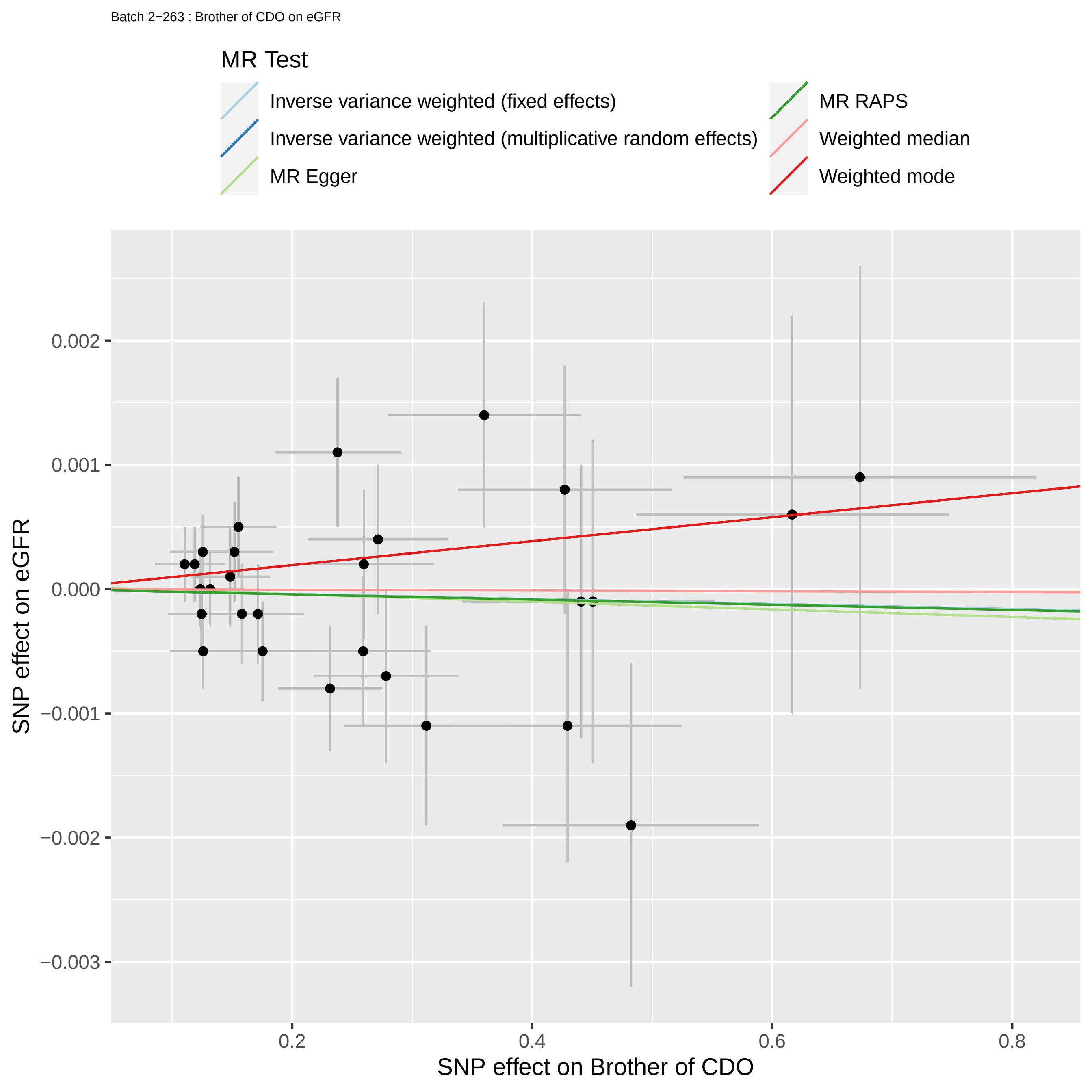

Batch 2−263 : Brother of CDO on eGFR
MR Test
Inverse variance weighted (fixed effects)
Inverse variance weighted (multiplicative random effects)
MR Egger
MR RAPS
Weighted median
Weighted mode
0.002
0.001
0.000
−0.001
−0.002
−0.003
SNP effect on eGFR
0.2
0.4
0.6
0.8
SNP effect on Brother of CDO

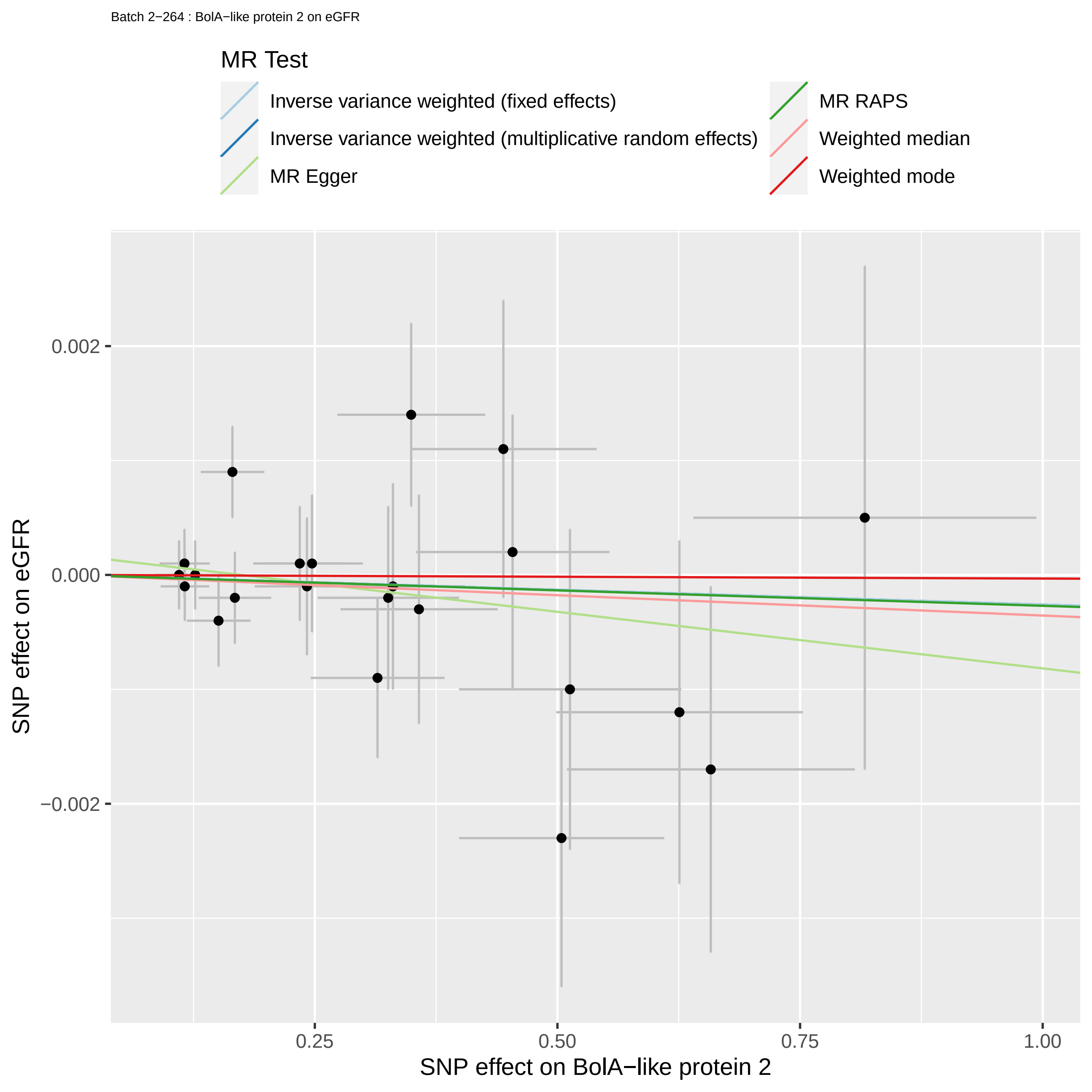

Batch 2−264 : BolA−like protein 2 on eGFR
MR Test
Inverse variance weighted (fixed effects)
Inverse variance weighted (multiplicative random effects)
MR Egger
MR RAPS
Weighted median
Weighted mode
0.002
0.000
−0.002
SNP effect on eGFR
0.25
0.50
0.75
1.00
SNP effect on BolA−like protein 2

Batch 2−265 : Bisphosphoglycerate mutase on eGFR

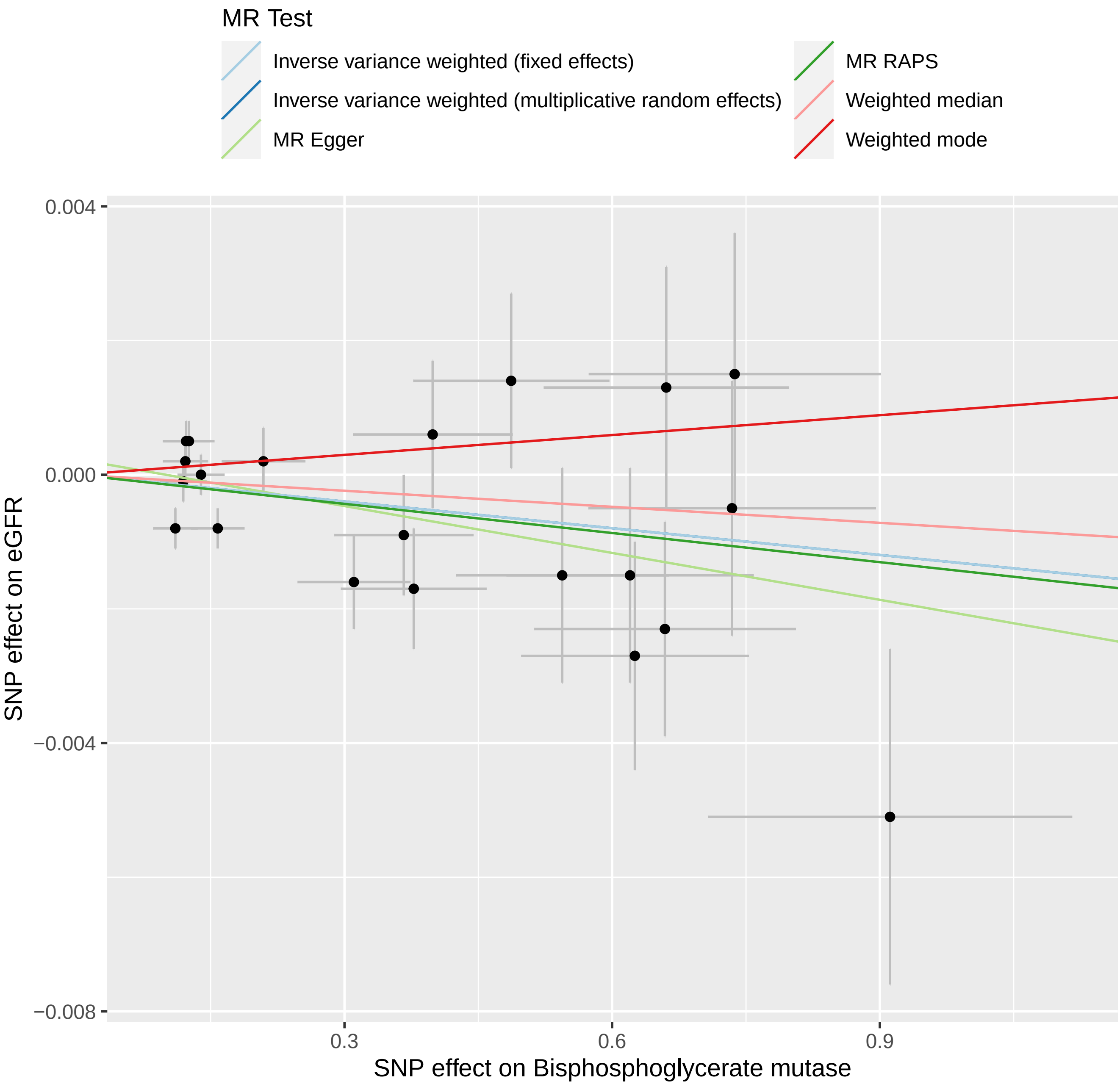

Batch 2−266 : Bactericidal permeability−increasing protein on eGFR

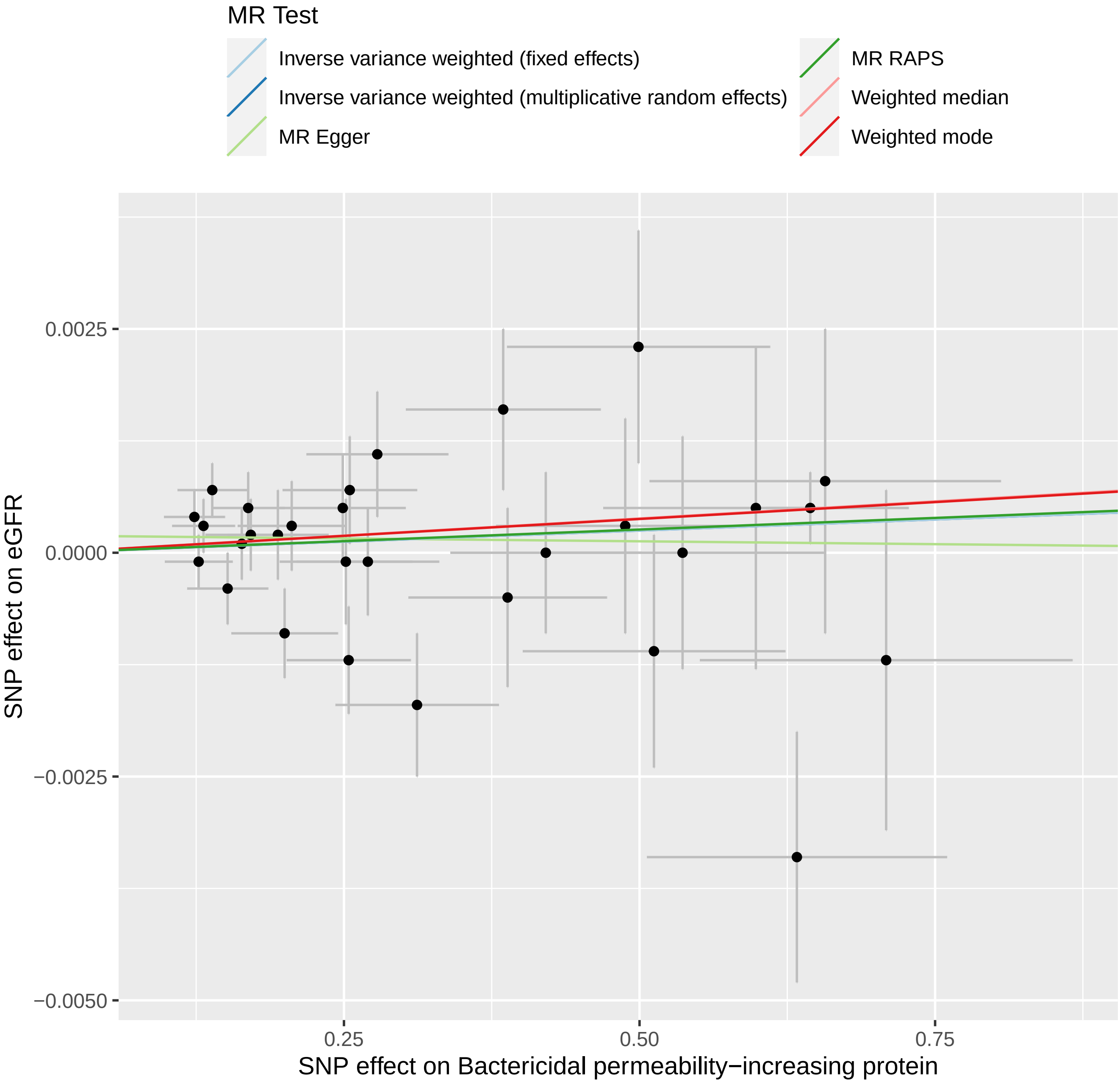

Batch 2−267 : BPI fold−containing family A member 1 on eGFR

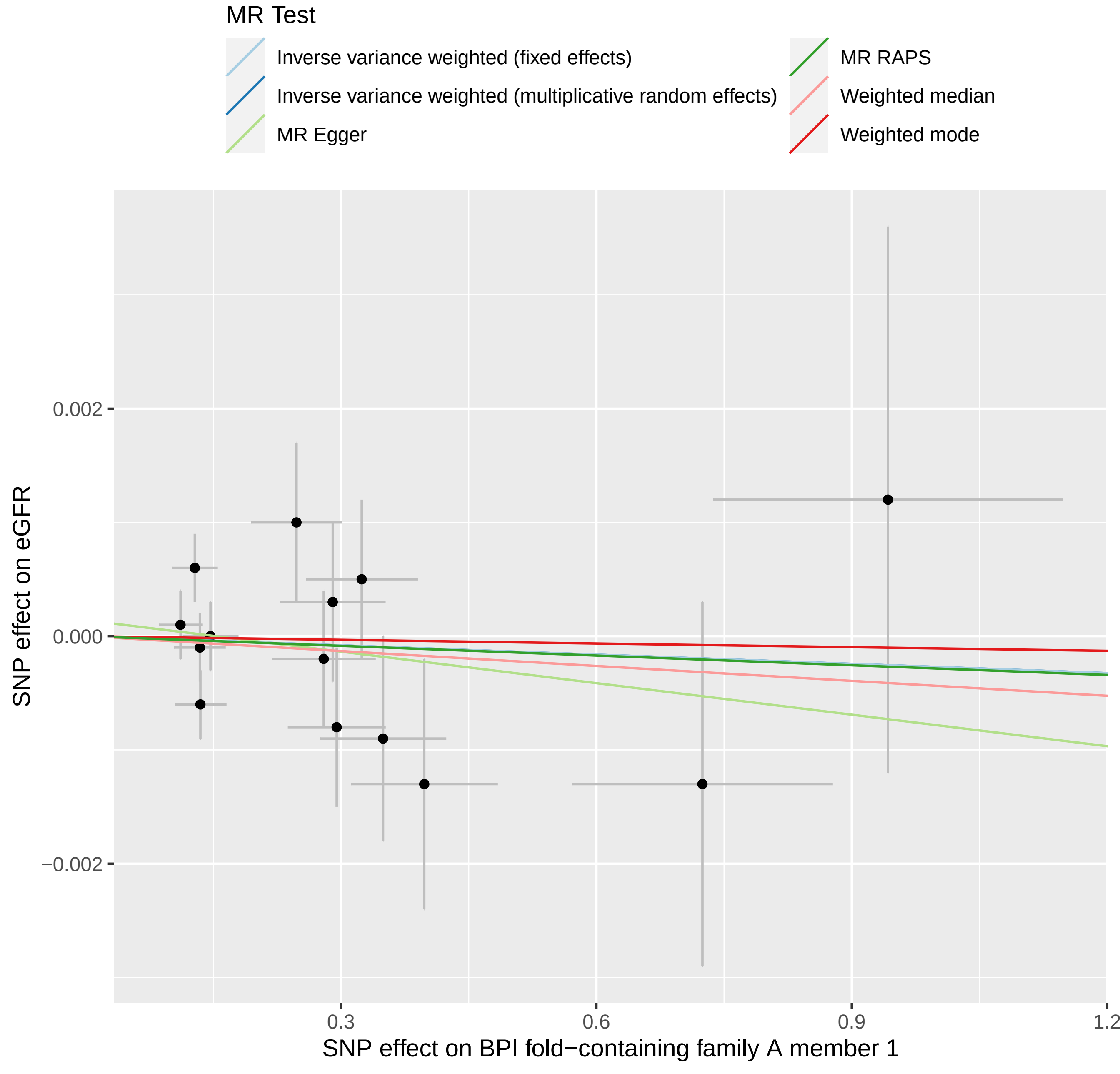

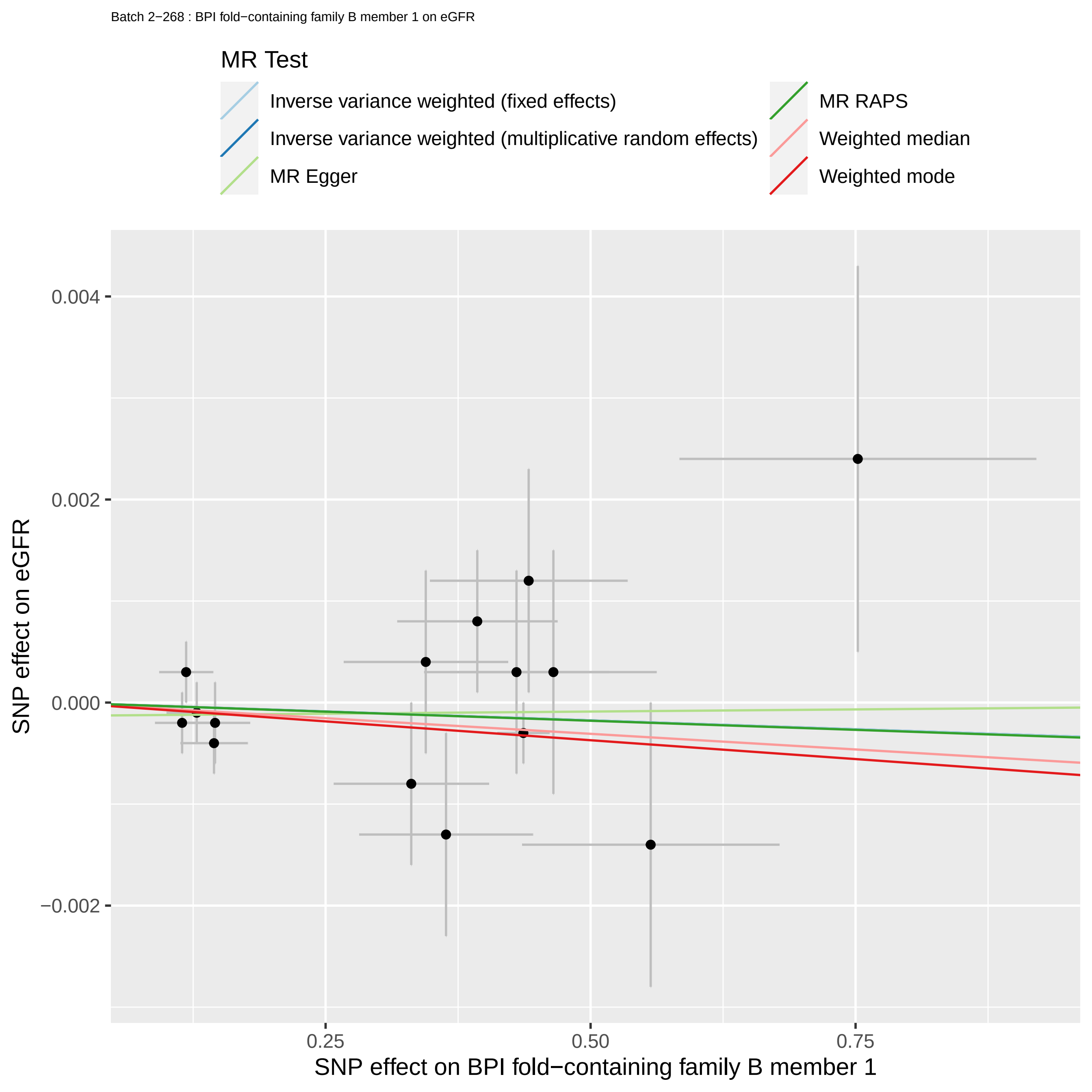

Batch 2−268 : BPI fold−containing family B member 1 on eGFR
MR Test
Inverse variance weighted (fixed effects)
Inverse variance weighted (multiplicative random effects)
MR Egger
MR RAPS
Weighted median
Weighted mode
0.004
0.002
0.000
−0.002
0.25
0.50
0.75
SNP effect on eGFR
SNP effect on BPI fold−containing family B member 1

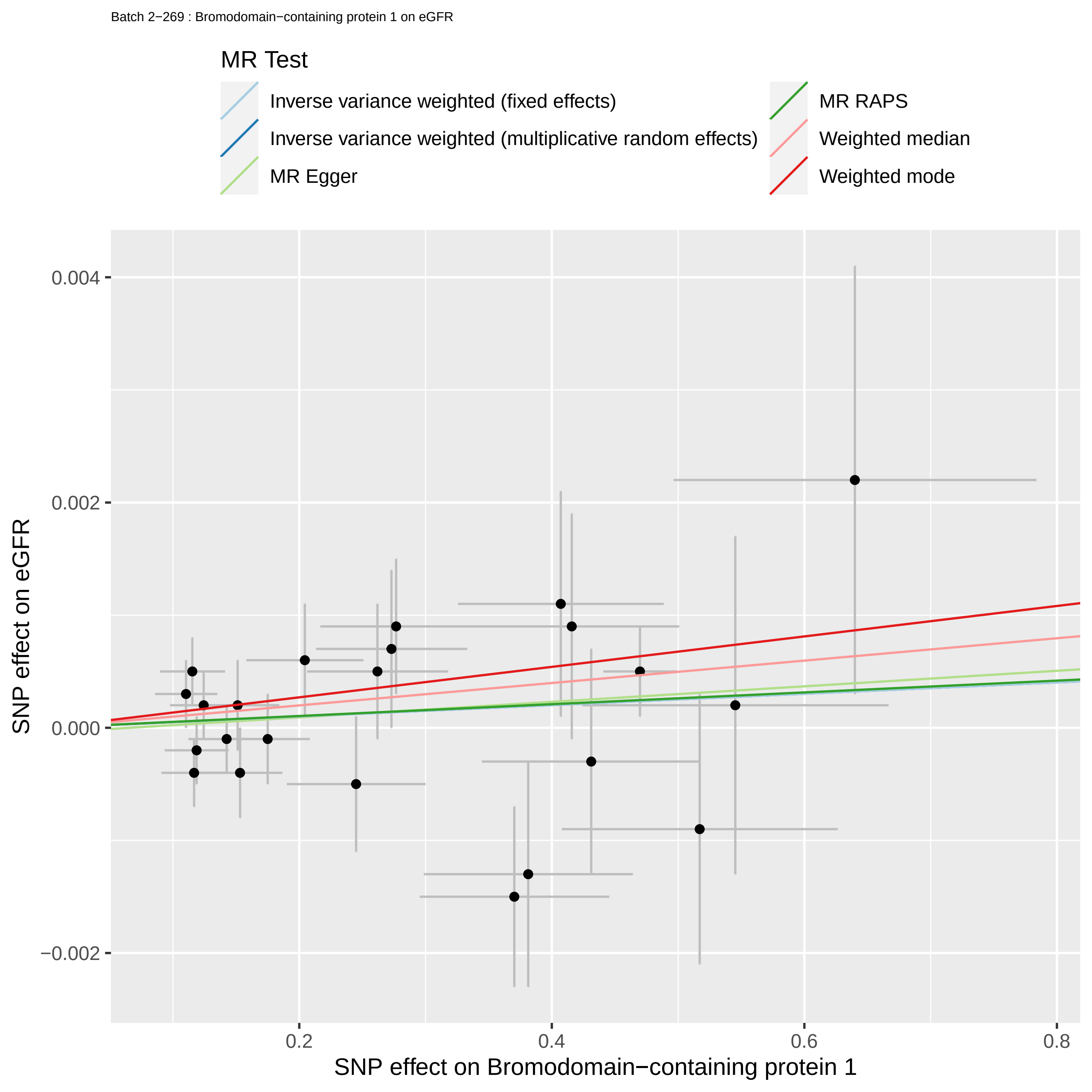
Batch 2−269 : Bromodomain−containing protein 1 on eGFR
MR Test
Inverse variance weighted (fixed effects)
Inverse variance weighted (multiplicative random effects)
MR Egger
MR RAPS
Weighted median
Weighted mode
0.004
0.002
0.000
−0.002
SNP effect on eGFR
0.2
0.4
0.6
0.8
SNP effect on Bromodomain−containing protein 1

Batch 2−270 : Bromodomain testis−specific protein on eGFR

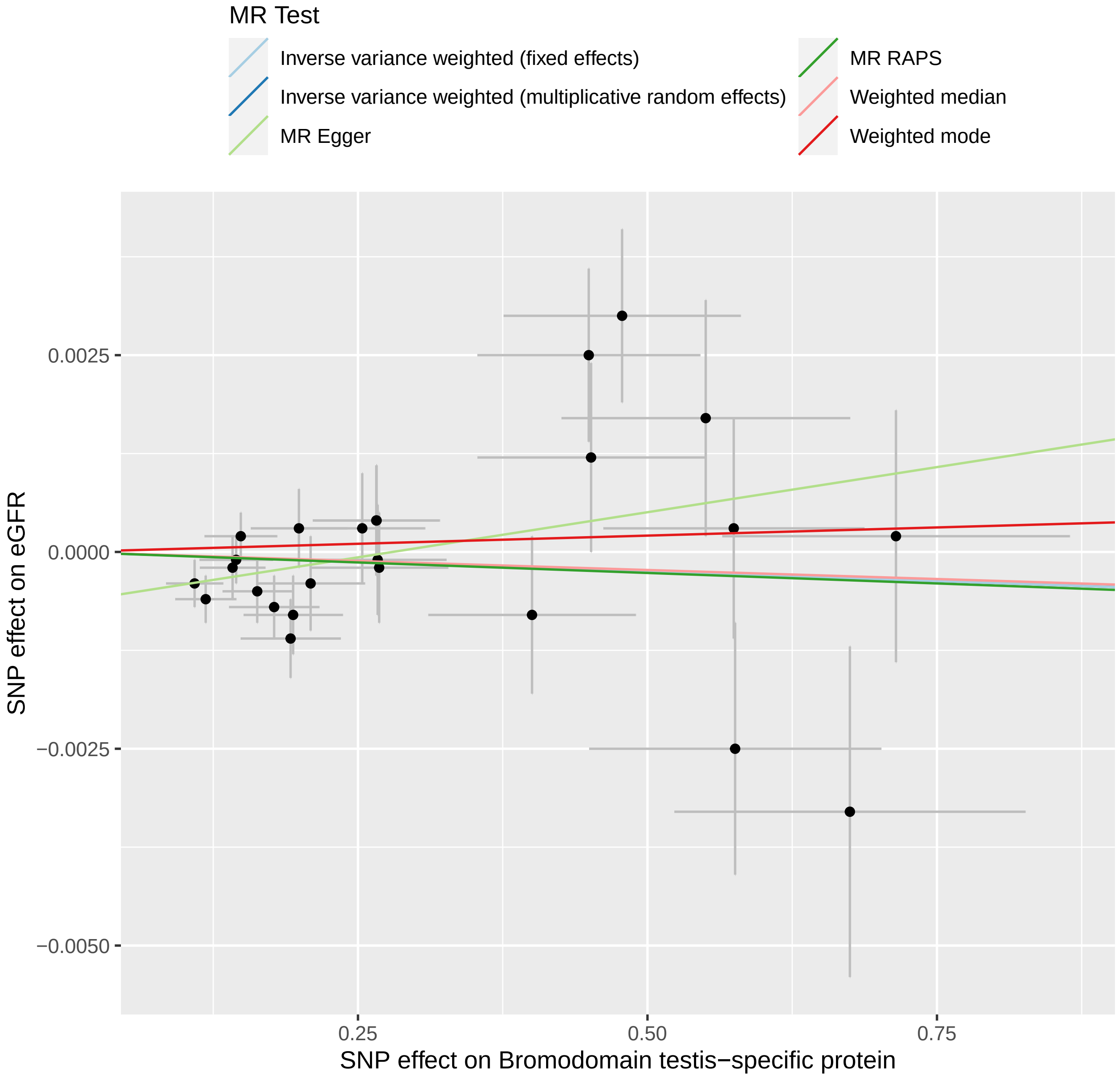

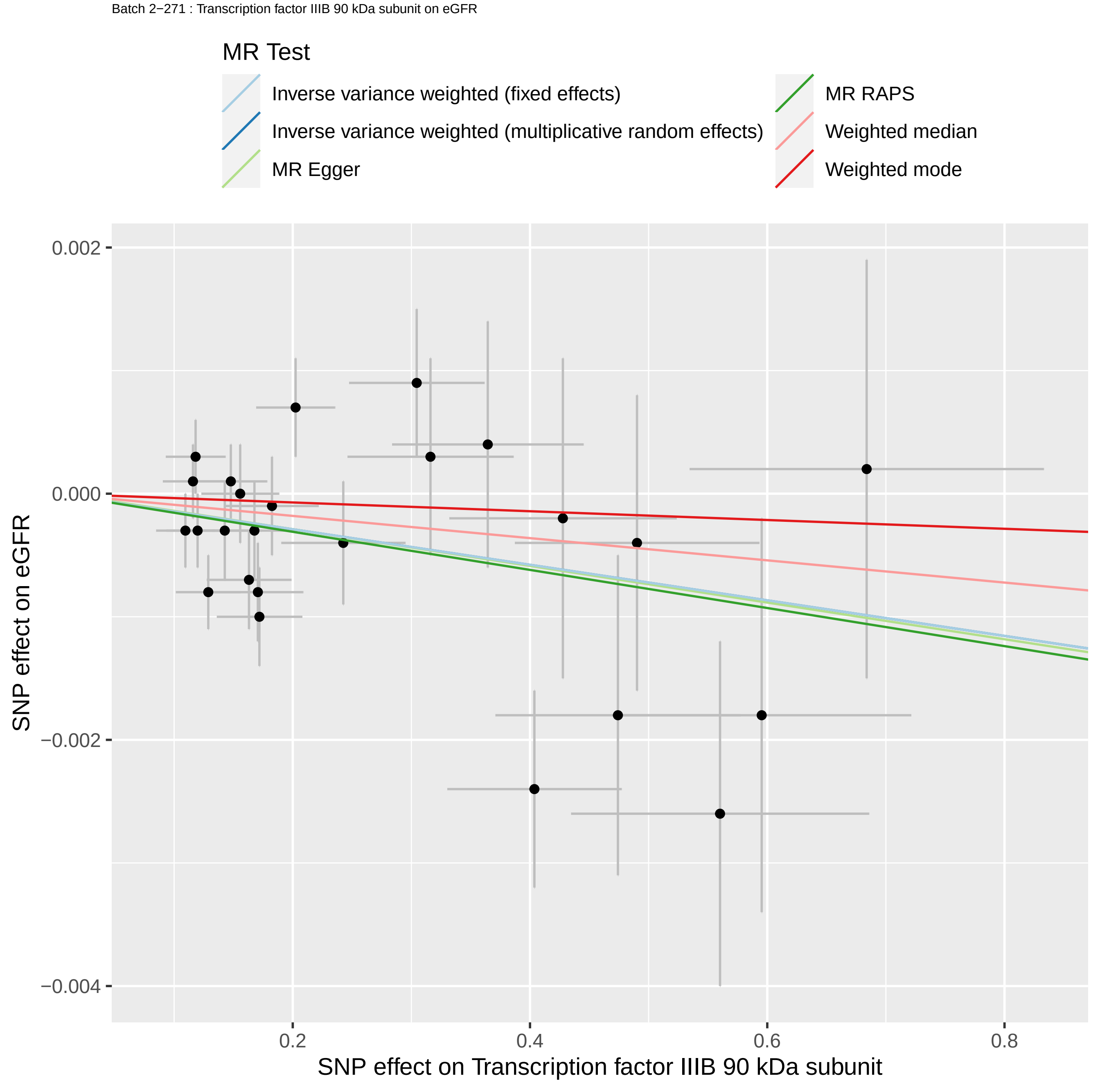

Batch 2−271 : Transcription factor IIIB 90 kDa subunit on eGFR
MR Test
Inverse variance weighted (fixed effects)
Inverse variance weighted (multiplicative random effects)
MR Egger
MR RAPS
Weighted median
Weighted mode
0.002
0.000
−0.002
−0.004
0.2
0.4
0.6
0.8
SNP effect on eGFR
SNP effect on Transcription factor IIIB 90 kDa subunit

Batch 2−272 : BRICHOS domain−containing protein 5 on eGFR

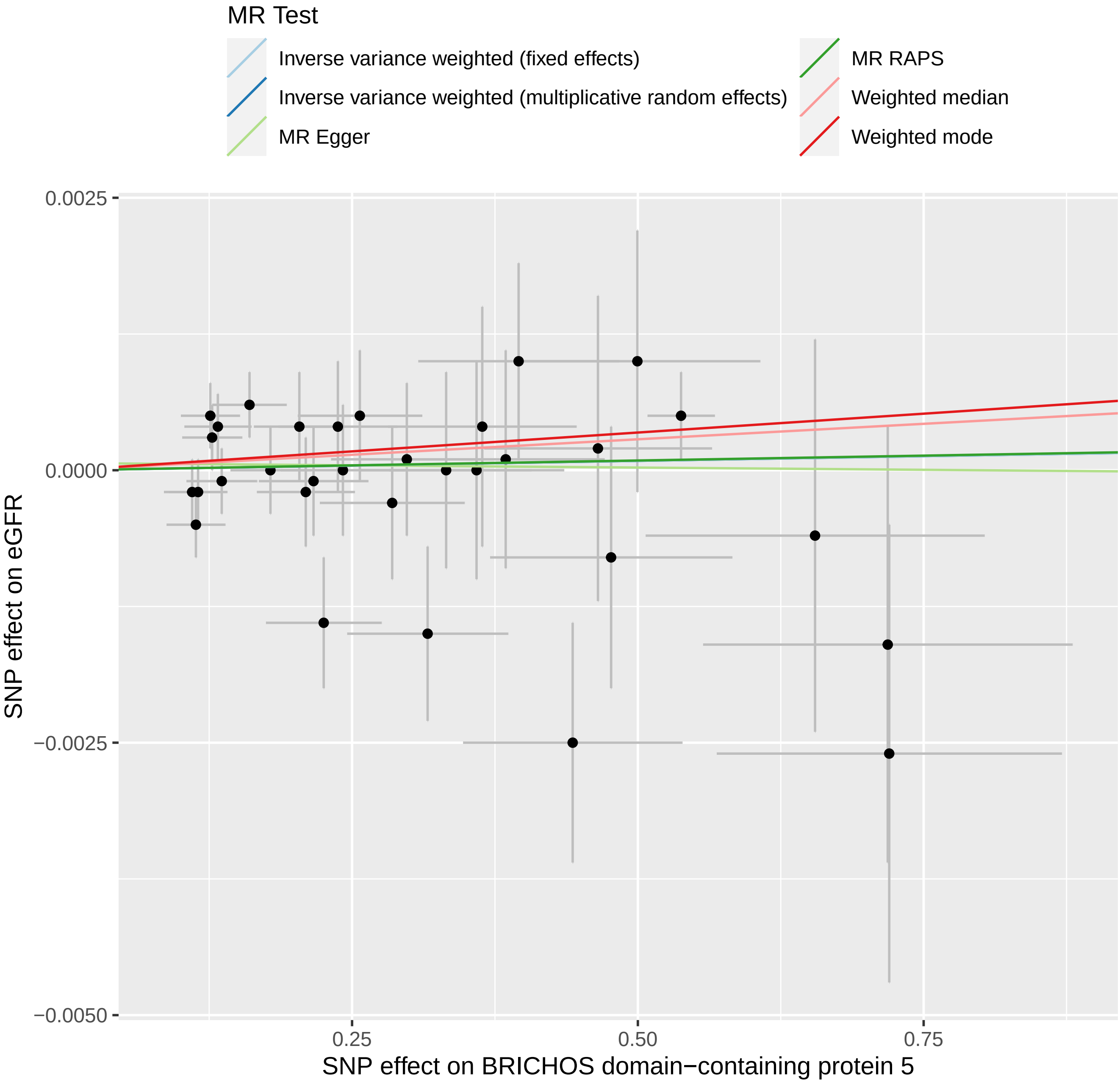

Batch 2−273 : Peregrin on eGFR

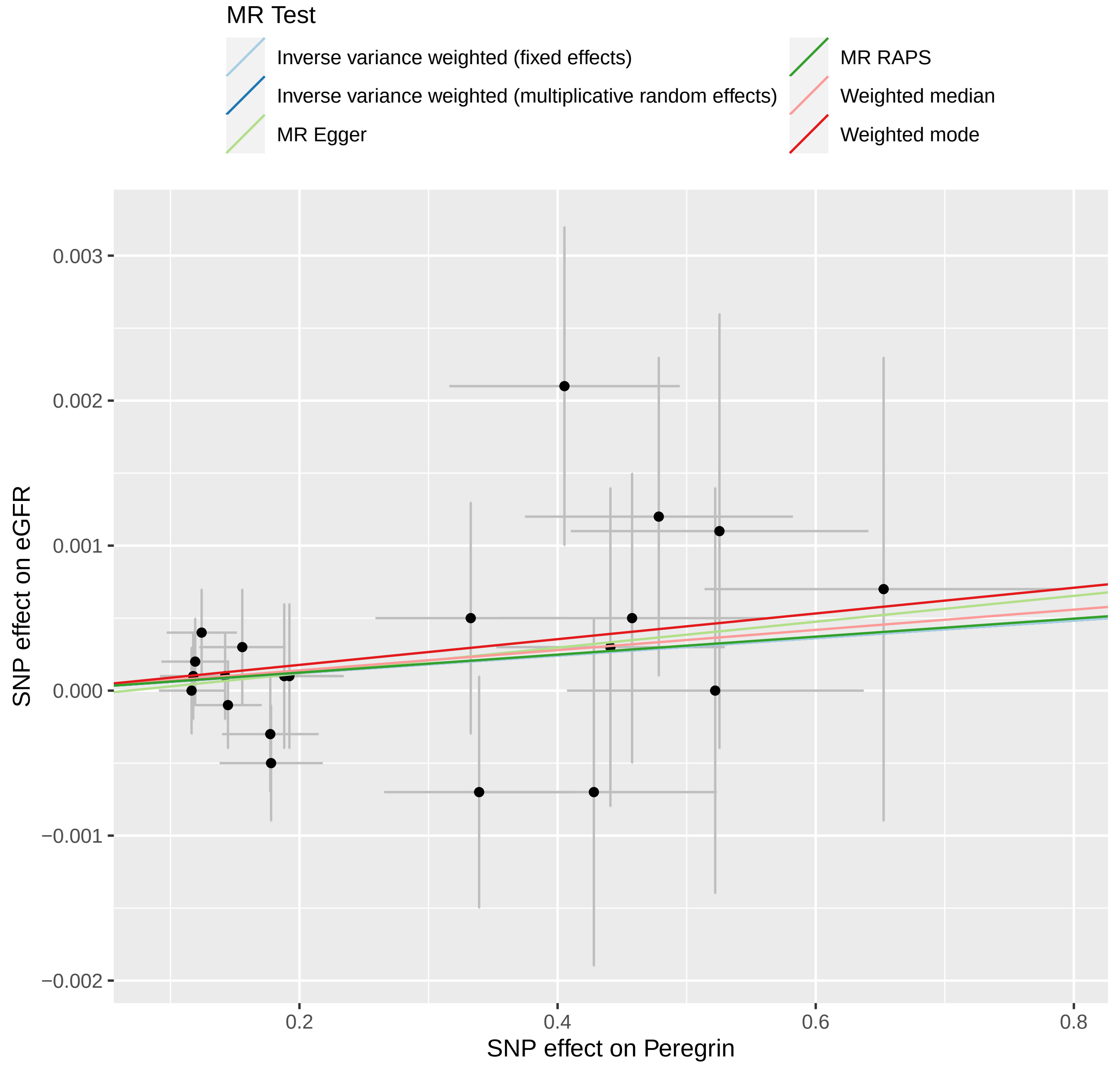

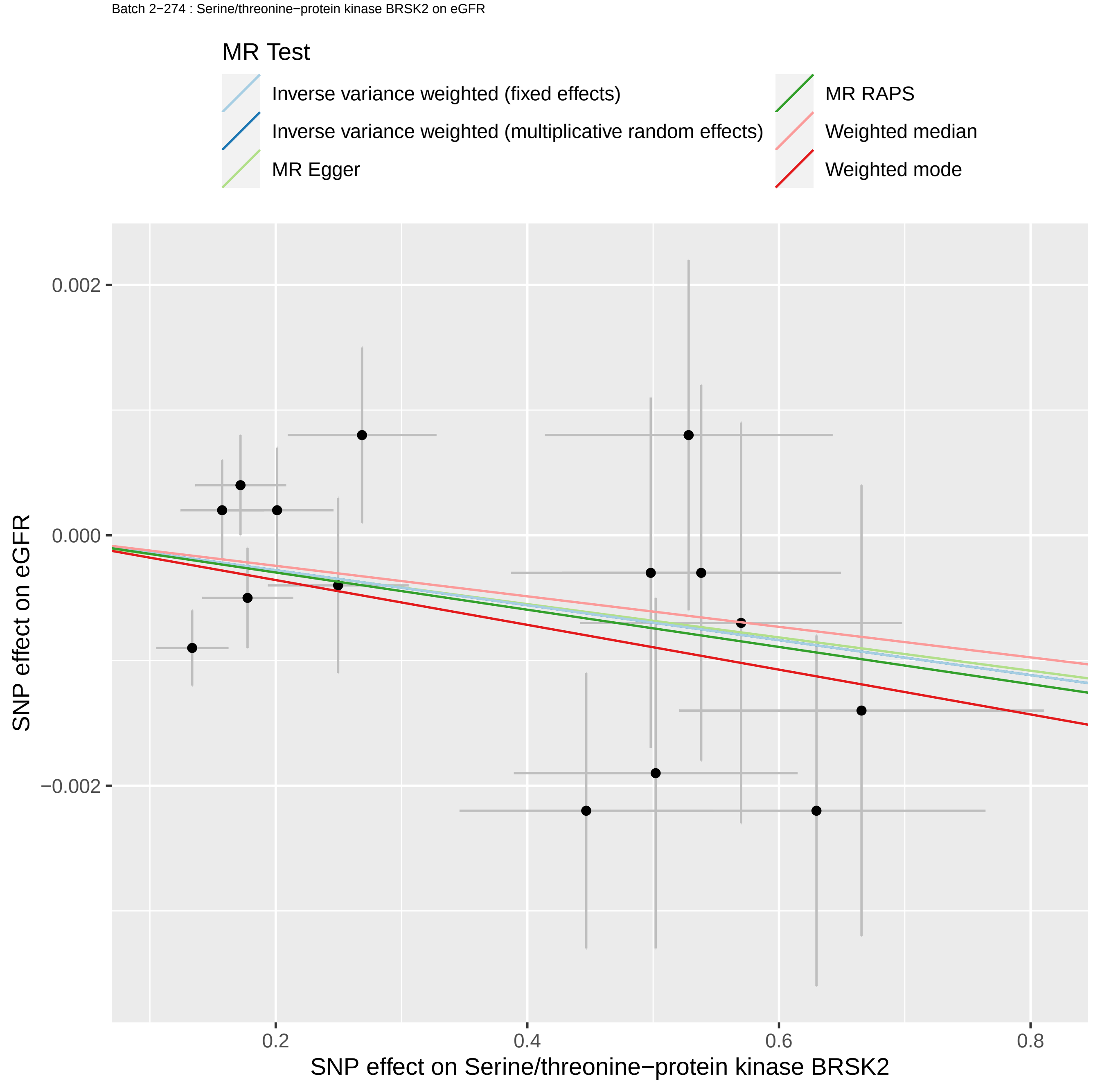
Batch 2−274 : Serine/threonine−protein kinase BRSK2 on eGFR
MR Test
Inverse variance weighted (fixed effects)
Inverse variance weighted (multiplicative random effects)
MR Egger
MR RAPS
Weighted median
Weighted mode
0.002
0.000
−0.002
0.2
0.4
0.6
0.8
SNP effect on eGFR
SNP effect on Serine/threonine−protein kinase BRSK2

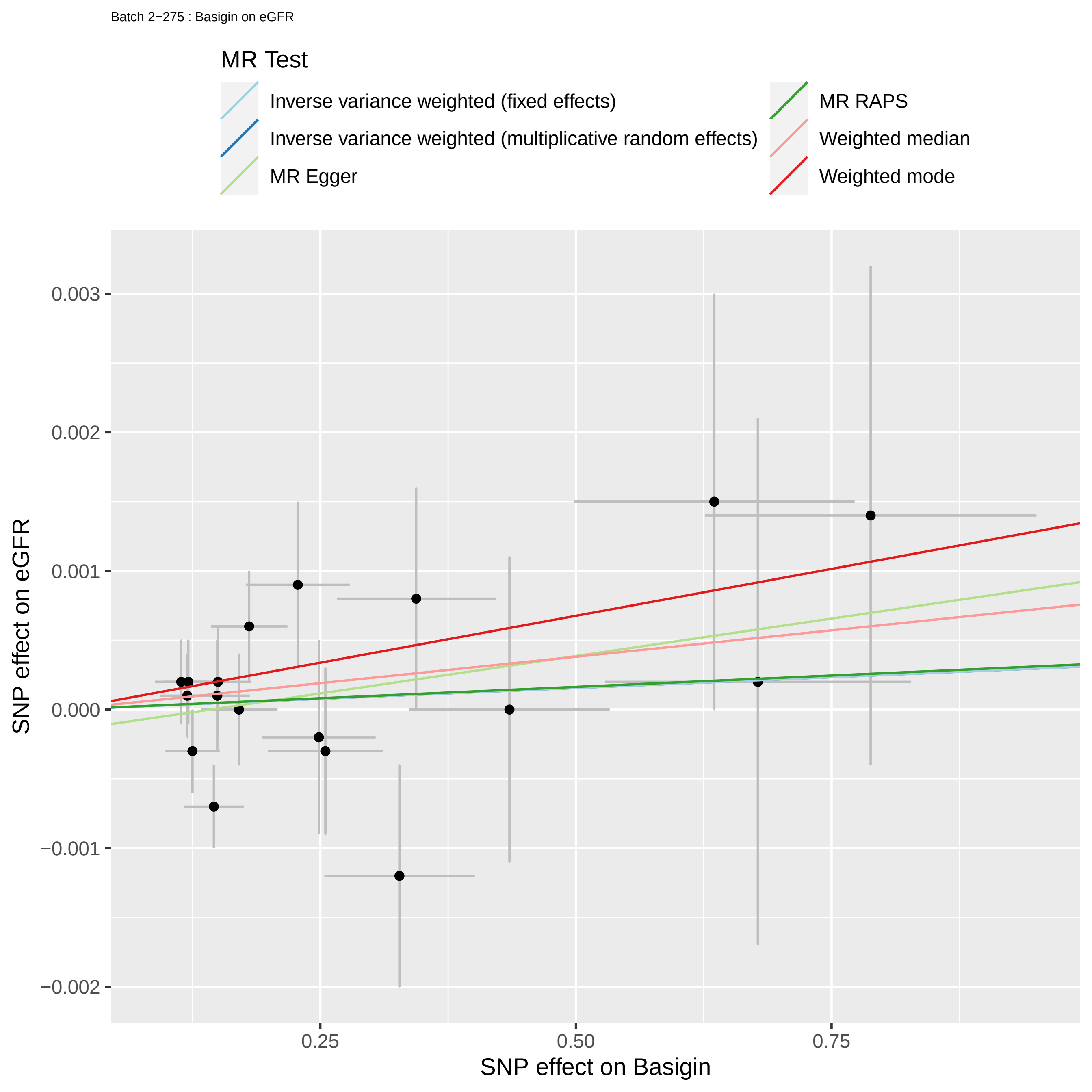
Batch 2−275 : Basigin on eGFR
MR Test
Inverse variance weighted (fixed effects)
Inverse variance weighted (multiplicative random effects)
MR Egger
MR RAPS
Weighted median
Weighted mode
SNP effect on eGFR
0.003
0.002
0.001
0.000
−0.001
−0.002
0.25
0.50
0.75
SNP effect on Basigin

Batch 2−276 : ADP−ribosyl cyclase/cyclic ADP−ribose hydrolase 2 on eGFR

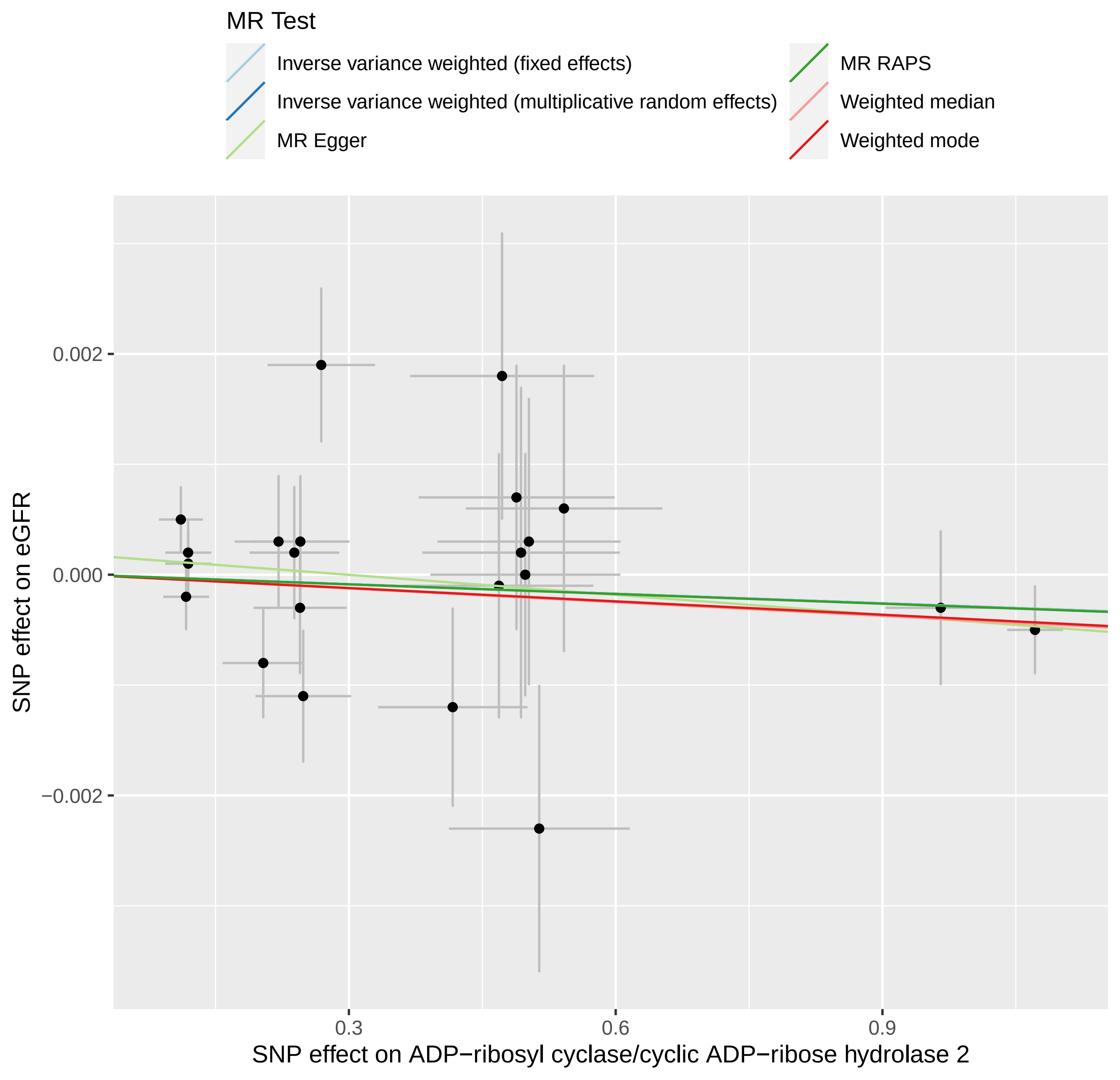

Batch 2−277 : Tyrosine−protein kinase BTK on eGFR

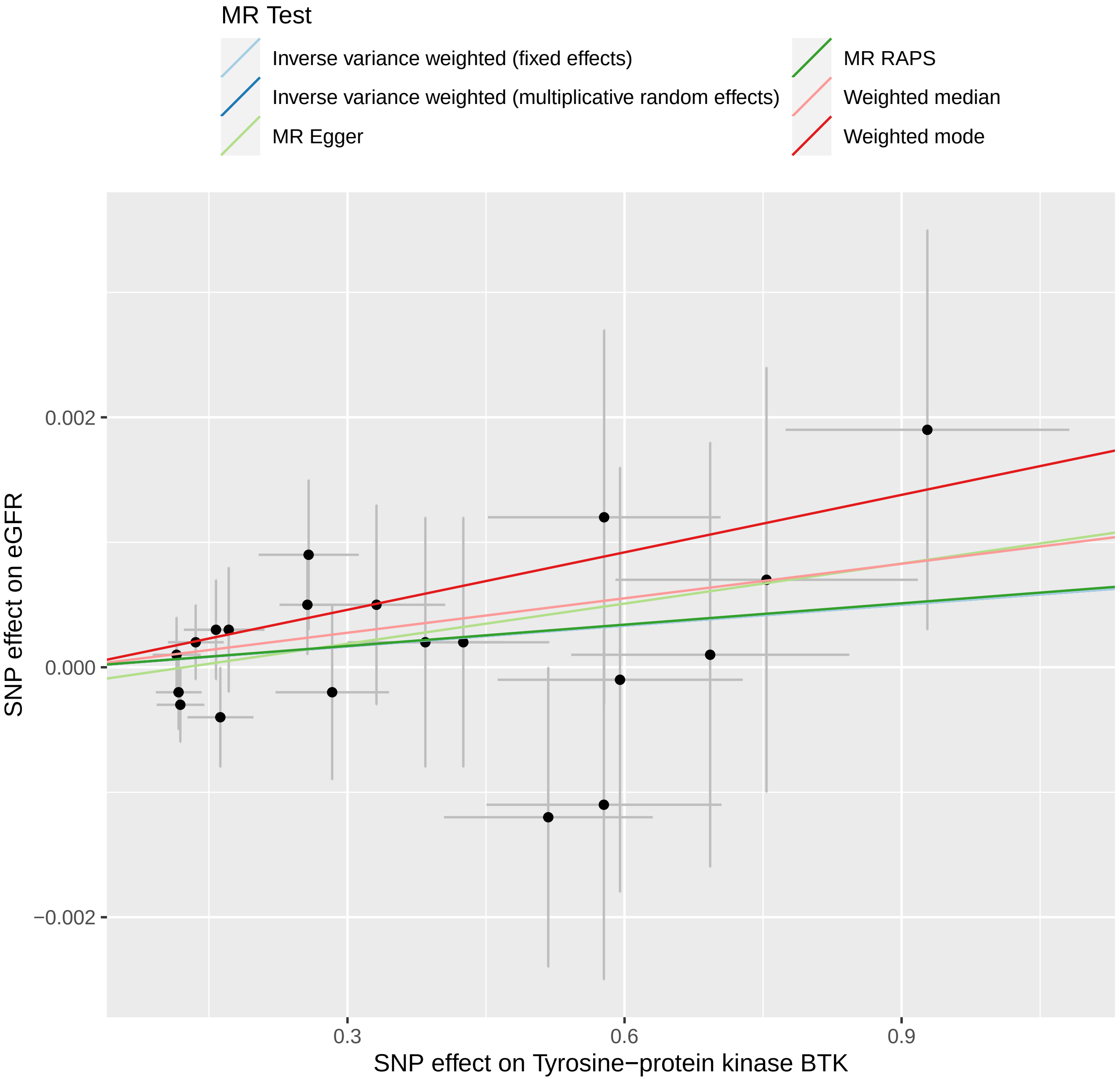

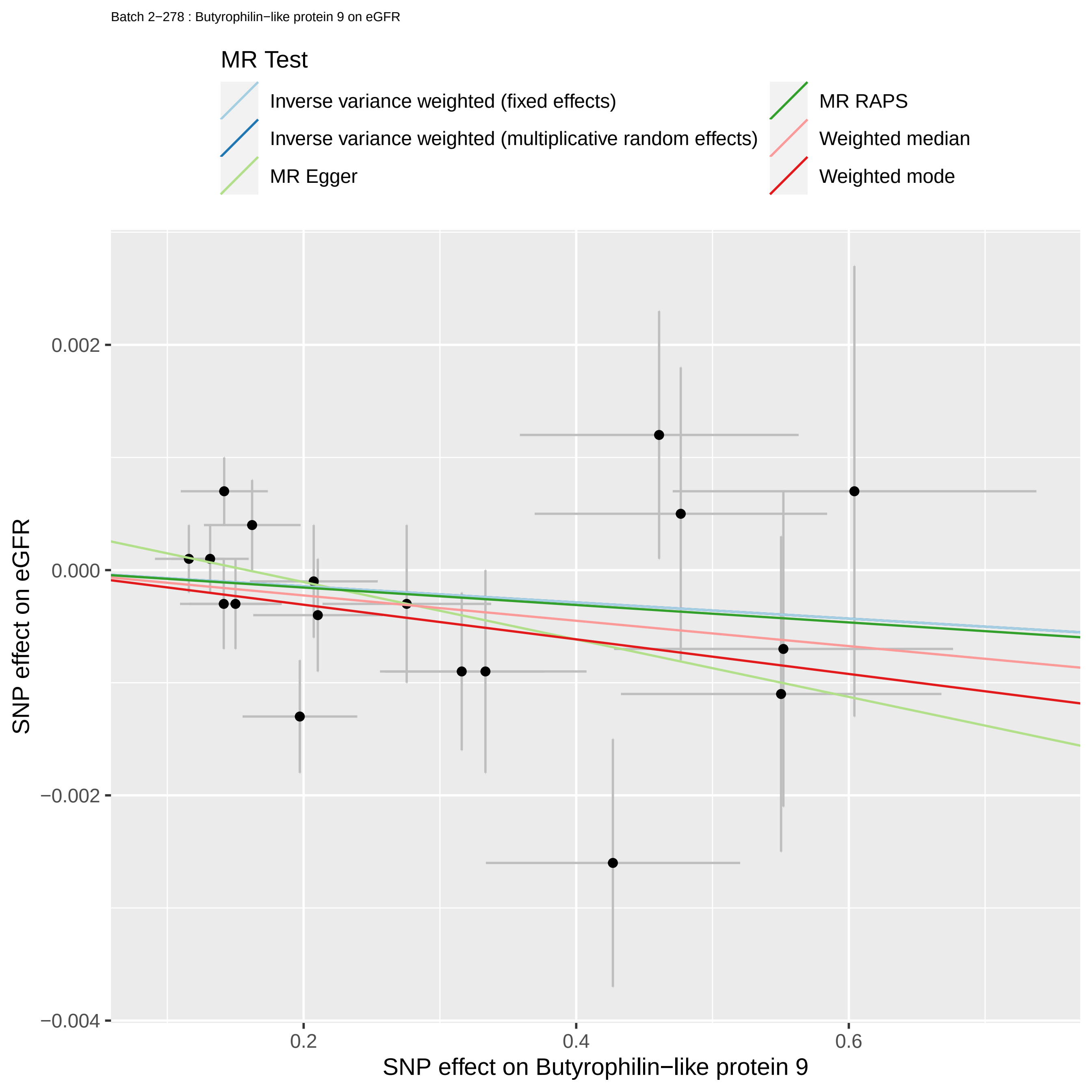
Batch 2−278 : Butyrophilin−like protein 9 on eGFR
MR Test
Inverse variance weighted (fixed effects)
Inverse variance weighted (multiplicative random effects)
MR Egger
MR RAPS
Weighted median
Weighted mode
0.002
0.000
−0.002
−0.004
SNP effect on eGFR
0.2
0.4
0.6
SNP effect on Butyrophilin−like protein 9

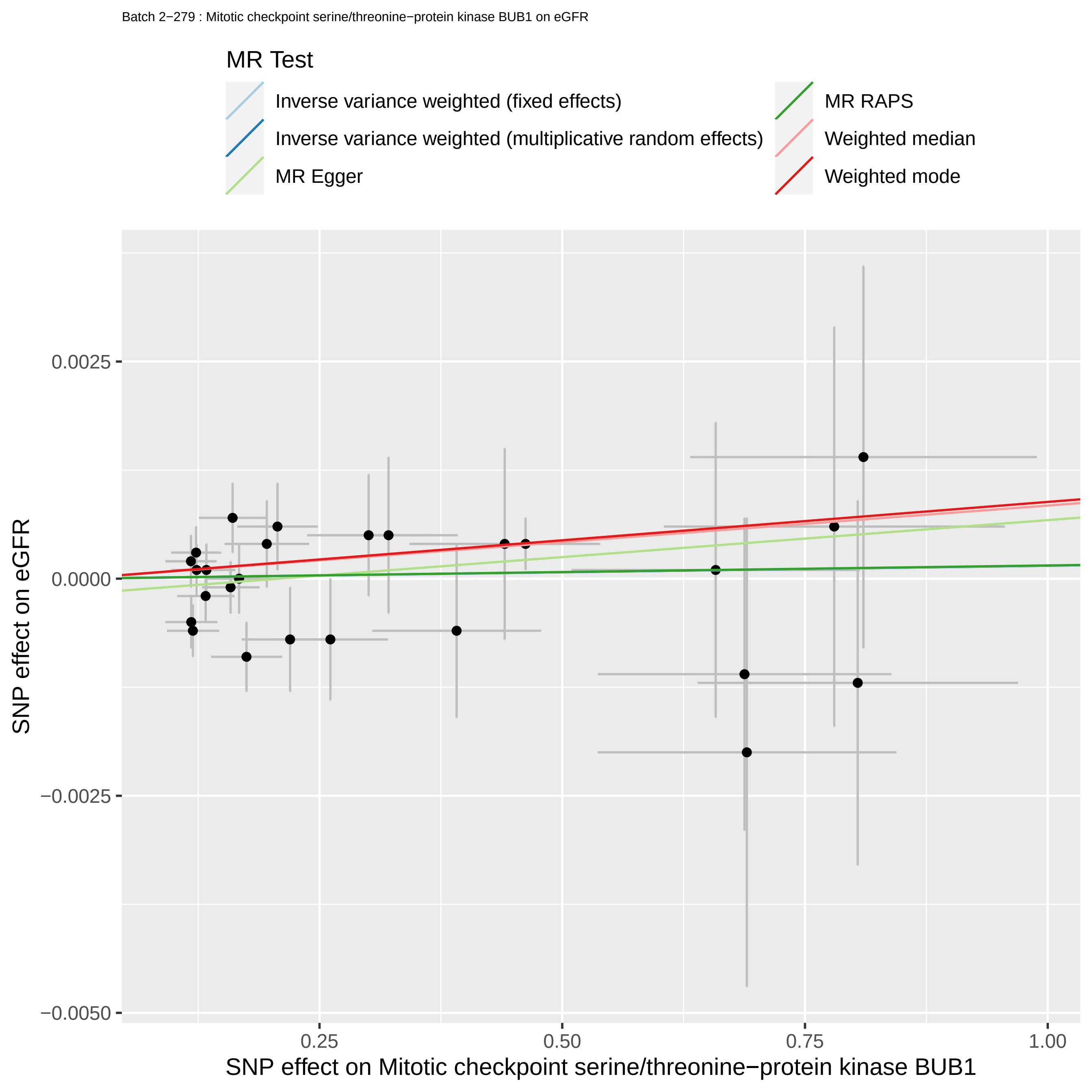

Batch 2−279 : Mitotic checkpoint serine/threonine−protein kinase BUB1 on eGFR
MR Test
Inverse variance weighted (fixed effects)
Inverse variance weighted (multiplicative random effects)
MR Egger
MR RAPS
Weighted median
Weighted mode
0.0025
0.0000
−0.0025
−0.0050
0.25
0.50
0.75
1.00
SNP effect on eGFR
SNP effect on Mitotic checkpoint serine/threonine−protein kinase BUB1

Batch 2−280 : Uncharacterized protein C10orf35 on eGFR

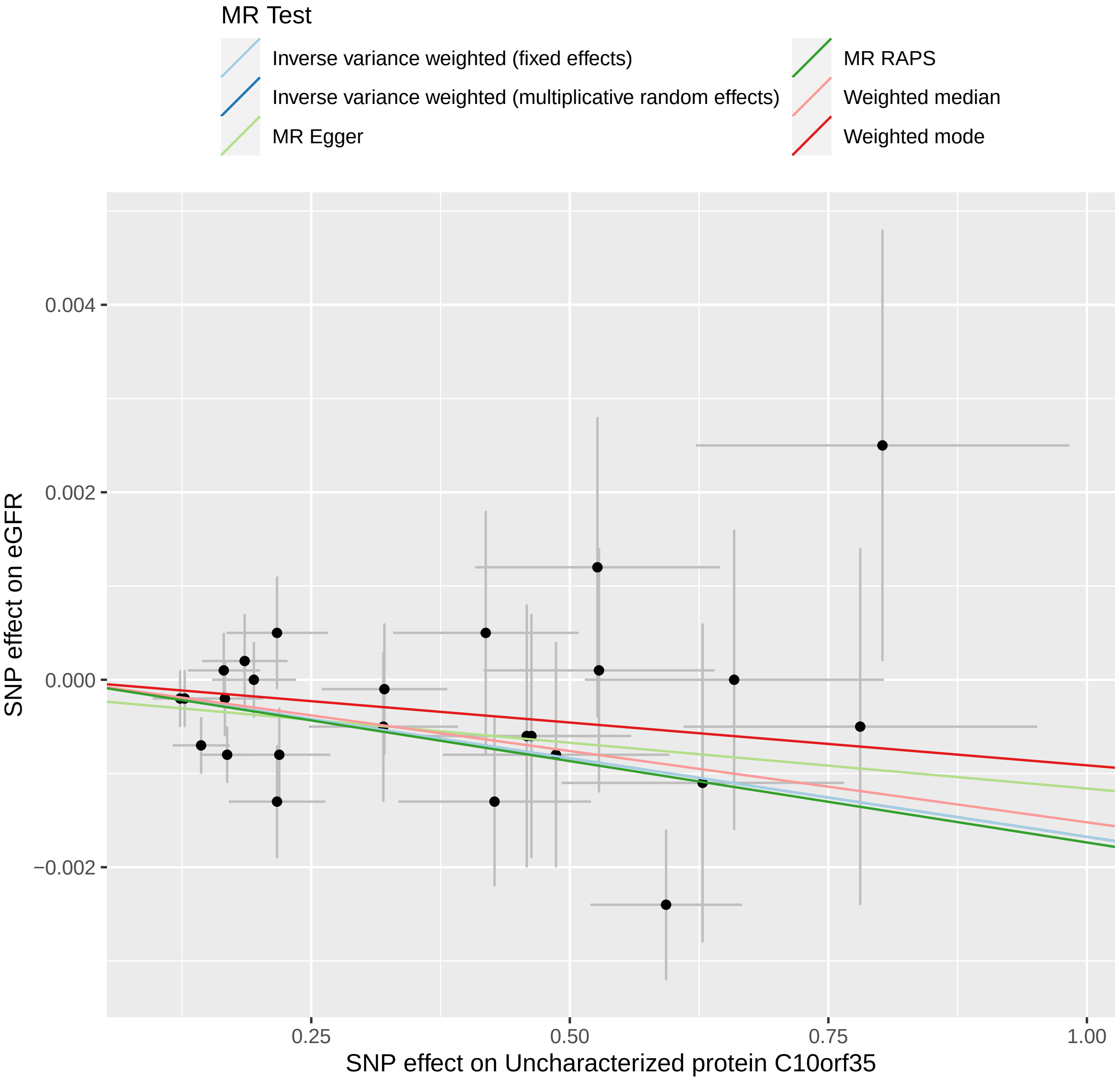

Batch 2−281 : Platelet receptor Gi24 on eGFR

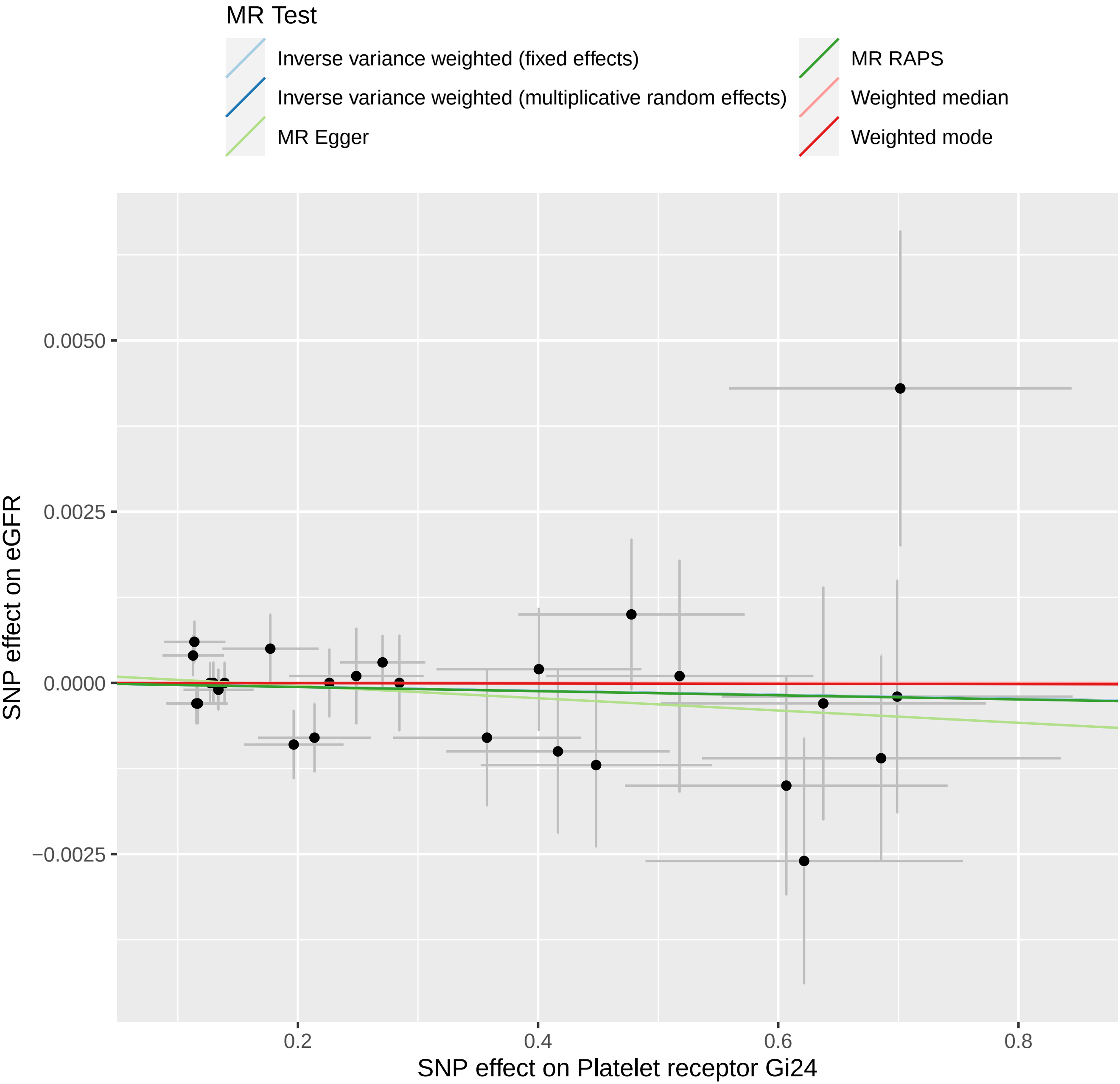

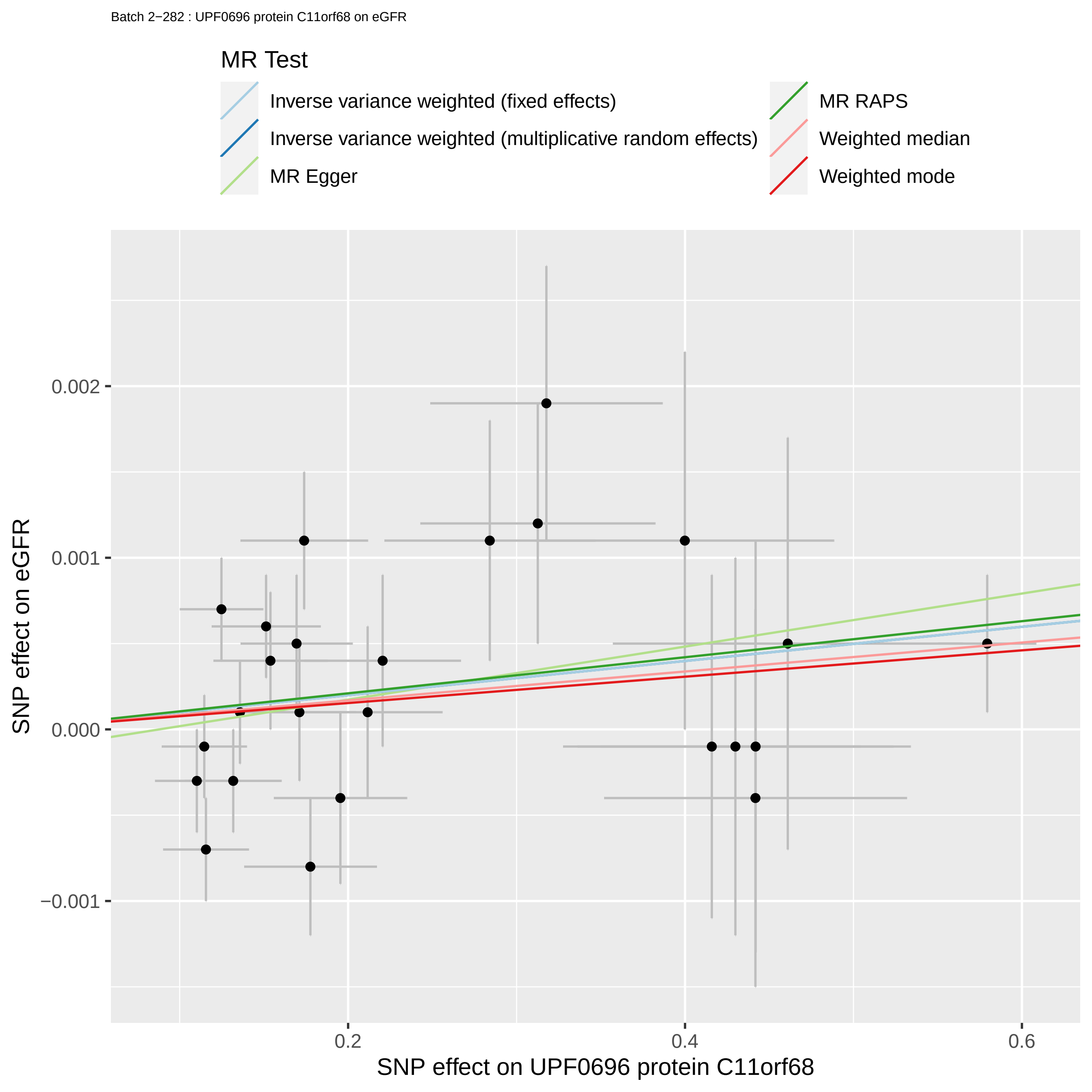

Batch 2−282 : UPF0696 protein C11orf68 on eGFR
MR Test
Inverse variance weighted (fixed effects)
Inverse variance weighted (multiplicative random effects)
MR Egger
MR RAPS
Weighted median
Weighted mode
0.002
0.001
0.000
−0.001
0.2
0.4
0.6
SNP effect on eGFR
SNP effect on UPF0696 protein C11orf68

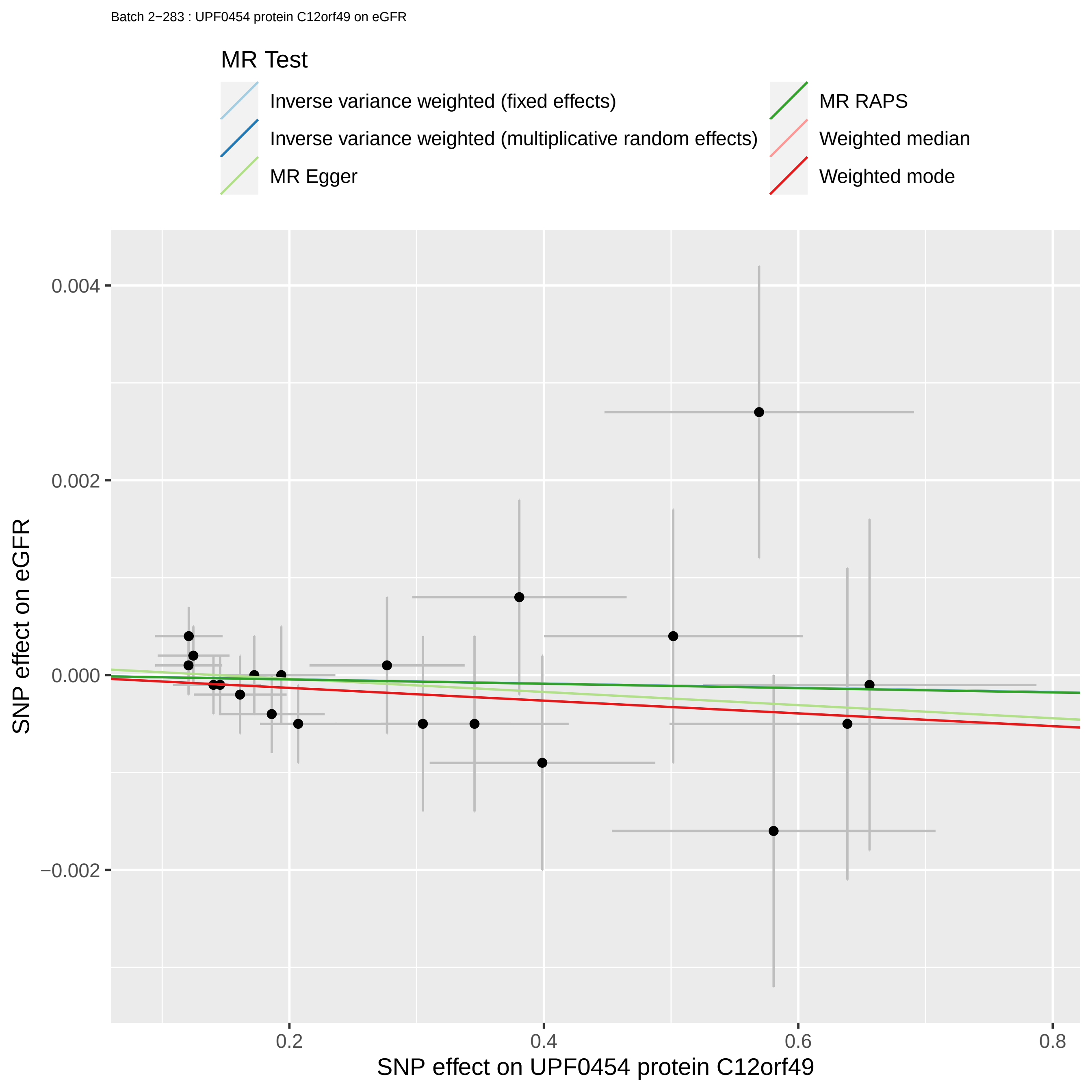

Batch 2−283 : UPF0454 protein C12orf49 on eGFR
MR Test
Inverse variance weighted (fixed effects)
Inverse variance weighted (multiplicative random effects)
MR Egger
MR RAPS
Weighted median
Weighted mode
0.004
0.002
0.000
−0.002
0.2
0.4
0.6
0.8
SNP effect on eGFR
SNP effect on UPF0454 protein C12orf49

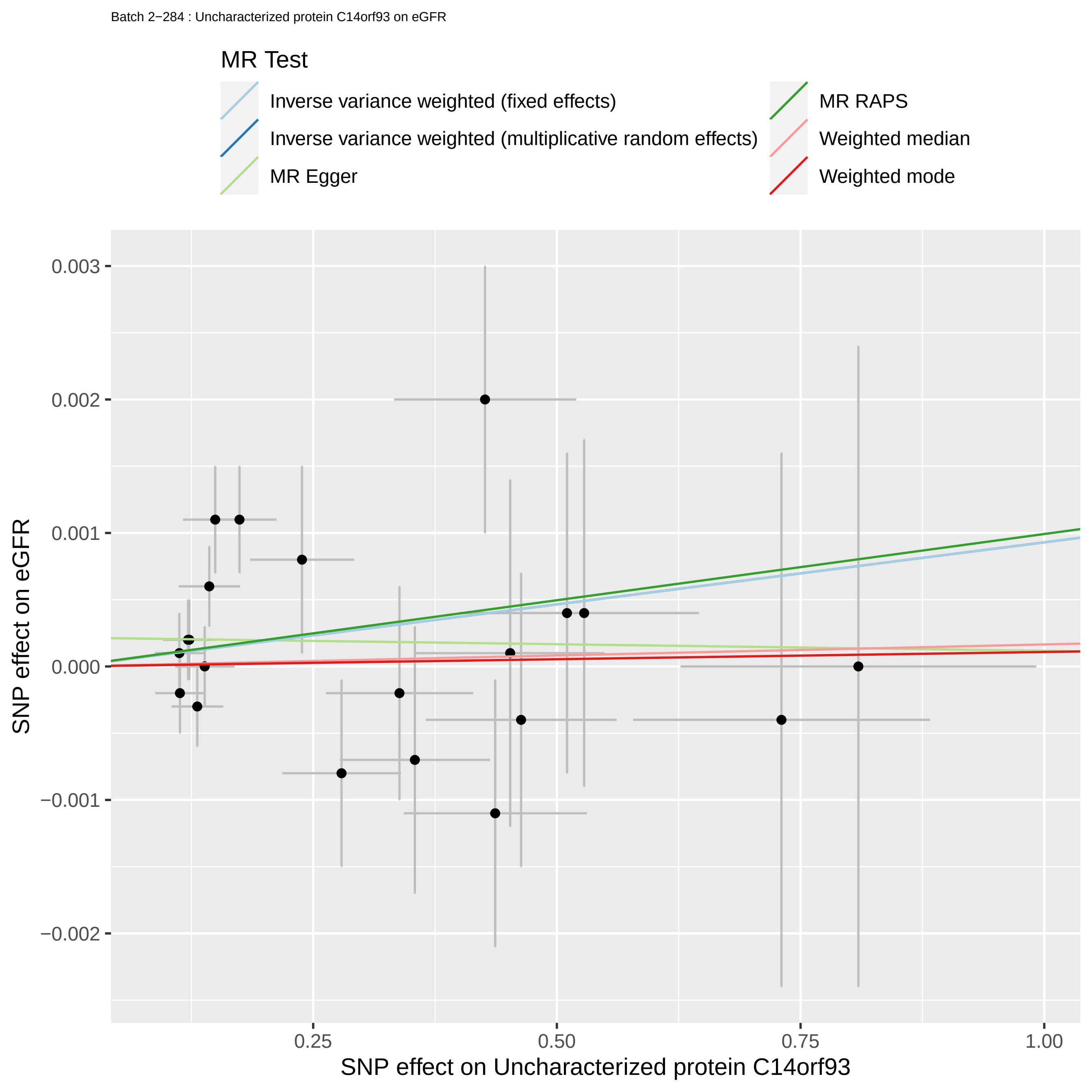

Batch 2−284 : Uncharacterized protein C14orf93 on eGFR
MR Test
Inverse variance weighted (fixed effects)
Inverse variance weighted (multiplicative random effects)
MR Egger
MR RAPS
Weighted median
Weighted mode
0.003
0.002
0.001
0.000
−0.001
−0.002
0.25
0.50
0.75
1.00
SNP effect on eGFR
SNP effect on Uncharacterized protein C14orf93

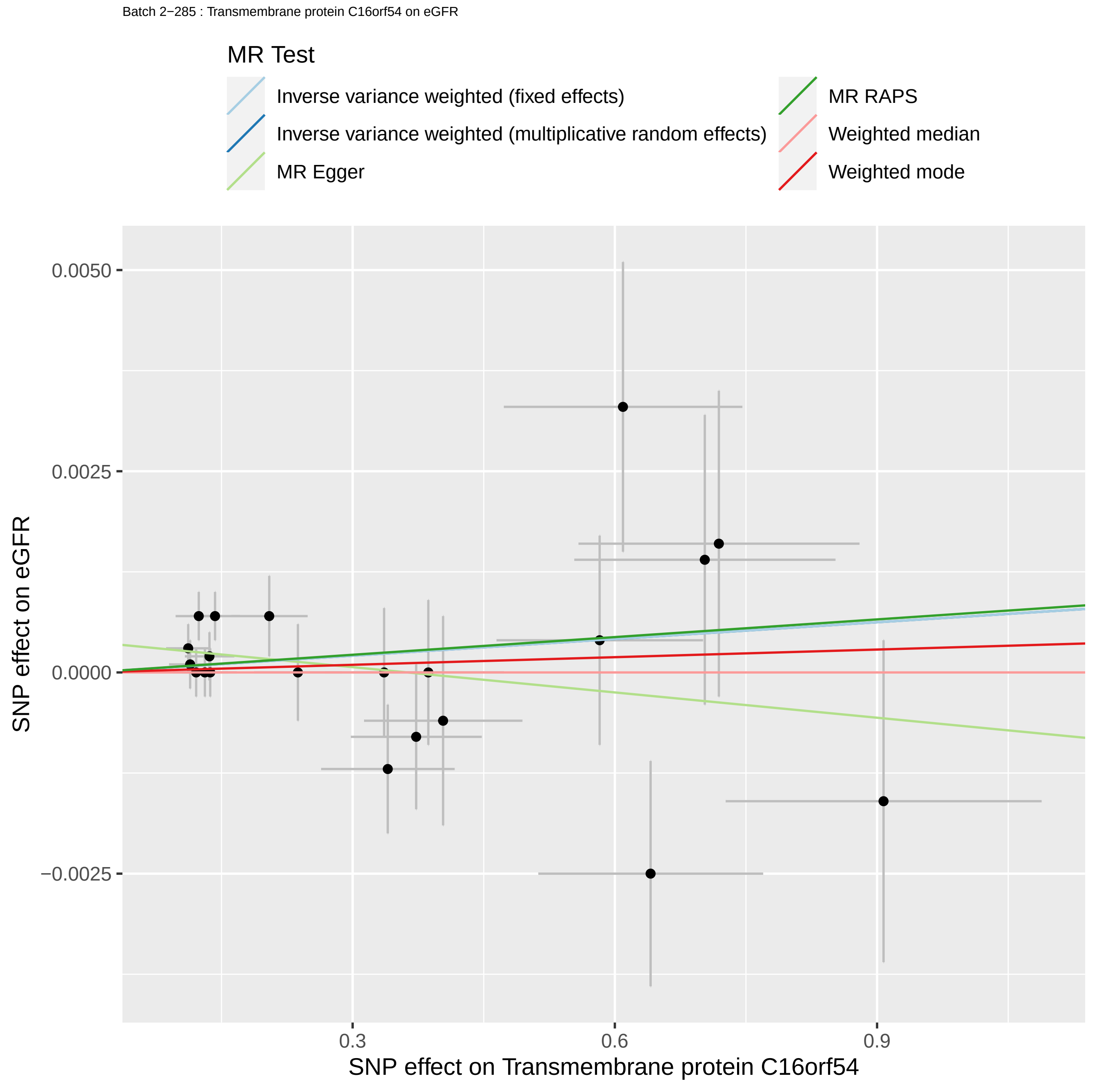

Batch 2−285 : Transmembrane protein C16orf54 on eGFR
MR Test
Inverse variance weighted (fixed effects)
Inverse variance weighted (multiplicative random effects)
MR Egger
MR RAPS
Weighted median
Weighted mode
0.0050
0.0025
0.0000
−0.0025
0.3
0.6
0.9
SNP effect on eGFR
SNP effect on Transmembrane protein C16orf54

Batch 2−286 : Uncharacterized protein C17orf67 on eGFR

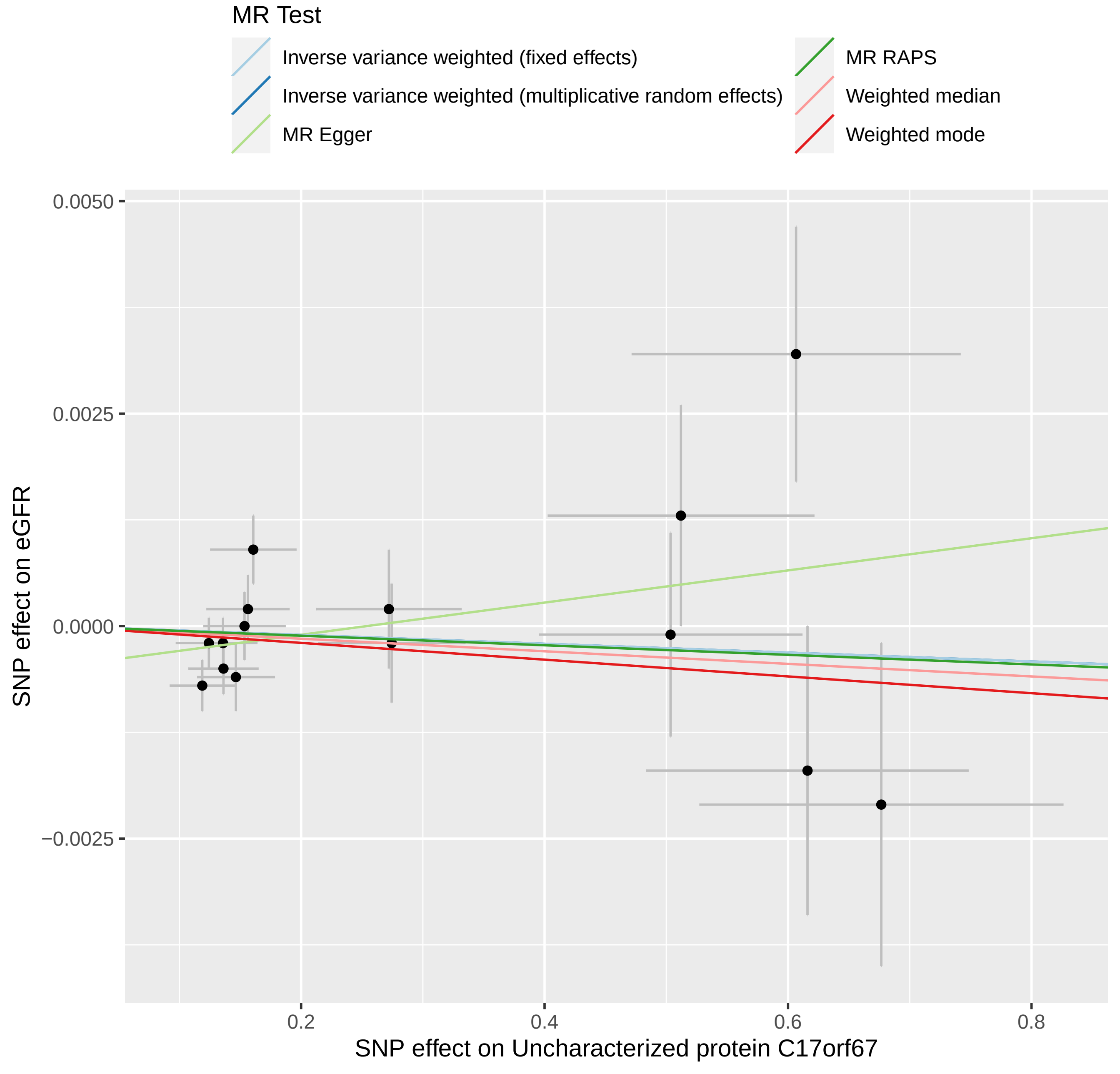

Batch 2−287 : Uncharacterized protein C17orf78 on eGFR

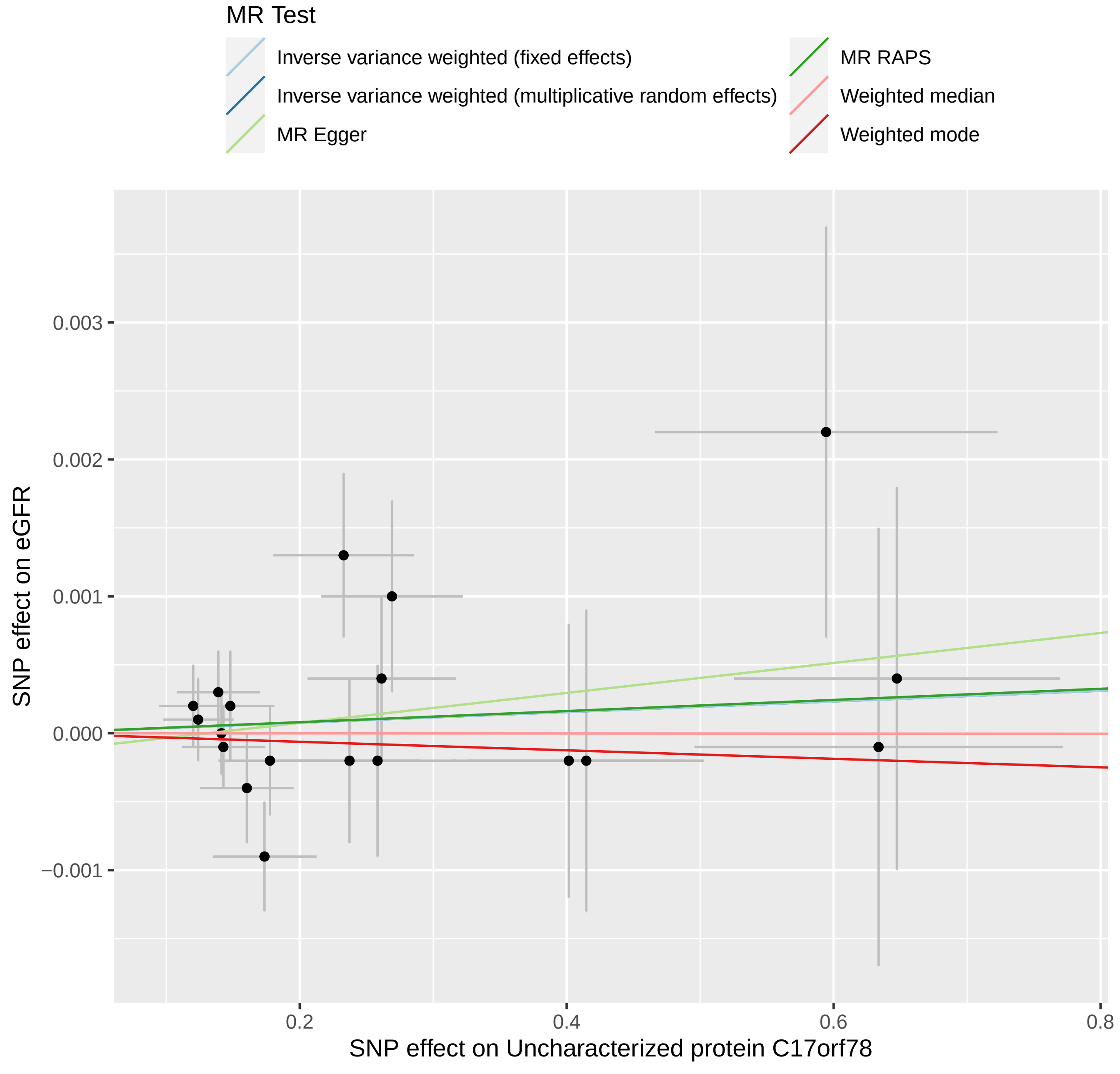

Batch 2−288 : Uncharacterized protein C17orf78 on eGFR

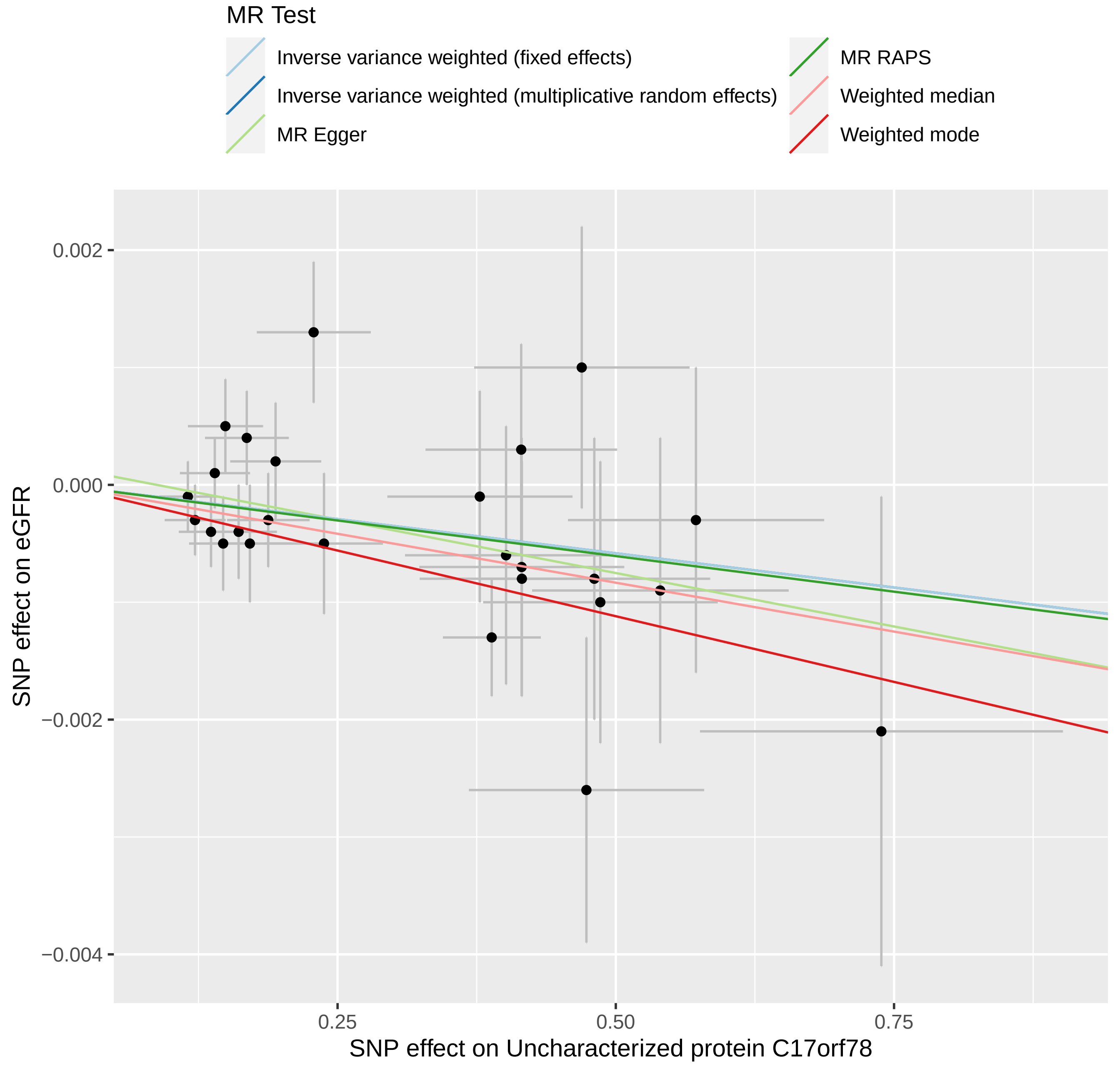

Batch 2−289 : Uncharacterized protein C17orf89 on eGFR

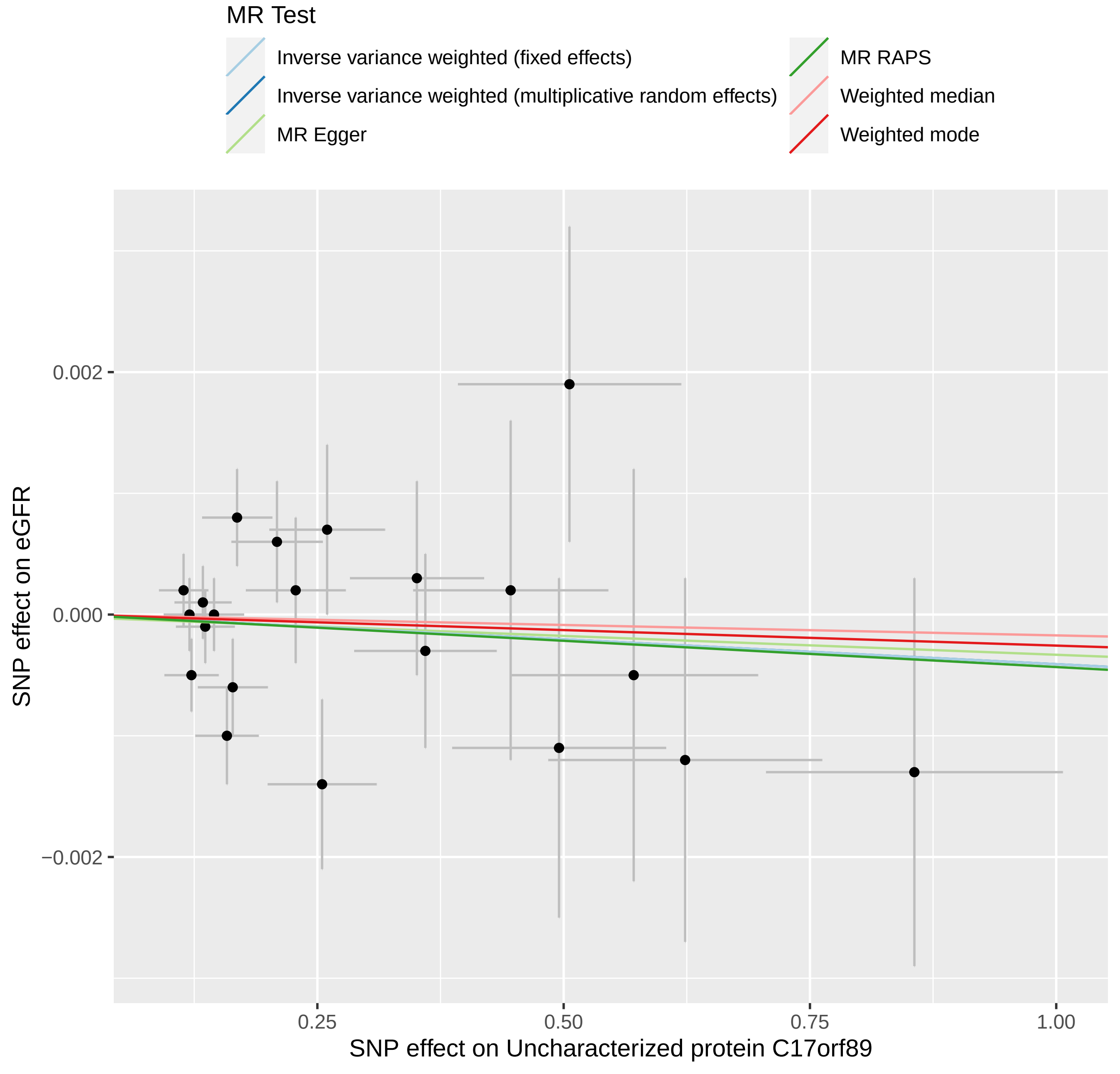

Batch 2−290 : UPF0729 protein C18orf32 on eGFR

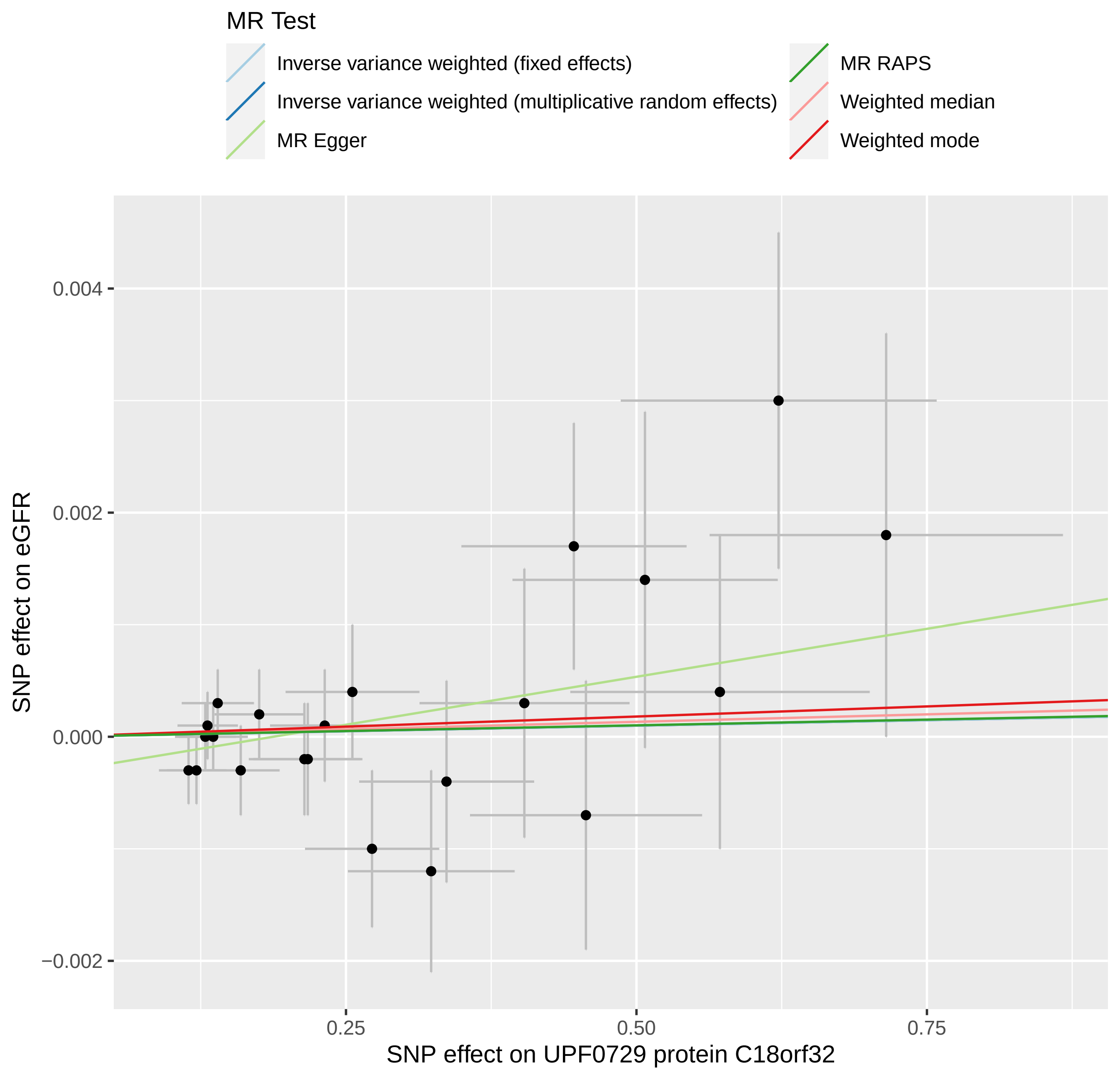

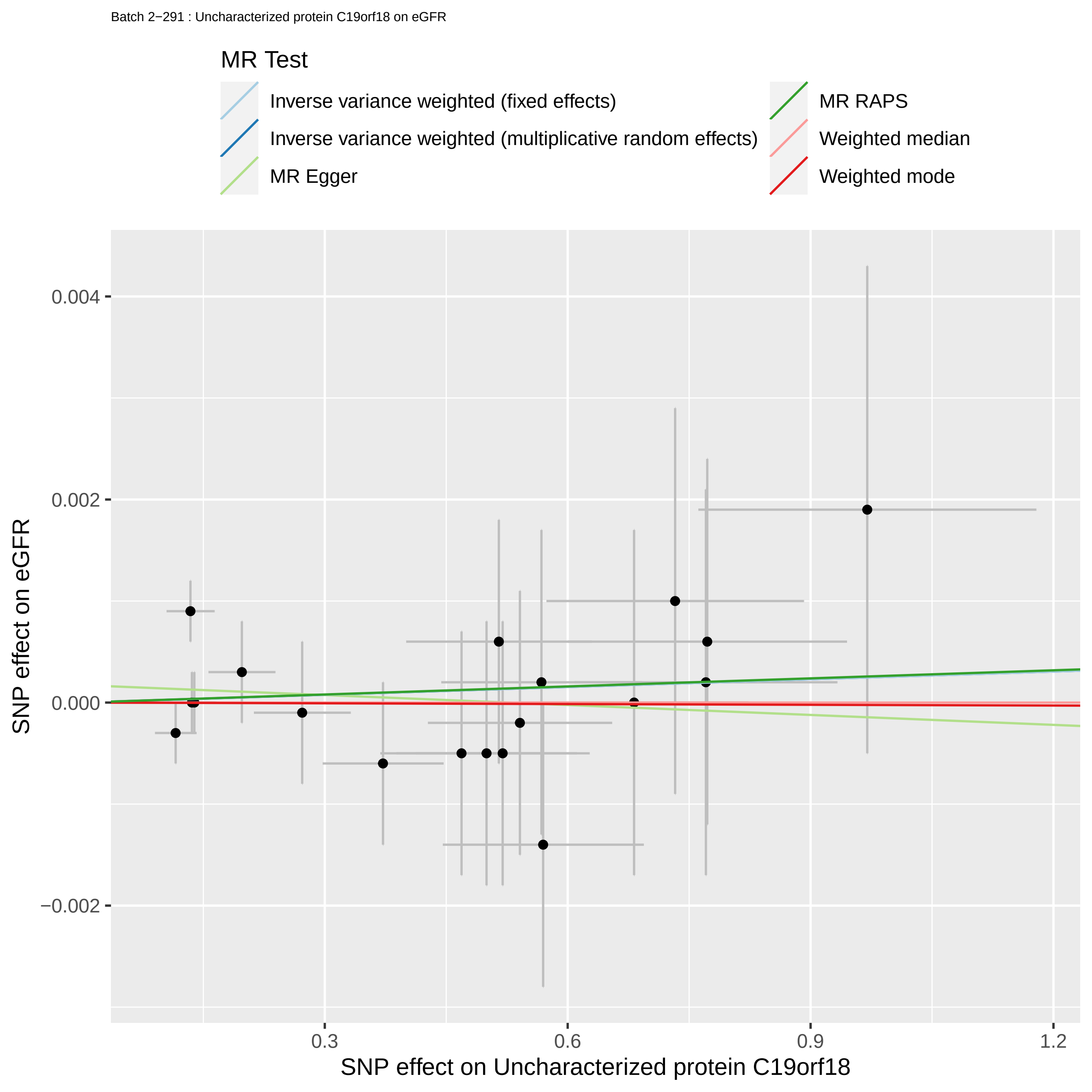
Batch 2−291 : Uncharacterized protein C19orf18 on eGFR
MR Test
Inverse variance weighted (fixed effects)
Inverse variance weighted (multiplicative random effects)
MR Egger
MR RAPS
Weighted median
Weighted mode
0.004
0.002
0.000
−0.002
0.3
0.6
0.9
1.2
SNP effect on eGFR
SNP effect on Uncharacterized protein C19orf18

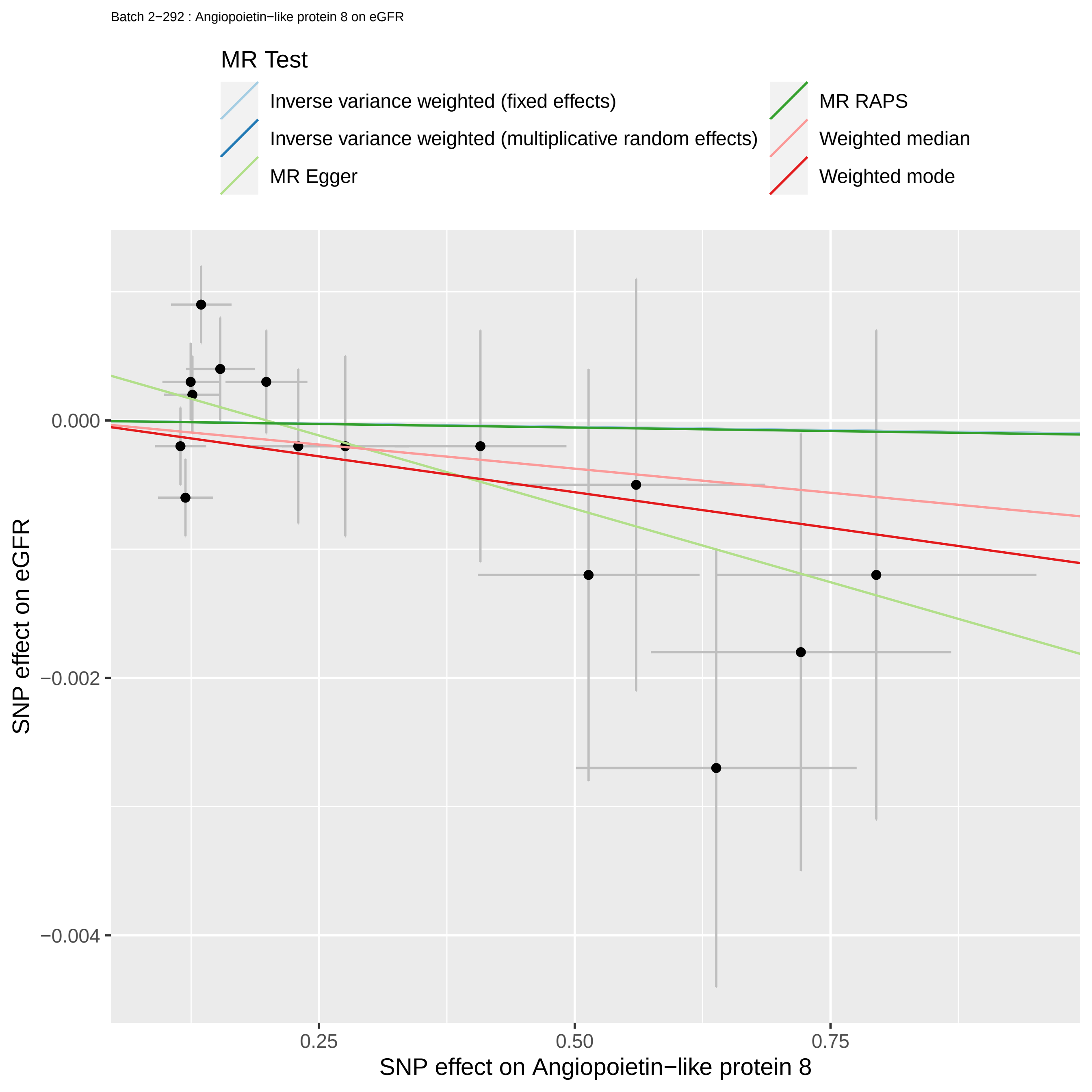

Batch 2−292 : Angiopoietin−like protein 8 on eGFR
MR Test
Inverse variance weighted (fixed effects)
Inverse variance weighted (multiplicative random effects)
MR Egger
MR RAPS
Weighted median
Weighted mode
0.000
−0.002
−0.004
0.25
0.50
0.75
SNP effect on eGFR
SNP effect on Angiopoietin−like protein 8

Batch 2−293 : Nuclear nucleic acid−binding protein C1D on eGFR

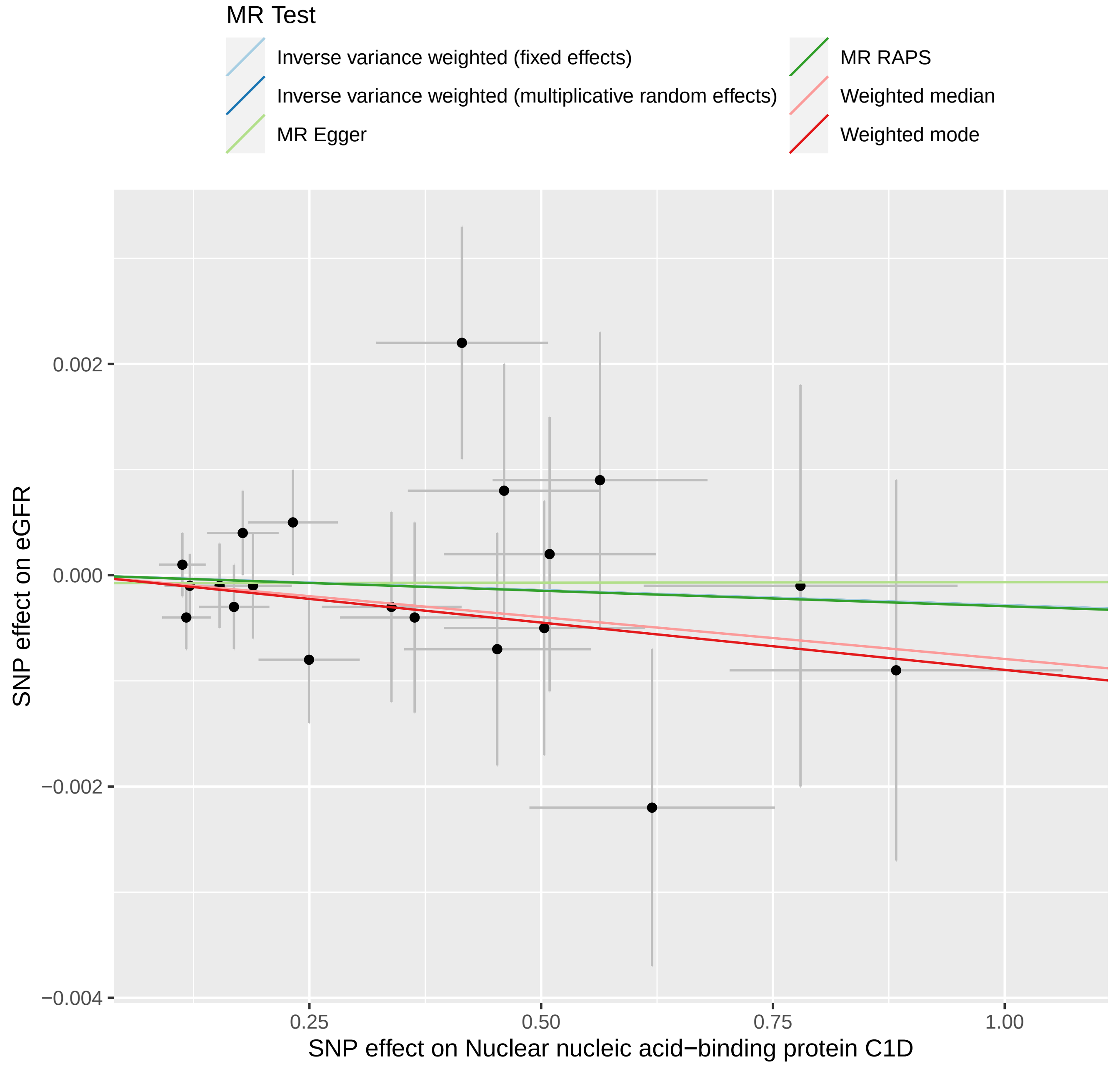

Batch 2−294 : C1GALT1−specific chaperone 1 on eGFR

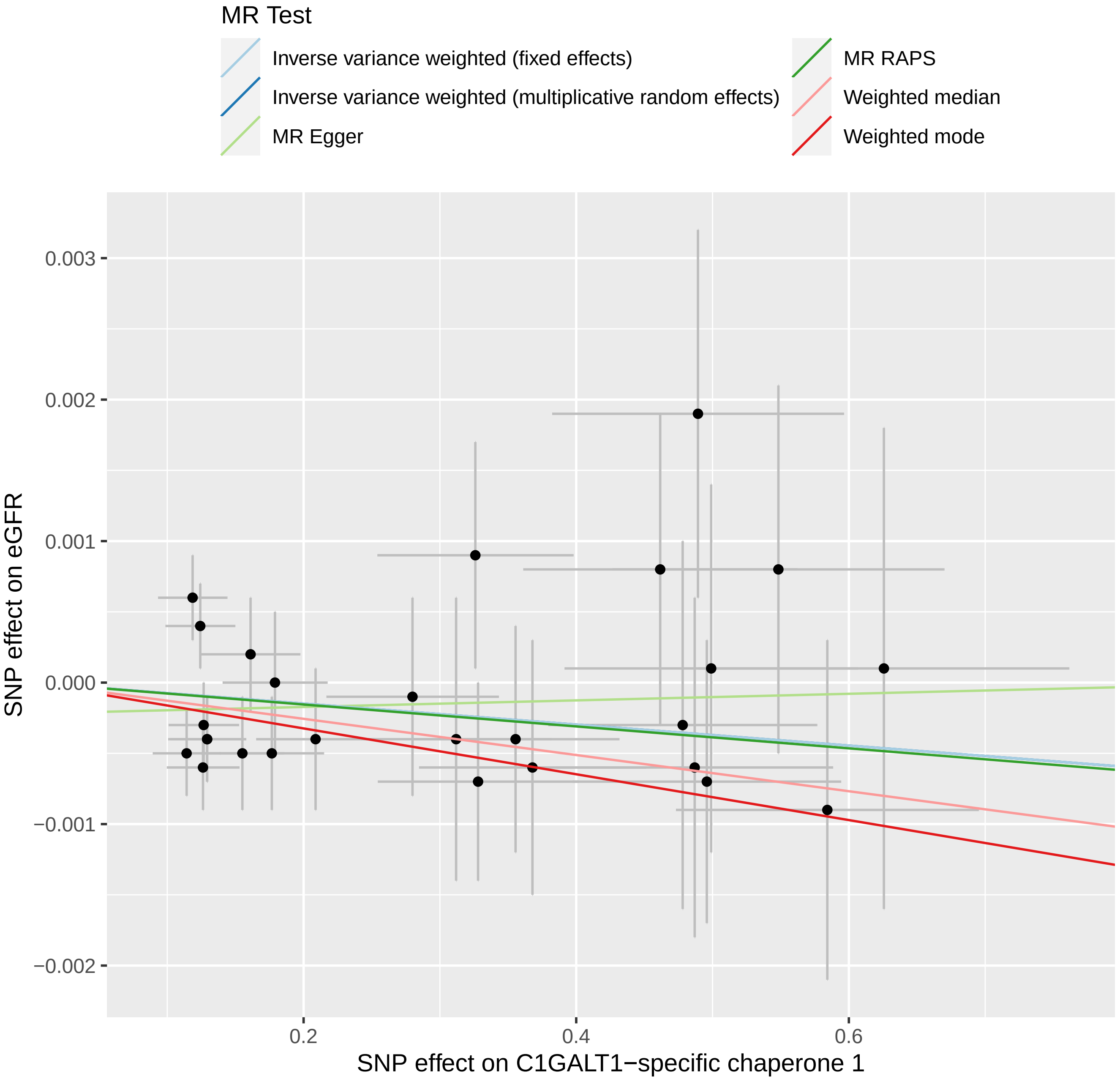

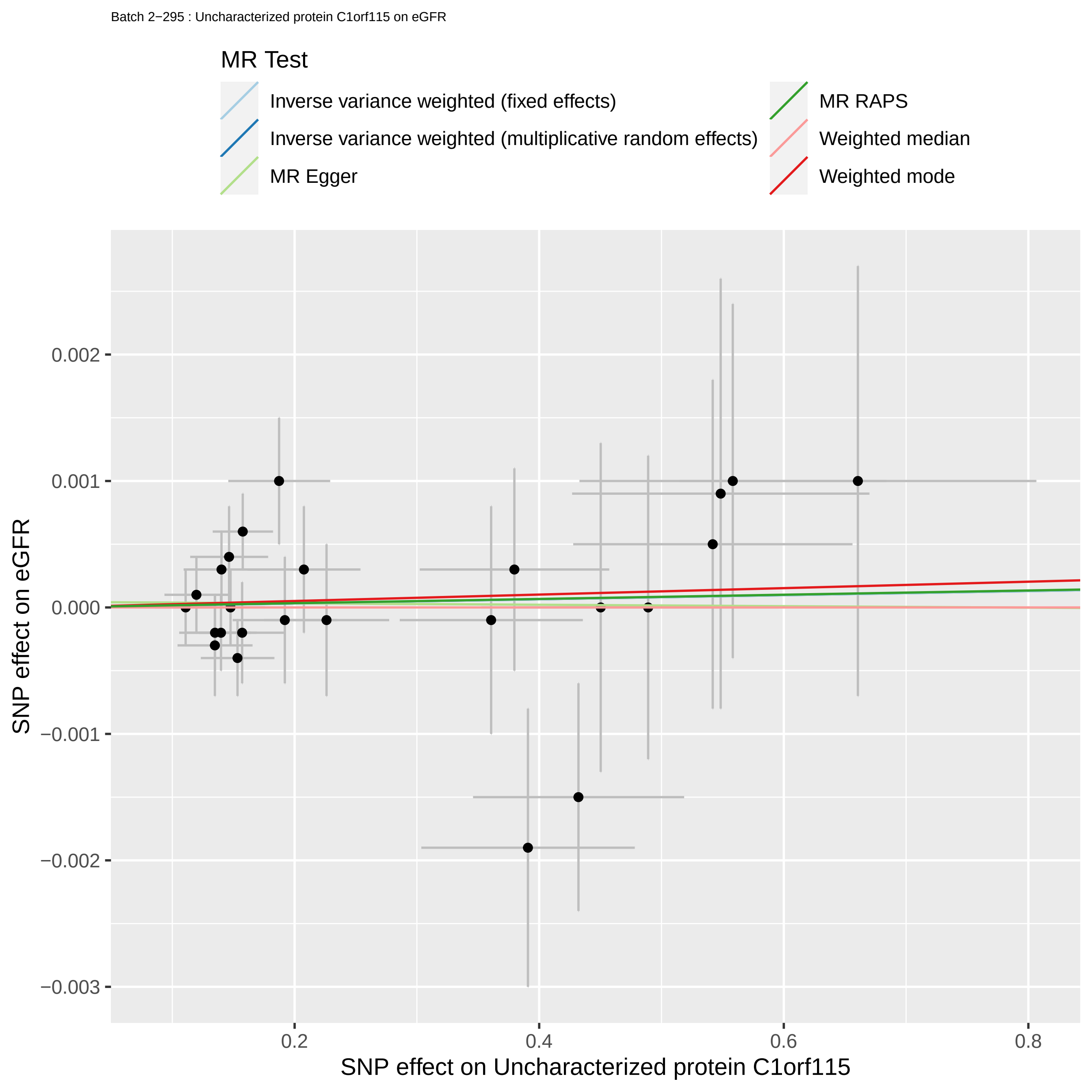
Batch 2−295 : Uncharacterized protein C1orf115 on eGFR
MR Test
Inverse variance weighted (fixed effects)
Inverse variance weighted (multiplicative random effects)
MR Egger
MR RAPS
Weighted median
Weighted mode
0.002
0.001
0.000
−0.001
−0.002
−0.003
SNP effect on eGFR
0.2
0.4
0.6
0.8
SNP effect on Uncharacterized protein C1orf115

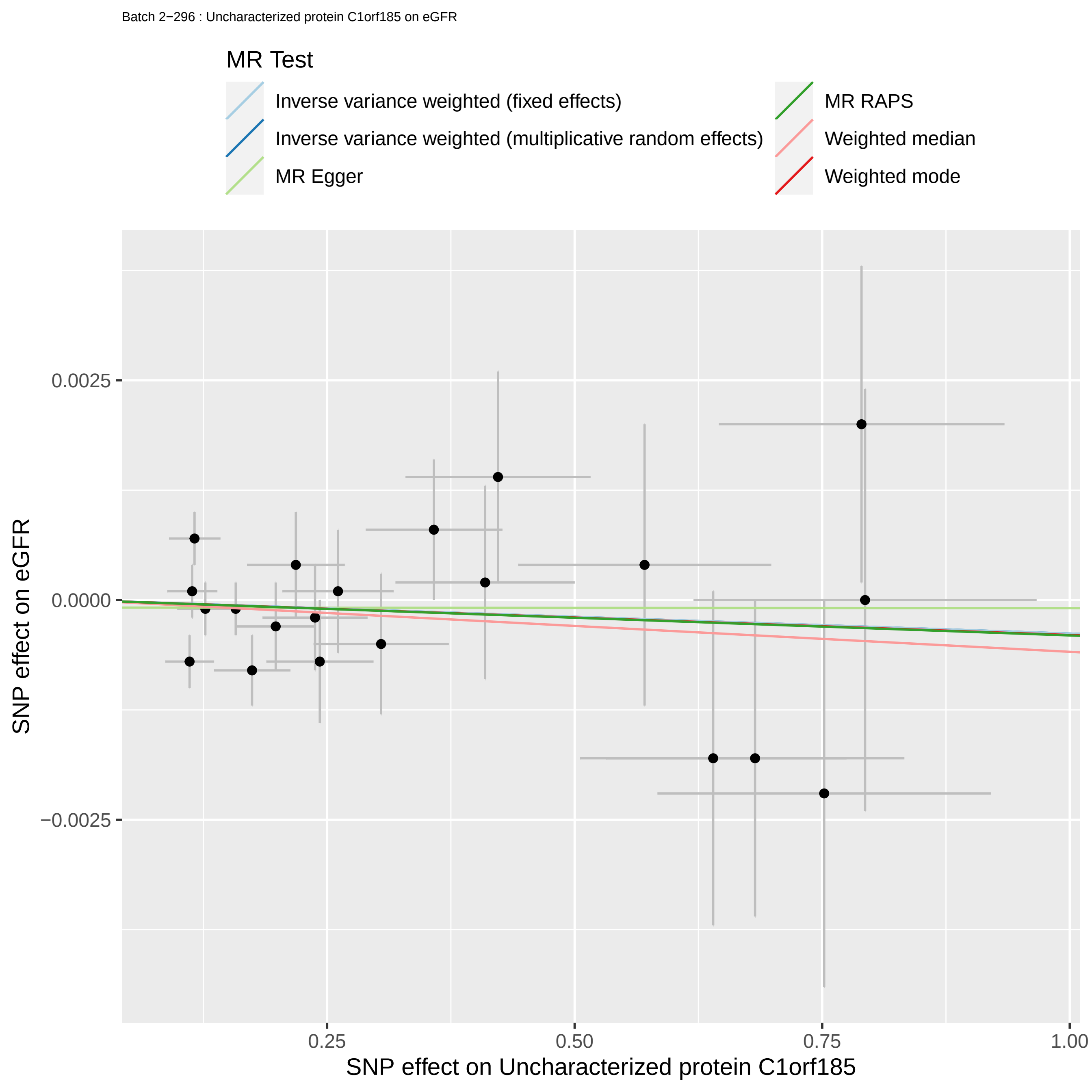
Batch 2−296 : Uncharacterized protein C1orf185 on eGFR
MR Test
Inverse variance weighted (fixed effects)
Inverse variance weighted (multiplicative random effects)
MR Egger
MR RAPS
Weighted median
Weighted mode
0.0025
0.0000
−0.0025
SNP effect on eGFR
0.25
0.50
0.75
1.00
SNP effect on Uncharacterized protein C1orf185

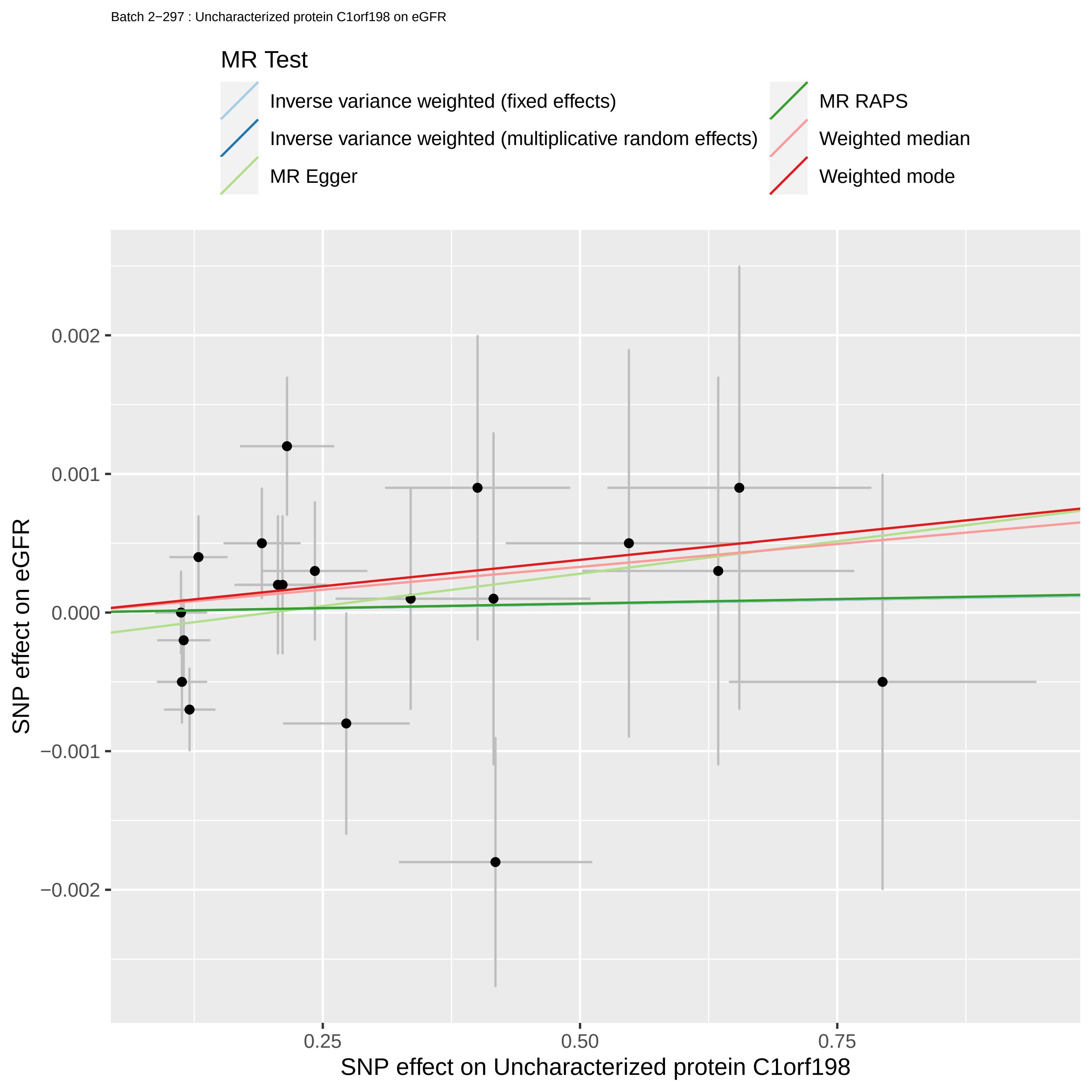

Batch 2−297 : Uncharacterized protein C1orf198 on eGFR
MR Test
Inverse variance weighted (fixed effects)
Inverse variance weighted (multiplicative random effects)
MR Egger
MR RAPS
Weighted median
Weighted mode
0.002
0.001
0.000
−0.001
−0.002
0.25
0.50
0.75
SNP effect on eGFR
SNP effect on Uncharacterized protein C1orf198

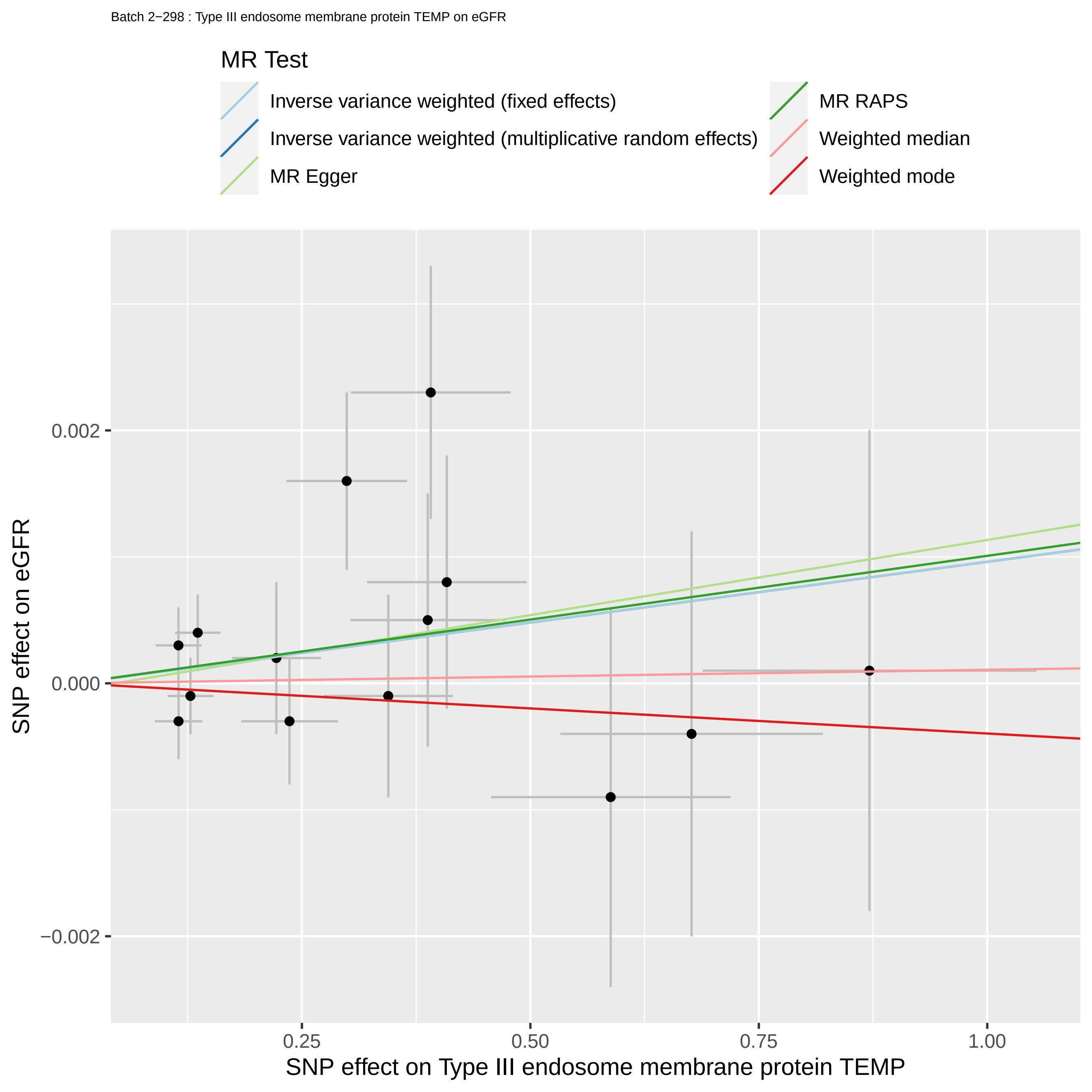
Batch 2−298 : Type III endosome membrane protein TEMP on eGFR
MR Test
Inverse variance weighted (fixed effects)
Inverse variance weighted (multiplicative random effects)
MR Egger
MR RAPS
Weighted median
Weighted mode
0.002
0.000
−0.002
0.25
0.50
0.75
1.00
SNP effect on eGFR
SNP effect on Type III endosome membrane protein TEMP

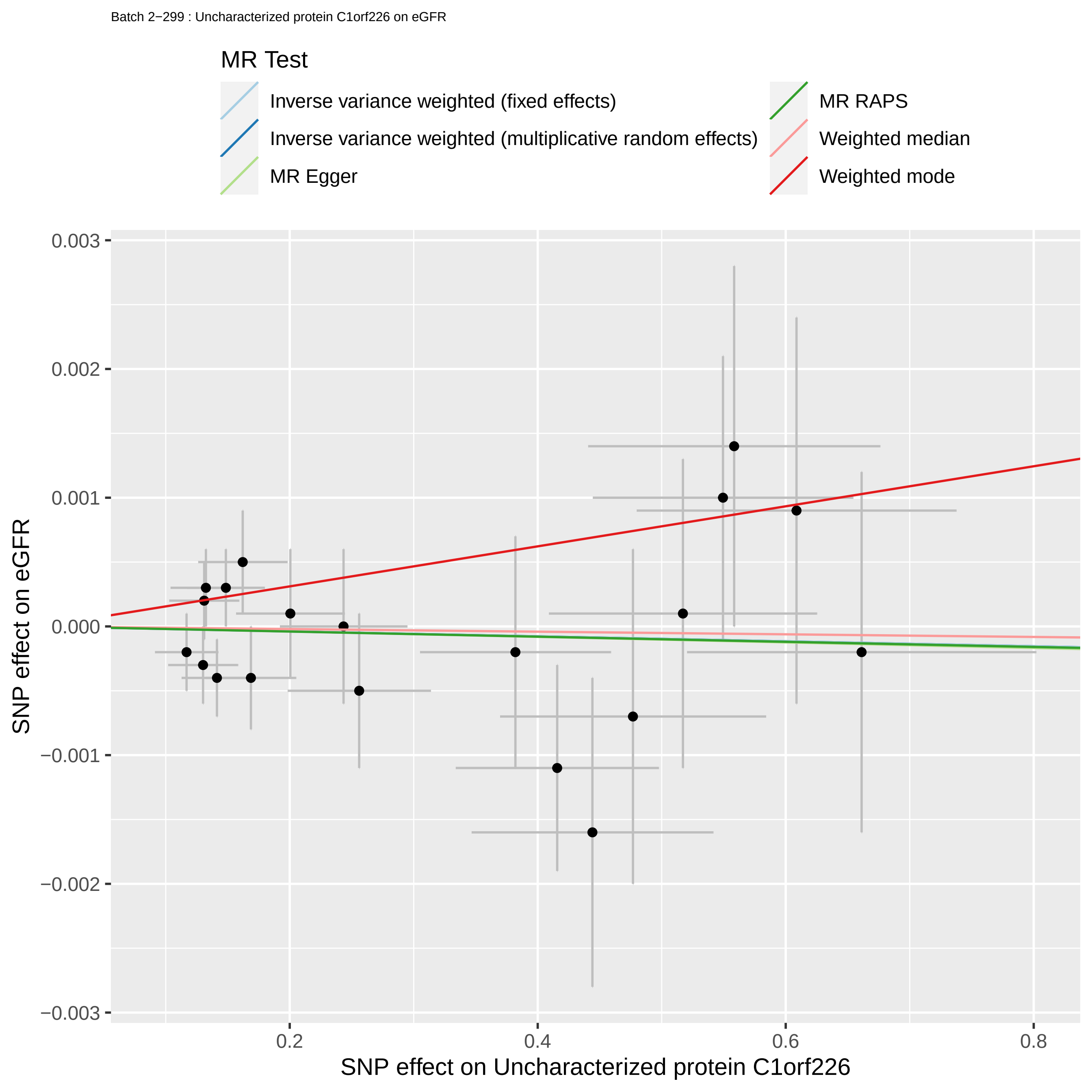

Batch 2−299 : Uncharacterized protein C1orf226 on eGFR
MR Test
Inverse variance weighted (fixed effects)
Inverse variance weighted (multiplicative random effects)
MR Egger
MR RAPS
Weighted median
Weighted mode
0.003
0.002
0.001
0.000
−0.001
−0.002
−0.003
0.2
0.4
0.6
0.8
SNP effect on eGFR
SNP effect on Uncharacterized protein C1orf226

Batch 2−300 : Complement component 1 Q subcomponent−binding protein, mitochondrial on eGFR

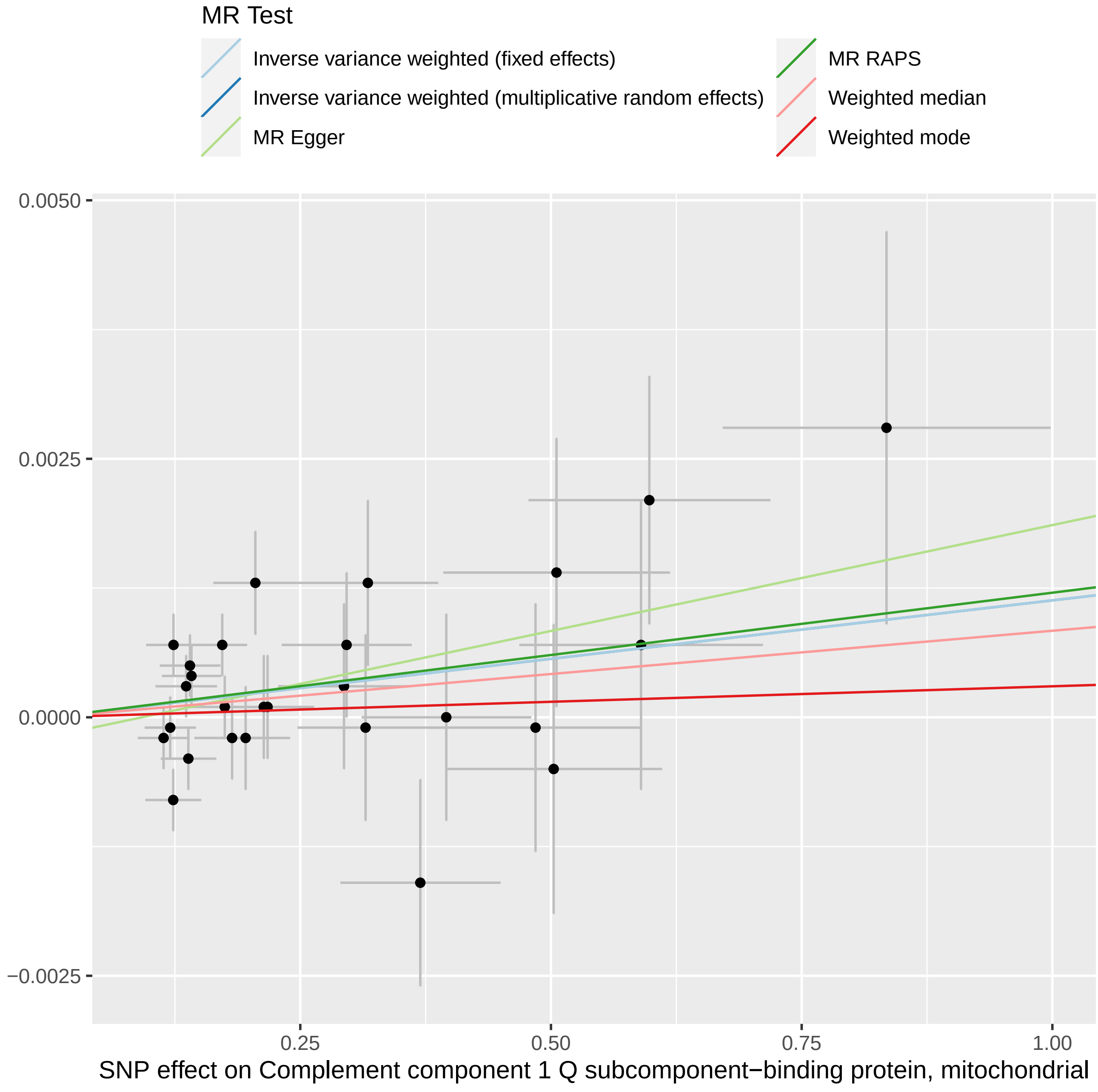

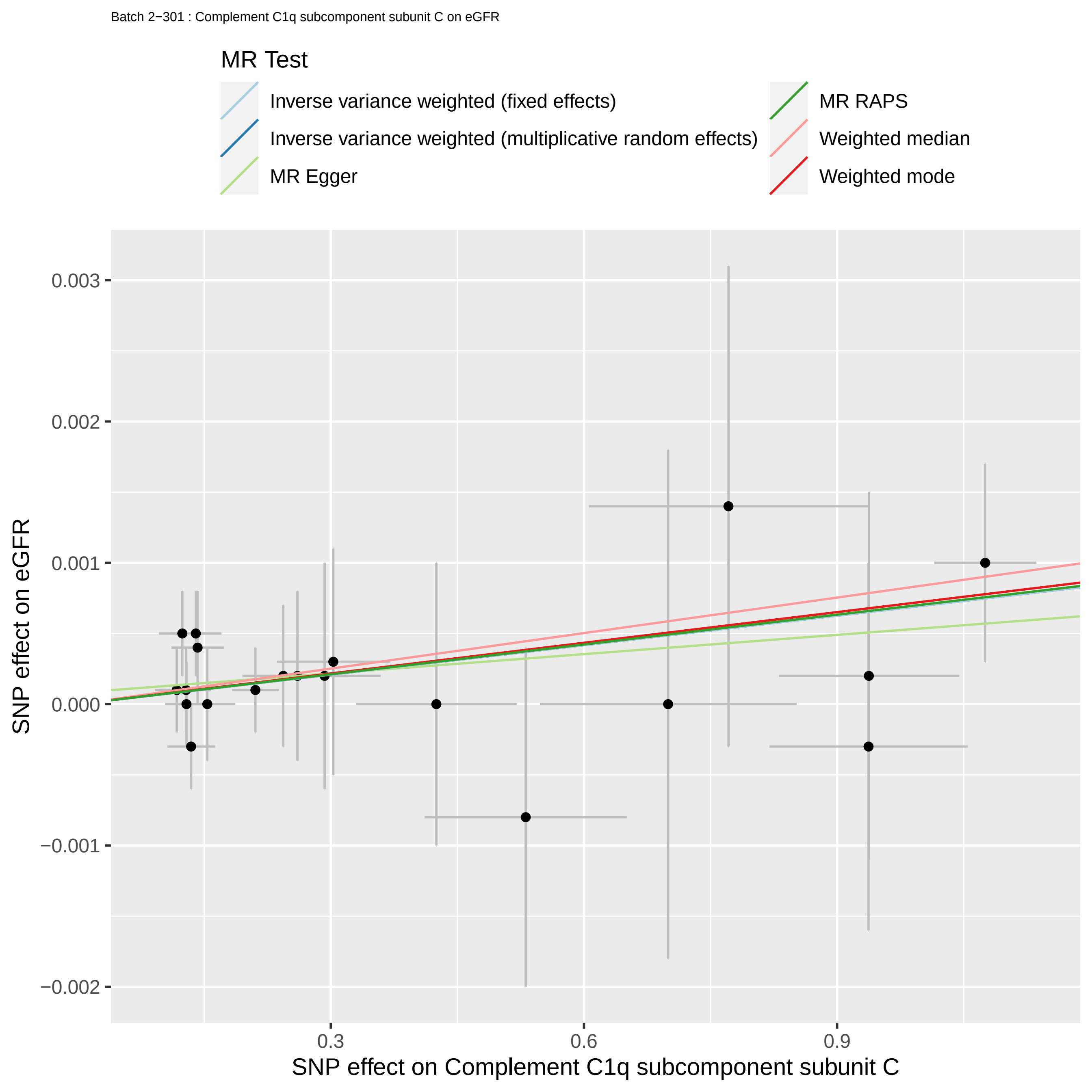

Batch 2−301 : Complement C1q subcomponent subunit C on eGFR
MR Test
Inverse variance weighted (fixed effects)
Inverse variance weighted (multiplicative random effects)
MR Egger
MR RAPS
Weighted median
Weighted mode
0.003
0.002
0.001
0.000
−0.001
−0.002
0.3
0.6
0.9
SNP effect on eGFR
SNP effect on Complement C1q subcomponent subunit C

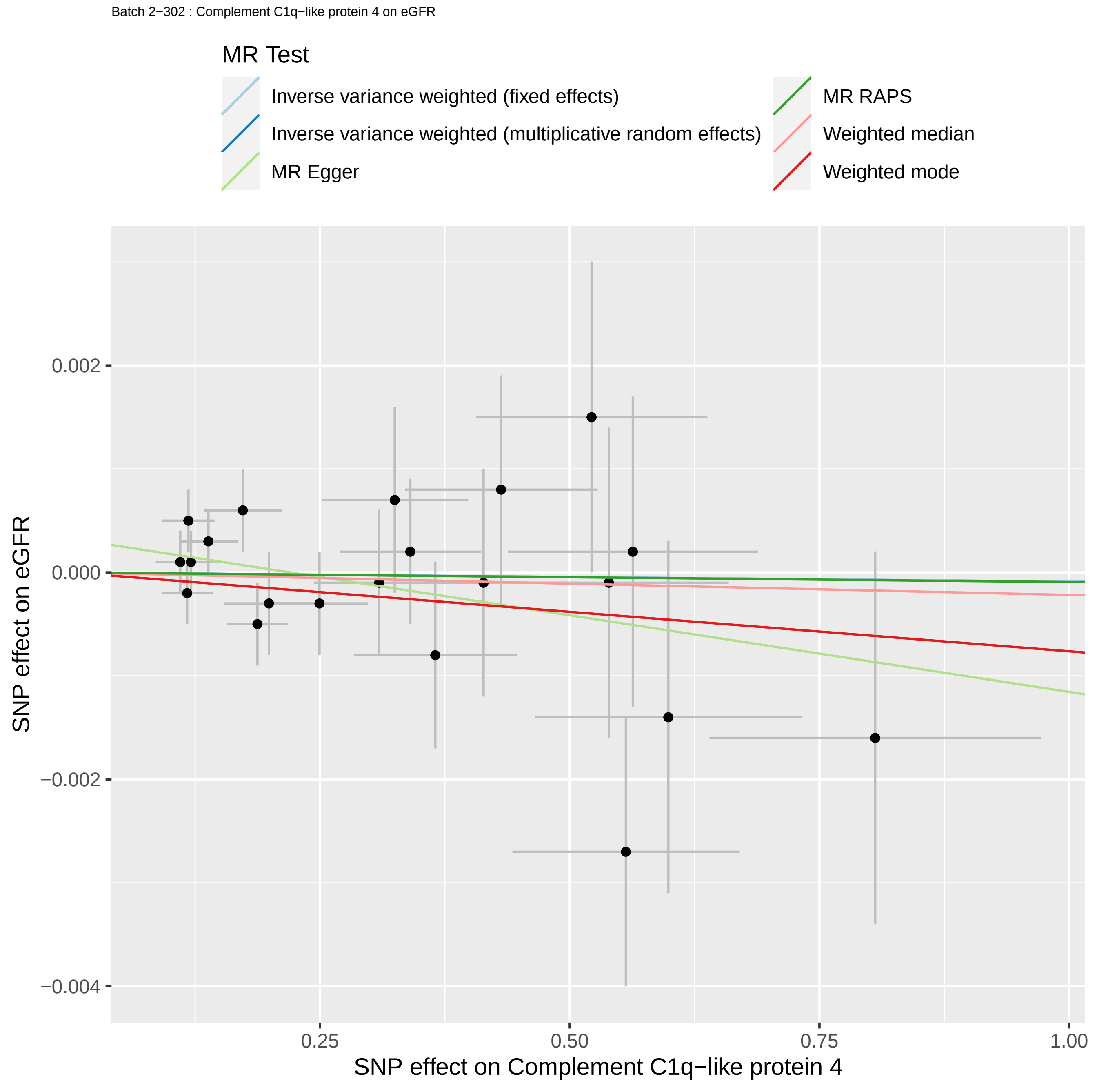

Batch 2−302 : Complement C1q−like protein 4 on eGFR
MR Test
Inverse variance weighted (fixed effects)
Inverse variance weighted (multiplicative random effects)
MR Egger
MR RAPS
Weighted median
Weighted mode
0.002
0.000
−0.002
−0.004
0.25
0.50
0.75
1.00
SNP effect on eGFR
SNP effect on Complement C1q−like protein 4

Batch 2−303 : Complement C1q tumor necrosis factor−related protein 1 on eGFR

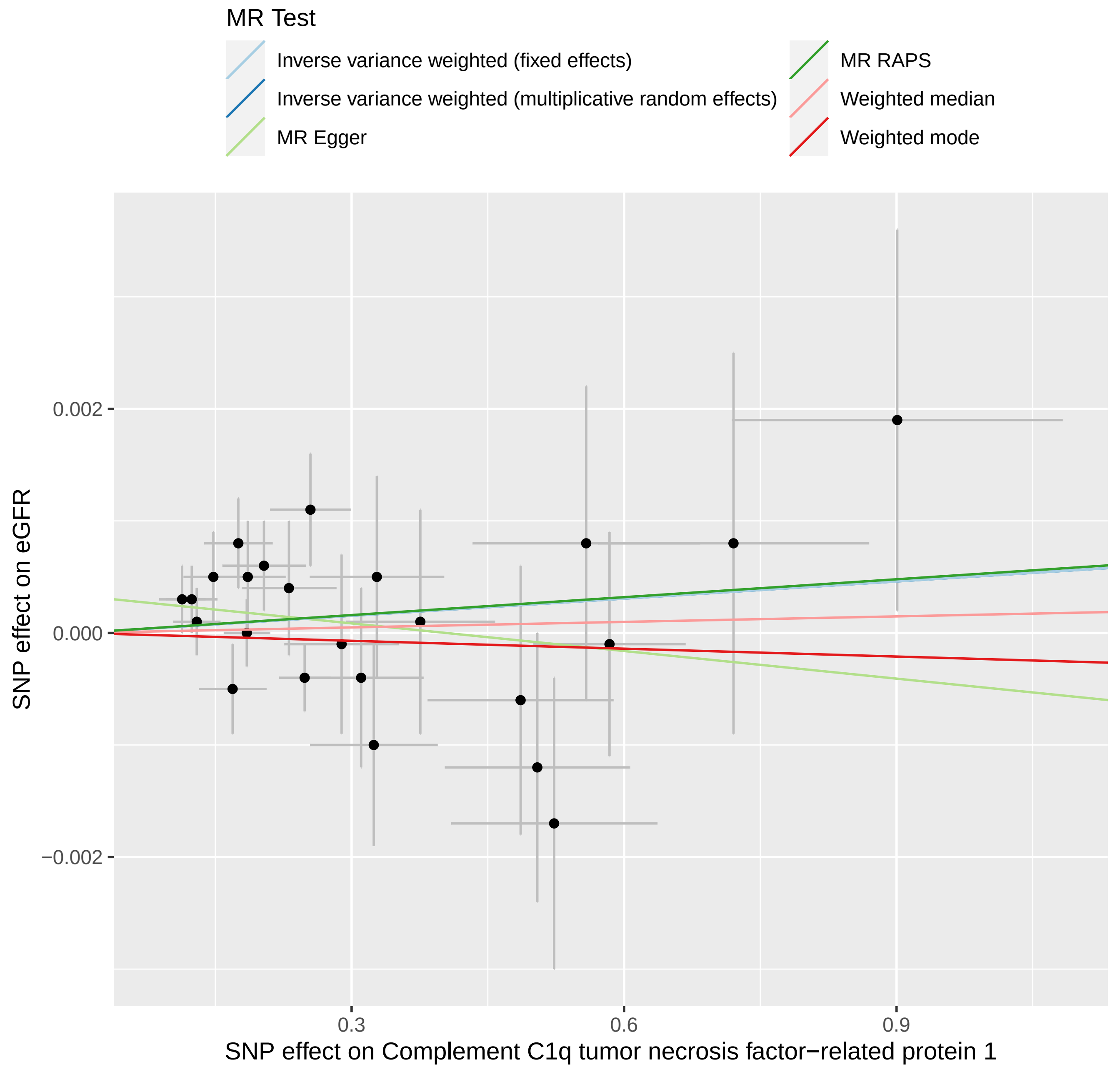

Batch 2−304 : Complement C1q tumor necrosis factor−related protein 3 on eGFR

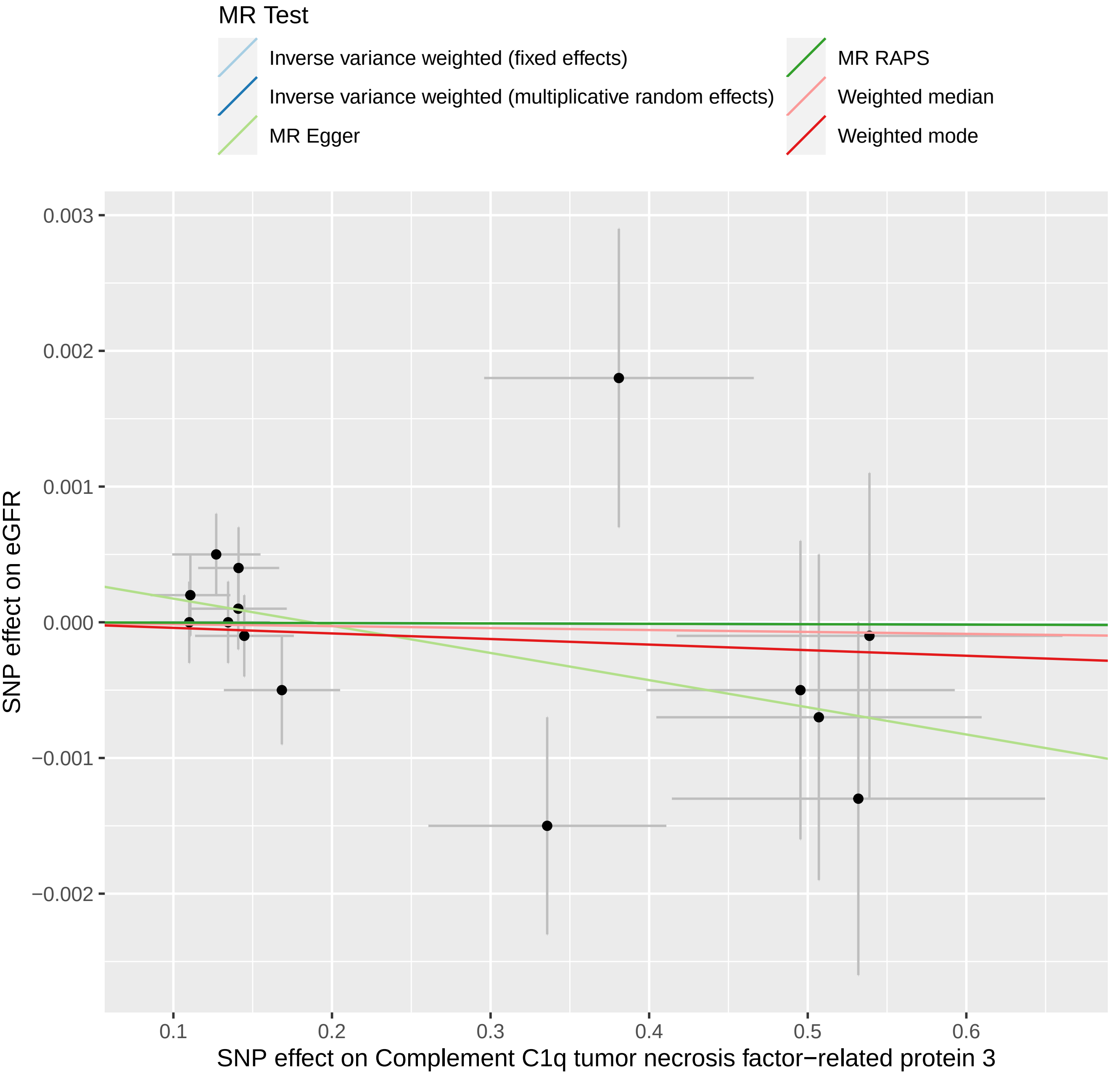

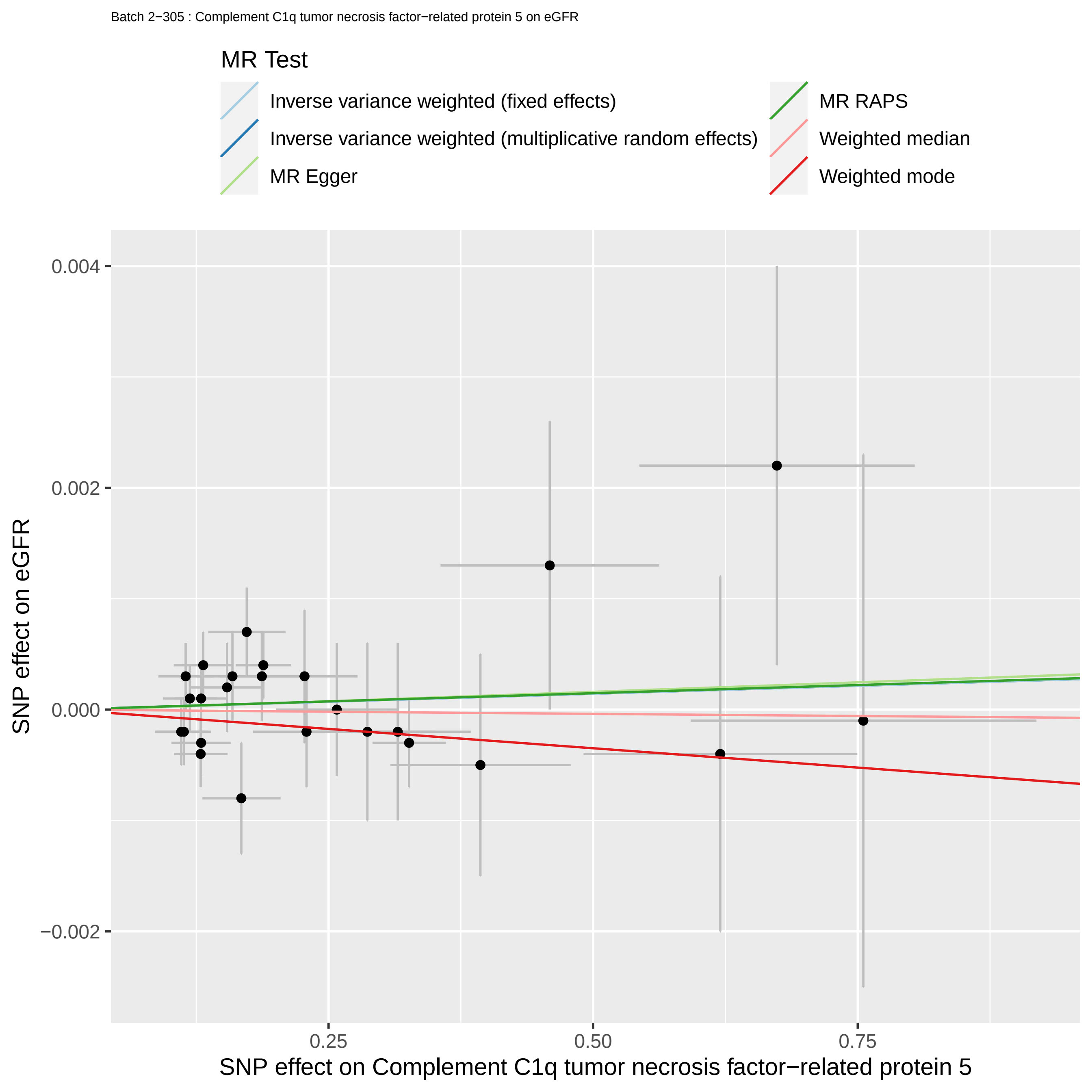

Batch 2−305 : Complement C1q tumor necrosis factor−related protein 5 on eGFR
MR Test
Inverse variance weighted (fixed effects)
Inverse variance weighted (multiplicative random effects)
MR Egger
MR RAPS
Weighted median
Weighted mode
0.004
0.002
0.000
−0.002
0.25
0.50
0.75
SNP effect on eGFR
SNP effect on Complement C1q tumor necrosis factor−related protein 5

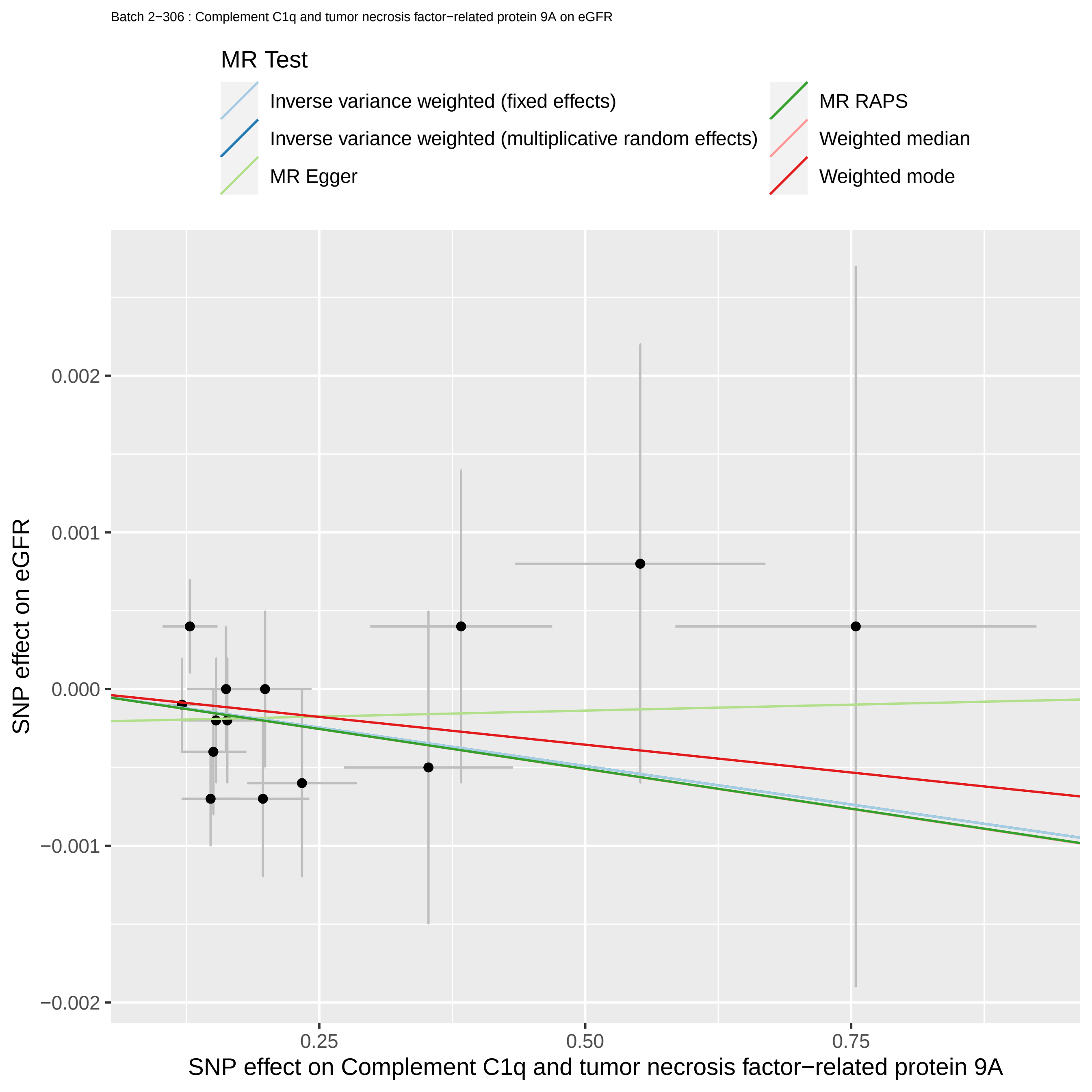
Batch 2−306 : Complement C1q and tumor necrosis factor−related protein 9A on eGFR
MR Test
Inverse variance weighted (fixed effects)
Inverse variance weighted (multiplicative random effects)
MR Egger
MR RAPS
Weighted median
Weighted mode
0.002
0.001
0.000
−0.001
−0.002
0.25
0.50
0.75
SNP effect on eGFR
SNP effect on Complement C1q and tumor necrosis factor−related protein 9A

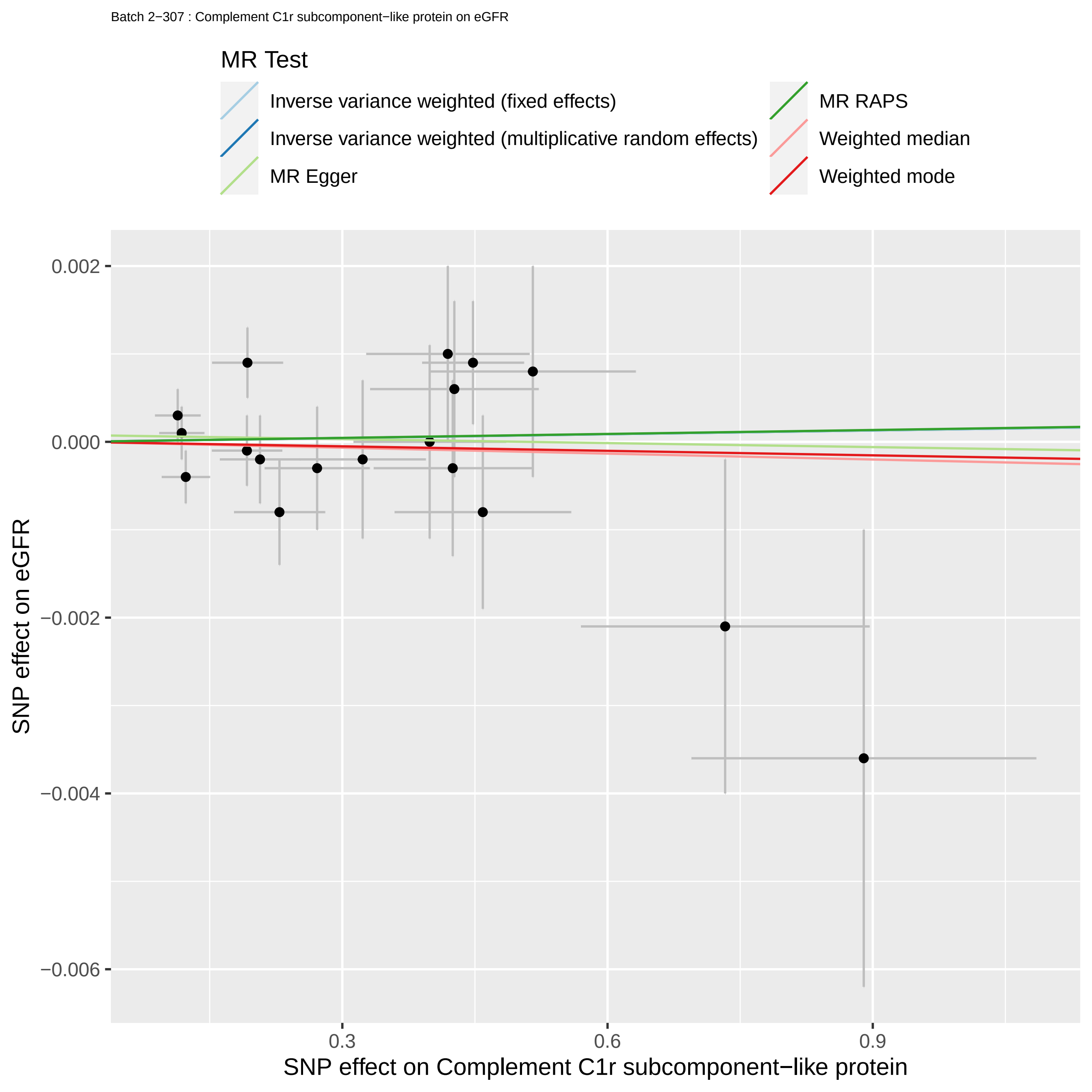

Batch 2−307 : Complement C1r subcomponent−like protein on eGFR
MR Test
Inverse variance weighted (fixed effects)
Inverse variance weighted (multiplicative random effects)
MR Egger
MR RAPS
Weighted median
Weighted mode
0.002
0.000
−0.002
−0.004
−0.006
0.3
0.6
0.9
SNP effect on eGFR
SNP effect on Complement C1r subcomponent−like protein

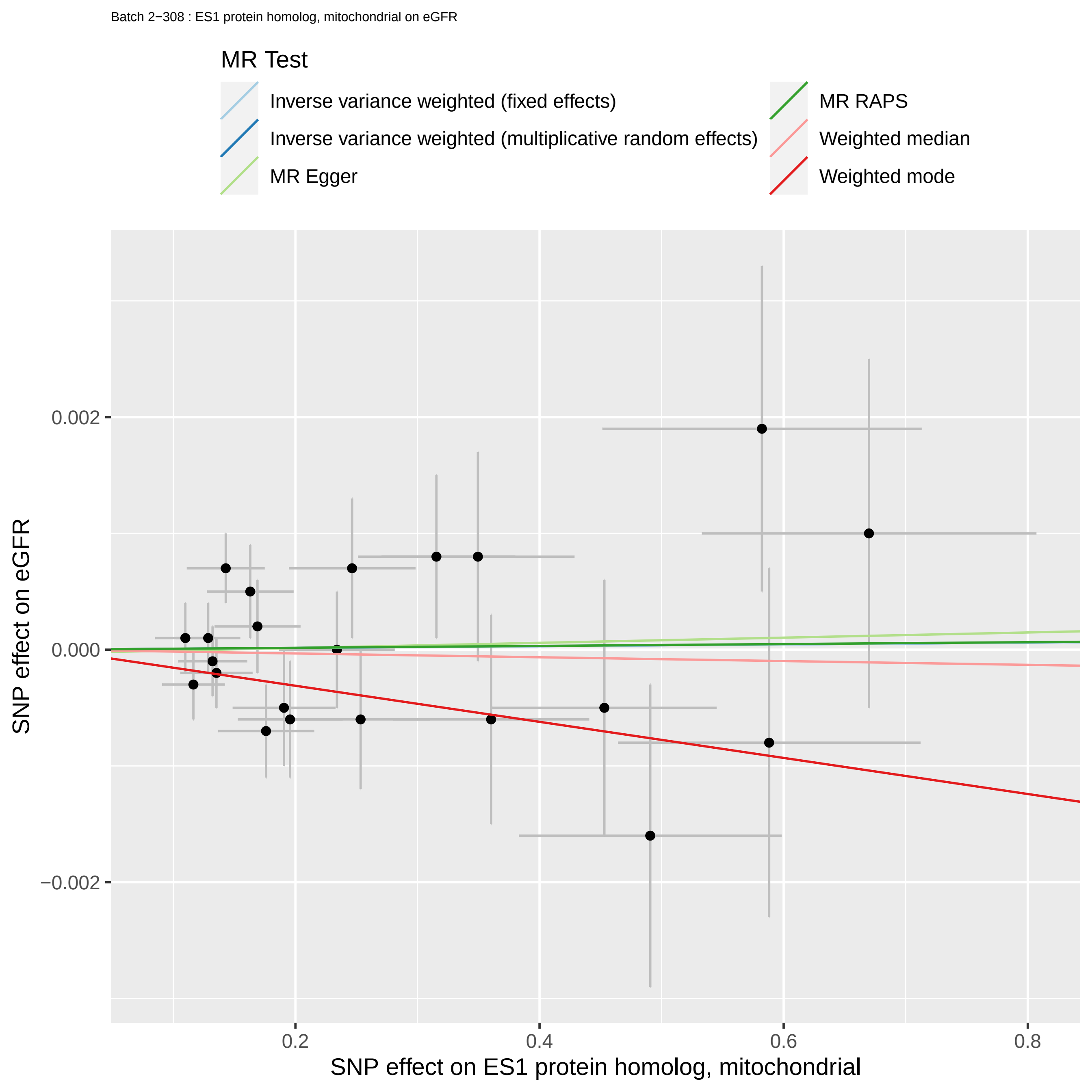

Batch 2−308 : ES1 protein homolog, mitochondrial on eGFR
MR Test
Inverse variance weighted (fixed effects)
Inverse variance weighted (multiplicative random effects)
MR Egger
MR RAPS
Weighted median
Weighted mode
0.002
0.000
−0.002
0.2
0.4
0.6
0.8
SNP effect on eGFR
SNP effect on ES1 protein homolog, mitochondrial

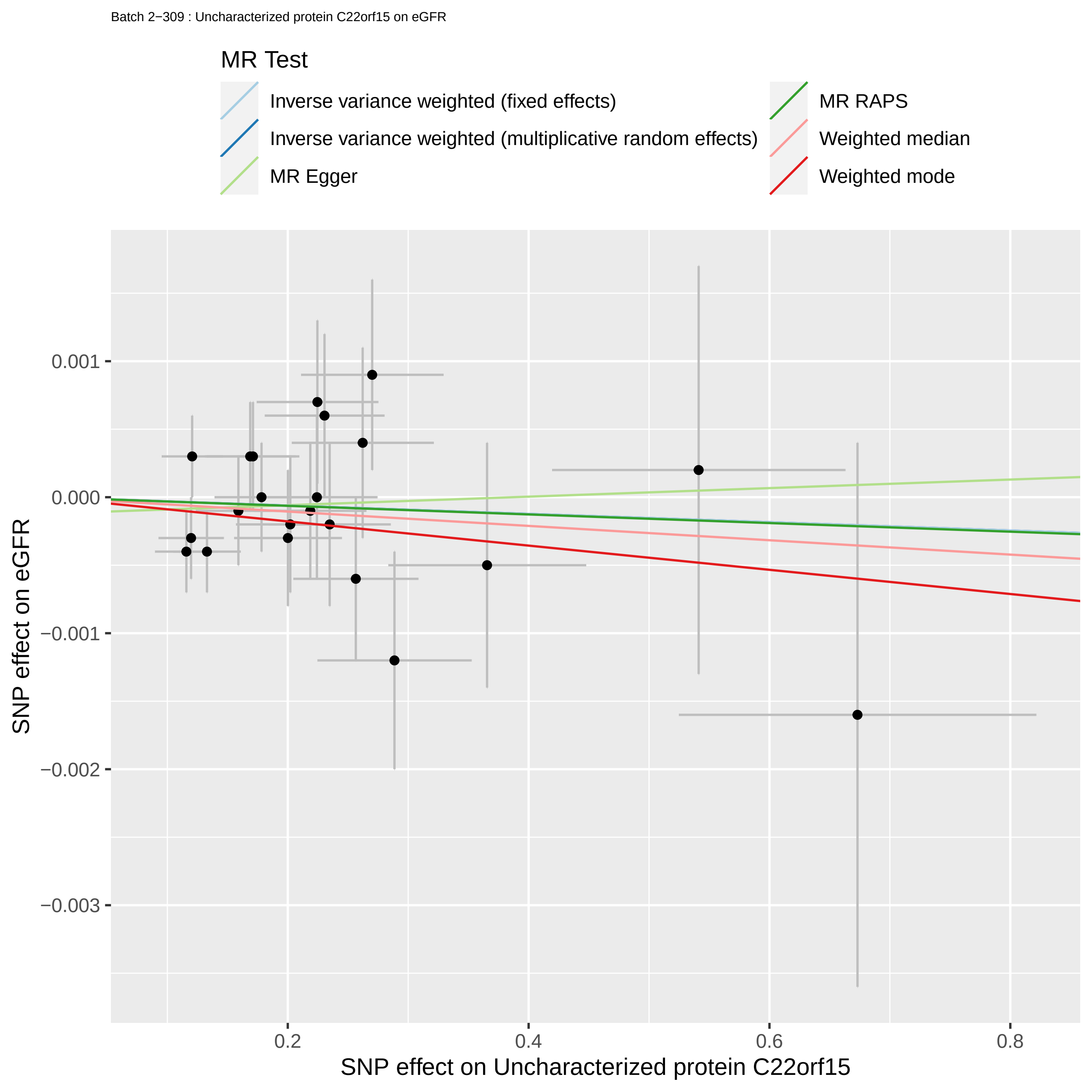
Batch 2−309 : Uncharacterized protein C22orf15 on eGFR
MR Test
Inverse variance weighted (fixed effects)
Inverse variance weighted (multiplicative random effects)
MR Egger
MR RAPS
Weighted median
Weighted mode
0.001
0.000
−0.001
−0.002
−0.003
0.2
0.4
0.6
0.8
SNP effect on eGFR
SNP effect on Uncharacterized protein C22orf15

Batch 2−310 : Augurin on eGFR

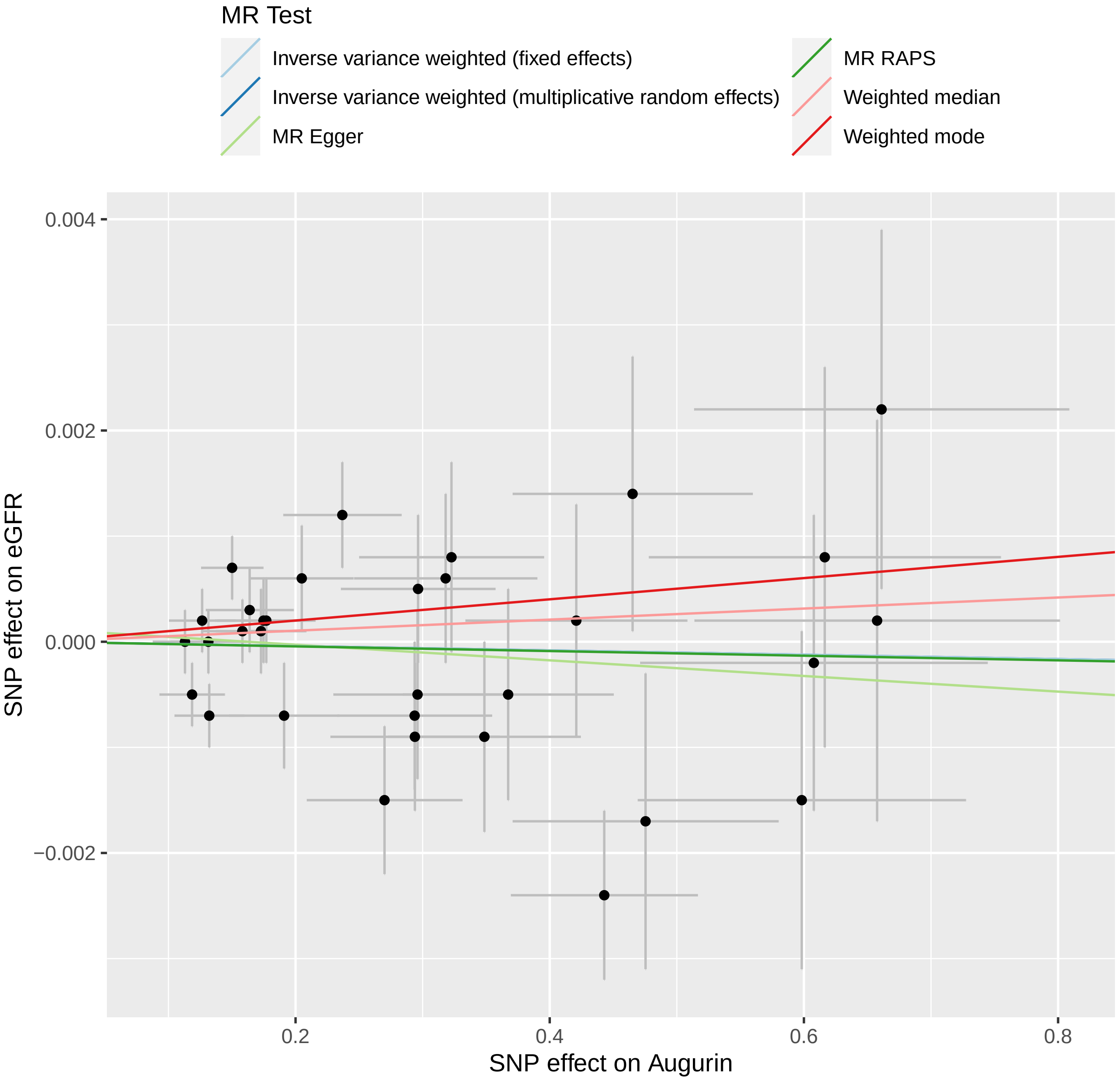

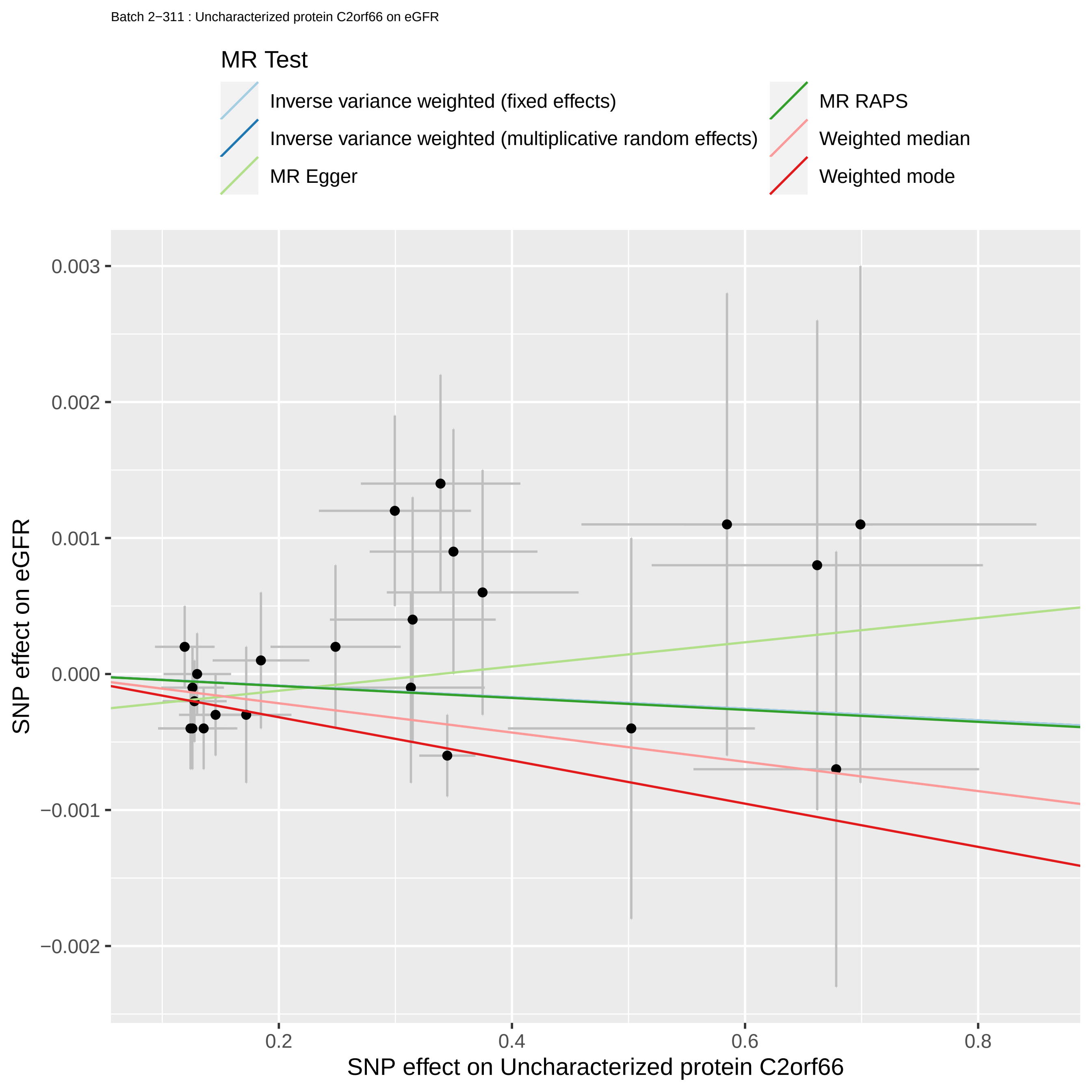

Batch 2−311 : Uncharacterized protein C2orf66 on eGFR
MR Test
Inverse variance weighted (fixed effects)
Inverse variance weighted (multiplicative random effects)
MR Egger
MR RAPS
Weighted median
Weighted mode
0.003
0.002
0.001
0.000
−0.001
−0.002
SNP effect on eGFR
0.2
0.4
0.6
0.8
SNP effect on Uncharacterized protein C2orf66

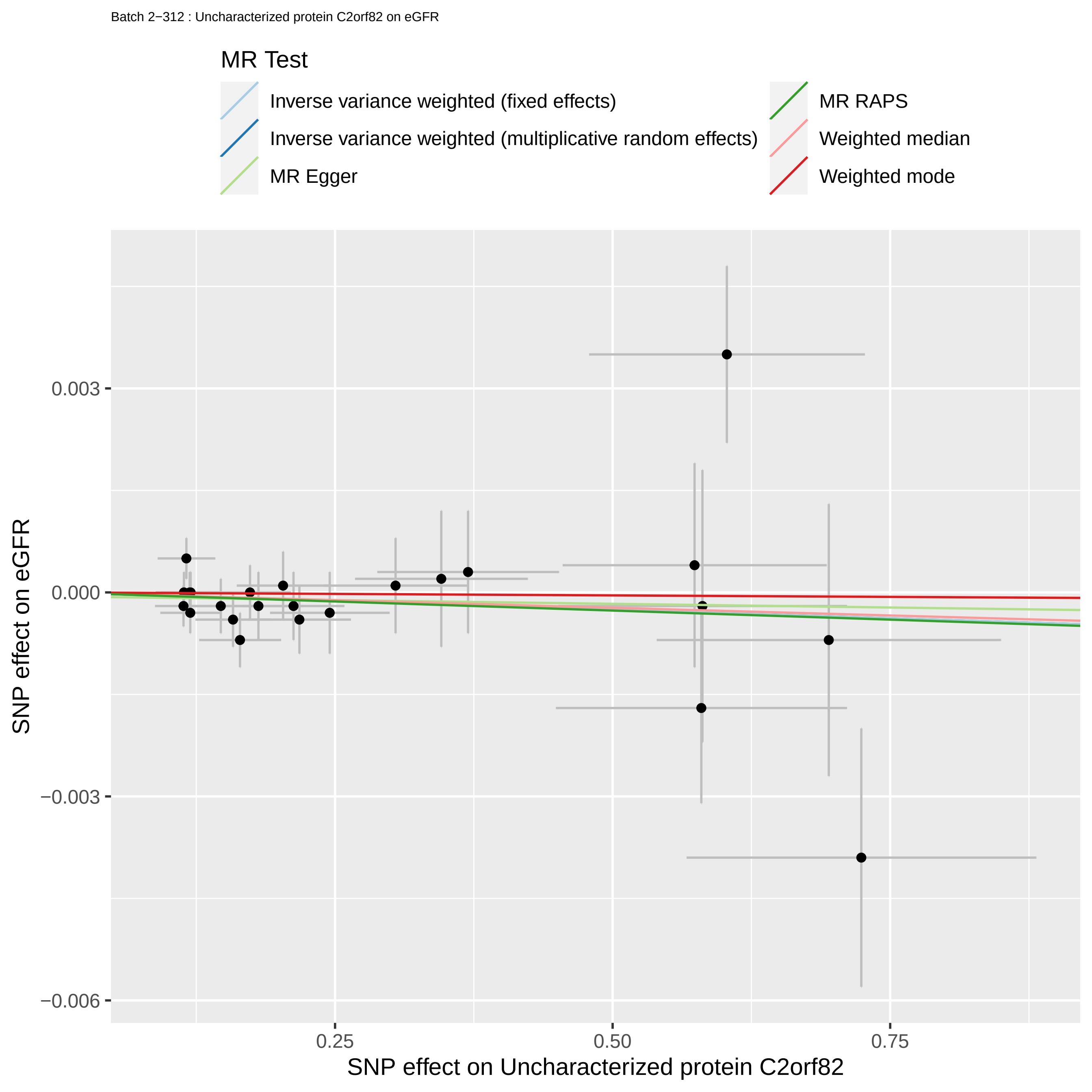
Batch 2−312 : Uncharacterized protein C2orf82 on eGFR
MR Test
Inverse variance weighted (fixed effects)
Inverse variance weighted (multiplicative random effects)
MR Egger
MR RAPS
Weighted median
Weighted mode
0.003
0.000
−0.003
−0.006
0.25
0.50
0.75
SNP effect on eGFR
SNP effect on Uncharacterized protein C2orf82

Batch 2−313 : C3a anaphylatoxin des Arginine on eGFR

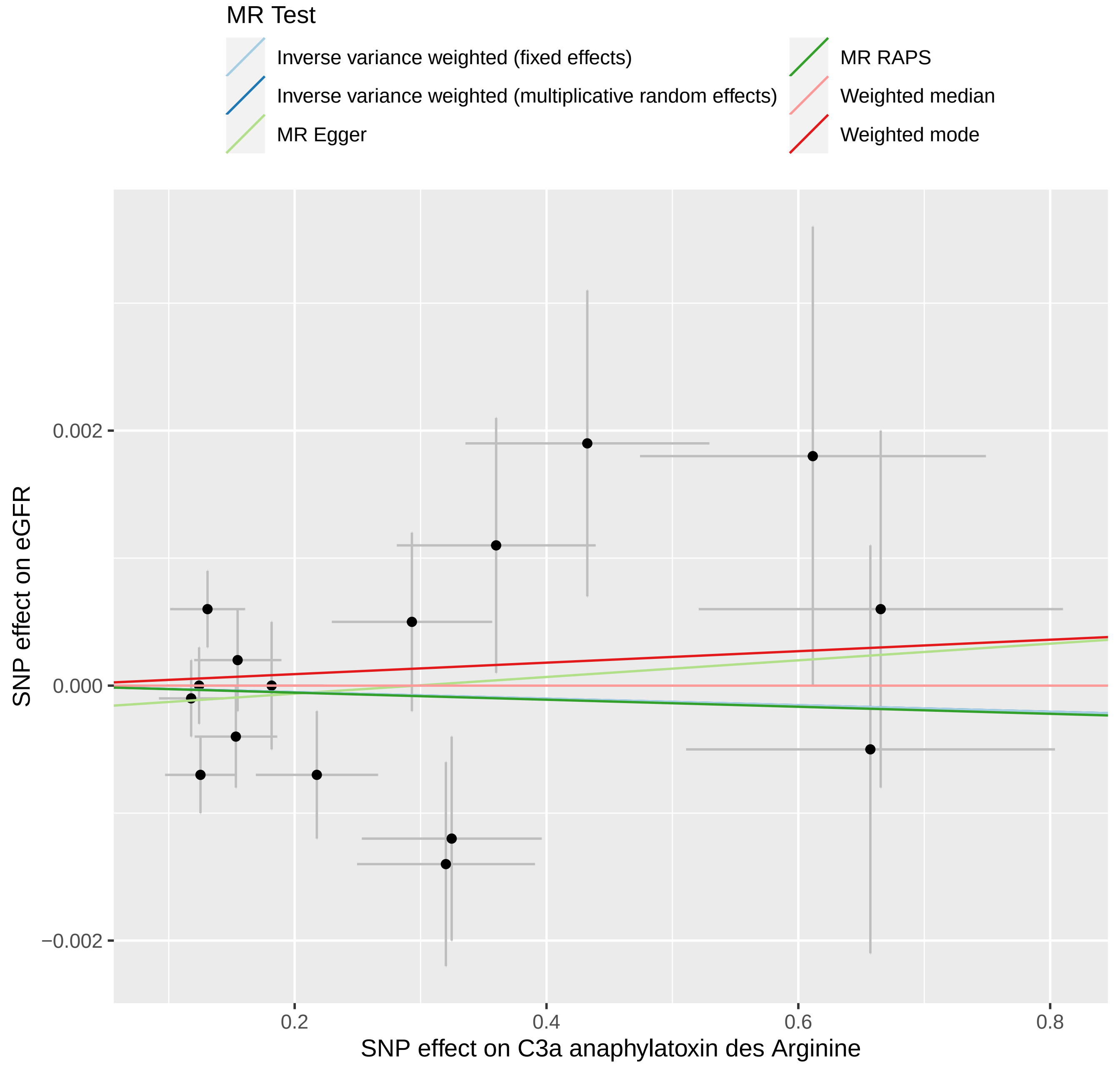

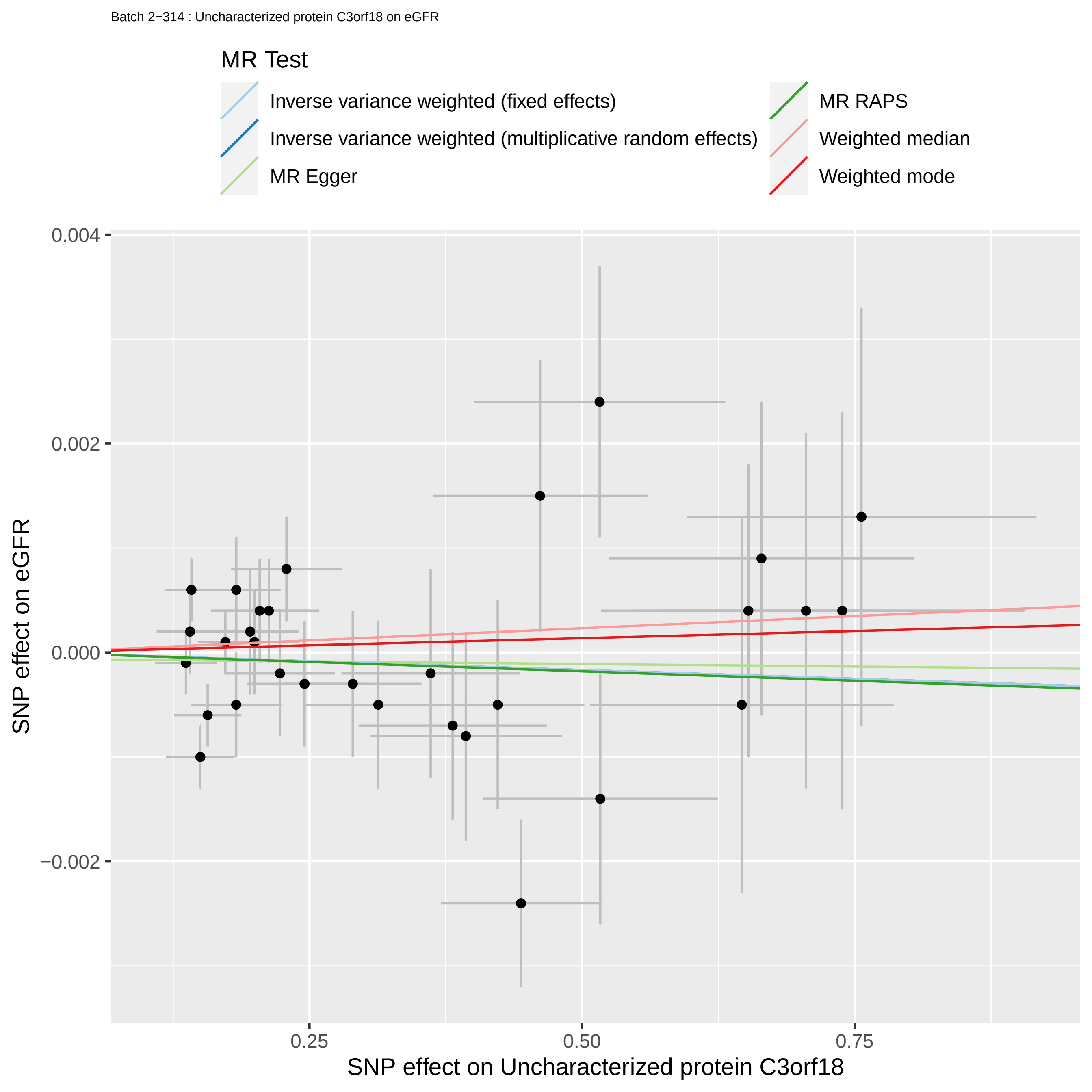
Batch 2−314 : Uncharacterized protein C3orf18 on eGFR
MR Test
Inverse variance weighted (fixed effects)
Inverse variance weighted (multiplicative random effects)
MR Egger
MR RAPS
Weighted median
Weighted mode
0.004
0.002
0.000
−0.002
0.25
0.50
0.75
SNP effect on eGFR
SNP effect on Uncharacterized protein C3orf18

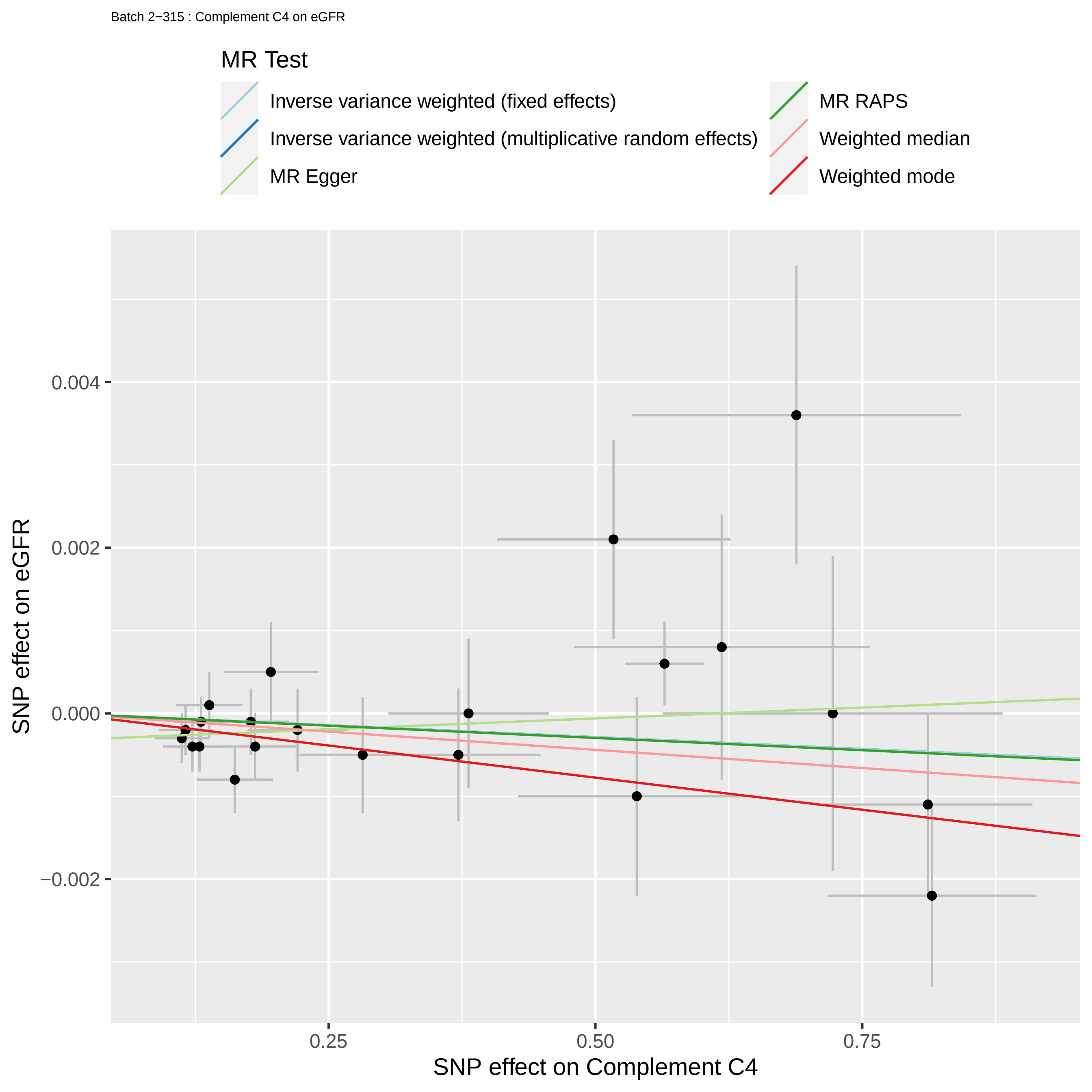

Batch 2−315 : Complement C4 on eGFR
MR Test
Inverse variance weighted (fixed effects)
Inverse variance weighted (multiplicative random effects)
MR Egger
MR RAPS
Weighted median
Weighted mode
0.004
0.002
0.000
−0.002
SNP effect on eGFR
0.25
0.50
0.75
SNP effect on Complement C4

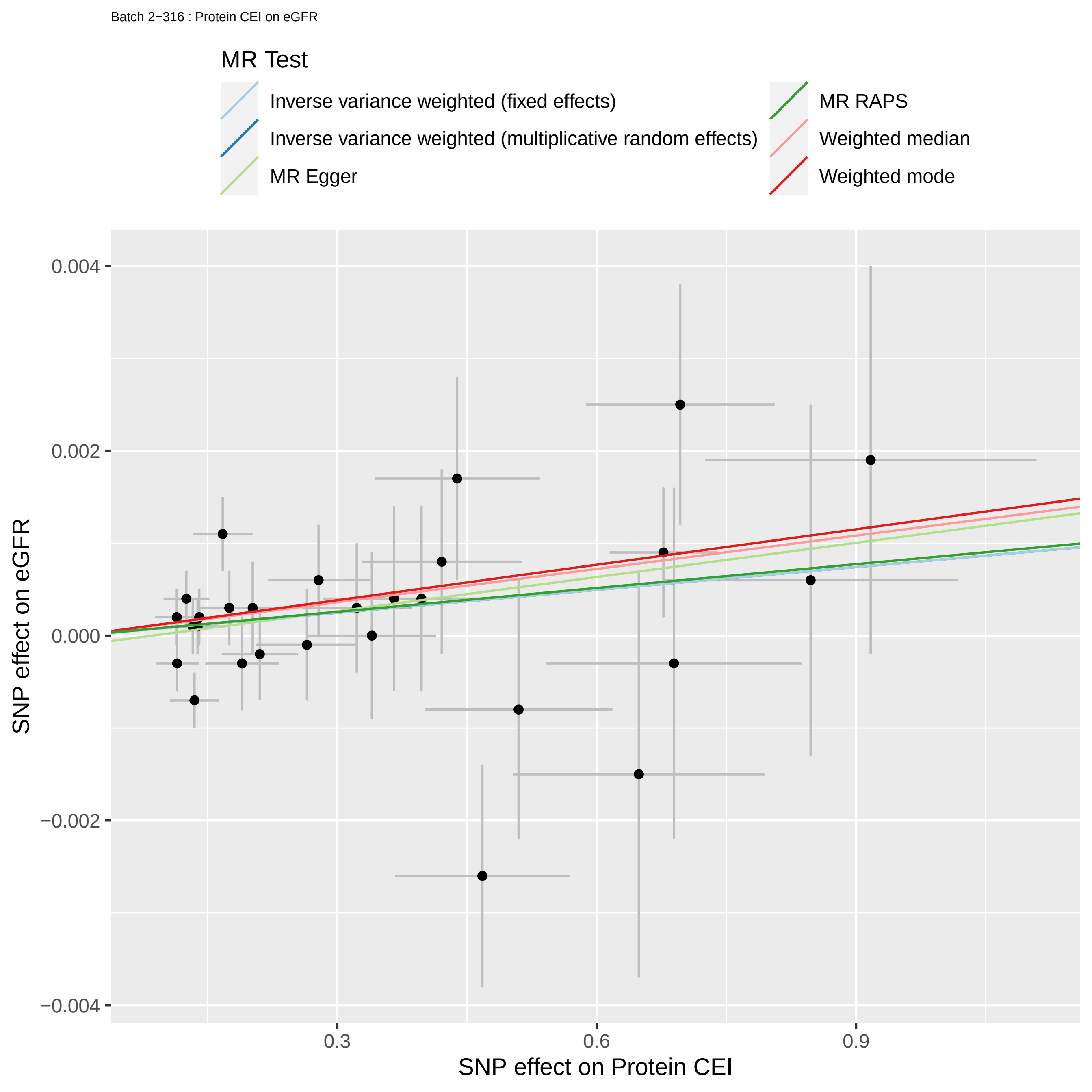

Batch 2−316 : Protein CEI on eGFR
MR Test
Inverse variance weighted (fixed effects)
Inverse variance weighted (multiplicative random effects)
MR Egger
MR RAPS
Weighted median
Weighted mode
0.004
0.002
0.000
−0.002
−0.004
0.3
0.6
0.9
SNP effect on eGFR
SNP effect on Protein CEI

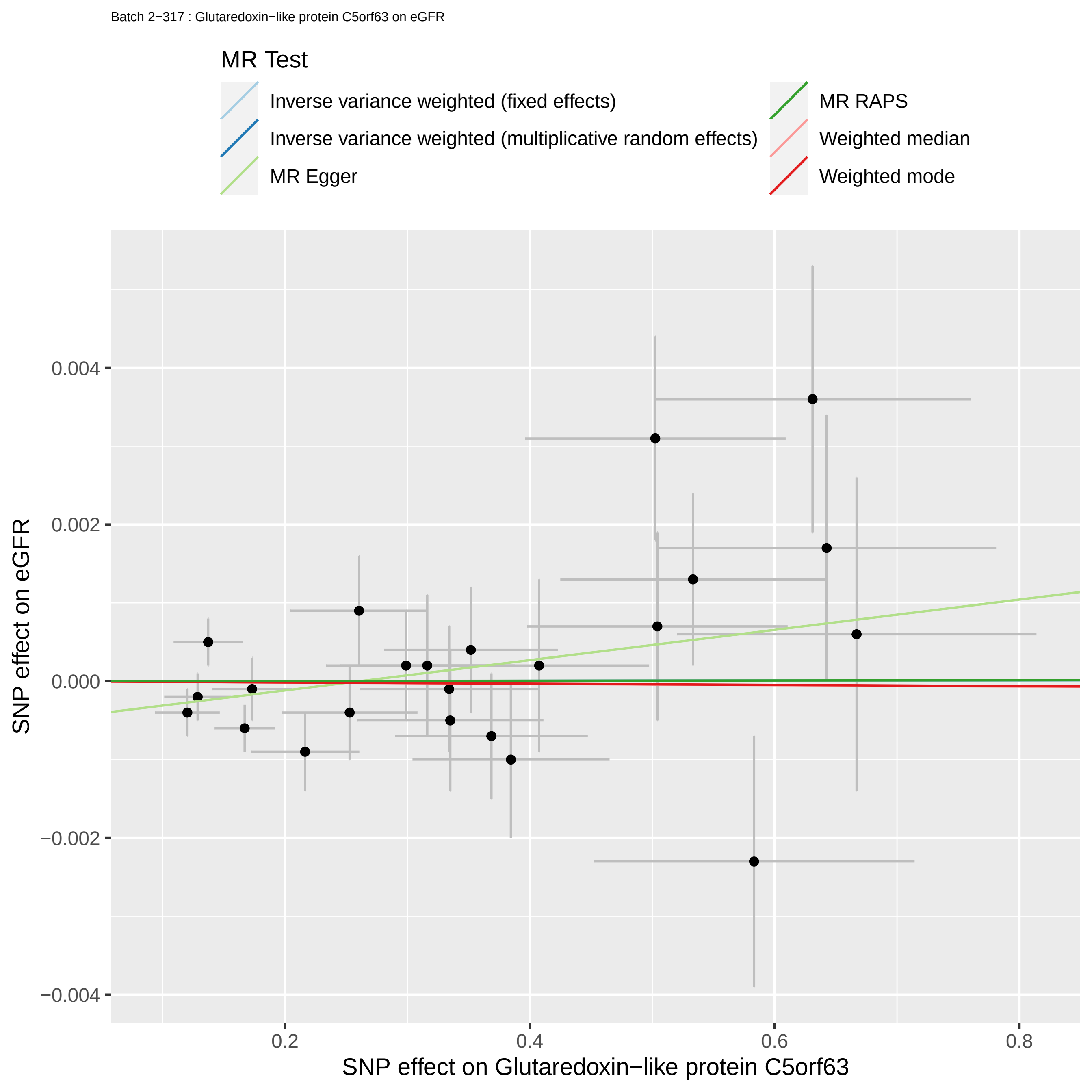
Batch 2−317 : Glutaredoxin−like protein C5orf63 on eGFR
MR Test
Inverse variance weighted (fixed effects)
Inverse variance weighted (multiplicative random effects)
MR Egger
MR RAPS
Weighted median
Weighted mode
0.004
0.002
0.000
−0.002
−0.004
SNP effect on eGFR
0.2
0.4
0.6
0.8
SNP effect on Glutaredoxin−like protein C5orf63

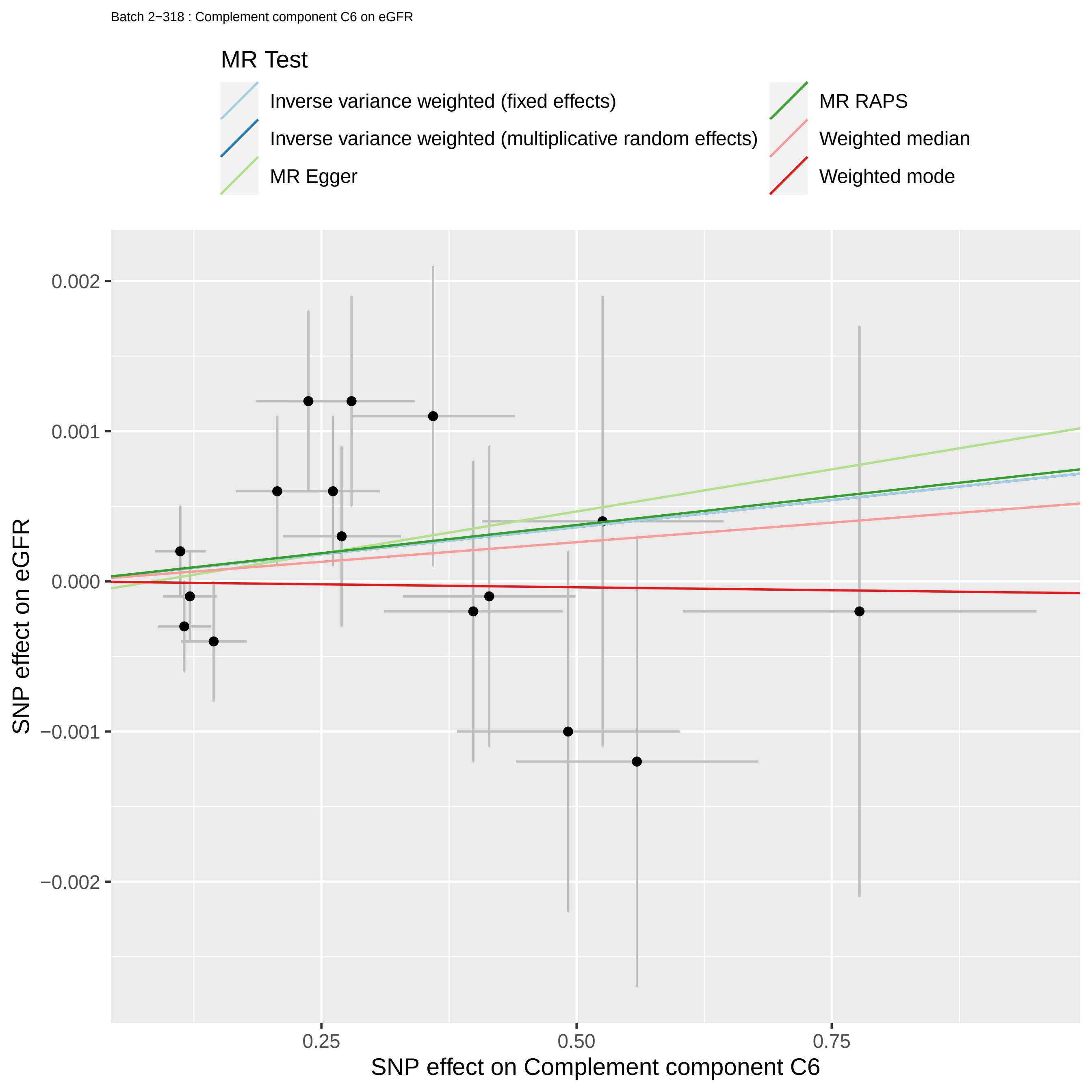
Batch 2−318 : Complement component C6 on eGFR
MR Test
Inverse variance weighted (fixed effects)
Inverse variance weighted (multiplicative random effects)
MR Egger
MR RAPS
Weighted median
Weighted mode
0.002
0.001
0.000
−0.001
−0.002
0.25
0.50
0.75
SNP effect on eGFR
SNP effect on Complement component C6

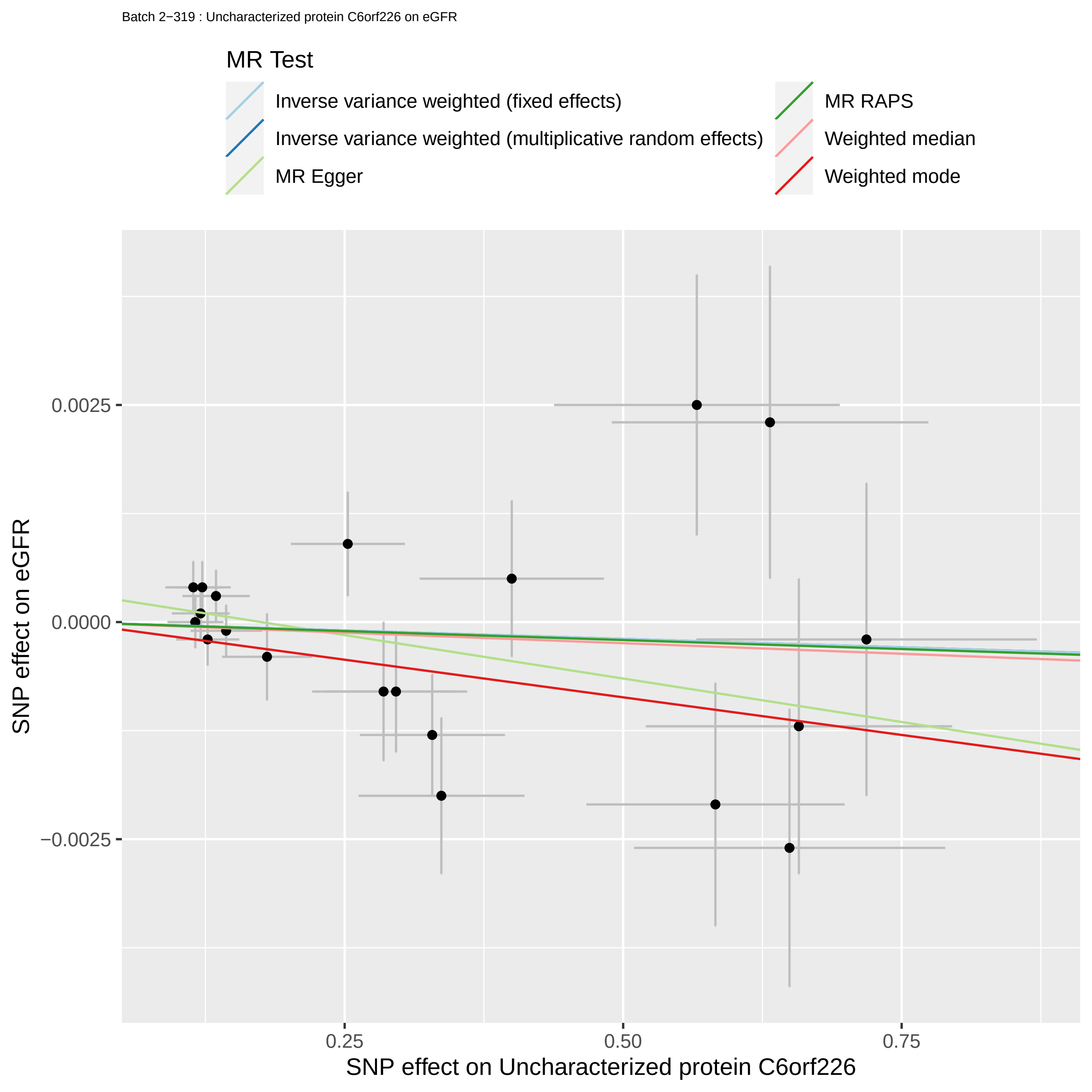

Batch 2−319 : Uncharacterized protein C6orf226 on eGFR
MR Test
Inverse variance weighted (fixed effects)
Inverse variance weighted (multiplicative random effects)
MR Egger
MR RAPS
Weighted median
Weighted mode
0.0025
0.0000
−0.0025
0.25
0.50
0.75
SNP effect on eGFR
SNP effect on Uncharacterized protein C6orf226

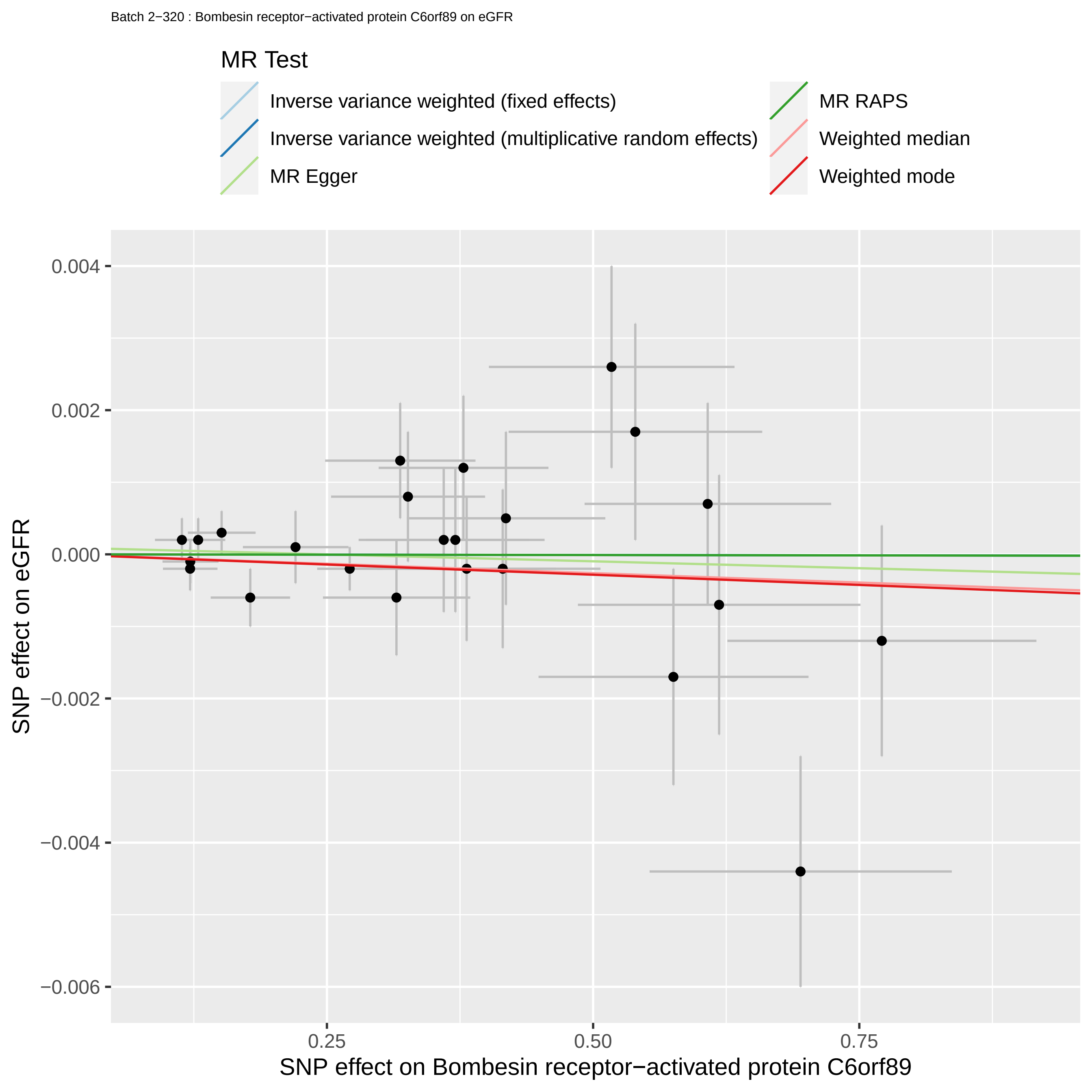
Batch 2−320 : Bombesin receptor−activated protein C6orf89 on eGFR
MR Test
Inverse variance weighted (fixed effects)
Inverse variance weighted (multiplicative random effects)
MR Egger
MR RAPS
Weighted median
Weighted mode
0.004
0.002
0.000
−0.002
−0.004
−0.006
0.25
0.50
0.75
SNP effect on eGFR
SNP effect on Bombesin receptor−activated protein C6orf89

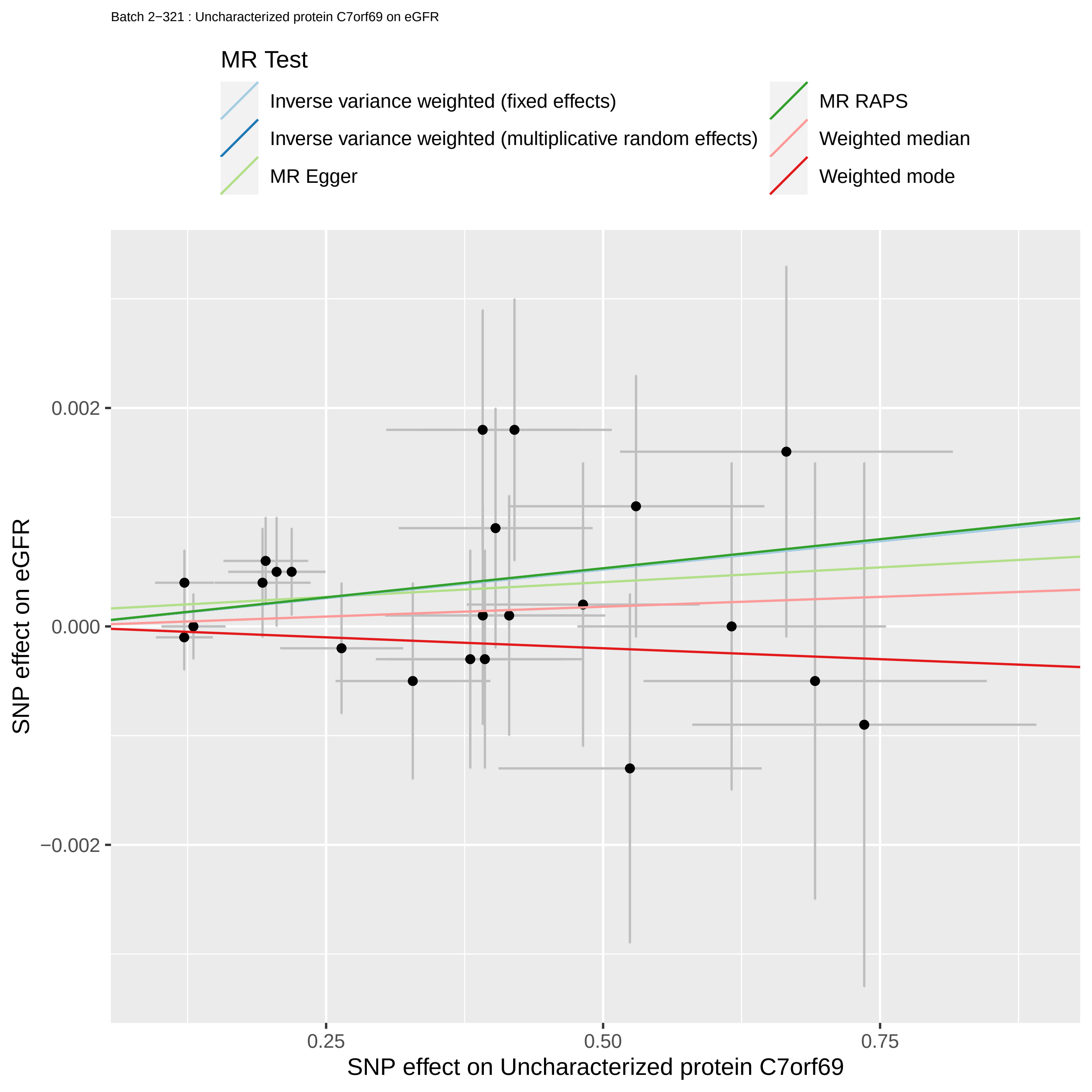
Batch 2−321 : Uncharacterized protein C7orf69 on eGFR
MR Test
Inverse variance weighted (fixed effects)
Inverse variance weighted (multiplicative random effects)
MR Egger
MR RAPS
Weighted median
Weighted mode
0.002
0.000
−0.002
0.25
0.50
0.75
SNP effect on eGFR
SNP effect on Uncharacterized protein C7orf69

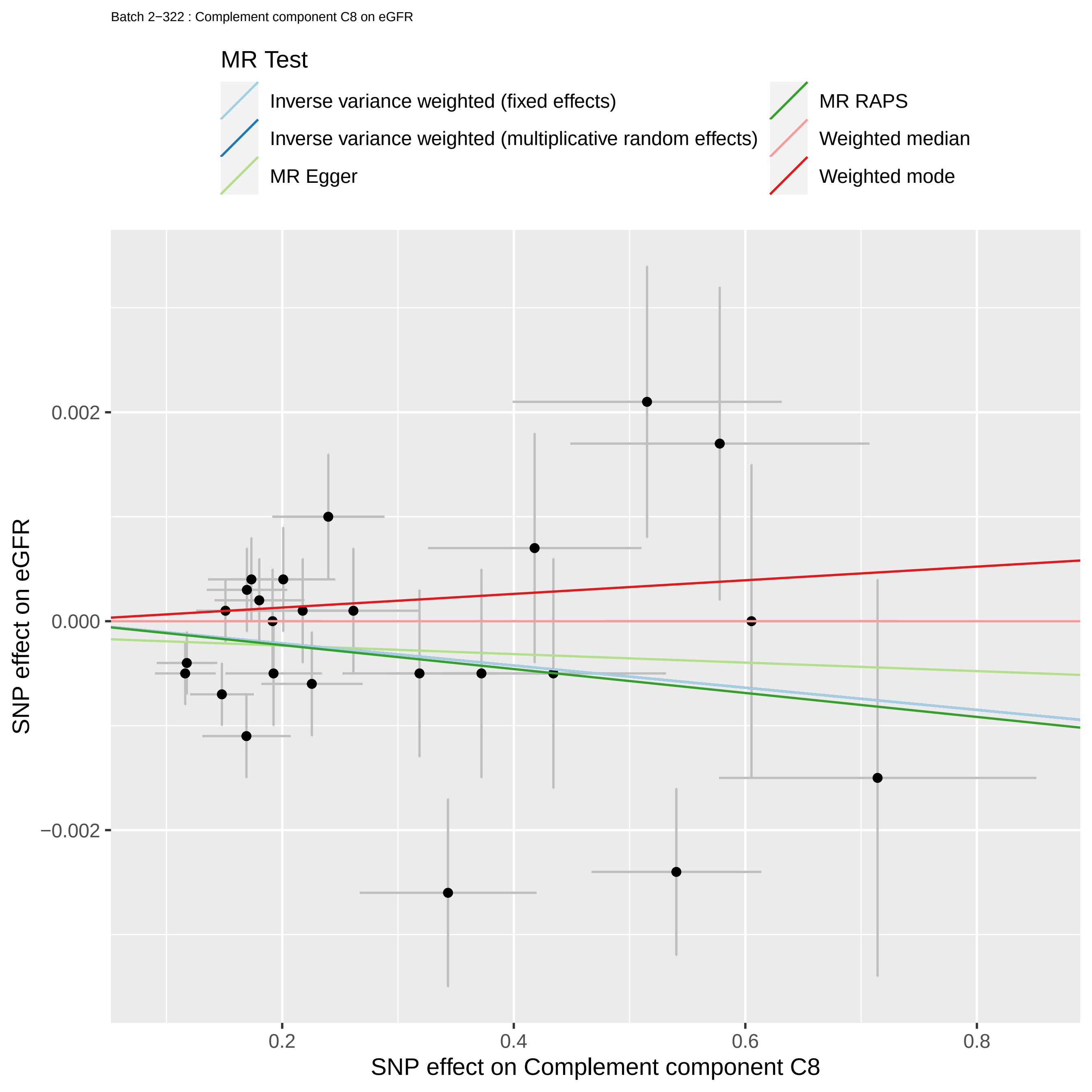
Batch 2−322 : Complement component C8 on eGFR
MR Test
Inverse variance weighted (fixed effects)
Inverse variance weighted (multiplicative random effects)
MR Egger
MR RAPS
Weighted median
Weighted mode
0.002
0.000
−0.002
0.2
0.4
0.6
0.8
SNP effect on eGFR
SNP effect on Complement component C8

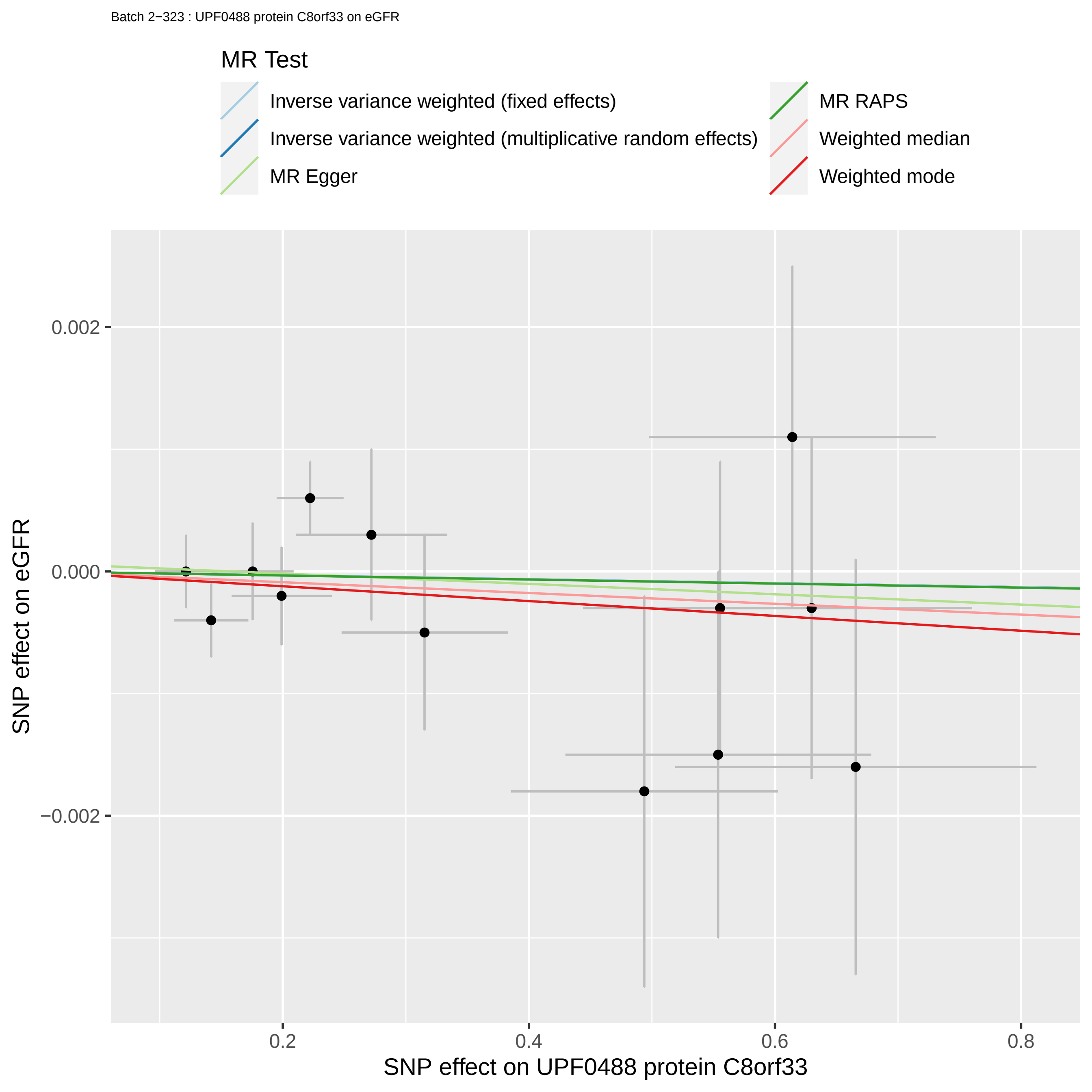

Batch 2−323 : UPF0488 protein C8orf33 on eGFR
MR Test
Inverse variance weighted (fixed effects)
Inverse variance weighted (multiplicative random effects)
MR Egger
MR RAPS
Weighted median
Weighted mode
0.002
0.000
−0.002
0.2
0.4
0.6
0.8
SNP effect on eGFR
SNP effect on UPF0488 protein C8orf33

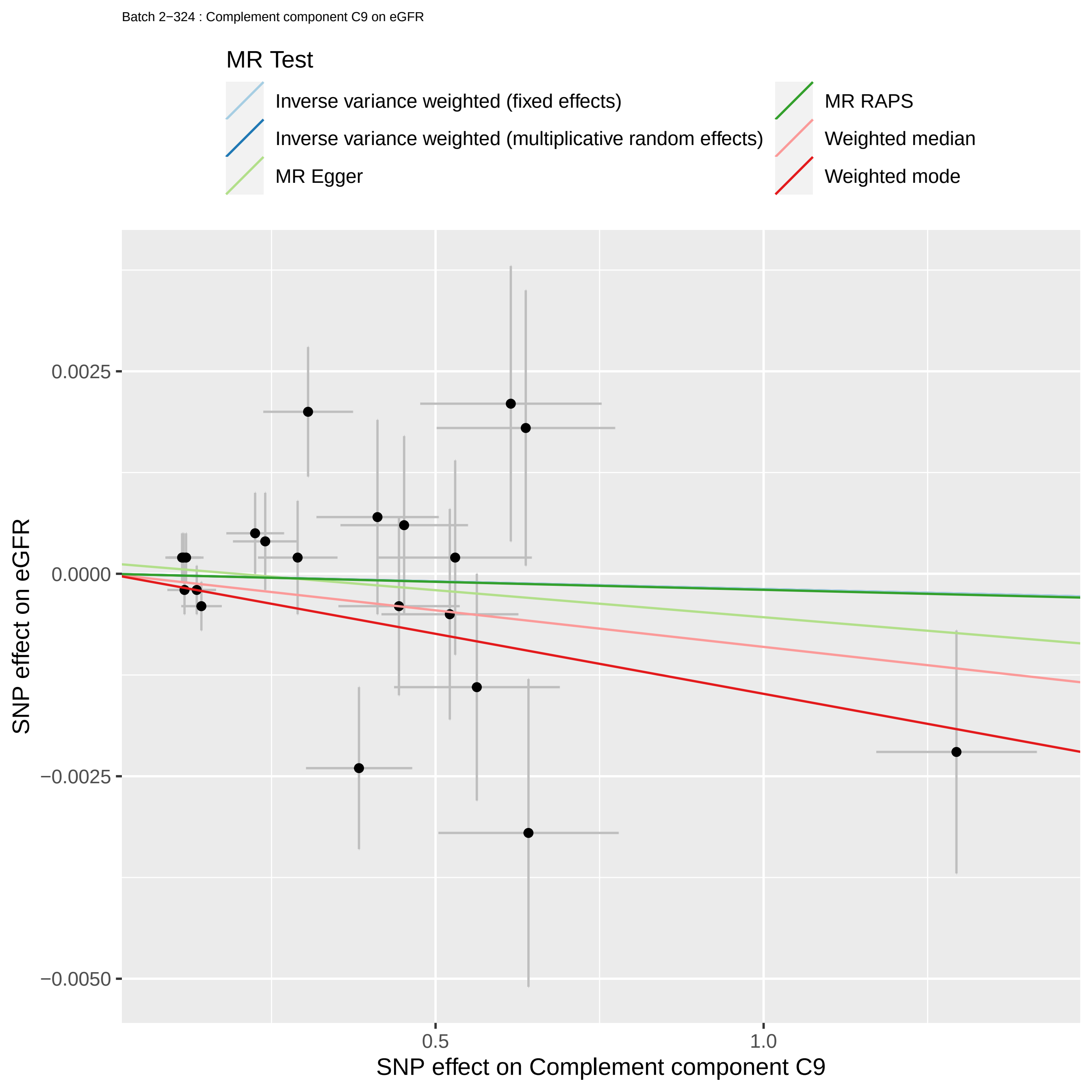
Batch 2−324 : Complement component C9 on eGFR
MR Test
Inverse variance weighted (fixed effects)
Inverse variance weighted (multiplicative random effects)
MR Egger
MR RAPS
Weighted median
Weighted mode
0.0025
0.0000
−0.0025
−0.0050
0.5
1.0
SNP effect on eGFR
SNP effect on Complement component C9

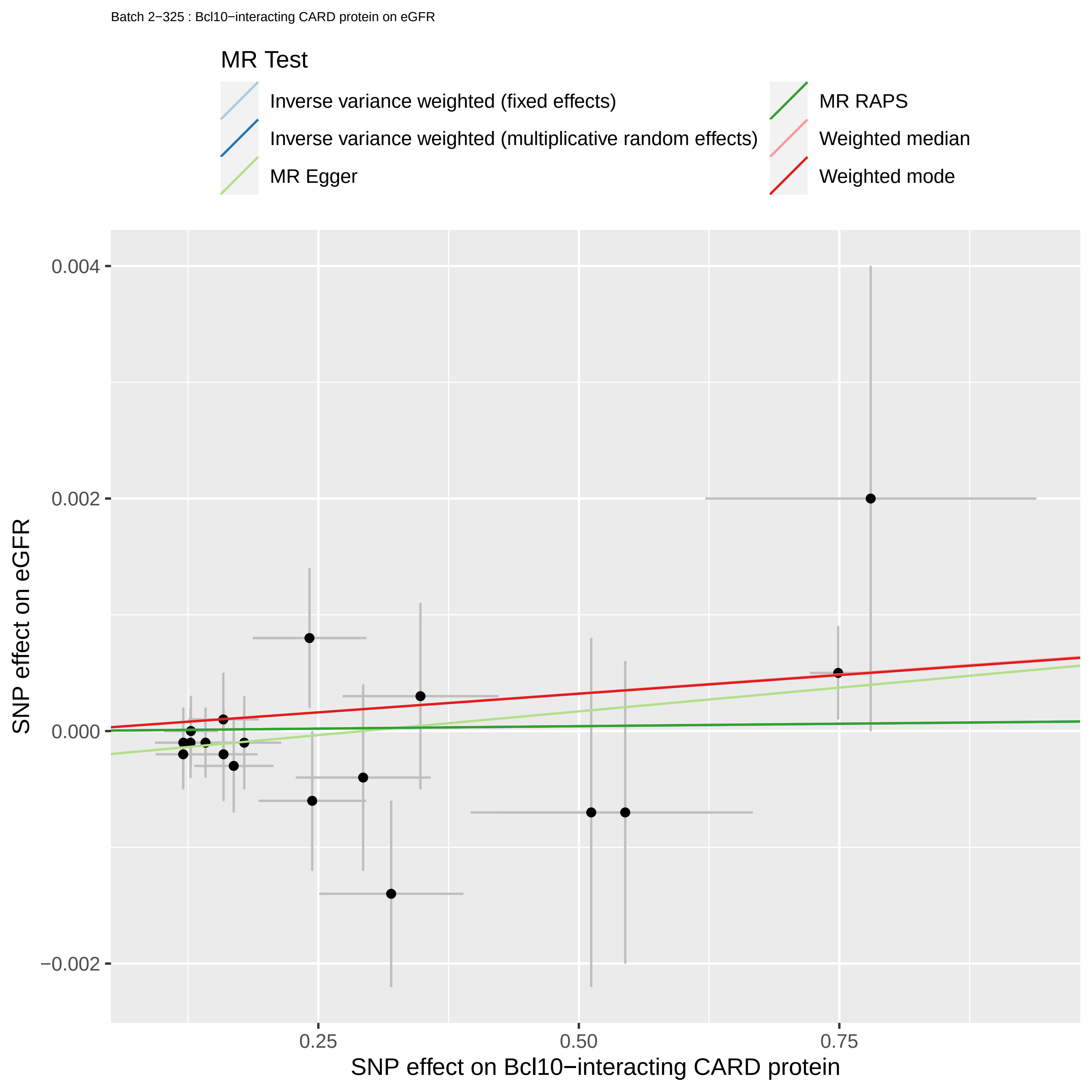
Batch 2−325 : Bcl10−interacting CARD protein on eGFR
MR Test
Inverse variance weighted (fixed effects)
Inverse variance weighted (multiplicative random effects)
MR Egger
MR RAPS
Weighted median
Weighted mode
0.004
0.002
0.000
−0.002
0.25
0.50
0.75
SNP effect on eGFR
SNP effect on Bcl10−interacting CARD protein

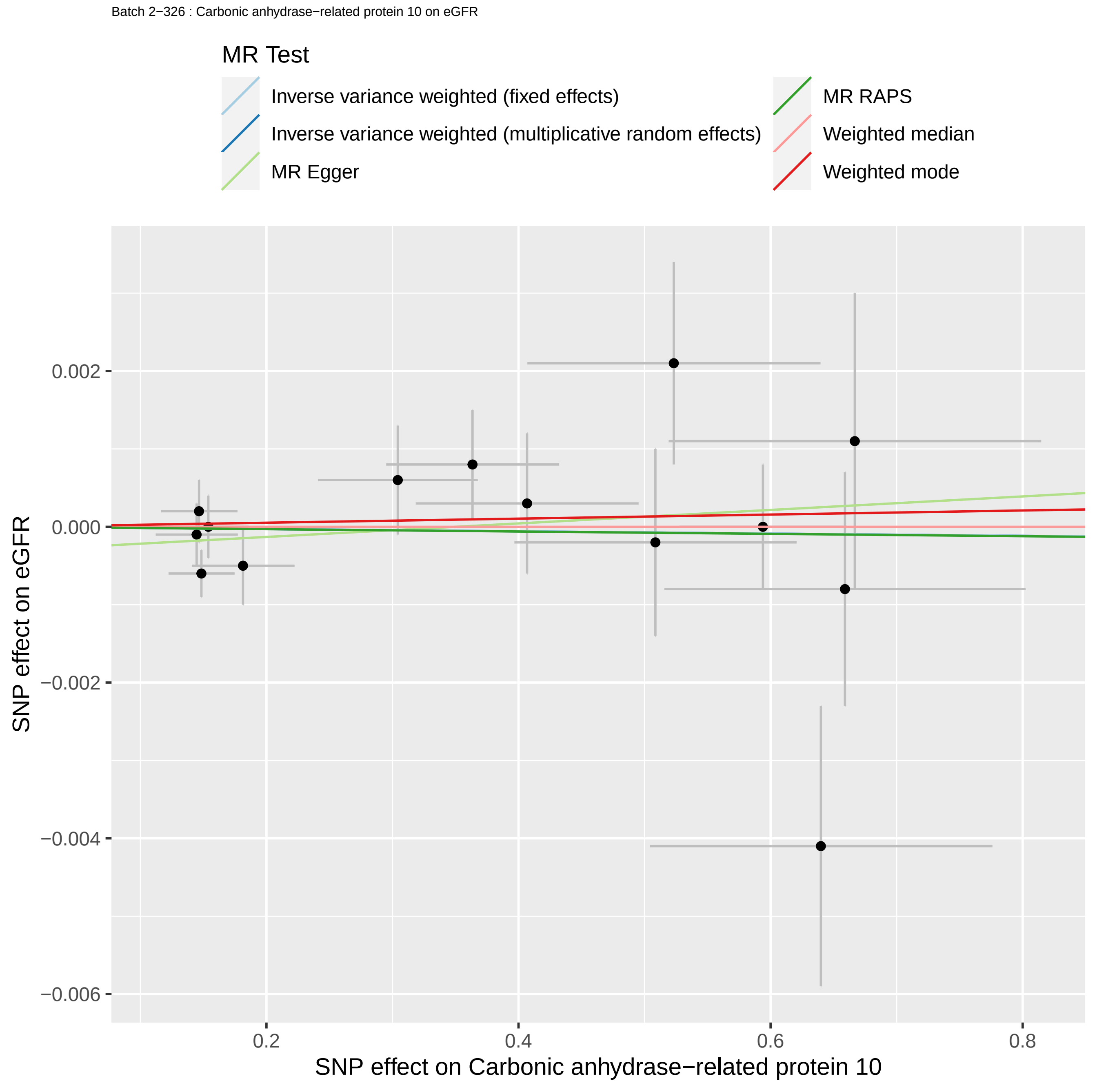

Batch 2−326 : Carbonic anhydrase−related protein 10 on eGFR
MR Test
Inverse variance weighted (fixed effects)
Inverse variance weighted (multiplicative random effects)
MR Egger
MR RAPS
Weighted median
Weighted mode
0.002
0.000
−0.002
−0.004
−0.006
0.2
0.4
0.6
0.8
SNP effect on eGFR
SNP effect on Carbonic anhydrase−related protein 10

Batch 2−327 : Carbonic anhydrase−related protein 10 on eGFR

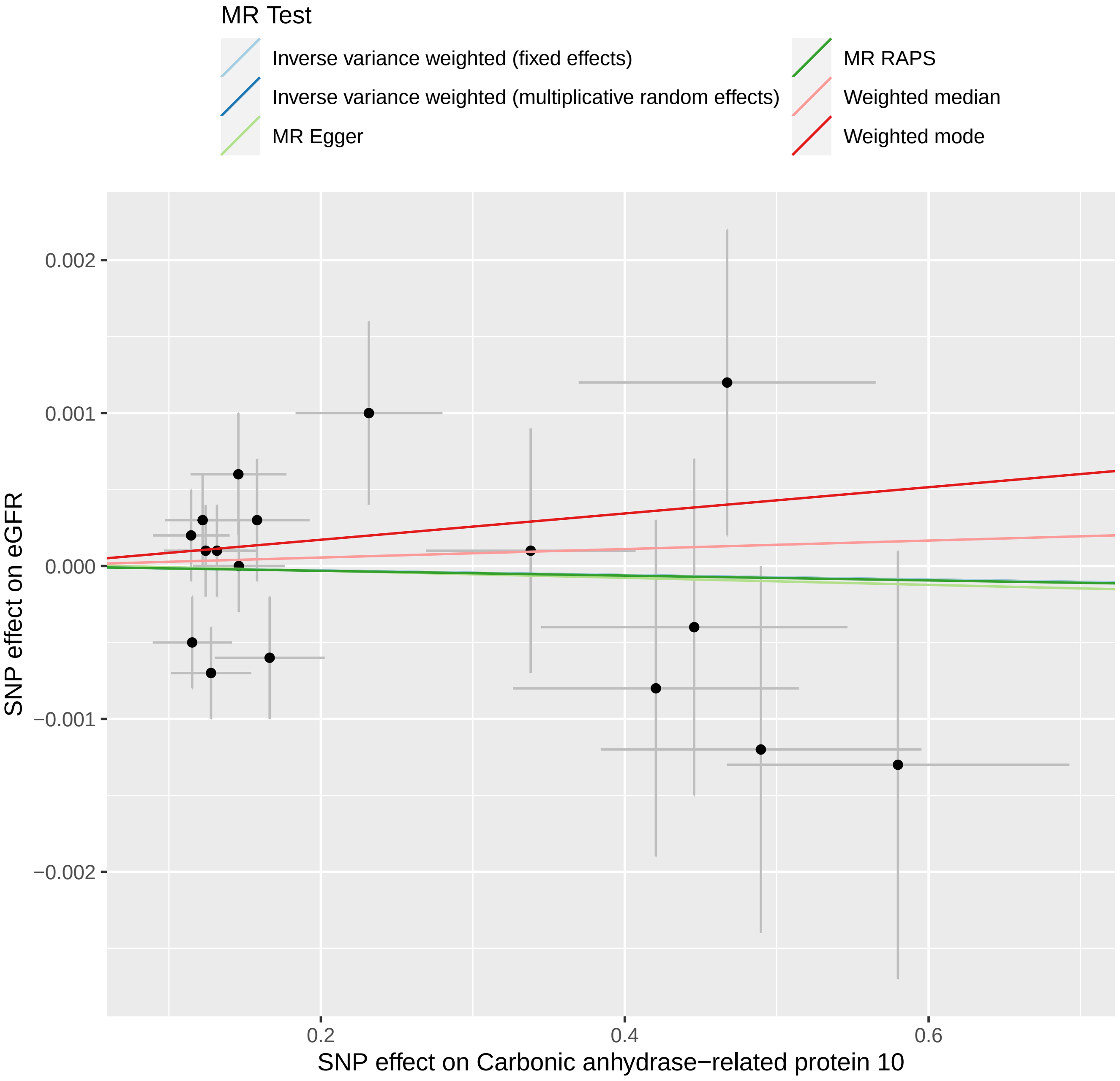

Batch 2−328 : Carbonic anhydrase 1 on eGFR

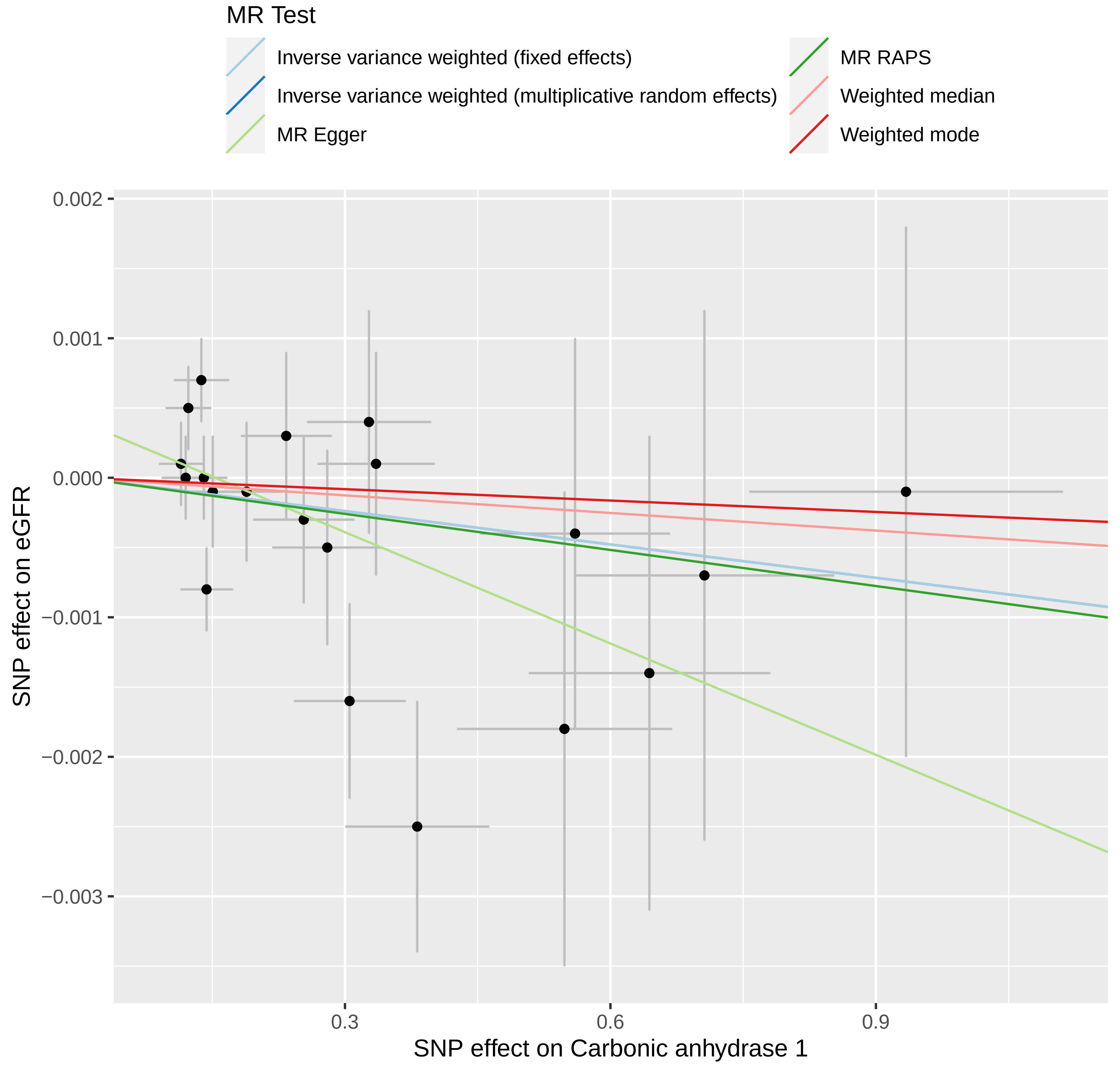

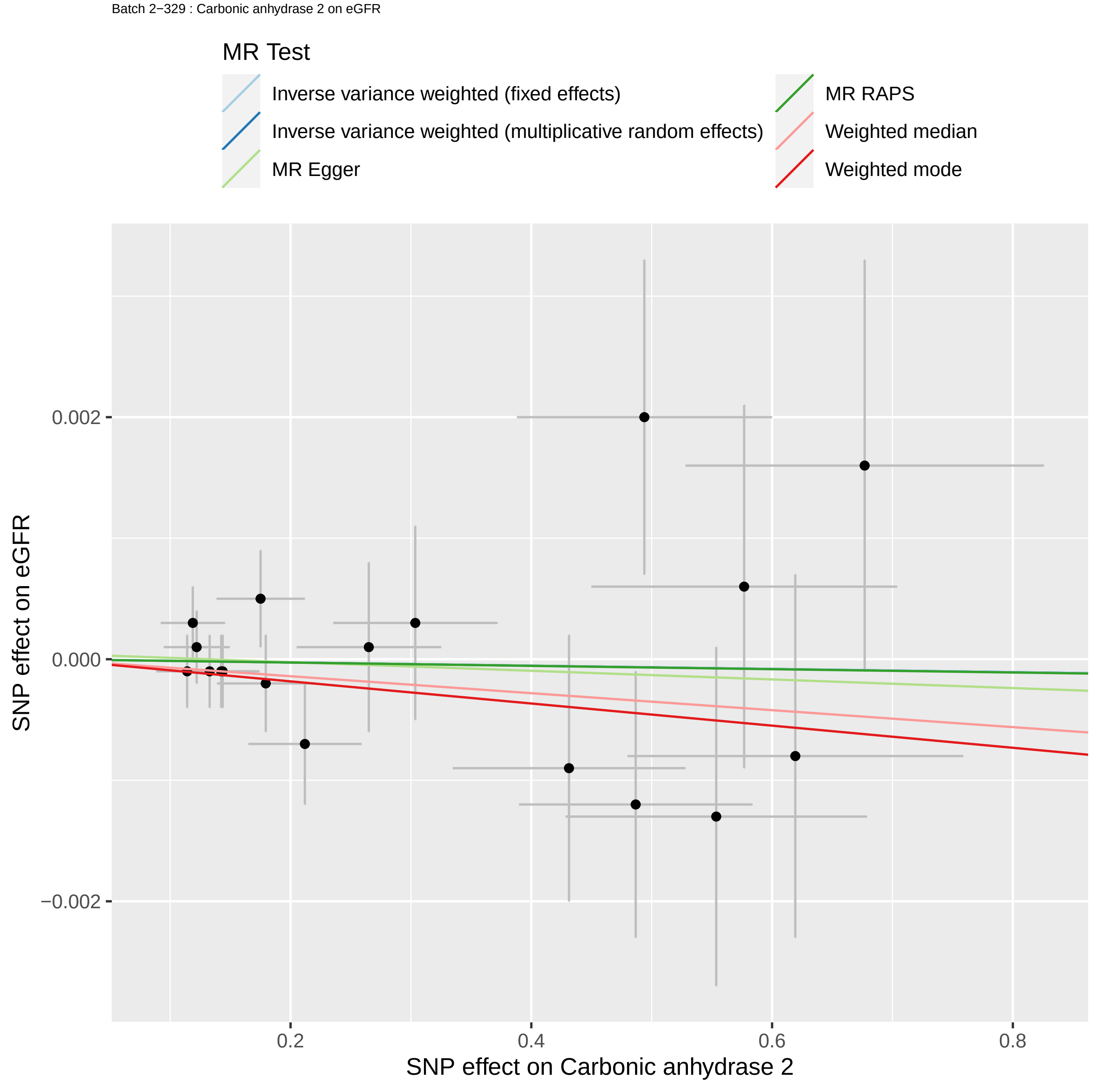

Batch 2−329 : Carbonic anhydrase 2 on eGFR
MR Test
Inverse variance weighted (fixed effects)
Inverse variance weighted (multiplicative random effects)
MR Egger
MR RAPS
Weighted median
Weighted mode
0.002
0.000
−0.002
0.2
0.4
0.6
0.8
SNP effect on eGFR
SNP effect on Carbonic anhydrase 2

Batch 2−330 : Carbonic anhydrase 3 on eGFR

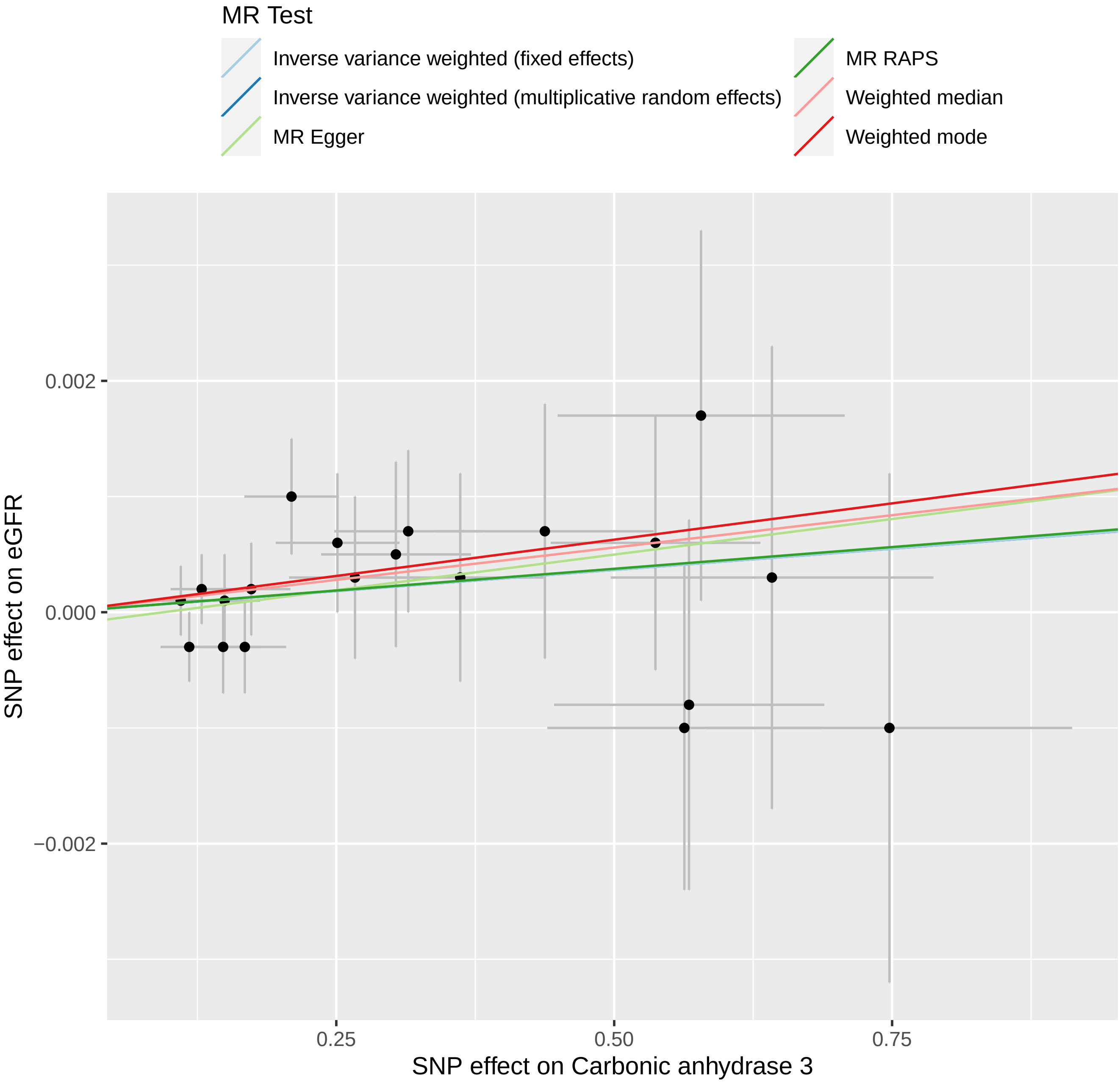

Batch 2−331 : Carbonic anhydrase 4 on eGFR

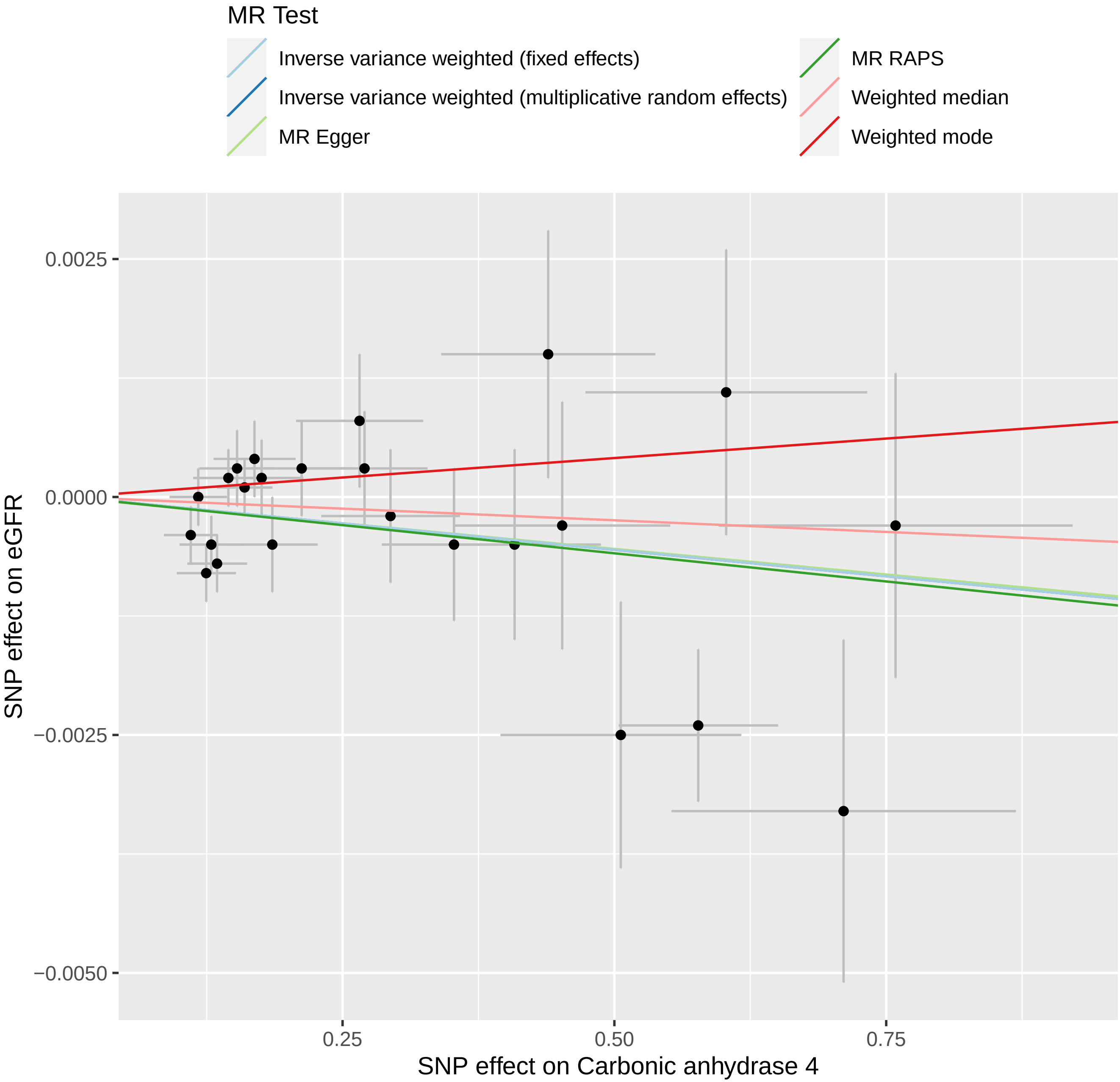

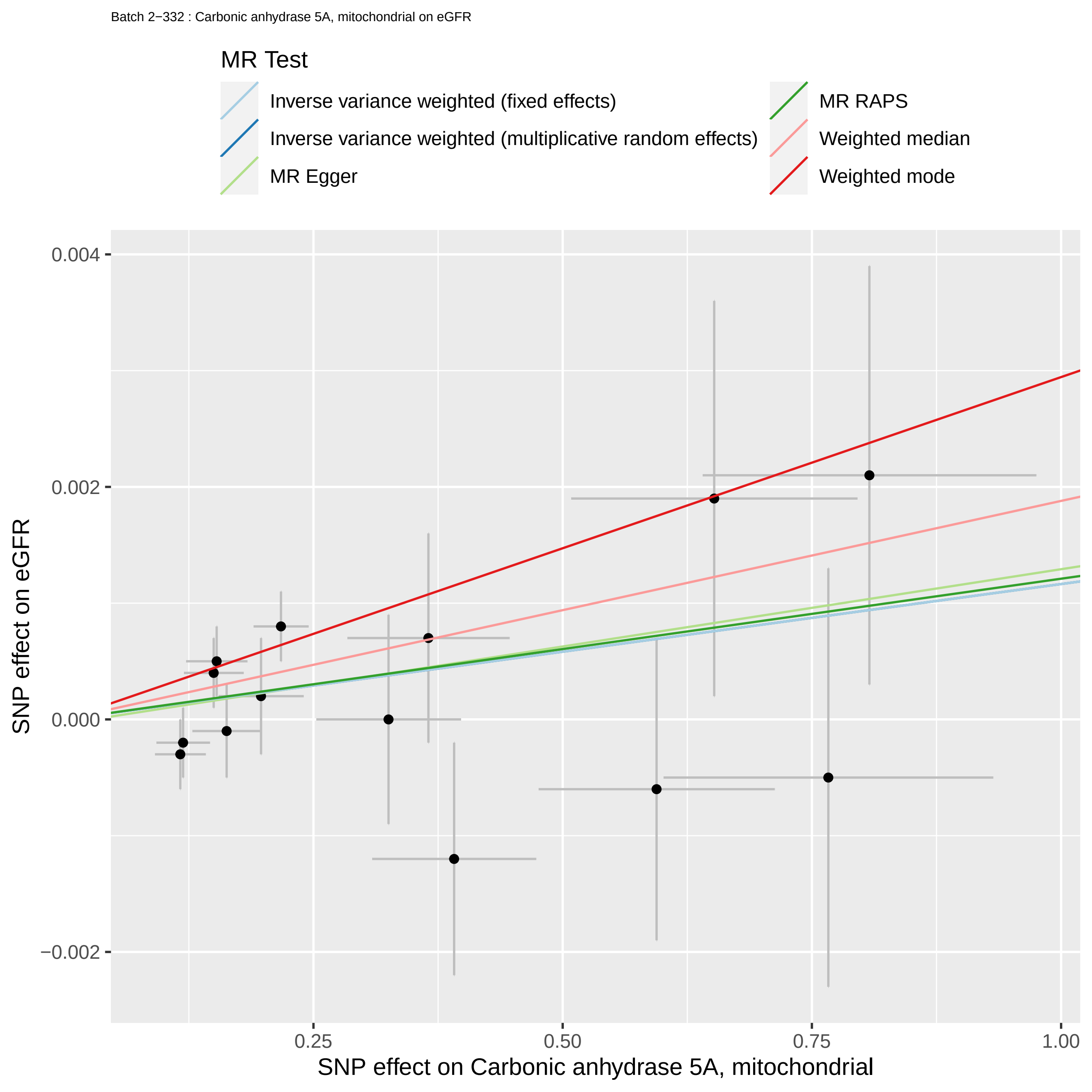
Batch 2−332 : Carbonic anhydrase 5A, mitochondrial on eGFR
MR Test
Inverse variance weighted (fixed effects)
Inverse variance weighted (multiplicative random effects)
MR Egger
MR RAPS
Weighted median
Weighted mode
0.004
0.002
0.000
−0.002
0.25
0.50
0.75
1.00
SNP effect on eGFR
SNP effect on Carbonic anhydrase 5A, mitochondrial

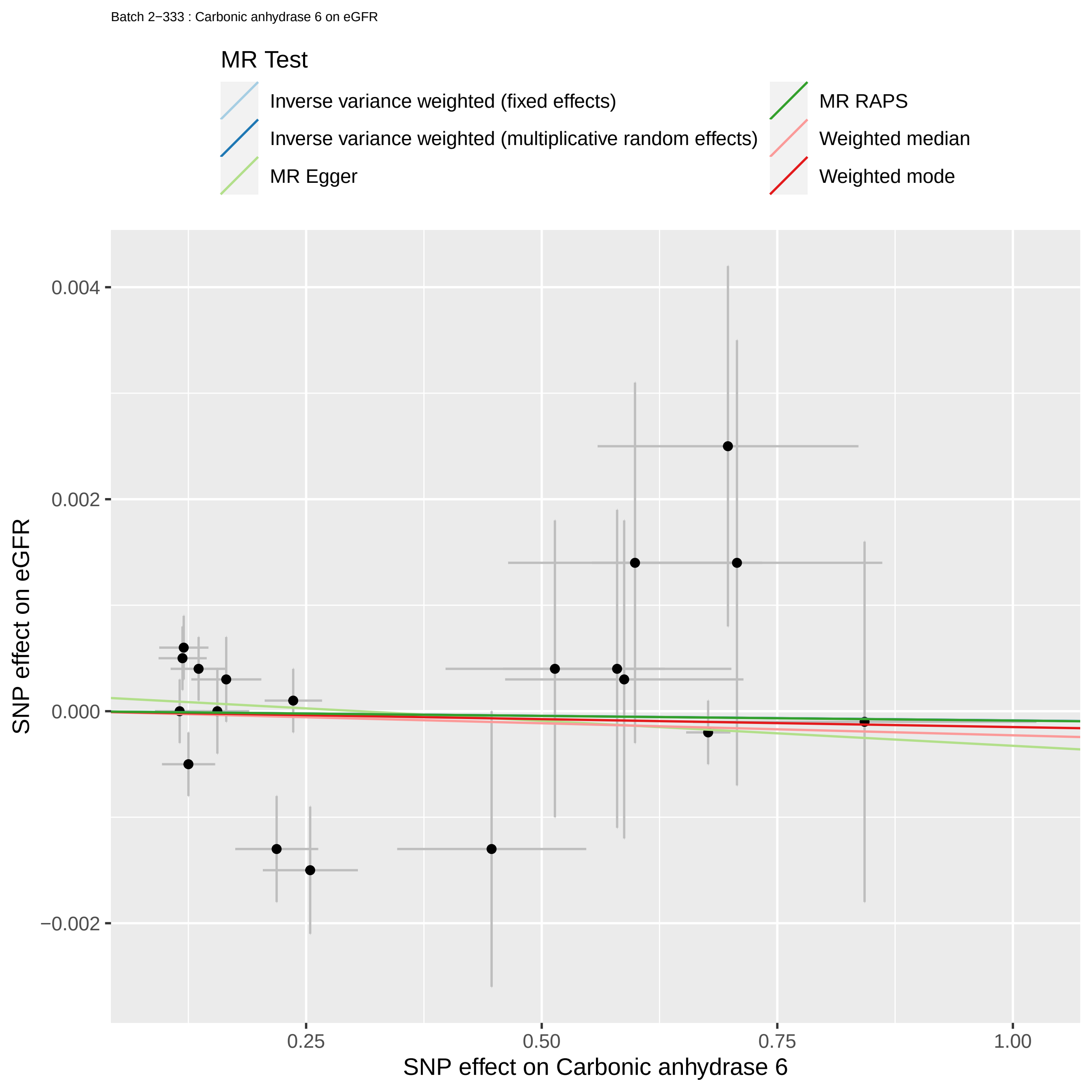

Batch 2−333 : Carbonic anhydrase 6 on eGFR
MR Test
Inverse variance weighted (fixed effects)
Inverse variance weighted (multiplicative random effects)
MR Egger
MR RAPS
Weighted median
Weighted mode
0.004
0.002
0.000
−0.002
0.25
0.50
0.75
1.00
SNP effect on eGFR
SNP effect on Carbonic anhydrase 6

Batch 2−334 : Carbonic anhydrase 9 on eGFR

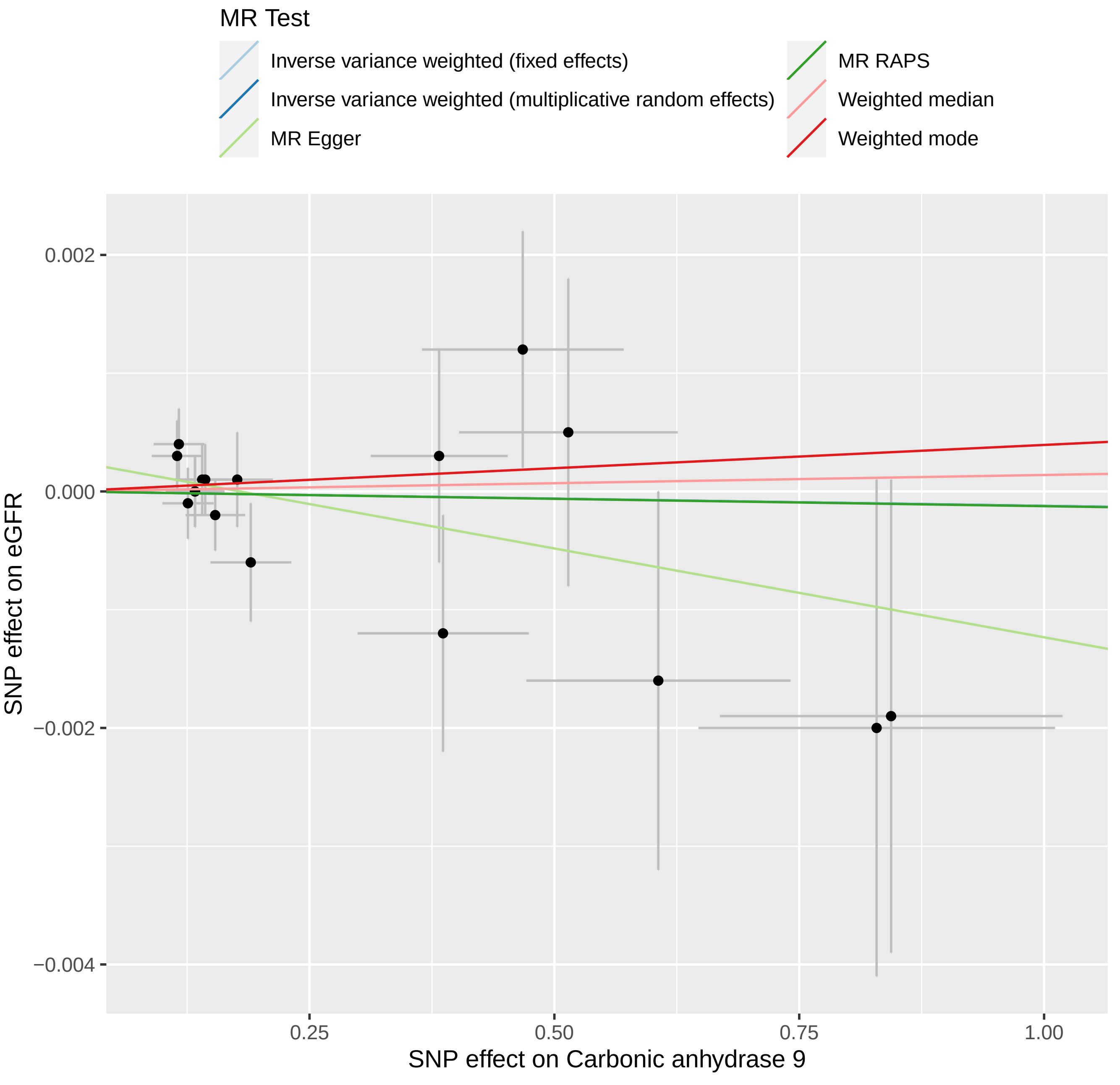

Batch 2−335 : Voltage−dependent calcium channel subunit alpha−2/delta−3 on eGFR

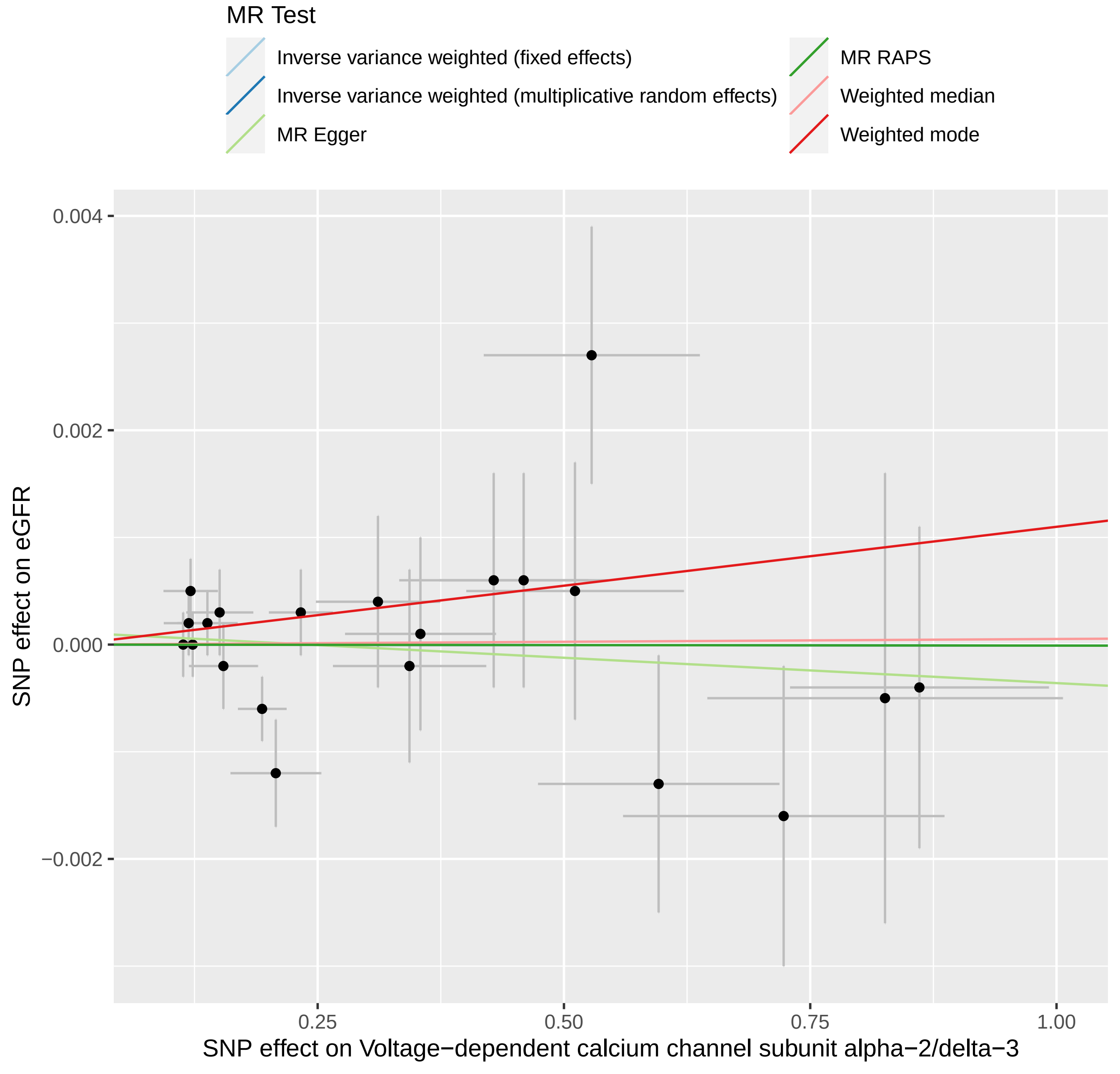

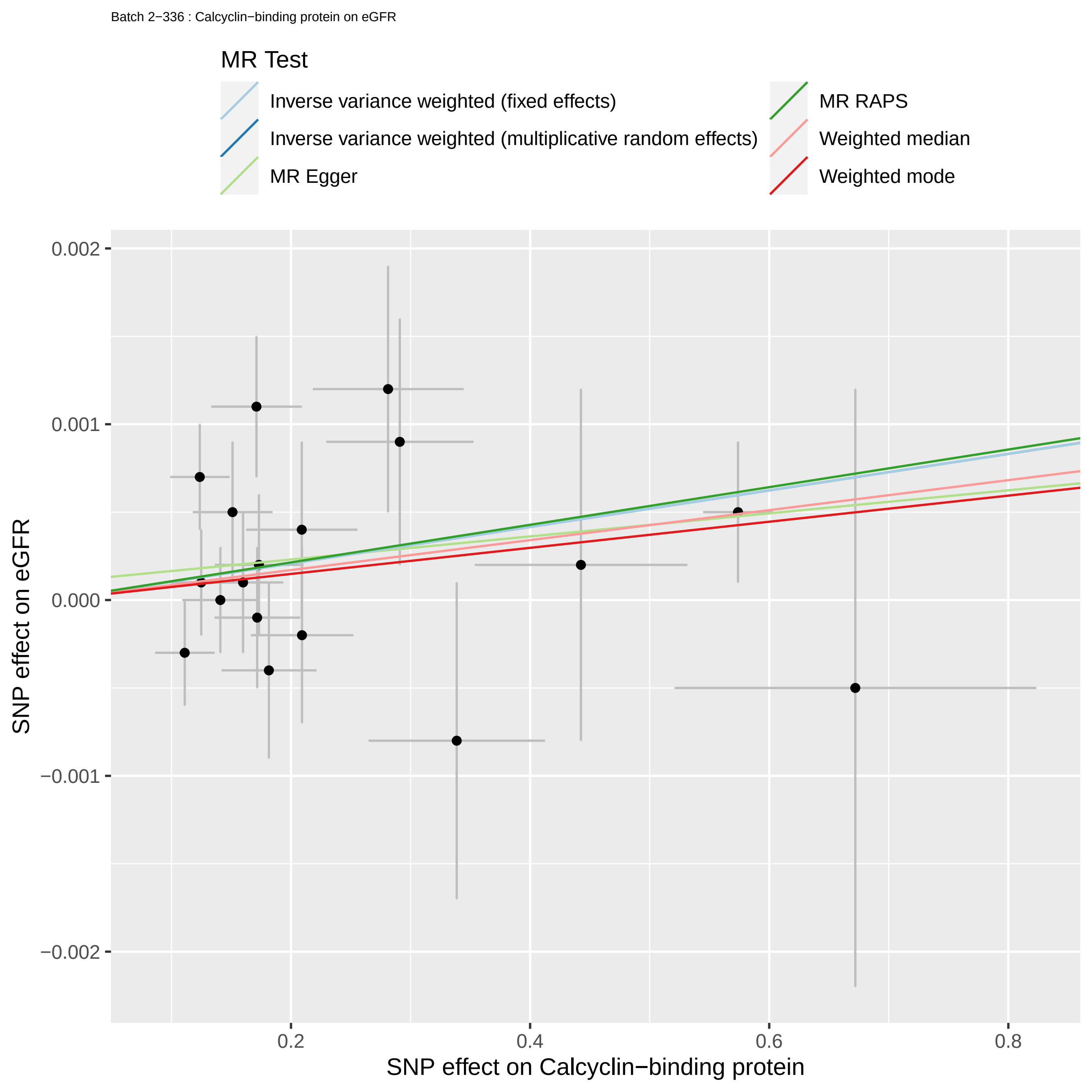

Batch 2−336 : Calcyclin−binding protein on eGFR
MR Test
Inverse variance weighted (fixed effects)
Inverse variance weighted (multiplicative random effects)
MR Egger
MR RAPS
Weighted median
Weighted mode
0.002
0.001
0.000
−0.001
−0.002
SNP effect on eGFR
0.2
0.4
0.6
0.8
SNP effect on Calcyclin−binding protein

Batch 2−337 : Cell adhesion molecule 1 on eGFR

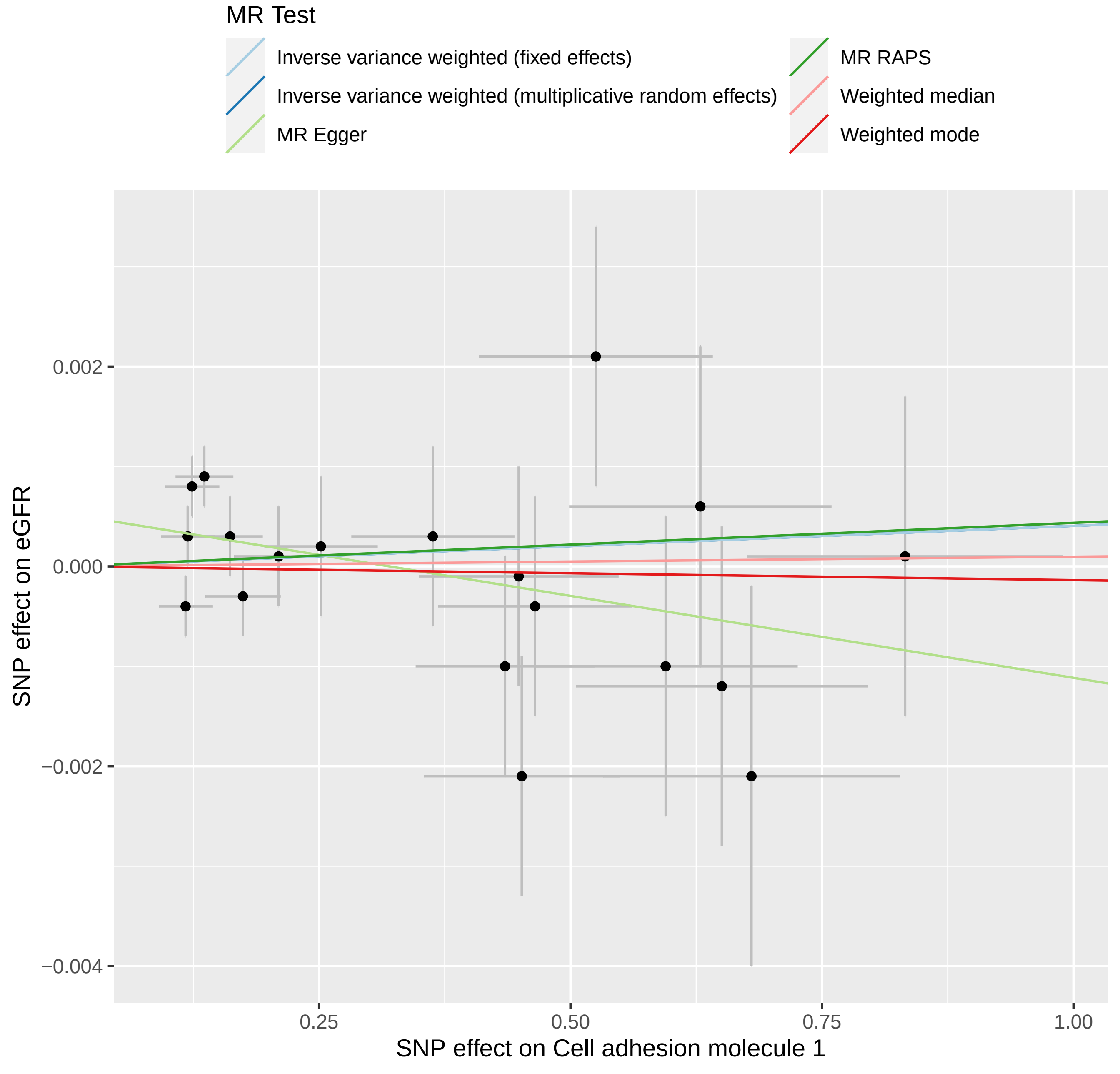

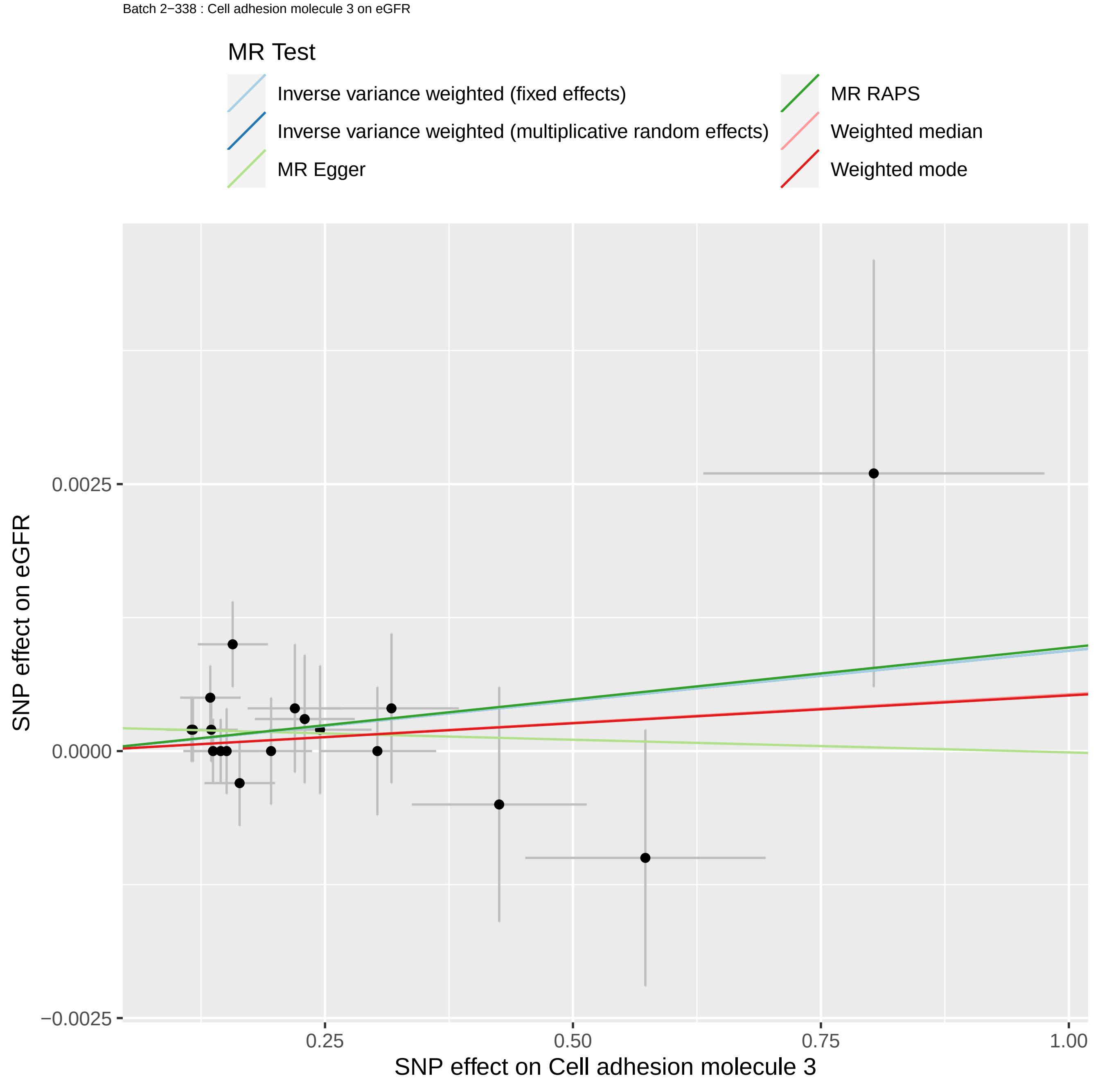

Batch 2−338 : Cell adhesion molecule 3 on eGFR
MR Test
Inverse variance weighted (fixed effects)
Inverse variance weighted (multiplicative random effects)
MR Egger
MR RAPS
Weighted median
Weighted mode
0.0025
0.0000
−0.0025
0.25
0.50
0.75
1.00
SNP effect on eGFR
SNP effect on Cell adhesion molecule 3

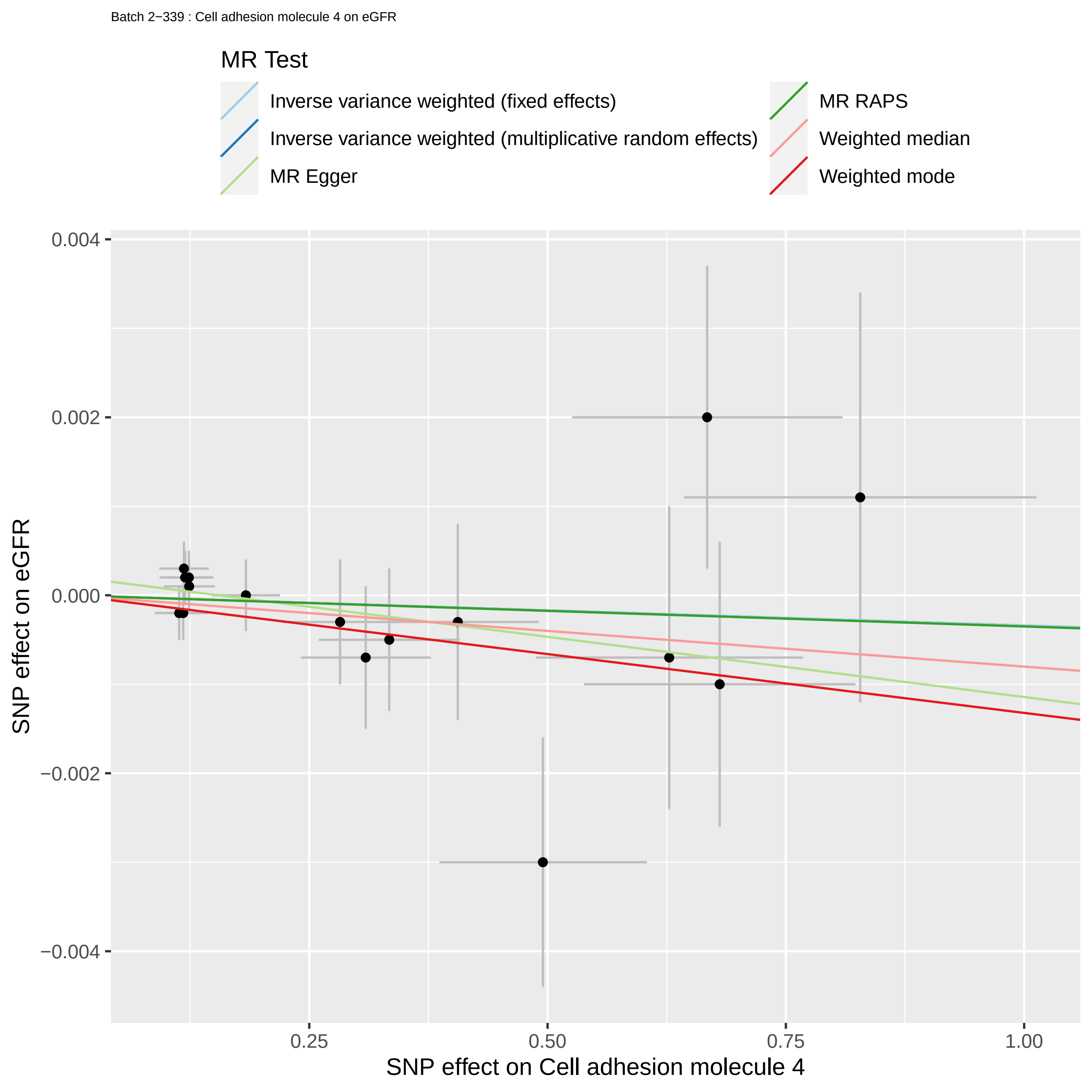

Batch 2−339 : Cell adhesion molecule 4 on eGFR
MR Test
Inverse variance weighted (fixed effects)
Inverse variance weighted (multiplicative random effects)
MR Egger
MR RAPS
Weighted median
Weighted mode
0.004
0.002
0.000
−0.002
−0.004
0.25
0.50
0.75
1.00
SNP effect on eGFR
SNP effect on Cell adhesion molecule 4

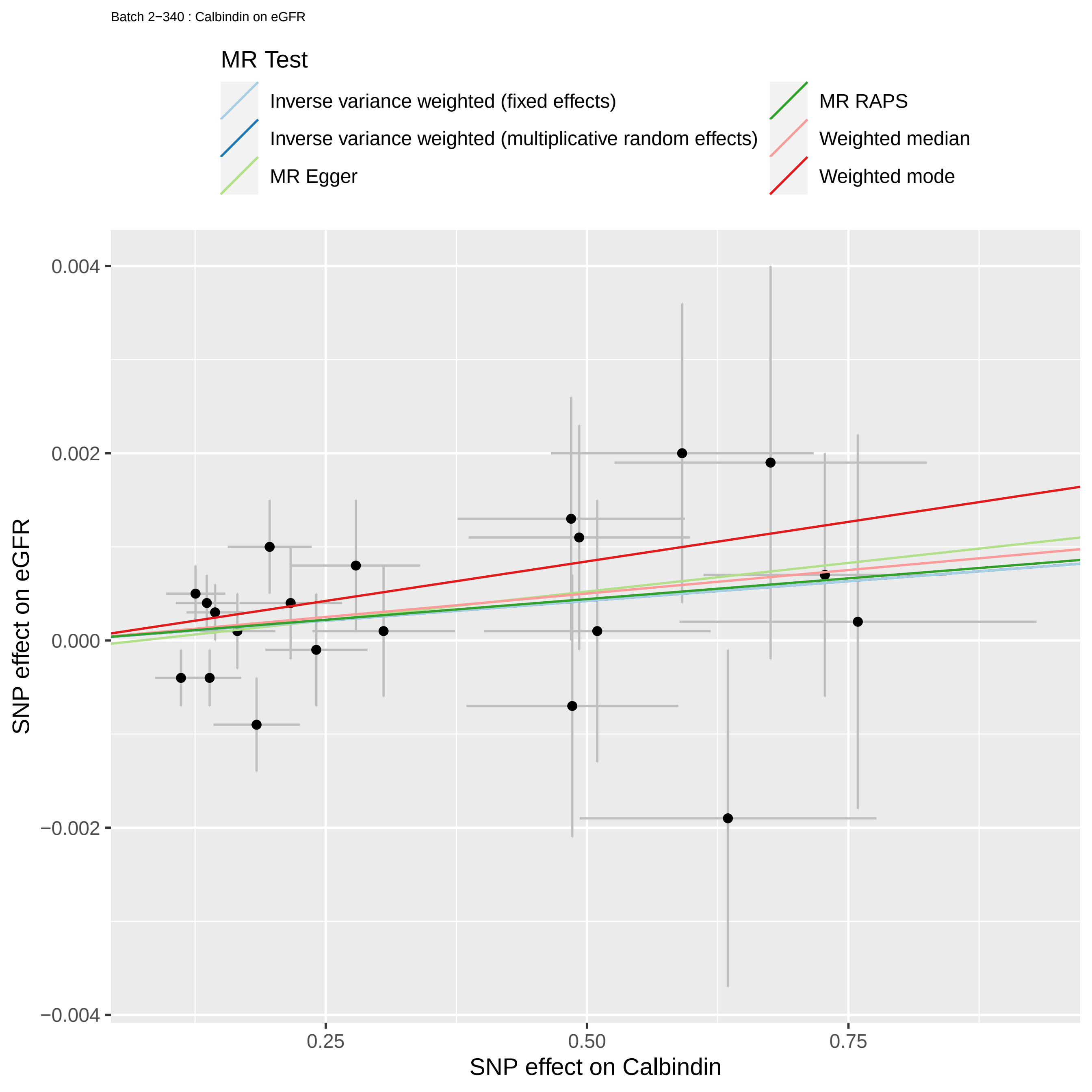

Batch 2−340 : Calbindin on eGFR
MR Test
Inverse variance weighted (fixed effects)
Inverse variance weighted (multiplicative random effects)
MR Egger
MR RAPS
Weighted median
Weighted mode
0.004
0.002
0.000
−0.002
−0.004
SNP effect on eGFR
0.25
0.50
0.75
SNP effect on Calbindin

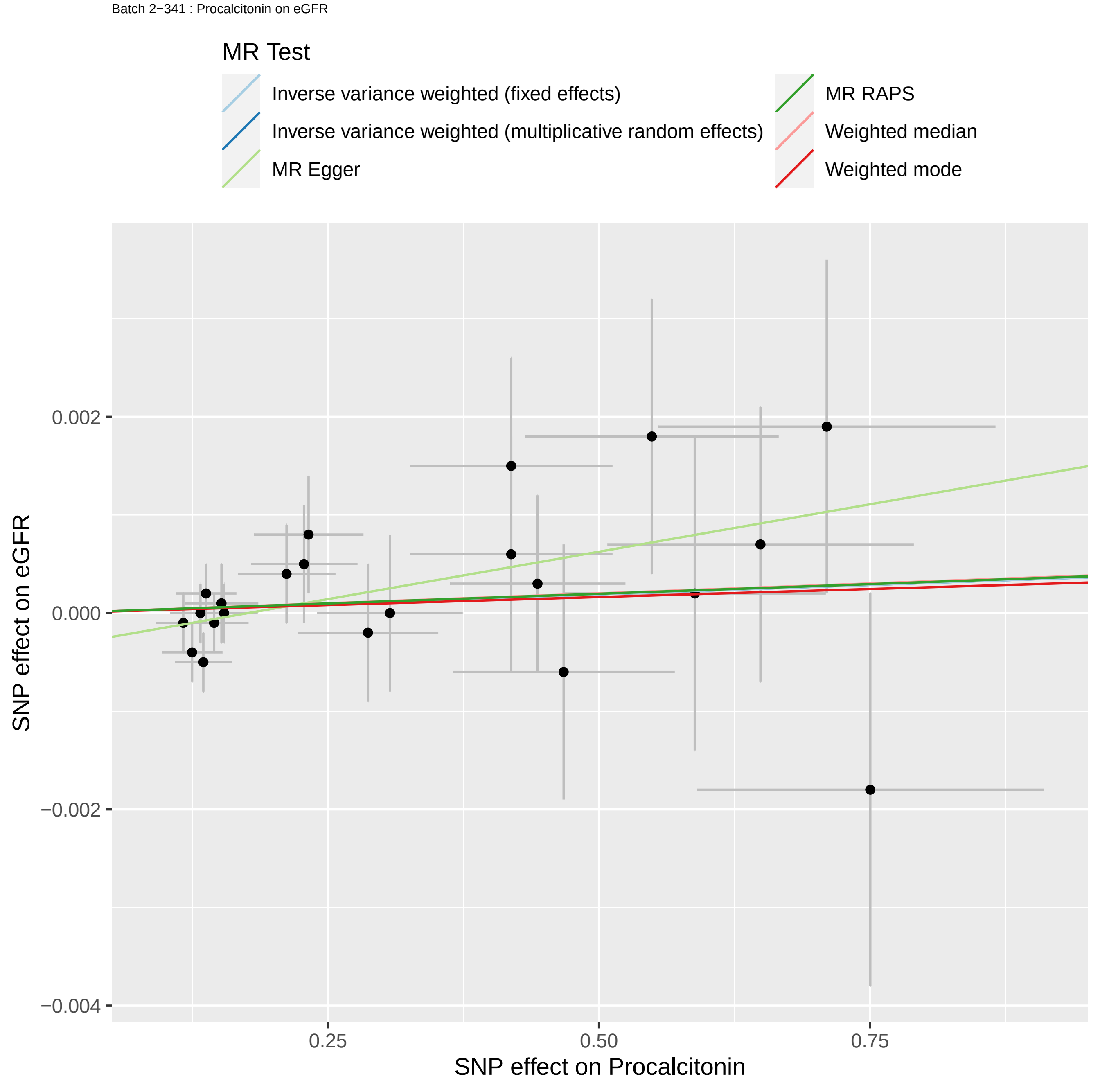
Batch 2−341 : Procalcitonin on eGFR
MR Test
Inverse variance weighted (fixed effects)
Inverse variance weighted (multiplicative random effects)
MR Egger
MR RAPS
Weighted median
Weighted mode
0.002
0.000
−0.002
−0.004
0.25
0.50
0.75
SNP effect on eGFR
SNP effect on Procalcitonin

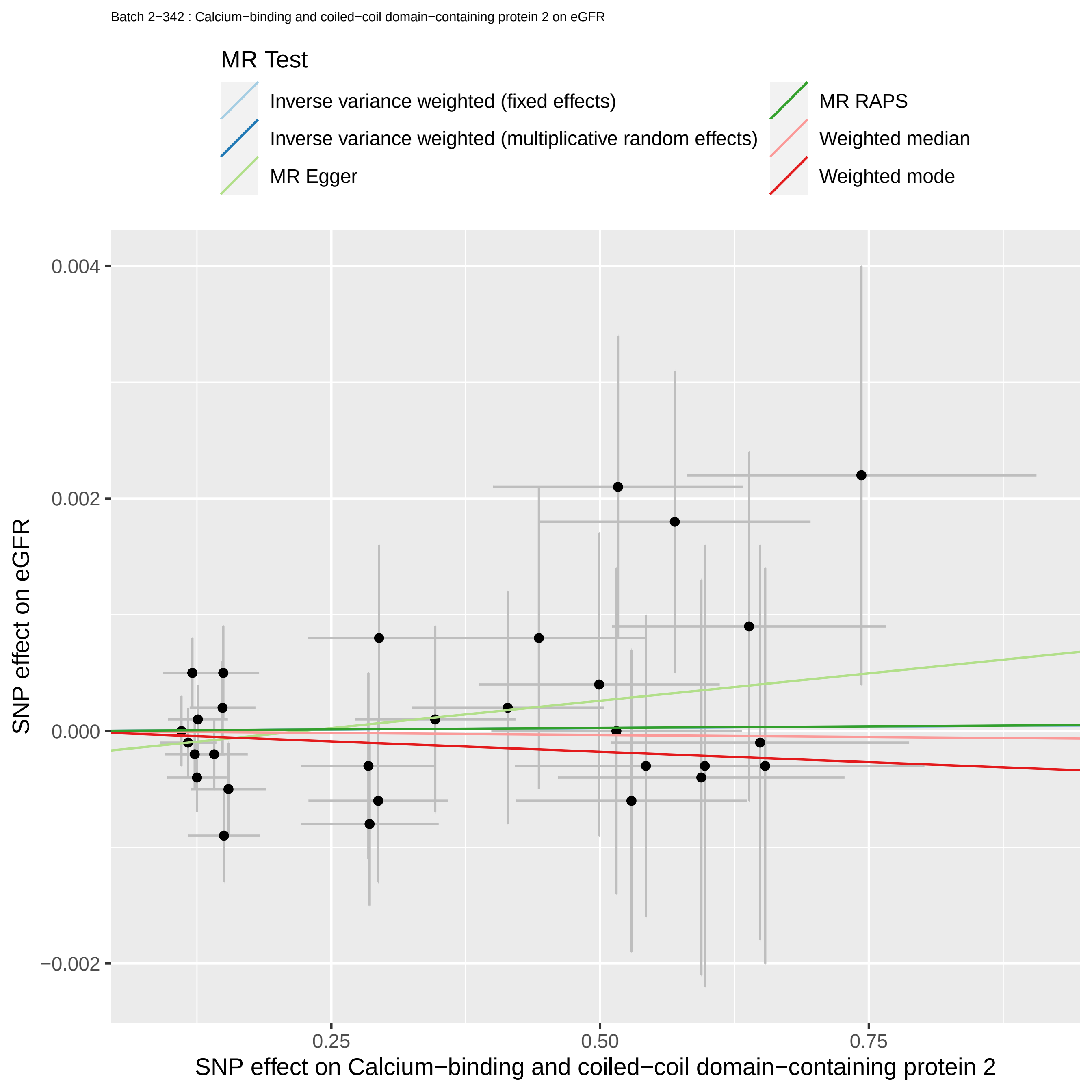
Batch 2−342 : Calcium−binding and coiled−coil domain−containing protein 2 on eGFR
MR Test
Inverse variance weighted (fixed effects)
Inverse variance weighted (multiplicative random effects)
MR Egger
MR RAPS
Weighted median
Weighted mode
0.004
0.002
0.000
−0.002
0.25
0.50
0.75
SNP effect on eGFR
SNP effect on Calcium−binding and coiled−coil domain−containing protein 2

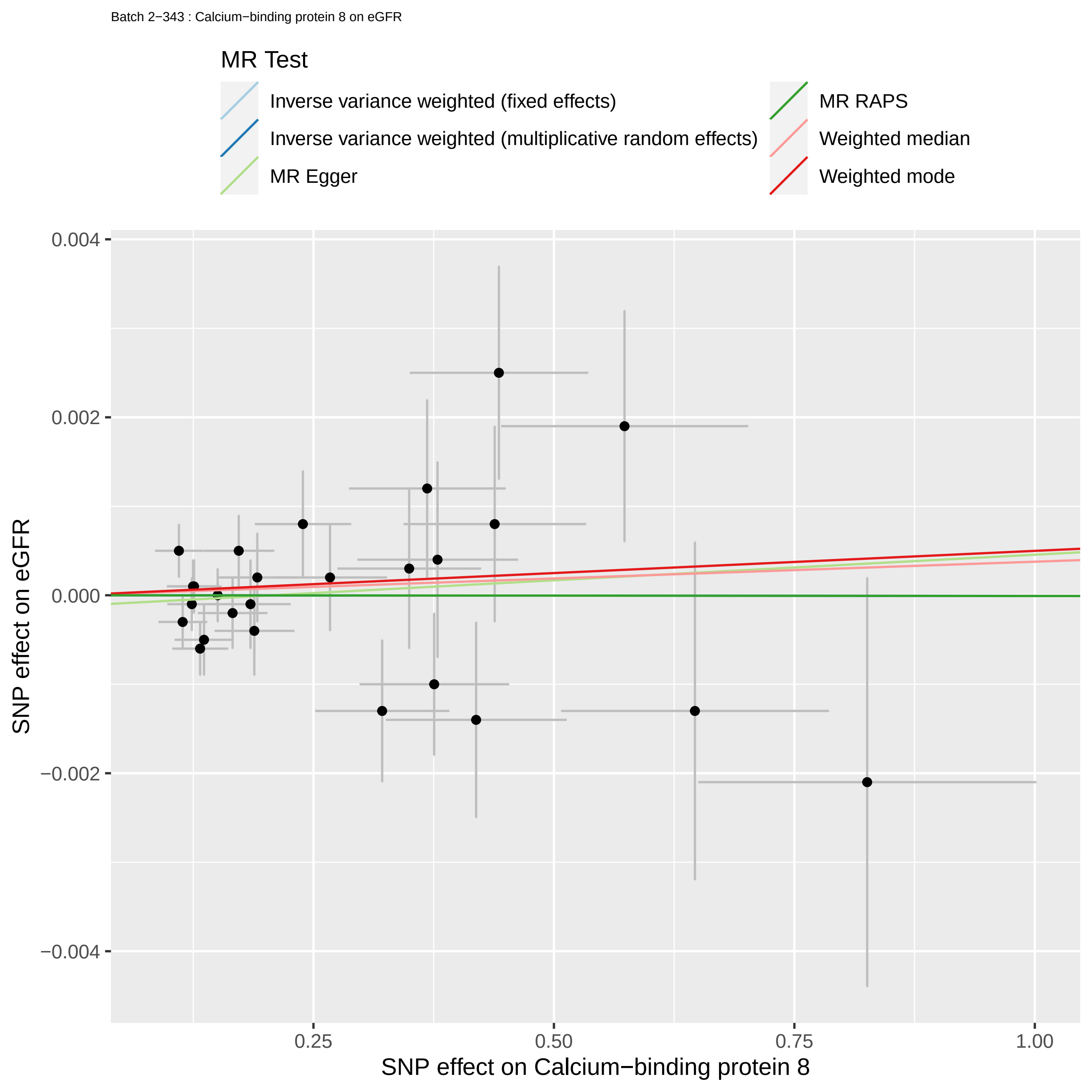
Batch 2−343 : Calcium−binding protein 8 on eGFR
MR Test
Inverse variance weighted (fixed effects)
Inverse variance weighted (multiplicative random effects)
MR Egger
MR RAPS
Weighted median
Weighted mode
0.004
0.002
0.000
−0.002
−0.004
0.25
0.50
0.75
1.00
SNP effect on eGFR
SNP effect on Calcium−binding protein 8

Batch 2−344 : Calreticulin on eGFR

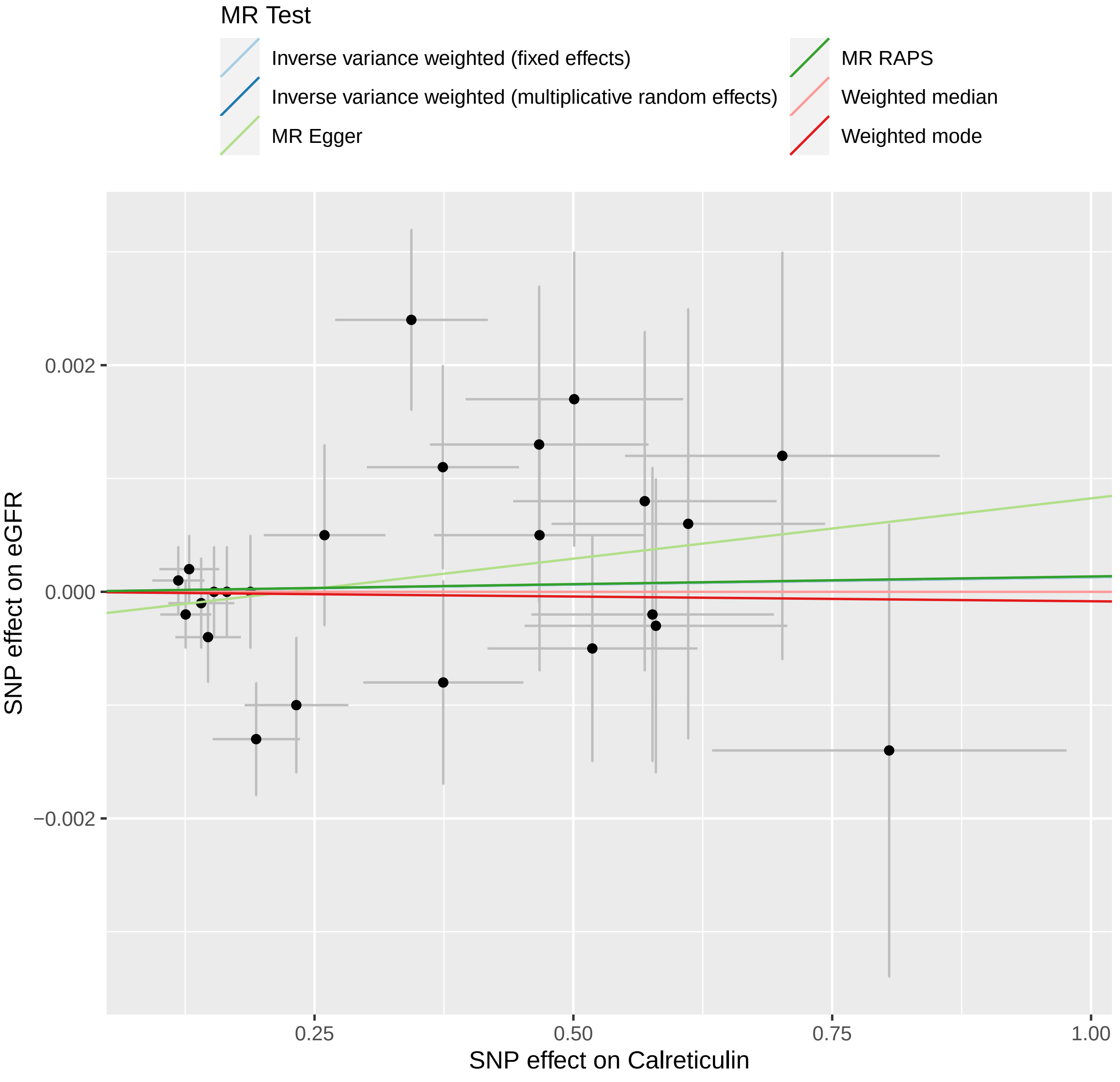

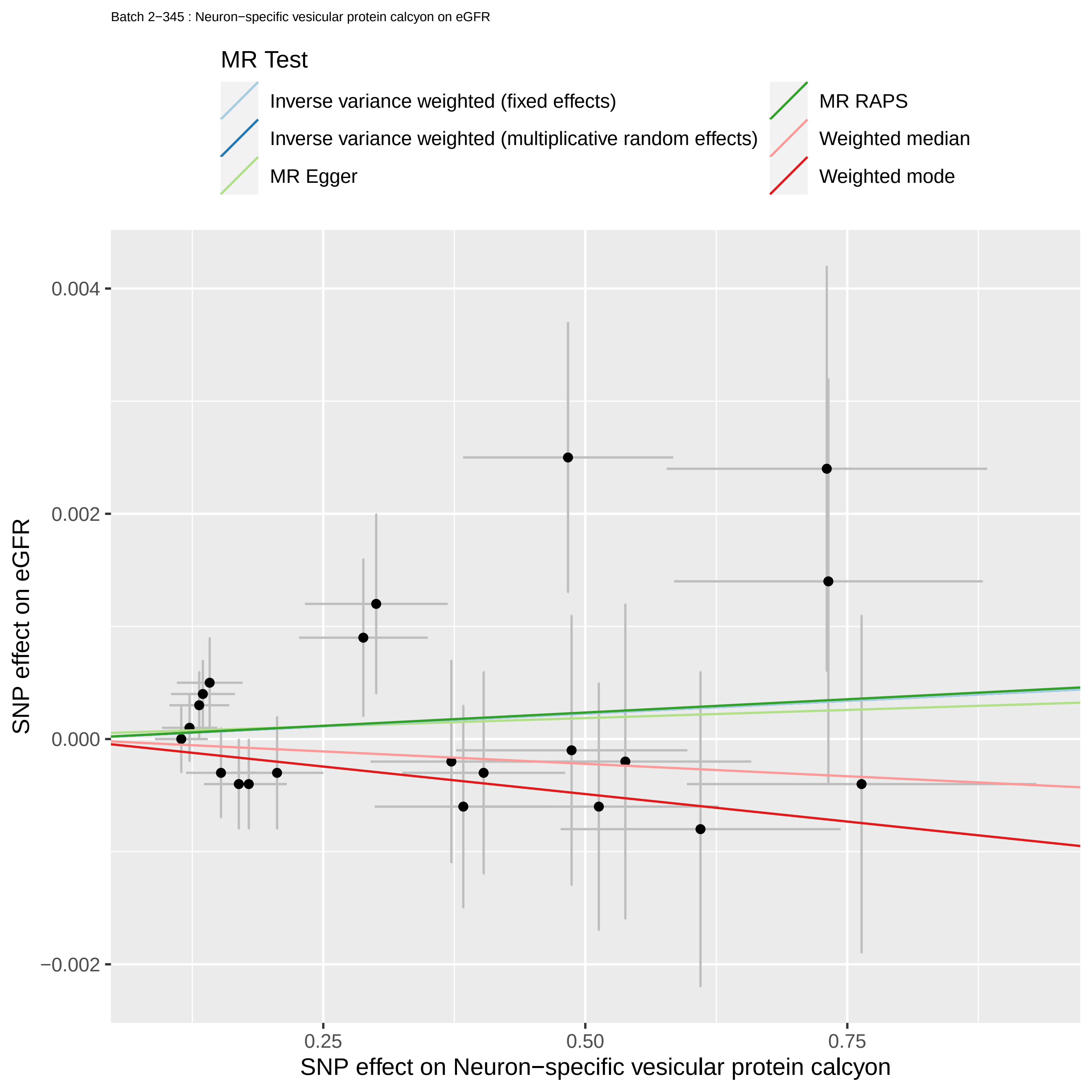
Batch 2−345 : Neuron−specific vesicular protein calcyon on eGFR
MR Test
Inverse variance weighted (fixed effects)
Inverse variance weighted (multiplicative random effects)
MR Egger
MR RAPS
Weighted median
Weighted mode
0.004
0.002
0.000
−0.002
SNP effect on eGFR
0.25
0.50
0.75
SNP effect on Neuron−specific vesicular protein calcyon

Batch 2−346 : Calcium/calmodulin−dependent protein kinase type 1 on eGFR

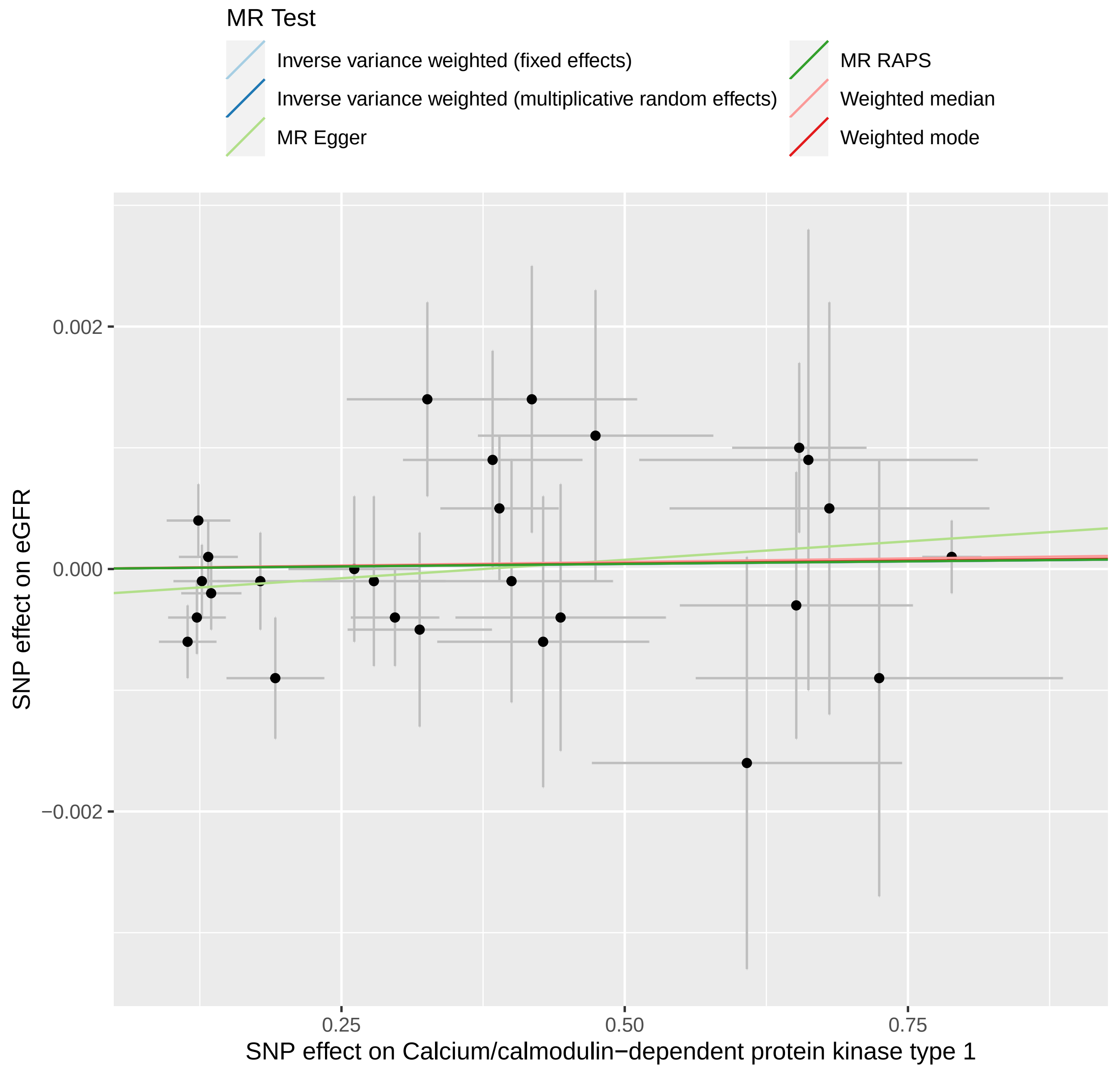

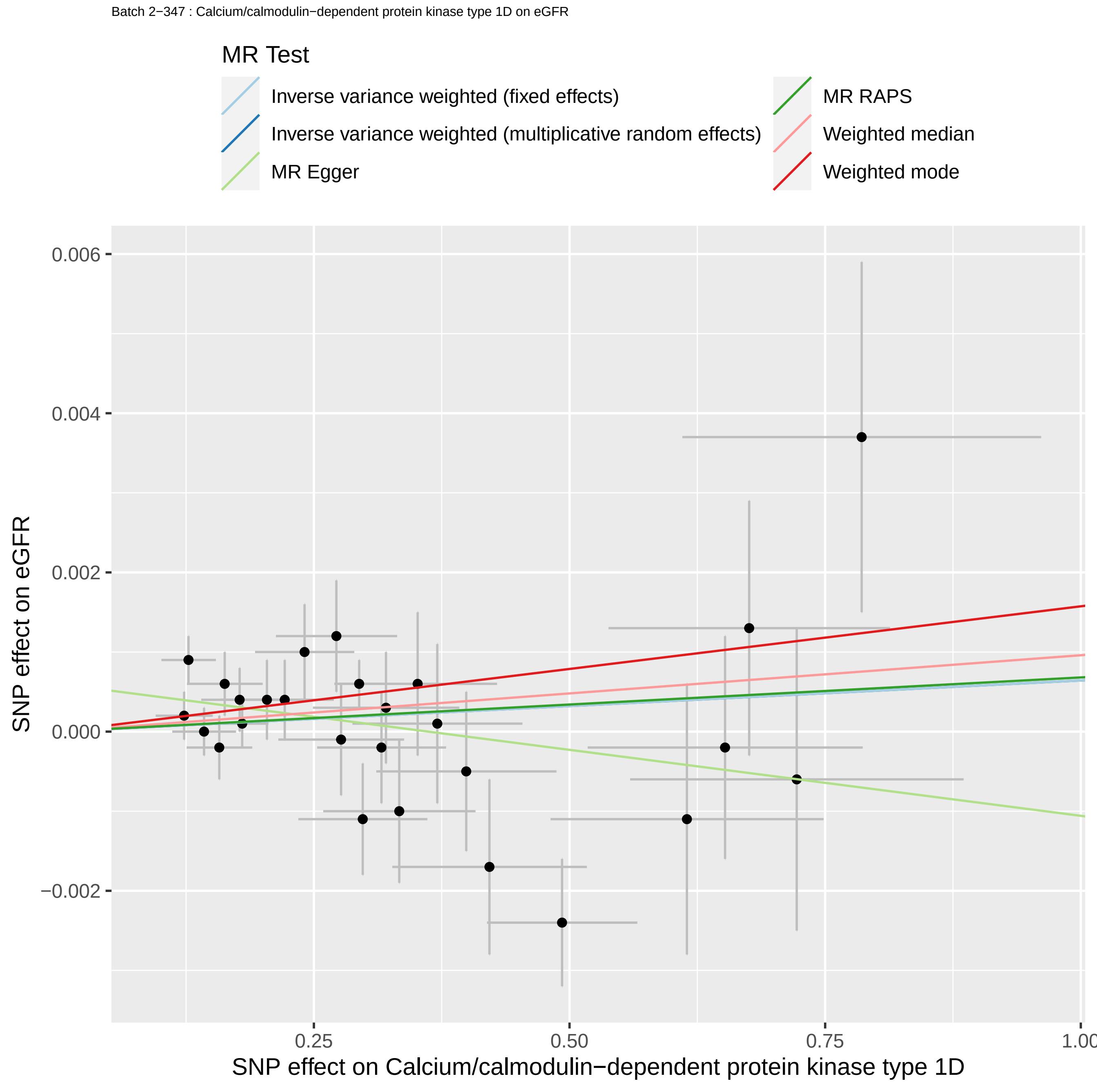

Batch 2−347 : Calcium/calmodulin−dependent protein kinase type 1D on eGFR
MR Test
Inverse variance weighted (fixed effects)
Inverse variance weighted (multiplicative random effects)
MR Egger
MR RAPS
Weighted median
Weighted mode
SNP effect on eGFR
0.006
0.004
0.002
0.000
−0.002
0.25
0.50
0.75
1.00
SNP effect on Calcium/calmodulin−dependent protein kinase type 1D

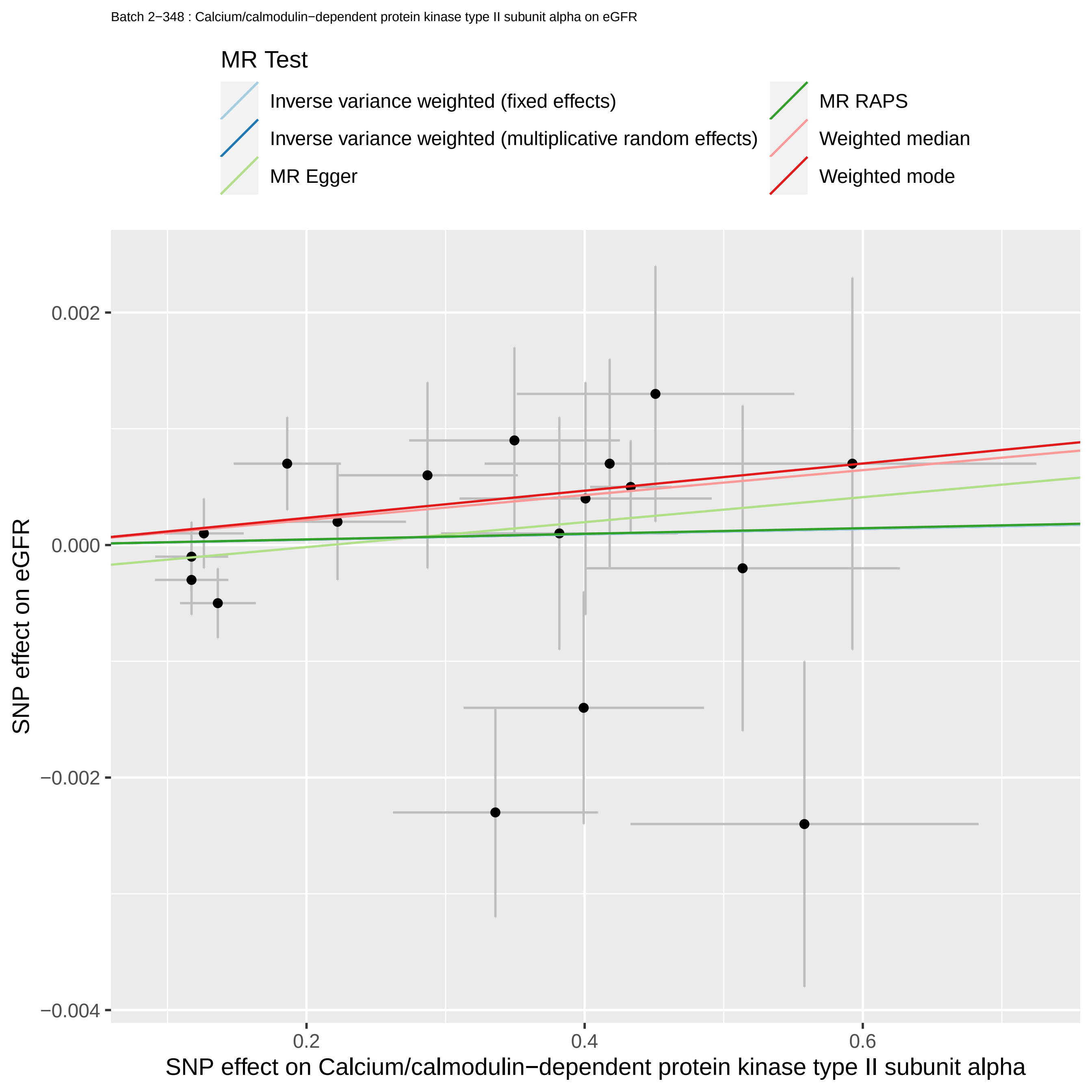
Batch 2−348 : Calcium/calmodulin−dependent protein kinase type II subunit alpha on eGFR
MR Test
Inverse variance weighted (fixed effects)
Inverse variance weighted (multiplicative random effects)
MR Egger
MR RAPS
Weighted median
Weighted mode
0.002
0.000
−0.002
−0.004
0.2
0.4
0.6
SNP effect on eGFR
SNP effect on Calcium/calmodulin−dependent protein kinase type II subunit alpha

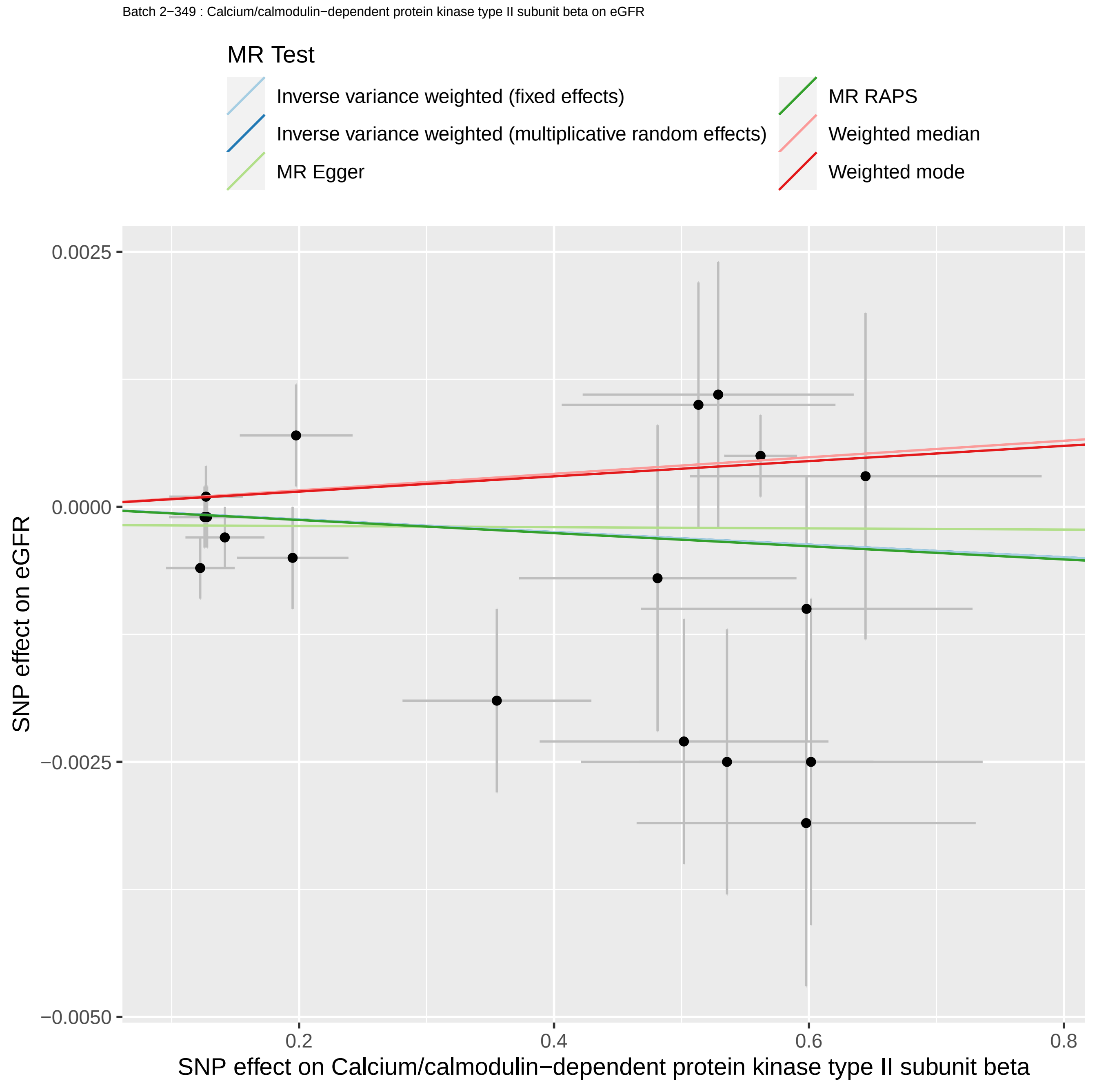

Batch 2−349 : Calcium/calmodulin−dependent protein kinase type II subunit beta on eGFR
MR Test
Inverse variance weighted (fixed effects)
Inverse variance weighted (multiplicative random effects)
MR Egger
MR RAPS
Weighted median
Weighted mode
0.0025
0.0000
−0.0025
−0.0050
0.2
0.4
0.6
0.8
SNP effect on eGFR
SNP effect on Calcium/calmodulin−dependent protein kinase type II subunit beta

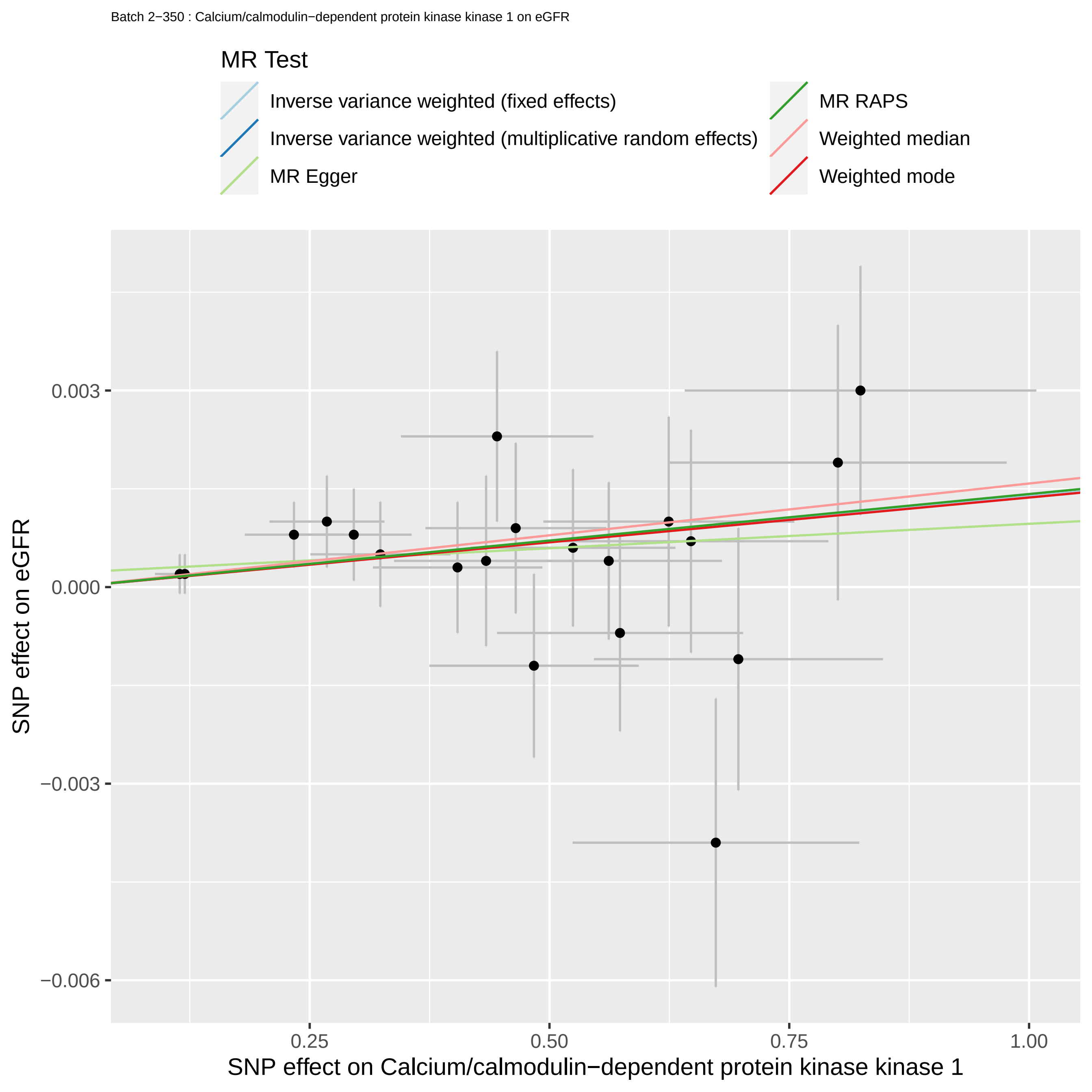
Batch 2−350 : Calcium/calmodulin−dependent protein kinase kinase 1 on eGFR
MR Test
Inverse variance weighted (fixed effects)
Inverse variance weighted (multiplicative random effects)
MR Egger
MR RAPS
Weighted median
Weighted mode
0.003
0.000
−0.003
−0.006
0.25
0.50
0.75
1.00
SNP effect on eGFR
SNP effect on Calcium/calmodulin−dependent protein kinase kinase 1

Batch 2−351 : Cullin−associated NEDD8−dissociated protein 1 on eGFR

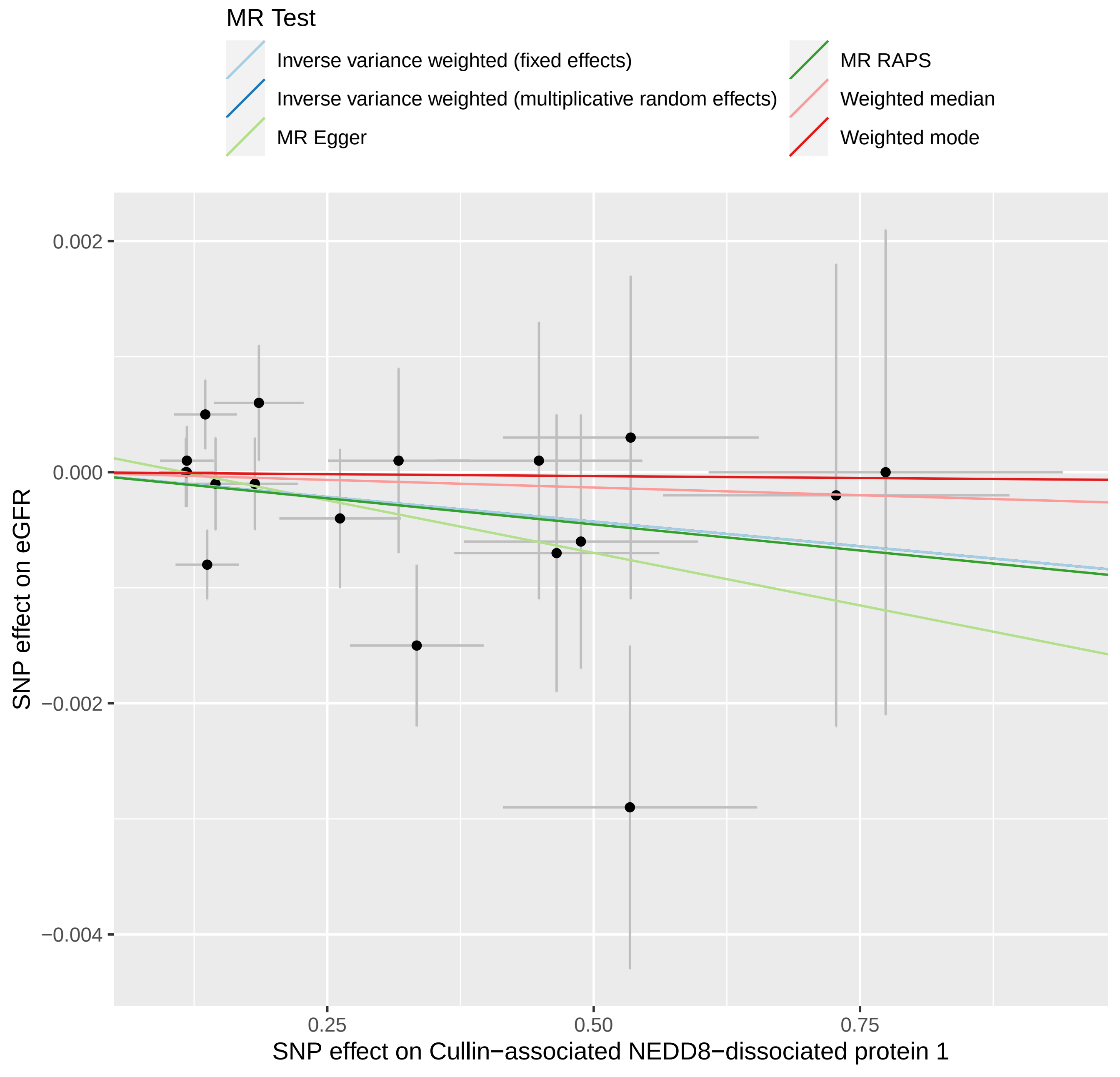

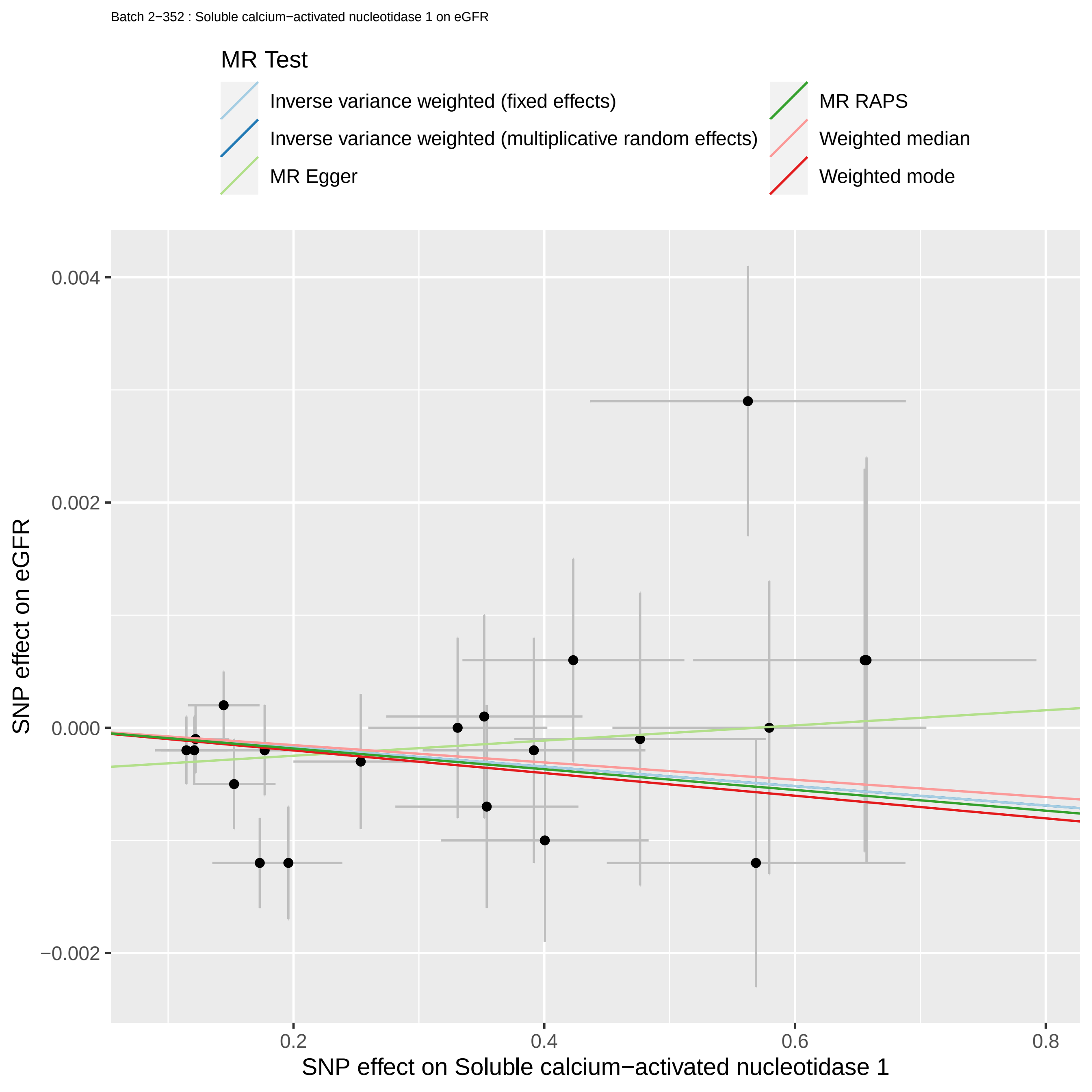

Batch 2−352 : Soluble calcium−activated nucleotidase 1 on eGFR
MR Test
Inverse variance weighted (fixed effects)
Inverse variance weighted (multiplicative random effects)
MR Egger
MR RAPS
Weighted median
Weighted mode
0.004
0.002
0.000
−0.002
0.2
0.4
0.6
0.8
SNP effect on eGFR
SNP effect on Soluble calcium−activated nucleotidase 1

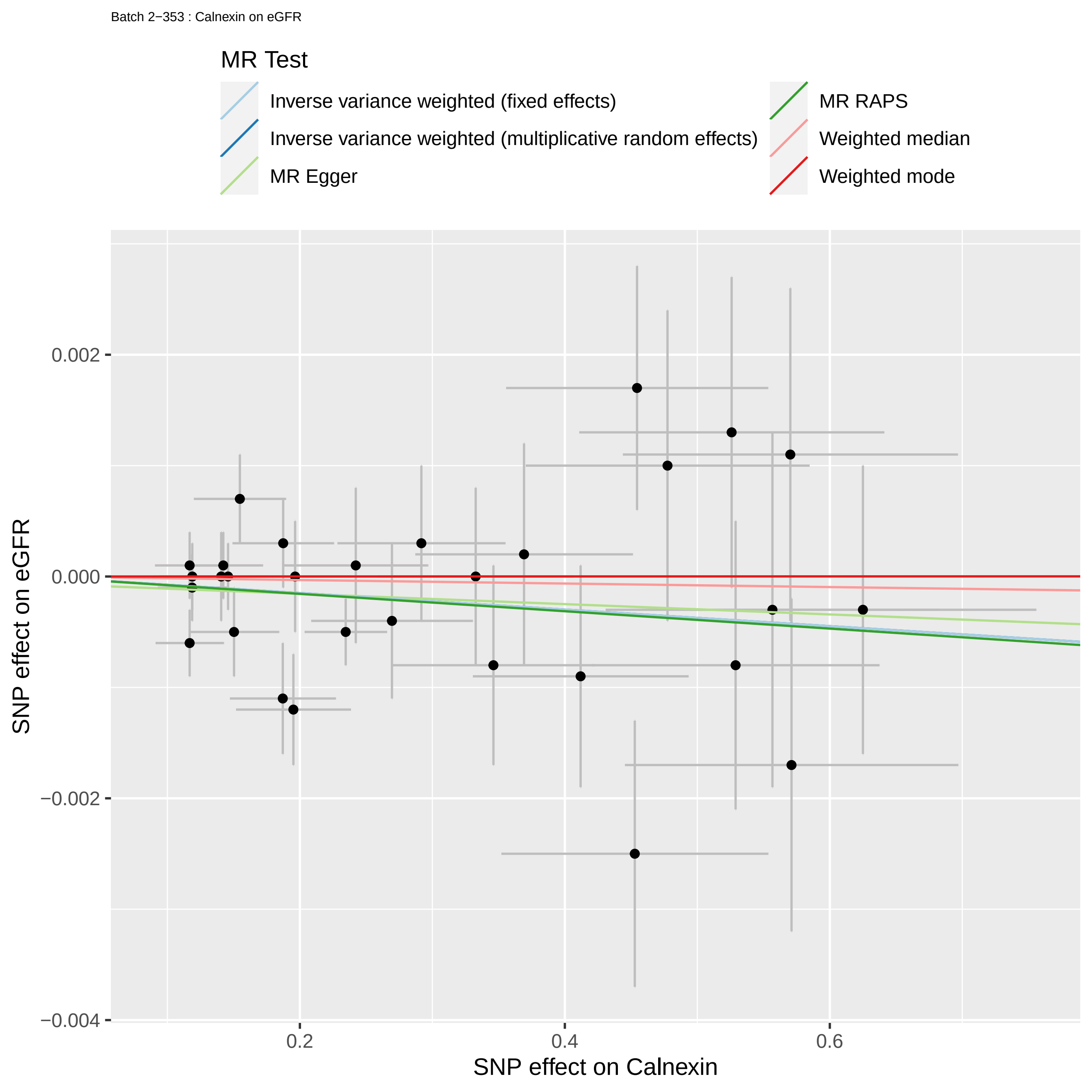

Batch 2−353 : Calnexin on eGFR
MR Test
Inverse variance weighted (fixed effects)
Inverse variance weighted (multiplicative random effects)
MR Egger
MR RAPS
Weighted median
Weighted mode
0.002
0.000
−0.002
−0.004
SNP effect on eGFR
0.2
0.4
0.6
SNP effect on Calnexin

Batch 2−354 : Calpain−2 catalytic subunit on eGFR

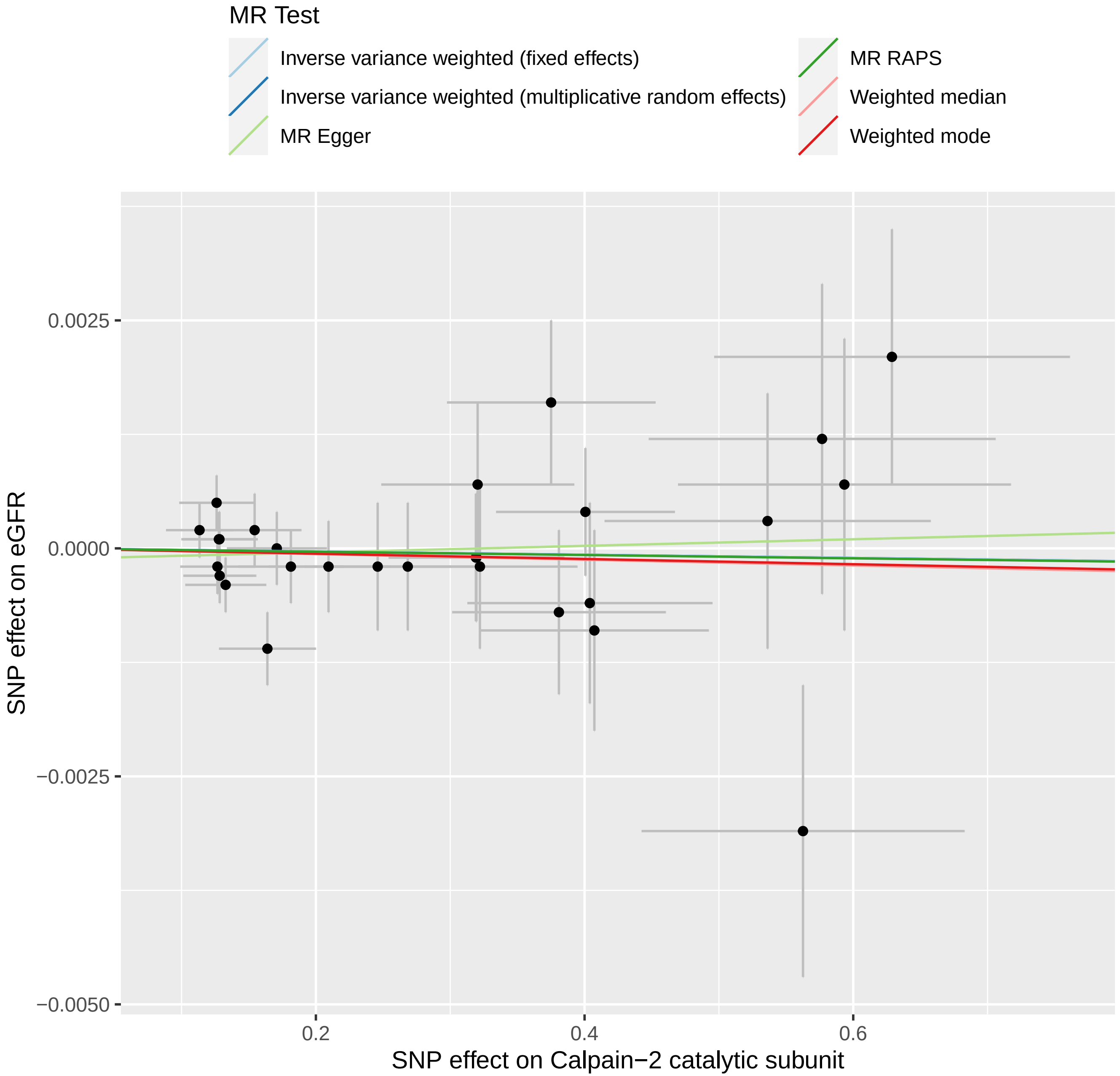

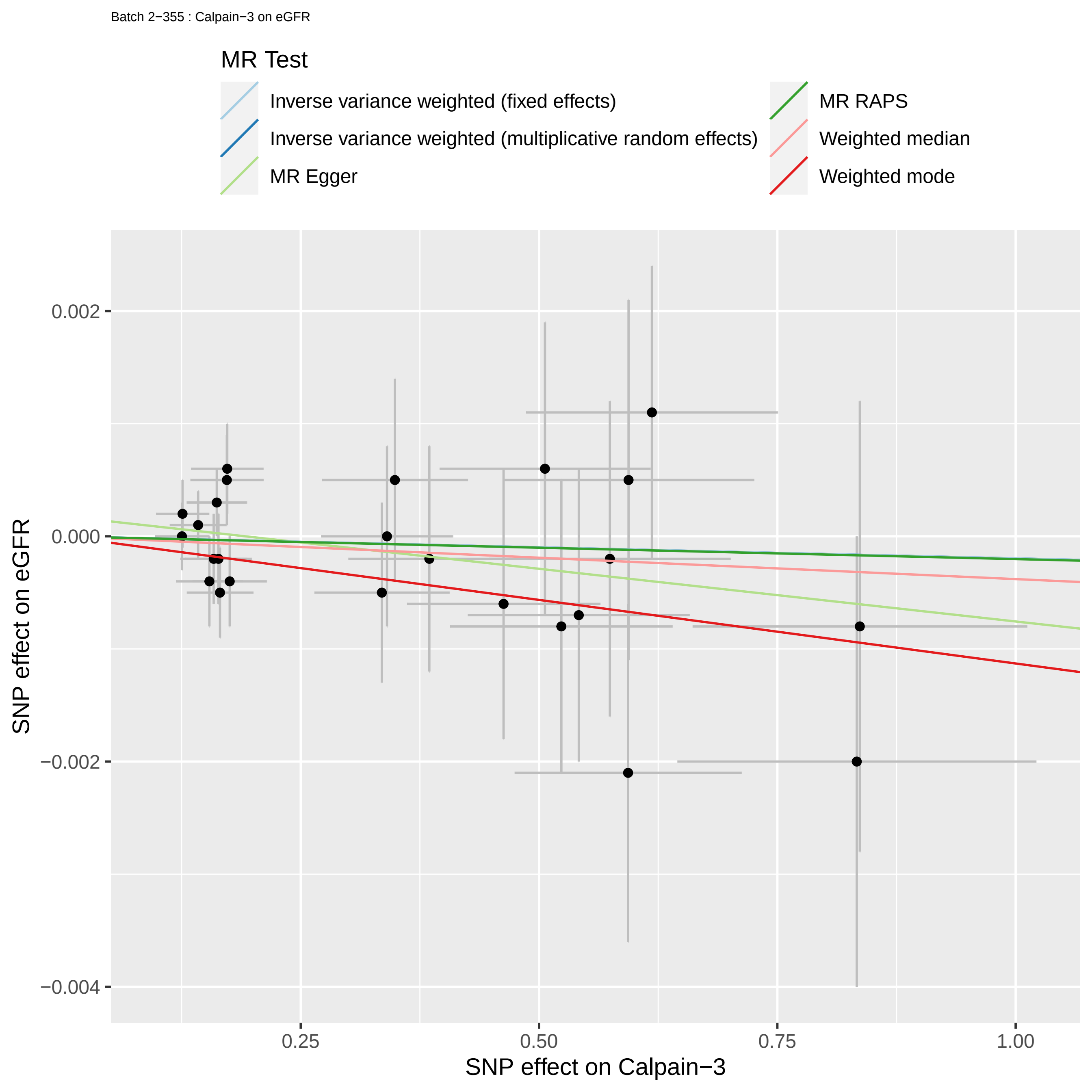
Batch 2−355 : Calpain−3 on eGFR
MR Test
Inverse variance weighted (fixed effects)
Inverse variance weighted (multiplicative random effects)
MR Egger
MR RAPS
Weighted median
Weighted mode
0.002
0.000
−0.002
−0.004
0.25
0.50
0.75
1.00
SNP effect on eGFR
SNP effect on Calpain−3

Batch 2−356 : Calcyphosin−like protein on eGFR

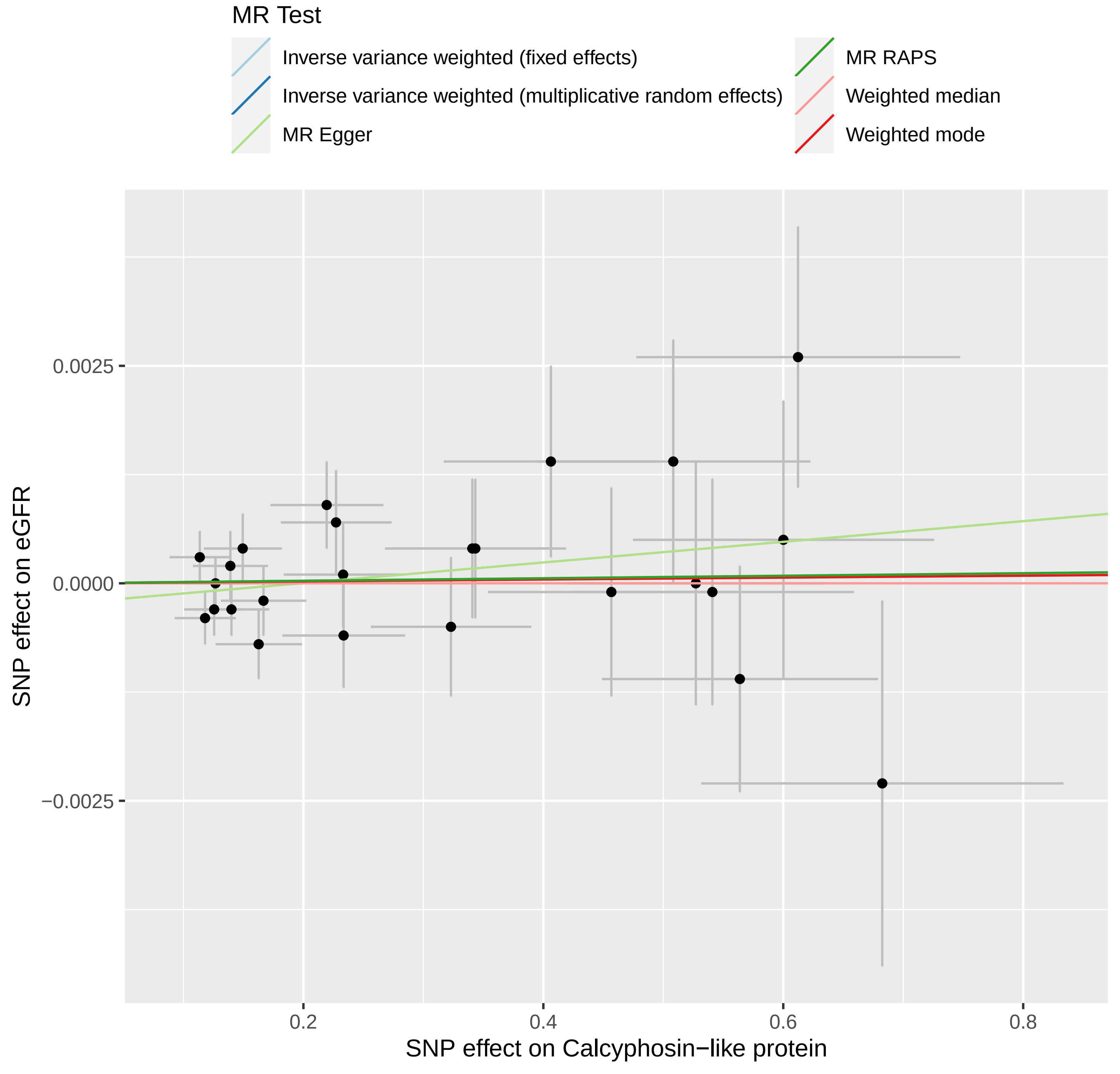

Batch 2−357 : Calcium−regulated heat stable protein 1 on eGFR

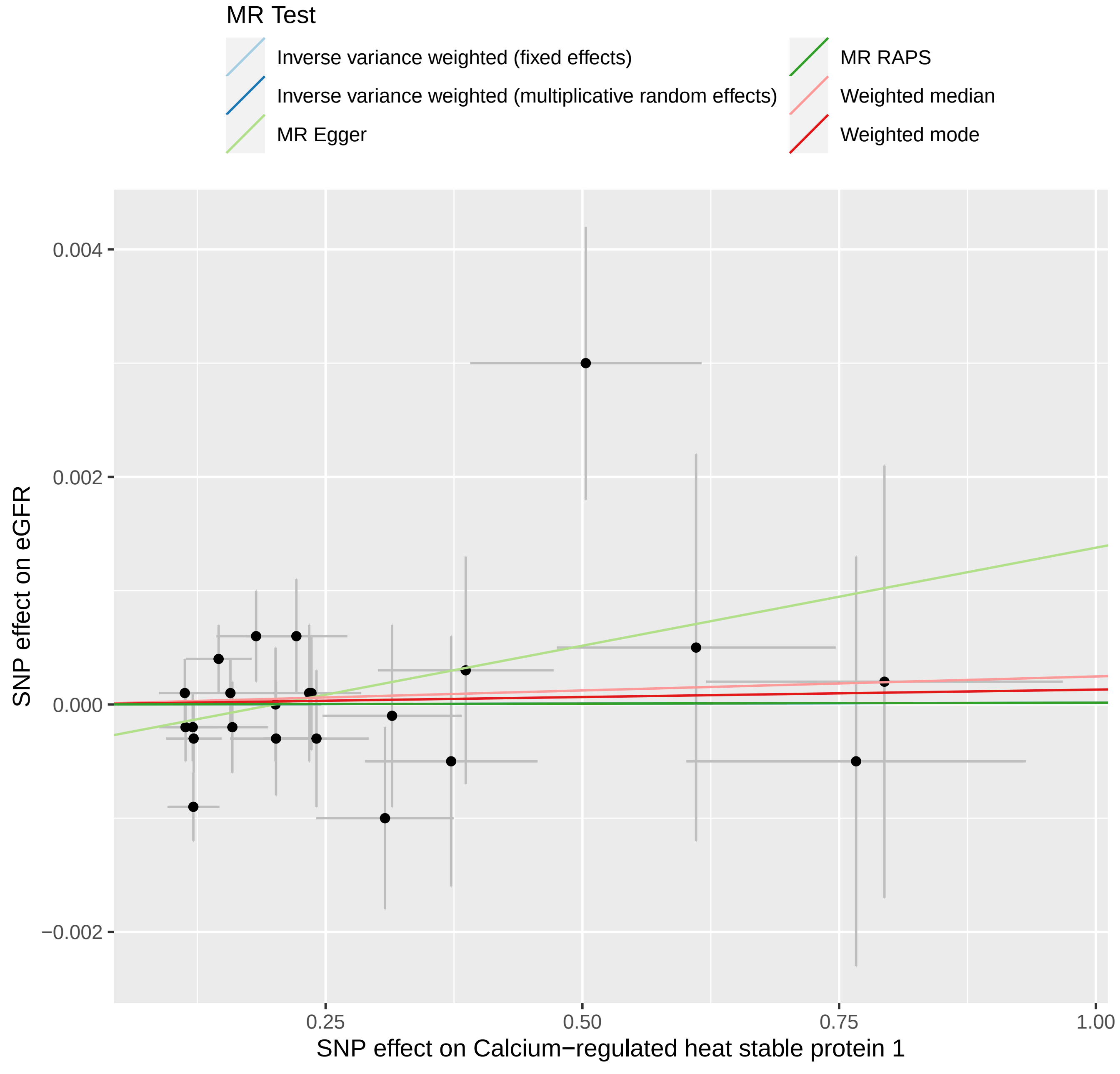

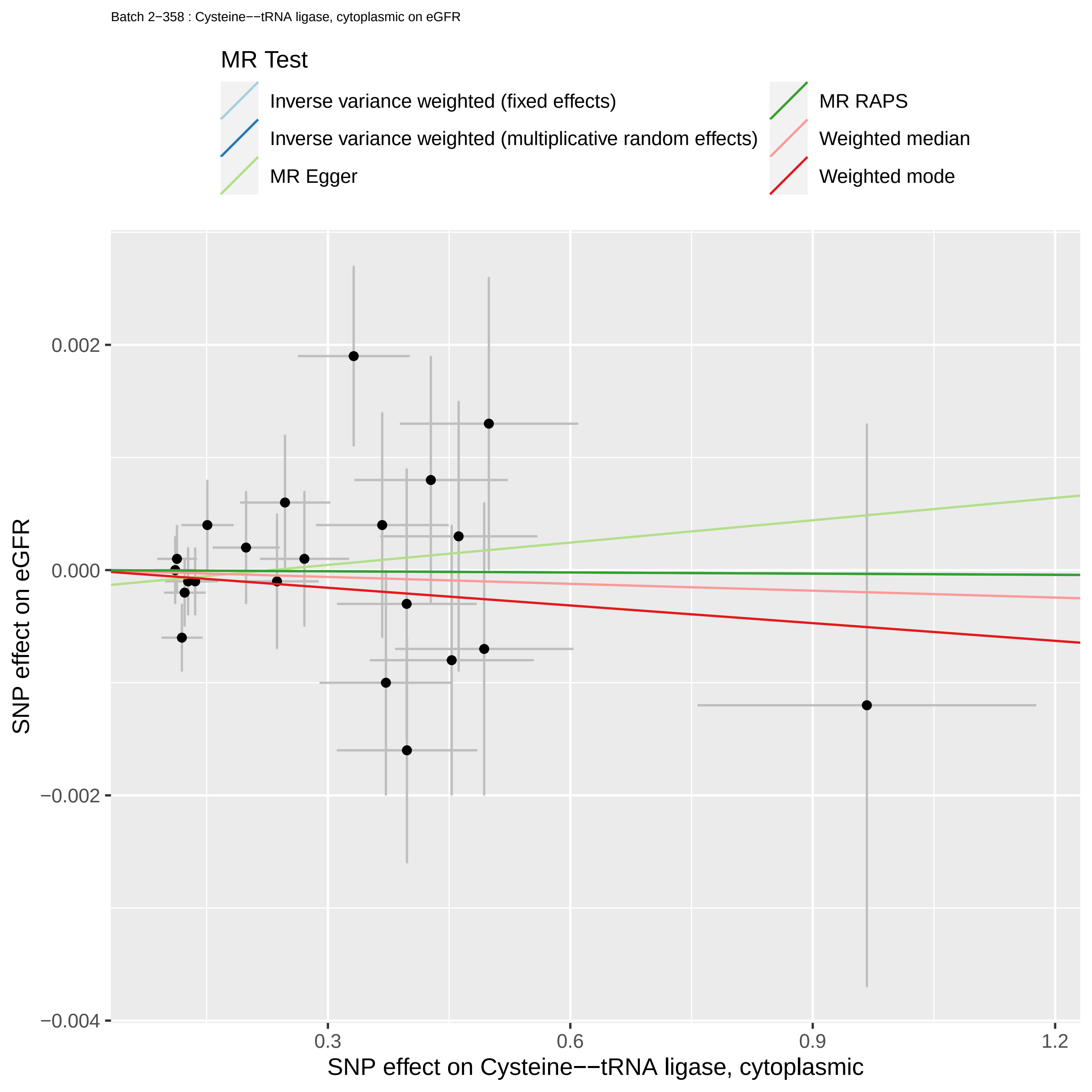
Batch 2−358 : Cysteine−−tRNA ligase, cytoplasmic on eGFR
MR Test
Inverse variance weighted (fixed effects)
Inverse variance weighted (multiplicative random effects)
MR Egger
MR RAPS
Weighted median
Weighted mode
0.002
0.000
−0.002
−0.004
0.3
0.6
0.9
1.2
SNP effect on eGFR
SNP effect on Cysteine−−tRNA ligase, cytoplasmic

Batch 2−359 : Cocaine− and amphetamine−regulated transcript protein on eGFR

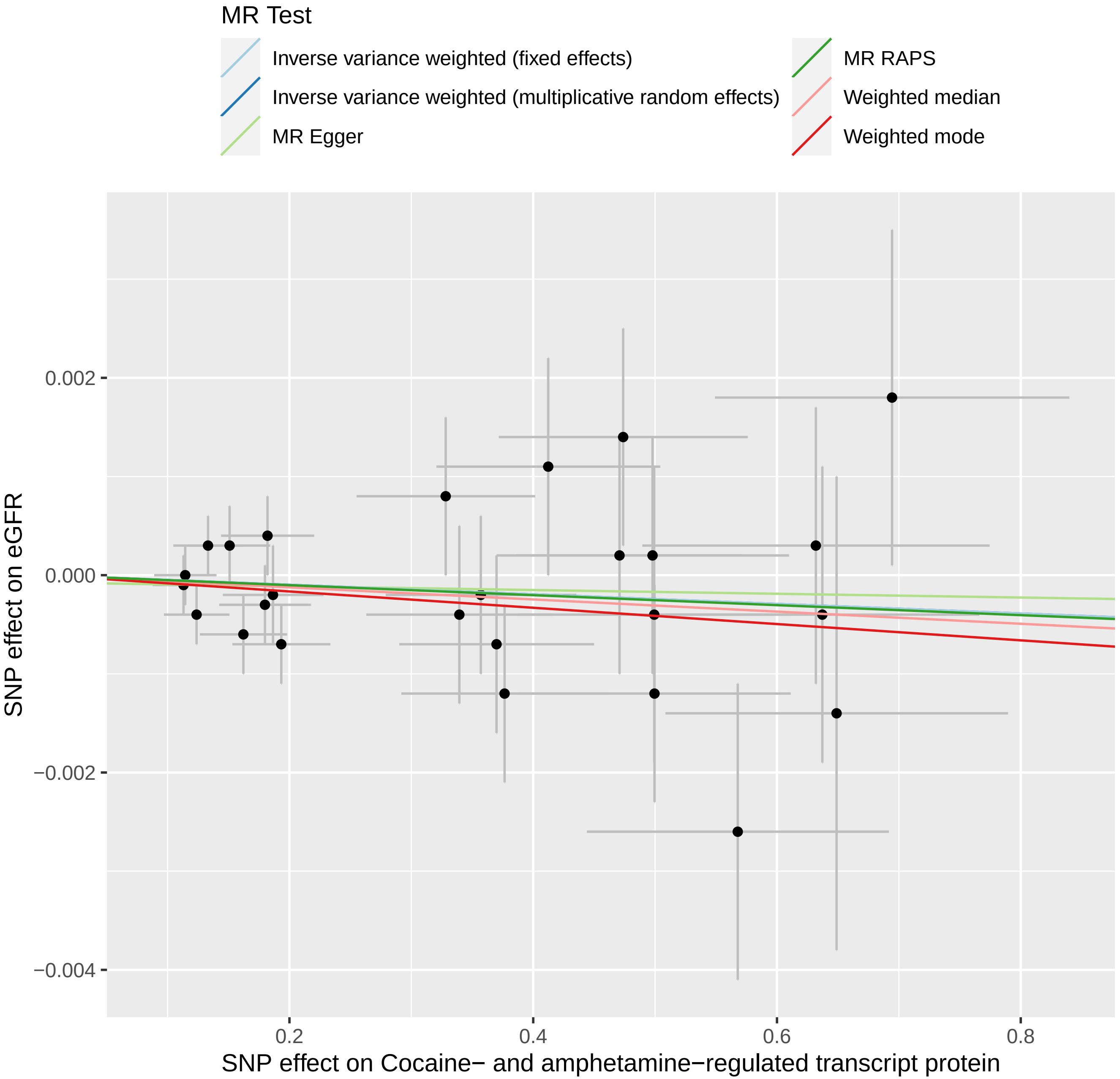

Batch 2−360 : Protein CASC4 on eGFR

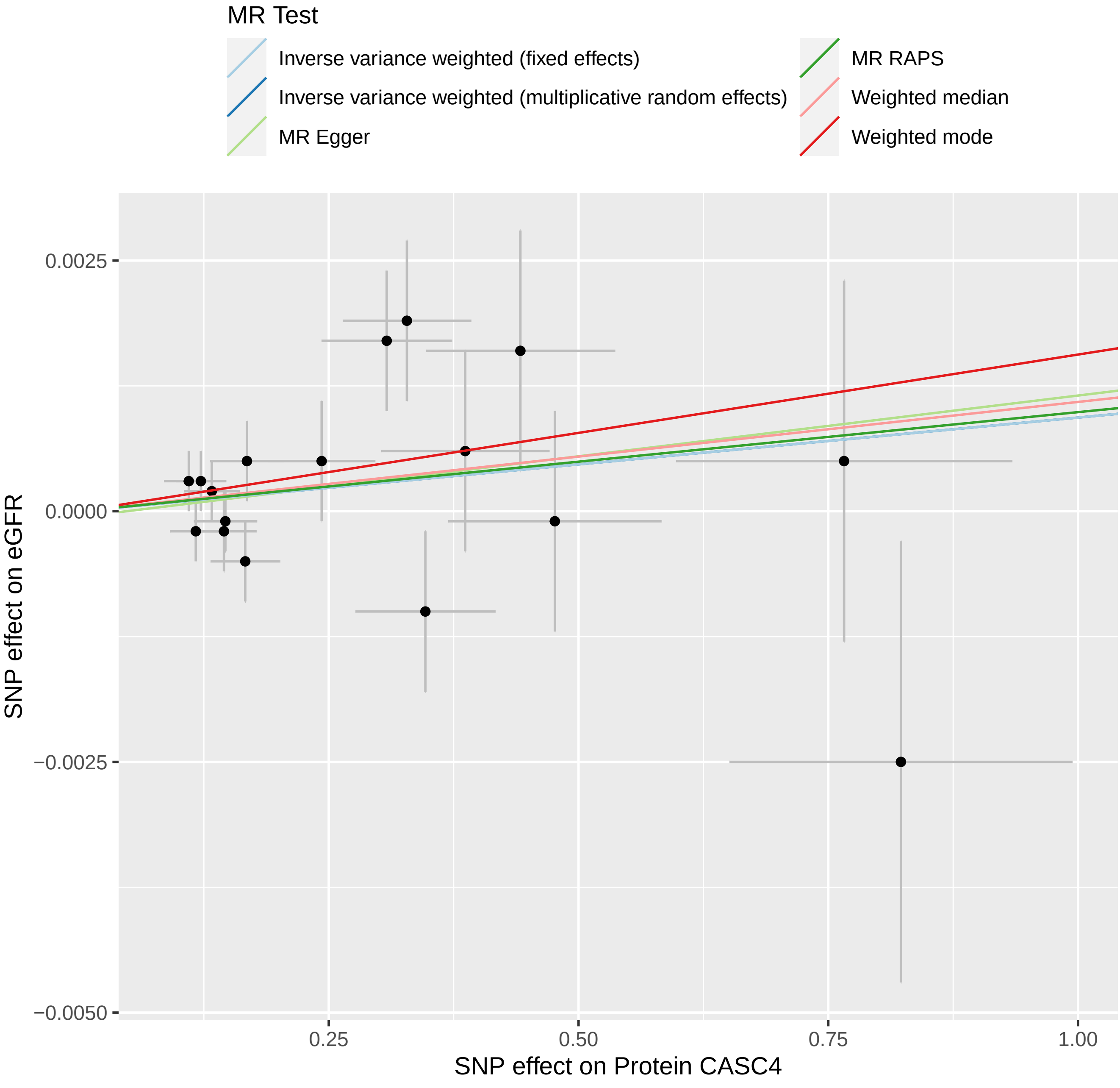

Batch 2−361 : Protein CASC4 on eGFR

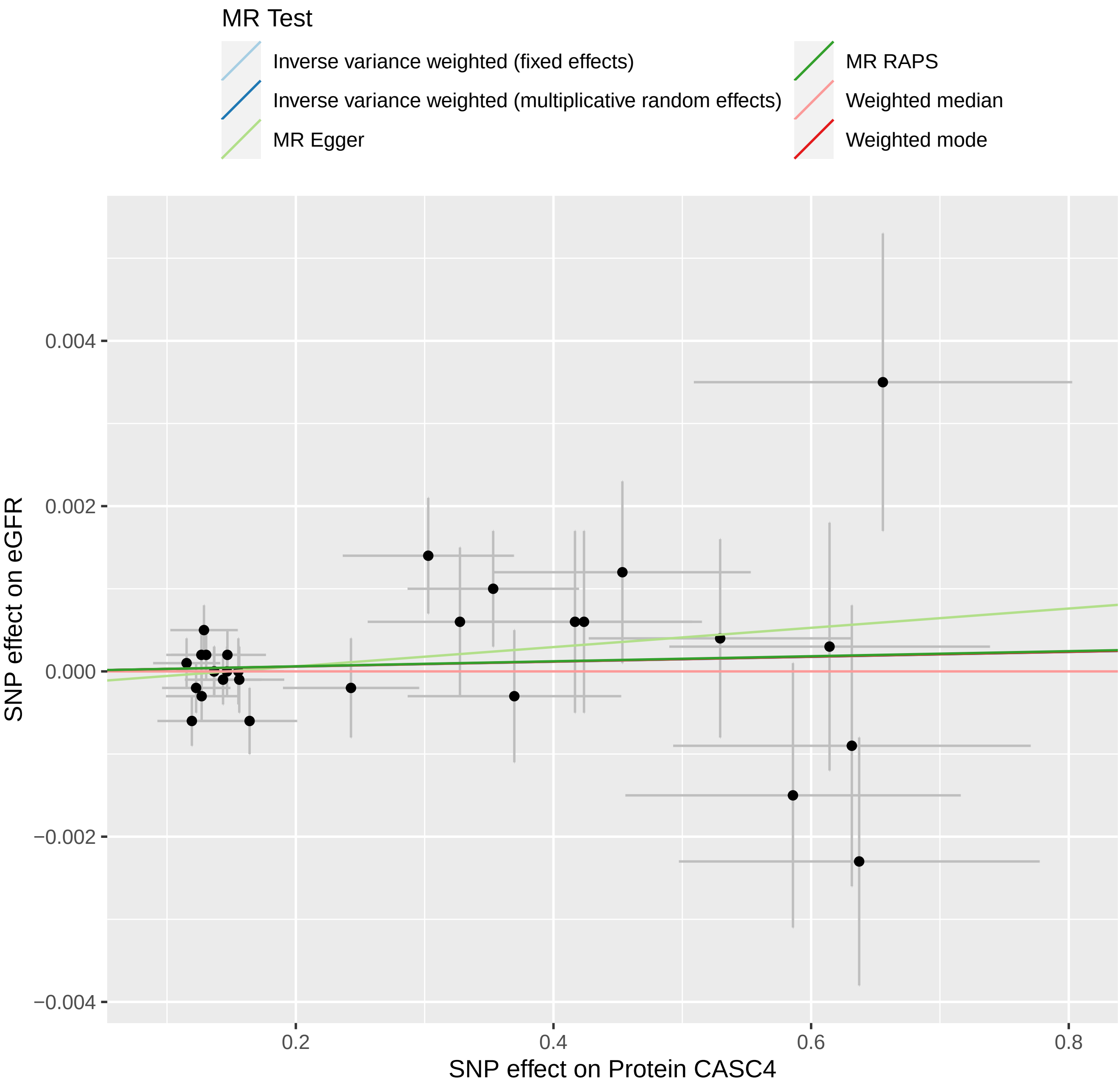

Batch 2−362 : Caspase−10 on eGFR

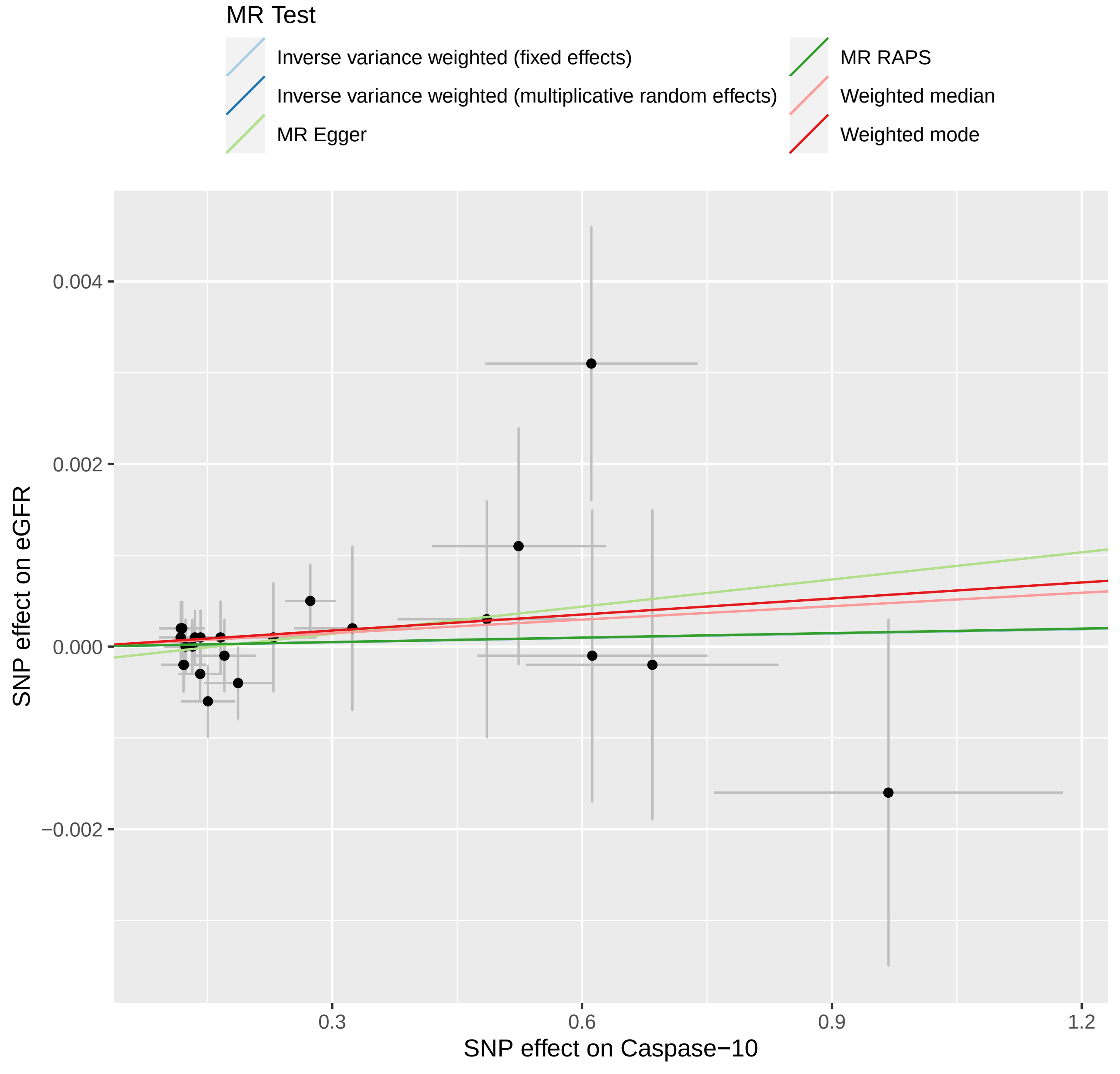

Batch 2−363 : Caspase−2 on eGFR

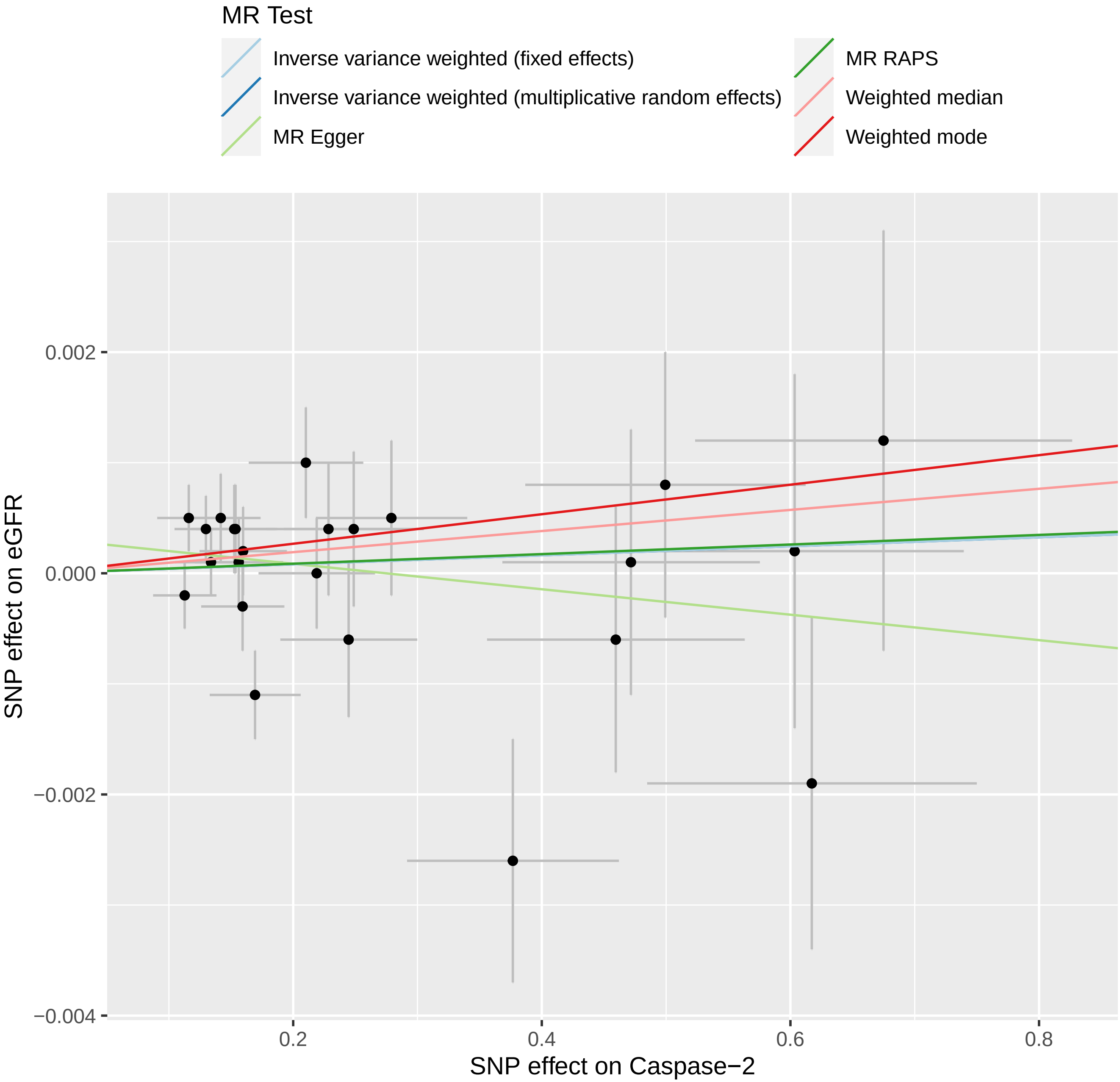

Batch 2−364 : Caspase−3 on eGFR

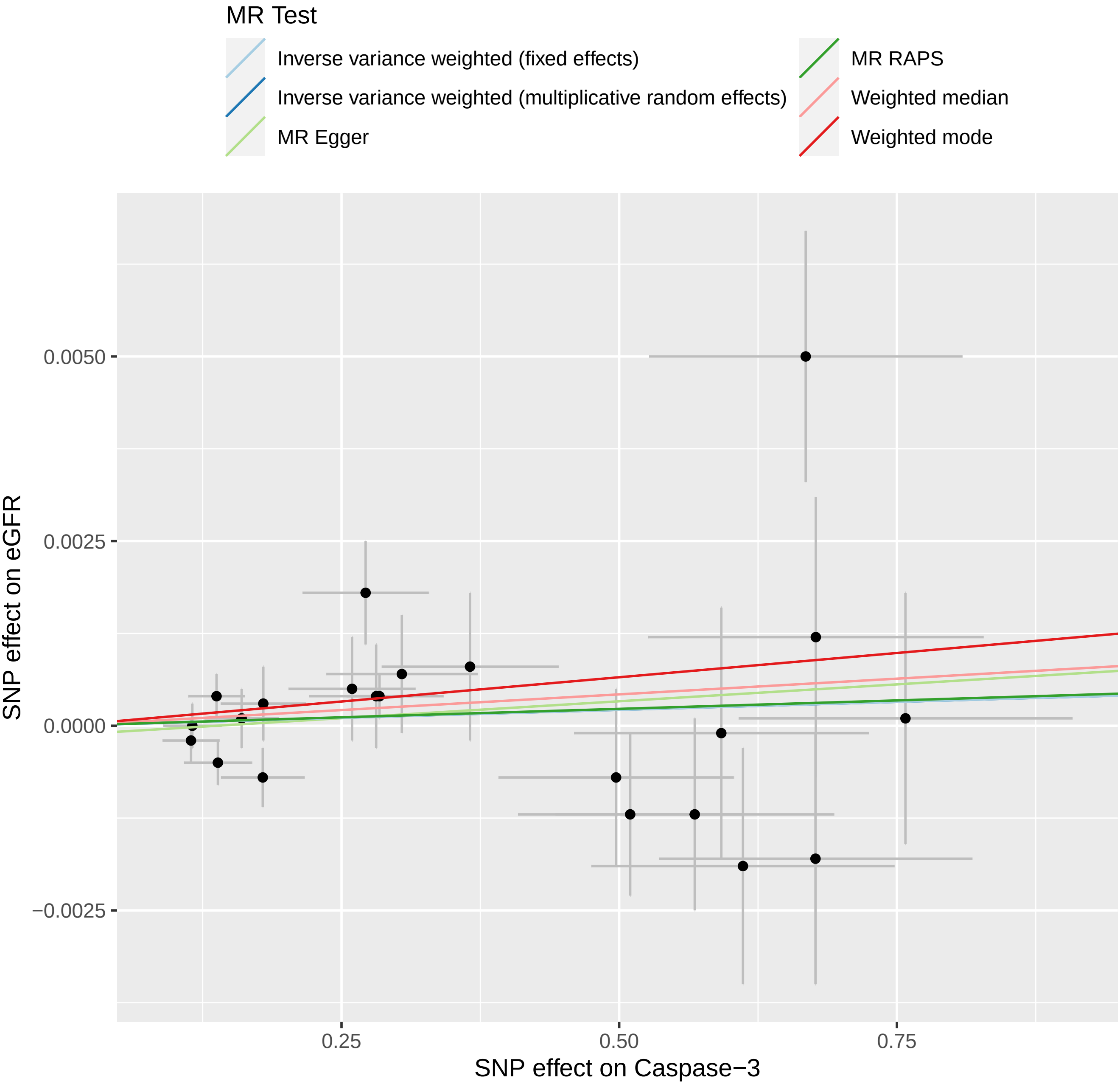

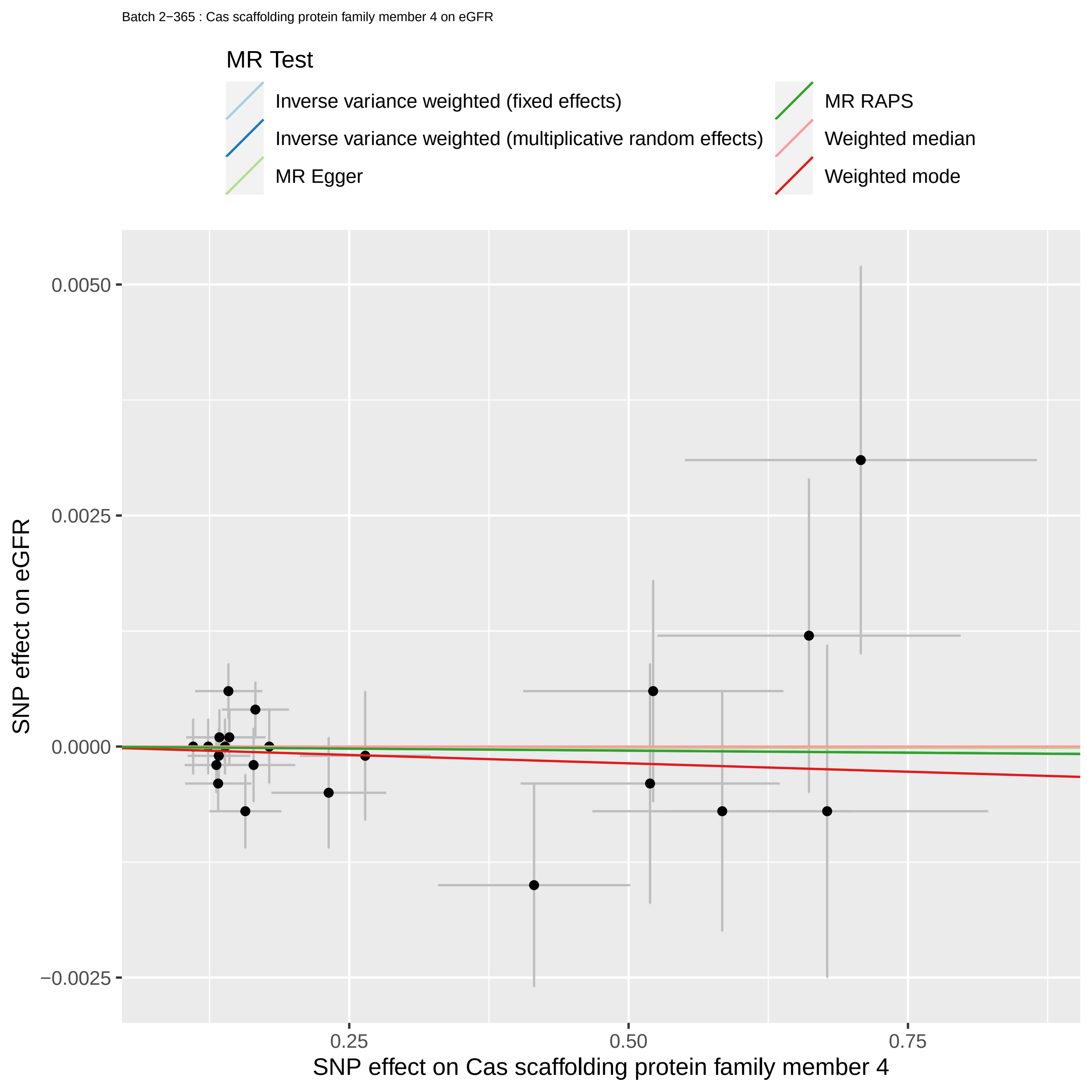

Batch 2−365 : Cas scaffolding protein family member 4 on eGFR
MR Test
Inverse variance weighted (fixed effects)
Inverse variance weighted (multiplicative random effects)
MR Egger
MR RAPS
Weighted median
Weighted mode
0.0050
0.0025
0.0000
−0.0025
0.25
0.50
0.75
SNP effect on eGFR
SNP effect on Cas scaffolding protein family member 4

Batch 2−366 : Calpastatin on eGFR

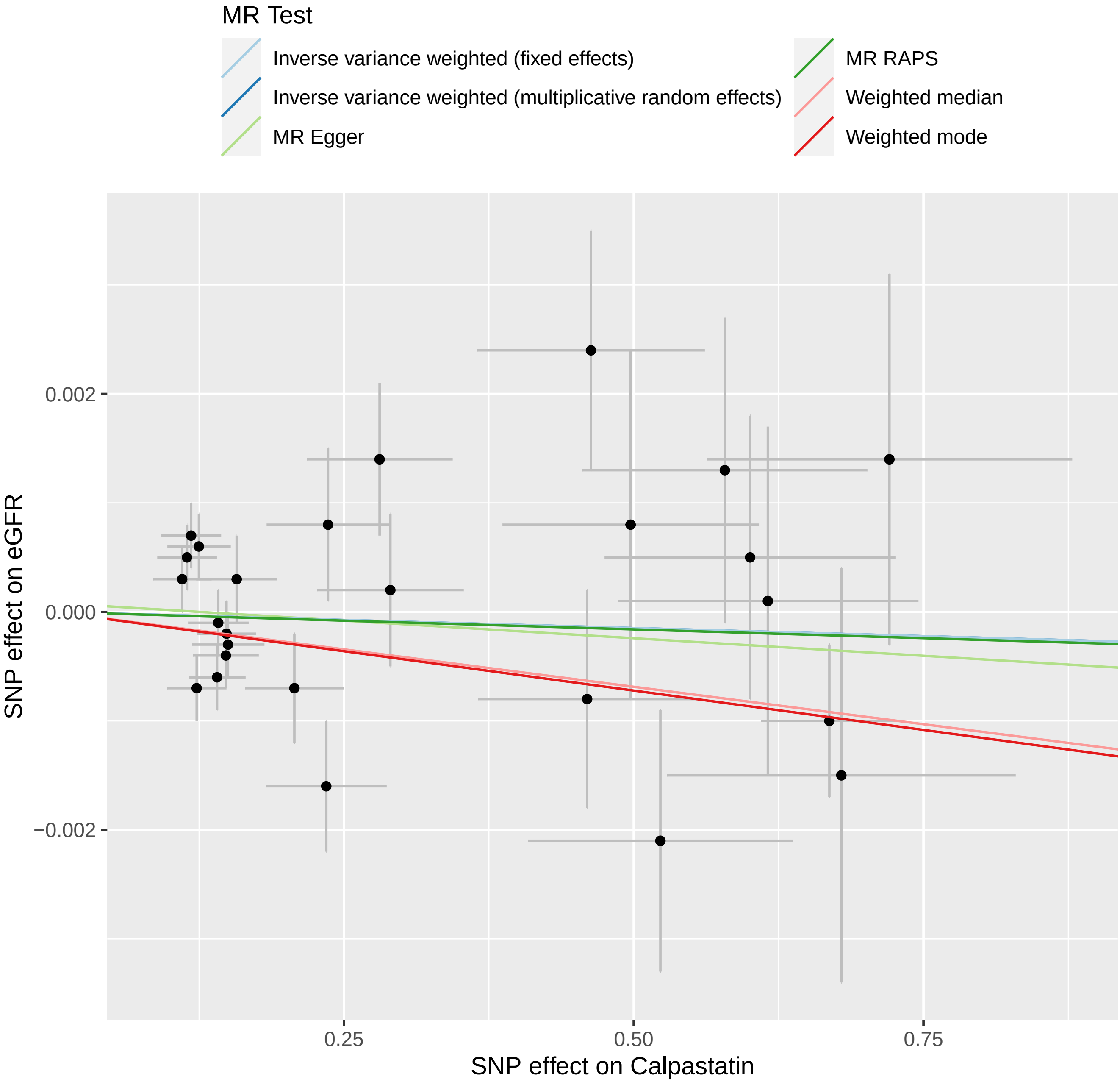

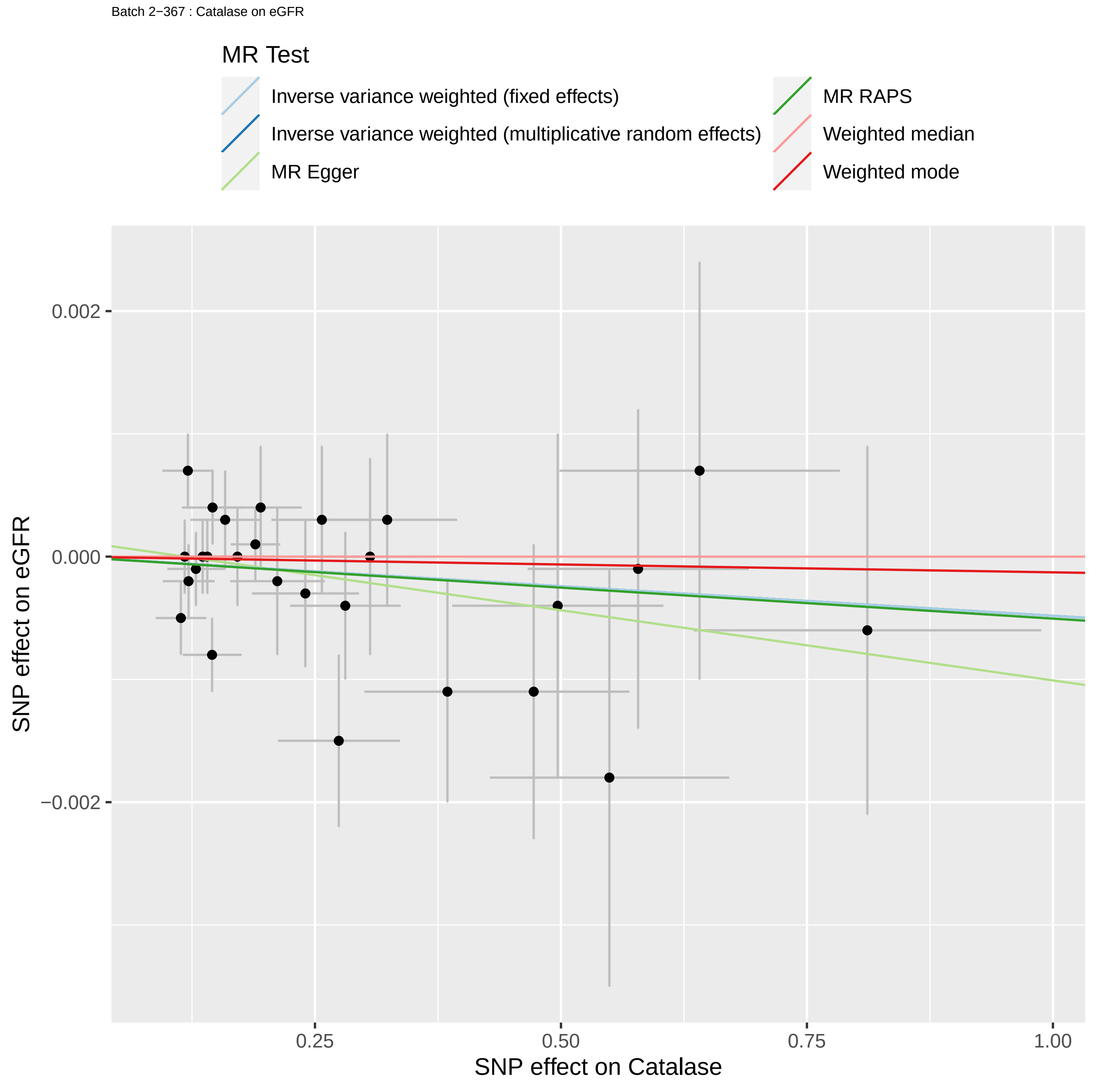

Batch 2−367 : Catalase on eGFR
MR Test
Inverse variance weighted (fixed effects)
Inverse variance weighted (multiplicative random effects)
MR Egger
MR RAPS
Weighted median
Weighted mode
0.002
0.000
−0.002
0.25
0.50
0.75
1.00
SNP effect on eGFR
SNP effect on Catalase

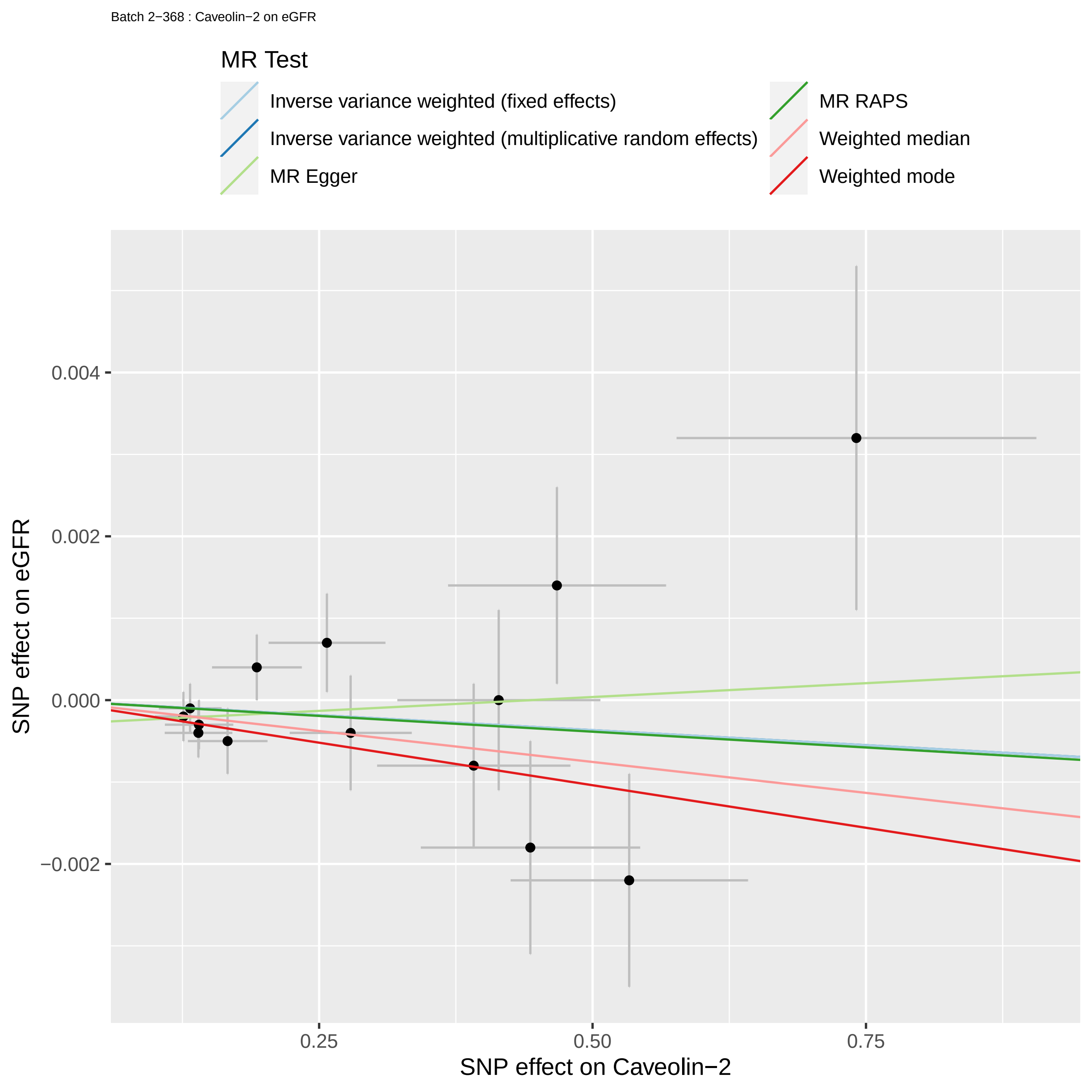

Batch 2−368 : Caveolin−2 on eGFR
MR Test
Inverse variance weighted (fixed effects)
Inverse variance weighted (multiplicative random effects)
MR Egger
MR RAPS
Weighted median
Weighted mode
0.004
0.002
0.000
−0.002
SNP effect on eGFR
0.25
0.50
0.75
SNP effect on Caveolin−2

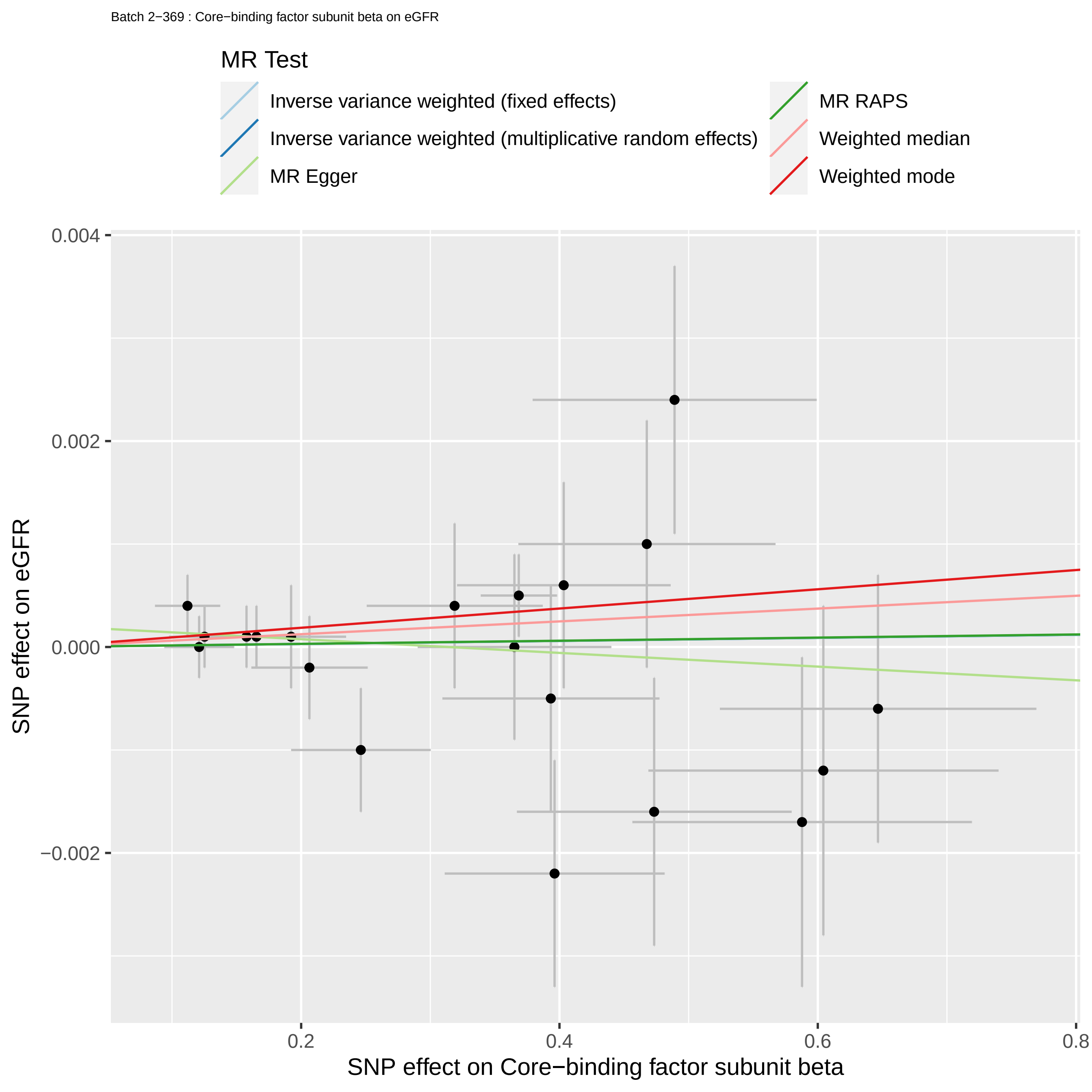

Batch 2−369 : Core−binding factor subunit beta on eGFR
MR Test
Inverse variance weighted (fixed effects)
Inverse variance weighted (multiplicative random effects)
MR Egger
MR RAPS
Weighted median
Weighted mode
0.004
0.002
0.000
−0.002
0.2
0.4
0.6
0.8
SNP effect on eGFR
SNP effect on Core−binding factor subunit beta

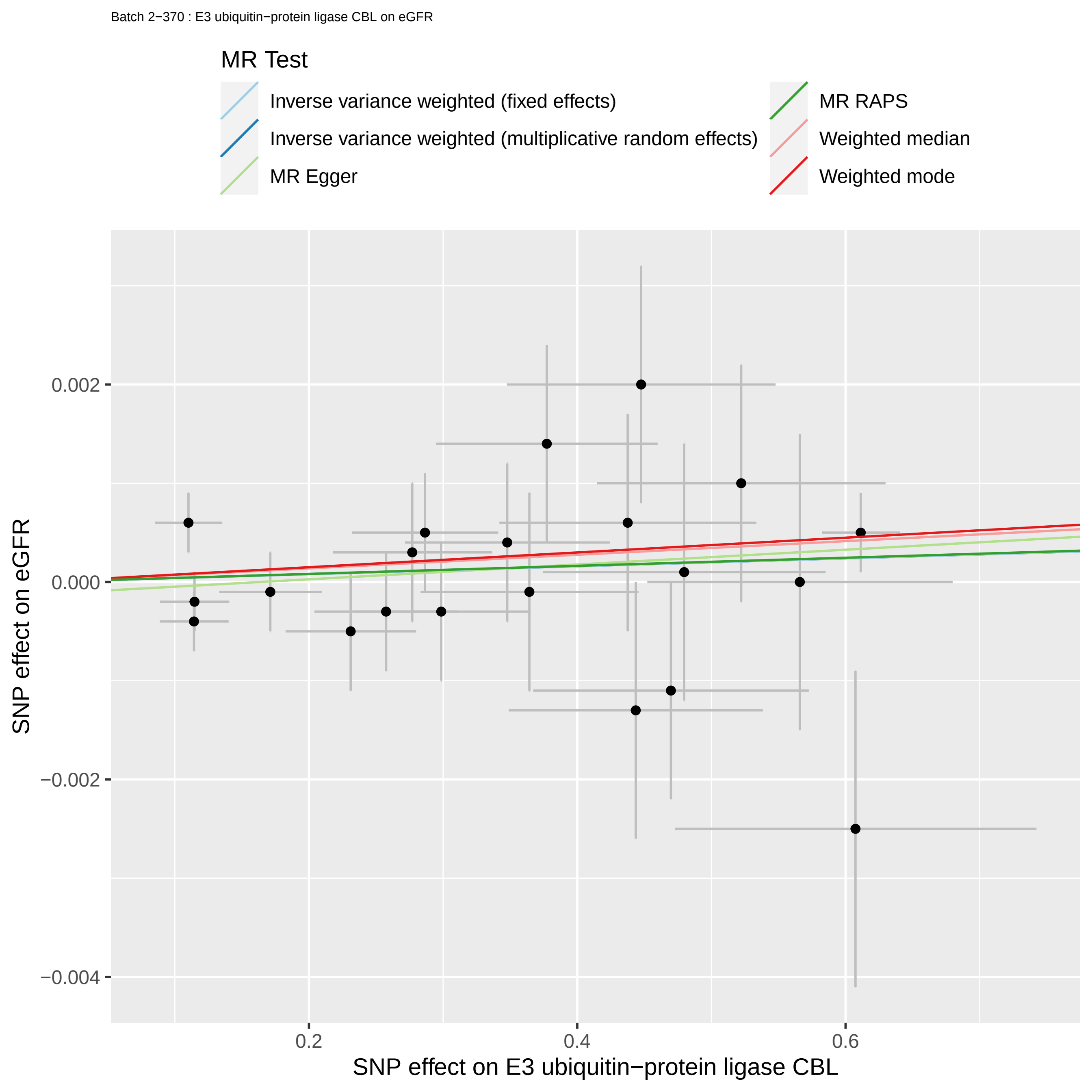
Batch 2−370 : E3 ubiquitin−protein ligase CBL on eGFR
MR Test
Inverse variance weighted (fixed effects)
Inverse variance weighted (multiplicative random effects)
MR Egger
MR RAPS
Weighted median
Weighted mode
0.002
0.000
−0.002
−0.004
0.2
0.4
0.6
SNP effect on eGFR
SNP effect on E3 ubiquitin−protein ligase CBL

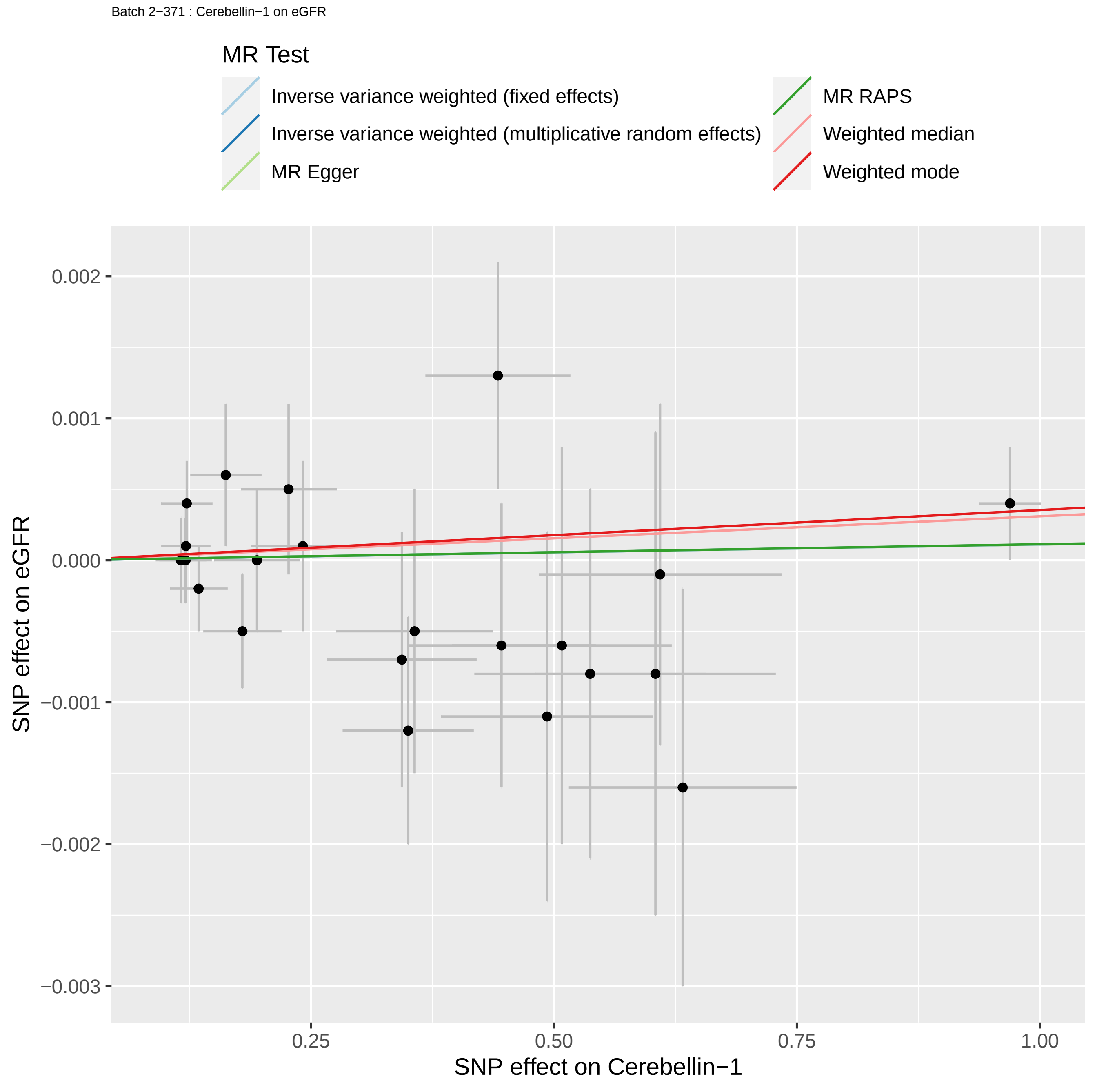

Batch 2−371 : Cerebellin−1 on eGFR
MR Test
Inverse variance weighted (fixed effects)
Inverse variance weighted (multiplicative random effects)
MR Egger
MR RAPS
Weighted median
Weighted mode
0.002
0.001
0.000
−0.001
−0.002
−0.003
SNP effect on eGFR
0.25
0.50
0.75
1.00
SNP effect on Cerebellin−1

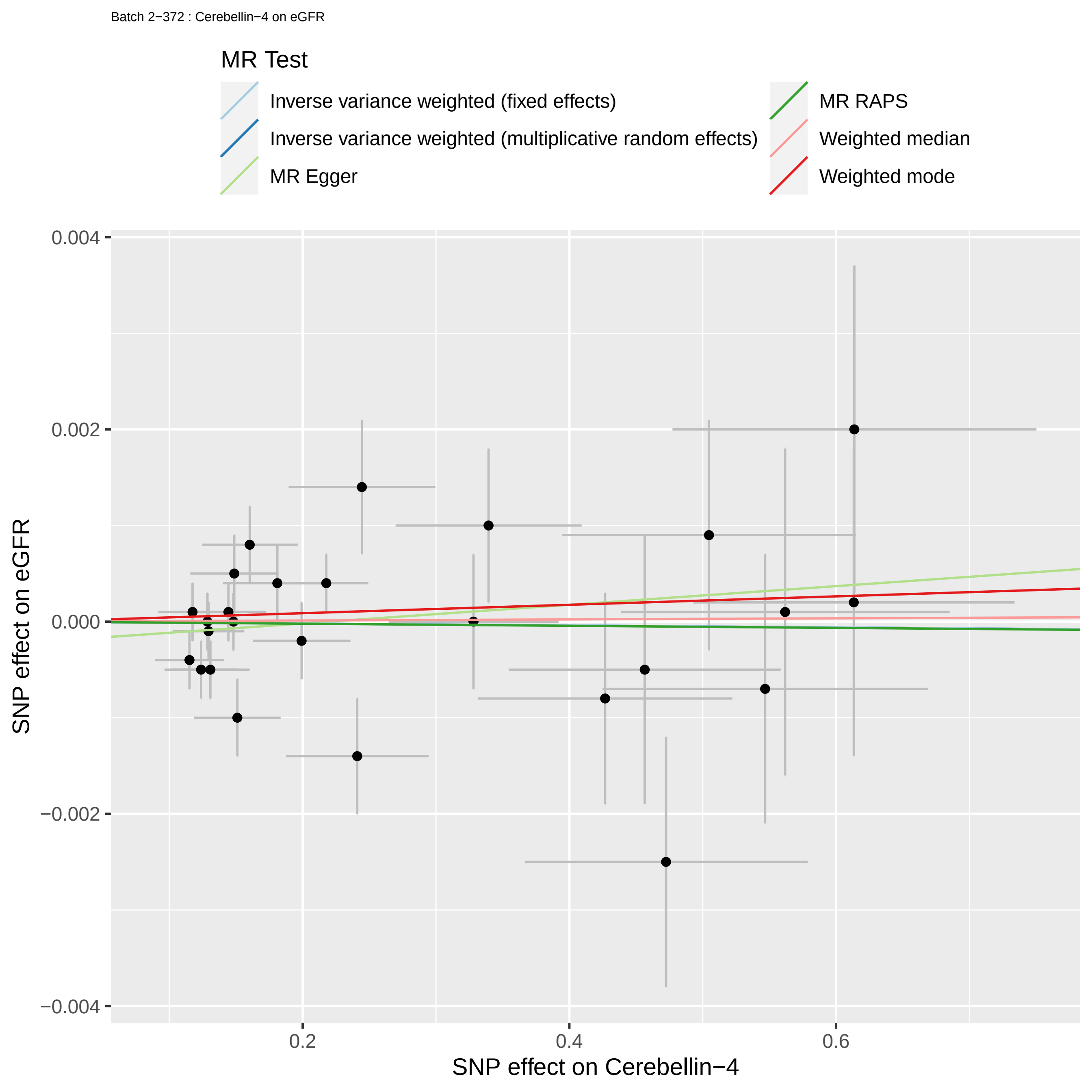
Batch 2−372 : Cerebellin−4 on eGFR
MR Test
Inverse variance weighted (fixed effects)
Inverse variance weighted (multiplicative random effects)
MR Egger
MR RAPS
Weighted median
Weighted mode
0.004
0.002
0.000
−0.002
−0.004
0.2
0.4
0.6
SNP effect on eGFR
SNP effect on Cerebellin−4

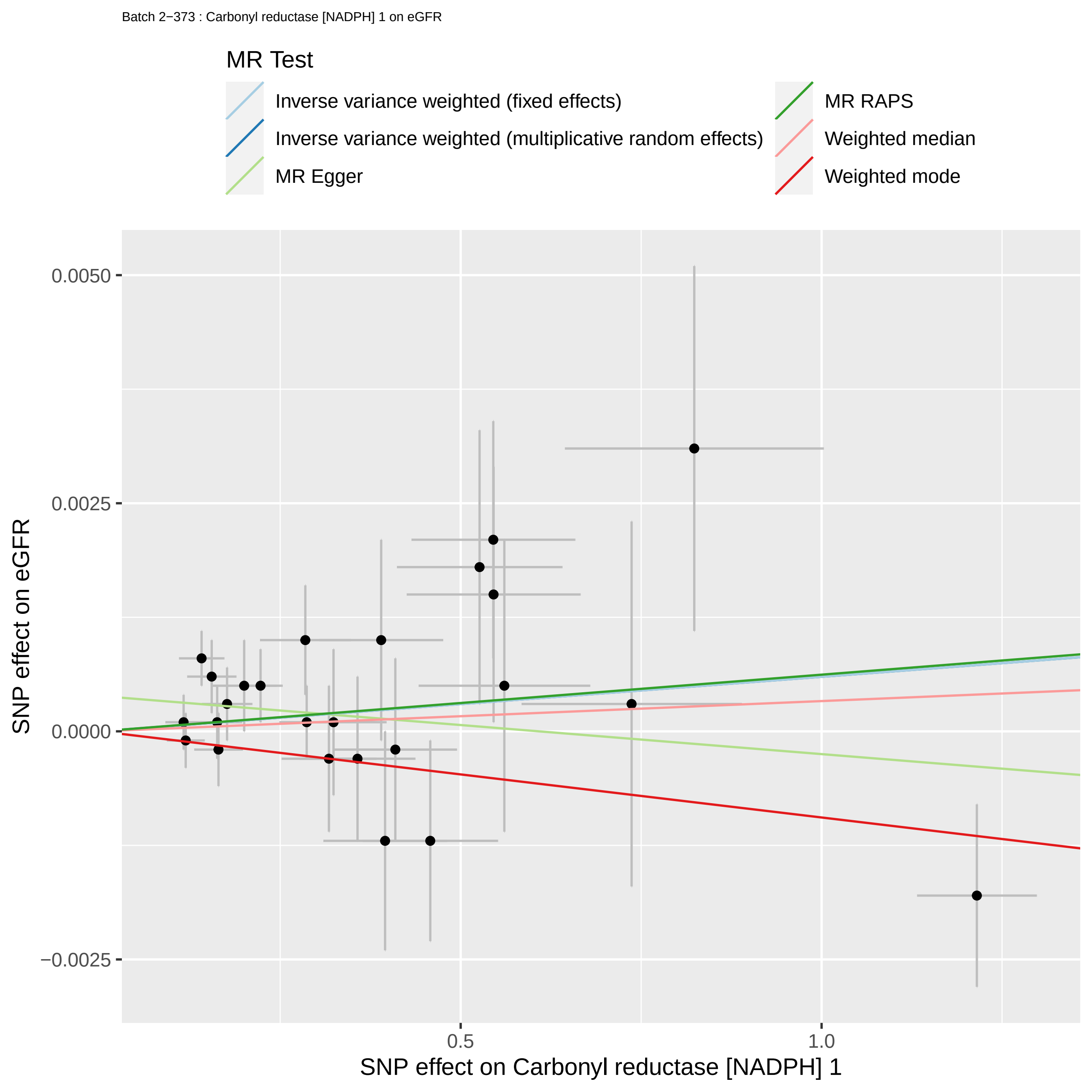

Batch 2−373 : Carbonyl reductase [NADPH] 1 on eGFR
MR Test
Inverse variance weighted (fixed effects)
Inverse variance weighted (multiplicative random effects)
MR Egger
MR RAPS
Weighted median
Weighted mode
0.0050
0.0025
0.0000
−0.0025
SNP effect on eGFR
0.5
1.0
SNP effect on Carbonyl reductase [NADPH] 1

Batch 2−374 : Carbonyl reductase [NADPH] 3 on eGFR

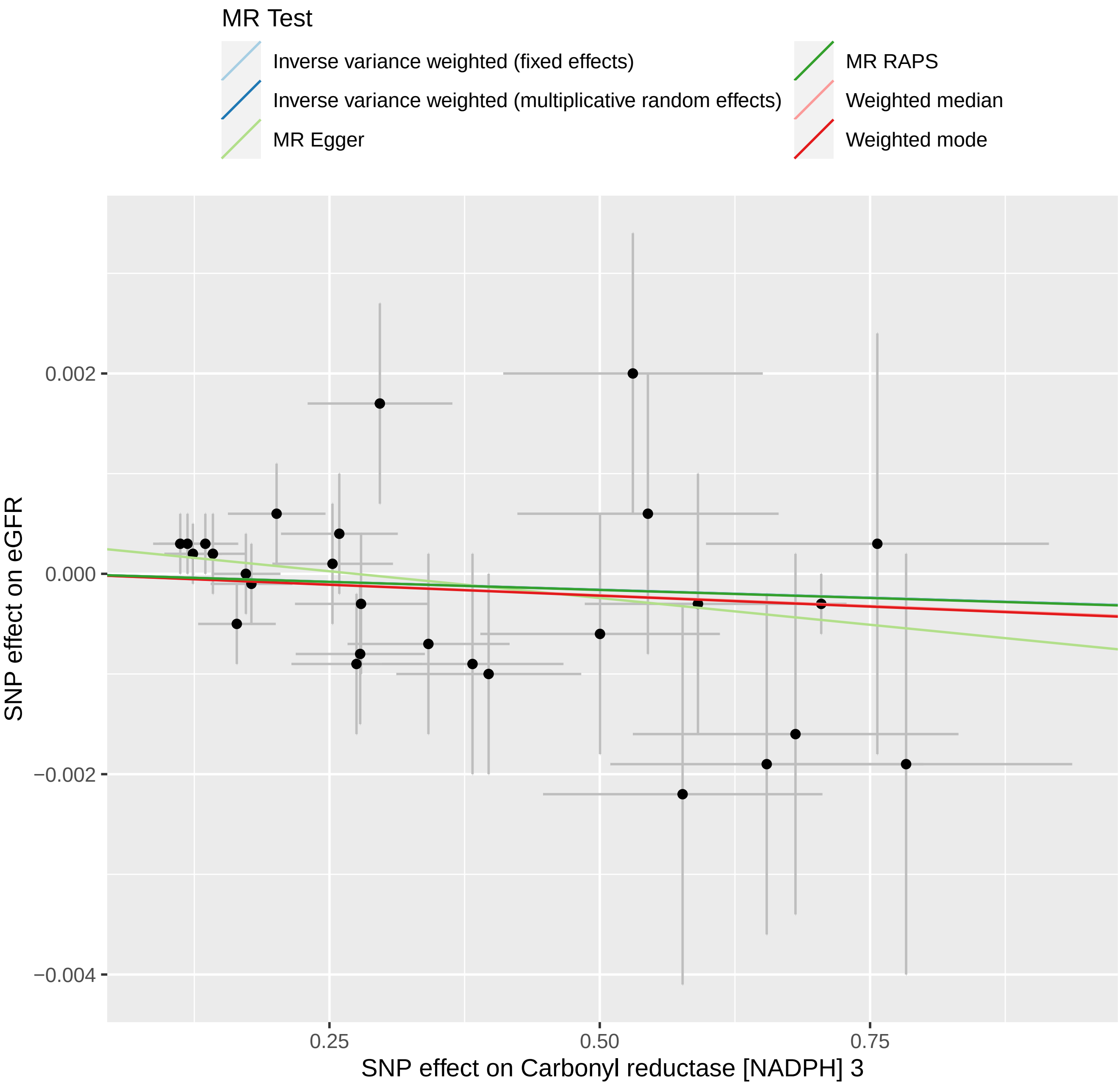

Batch 2−375 : Cystathionine beta−synthase on eGFR

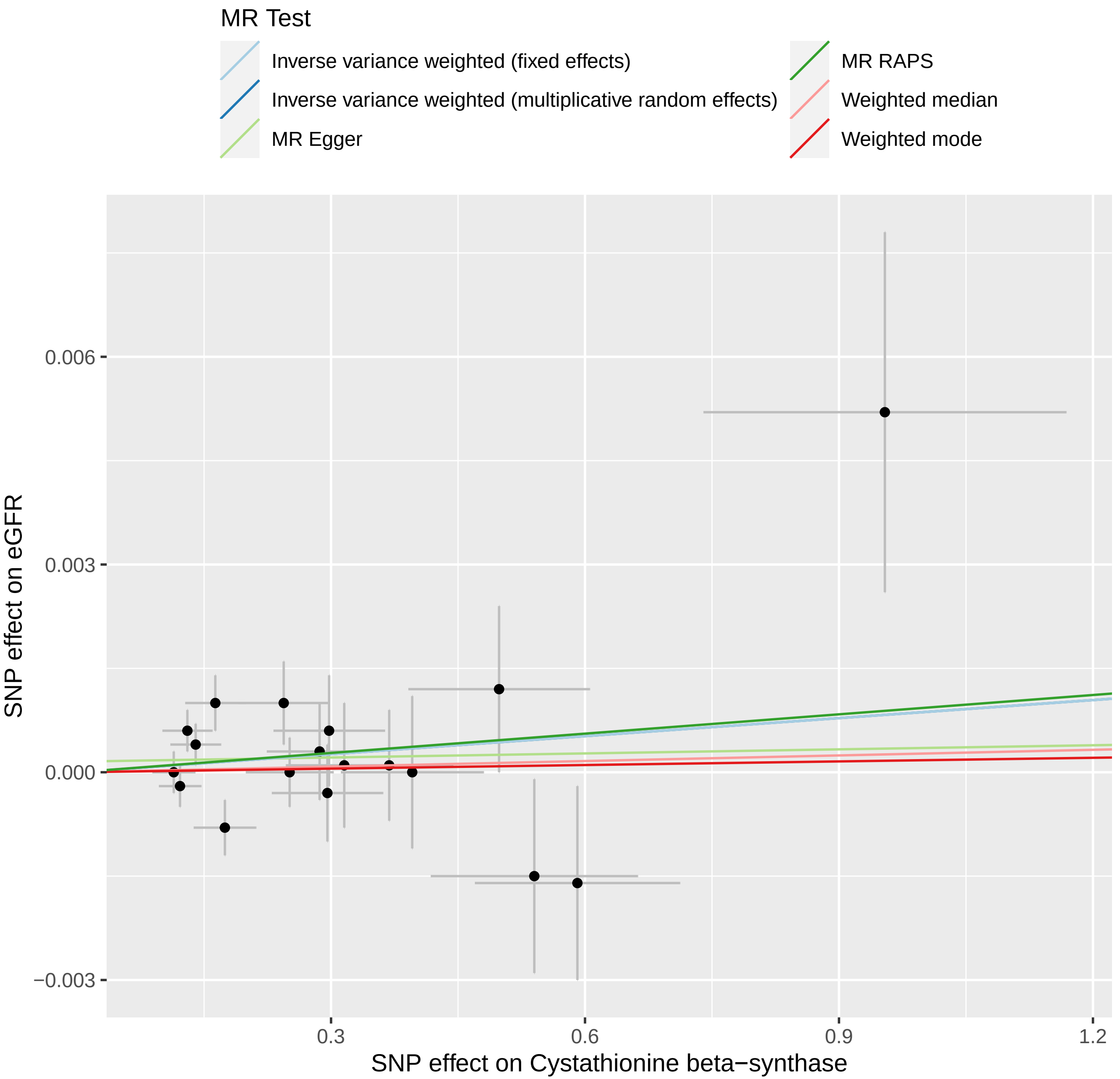

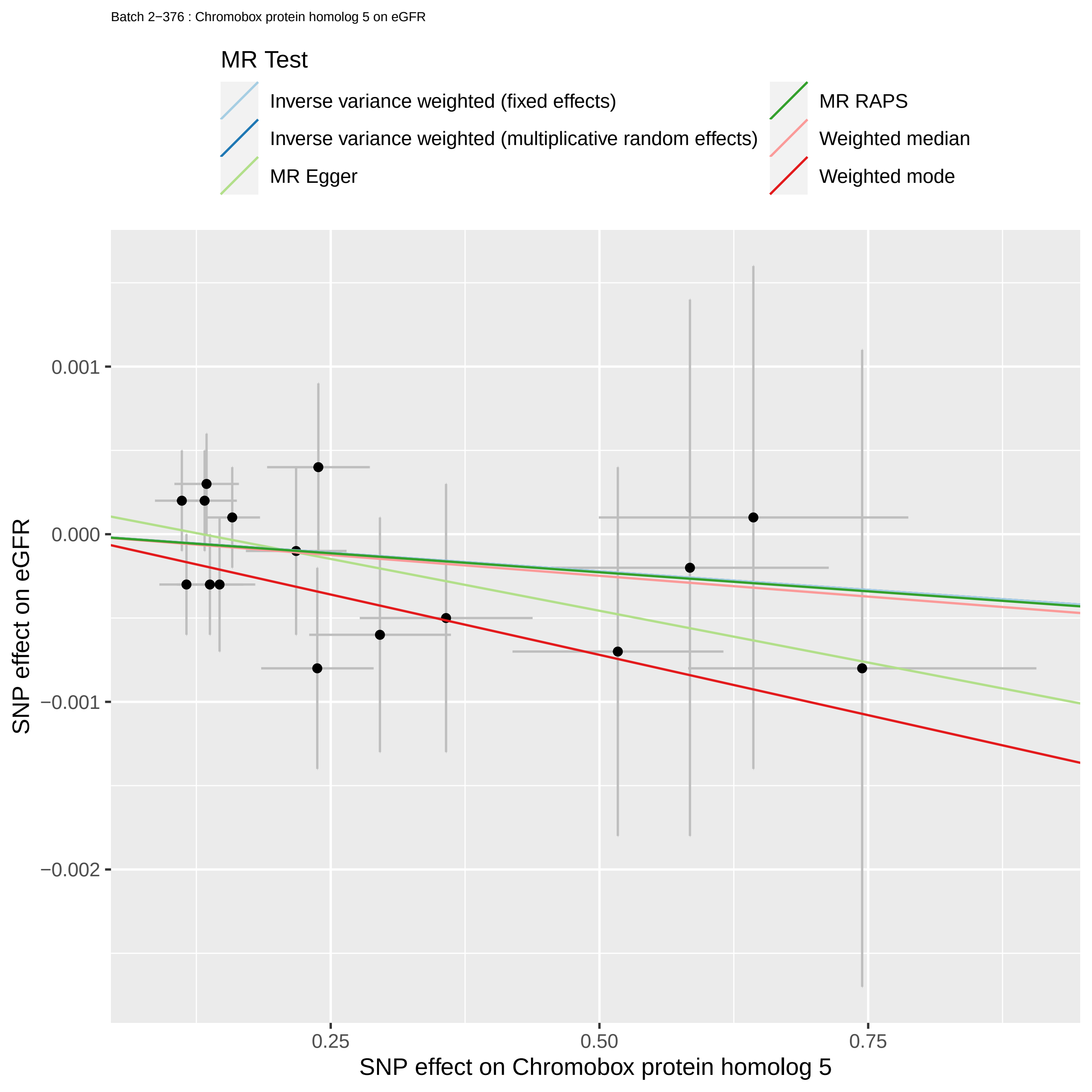
Batch 2−376 : Chromobox protein homolog 5 on eGFR
MR Test
Inverse variance weighted (fixed effects)
Inverse variance weighted (multiplicative random effects)
MR Egger
MR RAPS
Weighted median
Weighted mode
0.001
0.000
−0.001
−0.002
SNP effect on eGFR
0.25
0.50
0.75
SNP effect on Chromobox protein homolog 5

Batch 2−377 : Chromobox protein homolog 7 on eGFR

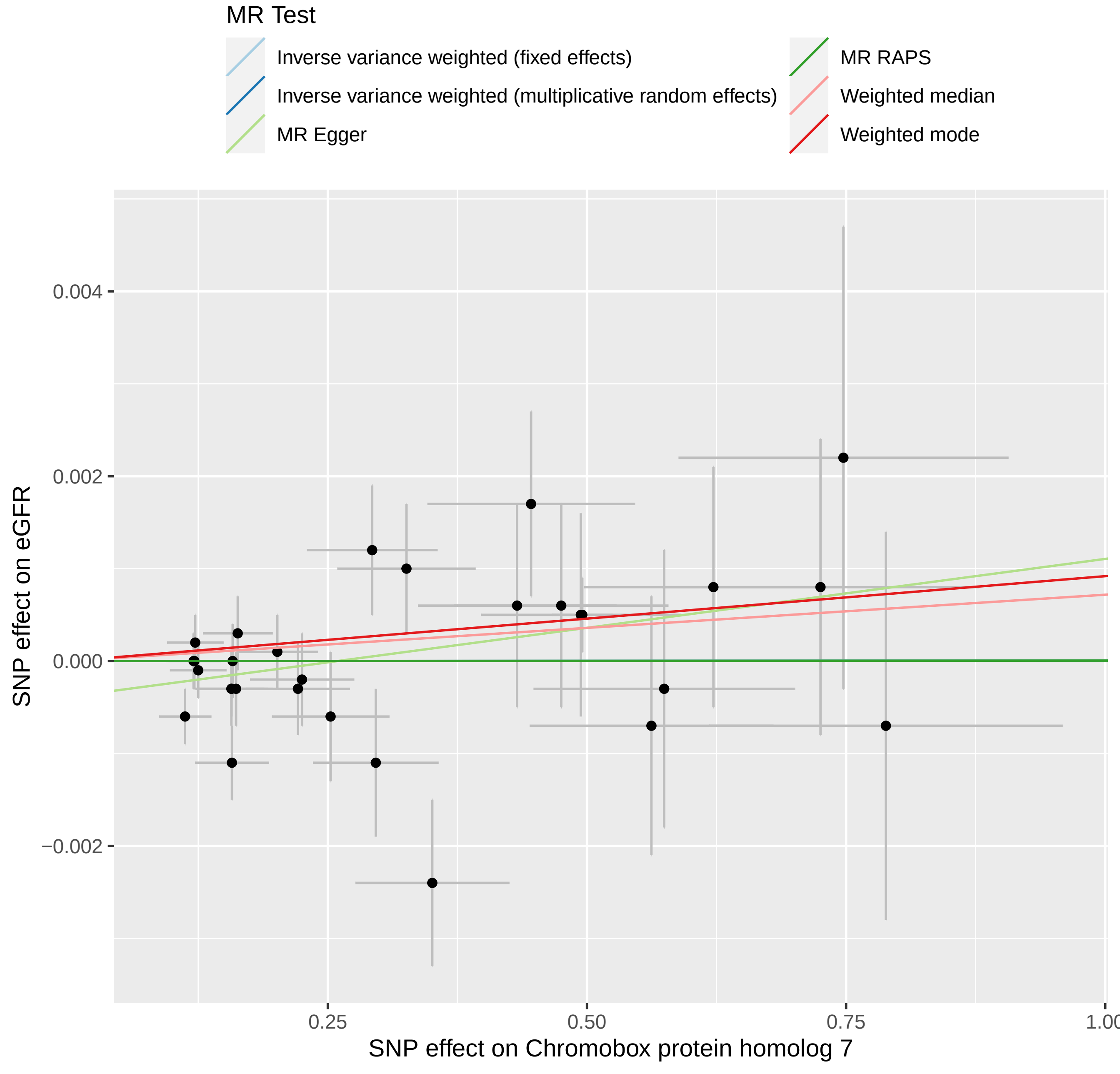

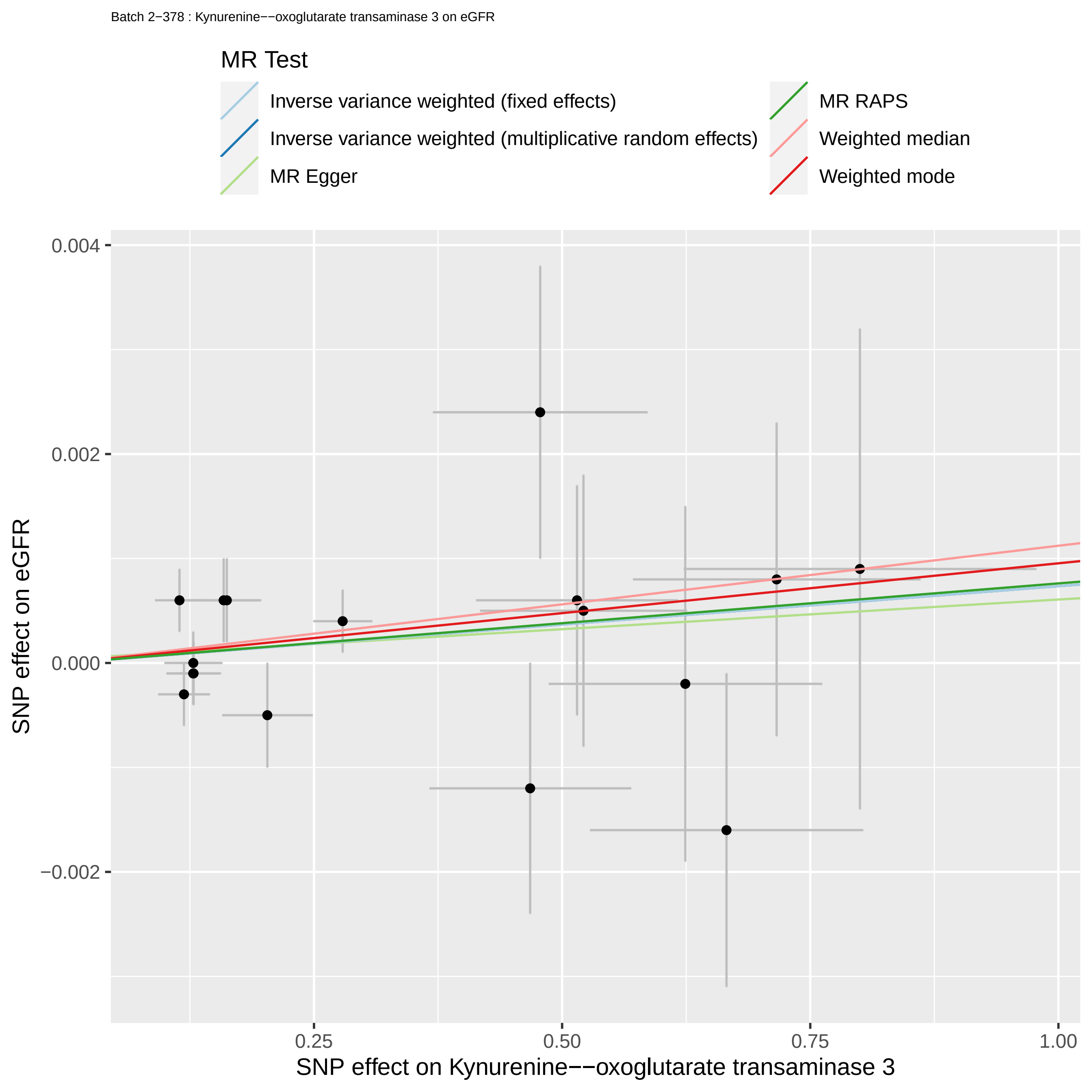

Batch 2−378 : Kynurenine−−oxoglutarate transaminase 3 on eGFR
MR Test
Inverse variance weighted (fixed effects)
Inverse variance weighted (multiplicative random effects)
MR Egger
MR RAPS
Weighted median
Weighted mode
0.004
0.002
0.000
−0.002
SNP effect on eGFR
0.25
0.50
0.75
1.00
SNP effect on Kynurenine−−oxoglutarate transaminase 3

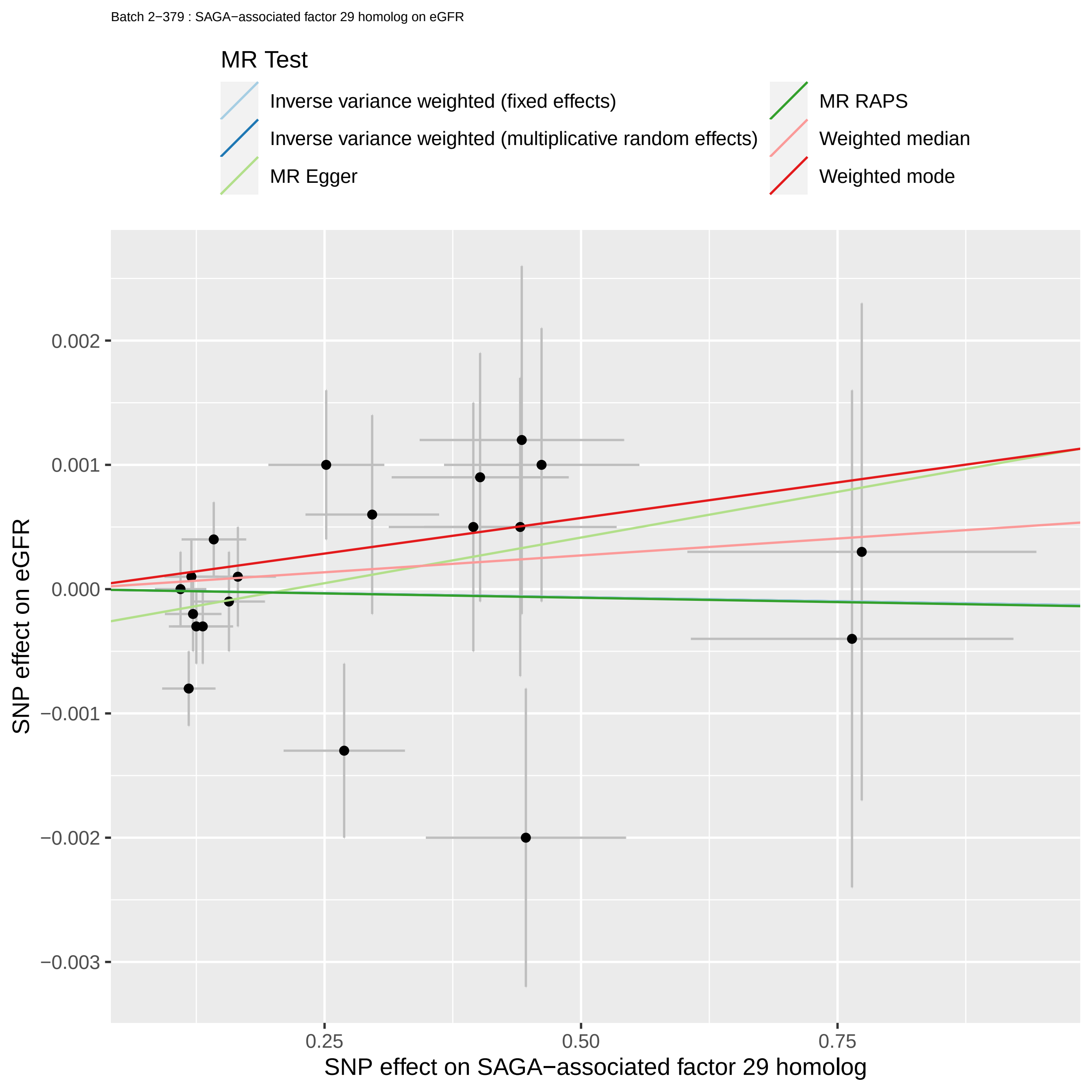
Batch 2−379 : SAGA−associated factor 29 homolog on eGFR
MR Test
Inverse variance weighted (fixed effects)
Inverse variance weighted (multiplicative random effects)
MR Egger
MR RAPS
Weighted median
Weighted mode
0.002
0.001
0.000
−0.001
−0.002
−0.003
SNP effect on eGFR
0.25
0.50
0.75
SNP effect on SAGA−associated factor 29 homolog

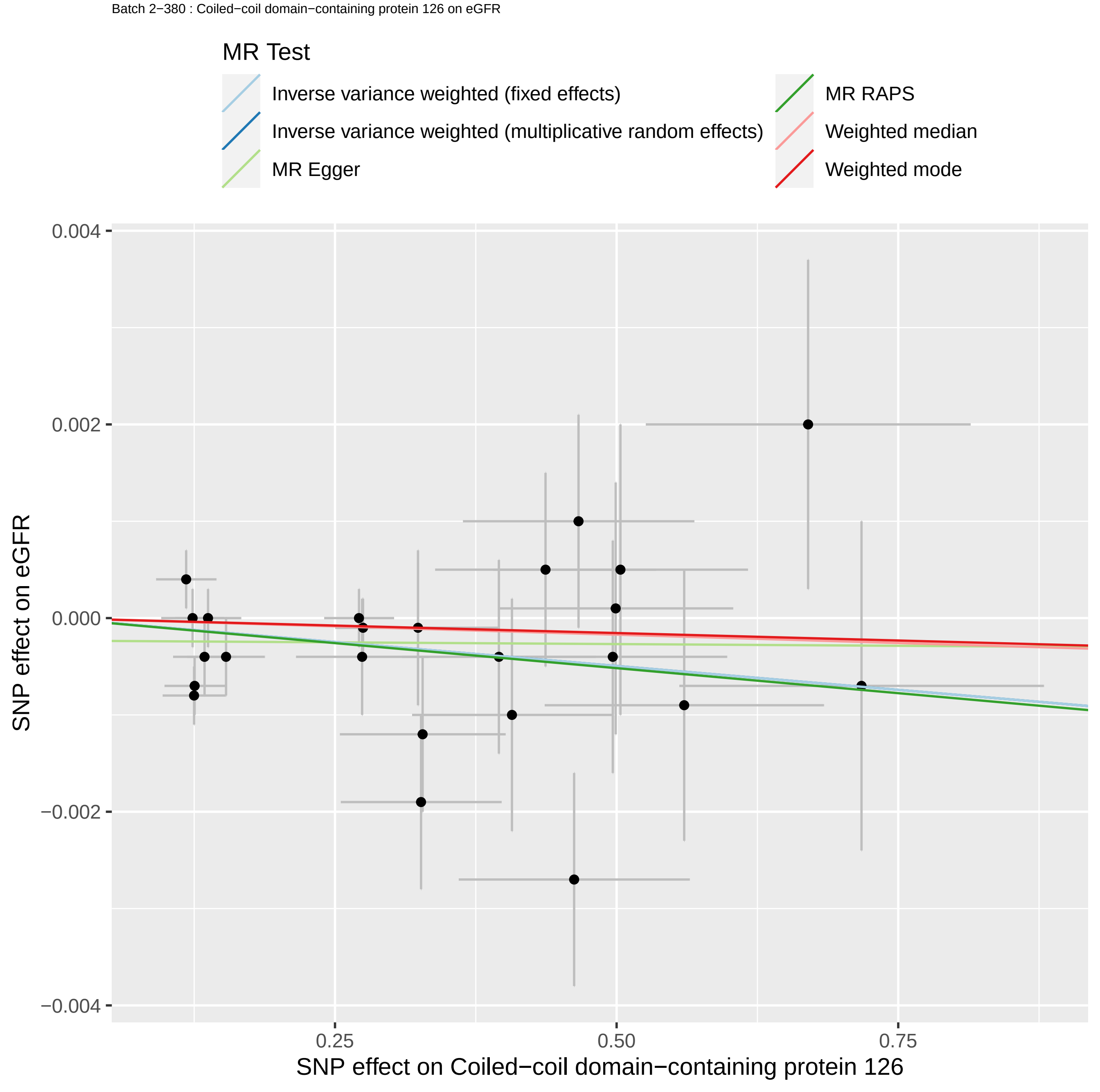

Batch 2−380 : Coiled−coil domain−containing protein 126 on eGFR
MR Test
Inverse variance weighted (fixed effects)
Inverse variance weighted (multiplicative random effects)
MR Egger
MR RAPS
Weighted median
Weighted mode
0.004
0.002
0.000
−0.002
−0.004
0.25
0.50
0.75
SNP effect on eGFR
SNP effect on Coiled−coil domain−containing protein 126

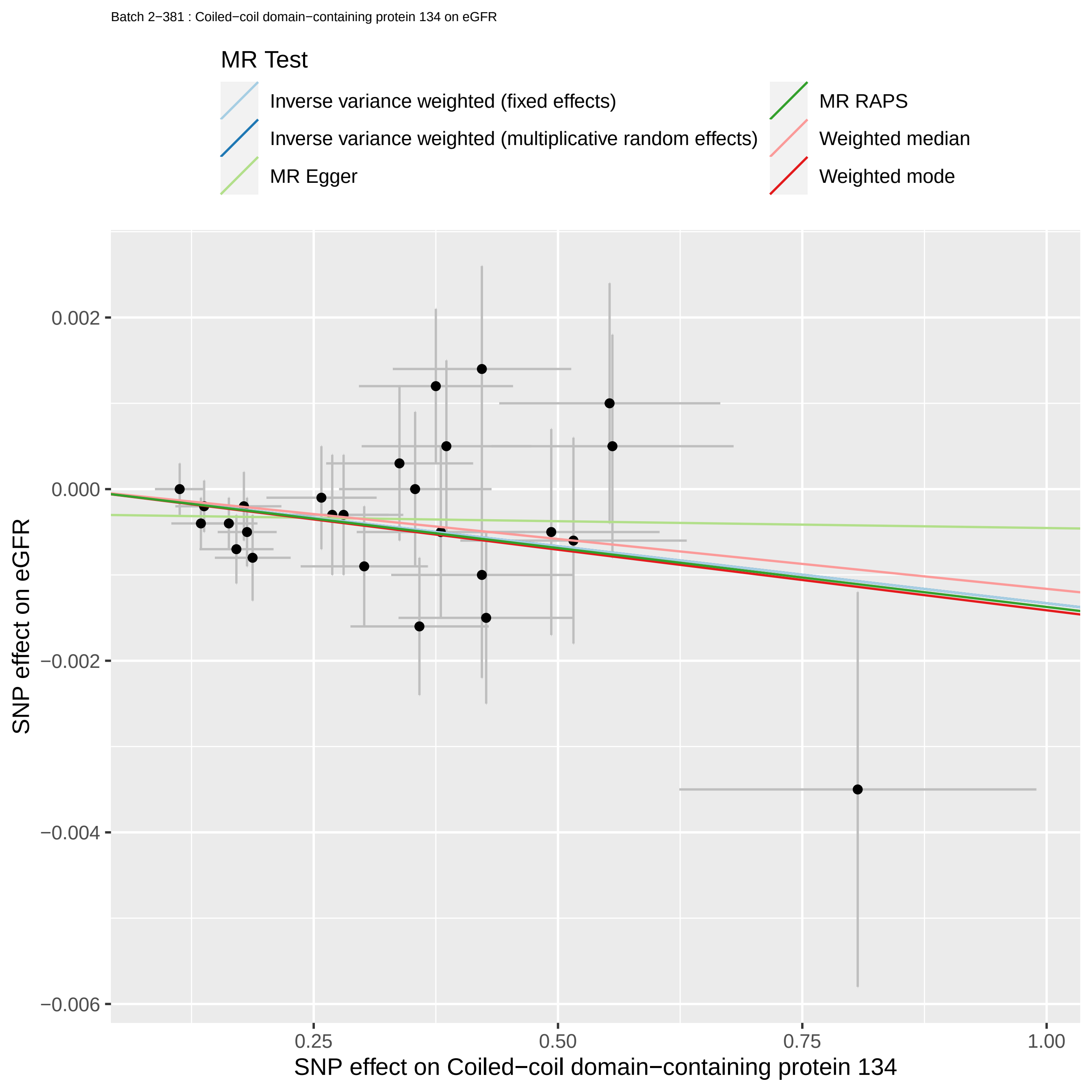

Batch 2−381 : Coiled−coil domain−containing protein 134 on eGFR
MR Test
Inverse variance weighted (fixed effects)
Inverse variance weighted (multiplicative random effects)
MR Egger
MR RAPS
Weighted median
Weighted mode
0.002
0.000
−0.002
−0.004
−0.006
SNP effect on eGFR
0.25
0.50
0.75
1.00
SNP effect on Coiled−coil domain−containing protein 134

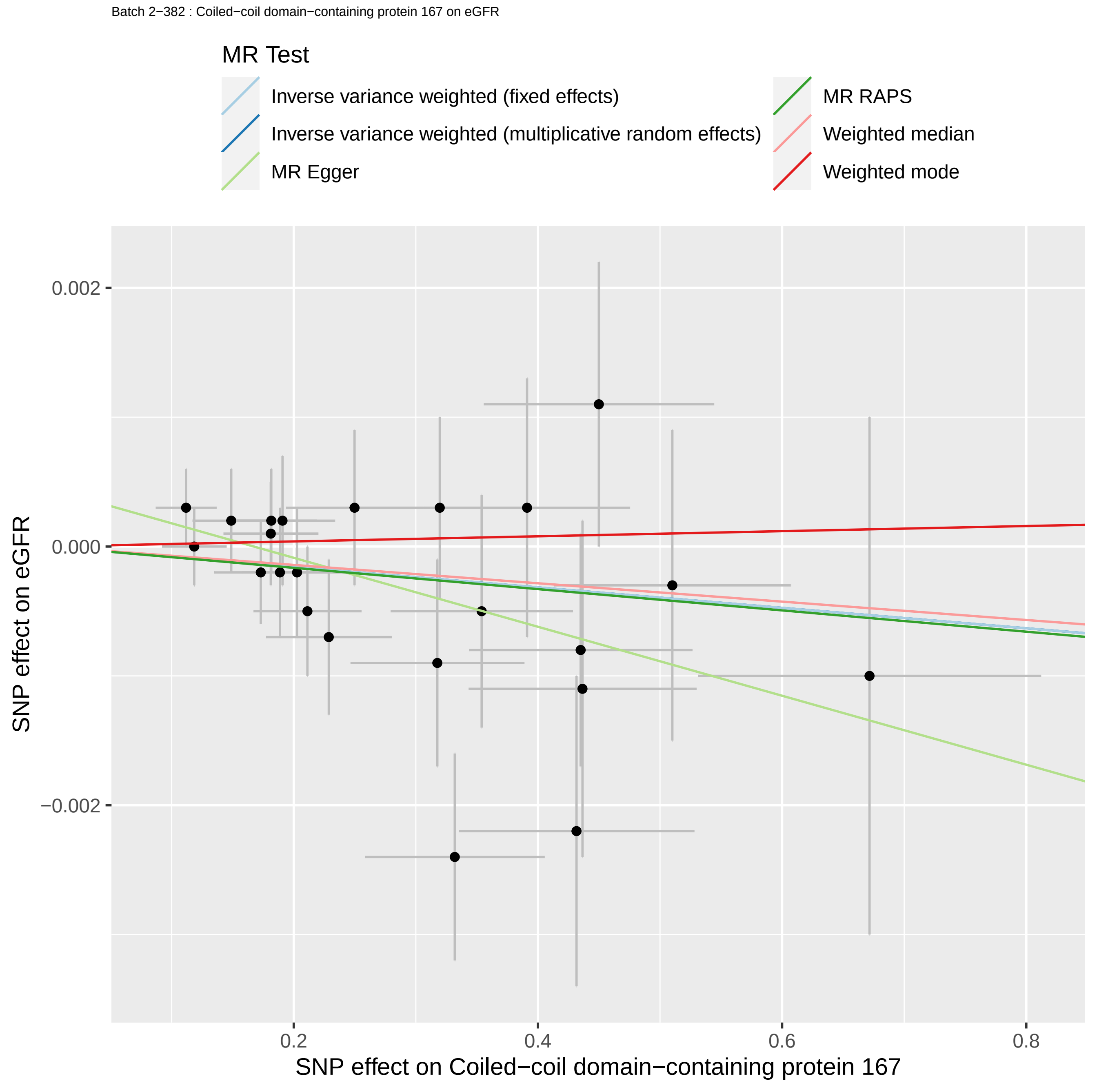
Batch 2−382 : Coiled−coil domain−containing protein 167 on eGFR
MR Test
Inverse variance weighted (fixed effects)
Inverse variance weighted (multiplicative random effects)
MR Egger
MR RAPS
Weighted median
Weighted mode
0.002
0.000
−0.002
0.2
0.4
0.6
0.8
SNP effect on eGFR
SNP effect on Coiled−coil domain−containing protein 167

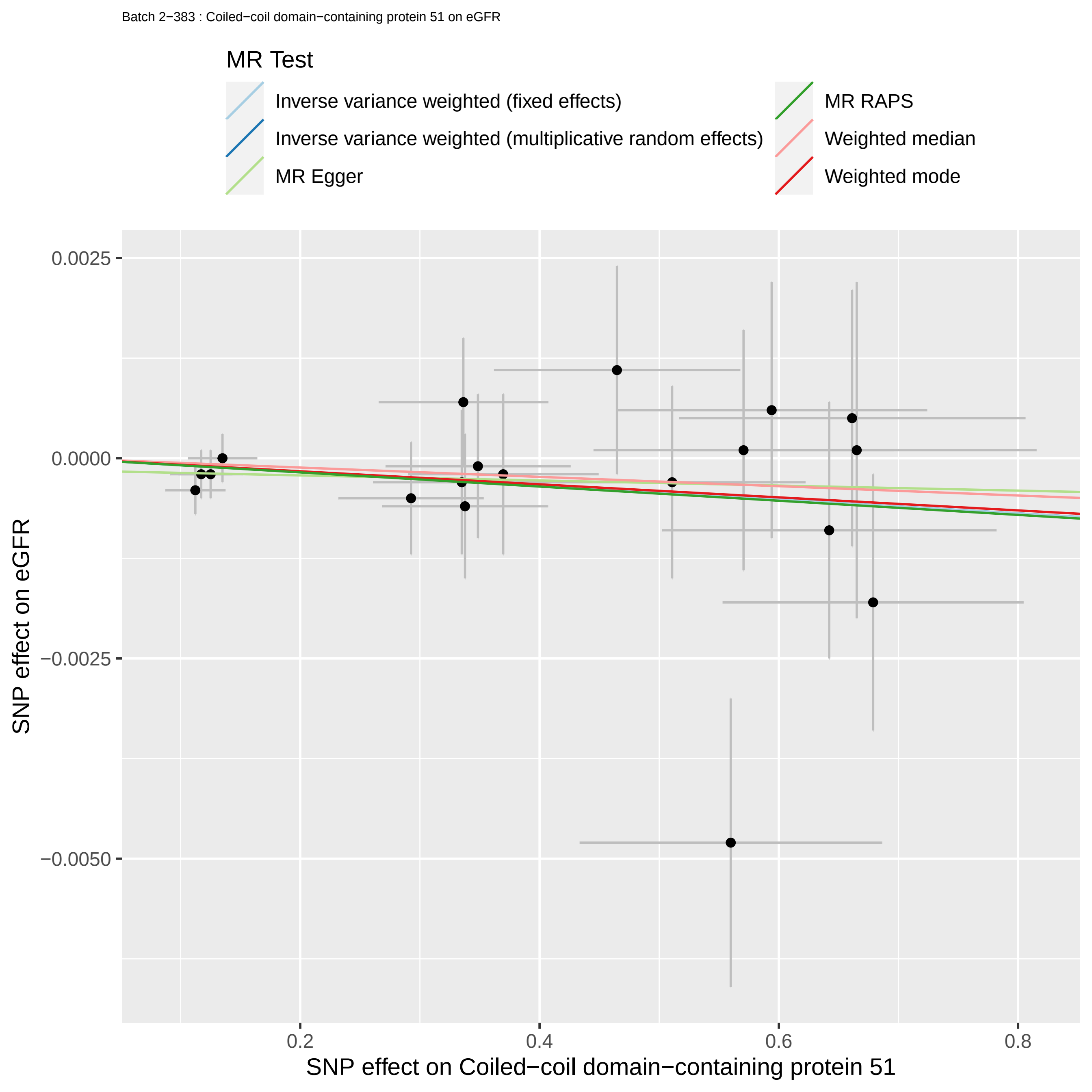

Batch 2−383 : Coiled−coil domain−containing protein 51 on eGFR
MR Test
Inverse variance weighted (fixed effects)
Inverse variance weighted (multiplicative random effects)
MR Egger
MR RAPS
Weighted median
Weighted mode
0.0025
0.0000
−0.0025
−0.0050
0.2
0.4
0.6
0.8
SNP effect on eGFR
SNP effect on Coiled−coil domain−containing protein 51

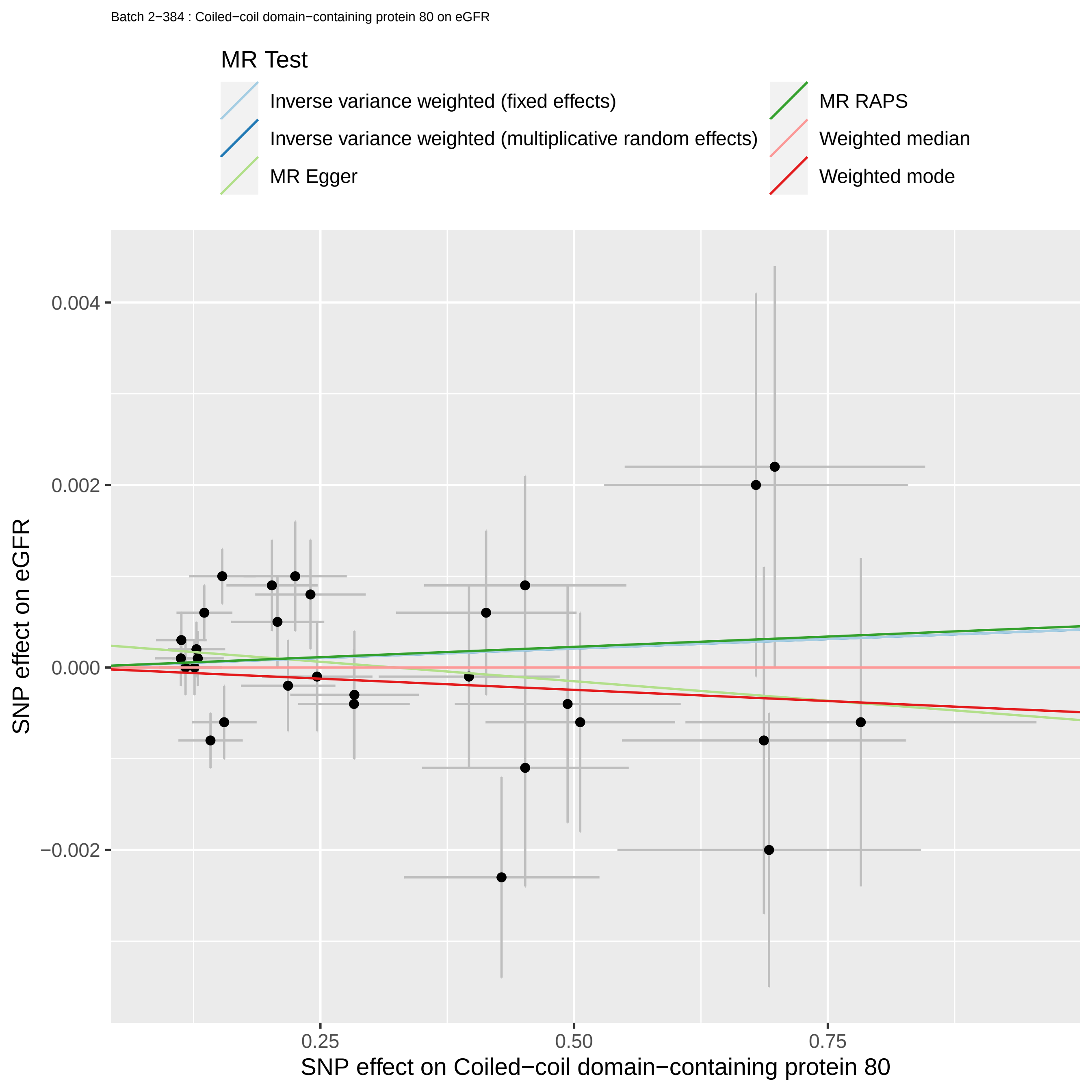

Batch 2−384 : Coiled−coil domain−containing protein 80 on eGFR
MR Test
Inverse variance weighted (fixed effects)
Inverse variance weighted (multiplicative random effects)
MR Egger
MR RAPS
Weighted median
Weighted mode
SNP effect on eGFR
0.004
0.002
0.000
−0.002
0.25
0.50
0.75
SNP effect on Coiled−coil domain−containing protein 80

Batch 2−385 : Coiled−coil domain−containing protein 90B, mitochondrial on eGFR

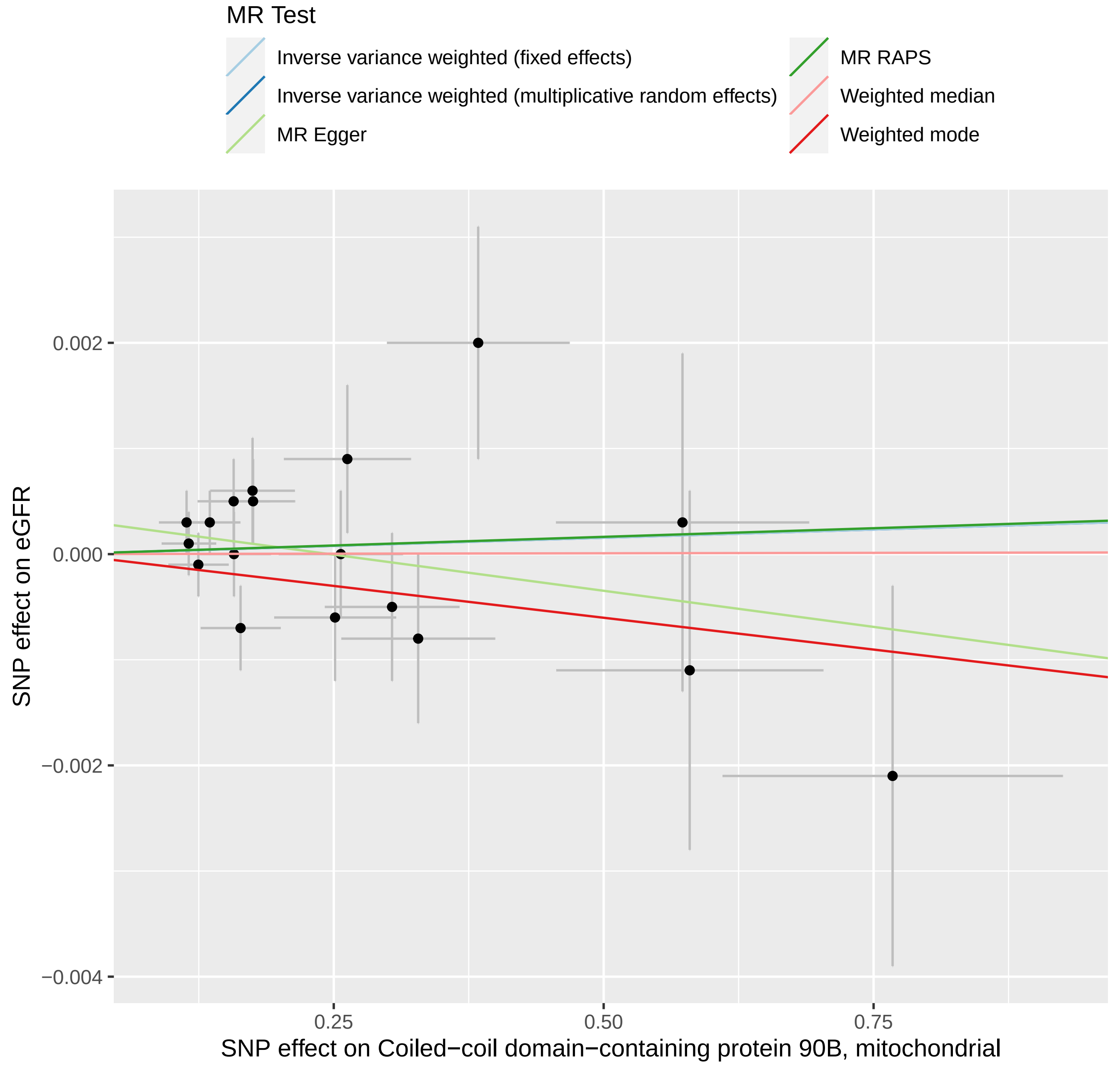

Batch 2−386 : C−C motif chemokine 1 on eGFR

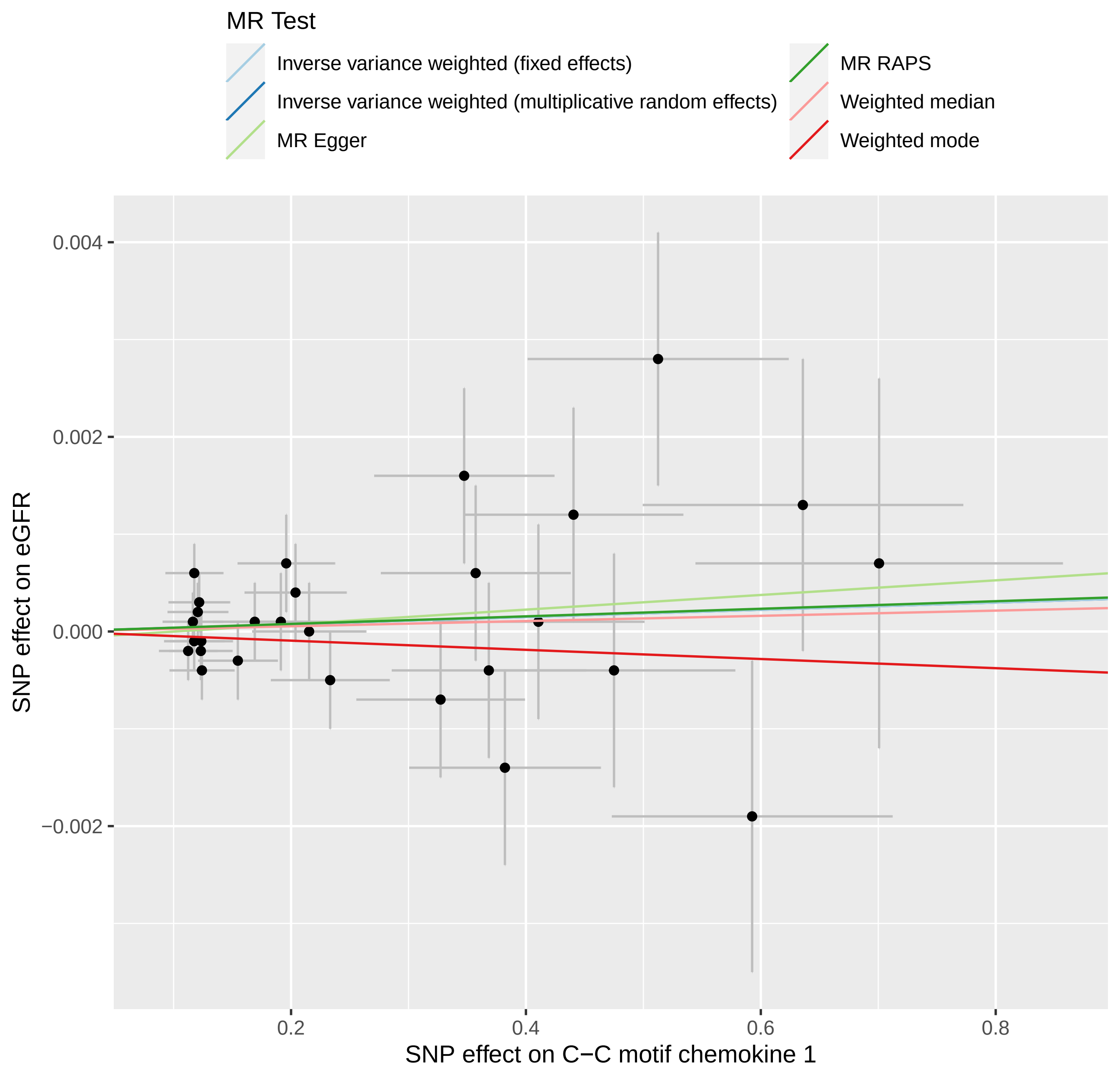

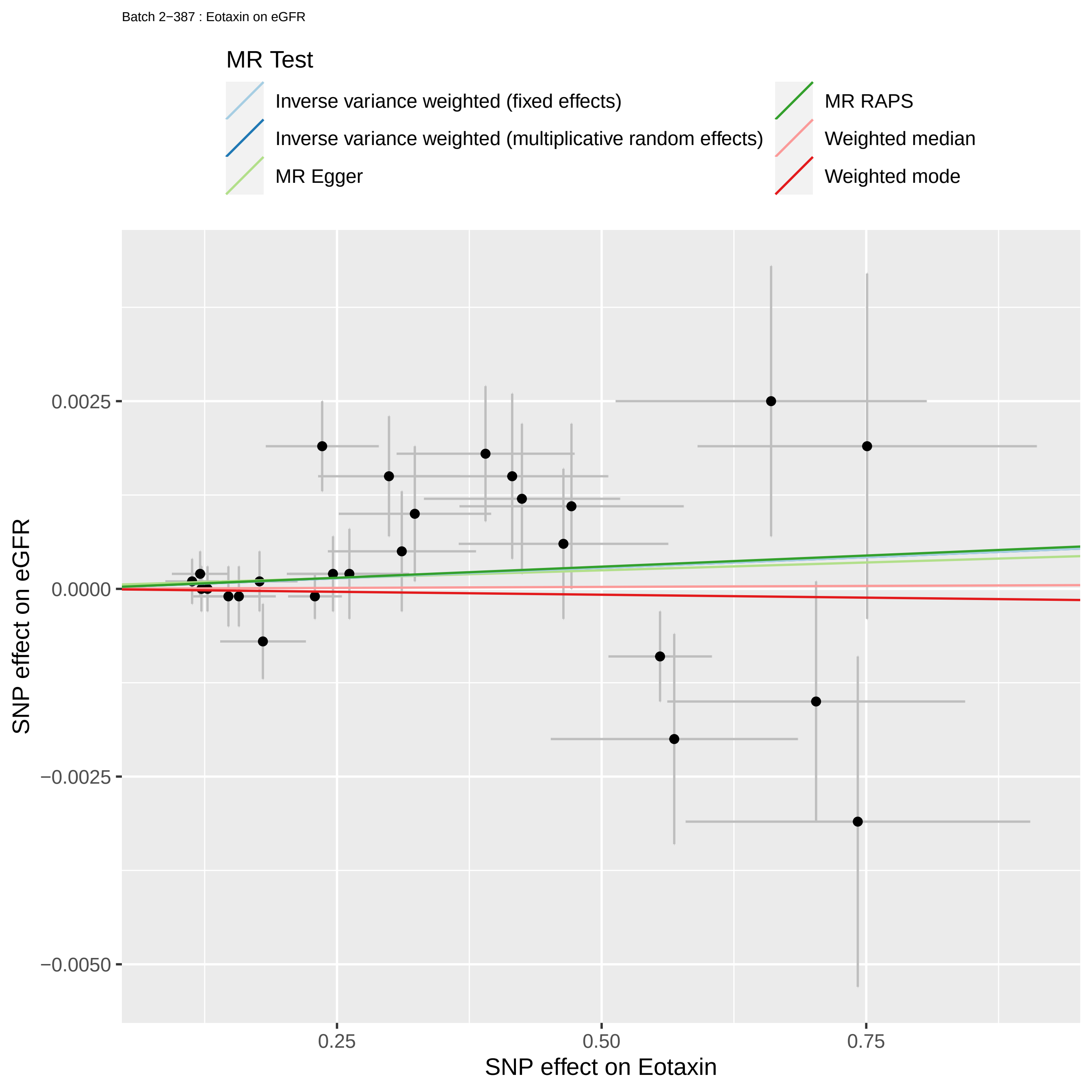

Batch 2−387 : Eotaxin on eGFR
MR Test
Inverse variance weighted (fixed effects)
Inverse variance weighted (multiplicative random effects)
MR Egger
MR RAPS
Weighted median
Weighted mode
0.0025
0.0000
−0.0025
−0.0050
SNP effect on eGFR
0.25
0.50
0.75
SNP effect on Eotaxin

Batch 2−388 : C−C motif chemokine 1 on eGFR

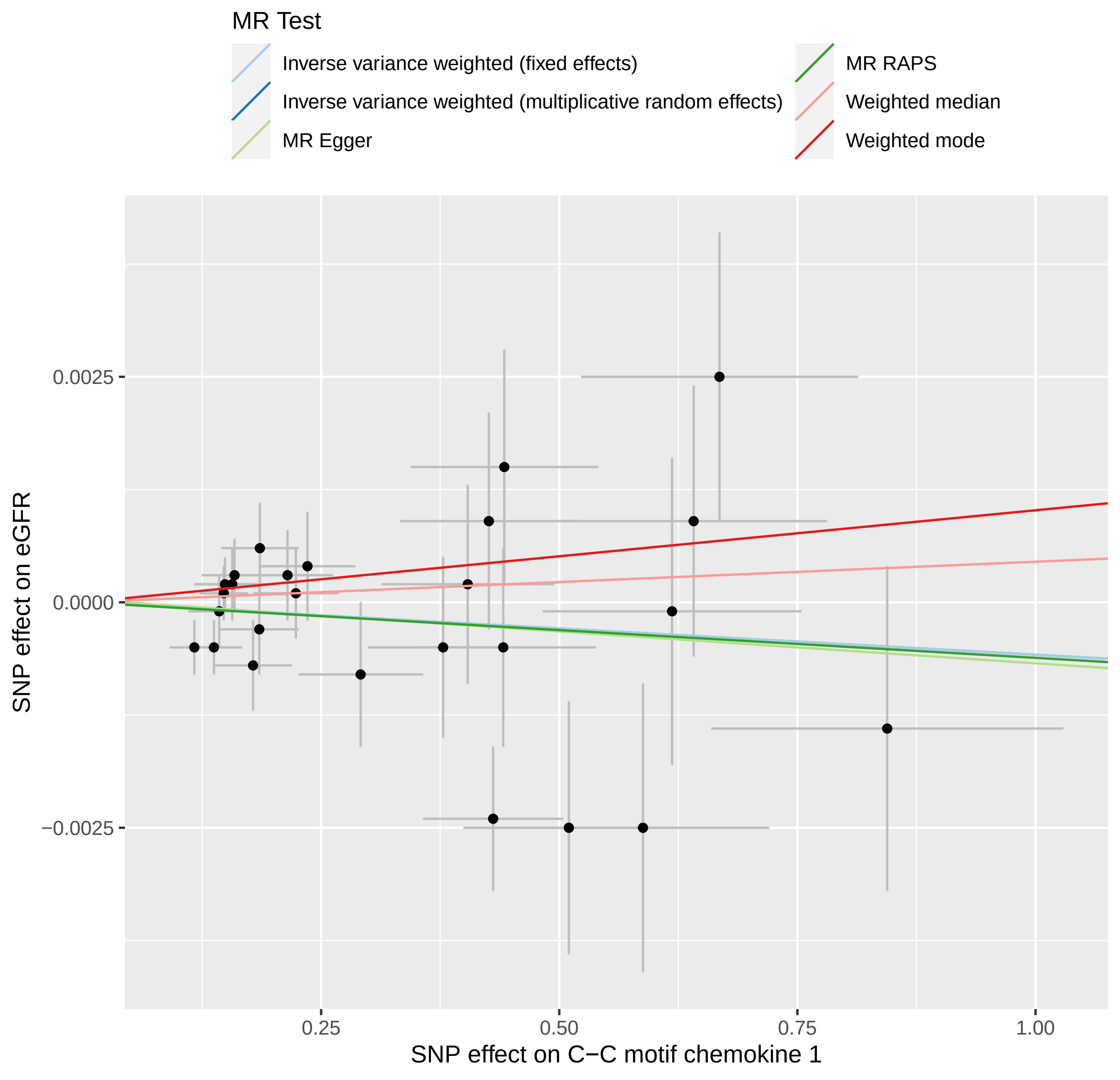

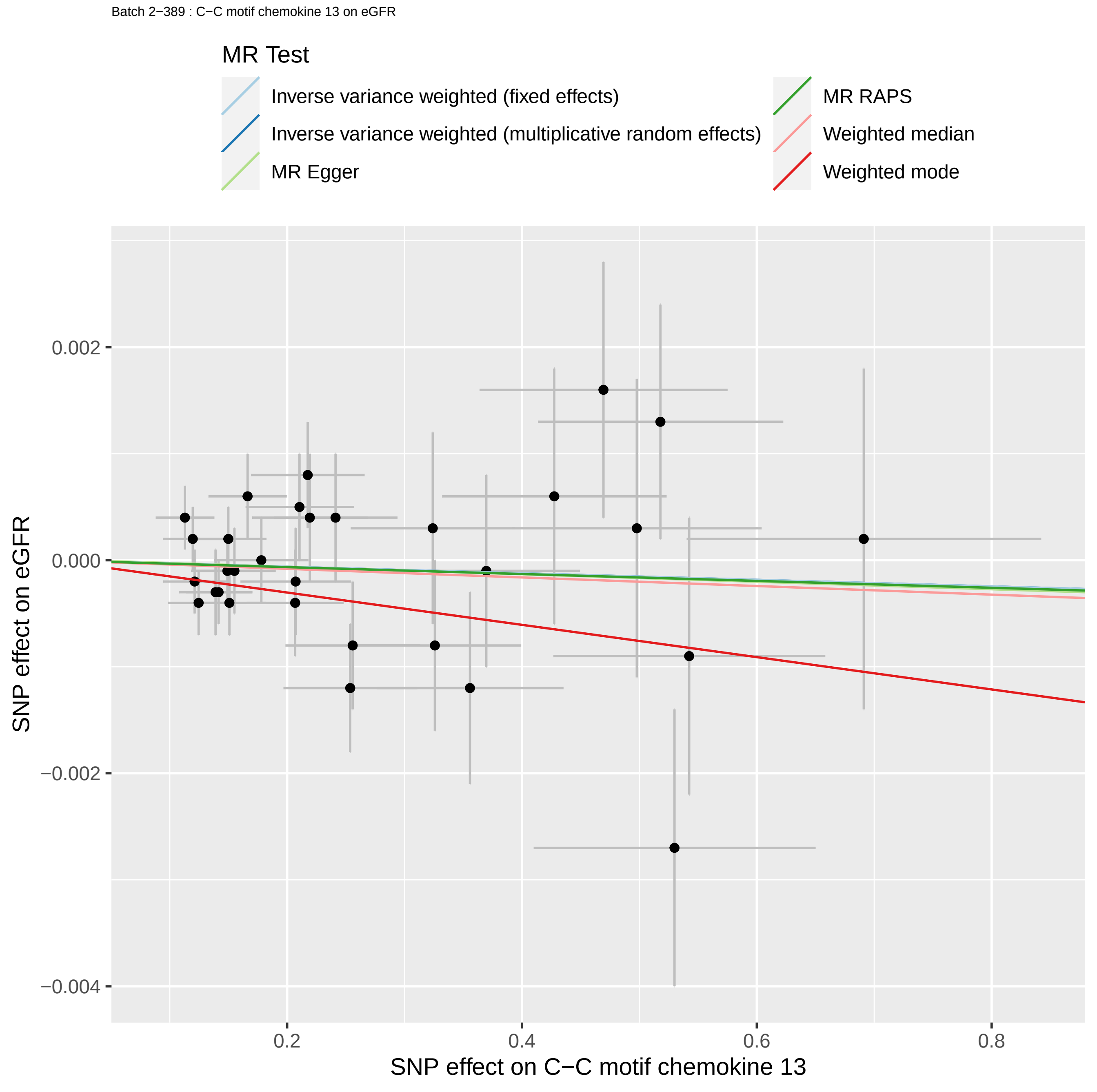

Batch 2−389 : C−C motif chemokine 13 on eGFR
MR Test
Inverse variance weighted (fixed effects)
Inverse variance weighted (multiplicative random effects)
MR Egger
MR RAPS
Weighted median
Weighted mode
0.002
0.000
−0.002
−0.004
0.2
0.4
0.6
0.8
SNP effect on eGFR
SNP effect on C−C motif chemokine 13

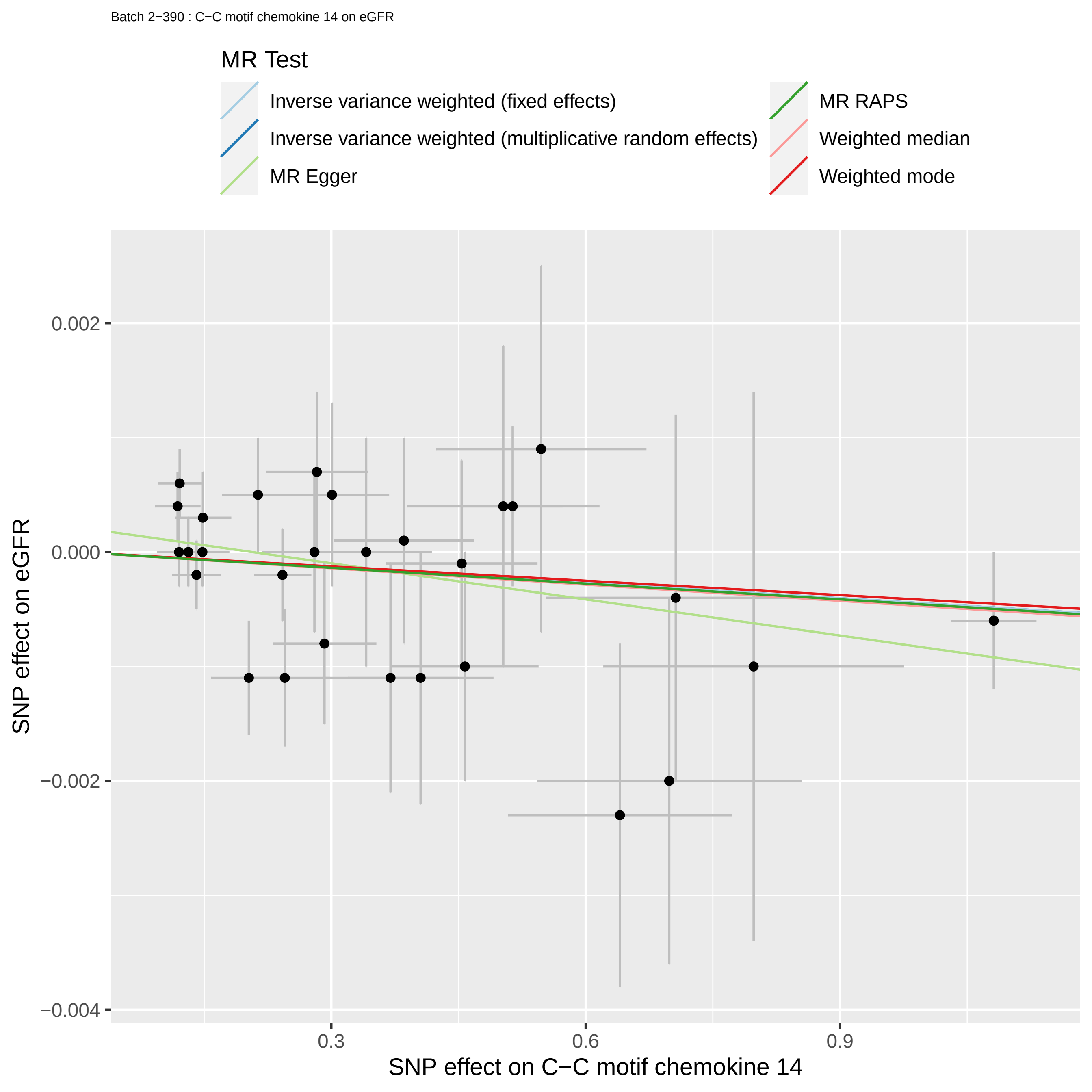
Batch 2−390 : C−C motif chemokine 14 on eGFR
MR Test
Inverse variance weighted (fixed effects)
Inverse variance weighted (multiplicative random effects)
MR Egger
MR RAPS
Weighted median
Weighted mode
0.002
0.000
−0.002
−0.004
0.3
0.6
0.9
SNP effect on eGFR
SNP effect on C−C motif chemokine 14

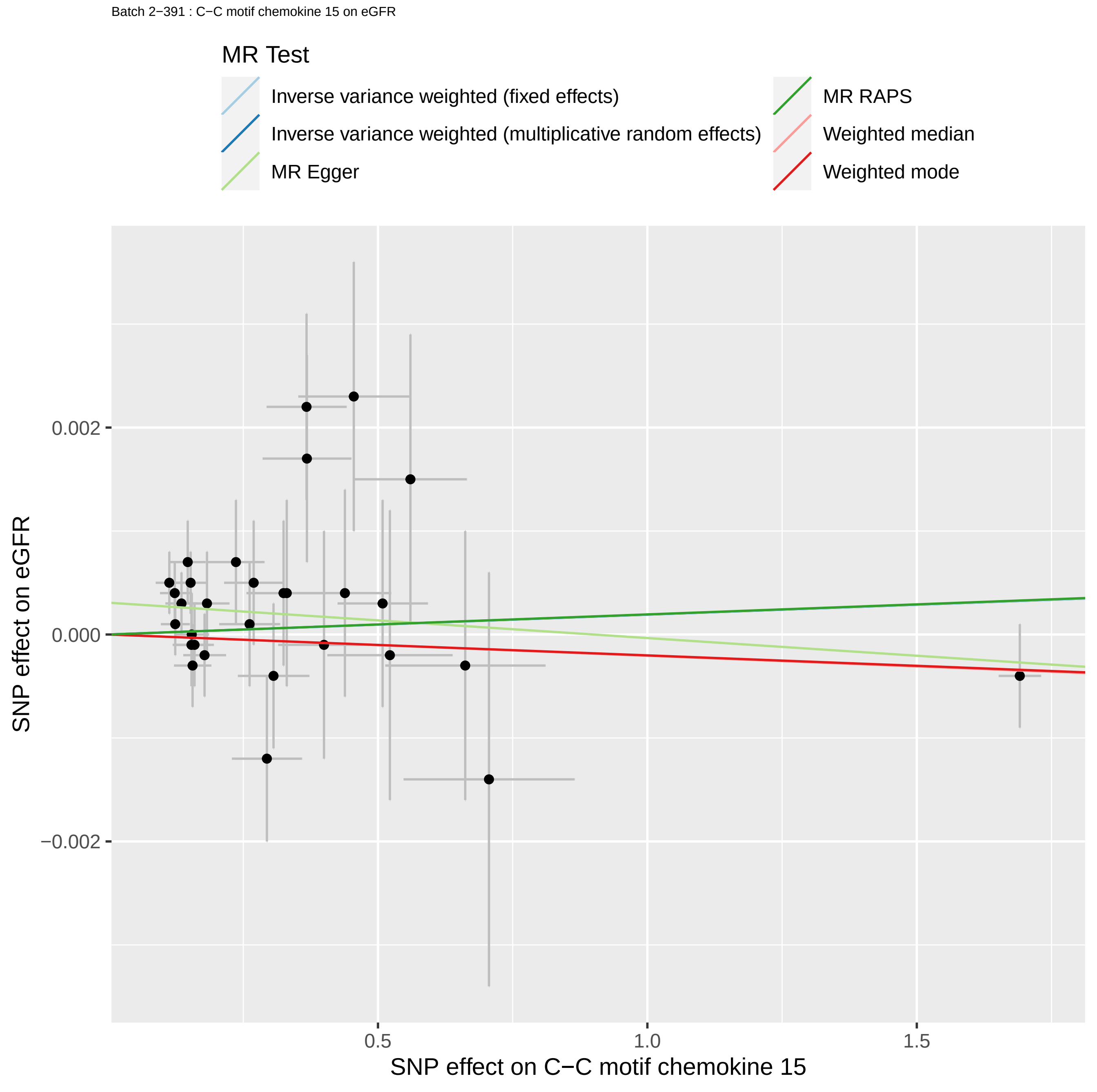

Batch 2−391 : C−C motif chemokine 15 on eGFR
MR Test
Inverse variance weighted (fixed effects)
Inverse variance weighted (multiplicative random effects)
MR Egger
MR RAPS
Weighted median
Weighted mode
0.002
0.000
−0.002
0.5
1.0
1.5
SNP effect on eGFR
SNP effect on C−C motif chemokine 15

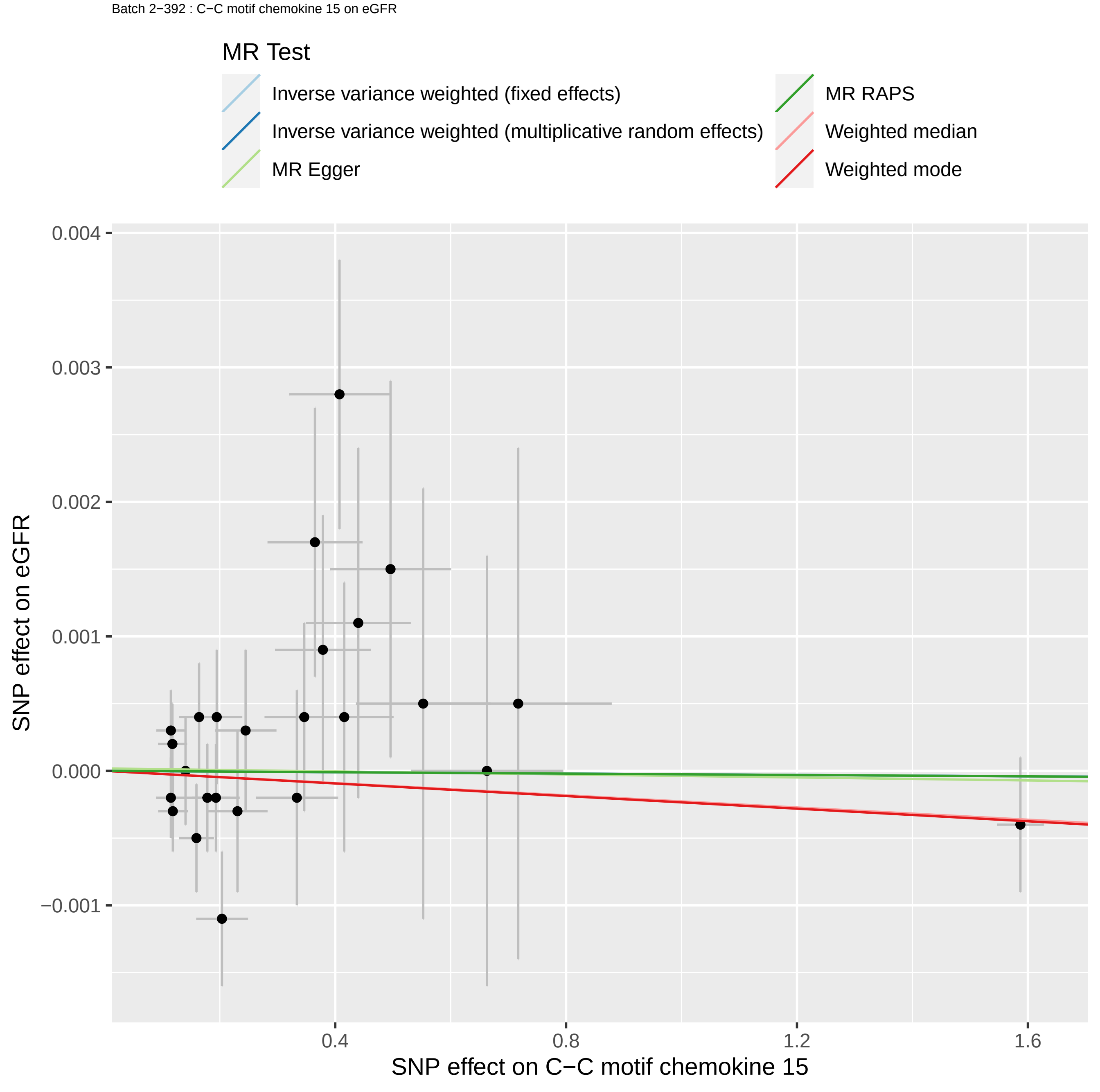

Batch 2−392 : C−C motif chemokine 15 on eGFR
MR Test
Inverse variance weighted (fixed effects)
Inverse variance weighted (multiplicative random effects)
MR Egger
MR RAPS
Weighted median
Weighted mode
0.004
0.003
0.002
0.001
0.000
−0.001
SNP effect on eGFR
0.4
0.8
1.2
1.6
SNP effect on C−C motif chemokine 15

Batch 2−393 : C−C motif chemokine 16 on eGFR

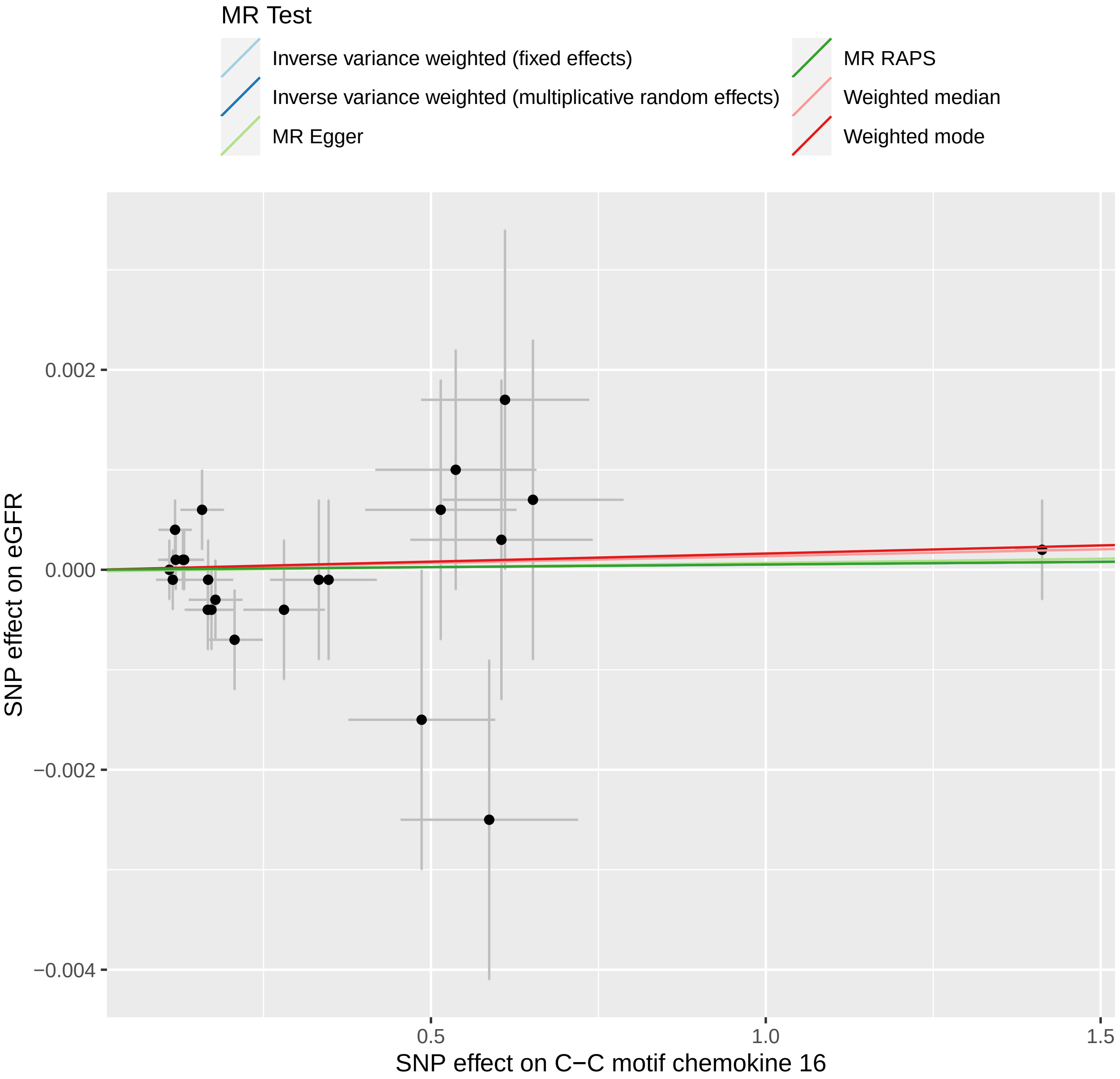

Batch 2−394 : C−C motif chemokine 17 on eGFR

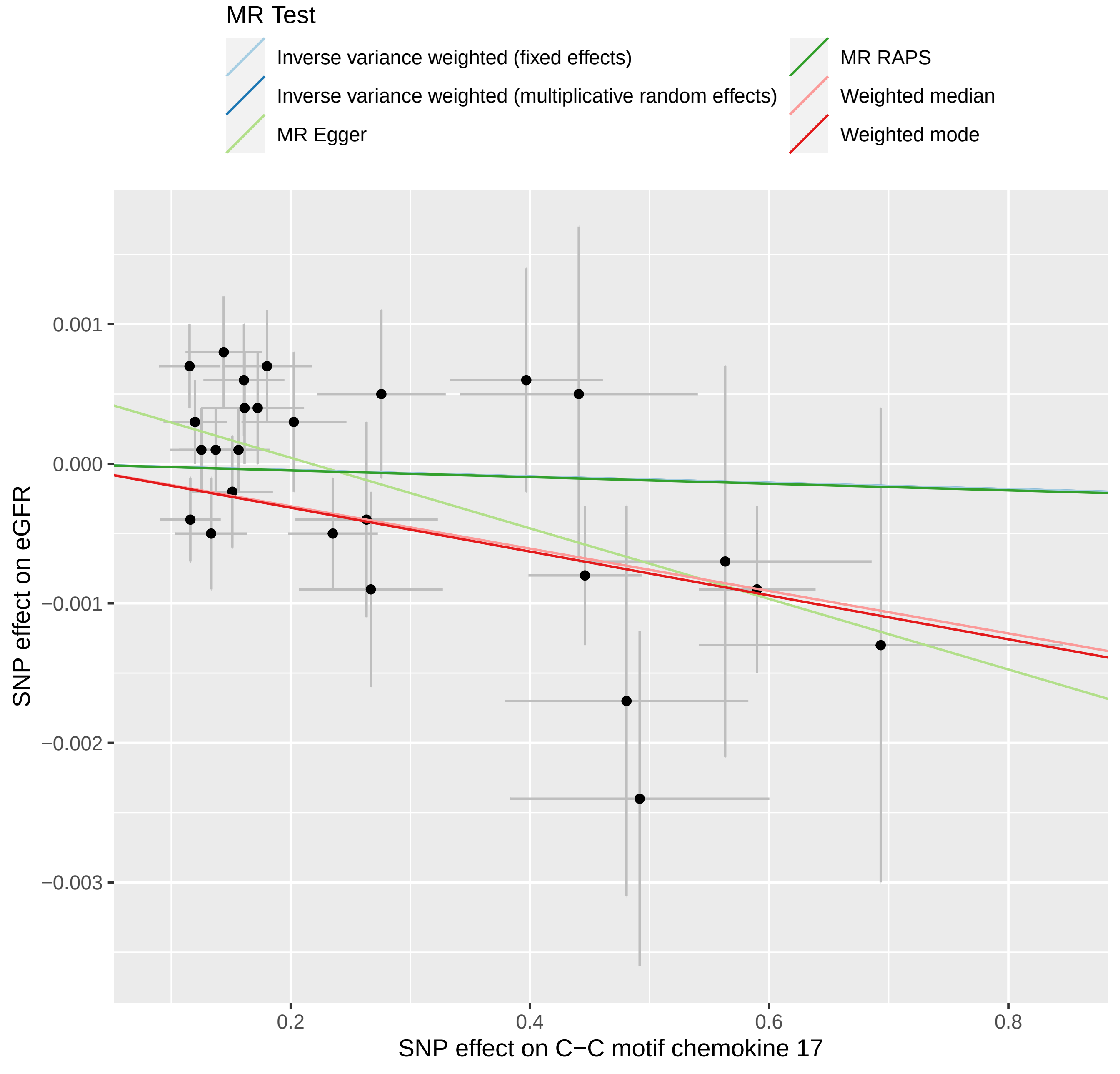

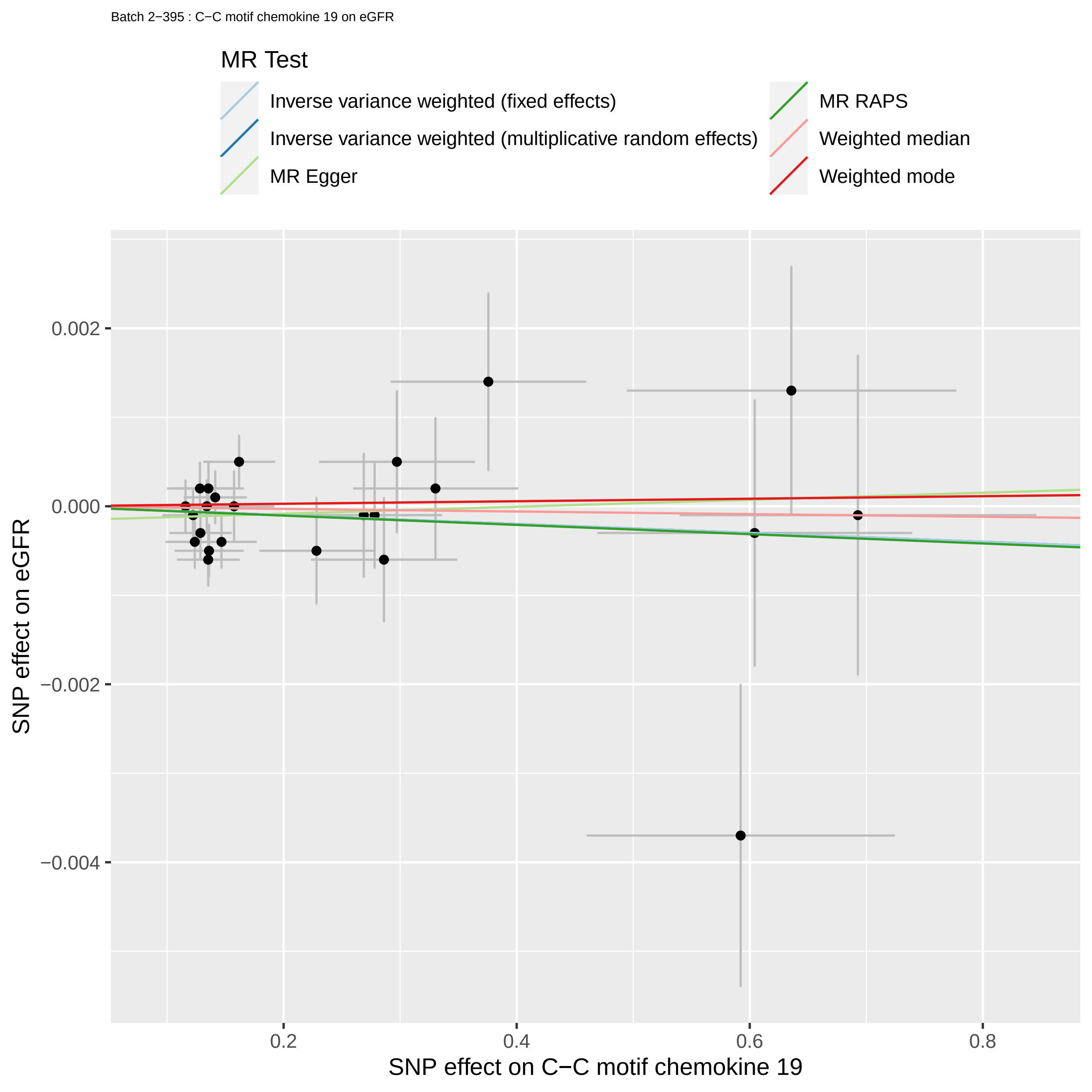
Batch 2−395 : C−C motif chemokine 19 on eGFR
MR Test
Inverse variance weighted (fixed effects)
Inverse variance weighted (multiplicative random effects)
MR Egger
MR RAPS
Weighted median
Weighted mode
0.002
0.000
−0.002
−0.004
0.2
0.4
0.6
0.8
SNP effect on eGFR
SNP effect on C−C motif chemokine 19

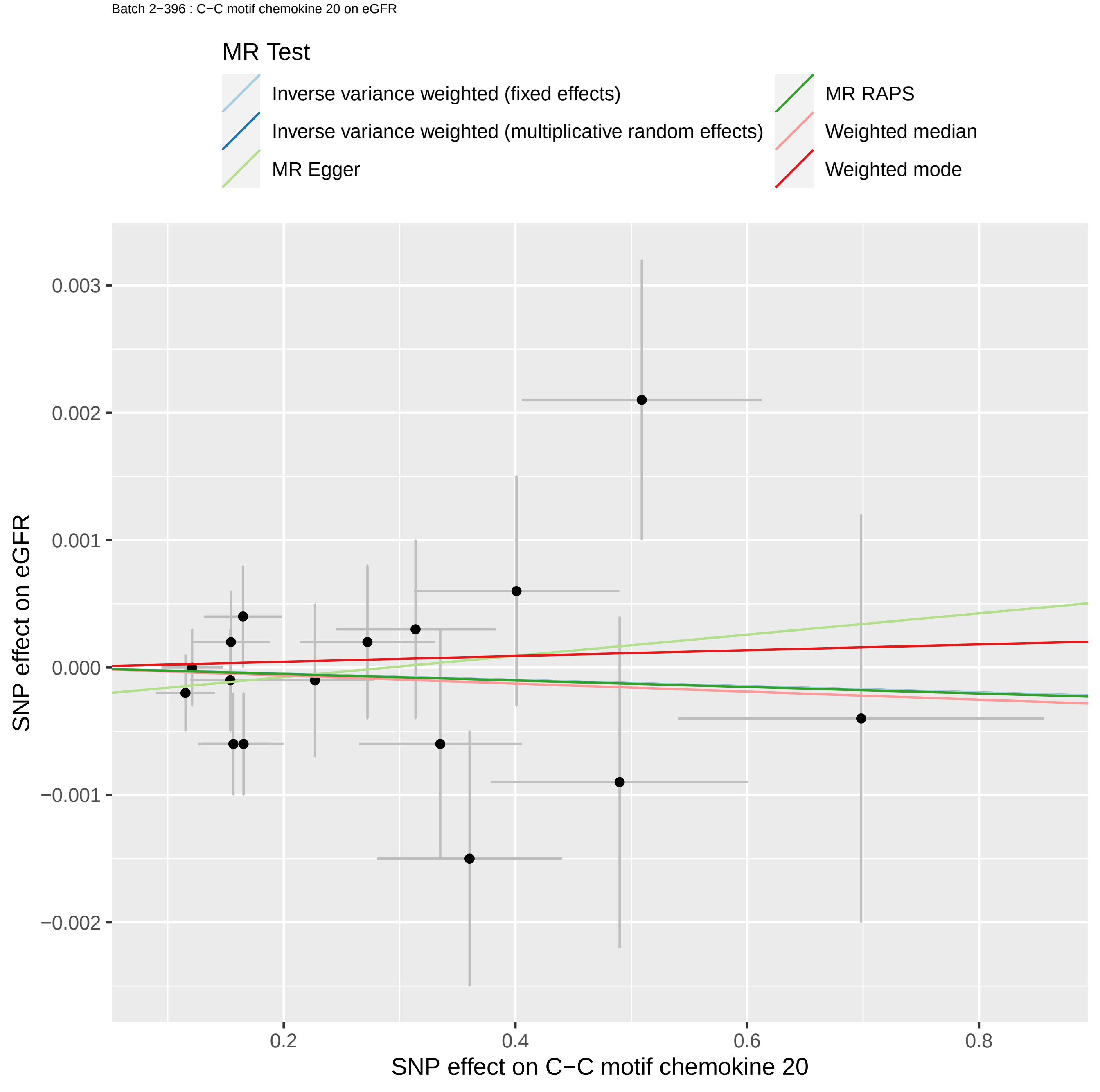

Batch 2−396 : C−C motif chemokine 20 on eGFR
MR Test
Inverse variance weighted (fixed effects)
Inverse variance weighted (multiplicative random effects)
MR Egger
MR RAPS
Weighted median
Weighted mode
SNP effect on eGFR
0.003
0.002
0.001
0.000
−0.001
−0.002
0.2
0.4
0.6
0.8
SNP effect on C−C motif chemokine 20

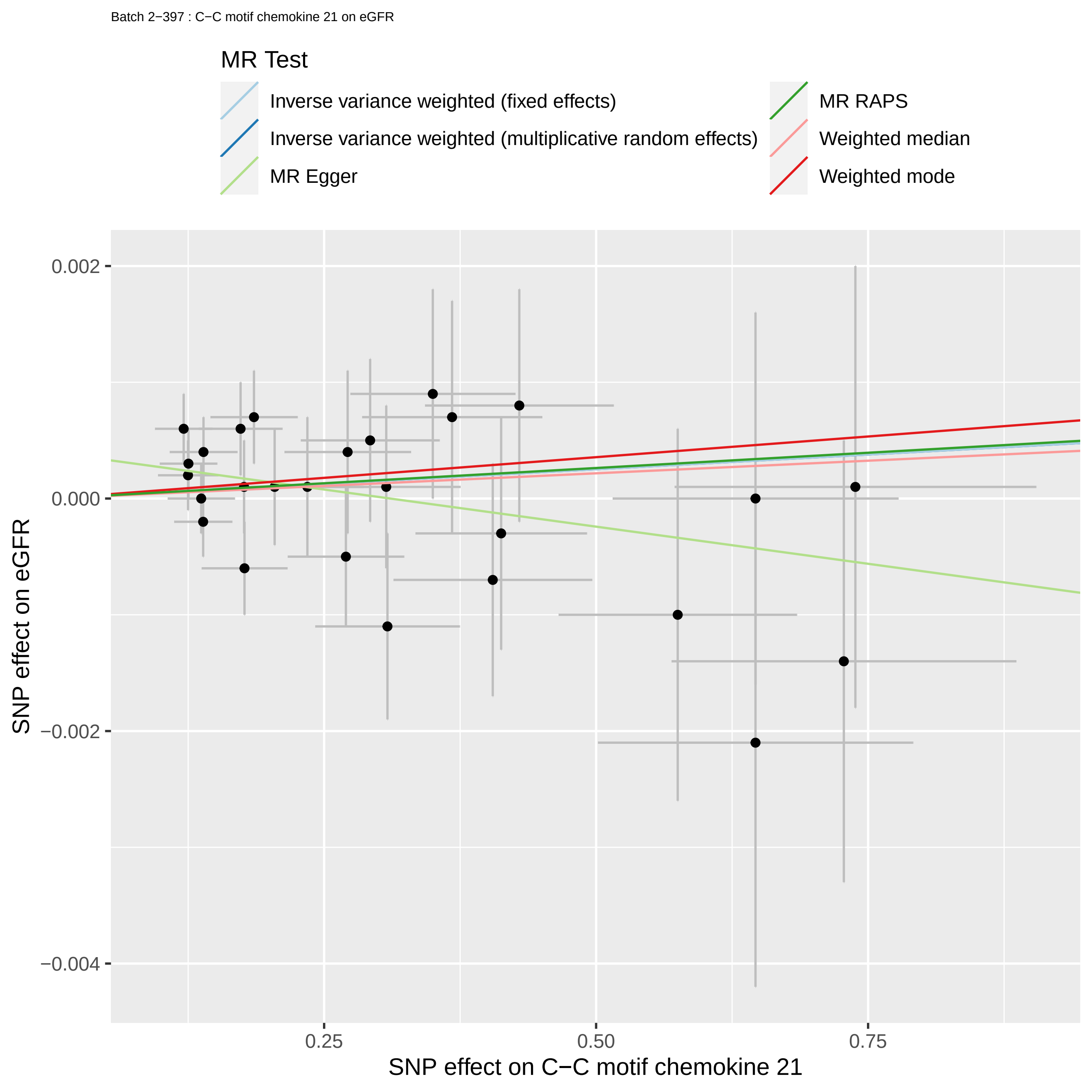
Batch 2−397 : C−C motif chemokine 21 on eGFR
MR Test
Inverse variance weighted (fixed effects)
Inverse variance weighted (multiplicative random effects)
MR Egger
MR RAPS
Weighted median
Weighted mode
0.002
0.000
−0.002
−0.004
SNP effect on eGFR
0.25
0.50
0.75
SNP effect on C−C motif chemokine 21

Batch 2−398 : C−C motif chemokine 22 on eGFR

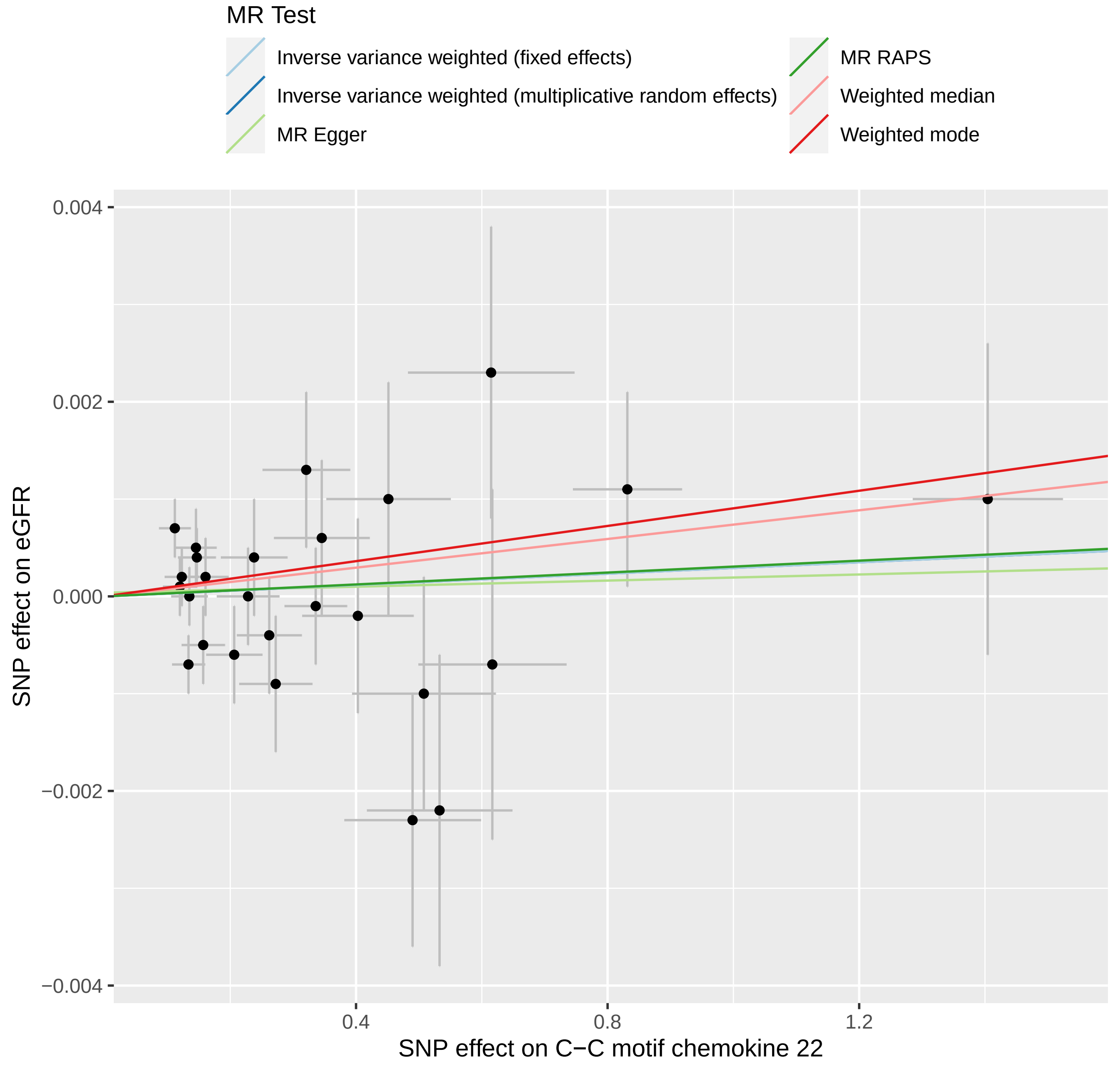

Batch 2−399 : C−C motif chemokine 23 on eGFR

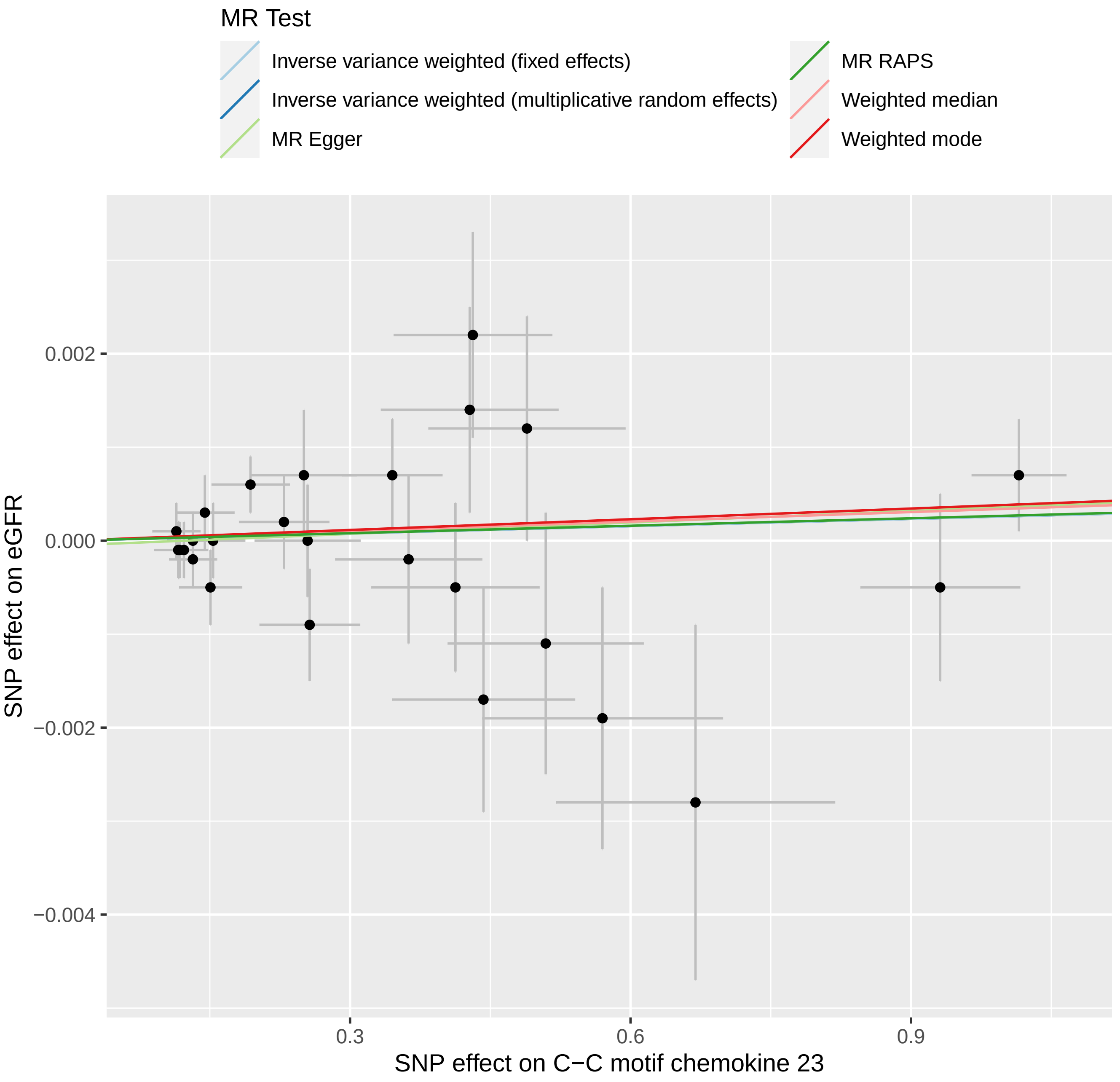

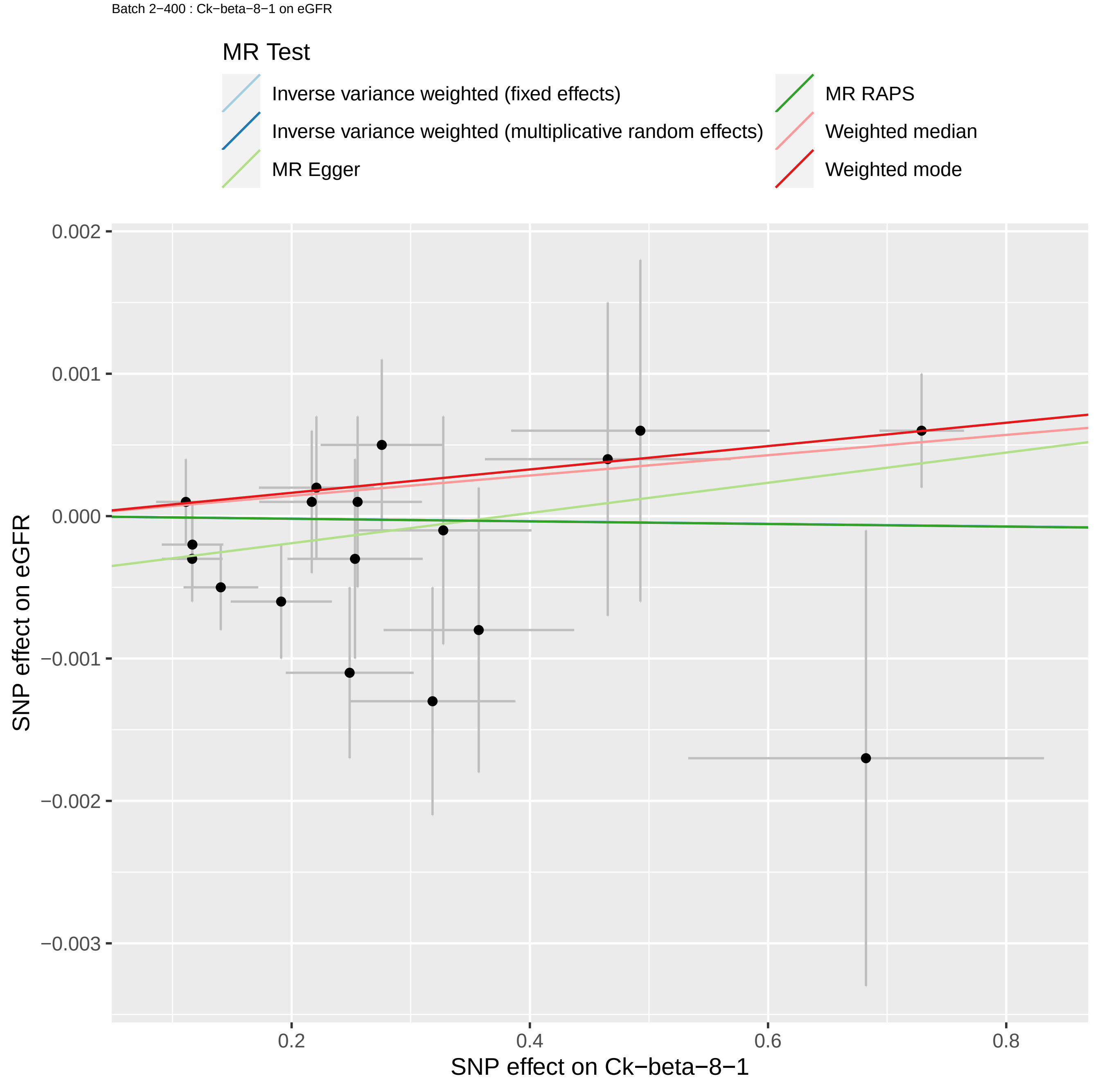

Batch 2−400 : Ck−beta−8−1 on eGFR
MR Test
Inverse variance weighted (fixed effects)
Inverse variance weighted (multiplicative random effects)
MR Egger
MR RAPS
Weighted median
Weighted mode
0.002
0.001
0.000
−0.001
−0.002
−0.003
0.2
0.4
0.6
0.8
SNP effect on Ck−beta−8−1
SNP effect on eGFR

Batch 2−401 : C−C motif chemokine 24 on eGFR

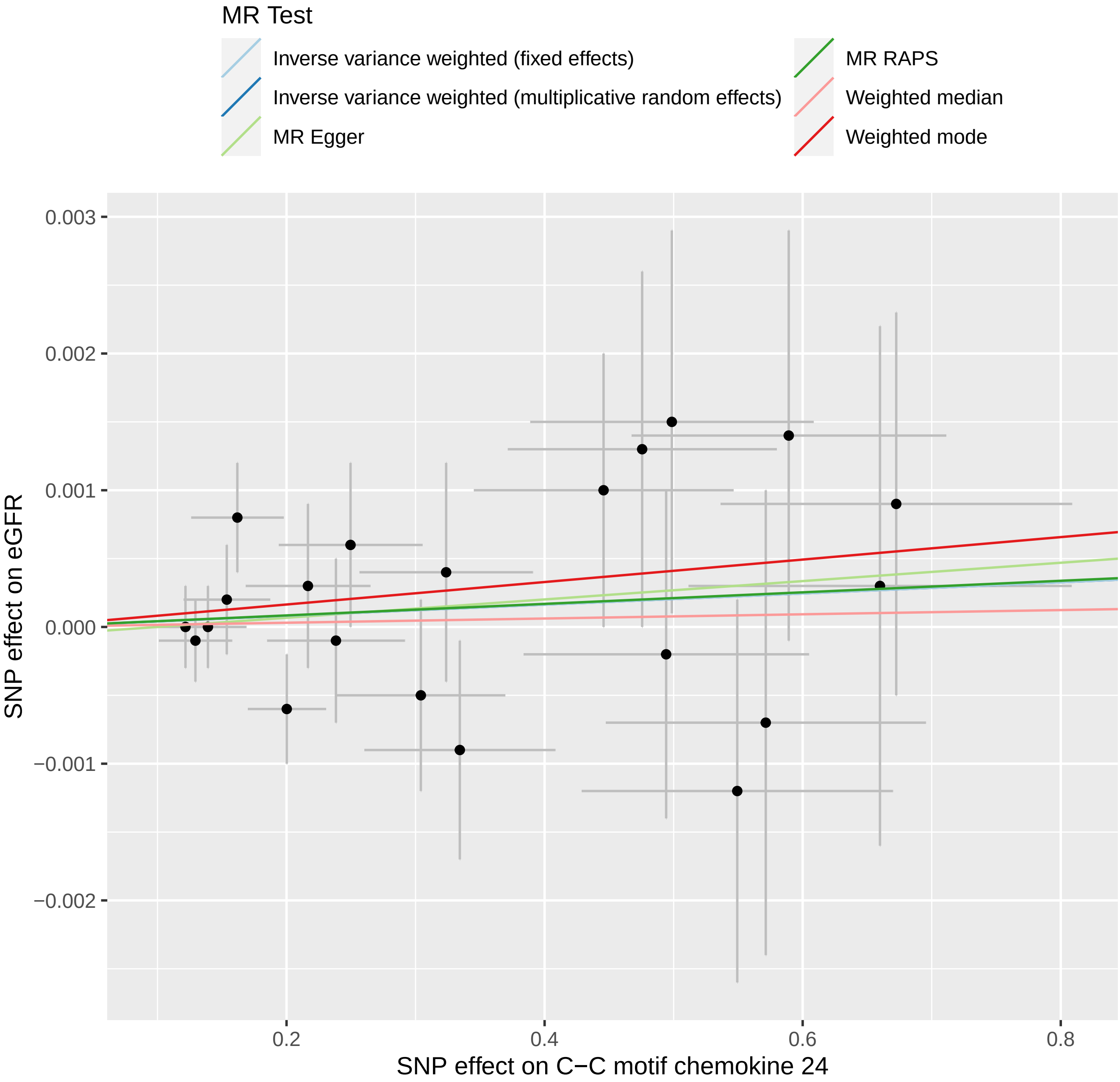

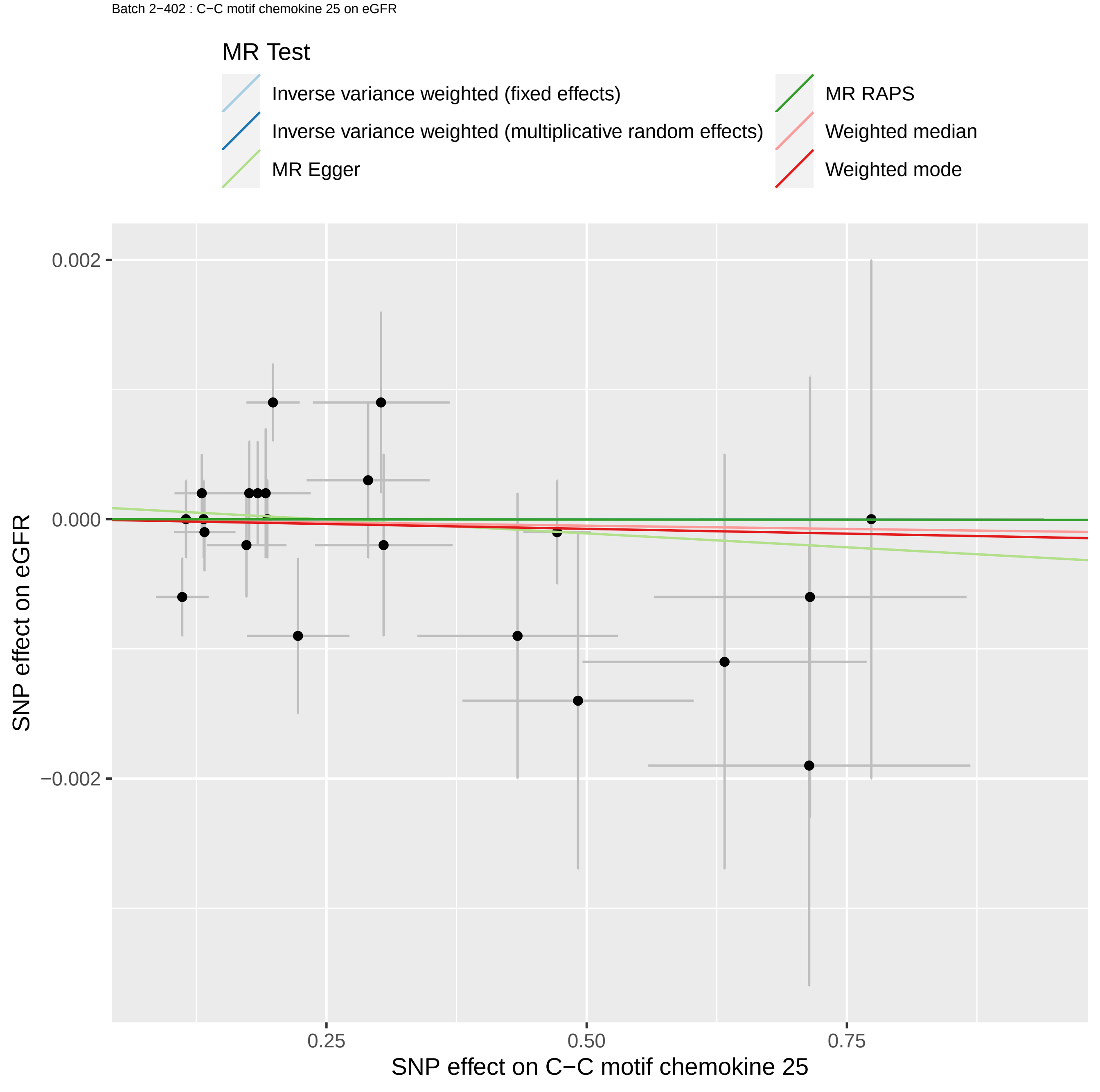

Batch 2−402 : C−C motif chemokine 25 on eGFR
MR Test
Inverse variance weighted (fixed effects)
Inverse variance weighted (multiplicative random effects)
MR Egger
MR RAPS
Weighted median
Weighted mode
0.002
0.000
−0.002
0.25
0.50
0.75
SNP effect on eGFR
SNP effect on C−C motif chemokine 25

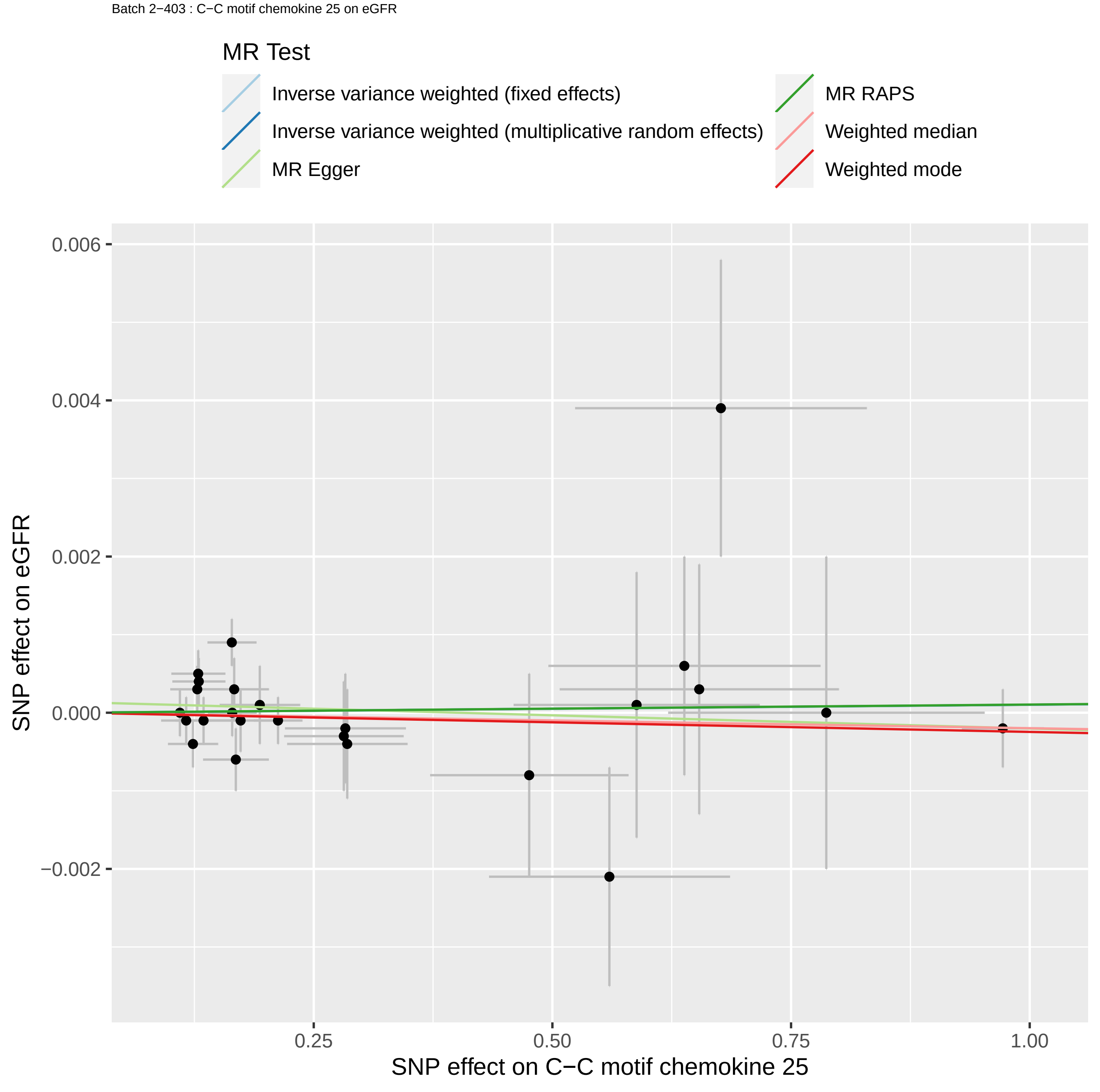

Batch 2−403 : C−C motif chemokine 25 on eGFR
MR Test
Inverse variance weighted (fixed effects)
Inverse variance weighted (multiplicative random effects)
MR Egger
MR RAPS
Weighted median
Weighted mode
0.006
0.004
0.002
0.000
−0.002
0.25
0.50
0.75
1.00
SNP effect on eGFR
SNP effect on C−C motif chemokine 25

Batch 2−404 : C−C motif chemokine 26 on eGFR

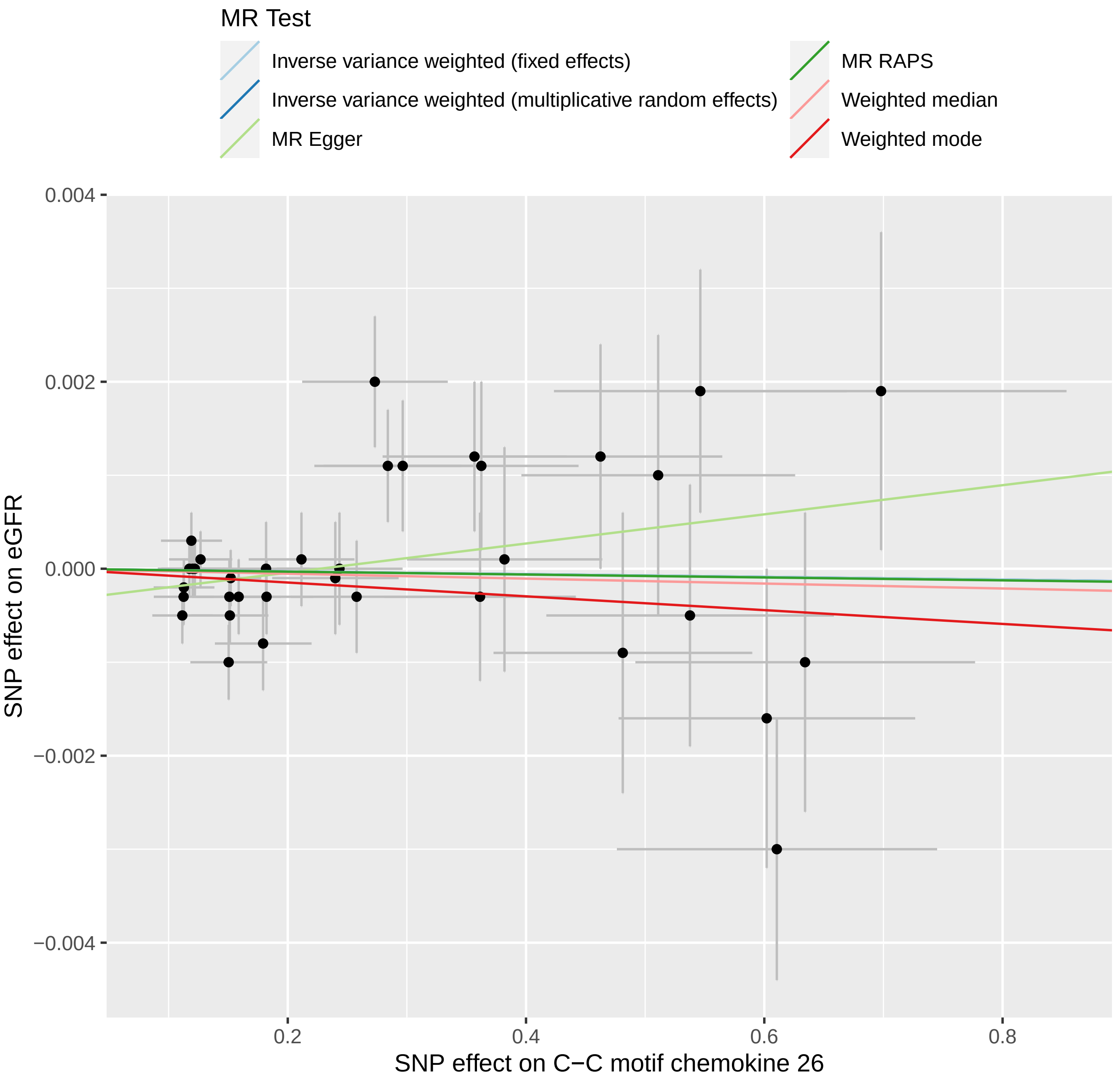

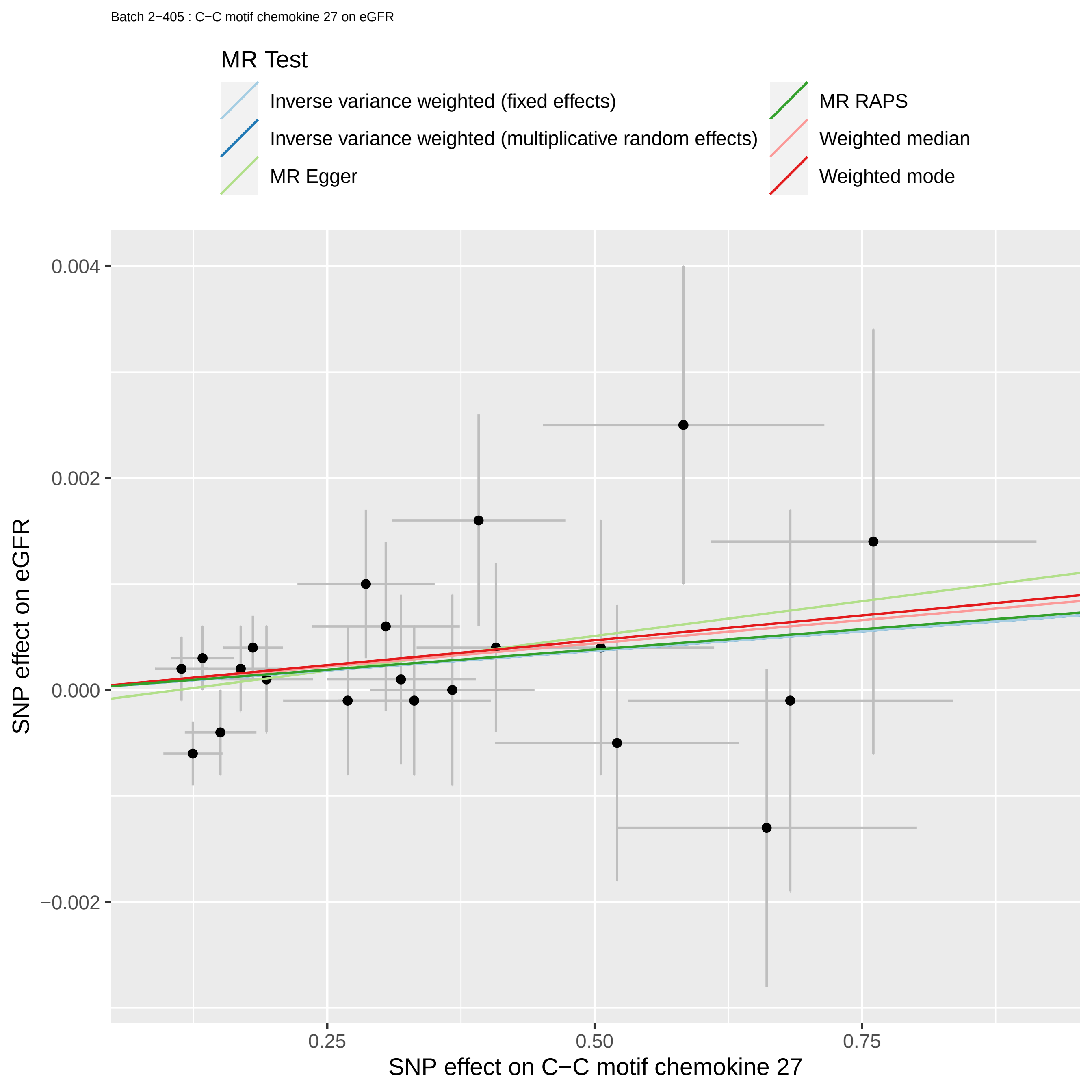
Batch 2−405 : C−C motif chemokine 27 on eGFR
MR Test
Inverse variance weighted (fixed effects)
Inverse variance weighted (multiplicative random effects)
MR Egger
MR RAPS
Weighted median
Weighted mode
0.004
0.002
0.000
−0.002
0.25
0.50
0.75
SNP effect on eGFR
SNP effect on C−C motif chemokine 27

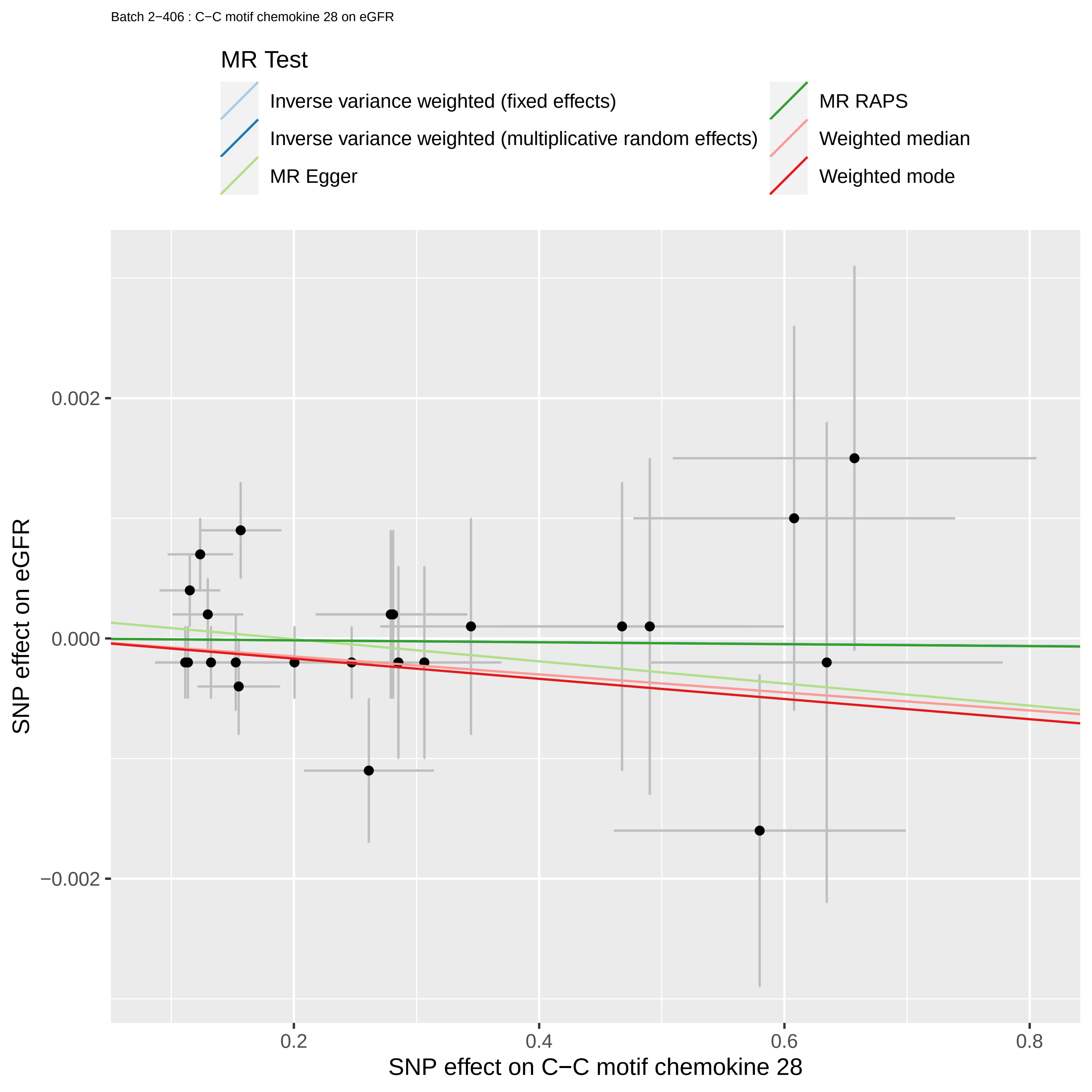

Batch 2−406 : C−C motif chemokine 28 on eGFR
MR Test
Inverse variance weighted (fixed effects)
Inverse variance weighted (multiplicative random effects)
MR Egger
MR RAPS
Weighted median
Weighted mode
SNP effect on eGFR
0.002
0.000
−0.002
0.2
0.4
0.6
0.8
SNP effect on C−C motif chemokine 28

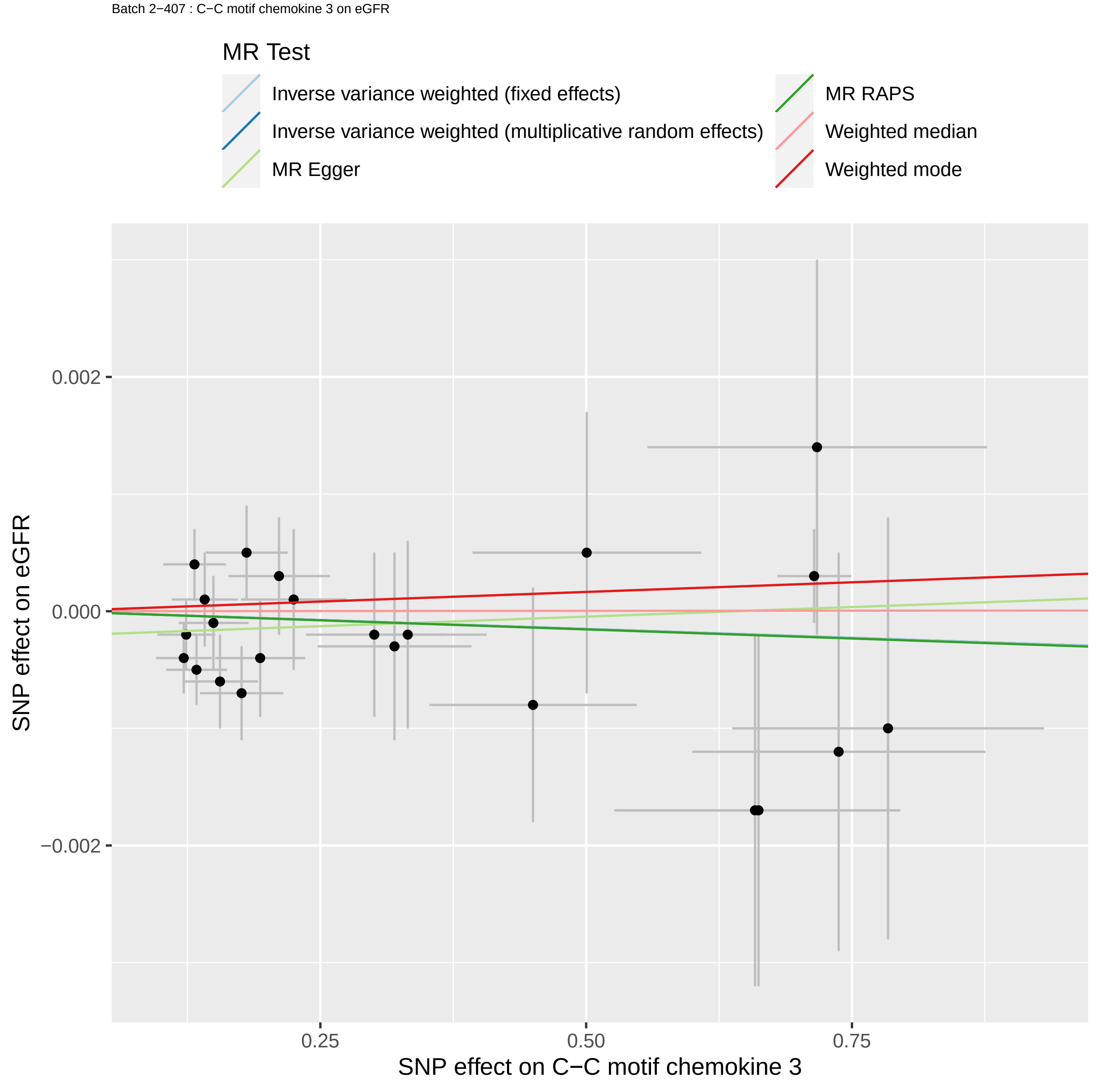

Batch 2−407 : C−C motif chemokine 3 on eGFR
MR Test
Inverse variance weighted (fixed effects)
Inverse variance weighted (multiplicative random effects)
MR Egger
MR RAPS
Weighted median
Weighted mode
0.002
0.000
−0.002
0.25
0.50
0.75
SNP effect on eGFR
SNP effect on C−C motif chemokine 3

Batch 2−408 : C−C motif chemokine 3−like 1 on eGFR

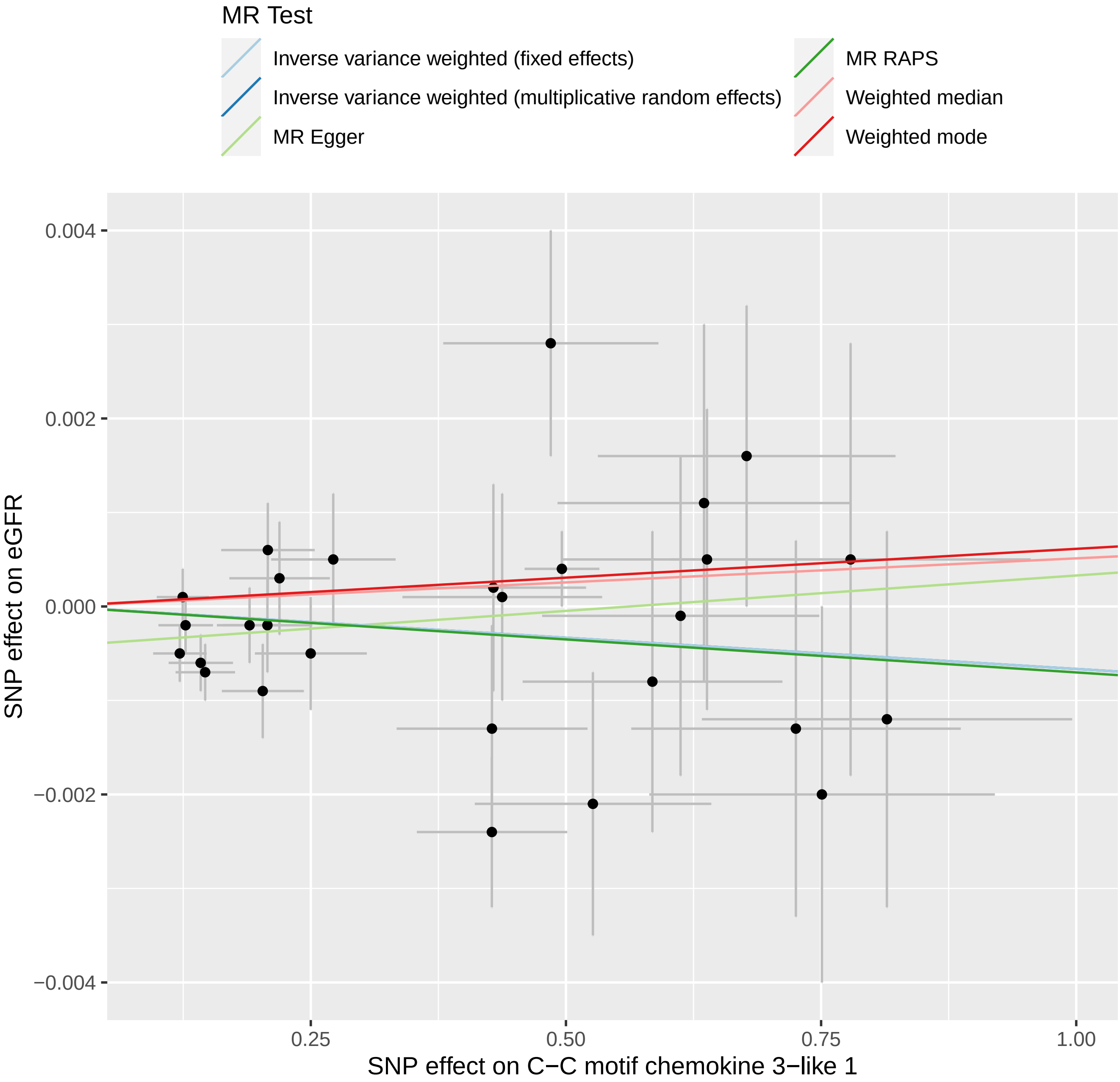

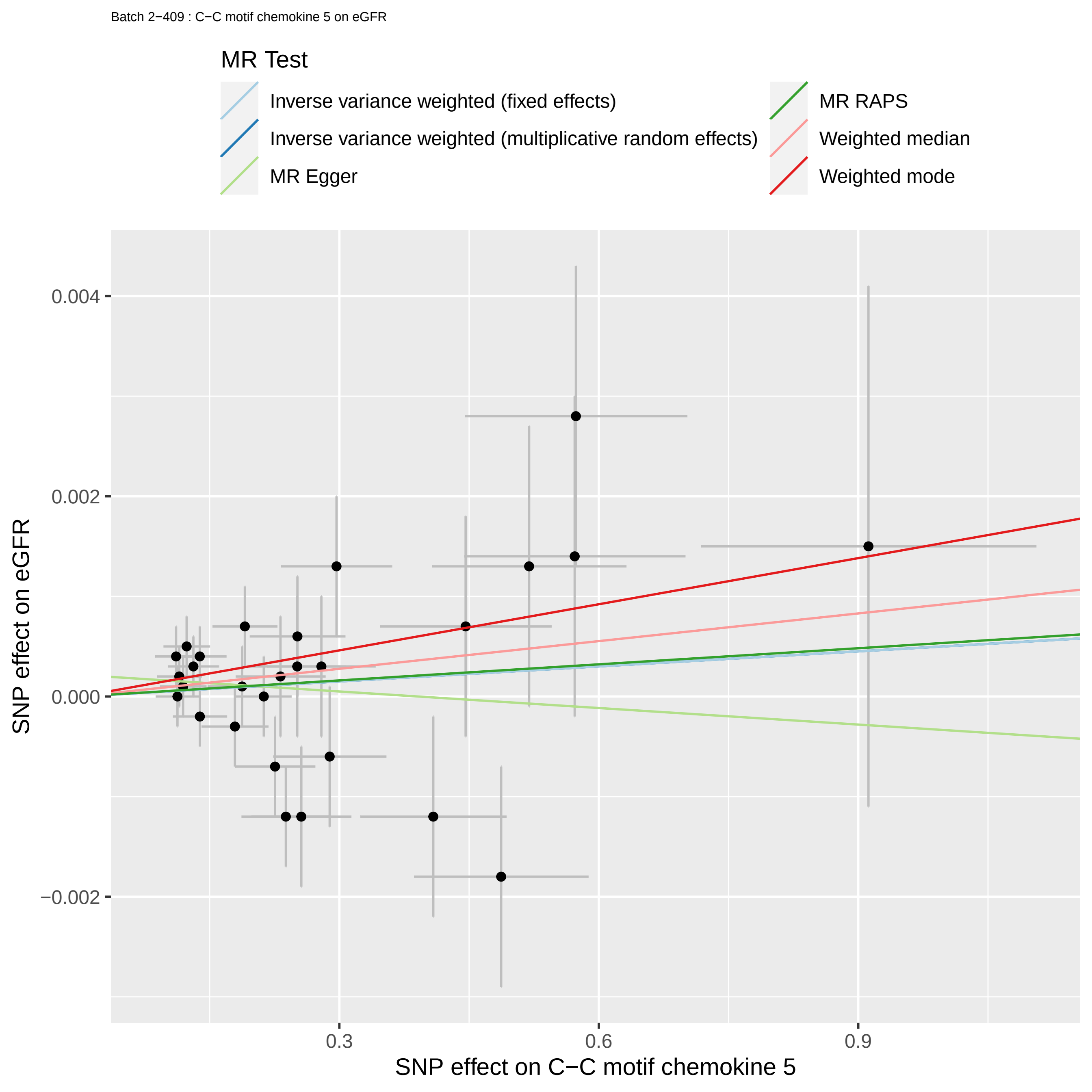

Batch 2−409 : C−C motif chemokine 5 on eGFR
MR Test
Inverse variance weighted (fixed effects)
Inverse variance weighted (multiplicative random effects)
MR Egger
MR RAPS
Weighted median
Weighted mode
0.004
0.002
0.000
−0.002
SNP effect on eGFR
0.3
0.6
0.9
SNP effect on C−C motif chemokine 5

Batch 2−410 : C−C motif chemokine 7 on eGFR

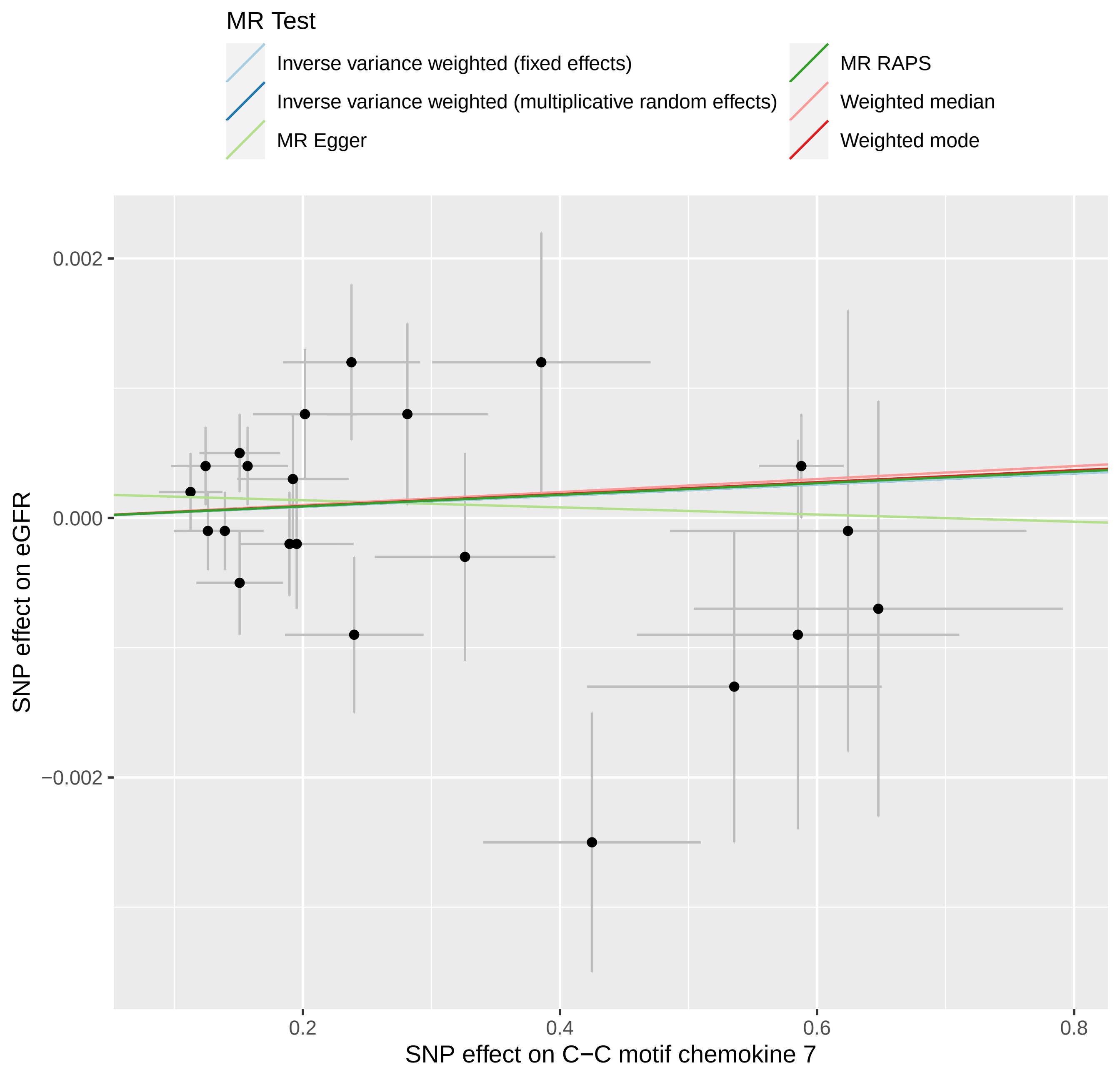

Batch 2−411 : C−C motif chemokine 8 on eGFR

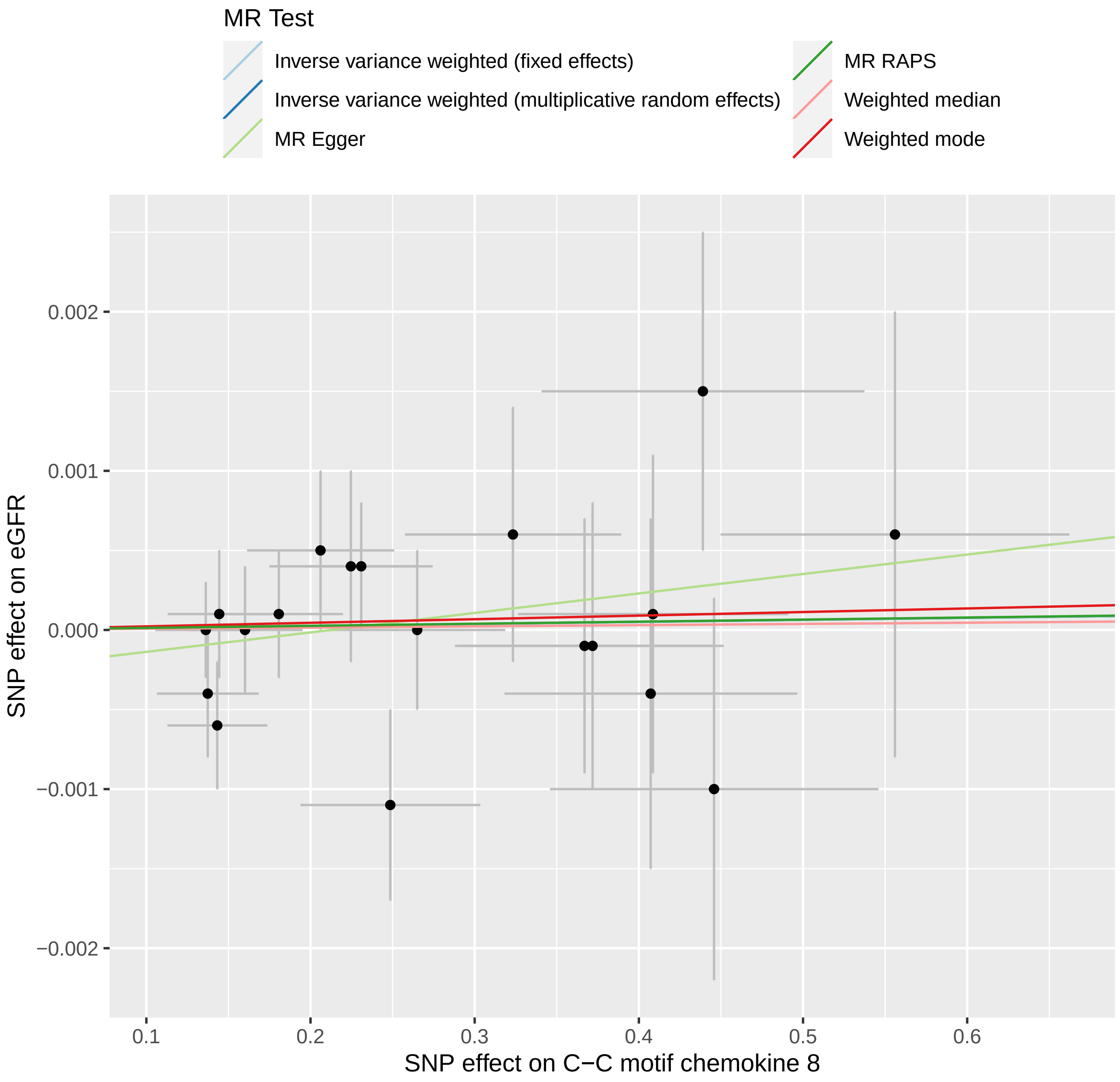

Batch 2−412 : Cerebral cavernous malformations 2 protein on eGFR

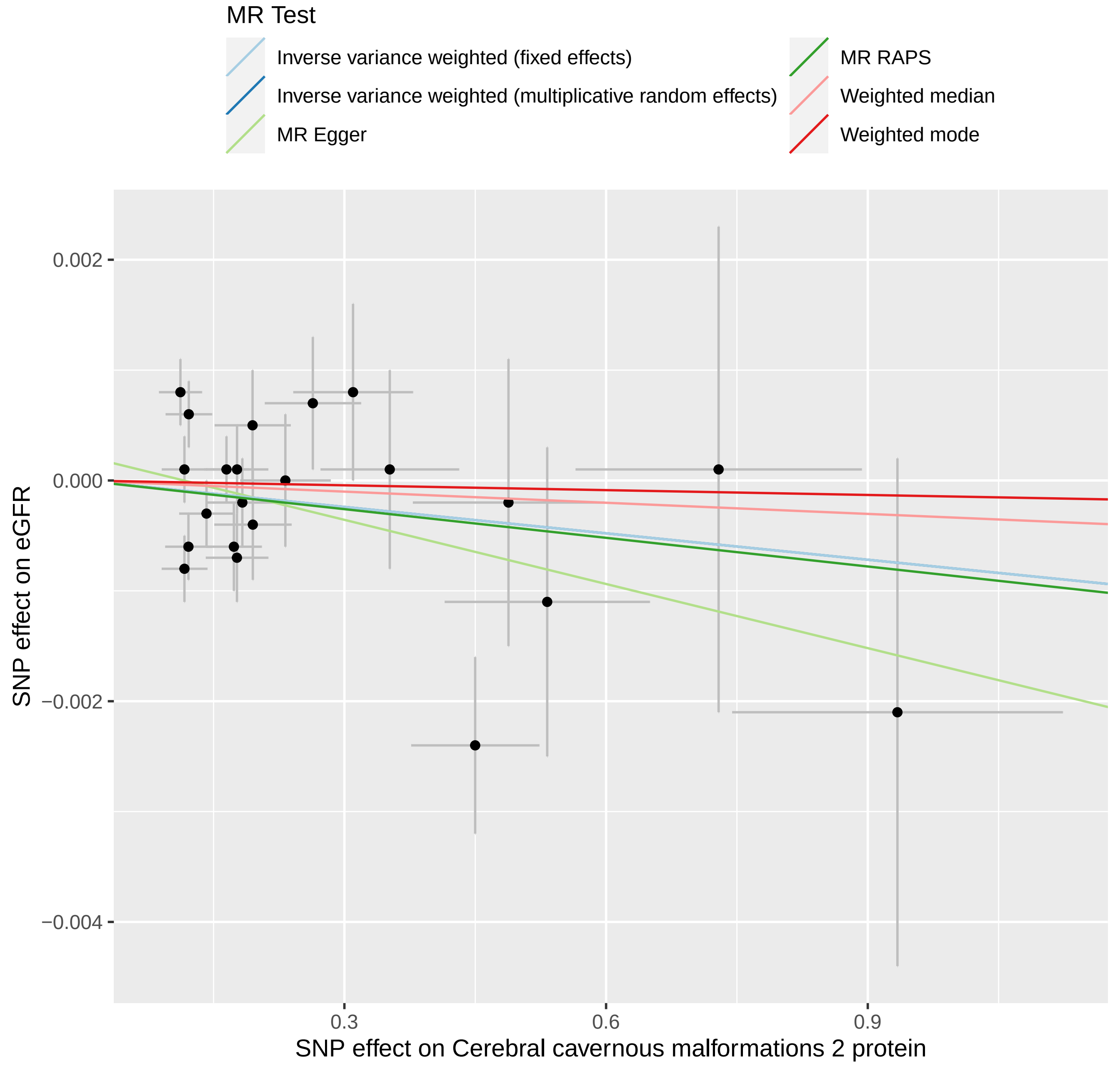

Batch 2−413 : G2/mitotic−specific cyclin−B1 on eGFR

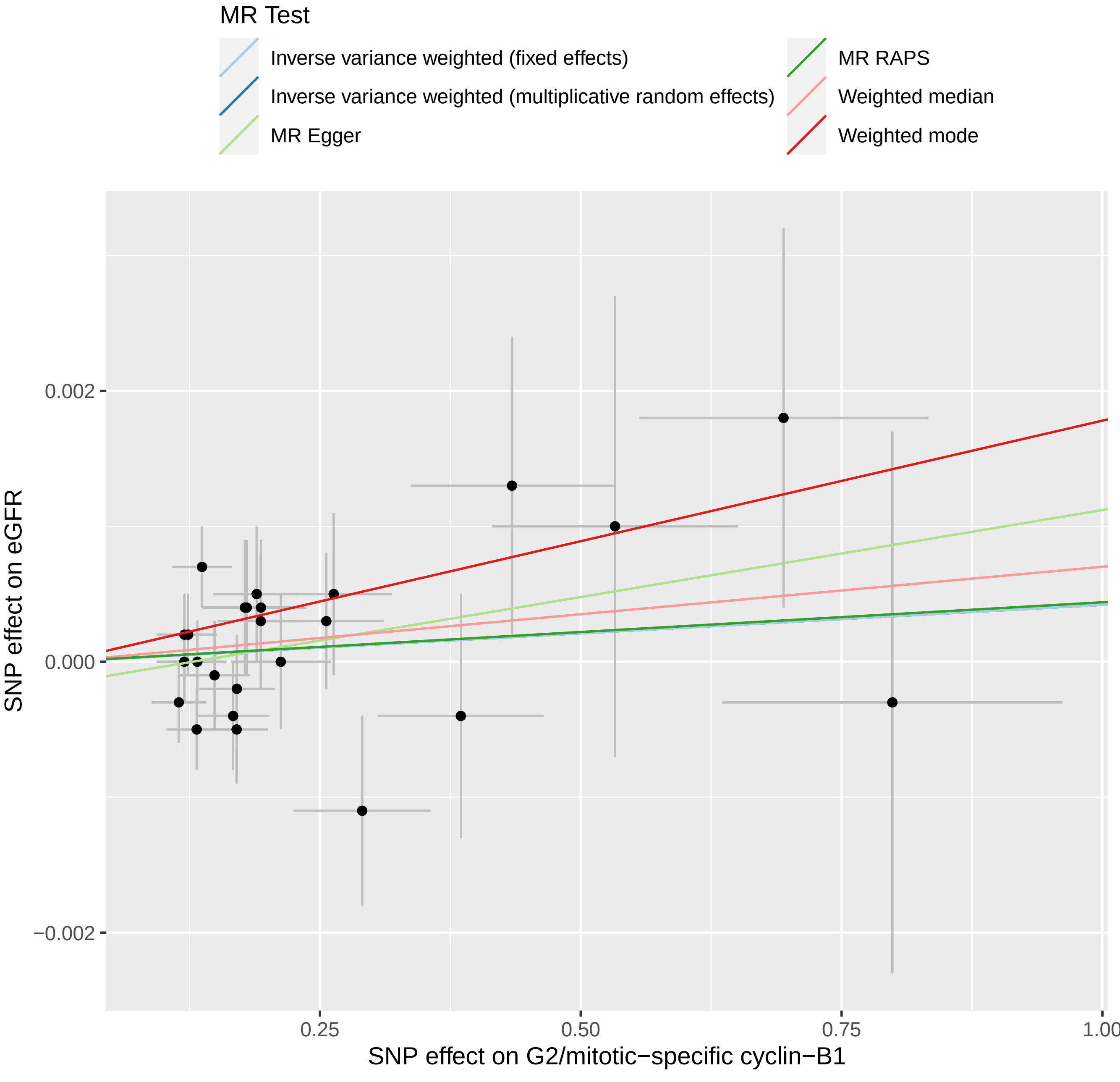

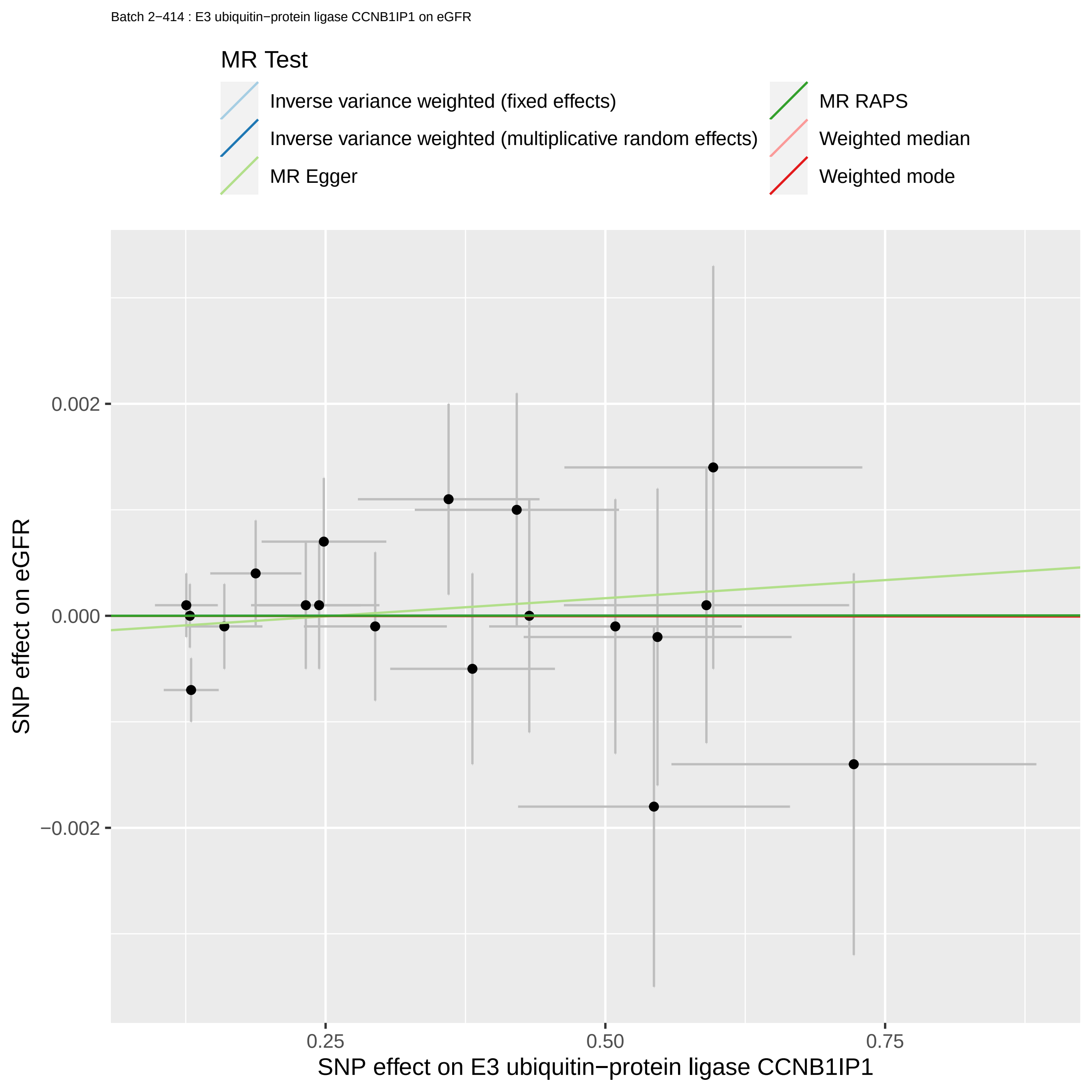
Batch 2−414 : E3 ubiquitin−protein ligase CCNB1IP1 on eGFR
MR Test
Inverse variance weighted (fixed effects)
Inverse variance weighted (multiplicative random effects)
MR Egger
MR RAPS
Weighted median
Weighted mode
0.002
0.000
−0.002
0.25
0.50
0.75
SNP effect on eGFR
SNP effect on E3 ubiquitin−protein ligase CCNB1IP1

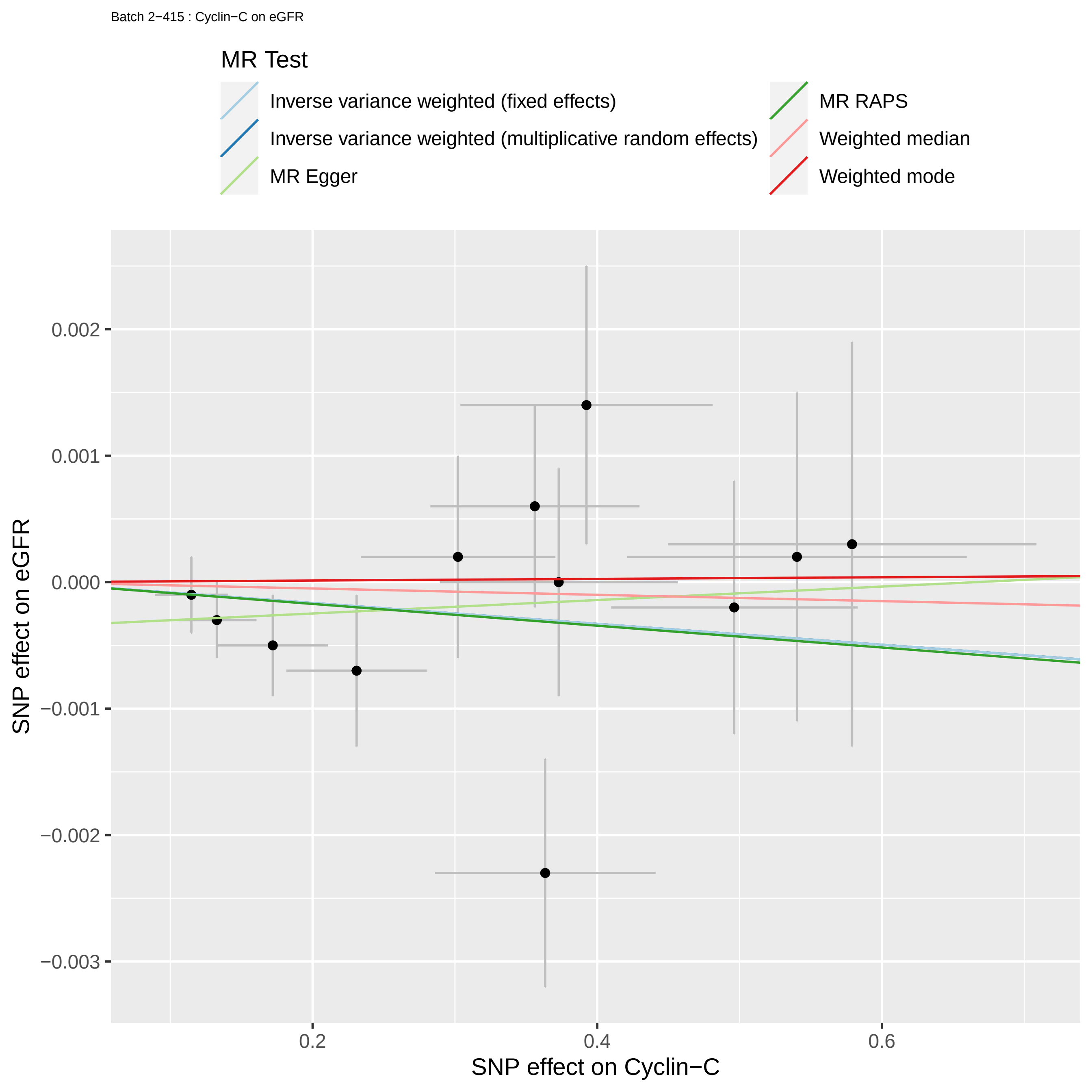

Batch 2−415 : Cyclin−C on eGFR
MR Test
Inverse variance weighted (fixed effects)
Inverse variance weighted (multiplicative random effects)
MR Egger
MR RAPS
Weighted median
Weighted mode
0.002
0.001
0.000
−0.001
−0.002
−0.003
0.2
0.4
0.6
SNP effect on eGFR
SNP effect on Cyclin−C

Batch 2−416 : Cyclin−H on eGFR

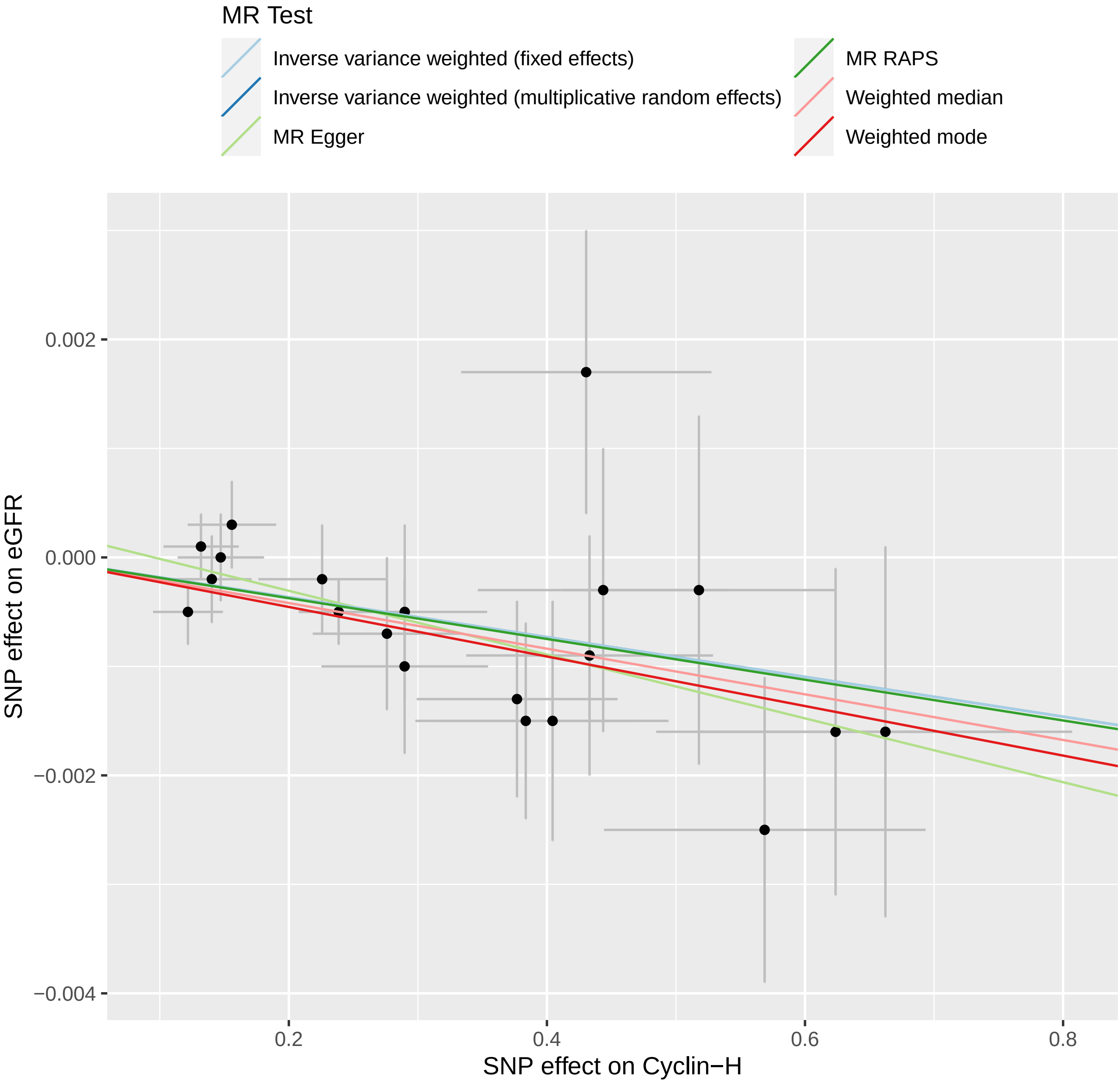

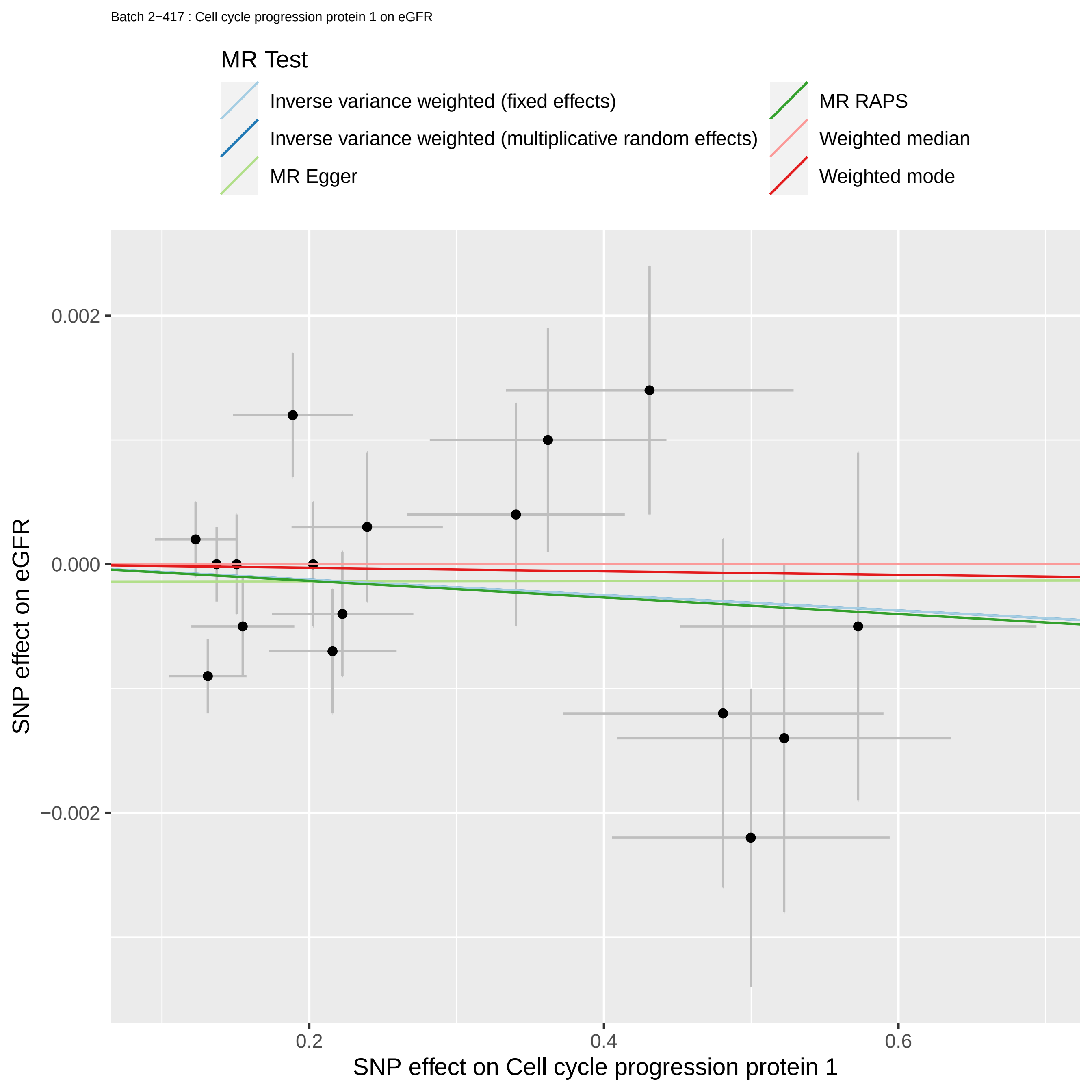
Batch 2−417 : Cell cycle progression protein 1 on eGFR
MR Test
Inverse variance weighted (fixed effects)
Inverse variance weighted (multiplicative random effects)
MR Egger
MR RAPS
Weighted median
Weighted mode
0.002
0.000
−0.002
0.2
0.4
0.6
SNP effect on eGFR
SNP effect on Cell cycle progression protein 1

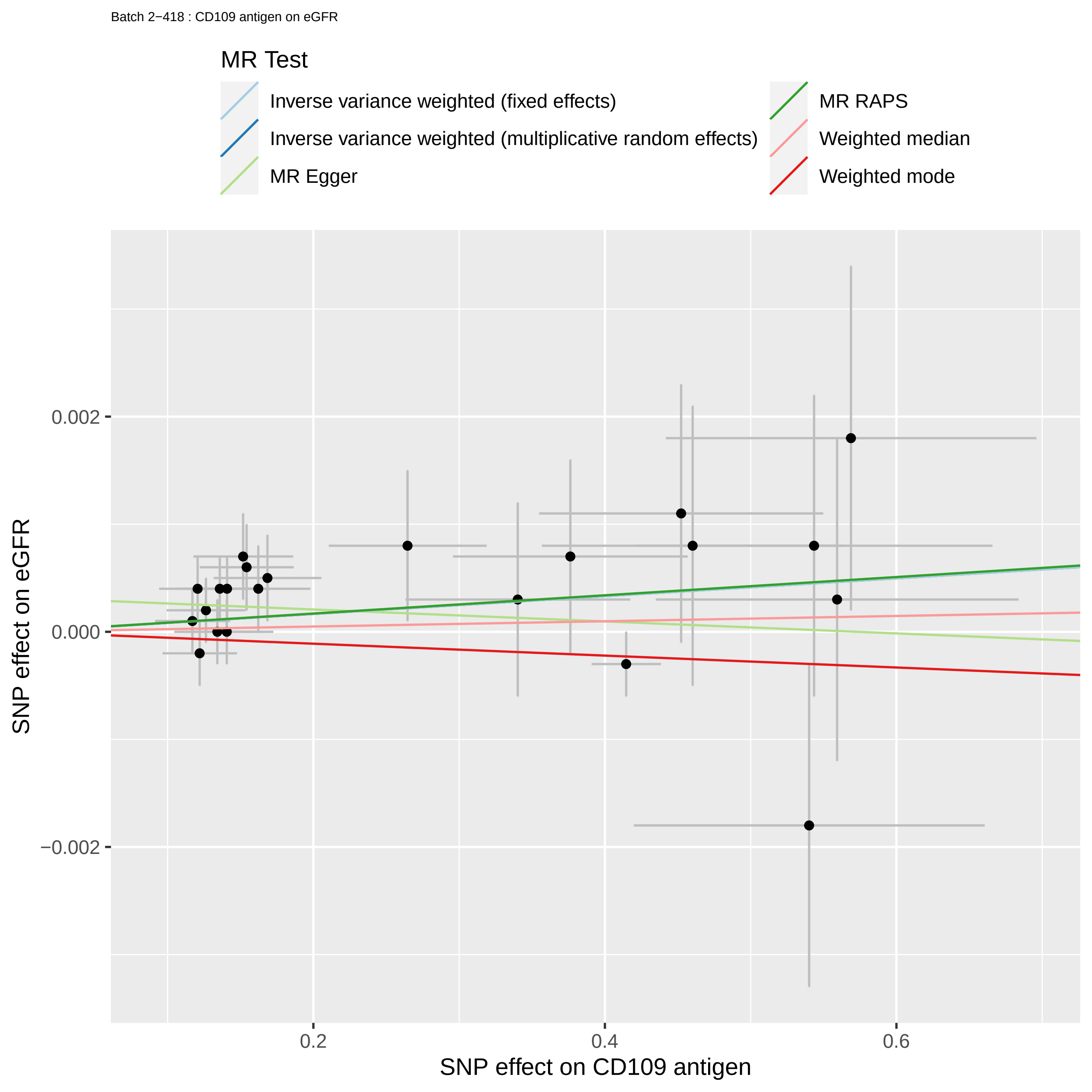
Batch 2−418 : CD109 antigen on eGFR
MR Test
Inverse variance weighted (fixed effects)
Inverse variance weighted (multiplicative random effects)
MR Egger
MR RAPS
Weighted median
Weighted mode
SNP effect on eGFR
0.002
0.000
−0.002
SNP effect on CD109 antigen
0.2
0.4
0.6

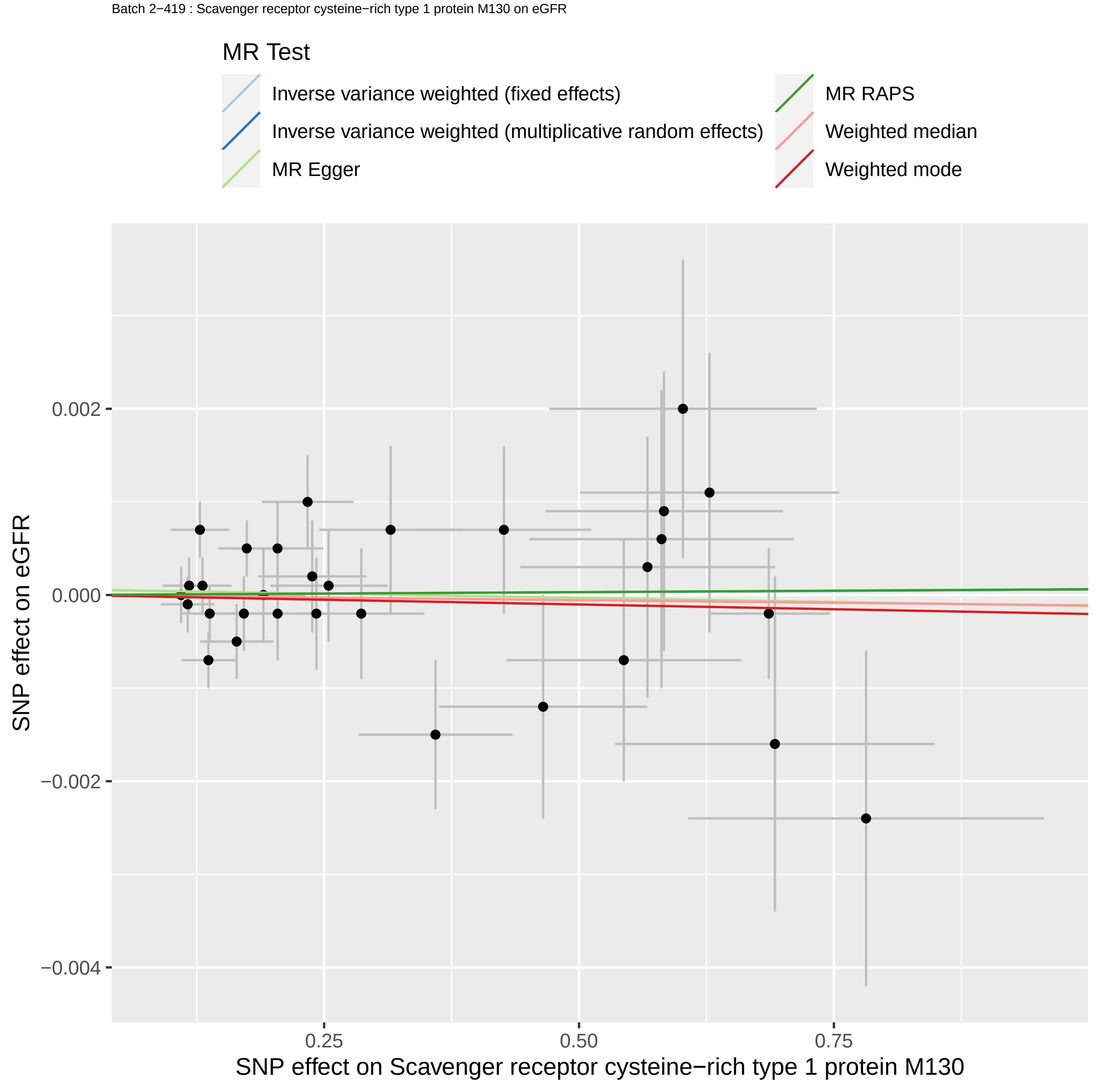
Batch 2−419 : Scavenger receptor cysteine−rich type 1 protein M130 on eGFR
MR Test
Inverse variance weighted (fixed effects)
Inverse variance weighted (multiplicative random effects)
MR Egger
MR RAPS
Weighted median
Weighted mode
0.002
0.000
−0.002
−0.004
SNP effect on eGFR
0.25
0.50
0.75
SNP effect on Scavenger receptor cysteine−rich type 1 protein M130

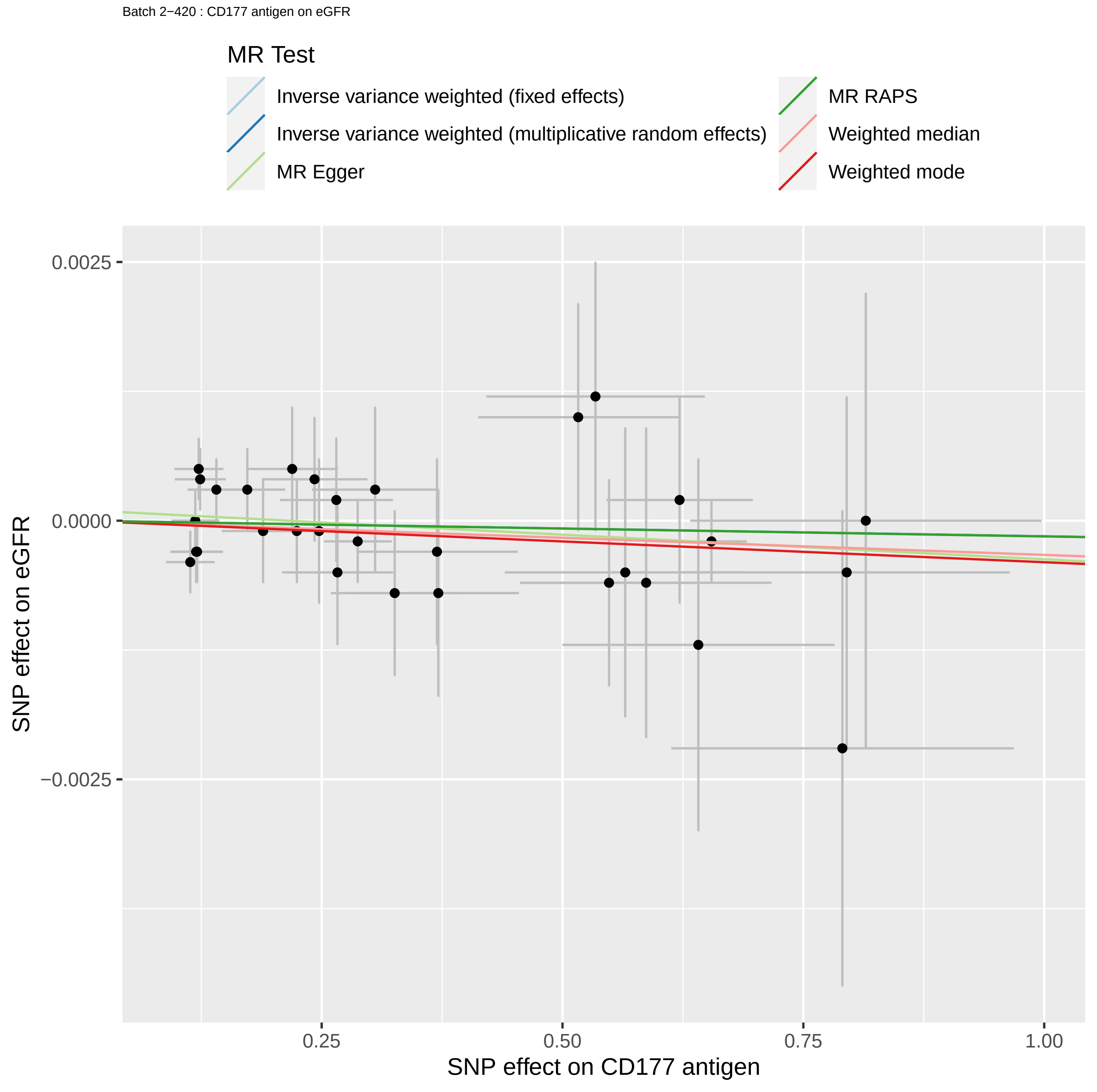
Batch 2−420 : CD177 antigen on eGFR
MR Test
Inverse variance weighted (fixed effects)
Inverse variance weighted (multiplicative random effects)
MR Egger
MR RAPS
Weighted median
Weighted mode
0.0025
0.0000
−0.0025
0.25
0.50
0.75
1.00
SNP effect on eGFR
SNP effect on CD177 antigen

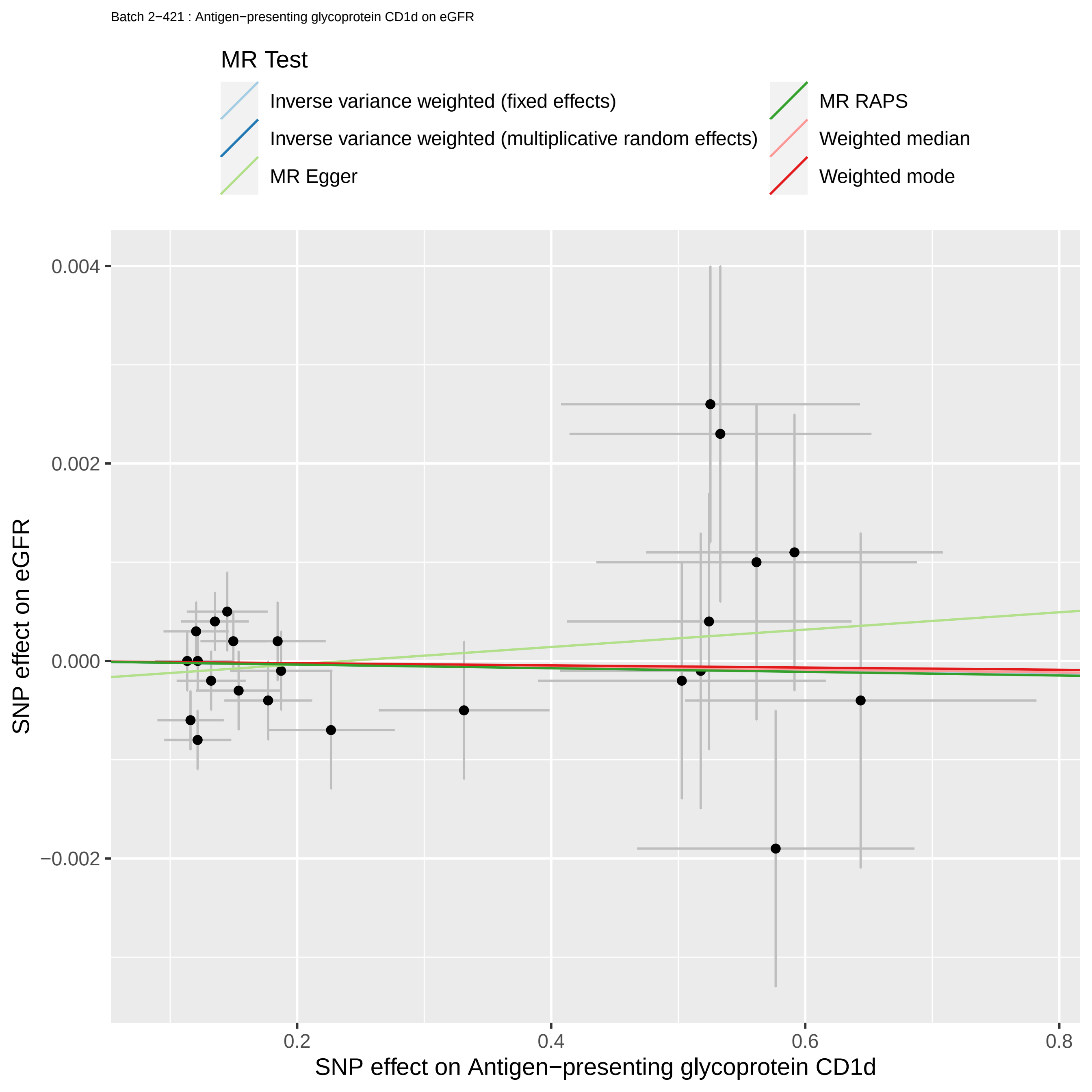

Batch 2−421 : Antigen−presenting glycoprotein CD1d on eGFR
MR Test
Inverse variance weighted (fixed effects)
Inverse variance weighted (multiplicative random effects)
MR Egger
MR RAPS
Weighted median
Weighted mode
0.004
0.002
0.000
−0.002
SNP effect on eGFR
0.2
0.4
0.6
0.8
SNP effect on Antigen−presenting glycoprotein CD1d

Batch 2−422 : OX−2 membrane glycoprotein on eGFR

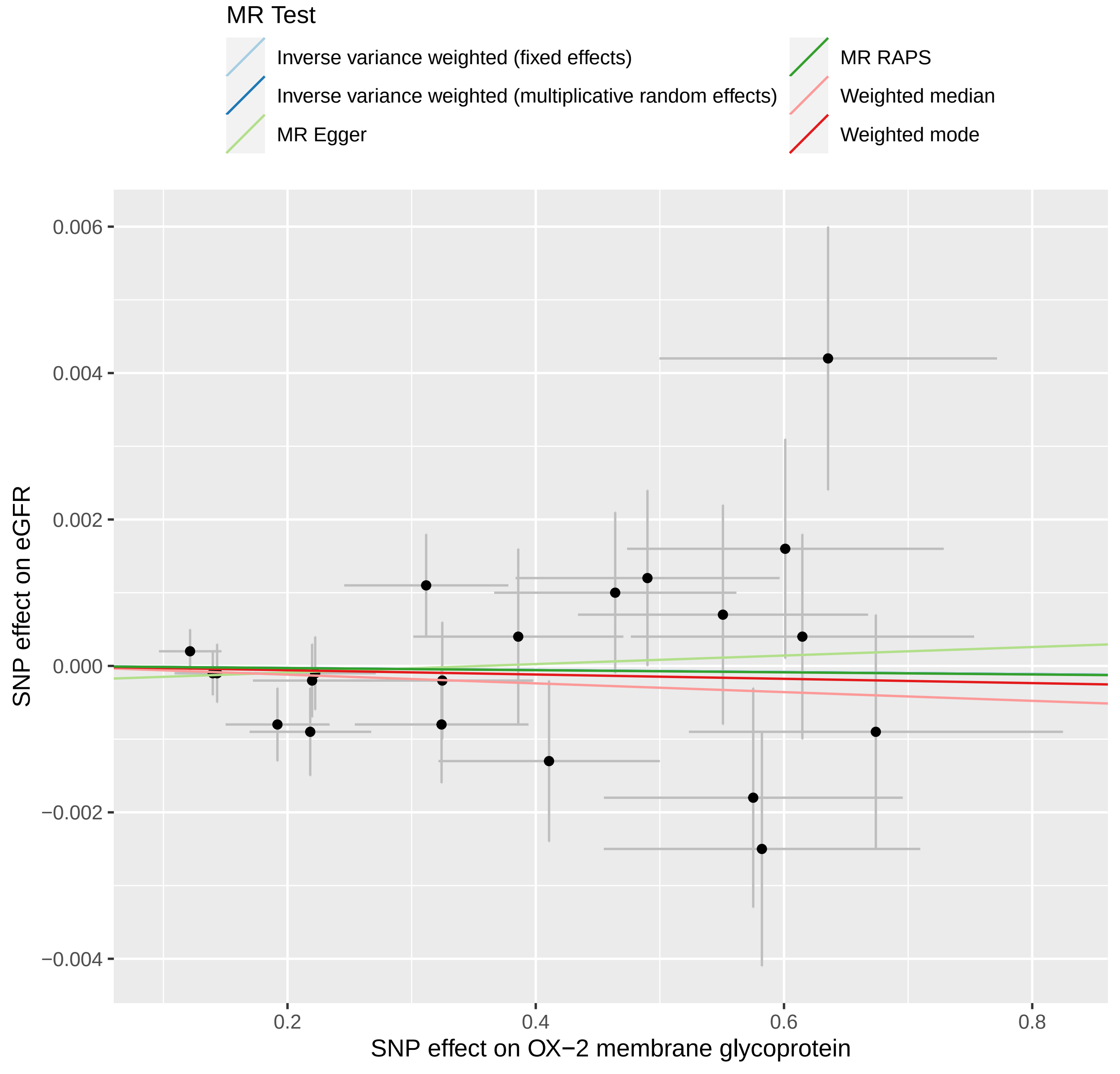

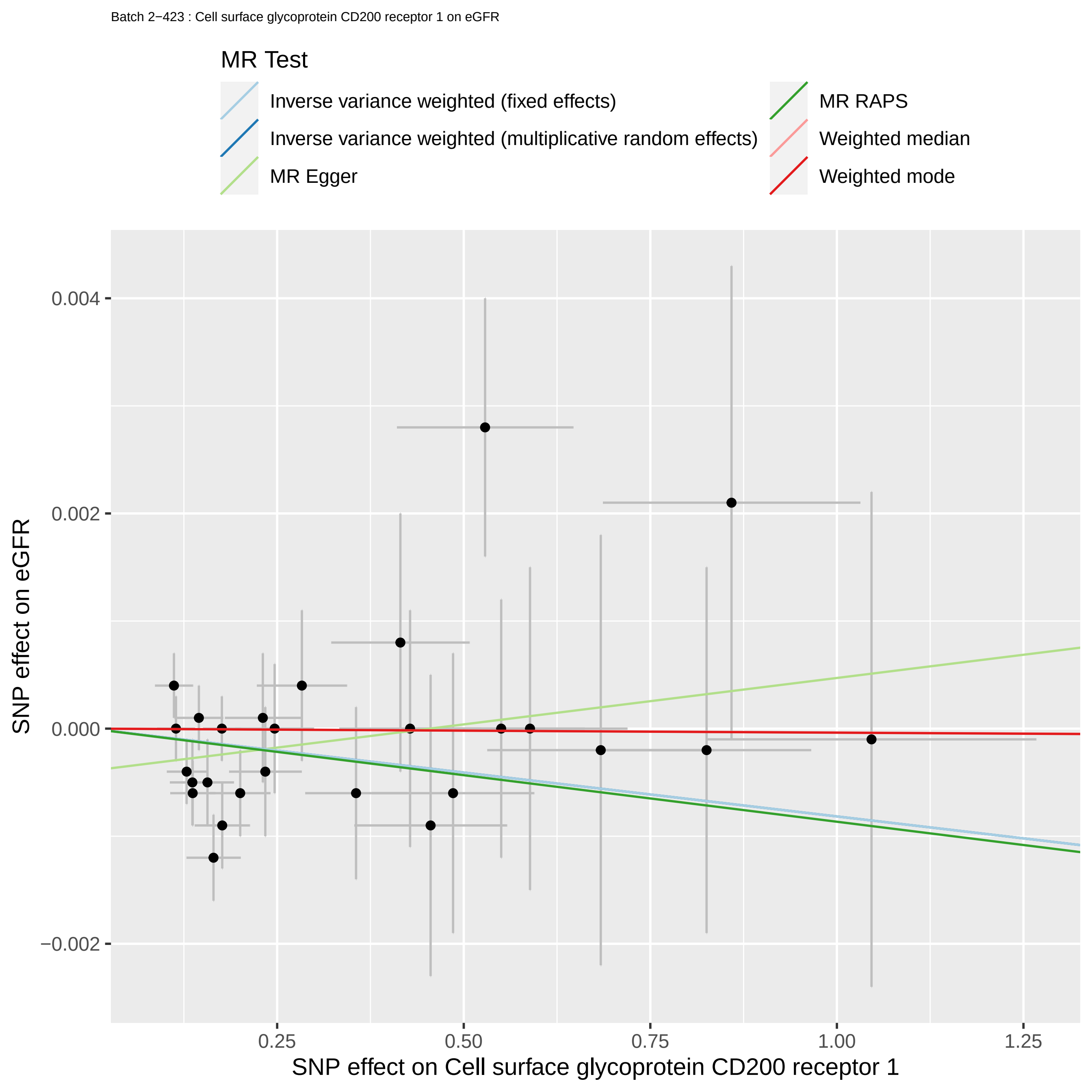

Batch 2−423 : Cell surface glycoprotein CD200 receptor 1 on eGFR
MR Test
Inverse variance weighted (fixed effects)
Inverse variance weighted (multiplicative random effects)
MR Egger
MR RAPS
Weighted median
Weighted mode
0.004
0.002
0.000
−0.002
0.25
0.50
0.75
1.00
1.25
SNP effect on eGFR
SNP effect on Cell surface glycoprotein CD200 receptor 1

Batch 2−424 : Cell surface glycoprotein CD200 receptor 2 on eGFR

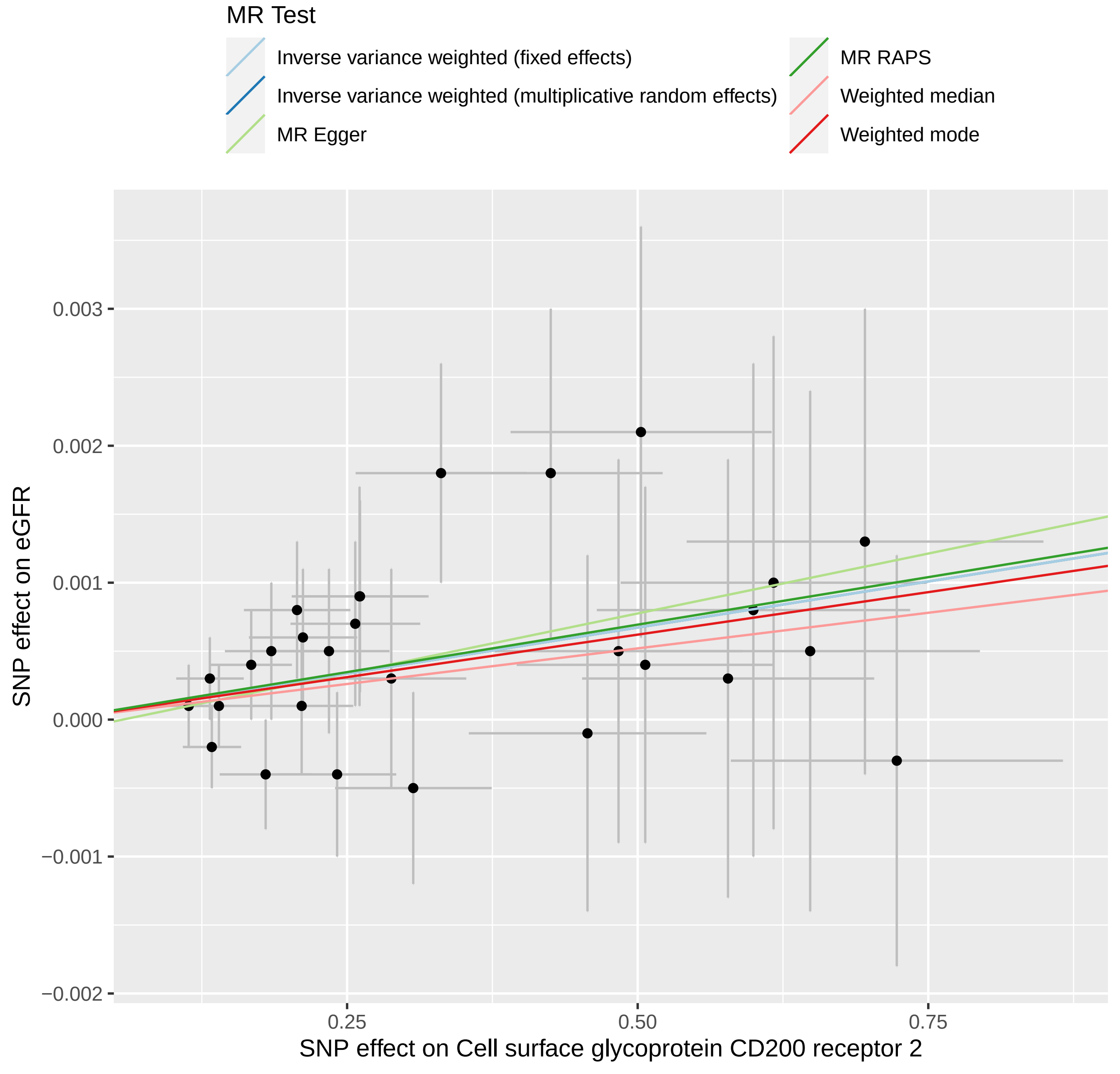

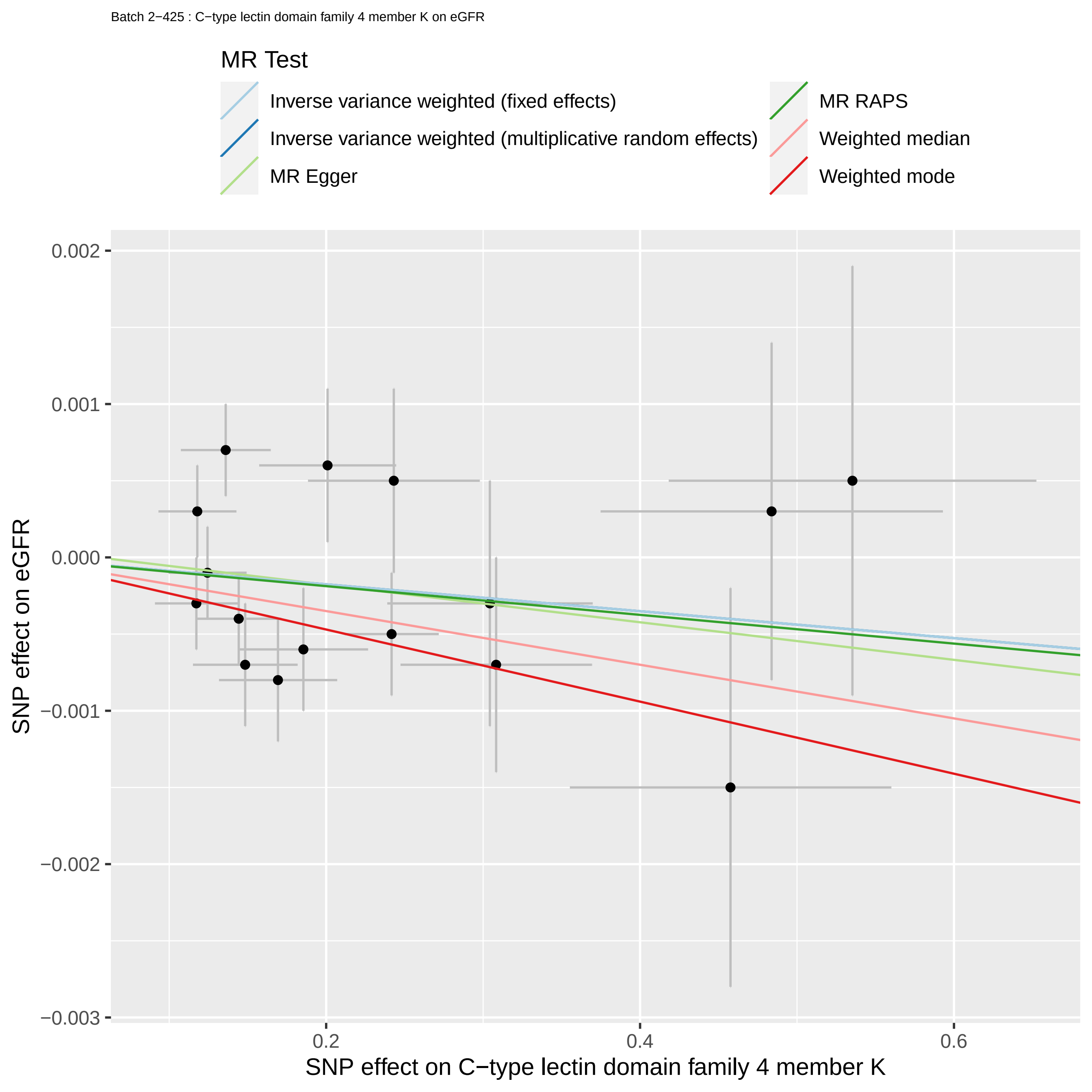

Batch 2−425 : C−type lectin domain family 4 member K on eGFR
MR Test
Inverse variance weighted (fixed effects)
Inverse variance weighted (multiplicative random effects)
MR Egger
MR RAPS
Weighted median
Weighted mode
0.002
0.001
0.000
−0.001
−0.002
−0.003
0.2
0.4
0.6
SNP effect on eGFR
SNP effect on C−type lectin domain family 4 member K

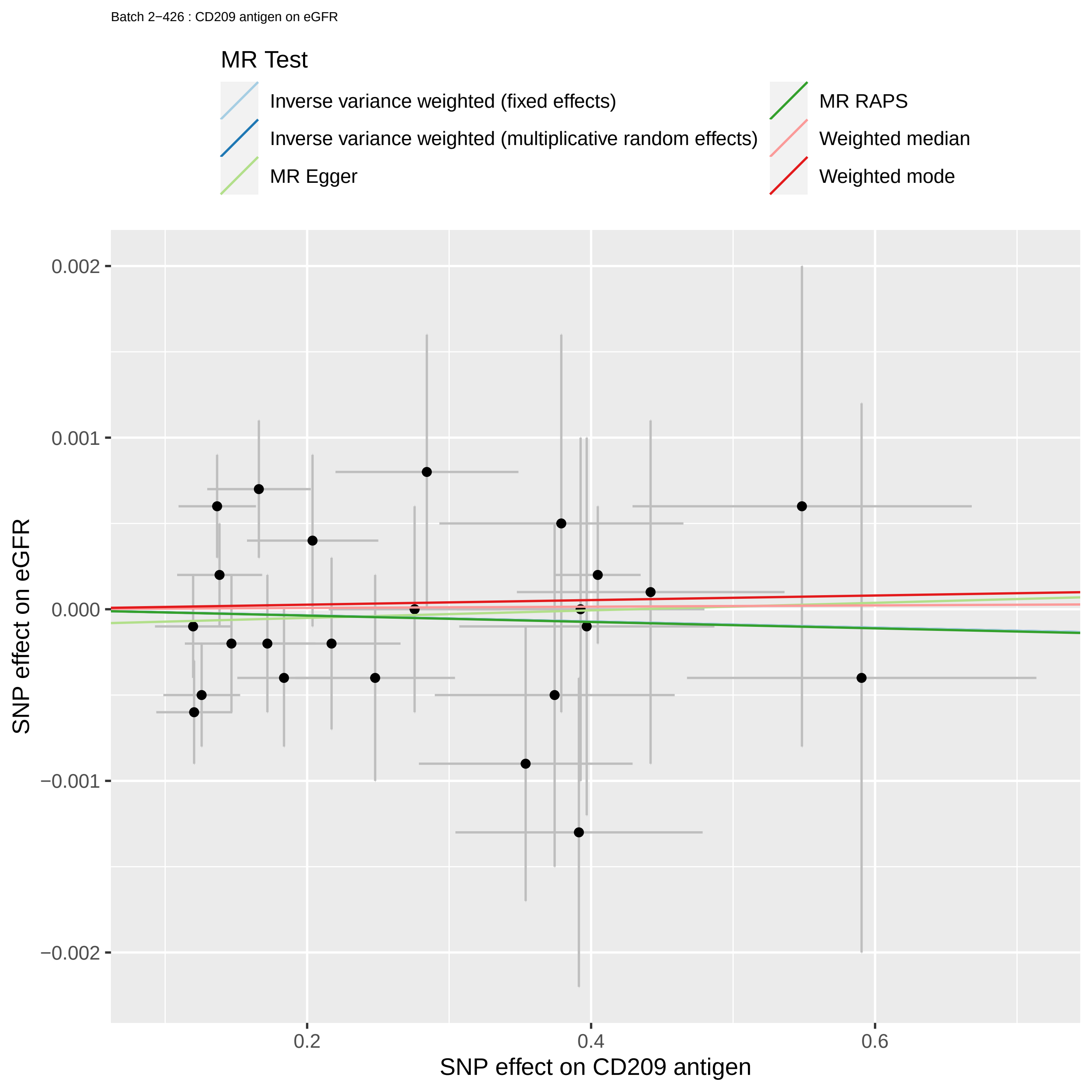

Batch 2−426 : CD209 antigen on eGFR
MR Test
Inverse variance weighted (fixed effects)
Inverse variance weighted (multiplicative random effects)
MR Egger
MR RAPS
Weighted median
Weighted mode
0.002
0.001
0.000
−0.001
−0.002
0.2
0.4
0.6
SNP effect on eGFR
SNP effect on CD209 antigen

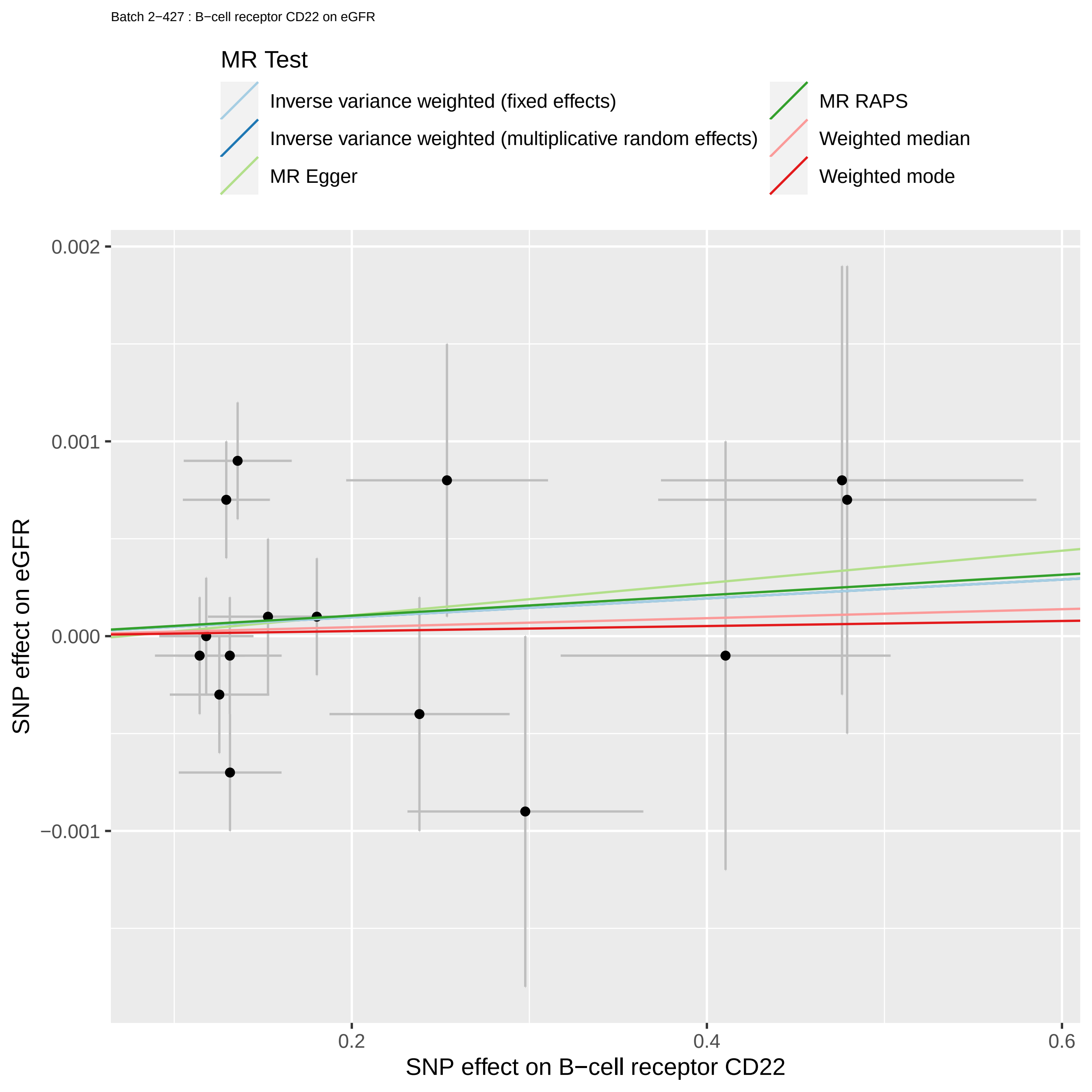
Batch 2−427 : B−cell receptor CD22 on eGFR
MR Test
Inverse variance weighted (fixed effects)
Inverse variance weighted (multiplicative random effects)
MR Egger
MR RAPS
Weighted median
Weighted mode
0.002
0.001
0.000
−0.001
0.2
0.4
0.6
SNP effect on eGFR
SNP effect on B−cell receptor CD22

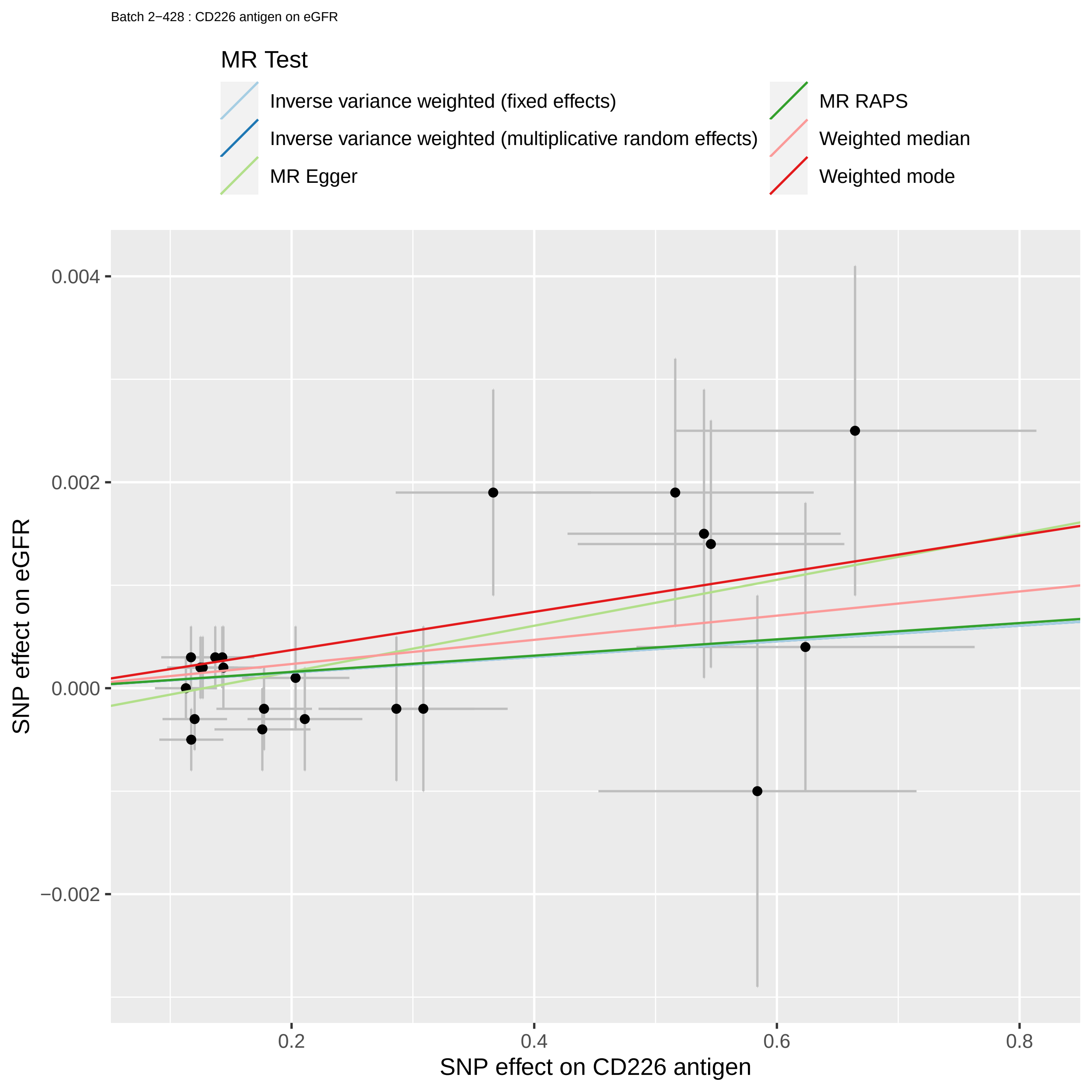
Batch 2−428 : CD226 antigen on eGFR
MR Test
Inverse variance weighted (fixed effects)
Inverse variance weighted (multiplicative random effects)
MR Egger
MR RAPS
Weighted median
Weighted mode
0.004
0.002
0.000
−0.002
0.2
0.4
0.6
0.8
SNP effect on CD226 antigen
SNP effect on eGFR

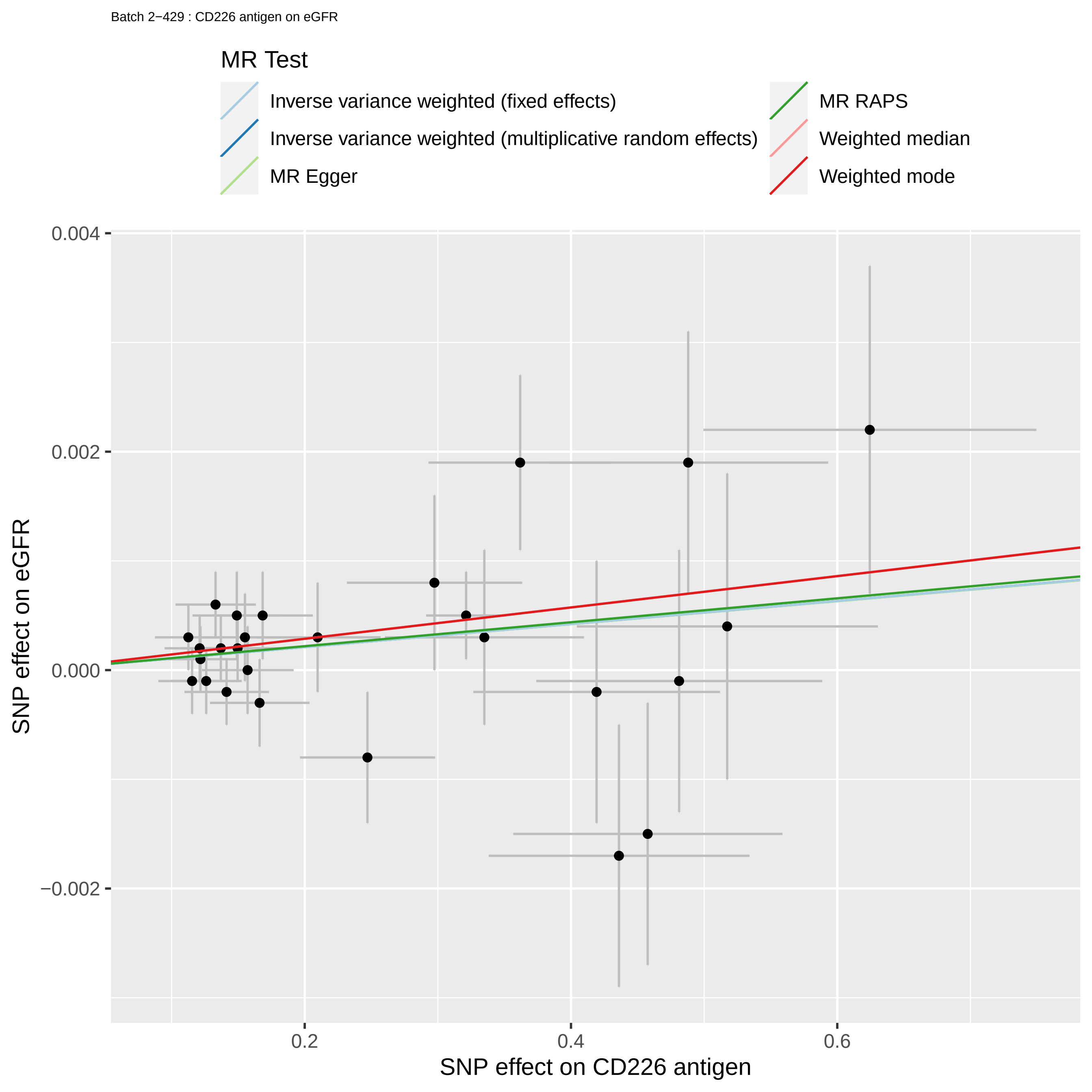

Batch 2−429 : CD226 antigen on eGFR
MR Test
Inverse variance weighted (fixed effects)
Inverse variance weighted (multiplicative random effects)
MR Egger
MR RAPS
Weighted median
Weighted mode
0.004
0.002
0.000
−0.002
0.2
0.4
0.6
SNP effect on eGFR
SNP effect on CD226 antigen

Batch 2−430 : T−cell surface antigen CD2 on eGFR

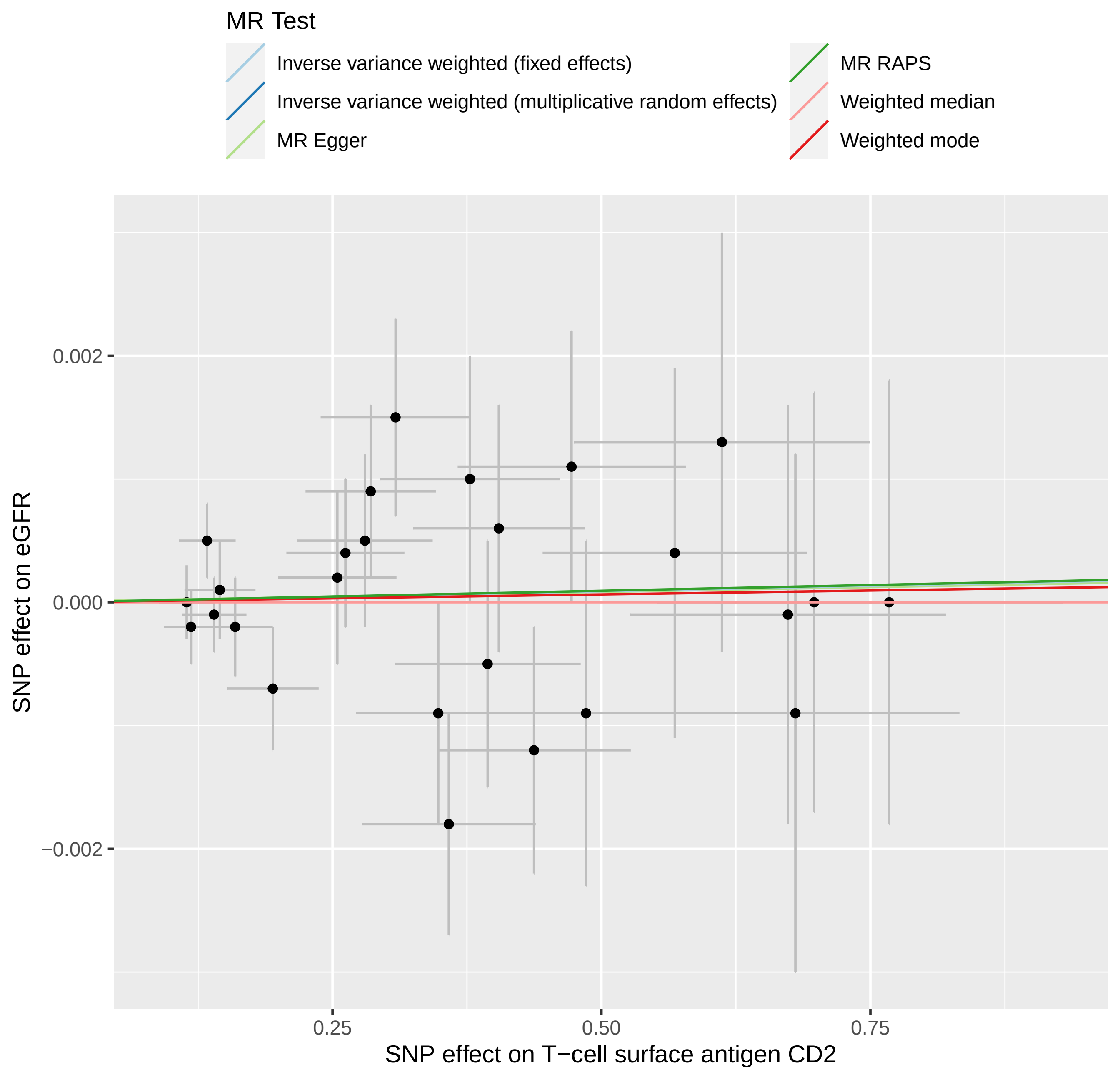

Batch 2−431 : Programmed cell death 1 ligand 1 on eGFR

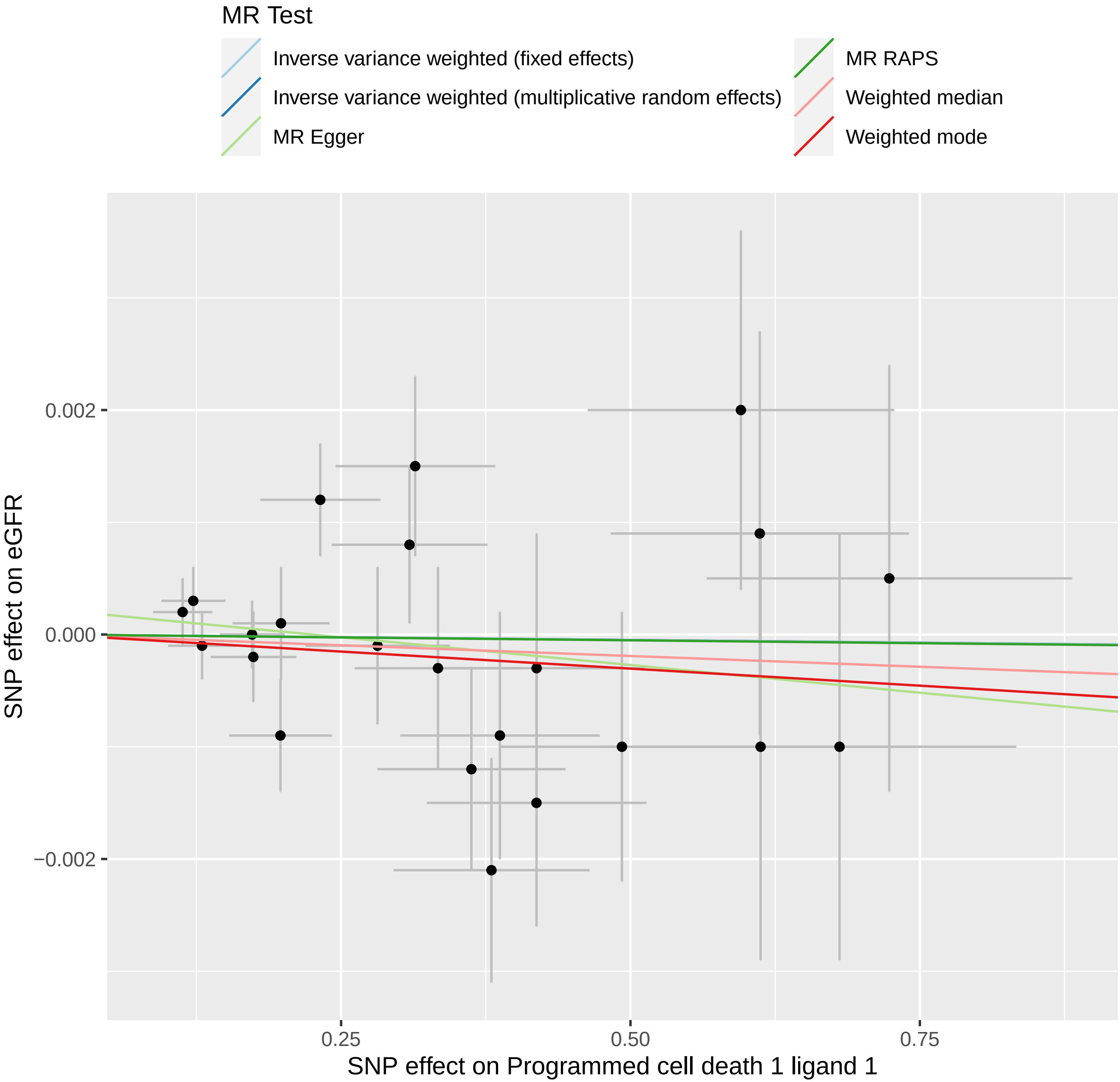

Batch 2−432 : CMRF35−like molecule 8 on eGFR

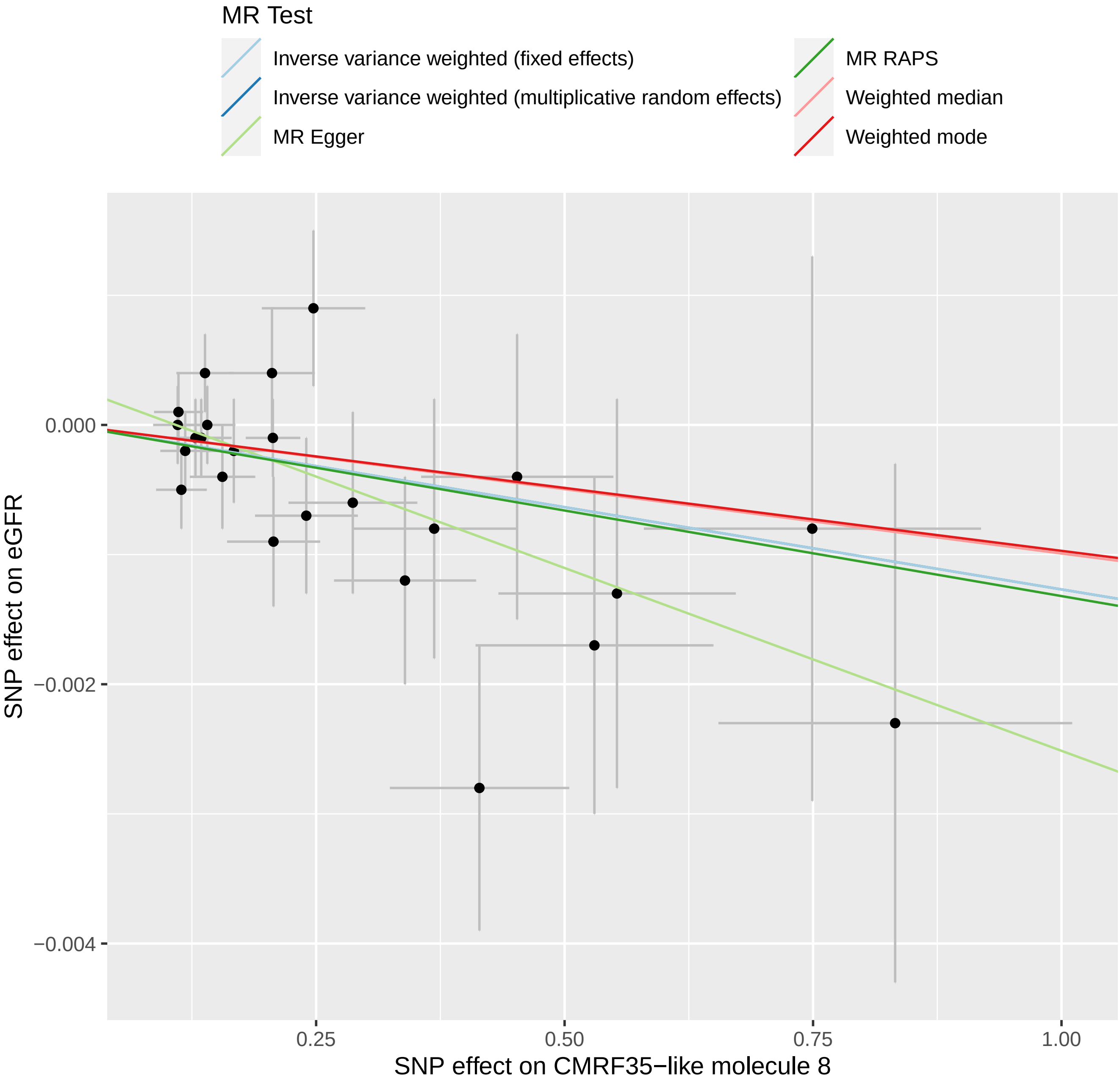

Batch 2−433 : CMRF35−like molecule 6 on eGFR

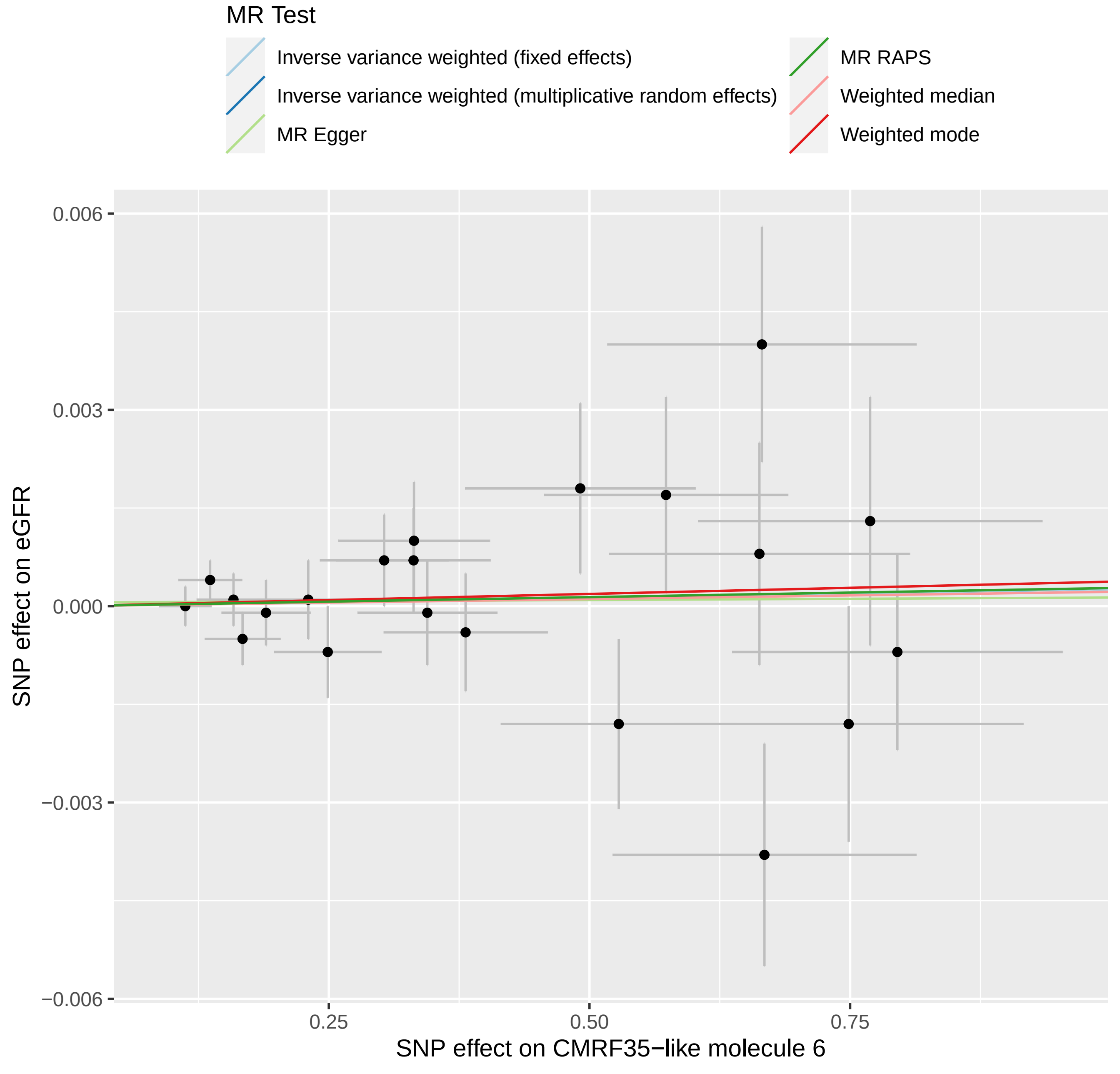

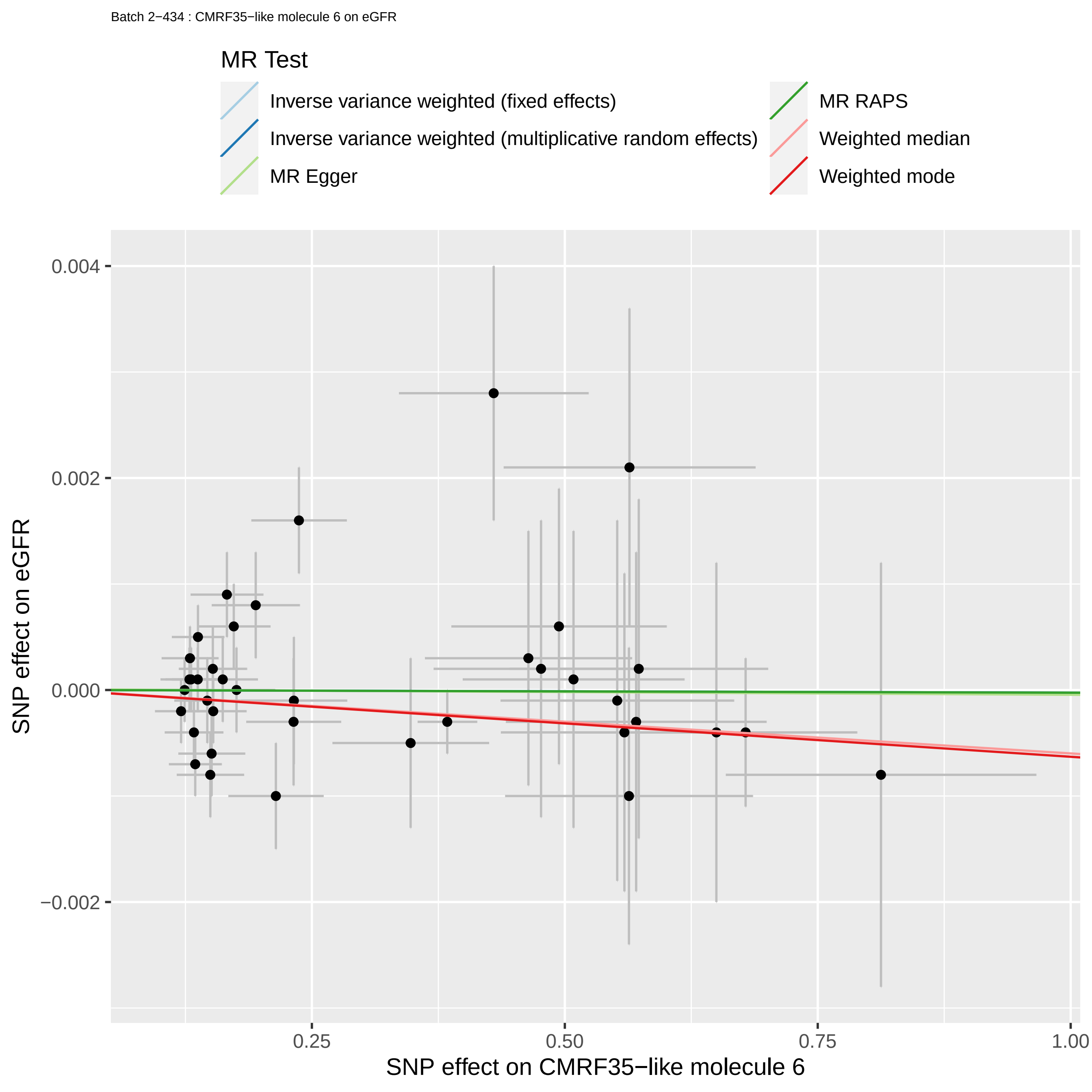

Batch 2−434 : CMRF35−like molecule 6 on eGFR
MR Test
Inverse variance weighted (fixed effects)
Inverse variance weighted (multiplicative random effects)
MR Egger
MR RAPS
Weighted median
Weighted mode
0.004
0.002
0.000
−0.002
0.25
0.50
0.75
1.00
SNP effect on eGFR
SNP effect on CMRF35−like molecule 6

Batch 2−435 : CMRF35−like molecule 2 on eGFR

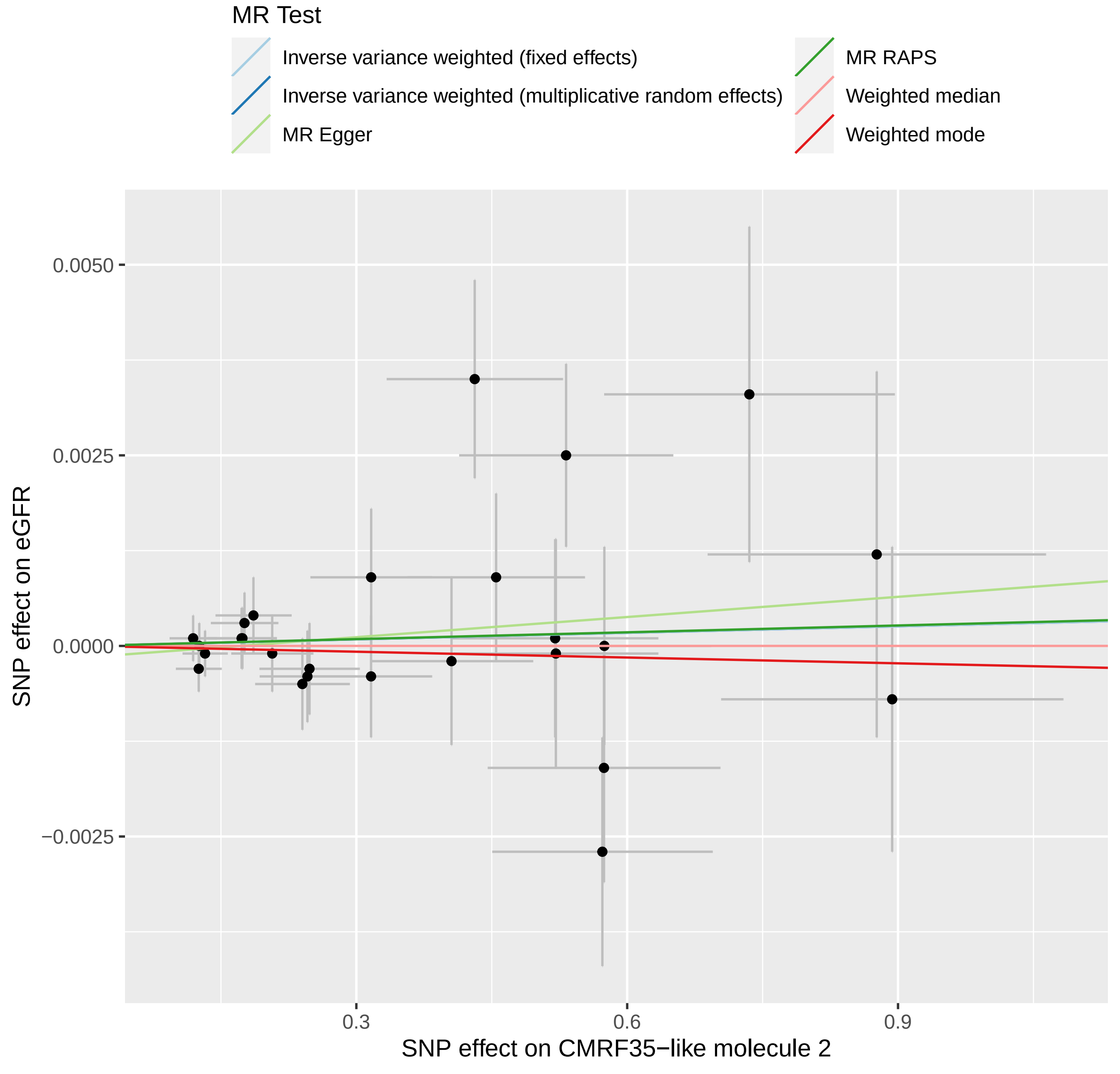

Batch 2−436 : CMRF35−like molecule 7 on eGFR

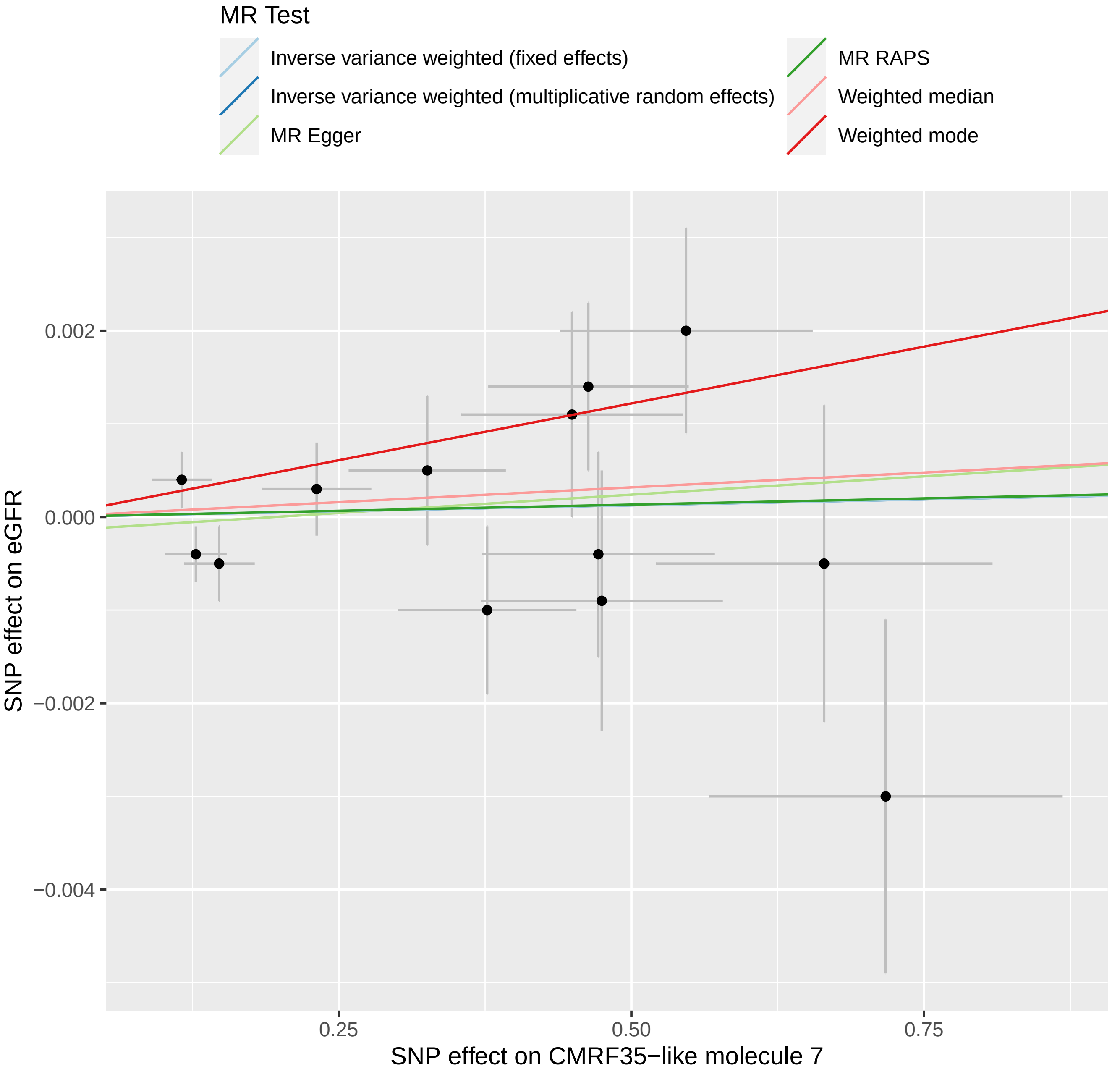

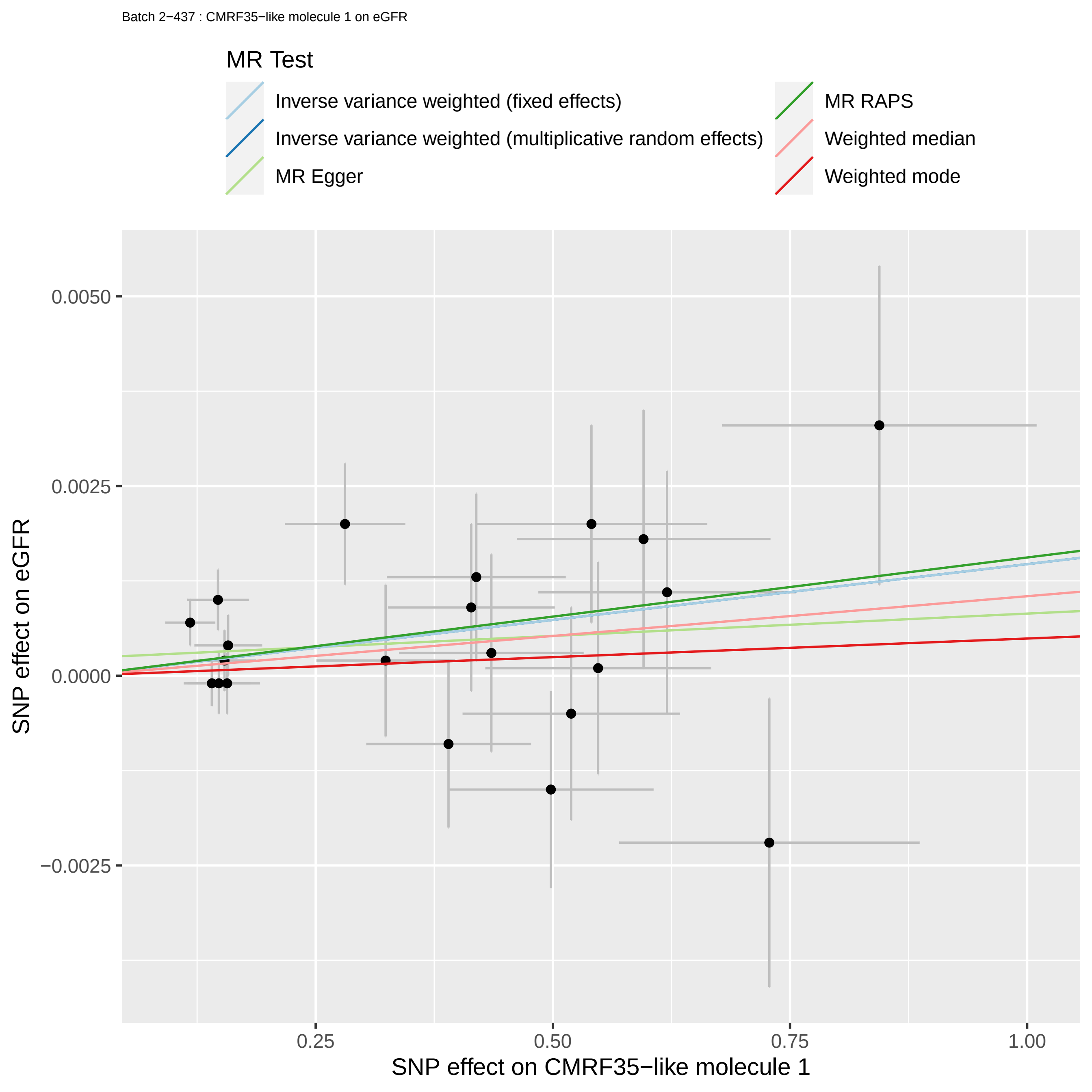
Batch 2−437 : CMRF35−like molecule 1 on eGFR
MR Test
Inverse variance weighted (fixed effects)
Inverse variance weighted (multiplicative random effects)
MR Egger
MR RAPS
Weighted median
Weighted mode
0.0050
0.0025
0.0000
−0.0025
0.25
0.50
0.75
1.00
SNP effect on eGFR
SNP effect on CMRF35−like molecule 1

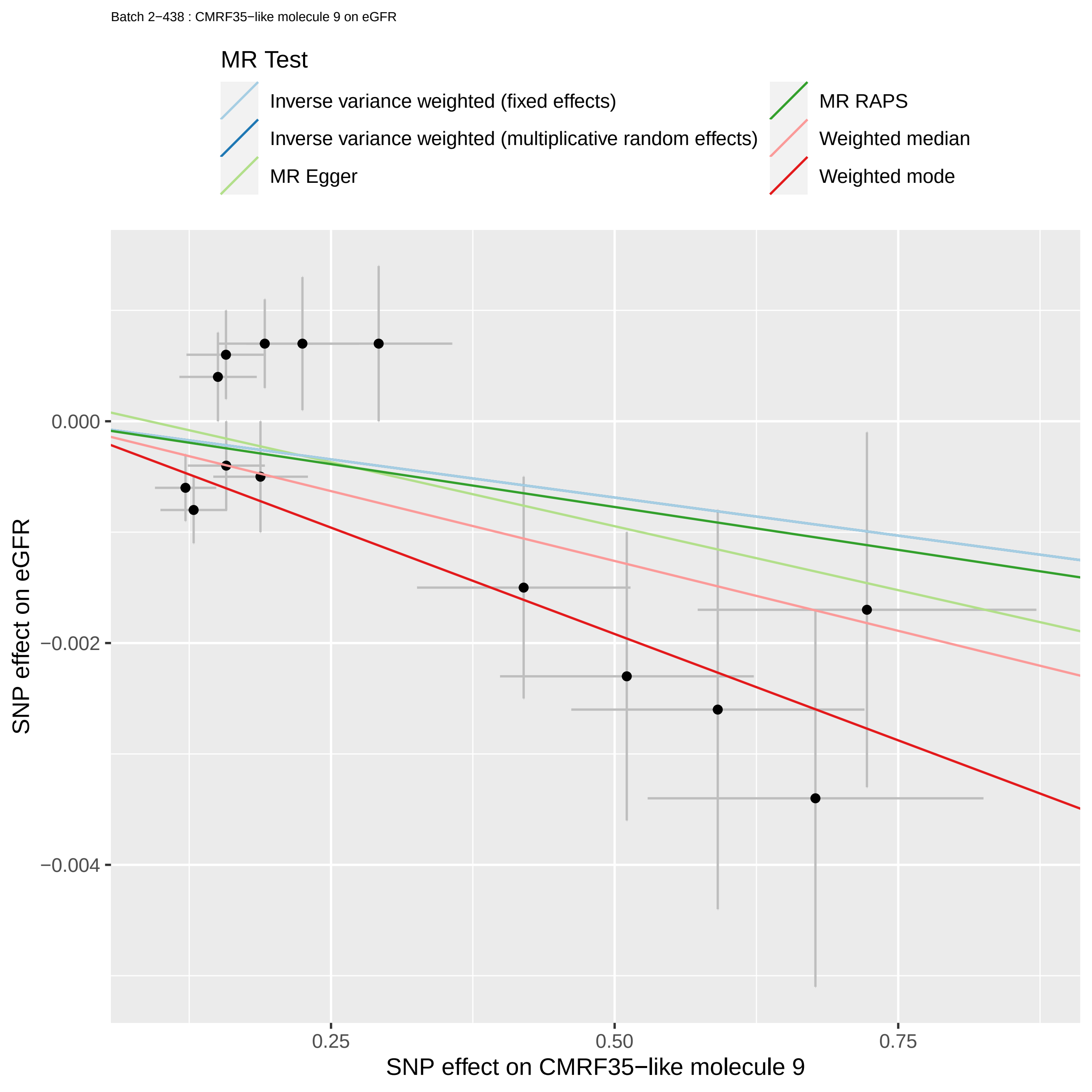
Batch 2−438 : CMRF35−like molecule 9 on eGFR
MR Test
Inverse variance weighted (fixed effects)
Inverse variance weighted (multiplicative random effects)
MR Egger
MR RAPS
Weighted median
Weighted mode
0.000
−0.002
−0.004
0.25
0.50
0.75
SNP effect on eGFR
SNP effect on CMRF35−like molecule 9

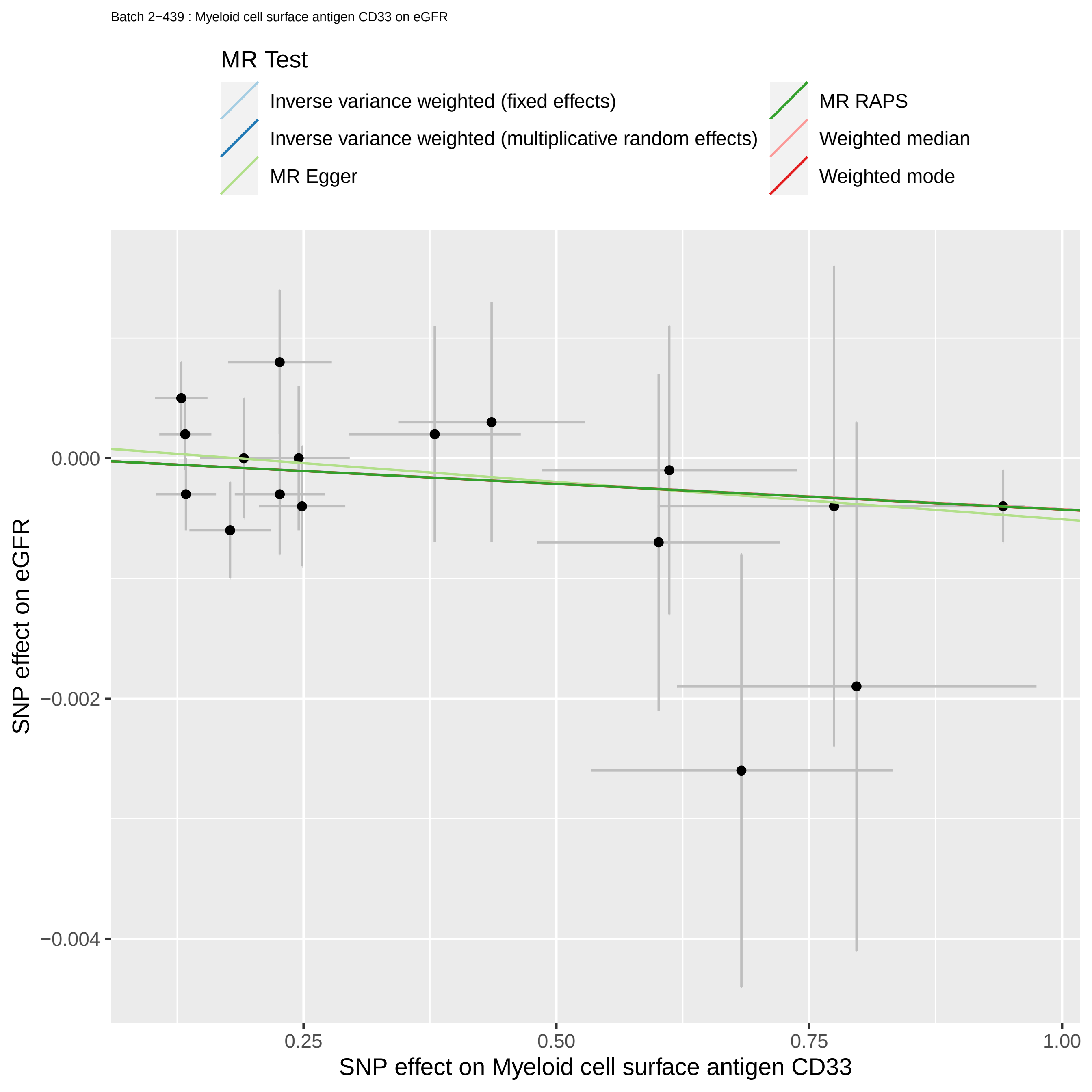

Batch 2−439 : Myeloid cell surface antigen CD33 on eGFR
MR Test
Inverse variance weighted (fixed effects)
Inverse variance weighted (multiplicative random effects)
MR Egger
MR RAPS
Weighted median
Weighted mode
0.000
−0.002
−0.004
SNP effect on eGFR
0.25
0.50
0.75
1.00
SNP effect on Myeloid cell surface antigen CD33

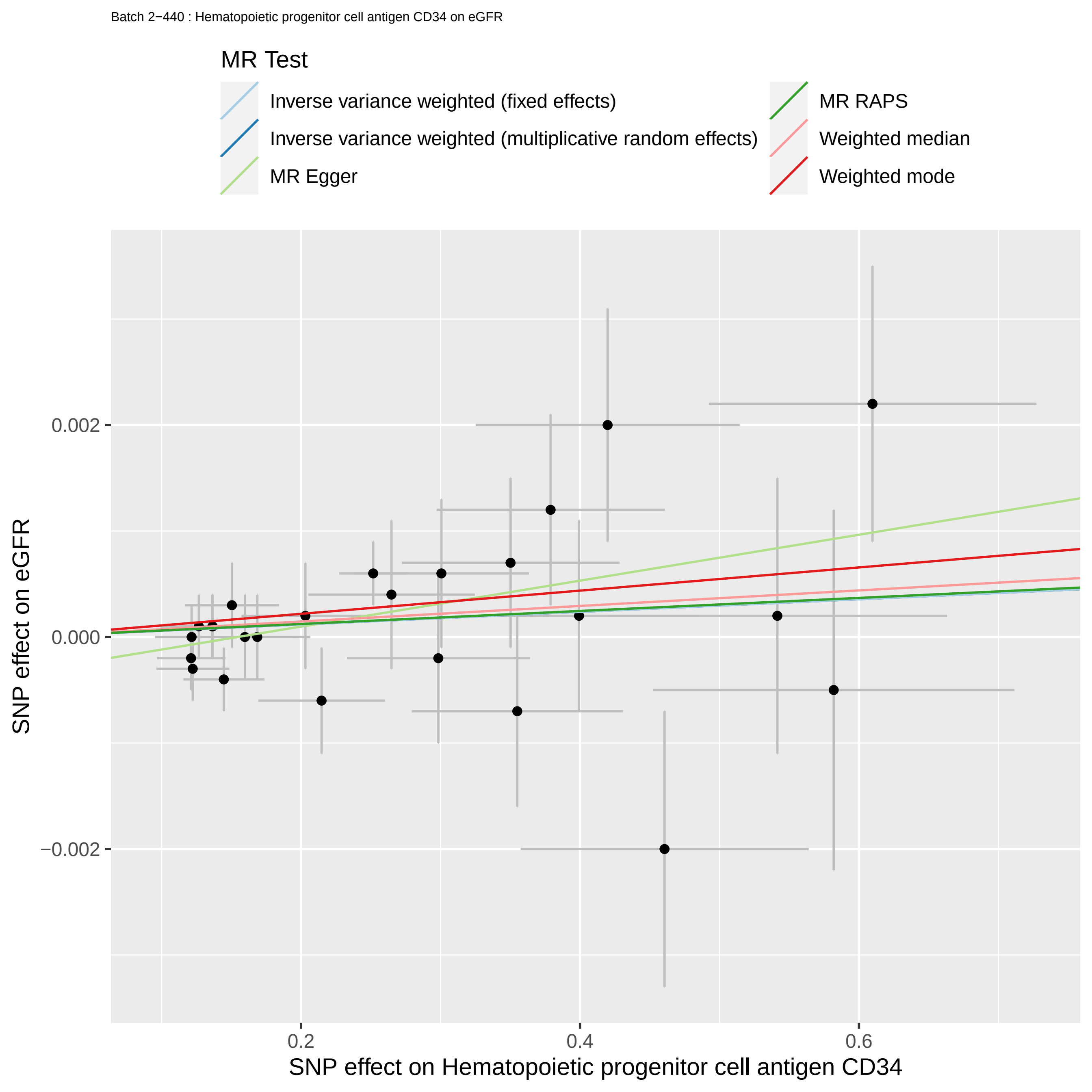

Batch 2−440 : Hematopoietic progenitor cell antigen CD34 on eGFR
MR Test
Inverse variance weighted (fixed effects)
Inverse variance weighted (multiplicative random effects)
MR Egger
MR RAPS
Weighted median
Weighted mode
0.002
0.000
−0.002
SNP effect on eGFR
0.2
0.4
0.6
SNP effect on Hematopoietic progenitor cell antigen CD34

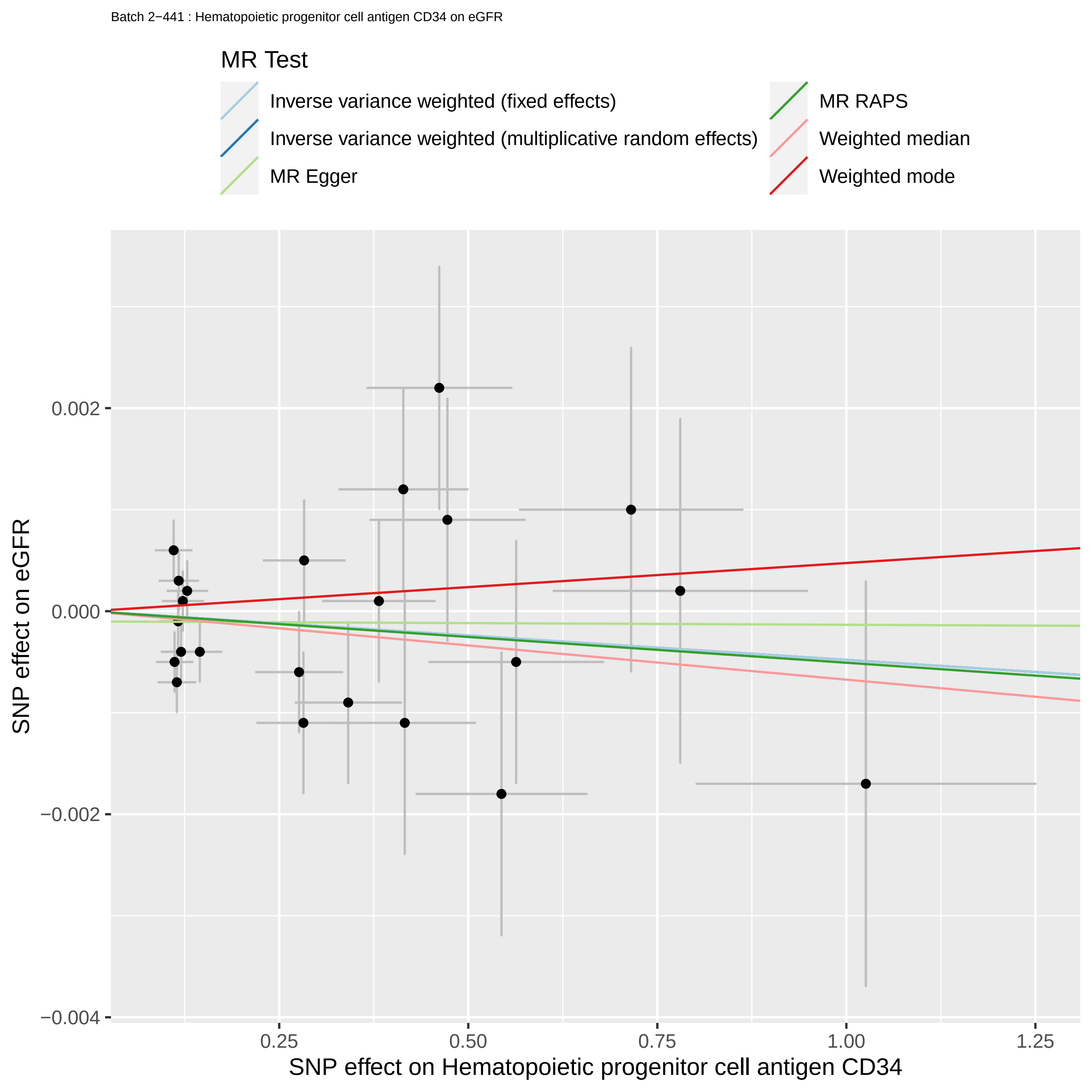

Batch 2−441 : Hematopoietic progenitor cell antigen CD34 on eGFR
MR Test
Inverse variance weighted (fixed effects)
Inverse variance weighted (multiplicative random effects)
MR Egger
MR RAPS
Weighted median
Weighted mode
SNP effect on eGFR
0.002
0.000
−0.002
−0.004
0.25
0.50
0.75
1.00
1.25
SNP effect on Hematopoietic progenitor cell antigen CD34

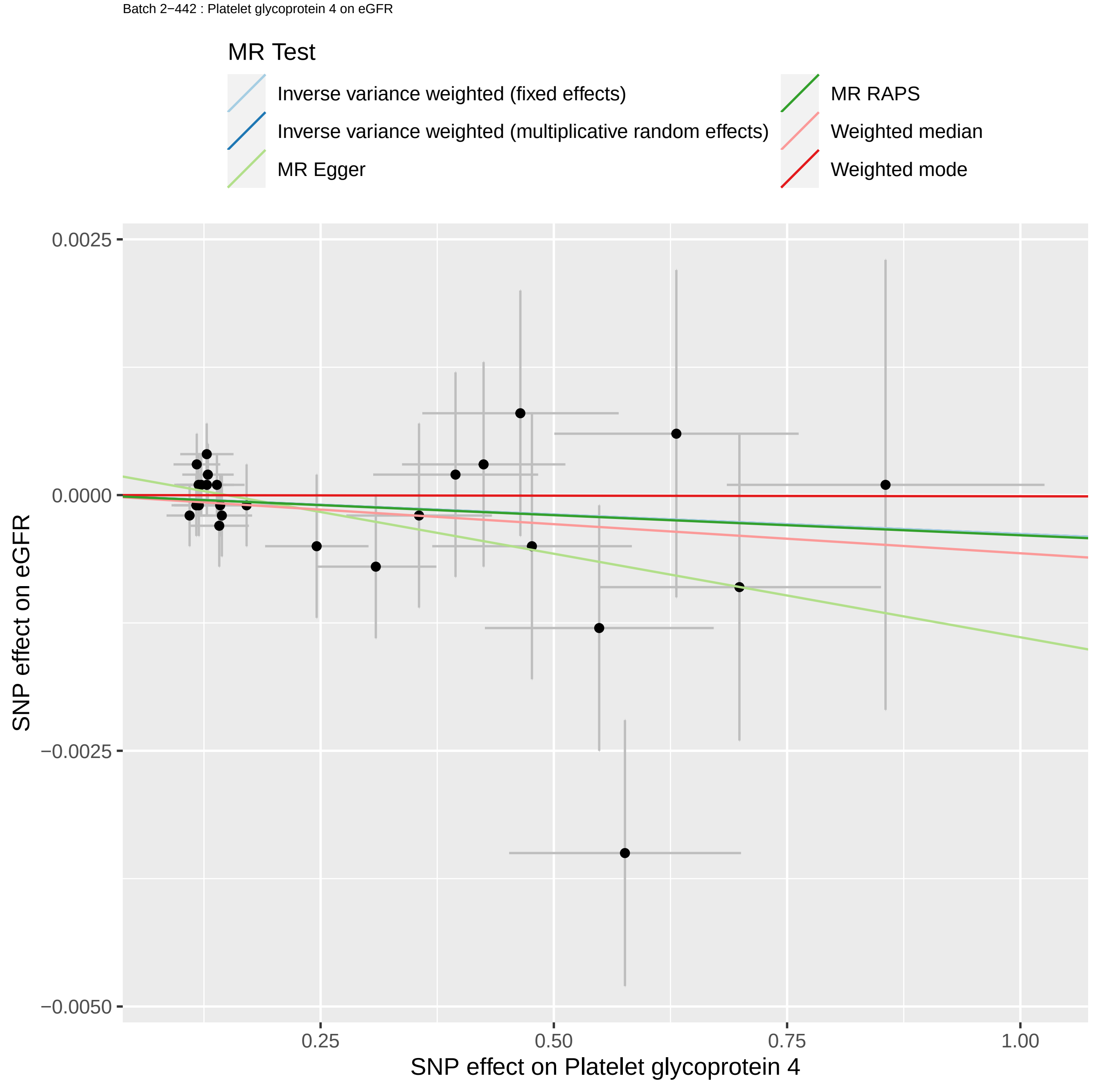
Batch 2−442 : Platelet glycoprotein 4 on eGFR
MR Test
Inverse variance weighted (fixed effects)
Inverse variance weighted (multiplicative random effects)
MR Egger
MR RAPS
Weighted median
Weighted mode
0.0025
0.0000
−0.0025
−0.0050
0.25
0.50
0.75
1.00
SNP effect on eGFR
SNP effect on Platelet glycoprotein 4

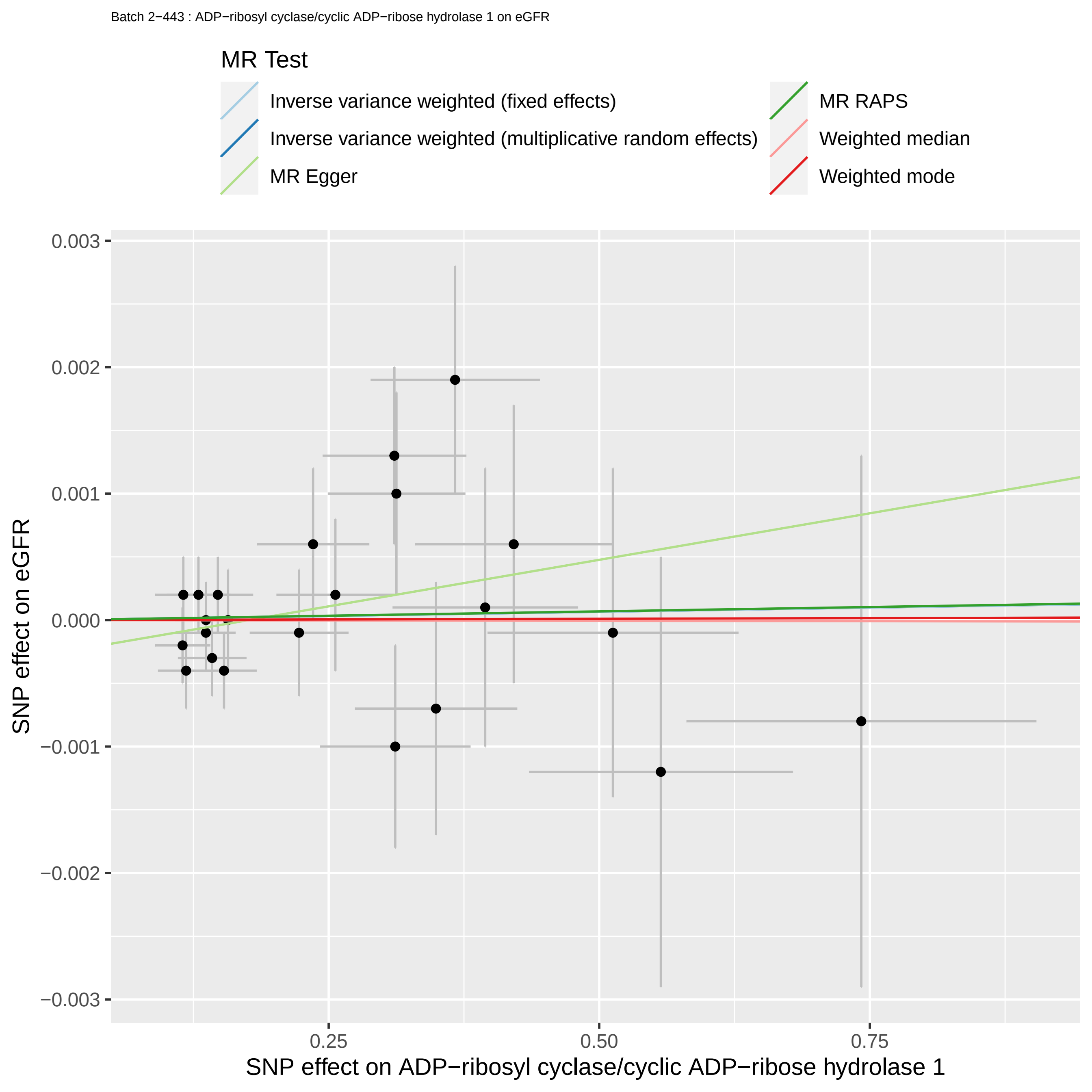

Batch 2−443 : ADP−ribosyl cyclase/cyclic ADP−ribose hydrolase 1 on eGFR
MR Test
Inverse variance weighted (fixed effects)
Inverse variance weighted (multiplicative random effects)
MR Egger
MR RAPS
Weighted median
Weighted mode
0.003
0.002
0.001
0.000
−0.001
−0.002
−0.003
SNP effect on eGFR
0.25
0.50
0.75
SNP effect on ADP−ribosyl cyclase/cyclic ADP−ribose hydrolase 1

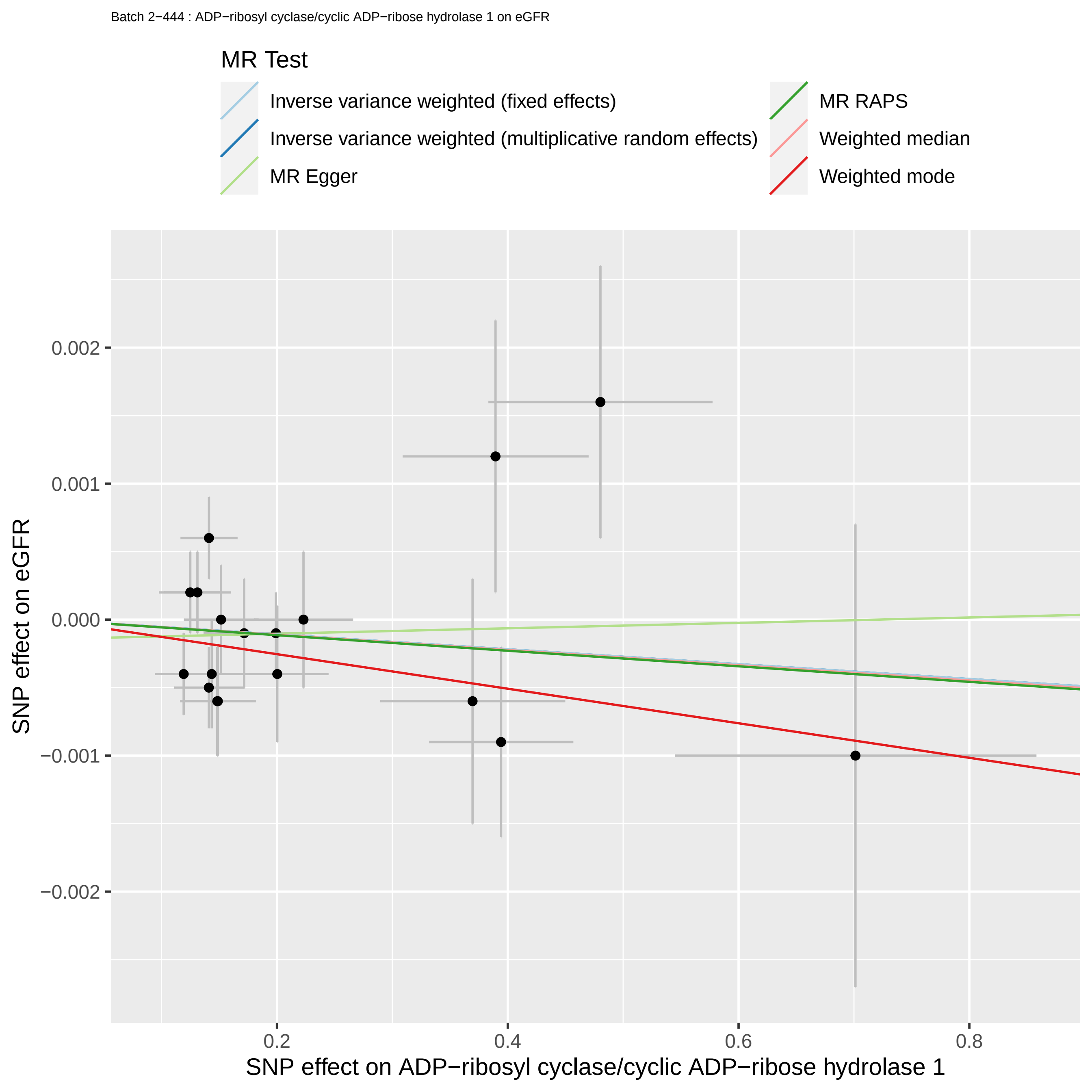
Batch 2−444 : ADP−ribosyl cyclase/cyclic ADP−ribose hydrolase 1 on eGFR
MR Test
Inverse variance weighted (fixed effects)
Inverse variance weighted (multiplicative random effects)
MR Egger
MR RAPS
Weighted median
Weighted mode
0.002
0.001
0.000
−0.001
−0.002
0.2
0.4
0.6
0.8
SNP effect on eGFR
SNP effect on ADP−ribosyl cyclase/cyclic ADP−ribose hydrolase 1

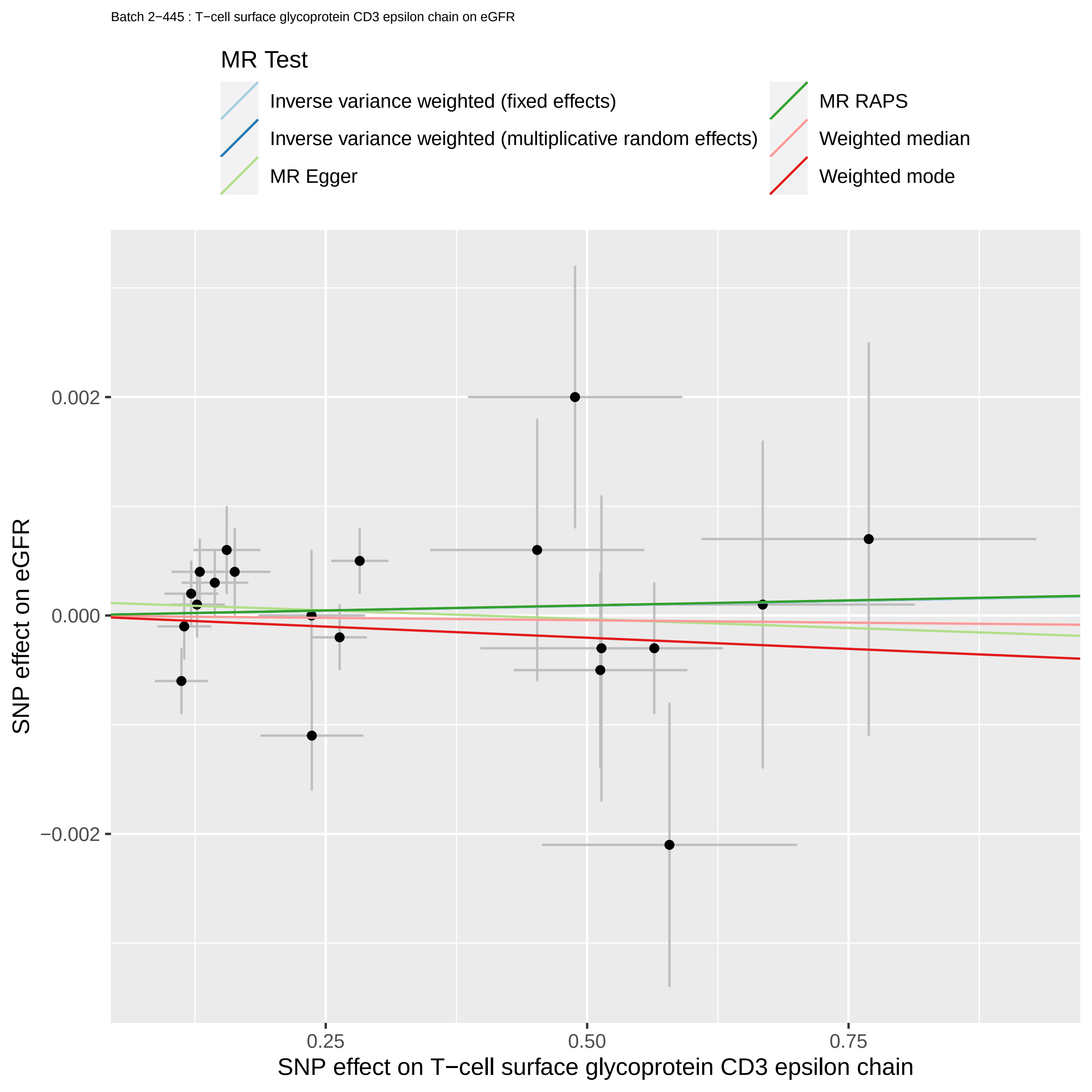

Batch 2−445 : T−cell surface glycoprotein CD3 epsilon chain on eGFR
MR Test
Inverse variance weighted (fixed effects)
Inverse variance weighted (multiplicative random effects)
MR Egger
MR RAPS
Weighted median
Weighted mode
0.002
0.000
−0.002
0.25
0.50
0.75
SNP effect on eGFR
SNP effect on T−cell surface glycoprotein CD3 epsilon chain

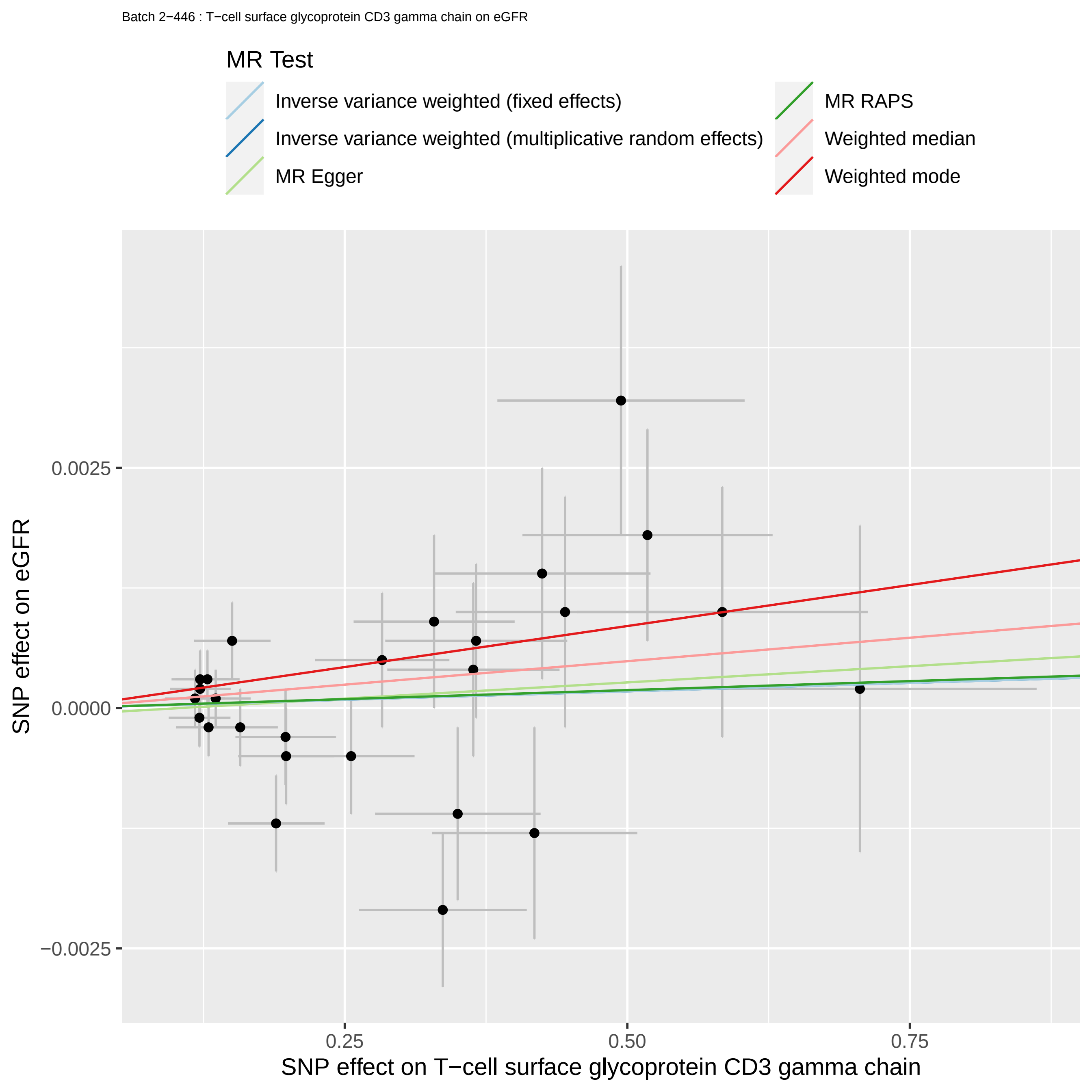
Batch 2−446 : T−cell surface glycoprotein CD3 gamma chain on eGFR
MR Test
Inverse variance weighted (fixed effects)
Inverse variance weighted (multiplicative random effects)
MR Egger
MR RAPS
Weighted median
Weighted mode
0.0025
0.0000
−0.0025
0.25
0.50
0.75
SNP effect on eGFR
SNP effect on T−cell surface glycoprotein CD3 gamma chain

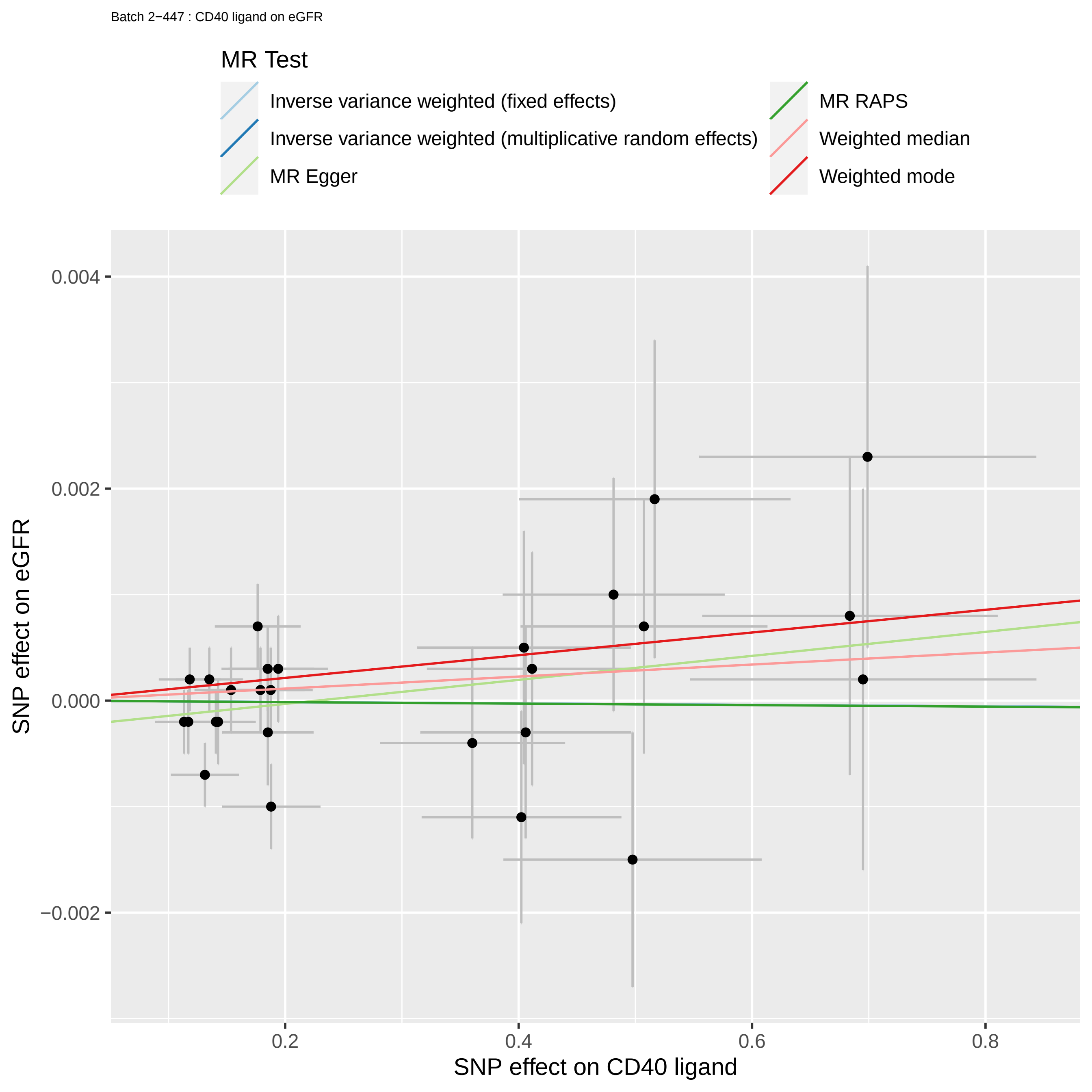

Batch 2−447 : CD40 ligand on eGFR
MR Test
Inverse variance weighted (fixed effects)
Inverse variance weighted (multiplicative random effects)
MR Egger
MR RAPS
Weighted median
Weighted mode
0.004
0.002
0.000
−0.002
0.2
0.4
0.6
0.8
SNP effect on eGFR
SNP effect on CD40 ligand

Batch 2−448 : T−cell surface glycoprotein CD4 on eGFR

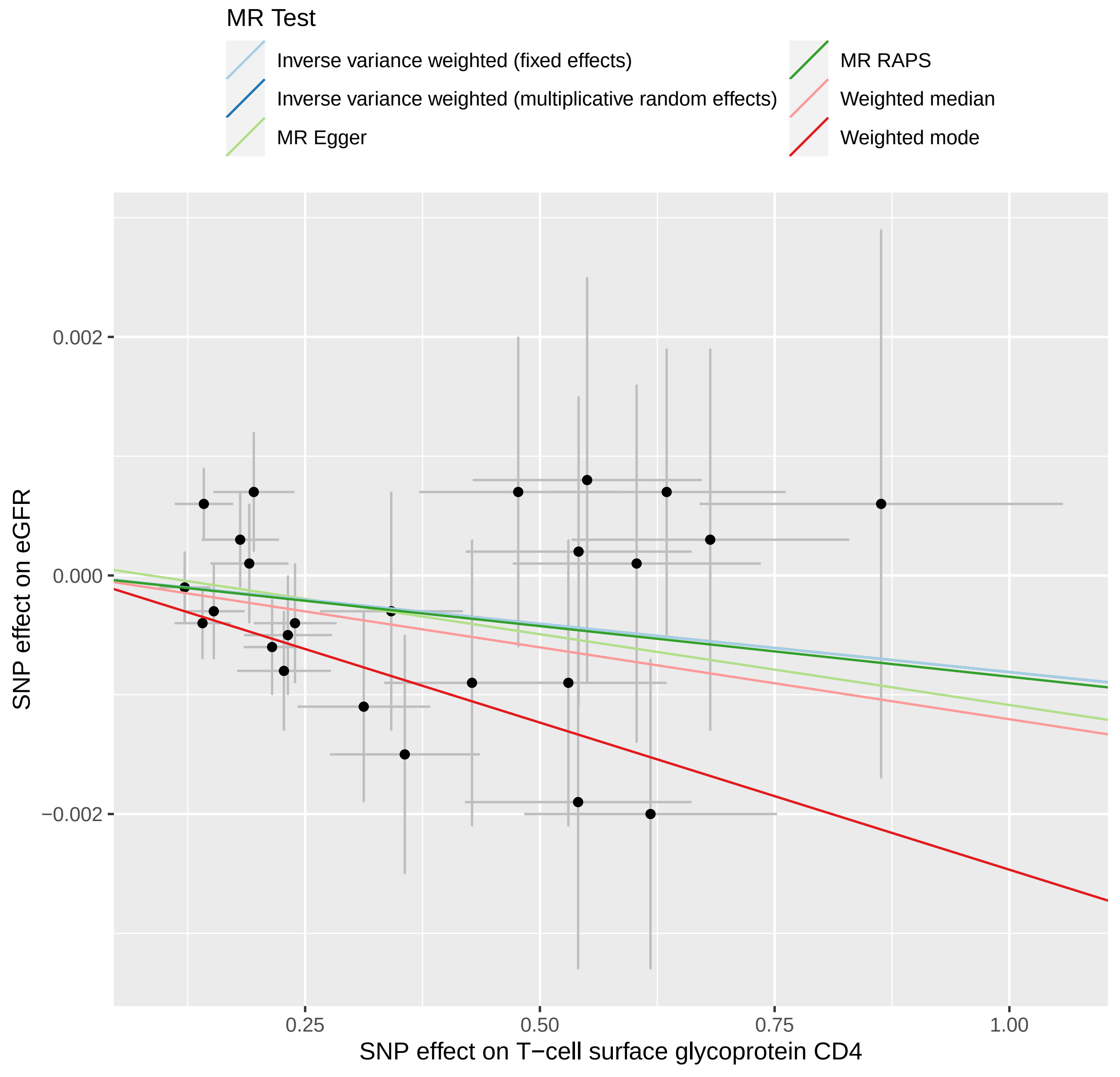

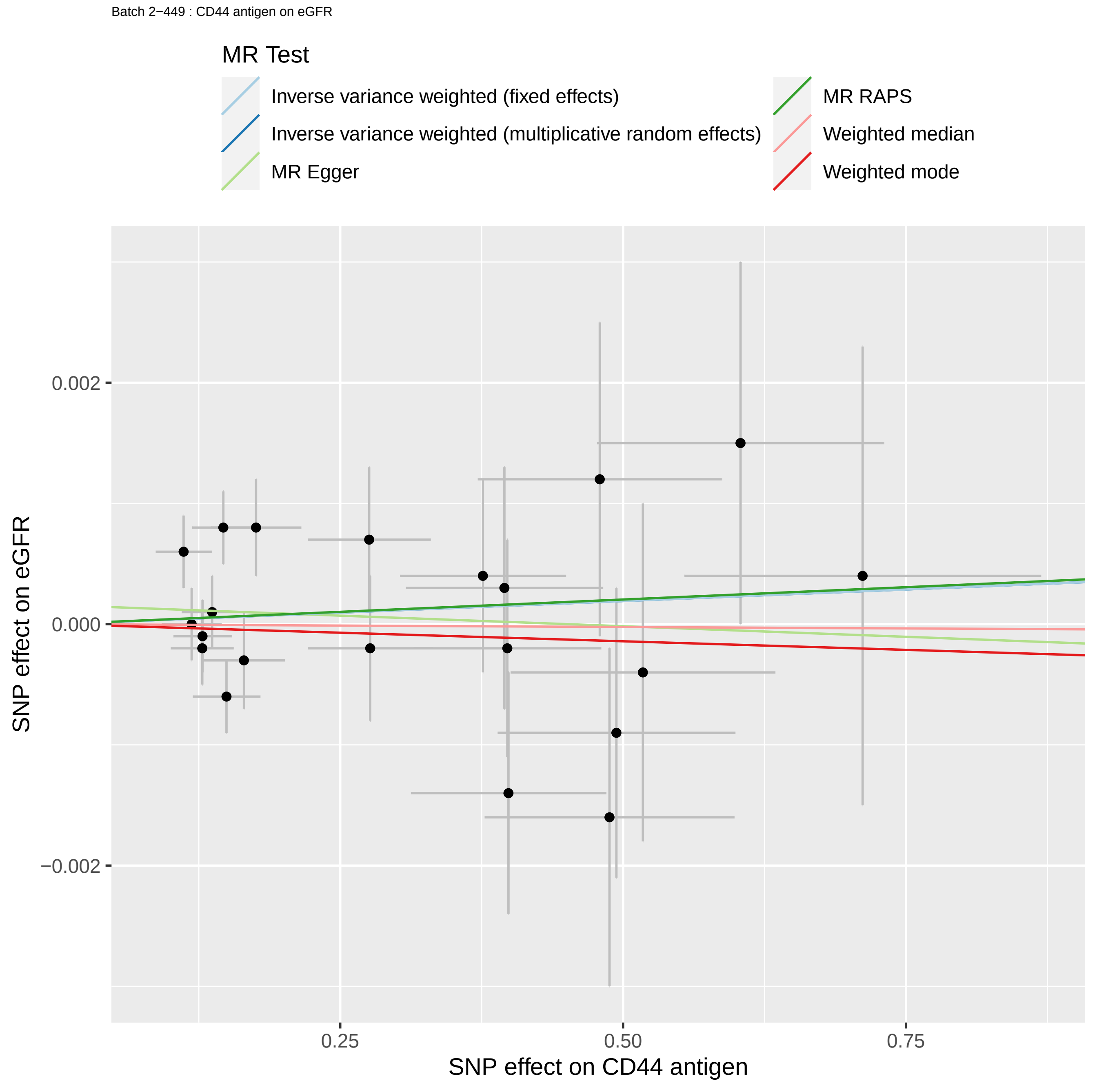
Batch 2−449 : CD44 antigen on eGFR
MR Test
Inverse variance weighted (fixed effects)
Inverse variance weighted (multiplicative random effects)
MR Egger
MR RAPS
Weighted median
Weighted mode
0.002
0.000
−0.002
0.25
0.50
0.75
SNP effect on CD44 antigen
SNP effect on eGFR

Batch 2−450 : Leukocyte surface antigen CD47 on eGFR

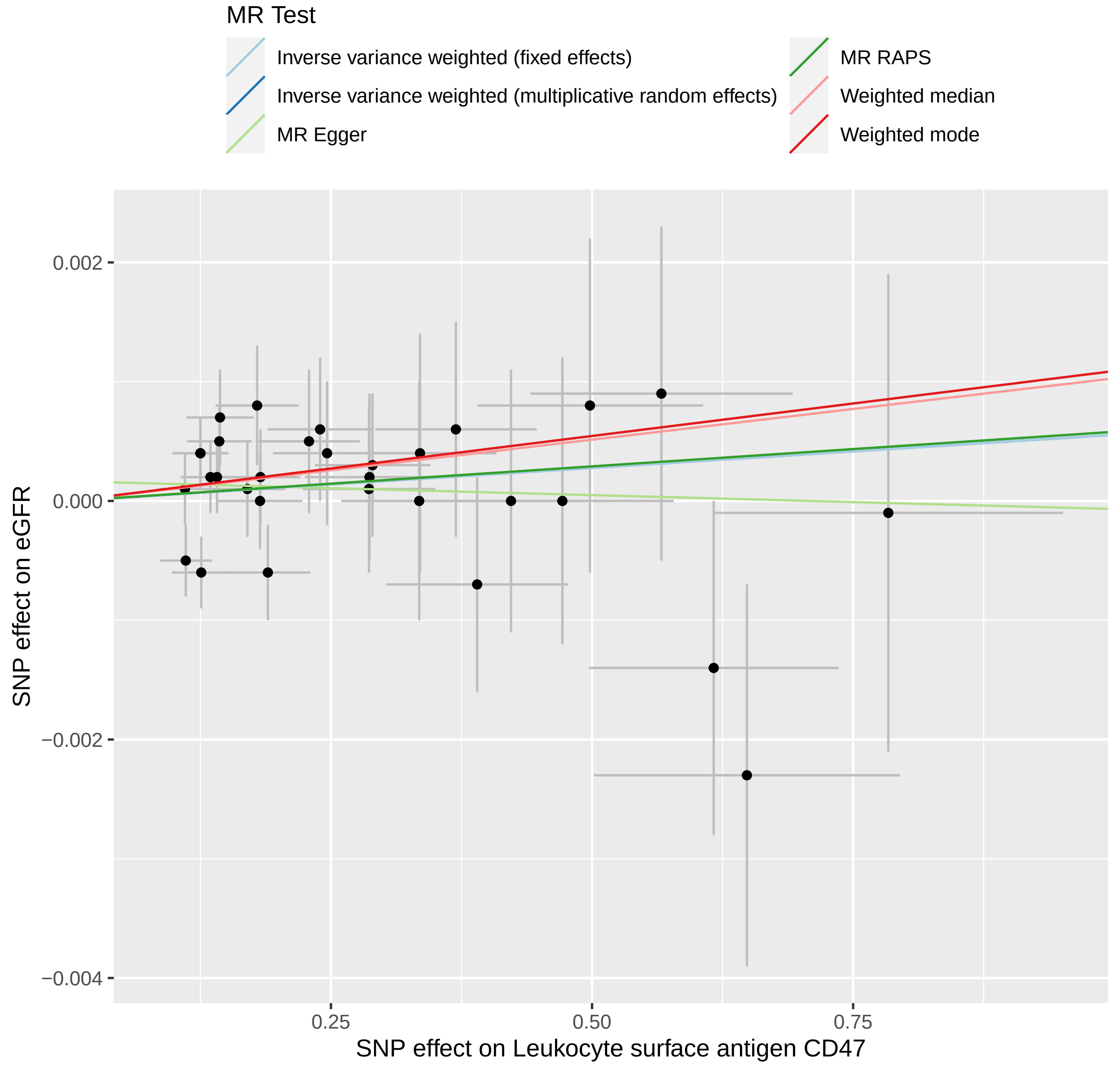

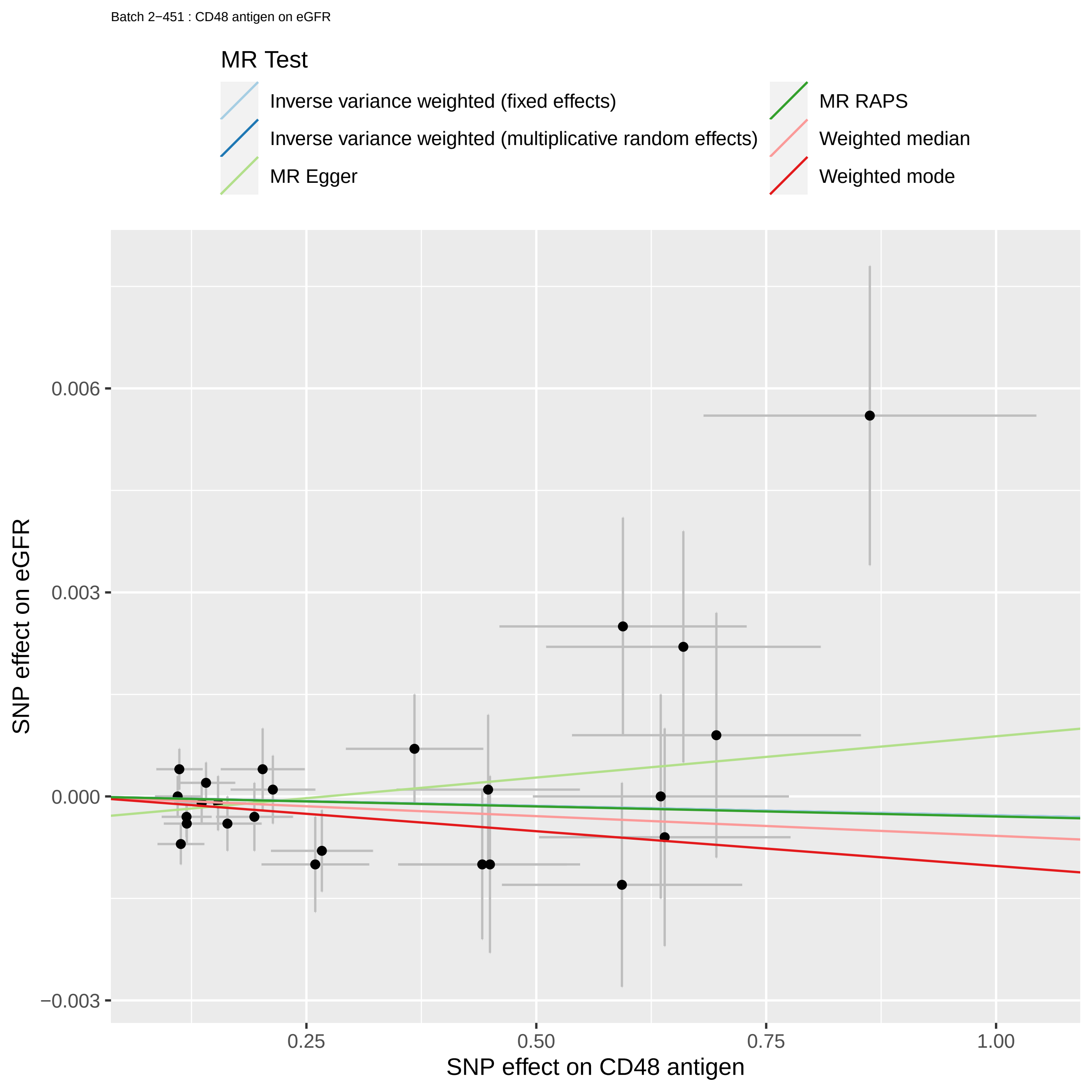
Batch 2−451 : CD48 antigen on eGFR
MR Test
Inverse variance weighted (fixed effects)
Inverse variance weighted (multiplicative random effects)
MR Egger
MR RAPS
Weighted median
Weighted mode
0.006
0.003
0.000
−0.003
SNP effect on eGFR
0.25
0.50
0.75
1.00
SNP effect on CD48 antigen

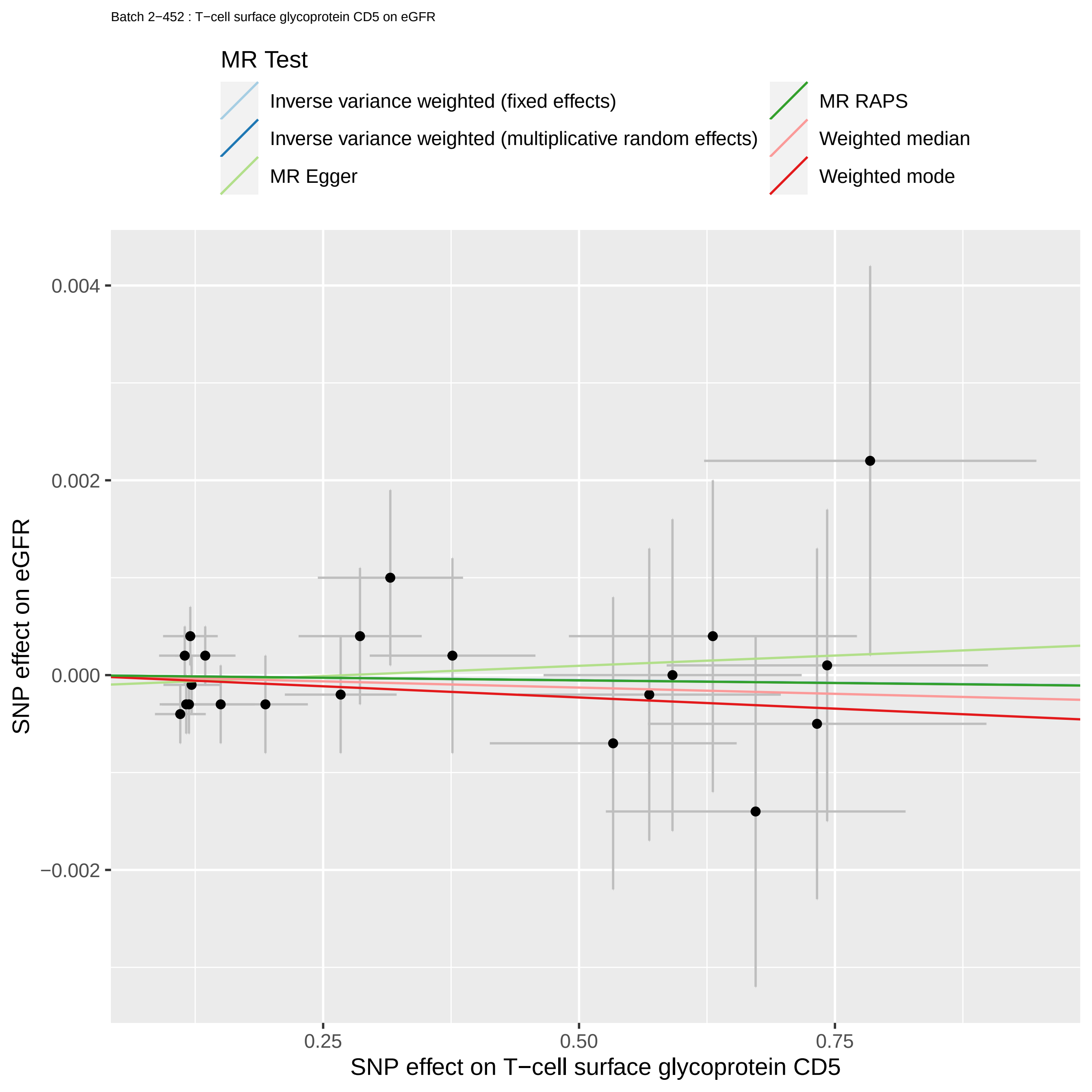

Batch 2−452 : T−cell surface glycoprotein CD5 on eGFR
MR Test
Inverse variance weighted (fixed effects)
Inverse variance weighted (multiplicative random effects)
MR Egger
MR RAPS
Weighted median
Weighted mode
0.004
0.002
0.000
−0.002
0.25
0.50
0.75
SNP effect on eGFR
SNP effect on T−cell surface glycoprotein CD5

Batch 2−453 : Complement decay−accelerating factor on eGFR

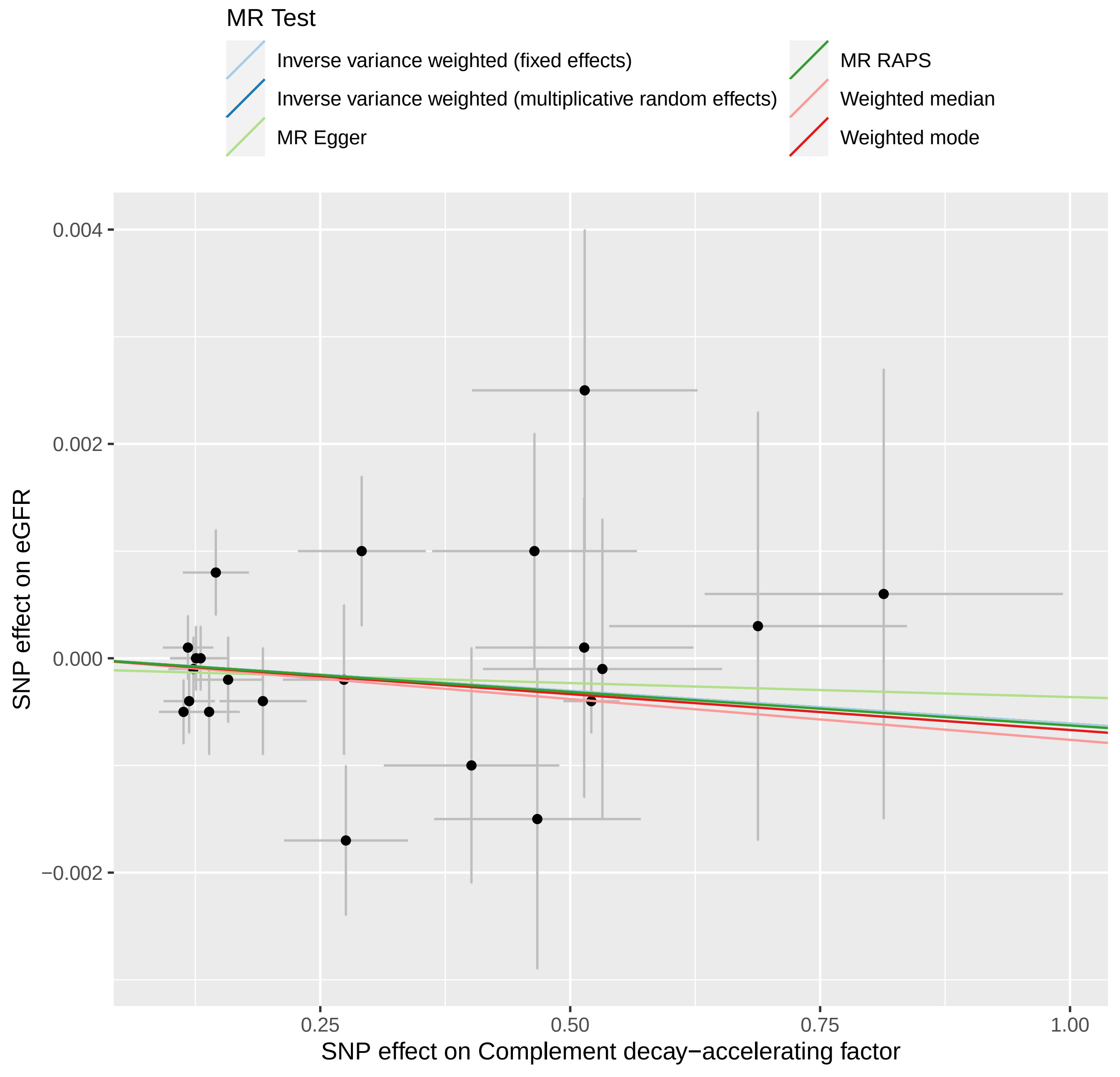

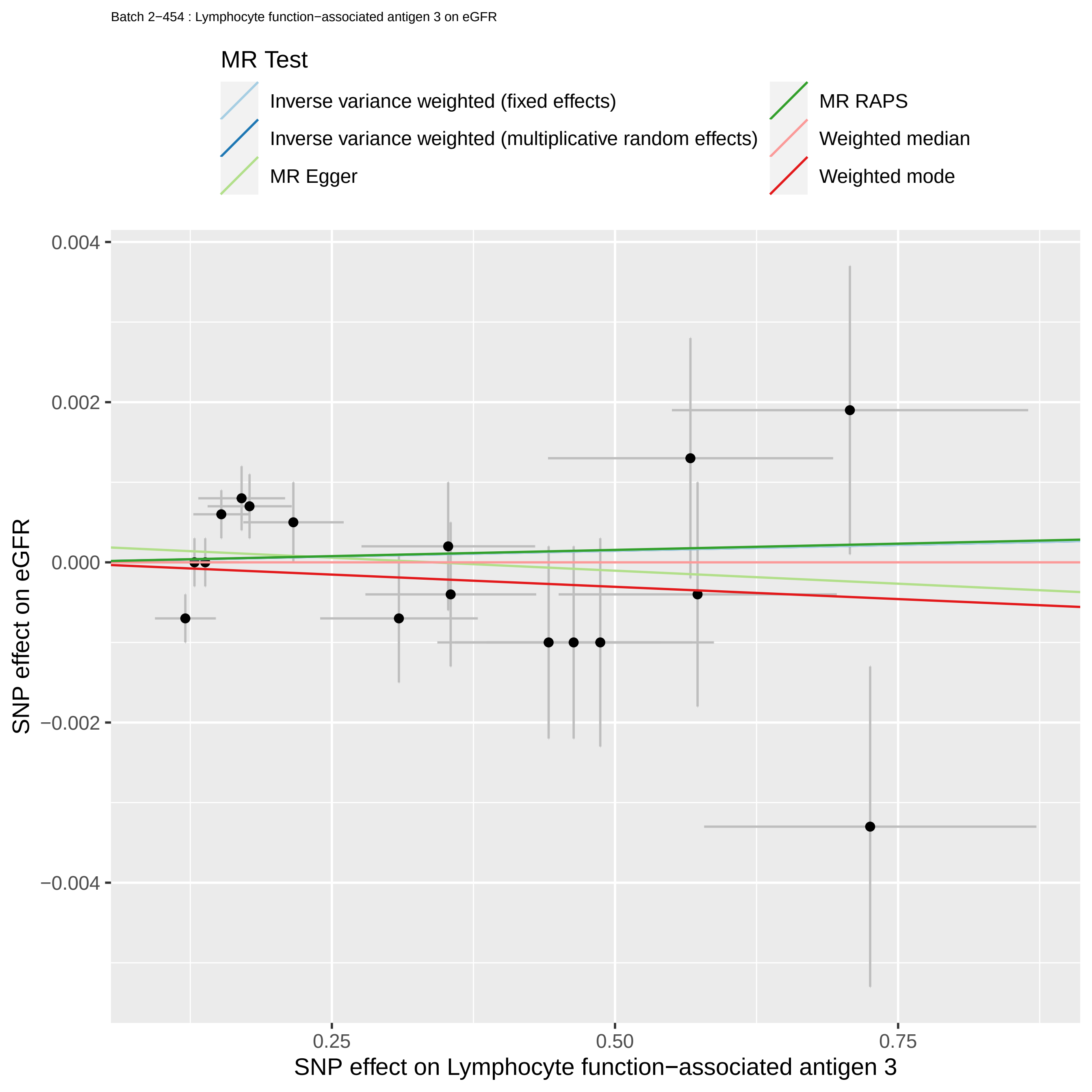

Batch 2−454 : Lymphocyte function−associated antigen 3 on eGFR
MR Test
Inverse variance weighted (fixed effects)
Inverse variance weighted (multiplicative random effects)
MR Egger
MR RAPS
Weighted median
Weighted mode
0.004
0.002
0.000
−0.002
−0.004
0.25
0.50
0.75
SNP effect on eGFR
SNP effect on Lymphocyte function−associated antigen 3

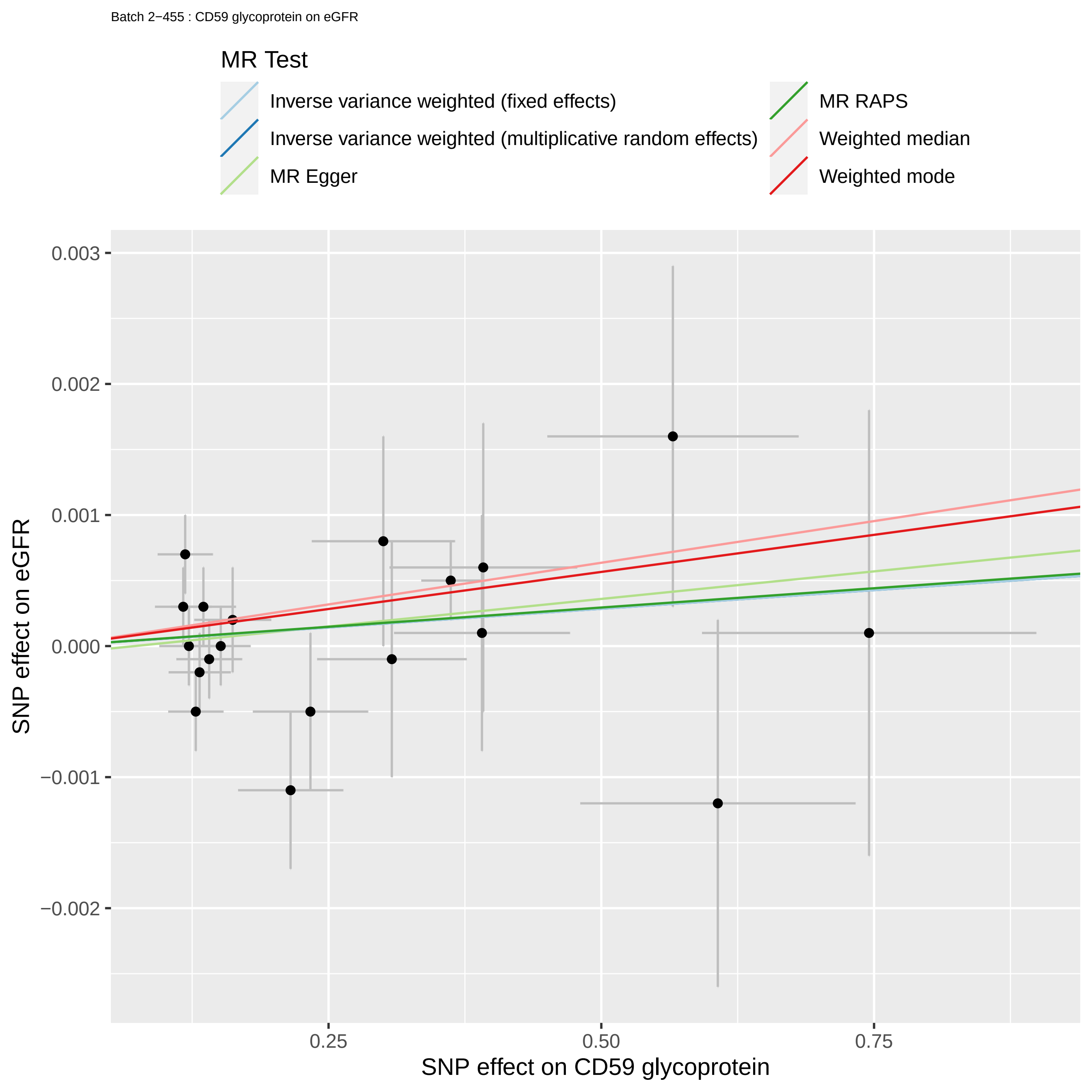
Batch 2−455 : CD59 glycoprotein on eGFR
MR Test
Inverse variance weighted (fixed effects)
Inverse variance weighted (multiplicative random effects)
MR Egger
MR RAPS
Weighted median
Weighted mode
0.003
0.002
0.001
0.000
−0.001
−0.002
SNP effect on eGFR
0.25
0.50
0.75
SNP effect on CD59 glycoprotein

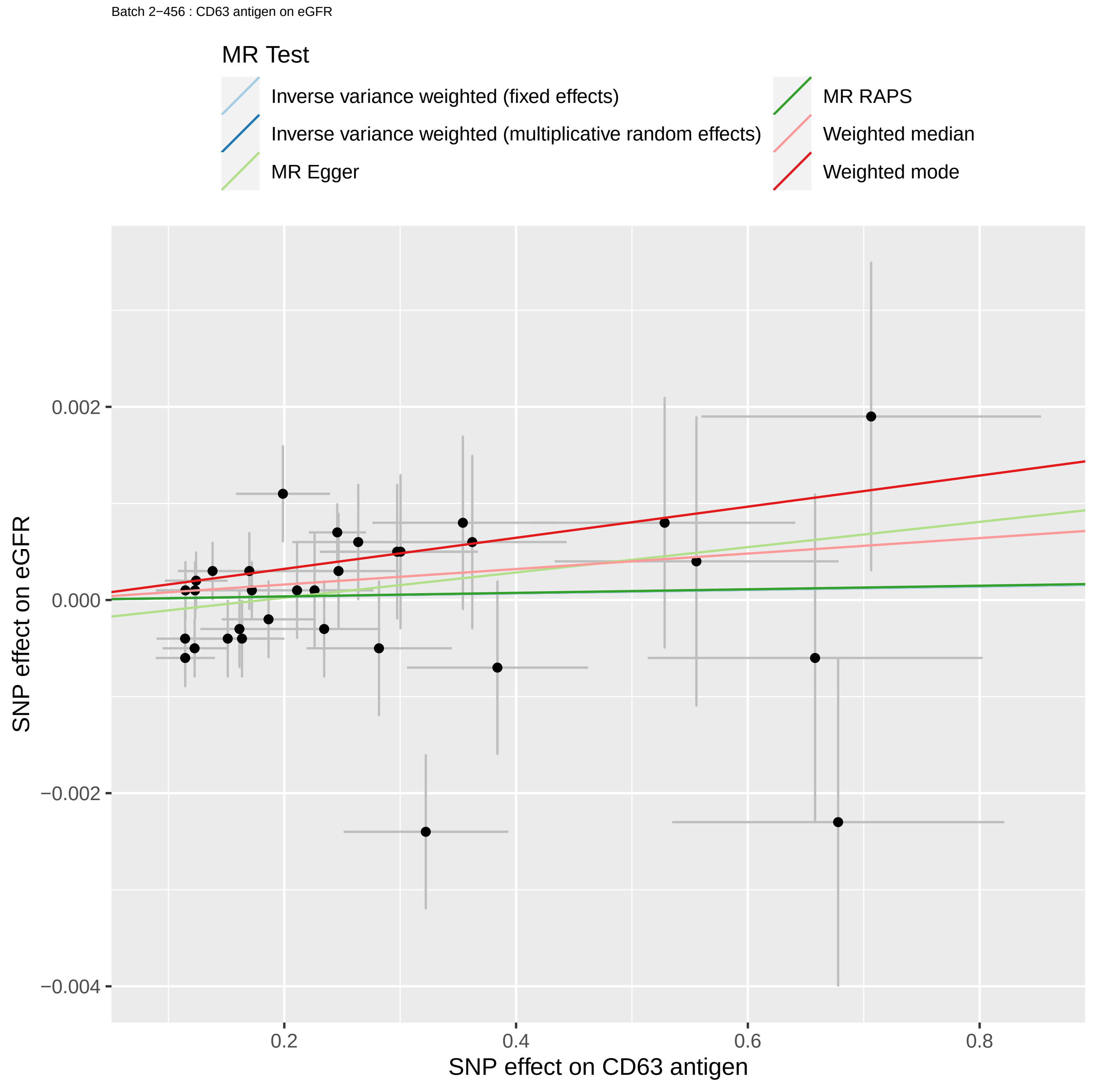

Batch 2−456 : CD63 antigen on eGFR
MR Test
Inverse variance weighted (fixed effects)
Inverse variance weighted (multiplicative random effects)
MR Egger
MR RAPS
Weighted median
Weighted mode
0.002
0.000
−0.002
−0.004
0.2
0.4
0.6
0.8
SNP effect on eGFR
SNP effect on CD63 antigen

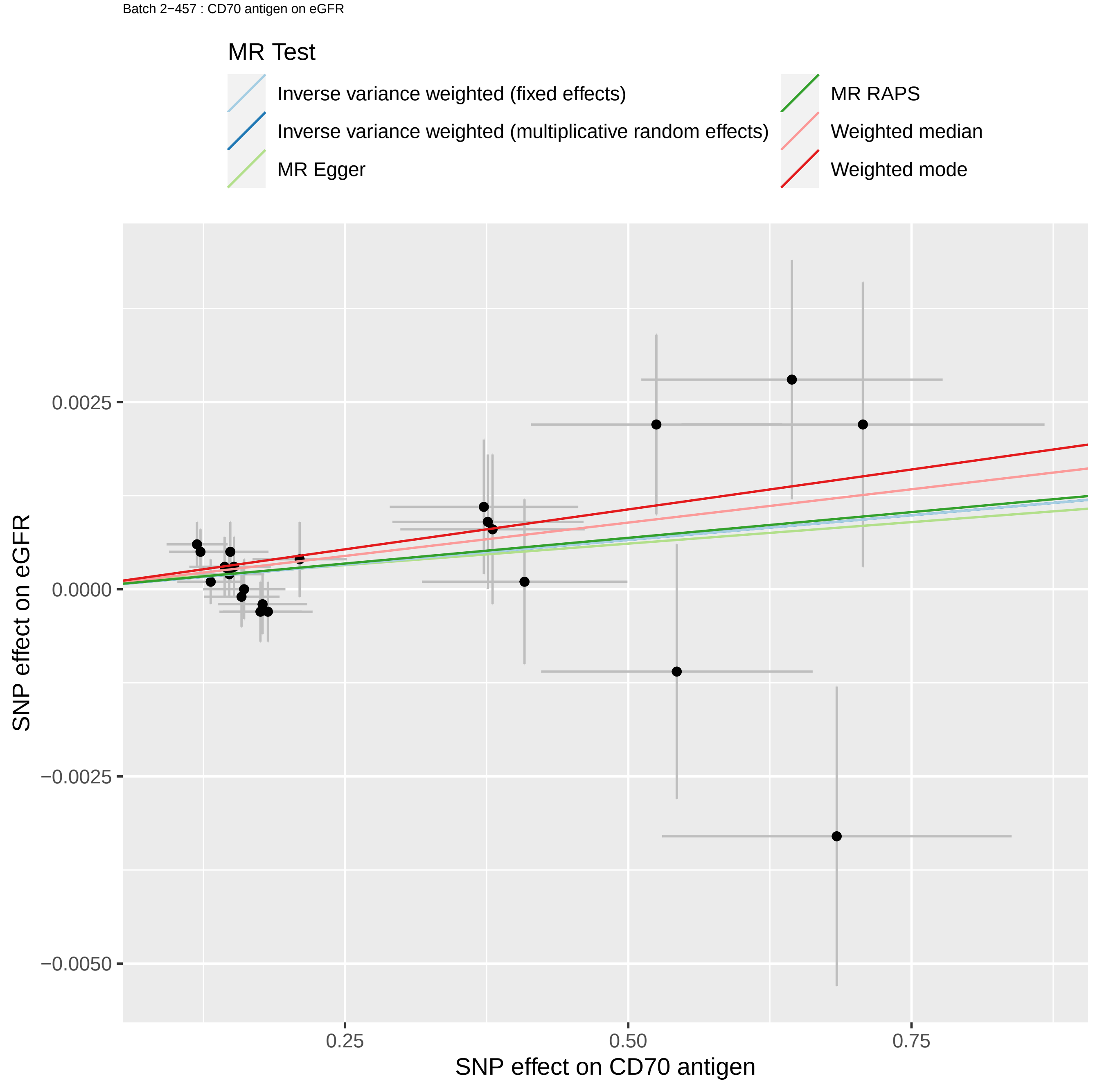
Batch 2−457 : CD70 antigen on eGFR
MR Test
Inverse variance weighted (fixed effects)
Inverse variance weighted (multiplicative random effects)
MR Egger
MR RAPS
Weighted median
Weighted mode
0.0025
0.0000
−0.0025
−0.0050
0.25
0.50
0.75
SNP effect on eGFR
SNP effect on CD70 antigen

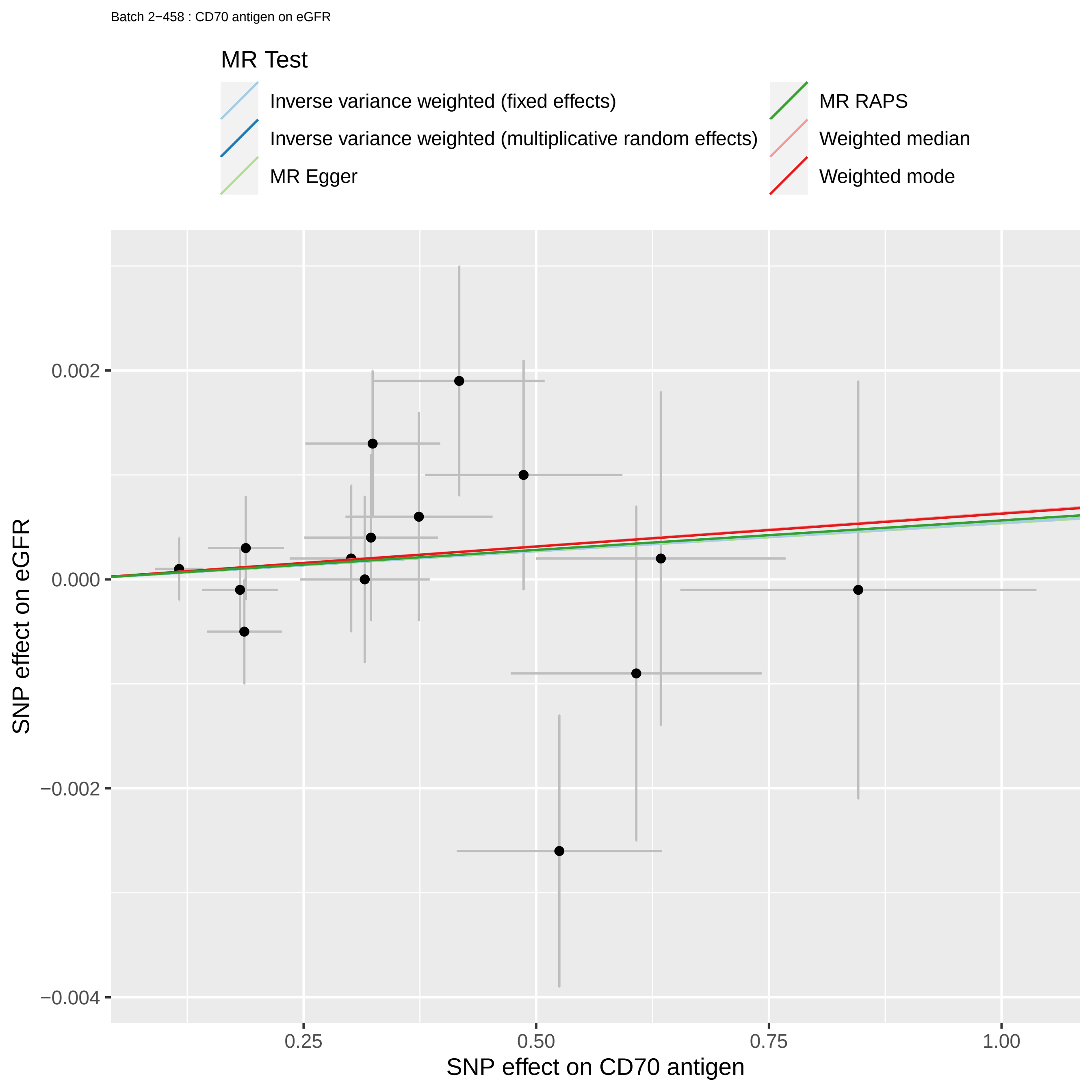

Batch 2−458 : CD70 antigen on eGFR
MR Test
Inverse variance weighted (fixed effects)
Inverse variance weighted (multiplicative random effects)
MR Egger
MR RAPS
Weighted median
Weighted mode
0.002
0.000
−0.002
−0.004
0.25
0.50
0.75
1.00
SNP effect on eGFR
SNP effect on CD70 antigen

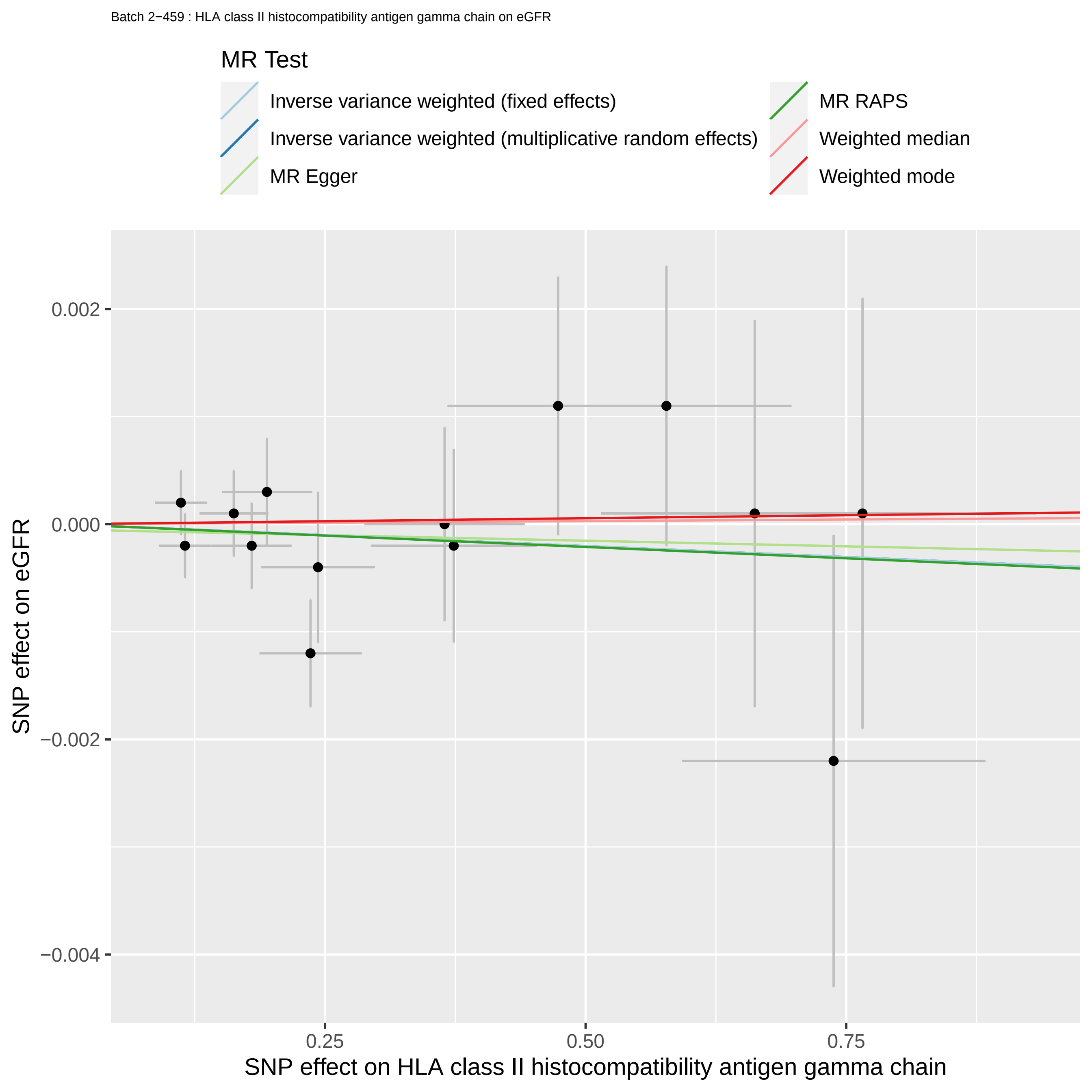
Batch 2−459 : HLA class II histocompatibility antigen gamma chain on eGFR
MR Test
Inverse variance weighted (fixed effects)
Inverse variance weighted (multiplicative random effects)
MR Egger
MR RAPS
Weighted median
Weighted mode
0.002
0.000
−0.002
−0.004
0.25
0.50
0.75
SNP effect on eGFR
SNP effect on HLA class II histocompatibility antigen gamma chain

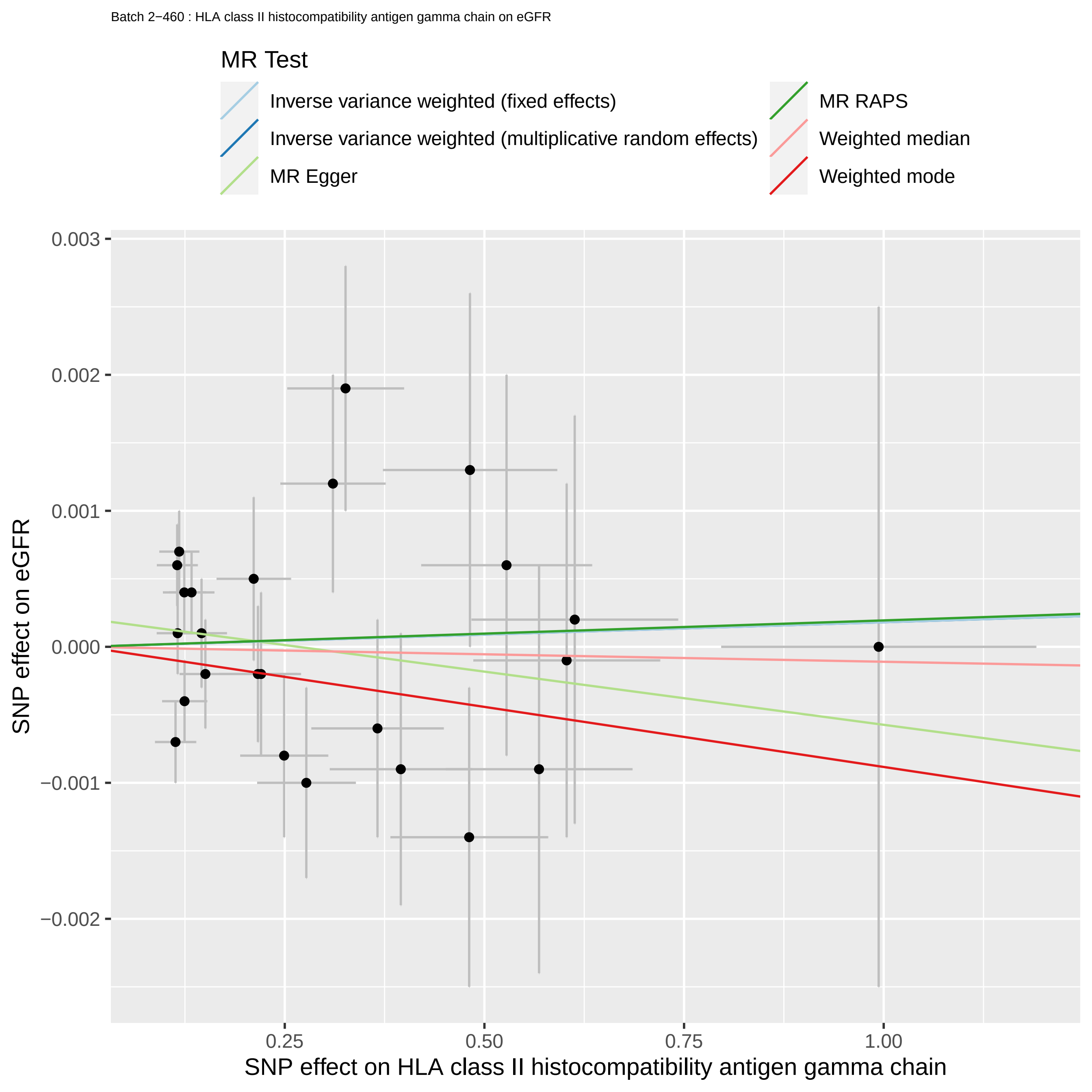

Batch 2−460 : HLA class II histocompatibility antigen gamma chain on eGFR
MR Test
Inverse variance weighted (fixed effects)
Inverse variance weighted (multiplicative random effects)
MR Egger
MR RAPS
Weighted median
Weighted mode
0.003
0.002
0.001
0.000
−0.001
−0.002
0.25
0.50
0.75
1.00
SNP effect on eGFR
SNP effect on HLA class II histocompatibility antigen gamma chain

Batch 2−461 : B−cell antigen receptor complex−associated protein alpha chain on eGFR

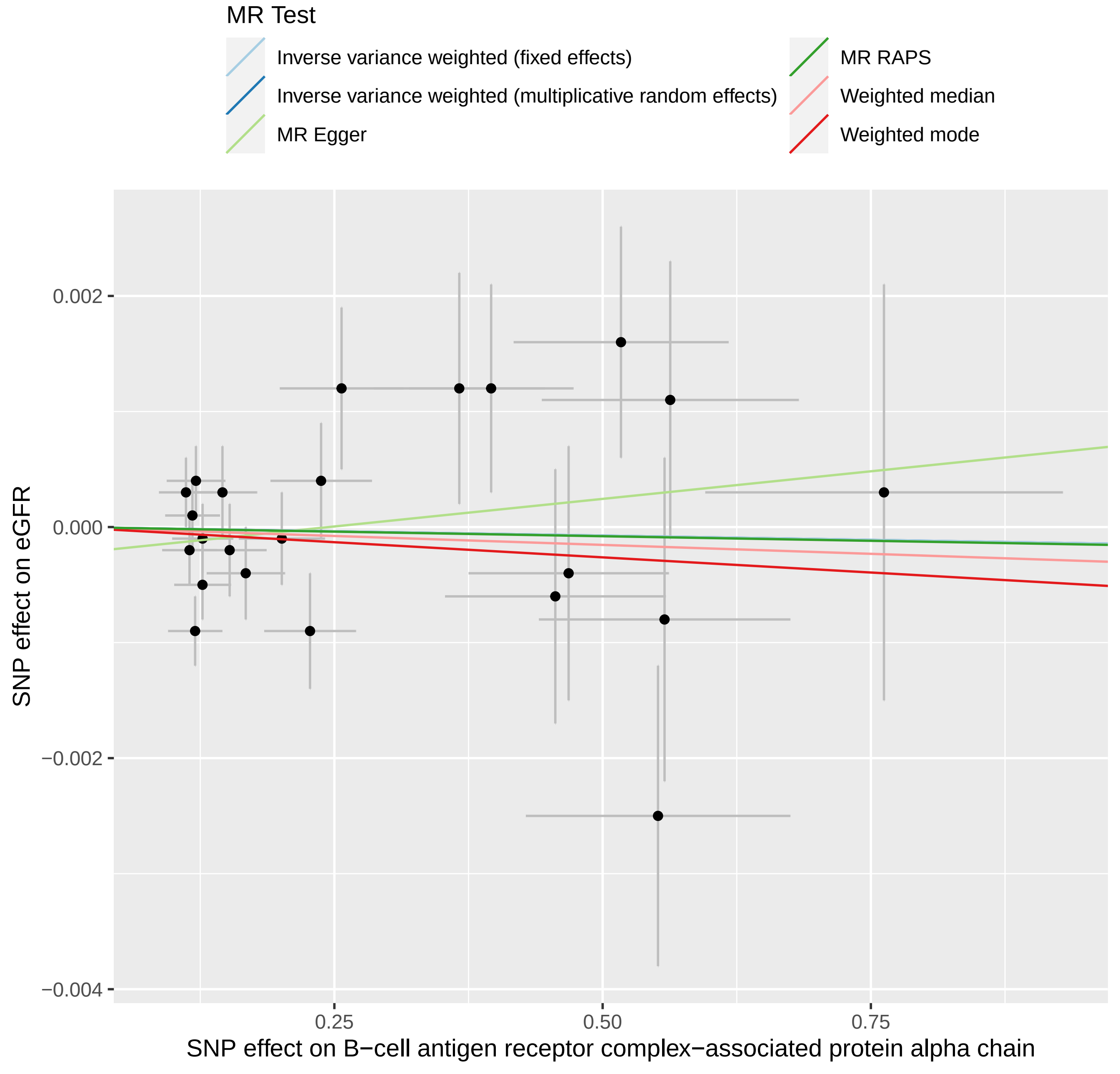

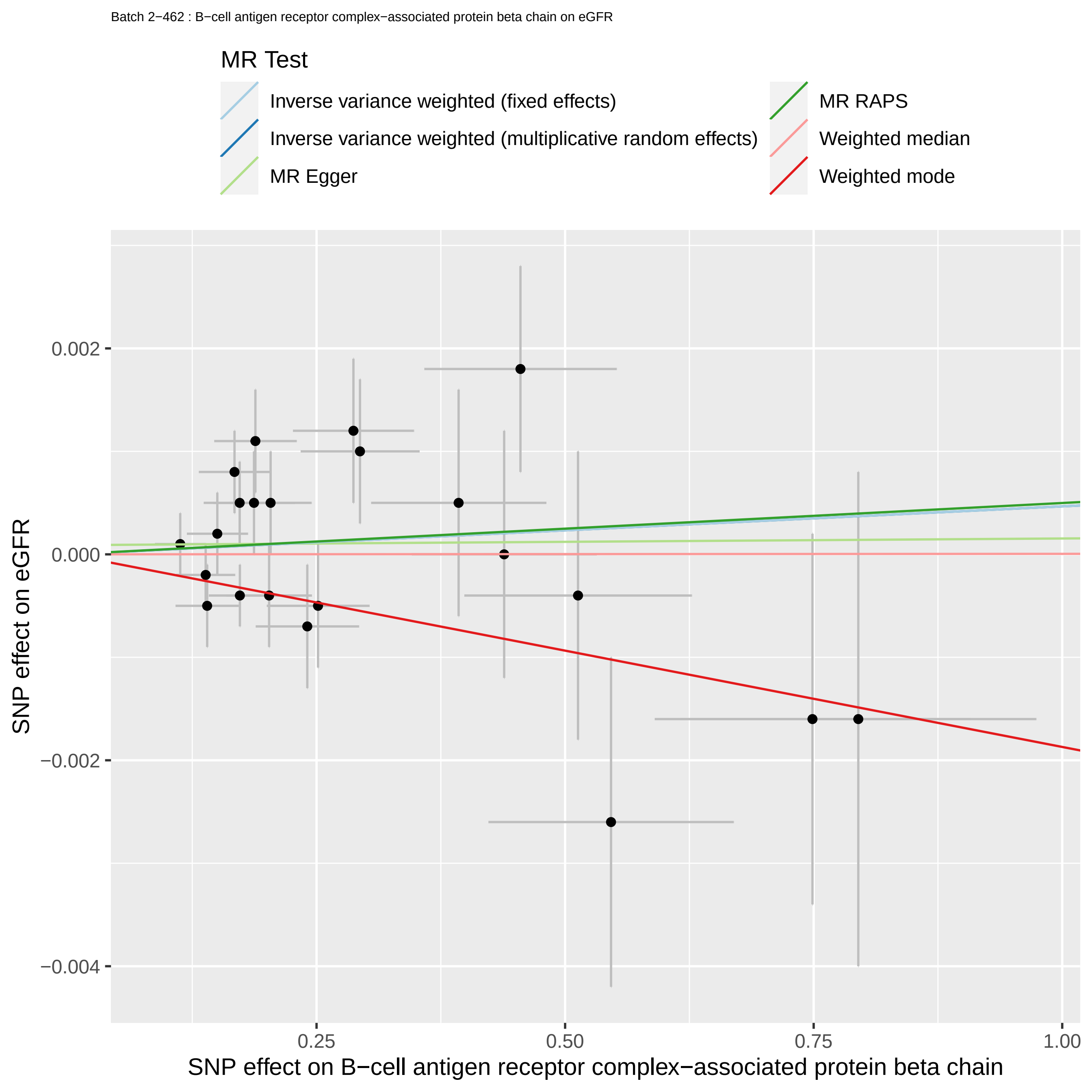

Batch 2−462 : B−cell antigen receptor complex−associated protein beta chain on eGFR
MR Test
Inverse variance weighted (fixed effects)
Inverse variance weighted (multiplicative random effects)
MR Egger
MR RAPS
Weighted median
Weighted mode
0.002
0.000
−0.002
−0.004
0.25
0.50
0.75
1.00
SNP effect on eGFR
SNP effect on B−cell antigen receptor complex−associated protein beta chain

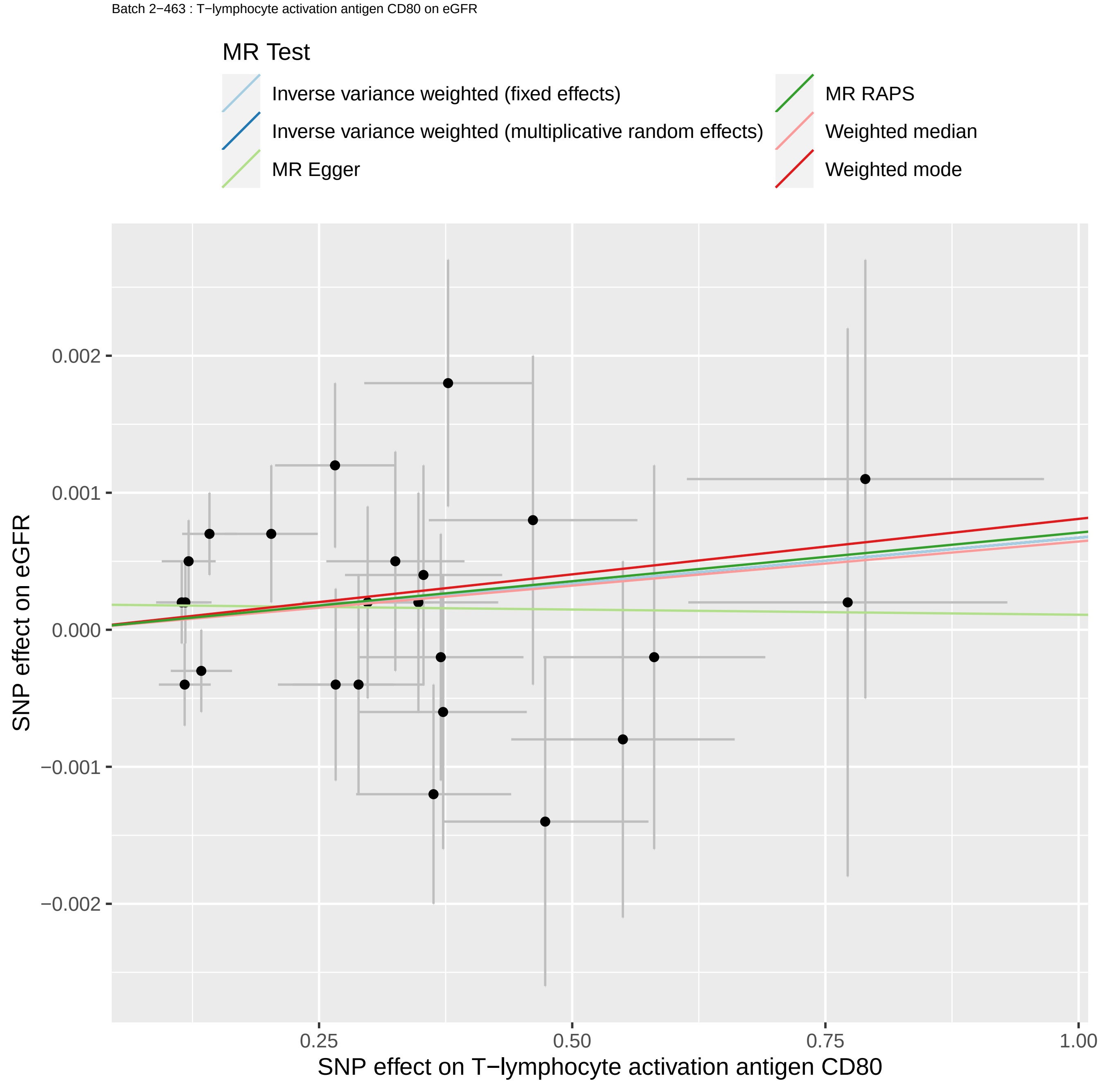

Batch 2−463 : T−lymphocyte activation antigen CD80 on eGFR
MR Test
Inverse variance weighted (fixed effects)
Inverse variance weighted (multiplicative random effects)
MR Egger
MR RAPS
Weighted median
Weighted mode
0.002
0.001
0.000
−0.001
−0.002
SNP effect on eGFR
0.25
0.50
0.75
1.00
SNP effect on T−lymphocyte activation antigen CD80

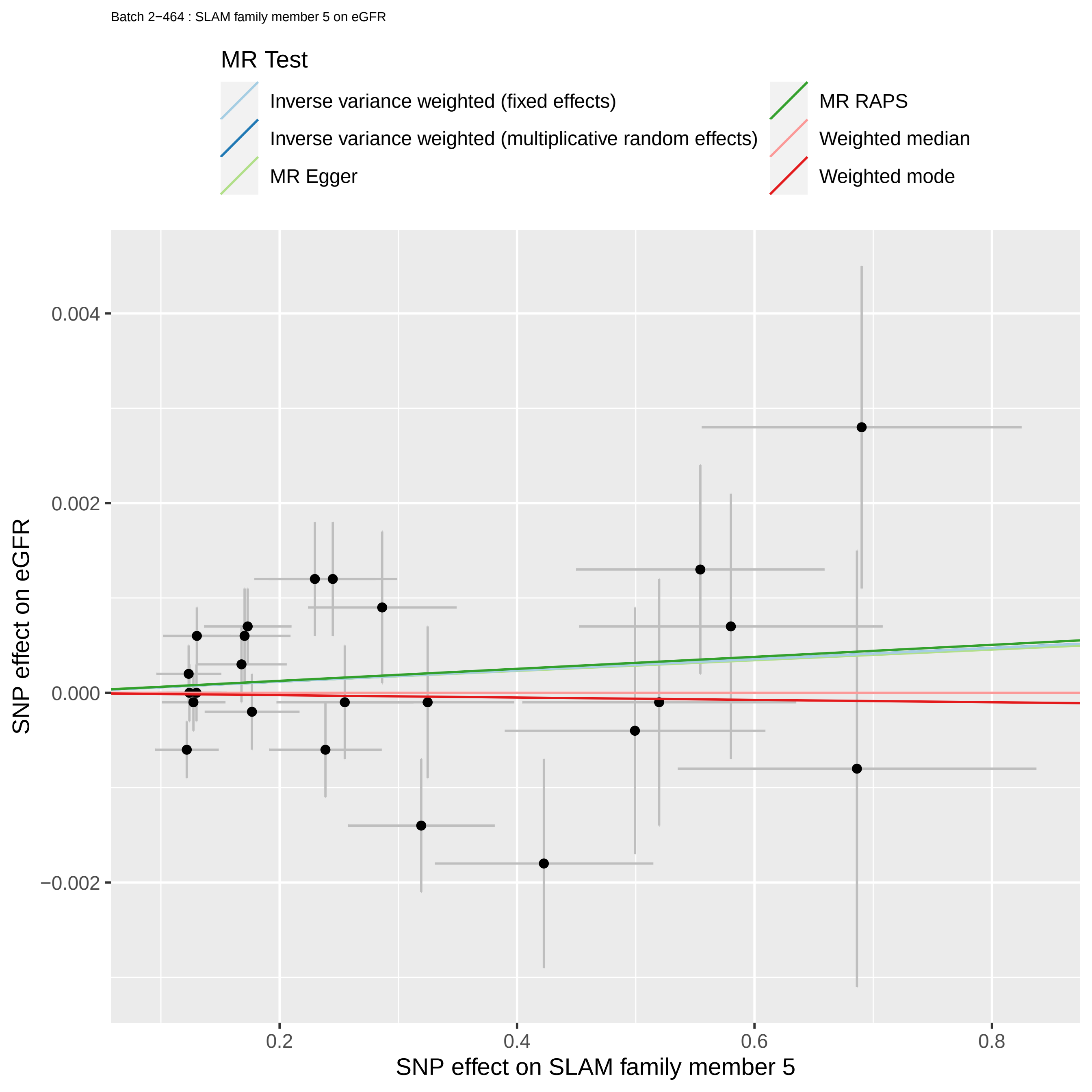
Batch 2−464 : SLAM family member 5 on eGFR
MR Test
Inverse variance weighted (fixed effects)
Inverse variance weighted (multiplicative random effects)
MR Egger
MR RAPS
Weighted median
Weighted mode
SNP effect on eGFR
0.004
0.002
0.000
−0.002
0.2
0.4
0.6
0.8
SNP effect on SLAM family member 5

Batch 2−465 : SLAM family member 5 on eGFR

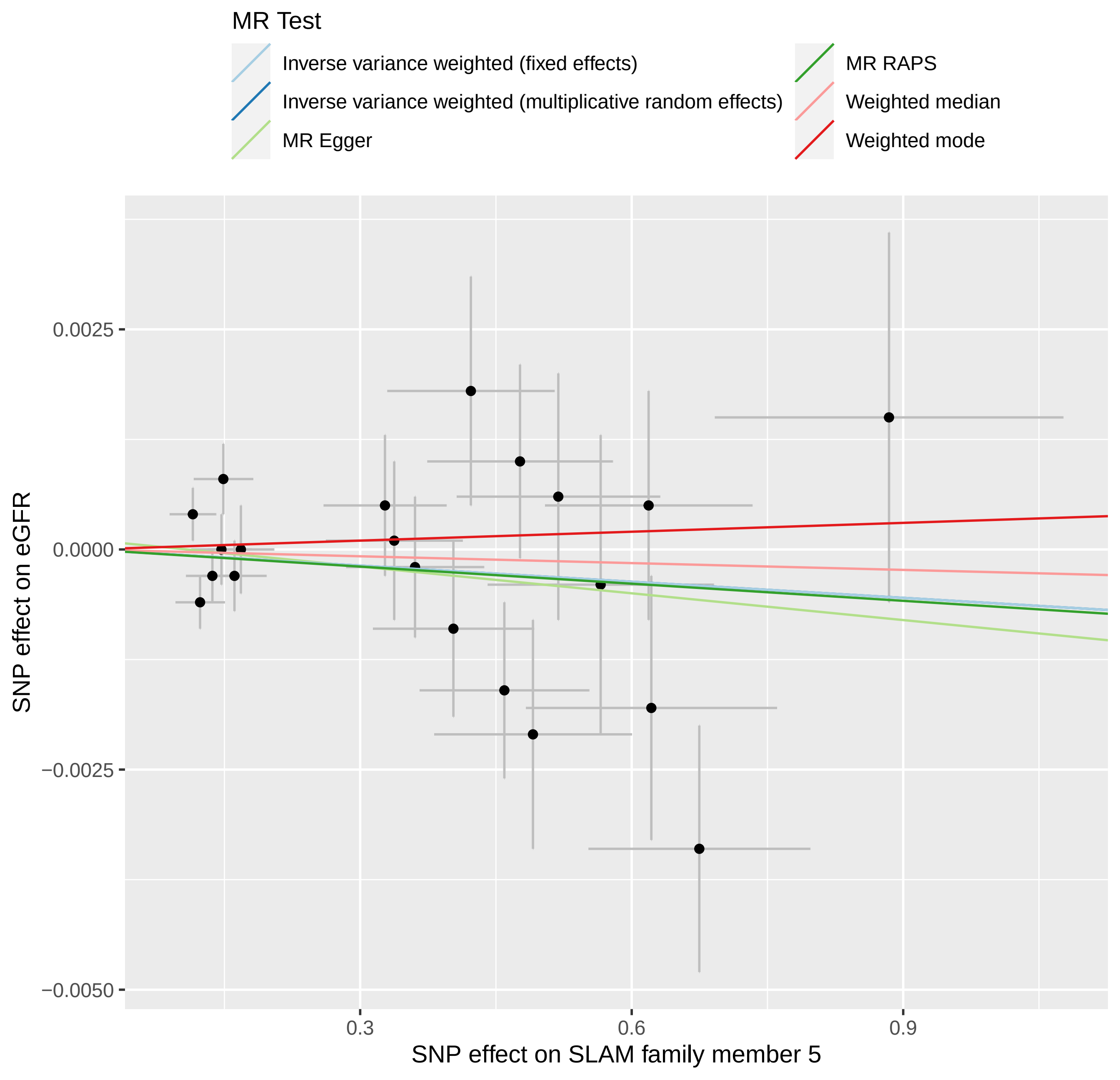

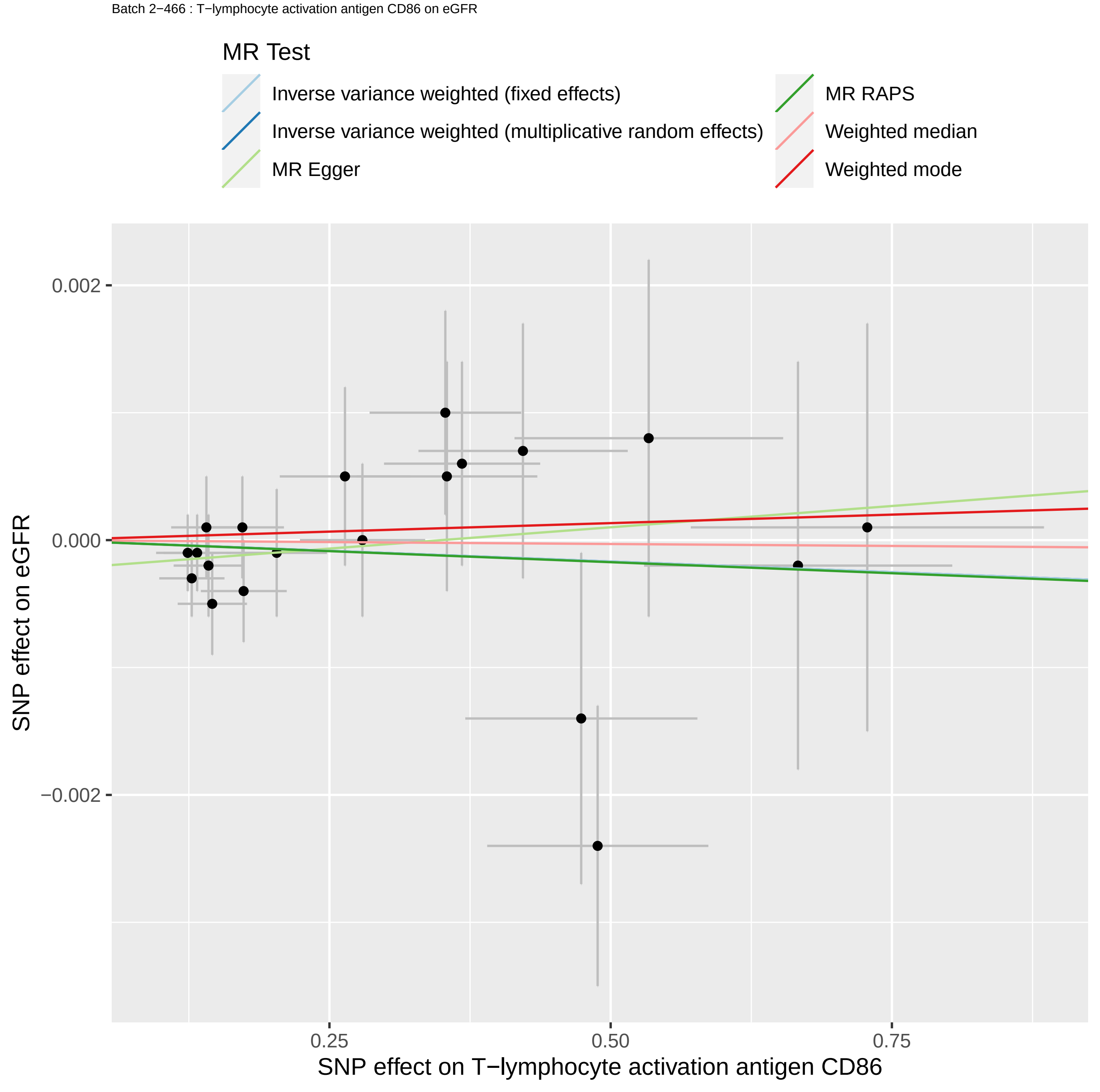

Batch 2−466 : T−lymphocyte activation antigen CD86 on eGFR
MR Test
Inverse variance weighted (fixed effects)
Inverse variance weighted (multiplicative random effects)
MR Egger
MR RAPS
Weighted median
Weighted mode
0.002
0.000
−0.002
0.25
0.50
0.75
SNP effect on eGFR
SNP effect on T−lymphocyte activation antigen CD86

Batch 2−467 : T−cell surface glycoprotein CD8 beta chain on eGFR

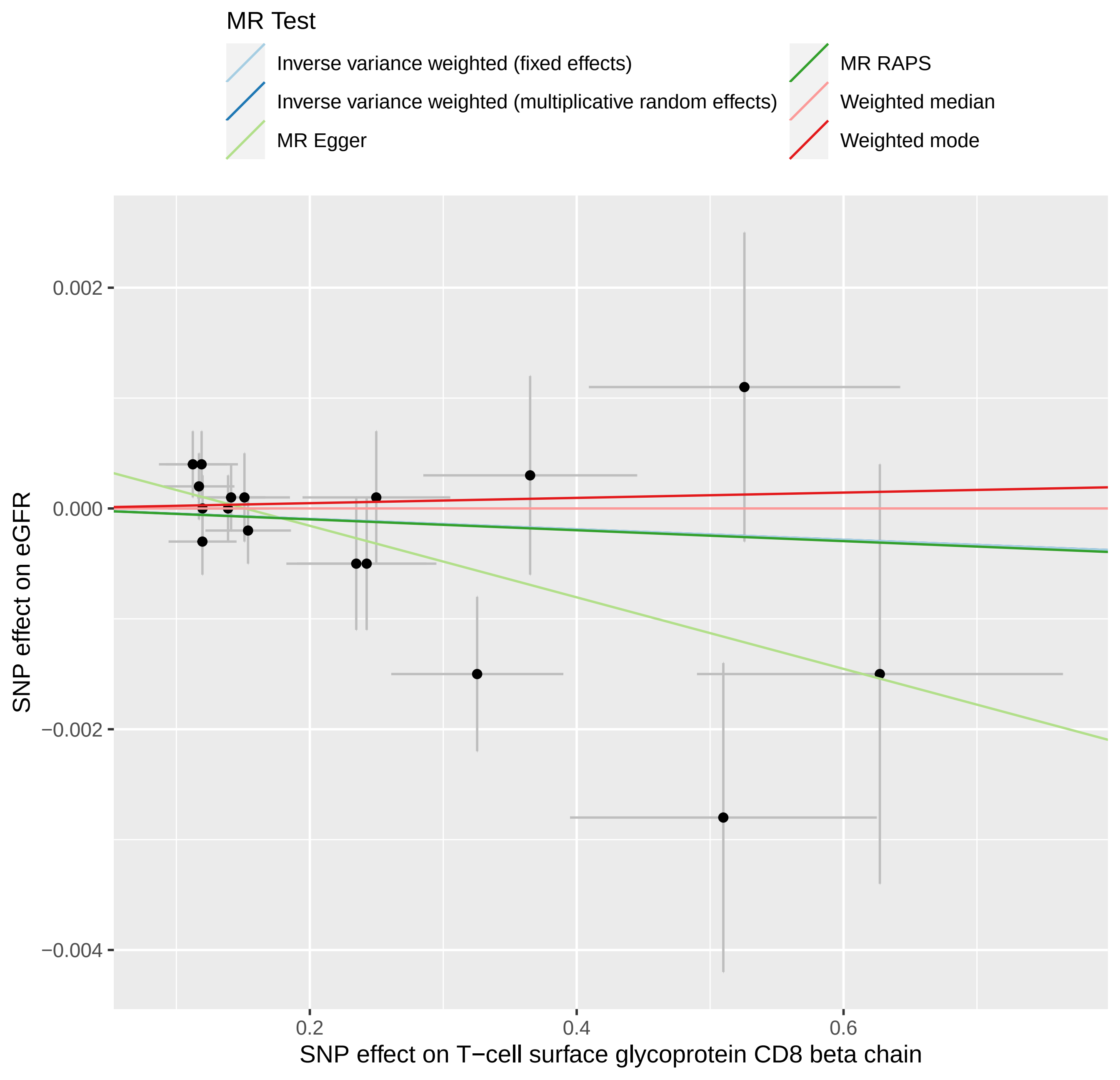

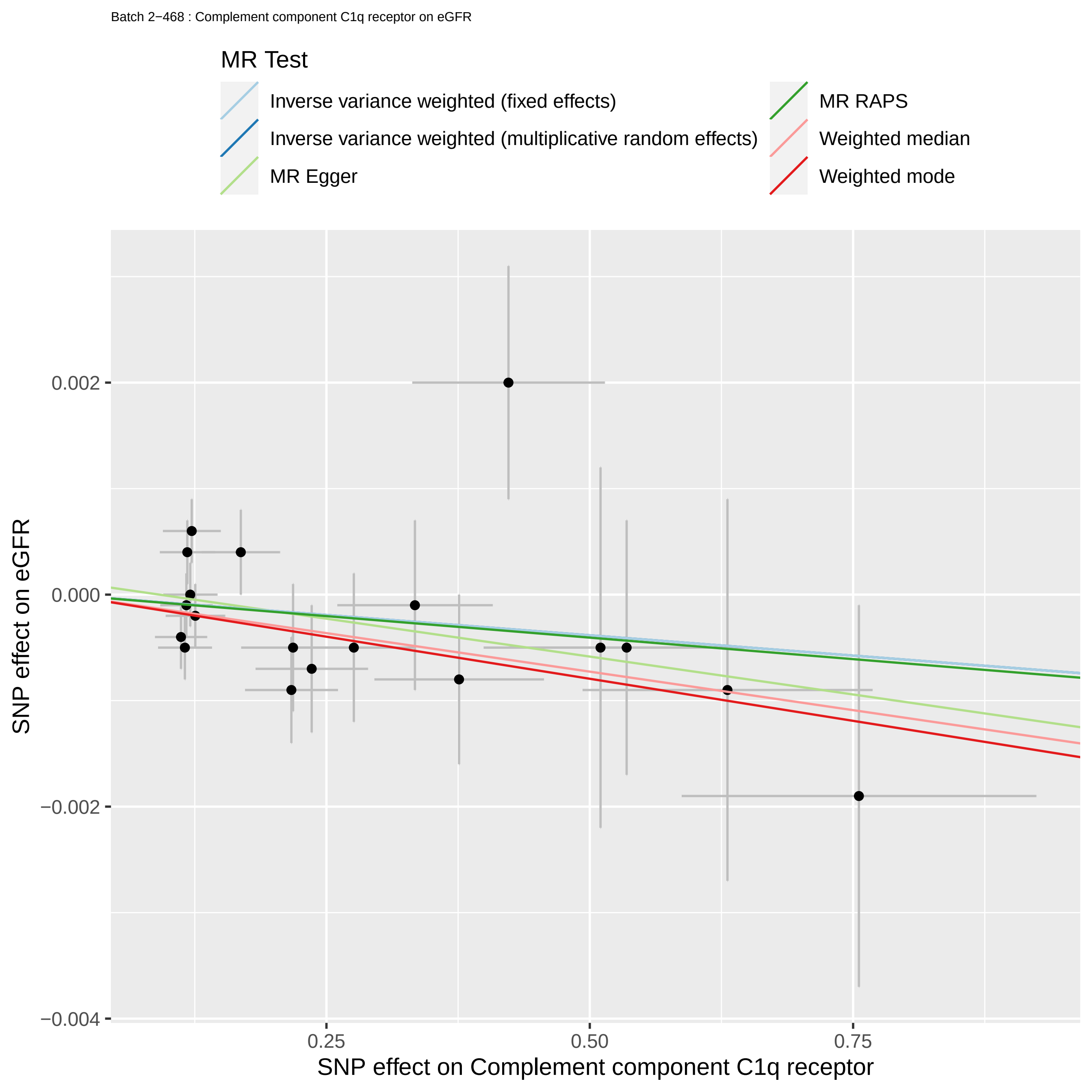
Batch 2−468 : Complement component C1q receptor on eGFR
MR Test
Inverse variance weighted (fixed effects)
Inverse variance weighted (multiplicative random effects)
MR Egger
MR RAPS
Weighted median
Weighted mode
0.002
0.000
−0.002
−0.004
0.25
0.50
0.75
SNP effect on eGFR
SNP effect on Complement component C1q receptor

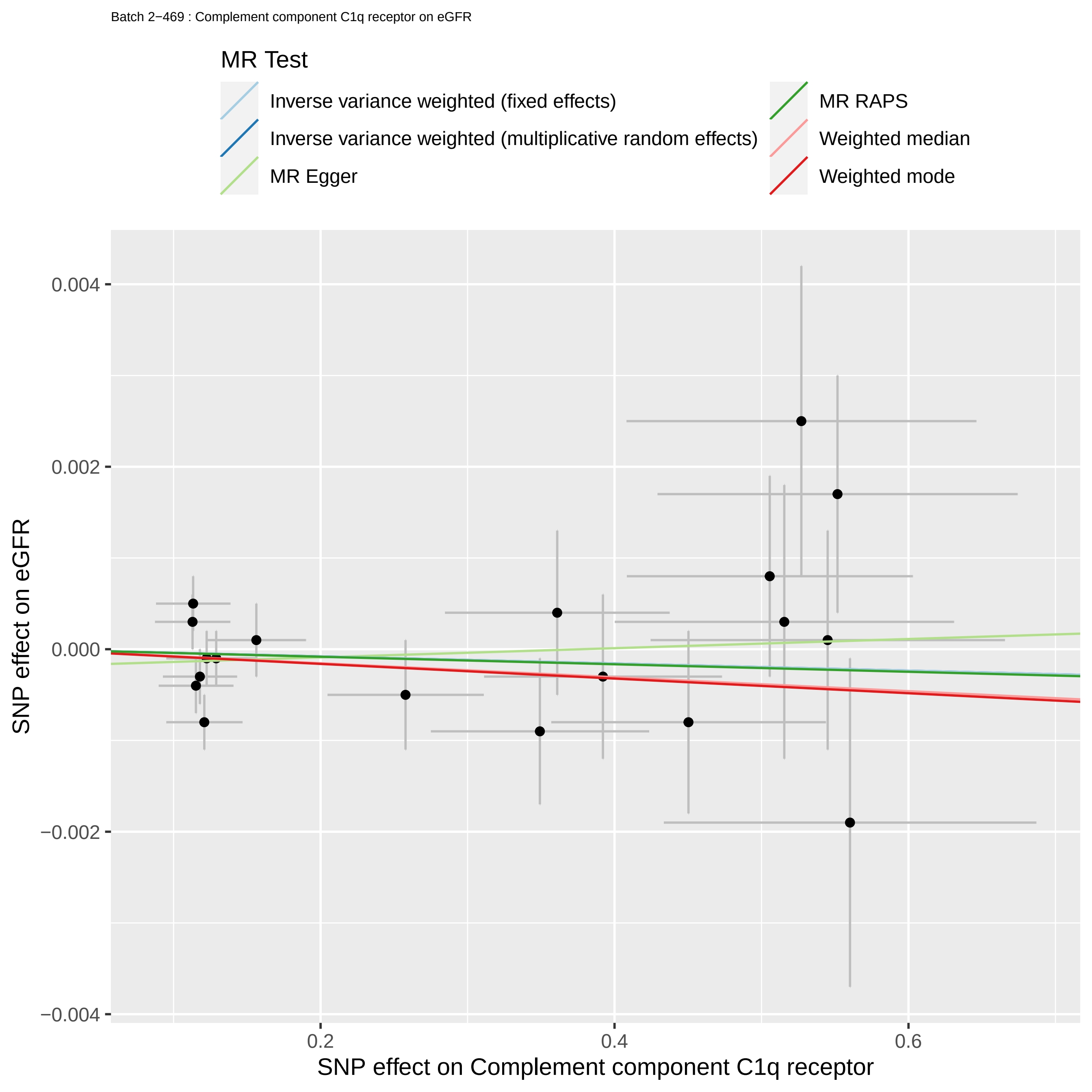

Batch 2−469 : Complement component C1q receptor on eGFR
MR Test
Inverse variance weighted (fixed effects)
Inverse variance weighted (multiplicative random effects)
MR Egger
MR RAPS
Weighted median
Weighted mode
SNP effect on eGFR
0.004
0.002
0.000
−0.002
−0.004
0.2
0.4
0.6
SNP effect on Complement component C1q receptor

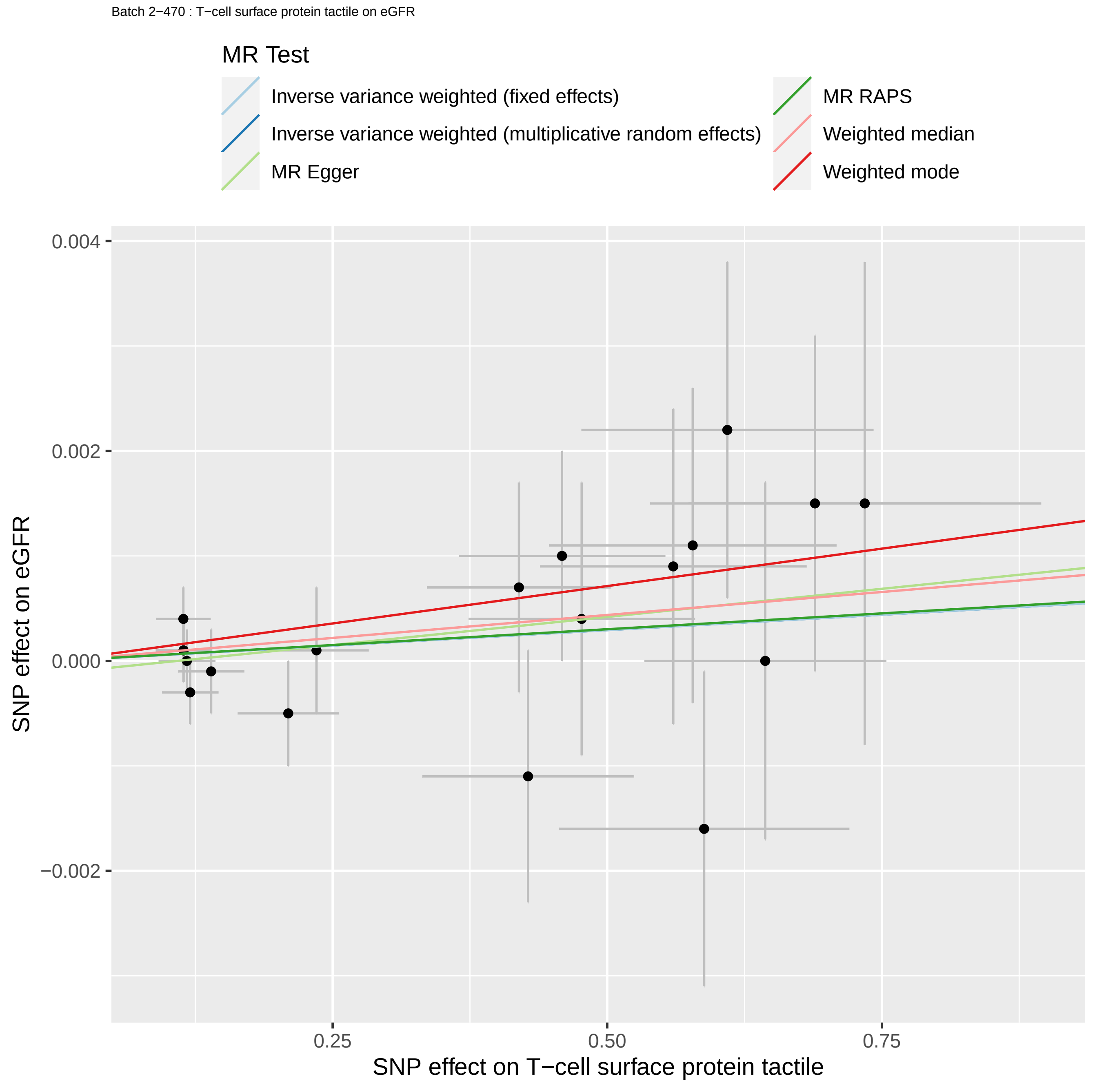
Batch 2−470 : T−cell surface protein tactile on eGFR
MR Test
Inverse variance weighted (fixed effects)
Inverse variance weighted (multiplicative random effects)
MR Egger
MR RAPS
Weighted median
Weighted mode
0.004
0.002
0.000
−0.002
SNP effect on eGFR
0.25
0.50
0.75
SNP effect on T−cell surface protein tactile

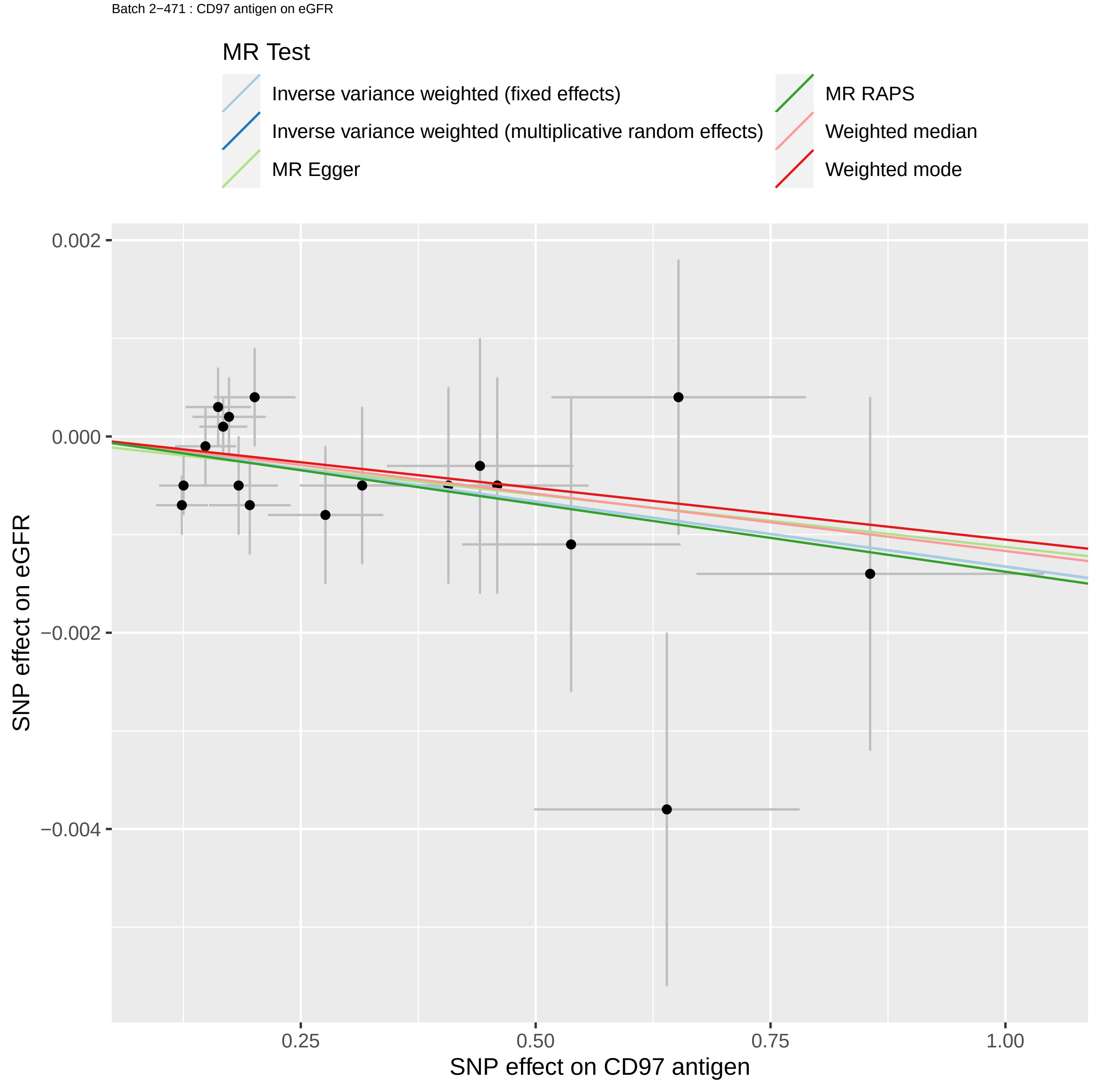

Batch 2−471 : CD97 antigen on eGFR
MR Test
Inverse variance weighted (fixed effects)
Inverse variance weighted (multiplicative random effects)
MR Egger
MR RAPS
Weighted median
Weighted mode
0.002
0.000
−0.002
−0.004
0.25
0.50
0.75
1.00
SNP effect on CD97 antigen
SNP effect on eGFR

Batch 2−472 : M−phase inducer phosphatase 2 on eGFR

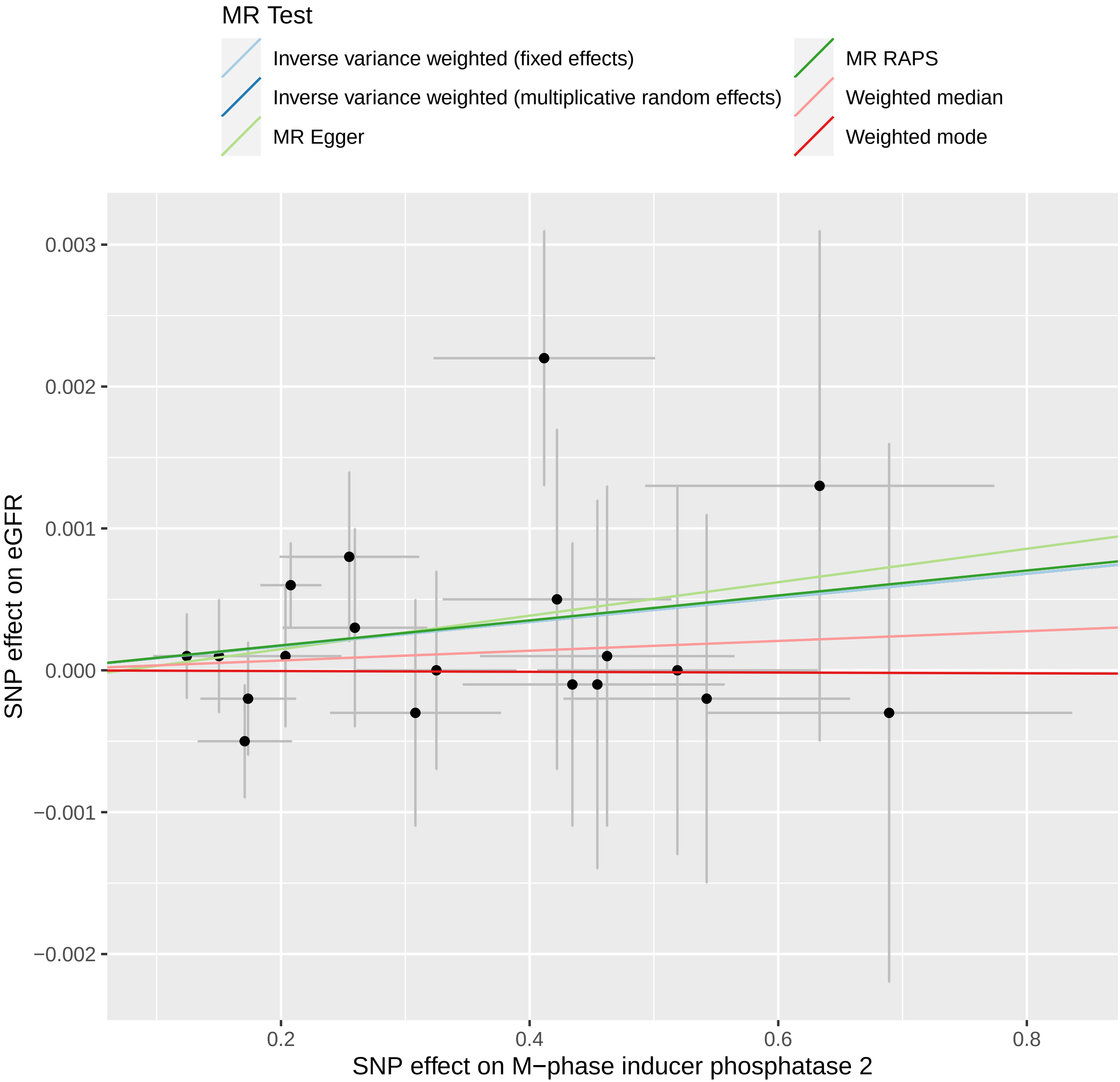

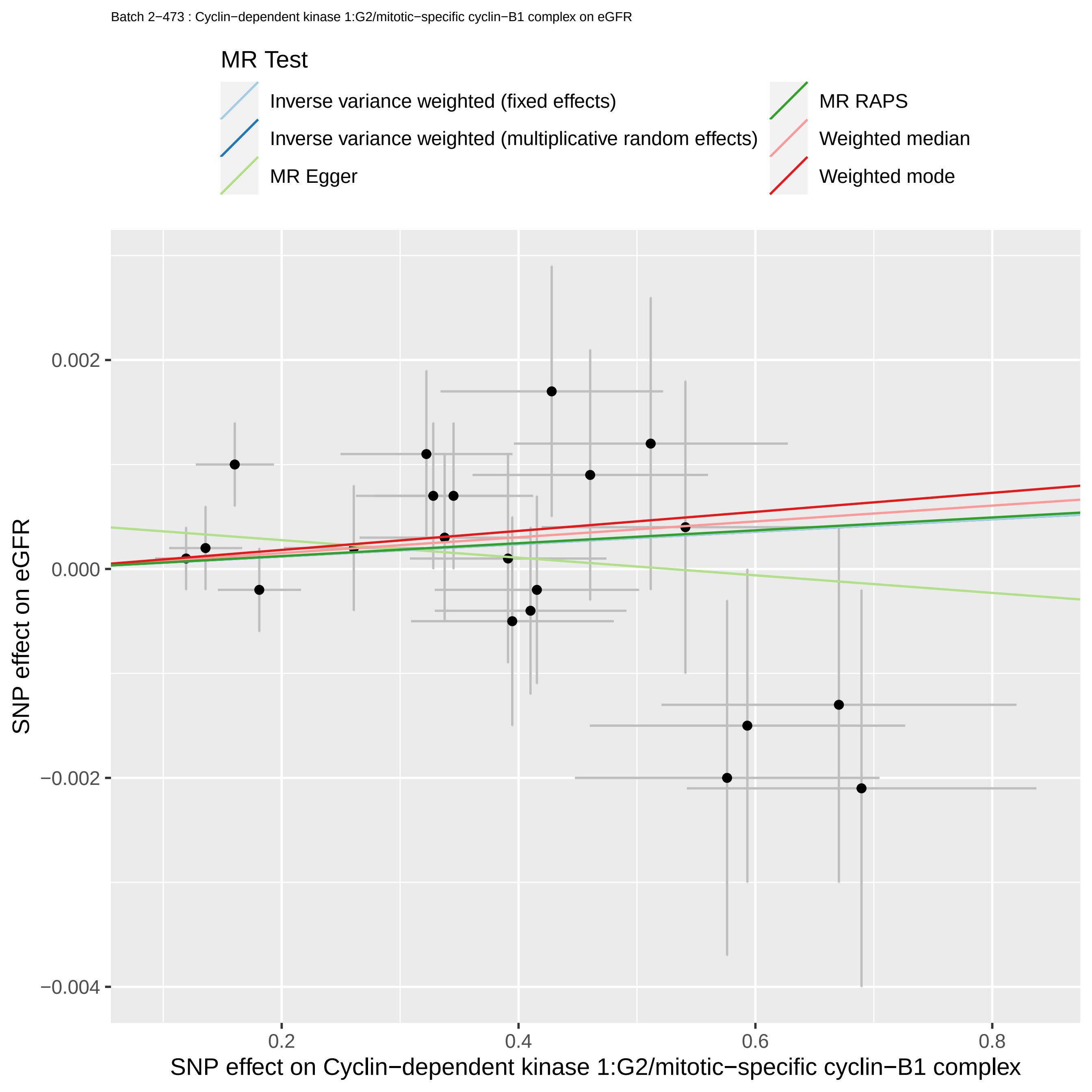
Batch 2−473 : Cyclin−dependent kinase 1:G2/mitotic−specific cyclin−B1 complex on eGFR
MR Test
Inverse variance weighted (fixed effects)
Inverse variance weighted (multiplicative random effects)
MR Egger
MR RAPS
Weighted median
Weighted mode
0.002
0.000
−0.002
−0.004
0.2
0.4
0.6
0.8
SNP effect on eGFR
SNP effect on Cyclin−dependent kinase 1:G2/mitotic−specific cyclin−B1 complex

Batch 2−474 : Cell division control protein 42 homolog on eGFR

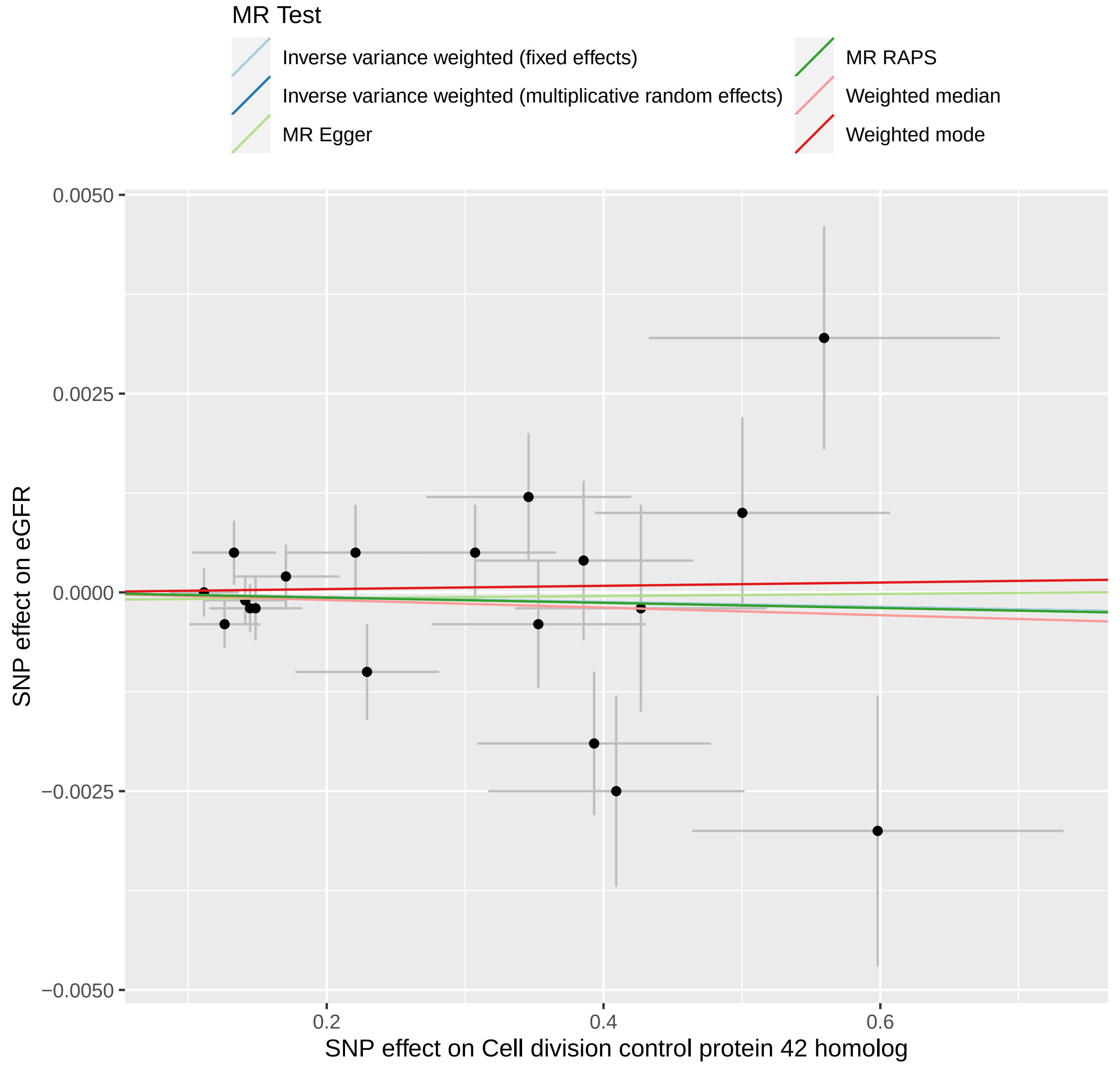

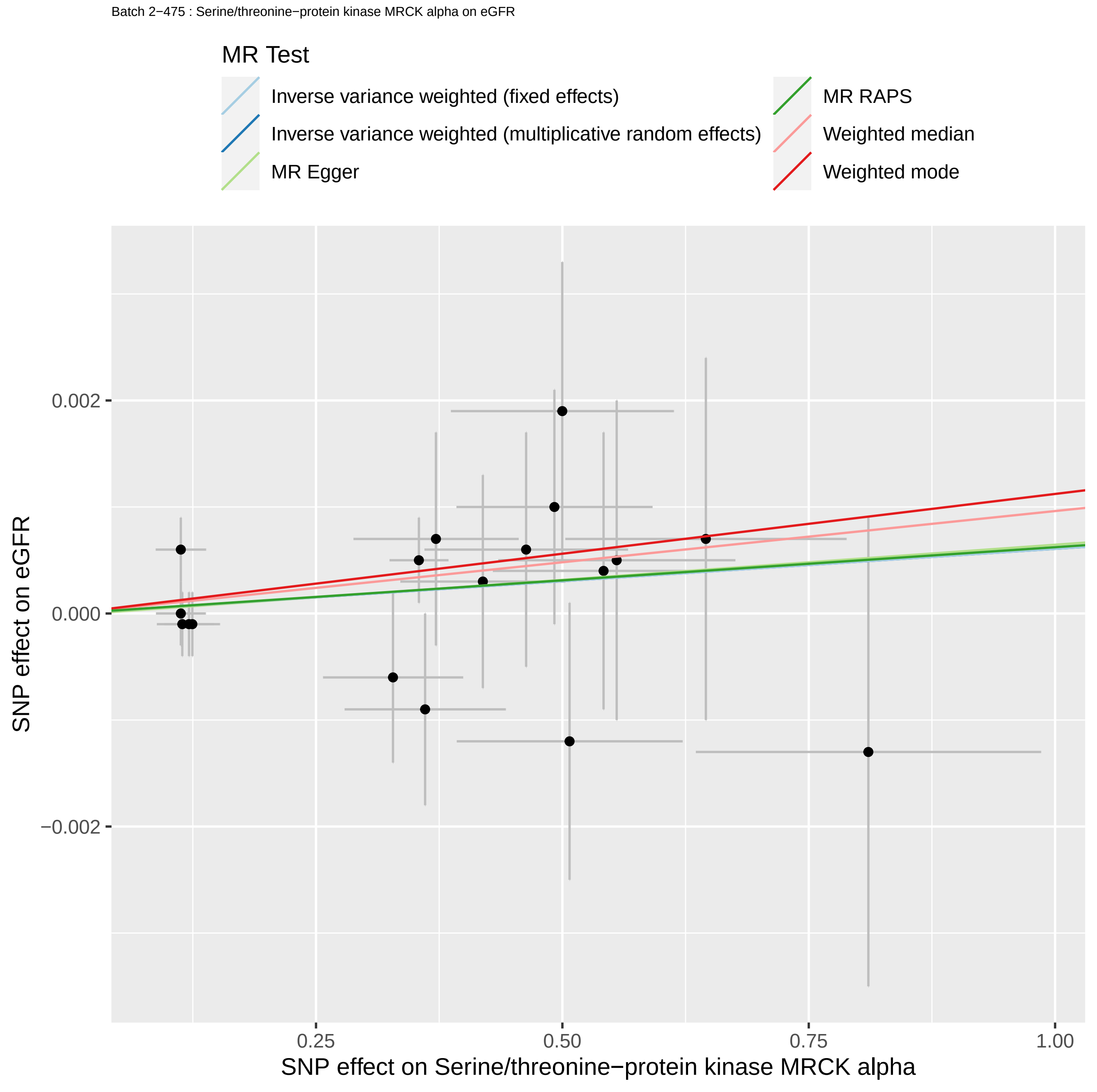

Batch 2−475 : Serine/threonine−protein kinase MRCK alpha on eGFR
MR Test
Inverse variance weighted (fixed effects)
Inverse variance weighted (multiplicative random effects)
MR Egger
MR RAPS
Weighted median
Weighted mode
0.002
0.000
−0.002
0.25
0.50
0.75
1.00
SNP effect on eGFR
SNP effect on Serine/threonine−protein kinase MRCK alpha

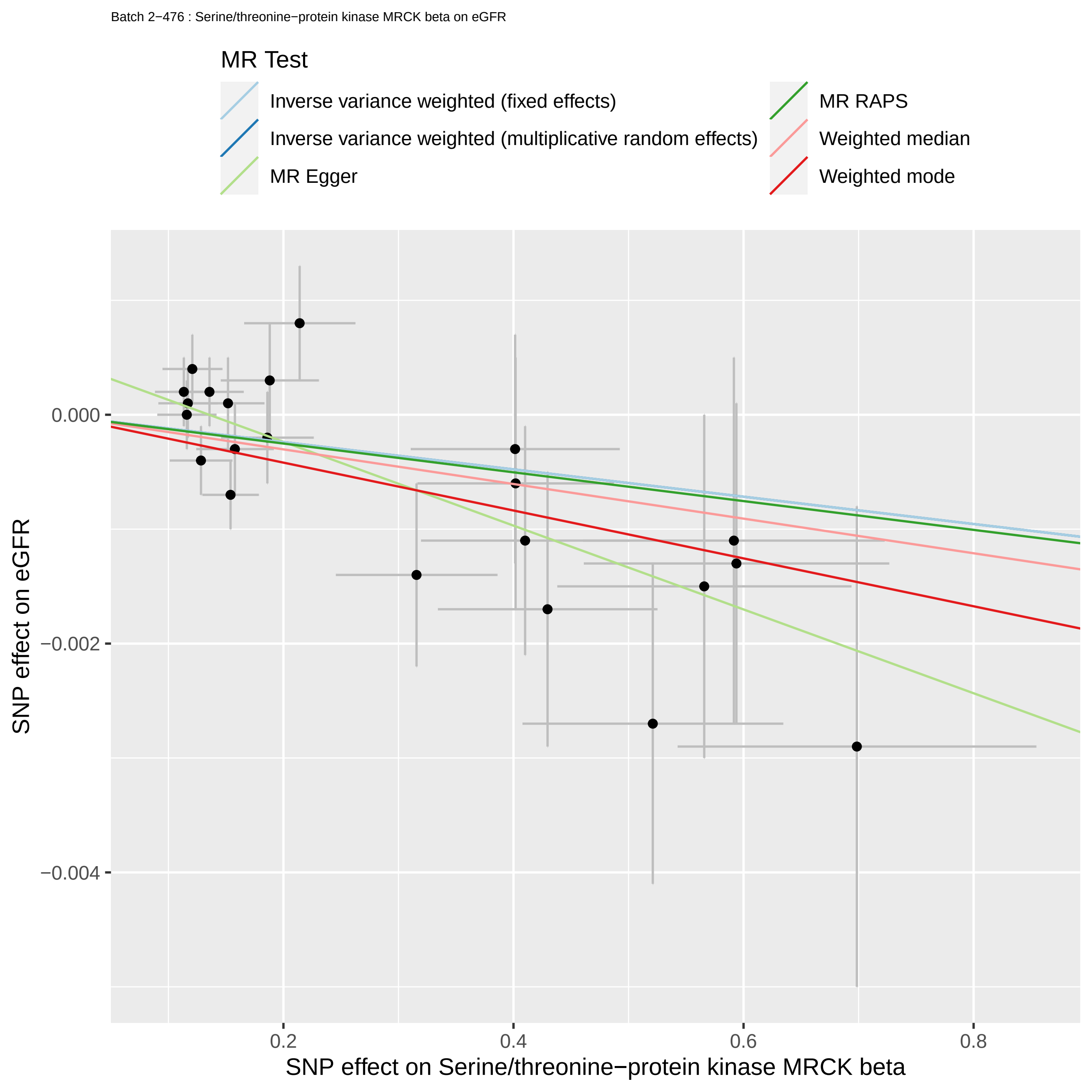
Batch 2−476 : Serine/threonine−protein kinase MRCK beta on eGFR
MR Test
Inverse variance weighted (fixed effects)
Inverse variance weighted (multiplicative random effects)
MR Egger
MR RAPS
Weighted median
Weighted mode
0.000
−0.002
−0.004
0.2
0.4
0.6
0.8
SNP effect on eGFR
SNP effect on Serine/threonine−protein kinase MRCK beta

Batch 2−477 : CUB domain−containing protein 1 on eGFR

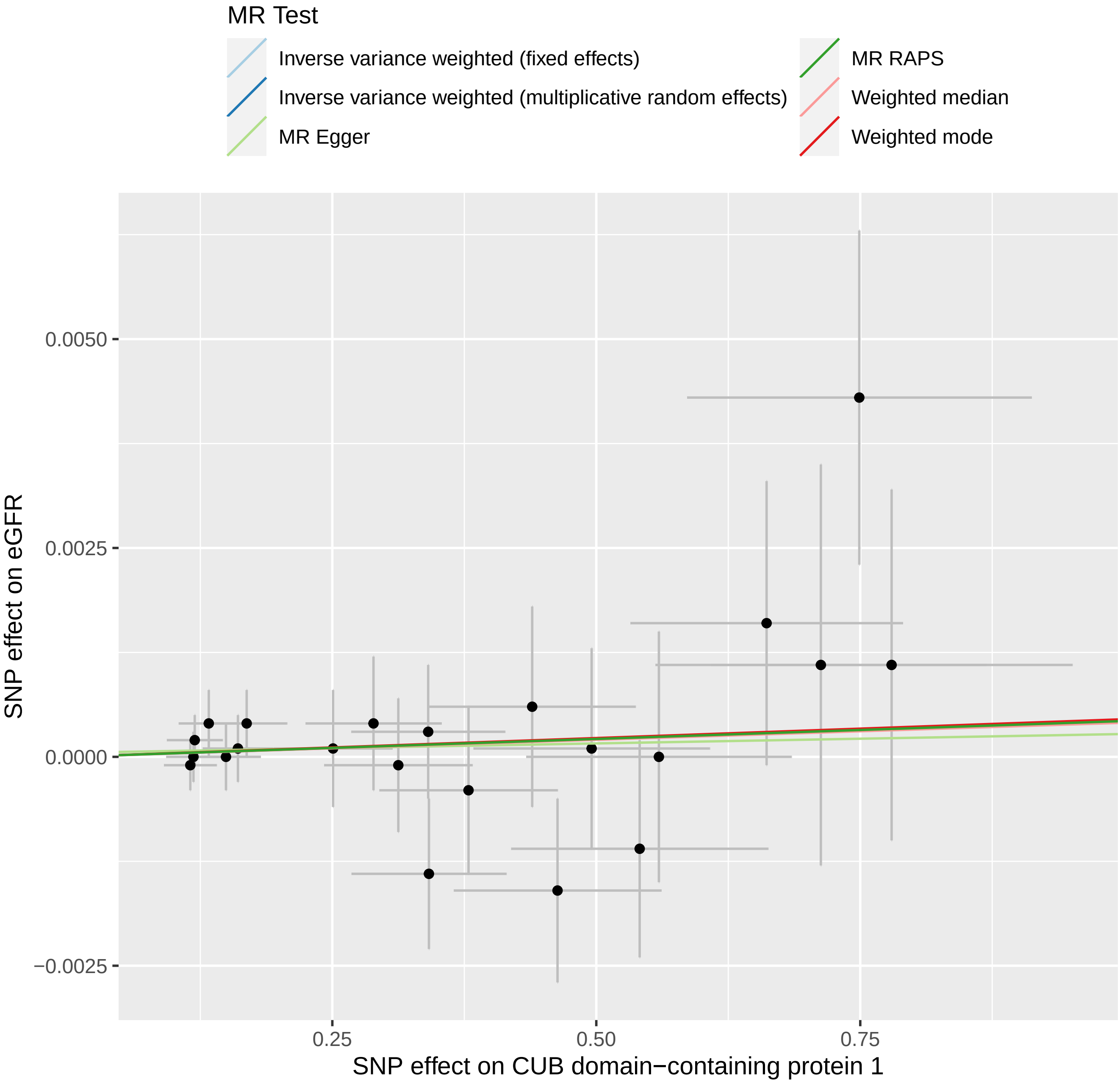

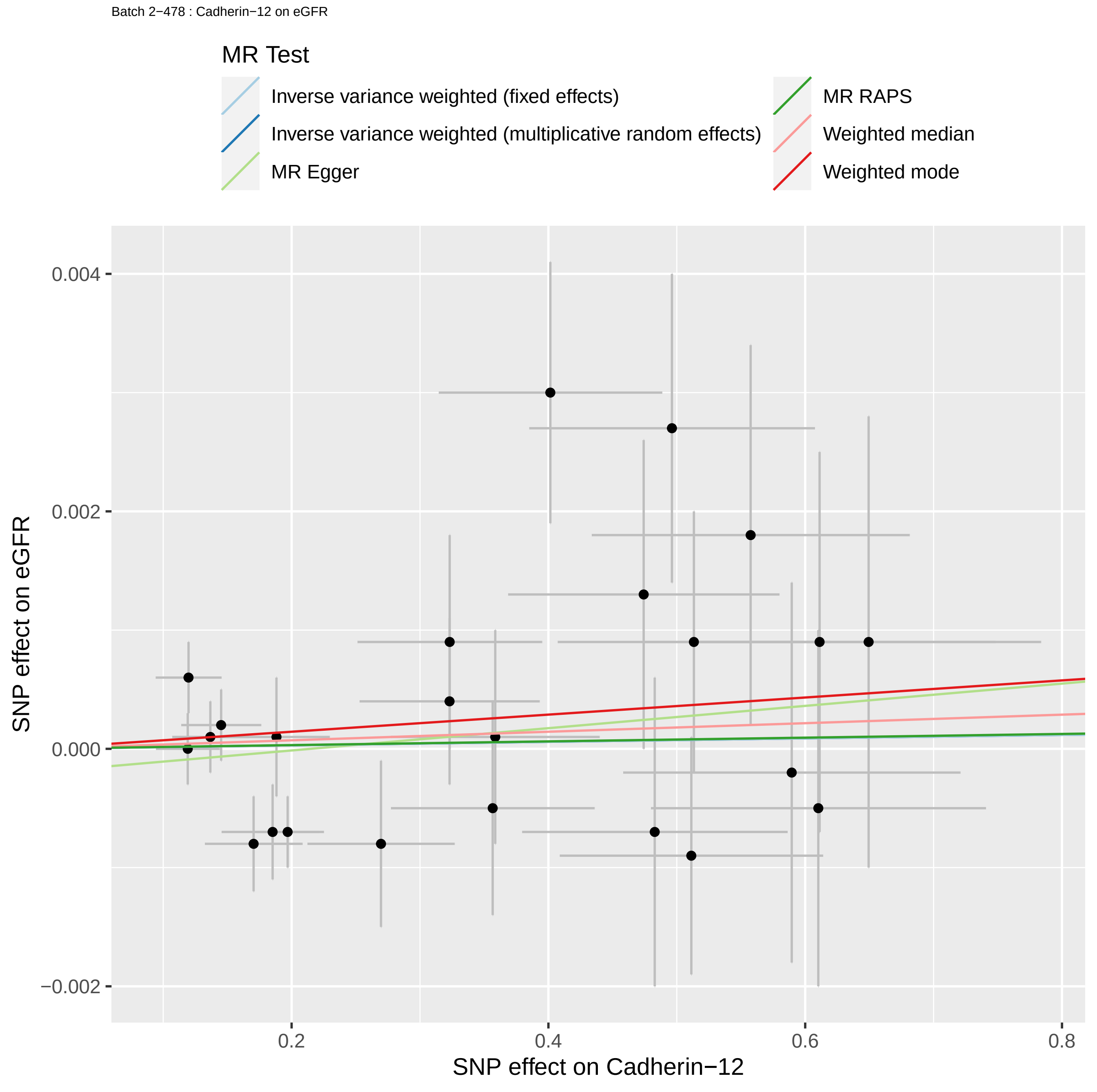
Batch 2−478 : Cadherin−12 on eGFR
MR Test
Inverse variance weighted (fixed effects)
Inverse variance weighted (multiplicative random effects)
MR Egger
MR RAPS
Weighted median
Weighted mode
0.004
0.002
0.000
−0.002
0.2
0.4
0.6
0.8
SNP effect on eGFR
SNP effect on Cadherin−12

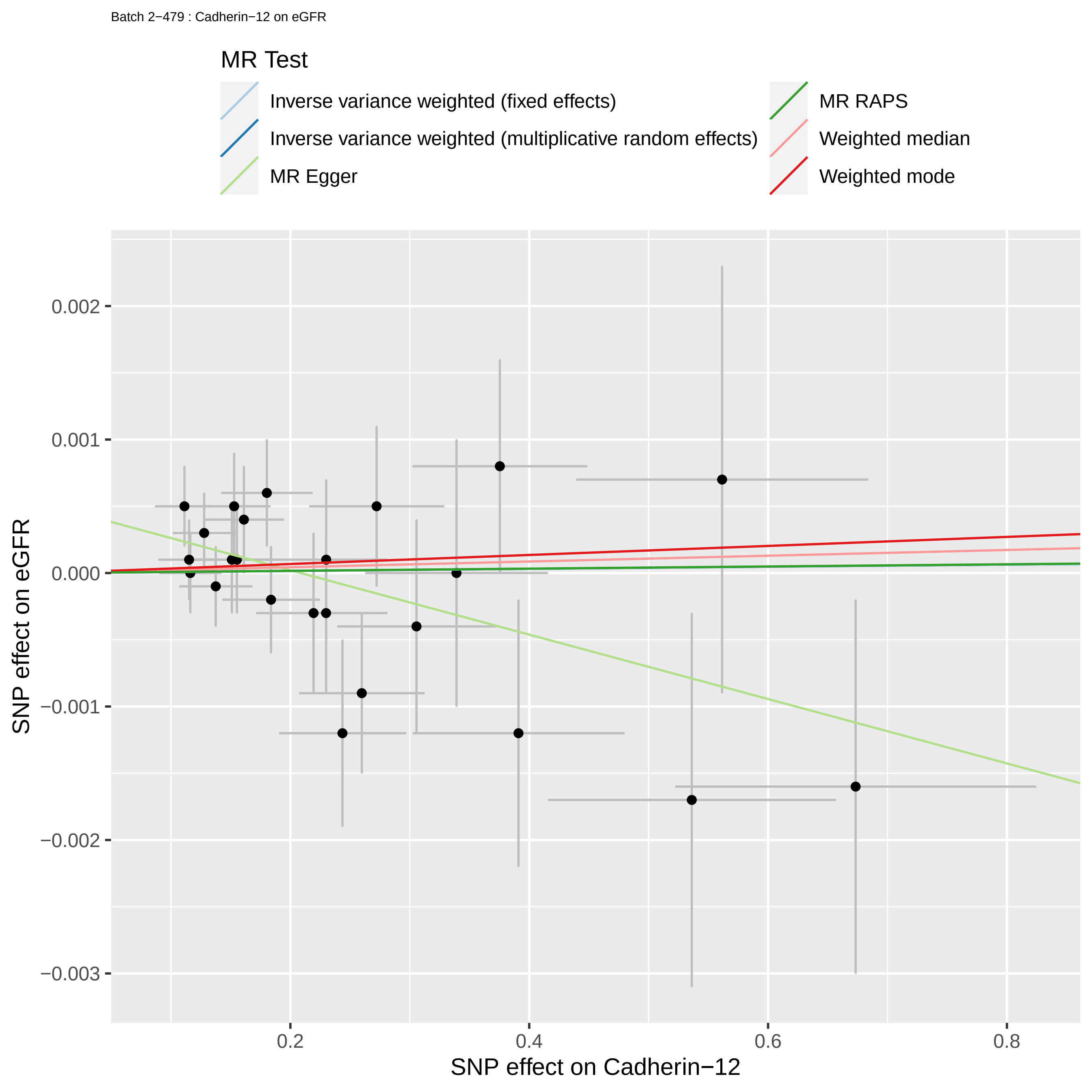

Batch 2−479 : Cadherin−12 on eGFR
MR Test
Inverse variance weighted (fixed effects)
Inverse variance weighted (multiplicative random effects)
MR Egger
MR RAPS
Weighted median
Weighted mode
0.002
0.001
0.000
−0.001
−0.002
−0.003
SNP effect on eGFR
0.2
0.4
0.6
0.8
SNP effect on Cadherin−12

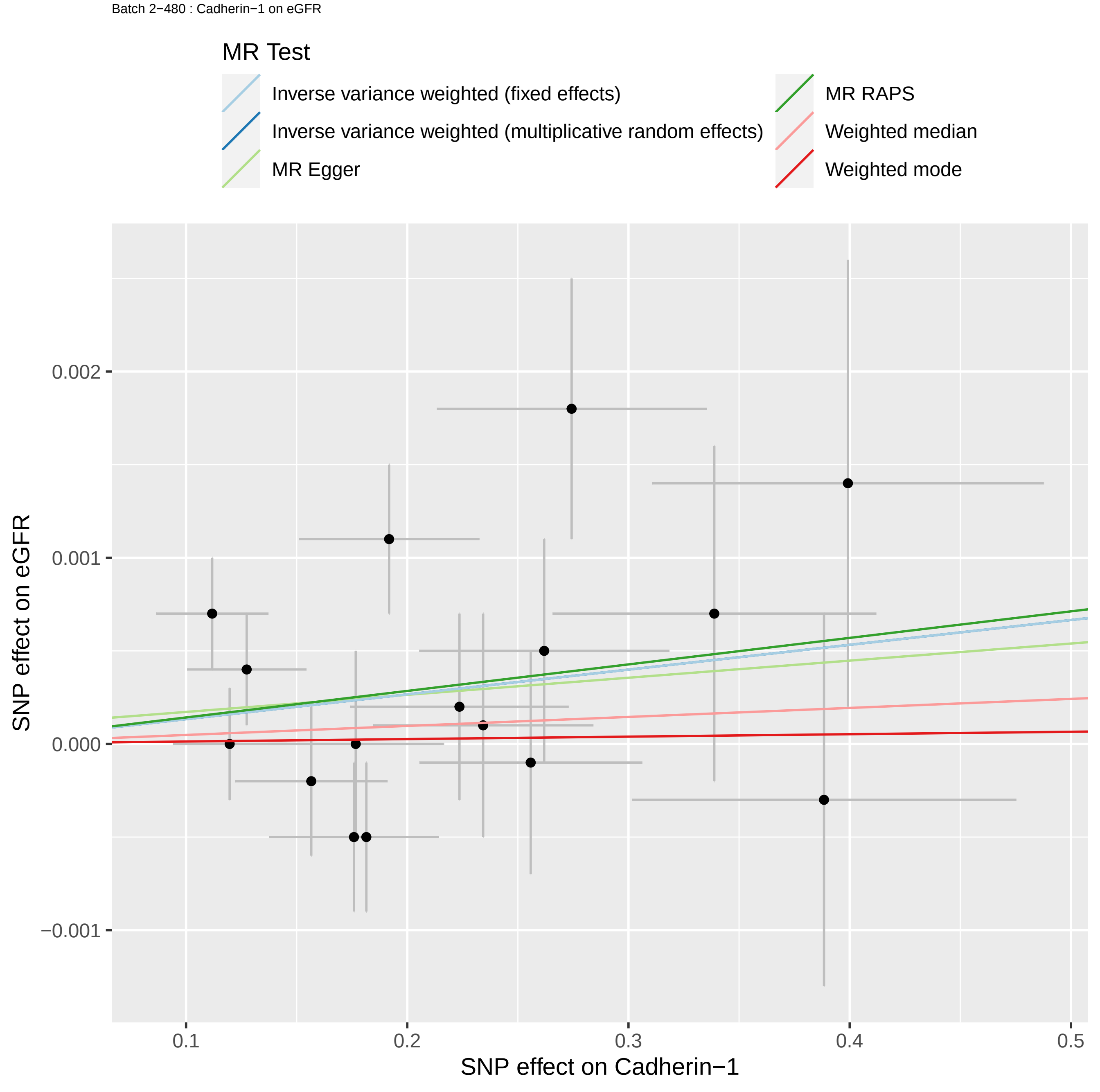
Batch 2−480 : Cadherin−1 on eGFR
MR Test
Inverse variance weighted (fixed effects)
Inverse variance weighted (multiplicative random effects)
MR Egger
MR RAPS
Weighted median
Weighted mode
0.002
0.001
0.000
−0.001
SNP effect on eGFR
0.1
0.2
0.3
0.4
0.5
SNP effect on Cadherin−1

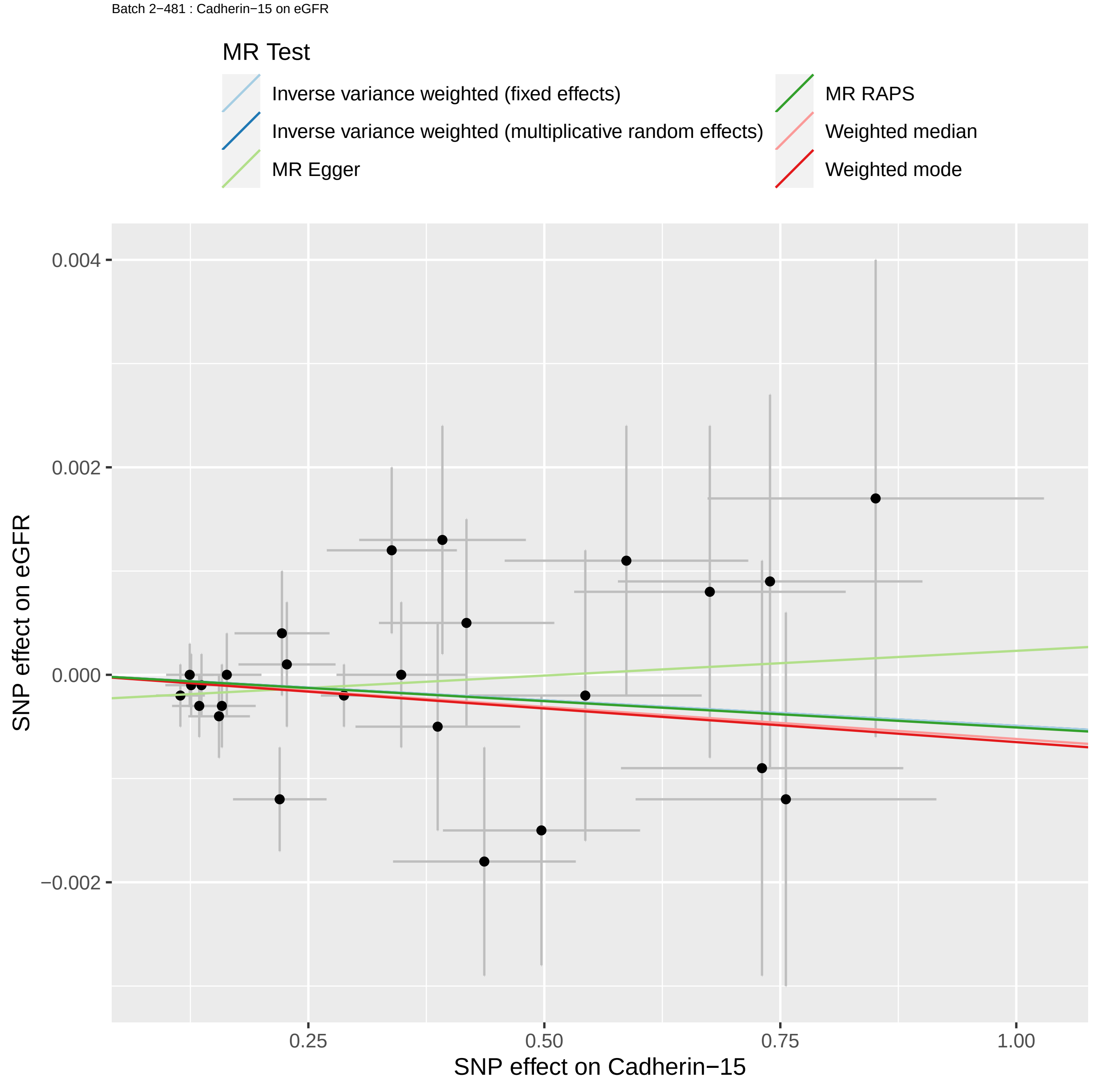

Batch 2−481 : Cadherin−15 on eGFR
MR Test
Inverse variance weighted (fixed effects)
Inverse variance weighted (multiplicative random effects)
MR Egger
MR RAPS
Weighted median
Weighted mode
0.004
0.002
0.000
−0.002
0.25
0.50
0.75
1.00
SNP effect on eGFR
SNP effect on Cadherin−15

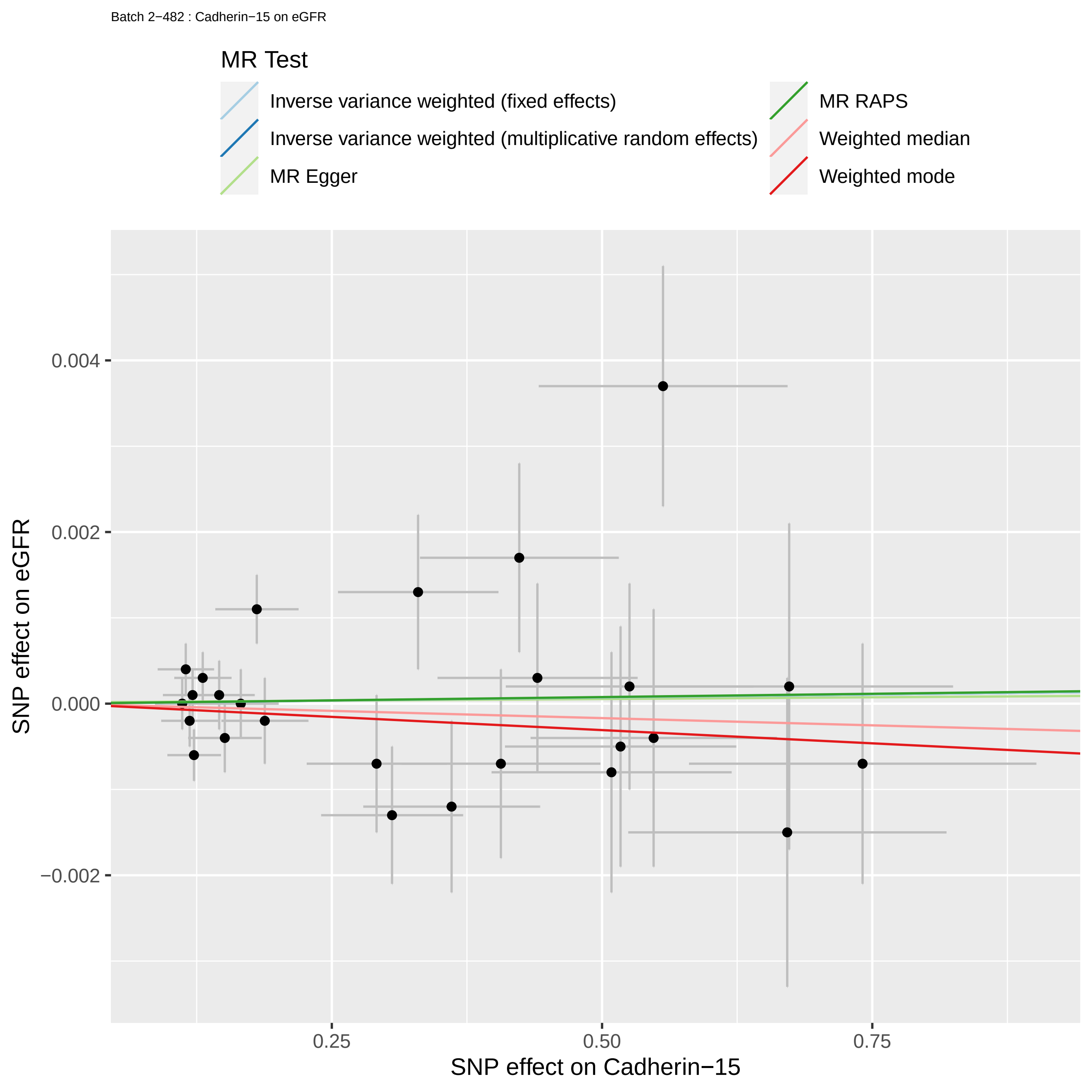

Batch 2−482 : Cadherin−15 on eGFR
MR Test
Inverse variance weighted (fixed effects)
Inverse variance weighted (multiplicative random effects)
MR Egger
MR RAPS
Weighted median
Weighted mode
0.004
0.002
0.000
−0.002
0.25
0.50
0.75
SNP effect on eGFR
SNP effect on Cadherin−15

Batch 2−483 : Cadherin−2 on eGFR

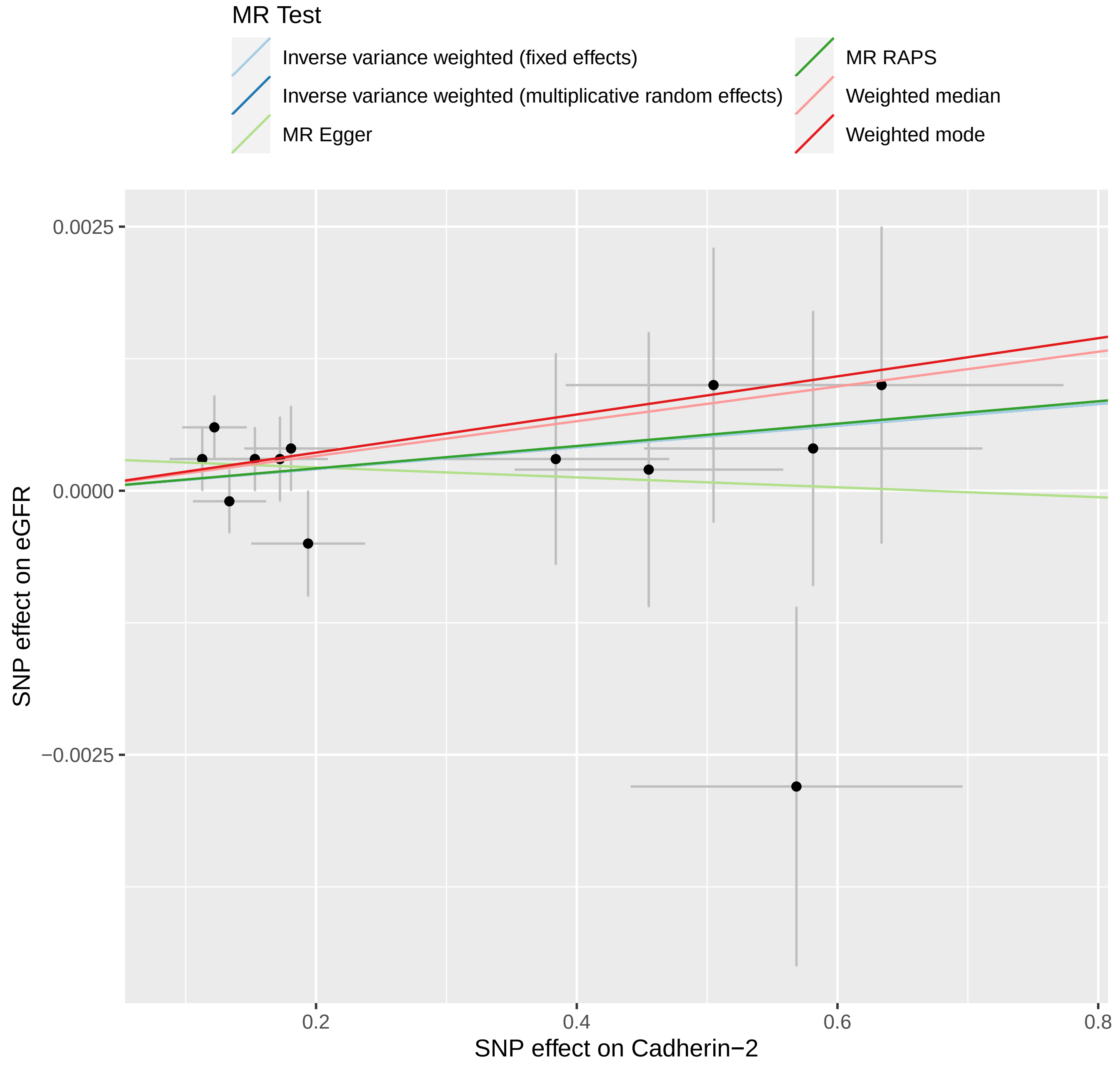

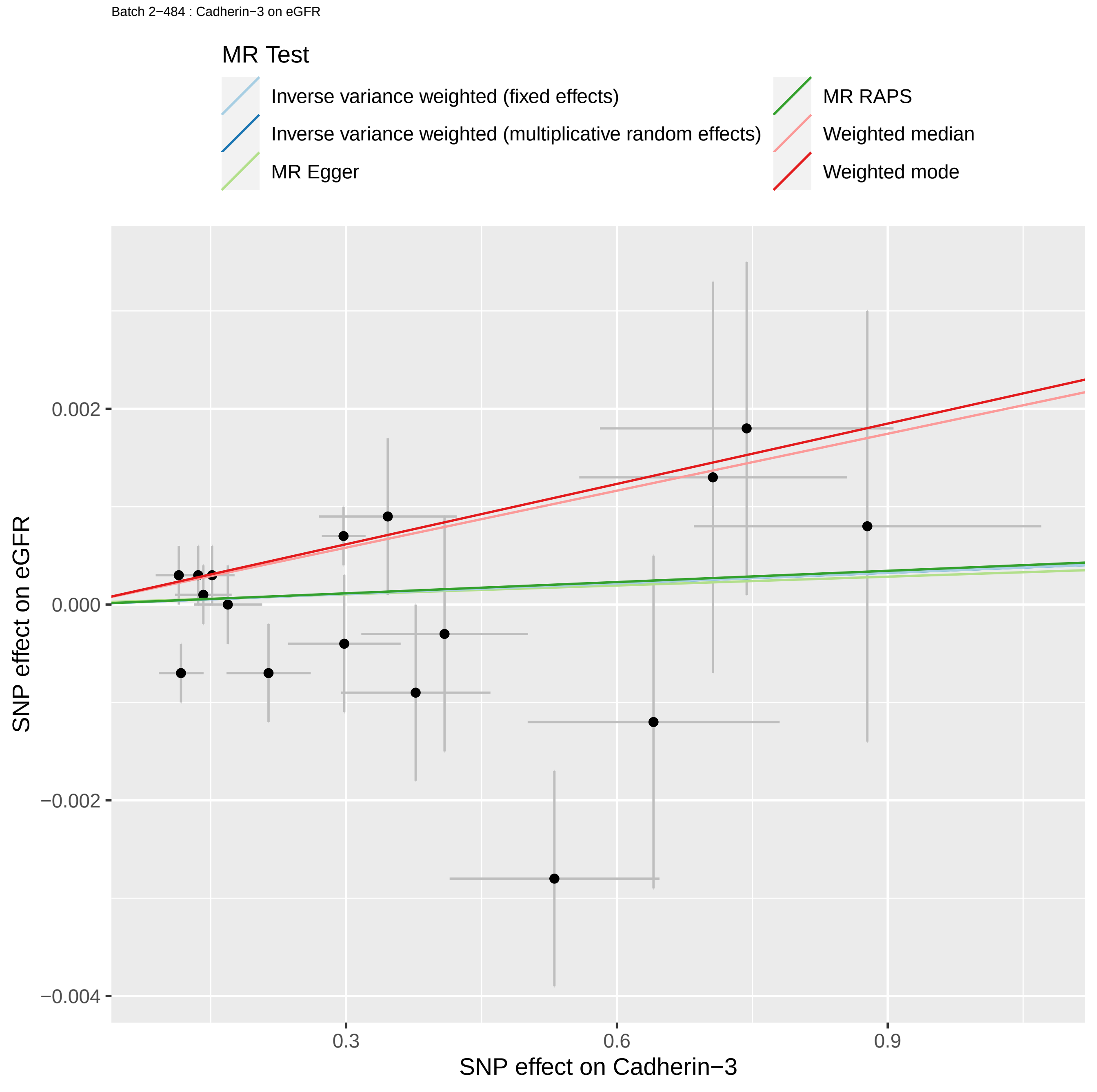
Batch 2−484 : Cadherin−3 on eGFR
MR Test
Inverse variance weighted (fixed effects)
Inverse variance weighted (multiplicative random effects)
MR Egger
MR RAPS
Weighted median
Weighted mode
0.002
0.000
−0.002
−0.004
0.3
0.6
0.9
SNP effect on eGFR
SNP effect on Cadherin−3

Batch 2−485 : Cadherin−5 on eGFR

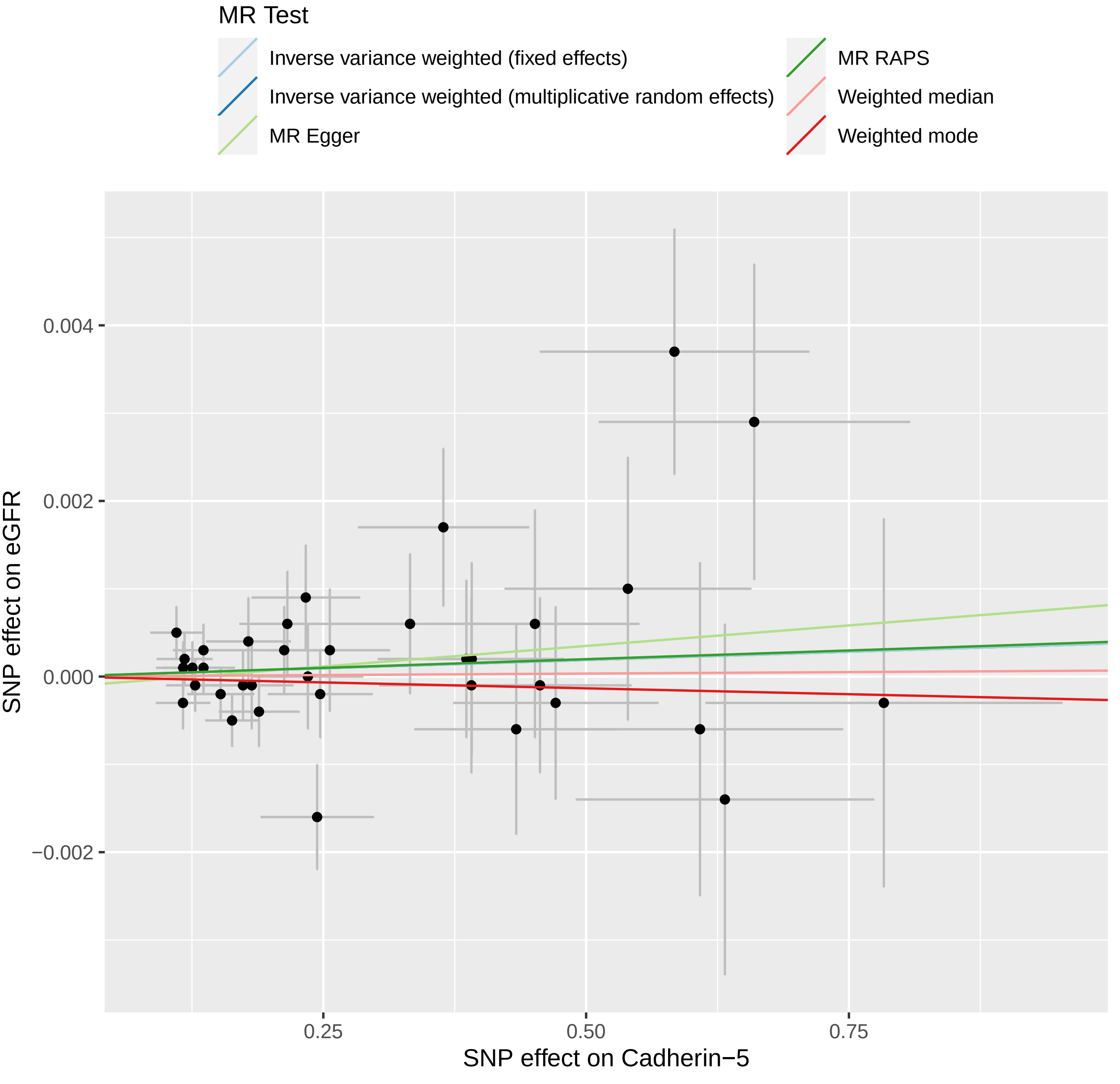

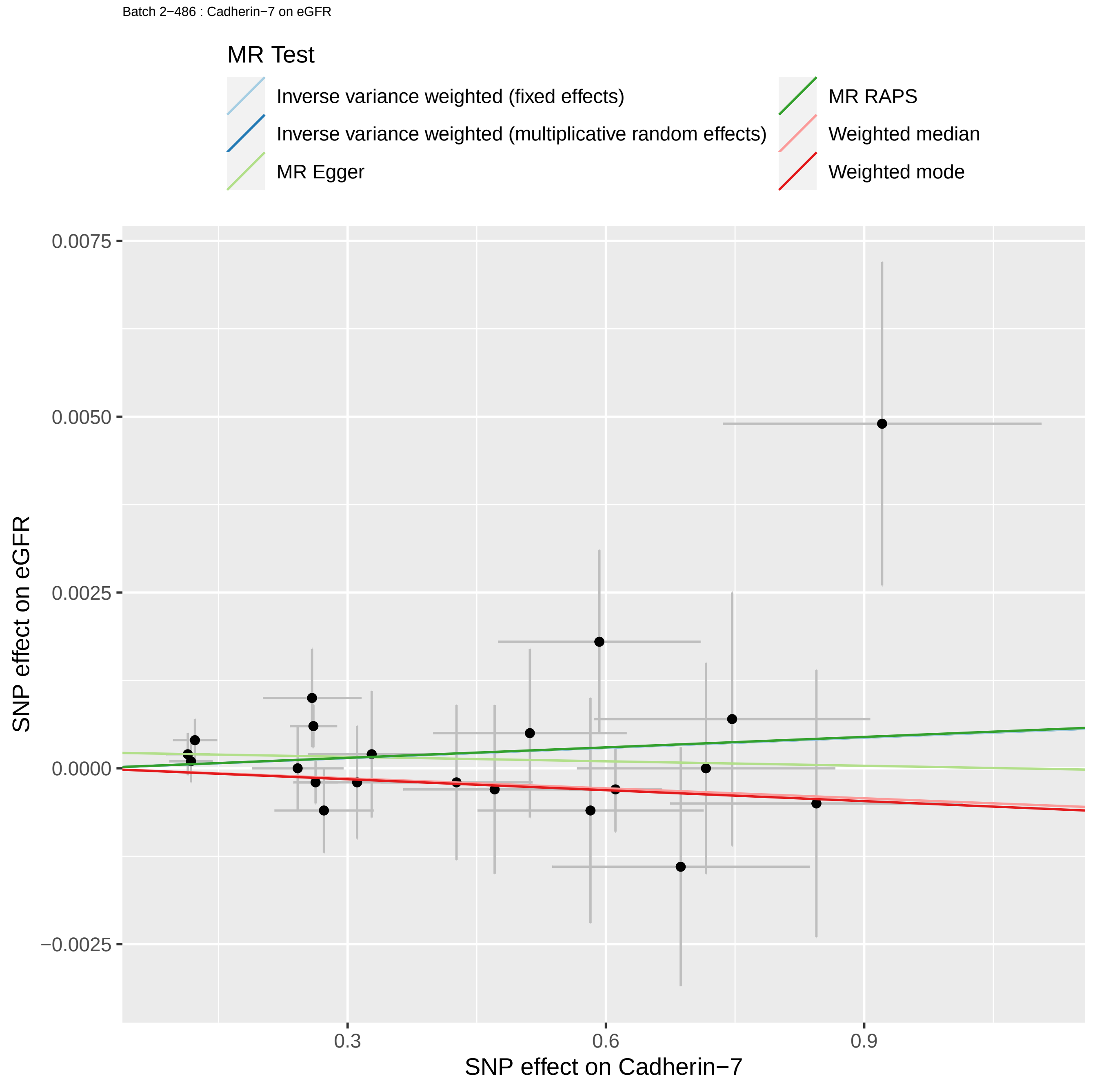

Batch 2−486 : Cadherin−7 on eGFR
MR Test
Inverse variance weighted (fixed effects)
Inverse variance weighted (multiplicative random effects)
MR Egger
MR RAPS
Weighted median
Weighted mode
0.0075
0.0050
0.0025
0.0000
−0.0025
0.3
0.6
0.9
SNP effect on eGFR
SNP effect on Cadherin−7

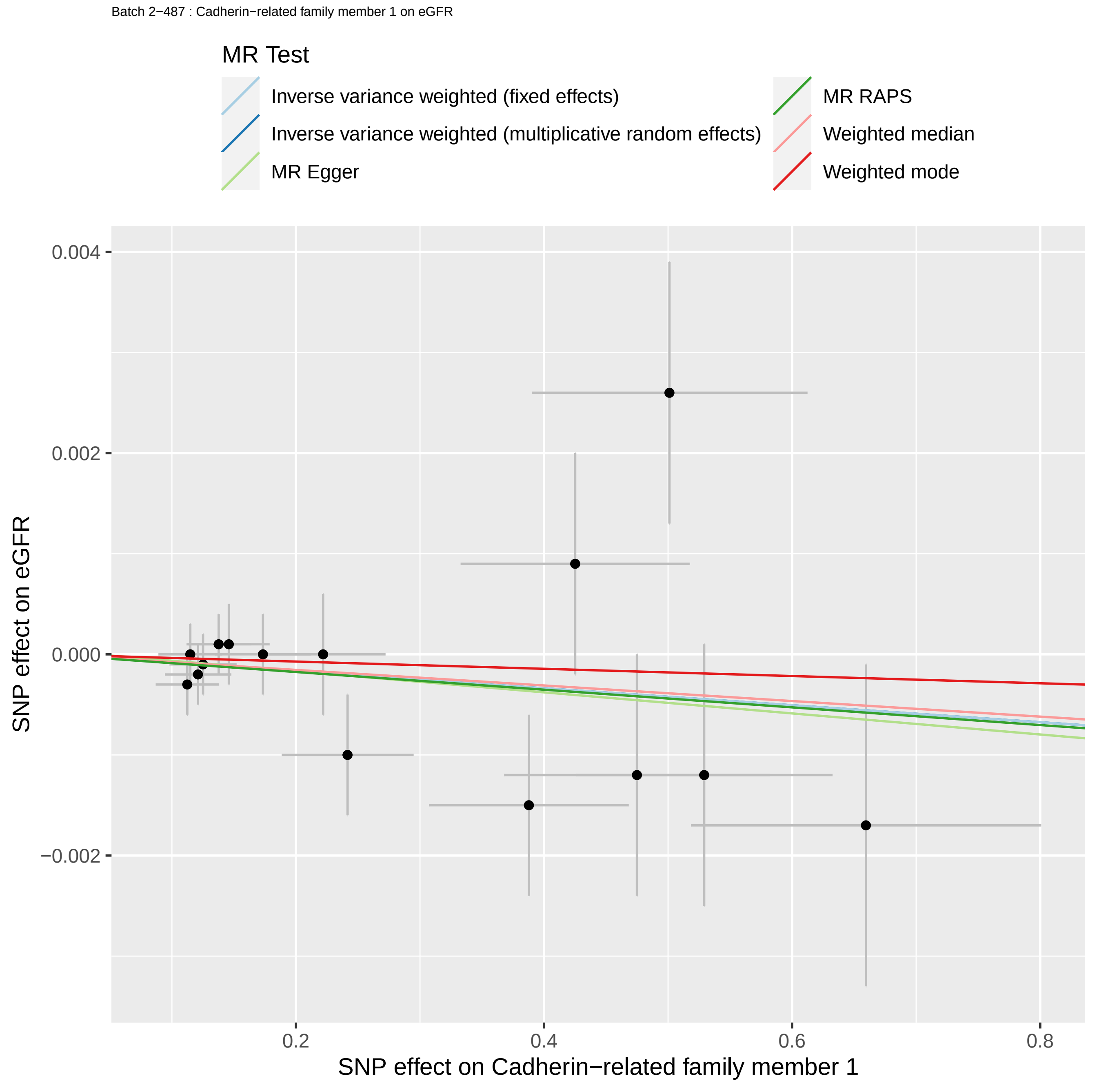

Batch 2−487 : Cadherin−related family member 1 on eGFR
MR Test
Inverse variance weighted (fixed effects)
Inverse variance weighted (multiplicative random effects)
MR Egger
MR RAPS
Weighted median
Weighted mode
0.004
0.002
0.000
−0.002
0.2
0.4
0.6
0.8
SNP effect on eGFR
SNP effect on Cadherin−related family member 1

Batch 2−488 : Cadherin−related family member 3 on eGFR

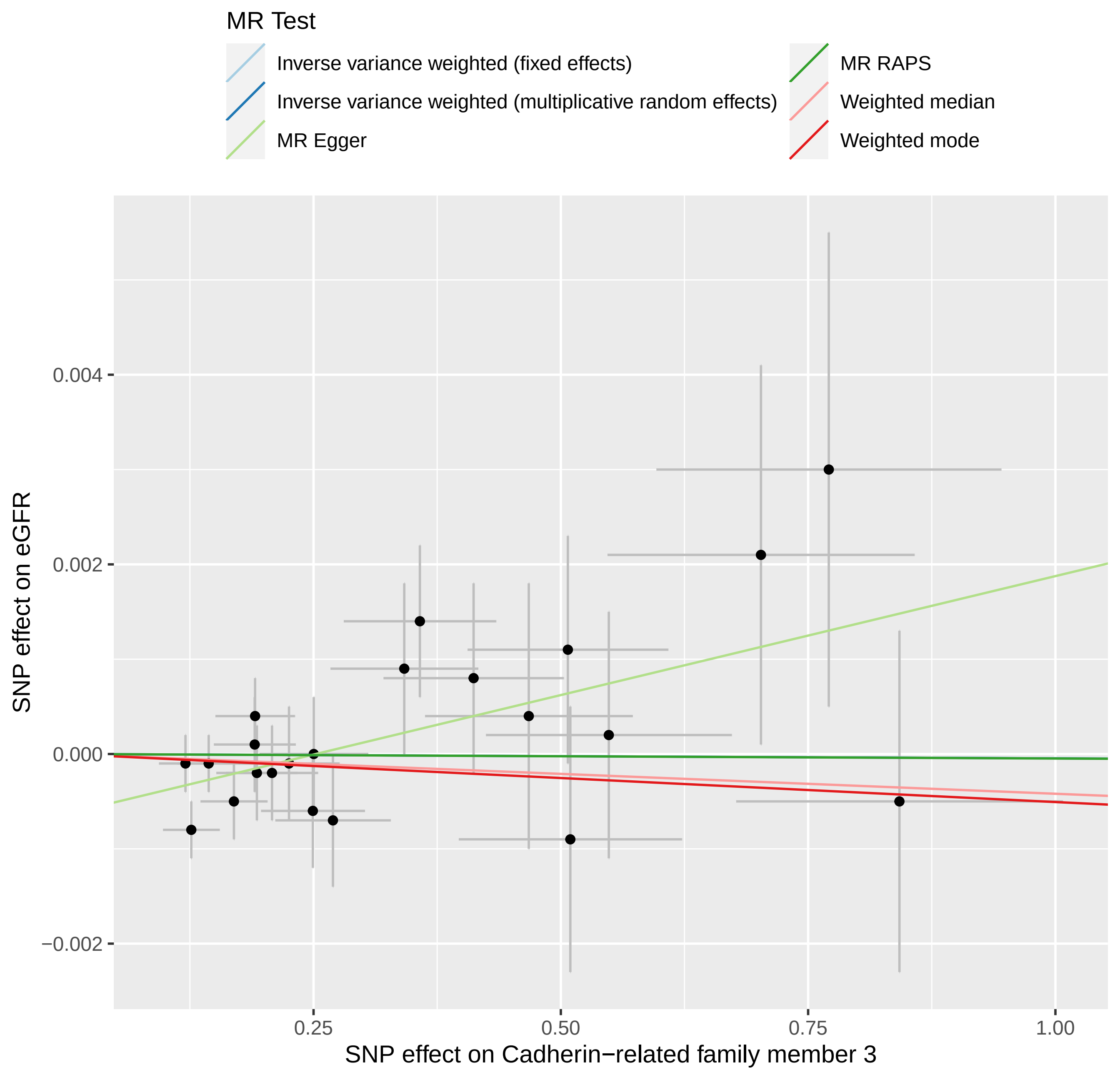

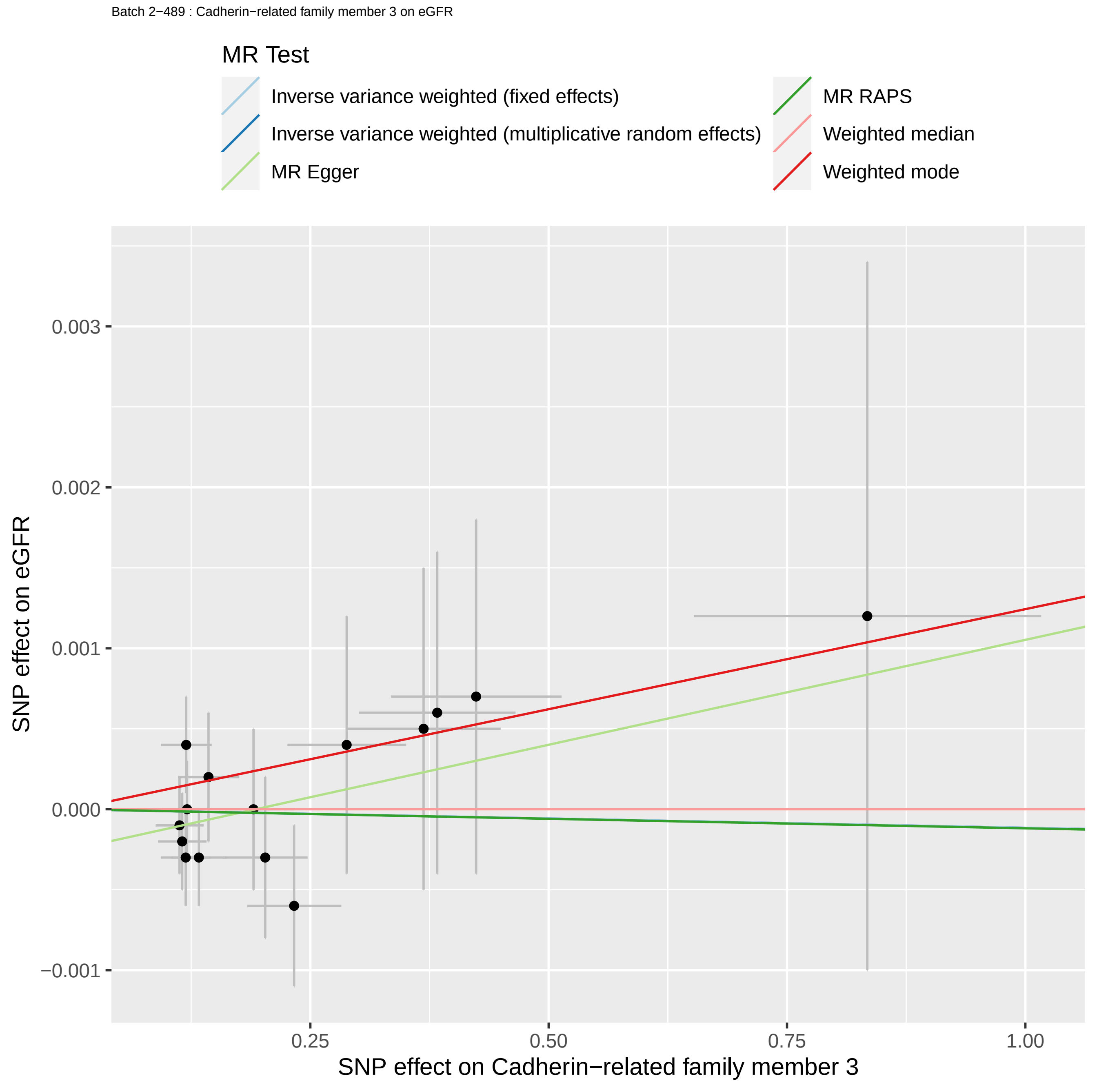

Batch 2−489 : Cadherin−related family member 3 on eGFR
MR Test
Inverse variance weighted (fixed effects)
Inverse variance weighted (multiplicative random effects)
MR Egger
MR RAPS
Weighted median
Weighted mode
0.003
0.002
0.001
0.000
−0.001
0.25
0.50
0.75
1.00
SNP effect on eGFR
SNP effect on Cadherin−related family member 3

Batch 2−490 : Cadherin−related family member 5 on eGFR

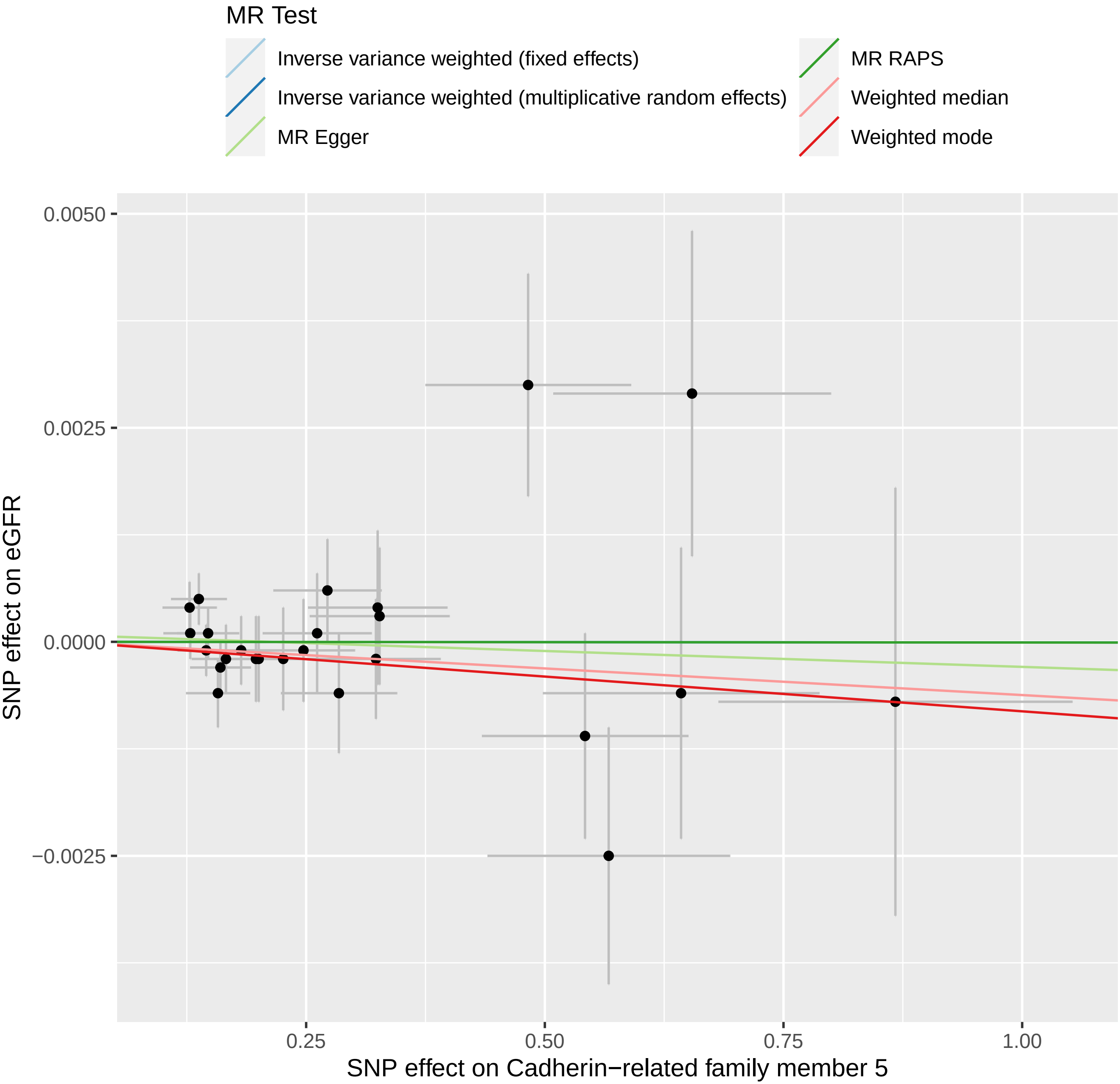

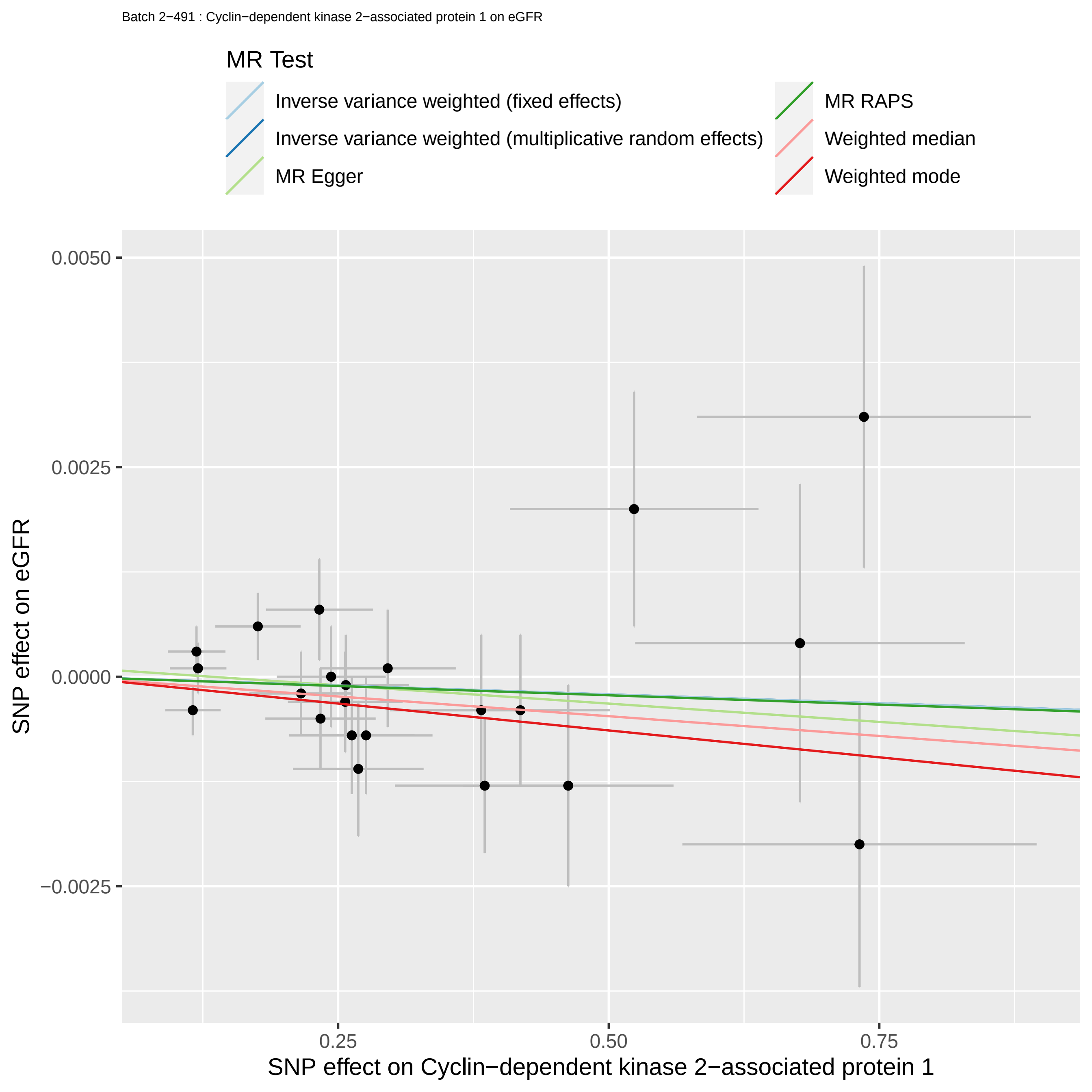

Batch 2−491 : Cyclin−dependent kinase 2−associated protein 1 on eGFR
MR Test
Inverse variance weighted (fixed effects)
Inverse variance weighted (multiplicative random effects)
MR Egger
MR RAPS
Weighted median
Weighted mode
0.0050
0.0025
0.0000
−0.0025
0.25
0.50
0.75
SNP effect on eGFR
SNP effect on Cyclin−dependent kinase 2−associated protein 1

Batch 2−492 : Cyclin−dependent kinase 2:Cyclin−A2 complex on eGFR

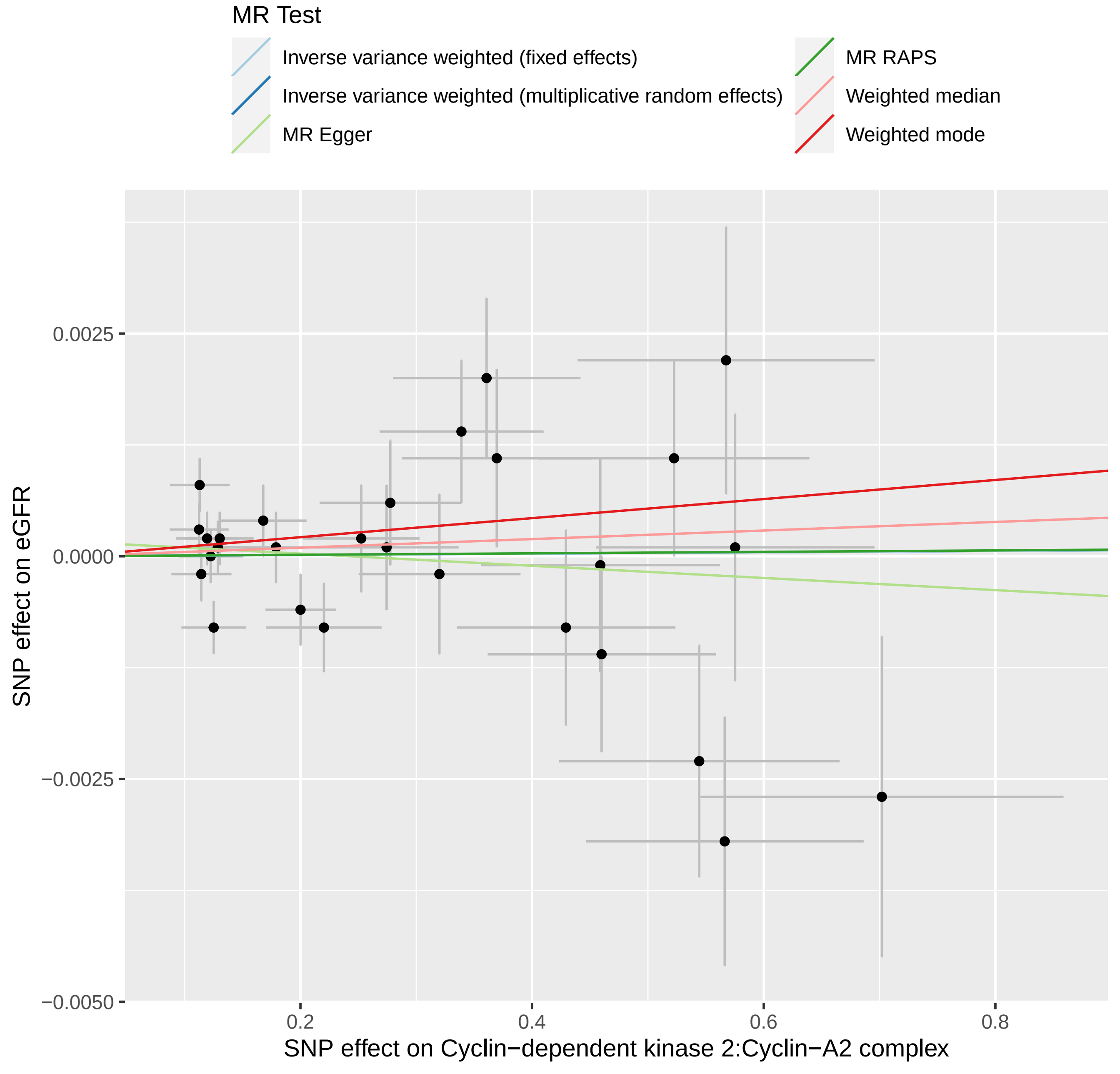

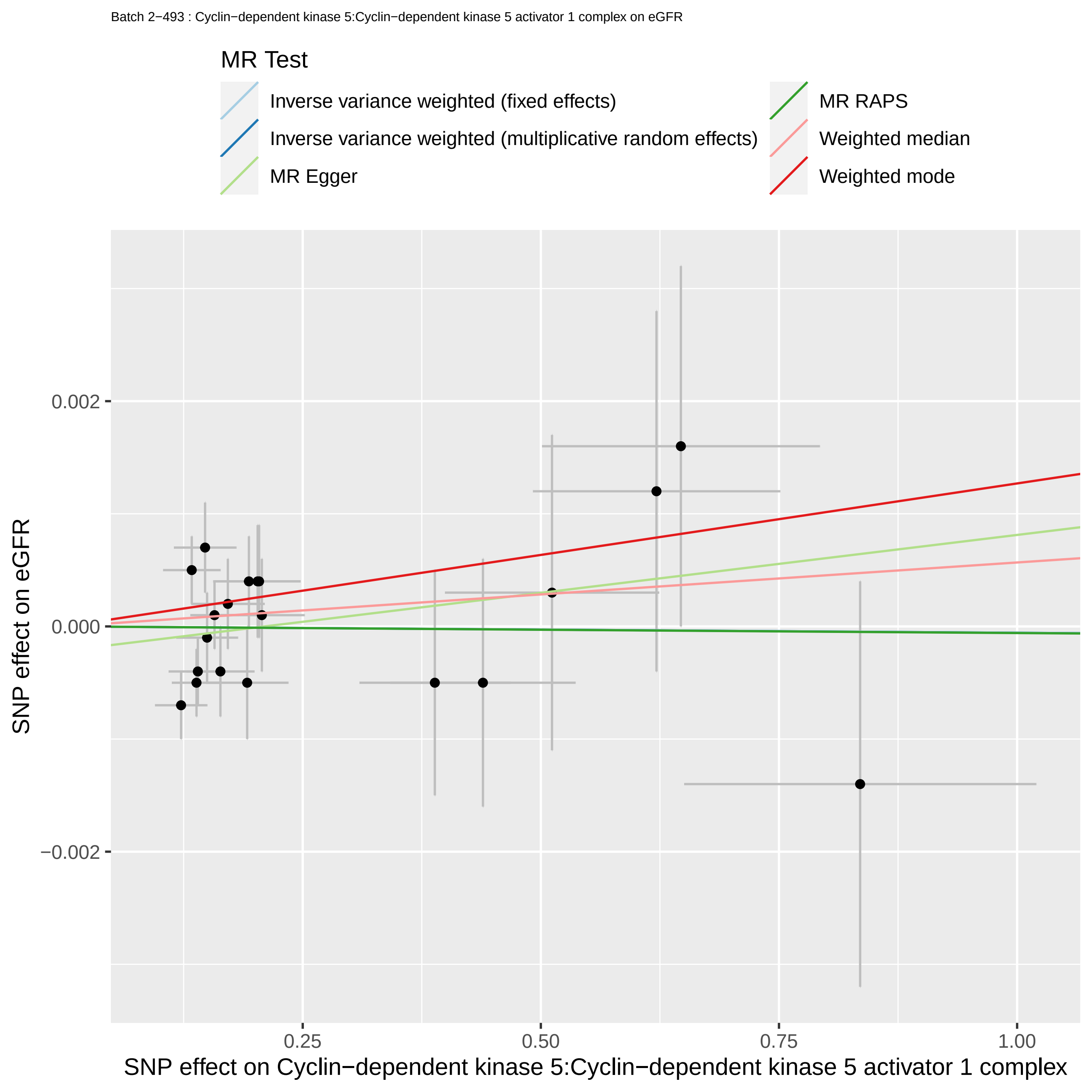

Batch 2−493 : Cyclin−dependent kinase 5:Cyclin−dependent kinase 5 activator 1 complex on eGFR
MR Test
Inverse variance weighted (fixed effects)
Inverse variance weighted (multiplicative random effects)
MR Egger
MR RAPS
Weighted median
Weighted mode
0.002
0.000
−0.002
0.25
0.50
0.75
1.00
SNP effect on eGFR
SNP effect on Cyclin−dependent kinase 5:Cyclin−dependent kinase 5 activator 1 complex

Batch 2−494 : Cyclin−dependent kinase 8:Cyclin−C complex on eGFR

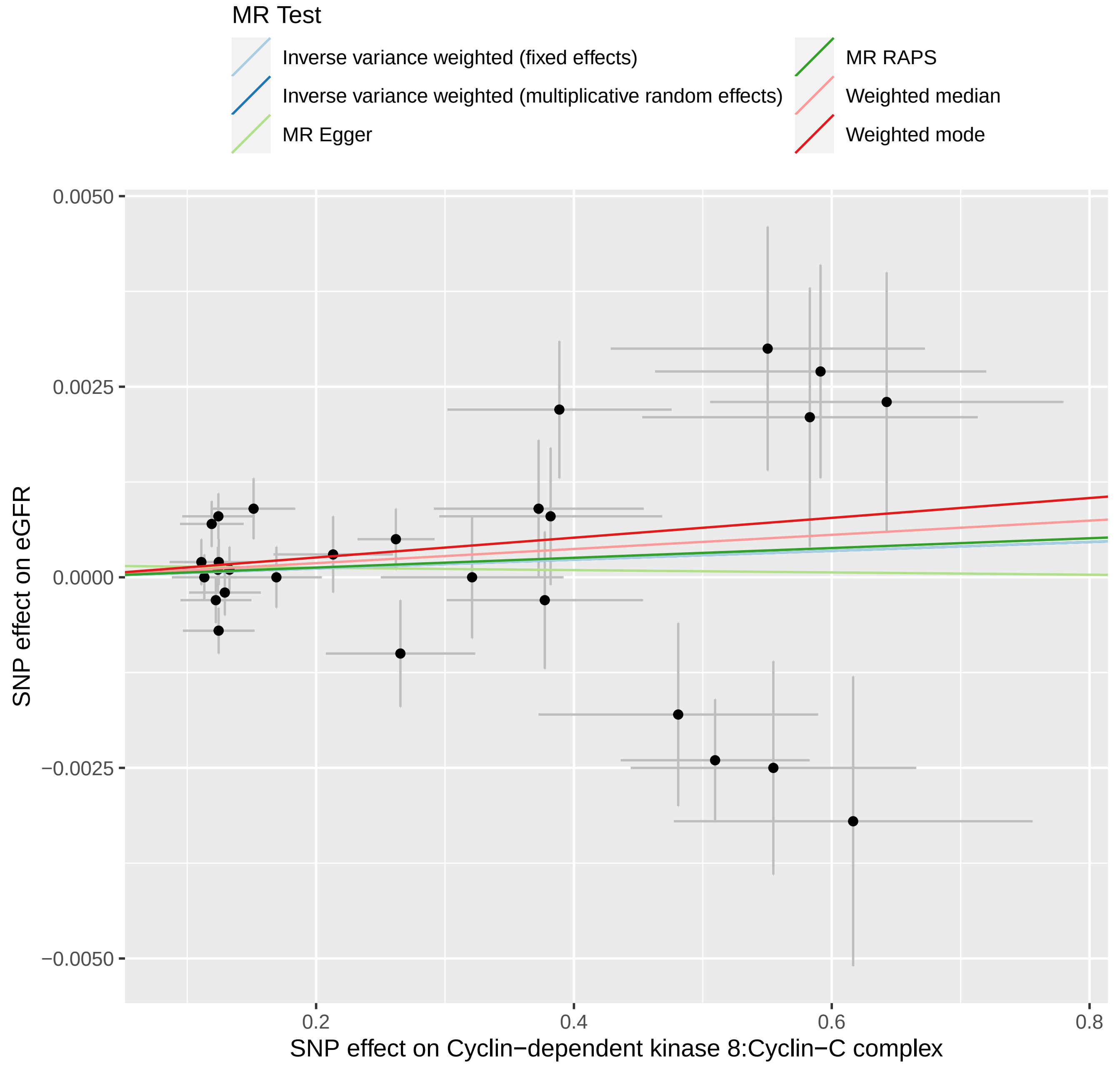

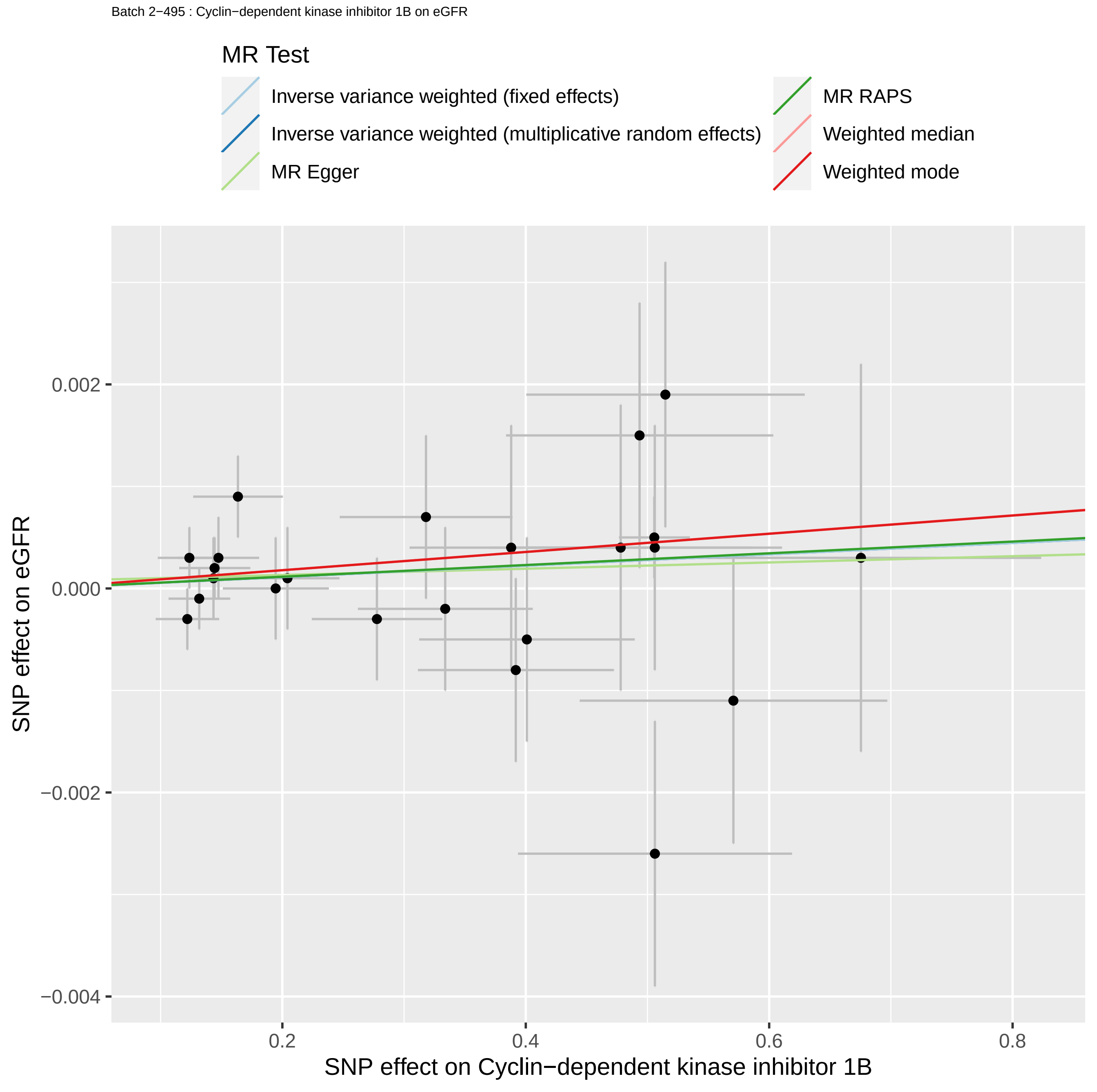
Batch 2−495 : Cyclin−dependent kinase inhibitor 1B on eGFR
MR Test
Inverse variance weighted (fixed effects)
Inverse variance weighted (multiplicative random effects)
MR Egger
MR RAPS
Weighted median
Weighted mode
0.002
0.000
−0.002
−0.004
0.2
0.4
0.6
0.8
SNP effect on eGFR
SNP effect on Cyclin−dependent kinase inhibitor 1B

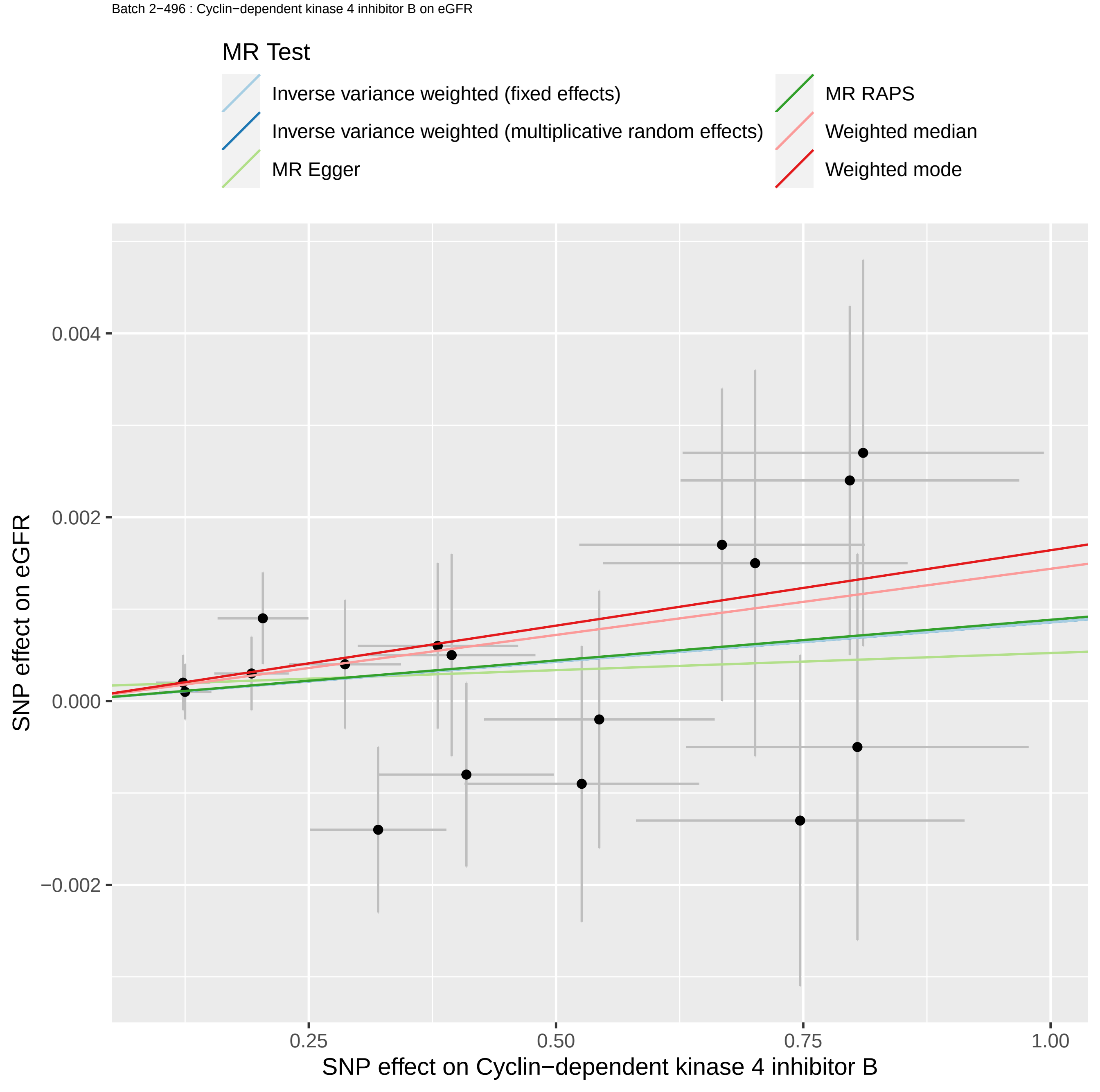
Batch 2−496 : Cyclin−dependent kinase 4 inhibitor B on eGFR
MR Test
Inverse variance weighted (fixed effects)
Inverse variance weighted (multiplicative random effects)
MR Egger
MR RAPS
Weighted median
Weighted mode
0.004
0.002
0.000
−0.002
0.25
0.50
0.75
1.00
SNP effect on eGFR
SNP effect on Cyclin−dependent kinase 4 inhibitor B

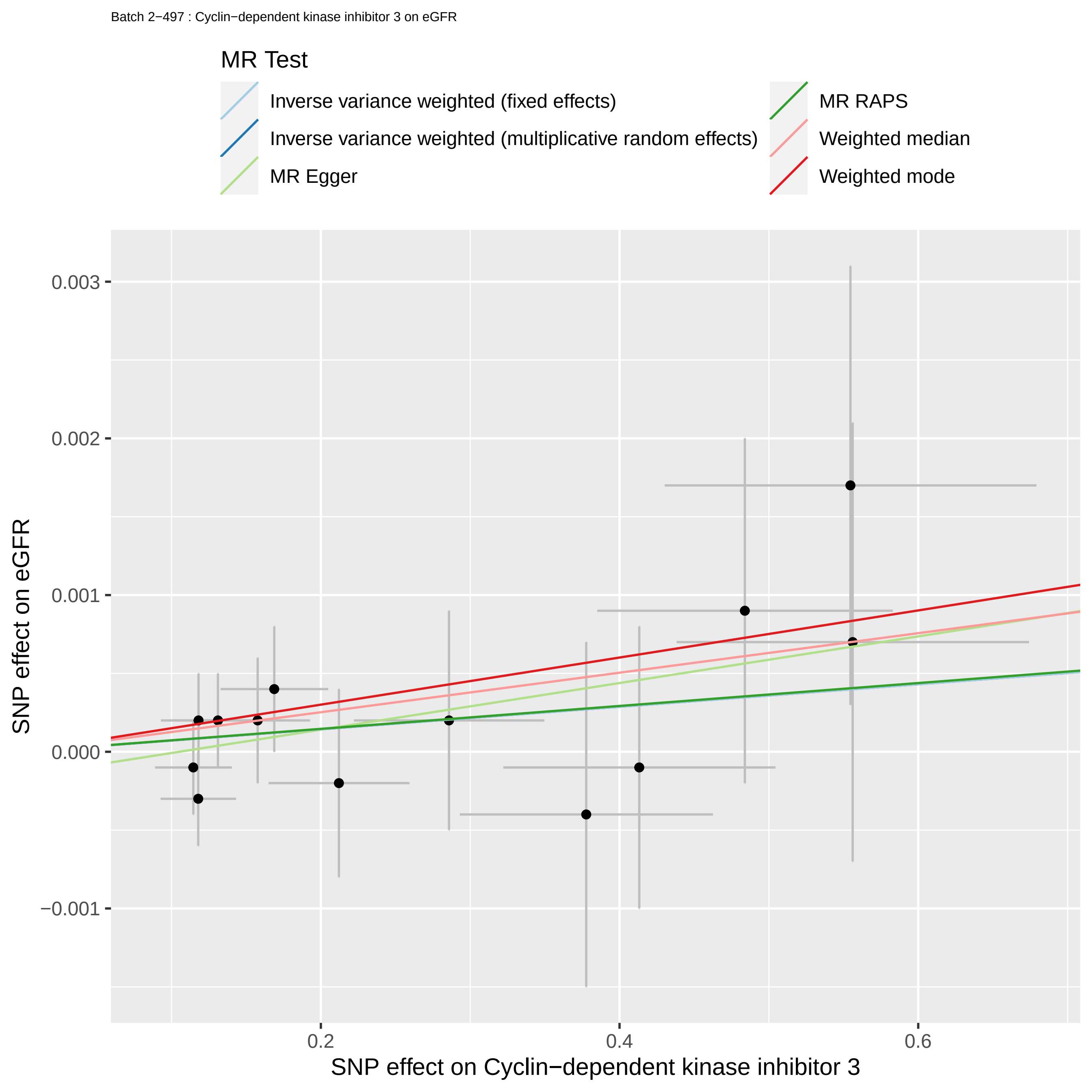

Batch 2−497 : Cyclin−dependent kinase inhibitor 3 on eGFR
MR Test
Inverse variance weighted (fixed effects)
Inverse variance weighted (multiplicative random effects)
MR Egger
MR RAPS
Weighted median
Weighted mode
0.003
0.002
0.001
0.000
−0.001
0.2
0.4
0.6
SNP effect on eGFR
SNP effect on Cyclin−dependent kinase inhibitor 3

Batch 2−498 : Cerebral dopamine neurotrophic factor on eGFR

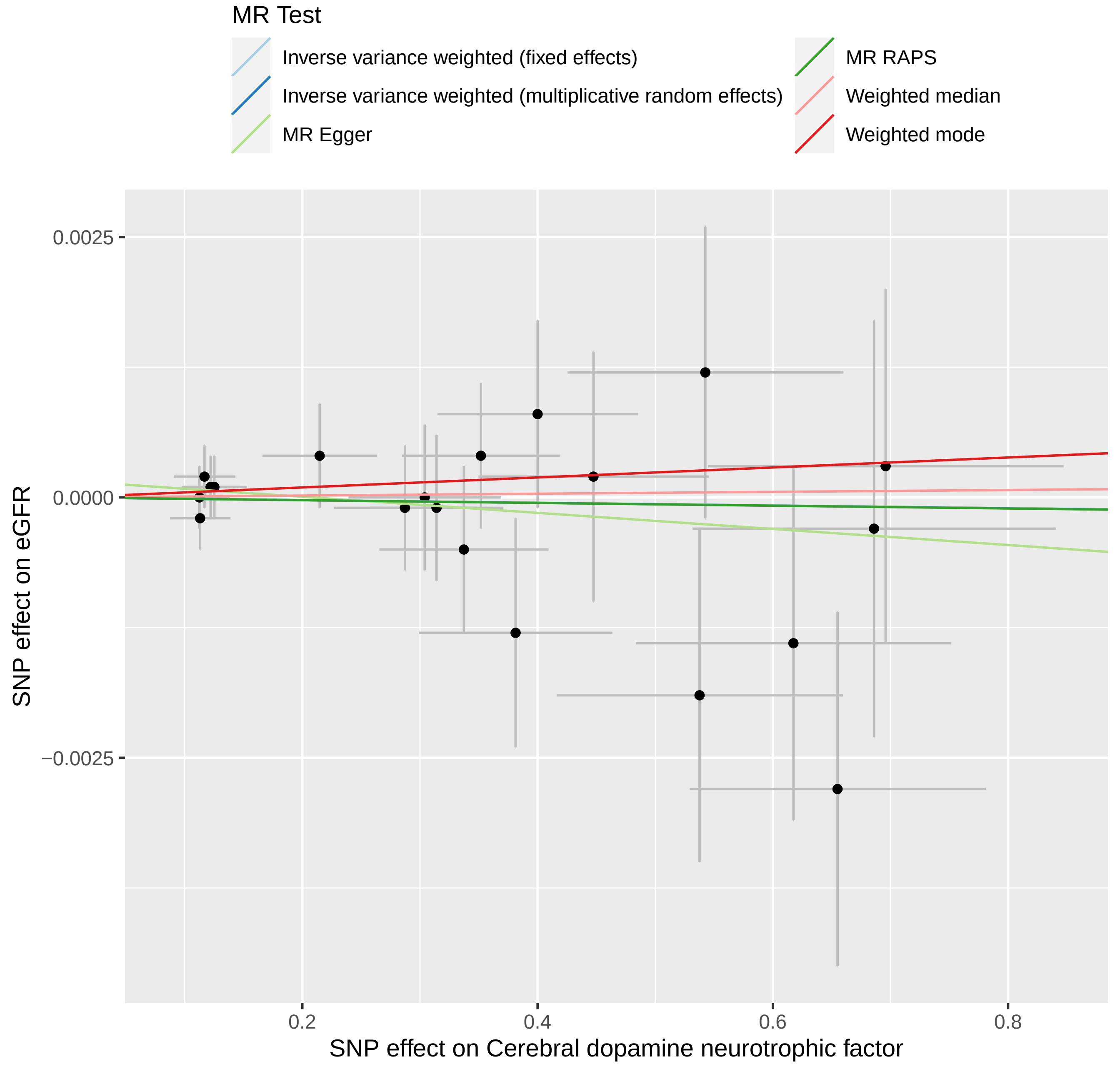

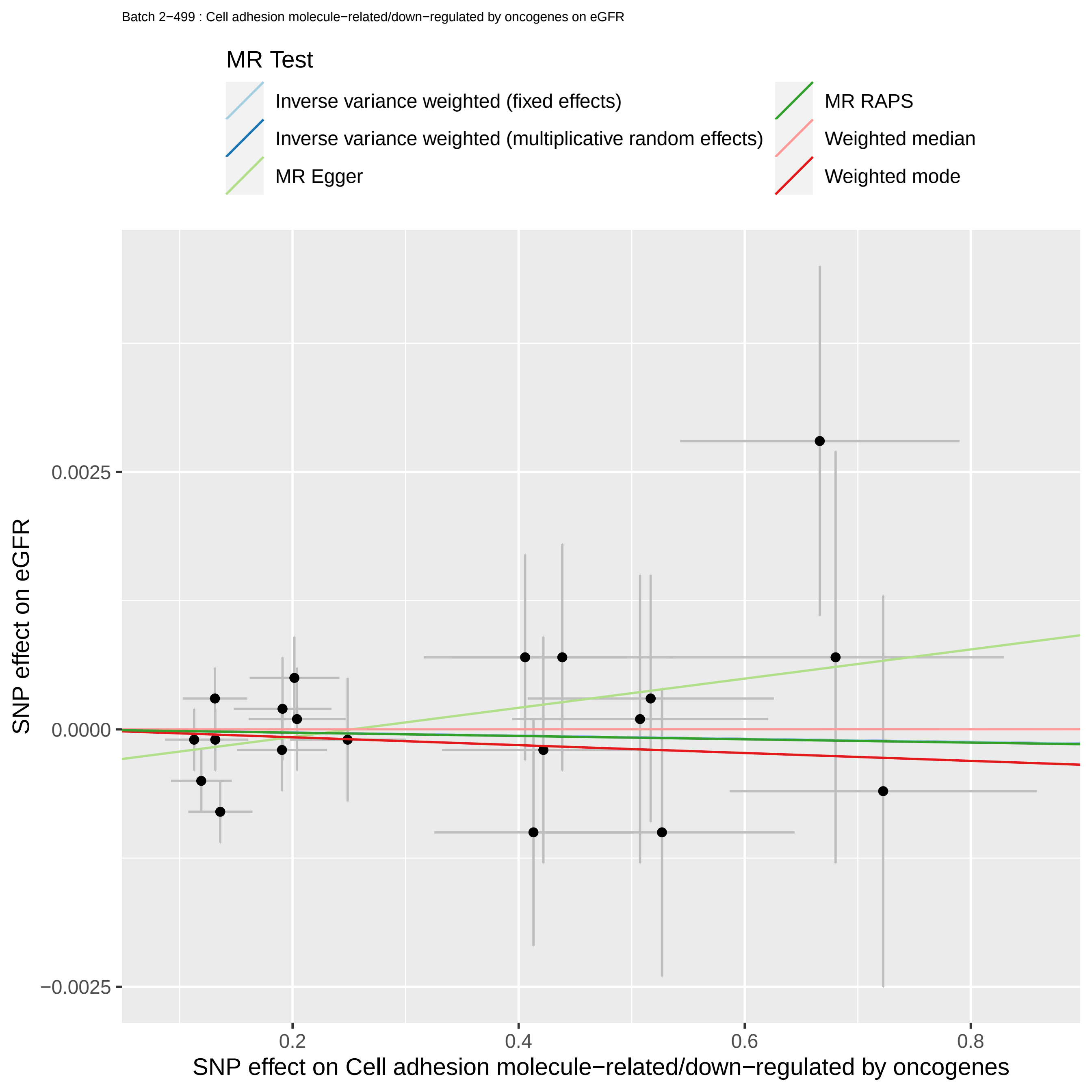

Batch 2−499 : Cell adhesion molecule−related/down−regulated by oncogenes on eGFR
MR Test
Inverse variance weighted (fixed effects)
Inverse variance weighted (multiplicative random effects)
MR Egger
MR RAPS
Weighted median
Weighted mode
SNP effect on eGFR
0.0025
0.0000
−0.0025
0.2
0.4
0.6
0.8
SNP effect on Cell adhesion molecule−related/down−regulated by oncogenes

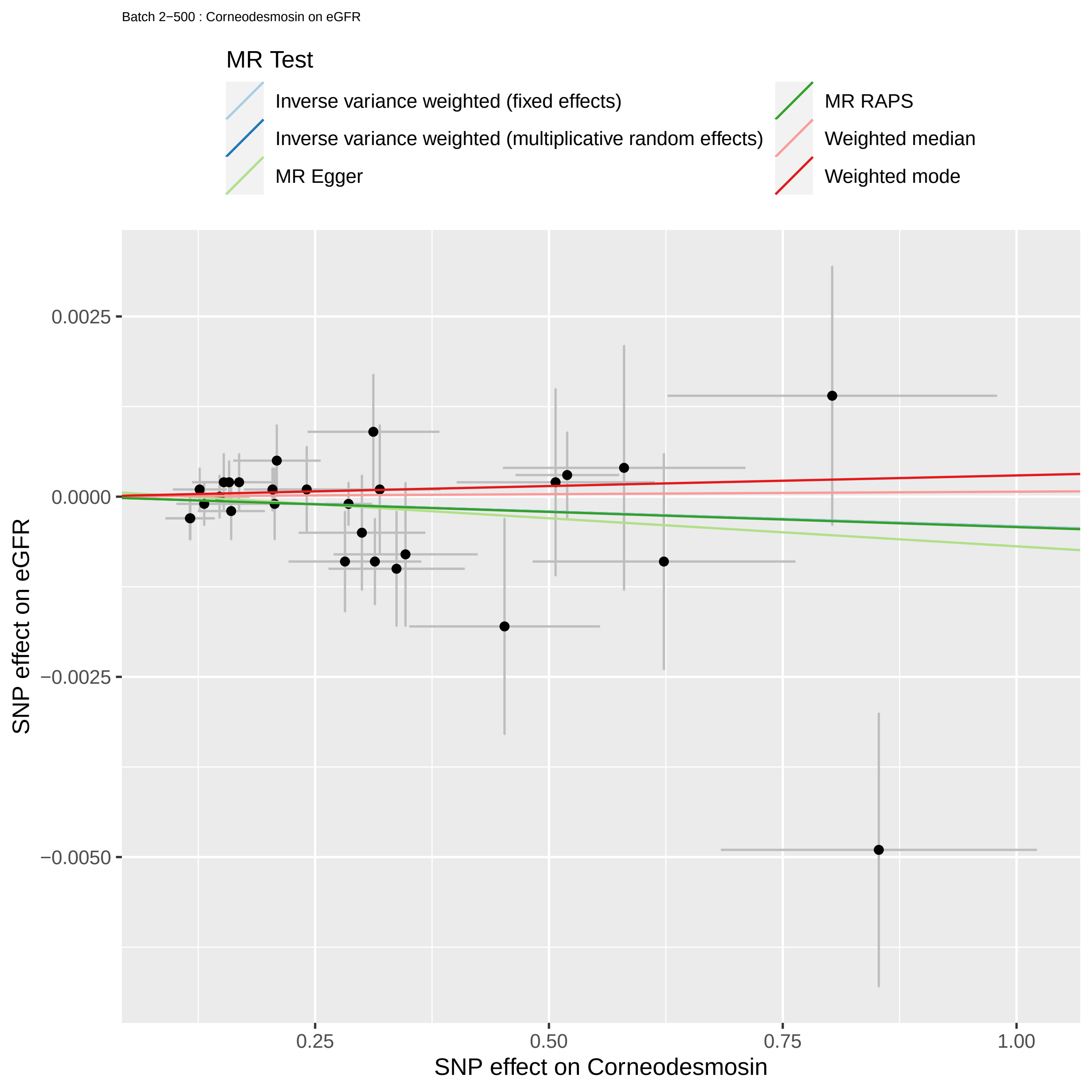
Batch 2−500 : Corneodesmosin on eGFR
MR Test
Inverse variance weighted (fixed effects)
Inverse variance weighted (multiplicative random effects)
MR Egger
MR RAPS
Weighted median
Weighted mode
0.0025
0.0000
−0.0025
−0.0050
SNP effect on eGFR
0.25
0.50
0.75
1.00
SNP effect on Corneodesmosin

Batch 2−501 : Testis−specific chromodomain protein Y 1 on eGFR

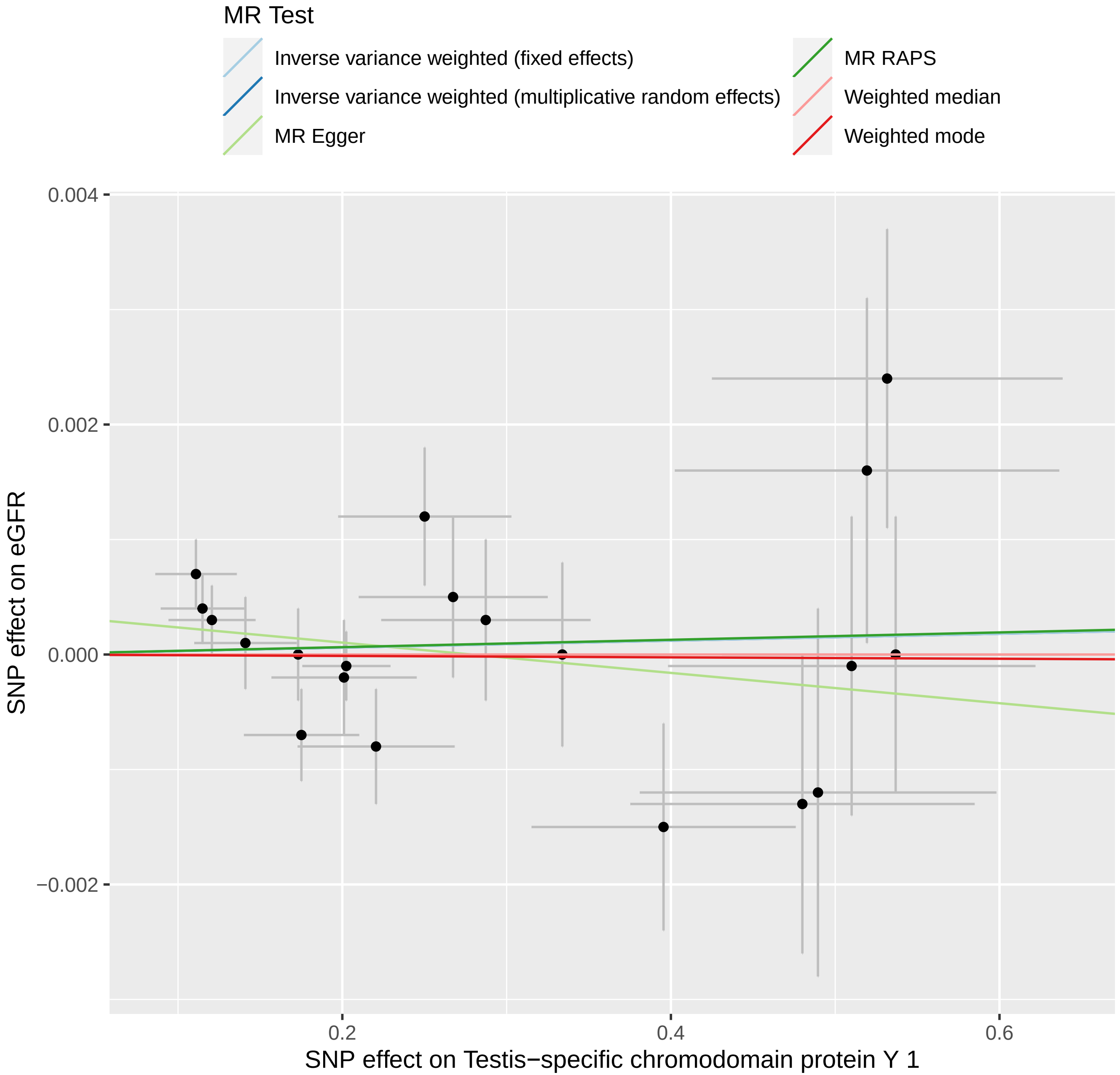

Batch 2−502 : Carcinoembryonic antigen−related cell adhesion molecule 1 on eGFR

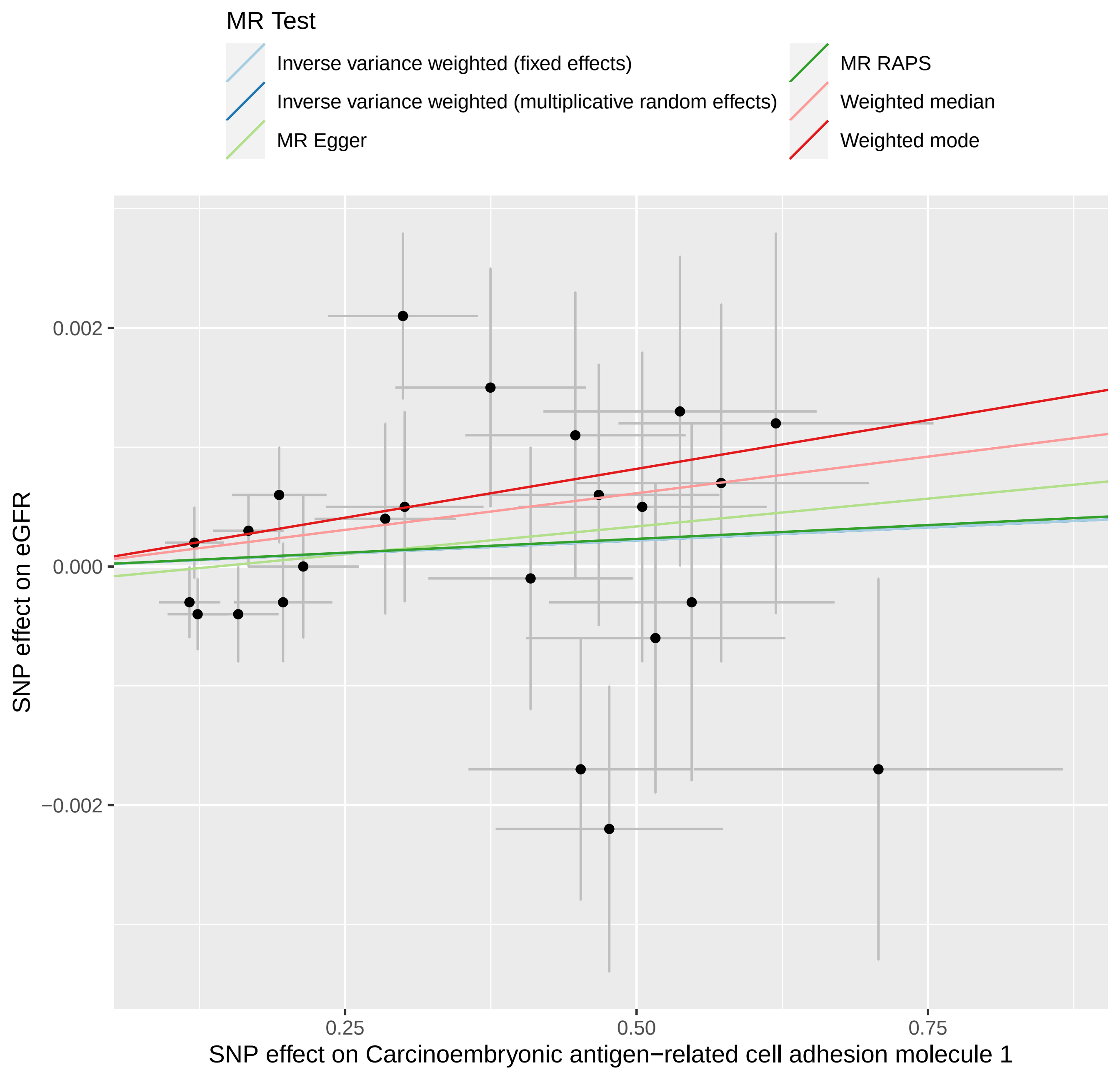

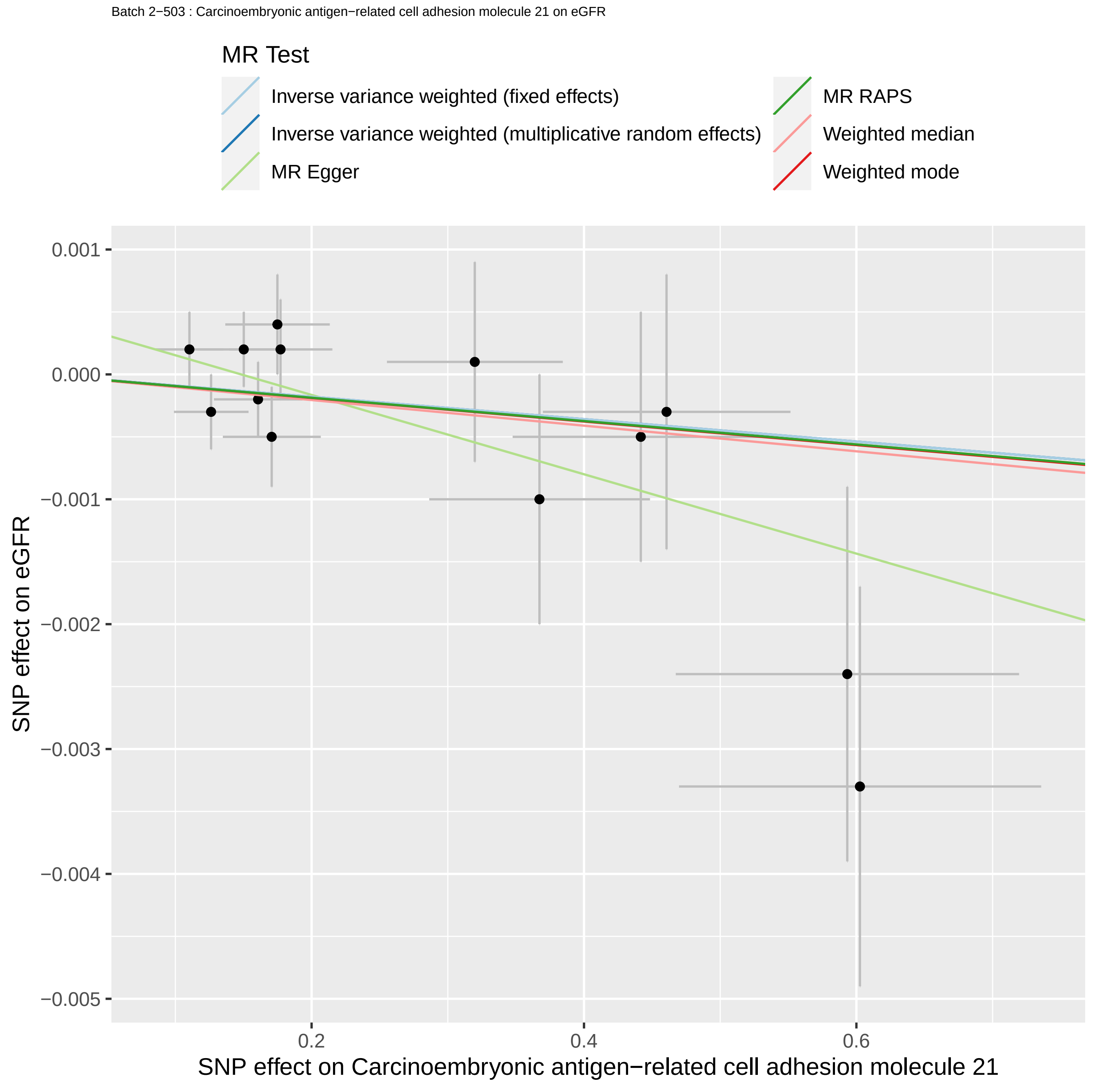
Batch 2−503 : Carcinoembryonic antigen−related cell adhesion molecule 21 on eGFR
MR Test
Inverse variance weighted (fixed effects)
Inverse variance weighted (multiplicative random effects)
MR Egger
MR RAPS
Weighted median
Weighted mode
0.001
0.000
−0.001
−0.002
−0.003
−0.004
−0.005
SNP effect on eGFR
0.2
0.4
0.6
SNP effect on Carcinoembryonic antigen−related cell adhesion molecule 21

Batch 2−504 : Carcinoembryonic antigen−related cell adhesion molecule 4 on eGFR

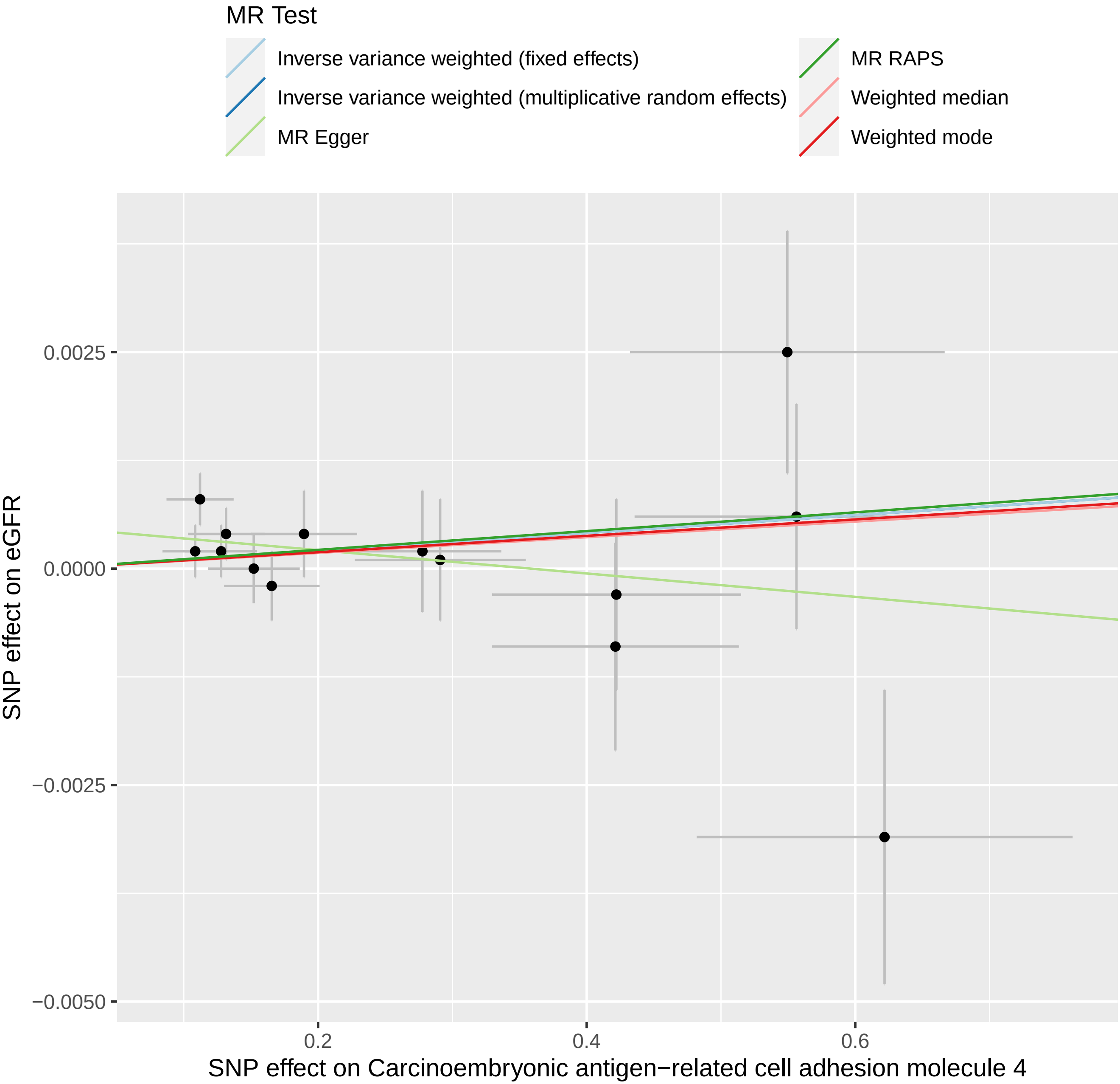

Batch 2−505 : CCAAT/enhancer−binding protein beta on eGFR

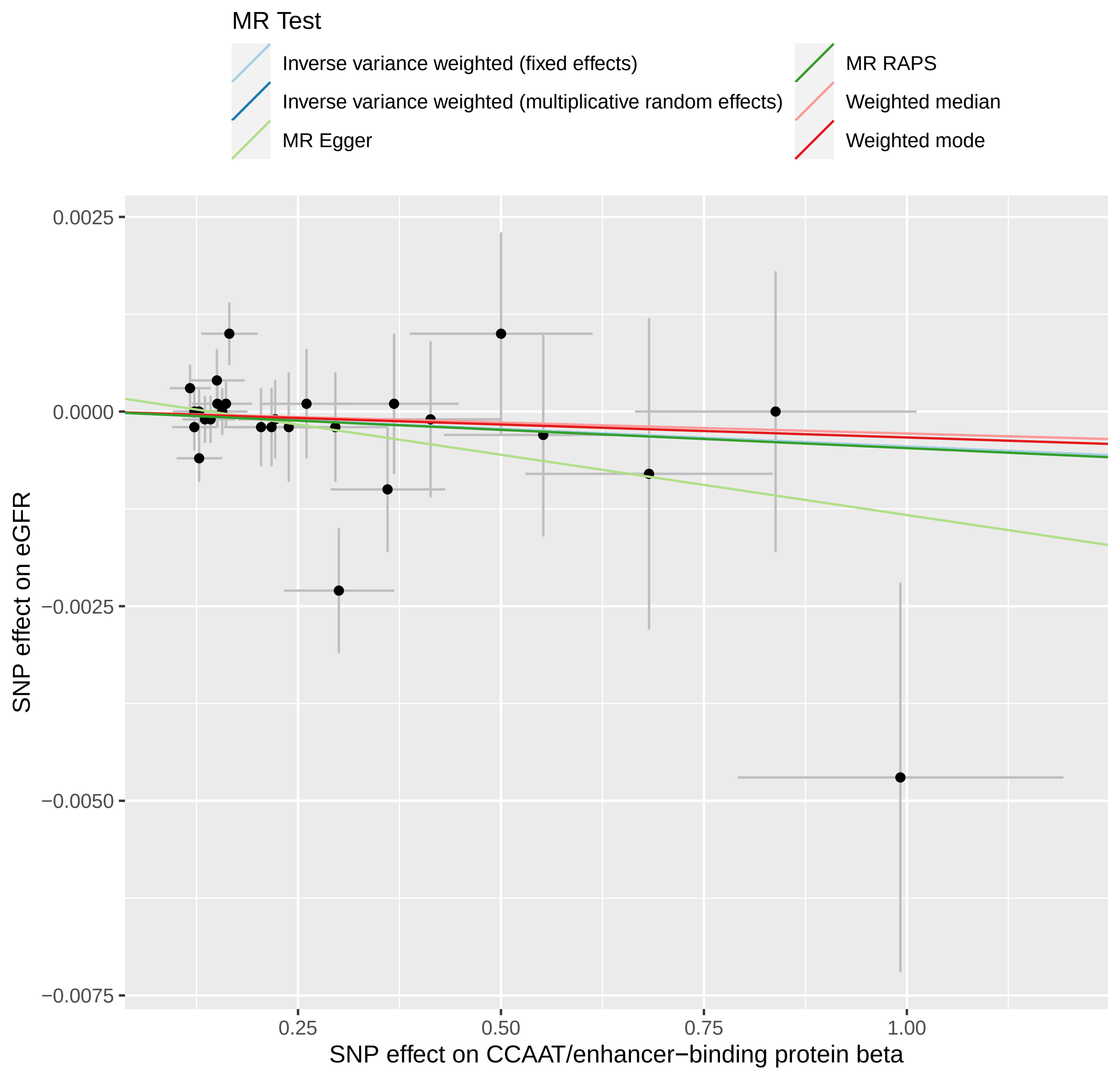

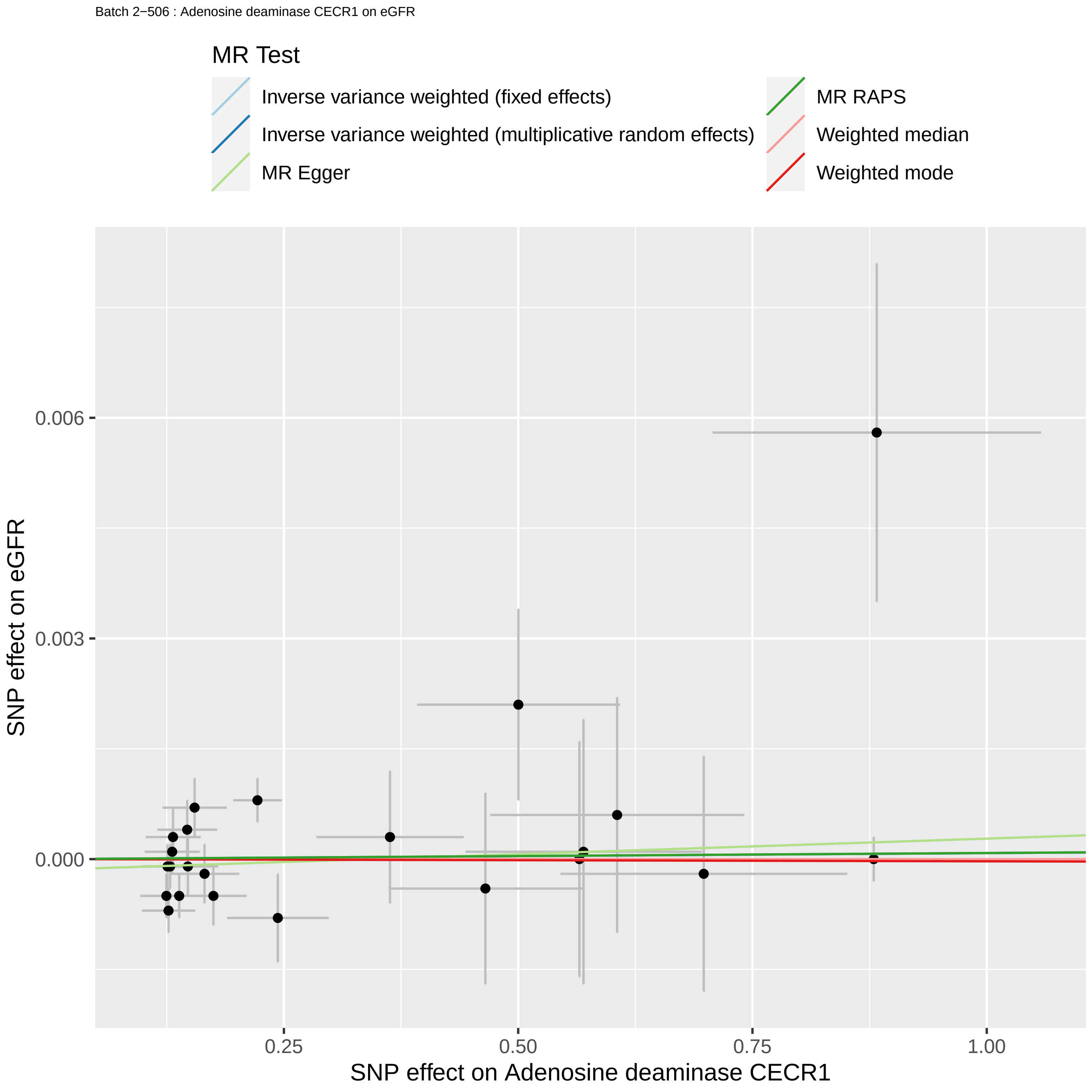
Batch 2−506 : Adenosine deaminase CECR1 on eGFR
MR Test
Inverse variance weighted (fixed effects)
Inverse variance weighted (multiplicative random effects)
MR Egger
MR RAPS
Weighted median
Weighted mode
0.006
0.003
0.000
0.25
0.50
0.75
1.00
SNP effect on eGFR
SNP effect on Adenosine deaminase CECR1

Batch 2−507 : Bile salt−activated lipase on eGFR

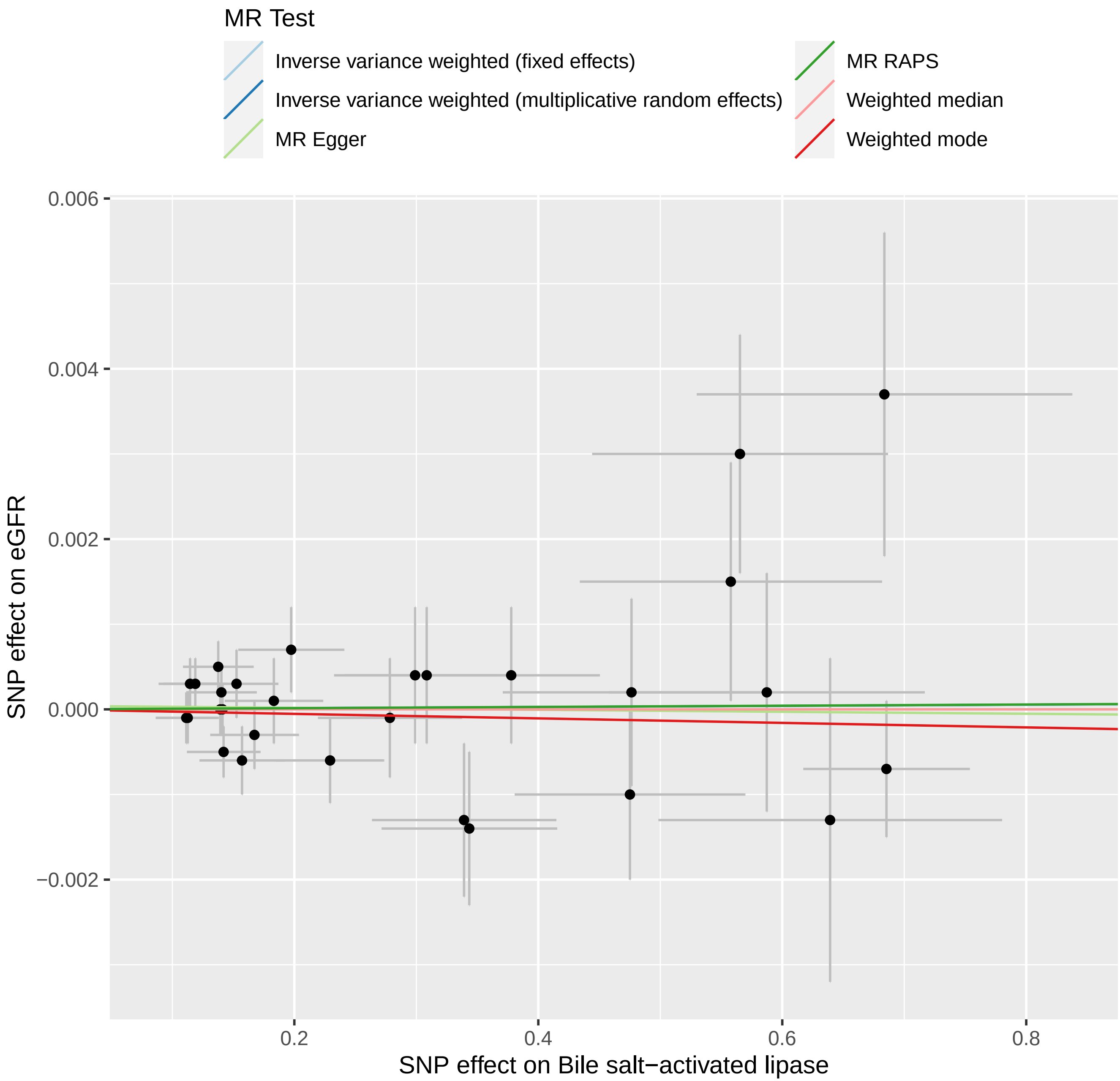

Batch 2−508 : Chymotrypsin−like elastase family member 2A on eGFR

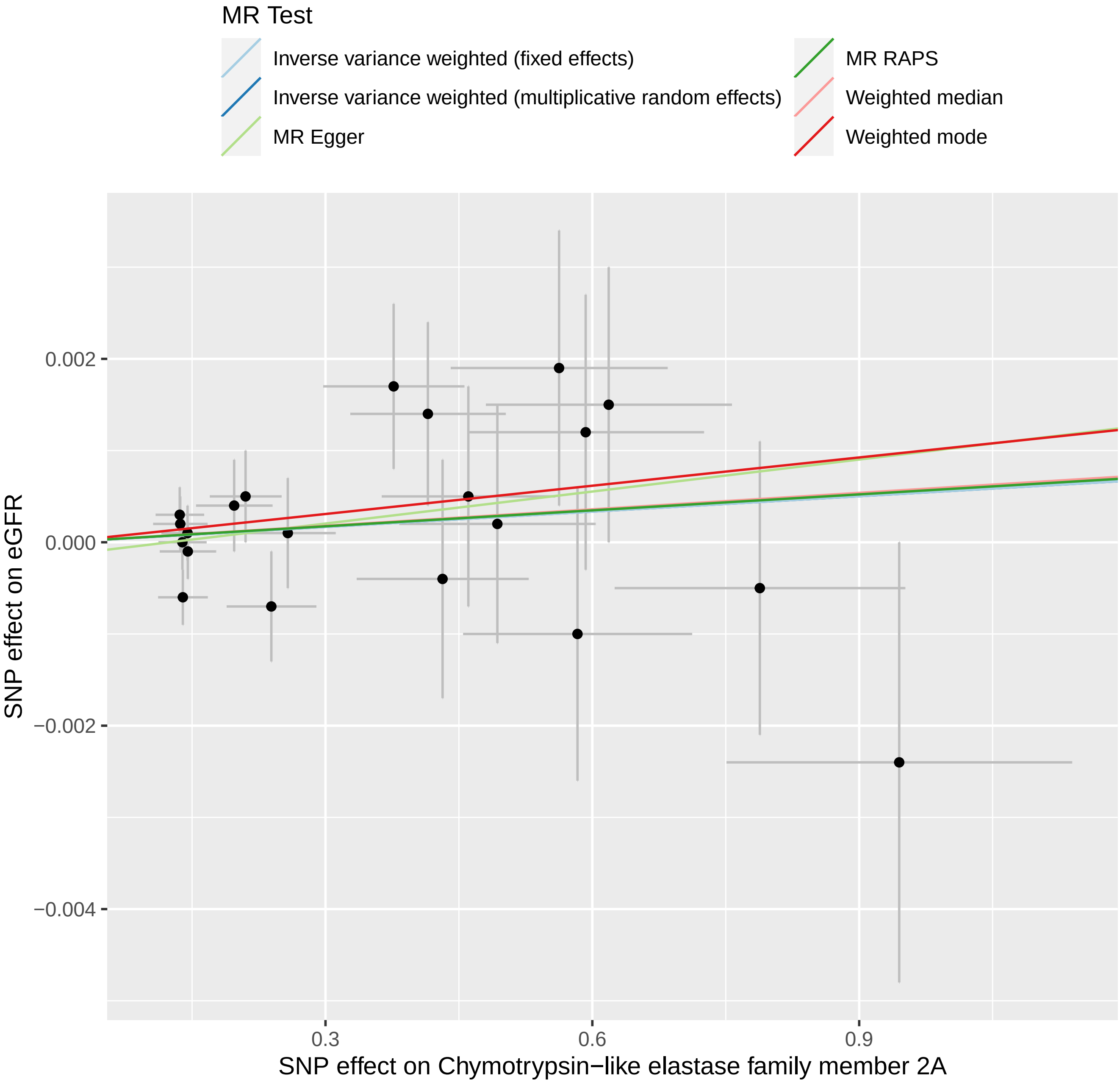

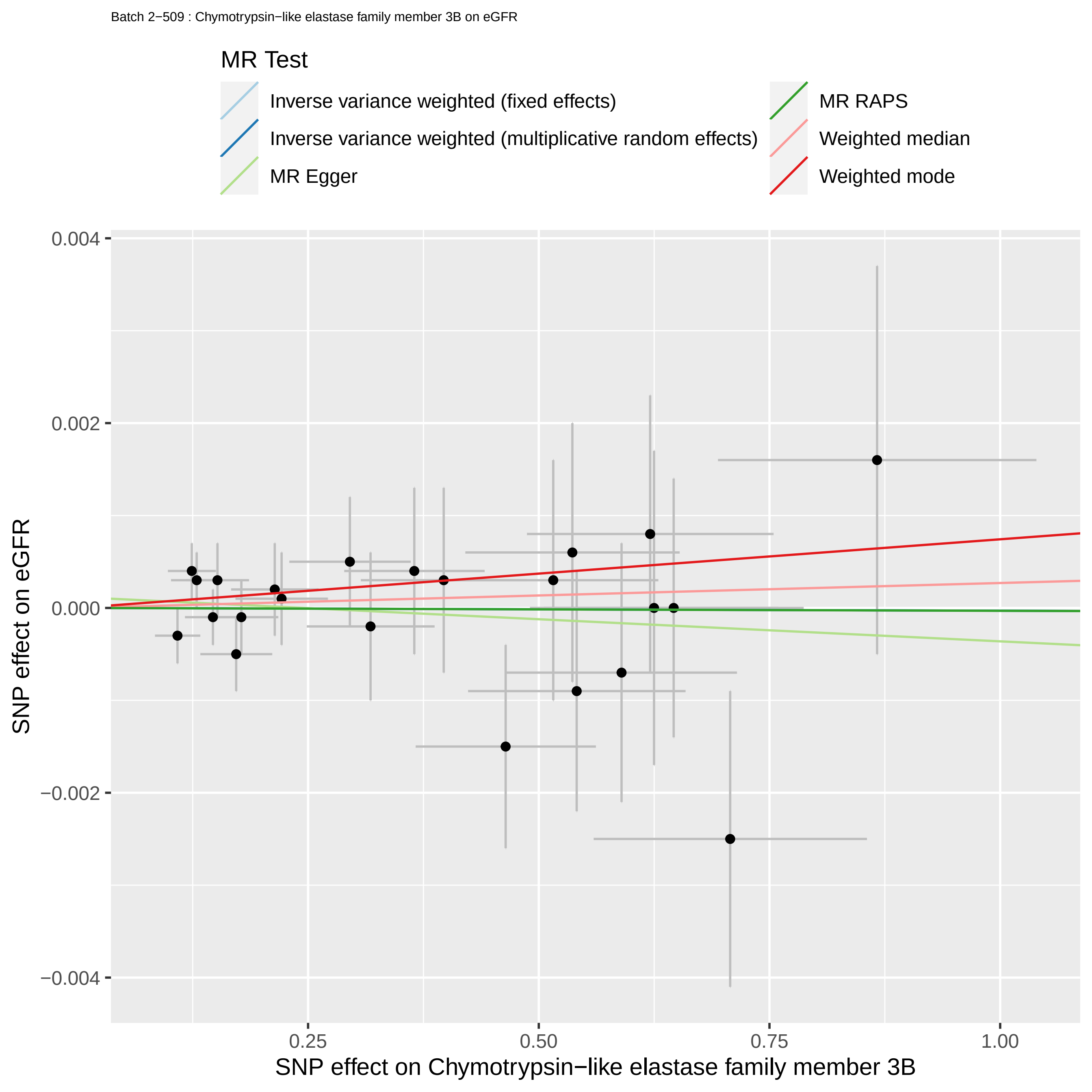
Batch 2−509 : Chymotrypsin−like elastase family member 3B on eGFR
MR Test
Inverse variance weighted (fixed effects)
Inverse variance weighted (multiplicative random effects)
MR Egger
MR RAPS
Weighted median
Weighted mode
0.004
0.002
0.000
−0.002
−0.004
0.25
0.50
0.75
1.00
SNP effect on eGFR
SNP effect on Chymotrypsin−like elastase family member 3B

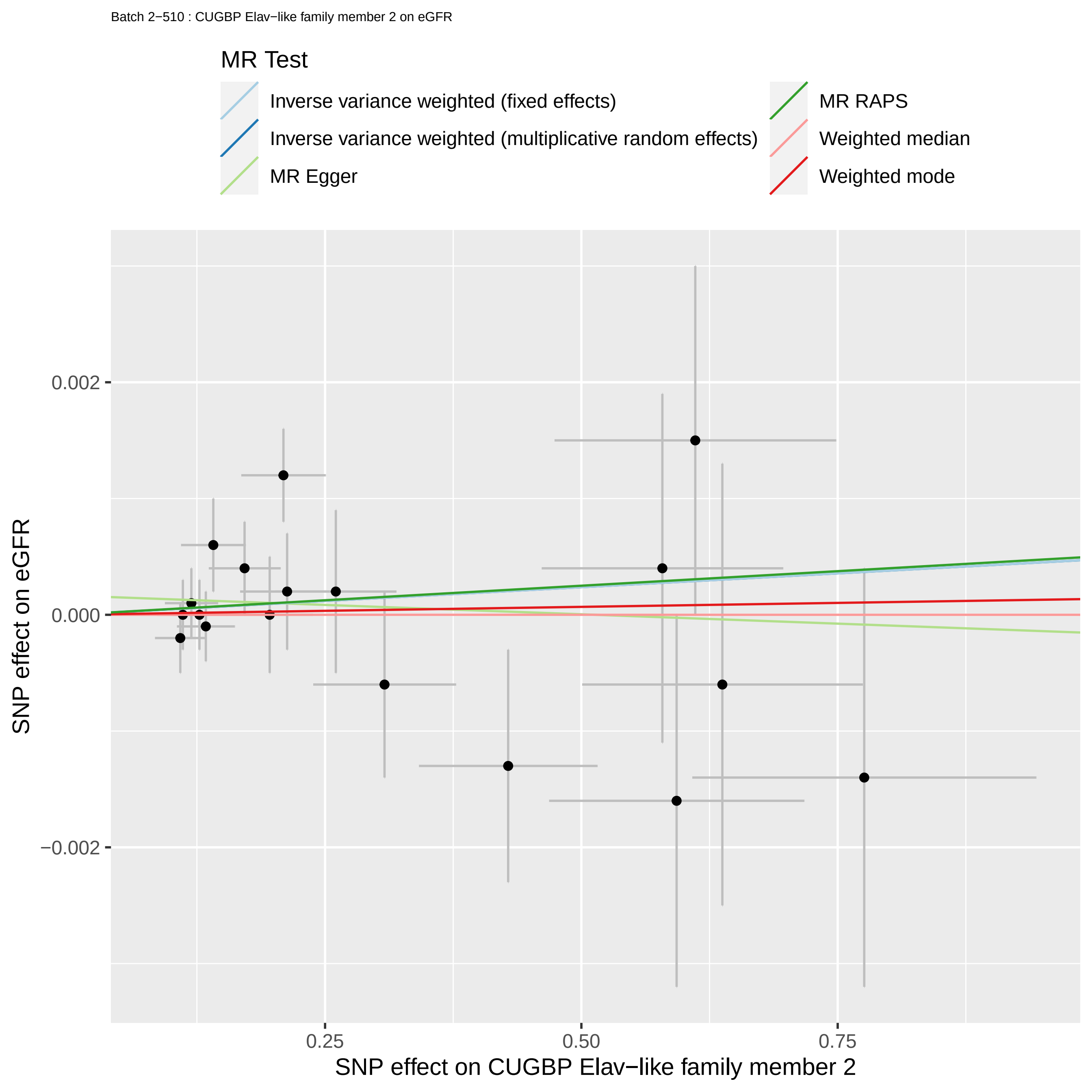

Batch 2−510 : CUGBP Elav−like family member 2 on eGFR
MR Test
Inverse variance weighted (fixed effects)
Inverse variance weighted (multiplicative random effects)
MR Egger
MR RAPS
Weighted median
Weighted mode
0.002
0.000
−0.002
0.25
0.50
0.75
SNP effect on eGFR
SNP effect on CUGBP Elav−like family member 2

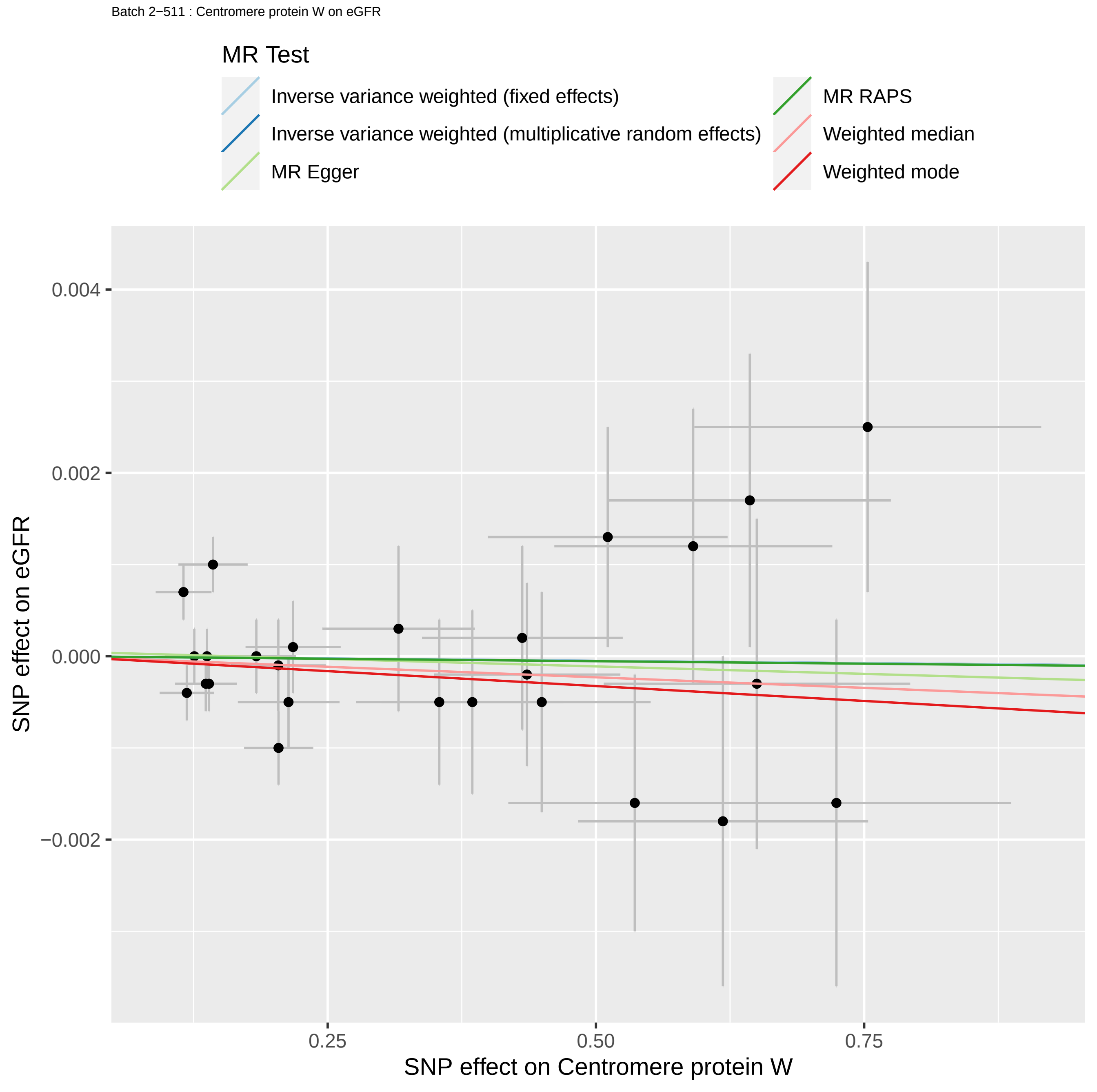
Batch 2−511 : Centromere protein W on eGFR
MR Test
Inverse variance weighted (fixed effects)
Inverse variance weighted (multiplicative random effects)
MR Egger
MR RAPS
Weighted median
Weighted mode
0.004
0.002
0.000
−0.002
SNP effect on eGFR
0.25
0.50
0.75
SNP effect on Centromere protein W

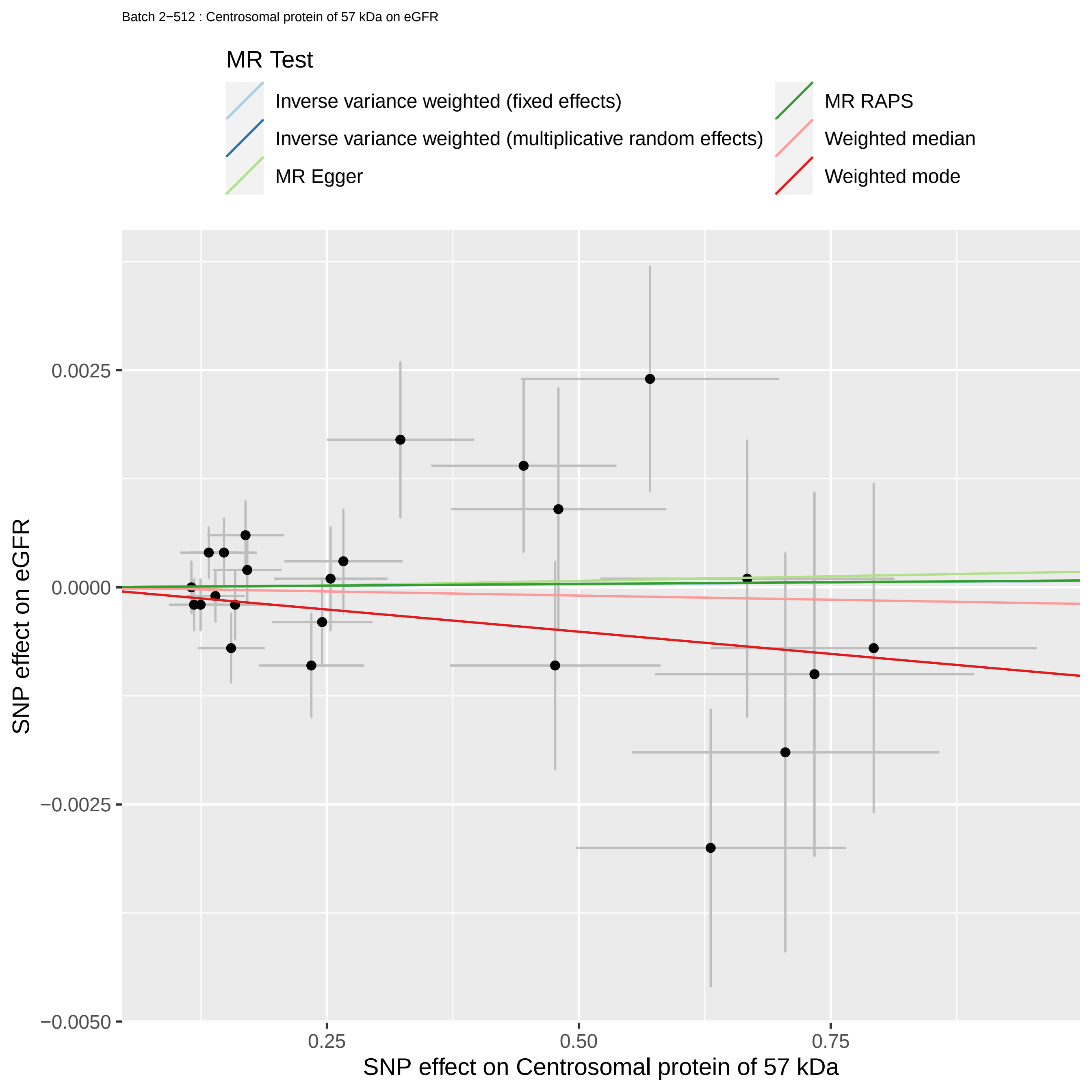
Batch 2−512 : Centrosomal protein of 57 kDa on eGFR
MR Test
Inverse variance weighted (fixed effects)
Inverse variance weighted (multiplicative random effects)
MR Egger
MR RAPS
Weighted median
Weighted mode
0.0025
0.0000
−0.0025
−0.0050
0.25
0.50
0.75
SNP effect on eGFR
SNP effect on Centrosomal protein of 57 kDa

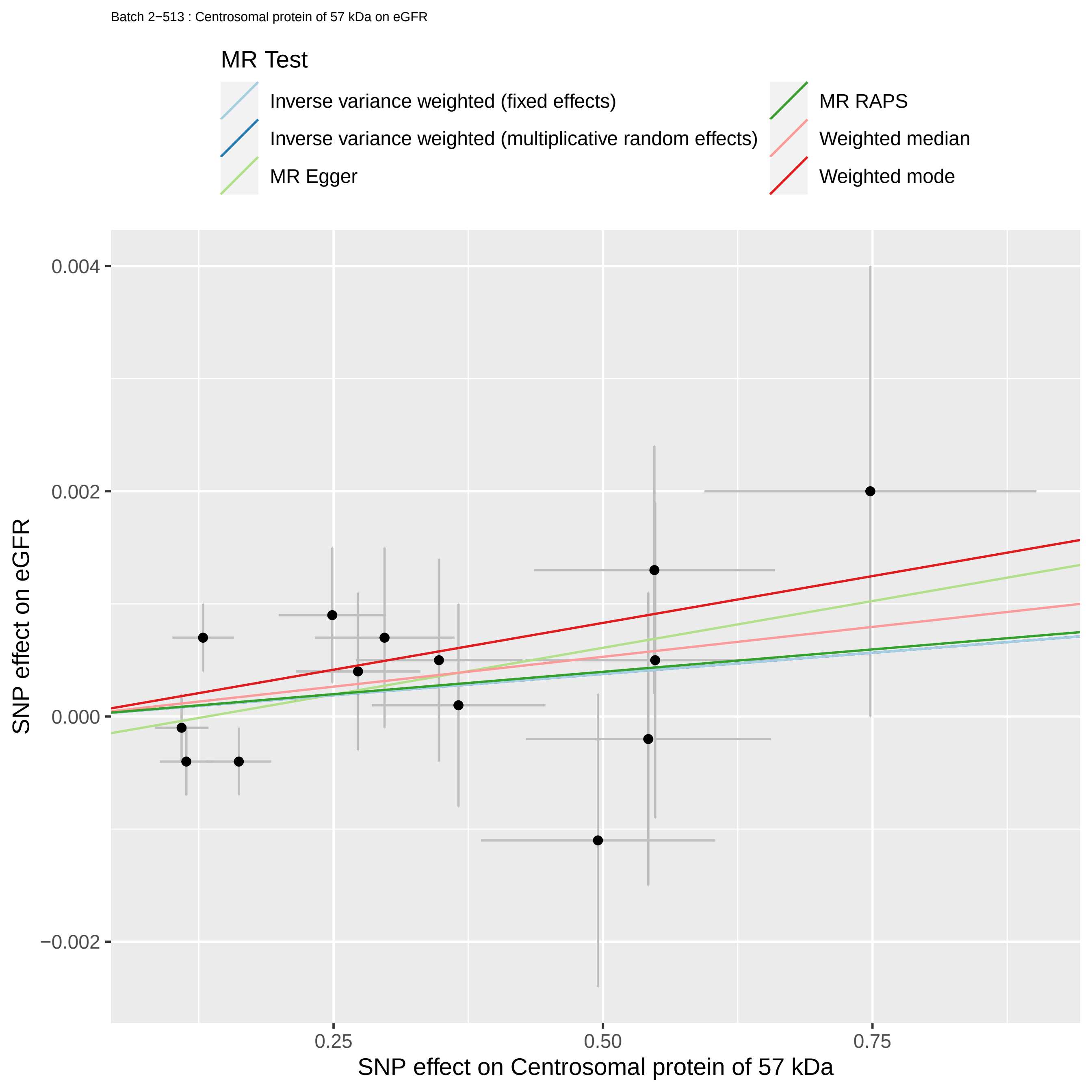

Batch 2−513 : Centrosomal protein of 57 kDa on eGFR
MR Test
Inverse variance weighted (fixed effects)
Inverse variance weighted (multiplicative random effects)
MR Egger
MR RAPS
Weighted median
Weighted mode
0.004
0.002
0.000
−0.002
SNP effect on eGFR
0.25
0.50
0.75
SNP effect on Centrosomal protein of 57 kDa

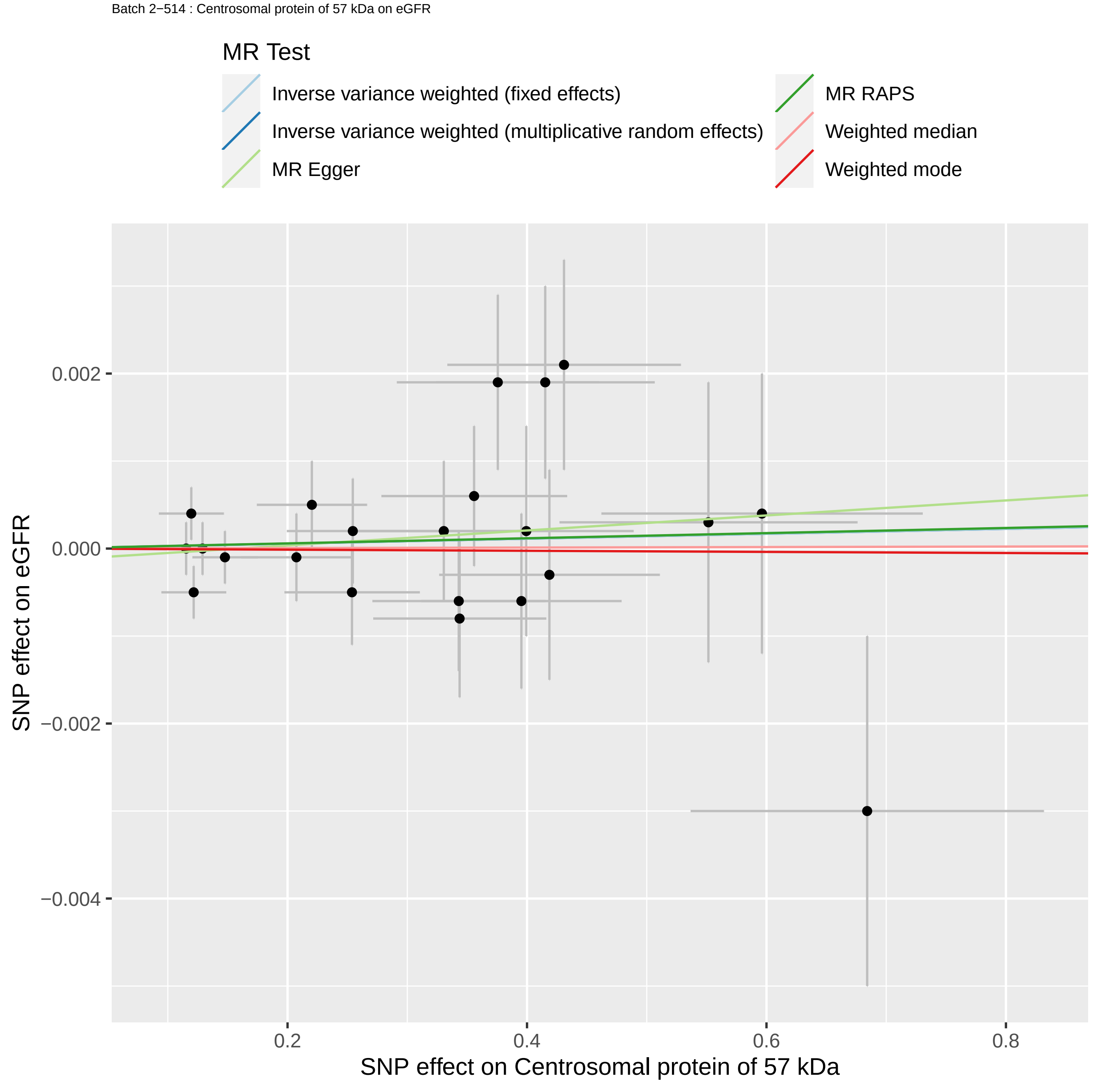

Batch 2−514 : Centrosomal protein of 57 kDa on eGFR
MR Test
Inverse variance weighted (fixed effects)
Inverse variance weighted (multiplicative random effects)
MR Egger
MR RAPS
Weighted median
Weighted mode
0.002
0.000
−0.002
−0.004
0.2
0.4
0.6
0.8
SNP effect on eGFR
SNP effect on Centrosomal protein of 57 kDa

Batch 2−515 : Ceramide synthase 5 on eGFR

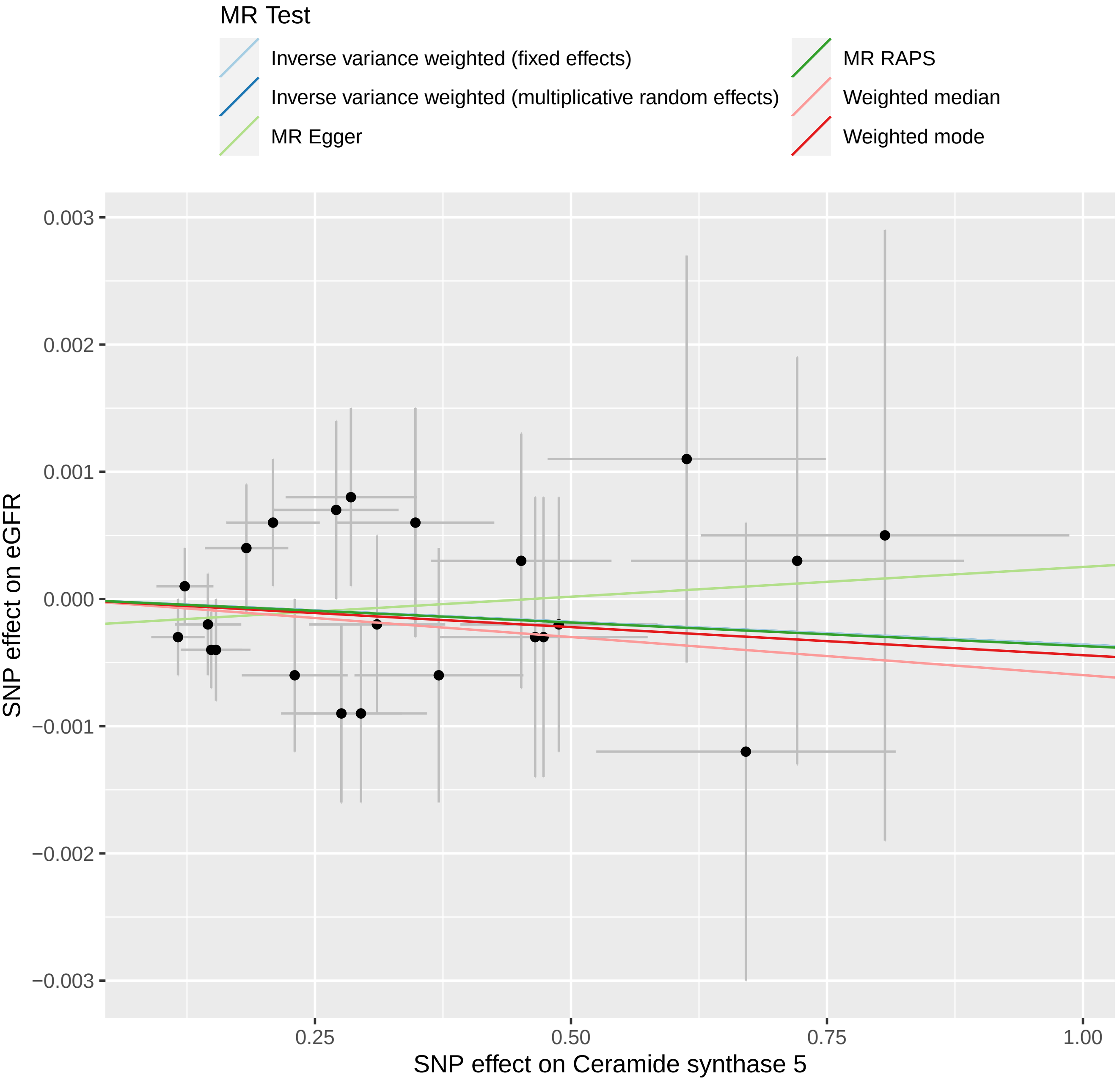

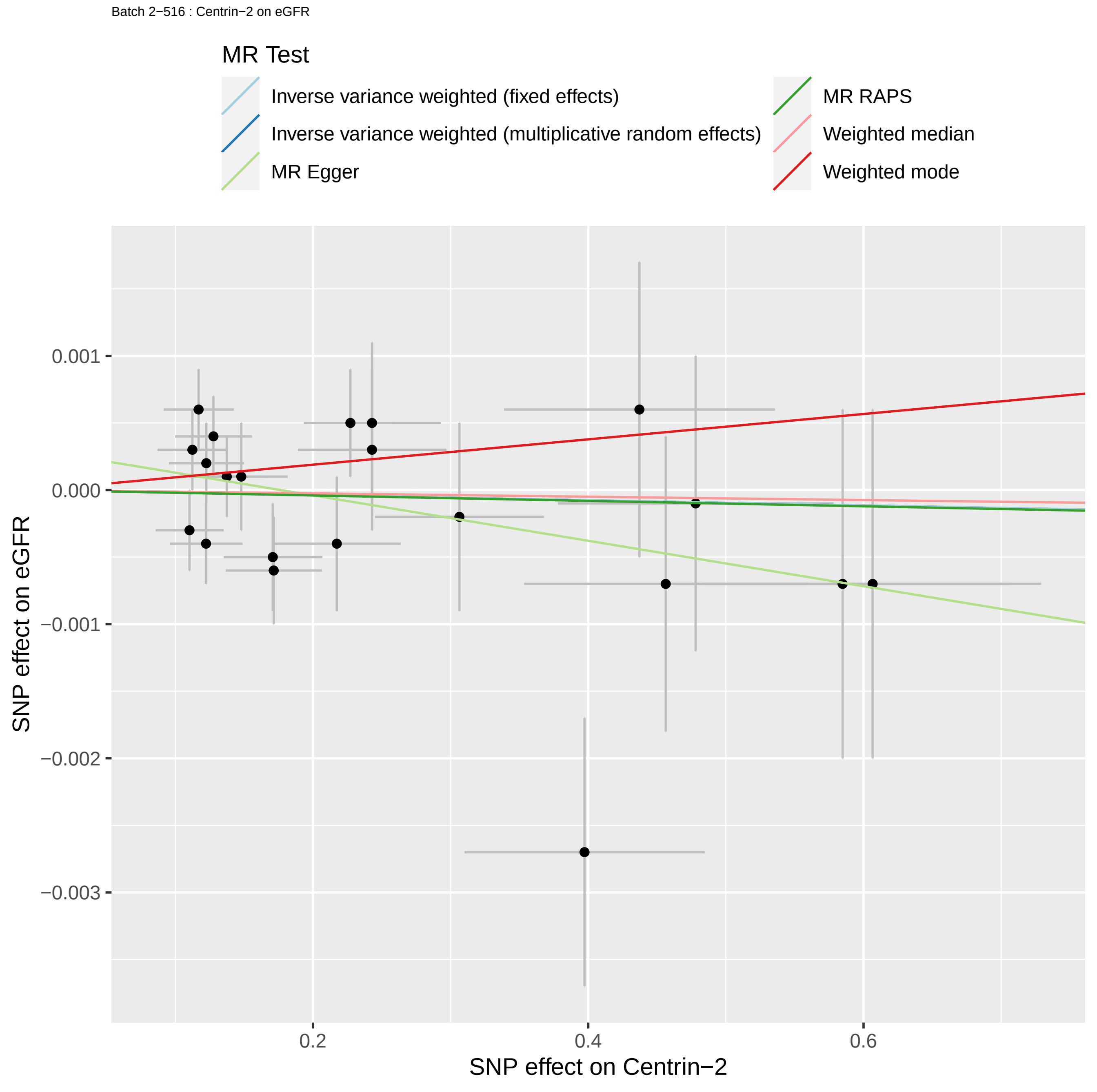
Batch 2−516 : Centrin−2 on eGFR
MR Test
Inverse variance weighted (fixed effects)
Inverse variance weighted (multiplicative random effects)
MR Egger
MR RAPS
Weighted median
Weighted mode
0.001
0.000
−0.001
−0.002
−0.003
SNP effect on eGFR
0.2
0.4
0.6
SNP effect on Centrin−2

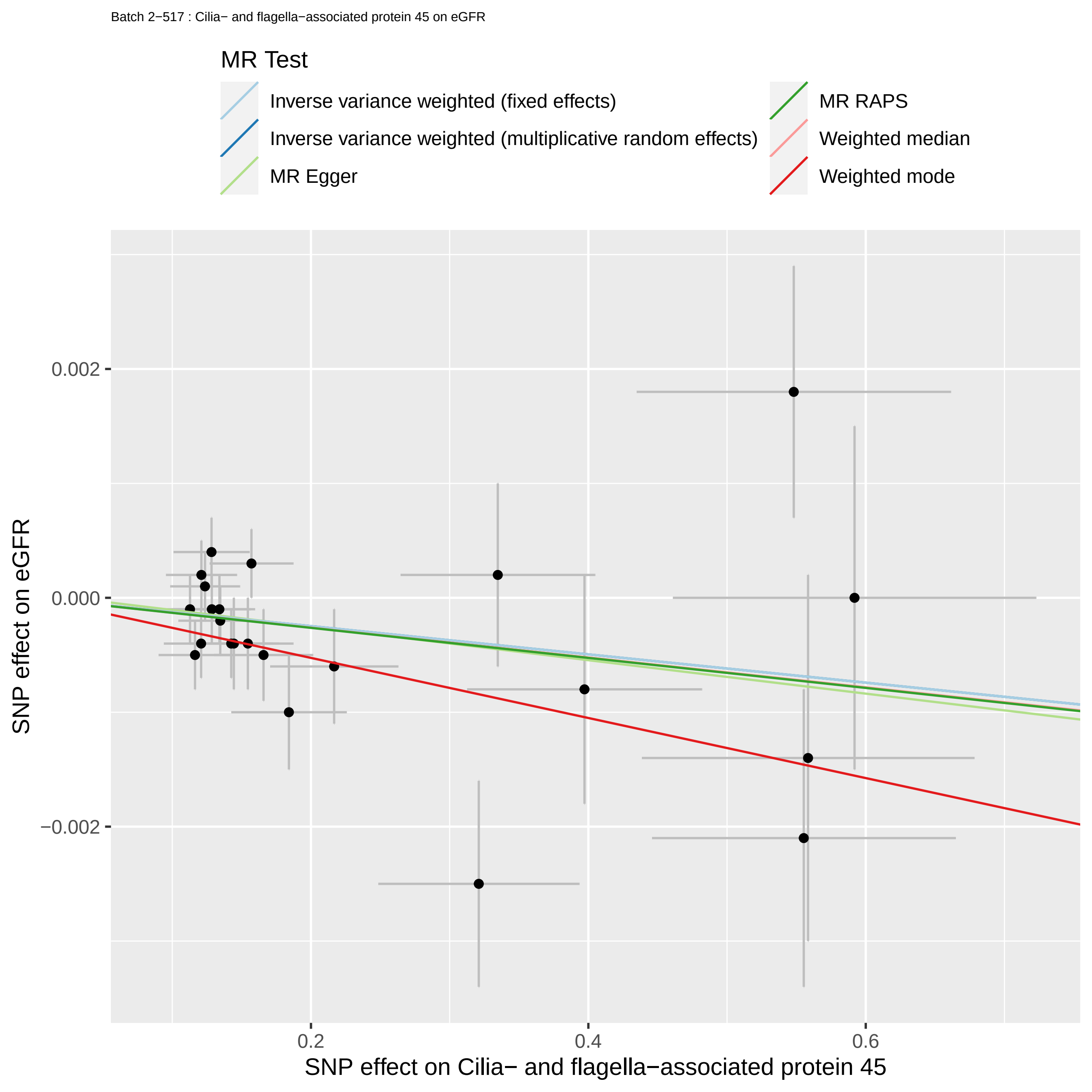
Batch 2−517 : Cilia− and flagella−associated protein 45 on eGFR
MR Test
Inverse variance weighted (fixed effects)
Inverse variance weighted (multiplicative random effects)
MR Egger
MR RAPS
Weighted median
Weighted mode
0.002
0.000
−0.002
0.2
0.4
0.6
SNP effect on eGFR
SNP effect on Cilia− and flagella−associated protein 45

Batch 2−518 : Complement factor B on eGFR

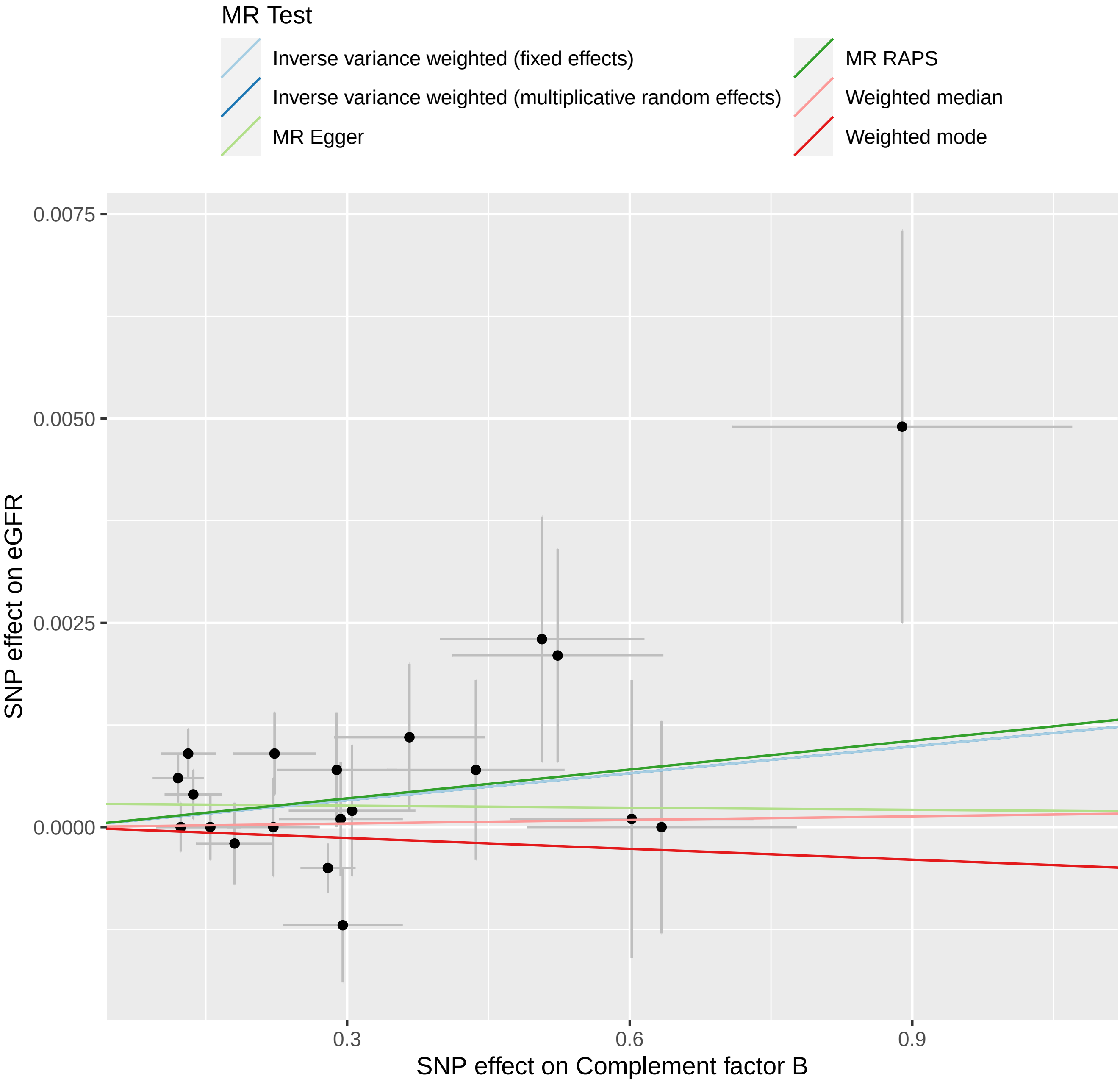

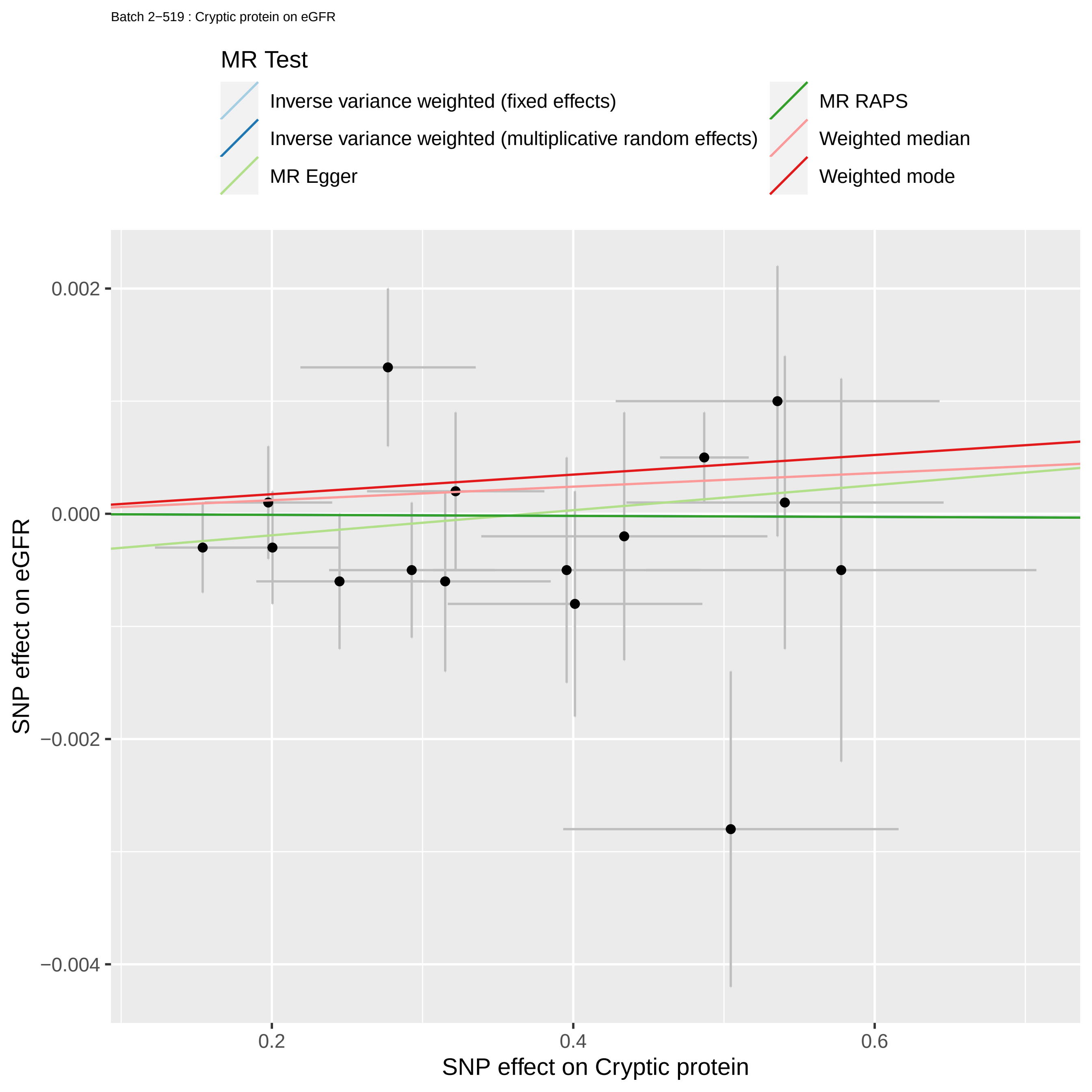

Batch 2−519 : Cryptic protein on eGFR
MR Test
Inverse variance weighted (fixed effects)
Inverse variance weighted (multiplicative random effects)
MR Egger
MR RAPS
Weighted median
Weighted mode
0.002
0.000
−0.002
−0.004
0.2
0.4
0.6
SNP effect on eGFR
SNP effect on Cryptic protein

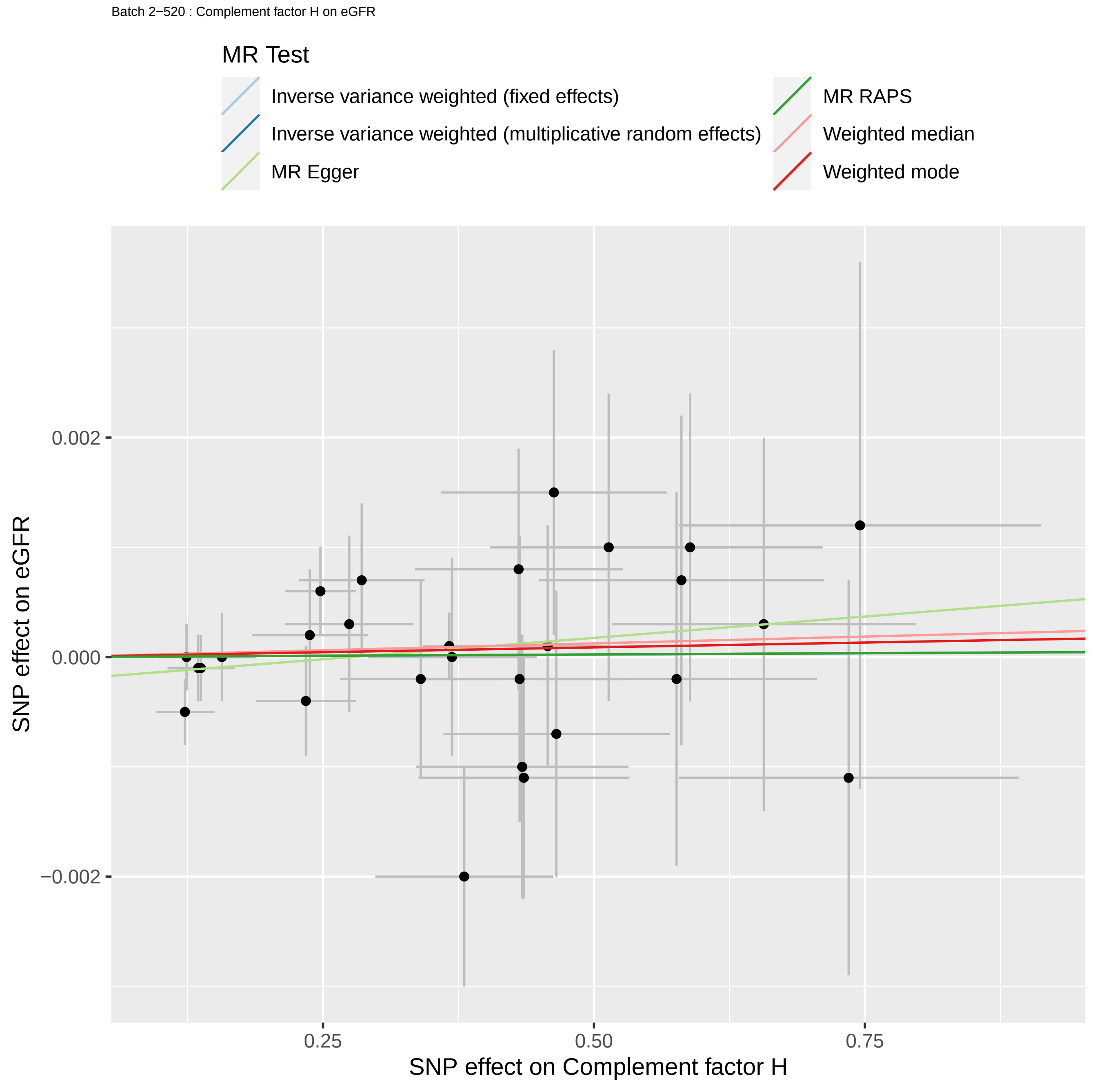

Batch 2−520 : Complement factor H on eGFR
MR Test
Inverse variance weighted (fixed effects)
Inverse variance weighted (multiplicative random effects)
MR Egger
MR RAPS
Weighted median
Weighted mode
0.002
0.000
−0.002
0.25
0.50
0.75
SNP effect on Complement factor H
SNP effect on eGFR

Batch 2−521 : Complement factor H−related protein 5 on eGFR

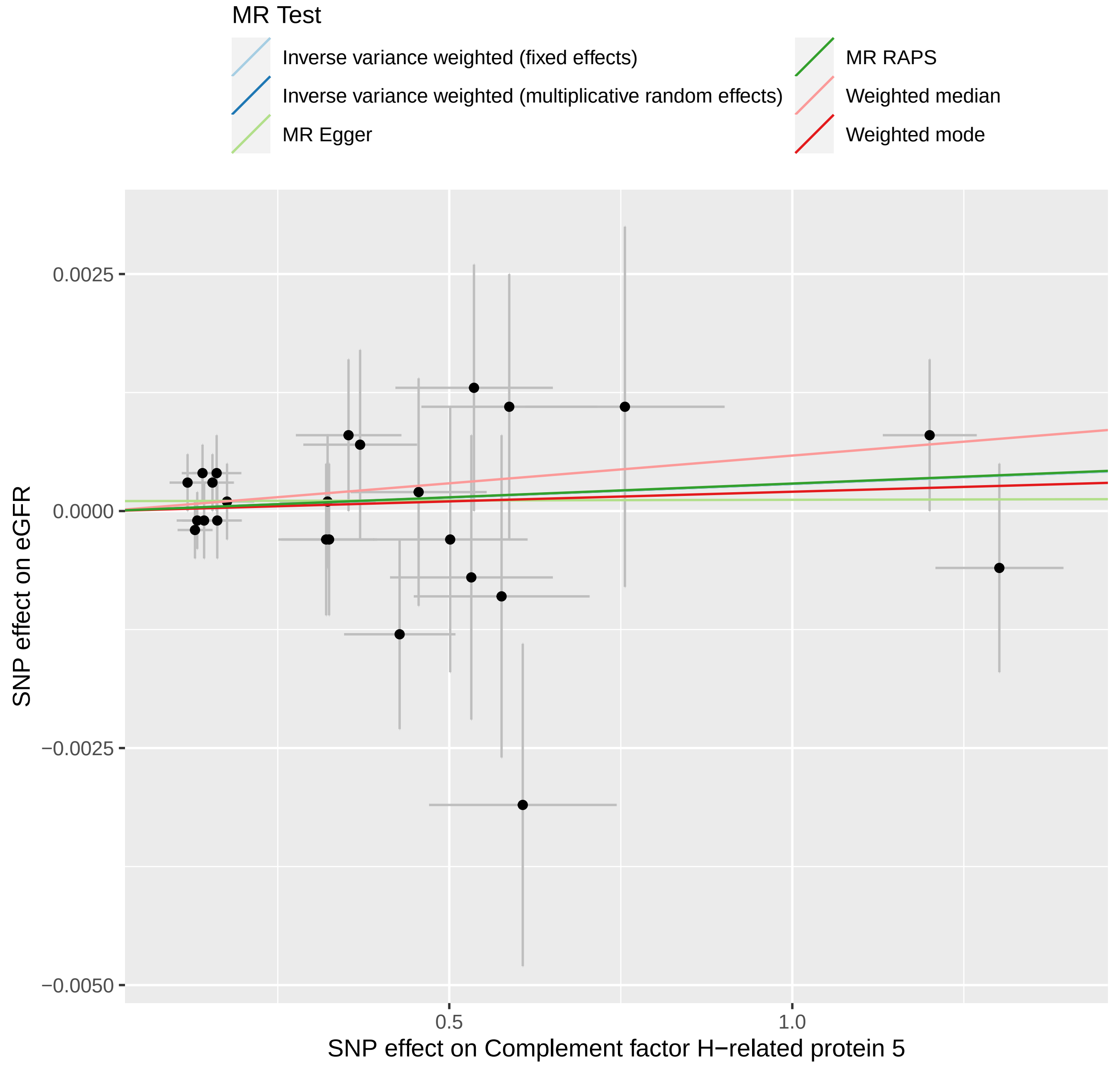

Batch 2−522 : Complement factor H−related protein 5 on eGFR

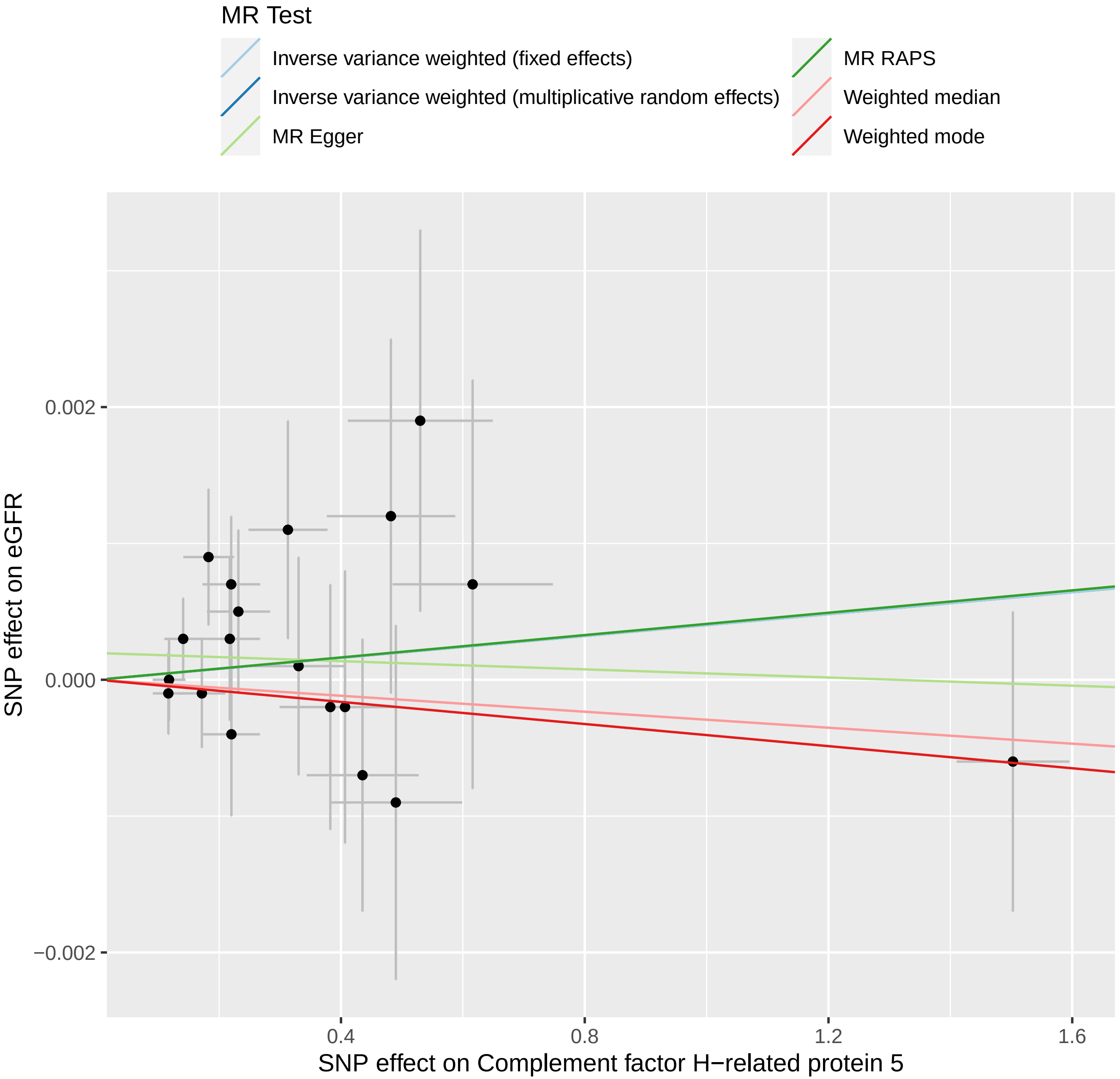

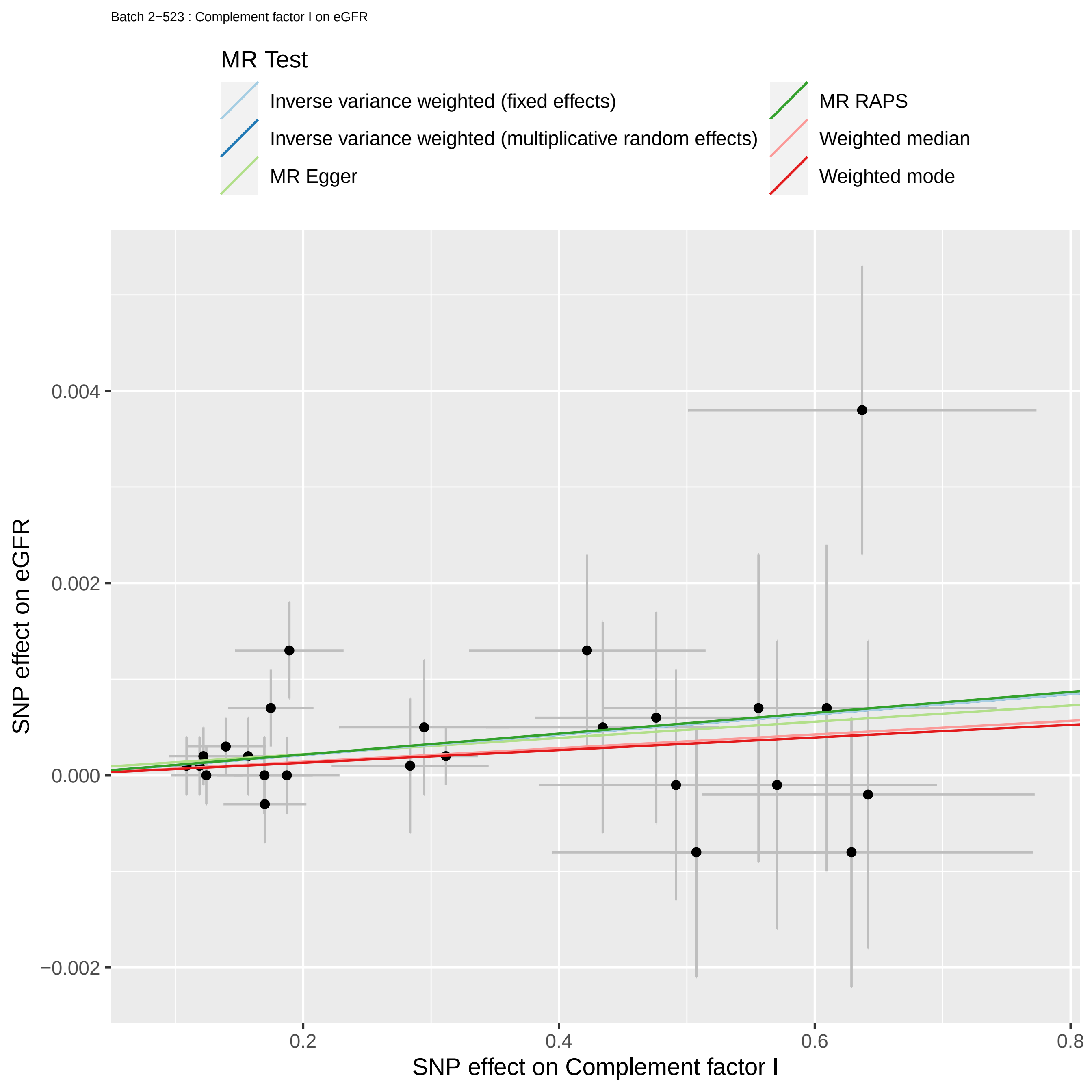

Batch 2−523 : Complement factor I on eGFR
MR Test
Inverse variance weighted (fixed effects)
Inverse variance weighted (multiplicative random effects)
MR Egger
MR RAPS
Weighted median
Weighted mode
0.004
0.002
0.000
−0.002
0.2
0.4
0.6
0.8
SNP effect on Complement factor I
SNP effect on eGFR

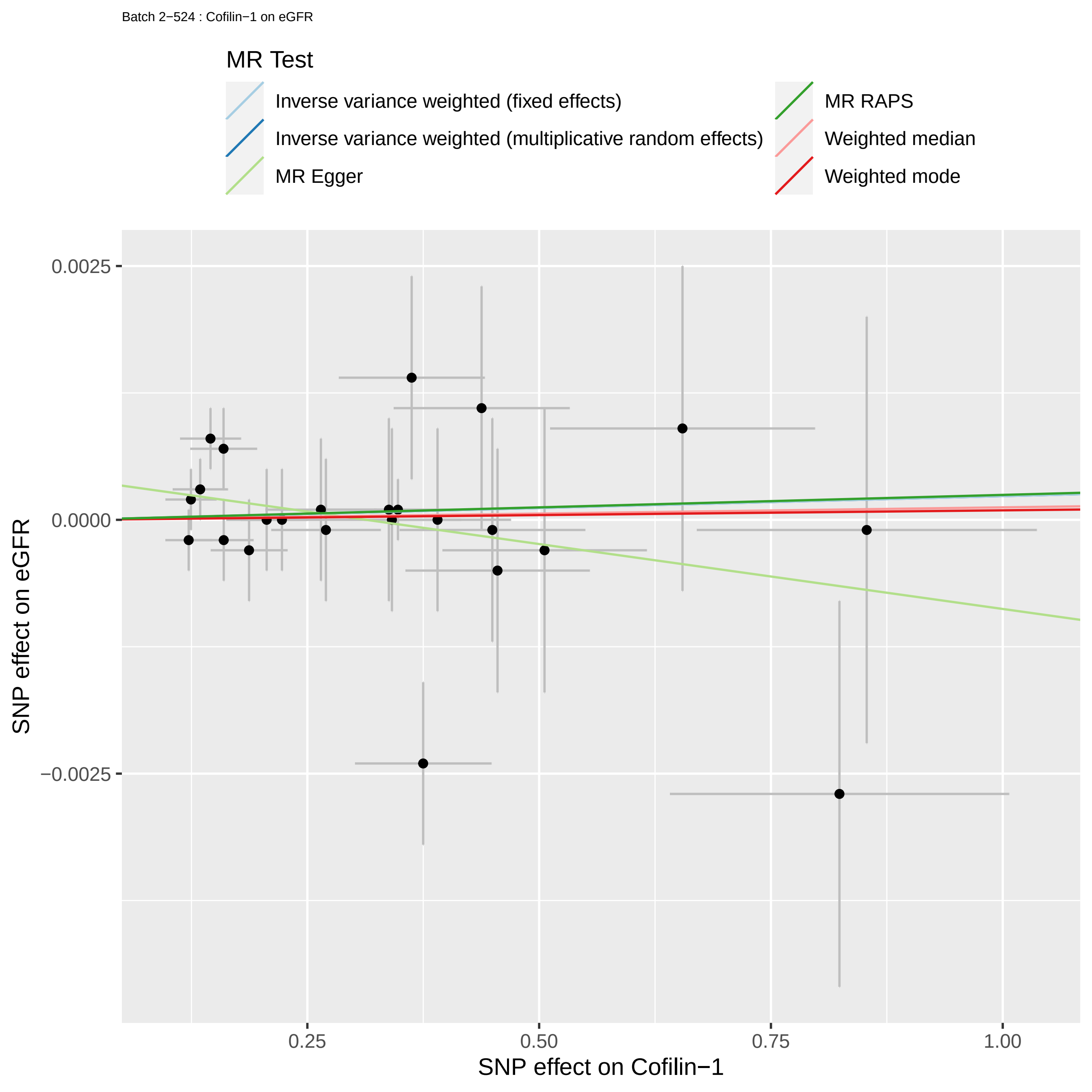

Batch 2−524 : Cofilin−1 on eGFR
MR Test
Inverse variance weighted (fixed effects)
Inverse variance weighted (multiplicative random effects)
MR Egger
MR RAPS
Weighted median
Weighted mode
0.0025
0.0000
−0.0025
SNP effect on eGFR
0.25
0.50
0.75
1.00
SNP effect on Cofilin−1

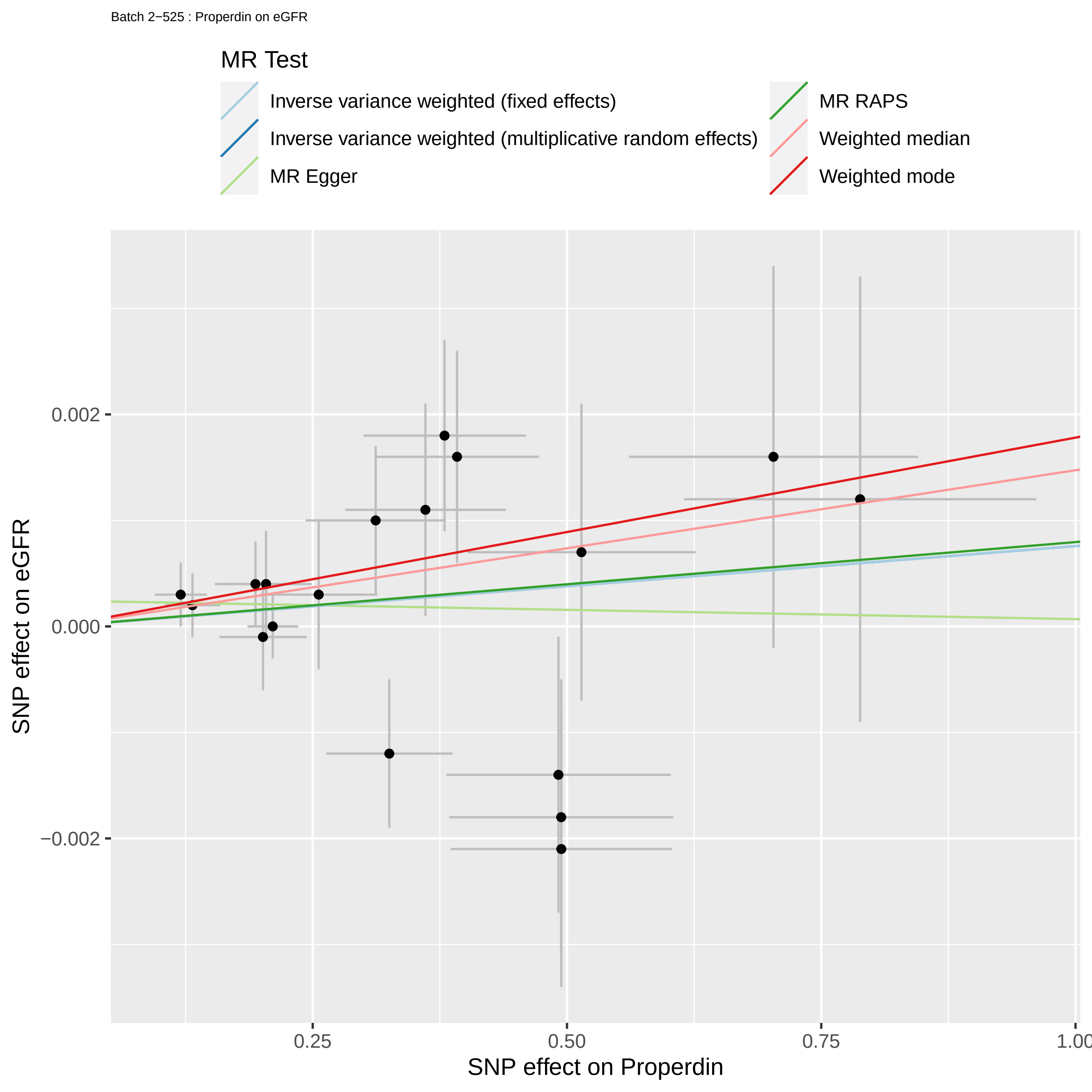
Batch 2−525 : Properdin on eGFR
MR Test
Inverse variance weighted (fixed effects)
Inverse variance weighted (multiplicative random effects)
MR Egger
MR RAPS
Weighted median
Weighted mode
0.002
0.000
−0.002
0.25
0.50
0.75
1.00
SNP effect on Properdin
SNP effect on eGFR

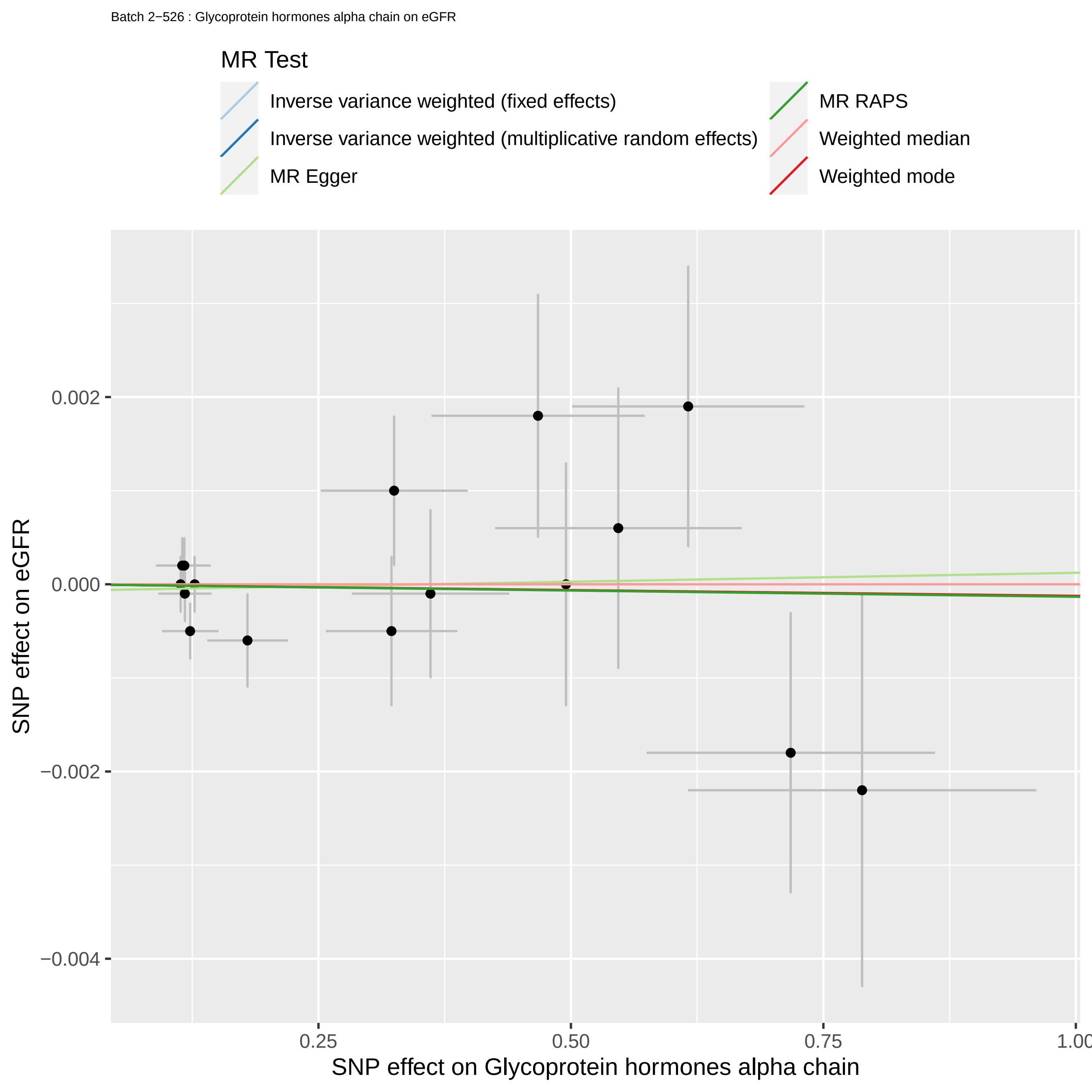

Batch 2−526 : Glycoprotein hormones alpha chain on eGFR
MR Test
Inverse variance weighted (fixed effects)
Inverse variance weighted (multiplicative random effects)
MR Egger
MR RAPS
Weighted median
Weighted mode
SNP effect on eGFR
0.002
0.000
−0.002
−0.004
0.25
0.50
0.75
1.00
SNP effect on Glycoprotein hormones alpha chain

Batch 2−527 : Human Chorionic Gonadotropin on eGFR

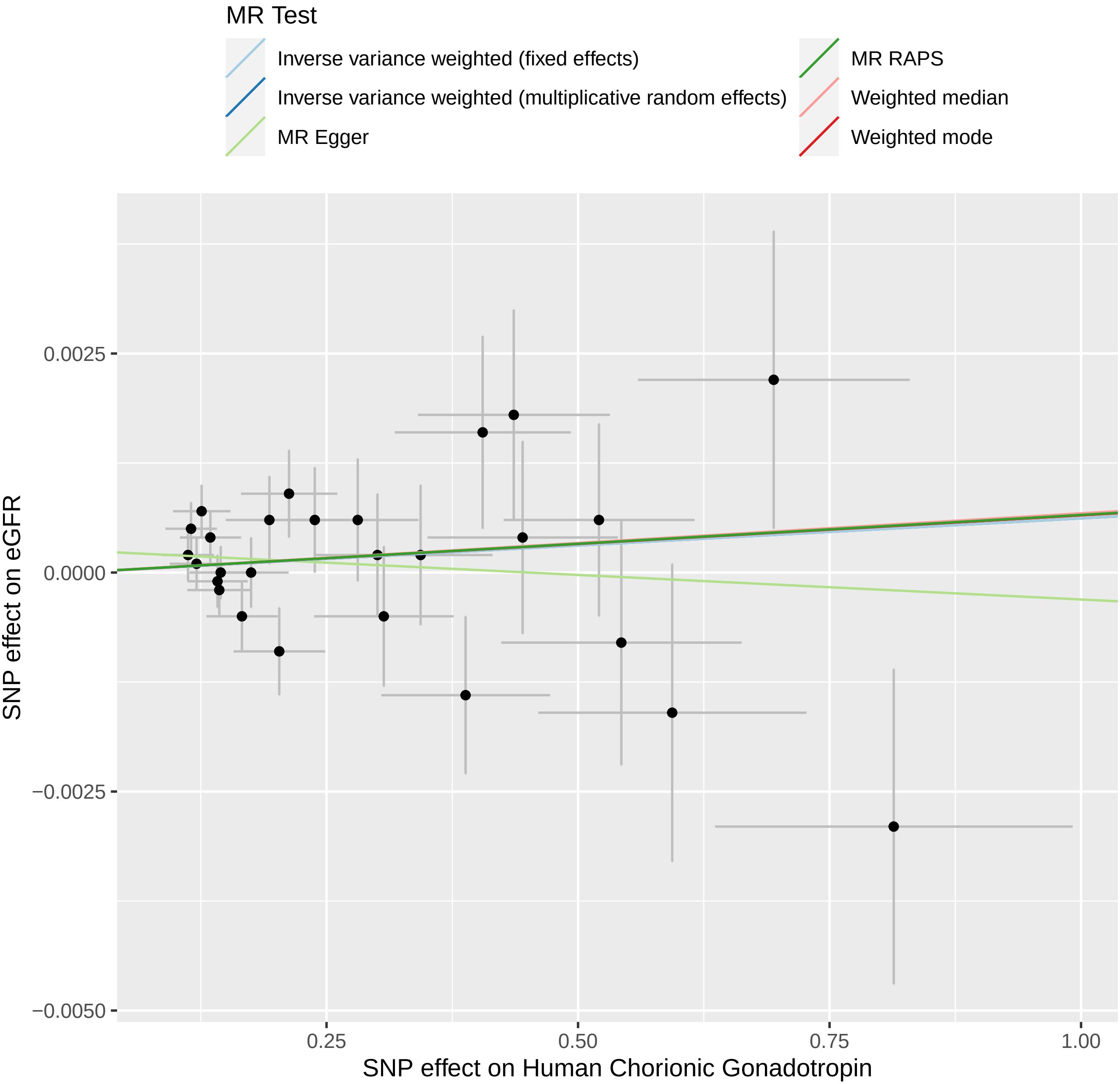

Batch 2−528 : Follicle stimulating hormone on eGFR

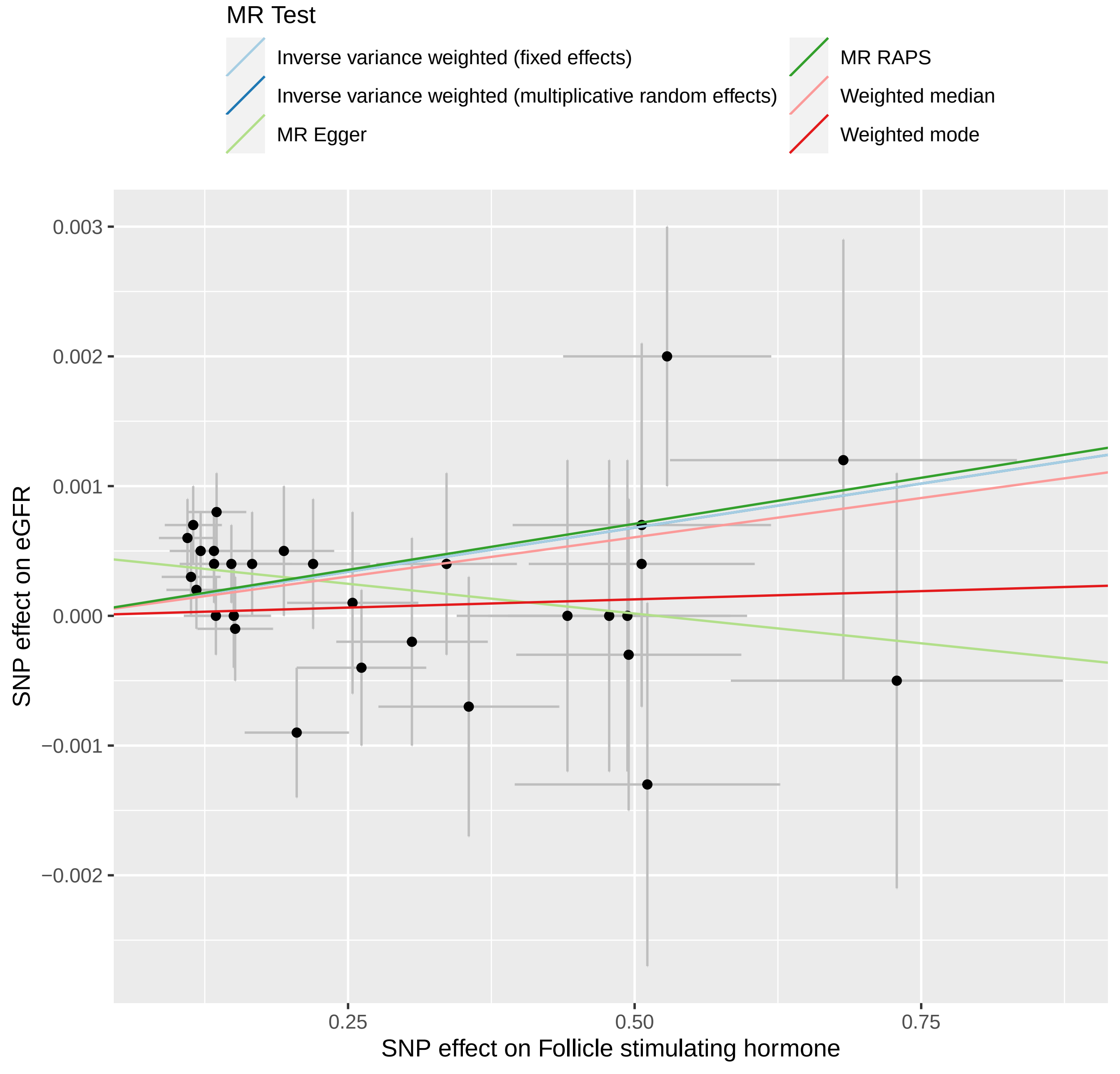

Batch 2−529 : Luteinizing hormone on eGFR

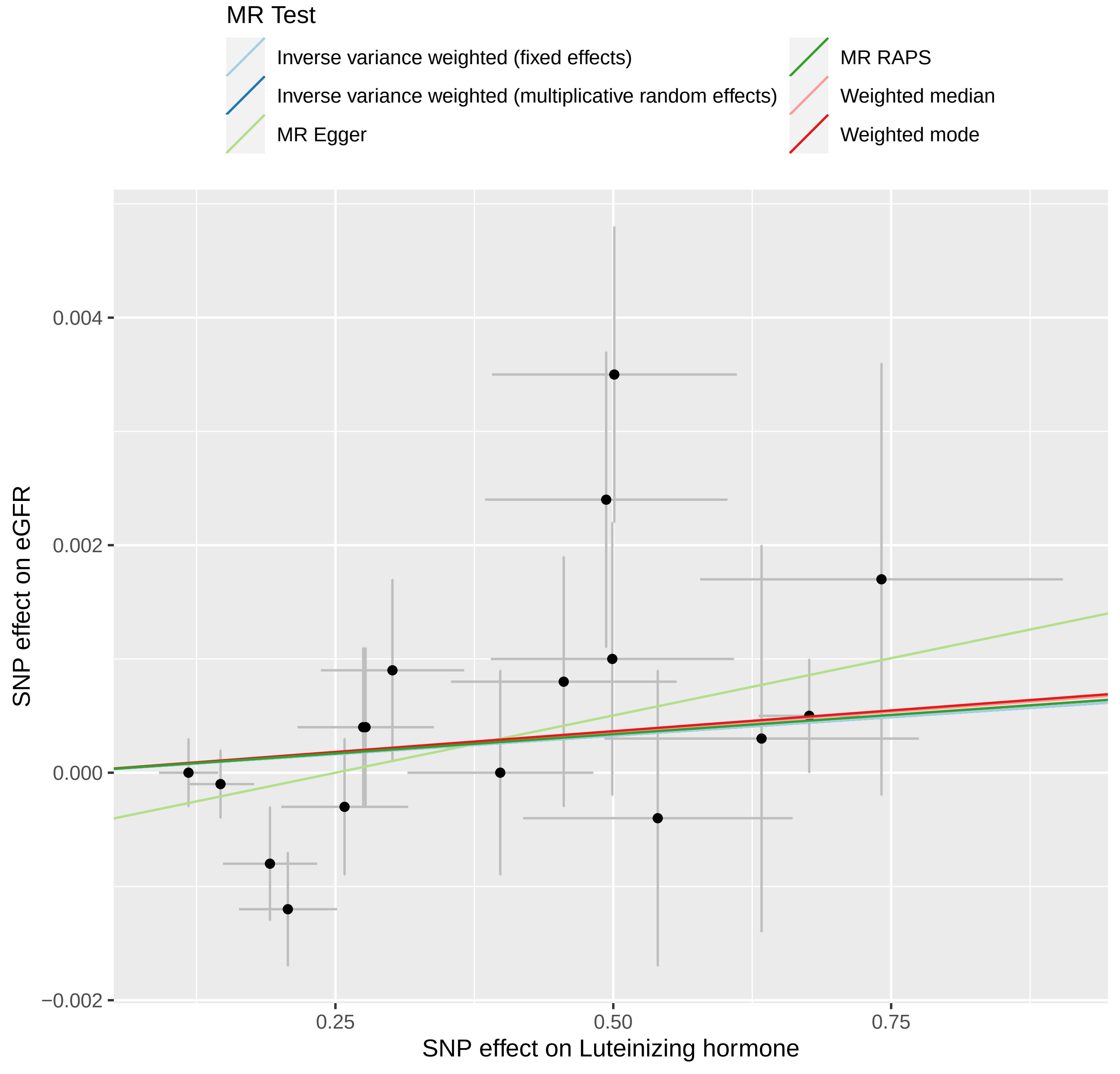

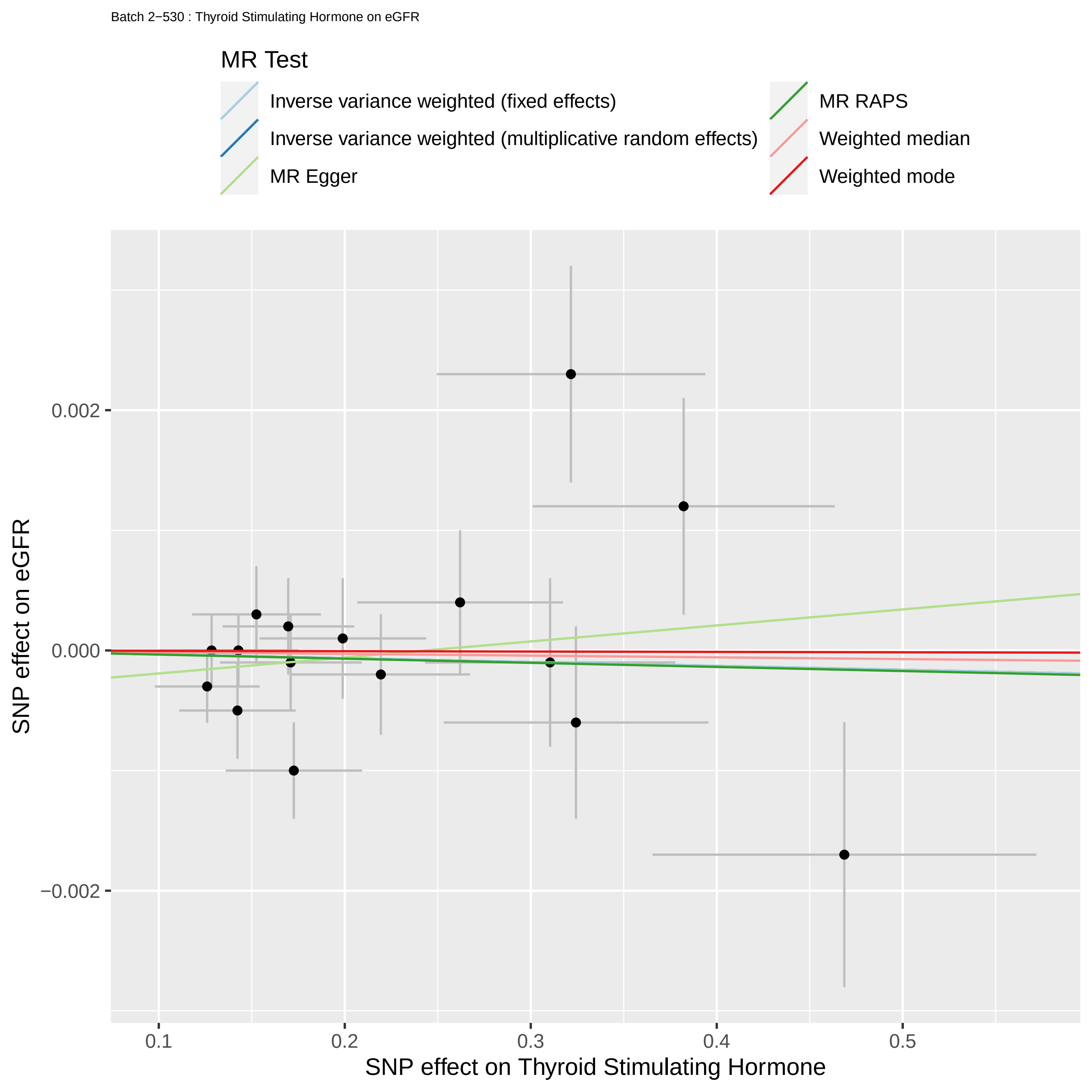

Batch 2−530 : Thyroid Stimulating Hormone on eGFR
MR Test
Inverse variance weighted (fixed effects)
Inverse variance weighted (multiplicative random effects)
MR Egger
MR RAPS
Weighted median
Weighted mode
0.002
0.000
−0.002
0.1
0.2
0.3
0.4
0.5
SNP effect on eGFR
SNP effect on Thyroid Stimulating Hormone

Batch 2−531 : Choriogonadotropin subunit beta variant 2 on eGFR

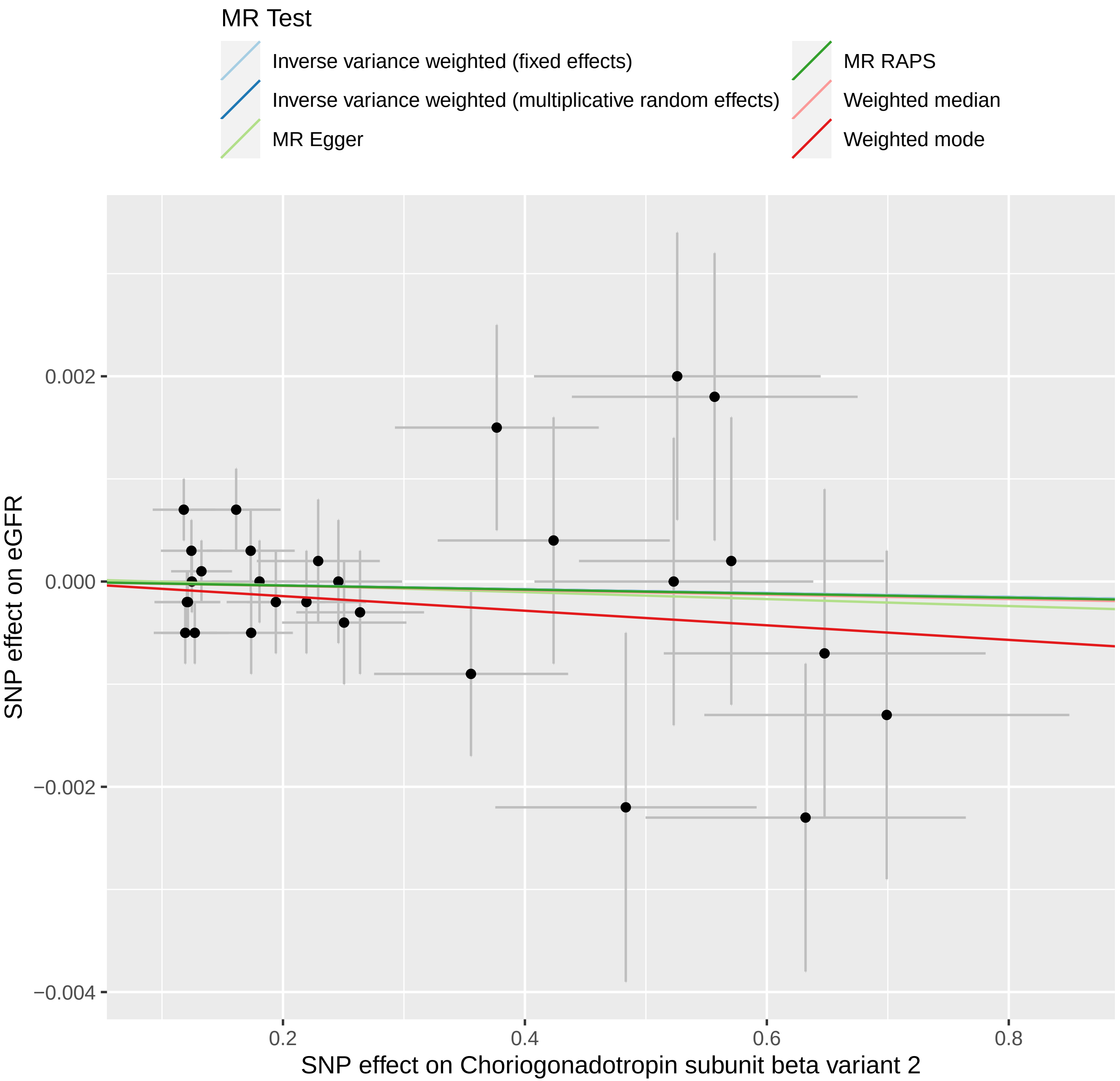

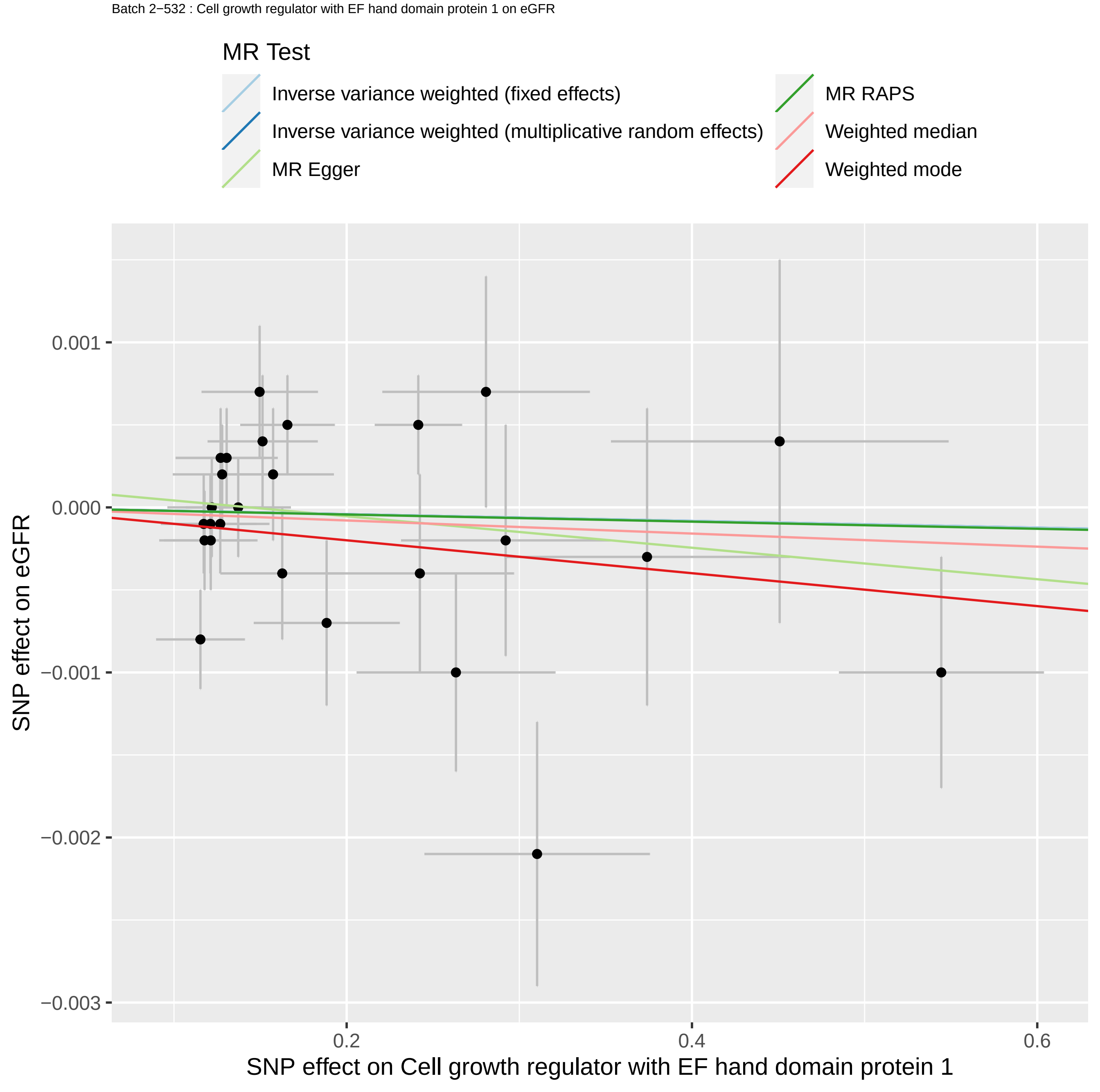
Batch 2−532 : Cell growth regulator with EF hand domain protein 1 on eGFR
MR Test
Inverse variance weighted (fixed effects)
Inverse variance weighted (multiplicative random effects)
MR Egger
MR RAPS
Weighted median
Weighted mode
0.001
0.000
−0.001
−0.002
−0.003
0.2
0.4
0.6
SNP effect on eGFR
SNP effect on Cell growth regulator with EF hand domain protein 1

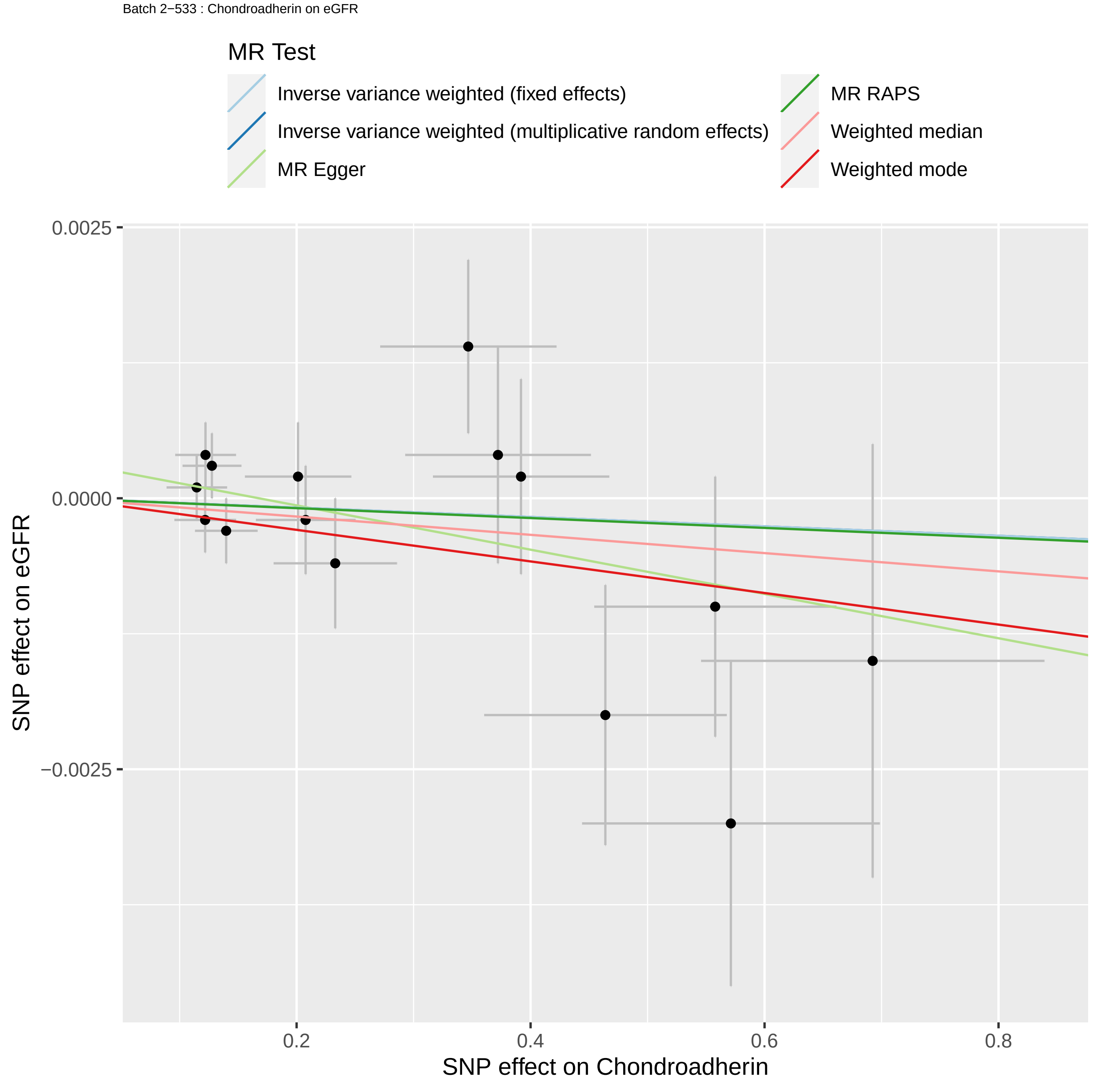

Batch 2−533 : Chondroadherin on eGFR
MR Test
Inverse variance weighted (fixed effects)
Inverse variance weighted (multiplicative random effects)
MR Egger
MR RAPS
Weighted median
Weighted mode
0.0025
0.0000
−0.0025
0.2
0.4
0.6
0.8
SNP effect on eGFR
SNP effect on Chondroadherin

Batch 2−534 : Coiled−coil−helix−coiled−coil−helix domain−containing protein 10, mitochondrial on eGFR

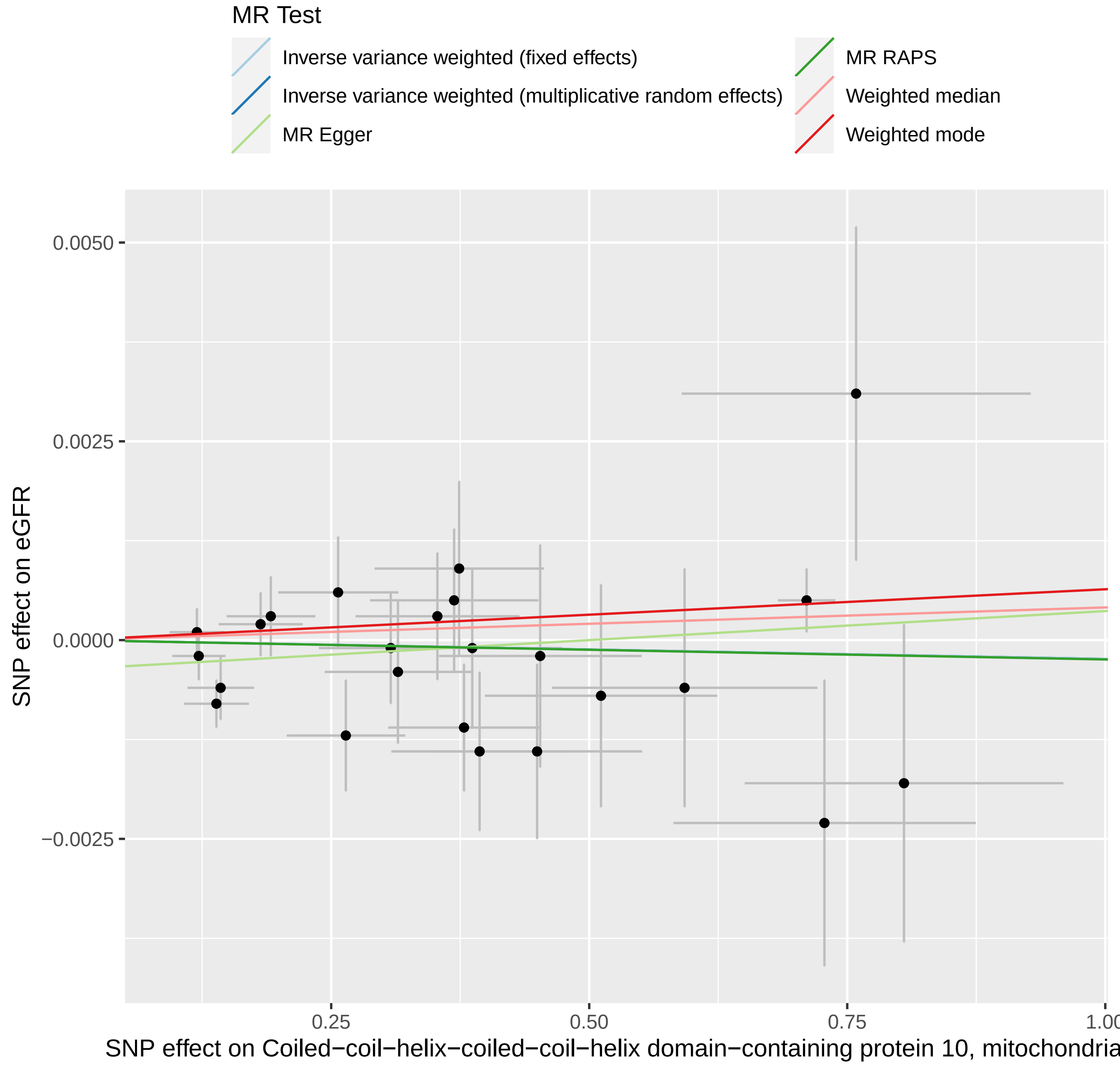

Batch 2−535 : Coiled−coil−helix−coiled−coil−helix domain−containing protein 2 on eGFR

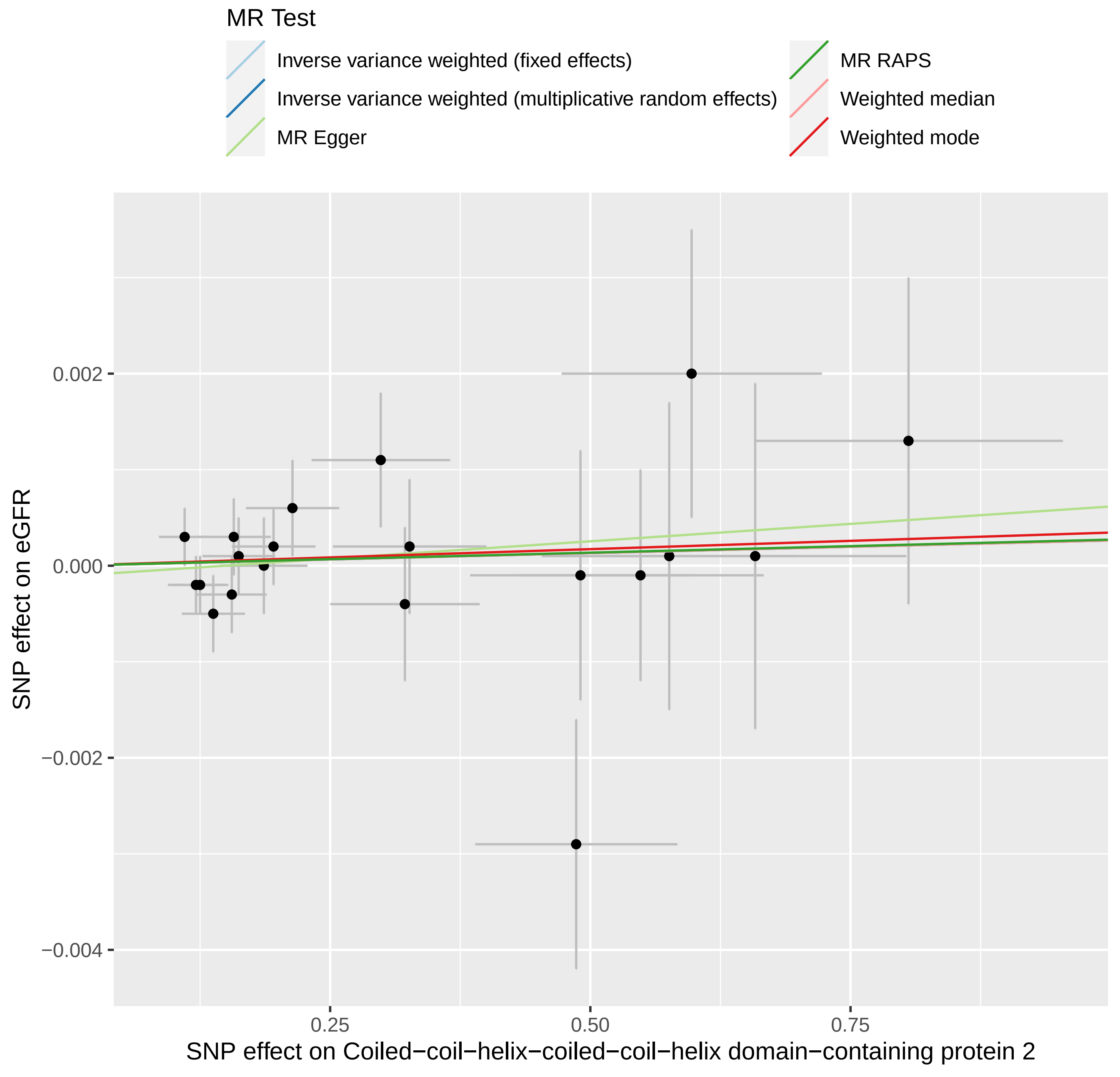

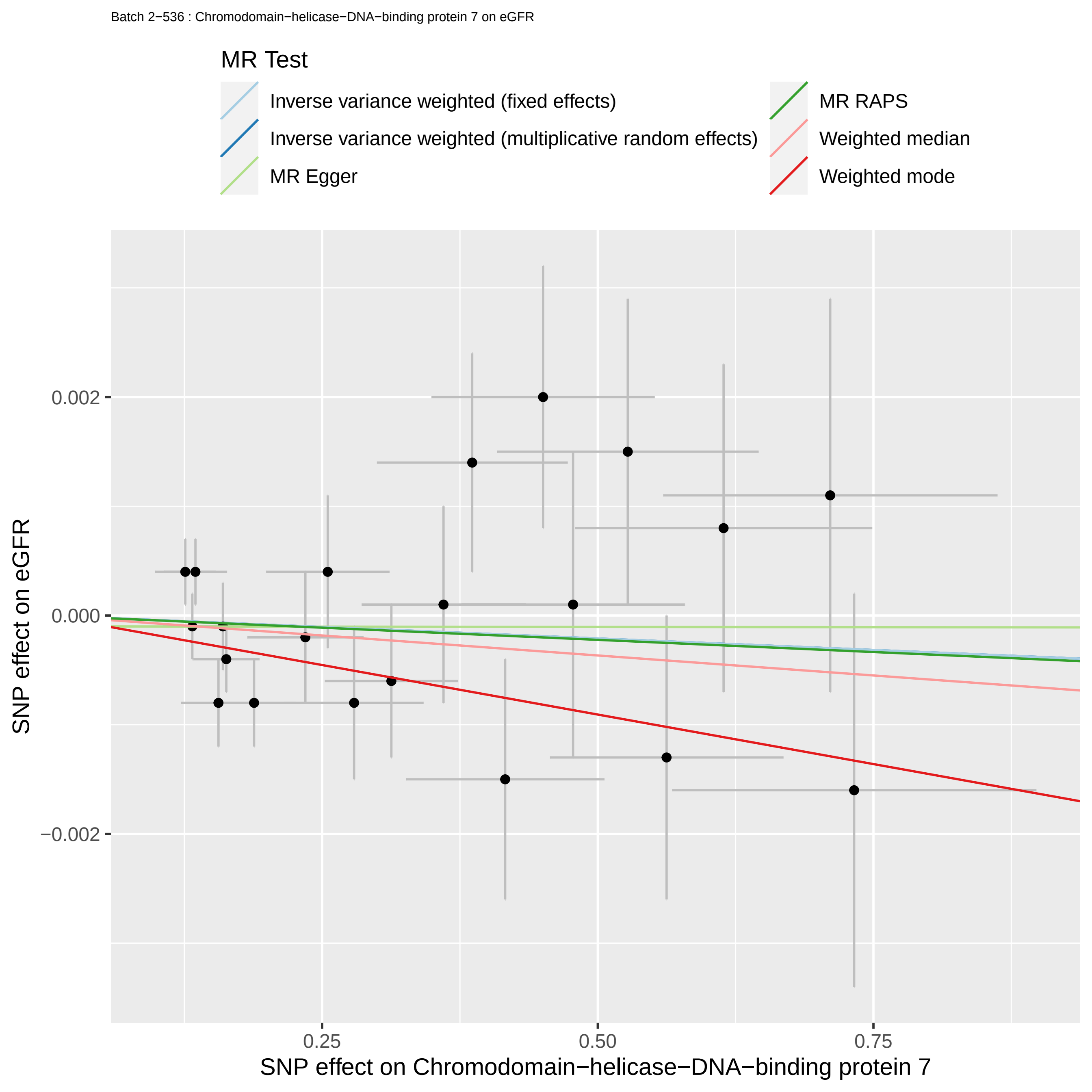

Batch 2−536 : Chromodomain−helicase−DNA−binding protein 7 on eGFR
MR Test
Inverse variance weighted (fixed effects)
Inverse variance weighted (multiplicative random effects)
MR Egger
MR RAPS
Weighted median
Weighted mode
0.002
0.000
−0.002
0.25
0.50
0.75
SNP effect on eGFR
SNP effect on Chromodomain−helicase−DNA−binding protein 7

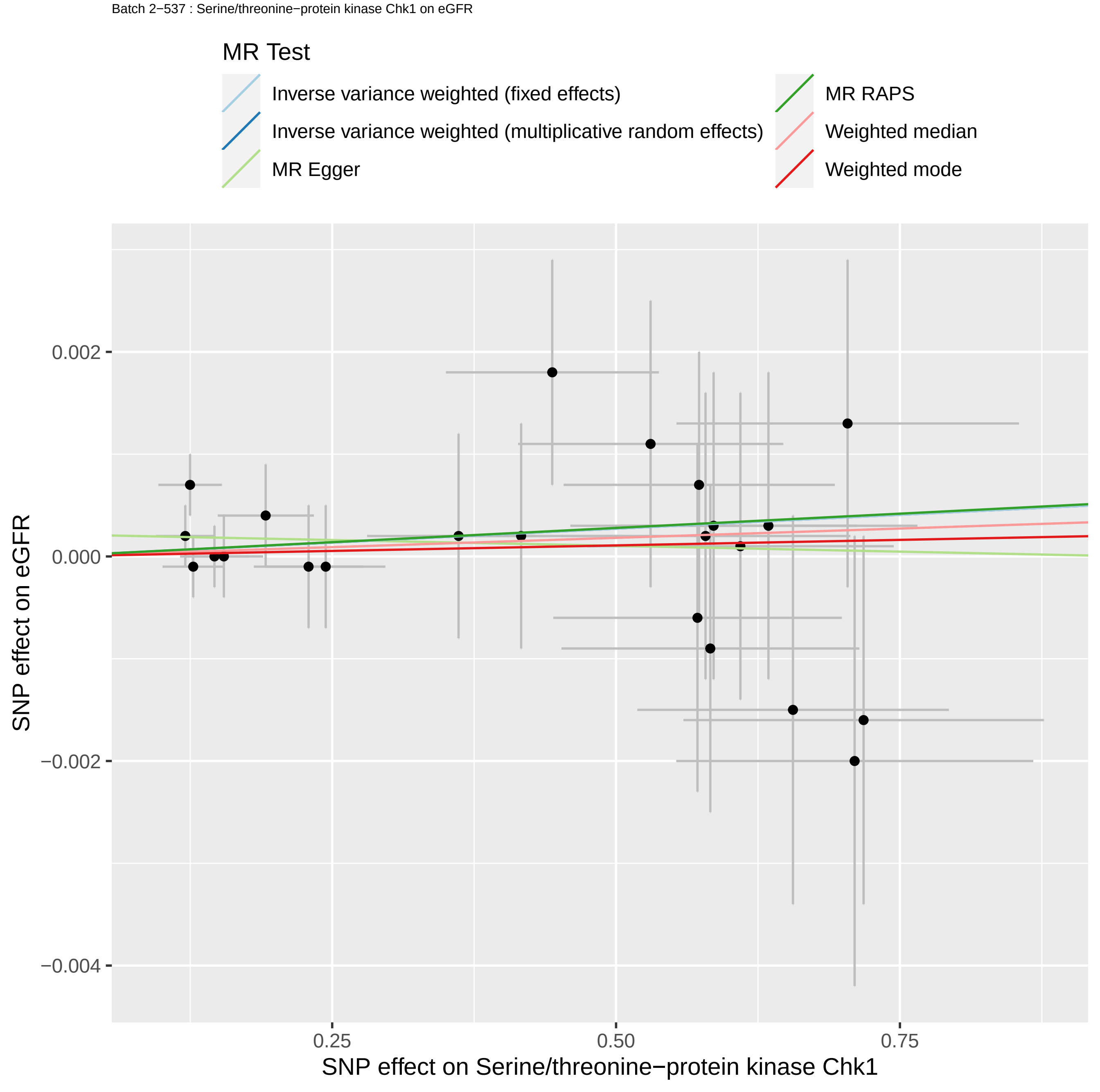

Batch 2−537 : Serine/threonine−protein kinase Chk1 on eGFR
MR Test
Inverse variance weighted (fixed effects)
Inverse variance weighted (multiplicative random effects)
MR Egger
MR RAPS
Weighted median
Weighted mode
0.002
0.000
−0.002
−0.004
0.25
0.50
0.75
SNP effect on eGFR
SNP effect on Serine/threonine−protein kinase Chk1

Batch 2−538 : Chromogranin−A on eGFR

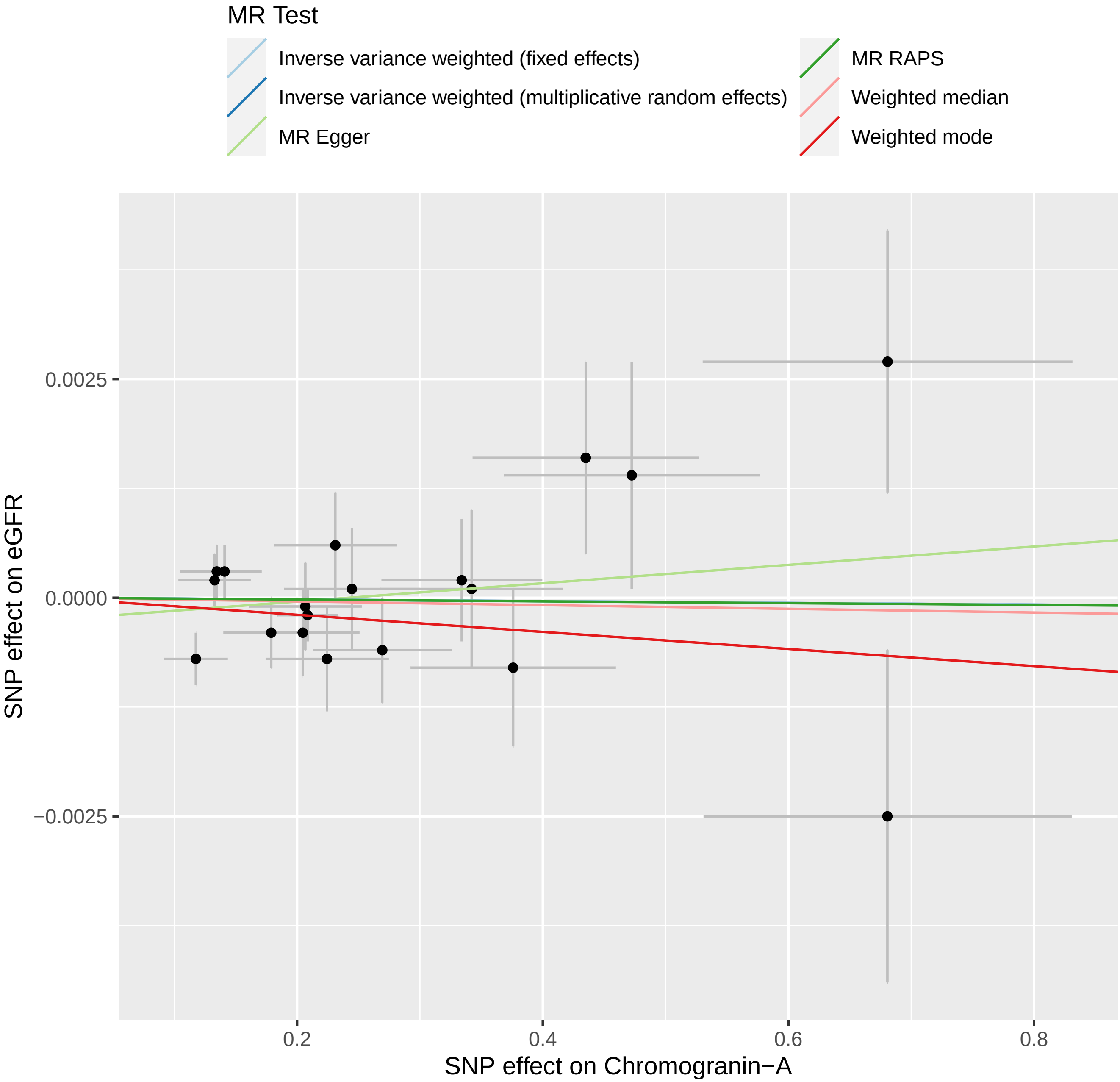

Batch 2−539 : Secretogranin−1 on eGFR

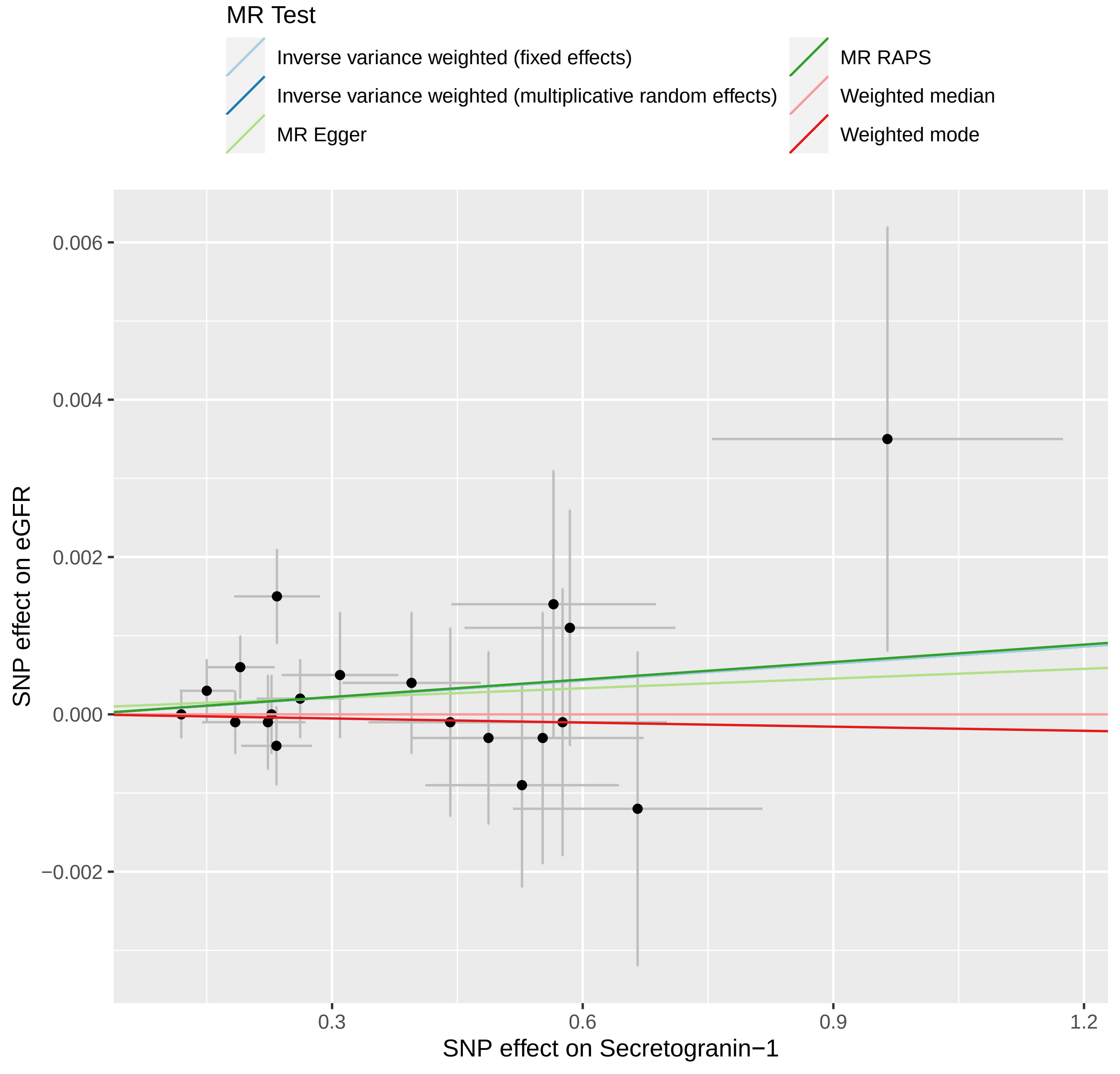

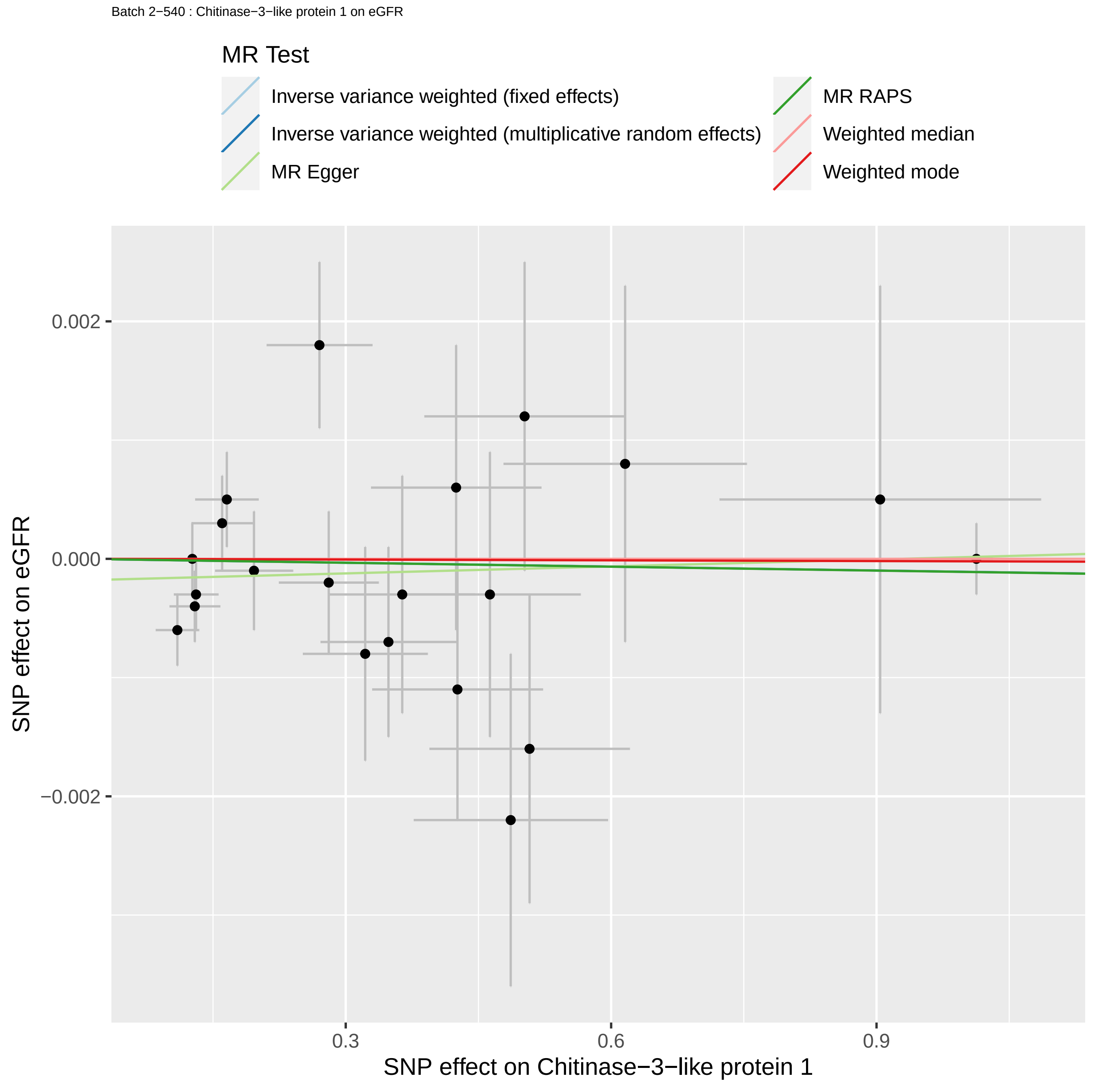

Batch 2−540 : Chitinase−3−like protein 1 on eGFR
MR Test
Inverse variance weighted (fixed effects)
Inverse variance weighted (multiplicative random effects)
MR Egger
MR RAPS
Weighted median
Weighted mode
0.002
0.000
−0.002
0.3
0.6
0.9
SNP effect on eGFR
SNP effect on Chitinase−3−like protein 1

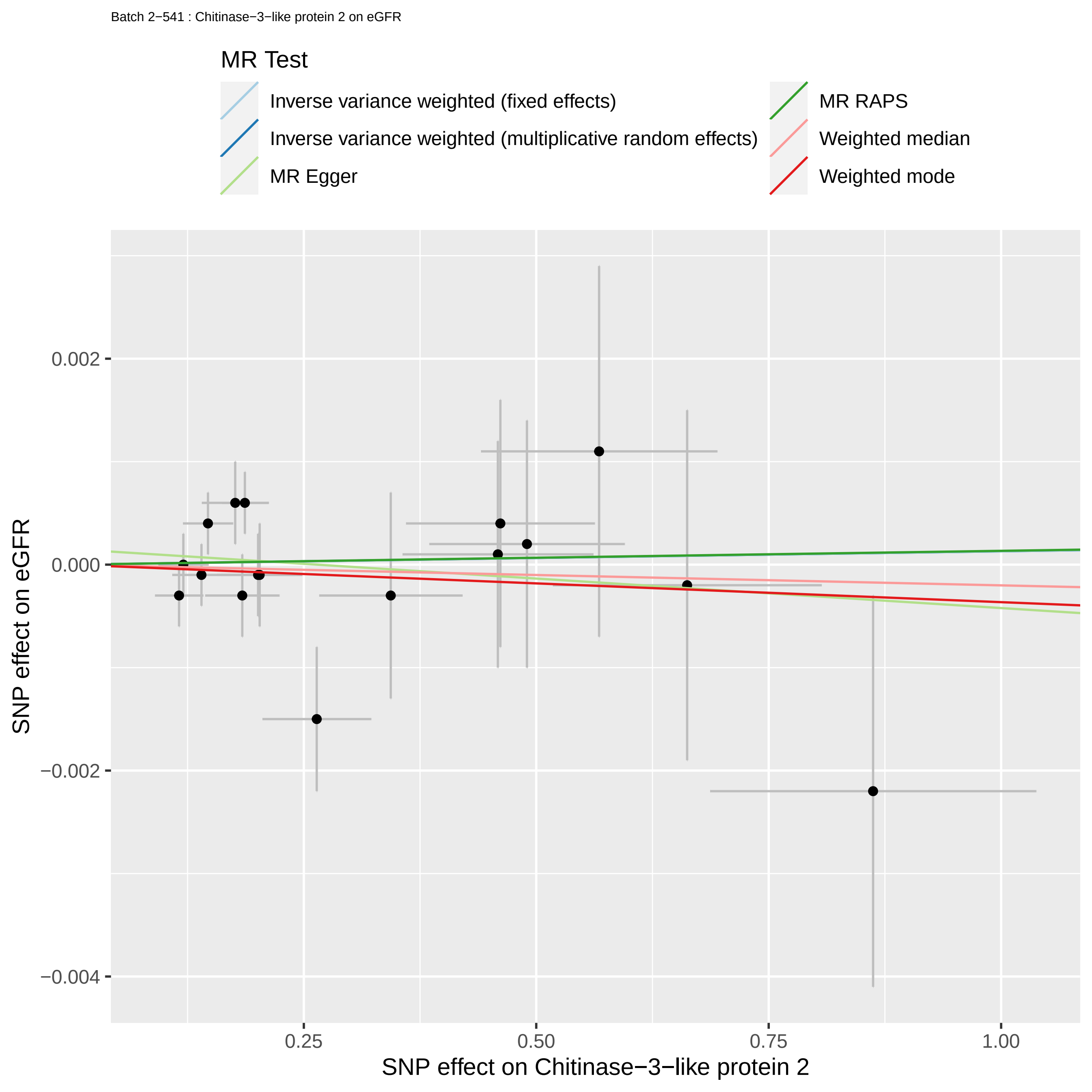

Batch 2−541 : Chitinase−3−like protein 2 on eGFR
MR Test
Inverse variance weighted (fixed effects)
Inverse variance weighted (multiplicative random effects)
MR Egger
MR RAPS
Weighted median
Weighted mode
0.002
0.000
−0.002
−0.004
0.25
0.50
0.75
1.00
SNP effect on Chitinase−3−like protein 2
SNP effect on eGFR

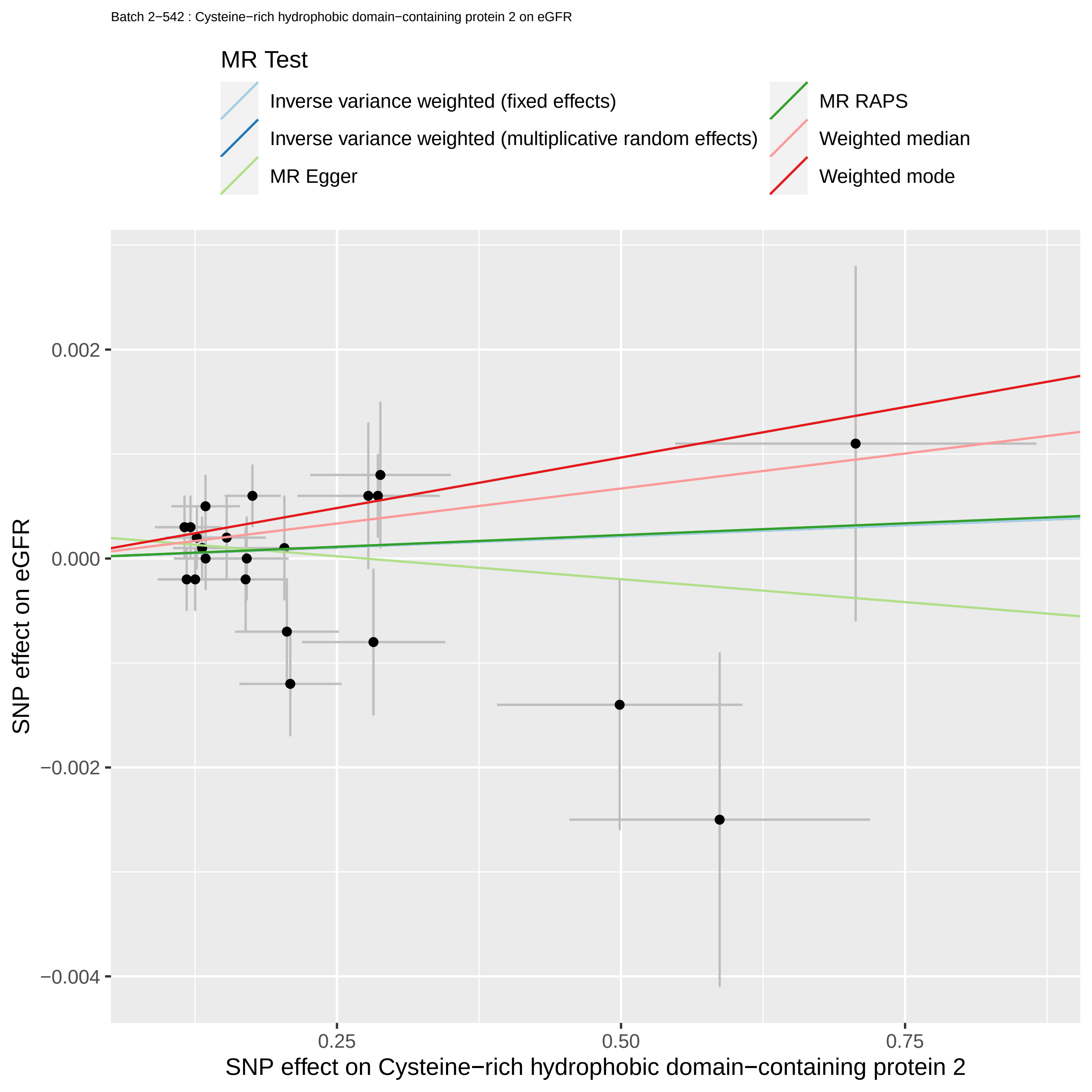
Batch 2−542 : Cysteine−rich hydrophobic domain−containing protein 2 on eGFR
MR Test
Inverse variance weighted (fixed effects)
Inverse variance weighted (multiplicative random effects)
MR Egger
MR RAPS
Weighted median
Weighted mode
0.002
0.000
−0.002
−0.004
0.25
0.50
0.75
SNP effect on eGFR
SNP effect on Cysteine−rich hydrophobic domain−containing protein 2

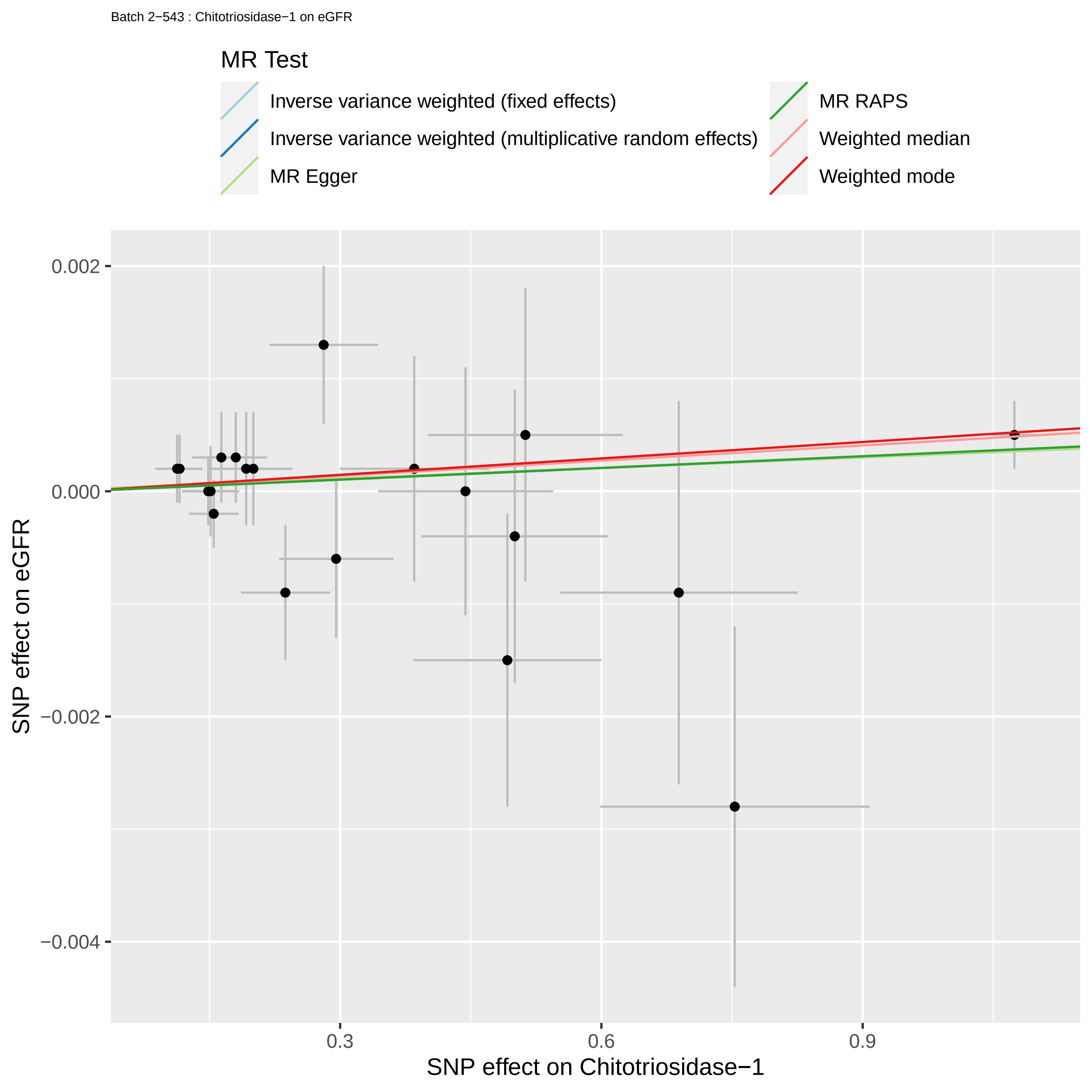
Batch 2−543 : Chitotriosidase−1 on eGFR
MR Test
Inverse variance weighted (fixed effects)
Inverse variance weighted (multiplicative random effects)
MR Egger
MR RAPS
Weighted median
Weighted mode
0.002
0.000
−0.002
−0.004
SNP effect on eGFR
0.3
0.6
0.9
SNP effect on Chitotriosidase−1

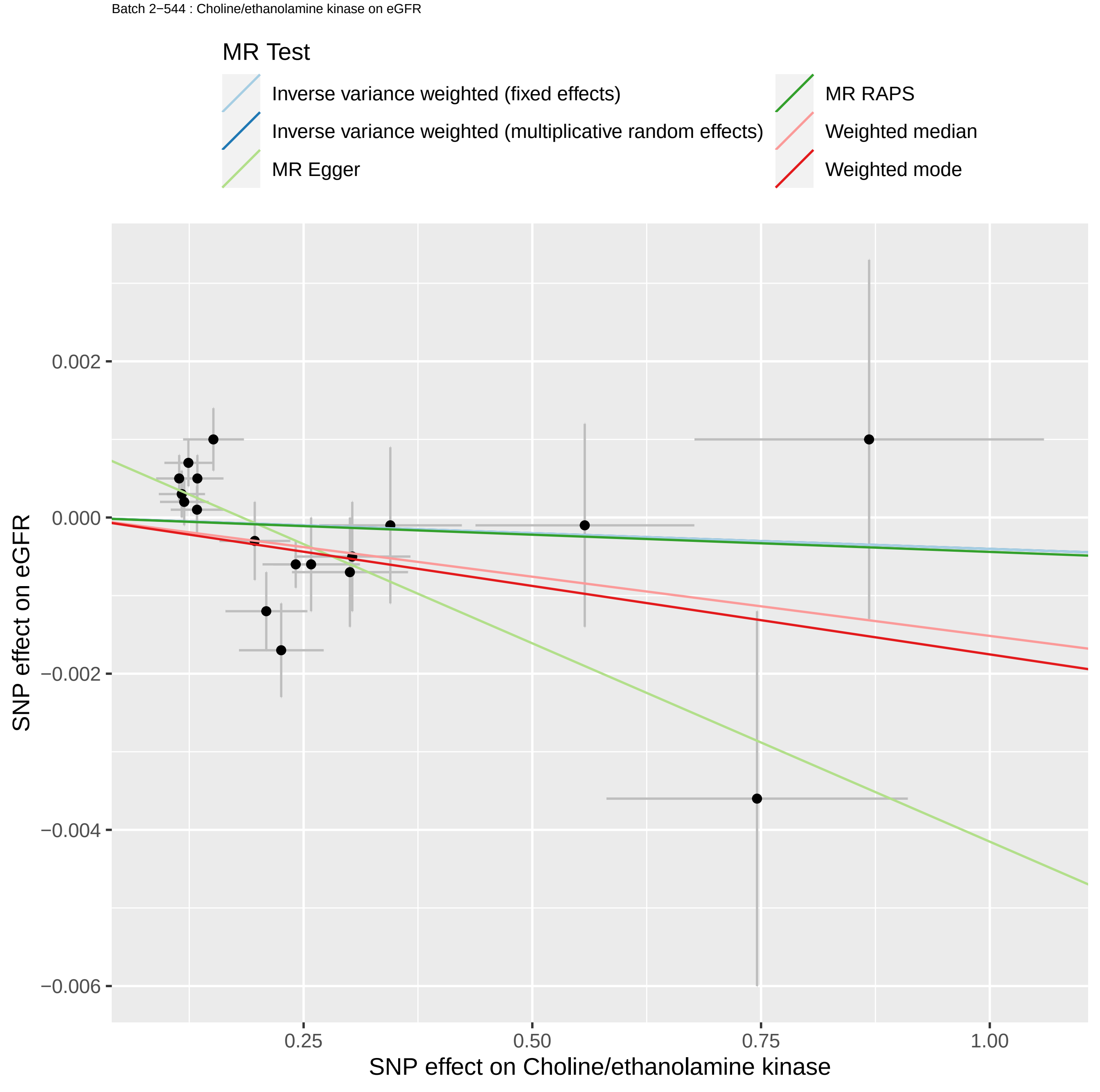

Batch 2−544 : Choline/ethanolamine kinase on eGFR
MR Test
Inverse variance weighted (fixed effects)
Inverse variance weighted (multiplicative random effects)
MR Egger
MR RAPS
Weighted median
Weighted mode
0.002
0.000
−0.002
−0.004
−0.006
0.25
0.50
0.75
1.00
SNP effect on eGFR
SNP effect on Choline/ethanolamine kinase

Batch 2−545 : Neural cell adhesion molecule L1−like protein on eGFR

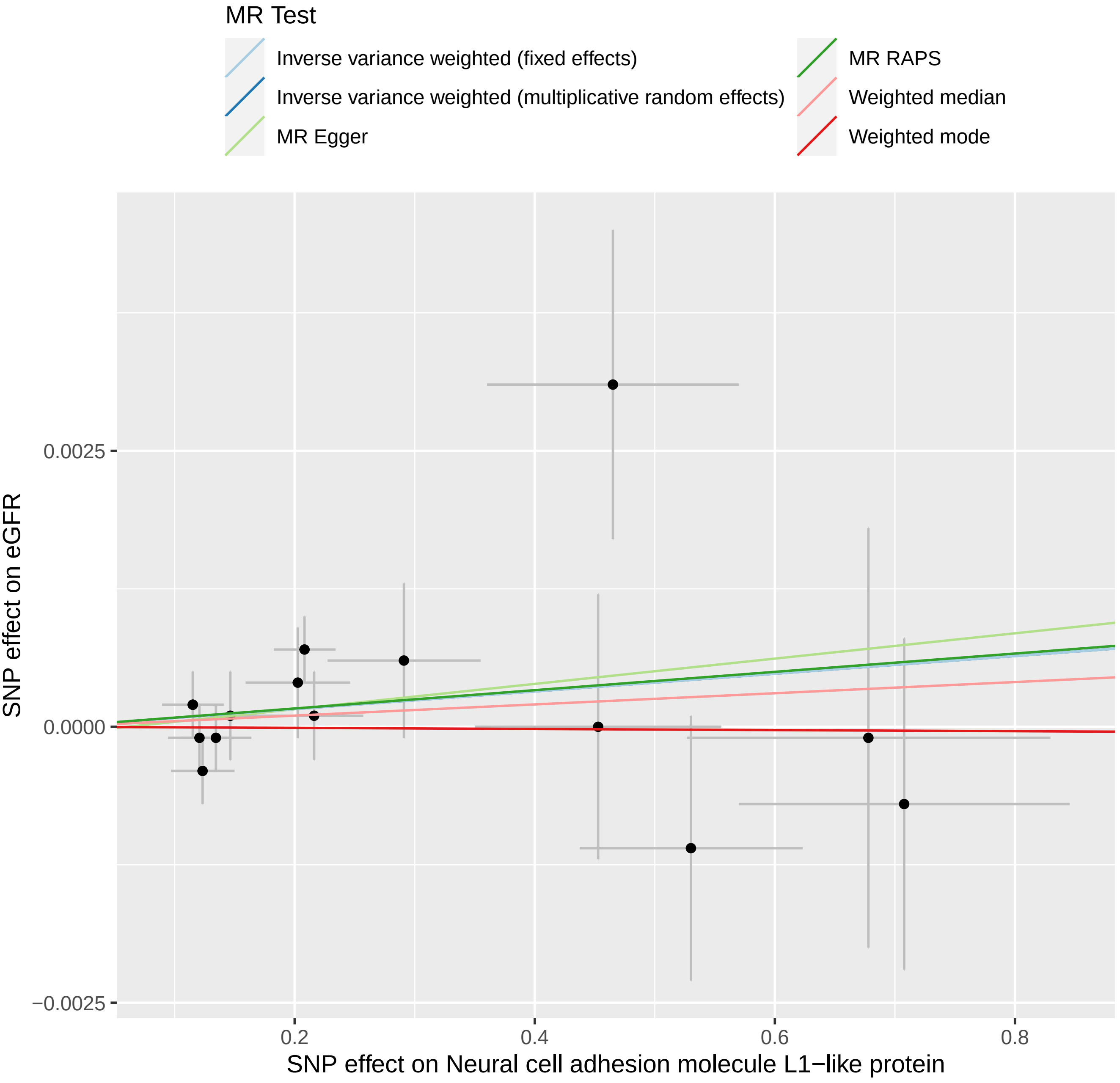

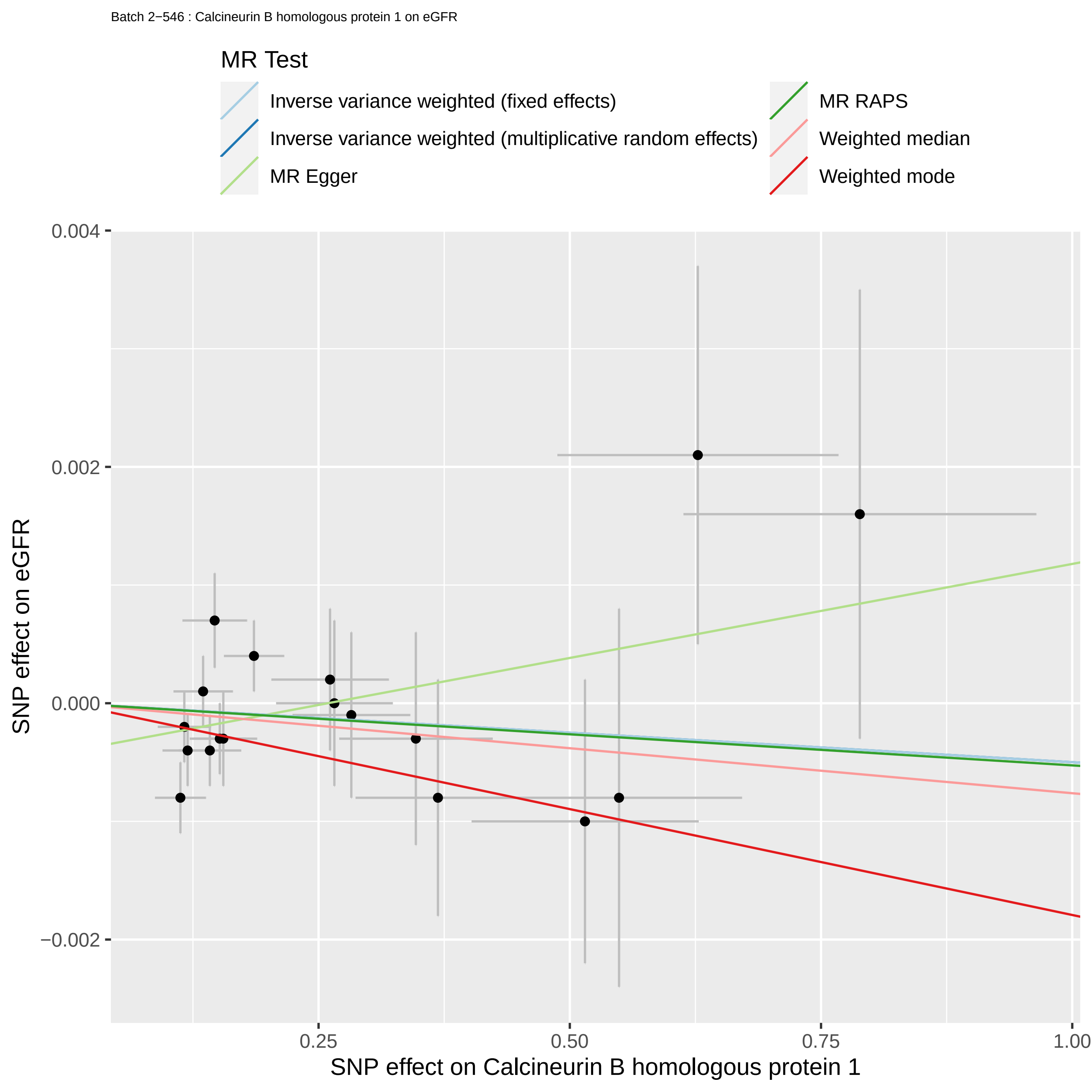

Batch 2−546 : Calcineurin B homologous protein 1 on eGFR
MR Test
Inverse variance weighted (fixed effects)
Inverse variance weighted (multiplicative random effects)
MR Egger
MR RAPS
Weighted median
Weighted mode
0.004
0.002
0.000
−0.002
0.25
0.50
0.75
1.00
SNP effect on eGFR
SNP effect on Calcineurin B homologous protein 1

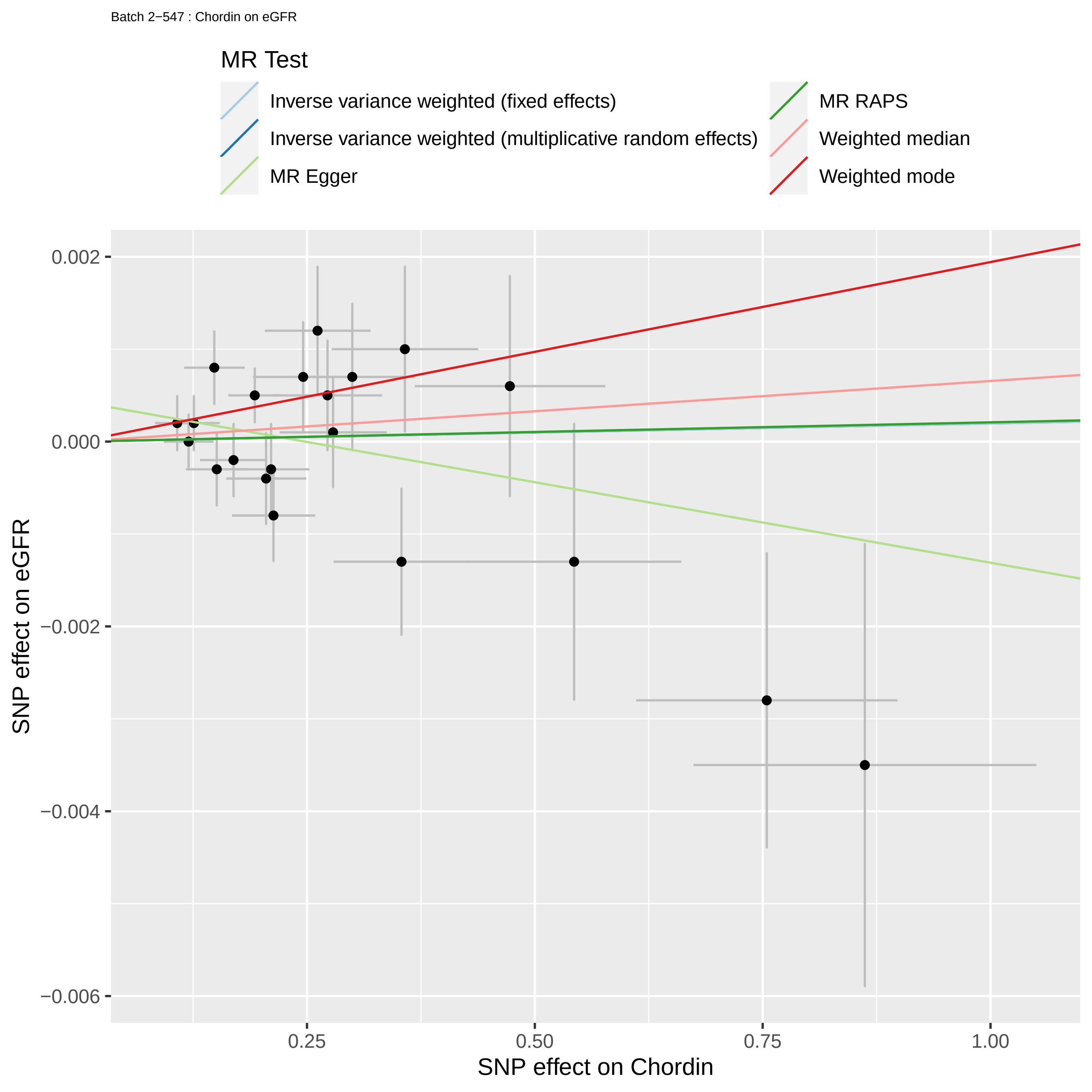

Batch 2−547 : Chordin on eGFR
MR Test
Inverse variance weighted (fixed effects)
Inverse variance weighted (multiplicative random effects)
MR Egger
MR RAPS
Weighted median
Weighted mode
0.002
0.000
−0.002
−0.004
−0.006
0.25
0.50
0.75
1.00
SNP effect on eGFR
SNP effect on Chordin

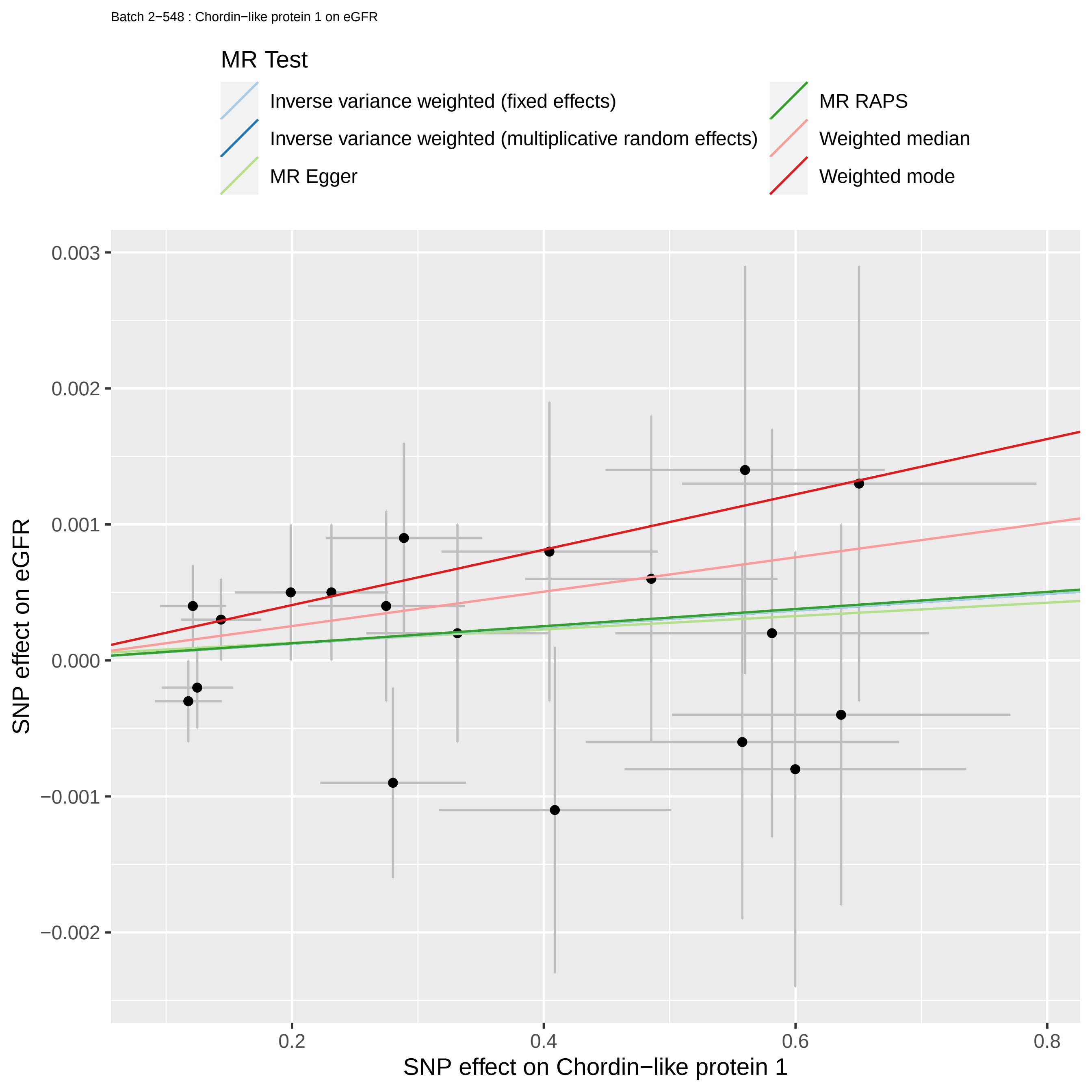

Batch 2−548 : Chordin−like protein 1 on eGFR
MR Test
Inverse variance weighted (fixed effects)
Inverse variance weighted (multiplicative random effects)
MR Egger
MR RAPS
Weighted median
Weighted mode
0.003
0.002
0.001
0.000
−0.001
−0.002
SNP effect on eGFR
0.2
0.4
0.6
0.8
SNP effect on Chordin−like protein 1

Batch 2−549 : Chordin−like protein 2 on eGFR

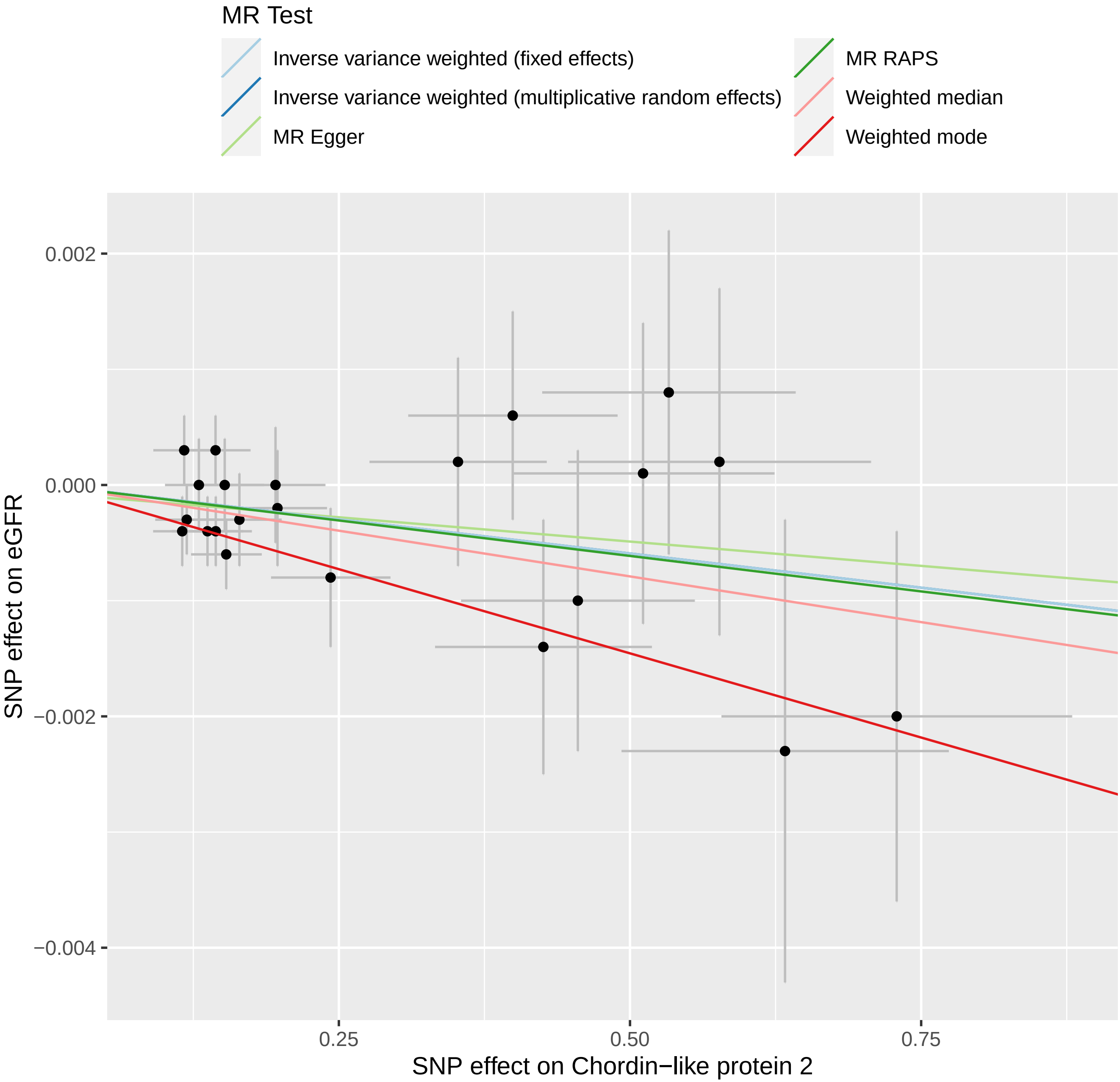

Batch 2−550 : Carbohydrate sulfotransferase 11 on eGFR

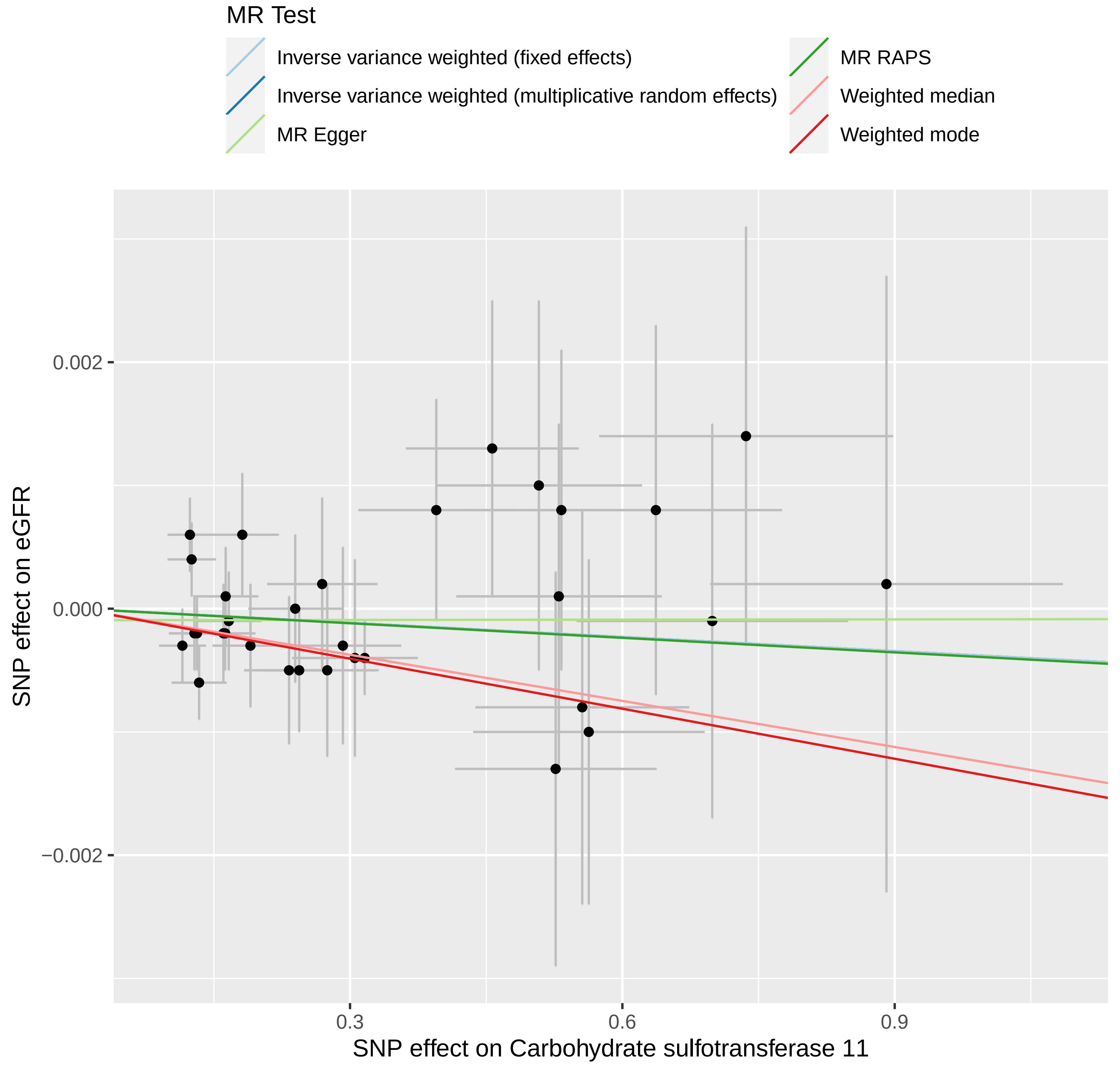

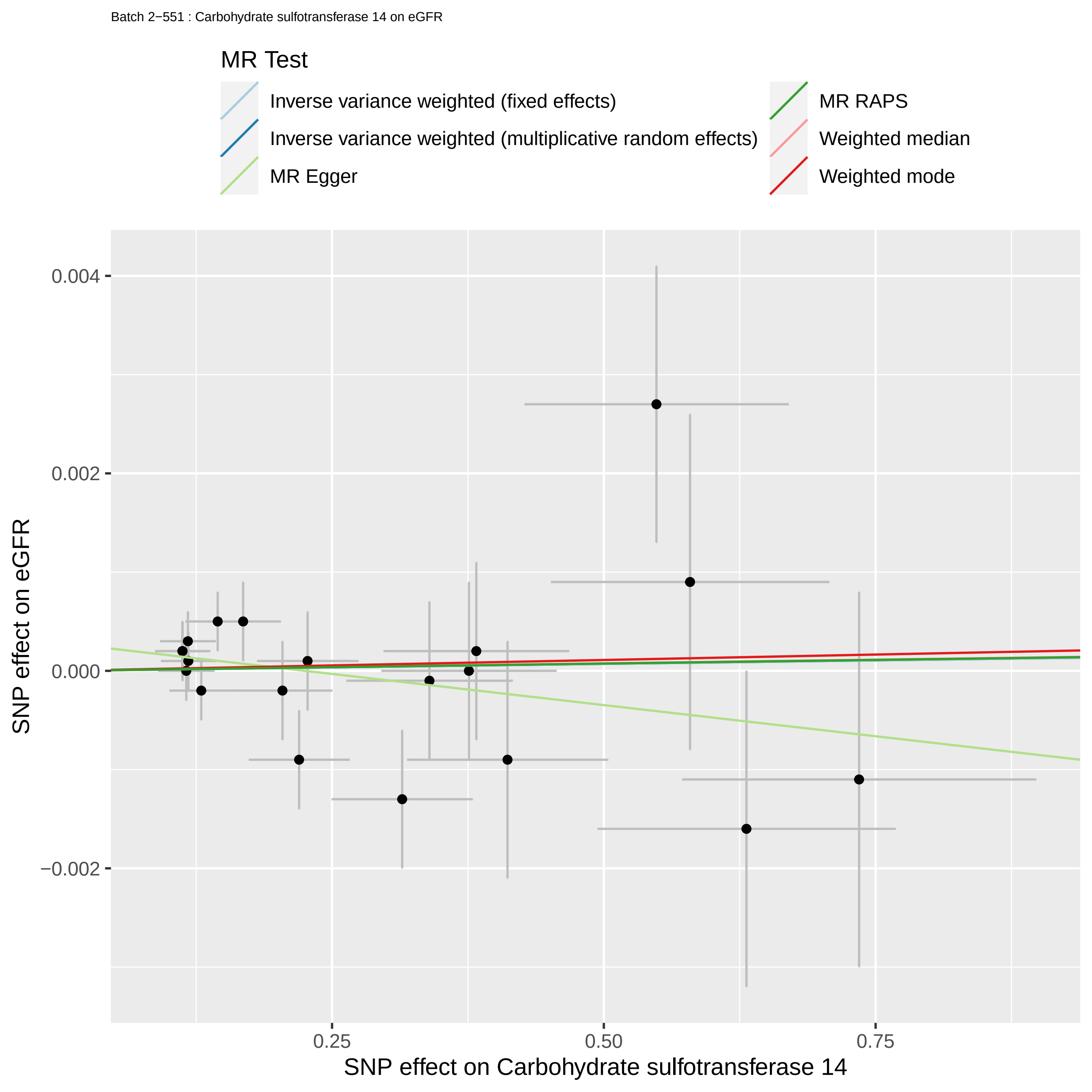
Batch 2−551 : Carbohydrate sulfotransferase 14 on eGFR
MR Test
Inverse variance weighted (fixed effects)
Inverse variance weighted (multiplicative random effects)
MR Egger
MR RAPS
Weighted median
Weighted mode
0.004
0.002
0.000
−0.002
SNP effect on eGFR
0.25
0.50
0.75
SNP effect on Carbohydrate sulfotransferase 14

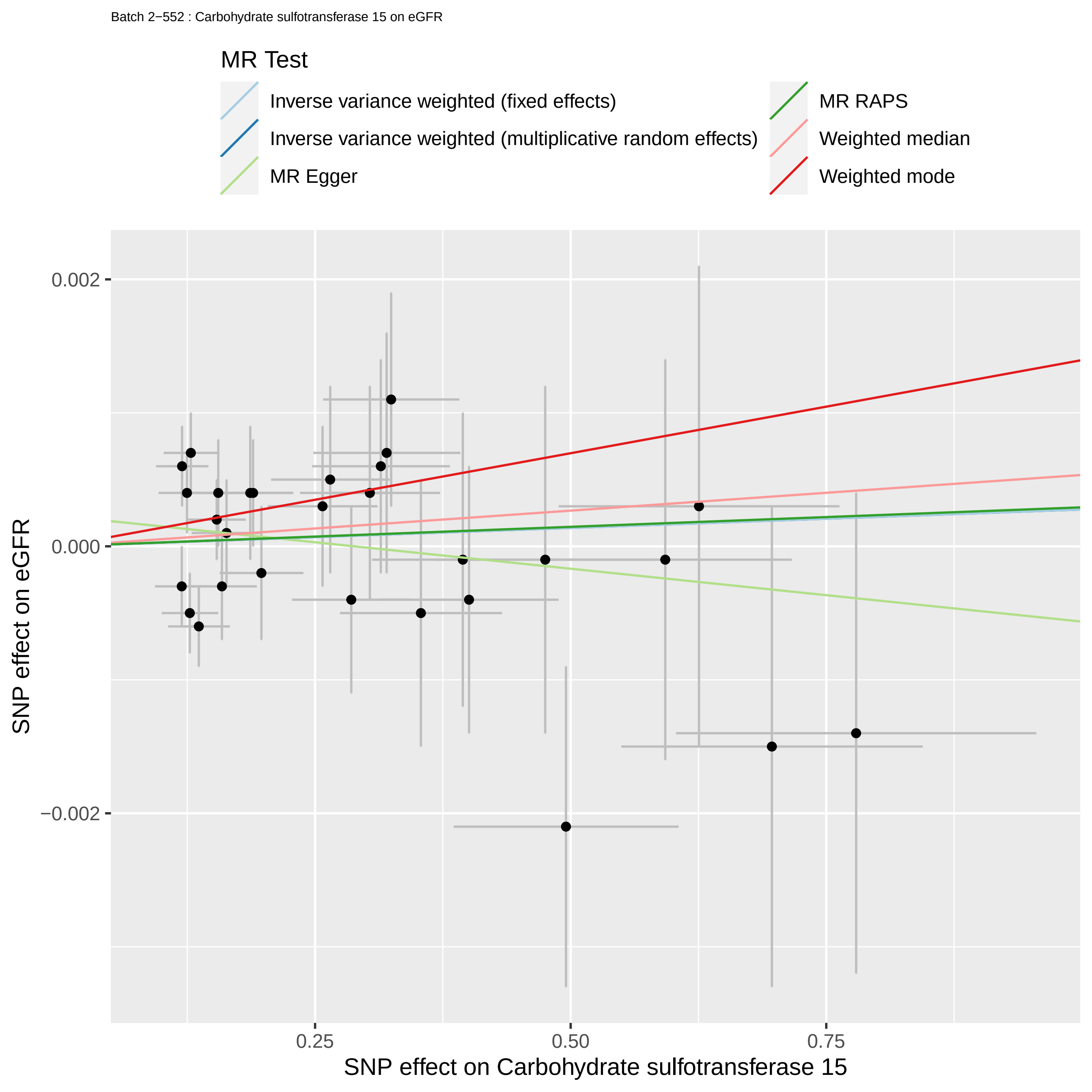
Batch 2−552 : Carbohydrate sulfotransferase 15 on eGFR
MR Test
Inverse variance weighted (fixed effects)
Inverse variance weighted (multiplicative random effects)
MR Egger
MR RAPS
Weighted median
Weighted mode
0.002
0.000
−0.002
0.25
0.50
0.75
SNP effect on eGFR
SNP effect on Carbohydrate sulfotransferase 15

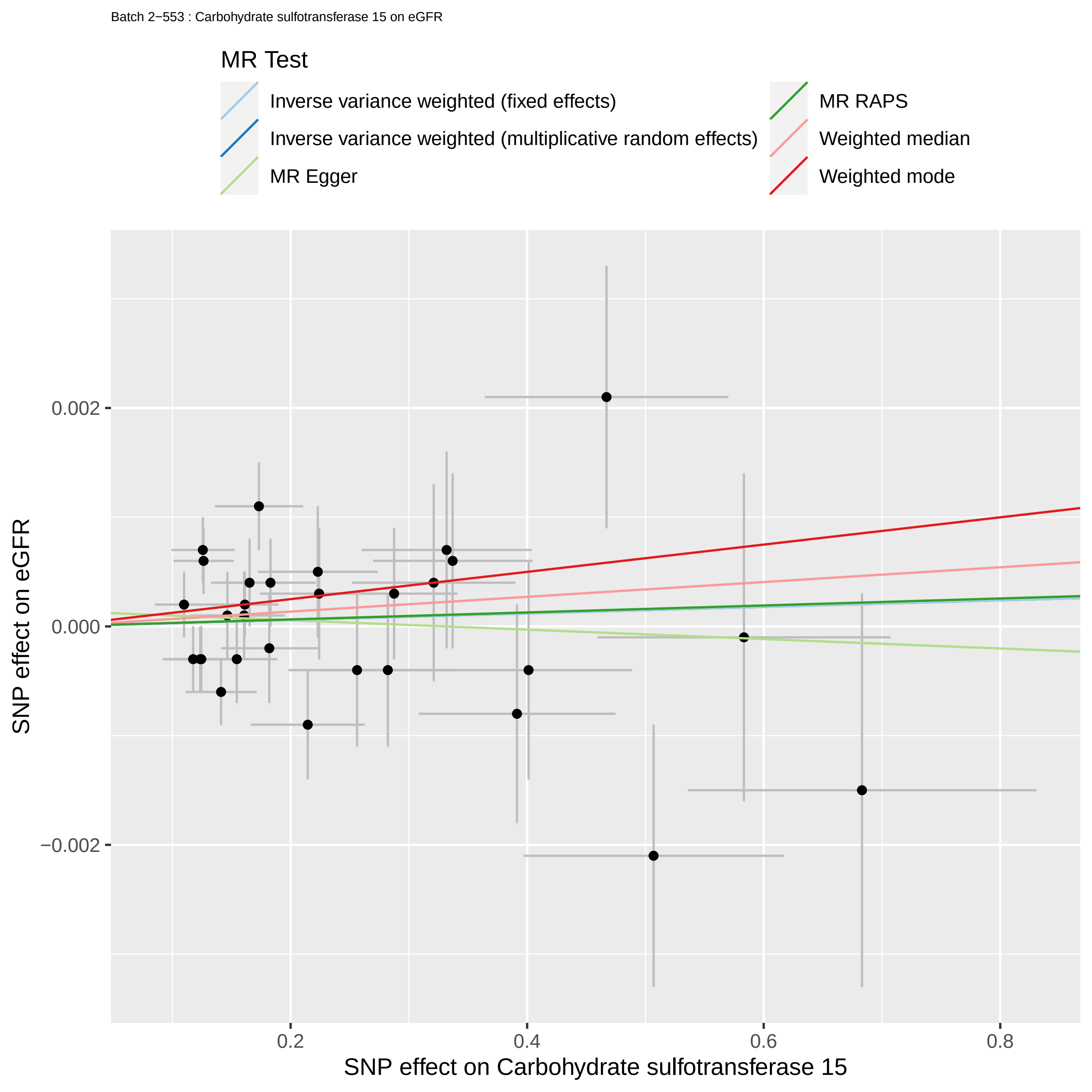
Batch 2−553 : Carbohydrate sulfotransferase 15 on eGFR
MR Test
Inverse variance weighted (fixed effects)
Inverse variance weighted (multiplicative random effects)
MR Egger
MR RAPS
Weighted median
Weighted mode
0.002
0.000
−0.002
SNP effect on eGFR
0.2
0.4
0.6
0.8
SNP effect on Carbohydrate sulfotransferase 15

Batch 2−554 : Carbohydrate sulfotransferase 1 on eGFR

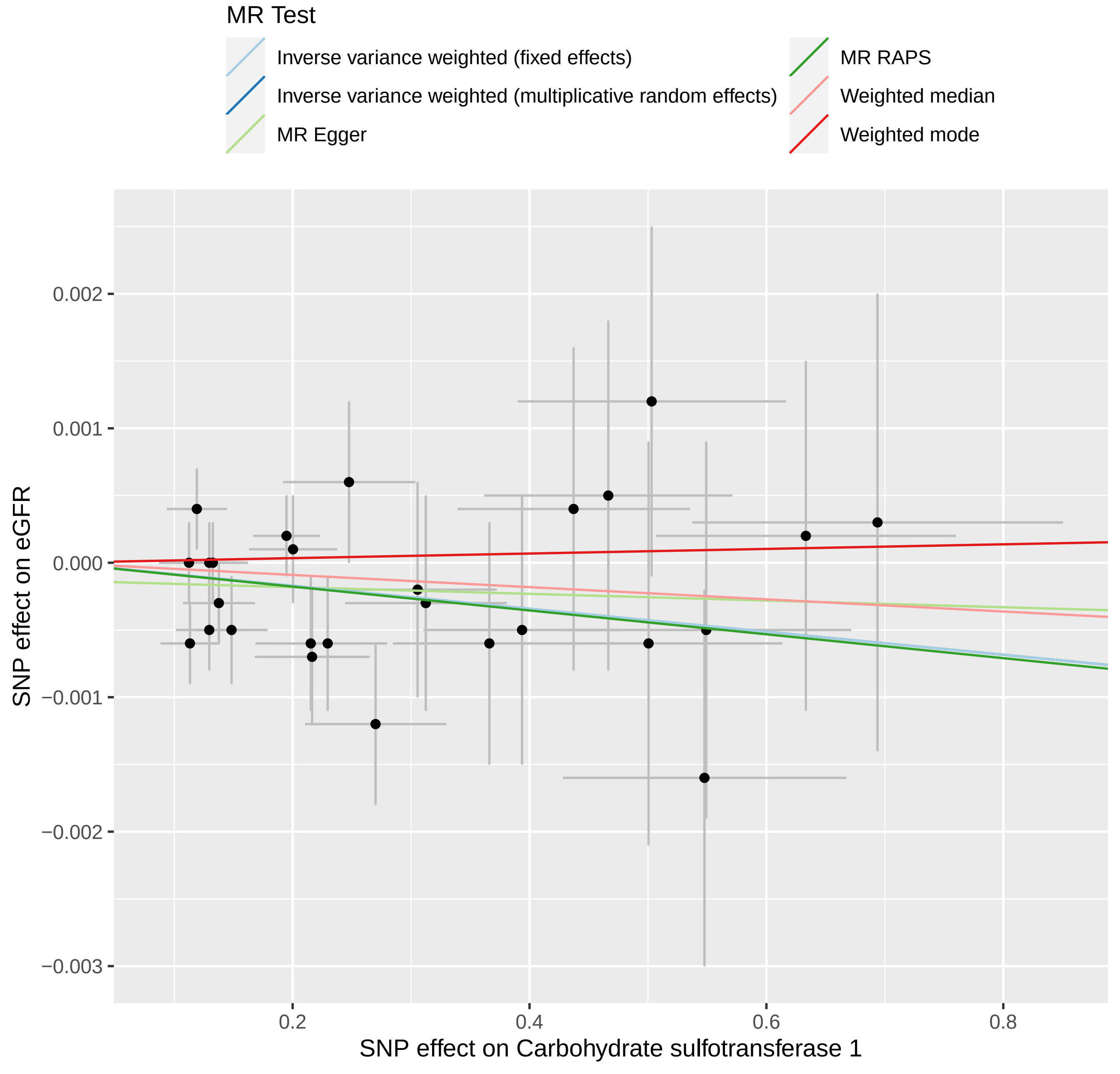

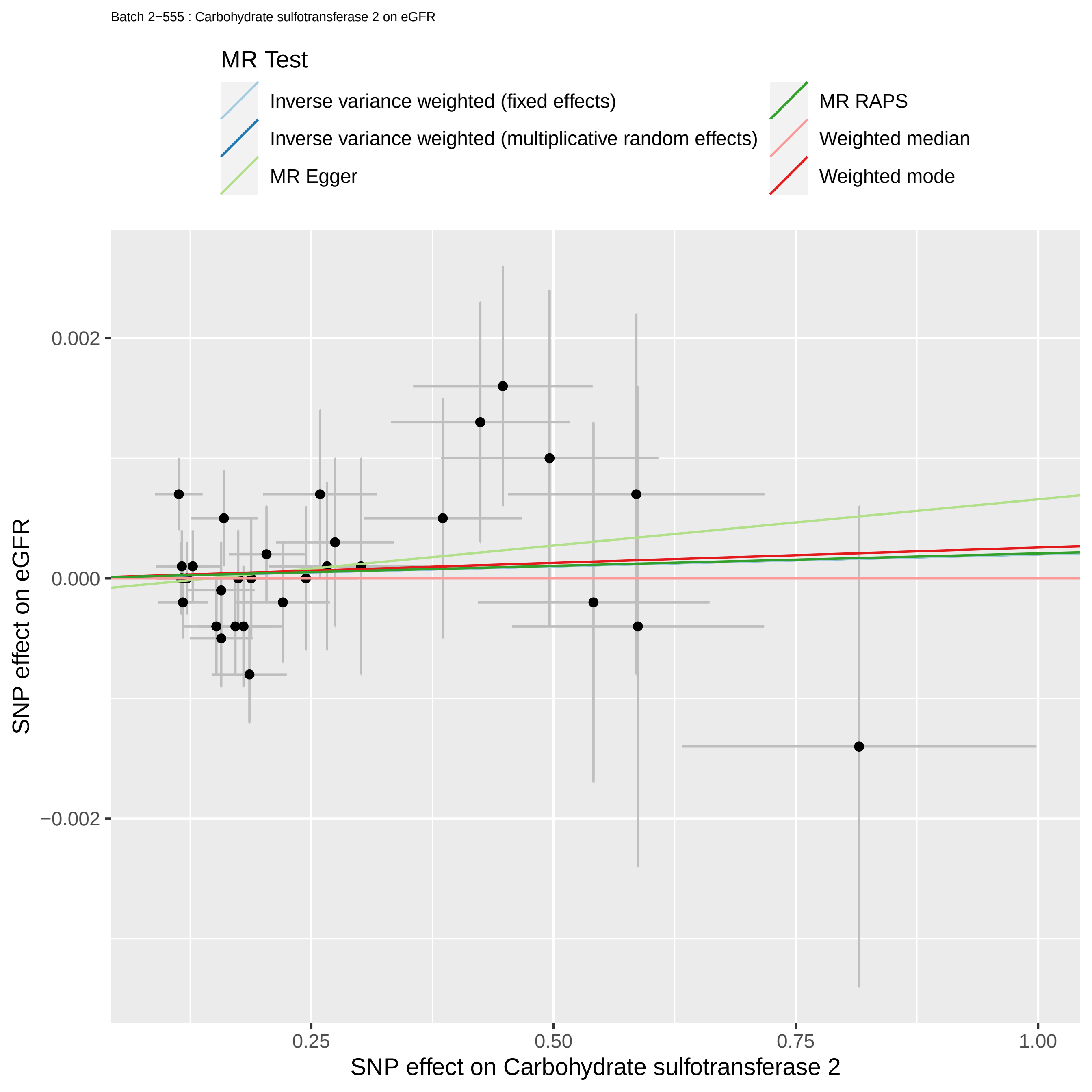

Batch 2−555 : Carbohydrate sulfotransferase 2 on eGFR
MR Test
Inverse variance weighted (fixed effects)
Inverse variance weighted (multiplicative random effects)
MR Egger
MR RAPS
Weighted median
Weighted mode
0.002
0.000
−0.002
0.25
0.50
0.75
1.00
SNP effect on eGFR
SNP effect on Carbohydrate sulfotransferase 2

Batch 2−556 : Carbohydrate sulfotransferase 3 on eGFR

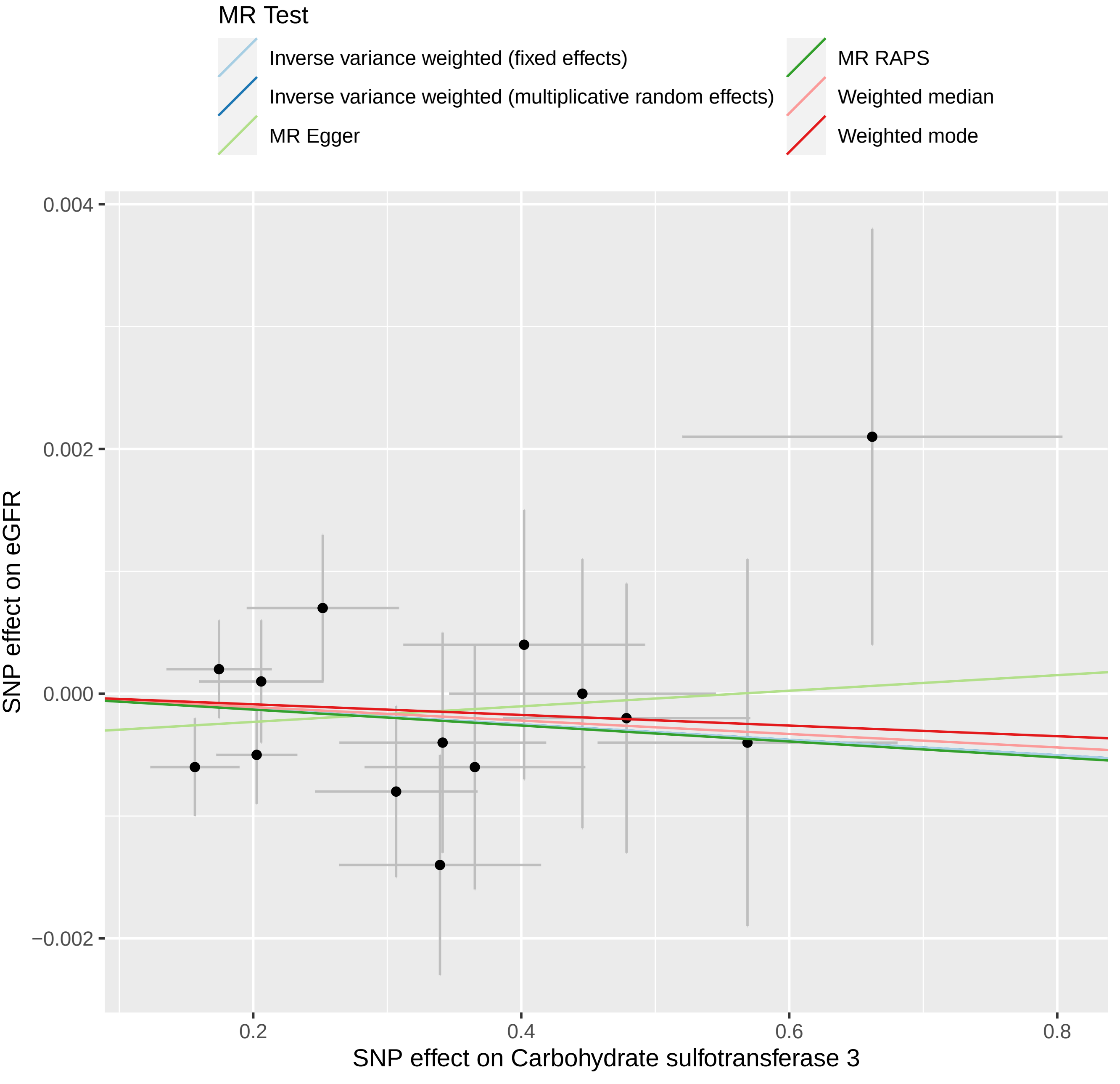

Batch 2−557 : Carbohydrate sulfotransferase 5 on eGFR

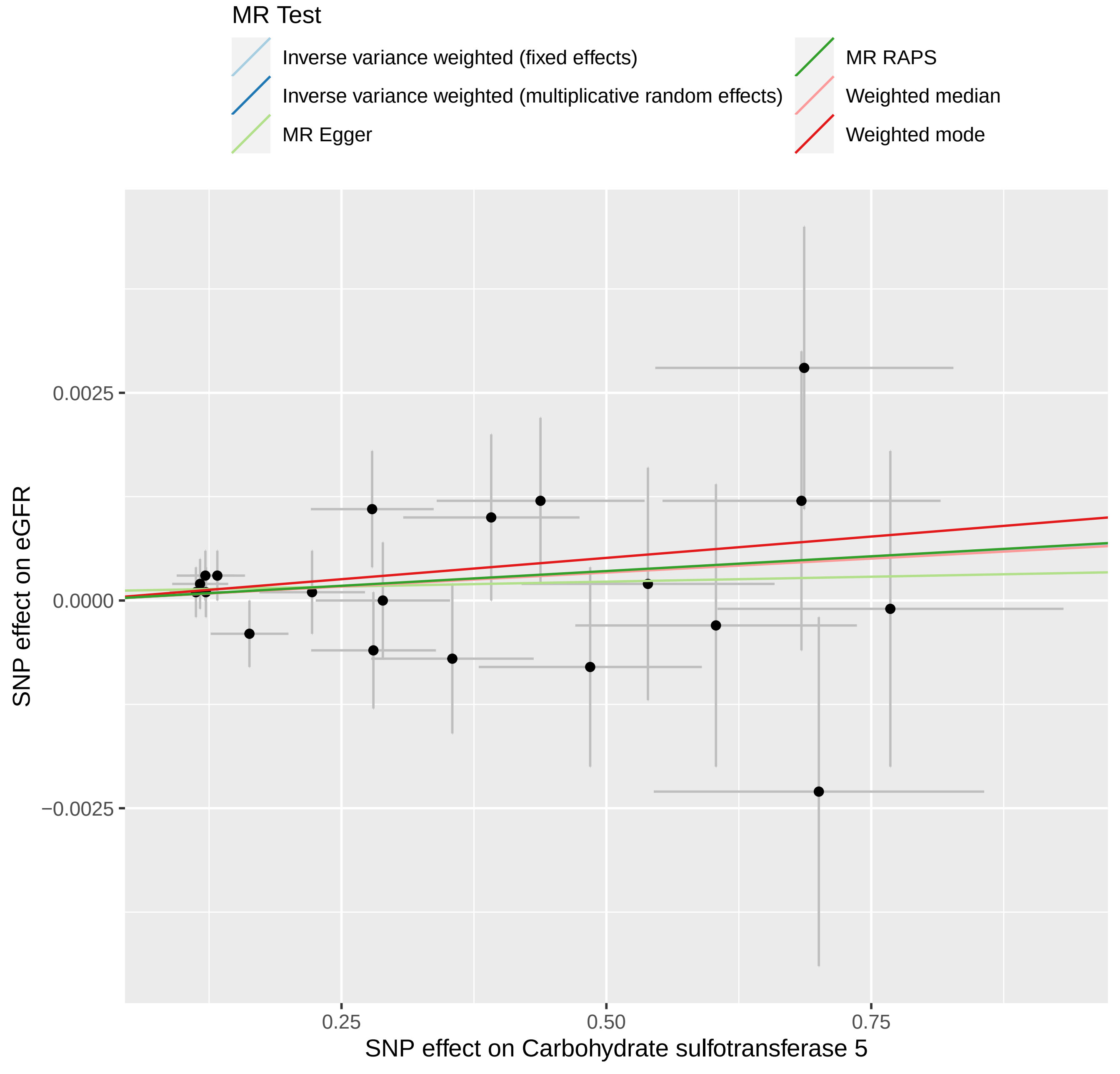

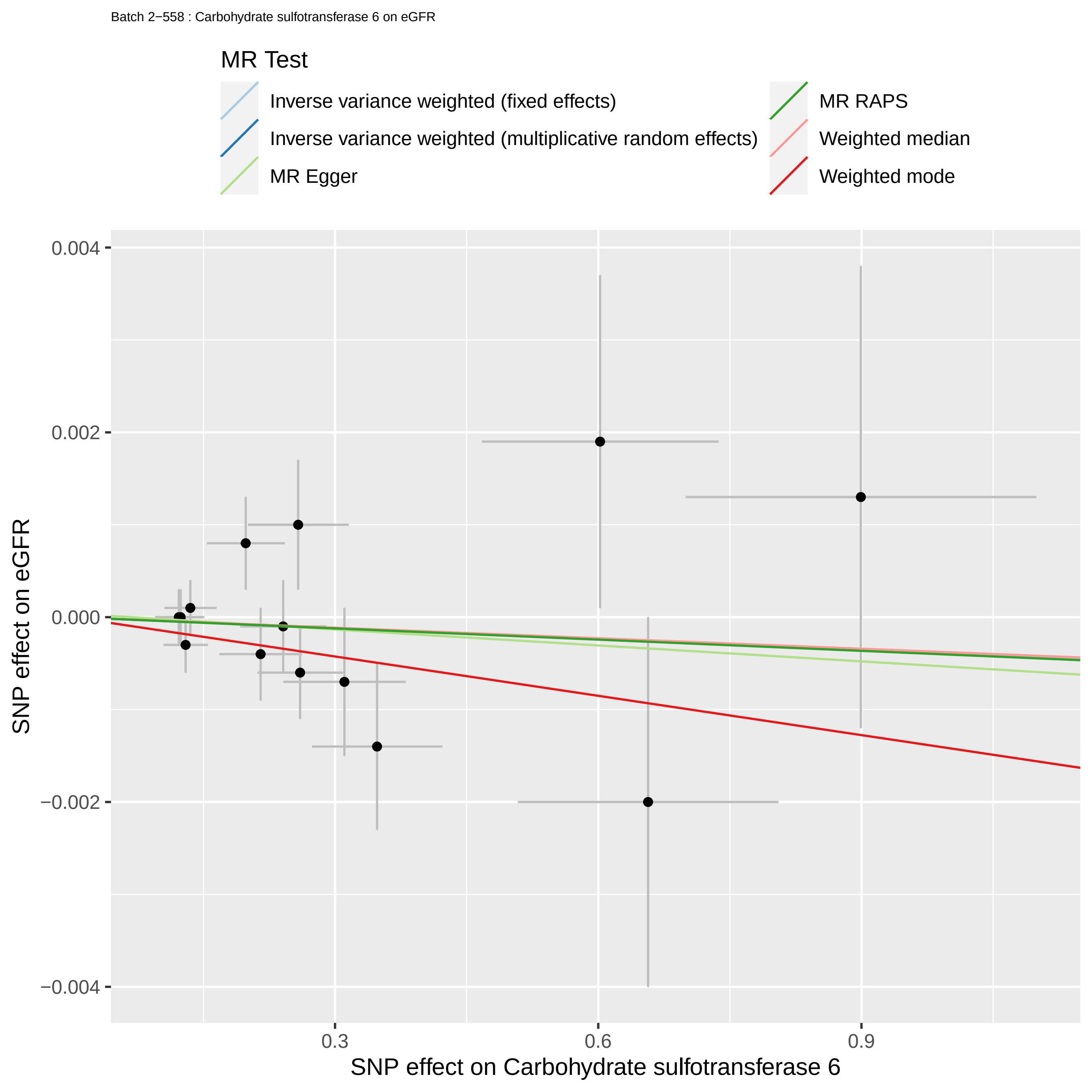

Batch 2−558 : Carbohydrate sulfotransferase 6 on eGFR
MR Test
Inverse variance weighted (fixed effects)
Inverse variance weighted (multiplicative random effects)
MR Egger
MR RAPS
Weighted median
Weighted mode
0.004
0.002
0.000
−0.002
−0.004
SNP effect on eGFR
0.3
0.6
0.9
SNP effect on Carbohydrate sulfotransferase 6

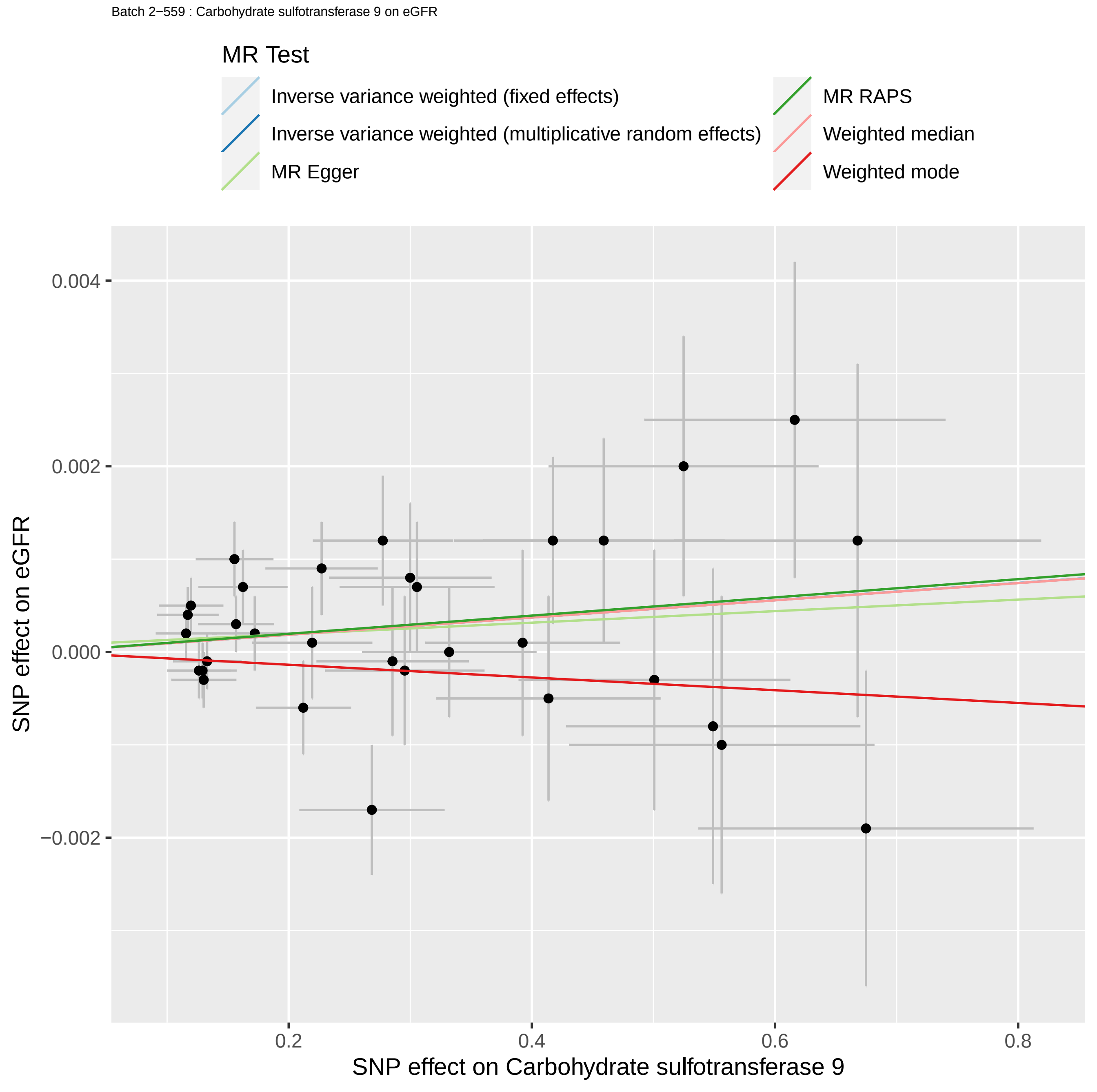

Batch 2−559 : Carbohydrate sulfotransferase 9 on eGFR
MR Test
Inverse variance weighted (fixed effects)
Inverse variance weighted (multiplicative random effects)
MR Egger
MR RAPS
Weighted median
Weighted mode
0.004
0.002
0.000
−0.002
0.2
0.4
0.6
0.8
SNP effect on eGFR
SNP effect on Carbohydrate sulfotransferase 9

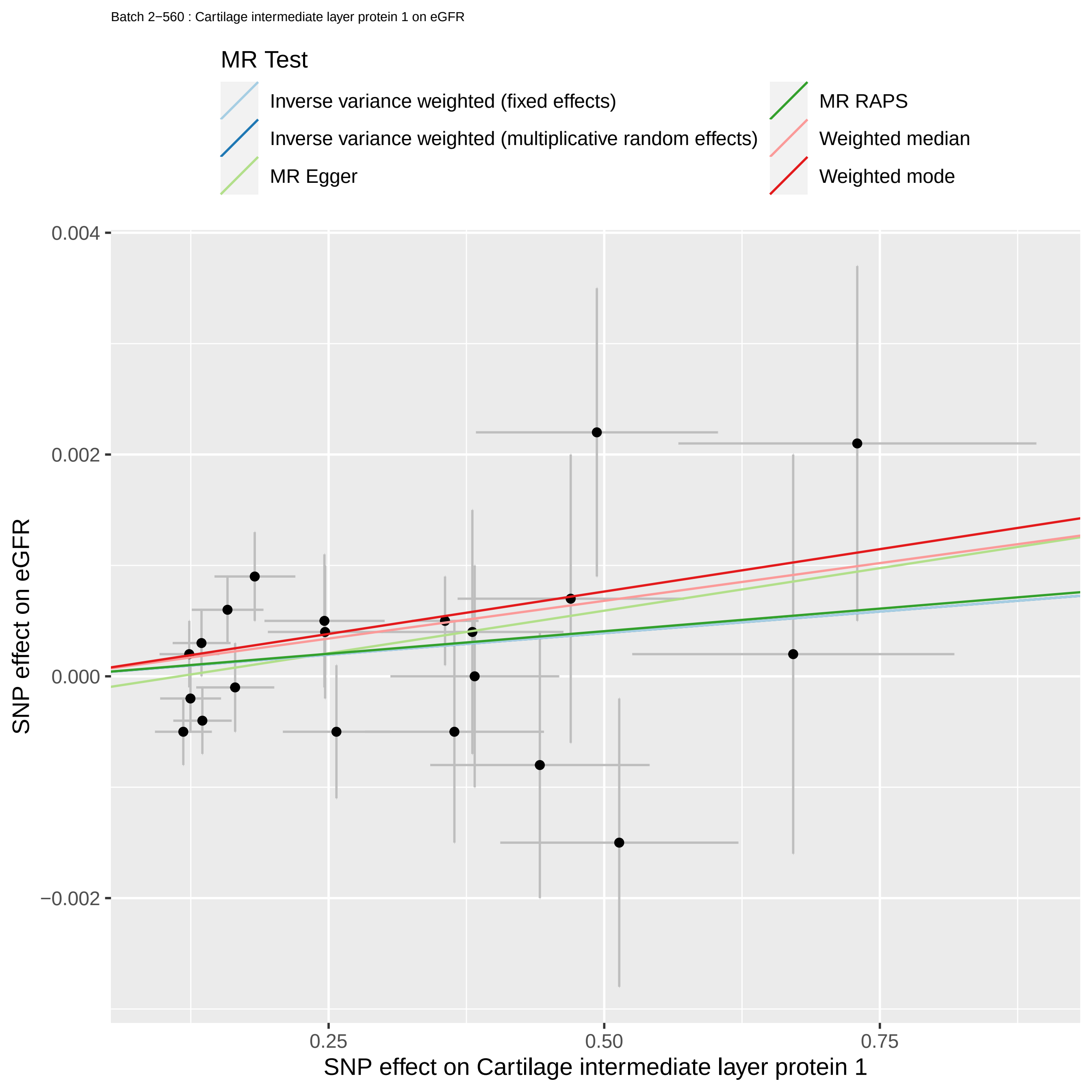

Batch 2−560 : Cartilage intermediate layer protein 1 on eGFR
MR Test
Inverse variance weighted (fixed effects)
Inverse variance weighted (multiplicative random effects)
MR Egger
MR RAPS
Weighted median
Weighted mode
0.004
0.002
0.000
−0.002
SNP effect on eGFR
0.25
0.50
0.75
SNP effect on Cartilage intermediate layer protein 1

Batch 2−561 : Cold−inducible RNA−binding protein on eGFR

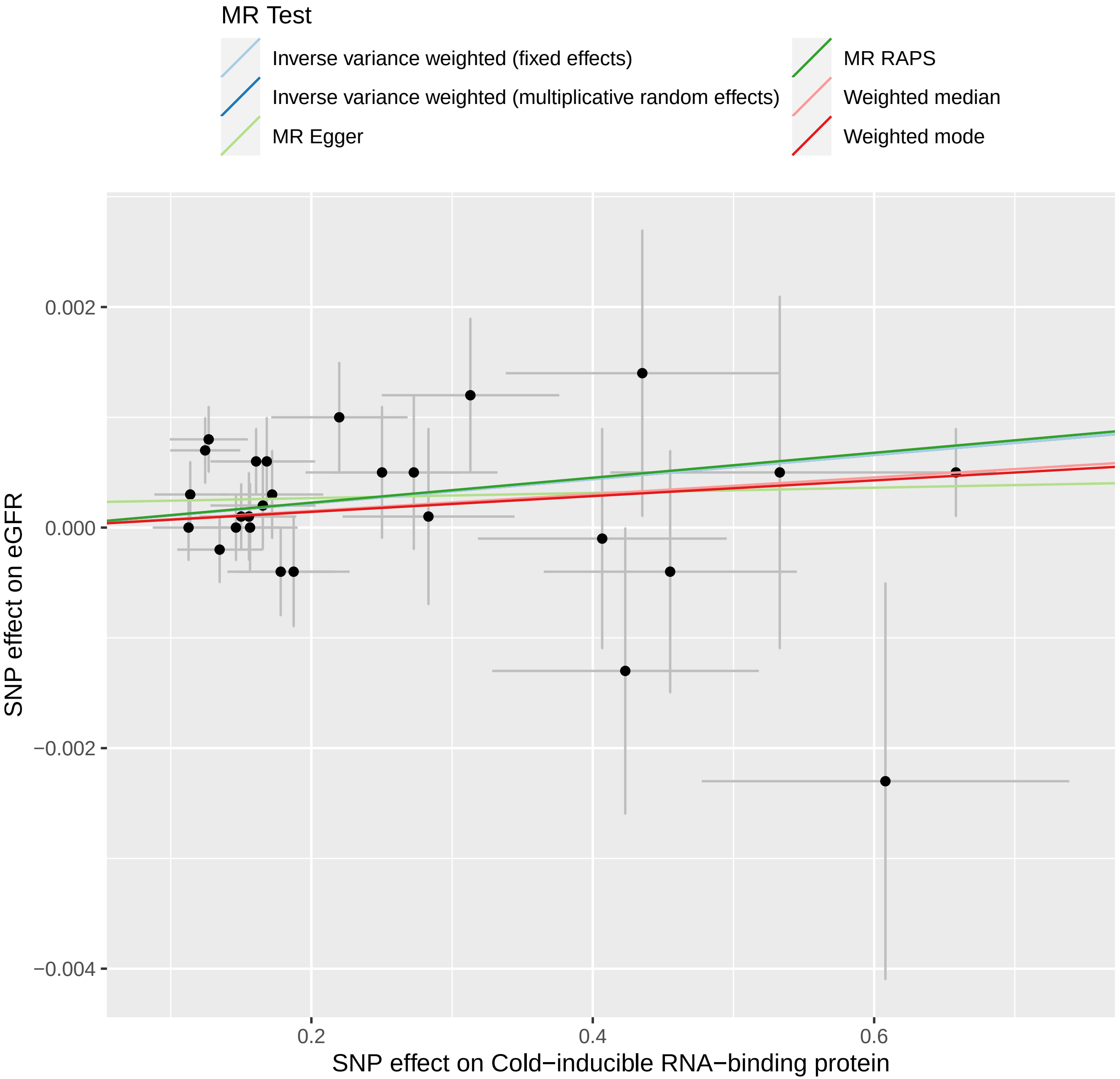

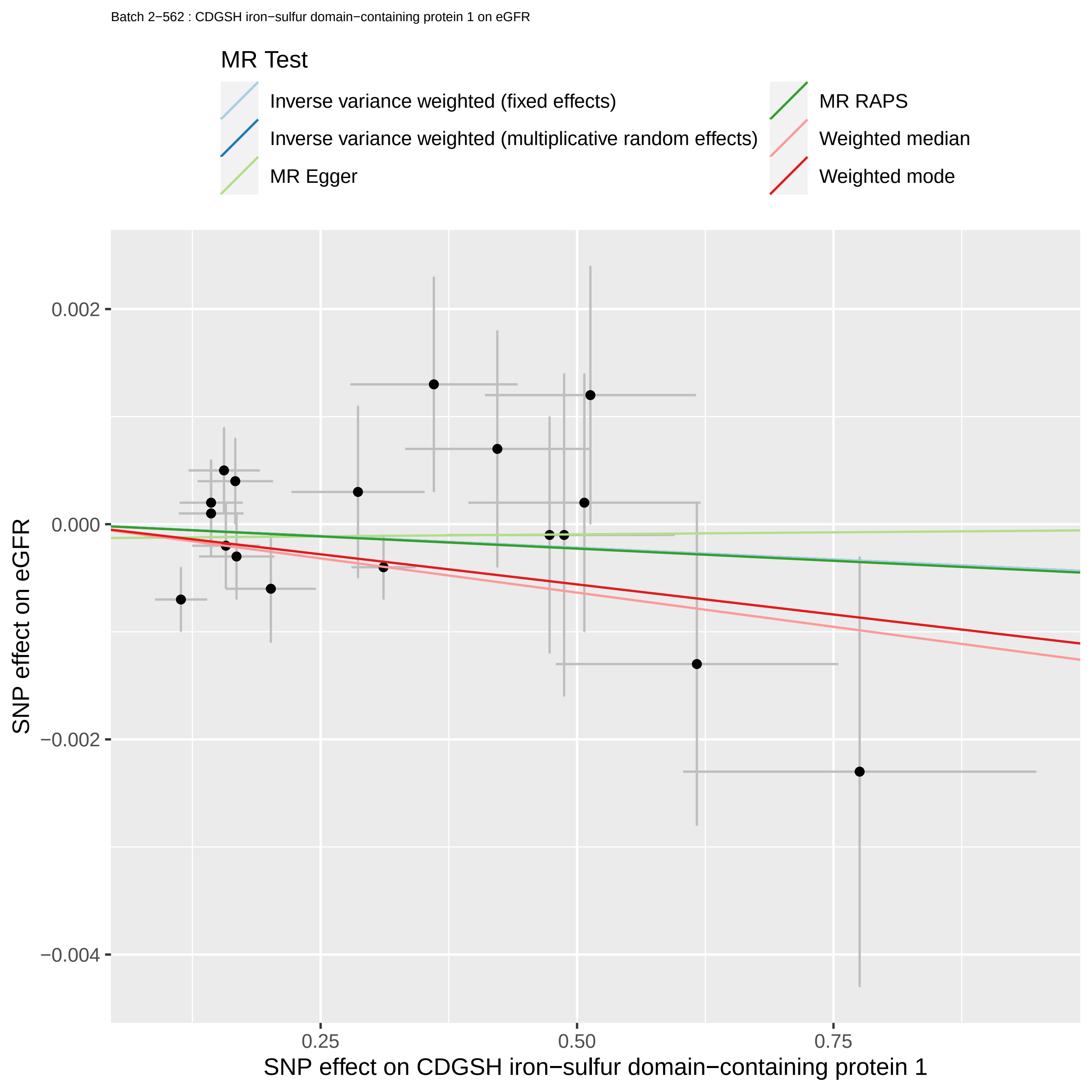
Batch 2−562 : CDGSH iron−sulfur domain−containing protein 1 on eGFR
MR Test
Inverse variance weighted (fixed effects)
Inverse variance weighted (multiplicative random effects)
MR Egger
MR RAPS
Weighted median
Weighted mode
0.002
0.000
−0.002
−0.004
SNP effect on eGFR
0.25
0.50
0.75
SNP effect on CDGSH iron−sulfur domain−containing protein 1

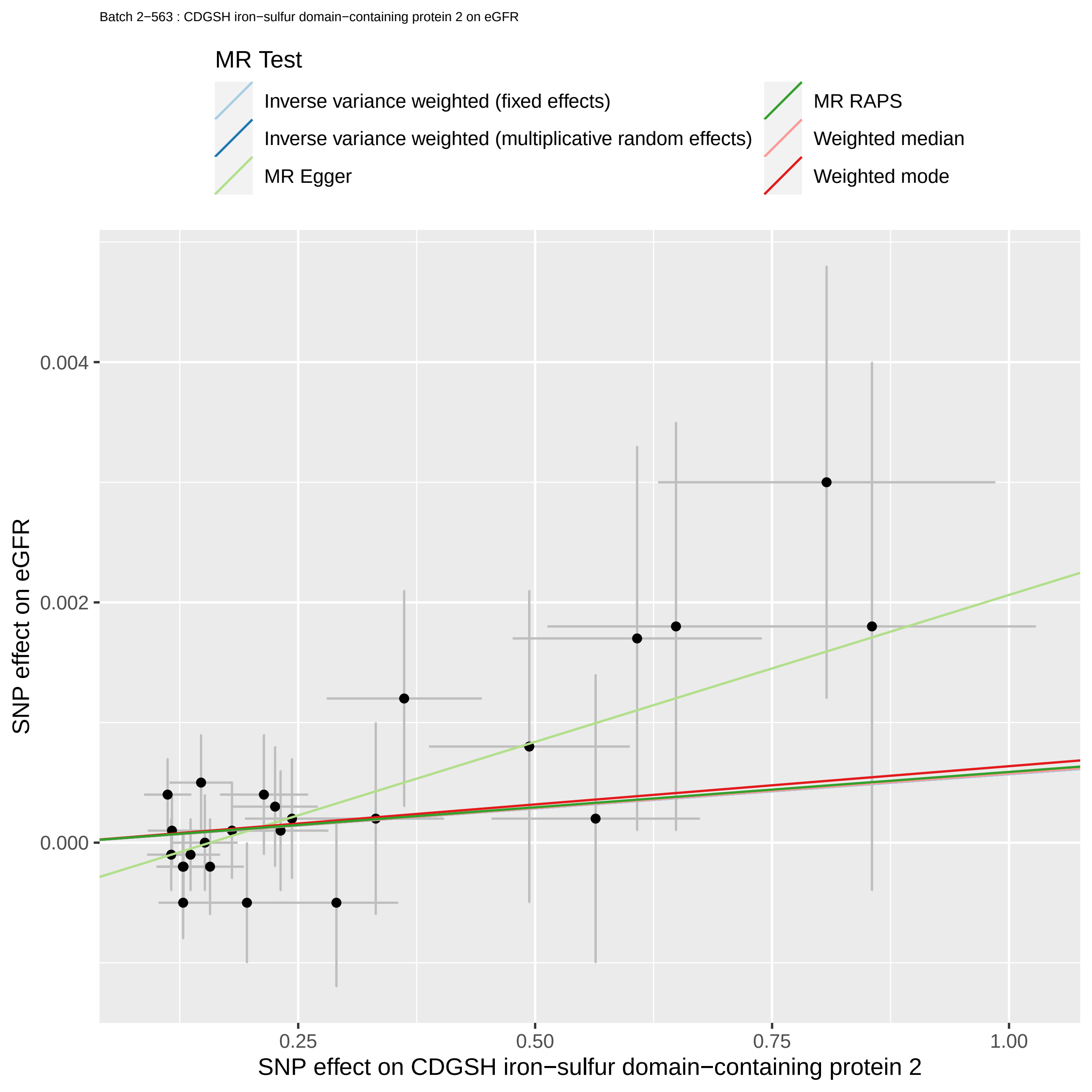
Batch 2−563 : CDGSH iron−sulfur domain−containing protein 2 on eGFR
MR Test
Inverse variance weighted (fixed effects)
Inverse variance weighted (multiplicative random effects)
MR Egger
MR RAPS
Weighted median
Weighted mode
0.004
0.002
0.000
SNP effect on eGFR
0.25
0.50
0.75
1.00
SNP effect on CDGSH iron−sulfur domain−containing protein 2

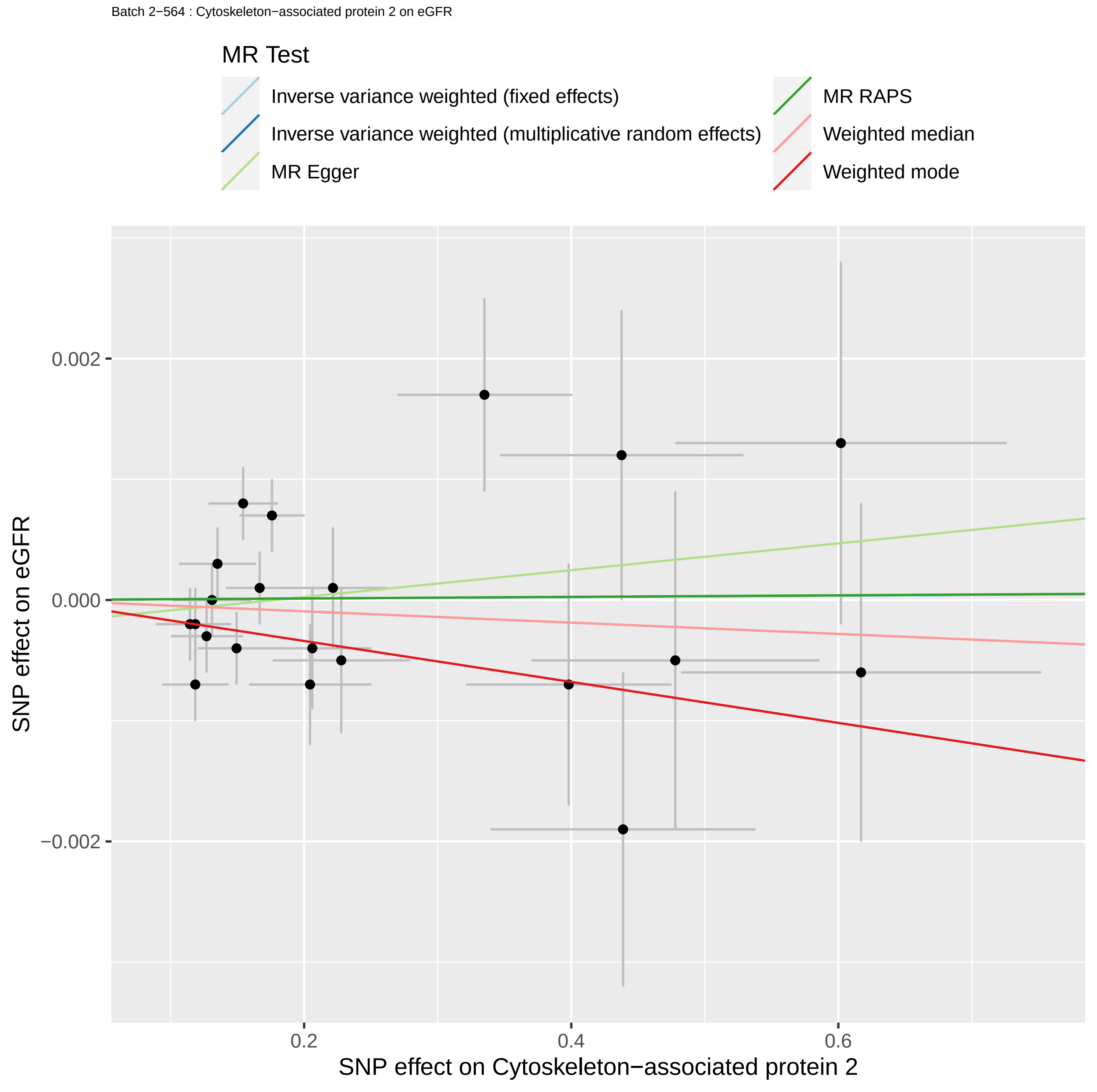
Batch 2−564 : Cytoskeleton−associated protein 2 on eGFR
MR Test
Inverse variance weighted (fixed effects)
Inverse variance weighted (multiplicative random effects)
MR Egger
MR RAPS
Weighted median
Weighted mode
0.002
0.000
−0.002
0.2
0.4
0.6
SNP effect on eGFR
SNP effect on Cytoskeleton−associated protein 2

Batch 2−565 : Creatine kinase B−type on eGFR

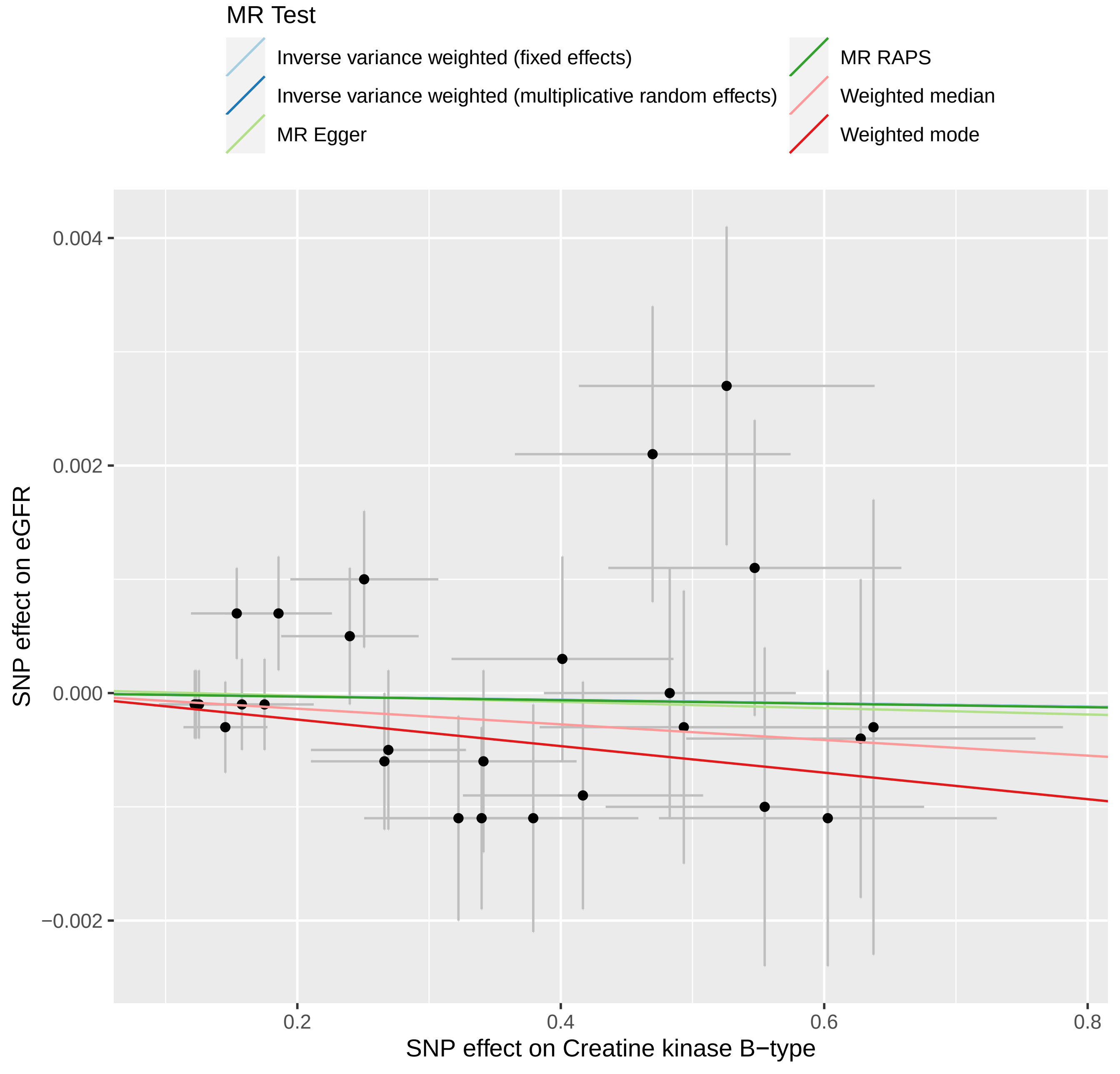

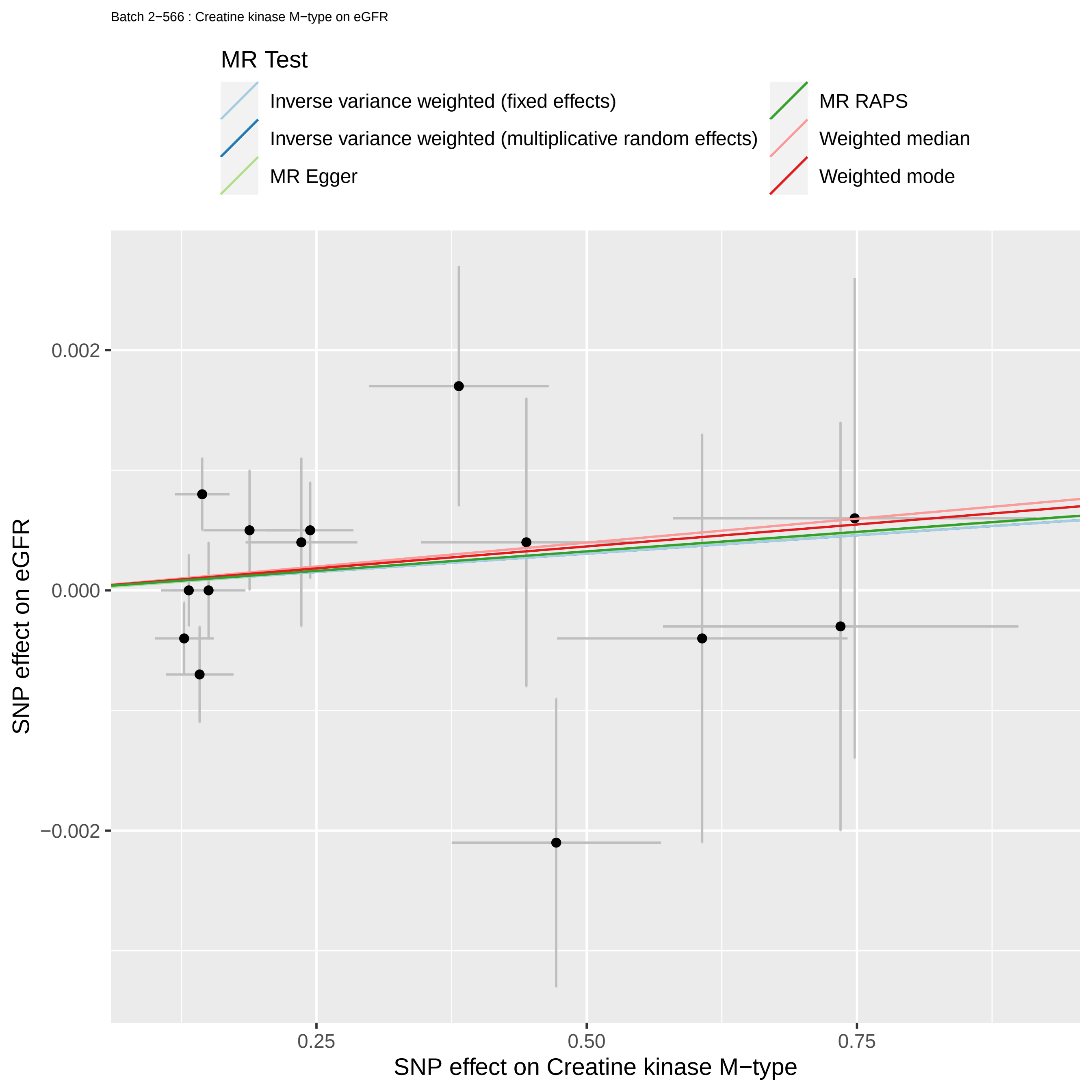

Batch 2−566 : Creatine kinase M−type on eGFR
MR Test
Inverse variance weighted (fixed effects)
Inverse variance weighted (multiplicative random effects)
MR Egger
MR RAPS
Weighted median
Weighted mode
0.002
0.000
−0.002
0.25
0.50
0.75
SNP effect on Creatine kinase M−type
SNP effect on eGFR

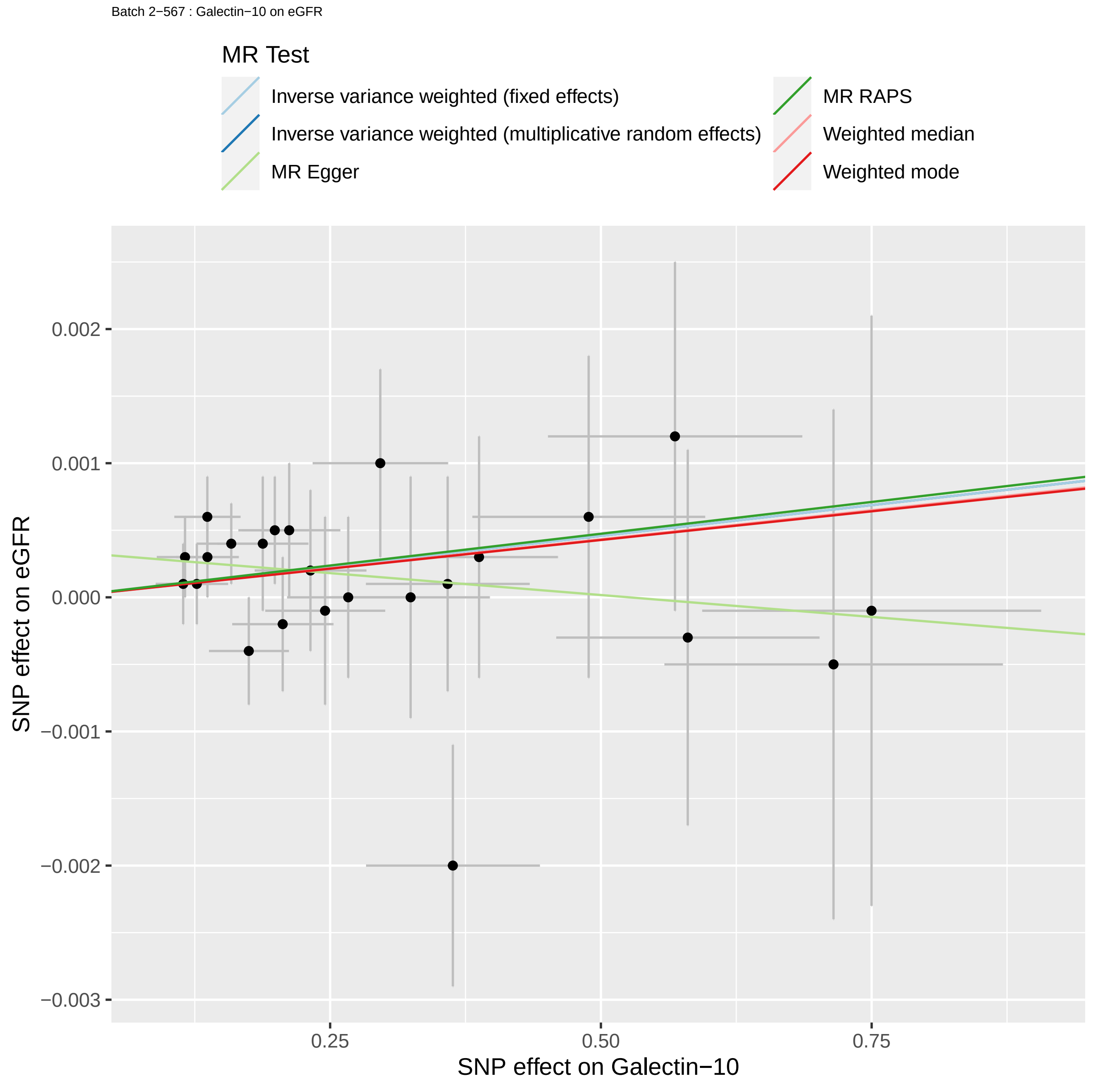
Batch 2−567 : Galectin−10 on eGFR
MR Test
Inverse variance weighted (fixed effects)
Inverse variance weighted (multiplicative random effects)
MR Egger
MR RAPS
Weighted median
Weighted mode
0.002
0.001
0.000
−0.001
−0.002
−0.003
0.25
0.50
0.75
SNP effect on Galectin−10
SNP effect on eGFR

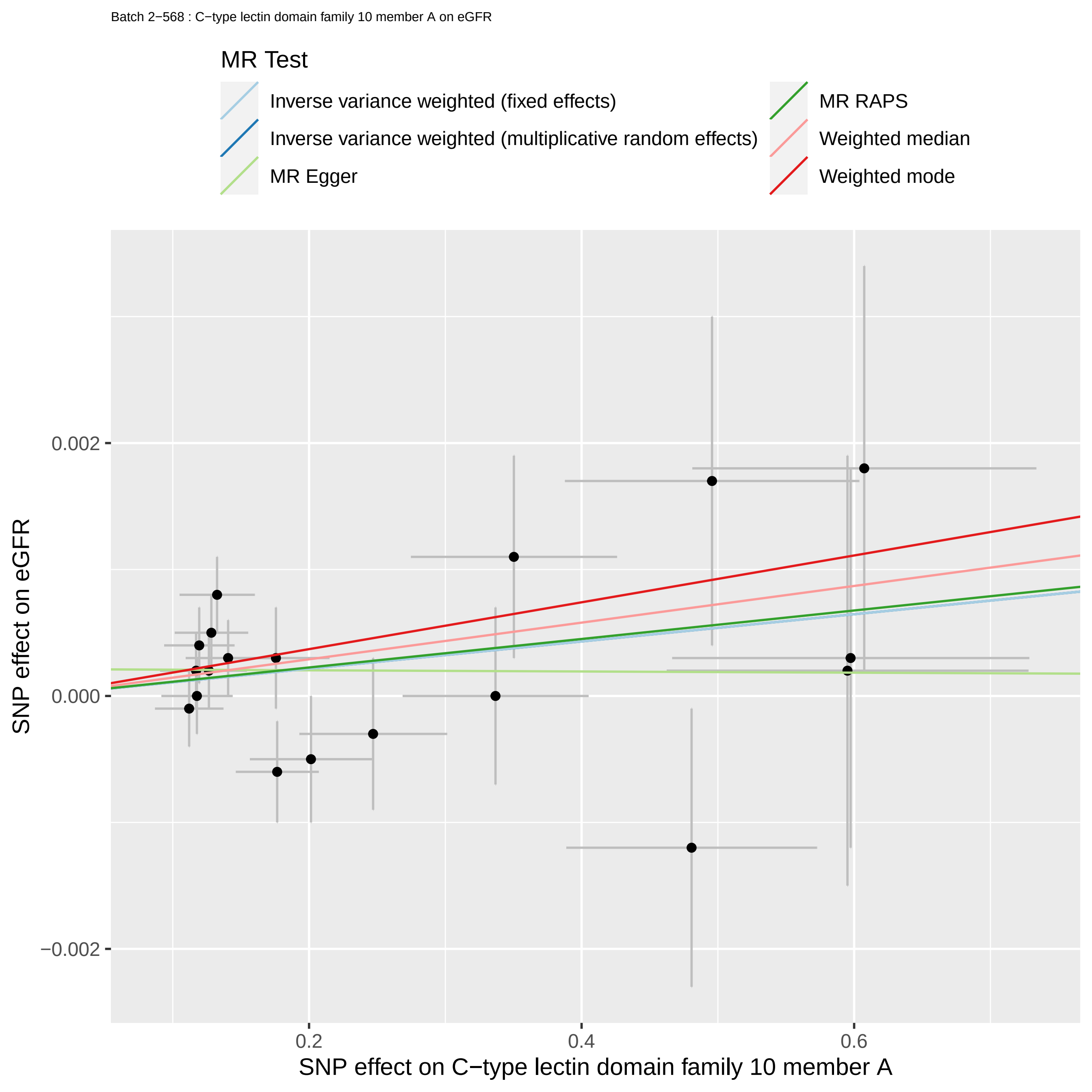

Batch 2−568 : C−type lectin domain family 10 member A on eGFR
MR Test
Inverse variance weighted (fixed effects)
Inverse variance weighted (multiplicative random effects)
MR Egger
MR RAPS
Weighted median
Weighted mode
0.002
0.000
−0.002
0.2
0.4
0.6
SNP effect on eGFR
SNP effect on C−type lectin domain family 10 member A

Batch 2−569 : Stem Cell Growth Factor−beta on eGFR

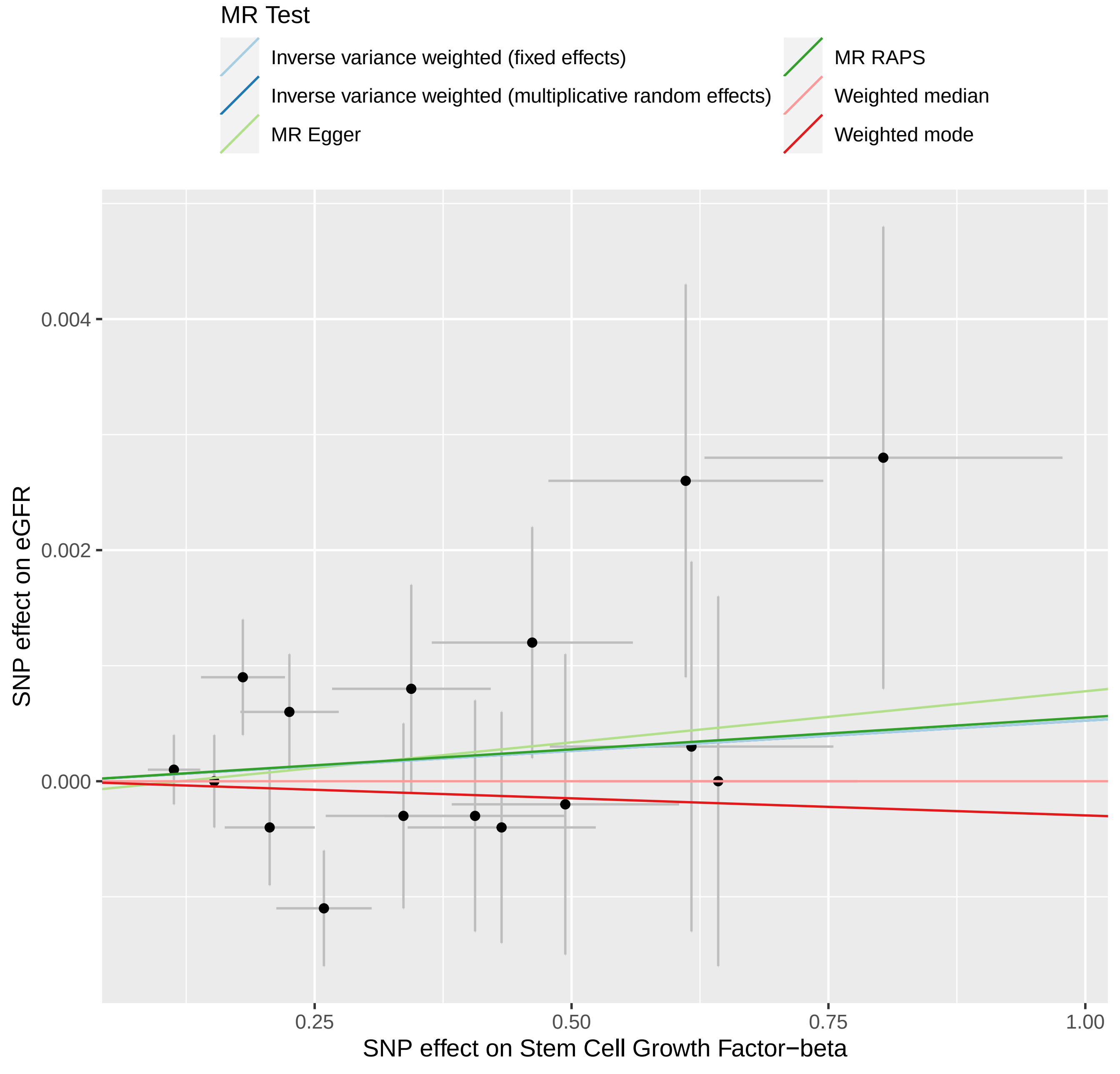

Batch 2−570 : C−type lectin domain family 12 member A on eGFR

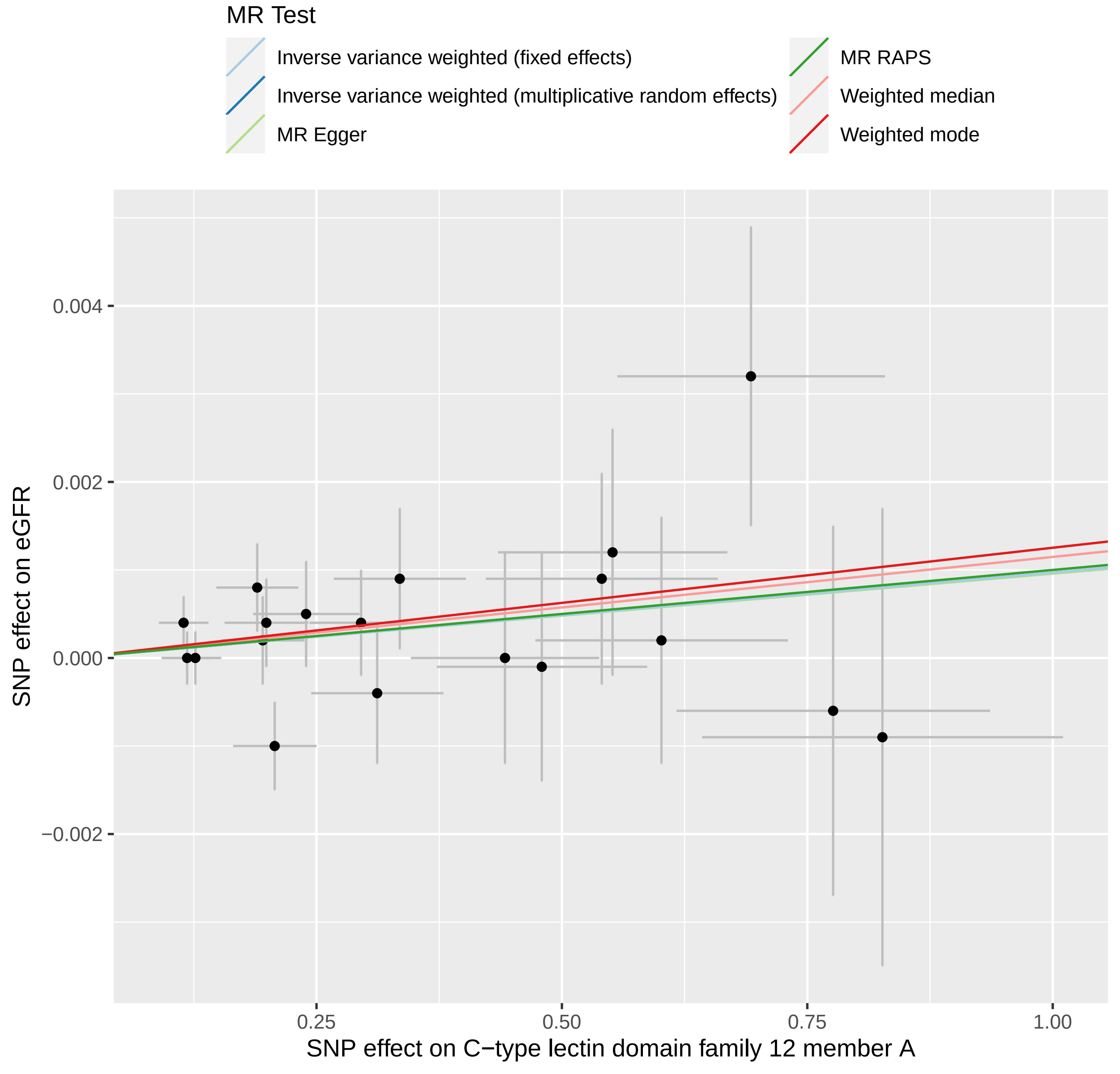

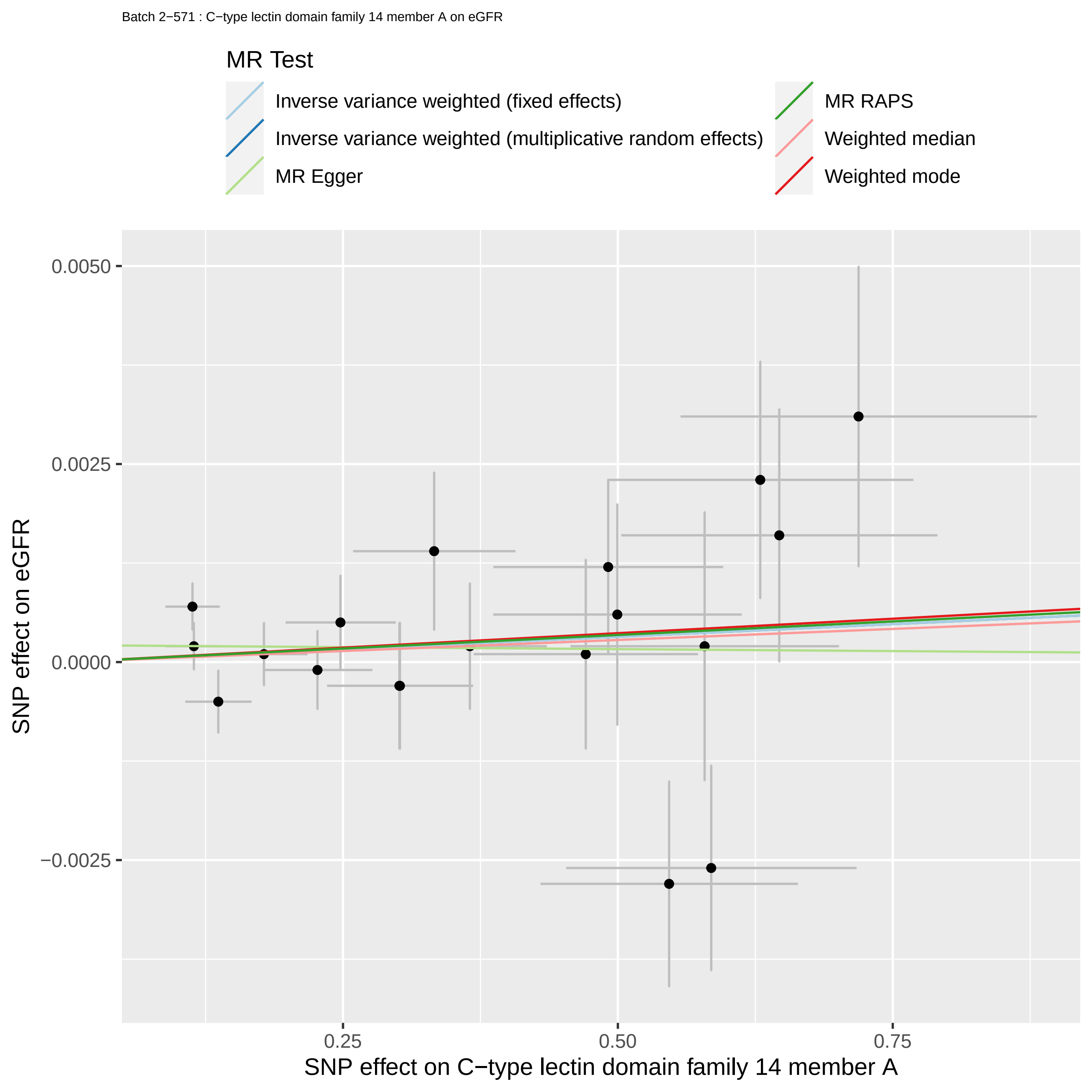
Batch 2−571 : C−type lectin domain family 14 member A on eGFR
MR Test
Inverse variance weighted (fixed effects)
Inverse variance weighted (multiplicative random effects)
MR Egger
MR RAPS
Weighted median
Weighted mode
0.0050
0.0025
0.0000
−0.0025
0.25
0.50
0.75
SNP effect on eGFR
SNP effect on C−type lectin domain family 14 member A

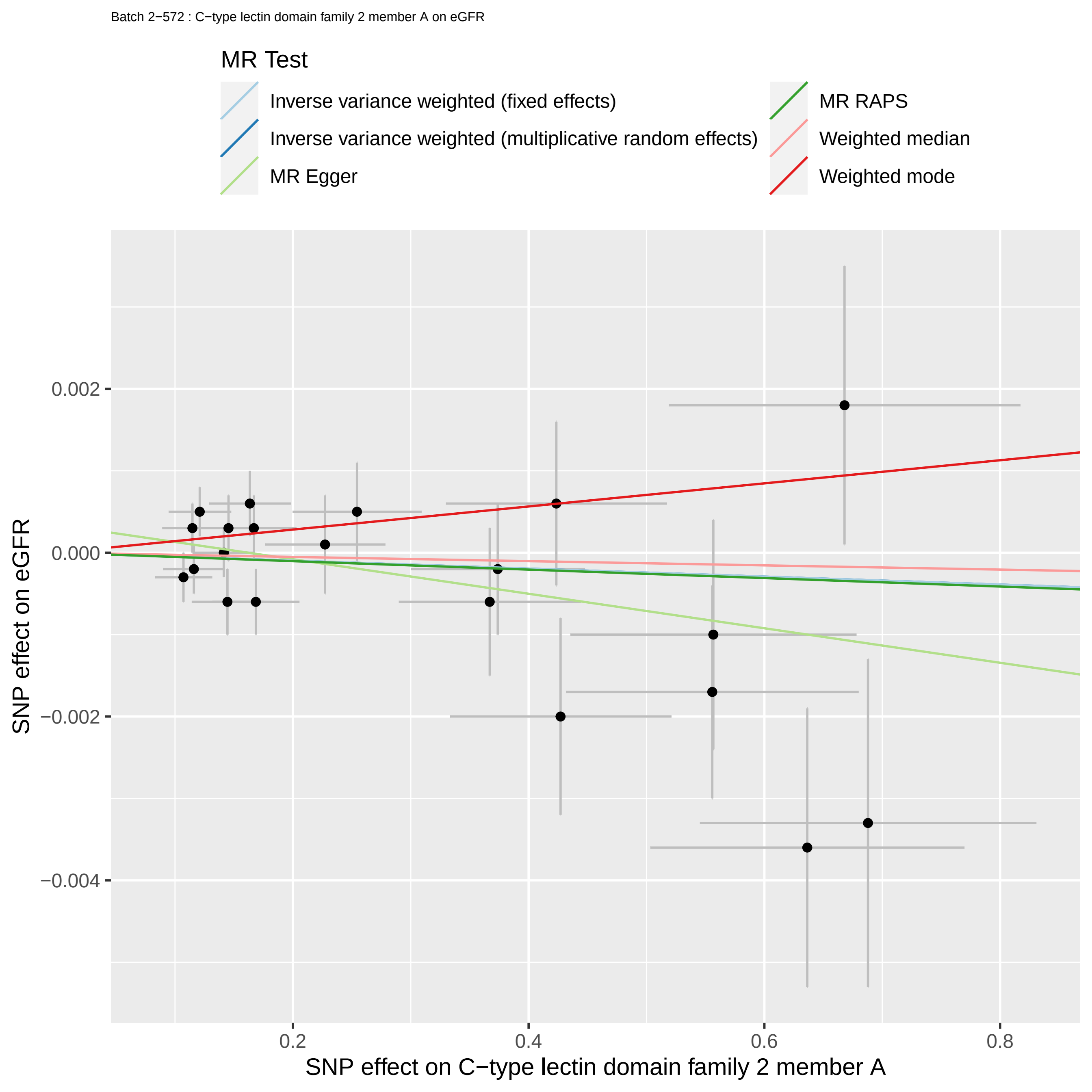
Batch 2−572 : C−type lectin domain family 2 member A on eGFR
MR Test
Inverse variance weighted (fixed effects)
Inverse variance weighted (multiplicative random effects)
MR Egger
MR RAPS
Weighted median
Weighted mode
0.002
0.000
−0.002
−0.004
0.2
0.4
0.6
0.8
SNP effect on eGFR
SNP effect on C−type lectin domain family 2 member A

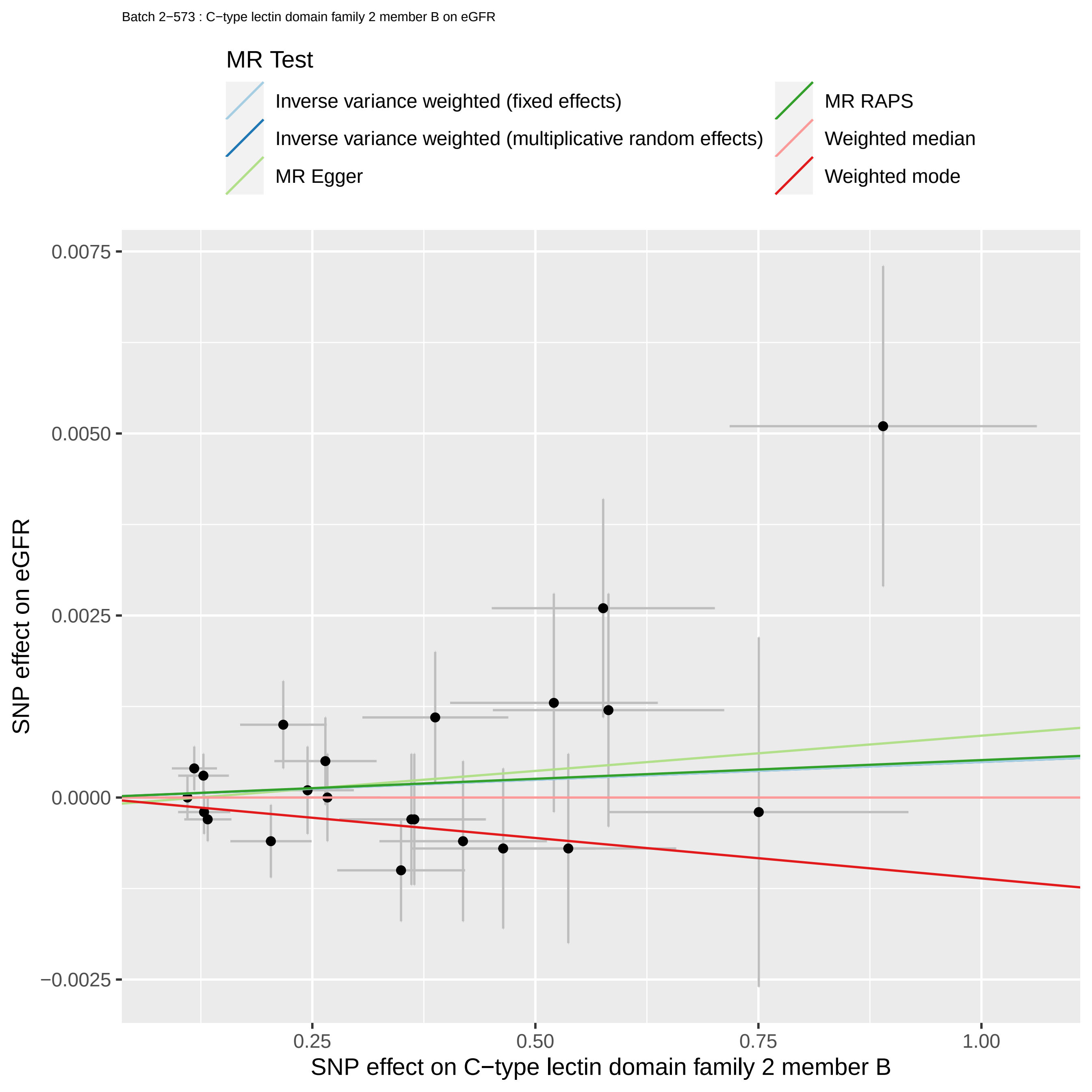
Batch 2−573 : C−type lectin domain family 2 member B on eGFR
MR Test
Inverse variance weighted (fixed effects)
Inverse variance weighted (multiplicative random effects)
MR Egger
MR RAPS
Weighted median
Weighted mode
0.0075
0.0050
0.0025
0.0000
−0.0025
SNP effect on eGFR
0.25
0.50
0.75
1.00
SNP effect on C−type lectin domain family 2 member B

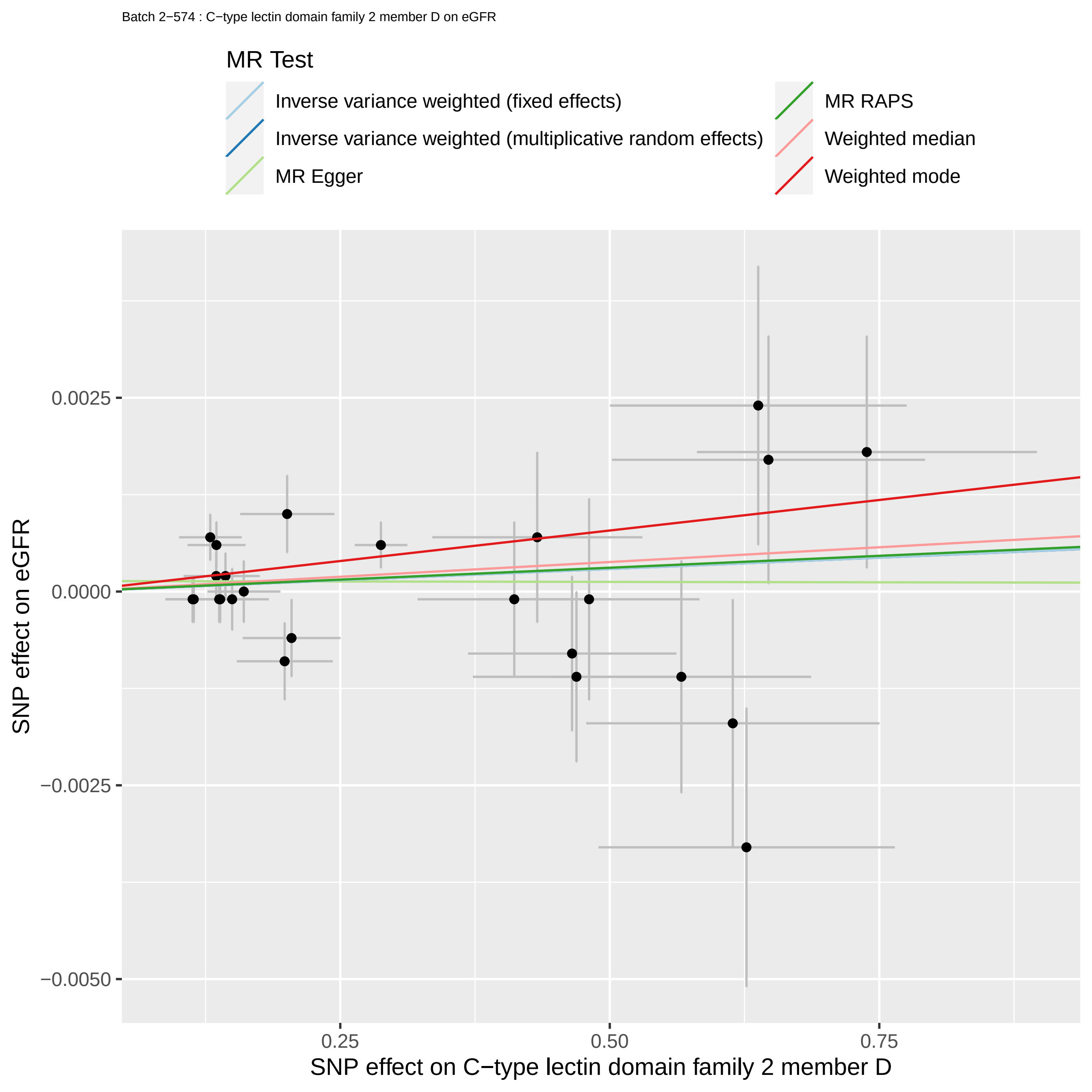

Batch 2−574 : C−type lectin domain family 2 member D on eGFR
MR Test
Inverse variance weighted (fixed effects)
Inverse variance weighted (multiplicative random effects)
MR Egger
MR RAPS
Weighted median
Weighted mode
0.0025
0.0000
−0.0025
−0.0050
0.25
0.50
0.75
SNP effect on eGFR
SNP effect on C−type lectin domain family 2 member D

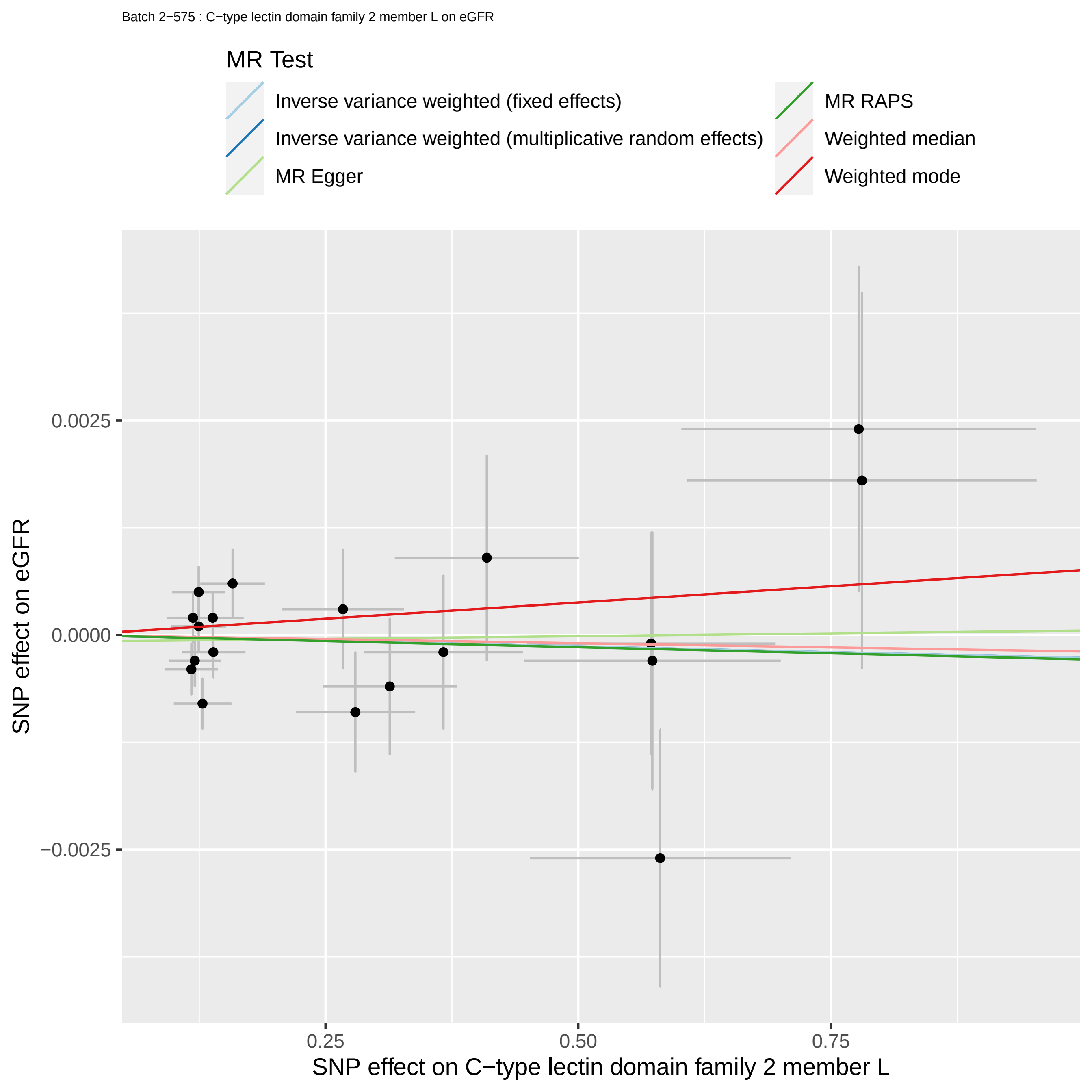
Batch 2−575 : C−type lectin domain family 2 member L on eGFR
MR Test
Inverse variance weighted (fixed effects)
Inverse variance weighted (multiplicative random effects)
MR Egger
MR RAPS
Weighted median
Weighted mode
0.0025
0.0000
−0.0025
0.25
0.50
0.75
SNP effect on eGFR
SNP effect on C−type lectin domain family 2 member L

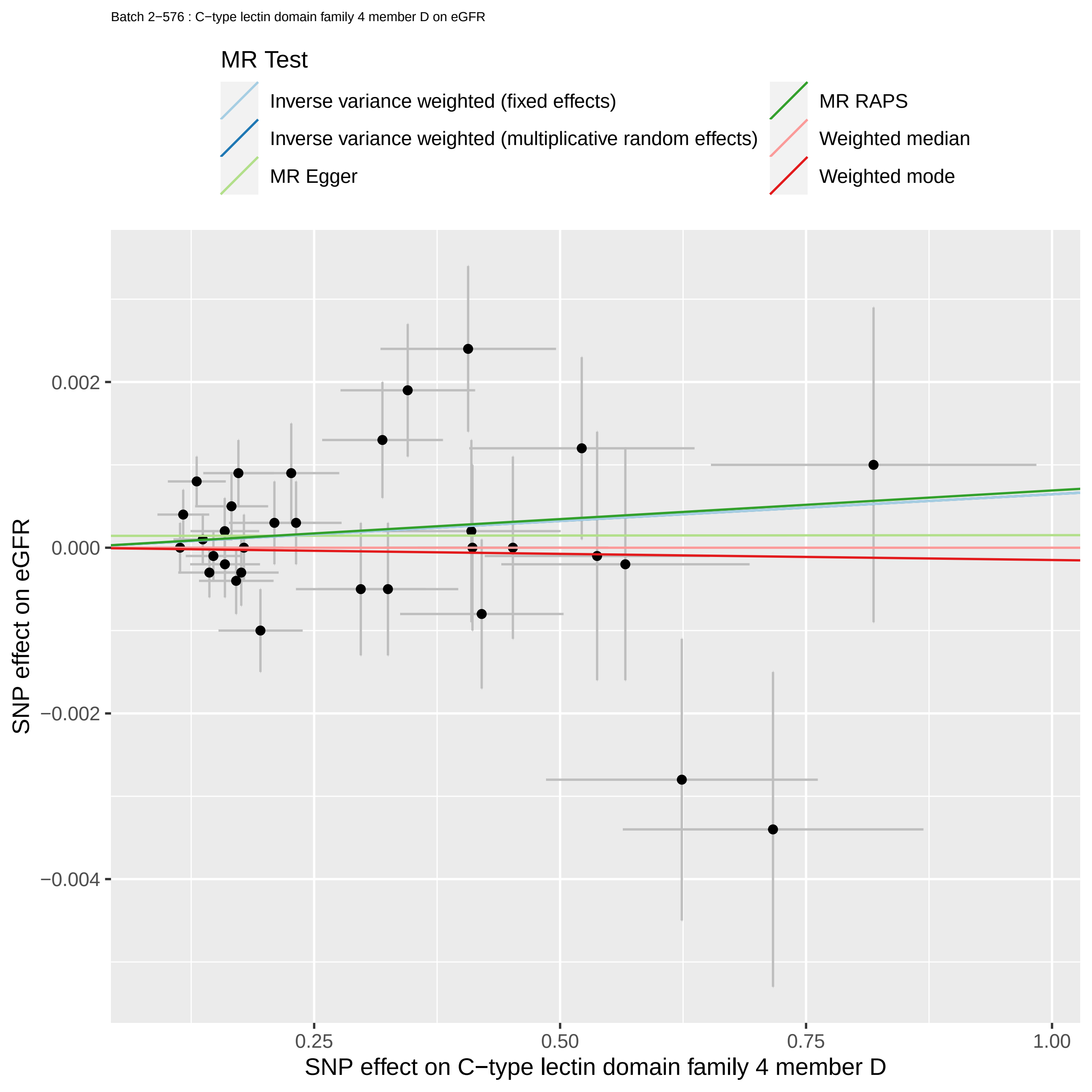
Batch 2−576 : C−type lectin domain family 4 member D on eGFR
MR Test
Inverse variance weighted (fixed effects)
Inverse variance weighted (multiplicative random effects)
MR Egger
MR RAPS
Weighted median
Weighted mode
0.002
0.000
−0.002
−0.004
SNP effect on eGFR
0.25
0.50
0.75
1.00
SNP effect on C−type lectin domain family 4 member D

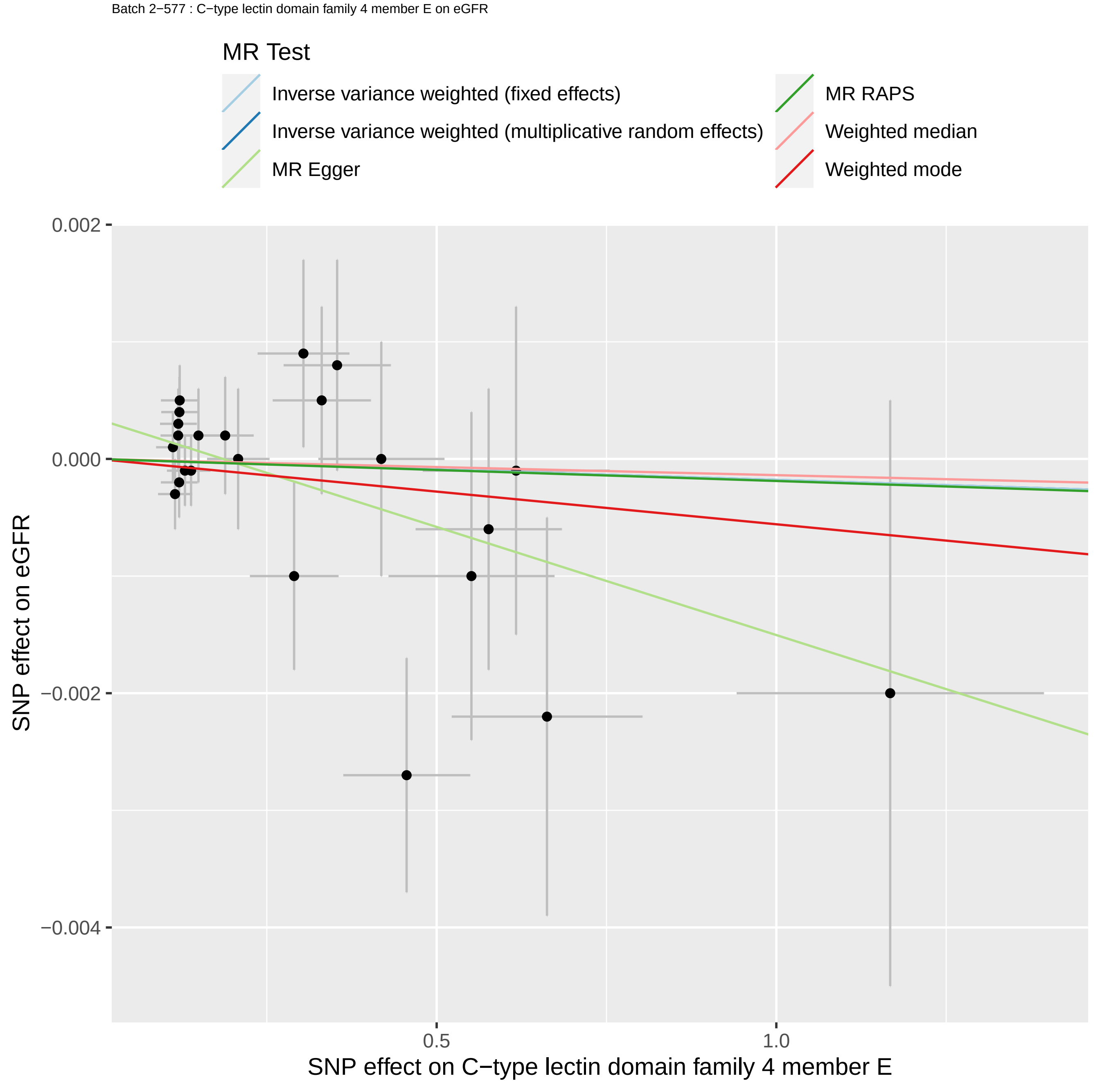

Batch 2−577 : C−type lectin domain family 4 member E on eGFR
MR Test
Inverse variance weighted (fixed effects)
Inverse variance weighted (multiplicative random effects)
MR Egger
MR RAPS
Weighted median
Weighted mode
0.002
0.000
−0.002
−0.004
0.5
1.0
SNP effect on eGFR
SNP effect on C−type lectin domain family 4 member E

Batch 2−578 : C−type lectin domain family 4 member M on eGFR

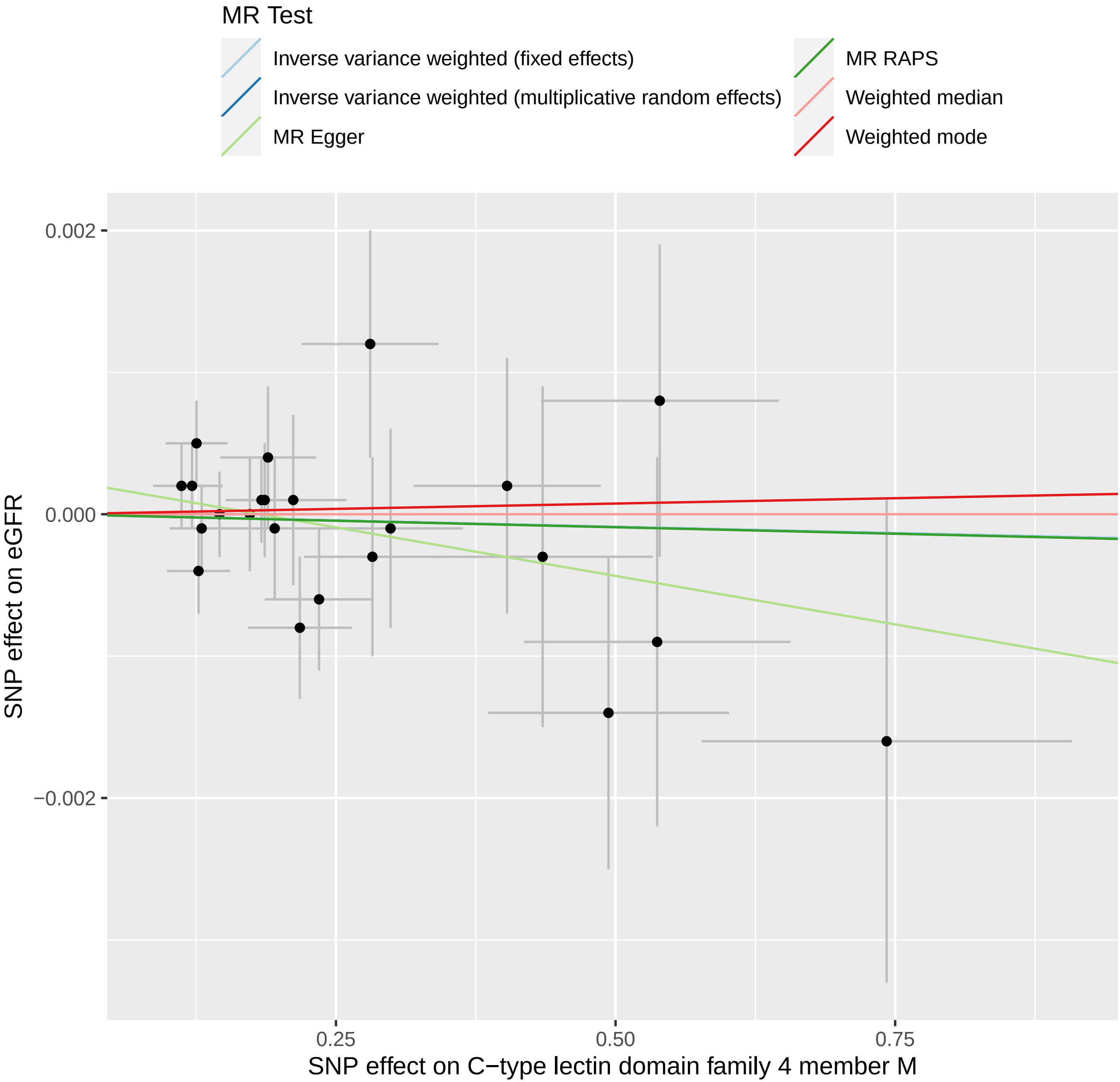

Batch 2−579 : C−type lectin domain family 5 member A on eGFR

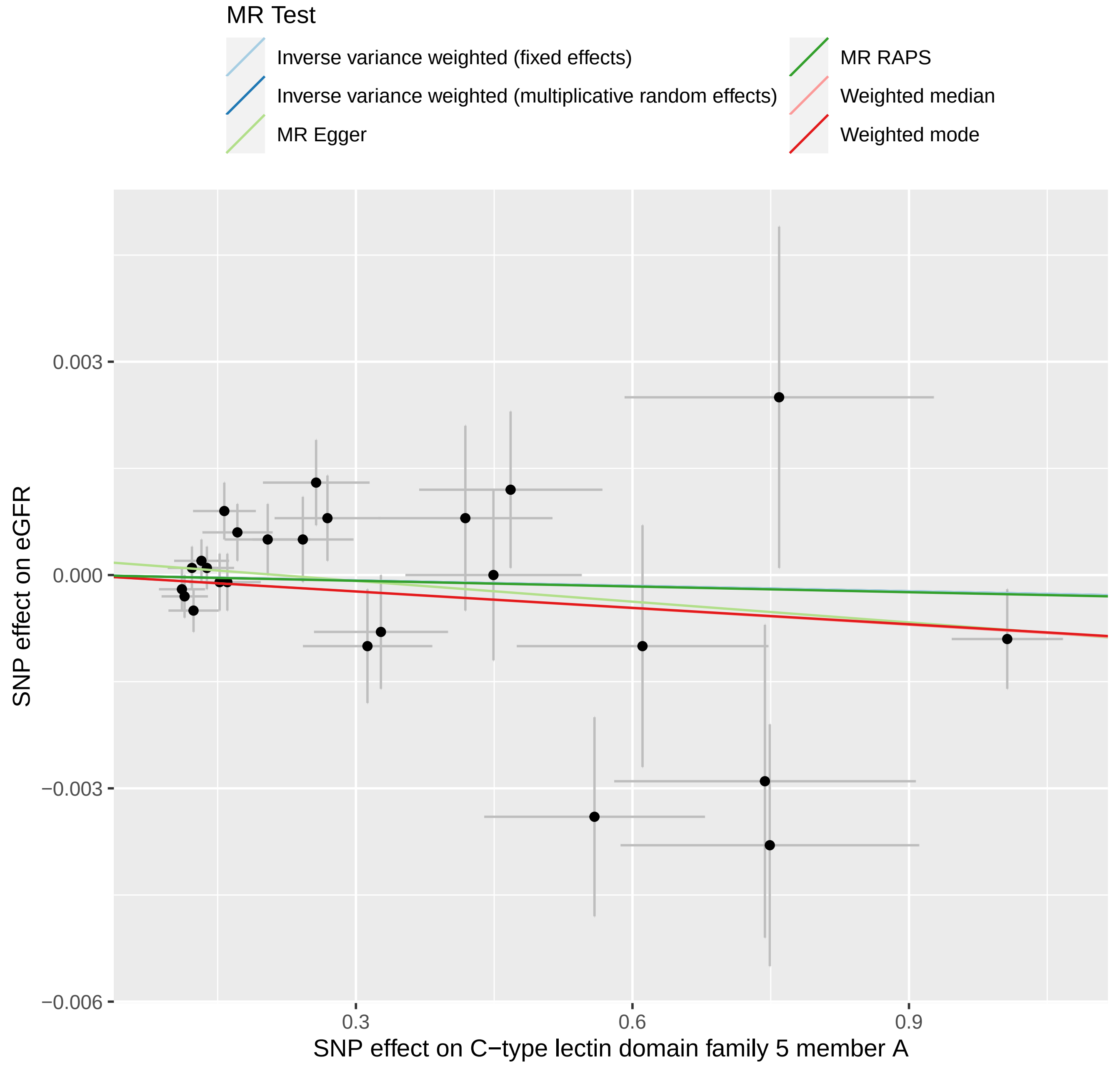

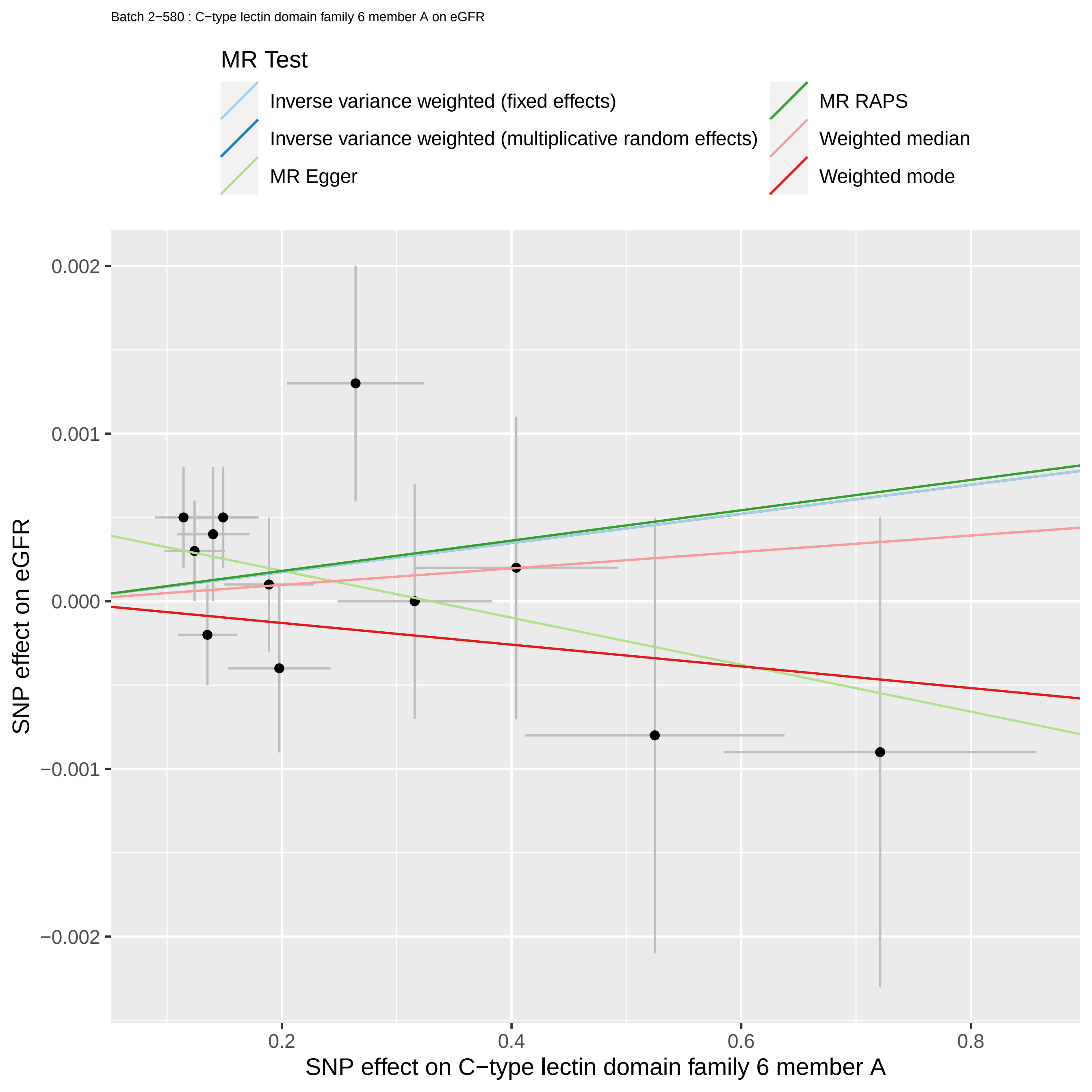

Batch 2−580 : C−type lectin domain family 6 member A on eGFR
MR Test
Inverse variance weighted (fixed effects)
Inverse variance weighted (multiplicative random effects)
MR Egger
MR RAPS
Weighted median
Weighted mode
0.002
0.001
0.000
−0.001
−0.002
SNP effect on eGFR
0.2
0.4
0.6
0.8
SNP effect on C−type lectin domain family 6 member A

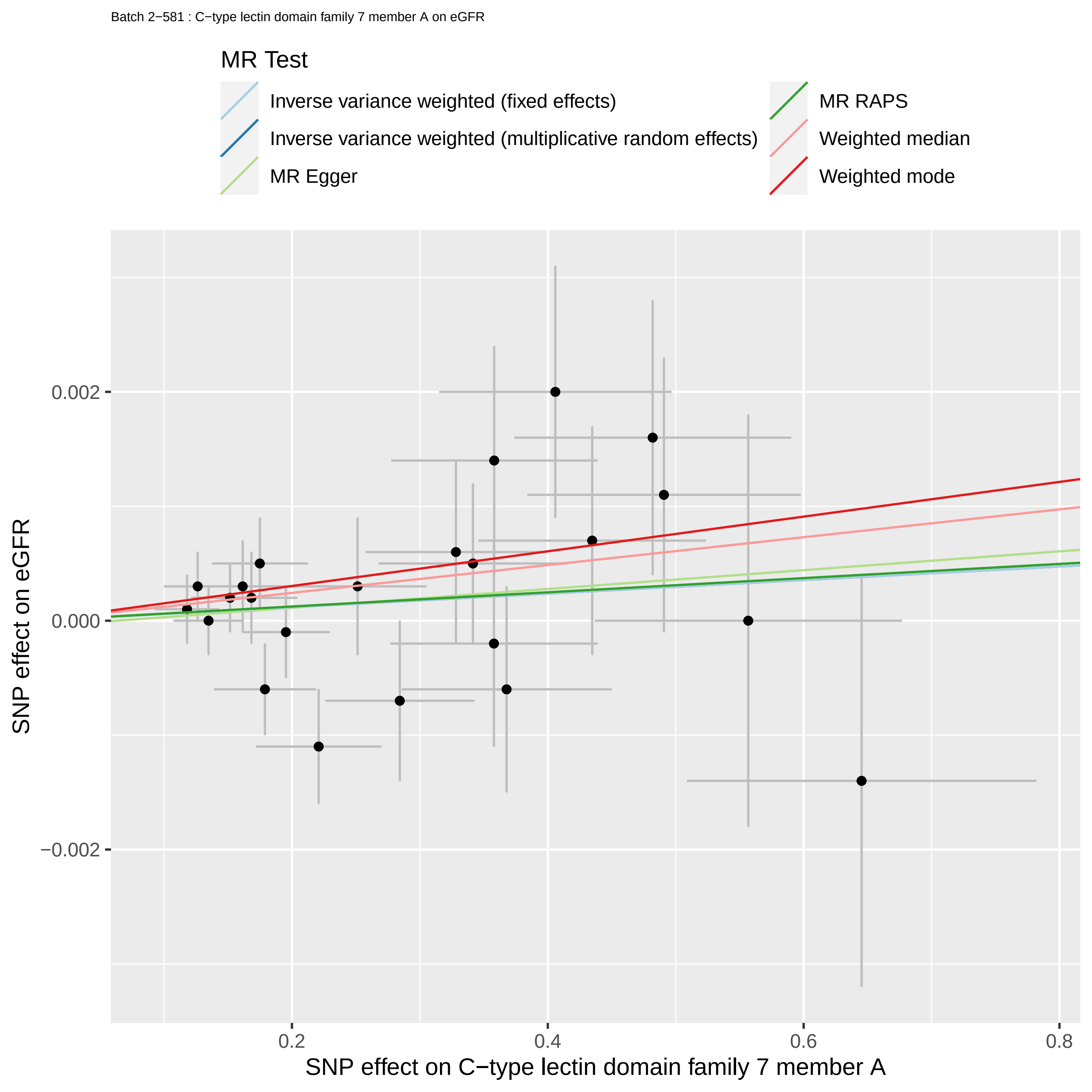

Batch 2−581 : C−type lectin domain family 7 member A on eGFR
MR Test
Inverse variance weighted (fixed effects)
Inverse variance weighted (multiplicative random effects)
MR Egger
MR RAPS
Weighted median
Weighted mode
0.002
0.000
−0.002
0.2
0.4
0.6
0.8
SNP effect on eGFR
SNP effect on C−type lectin domain family 7 member A

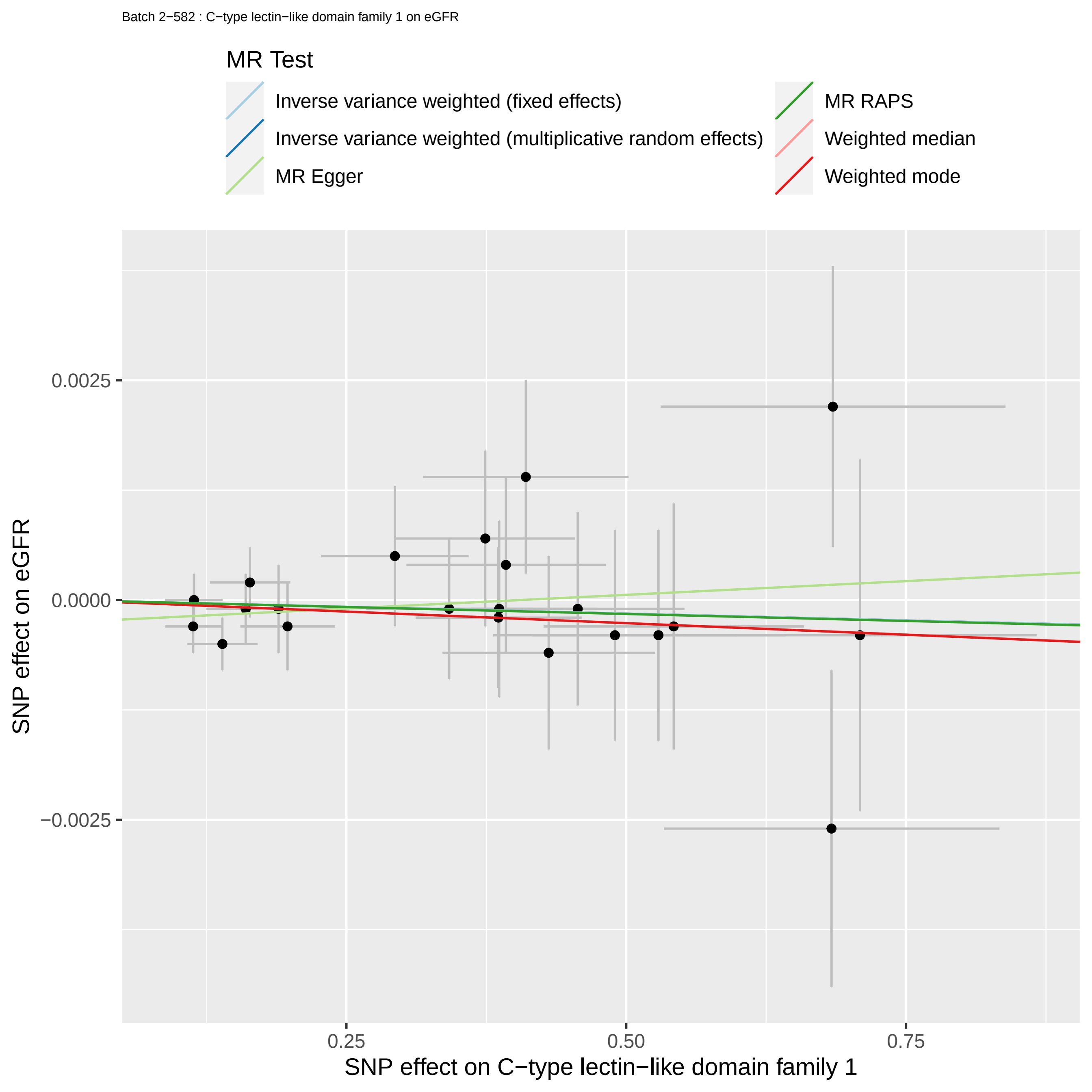

Batch 2−582 : C−type lectin−like domain family 1 on eGFR
MR Test
Inverse variance weighted (fixed effects)
Inverse variance weighted (multiplicative random effects)
MR Egger
MR RAPS
Weighted median
Weighted mode
0.0025
0.0000
−0.0025
0.25
0.50
0.75
SNP effect on eGFR
SNP effect on C−type lectin−like domain family 1

Batch 2−583 : Chloride intracellular channel protein 4 on eGFR

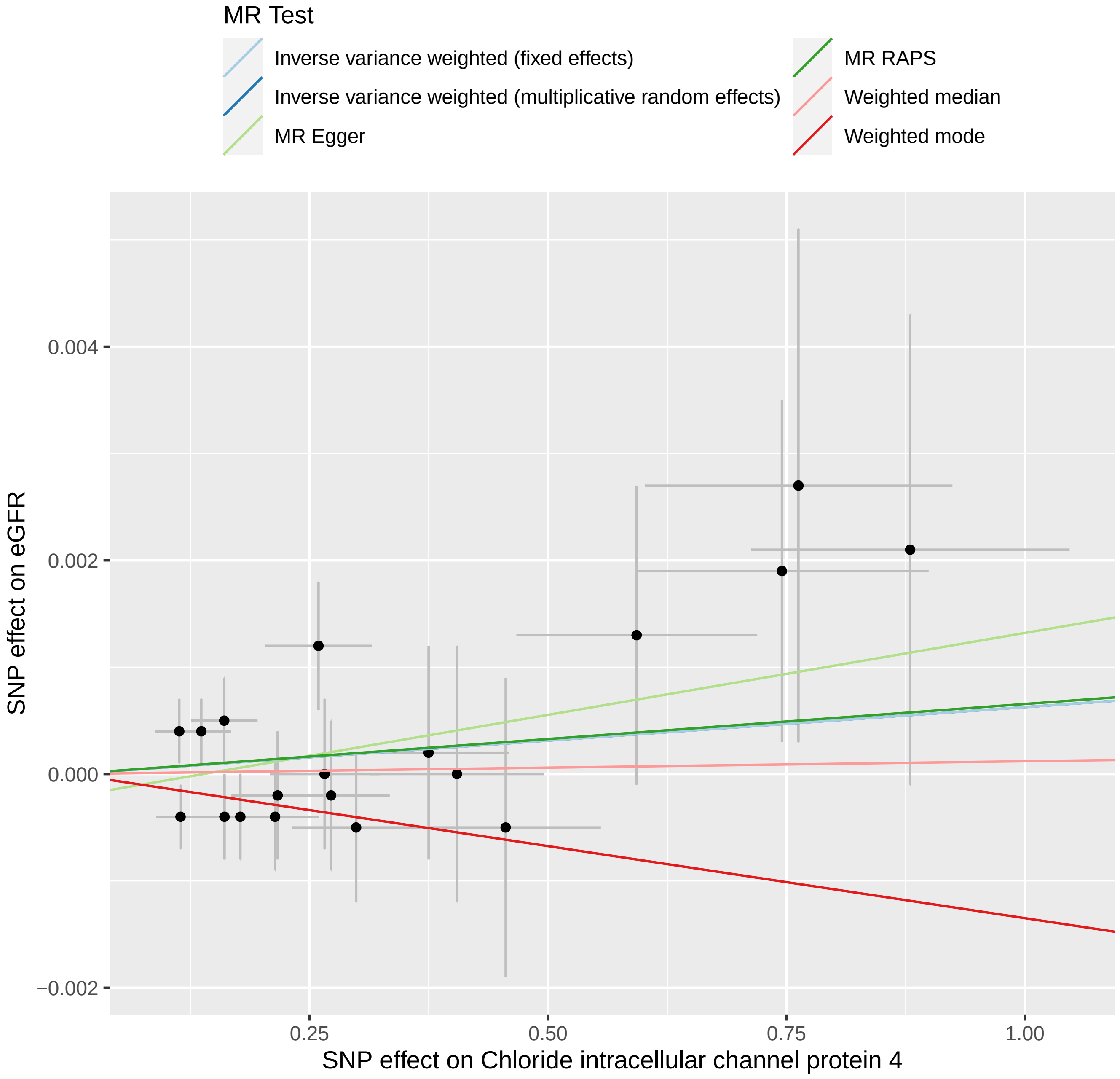

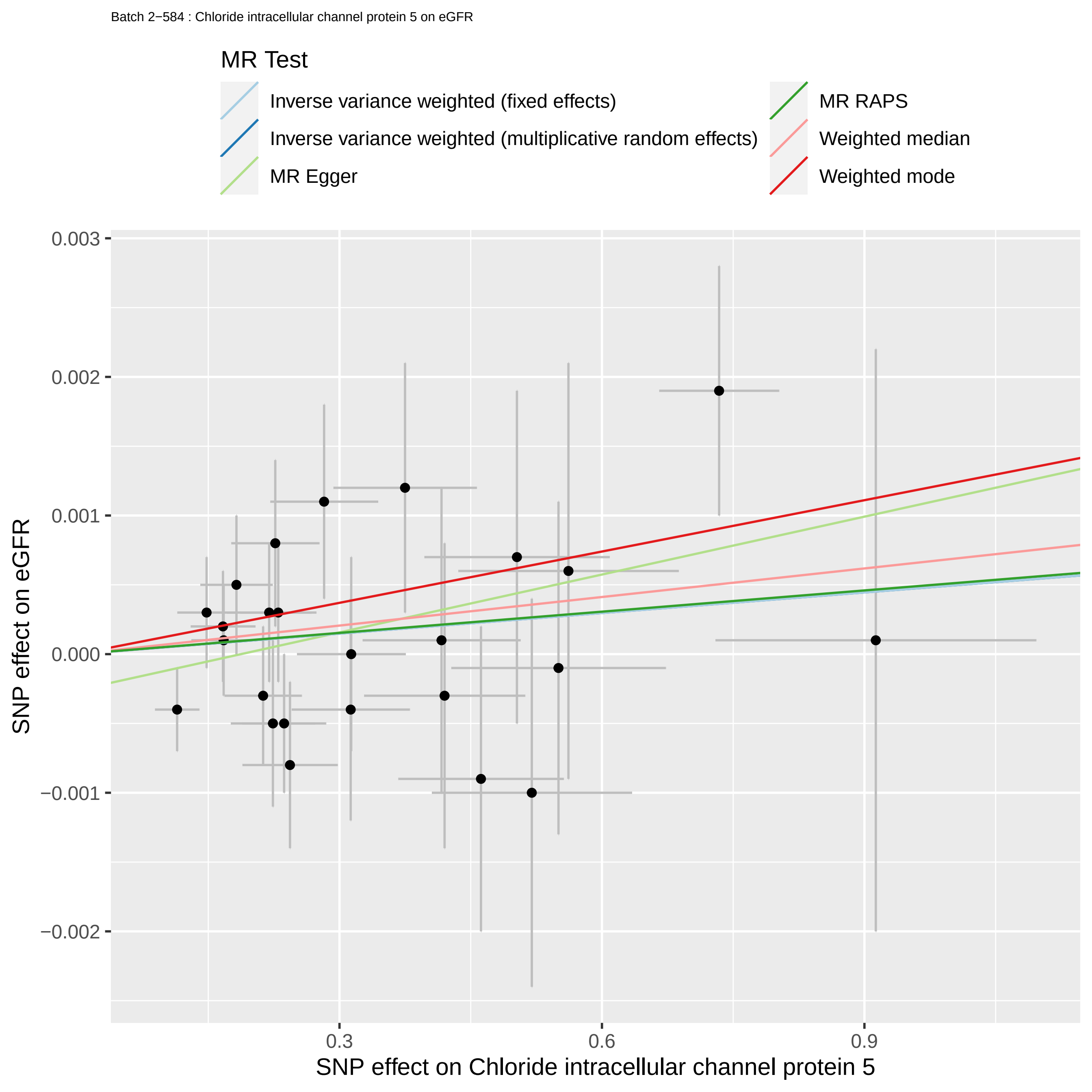

Batch 2−584 : Chloride intracellular channel protein 5 on eGFR
MR Test
Inverse variance weighted (fixed effects)
Inverse variance weighted (multiplicative random effects)
MR Egger
MR RAPS
Weighted median
Weighted mode
0.003
0.002
0.001
0.000
−0.001
−0.002
SNP effect on eGFR
0.3
0.6
0.9
SNP effect on Chloride intracellular channel protein 5

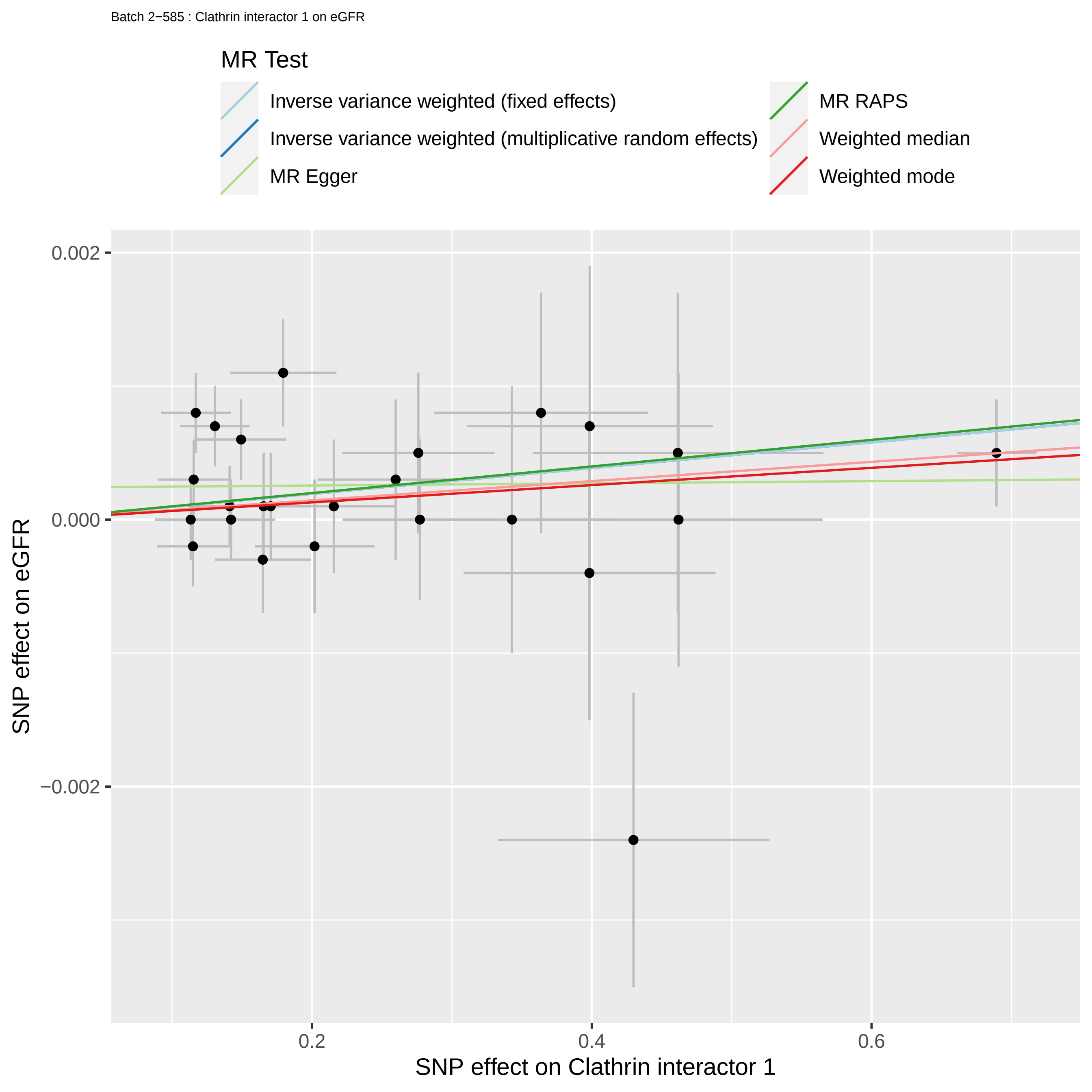
Batch 2−585 : Clathrin interactor 1 on eGFR
MR Test
Inverse variance weighted (fixed effects)
Inverse variance weighted (multiplicative random effects)
MR Egger
MR RAPS
Weighted median
Weighted mode
0.002
0.000
−0.002
0.2
0.4
0.6
SNP effect on eGFR
SNP effect on Clathrin interactor 1

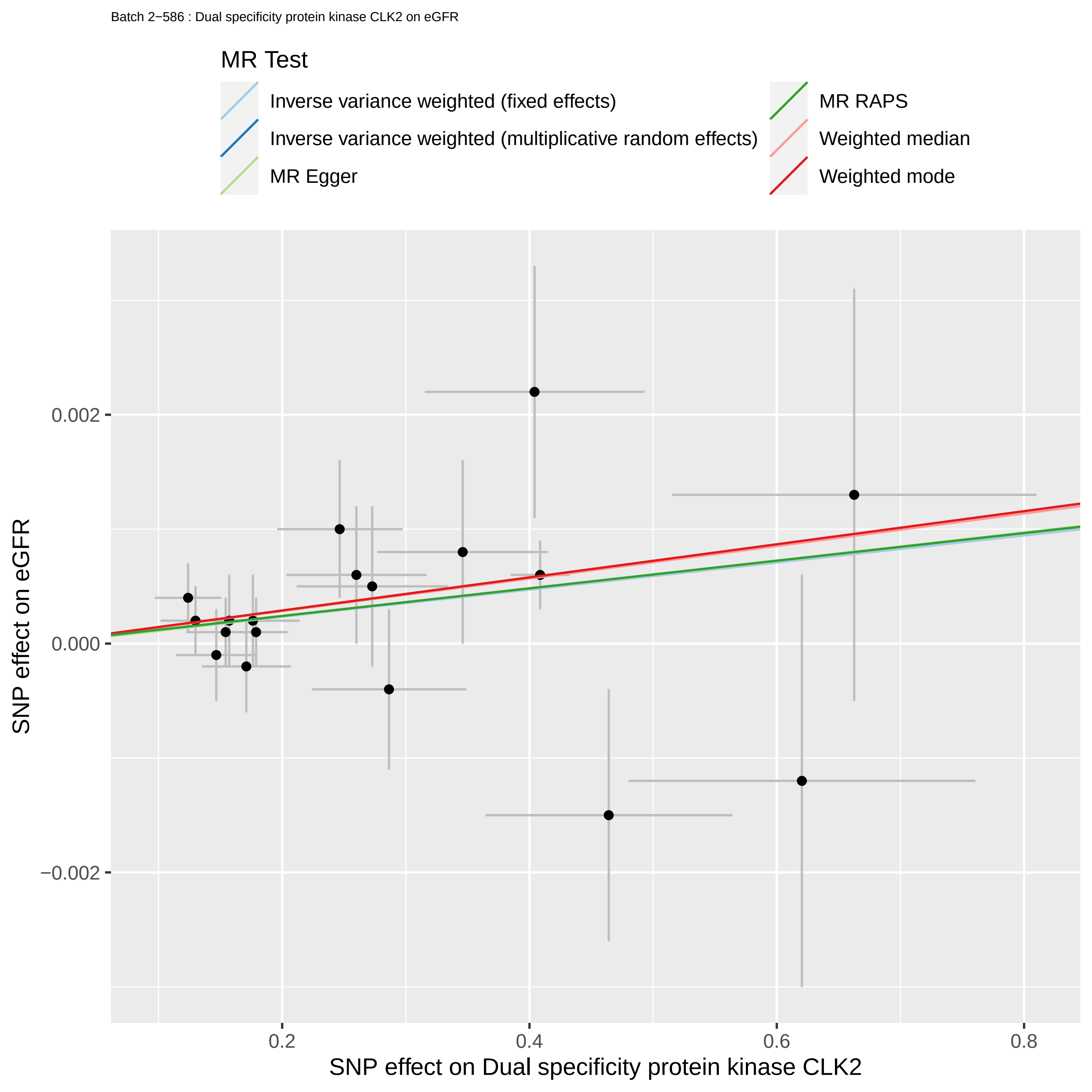

Batch 2−586 : Dual specificity protein kinase CLK2 on eGFR
MR Test
Inverse variance weighted (fixed effects)
Inverse variance weighted (multiplicative random effects)
MR Egger
MR RAPS
Weighted median
Weighted mode
0.002
0.000
−0.002
0.2
0.4
0.6
0.8
SNP effect on eGFR
SNP effect on Dual specificity protein kinase CLK2

Batch 2−587 : CXADR−like membrane protein on eGFR

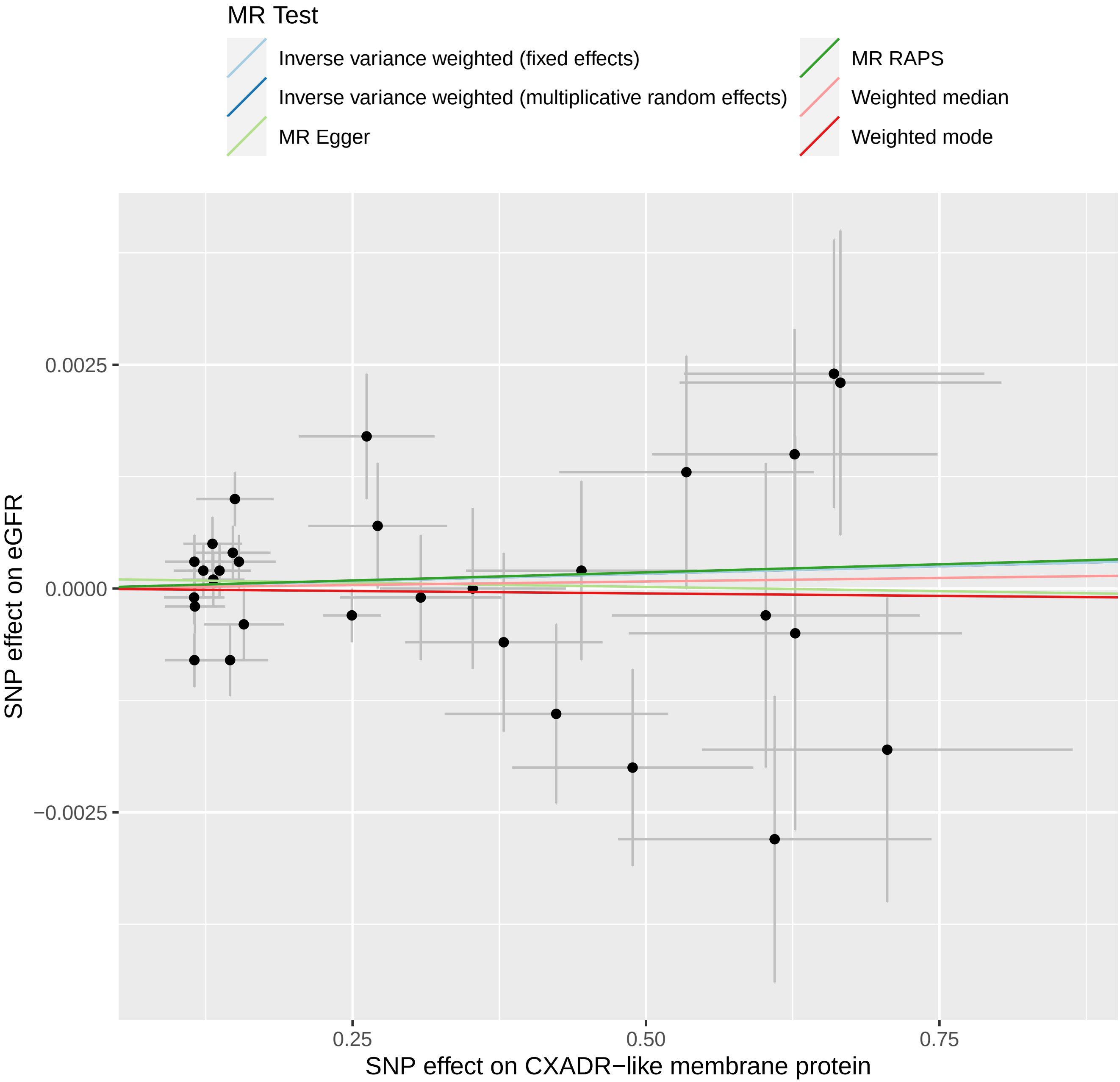

Batch 2−588 : CXADR−like membrane protein on eGFR

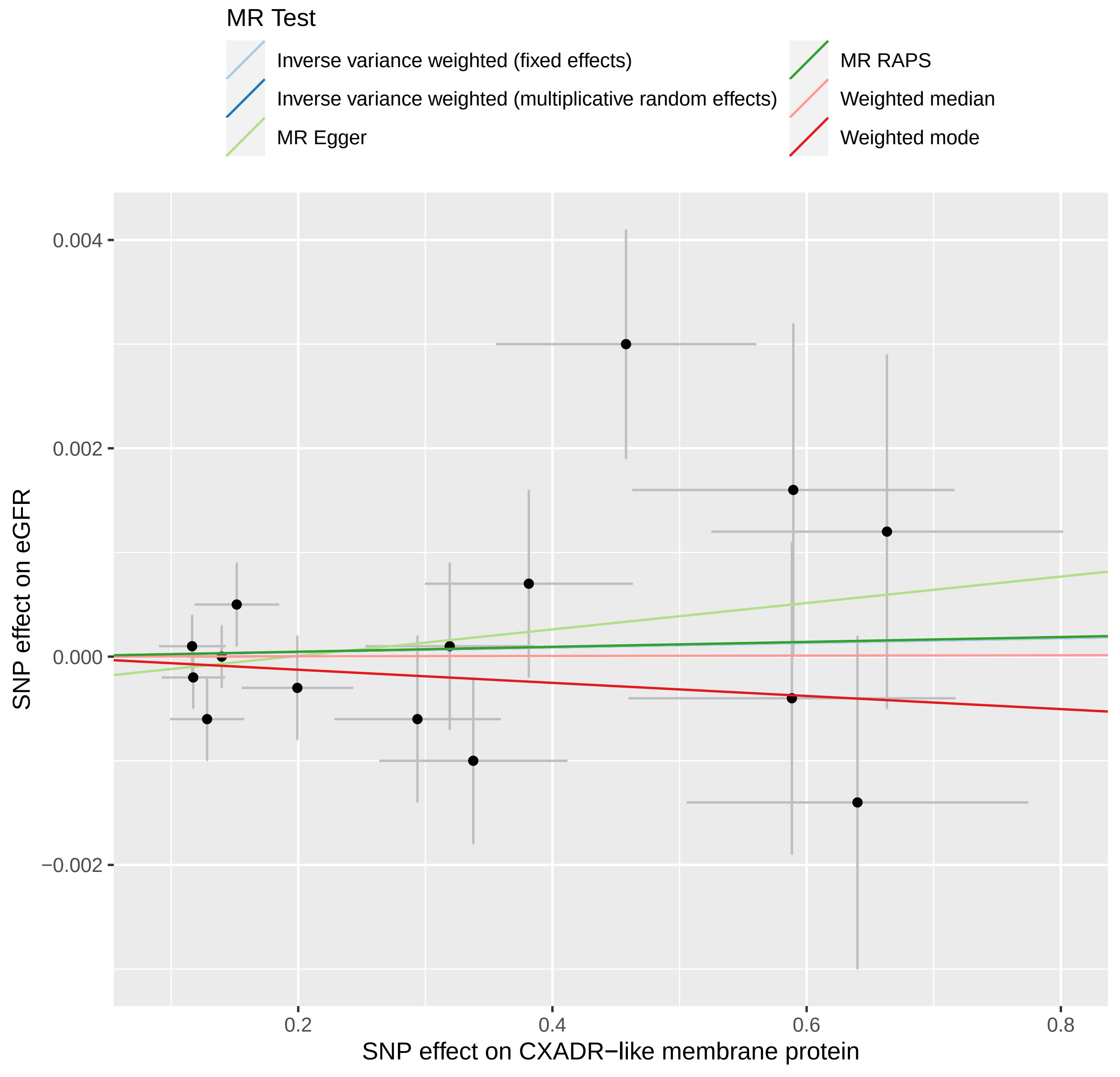

Batch 2−589 : Ceroid−lipofuscinosis neuronal protein 5 on eGFR

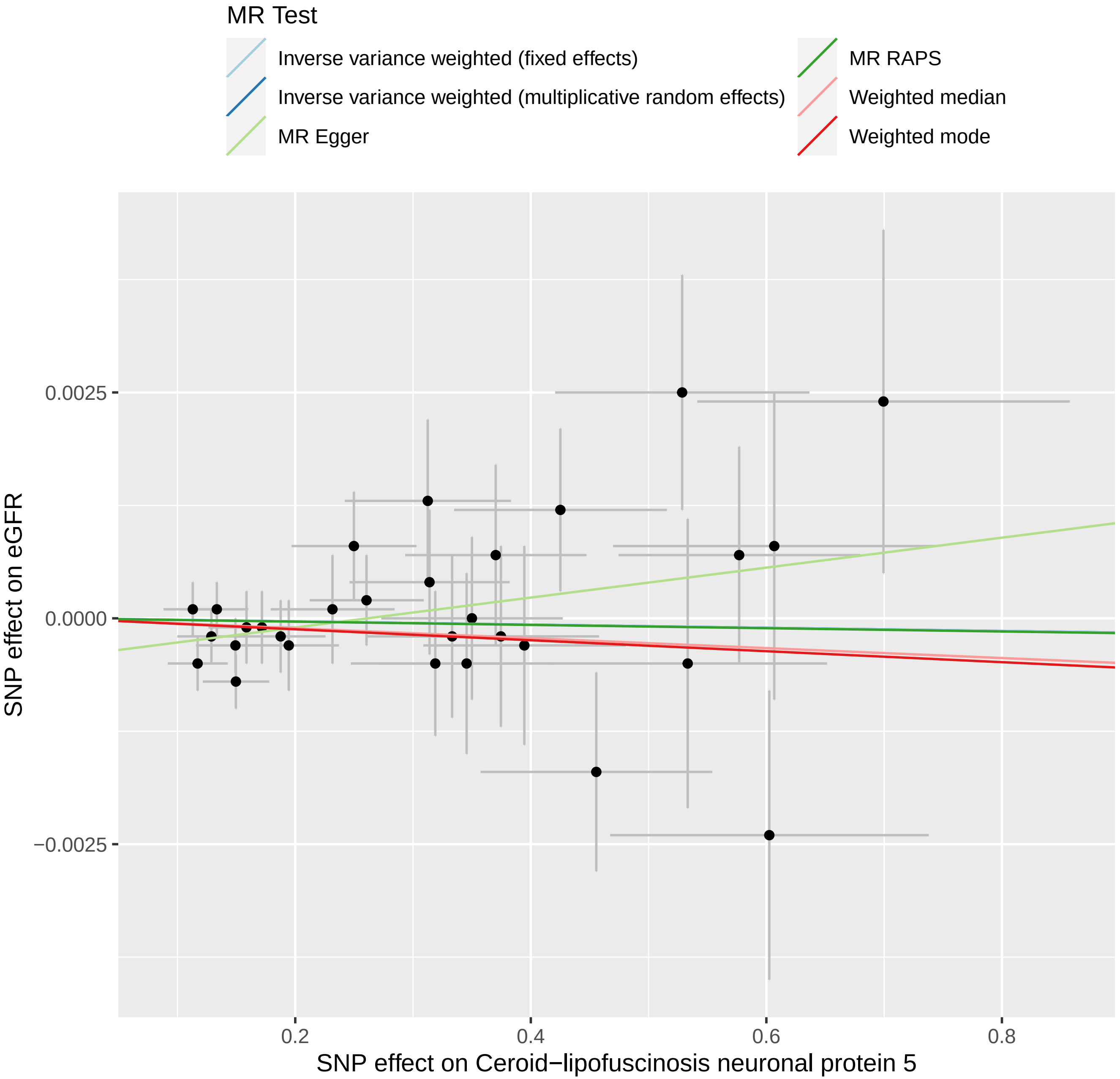

Batch 2−590 : Ceroid−lipofuscinosis neuronal protein 5 on eGFR

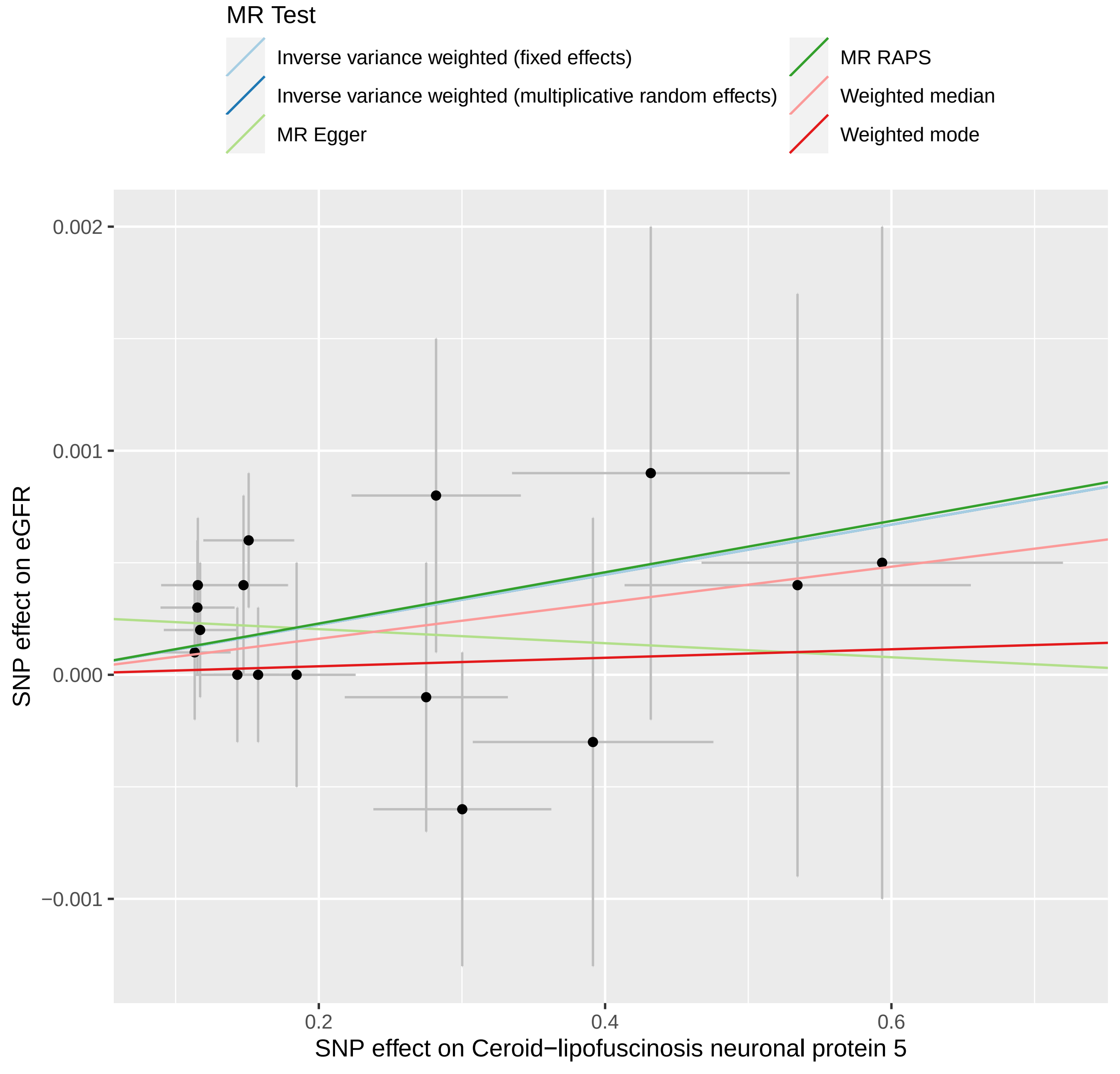

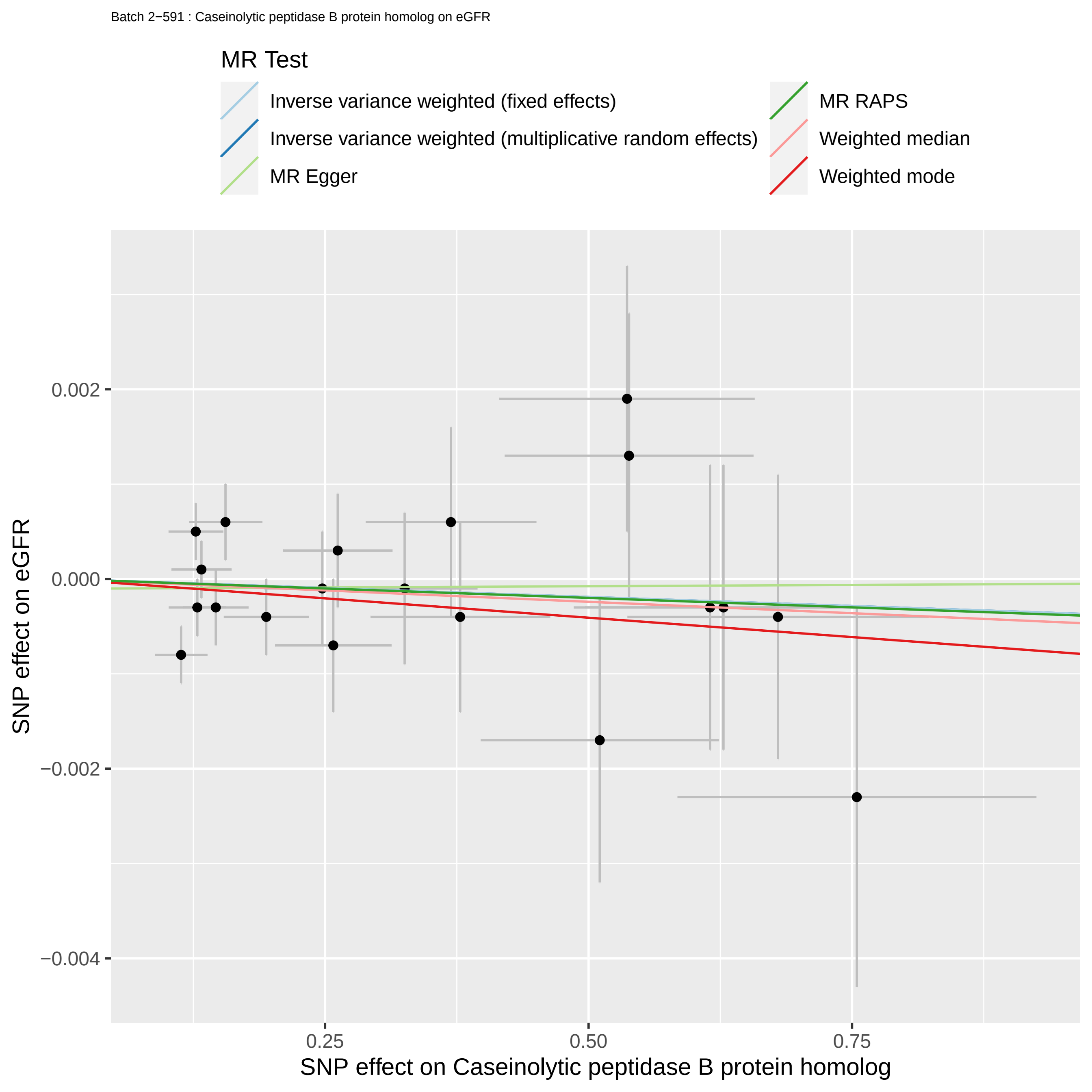
Batch 2−591 : Caseinolytic peptidase B protein homolog on eGFR
MR Test
Inverse variance weighted (fixed effects)
Inverse variance weighted (multiplicative random effects)
MR Egger
MR RAPS
Weighted median
Weighted mode
0.002
0.000
−0.002
−0.004
0.25
0.50
0.75
SNP effect on eGFR
SNP effect on Caseinolytic peptidase B protein homolog

Batch 2−592 : Colipase on eGFR

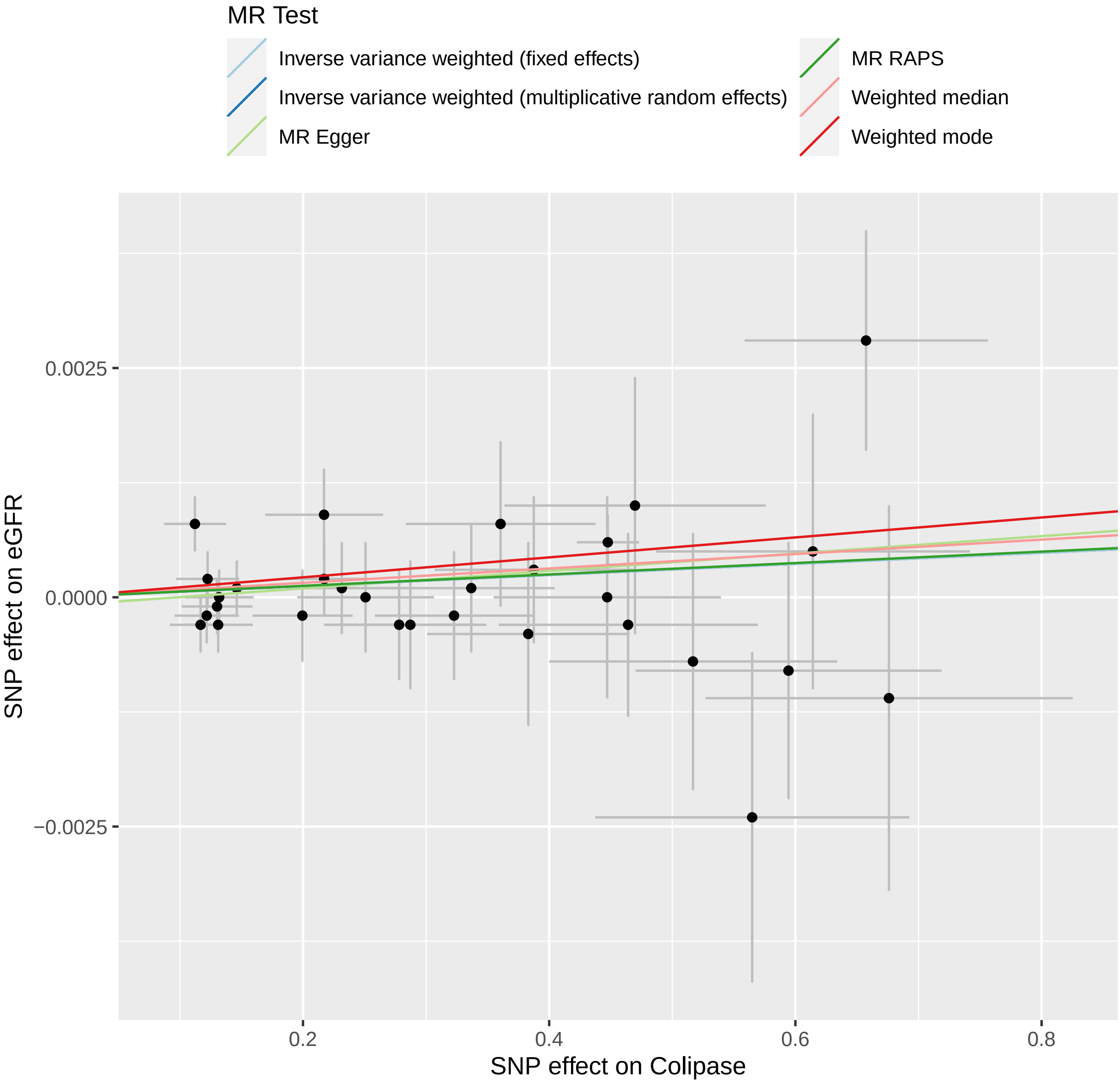

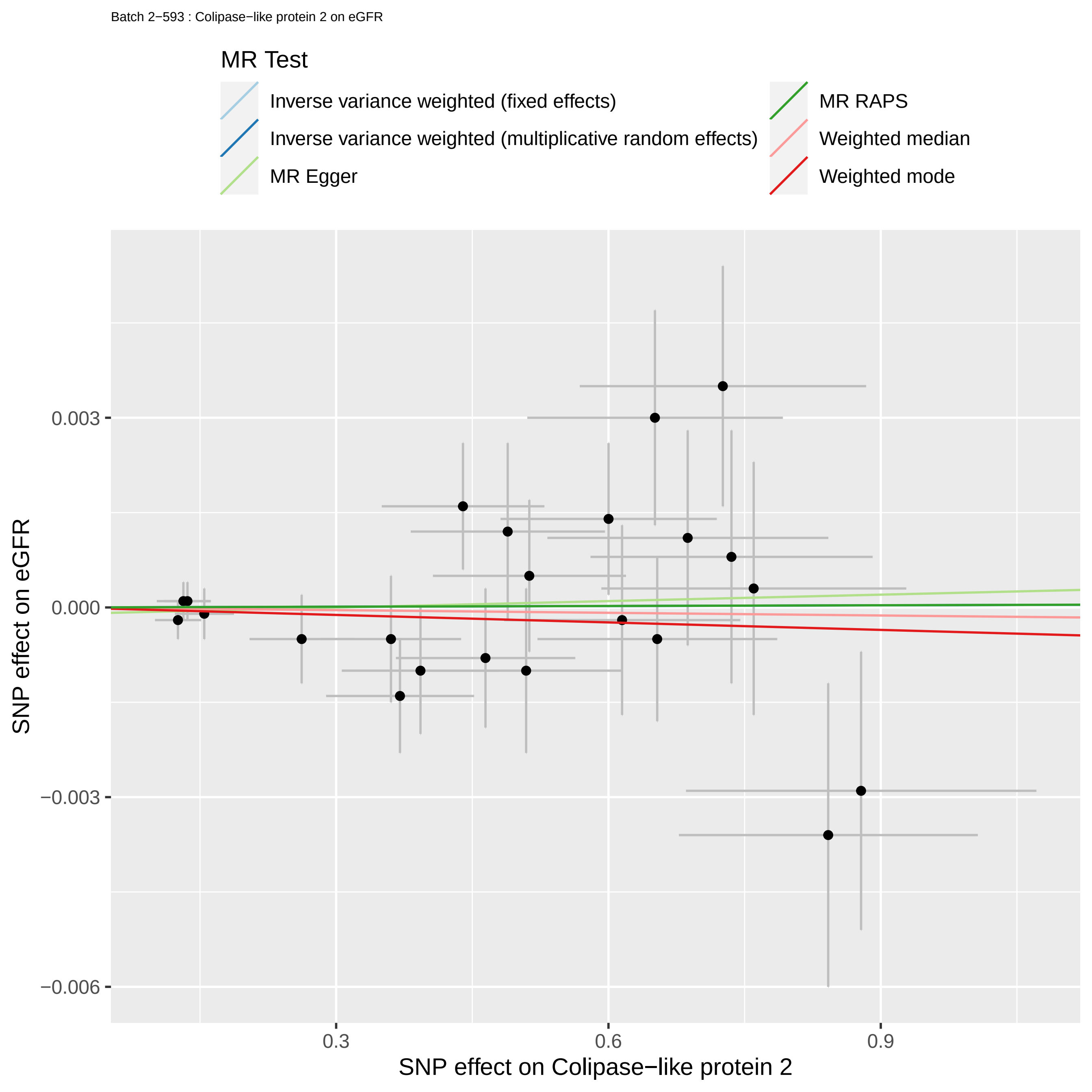

Batch 2−593 : Colipase−like protein 2 on eGFR
MR Test
Inverse variance weighted (fixed effects)
Inverse variance weighted (multiplicative random effects)
MR Egger
MR RAPS
Weighted median
Weighted mode
0.003
0.000
−0.003
−0.006
0.3
0.6
0.9
SNP effect on eGFR
SNP effect on Colipase−like protein 2

Batch 2−594 : Calsyntenin−3 on eGFR

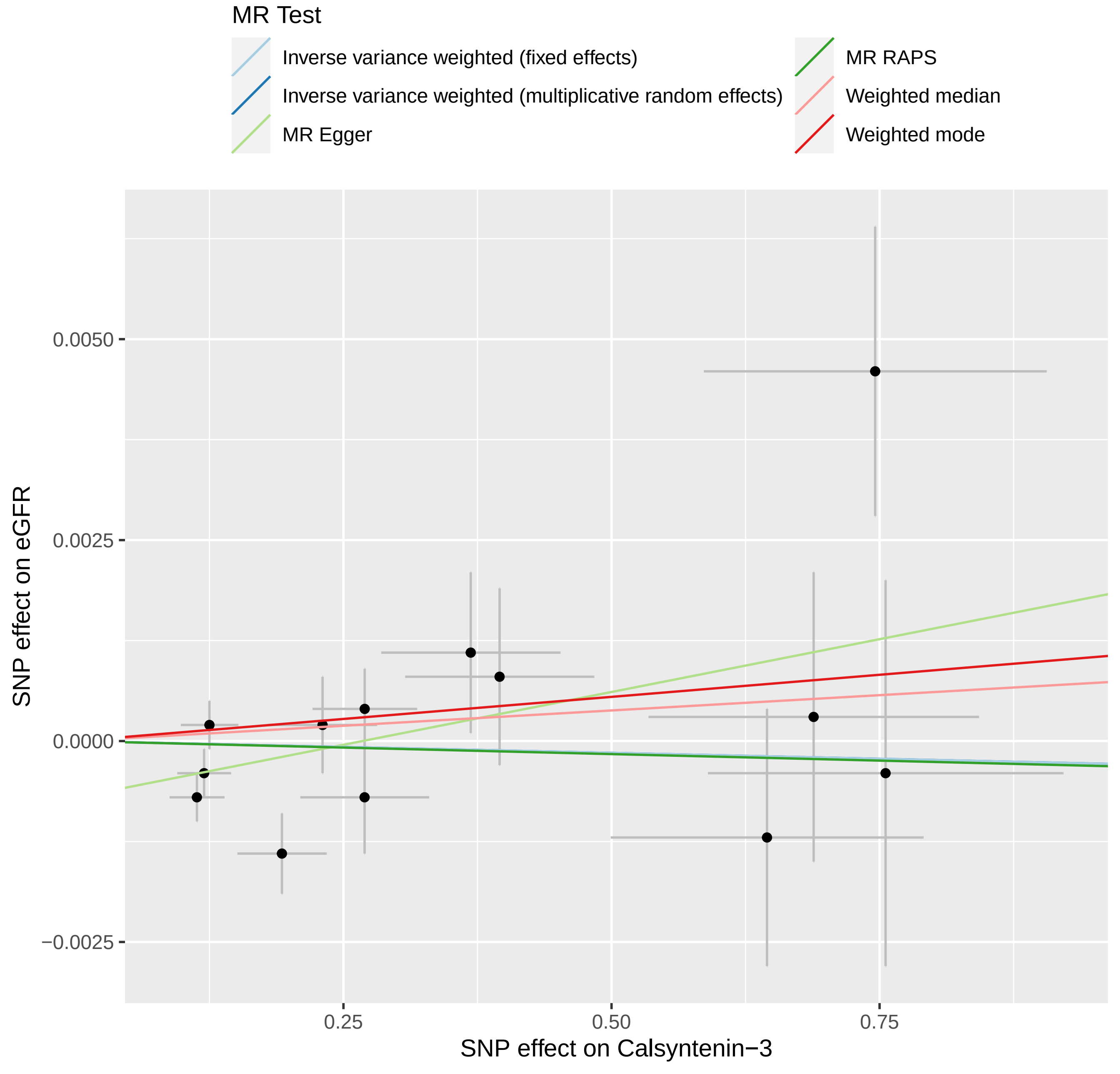

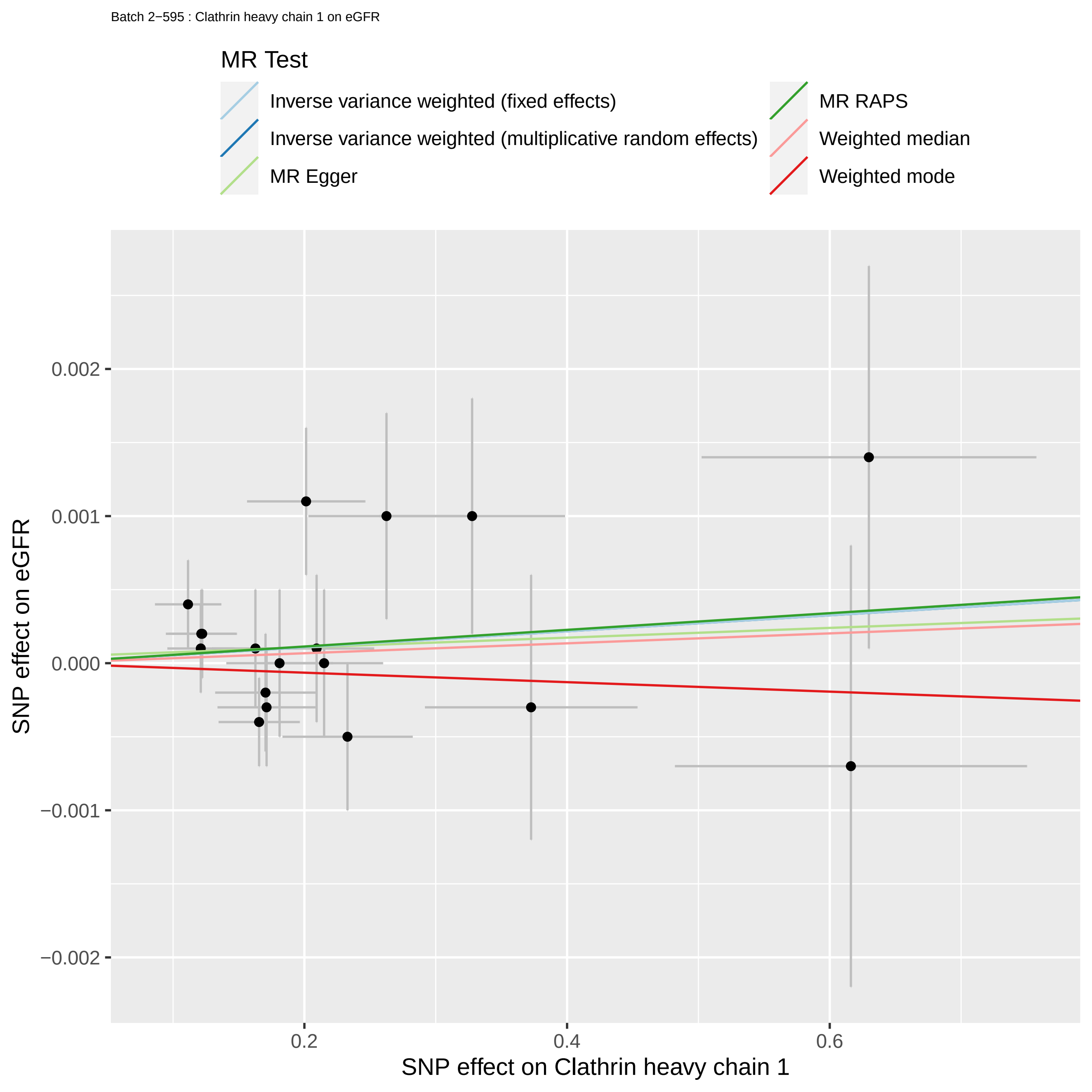

Batch 2−595 : Clathrin heavy chain 1 on eGFR
MR Test
Inverse variance weighted (fixed effects)
Inverse variance weighted (multiplicative random effects)
MR Egger
MR RAPS
Weighted median
Weighted mode
0.002
0.001
0.000
−0.001
−0.002
0.2
0.4
0.6
SNP effect on eGFR
SNP effect on Clathrin heavy chain 1

Batch 2−596 : Clusterin on eGFR

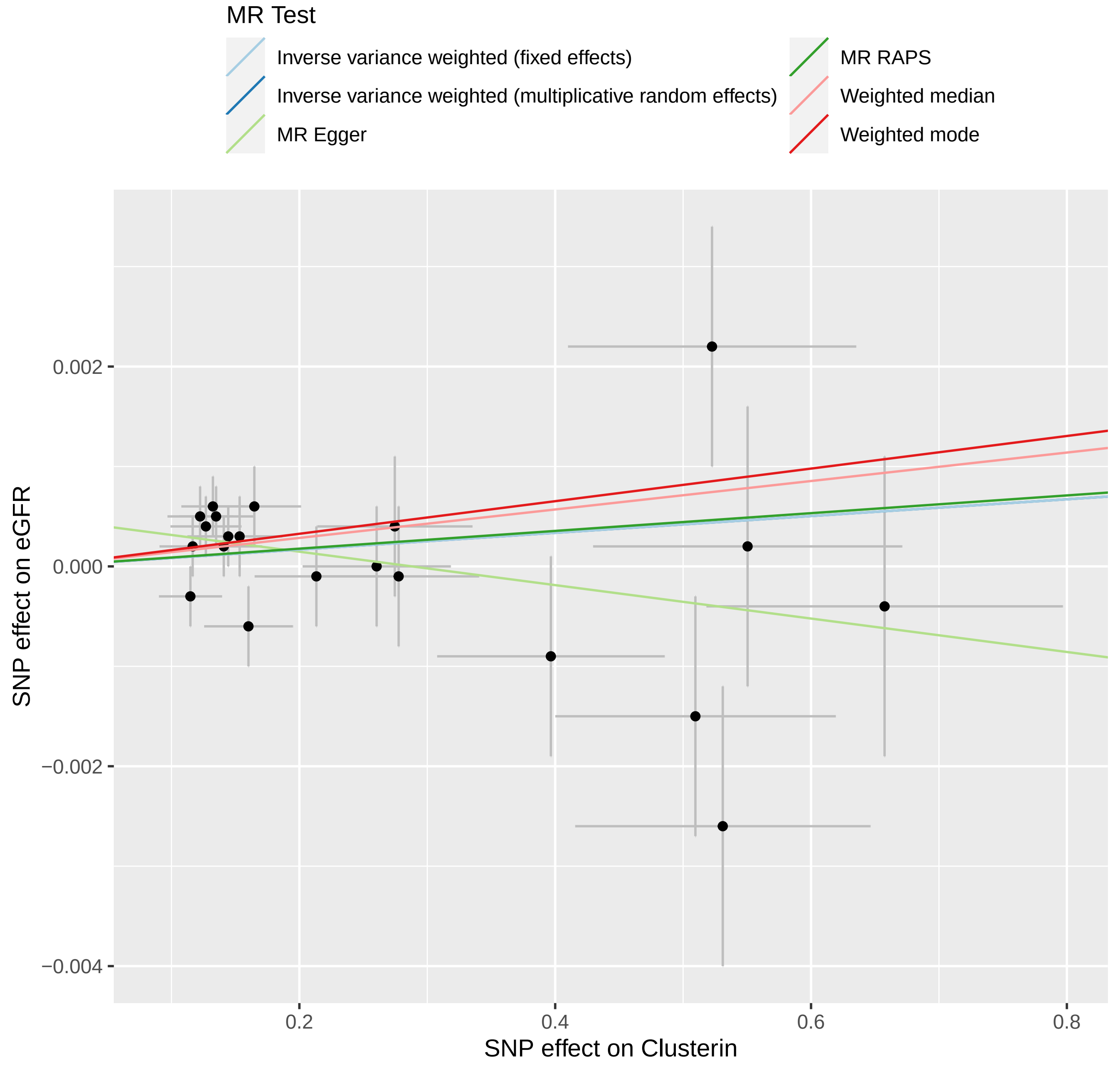

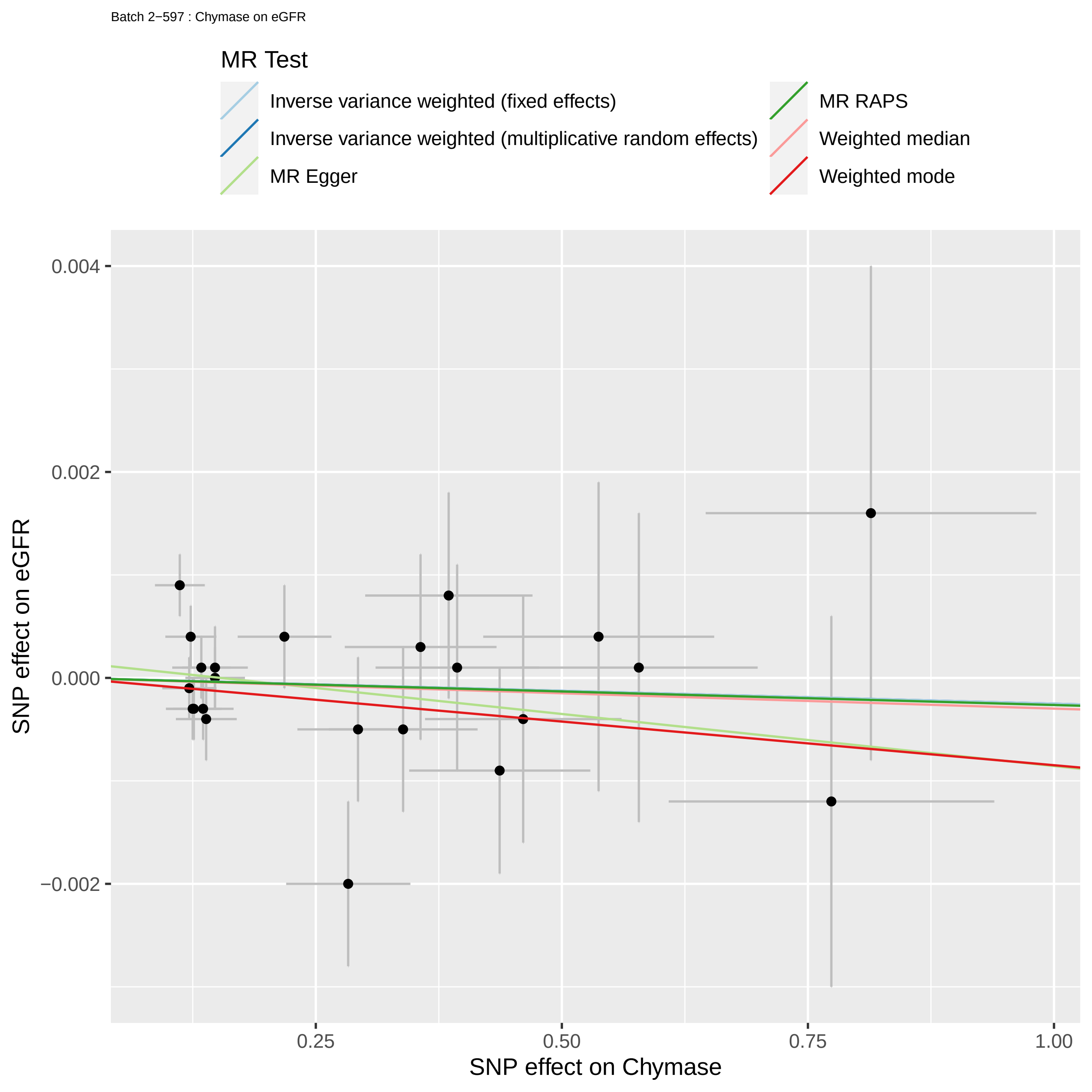
Batch 2−597 : Chymase on eGFR
MR Test
Inverse variance weighted (fixed effects)
Inverse variance weighted (multiplicative random effects)
MR Egger
MR RAPS
Weighted median
Weighted mode
0.004
0.002
0.000
−0.002
SNP effect on eGFR
0.25
0.50
0.75
1.00
SNP effect on Chymase

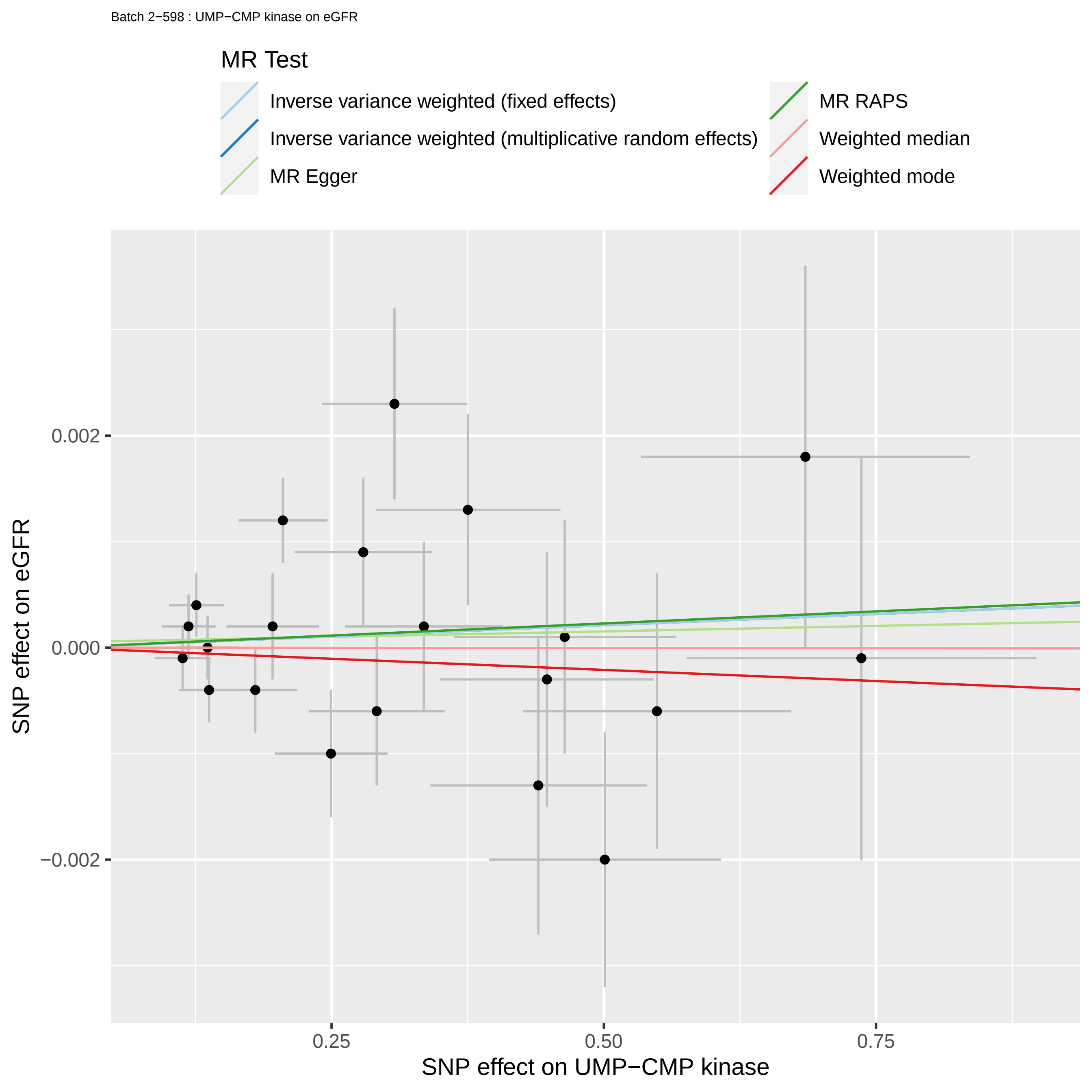

Batch 2−598 : UMP−CMP kinase on eGFR
MR Test
Inverse variance weighted (fixed effects)
Inverse variance weighted (multiplicative random effects)
MR Egger
MR RAPS
Weighted median
Weighted mode
0.002
0.000
−0.002
0.25
0.50
0.75
SNP effect on eGFR
SNP effect on UMP−CMP kinase

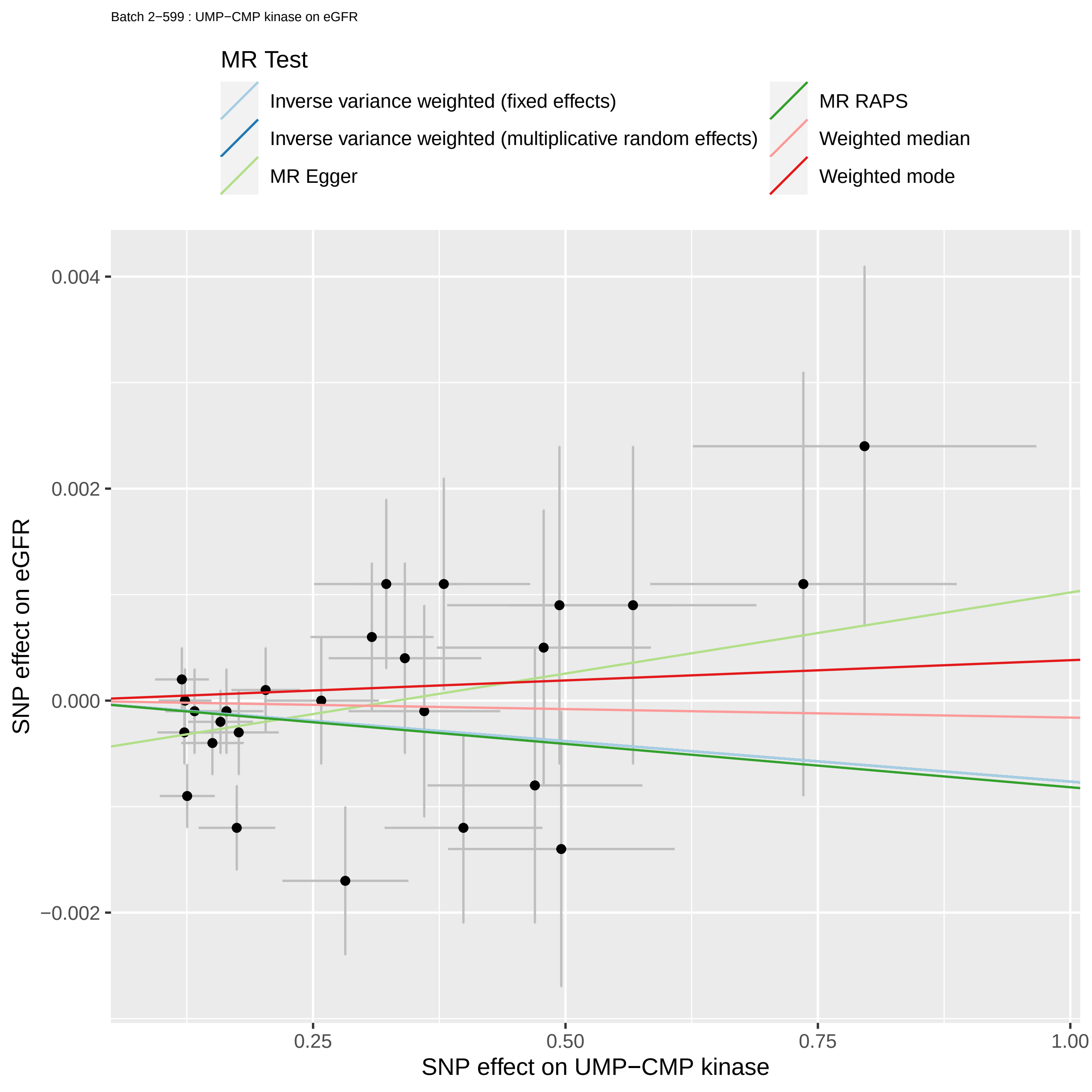

Batch 2−599 : UMP−CMP kinase on eGFR
MR Test
Inverse variance weighted (fixed effects)
Inverse variance weighted (multiplicative random effects)
MR Egger
MR RAPS
Weighted median
Weighted mode
0.004
0.002
0.000
−0.002
0.25
0.50
0.75
1.00
SNP effect on eGFR
SNP effect on UMP−CMP kinase

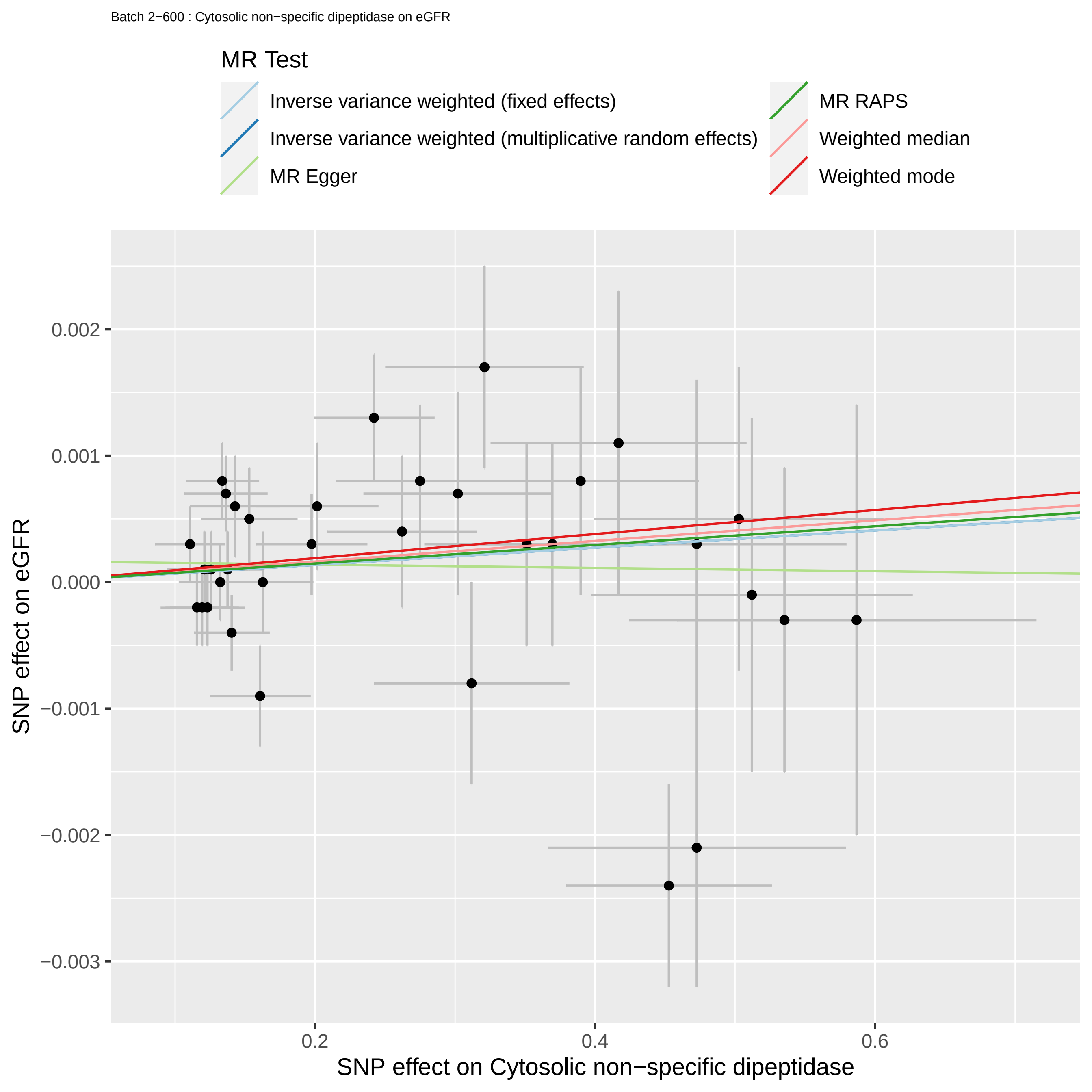

Batch 2−600 : Cytosolic non−specific dipeptidase on eGFR
MR Test
Inverse variance weighted (fixed effects)
Inverse variance weighted (multiplicative random effects)
MR Egger
MR RAPS
Weighted median
Weighted mode
0.002
0.001
0.000
−0.001
−0.002
−0.003
SNP effect on eGFR
0.2
0.4
0.6
SNP effect on Cytosolic non−specific dipeptidase

Batch 2−601 : Nuclear envelope phosphatase−regulatory subunit 1 on eGFR

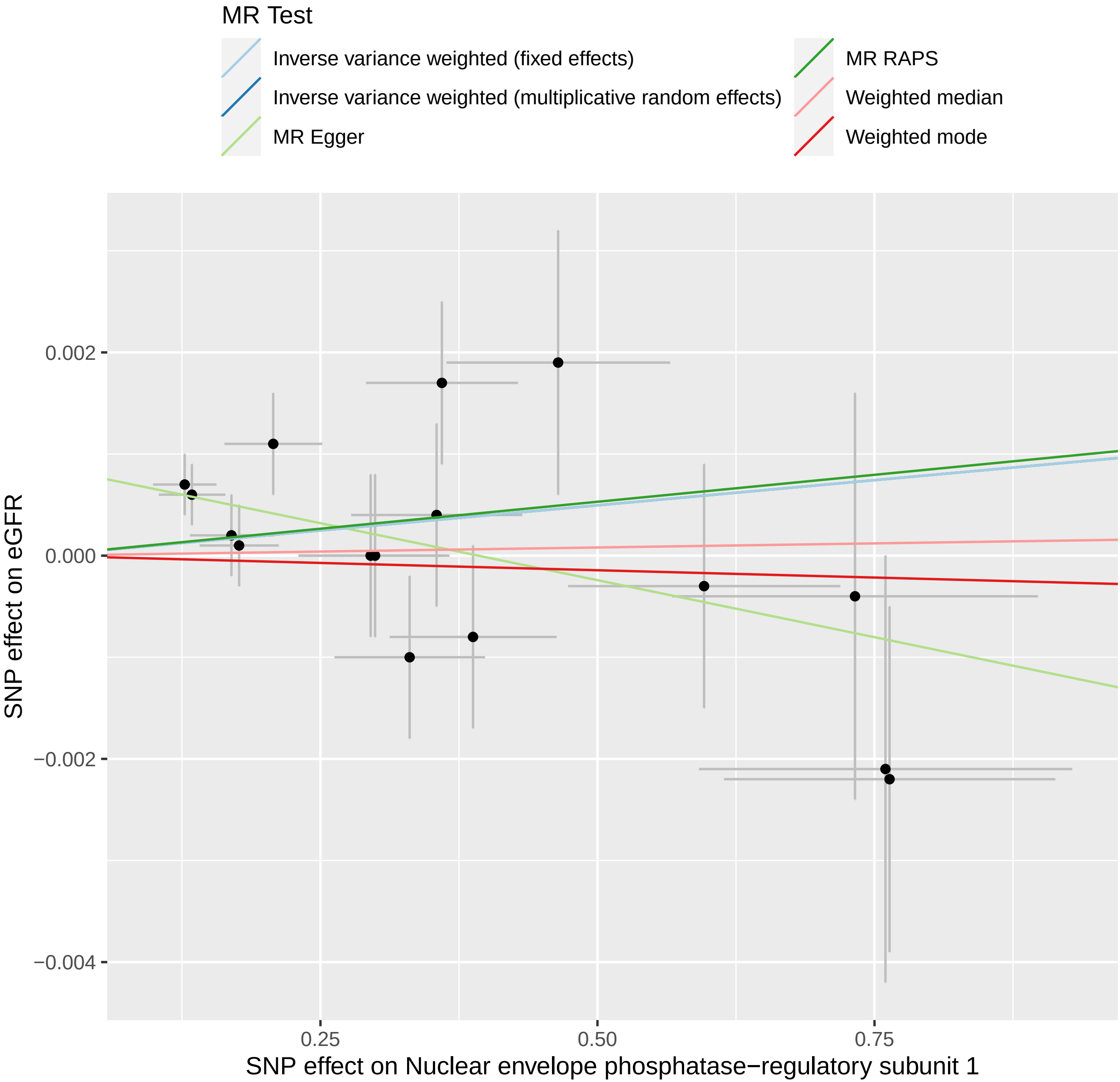

Batch 2−602 : CCR4−NOT transcription complex subunit 1 on eGFR

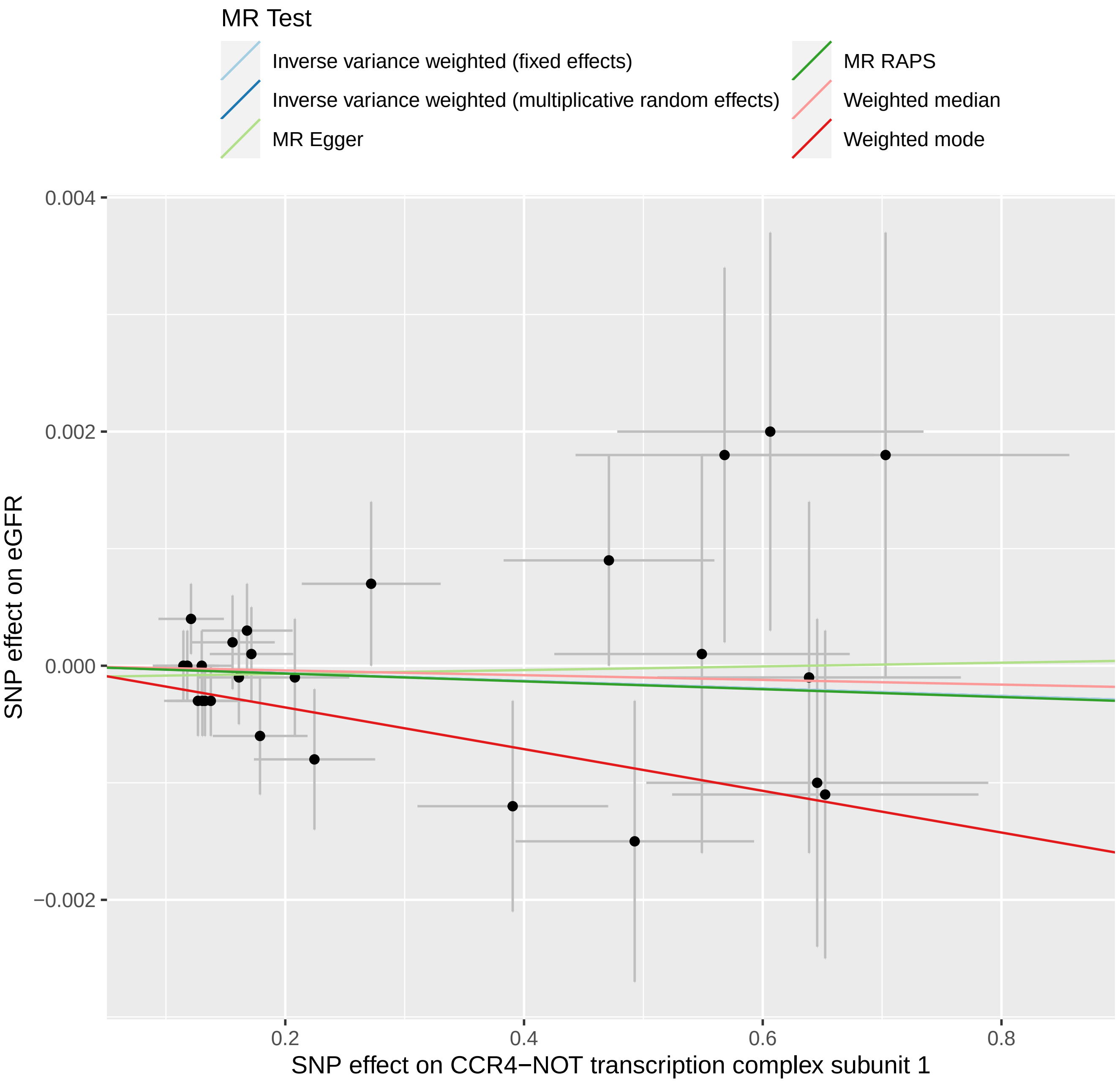

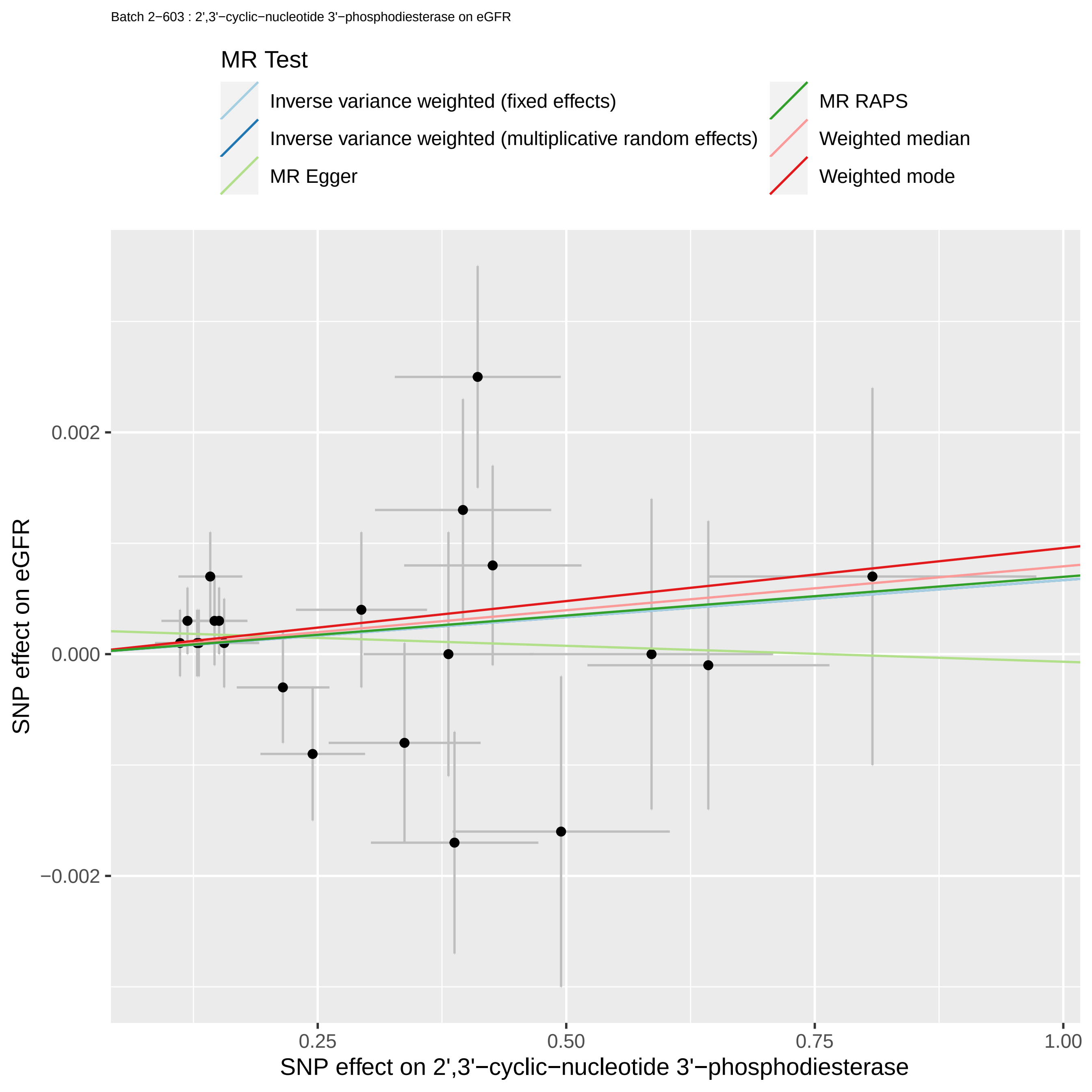

Batch 2−603 : 2',3'−cyclic−nucleotide 3'−phosphodiesterase on eGFR
MR Test
Inverse variance weighted (fixed effects)
Inverse variance weighted (multiplicative random effects)
MR Egger
MR RAPS
Weighted median
Weighted mode
0.002
0.000
−0.002
0.25
0.50
0.75
1.00
SNP effect on eGFR
SNP effect on 2',3'−cyclic−nucleotide 3'−phosphodiesterase

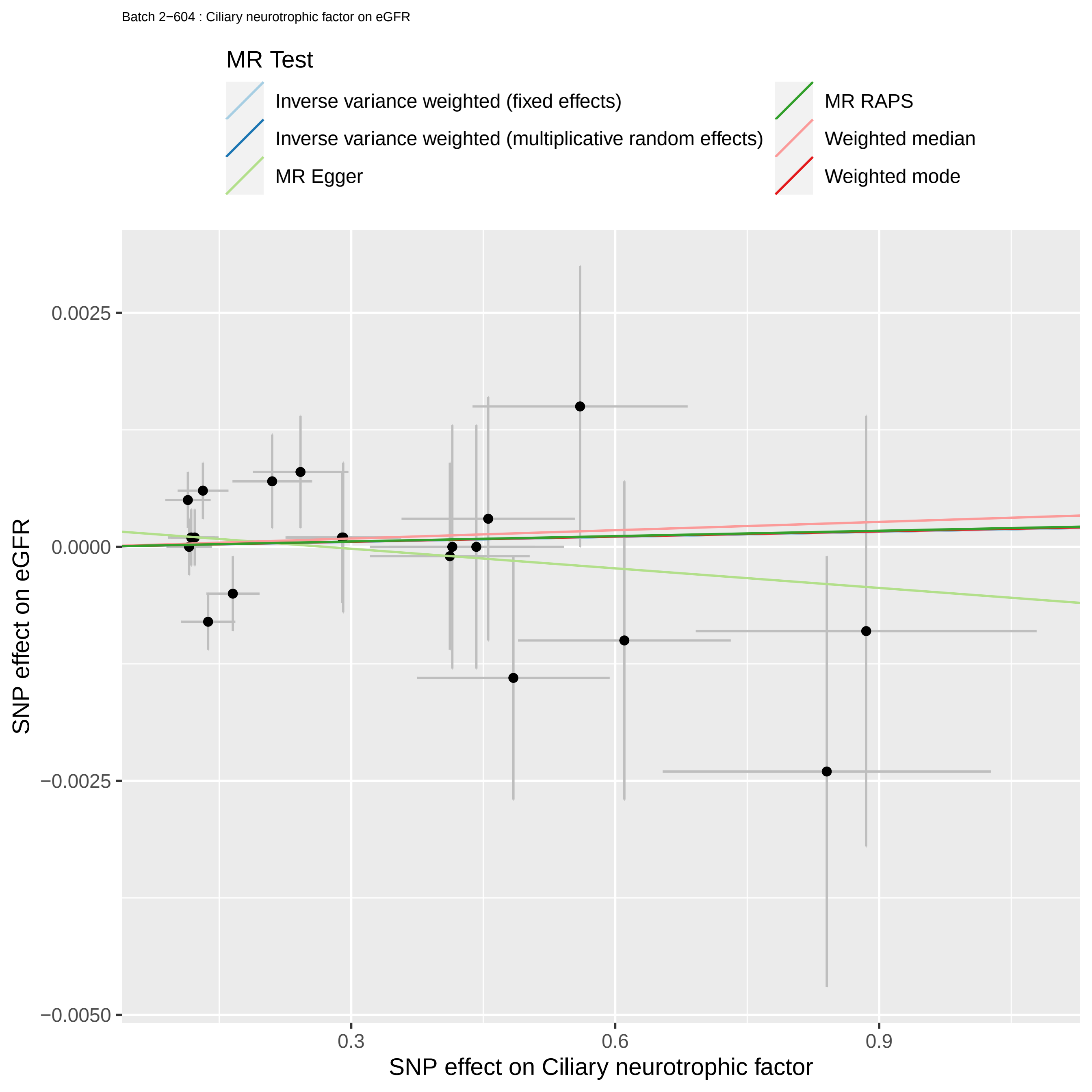

Batch 2−604 : Ciliary neurotrophic factor on eGFR
MR Test
Inverse variance weighted (fixed effects)
Inverse variance weighted (multiplicative random effects)
MR Egger
MR RAPS
Weighted median
Weighted mode
0.0025
0.0000
−0.0025
−0.0050
0.3
0.6
0.9
SNP effect on eGFR
SNP effect on Ciliary neurotrophic factor

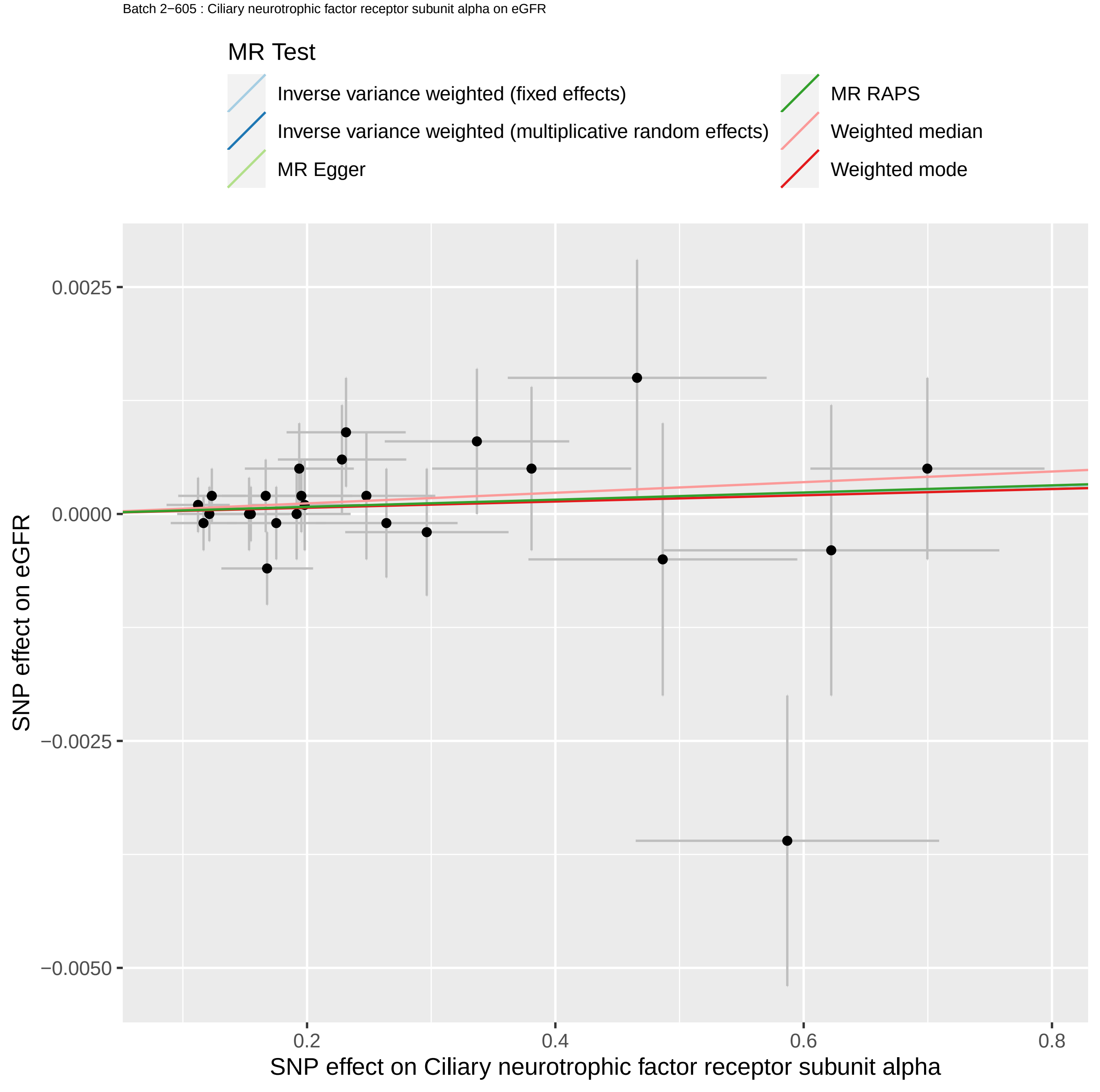
Batch 2−605 : Ciliary neurotrophic factor receptor subunit alpha on eGFR
MR Test
Inverse variance weighted (fixed effects)
Inverse variance weighted (multiplicative random effects)
MR Egger
MR RAPS
Weighted median
Weighted mode
0.0025
0.0000
−0.0025
−0.0050
0.2
0.4
0.6
0.8
SNP effect on eGFR
SNP effect on Ciliary neurotrophic factor receptor subunit alpha

Batch 2−606 : Ciliary neurotrophic factor receptor subunit alpha on eGFR

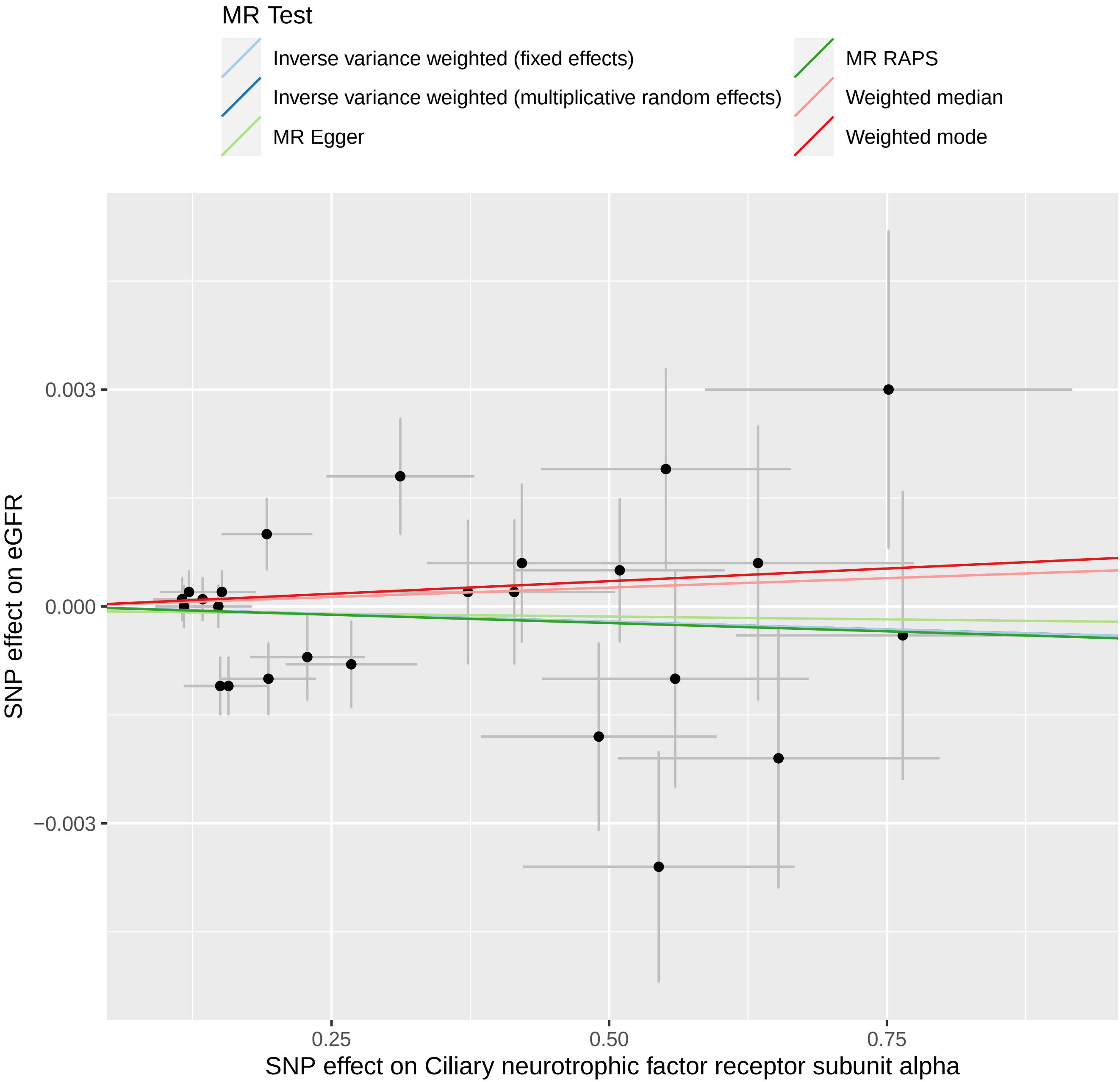

Batch 2−607 : Contactin−2 on eGFR

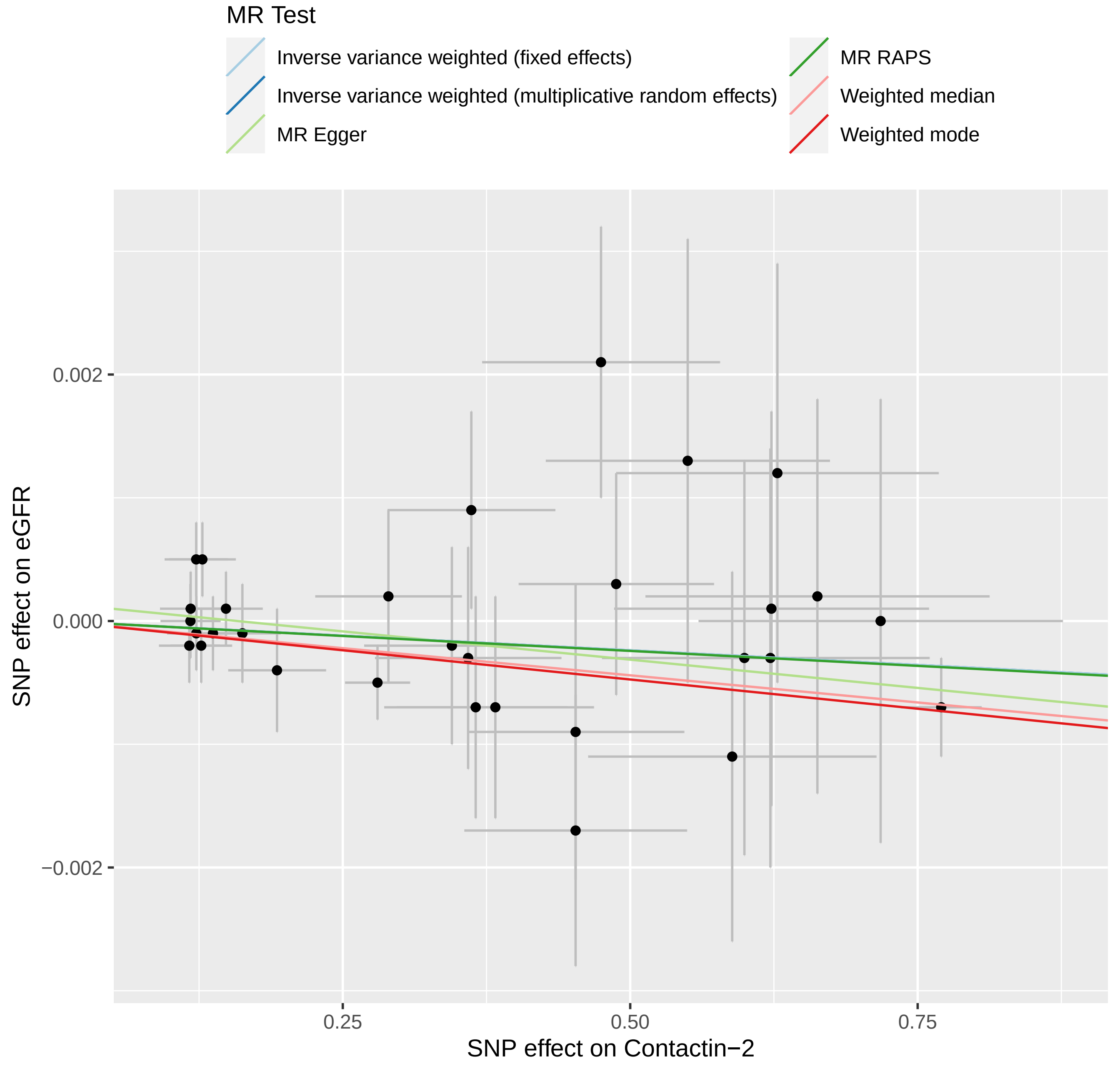

Batch 2−608 : Contactin−4 on eGFR

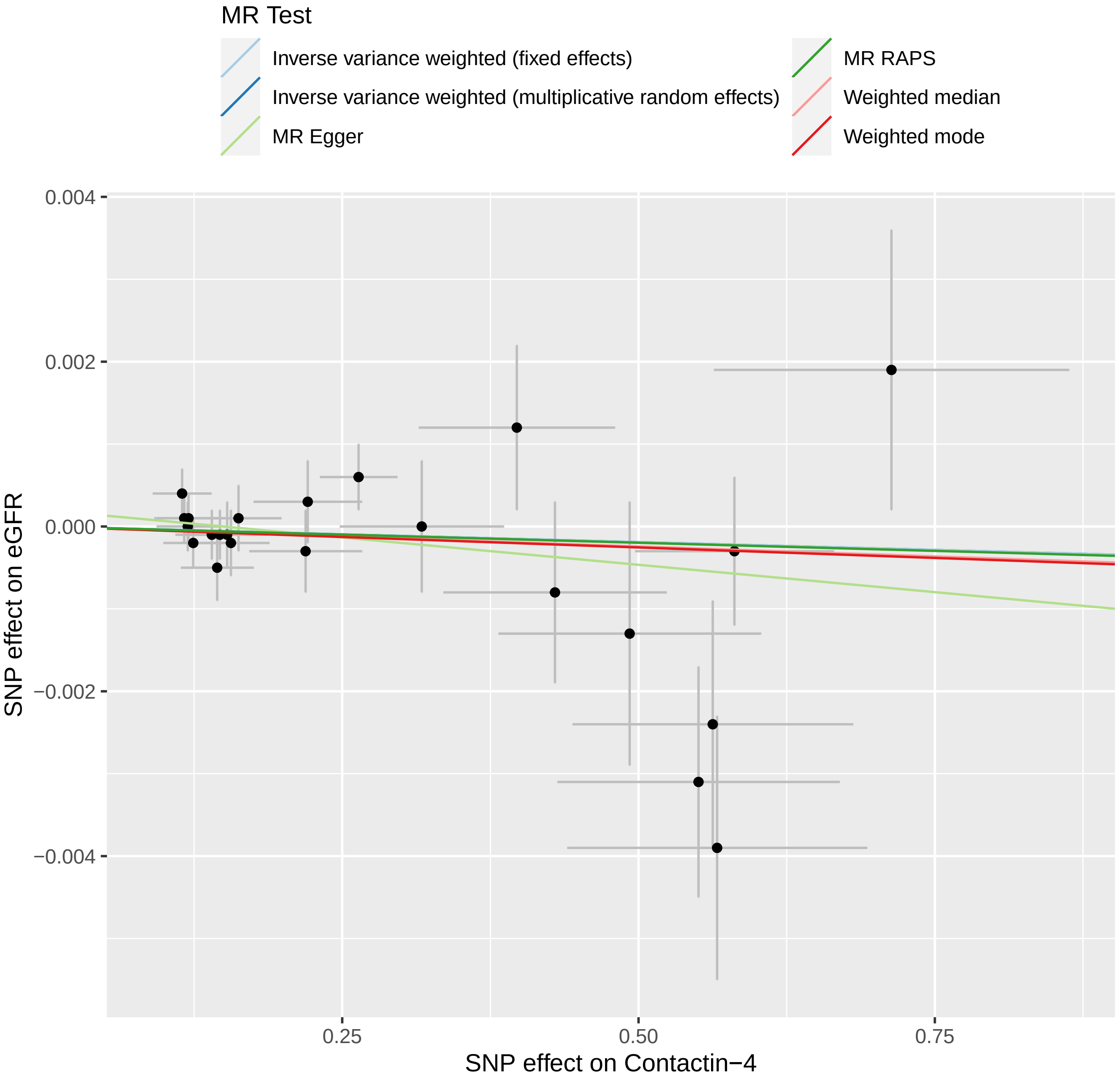

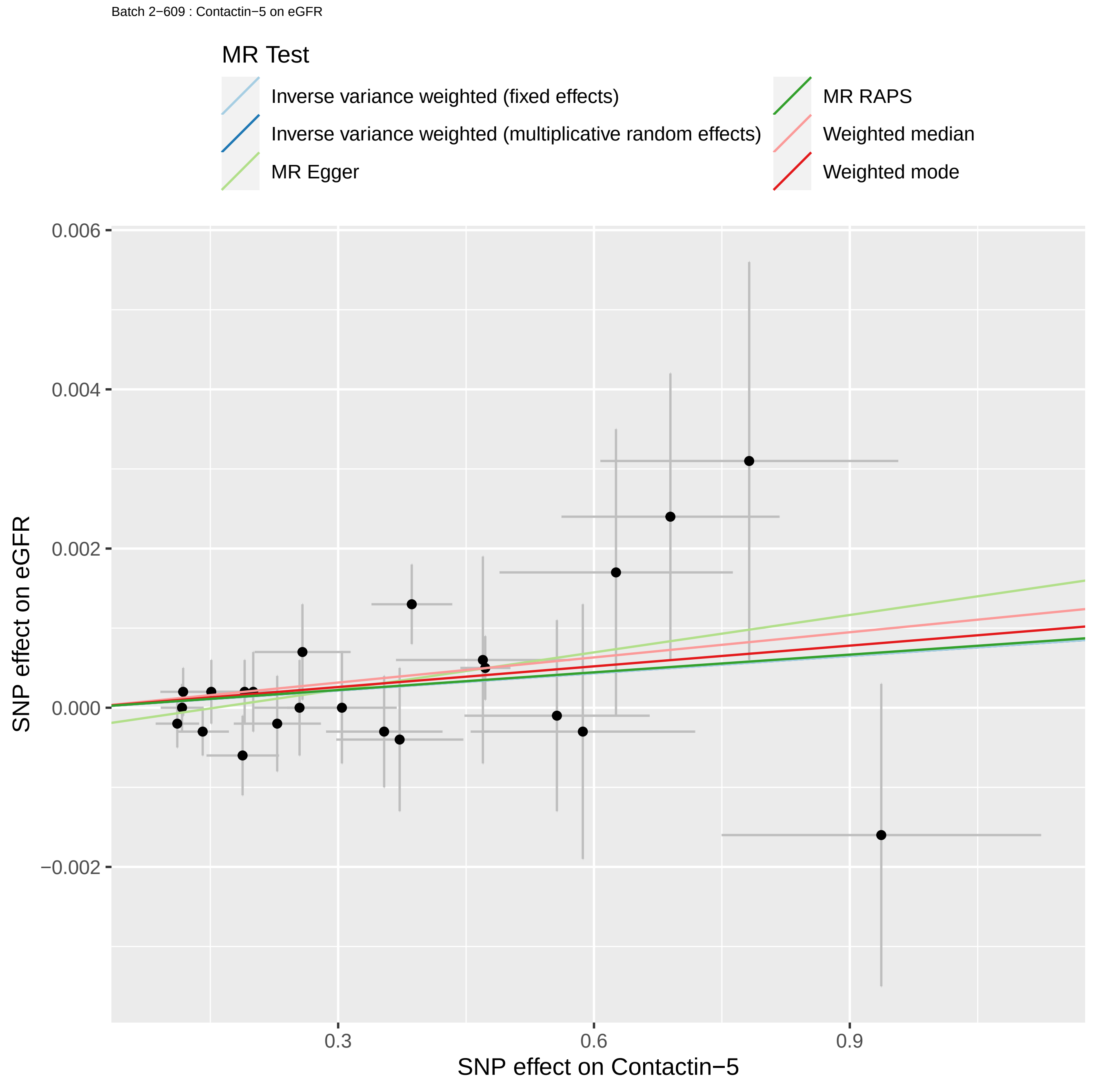

Batch 2−609 : Contactin−5 on eGFR
MR Test
Inverse variance weighted (fixed effects)
Inverse variance weighted (multiplicative random effects)
MR Egger
MR RAPS
Weighted median
Weighted mode
0.006
0.004
0.002
0.000
−0.002
0.3
0.6
0.9
SNP effect on eGFR
SNP effect on Contactin−5

Batch 2−610 : Contactin−associated protein 1 on eGFR

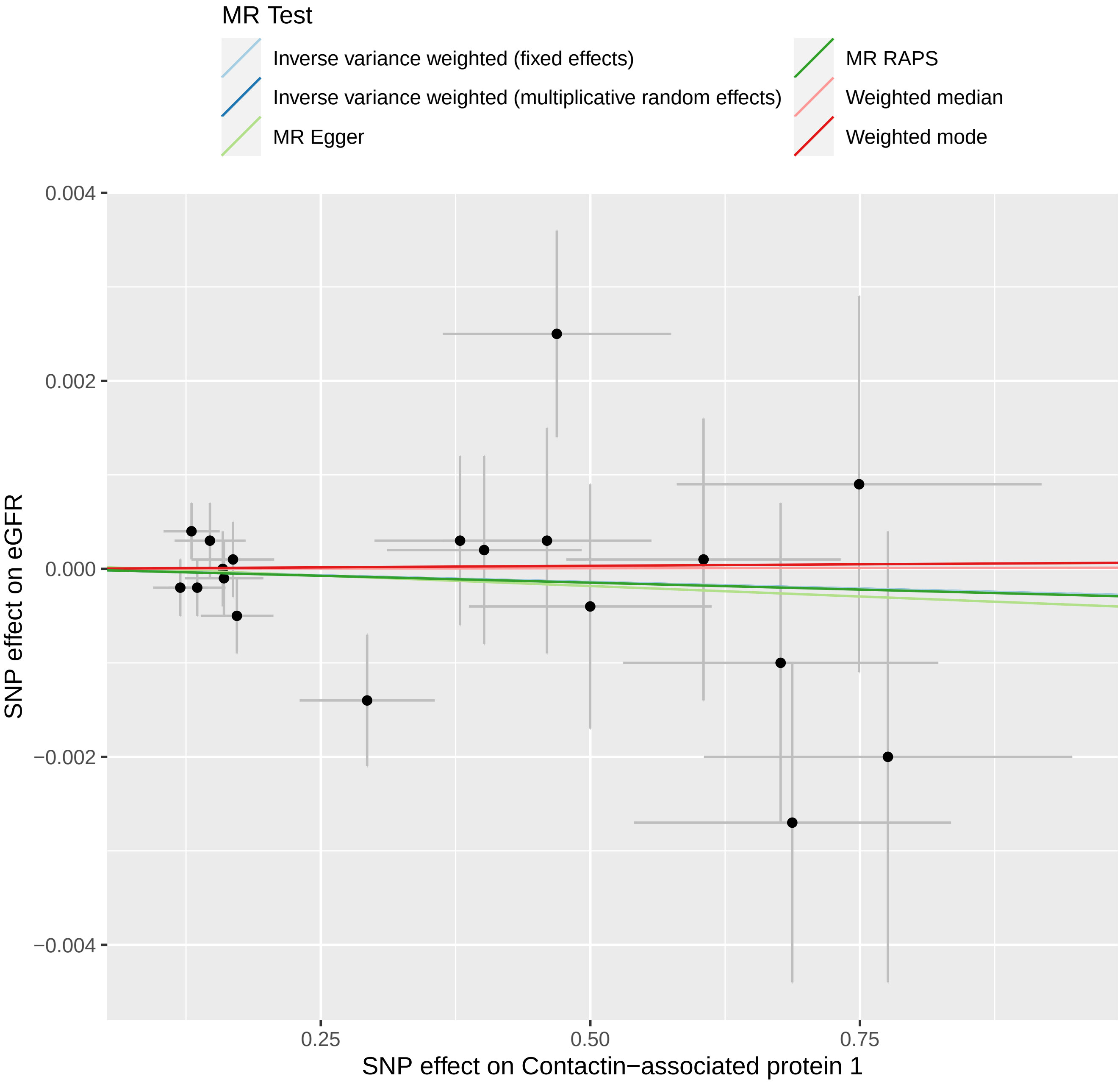

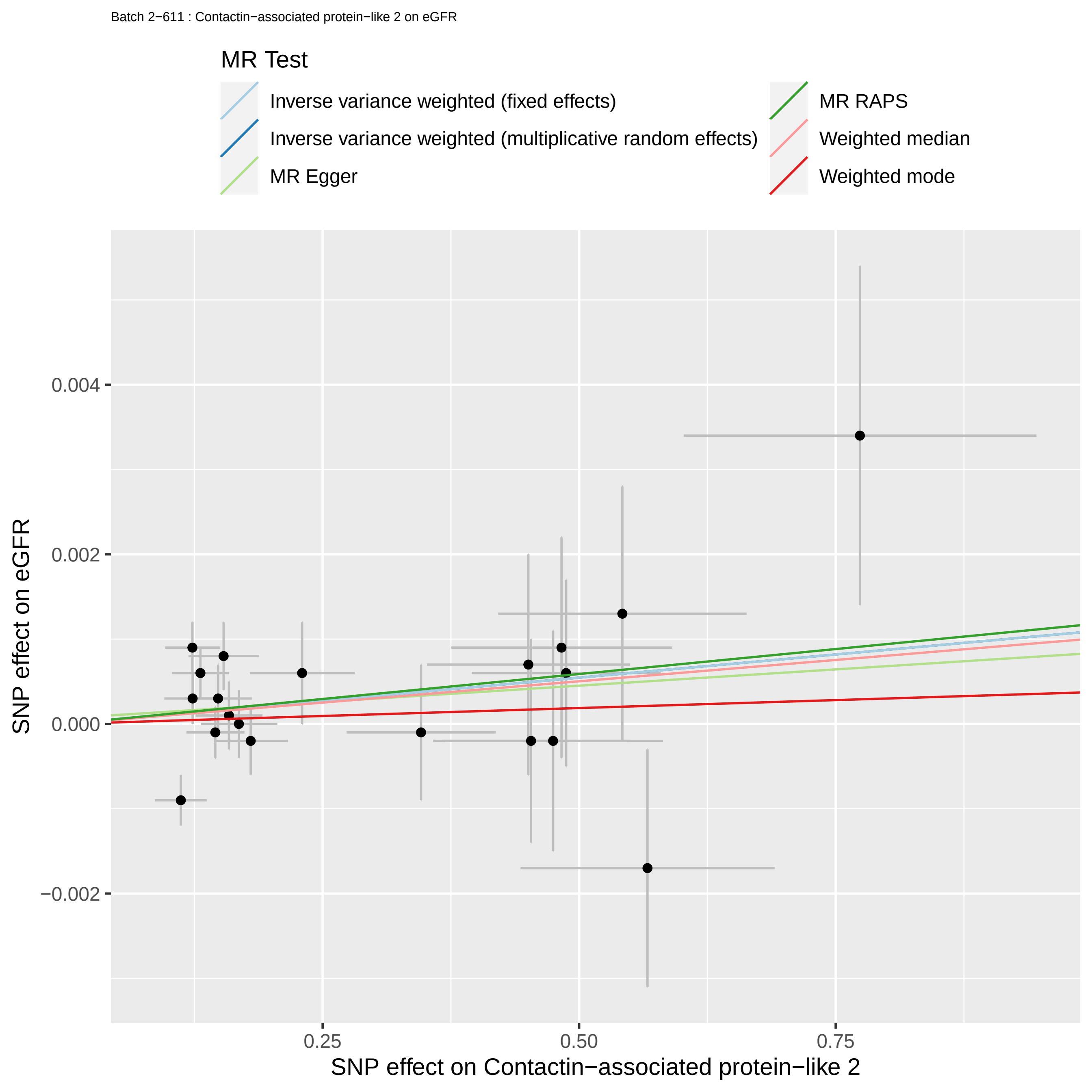
Batch 2−611 : Contactin−associated protein−like 2 on eGFR
MR Test
Inverse variance weighted (fixed effects)
Inverse variance weighted (multiplicative random effects)
MR Egger
MR RAPS
Weighted median
Weighted mode
0.004
0.002
0.000
−0.002
SNP effect on eGFR
0.25
0.50
0.75
SNP effect on Contactin−associated protein−like 2

Batch 2−612 : Cytochrome c oxidase assembly factor 3 homolog, mitochondrial on eGFR

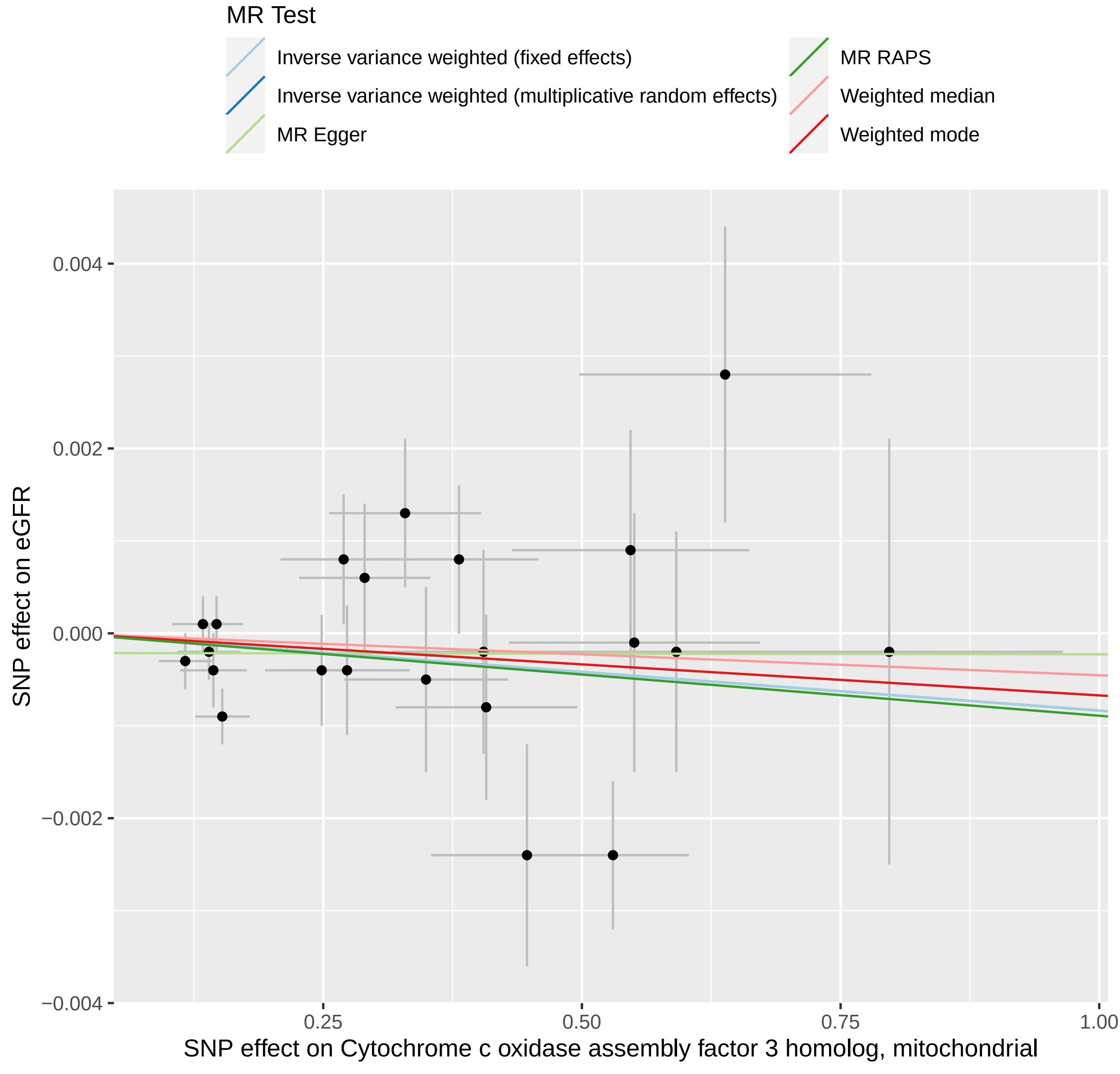

Batch 2−613 : Cochlin on eGFR

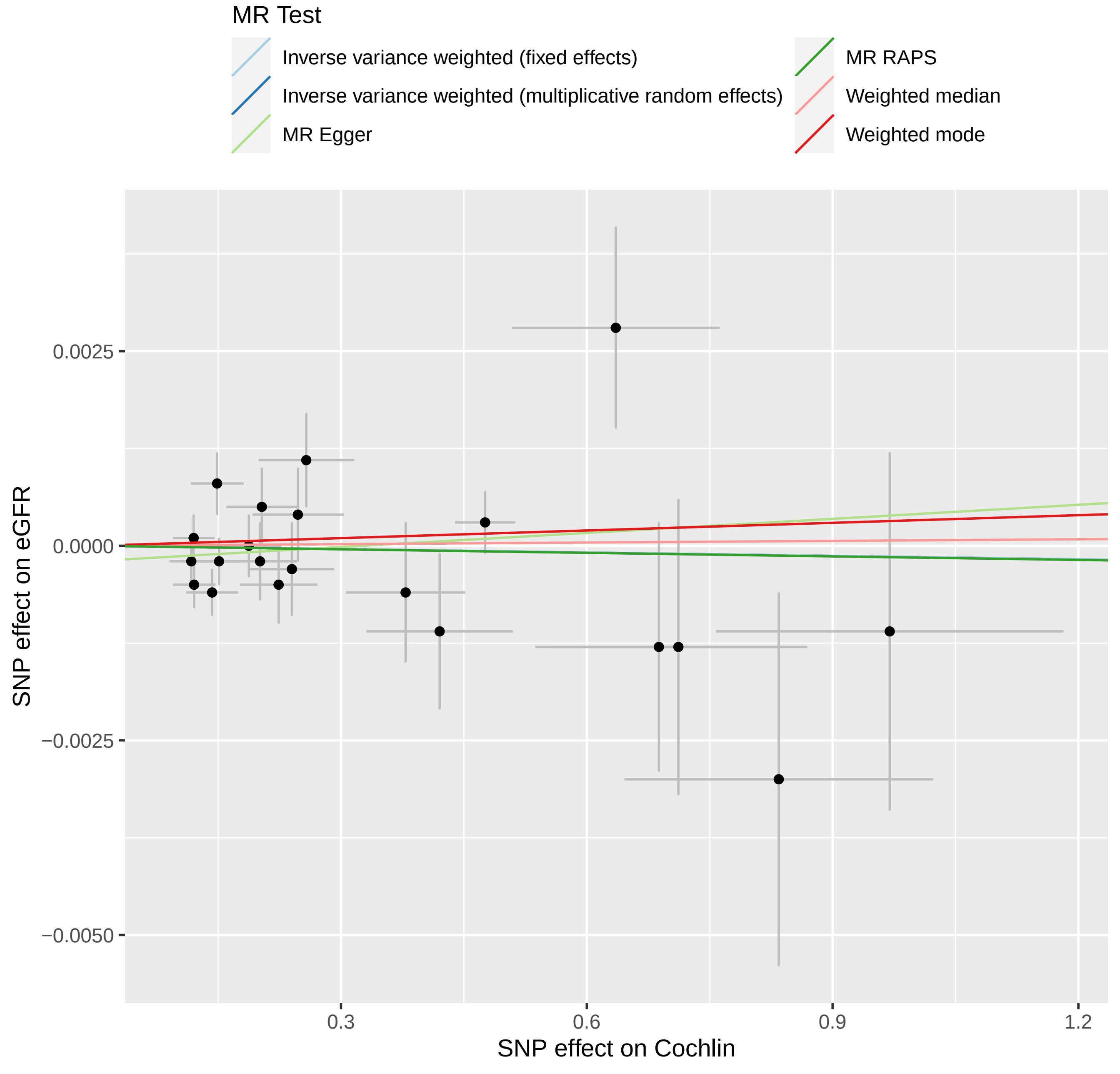

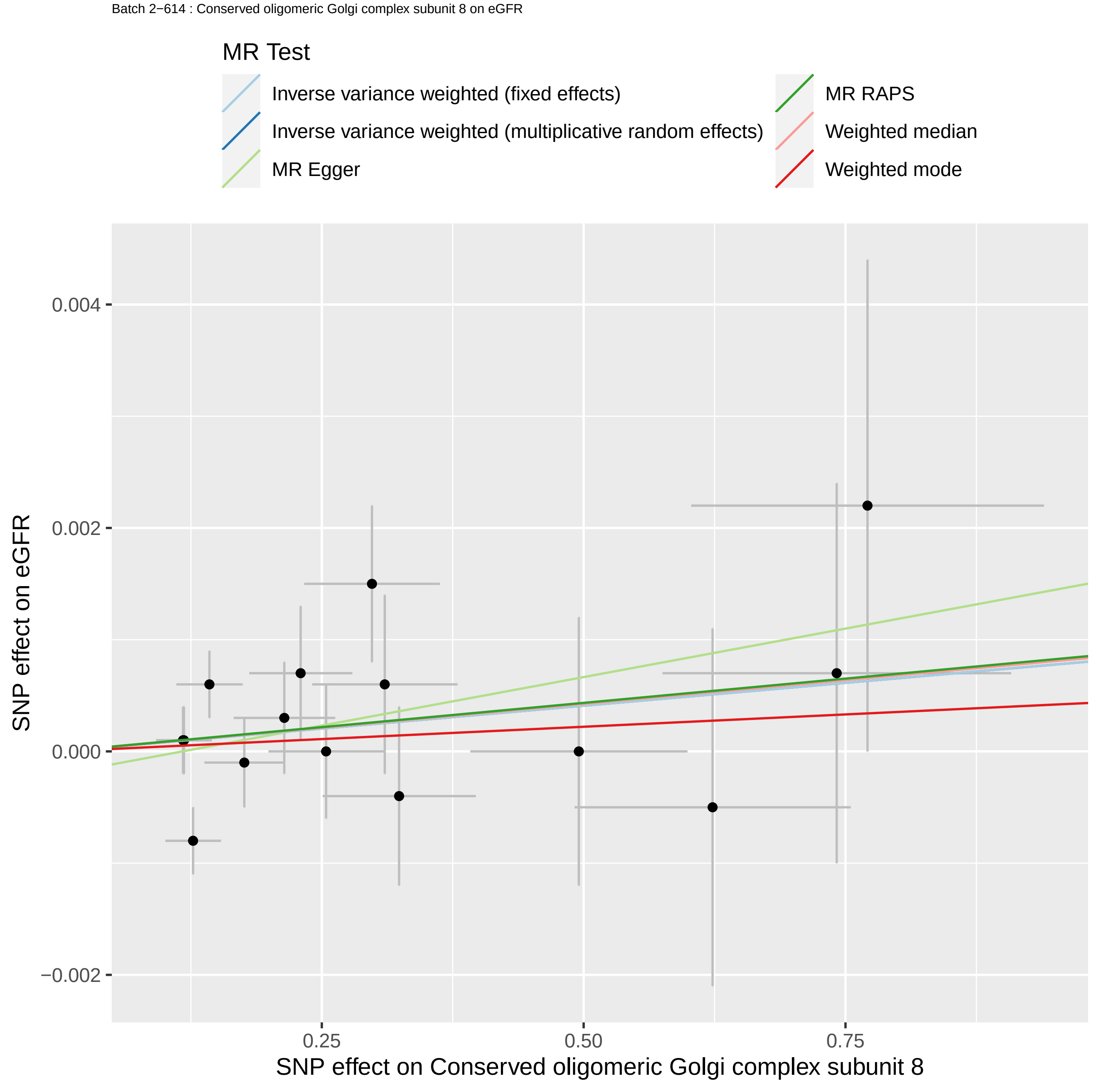
Batch 2−614 : Conserved oligomeric Golgi complex subunit 8 on eGFR
MR Test
Inverse variance weighted (fixed effects)
Inverse variance weighted (multiplicative random effects)
MR Egger
MR RAPS
Weighted median
Weighted mode
0.004
0.002
0.000
−0.002
0.25
0.50
0.75
SNP effect on eGFR
SNP effect on Conserved oligomeric Golgi complex subunit 8

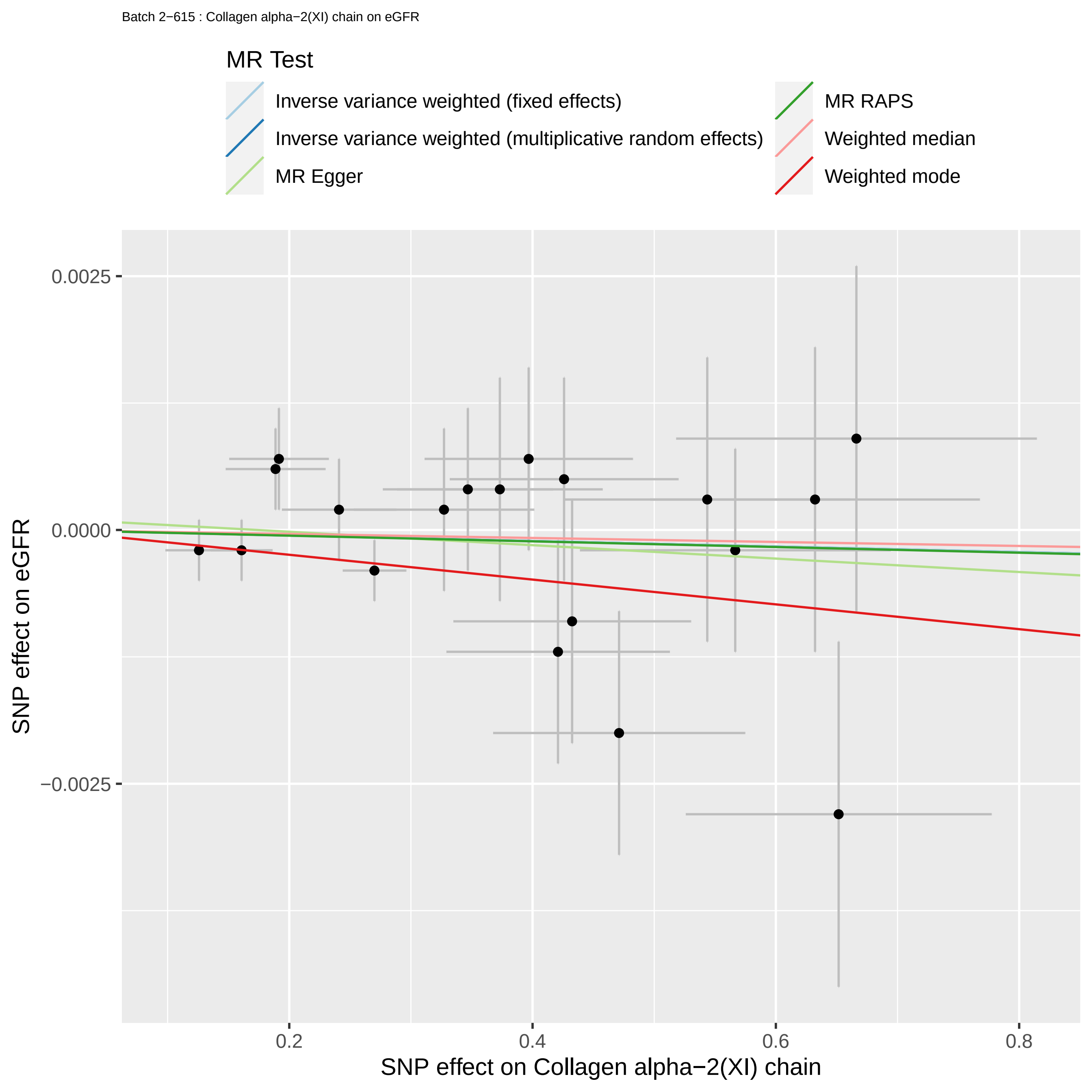

Batch 2−615 : Collagen alpha−2(XI) chain on eGFR
MR Test
Inverse variance weighted (fixed effects)
Inverse variance weighted (multiplicative random effects)
MR Egger
MR RAPS
Weighted median
Weighted mode
0.0025
0.0000
−0.0025
0.2
0.4
0.6
0.8
SNP effect on eGFR
SNP effect on Collagen alpha−2(XI) chain

Batch 2−616 : Collagen alpha−1(XV) chain on eGFR

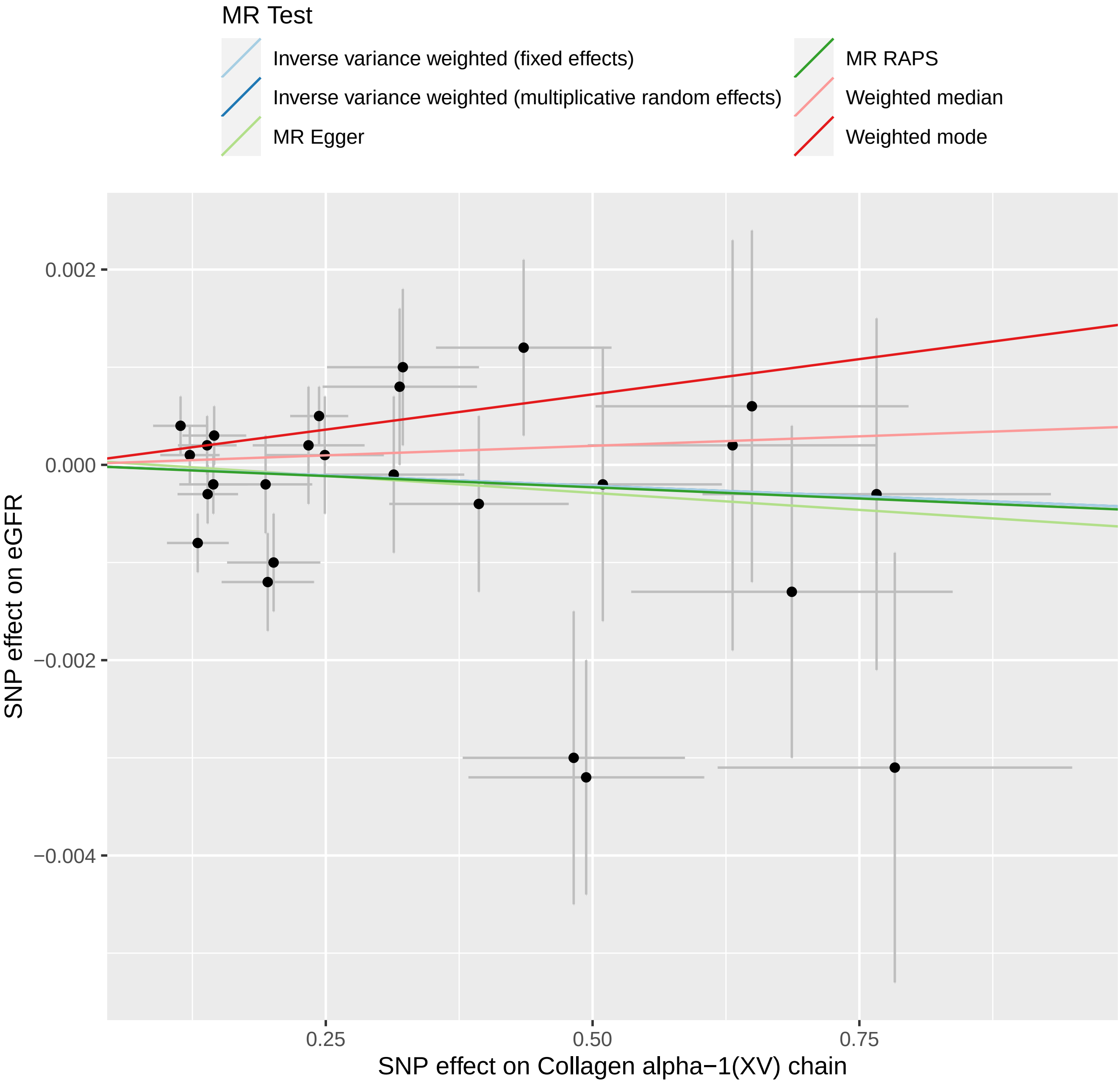

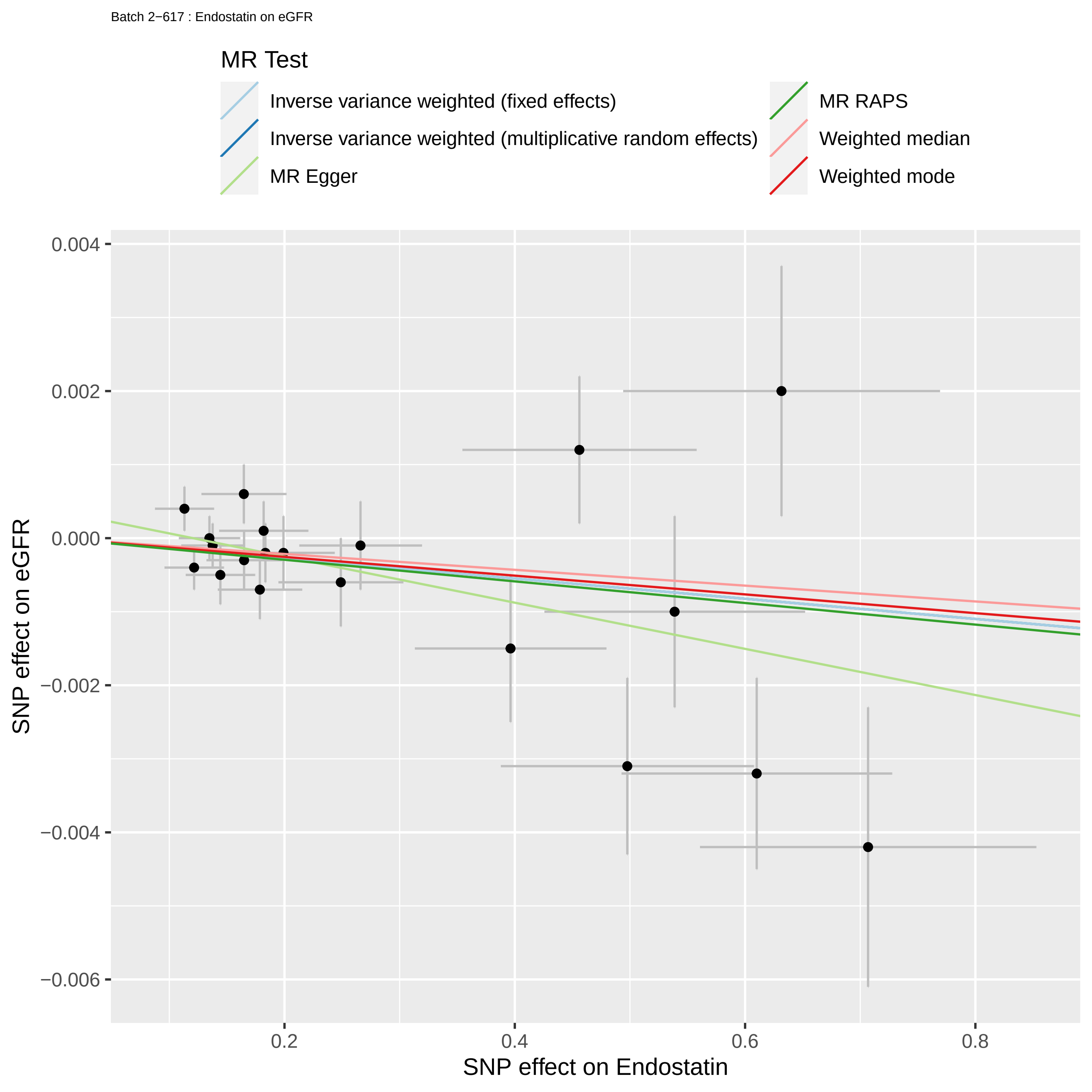

Batch 2−617 : Endostatin on eGFR
MR Test
Inverse variance weighted (fixed effects)
Inverse variance weighted (multiplicative random effects)
MR Egger
MR RAPS
Weighted median
Weighted mode
0.004
0.002
0.000
−0.002
−0.004
−0.006
0.2
0.4
0.6
0.8
SNP effect on eGFR
SNP effect on Endostatin

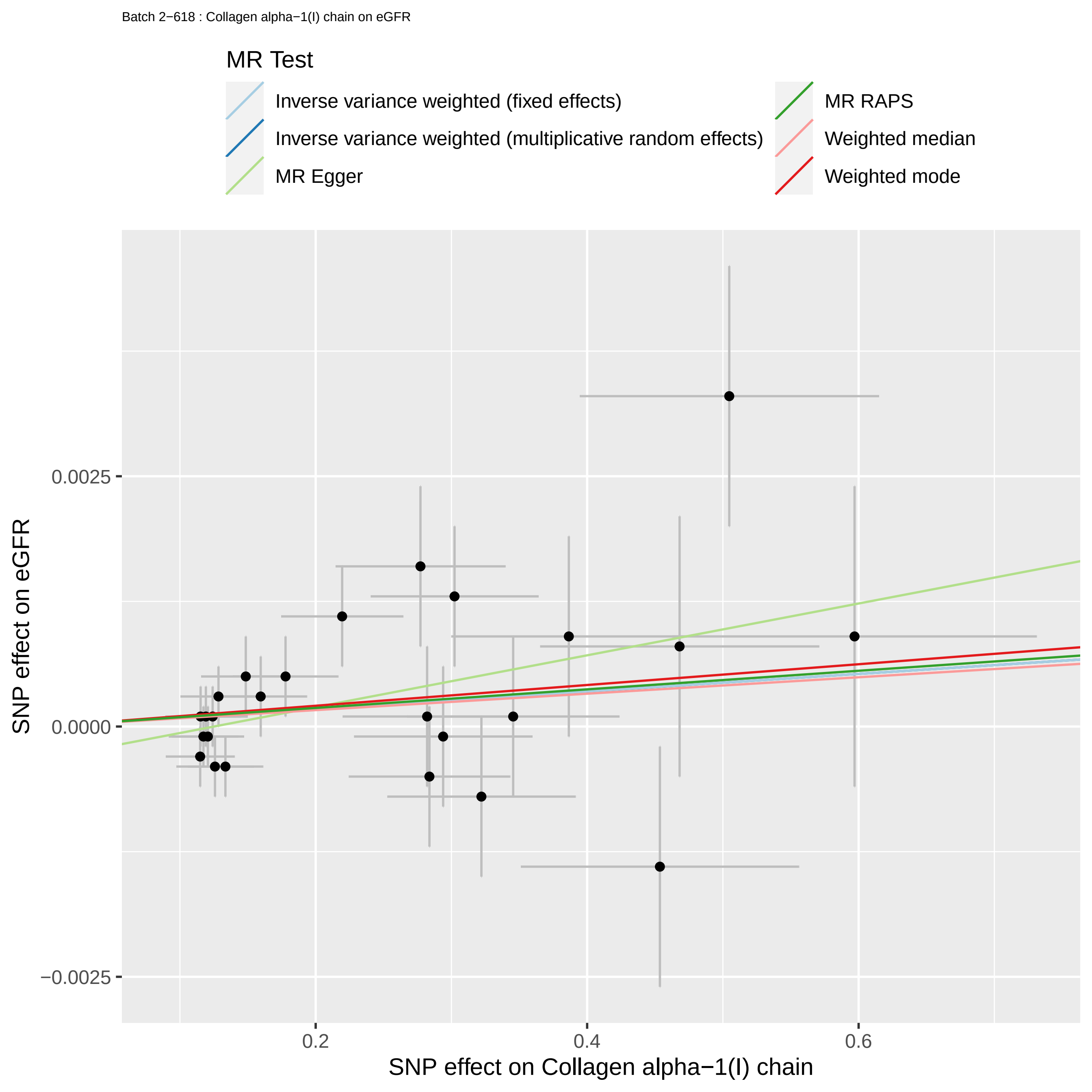
Batch 2−618 : Collagen alpha−1(I) chain on eGFR
MR Test
Inverse variance weighted (fixed effects)
Inverse variance weighted (multiplicative random effects)
MR Egger
MR RAPS
Weighted median
Weighted mode
0.0025
0.0000
−0.0025
0.2
0.4
0.6
SNP effect on eGFR
SNP effect on Collagen alpha−1(I) chain

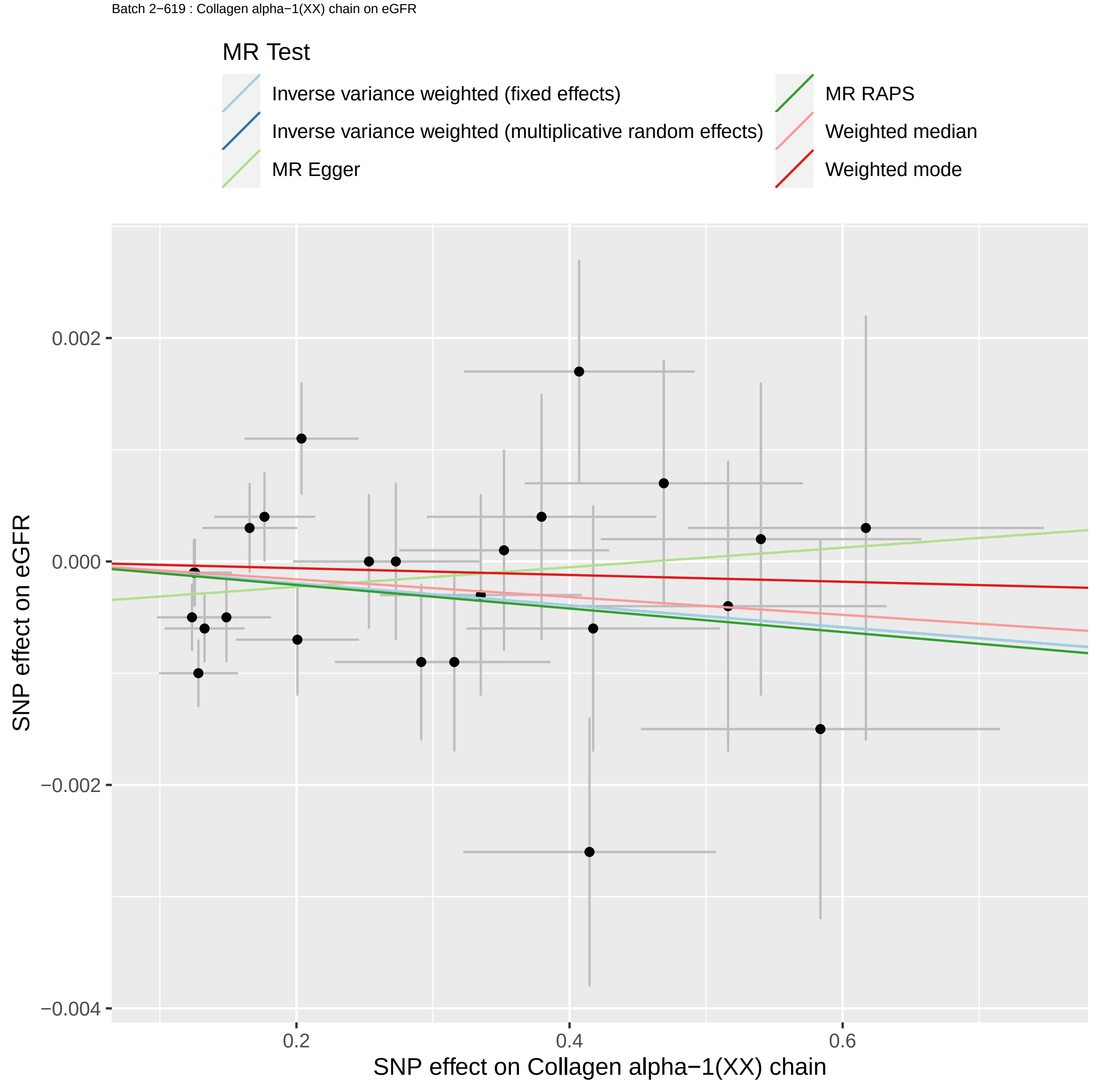
Batch 2−619 : Collagen alpha−1(XX) chain on eGFR
MR Test
Inverse variance weighted (fixed effects)
Inverse variance weighted (multiplicative random effects)
MR Egger
MR RAPS
Weighted median
Weighted mode
SNP effect on eGFR
0.002
0.000
−0.002
−0.004
0.2
0.4
0.6
SNP effect on Collagen alpha−1(XX) chain

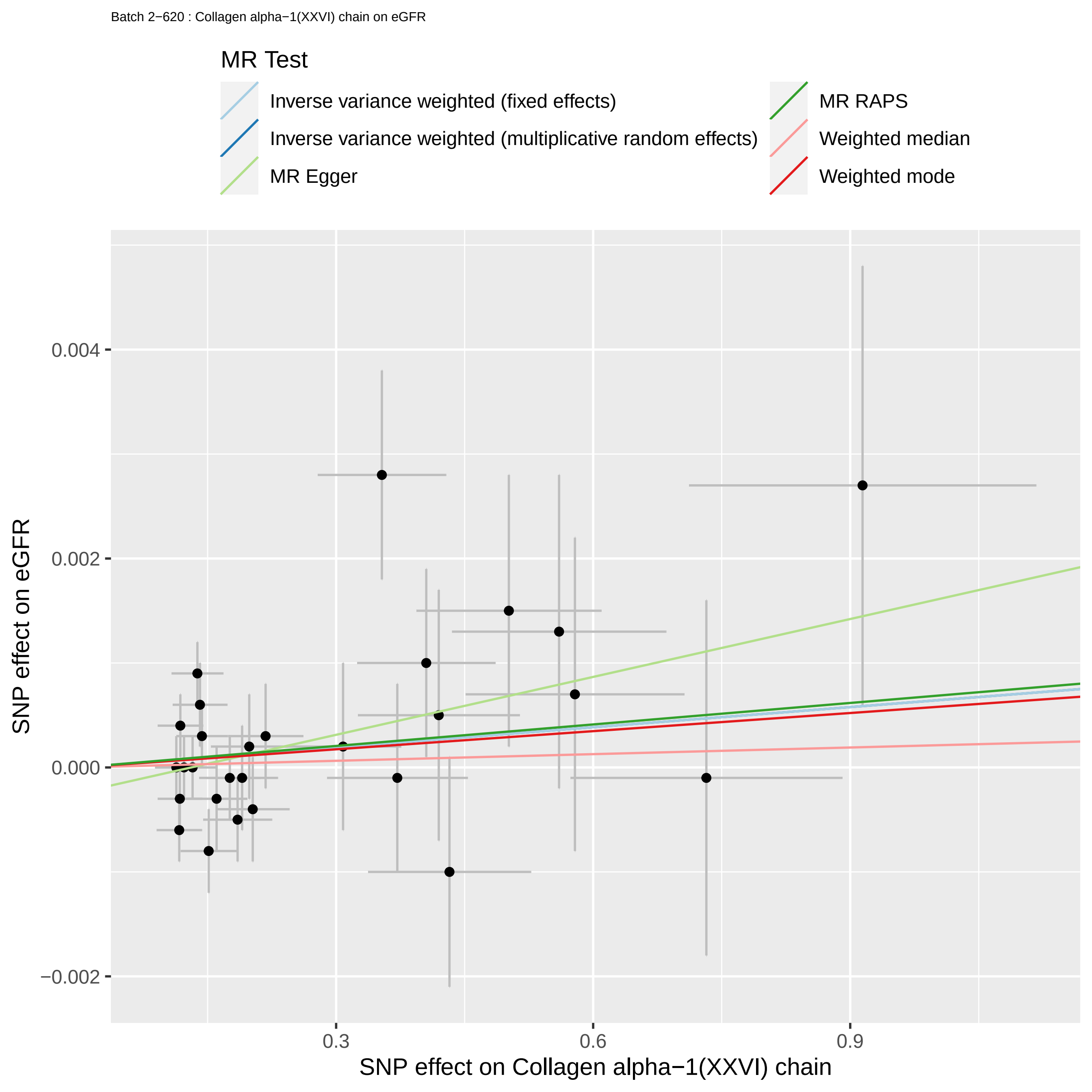

Batch 2−620 : Collagen alpha−1(XXVI) chain on eGFR
MR Test
Inverse variance weighted (fixed effects)
Inverse variance weighted (multiplicative random effects)
MR Egger
MR RAPS
Weighted median
Weighted mode
0.004
0.002
0.000
−0.002
0.3
0.6
0.9
SNP effect on eGFR
SNP effect on Collagen alpha−1(XXVI) chain

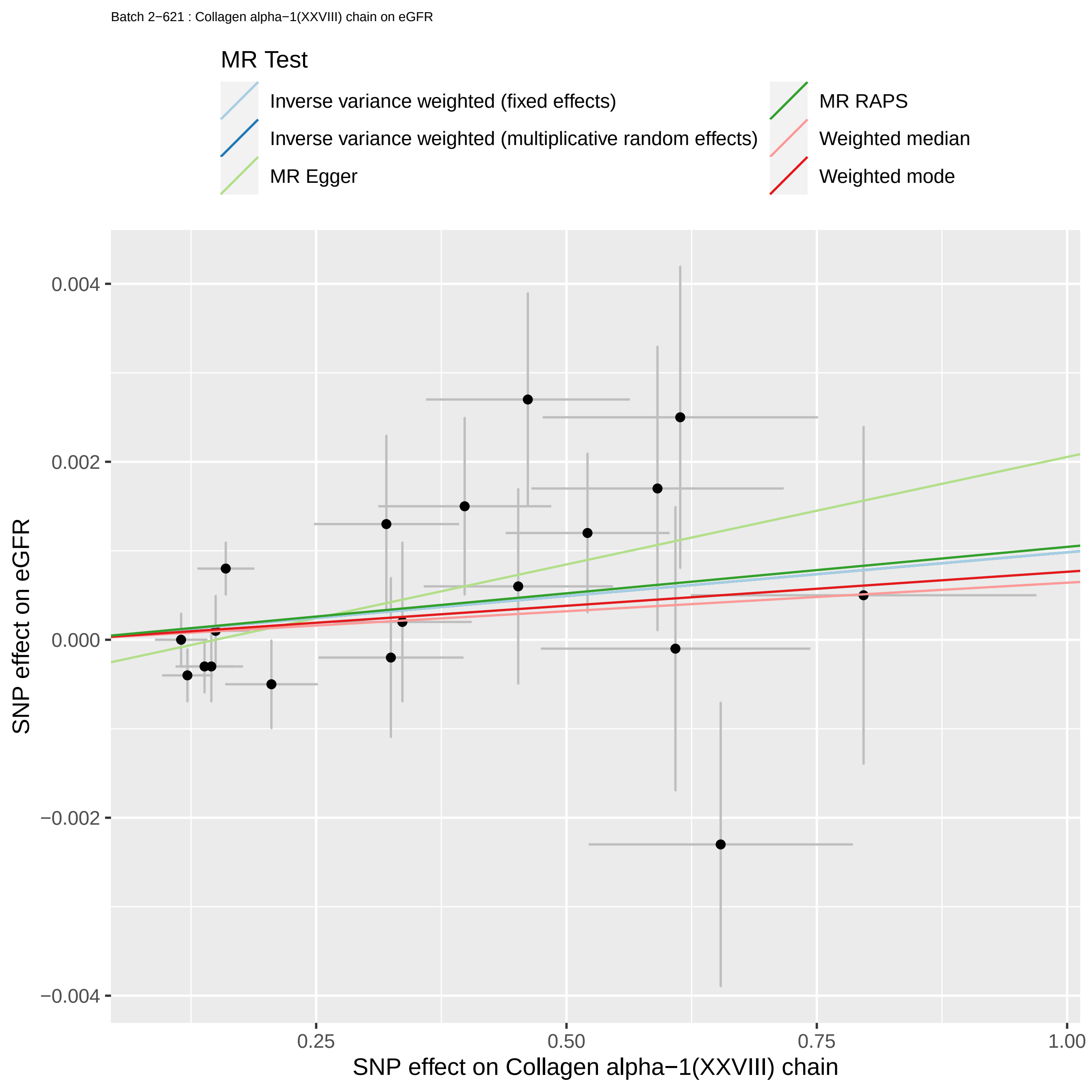

Batch 2−621 : Collagen alpha−1(XXVIII) chain on eGFR
MR Test
Inverse variance weighted (fixed effects)
Inverse variance weighted (multiplicative random effects)
MR Egger
MR RAPS
Weighted median
Weighted mode
0.004
0.002
0.000
−0.002
−0.004
0.25
0.50
0.75
1.00
SNP effect on eGFR
SNP effect on Collagen alpha−1(XXVIII) chain

Batch 2−622 : Collagen alpha−1(VI) chain on eGFR

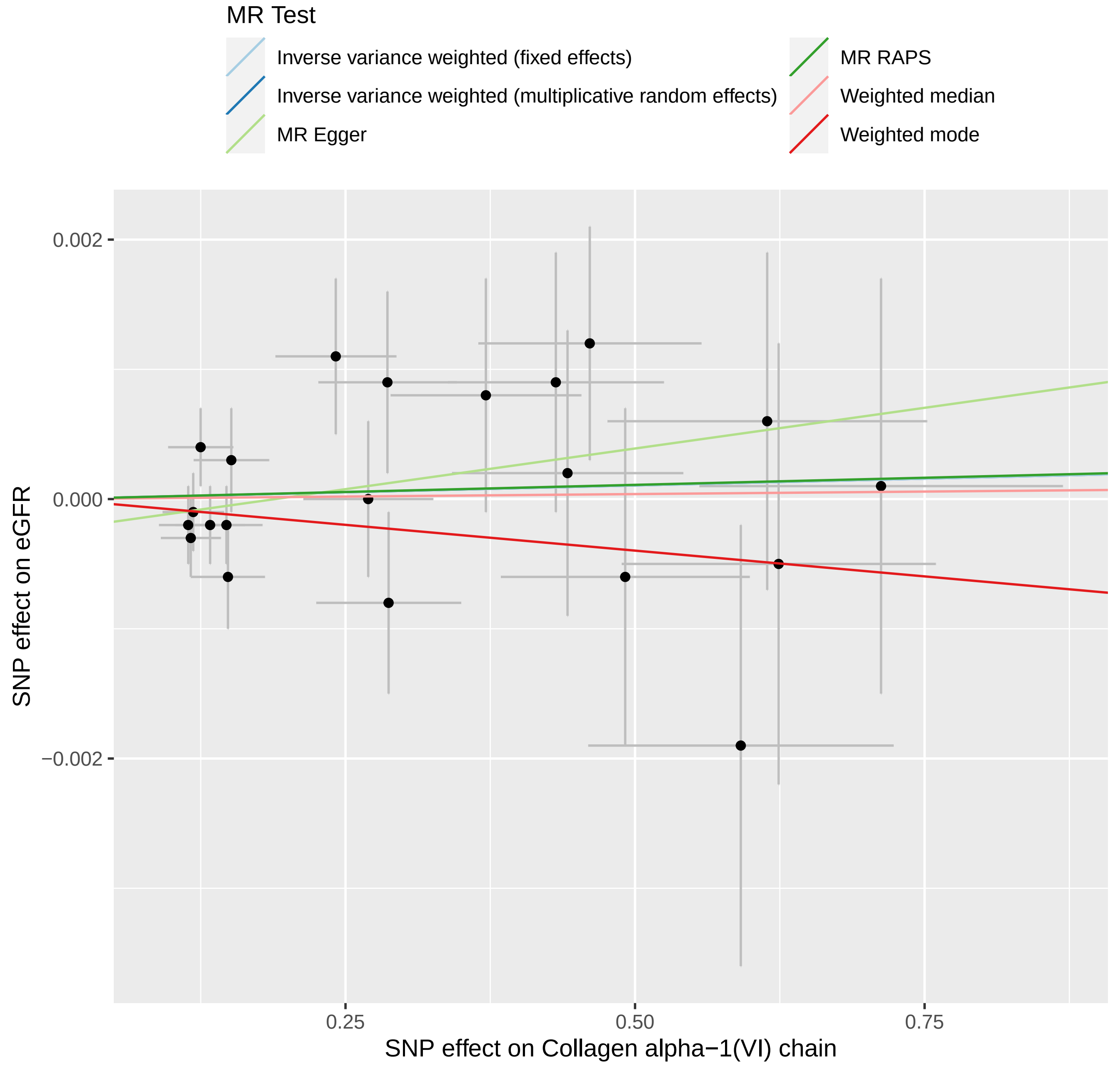

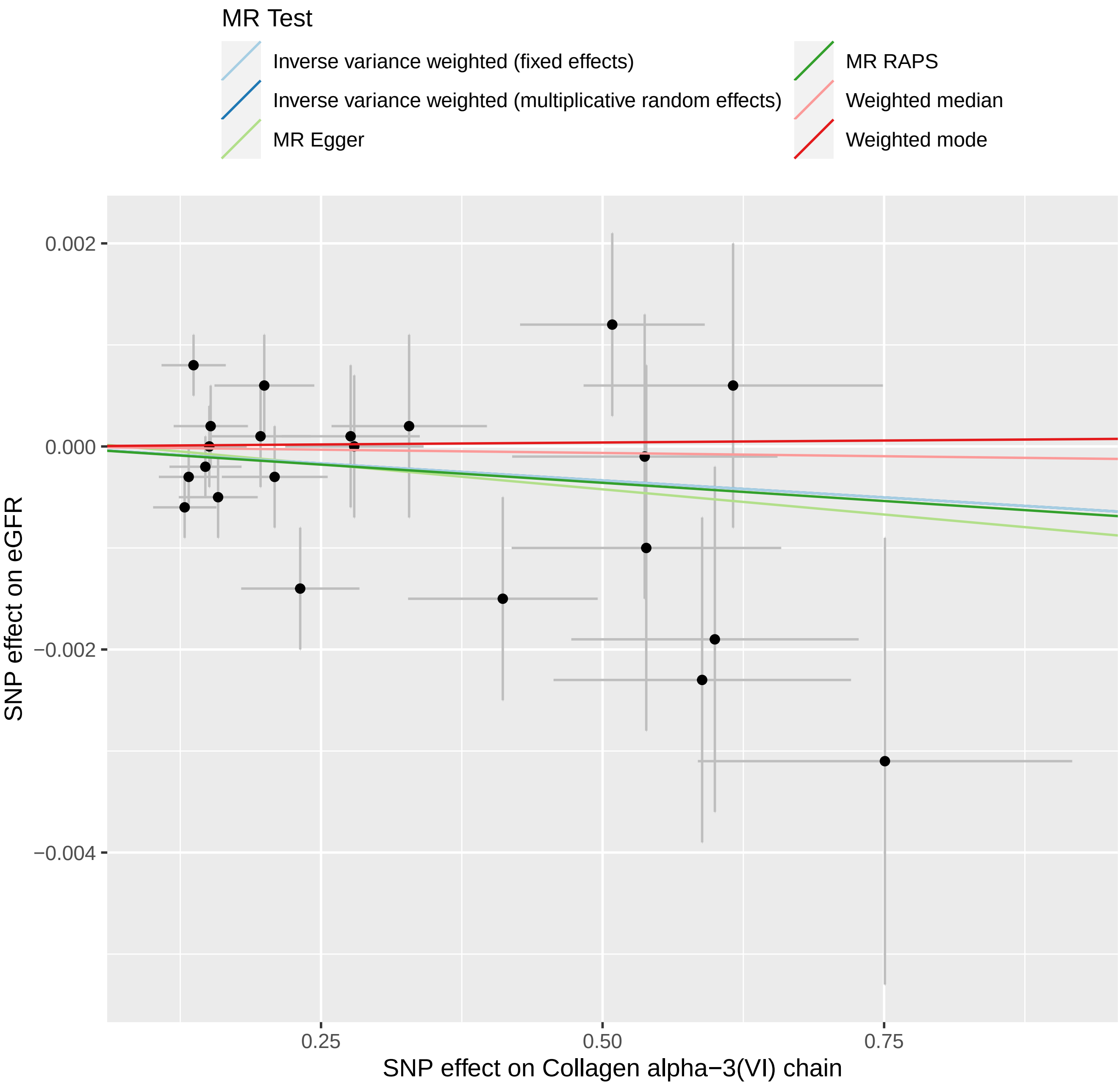
MR Test
Inverse variance weighted (fixed effects)
Inverse variance weighted (multiplicative random effects)
MR Egger
MR RAPS
Weighted median
Weighted mode
0.002
0.000
−0.002
−0.004
SNP effect on eGFR
0.25
0.50
0.75
SNP effect on Collagen alpha−3(VI) chain

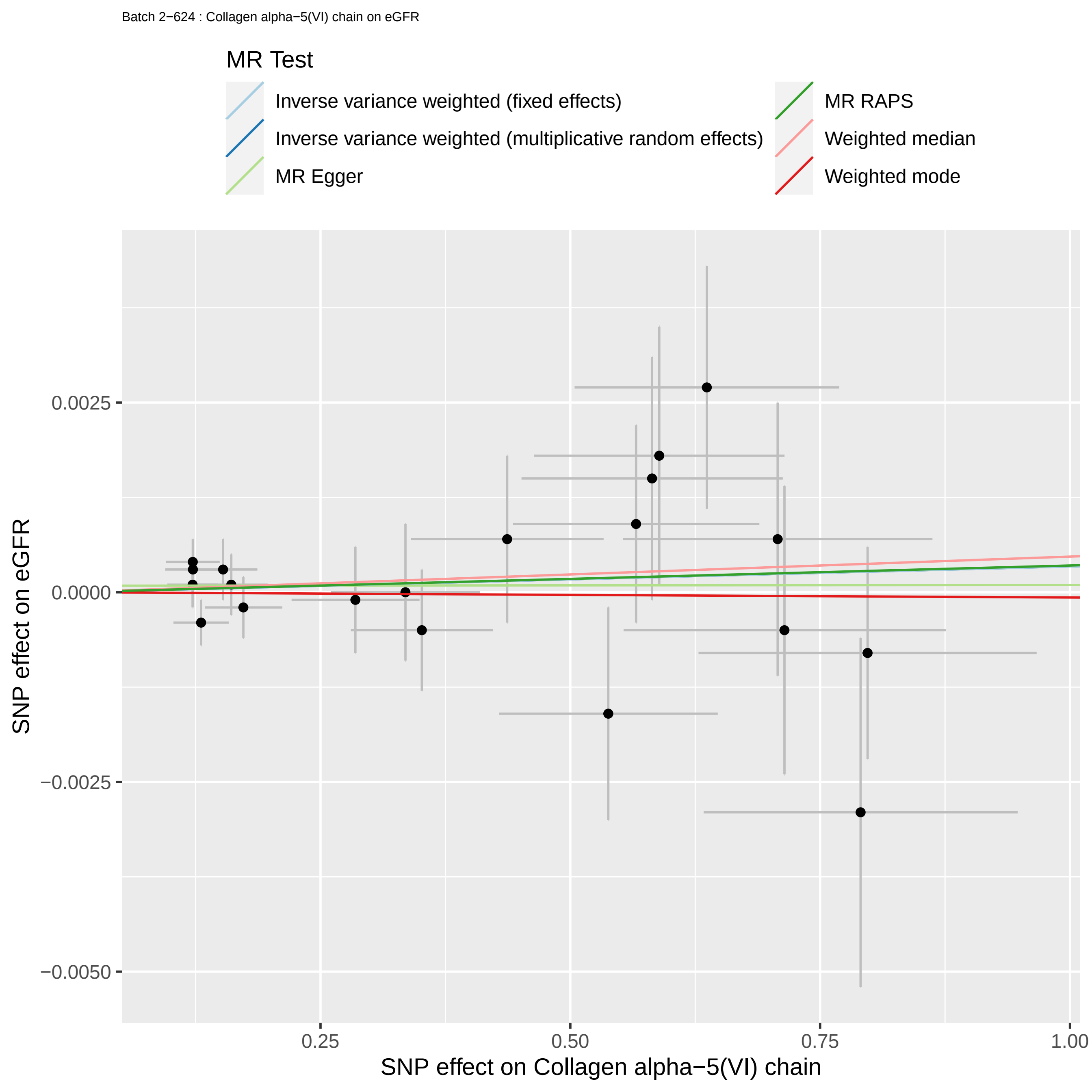

Batch 2−624 : Collagen alpha−5(VI) chain on eGFR
MR Test
Inverse variance weighted (fixed effects)
Inverse variance weighted (multiplicative random effects)
MR Egger
MR RAPS
Weighted median
Weighted mode
0.0025
0.0000
−0.0025
−0.0050
0.25
0.50
0.75
1.00
SNP effect on eGFR
SNP effect on Collagen alpha−5(VI) chain

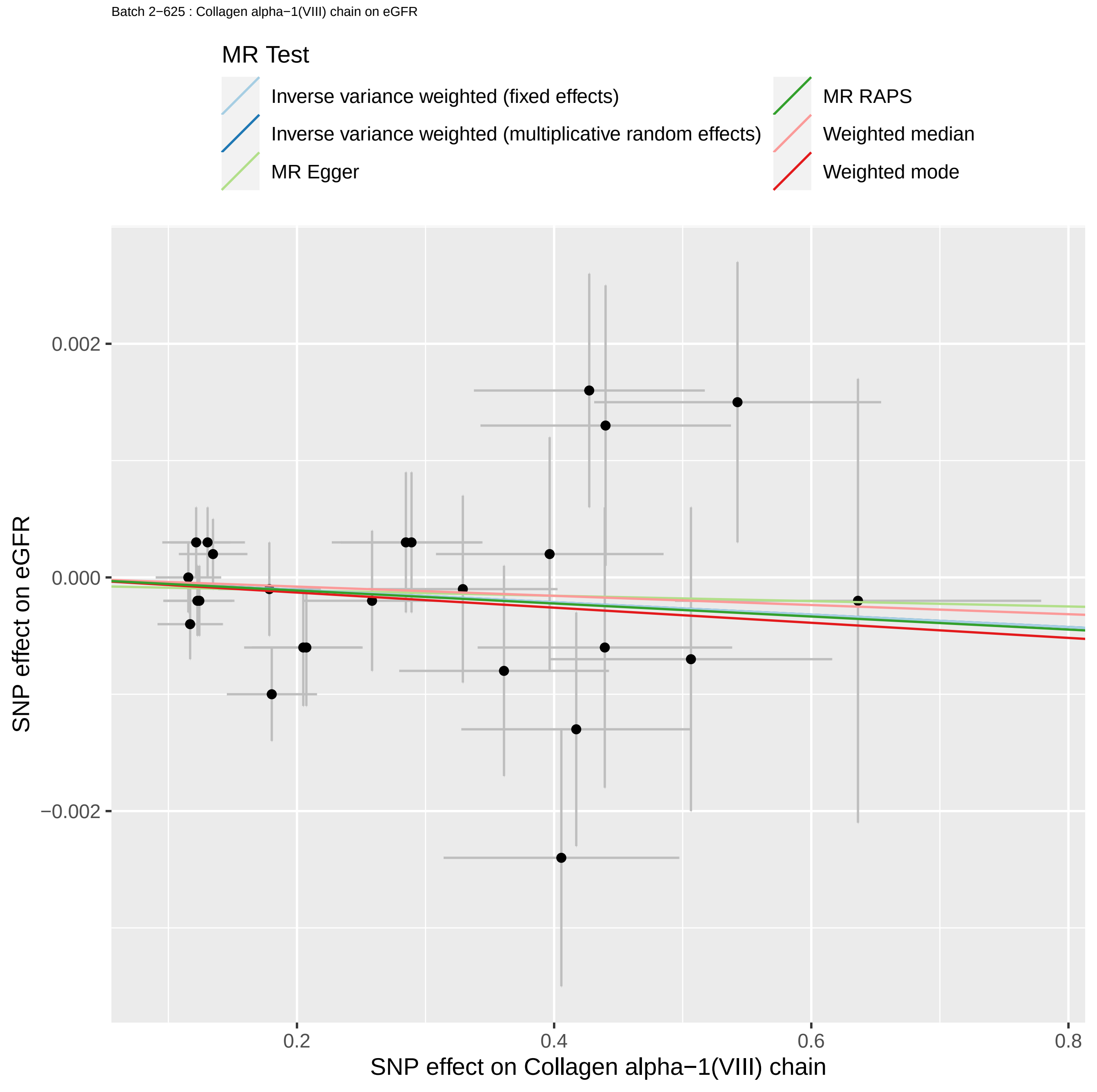
Batch 2−625 : Collagen alpha−1(VIII) chain on eGFR
MR Test
Inverse variance weighted (fixed effects)
Inverse variance weighted (multiplicative random effects)
MR Egger
MR RAPS
Weighted median
Weighted mode
0.002
0.000
−0.002
0.2
0.4
0.6
0.8
SNP effect on eGFR
SNP effect on Collagen alpha−1(VIII) chain

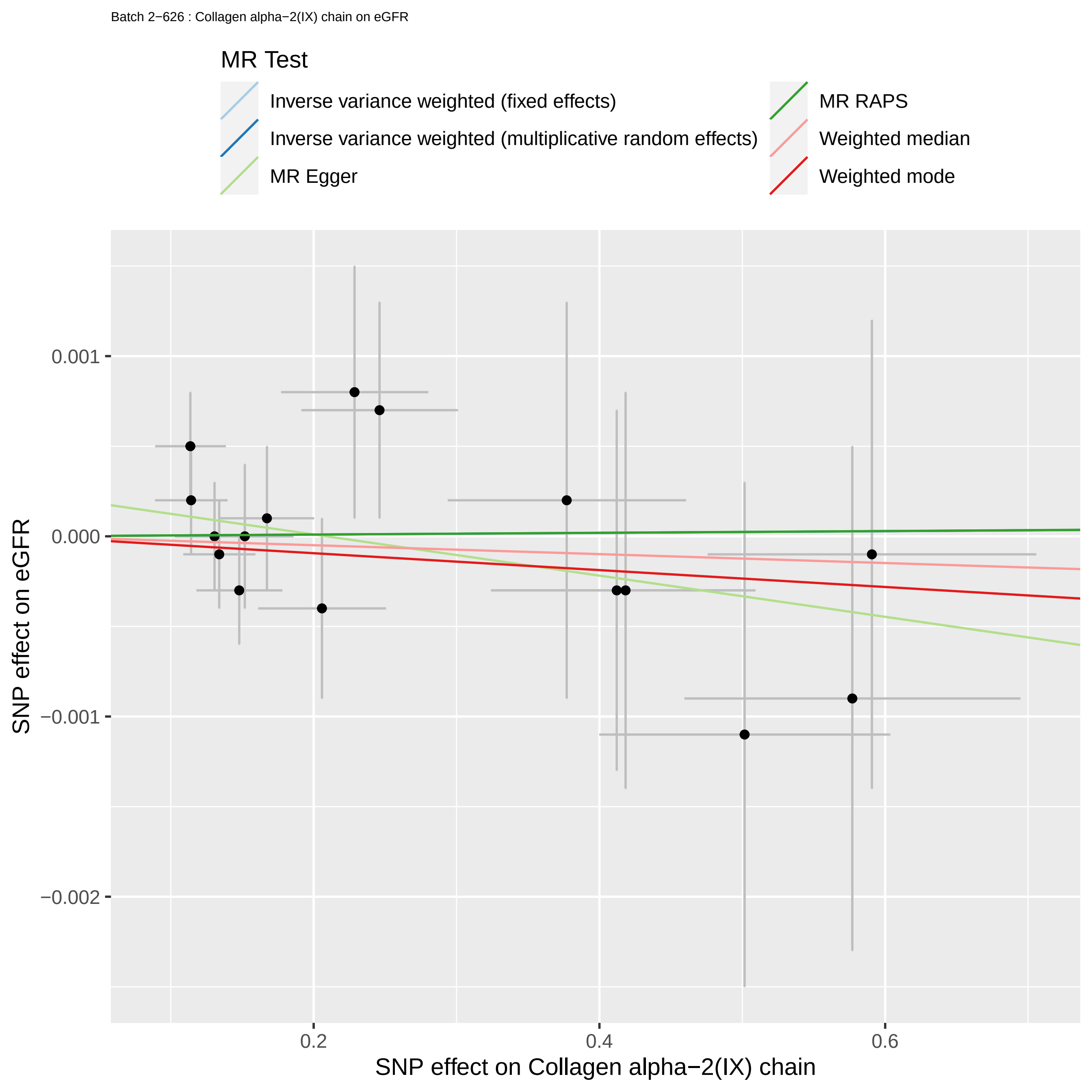

Batch 2−626 : Collagen alpha−2(IX) chain on eGFR
MR Test
Inverse variance weighted (fixed effects)
Inverse variance weighted (multiplicative random effects)
MR Egger
MR RAPS
Weighted median
Weighted mode
0.001
0.000
−0.001
−0.002
0.2
0.4
0.6
SNP effect on eGFR
SNP effect on Collagen alpha−2(IX) chain

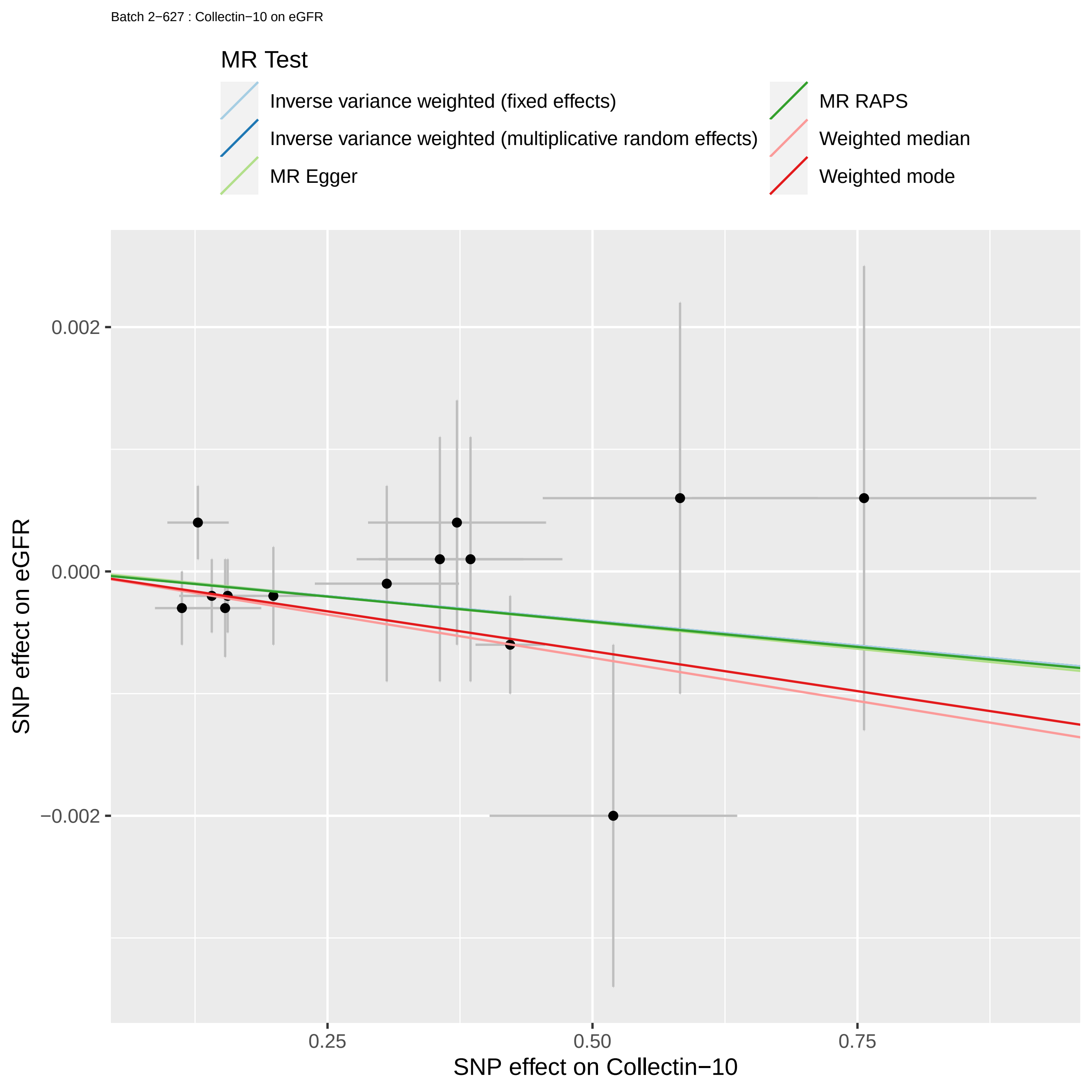

Batch 2−627 : Collectin−10 on eGFR
MR Test
Inverse variance weighted (fixed effects)
Inverse variance weighted (multiplicative random effects)
MR Egger
MR RAPS
Weighted median
Weighted mode
0.002
0.000
−0.002
0.25
0.50
0.75
SNP effect on eGFR
SNP effect on Collectin−10

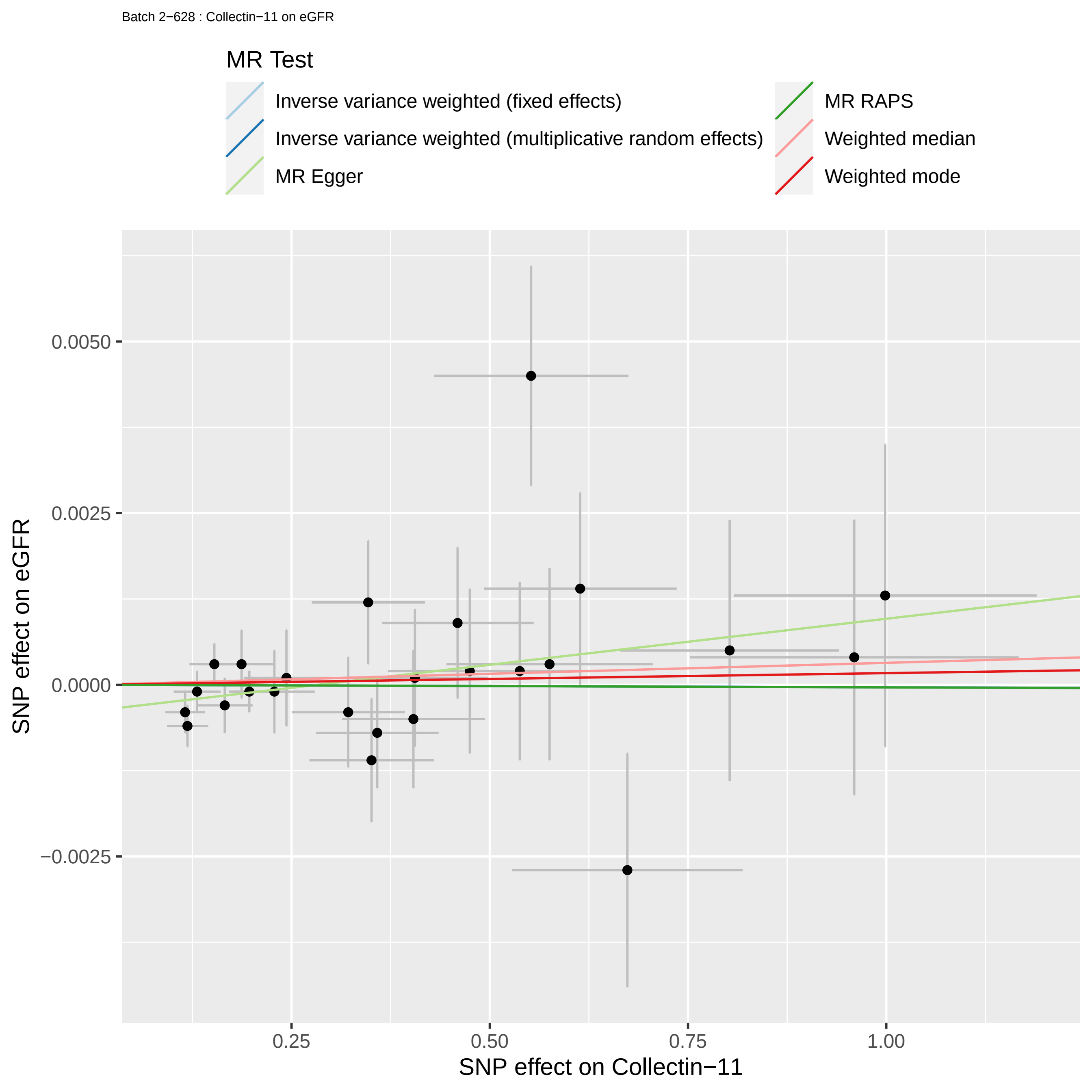
Batch 2−628 : Collectin−11 on eGFR
MR Test
Inverse variance weighted (fixed effects)
Inverse variance weighted (multiplicative random effects)
MR Egger
MR RAPS
Weighted median
Weighted mode
SNP effect on eGFR
0.0050
0.0025
0.0000
−0.0025
0.25
0.50
0.75
1.00
SNP effect on Collectin−11

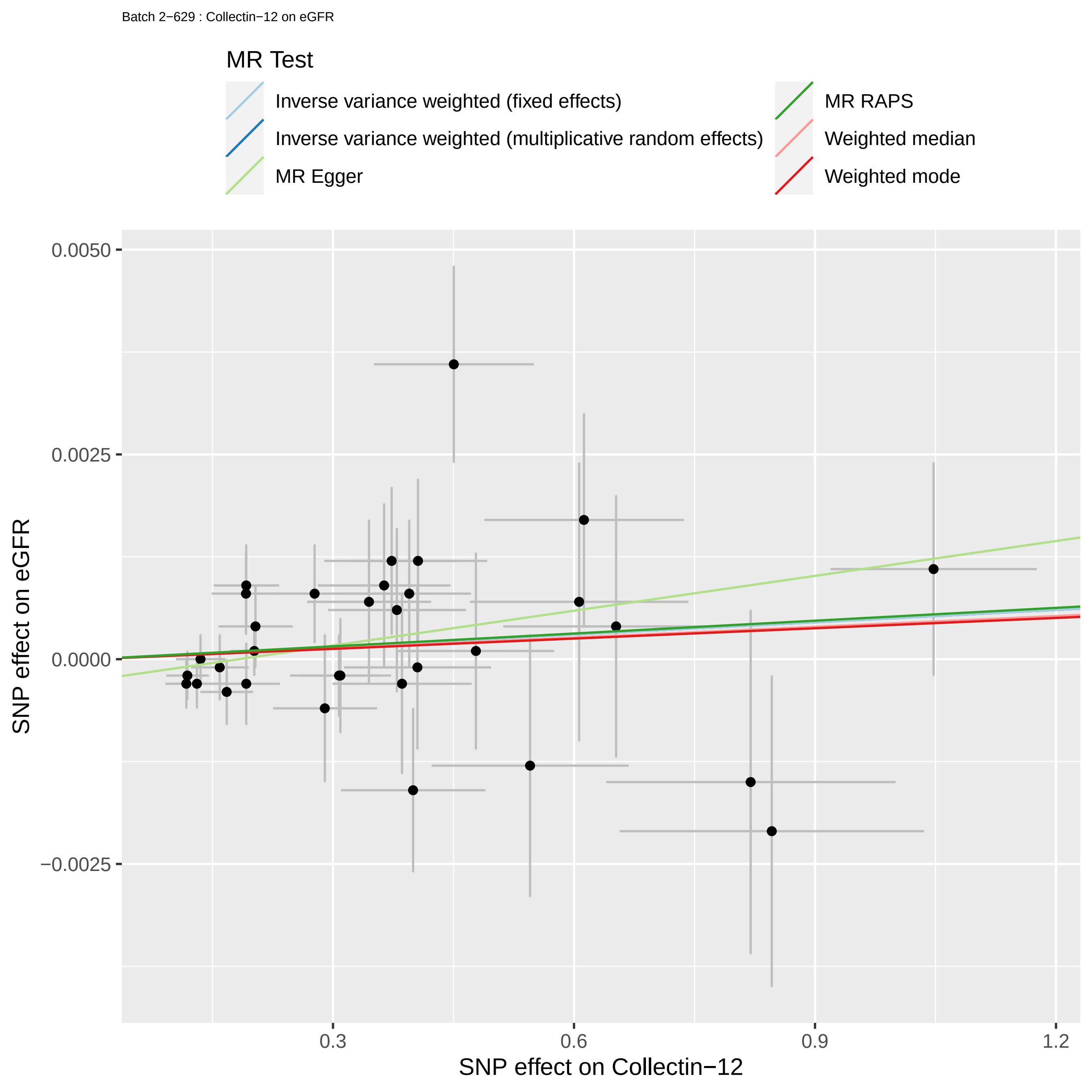

Batch 2−629 : Collectin−12 on eGFR
MR Test
Inverse variance weighted (fixed effects)
Inverse variance weighted (multiplicative random effects)
MR Egger
MR RAPS
Weighted median
Weighted mode
0.0050
0.0025
0.0000
−0.0025
0.3
0.6
0.9
1.2
SNP effect on eGFR
SNP effect on Collectin−12

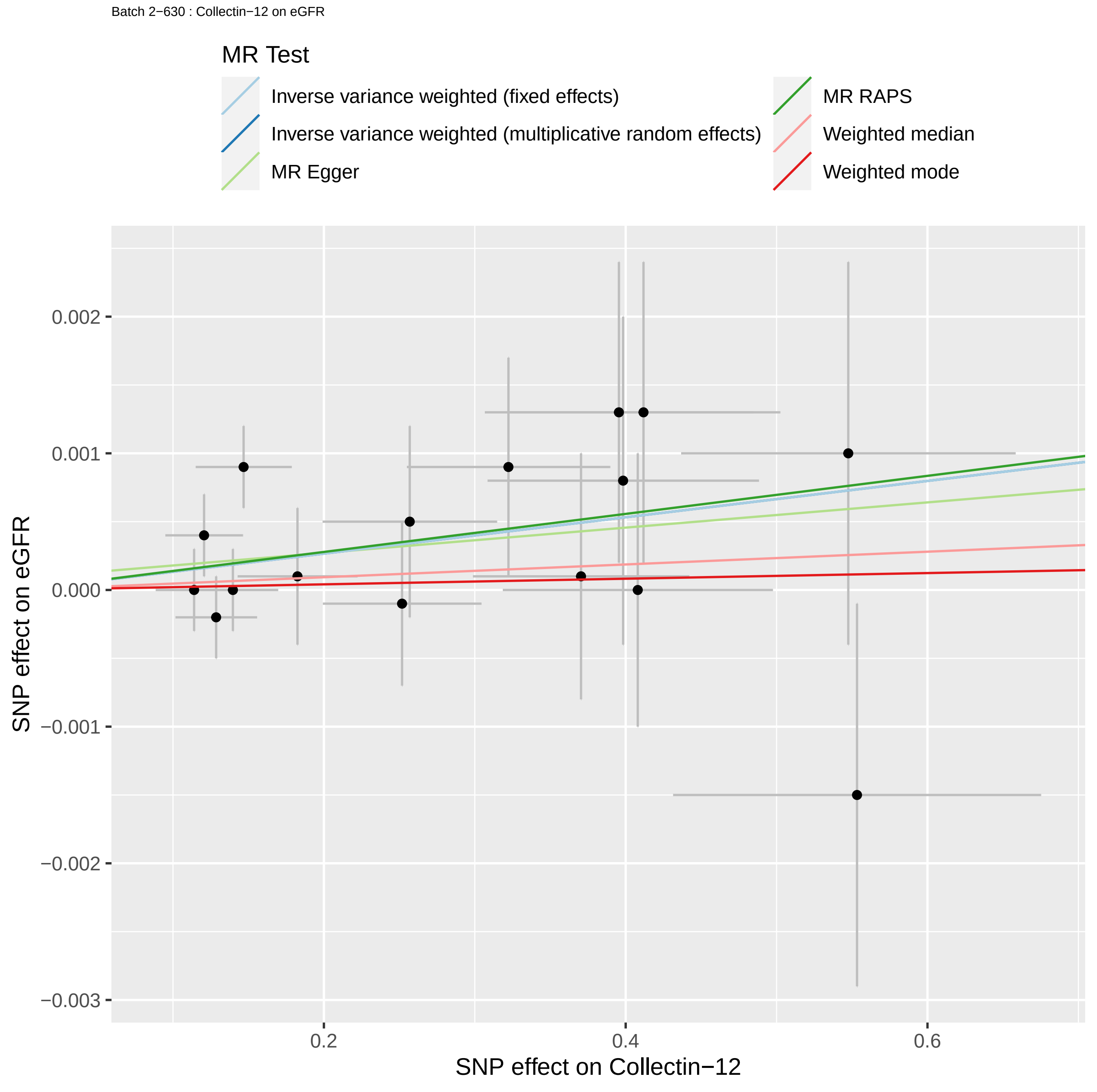

Batch 2−630 : Collectin−12 on eGFR
MR Test
Inverse variance weighted (fixed effects)
Inverse variance weighted (multiplicative random effects)
MR Egger
MR RAPS
Weighted median
Weighted mode
0.002
0.001
0.000
−0.001
−0.002
−0.003
0.2
0.4
0.6
SNP effect on eGFR
SNP effect on Collectin−12

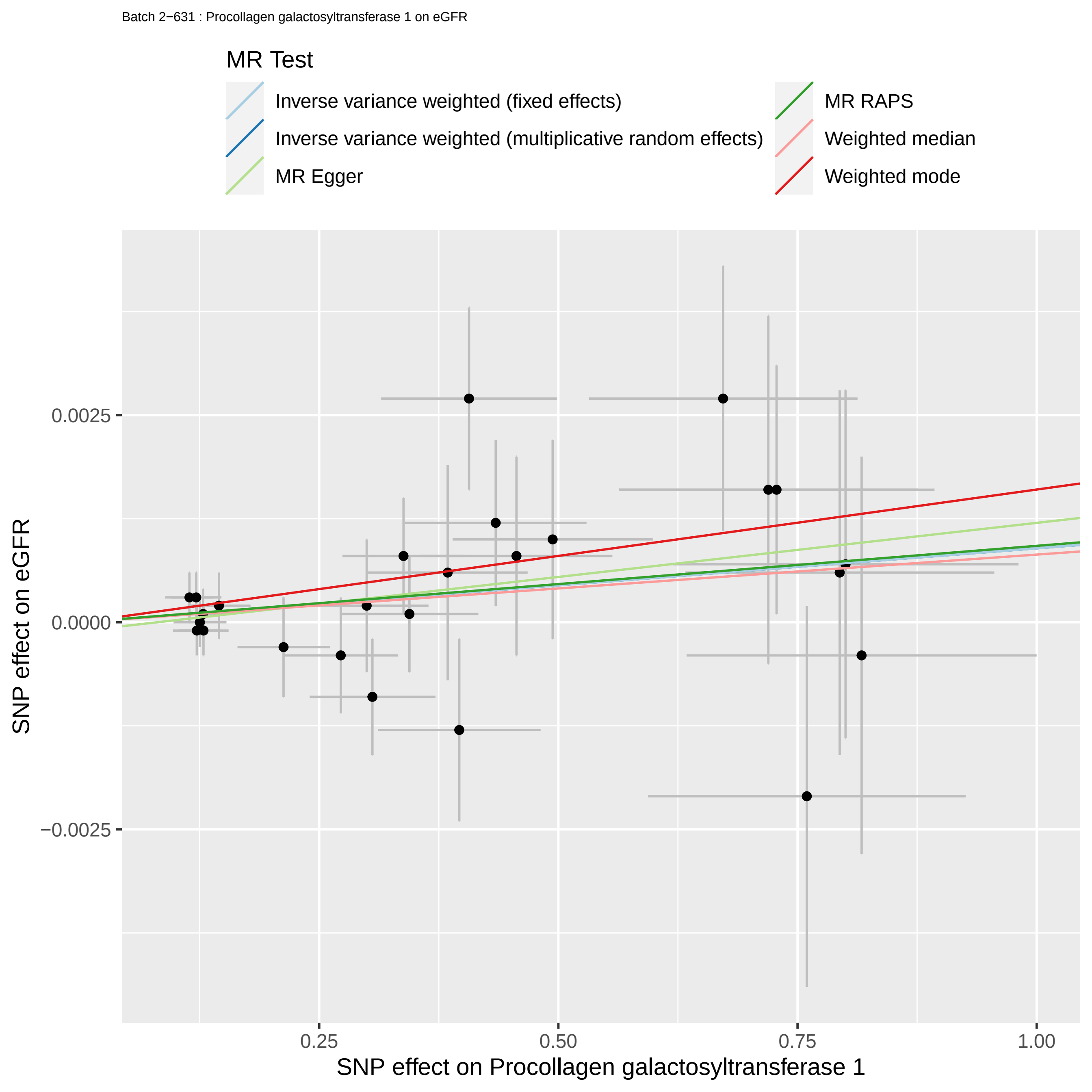

Batch 2−631 : Procollagen galactosyltransferase 1 on eGFR
MR Test
Inverse variance weighted (fixed effects)
Inverse variance weighted (multiplicative random effects)
MR Egger
MR RAPS
Weighted median
Weighted mode
0.0025
0.0000
−0.0025
SNP effect on eGFR
0.25
0.50
0.75
1.00
SNP effect on Procollagen galactosyltransferase 1

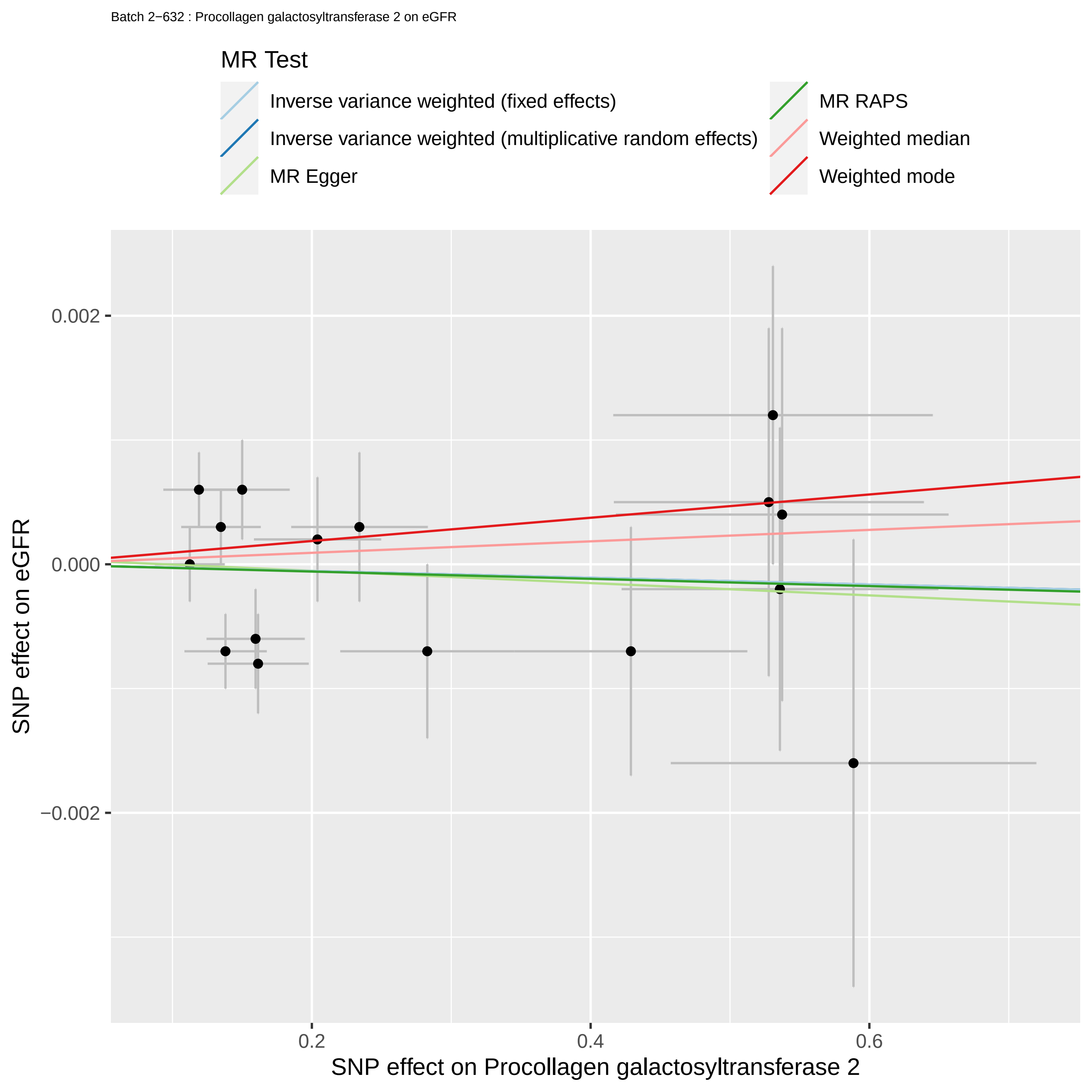
Batch 2−632 : Procollagen galactosyltransferase 2 on eGFR
MR Test
Inverse variance weighted (fixed effects)
Inverse variance weighted (multiplicative random effects)
MR Egger
MR RAPS
Weighted median
Weighted mode
0.002
0.000
−0.002
0.2
0.4
0.6
SNP effect on eGFR
SNP effect on Procollagen galactosyltransferase 2

Batch 2−633 : COMM domain−containing protein 1 on eGFR

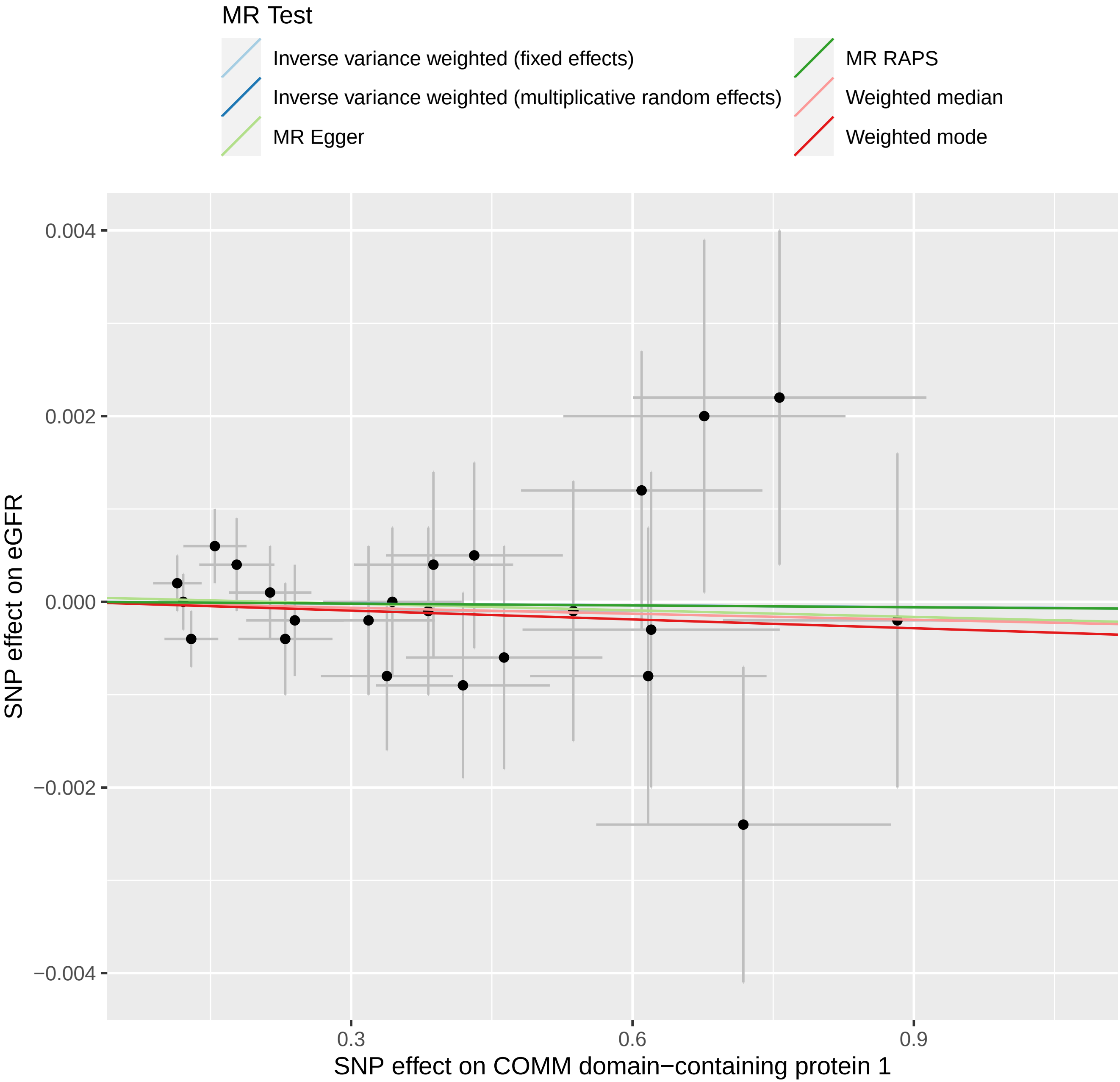

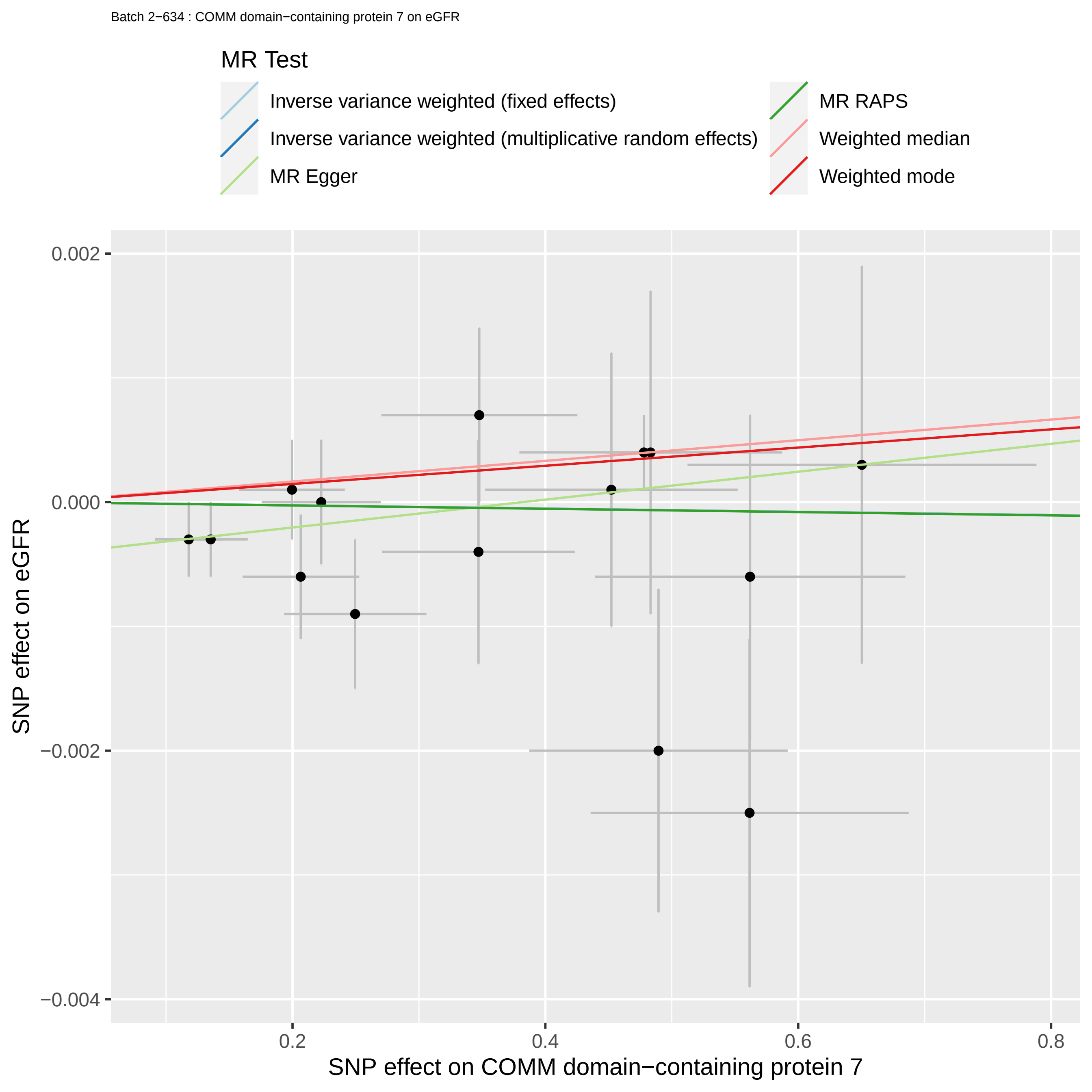
Batch 2−634 : COMM domain−containing protein 7 on eGFR
MR Test
Inverse variance weighted (fixed effects)
Inverse variance weighted (multiplicative random effects)
MR Egger
MR RAPS
Weighted median
Weighted mode
0.002
0.000
−0.002
−0.004
0.2
0.4
0.6
0.8
SNP effect on eGFR
SNP effect on COMM domain−containing protein 7

Batch 2−635 : COP9 signalosome complex subunit 2 on eGFR

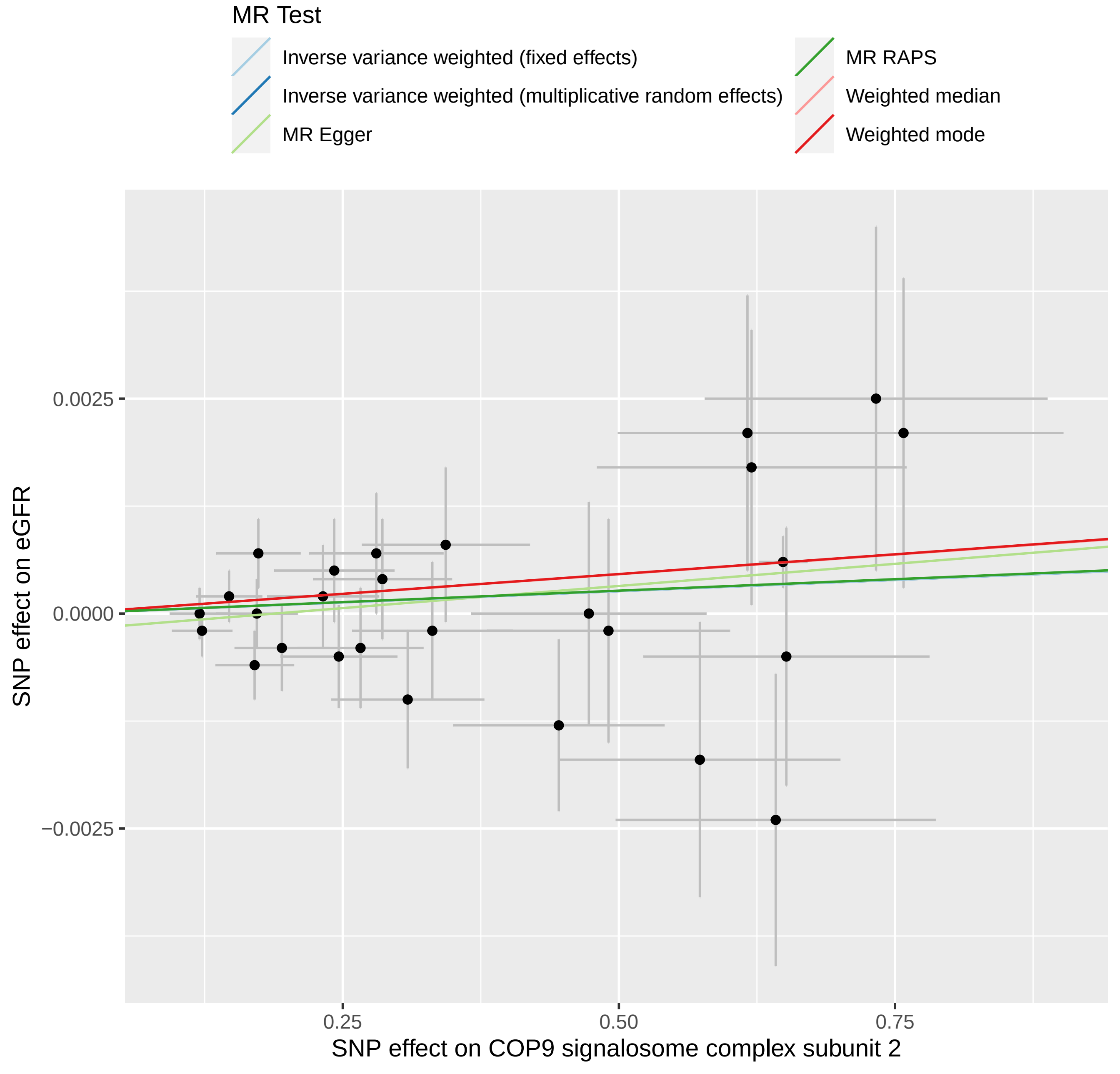

Batch 2−636 : Coactosin−like protein on eGFR

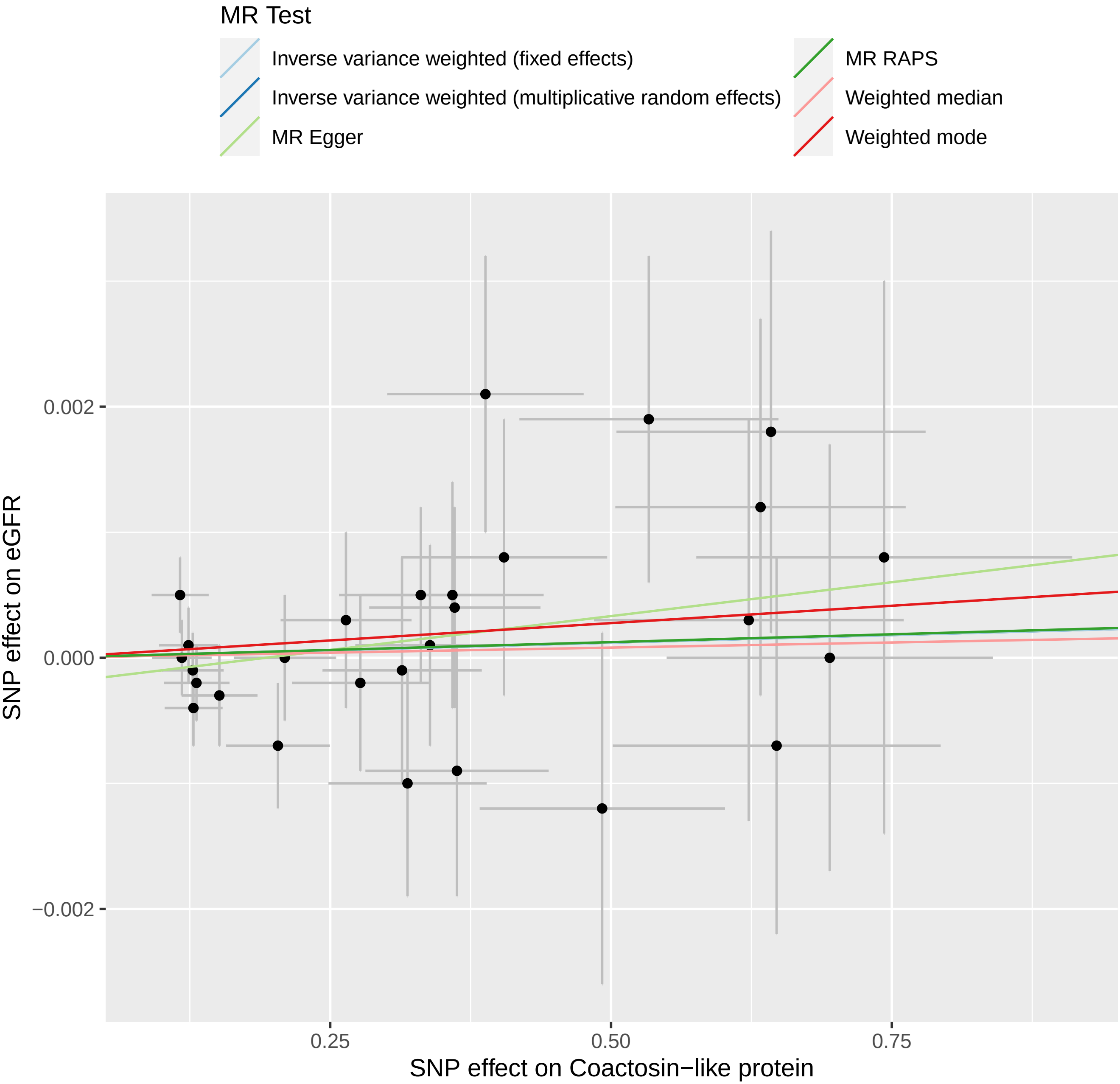

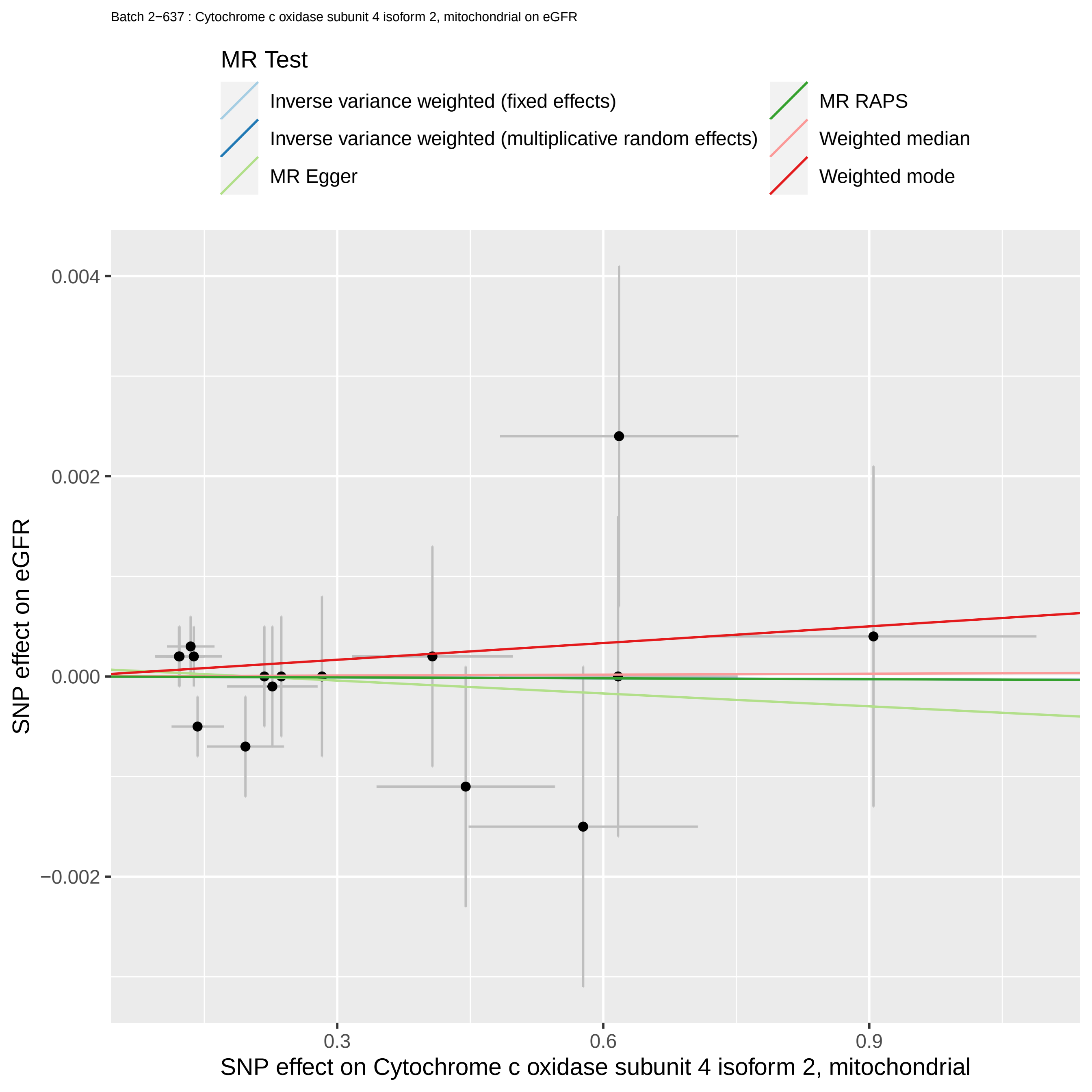

Batch 2−637 : Cytochrome c oxidase subunit 4 isoform 2, mitochondrial on eGFR
MR Test
Inverse variance weighted (fixed effects)
Inverse variance weighted (multiplicative random effects)
MR Egger
MR RAPS
Weighted median
Weighted mode
0.004
0.002
0.000
−0.002
SNP effect on eGFR
0.3
0.6
0.9
SNP effect on Cytochrome c oxidase subunit 4 isoform 2, mitochondrial

Batch 2−638 : Cytochrome c oxidase subunit 5B, mitochondrial on eGFR

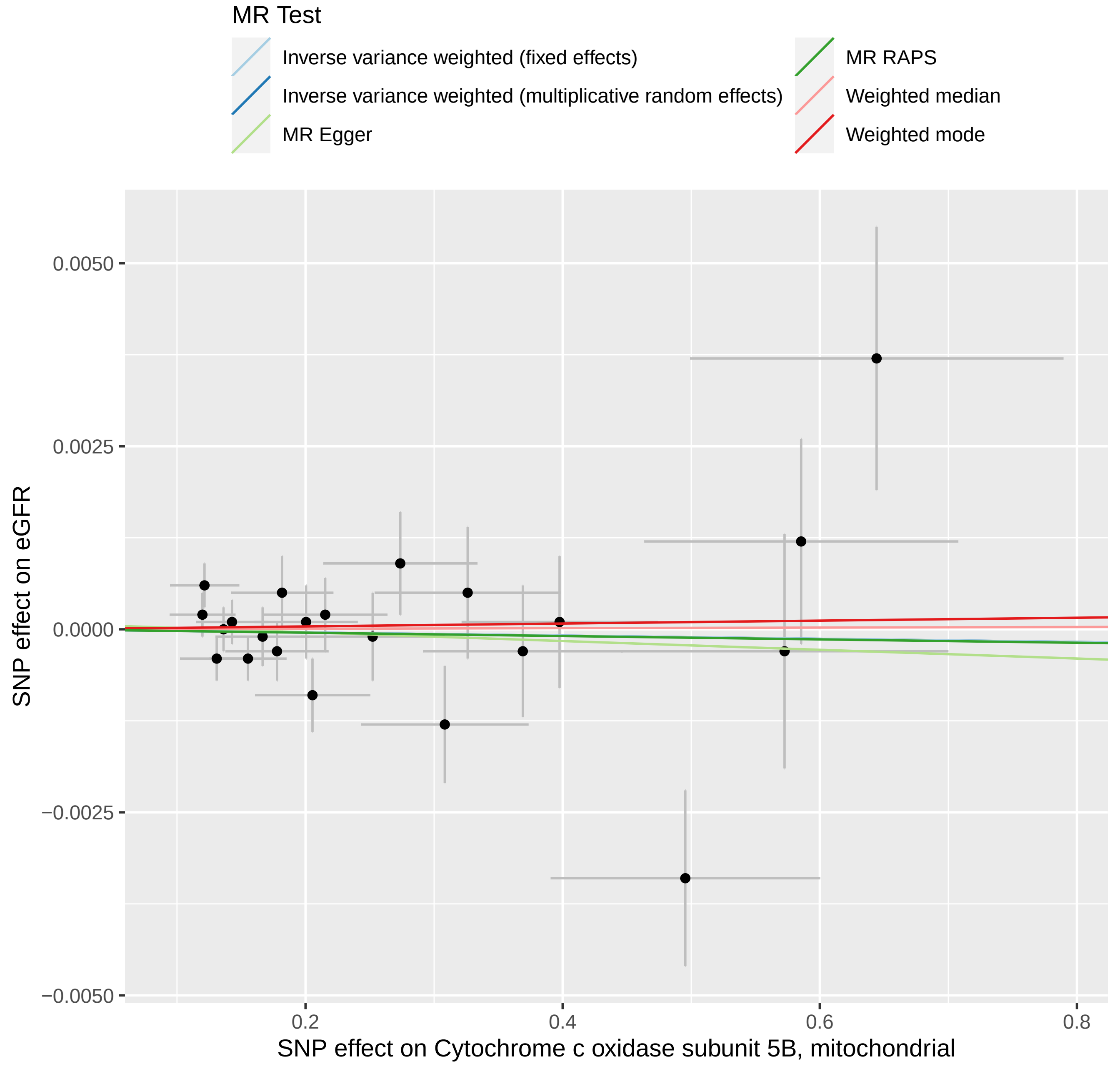

Batch 2−639 : Cytochrome c oxidase subunit 6C on eGFR

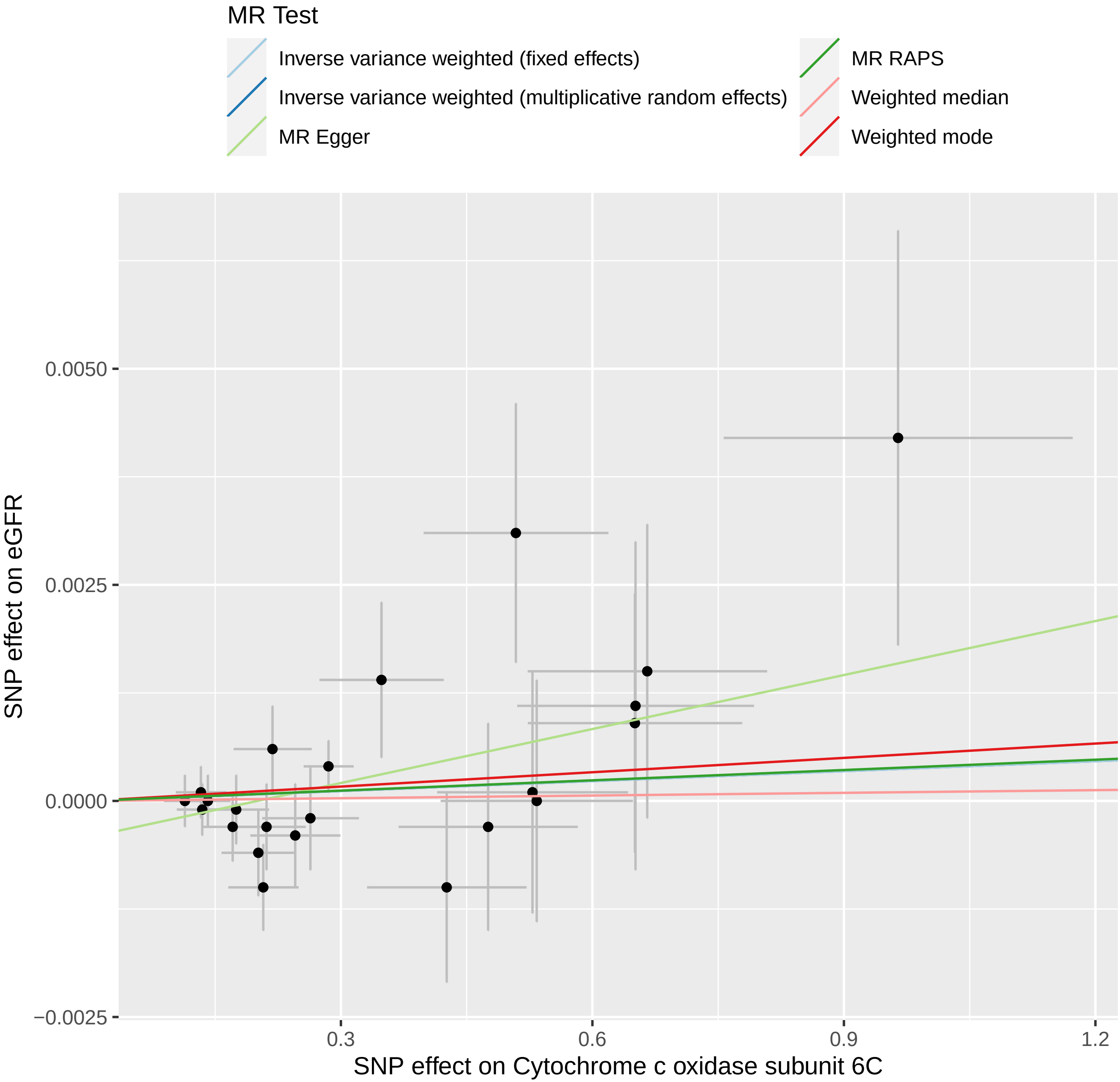

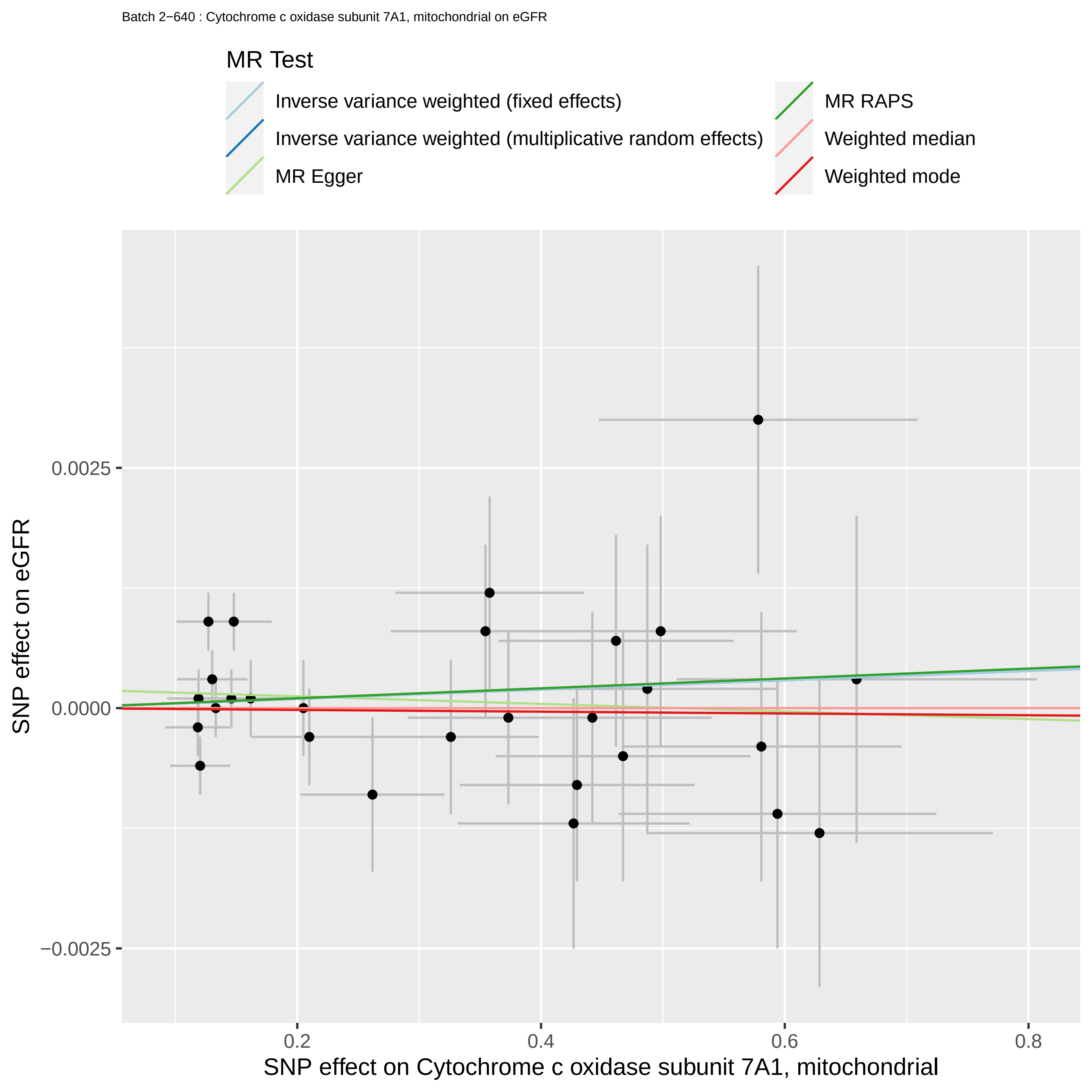

Batch 2−640 : Cytochrome c oxidase subunit 7A1, mitochondrial on eGFR
MR Test
Inverse variance weighted (fixed effects)
Inverse variance weighted (multiplicative random effects)
MR Egger
MR RAPS
Weighted median
Weighted mode
0.0025
0.0000
−0.0025
SNP effect on eGFR
0.2
0.4
0.6
0.8
SNP effect on Cytochrome c oxidase subunit 7A1, mitochondrial

Batch 2−641 : Cytochrome c oxidase subunit 8A, mitochondrial on eGFR

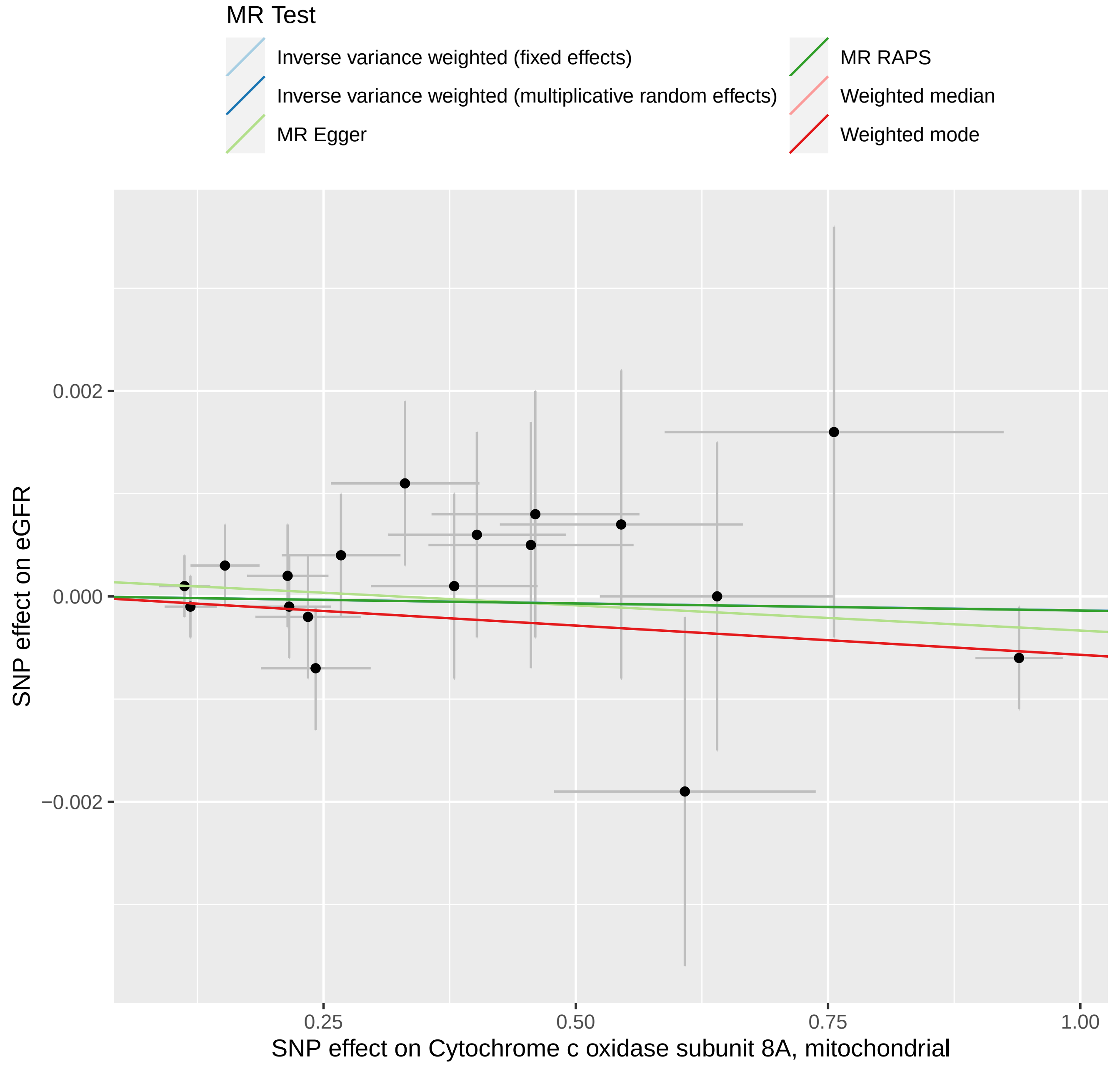

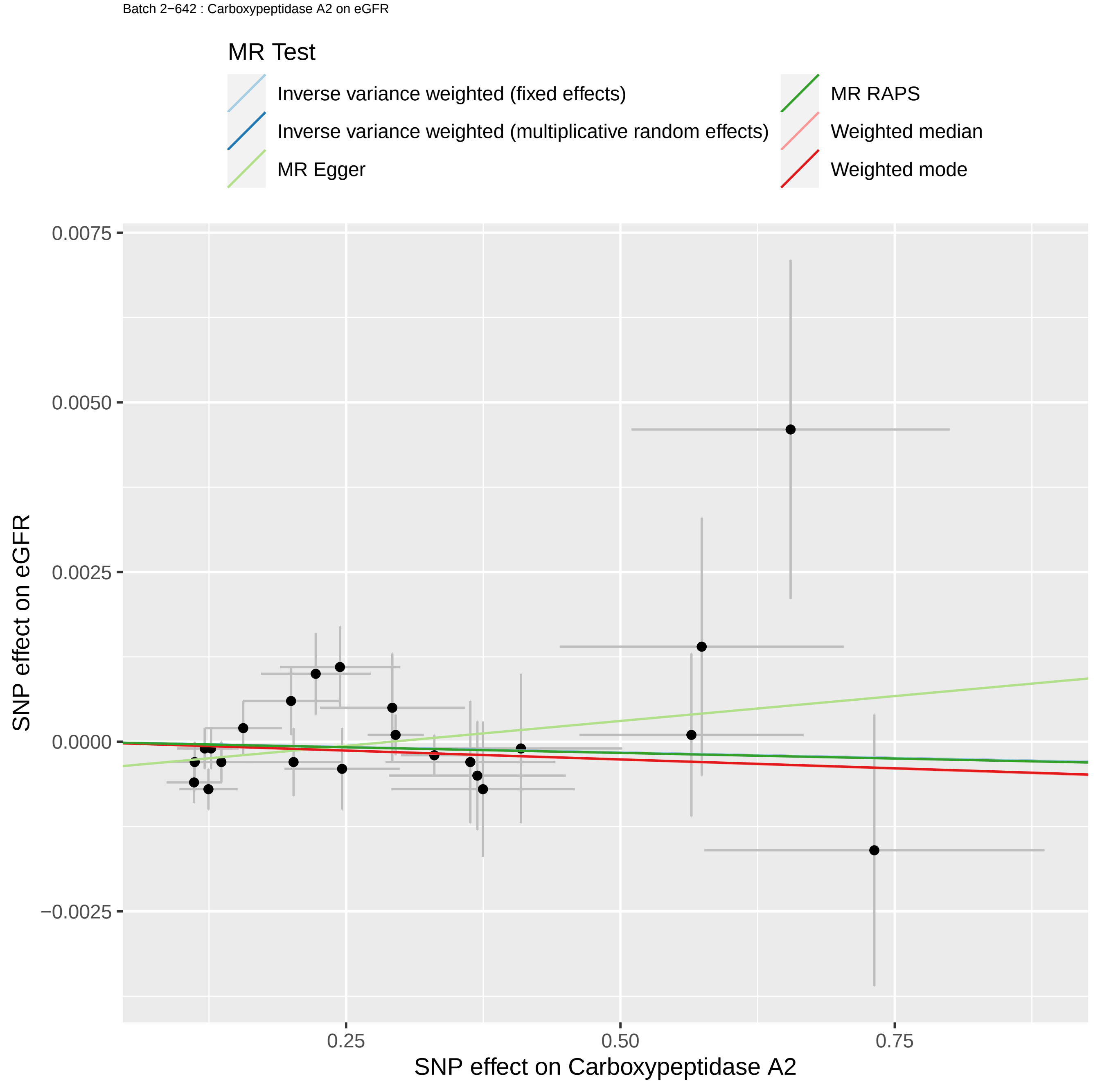

Batch 2−642 : Carboxypeptidase A2 on eGFR
MR Test
Inverse variance weighted (fixed effects)
Inverse variance weighted (multiplicative random effects)
MR Egger
MR RAPS
Weighted median
Weighted mode
0.0075
0.0050
0.0025
0.0000
−0.0025
SNP effect on eGFR
0.25
0.50
0.75
SNP effect on Carboxypeptidase A2

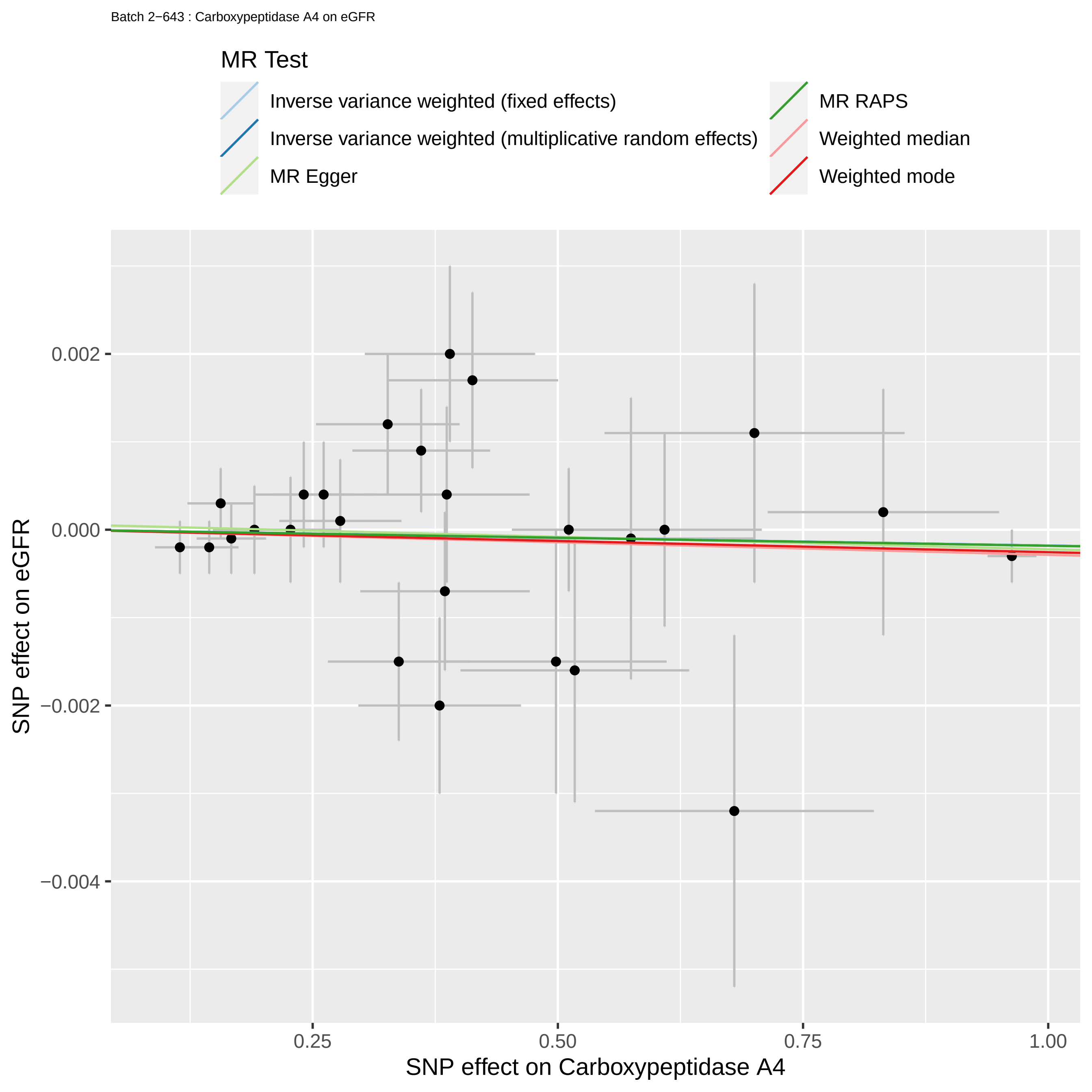

Batch 2−643 : Carboxypeptidase A4 on eGFR
MR Test
Inverse variance weighted (fixed effects)
Inverse variance weighted (multiplicative random effects)
MR Egger
MR RAPS
Weighted median
Weighted mode
0.002
0.000
−0.002
−0.004
SNP effect on eGFR
0.25
0.50
0.75
1.00
SNP effect on Carboxypeptidase A4

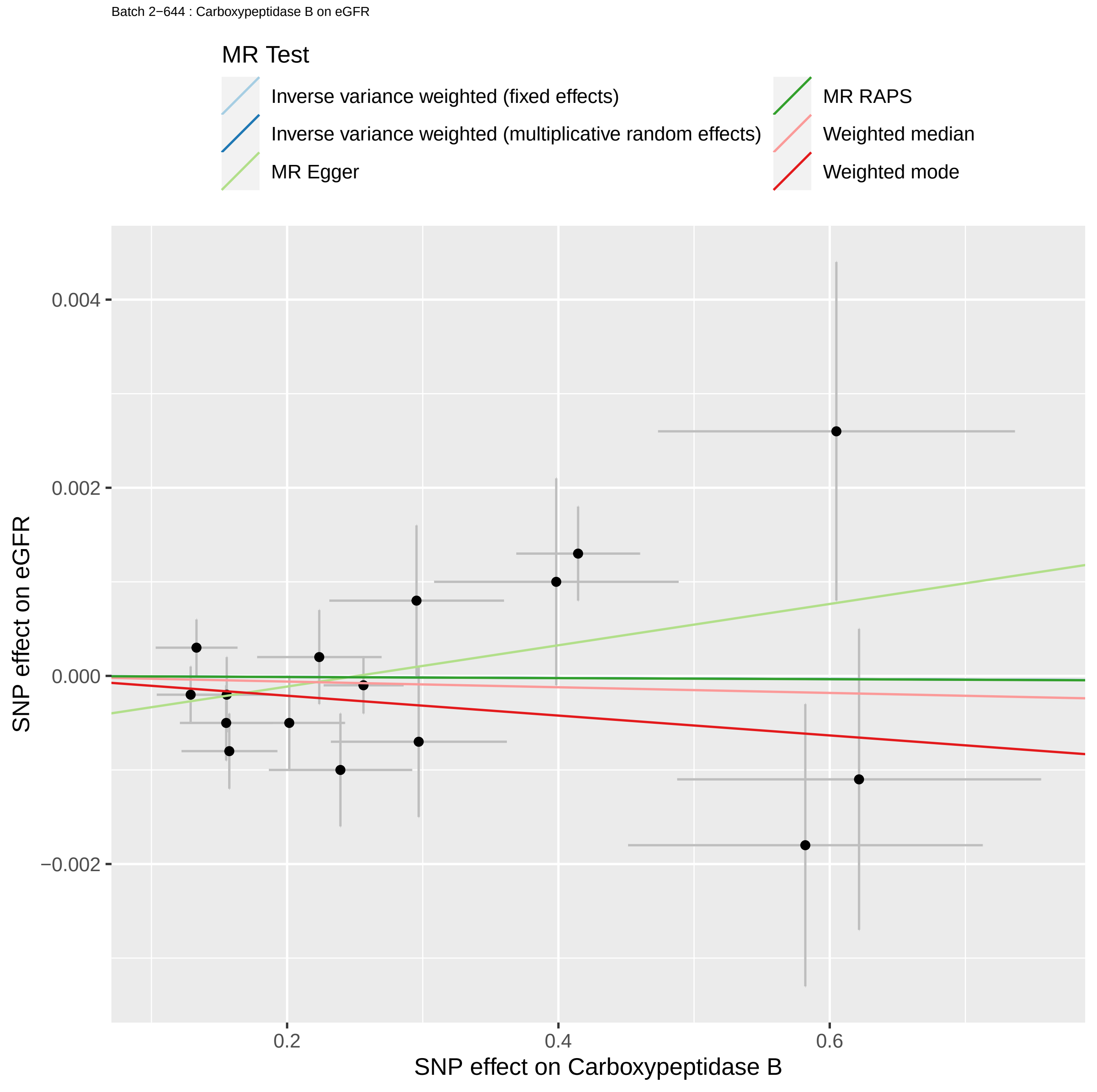
Batch 2−644 : Carboxypeptidase B on eGFR
MR Test
Inverse variance weighted (fixed effects)
Inverse variance weighted (multiplicative random effects)
MR Egger
MR RAPS
Weighted median
Weighted mode
0.004
0.002
0.000
−0.002
0.2
0.4
0.6
SNP effect on eGFR
SNP effect on Carboxypeptidase B

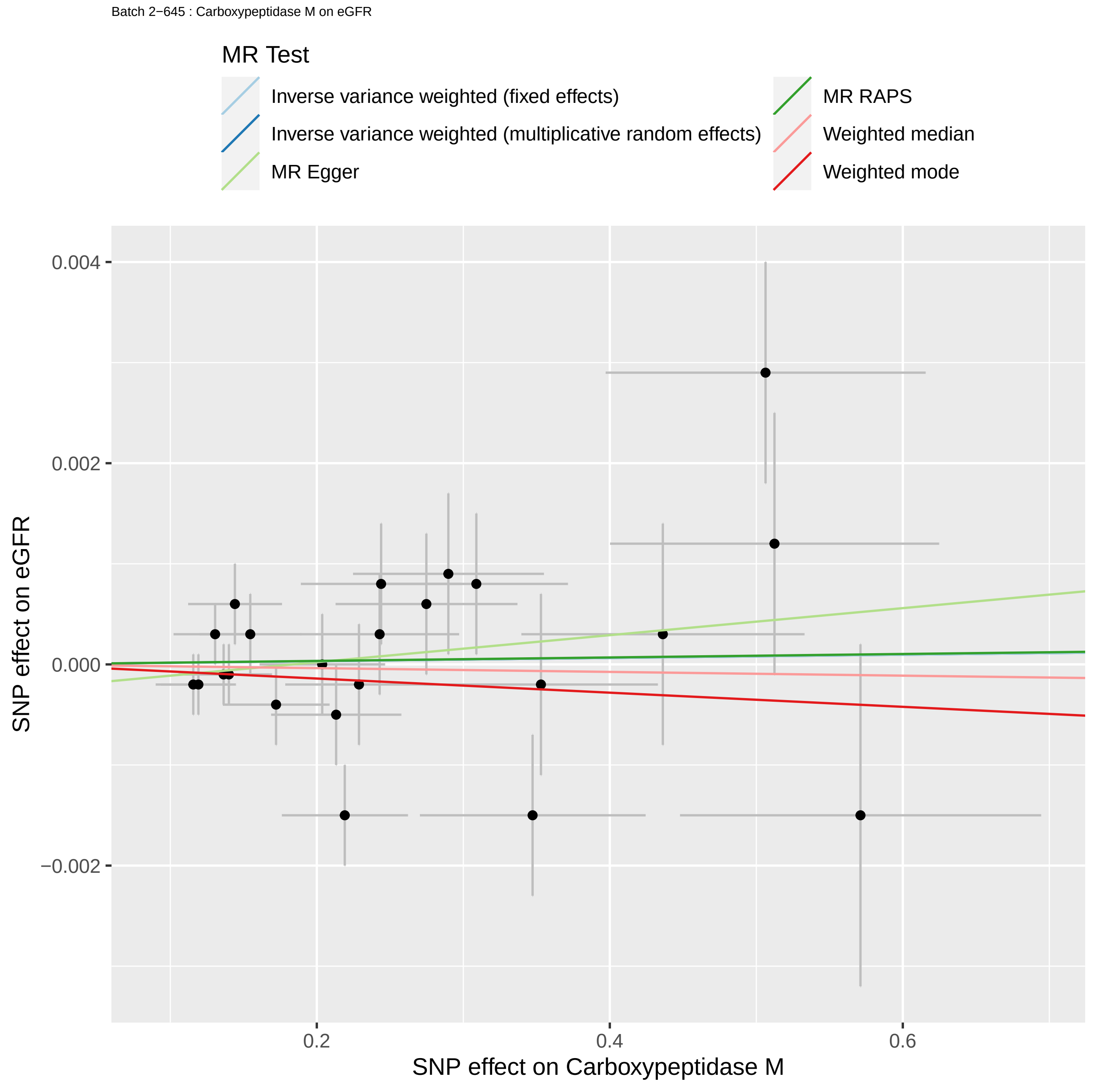
Batch 2−645 : Carboxypeptidase M on eGFR
MR Test
Inverse variance weighted (fixed effects)
Inverse variance weighted (multiplicative random effects)
MR Egger
MR RAPS
Weighted median
Weighted mode
0.004
0.002
0.000
−0.002
0.2
0.4
0.6
SNP effect on eGFR
SNP effect on Carboxypeptidase M

Batch 2−646 : Copine−1 on eGFR

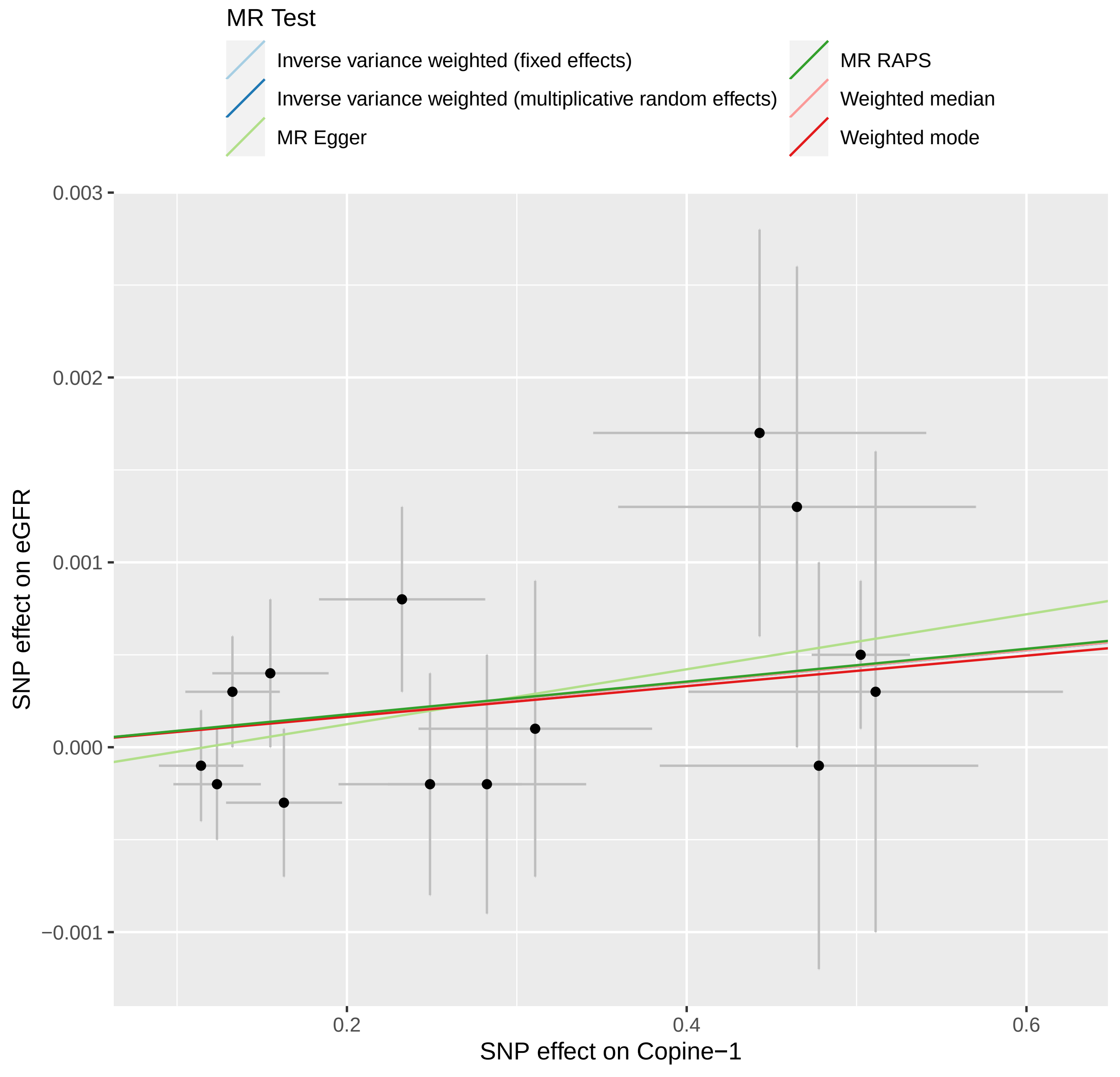

Batch 2−647 : Probable serine carboxypeptidase CPVL on eGFR

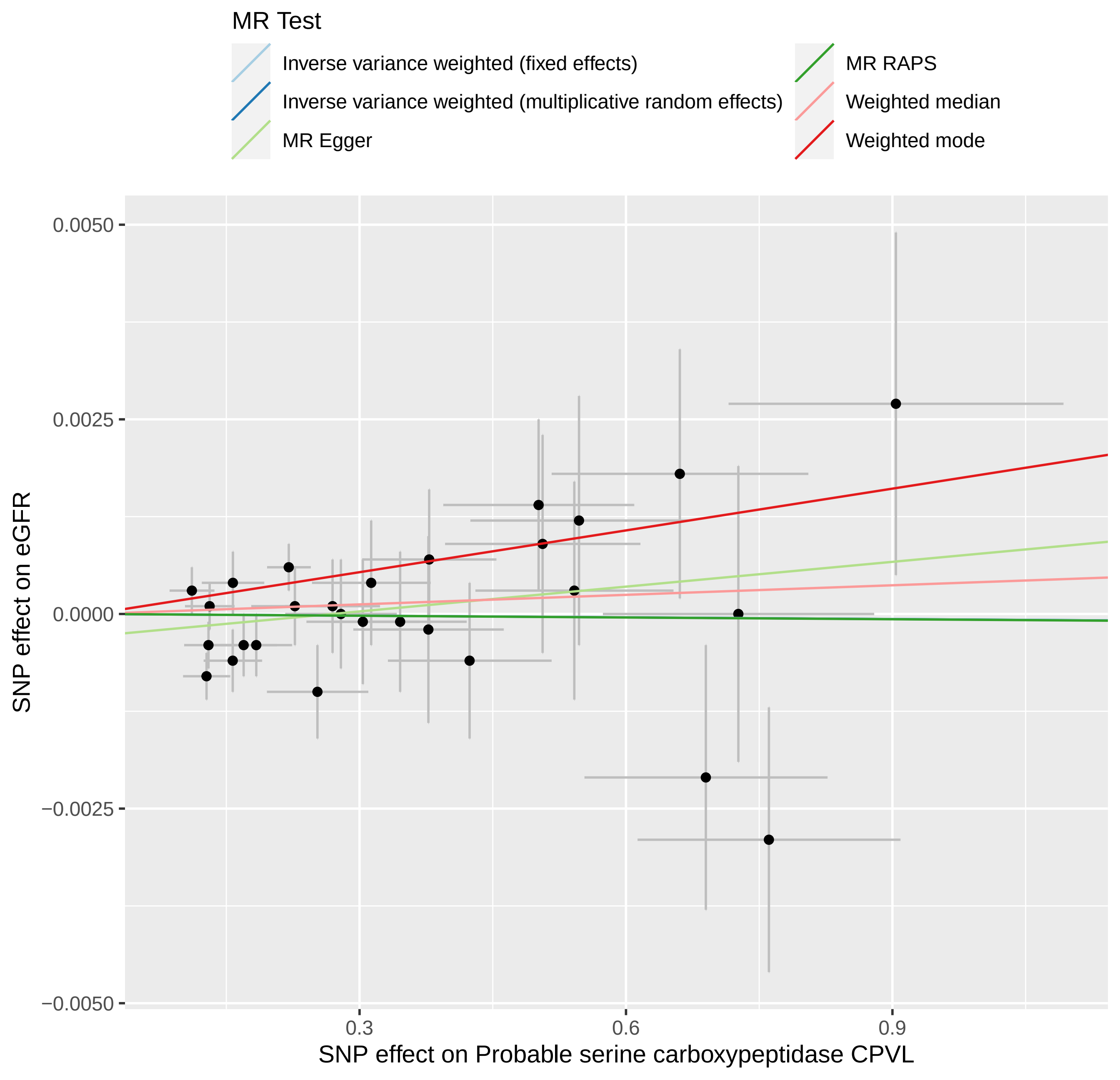

Batch 2−648 : Probable carboxypeptidase X1 on eGFR

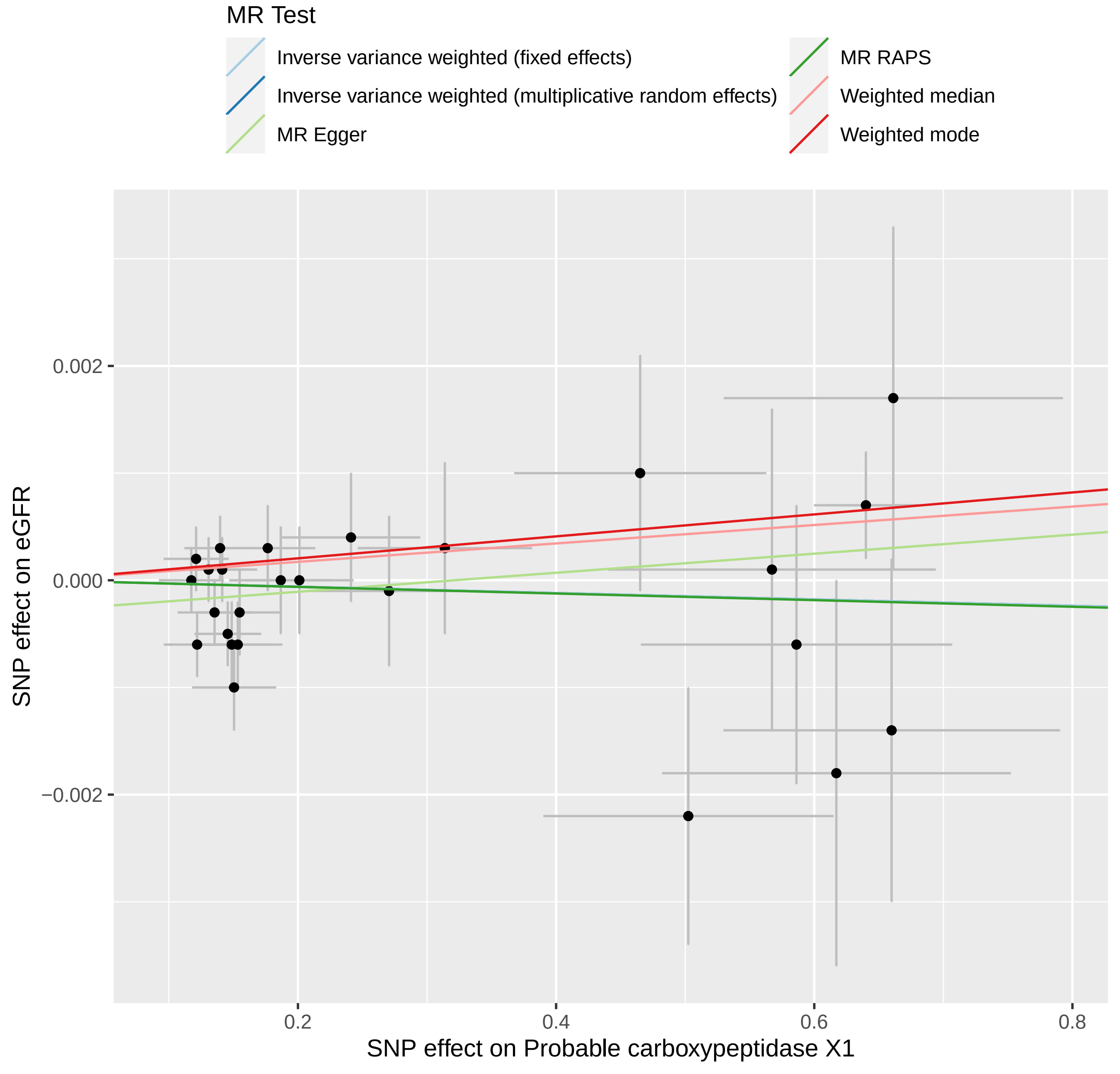

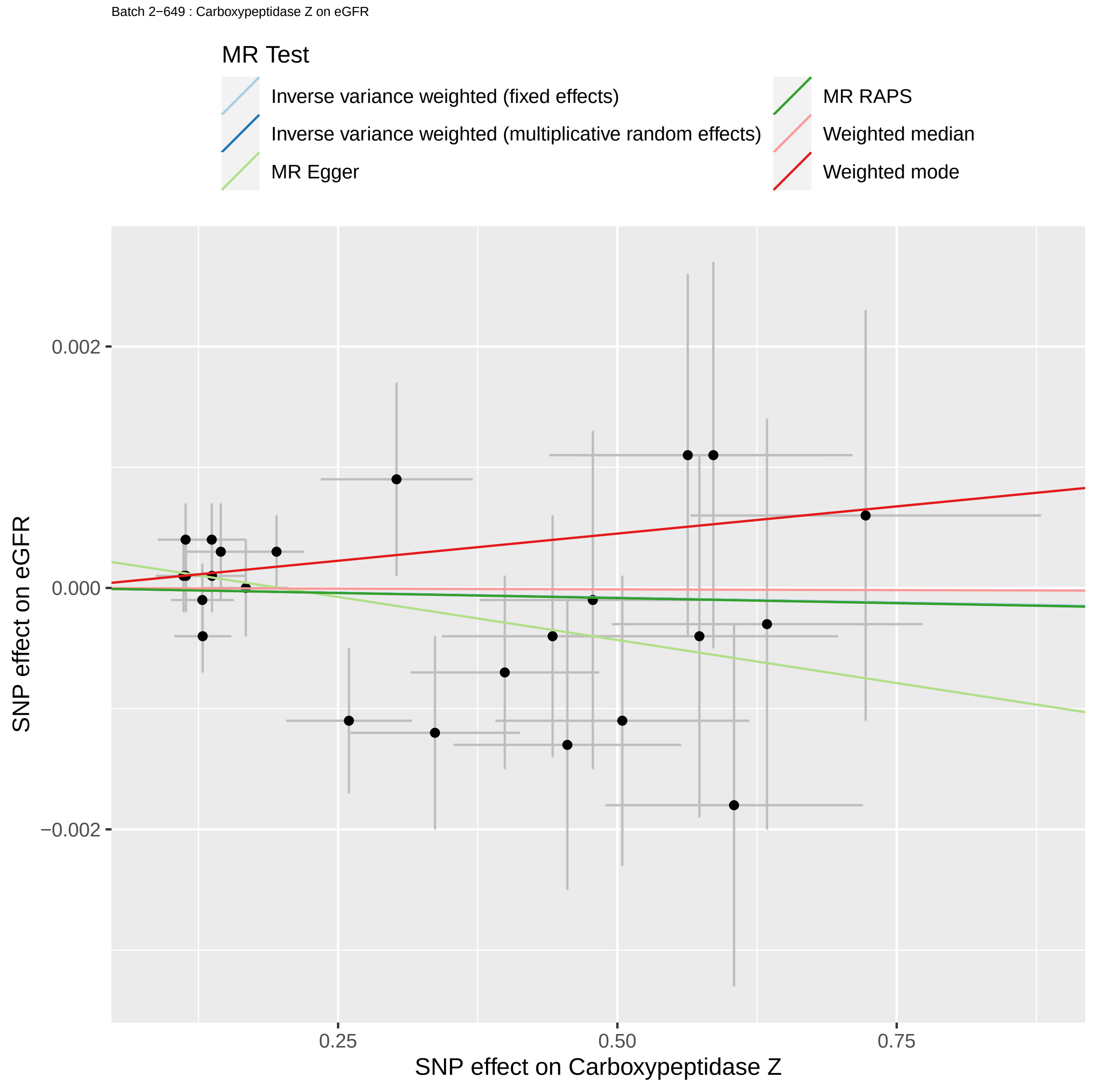

Batch 2−649 : Carboxypeptidase Z on eGFR
MR Test
Inverse variance weighted (fixed effects)
Inverse variance weighted (multiplicative random effects)
MR Egger
MR RAPS
Weighted median
Weighted mode
0.002
0.000
−0.002
SNP effect on eGFR
0.25
0.50
0.75
SNP effect on Carboxypeptidase Z

Batch 2−650 : Cellular retinoic acid−binding protein 1 on eGFR

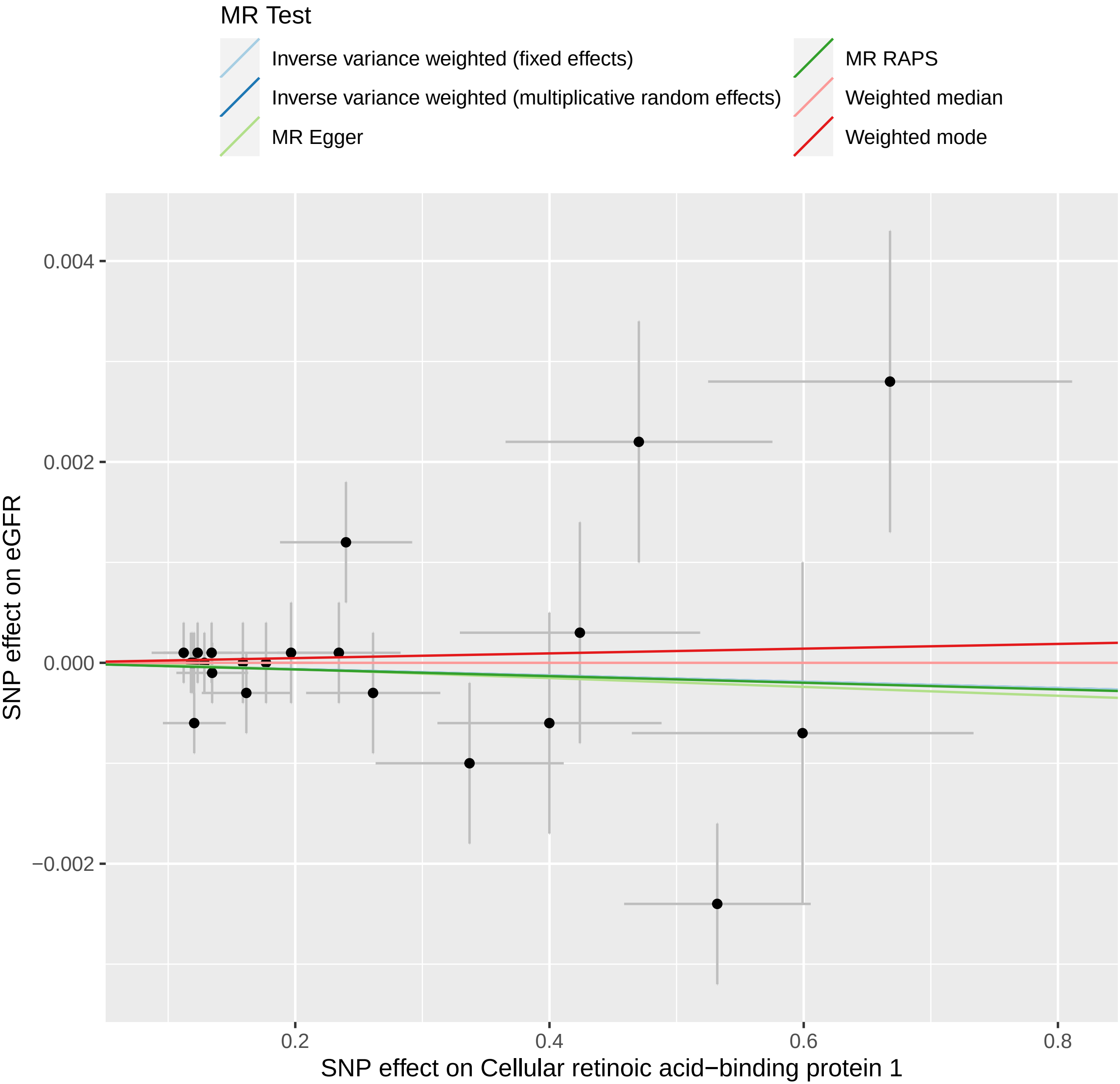

Batch 2−651 : Cellular retinoic acid−binding protein 2 on eGFR

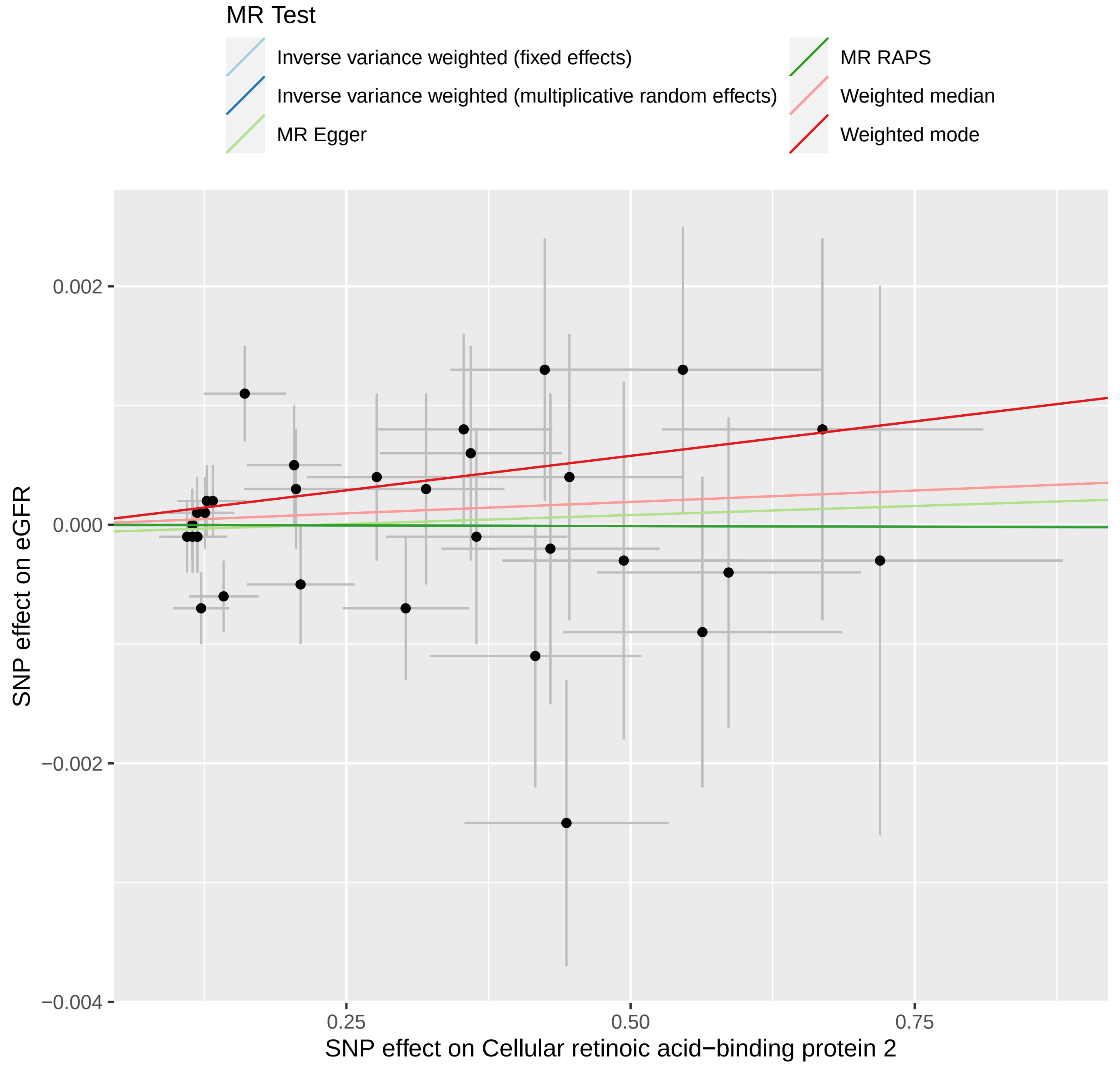

Batch 2−652 : Protein crumbs homolog 1 on eGFR

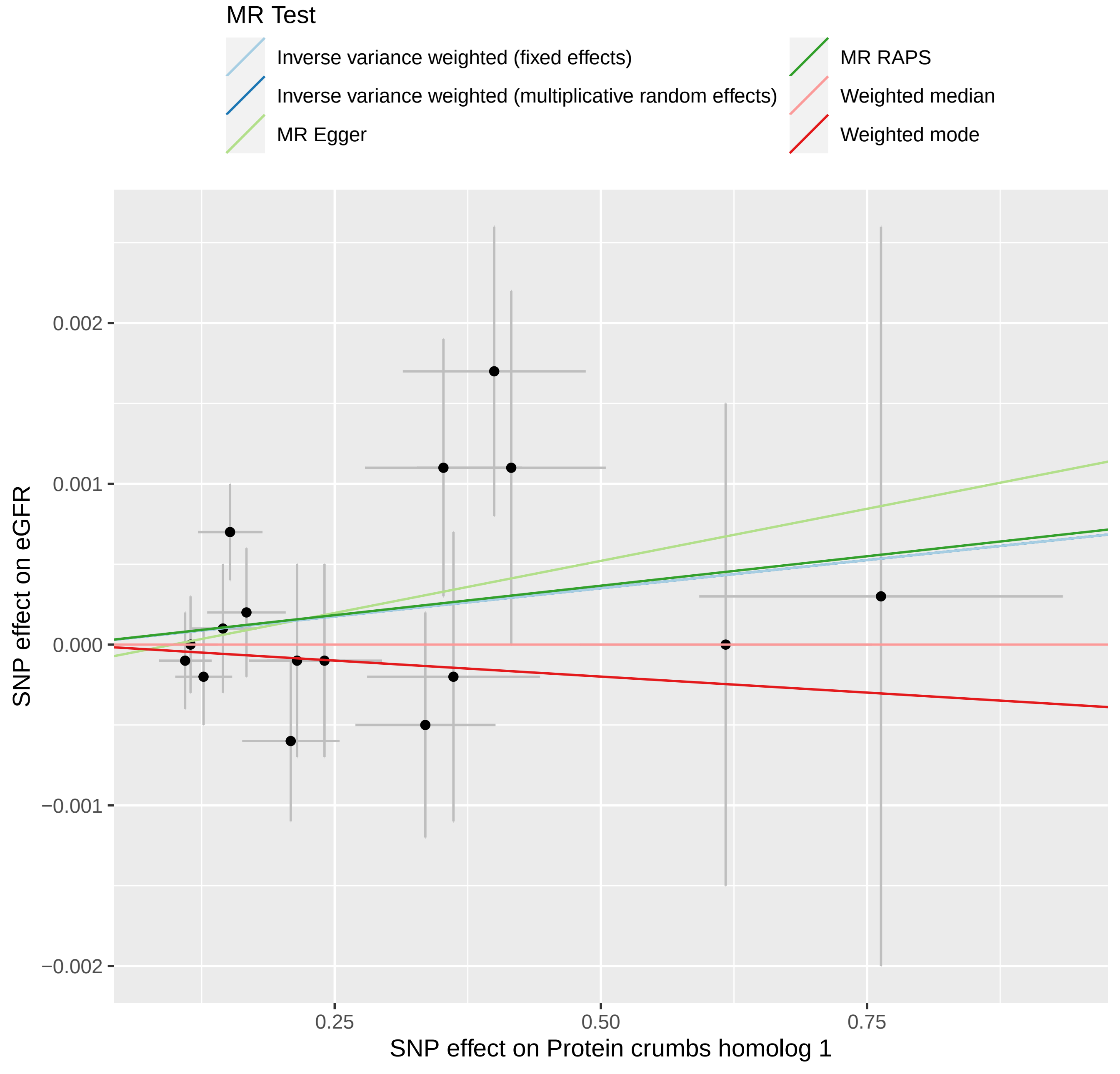

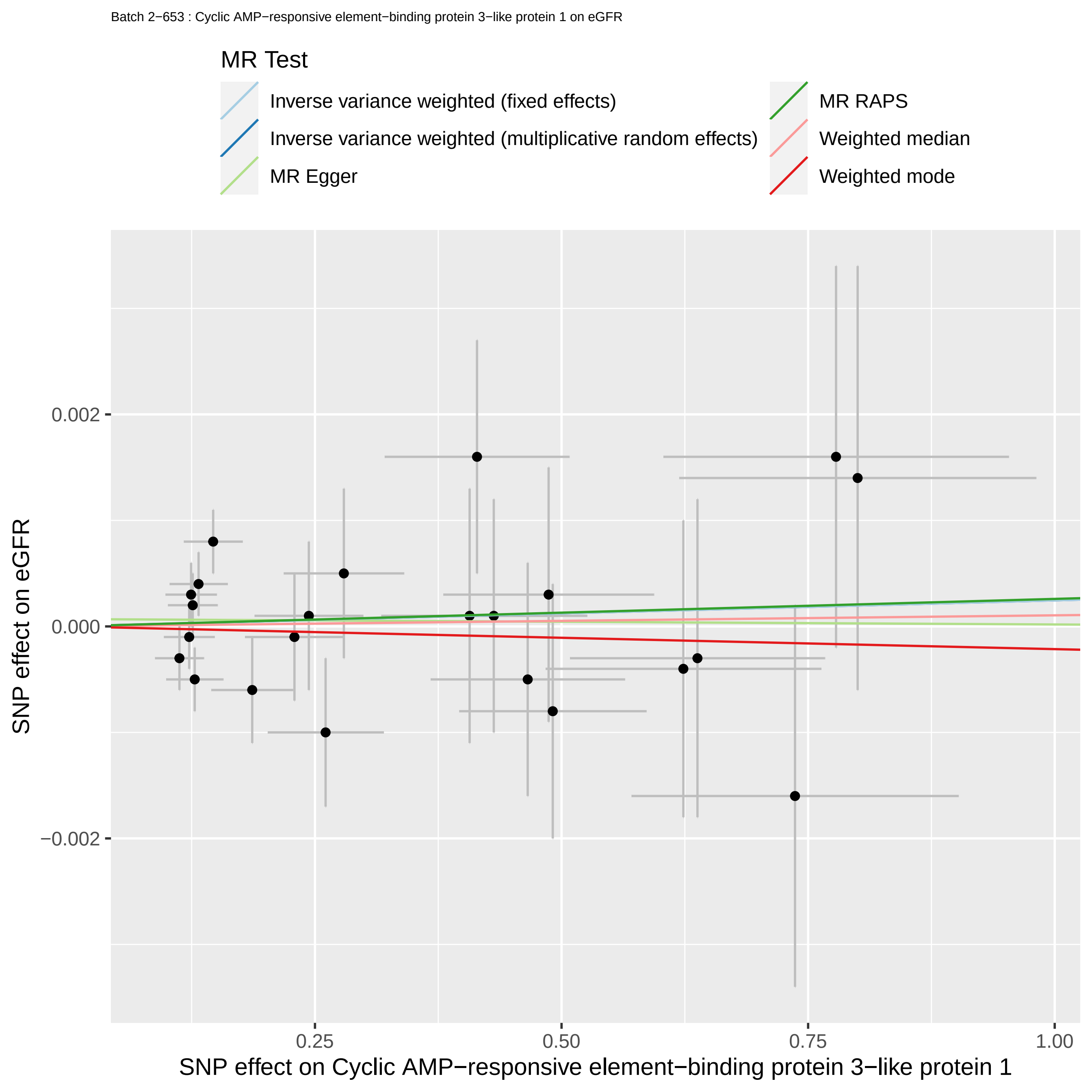

Batch 2−653 : Cyclic AMP−responsive element−binding protein 3−like protein 1 on eGFR
MR Test
Inverse variance weighted (fixed effects)
Inverse variance weighted (multiplicative random effects)
MR Egger
MR RAPS
Weighted median
Weighted mode
0.002
0.000
−0.002
0.25
0.50
0.75
1.00
SNP effect on eGFR
SNP effect on Cyclic AMP−responsive element−binding protein 3−like protein 1

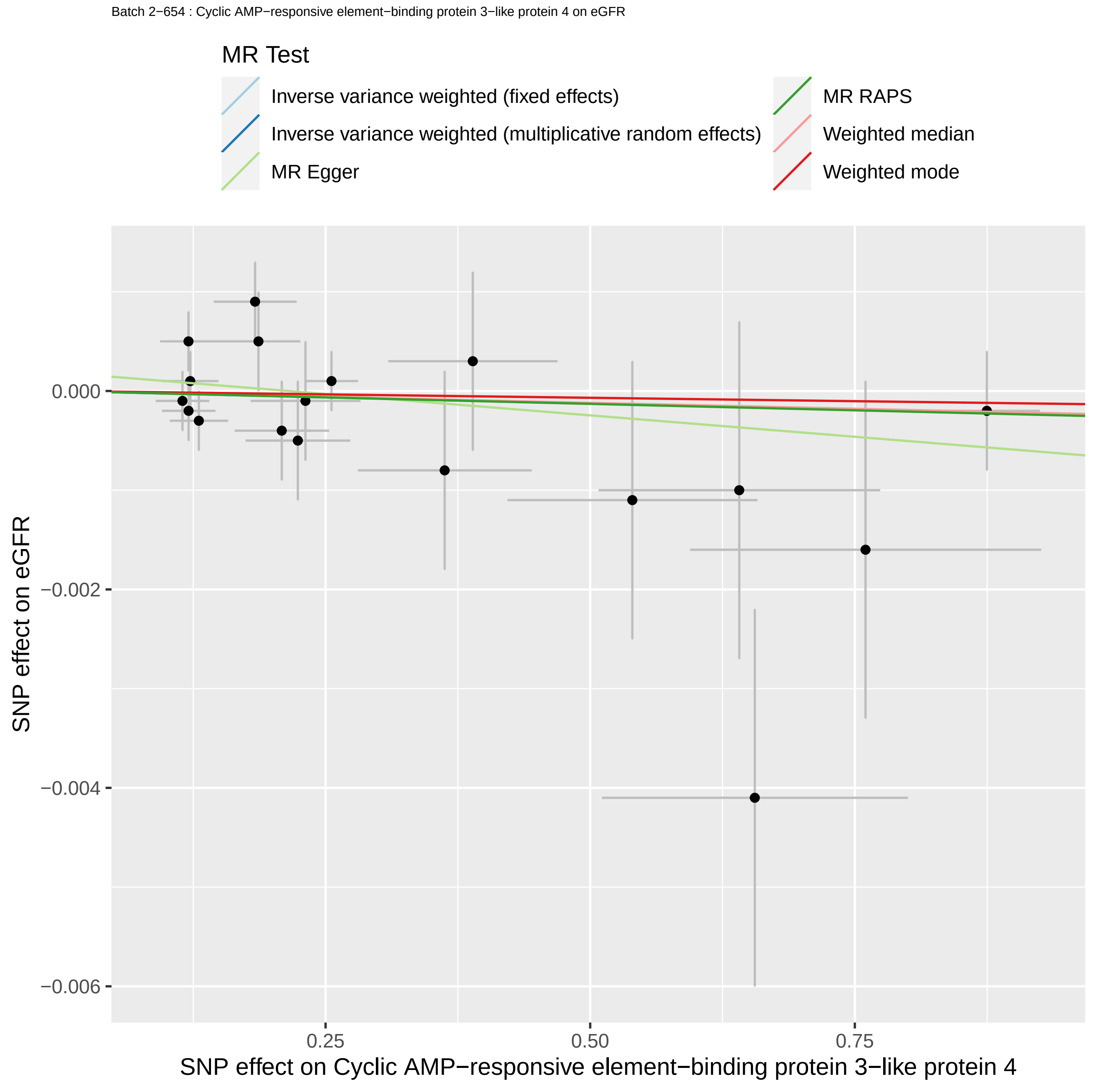

Batch 2−654 : Cyclic AMP−responsive element−binding protein 3−like protein 4 on eGFR
MR Test
Inverse variance weighted (fixed effects)
Inverse variance weighted (multiplicative random effects)
MR Egger
MR RAPS
Weighted median
Weighted mode
0.000
−0.002
−0.004
−0.006
0.25
0.50
0.75
SNP effect on eGFR
SNP effect on Cyclic AMP−responsive element−binding protein 3−like protein 4

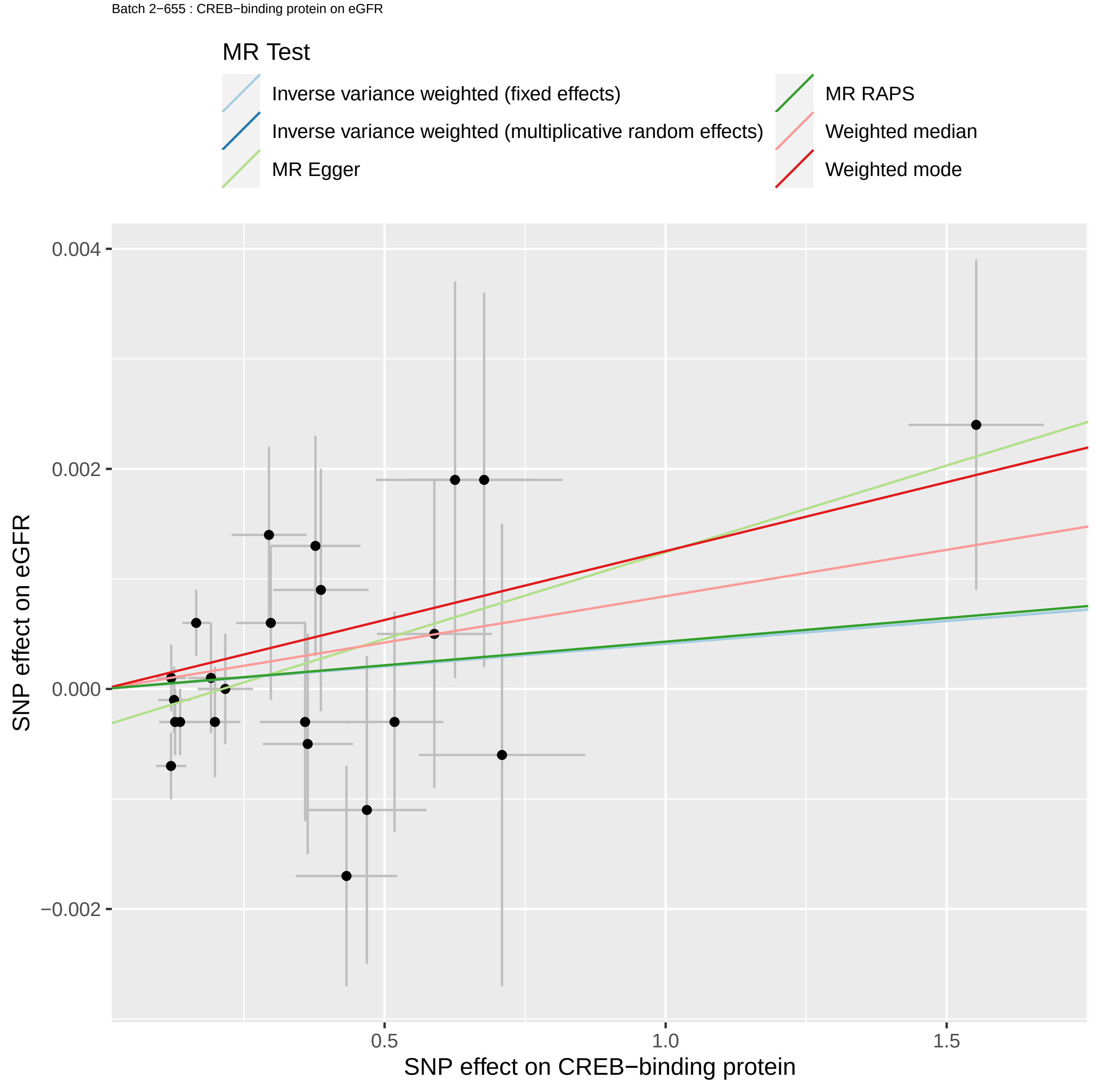
Batch 2−655 : CREB−binding protein on eGFR
MR Test
Inverse variance weighted (fixed effects)
Inverse variance weighted (multiplicative random effects)
MR Egger
MR RAPS
Weighted median
Weighted mode
0.004
0.002
0.000
−0.002
0.5
1.0
1.5
SNP effect on eGFR
SNP effect on CREB−binding protein

Batch 2−656 : Protein CREG1 on eGFR

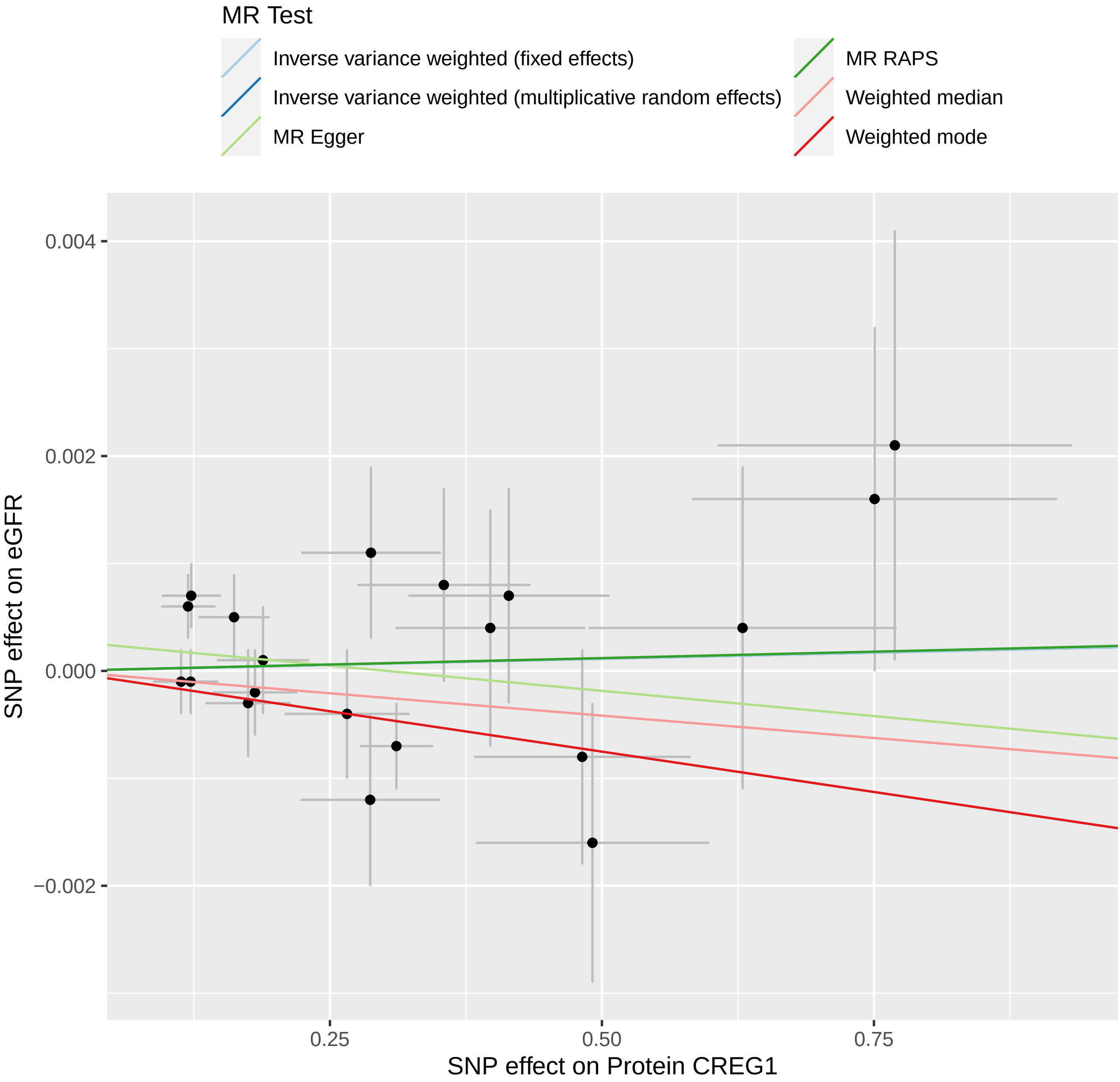

Batch 2−657 : Cysteine−rich with EGF−like domain protein 1 on eGFR

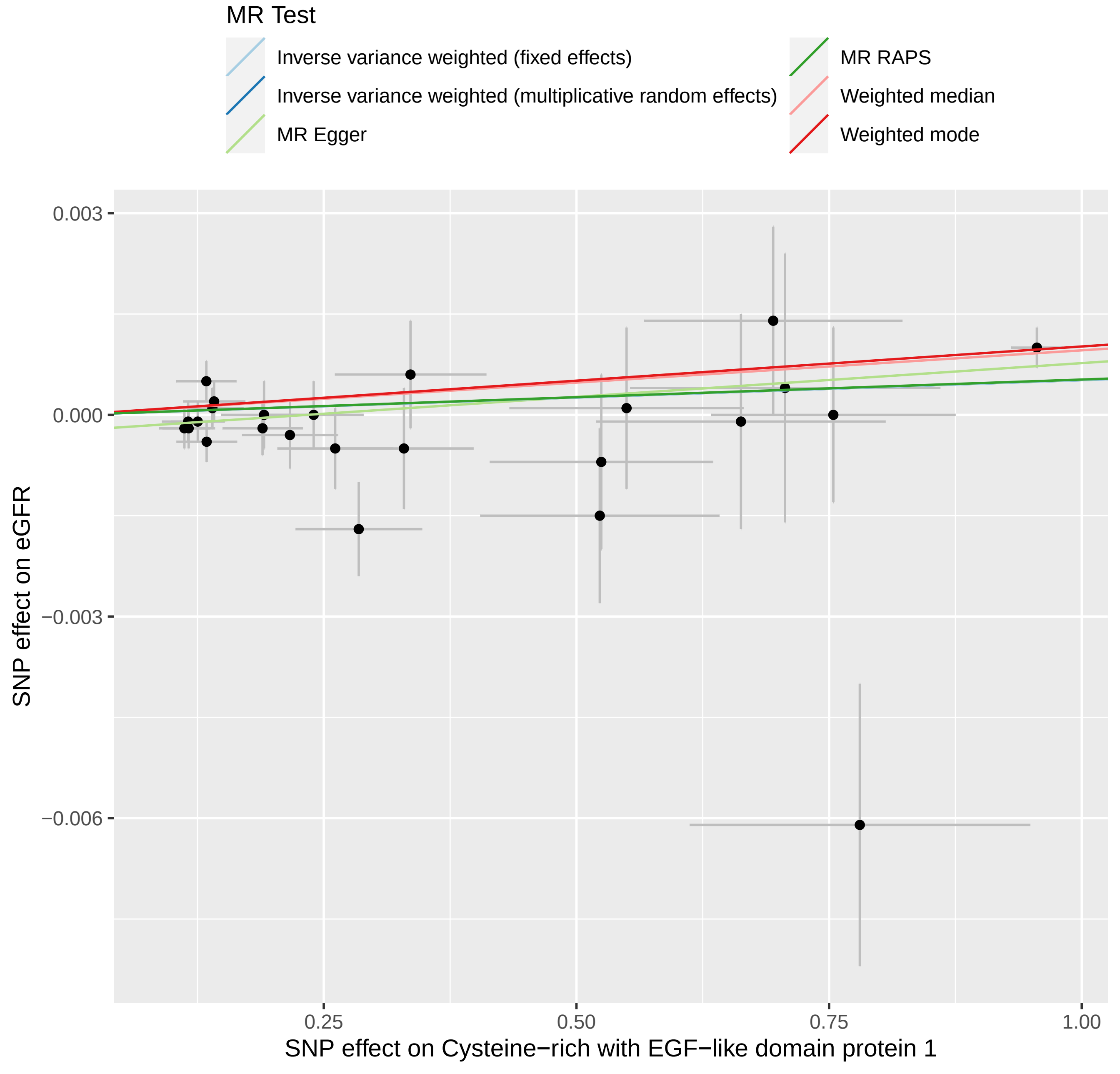

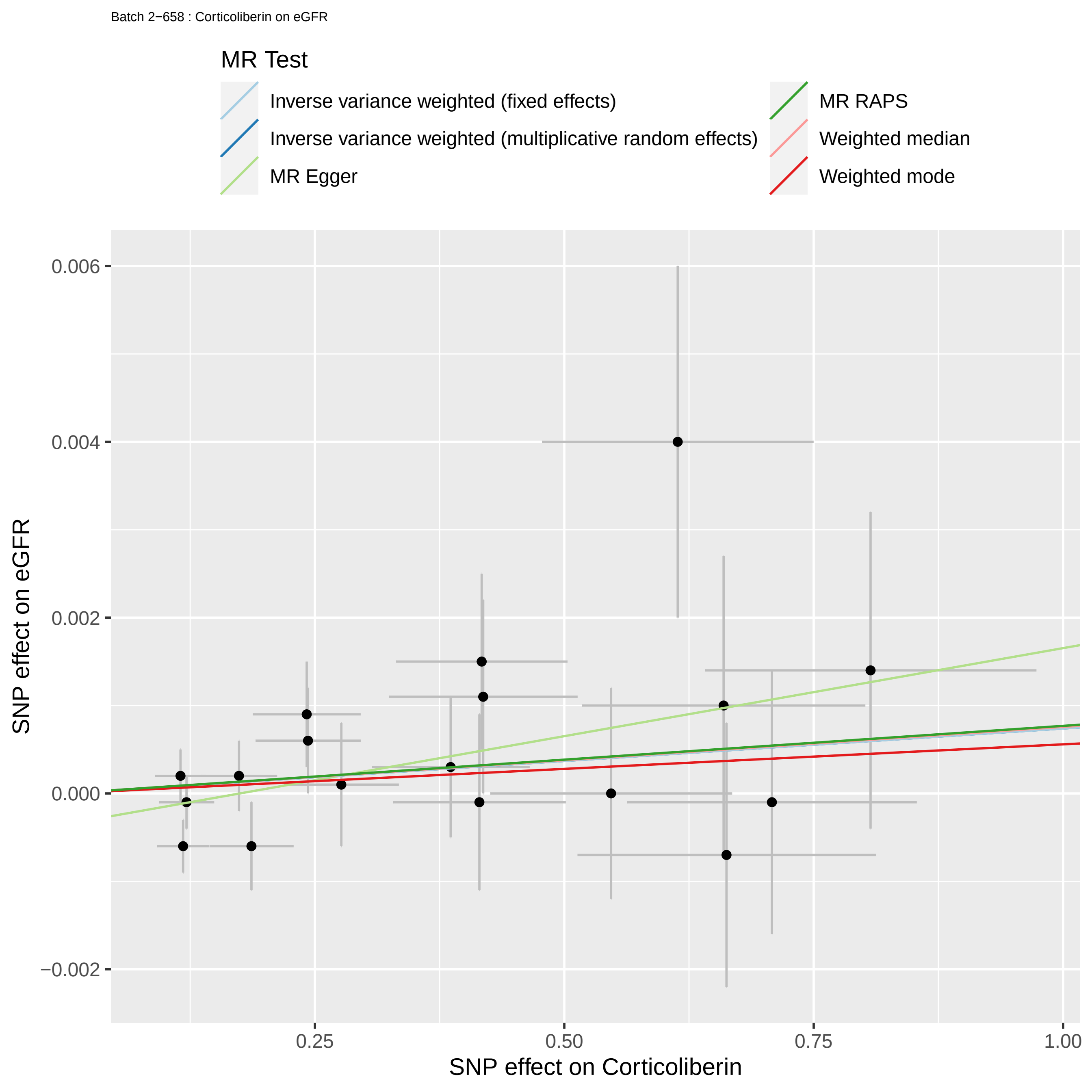

Batch 2−658 : Corticoliberin on eGFR
MR Test
Inverse variance weighted (fixed effects)
Inverse variance weighted (multiplicative random effects)
MR Egger
MR RAPS
Weighted median
Weighted mode
0.006
0.004
0.002
0.000
−0.002
0.25
0.50
0.75
1.00
SNP effect on eGFR
SNP effect on Corticoliberin

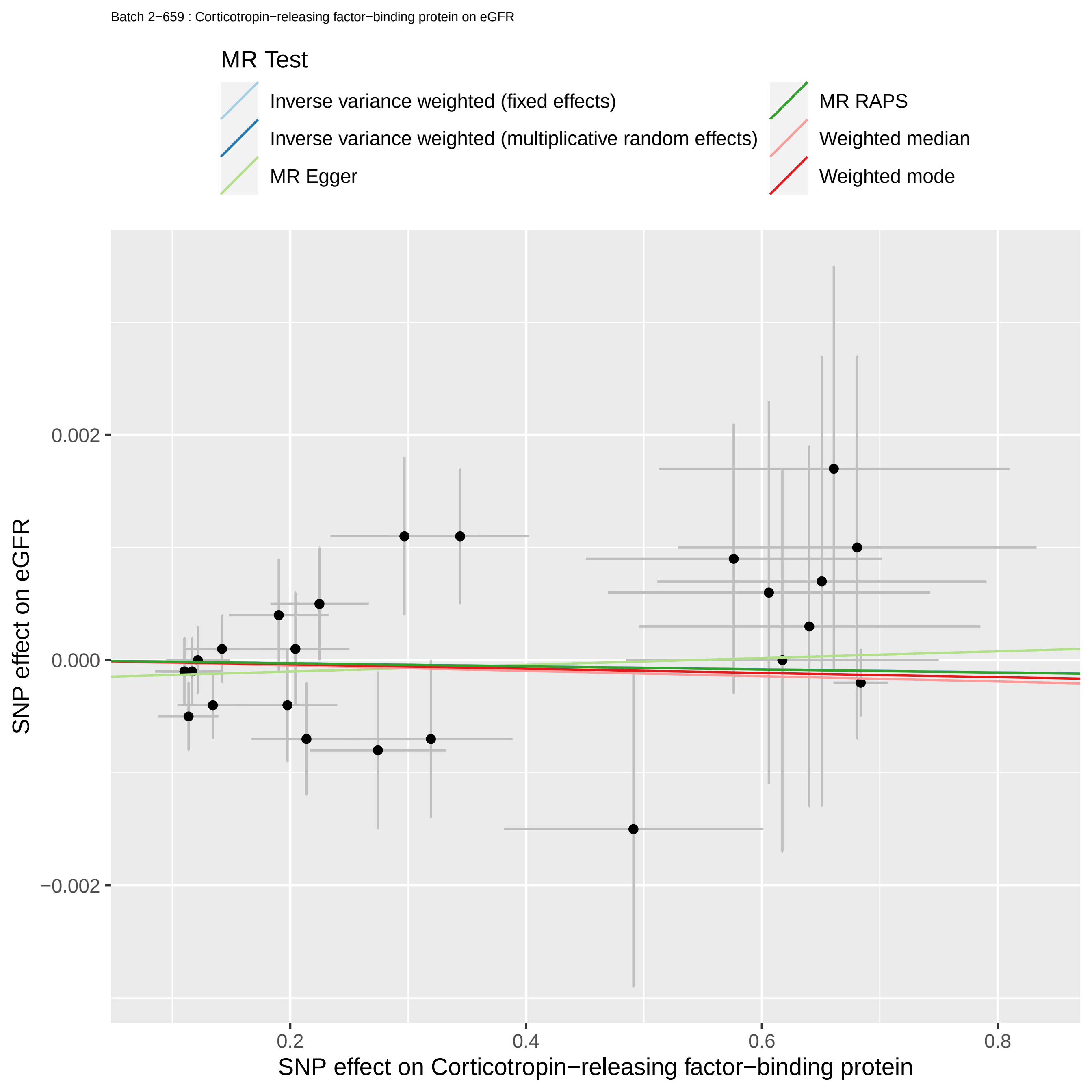

Batch 2−659 : Corticotropin−releasing factor−binding protein on eGFR
MR Test
Inverse variance weighted (fixed effects)
Inverse variance weighted (multiplicative random effects)
MR Egger
MR RAPS
Weighted median
Weighted mode
0.002
0.000
−0.002
0.2
0.4
0.6
0.8
SNP effect on eGFR
SNP effect on Corticotropin−releasing factor−binding protein

Batch 2−660 : Cysteine−rich motor neuron 1 protein on eGFR

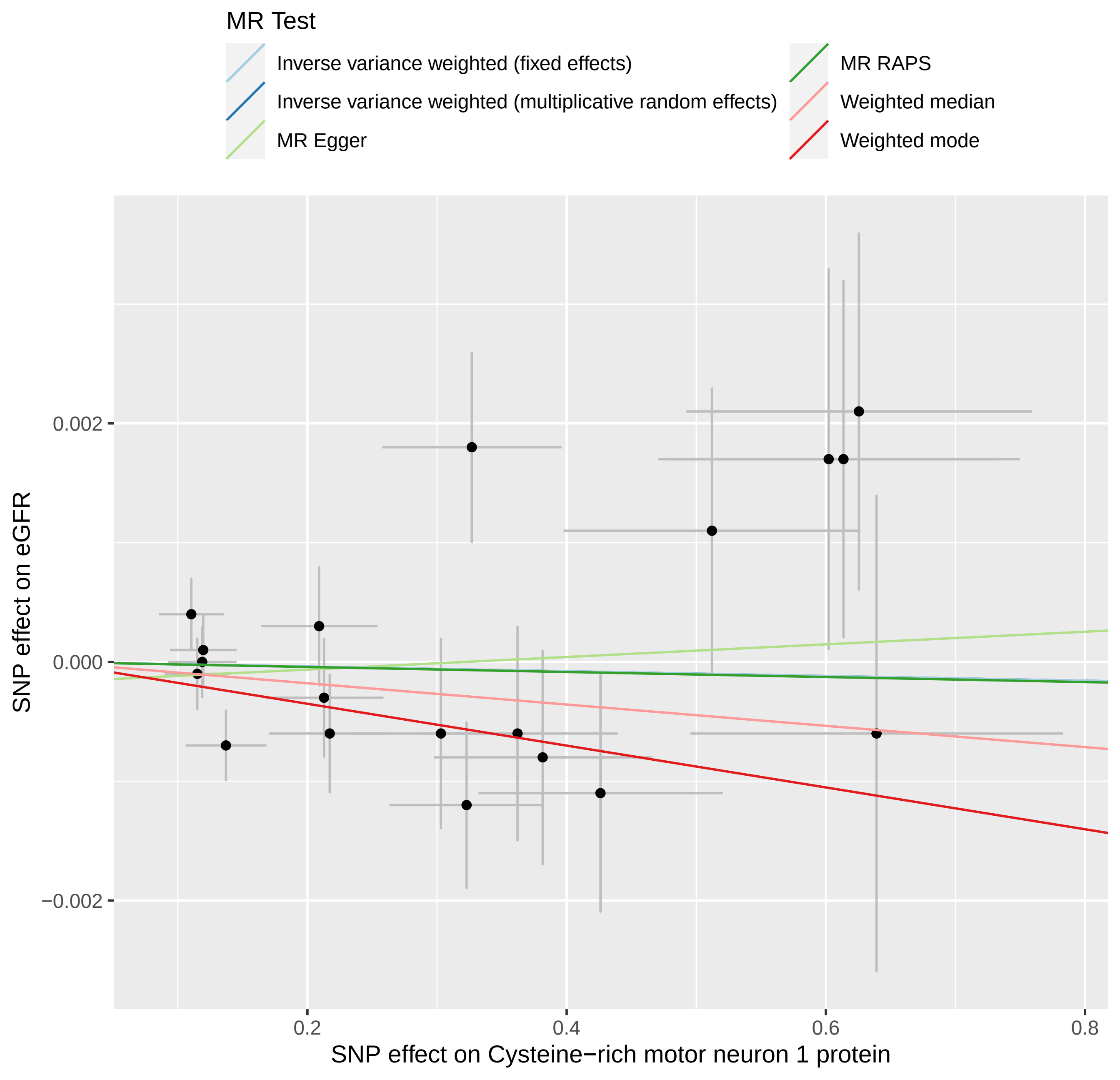

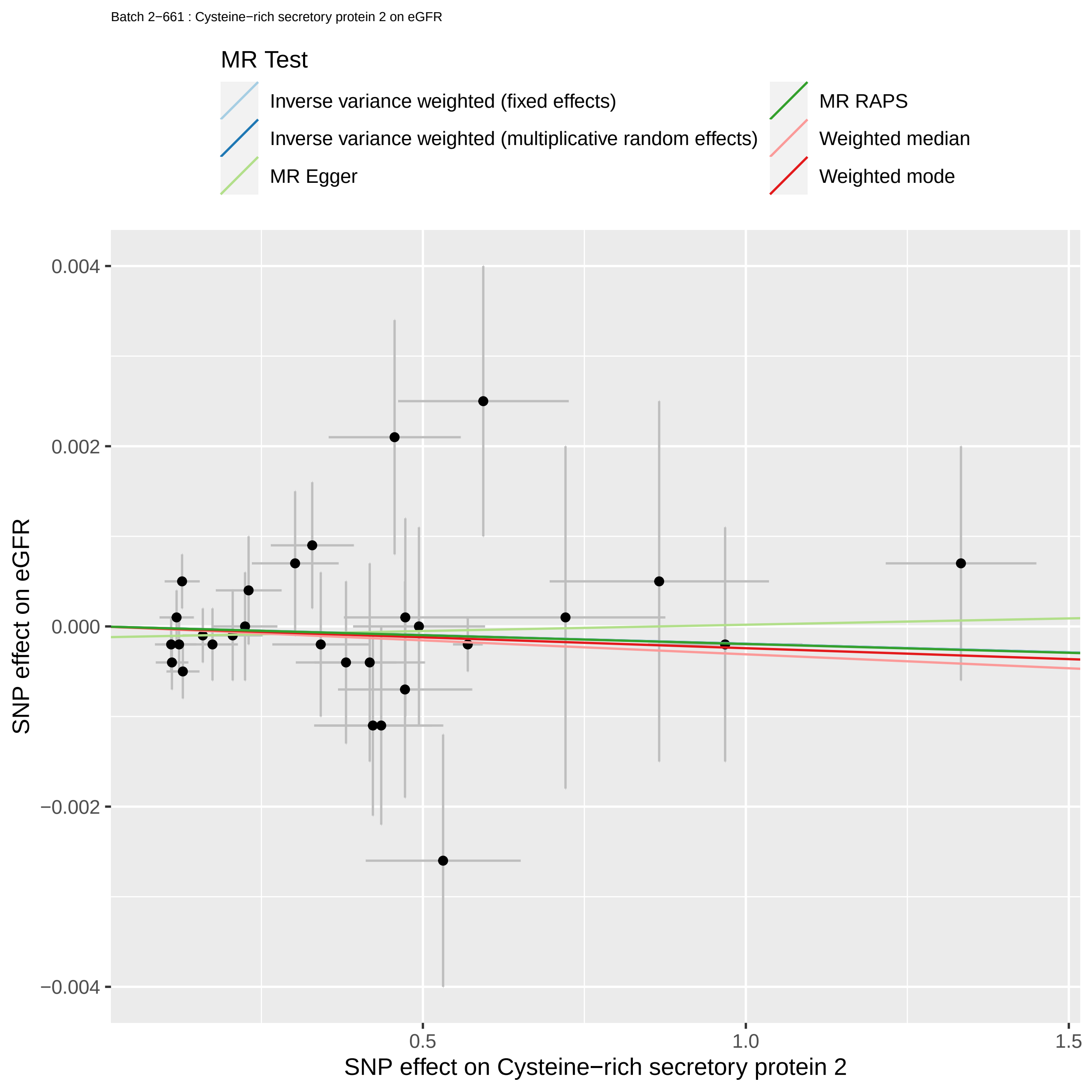
Batch 2−661 : Cysteine−rich secretory protein 2 on eGFR
MR Test
Inverse variance weighted (fixed effects)
Inverse variance weighted (multiplicative random effects)
MR Egger
MR RAPS
Weighted median
Weighted mode
0.004
0.002
0.000
−0.002
−0.004
0.5
1.0
1.5
SNP effect on eGFR
SNP effect on Cysteine−rich secretory protein 2

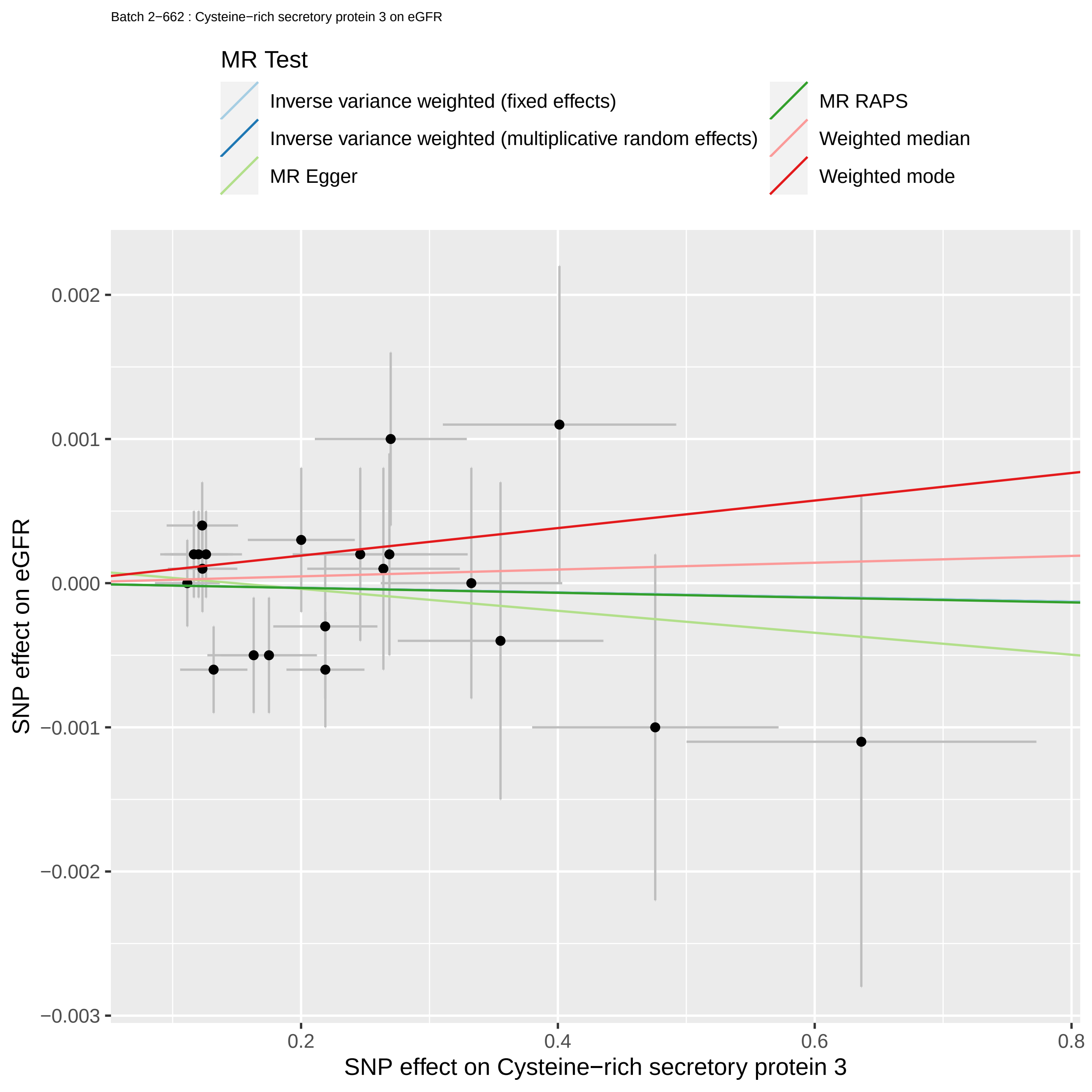
Batch 2−662 : Cysteine−rich secretory protein 3 on eGFR
MR Test
Inverse variance weighted (fixed effects)
Inverse variance weighted (multiplicative random effects)
MR Egger
MR RAPS
Weighted median
Weighted mode
0.002
0.001
0.000
−0.001
−0.002
−0.003
SNP effect on eGFR
0.2
0.4
0.6
0.8
SNP effect on Cysteine−rich secretory protein 3

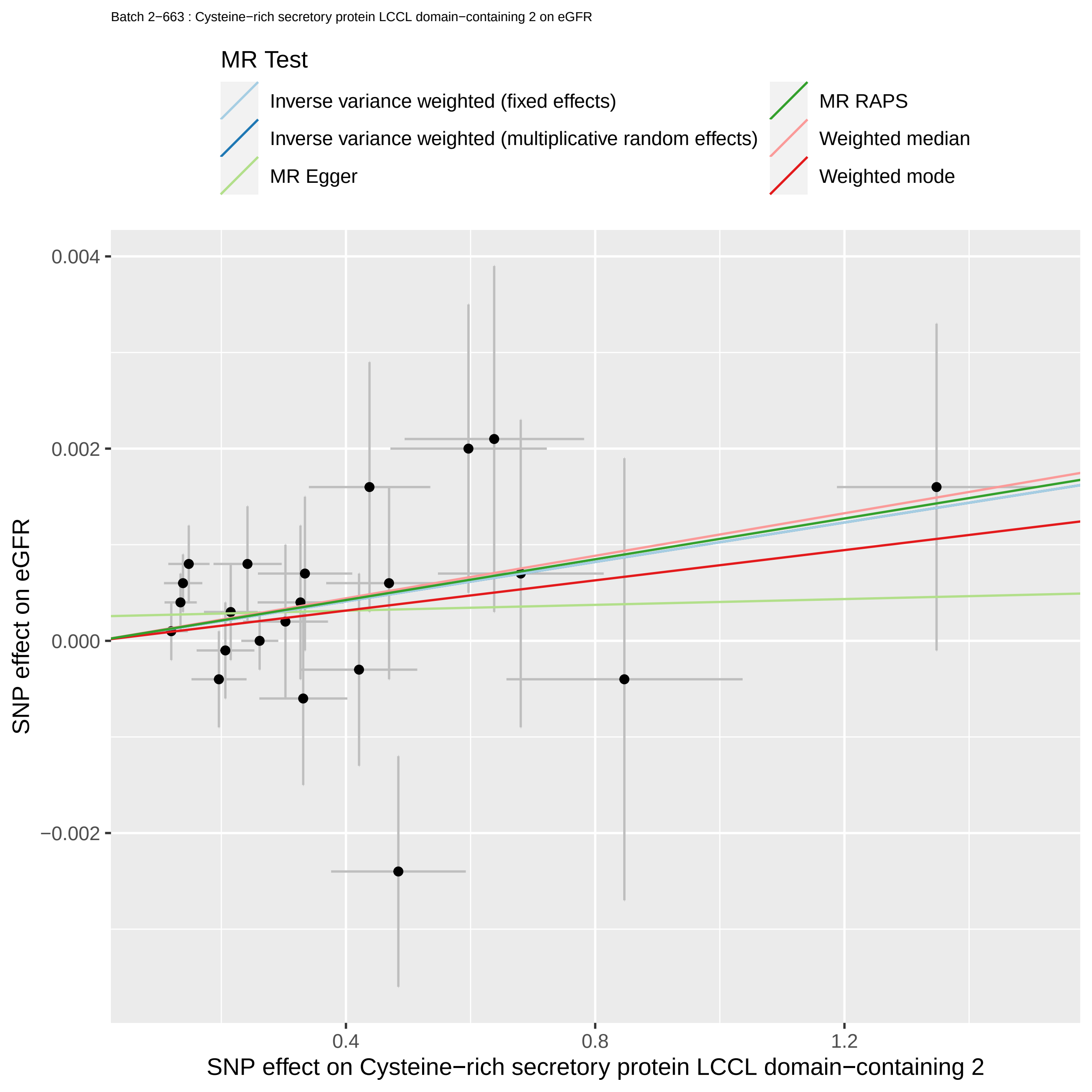
Batch 2−663 : Cysteine−rich secretory protein LCCL domain−containing 2 on eGFR
MR Test
Inverse variance weighted (fixed effects)
Inverse variance weighted (multiplicative random effects)
MR Egger
MR RAPS
Weighted median
Weighted mode
0.004
0.002
0.000
−0.002
0.4
0.8
1.2
SNP effect on eGFR
SNP effect on Cysteine−rich secretory protein LCCL domain−containing 2

Batch 2−664 : Adapter molecule crk on eGFR

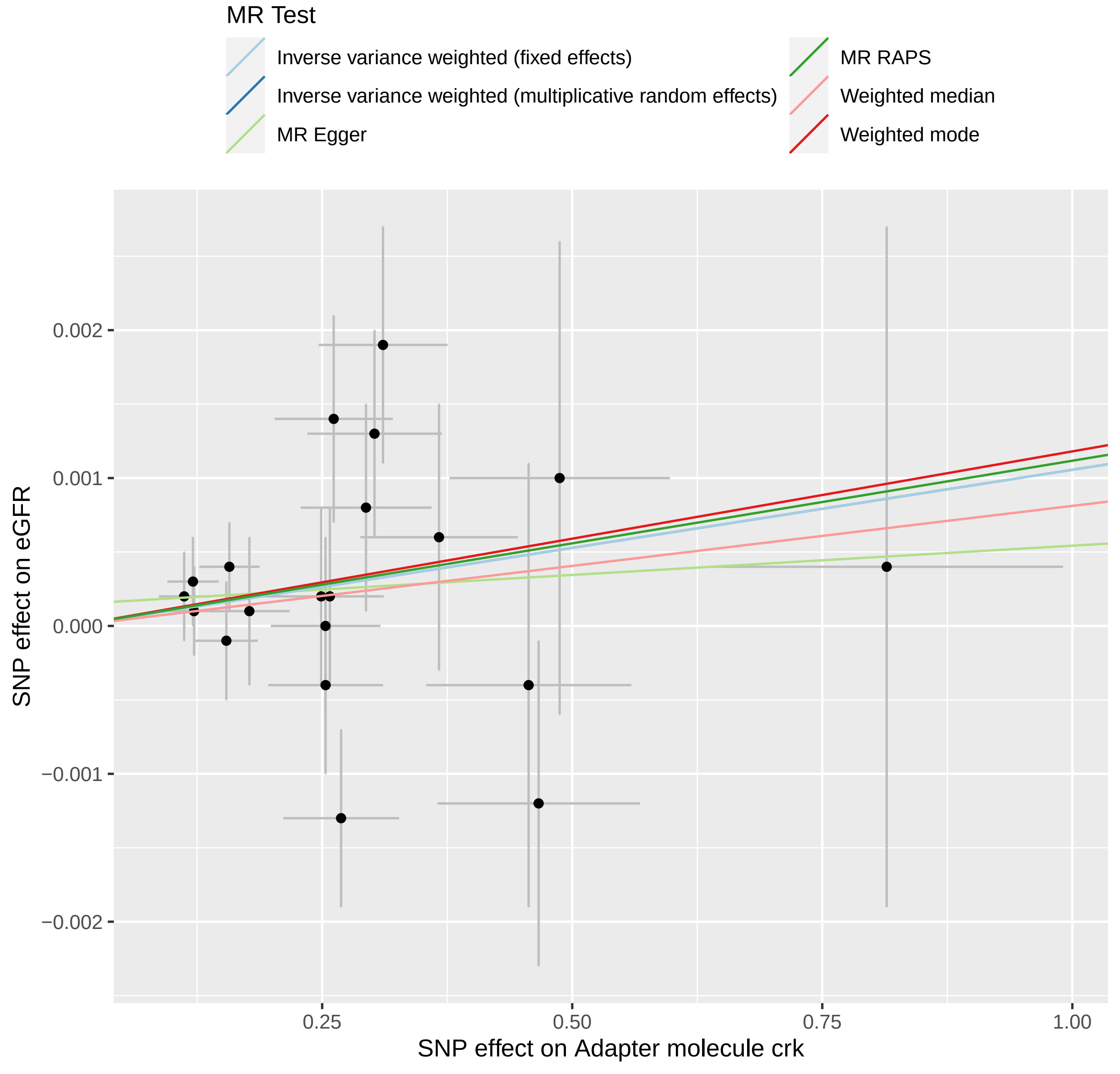

Batch 2−665 : Crk−like protein on eGFR

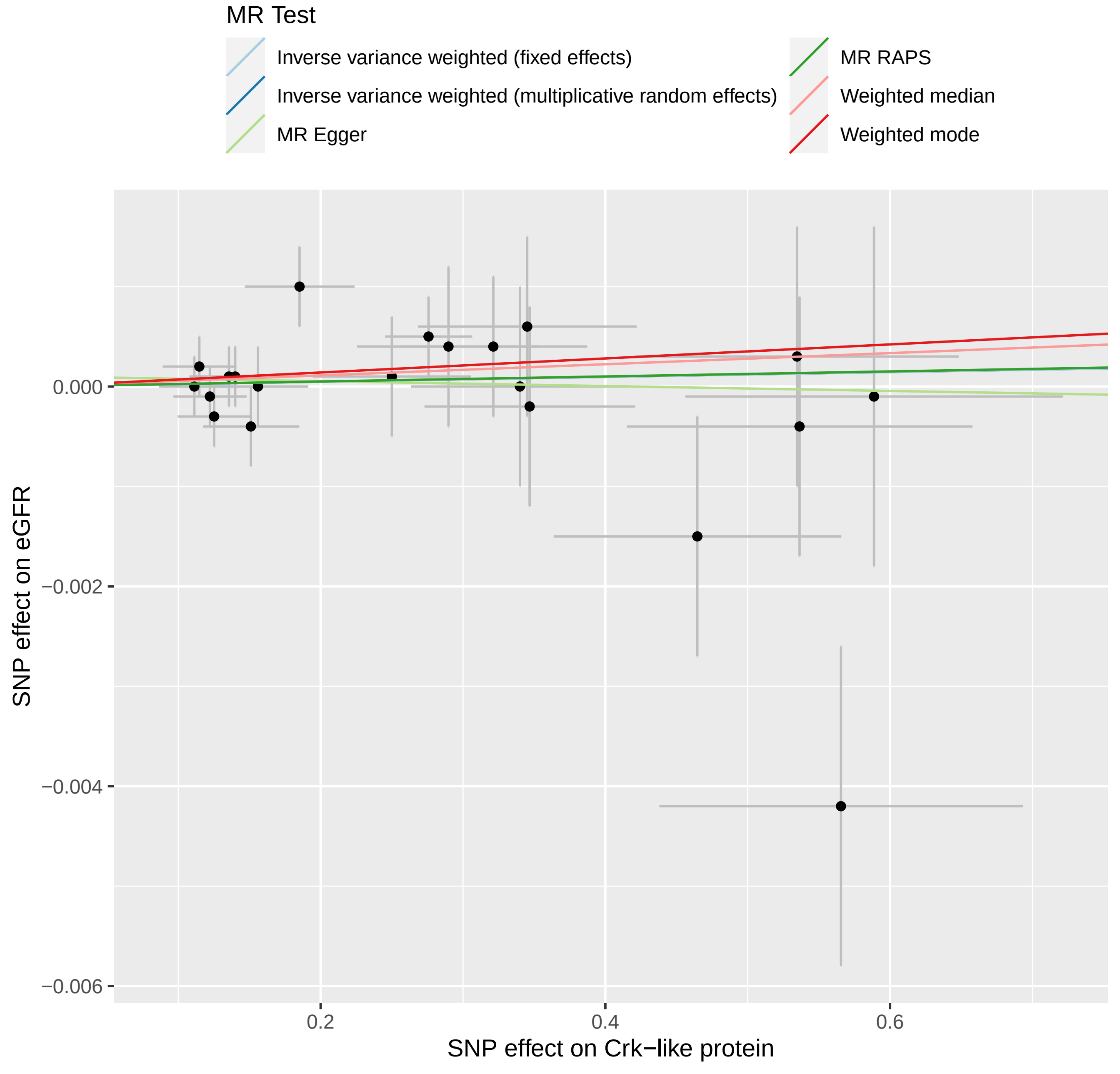

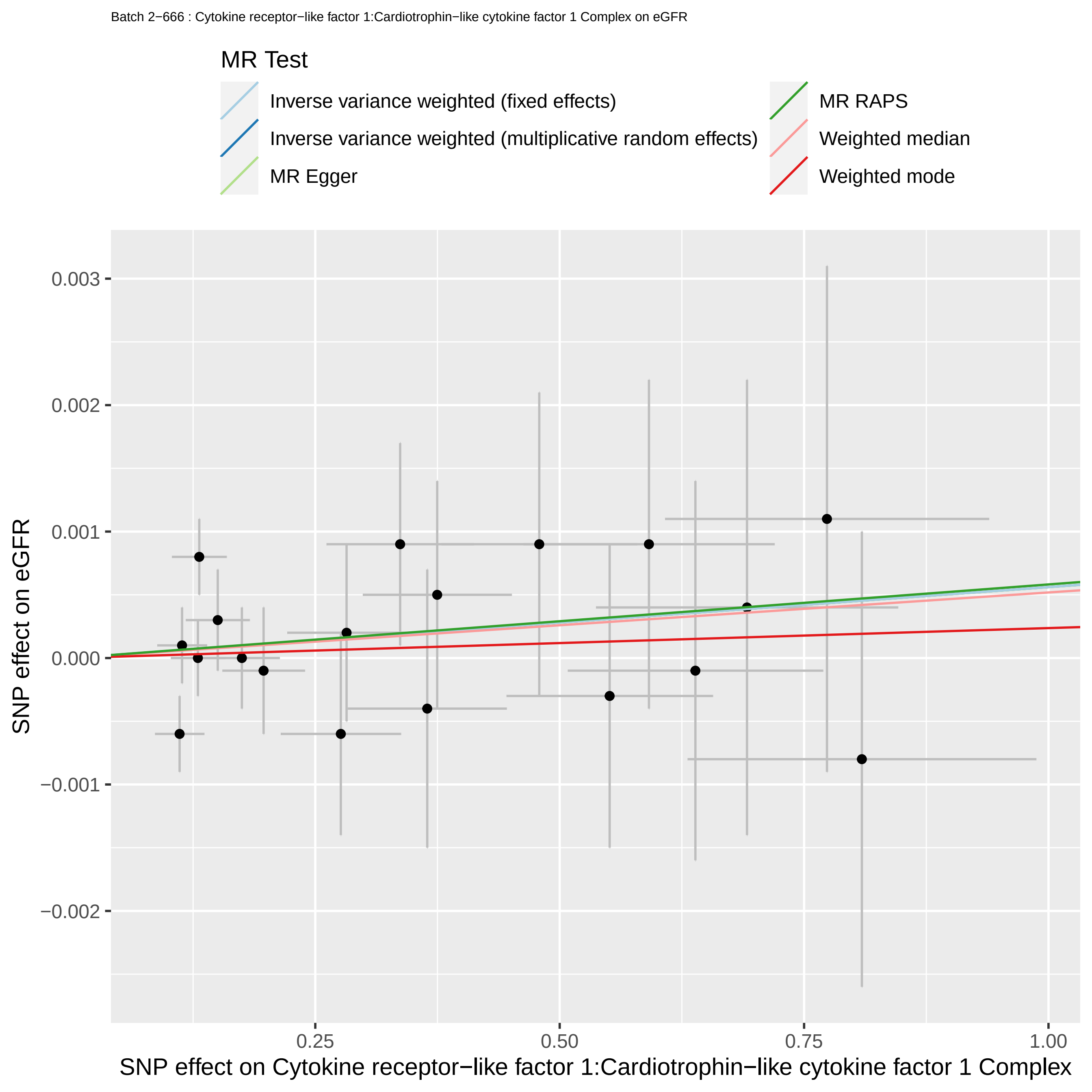
Batch 2−666 : Cytokine receptor−like factor 1:Cardiotrophin−like cytokine factor 1 Complex on eGFR
MR Test
Inverse variance weighted (fixed effects)
Inverse variance weighted (multiplicative random effects)
MR Egger
MR RAPS
Weighted median
Weighted mode
0.003
0.002
0.001
0.000
−0.001
−0.002
0.25
0.50
0.75
1.00
SNP effect on eGFR
SNP effect on Cytokine receptor−like factor 1:Cardiotrophin−like cytokine factor 1 Complex

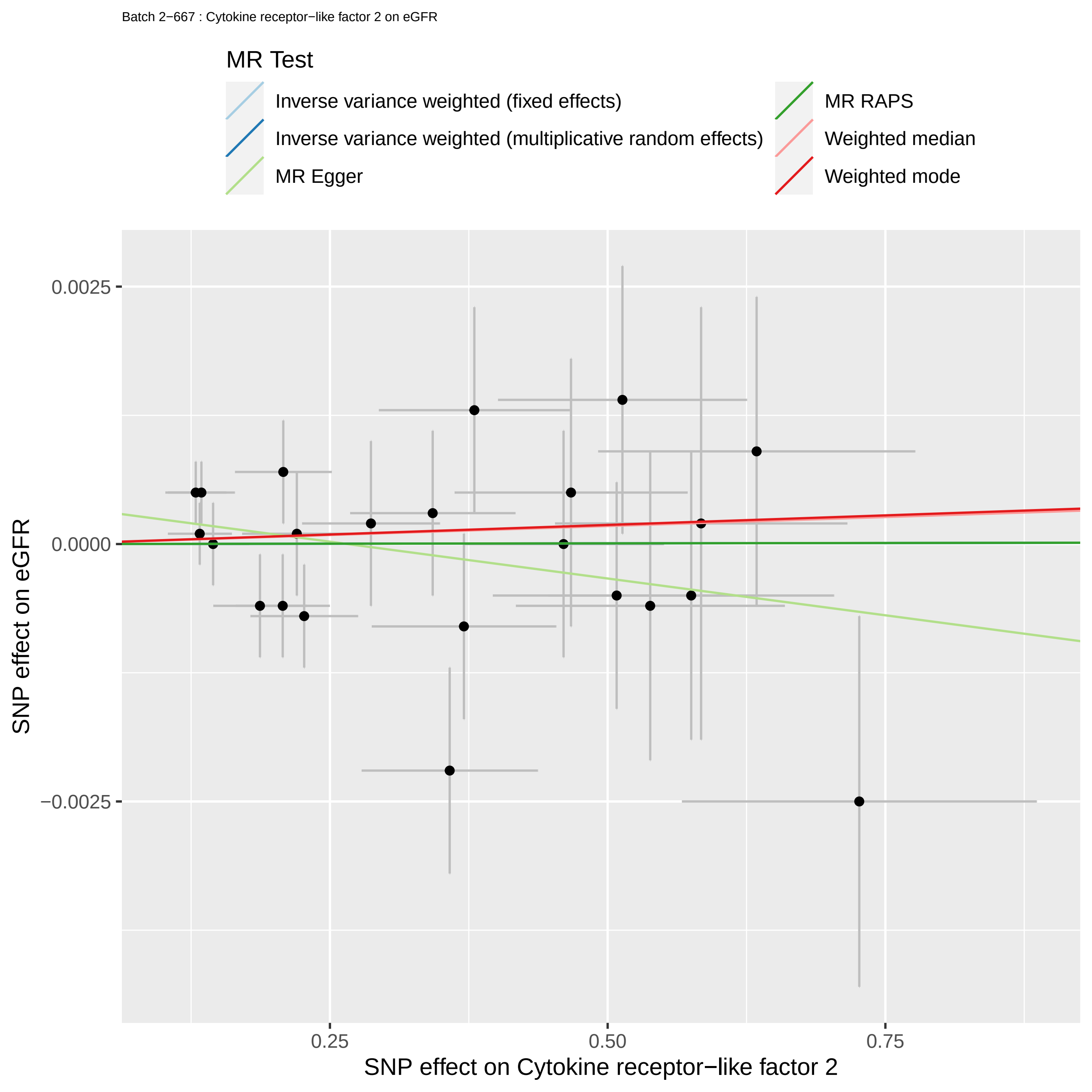

Batch 2−667 : Cytokine receptor−like factor 2 on eGFR
MR Test
Inverse variance weighted (fixed effects)
Inverse variance weighted (multiplicative random effects)
MR Egger
MR RAPS
Weighted median
Weighted mode
0.0025
0.0000
−0.0025
0.25
0.50
0.75
SNP effect on eGFR
SNP effect on Cytokine receptor−like factor 2

Batch 2−668 : Cytokine receptor−like factor 2 on eGFR

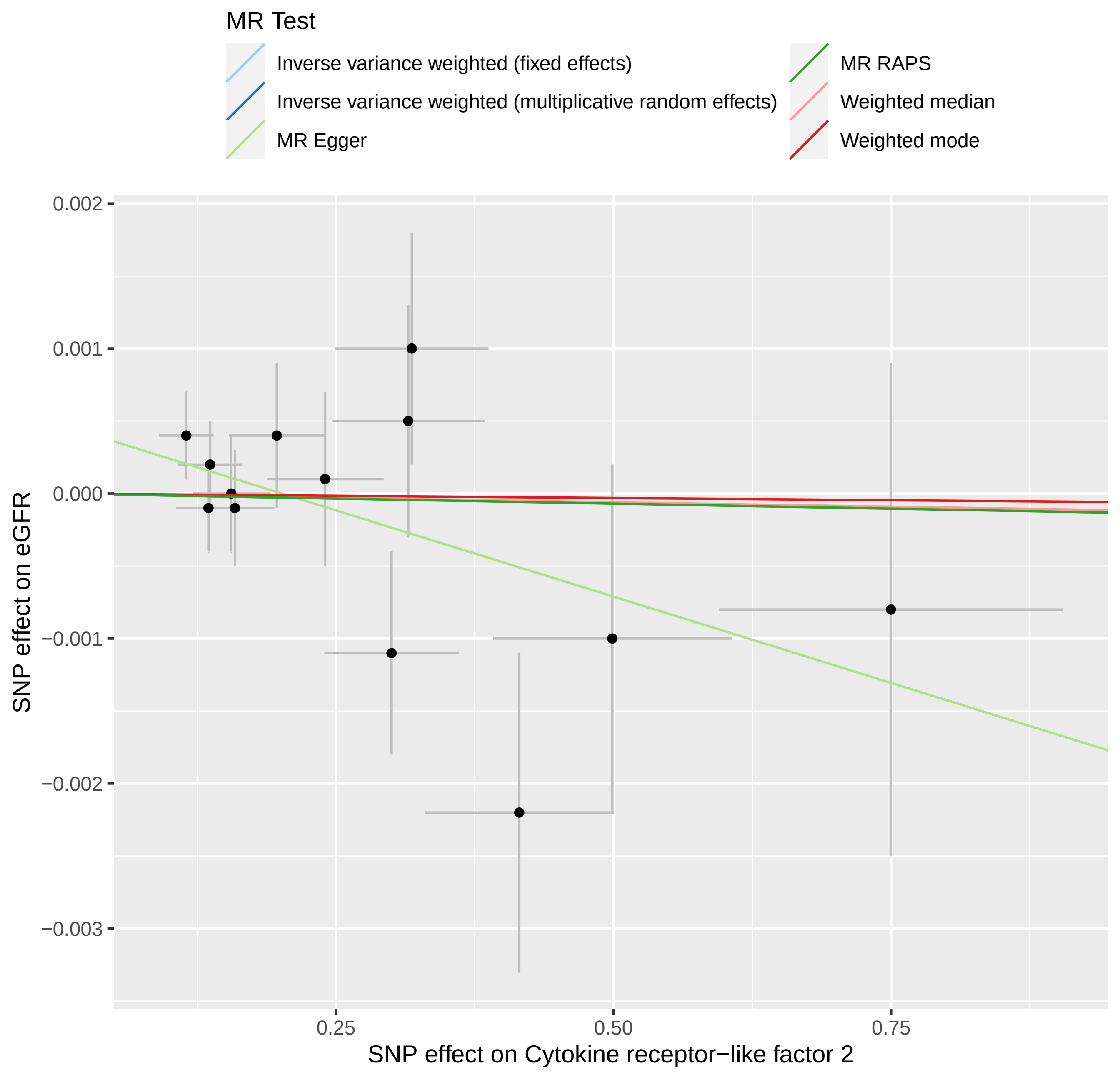

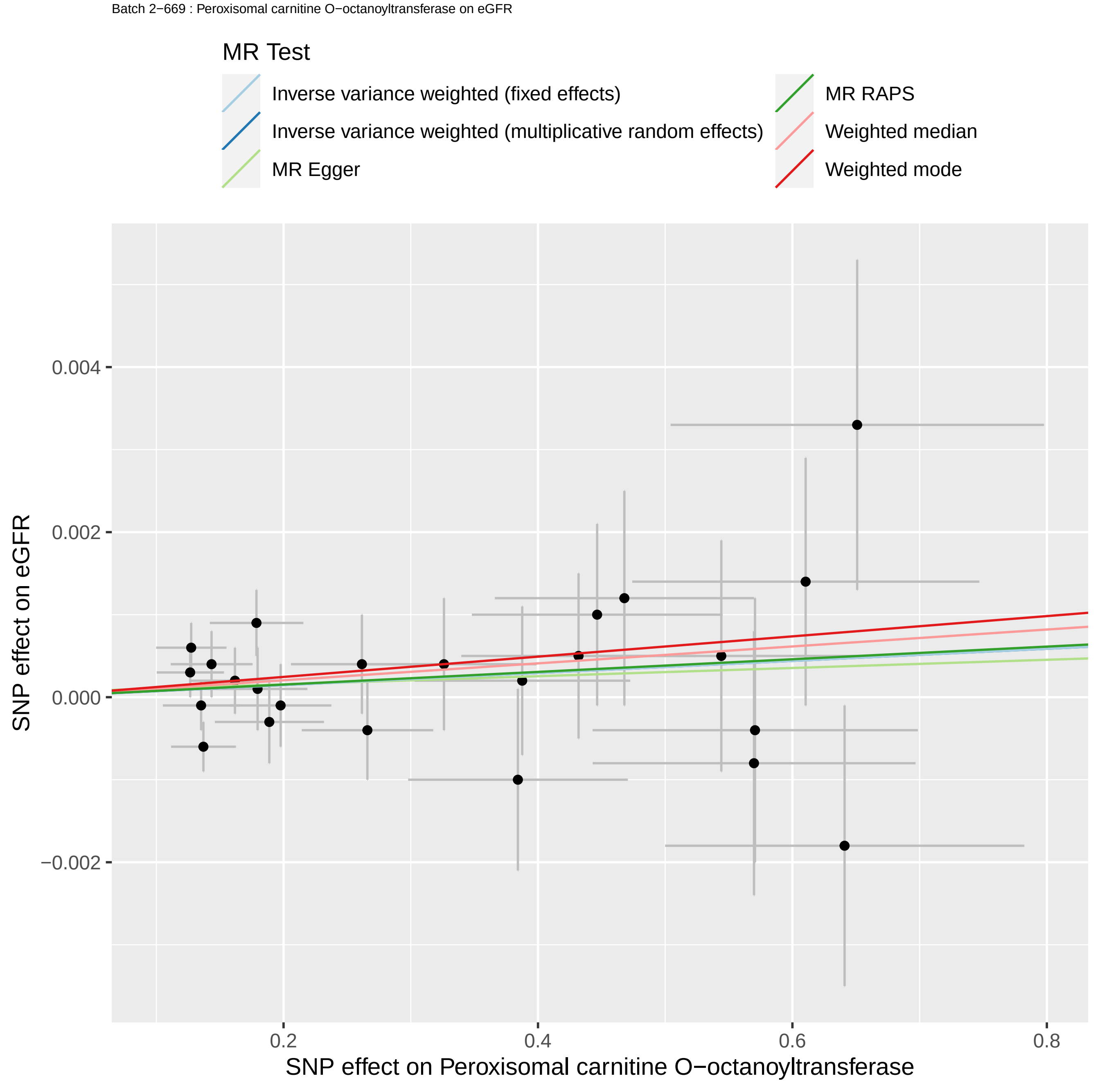
Batch 2−669 : Peroxisomal carnitine O−octanoyltransferase on eGFR
MR Test
Inverse variance weighted (fixed effects)
Inverse variance weighted (multiplicative random effects)
MR Egger
MR RAPS
Weighted median
Weighted mode
0.004
0.002
0.000
−0.002
0.2
0.4
0.6
0.8
SNP effect on eGFR
SNP effect on Peroxisomal carnitine O−octanoyltransferase

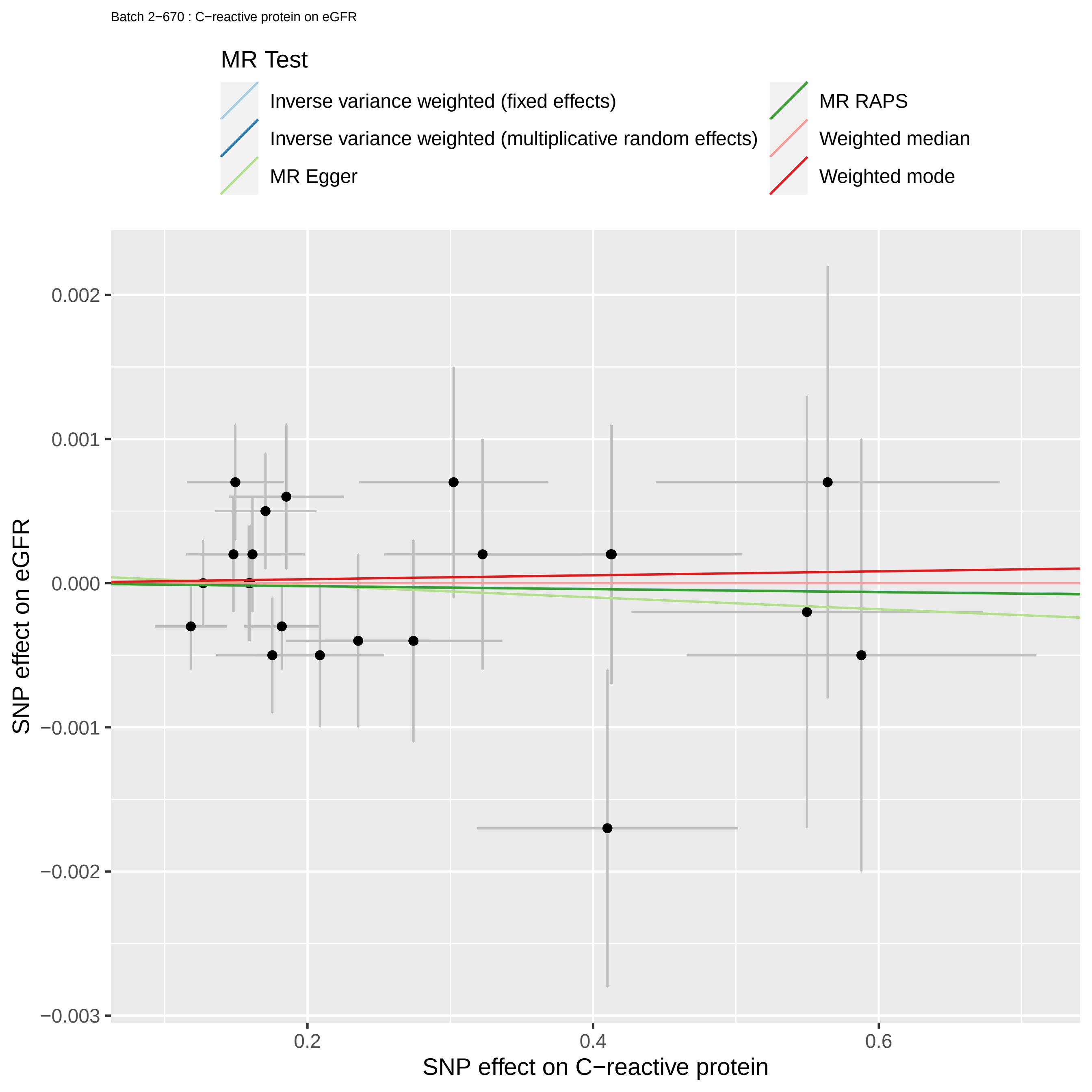
Batch 2−670 : C−reactive protein on eGFR
MR Test
Inverse variance weighted (fixed effects)
Inverse variance weighted (multiplicative random effects)
MR Egger
MR RAPS
Weighted median
Weighted mode
0.002
0.001
0.000
−0.001
−0.002
−0.003
SNP effect on eGFR
0.2
0.4
0.6
SNP effect on C−reactive protein

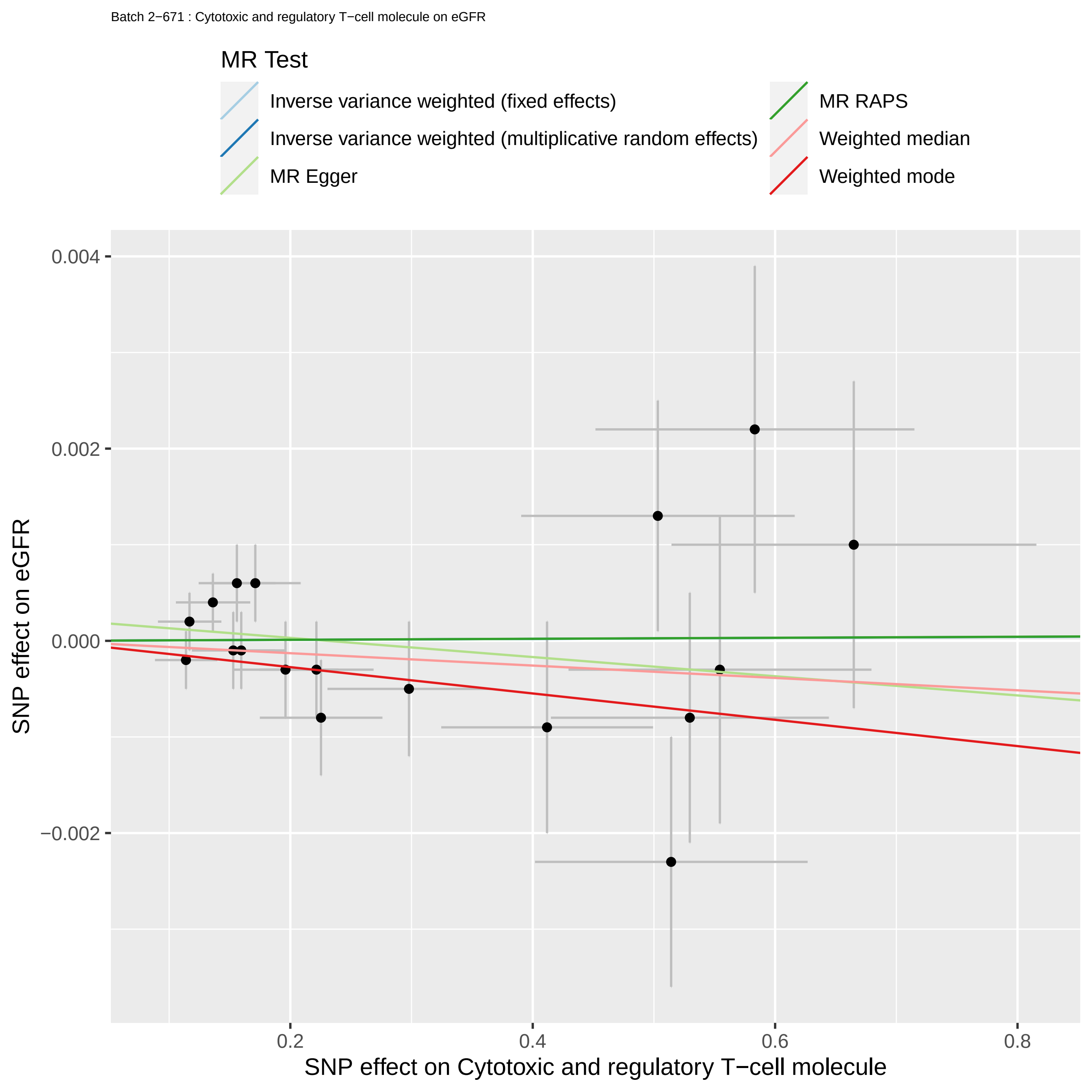
Batch 2−671 : Cytotoxic and regulatory T−cell molecule on eGFR
MR Test
Inverse variance weighted (fixed effects)
Inverse variance weighted (multiplicative random effects)
MR Egger
MR RAPS
Weighted median
Weighted mode
0.004
0.002
0.000
−0.002
0.2
0.4
0.6
0.8
SNP effect on eGFR
SNP effect on Cytotoxic and regulatory T−cell molecule

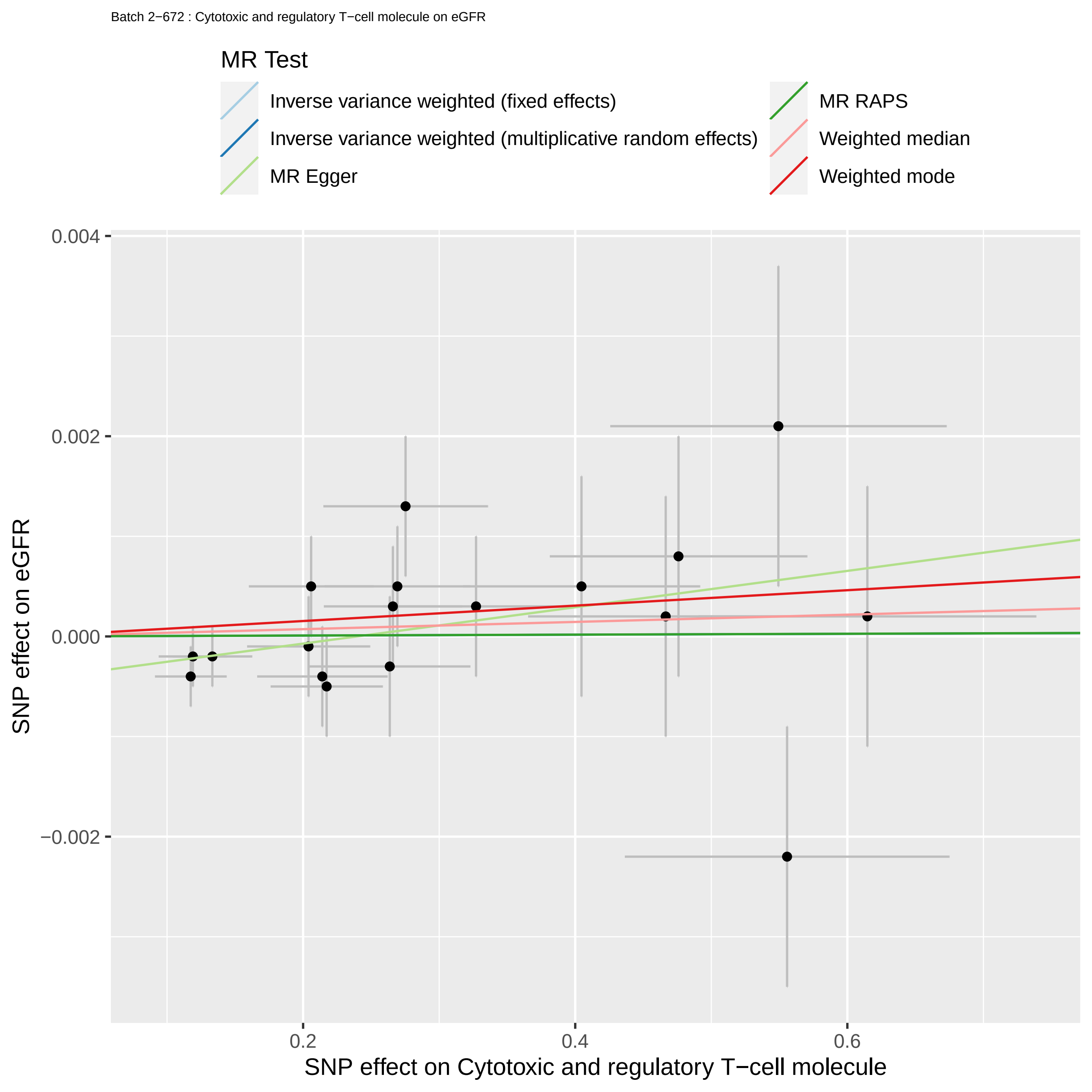

Batch 2−672 : Cytotoxic and regulatory T−cell molecule on eGFR
MR Test
Inverse variance weighted (fixed effects)
Inverse variance weighted (multiplicative random effects)
MR Egger
MR RAPS
Weighted median
Weighted mode
0.004
0.002
0.000
−0.002
SNP effect on eGFR
0.2
0.4
0.6
SNP effect on Cytotoxic and regulatory T−cell molecule

Batch 2−673 : Alpha−crystallin A chain on eGFR

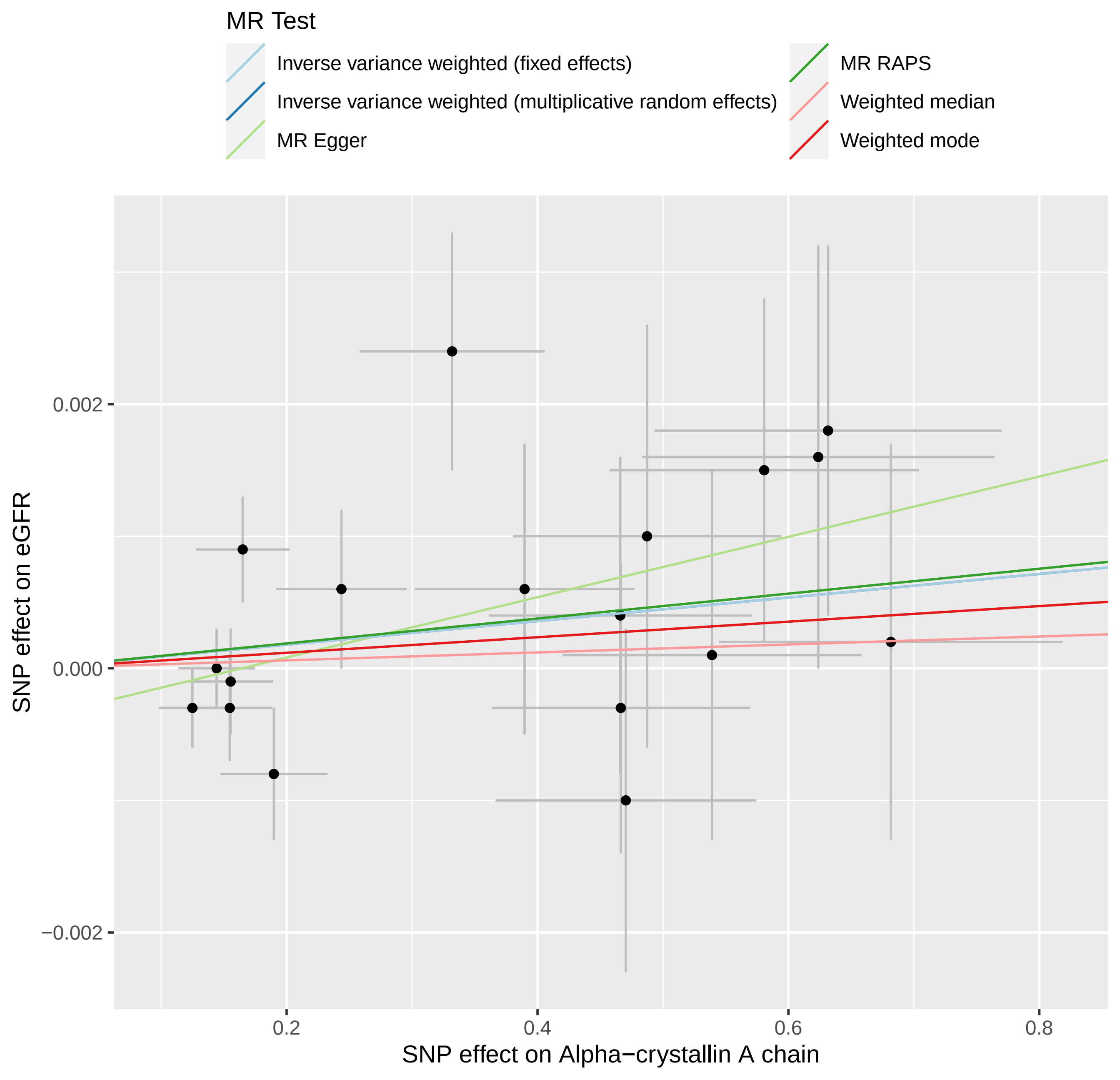

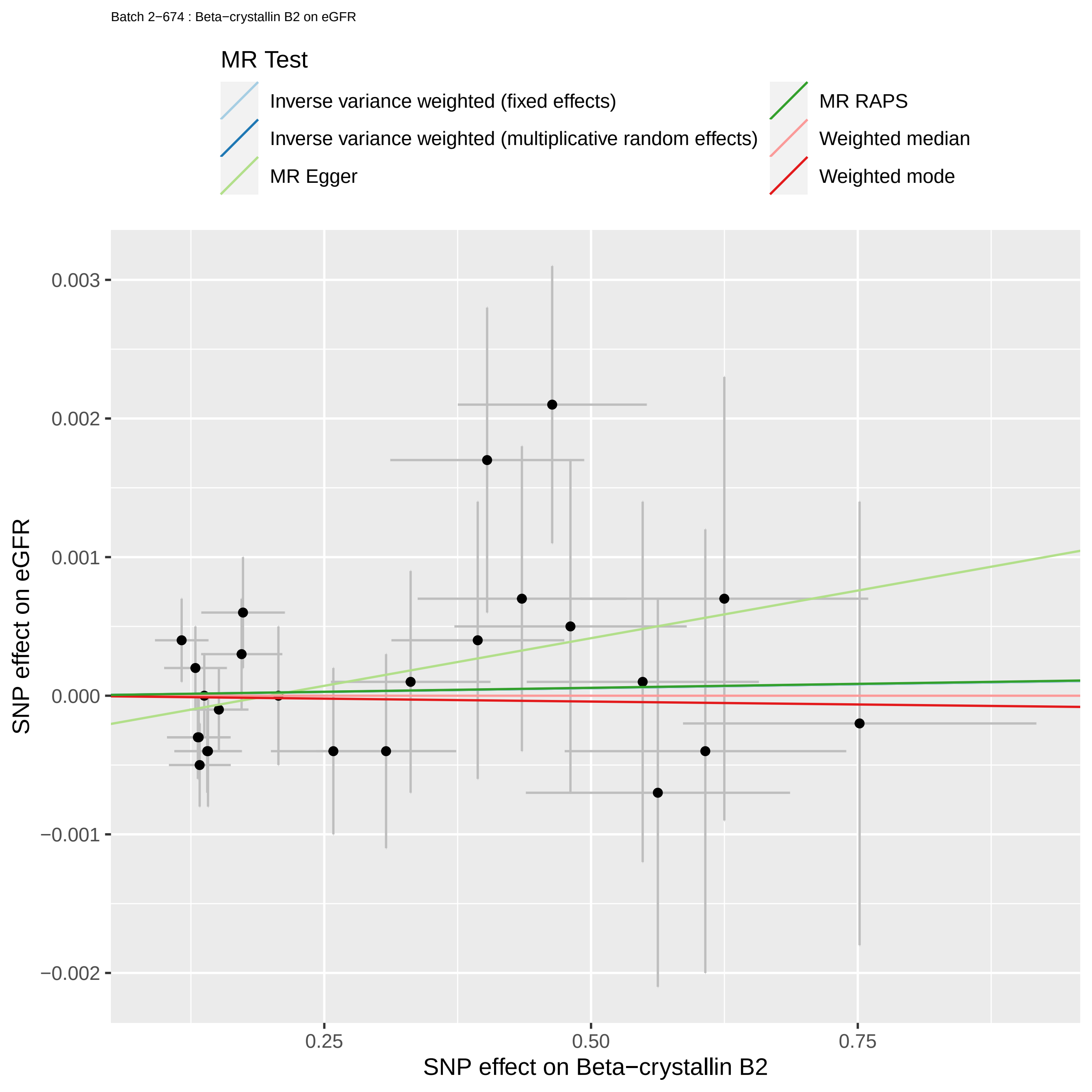
Batch 2−674 : Beta−crystallin B2 on eGFR
MR Test
Inverse variance weighted (fixed effects)
Inverse variance weighted (multiplicative random effects)
MR Egger
MR RAPS
Weighted median
Weighted mode
0.003
0.002
0.001
0.000
−0.001
−0.002
0.25
0.50
0.75
SNP effect on eGFR
SNP effect on Beta−crystallin B2

Batch 2−675 : Gamma−crystallin D on eGFR

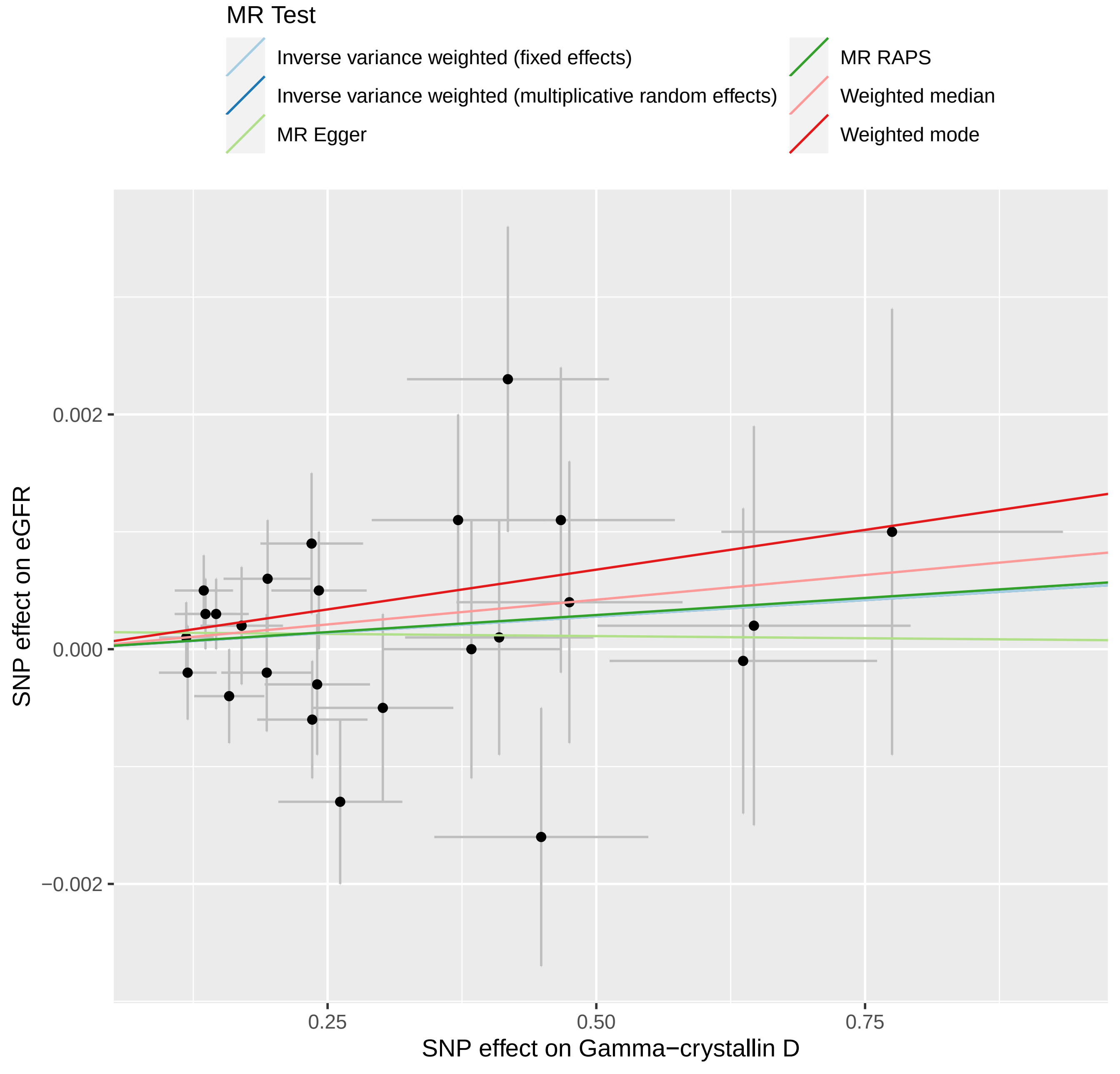

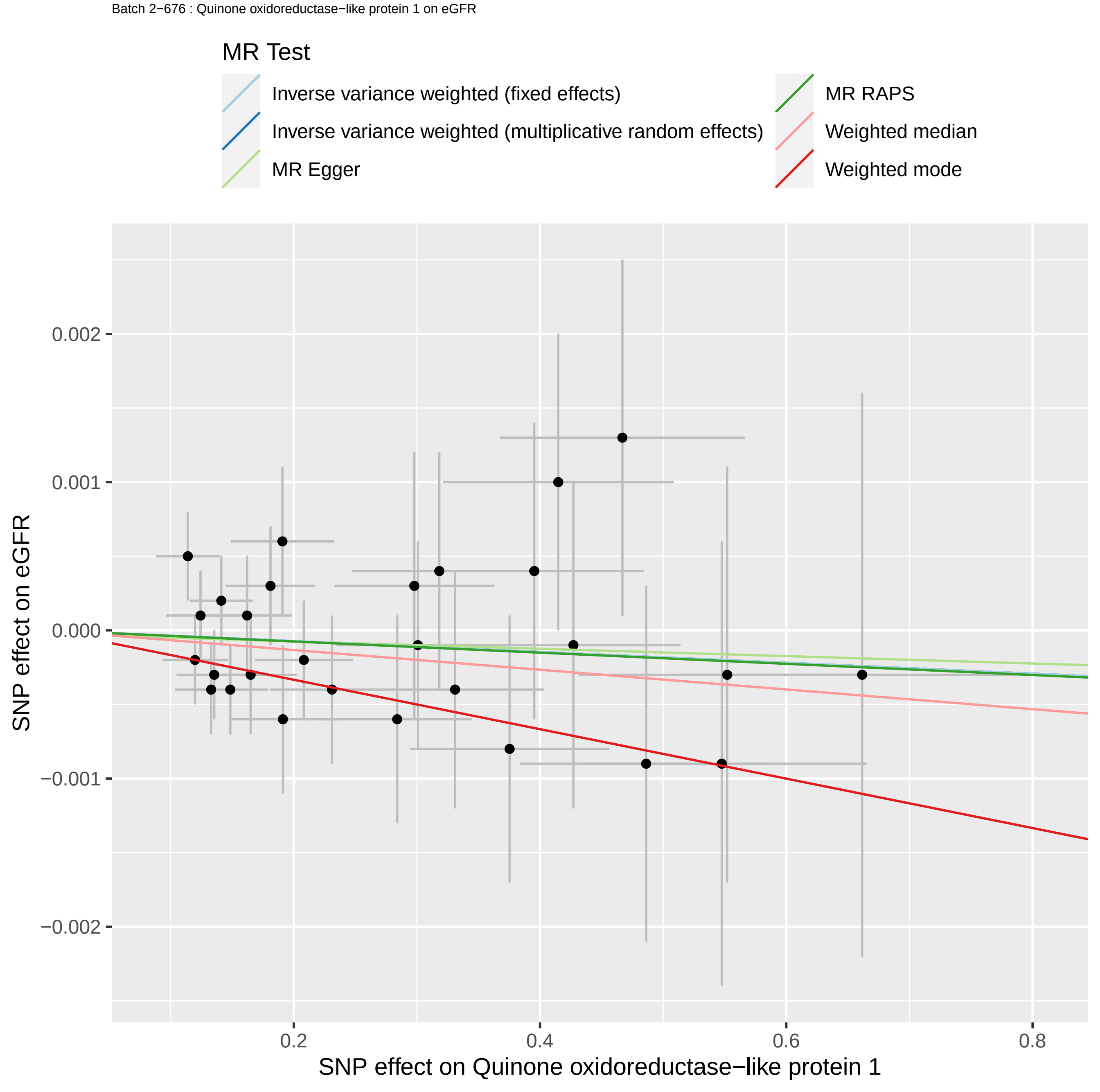

Batch 2−676 : Quinone oxidoreductase−like protein 1 on eGFR
MR Test
Inverse variance weighted (fixed effects)
Inverse variance weighted (multiplicative random effects)
MR Egger
MR RAPS
Weighted median
Weighted mode
0.002
0.001
0.000
−0.001
−0.002
SNP effect on eGFR
0.2
0.4
0.6
0.8
SNP effect on Quinone oxidoreductase−like protein 1

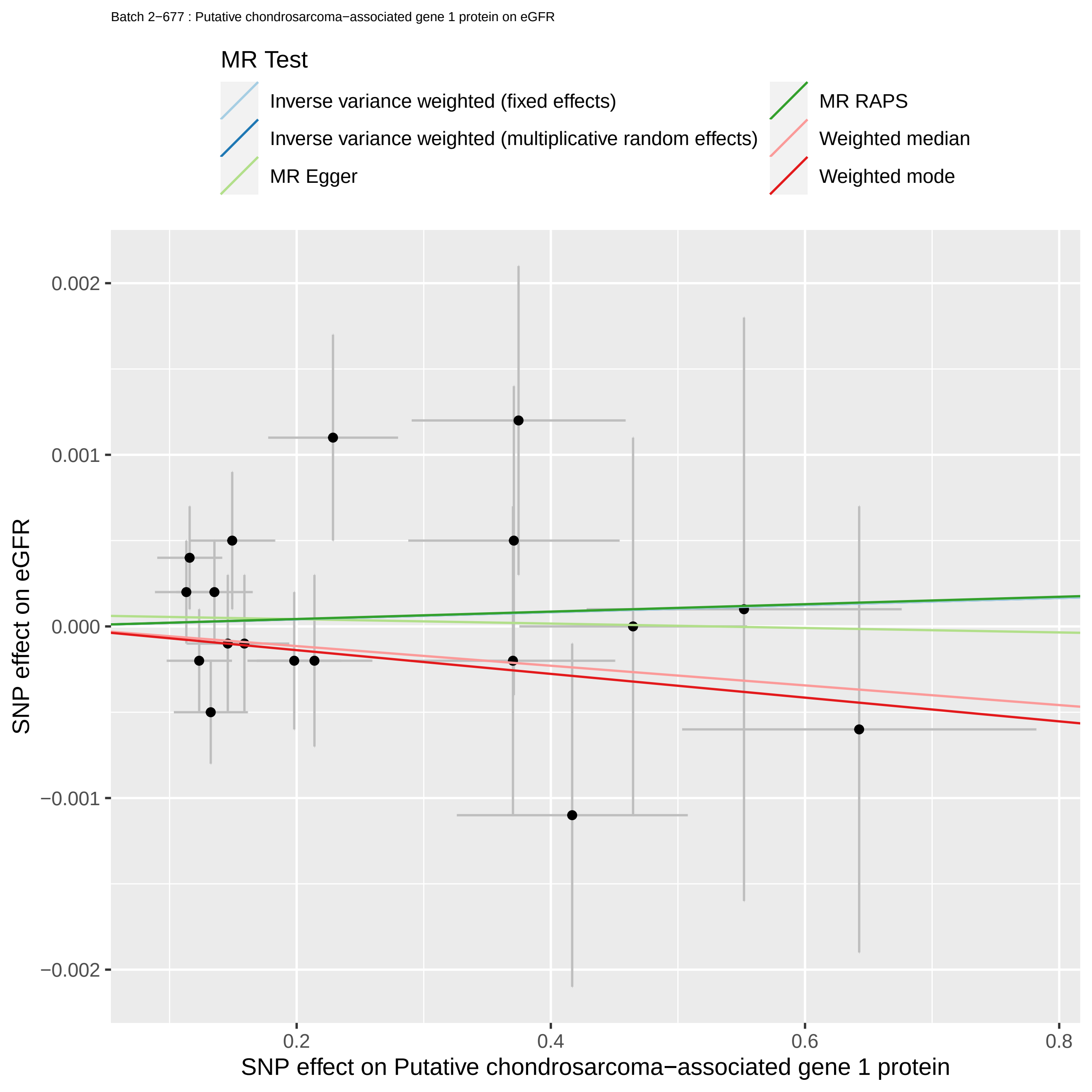
Batch 2−677 : Putative chondrosarcoma−associated gene 1 protein on eGFR
MR Test
Inverse variance weighted (fixed effects)
Inverse variance weighted (multiplicative random effects)
MR Egger
MR RAPS
Weighted median
Weighted mode
0.002
0.001
0.000
−0.001
−0.002
SNP effect on eGFR
0.2
0.4
0.6
0.8
SNP effect on Putative chondrosarcoma−associated gene 1 protein

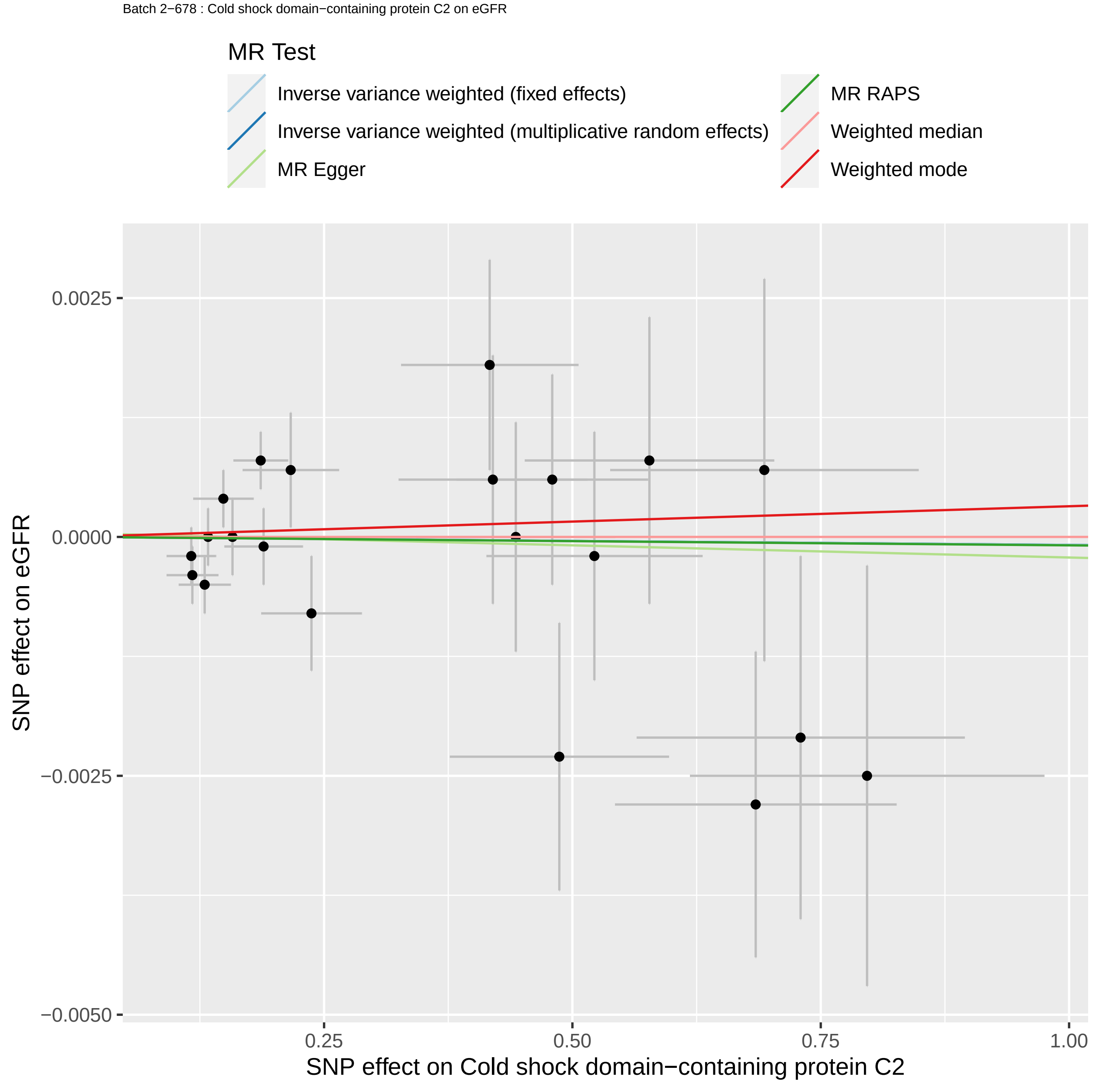
Batch 2−678 : Cold shock domain−containing protein C2 on eGFR
MR Test
Inverse variance weighted (fixed effects)
Inverse variance weighted (multiplicative random effects)
MR Egger
MR RAPS
Weighted median
Weighted mode
0.0025
0.0000
−0.0025
−0.0050
0.25
0.50
0.75
1.00
SNP effect on eGFR
SNP effect on Cold shock domain−containing protein C2

Batch 2−679 : Cold shock domain−containing protein E1 on eGFR

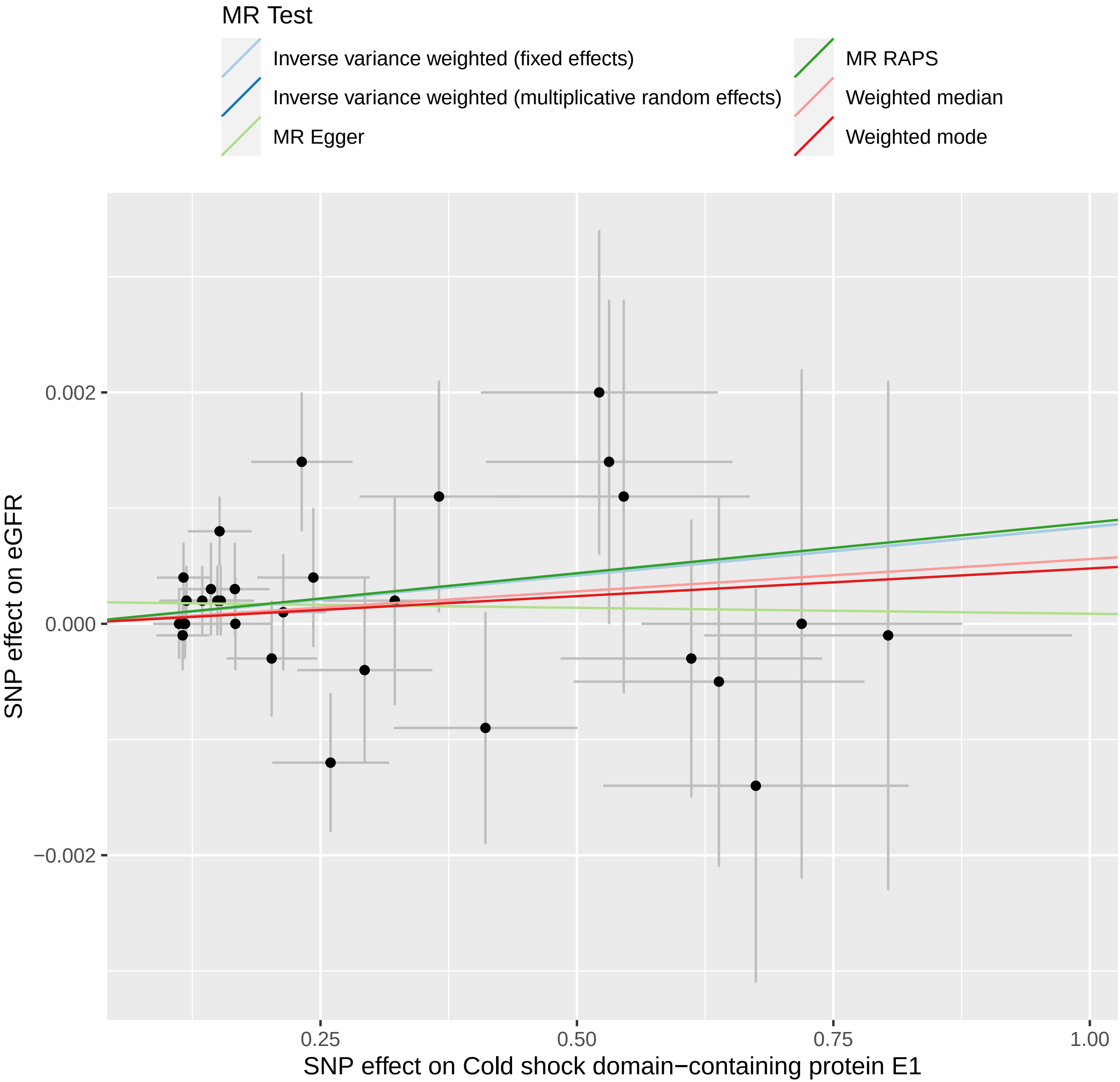

Batch 2−680 : Macrophage colony−stimulating factor 1 on eGFR

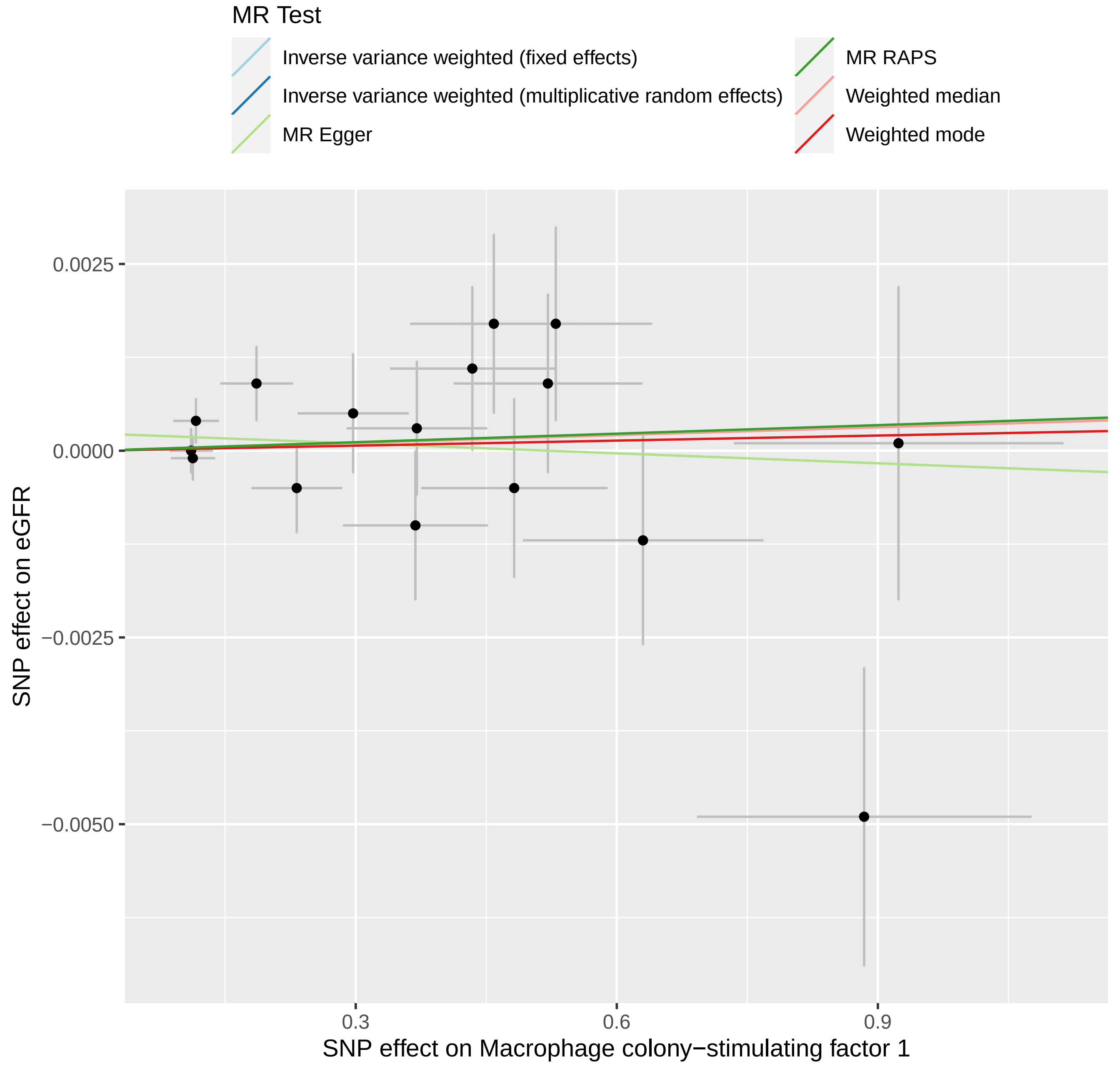

Batch 2−681 : Granulocyte−macrophage colony−stimulating factor on eGFR

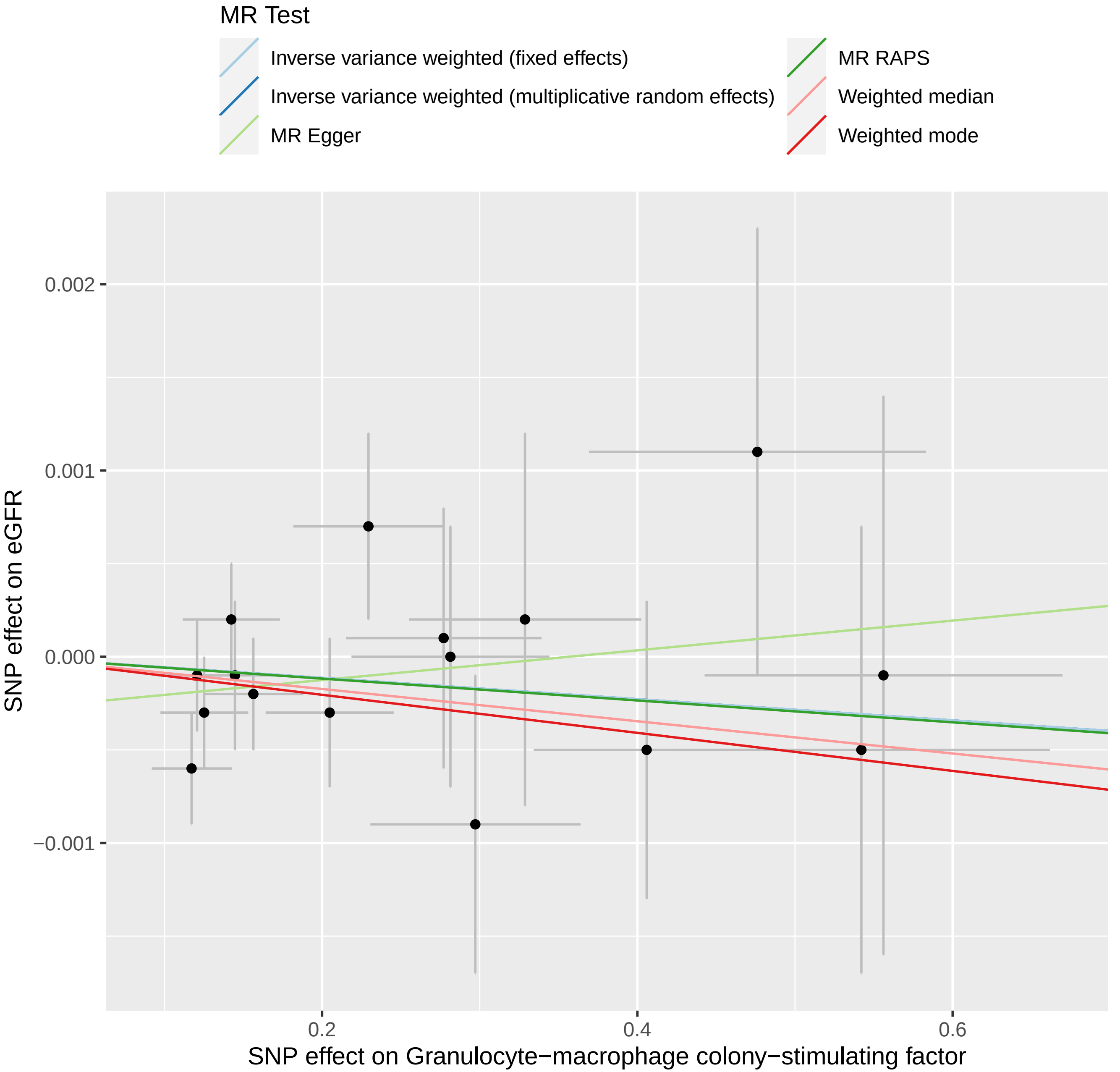

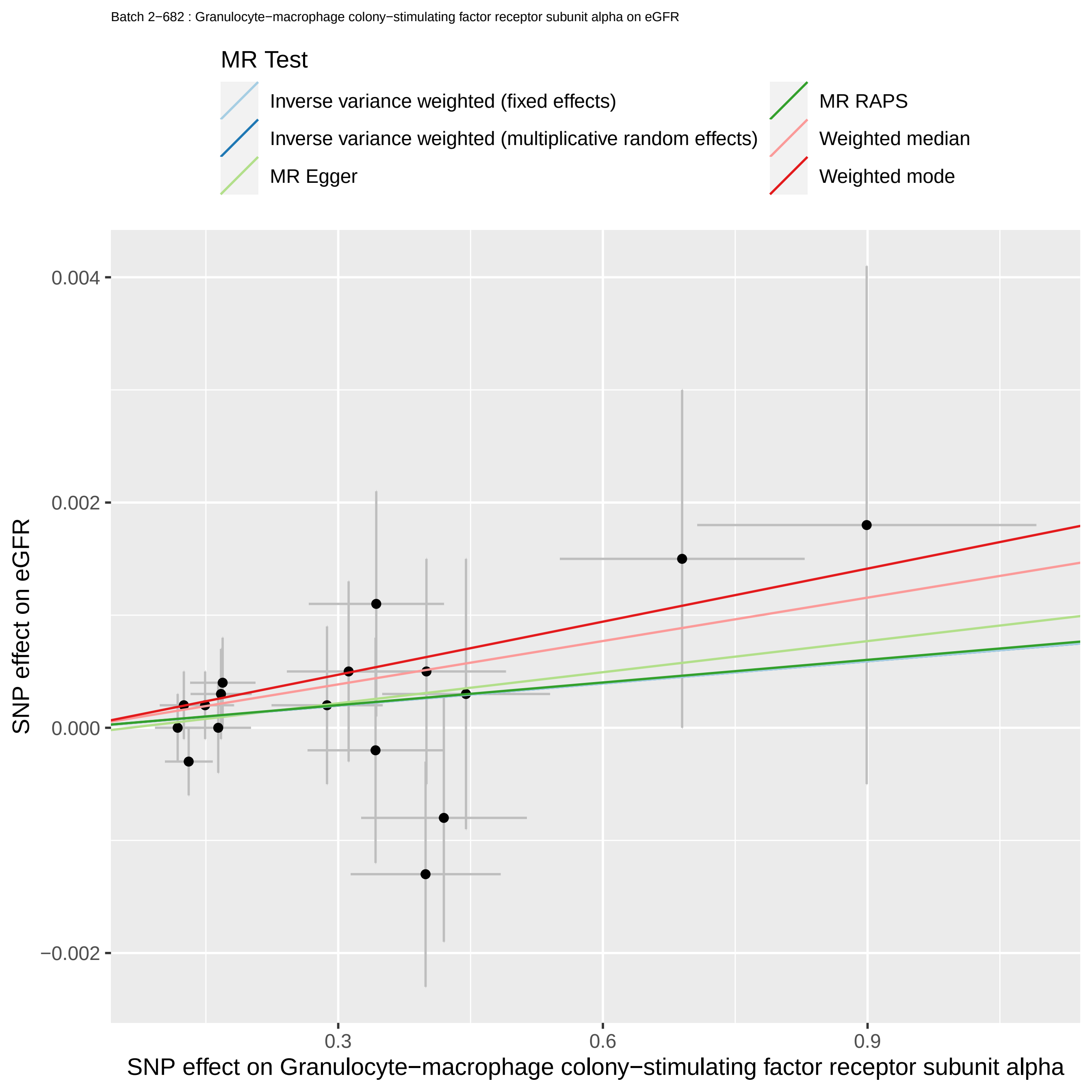
Batch 2−682 : Granulocyte−macrophage colony−stimulating factor receptor subunit alpha on eGFR
MR Test
Inverse variance weighted (fixed effects)
Inverse variance weighted (multiplicative random effects)
MR Egger
MR RAPS
Weighted median
Weighted mode
0.004
0.002
0.000
−0.002
0.3
0.6
0.9
SNP effect on eGFR
SNP effect on Granulocyte−macrophage colony−stimulating factor receptor subunit alpha

Batch 2−683 : Cytokine receptor common subunit beta on eGFR

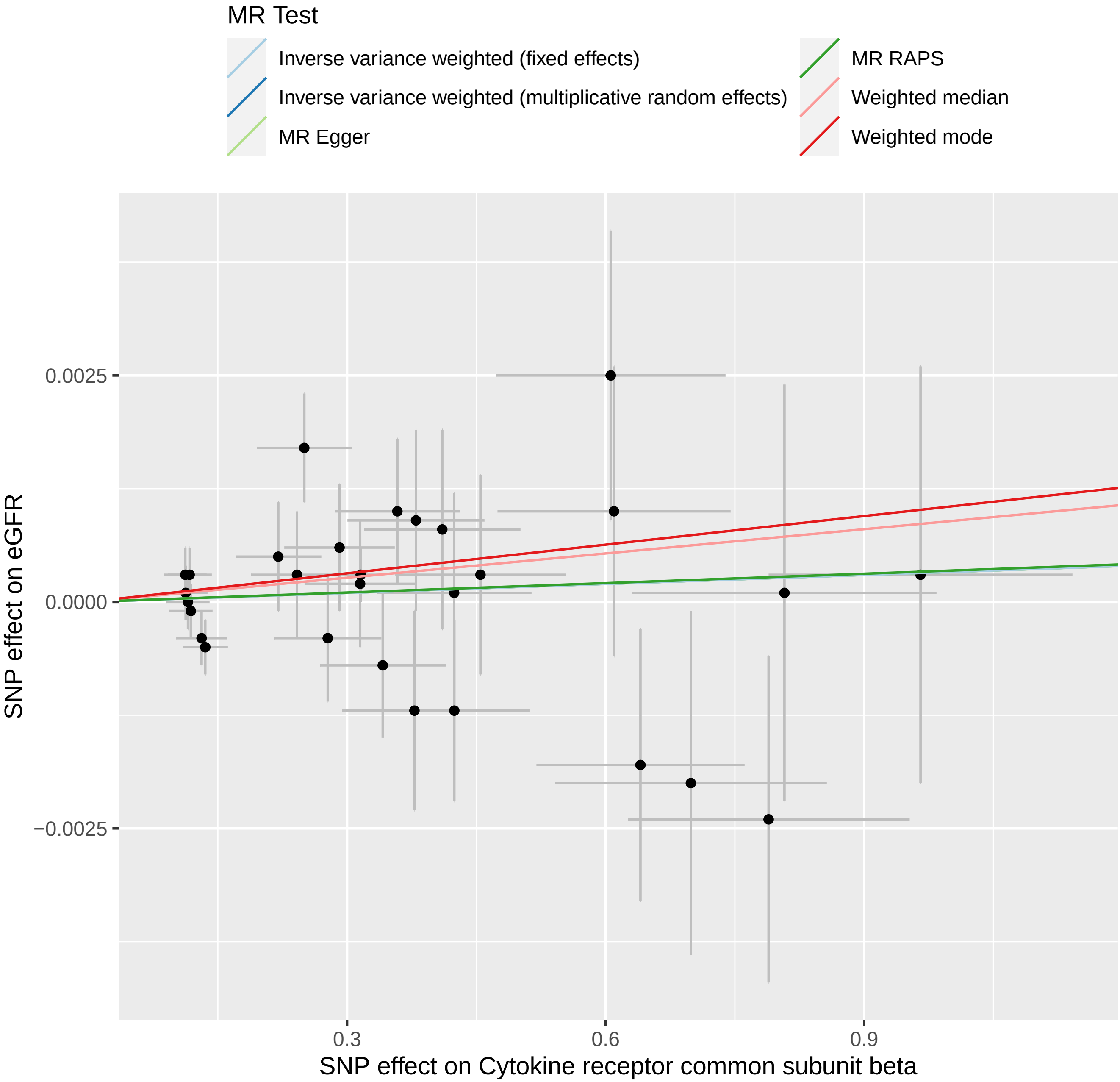

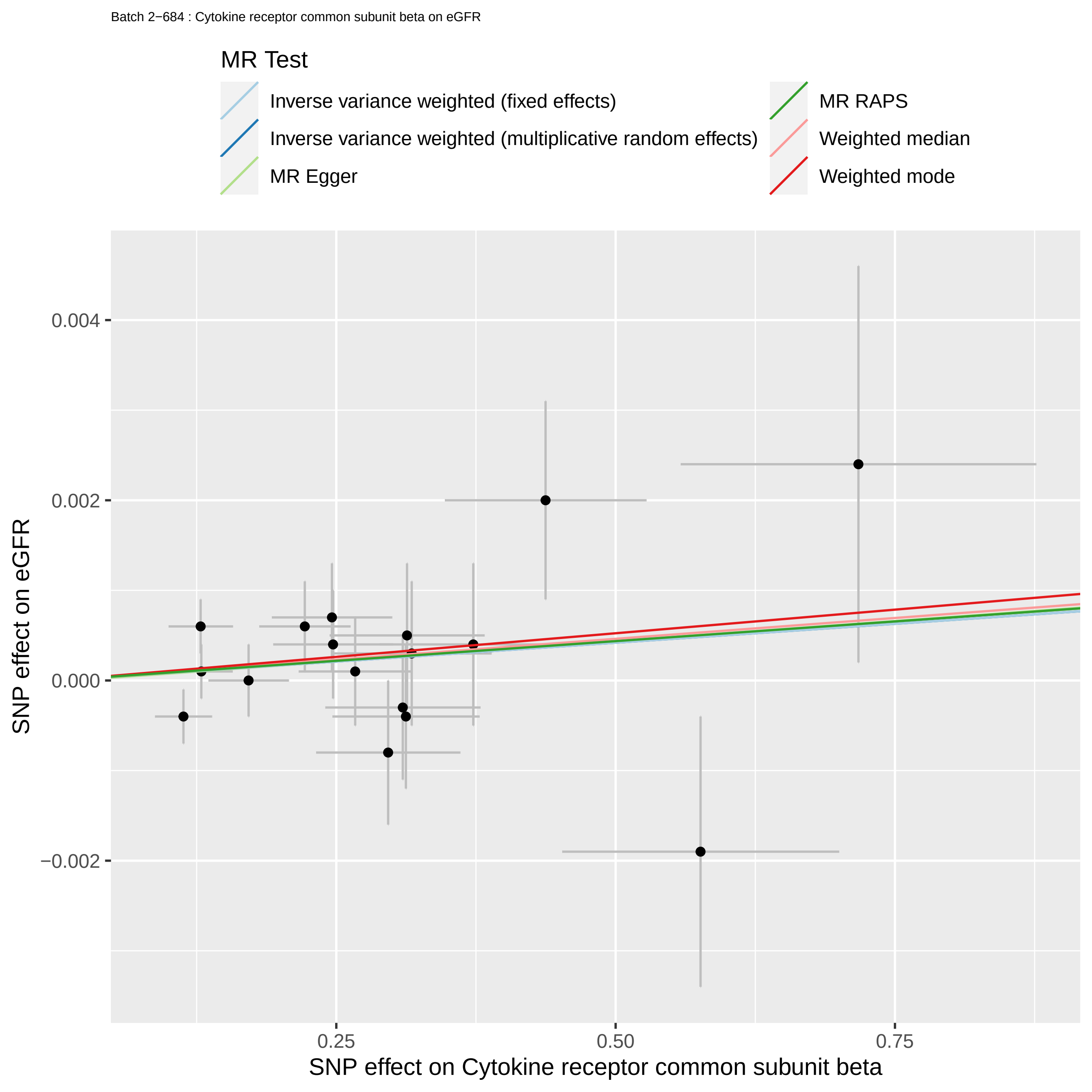

Batch 2−684 : Cytokine receptor common subunit beta on eGFR
MR Test
Inverse variance weighted (fixed effects)
Inverse variance weighted (multiplicative random effects)
MR Egger
MR RAPS
Weighted median
Weighted mode
0.004
0.002
0.000
−0.002
0.25
0.50
0.75
SNP effect on eGFR
SNP effect on Cytokine receptor common subunit beta

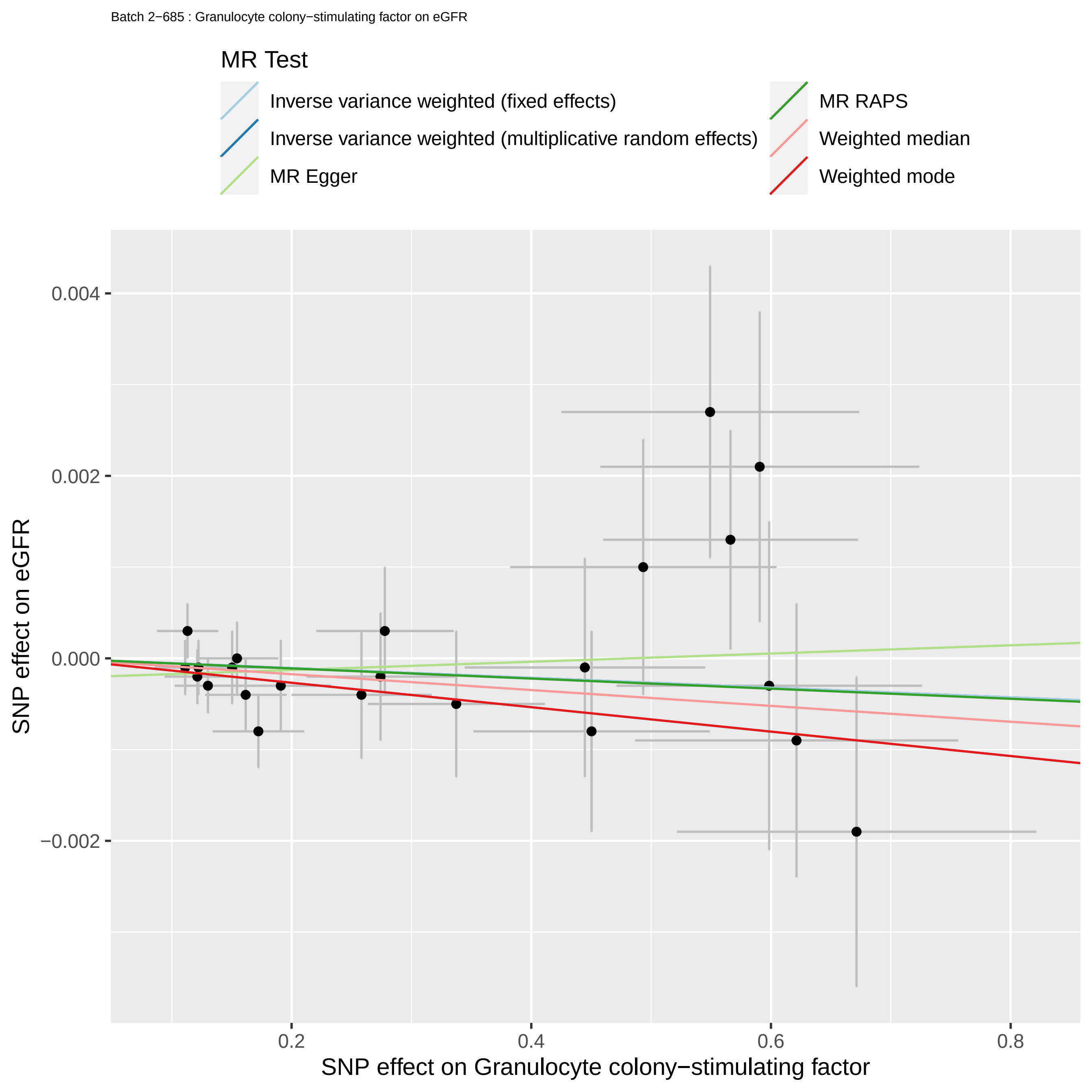

Batch 2−685 : Granulocyte colony−stimulating factor on eGFR
MR Test
Inverse variance weighted (fixed effects)
Inverse variance weighted (multiplicative random effects)
MR Egger
MR RAPS
Weighted median
Weighted mode
0.004
0.002
0.000
−0.002
0.2
0.4
0.6
0.8
SNP effect on eGFR
SNP effect on Granulocyte colony−stimulating factor

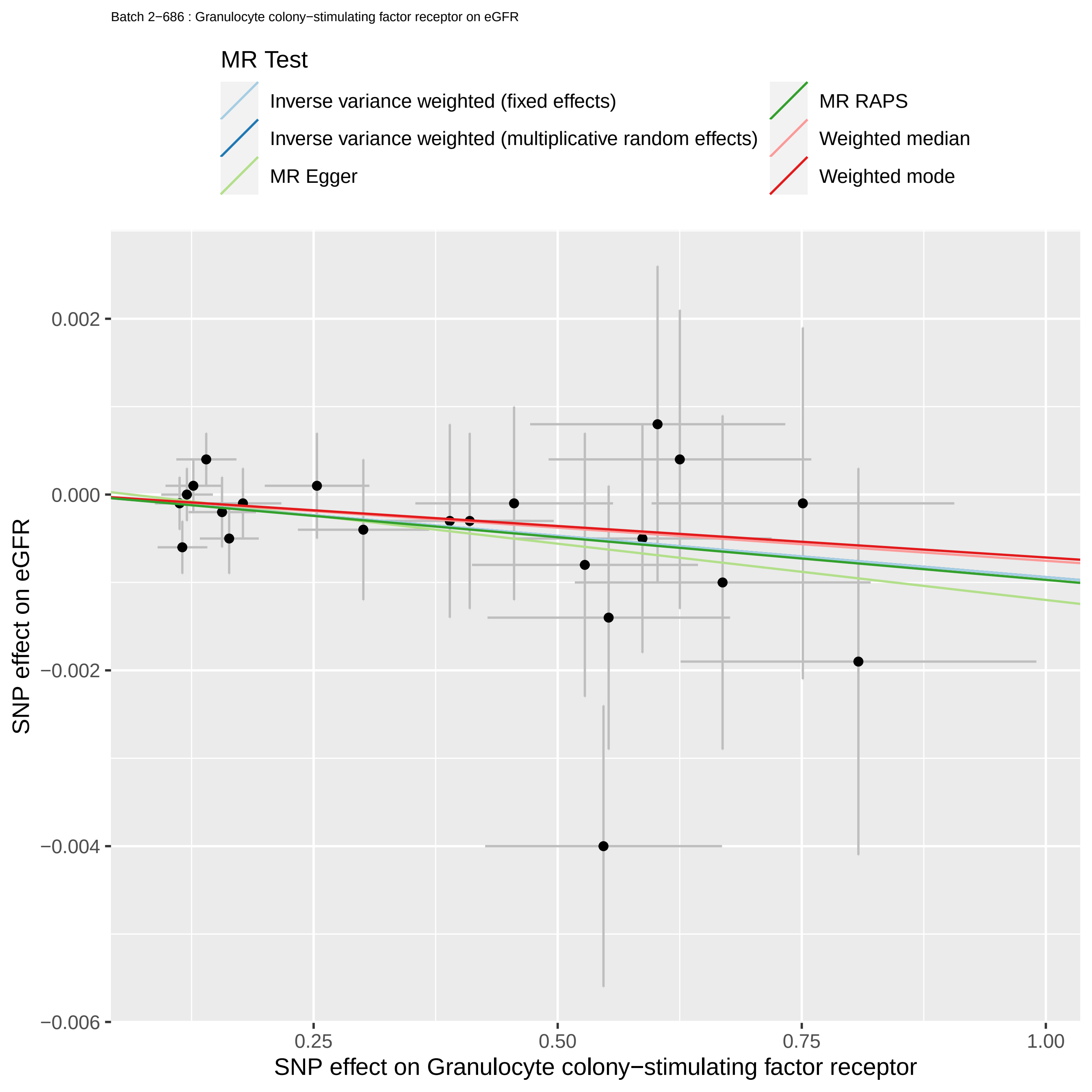

Batch 2−686 : Granulocyte colony−stimulating factor receptor on eGFR
MR Test
Inverse variance weighted (fixed effects)
Inverse variance weighted (multiplicative random effects)
MR Egger
MR RAPS
Weighted median
Weighted mode
0.002
0.000
−0.002
−0.004
−0.006
SNP effect on eGFR
0.25
0.50
0.75
1.00
SNP effect on Granulocyte colony−stimulating factor receptor

Batch 2−687 : Chondroitin sulfate N−acetylgalactosaminyltransferase 2 on eGFR

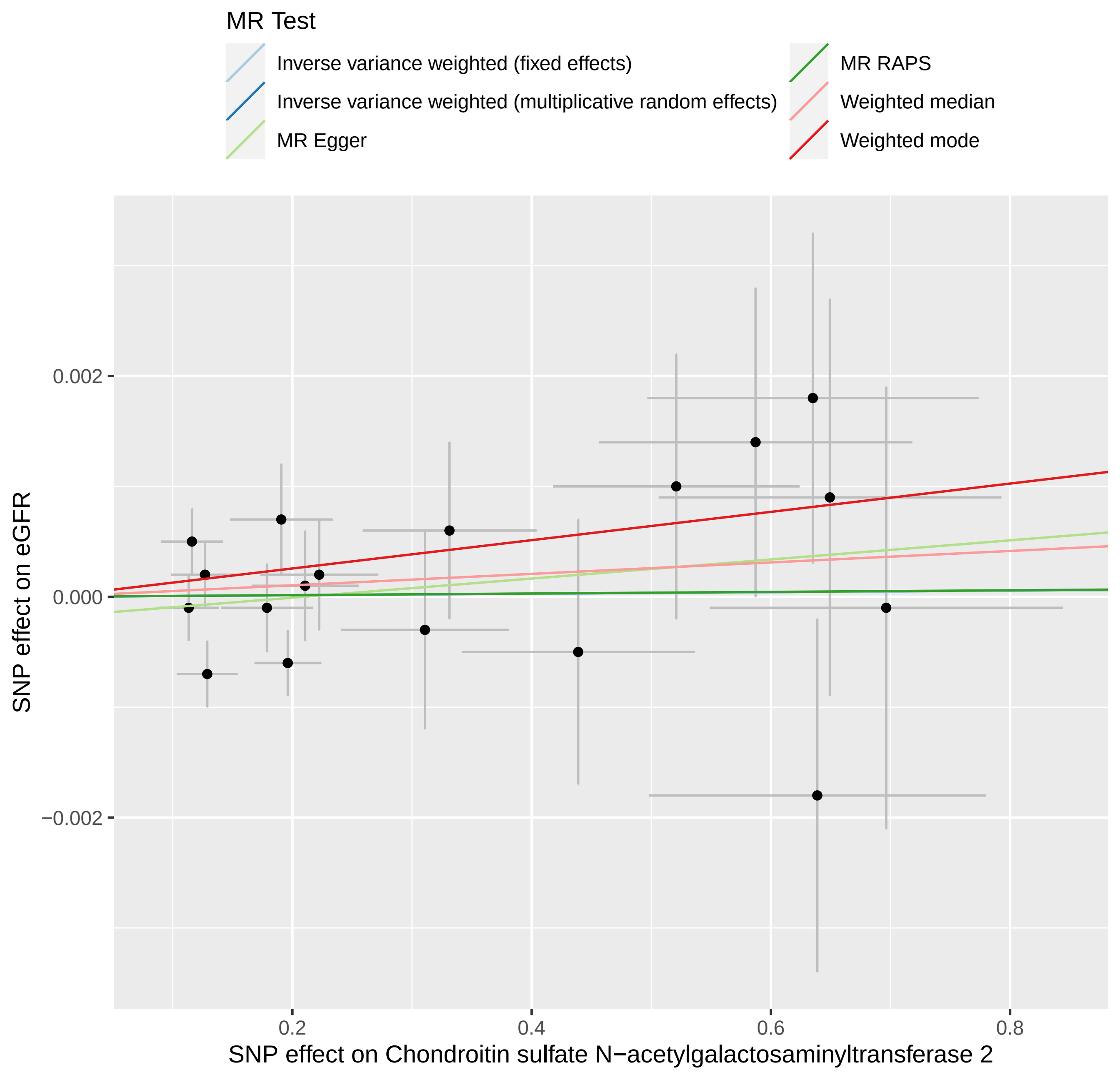

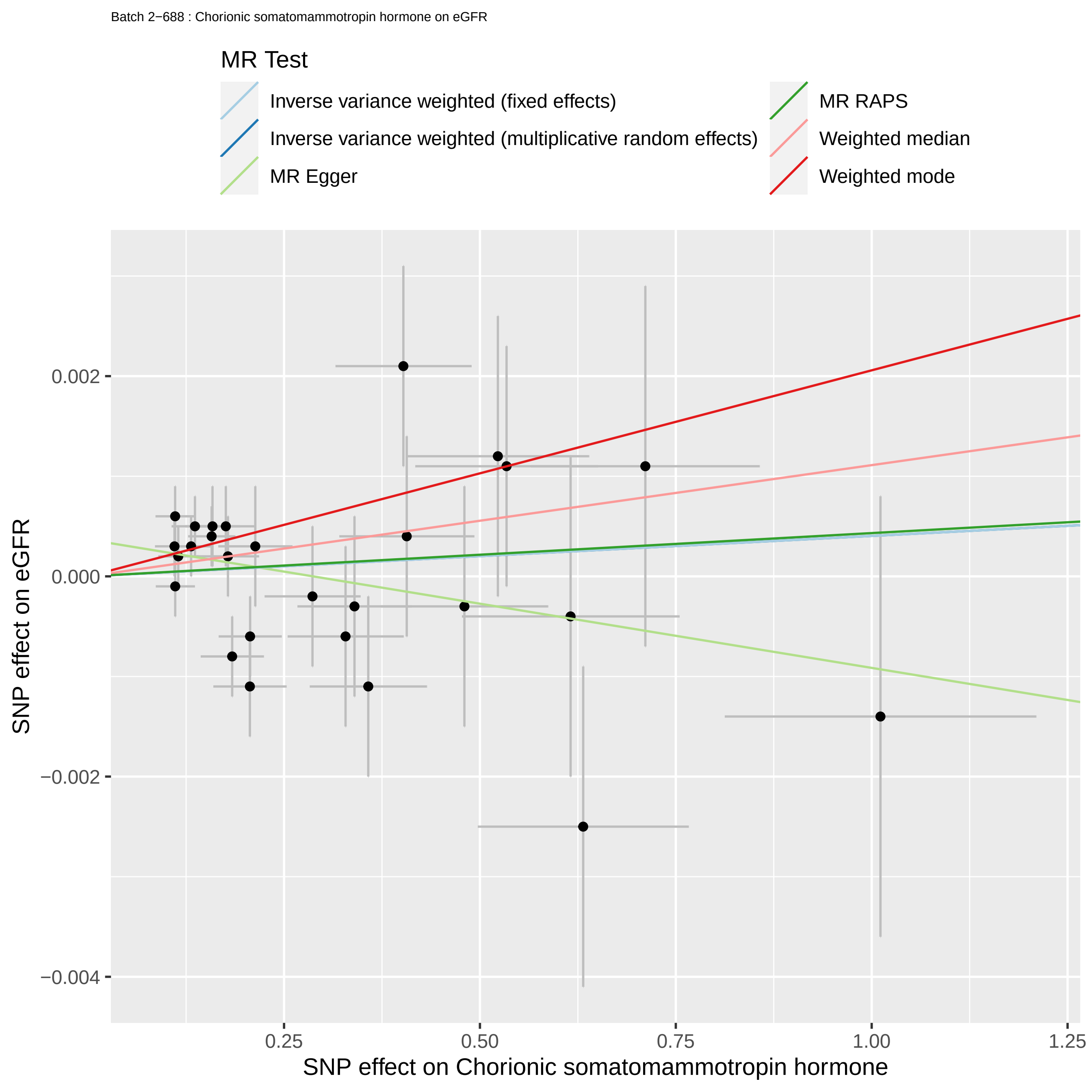
Batch 2−688 : Chorionic somatomammotropin hormone on eGFR
MR Test
Inverse variance weighted (fixed effects)
Inverse variance weighted (multiplicative random effects)
MR Egger
MR RAPS
Weighted median
Weighted mode
0.002
0.000
−0.002
−0.004
0.25
0.50
0.75
1.00
1.25
SNP effect on eGFR
SNP effect on Chorionic somatomammotropin hormone

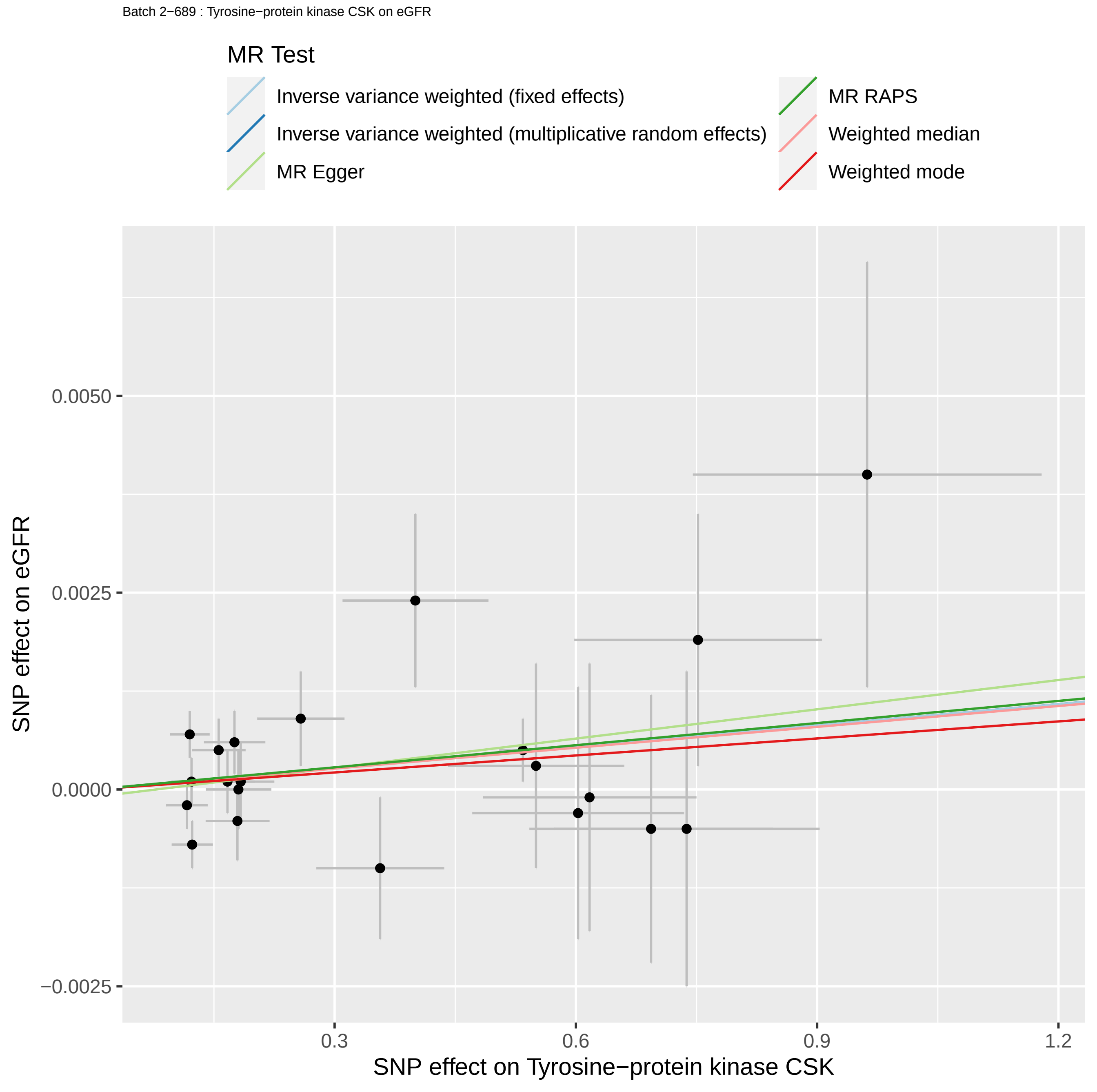
Batch 2−689 : Tyrosine−protein kinase CSK on eGFR
MR Test
Inverse variance weighted (fixed effects)
Inverse variance weighted (multiplicative random effects)
MR Egger
MR RAPS
Weighted median
Weighted mode
0.0050
0.0025
0.0000
−0.0025
SNP effect on eGFR
0.3
0.6
0.9
1.2
SNP effect on Tyrosine−protein kinase CSK

Batch 2−690 : CUB and sushi domain−containing protein 1 on eGFR

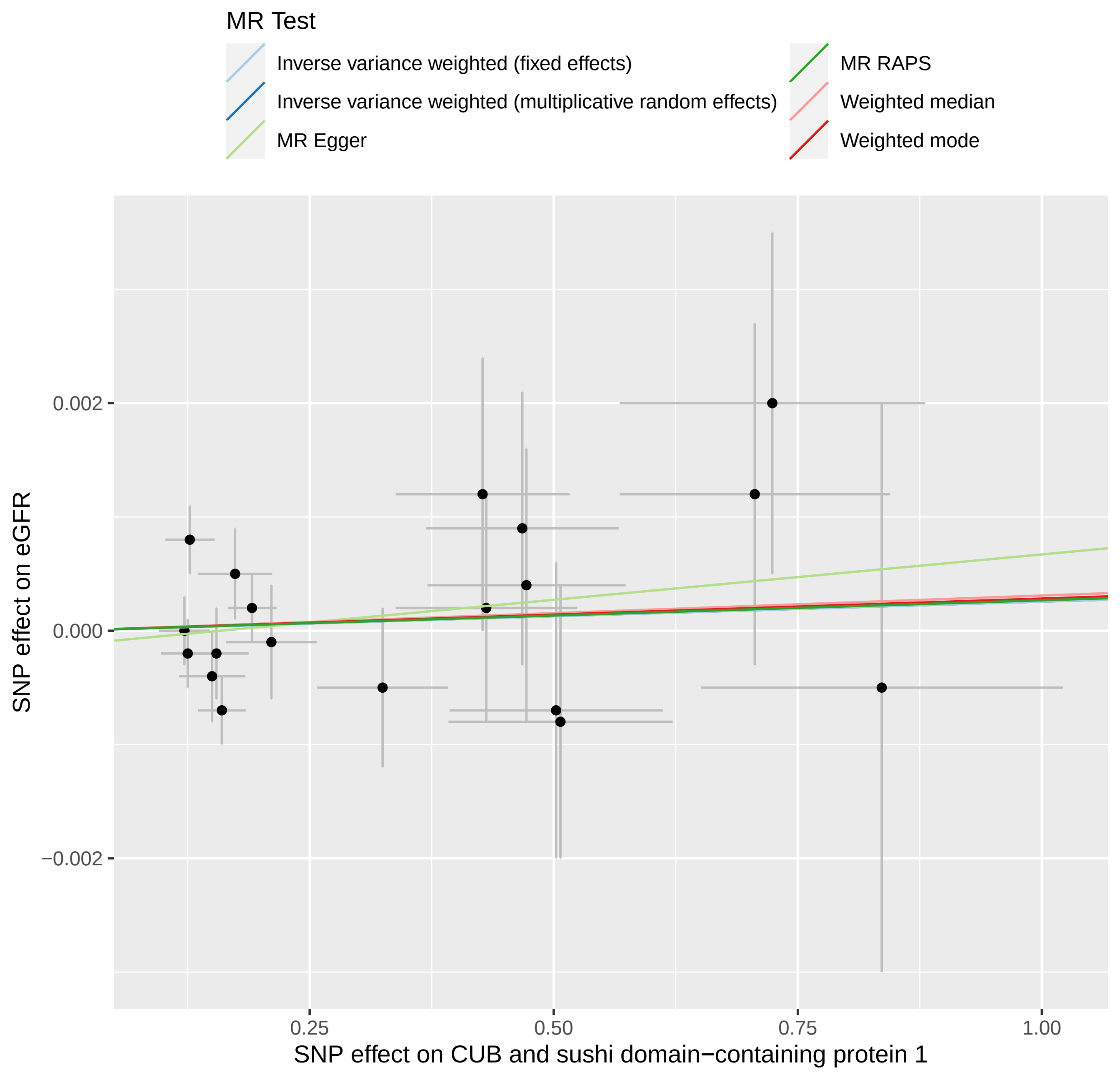

Batch 2−691 : CUB and sushi domain−containing protein 2 on eGFR

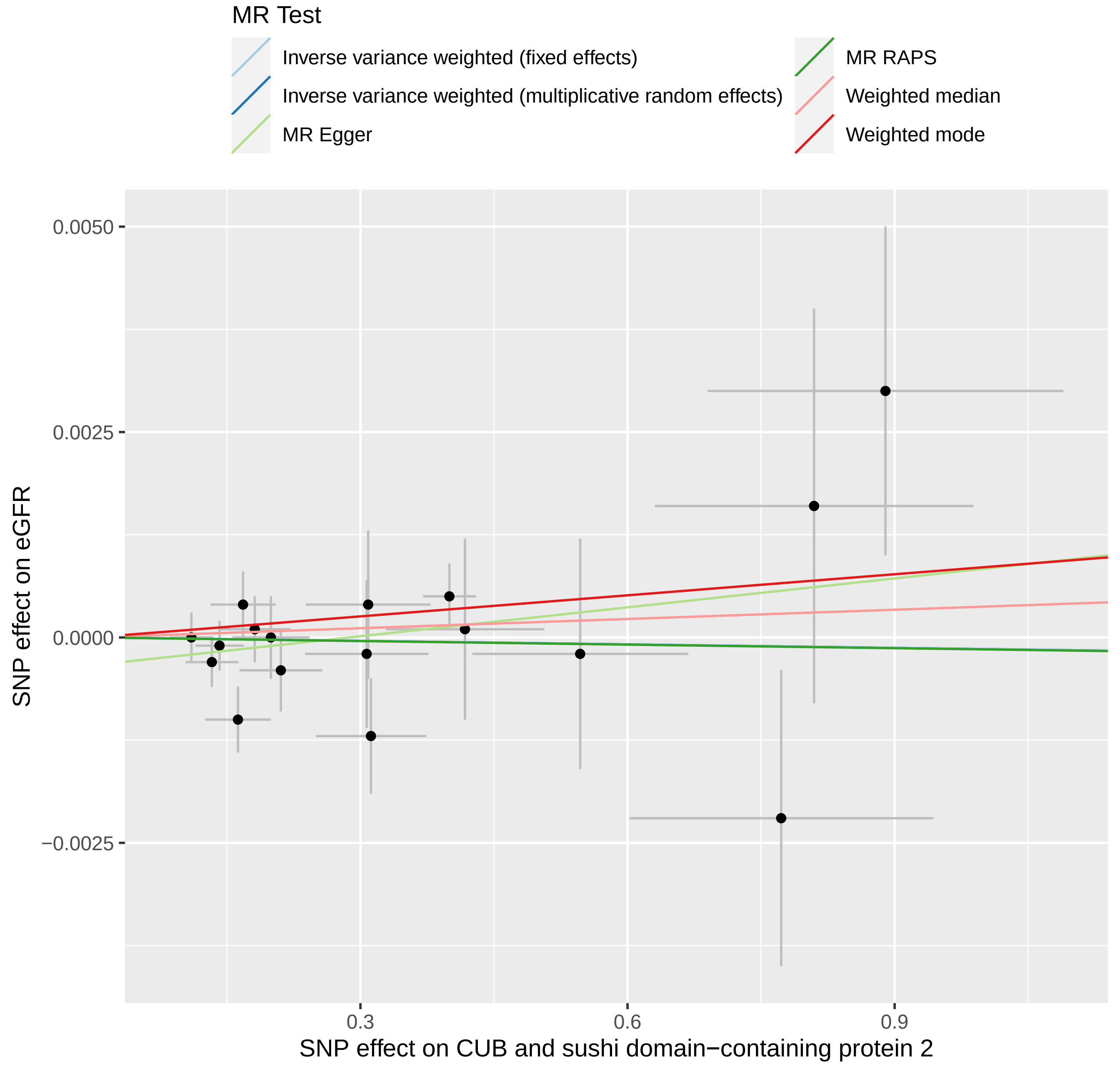

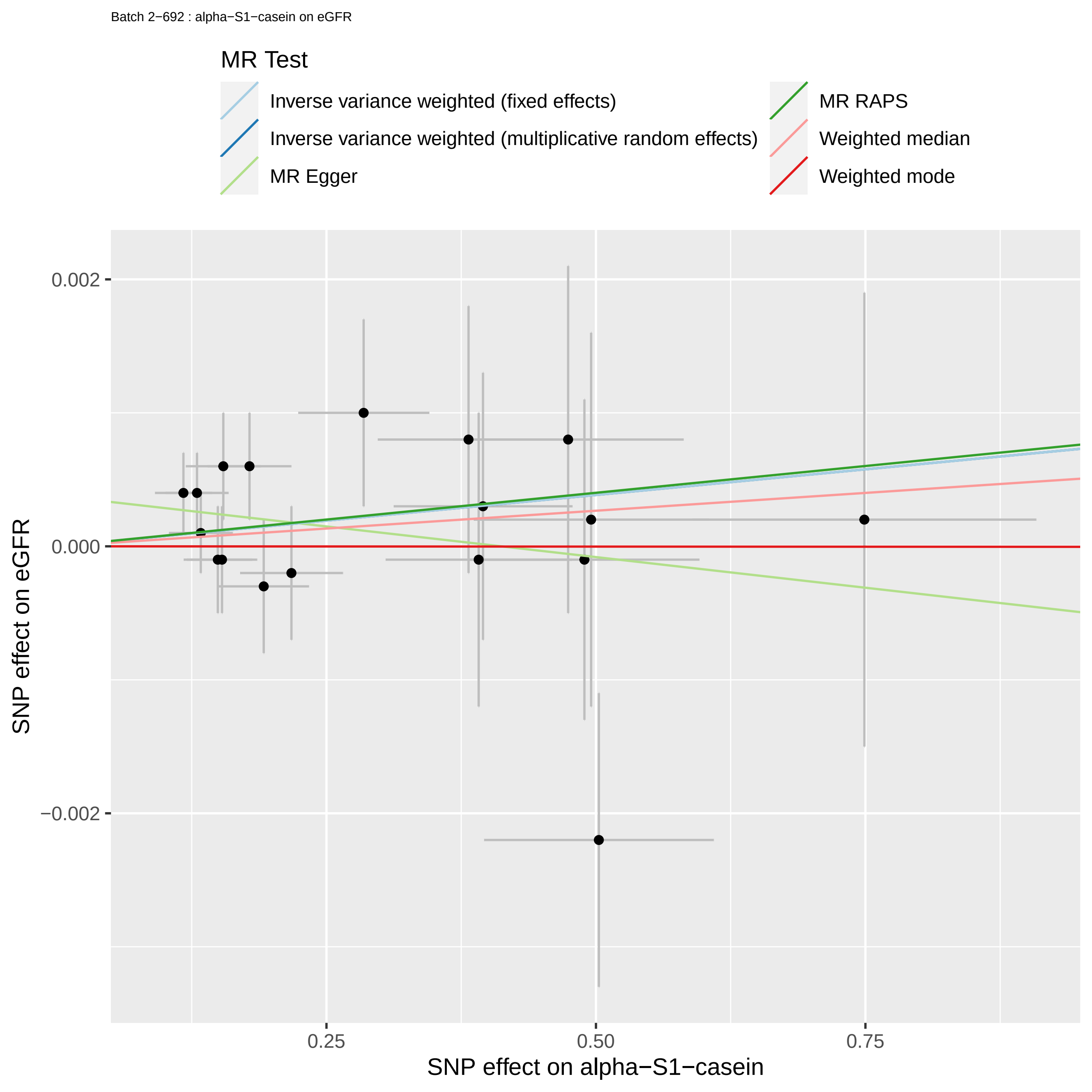

Batch 2−692 : alpha−S1−casein on eGFR
MR Test
Inverse variance weighted (fixed effects)
Inverse variance weighted (multiplicative random effects)
MR Egger
MR RAPS
Weighted median
Weighted mode
0.002
0.000
−0.002
0.25
0.50
0.75
SNP effect on eGFR
SNP effect on alpha−S1−casein

Batch 2−693 : Casein kinase I isoform delta on eGFR

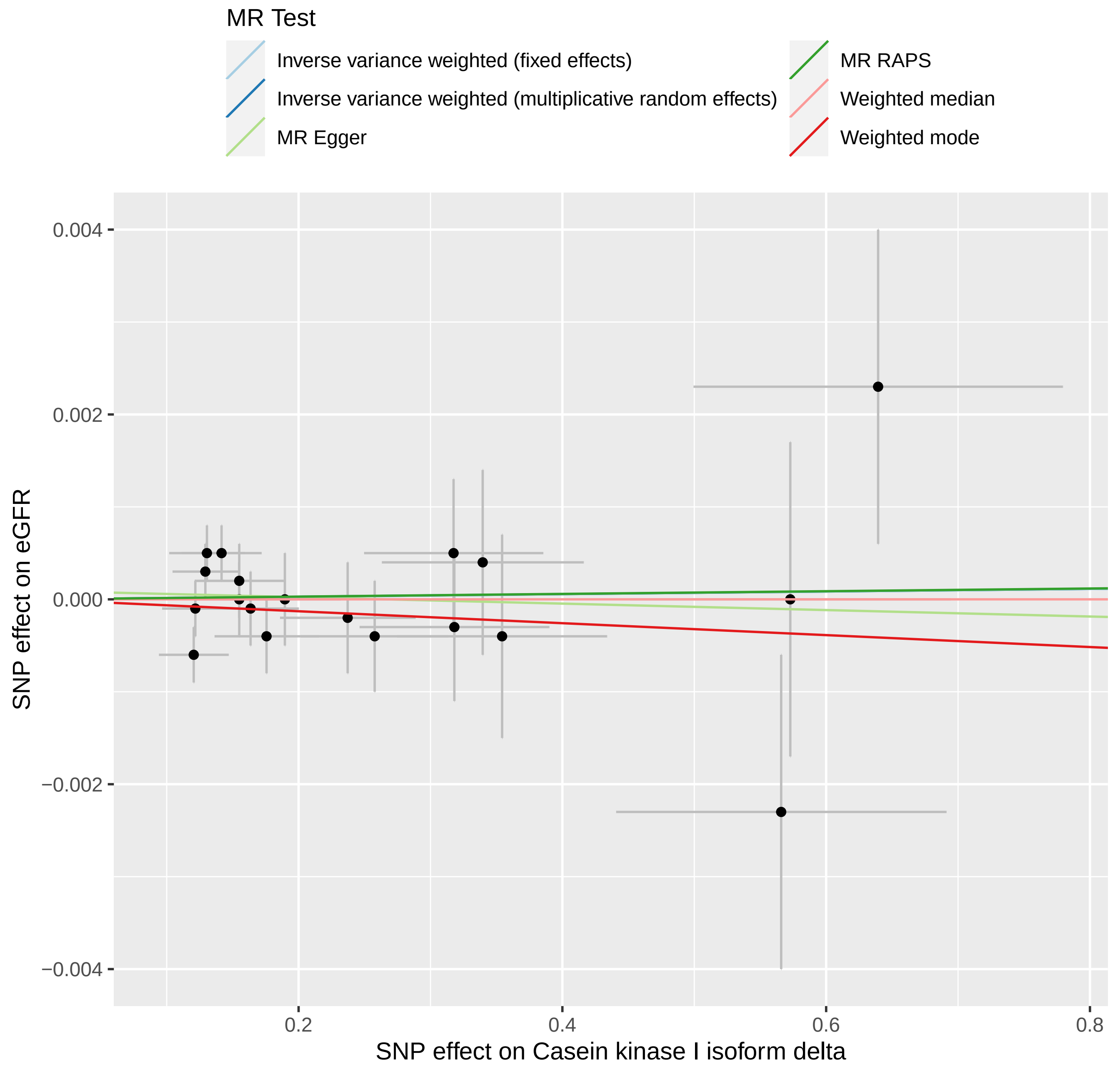

Batch 2−694 : Casein kinase I isoform gamma−2 on eGFR

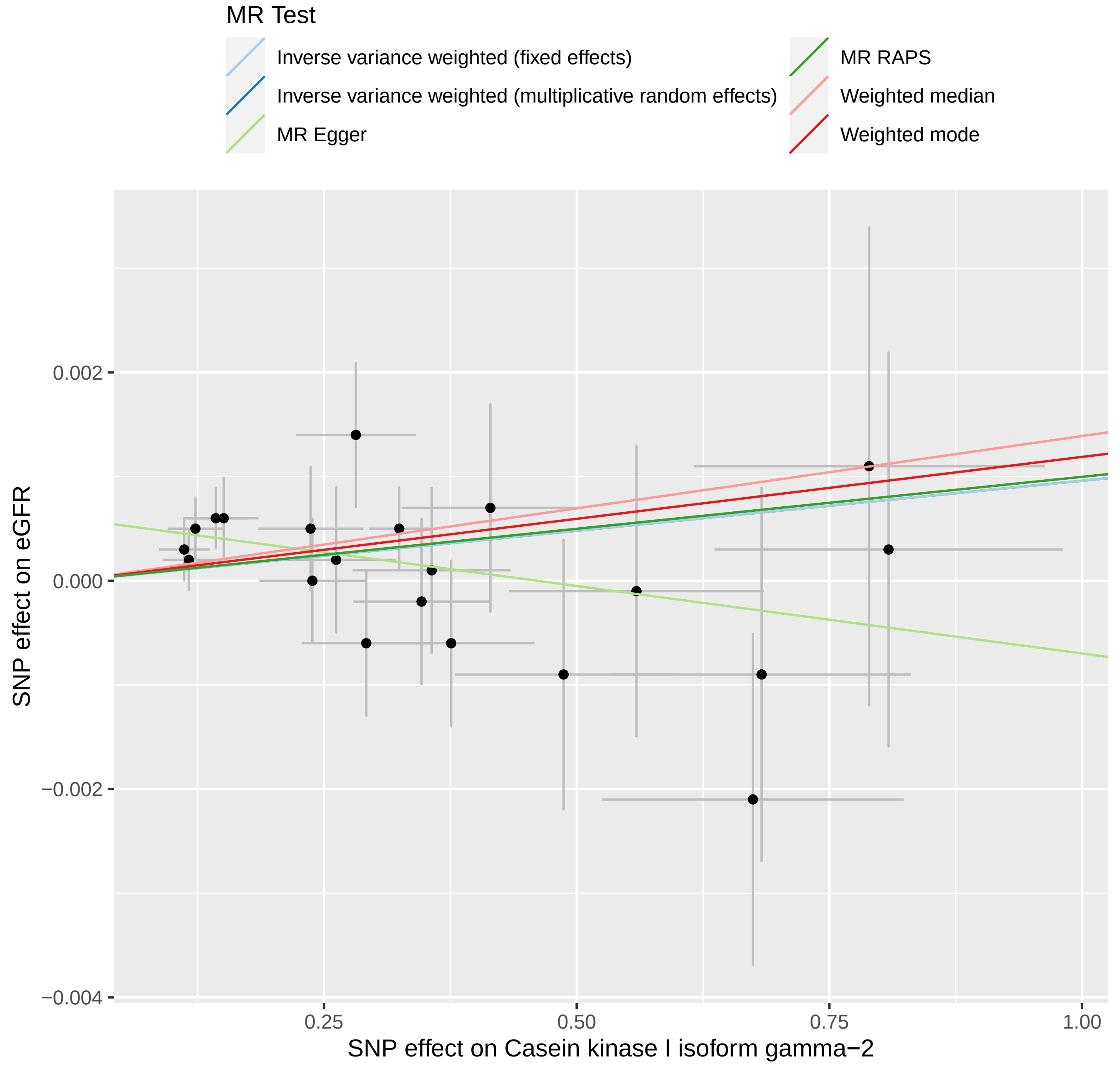

Batch 2−695 : Casein kinase II subunit alpha on eGFR

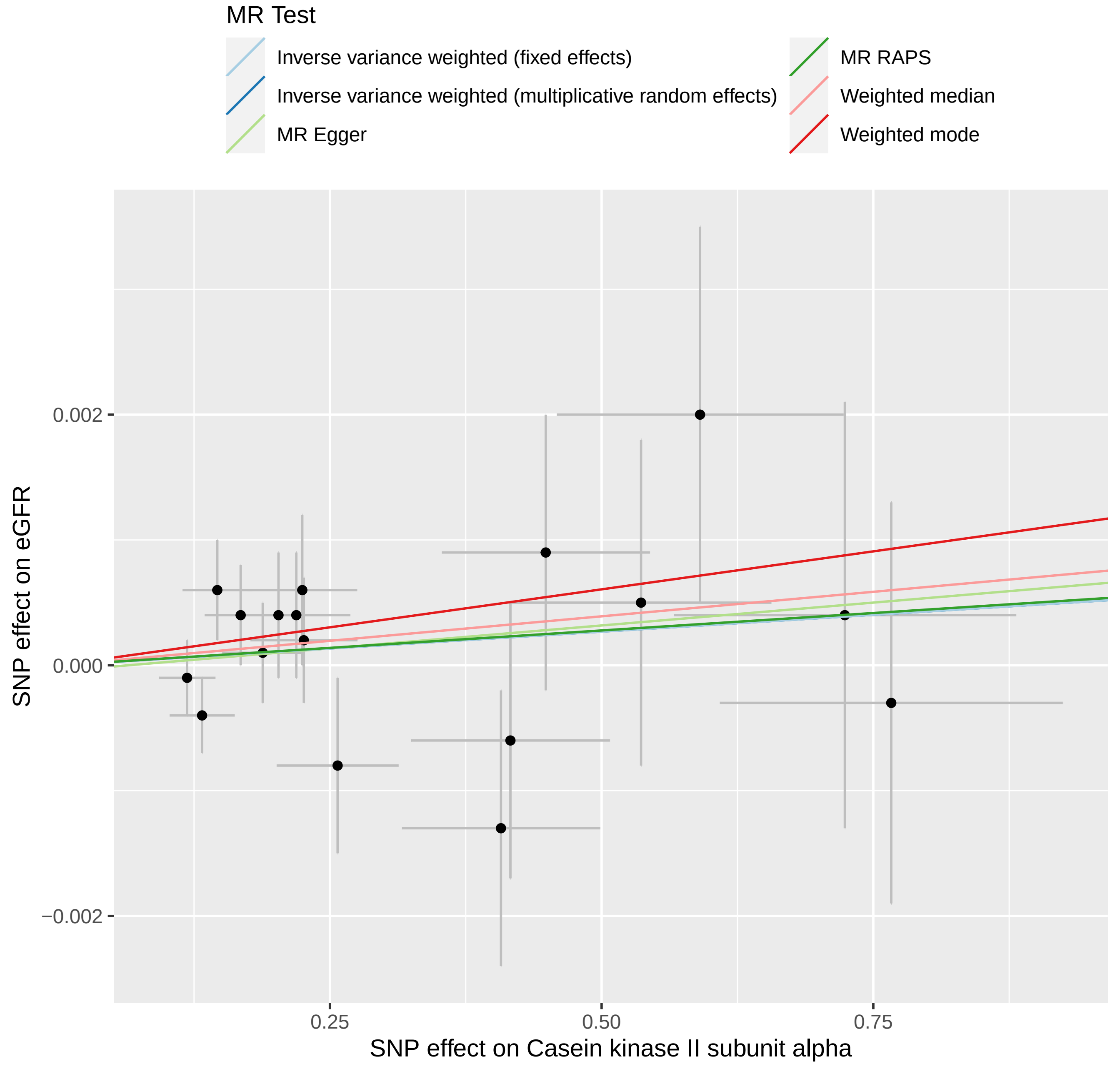

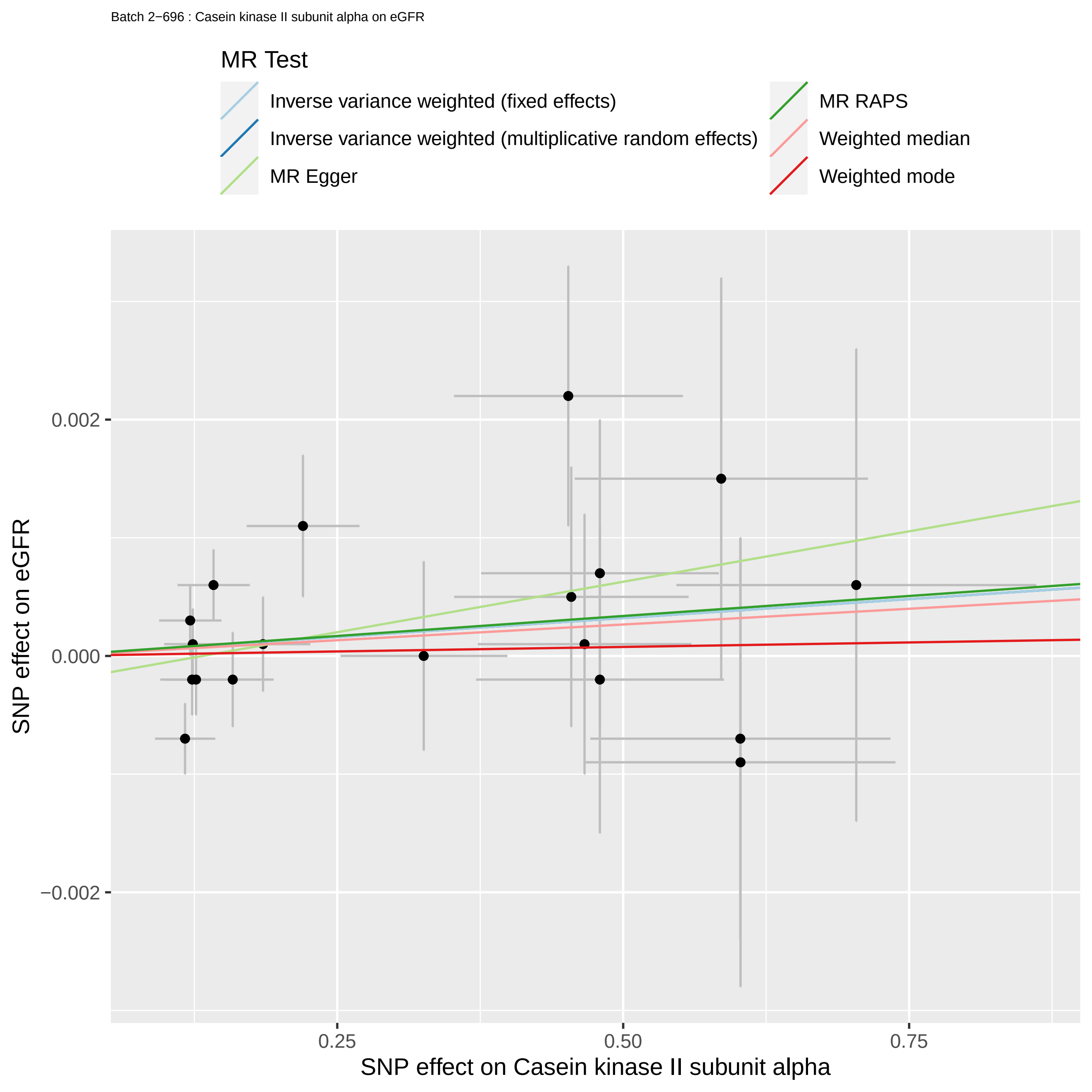

Batch 2−696 : Casein kinase II subunit alpha on eGFR
MR Test
Inverse variance weighted (fixed effects)
Inverse variance weighted (multiplicative random effects)
MR Egger
MR RAPS
Weighted median
Weighted mode
0.002
0.000
−0.002
0.25
0.50
0.75
SNP effect on eGFR
SNP effect on Casein kinase II subunit alpha

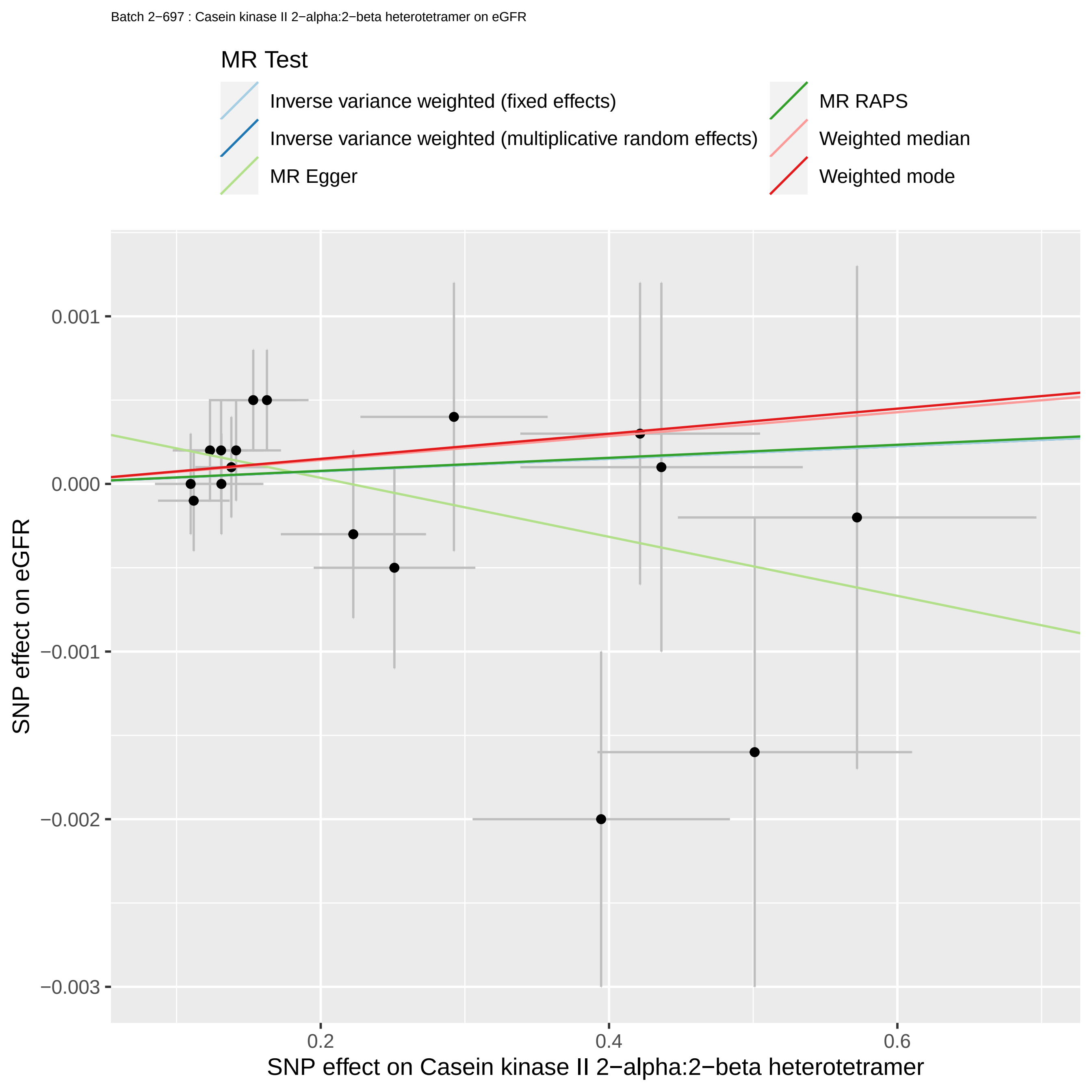

Batch 2−697 : Casein kinase II 2−alpha:2−beta heterotetramer on eGFR
MR Test
Inverse variance weighted (fixed effects)
Inverse variance weighted (multiplicative random effects)
MR Egger
MR RAPS
Weighted median
Weighted mode
0.001
0.000
−0.001
−0.002
−0.003
0.2
0.4
0.6
SNP effect on eGFR
SNP effect on Casein kinase II 2−alpha:2−beta heterotetramer

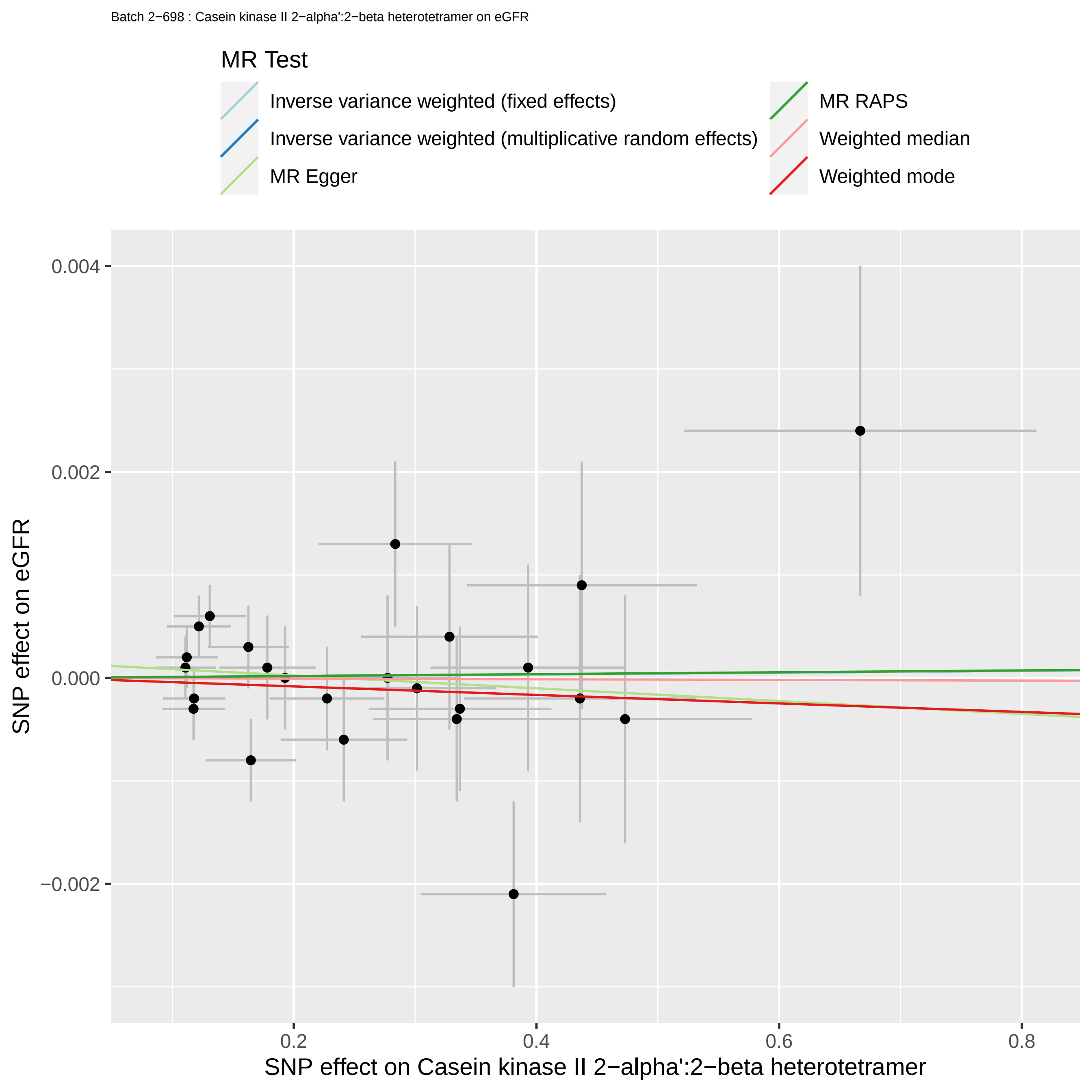
Batch 2−698 : Casein kinase II 2−alpha':2−beta heterotetramer on eGFR
MR Test
Inverse variance weighted (fixed effects)
Inverse variance weighted (multiplicative random effects)
MR Egger
MR RAPS
Weighted median
Weighted mode
SNP effect on eGFR
0.004
0.002
0.000
−0.002
0.2
0.4
0.6
0.8
SNP effect on Casein kinase II 2−alpha':2−beta heterotetramer

Batch 2−699 : Chondroitin sulfate proteoglycan 4 on eGFR

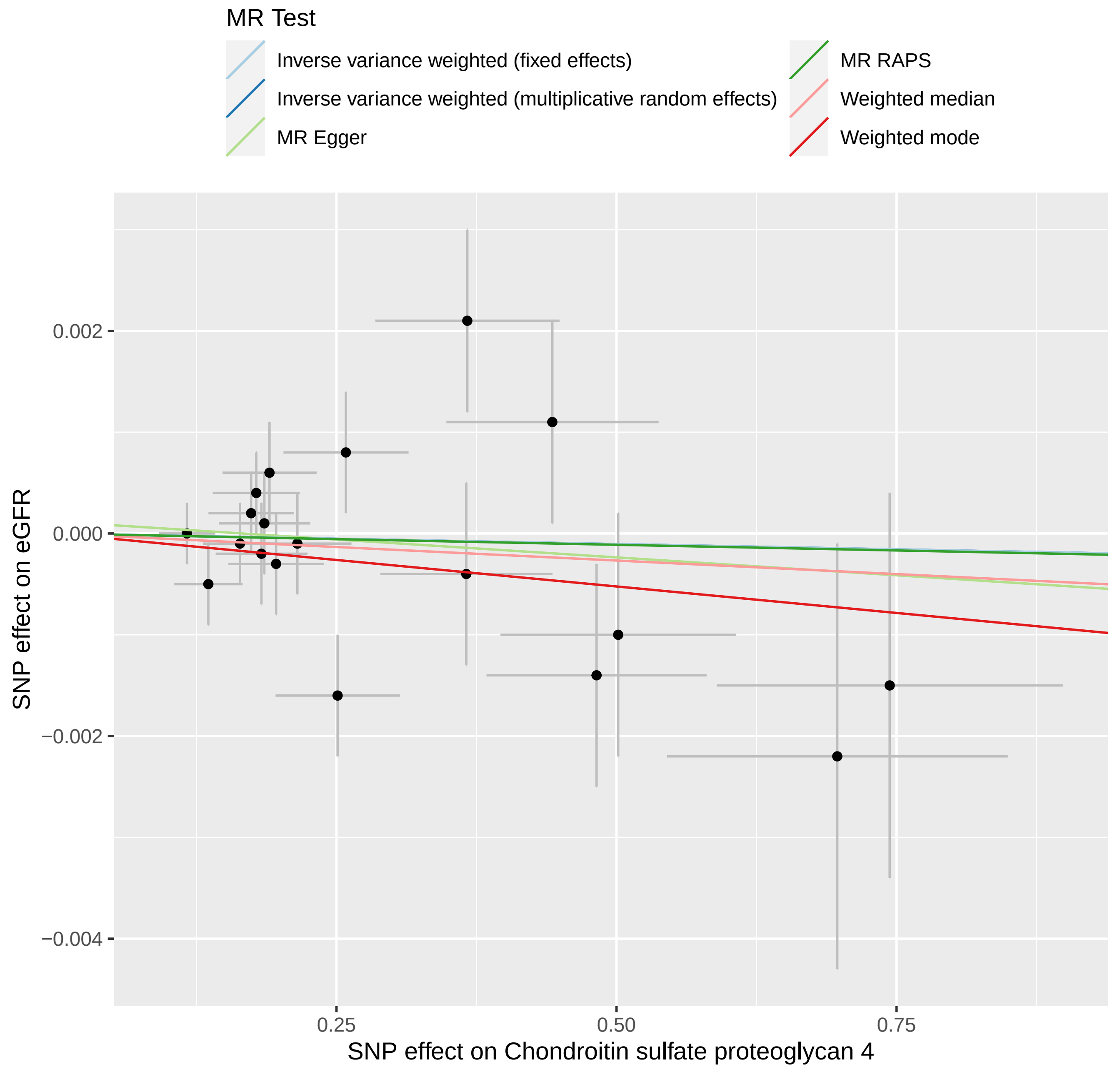

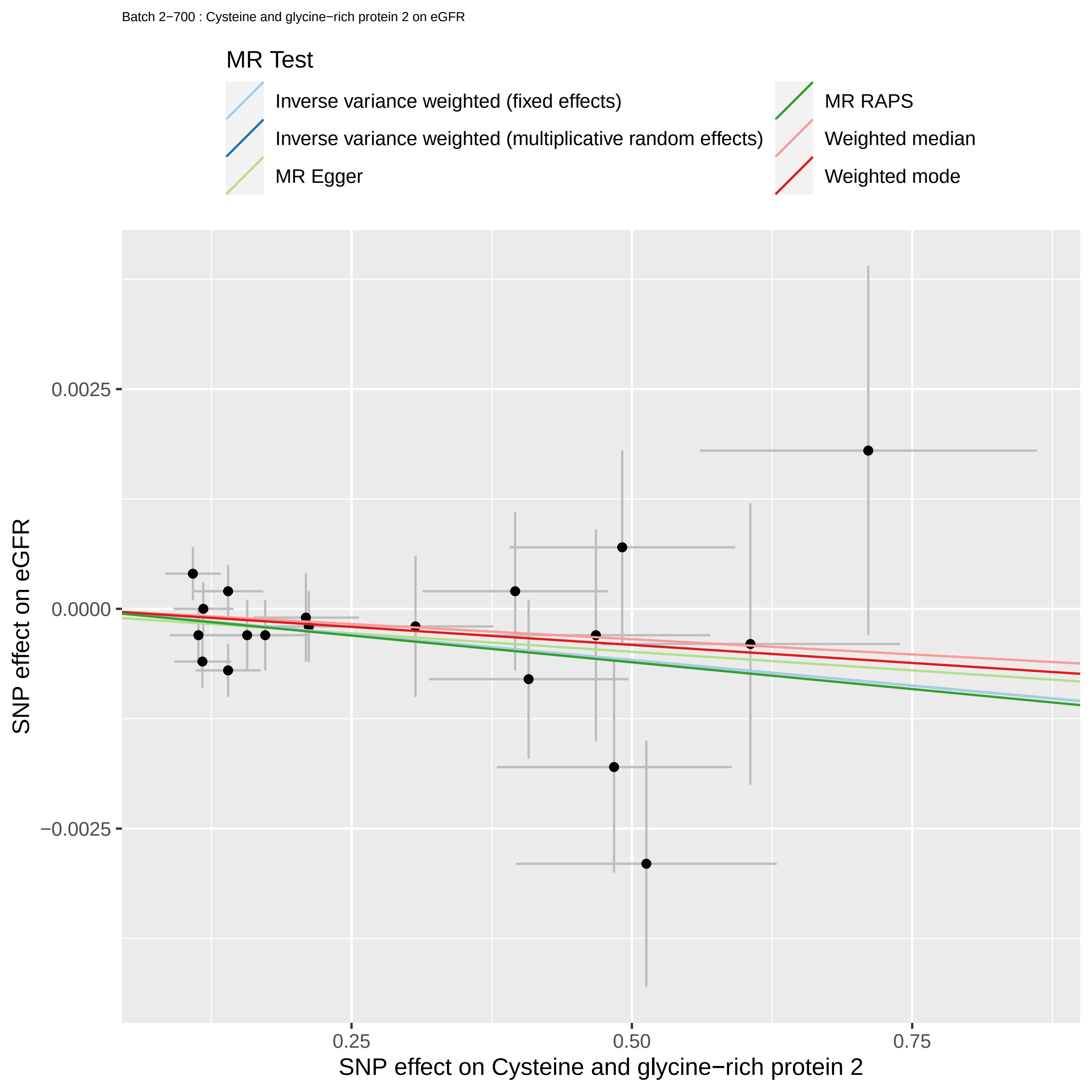
Batch 2−700 : Cysteine and glycine−rich protein 2 on eGFR
MR Test
Inverse variance weighted (fixed effects)
Inverse variance weighted (multiplicative random effects)
MR Egger
MR RAPS
Weighted median
Weighted mode
0.0025
0.0000
−0.0025
0.25
0.50
0.75
SNP effect on eGFR
SNP effect on Cysteine and glycine−rich protein 2

Batch 2−701 : Cysteine and glycine−rich protein 3 on eGFR

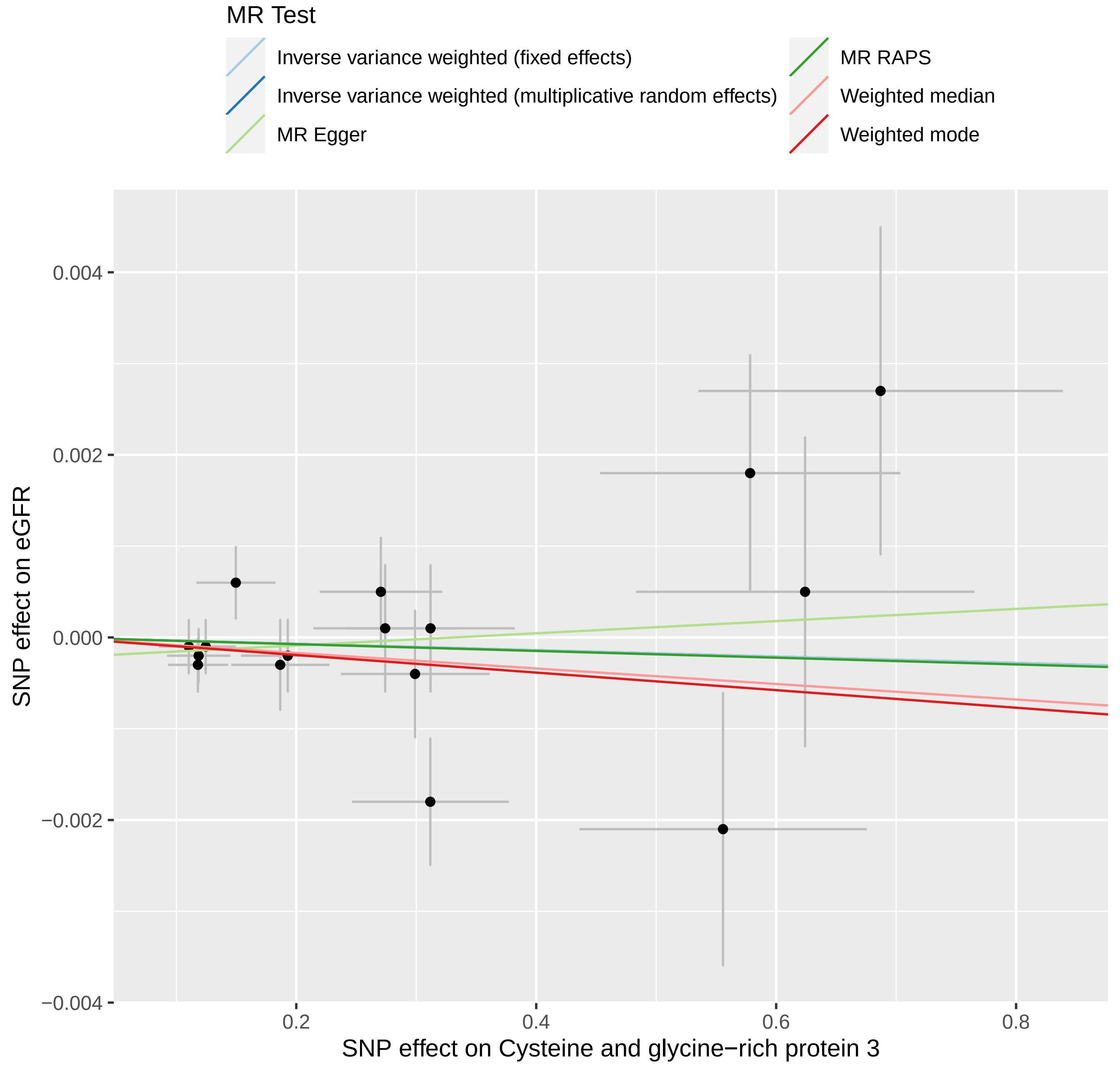

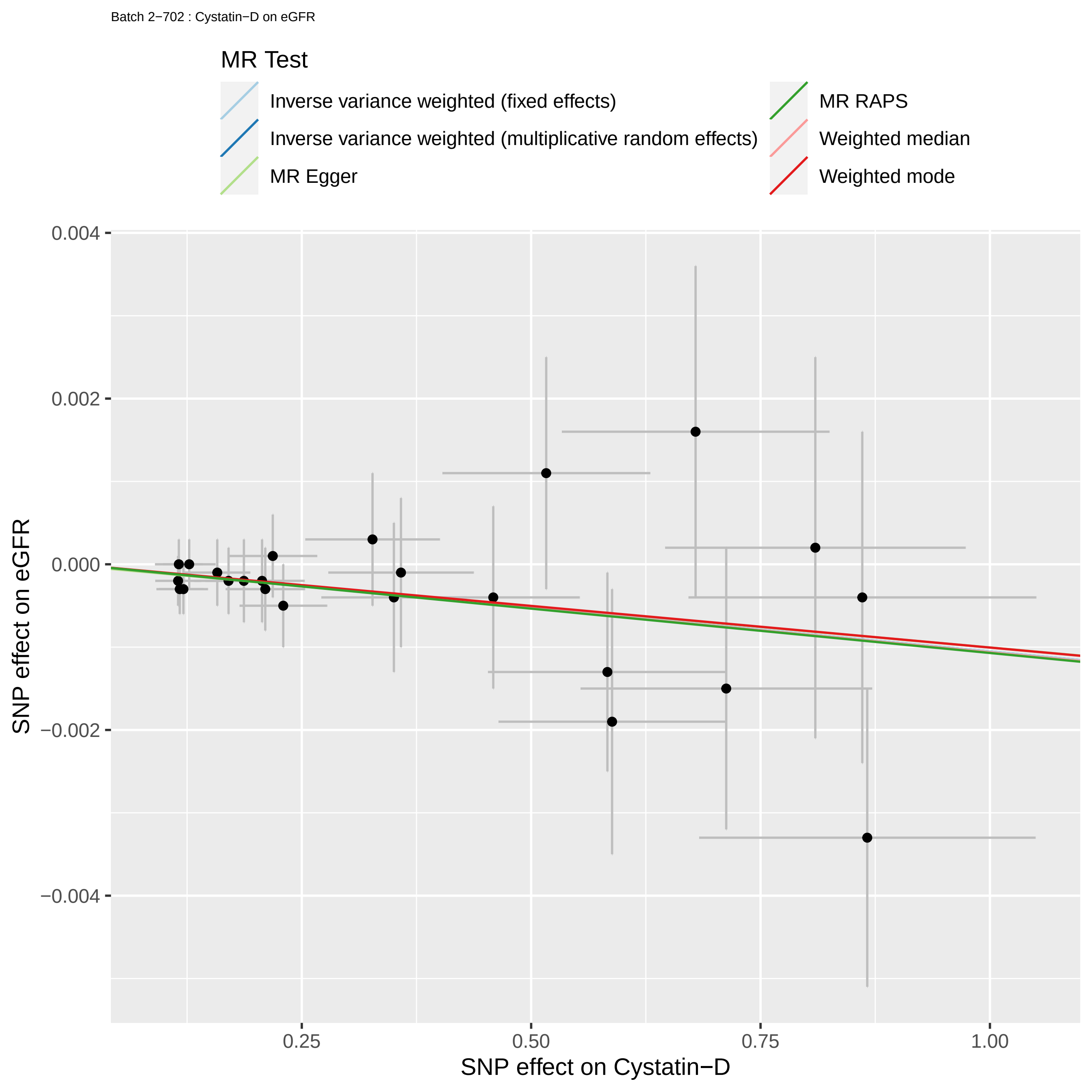
Batch 2−702 : Cystatin−D on eGFR
MR Test
Inverse variance weighted (fixed effects)
Inverse variance weighted (multiplicative random effects)
MR Egger
MR RAPS
Weighted median
Weighted mode
0.004
0.002
0.000
−0.002
−0.004
SNP effect on eGFR
0.25
0.50
0.75
1.00
SNP effect on Cystatin−D

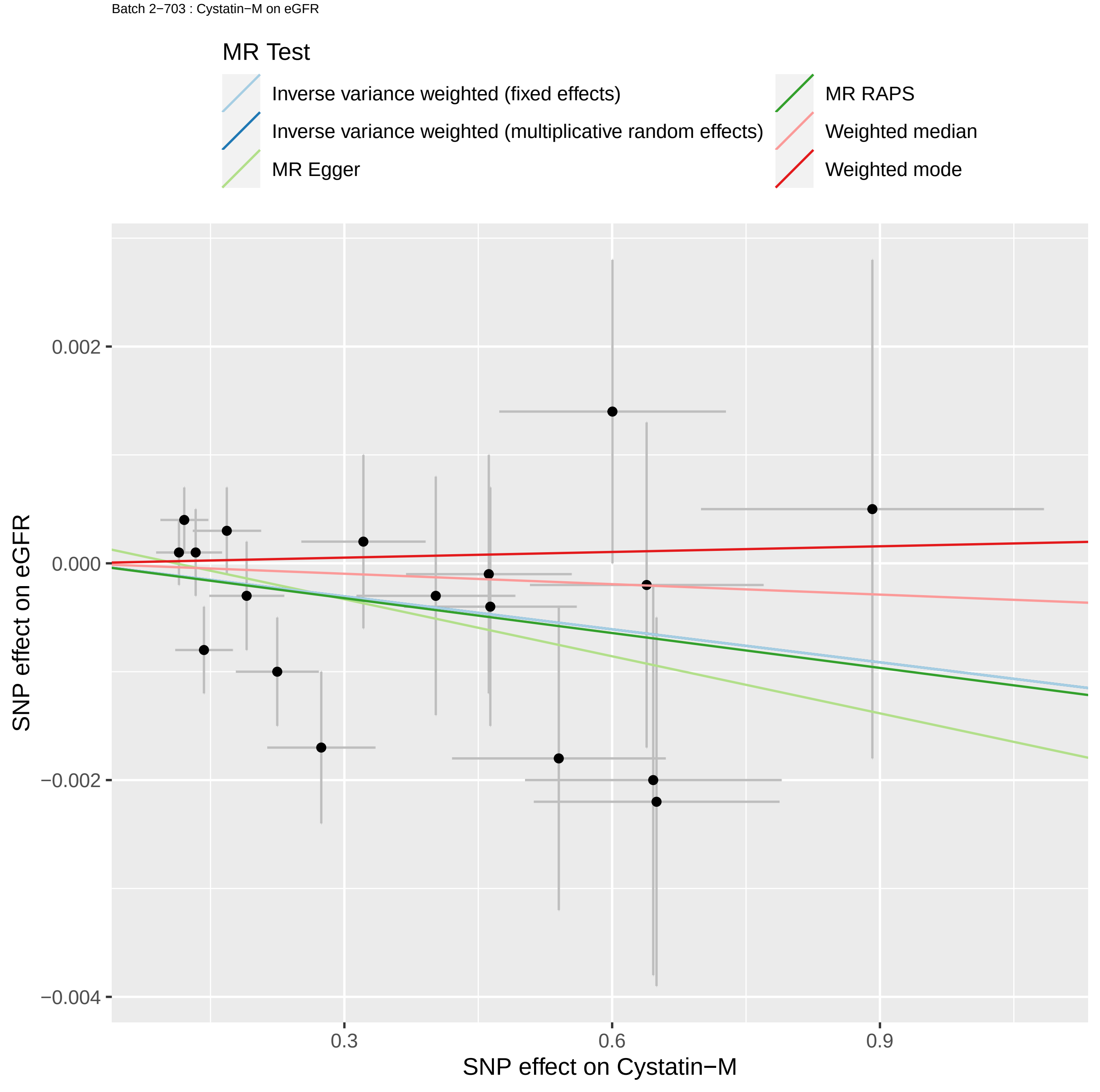

Batch 2−703 : Cystatin−M on eGFR
MR Test
Inverse variance weighted (fixed effects)
Inverse variance weighted (multiplicative random effects)
MR Egger
MR RAPS
Weighted median
Weighted mode
0.002
0.000
−0.002
−0.004
0.3
0.6
0.9
SNP effect on eGFR
SNP effect on Cystatin−M

Batch 2−704 : Cystatin−M on eGFR

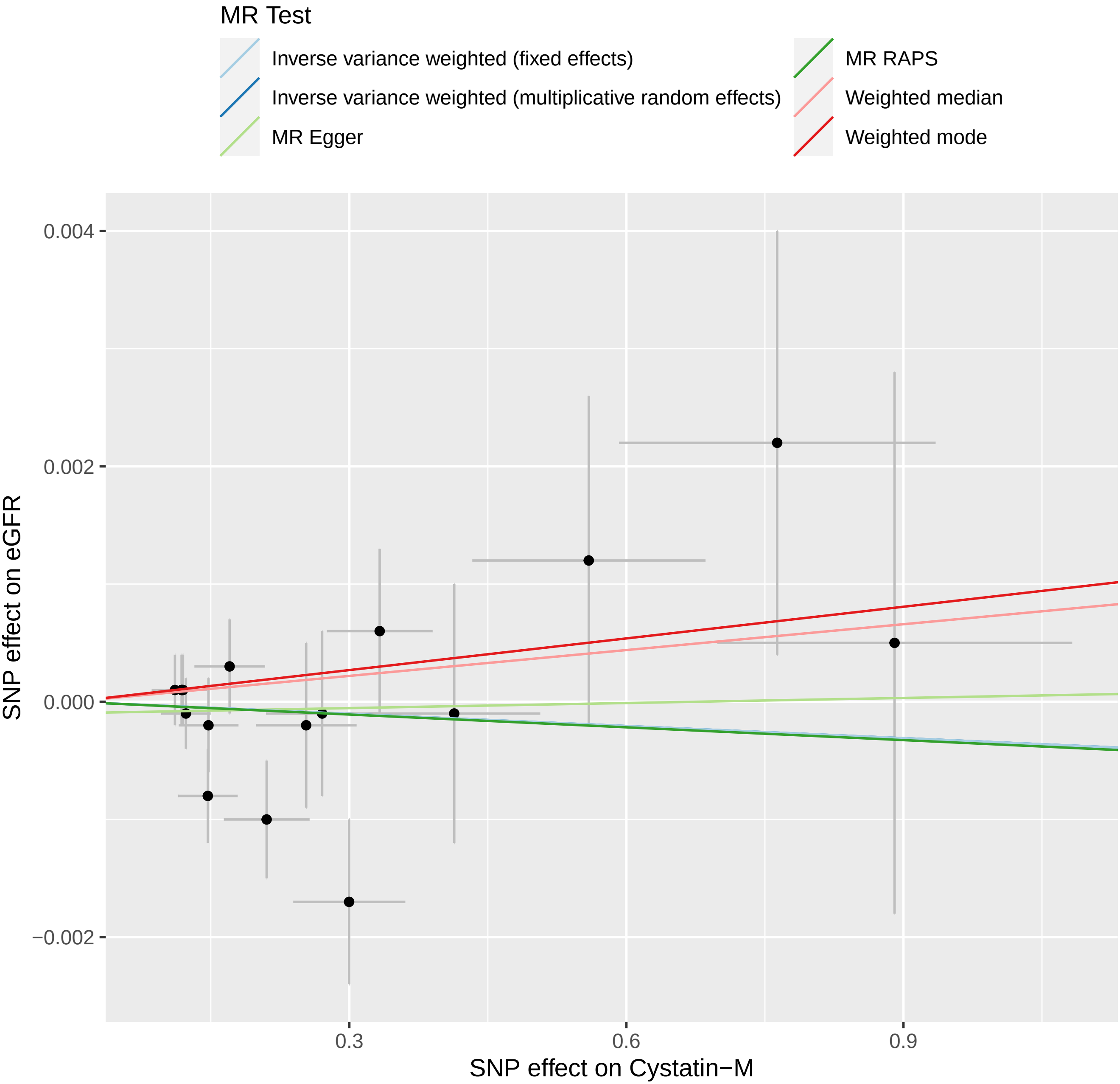

Batch 2−705 : Cystatin−F on eGFR

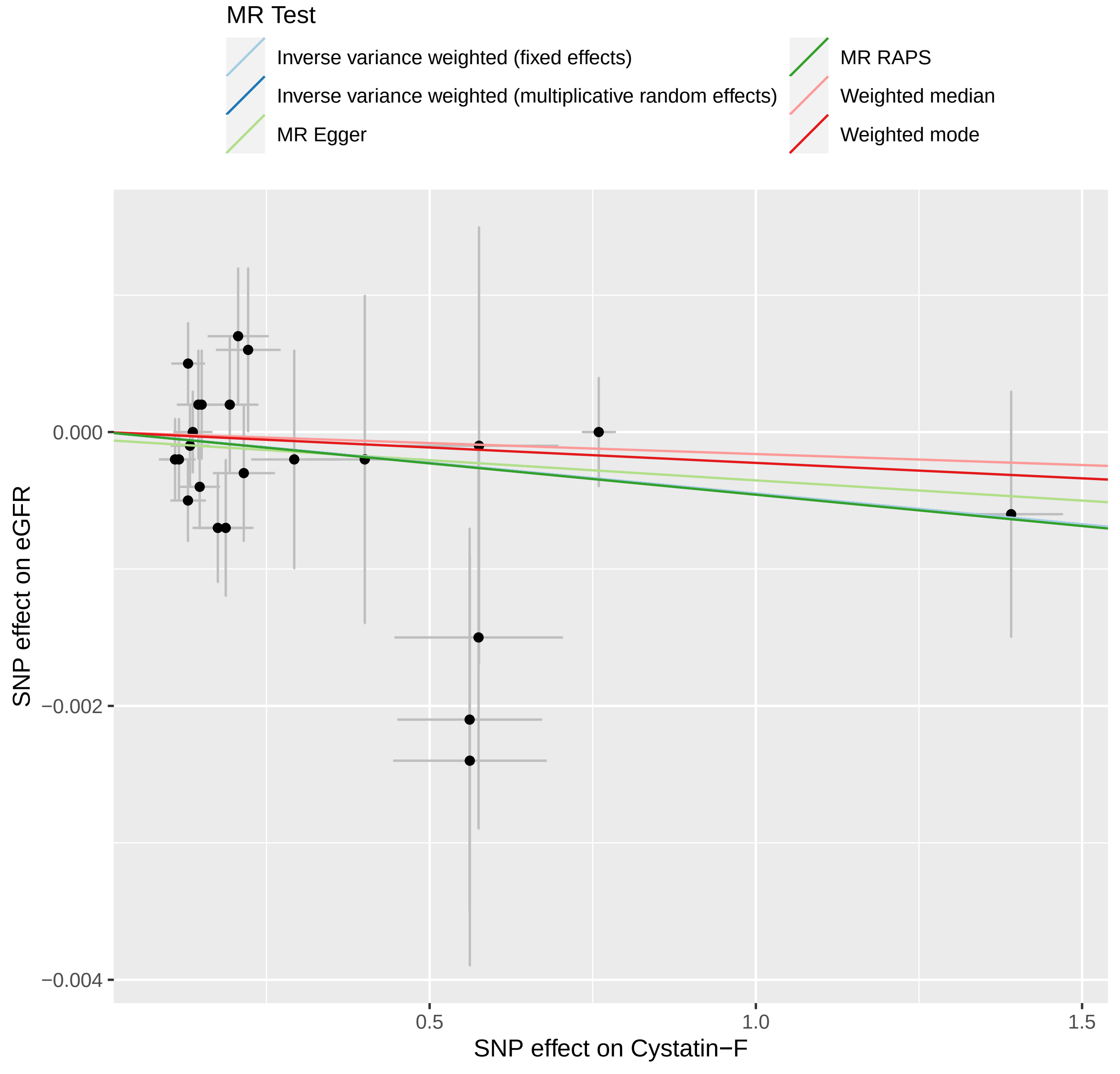

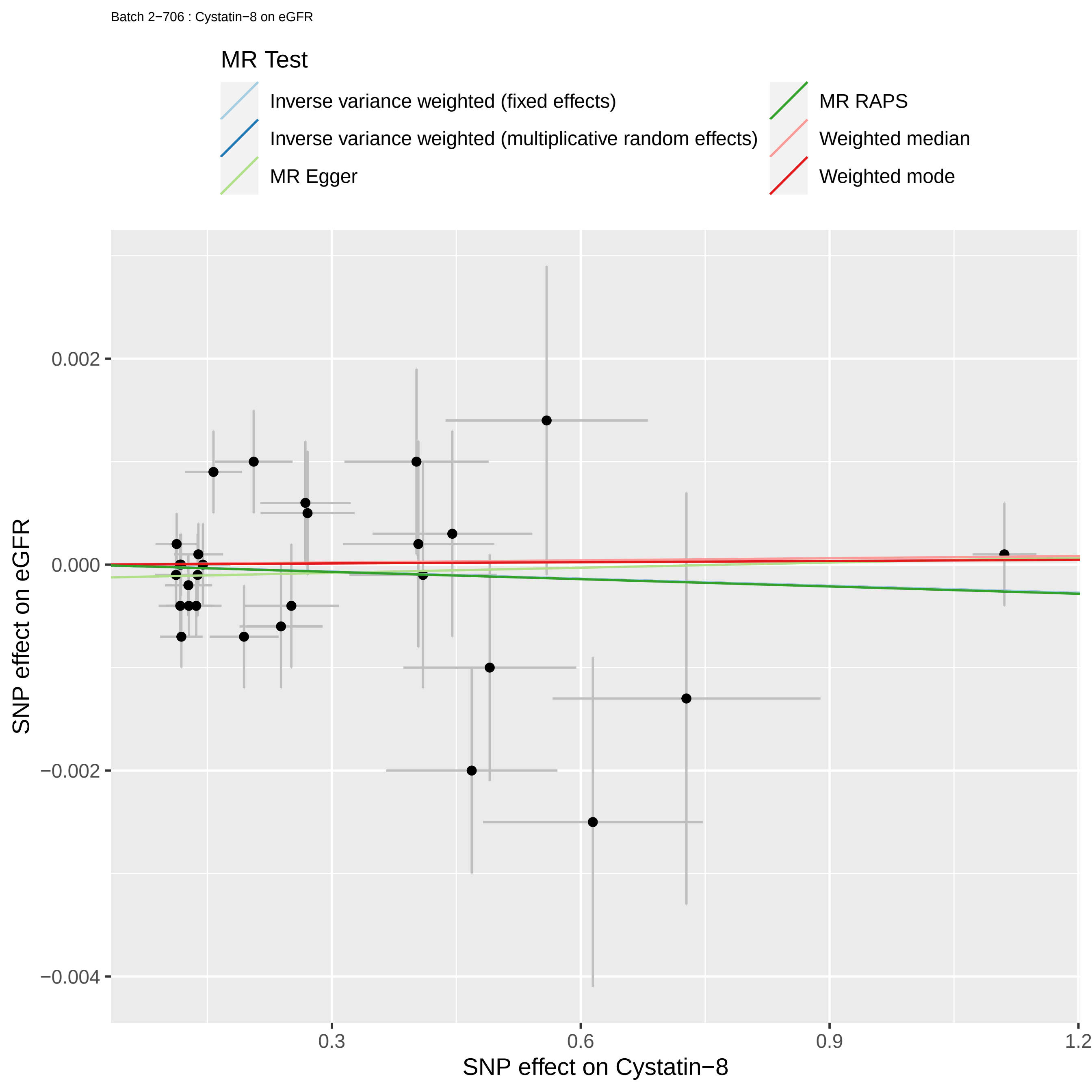

Batch 2−706 : Cystatin−8 on eGFR
MR Test
Inverse variance weighted (fixed effects)
Inverse variance weighted (multiplicative random effects)
MR Egger
MR RAPS
Weighted median
Weighted mode
0.002
0.000
−0.002
−0.004
SNP effect on eGFR
0.3
0.6
0.9
1.2
SNP effect on Cystatin−8

Batch 2−707 : Cancer/testis antigen 55 on eGFR

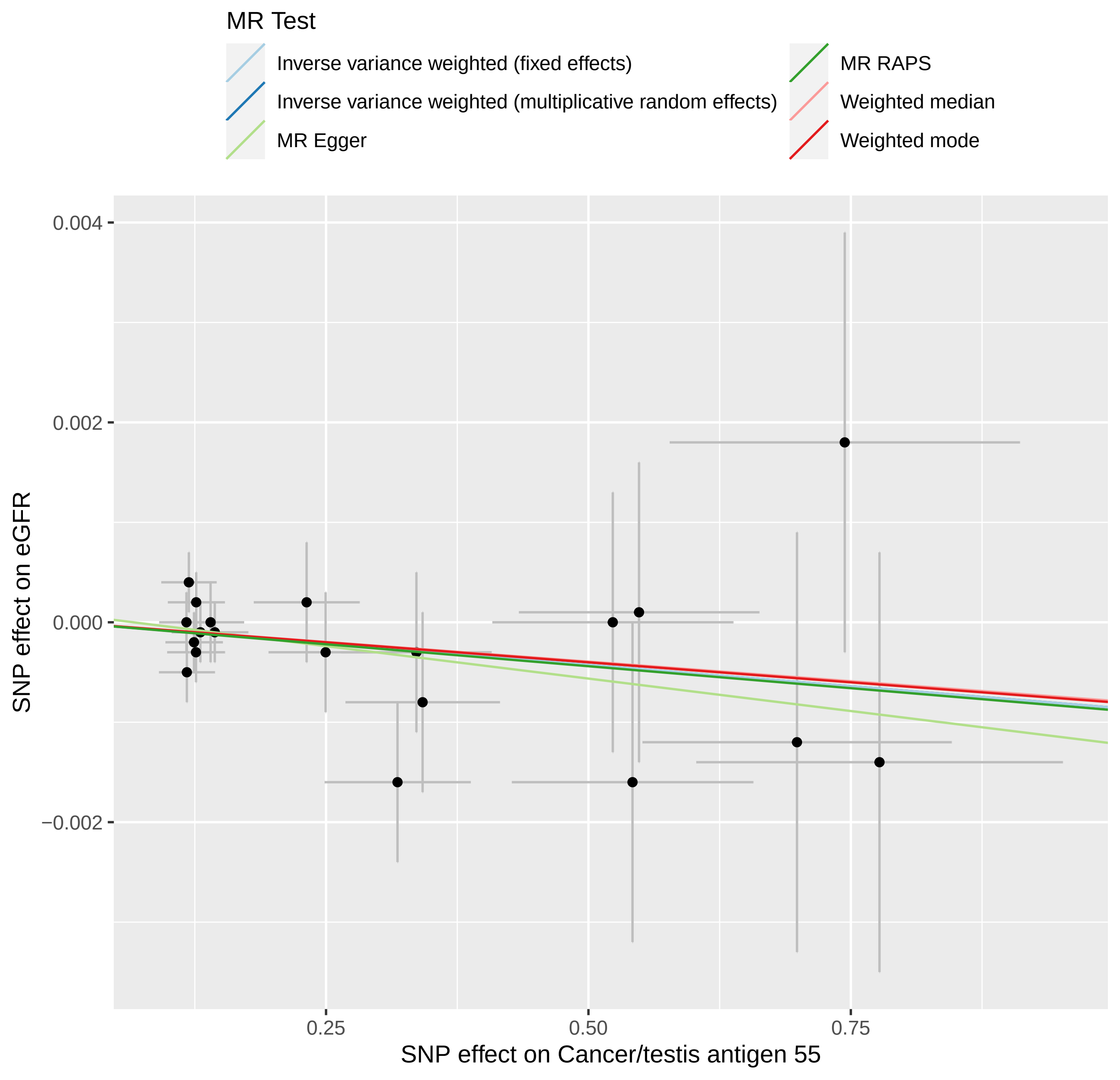

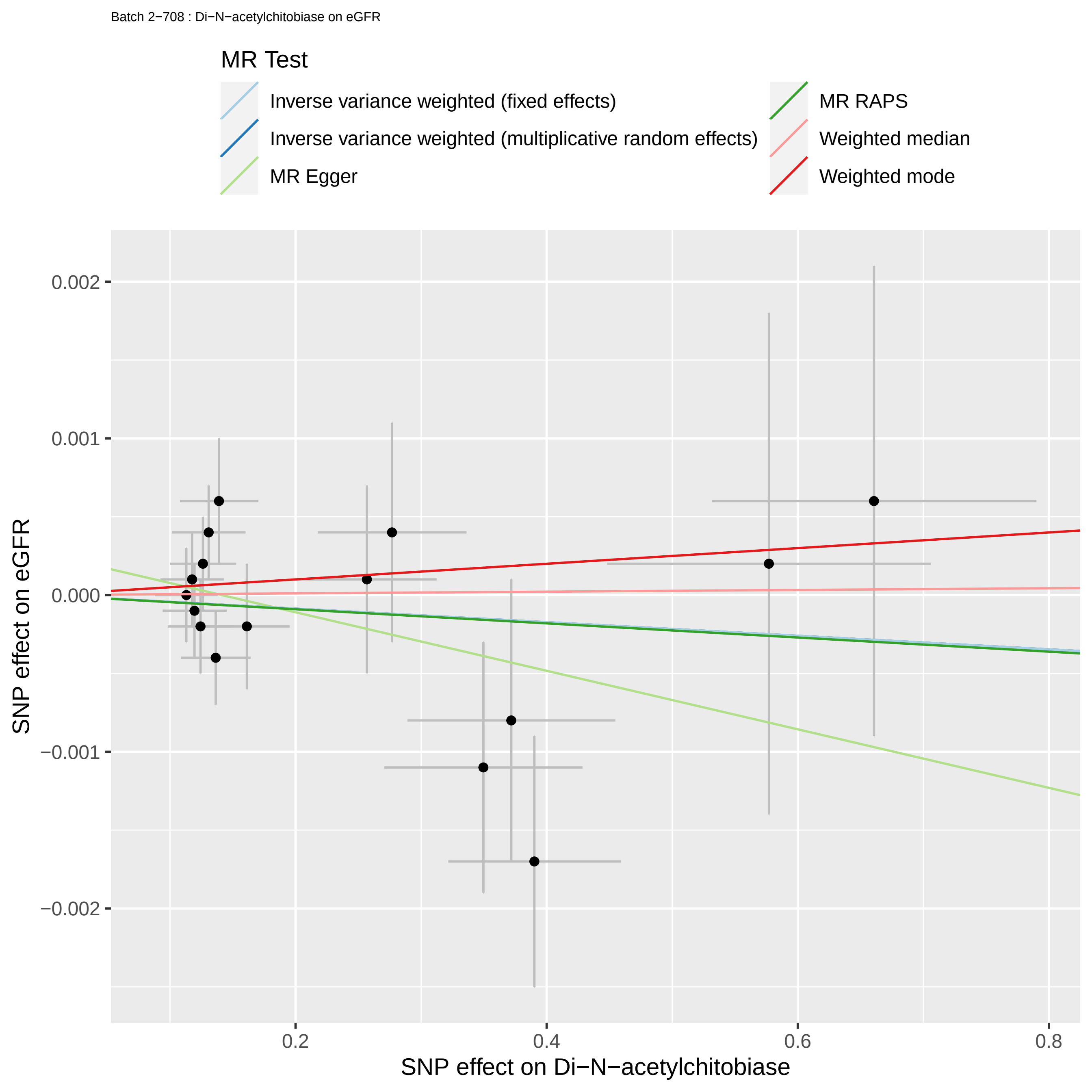

Batch 2−708 : Di−N−acetylchitobiase on eGFR
MR Test
Inverse variance weighted (fixed effects)
Inverse variance weighted (multiplicative random effects)
MR Egger
MR RAPS
Weighted median
Weighted mode
0.002
0.001
0.000
−0.001
−0.002
SNP effect on eGFR
0.2
0.4
0.6
0.8
SNP effect on Di−N−acetylchitobiase

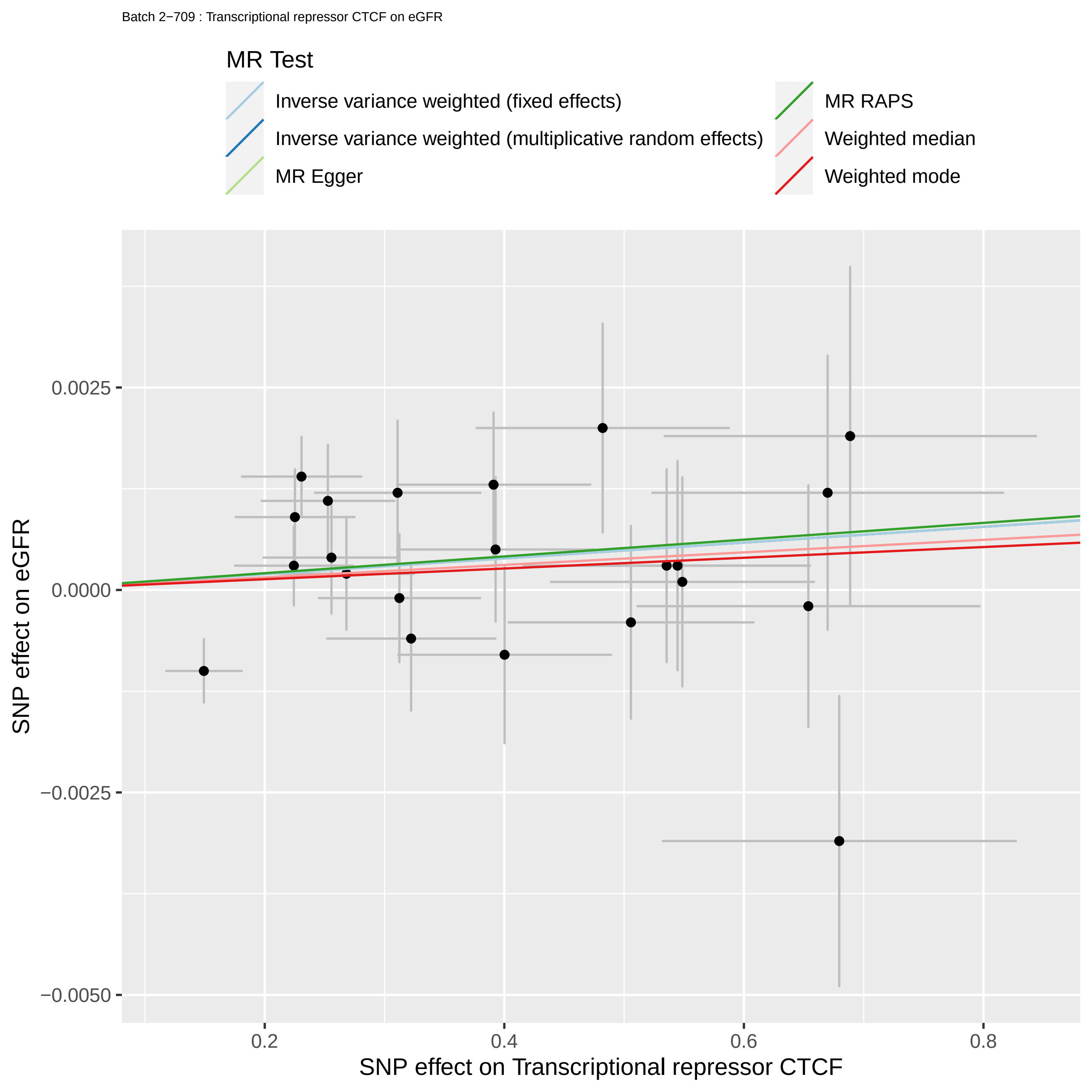
Batch 2−709 : Transcriptional repressor CTCF on eGFR
MR Test
Inverse variance weighted (fixed effects)
Inverse variance weighted (multiplicative random effects)
MR Egger
MR RAPS
Weighted median
Weighted mode
0.0025
0.0000
−0.0025
−0.0050
0.2
0.4
0.6
0.8
SNP effect on eGFR
SNP effect on Transcriptional repressor CTCF

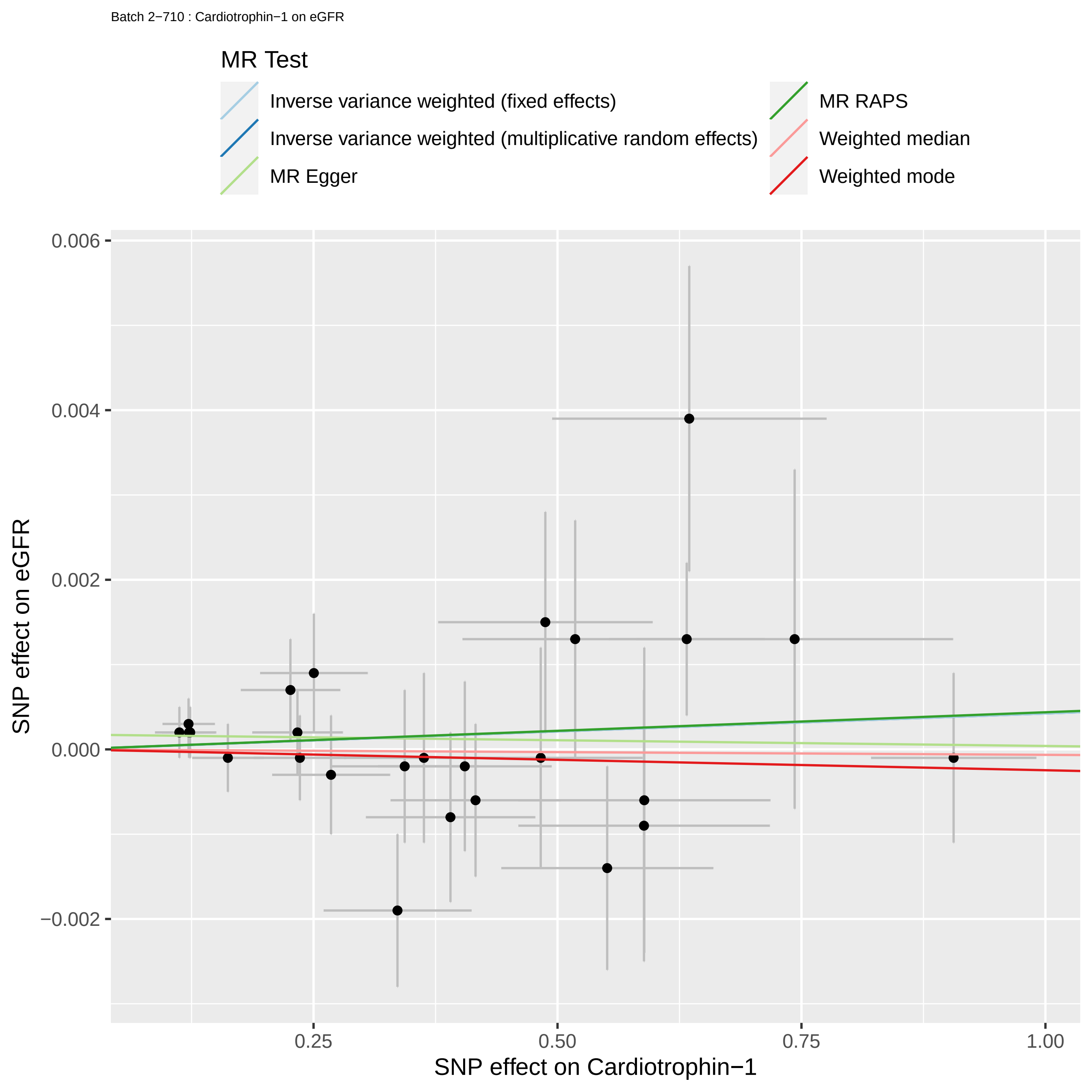

Batch 2−710 : Cardiotrophin−1 on eGFR
MR Test
Inverse variance weighted (fixed effects)
Inverse variance weighted (multiplicative random effects)
MR Egger
MR RAPS
Weighted median
Weighted mode
0.006
0.004
0.002
0.000
−0.002
0.25
0.50
0.75
1.00
SNP effect on eGFR
SNP effect on Cardiotrophin−1

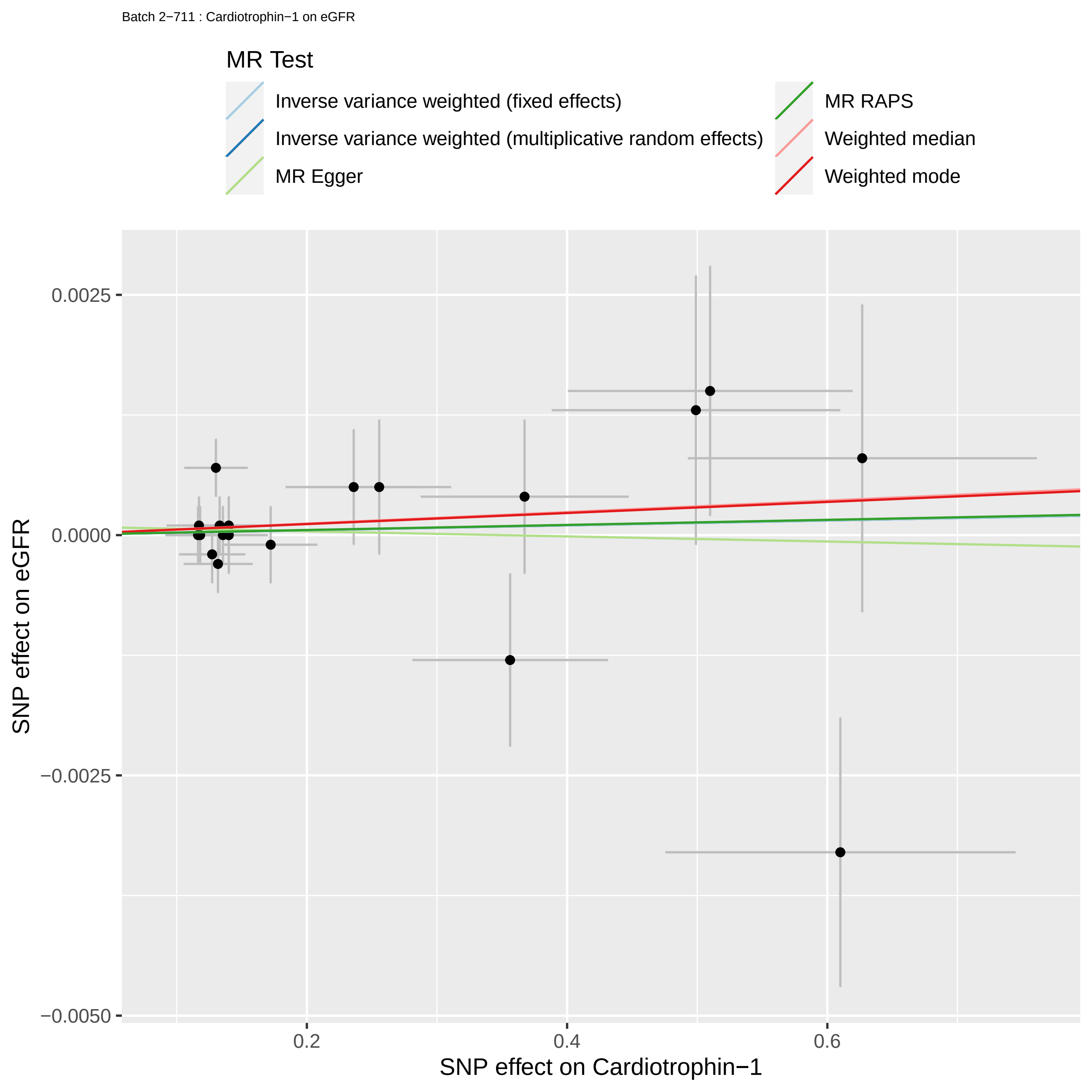
Batch 2−711 : Cardiotrophin−1 on eGFR
MR Test
Inverse variance weighted (fixed effects)
Inverse variance weighted (multiplicative random effects)
MR Egger
MR RAPS
Weighted median
Weighted mode
0.0025
0.0000
−0.0025
−0.0050
0.2
0.4
0.6
SNP effect on eGFR
SNP effect on Cardiotrophin−1

Batch 2−712 : Connective tissue growth factor on eGFR

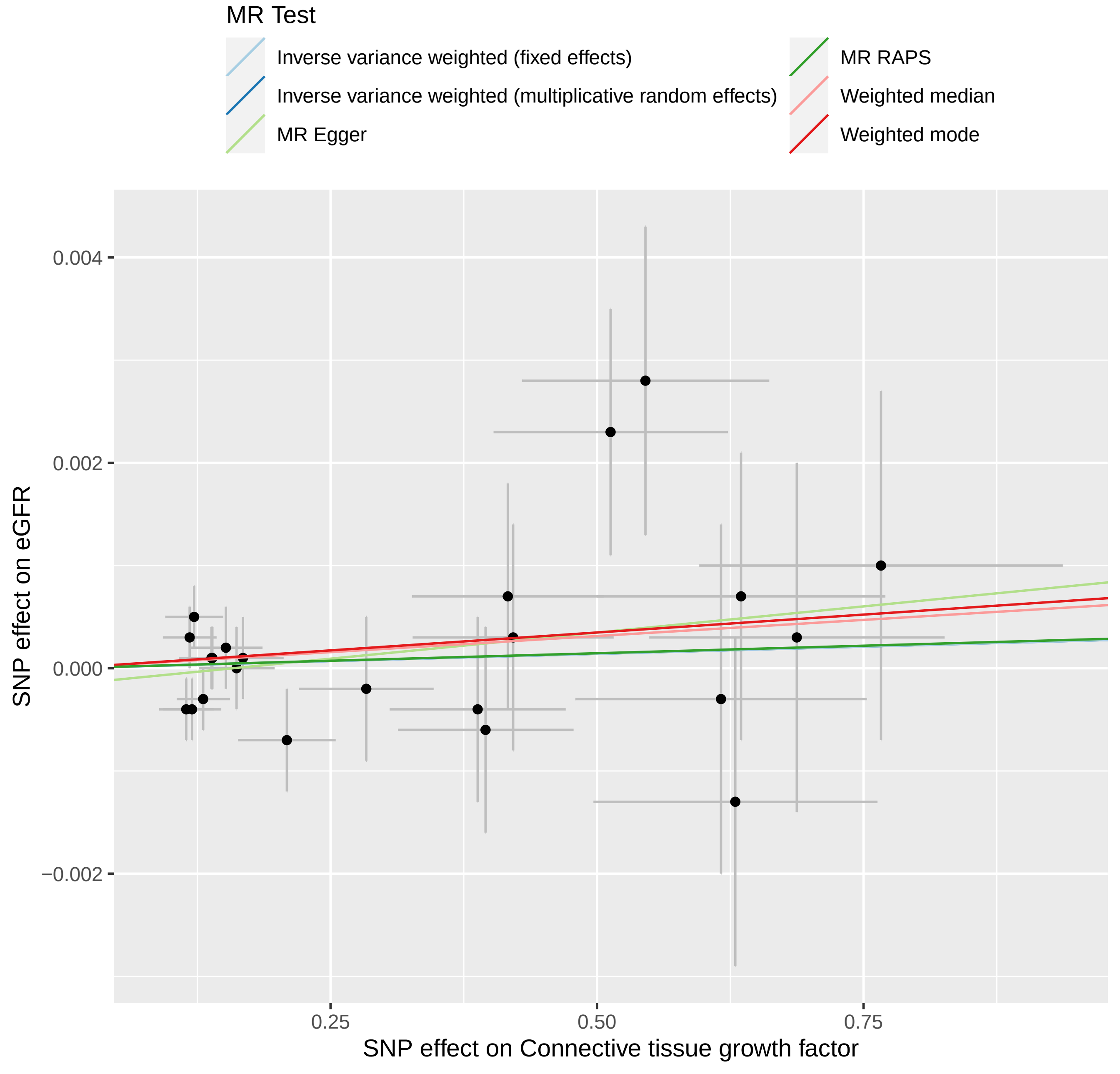

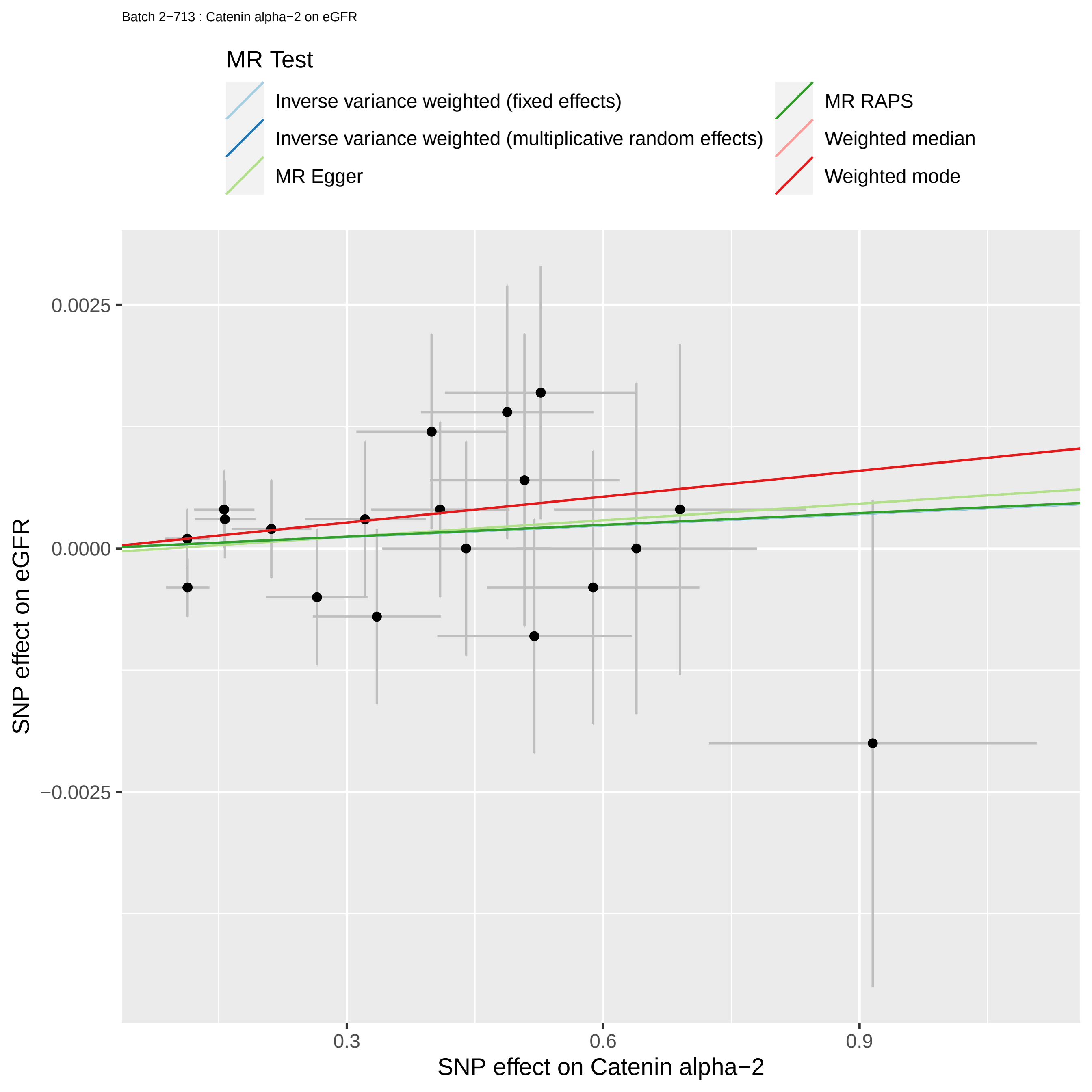
Batch 2−713 : Catenin alpha−2 on eGFR
MR Test
Inverse variance weighted (fixed effects)
Inverse variance weighted (multiplicative random effects)
MR Egger
MR RAPS
Weighted median
Weighted mode
0.0025
0.0000
−0.0025
0.3
0.6
0.9
SNP effect on eGFR
SNP effect on Catenin alpha−2

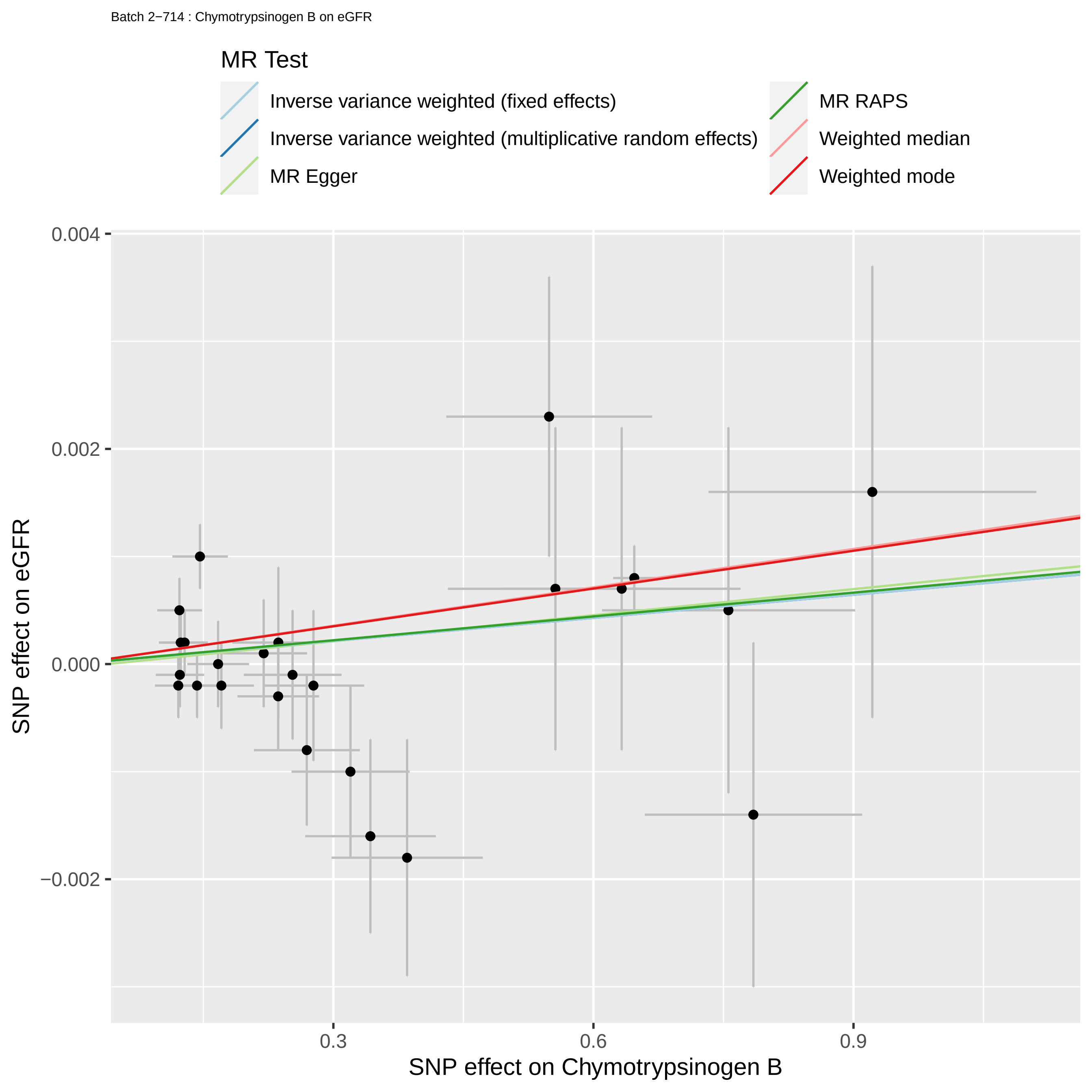
Batch 2−714 : Chymotrypsinogen B on eGFR
MR Test
Inverse variance weighted (fixed effects)
Inverse variance weighted (multiplicative random effects)
MR Egger
MR RAPS
Weighted median
Weighted mode
0.004
0.002
0.000
−0.002
0.3
0.6
0.9
SNP effect on eGFR
SNP effect on Chymotrypsinogen B

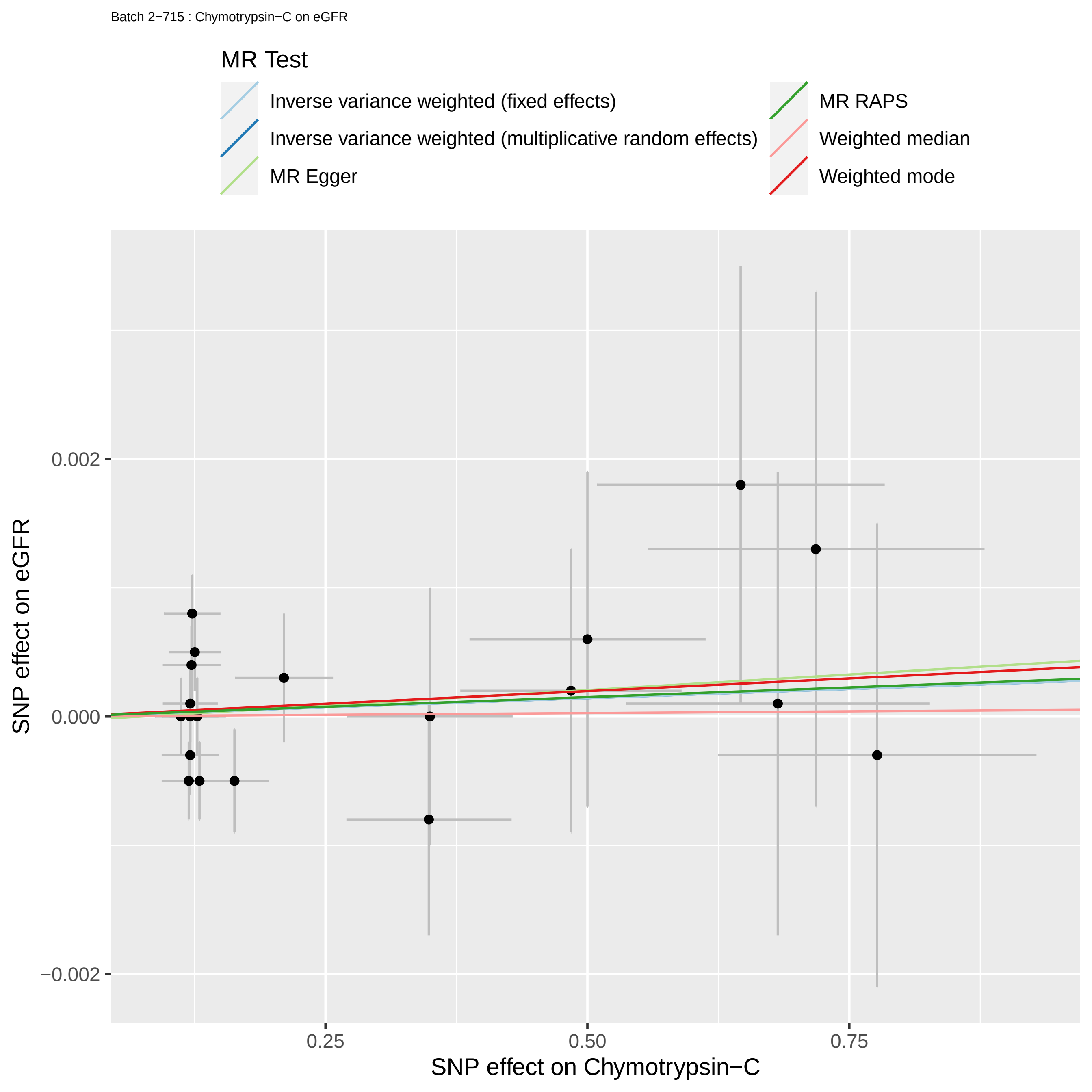
Batch 2−715 : Chymotrypsin−C on eGFR
MR Test
Inverse variance weighted (fixed effects)
Inverse variance weighted (multiplicative random effects)
MR Egger
MR RAPS
Weighted median
Weighted mode
0.002
0.000
−0.002
0.25
0.50
0.75
SNP effect on eGFR
SNP effect on Chymotrypsin−C

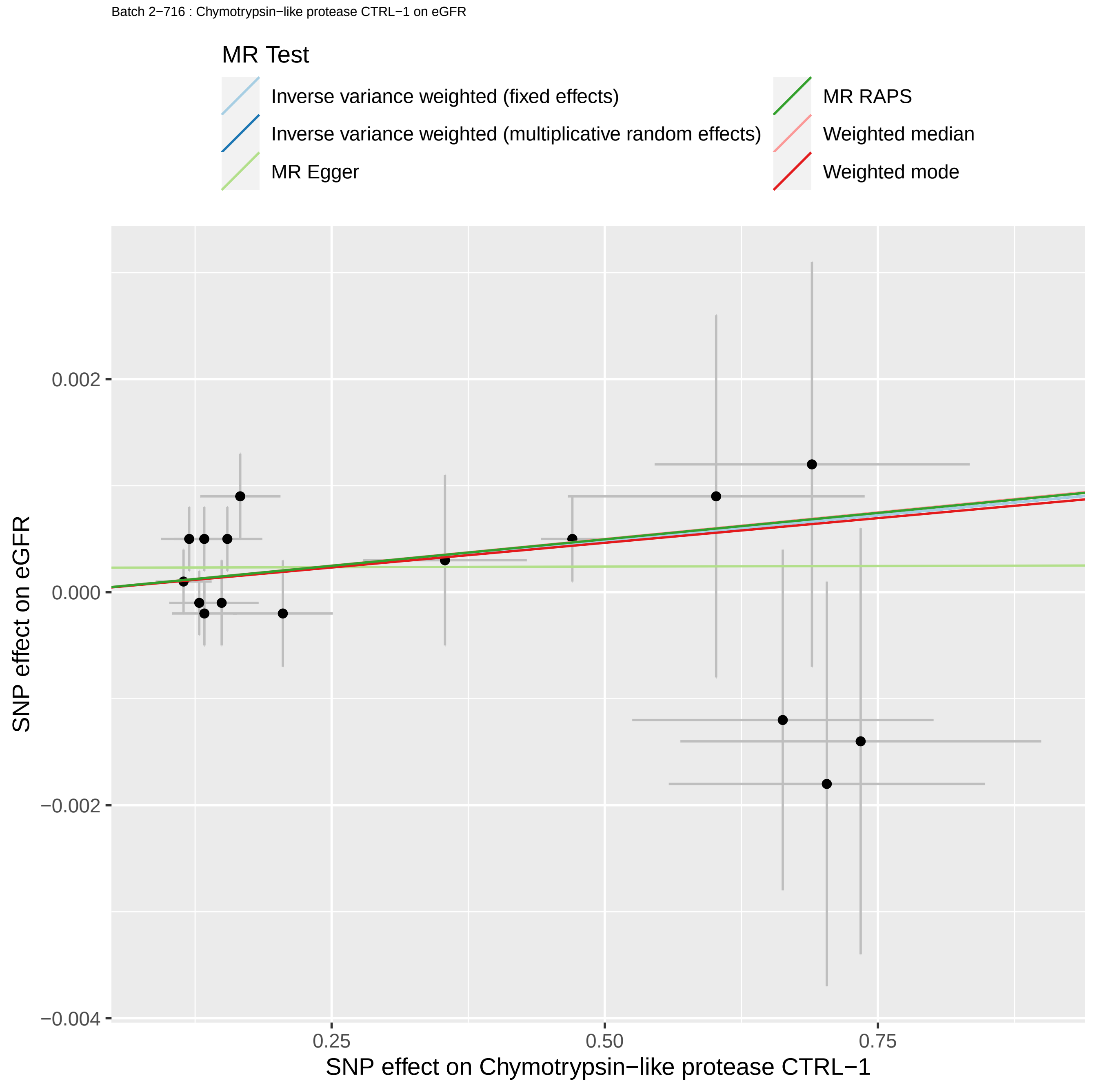

Batch 2−716 : Chymotrypsin−like protease CTRL−1 on eGFR
MR Test
Inverse variance weighted (fixed effects)
Inverse variance weighted (multiplicative random effects)
MR Egger
MR RAPS
Weighted median
Weighted mode
0.002
0.000
−0.002
−0.004
0.25
0.50
0.75
SNP effect on eGFR
SNP effect on Chymotrypsin−like protease CTRL−1

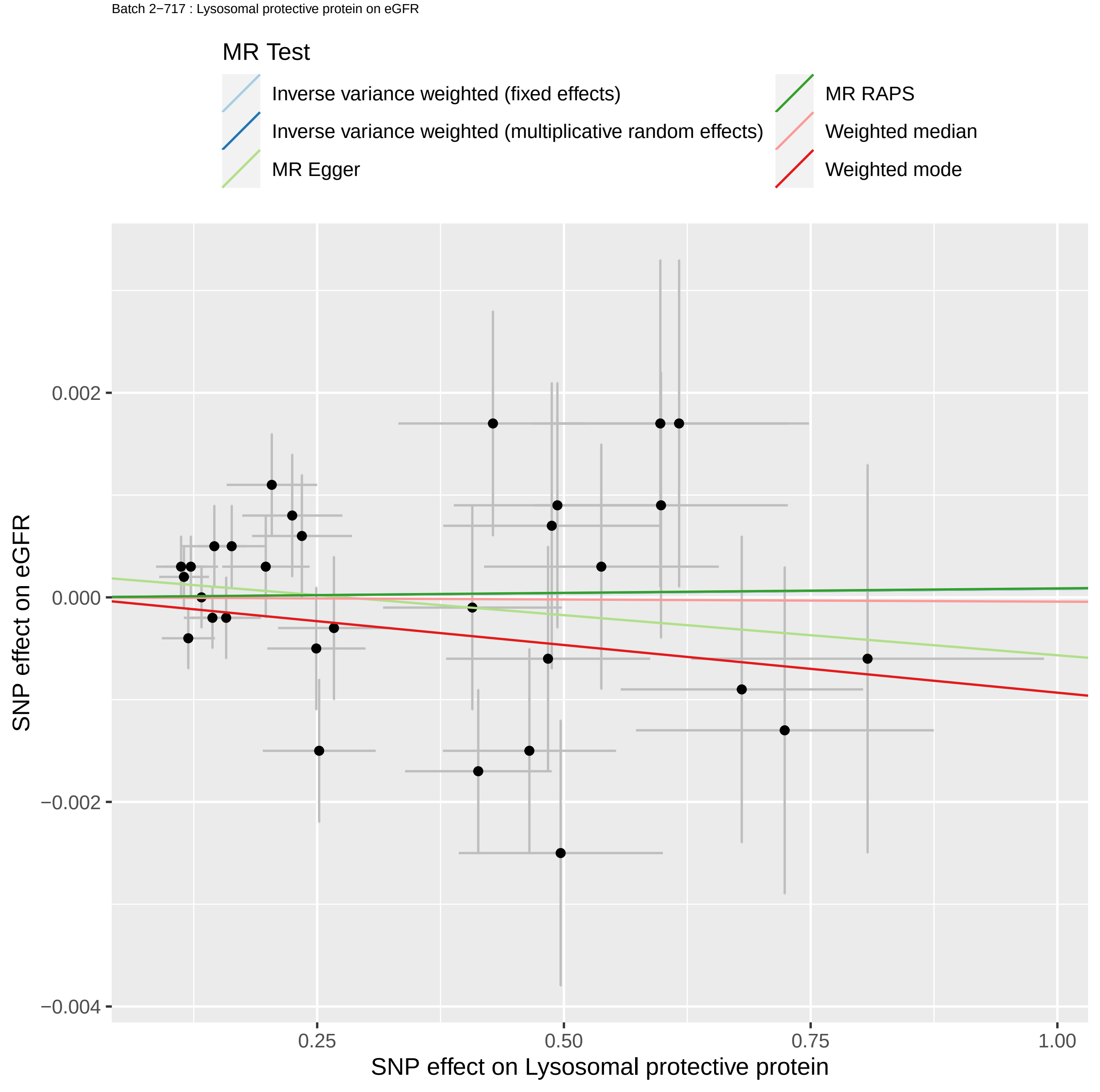

Batch 2−717 : Lysosomal protective protein on eGFR
MR Test
Inverse variance weighted (fixed effects)
Inverse variance weighted (multiplicative random effects)
MR Egger
MR RAPS
Weighted median
Weighted mode
0.002
0.000
−0.002
−0.004
SNP effect on eGFR
0.25
0.50
0.75
1.00
SNP effect on Lysosomal protective protein

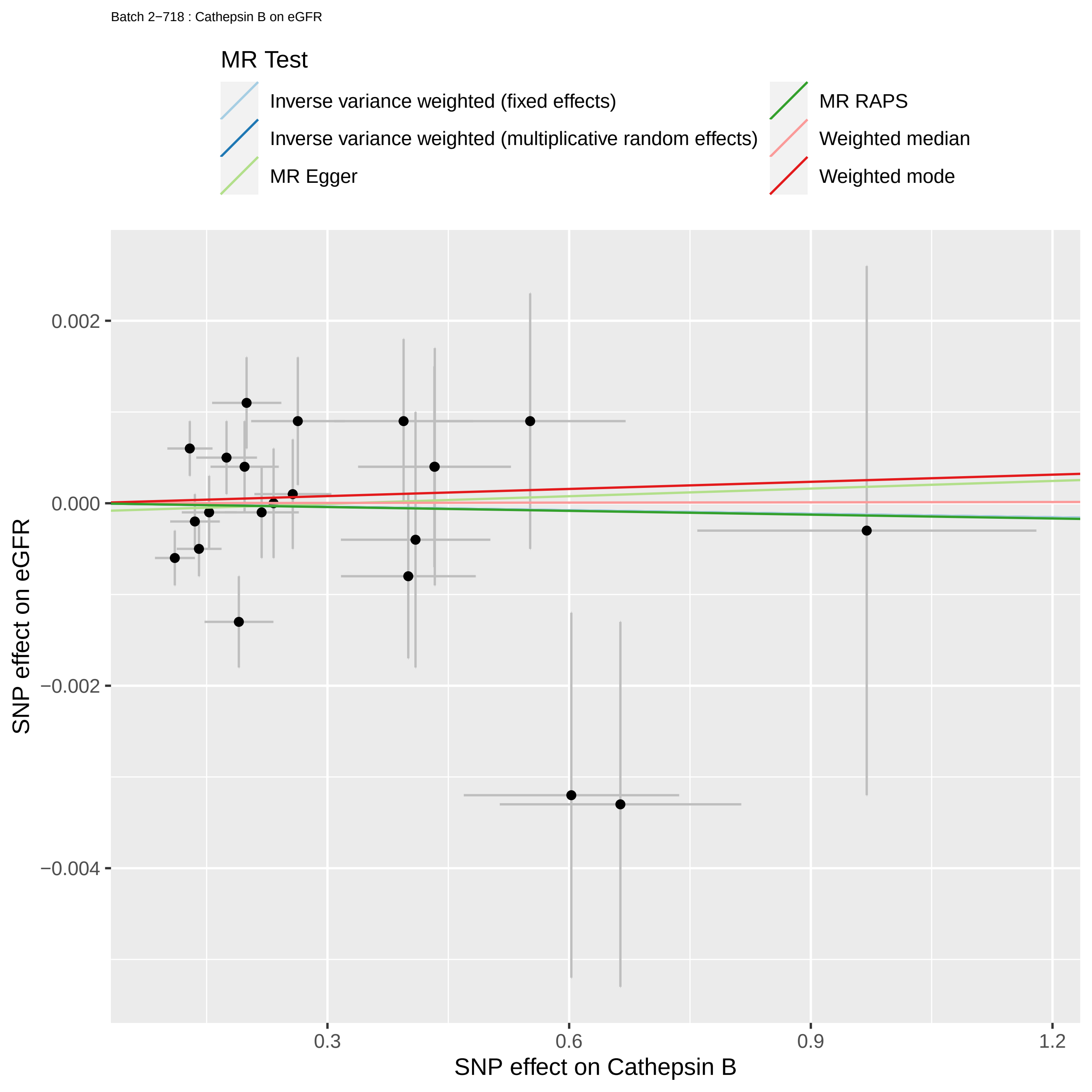
Batch 2−718 : Cathepsin B on eGFR
MR Test
Inverse variance weighted (fixed effects)
Inverse variance weighted (multiplicative random effects)
MR Egger
MR RAPS
Weighted median
Weighted mode
0.002
0.000
−0.002
−0.004
0.3
0.6
0.9
1.2
SNP effect on Cathepsin B
SNP effect on eGFR

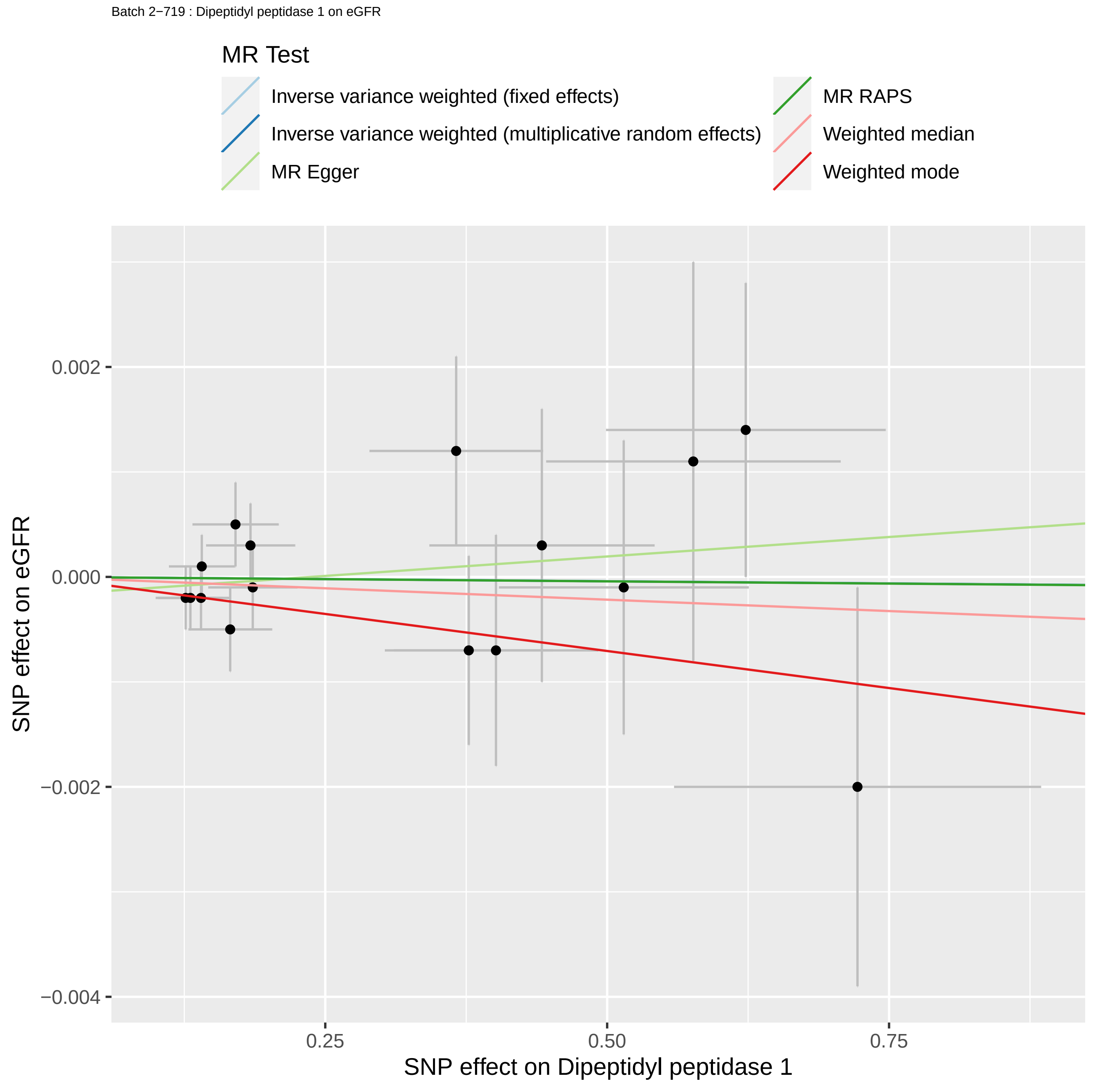

Batch 2−719 : Dipeptidyl peptidase 1 on eGFR
MR Test
Inverse variance weighted (fixed effects)
Inverse variance weighted (multiplicative random effects)
MR Egger
MR RAPS
Weighted median
Weighted mode
0.002
0.000
−0.002
−0.004
0.25
0.50
0.75
SNP effect on eGFR
SNP effect on Dipeptidyl peptidase 1

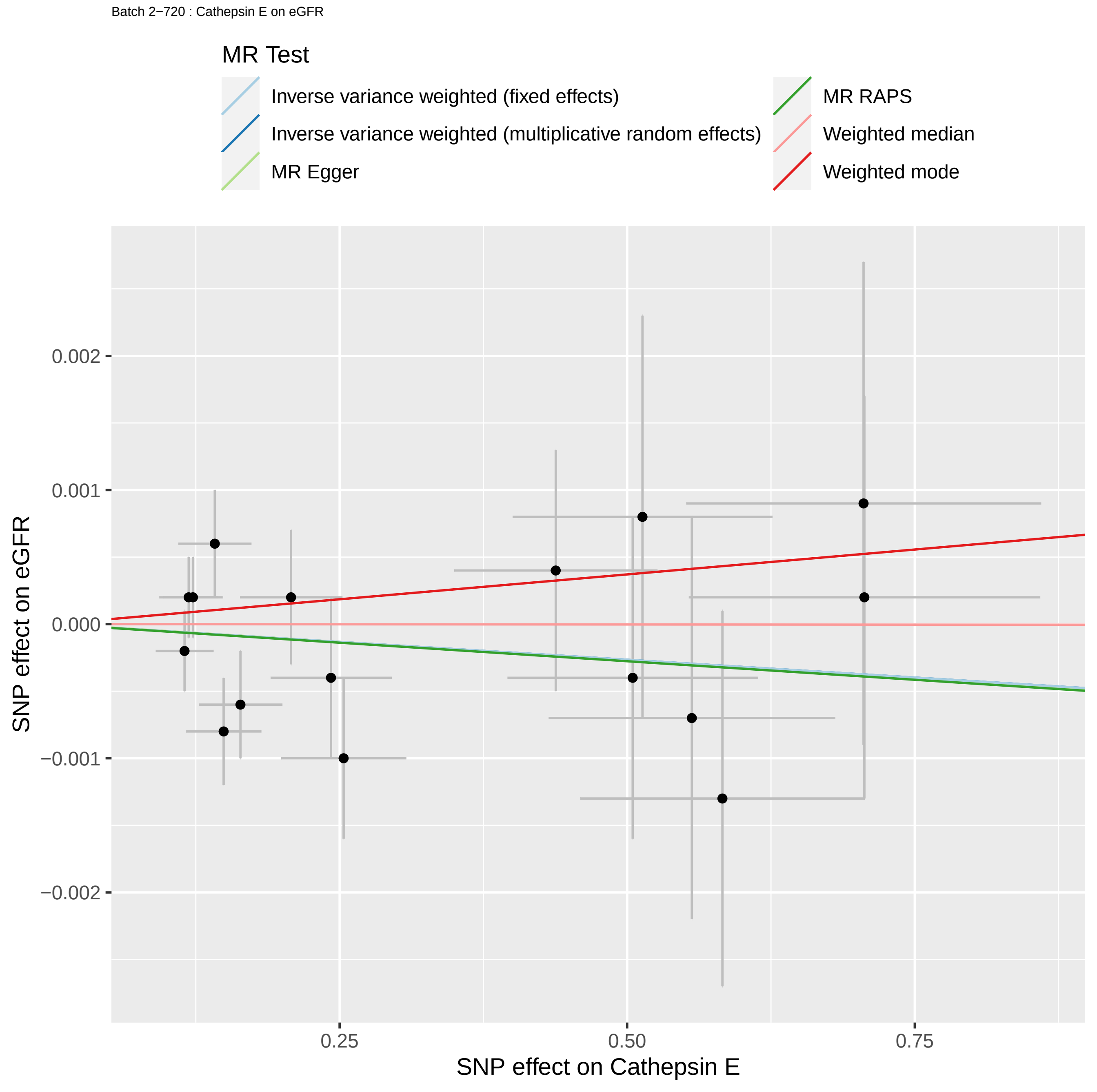

Batch 2−720 : Cathepsin E on eGFR
MR Test
Inverse variance weighted (fixed effects)
Inverse variance weighted (multiplicative random effects)
MR Egger
MR RAPS
Weighted median
Weighted mode
0.002
0.001
0.000
−0.001
−0.002
0.25
0.50
0.75
SNP effect on eGFR
SNP effect on Cathepsin E

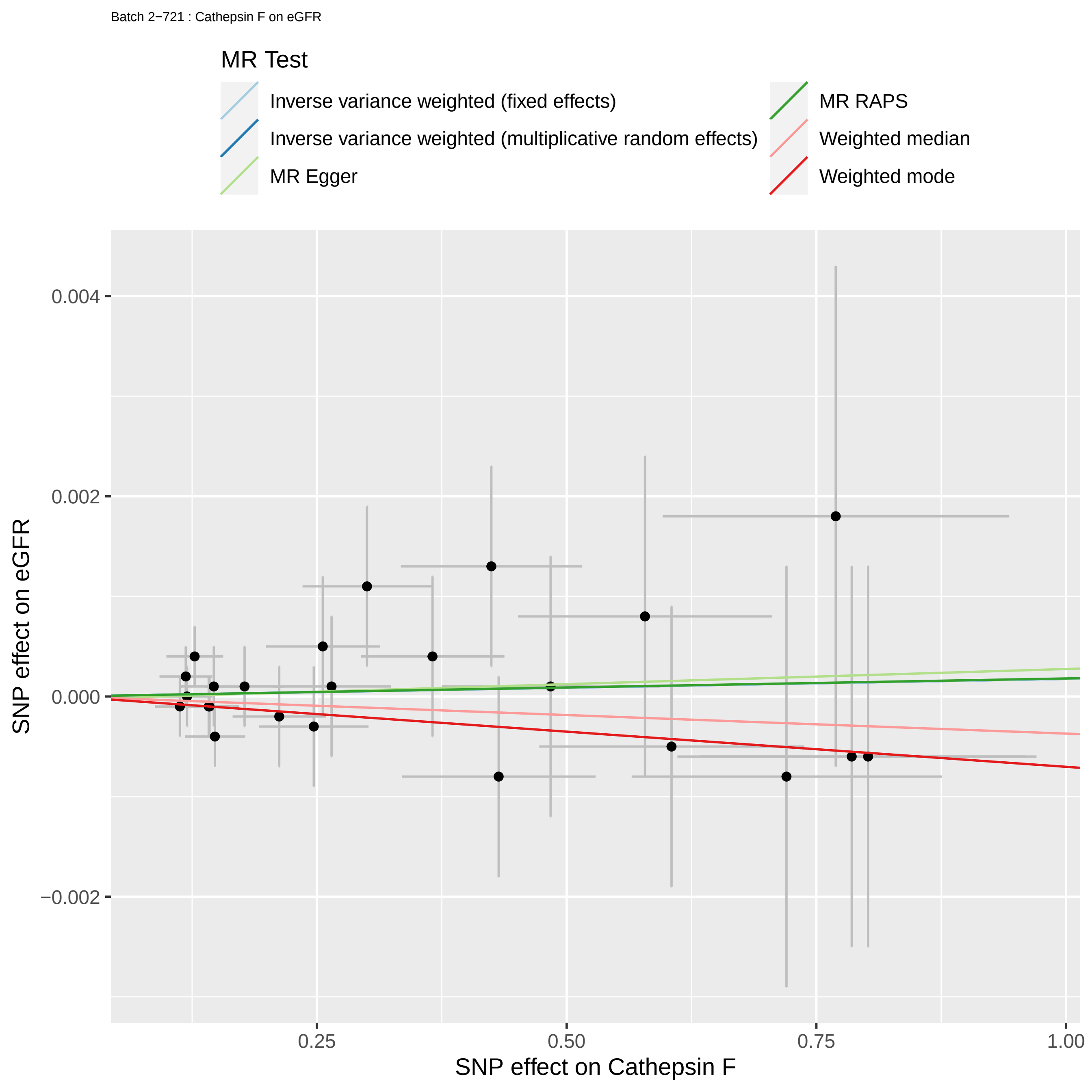
Batch 2−721 : Cathepsin F on eGFR
MR Test
Inverse variance weighted (fixed effects)
Inverse variance weighted (multiplicative random effects)
MR Egger
MR RAPS
Weighted median
Weighted mode
0.004
0.002
0.000
−0.002
SNP effect on eGFR
0.25
0.50
0.75
1.00
SNP effect on Cathepsin F

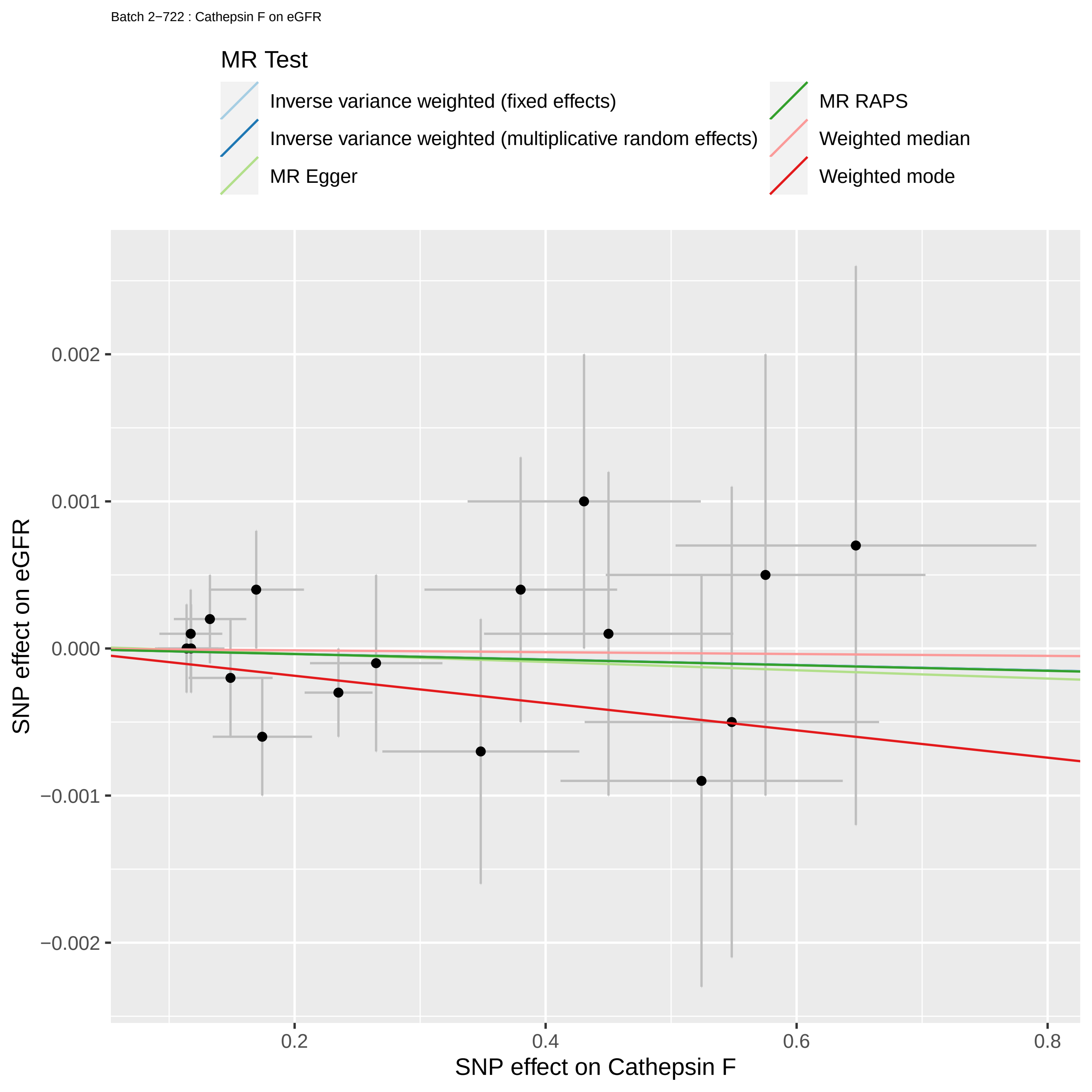
Batch 2−722 : Cathepsin F on eGFR
MR Test
Inverse variance weighted (fixed effects)
Inverse variance weighted (multiplicative random effects)
MR Egger
MR RAPS
Weighted median
Weighted mode
0.002
0.001
0.000
−0.001
−0.002
SNP effect on eGFR
0.2
0.4
0.6
0.8
SNP effect on Cathepsin F

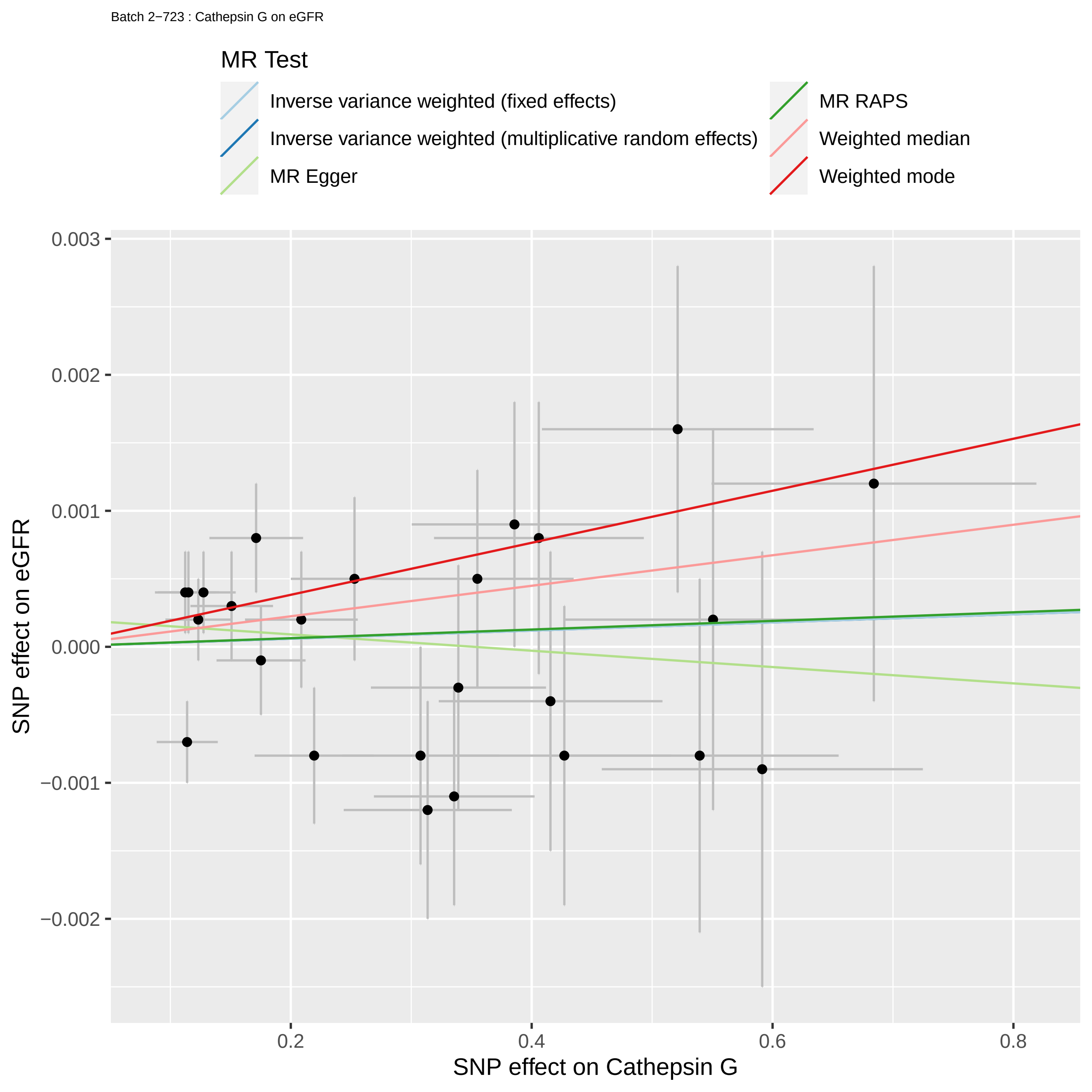

Batch 2−723 : Cathepsin G on eGFR
MR Test
Inverse variance weighted (fixed effects)
Inverse variance weighted (multiplicative random effects)
MR Egger
MR RAPS
Weighted median
Weighted mode
0.003
0.002
0.001
0.000
−0.001
−0.002
0.2
0.4
0.6
0.8
SNP effect on eGFR
SNP effect on Cathepsin G

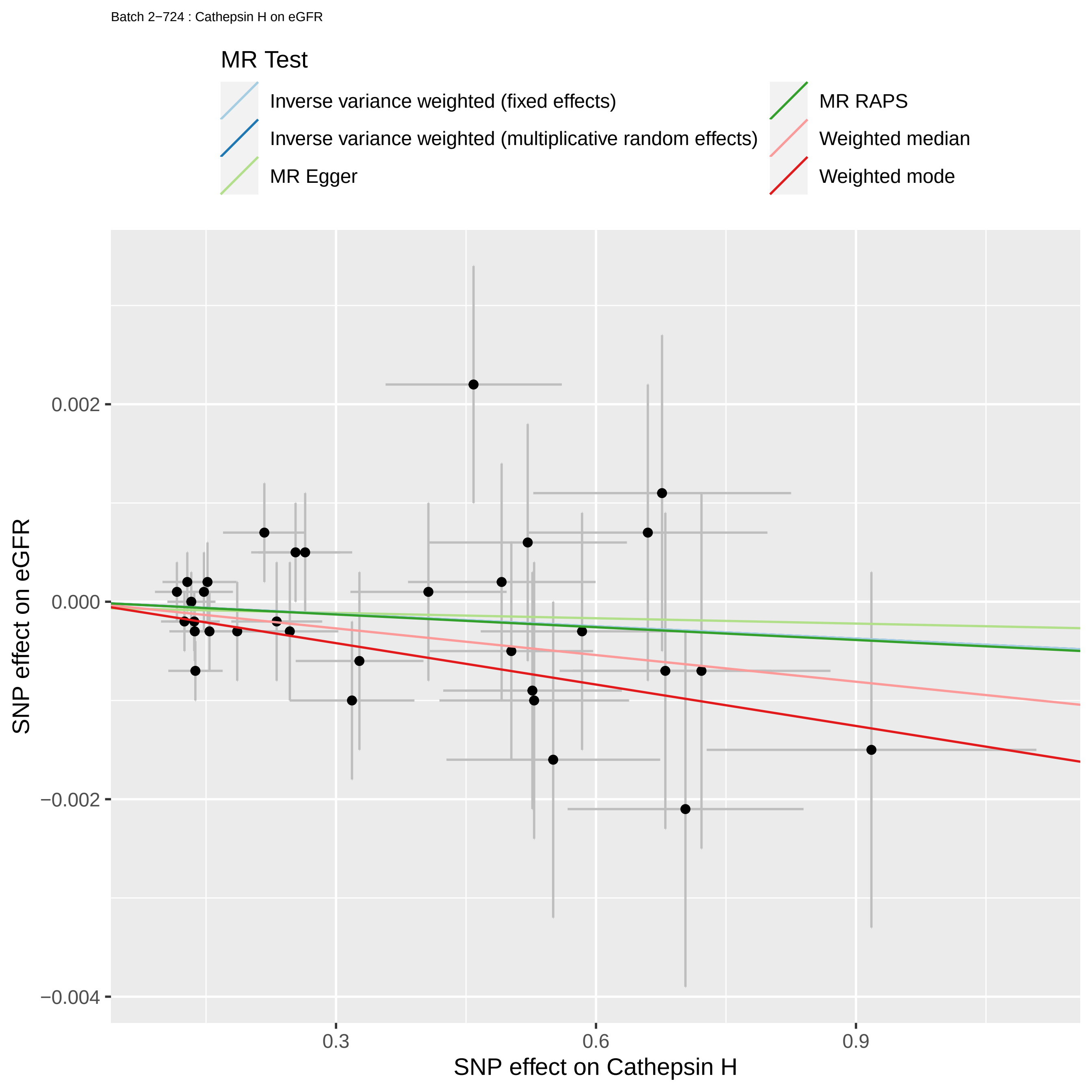
Batch 2−724 : Cathepsin H on eGFR
MR Test
Inverse variance weighted (fixed effects)
Inverse variance weighted (multiplicative random effects)
MR Egger
MR RAPS
Weighted median
Weighted mode
0.002
0.000
−0.002
−0.004
0.3
0.6
0.9
SNP effect on eGFR
SNP effect on Cathepsin H

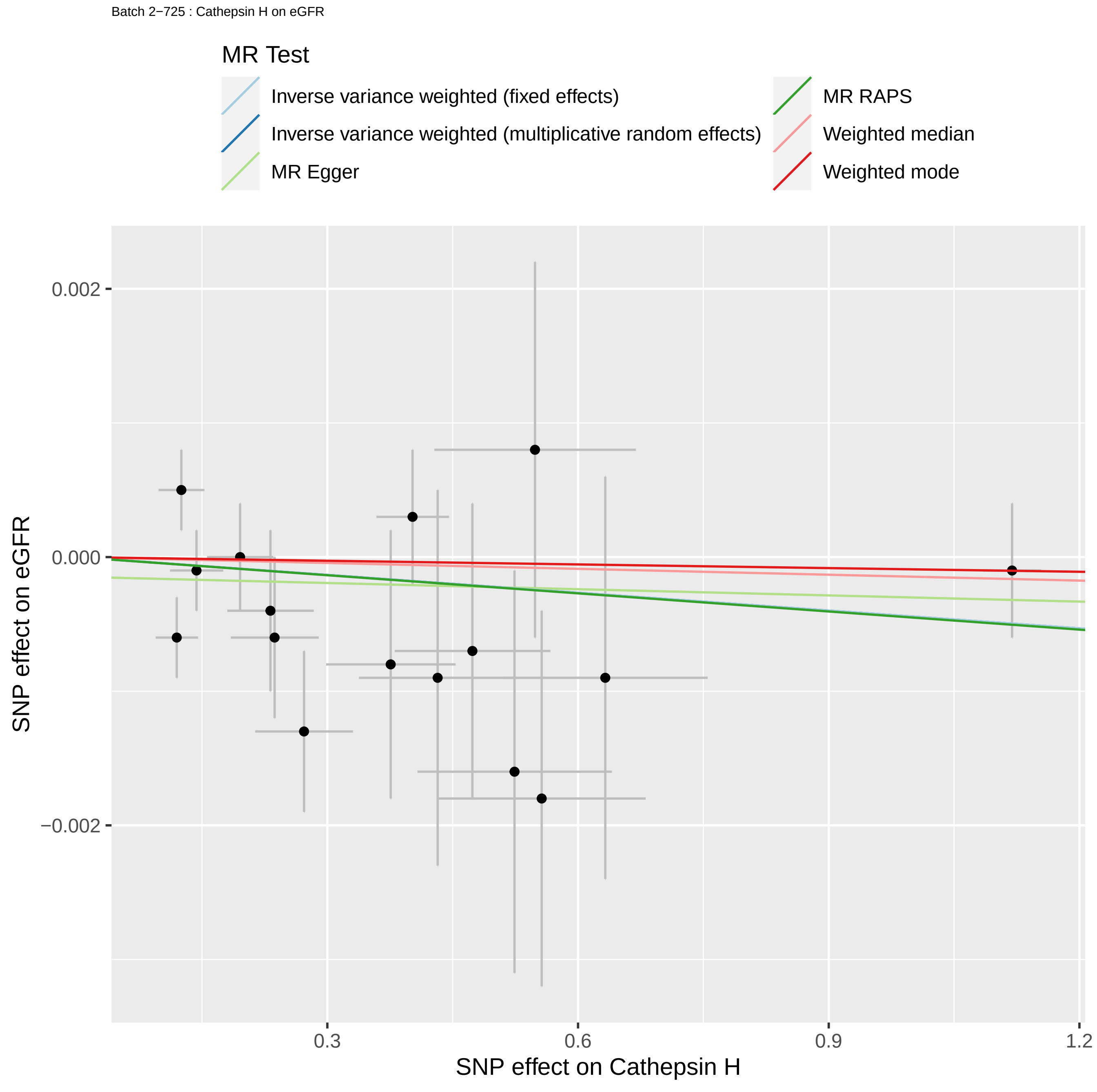
Batch 2−725 : Cathepsin H on eGFR
MR Test
Inverse variance weighted (fixed effects)
Inverse variance weighted (multiplicative random effects)
MR Egger
MR RAPS
Weighted median
Weighted mode
0.002
0.000
−0.002
SNP effect on eGFR
0.3
0.6
0.9
1.2
SNP effect on Cathepsin H

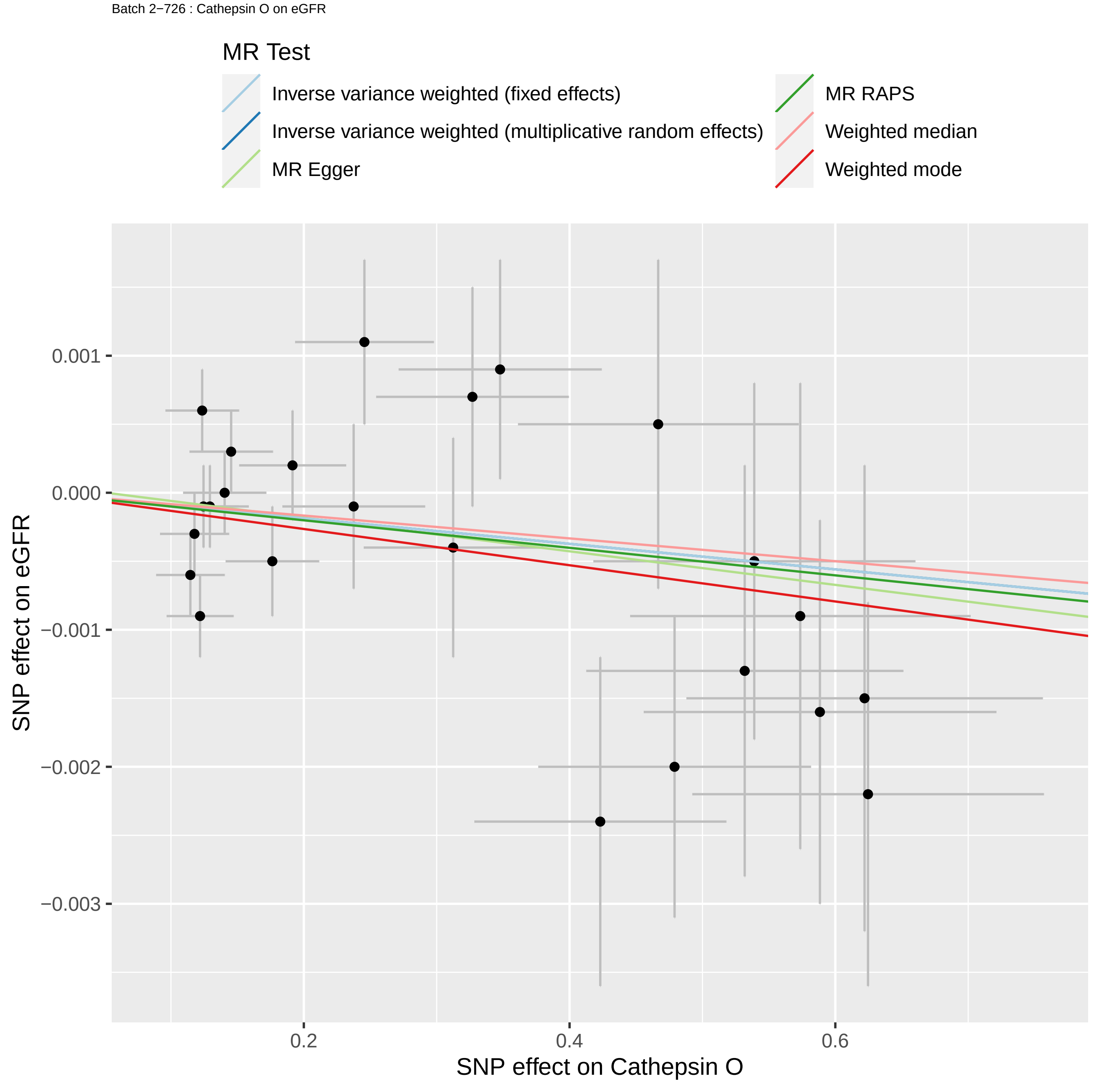

Batch 2−726 : Cathepsin O on eGFR
MR Test
Inverse variance weighted (fixed effects)
Inverse variance weighted (multiplicative random effects)
MR Egger
MR RAPS
Weighted median
Weighted mode
0.001
0.000
−0.001
−0.002
−0.003
SNP effect on eGFR
0.2
0.4
0.6
SNP effect on Cathepsin O

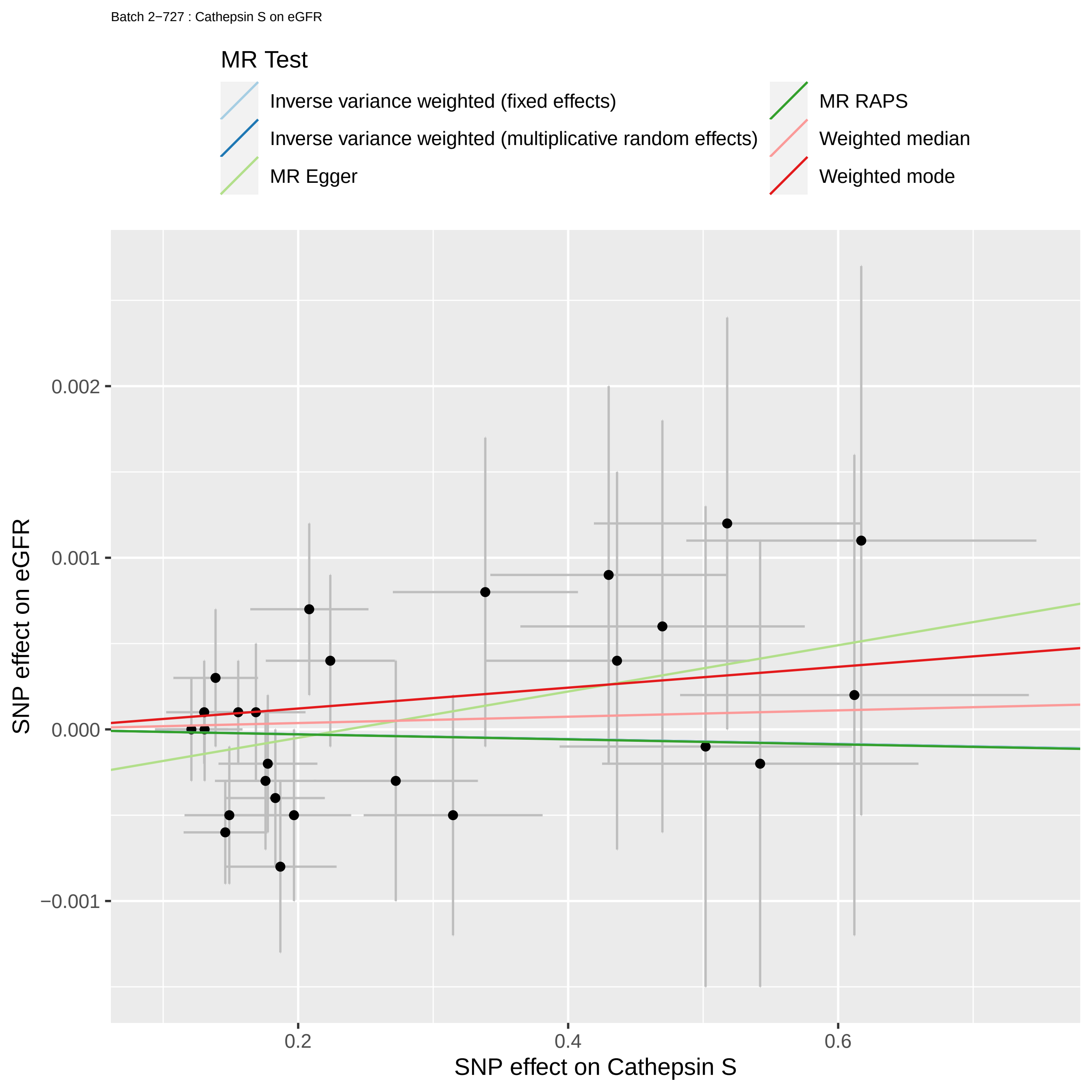

Batch 2−727 : Cathepsin S on eGFR
MR Test
Inverse variance weighted (fixed effects)
Inverse variance weighted (multiplicative random effects)
MR Egger
MR RAPS
Weighted median
Weighted mode
0.002
0.001
0.000
−0.001
0.2
0.4
0.6
SNP effect on eGFR
SNP effect on Cathepsin S

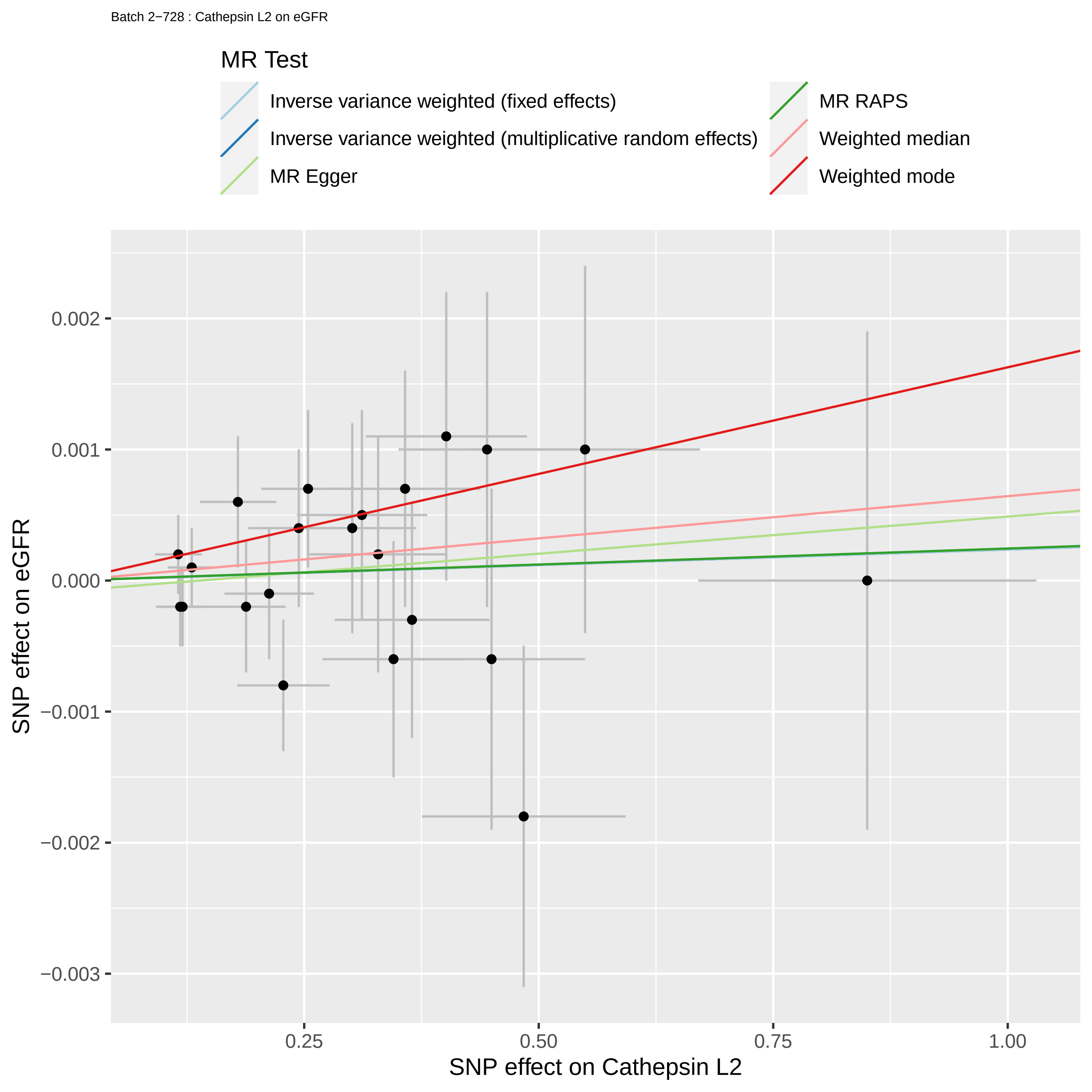

Batch 2−728 : Cathepsin L2 on eGFR
MR Test
Inverse variance weighted (fixed effects)
Inverse variance weighted (multiplicative random effects)
MR Egger
MR RAPS
Weighted median
Weighted mode
0.002
0.001
0.000
−0.001
−0.002
−0.003
SNP effect on eGFR
0.25
0.50
0.75
1.00
SNP effect on Cathepsin L2

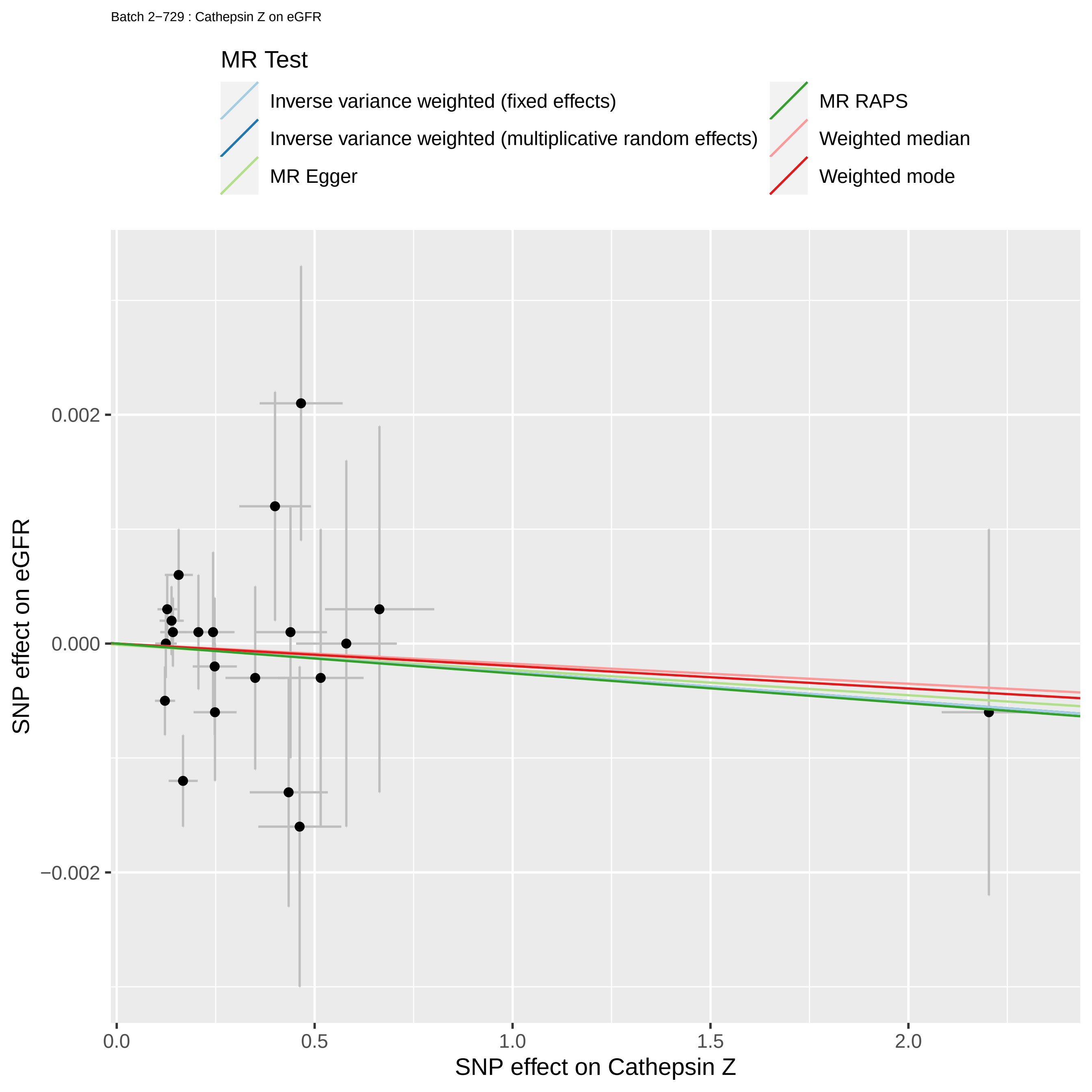
Batch 2−729 : Cathepsin Z on eGFR
MR Test
Inverse variance weighted (fixed effects)
Inverse variance weighted (multiplicative random effects)
MR Egger
MR RAPS
Weighted median
Weighted mode
0.002
0.000
−0.002
0.0
0.5
1.0
1.5
2.0
SNP effect on eGFR
SNP effect on Cathepsin Z

Batch 2−730 : Cortexin−3 on eGFR

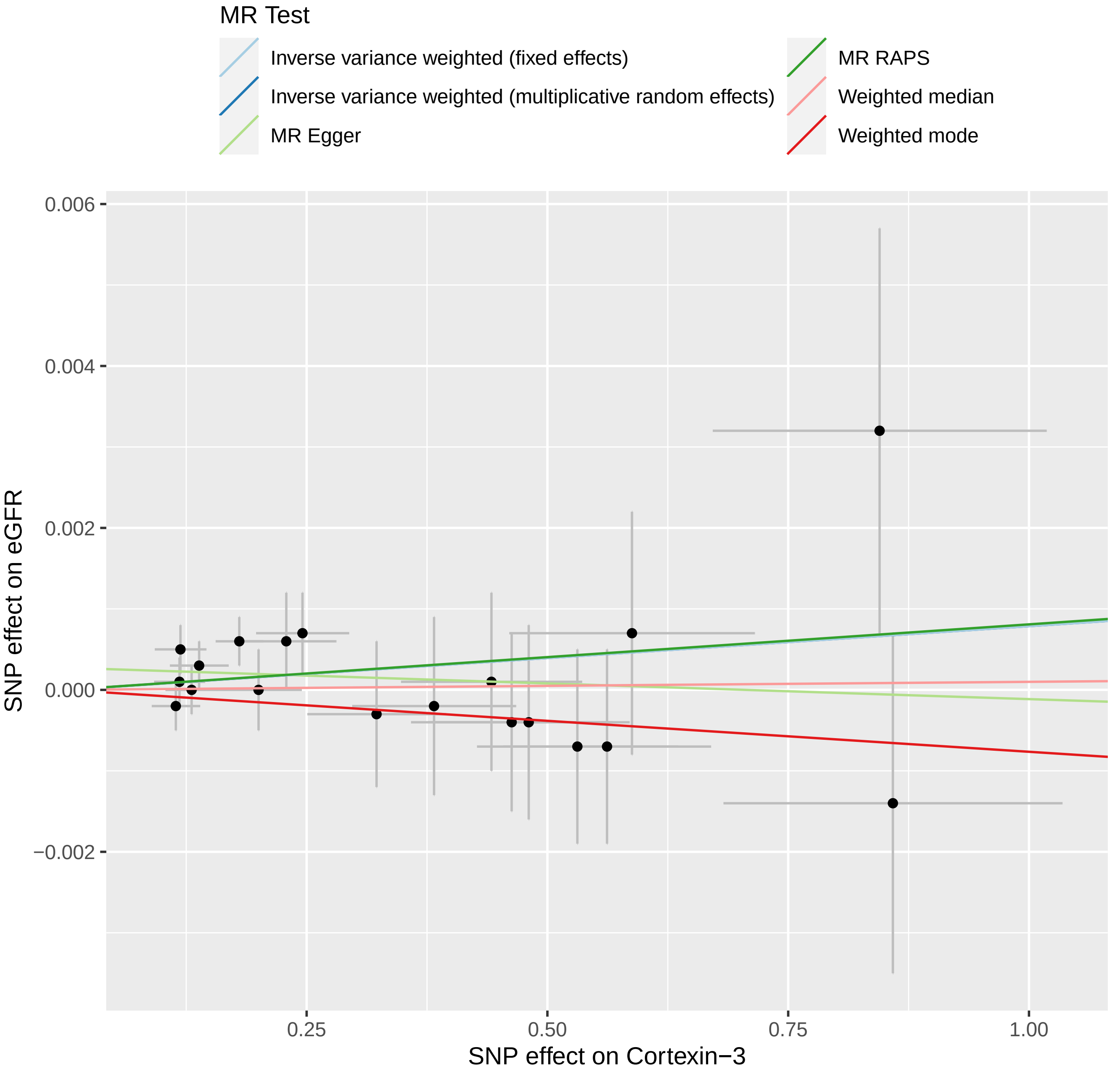

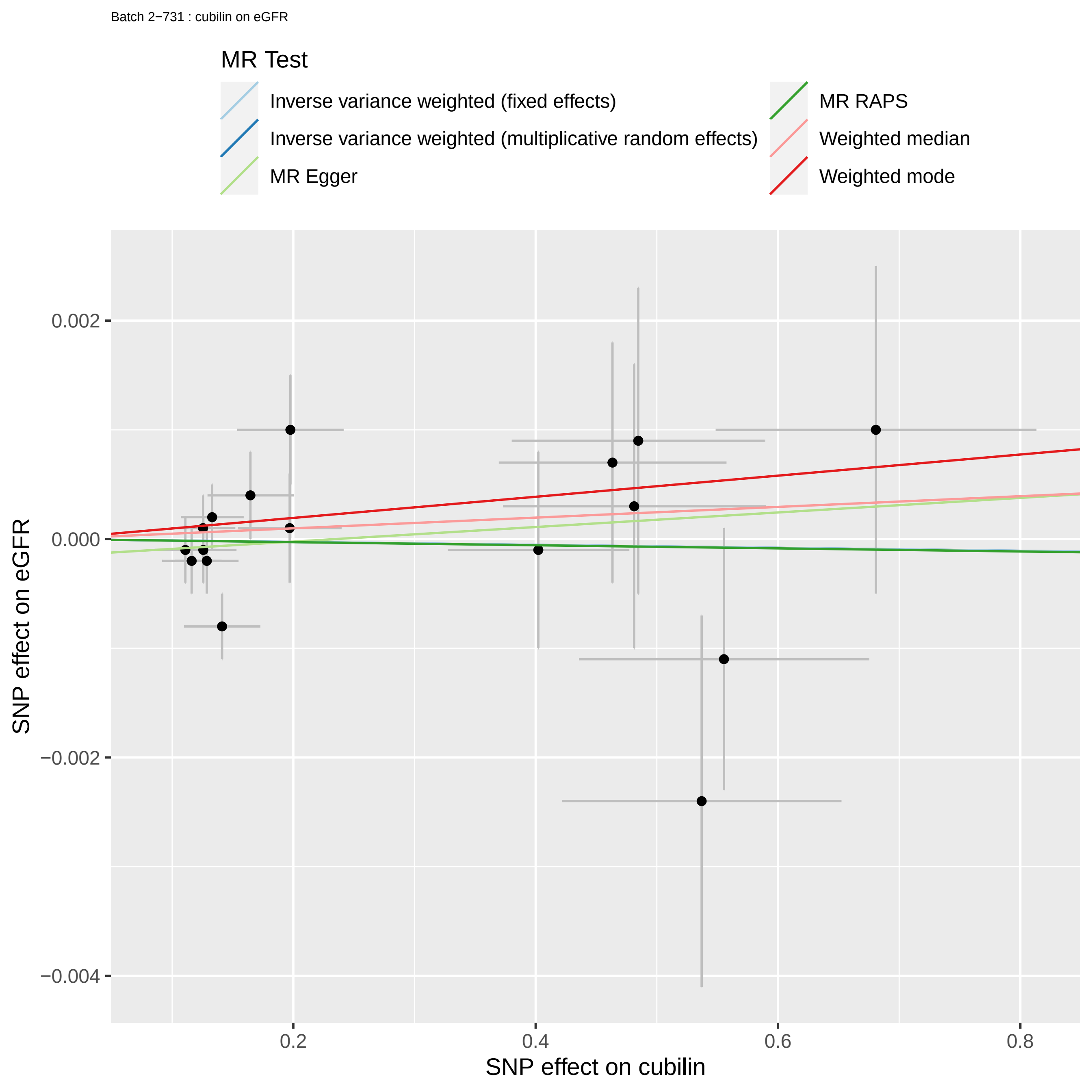

Batch 2−731 : cubilin on eGFR
MR Test
Inverse variance weighted (fixed effects)
Inverse variance weighted (multiplicative random effects)
MR Egger
MR RAPS
Weighted median
Weighted mode
0.002
0.000
−0.002
−0.004
0.2
0.4
0.6
0.8
SNP effect on cubilin
SNP effect on eGFR

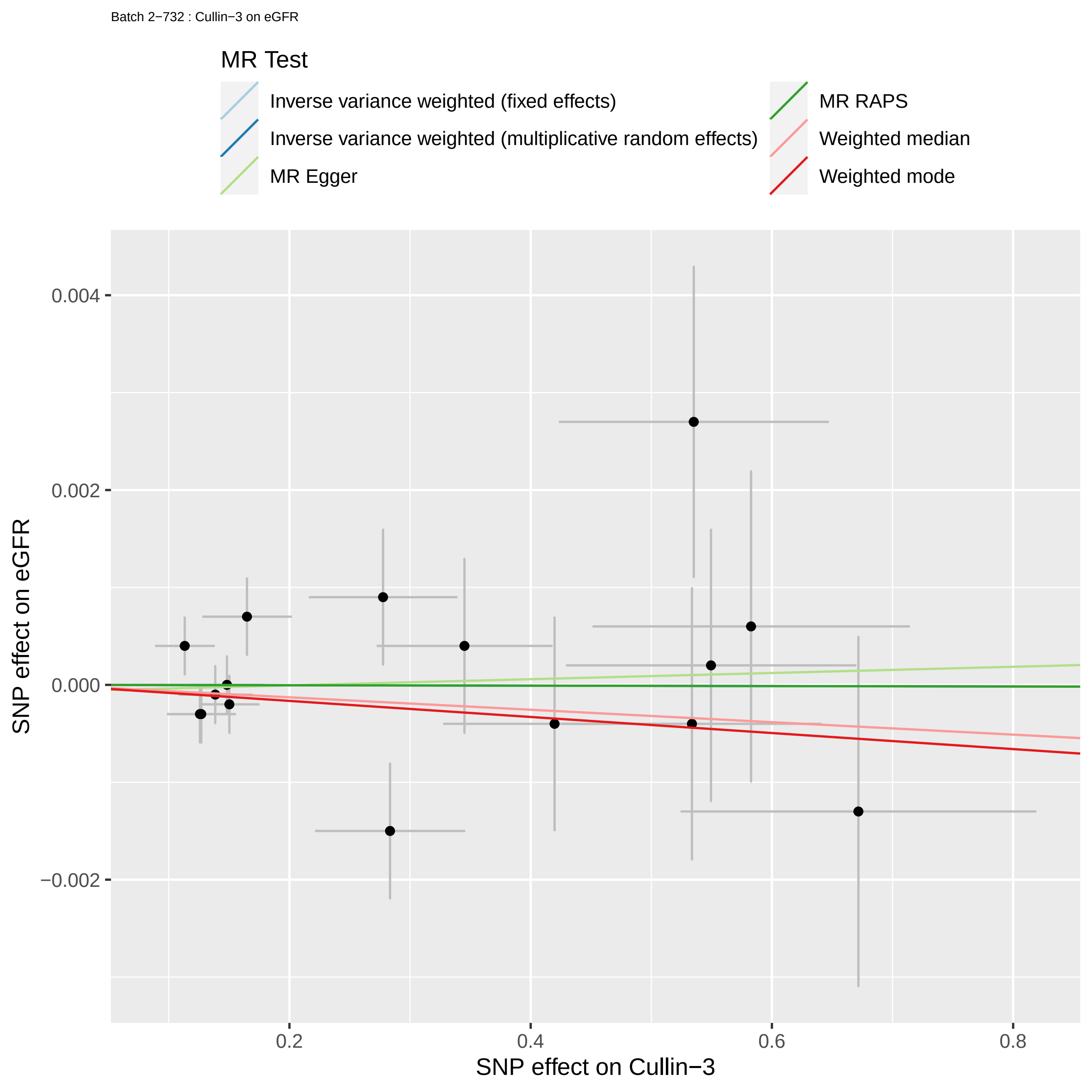
Batch 2−732 : Cullin−3 on eGFR
MR Test
Inverse variance weighted (fixed effects)
Inverse variance weighted (multiplicative random effects)
MR Egger
MR RAPS
Weighted median
Weighted mode
0.004
0.002
0.000
−0.002
0.2
0.4
0.6
0.8
SNP effect on eGFR
SNP effect on Cullin−3

Batch 2−733 : Cullin−4B on eGFR

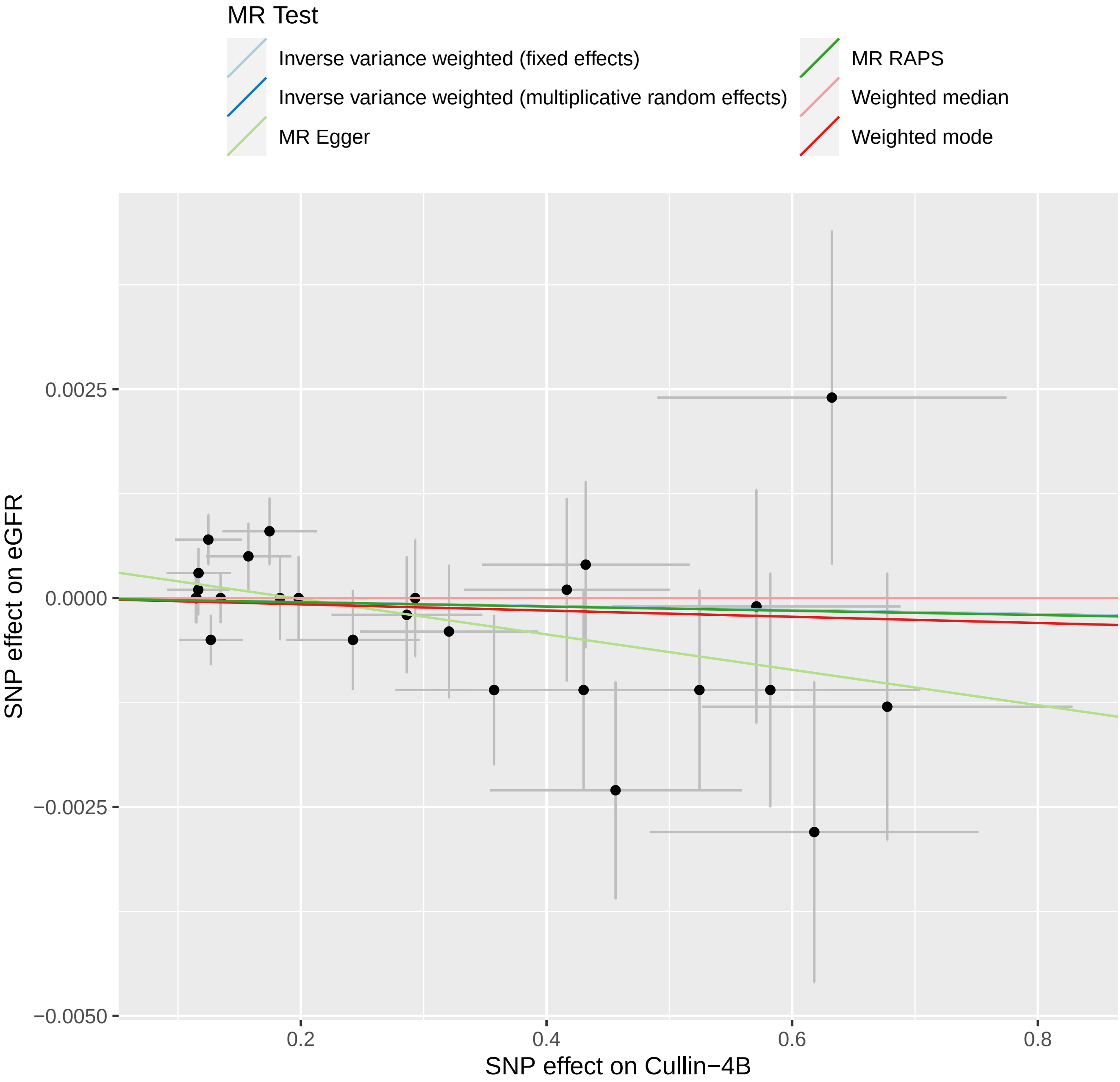

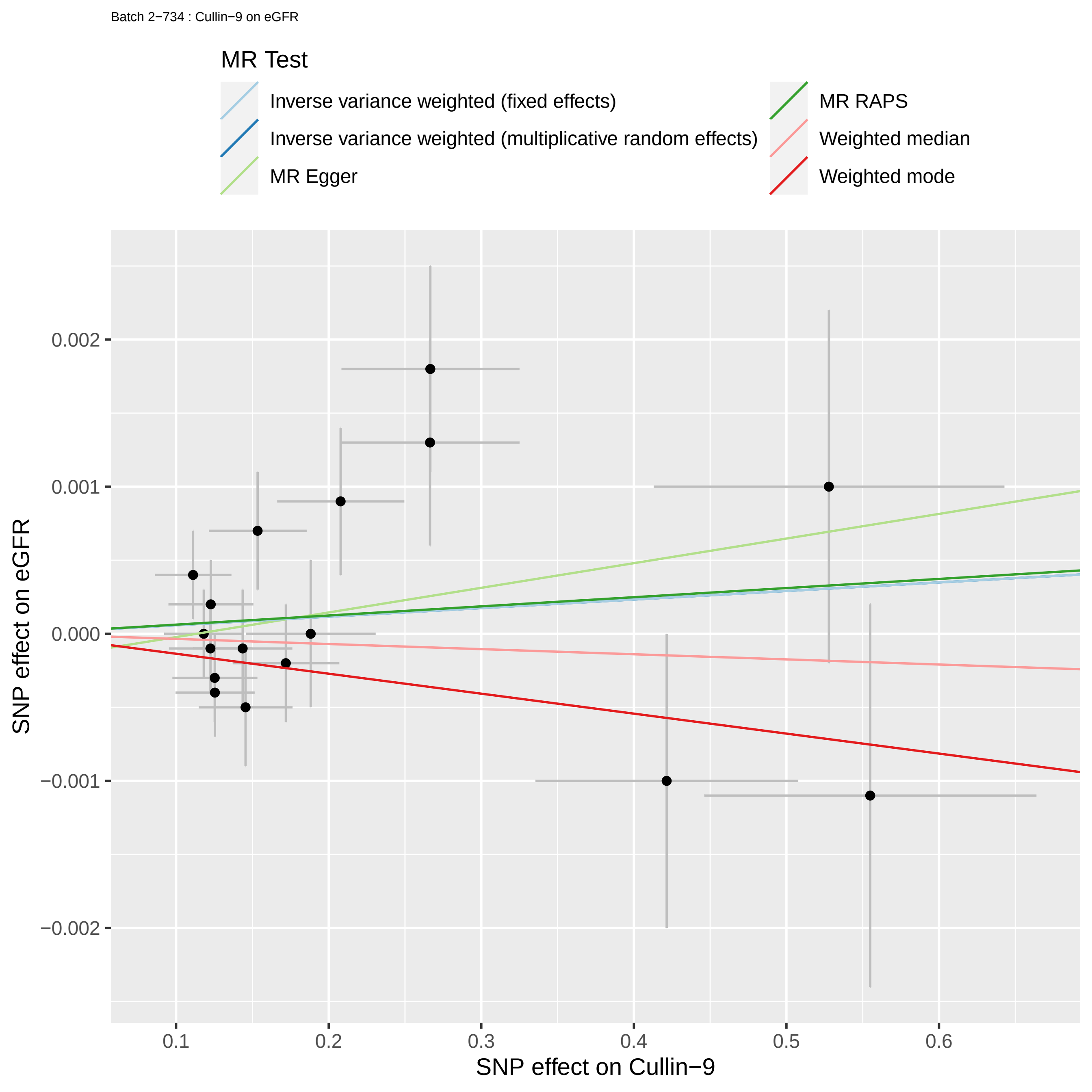

Batch 2−734 : Cullin−9 on eGFR
MR Test
Inverse variance weighted (fixed effects)
Inverse variance weighted (multiplicative random effects)
MR Egger
MR RAPS
Weighted median
Weighted mode
0.002
0.001
0.000
−0.001
−0.002
0.1
0.2
0.3
0.4
0.5
0.6
SNP effect on eGFR
SNP effect on Cullin−9

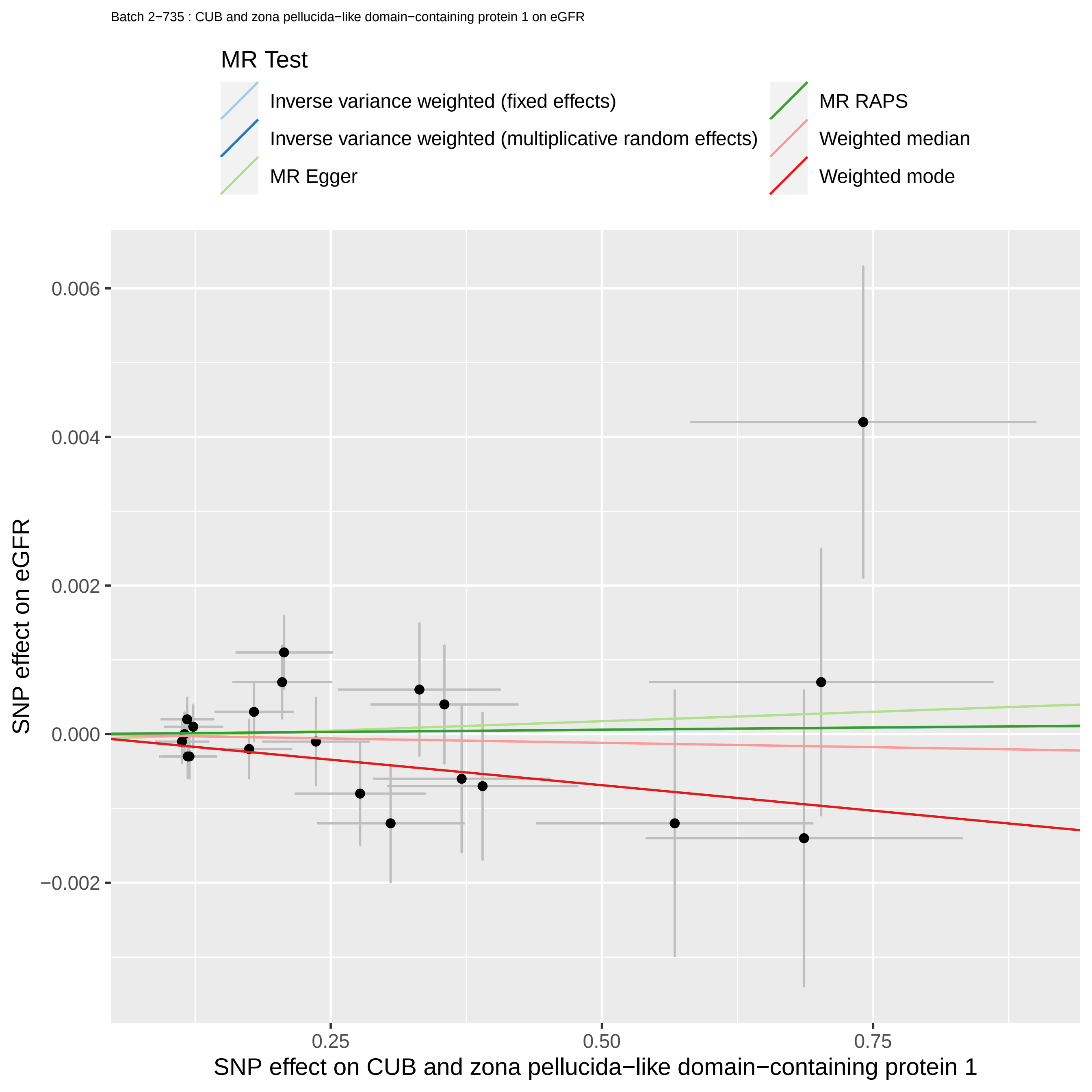

Batch 2−735 : CUB and zona pellucida−like domain−containing protein 1 on eGFR
MR Test
Inverse variance weighted (fixed effects)
Inverse variance weighted (multiplicative random effects)
MR Egger
MR RAPS
Weighted median
Weighted mode
0.006
0.004
0.002
0.000
−0.002
SNP effect on eGFR
0.25
0.50
0.75
SNP effect on CUB and zona pellucida−like domain−containing protein 1

Batch 2−736 : Fractalkine on eGFR

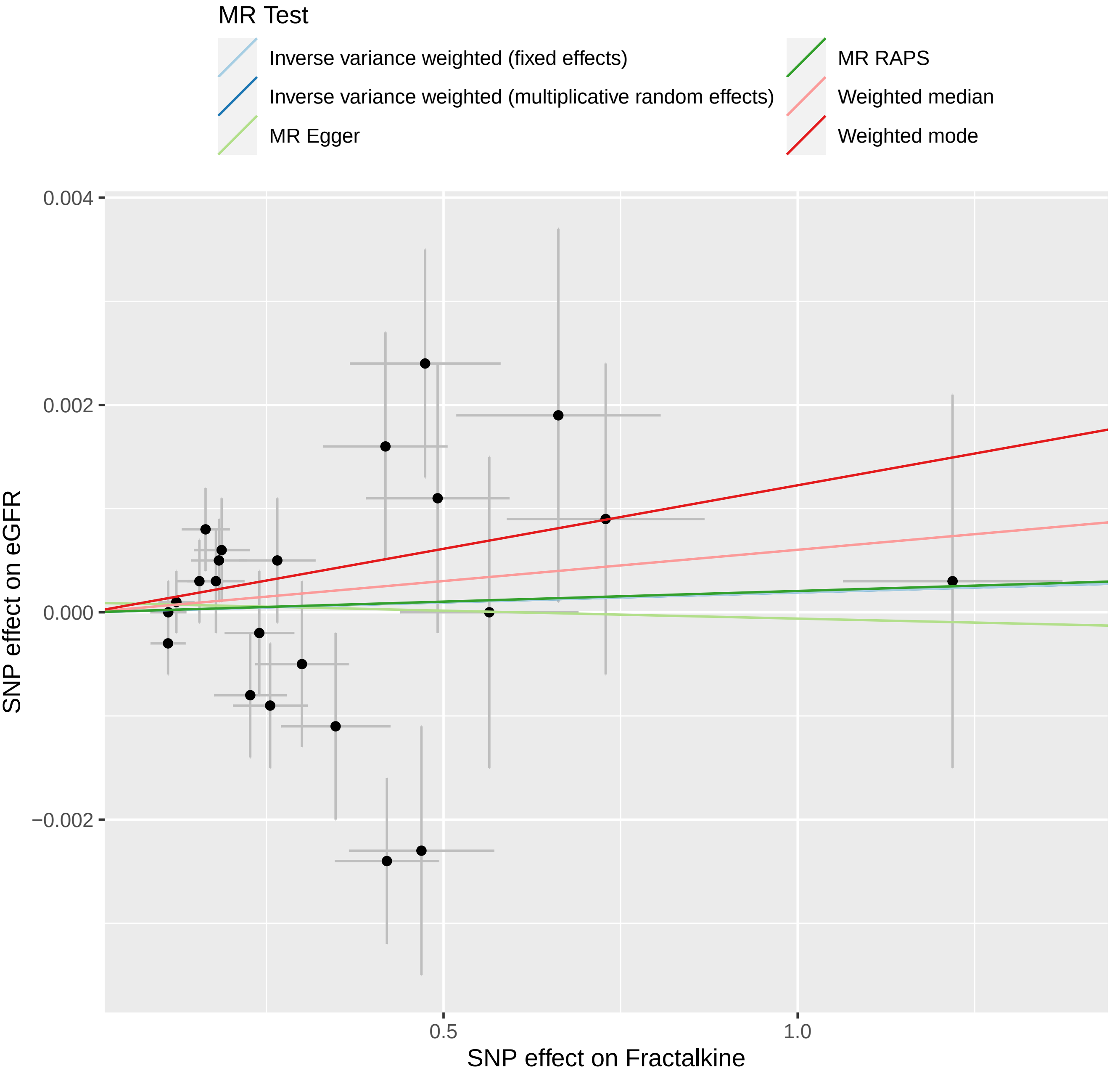

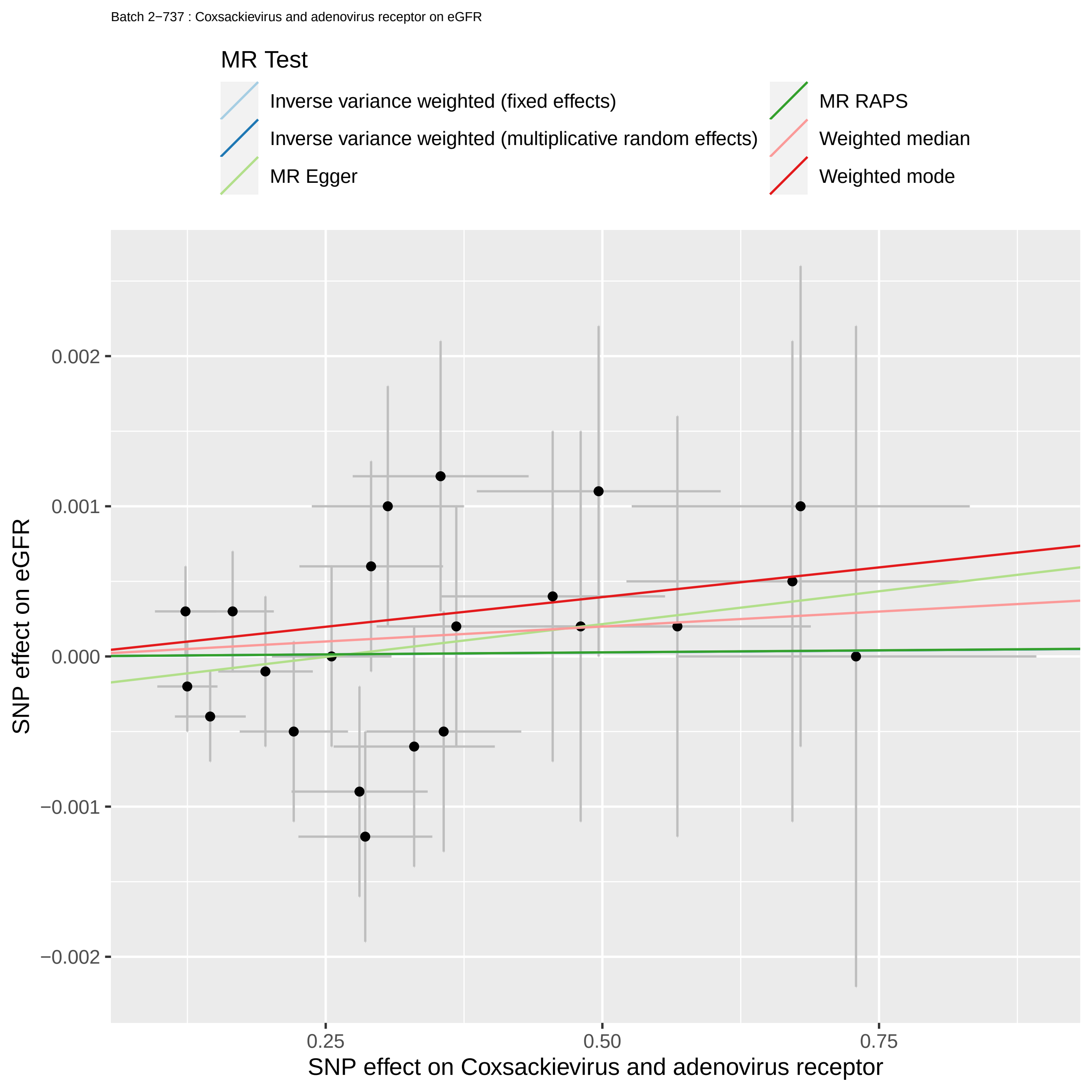
Batch 2−737 : Coxsackievirus and adenovirus receptor on eGFR
MR Test
Inverse variance weighted (fixed effects)
Inverse variance weighted (multiplicative random effects)
MR Egger
MR RAPS
Weighted median
Weighted mode
0.002
0.001
0.000
−0.001
−0.002
SNP effect on eGFR
0.25
0.50
0.75
SNP effect on Coxsackievirus and adenovirus receptor

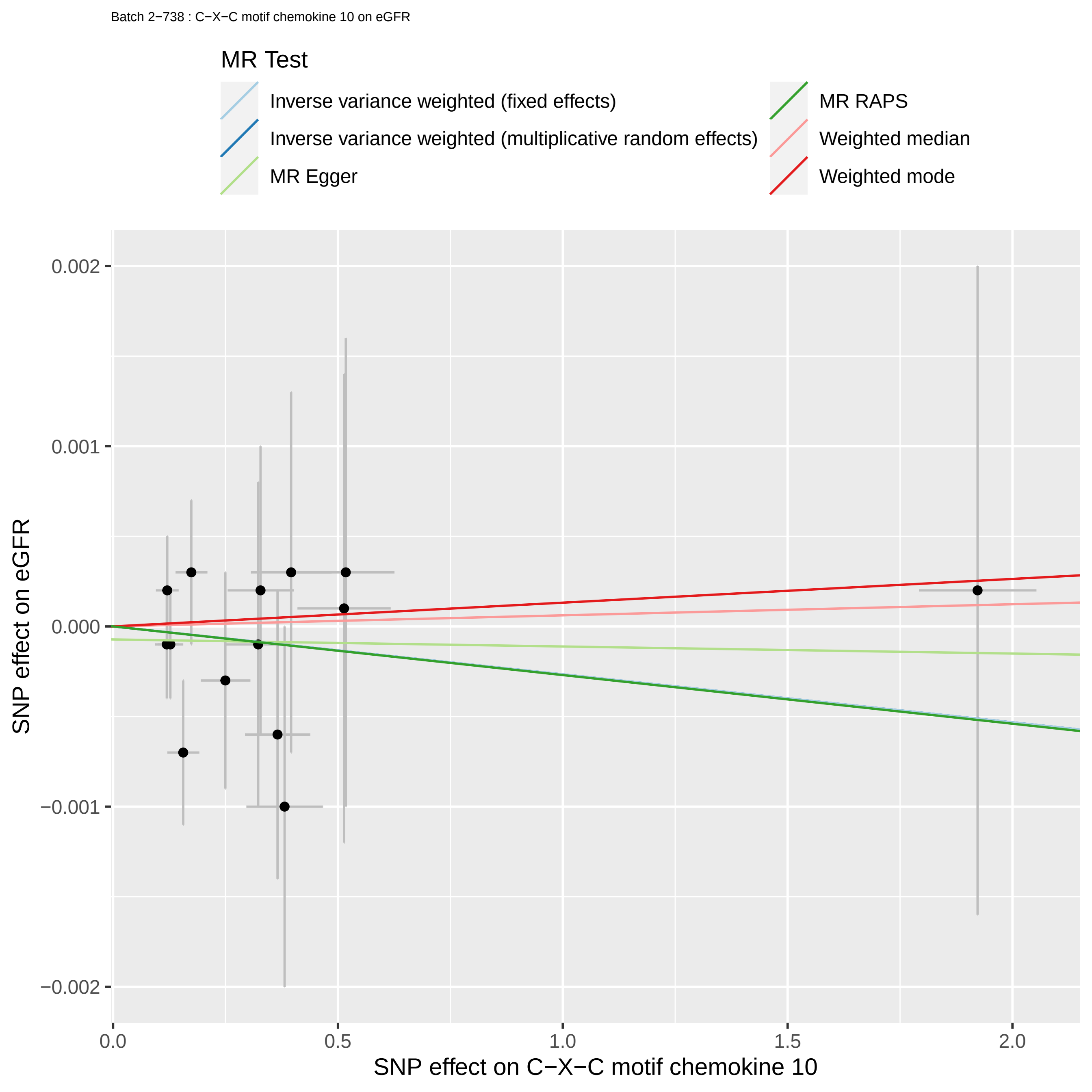

Batch 2−738 : C−X−C motif chemokine 10 on eGFR
MR Test
Inverse variance weighted (fixed effects)
Inverse variance weighted (multiplicative random effects)
MR Egger
MR RAPS
Weighted median
Weighted mode
0.002
0.001
0.000
−0.001
−0.002
SNP effect on eGFR
0.0
0.5
1.0
1.5
2.0
SNP effect on C−X−C motif chemokine 10

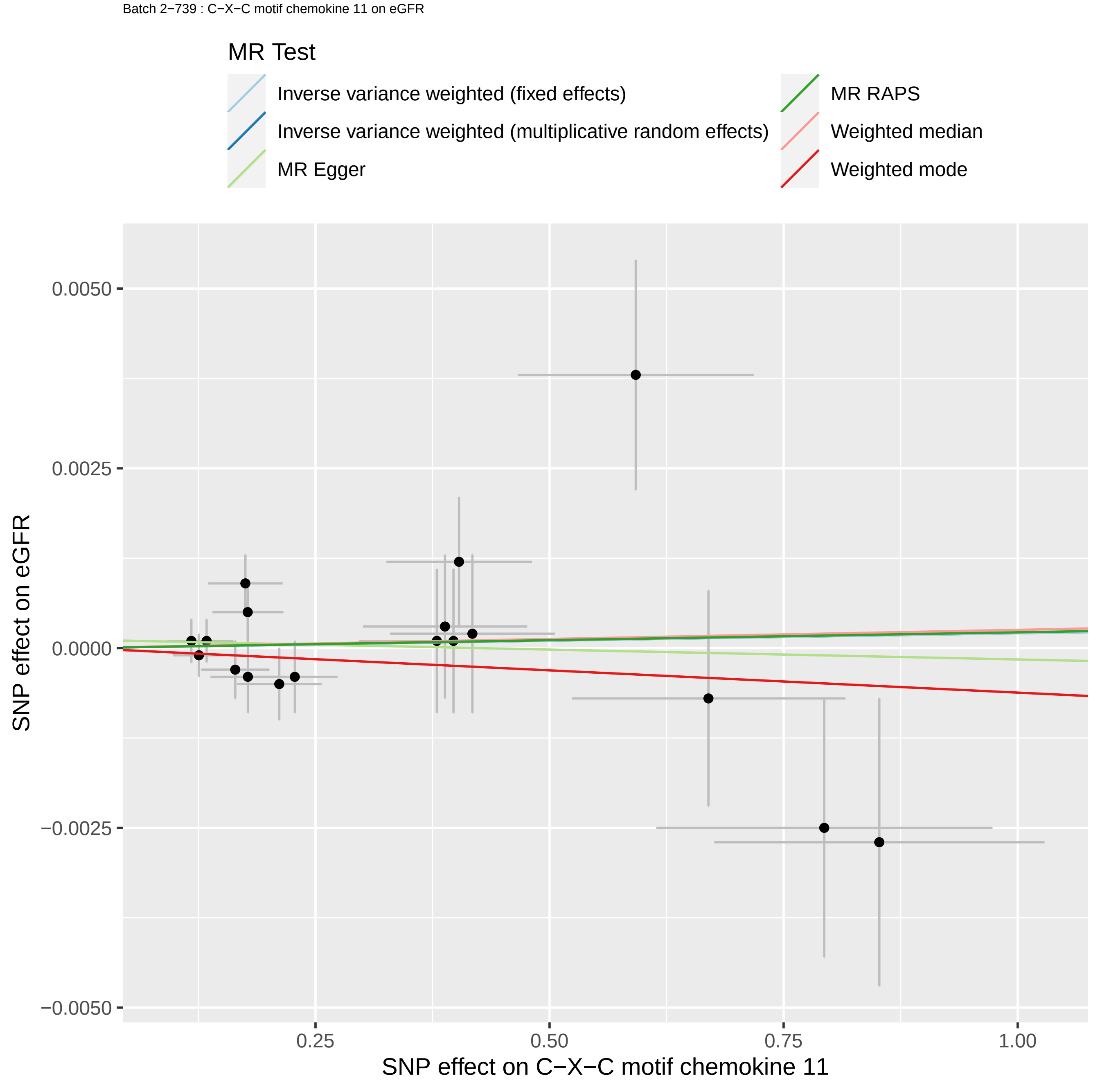

Batch 2−739 : C−X−C motif chemokine 11 on eGFR
MR Test
Inverse variance weighted (fixed effects)
Inverse variance weighted (multiplicative random effects)
MR Egger
MR RAPS
Weighted median
Weighted mode
0.0050
0.0025
0.0000
−0.0025
−0.0050
SNP effect on eGFR
0.25
0.50
0.75
1.00
SNP effect on C−X−C motif chemokine 11

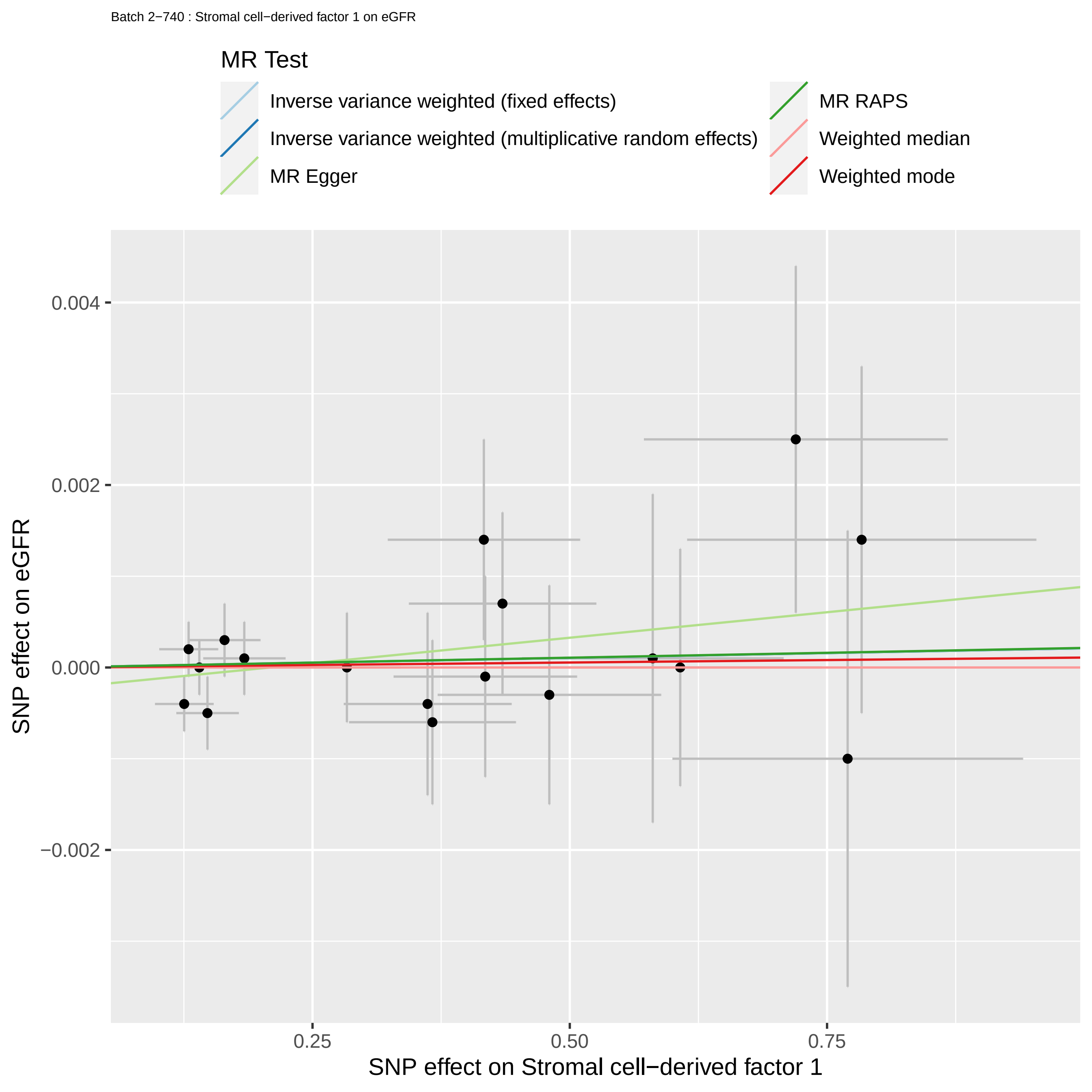

Batch 2−740 : Stromal cell−derived factor 1 on eGFR
MR Test
Inverse variance weighted (fixed effects)
Inverse variance weighted (multiplicative random effects)
MR Egger
MR RAPS
Weighted median
Weighted mode
0.004
0.002
0.000
−0.002
0.25
0.50
0.75
SNP effect on eGFR
SNP effect on Stromal cell−derived factor 1

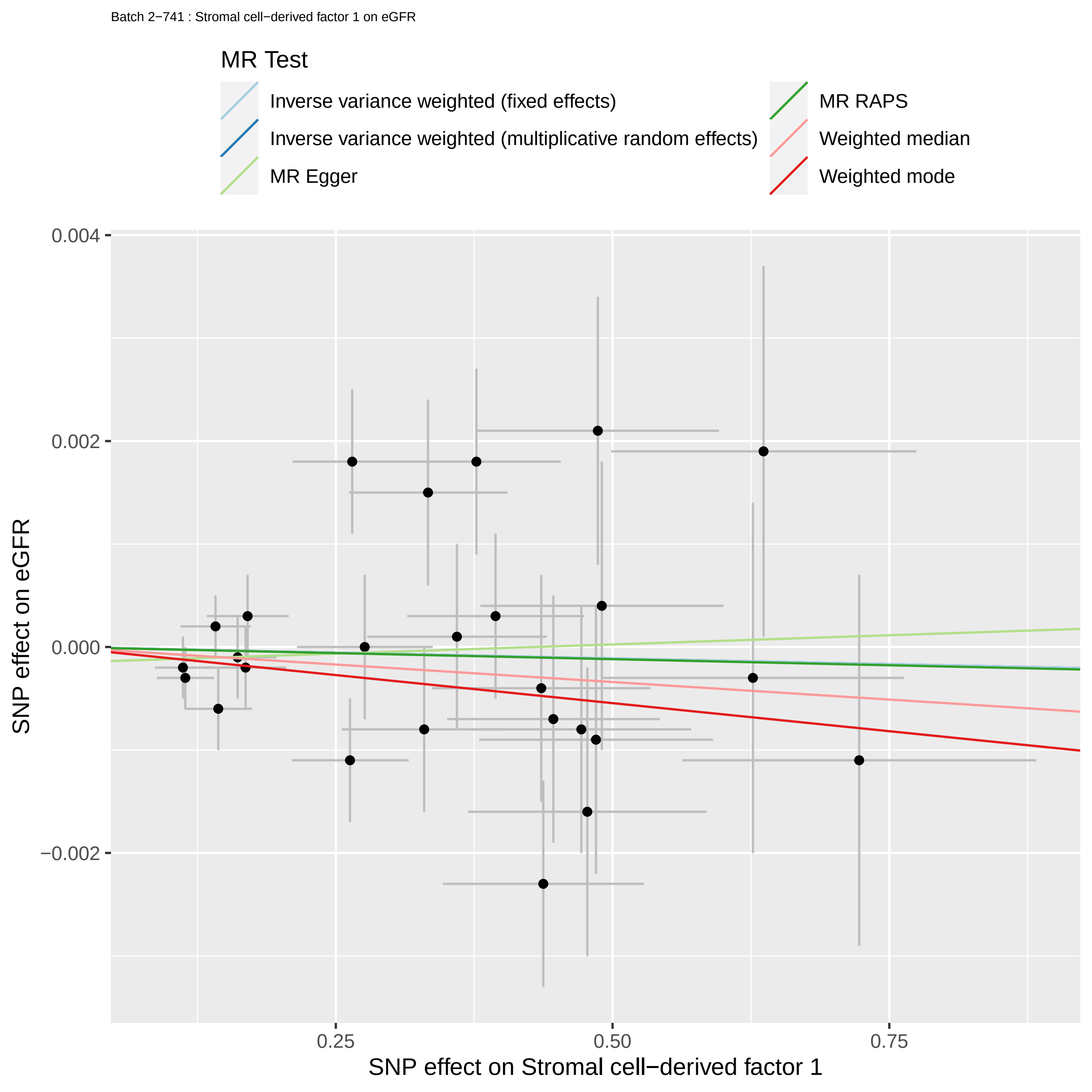
Batch 2−741 : Stromal cell−derived factor 1 on eGFR
MR Test
Inverse variance weighted (fixed effects)
Inverse variance weighted (multiplicative random effects)
MR Egger
MR RAPS
Weighted median
Weighted mode
0.004
0.002
0.000
−0.002
SNP effect on eGFR
0.25
0.50
0.75
SNP effect on Stromal cell−derived factor 1

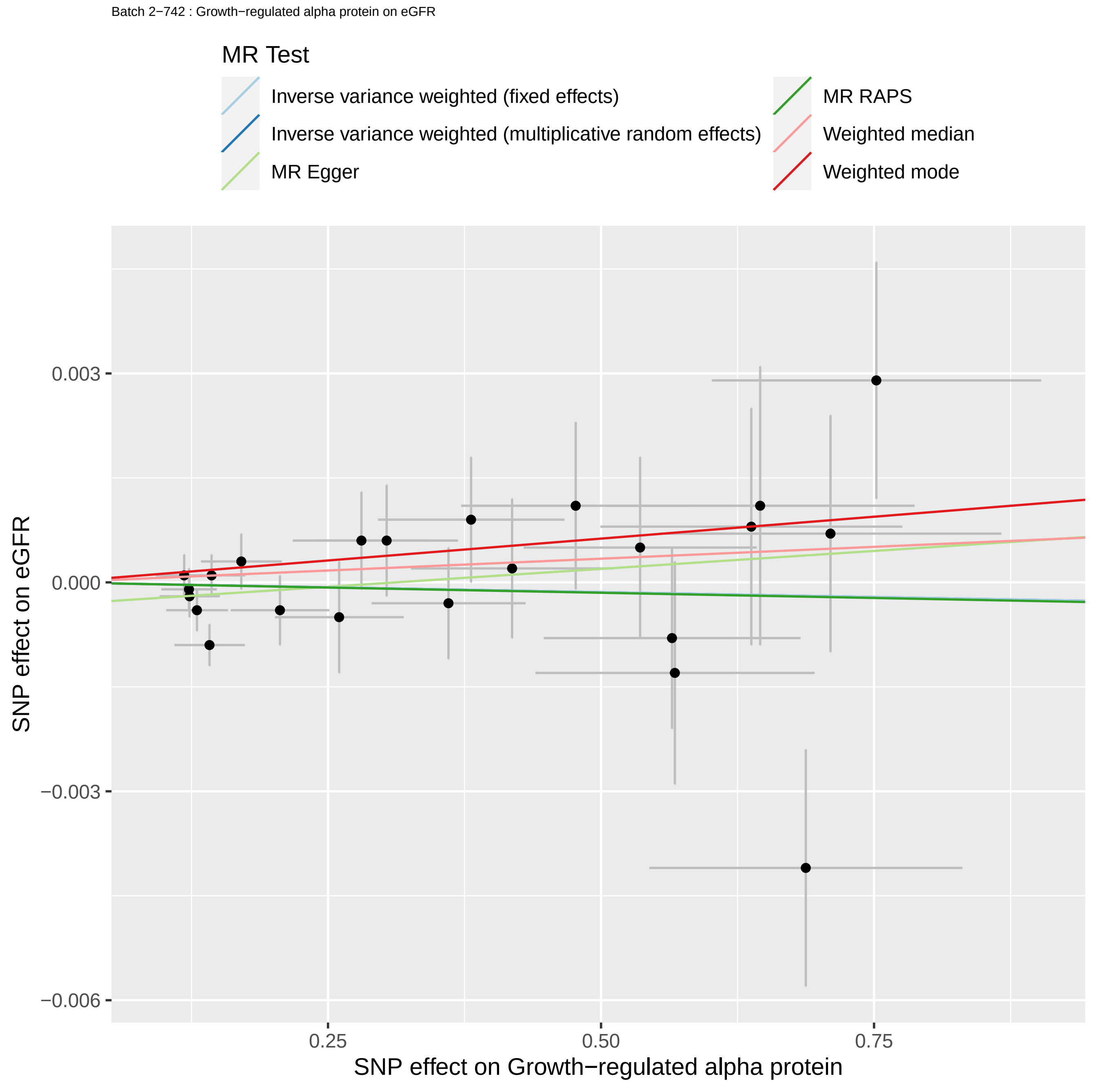

Batch 2−742 : Growth−regulated alpha protein on eGFR
MR Test
Inverse variance weighted (fixed effects)
Inverse variance weighted (multiplicative random effects)
MR Egger
MR RAPS
Weighted median
Weighted mode
0.003
0.000
−0.003
−0.006
0.25
0.50
0.75
SNP effect on eGFR
SNP effect on Growth−regulated alpha protein

Batch 2−743 : C−X−C motif chemokine 13 on eGFR

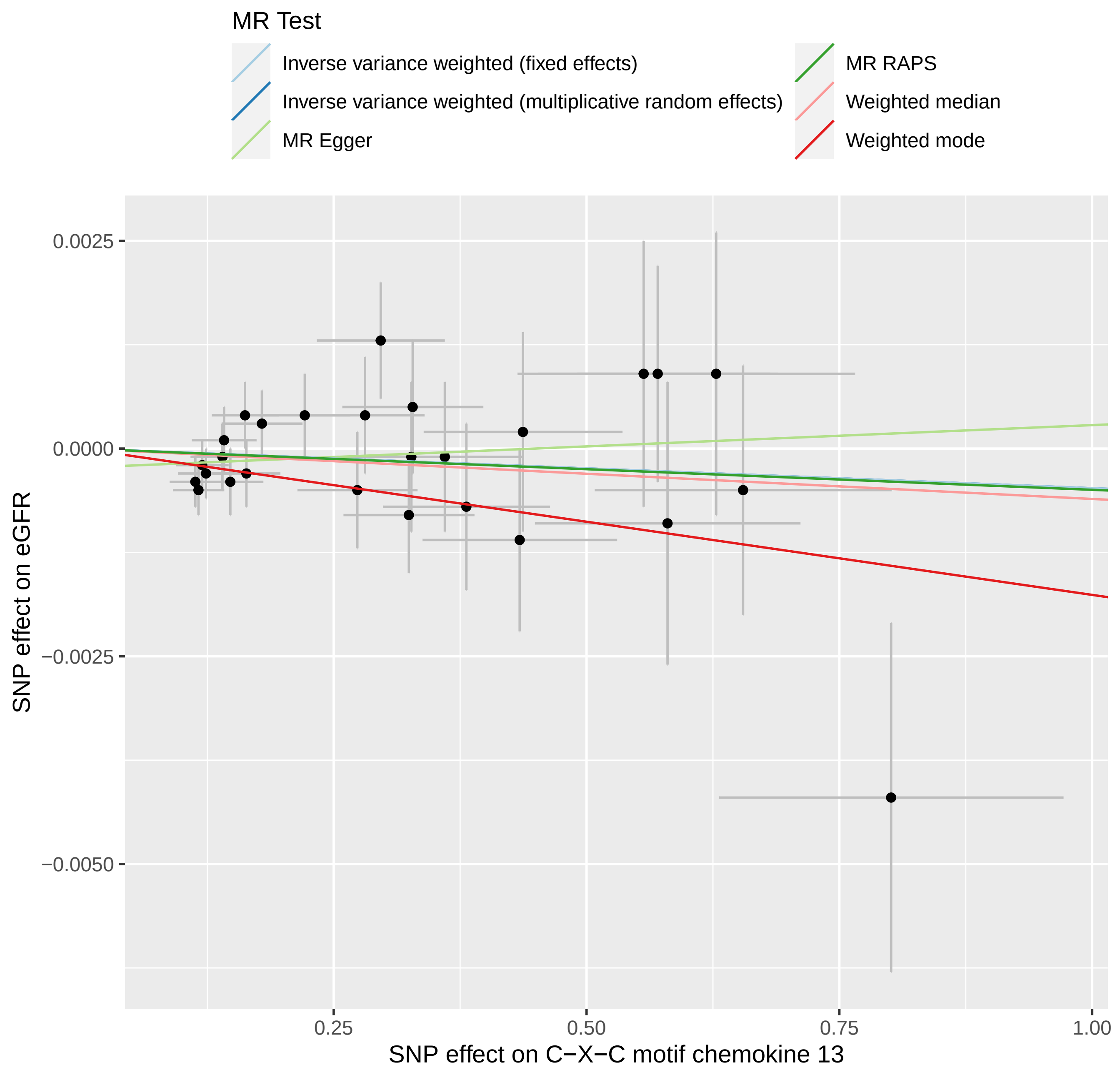

Batch 2−744 : C−X−C motif chemokine 14 on eGFR

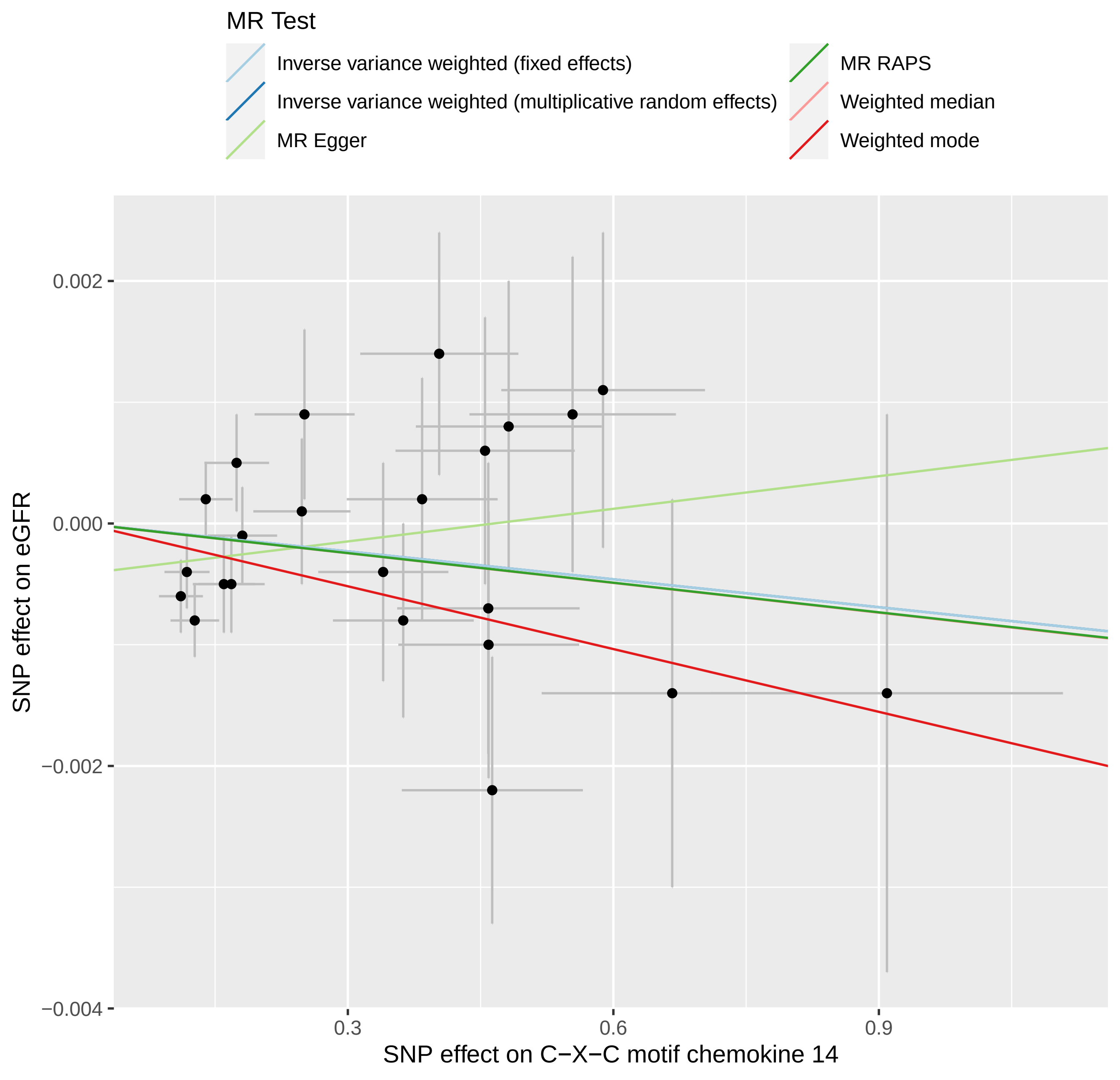

Batch 2−745 : C−X−C motif chemokine 16 on eGFR

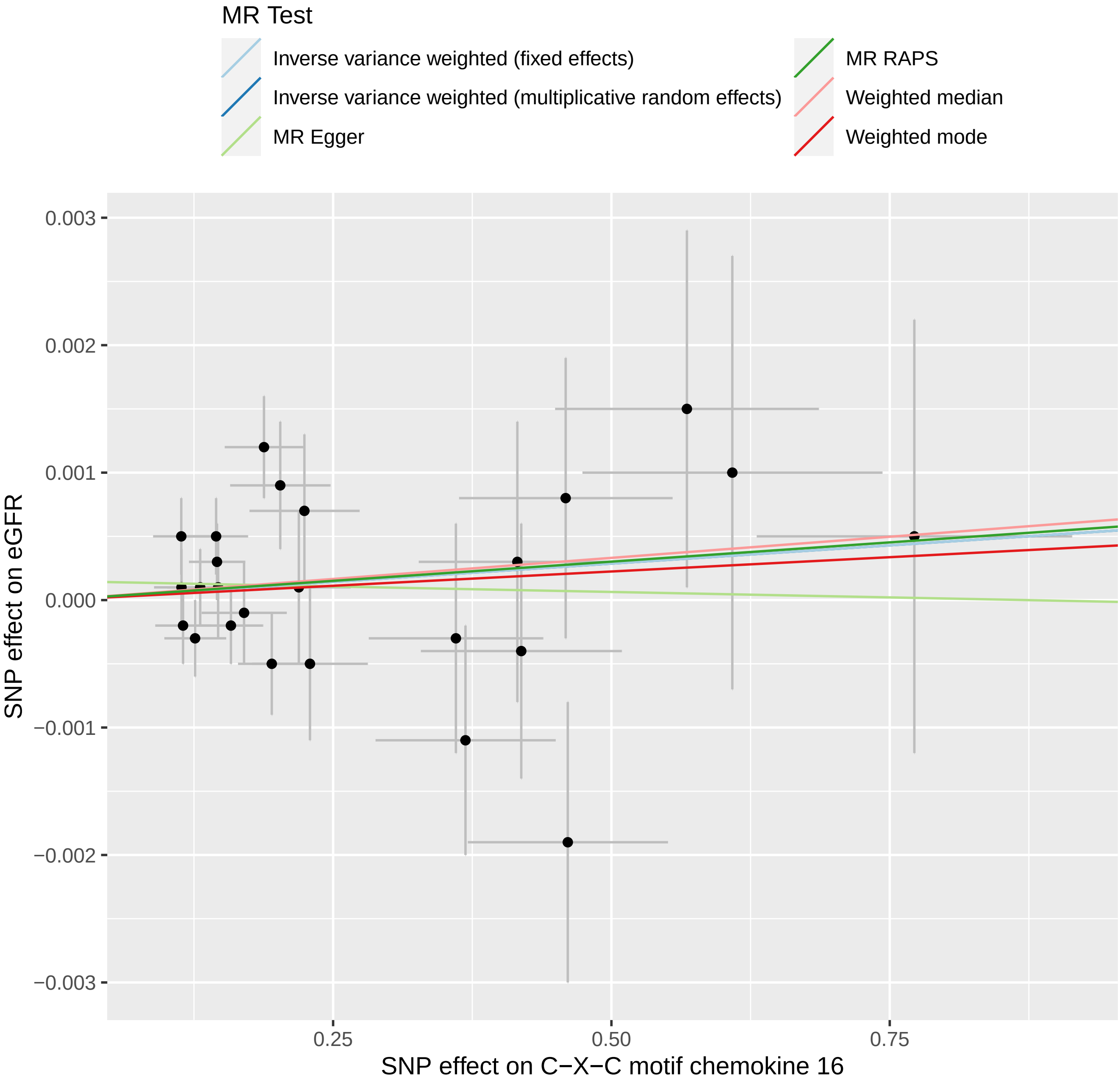

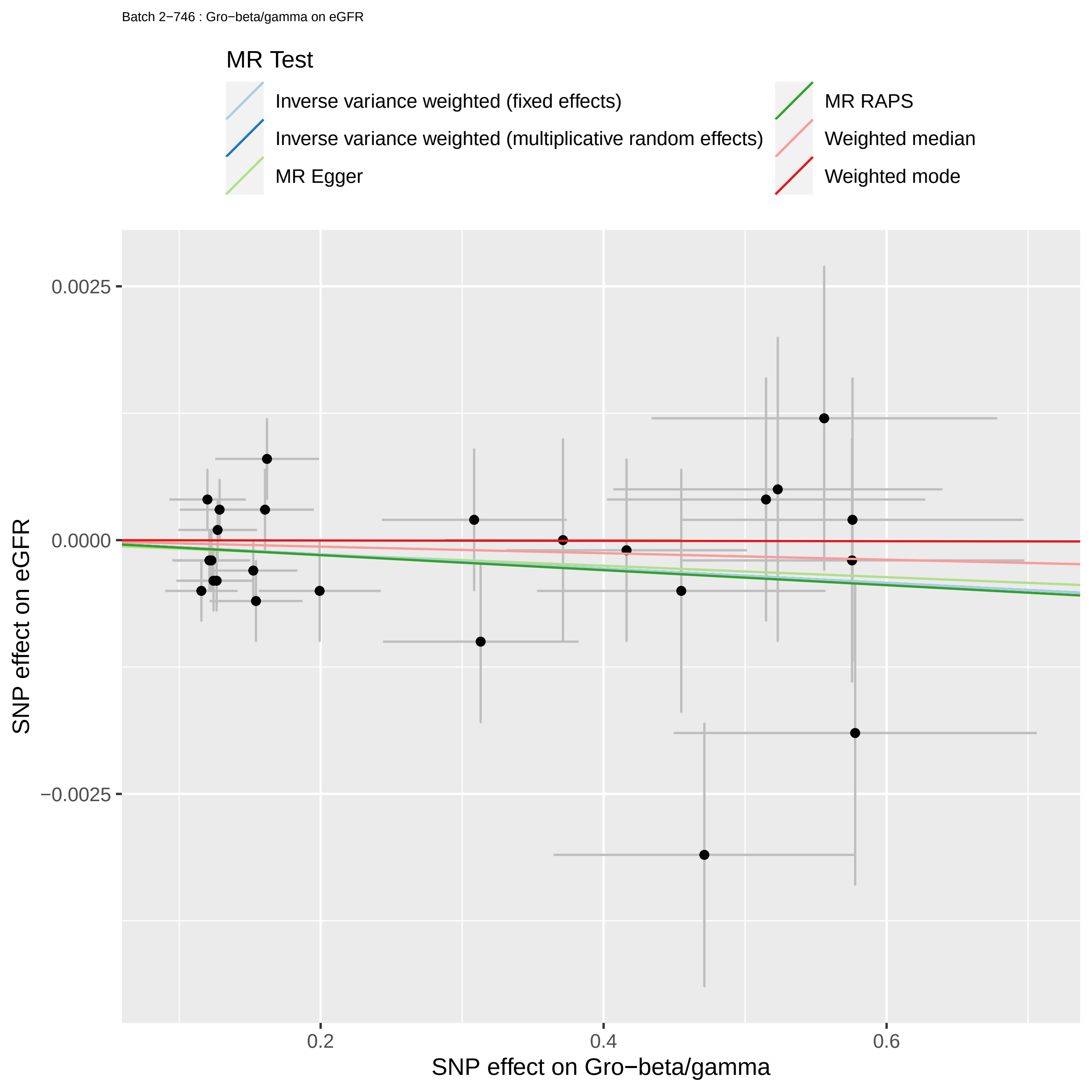
Batch 2−746 : Gro−beta/gamma on eGFR
MR Test
Inverse variance weighted (fixed effects)
Inverse variance weighted (multiplicative random effects)
MR Egger
MR RAPS
Weighted median
Weighted mode
0.0025
0.0000
−0.0025
SNP effect on eGFR
0.2
0.4
0.6
SNP effect on Gro−beta/gamma

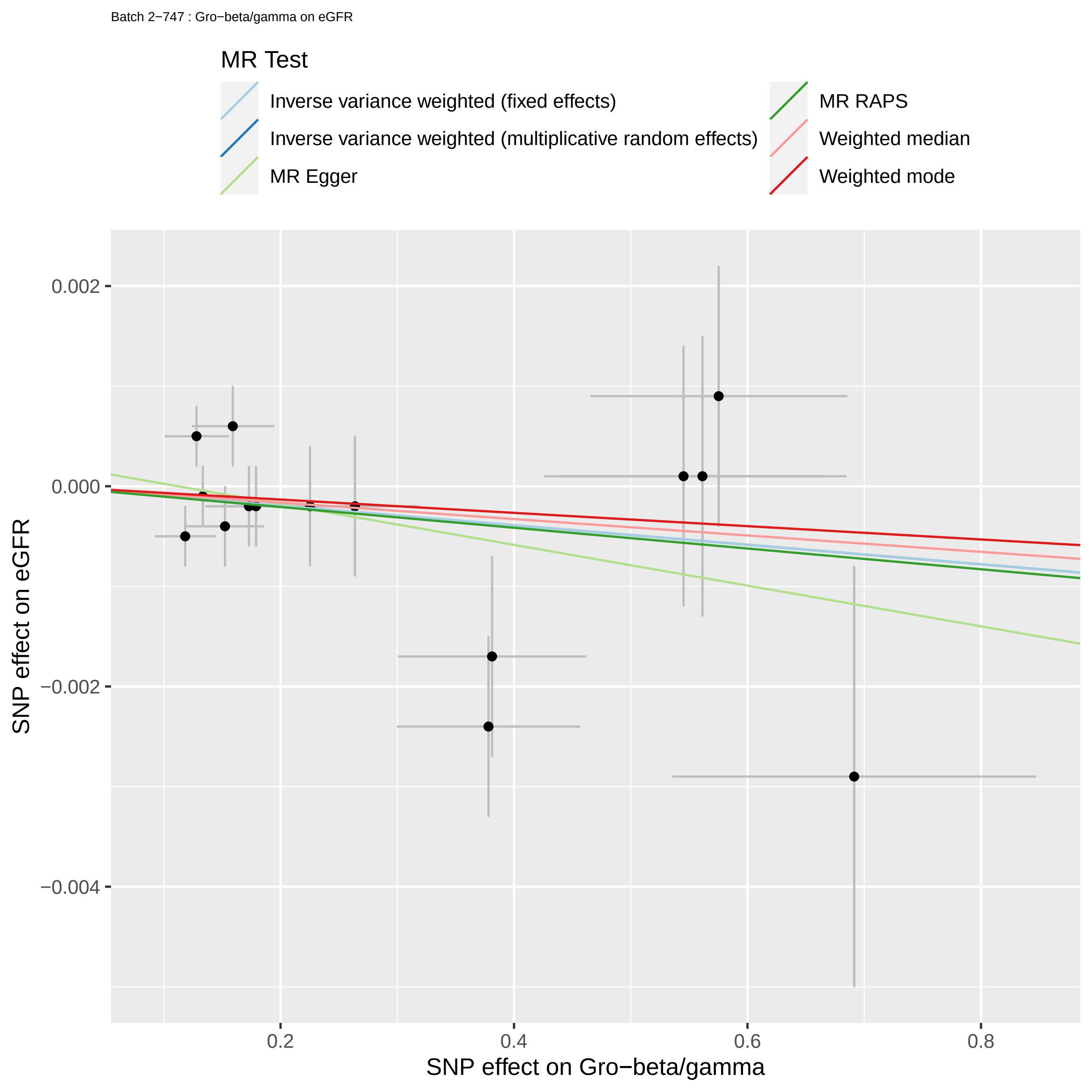

Batch 2−747 : Gro−beta/gamma on eGFR
MR Test
Inverse variance weighted (fixed effects)
Inverse variance weighted (multiplicative random effects)
MR Egger
MR RAPS
Weighted median
Weighted mode
0.002
0.000
−0.002
−0.004
0.2
0.4
0.6
0.8
SNP effect on eGFR
SNP effect on Gro−beta/gamma

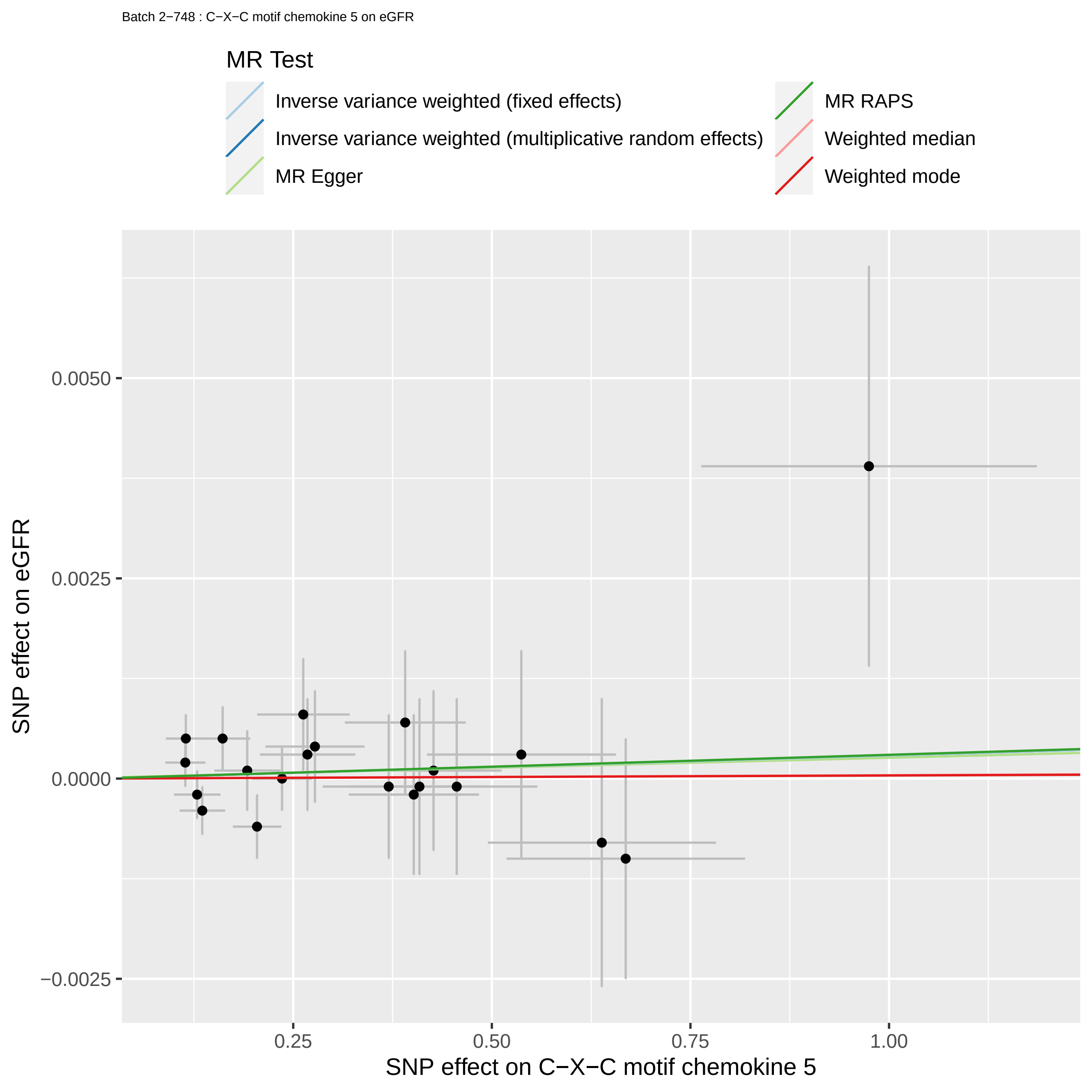

Batch 2−748 : C−X−C motif chemokine 5 on eGFR
MR Test
Inverse variance weighted (fixed effects)
Inverse variance weighted (multiplicative random effects)
MR Egger
MR RAPS
Weighted median
Weighted mode
0.0050
0.0025
0.0000
−0.0025
0.25
0.50
0.75
1.00
SNP effect on eGFR
SNP effect on C−X−C motif chemokine 5

Batch 2−749 : Interleukin−8 on eGFR

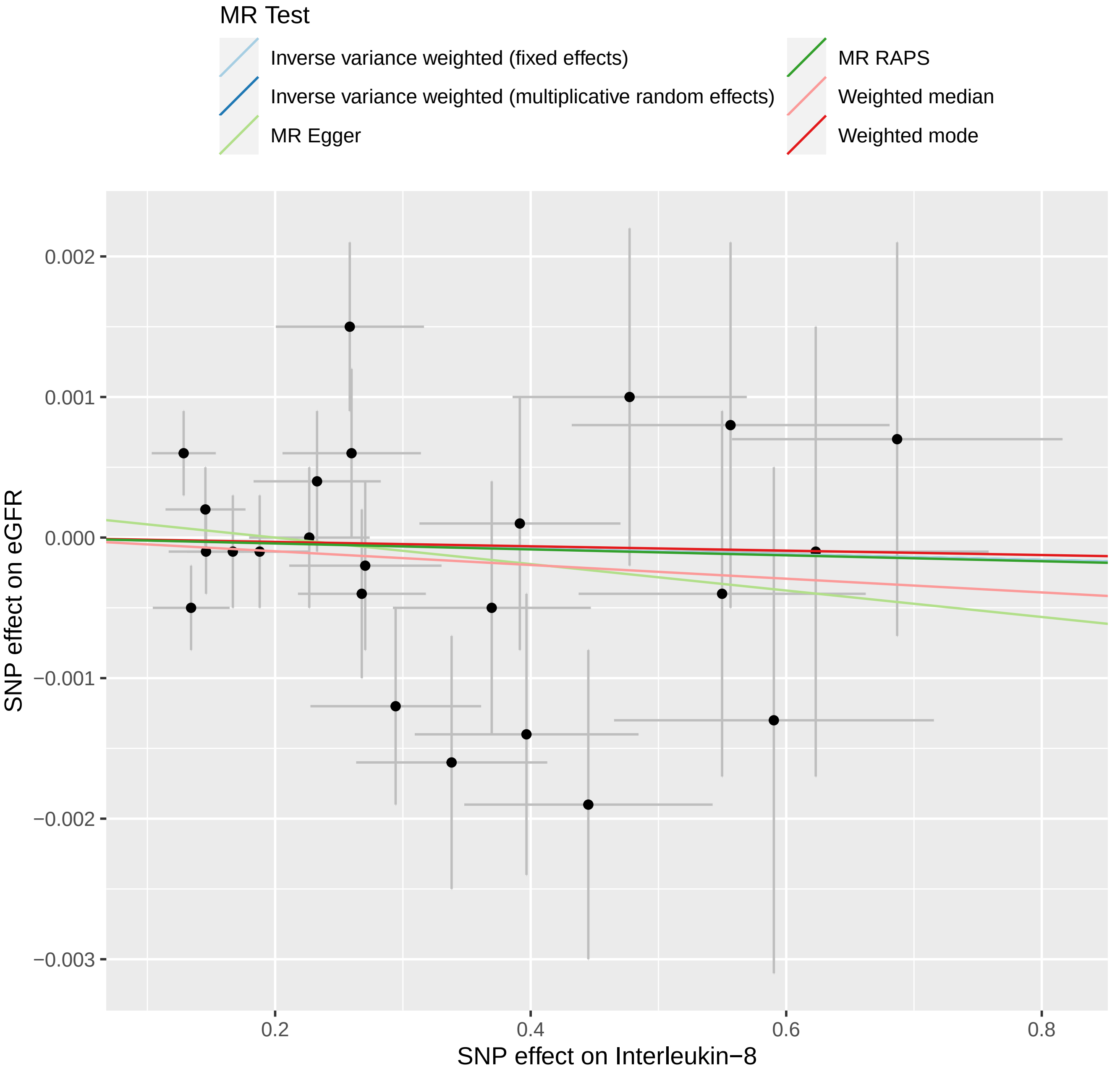

Batch 2−750 : C−X−C motif chemokine 9 on eGFR

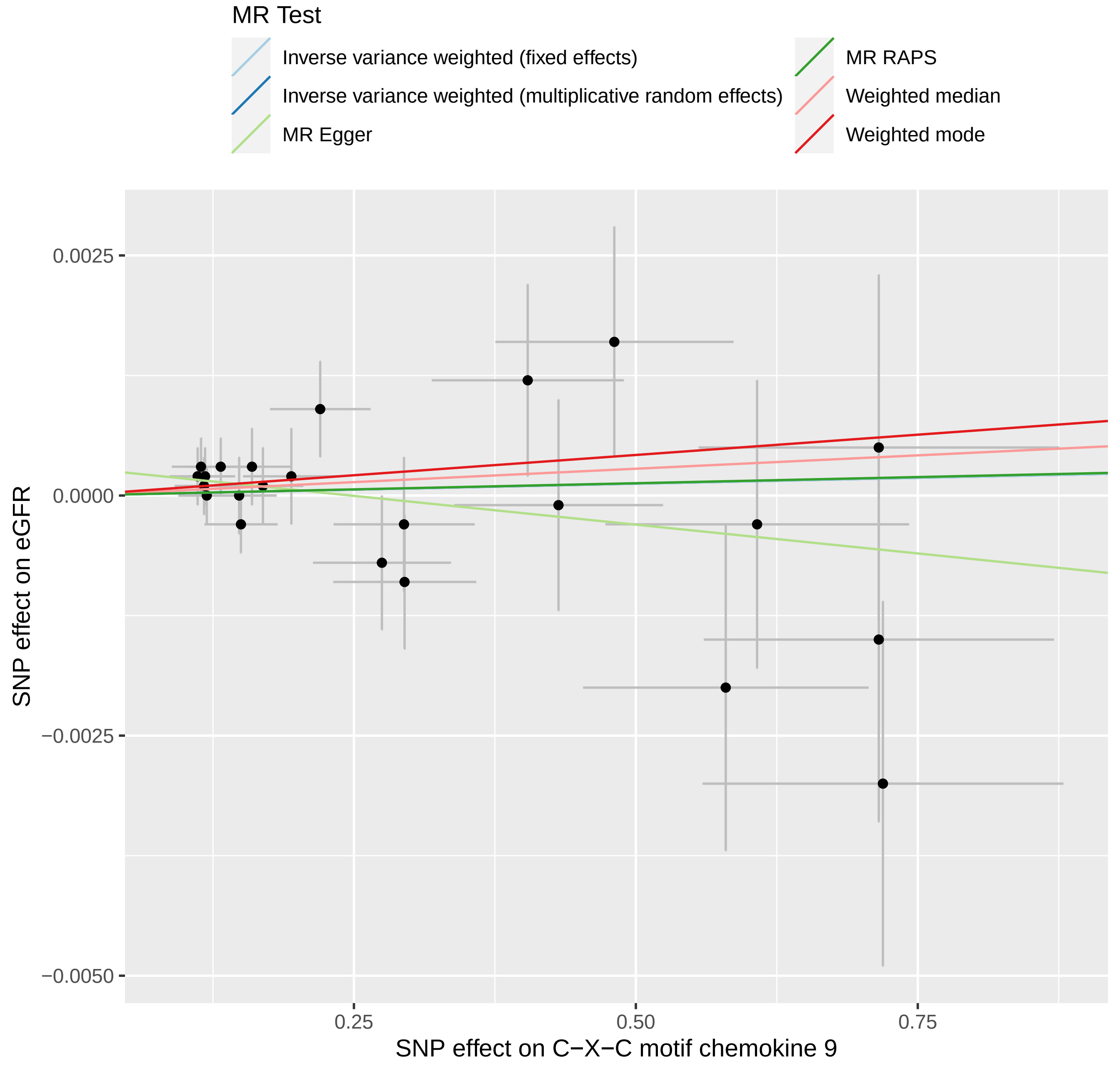

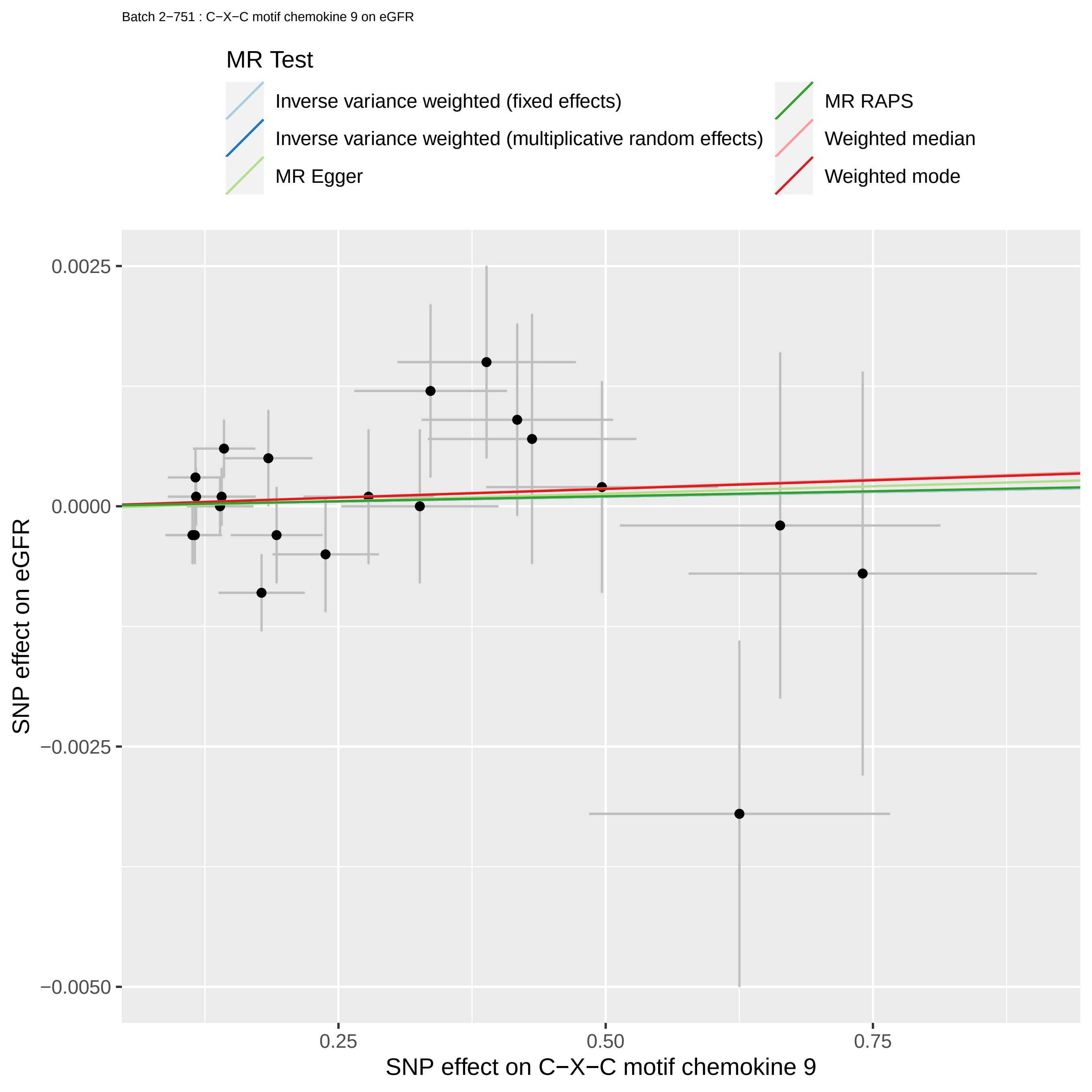
Batch 2−751 : C−X−C motif chemokine 9 on eGFR
MR Test
Inverse variance weighted (fixed effects)
Inverse variance weighted (multiplicative random effects)
MR Egger
MR RAPS
Weighted median
Weighted mode
0.0025
0.0000
−0.0025
−0.0050
0.25
0.50
0.75
SNP effect on eGFR
SNP effect on C−X−C motif chemokine 9

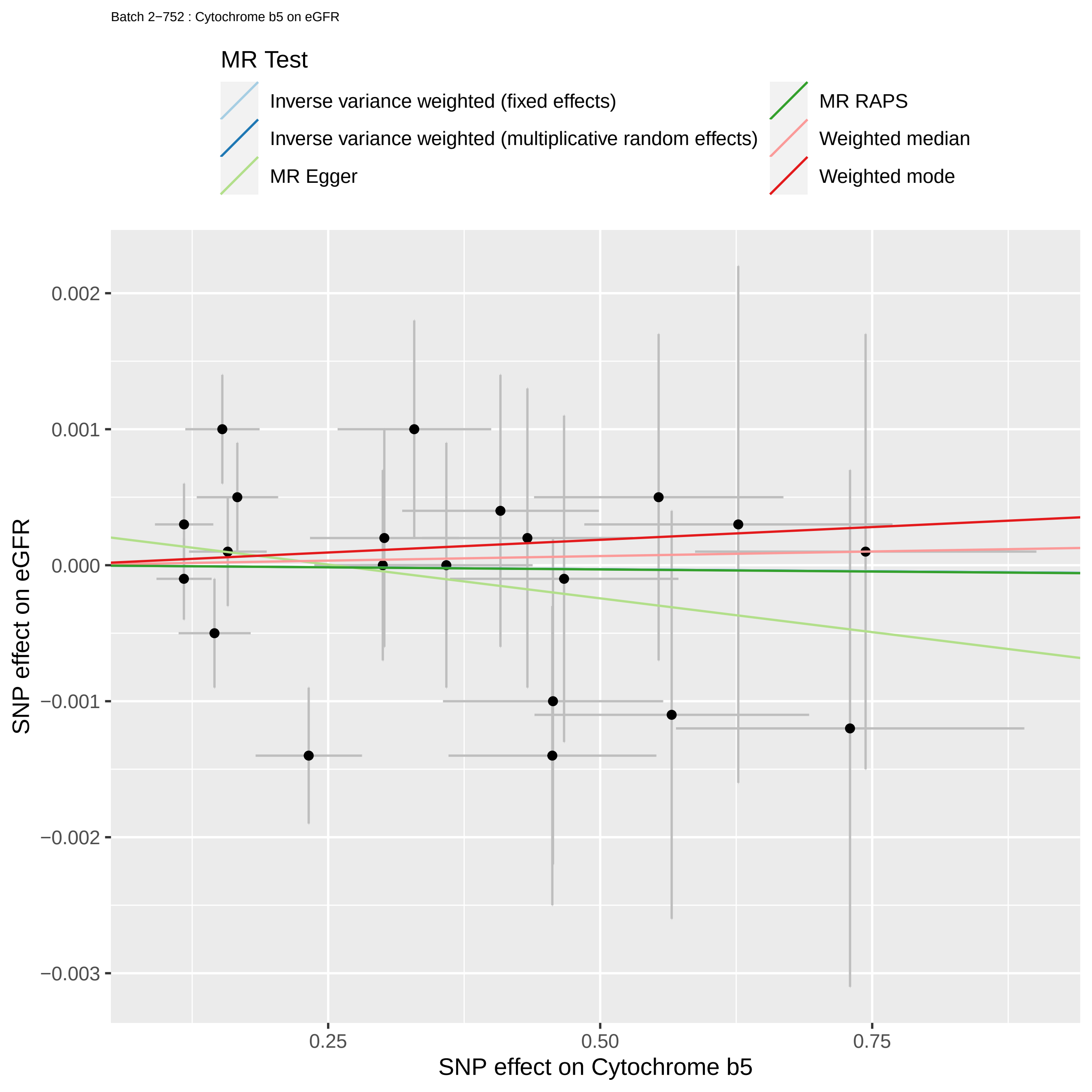

Batch 2−752 : Cytochrome b5 on eGFR
MR Test
Inverse variance weighted (fixed effects)
Inverse variance weighted (multiplicative random effects)
MR Egger
MR RAPS
Weighted median
Weighted mode
0.002
0.001
0.000
−0.001
−0.002
−0.003
SNP effect on eGFR
0.25
0.50
0.75
SNP effect on Cytochrome b5

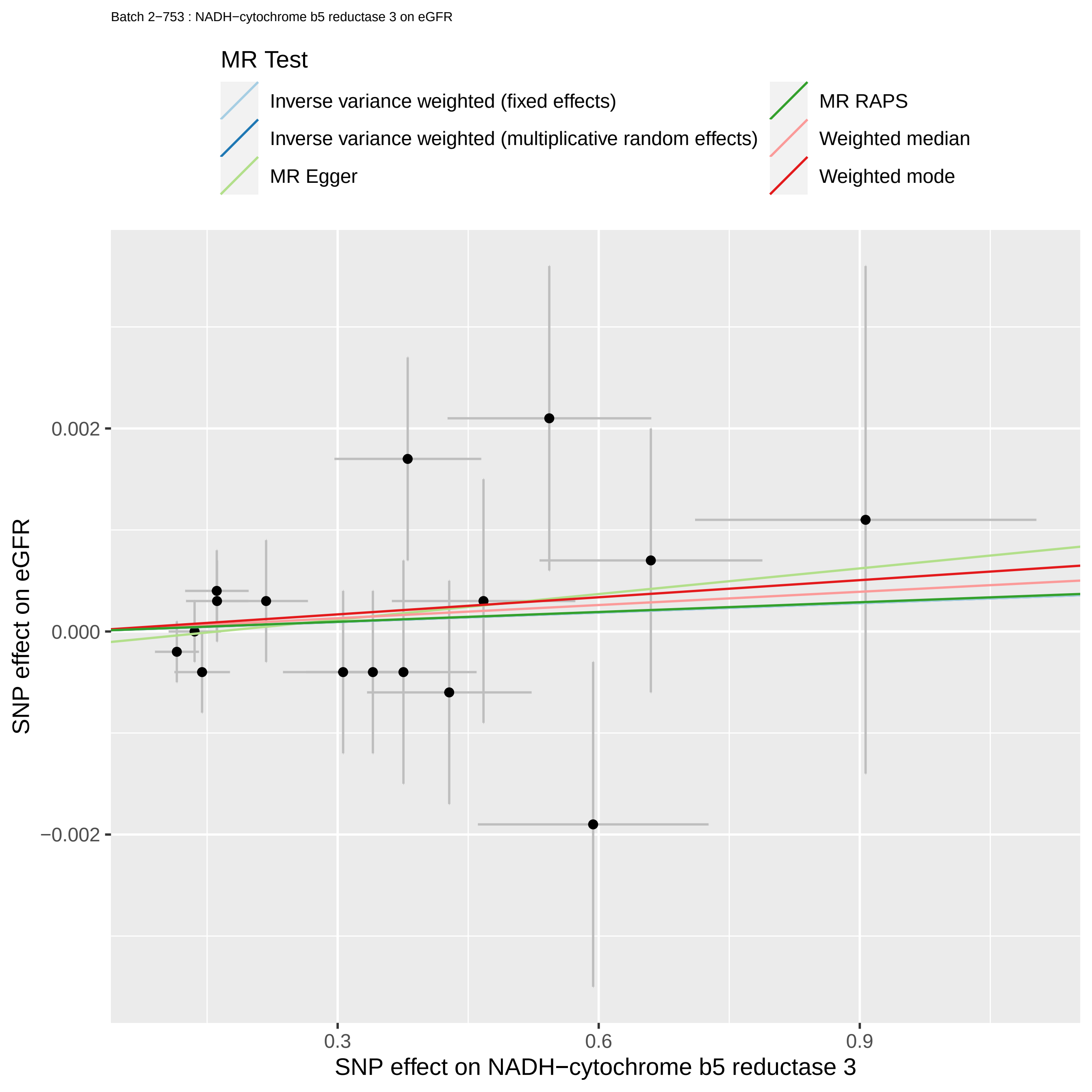

Batch 2−753 : NADH−cytochrome b5 reductase 3 on eGFR
MR Test
Inverse variance weighted (fixed effects)
Inverse variance weighted (multiplicative random effects)
MR Egger
MR RAPS
Weighted median
Weighted mode
0.002
0.000
−0.002
0.3
0.6
0.9
SNP effect on eGFR
SNP effect on NADH−cytochrome b5 reductase 3

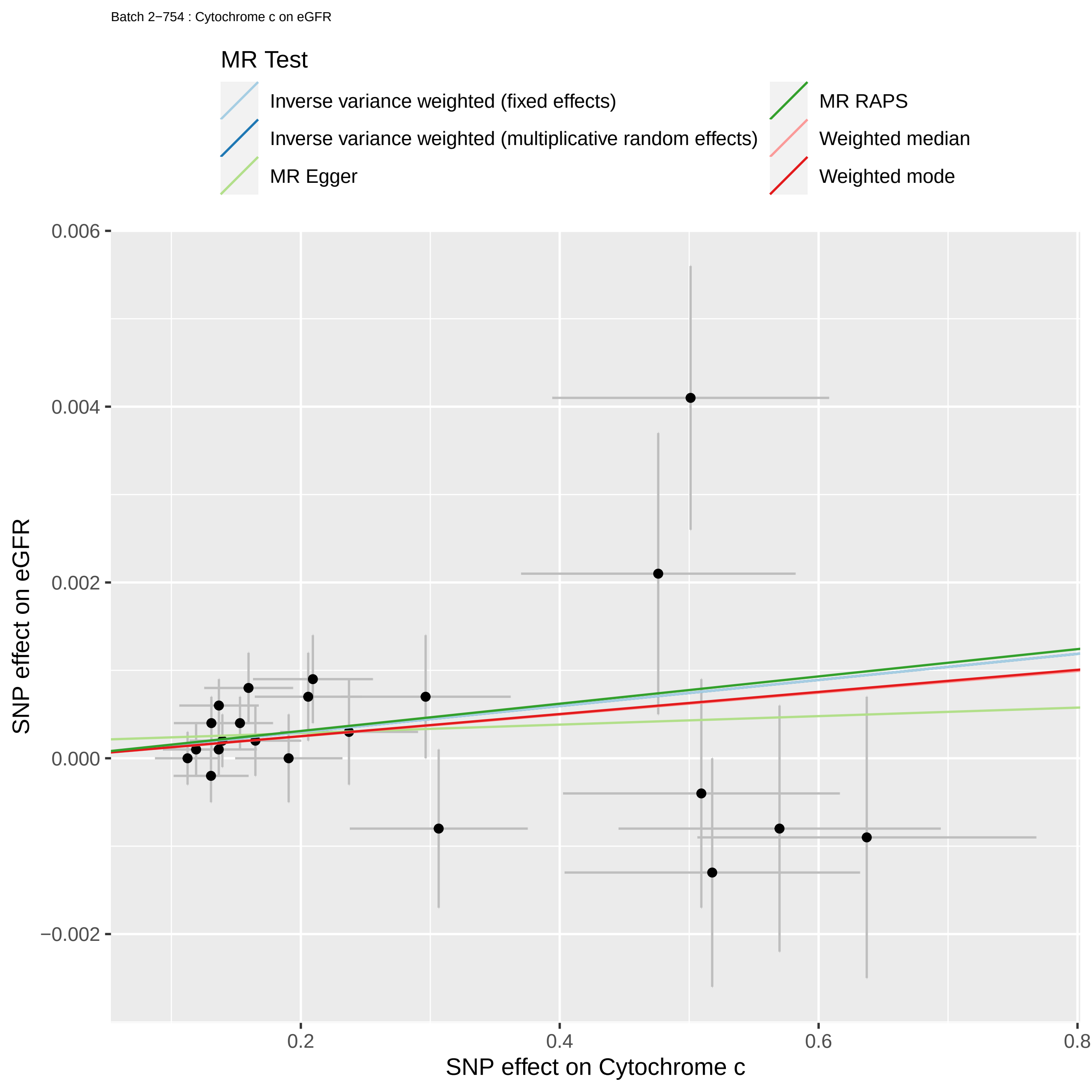

Batch 2−754 : Cytochrome c on eGFR
MR Test
Inverse variance weighted (fixed effects)
Inverse variance weighted (multiplicative random effects)
MR Egger
MR RAPS
Weighted median
Weighted mode
0.006
0.004
0.002
0.000
−0.002
0.2
0.4
0.6
0.8
SNP effect on Cytochrome c
SNP effect on eGFR

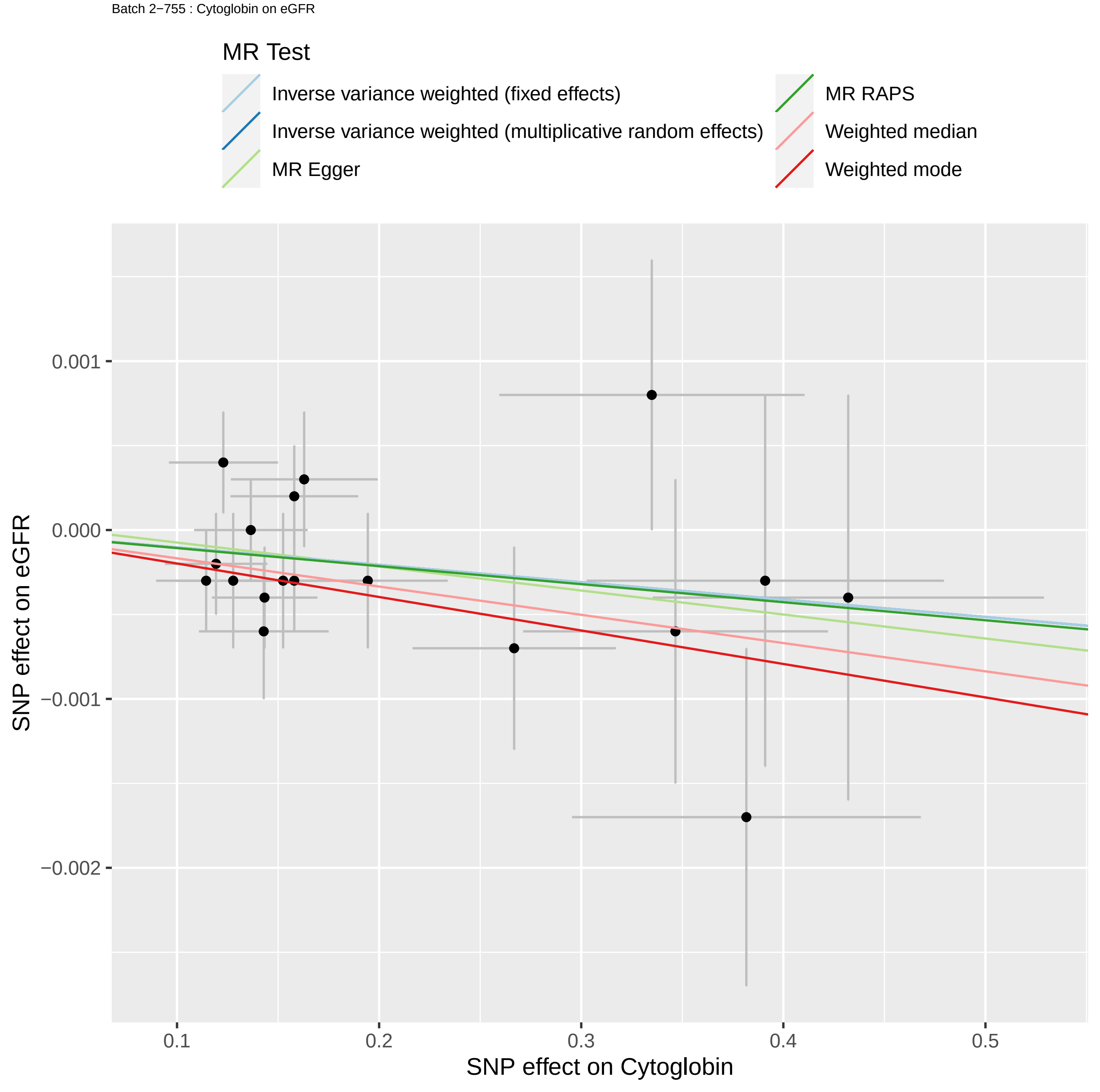
Batch 2−755 : Cytoglobin on eGFR
MR Test
Inverse variance weighted (fixed effects)
Inverse variance weighted (multiplicative random effects)
MR Egger
MR RAPS
Weighted median
Weighted mode
0.001
0.000
−0.001
−0.002
0.1
0.2
0.3
0.4
0.5
SNP effect on eGFR
SNP effect on Cytoglobin

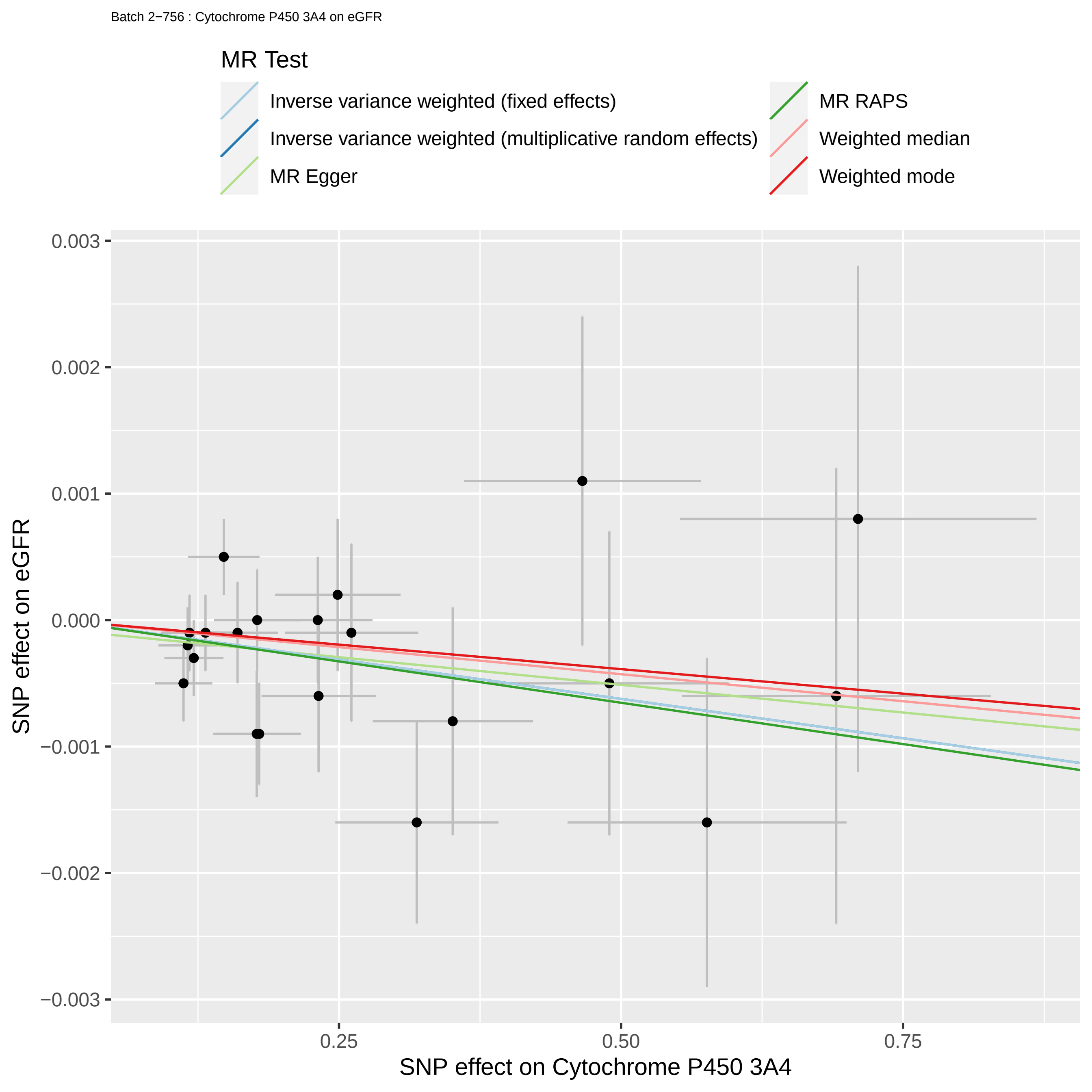

Batch 2−756 : Cytochrome P450 3A4 on eGFR
MR Test
Inverse variance weighted (fixed effects)
Inverse variance weighted (multiplicative random effects)
MR Egger
MR RAPS
Weighted median
Weighted mode
0.003
0.002
0.001
0.000
−0.001
−0.002
−0.003
SNP effect on eGFR
0.25
0.50
0.75
SNP effect on Cytochrome P450 3A4

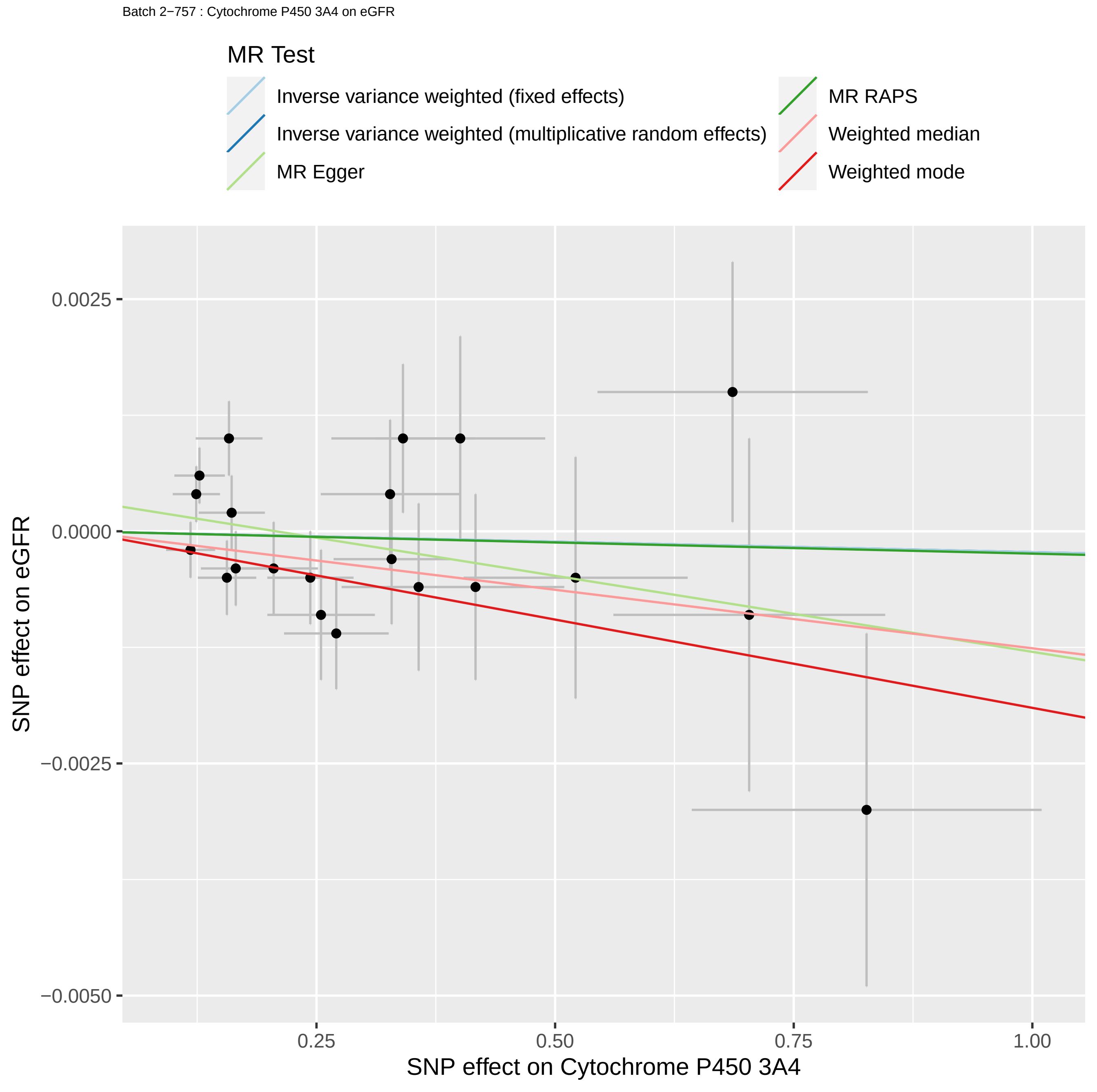
Batch 2−757 : Cytochrome P450 3A4 on eGFR
MR Test
Inverse variance weighted (fixed effects)
Inverse variance weighted (multiplicative random effects)
MR Egger
MR RAPS
Weighted median
Weighted mode
0.0025
0.0000
−0.0025
−0.0050
0.25
0.50
0.75
1.00
SNP effect on eGFR
SNP effect on Cytochrome P450 3A4

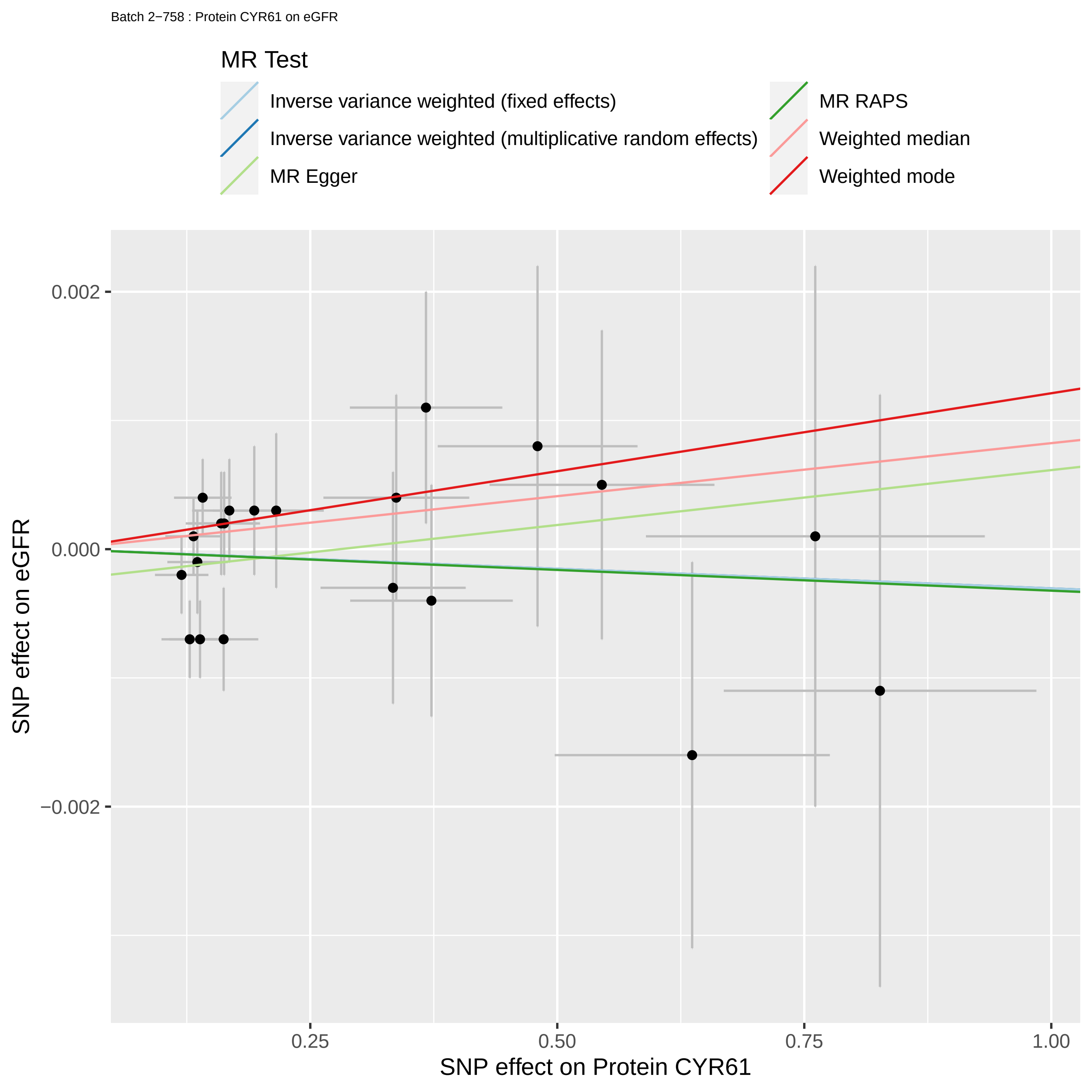

Batch 2−758 : Protein CYR61 on eGFR
MR Test
Inverse variance weighted (fixed effects)
Inverse variance weighted (multiplicative random effects)
MR Egger
MR RAPS
Weighted median
Weighted mode
0.002
0.000
−0.002
SNP effect on eGFR
0.25
0.50
0.75
1.00
SNP effect on Protein CYR61

Batch 2−759 : Cytohesin−2 on eGFR

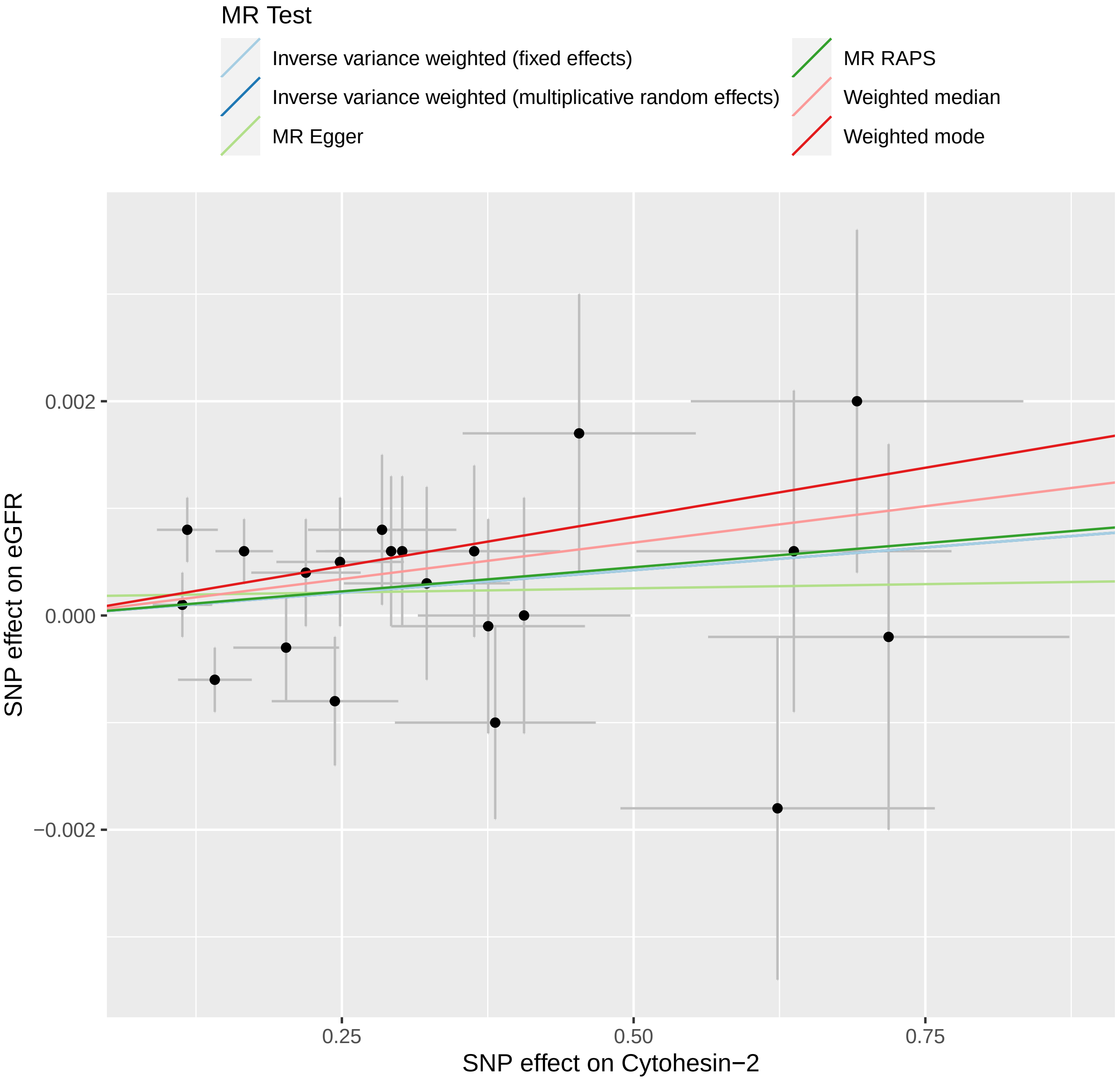

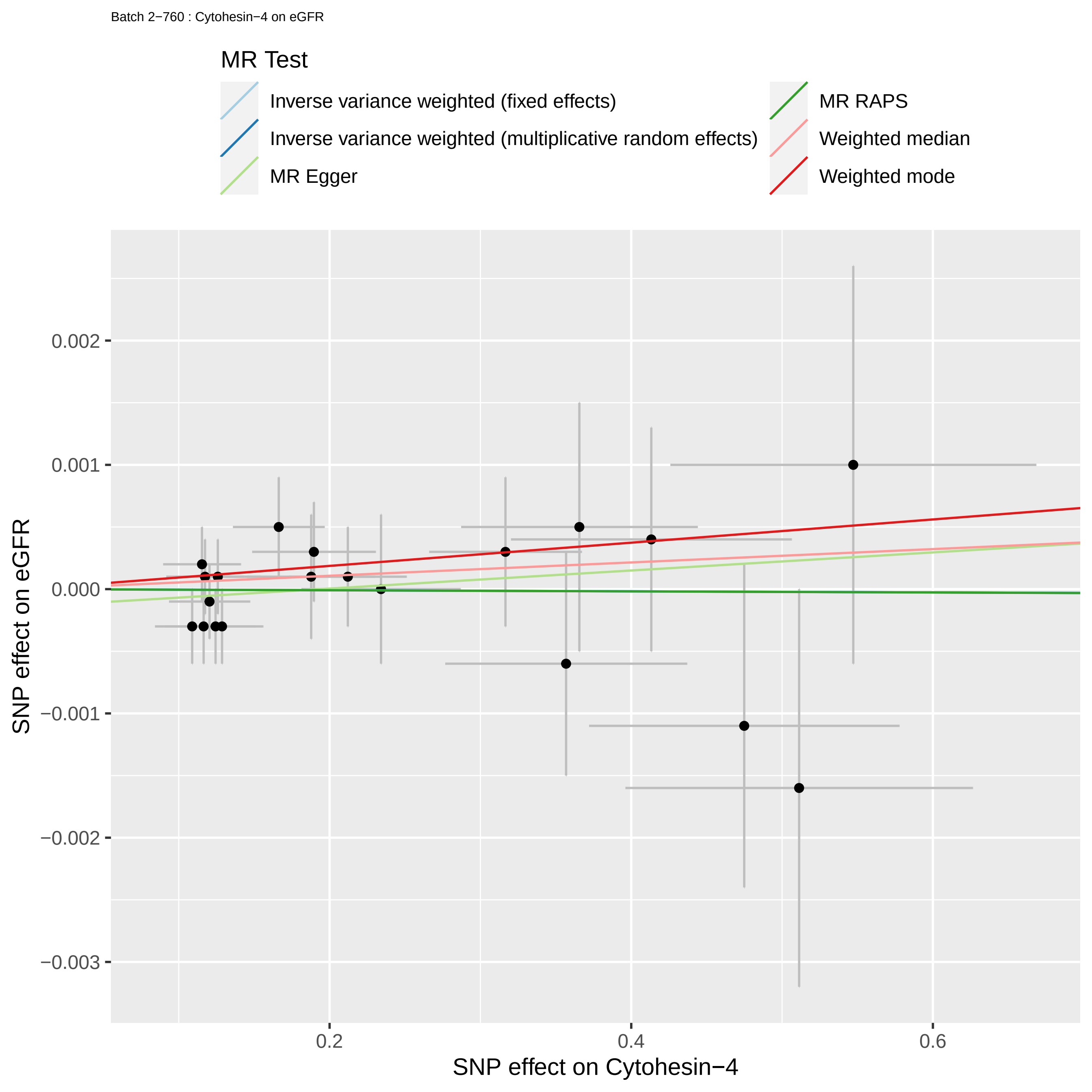
Batch 2−760 : Cytohesin−4 on eGFR
MR Test
Inverse variance weighted (fixed effects)
Inverse variance weighted (multiplicative random effects)
MR Egger
MR RAPS
Weighted median
Weighted mode
0.002
0.001
0.000
−0.001
−0.002
−0.003
SNP effect on eGFR
0.2
0.4
0.6
SNP effect on Cytohesin−4

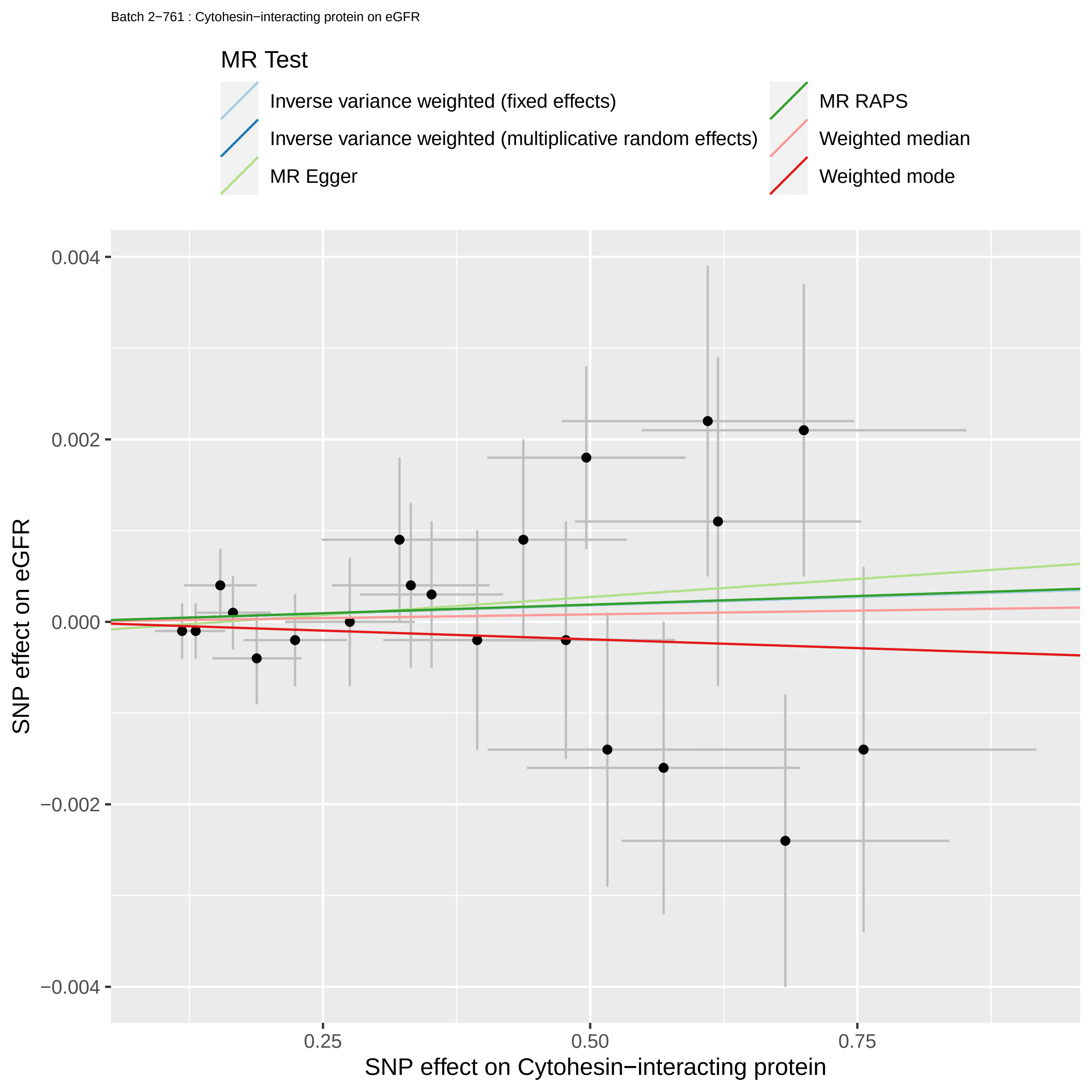
Batch 2−761 : Cytohesin−interacting protein on eGFR
MR Test
Inverse variance weighted (fixed effects)
Inverse variance weighted (multiplicative random effects)
MR Egger
MR RAPS
Weighted median
Weighted mode
0.004
0.002
0.000
−0.002
−0.004
SNP effect on eGFR
0.25
0.50
0.75
SNP effect on Cytohesin−interacting protein

Batch 2−762 : Dystroglycan on eGFR

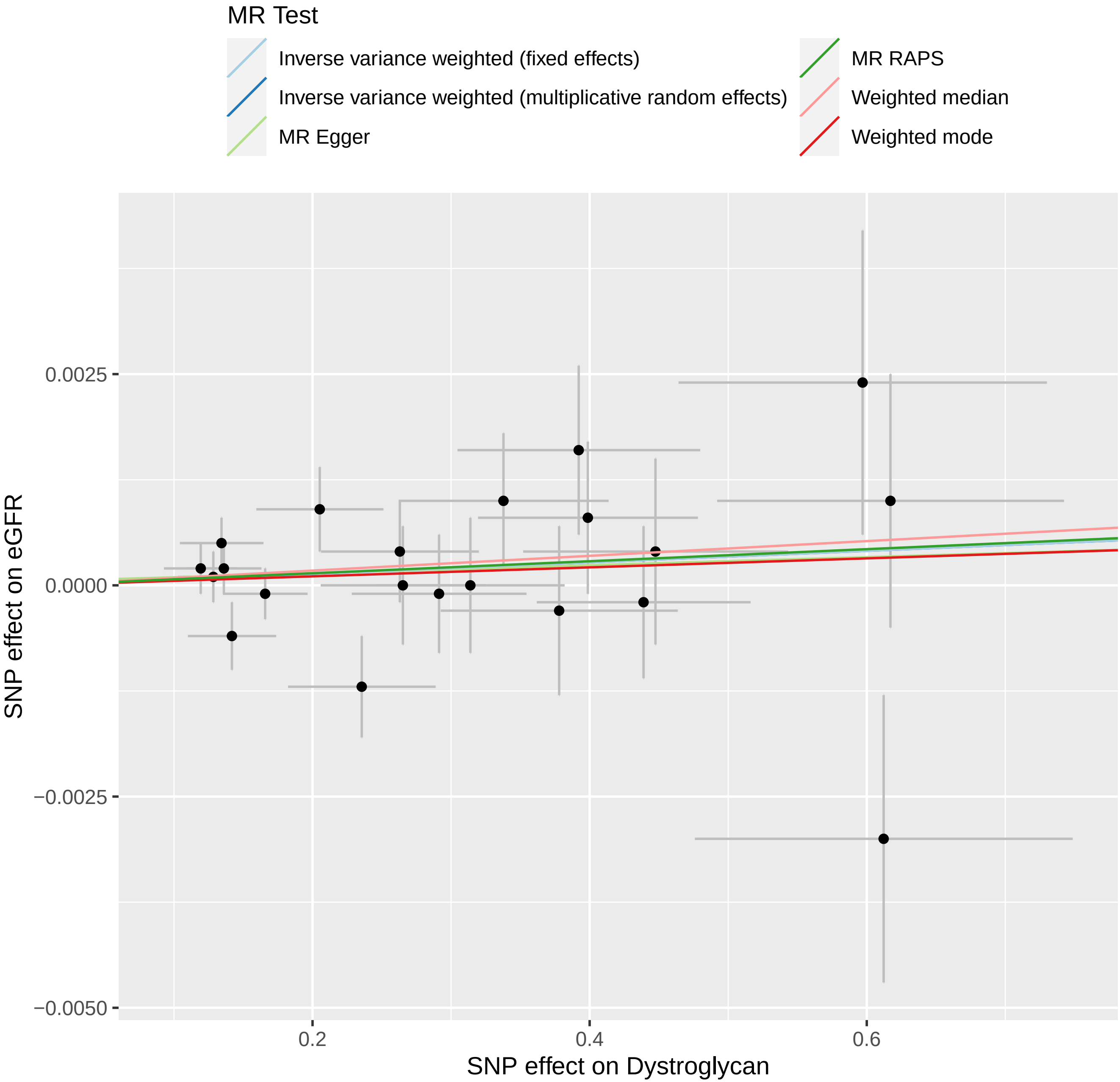

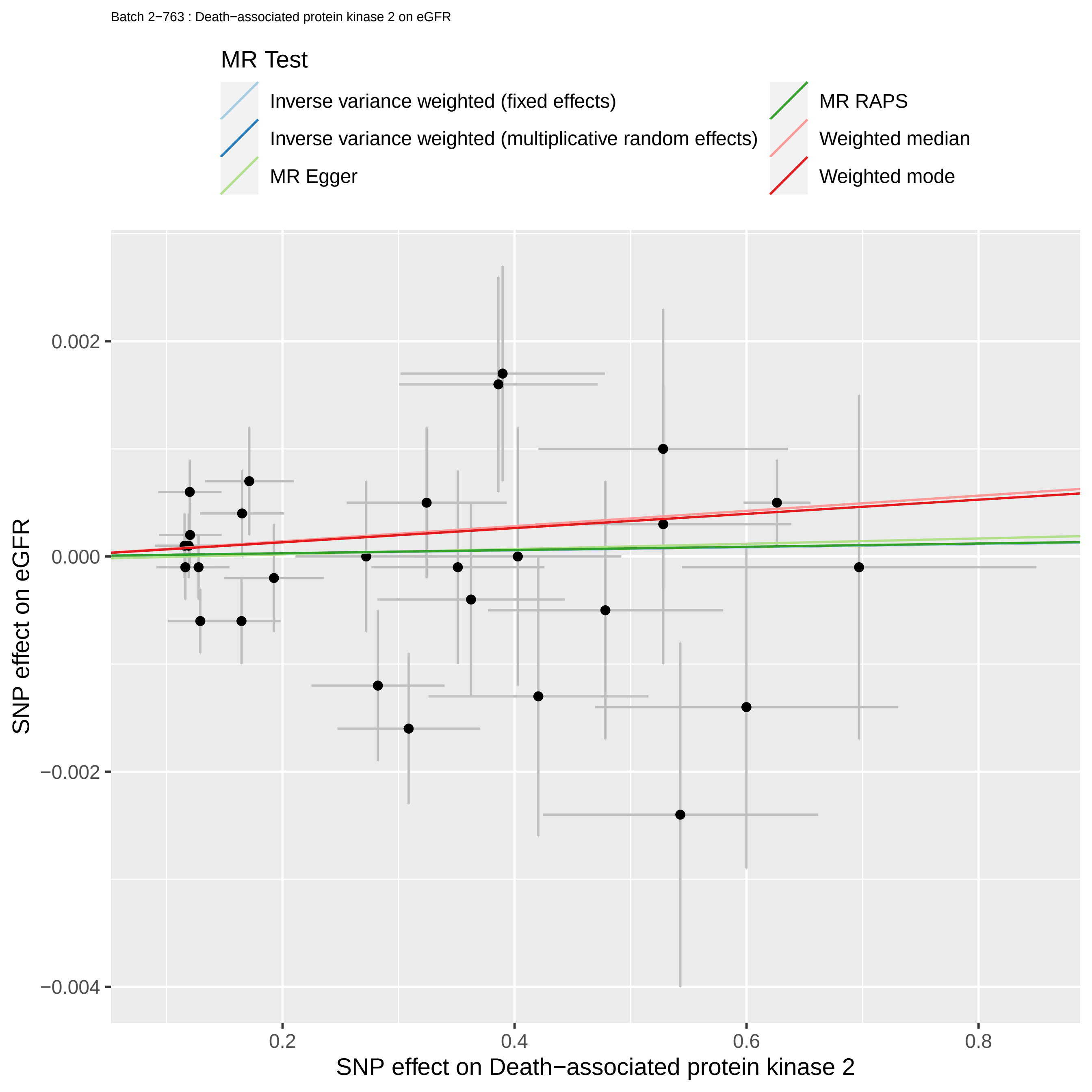

Batch 2−763 : Death−associated protein kinase 2 on eGFR
MR Test
Inverse variance weighted (fixed effects)
Inverse variance weighted (multiplicative random effects)
MR Egger
MR RAPS
Weighted median
Weighted mode
0.002
0.000
−0.002
−0.004
0.2
0.4
0.6
0.8
SNP effect on eGFR
SNP effect on Death−associated protein kinase 2

Batch 2−764 : Drebrin−like protein on eGFR

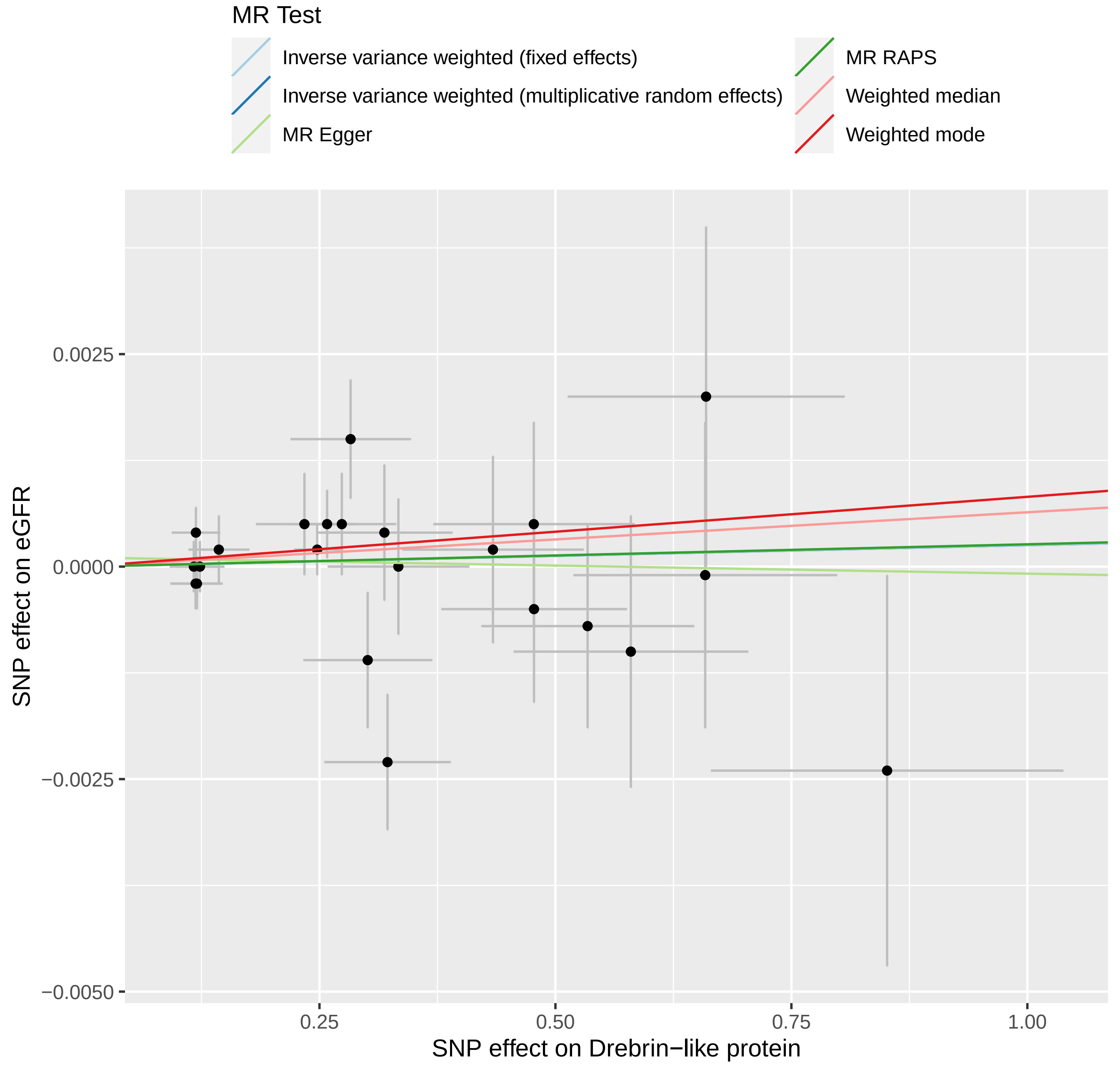

Batch 2−765 : DDB1− and CUL4−associated factor 5 on eGFR

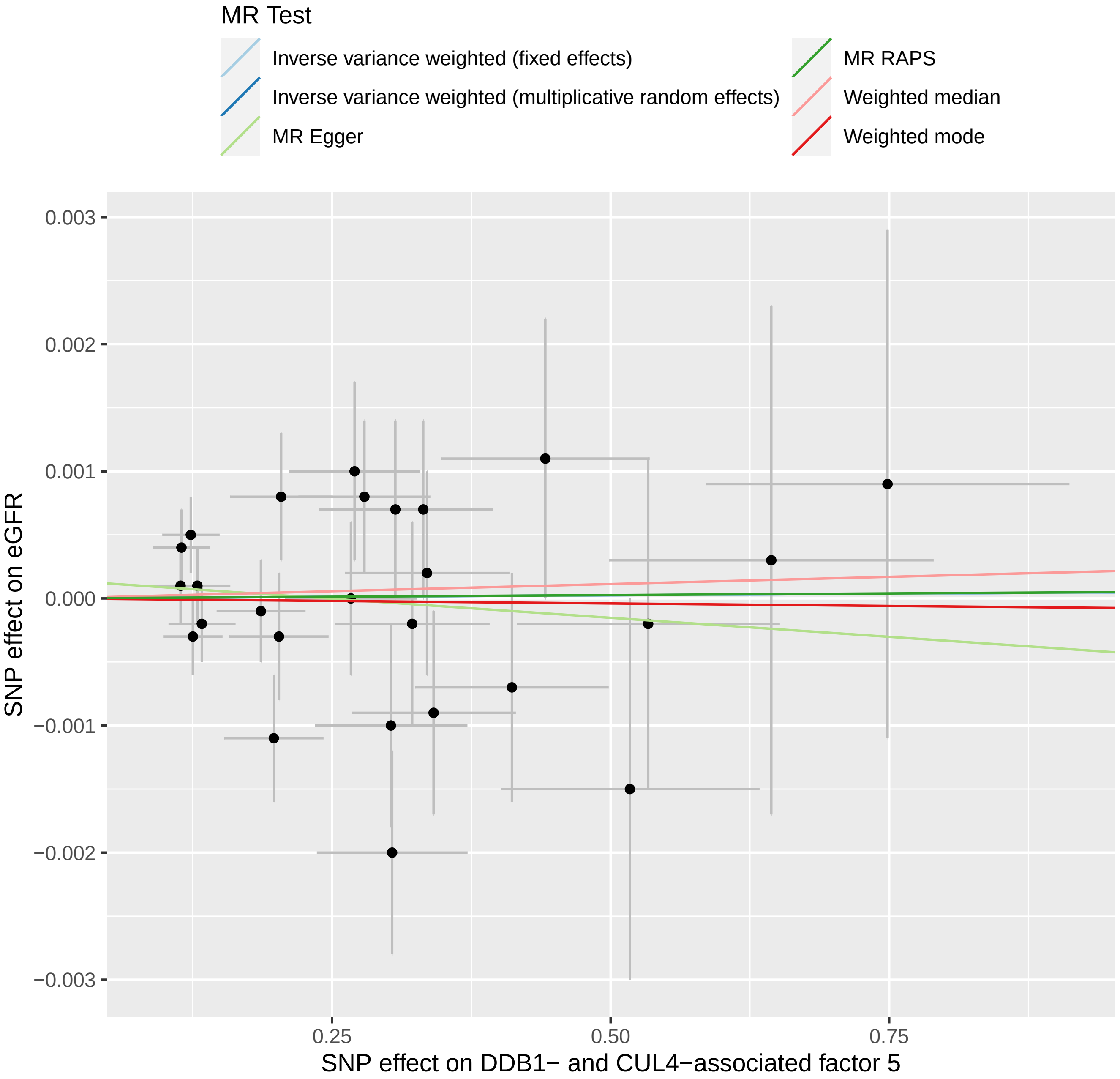

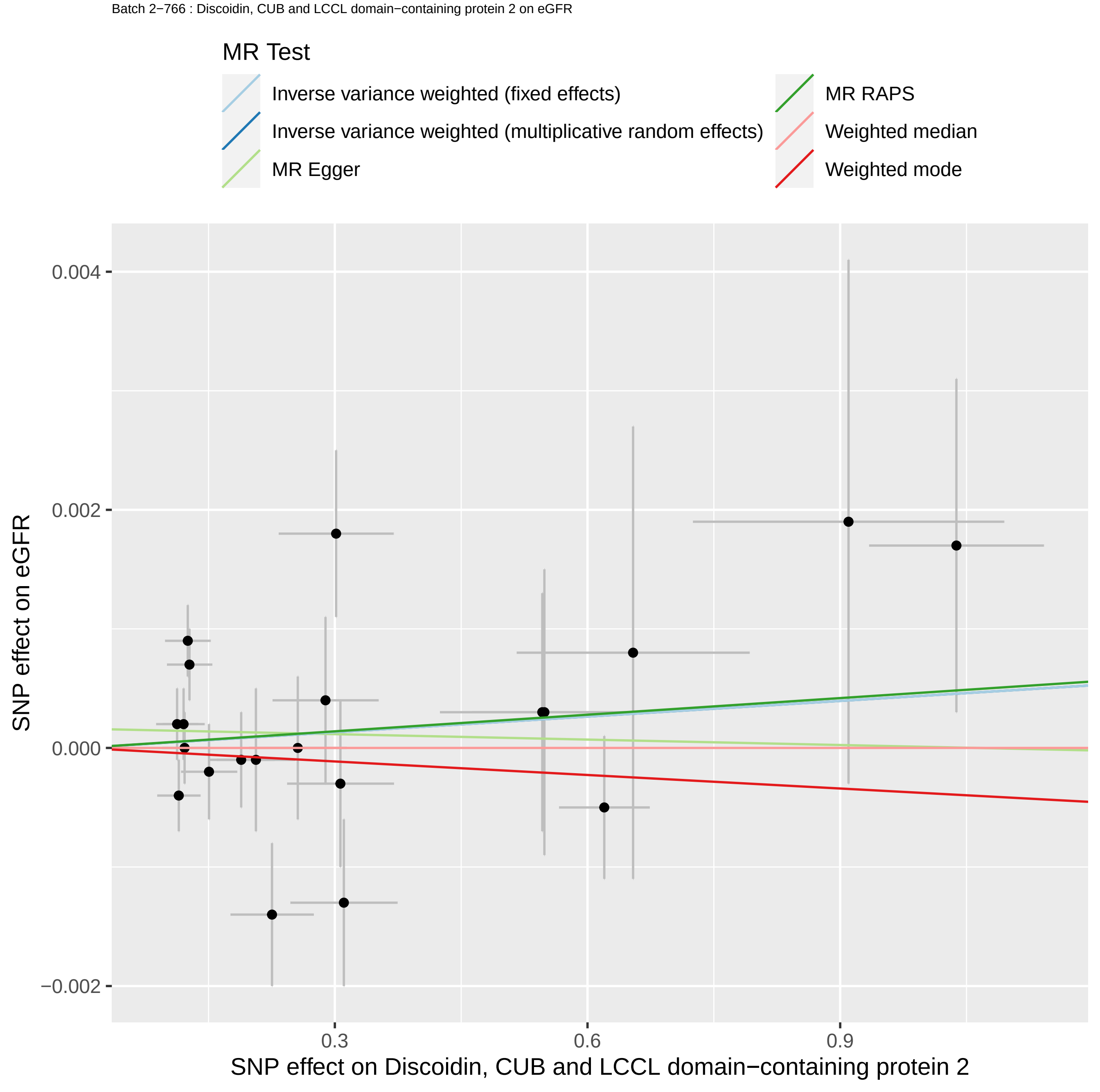

Batch 2−766 : Discoidin, CUB and LCCL domain−containing protein 2 on eGFR
MR Test
Inverse variance weighted (fixed effects)
Inverse variance weighted (multiplicative random effects)
MR Egger
MR RAPS
Weighted median
Weighted mode
SNP effect on eGFR
0.004
0.002
0.000
−0.002
0.3
0.6
0.9
SNP effect on Discoidin, CUB and LCCL domain−containing protein 2

Batch 2−767 : Deoxycytidine kinase on eGFR

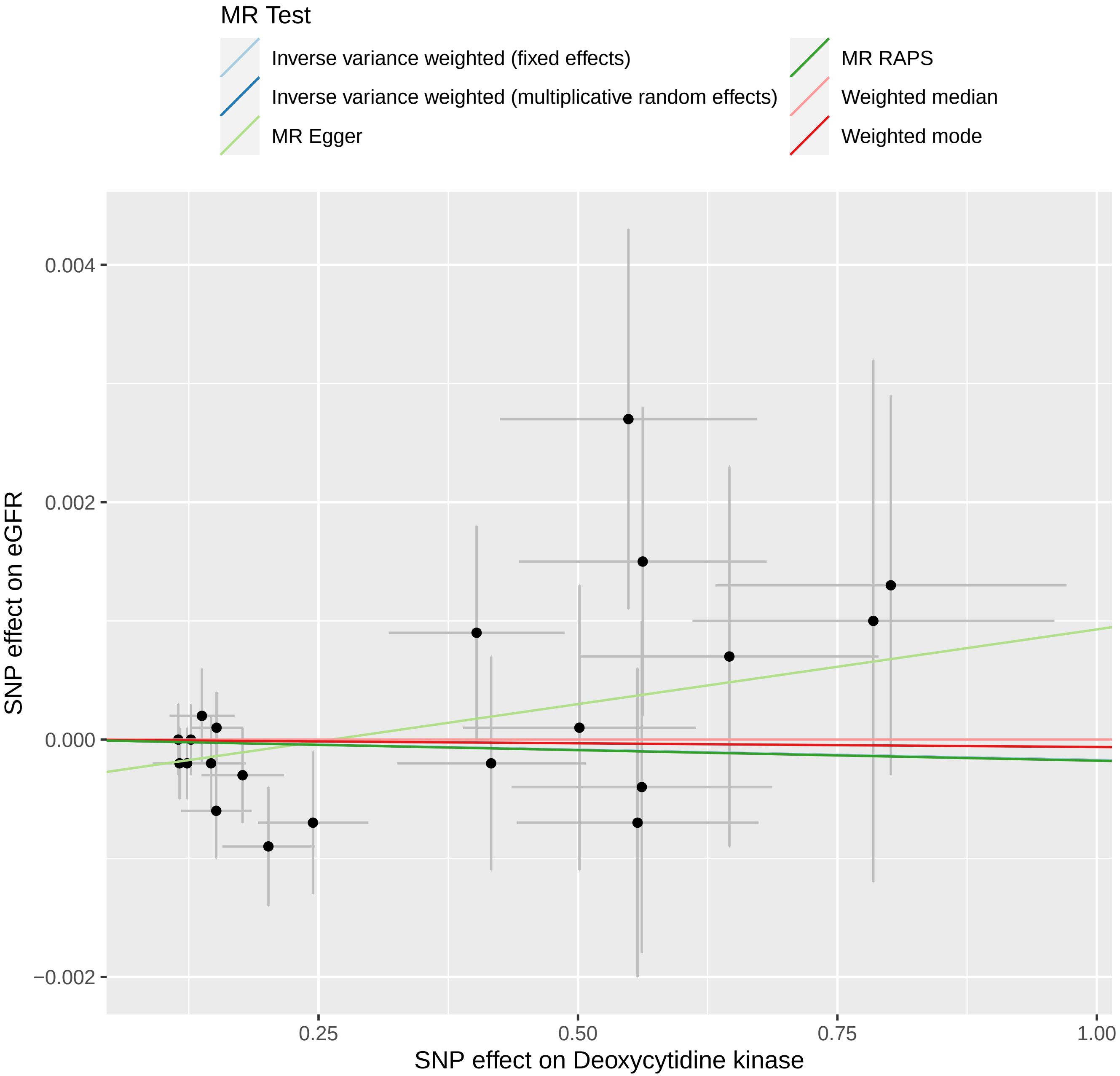

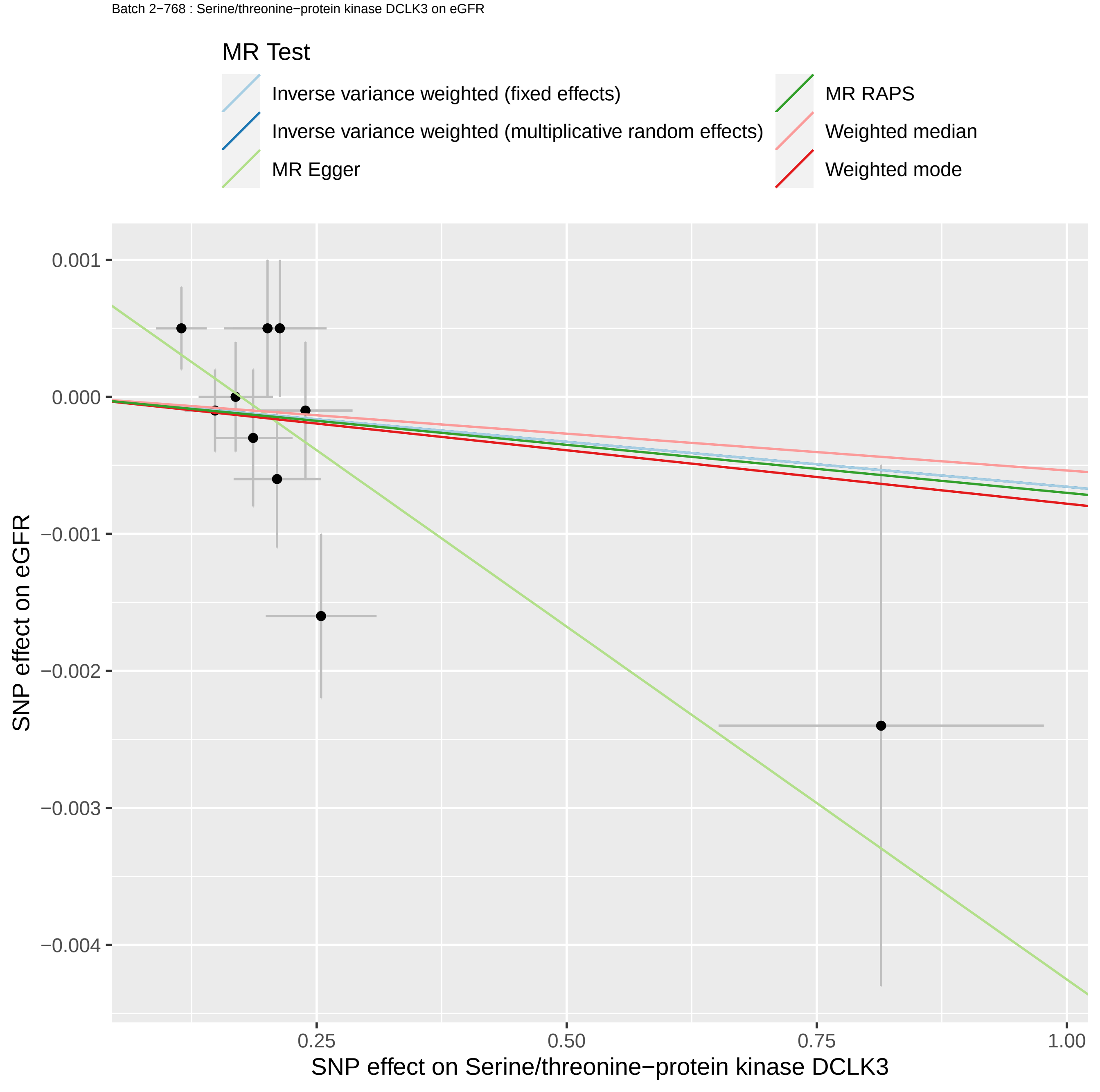
Batch 2−768 : Serine/threonine−protein kinase DCLK3 on eGFR
MR Test
Inverse variance weighted (fixed effects)
Inverse variance weighted (multiplicative random effects)
MR Egger
MR RAPS
Weighted median
Weighted mode
0.001
0.000
−0.001
−0.002
−0.003
−0.004
0.25
0.50
0.75
1.00
SNP effect on eGFR
SNP effect on Serine/threonine−protein kinase DCLK3

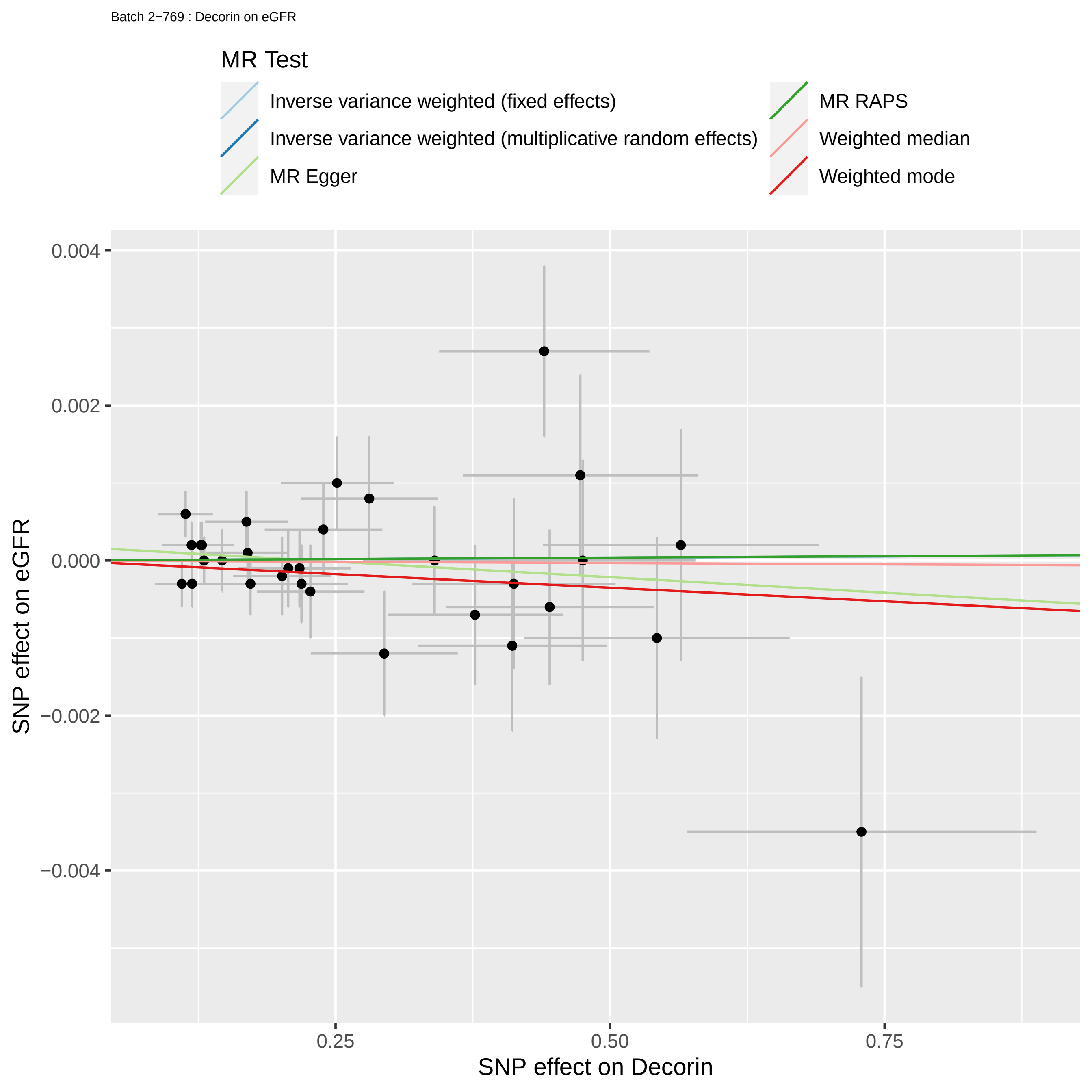
Batch 2−769 : Decorin on eGFR
MR Test
Inverse variance weighted (fixed effects)
Inverse variance weighted (multiplicative random effects)
MR Egger
MR RAPS
Weighted median
Weighted mode
0.004
0.002
0.000
−0.002
−0.004
0.25
0.50
0.75
SNP effect on eGFR
SNP effect on Decorin

Batch 2−770 : mRNA−decapping enzyme 1A on eGFR

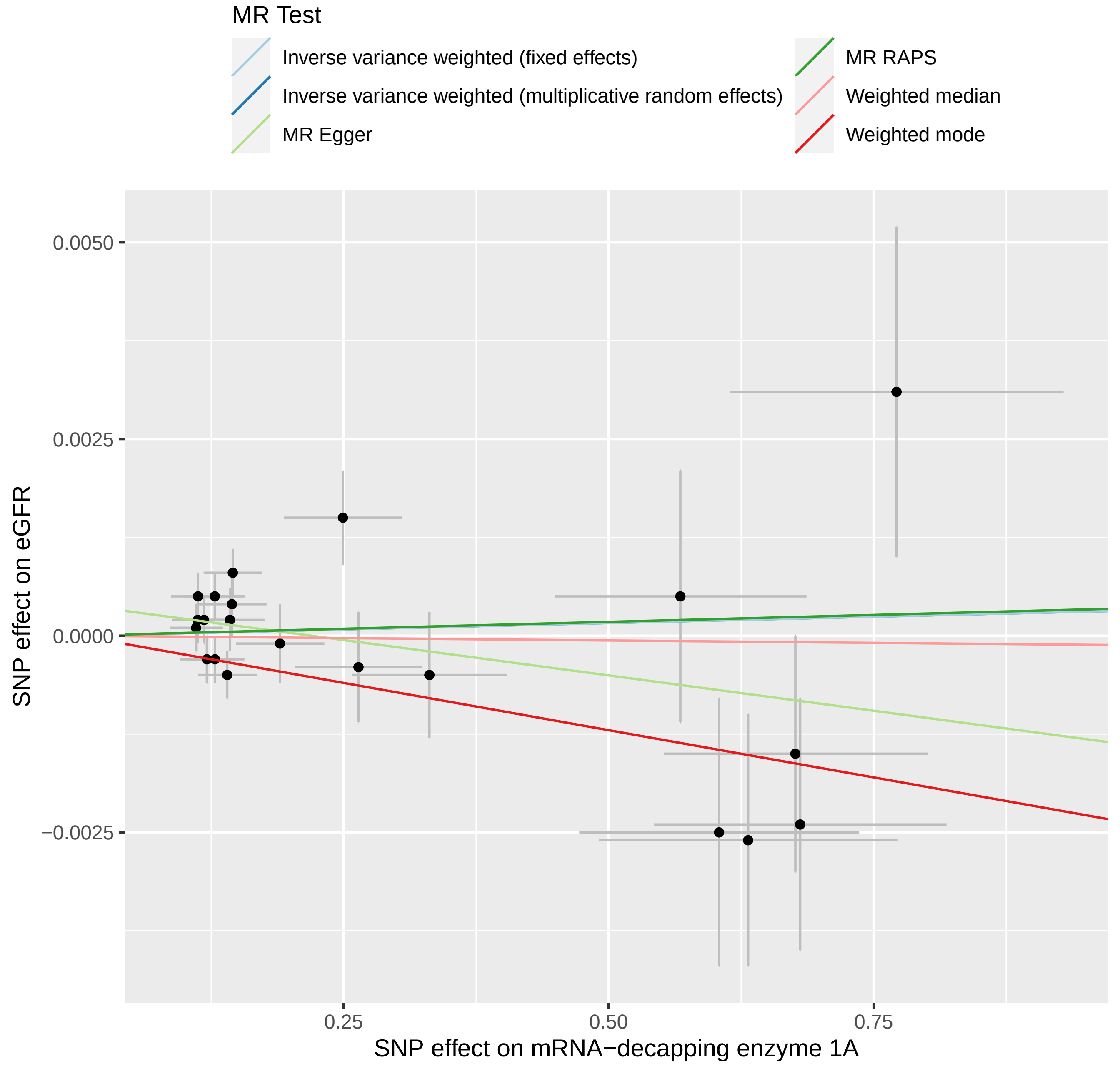

Batch 2−771 : Dynactin subunit 2 on eGFR

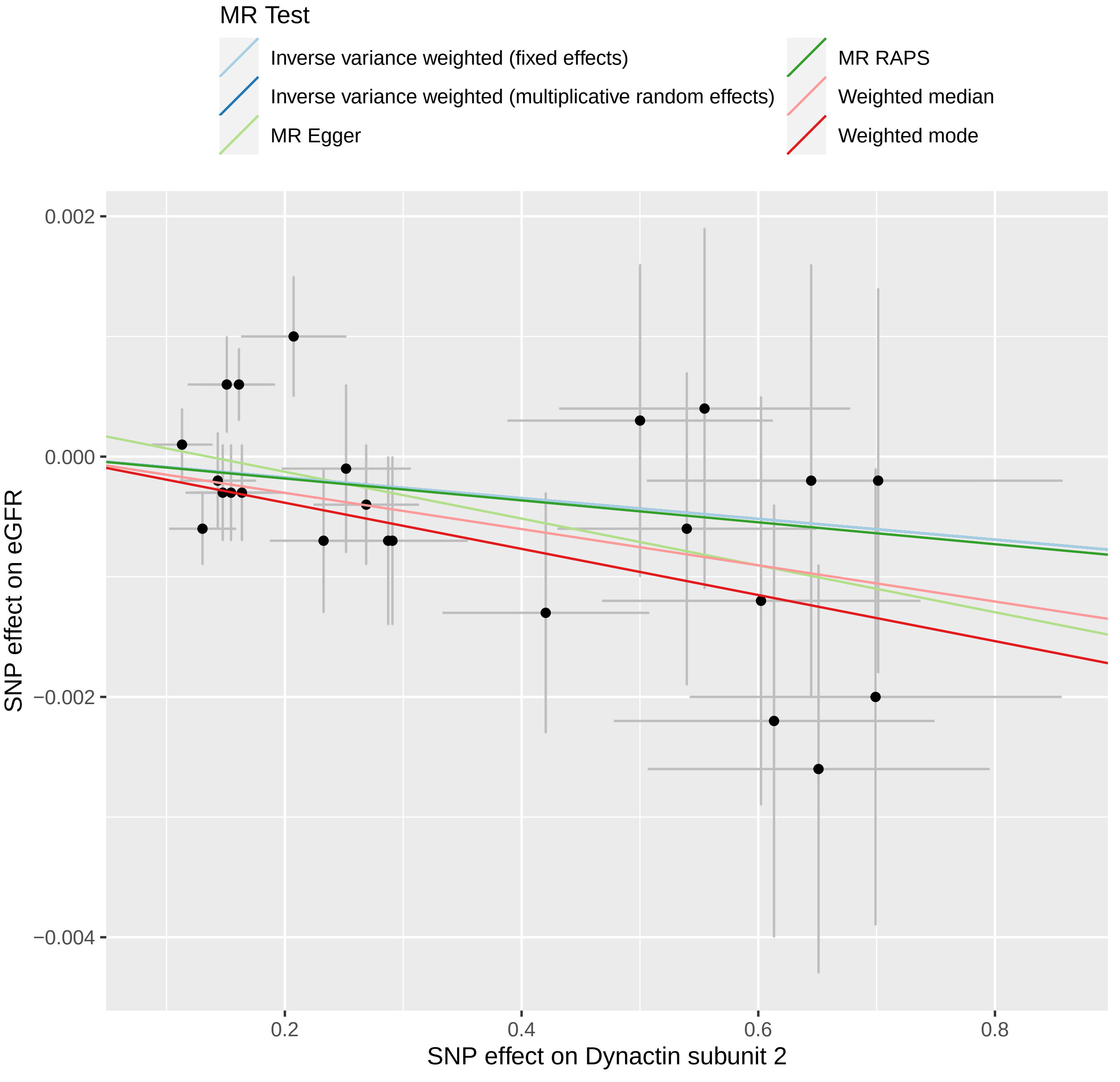

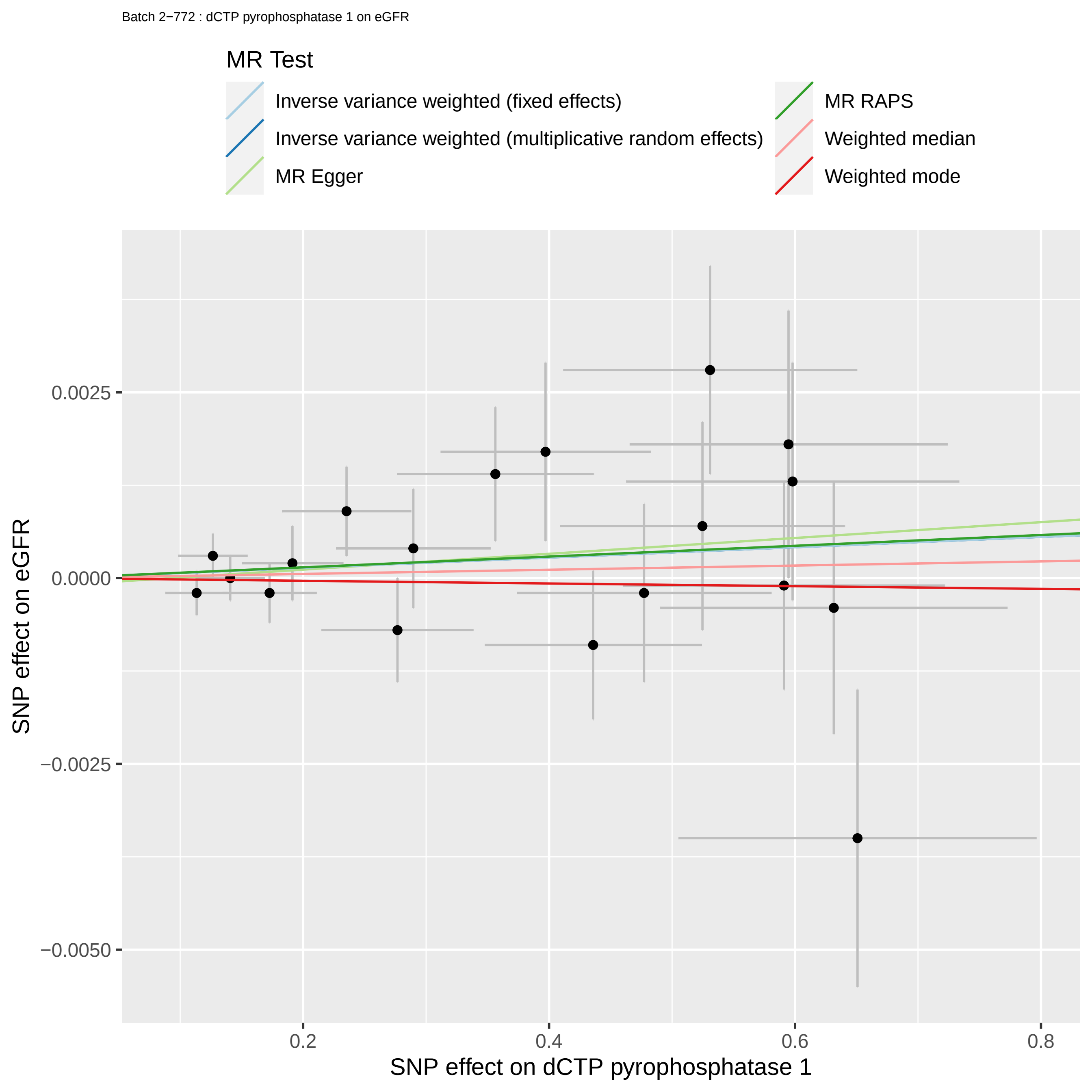

Batch 2−772 : dCTP pyrophosphatase 1 on eGFR
MR Test
Inverse variance weighted (fixed effects)
Inverse variance weighted (multiplicative random effects)
MR Egger
MR RAPS
Weighted median
Weighted mode
0.0025
0.0000
−0.0025
−0.0050
0.2
0.4
0.6
0.8
SNP effect on eGFR
SNP effect on dCTP pyrophosphatase 1

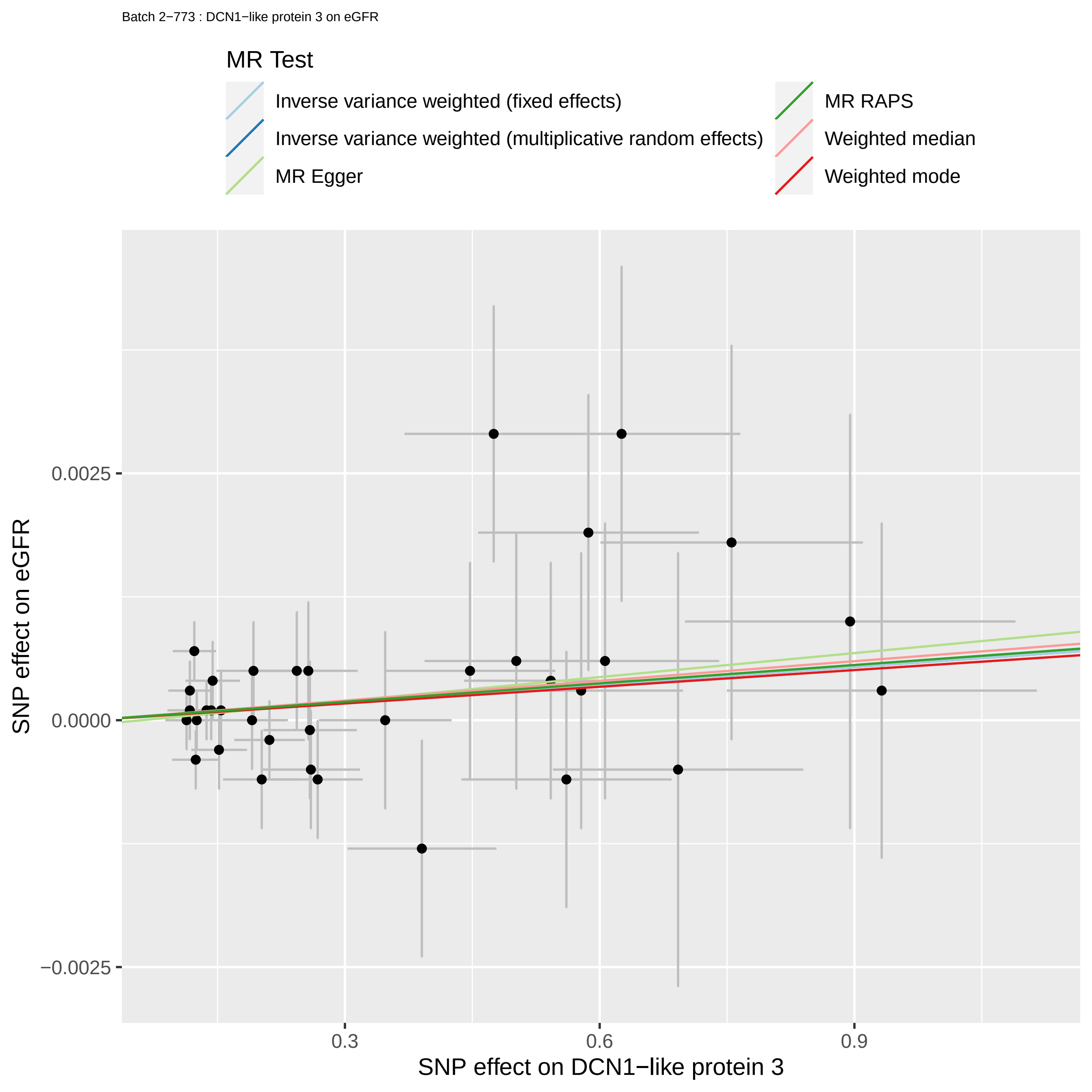

Batch 2−773 : DCN1−like protein 3 on eGFR
MR Test
Inverse variance weighted (fixed effects)
Inverse variance weighted (multiplicative random effects)
MR Egger
MR RAPS
Weighted median
Weighted mode
0.0025
0.0000
−0.0025
0.3
0.6
0.9
SNP effect on DCN1−like protein 3
SNP effect on eGFR

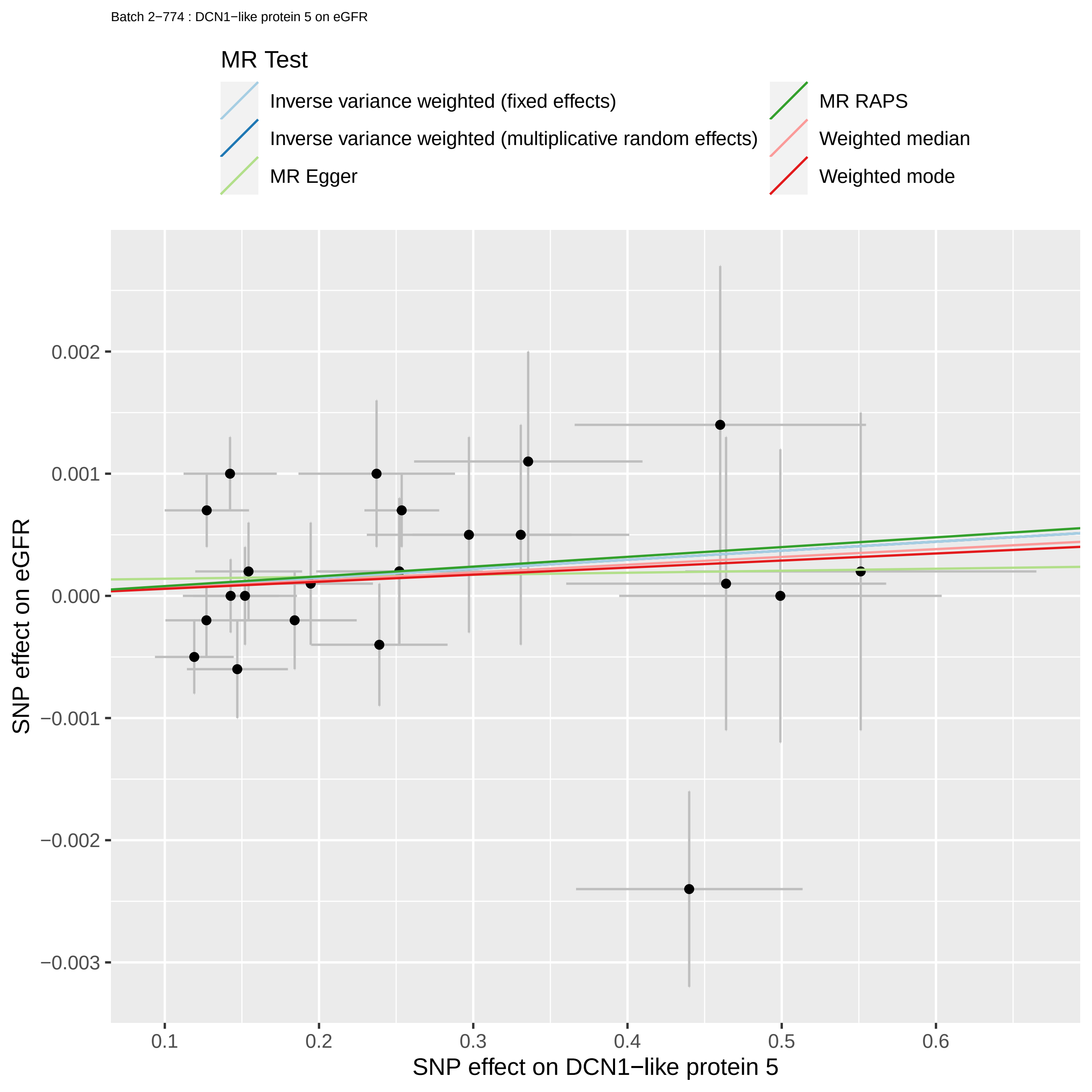

Batch 2−774 : DCN1−like protein 5 on eGFR
MR Test
Inverse variance weighted (fixed effects)
Inverse variance weighted (multiplicative random effects)
MR Egger
MR RAPS
Weighted median
Weighted mode
0.002
0.001
0.000
−0.001
−0.002
−0.003
0.1
0.2
0.3
0.4
0.5
0.6
SNP effect on eGFR
SNP effect on DCN1−like protein 5

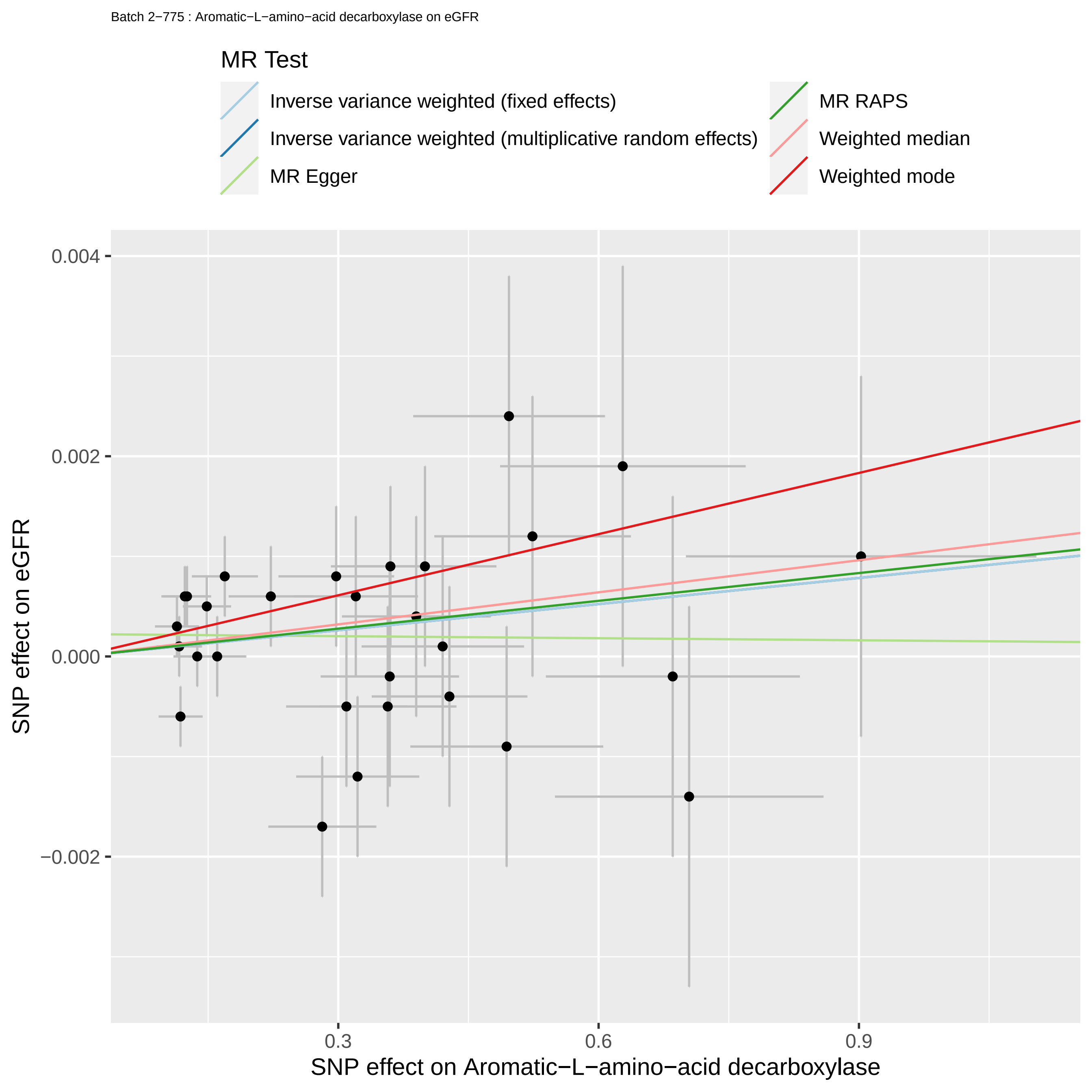
Batch 2−775 : Aromatic−L−amino−acid decarboxylase on eGFR
MR Test
Inverse variance weighted (fixed effects)
Inverse variance weighted (multiplicative random effects)
MR Egger
MR RAPS
Weighted median
Weighted mode
0.004
0.002
0.000
−0.002
0.3
0.6
0.9
SNP effect on eGFR
SNP effect on Aromatic−L−amino−acid decarboxylase

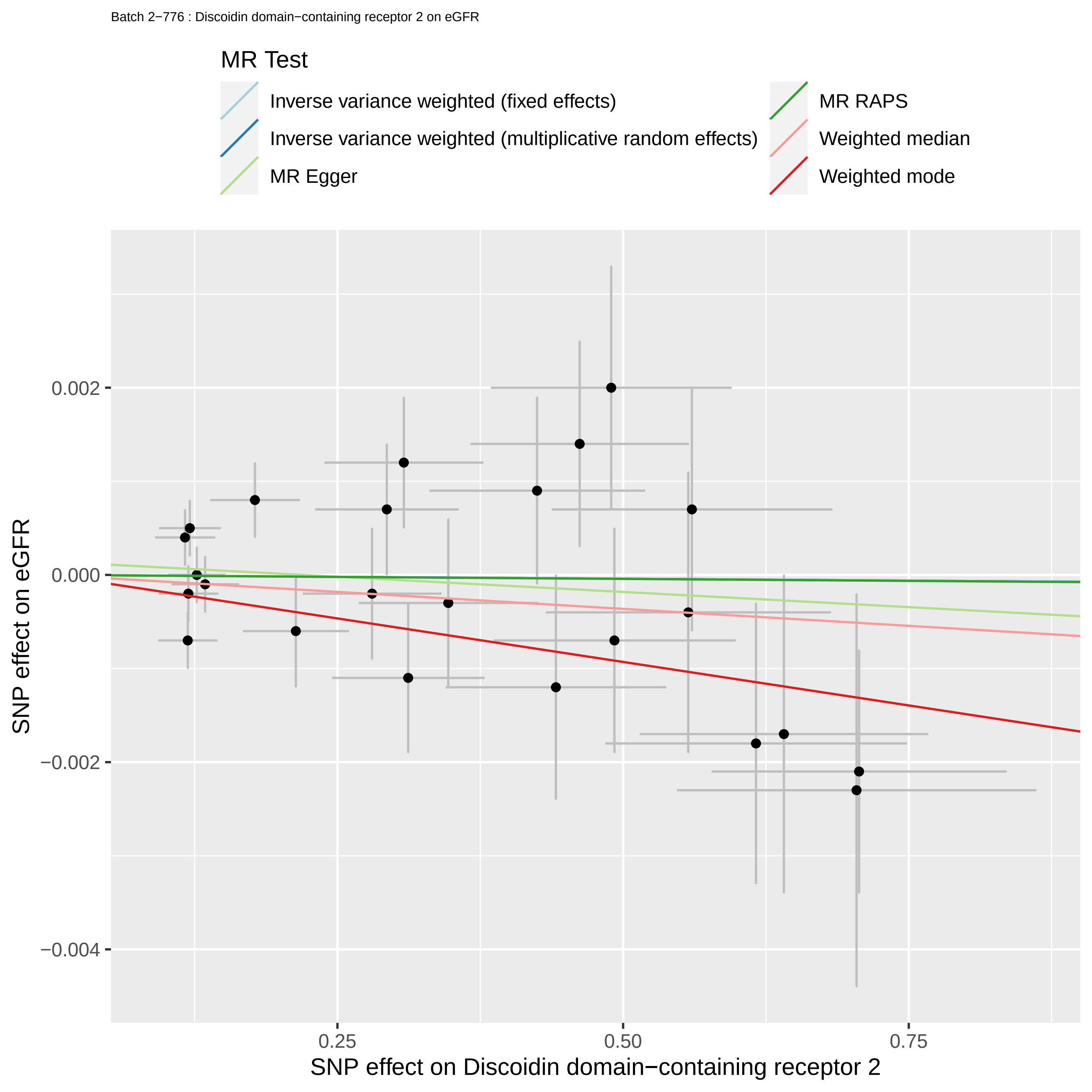
Batch 2−776 : Discoidin domain−containing receptor 2 on eGFR
MR Test
Inverse variance weighted (fixed effects)
Inverse variance weighted (multiplicative random effects)
MR Egger
MR RAPS
Weighted median
Weighted mode
0.002
0.000
−0.002
−0.004
0.25
0.50
0.75
SNP effect on eGFR
SNP effect on Discoidin domain−containing receptor 2

Batch 2−777 : ATP−dependent RNA helicase DDX25 on eGFR

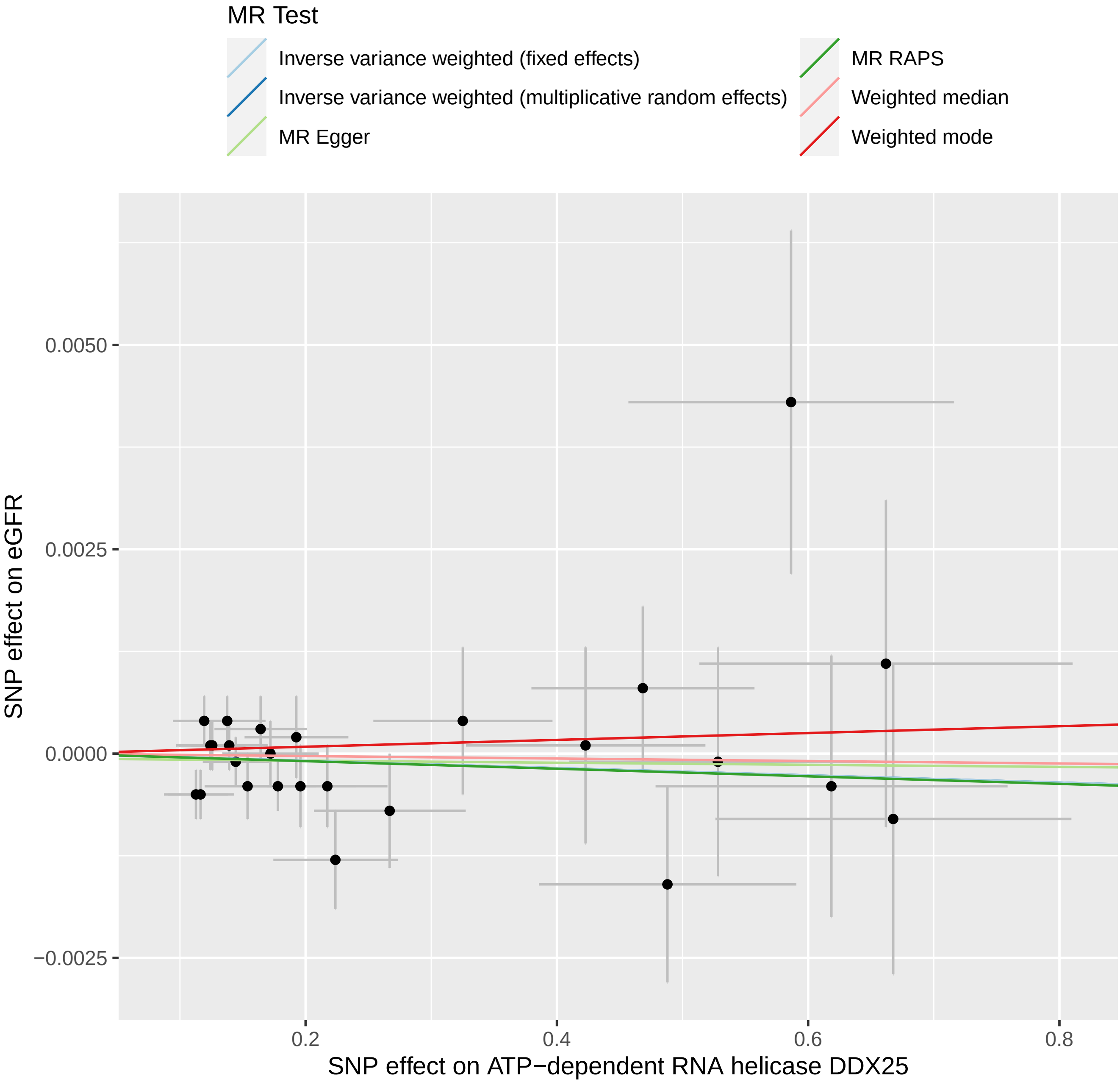

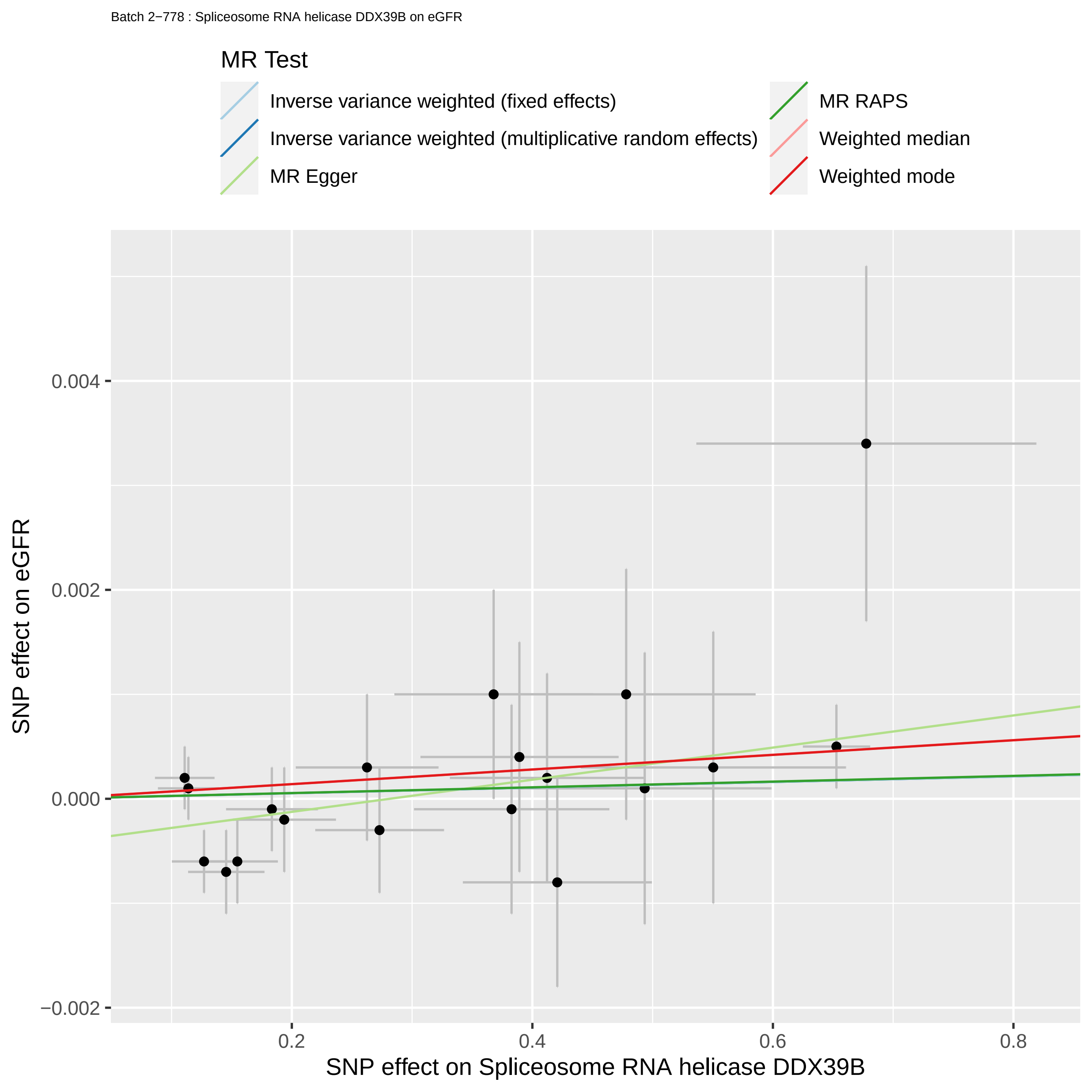

Batch 2−778 : Spliceosome RNA helicase DDX39B on eGFR
MR Test
Inverse variance weighted (fixed effects)
Inverse variance weighted (multiplicative random effects)
MR Egger
MR RAPS
Weighted median
Weighted mode
SNP effect on eGFR
0.004
0.002
0.000
−0.002
0.2
0.4
0.6
0.8
SNP effect on Spliceosome RNA helicase DDX39B

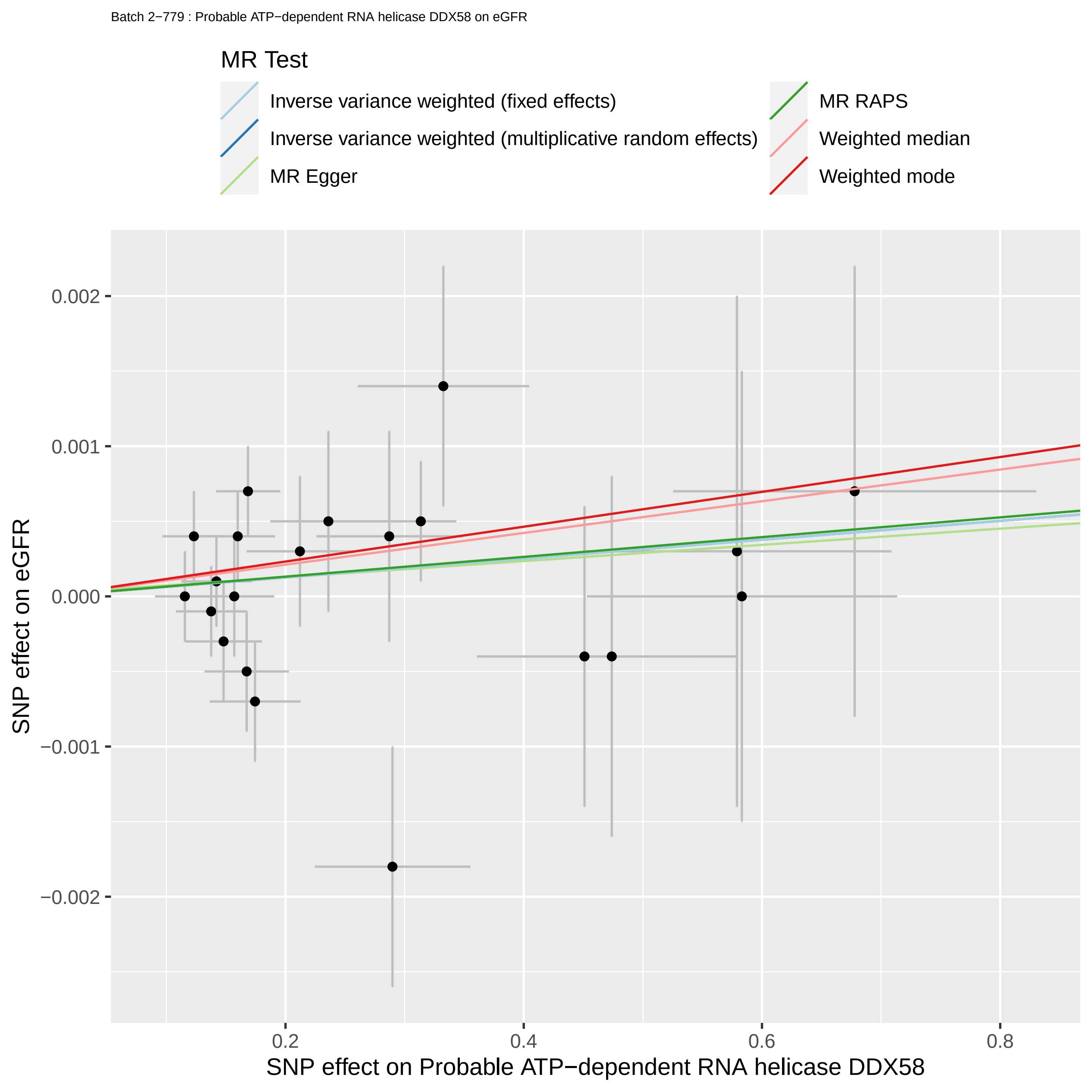
Batch 2−779 : Probable ATP−dependent RNA helicase DDX58 on eGFR
MR Test
Inverse variance weighted (fixed effects)
Inverse variance weighted (multiplicative random effects)
MR Egger
MR RAPS
Weighted median
Weighted mode
0.002
0.001
0.000
−0.001
−0.002
0.2
0.4
0.6
0.8
SNP effect on eGFR
SNP effect on Probable ATP−dependent RNA helicase DDX58

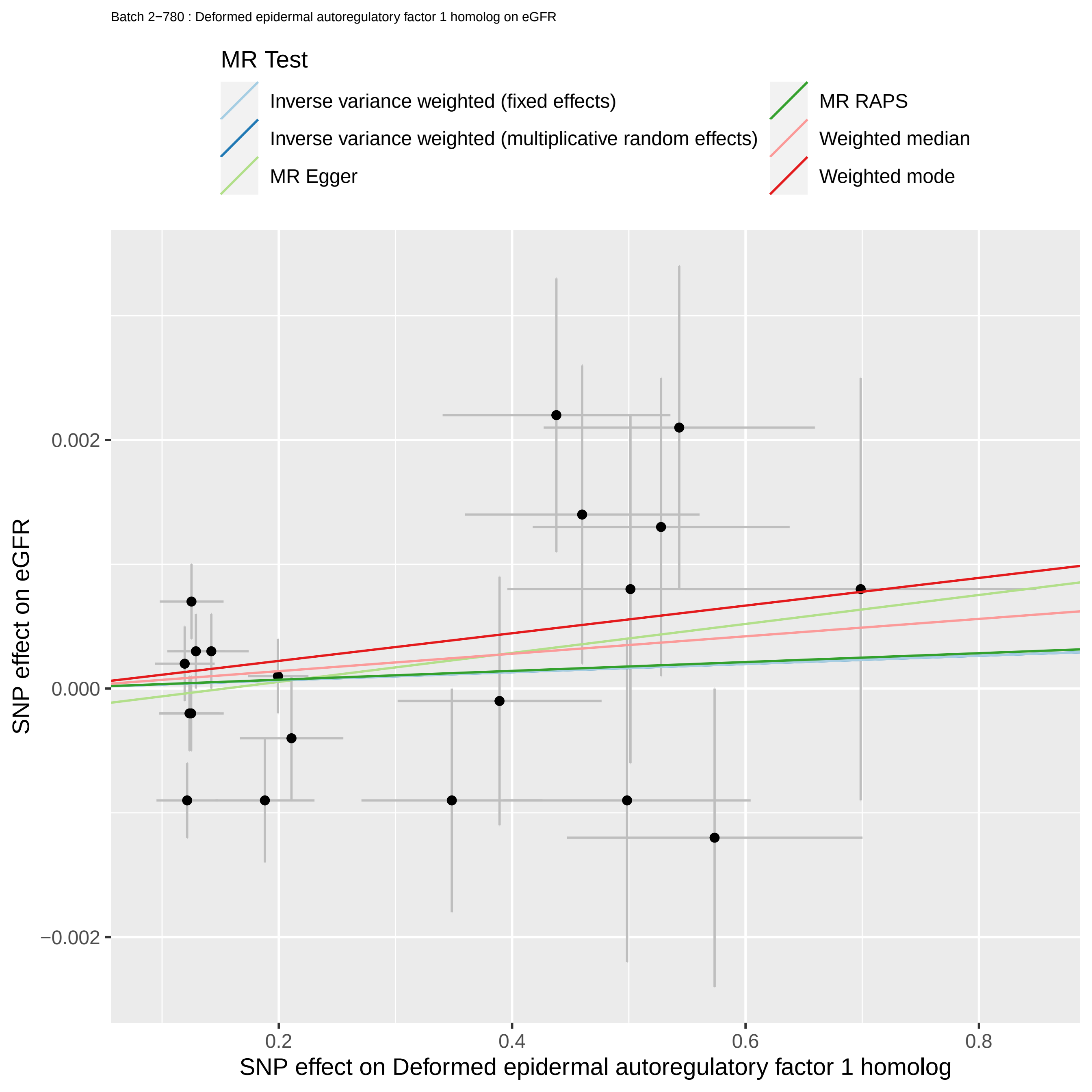
Batch 2−780 : Deformed epidermal autoregulatory factor 1 homolog on eGFR
MR Test
Inverse variance weighted (fixed effects)
Inverse variance weighted (multiplicative random effects)
MR Egger
MR RAPS
Weighted median
Weighted mode
0.002
0.000
−0.002
0.2
0.4
0.6
0.8
SNP effect on eGFR
SNP effect on Deformed epidermal autoregulatory factor 1 homolog

Batch 2−781 : Beta−defensin 103 on eGFR

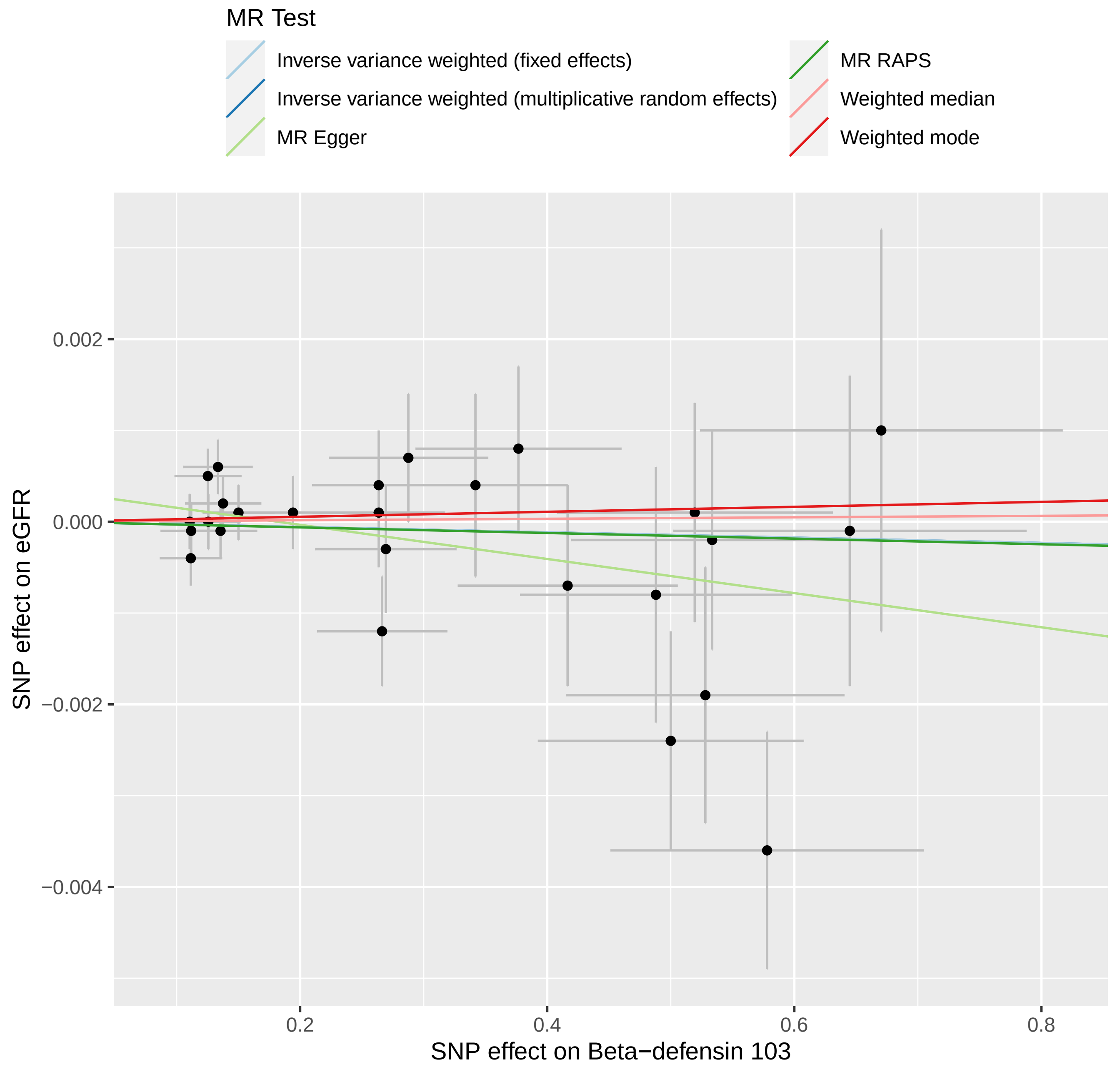

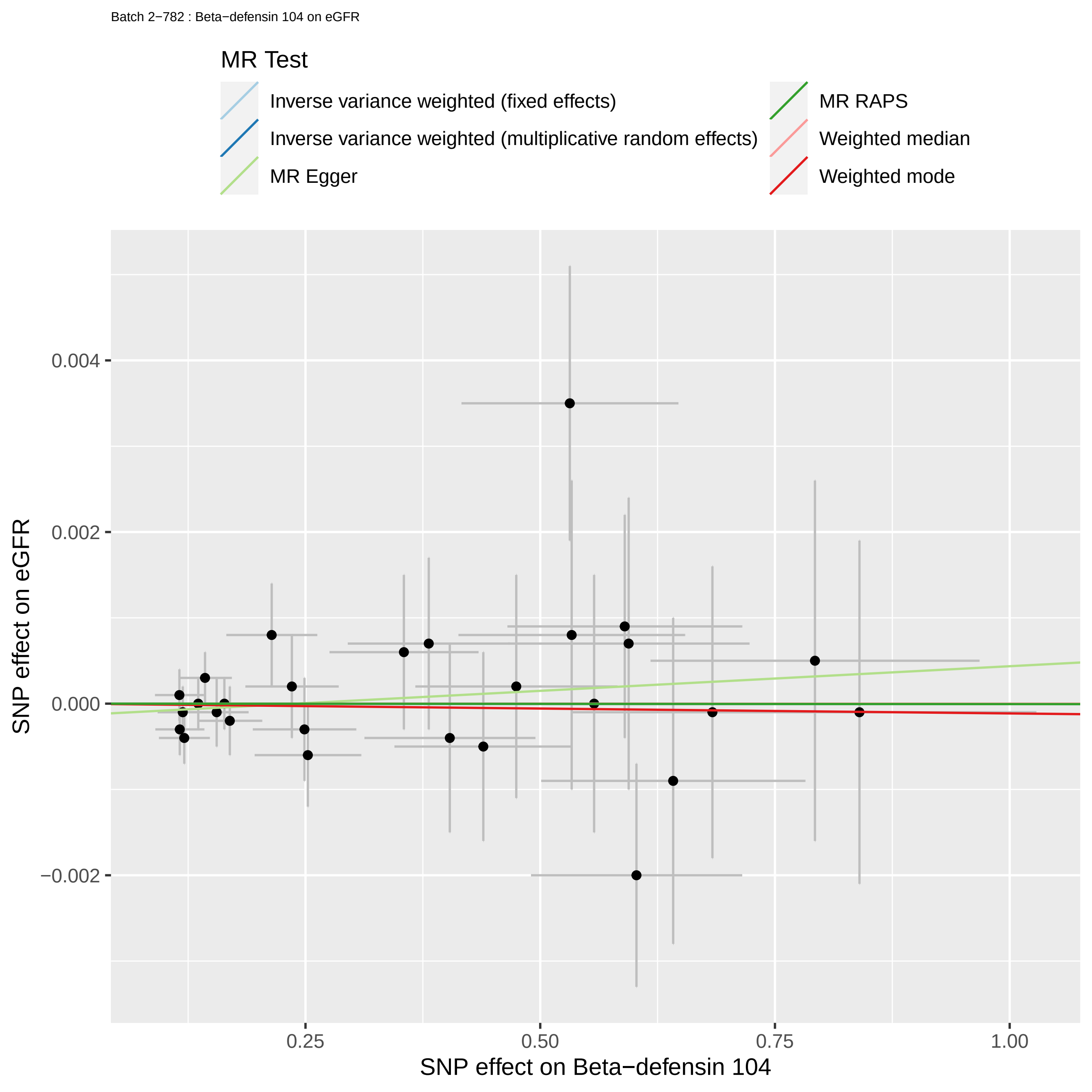
Batch 2−782 : Beta−defensin 104 on eGFR
MR Test
Inverse variance weighted (fixed effects)
Inverse variance weighted (multiplicative random effects)
MR Egger
MR RAPS
Weighted median
Weighted mode
0.004
0.002
0.000
−0.002
SNP effect on eGFR
0.25
0.50
0.75
1.00
SNP effect on Beta−defensin 104

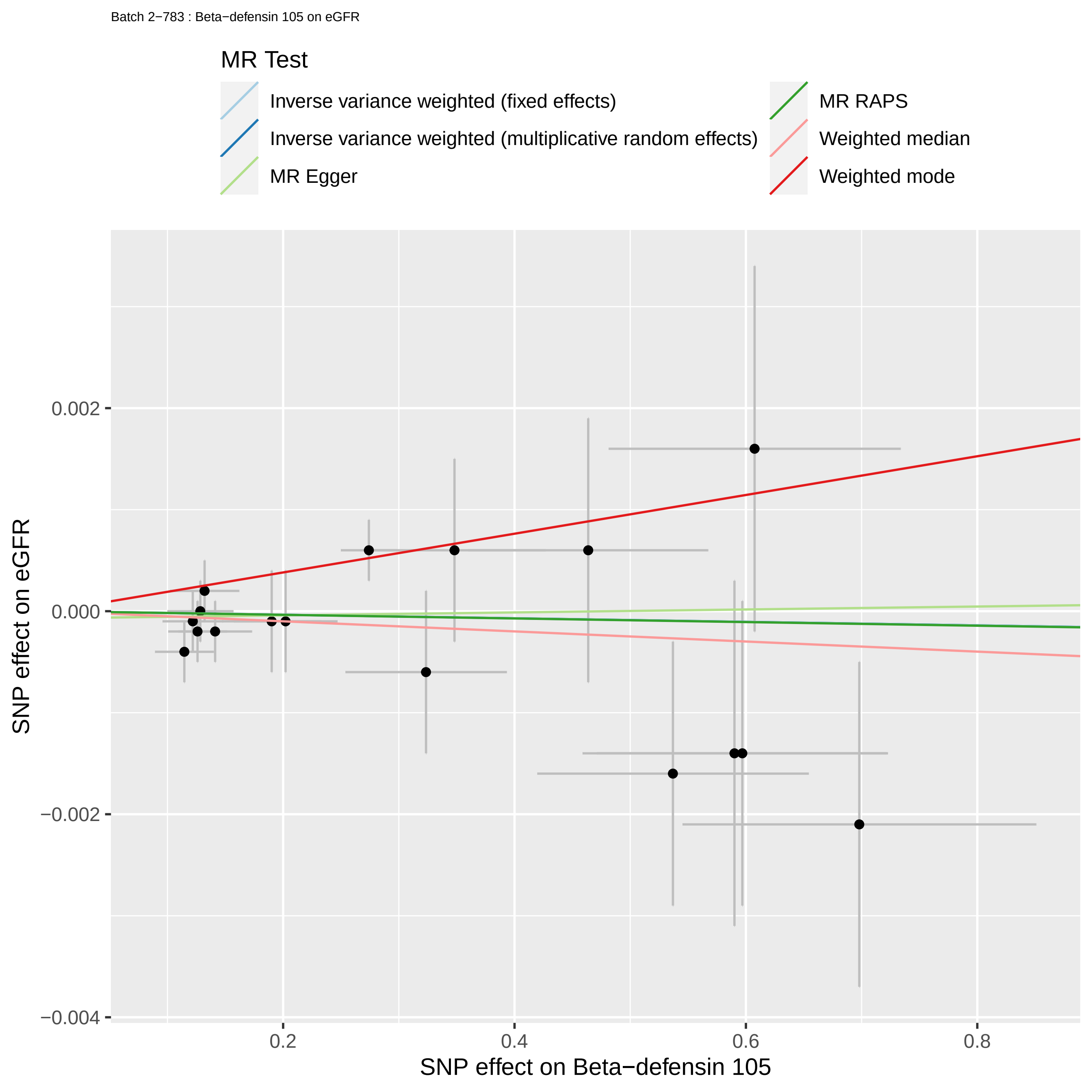

Batch 2−783 : Beta−defensin 105 on eGFR
MR Test
Inverse variance weighted (fixed effects)
Inverse variance weighted (multiplicative random effects)
MR Egger
MR RAPS
Weighted median
Weighted mode
0.002
0.000
−0.002
−0.004
0.2
0.4
0.6
0.8
SNP effect on eGFR
SNP effect on Beta−defensin 105

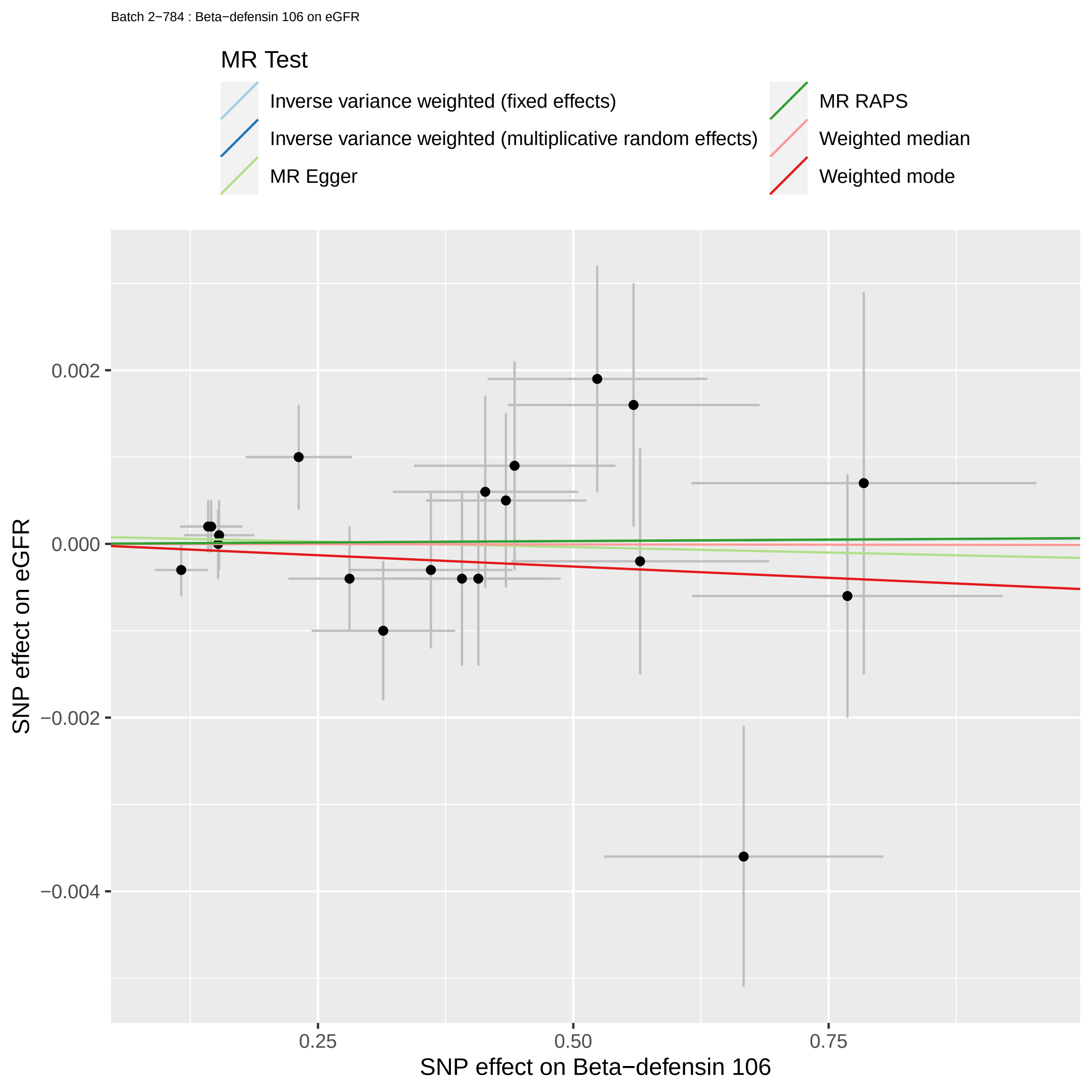

Batch 2−784 : Beta−defensin 106 on eGFR
MR Test
Inverse variance weighted (fixed effects)
Inverse variance weighted (multiplicative random effects)
MR Egger
MR RAPS
Weighted median
Weighted mode
0.002
0.000
−0.002
−0.004
0.25
0.50
0.75
SNP effect on eGFR
SNP effect on Beta−defensin 106

Batch 2−785 : Beta−defensin 107 on eGFR

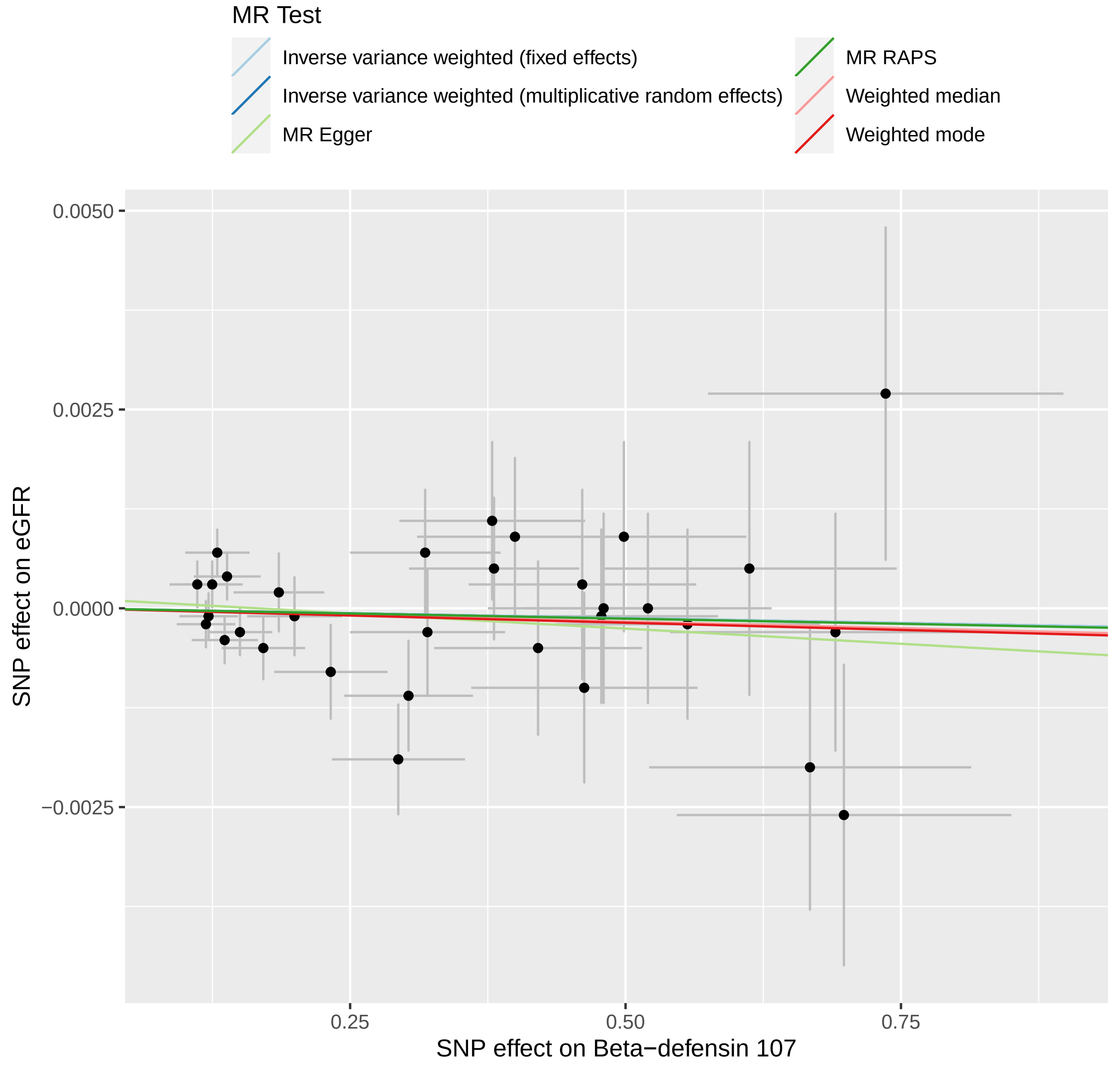

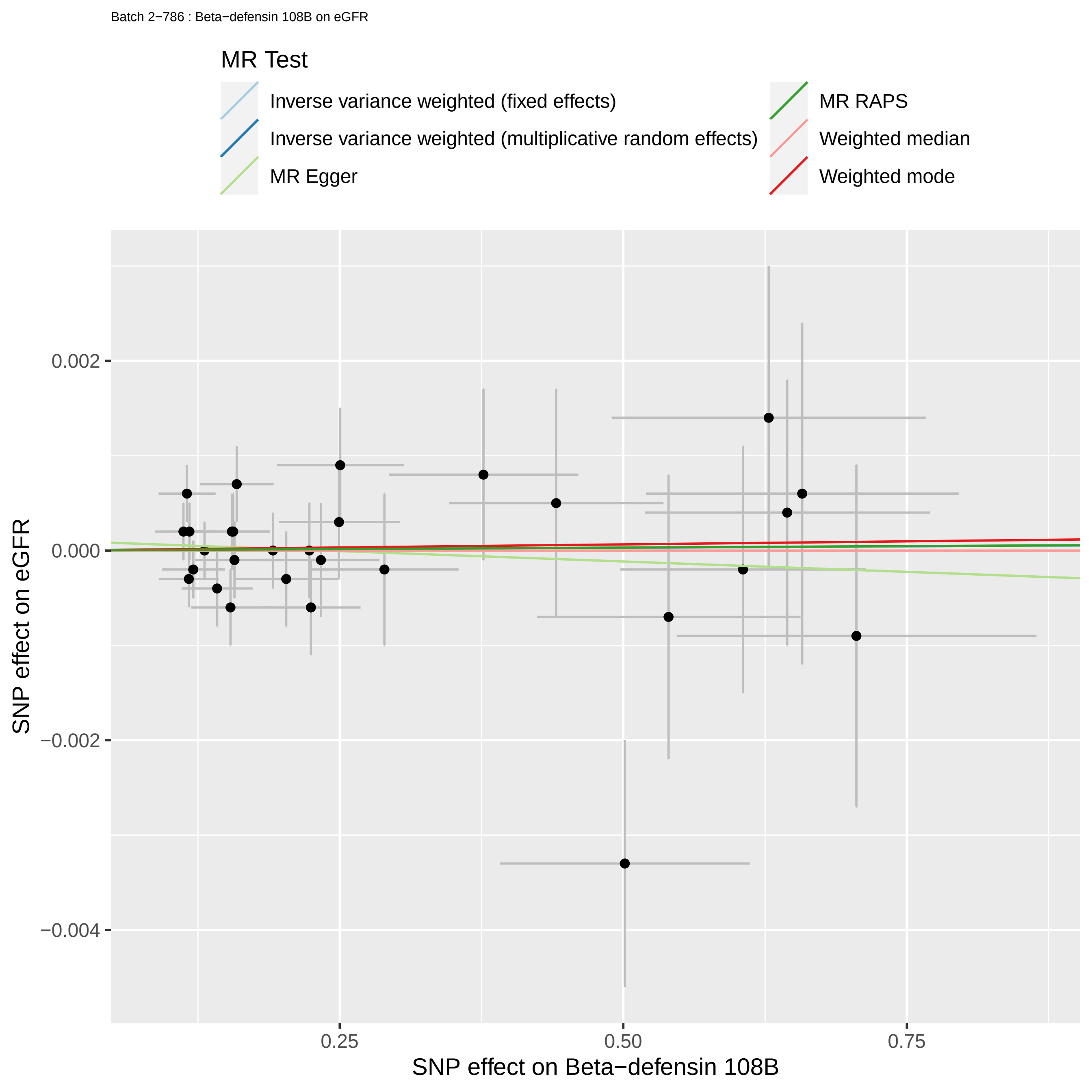

Batch 2−786 : Beta−defensin 108B on eGFR
MR Test
Inverse variance weighted (fixed effects)
Inverse variance weighted (multiplicative random effects)
MR Egger
MR RAPS
Weighted median
Weighted mode
0.002
0.000
−0.002
−0.004
0.25
0.50
0.75
SNP effect on eGFR
SNP effect on Beta−defensin 108B

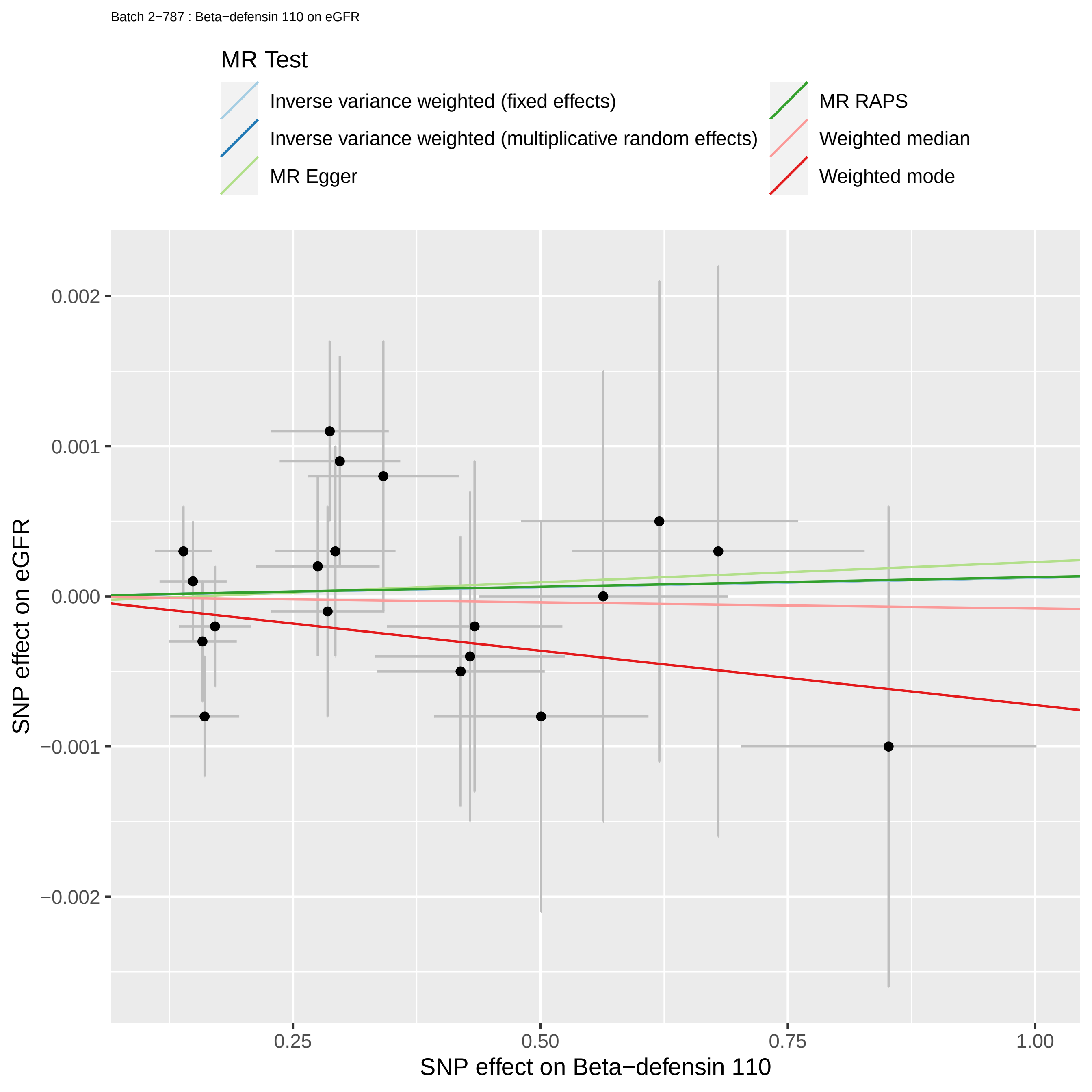

Batch 2−787 : Beta−defensin 110 on eGFR
MR Test
Inverse variance weighted (fixed effects)
Inverse variance weighted (multiplicative random effects)
MR Egger
MR RAPS
Weighted median
Weighted mode
0.002
0.001
0.000
−0.001
−0.002
SNP effect on eGFR
0.25
0.50
0.75
1.00
SNP effect on Beta−defensin 110

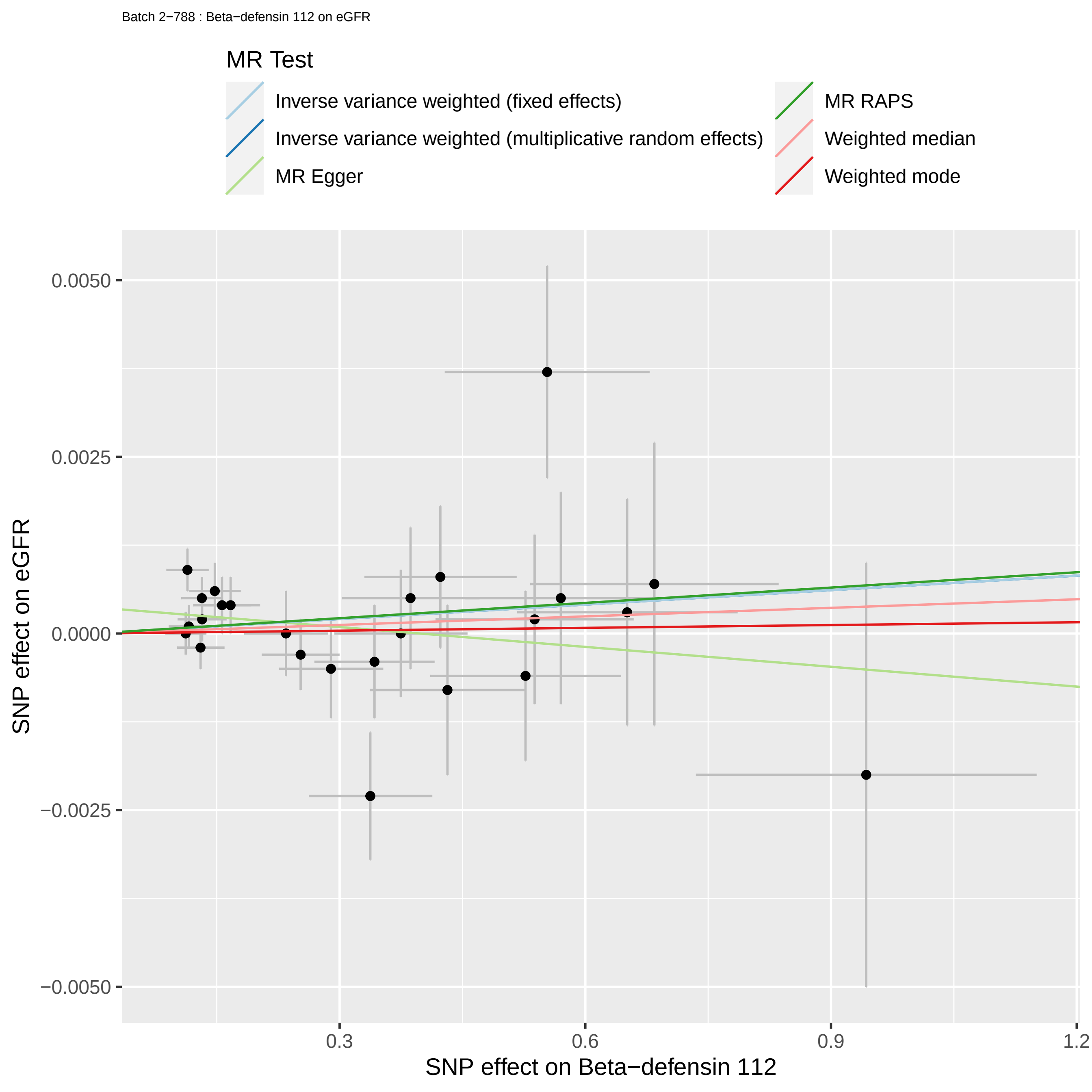

Batch 2−788 : Beta−defensin 112 on eGFR
MR Test
Inverse variance weighted (fixed effects)
Inverse variance weighted (multiplicative random effects)
MR Egger
MR RAPS
Weighted median
Weighted mode
0.0050
0.0025
0.0000
−0.0025
−0.0050
SNP effect on eGFR
0.3
0.6
0.9
1.2
SNP effect on Beta−defensin 112

Batch 2−789 : Beta−defensin 113 on eGFR

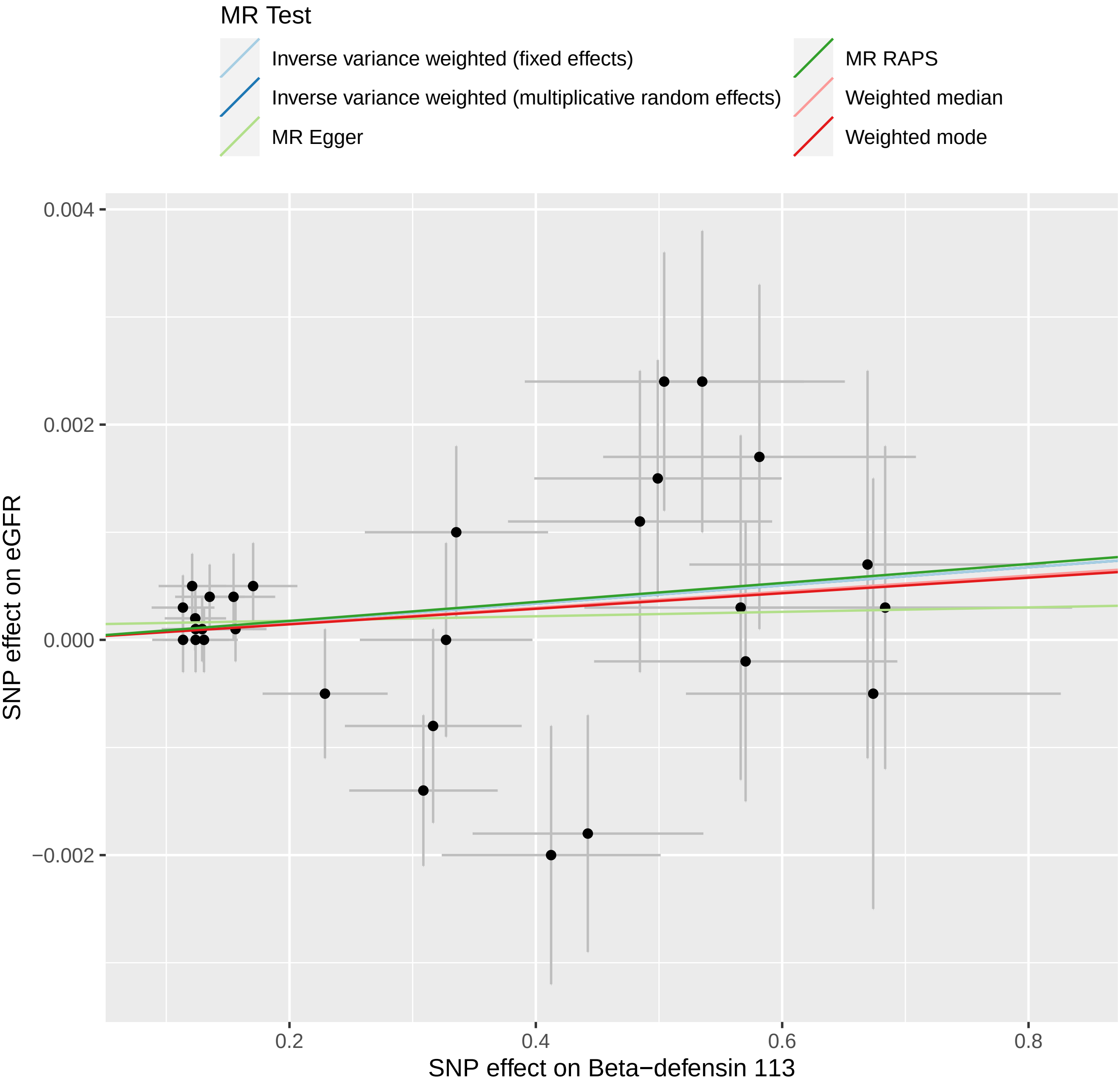

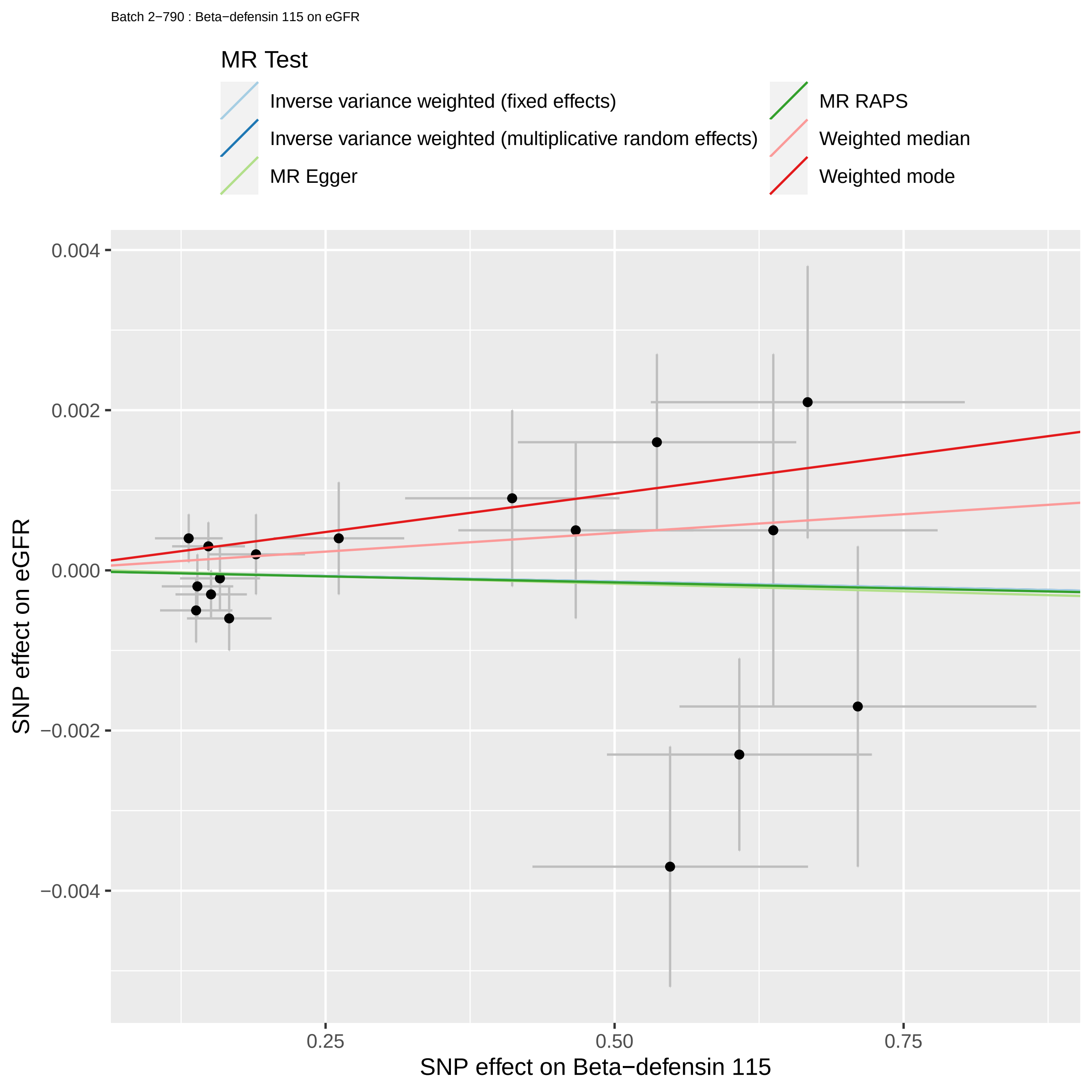

Batch 2−790 : Beta−defensin 115 on eGFR
MR Test
Inverse variance weighted (fixed effects)
Inverse variance weighted (multiplicative random effects)
MR Egger
MR RAPS
Weighted median
Weighted mode
0.004
0.002
0.000
−0.002
−0.004
0.25
0.50
0.75
SNP effect on Beta−defensin 115
SNP effect on eGFR

Batch 2−791 : Beta−defensin 116 on eGFR

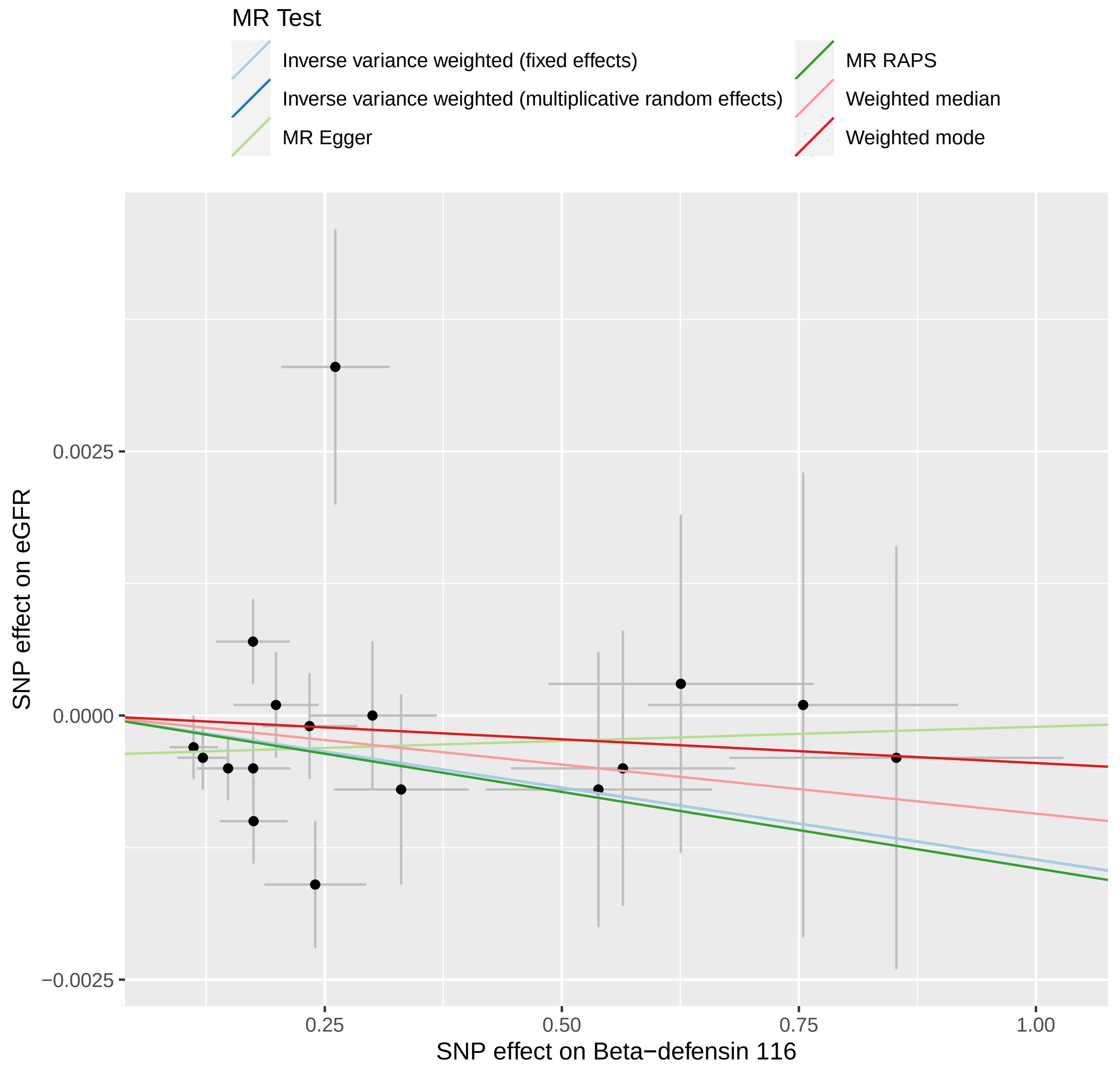

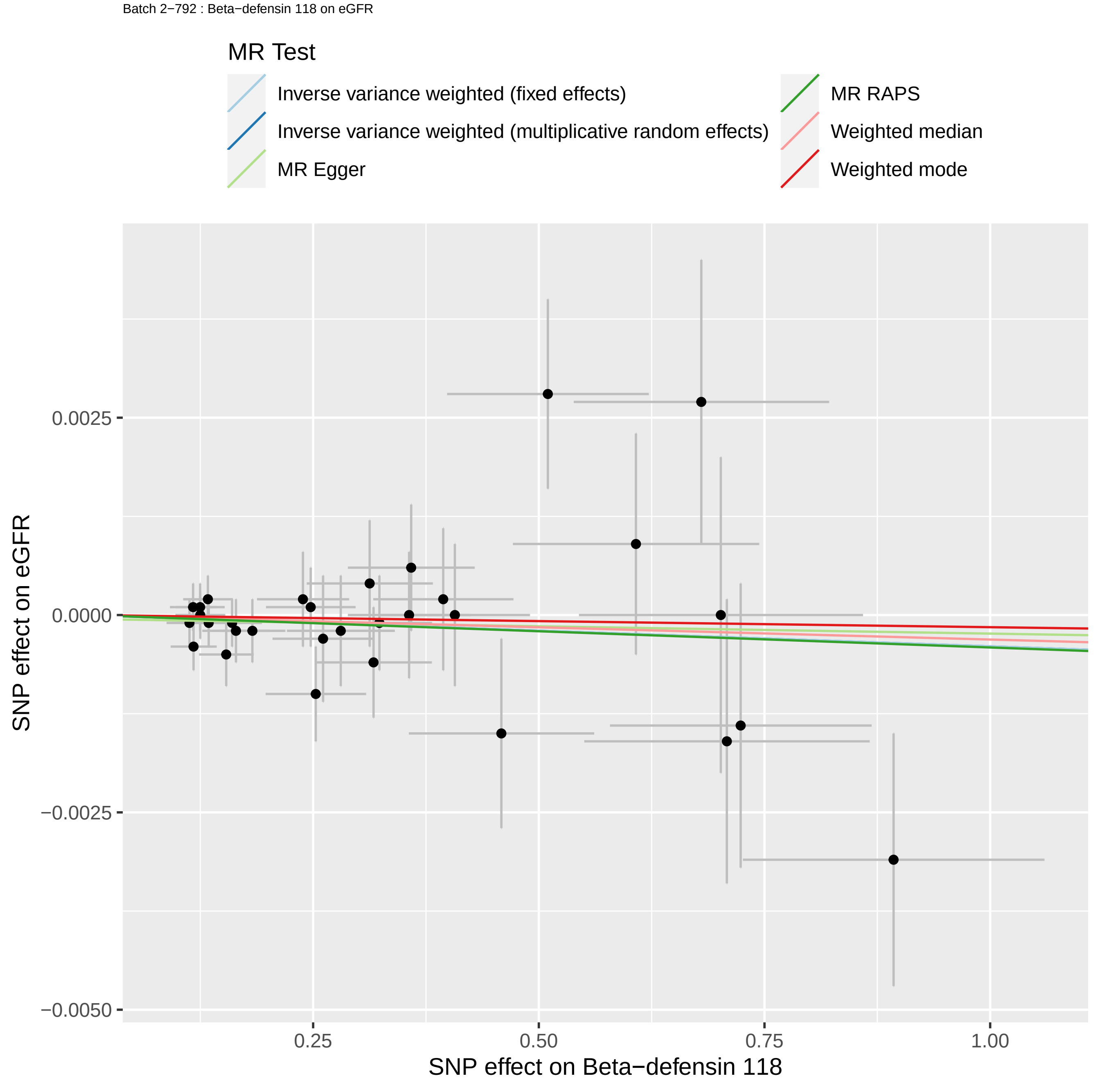

Batch 2−792 : Beta−defensin 118 on eGFR
MR Test
Inverse variance weighted (fixed effects)
Inverse variance weighted (multiplicative random effects)
MR Egger
MR RAPS
Weighted median
Weighted mode
0.0025
0.0000
−0.0025
−0.0050
0.25
0.50
0.75
1.00
SNP effect on eGFR
SNP effect on Beta−defensin 118

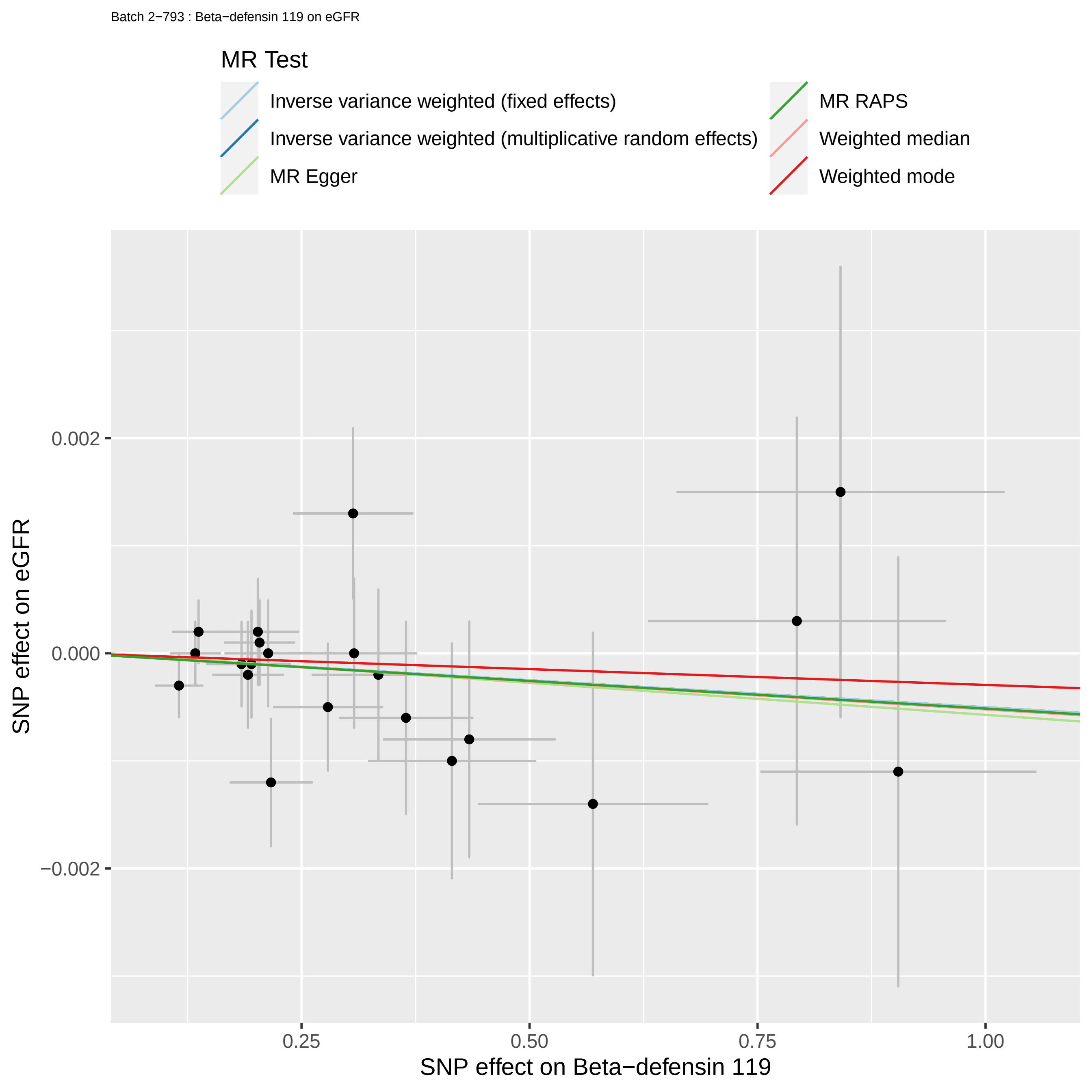

Batch 2−793 : Beta−defensin 119 on eGFR
MR Test
Inverse variance weighted (fixed effects)
Inverse variance weighted (multiplicative random effects)
MR Egger
MR RAPS
Weighted median
Weighted mode
0.002
0.000
−0.002
0.25
0.50
0.75
1.00
SNP effect on Beta−defensin 119
SNP effect on eGFR

Batch 2−794 : Beta−defensin 119 on eGFR

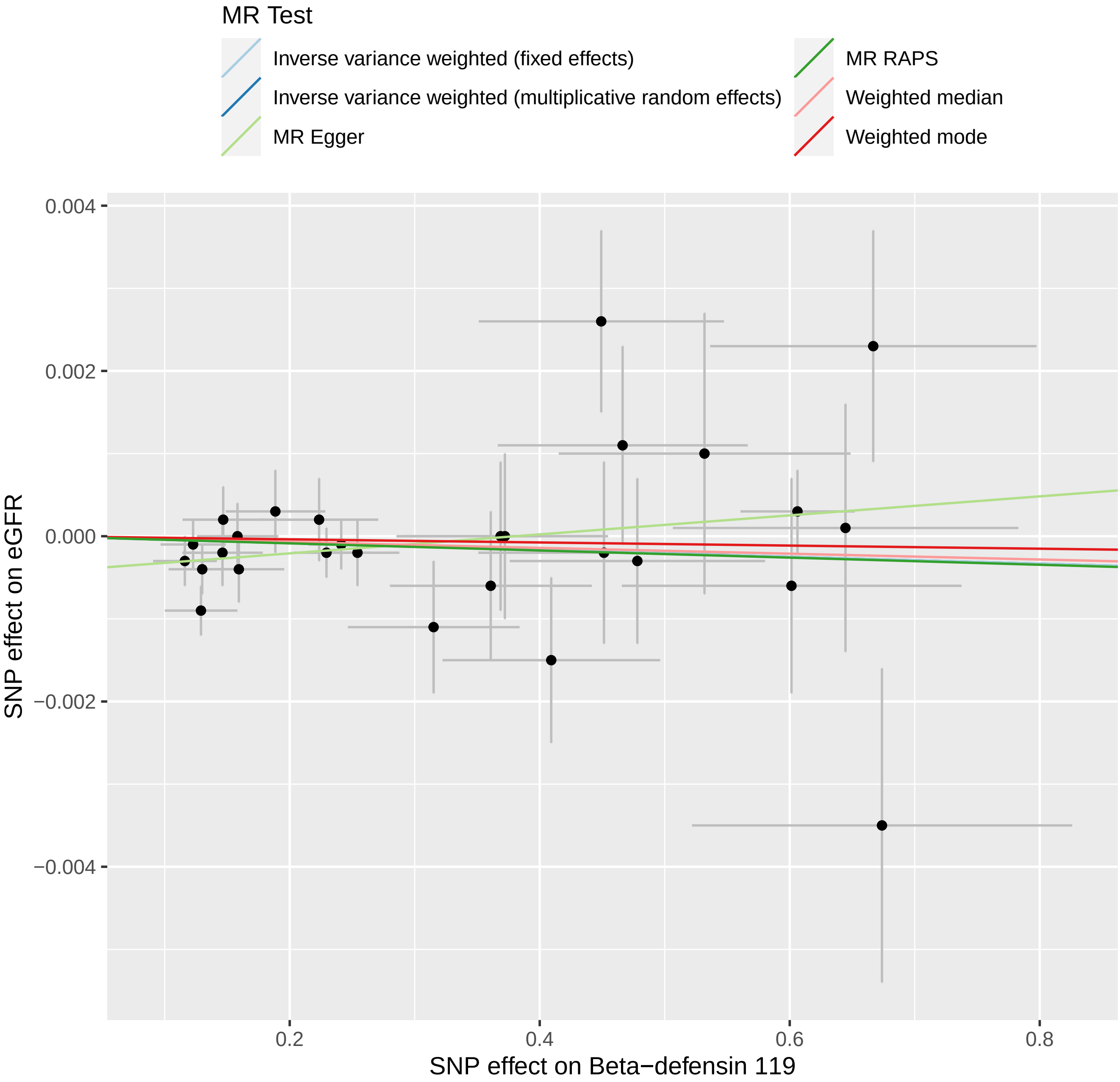

Batch 2−795 : Beta−defensin 119 on eGFR

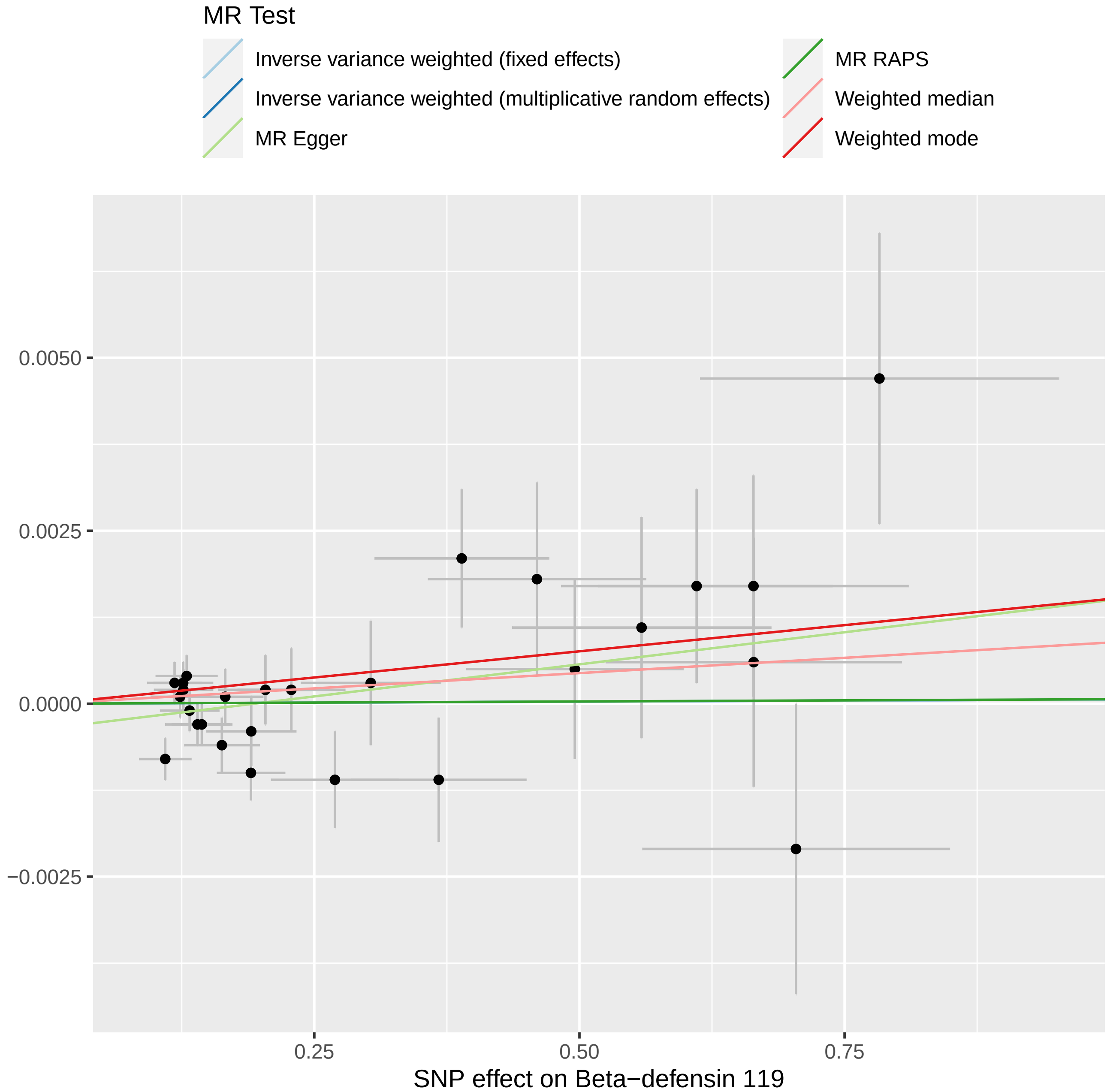

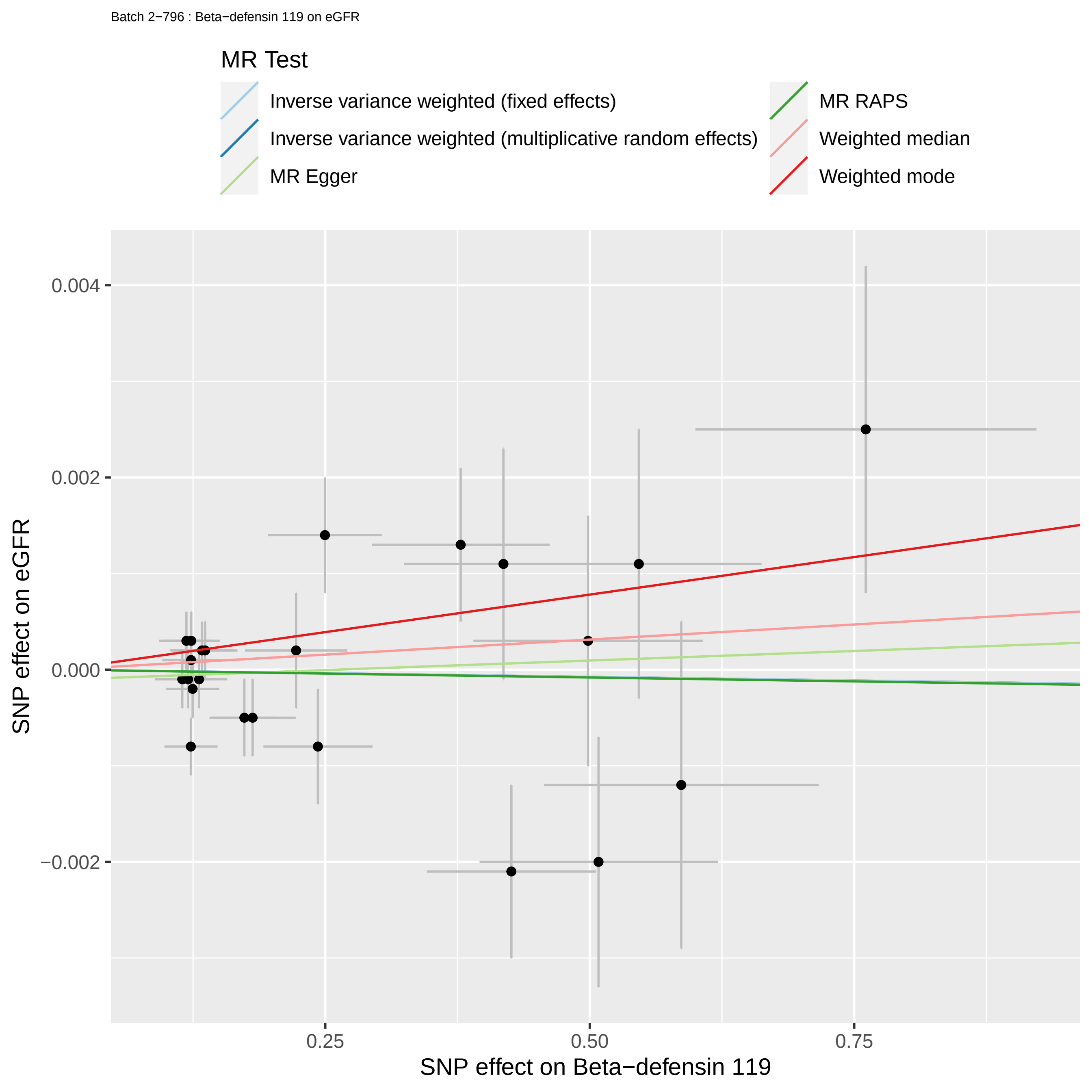
Batch 2−796 : Beta−defensin 119 on eGFR
MR Test
Inverse variance weighted (fixed effects)
Inverse variance weighted (multiplicative random effects)
MR Egger
MR RAPS
Weighted median
Weighted mode
0.004
0.002
0.000
−0.002
SNP effect on eGFR
0.25
0.50
0.75
SNP effect on Beta−defensin 119

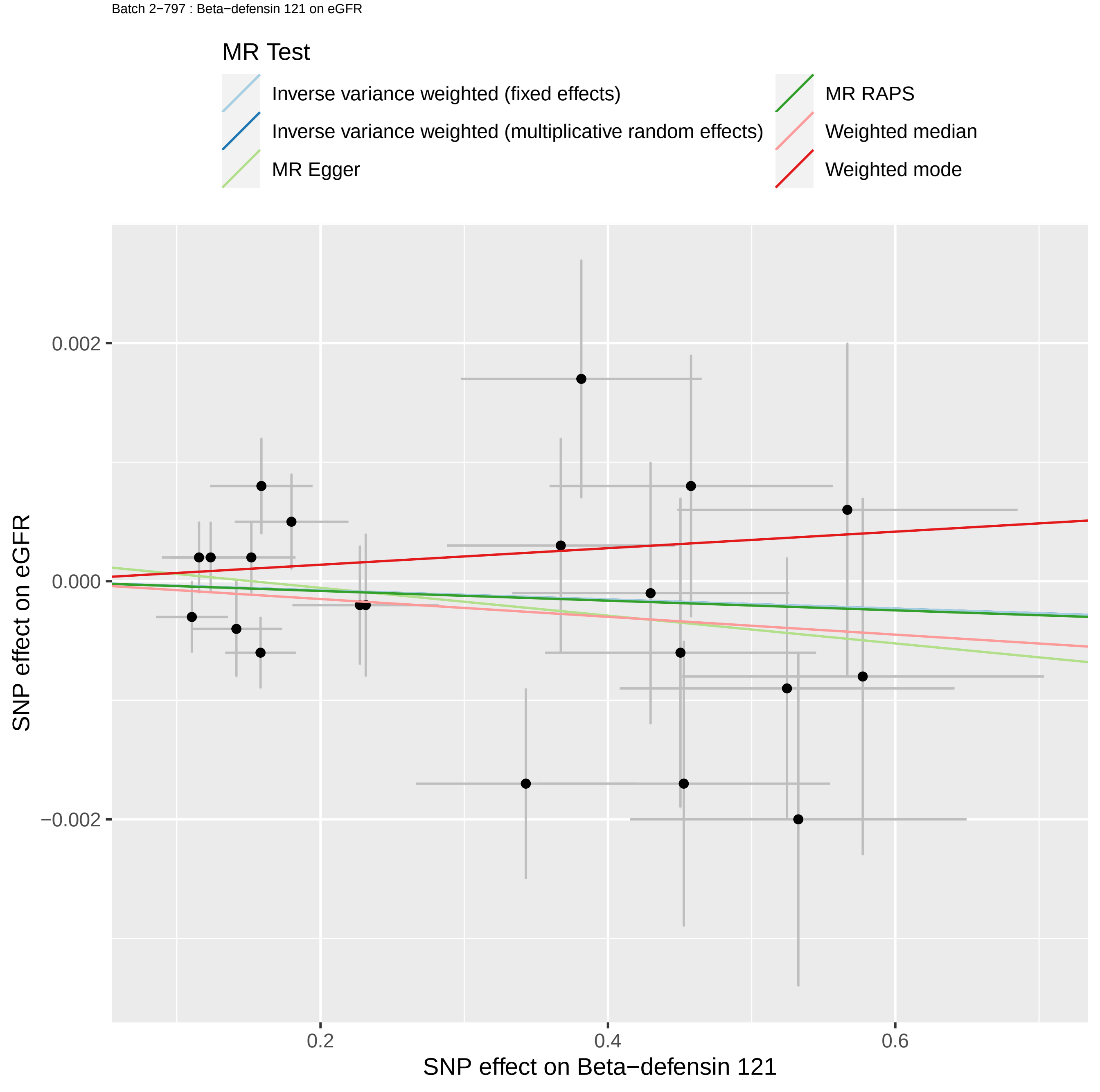
Batch 2−797 : Beta−defensin 121 on eGFR
MR Test
Inverse variance weighted (fixed effects)
Inverse variance weighted (multiplicative random effects)
MR Egger
MR RAPS
Weighted median
Weighted mode
0.002
0.000
−0.002
0.2
0.4
0.6
SNP effect on eGFR
SNP effect on Beta−defensin 121

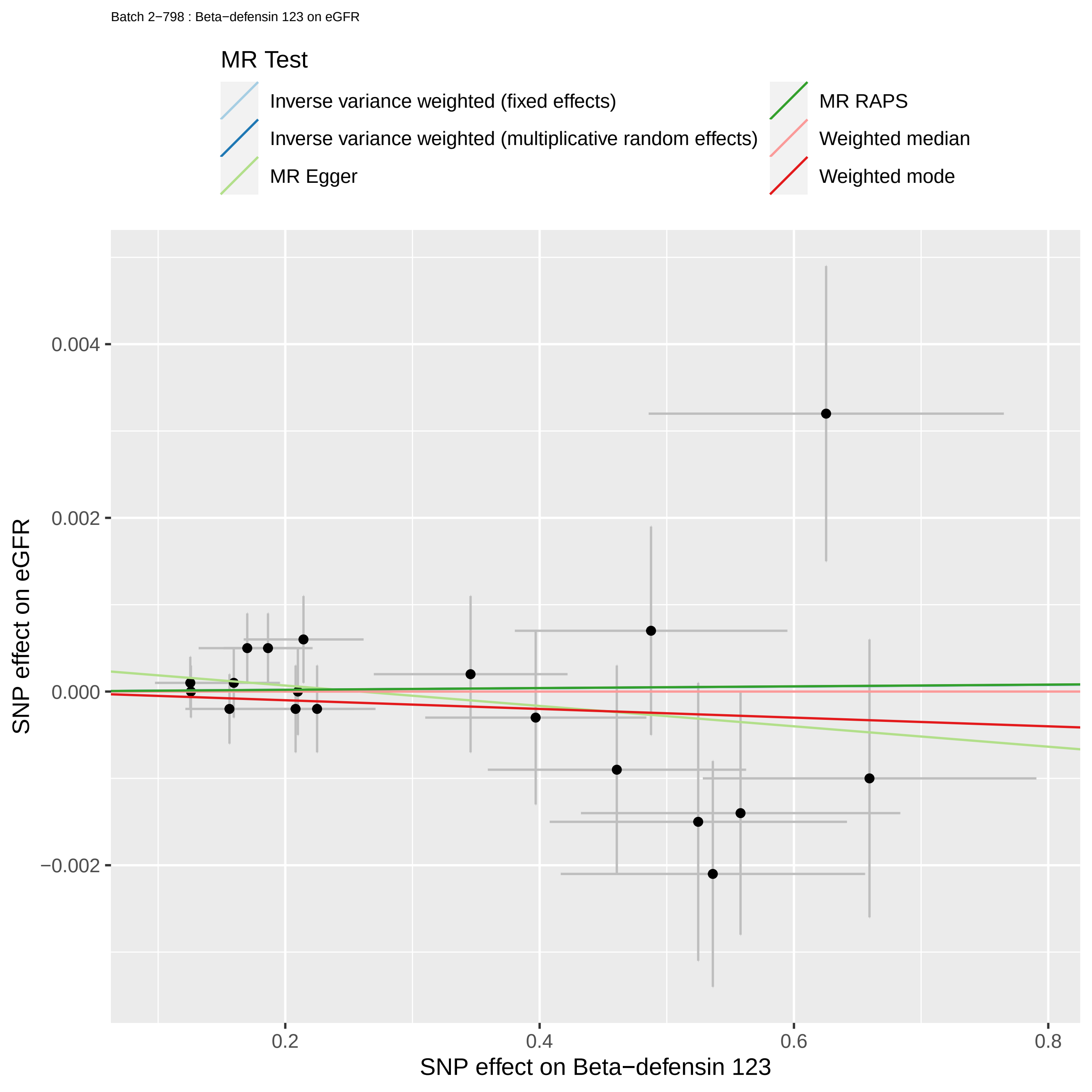
Batch 2−798 : Beta−defensin 123 on eGFR
MR Test
Inverse variance weighted (fixed effects)
Inverse variance weighted (multiplicative random effects)
MR Egger
MR RAPS
Weighted median
Weighted mode
0.004
0.002
0.000
−0.002
0.2
0.4
0.6
0.8
SNP effect on eGFR
SNP effect on Beta−defensin 123

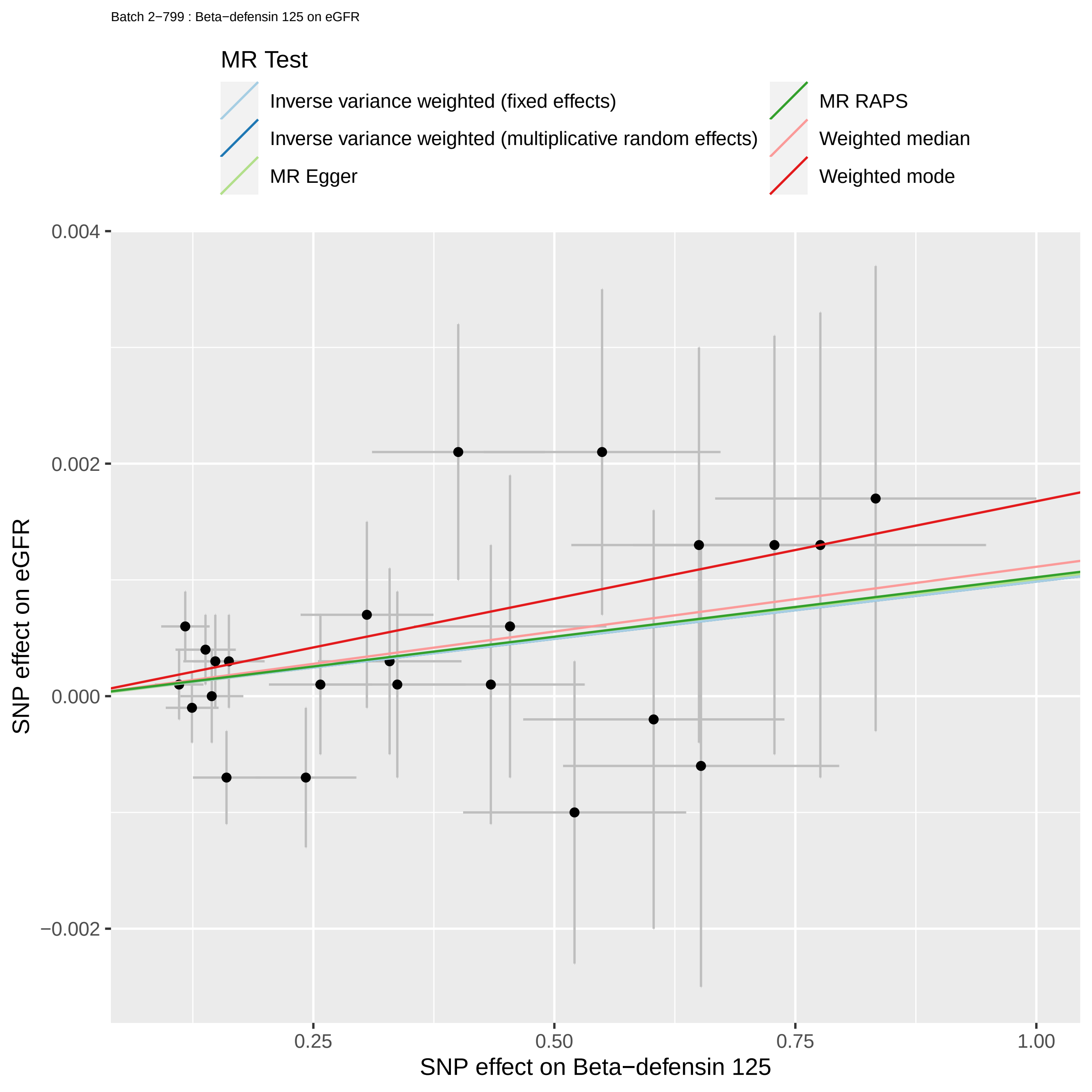
Batch 2−799 : Beta−defensin 125 on eGFR
MR Test
Inverse variance weighted (fixed effects)
Inverse variance weighted (multiplicative random effects)
MR Egger
MR RAPS
Weighted median
Weighted mode
0.004
0.002
0.000
−0.002
0.25
0.50
0.75
1.00
SNP effect on eGFR
SNP effect on Beta−defensin 125

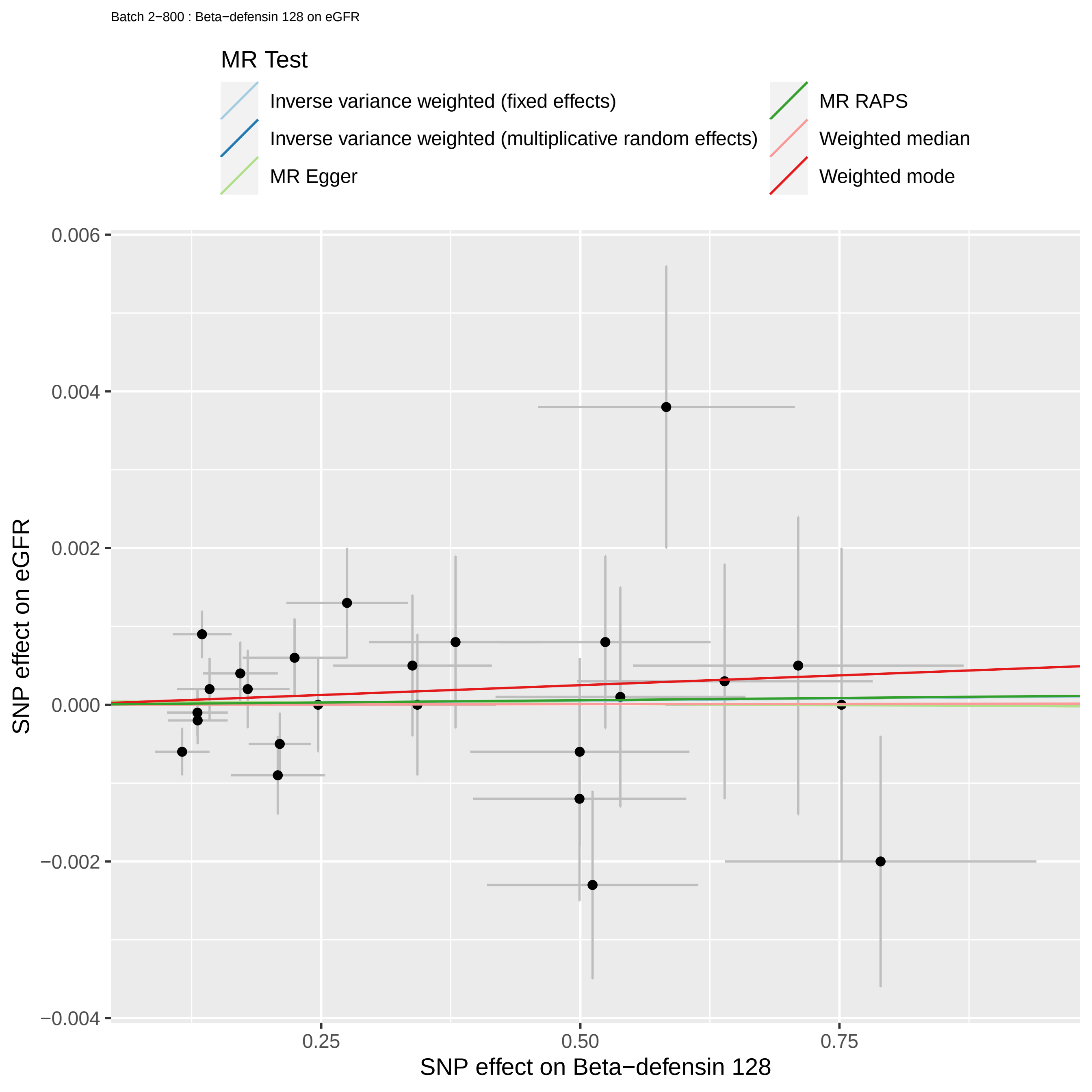

Batch 2−800 : Beta−defensin 128 on eGFR
MR Test
Inverse variance weighted (fixed effects)
Inverse variance weighted (multiplicative random effects)
MR Egger
MR RAPS
Weighted median
Weighted mode
0.006
0.004
0.002
0.000
−0.002
−0.004
0.25
0.50
0.75
SNP effect on eGFR
SNP effect on Beta−defensin 128

Batch 2−801 : Beta−defensin 134 on eGFR

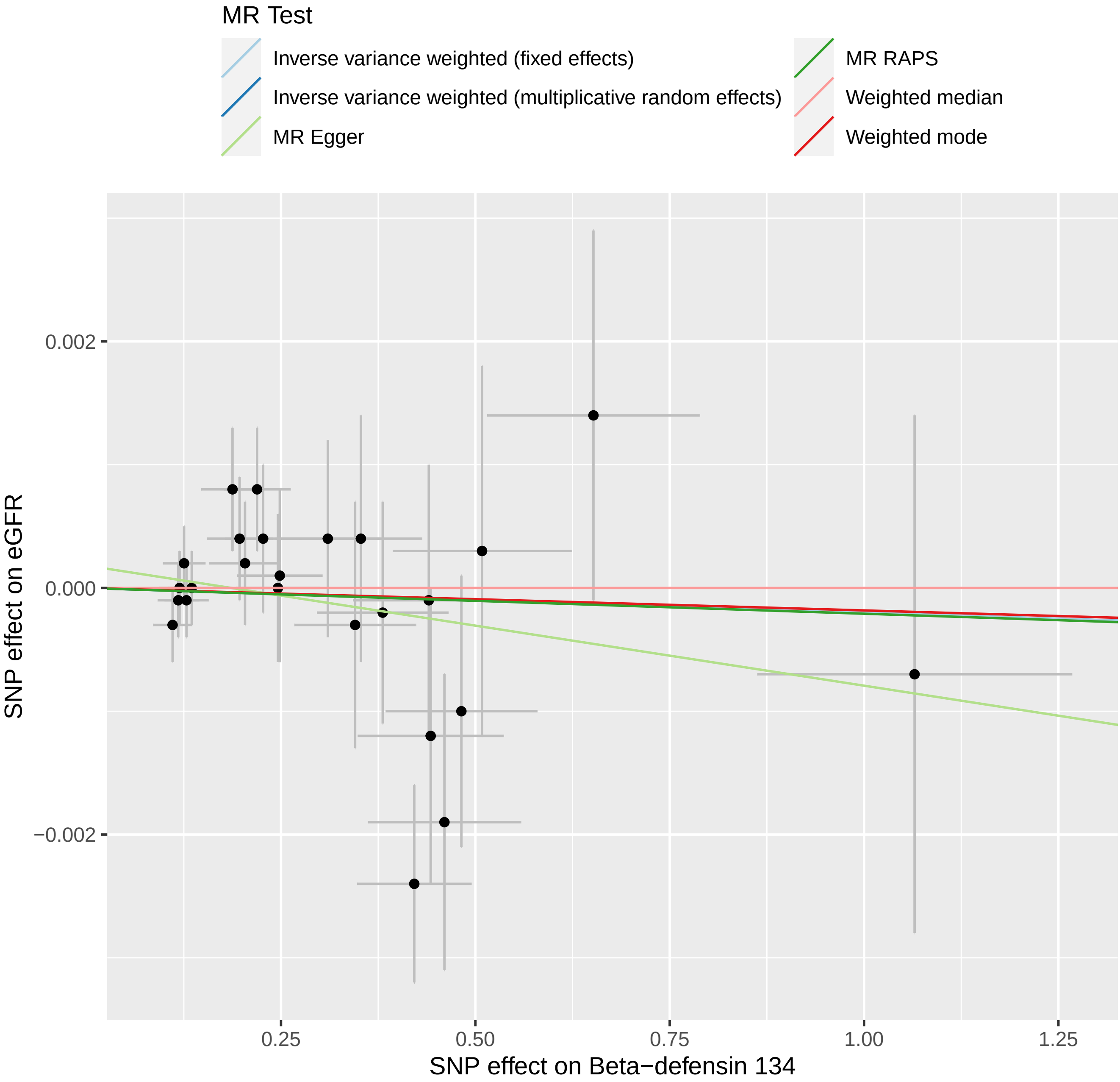

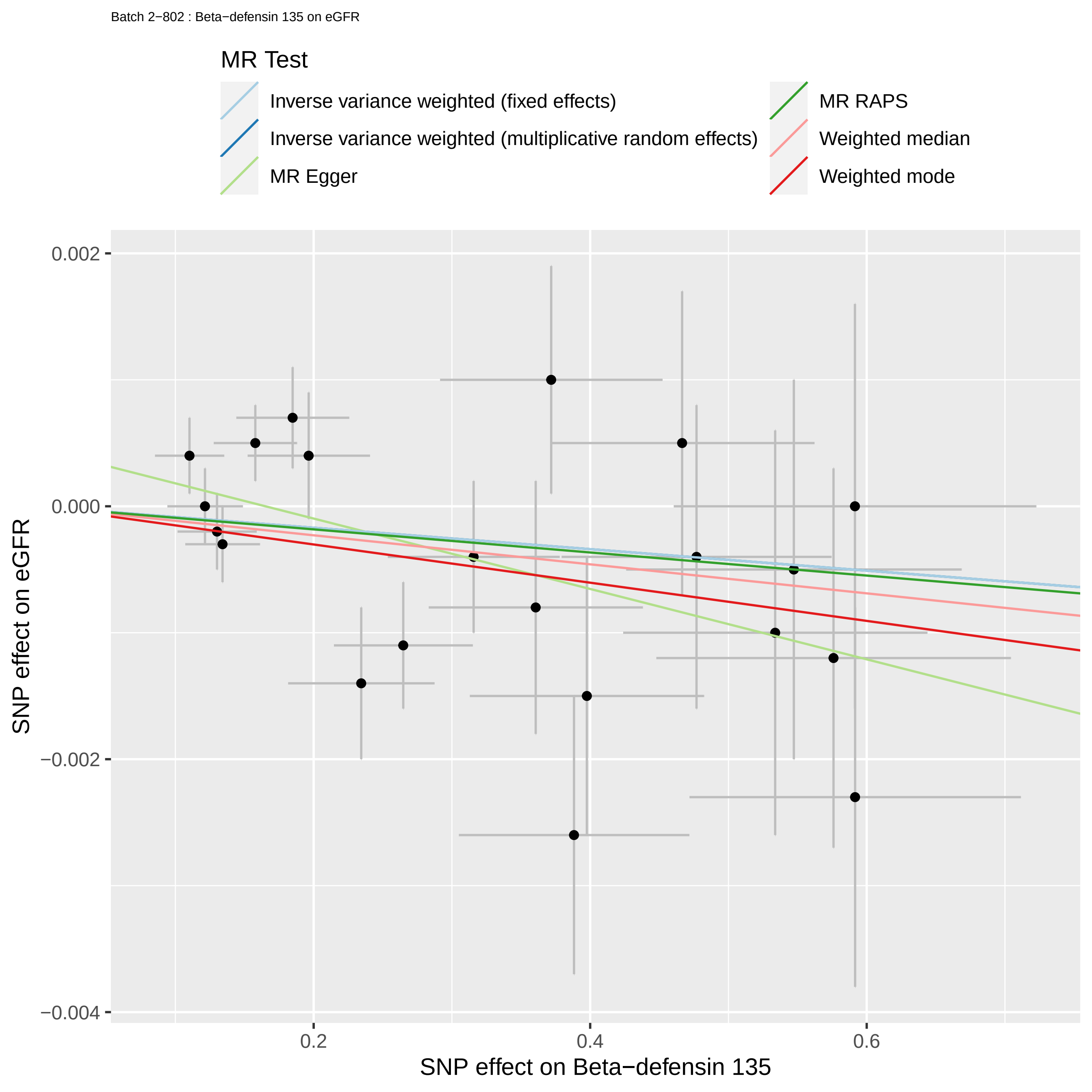

Batch 2−802 : Beta−defensin 135 on eGFR
MR Test
Inverse variance weighted (fixed effects)
Inverse variance weighted (multiplicative random effects)
MR Egger
MR RAPS
Weighted median
Weighted mode
0.002
0.000
−0.002
−0.004
SNP effect on eGFR
0.2
0.4
0.6
SNP effect on Beta−defensin 135

Batch 2−803 : Beta−defensin 136 on eGFR

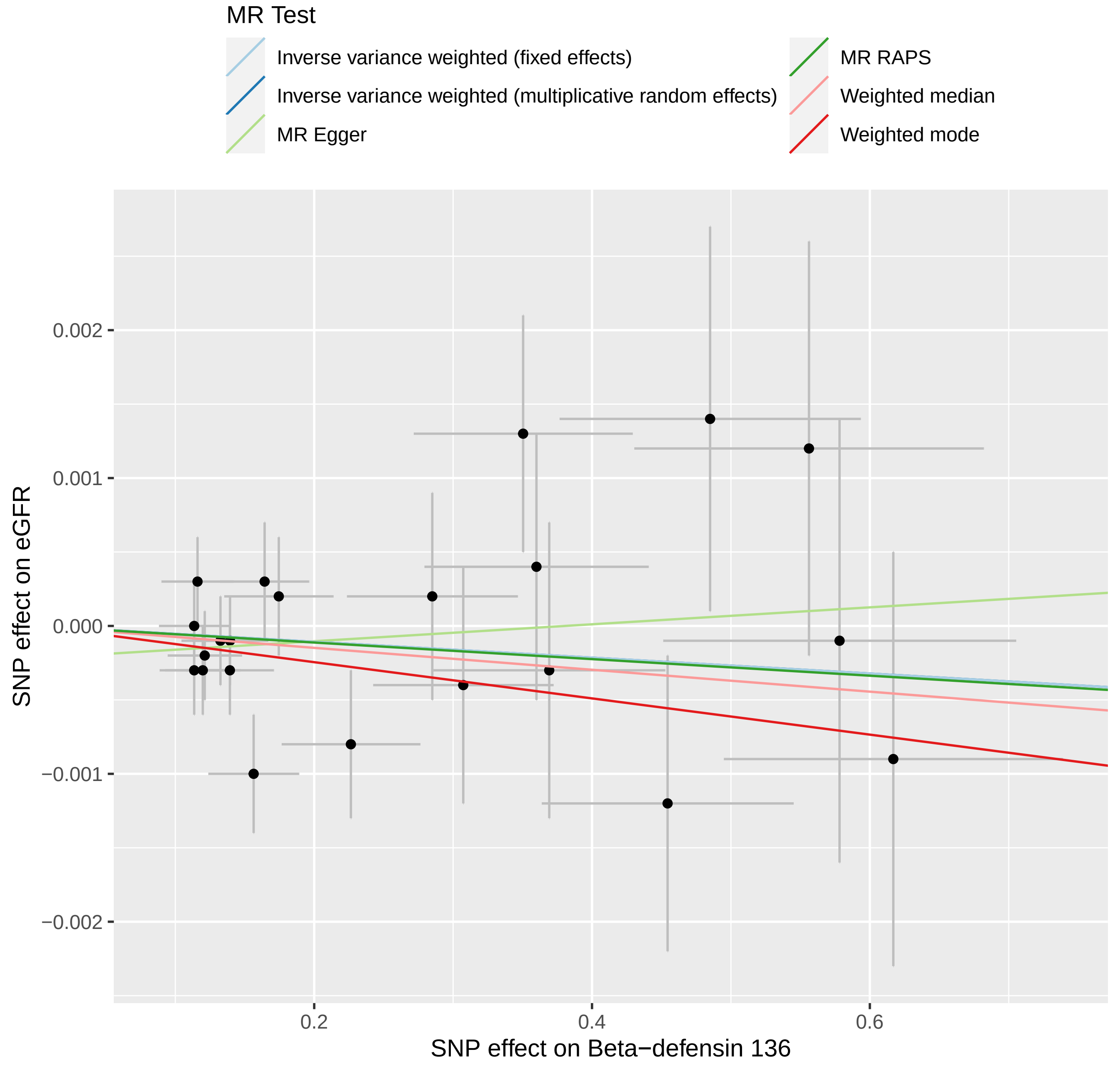

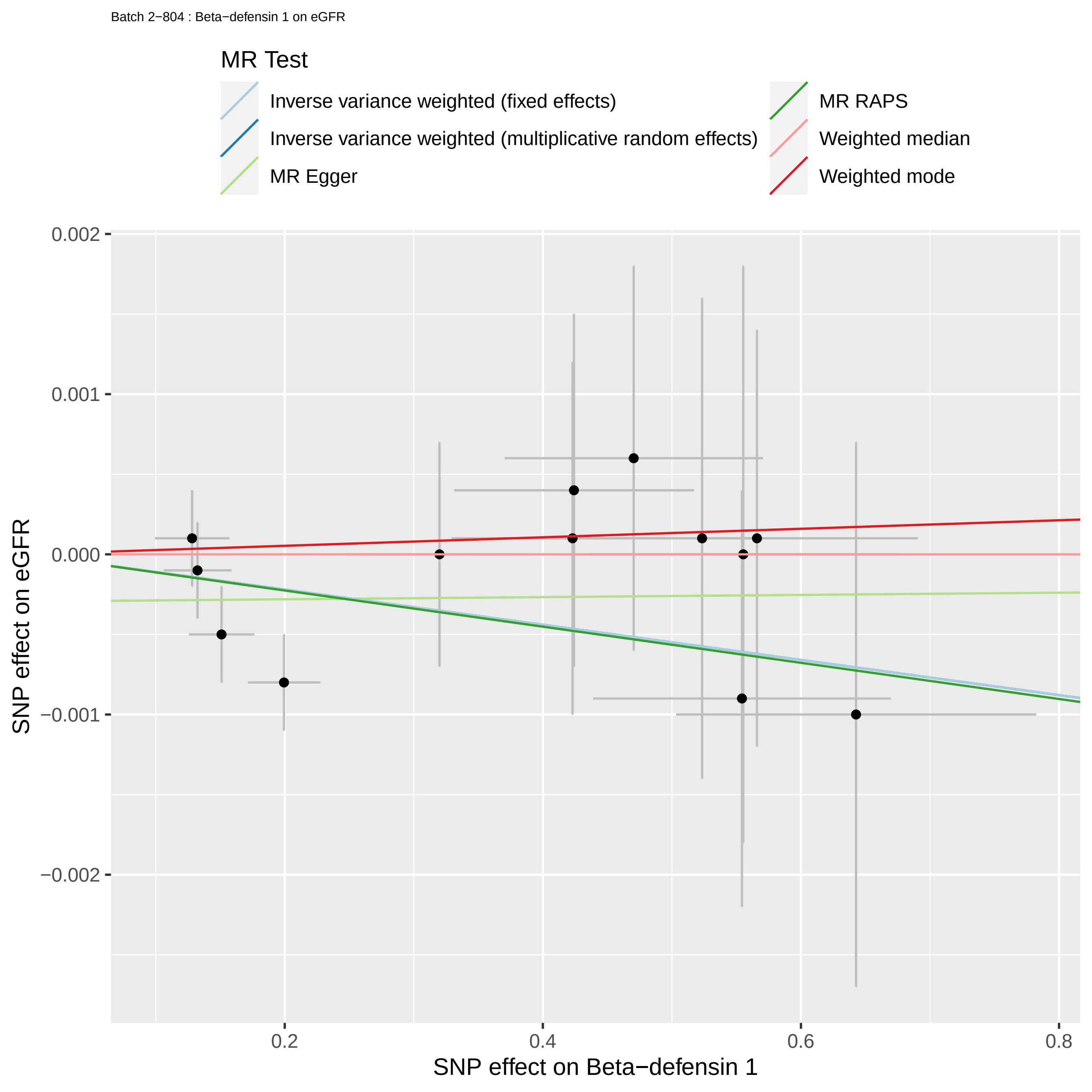

Batch 2−804 : Beta−defensin 1 on eGFR
MR Test
Inverse variance weighted (fixed effects)
Inverse variance weighted (multiplicative random effects)
MR Egger
MR RAPS
Weighted median
Weighted mode
0.002
0.001
0.000
−0.001
−0.002
SNP effect on eGFR
0.2
0.4
0.6
0.8
SNP effect on Beta−defensin 1

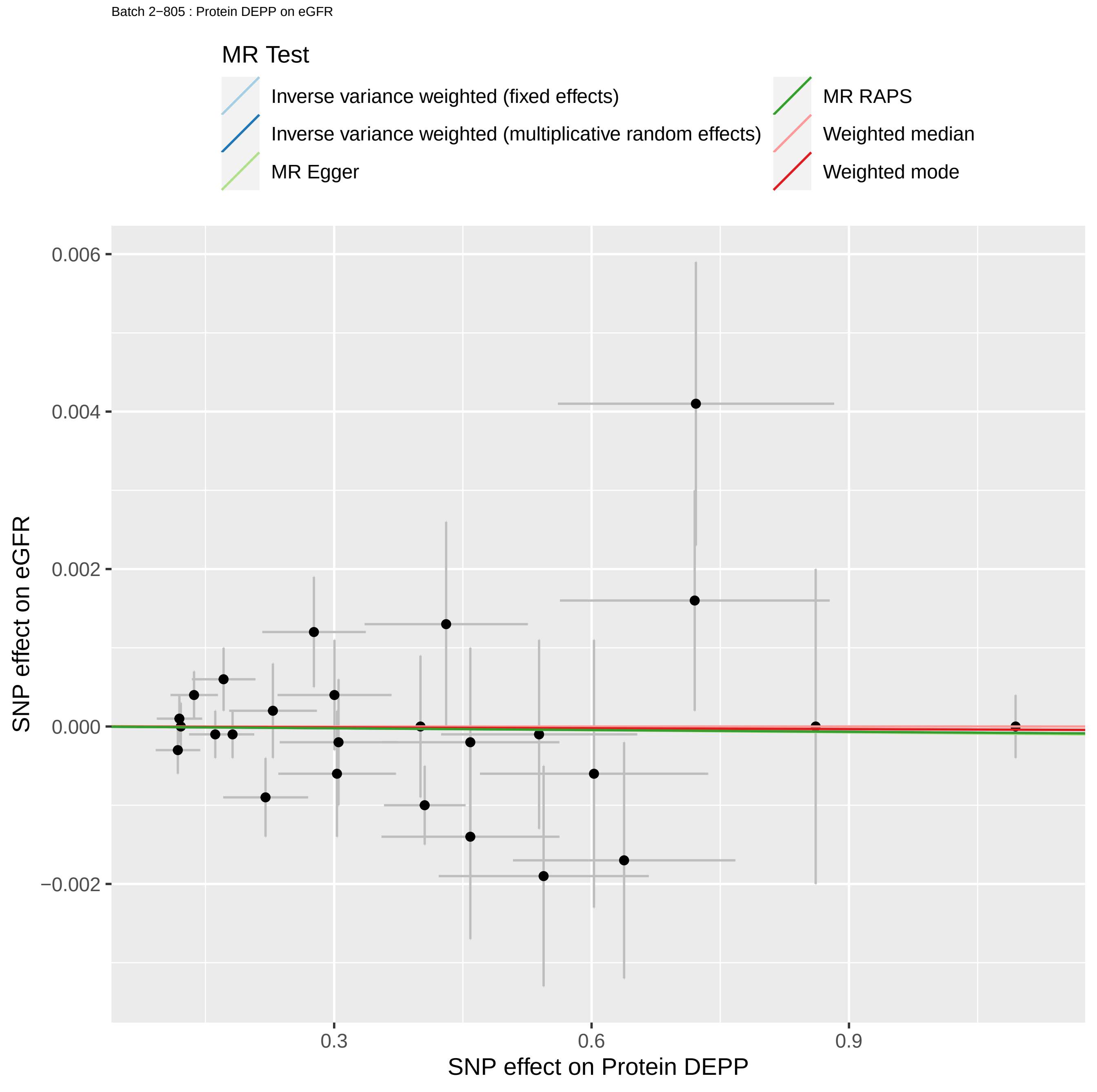

Batch 2−805 : Protein DEPP on eGFR
MR Test
Inverse variance weighted (fixed effects)
Inverse variance weighted (multiplicative random effects)
MR Egger
MR RAPS
Weighted median
Weighted mode
0.006
0.004
0.002
0.000
−0.002
0.3
0.6
0.9
SNP effect on eGFR
SNP effect on Protein DEPP

Batch 2−806 : Derlin−1 on eGFR

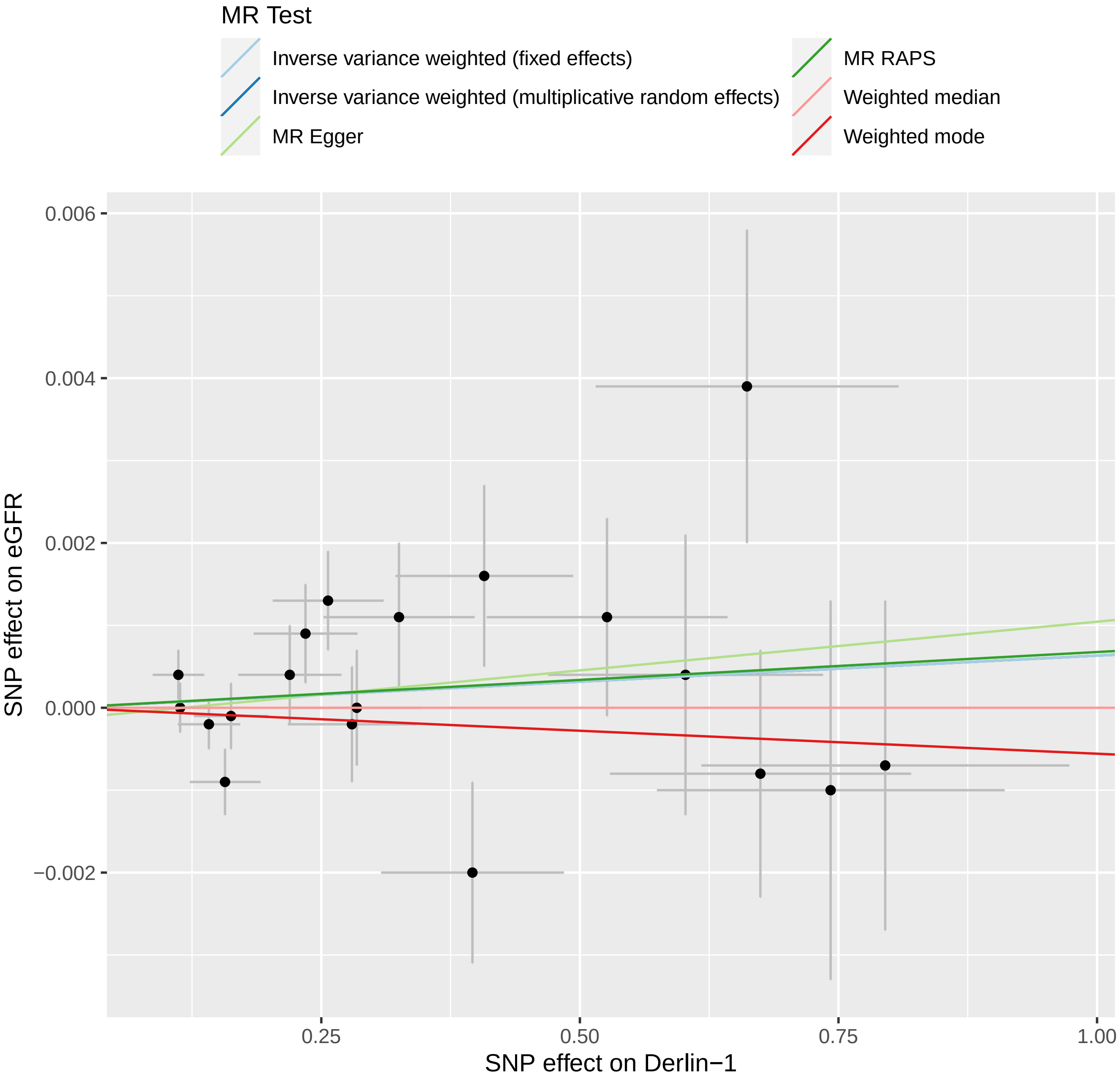

Batch 2−807 : Desmin on eGFR

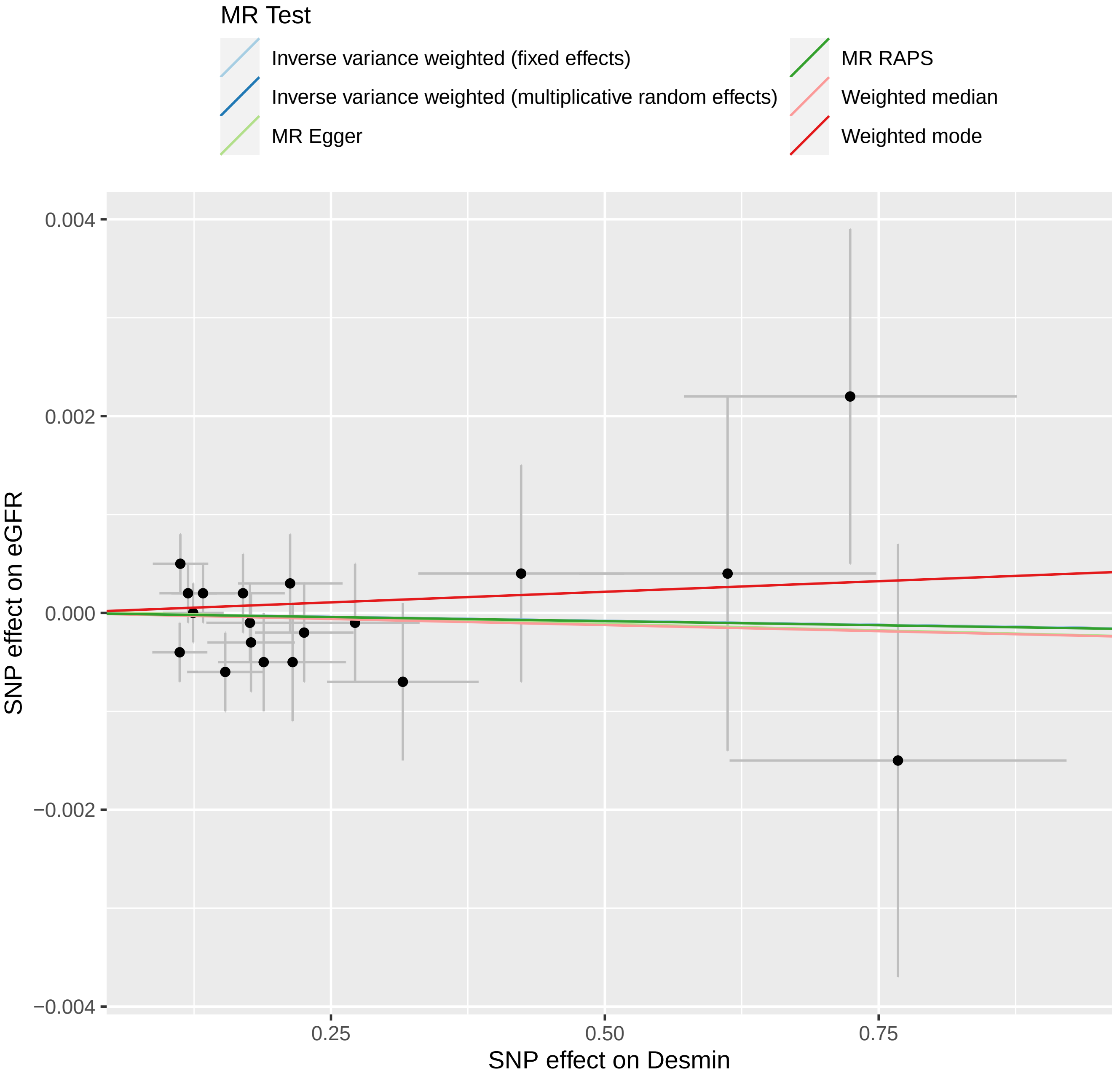

Batch 2−808 : Protein DGCR14 on eGFR

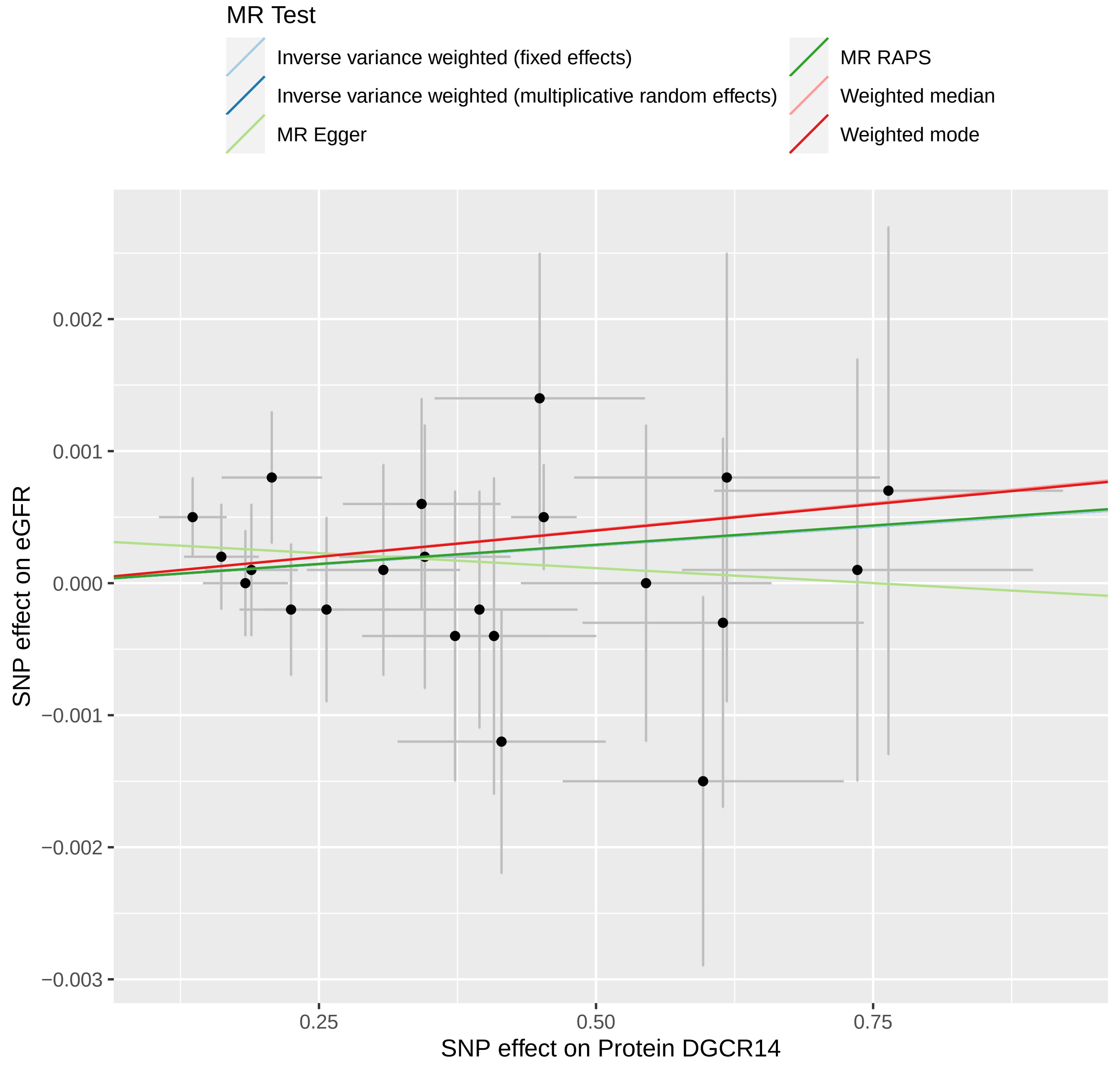

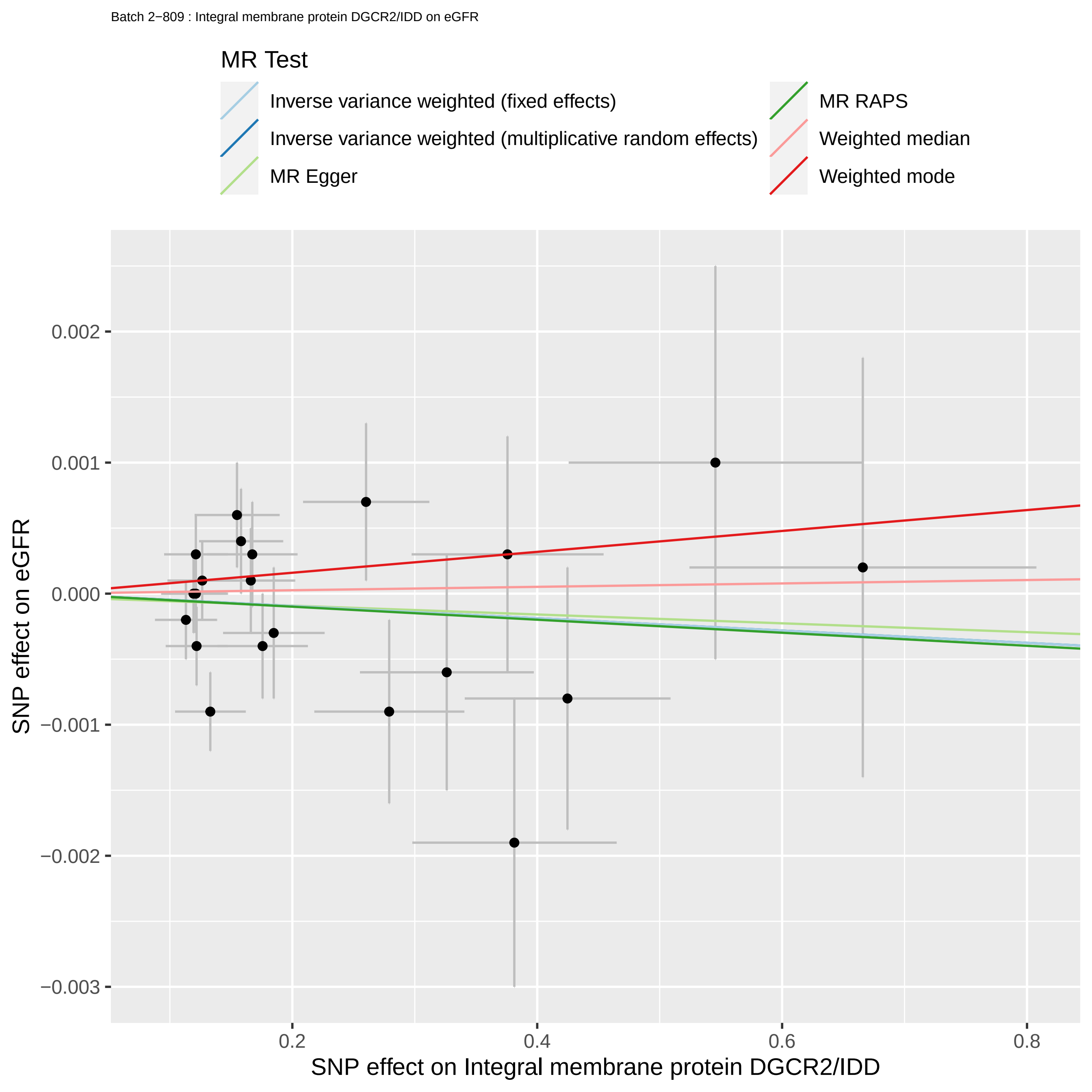

Batch 2−809 : Integral membrane protein DGCR2/IDD on eGFR
MR Test
Inverse variance weighted (fixed effects)
Inverse variance weighted (multiplicative random effects)
MR Egger
MR RAPS
Weighted median
Weighted mode
0.002
0.001
0.000
−0.001
−0.002
−0.003
0.2
0.4
0.6
0.8
SNP effect on eGFR
SNP effect on Integral membrane protein DGCR2/IDD

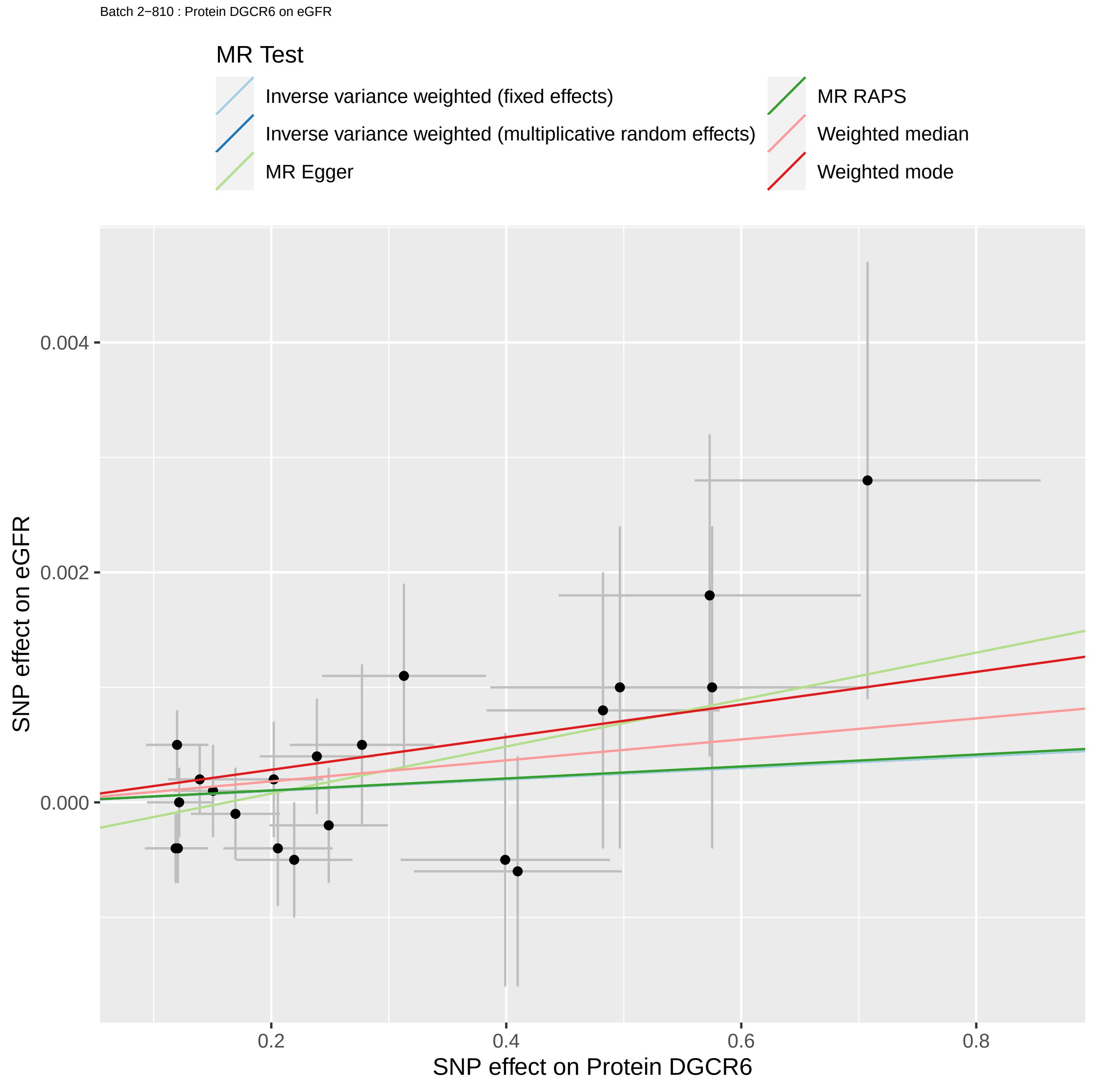
Batch 2−810 : Protein DGCR6 on eGFR
MR Test
Inverse variance weighted (fixed effects)
Inverse variance weighted (multiplicative random effects)
MR Egger
MR RAPS
Weighted median
Weighted mode
SNP effect on eGFR
0.004
0.002
0.000
0.2
0.4
0.6
0.8
SNP effect on Protein DGCR6

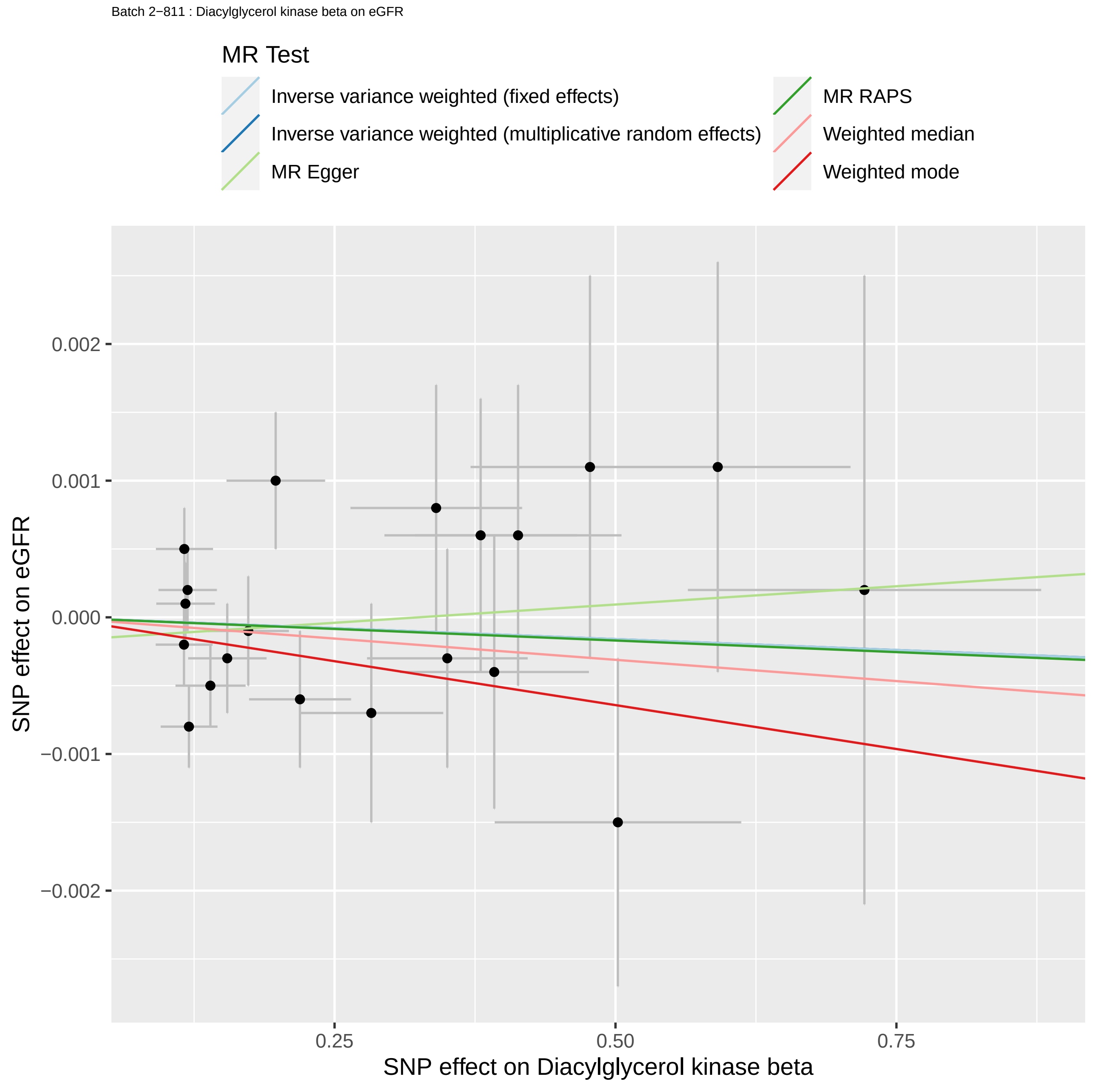
Batch 2−811 : Diacylglycerol kinase beta on eGFR
MR Test
Inverse variance weighted (fixed effects)
Inverse variance weighted (multiplicative random effects)
MR Egger
MR RAPS
Weighted median
Weighted mode
0.002
0.001
0.000
−0.001
−0.002
SNP effect on eGFR
0.25
0.50
0.75
SNP effect on Diacylglycerol kinase beta

Batch 2−812 : Dihydrofolate reductase on eGFR

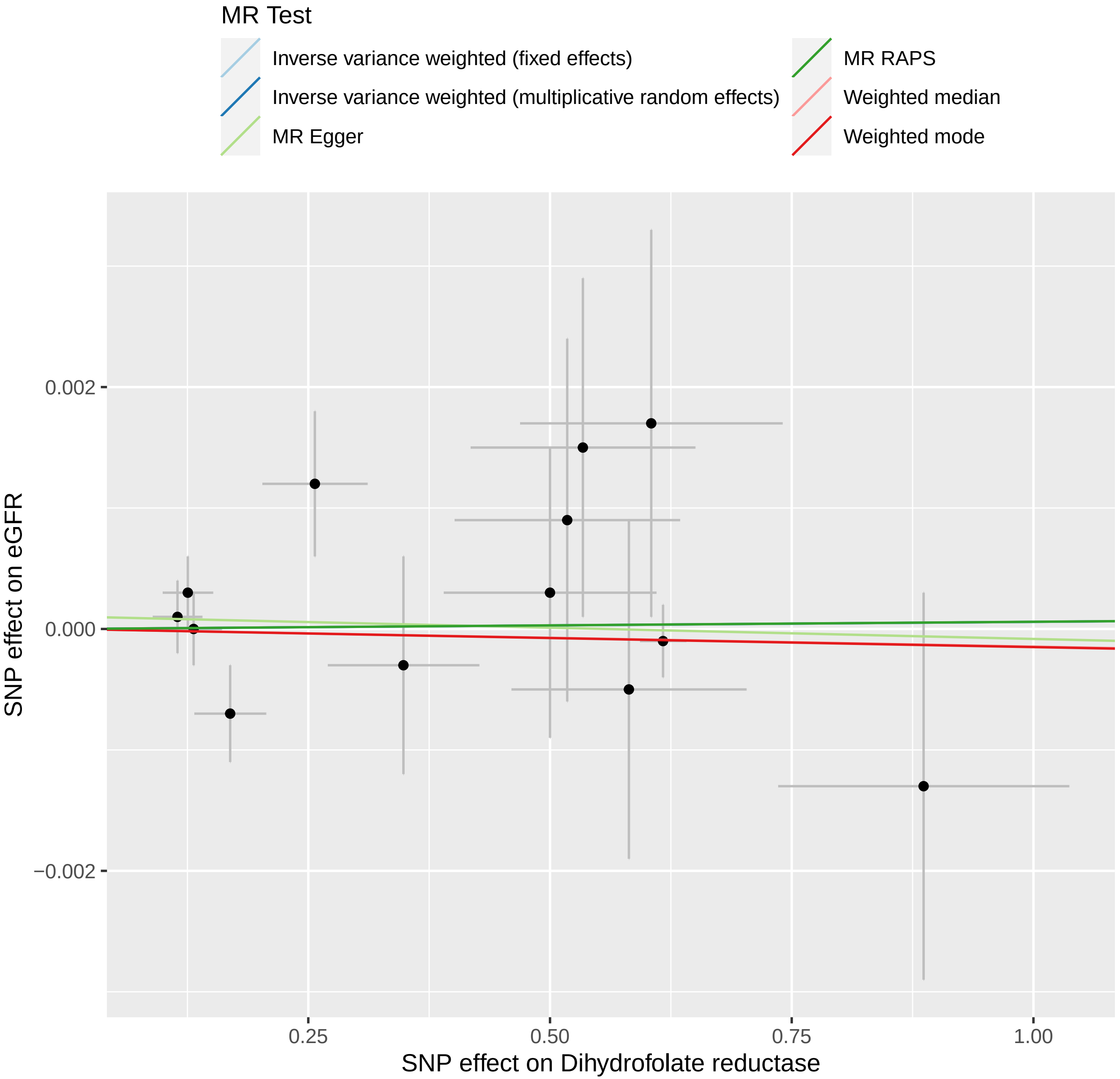

Batch 2−813 : Desert hedgehog protein N−product on eGFR

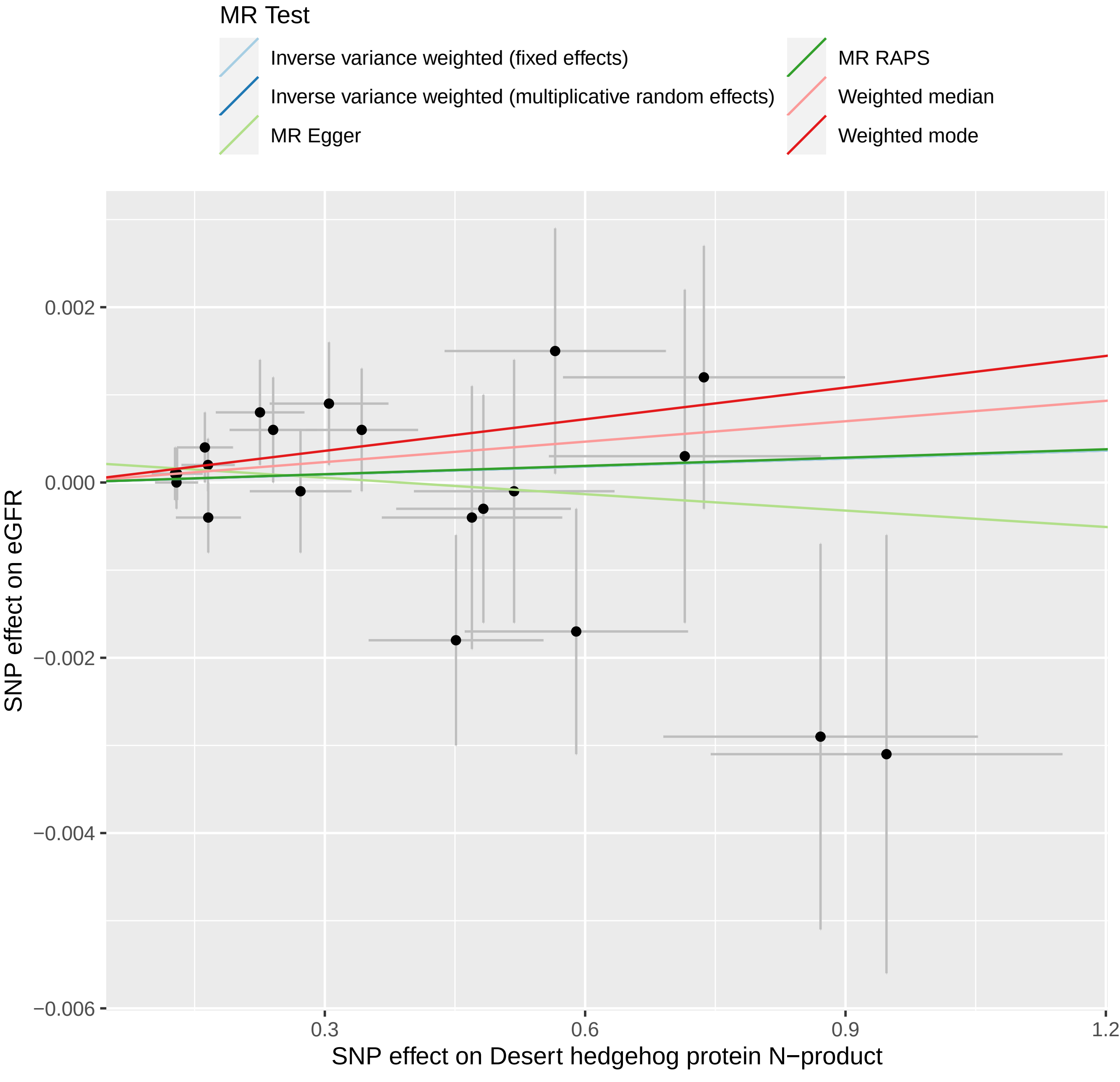

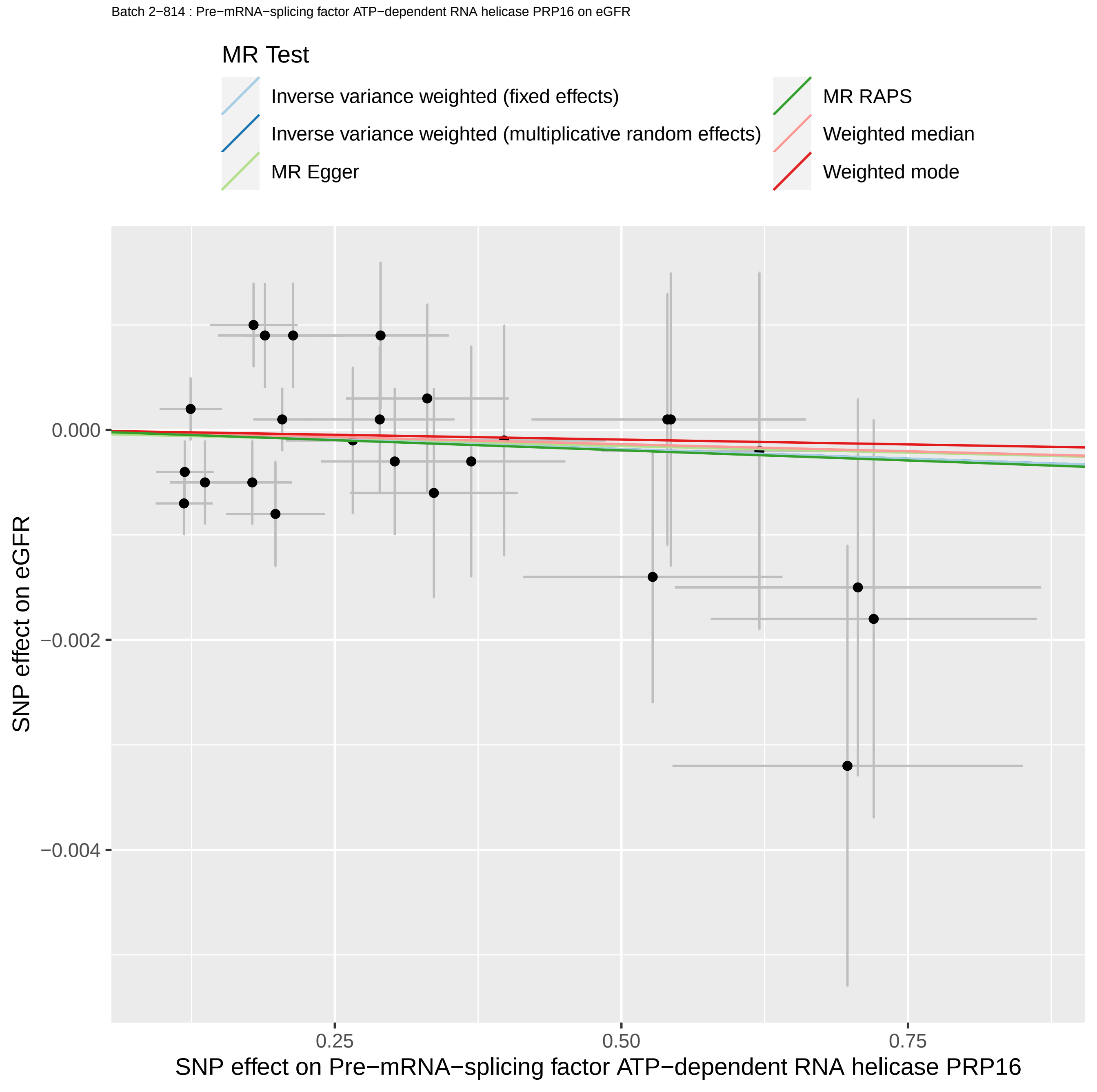
Batch 2−814 : Pre−mRNA−splicing factor ATP−dependent RNA helicase PRP16 on eGFR
MR Test
Inverse variance weighted (fixed effects)
Inverse variance weighted (multiplicative random effects)
MR Egger
MR RAPS
Weighted median
Weighted mode
0.000
−0.002
−0.004
0.25
0.50
0.75
SNP effect on eGFR
SNP effect on Pre−mRNA−splicing factor ATP−dependent RNA helicase PRP16

Batch 2−815 : Probable ATP−dependent RNA helicase DHX58 on eGFR

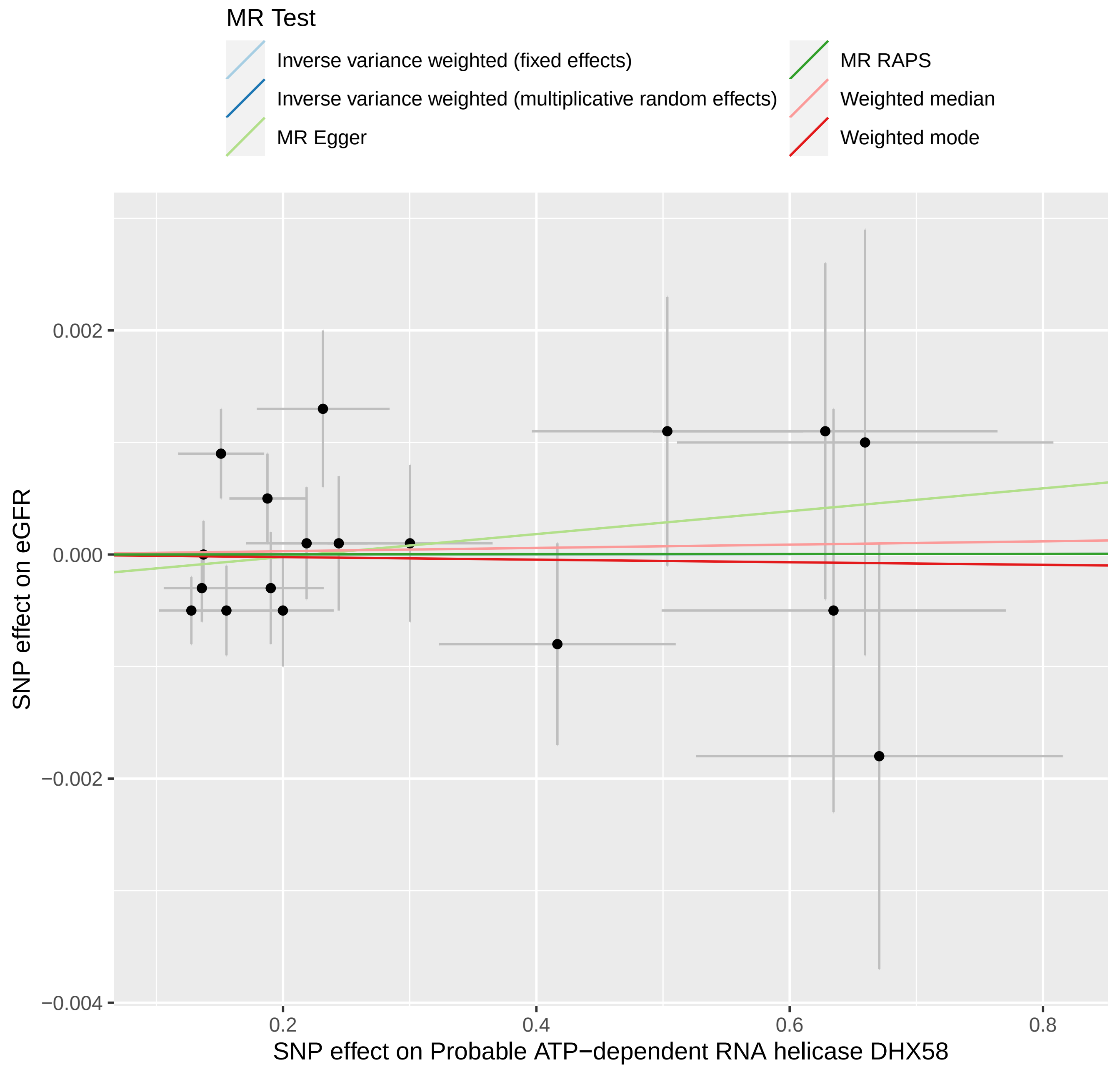

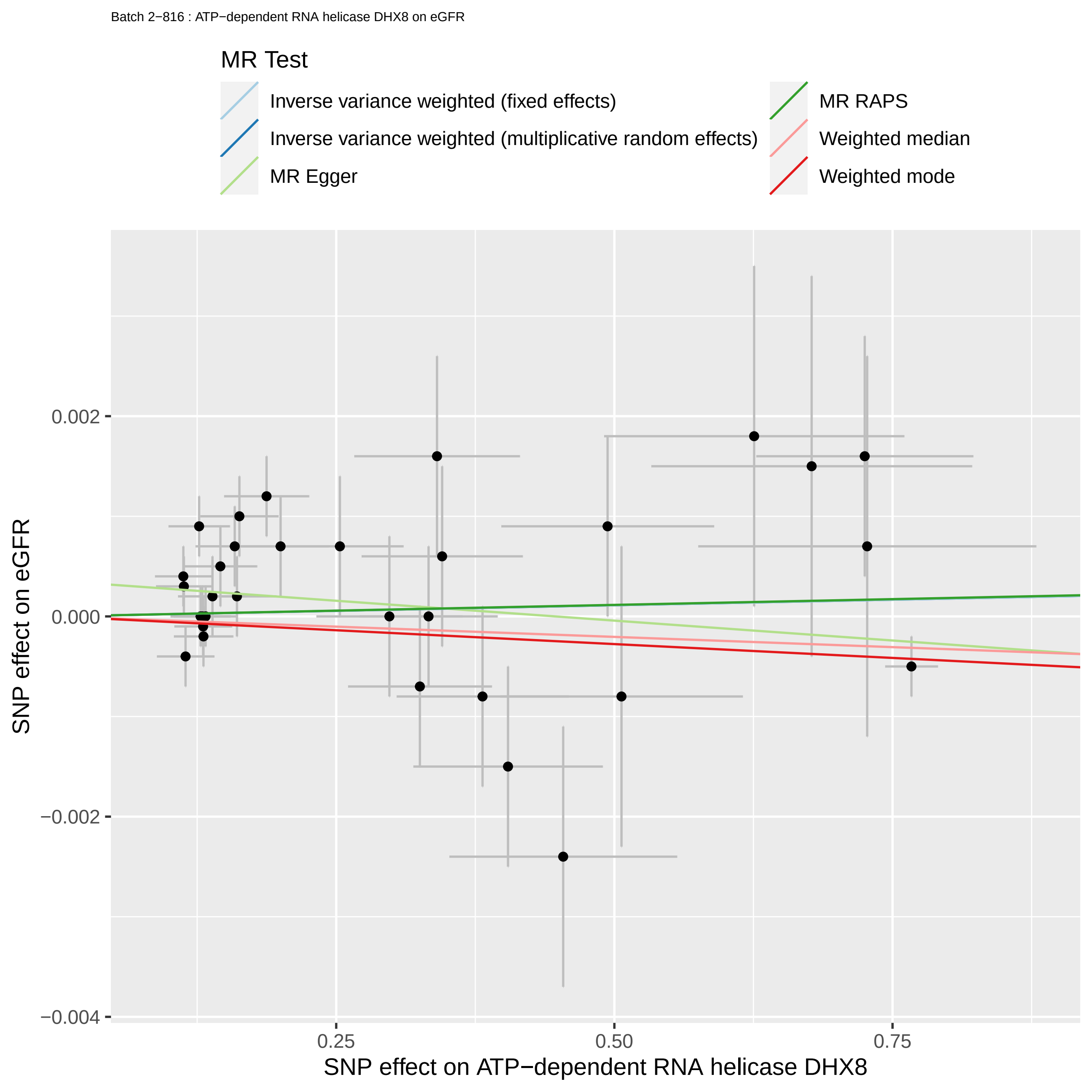

Batch 2−816 : ATP−dependent RNA helicase DHX8 on eGFR
MR Test
Inverse variance weighted (fixed effects)
Inverse variance weighted (multiplicative random effects)
MR Egger
MR RAPS
Weighted median
Weighted mode
0.002
0.000
−0.002
−0.004
0.25
0.50
0.75
SNP effect on eGFR
SNP effect on ATP−dependent RNA helicase DHX8

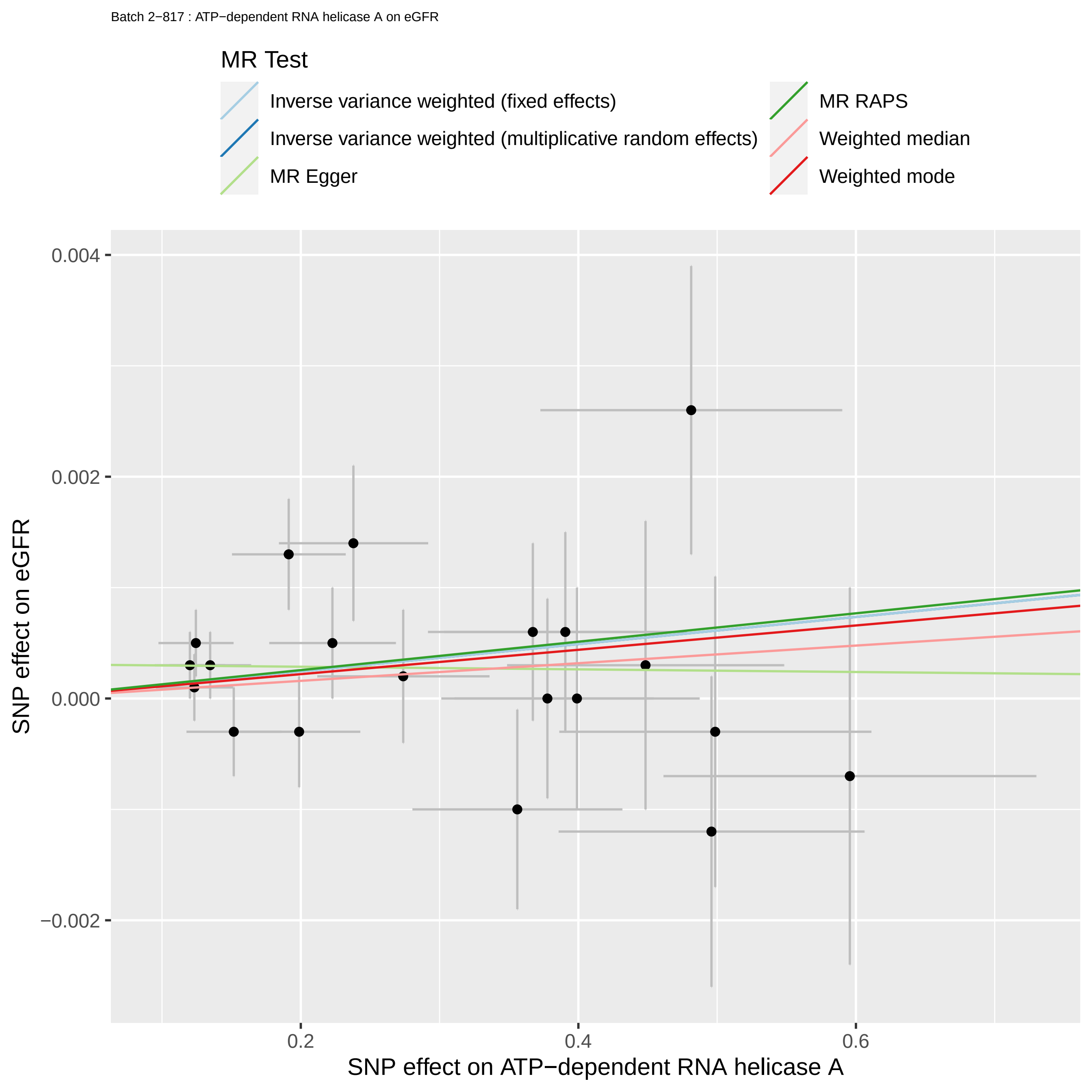

Batch 2−817 : ATP−dependent RNA helicase A on eGFR
MR Test
Inverse variance weighted (fixed effects)
Inverse variance weighted (multiplicative random effects)
MR Egger
MR RAPS
Weighted median
Weighted mode
0.004
0.002
0.000
−0.002
SNP effect on eGFR
0.2
0.4
0.6
SNP effect on ATP−dependent RNA helicase A

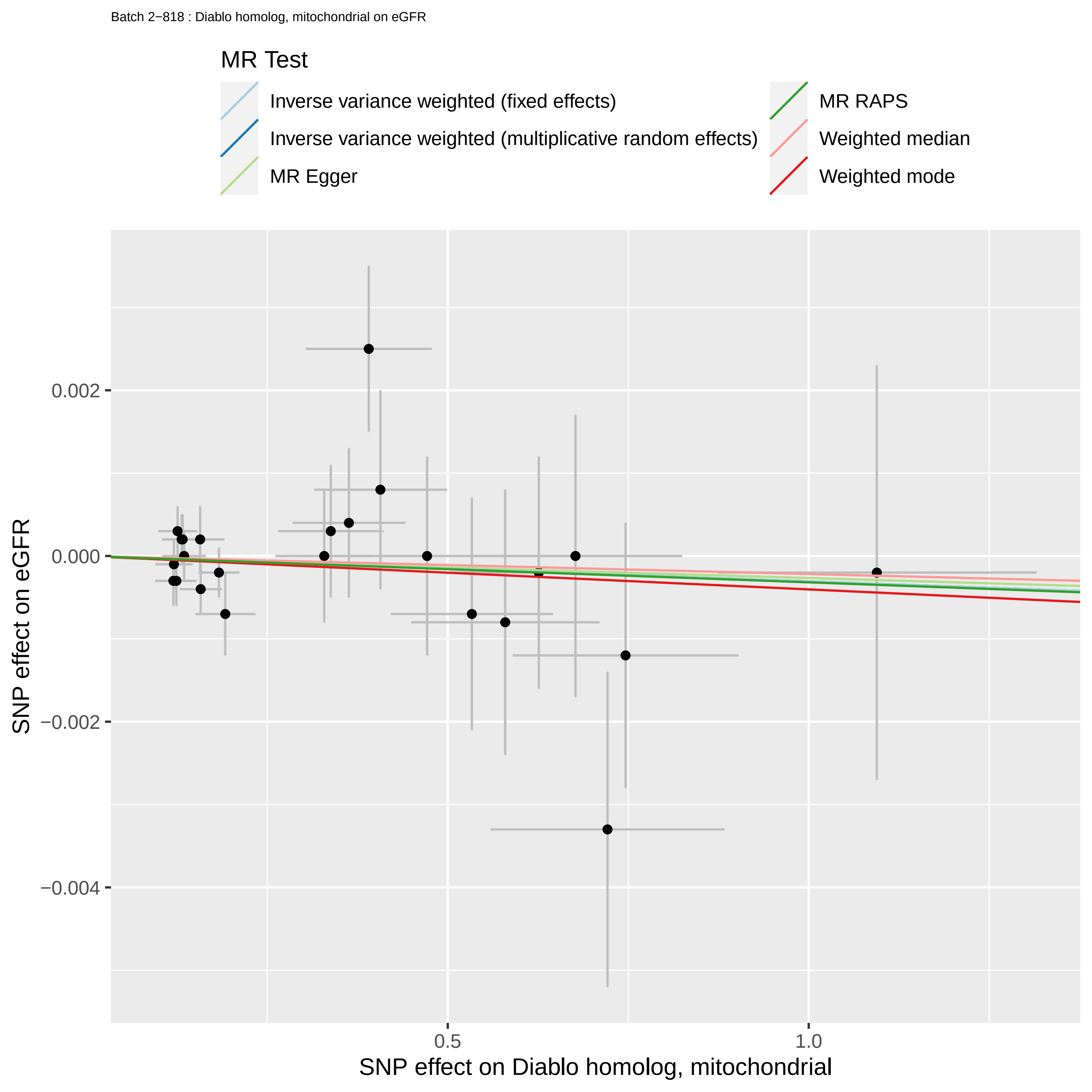

Batch 2−818 : Diablo homolog, mitochondrial on eGFR
MR Test
Inverse variance weighted (fixed effects)
Inverse variance weighted (multiplicative random effects)
MR Egger
MR RAPS
Weighted median
Weighted mode
0.002
0.000
−0.002
−0.004
SNP effect on eGFR
0.5
1.0
SNP effect on Diablo homolog, mitochondrial

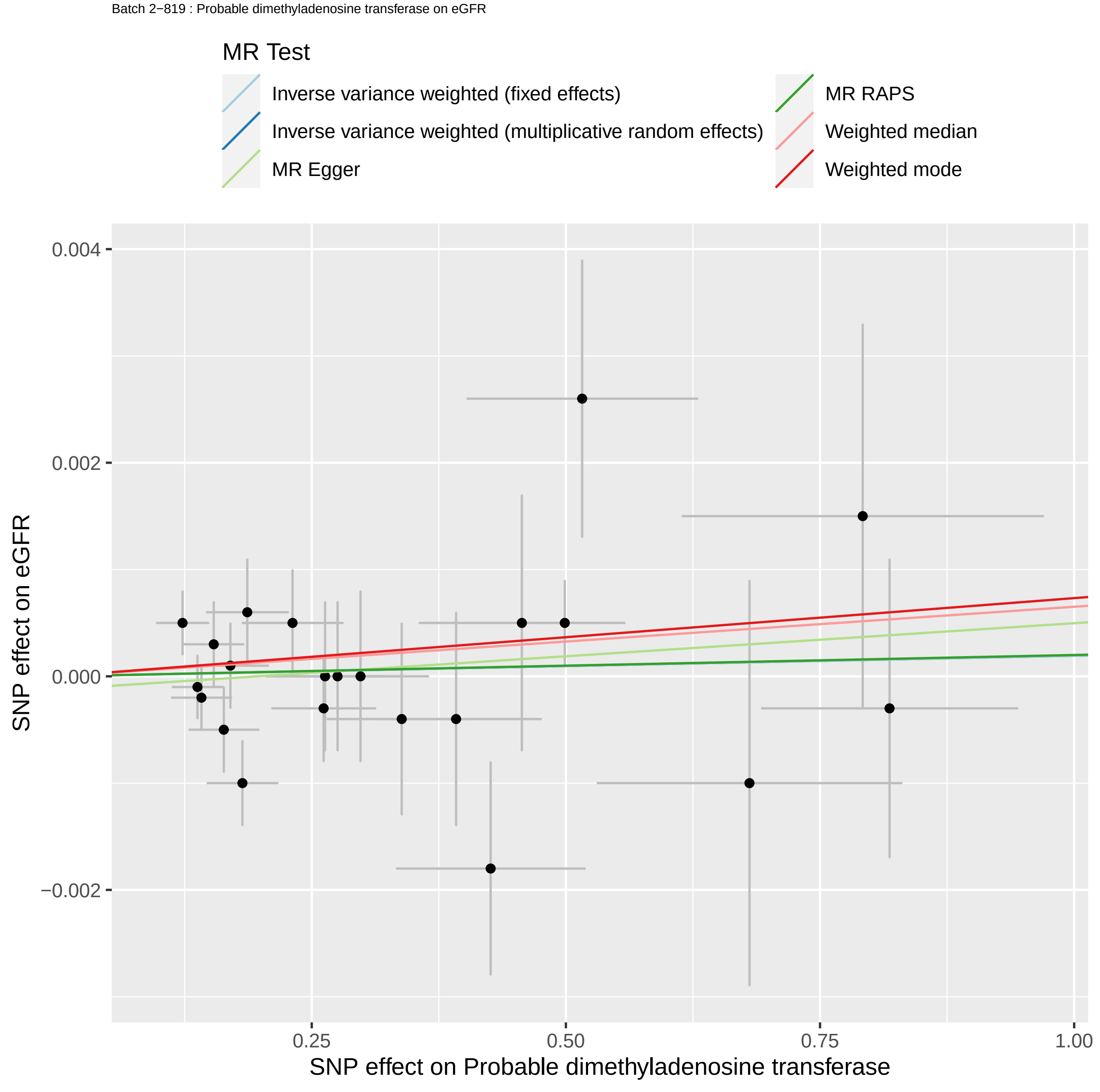

Batch 2−819 : Probable dimethyladenosine transferase on eGFR
MR Test
Inverse variance weighted (fixed effects)
Inverse variance weighted (multiplicative random effects)
MR Egger
MR RAPS
Weighted median
Weighted mode
0.004
0.002
0.000
−0.002
SNP effect on eGFR
0.25
0.50
0.75
1.00
SNP effect on Probable dimethyladenosine transferase

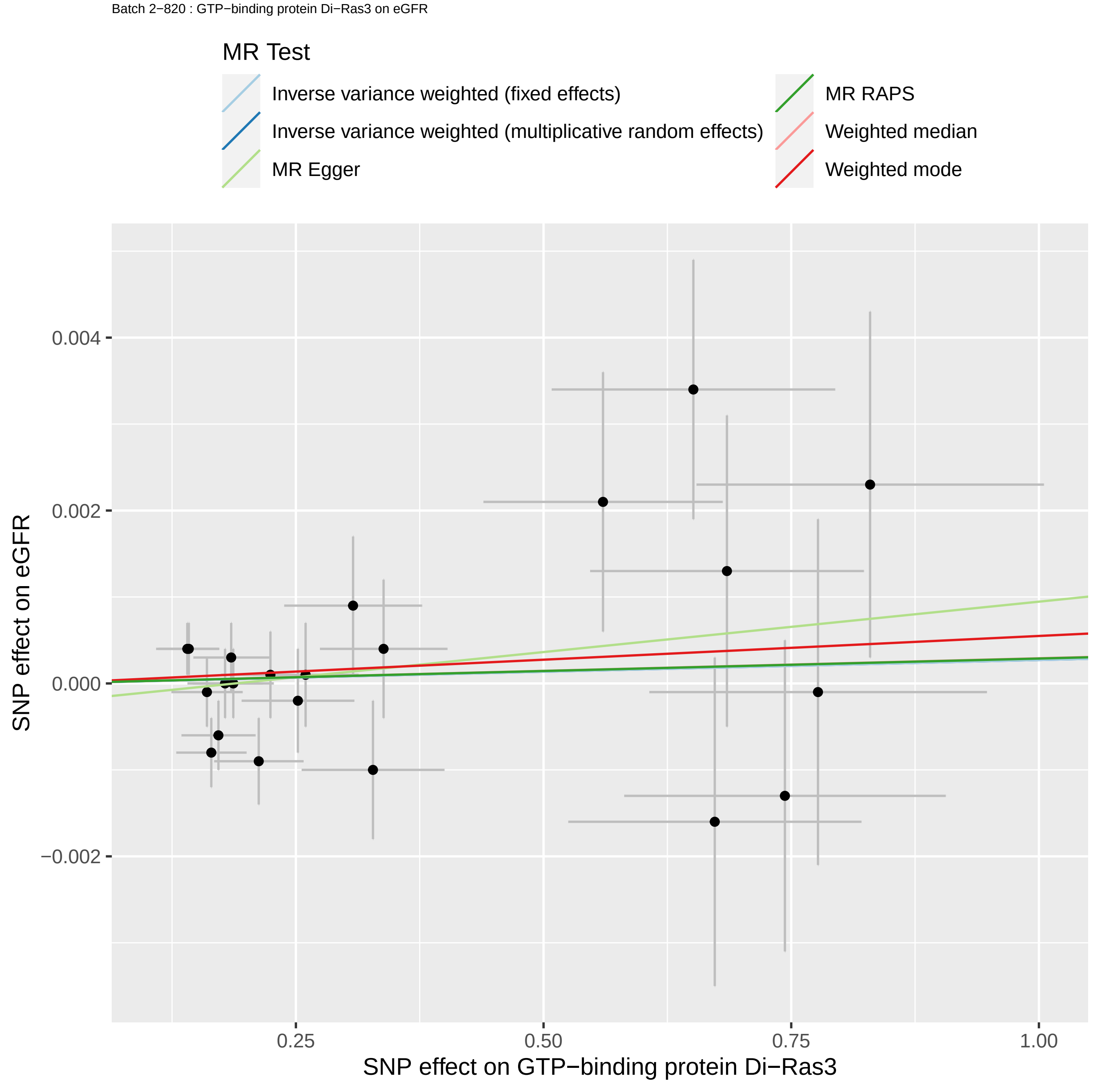
Batch 2−820 : GTP−binding protein Di−Ras3 on eGFR
MR Test
Inverse variance weighted (fixed effects)
Inverse variance weighted (multiplicative random effects)
MR Egger
MR RAPS
Weighted median
Weighted mode
0.004
0.002
0.000
−0.002
SNP effect on eGFR
0.25
0.50
0.75
1.00
SNP effect on GTP−binding protein Di−Ras3

Batch 2−821 : Dickkopf−related protein 1 on eGFR

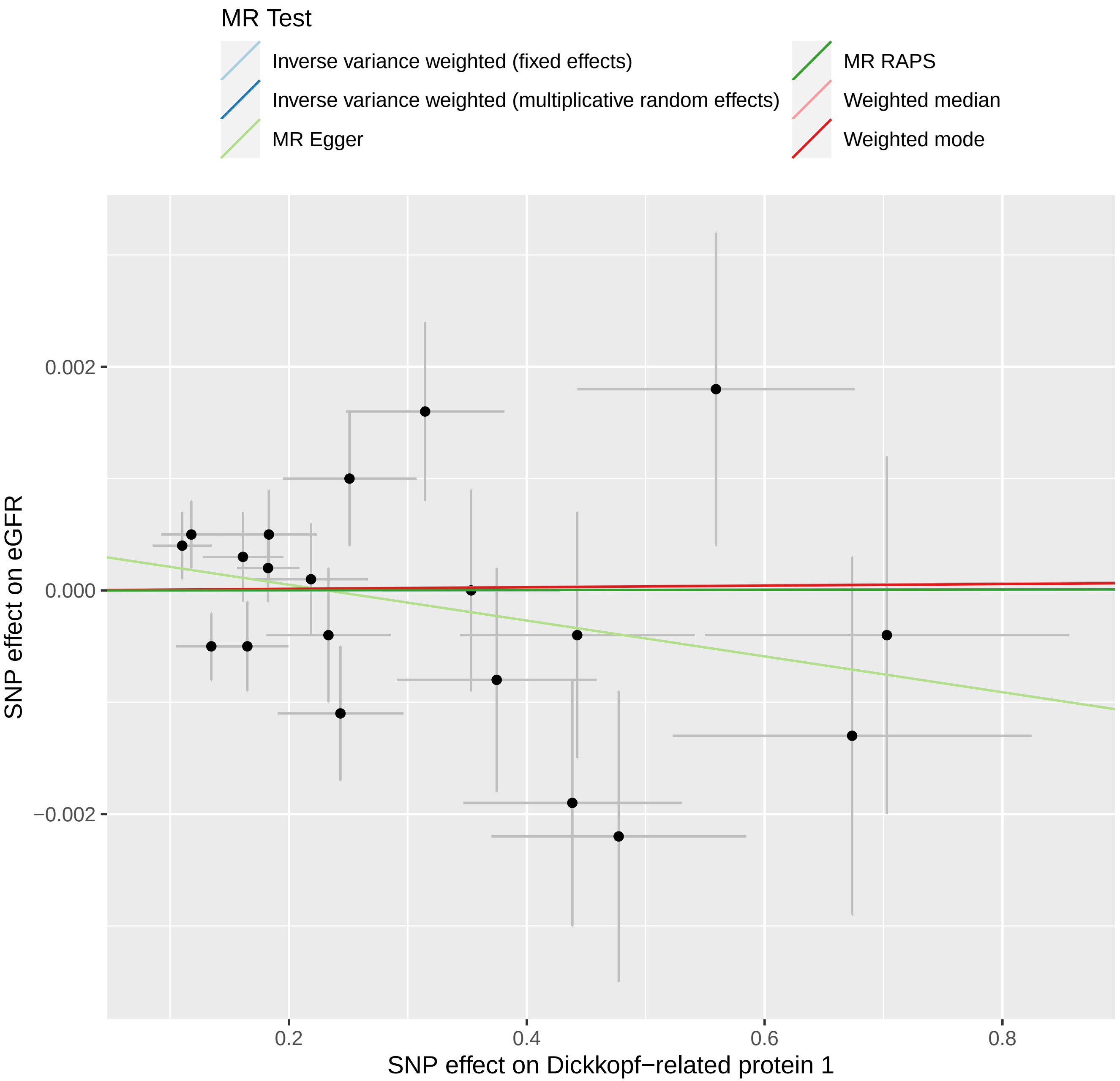

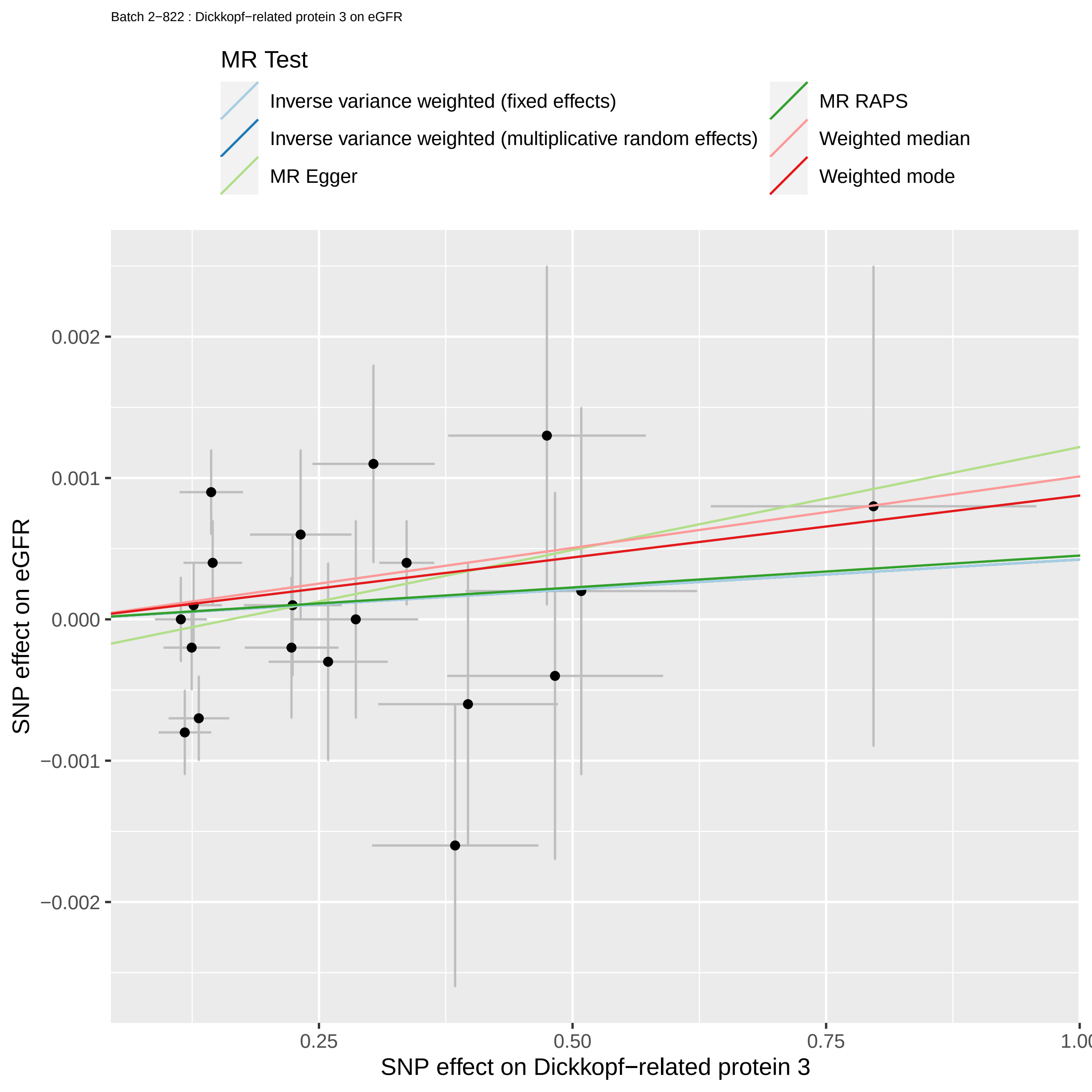

Batch 2−822 : Dickkopf−related protein 3 on eGFR
MR Test
Inverse variance weighted (fixed effects)
Inverse variance weighted (multiplicative random effects)
MR Egger
MR RAPS
Weighted median
Weighted mode
0.002
0.001
0.000
−0.001
−0.002
SNP effect on eGFR
0.25
0.50
0.75
SNP effect on Dickkopf−related protein 3

Batch 2−823 : Dickkopf−related protein 4 on eGFR

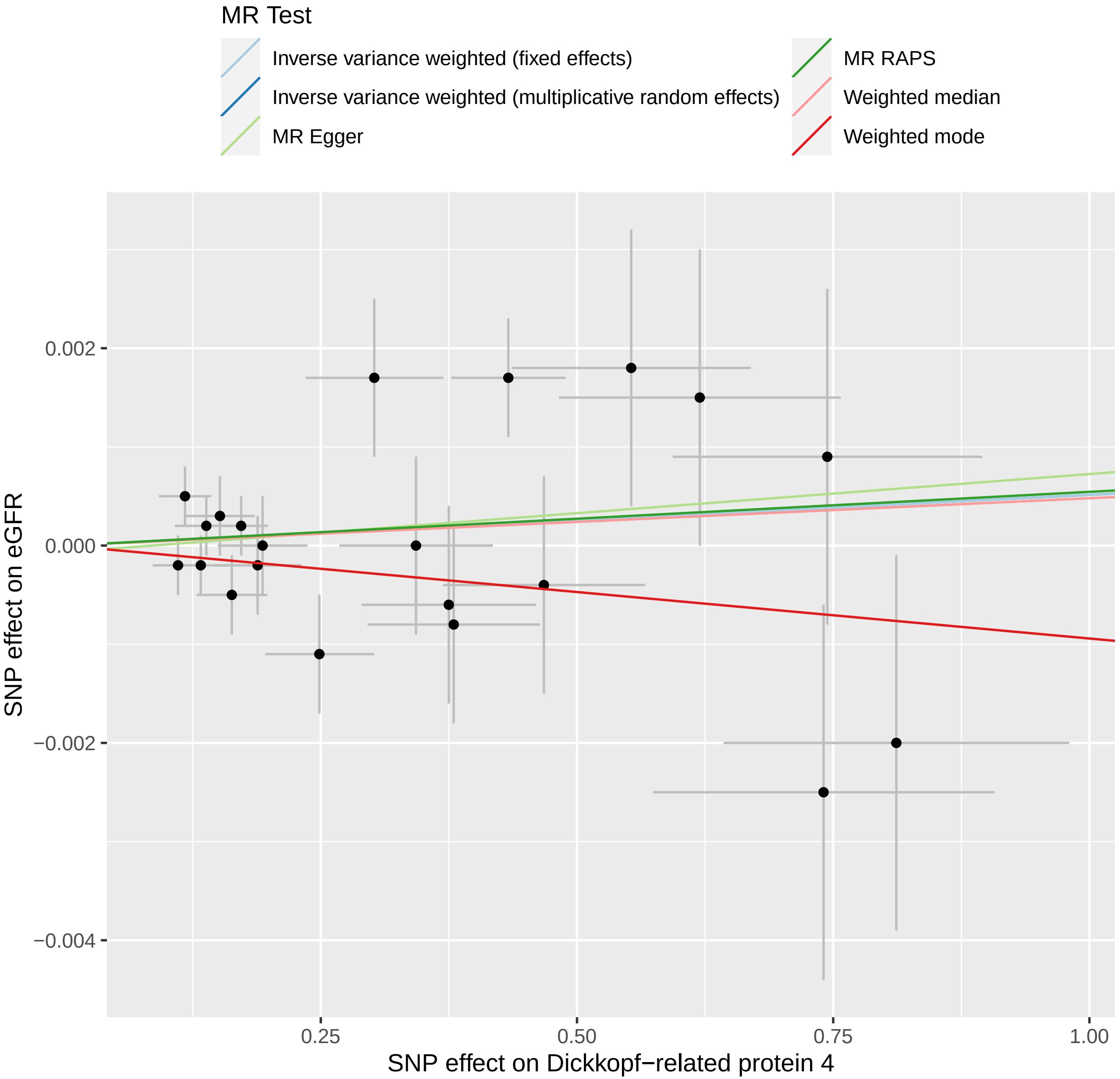

Batch 2−824 : Dickkopf−like protein 1 on eGFR

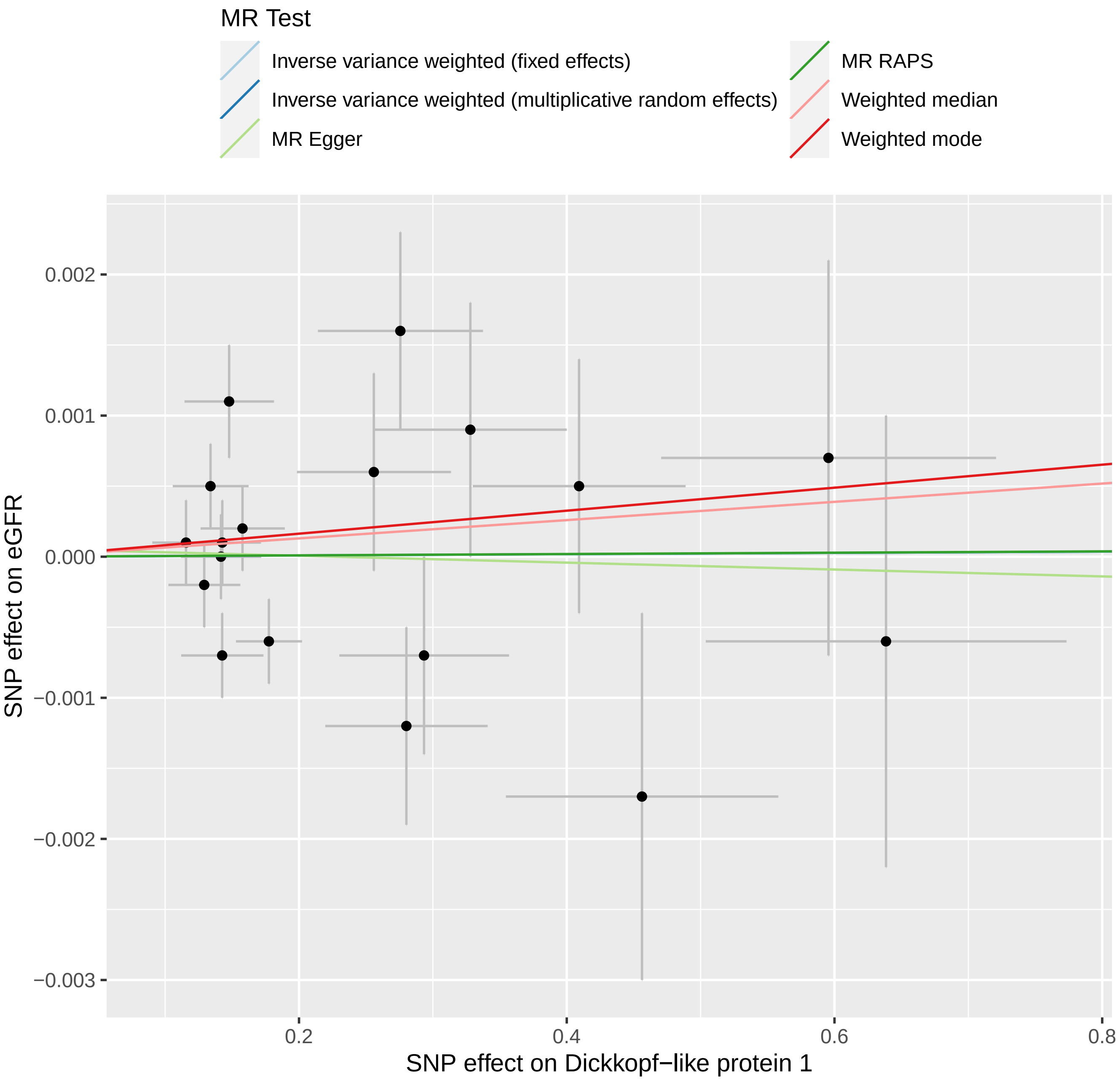

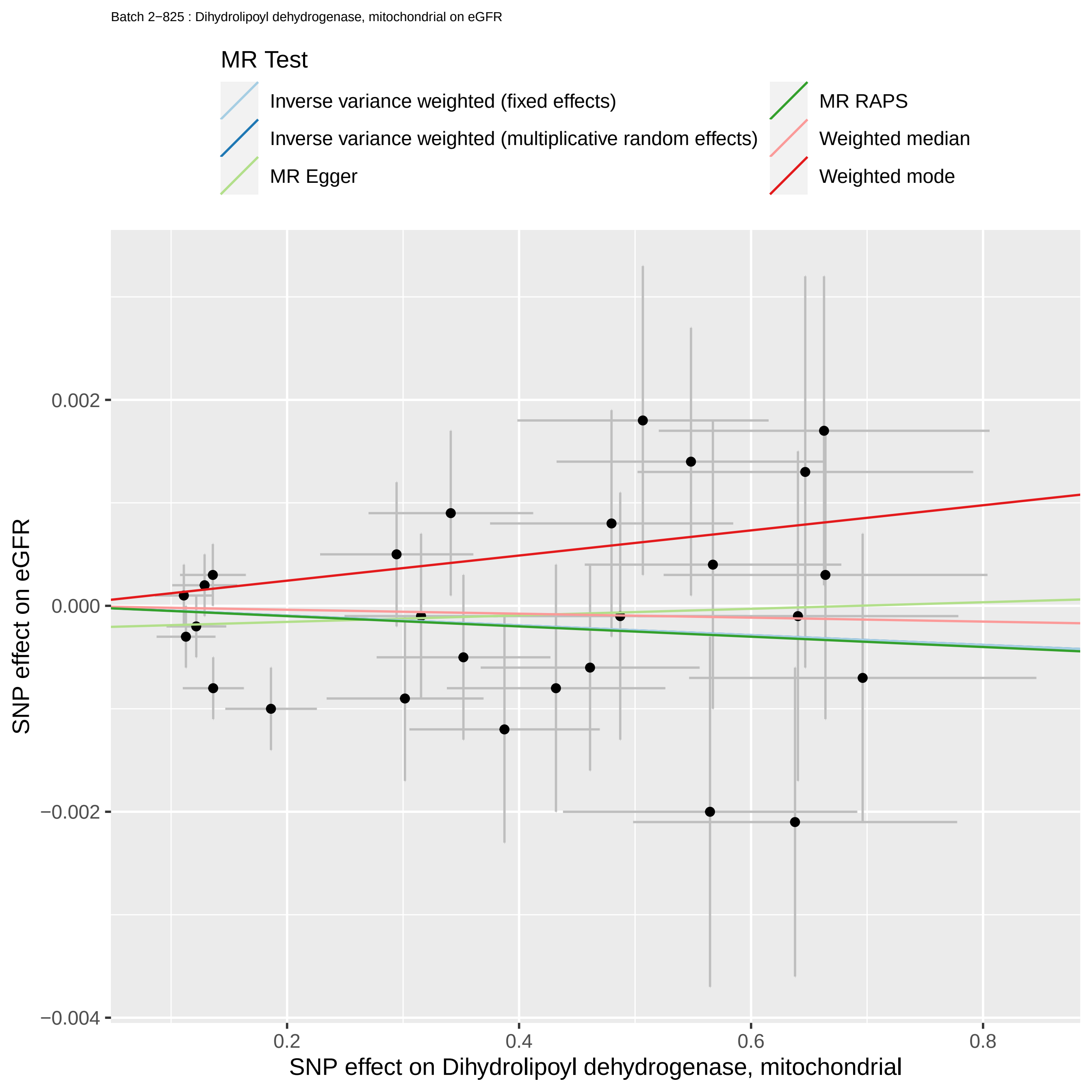

Batch 2−825 : Dihydrolipoyl dehydrogenase, mitochondrial on eGFR
MR Test
Inverse variance weighted (fixed effects)
Inverse variance weighted (multiplicative random effects)
MR Egger
MR RAPS
Weighted median
Weighted mode
0.002
0.000
−0.002
−0.004
0.2
0.4
0.6
0.8
SNP effect on eGFR
SNP effect on Dihydrolipoyl dehydrogenase, mitochondrial

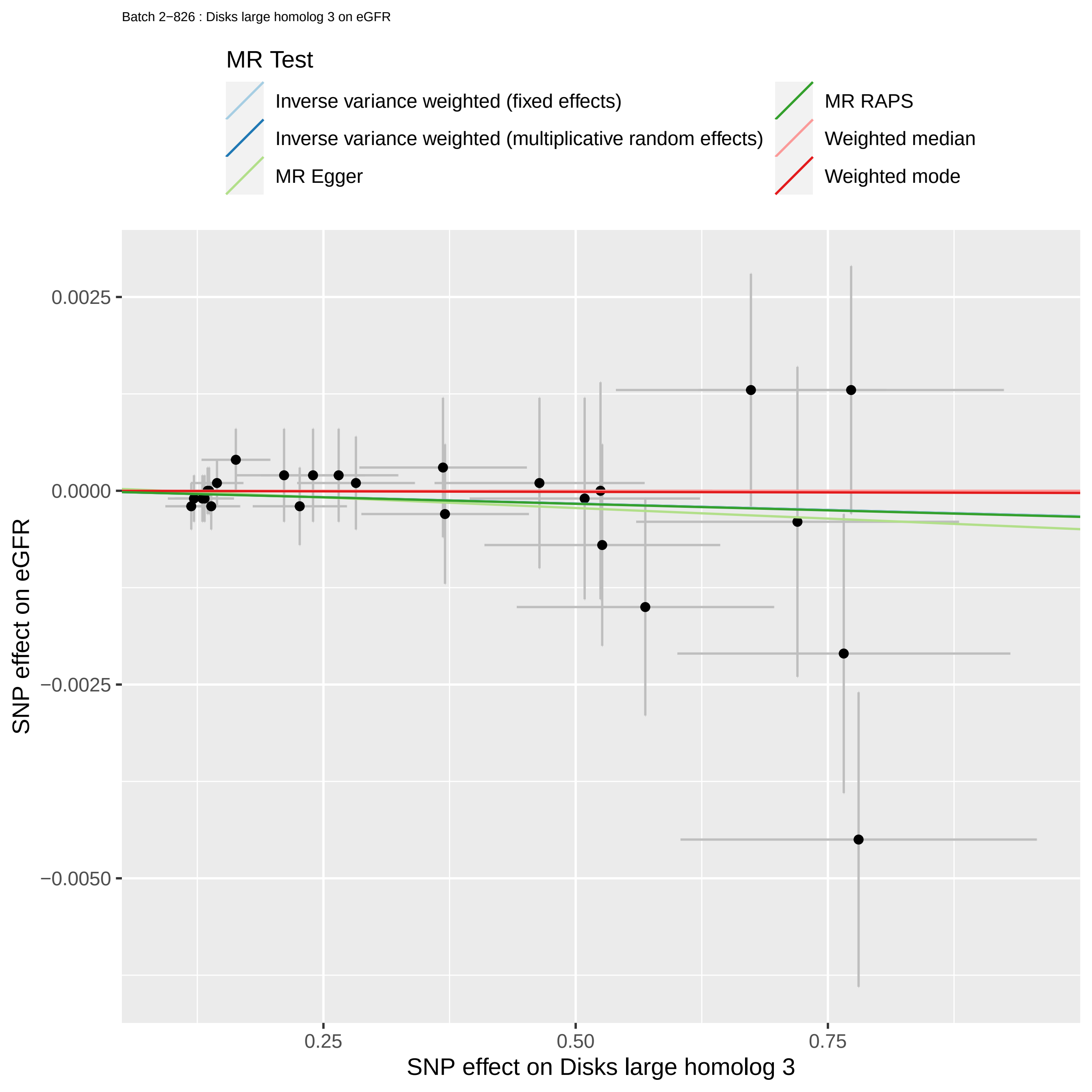

Batch 2−826 : Disks large homolog 3 on eGFR
MR Test
Inverse variance weighted (fixed effects)
Inverse variance weighted (multiplicative random effects)
MR Egger
MR RAPS
Weighted median
Weighted mode
0.0025
0.0000
−0.0025
−0.0050
0.25
0.50
0.75
SNP effect on eGFR
SNP effect on Disks large homolog 3

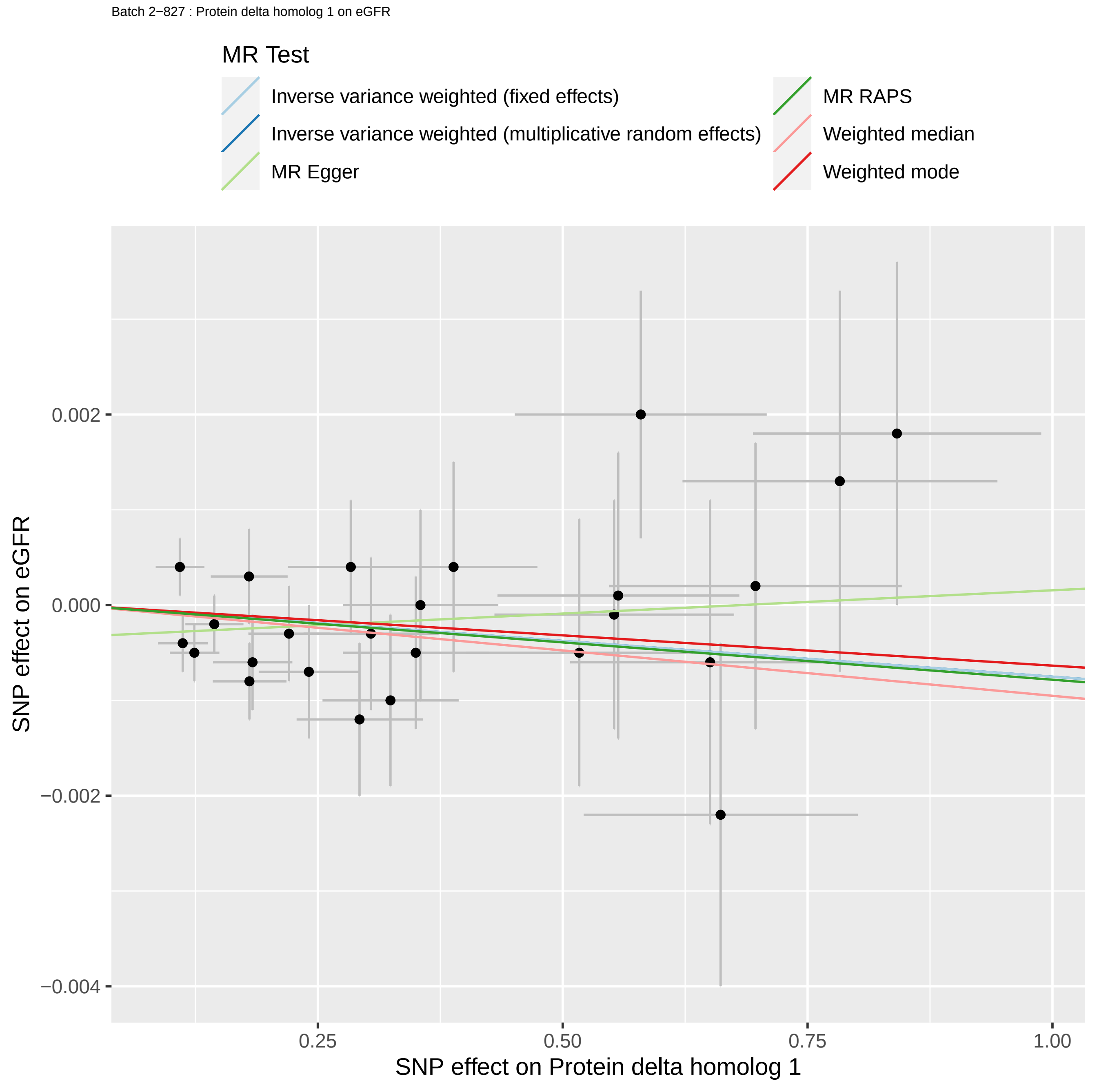

Batch 2−827 : Protein delta homolog 1 on eGFR
MR Test
Inverse variance weighted (fixed effects)
Inverse variance weighted (multiplicative random effects)
MR Egger
MR RAPS
Weighted median
Weighted mode
0.002
0.000
−0.002
−0.004
SNP effect on eGFR
0.25
0.50
0.75
1.00
SNP effect on Protein delta homolog 1

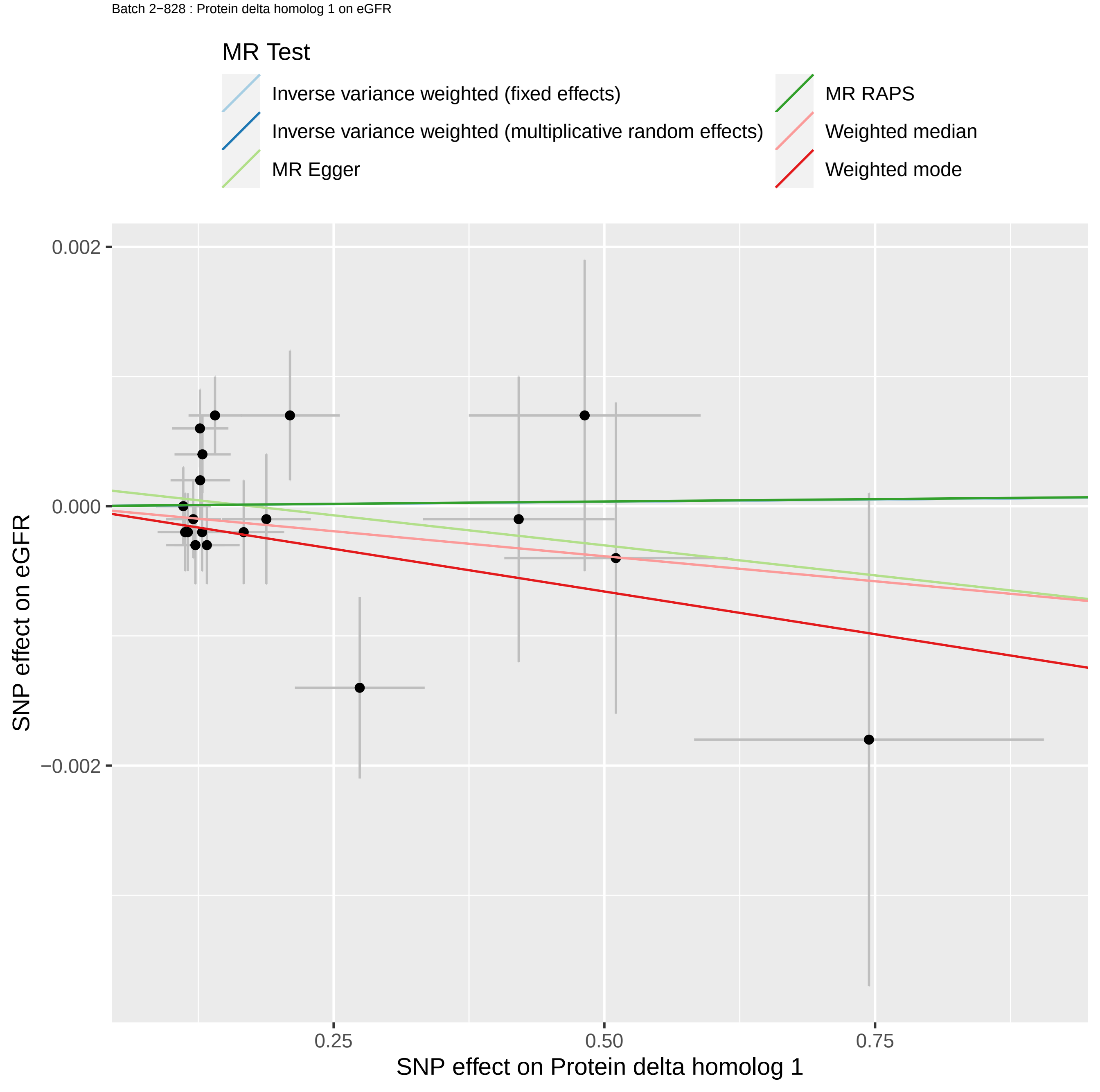

Batch 2−828 : Protein delta homolog 1 on eGFR
MR Test
Inverse variance weighted (fixed effects)
Inverse variance weighted (multiplicative random effects)
MR Egger
MR RAPS
Weighted median
Weighted mode
0.002
0.000
−0.002
0.25
0.50
0.75
SNP effect on eGFR
SNP effect on Protein delta homolog 1

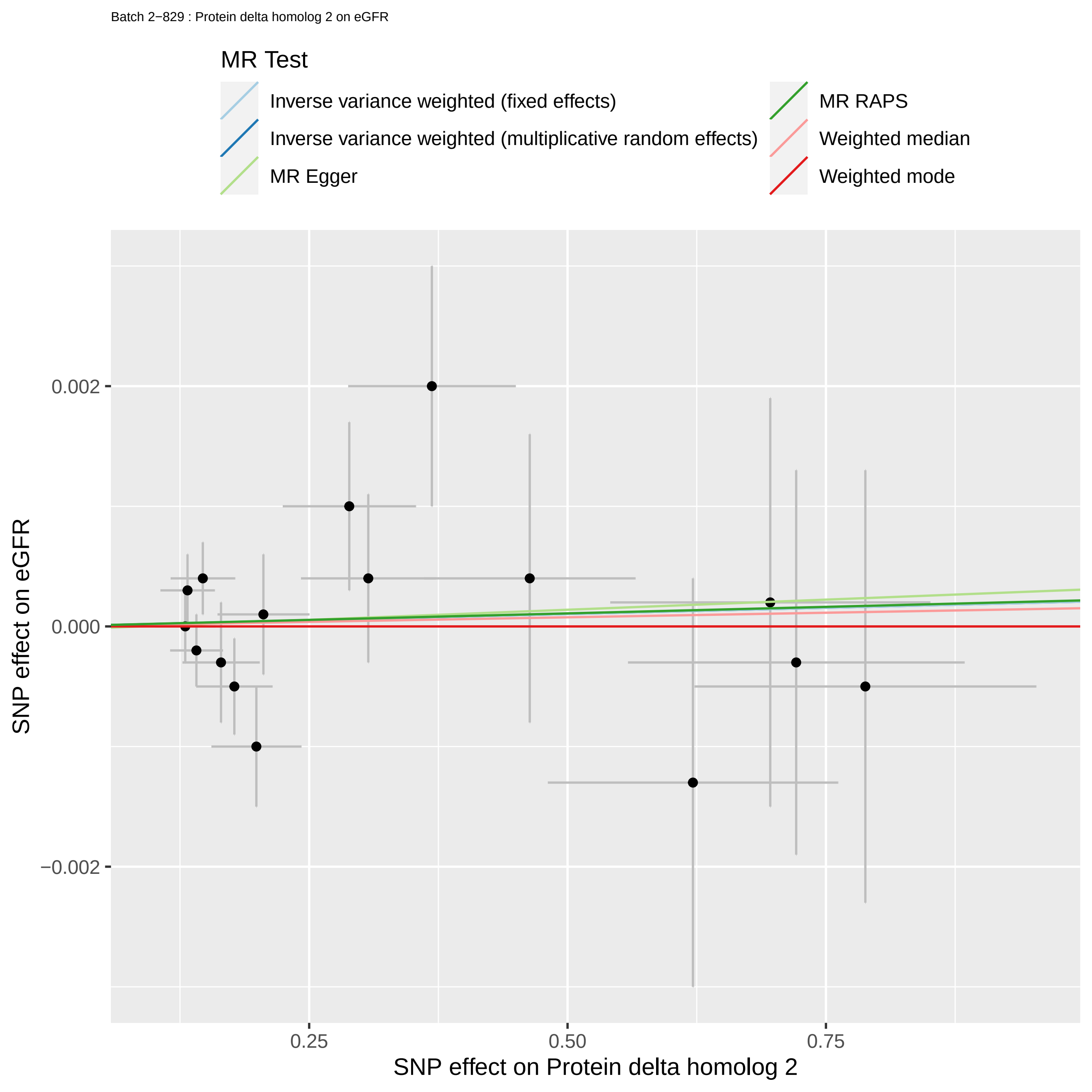

Batch 2−829 : Protein delta homolog 2 on eGFR
MR Test
Inverse variance weighted (fixed effects)
Inverse variance weighted (multiplicative random effects)
MR Egger
MR RAPS
Weighted median
Weighted mode
0.002
0.000
−0.002
0.25
0.50
0.75
SNP effect on Protein delta homolog 2
SNP effect on eGFR

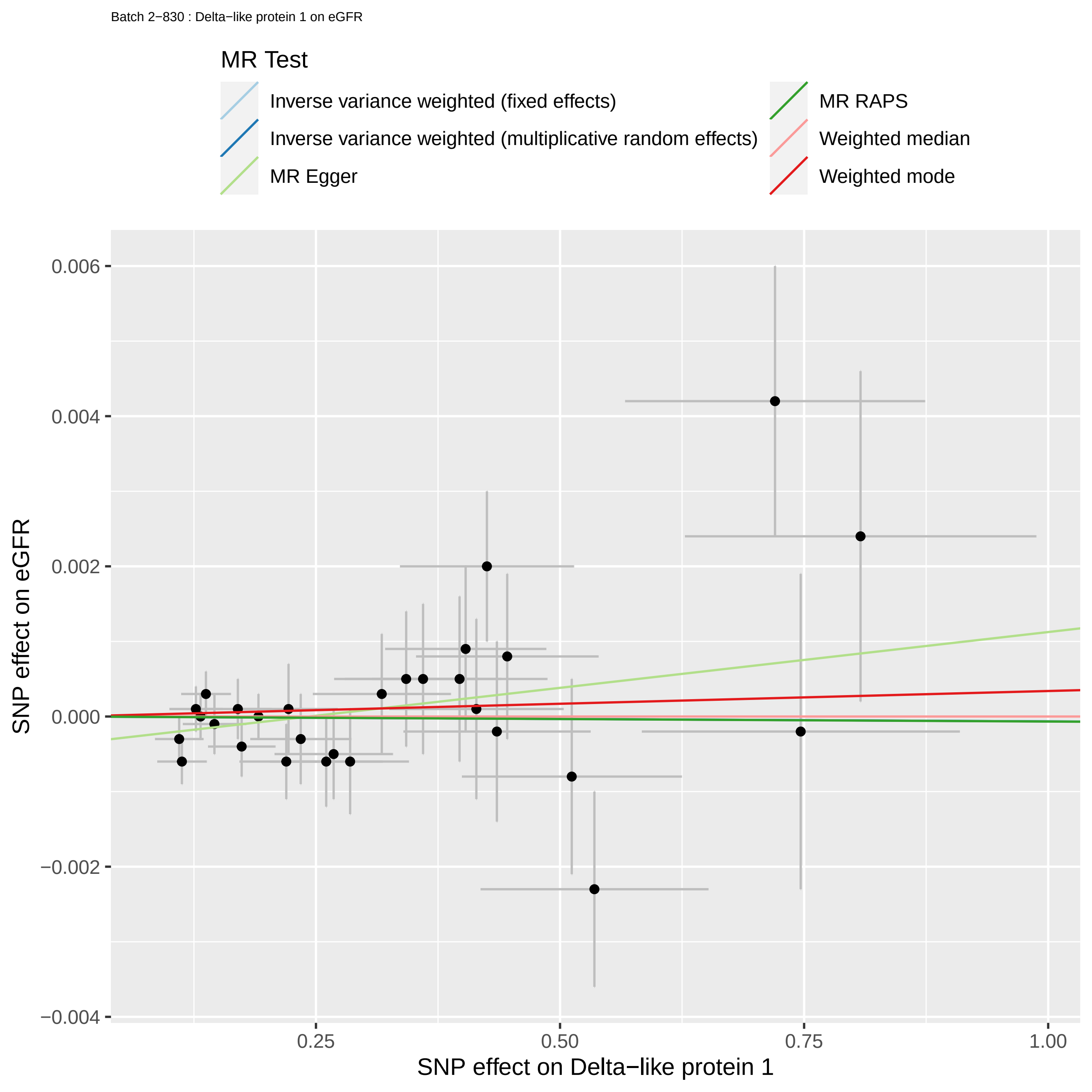
Batch 2−830 : Delta−like protein 1 on eGFR
MR Test
Inverse variance weighted (fixed effects)
Inverse variance weighted (multiplicative random effects)
MR Egger
MR RAPS
Weighted median
Weighted mode
SNP effect on eGFR
0.006
0.004
0.002
0.000
−0.002
−0.004
0.25
0.50
0.75
1.00
SNP effect on Delta−like protein 1

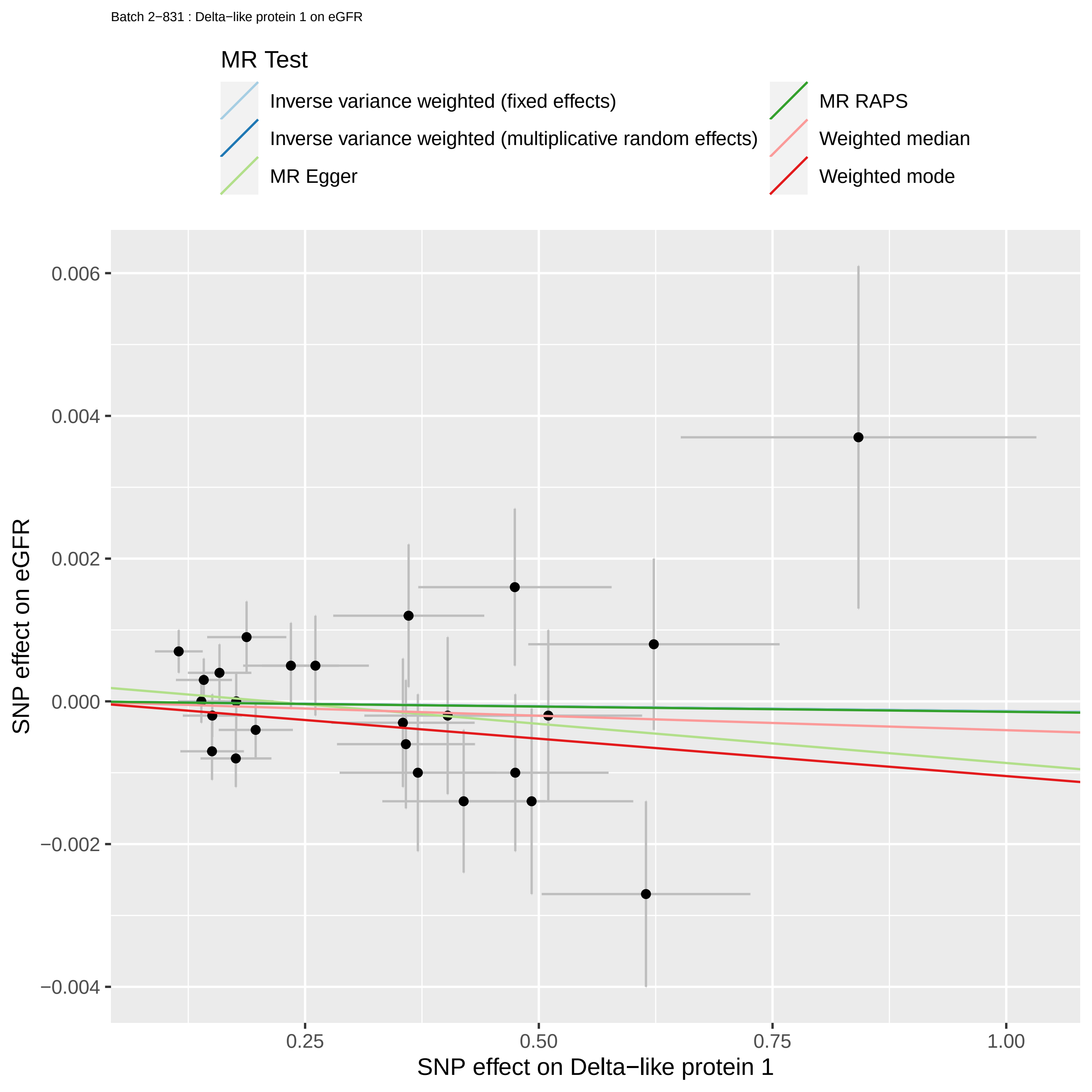

Batch 2−831 : Delta−like protein 1 on eGFR
MR Test
Inverse variance weighted (fixed effects)
Inverse variance weighted (multiplicative random effects)
MR Egger
MR RAPS
Weighted median
Weighted mode
0.006
0.004
0.002
0.000
−0.002
−0.004
SNP effect on eGFR
0.25
0.50
0.75
1.00
SNP effect on Delta−like protein 1

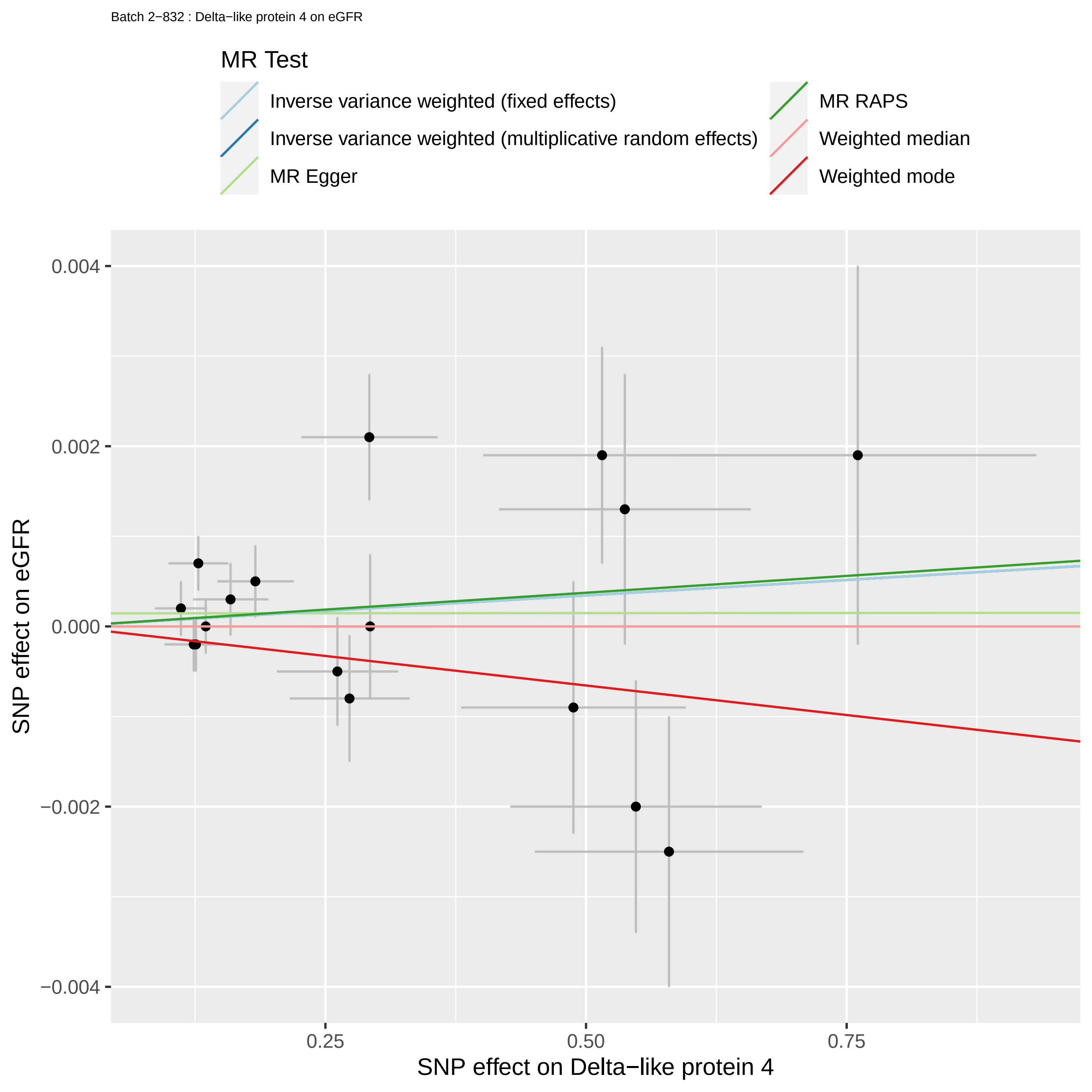
Batch 2−832 : Delta−like protein 4 on eGFR
MR Test
Inverse variance weighted (fixed effects)
Inverse variance weighted (multiplicative random effects)
MR Egger
MR RAPS
Weighted median
Weighted mode
0.004
0.002
0.000
−0.002
−0.004
SNP effect on eGFR
0.25
0.50
0.75
SNP effect on Delta−like protein 4

Batch 2−833 : Dermokine on eGFR

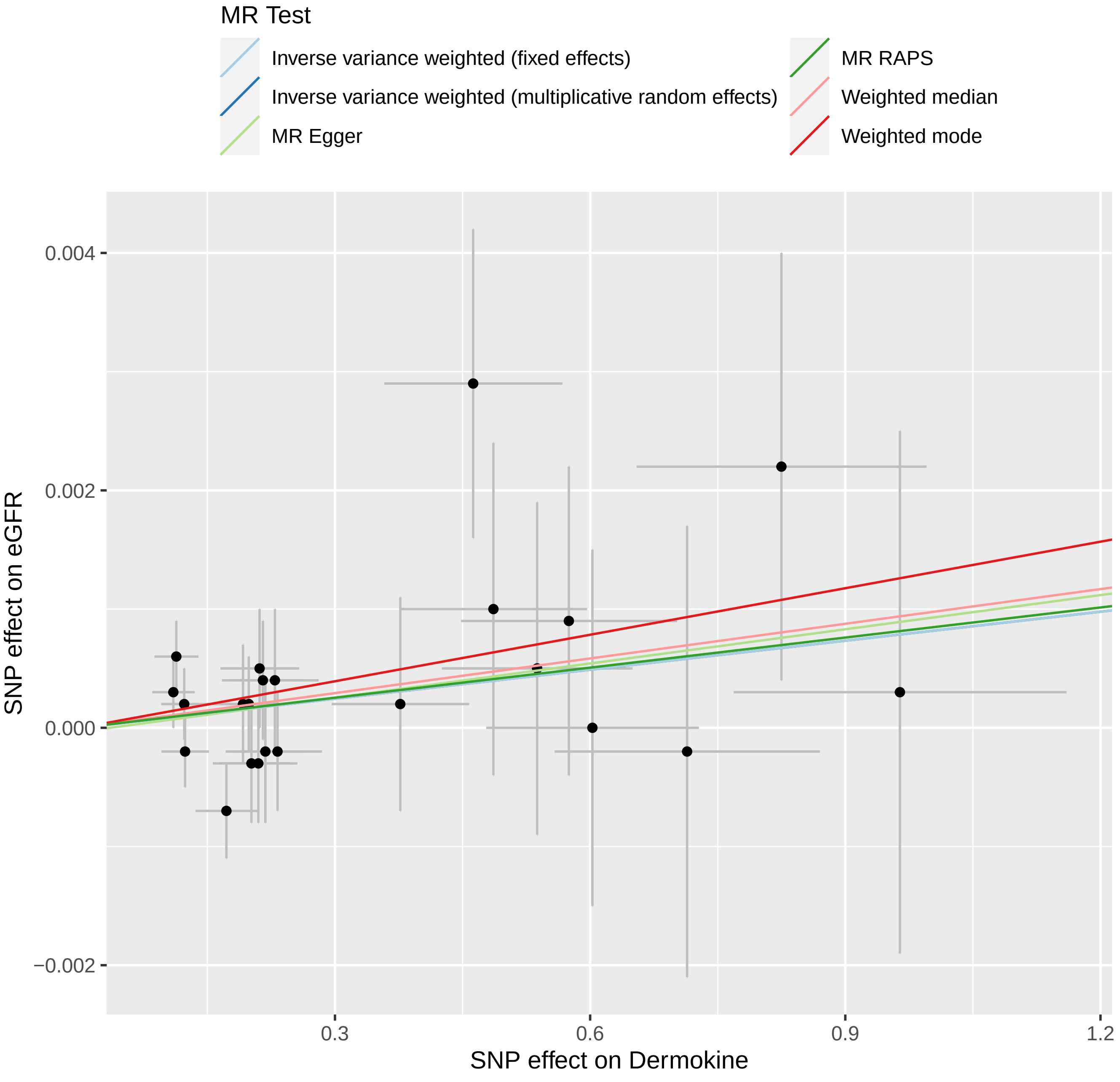

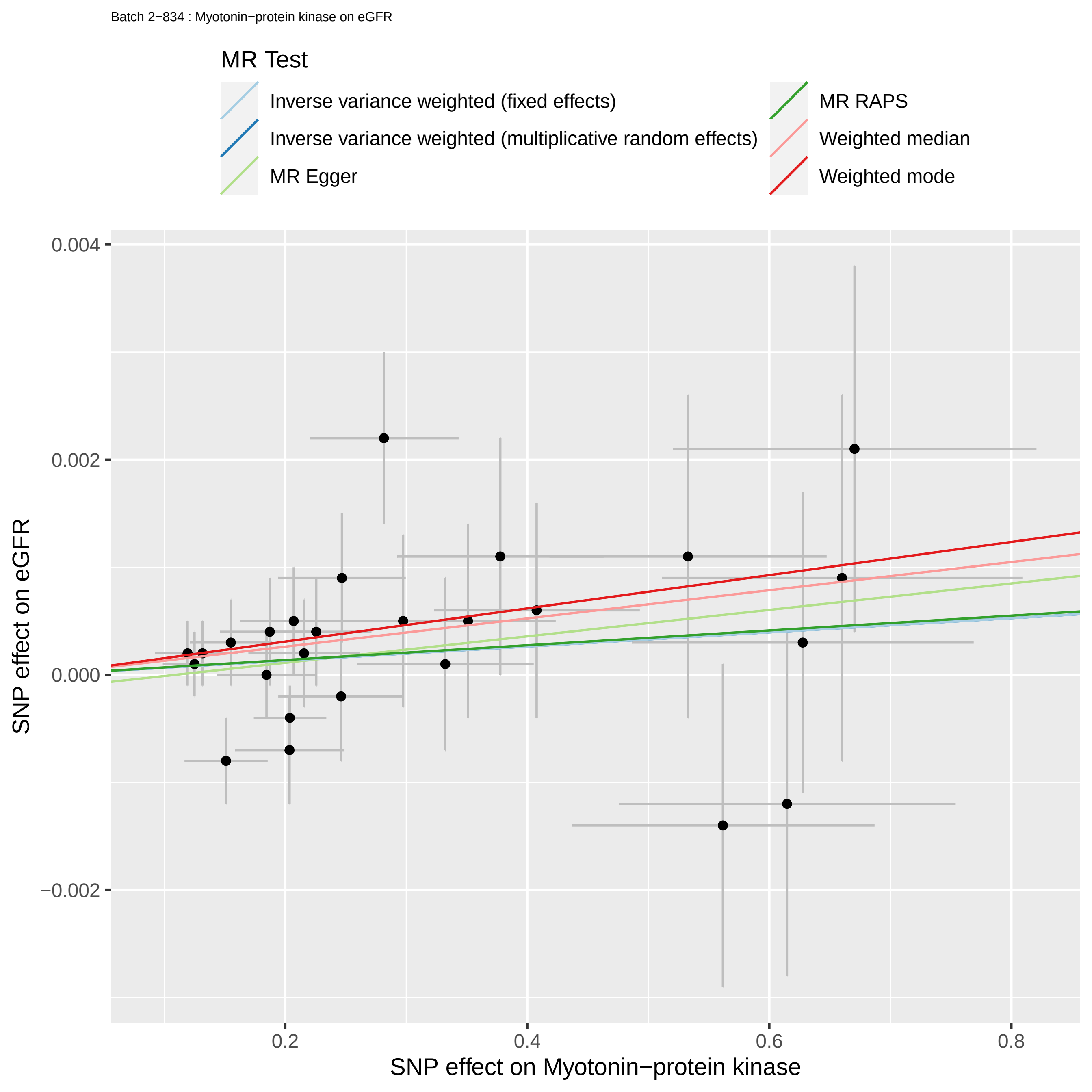

Batch 2−834 : Myotonin−protein kinase on eGFR
MR Test
Inverse variance weighted (fixed effects)
Inverse variance weighted (multiplicative random effects)
MR Egger
MR RAPS
Weighted median
Weighted mode
0.004
0.002
0.000
−0.002
SNP effect on eGFR
0.2
0.4
0.6
0.8
SNP effect on Myotonin−protein kinase

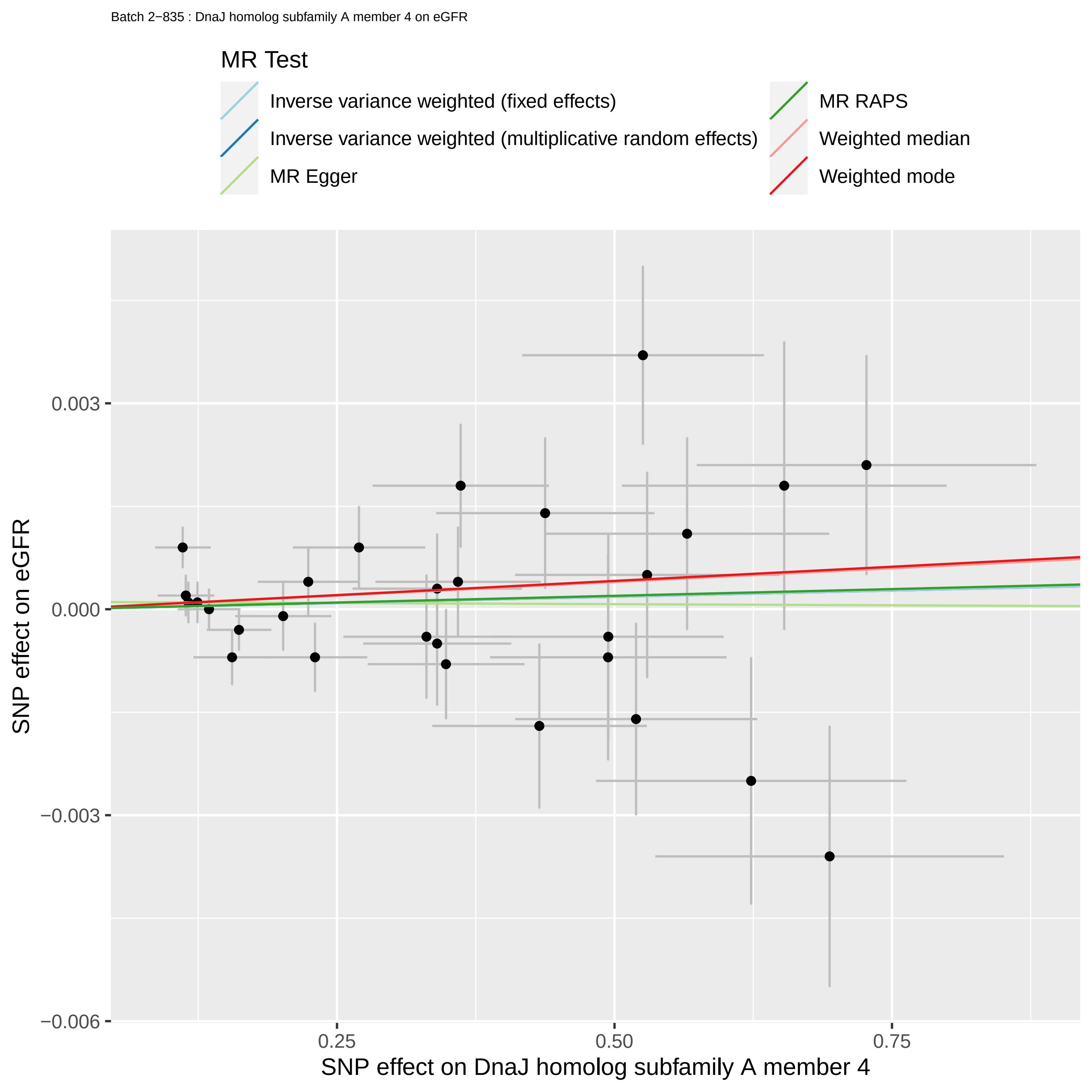
Batch 2−835 : DnaJ homolog subfamily A member 4 on eGFR
MR Test
Inverse variance weighted (fixed effects)
Inverse variance weighted (multiplicative random effects)
MR Egger
MR RAPS
Weighted median
Weighted mode
0.003
0.000
−0.003
−0.006
0.25
0.50
0.75
SNP effect on eGFR
SNP effect on DnaJ homolog subfamily A member 4

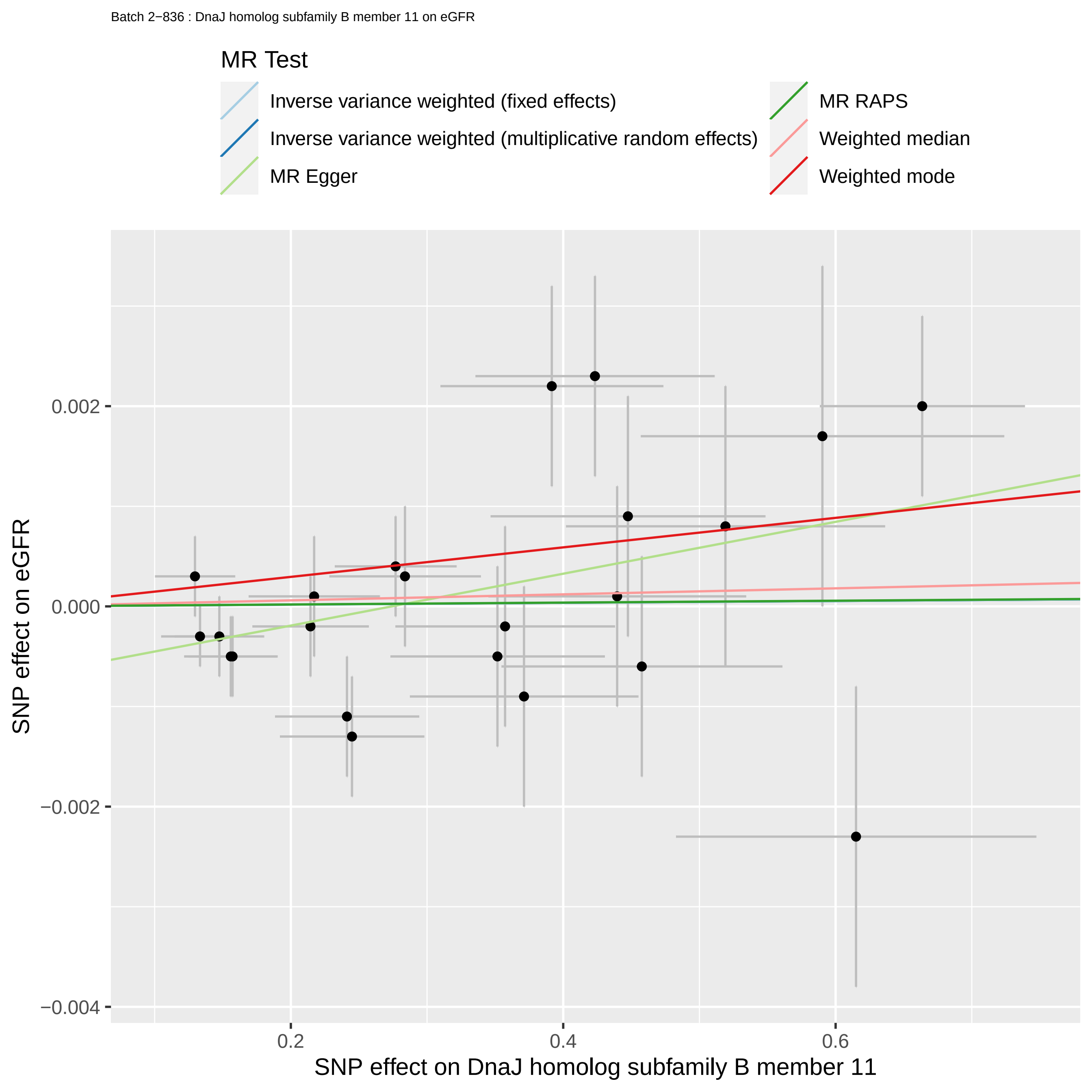
Batch 2−836 : DnaJ homolog subfamily B member 11 on eGFR
MR Test
Inverse variance weighted (fixed effects)
Inverse variance weighted (multiplicative random effects)
MR Egger
MR RAPS
Weighted median
Weighted mode
0.002
0.000
−0.002
−0.004
0.2
0.4
0.6
SNP effect on eGFR
SNP effect on DnaJ homolog subfamily B member 11

Batch 2−837 : DnaJ homolog subfamily B member 12 on eGFR

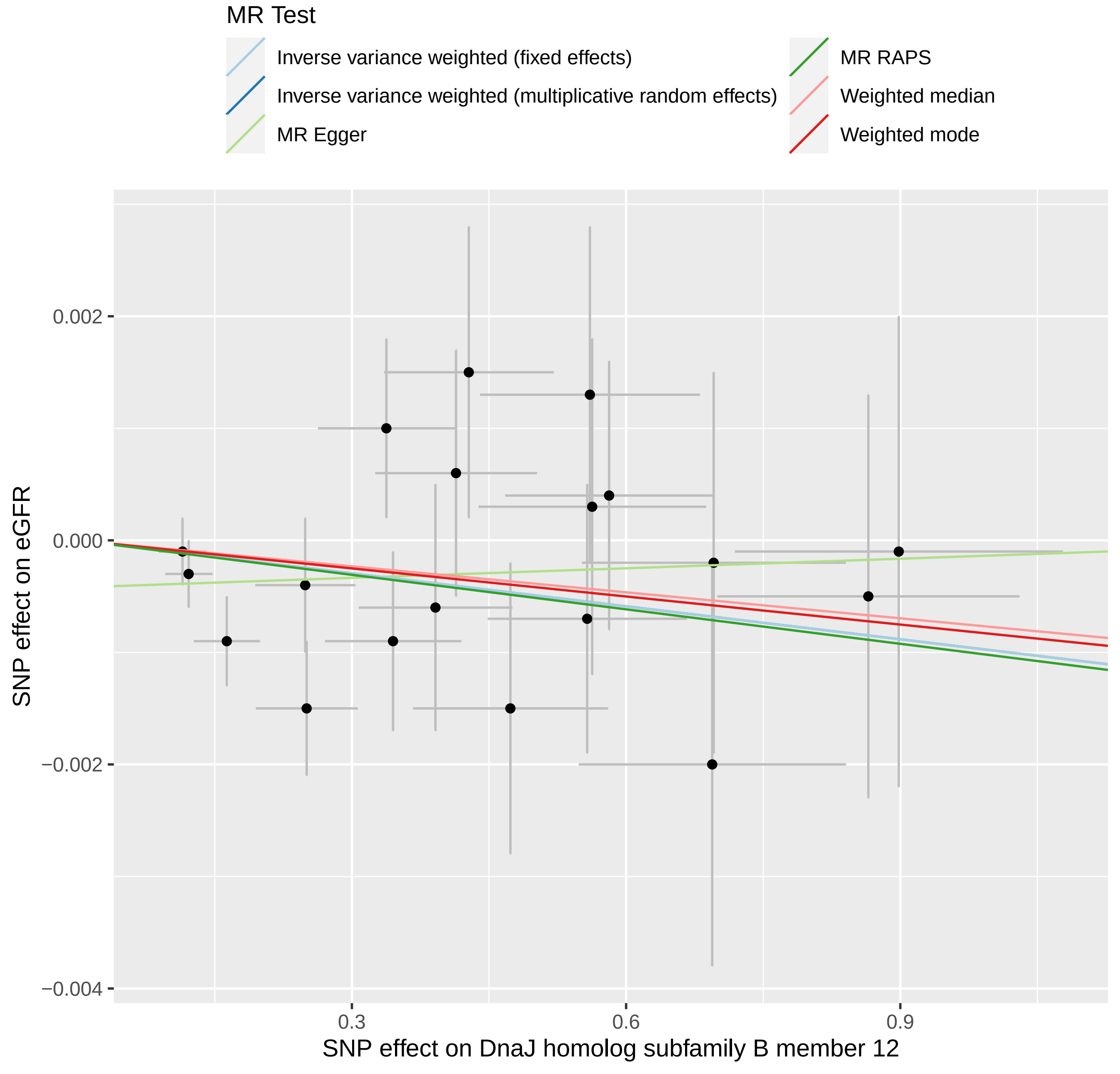

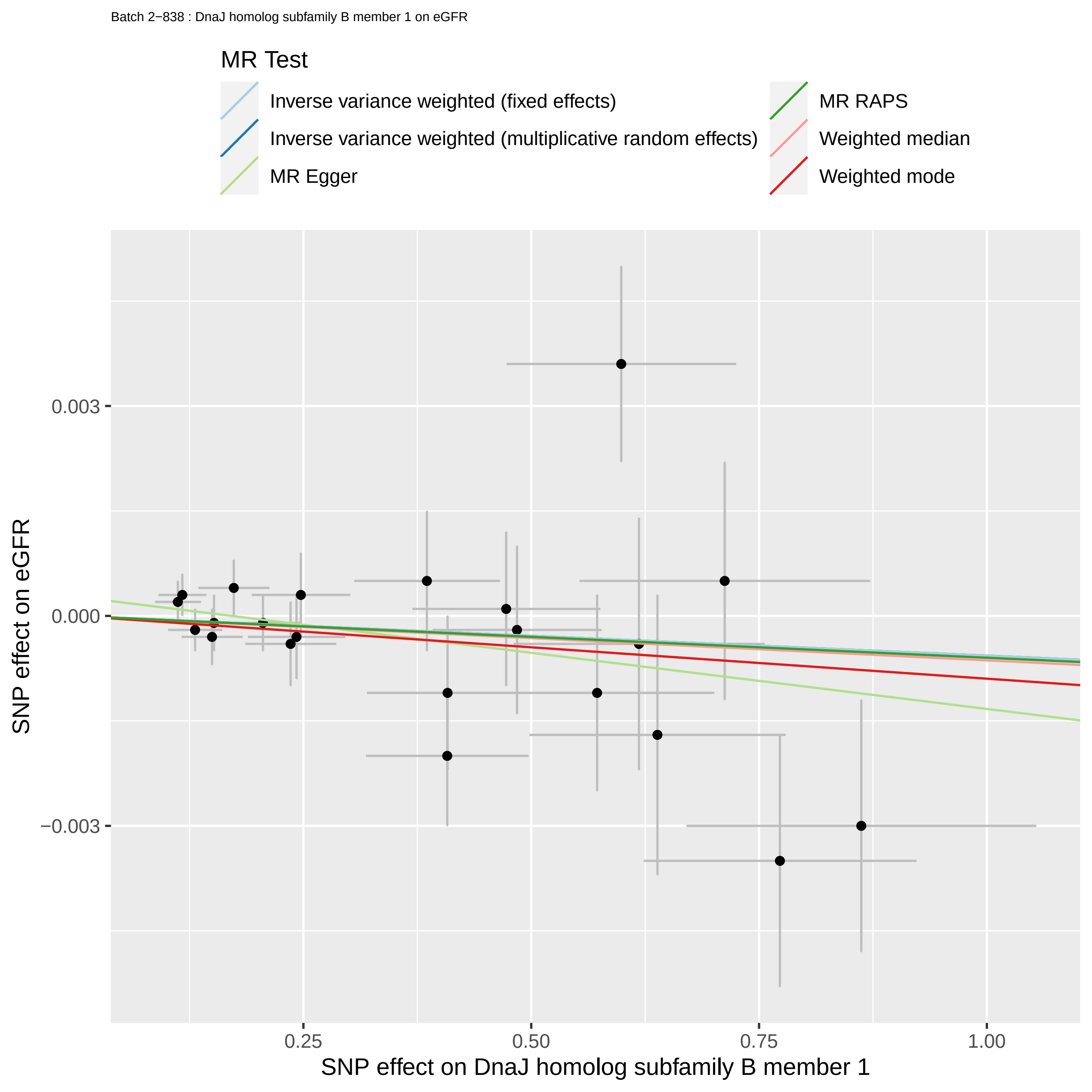

Batch 2−838 : DnaJ homolog subfamily B member 1 on eGFR
MR Test
Inverse variance weighted (fixed effects)
Inverse variance weighted (multiplicative random effects)
MR Egger
MR RAPS
Weighted median
Weighted mode
0.003
0.000
−0.003
0.25
0.50
0.75
1.00
SNP effect on eGFR
SNP effect on DnaJ homolog subfamily B member 1

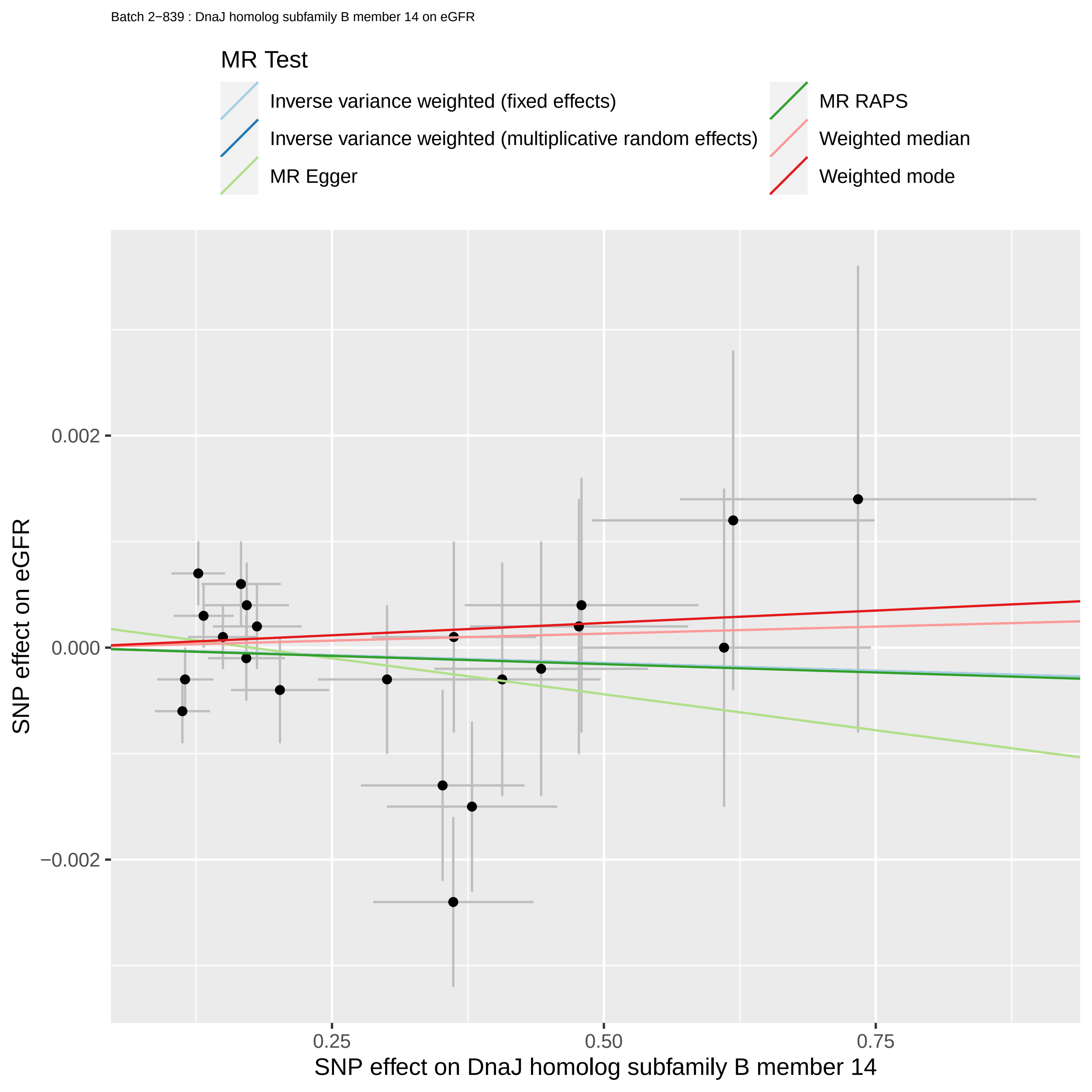

Batch 2−839 : DnaJ homolog subfamily B member 14 on eGFR
MR Test
Inverse variance weighted (fixed effects)
Inverse variance weighted (multiplicative random effects)
MR Egger
MR RAPS
Weighted median
Weighted mode
0.002
0.000
−0.002
0.25
0.50
0.75
SNP effect on eGFR
SNP effect on DnaJ homolog subfamily B member 14

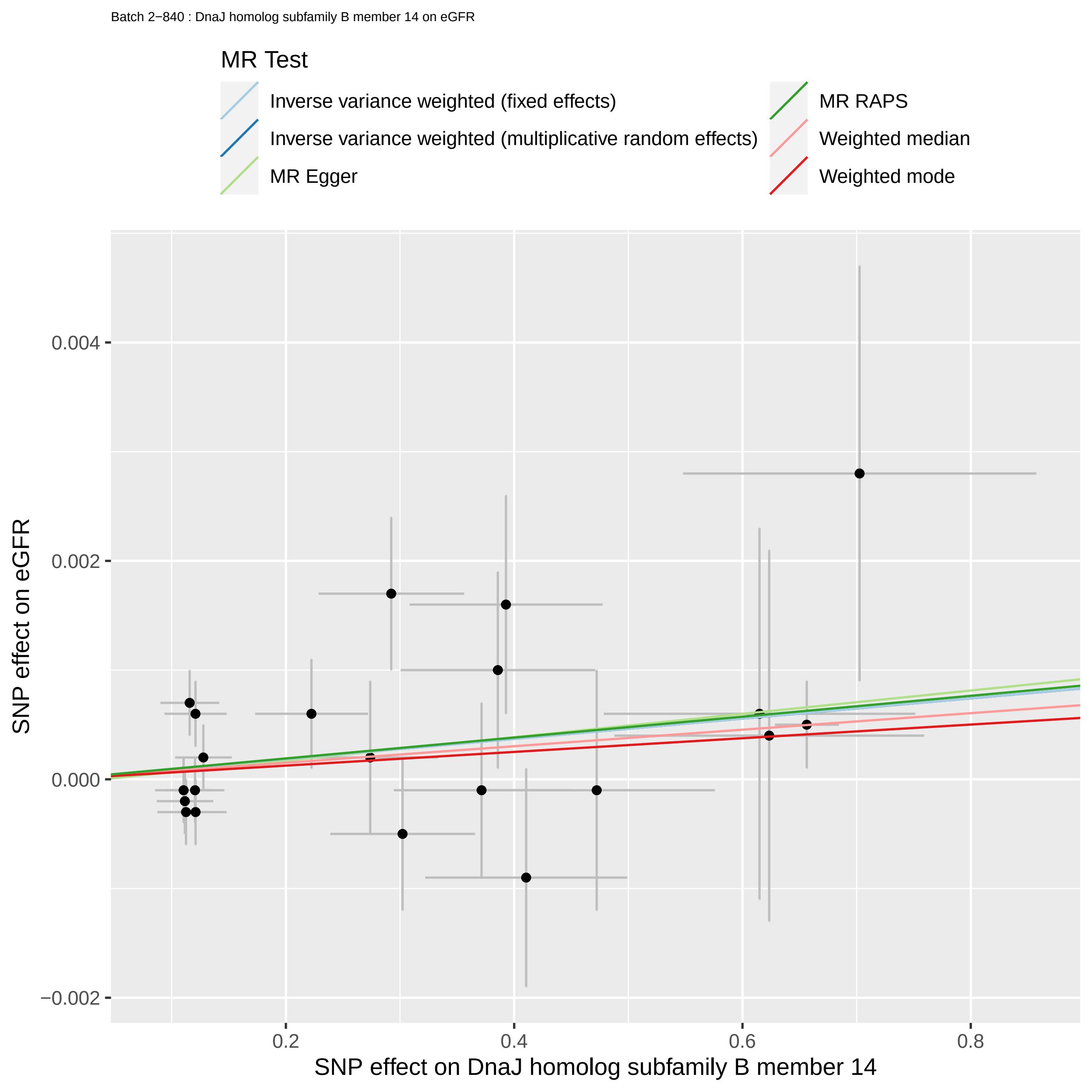

Batch 2−840 : DnaJ homolog subfamily B member 14 on eGFR
MR Test
Inverse variance weighted (fixed effects)
Inverse variance weighted (multiplicative random effects)
MR Egger
MR RAPS
Weighted median
Weighted mode
0.004
0.002
0.000
−0.002
0.2
0.4
0.6
0.8
SNP effect on eGFR
SNP effect on DnaJ homolog subfamily B member 14

Batch 2−841 : DnaJ homolog subfamily B member 2 on eGFR

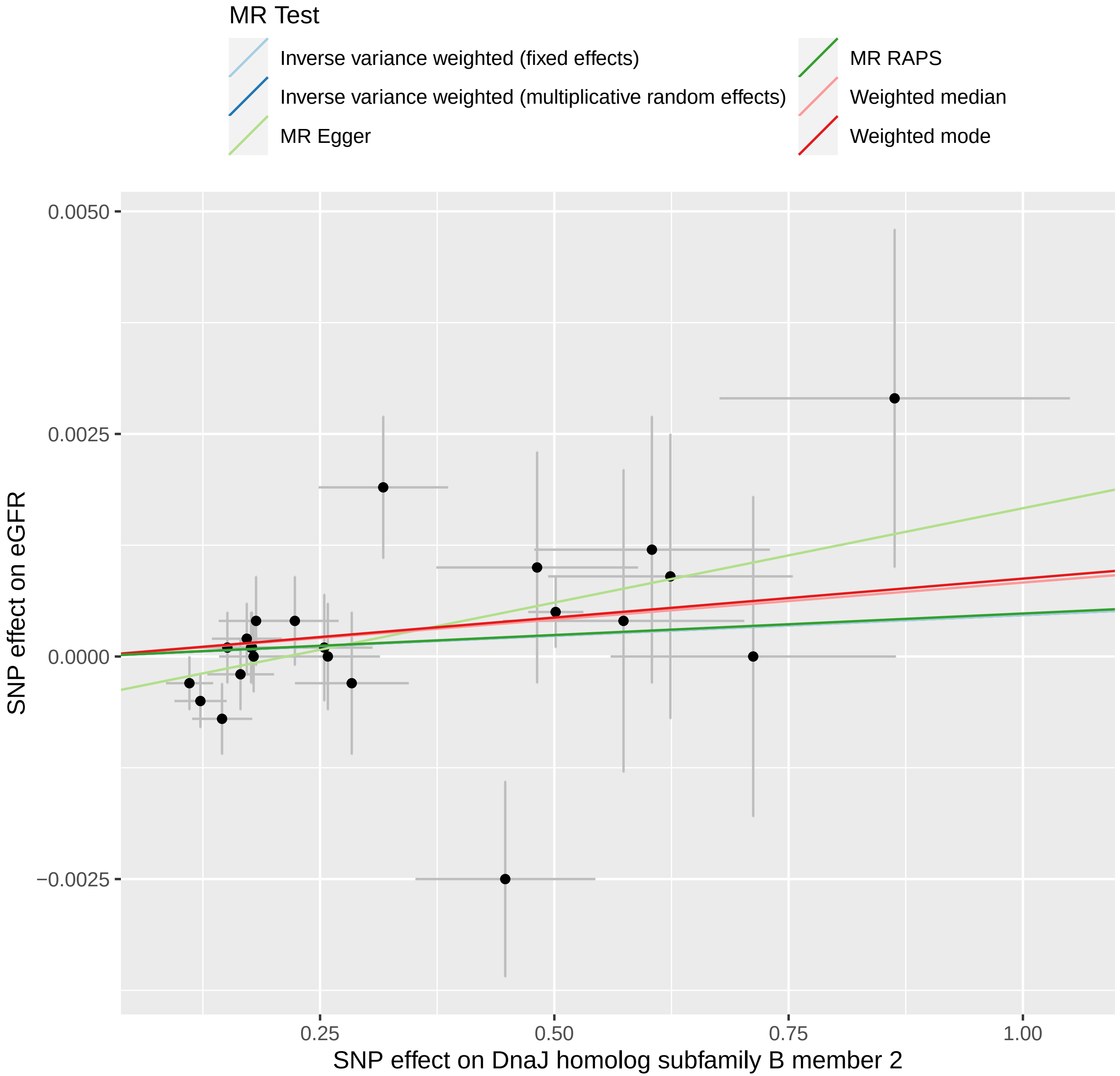

Batch 2−842 : DnaJ homolog subfamily B member 9 on eGFR

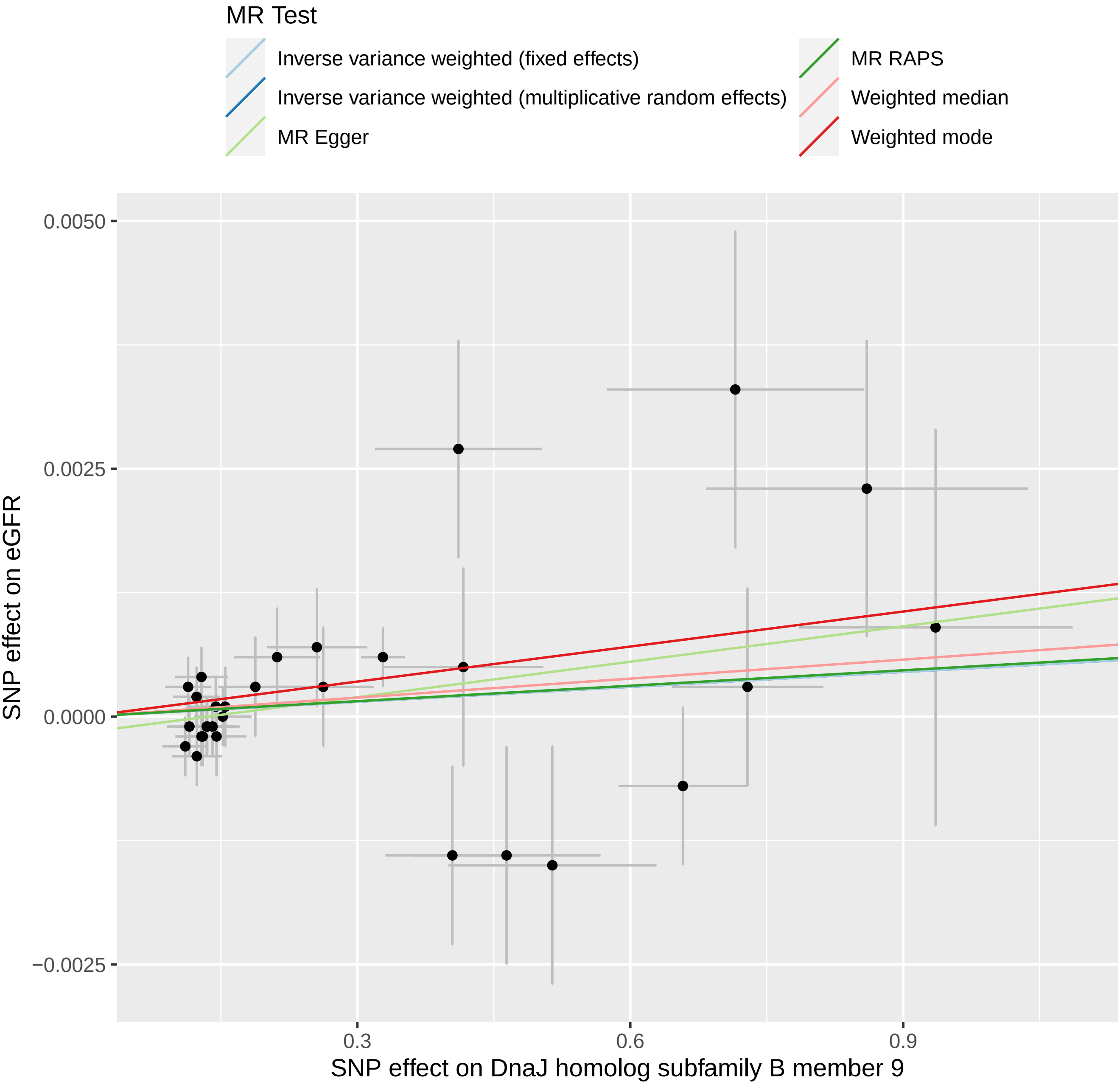

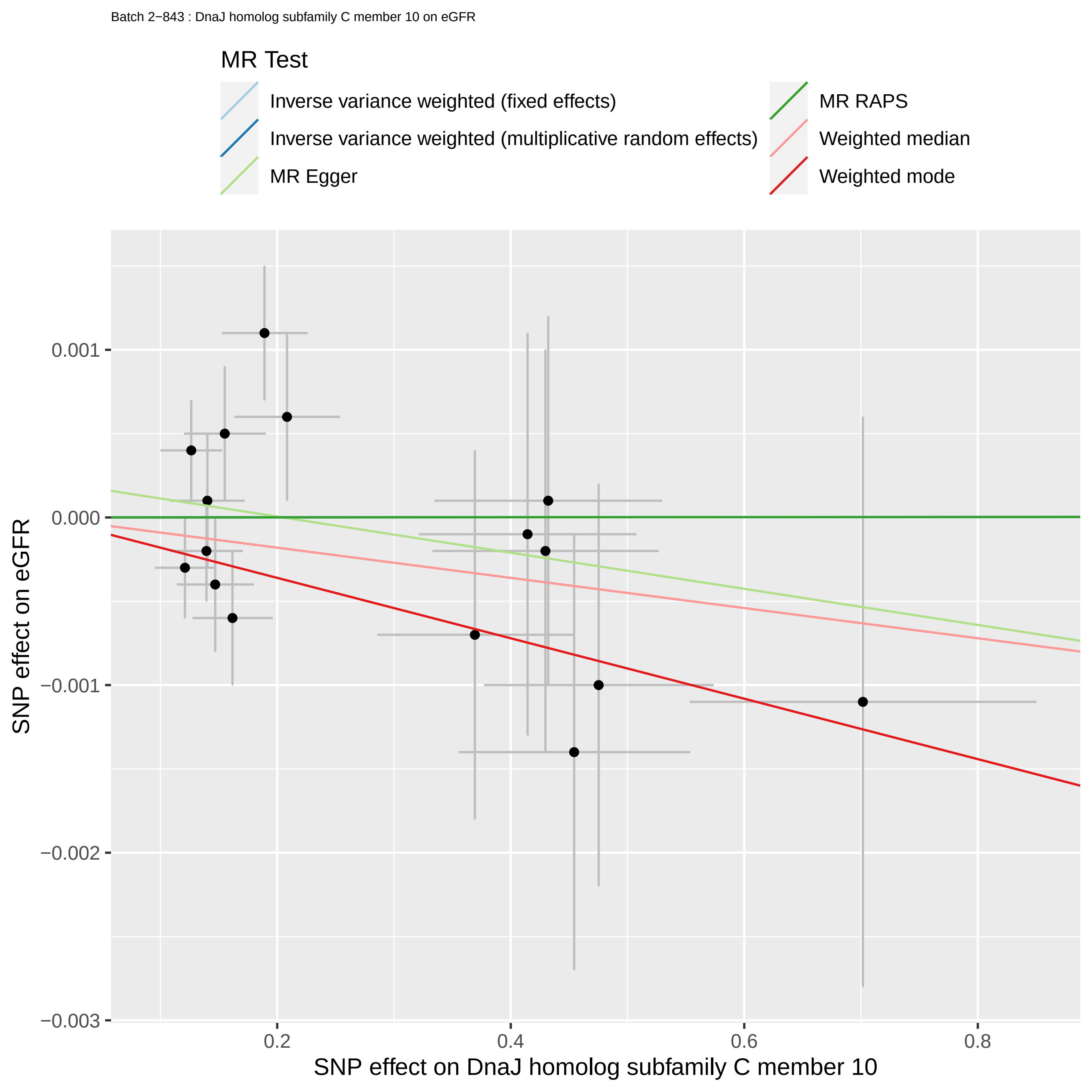

Batch 2−843 : DnaJ homolog subfamily C member 10 on eGFR
MR Test
Inverse variance weighted (fixed effects)
Inverse variance weighted (multiplicative random effects)
MR Egger
MR RAPS
Weighted median
Weighted mode
0.001
0.000
−0.001
−0.002
−0.003
0.2
0.4
0.6
0.8
SNP effect on eGFR
SNP effect on DnaJ homolog subfamily C member 10

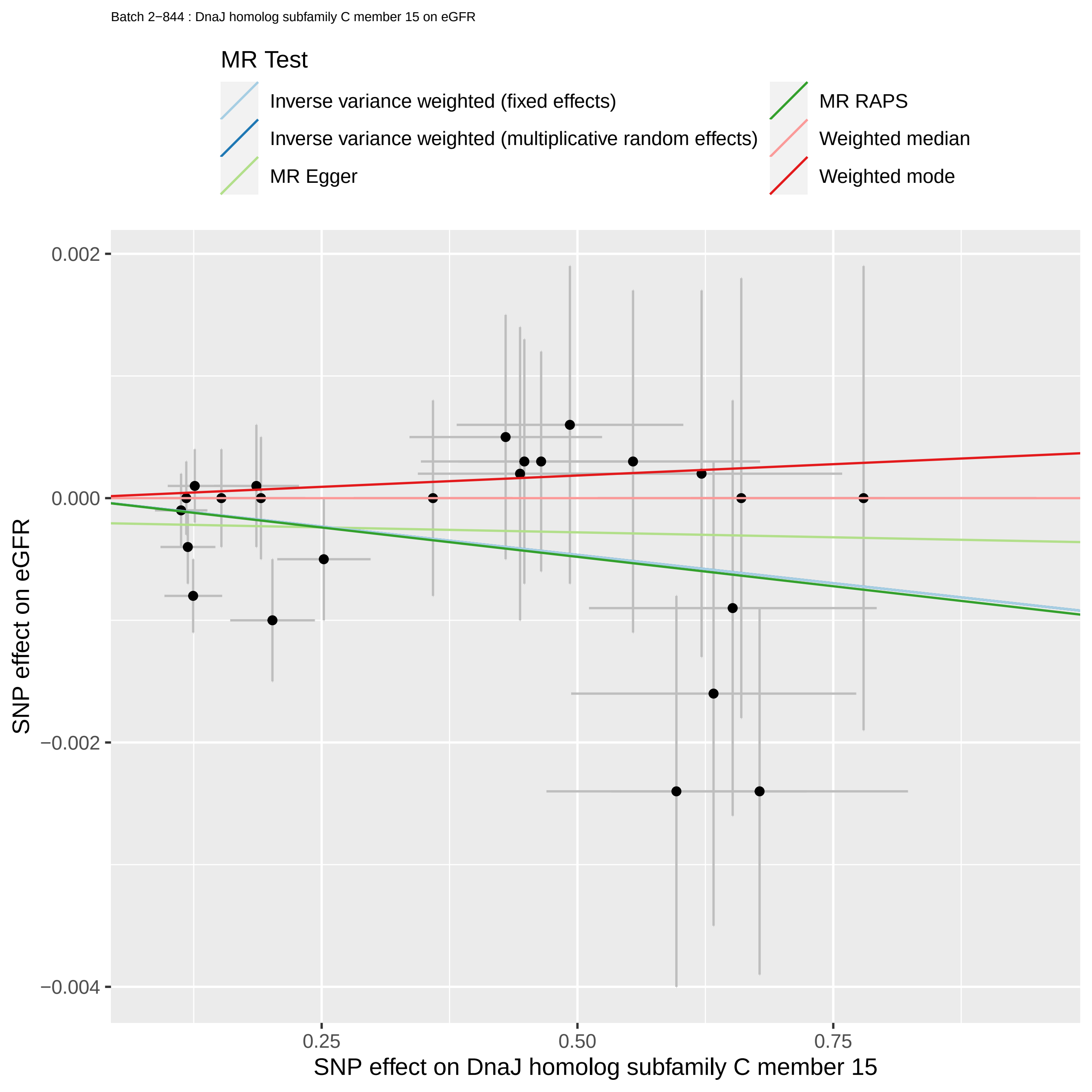

Batch 2−844 : DnaJ homolog subfamily C member 15 on eGFR
MR Test
Inverse variance weighted (fixed effects)
Inverse variance weighted (multiplicative random effects)
MR Egger
MR RAPS
Weighted median
Weighted mode
0.002
0.000
−0.002
−0.004
SNP effect on eGFR
0.25
0.50
0.75
SNP effect on DnaJ homolog subfamily C member 15

Batch 2−845 : DnaJ homolog subfamily C member 17 on eGFR

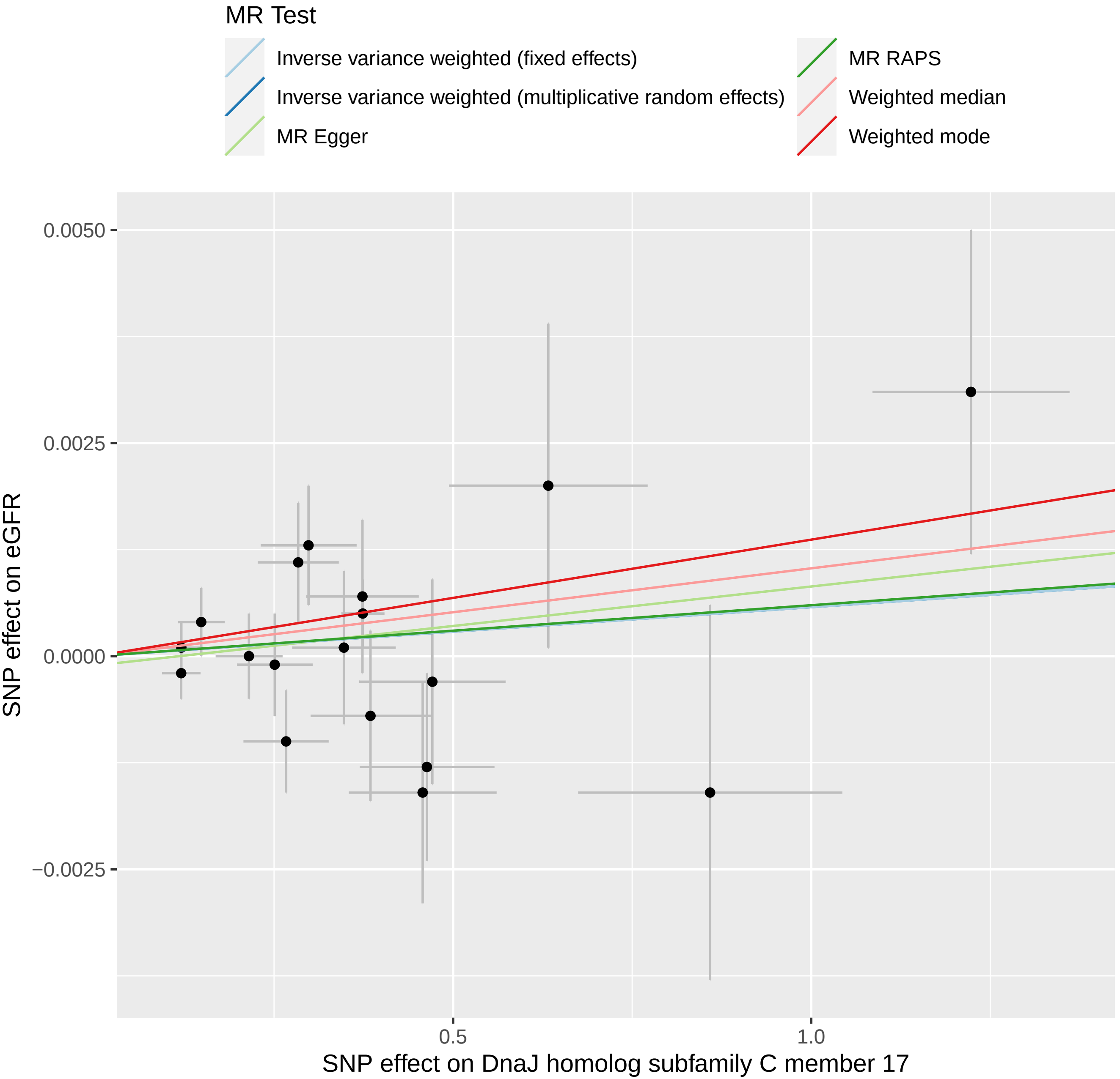

Batch 2−846 : DnaJ homolog subfamily C member 18 on eGFR

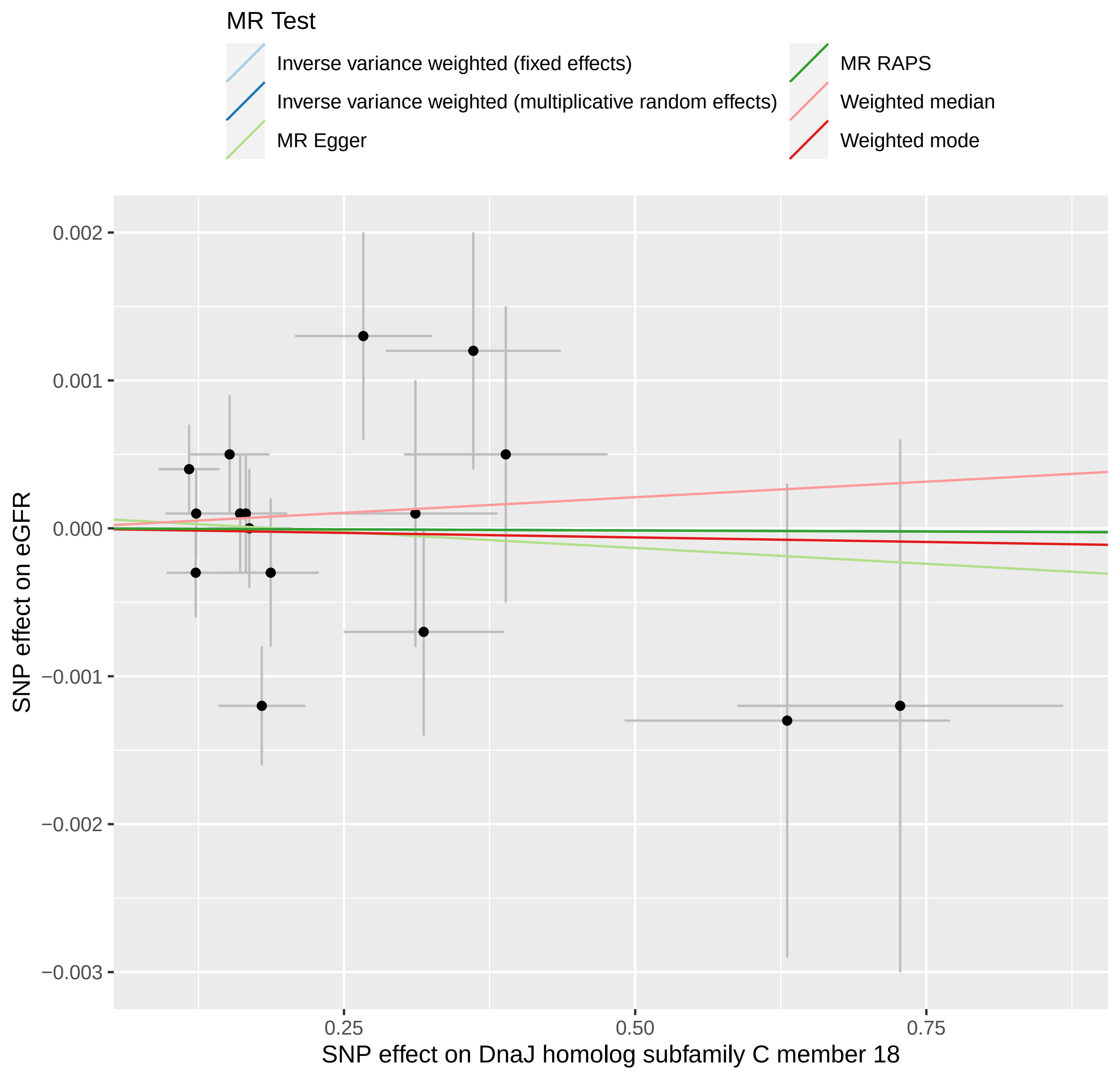

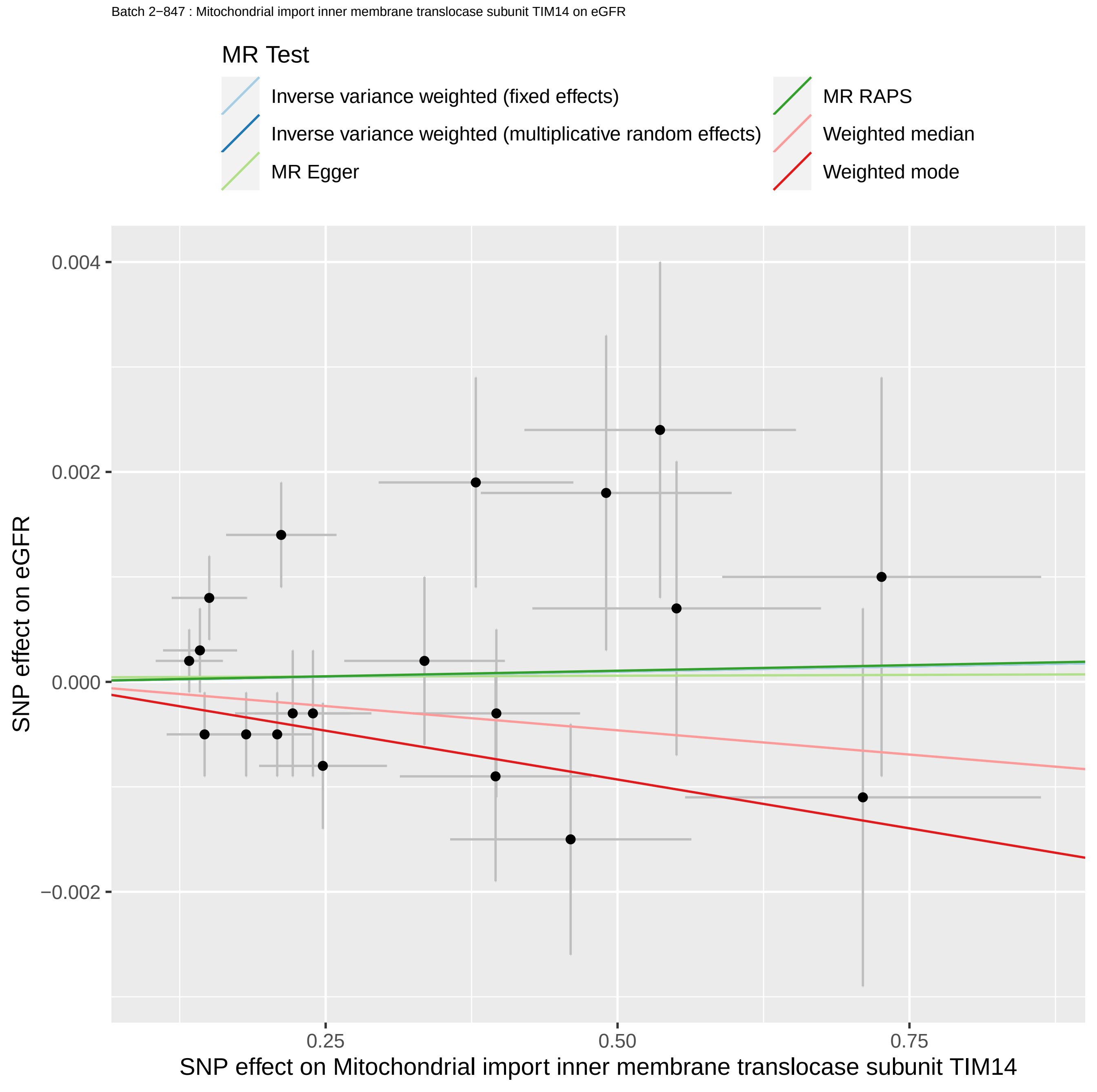

Batch 2−847 : Mitochondrial import inner membrane translocase subunit TIM14 on eGFR
MR Test
Inverse variance weighted (fixed effects)
Inverse variance weighted (multiplicative random effects)
MR Egger
MR RAPS
Weighted median
Weighted mode
0.004
0.002
0.000
−0.002
0.25
0.50
0.75
SNP effect on eGFR
SNP effect on Mitochondrial import inner membrane translocase subunit TIM14

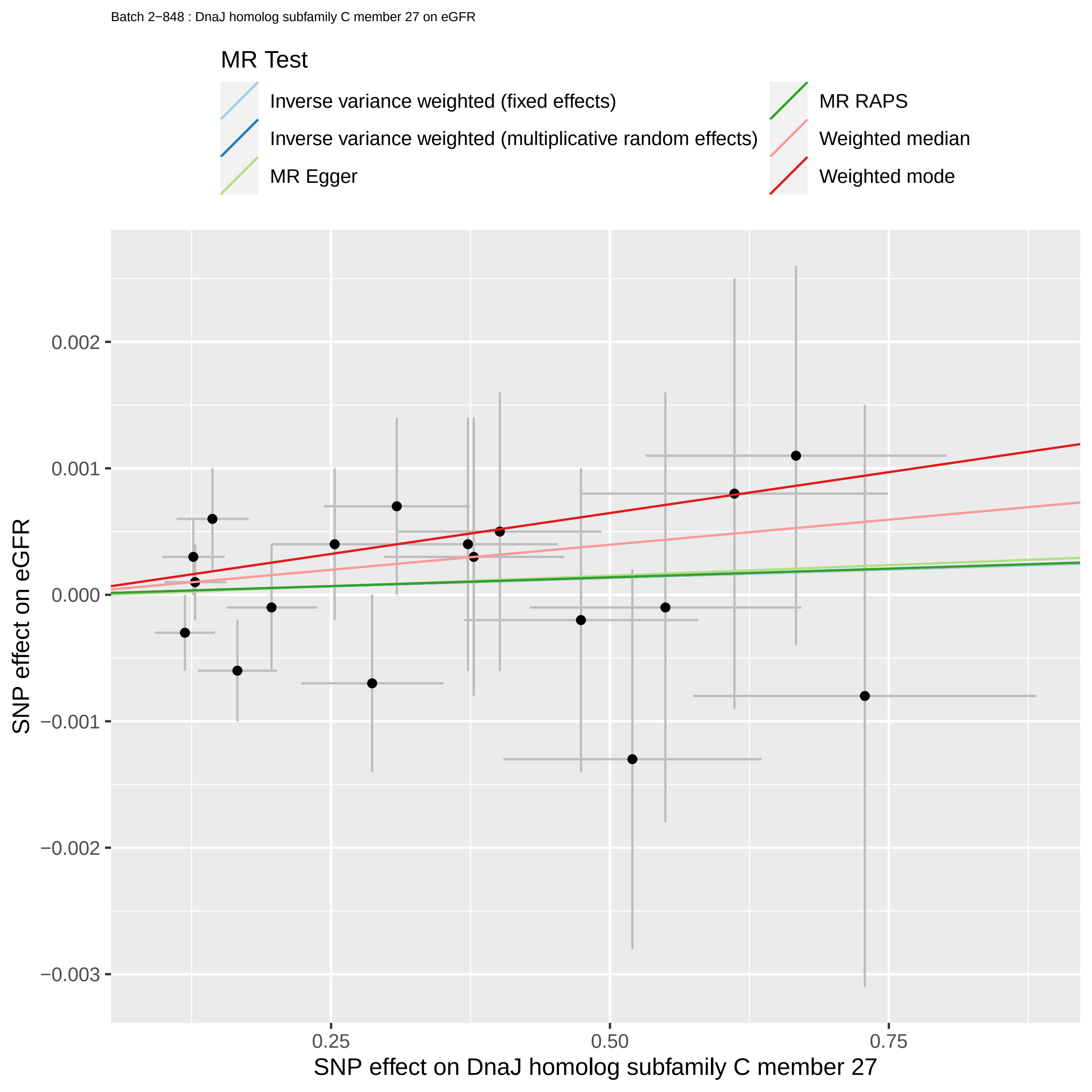

Batch 2−848 : DnaJ homolog subfamily C member 27 on eGFR
MR Test
Inverse variance weighted (fixed effects)
Inverse variance weighted (multiplicative random effects)
MR Egger
MR RAPS
Weighted median
Weighted mode
0.002
0.001
0.000
−0.001
−0.002
−0.003
SNP effect on eGFR
0.25
0.50
0.75
SNP effect on DnaJ homolog subfamily C member 27

Batch 2−849 : DnaJ homolog subfamily C member 30 on eGFR

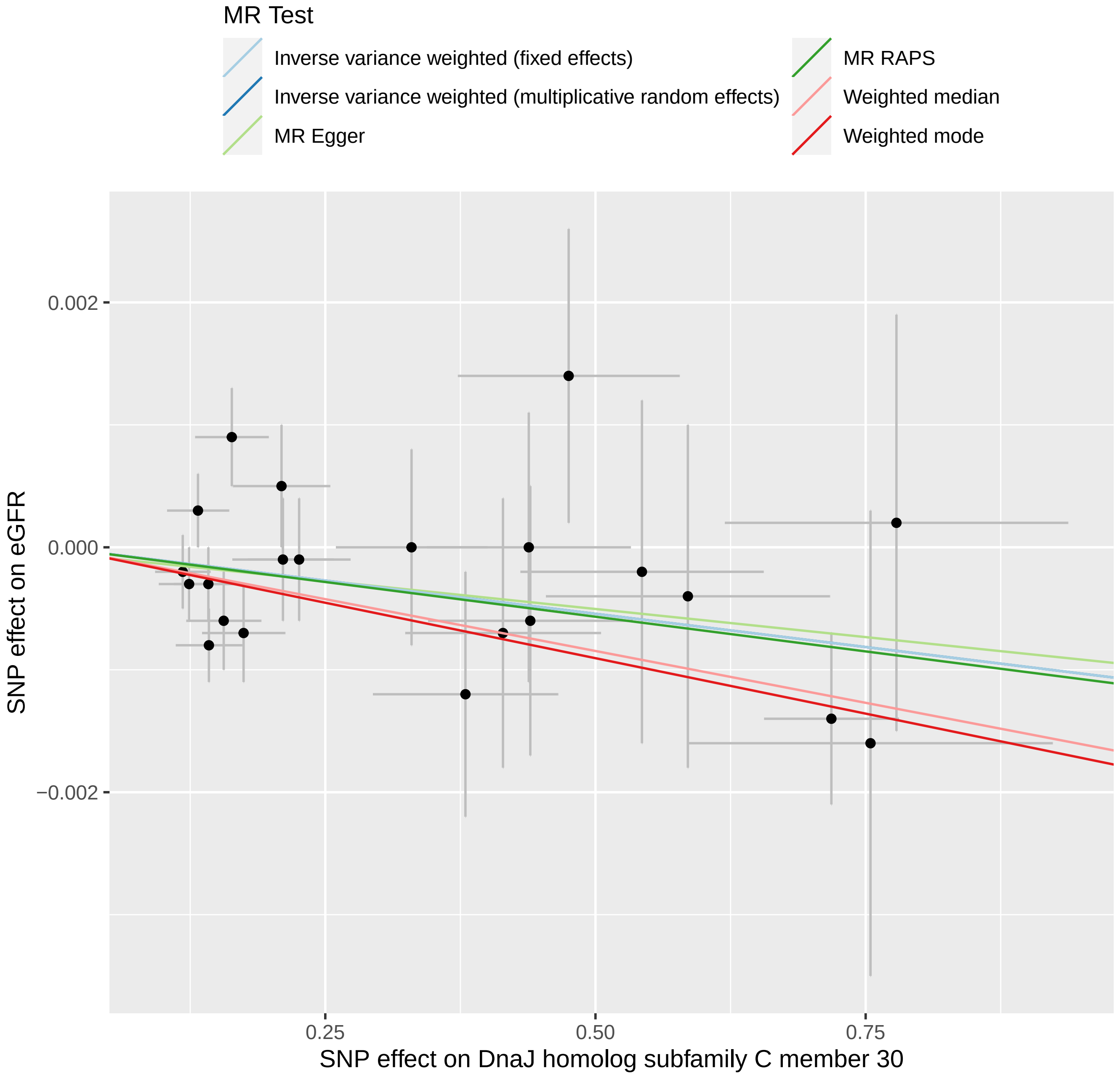

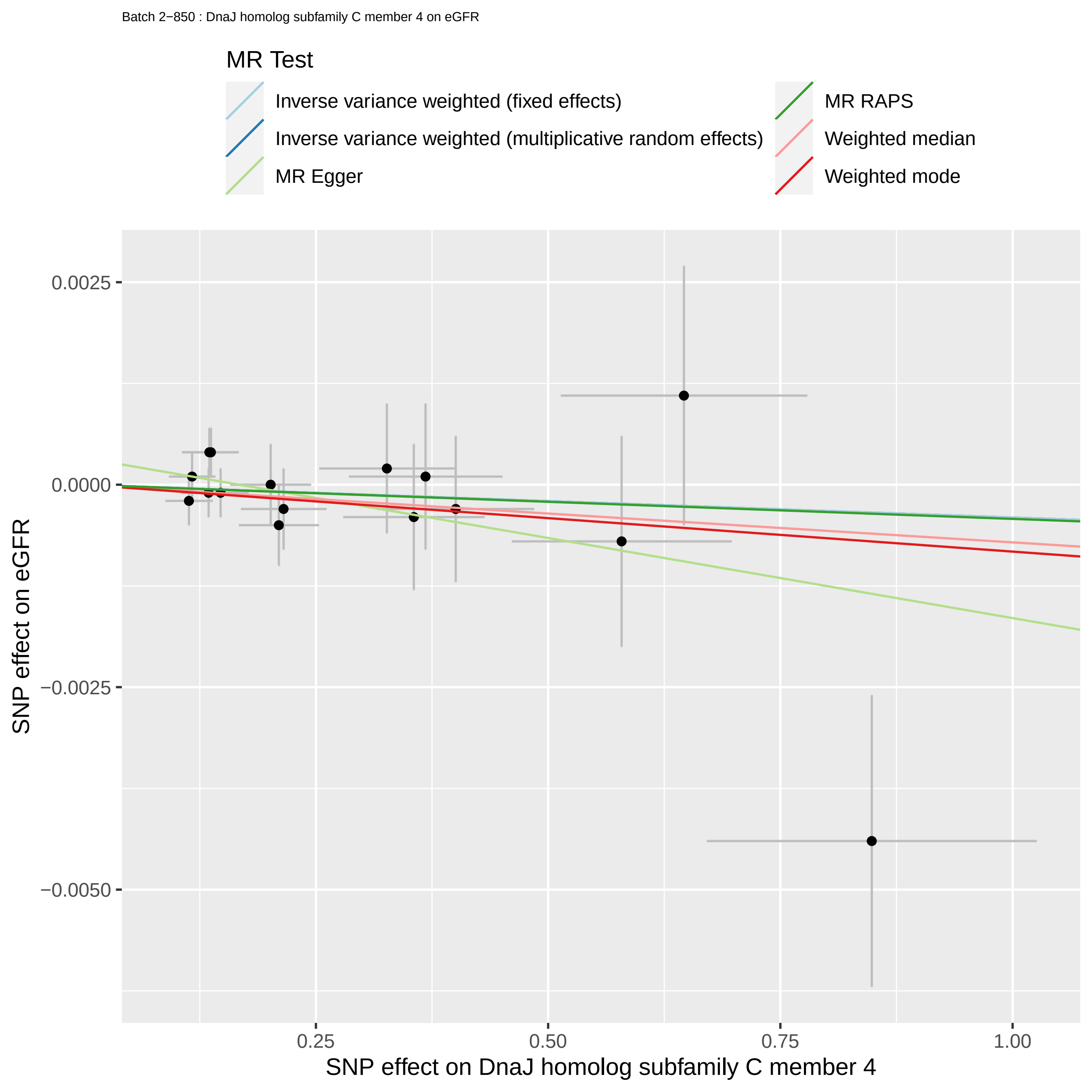
Batch 2−850 : DnaJ homolog subfamily C member 4 on eGFR
MR Test
Inverse variance weighted (fixed effects)
Inverse variance weighted (multiplicative random effects)
MR Egger
MR RAPS
Weighted median
Weighted mode
0.0025
0.0000
−0.0025
−0.0050
SNP effect on eGFR
0.25
0.50
0.75
1.00
SNP effect on DnaJ homolog subfamily C member 4

Batch 2−851 : Deoxyribonuclease−2−beta on eGFR

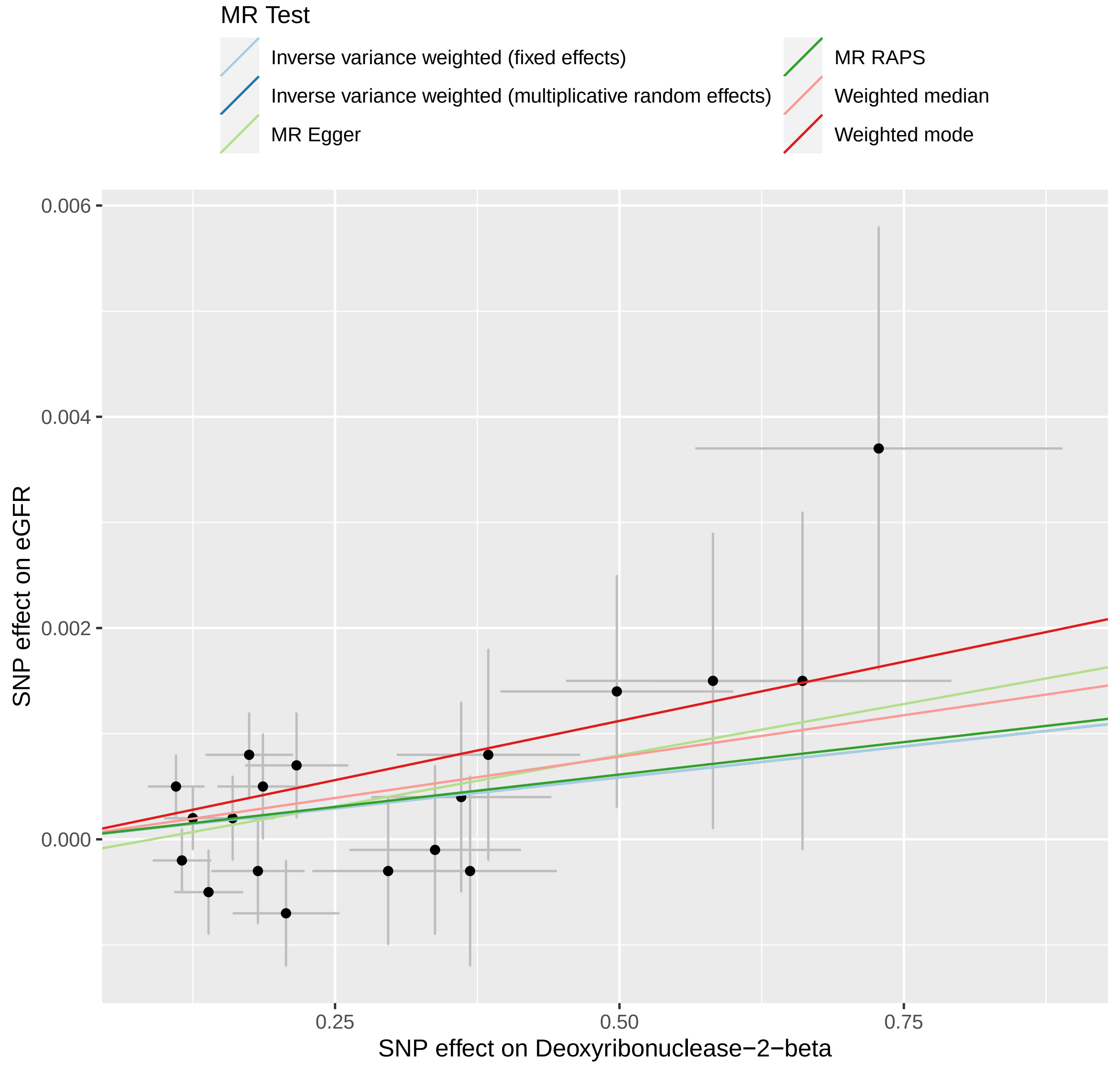

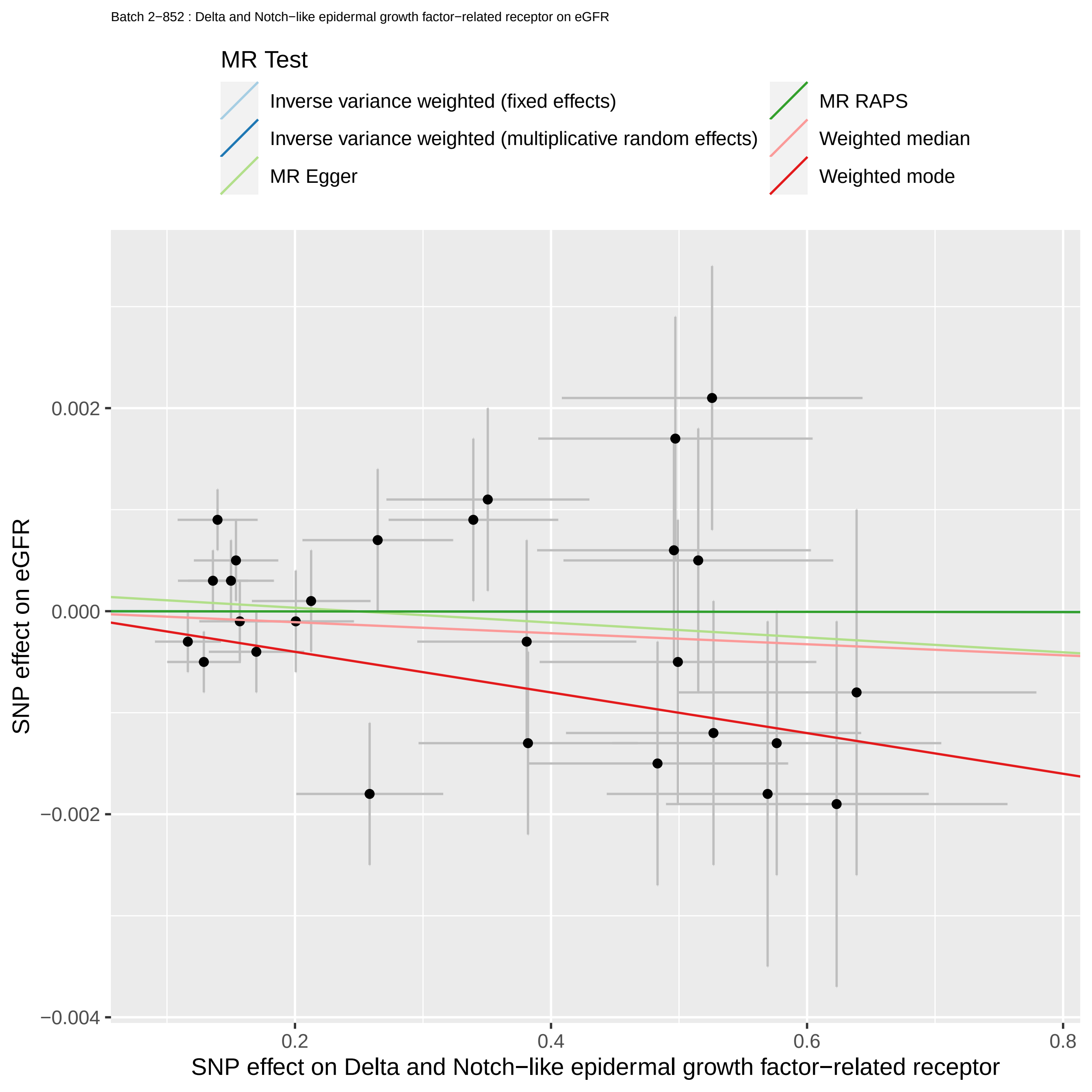

Batch 2−852 : Delta and Notch−like epidermal growth factor−related receptor on eGFR
MR Test
Inverse variance weighted (fixed effects)
Inverse variance weighted (multiplicative random effects)
MR Egger
MR RAPS
Weighted median
Weighted mode
0.002
0.000
−0.002
−0.004
0.2
0.4
0.6
0.8
SNP effect on eGFR
SNP effect on Delta and Notch−like epidermal growth factor−related receptor

Batch 2−853 : Delta and Notch−like epidermal growth factor−related receptor on eGFR

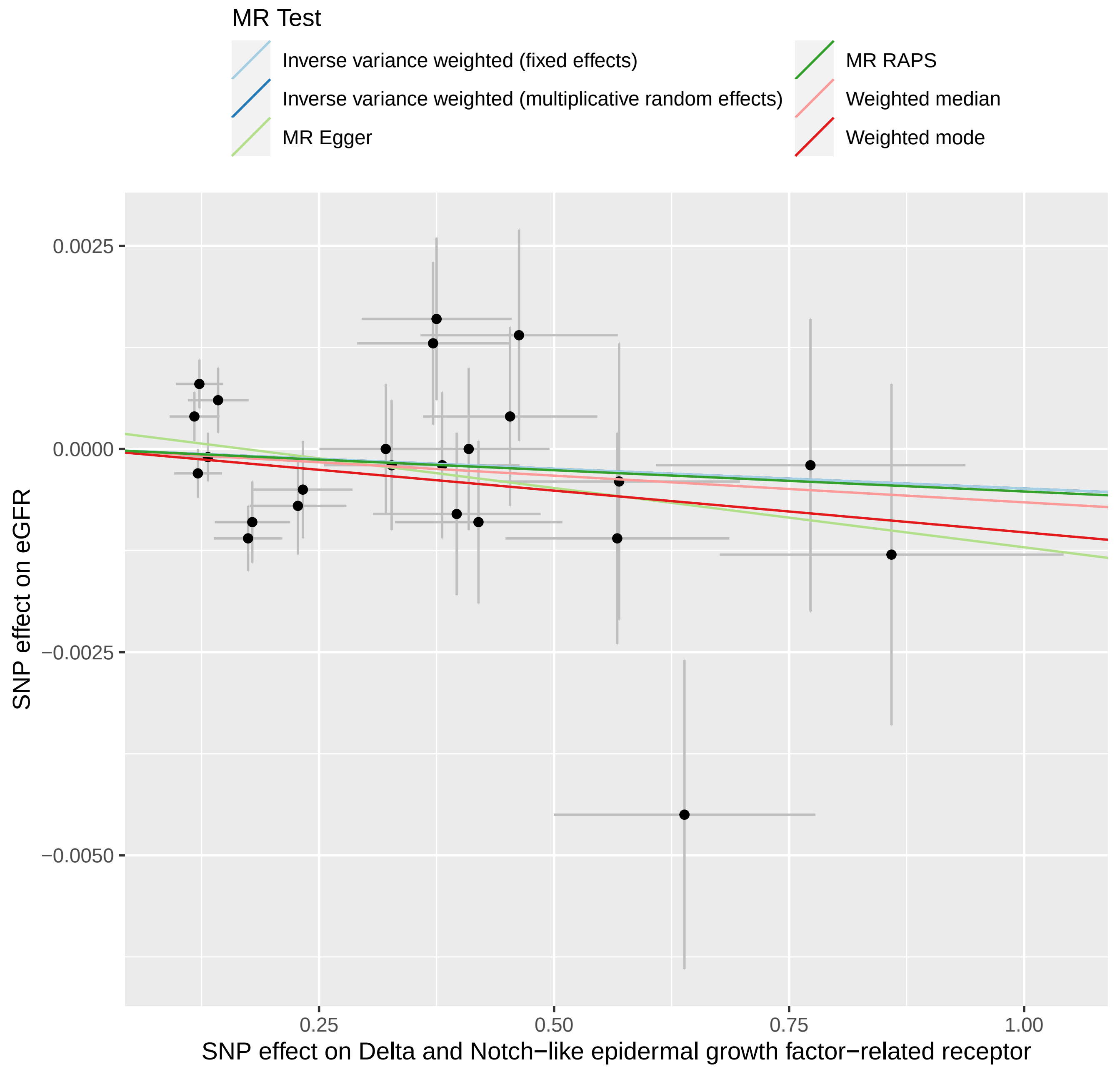

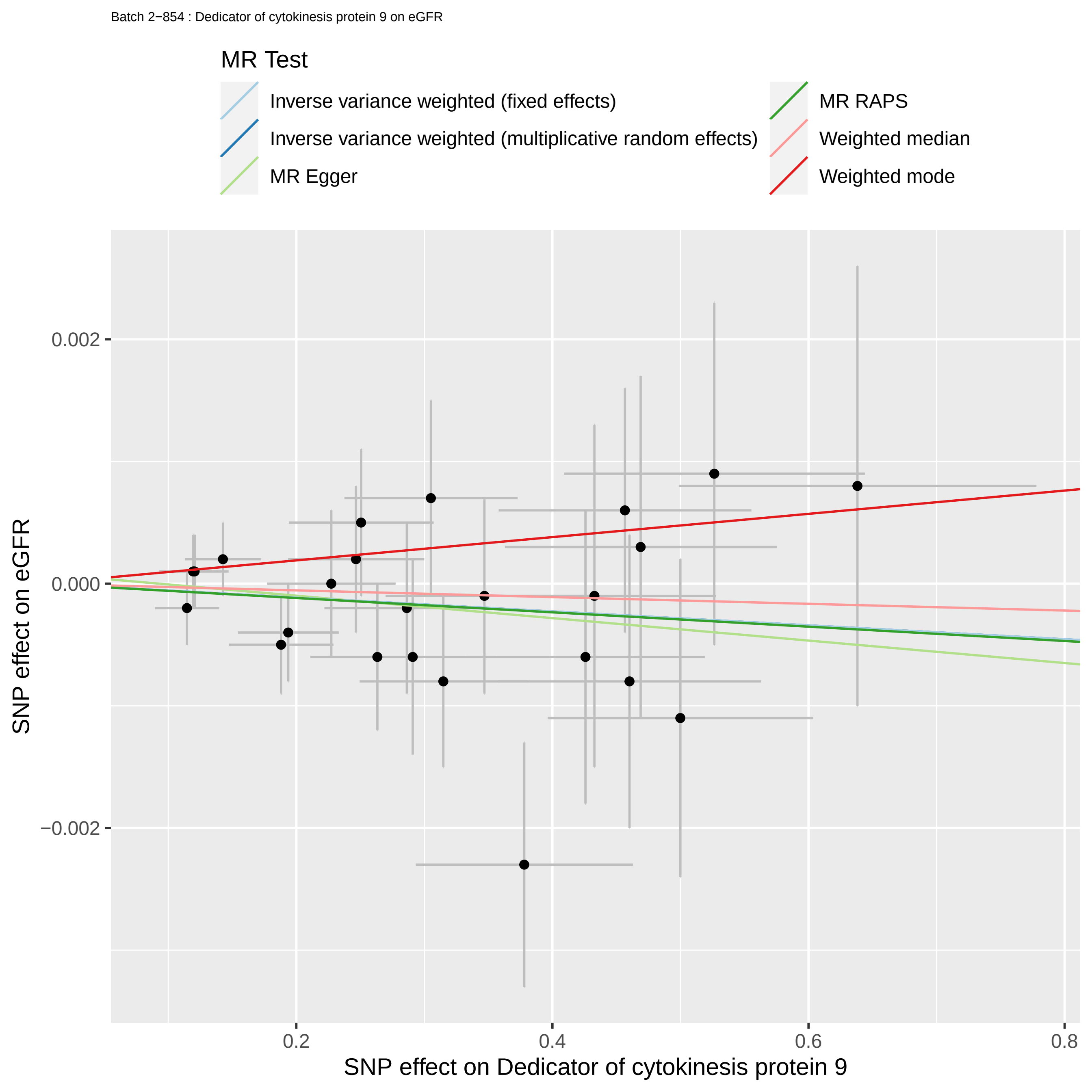
Batch 2−854 : Dedicator of cytokinesis protein 9 on eGFR
MR Test
Inverse variance weighted (fixed effects)
Inverse variance weighted (multiplicative random effects)
MR Egger
MR RAPS
Weighted median
Weighted mode
SNP effect on eGFR
0.002
0.000
−0.002
0.2
0.4
0.6
0.8
SNP effect on Dedicator of cytokinesis protein 9

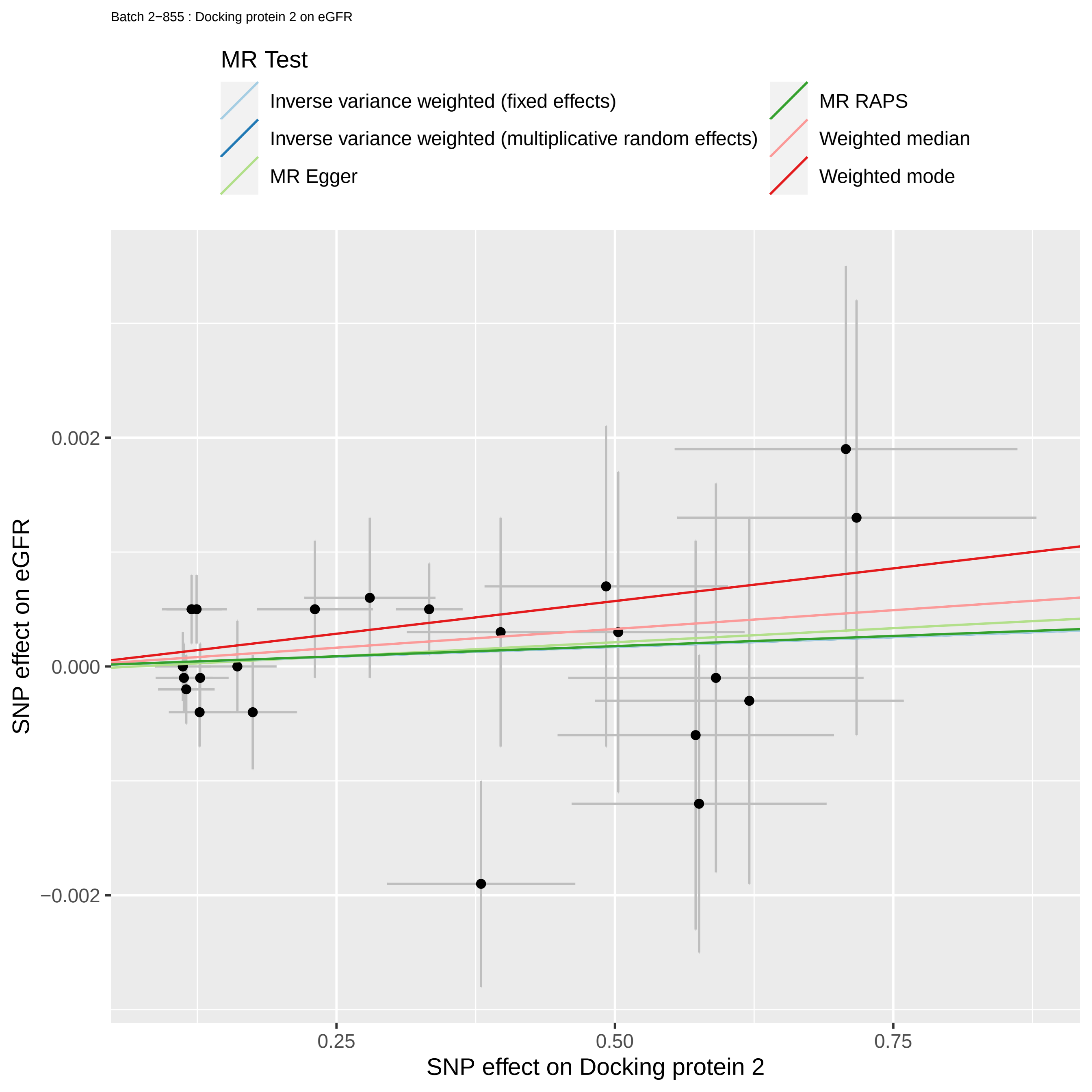

Batch 2−855 : Docking protein 2 on eGFR
MR Test
Inverse variance weighted (fixed effects)
Inverse variance weighted (multiplicative random effects)
MR Egger
MR RAPS
Weighted median
Weighted mode
0.002
0.000
−0.002
0.25
0.50
0.75
SNP effect on eGFR
SNP effect on Docking protein 2

Batch 2−856 : Dipeptidase 1 on eGFR

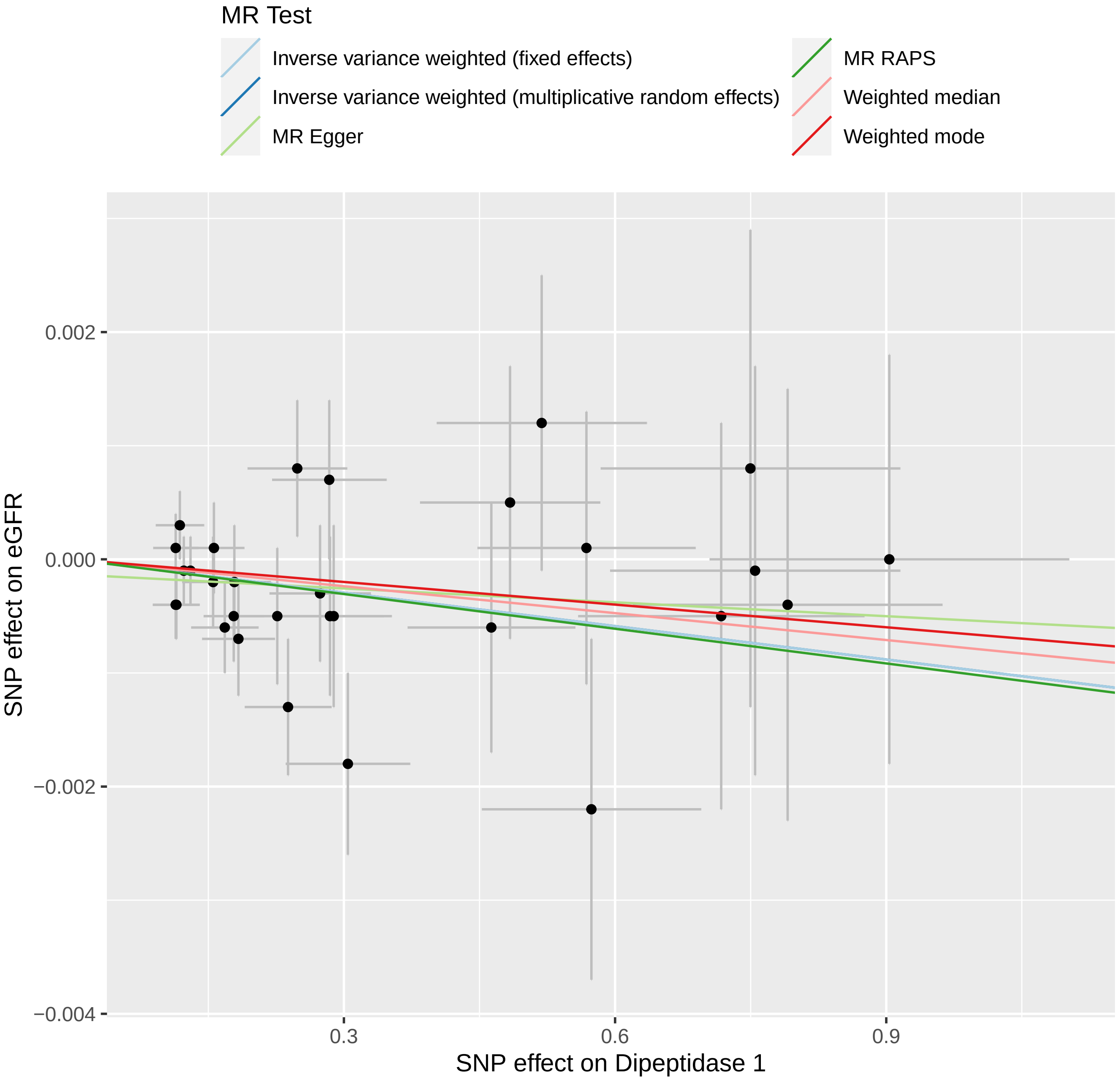

Batch 2−857 : Dipeptidase 2 on eGFR

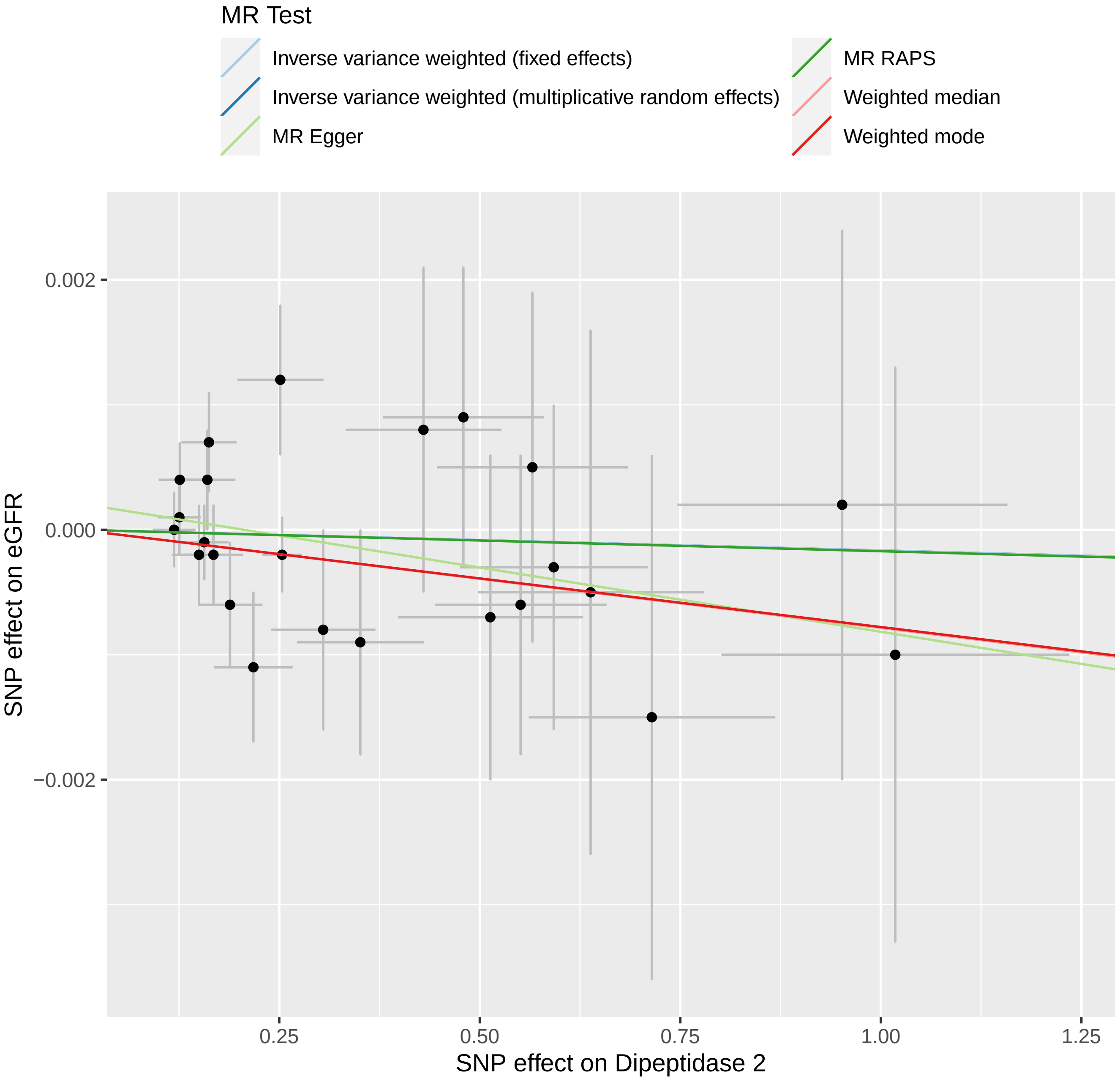

Batch 2−858 : Inactive dipeptidyl peptidase 10 on eGFR

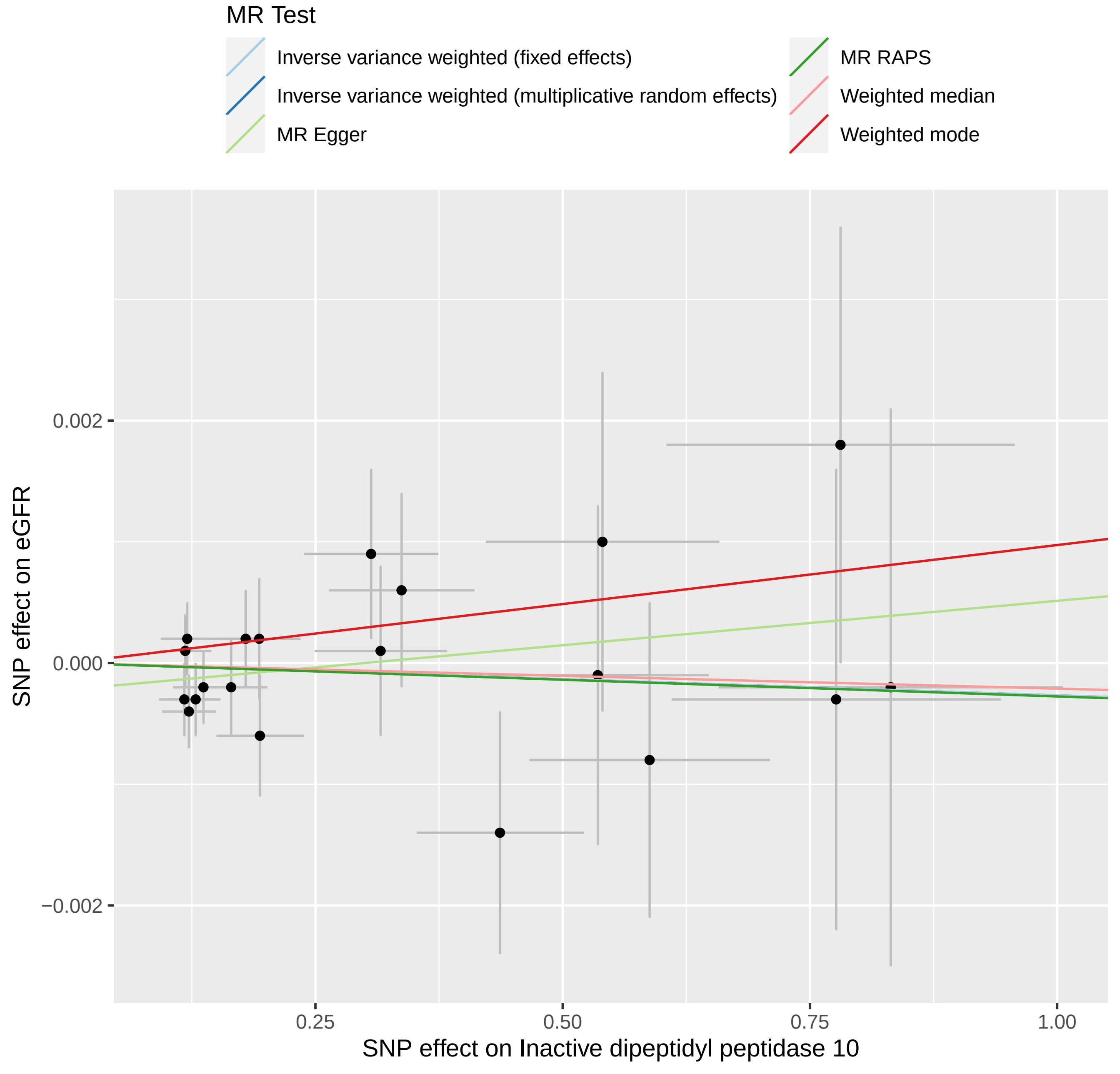

Batch 2−859 : Dipeptidyl peptidase 2 on eGFR

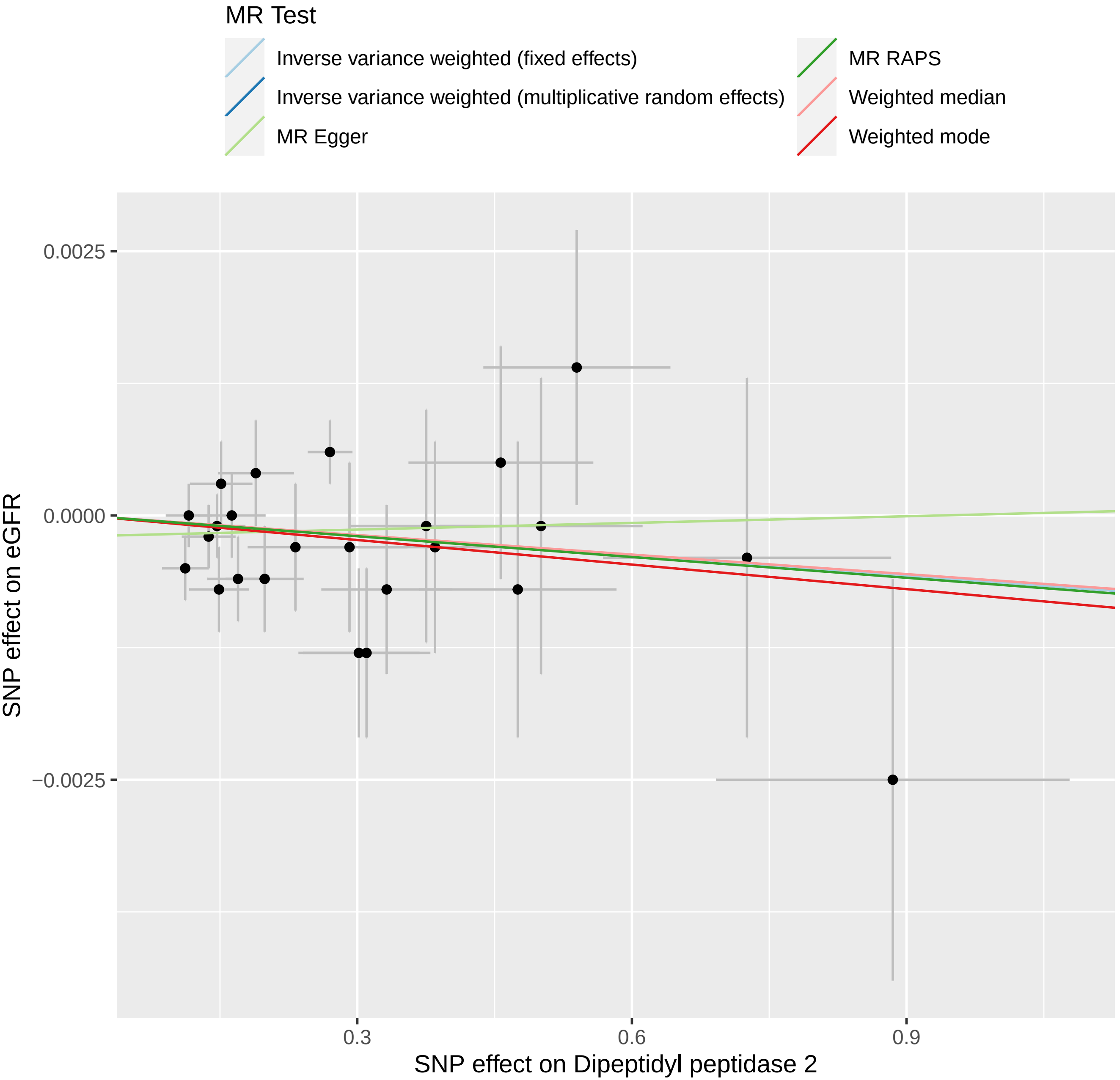

Batch 2−860 : Dipeptidyl peptidase 2 on eGFR

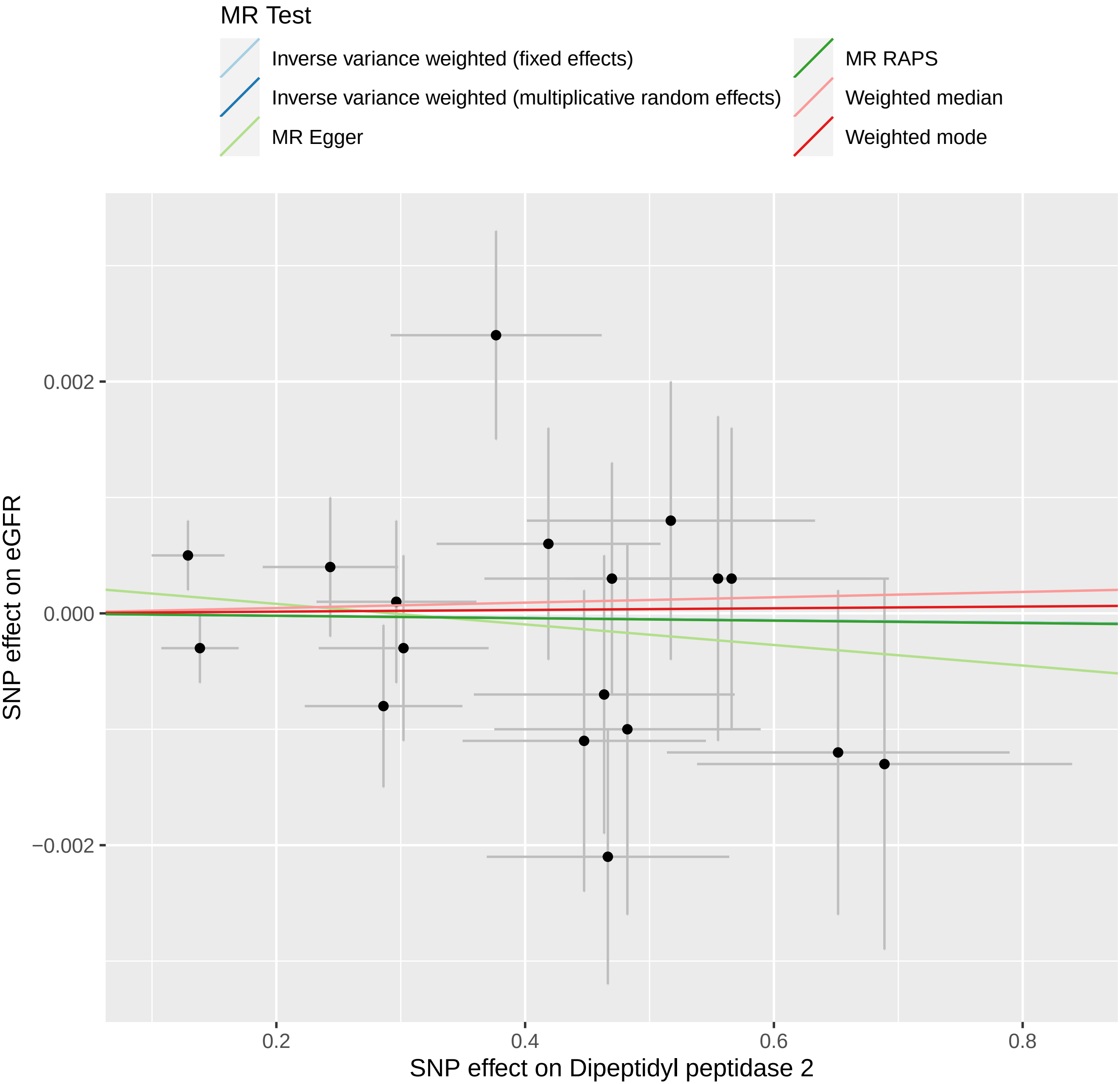

Batch 2−861 : Dermatopontin on eGFR

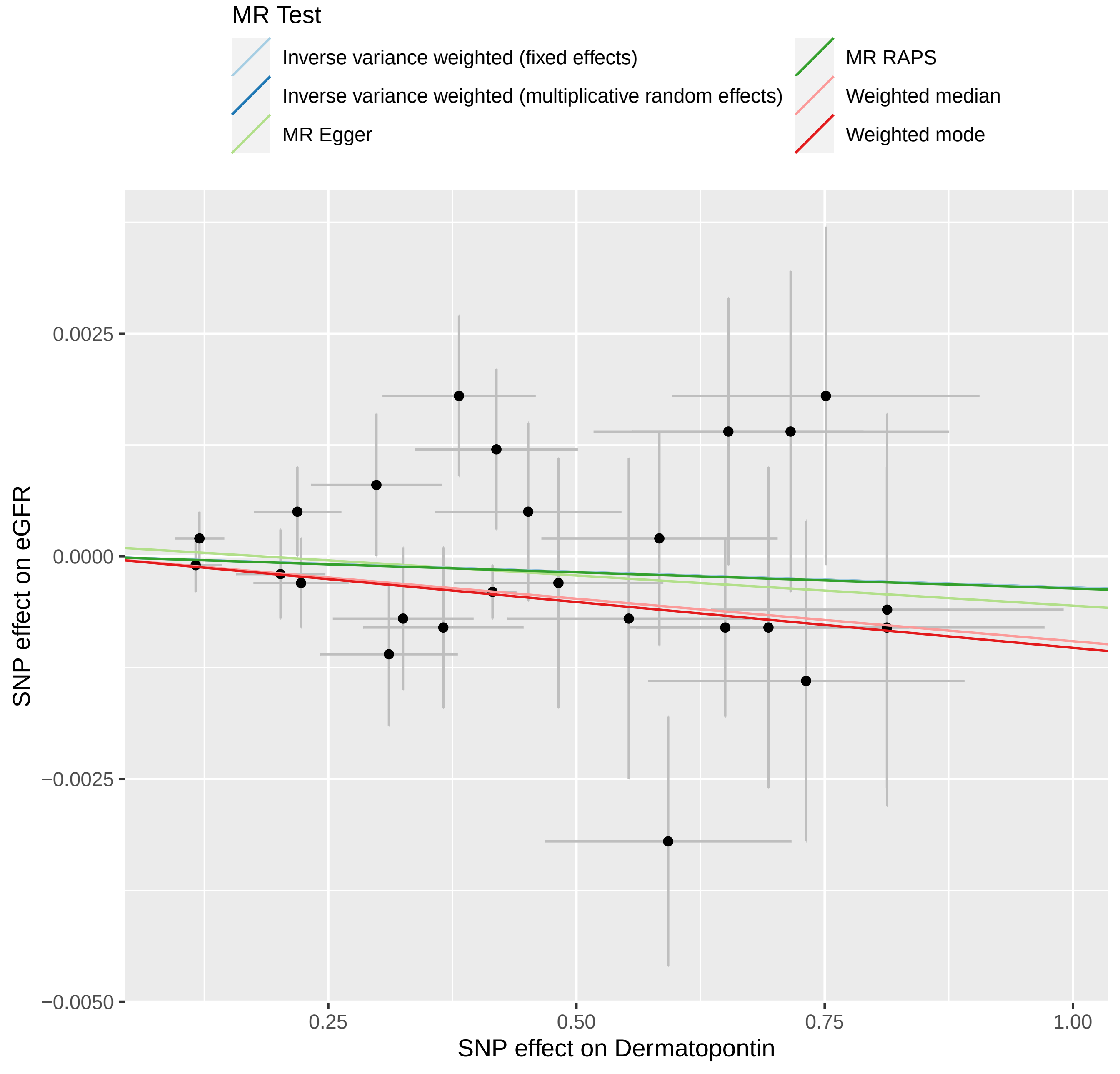

Batch 2−862 : Protein dpy−30 homolog on eGFR

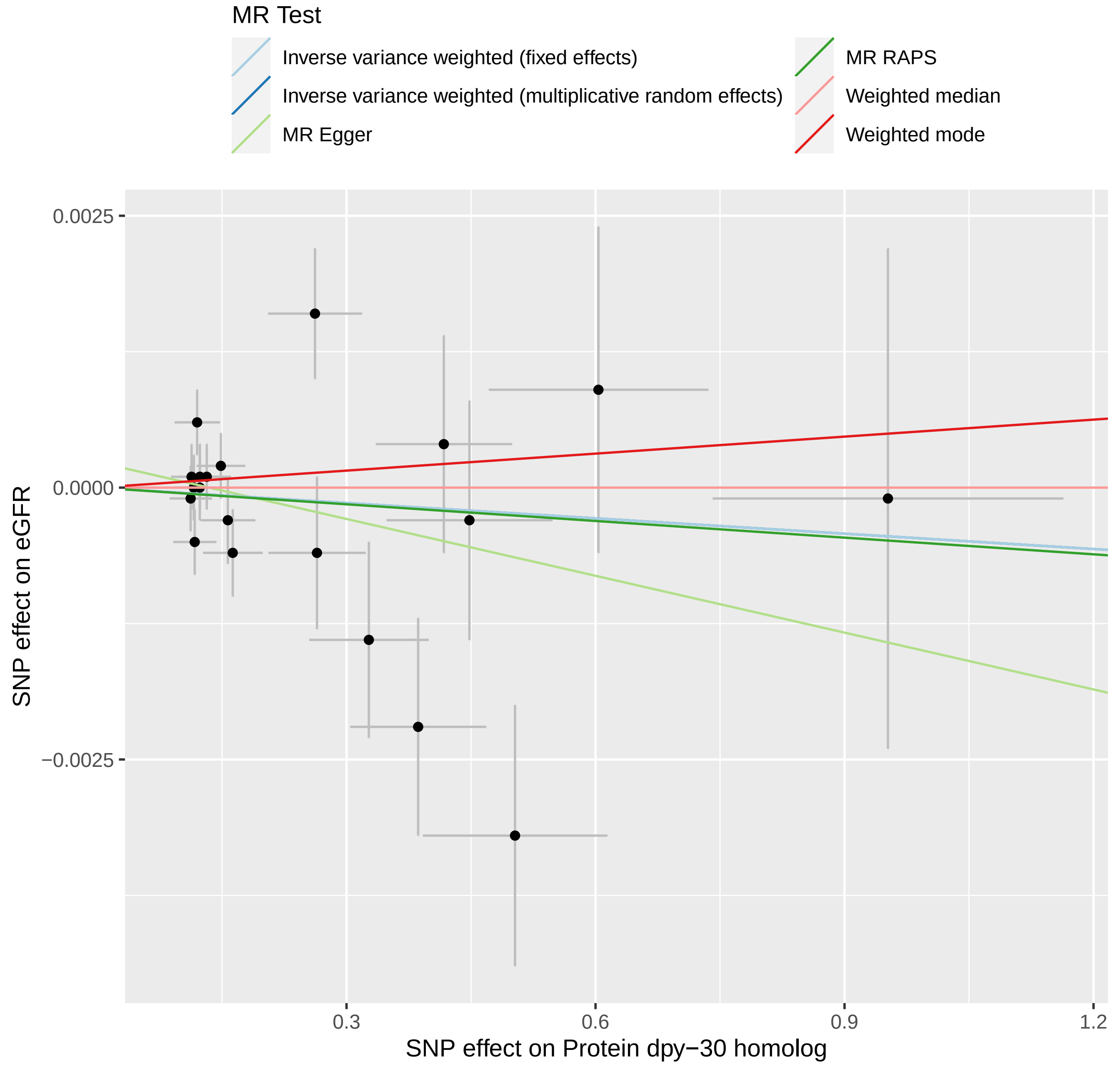

Batch 2−863 : Dihydropyrimidinase−related protein 3 on eGFR

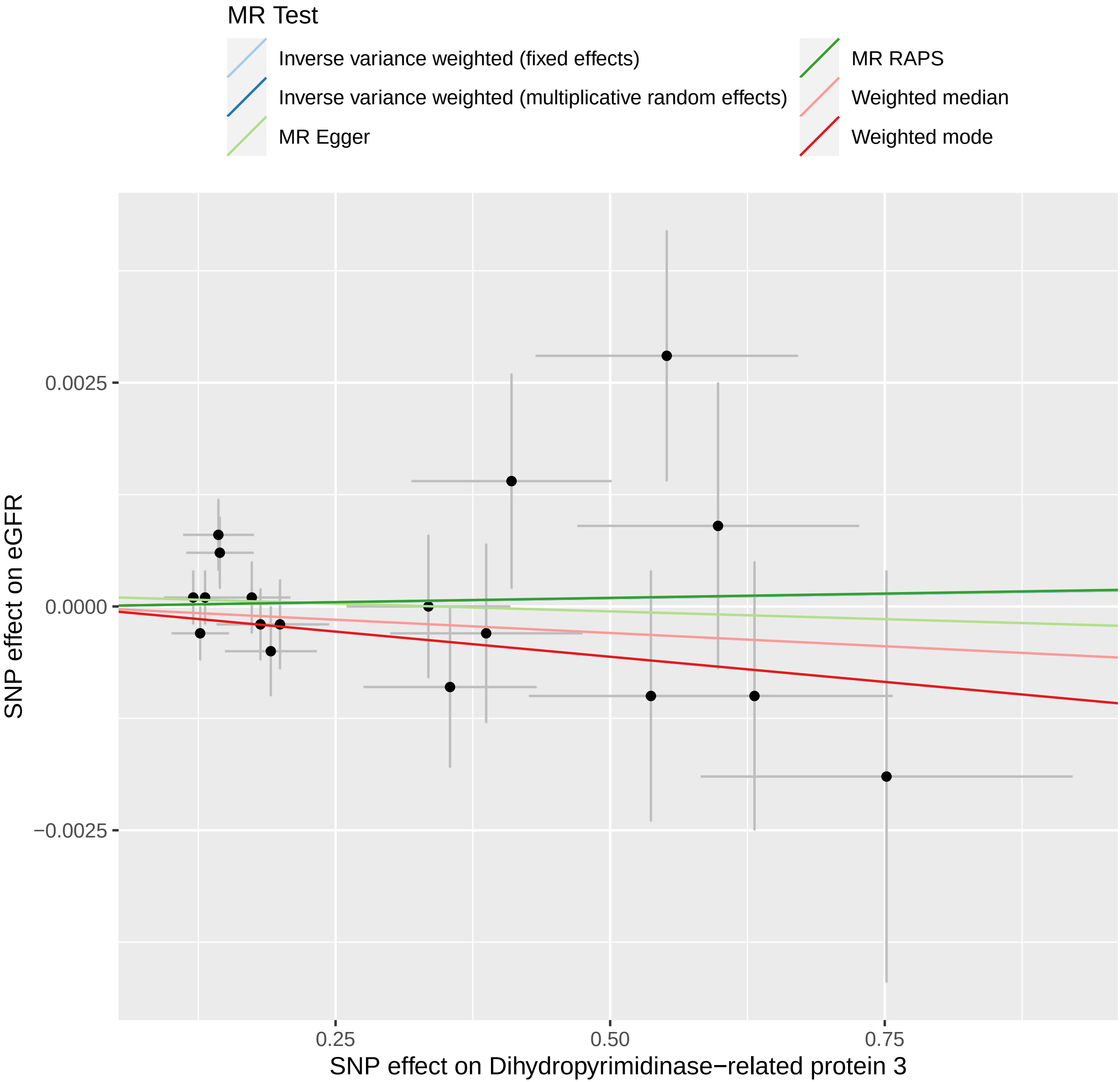

Batch 2−864 : Dihydropyrimidinase−related protein 5 on eGFR

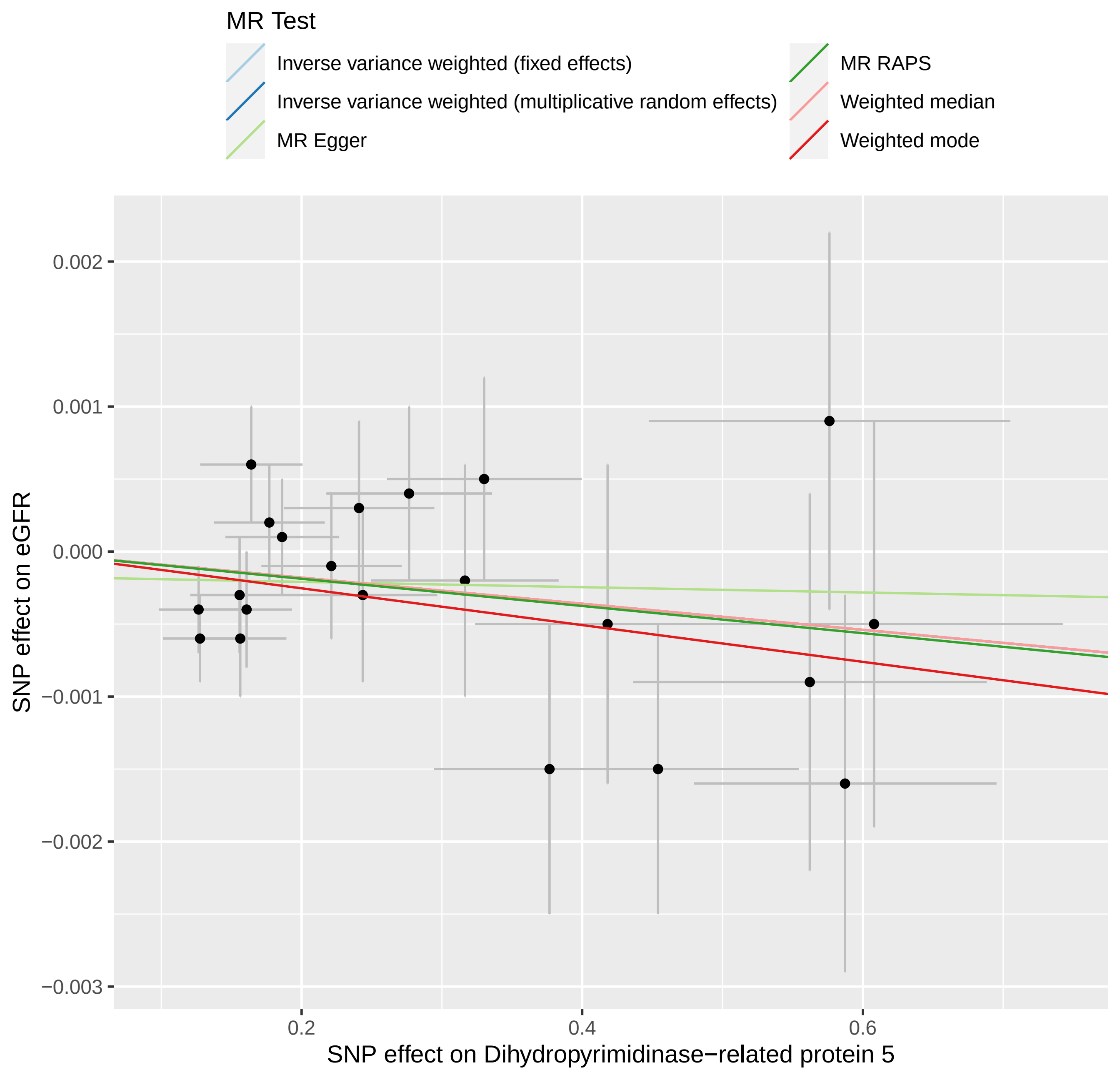

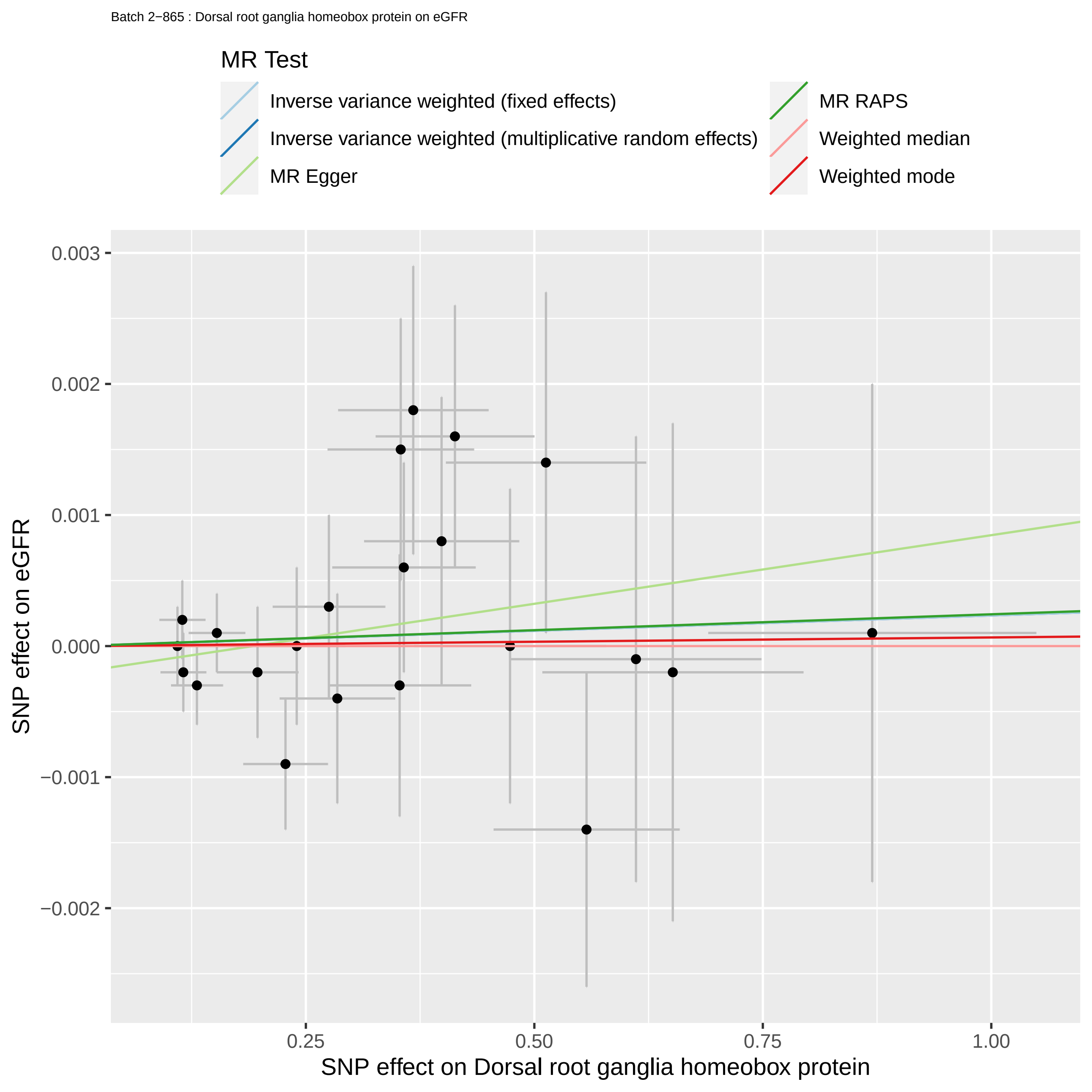
Batch 2−865 : Dorsal root ganglia homeobox protein on eGFR
MR Test
Inverse variance weighted (fixed effects)
Inverse variance weighted (multiplicative random effects)
MR Egger
MR RAPS
Weighted median
Weighted mode
0.003
0.002
0.001
0.000
−0.001
−0.002
SNP effect on eGFR
0.25
0.50
0.75
1.00
SNP effect on Dorsal root ganglia homeobox protein

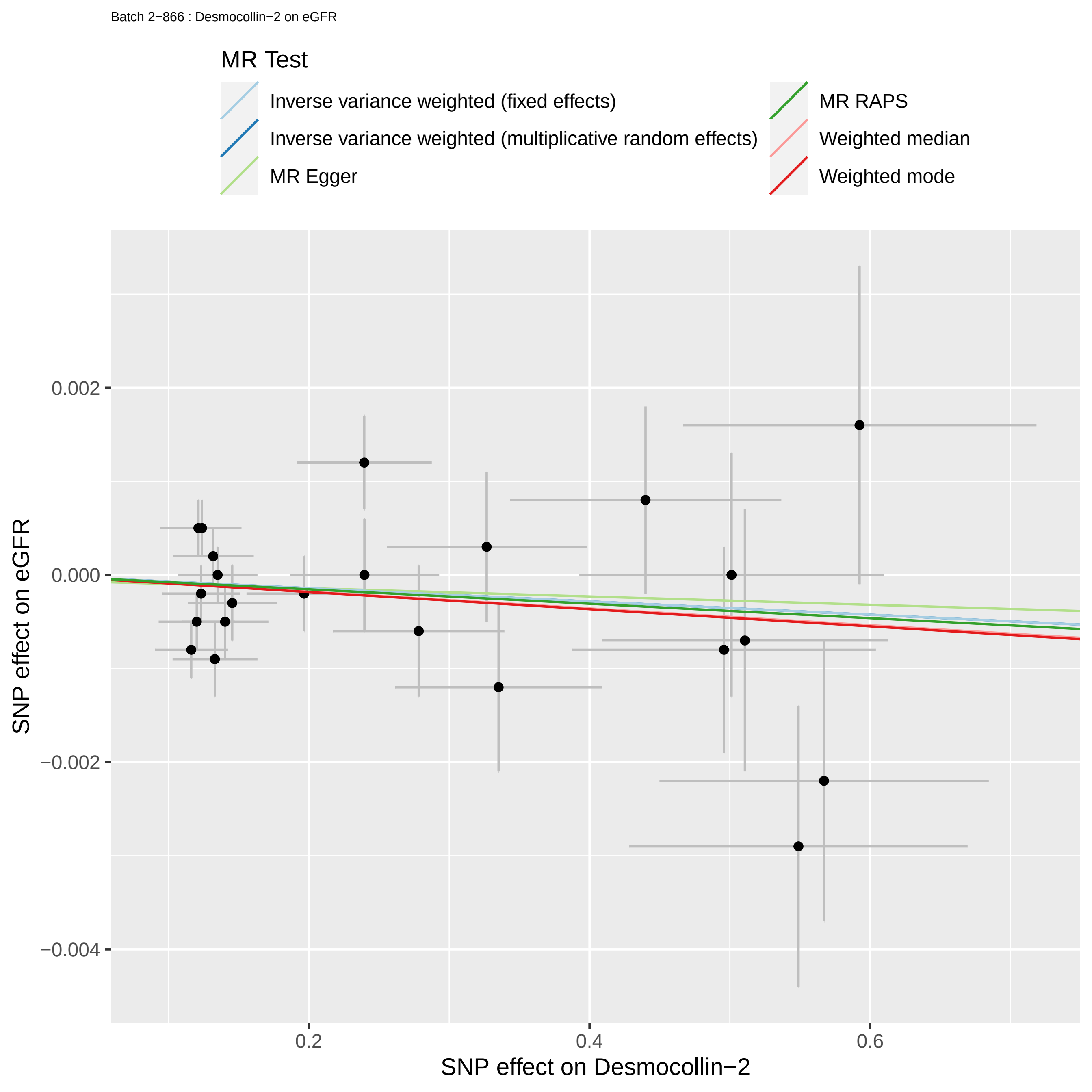

Batch 2−866 : Desmocollin−2 on eGFR
MR Test
Inverse variance weighted (fixed effects)
Inverse variance weighted (multiplicative random effects)
MR Egger
MR RAPS
Weighted median
Weighted mode
0.002
0.000
−0.002
−0.004
SNP effect on eGFR
0.2
0.4
0.6
SNP effect on Desmocollin−2

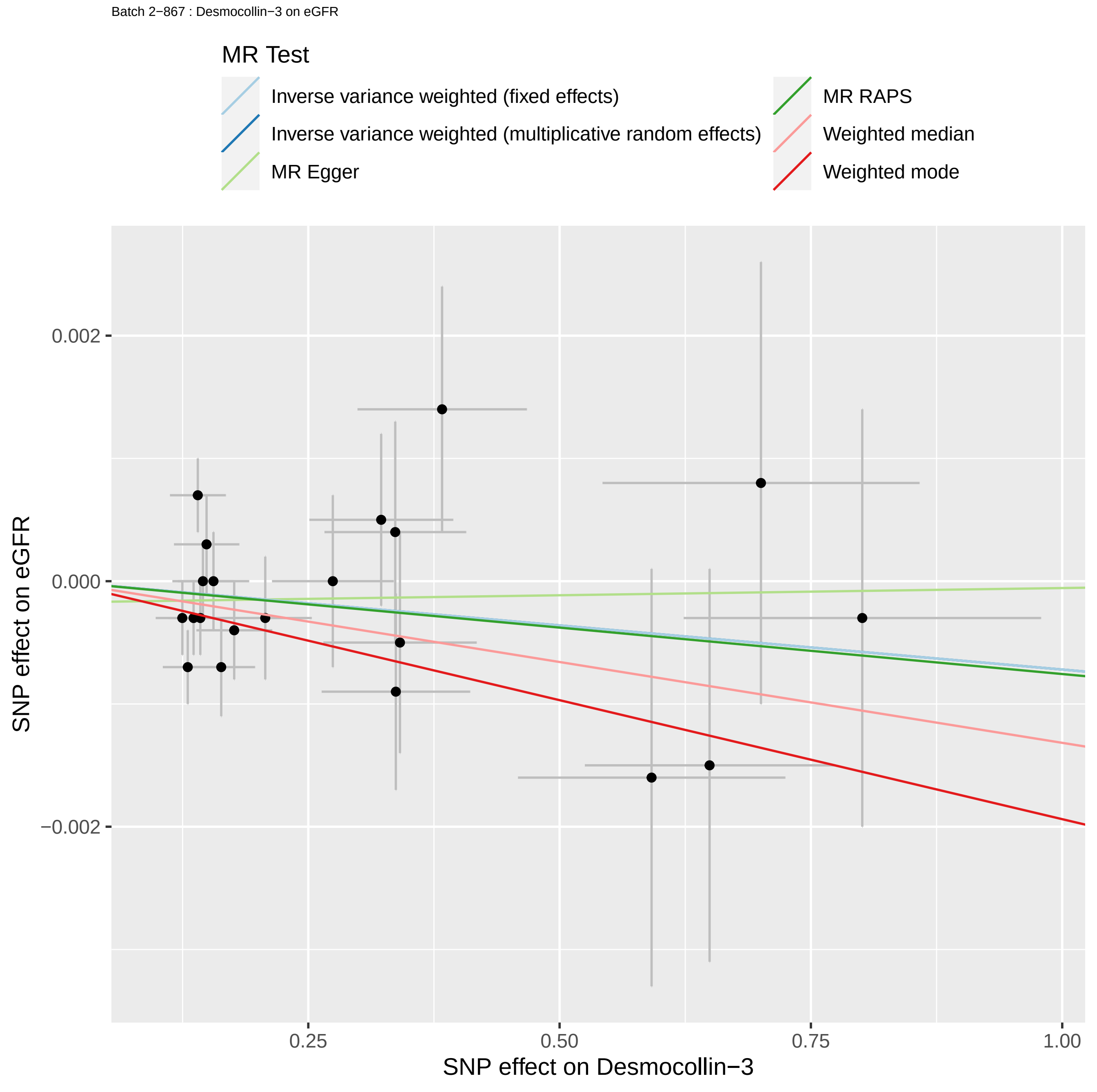

Batch 2−867 : Desmocollin−3 on eGFR
MR Test
Inverse variance weighted (fixed effects)
Inverse variance weighted (multiplicative random effects)
MR Egger
MR RAPS
Weighted median
Weighted mode
0.002
0.000
−0.002
0.25
0.50
0.75
1.00
SNP effect on eGFR
SNP effect on Desmocollin−3

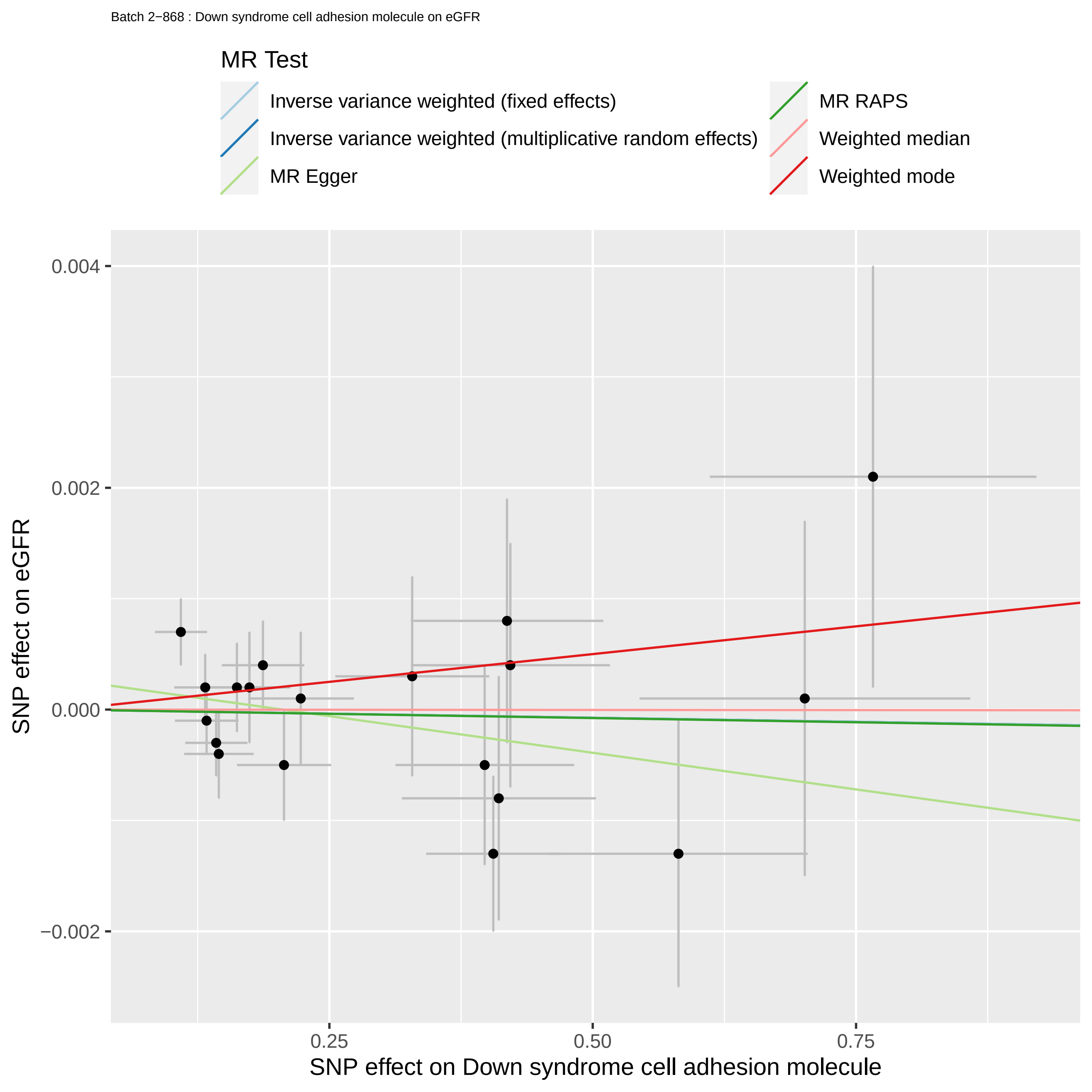
Batch 2−868 : Down syndrome cell adhesion molecule on eGFR
MR Test
Inverse variance weighted (fixed effects)
Inverse variance weighted (multiplicative random effects)
MR Egger
MR RAPS
Weighted median
Weighted mode
0.004
0.002
0.000
−0.002
0.25
0.50
0.75
SNP effect on eGFR
SNP effect on Down syndrome cell adhesion molecule

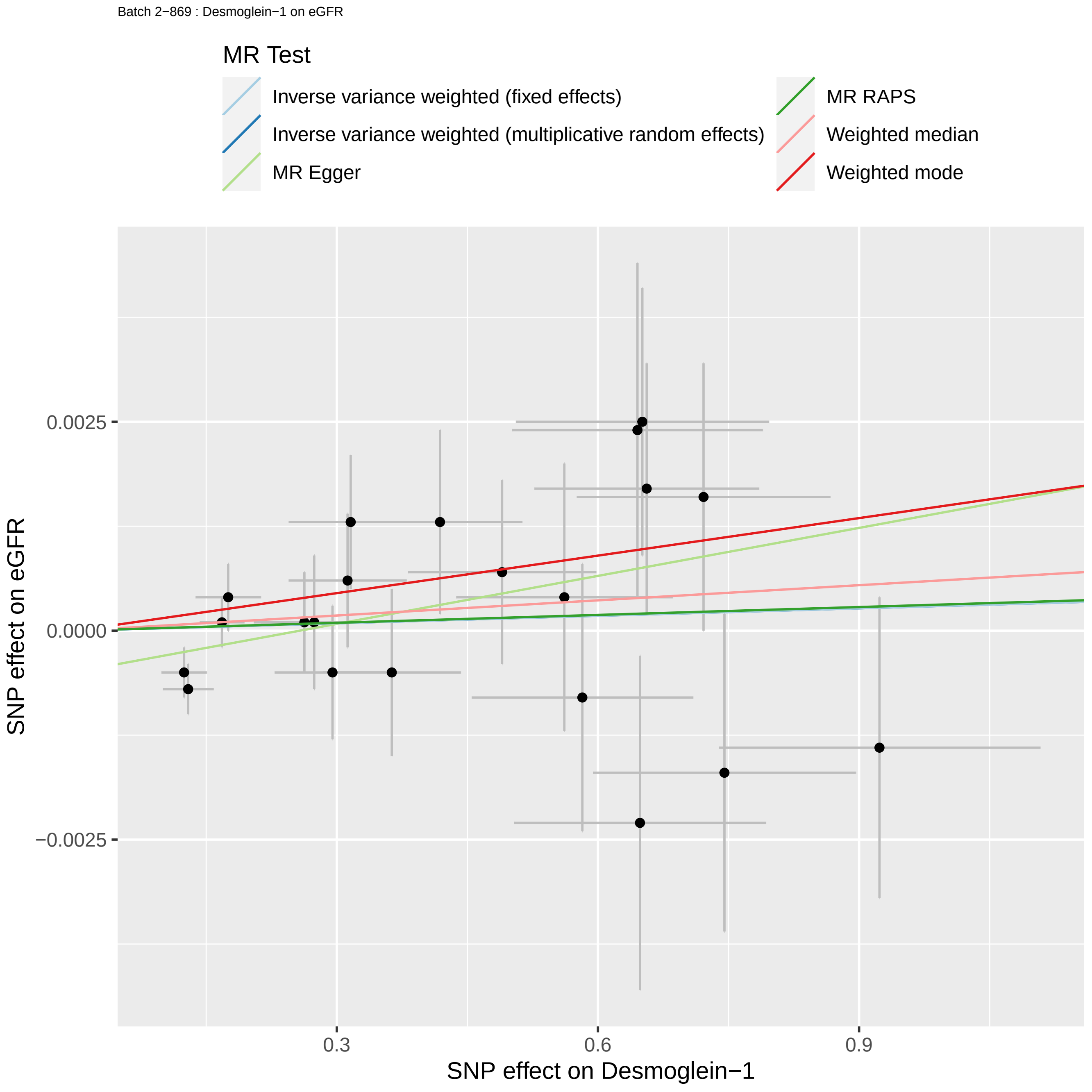

Batch 2−869 : Desmoglein−1 on eGFR
MR Test
Inverse variance weighted (fixed effects)
Inverse variance weighted (multiplicative random effects)
MR Egger
MR RAPS
Weighted median
Weighted mode
SNP effect on eGFR
0.0025
0.0000
−0.0025
0.3
0.6
0.9
SNP effect on Desmoglein−1

Batch 2−870 : Desmoglein−2 on eGFR

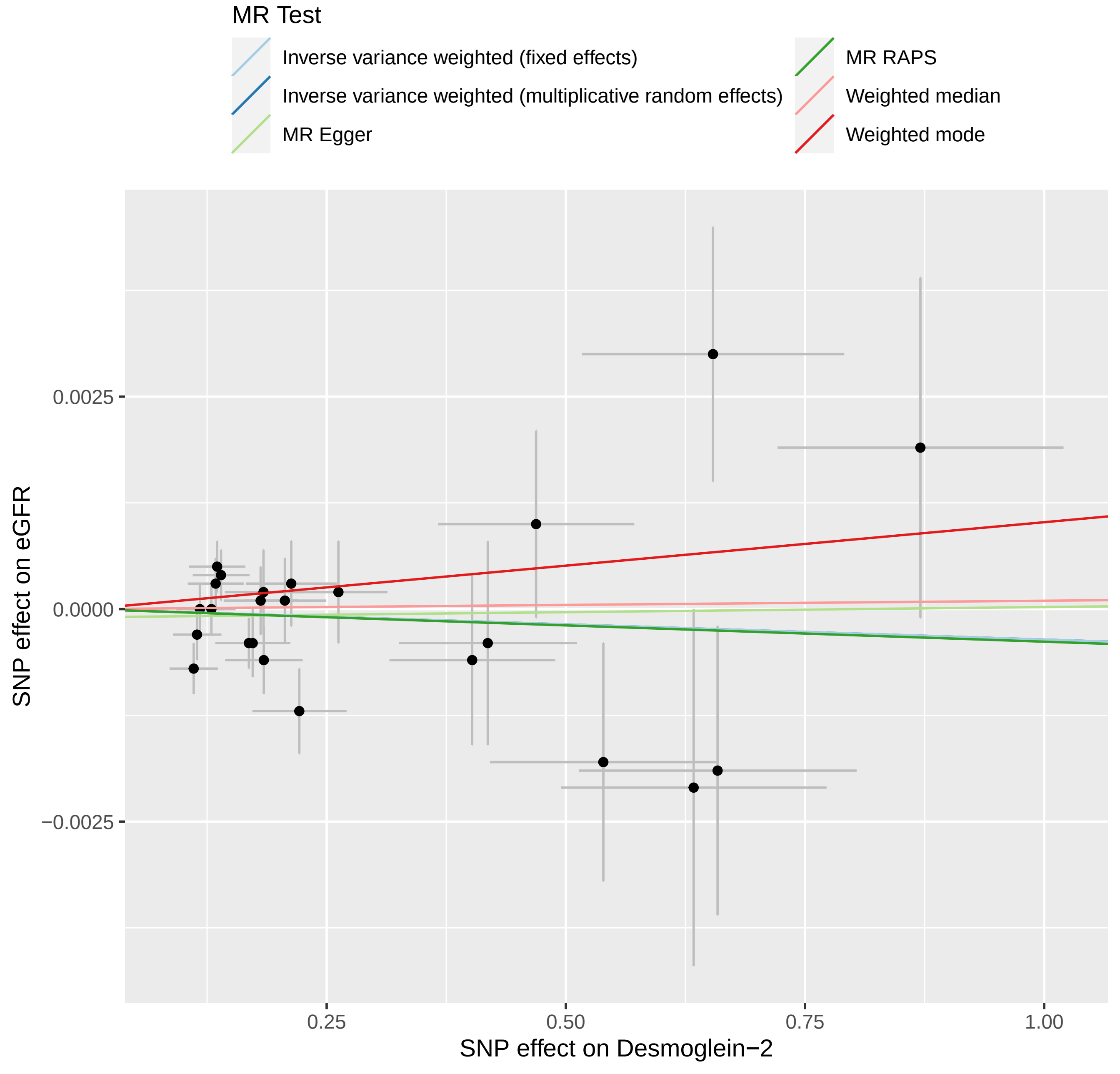

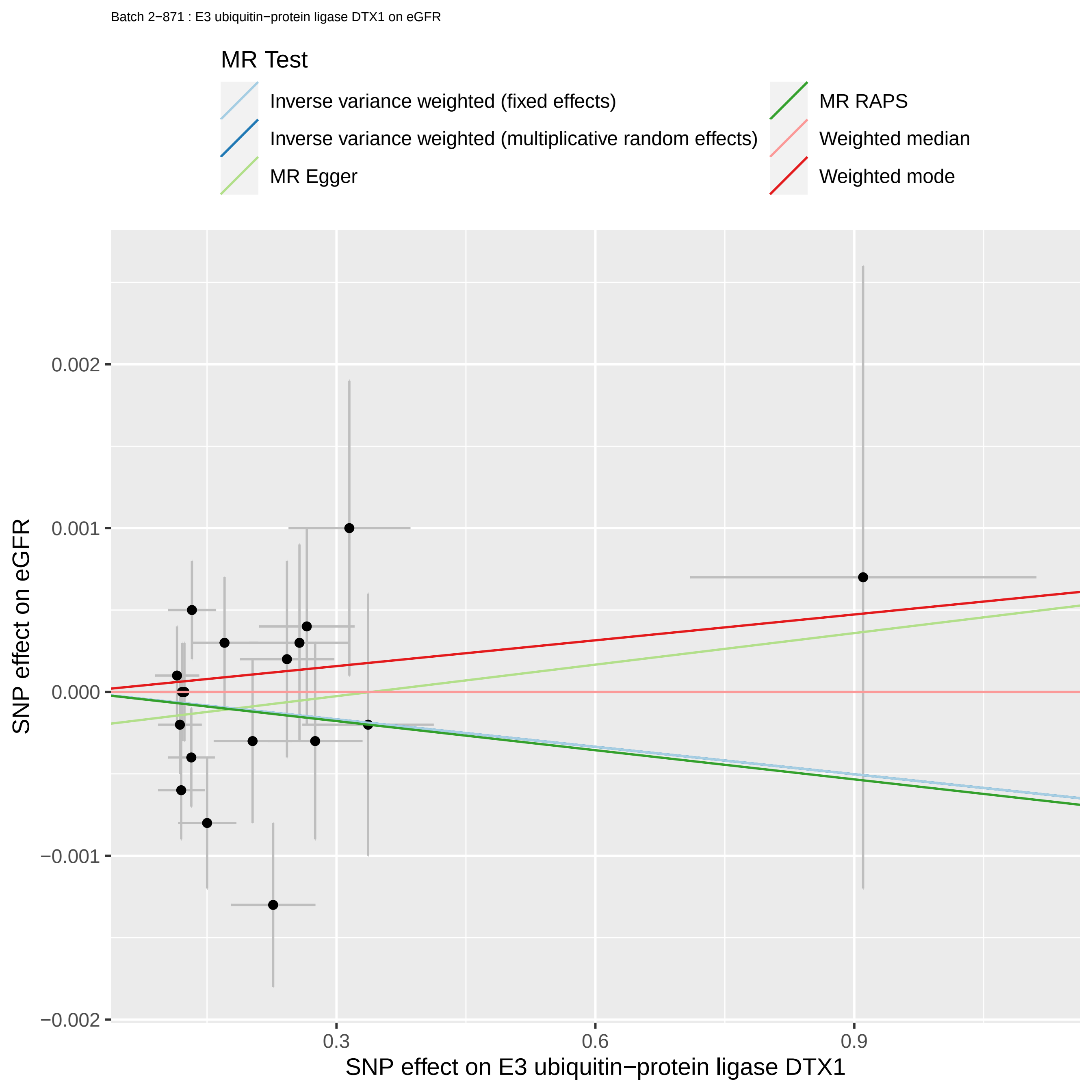

Batch 2−871 : E3 ubiquitin−protein ligase DTX1 on eGFR
MR Test
Inverse variance weighted (fixed effects)
Inverse variance weighted (multiplicative random effects)
MR Egger
MR RAPS
Weighted median
Weighted mode
0.002
0.001
0.000
−0.001
−0.002
0.3
0.6
0.9
SNP effect on eGFR
SNP effect on E3 ubiquitin−protein ligase DTX1

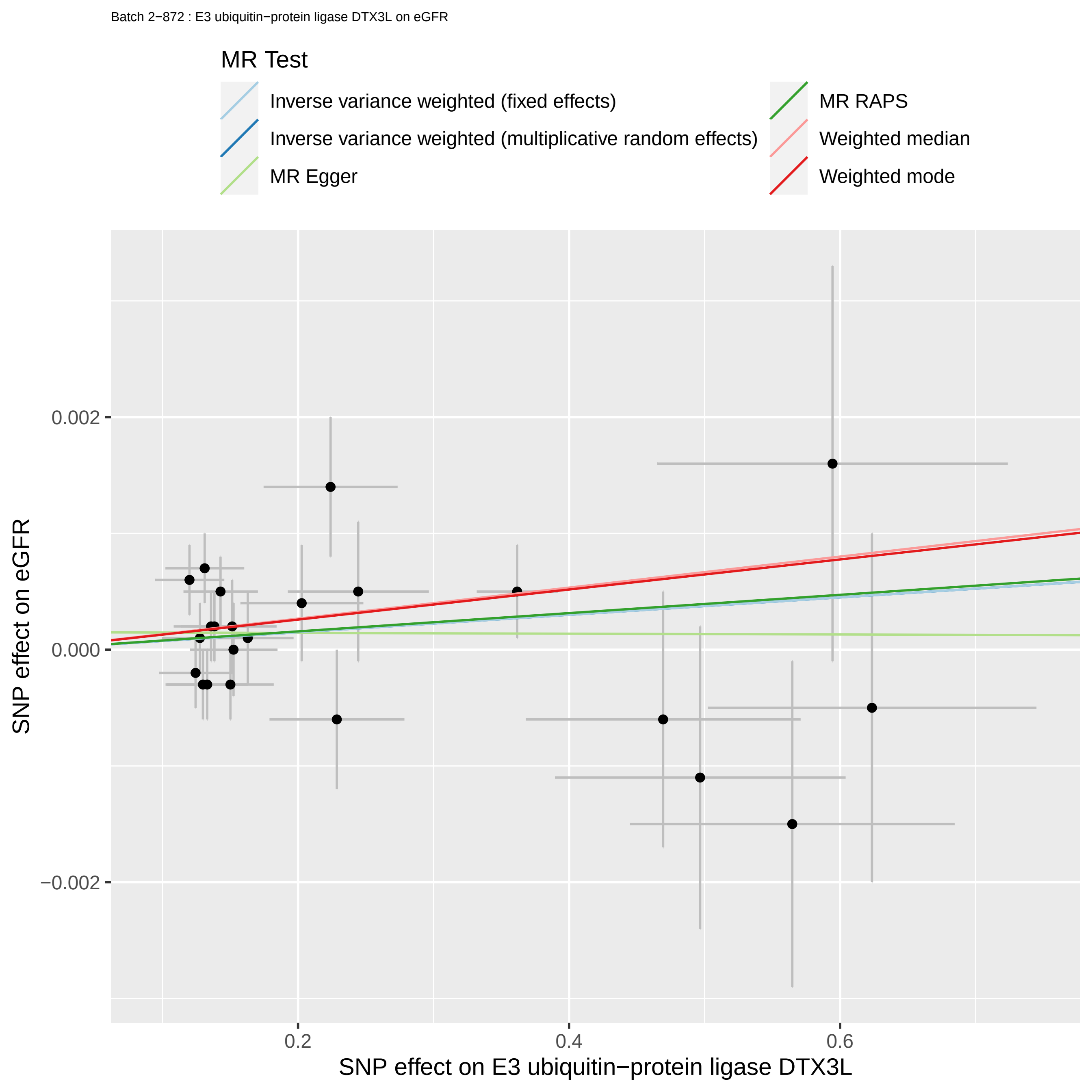

Batch 2−872 : E3 ubiquitin−protein ligase DTX3L on eGFR
MR Test
Inverse variance weighted (fixed effects)
Inverse variance weighted (multiplicative random effects)
MR Egger
MR RAPS
Weighted median
Weighted mode
0.002
0.000
−0.002
0.2
0.4
0.6
SNP effect on eGFR
SNP effect on E3 ubiquitin−protein ligase DTX3L

Batch 2−873 : Dual specificity protein phosphatase 13 isoform A on eGFR

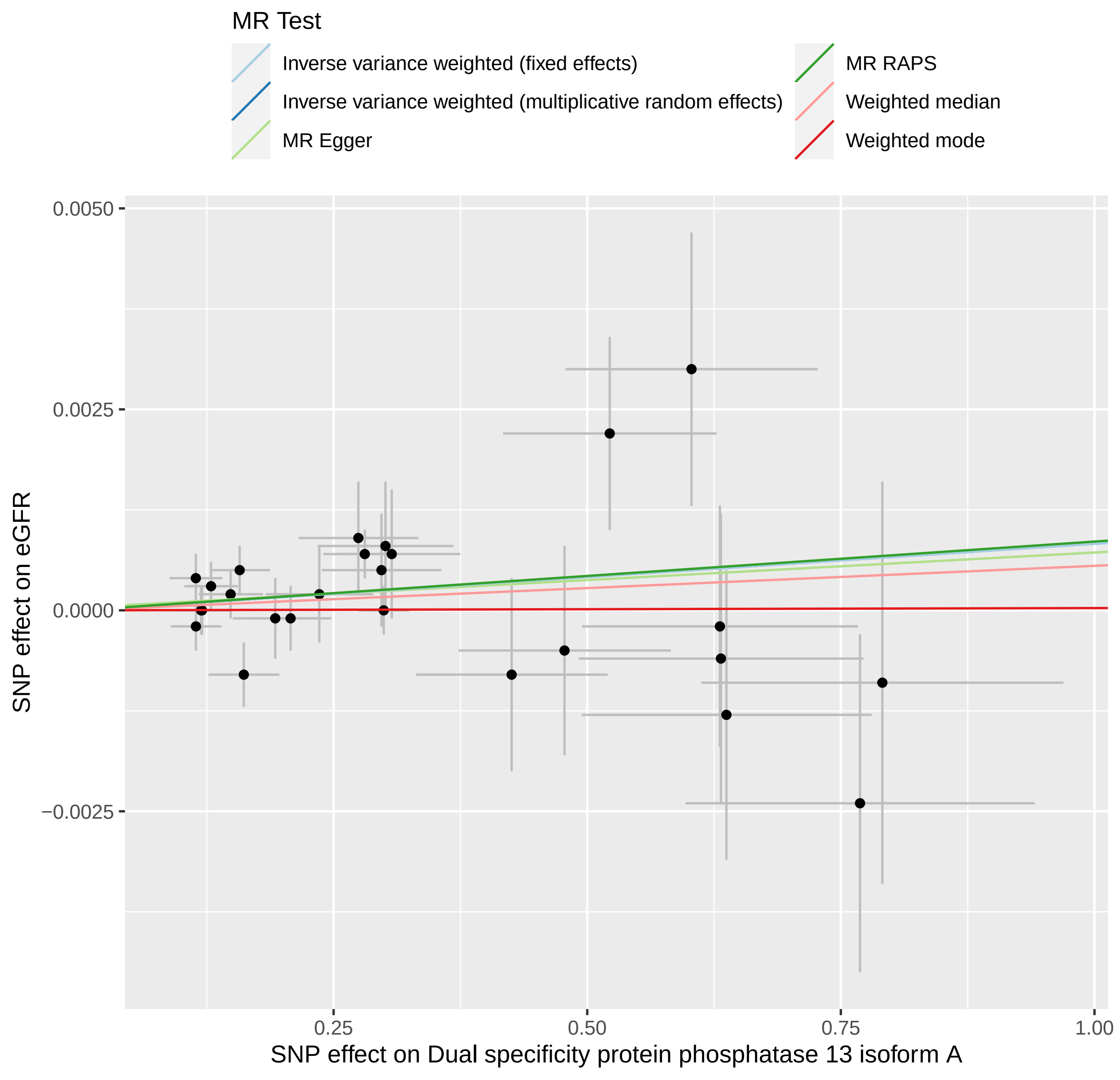

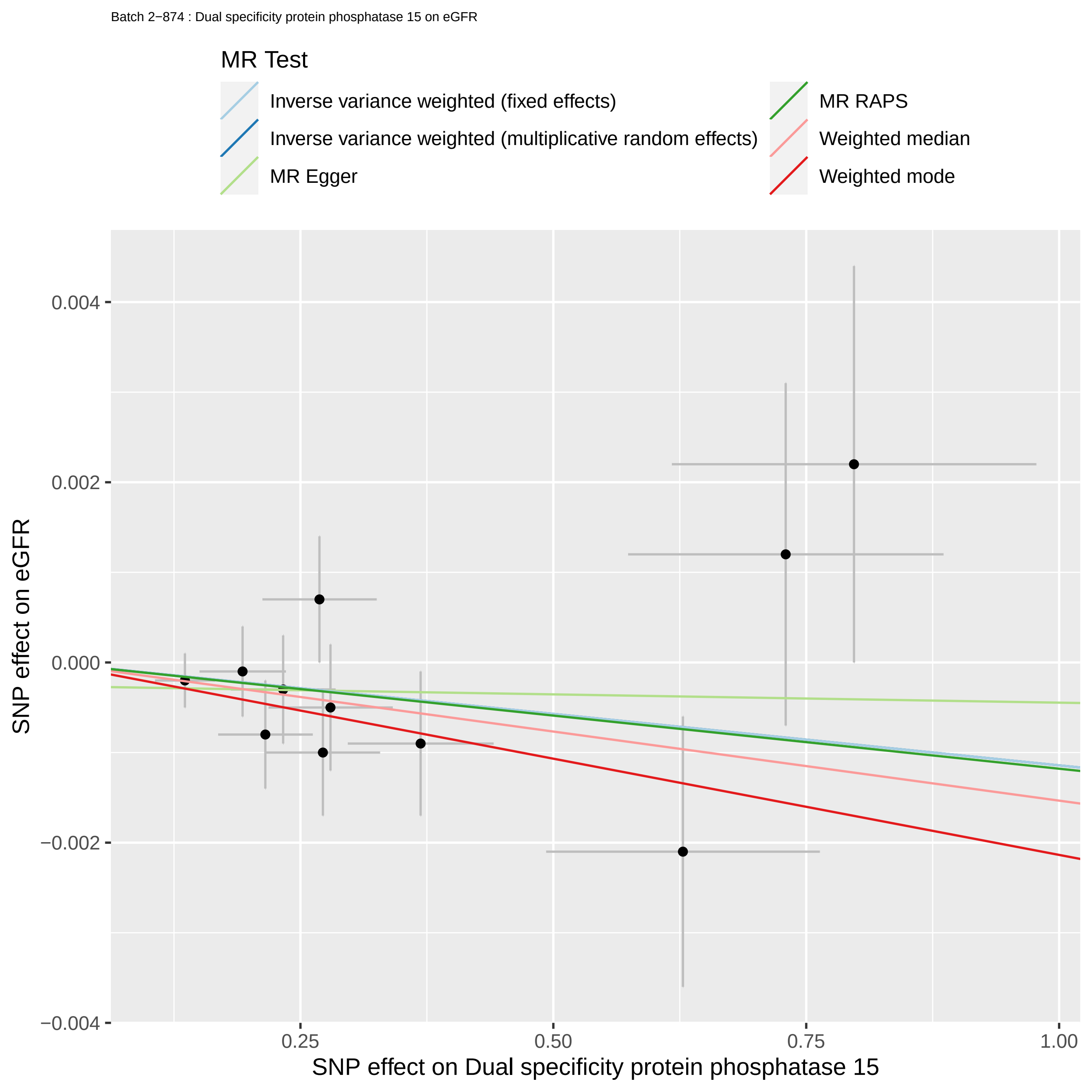

Batch 2−874 : Dual specificity protein phosphatase 15 on eGFR
MR Test
Inverse variance weighted (fixed effects)
Inverse variance weighted (multiplicative random effects)
MR Egger
MR RAPS
Weighted median
Weighted mode
0.004
0.002
0.000
−0.002
−0.004
0.25
0.50
0.75
1.00
SNP effect on eGFR
SNP effect on Dual specificity protein phosphatase 15

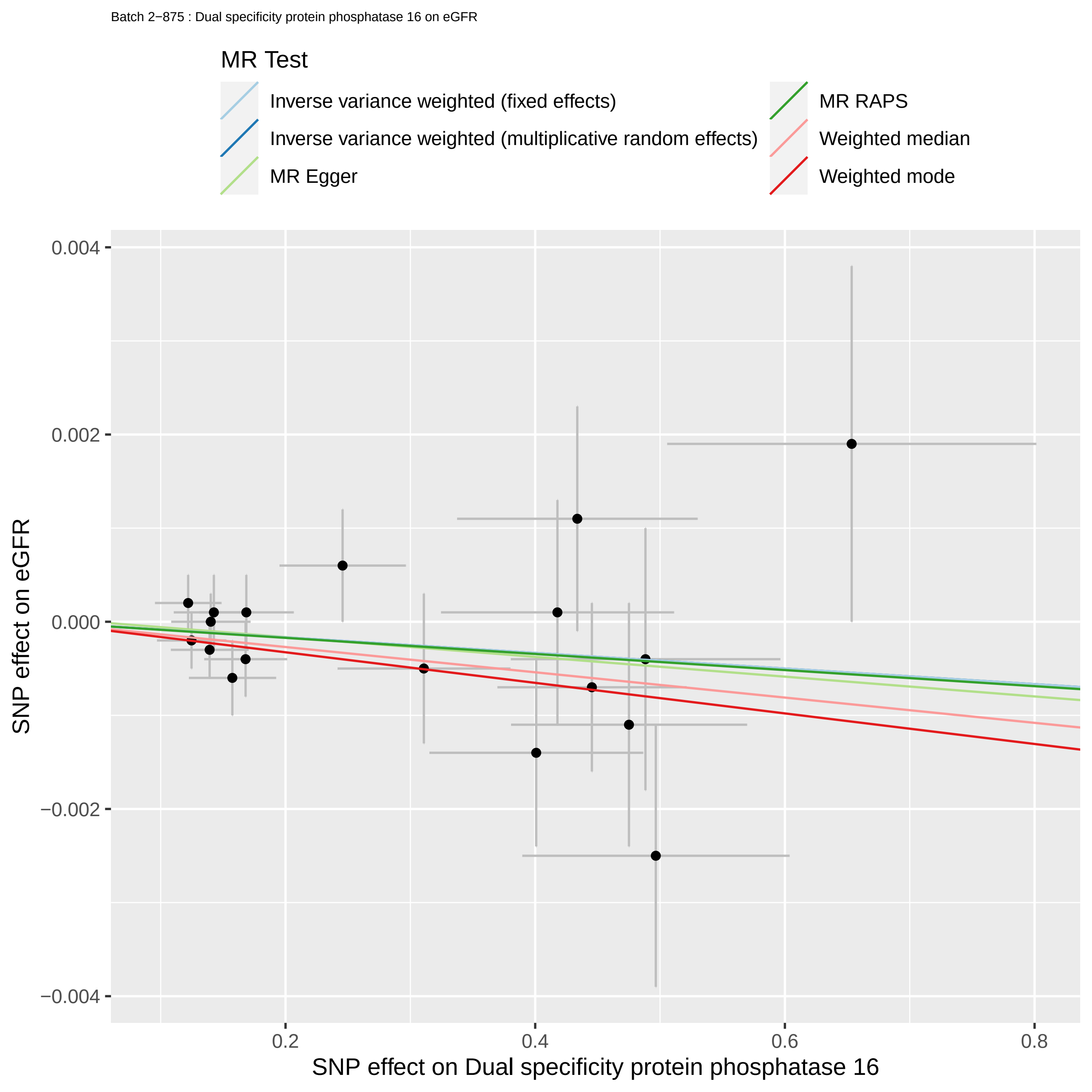

Batch 2−875 : Dual specificity protein phosphatase 16 on eGFR
MR Test
Inverse variance weighted (fixed effects)
Inverse variance weighted (multiplicative random effects)
MR Egger
MR RAPS
Weighted median
Weighted mode
0.004
0.002
0.000
−0.002
−0.004
0.2
0.4
0.6
0.8
SNP effect on eGFR
SNP effect on Dual specificity protein phosphatase 16

Batch 2−876 : Dual specificity protein phosphatase 4 on eGFR

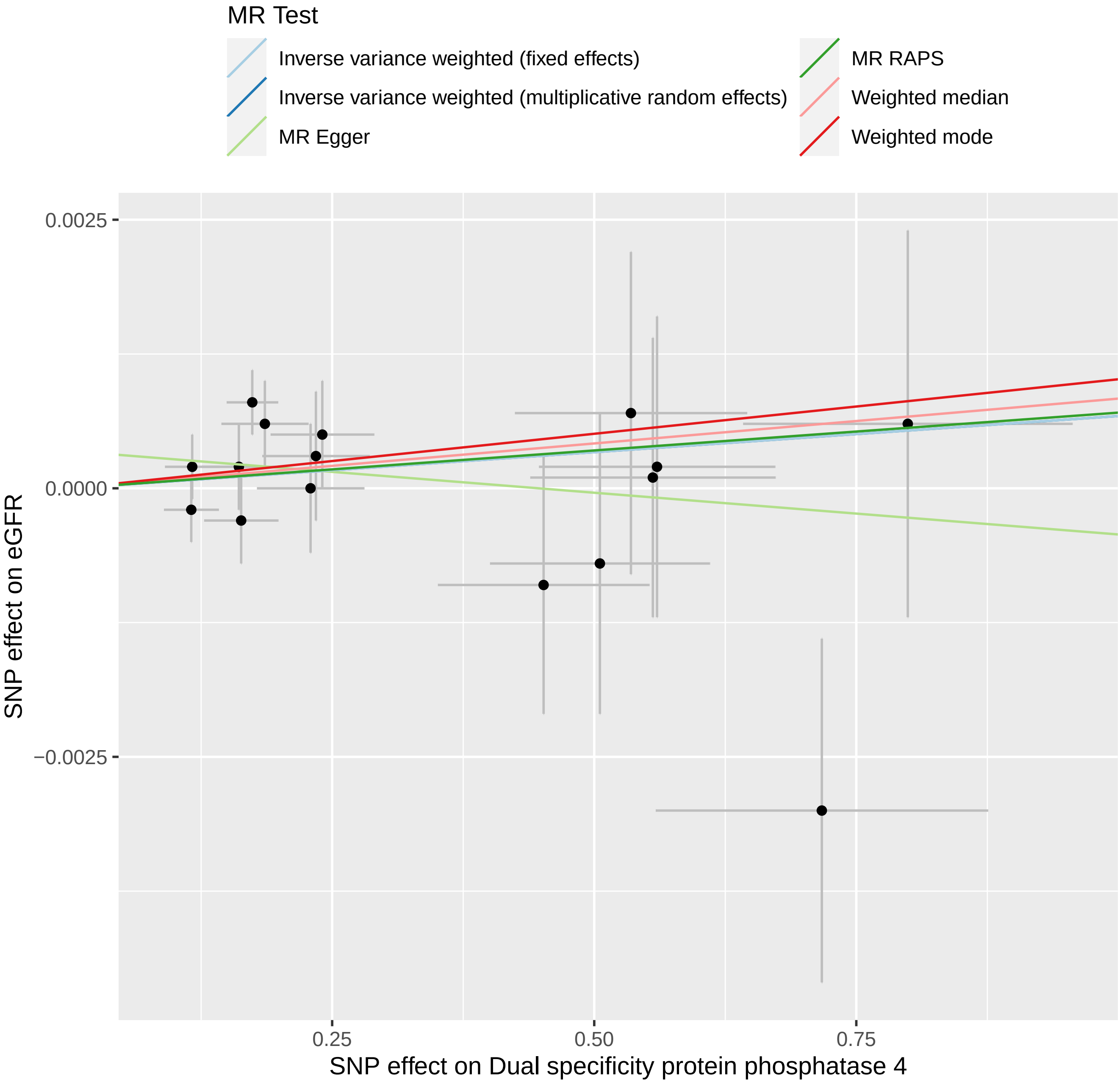

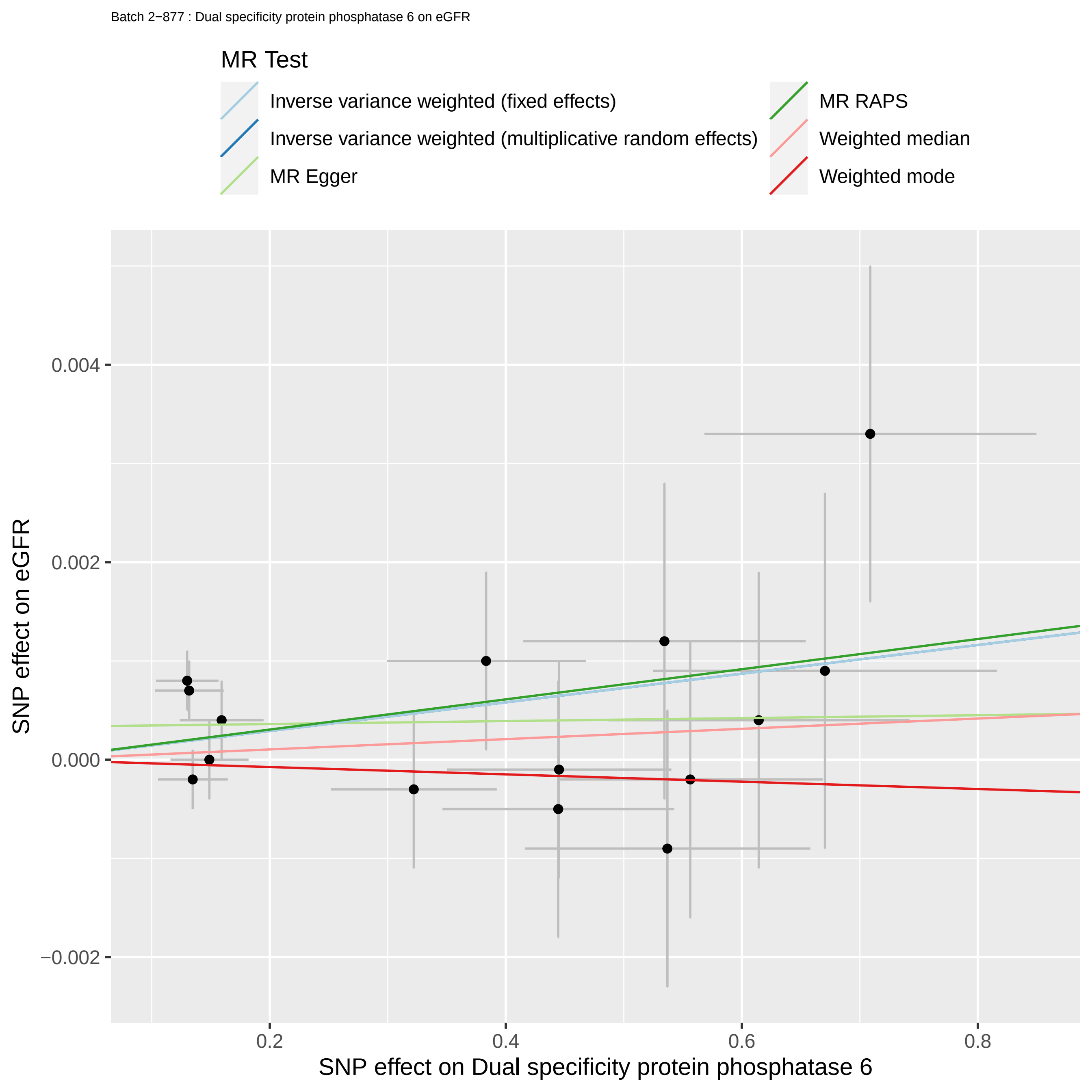

Batch 2−877 : Dual specificity protein phosphatase 6 on eGFR
MR Test
Inverse variance weighted (fixed effects)
Inverse variance weighted (multiplicative random effects)
MR Egger
MR RAPS
Weighted median
Weighted mode
0.004
0.002
0.000
−0.002
SNP effect on eGFR
0.2
0.4
0.6
0.8
SNP effect on Dual specificity protein phosphatase 6

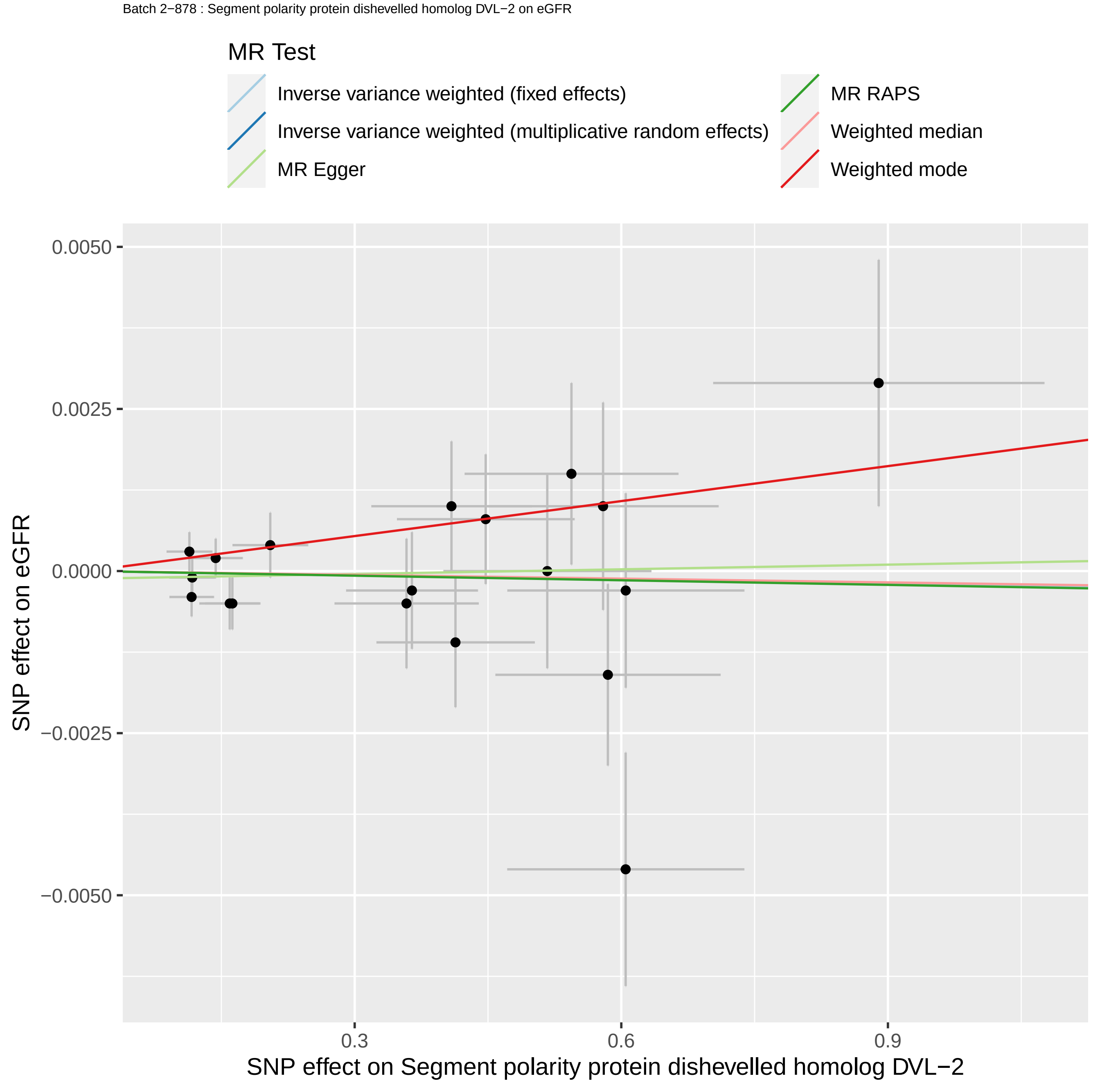

Batch 2−878 : Segment polarity protein dishevelled homolog DVL−2 on eGFR
MR Test
Inverse variance weighted (fixed effects)
Inverse variance weighted (multiplicative random effects)
MR Egger
MR RAPS
Weighted median
Weighted mode
0.0050
0.0025
0.0000
−0.0025
−0.0050
SNP effect on eGFR
0.3
0.6
0.9
SNP effect on Segment polarity protein dishevelled homolog DVL−2

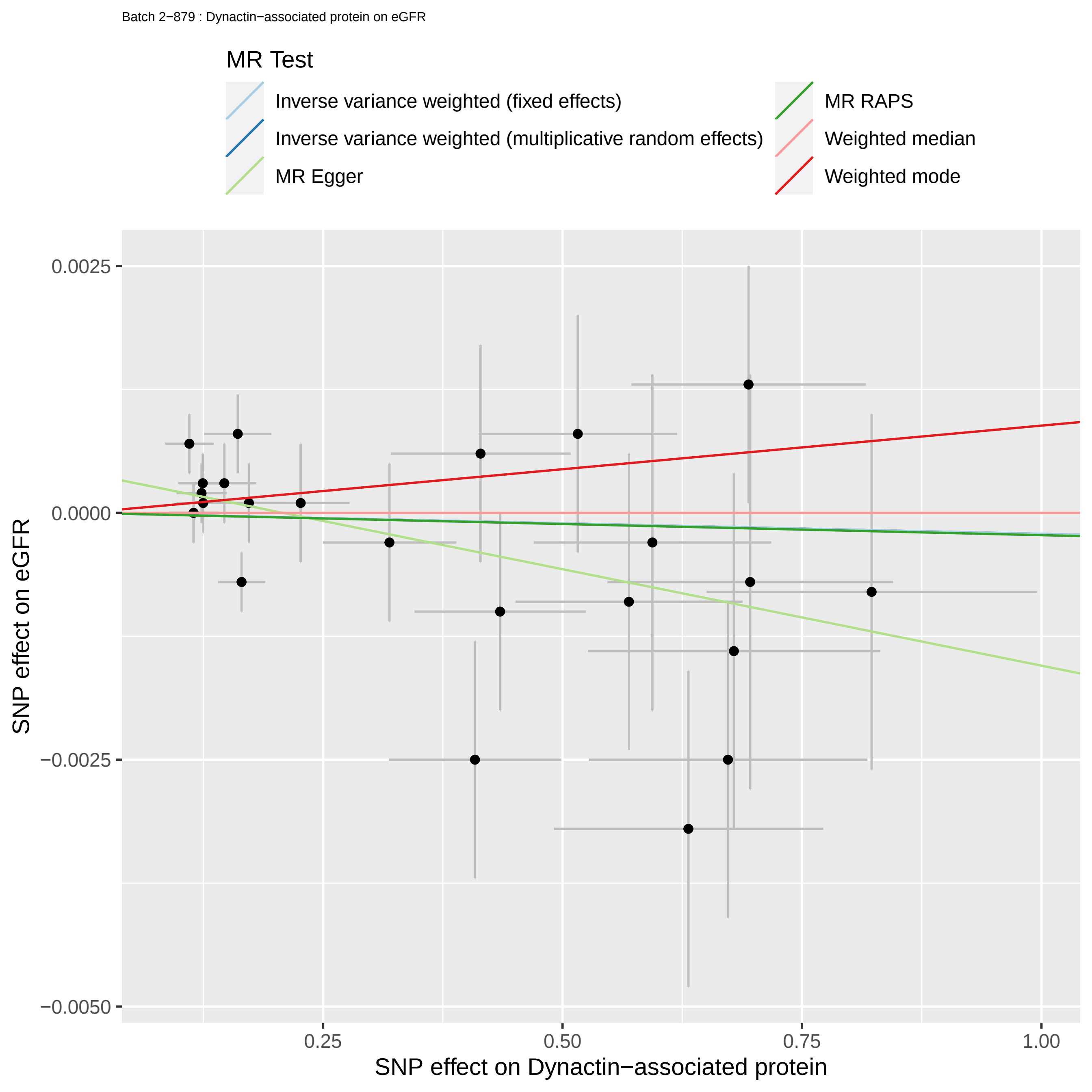
Batch 2−879 : Dynactin−associated protein on eGFR
MR Test
Inverse variance weighted (fixed effects)
Inverse variance weighted (multiplicative random effects)
MR Egger
MR RAPS
Weighted median
Weighted mode
0.0025
0.0000
−0.0025
−0.0050
0.25
0.50
0.75
1.00
SNP effect on eGFR
SNP effect on Dynactin−associated protein

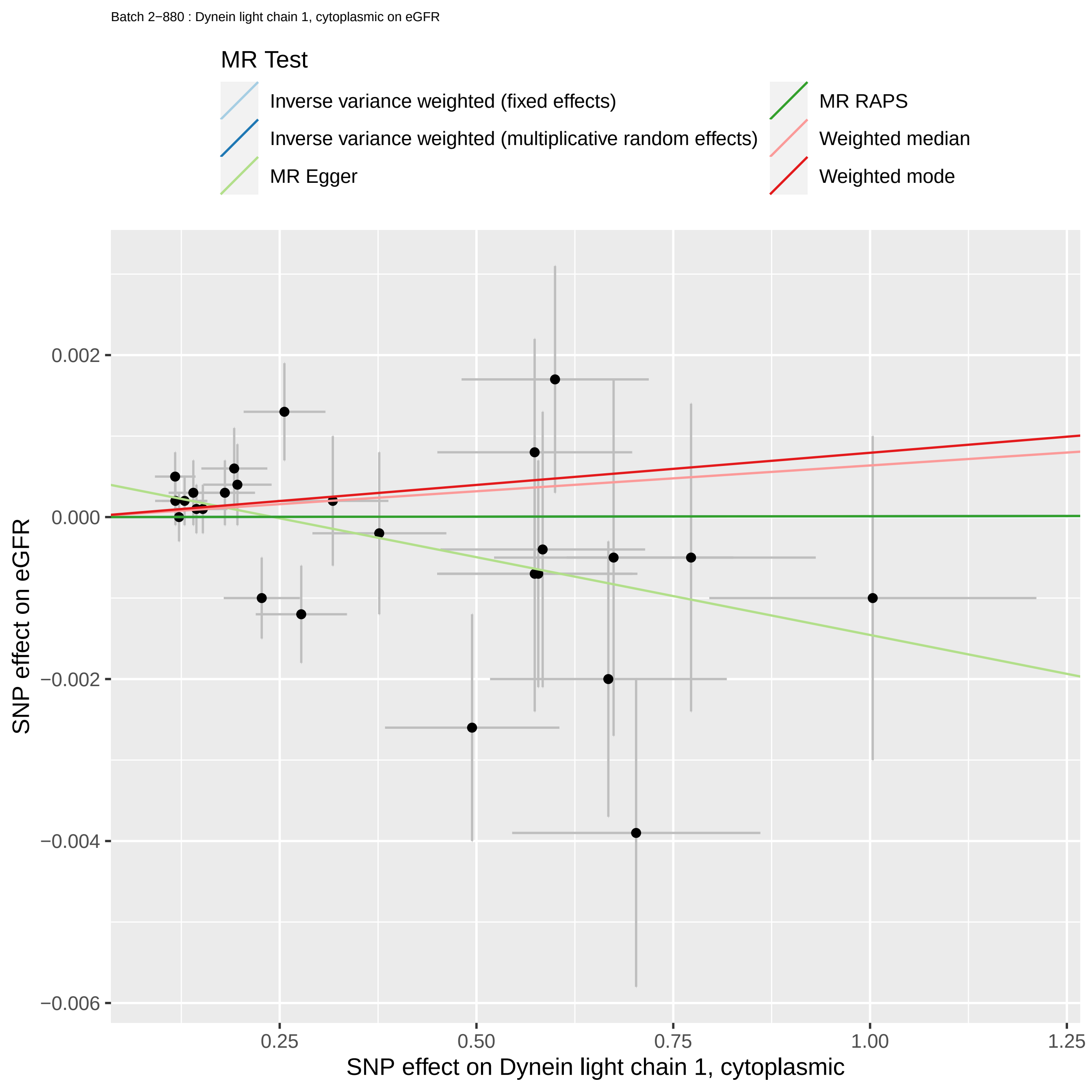

Batch 2−880 : Dynein light chain 1, cytoplasmic on eGFR
MR Test
Inverse variance weighted (fixed effects)
Inverse variance weighted (multiplicative random effects)
MR Egger
MR RAPS
Weighted median
Weighted mode
0.002
0.000
−0.002
−0.004
−0.006
SNP effect on eGFR
0.25
0.50
0.75
1.00
1.25
SNP effect on Dynein light chain 1, cytoplasmic

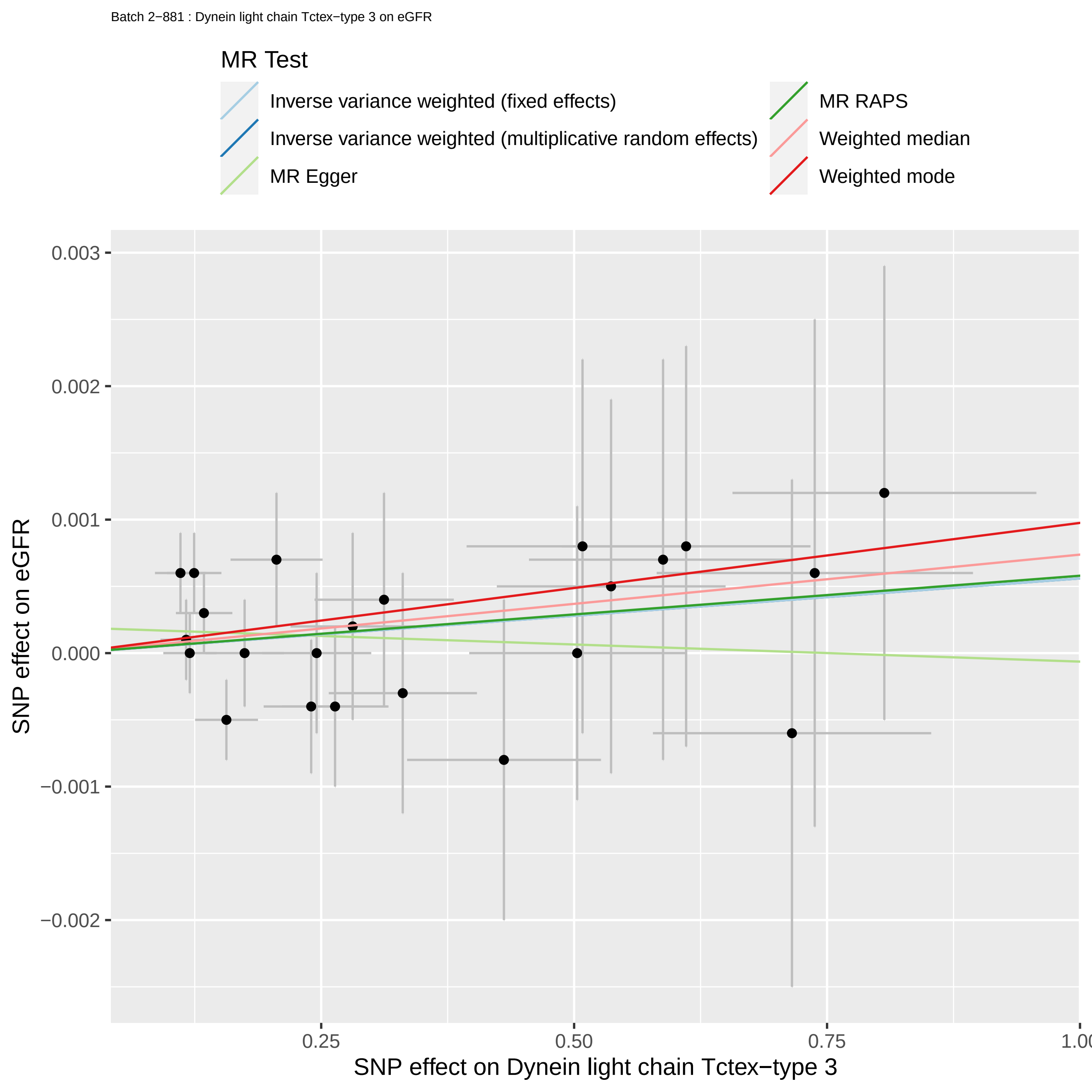
Batch 2−881 : Dynein light chain Tctex−type 3 on eGFR
MR Test
Inverse variance weighted (fixed effects)
Inverse variance weighted (multiplicative random effects)
MR Egger
MR RAPS
Weighted median
Weighted mode
0.003
0.002
0.001
0.000
−0.001
−0.002
0.25
0.50
0.75
1.00
SNP effect on eGFR
SNP effect on Dynein light chain Tctex−type 3

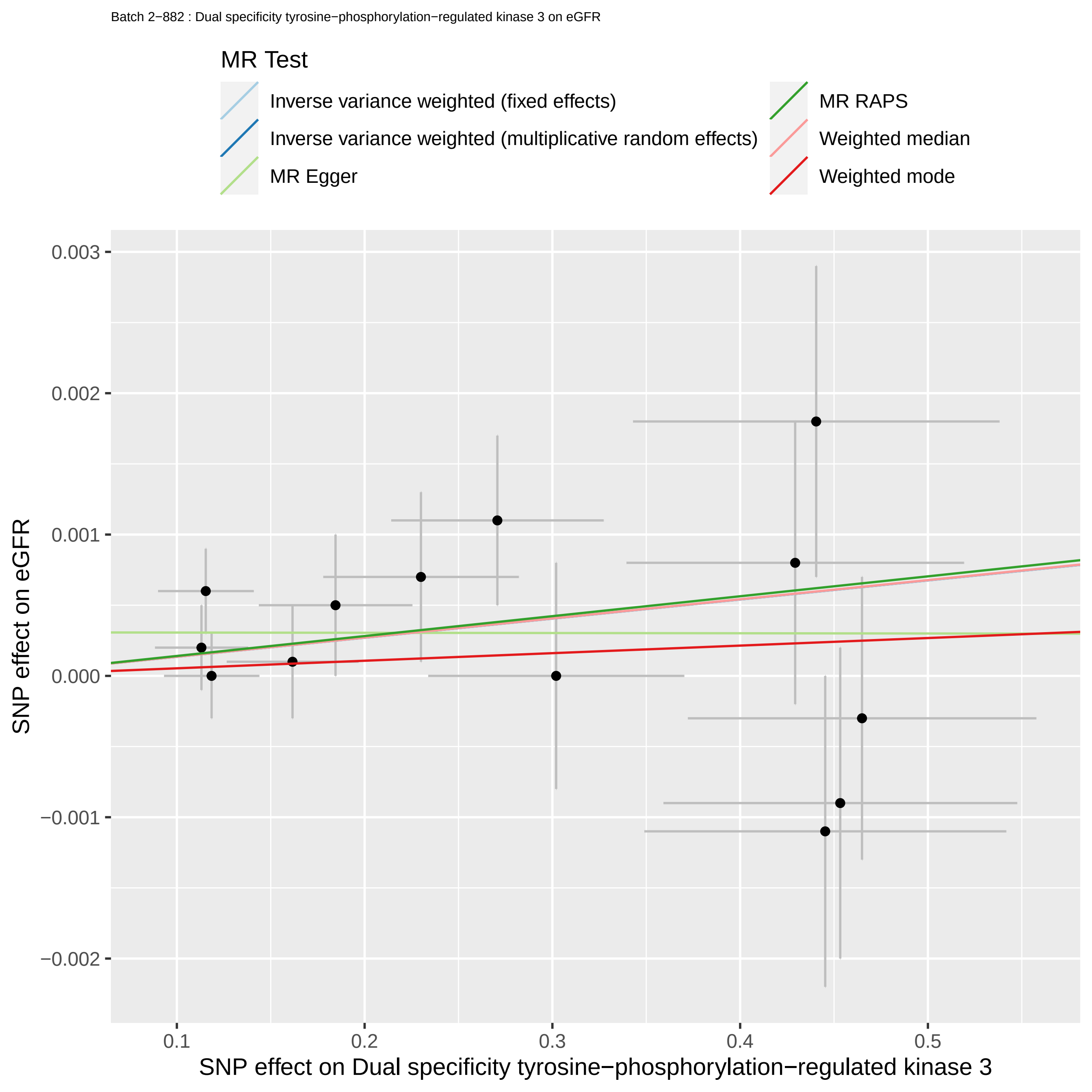
Batch 2−882 : Dual specificity tyrosine−phosphorylation−regulated kinase 3 on eGFR
MR Test
Inverse variance weighted (fixed effects)
Inverse variance weighted (multiplicative random effects)
MR Egger
MR RAPS
Weighted median
Weighted mode
0.003
0.002
0.001
0.000
−0.001
−0.002
SNP effect on eGFR
0.1
0.2
0.3
0.4
0.5
SNP effect on Dual specificity tyrosine−phosphorylation−regulated kinase 3

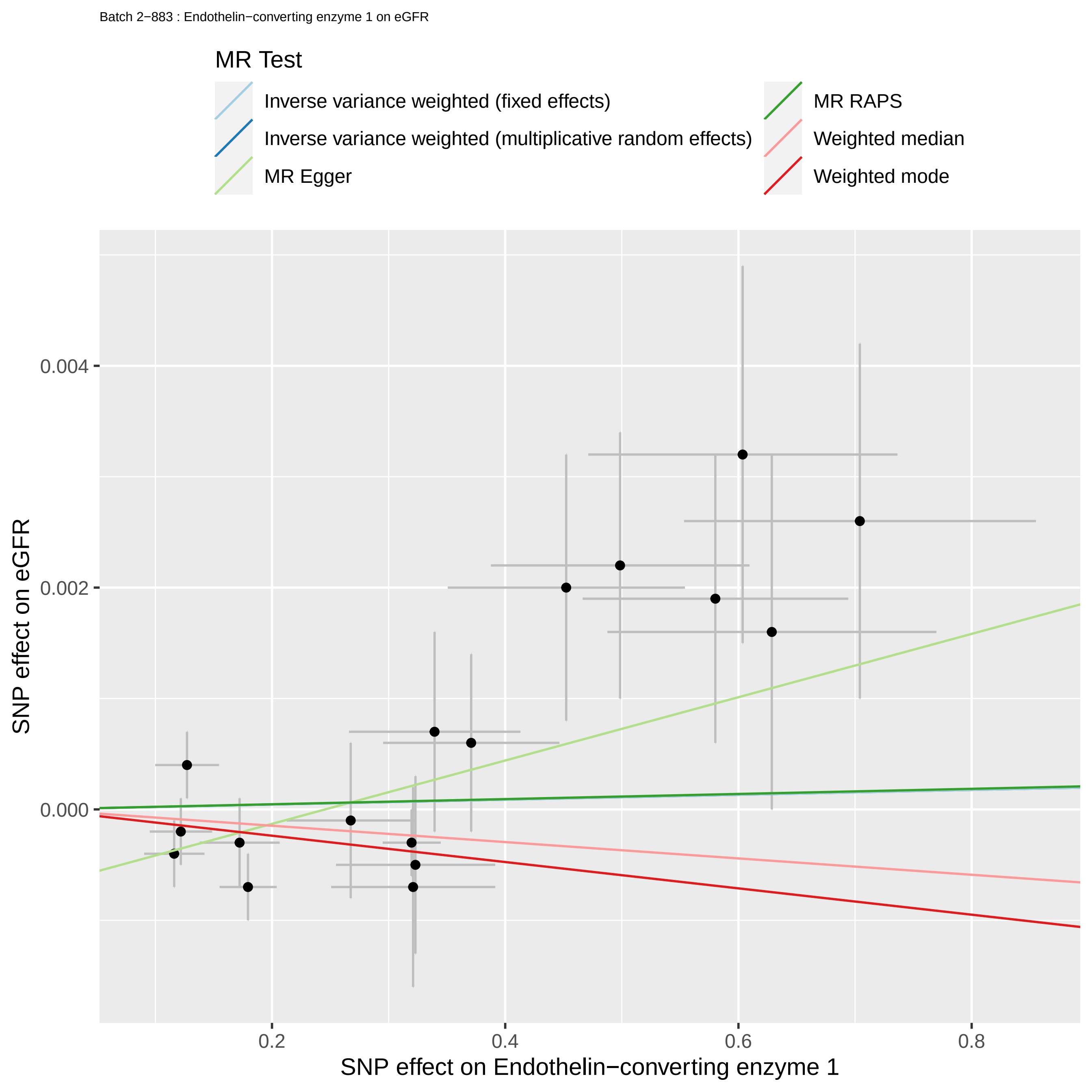
Batch 2−883 : Endothelin−converting enzyme 1 on eGFR
MR Test
Inverse variance weighted (fixed effects)
Inverse variance weighted (multiplicative random effects)
MR Egger
MR RAPS
Weighted median
Weighted mode
0.004
0.002
0.000
0.2
0.4
0.6
0.8
SNP effect on eGFR
SNP effect on Endothelin−converting enzyme 1

Batch 2−884 : Endothelin−converting enzyme 1 on eGFR

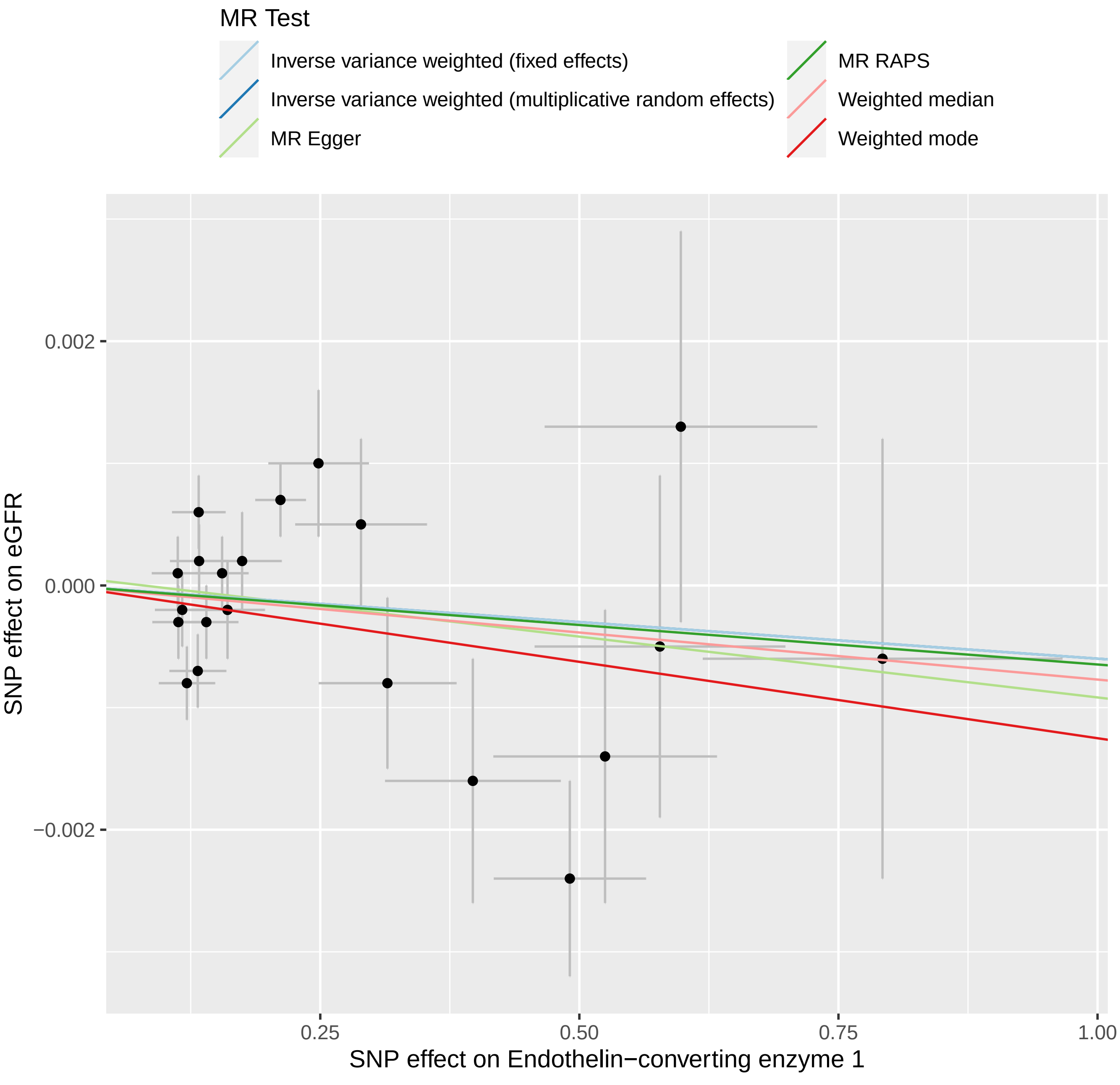

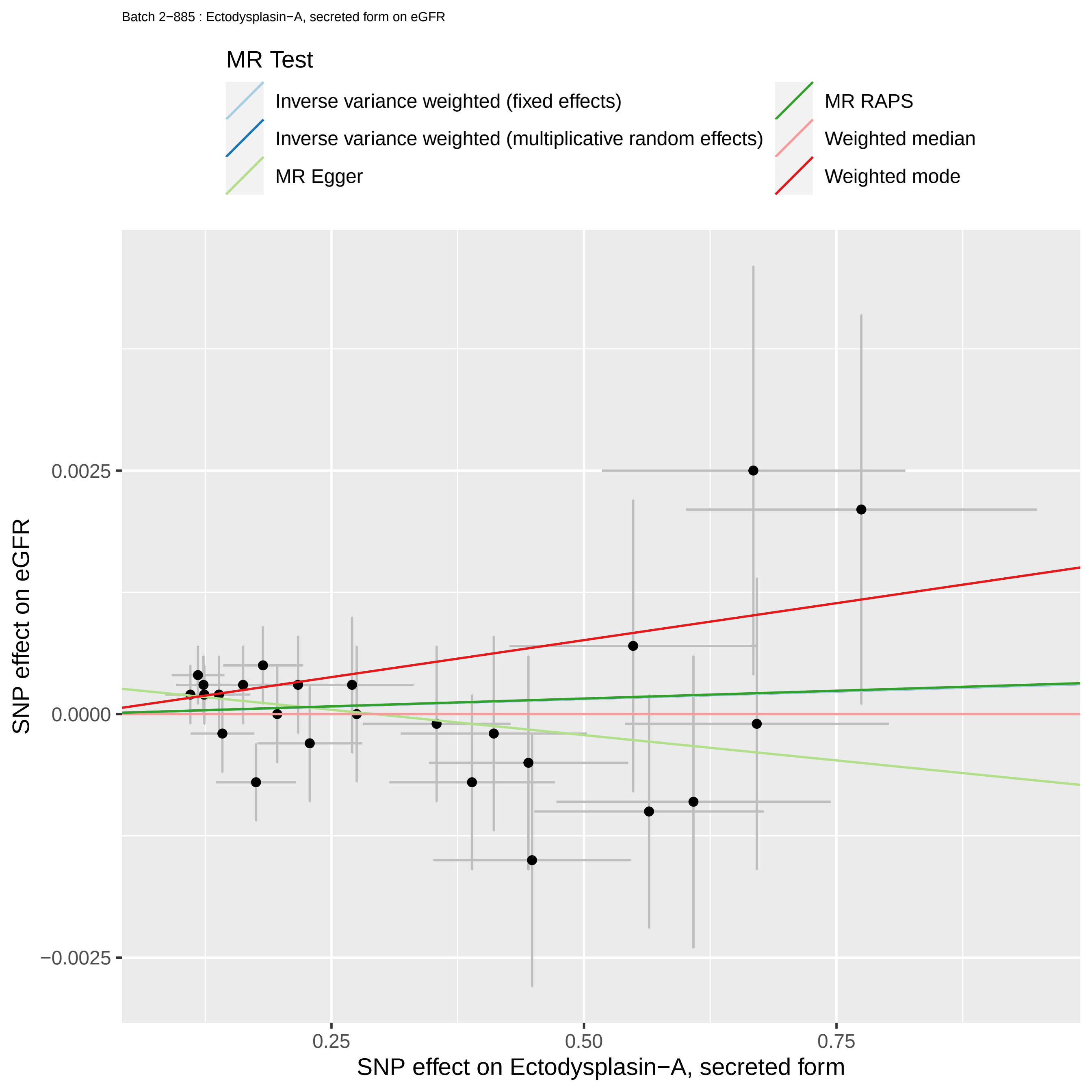
Batch 2−885 : Ectodysplasin−A, secreted form on eGFR
MR Test
Inverse variance weighted (fixed effects)
Inverse variance weighted (multiplicative random effects)
MR Egger
MR RAPS
Weighted median
Weighted mode
0.0025
0.0000
−0.0025
SNP effect on eGFR
0.25
0.50
0.75
SNP effect on Ectodysplasin−A, secreted form

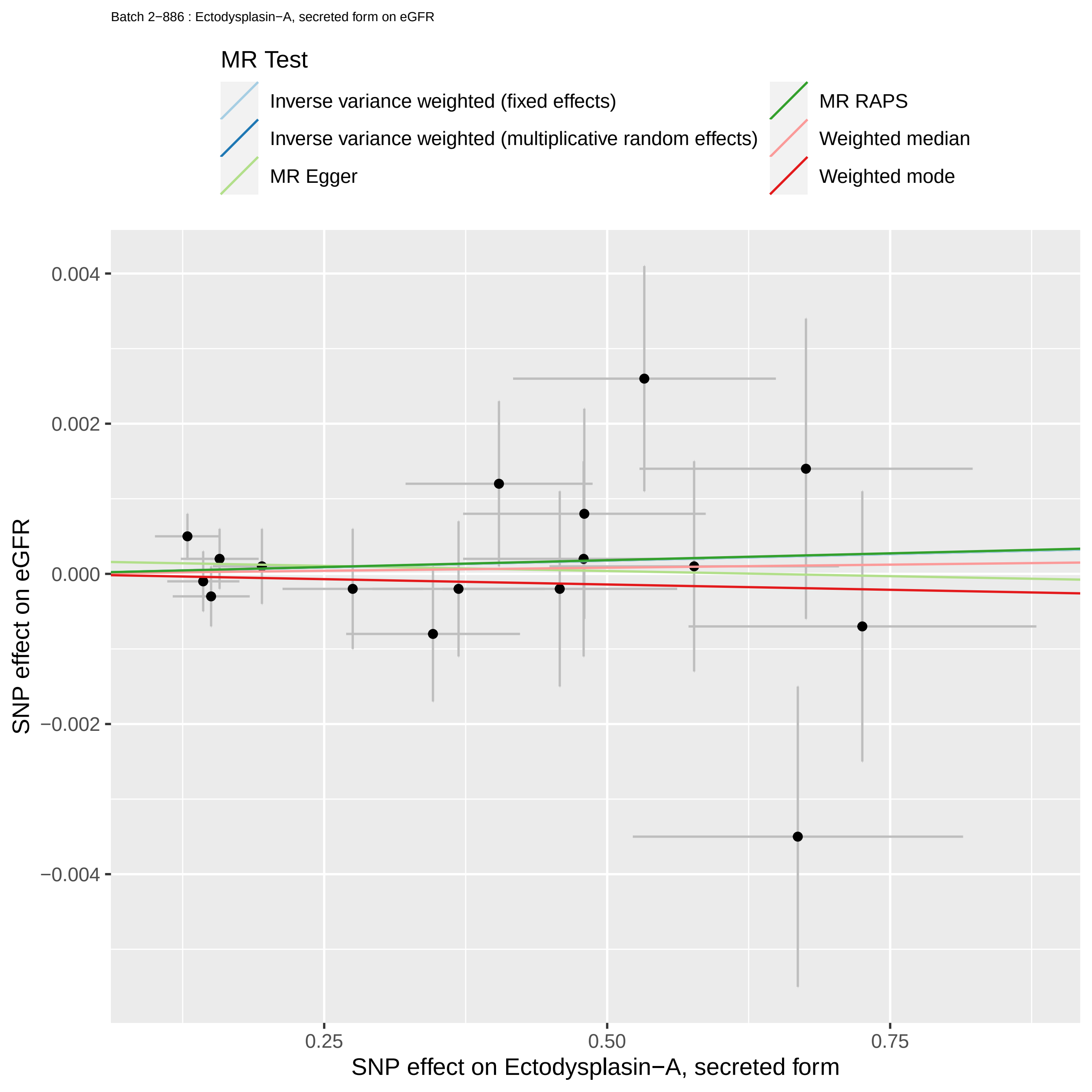

Batch 2−886 : Ectodysplasin−A, secreted form on eGFR
MR Test
Inverse variance weighted (fixed effects)
Inverse variance weighted (multiplicative random effects)
MR Egger
MR RAPS
Weighted median
Weighted mode
0.004
0.002
0.000
−0.002
−0.004
0.25
0.50
0.75
SNP effect on eGFR
SNP effect on Ectodysplasin−A, secreted form

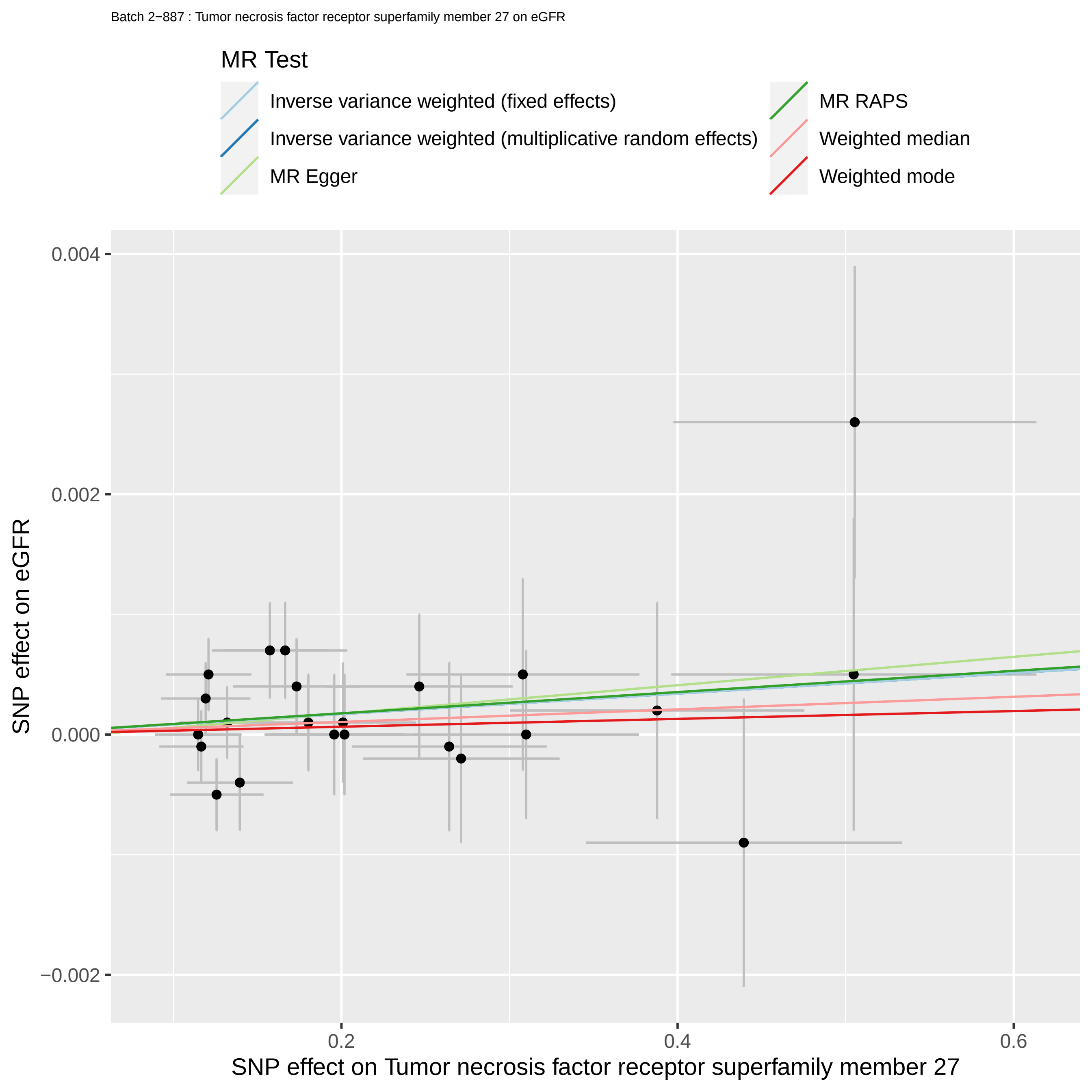

Batch 2−887 : Tumor necrosis factor receptor superfamily member 27 on eGFR
MR Test
Inverse variance weighted (fixed effects)
Inverse variance weighted (multiplicative random effects)
MR Egger
MR RAPS
Weighted median
Weighted mode
0.004
0.002
0.000
−0.002
0.2
0.4
0.6
SNP effect on eGFR
SNP effect on Tumor necrosis factor receptor superfamily member 27

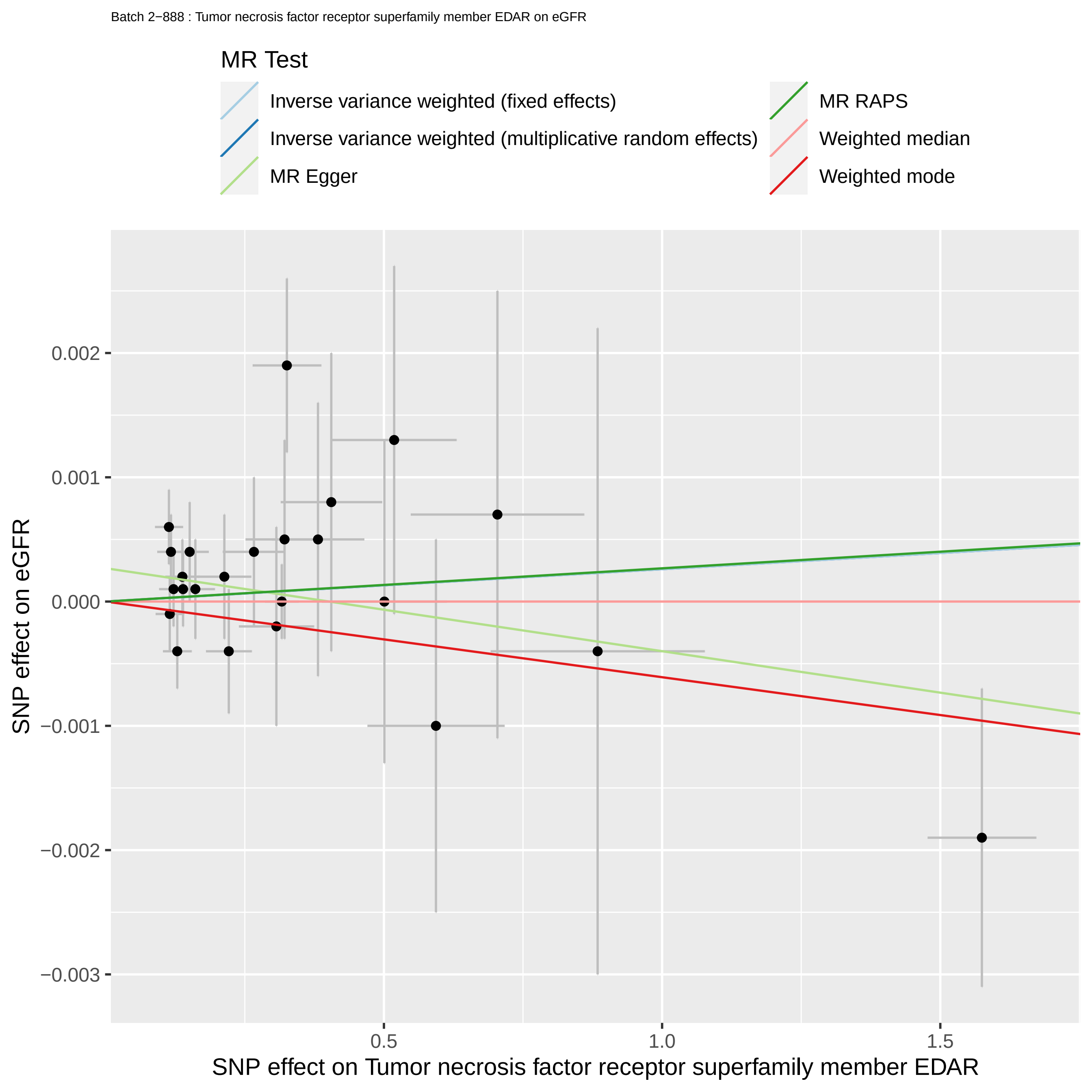
Batch 2−888 : Tumor necrosis factor receptor superfamily member EDAR on eGFR
MR Test
Inverse variance weighted (fixed effects)
Inverse variance weighted (multiplicative random effects)
MR Egger
MR RAPS
Weighted median
Weighted mode
0.002
0.001
0.000
−0.001
−0.002
−0.003
0.5
1.0
1.5
SNP effect on eGFR
SNP effect on Tumor necrosis factor receptor superfamily member EDAR

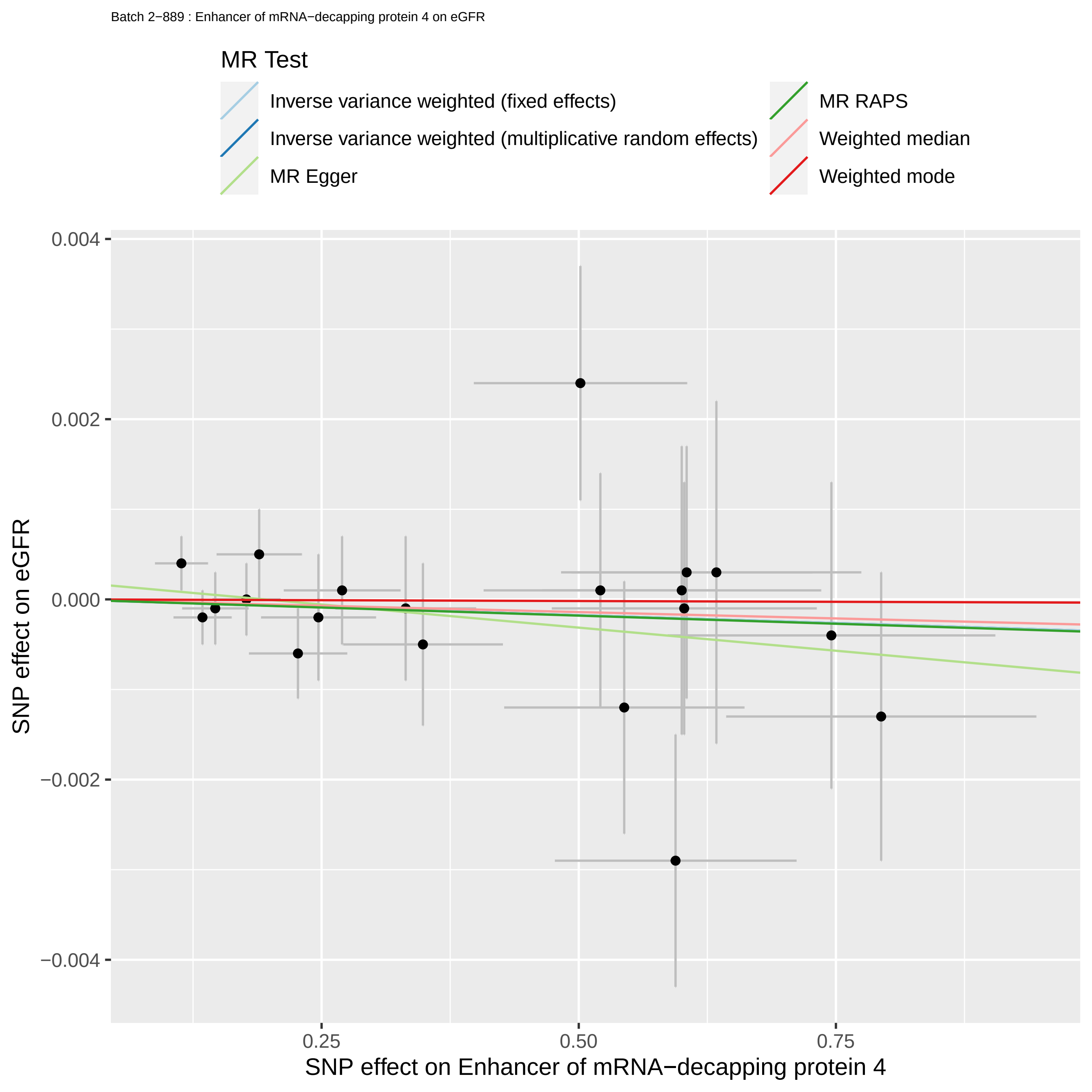

Batch 2−889 : Enhancer of mRNA−decapping protein 4 on eGFR
MR Test
Inverse variance weighted (fixed effects)
Inverse variance weighted (multiplicative random effects)
MR Egger
MR RAPS
Weighted median
Weighted mode
0.004
0.002
0.000
−0.002
−0.004
SNP effect on eGFR
0.25
0.50
0.75
SNP effect on Enhancer of mRNA−decapping protein 4

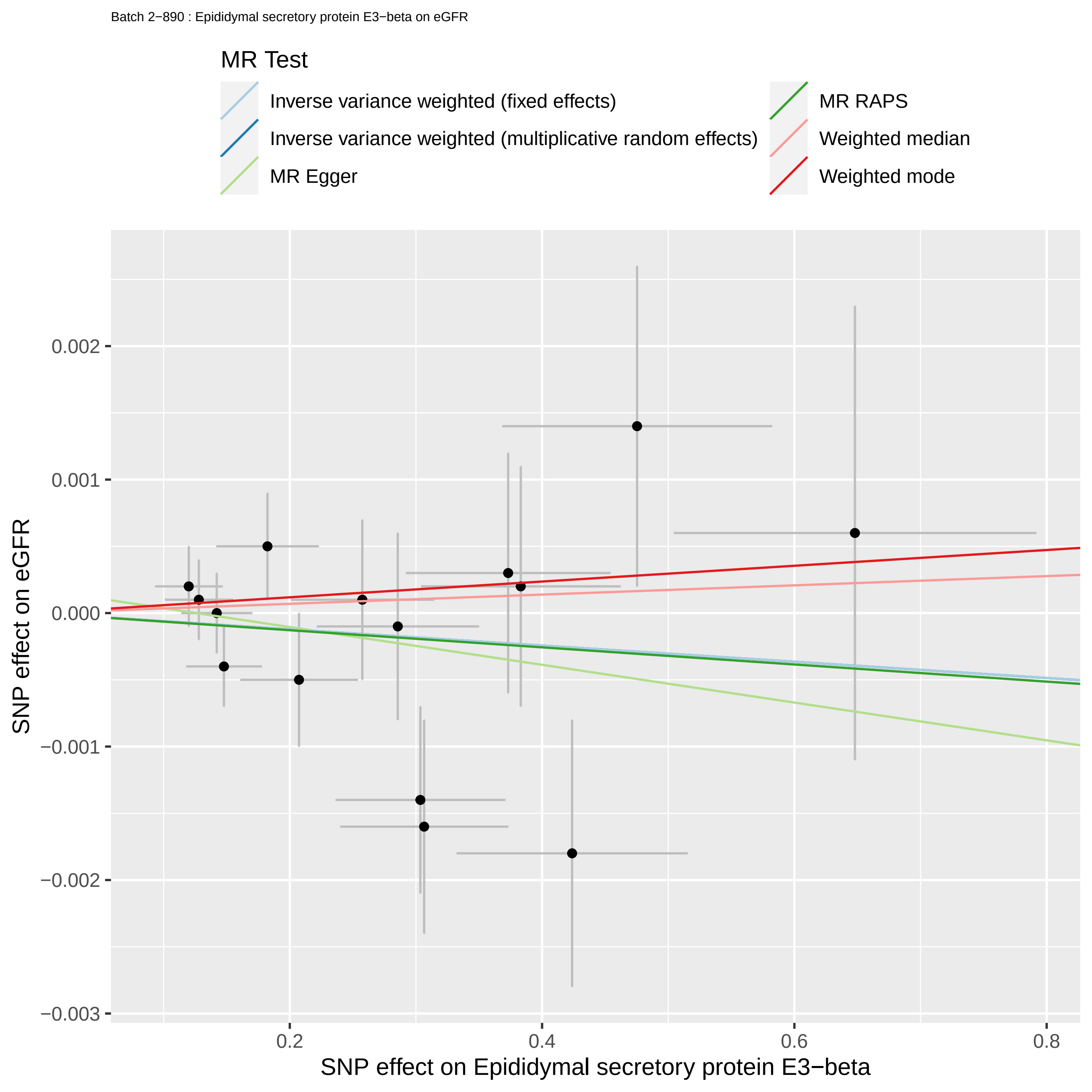

Batch 2−890 : Epididymal secretory protein E3−beta on eGFR
MR Test
Inverse variance weighted (fixed effects)
Inverse variance weighted (multiplicative random effects)
MR Egger
MR RAPS
Weighted median
Weighted mode
0.002
0.001
0.000
−0.001
−0.002
−0.003
0.2
0.4
0.6
0.8
SNP effect on eGFR
SNP effect on Epididymal secretory protein E3−beta

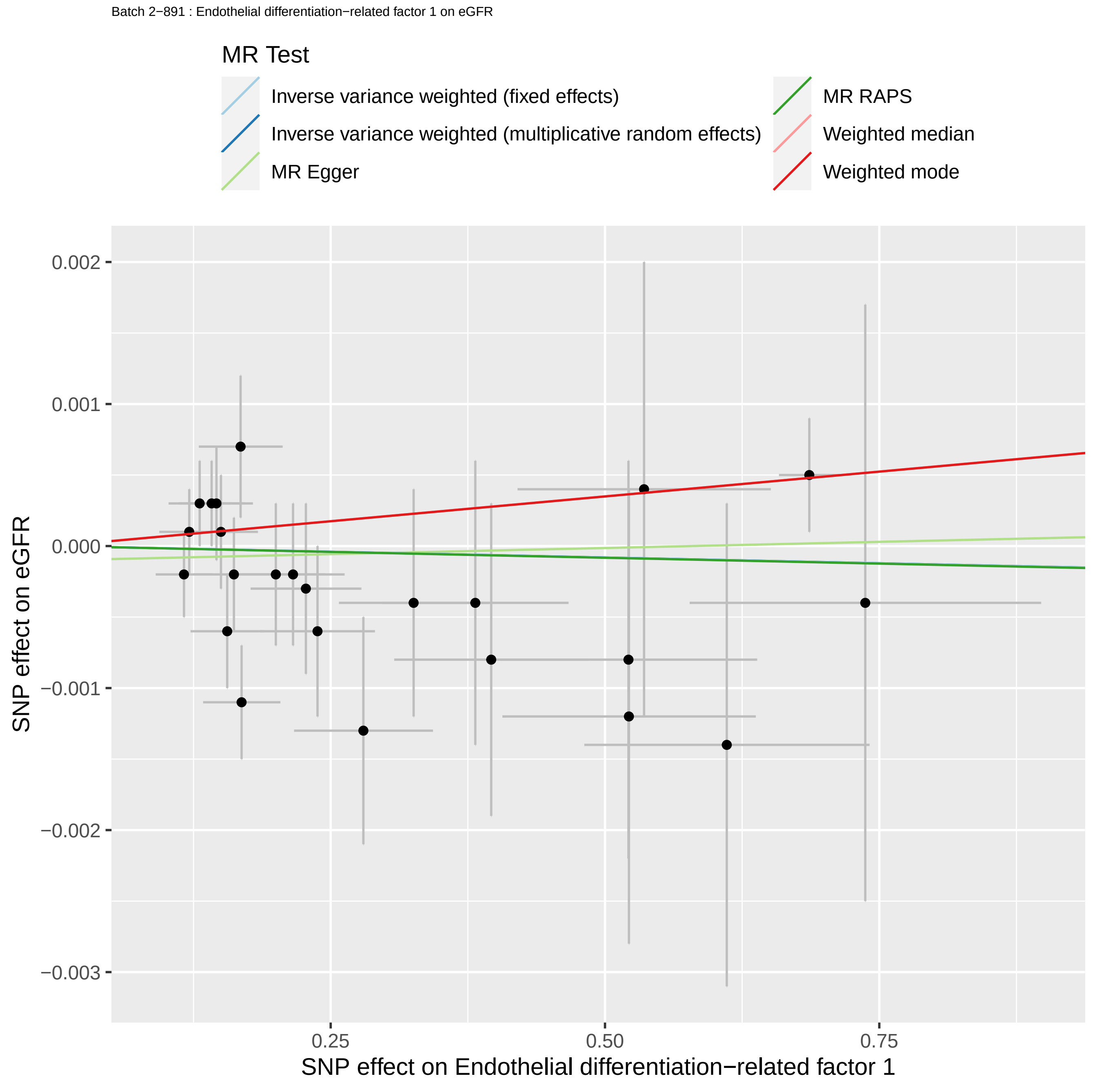

Batch 2−891 : Endothelial differentiation−related factor 1 on eGFR
MR Test
Inverse variance weighted (fixed effects)
Inverse variance weighted (multiplicative random effects)
MR Egger
MR RAPS
Weighted median
Weighted mode
0.002
0.001
0.000
−0.001
−0.002
−0.003
0.25
0.50
0.75
SNP effect on eGFR
SNP effect on Endothelial differentiation−related factor 1

Batch 2−892 : Endothelin−2 on eGFR

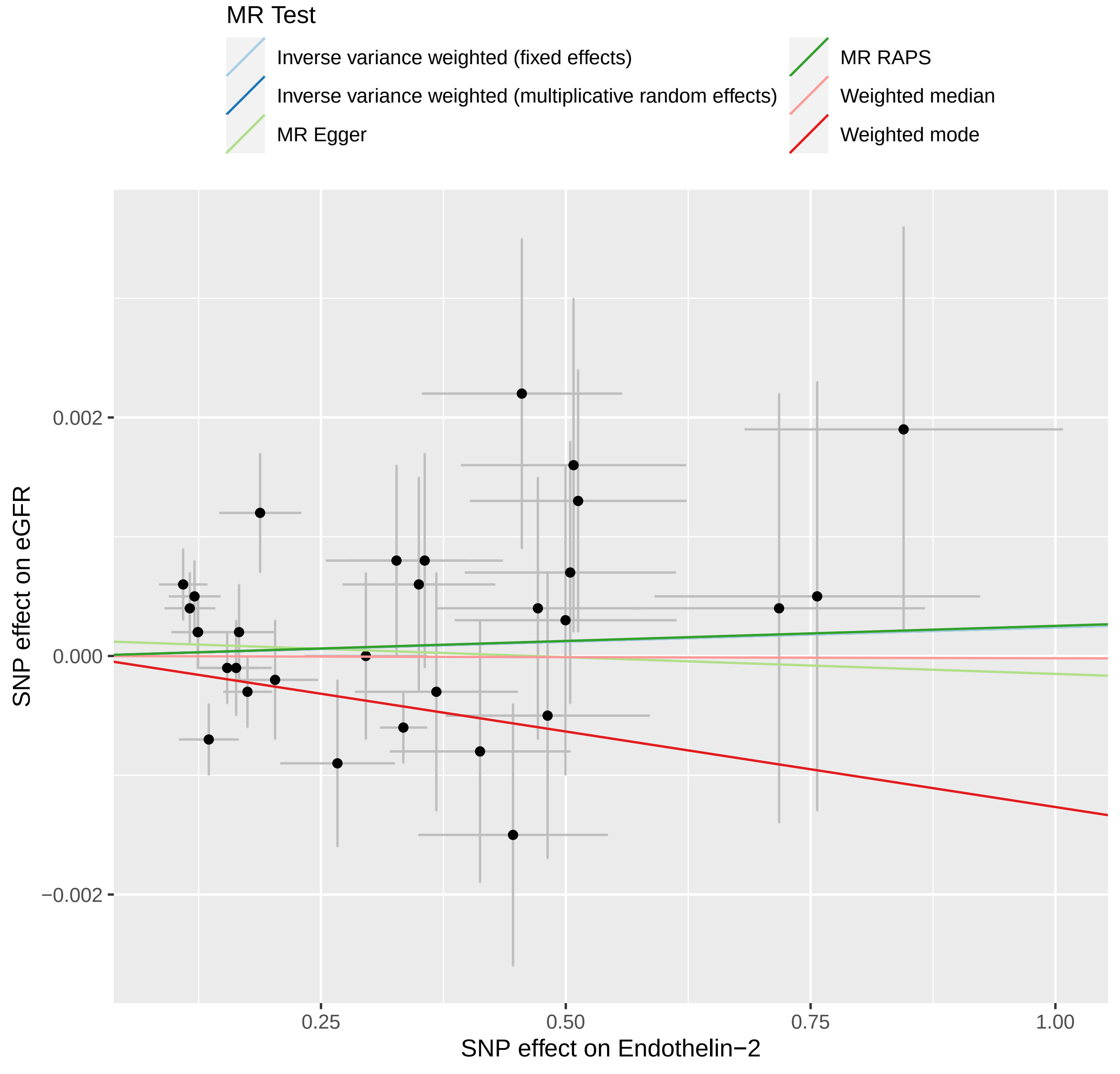

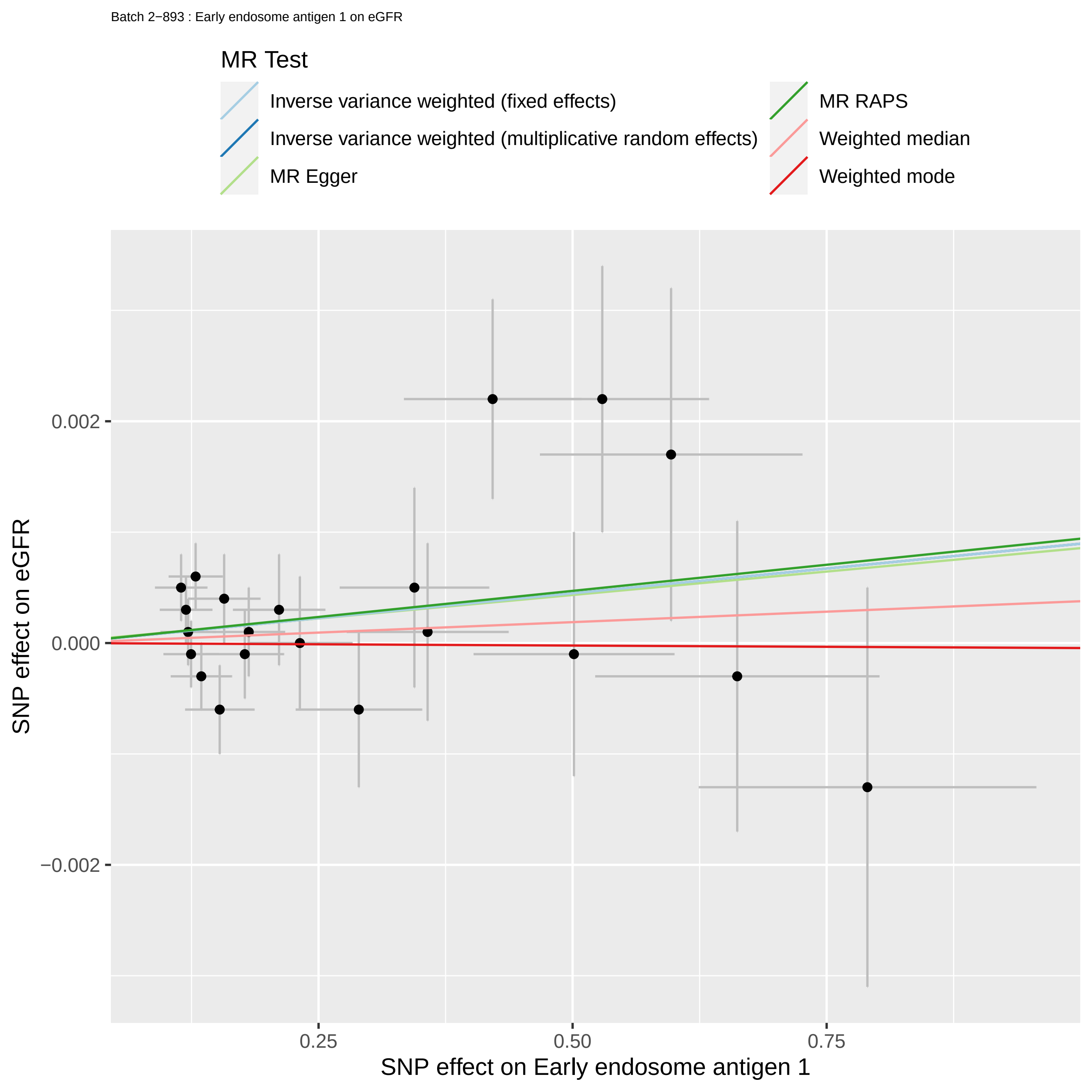
Batch 2−893 : Early endosome antigen 1 on eGFR
MR Test
Inverse variance weighted (fixed effects)
Inverse variance weighted (multiplicative random effects)
MR Egger
MR RAPS
Weighted median
Weighted mode
0.002
0.000
−0.002
0.25
0.50
0.75
SNP effect on Early endosome antigen 1
SNP effect on eGFR

Batch 2−894 : Elongation factor 1−beta on eGFR

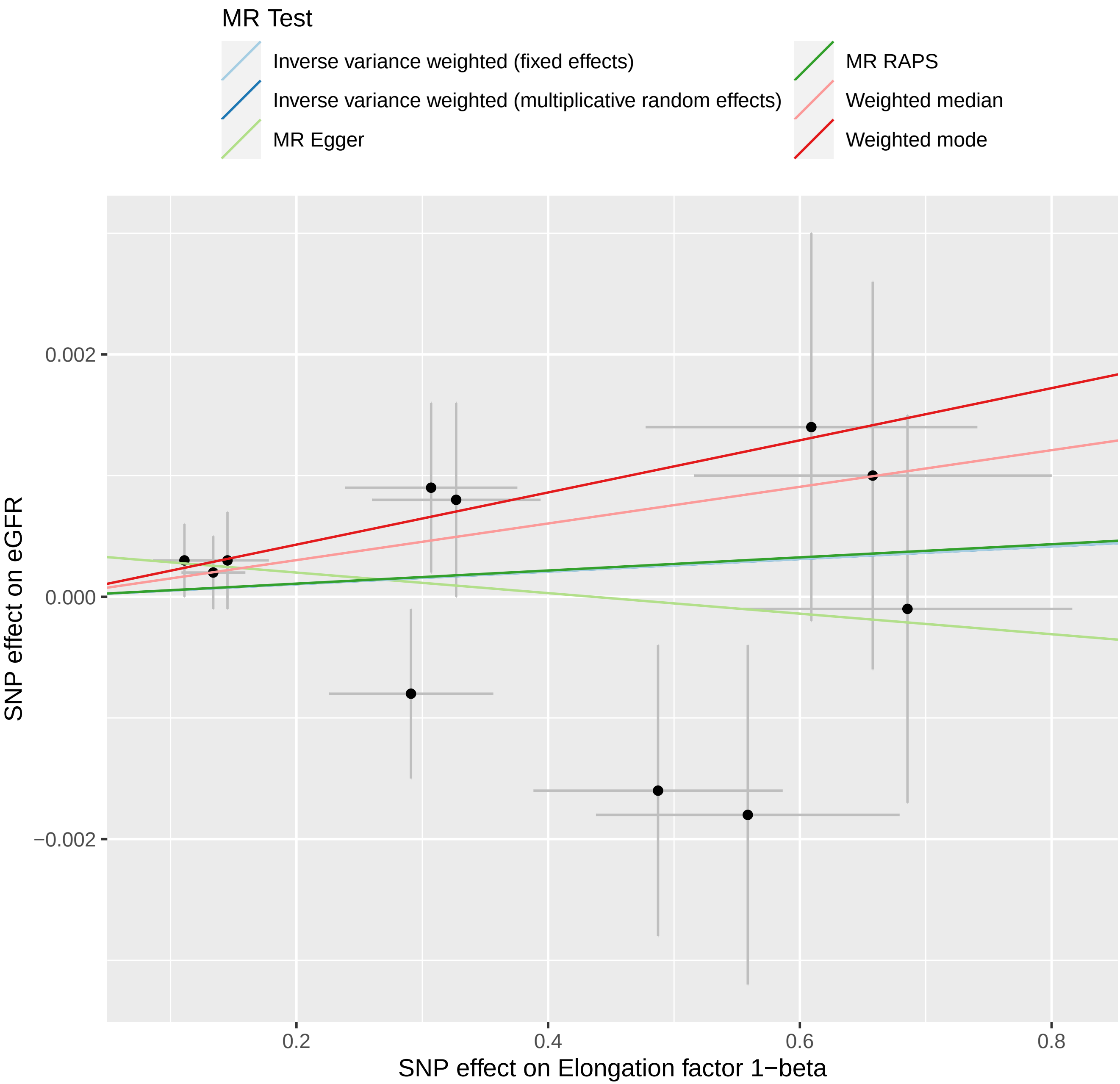

Batch 2−895 : EF−hand calcium−binding domain−containing protein 14 on eGFR

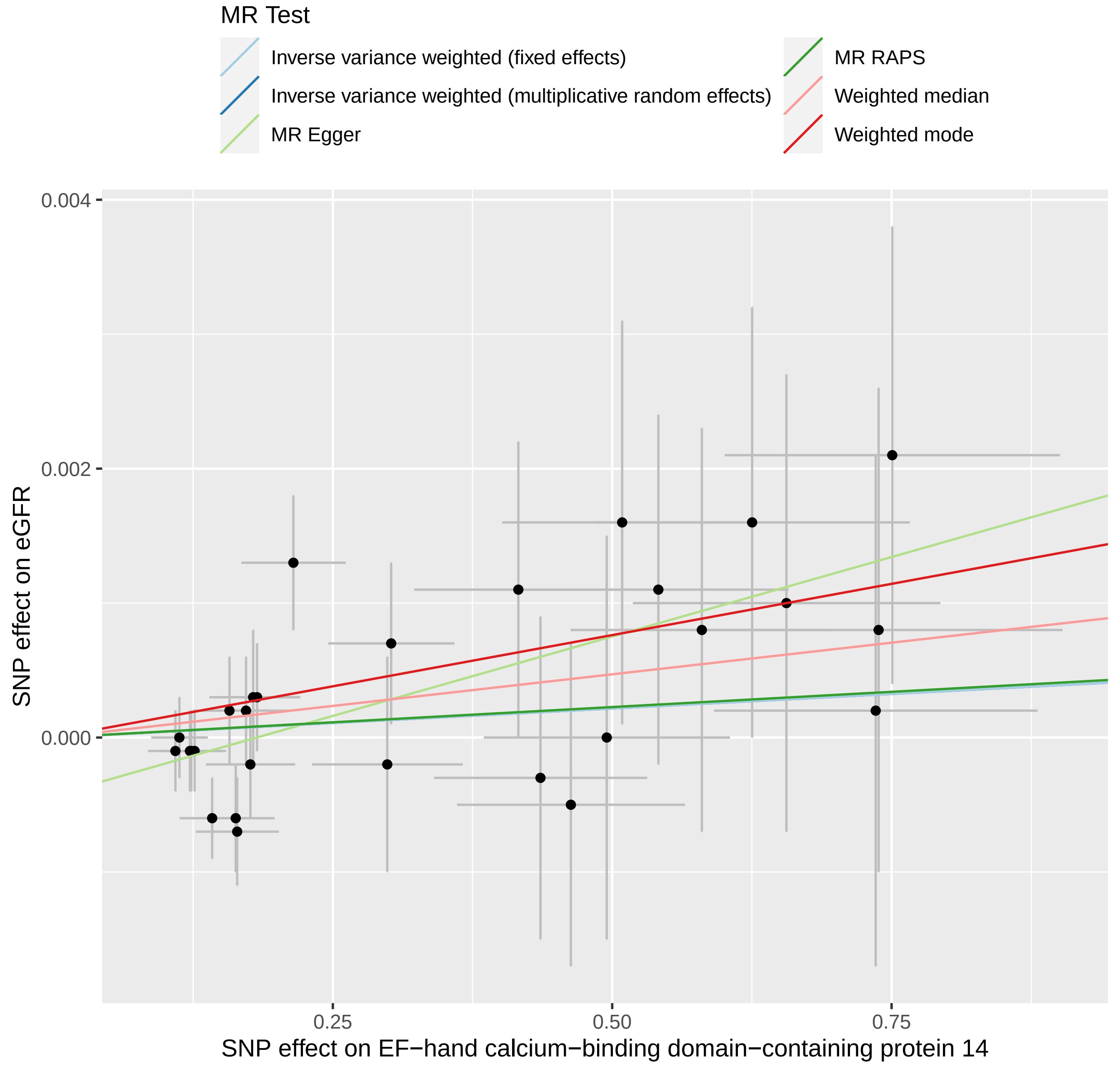

Batch 2−896 : Calcium uptake protein 3, mitochondrial on eGFR

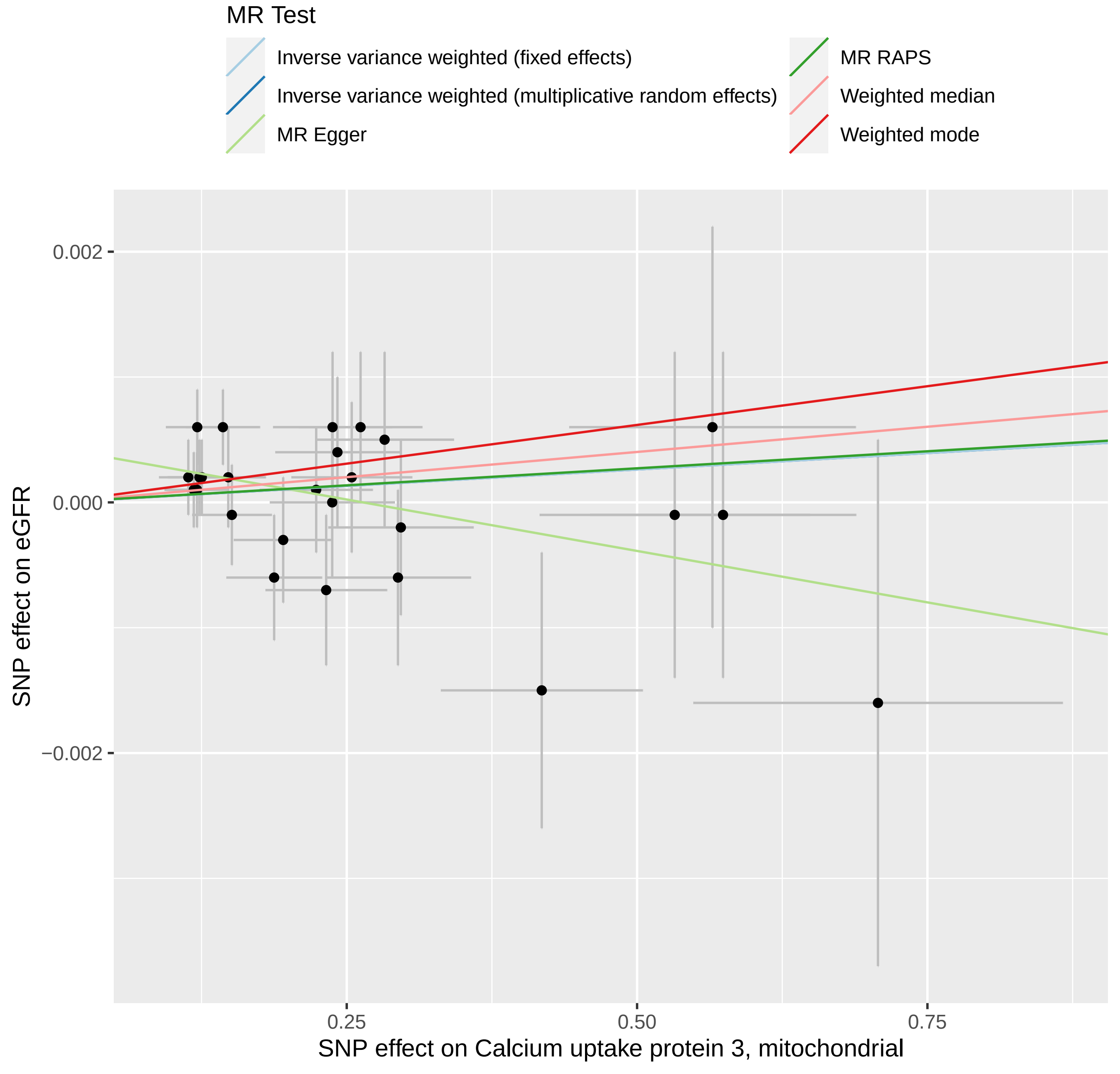

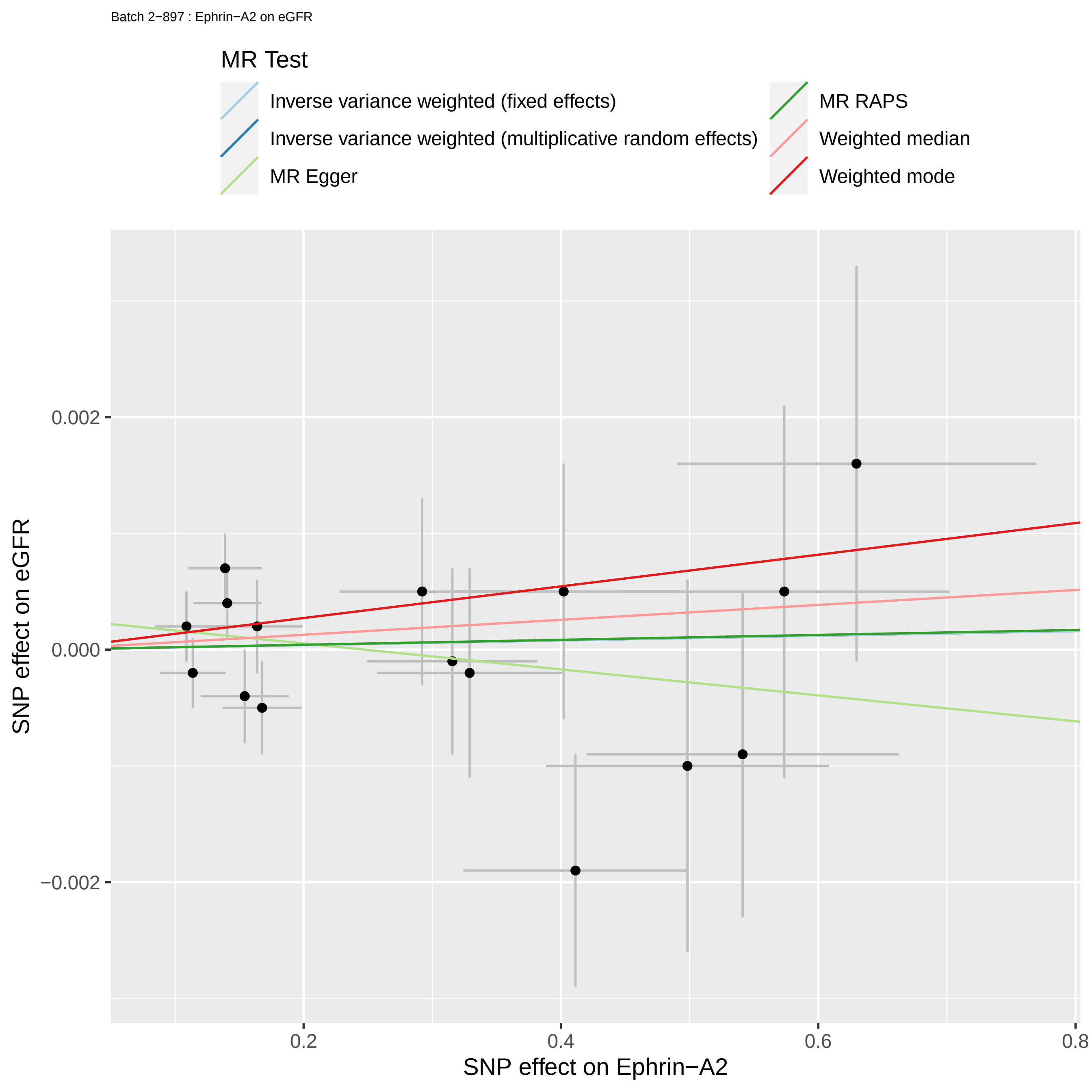

Batch 2−897 : Ephrin−A2 on eGFR
MR Test
Inverse variance weighted (fixed effects)
Inverse variance weighted (multiplicative random effects)
MR Egger
MR RAPS
Weighted median
Weighted mode
0.002
0.000
−0.002
0.2
0.4
0.6
0.8
SNP effect on eGFR
SNP effect on Ephrin−A2

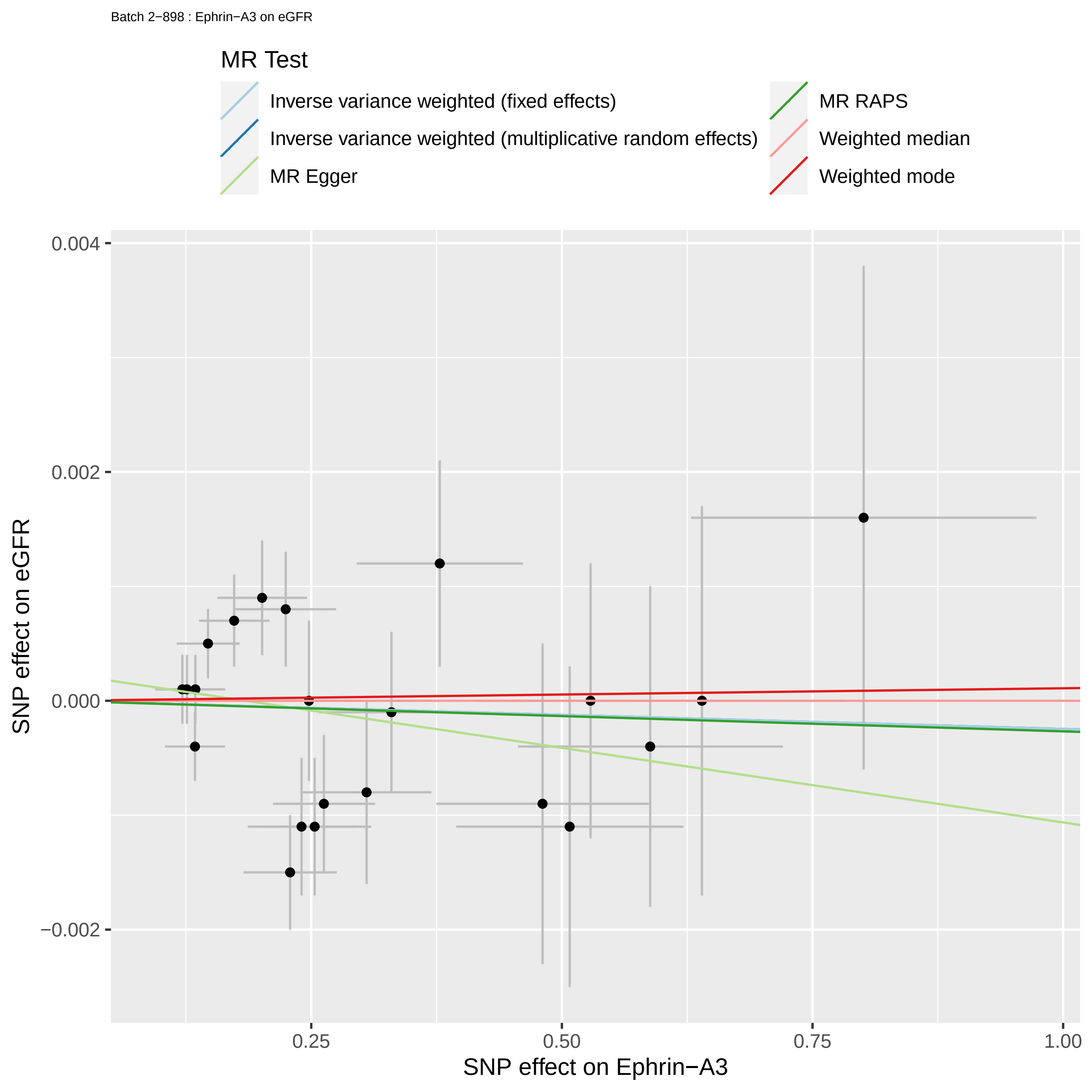

Batch 2−898 : Ephrin−A3 on eGFR
MR Test
Inverse variance weighted (fixed effects)
Inverse variance weighted (multiplicative random effects)
MR Egger
MR RAPS
Weighted median
Weighted mode
0.004
0.002
0.000
−0.002
0.25
0.50
0.75
1.00
SNP effect on eGFR
SNP effect on Ephrin−A3

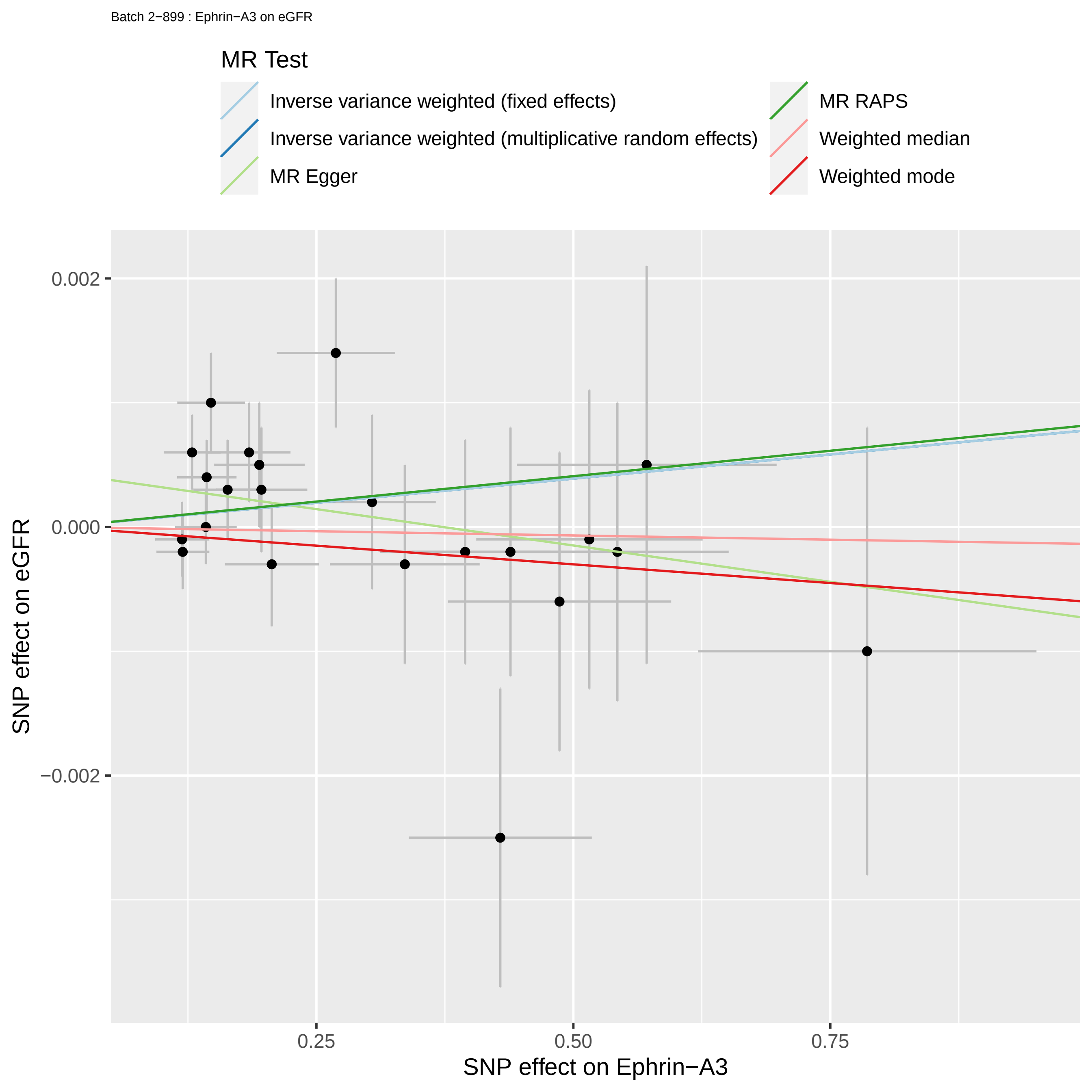

Batch 2−899 : Ephrin−A3 on eGFR
MR Test
Inverse variance weighted (fixed effects)
Inverse variance weighted (multiplicative random effects)
MR Egger
MR RAPS
Weighted median
Weighted mode
0.002
0.000
−0.002
SNP effect on eGFR
0.25
0.50
0.75
SNP effect on Ephrin−A3

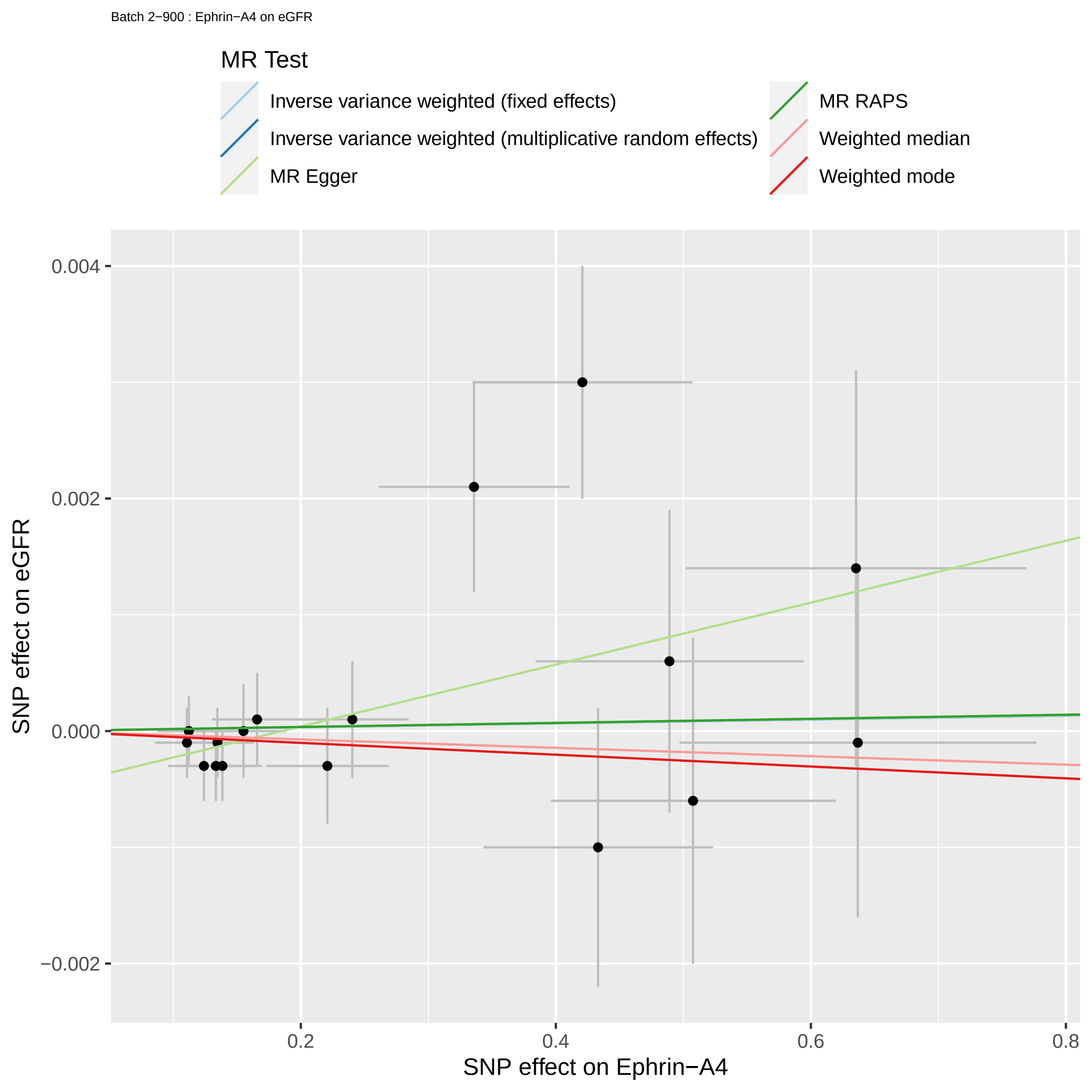

Batch 2−900 : Ephrin−A4 on eGFR
MR Test
Inverse variance weighted (fixed effects)
Inverse variance weighted (multiplicative random effects)
MR Egger
MR RAPS
Weighted median
Weighted mode
0.004
0.002
0.000
−0.002
SNP effect on eGFR
0.2
0.4
0.6
0.8
SNP effect on Ephrin−A4

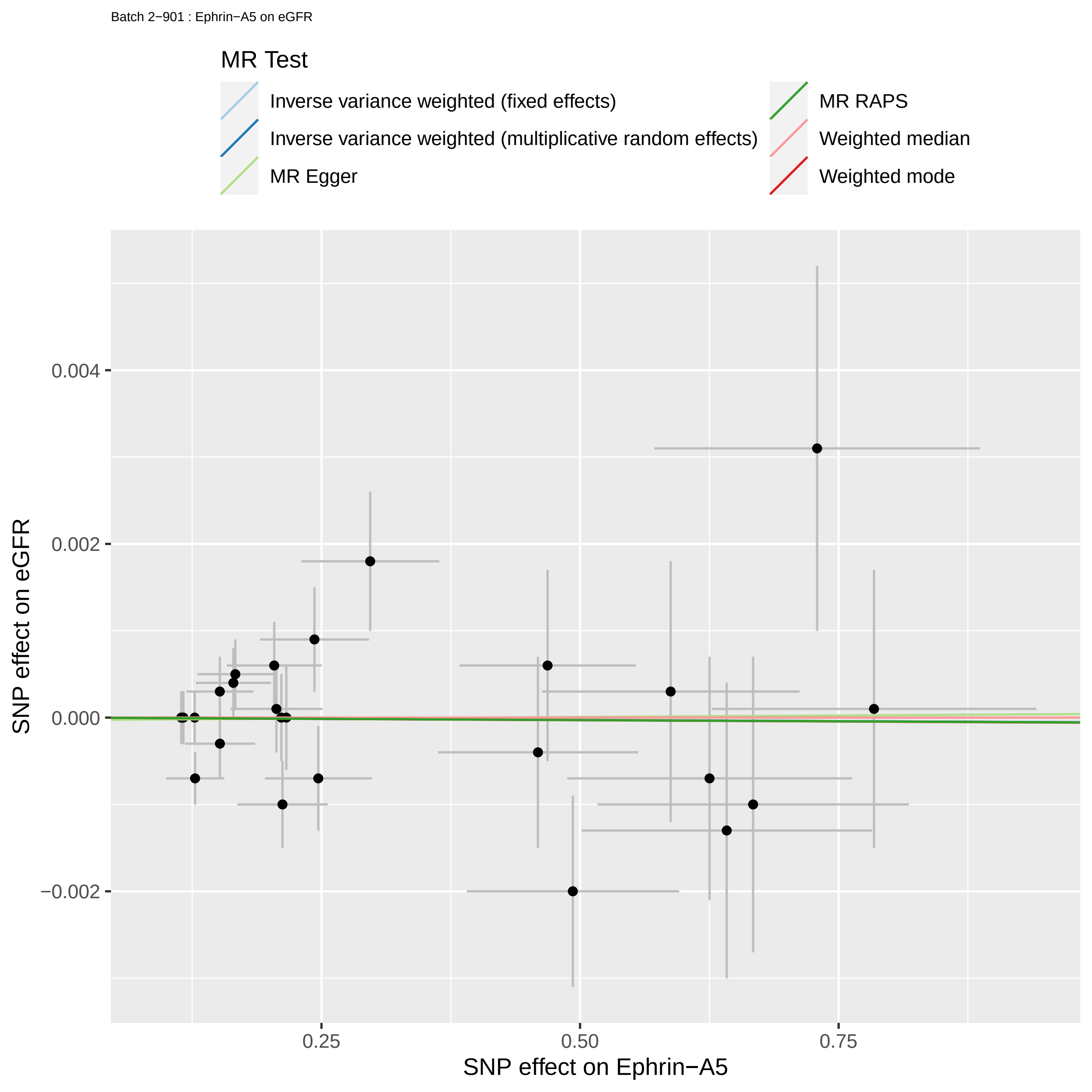
Batch 2−901 : Ephrin−A5 on eGFR
MR Test
Inverse variance weighted (fixed effects)
Inverse variance weighted (multiplicative random effects)
MR Egger
MR RAPS
Weighted median
Weighted mode
0.004
0.002
0.000
−0.002
0.25
0.50
0.75
SNP effect on eGFR
SNP effect on Ephrin−A5

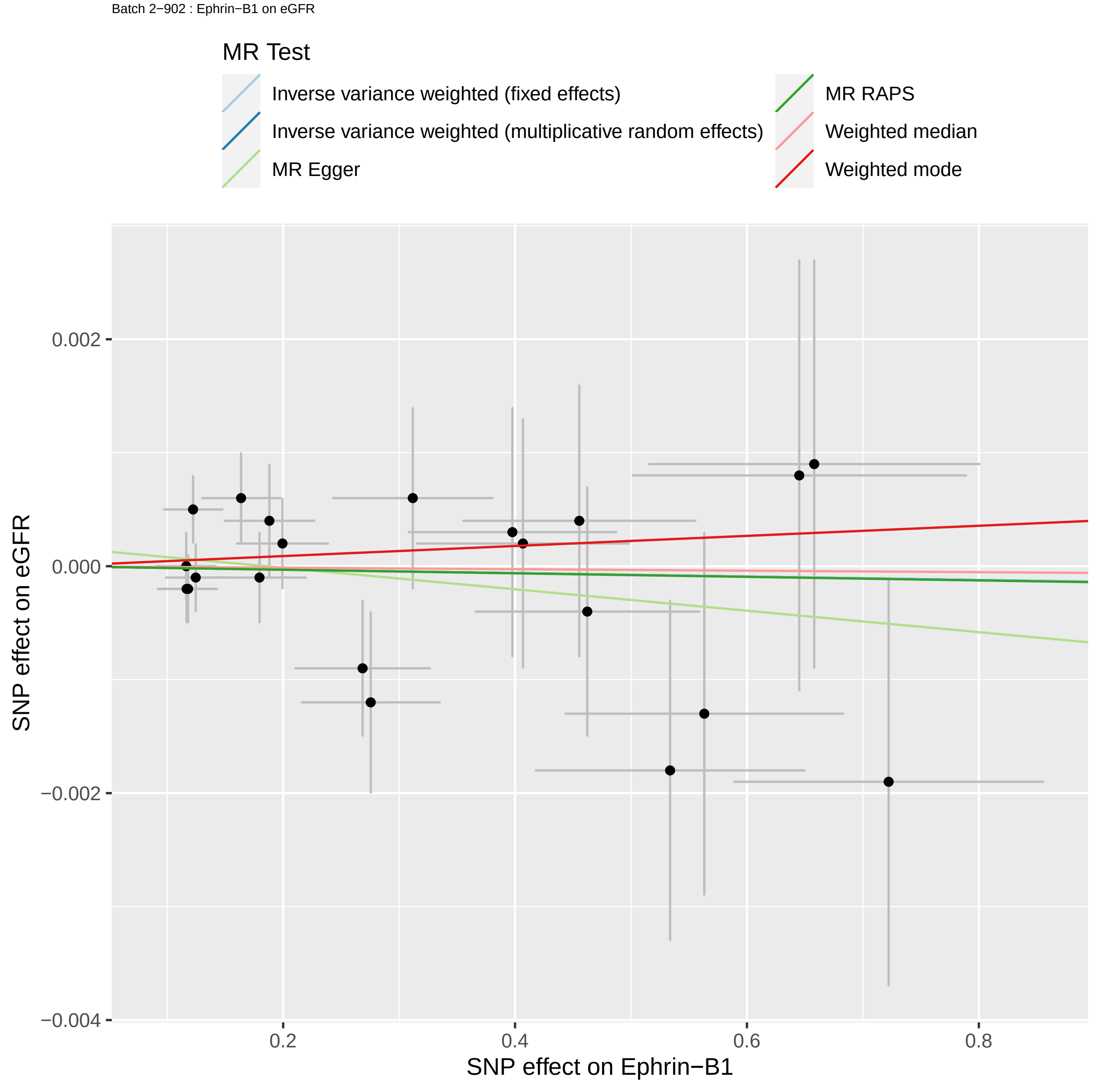
Batch 2−902 : Ephrin−B1 on eGFR
MR Test
Inverse variance weighted (fixed effects)
Inverse variance weighted (multiplicative random effects)
MR Egger
MR RAPS
Weighted median
Weighted mode
SNP effect on eGFR
0.002
0.000
−0.002
−0.004
SNP effect on Ephrin−B1
0.2
0.4
0.6
0.8

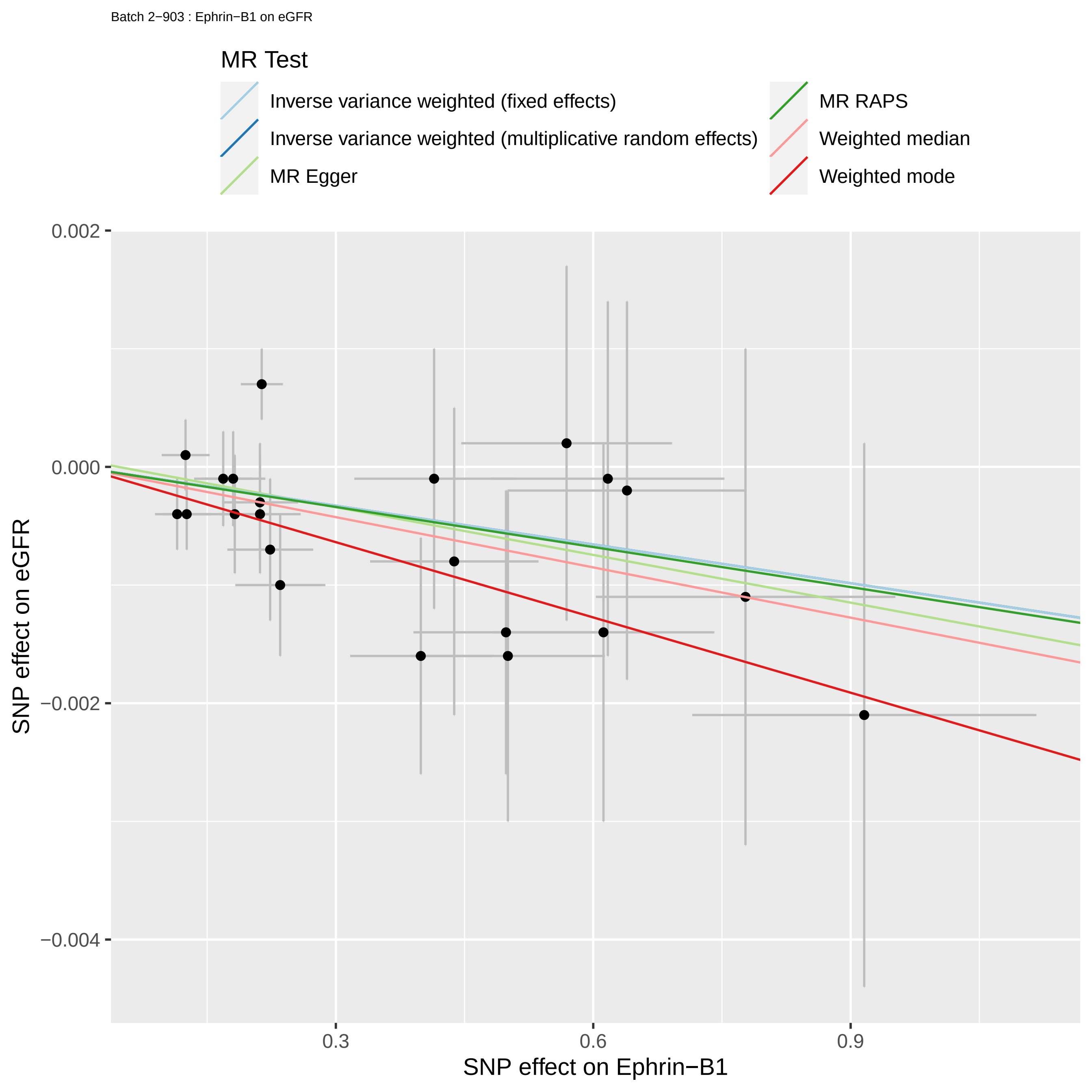

Batch 2−903 : Ephrin−B1 on eGFR
MR Test
Inverse variance weighted (fixed effects)
Inverse variance weighted (multiplicative random effects)
MR Egger
MR RAPS
Weighted median
Weighted mode
0.002
0.000
−0.002
−0.004
0.3
0.6
0.9
SNP effect on eGFR
SNP effect on Ephrin−B1

Batch 2−904 : Ephrin−B2 on eGFR

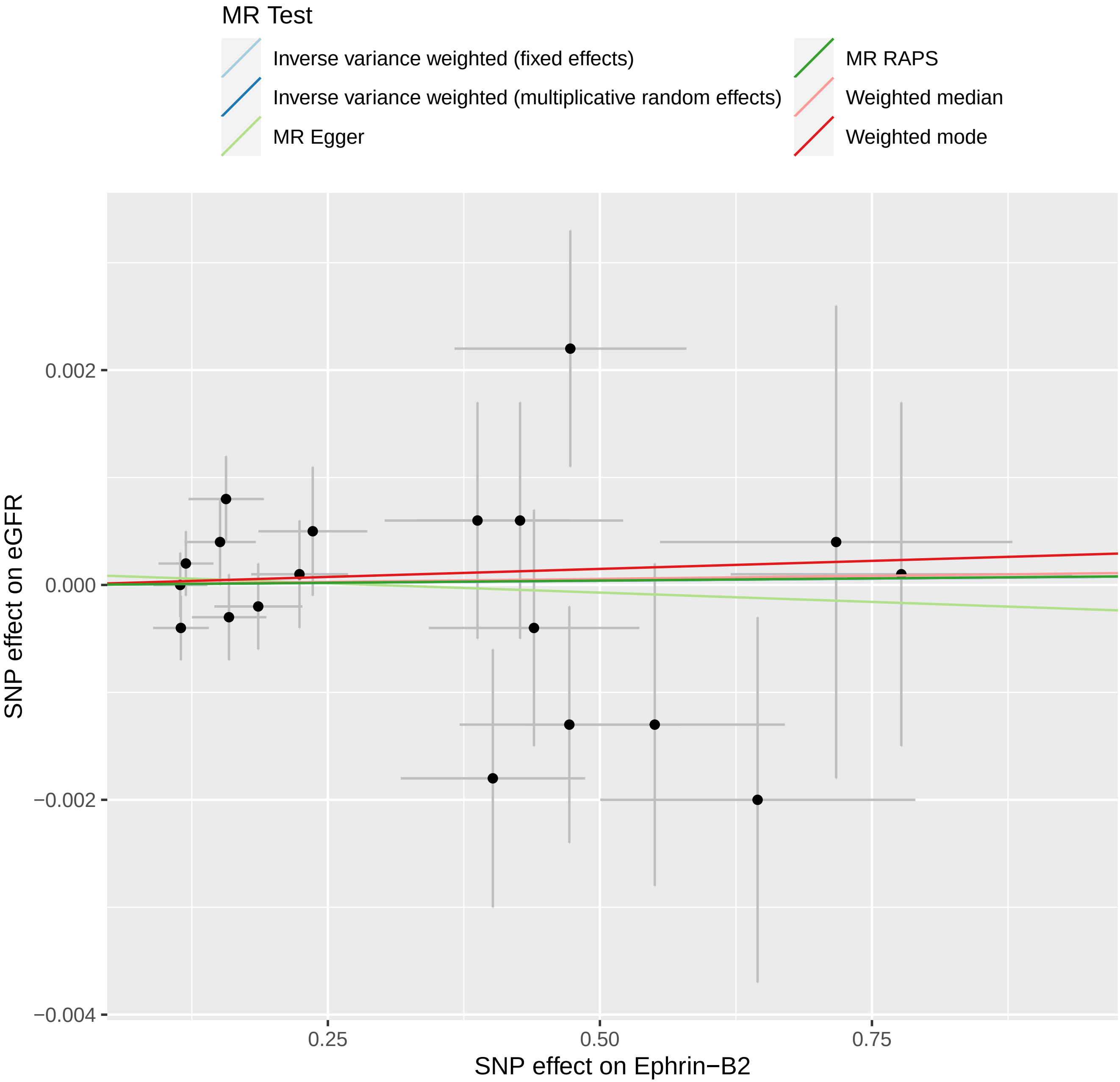

Batch 2−905 : Ephrin−B2 on eGFR

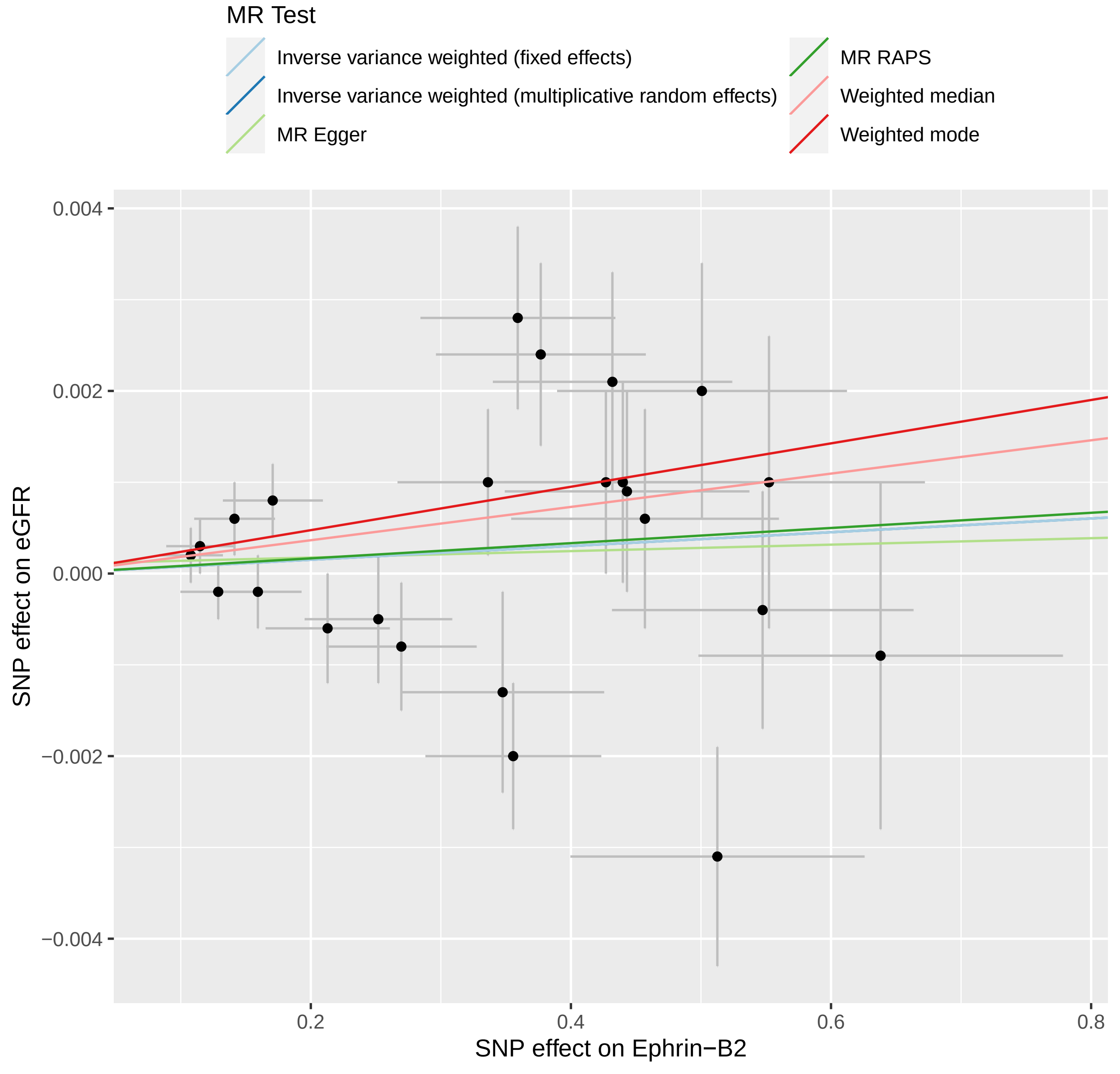

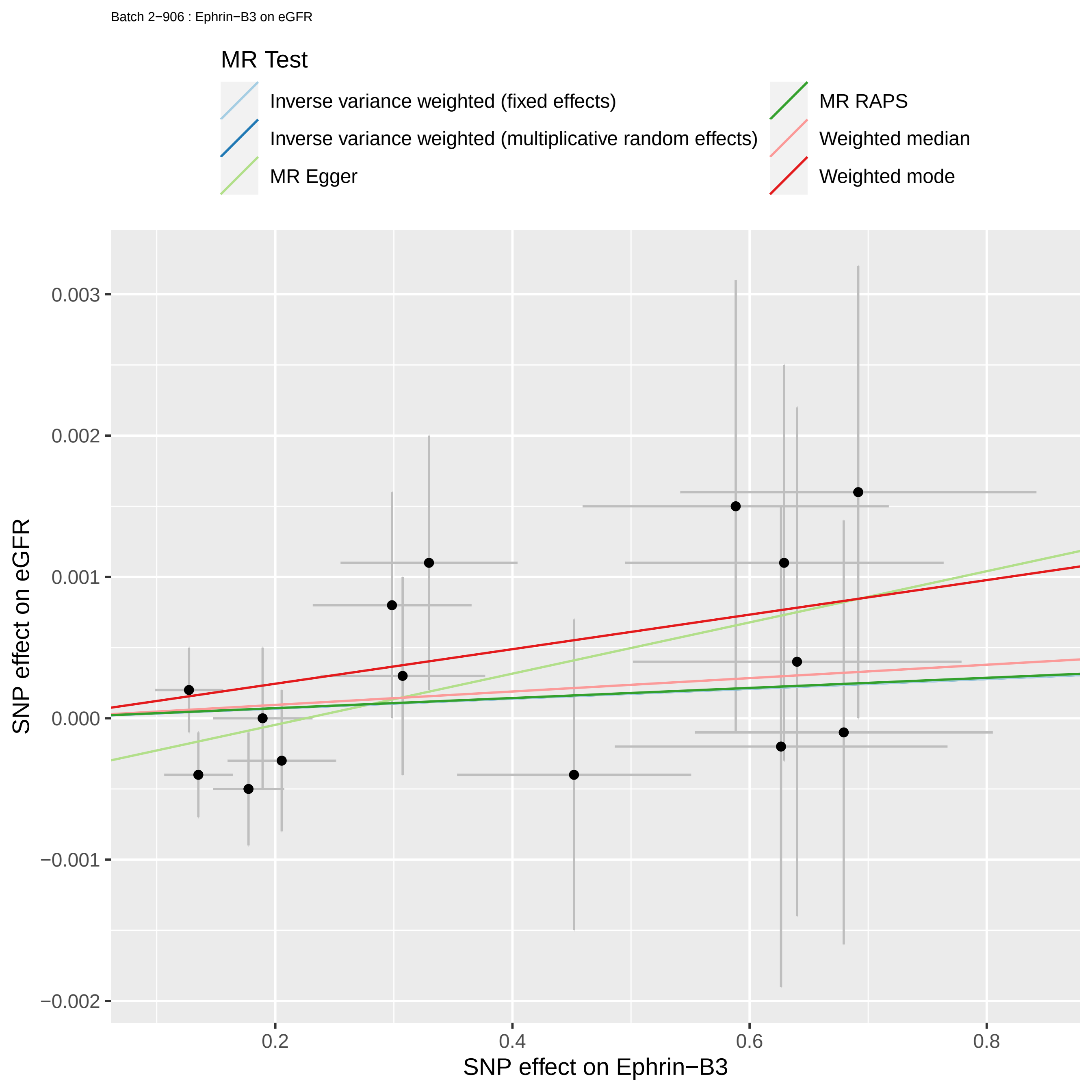
Batch 2−906 : Ephrin−B3 on eGFR
MR Test
Inverse variance weighted (fixed effects)
Inverse variance weighted (multiplicative random effects)
MR Egger
MR RAPS
Weighted median
Weighted mode
0.003
0.002
0.001
0.000
−0.001
−0.002
0.2
0.4
0.6
0.8
SNP effect on eGFR
SNP effect on Ephrin−B3

Batch 2−907 : Ephrin−B3 on eGFR

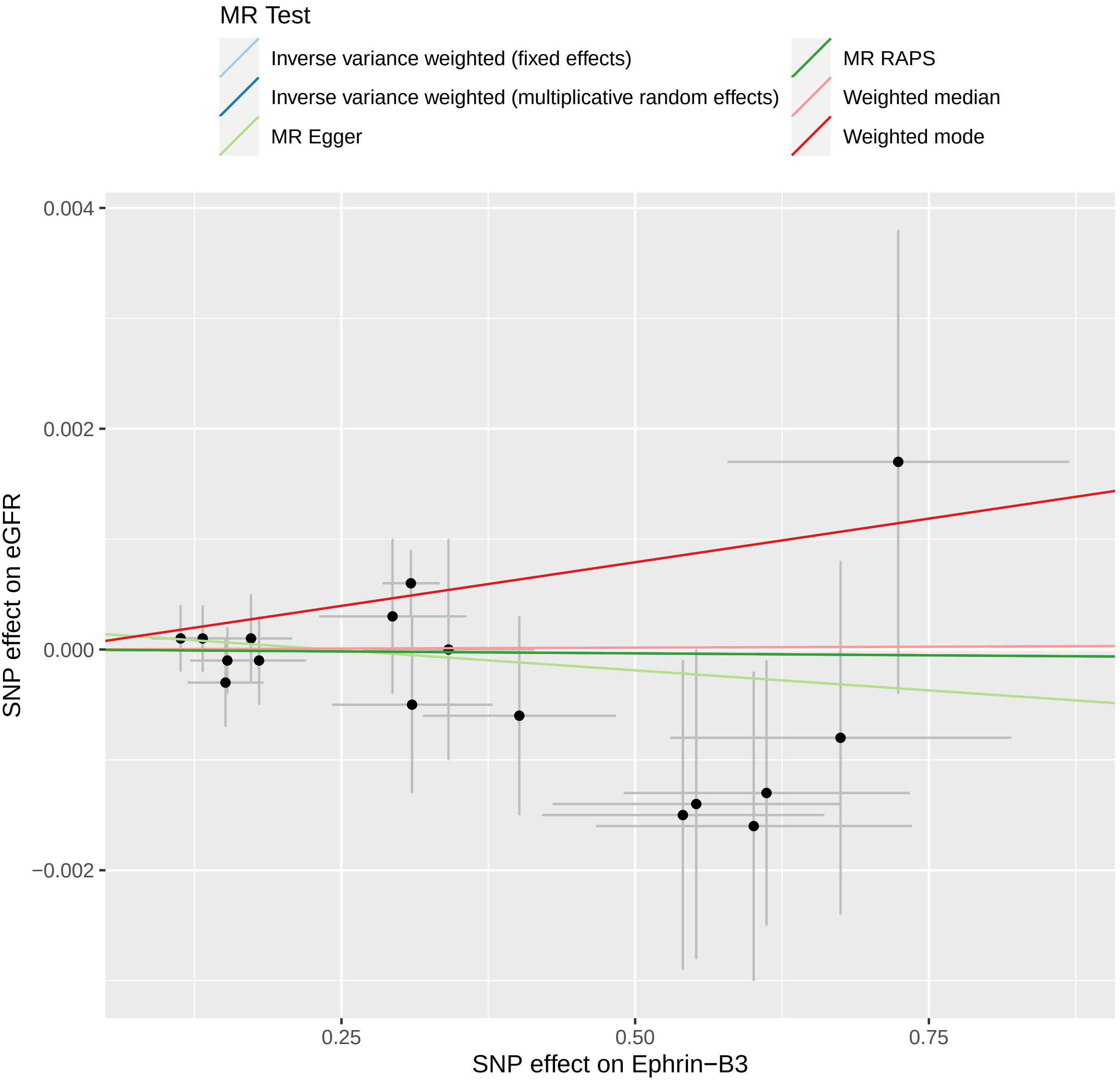

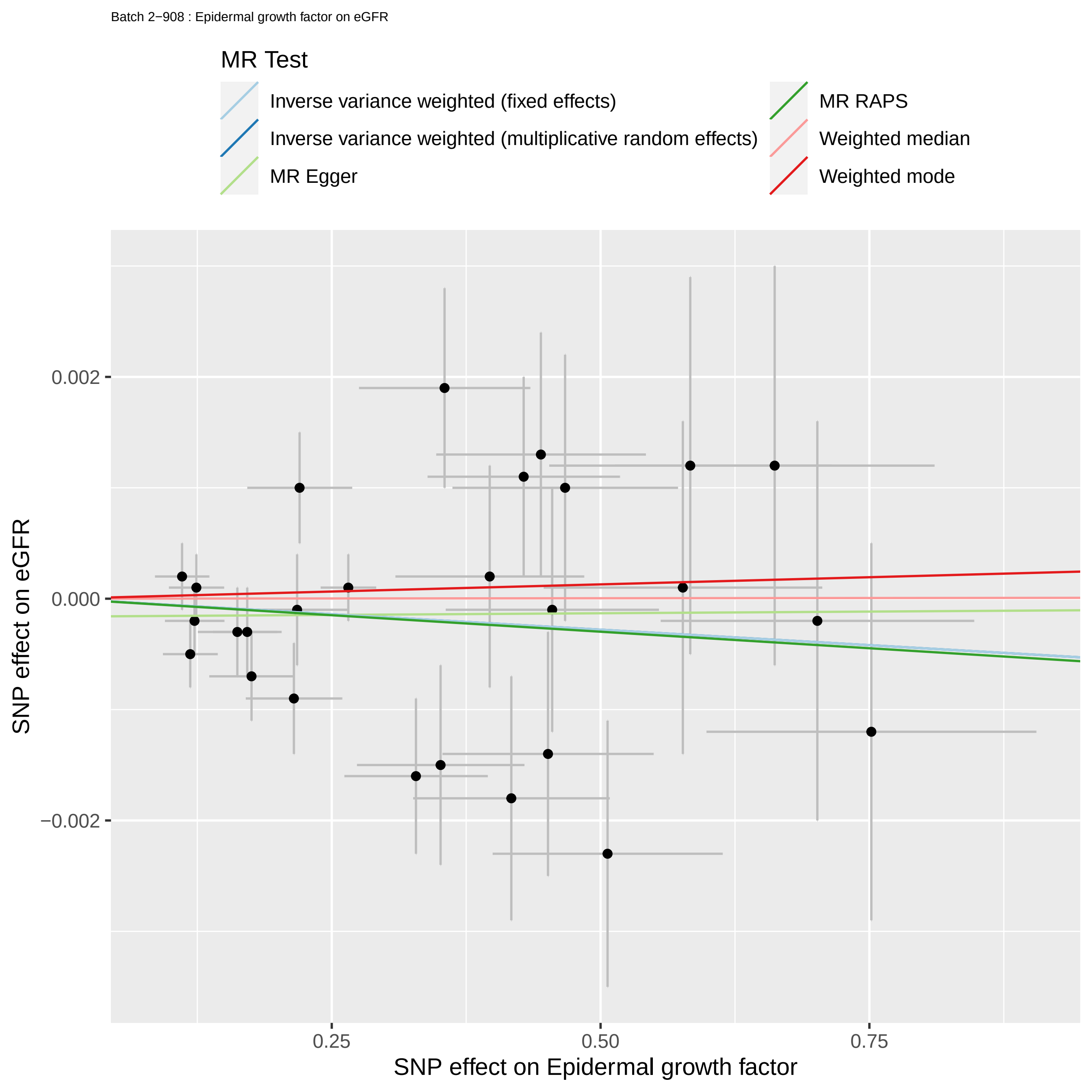

Batch 2−908 : Epidermal growth factor on eGFR
MR Test
Inverse variance weighted (fixed effects)
Inverse variance weighted (multiplicative random effects)
MR Egger
MR RAPS
Weighted median
Weighted mode
0.002
0.000
−0.002
0.25
0.50
0.75
SNP effect on Epidermal growth factor
SNP effect on eGFR

Batch 2−909 : Epidermal growth factor receptor on eGFR

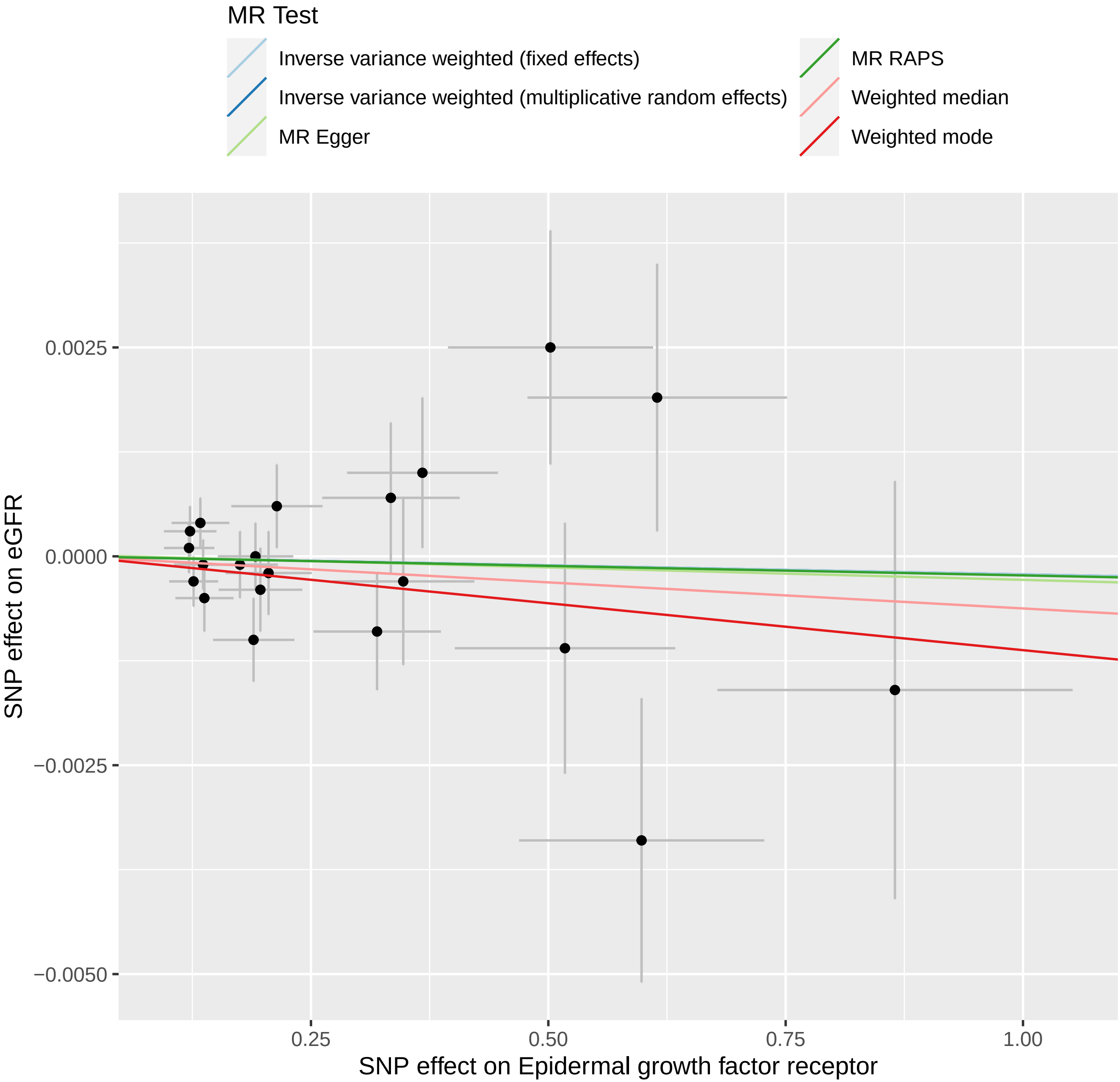

Batch 2−910 : Epidermal growth factor receptor variant III on eGFR

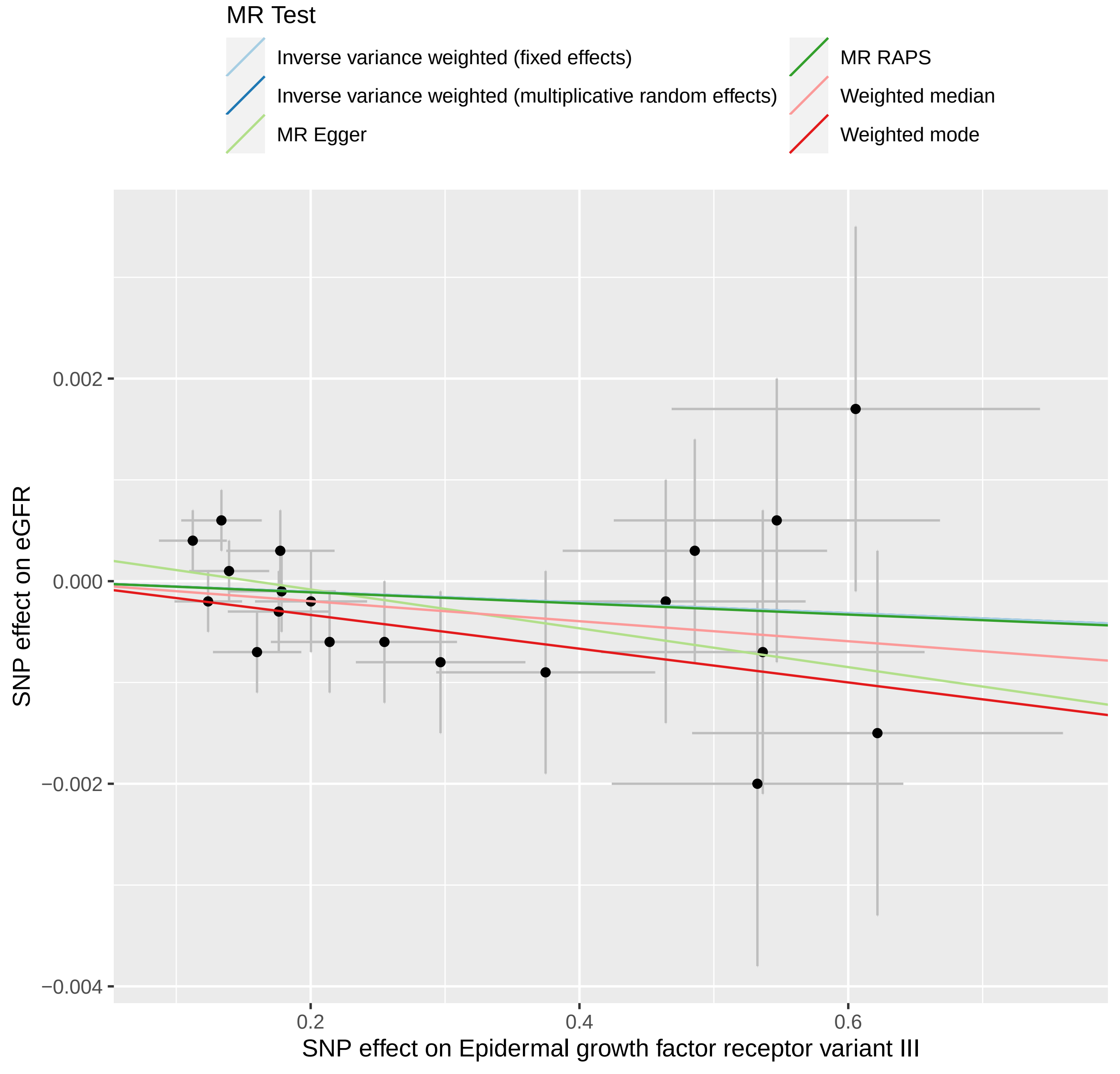

Batch 2−911 : EH domain−binding protein 1 on eGFR

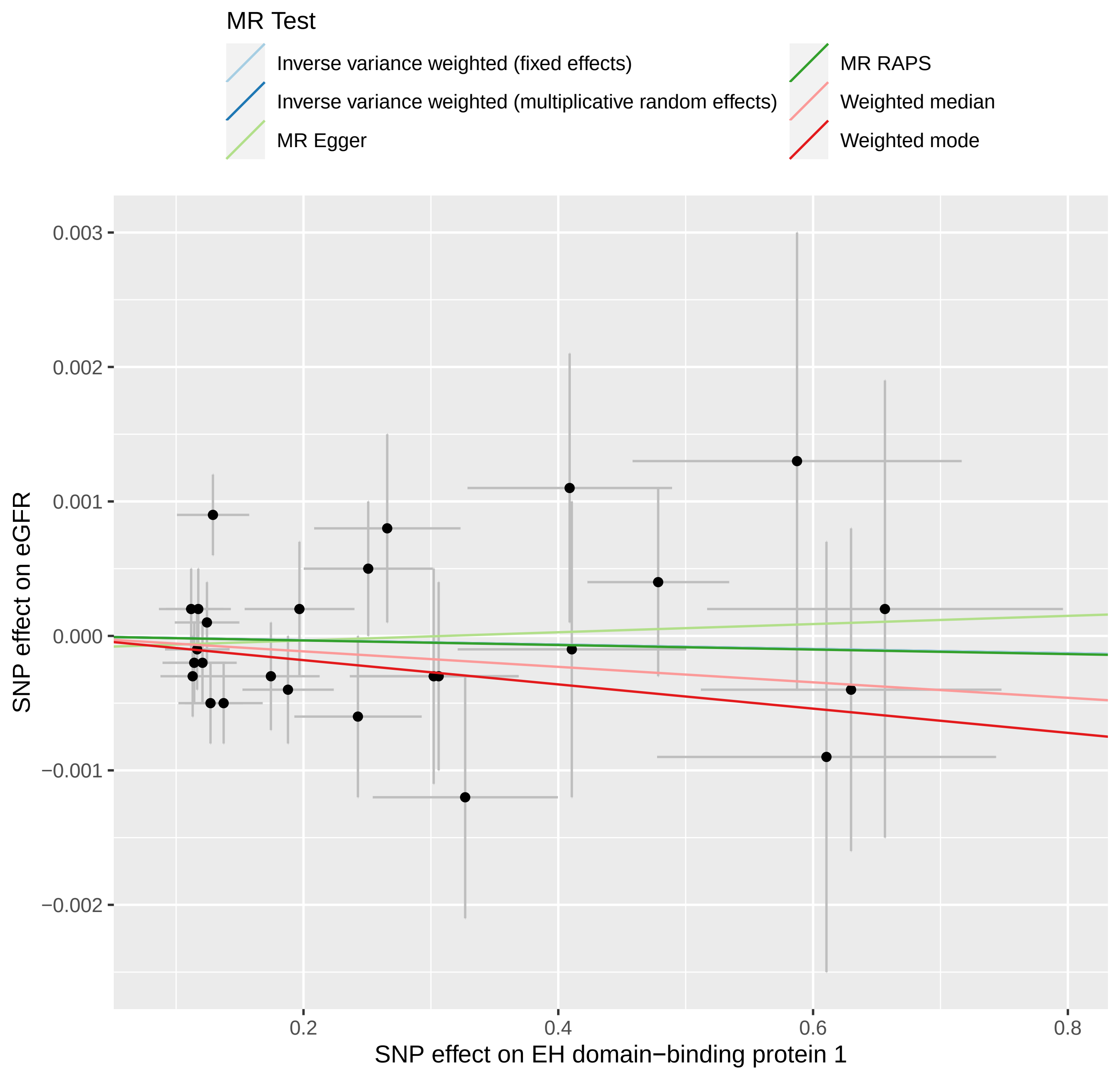

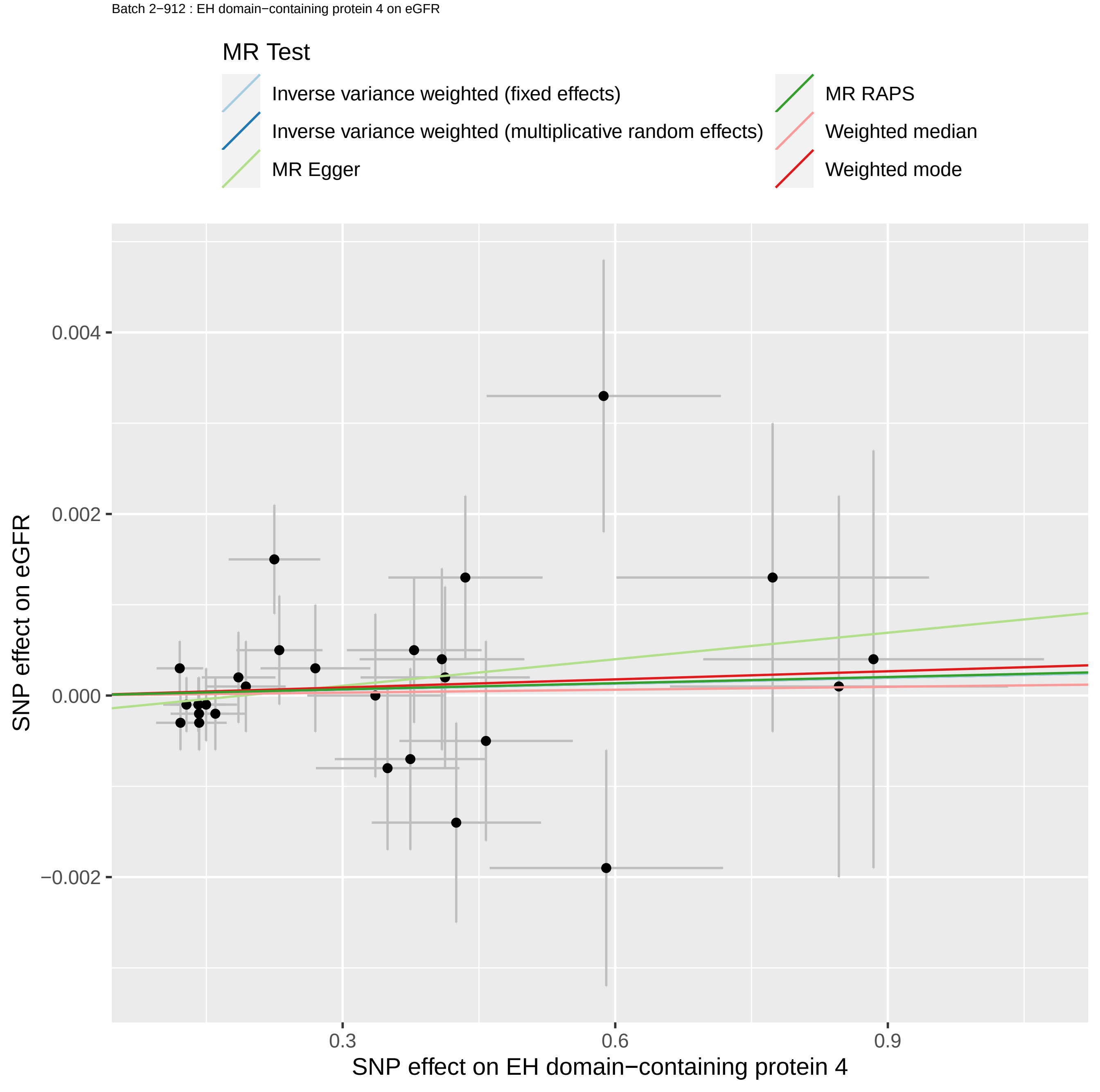

Batch 2−912 : EH domain−containing protein 4 on eGFR
MR Test
Inverse variance weighted (fixed effects)
Inverse variance weighted (multiplicative random effects)
MR Egger
MR RAPS
Weighted median
Weighted mode
0.004
0.002
0.000
−0.002
0.3
0.6
0.9
SNP effect on eGFR
SNP effect on EH domain−containing protein 4

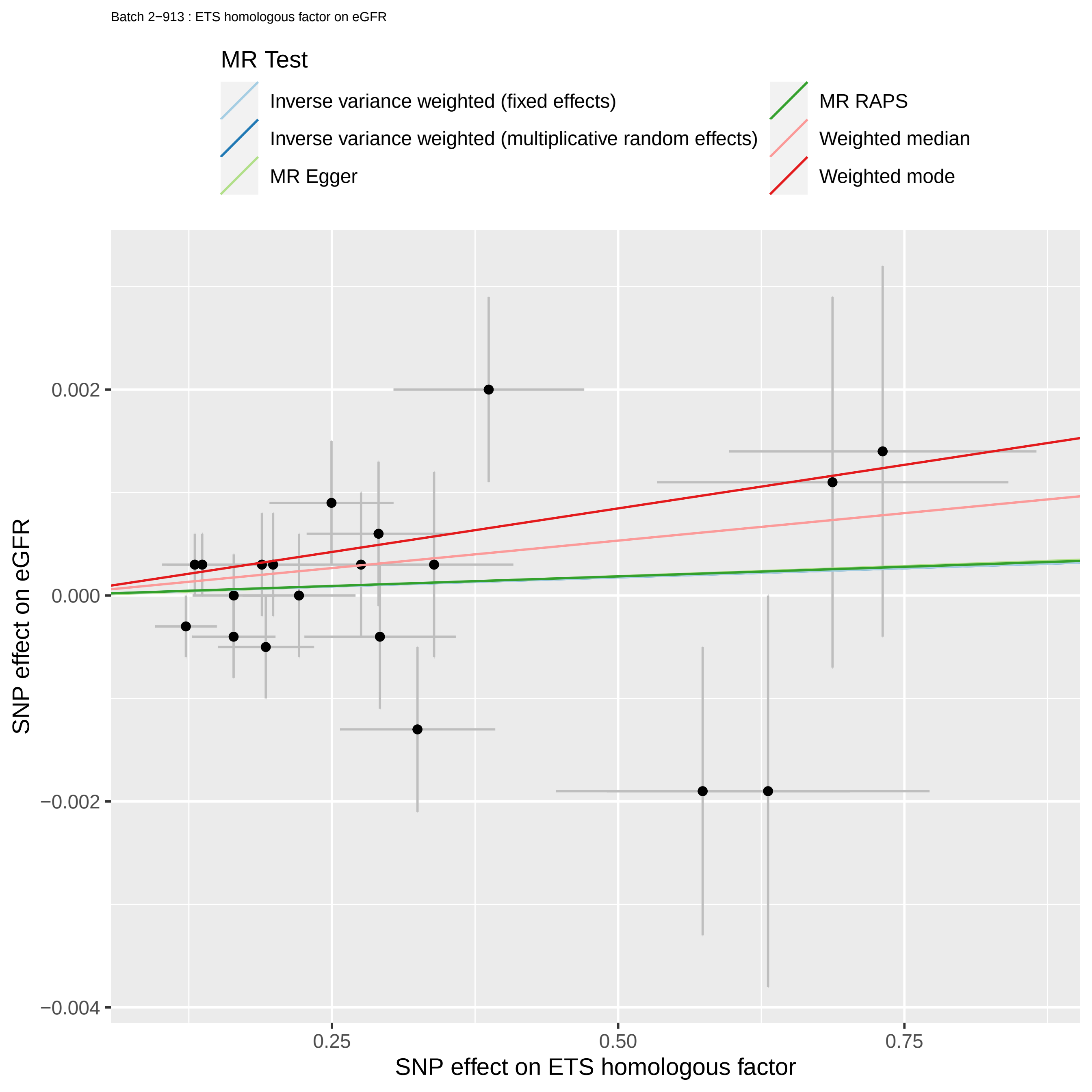

Batch 2−913 : ETS homologous factor on eGFR
MR Test
Inverse variance weighted (fixed effects)
Inverse variance weighted (multiplicative random effects)
MR Egger
MR RAPS
Weighted median
Weighted mode
0.002
0.000
−0.002
−0.004
0.25
0.50
0.75
SNP effect on eGFR
SNP effect on ETS homologous factor

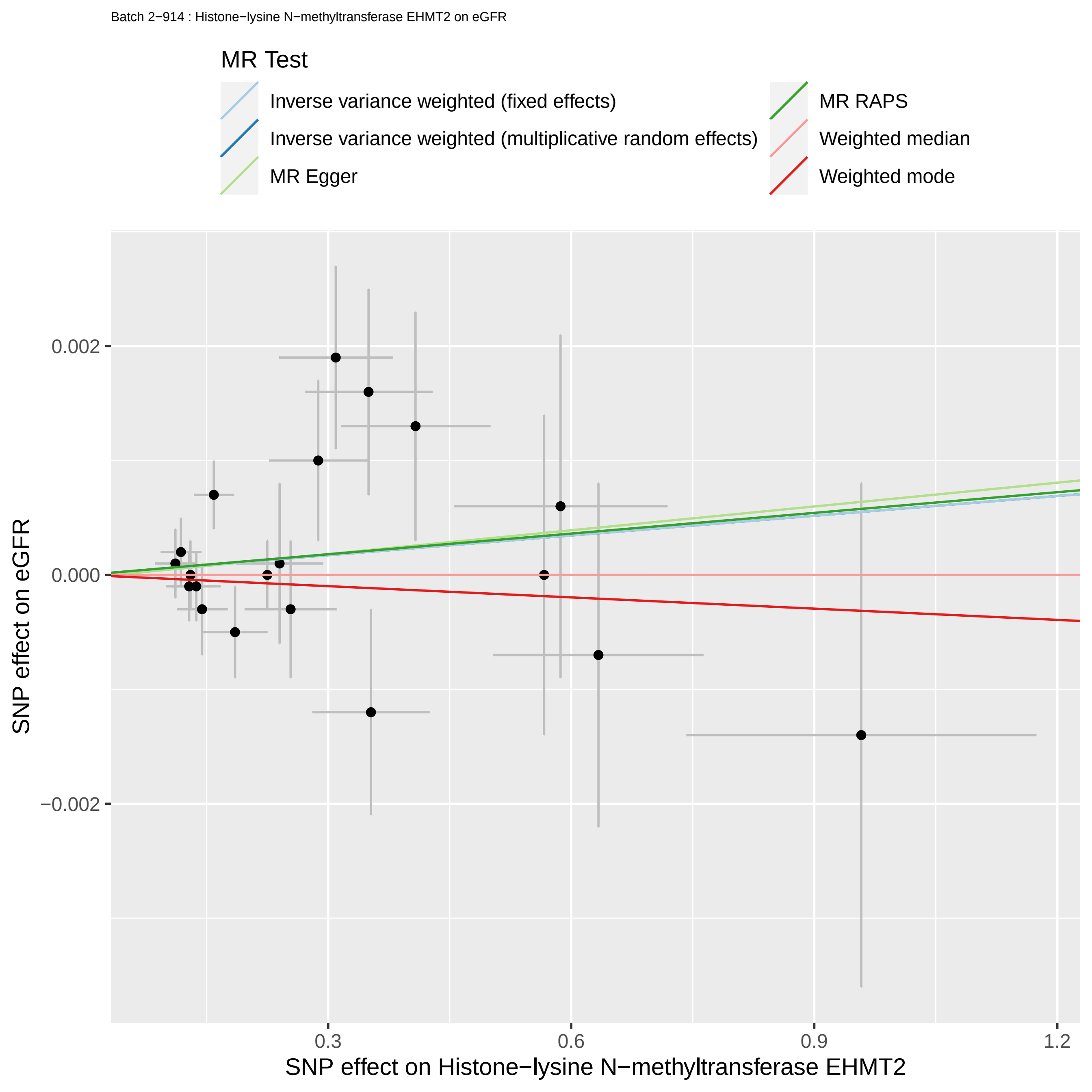

Batch 2−914 : Histone−lysine N−methyltransferase EHMT2 on eGFR
MR Test
Inverse variance weighted (fixed effects)
Inverse variance weighted (multiplicative random effects)
MR Egger
MR RAPS
Weighted median
Weighted mode
SNP effect on eGFR
0.002
0.000
−0.002
0.3
0.6
0.9
1.2
SNP effect on Histone−lysine N−methyltransferase EHMT2

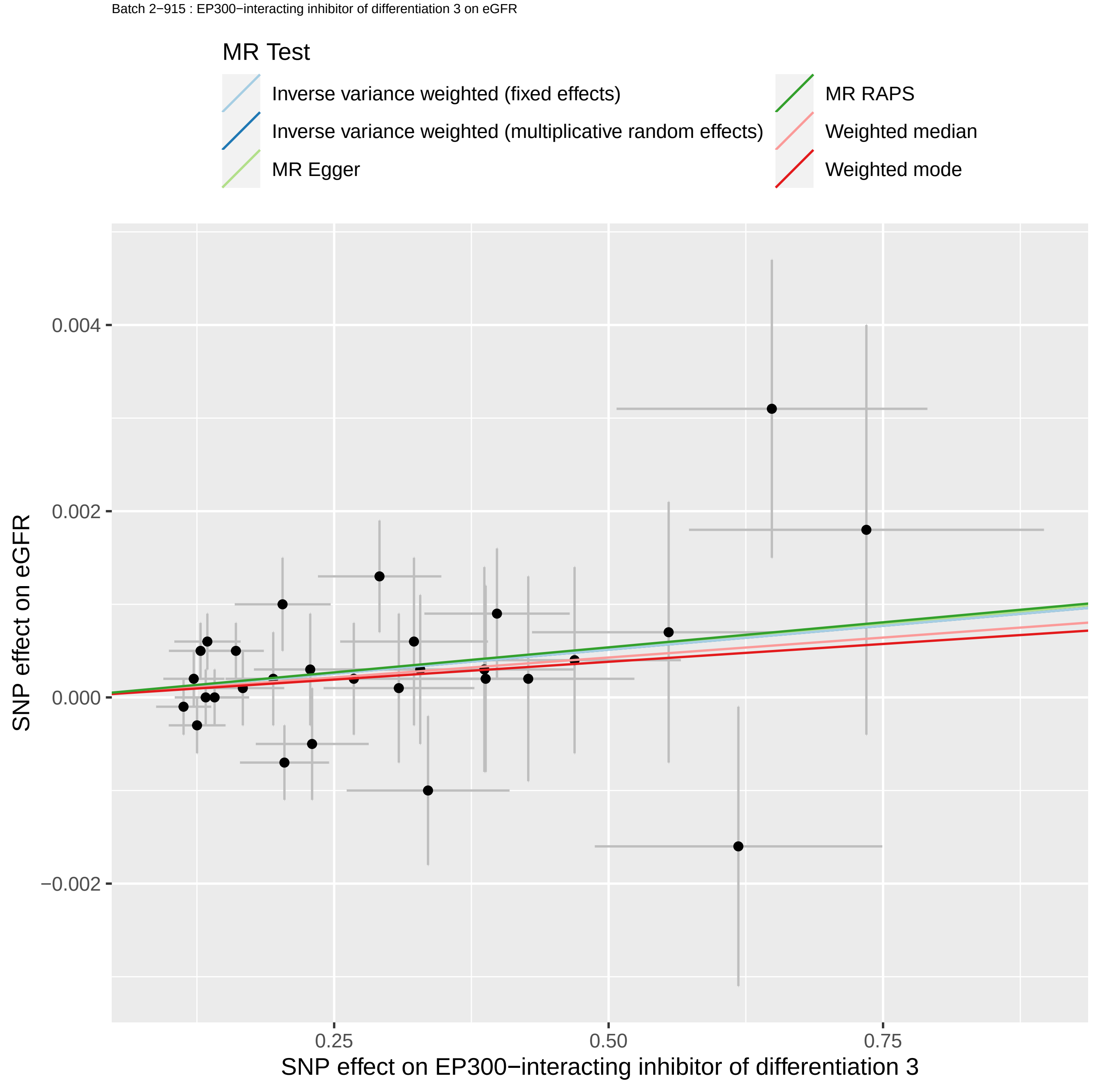

Batch 2−915 : EP300−interacting inhibitor of differentiation 3 on eGFR
MR Test
Inverse variance weighted (fixed effects)
Inverse variance weighted (multiplicative random effects)
MR Egger
MR RAPS
Weighted median
Weighted mode
SNP effect on eGFR
0.004
0.002
0.000
−0.002
0.25
0.50
0.75
SNP effect on EP300−interacting inhibitor of differentiation 3

Batch 2−916 : Probable RNA−binding protein EIF1AD on eGFR

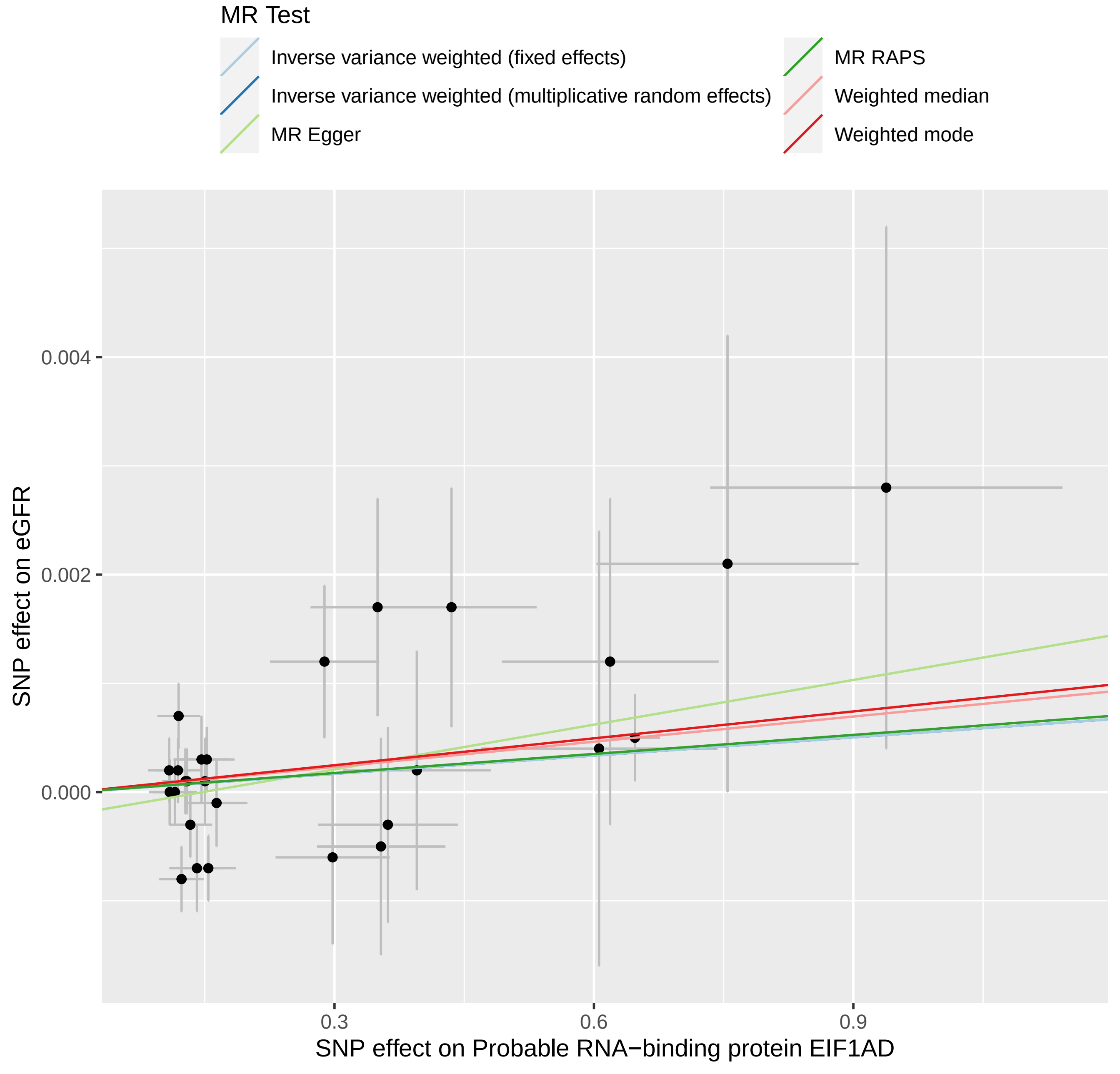

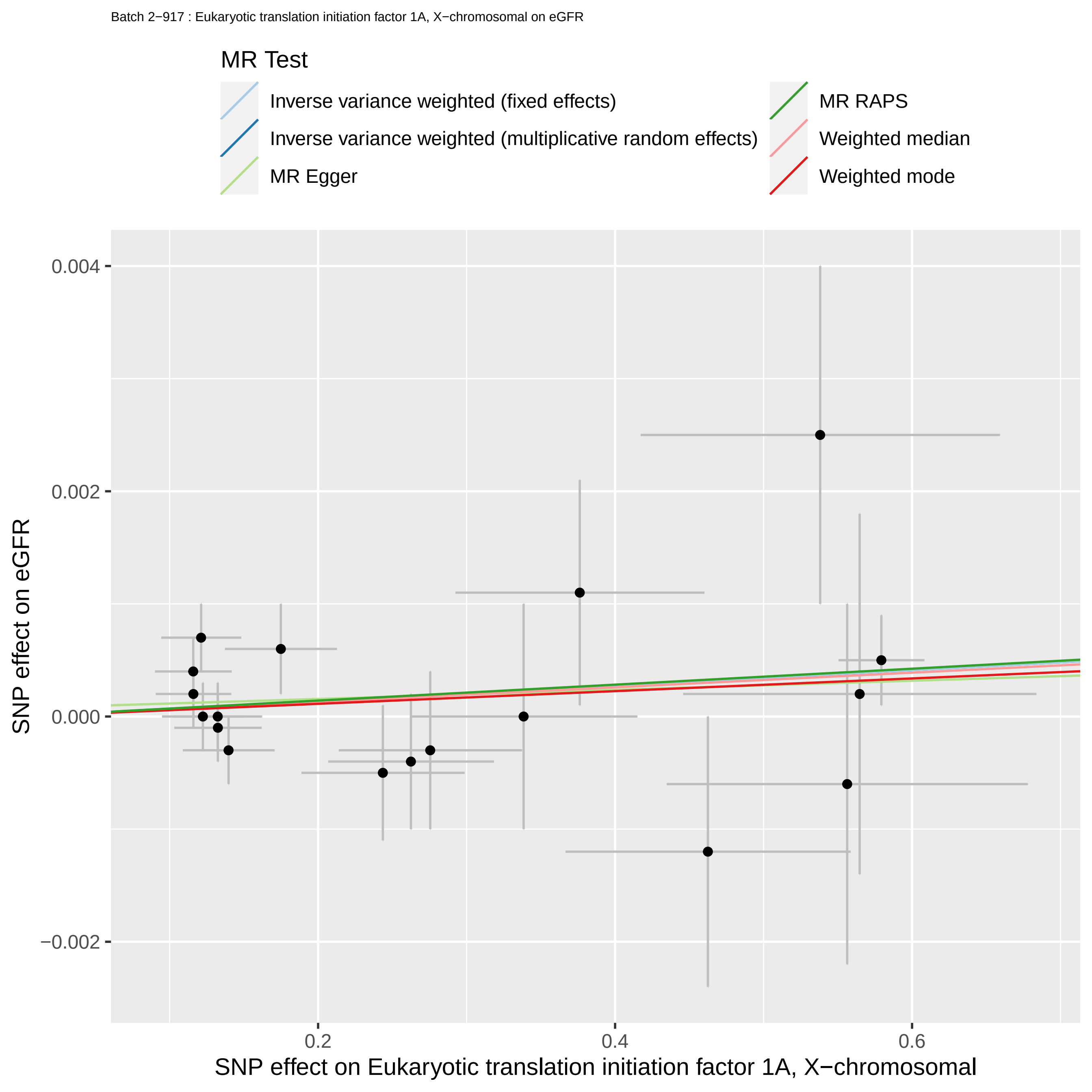

Batch 2−917 : Eukaryotic translation initiation factor 1A, X−chromosomal on eGFR
MR Test
Inverse variance weighted (fixed effects)
Inverse variance weighted (multiplicative random effects)
MR Egger
MR RAPS
Weighted median
Weighted mode
0.004
0.002
0.000
−0.002
0.2
0.4
0.6
SNP effect on eGFR
SNP effect on Eukaryotic translation initiation factor 1A, X−chromosomal

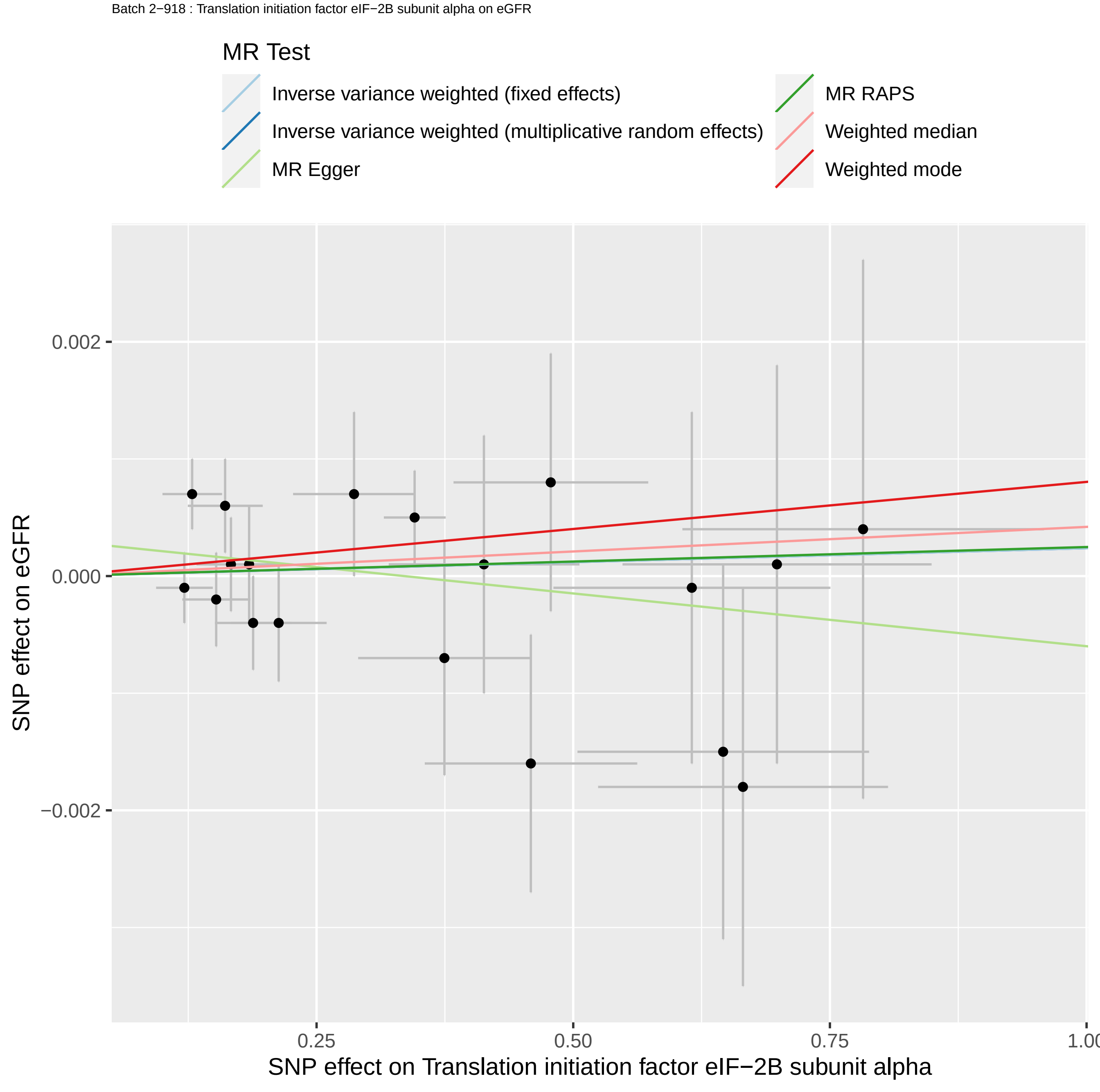

Batch 2−918 : Translation initiation factor eIF−2B subunit alpha on eGFR
MR Test
Inverse variance weighted (fixed effects)
Inverse variance weighted (multiplicative random effects)
MR Egger
MR RAPS
Weighted median
Weighted mode
0.002
0.000
−0.002
0.25
0.50
0.75
1.00
SNP effect on eGFR
SNP effect on Translation initiation factor eIF−2B subunit alpha

Batch 2−919 : Eukaryotic translation initiation factor 3 subunit G on eGFR

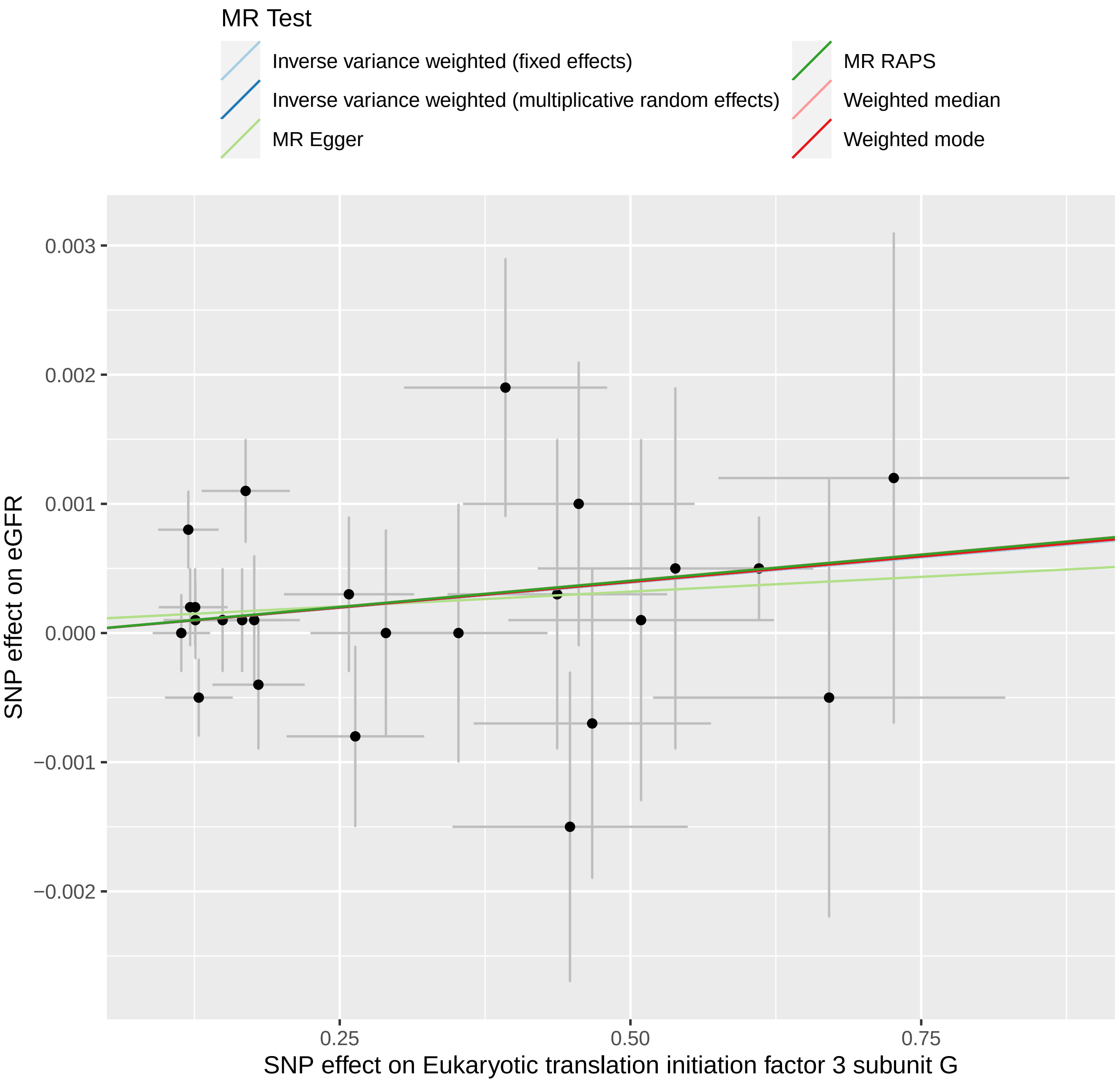

Batch 2−920 : Eukaryotic translation initiation factor 3 subunit J on eGFR

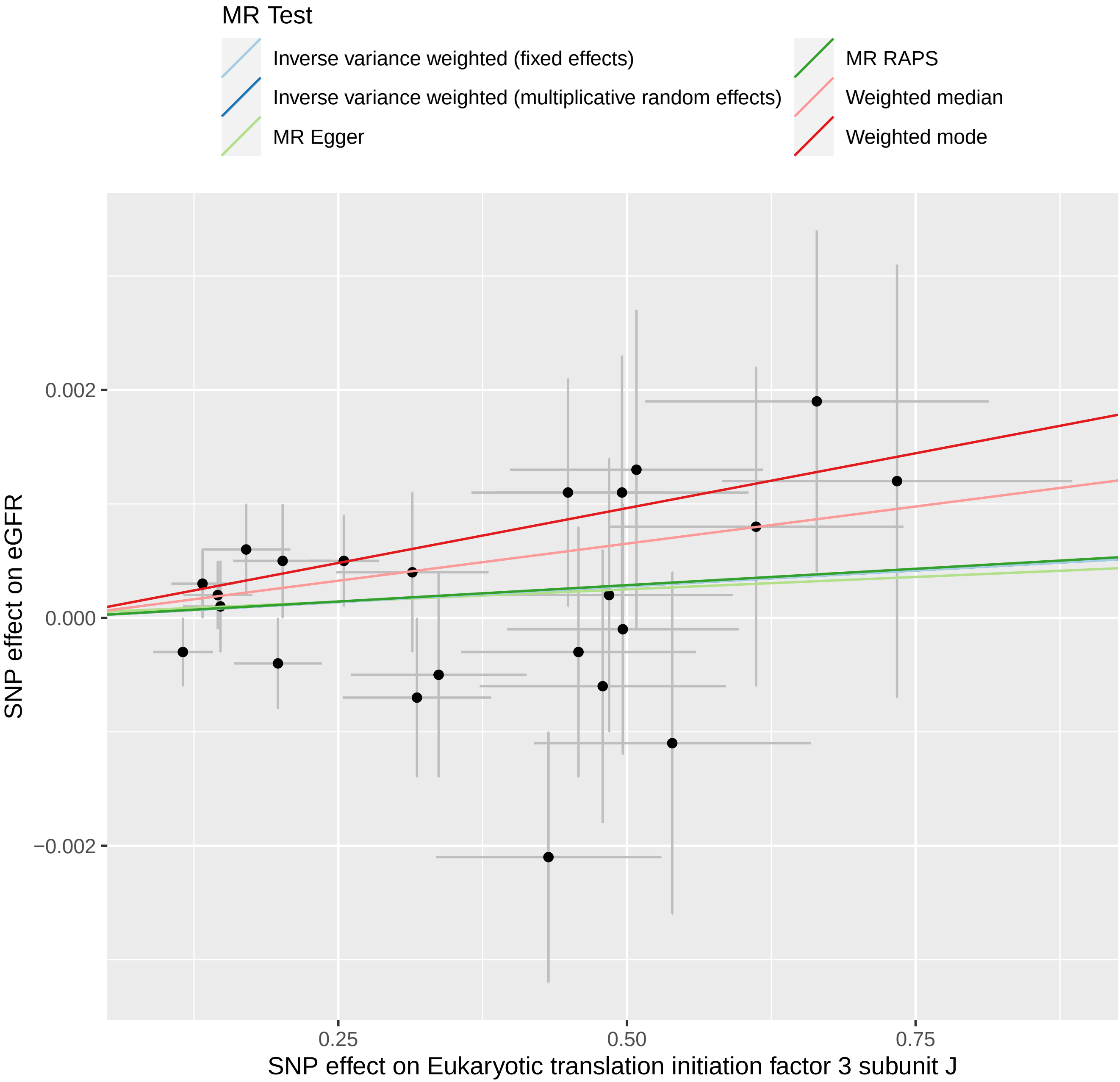

Batch 2−921 : Eukaryotic initiation factor 4A−III on eGFR

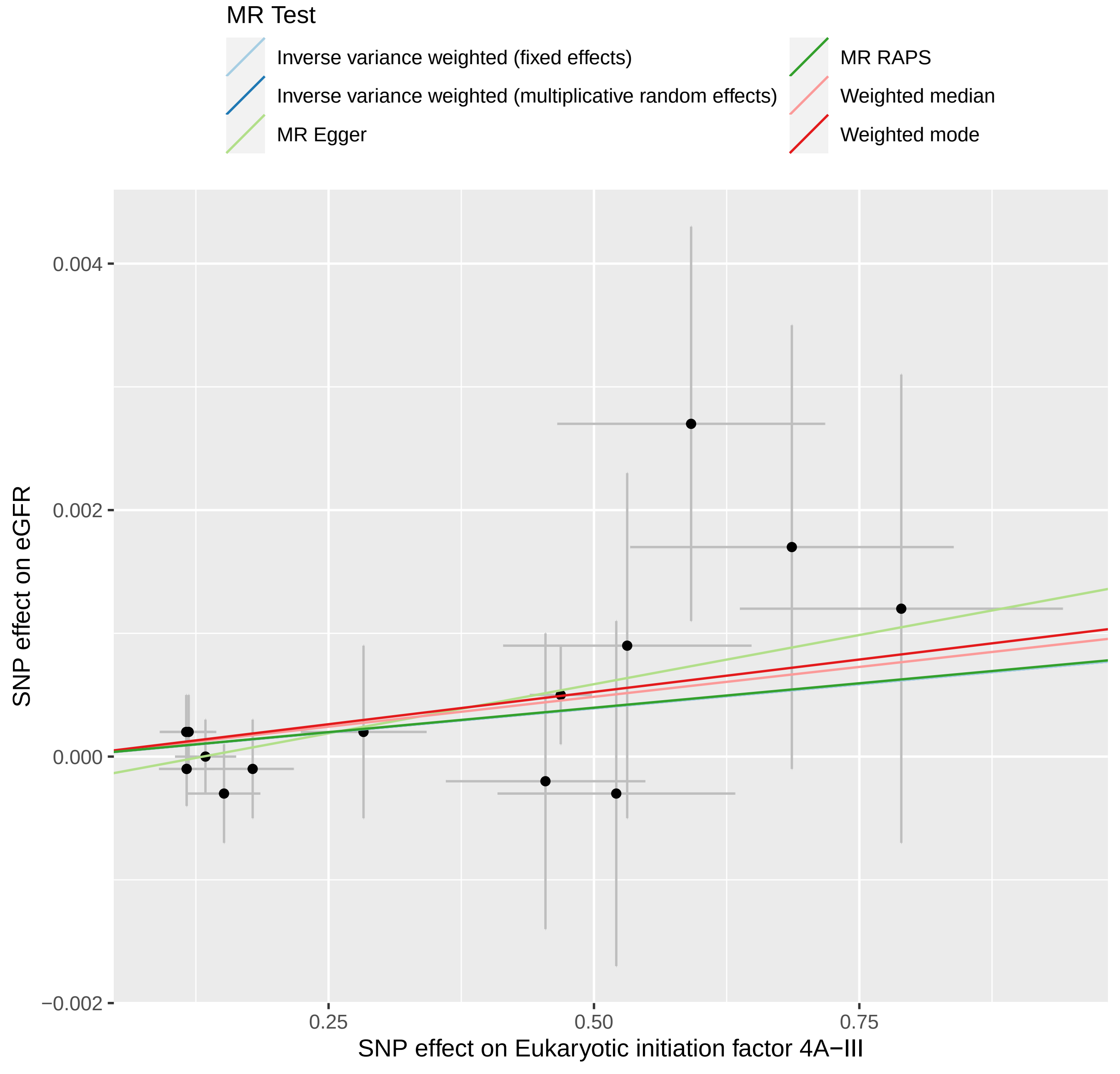

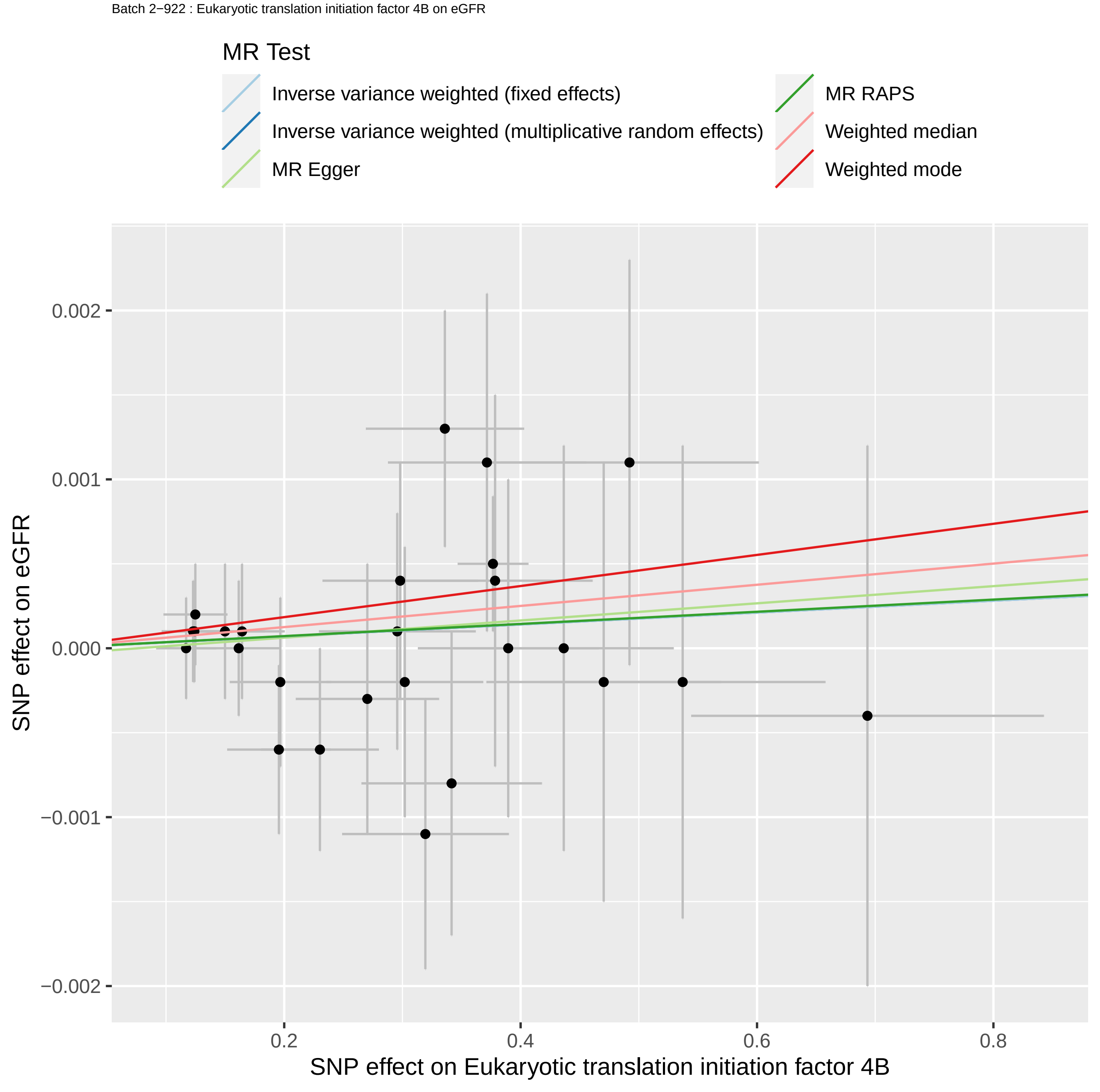
Batch 2−922 : Eukaryotic translation initiation factor 4B on eGFR
MR Test
Inverse variance weighted (fixed effects)
Inverse variance weighted (multiplicative random effects)
MR Egger
MR RAPS
Weighted median
Weighted mode
0.002
0.001
0.000
−0.001
−0.002
0.2
0.4
0.6
0.8
SNP effect on eGFR
SNP effect on Eukaryotic translation initiation factor 4B

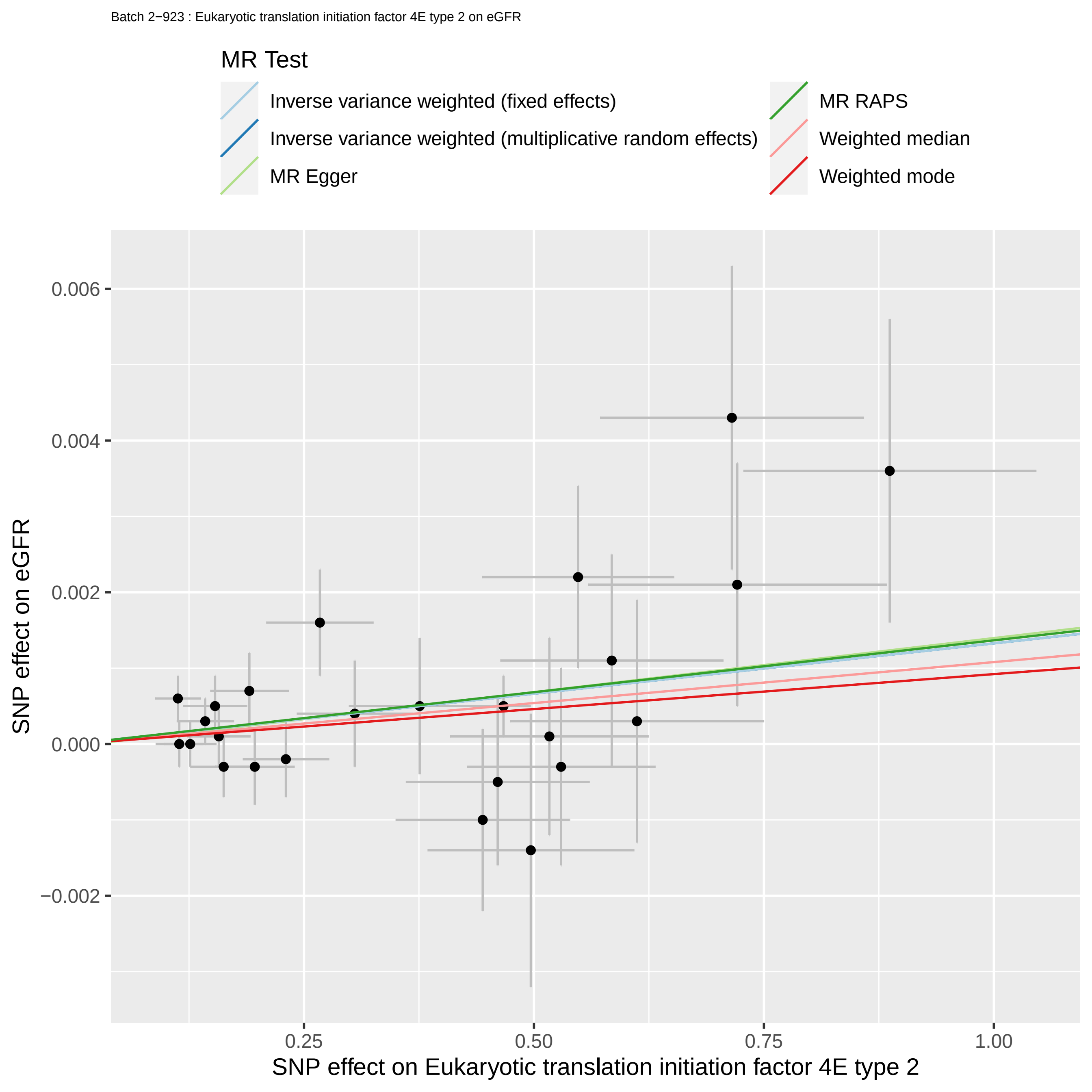
Batch 2−923 : Eukaryotic translation initiation factor 4E type 2 on eGFR
MR Test
Inverse variance weighted (fixed effects)
Inverse variance weighted (multiplicative random effects)
MR Egger
MR RAPS
Weighted median
Weighted mode
0.006
0.004
0.002
0.000
−0.002
SNP effect on eGFR
0.25
0.50
0.75
1.00
SNP effect on Eukaryotic translation initiation factor 4E type 2

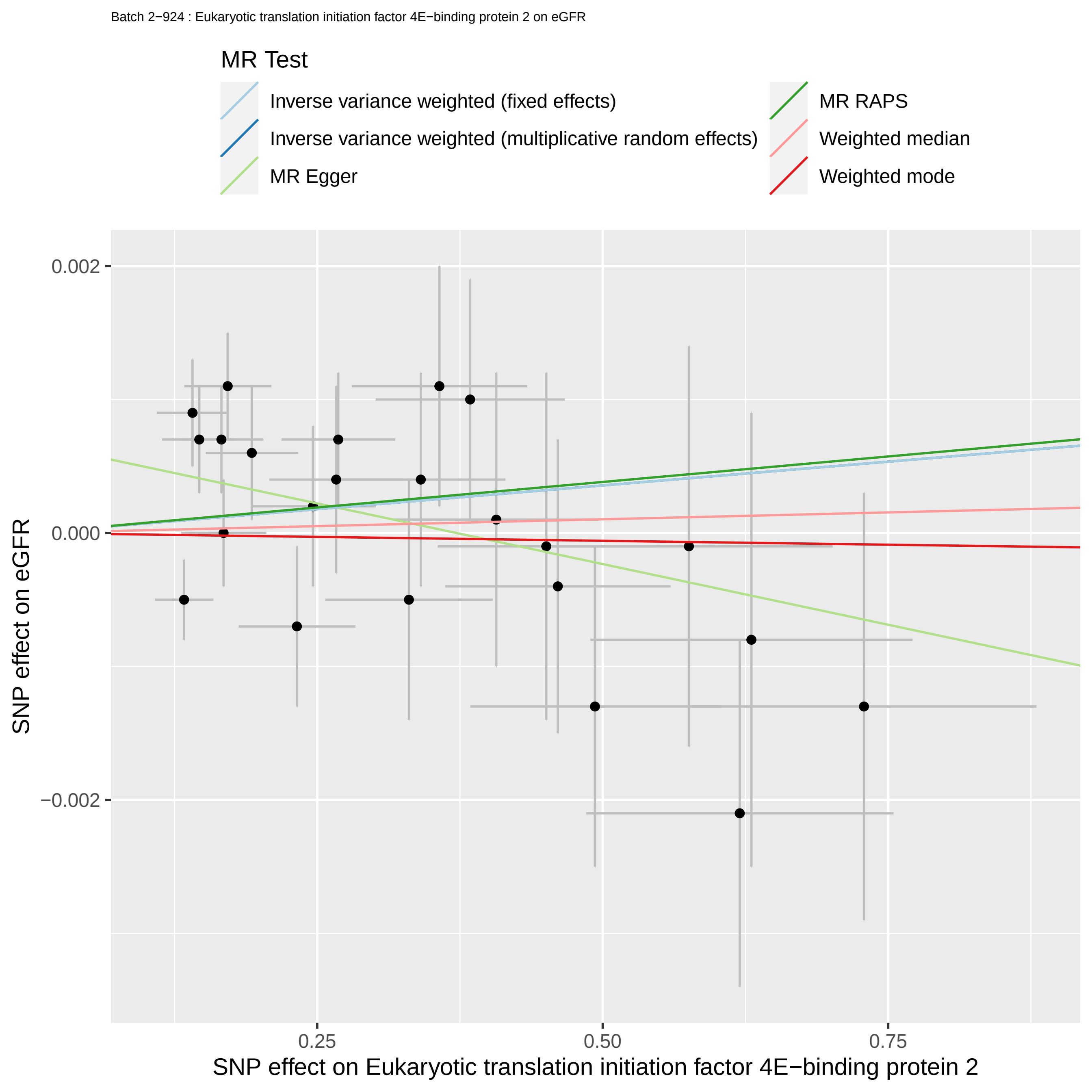
Batch 2−924 : Eukaryotic translation initiation factor 4E−binding protein 2 on eGFR
MR Test
Inverse variance weighted (fixed effects)
Inverse variance weighted (multiplicative random effects)
MR Egger
MR RAPS
Weighted median
Weighted mode
0.002
0.000
−0.002
0.25
0.50
0.75
SNP effect on eGFR
SNP effect on Eukaryotic translation initiation factor 4E−binding protein 2

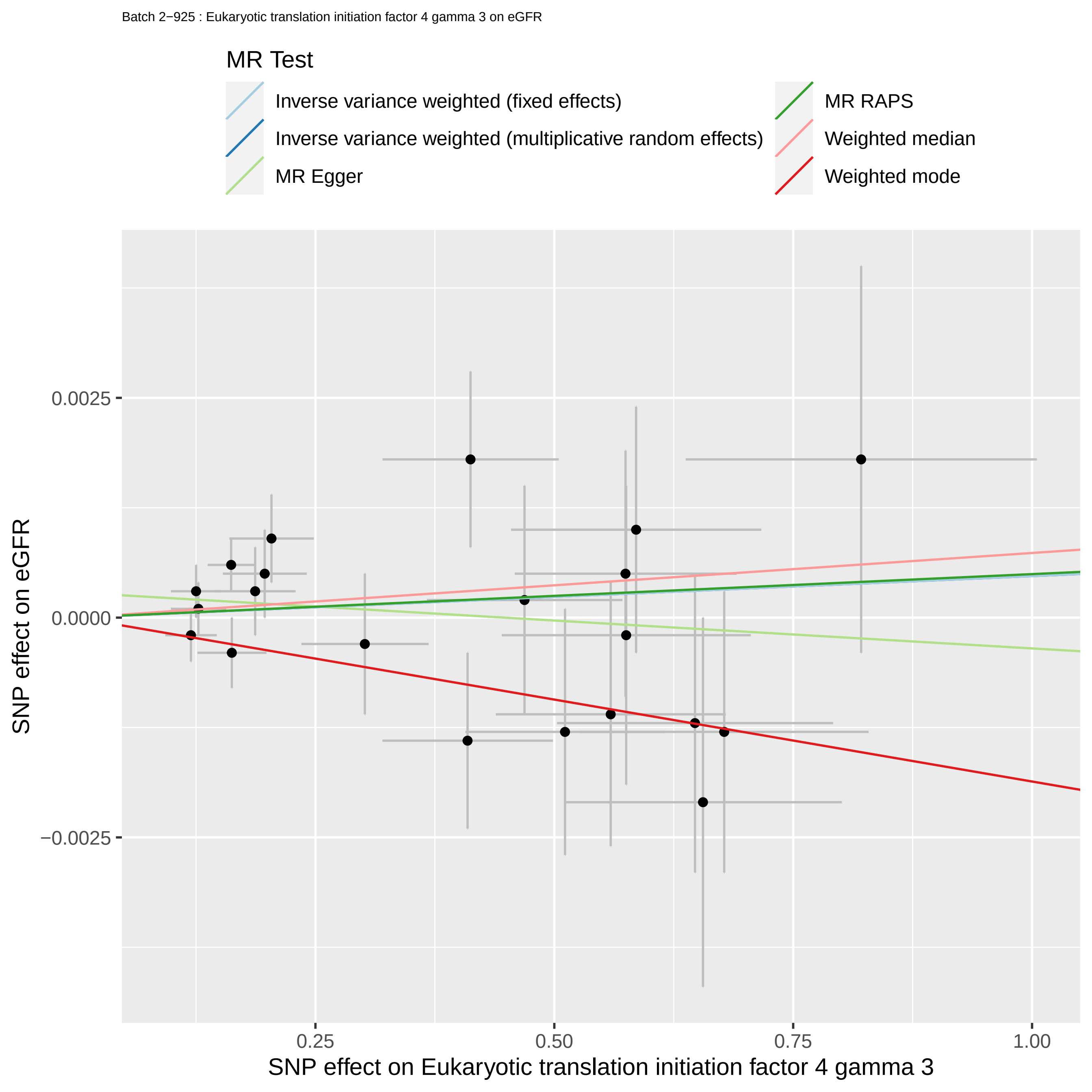
Batch 2−925 : Eukaryotic translation initiation factor 4 gamma 3 on eGFR
MR Test
Inverse variance weighted (fixed effects)
Inverse variance weighted (multiplicative random effects)
MR Egger
MR RAPS
Weighted median
Weighted mode
0.0025
0.0000
−0.0025
0.25
0.50
0.75
1.00
SNP effect on eGFR
SNP effect on Eukaryotic translation initiation factor 4 gamma 3

Batch 2−926 : Eukaryotic translation initiation factor 5 on eGFR

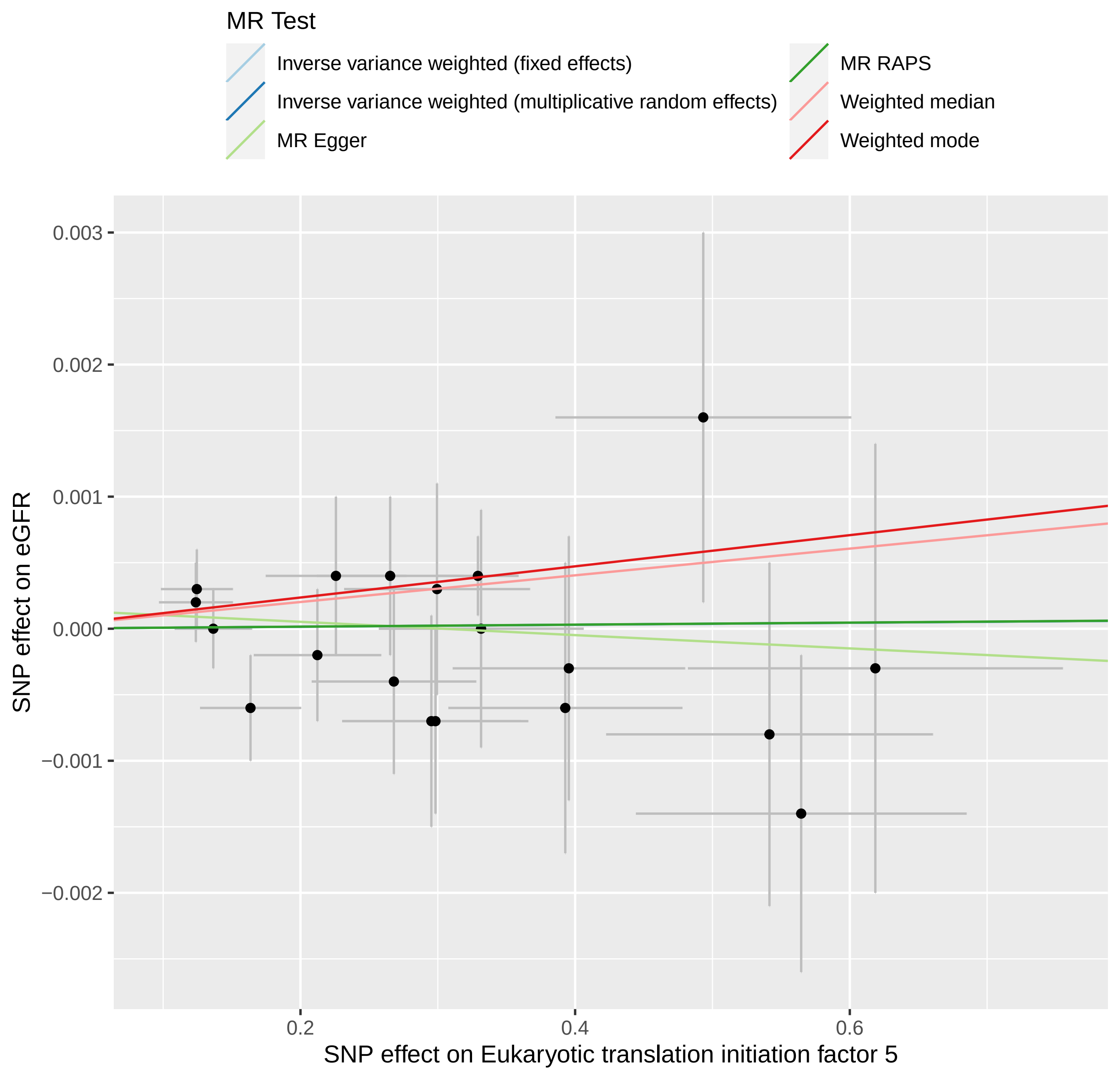

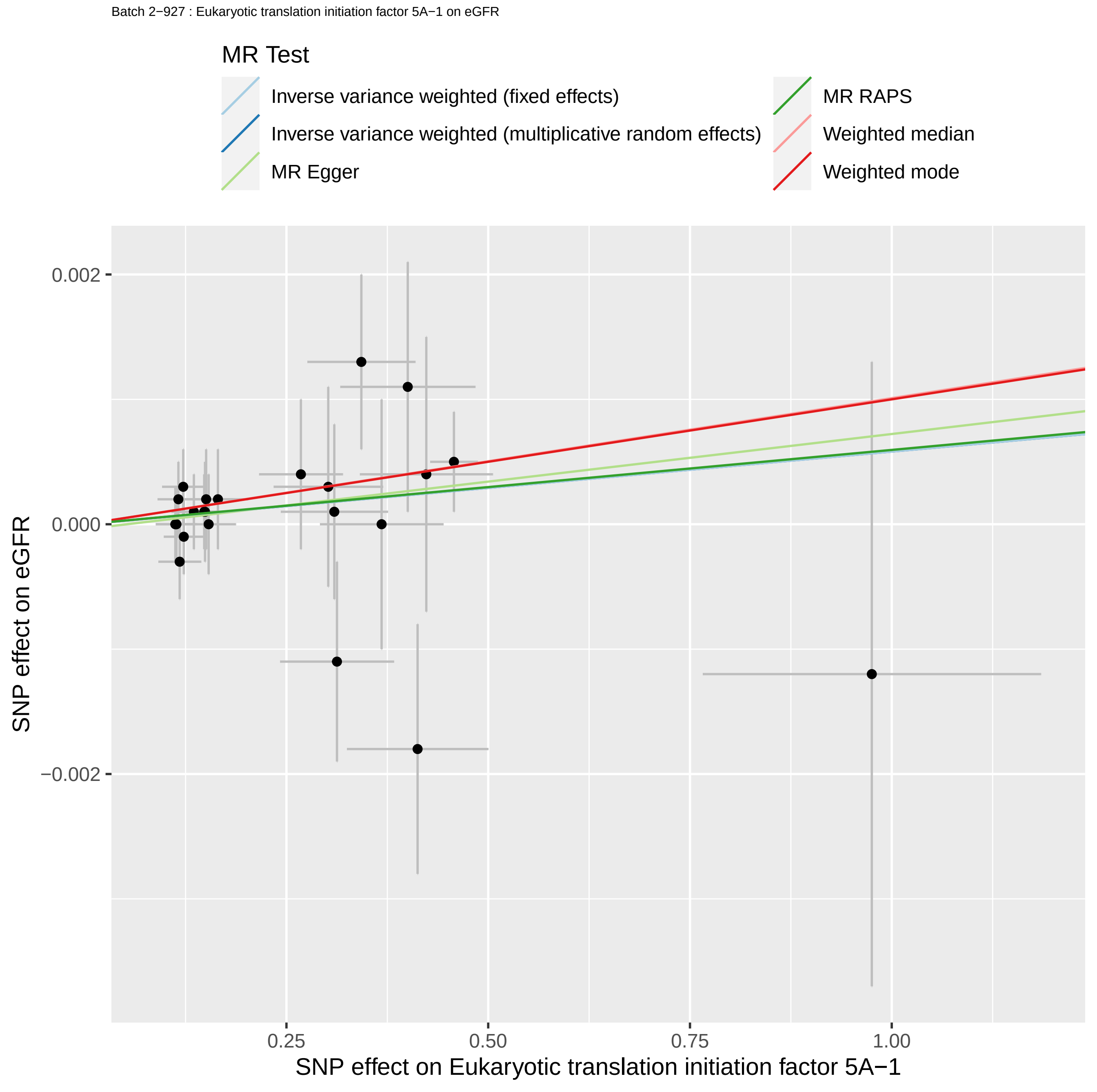

Batch 2−927 : Eukaryotic translation initiation factor 5A−1 on eGFR
MR Test
Inverse variance weighted (fixed effects)
Inverse variance weighted (multiplicative random effects)
MR Egger
MR RAPS
Weighted median
Weighted mode
0.002
0.000
−0.002
0.25
0.50
0.75
1.00
SNP effect on eGFR
SNP effect on Eukaryotic translation initiation factor 5A−1

Batch 2−928 : ELAV−like protein 1 on eGFR

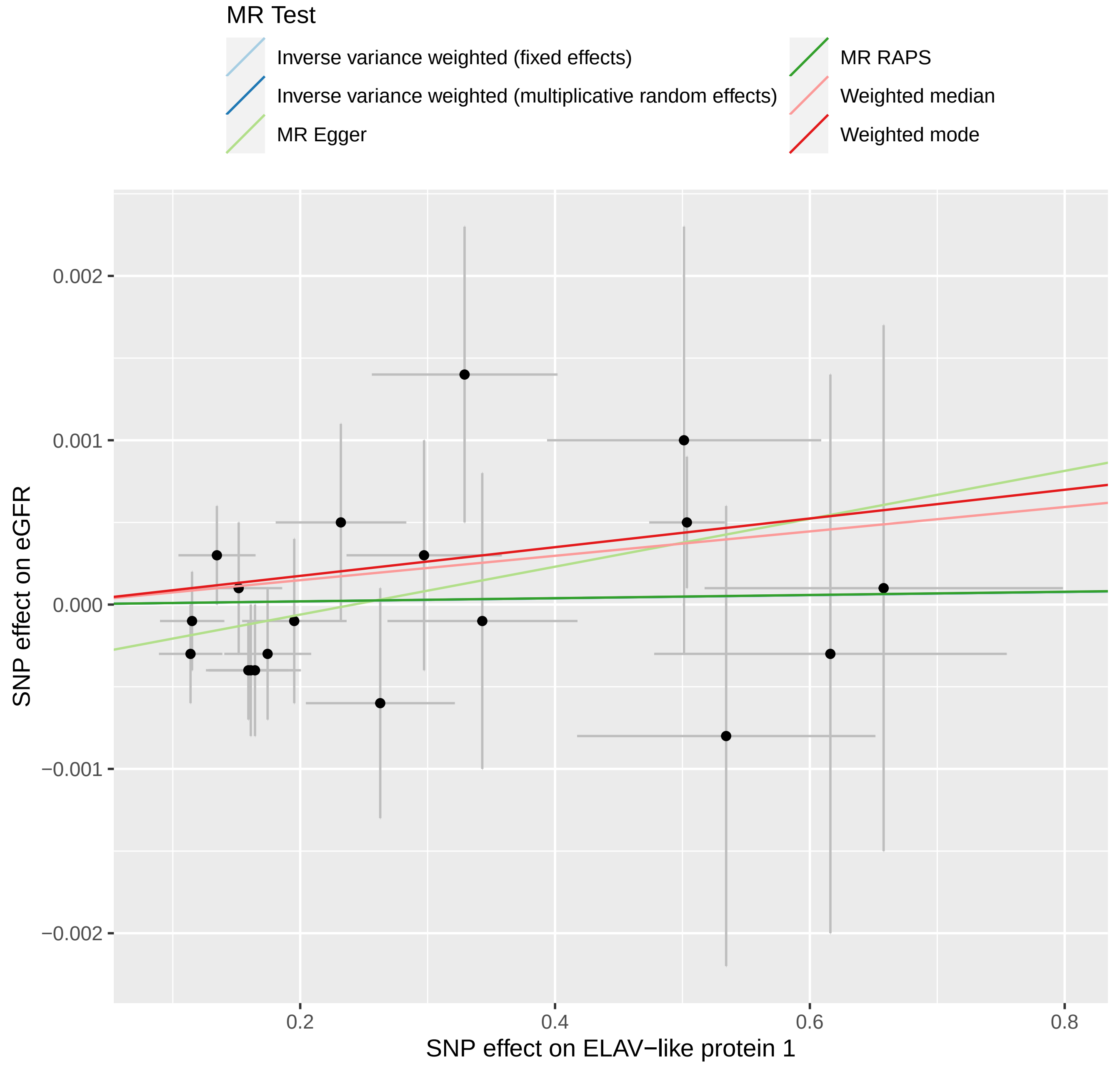

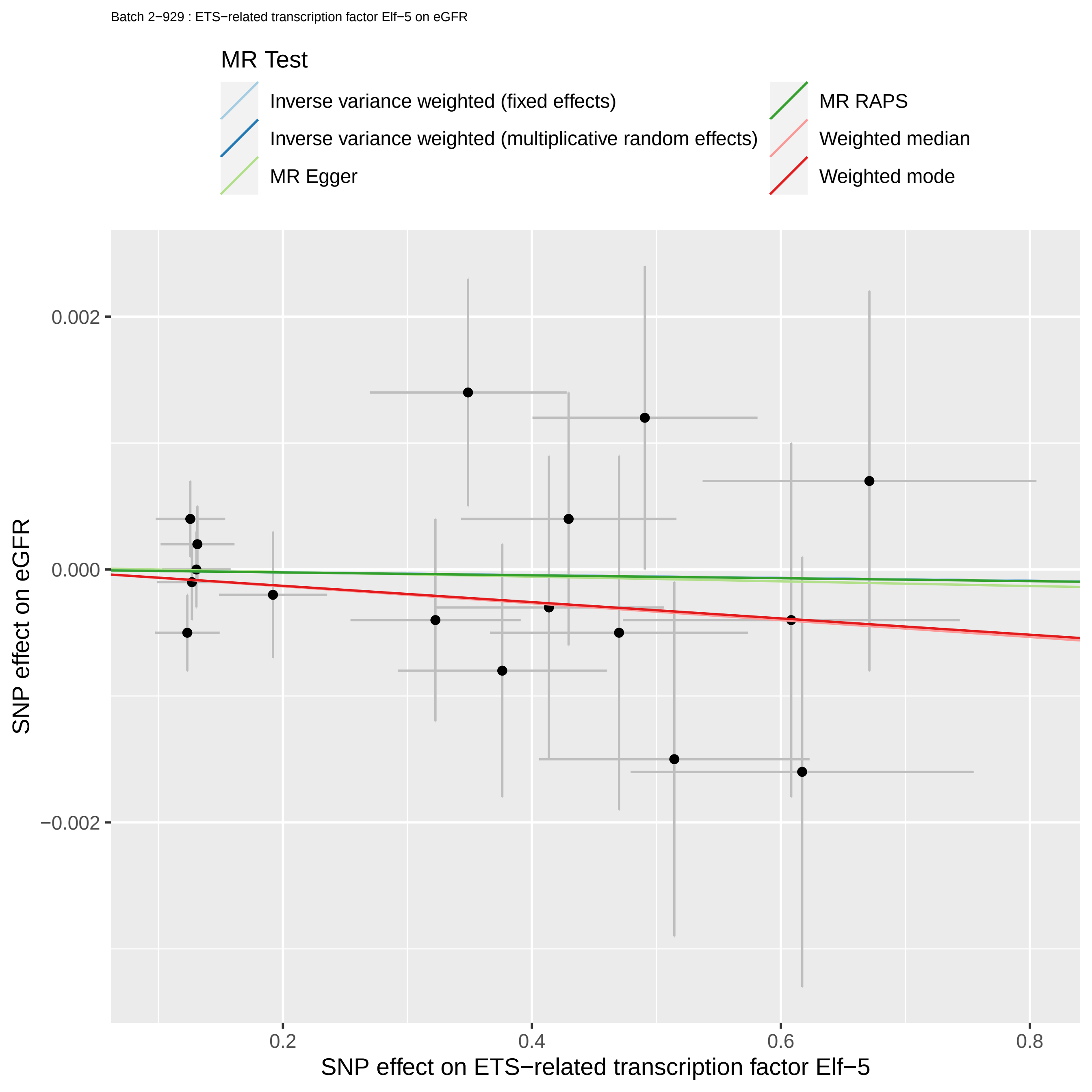
Batch 2−929 : ETS−related transcription factor Elf−5 on eGFR
MR Test
Inverse variance weighted (fixed effects)
Inverse variance weighted (multiplicative random effects)
MR Egger
MR RAPS
Weighted median
Weighted mode
0.002
0.000
−0.002
0.2
0.4
0.6
0.8
SNP effect on eGFR
SNP effect on ETS−related transcription factor Elf−5

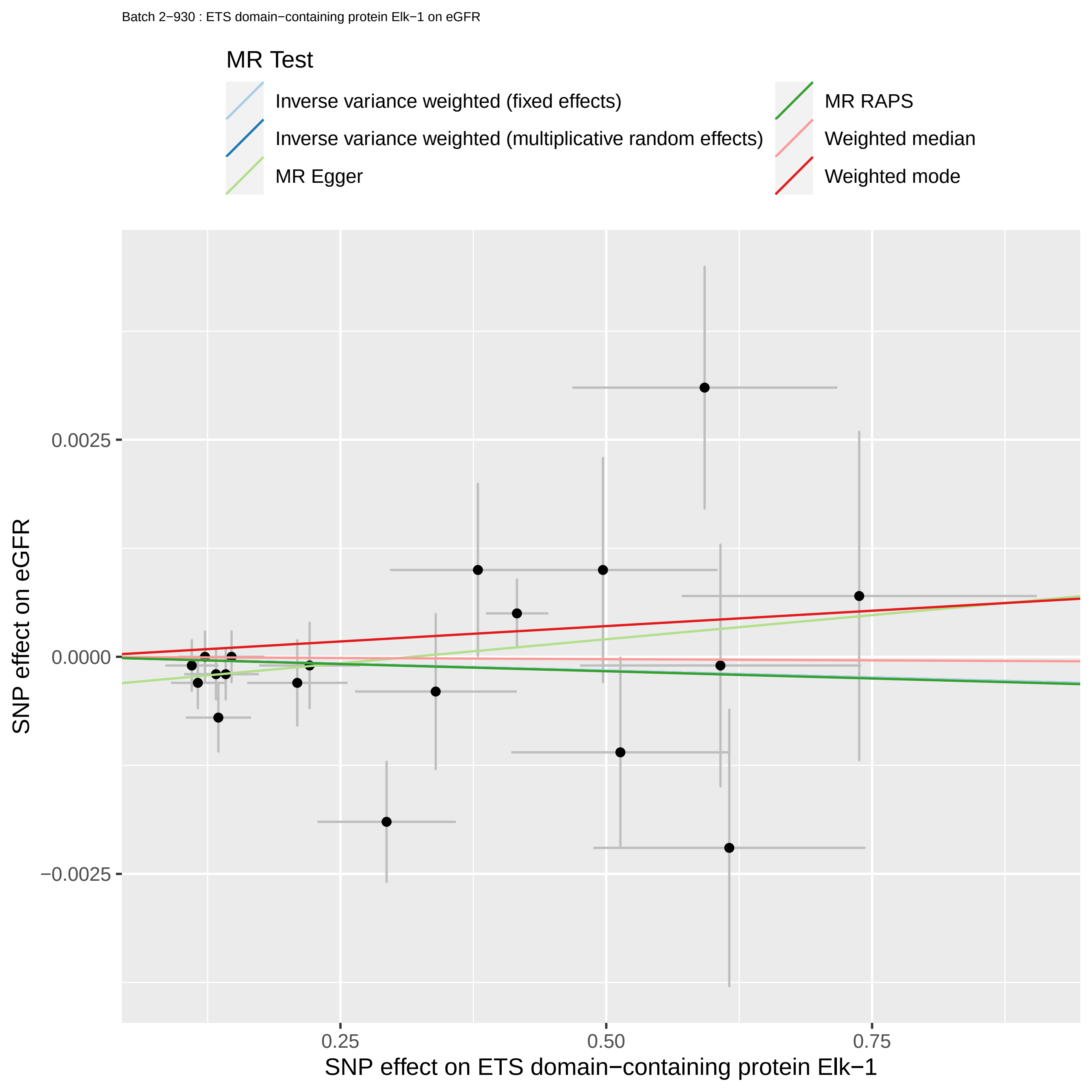

Batch 2−930 : ETS domain−containing protein Elk−1 on eGFR
MR Test
Inverse variance weighted (fixed effects)
Inverse variance weighted (multiplicative random effects)
MR Egger
MR RAPS
Weighted median
Weighted mode
0.0025
0.0000
−0.0025
0.25
0.50
0.75
SNP effect on eGFR
SNP effect on ETS domain−containing protein Elk−1

Batch 2−931 : ETS domain−containing protein Elk−3 on eGFR

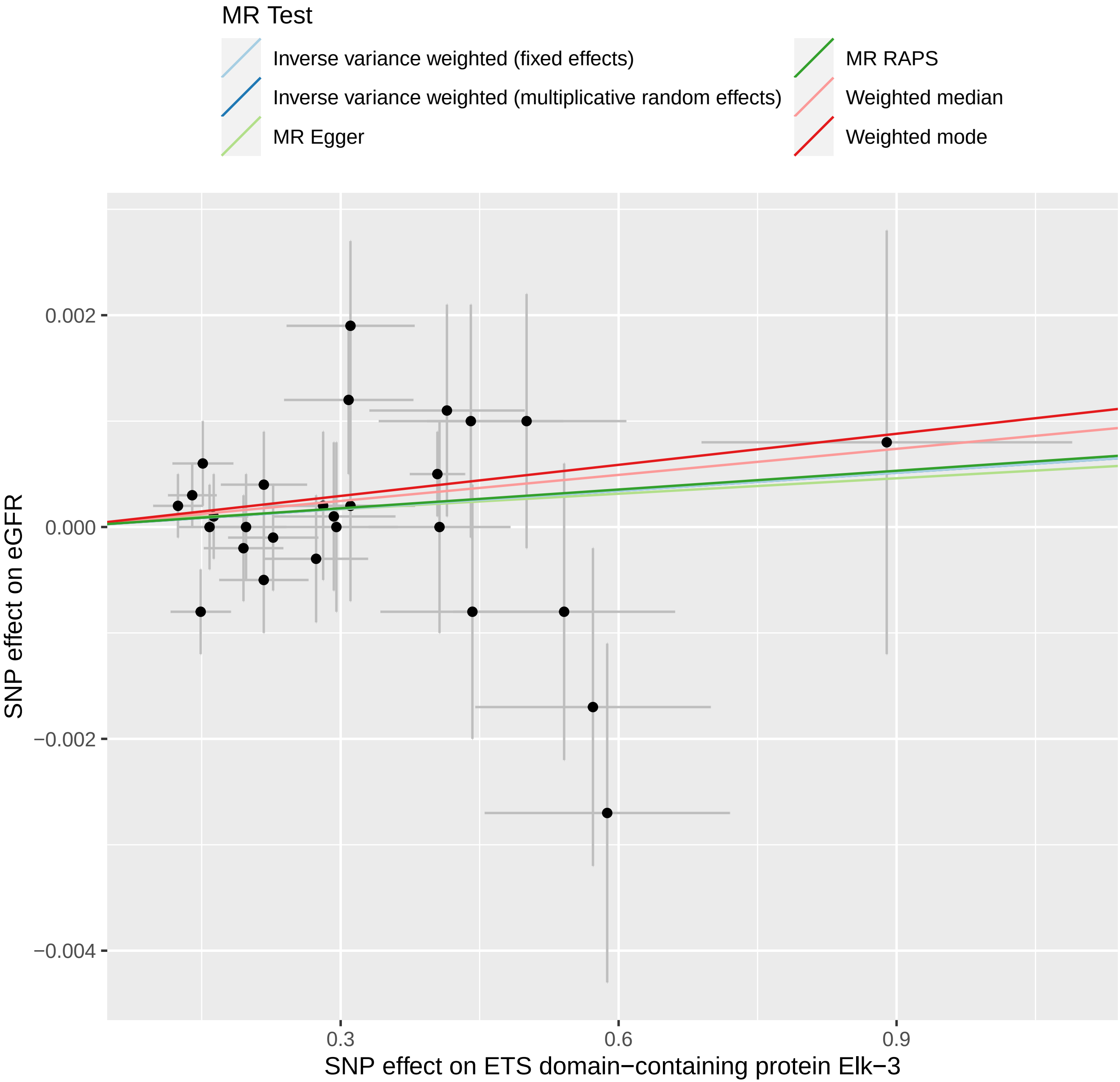

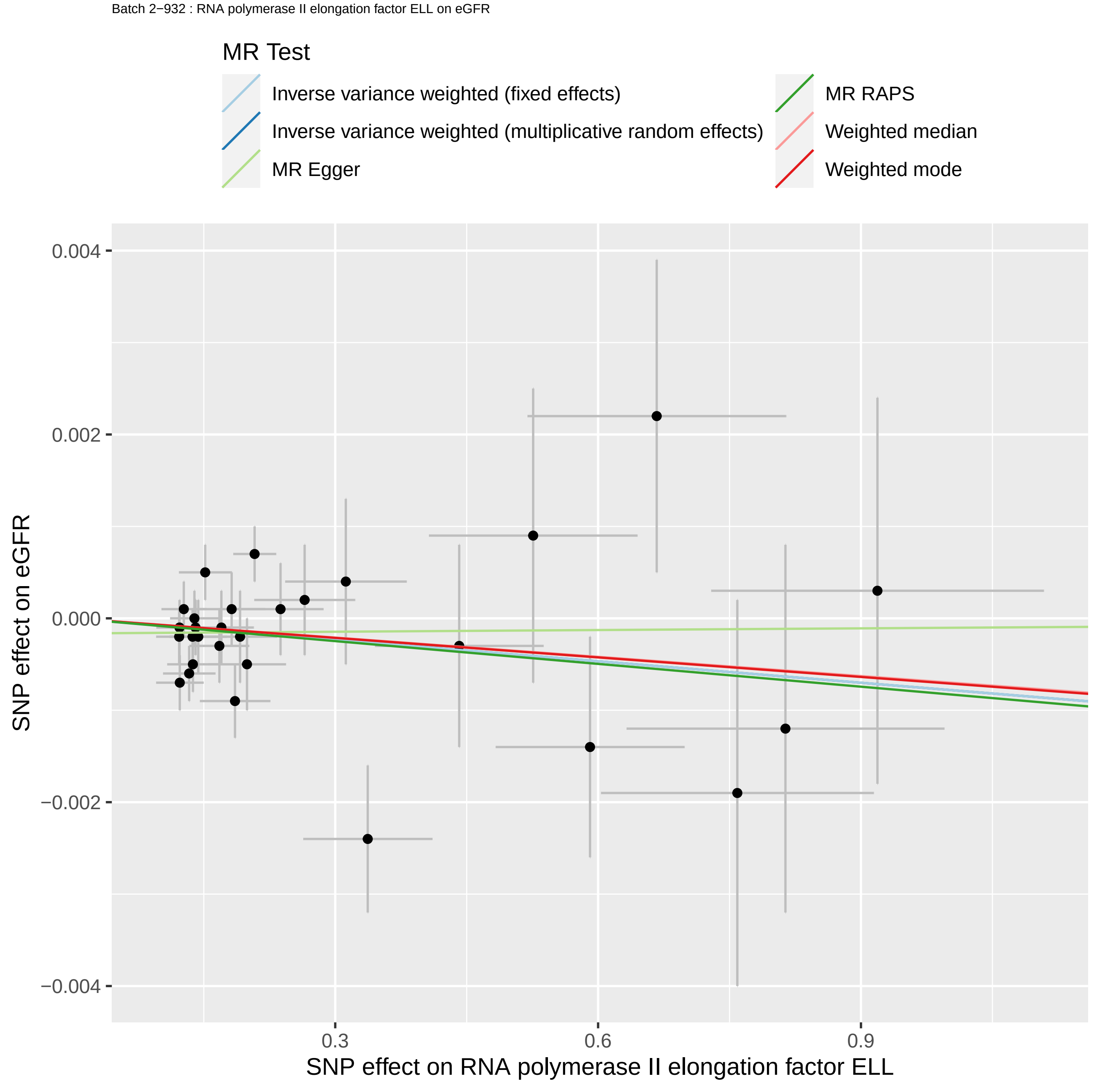
Batch 2−932 : RNA polymerase II elongation factor ELL on eGFR
MR Test
Inverse variance weighted (fixed effects)
Inverse variance weighted (multiplicative random effects)
MR Egger
MR RAPS
Weighted median
Weighted mode
0.004
0.002
0.000
−0.002
−0.004
SNP effect on eGFR
0.3
0.6
0.9
SNP effect on RNA polymerase II elongation factor ELL

Batch 2−933 : RNA polymerase II elongation factor ELL2 on eGFR

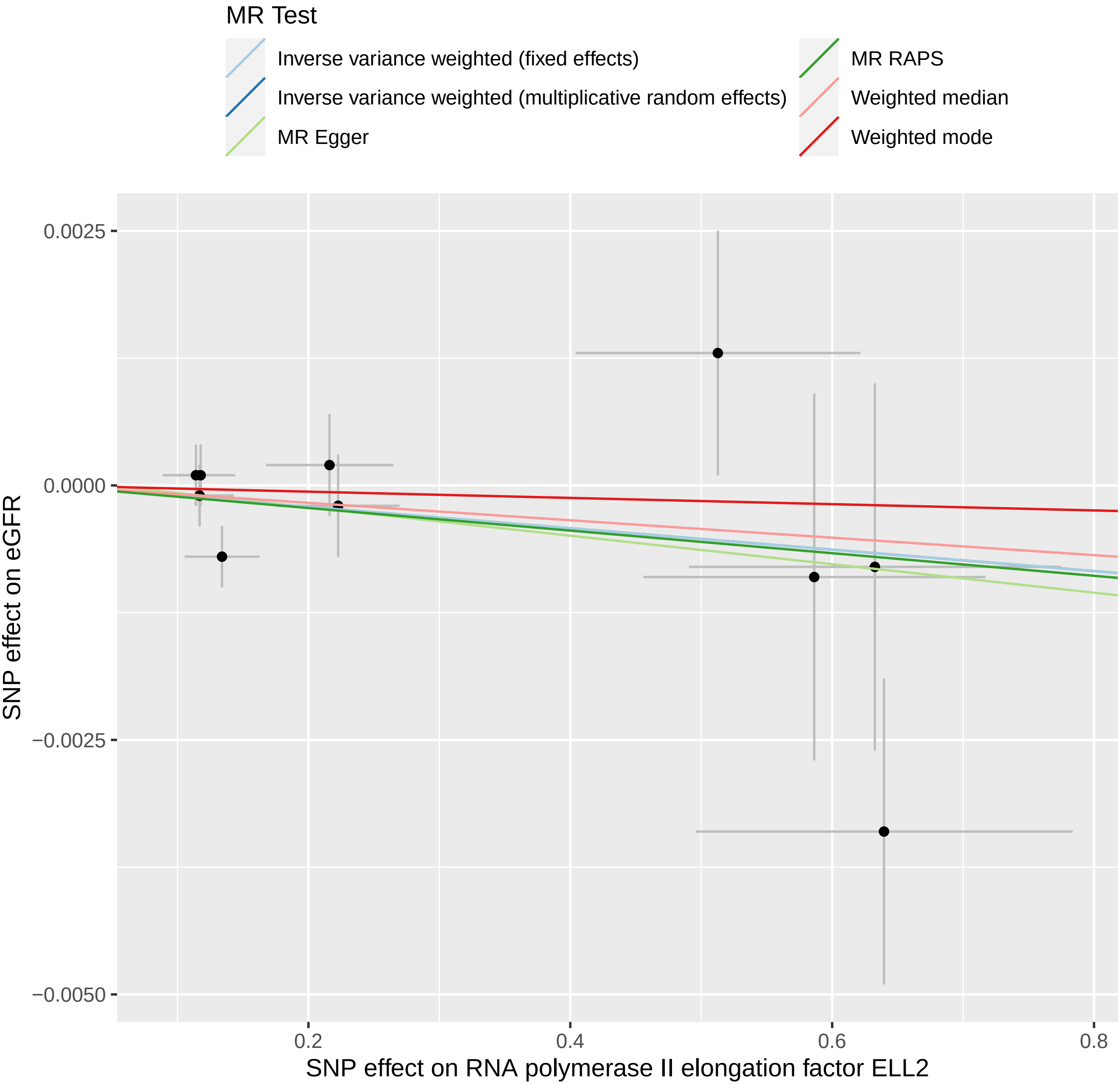

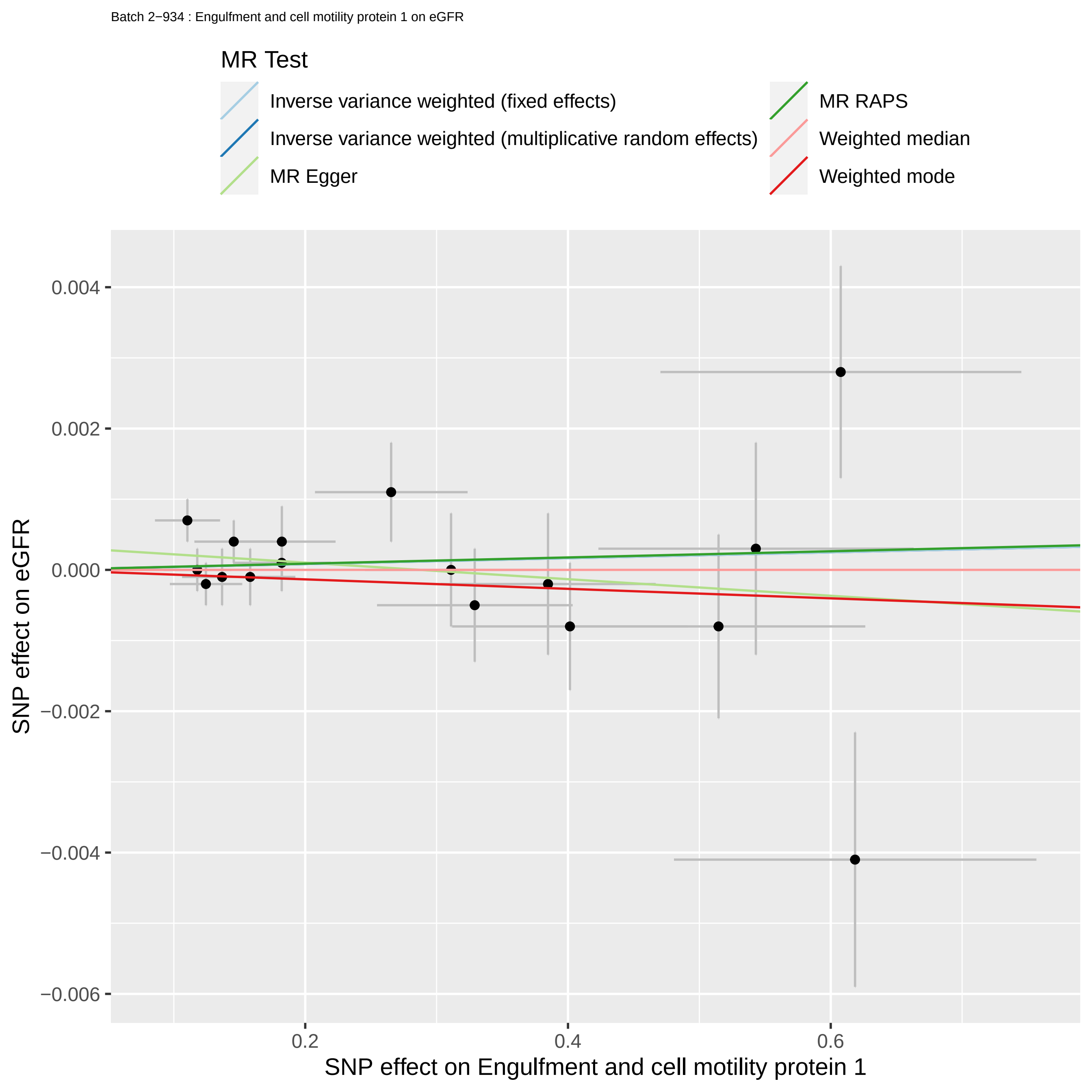

Batch 2−934 : Engulfment and cell motility protein 1 on eGFR
MR Test
Inverse variance weighted (fixed effects)
Inverse variance weighted (multiplicative random effects)
MR Egger
MR RAPS
Weighted median
Weighted mode
0.004
0.002
0.000
−0.002
−0.004
−0.006
SNP effect on eGFR
0.2
0.4
0.6
SNP effect on Engulfment and cell motility protein 1

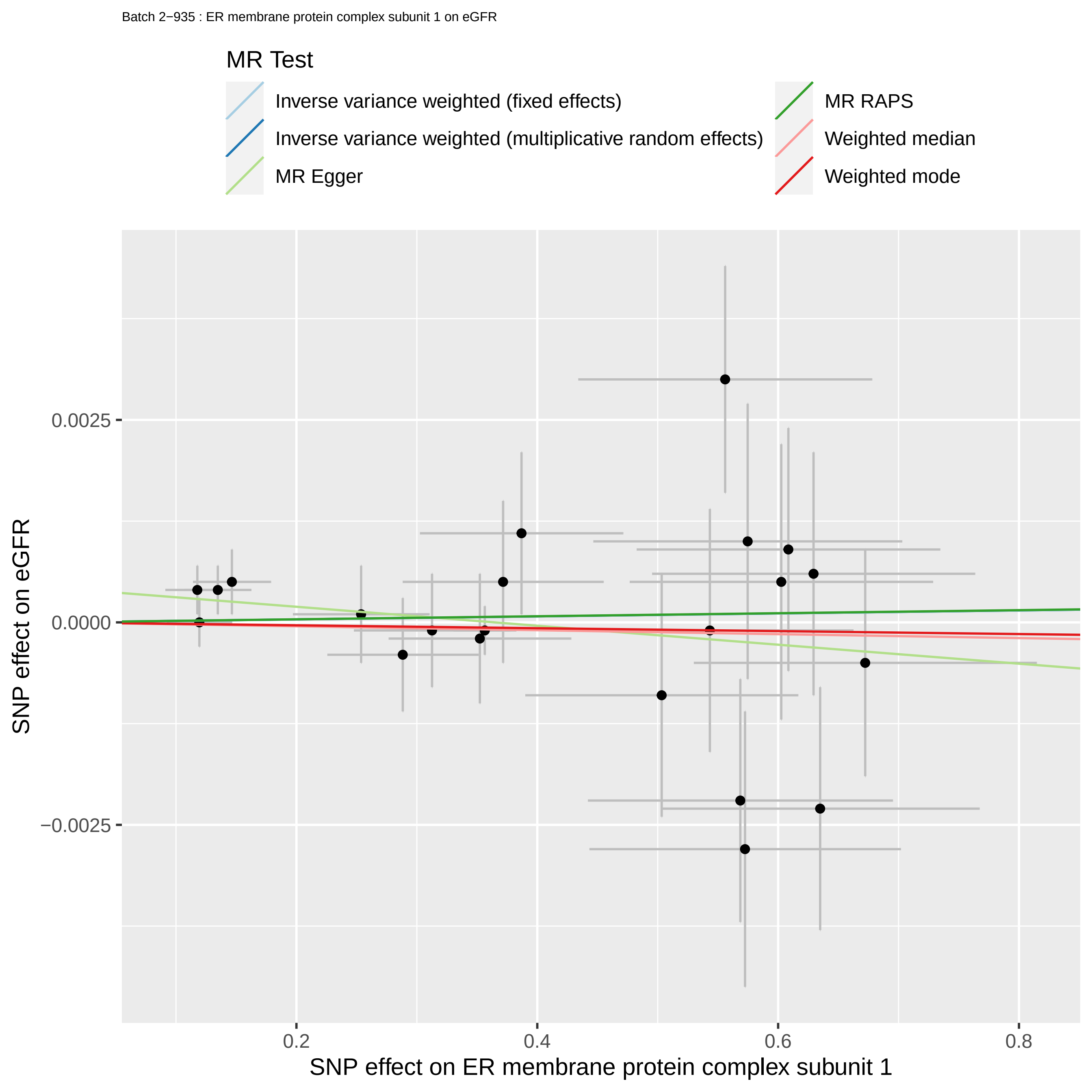
Batch 2−935 : ER membrane protein complex subunit 1 on eGFR
MR Test
Inverse variance weighted (fixed effects)
Inverse variance weighted (multiplicative random effects)
MR Egger
MR RAPS
Weighted median
Weighted mode
SNP effect on eGFR
0.0025
0.0000
−0.0025
0.2
0.4
0.6
0.8
SNP effect on ER membrane protein complex subunit 1

Batch 2−936 : ER membrane protein complex subunit 4 on eGFR

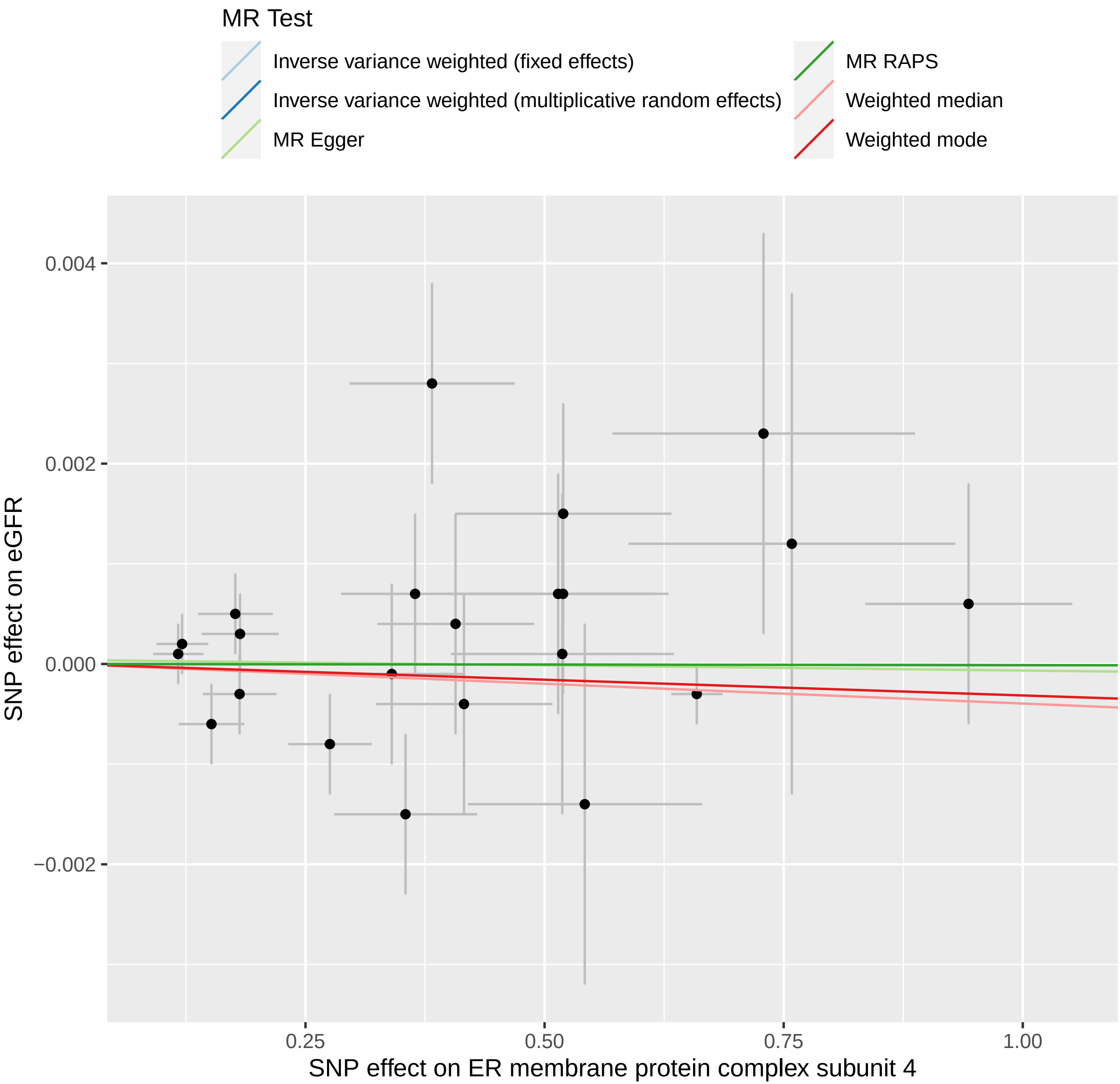

Batch 2−937 : EMI domain−containing protein 1 on eGFR

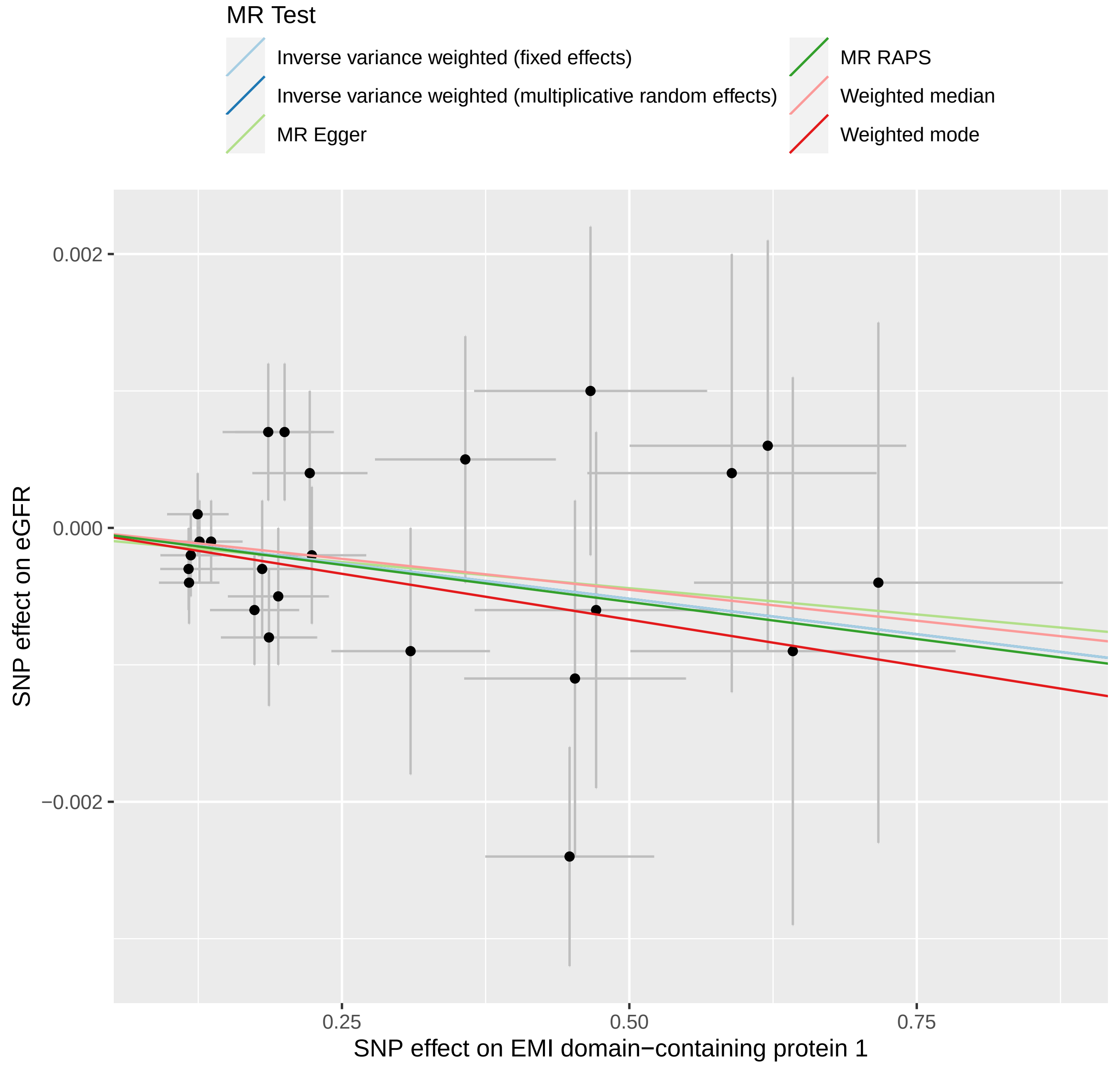

Batch 2−938 : EMILIN−3 on eGFR

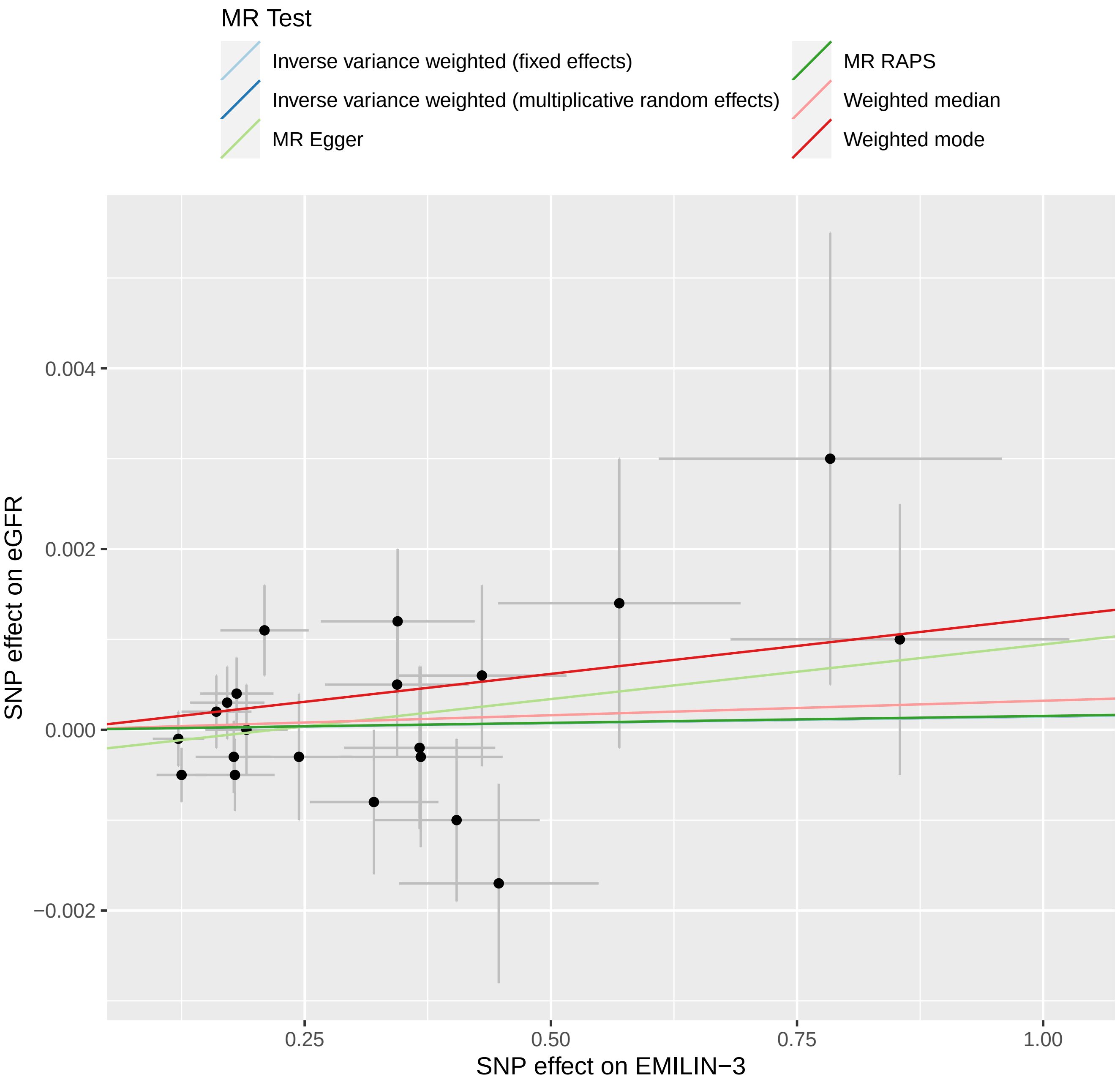

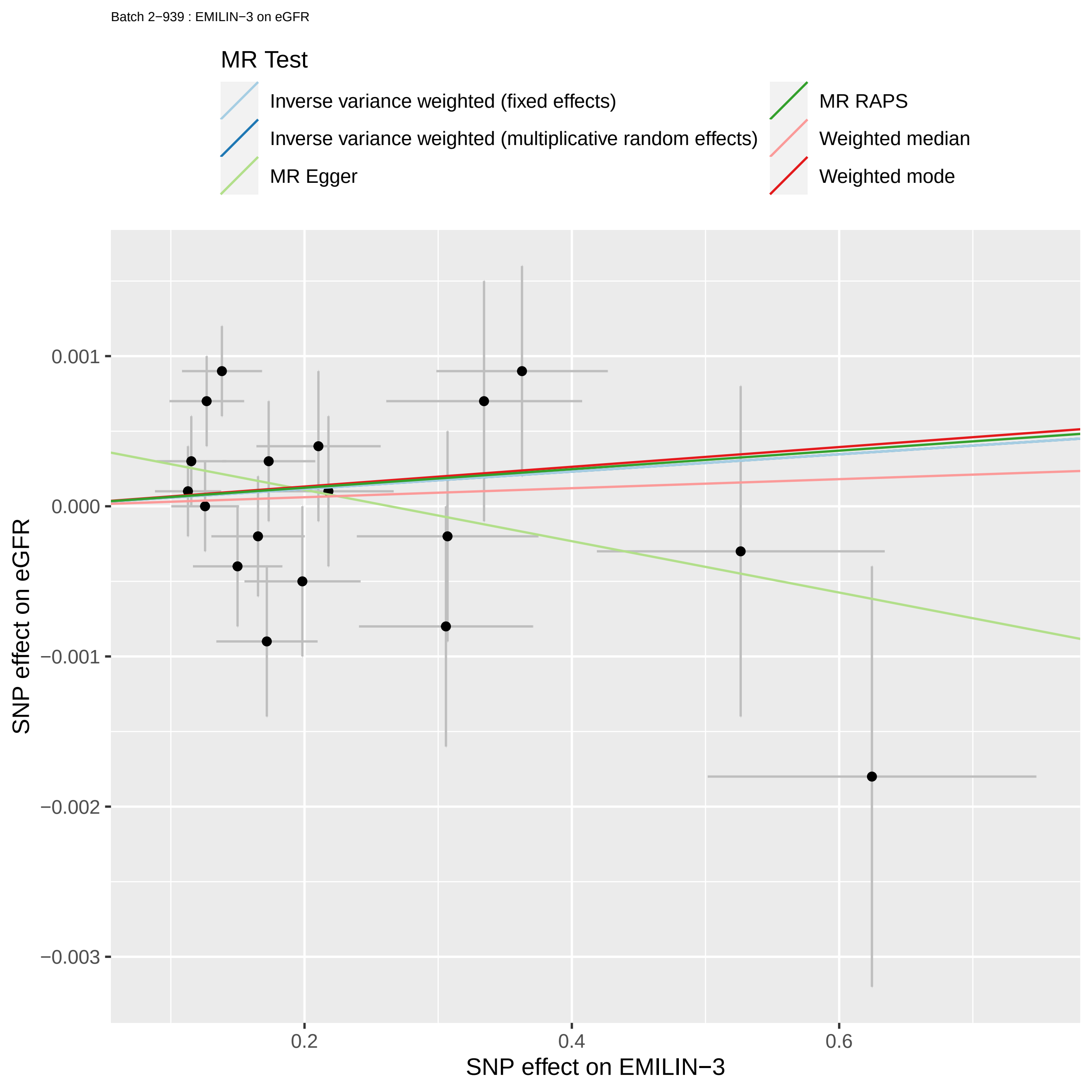

Batch 2−939 : EMILIN−3 on eGFR
MR Test
Inverse variance weighted (fixed effects)
Inverse variance weighted (multiplicative random effects)
MR Egger
MR RAPS
Weighted median
Weighted mode
0.001
0.000
−0.001
−0.002
−0.003
0.2
0.4
0.6
SNP effect on eGFR
SNP effect on EMILIN−3

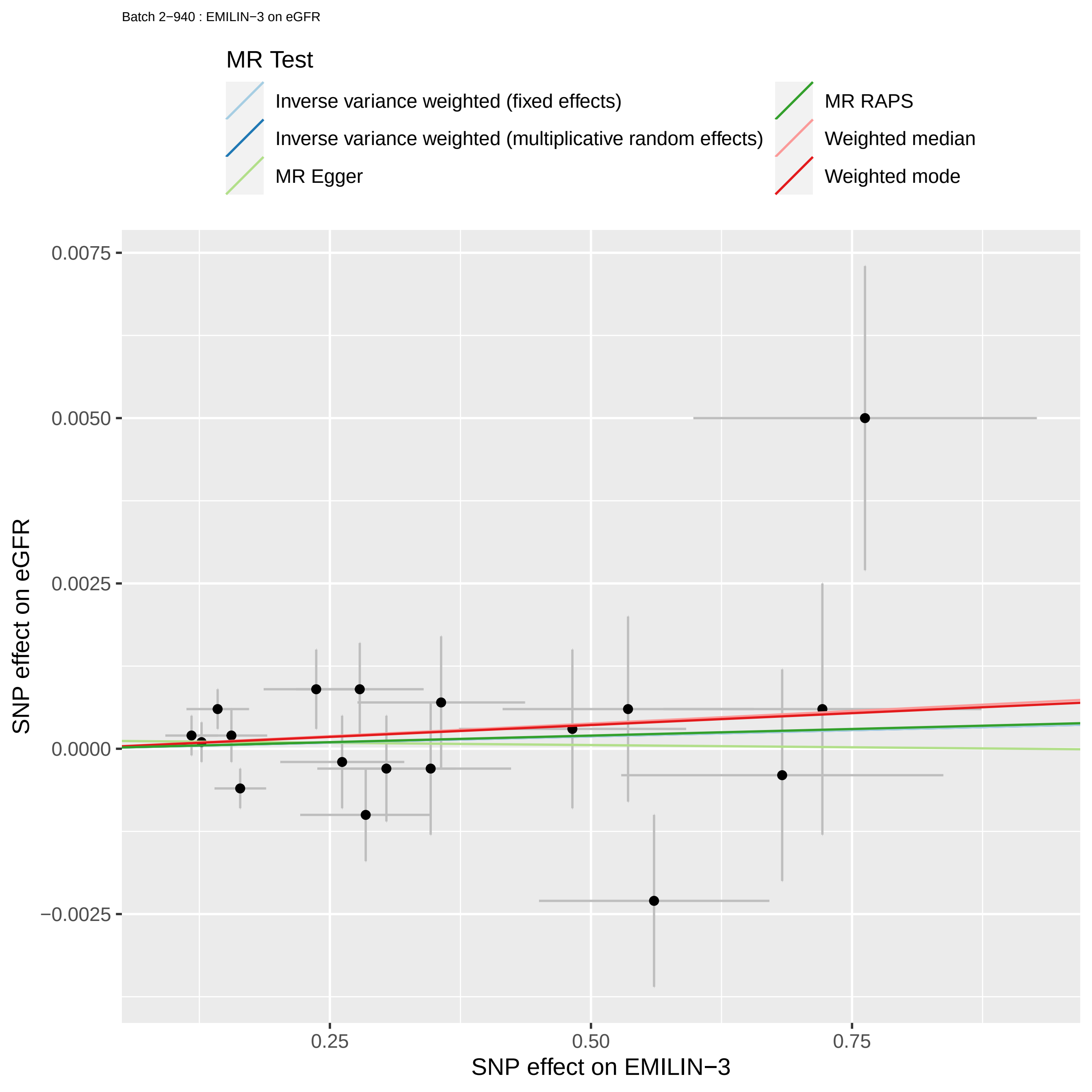

Batch 2−940 : EMILIN−3 on eGFR
MR Test
Inverse variance weighted (fixed effects)
Inverse variance weighted (multiplicative random effects)
MR Egger
MR RAPS
Weighted median
Weighted mode
0.0075
0.0050
0.0025
0.0000
−0.0025
0.25
0.50
0.75
SNP effect on eGFR
SNP effect on EMILIN−3

Batch 2−941 : Protein enabled homolog on eGFR

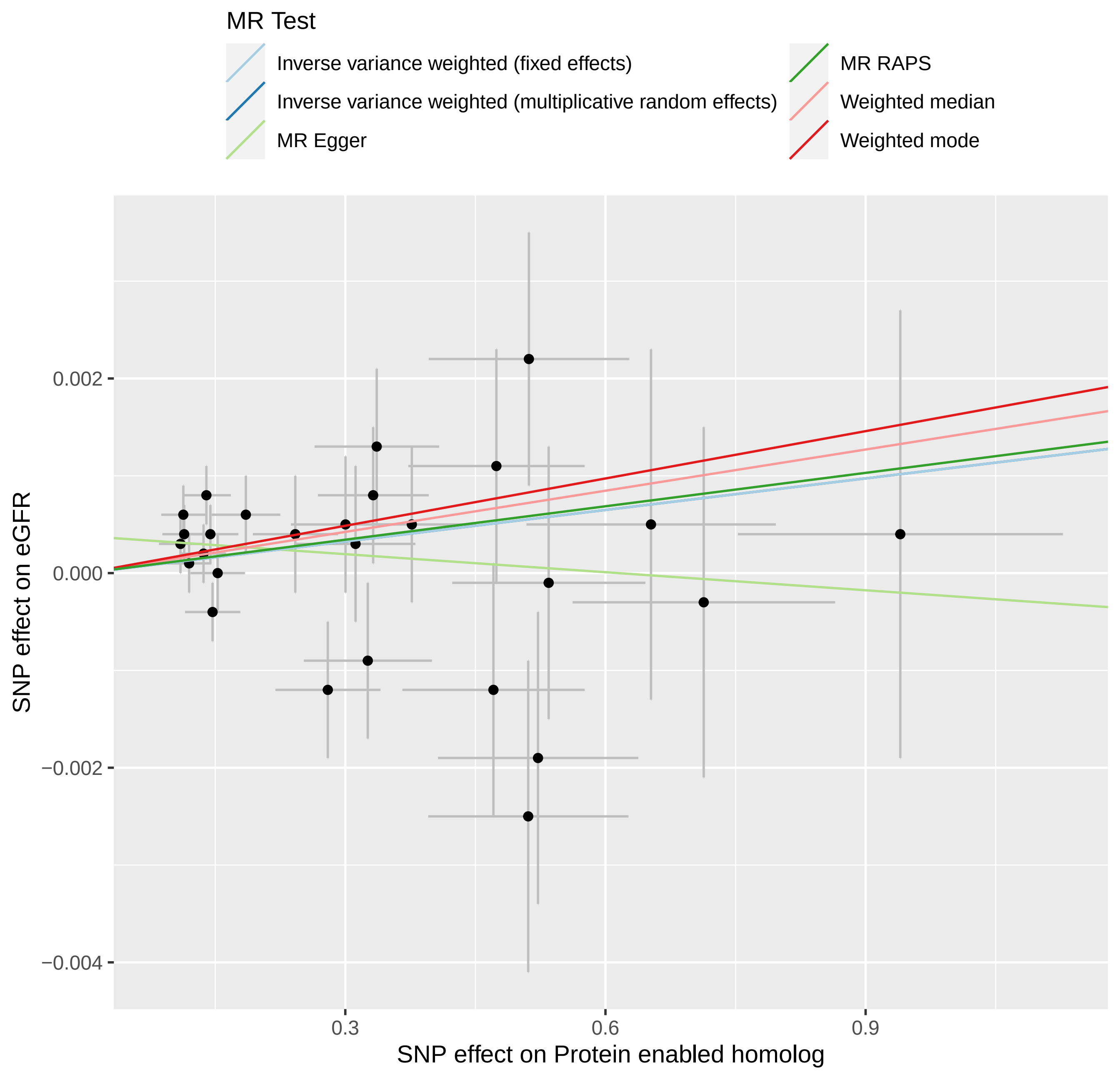

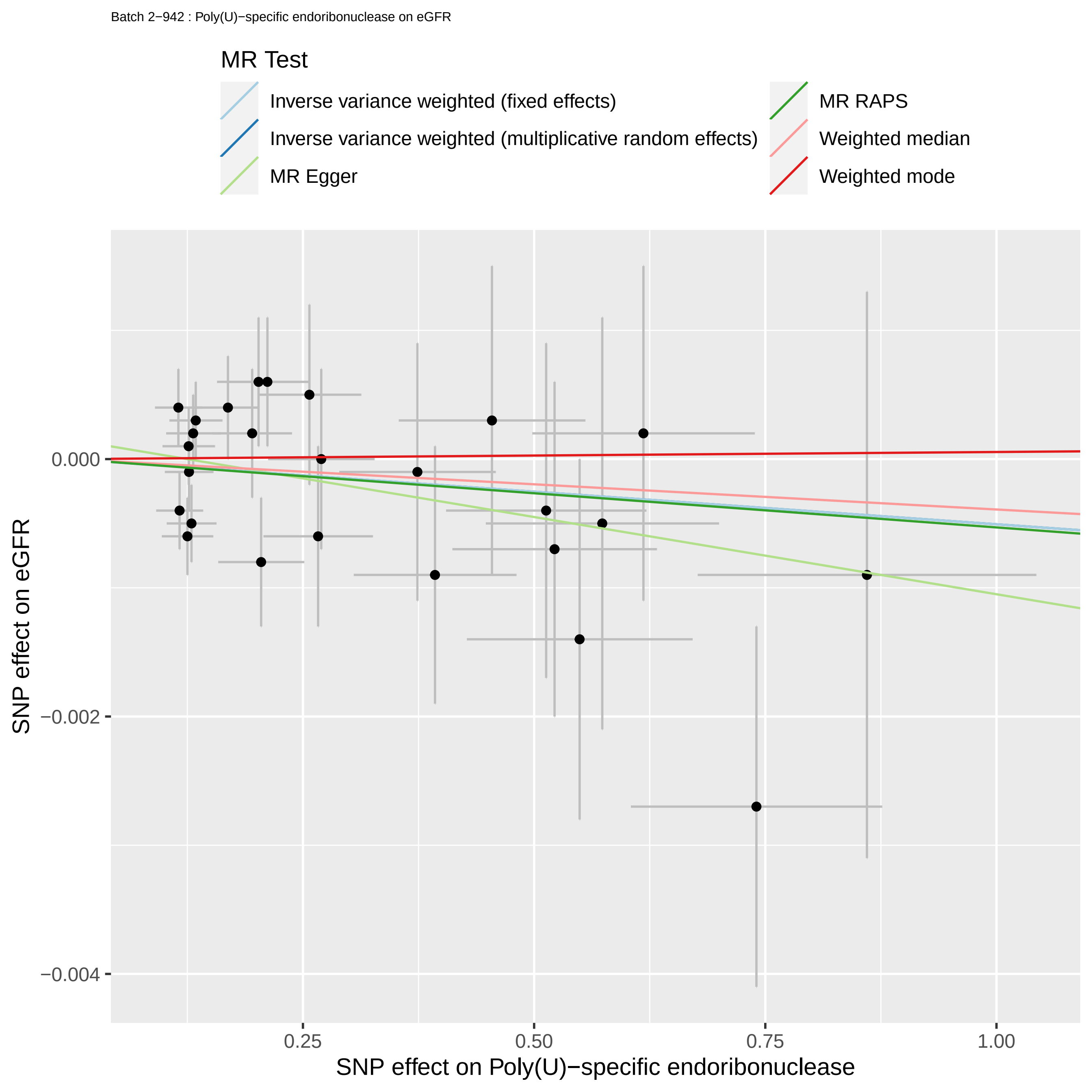

Batch 2−942 : Poly(U)−specific endoribonuclease on eGFR
MR Test
Inverse variance weighted (fixed effects)
Inverse variance weighted (multiplicative random effects)
MR Egger
MR RAPS
Weighted median
Weighted mode
0.000
−0.002
−0.004
0.25
0.50
0.75
1.00
SNP effect on eGFR
SNP effect on Poly(U)−specific endoribonuclease

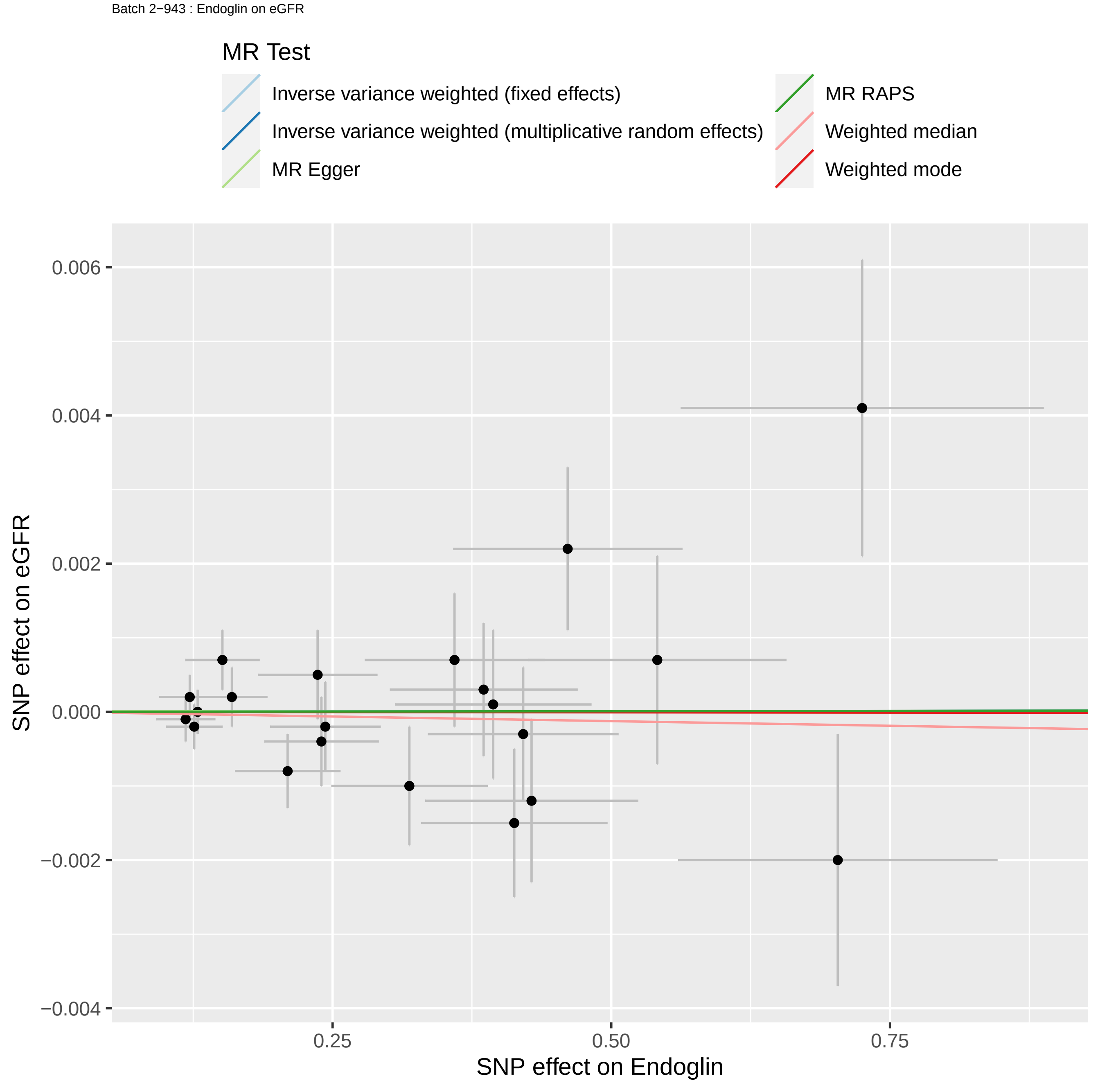

Batch 2−943 : Endoglin on eGFR
MR Test
Inverse variance weighted (fixed effects)
Inverse variance weighted (multiplicative random effects)
MR Egger
MR RAPS
Weighted median
Weighted mode
0.006
0.004
0.002
0.000
−0.002
−0.004
0.25
0.50
0.75
SNP effect on eGFR
SNP effect on Endoglin

Batch 2−944 : Gamma−enolase on eGFR

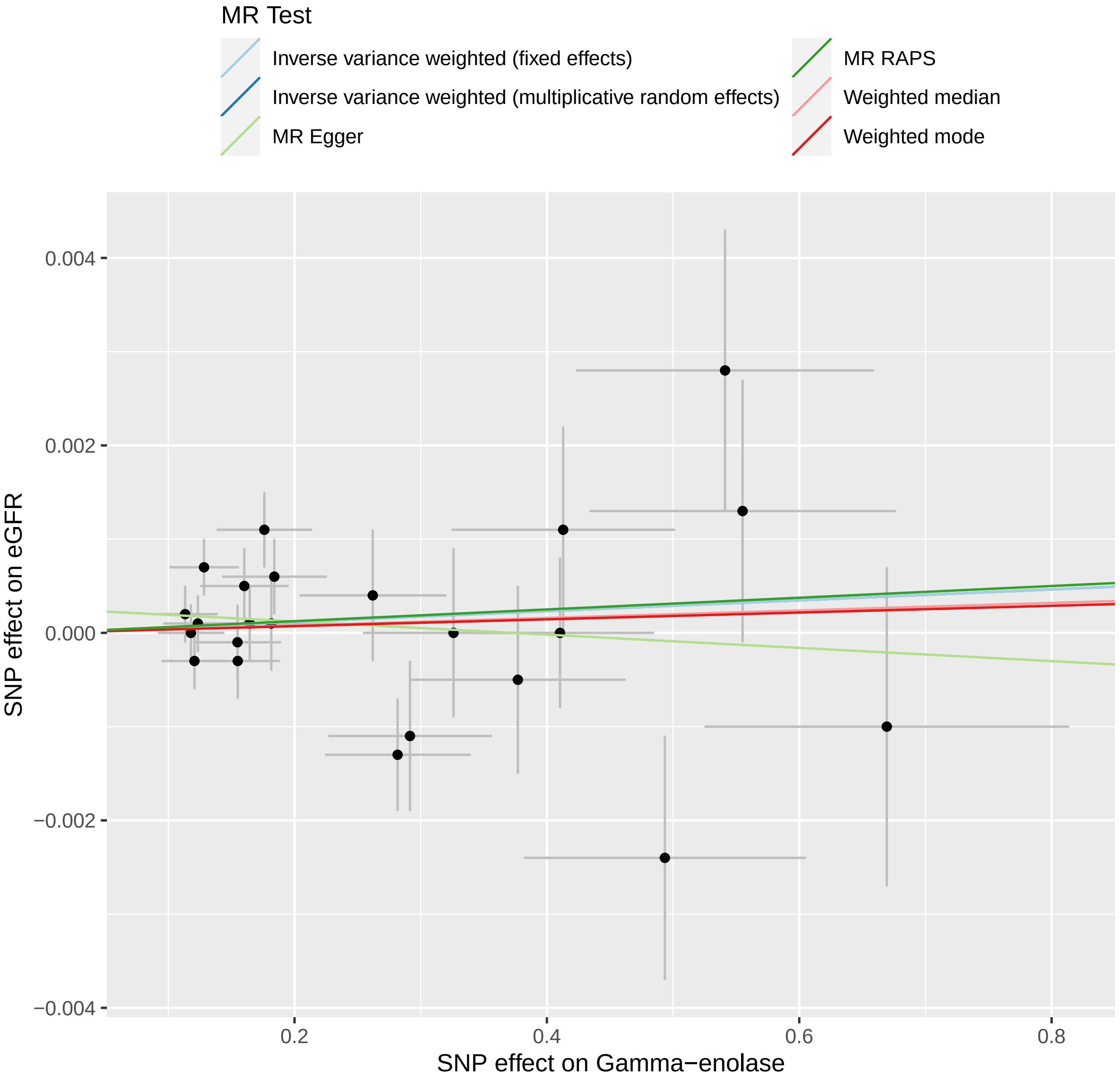

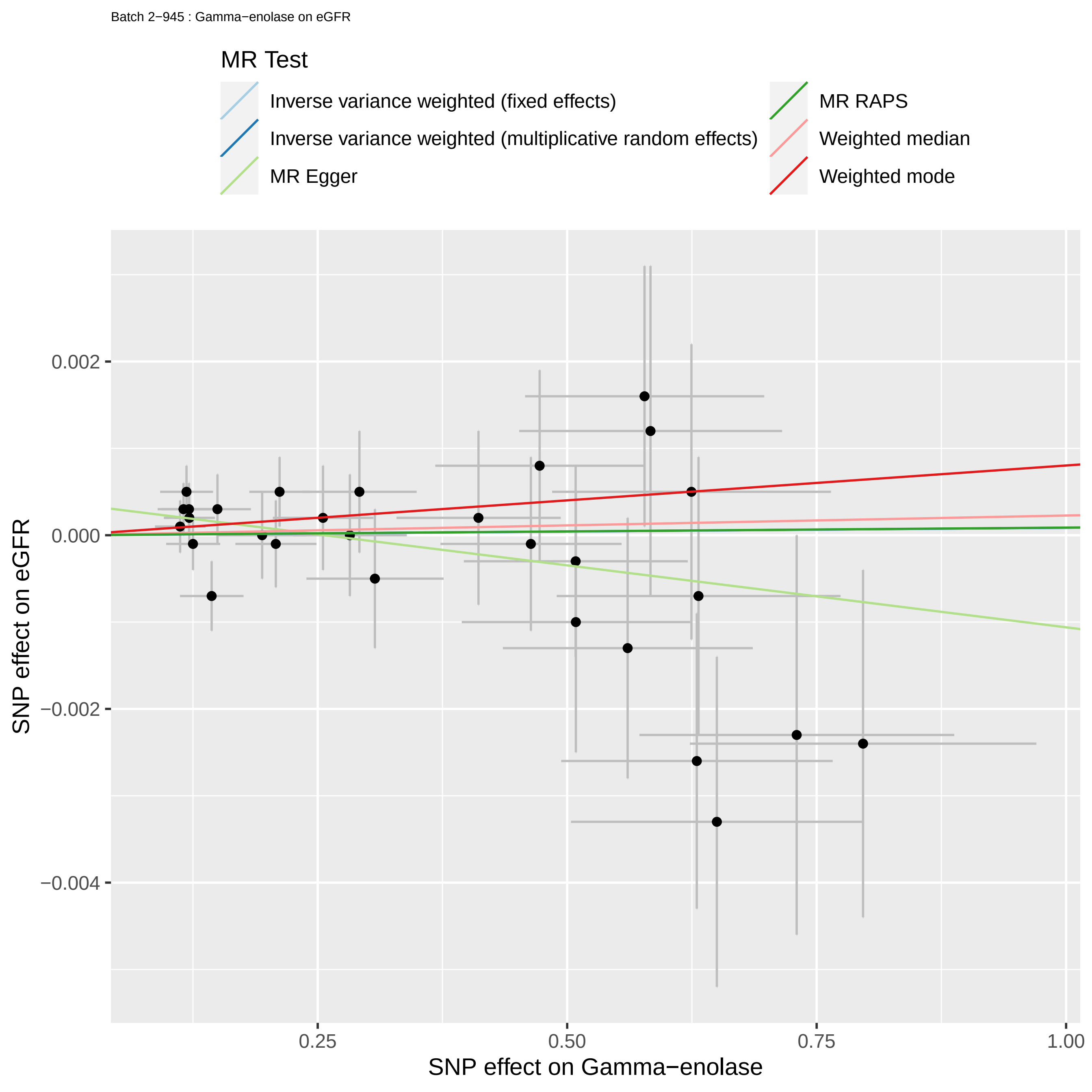

Batch 2−945 : Gamma−enolase on eGFR
MR Test
Inverse variance weighted (fixed effects)
Inverse variance weighted (multiplicative random effects)
MR Egger
MR RAPS
Weighted median
Weighted mode
0.002
0.000
−0.002
−0.004
0.25
0.50
0.75
1.00
SNP effect on eGFR
SNP effect on Gamma−enolase

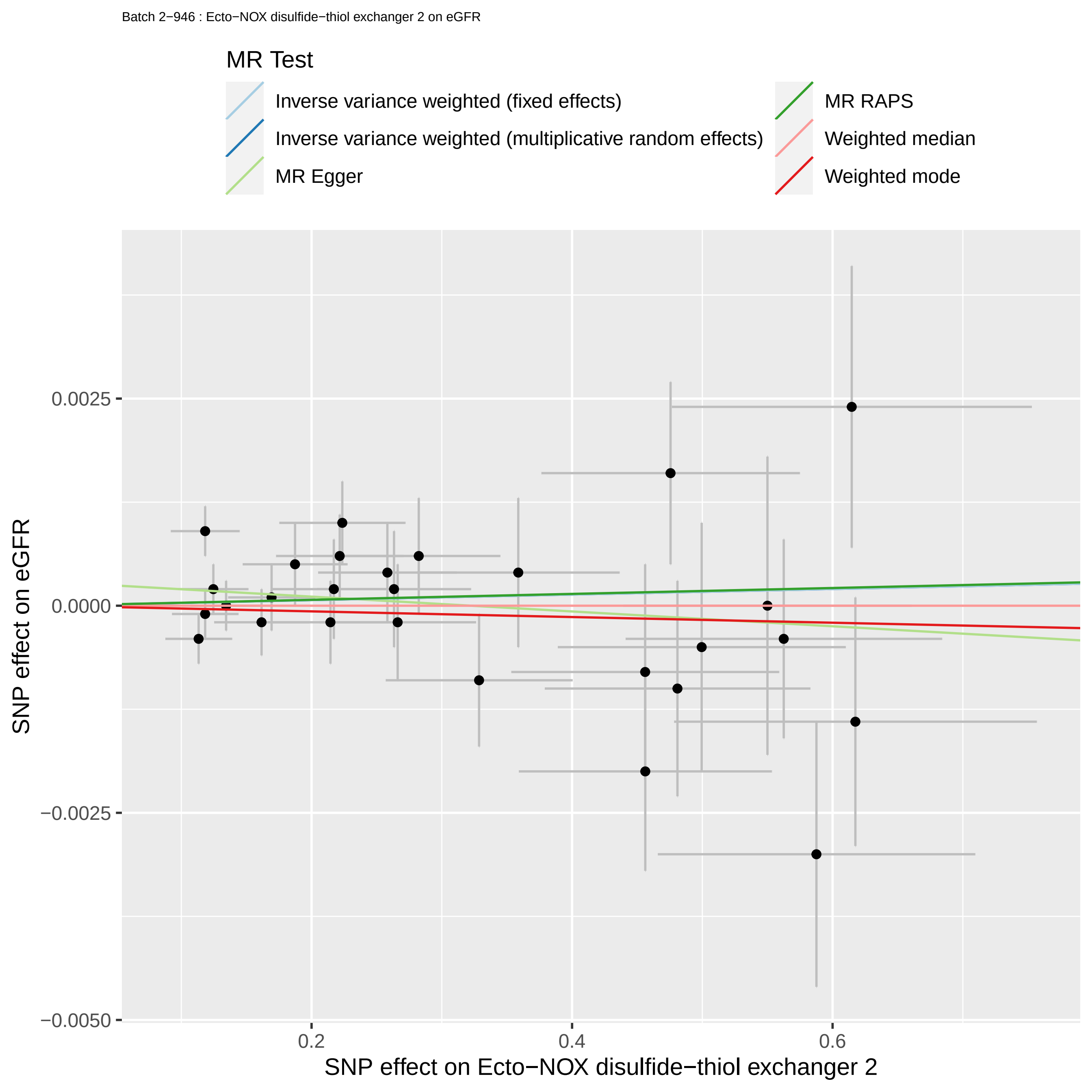

Batch 2−946 : Ecto−NOX disulfide−thiol exchanger 2 on eGFR
MR Test
Inverse variance weighted (fixed effects)
Inverse variance weighted (multiplicative random effects)
MR Egger
MR RAPS
Weighted median
Weighted mode
0.0025
0.0000
−0.0025
−0.0050
0.2
0.4
0.6
SNP effect on eGFR
SNP effect on Ecto−NOX disulfide−thiol exchanger 2

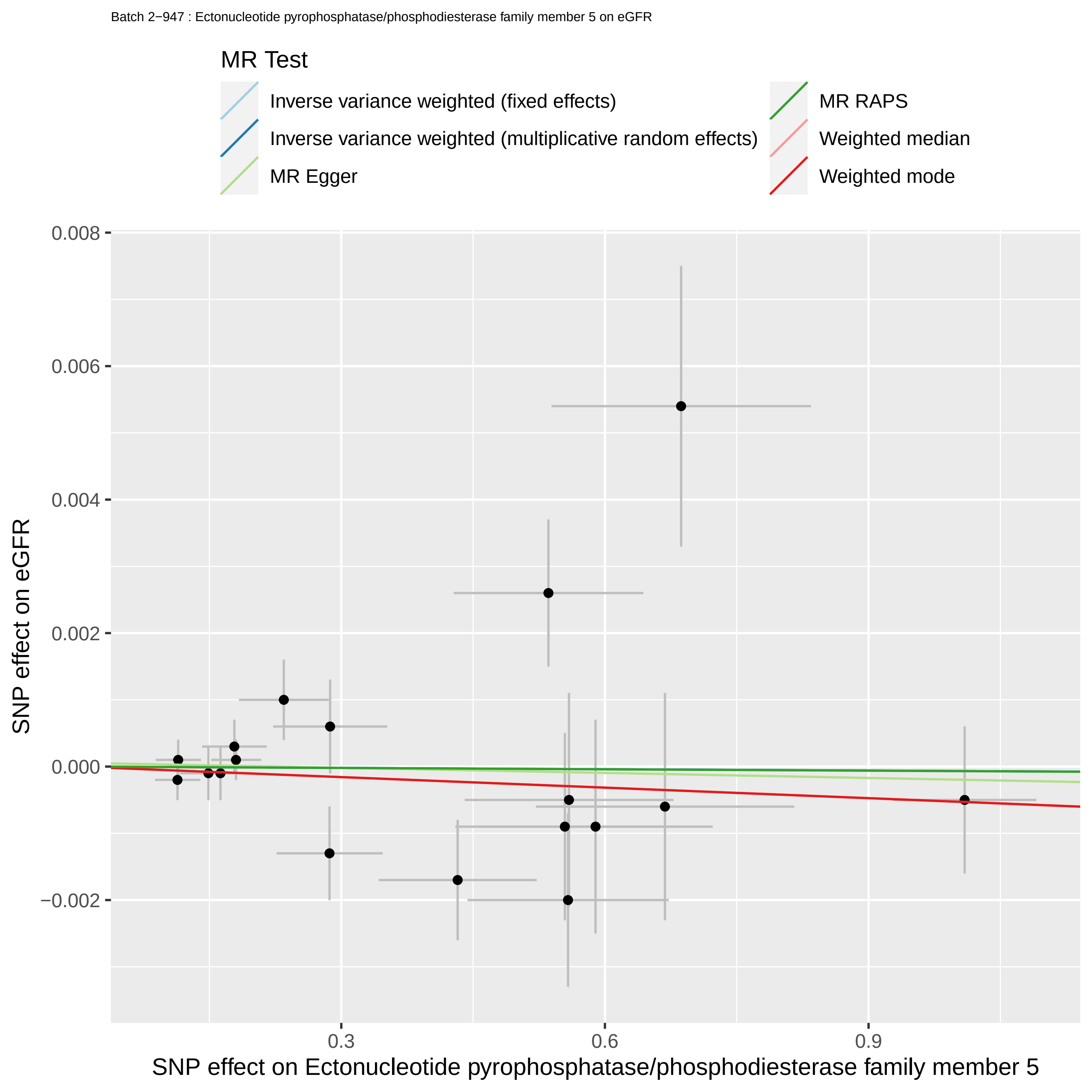

Batch 2−947 : Ectonucleotide pyrophosphatase/phosphodiesterase family member 5 on eGFR
MR Test
Inverse variance weighted (fixed effects)
Inverse variance weighted (multiplicative random effects)
MR Egger
MR RAPS
Weighted median
Weighted mode
0.008
0.006
0.004
0.002
0.000
−0.002
0.3
0.6
0.9
SNP effect on eGFR
SNP effect on Ectonucleotide pyrophosphatase/phosphodiesterase family member 5

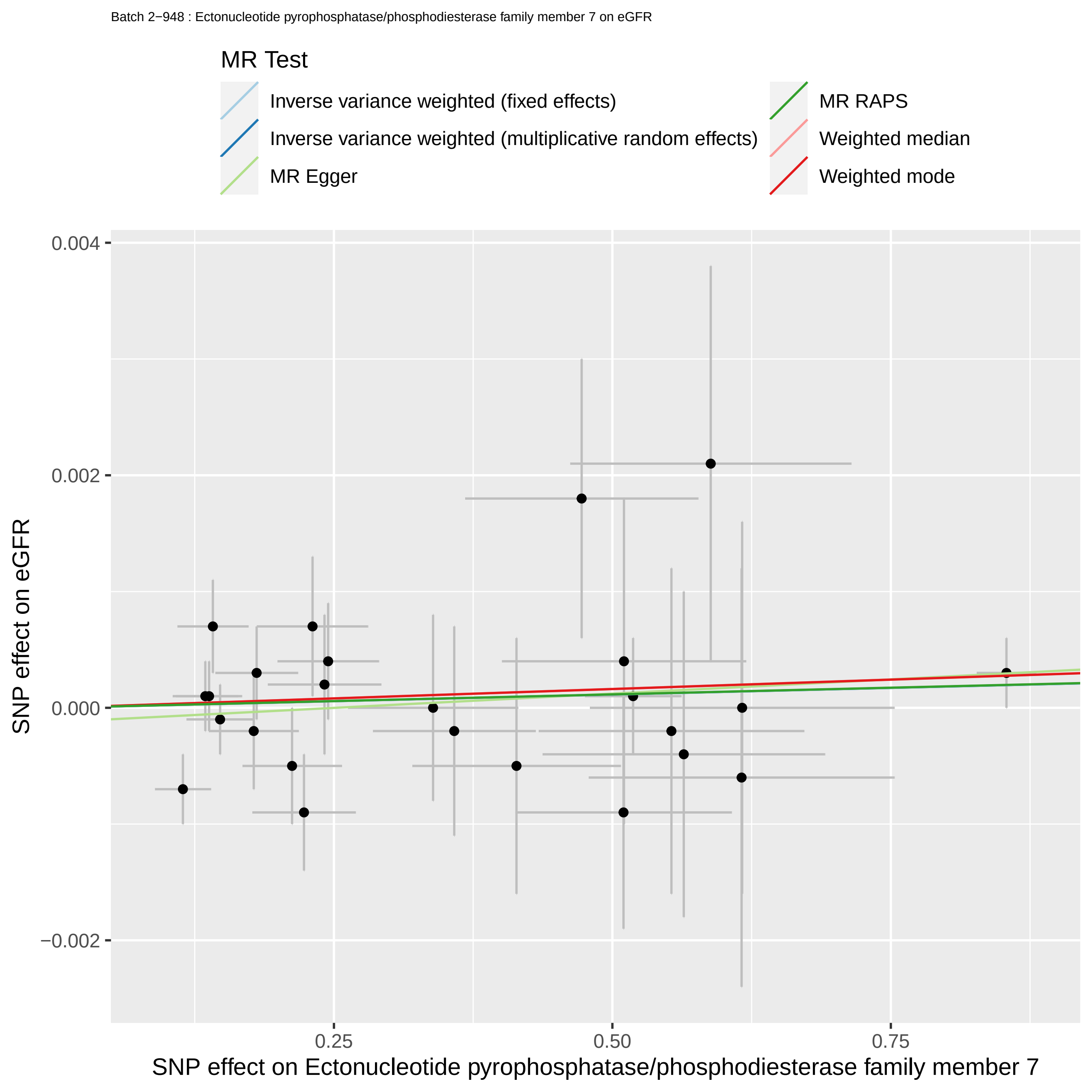
Batch 2−948 : Ectonucleotide pyrophosphatase/phosphodiesterase family member 7 on eGFR
MR Test
Inverse variance weighted (fixed effects)
Inverse variance weighted (multiplicative random effects)
MR Egger
MR RAPS
Weighted median
Weighted mode
0.004
0.002
0.000
−0.002
0.25
0.50
0.75
SNP effect on eGFR
SNP effect on Ectonucleotide pyrophosphatase/phosphodiesterase family member 7

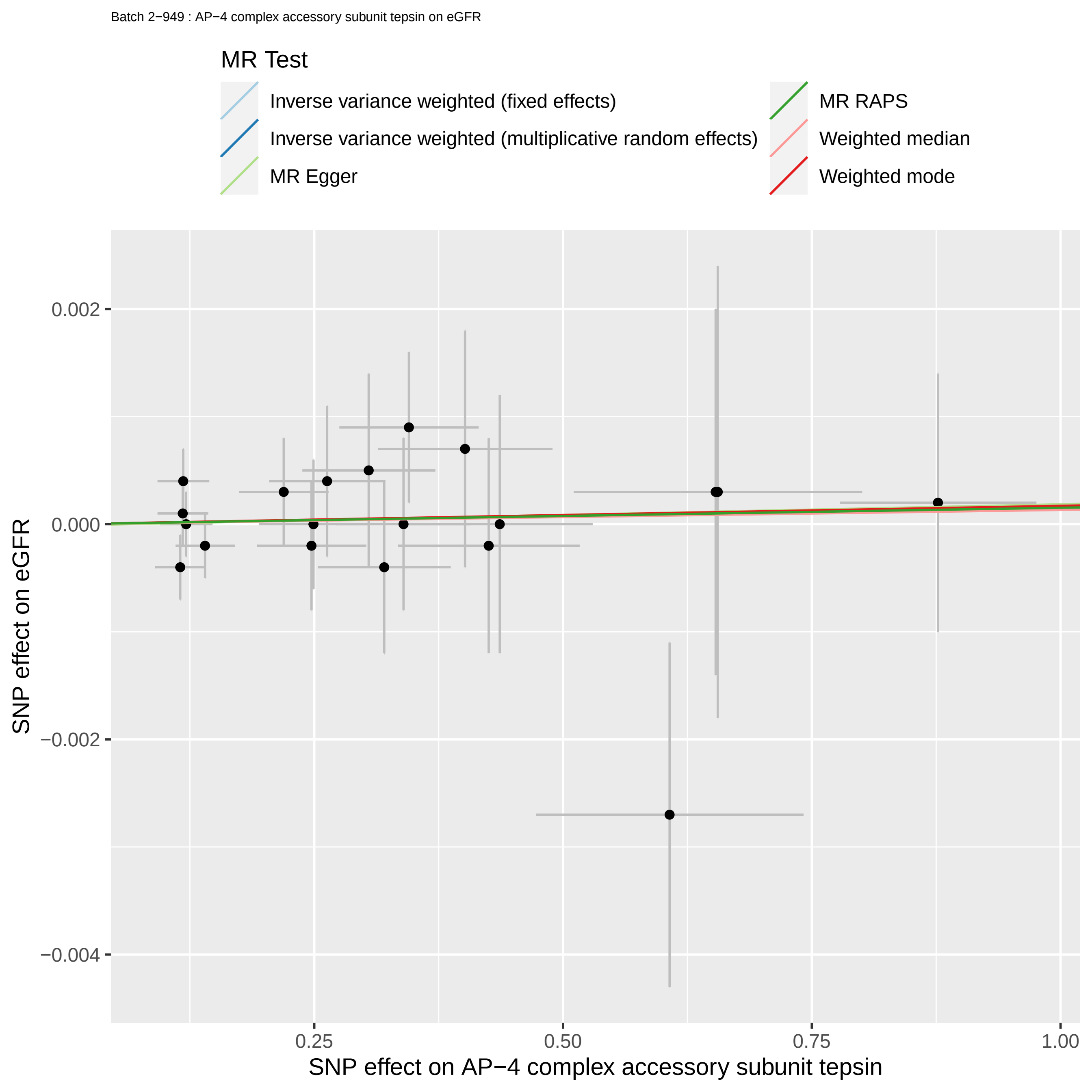
Batch 2−949 : AP−4 complex accessory subunit tepsin on eGFR
MR Test
Inverse variance weighted (fixed effects)
Inverse variance weighted (multiplicative random effects)
MR Egger
MR RAPS
Weighted median
Weighted mode
0.002
0.000
−0.002
−0.004
0.25
0.50
0.75
1.00
SNP effect on eGFR
SNP effect on AP−4 complex accessory subunit tepsin

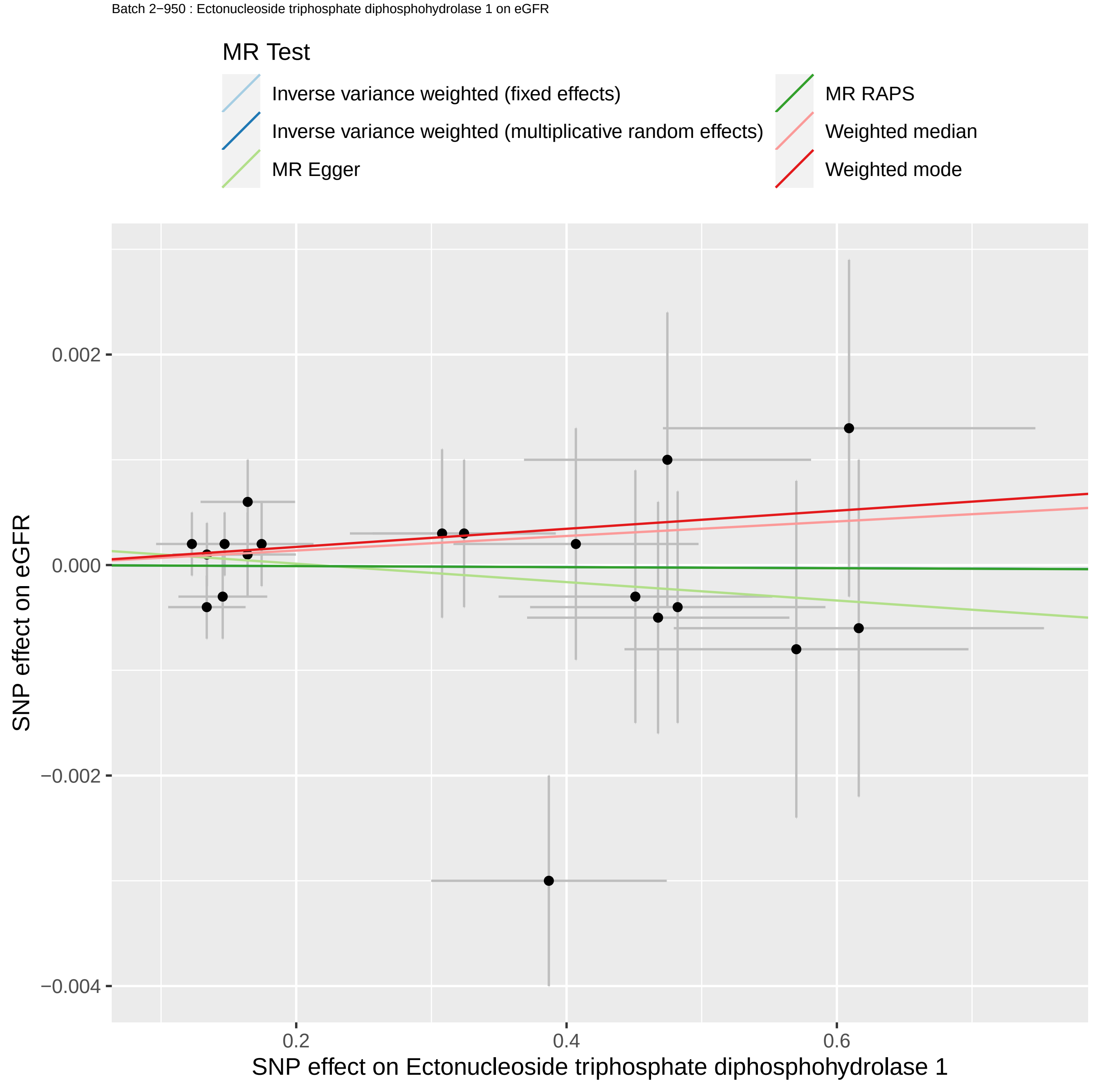
Batch 2−950 : Ectonucleoside triphosphate diphosphohydrolase 1 on eGFR
MR Test
Inverse variance weighted (fixed effects)
Inverse variance weighted (multiplicative random effects)
MR Egger
MR RAPS
Weighted median
Weighted mode
0.002
0.000
−0.002
−0.004
0.2
0.4
0.6
SNP effect on eGFR
SNP effect on Ectonucleoside triphosphate diphosphohydrolase 1

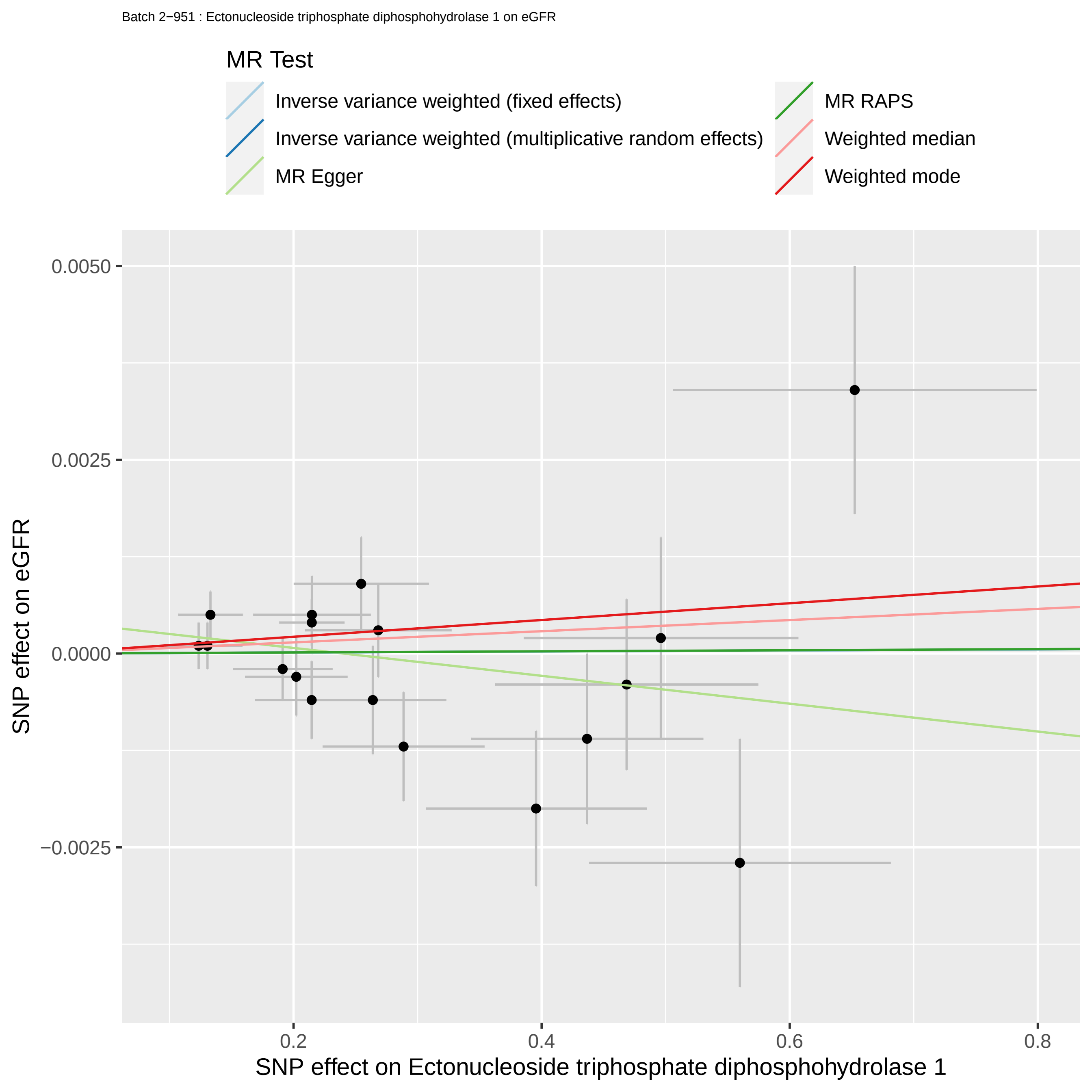
Batch 2−951 : Ectonucleoside triphosphate diphosphohydrolase 1 on eGFR
MR Test
Inverse variance weighted (fixed effects)
Inverse variance weighted (multiplicative random effects)
MR Egger
MR RAPS
Weighted median
Weighted mode
0.0050
0.0025
0.0000
−0.0025
0.2
0.4
0.6
0.8
SNP effect on eGFR
SNP effect on Ectonucleoside triphosphate diphosphohydrolase 1

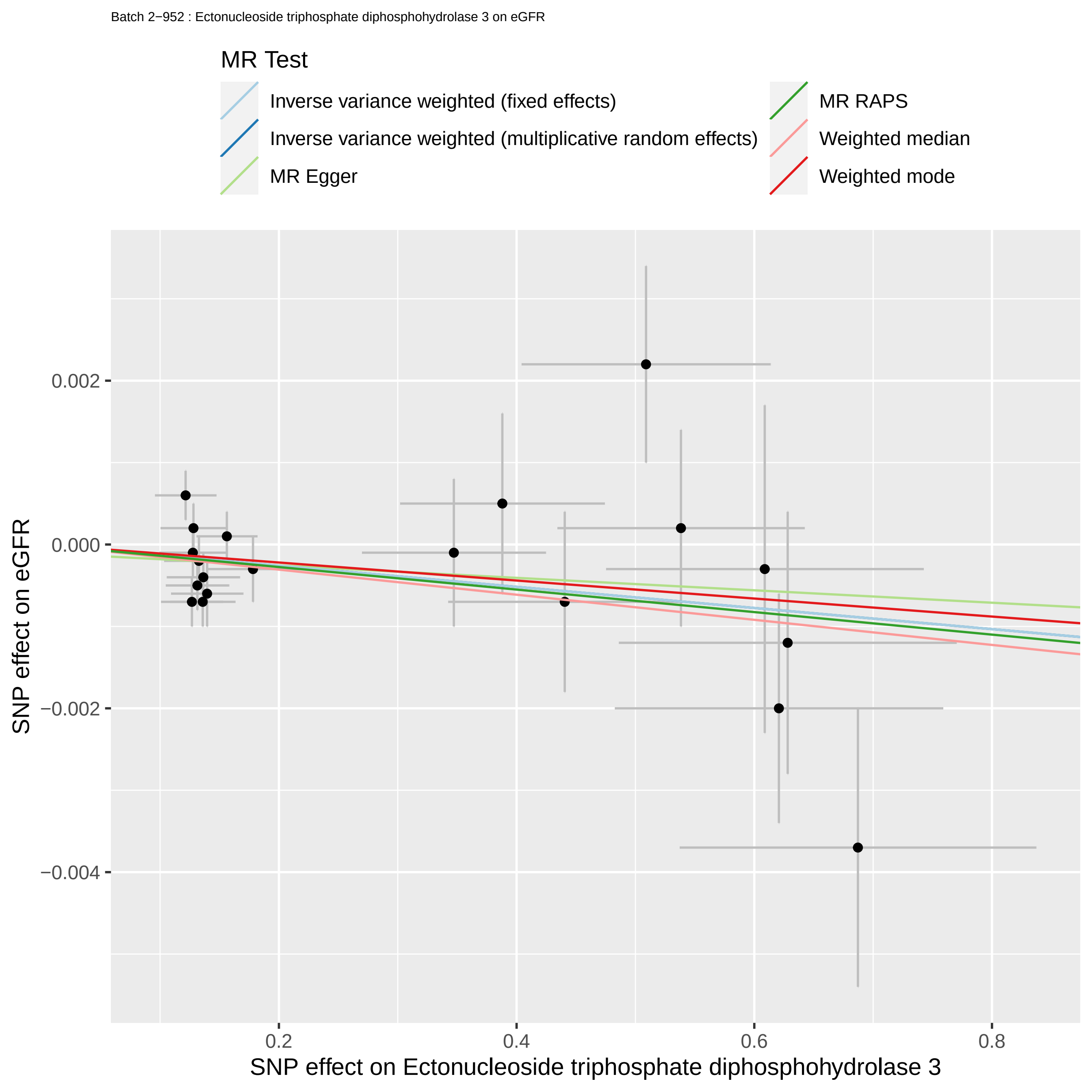

Batch 2−952 : Ectonucleoside triphosphate diphosphohydrolase 3 on eGFR
MR Test
Inverse variance weighted (fixed effects)
Inverse variance weighted (multiplicative random effects)
MR Egger
MR RAPS
Weighted median
Weighted mode
0.002
0.000
−0.002
−0.004
0.2
0.4
0.6
0.8
SNP effect on eGFR
SNP effect on Ectonucleoside triphosphate diphosphohydrolase 3

Batch 2−953 : Ectonucleoside triphosphate diphosphohydrolase 5 on eGFR

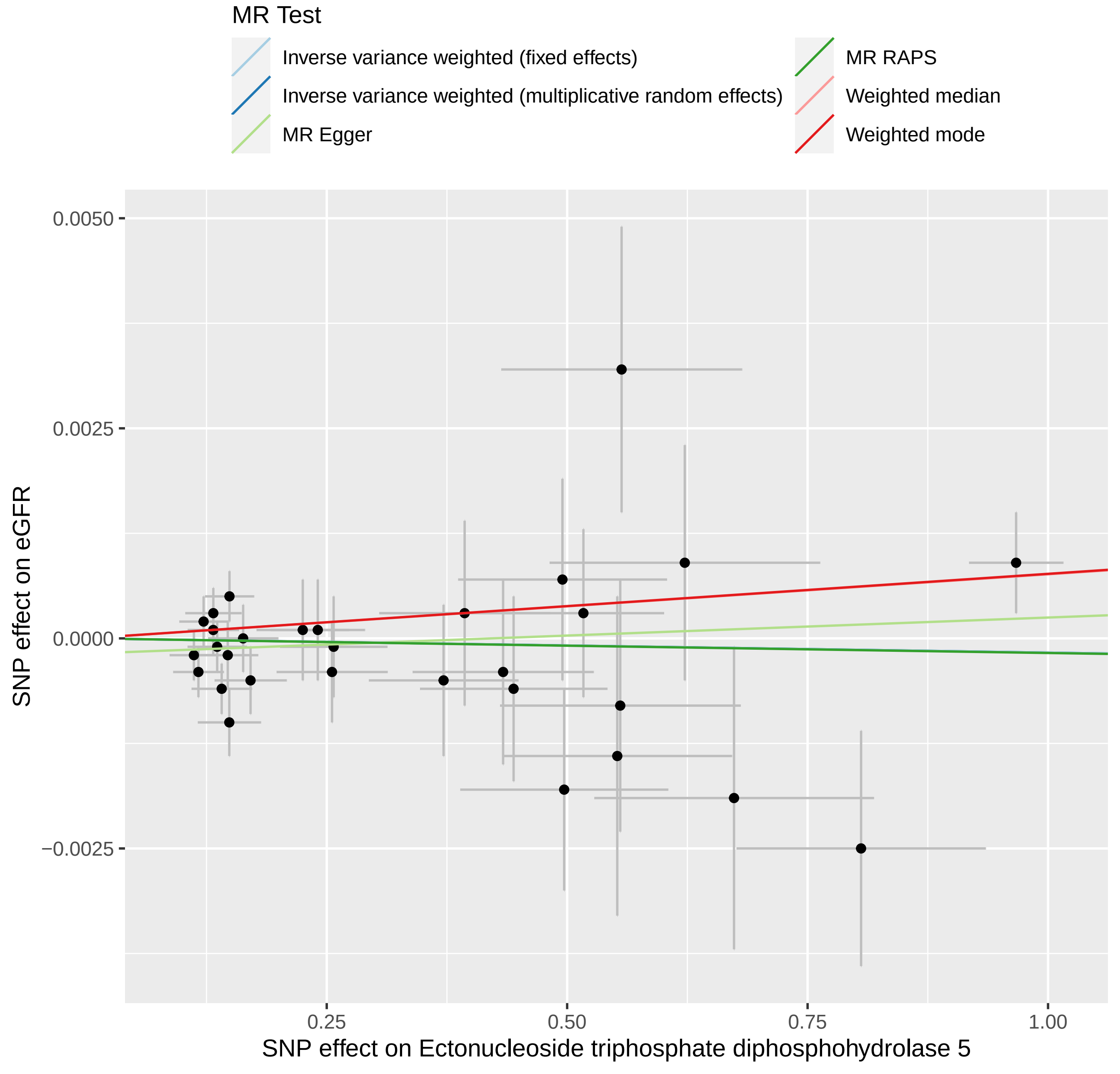

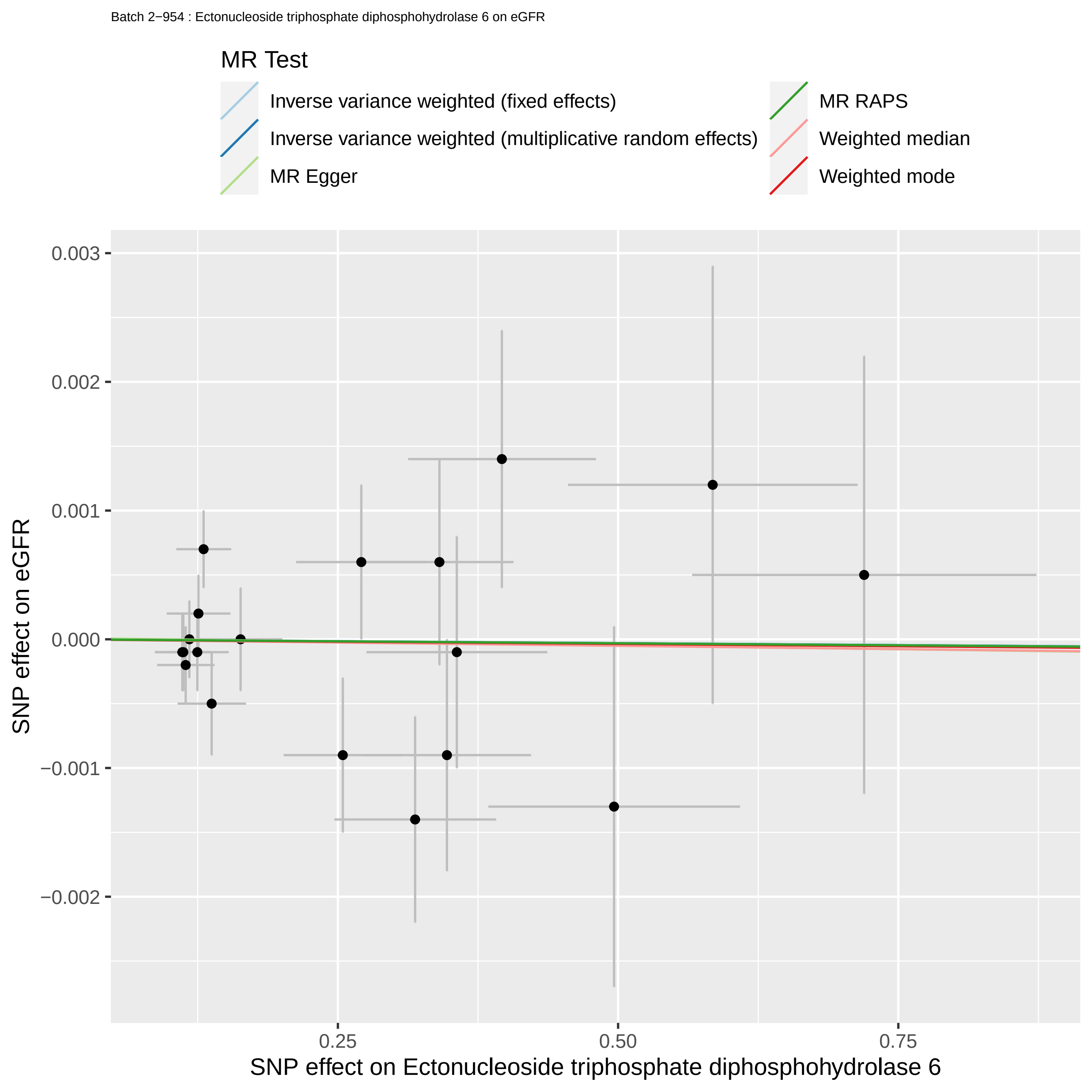
Batch 2−954 : Ectonucleoside triphosphate diphosphohydrolase 6 on eGFR
MR Test
Inverse variance weighted (fixed effects)
Inverse variance weighted (multiplicative random effects)
MR Egger
MR RAPS
Weighted median
Weighted mode
0.003
0.002
0.001
0.000
−0.001
−0.002
0.25
0.50
0.75
SNP effect on eGFR
SNP effect on Ectonucleoside triphosphate diphosphohydrolase 6

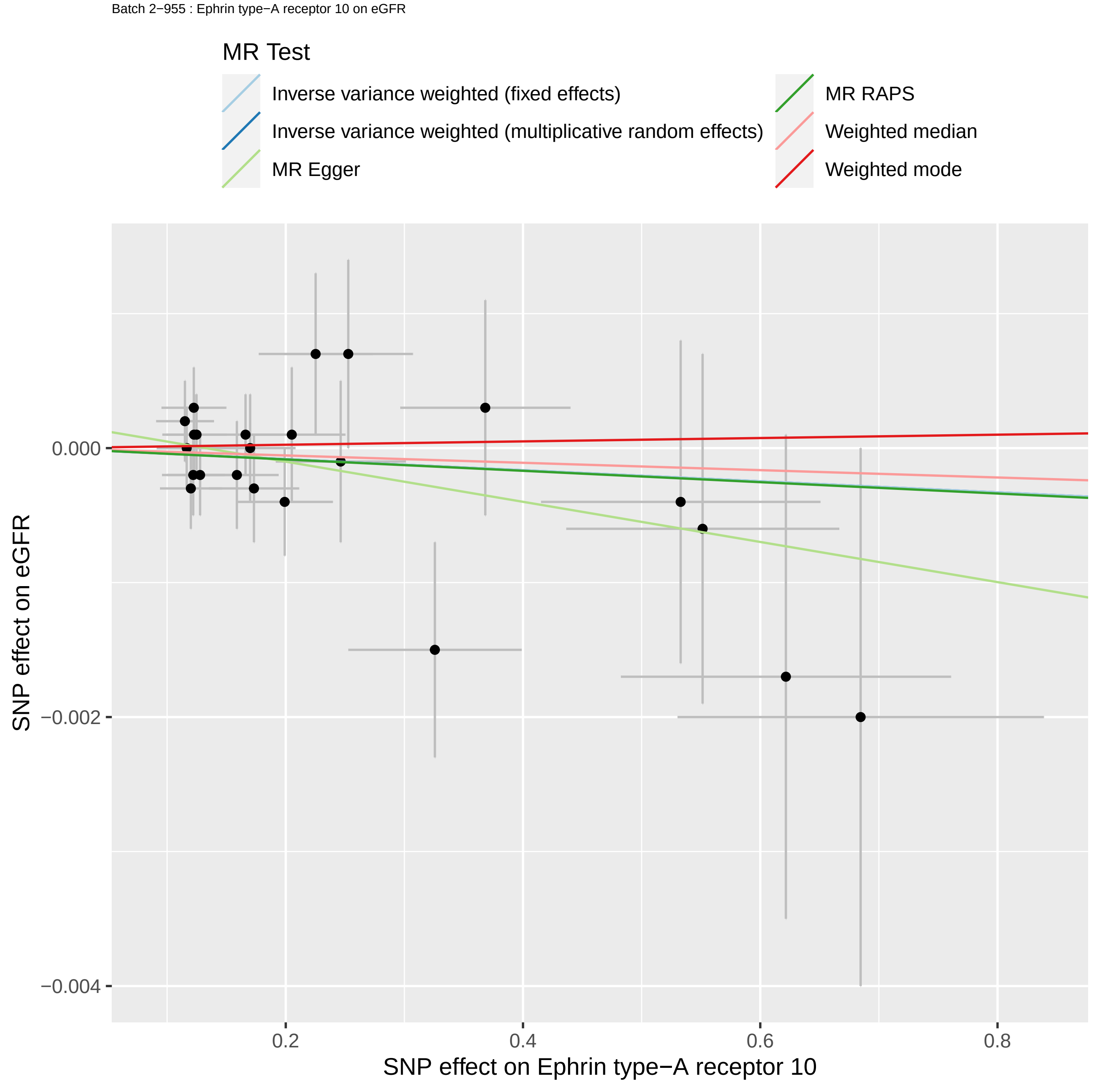
Batch 2−955 : Ephrin type−A receptor 10 on eGFR
MR Test
Inverse variance weighted (fixed effects)
Inverse variance weighted (multiplicative random effects)
MR Egger
MR RAPS
Weighted median
Weighted mode
0.000
−0.002
−0.004
0.2
0.4
0.6
0.8
SNP effect on eGFR
SNP effect on Ephrin type−A receptor 10

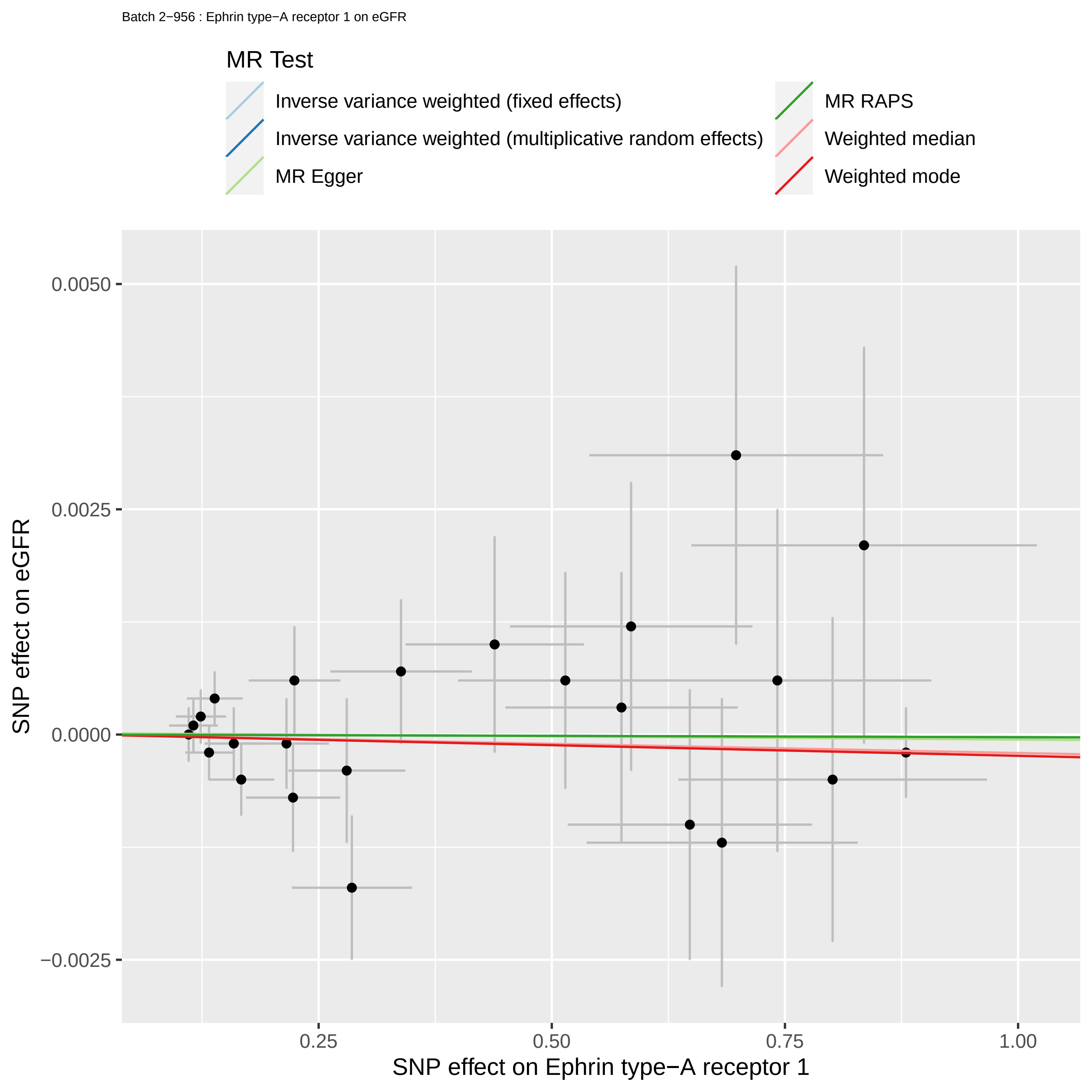

Batch 2−956 : Ephrin type−A receptor 1 on eGFR
MR Test
Inverse variance weighted (fixed effects)
Inverse variance weighted (multiplicative random effects)
MR Egger
MR RAPS
Weighted median
Weighted mode
0.0050
0.0025
0.0000
−0.0025
0.25
0.50
0.75
1.00
SNP effect on eGFR
SNP effect on Ephrin type−A receptor 1

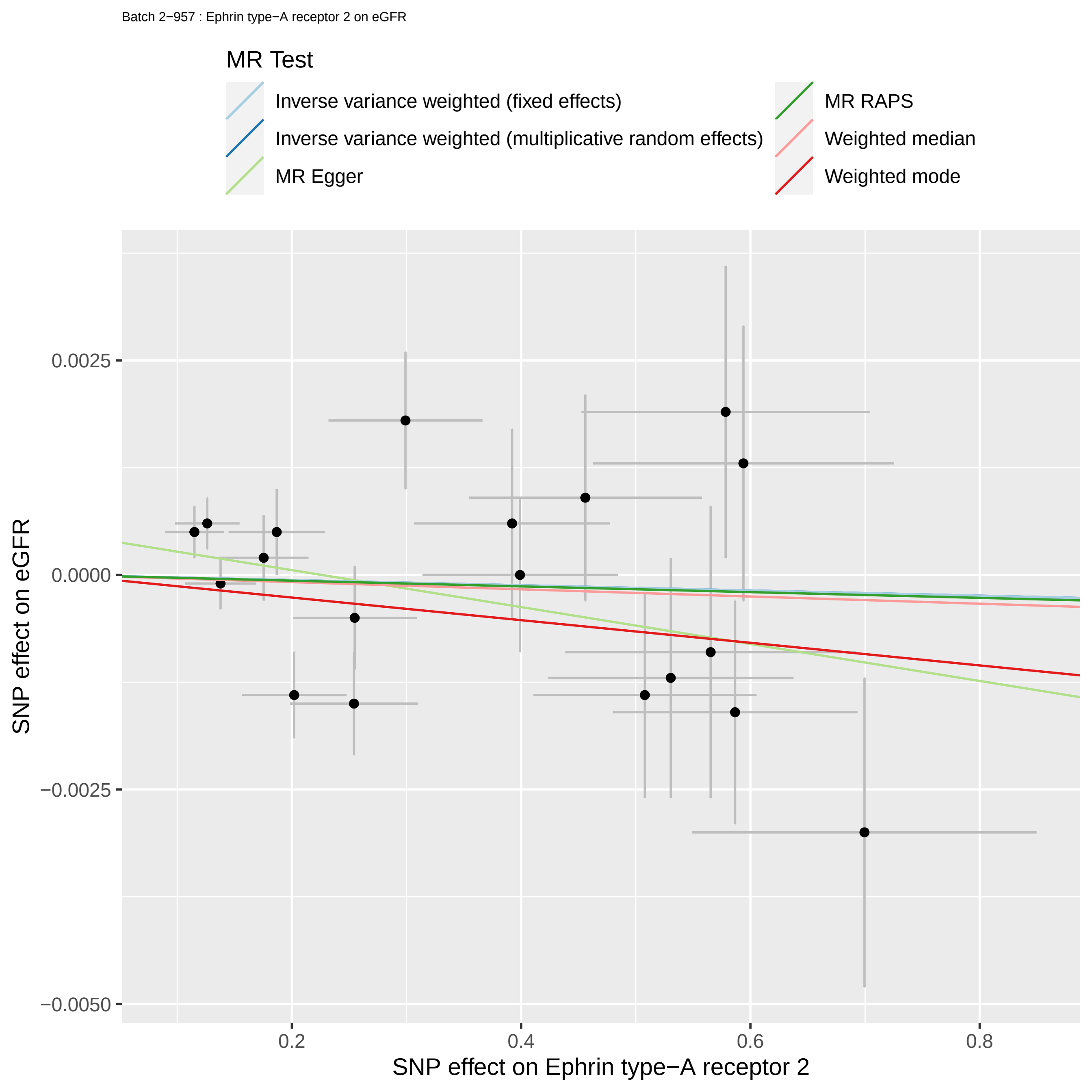

Batch 2−957 : Ephrin type−A receptor 2 on eGFR
MR Test
Inverse variance weighted (fixed effects)
Inverse variance weighted (multiplicative random effects)
MR Egger
MR RAPS
Weighted median
Weighted mode
0.0025
0.0000
−0.0025
−0.0050
0.2
0.4
0.6
0.8
SNP effect on eGFR
SNP effect on Ephrin type−A receptor 2

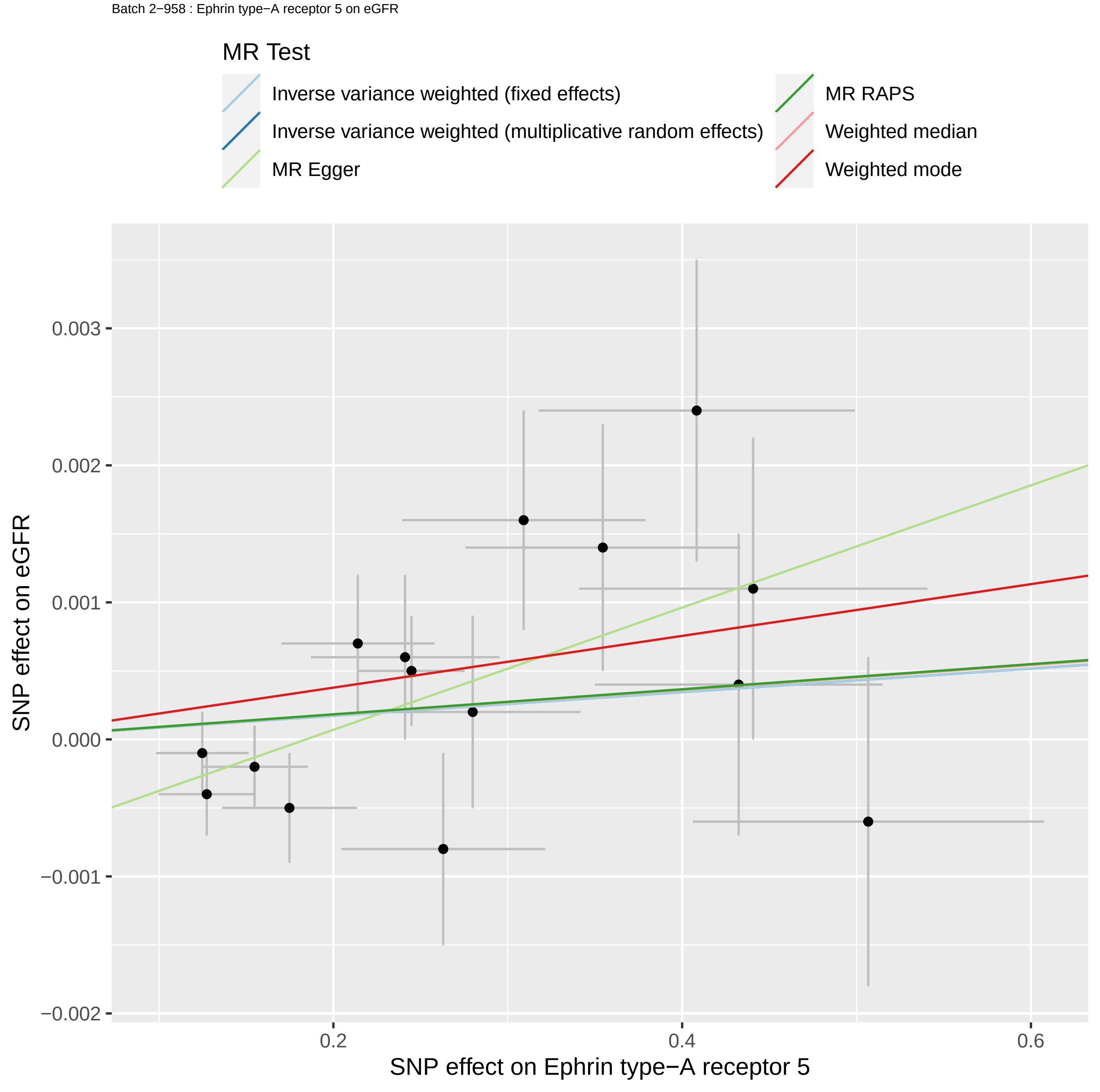
Batch 2−958 : Ephrin type−A receptor 5 on eGFR
MR Test
Inverse variance weighted (fixed effects)
Inverse variance weighted (multiplicative random effects)
MR Egger
MR RAPS
Weighted median
Weighted mode
0.003
0.002
0.001
0.000
−0.001
−0.002
SNP effect on eGFR
0.2
0.4
0.6
SNP effect on Ephrin type−A receptor 5

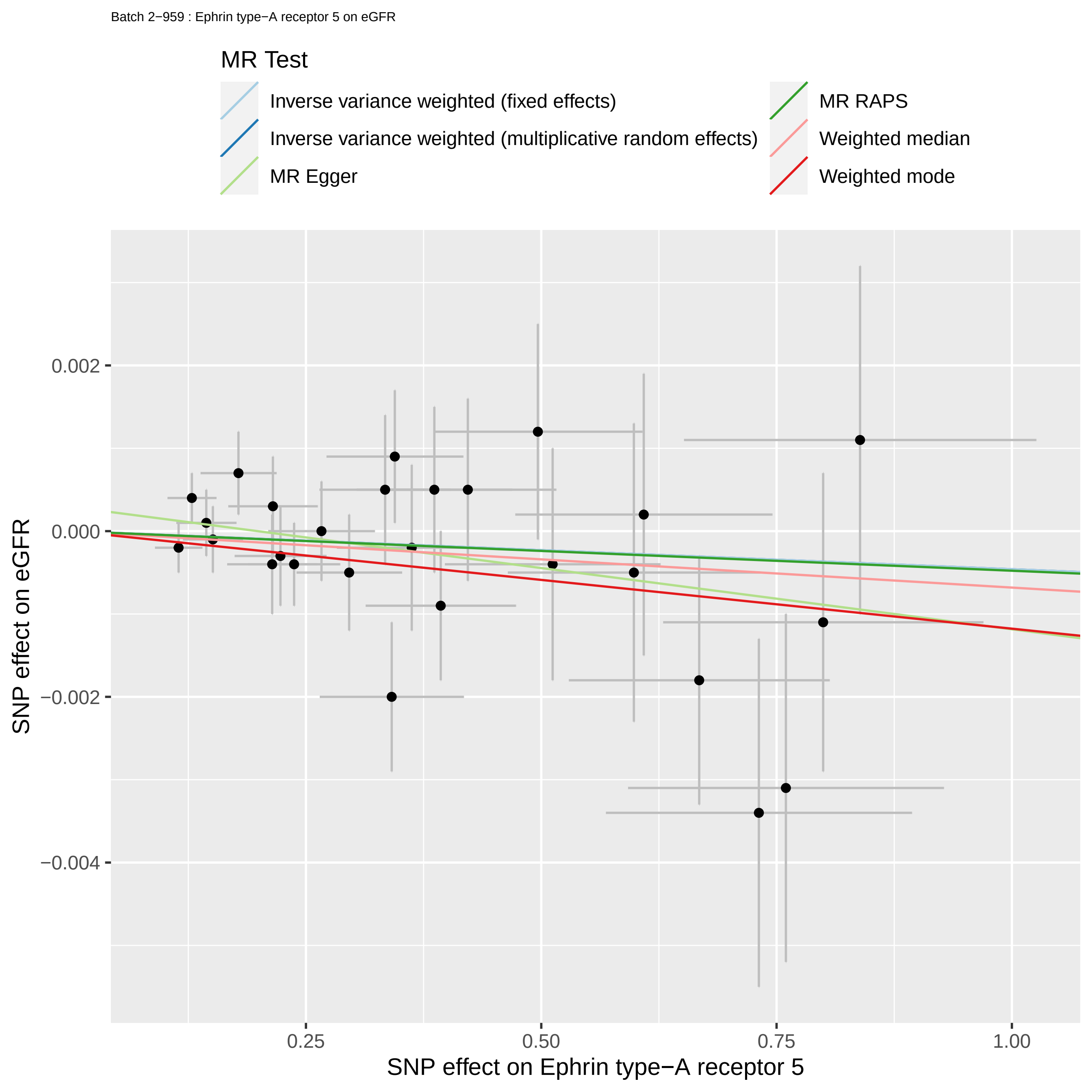
Batch 2−959 : Ephrin type−A receptor 5 on eGFR
MR Test
Inverse variance weighted (fixed effects)
Inverse variance weighted (multiplicative random effects)
MR Egger
MR RAPS
Weighted median
Weighted mode
0.002
0.000
−0.002
−0.004
SNP effect on eGFR
0.25
0.50
0.75
1.00
SNP effect on Ephrin type−A receptor 5

Batch 2−960 : Ephrin type−B receptor 2 on eGFR

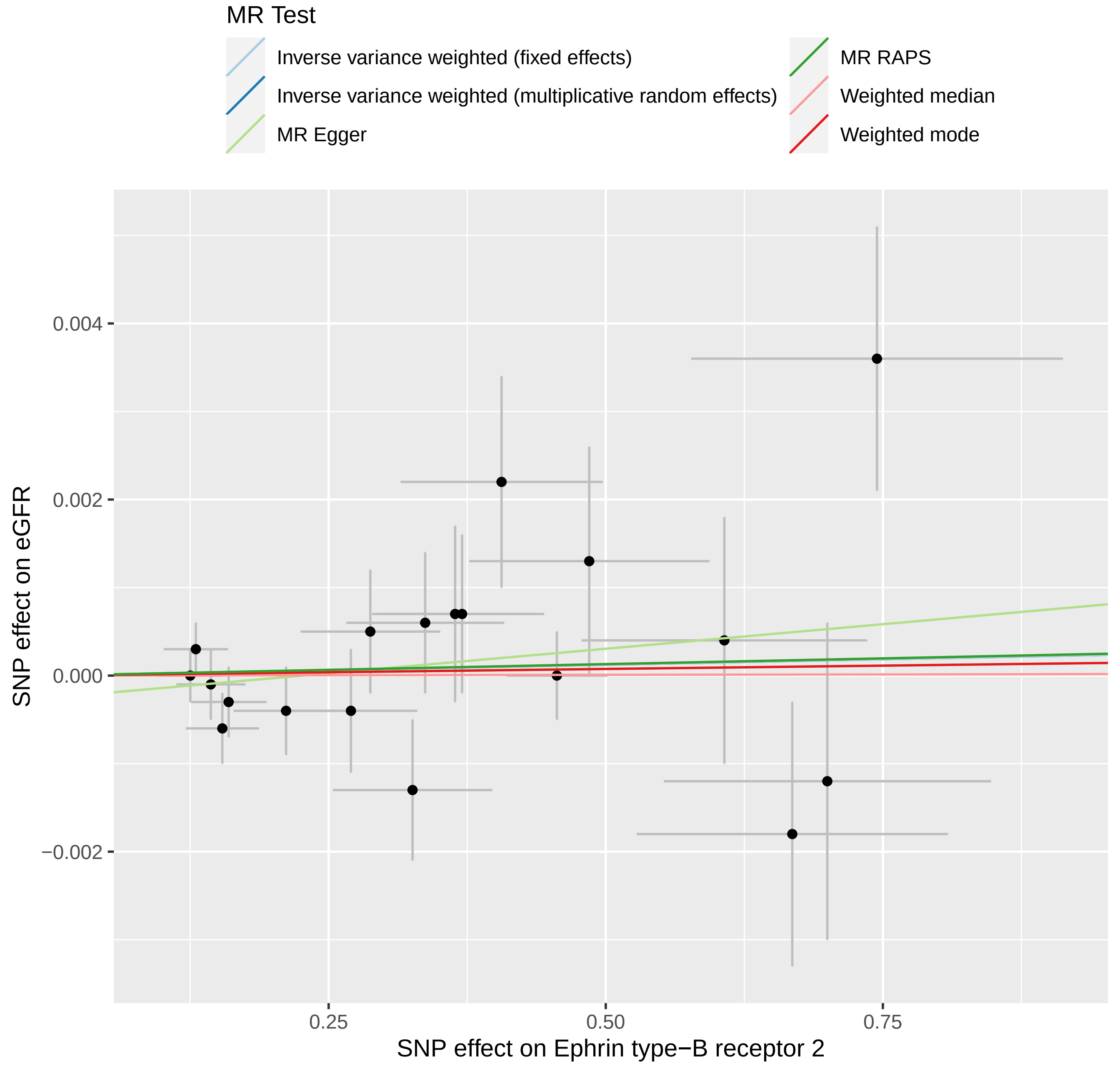

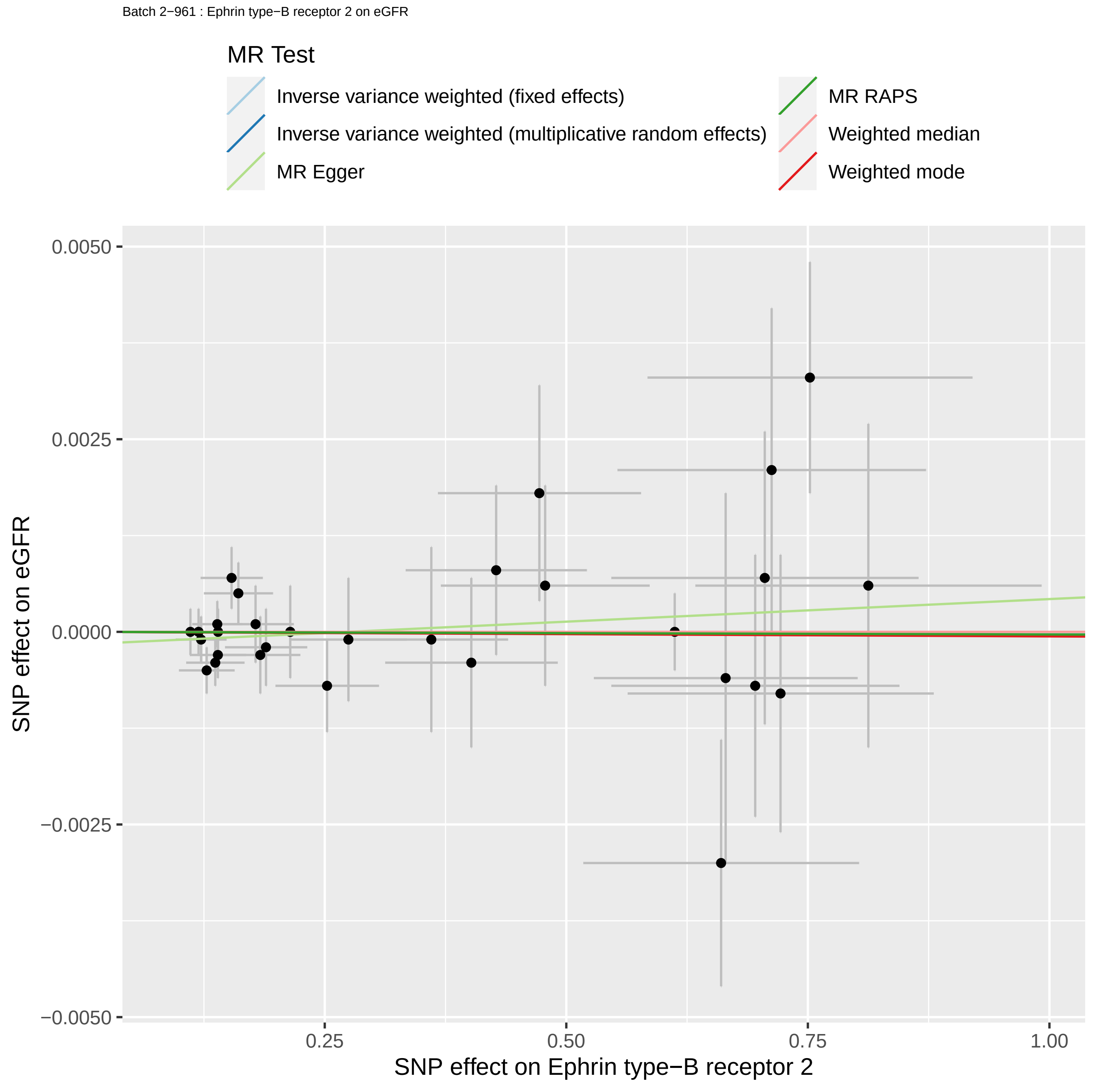
Batch 2−961 : Ephrin type−B receptor 2 on eGFR
MR Test
Inverse variance weighted (fixed effects)
Inverse variance weighted (multiplicative random effects)
MR Egger
MR RAPS
Weighted median
Weighted mode
SNP effect on eGFR
0.0050
0.0025
0.0000
−0.0025
−0.0050
0.25
0.50
0.75
1.00
SNP effect on Ephrin type−B receptor 2

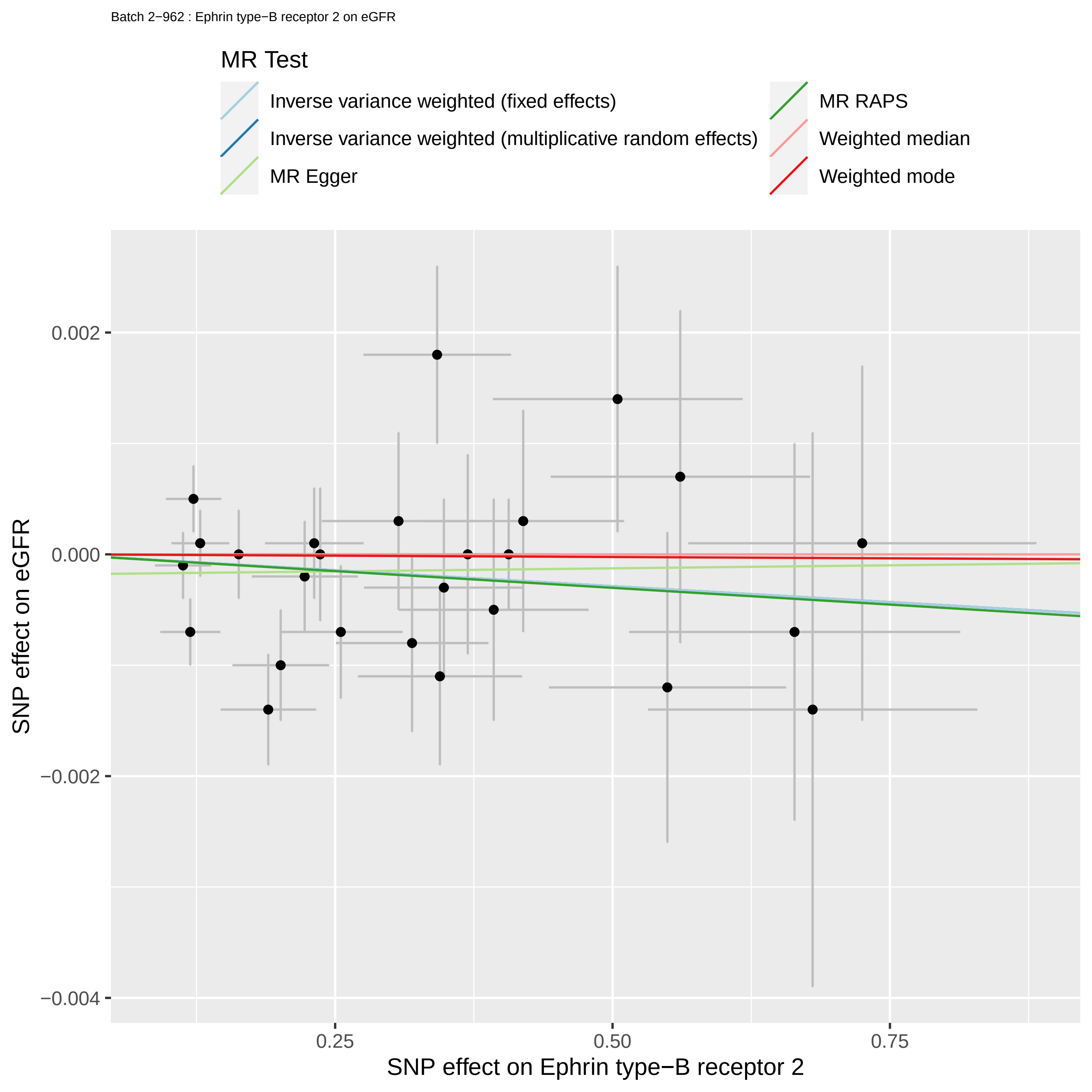
Batch 2−962 : Ephrin type−B receptor 2 on eGFR
MR Test
Inverse variance weighted (fixed effects)
Inverse variance weighted (multiplicative random effects)
MR Egger
MR RAPS
Weighted median
Weighted mode
0.002
0.000
−0.002
−0.004
0.25
0.50
0.75
SNP effect on eGFR
SNP effect on Ephrin type−B receptor 2

Batch 2−963 : Ephrin type−B receptor 3 on eGFR

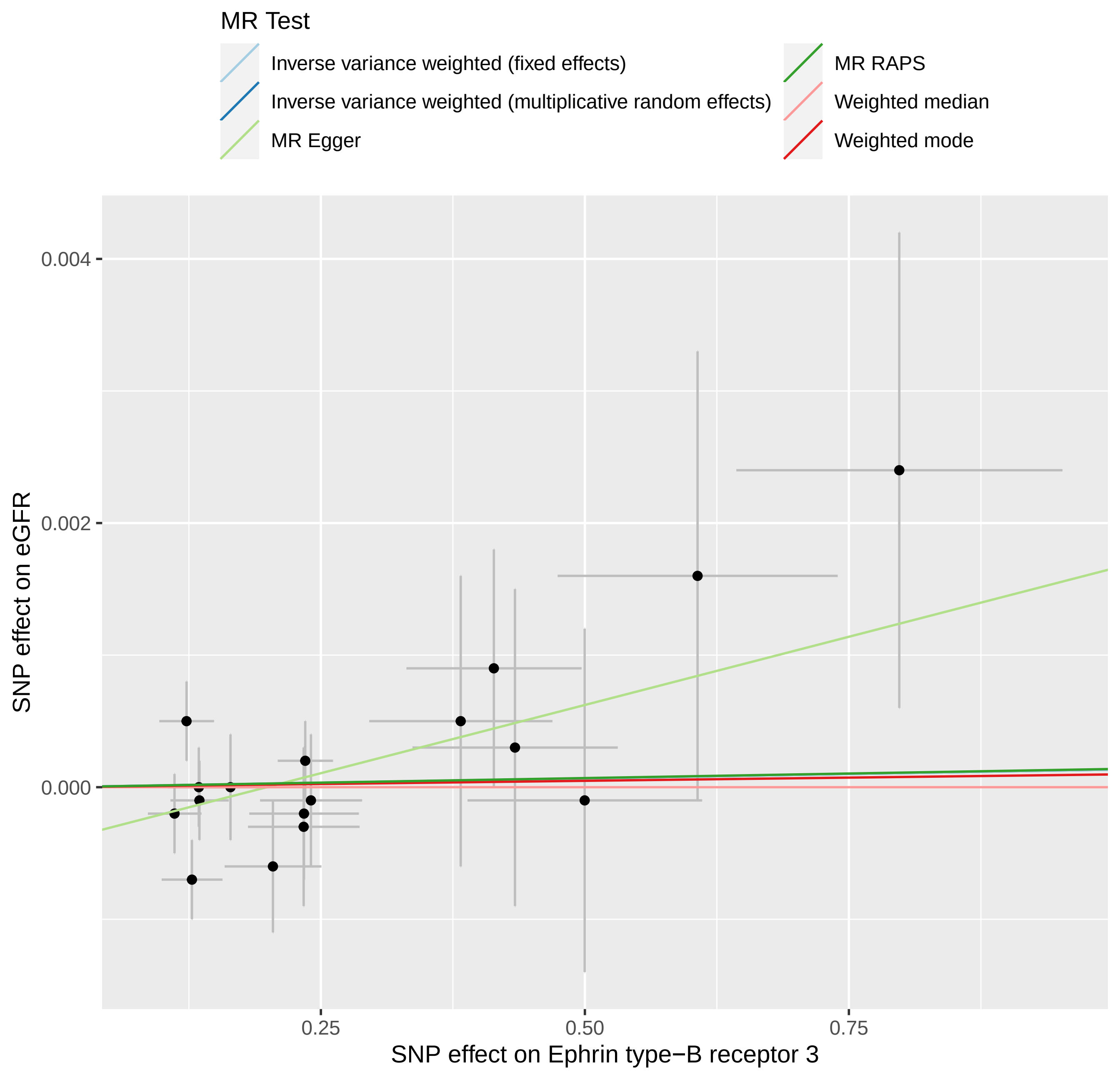

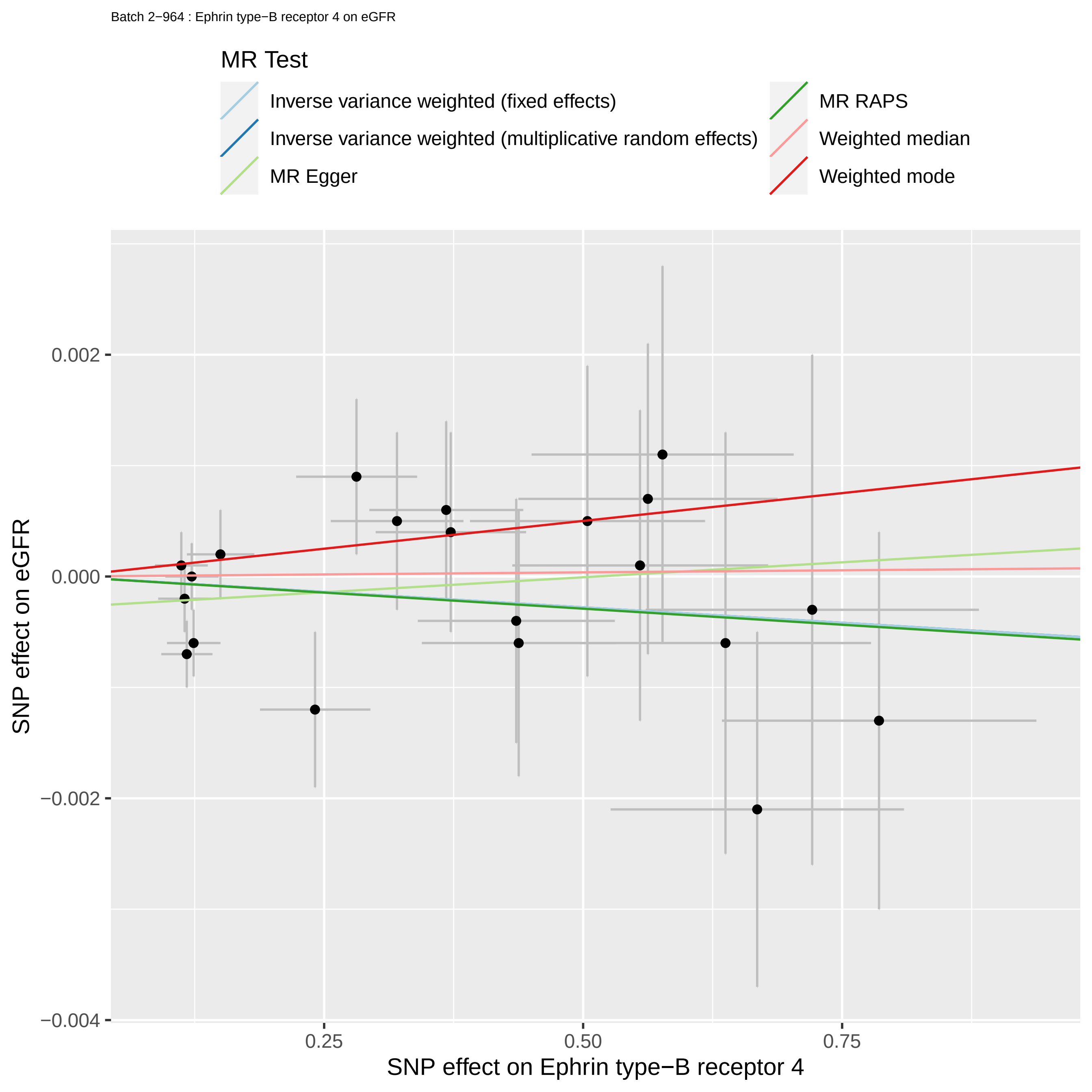

Batch 2−964 : Ephrin type−B receptor 4 on eGFR
MR Test
Inverse variance weighted (fixed effects)
Inverse variance weighted (multiplicative random effects)
MR Egger
MR RAPS
Weighted median
Weighted mode
0.002
0.000
−0.002
−0.004
0.25
0.50
0.75
SNP effect on Ephrin type−B receptor 4
SNP effect on eGFR

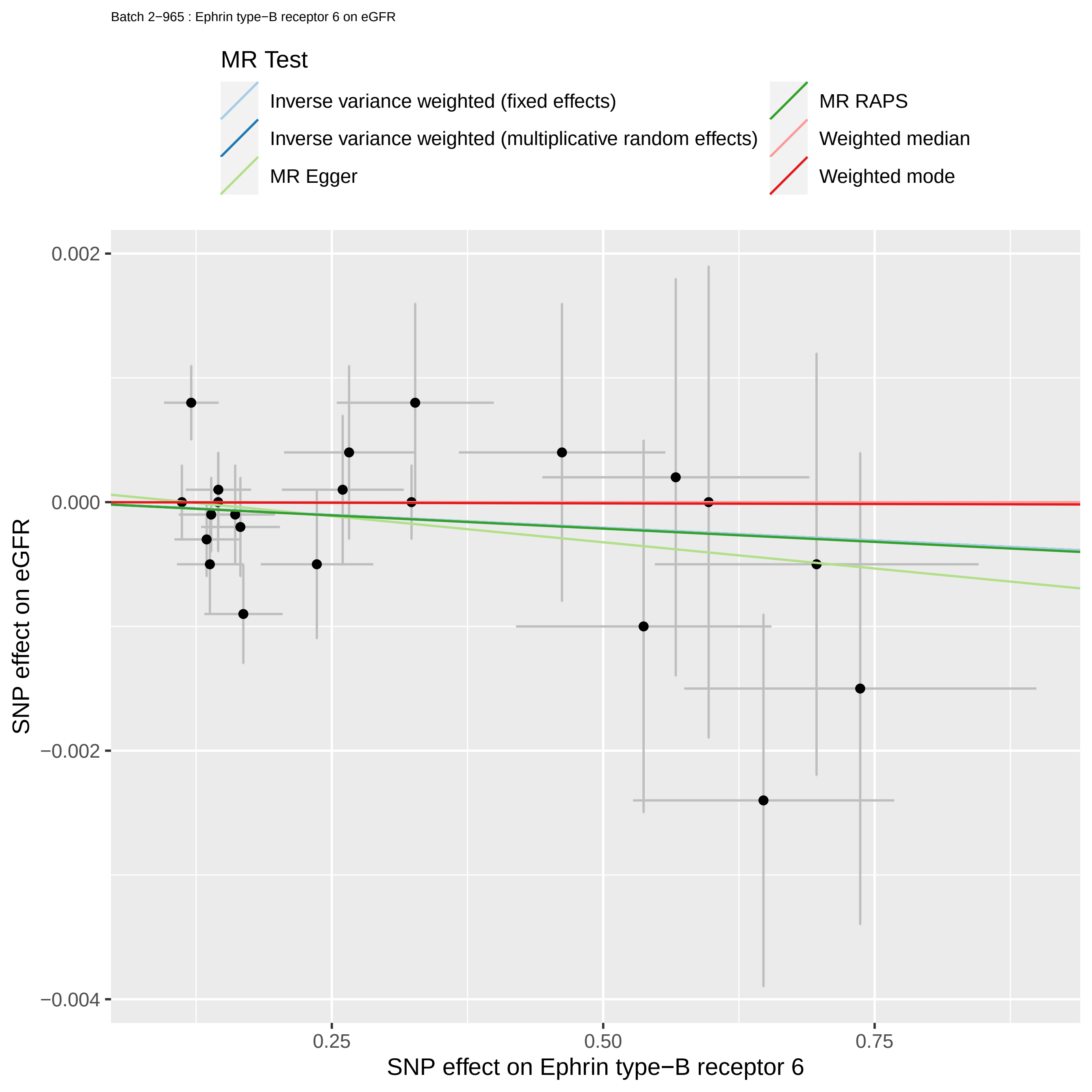
Batch 2−965 : Ephrin type−B receptor 6 on eGFR
MR Test
Inverse variance weighted (fixed effects)
Inverse variance weighted (multiplicative random effects)
MR Egger
MR RAPS
Weighted median
Weighted mode
0.002
0.000
−0.002
−0.004
0.25
0.50
0.75
SNP effect on eGFR
SNP effect on Ephrin type−B receptor 6

Batch 2−966 : Ephrin type−B receptor 6 on eGFR

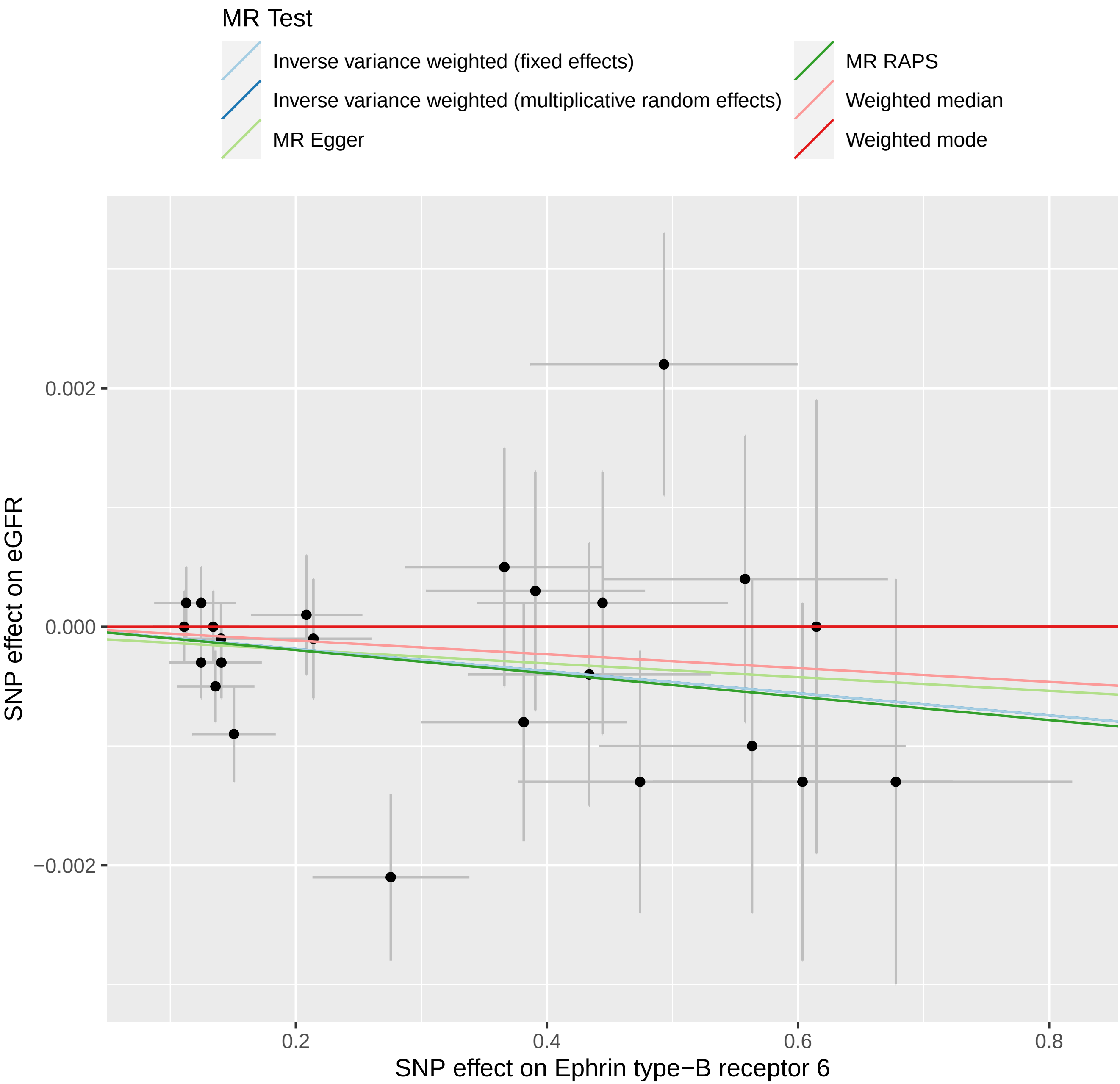

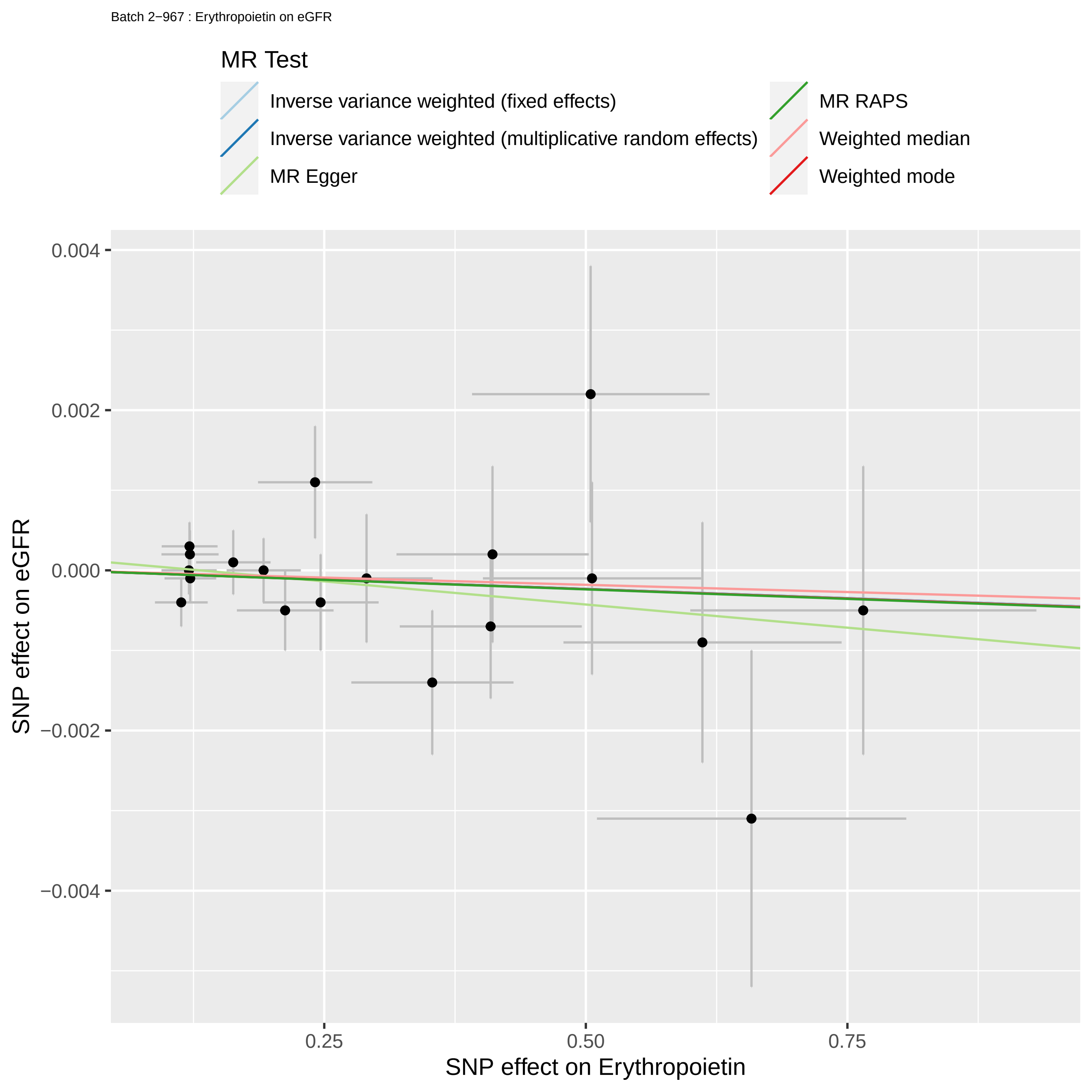
Batch 2−967 : Erythropoietin on eGFR
MR Test
Inverse variance weighted (fixed effects)
Inverse variance weighted (multiplicative random effects)
MR Egger
MR RAPS
Weighted median
Weighted mode
0.004
0.002
0.000
−0.002
−0.004
SNP effect on eGFR
0.25
0.50
0.75
SNP effect on Erythropoietin

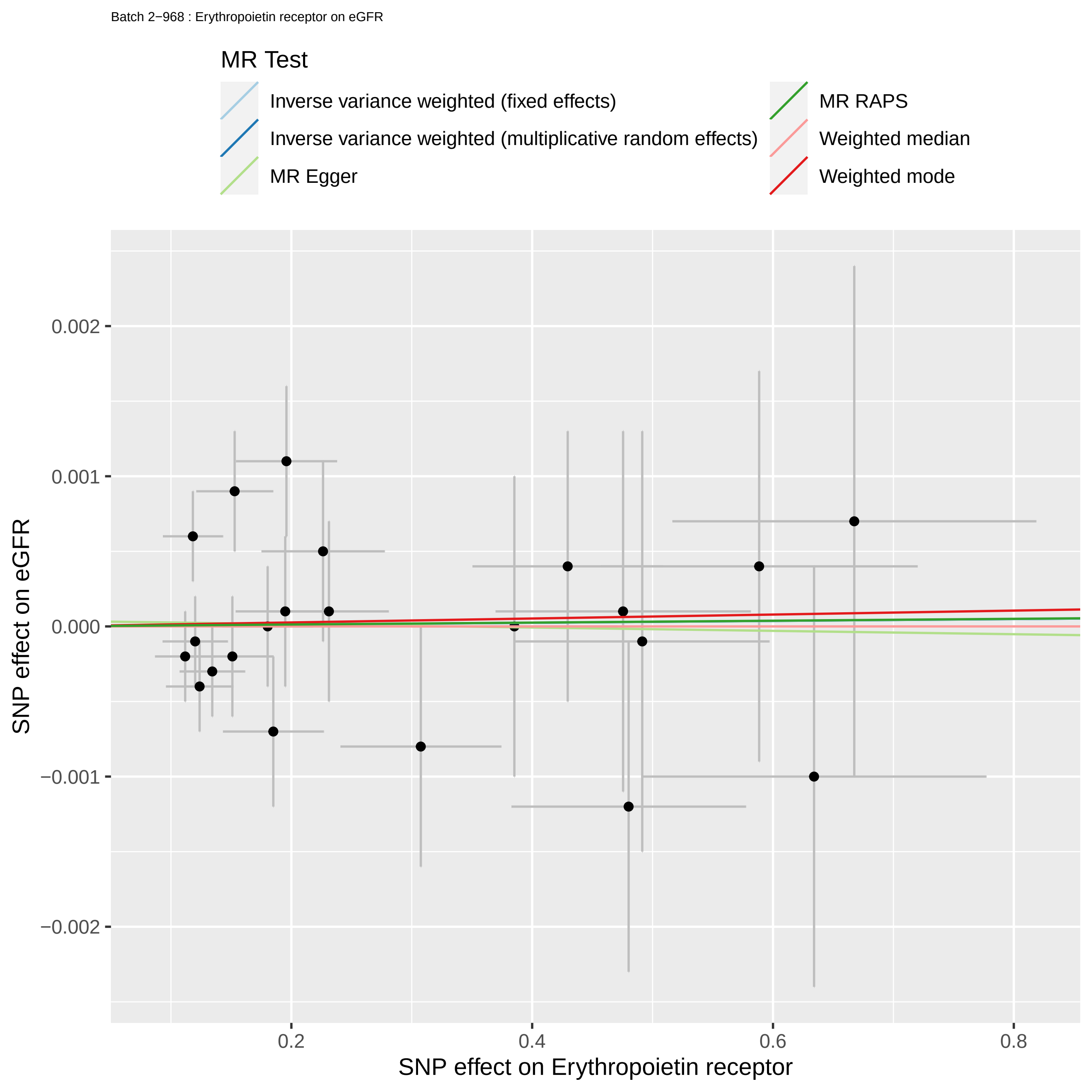

Batch 2−968 : Erythropoietin receptor on eGFR
MR Test
Inverse variance weighted (fixed effects)
Inverse variance weighted (multiplicative random effects)
MR Egger
MR RAPS
Weighted median
Weighted mode
0.002
0.001
0.000
−0.001
−0.002
0.2
0.4
0.6
0.8
SNP effect on eGFR
SNP effect on Erythropoietin receptor

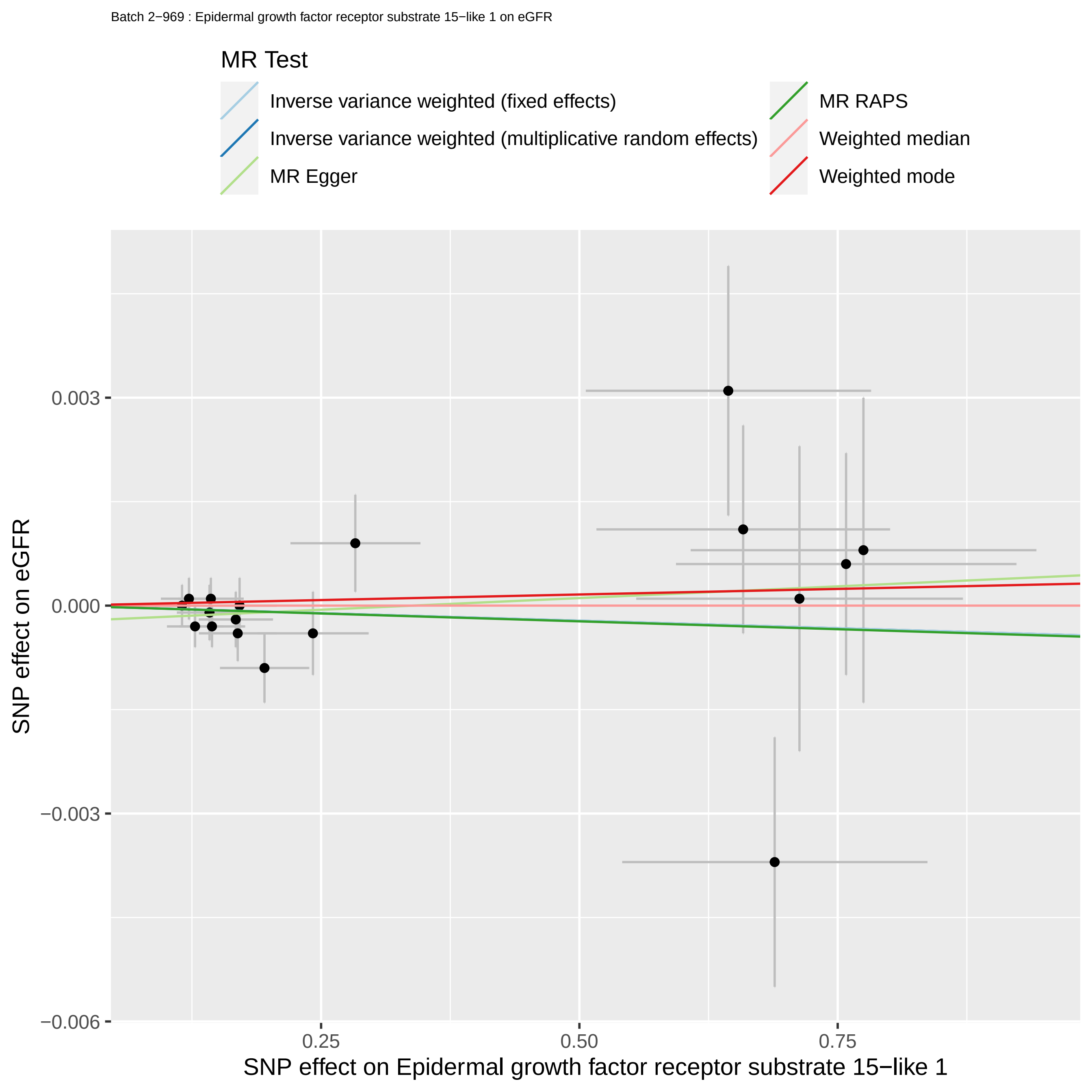

Batch 2−969 : Epidermal growth factor receptor substrate 15−like 1 on eGFR
MR Test
Inverse variance weighted (fixed effects)
Inverse variance weighted (multiplicative random effects)
MR Egger
MR RAPS
Weighted median
Weighted mode
0.003
0.000
−0.003
−0.006
0.25
0.50
0.75
SNP effect on eGFR
SNP effect on Epidermal growth factor receptor substrate 15−like 1

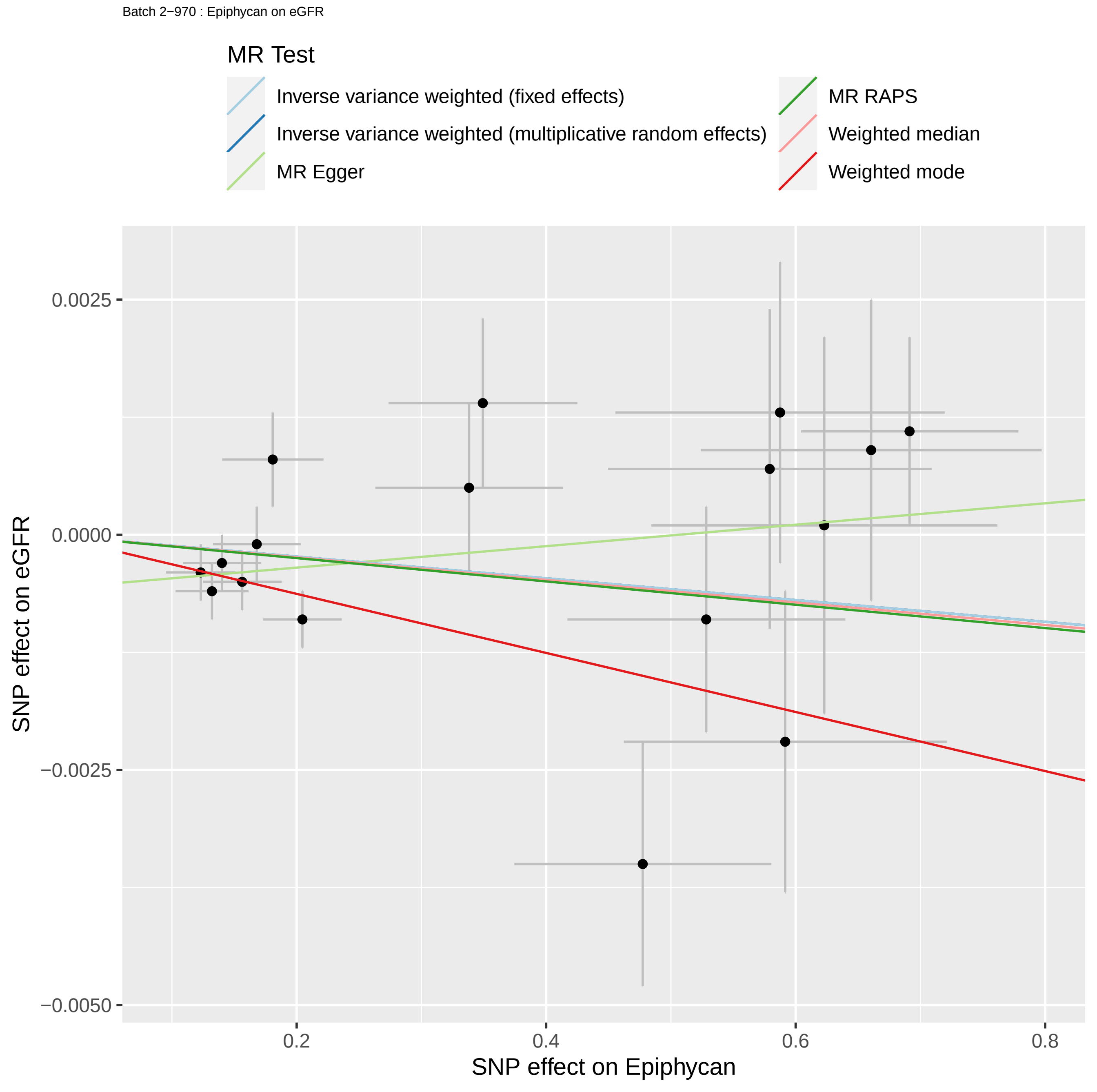
Batch 2−970 : Epiphycan on eGFR
MR Test
Inverse variance weighted (fixed effects)
Inverse variance weighted (multiplicative random effects)
MR Egger
MR RAPS
Weighted median
Weighted mode
0.0025
0.0000
−0.0025
−0.0050
0.2
0.4
0.6
0.8
SNP effect on eGFR
SNP effect on Epiphycan

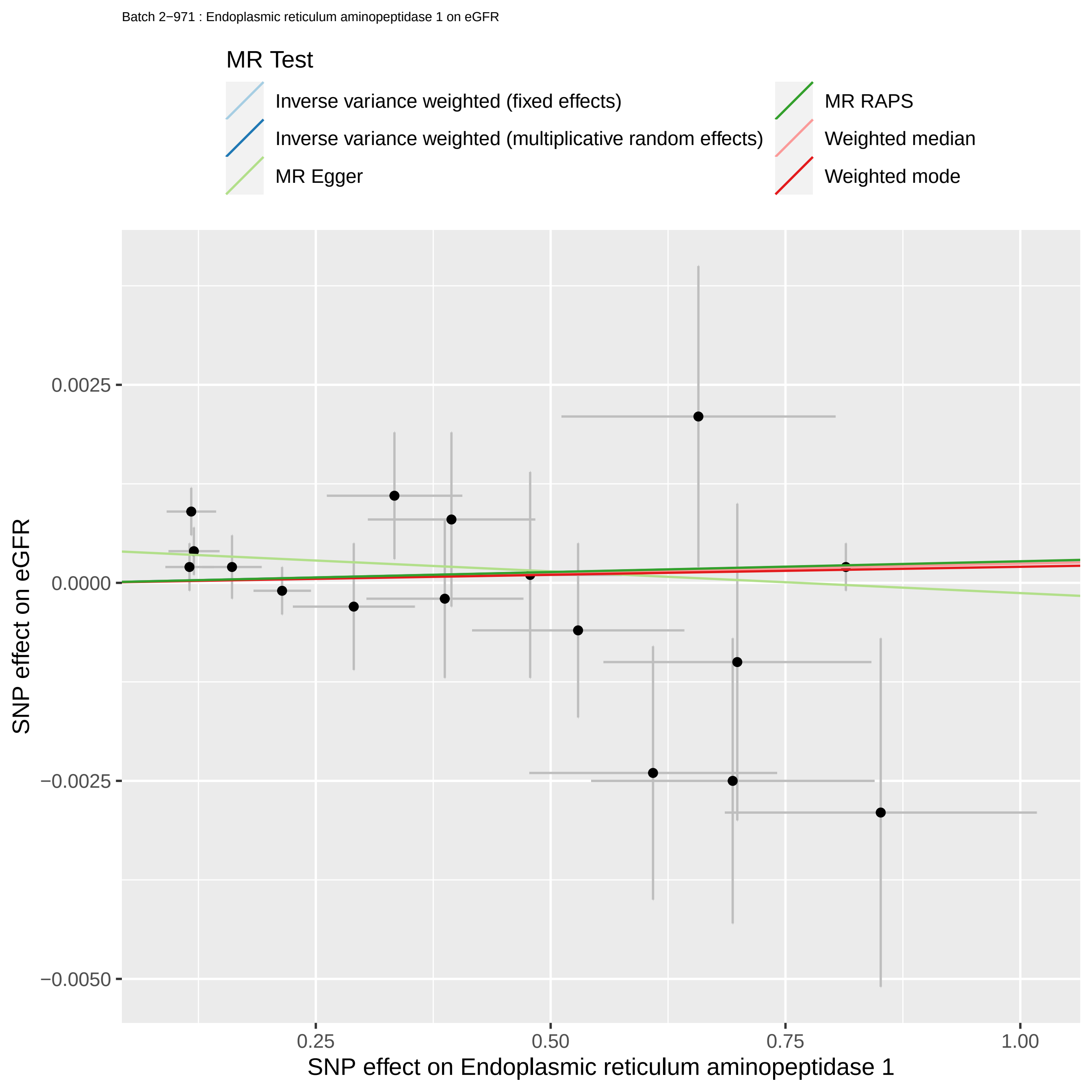

Batch 2−971 : Endoplasmic reticulum aminopeptidase 1 on eGFR
MR Test
Inverse variance weighted (fixed effects)
Inverse variance weighted (multiplicative random effects)
MR Egger
MR RAPS
Weighted median
Weighted mode
0.0025
0.0000
−0.0025
−0.0050
0.25
0.50
0.75
1.00
SNP effect on eGFR
SNP effect on Endoplasmic reticulum aminopeptidase 1

Batch 2−972 : Endoplasmic reticulum aminopeptidase 2 on eGFR

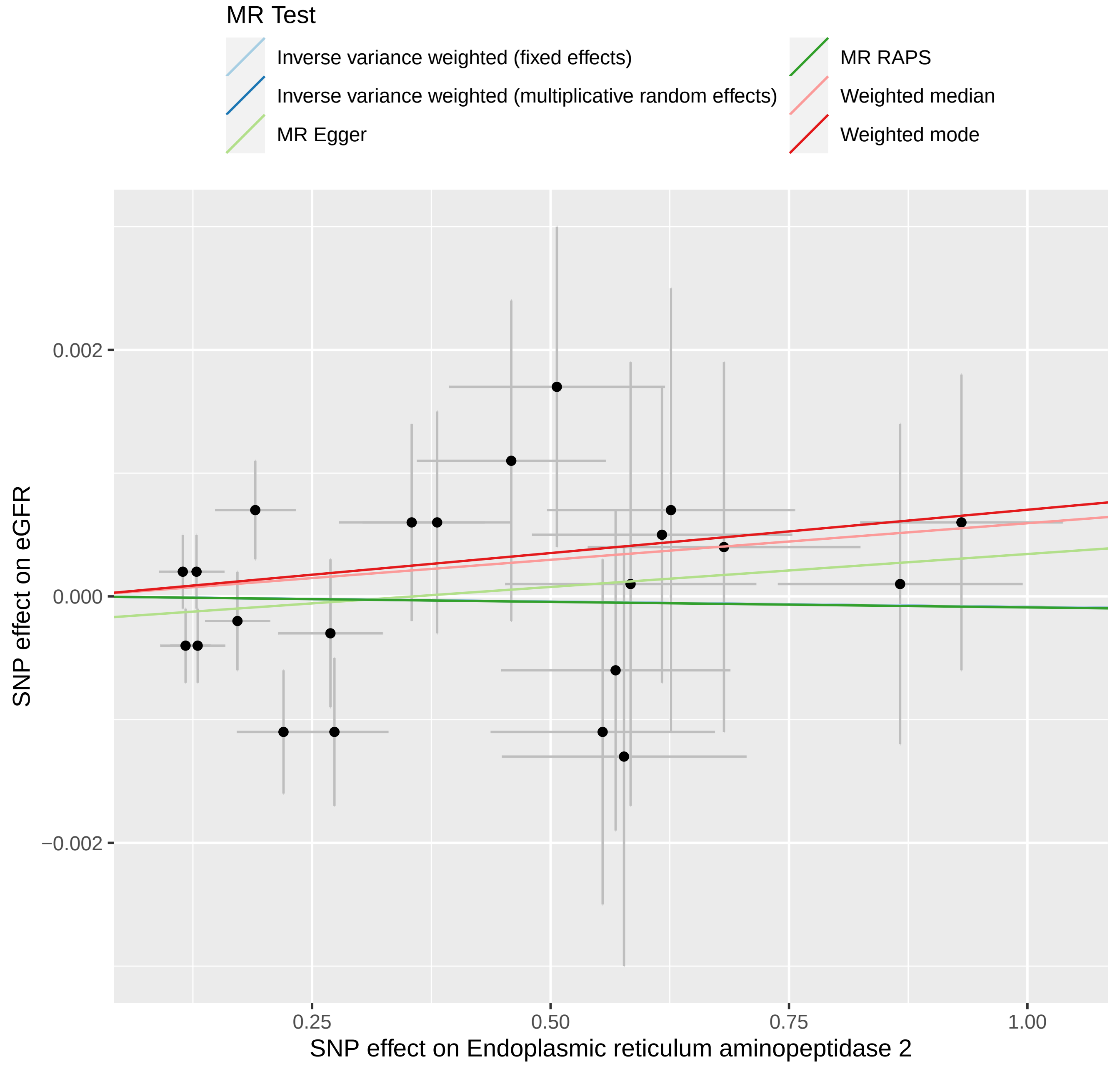

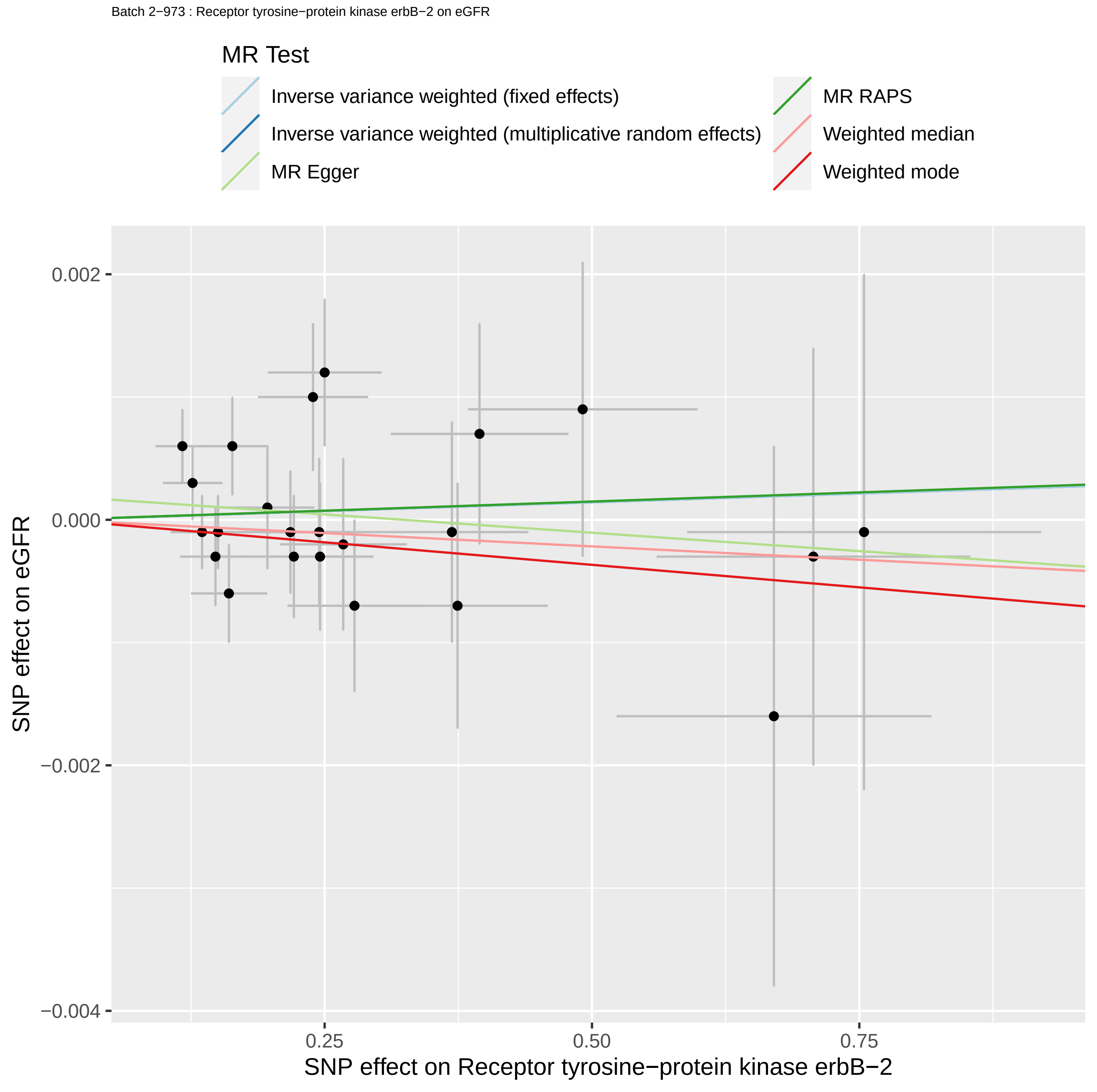

Batch 2−973 : Receptor tyrosine−protein kinase erbB−2 on eGFR
MR Test
Inverse variance weighted (fixed effects)
Inverse variance weighted (multiplicative random effects)
MR Egger
MR RAPS
Weighted median
Weighted mode
0.002
0.000
−0.002
−0.004
0.25
0.50
0.75
SNP effect on eGFR
SNP effect on Receptor tyrosine−protein kinase erbB−2

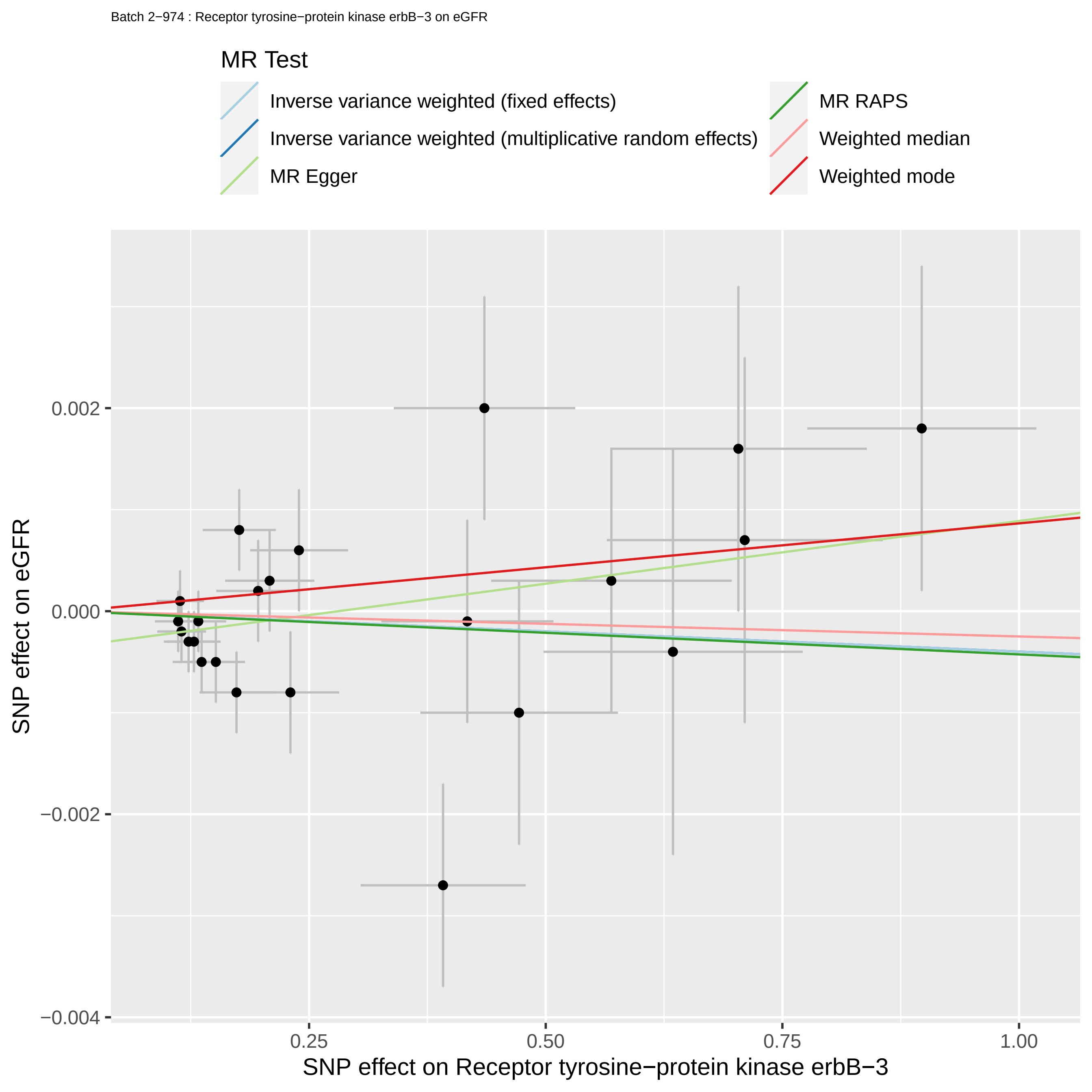

Batch 2−974 : Receptor tyrosine−protein kinase erbB−3 on eGFR
MR Test
Inverse variance weighted (fixed effects)
Inverse variance weighted (multiplicative random effects)
MR Egger
MR RAPS
Weighted median
Weighted mode
0.002
0.000
−0.002
−0.004
SNP effect on eGFR
0.25
0.50
0.75
1.00
SNP effect on Receptor tyrosine−protein kinase erbB−3

Batch 2−975 : DNA excision repair protein ERCC−1 on eGFR

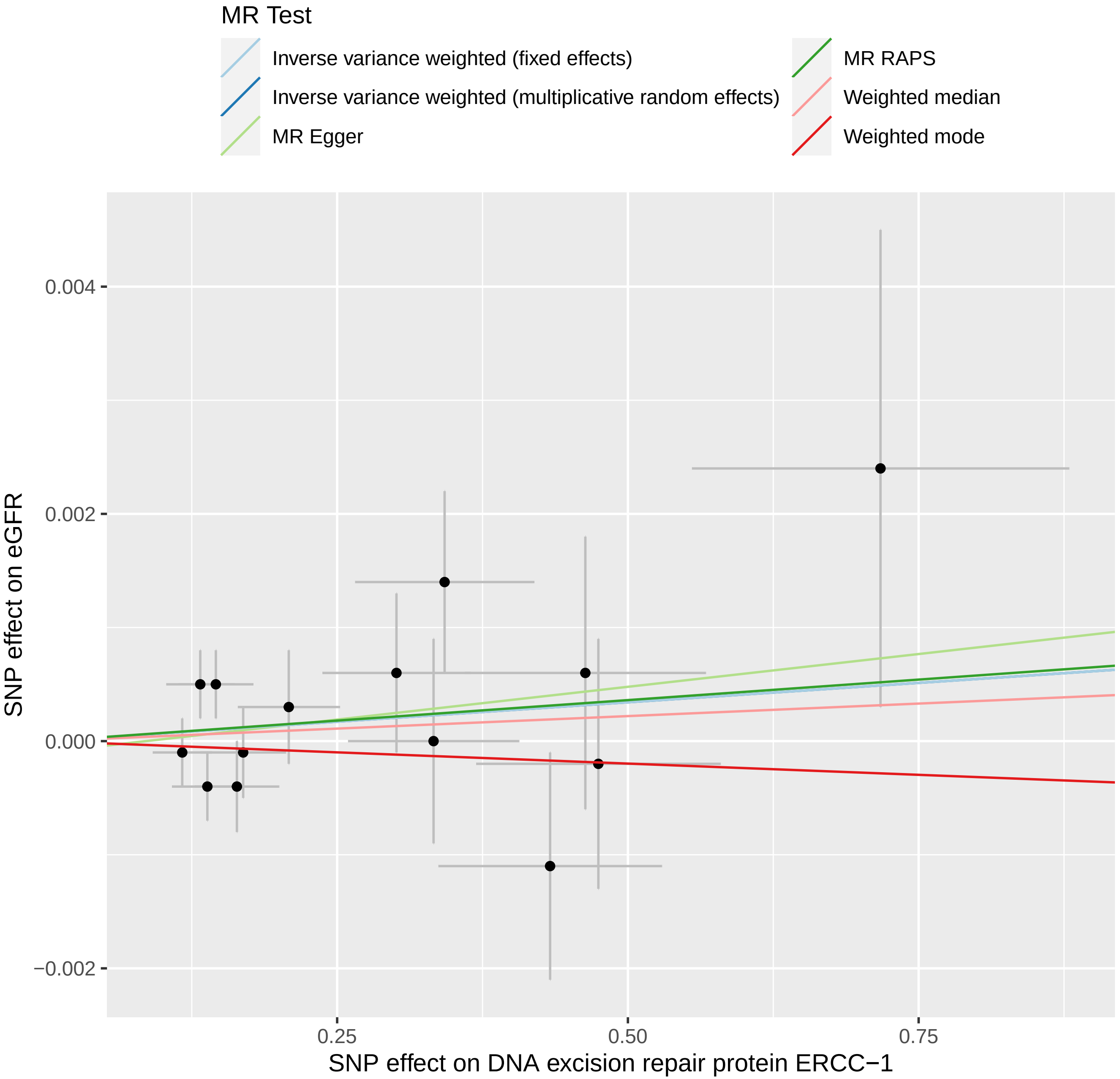

Batch 2−976 : DNA repair endonuclease XPF on eGFR

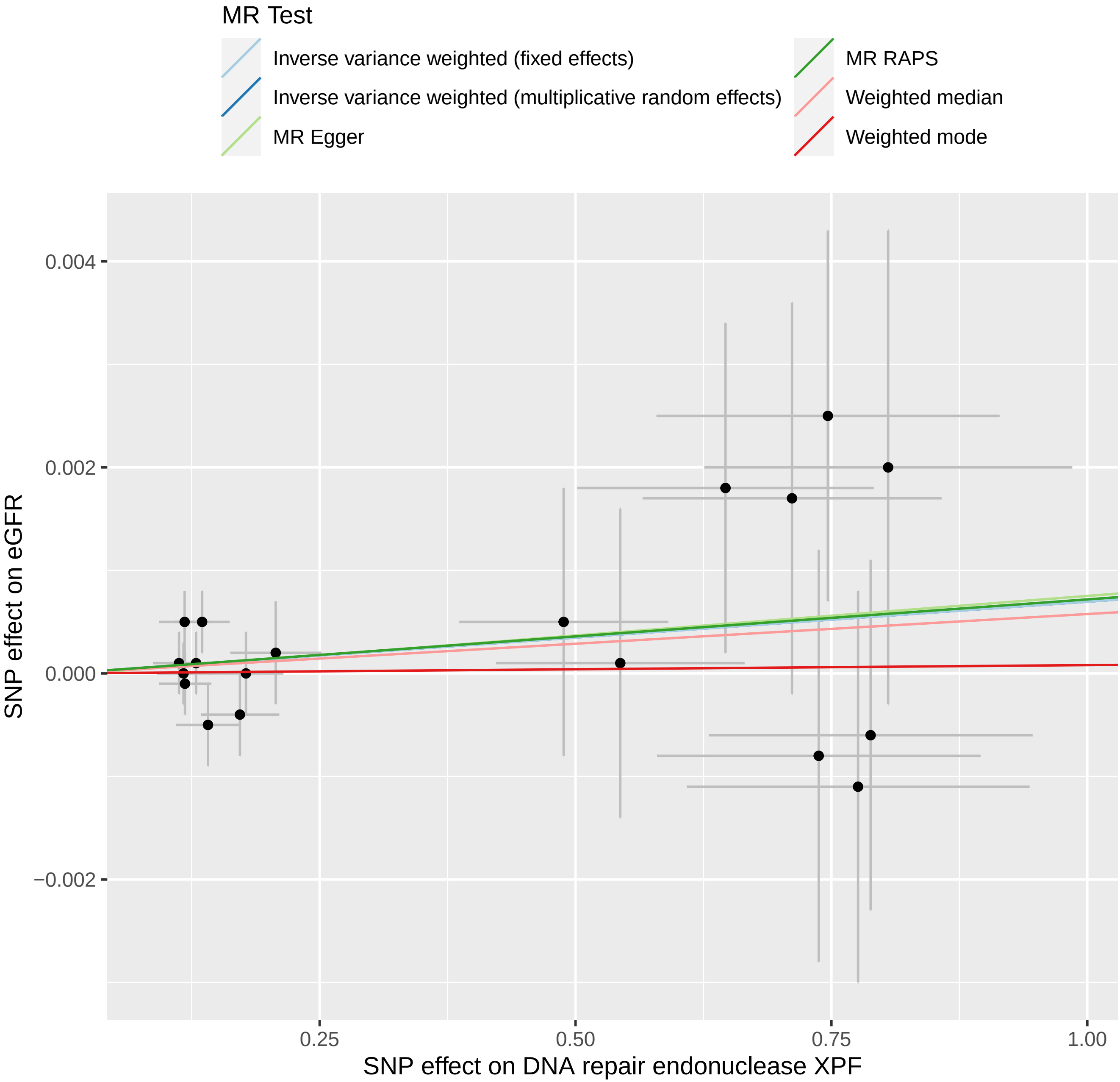

Batch 2−977 : Epiregulin on eGFR

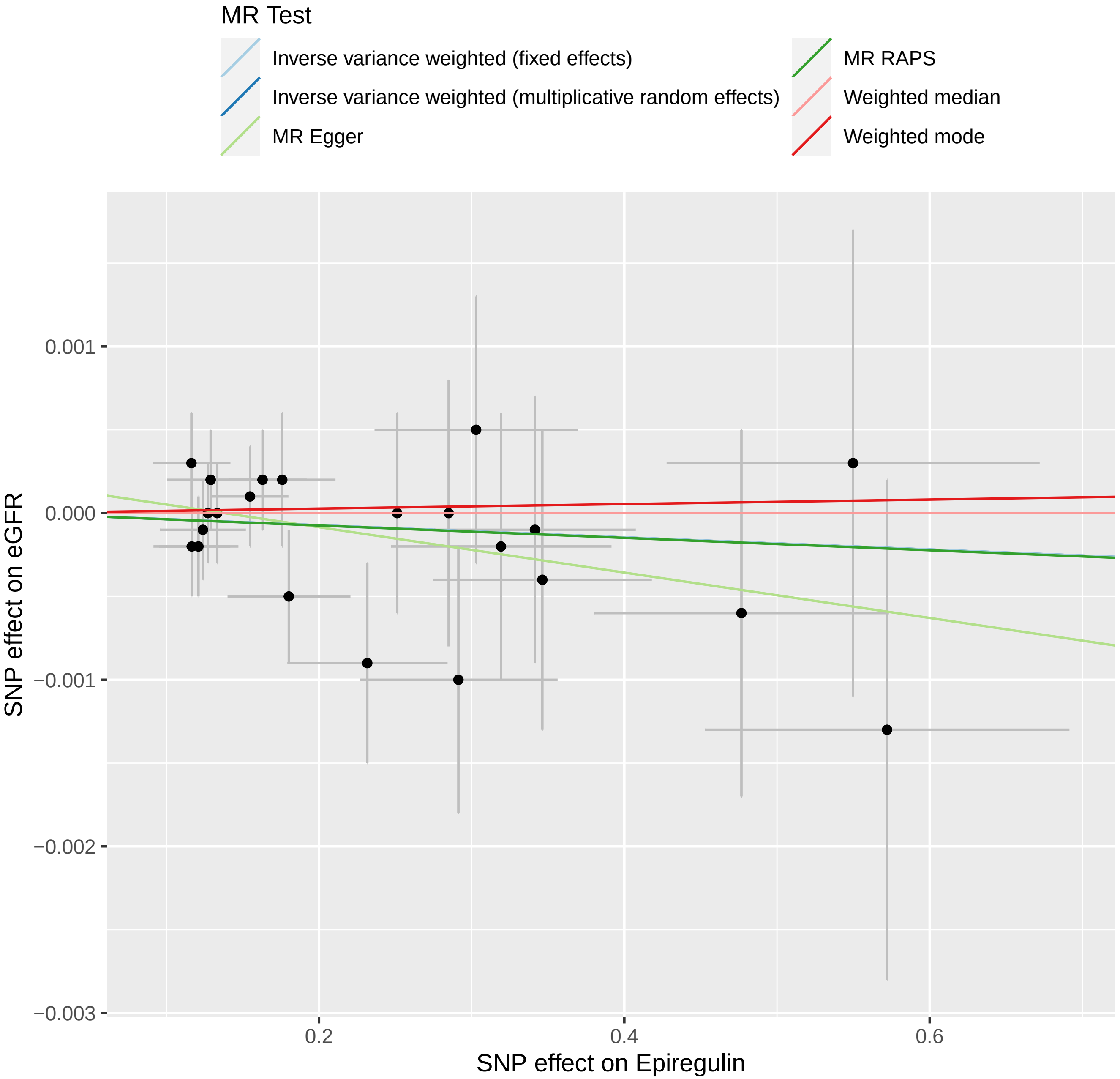

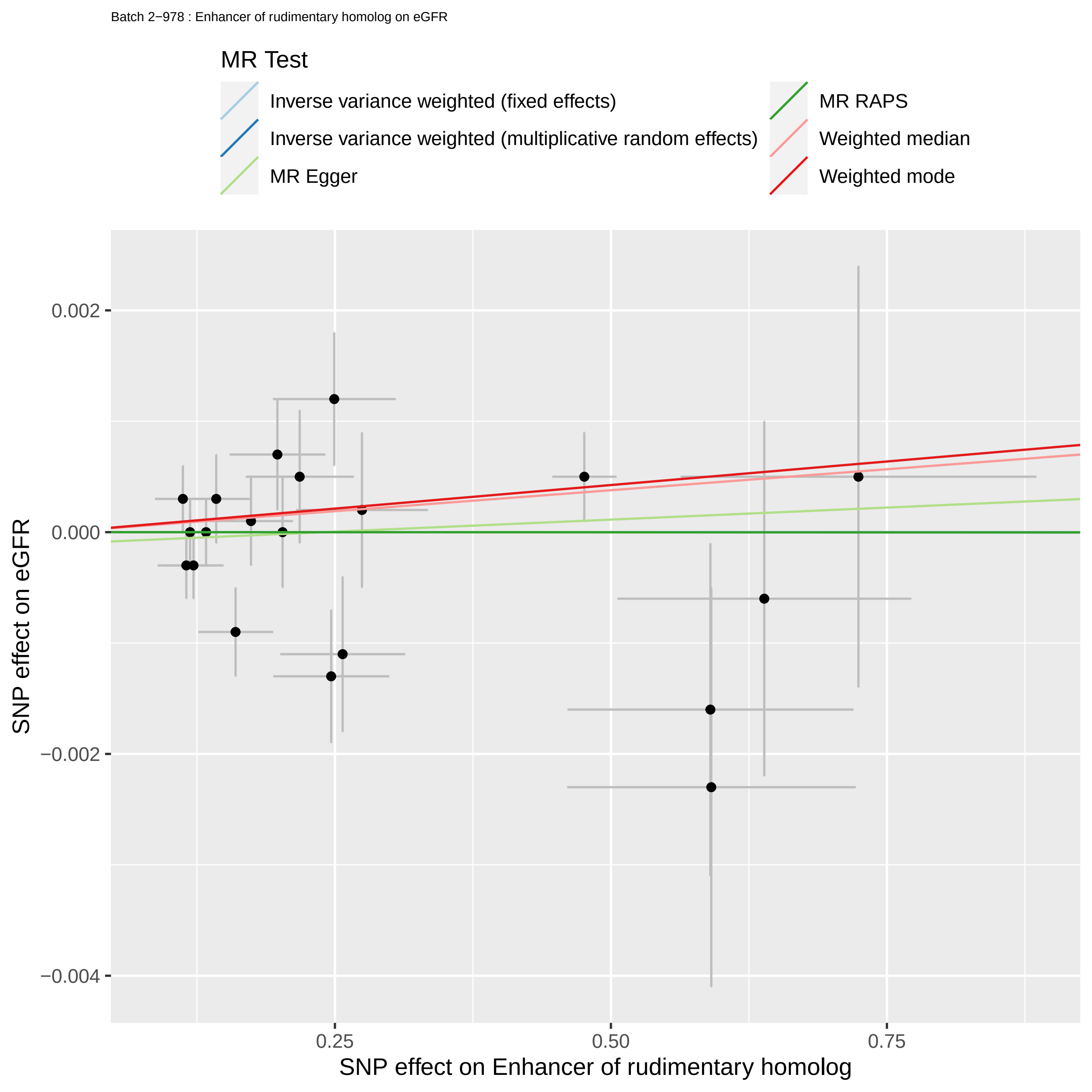
Batch 2−978 : Enhancer of rudimentary homolog on eGFR
MR Test
Inverse variance weighted (fixed effects)
Inverse variance weighted (multiplicative random effects)
MR Egger
MR RAPS
Weighted median
Weighted mode
0.002
0.000
−0.002
−0.004
0.25
0.50
0.75
SNP effect on eGFR
SNP effect on Enhancer of rudimentary homolog

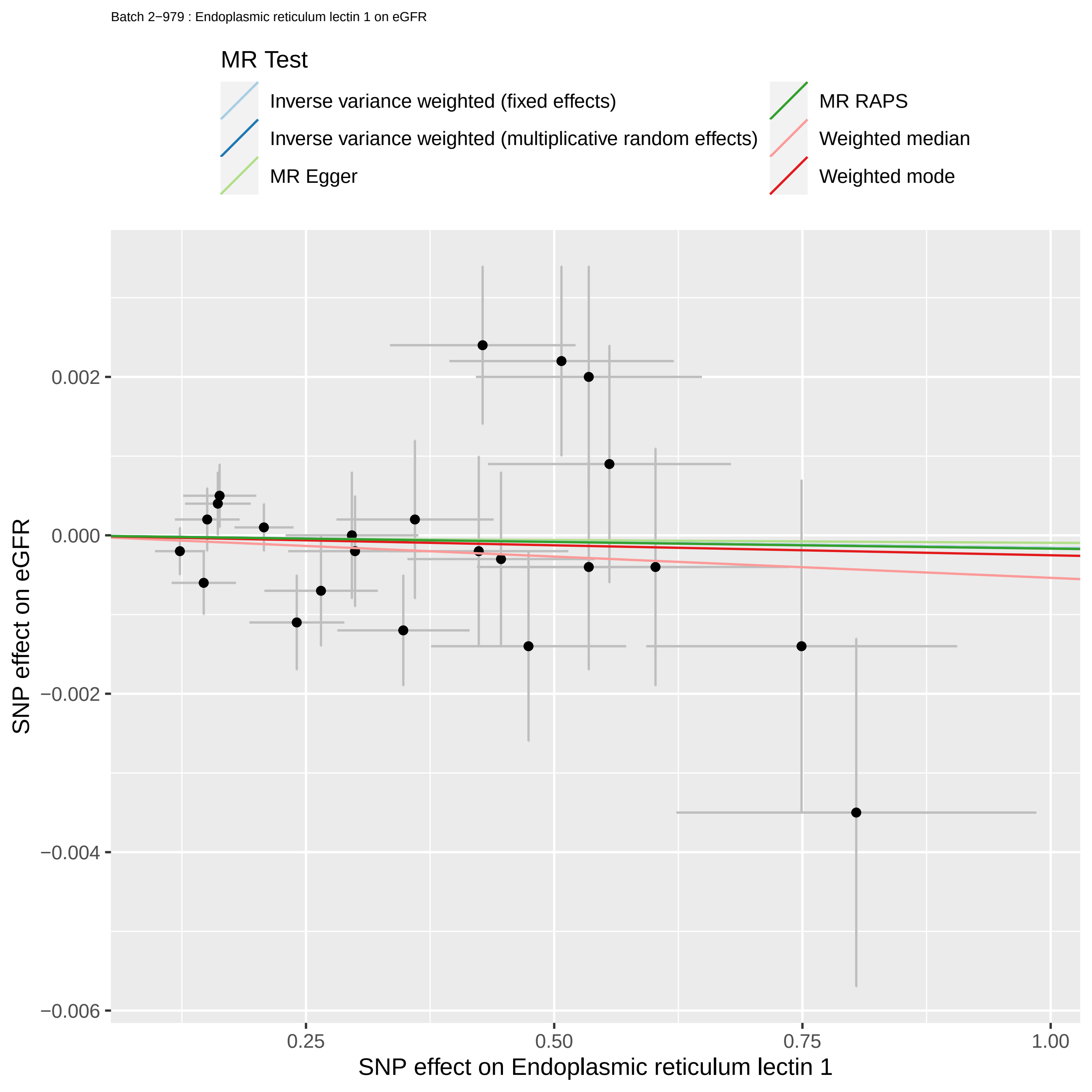
Batch 2−979 : Endoplasmic reticulum lectin 1 on eGFR
MR Test
Inverse variance weighted (fixed effects)
Inverse variance weighted (multiplicative random effects)
MR Egger
MR RAPS
Weighted median
Weighted mode
0.002
0.000
−0.002
−0.004
−0.006
0.25
0.50
0.75
1.00
SNP effect on Endoplasmic reticulum lectin 1
SNP effect on eGFR

Batch 2−980 : Erlin−1 on eGFR

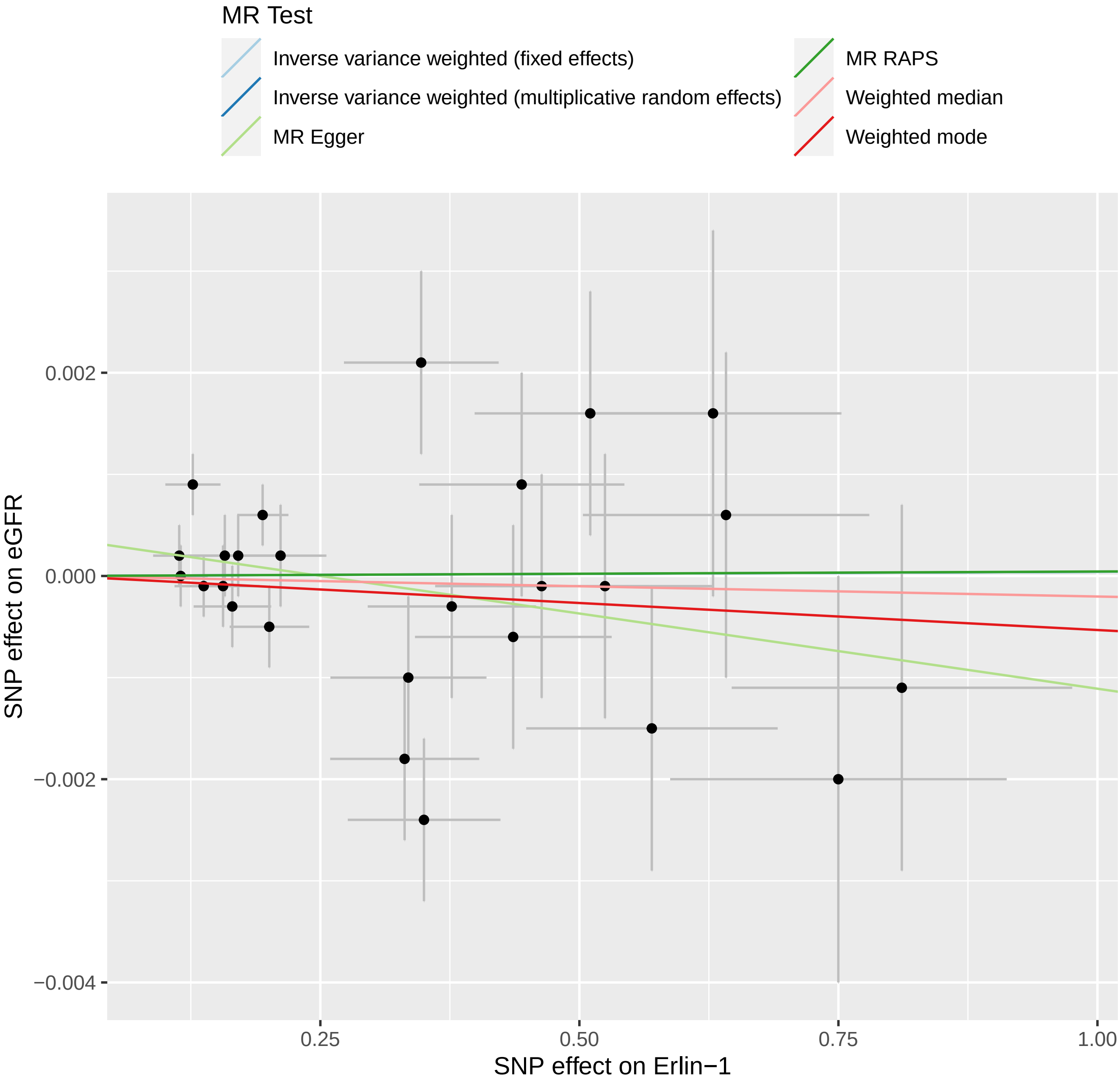

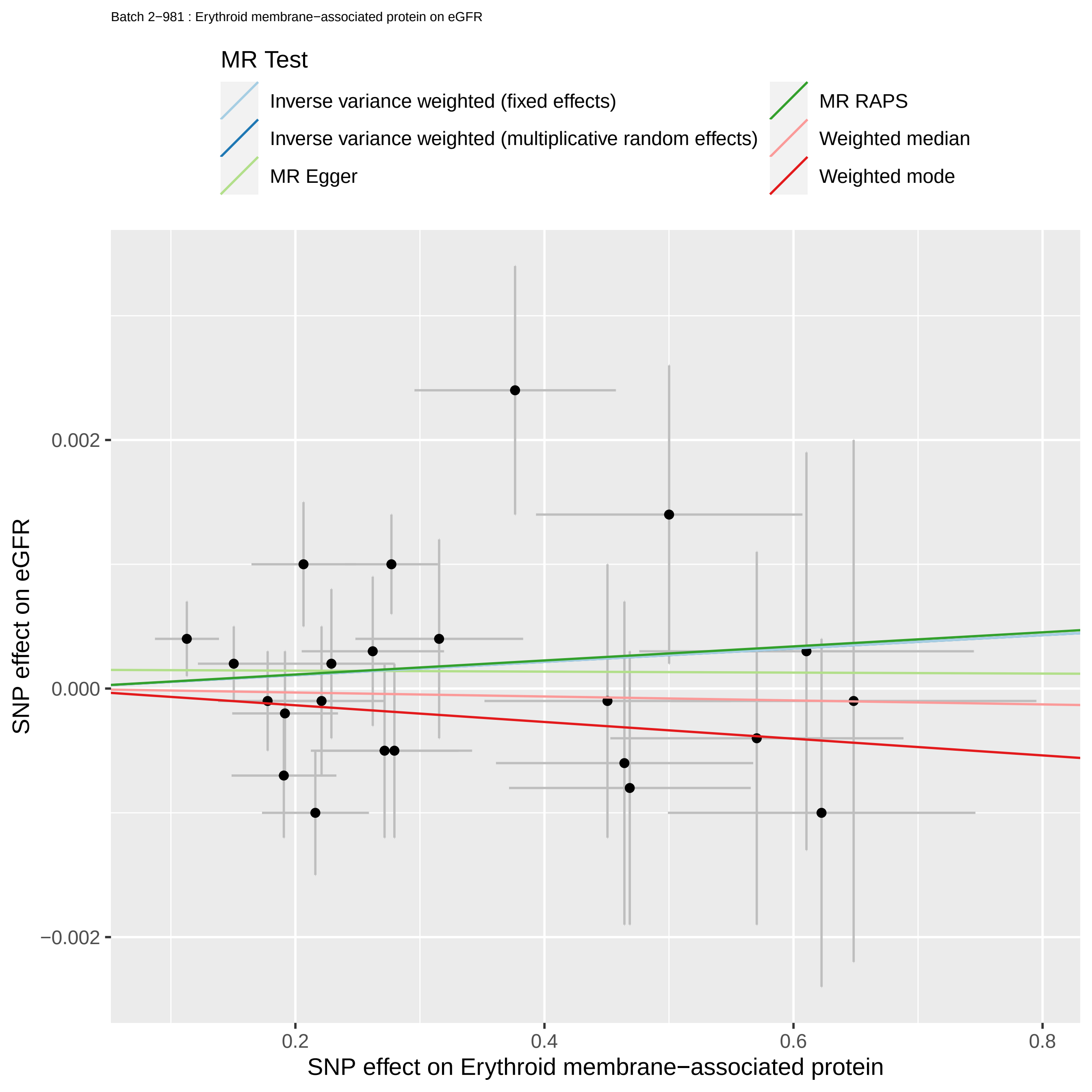
Batch 2−981 : Erythroid membrane−associated protein on eGFR
MR Test
Inverse variance weighted (fixed effects)
Inverse variance weighted (multiplicative random effects)
MR Egger
MR RAPS
Weighted median
Weighted mode
0.002
0.000
−0.002
0.2
0.4
0.6
0.8
SNP effect on eGFR
SNP effect on Erythroid membrane−associated protein

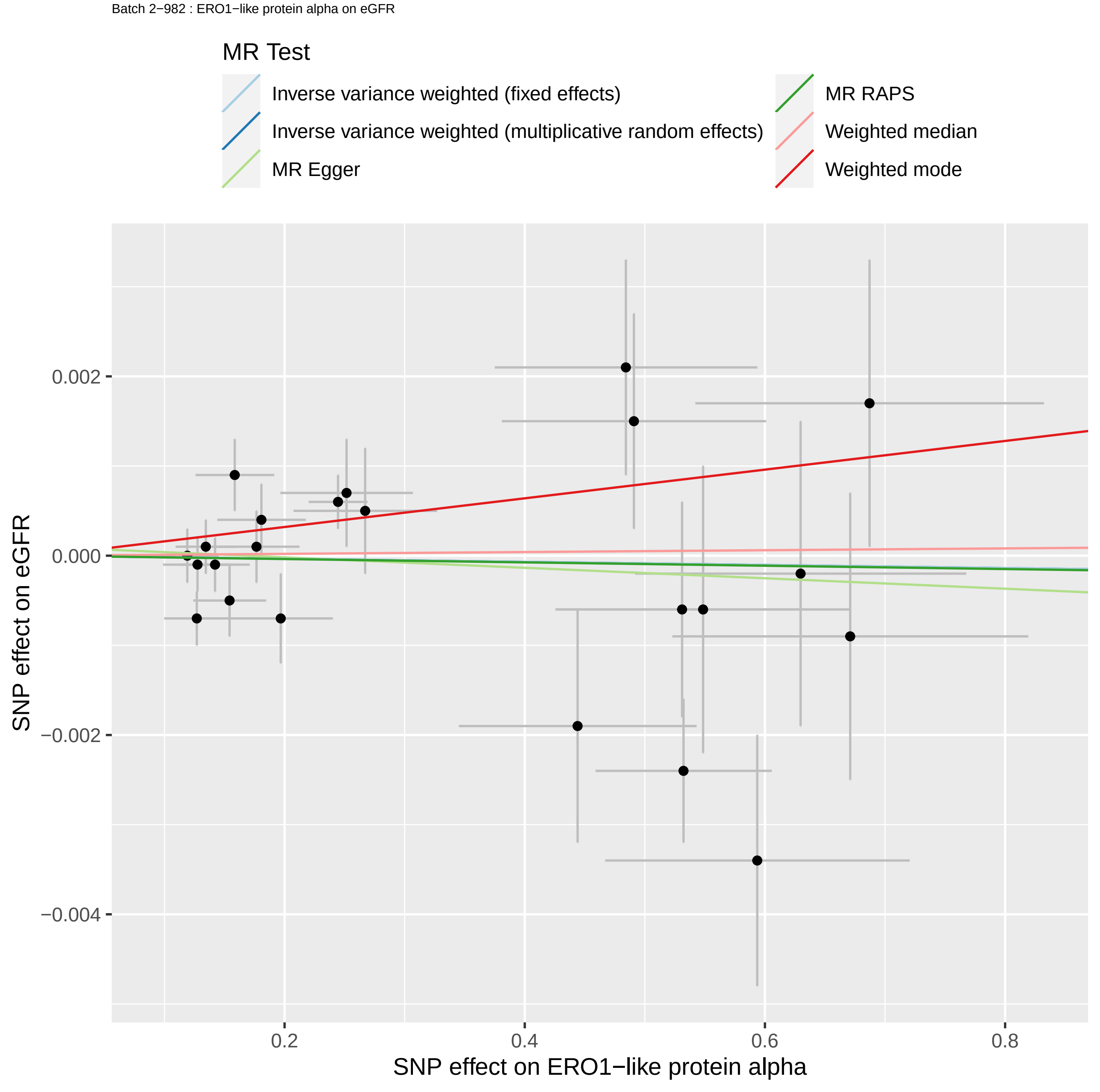

Batch 2−982 : ERO1−like protein alpha on eGFR
MR Test
Inverse variance weighted (fixed effects)
Inverse variance weighted (multiplicative random effects)
MR Egger
MR RAPS
Weighted median
Weighted mode
0.002
0.000
−0.002
−0.004
0.2
0.4
0.6
0.8
SNP effect on eGFR
SNP effect on ERO1−like protein alpha

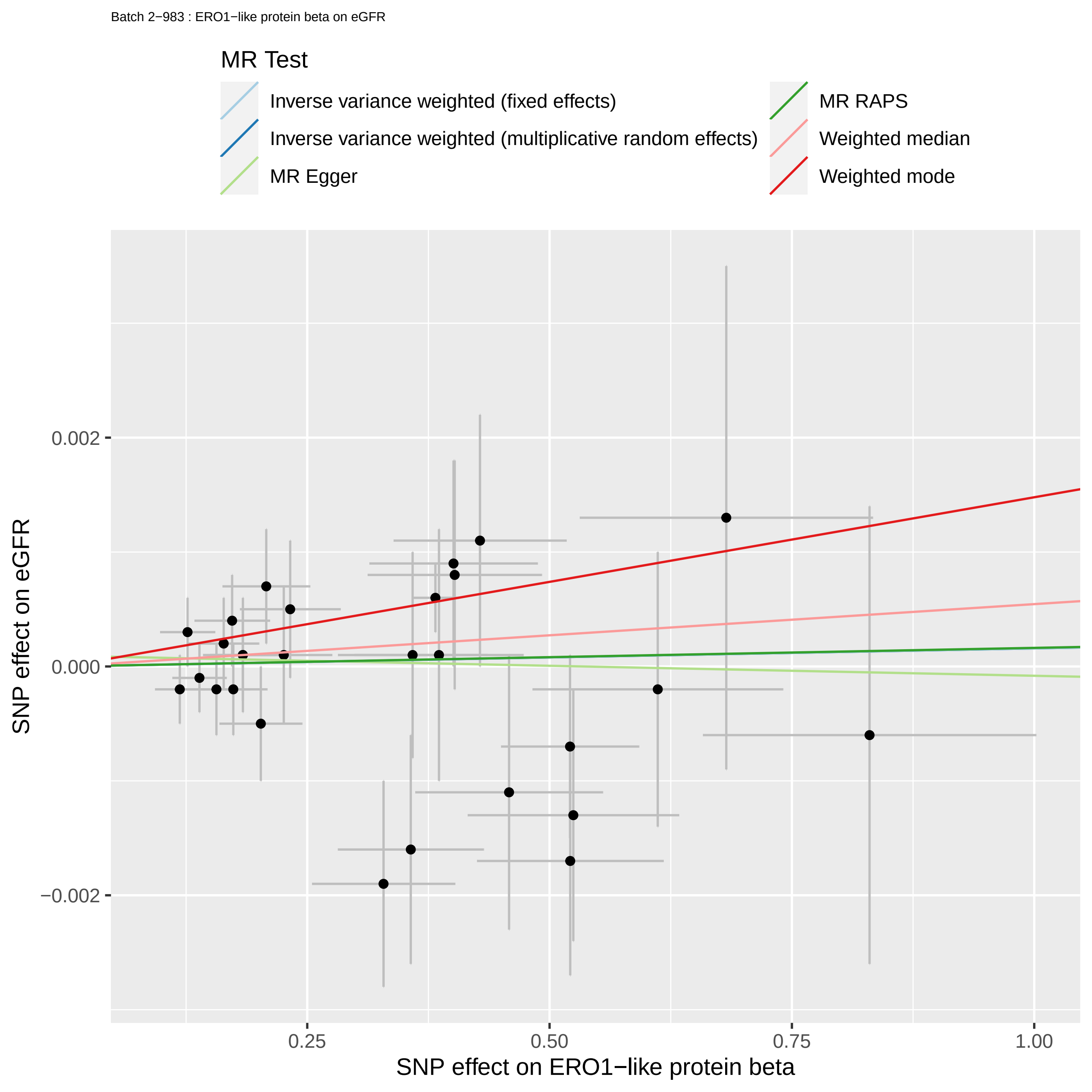

Batch 2−983 : ERO1−like protein beta on eGFR
MR Test
Inverse variance weighted (fixed effects)
Inverse variance weighted (multiplicative random effects)
MR Egger
MR RAPS
Weighted median
Weighted mode
0.002
0.000
−0.002
SNP effect on eGFR
0.25
0.50
0.75
1.00
SNP effect on ERO1−like protein beta

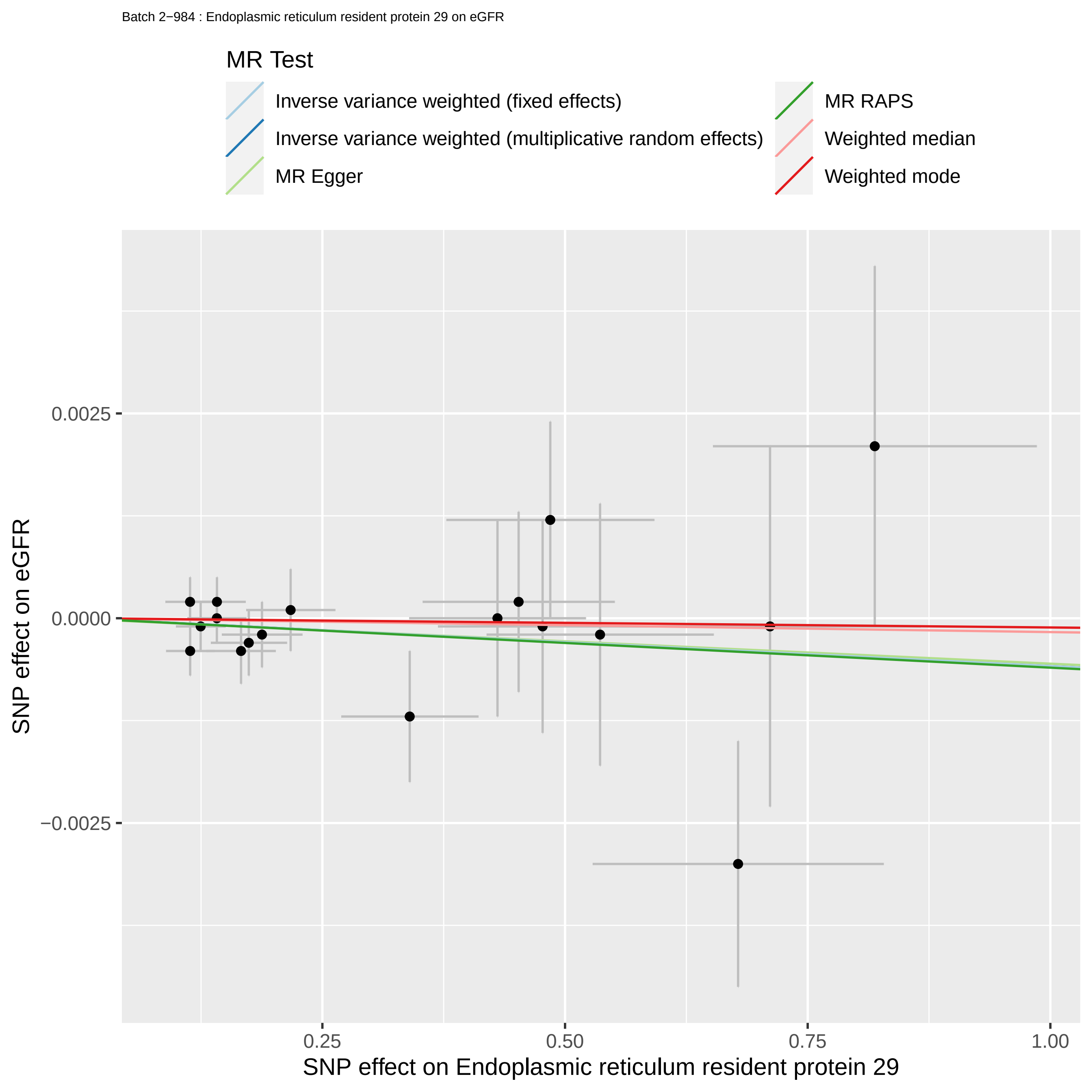

Batch 2−984 : Endoplasmic reticulum resident protein 29 on eGFR
MR Test
Inverse variance weighted (fixed effects)
Inverse variance weighted (multiplicative random effects)
MR Egger
MR RAPS
Weighted median
Weighted mode
0.0025
0.0000
−0.0025
0.25
0.50
0.75
1.00
SNP effect on eGFR
SNP effect on Endoplasmic reticulum resident protein 29

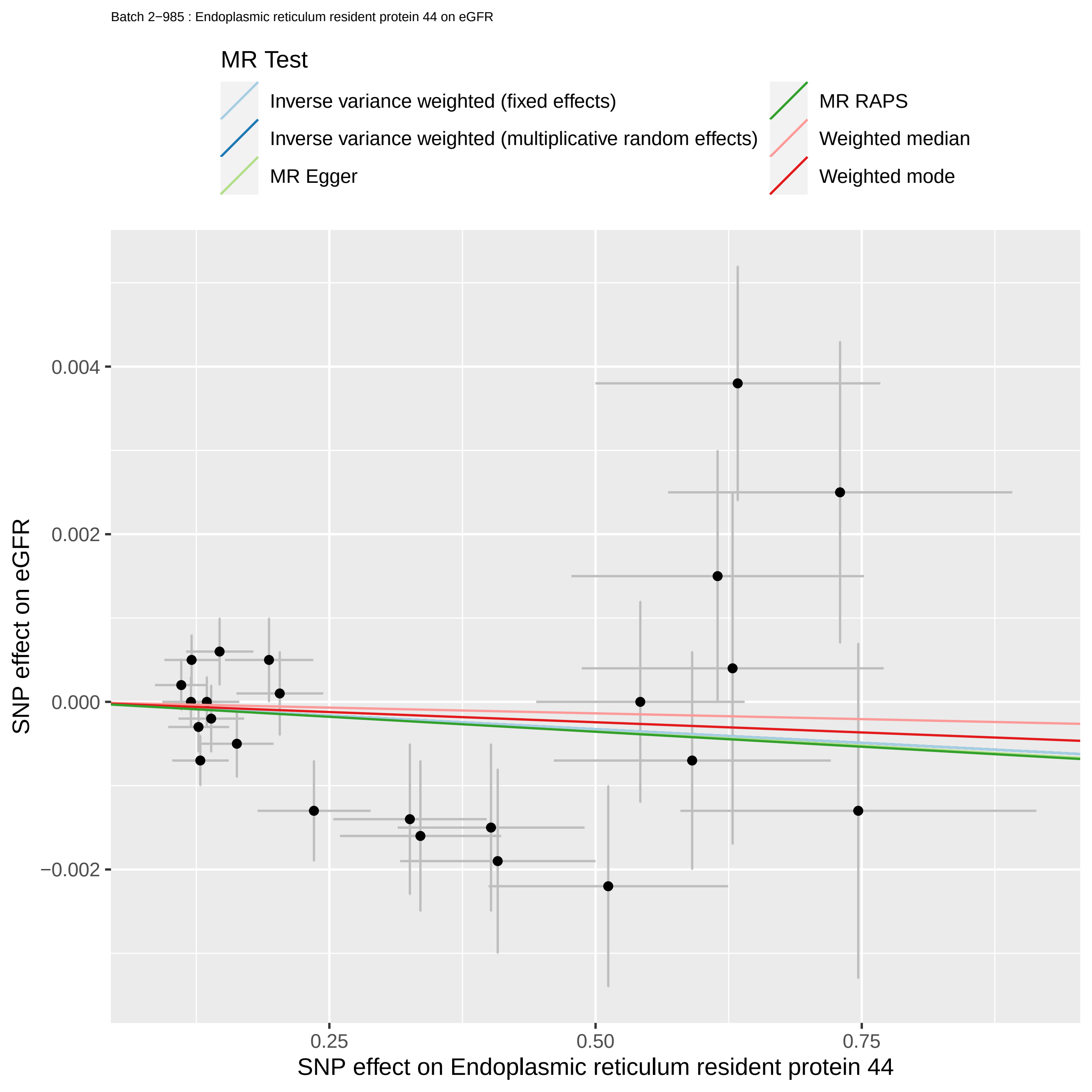
Batch 2−985 : Endoplasmic reticulum resident protein 44 on eGFR
MR Test
Inverse variance weighted (fixed effects)
Inverse variance weighted (multiplicative random effects)
MR Egger
MR RAPS
Weighted median
Weighted mode
SNP effect on eGFR
0.004
0.002
0.000
−0.002
0.25
0.50
0.75
SNP effect on Endoplasmic reticulum resident protein 44

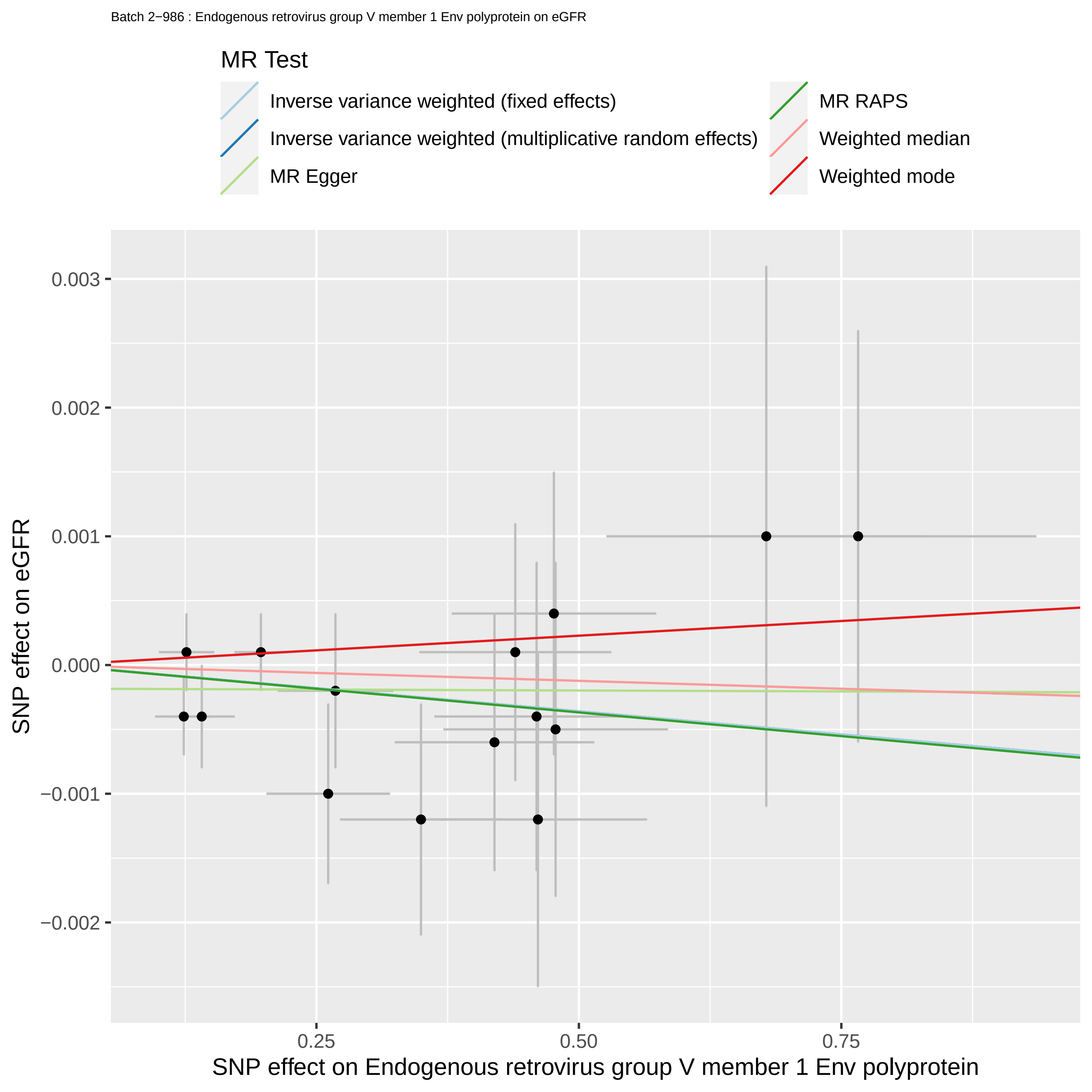

Batch 2−986 : Endogenous retrovirus group V member 1 Env polyprotein on eGFR
MR Test
Inverse variance weighted (fixed effects)
Inverse variance weighted (multiplicative random effects)
MR Egger
MR RAPS
Weighted median
Weighted mode
0.003
0.002
0.001
0.000
−0.001
−0.002
SNP effect on eGFR
0.25
0.50
0.75
SNP effect on Endogenous retrovirus group V member 1 Env polyprotein

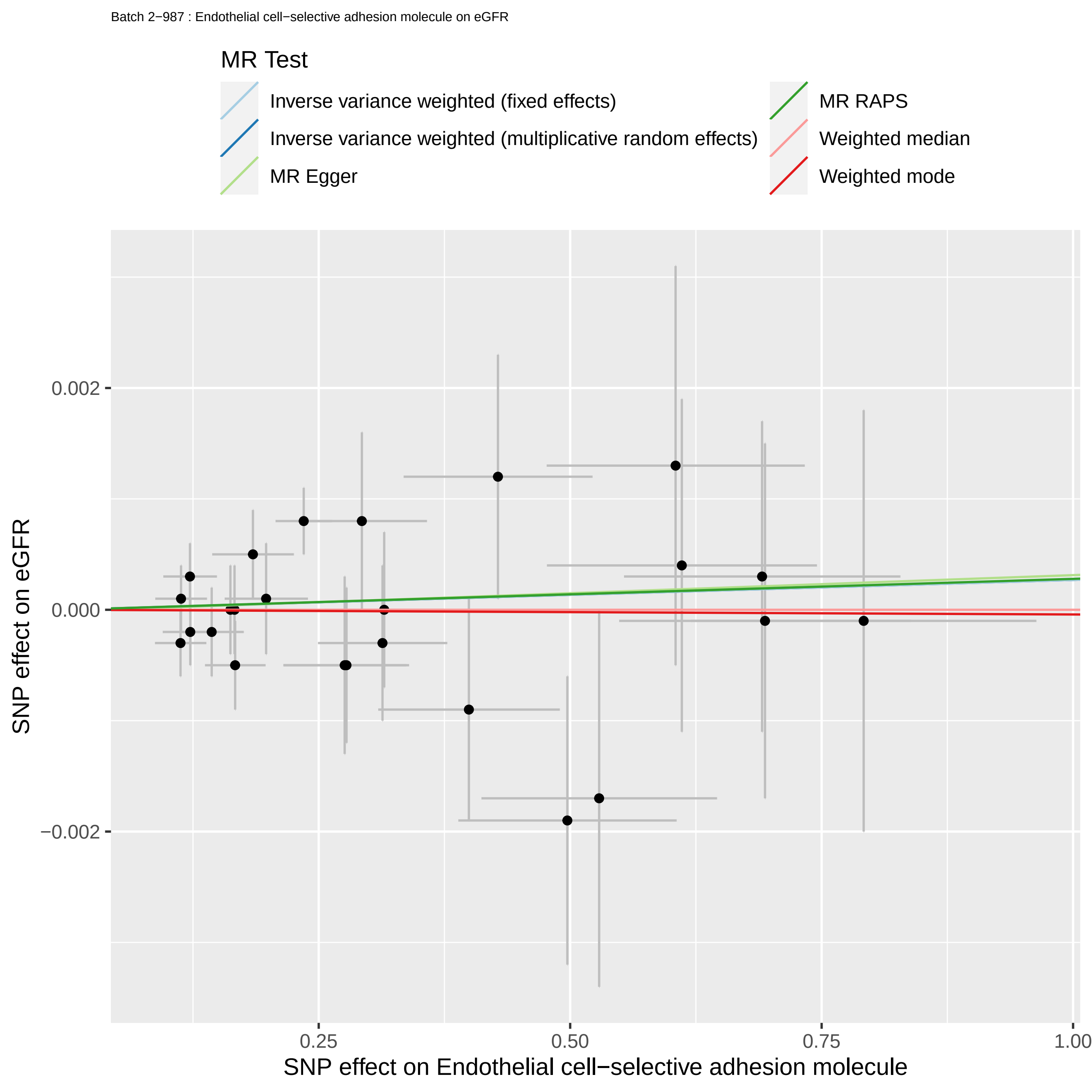

Batch 2−987 : Endothelial cell−selective adhesion molecule on eGFR
MR Test
Inverse variance weighted (fixed effects)
Inverse variance weighted (multiplicative random effects)
MR Egger
MR RAPS
Weighted median
Weighted mode
0.002
0.000
−0.002
SNP effect on eGFR
0.25
0.50
0.75
1.00
SNP effect on Endothelial cell−selective adhesion molecule

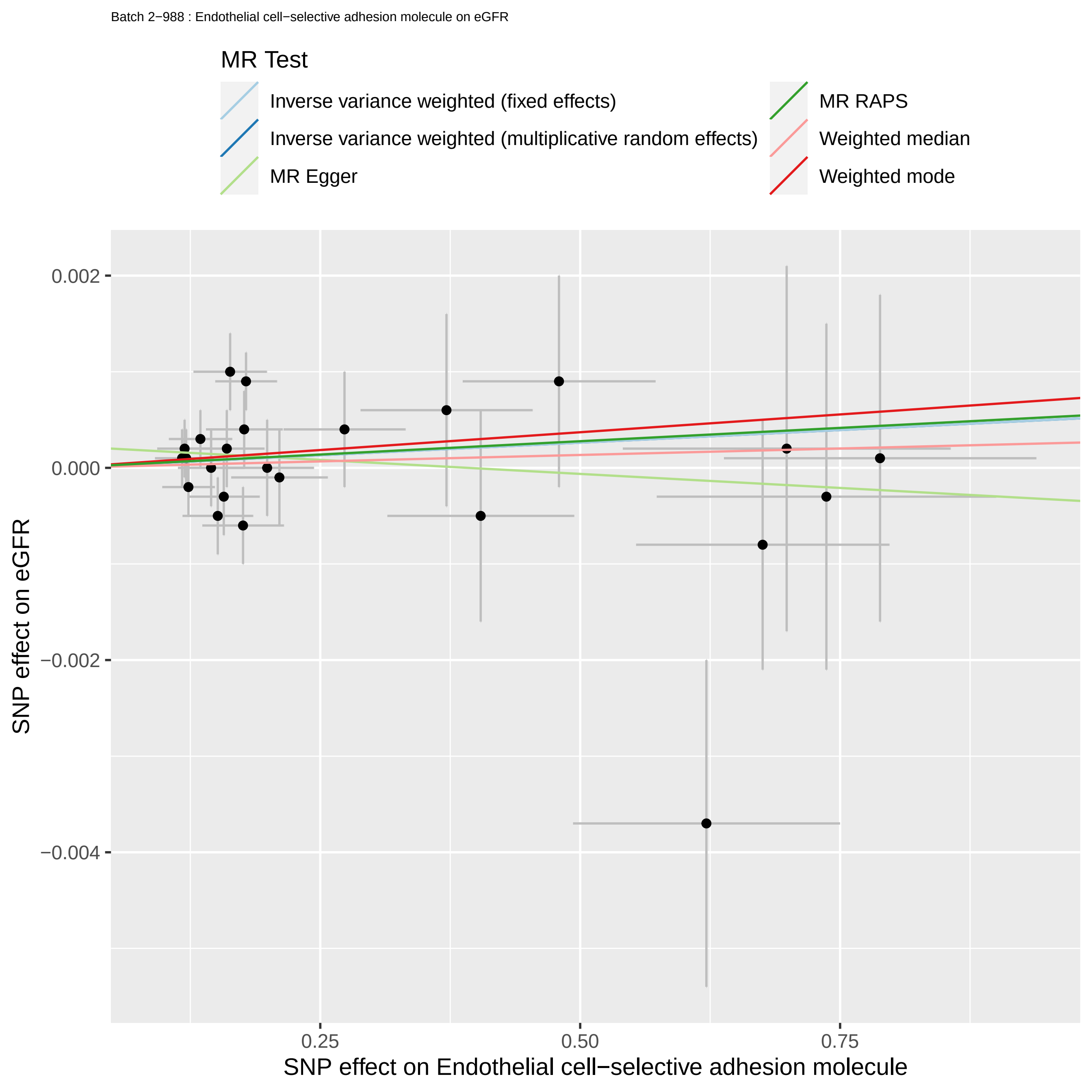

Batch 2−988 : Endothelial cell−selective adhesion molecule on eGFR
MR Test
Inverse variance weighted (fixed effects)
Inverse variance weighted (multiplicative random effects)
MR Egger
MR RAPS
Weighted median
Weighted mode
0.002
0.000
−0.002
−0.004
0.25
0.50
0.75
SNP effect on eGFR
SNP effect on Endothelial cell−selective adhesion molecule

Batch 2−989 : S−formylglutathione hydrolase on eGFR

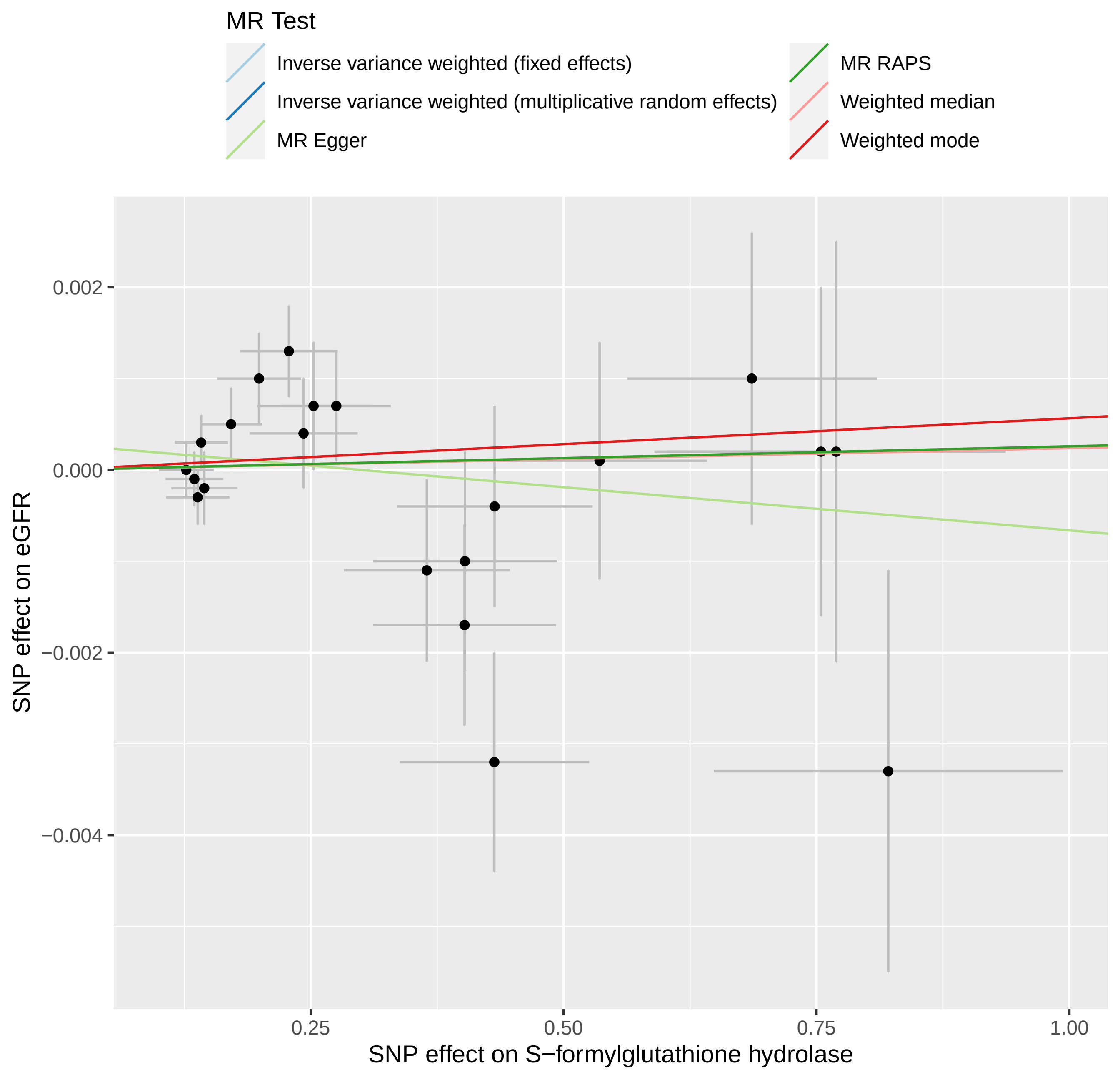

Batch 2−990 : Endothelial cell−specific molecule 1 on eGFR

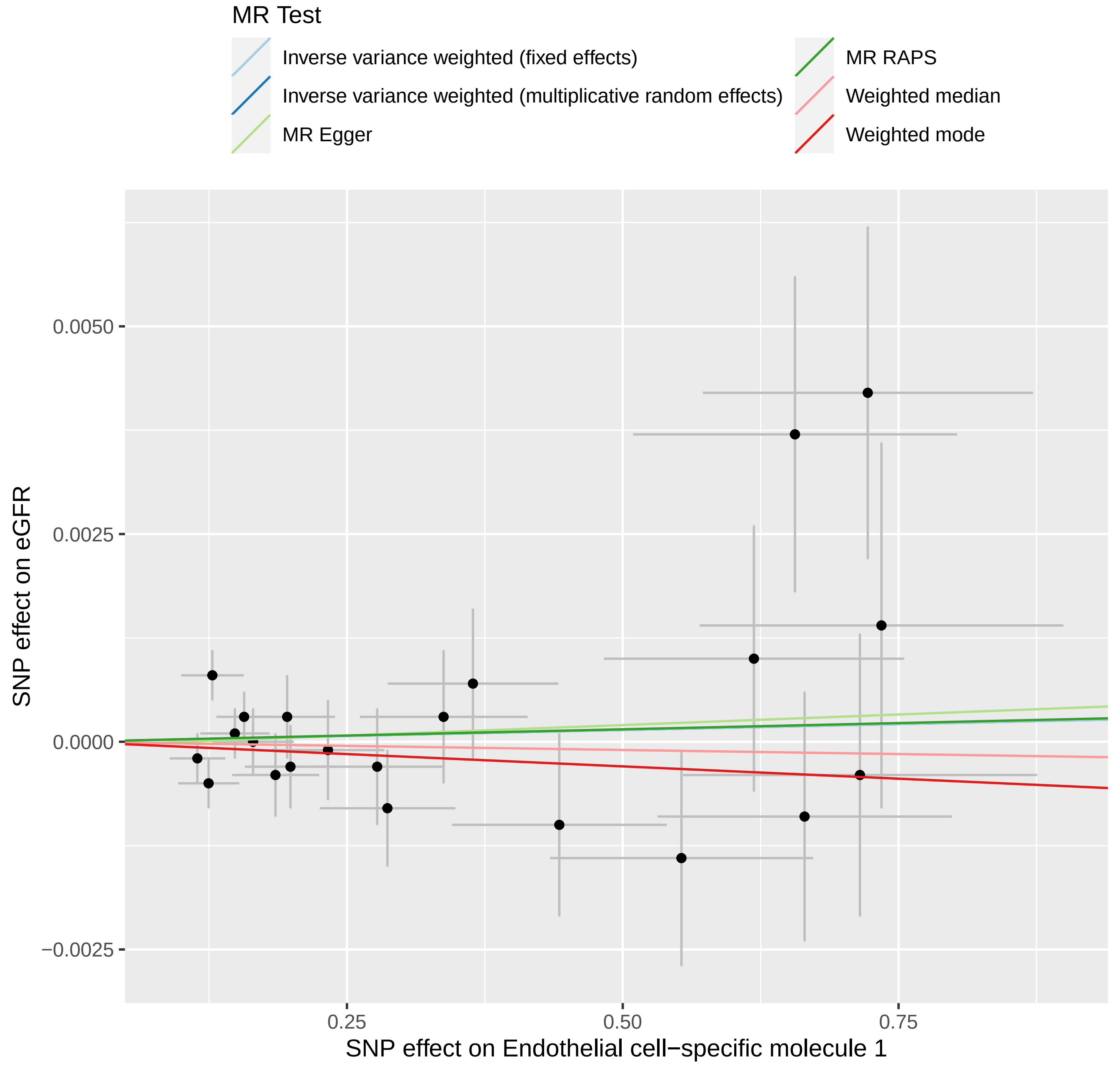

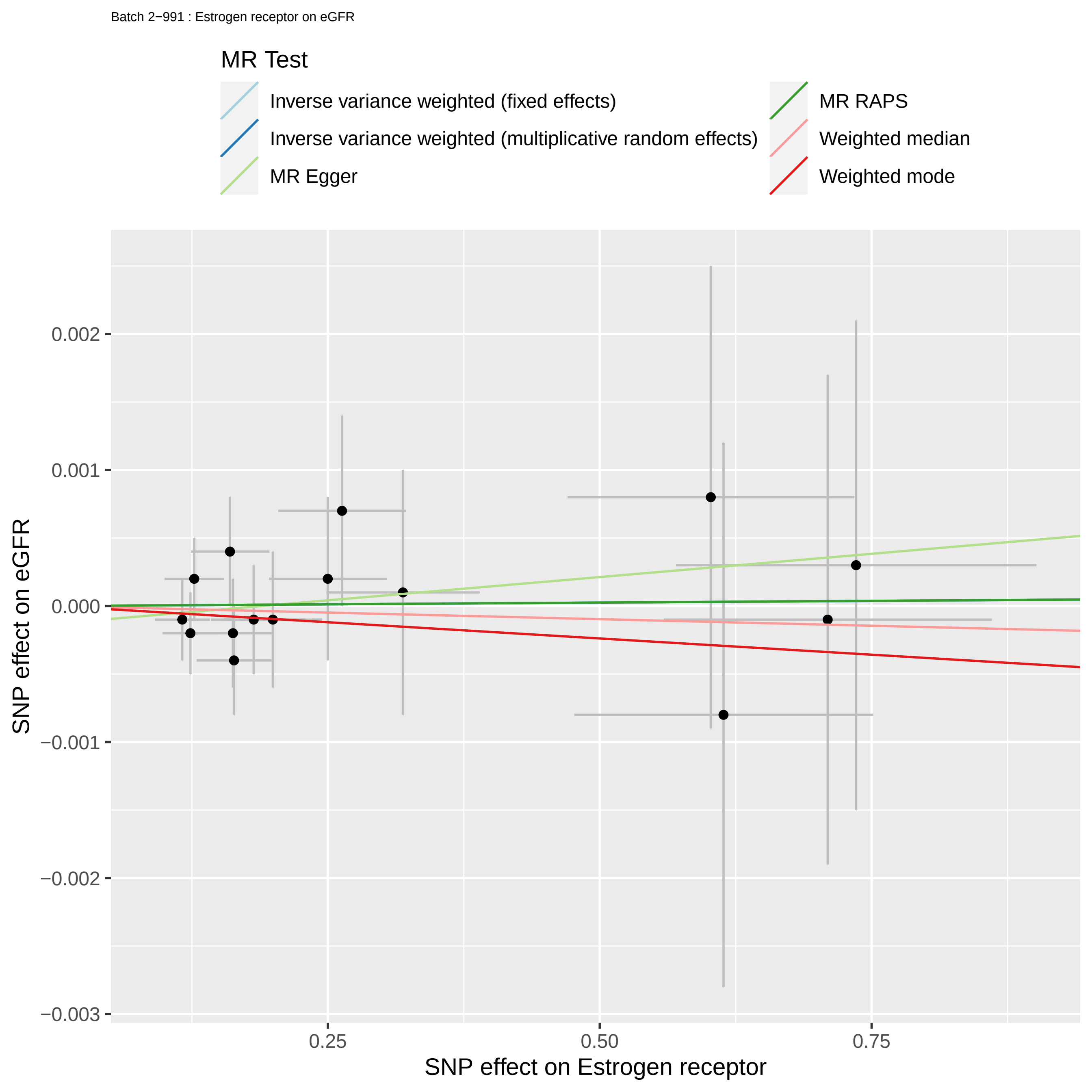

Batch 2−991 : Estrogen receptor on eGFR
MR Test
Inverse variance weighted (fixed effects)
Inverse variance weighted (multiplicative random effects)
MR Egger
MR RAPS
Weighted median
Weighted mode
0.002
0.001
0.000
−0.001
−0.002
−0.003
0.25
0.50
0.75
SNP effect on eGFR
SNP effect on Estrogen receptor

Batch 2−992 : Persulfide dioxygenase ETHE1, mitochondrial on eGFR

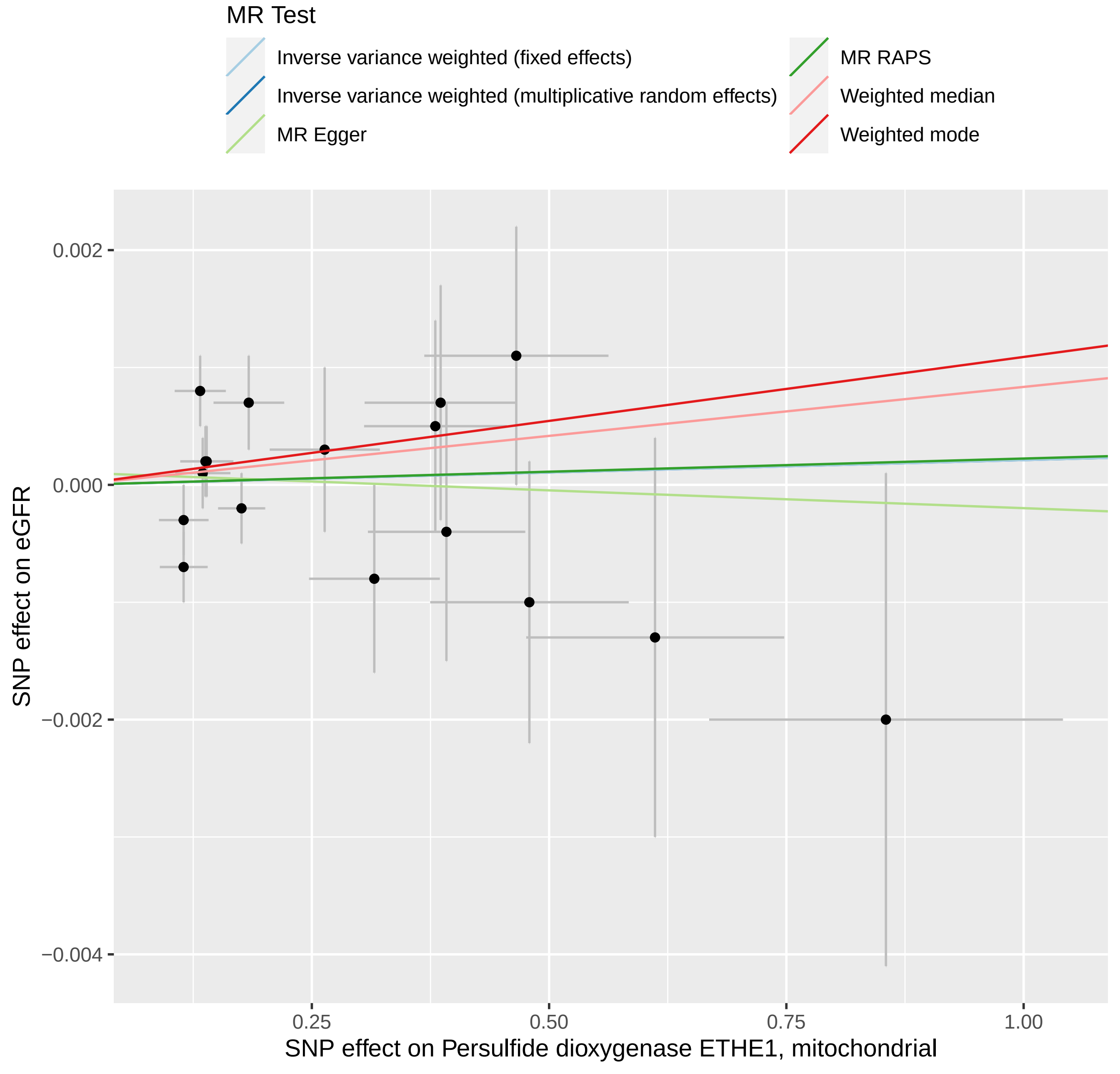

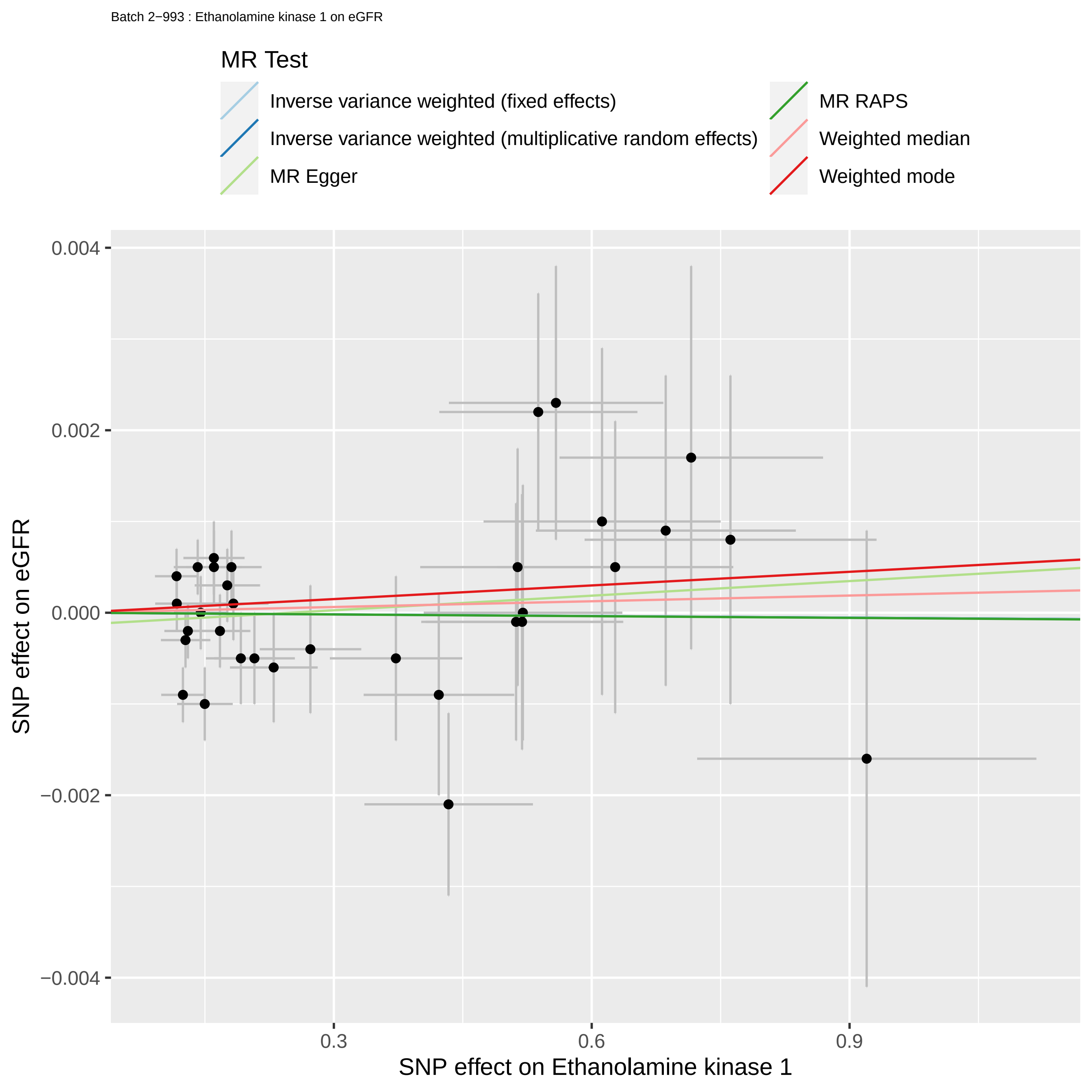

Batch 2−993 : Ethanolamine kinase 1 on eGFR
MR Test
Inverse variance weighted (fixed effects)
Inverse variance weighted (multiplicative random effects)
MR Egger
MR RAPS
Weighted median
Weighted mode
0.004
0.002
0.000
−0.002
−0.004
0.3
0.6
0.9
SNP effect on eGFR
SNP effect on Ethanolamine kinase 1

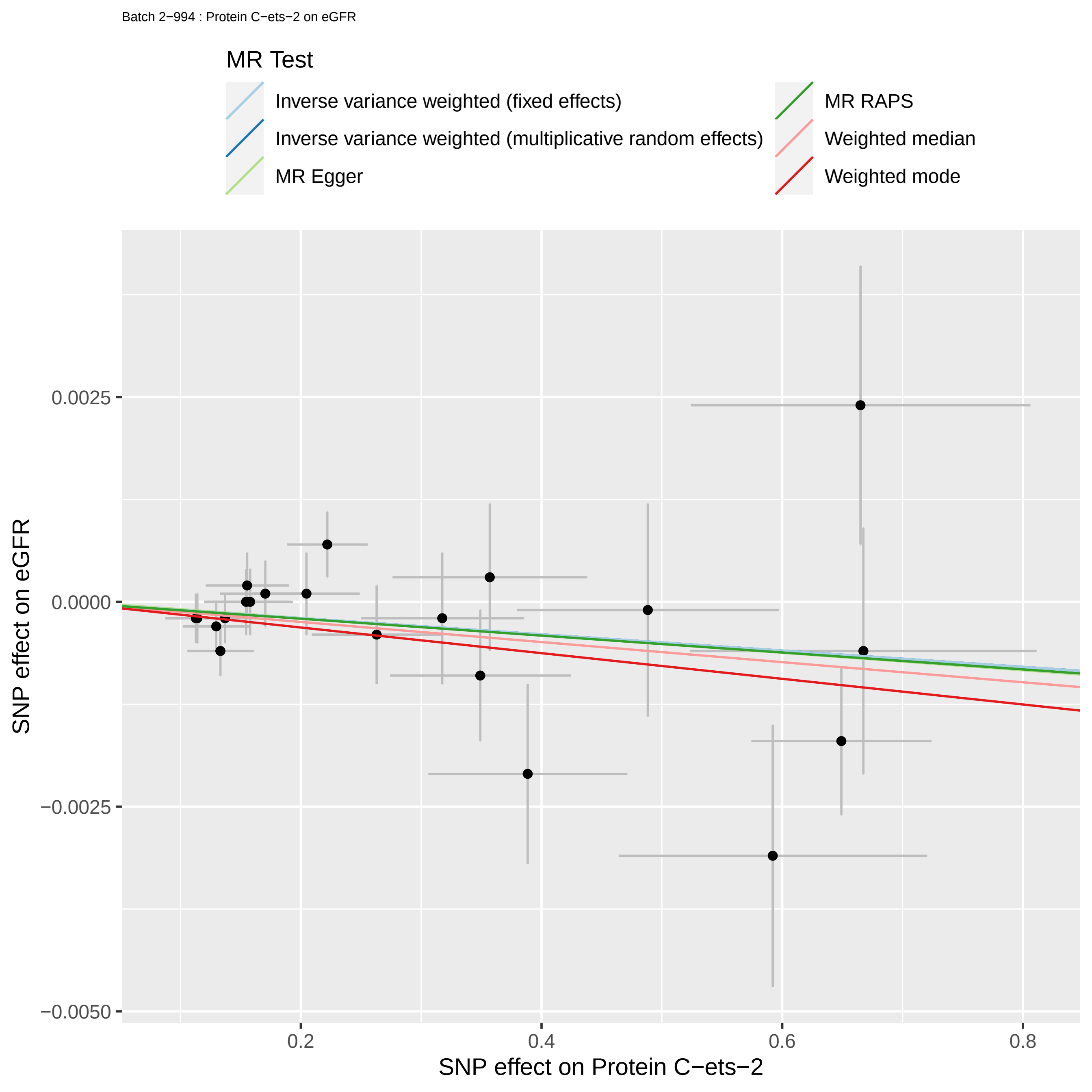
Batch 2−994 : Protein C−ets−2 on eGFR
MR Test
Inverse variance weighted (fixed effects)
Inverse variance weighted (multiplicative random effects)
MR Egger
MR RAPS
Weighted median
Weighted mode
0.0025
0.0000
−0.0025
−0.0050
0.2
0.4
0.6
0.8
SNP effect on eGFR
SNP effect on Protein C−ets−2

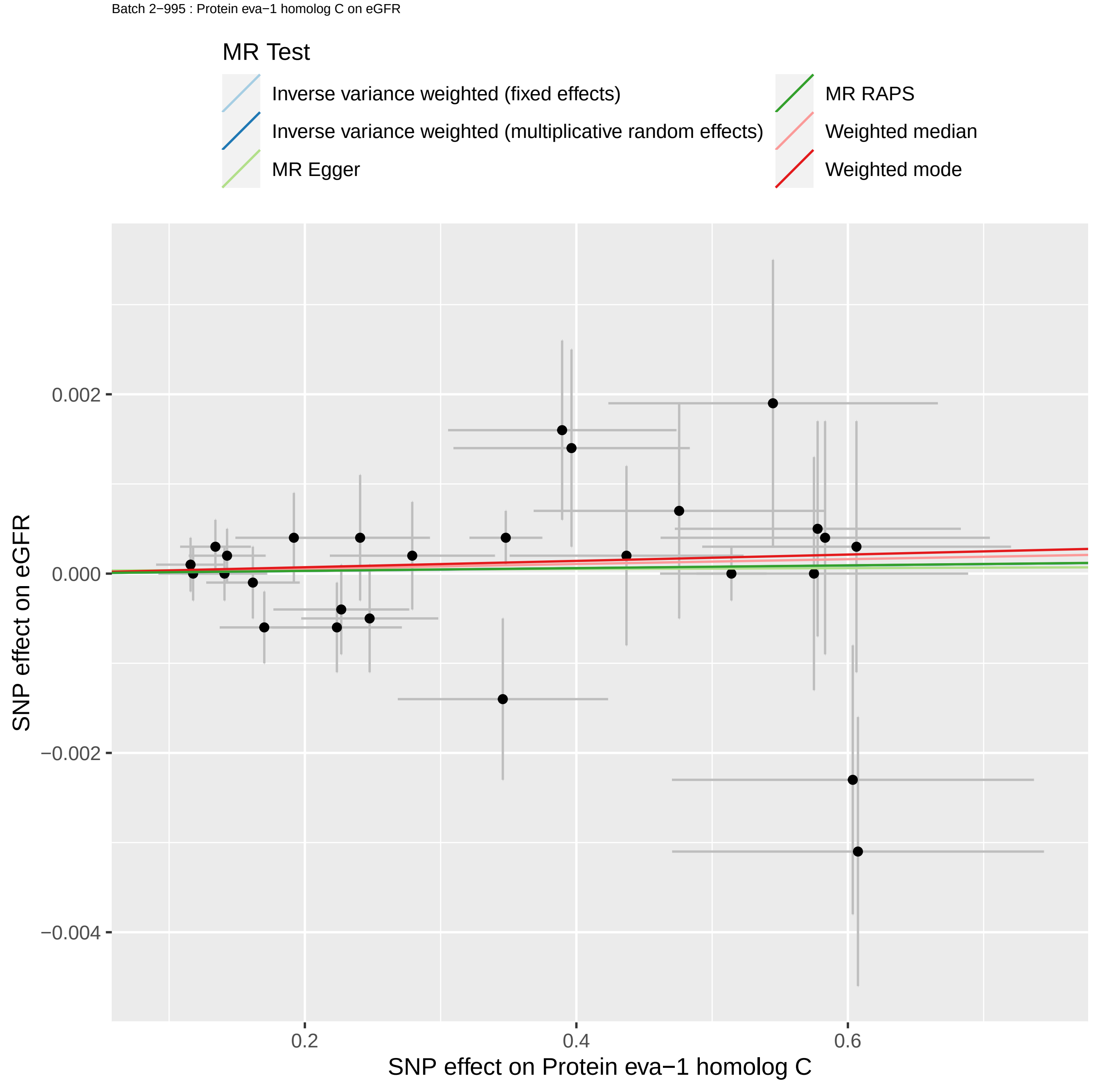

Batch 2−995 : Protein eva−1 homolog C on eGFR
MR Test
Inverse variance weighted (fixed effects)
Inverse variance weighted (multiplicative random effects)
MR Egger
MR RAPS
Weighted median
Weighted mode
0.002
0.000
−0.002
−0.004
0.2
0.4
0.6
SNP effect on eGFR
SNP effect on Protein eva−1 homolog C

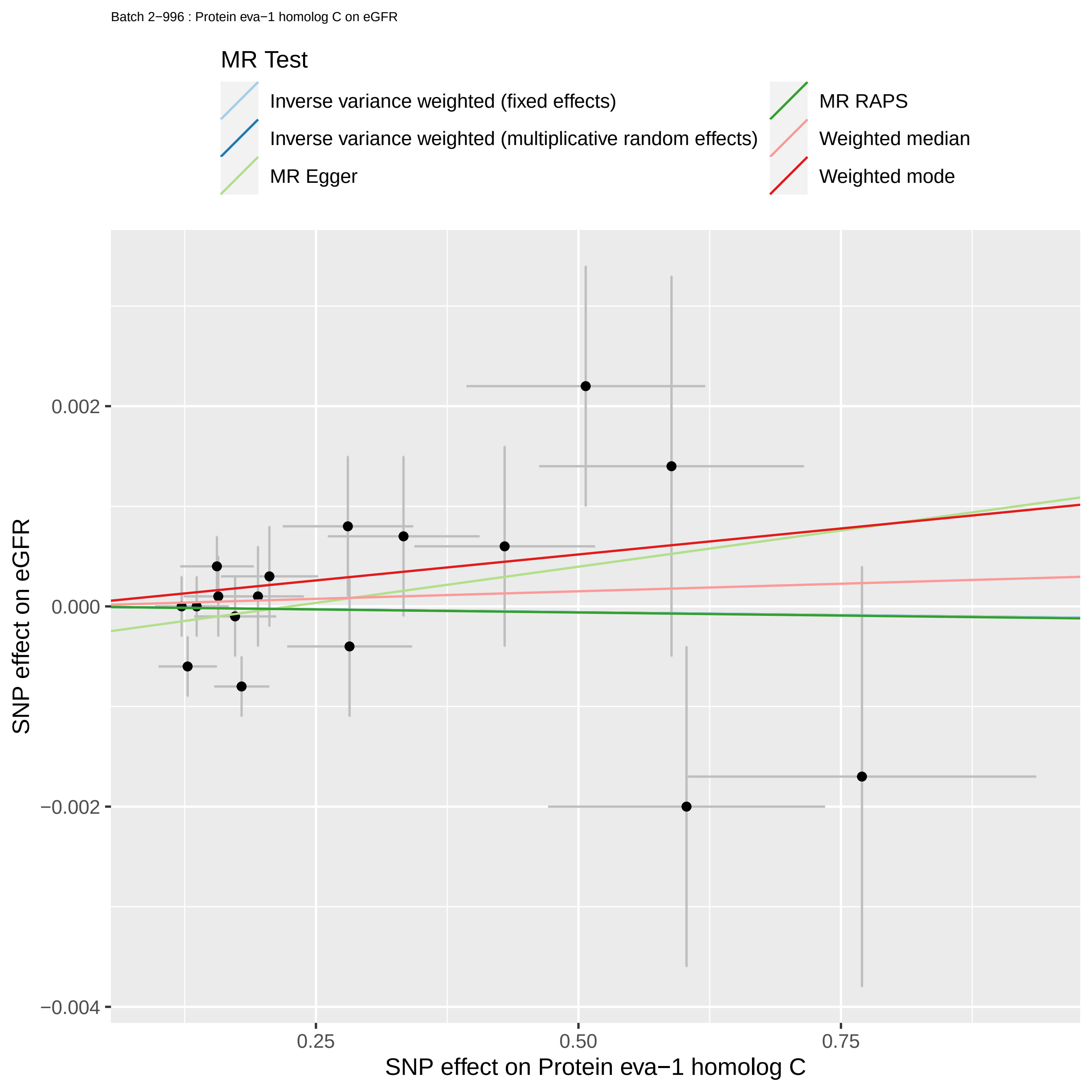
Batch 2−996 : Protein eva−1 homolog C on eGFR
MR Test
Inverse variance weighted (fixed effects)
Inverse variance weighted (multiplicative random effects)
MR Egger
MR RAPS
Weighted median
Weighted mode
0.002
0.000
−0.002
−0.004
0.25
0.50
0.75
SNP effect on eGFR
SNP effect on Protein eva−1 homolog C

Batch 2−997 : Protein EVI2B on eGFR

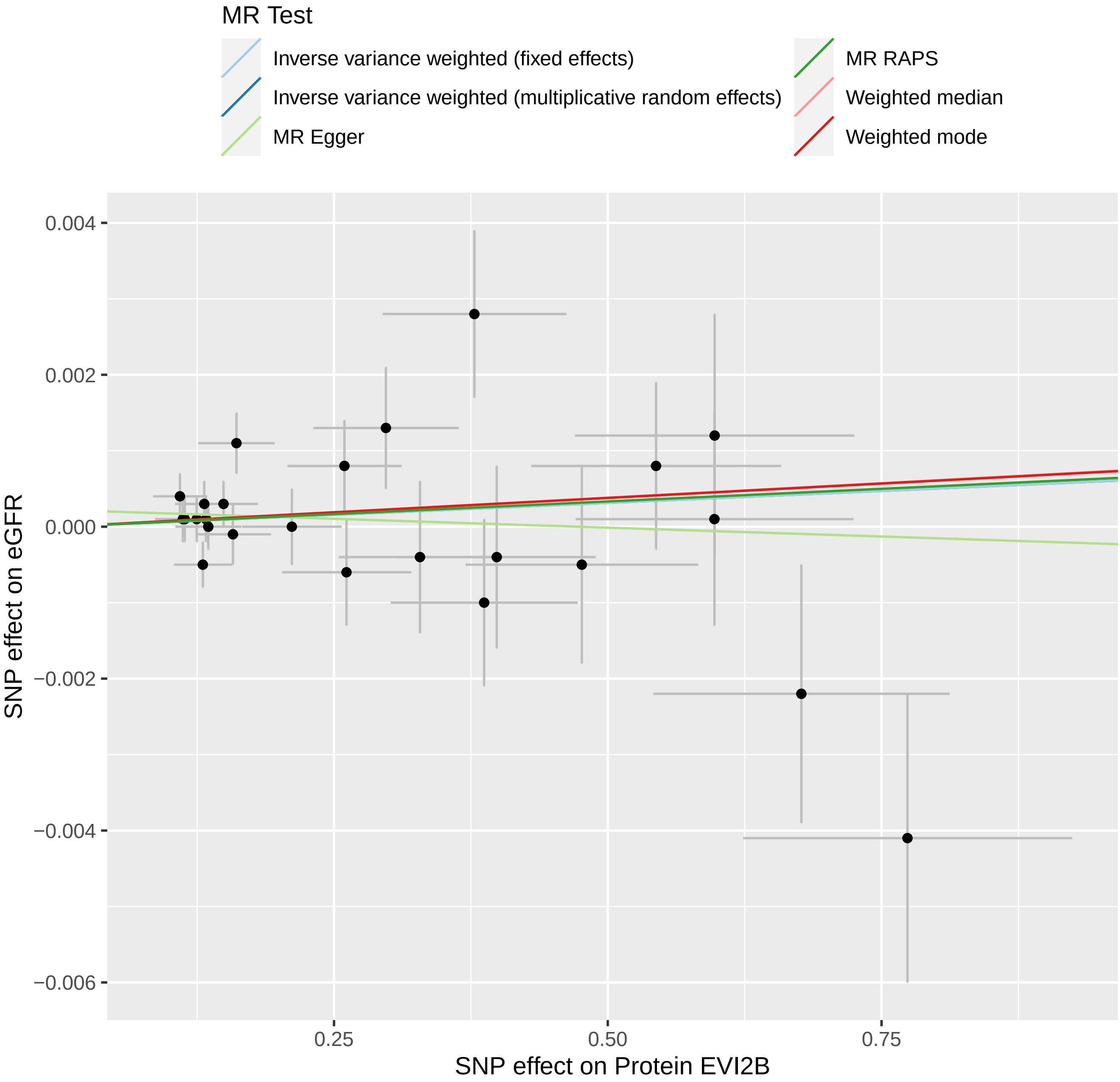

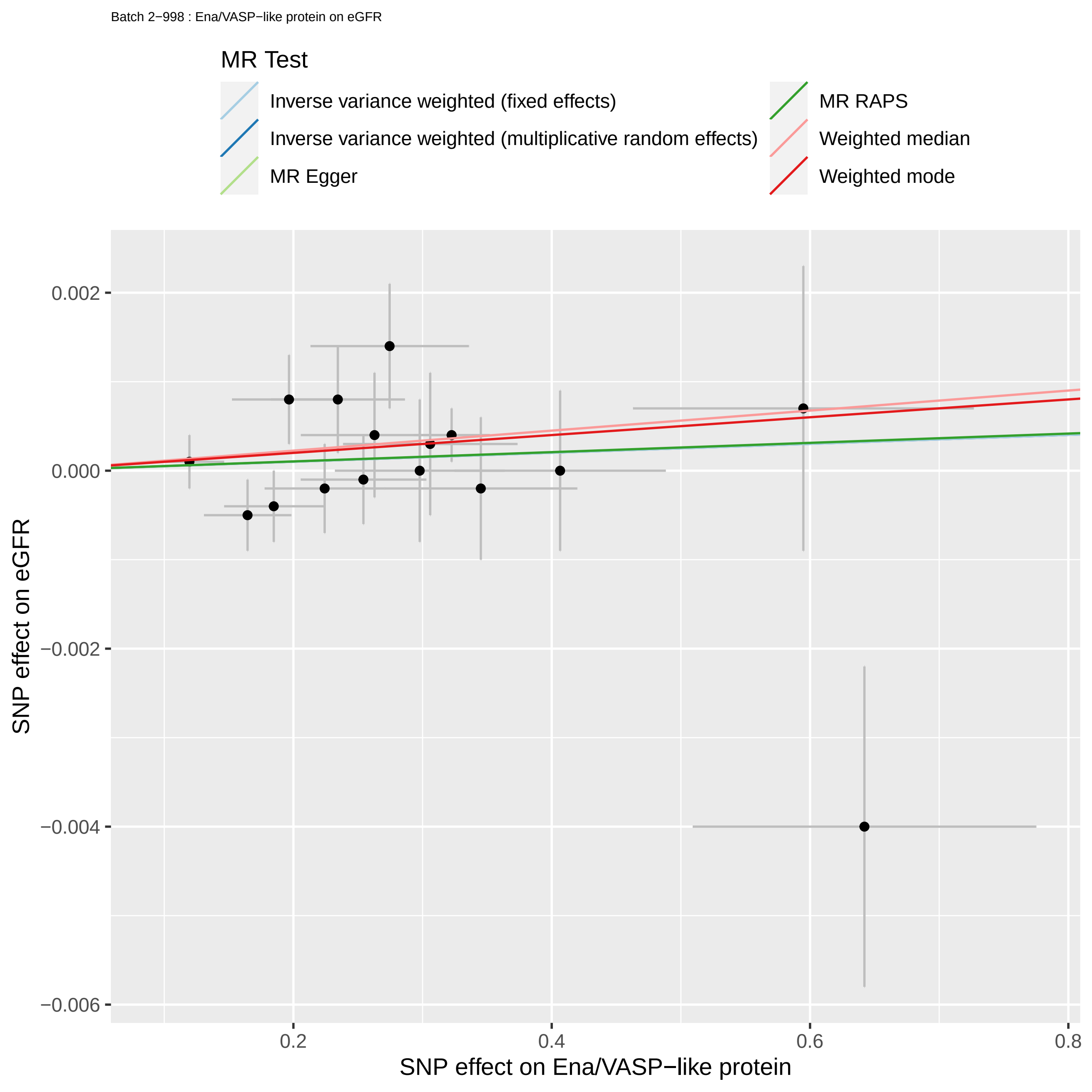
Batch 2−998 : Ena/VASP−like protein on eGFR
MR Test
Inverse variance weighted (fixed effects)
Inverse variance weighted (multiplicative random effects)
MR Egger
MR RAPS
Weighted median
Weighted mode
0.002
0.000
−0.002
−0.004
−0.006
0.2
0.4
0.6
0.8
SNP effect on eGFR
SNP effect on Ena/VASP−like protein

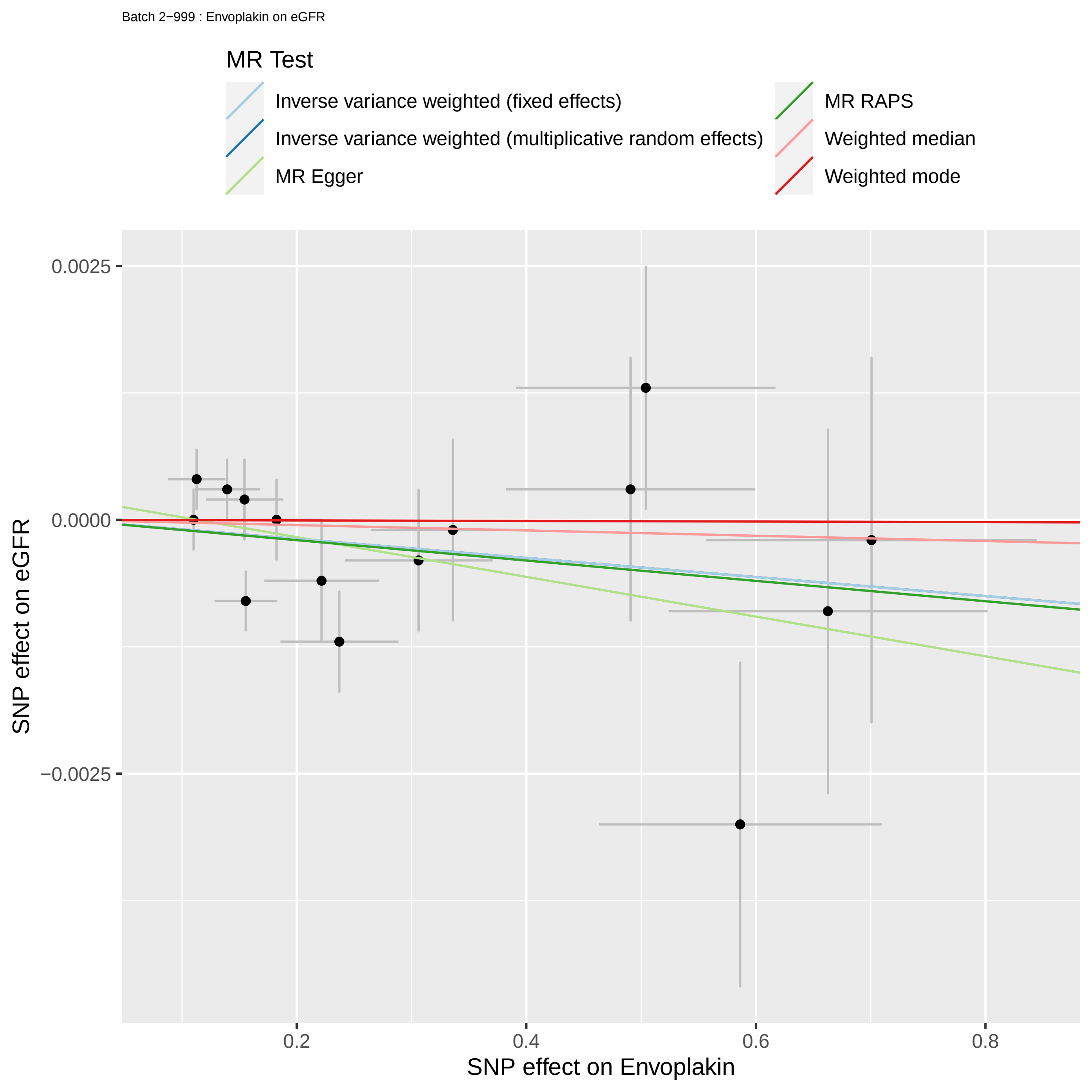
Batch 2−999 : Envoplakin on eGFR
MR Test
Inverse variance weighted (fixed effects)
Inverse variance weighted (multiplicative random effects)
MR Egger
MR RAPS
Weighted median
Weighted mode
0.0025
0.0000
−0.0025
SNP effect on eGFR
0.2
0.4
0.6
0.8
SNP effect on Envoplakin

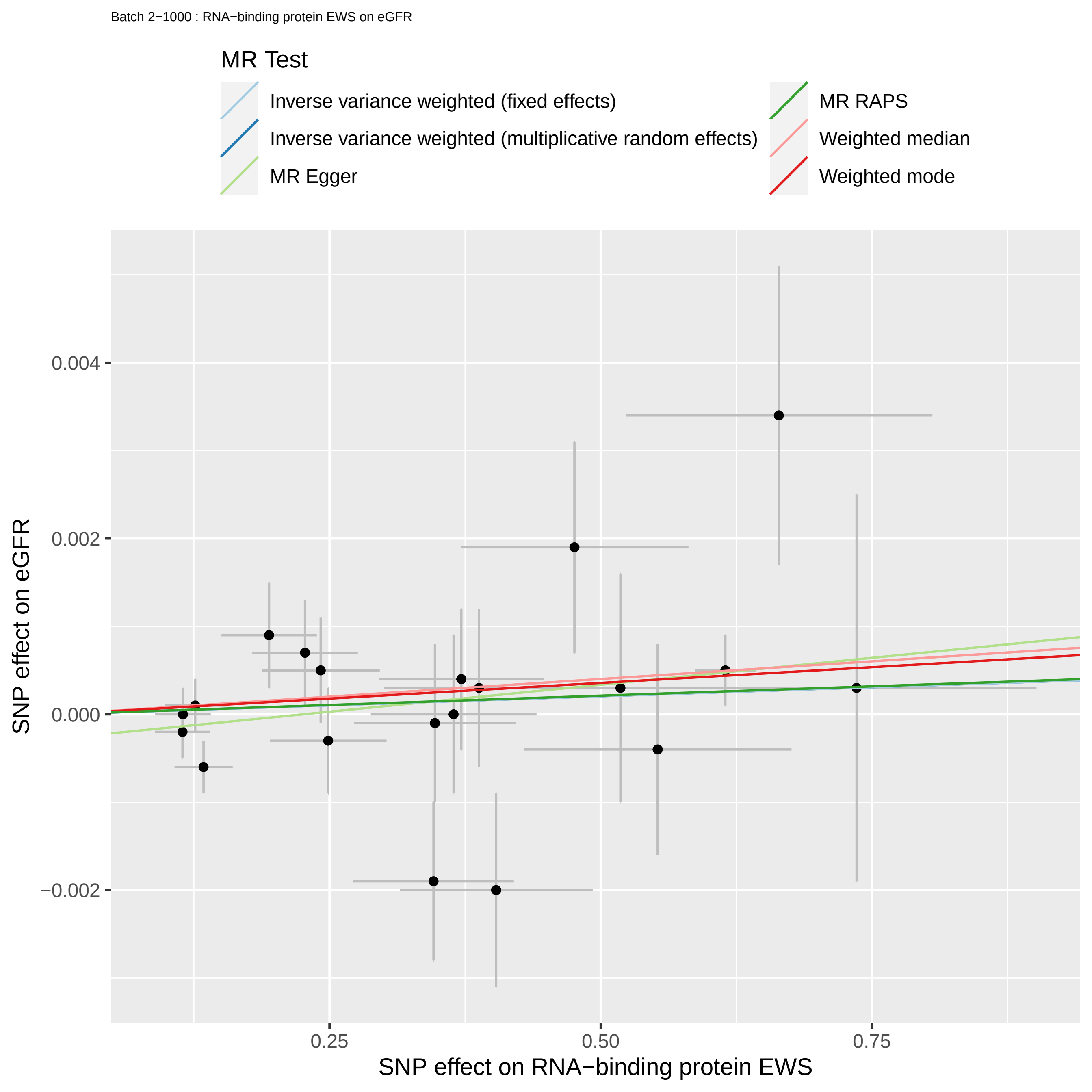
Batch 2−1000 : RNA−binding protein EWS on eGFR
MR Test
Inverse variance weighted (fixed effects)
Inverse variance weighted (multiplicative random effects)
MR Egger
MR RAPS
Weighted median
Weighted mode
0.004
0.002
0.000
−0.002
0.25
0.50
0.75
SNP effect on eGFR
SNP effect on RNA−binding protein EWS

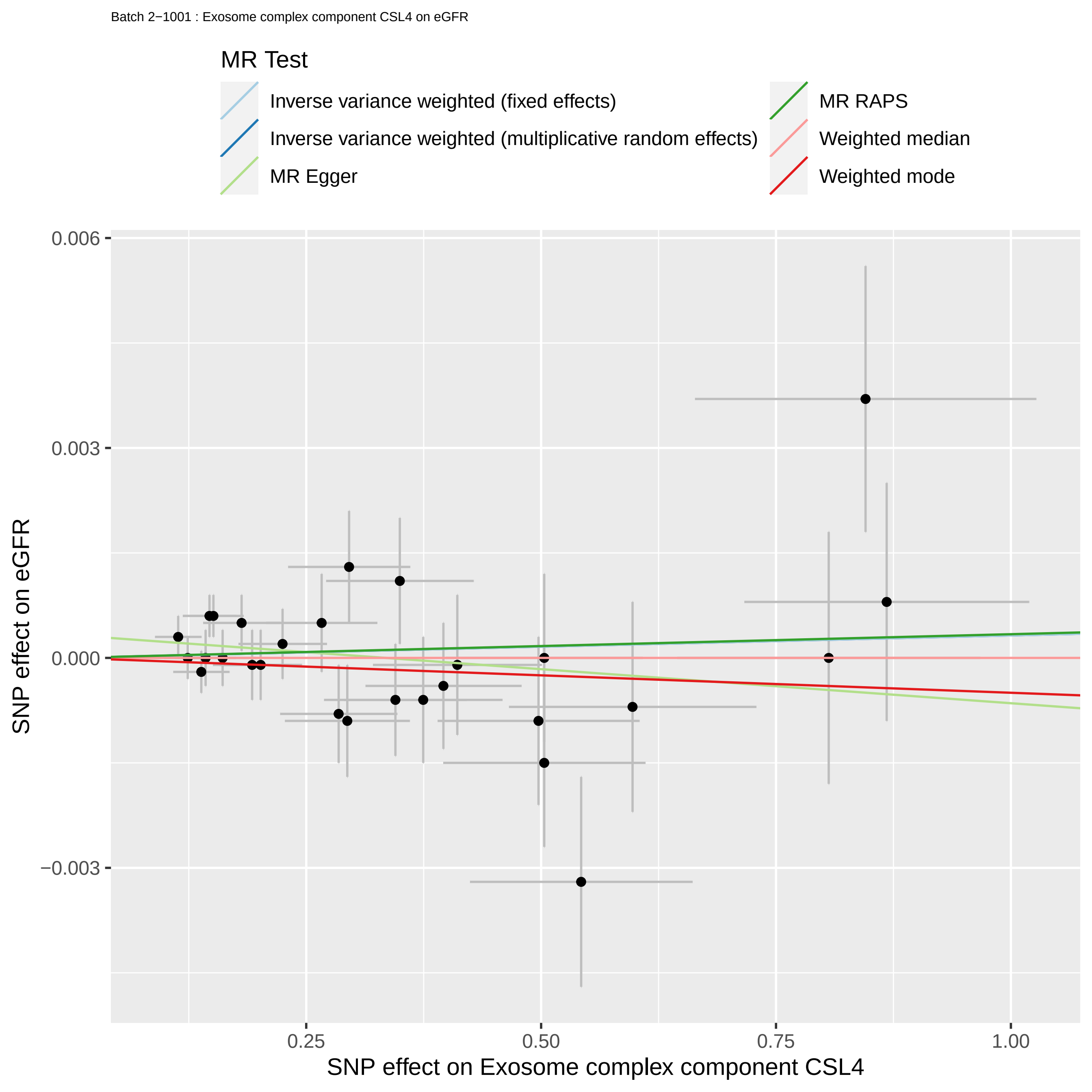

Batch 2−1001 : Exosome complex component CSL4 on eGFR
MR Test
Inverse variance weighted (fixed effects)
Inverse variance weighted (multiplicative random effects)
MR Egger
MR RAPS
Weighted median
Weighted mode
0.006
0.003
0.000
−0.003
SNP effect on eGFR
0.25
0.50
0.75
1.00
SNP effect on Exosome complex component CSL4

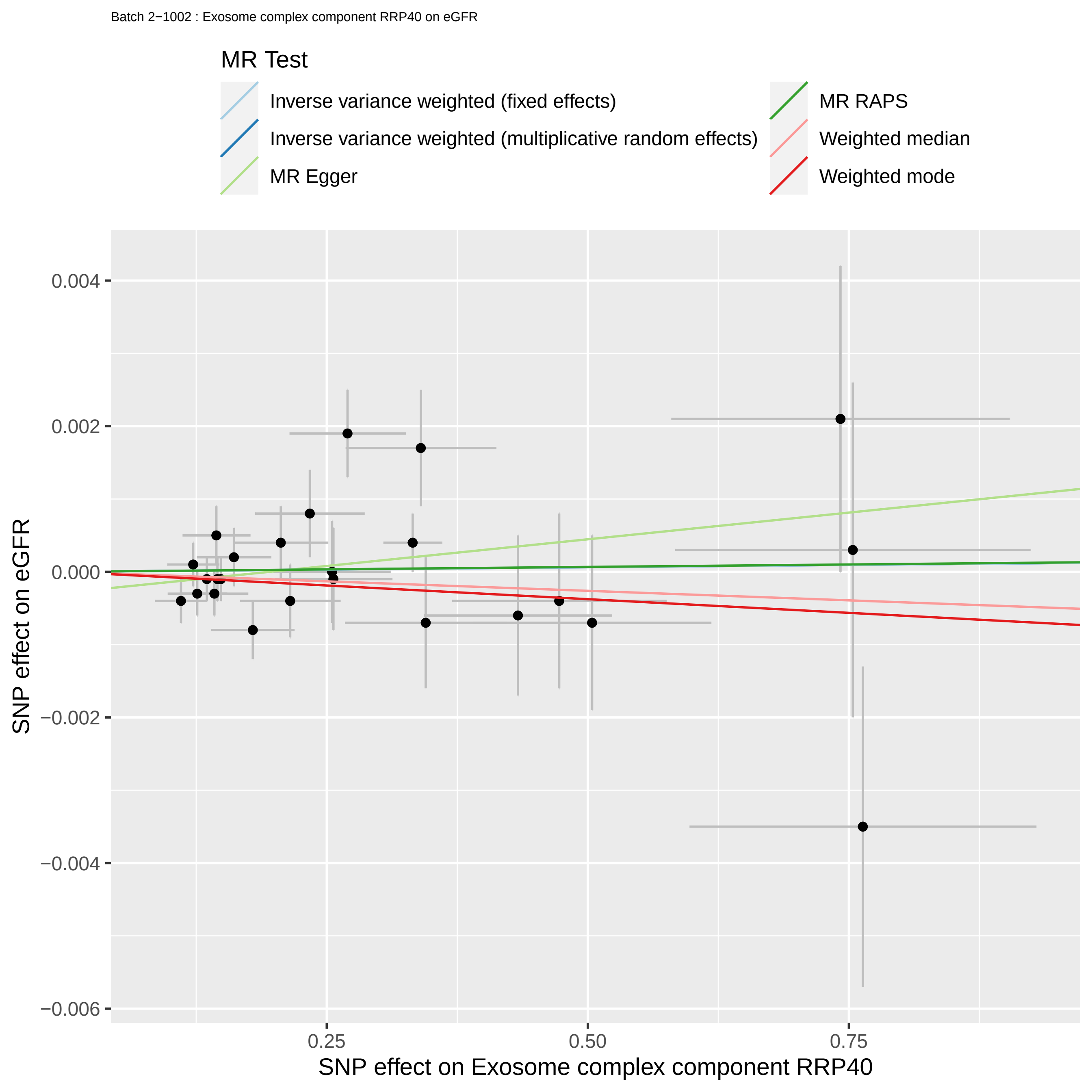

Batch 2−1002 : Exosome complex component RRP40 on eGFR
MR Test
Inverse variance weighted (fixed effects)
Inverse variance weighted (multiplicative random effects)
MR Egger
MR RAPS
Weighted median
Weighted mode
0.004
0.002
0.000
−0.002
−0.004
−0.006
0.25
0.50
0.75
SNP effect on eGFR
SNP effect on Exosome complex component RRP40

Batch 2−1003 : Exostosin−like 2 on eGFR

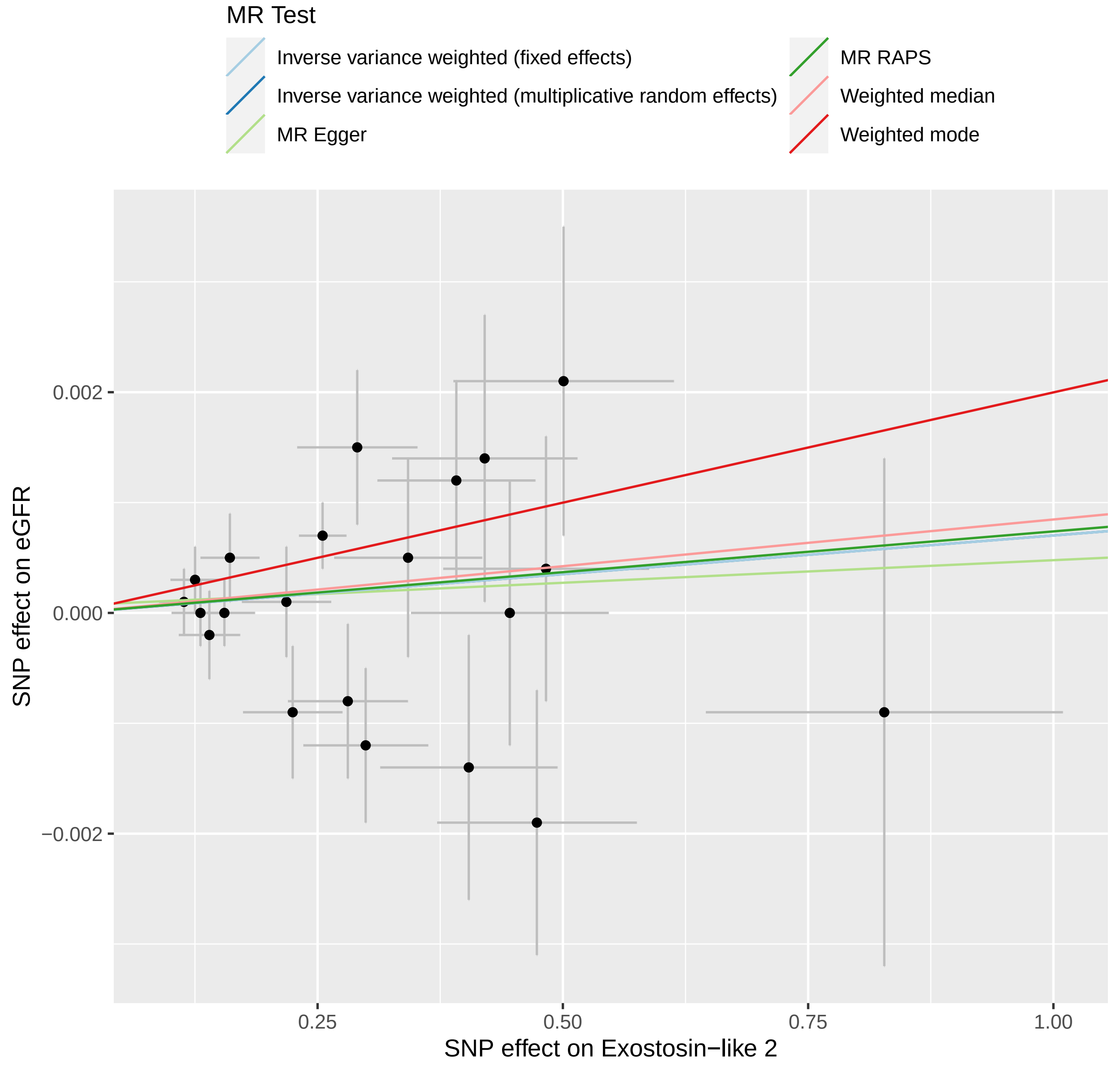

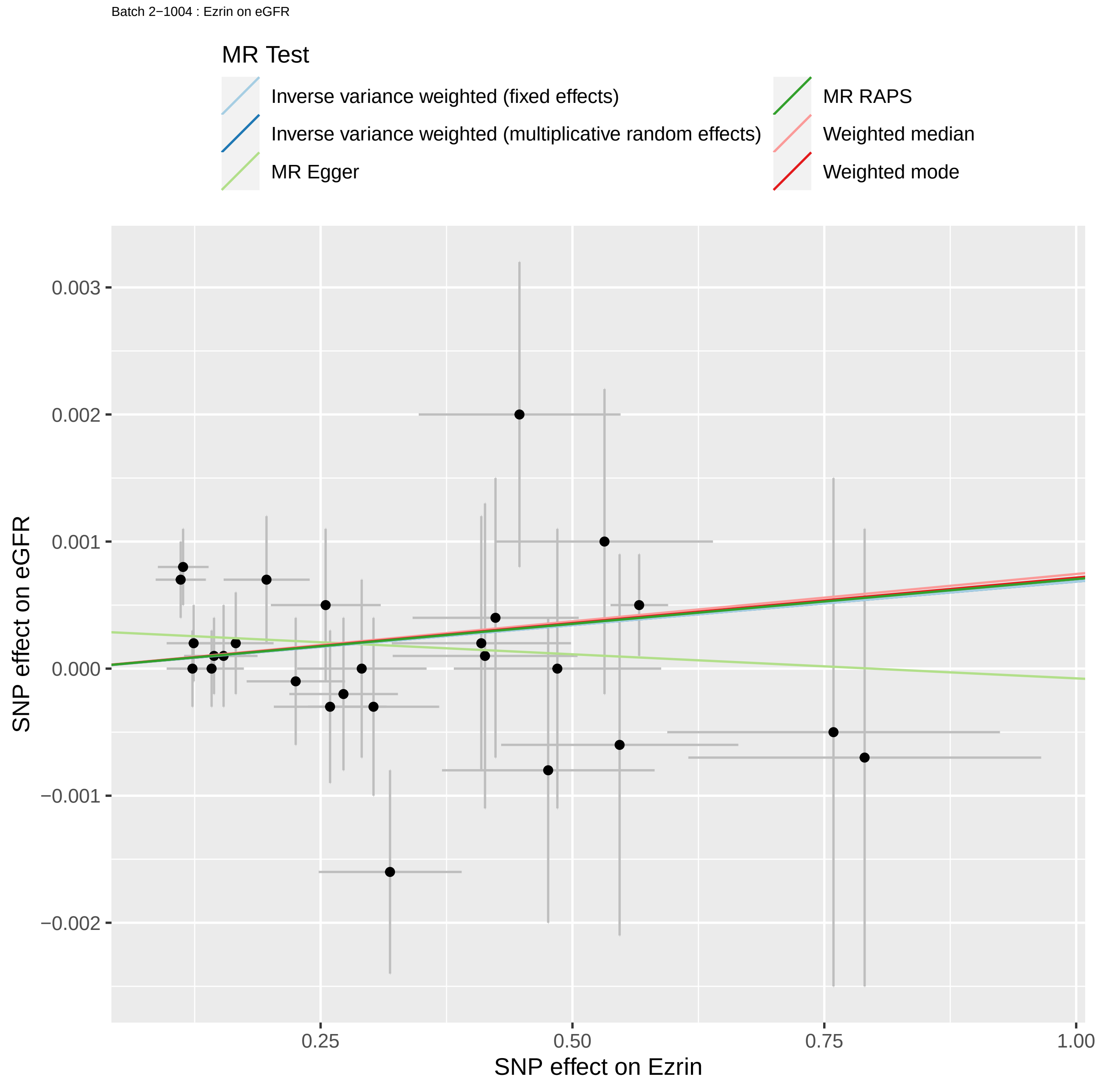

Batch 2−1004 : Ezrin on eGFR
MR Test
Inverse variance weighted (fixed effects)
Inverse variance weighted (multiplicative random effects)
MR Egger
MR RAPS
Weighted median
Weighted mode
0.003
0.002
0.001
0.000
−0.001
−0.002
0.25
0.50
0.75
1.00
SNP effect on eGFR
SNP effect on Ezrin

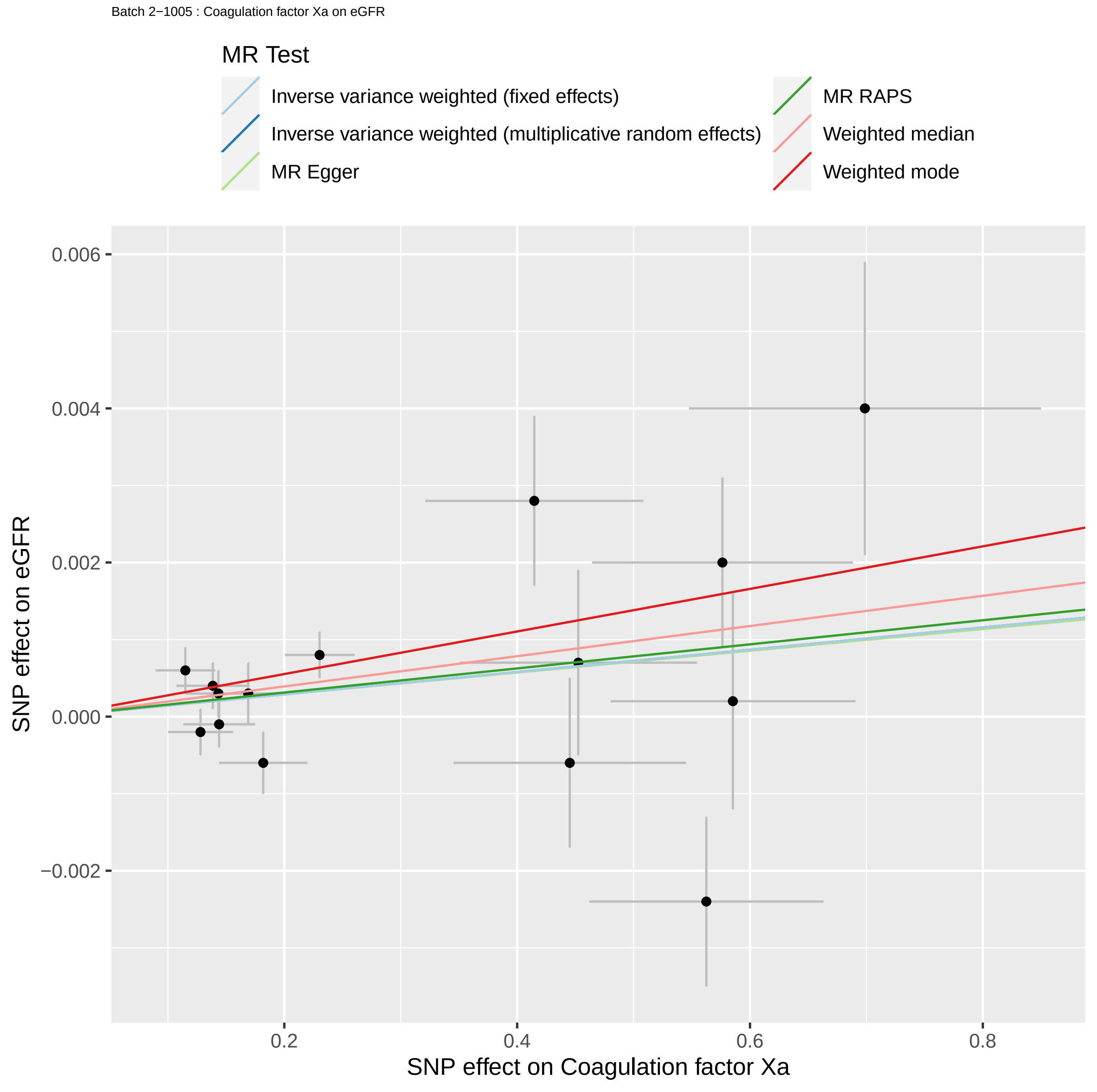

Batch 2−1005 : Coagulation factor Xa on eGFR
MR Test
Inverse variance weighted (fixed effects)
Inverse variance weighted (multiplicative random effects)
MR Egger
MR RAPS
Weighted median
Weighted mode
0.006
0.004
0.002
0.000
−0.002
0.2
0.4
0.6
0.8
SNP effect on eGFR
SNP effect on Coagulation factor Xa

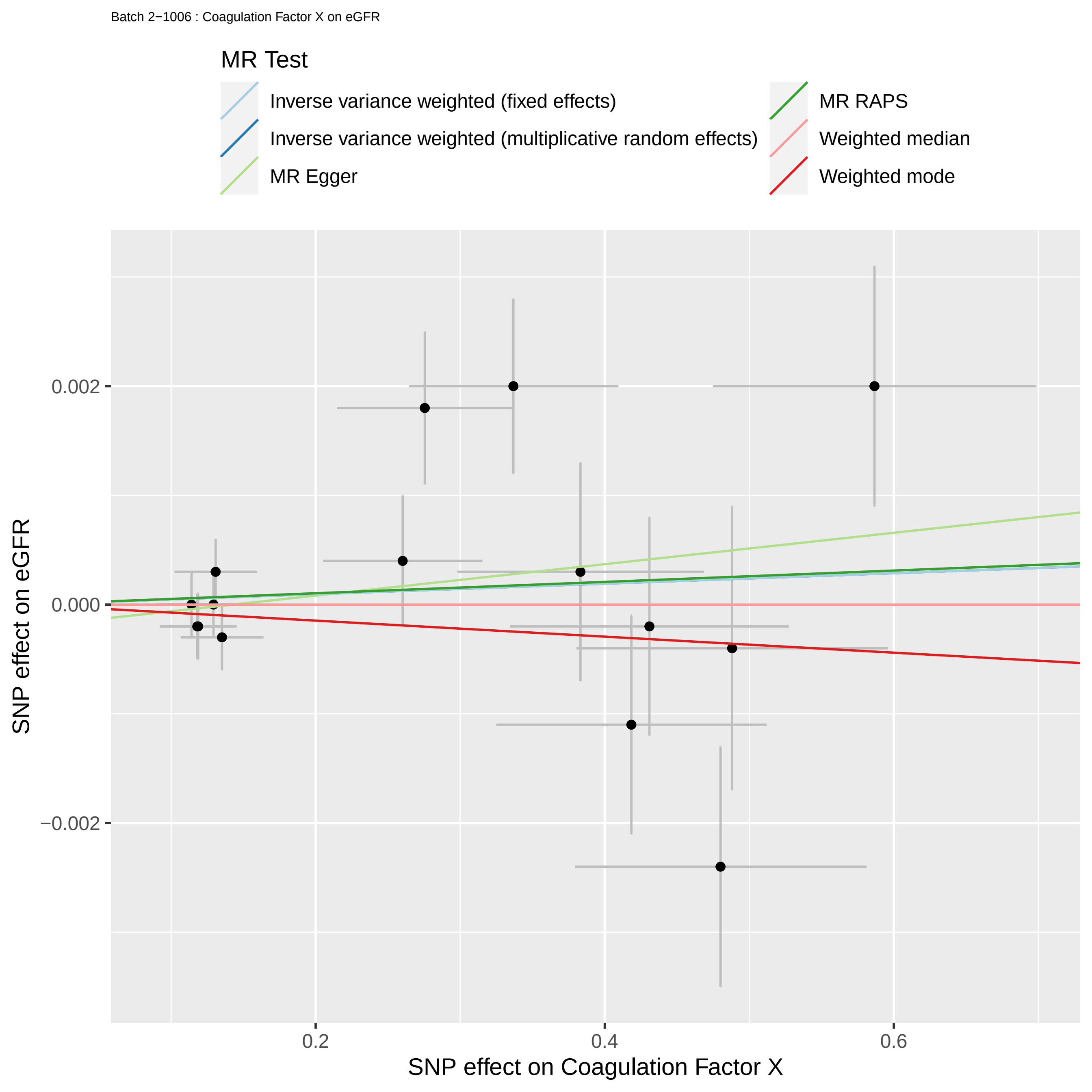

Batch 2−1006 : Coagulation Factor X on eGFR
MR Test
Inverse variance weighted (fixed effects)
Inverse variance weighted (multiplicative random effects)
MR Egger
MR RAPS
Weighted median
Weighted mode
0.002
0.000
−0.002
0.2
0.4
0.6
SNP effect on eGFR
SNP effect on Coagulation Factor X

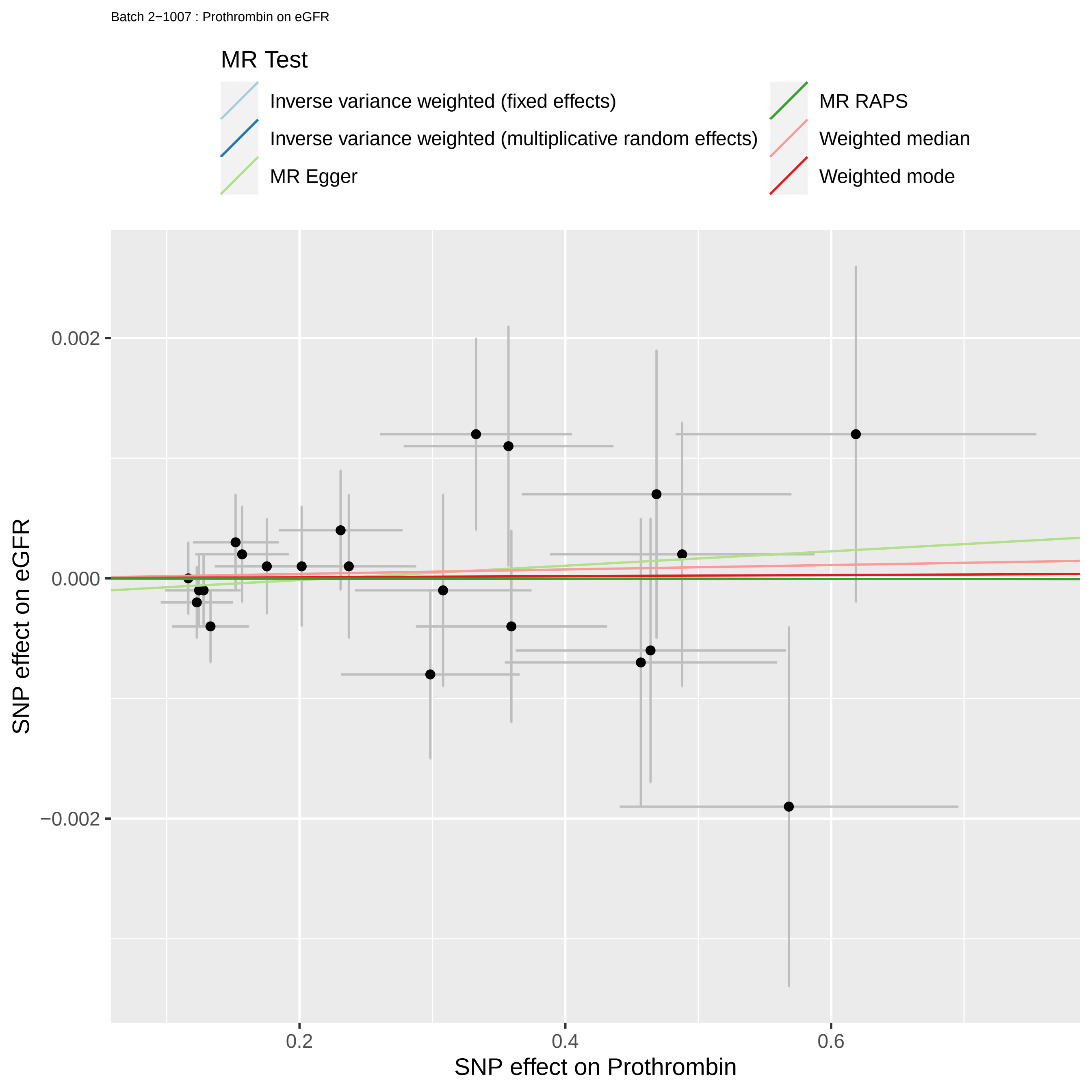

Batch 2−1007 : Prothrombin on eGFR
MR Test
Inverse variance weighted (fixed effects)
Inverse variance weighted (multiplicative random effects)
MR Egger
MR RAPS
Weighted median
Weighted mode
0.002
0.000
−0.002
SNP effect on eGFR
0.2
0.4
0.6
SNP effect on Prothrombin

Batch 2−1008 : Tissue Factor on eGFR

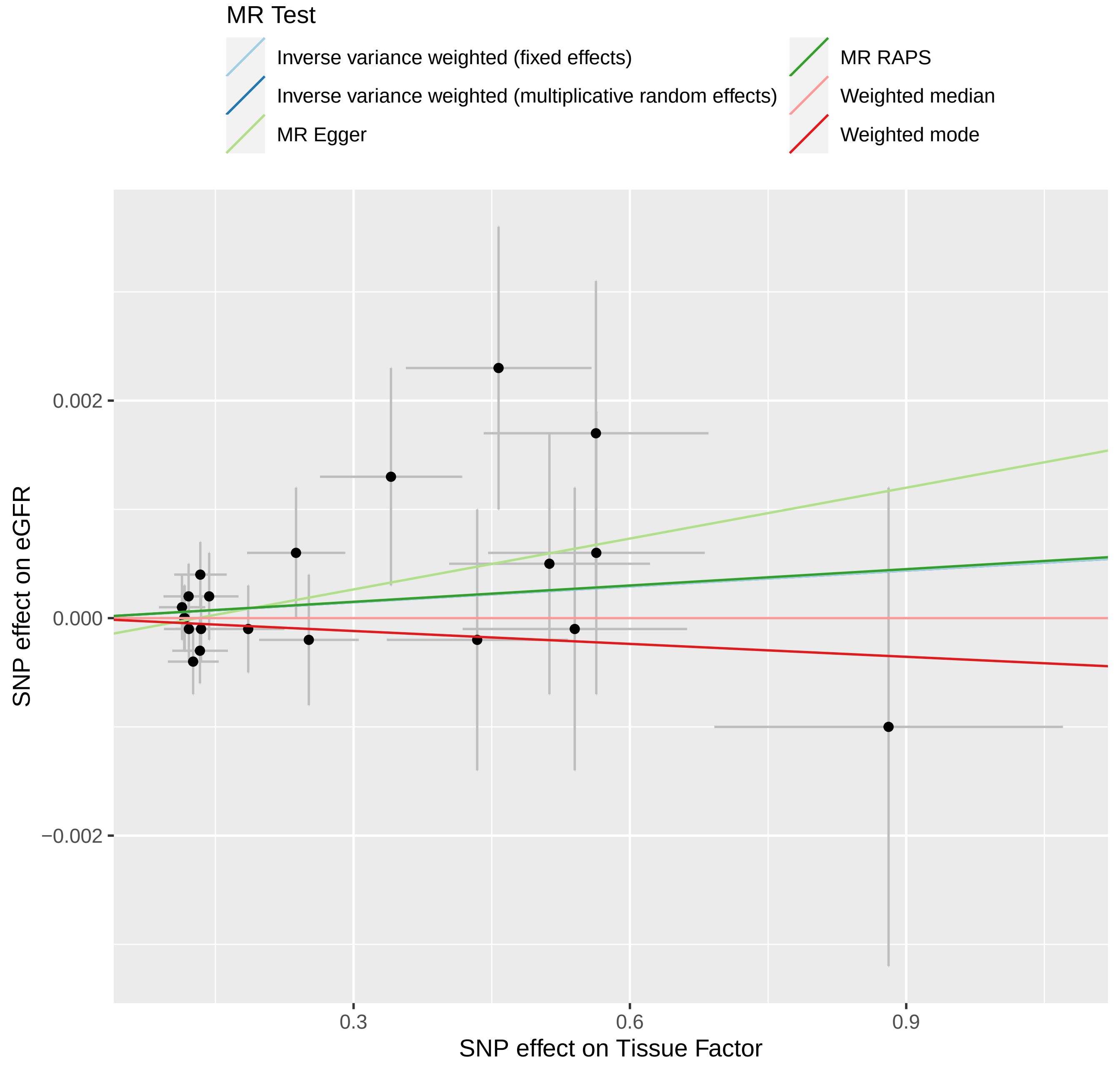

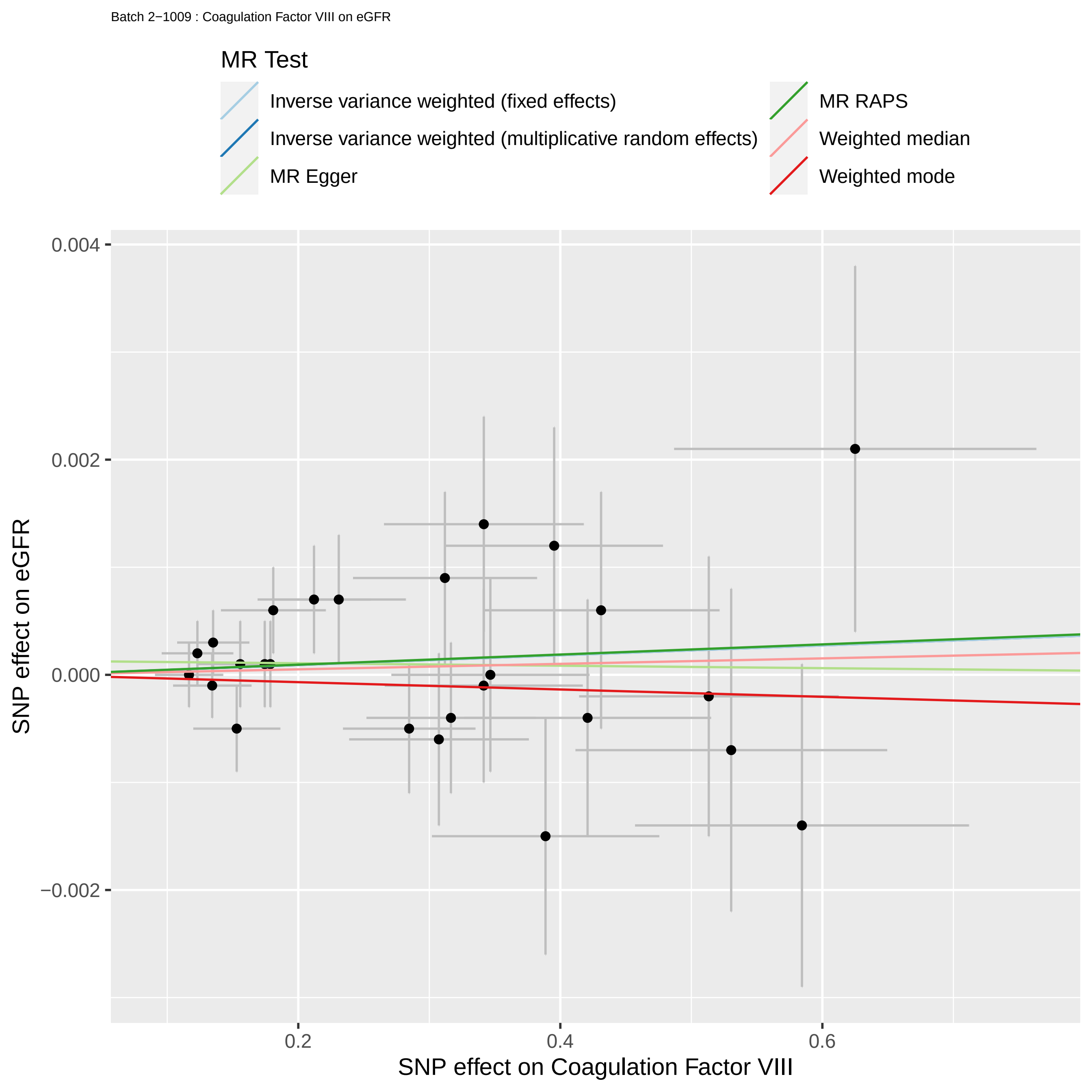
Batch 2−1009 : Coagulation Factor VIII on eGFR
MR Test
Inverse variance weighted (fixed effects)
Inverse variance weighted (multiplicative random effects)
MR Egger
MR RAPS
Weighted median
Weighted mode
0.004
0.002
0.000
−0.002
SNP effect on eGFR
0.2
0.4
0.6
SNP effect on Coagulation Factor VIII

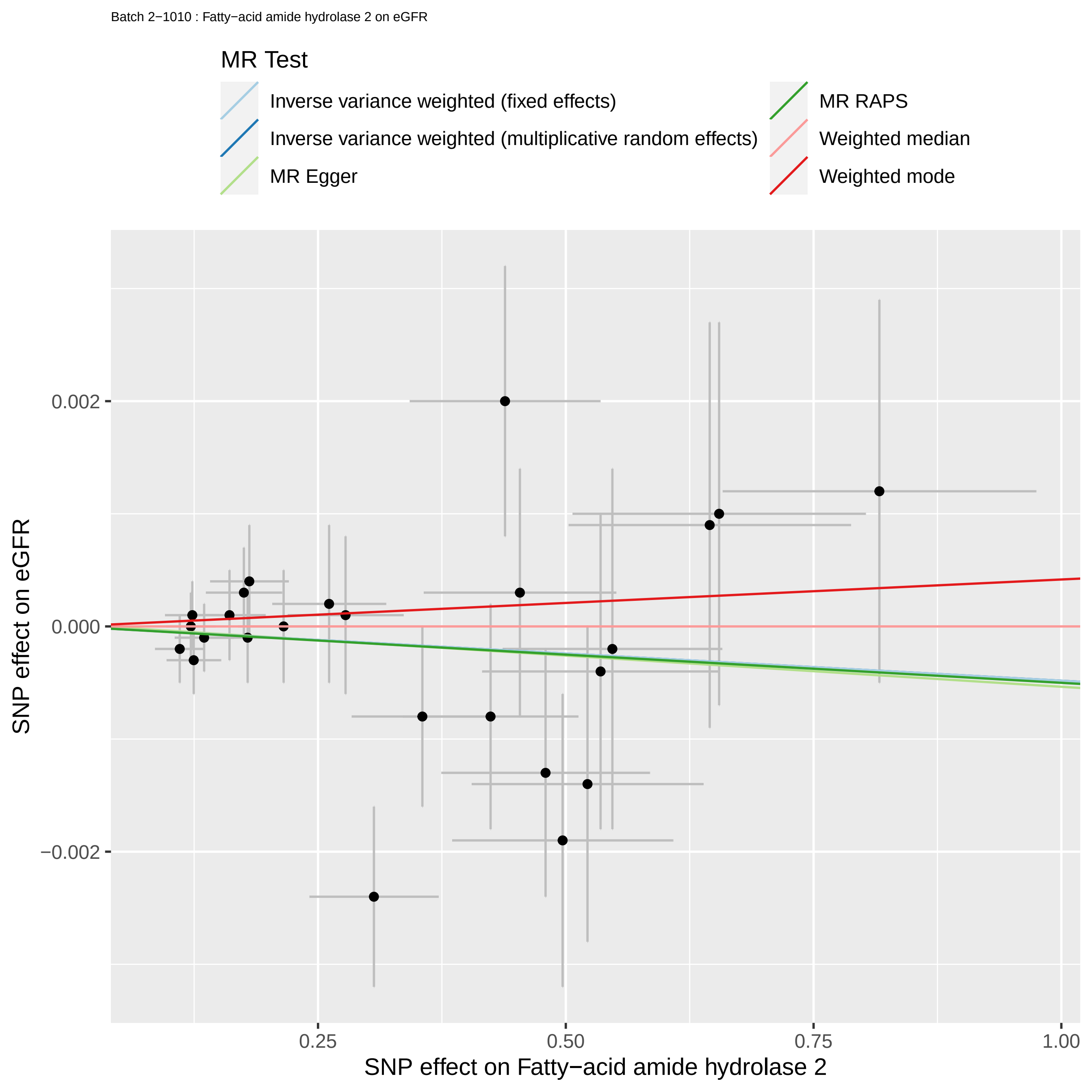
Batch 2−1010 : Fatty−acid amide hydrolase 2 on eGFR
MR Test
Inverse variance weighted (fixed effects)
Inverse variance weighted (multiplicative random effects)
MR Egger
MR RAPS
Weighted median
Weighted mode
0.002
0.000
−0.002
SNP effect on eGFR
0.25
0.50
0.75
1.00
SNP effect on Fatty−acid amide hydrolase 2

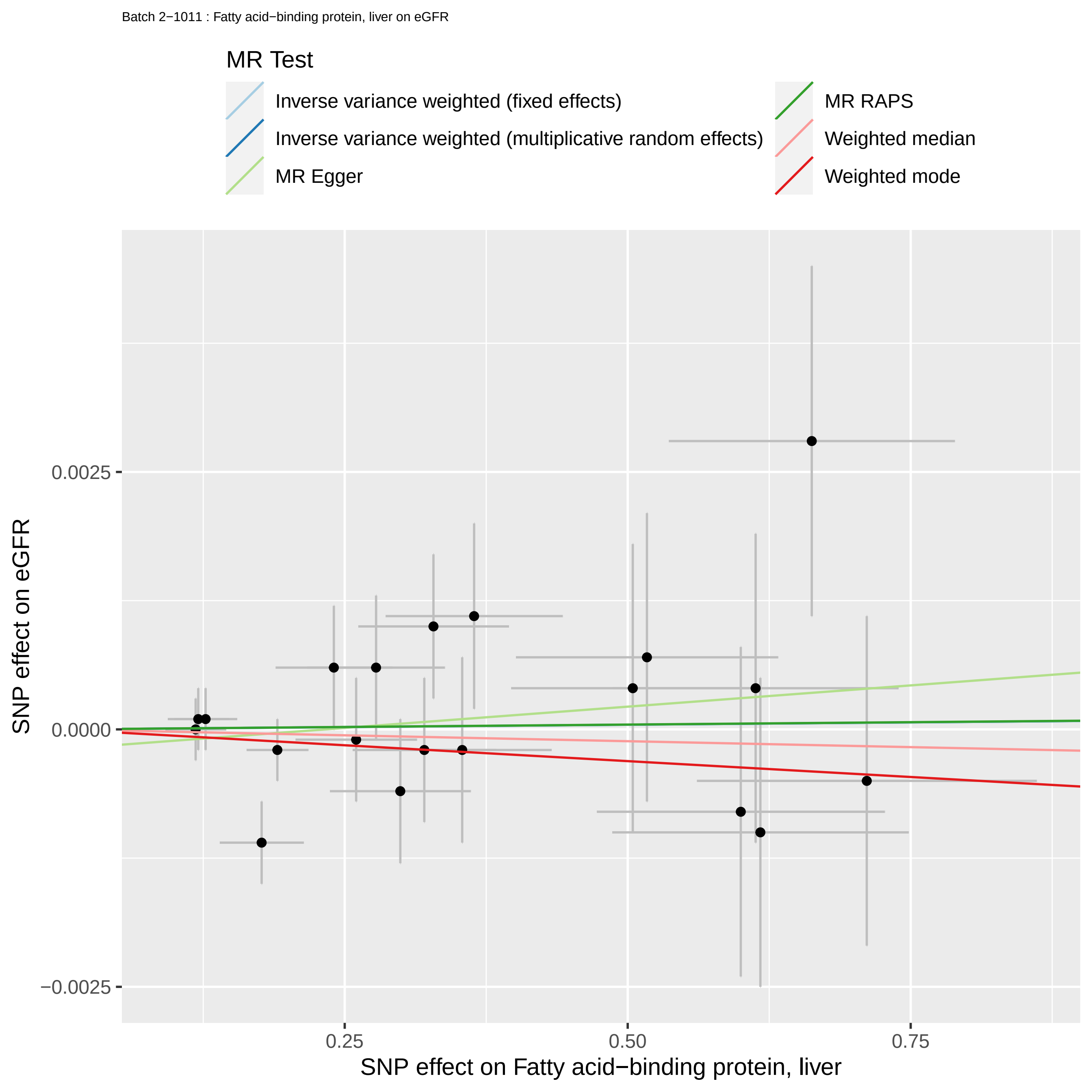
Batch 2−1011 : Fatty acid−binding protein, liver on eGFR
MR Test
Inverse variance weighted (fixed effects)
Inverse variance weighted (multiplicative random effects)
MR Egger
MR RAPS
Weighted median
Weighted mode
SNP effect on eGFR
0.0025
0.0000
−0.0025
0.25
0.50
0.75
SNP effect on Fatty acid−binding protein, liver

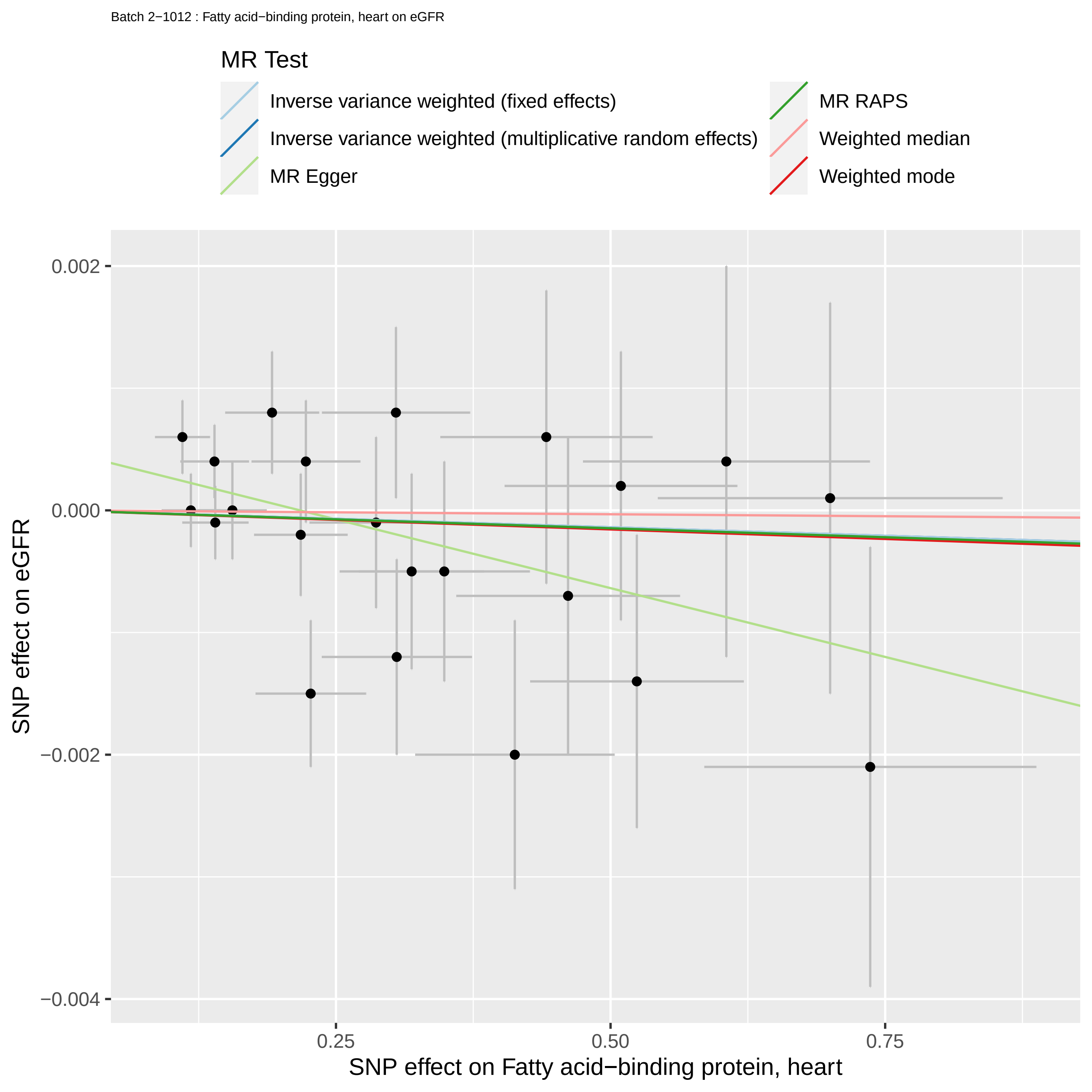
Batch 2−1012 : Fatty acid−binding protein, heart on eGFR
MR Test
Inverse variance weighted (fixed effects)
Inverse variance weighted (multiplicative random effects)
MR Egger
MR RAPS
Weighted median
Weighted mode
0.002
0.000
−0.002
−0.004
SNP effect on eGFR
0.25
0.50
0.75
SNP effect on Fatty acid−binding protein, heart

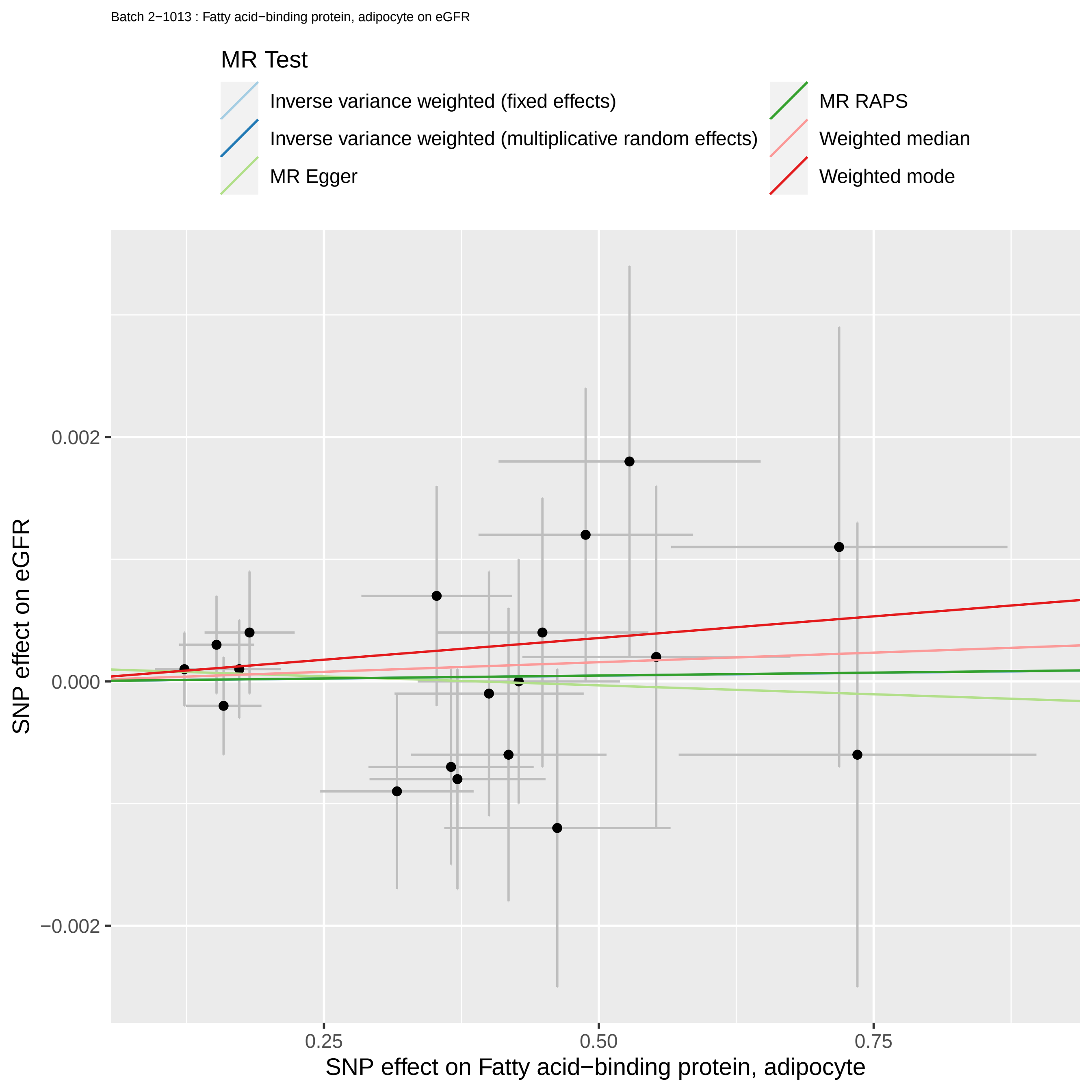
Batch 2−1013 : Fatty acid−binding protein, adipocyte on eGFR
MR Test
Inverse variance weighted (fixed effects)
Inverse variance weighted (multiplicative random effects)
MR Egger
MR RAPS
Weighted median
Weighted mode
0.002
0.000
−0.002
SNP effect on eGFR
0.25
0.50
0.75
SNP effect on Fatty acid−binding protein, adipocyte

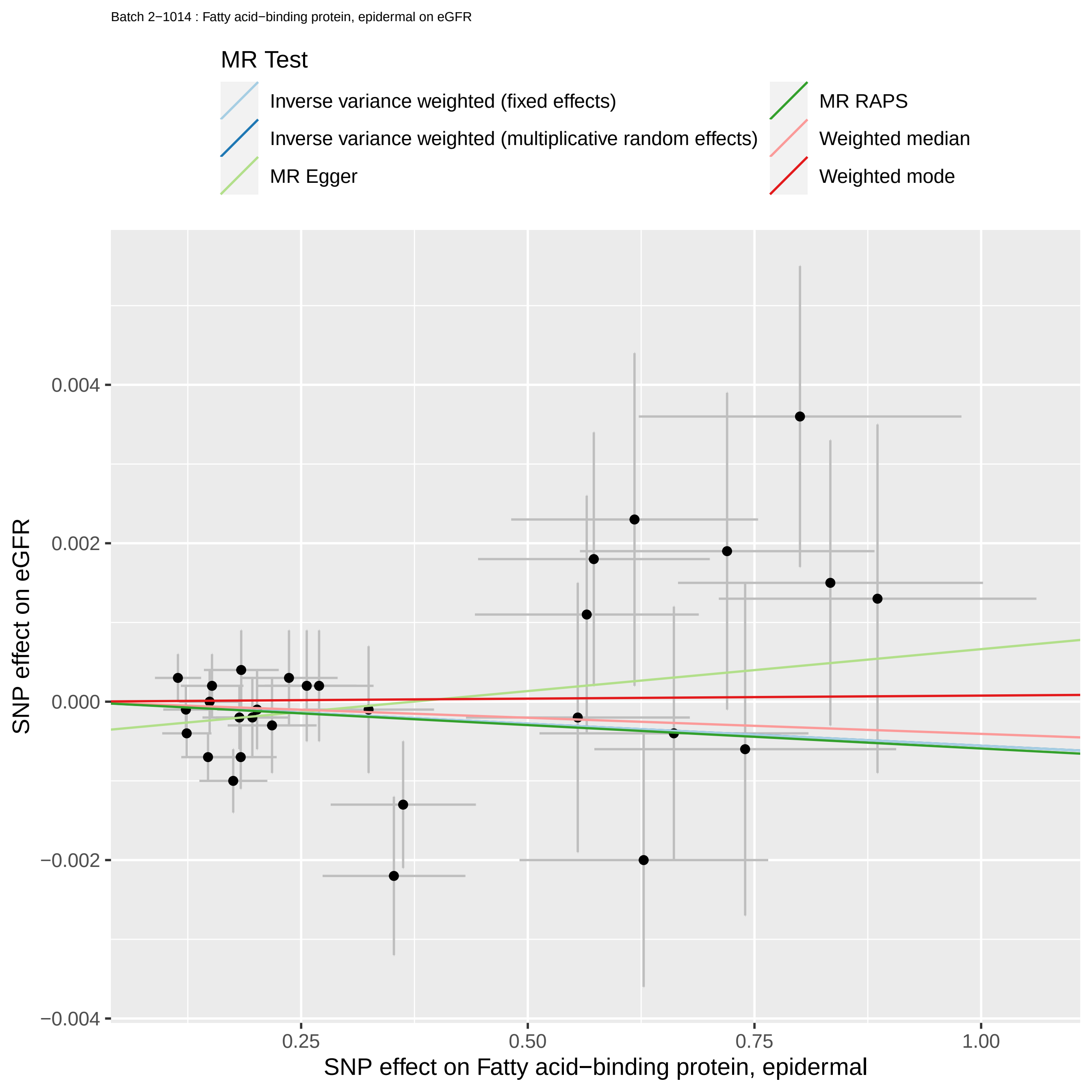

Batch 2−1014 : Fatty acid−binding protein, epidermal on eGFR
MR Test
Inverse variance weighted (fixed effects)
Inverse variance weighted (multiplicative random effects)
MR Egger
MR RAPS
Weighted median
Weighted mode
0.004
0.002
0.000
−0.002
−0.004
SNP effect on eGFR
0.25
0.50
0.75
1.00
SNP effect on Fatty acid−binding protein, epidermal

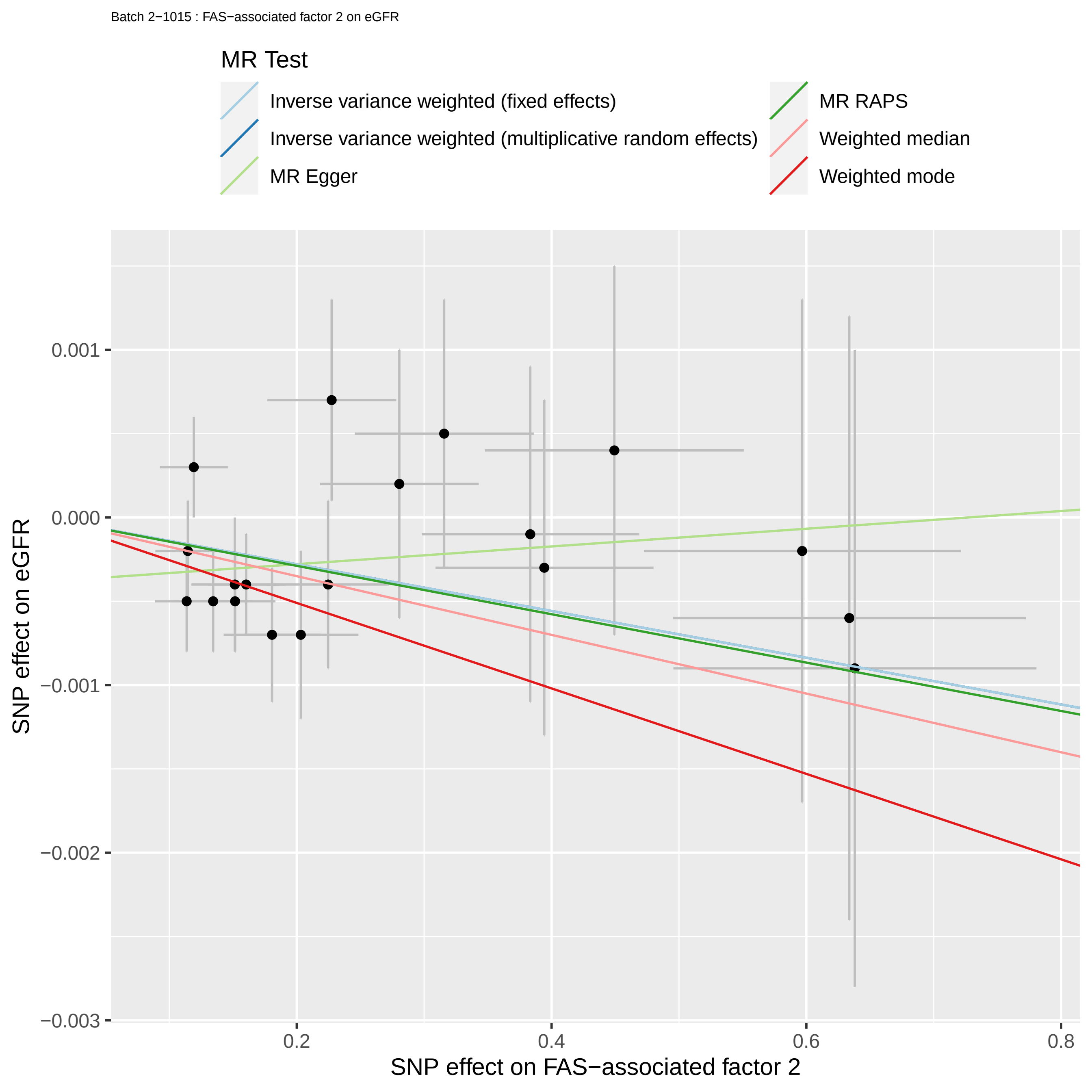

Batch 2−1015 : FAS−associated factor 2 on eGFR
MR Test
Inverse variance weighted (fixed effects)
Inverse variance weighted (multiplicative random effects)
MR Egger
MR RAPS
Weighted median
Weighted mode
0.001
0.000
−0.001
−0.002
−0.003
0.2
0.4
0.6
0.8
SNP effect on eGFR
SNP effect on FAS−associated factor 2

Batch 2−1016 : FAS−associated factor 2 on eGFR

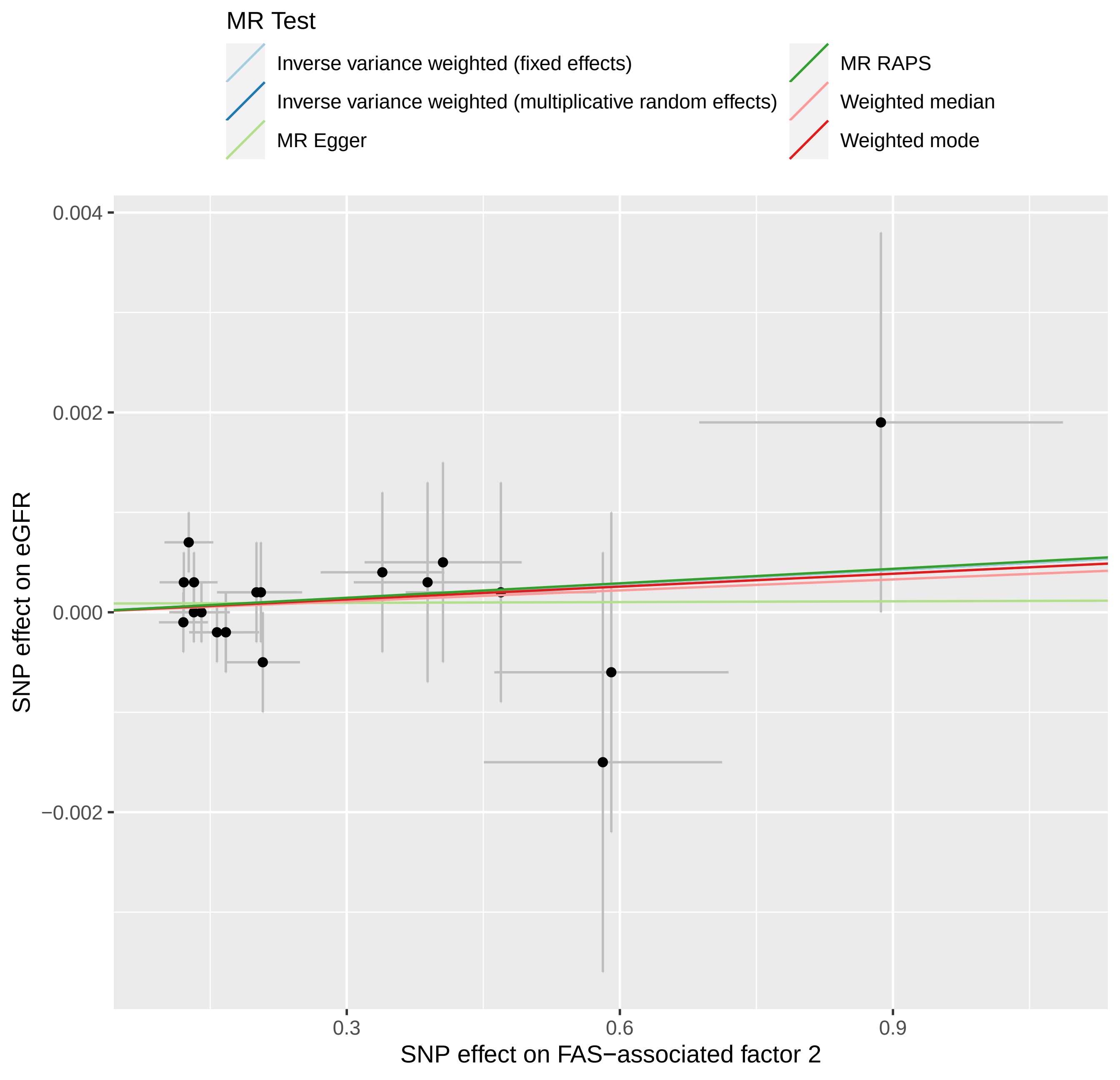

Batch 2−1017 : FAS−associated factor 2 on eGFR

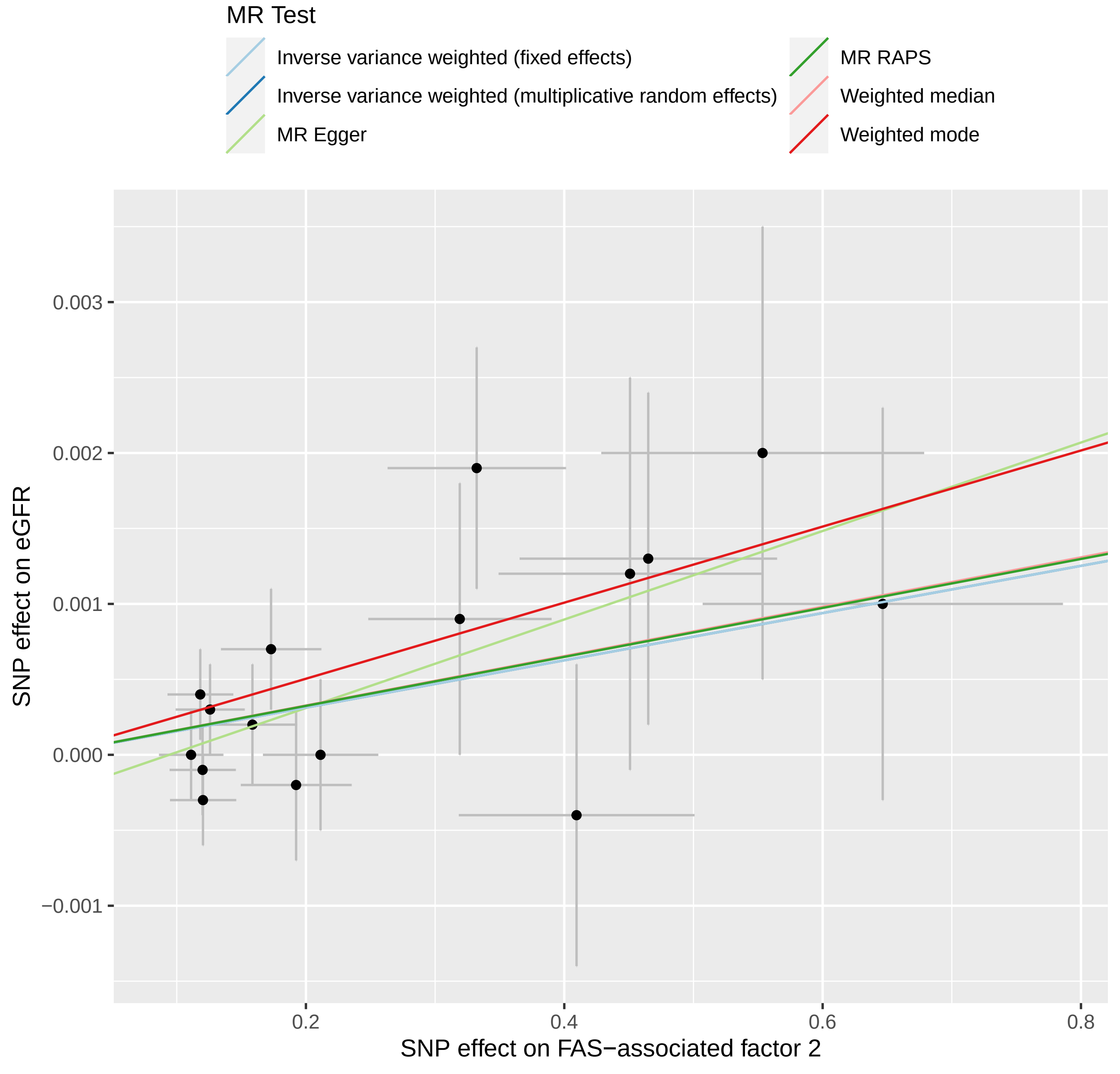

Batch 2−1018 : Fumarylacetoacetase on eGFR

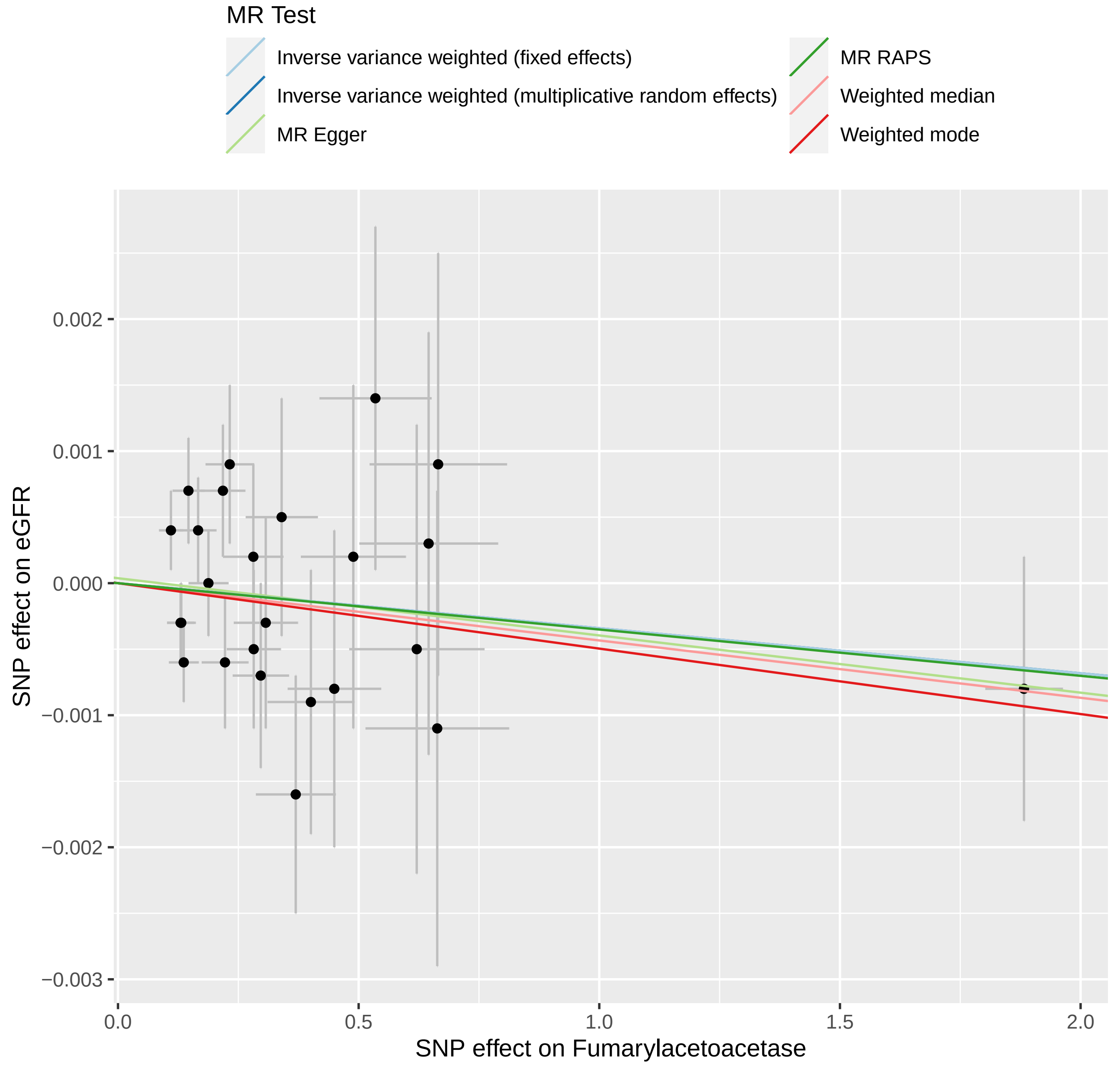

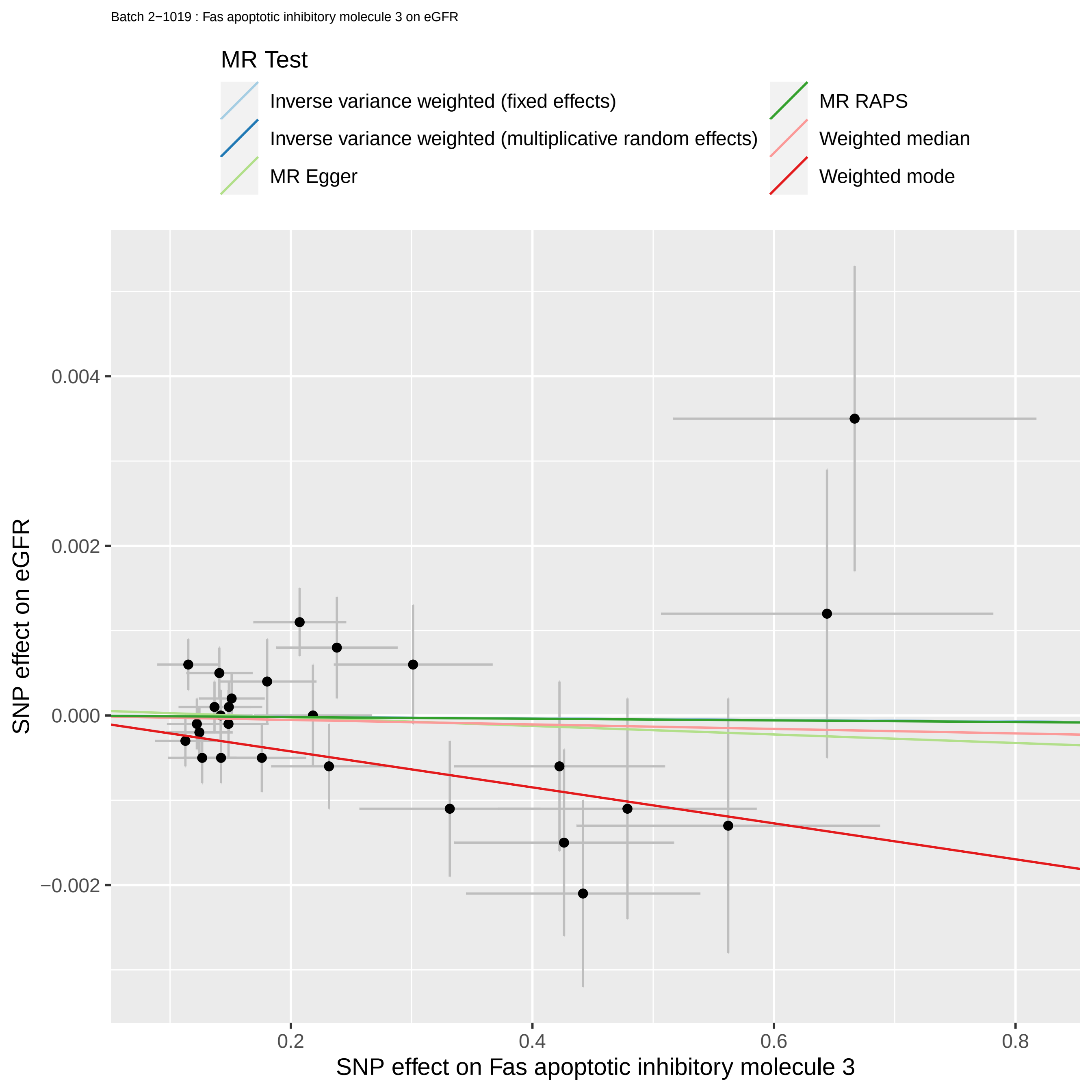

Batch 2−1019 : Fas apoptotic inhibitory molecule 3 on eGFR
MR Test
Inverse variance weighted (fixed effects)
Inverse variance weighted (multiplicative random effects)
MR Egger
MR RAPS
Weighted median
Weighted mode
0.004
0.002
0.000
−0.002
0.2
0.4
0.6
0.8
SNP effect on eGFR
SNP effect on Fas apoptotic inhibitory molecule 3

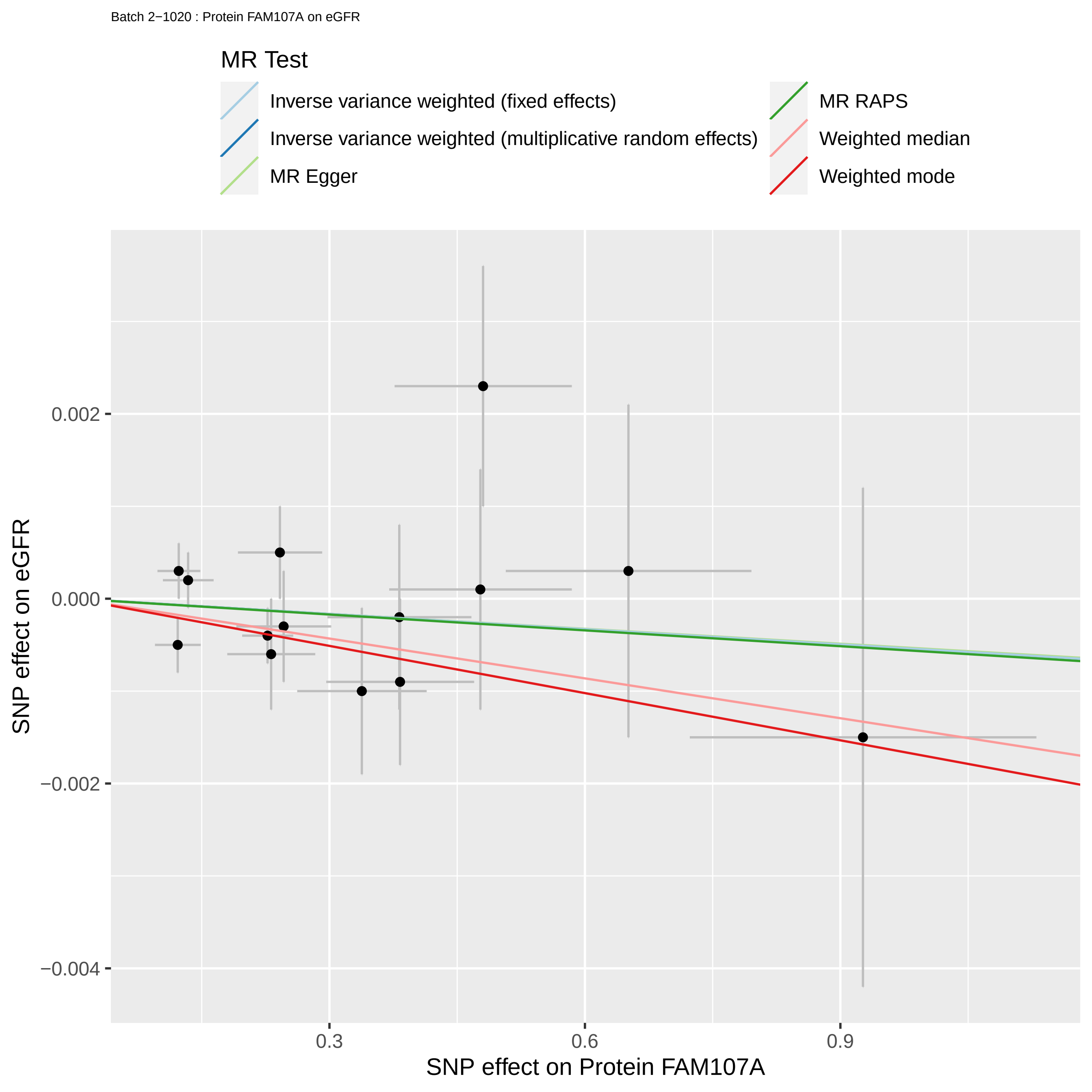

Batch 2−1020 : Protein FAM107A on eGFR
MR Test
Inverse variance weighted (fixed effects)
Inverse variance weighted (multiplicative random effects)
MR Egger
MR RAPS
Weighted median
Weighted mode
0.002
0.000
−0.002
−0.004
0.3
0.6
0.9
SNP effect on eGFR
SNP effect on Protein FAM107A

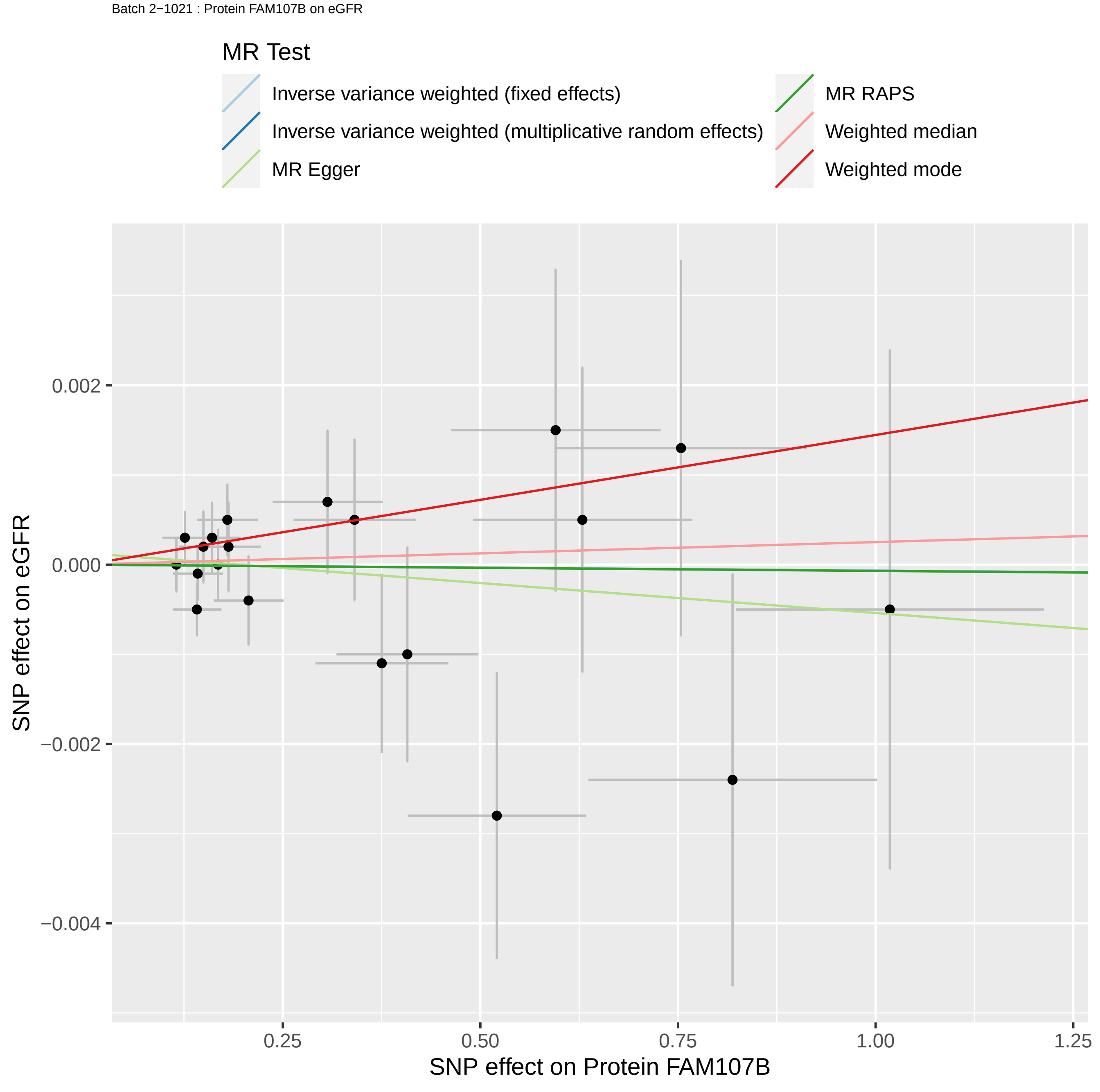

Batch 2−1021 : Protein FAM107B on eGFR
MR Test
Inverse variance weighted (fixed effects)
Inverse variance weighted (multiplicative random effects)
MR Egger
MR RAPS
Weighted median
Weighted mode
0.002
0.000
−0.002
−0.004
SNP effect on eGFR
0.25
0.50
0.75
1.00
1.25
SNP effect on Protein FAM107B

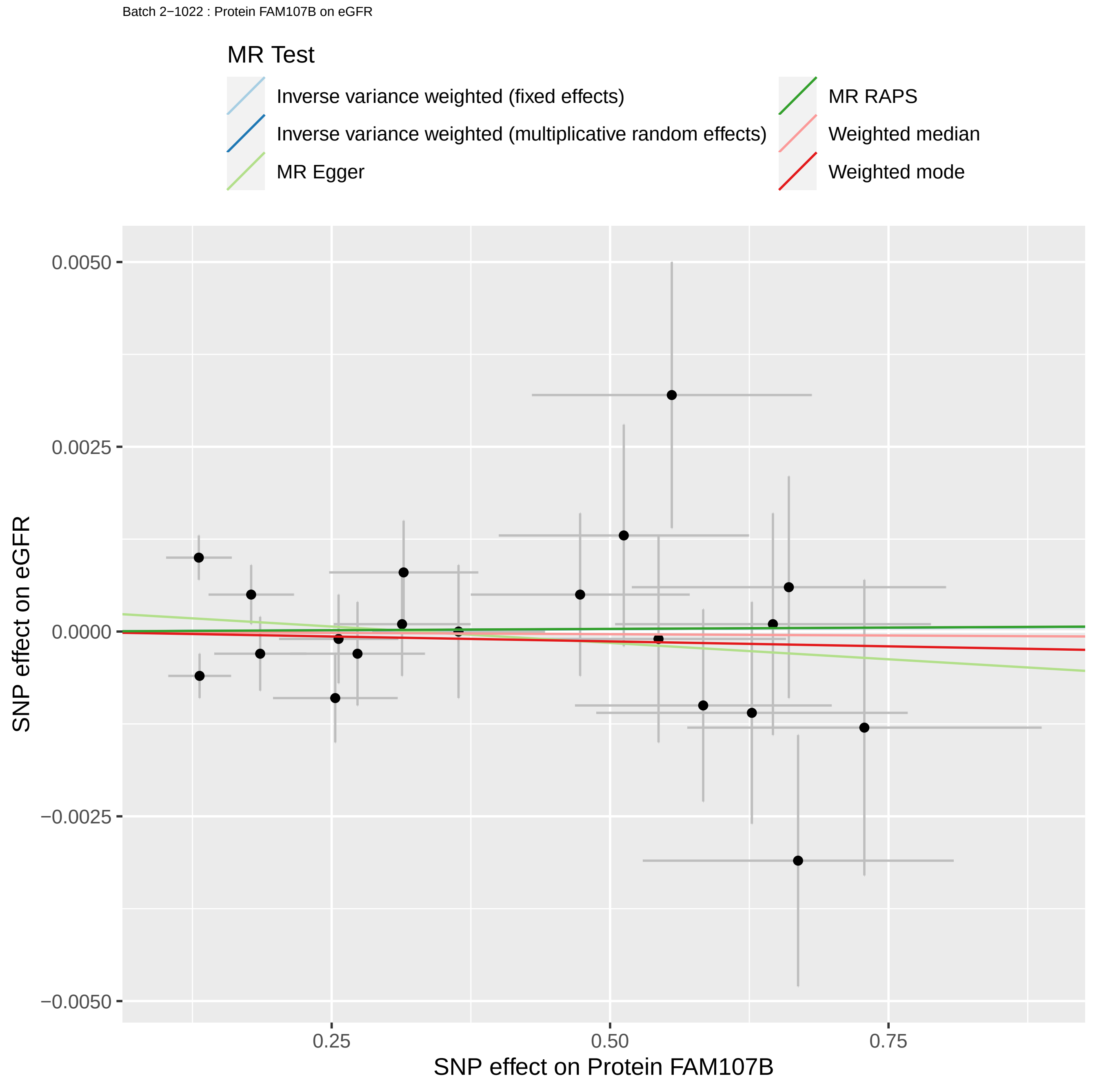

Batch 2−1022 : Protein FAM107B on eGFR
MR Test
Inverse variance weighted (fixed effects)
Inverse variance weighted (multiplicative random effects)
MR Egger
MR RAPS
Weighted median
Weighted mode
0.0050
0.0025
0.0000
−0.0025
−0.0050
SNP effect on eGFR
0.25
0.50
0.75
SNP effect on Protein FAM107B

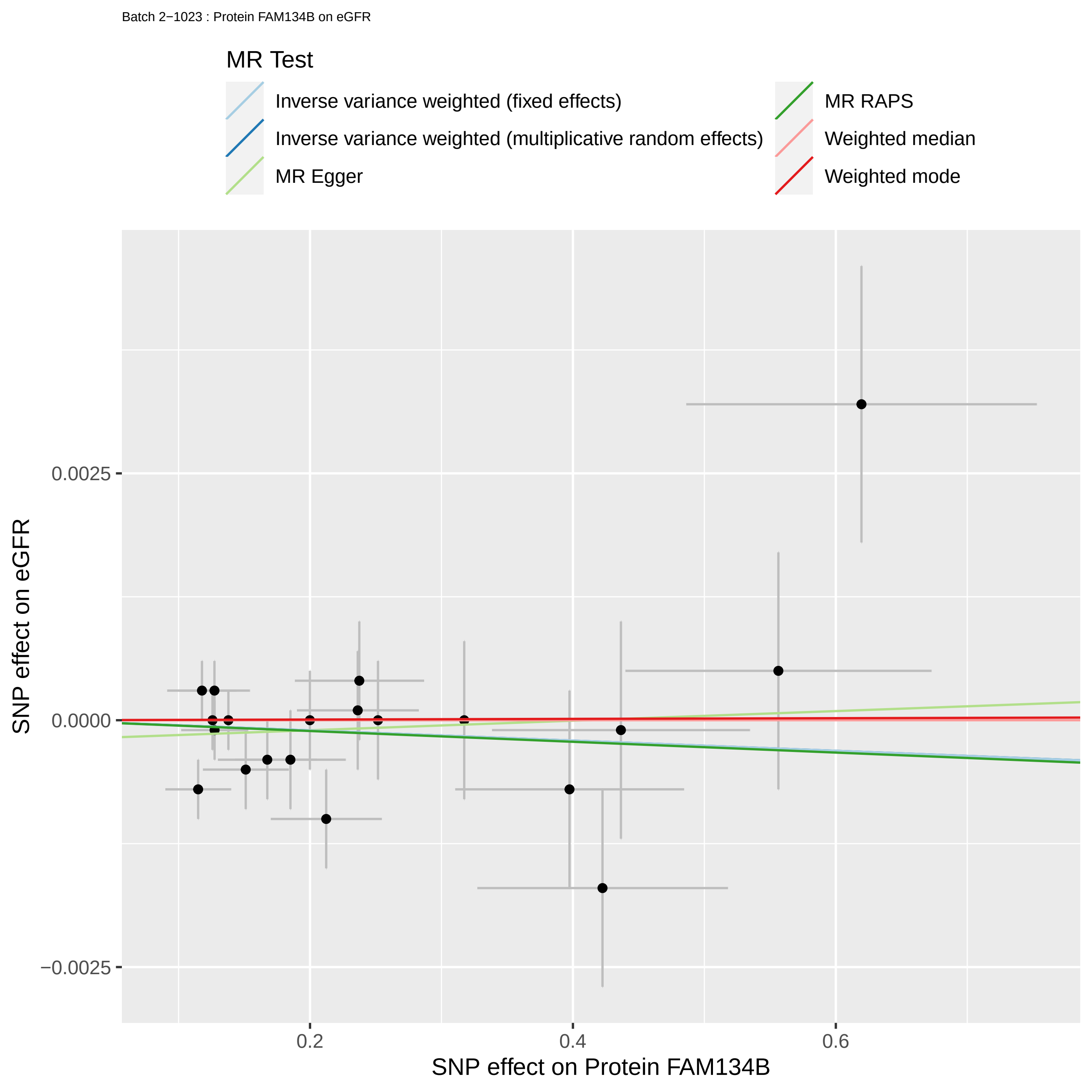

Batch 2−1023 : Protein FAM134B on eGFR
MR Test
Inverse variance weighted (fixed effects)
Inverse variance weighted (multiplicative random effects)
MR Egger
MR RAPS
Weighted median
Weighted mode
0.0025
0.0000
−0.0025
SNP effect on eGFR
0.2
0.4
0.6
SNP effect on Protein FAM134B

Batch 2−1024 : Protein FAM150B on eGFR

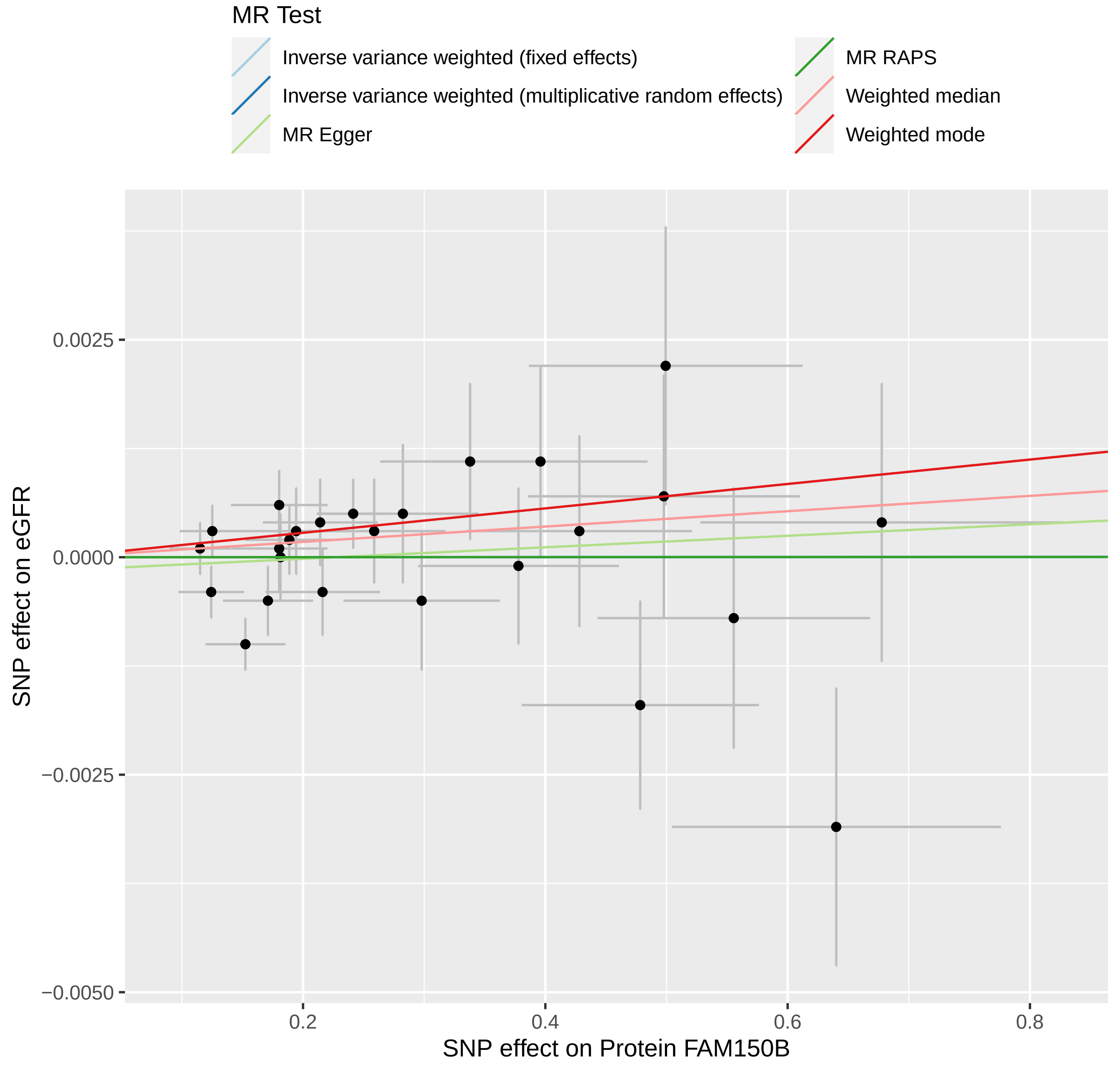

Batch 2−1025 : Protein FAM151A on eGFR

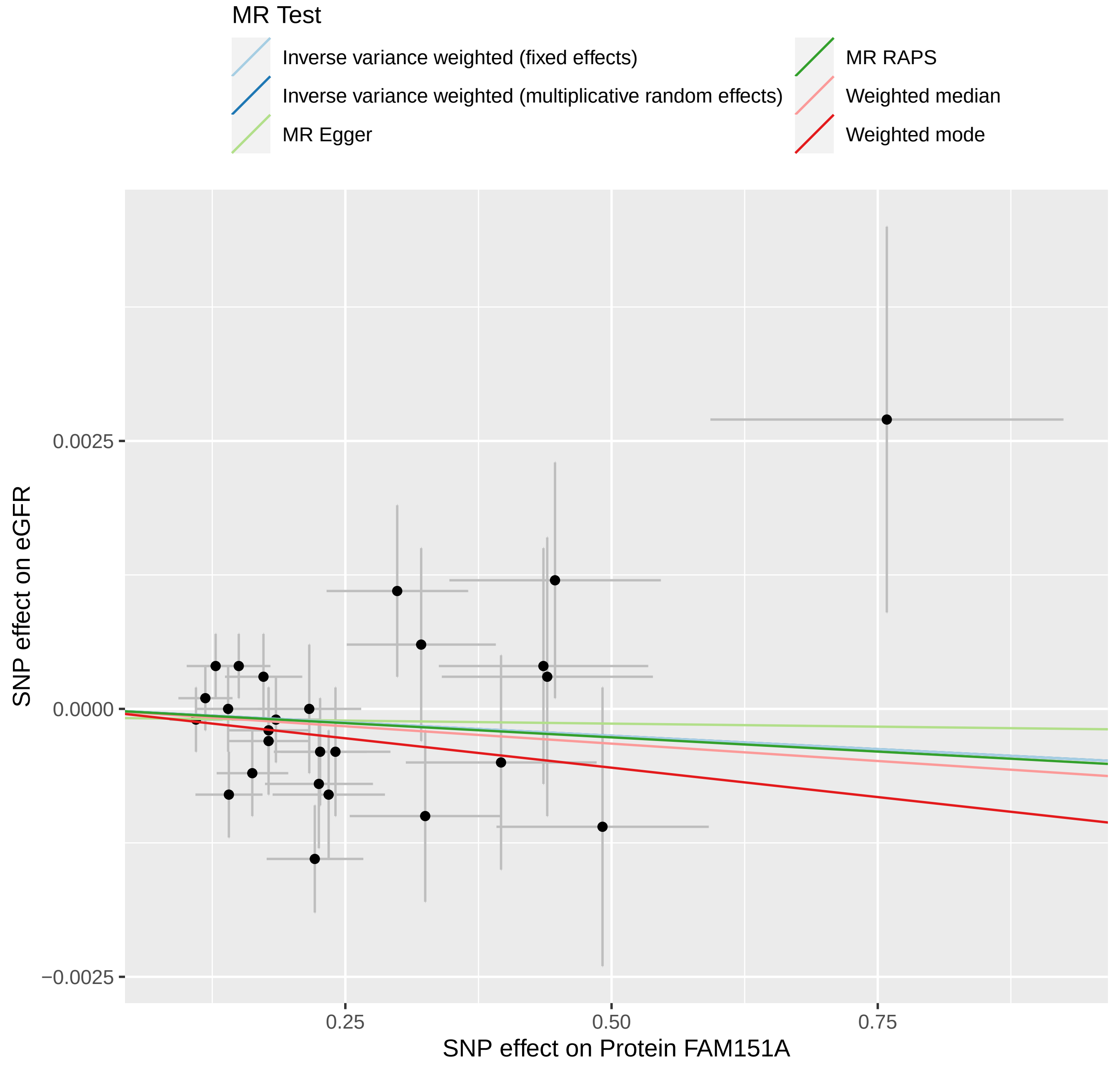

Batch 2−1026 : Membrane protein FAM159B on eGFR

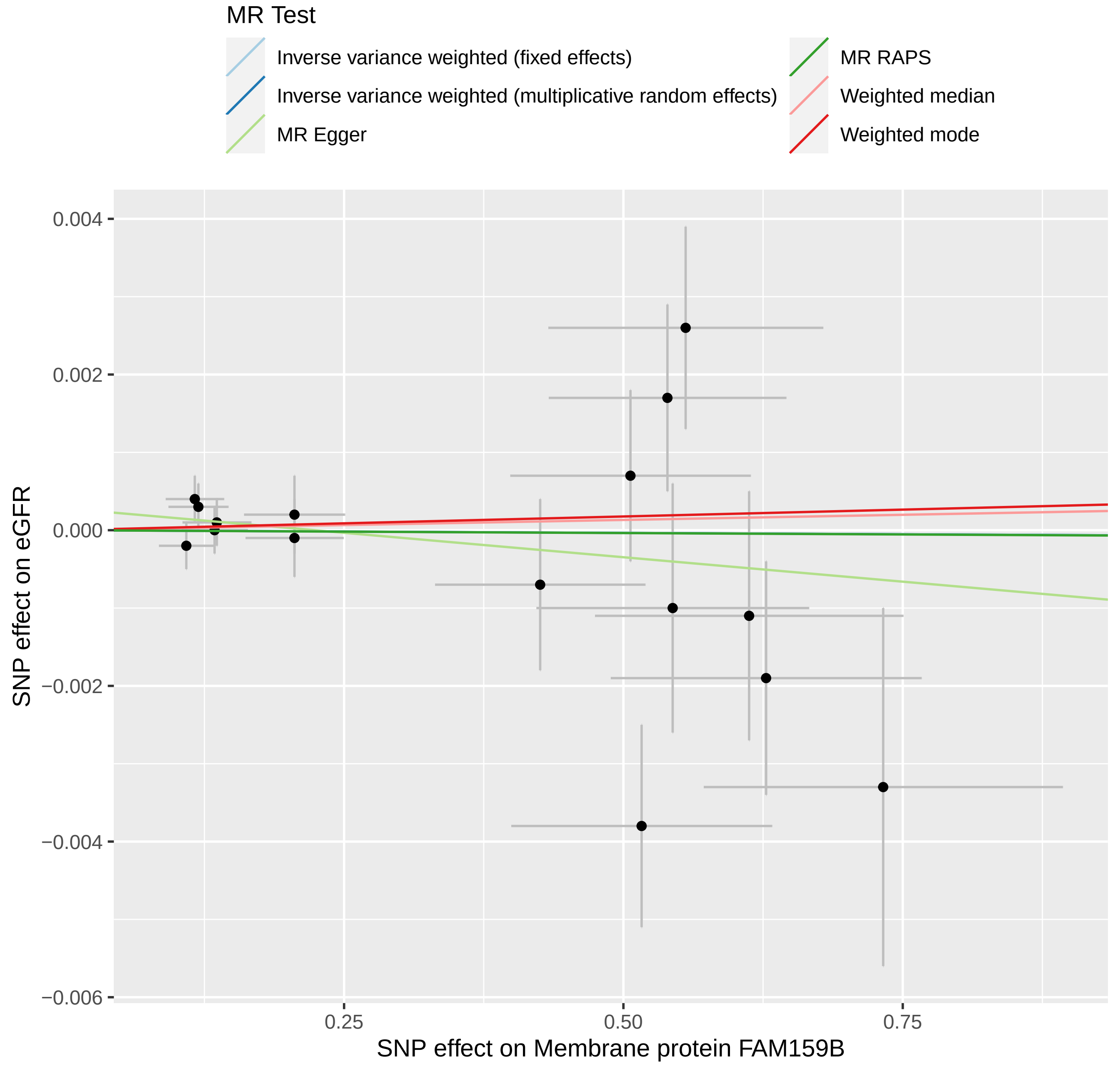

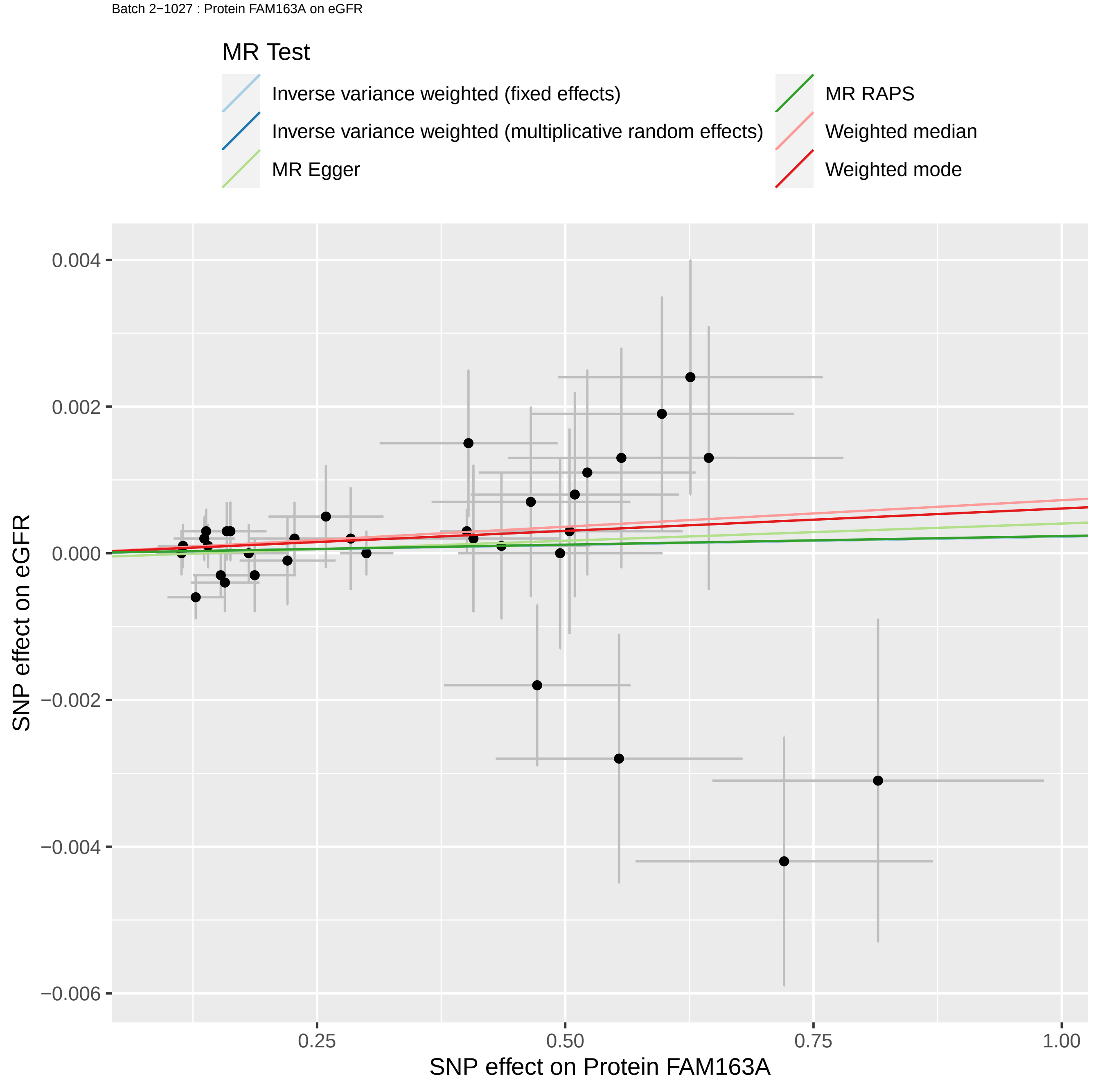

Batch 2−1027 : Protein FAM163A on eGFR
MR Test
Inverse variance weighted (fixed effects)
Inverse variance weighted (multiplicative random effects)
MR Egger
MR RAPS
Weighted median
Weighted mode
0.004
0.002
0.000
−0.002
−0.004
−0.006
SNP effect on eGFR
0.25
0.50
0.75
1.00
SNP effect on Protein FAM163A

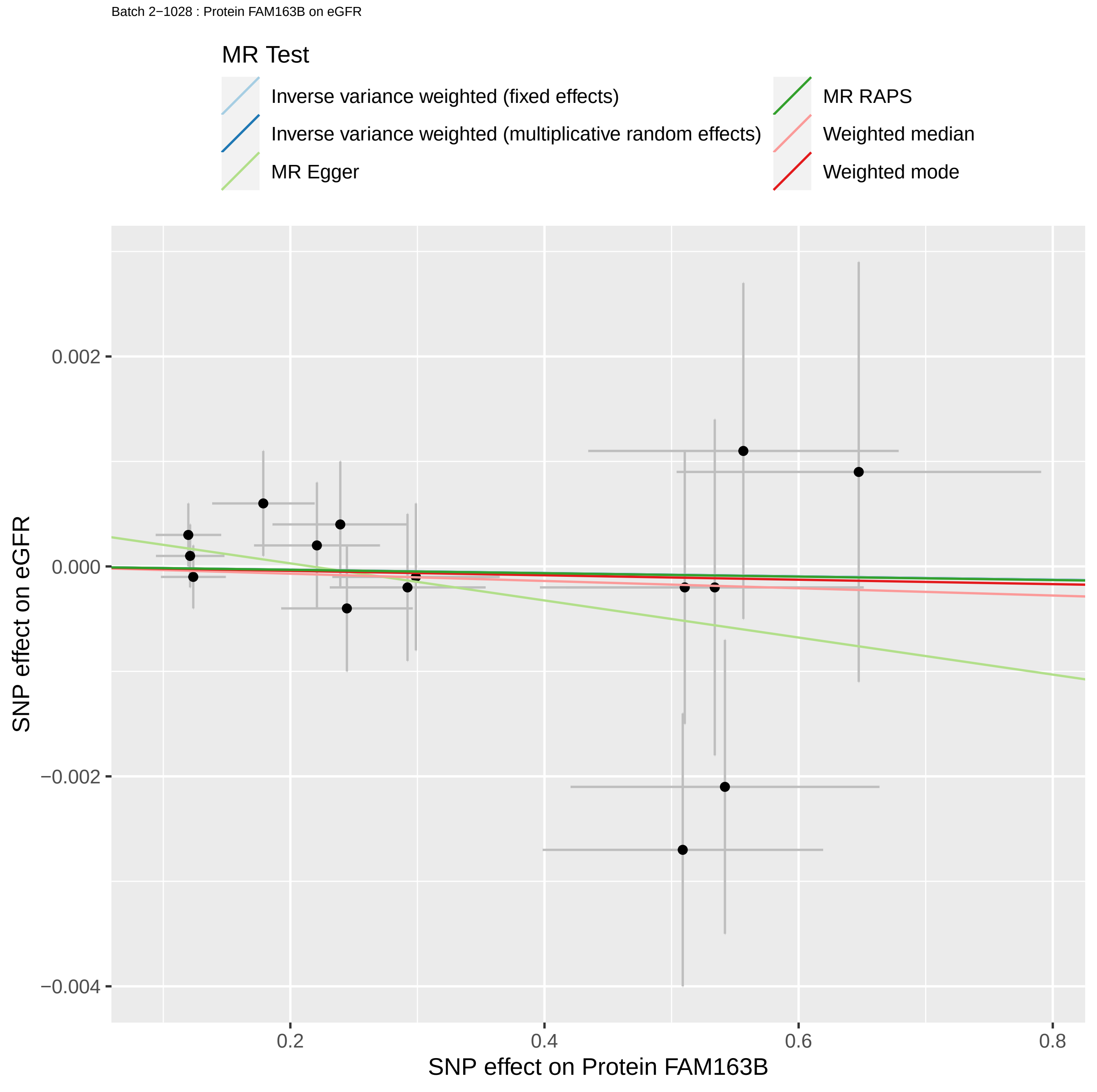
Batch 2−1028 : Protein FAM163B on eGFR
MR Test
Inverse variance weighted (fixed effects)
Inverse variance weighted (multiplicative random effects)
MR Egger
MR RAPS
Weighted median
Weighted mode
0.002
0.000
−0.002
−0.004
0.2
0.4
0.6
0.8
SNP effect on eGFR
SNP effect on Protein FAM163B

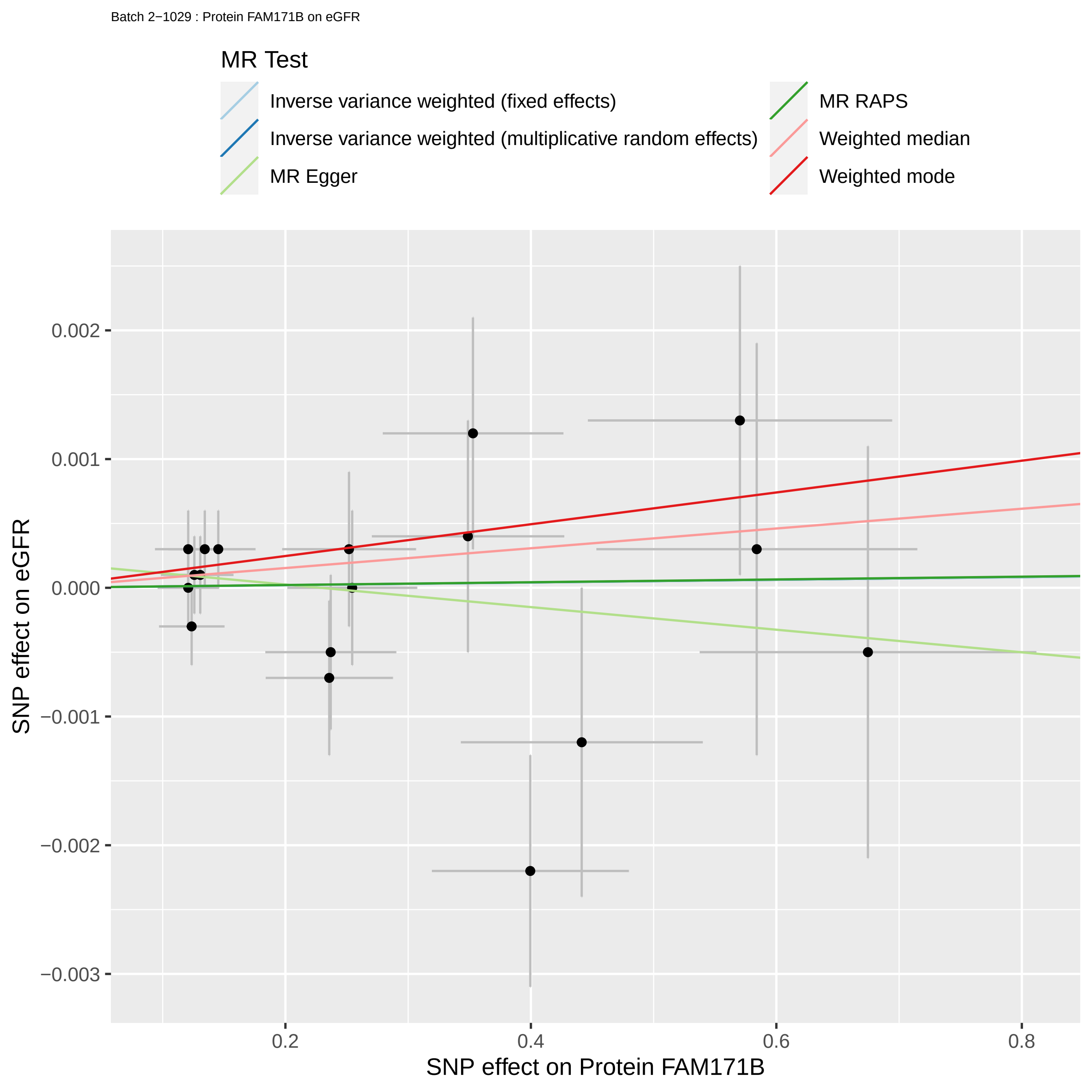

Batch 2−1029 : Protein FAM171B on eGFR
MR Test
Inverse variance weighted (fixed effects)
Inverse variance weighted (multiplicative random effects)
MR Egger
MR RAPS
Weighted median
Weighted mode
0.002
0.001
0.000
−0.001
−0.002
−0.003
0.2
0.4
0.6
0.8
SNP effect on eGFR
SNP effect on Protein FAM171B

Batch 2−1030 : Protein FAM171B on eGFR

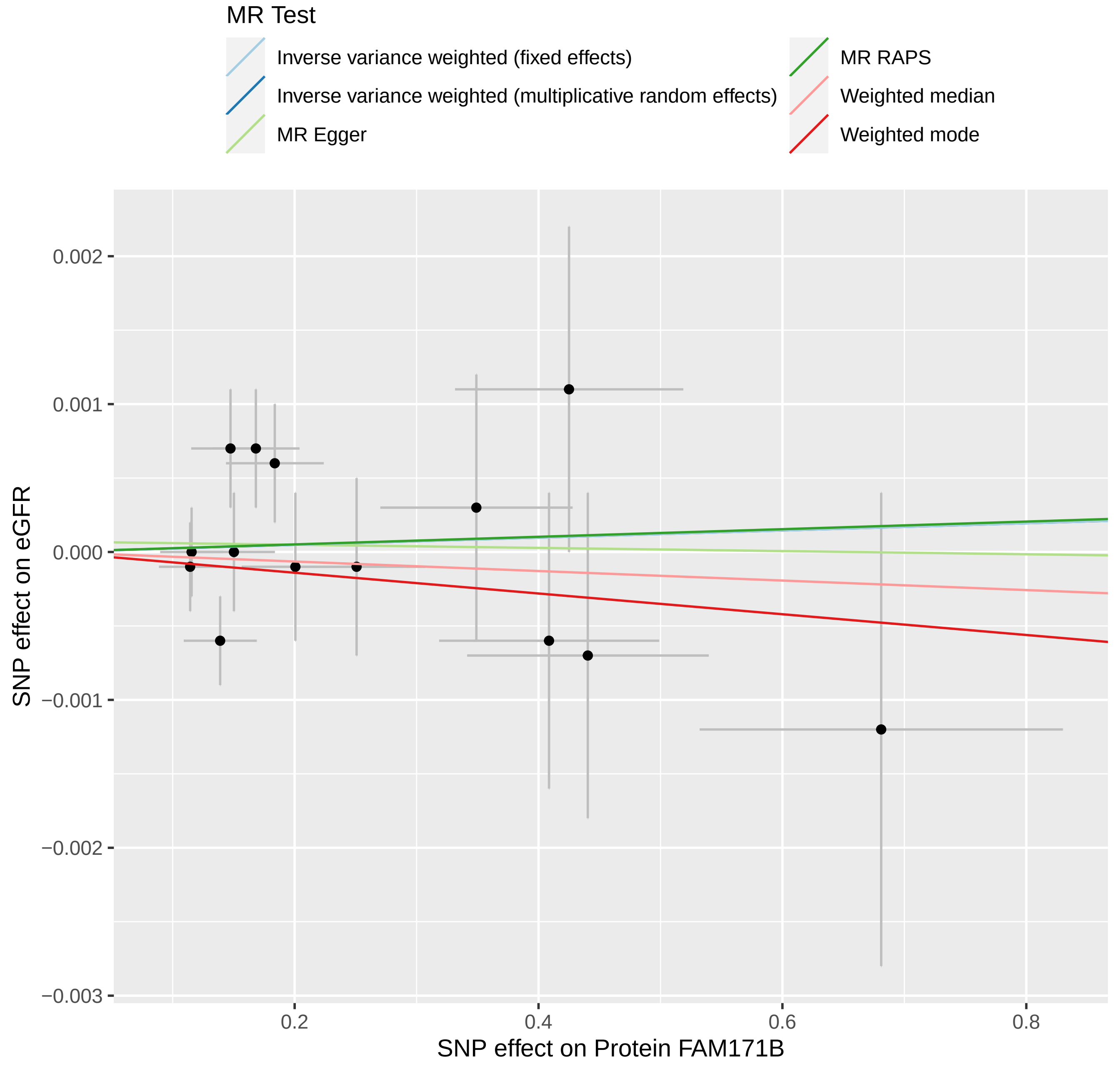

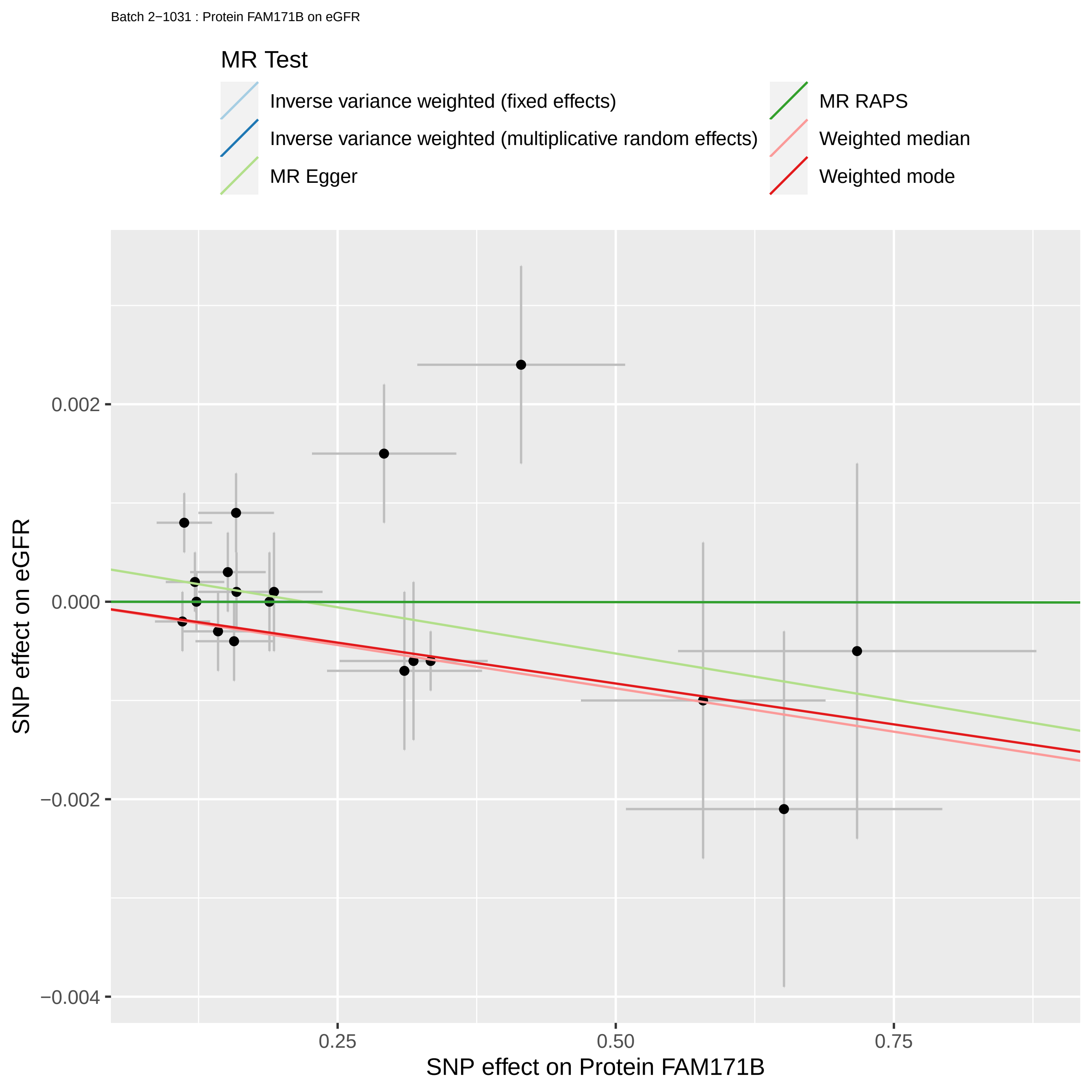

Batch 2−1031 : Protein FAM171B on eGFR
MR Test
Inverse variance weighted (fixed effects)
Inverse variance weighted (multiplicative random effects)
MR Egger
MR RAPS
Weighted median
Weighted mode
0.002
0.000
−0.002
−0.004
0.25
0.50
0.75
SNP effect on eGFR
SNP effect on Protein FAM171B

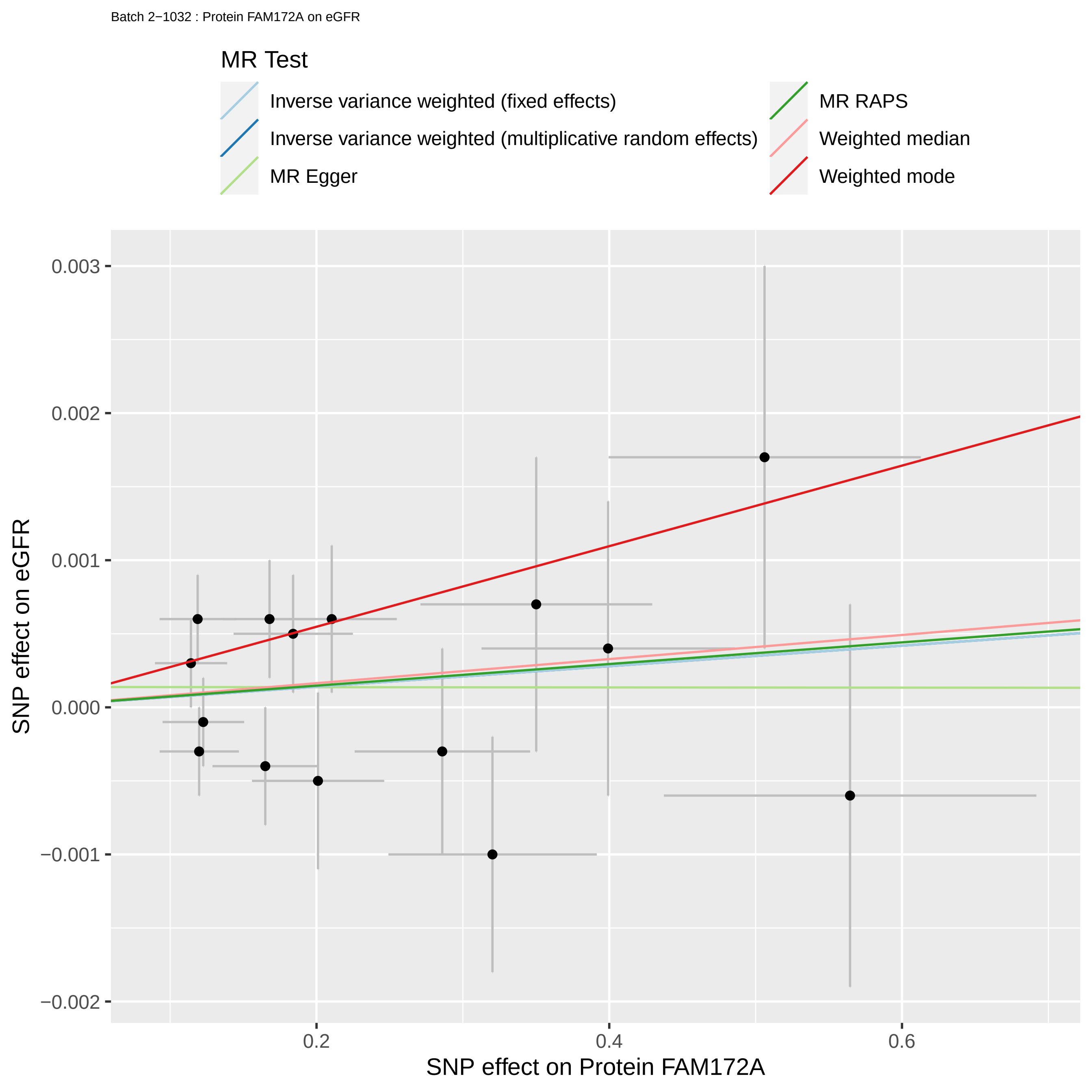
Batch 2−1032 : Protein FAM172A on eGFR
MR Test
Inverse variance weighted (fixed effects)
Inverse variance weighted (multiplicative random effects)
MR Egger
MR RAPS
Weighted median
Weighted mode
0.003
0.002
0.001
0.000
−0.001
−0.002
SNP effect on eGFR
0.2
0.4
0.6
SNP effect on Protein FAM172A

Batch 2−1033 : Protein FAM173A on eGFR

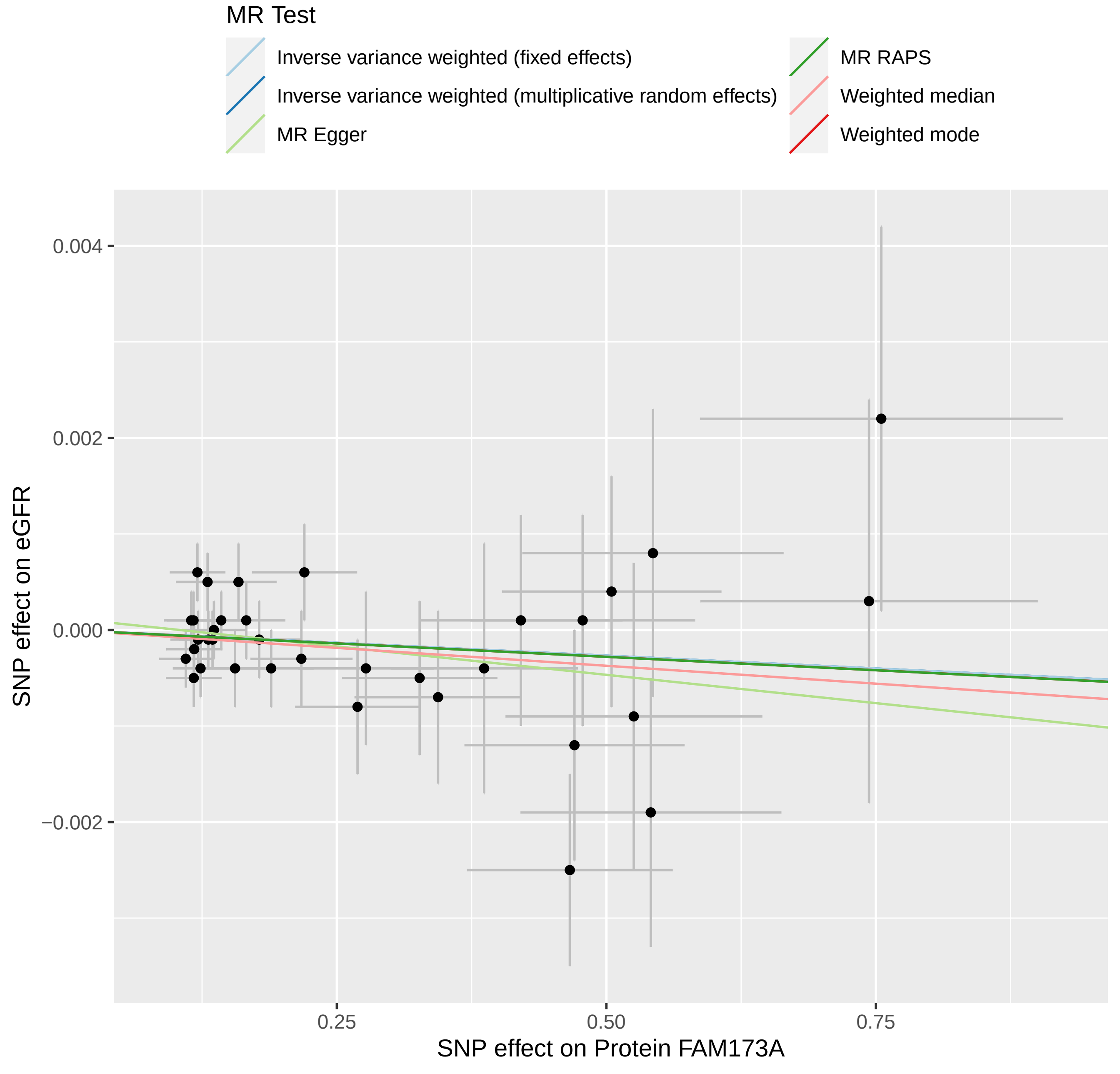

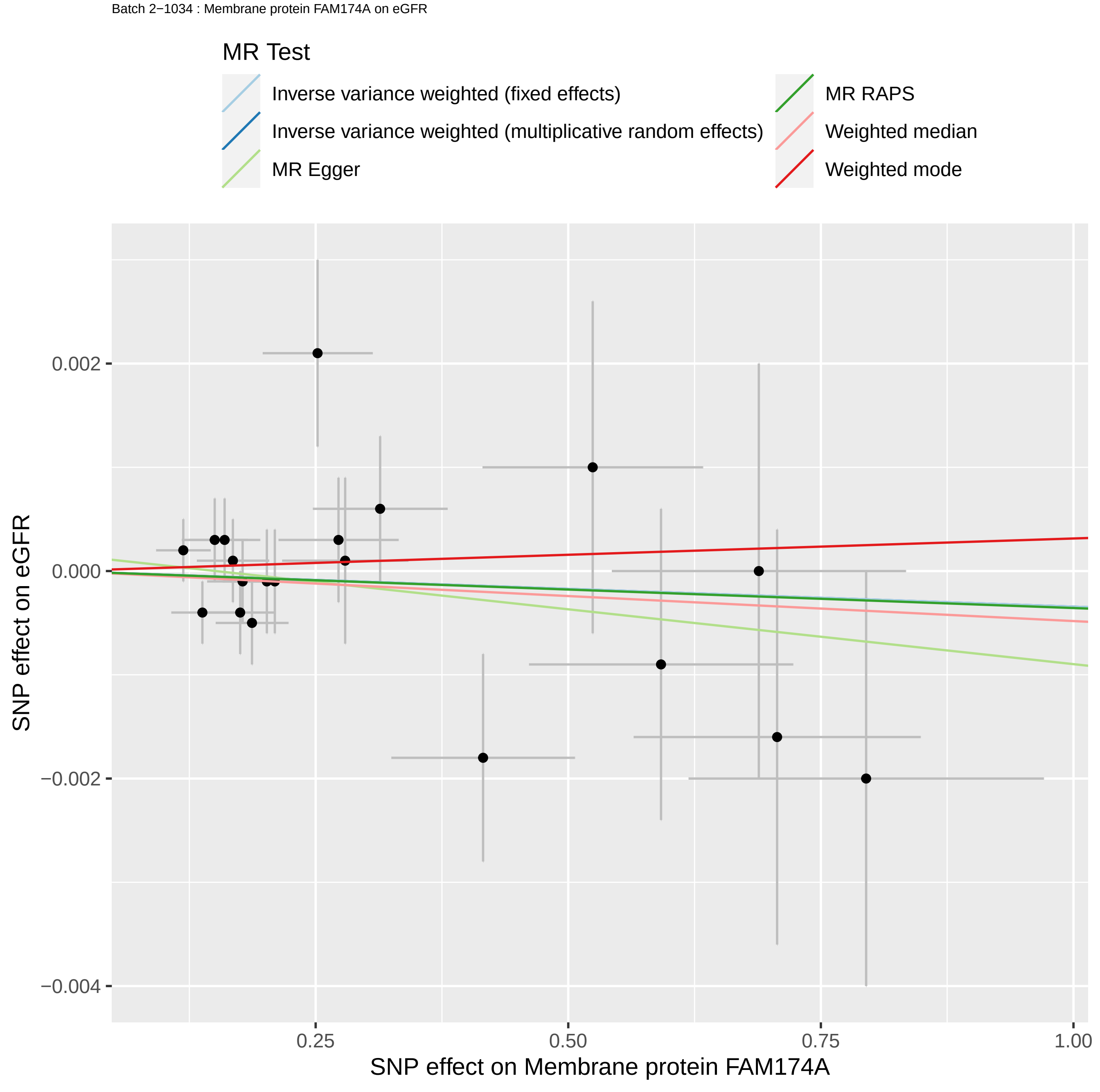

Batch 2−1034 : Membrane protein FAM174A on eGFR
MR Test
Inverse variance weighted (fixed effects)
Inverse variance weighted (multiplicative random effects)
MR Egger
MR RAPS
Weighted median
Weighted mode
0.002
0.000
−0.002
−0.004
0.25
0.50
0.75
1.00
SNP effect on eGFR
SNP effect on Membrane protein FAM174A

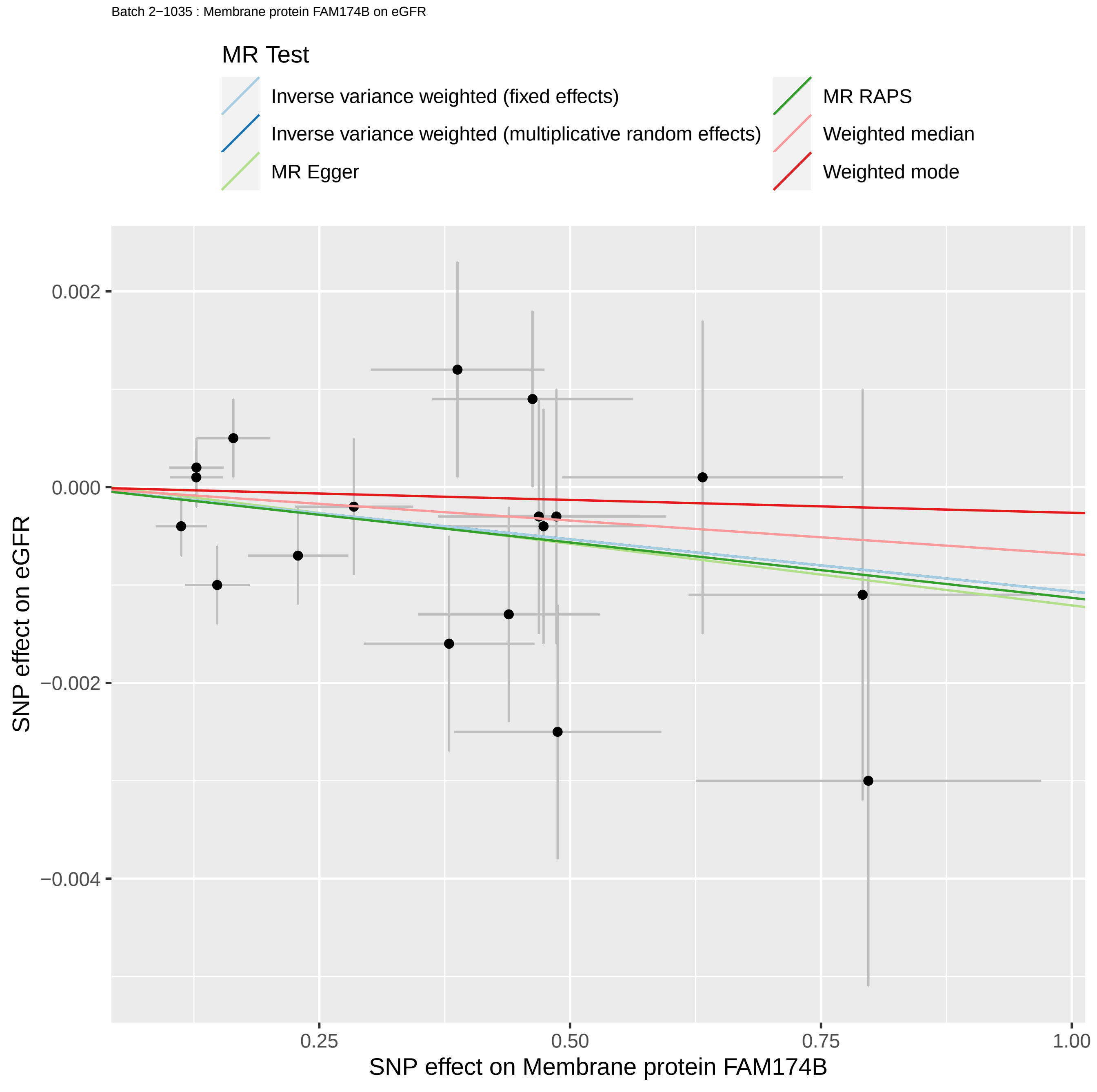

Batch 2−1035 : Membrane protein FAM174B on eGFR
MR Test
Inverse variance weighted (fixed effects)
Inverse variance weighted (multiplicative random effects)
MR Egger
MR RAPS
Weighted median
Weighted mode
0.002
0.000
−0.002
−0.004
SNP effect on eGFR
0.25
0.50
0.75
1.00
SNP effect on Membrane protein FAM174B

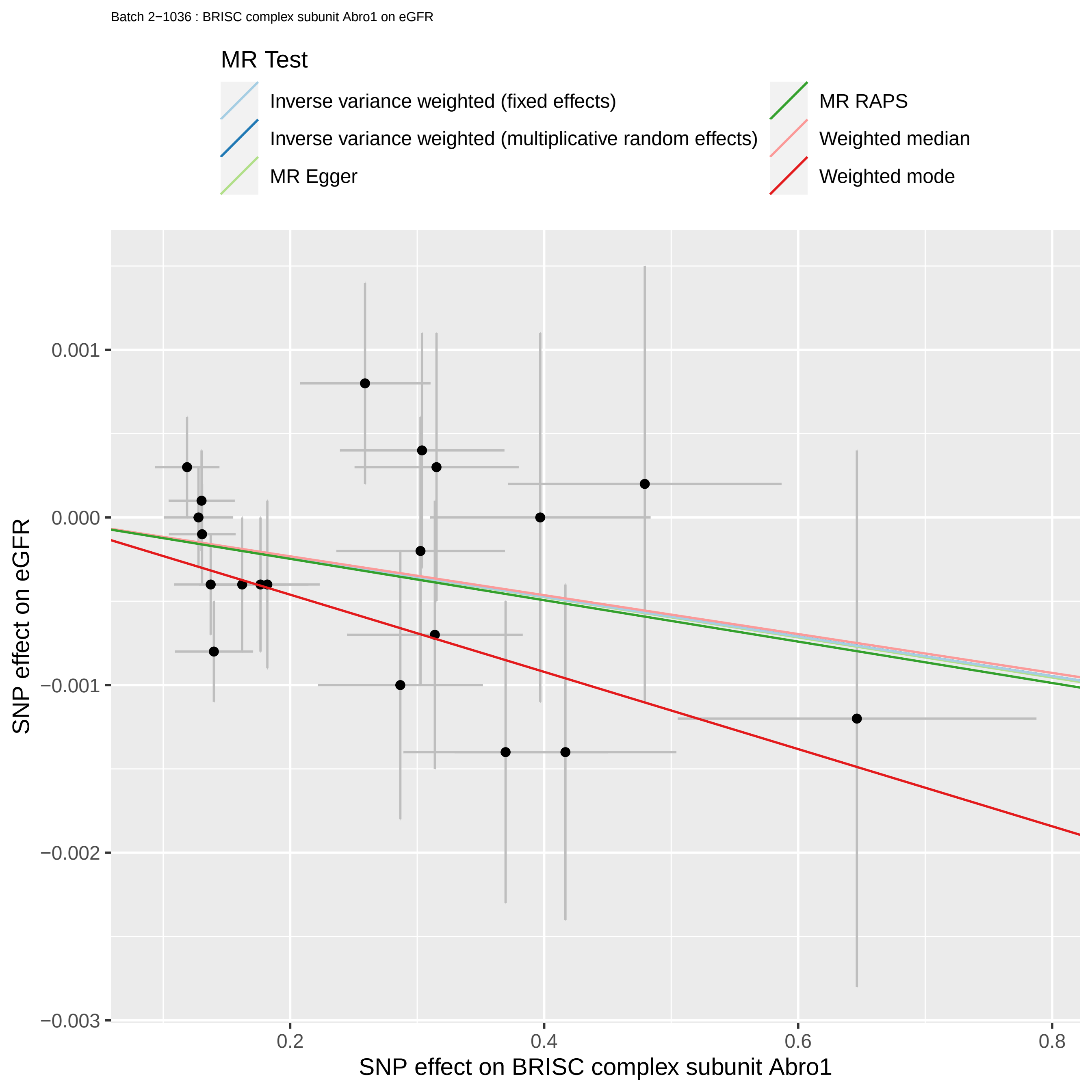

Batch 2−1036 : BRISC complex subunit Abro1 on eGFR
MR Test
Inverse variance weighted (fixed effects)
Inverse variance weighted (multiplicative random effects)
MR Egger
MR RAPS
Weighted median
Weighted mode
0.001
0.000
−0.001
−0.002
−0.003
0.2
0.4
0.6
0.8
SNP effect on eGFR
SNP effect on BRISC complex subunit Abro1

Batch 2−1037 : Protein FAM177A1 on eGFR

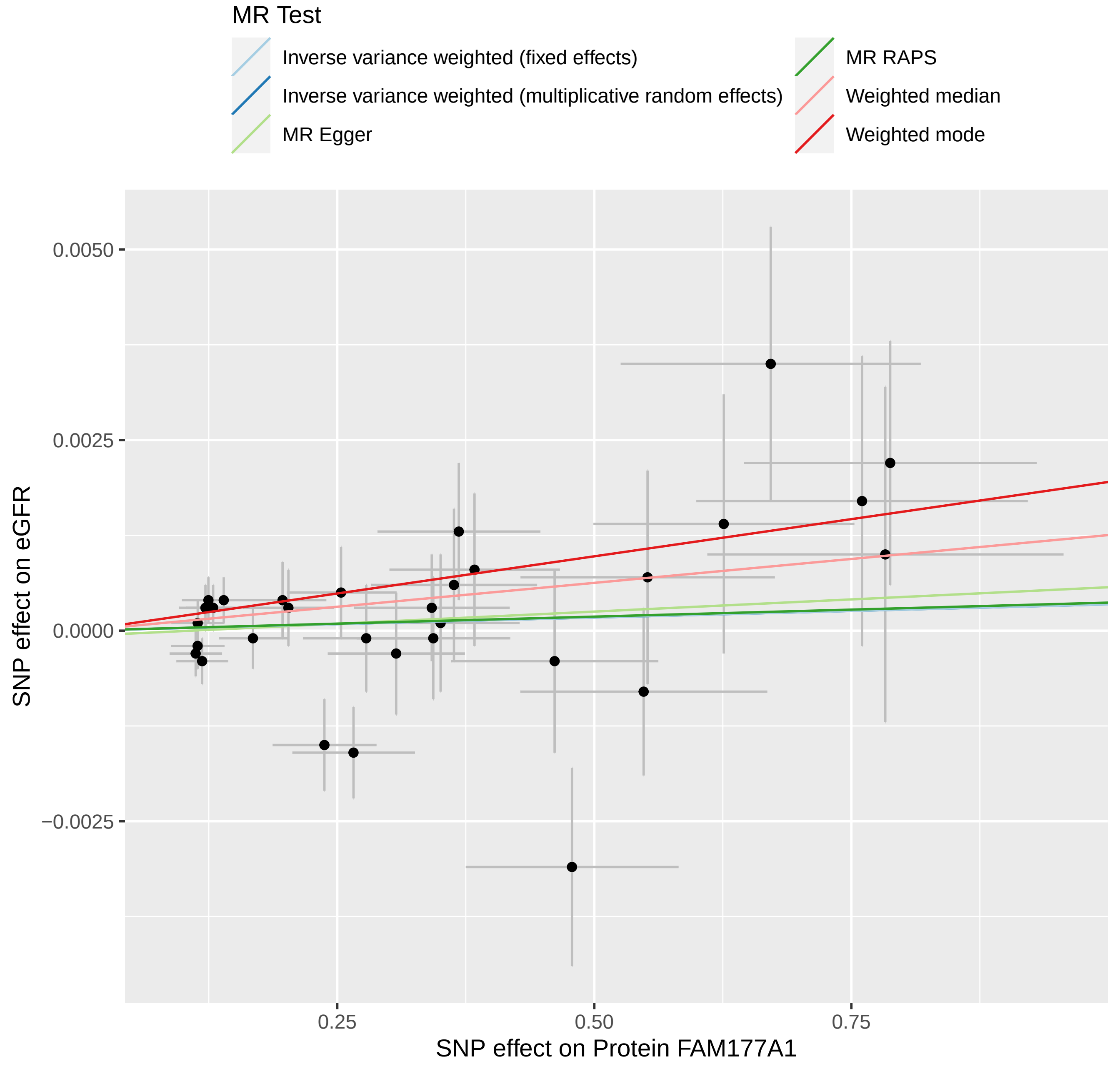

Batch 2−1038 : Protein FAM189A2 on eGFR

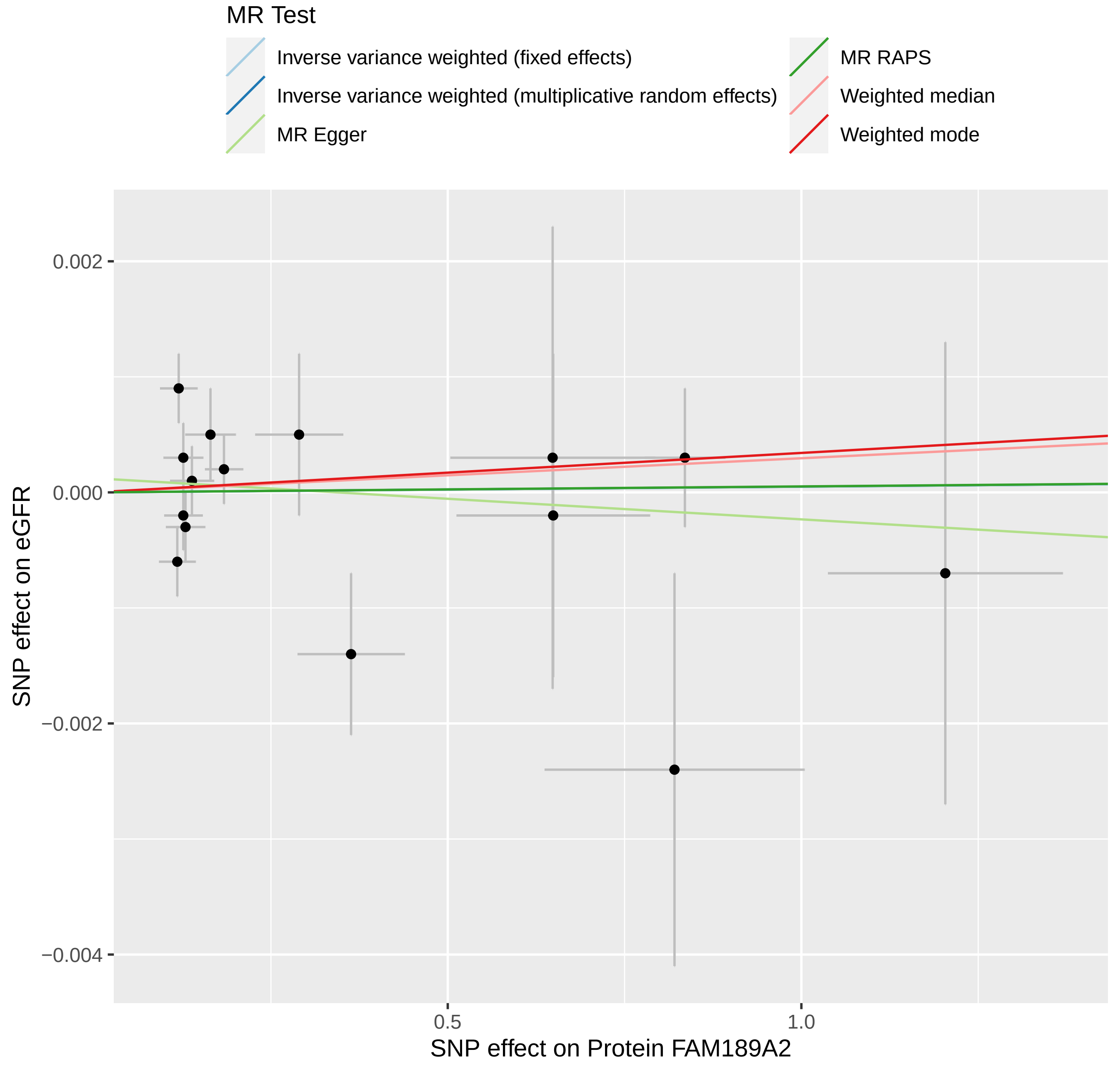

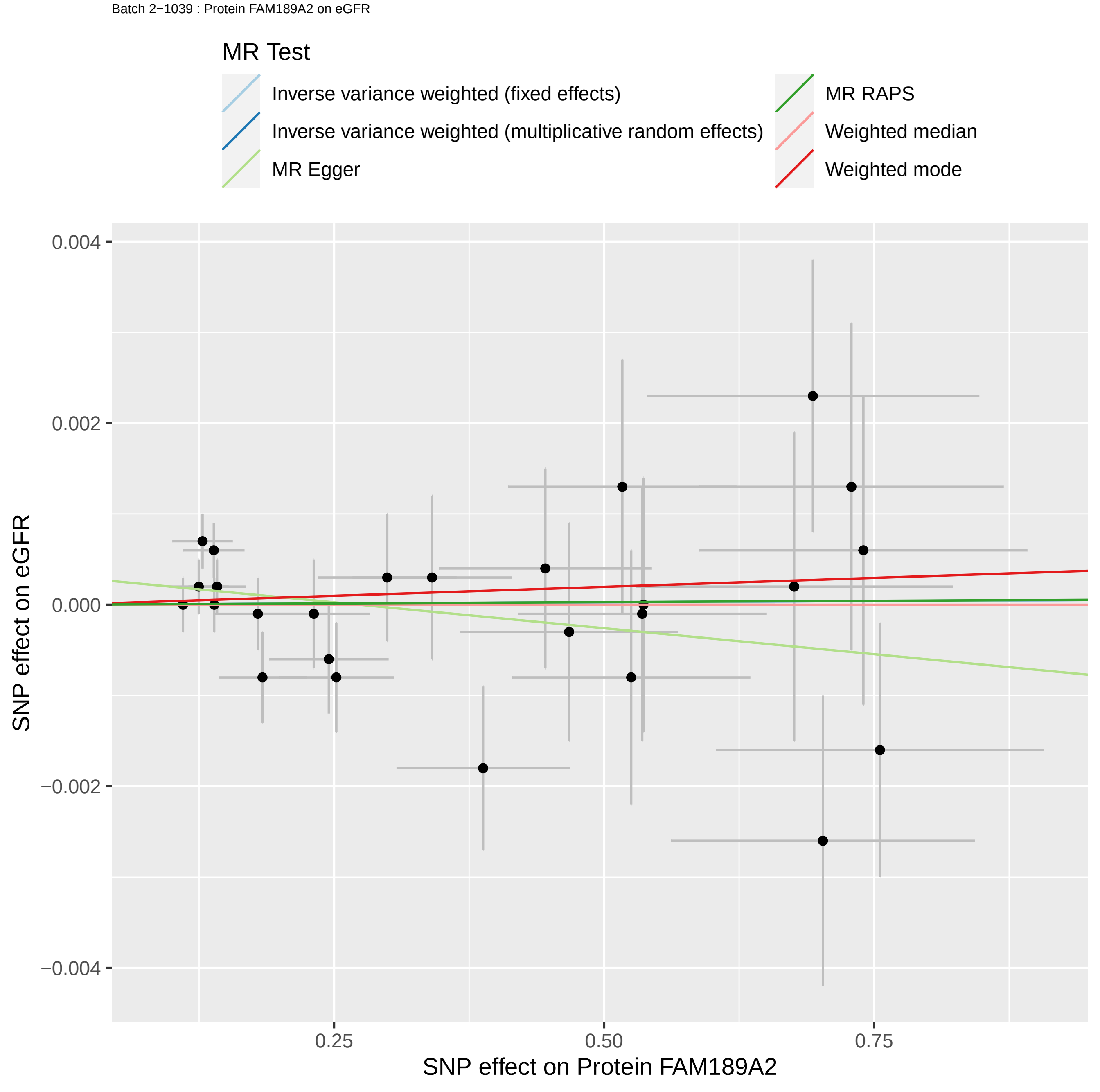
Batch 2−1039 : Protein FAM189A2 on eGFR
MR Test
Inverse variance weighted (fixed effects)
Inverse variance weighted (multiplicative random effects)
MR Egger
MR RAPS
Weighted median
Weighted mode
0.004
0.002
0.000
−0.002
−0.004
0.25
0.50
0.75
SNP effect on eGFR
SNP effect on Protein FAM189A2

Batch 2−1040 : Protein FAM19A2 on eGFR

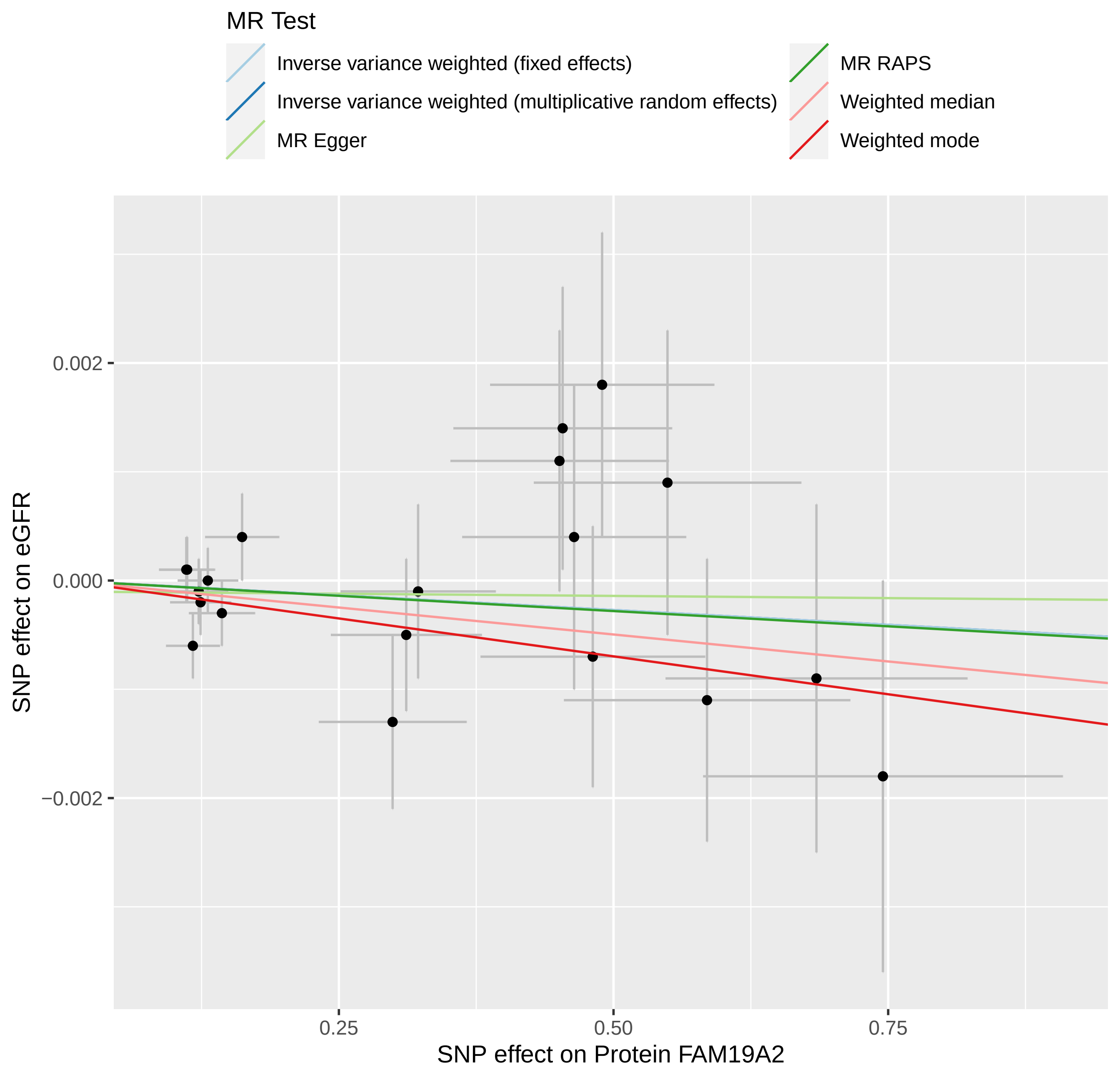

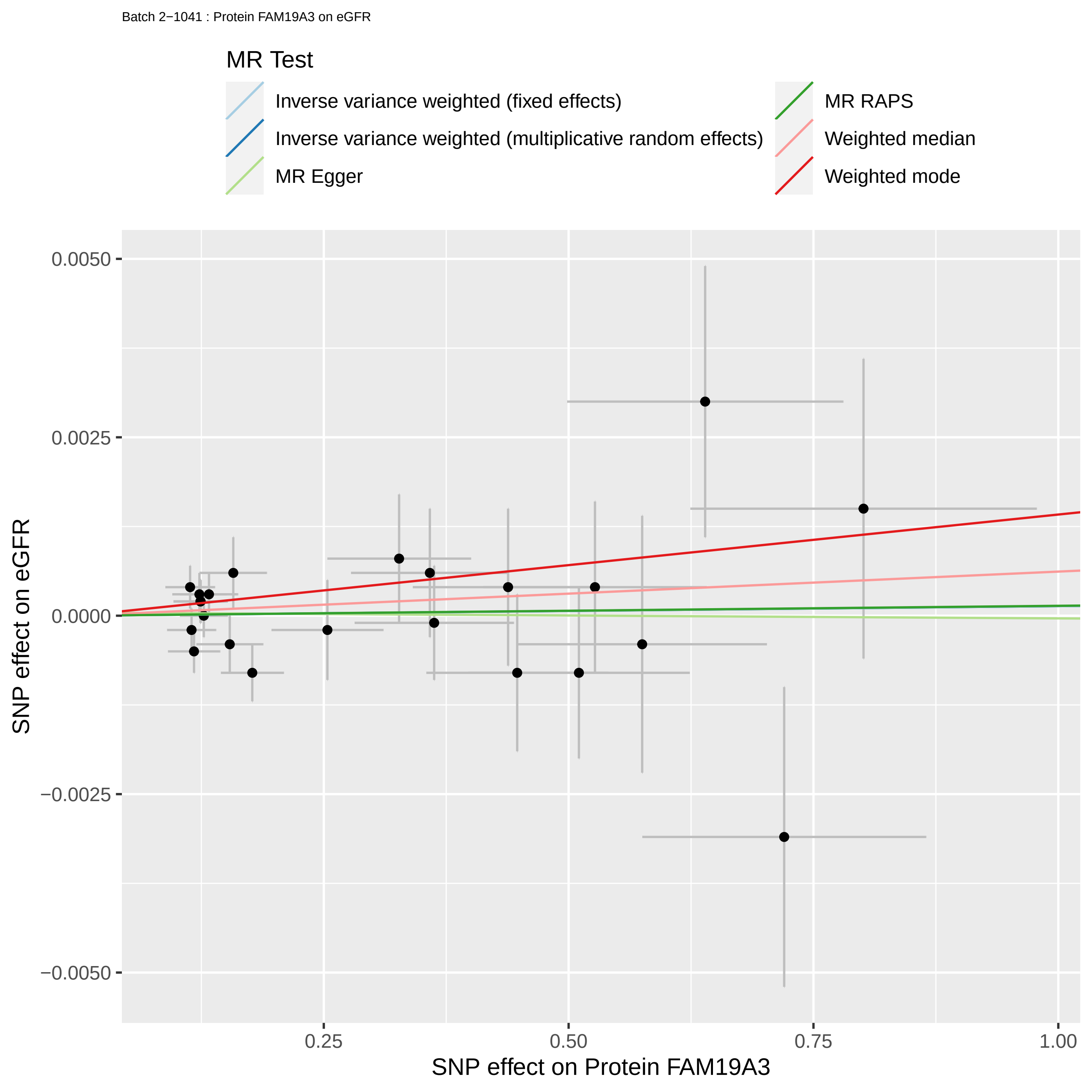

Batch 2−1041 : Protein FAM19A3 on eGFR
MR Test
Inverse variance weighted (fixed effects)
Inverse variance weighted (multiplicative random effects)
MR Egger
MR RAPS
Weighted median
Weighted mode
0.0050
0.0025
0.0000
−0.0025
−0.0050
0.25
0.50
0.75
1.00
SNP effect on eGFR
SNP effect on Protein FAM19A3

Batch 2−1042 : Protein FAM19A4 on eGFR

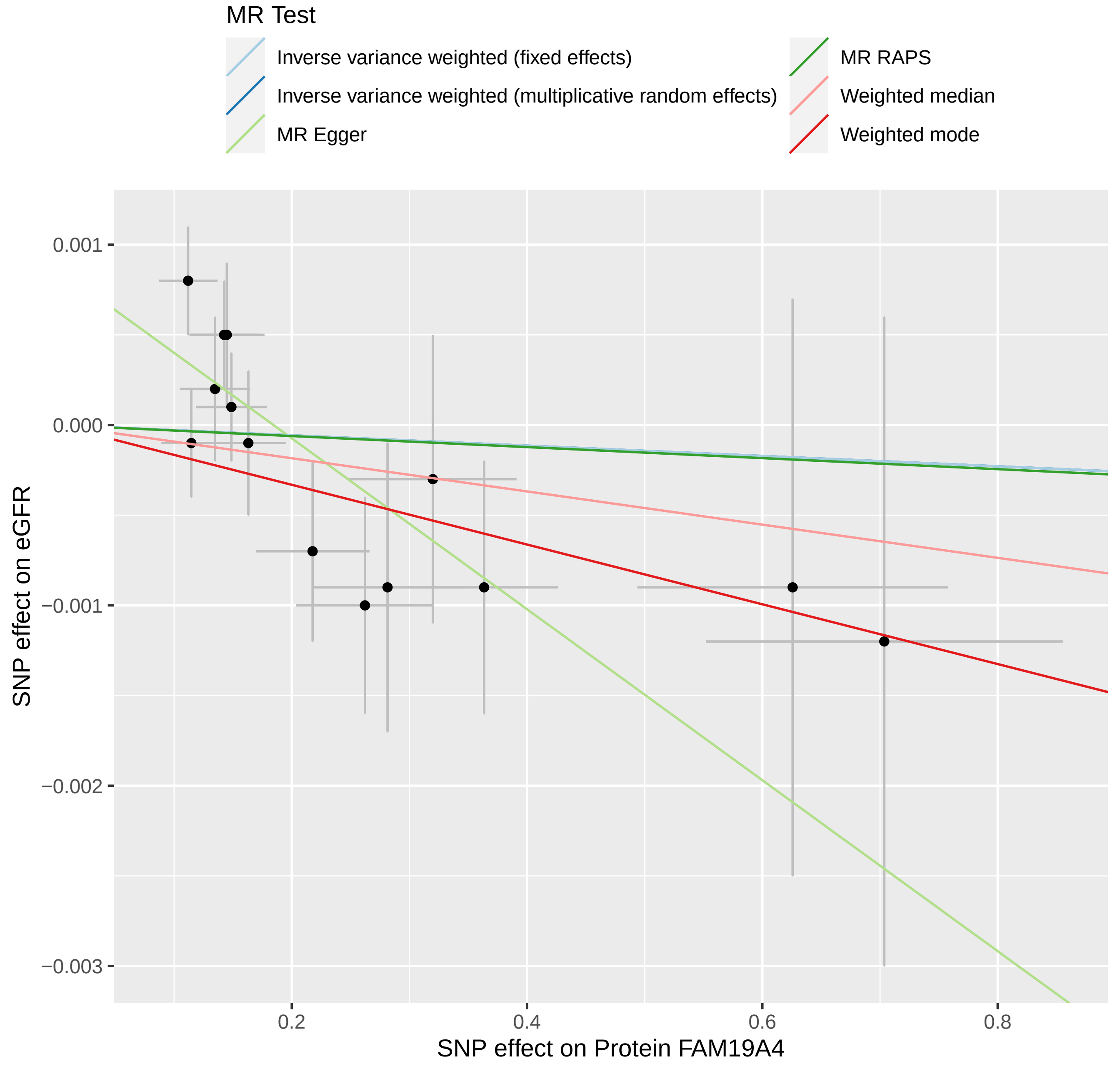

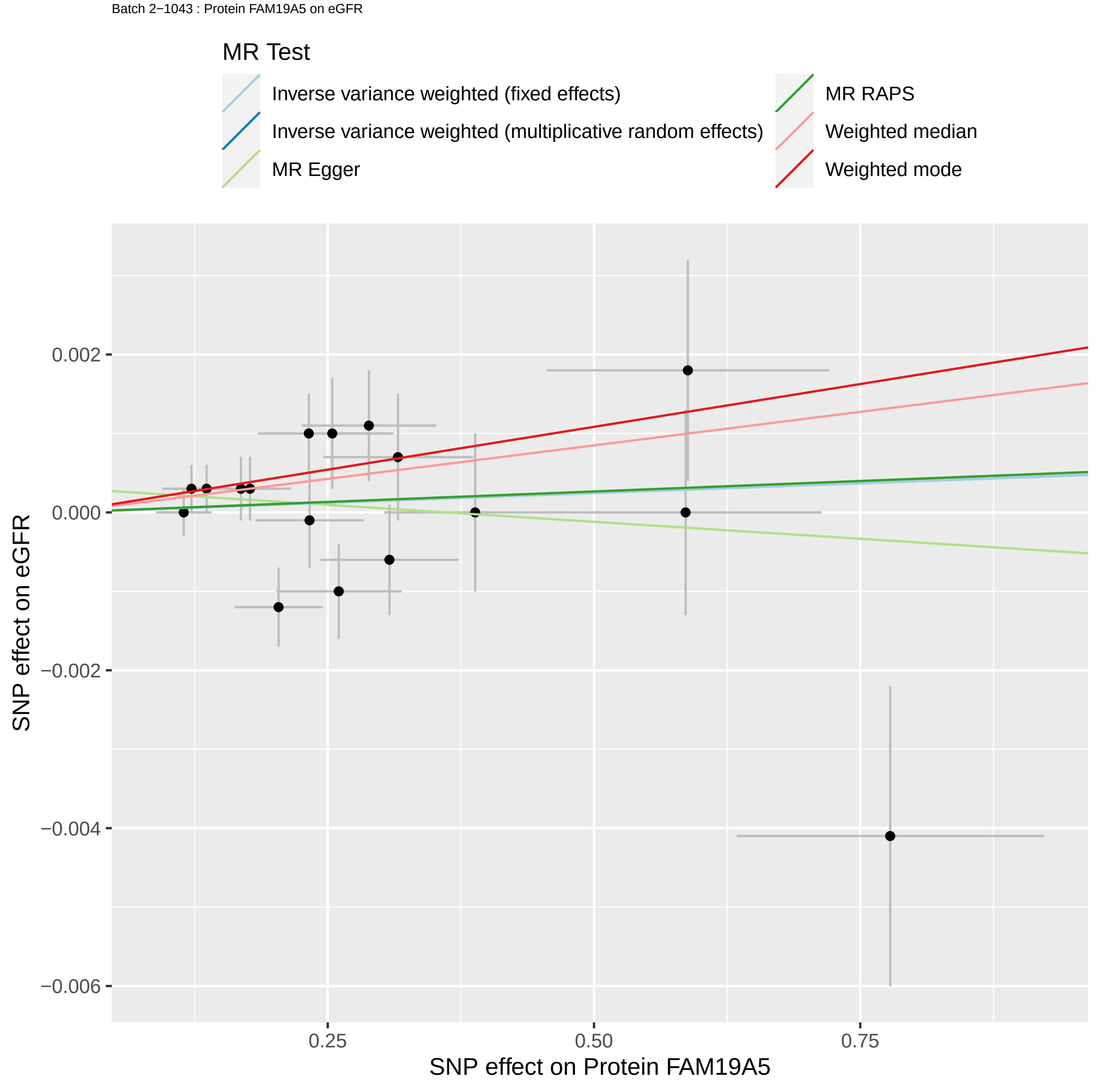
Batch 2−1043 : Protein FAM19A5 on eGFR
MR Test
Inverse variance weighted (fixed effects)
Inverse variance weighted (multiplicative random effects)
MR Egger
MR RAPS
Weighted median
Weighted mode
0.002
0.000
−0.002
−0.004
−0.006
0.25
0.50
0.75
SNP effect on eGFR
SNP effect on Protein FAM19A5

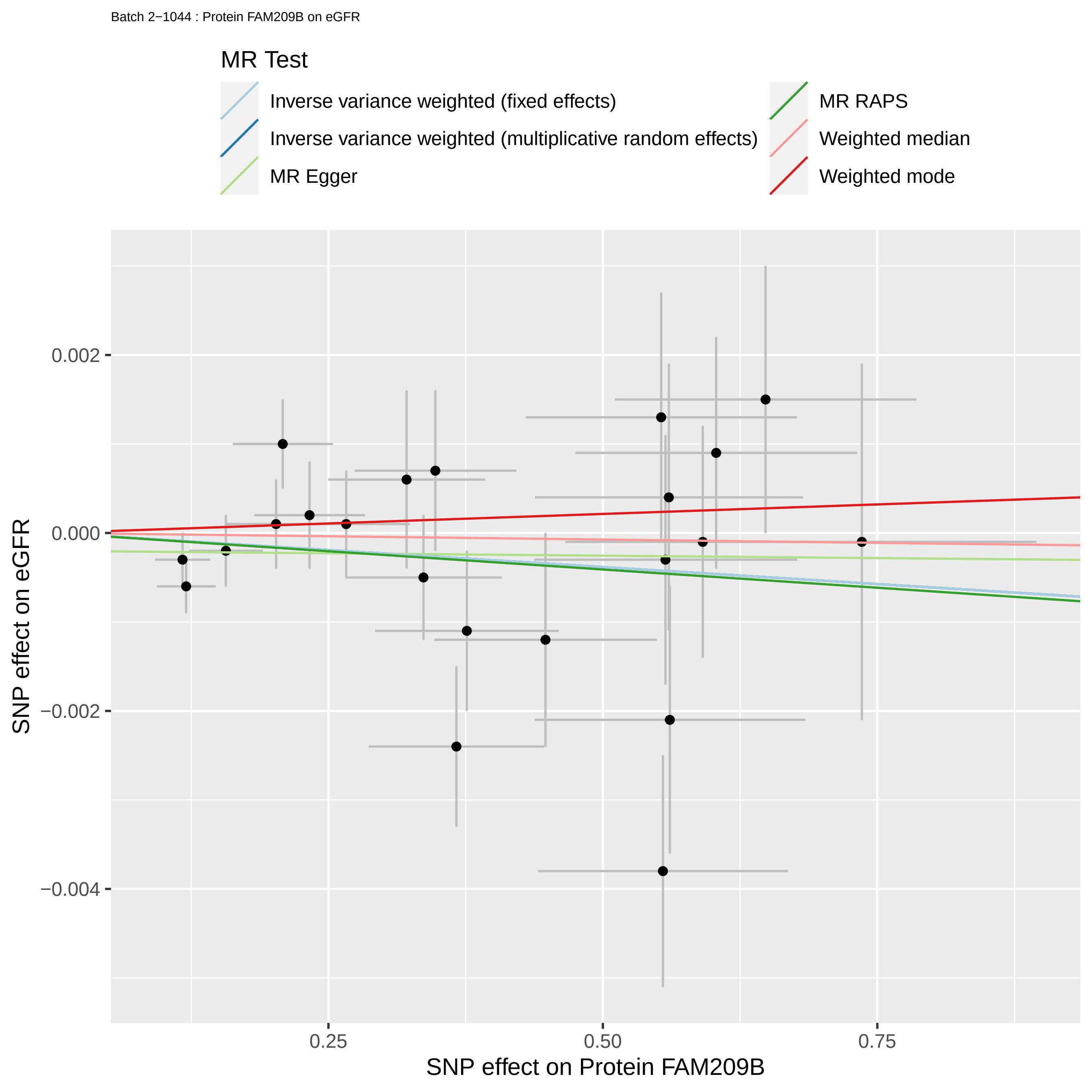

Batch 2−1044 : Protein FAM209B on eGFR
MR Test
Inverse variance weighted (fixed effects)
Inverse variance weighted (multiplicative random effects)
MR Egger
MR RAPS
Weighted median
Weighted mode
0.002
0.000
−0.002
−0.004
0.25
0.50
0.75
SNP effect on eGFR
SNP effect on Protein FAM209B

Batch 2−1045 : Pseudokinase FAM20A on eGFR

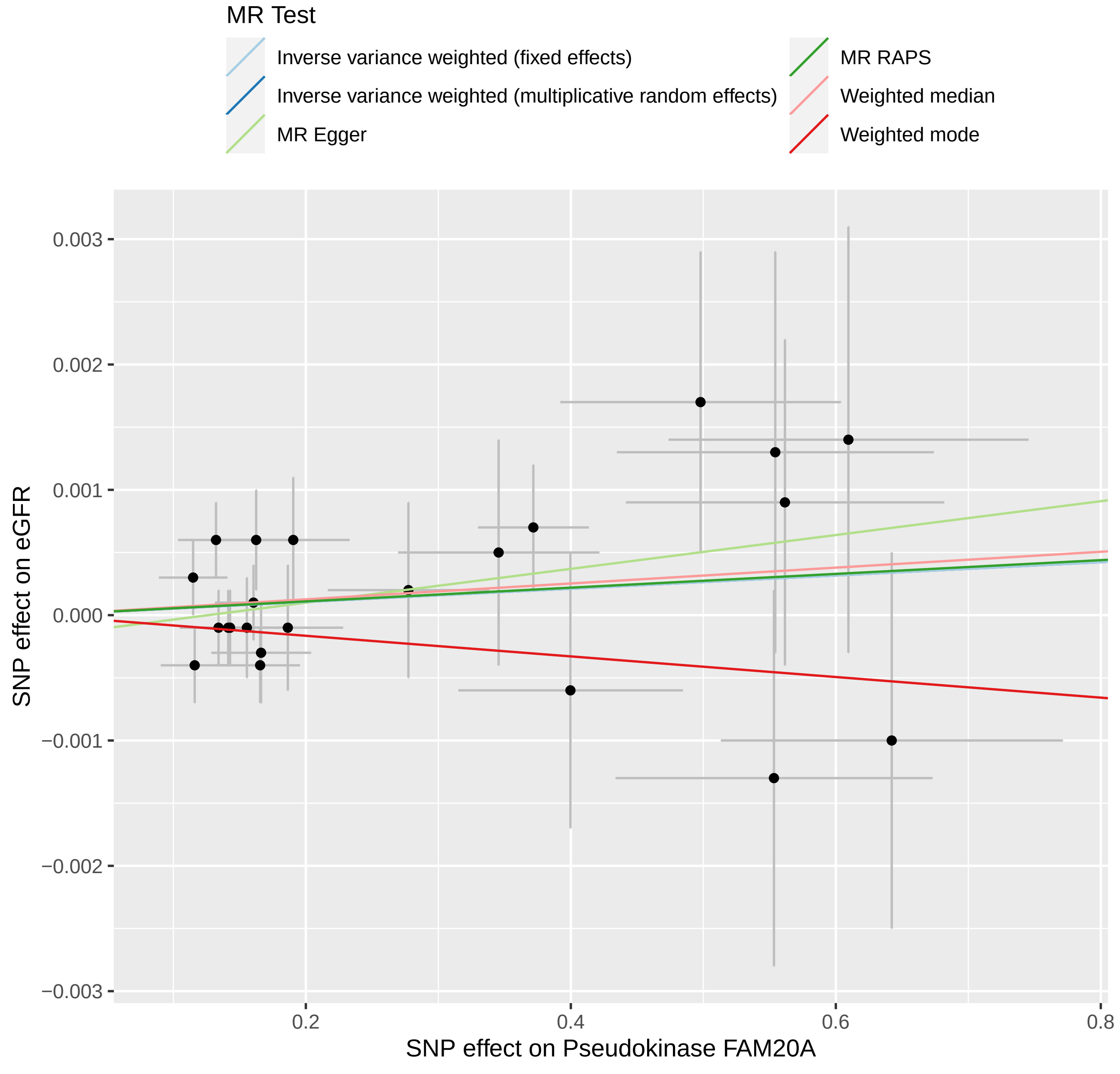

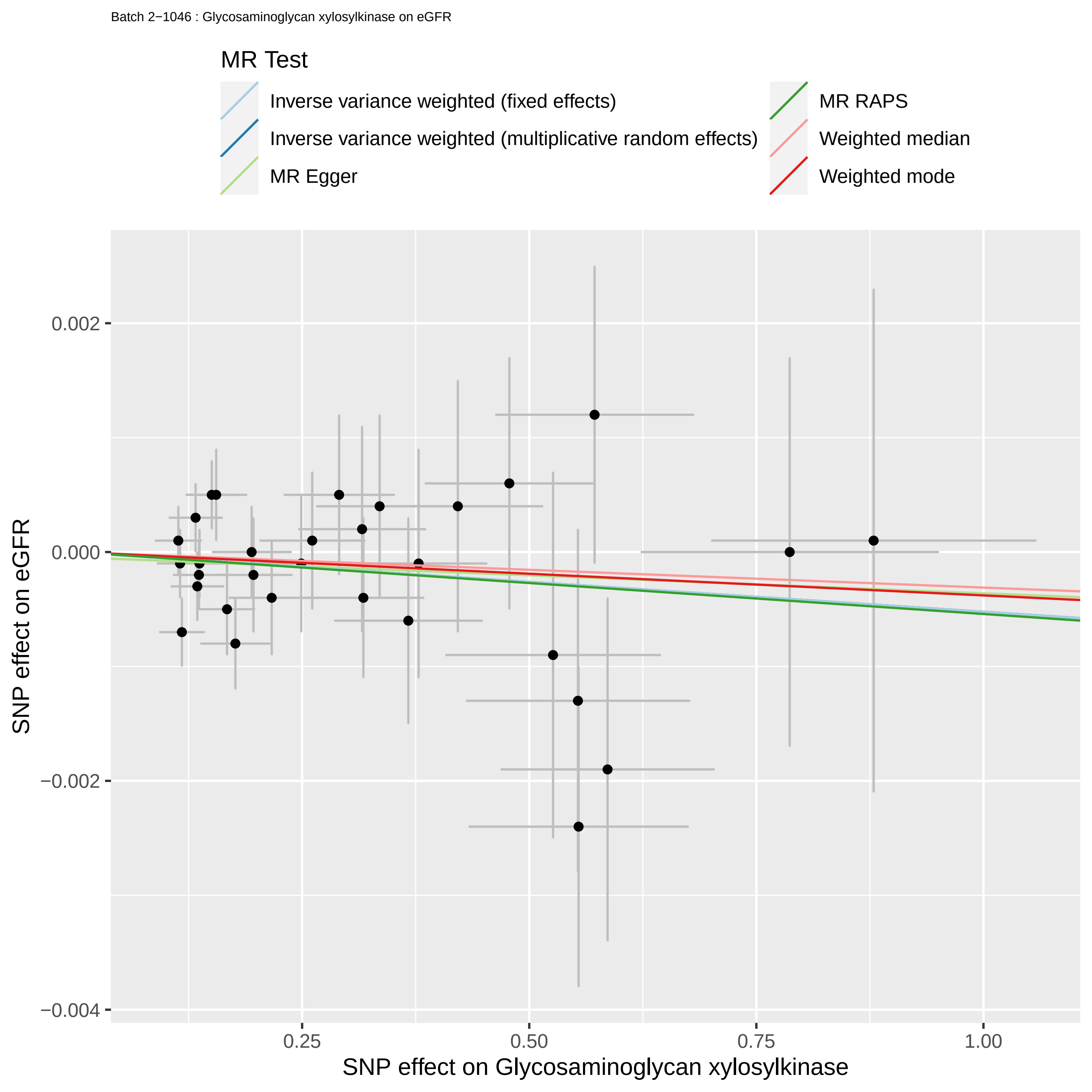
Batch 2−1046 : Glycosaminoglycan xylosylkinase on eGFR
MR Test
Inverse variance weighted (fixed effects)
Inverse variance weighted (multiplicative random effects)
MR Egger
MR RAPS
Weighted median
Weighted mode
0.002
0.000
−0.002
−0.004
0.25
0.50
0.75
1.00
SNP effect on eGFR
SNP effect on Glycosaminoglycan xylosylkinase

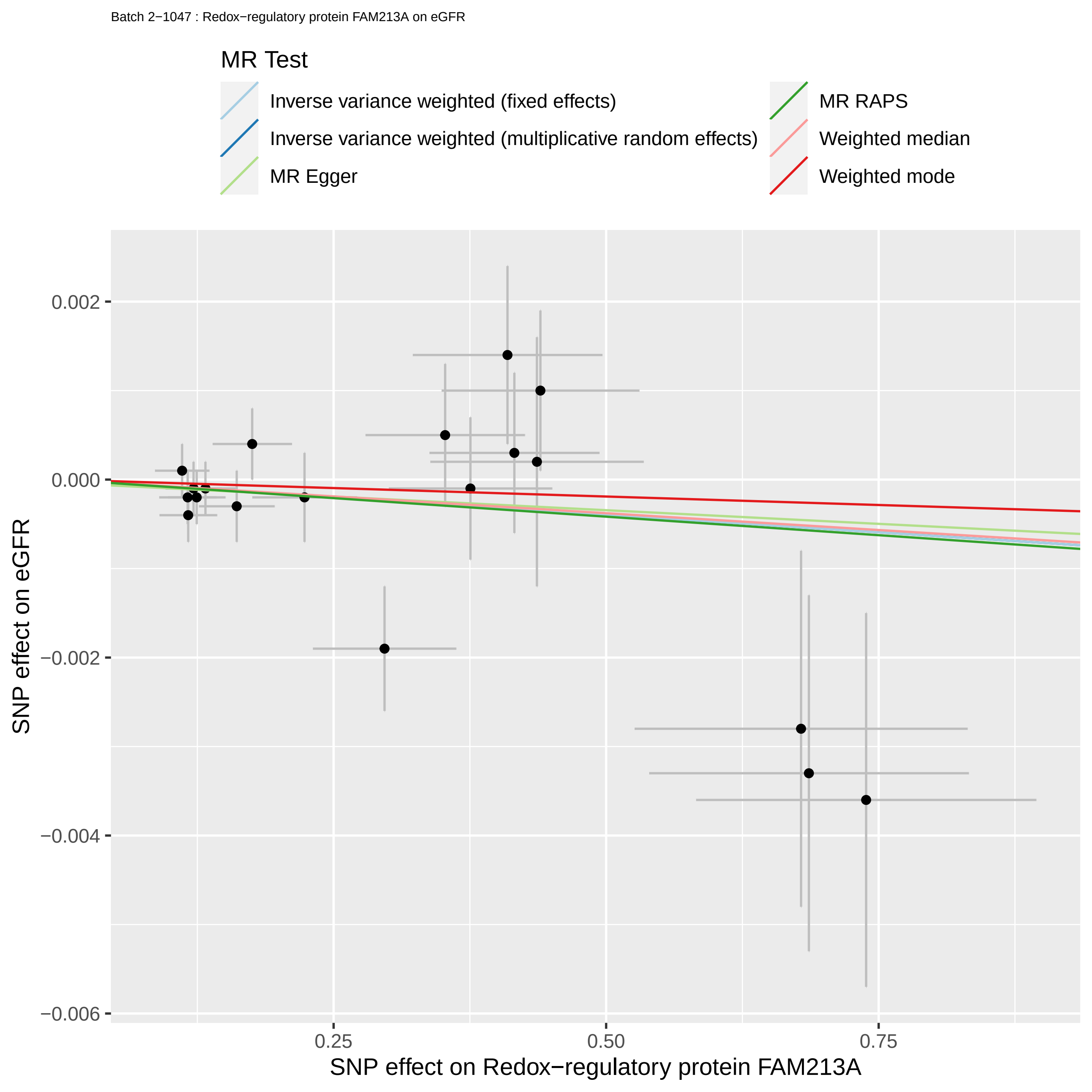

Batch 2−1047 : Redox−regulatory protein FAM213A on eGFR
MR Test
Inverse variance weighted (fixed effects)
Inverse variance weighted (multiplicative random effects)
MR Egger
MR RAPS
Weighted median
Weighted mode
0.002
0.000
−0.002
−0.004
−0.006
0.25
0.50
0.75
SNP effect on eGFR
SNP effect on Redox−regulatory protein FAM213A

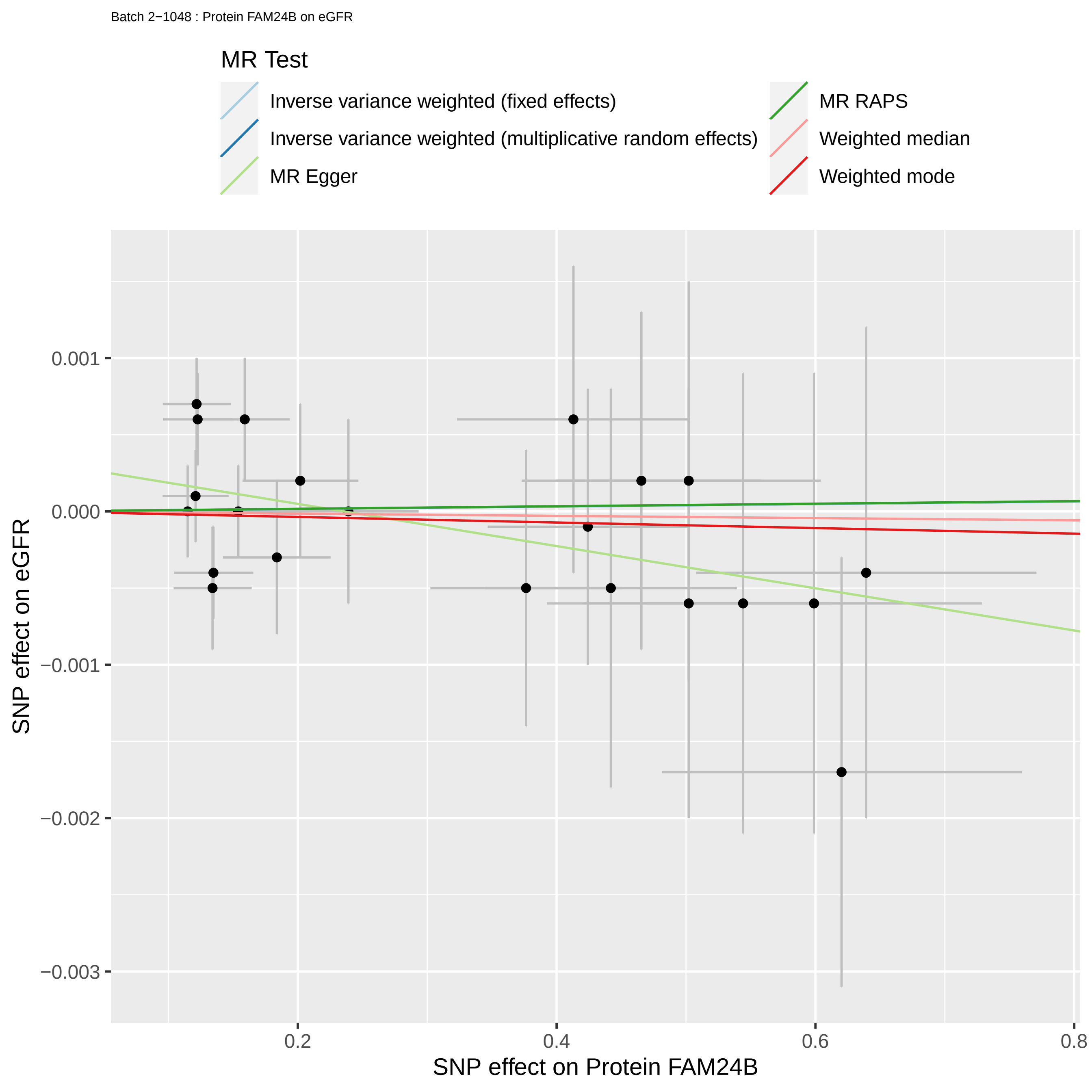
Batch 2−1048 : Protein FAM24B on eGFR
MR Test
Inverse variance weighted (fixed effects)
Inverse variance weighted (multiplicative random effects)
MR Egger
MR RAPS
Weighted median
Weighted mode
0.001
0.000
−0.001
−0.002
−0.003
0.2
0.4
0.6
0.8
SNP effect on eGFR
SNP effect on Protein FAM24B

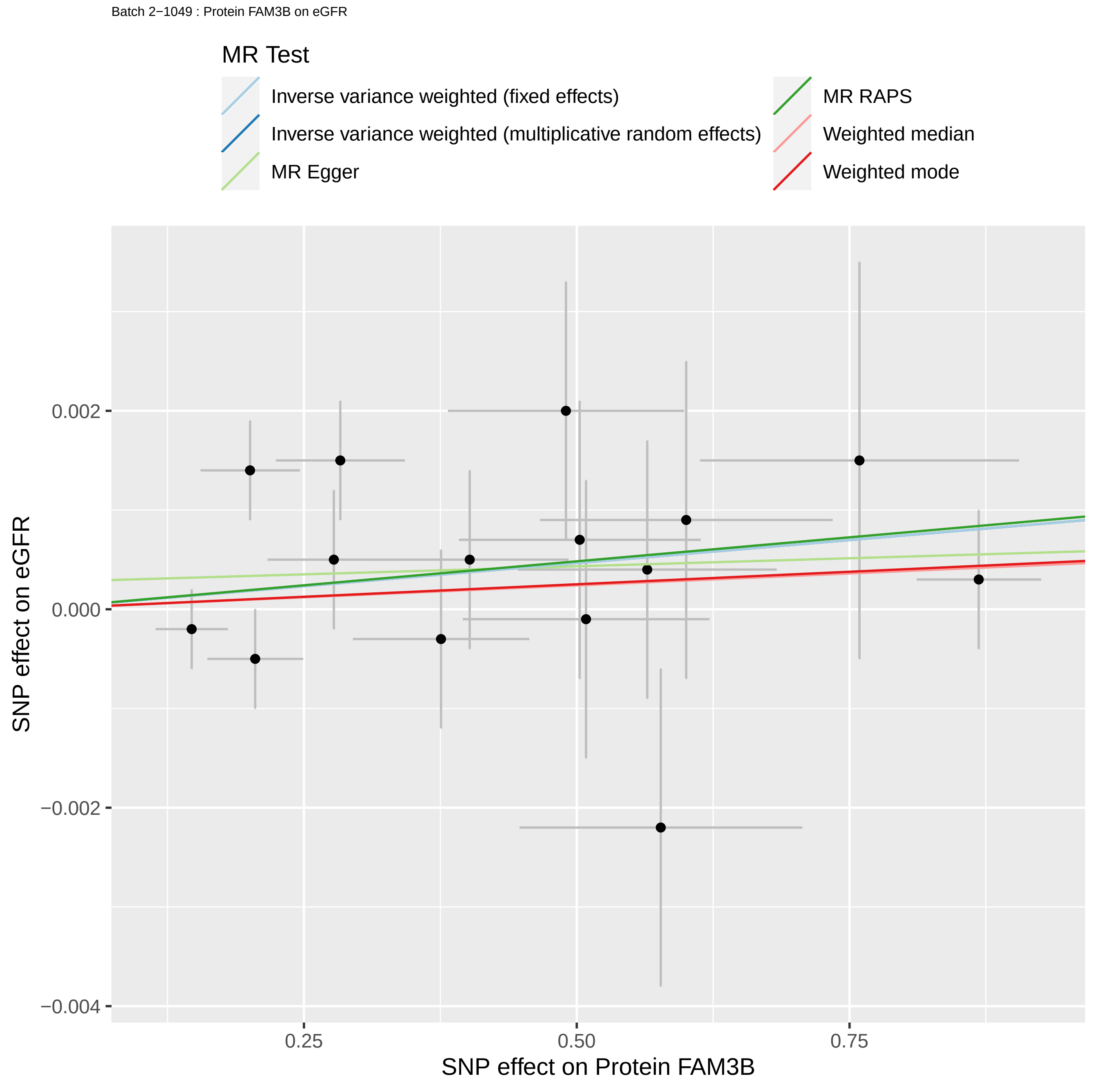

Batch 2−1049 : Protein FAM3B on eGFR
MR Test
Inverse variance weighted (fixed effects)
Inverse variance weighted (multiplicative random effects)
MR Egger
MR RAPS
Weighted median
Weighted mode
0.002
0.000
−0.002
−0.004
SNP effect on eGFR
0.25
0.50
0.75
SNP effect on Protein FAM3B

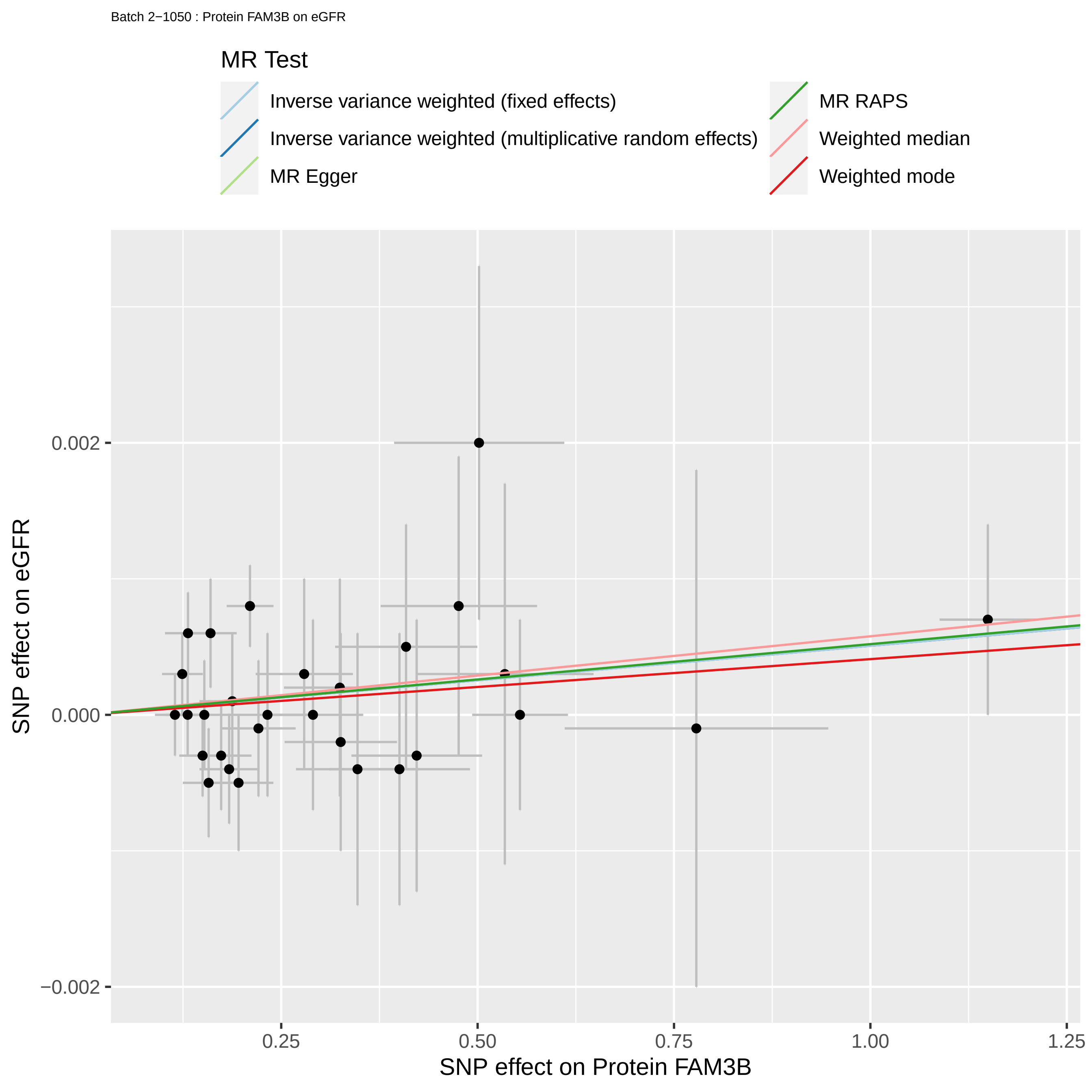

Batch 2−1050 : Protein FAM3B on eGFR
MR Test
Inverse variance weighted (fixed effects)
Inverse variance weighted (multiplicative random effects)
MR Egger
MR RAPS
Weighted median
Weighted mode
0.002
0.000
−0.002
SNP effect on eGFR
0.25
0.50
0.75
1.00
1.25
SNP effect on Protein FAM3B

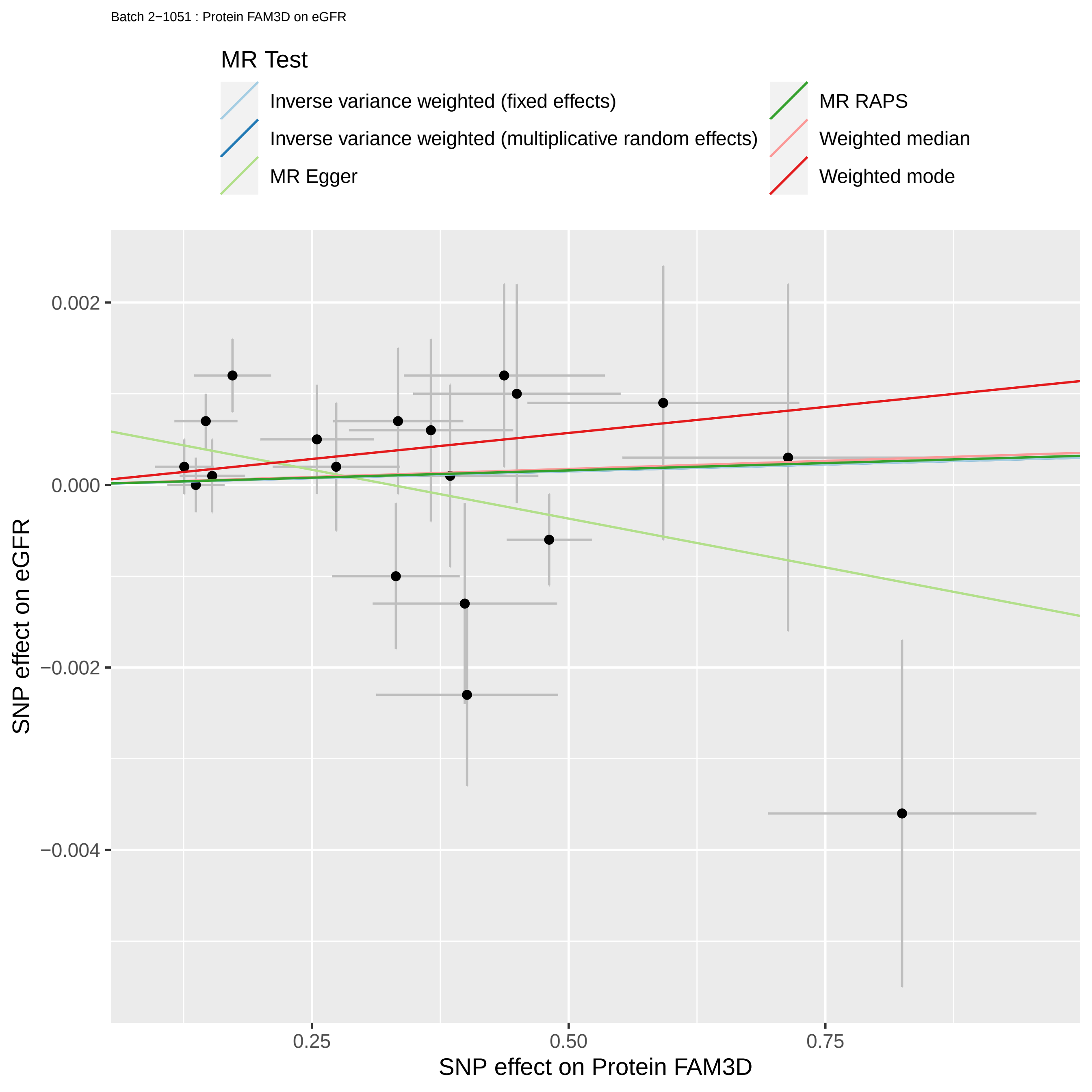

Batch 2−1051 : Protein FAM3D on eGFR
MR Test
Inverse variance weighted (fixed effects)
Inverse variance weighted (multiplicative random effects)
MR Egger
MR RAPS
Weighted median
Weighted mode
0.002
0.000
−0.002
−0.004
0.25
0.50
0.75
SNP effect on eGFR
SNP effect on Protein FAM3D

Batch 2−1052 : MIP18 family protein FAM96A on eGFR

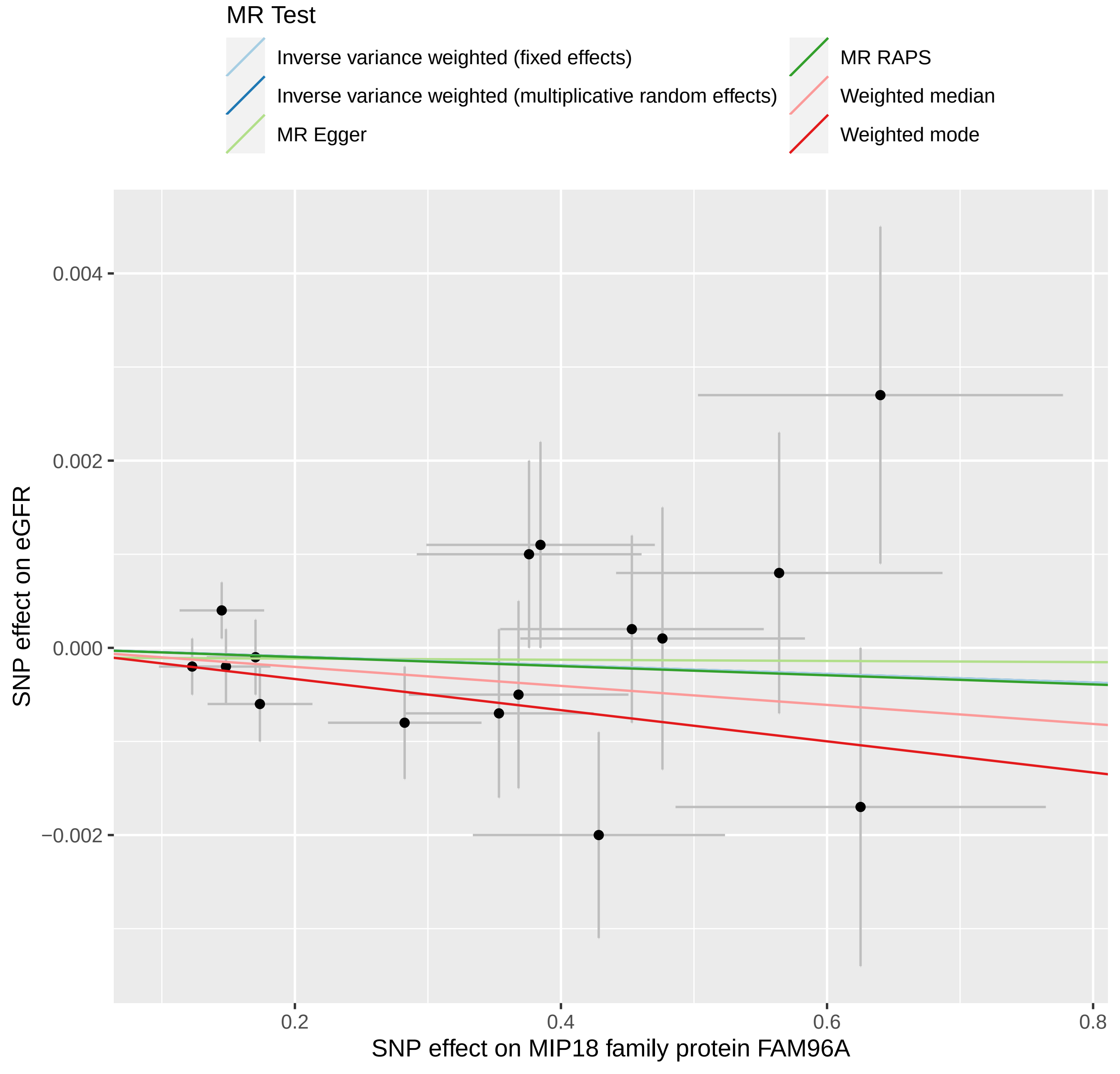

Batch 2−1053 : E3 ubiquitin−protein ligase FANCL on eGFR

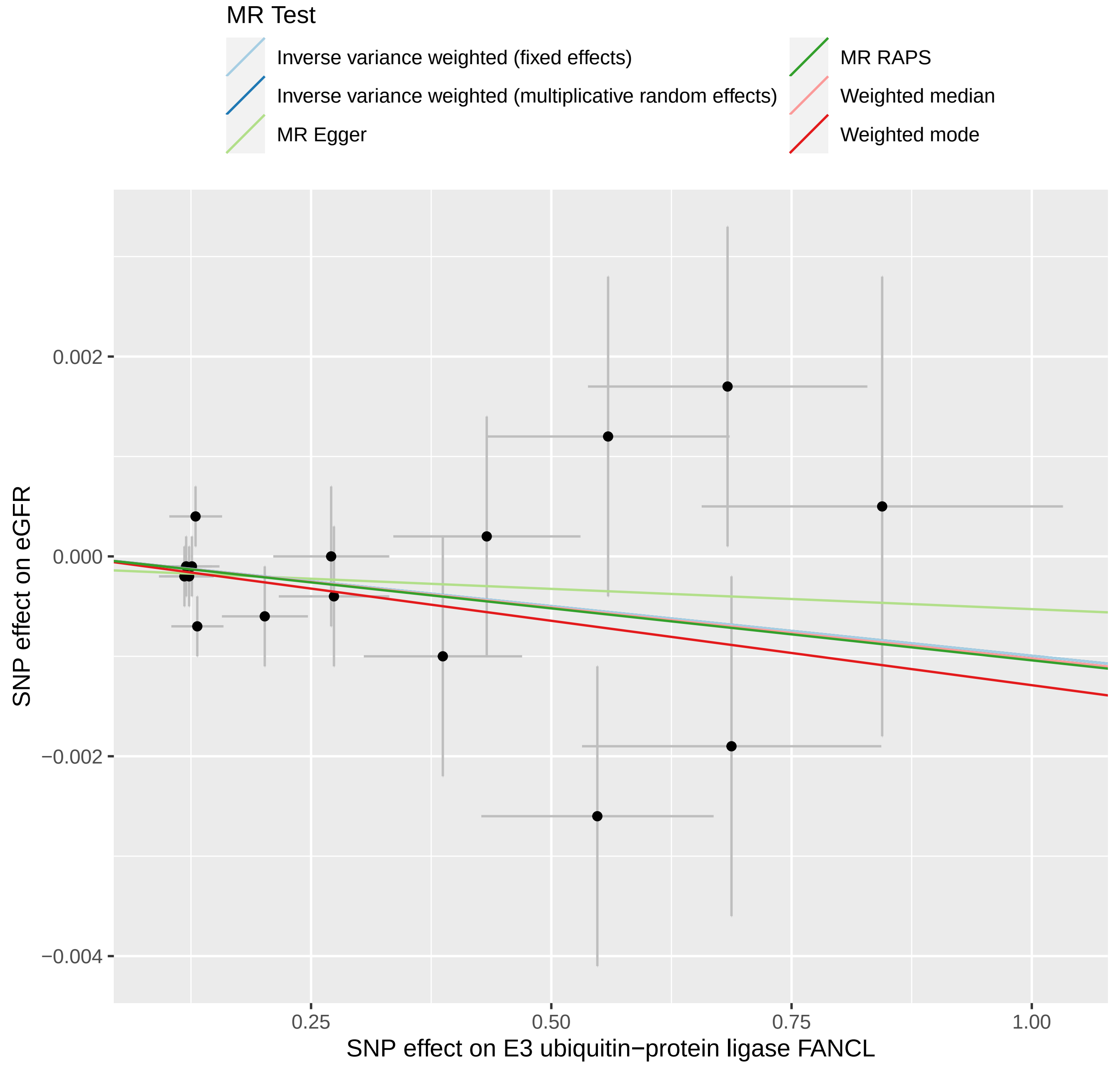

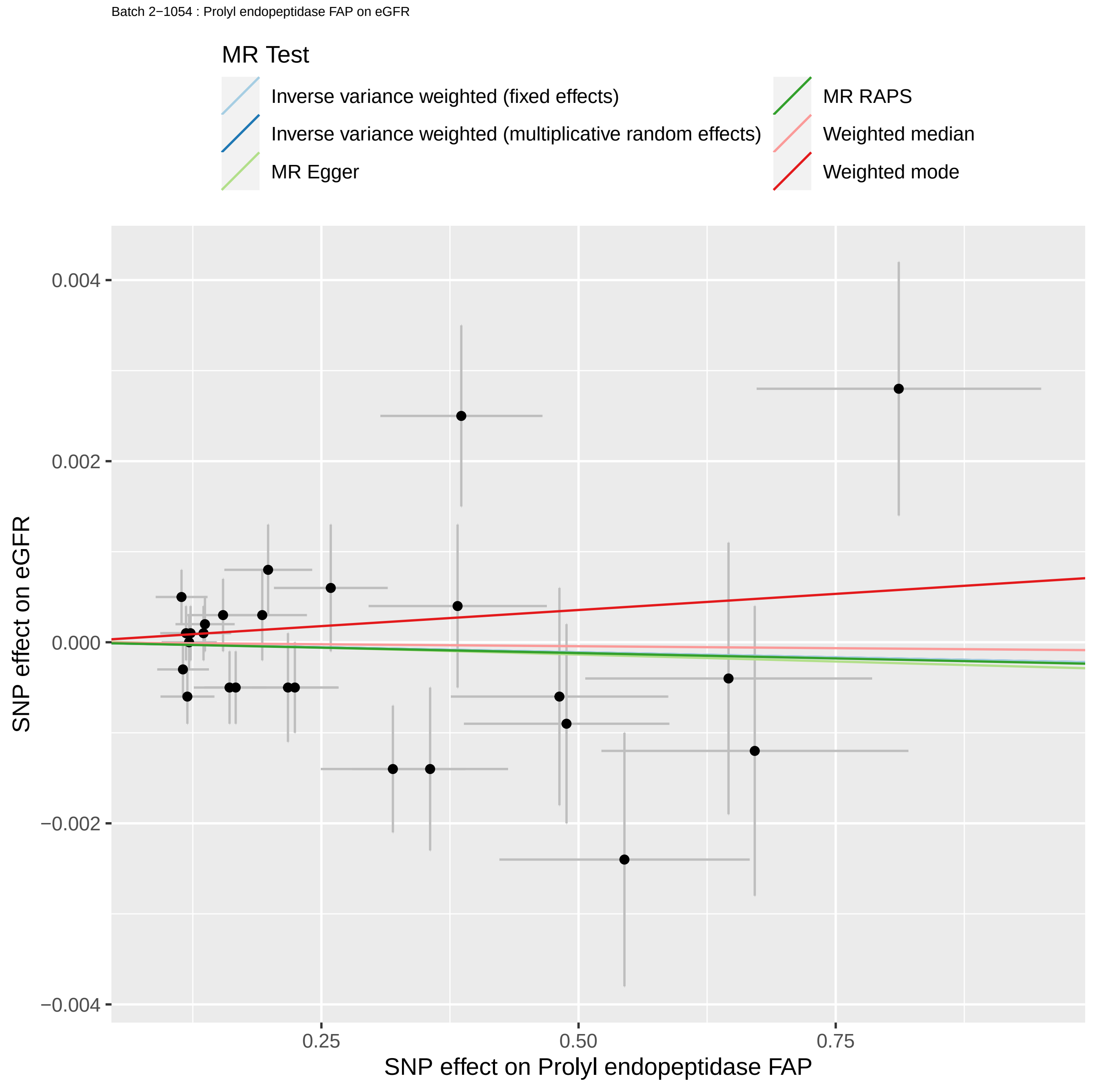
Batch 2−1054 : Prolyl endopeptidase FAP on eGFR
MR Test
Inverse variance weighted (fixed effects)
Inverse variance weighted (multiplicative random effects)
MR Egger
MR RAPS
Weighted median
Weighted mode
0.004
0.002
0.000
−0.002
−0.004
0.25
0.50
0.75
SNP effect on eGFR
SNP effect on Prolyl endopeptidase FAP

Batch 2−1055 : Phenylalanine−−tRNA ligase, mitochondrial on eGFR

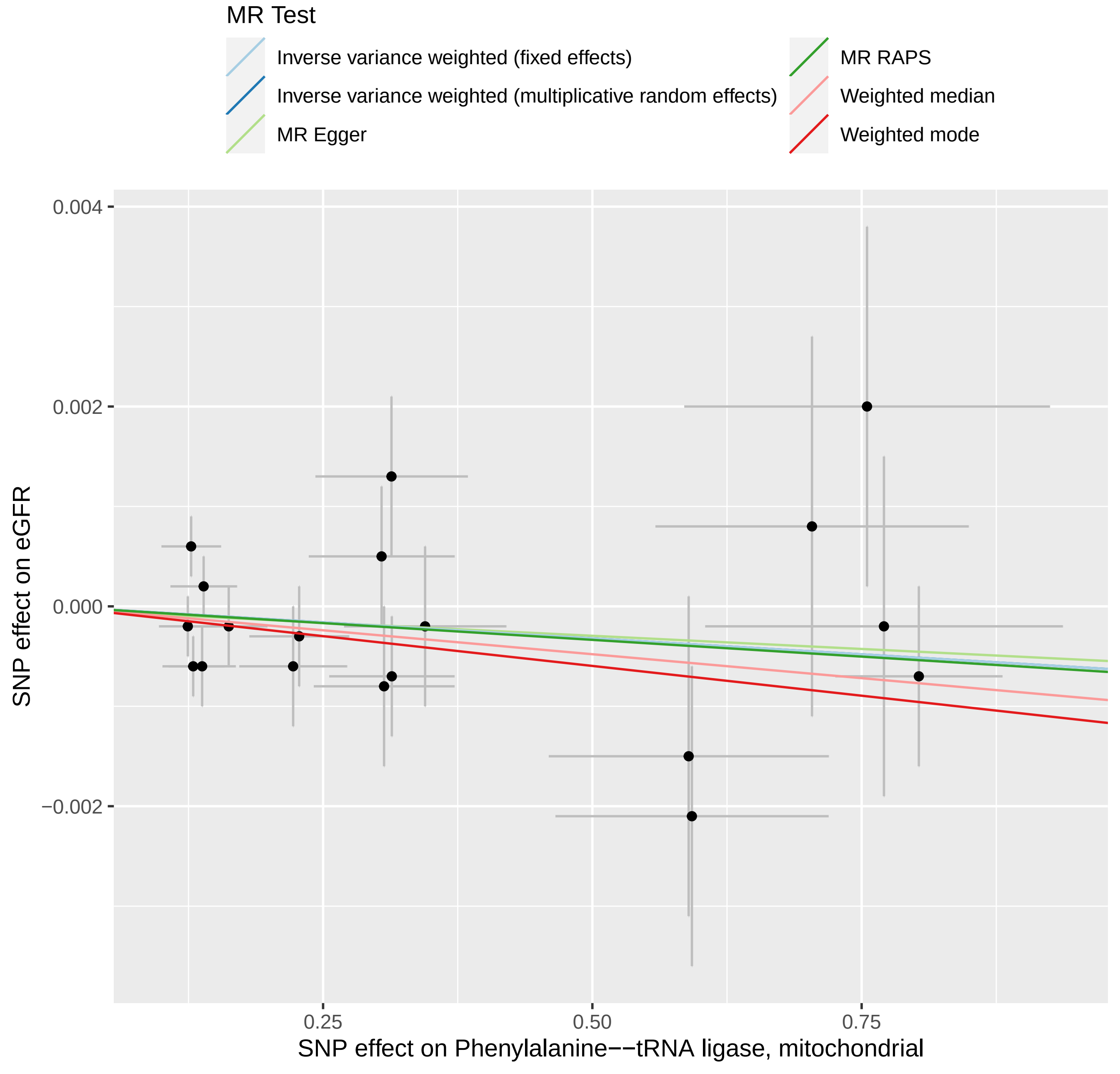

Batch 2−1056 : Tumor necrosis factor receptor superfamily member 6 on eGFR

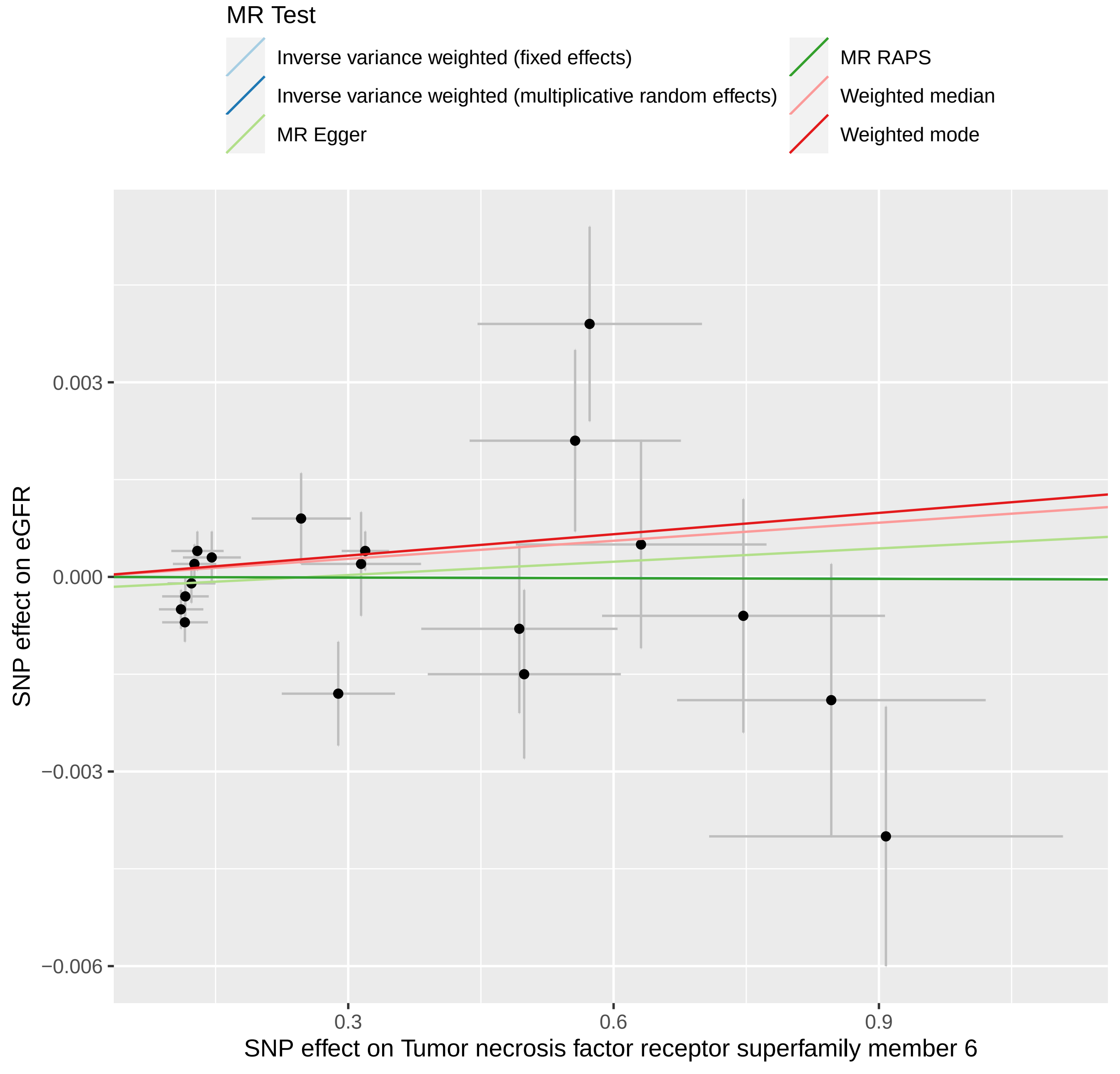

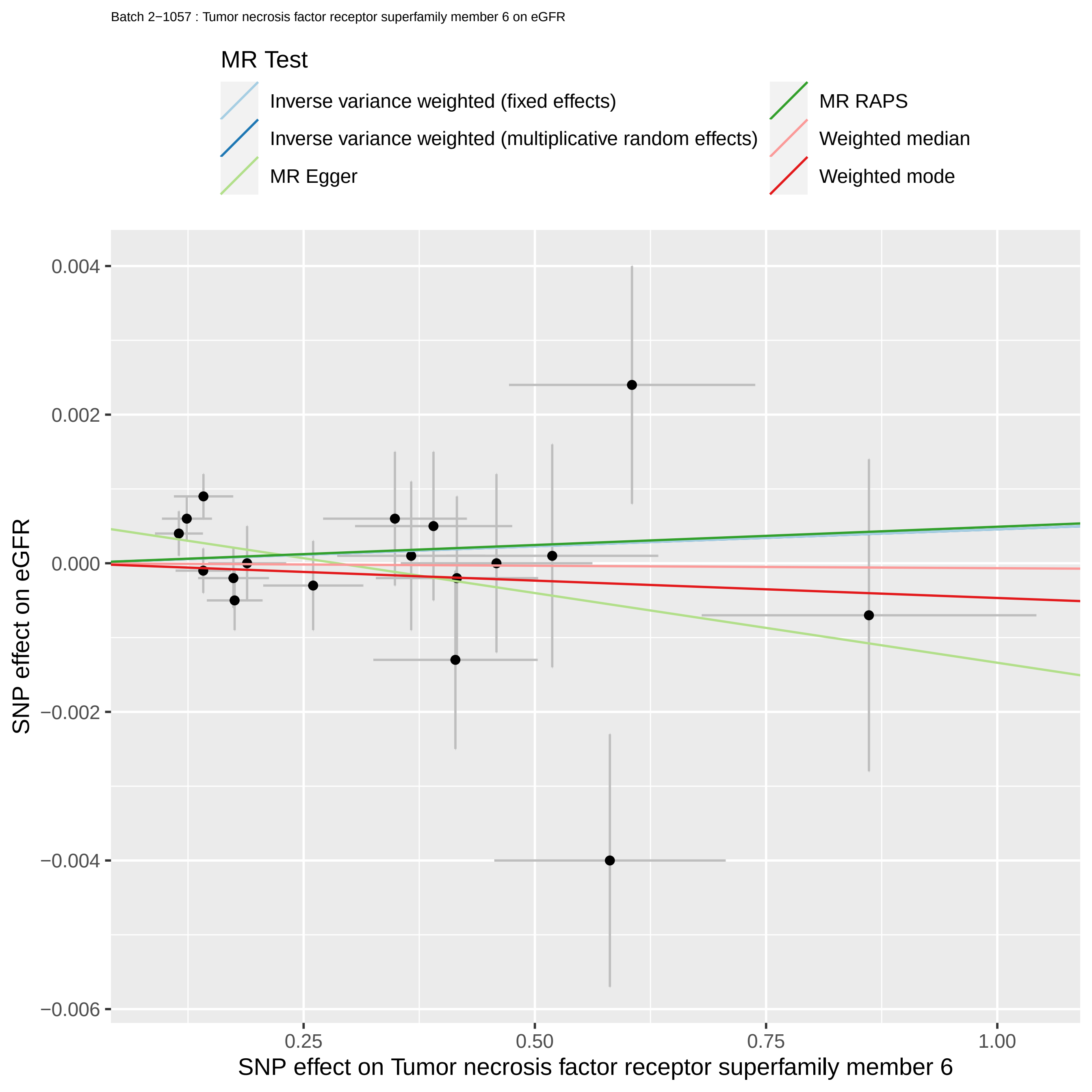

Batch 2−1057 : Tumor necrosis factor receptor superfamily member 6 on eGFR
MR Test
Inverse variance weighted (fixed effects)
Inverse variance weighted (multiplicative random effects)
MR Egger
MR RAPS
Weighted median
Weighted mode
0.004
0.002
0.000
−0.002
−0.004
−0.006
SNP effect on eGFR
0.25
0.50
0.75
1.00
SNP effect on Tumor necrosis factor receptor superfamily member 6

Batch 2−1058 : Tumor necrosis factor receptor superfamily member 6 on eGFR

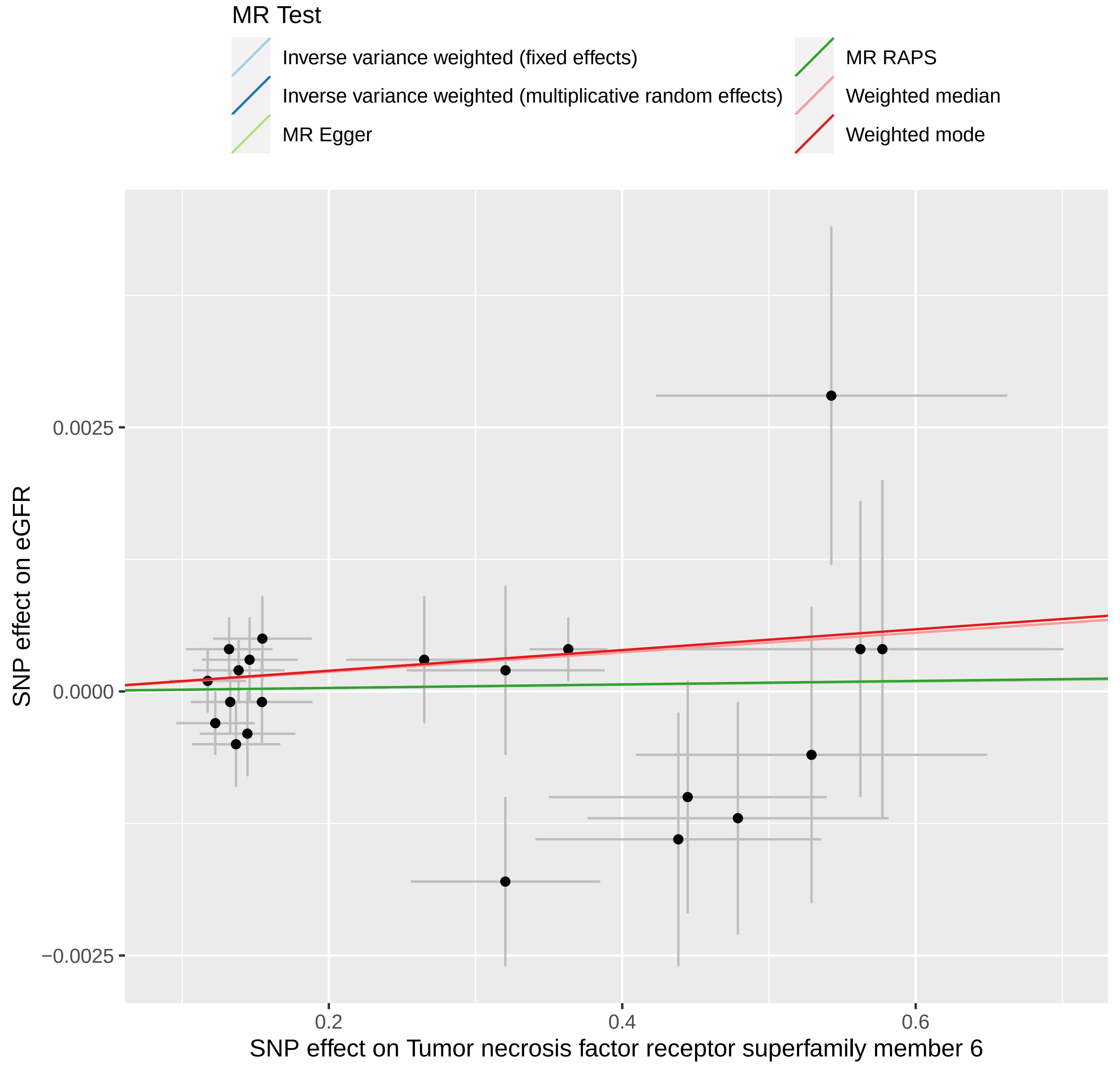

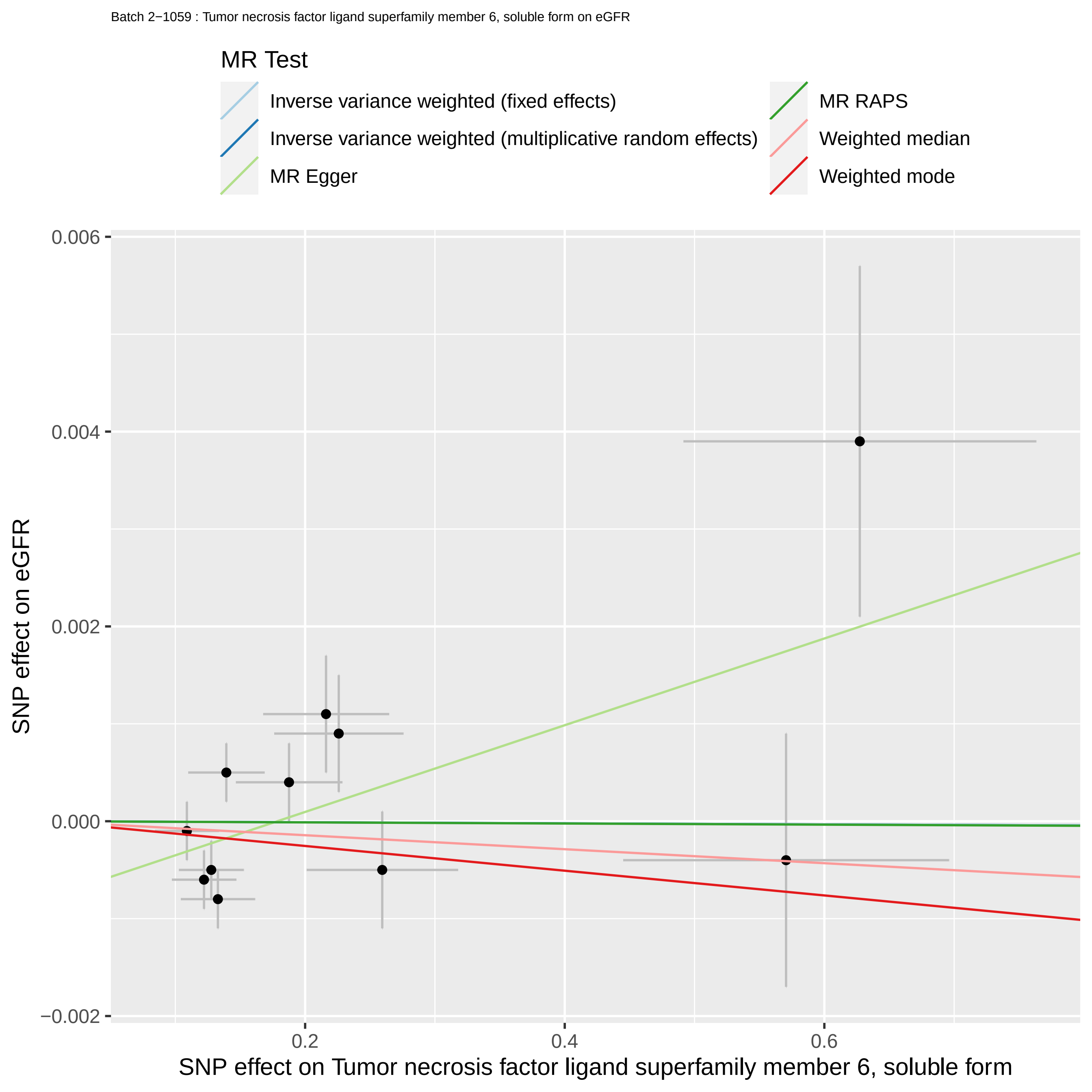
Batch 2−1059 : Tumor necrosis factor ligand superfamily member 6, soluble form on eGFR
MR Test
Inverse variance weighted (fixed effects)
Inverse variance weighted (multiplicative random effects)
MR Egger
MR RAPS
Weighted median
Weighted mode
0.006
0.004
0.002
0.000
−0.002
0.2
0.4
0.6
SNP effect on eGFR
SNP effect on Tumor necrosis factor ligand superfamily member 6, soluble form

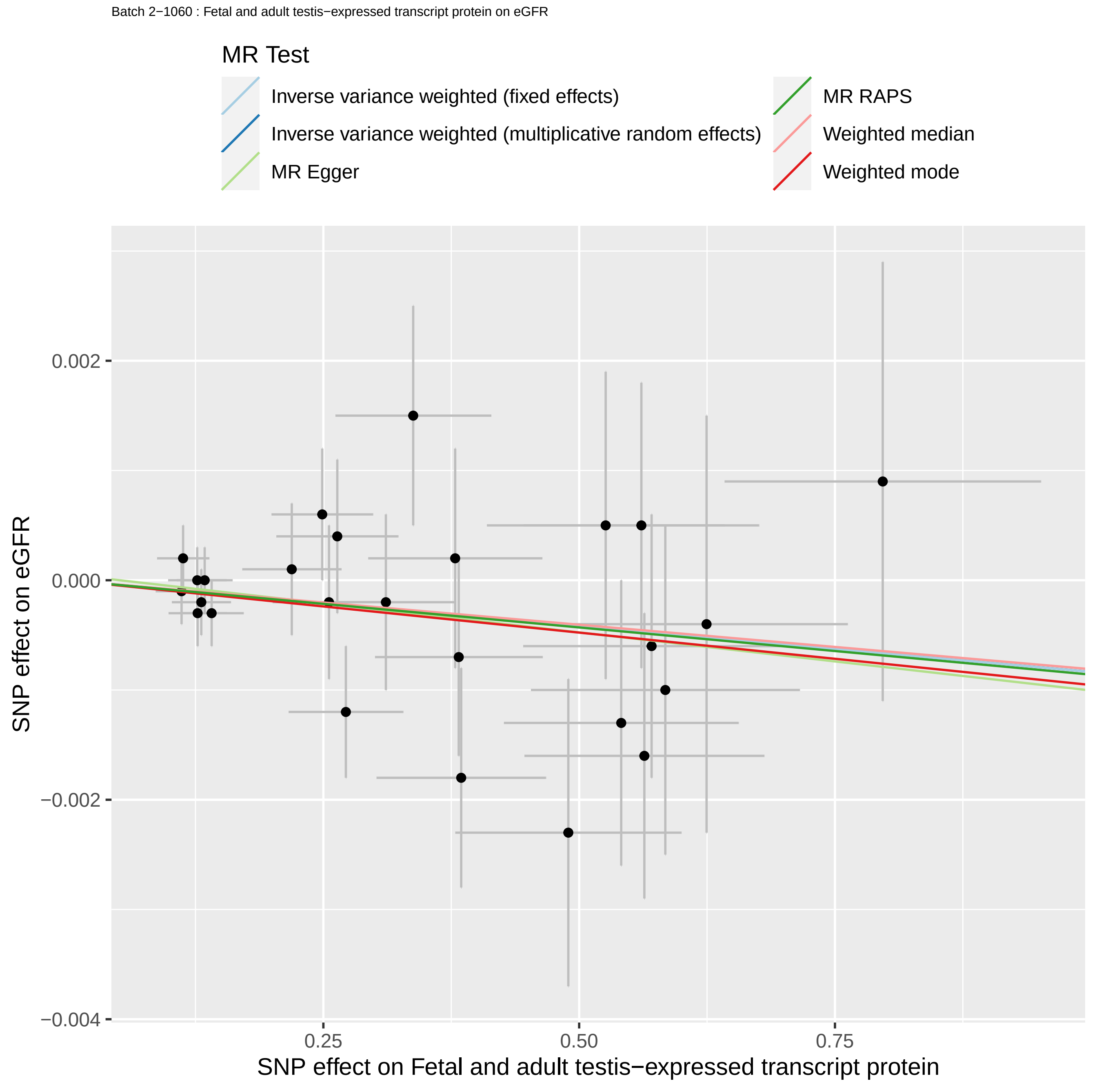

Batch 2−1060 : Fetal and adult testis−expressed transcript protein on eGFR
MR Test
Inverse variance weighted (fixed effects)
Inverse variance weighted (multiplicative random effects)
MR Egger
MR RAPS
Weighted median
Weighted mode
0.002
0.000
−0.002
−0.004
0.25
0.50
0.75
SNP effect on eGFR
SNP effect on Fetal and adult testis−expressed transcript protein

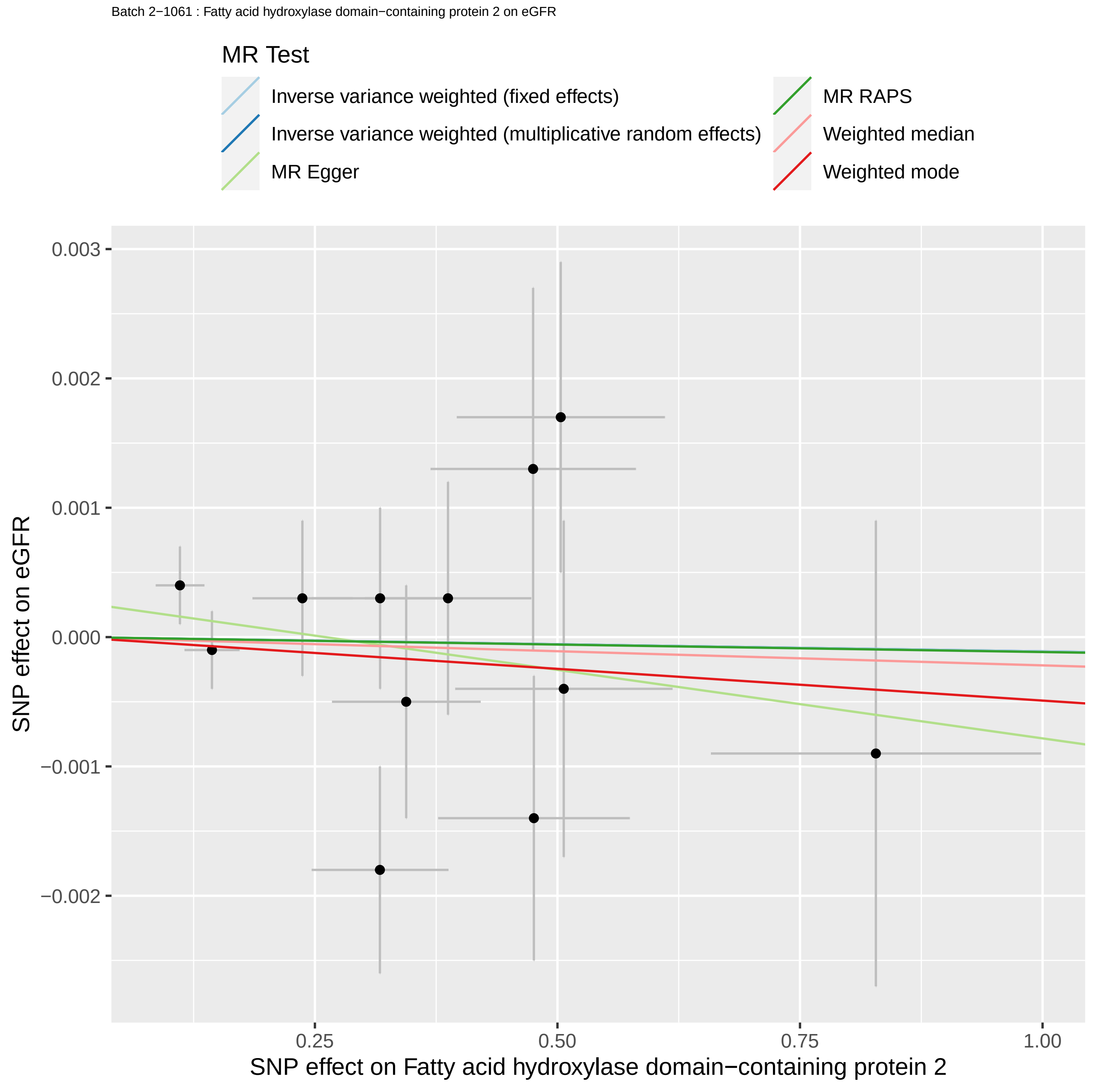
Batch 2−1061 : Fatty acid hydroxylase domain−containing protein 2 on eGFR
MR Test
Inverse variance weighted (fixed effects)
Inverse variance weighted (multiplicative random effects)
MR Egger
MR RAPS
Weighted median
Weighted mode
0.003
0.002
0.001
0.000
−0.001
−0.002
SNP effect on eGFR
0.25
0.50
0.75
1.00
SNP effect on Fatty acid hydroxylase domain−containing protein 2

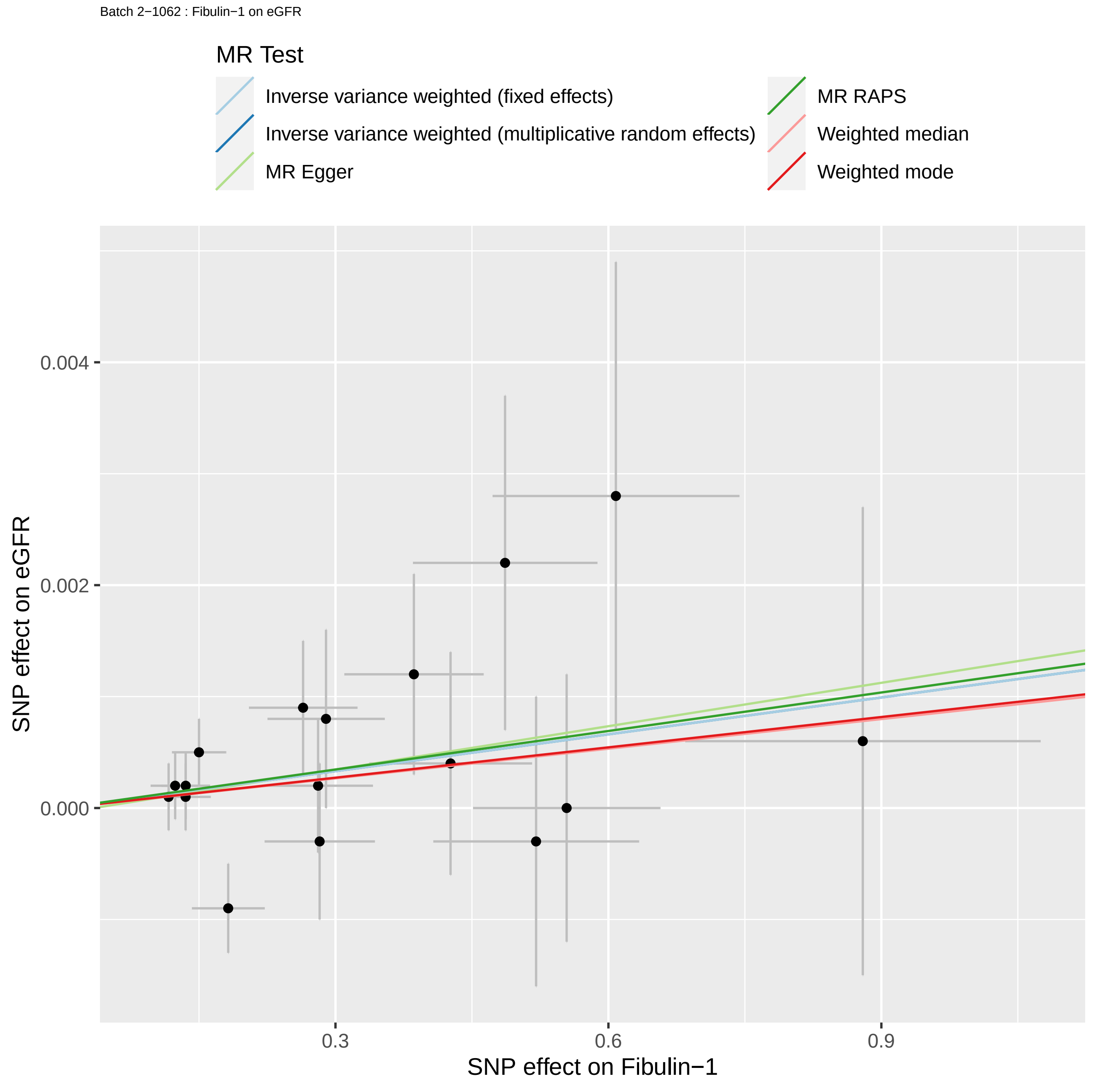
Batch 2−1062 : Fibulin−1 on eGFR
MR Test
Inverse variance weighted (fixed effects)
Inverse variance weighted (multiplicative random effects)
MR Egger
MR RAPS
Weighted median
Weighted mode
0.004
0.002
0.000
SNP effect on eGFR
0.3
0.6
0.9
SNP effect on Fibulin−1

Batch 2−1063 : Fructose−1,6−bisphosphatase 1 on eGFR

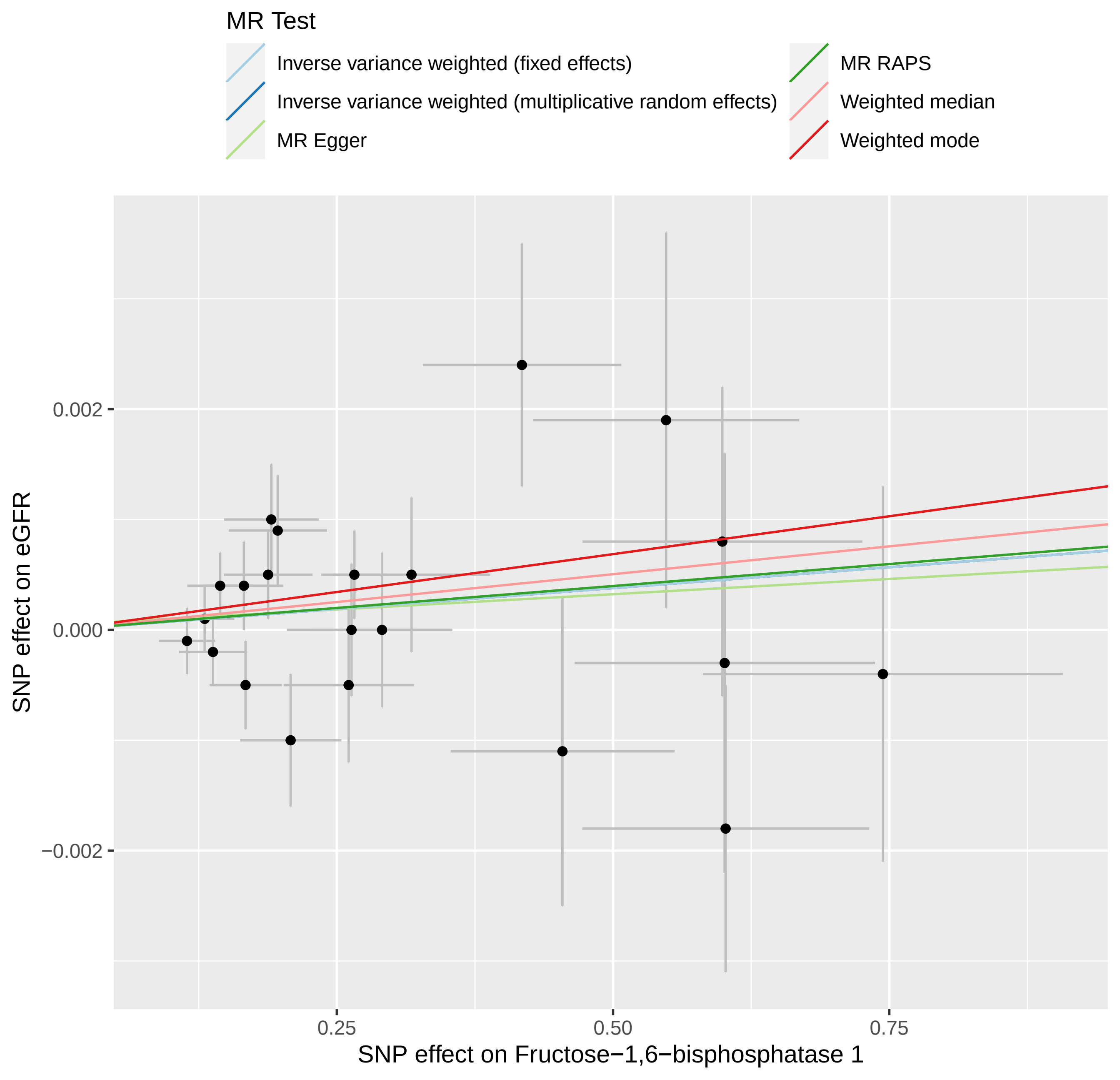

Batch 2−1064 : Fructose−1,6−bisphosphatase isozyme 2 on eGFR

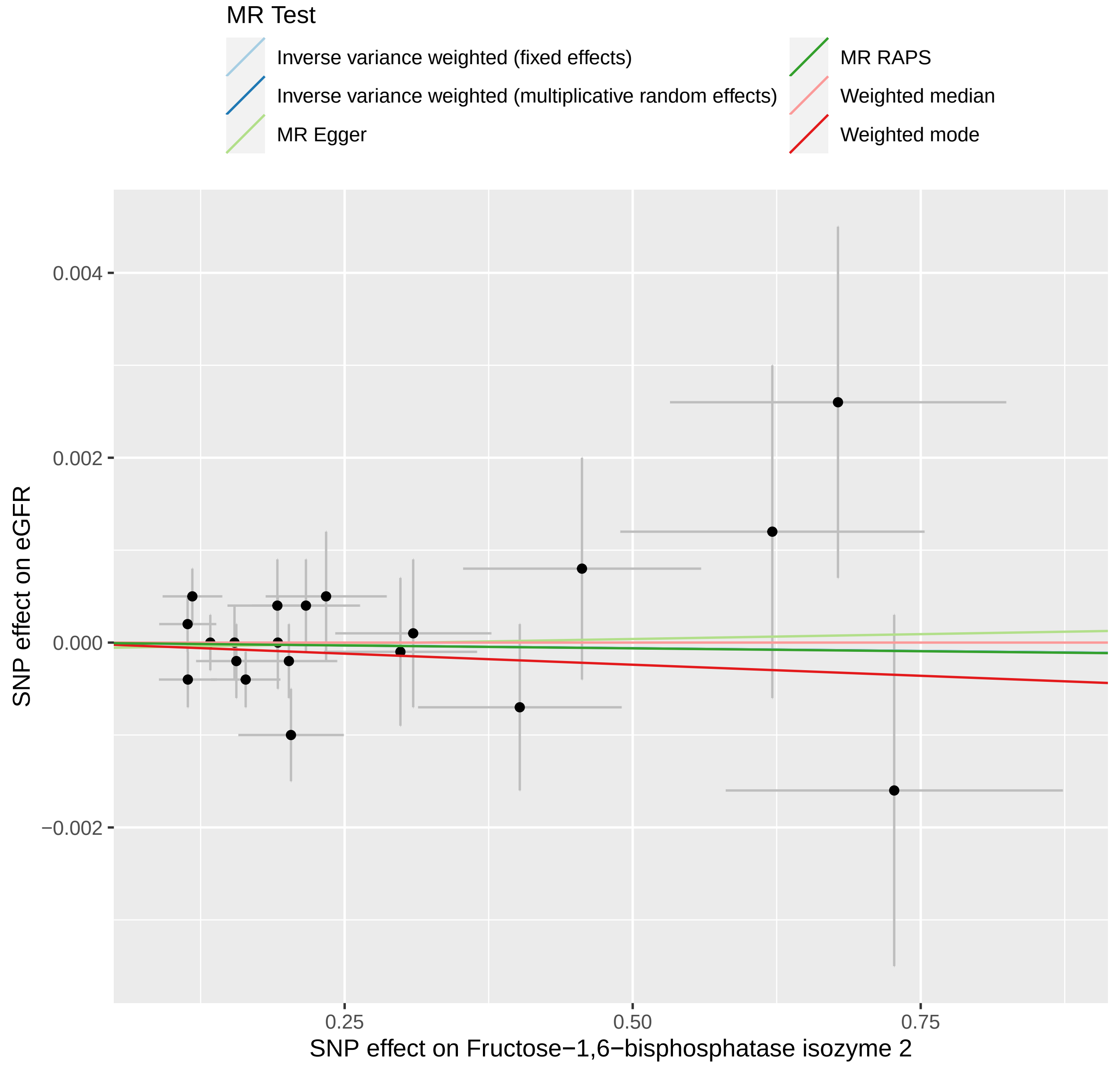

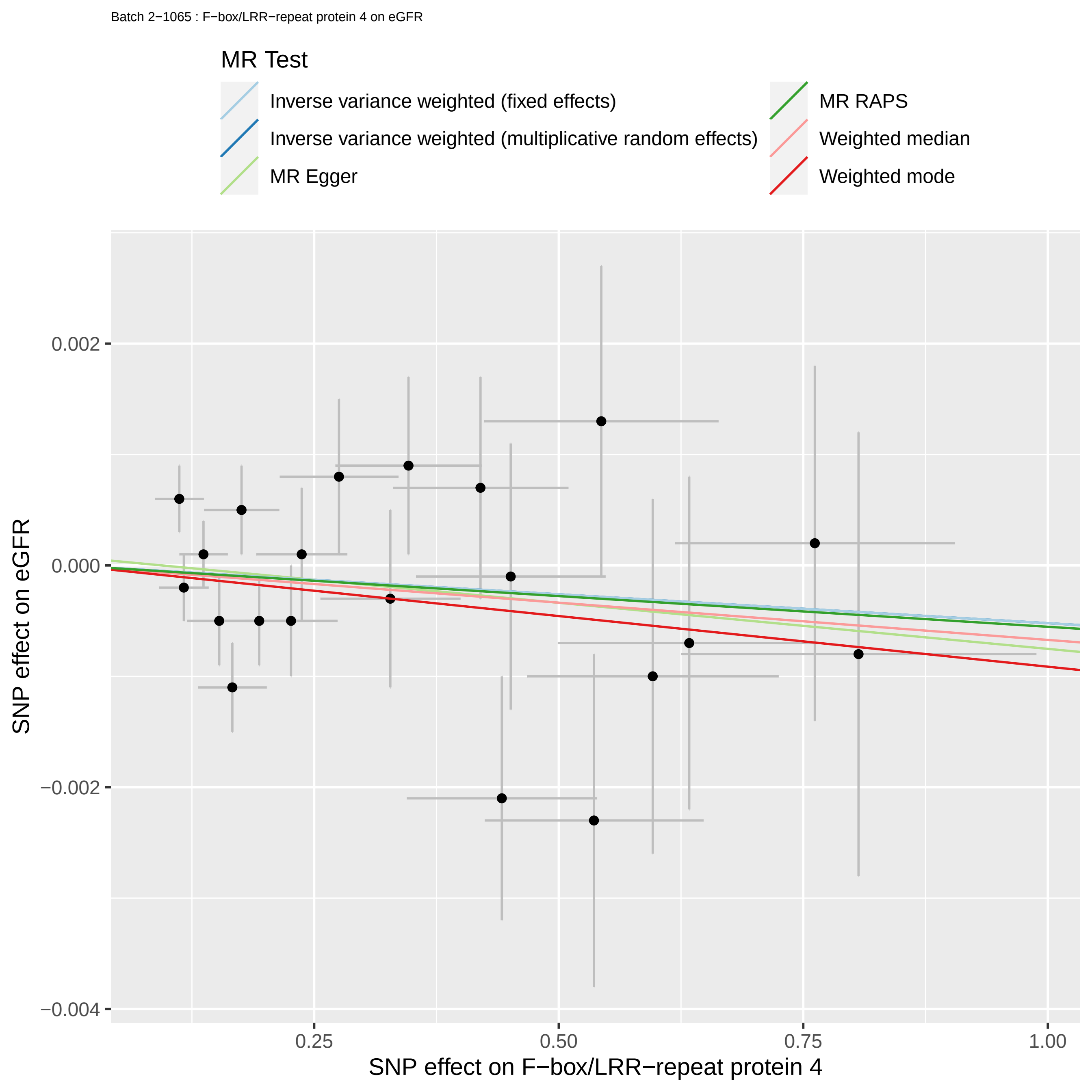

Batch 2−1065 : F−box/LRR−repeat protein 4 on eGFR
MR Test
Inverse variance weighted (fixed effects)
Inverse variance weighted (multiplicative random effects)
MR Egger
MR RAPS
Weighted median
Weighted mode
0.002
0.000
−0.002
−0.004
0.25
0.50
0.75
1.00
SNP effect on eGFR
SNP effect on F−box/LRR−repeat protein 4

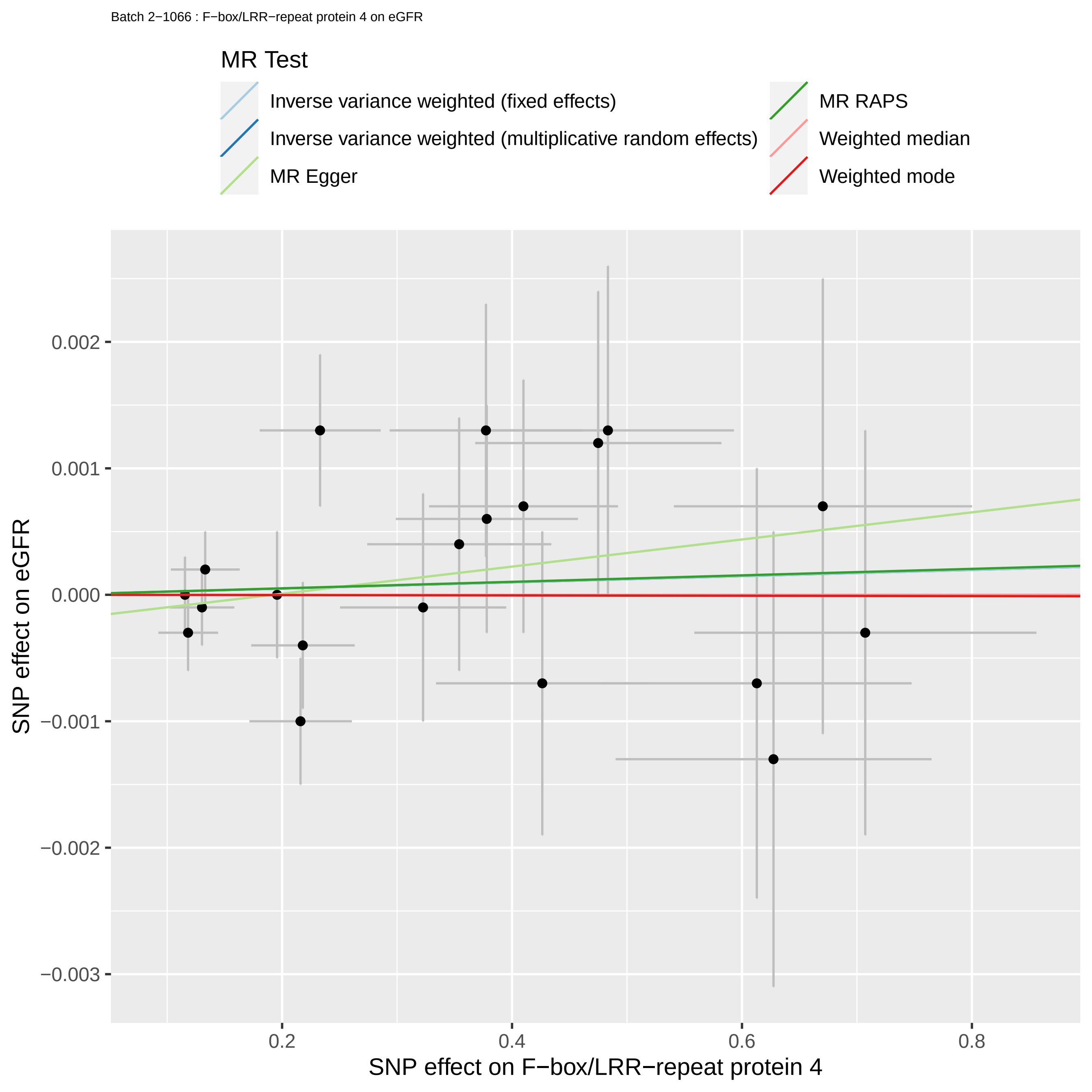
Batch 2−1066 : F−box/LRR−repeat protein 4 on eGFR
MR Test
Inverse variance weighted (fixed effects)
Inverse variance weighted (multiplicative random effects)
MR Egger
MR RAPS
Weighted median
Weighted mode
SNP effect on eGFR
0.002
0.001
0.000
−0.001
−0.002
−0.003
0.2
0.4
0.6
0.8
SNP effect on F−box/LRR−repeat protein 4

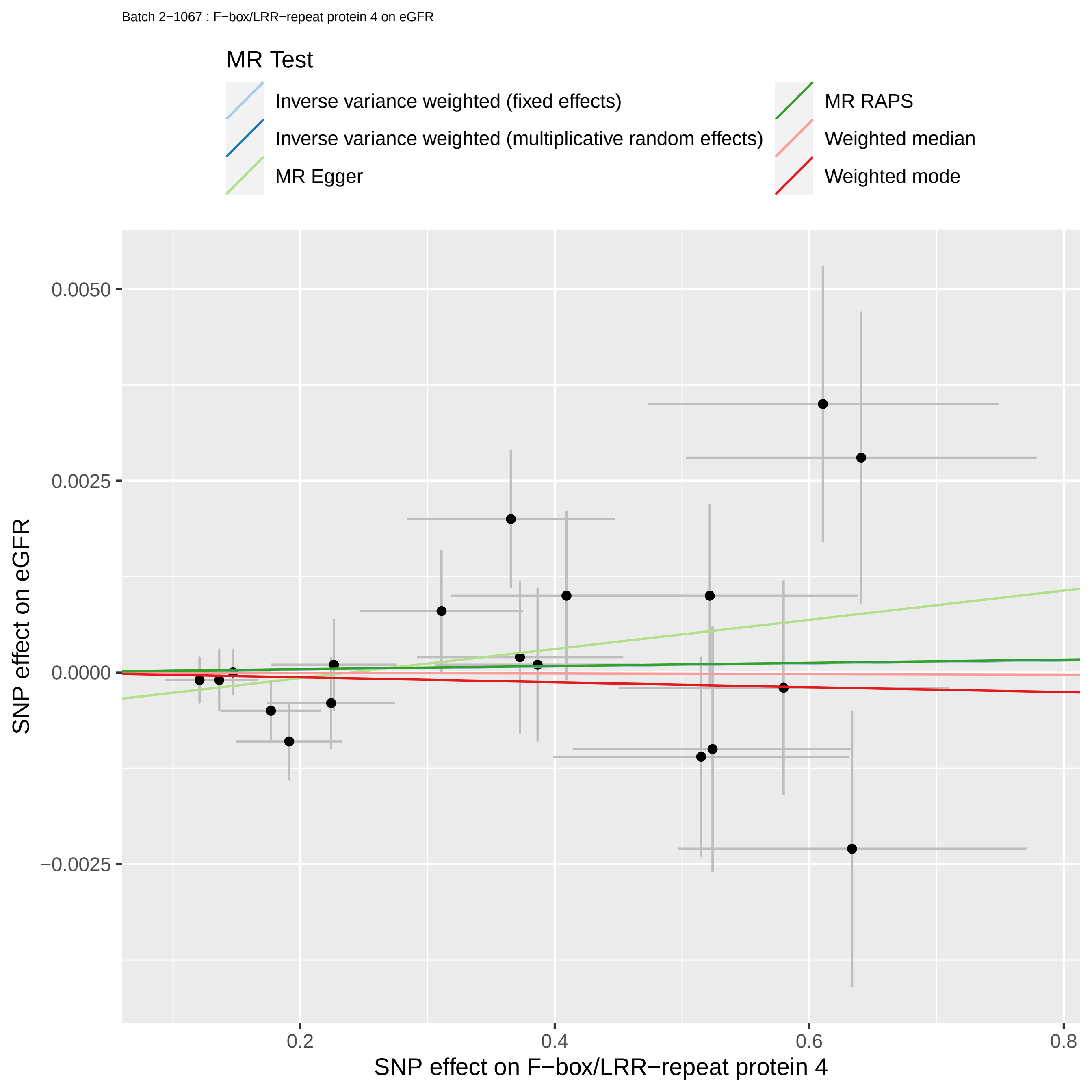

Batch 2−1067 : F−box/LRR−repeat protein 4 on eGFR
MR Test
Inverse variance weighted (fixed effects)
Inverse variance weighted (multiplicative random effects)
MR Egger
MR RAPS
Weighted median
Weighted mode
0.0050
0.0025
0.0000
−0.0025
SNP effect on eGFR
0.2
0.4
0.6
0.8
SNP effect on F−box/LRR−repeat protein 4

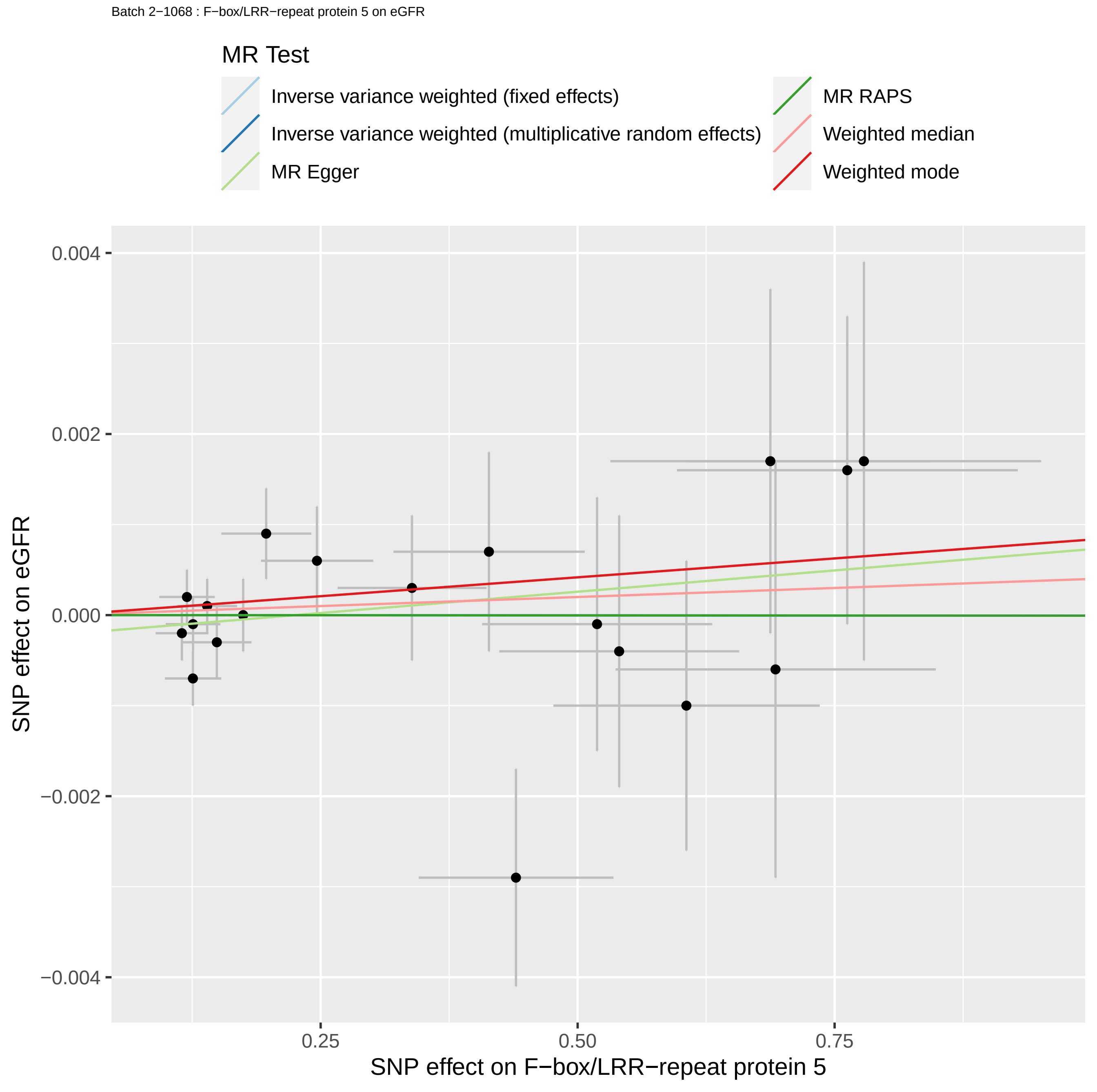
Batch 2−1068 : F−box/LRR−repeat protein 5 on eGFR
MR Test
Inverse variance weighted (fixed effects)
Inverse variance weighted (multiplicative random effects)
MR Egger
MR RAPS
Weighted median
Weighted mode
0.004
0.002
0.000
−0.002
−0.004
0.25
0.50
0.75
SNP effect on eGFR
SNP effect on F−box/LRR−repeat protein 5

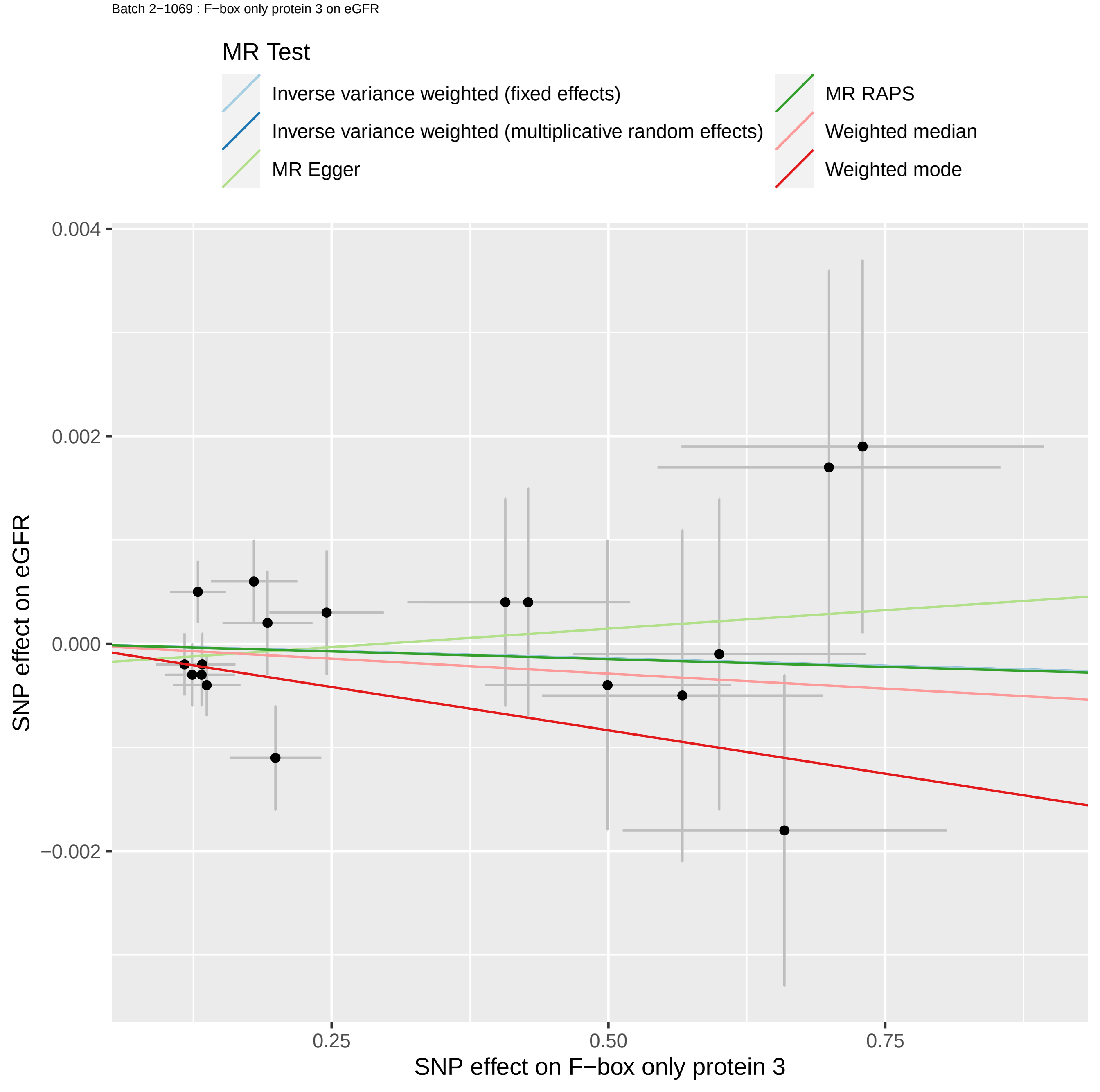

Batch 2−1069 : F−box only protein 3 on eGFR
MR Test
Inverse variance weighted (fixed effects)
Inverse variance weighted (multiplicative random effects)
MR Egger
MR RAPS
Weighted median
Weighted mode
0.004
0.002
0.000
−0.002
SNP effect on eGFR
0.25
0.50
0.75
SNP effect on F−box only protein 3

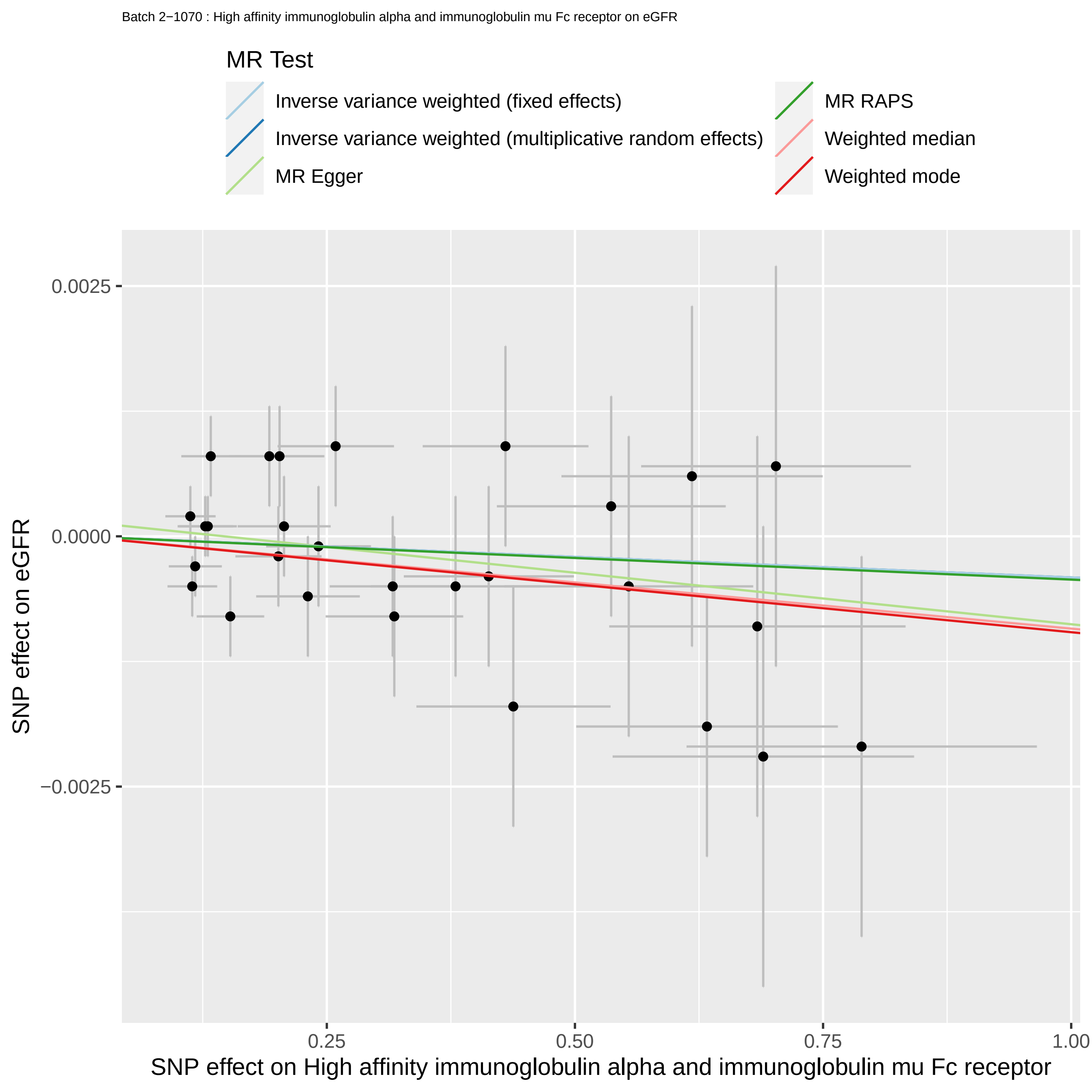

Batch 2−1070 : High affinity immunoglobulin alpha and immunoglobulin mu Fc receptor on eGFR
MR Test
Inverse variance weighted (fixed effects)
Inverse variance weighted (multiplicative random effects)
MR Egger
MR RAPS
Weighted median
Weighted mode
0.0025
0.0000
−0.0025
0.25
0.50
0.75
1.00
SNP effect on eGFR
SNP effect on High affinity immunoglobulin alpha and immunoglobulin mu Fc receptor

Batch 2−1071 : Immunoglobulin alpha Fc receptor on eGFR

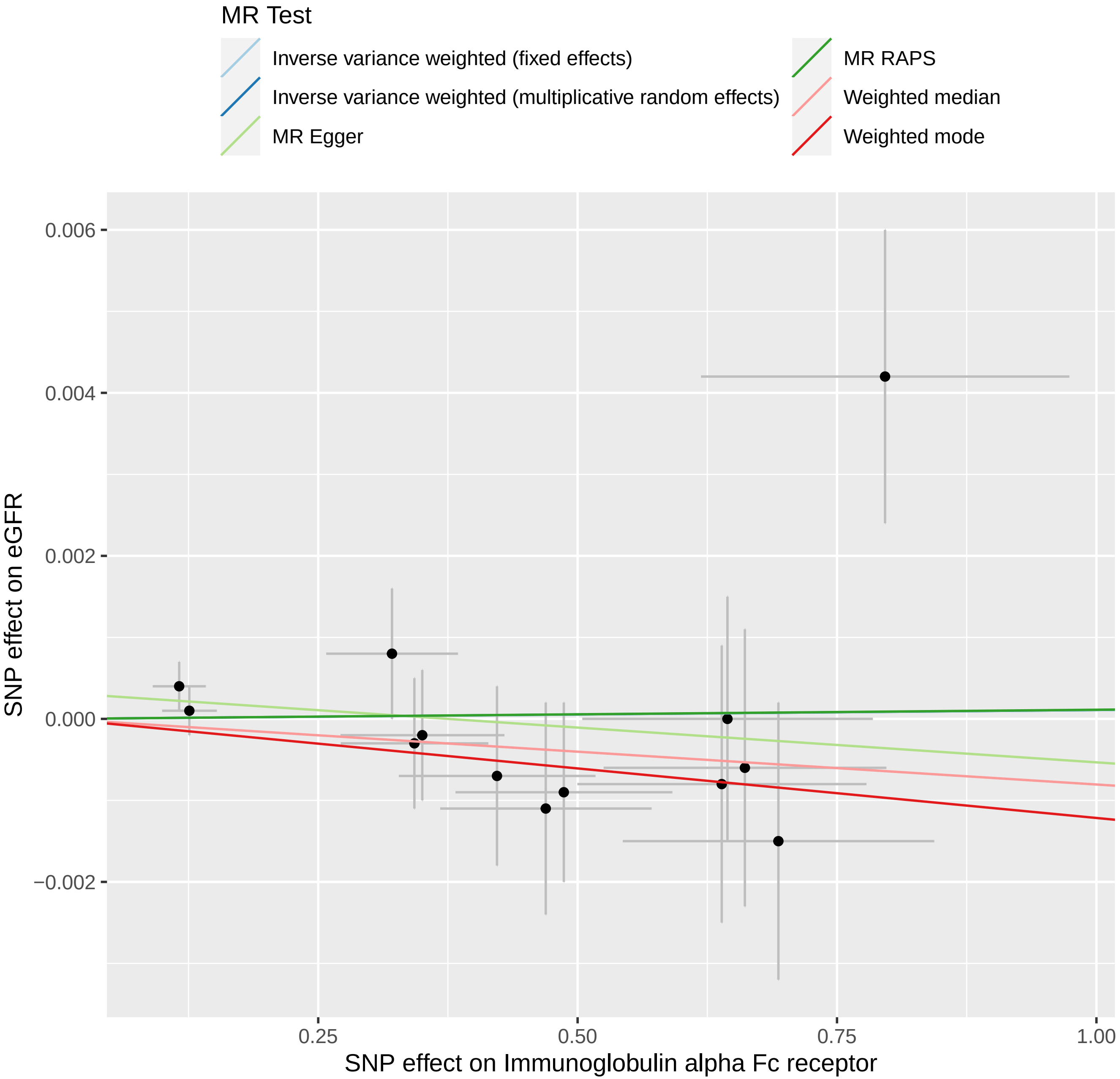

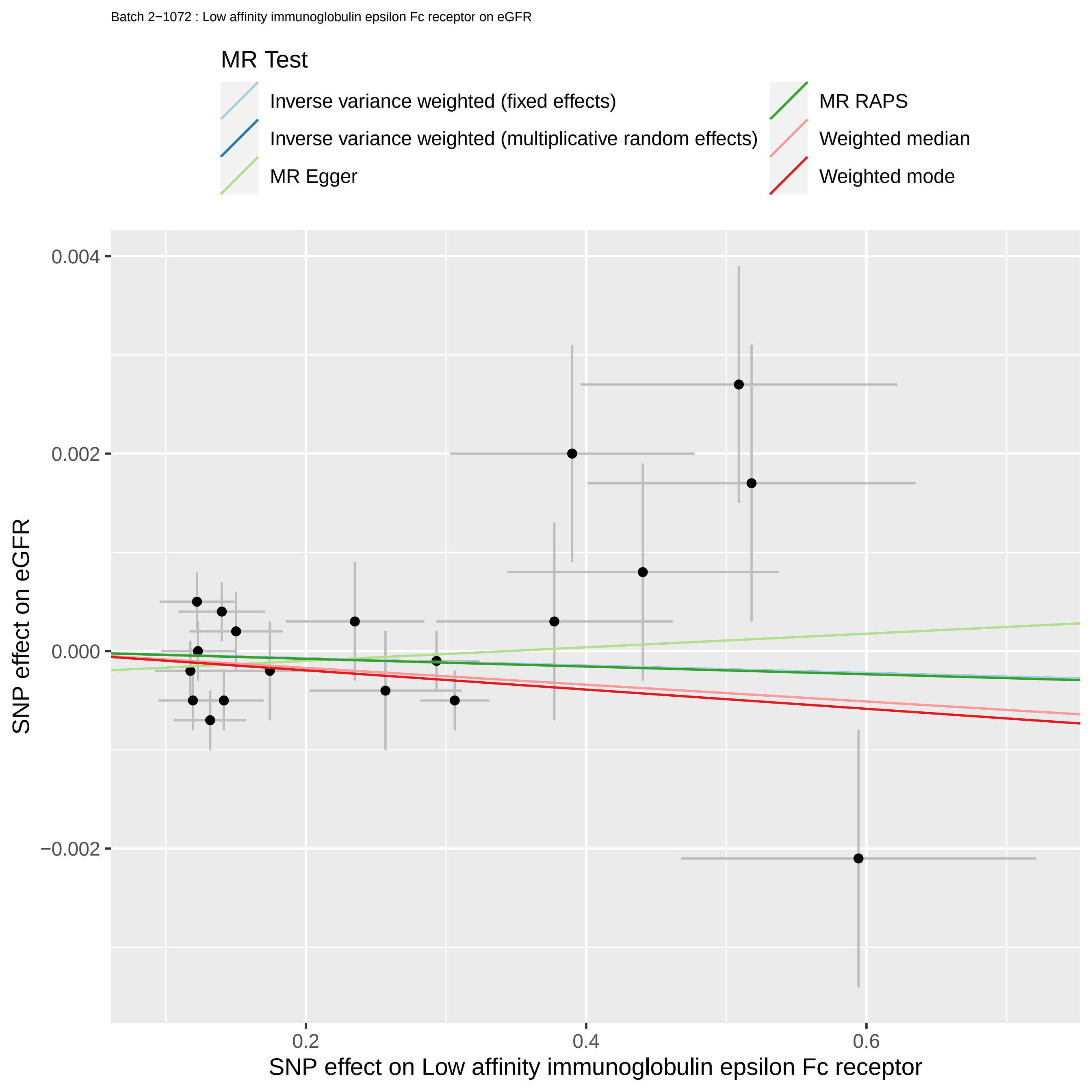
Batch 2−1072 : Low affinity immunoglobulin epsilon Fc receptor on eGFR
MR Test
Inverse variance weighted (fixed effects)
Inverse variance weighted (multiplicative random effects)
MR Egger
MR RAPS
Weighted median
Weighted mode
0.004
0.002
0.000
−0.002
0.2
0.4
0.6
SNP effect on eGFR
SNP effect on Low affinity immunoglobulin epsilon Fc receptor

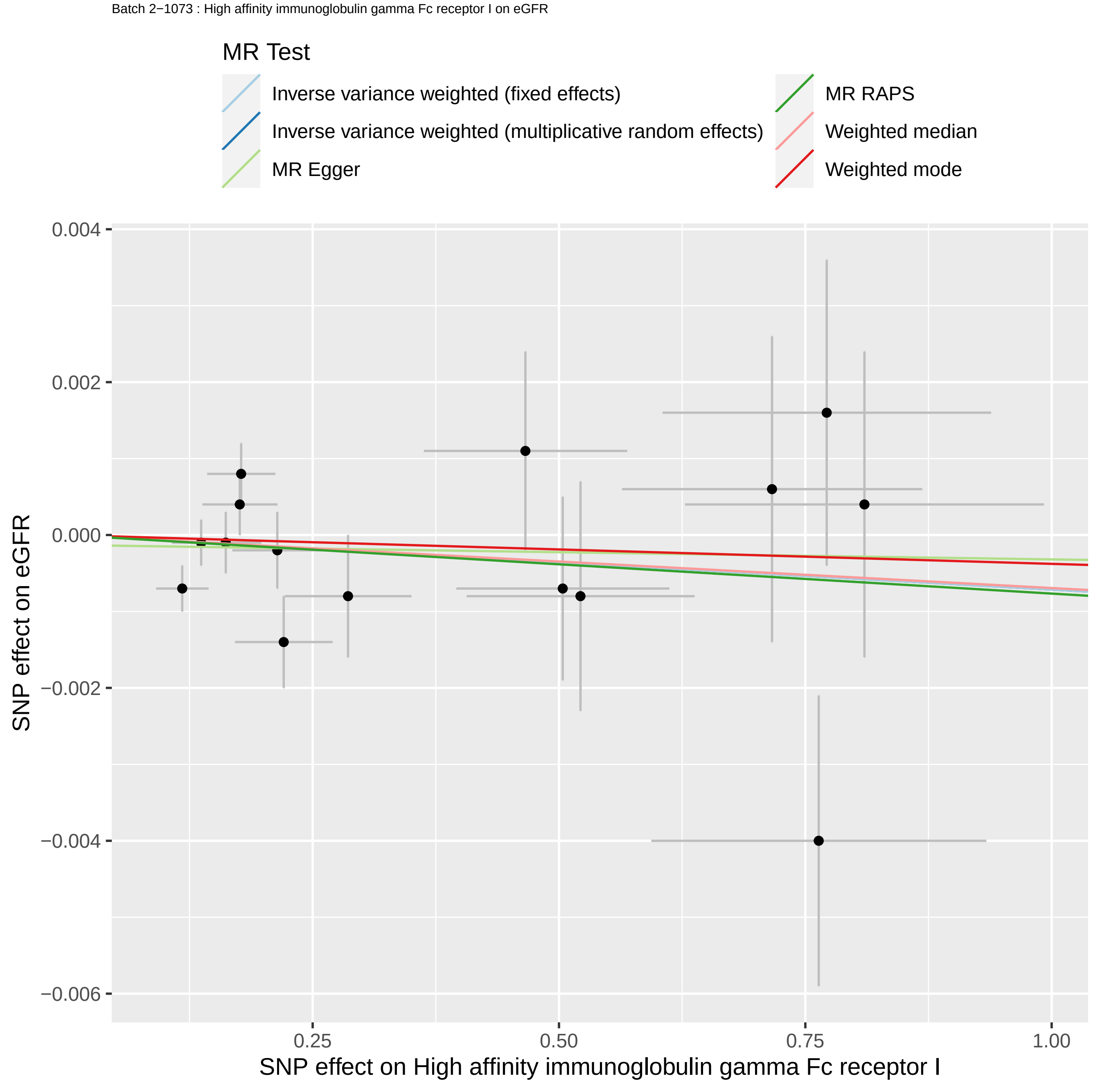
Batch 2−1073 : High affinity immunoglobulin gamma Fc receptor I on eGFR
MR Test
Inverse variance weighted (fixed effects)
Inverse variance weighted (multiplicative random effects)
MR Egger
MR RAPS
Weighted median
Weighted mode
0.004
0.002
0.000
−0.002
−0.004
−0.006
0.25
0.50
0.75
1.00
SNP effect on eGFR
SNP effect on High affinity immunoglobulin gamma Fc receptor I

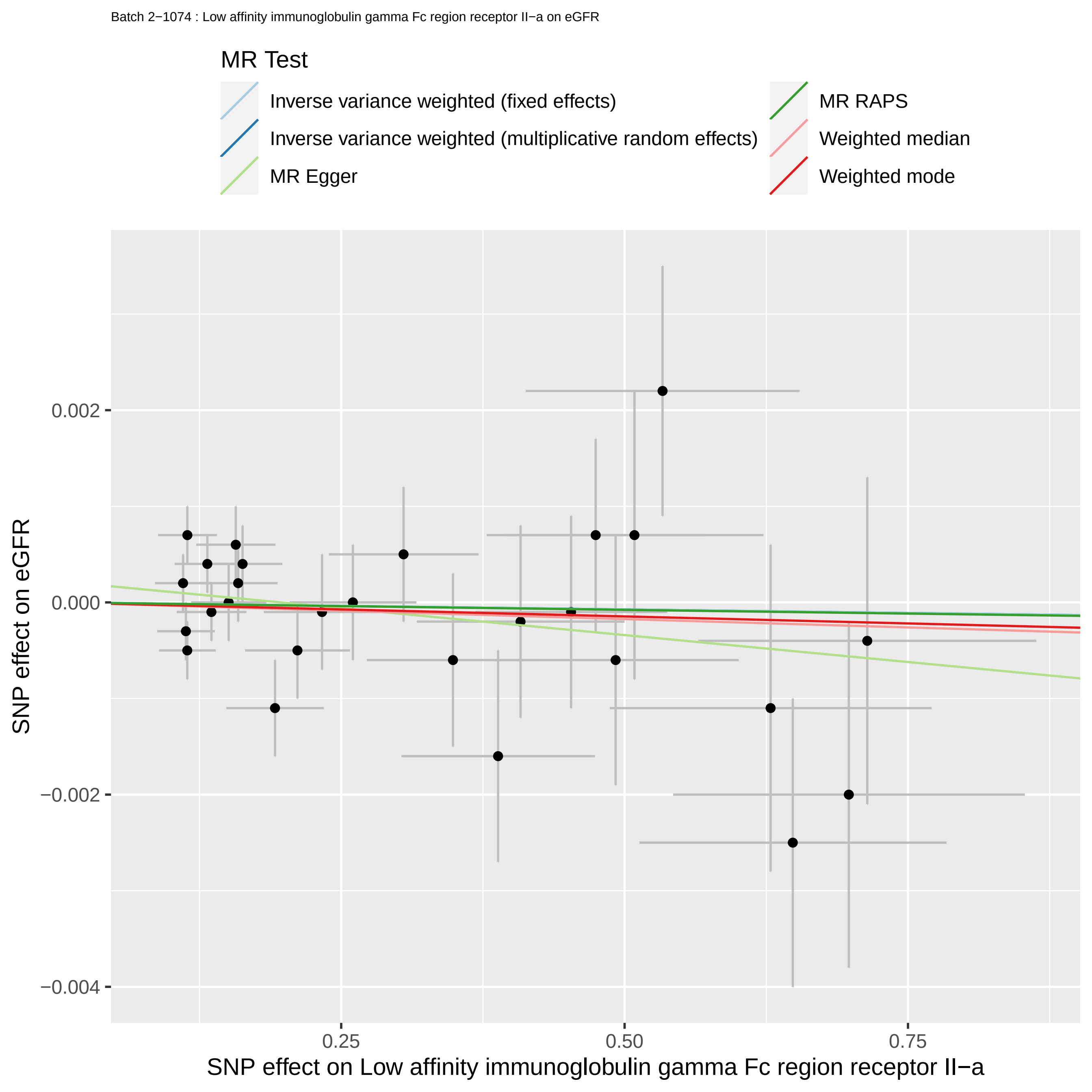
Batch 2−1074 : Low affinity immunoglobulin gamma Fc region receptor II−a on eGFR
MR Test
Inverse variance weighted (fixed effects)
Inverse variance weighted (multiplicative random effects)
MR Egger
MR RAPS
Weighted median
Weighted mode
0.002
0.000
−0.002
−0.004
SNP effect on eGFR
0.25
0.50
0.75
SNP effect on Low affinity immunoglobulin gamma Fc region receptor II−a

Batch 2−1075 : Low affinity immunoglobulin gamma Fc region receptor II−b on eGFR

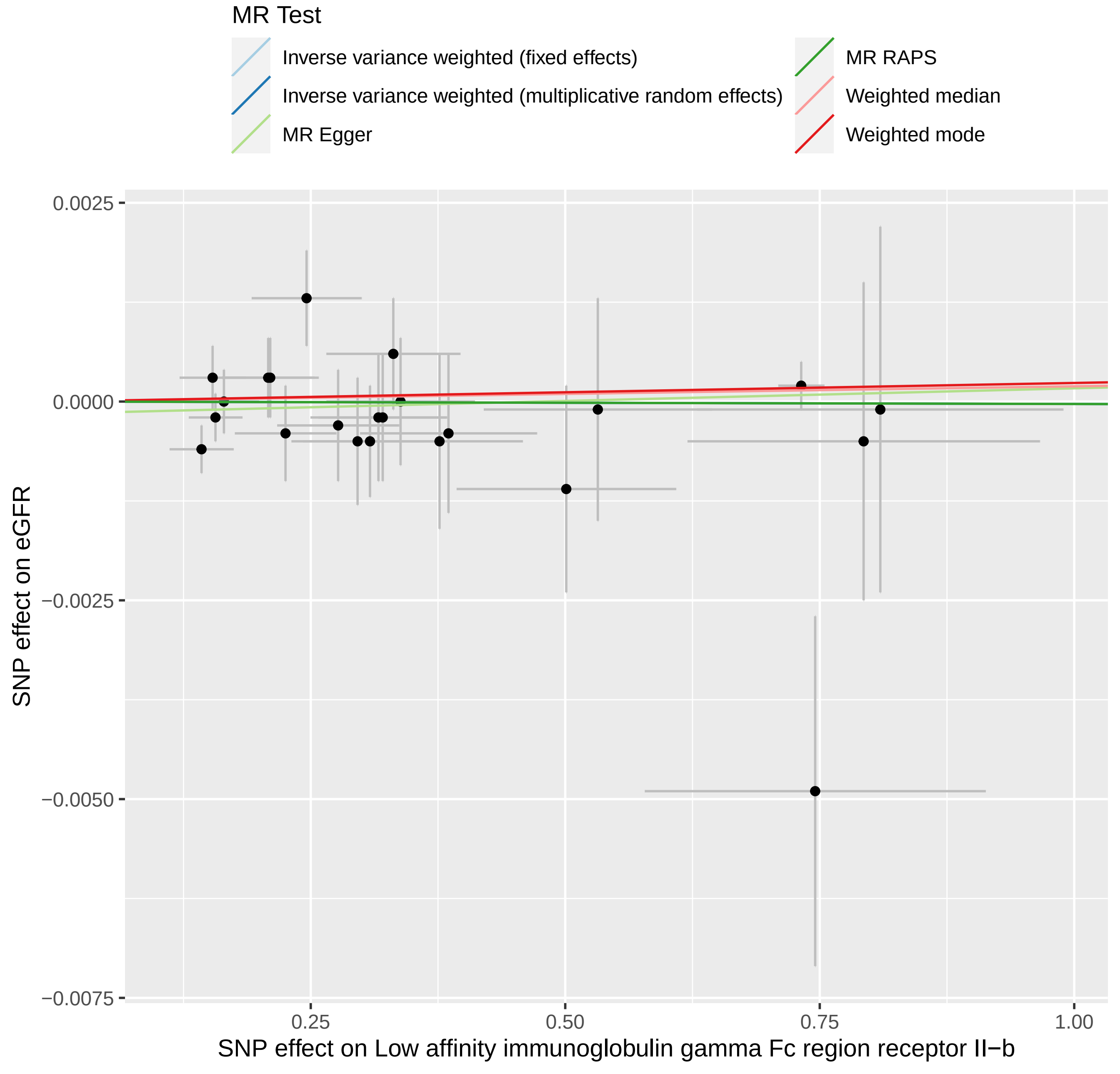

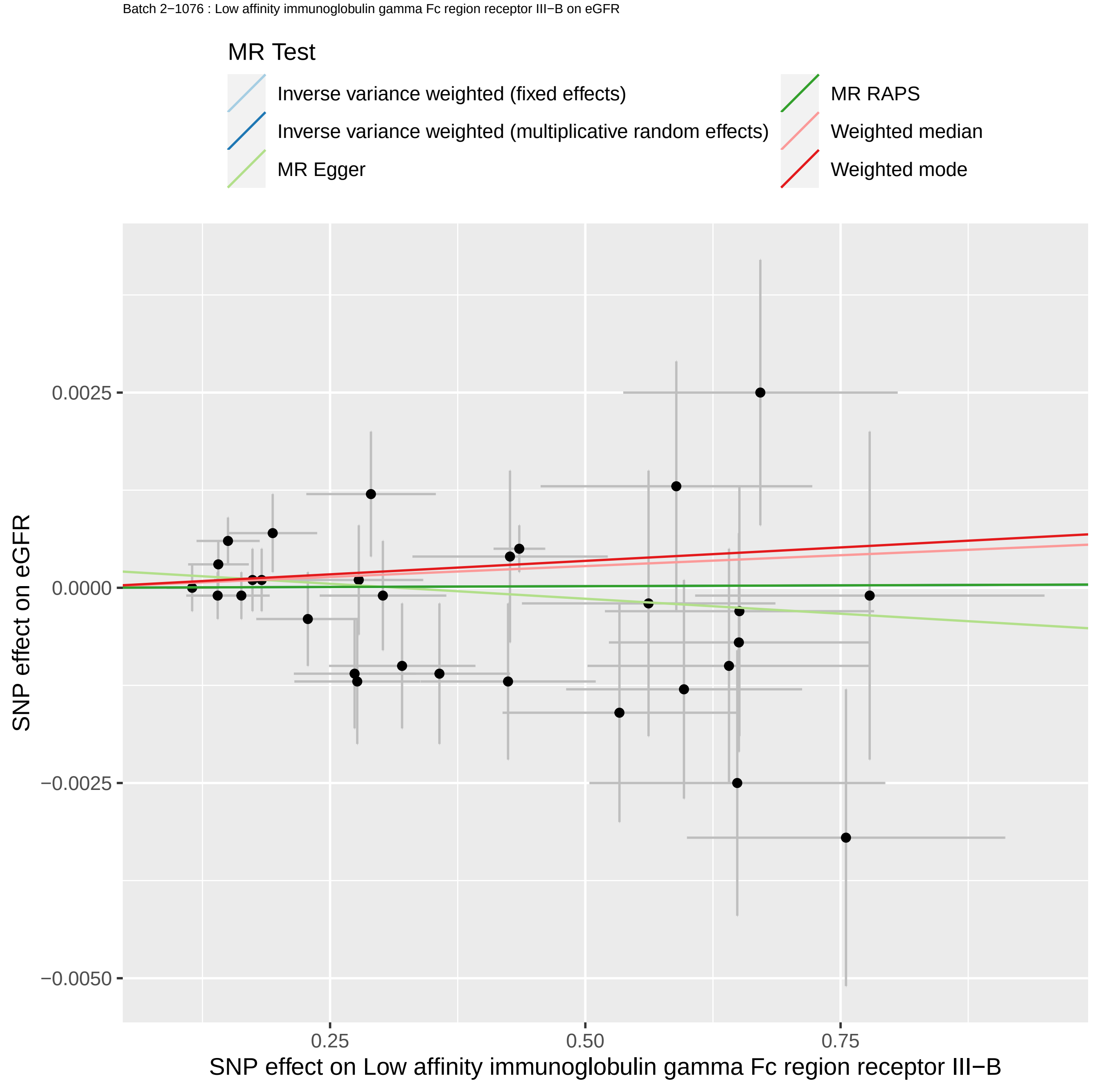

Batch 2−1076 : Low affinity immunoglobulin gamma Fc region receptor III−B on eGFR
MR Test
Inverse variance weighted (fixed effects)
Inverse variance weighted (multiplicative random effects)
MR Egger
MR RAPS
Weighted median
Weighted mode
0.0025
0.0000
−0.0025
−0.0050
0.25
0.50
0.75
SNP effect on eGFR
SNP effect on Low affinity immunoglobulin gamma Fc region receptor III−B

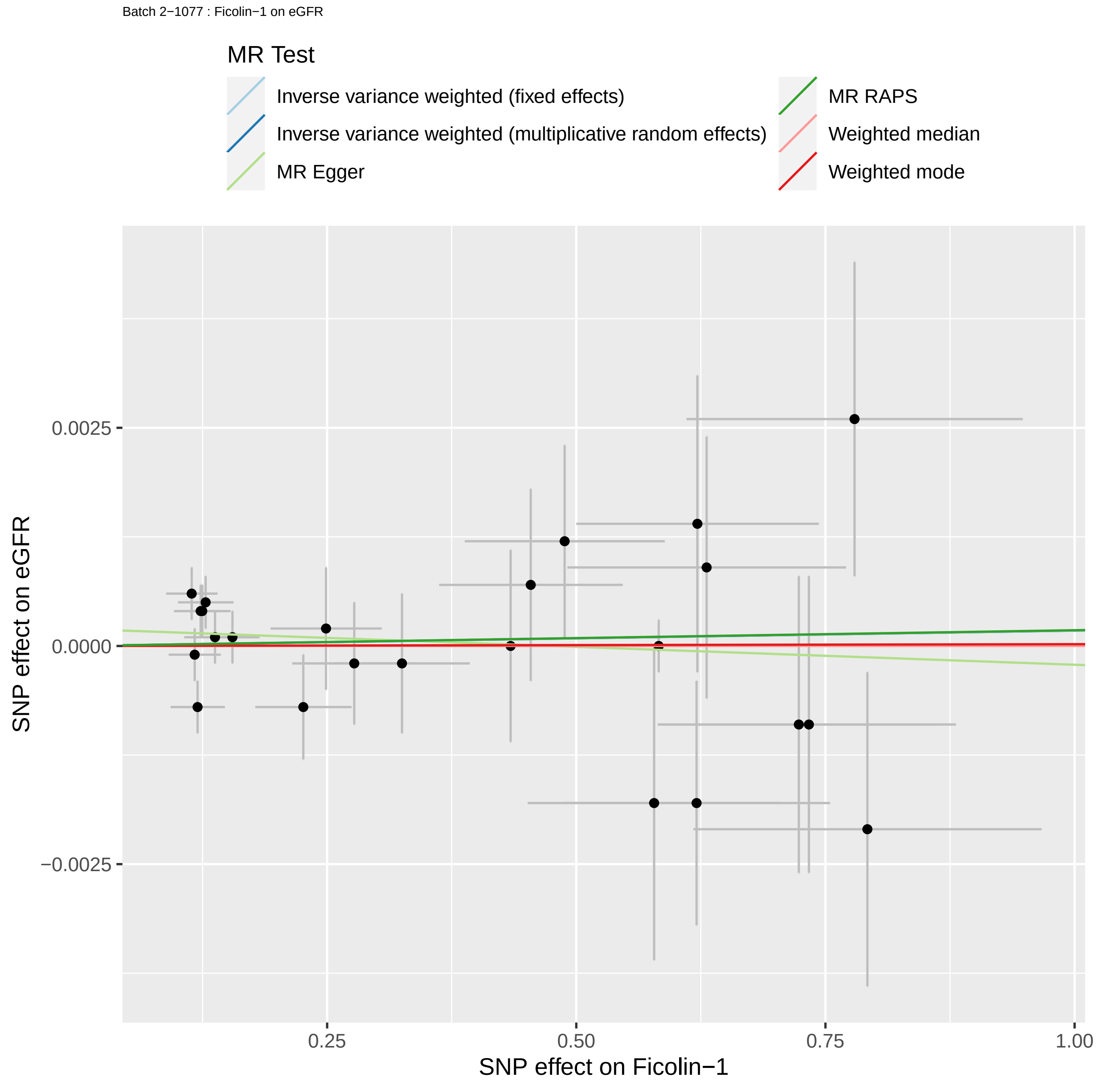

Batch 2−1077 : Ficolin−1 on eGFR
MR Test
Inverse variance weighted (fixed effects)
Inverse variance weighted (multiplicative random effects)
MR Egger
MR RAPS
Weighted median
Weighted mode
0.0025
0.0000
−0.0025
0.25
0.50
0.75
1.00
SNP effect on Ficolin−1
SNP effect on eGFR

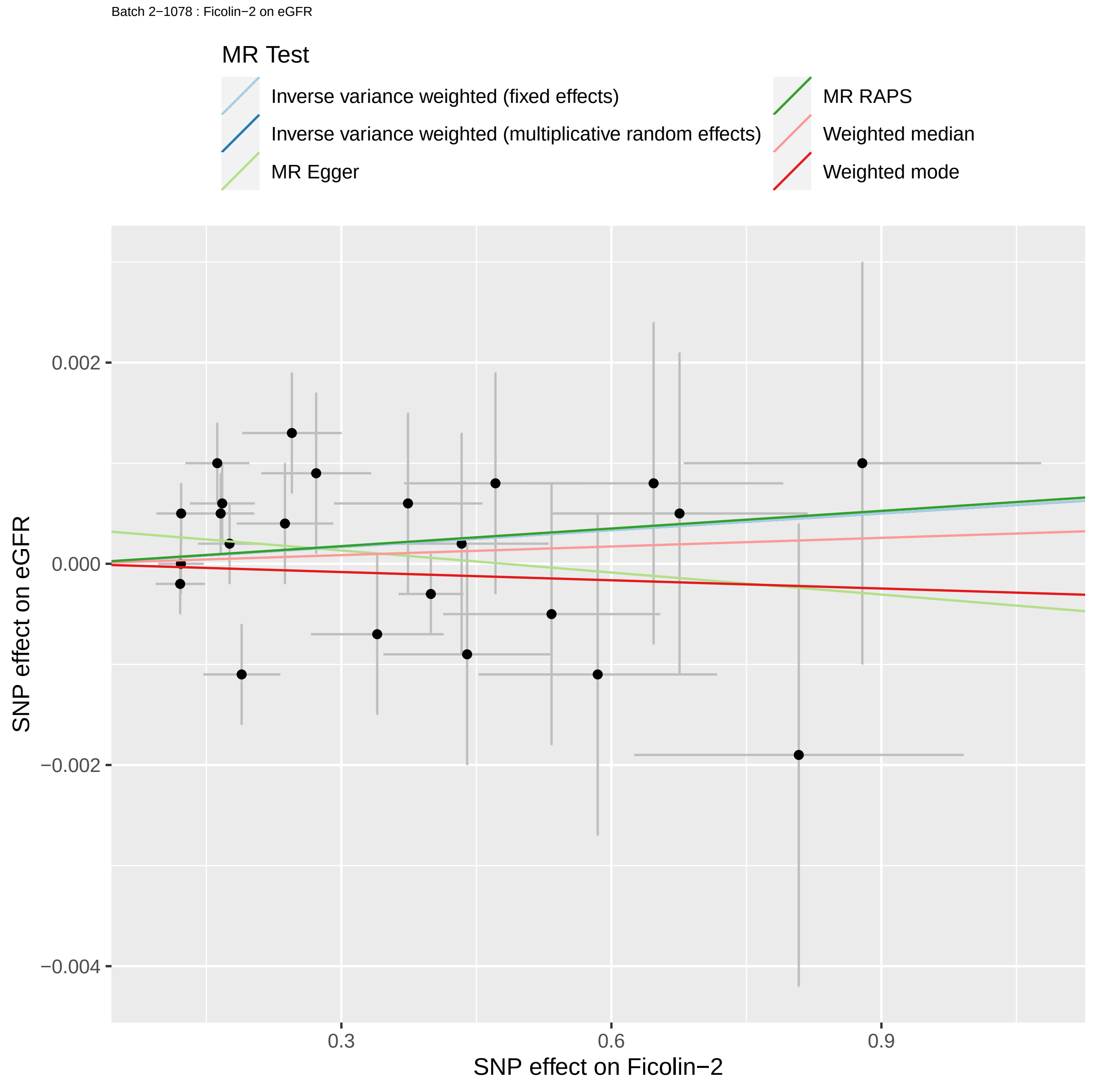
Batch 2−1078 : Ficolin−2 on eGFR
MR Test
Inverse variance weighted (fixed effects)
Inverse variance weighted (multiplicative random effects)
MR Egger
MR RAPS
Weighted median
Weighted mode
SNP effect on eGFR
0.002
0.000
−0.002
−0.004
0.3
0.6
0.9
SNP effect on Ficolin−2

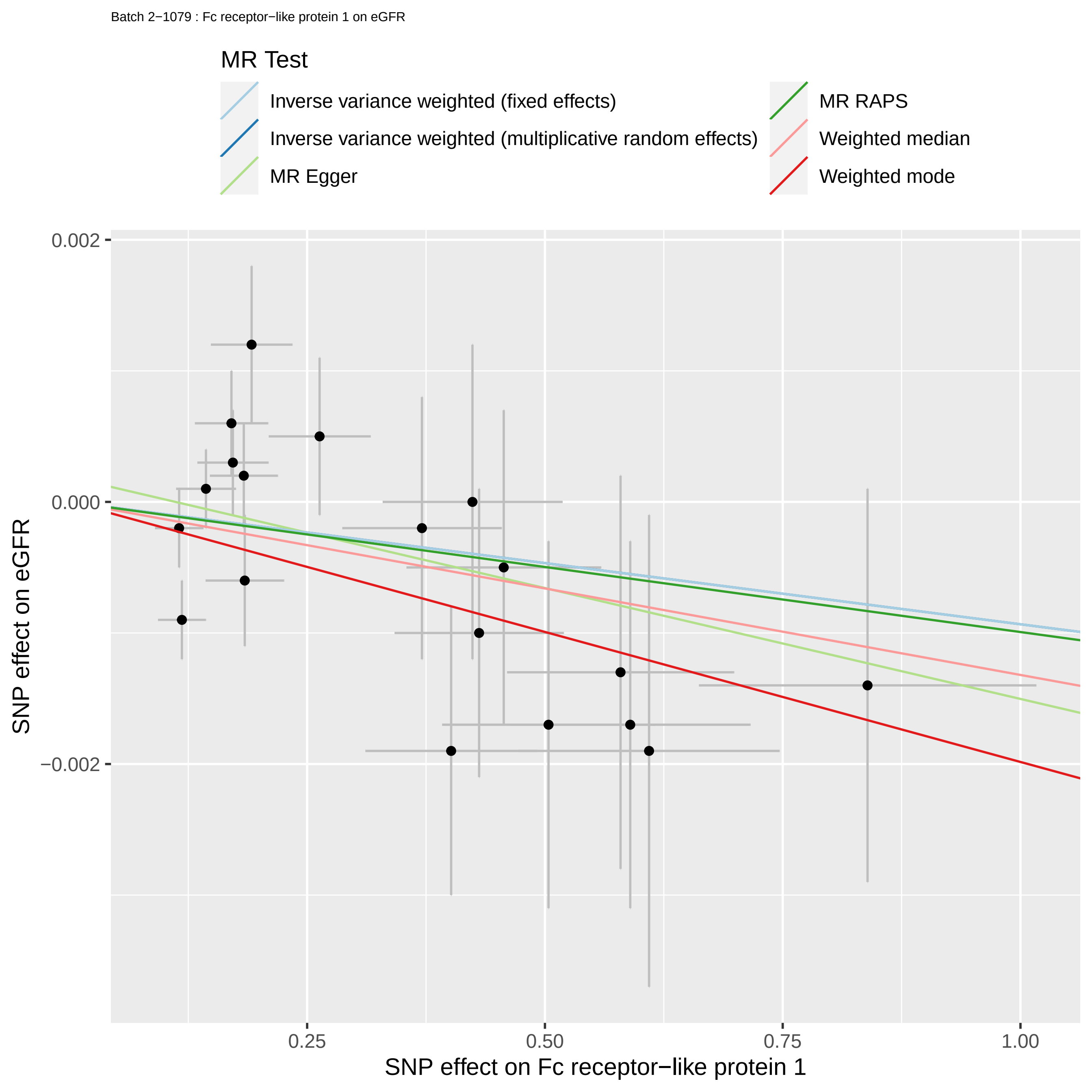
Batch 2−1079 : Fc receptor−like protein 1 on eGFR
MR Test
Inverse variance weighted (fixed effects)
Inverse variance weighted (multiplicative random effects)
MR Egger
MR RAPS
Weighted median
Weighted mode
0.002
0.000
−0.002
0.25
0.50
0.75
1.00
SNP effect on eGFR
SNP effect on Fc receptor−like protein 1

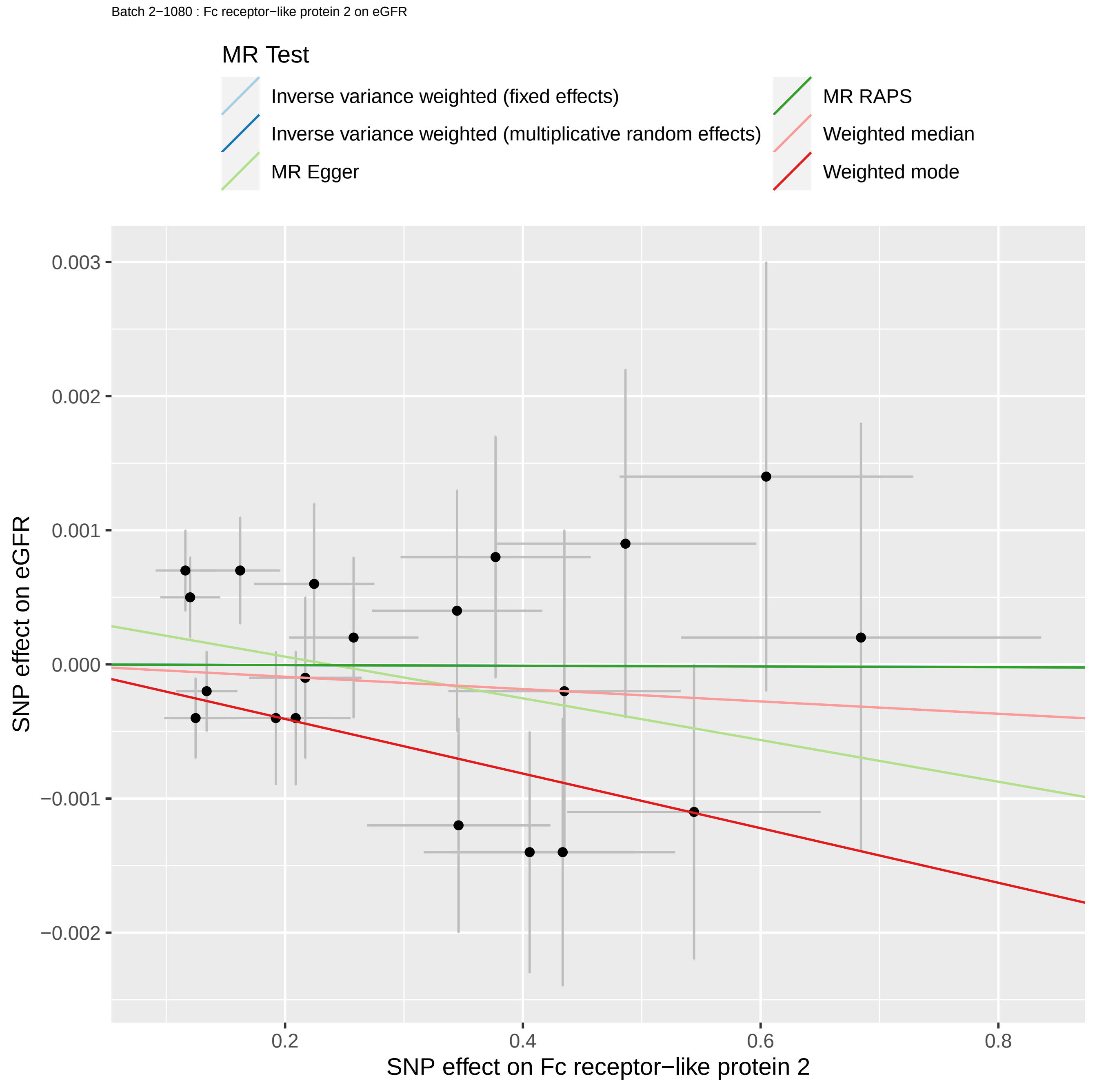
Batch 2−1080 : Fc receptor−like protein 2 on eGFR
MR Test
Inverse variance weighted (fixed effects)
Inverse variance weighted (multiplicative random effects)
MR Egger
MR RAPS
Weighted median
Weighted mode
SNP effect on eGFR
0.003
0.002
0.001
0.000
−0.001
−0.002
0.2
0.4
0.6
0.8
SNP effect on Fc receptor−like protein 2

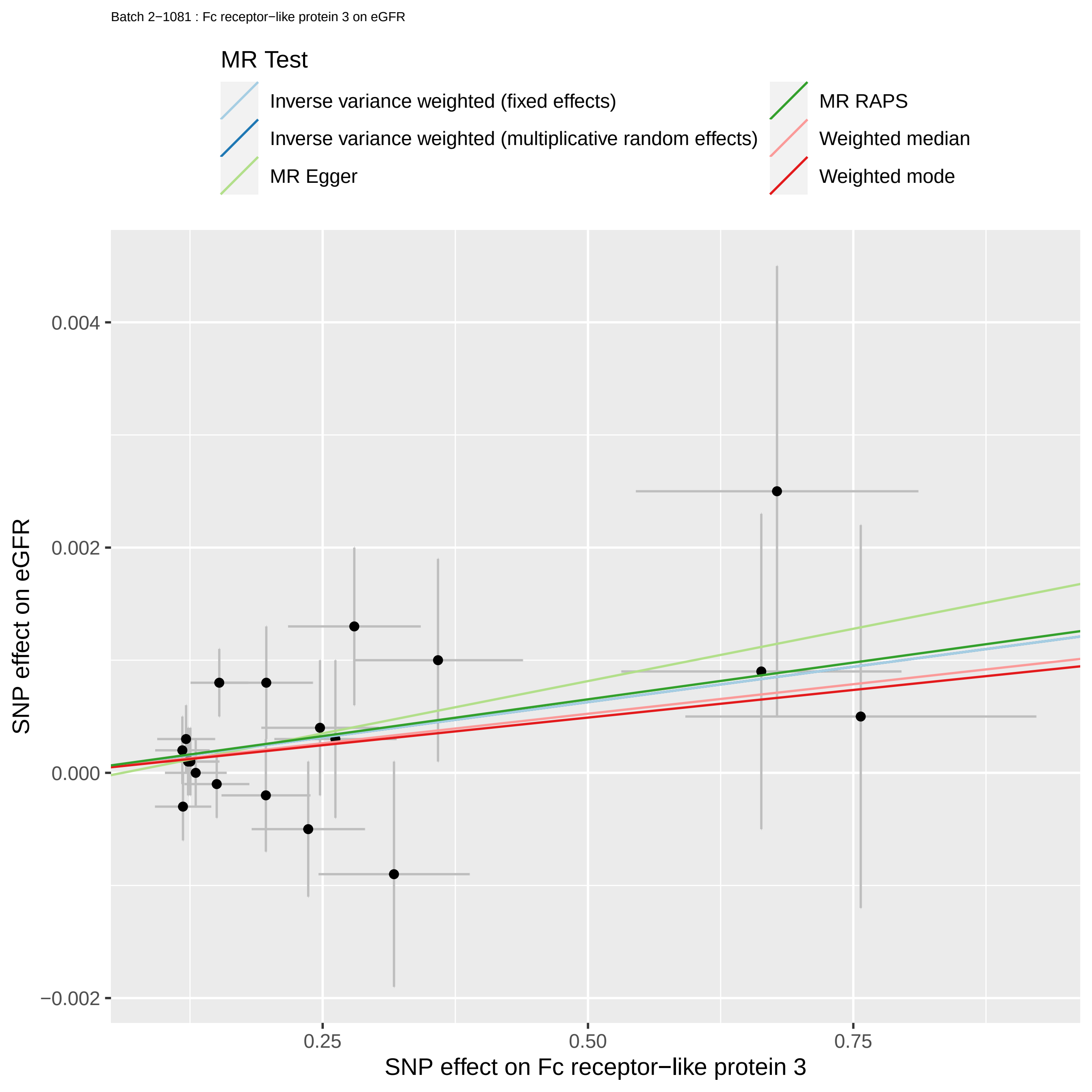
Batch 2−1081 : Fc receptor−like protein 3 on eGFR
MR Test
Inverse variance weighted (fixed effects)
Inverse variance weighted (multiplicative random effects)
MR Egger
MR RAPS
Weighted median
Weighted mode
0.004
0.002
0.000
−0.002
SNP effect on eGFR
0.25
0.50
0.75
SNP effect on Fc receptor−like protein 3

Batch 2−1082 : Fc receptor−like protein 4 on eGFR

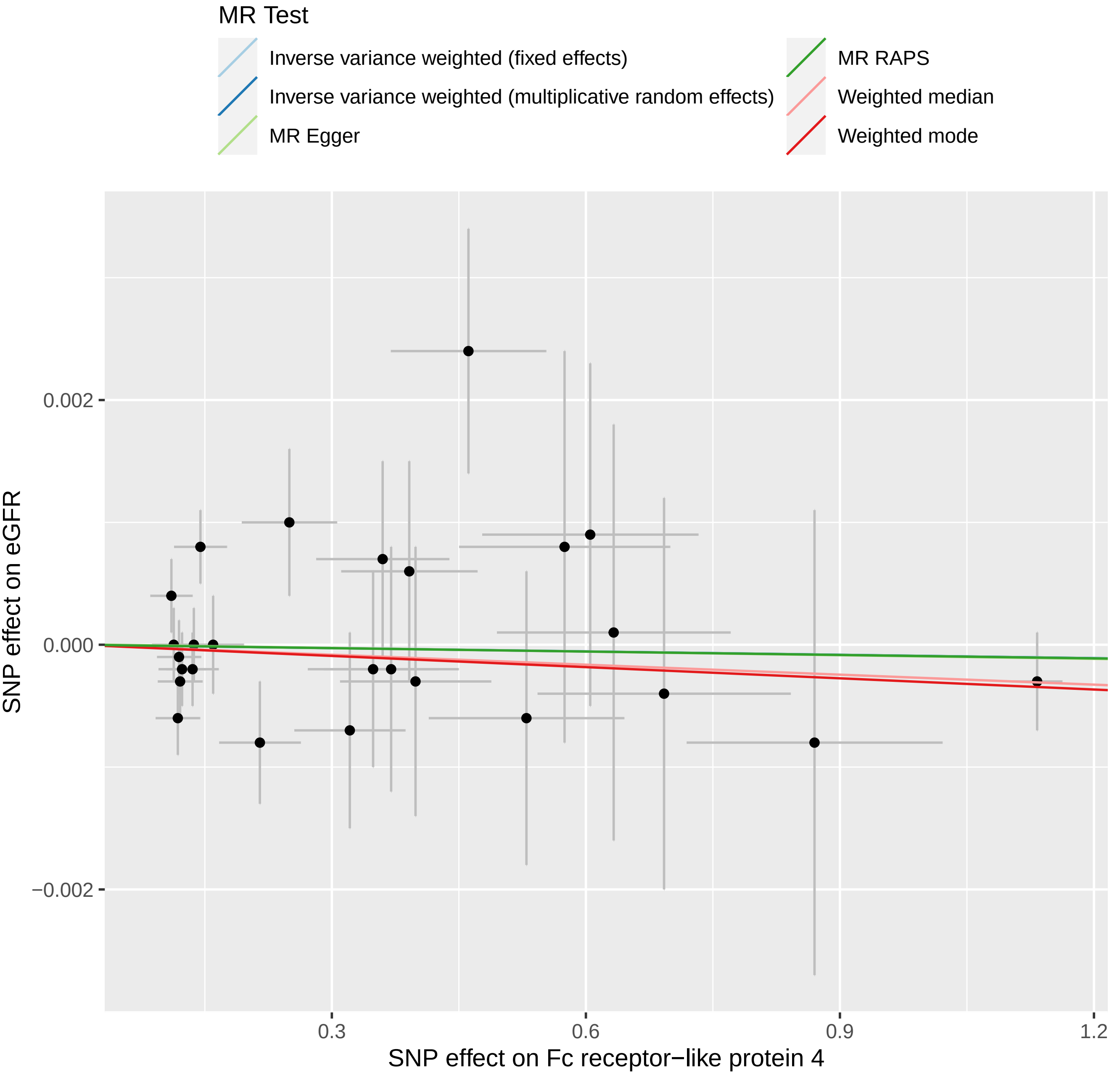

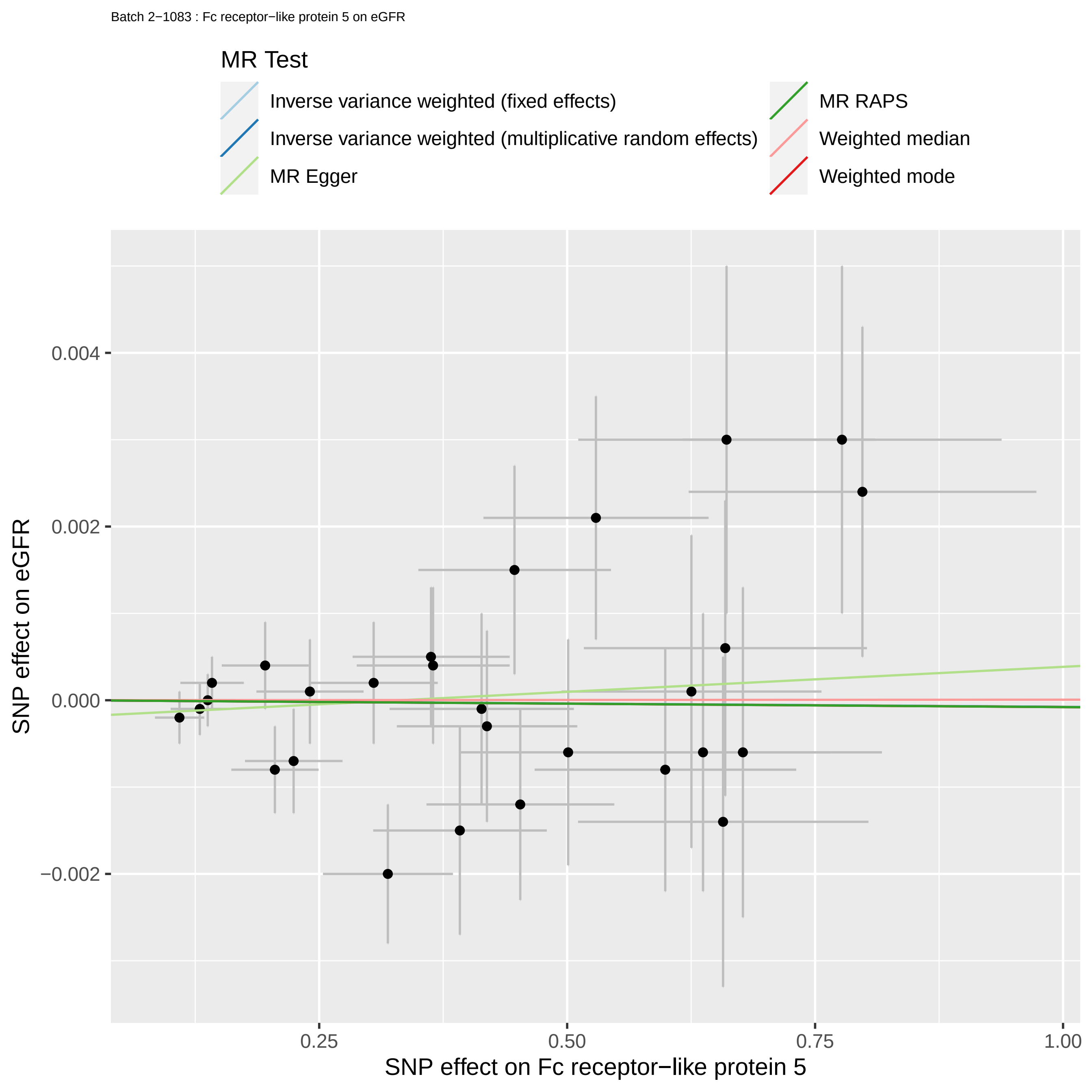
Batch 2−1083 : Fc receptor−like protein 5 on eGFR
MR Test
Inverse variance weighted (fixed effects)
Inverse variance weighted (multiplicative random effects)
MR Egger
MR RAPS
Weighted median
Weighted mode
0.004
0.002
0.000
−0.002
SNP effect on eGFR
0.25
0.50
0.75
1.00
SNP effect on Fc receptor−like protein 5

Batch 2−1084 : Fc receptor−like protein 6 on eGFR

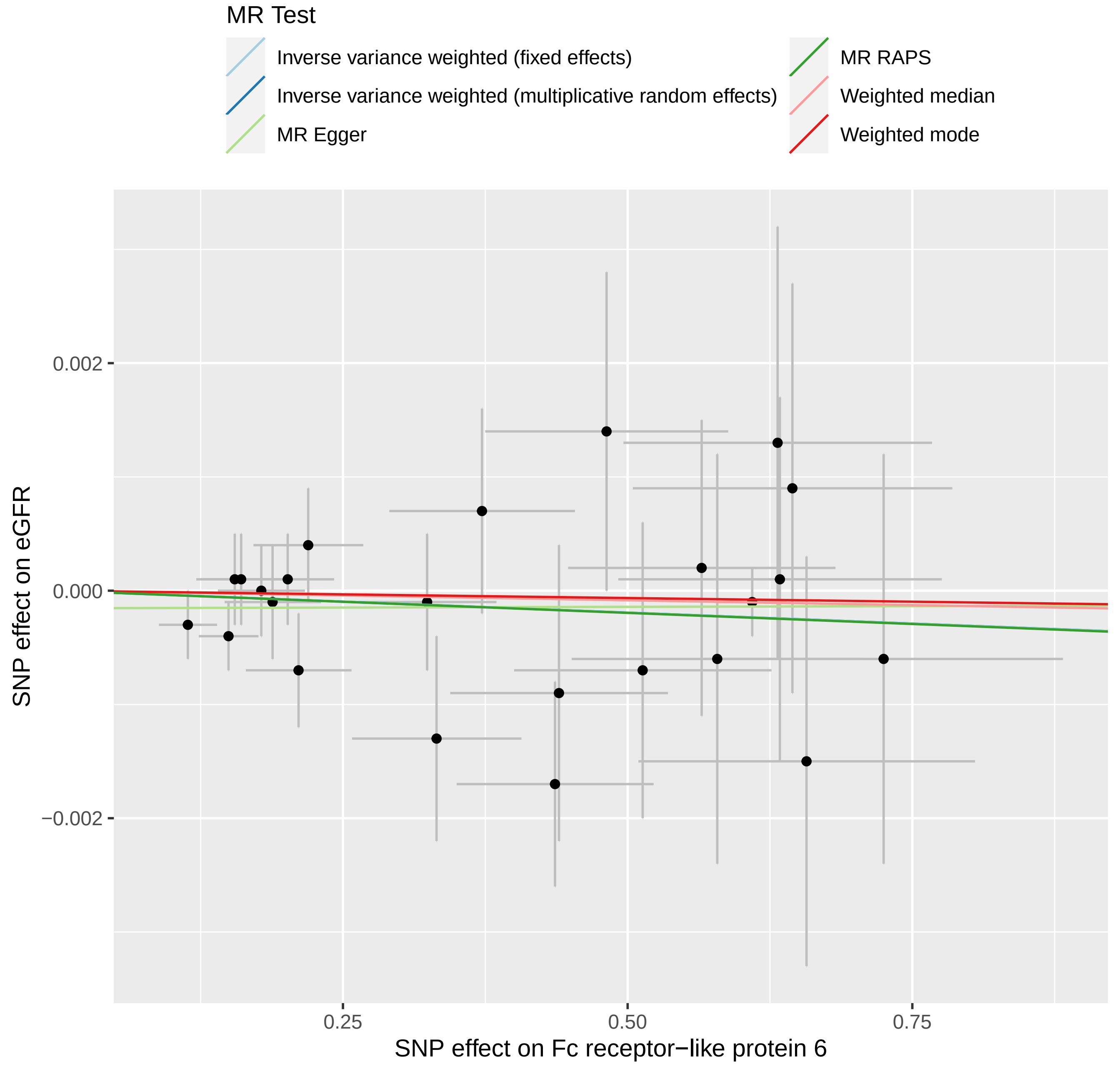

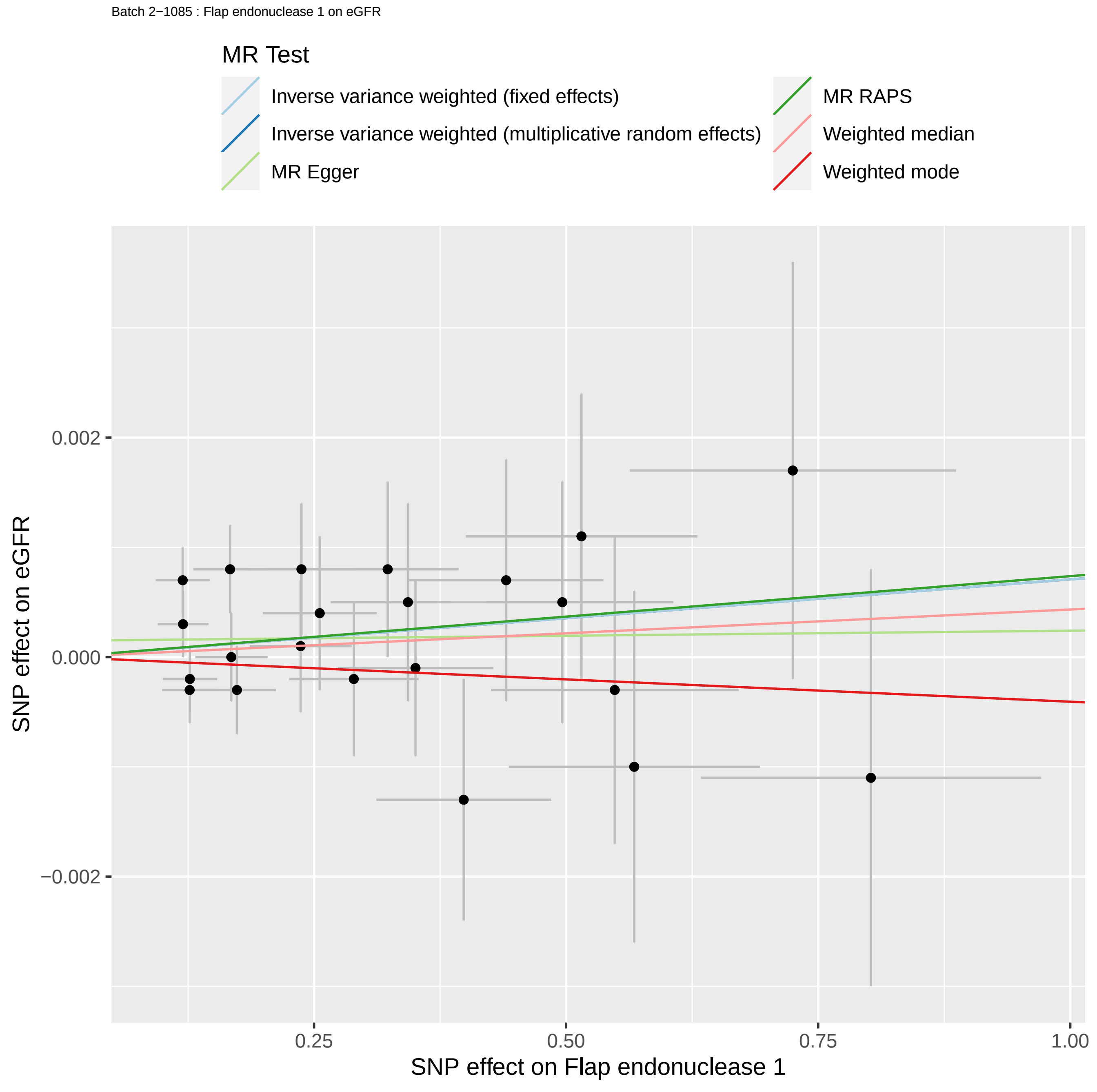

Batch 2−1085 : Flap endonuclease 1 on eGFR
MR Test
Inverse variance weighted (fixed effects)
Inverse variance weighted (multiplicative random effects)
MR Egger
MR RAPS
Weighted median
Weighted mode
0.002
0.000
−0.002
0.25
0.50
0.75
1.00
SNP effect on Flap endonuclease 1
SNP effect on eGFR

Batch 2−1086 : D−dimer on eGFR

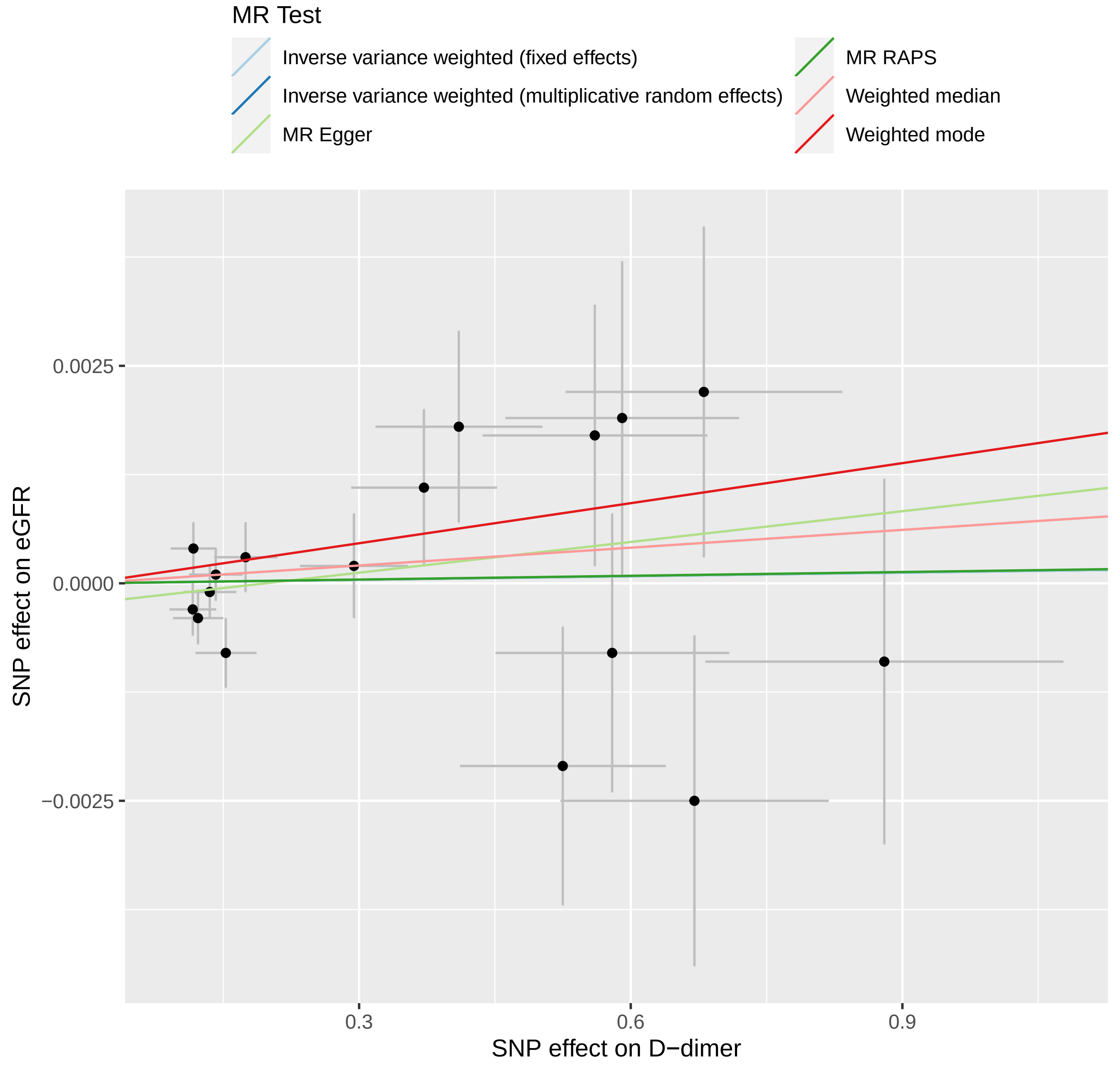

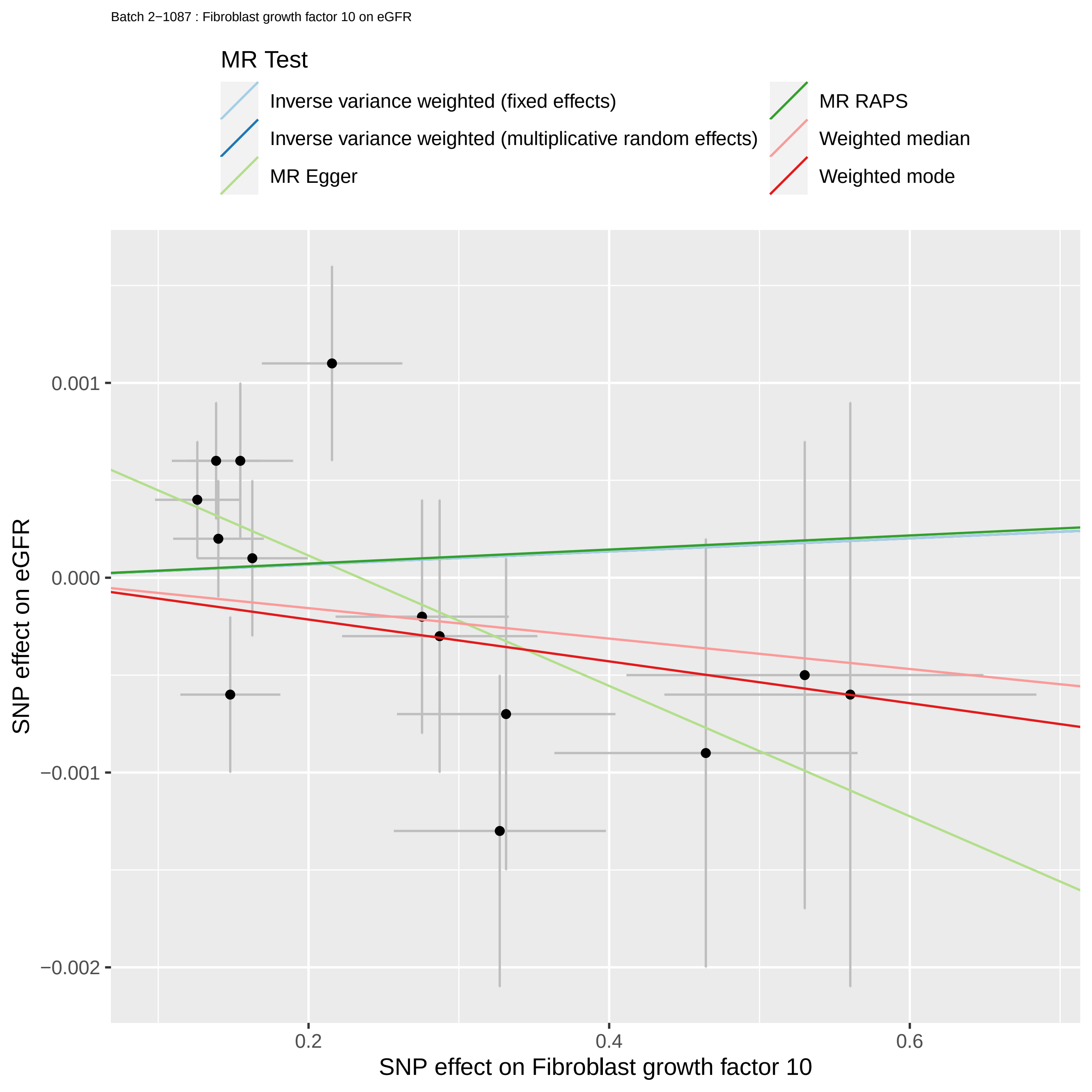
Batch 2−1087 : Fibroblast growth factor 10 on eGFR
MR Test
Inverse variance weighted (fixed effects)
Inverse variance weighted (multiplicative random effects)
MR Egger
MR RAPS
Weighted median
Weighted mode
0.001
0.000
−0.001
−0.002
0.2
0.4
0.6
SNP effect on eGFR
SNP effect on Fibroblast growth factor 10

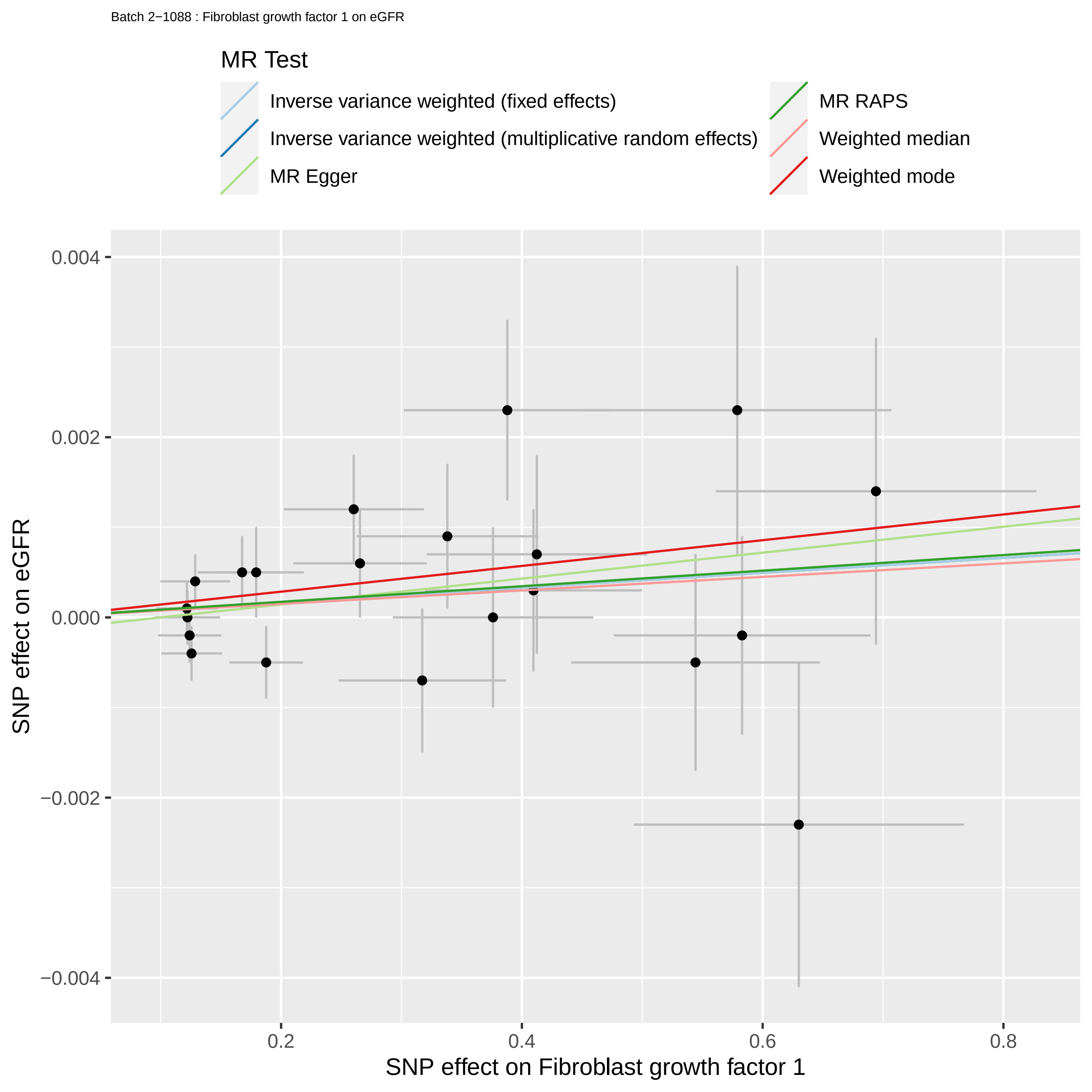

Batch 2−1088 : Fibroblast growth factor 1 on eGFR
MR Test
Inverse variance weighted (fixed effects)
Inverse variance weighted (multiplicative random effects)
MR Egger
MR RAPS
Weighted median
Weighted mode
0.004
0.002
0.000
−0.002
−0.004
SNP effect on eGFR
0.2
0.4
0.6
0.8
SNP effect on Fibroblast growth factor 1

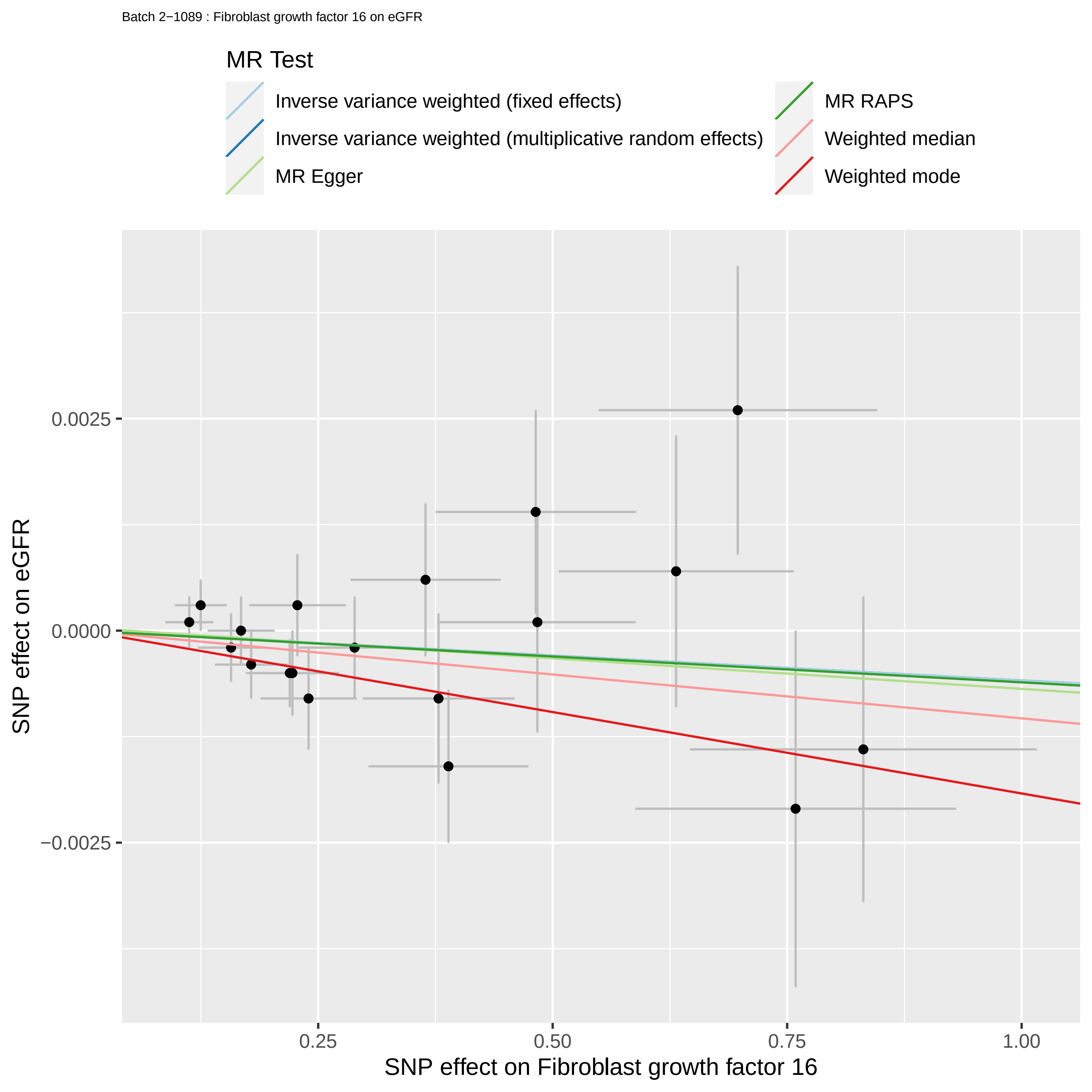

Batch 2−1089 : Fibroblast growth factor 16 on eGFR
MR Test
Inverse variance weighted (fixed effects)
Inverse variance weighted (multiplicative random effects)
MR Egger
MR RAPS
Weighted median
Weighted mode
0.0025
0.0000
−0.0025
0.25
0.50
0.75
1.00
SNP effect on eGFR
SNP effect on Fibroblast growth factor 16

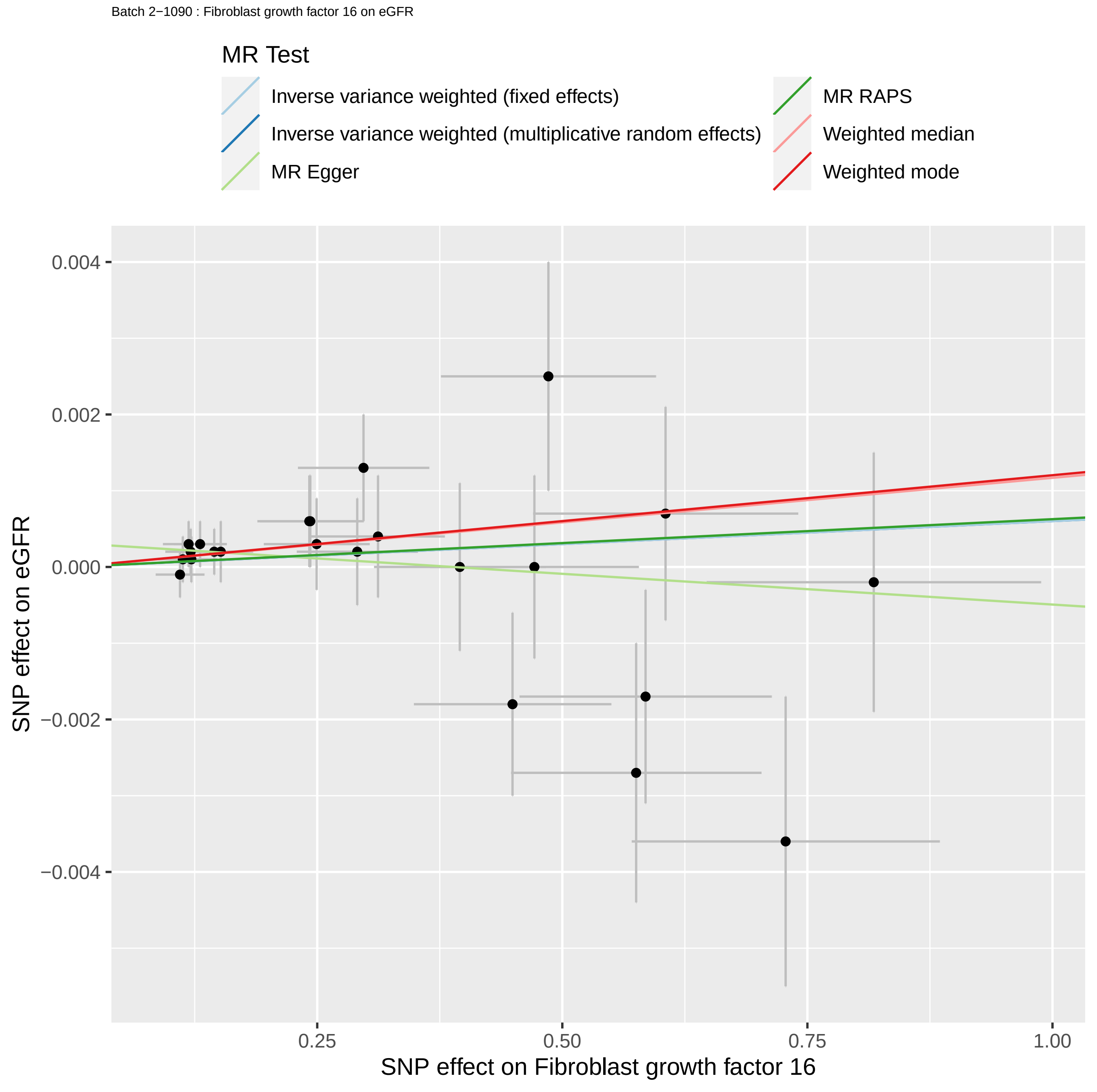

Batch 2−1090 : Fibroblast growth factor 16 on eGFR
MR Test
Inverse variance weighted (fixed effects)
Inverse variance weighted (multiplicative random effects)
MR Egger
MR RAPS
Weighted median
Weighted mode
0.004
0.002
0.000
−0.002
−0.004
0.25
0.50
0.75
1.00
SNP effect on eGFR
SNP effect on Fibroblast growth factor 16

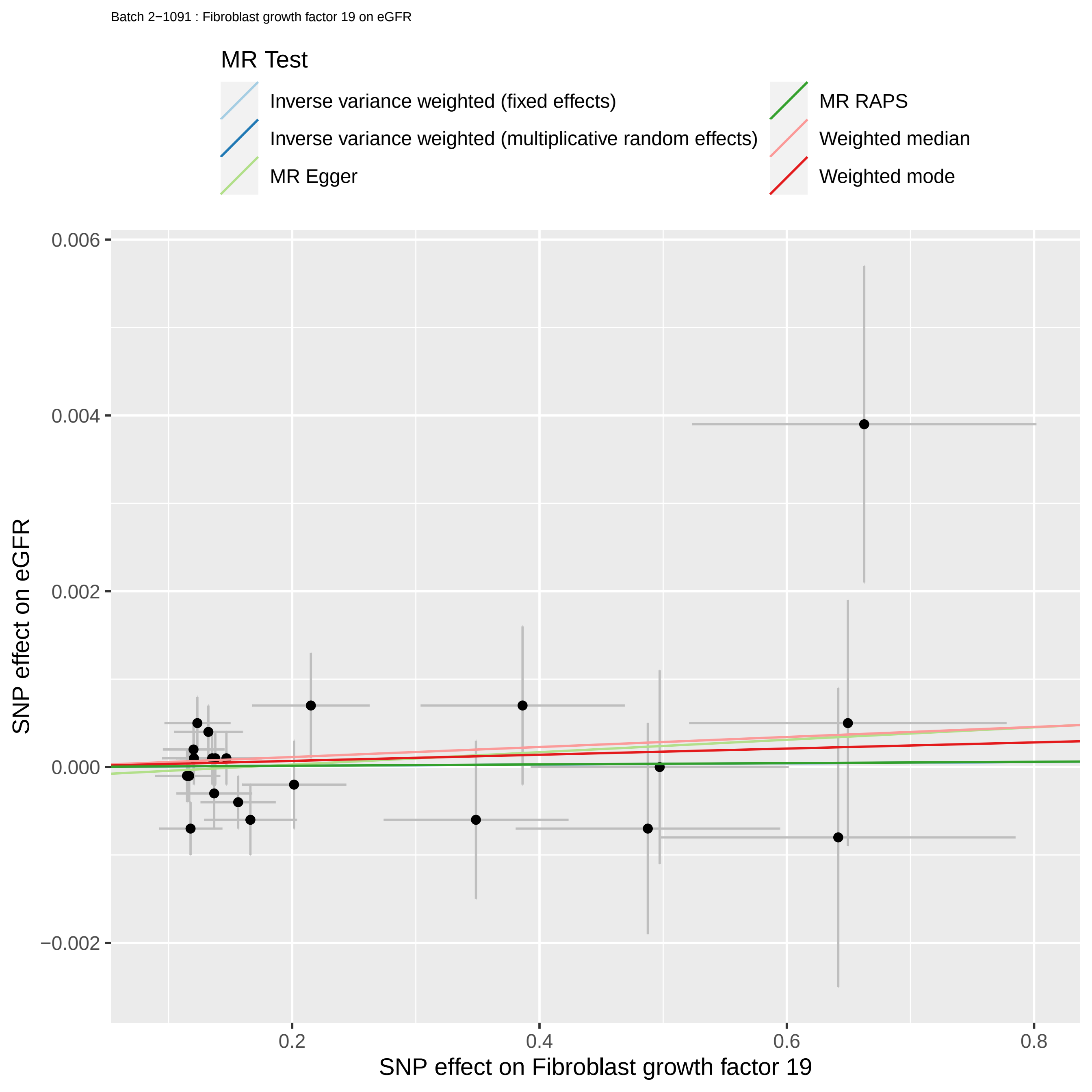

Batch 2−1091 : Fibroblast growth factor 19 on eGFR
MR Test
Inverse variance weighted (fixed effects)
Inverse variance weighted (multiplicative random effects)
MR Egger
MR RAPS
Weighted median
Weighted mode
0.006
0.004
0.002
0.000
−0.002
0.2
0.4
0.6
0.8
SNP effect on eGFR
SNP effect on Fibroblast growth factor 19

Batch 2−1092 : Fibroblast growth factor 20 on eGFR

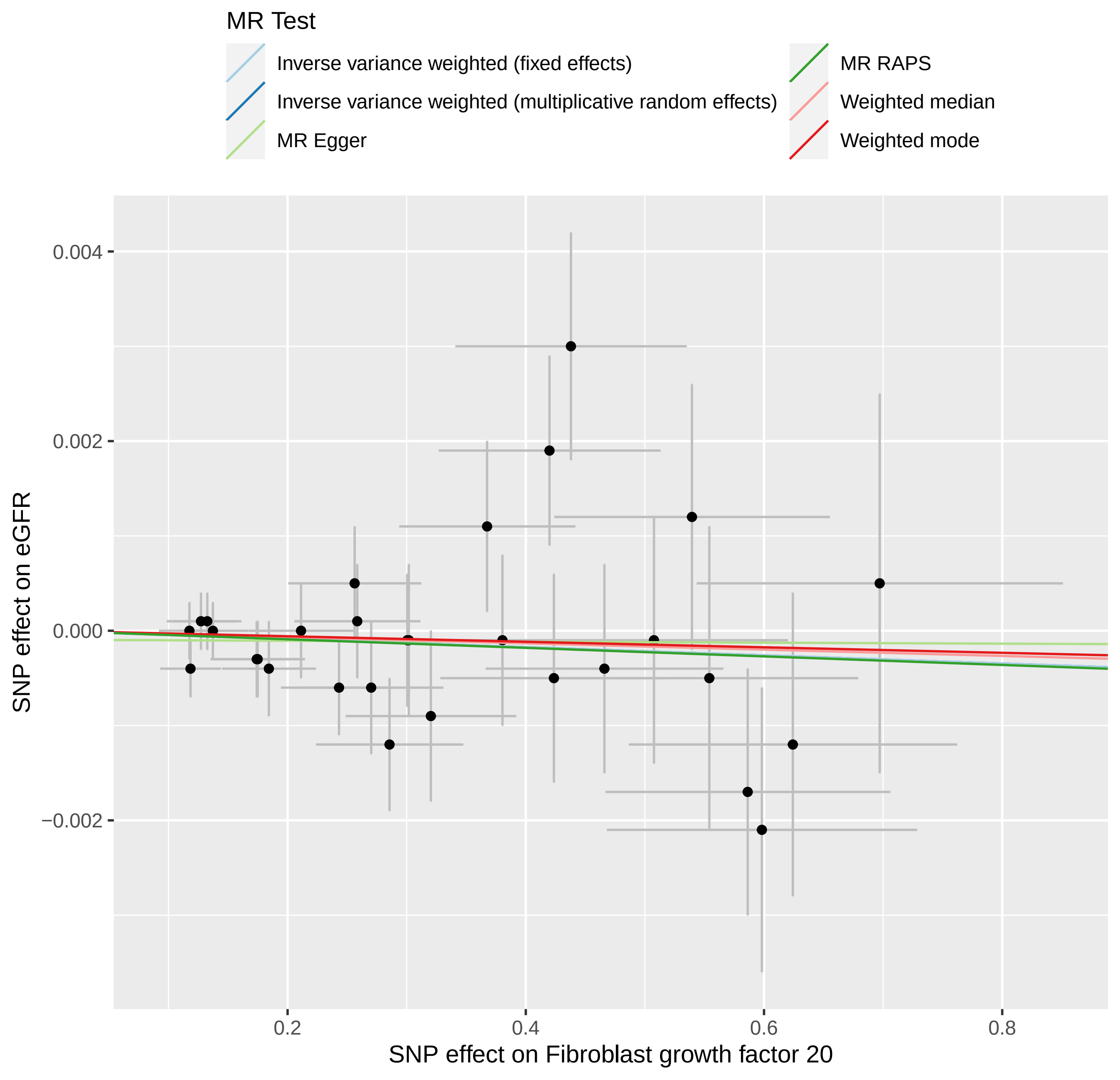

Batch 2−1093 : Fibroblast growth factor 23 on eGFR

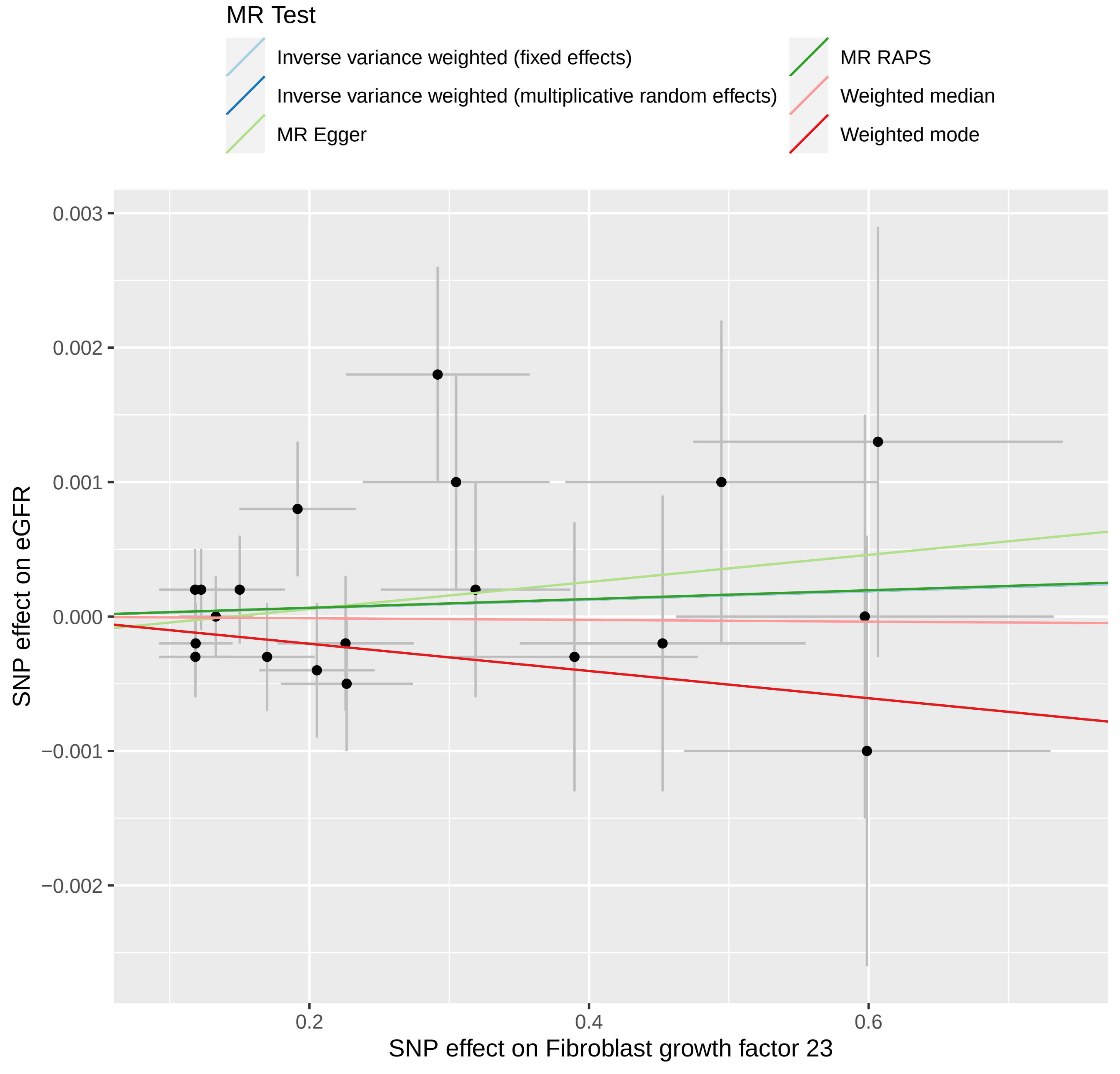

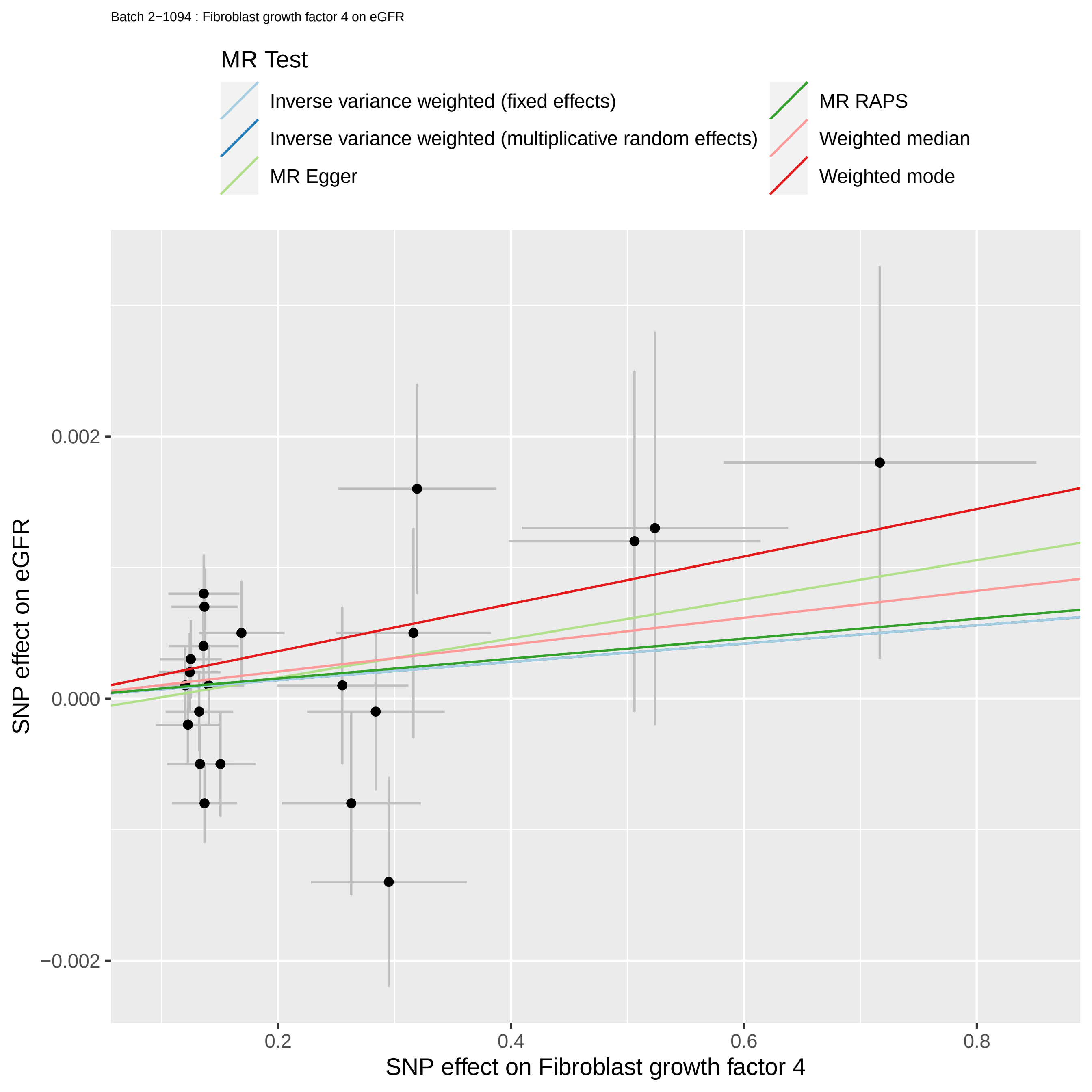

Batch 2−1094 : Fibroblast growth factor 4 on eGFR
MR Test
Inverse variance weighted (fixed effects)
Inverse variance weighted (multiplicative random effects)
MR Egger
MR RAPS
Weighted median
Weighted mode
0.002
0.000
−0.002
0.2
0.4
0.6
0.8
SNP effect on eGFR
SNP effect on Fibroblast growth factor 4

Batch 2−1095 : Fibroblast growth factor 5 on eGFR

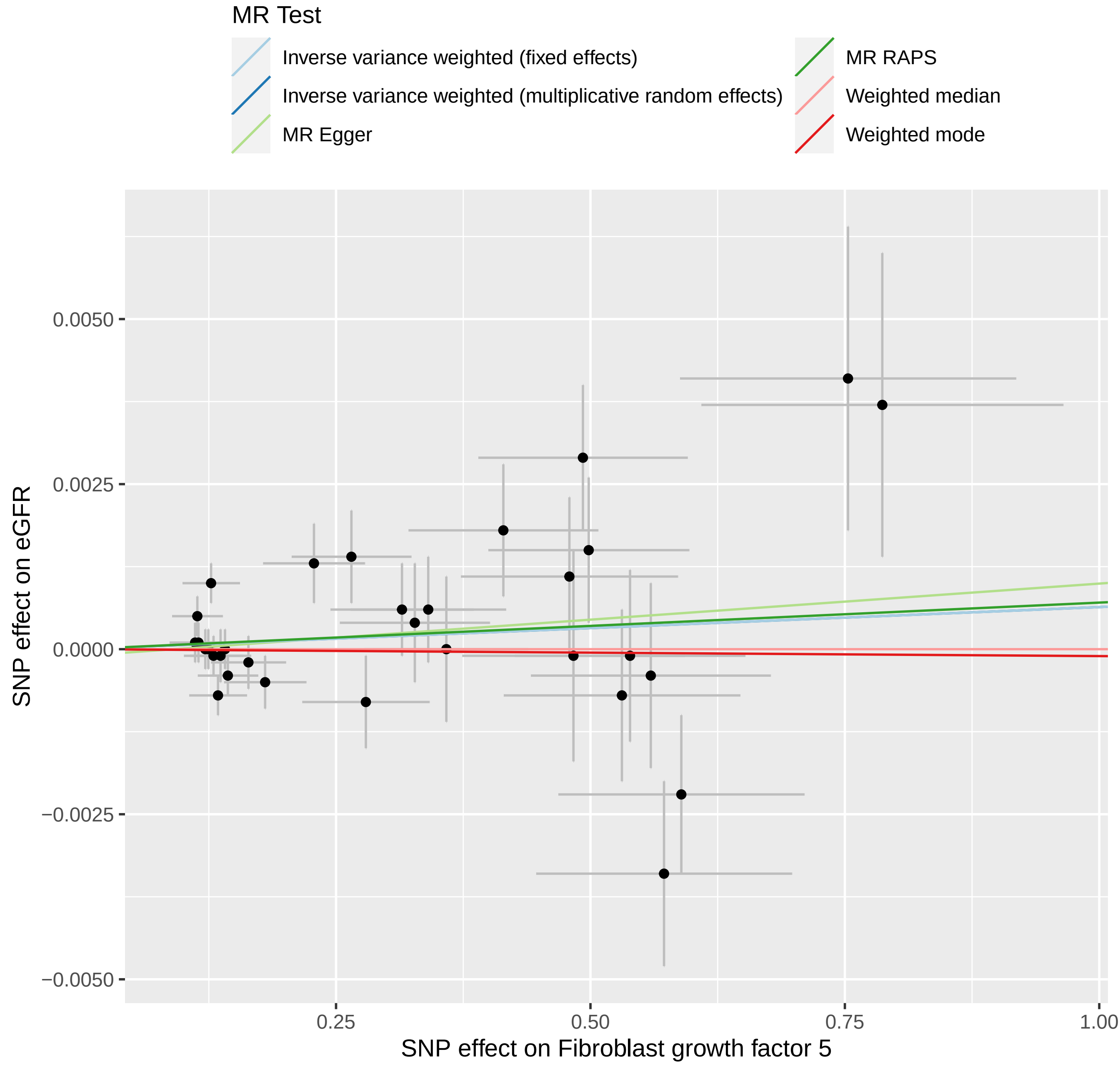

Batch 2−1096 : Fibroblast growth factor 6 on eGFR

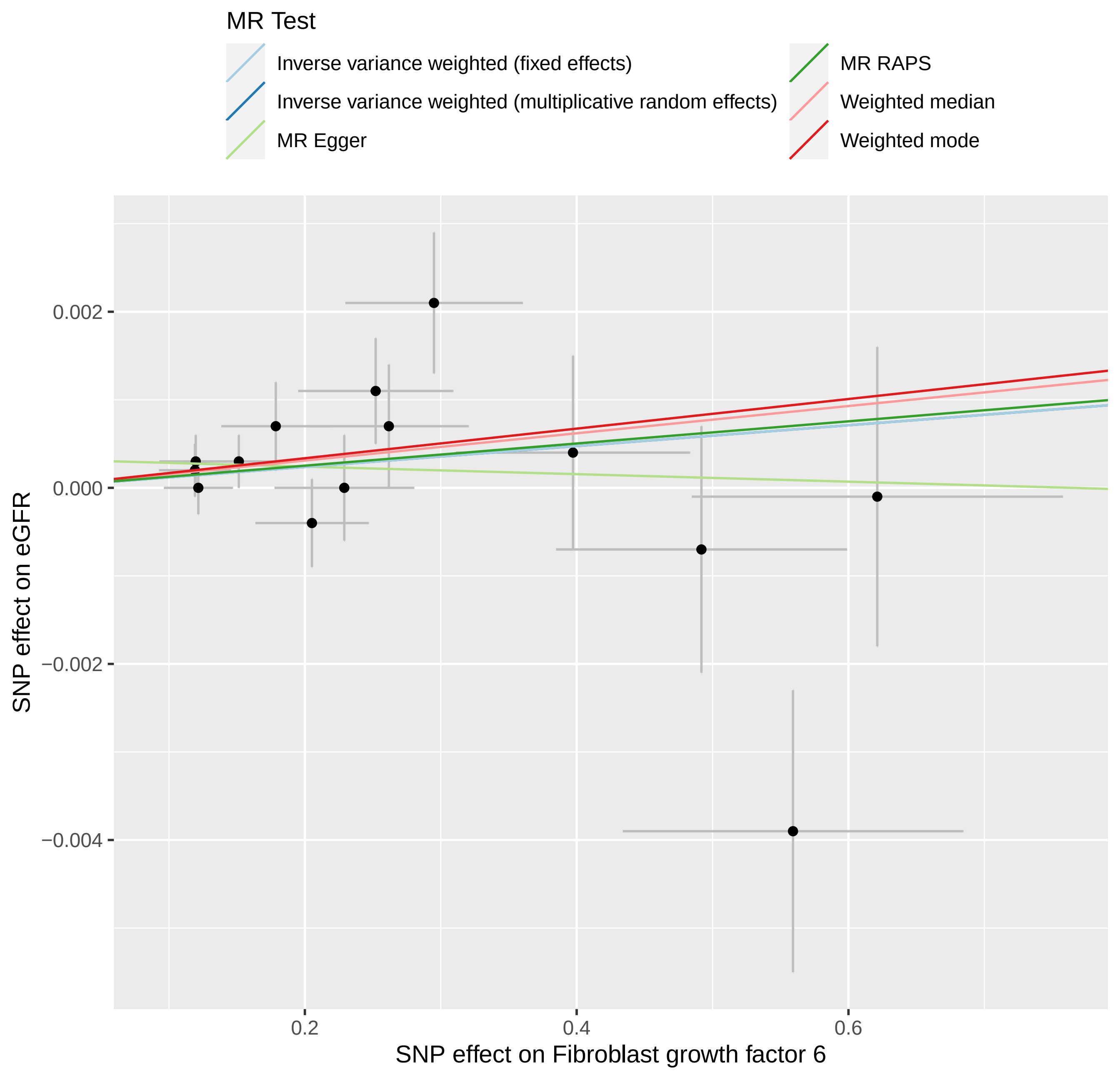

Batch 2−1097 : Fibroblast growth factor 7 on eGFR

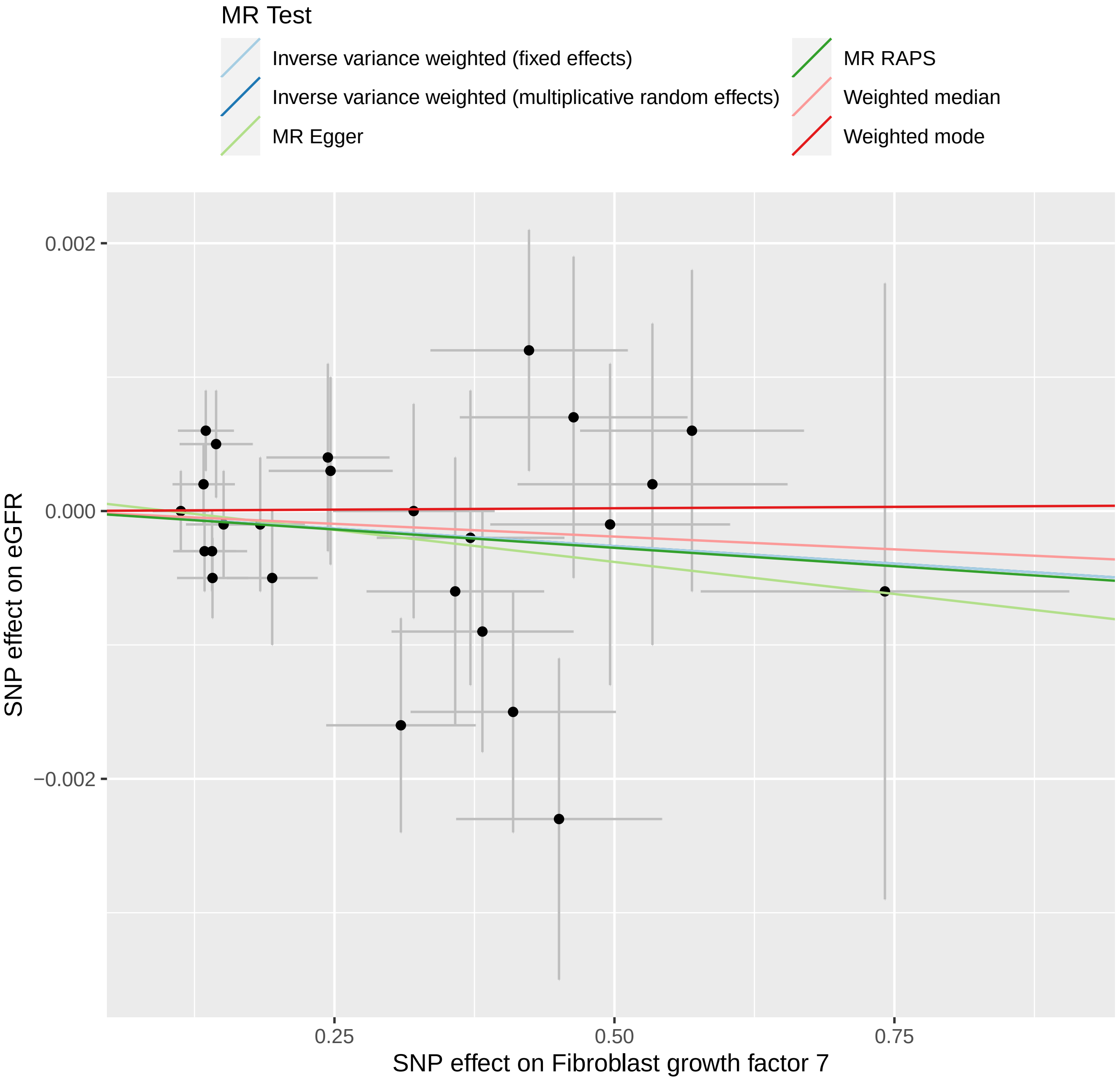

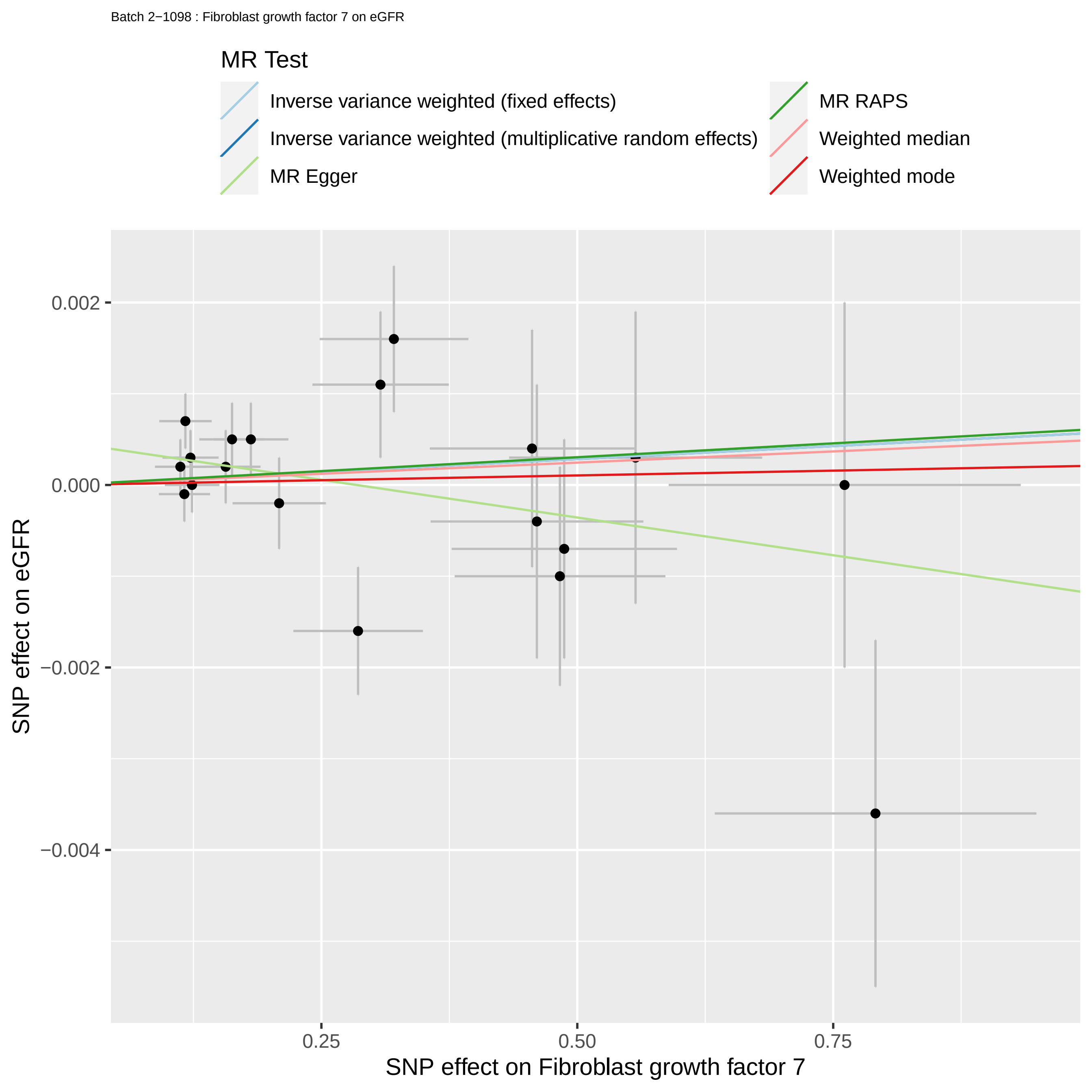
Batch 2−1098 : Fibroblast growth factor 7 on eGFR
MR Test
Inverse variance weighted (fixed effects)
Inverse variance weighted (multiplicative random effects)
MR Egger
MR RAPS
Weighted median
Weighted mode
0.002
0.000
−0.002
−0.004
0.25
0.50
0.75
SNP effect on eGFR
SNP effect on Fibroblast growth factor 7

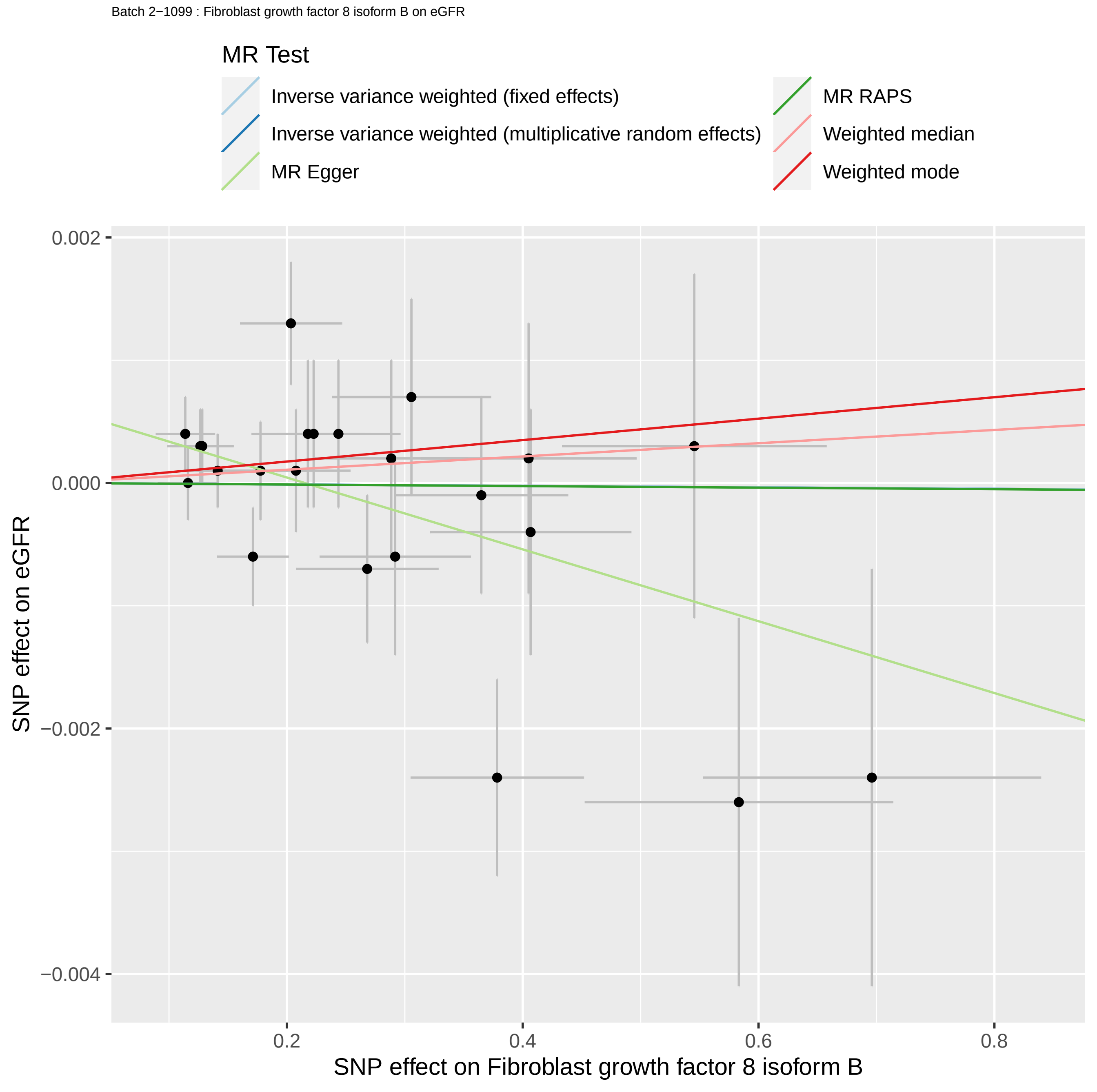

Batch 2−1099 : Fibroblast growth factor 8 isoform B on eGFR
MR Test
Inverse variance weighted (fixed effects)
Inverse variance weighted (multiplicative random effects)
MR Egger
MR RAPS
Weighted median
Weighted mode
0.002
0.000
−0.002
−0.004
SNP effect on eGFR
0.2
0.4
0.6
0.8
SNP effect on Fibroblast growth factor 8 isoform B

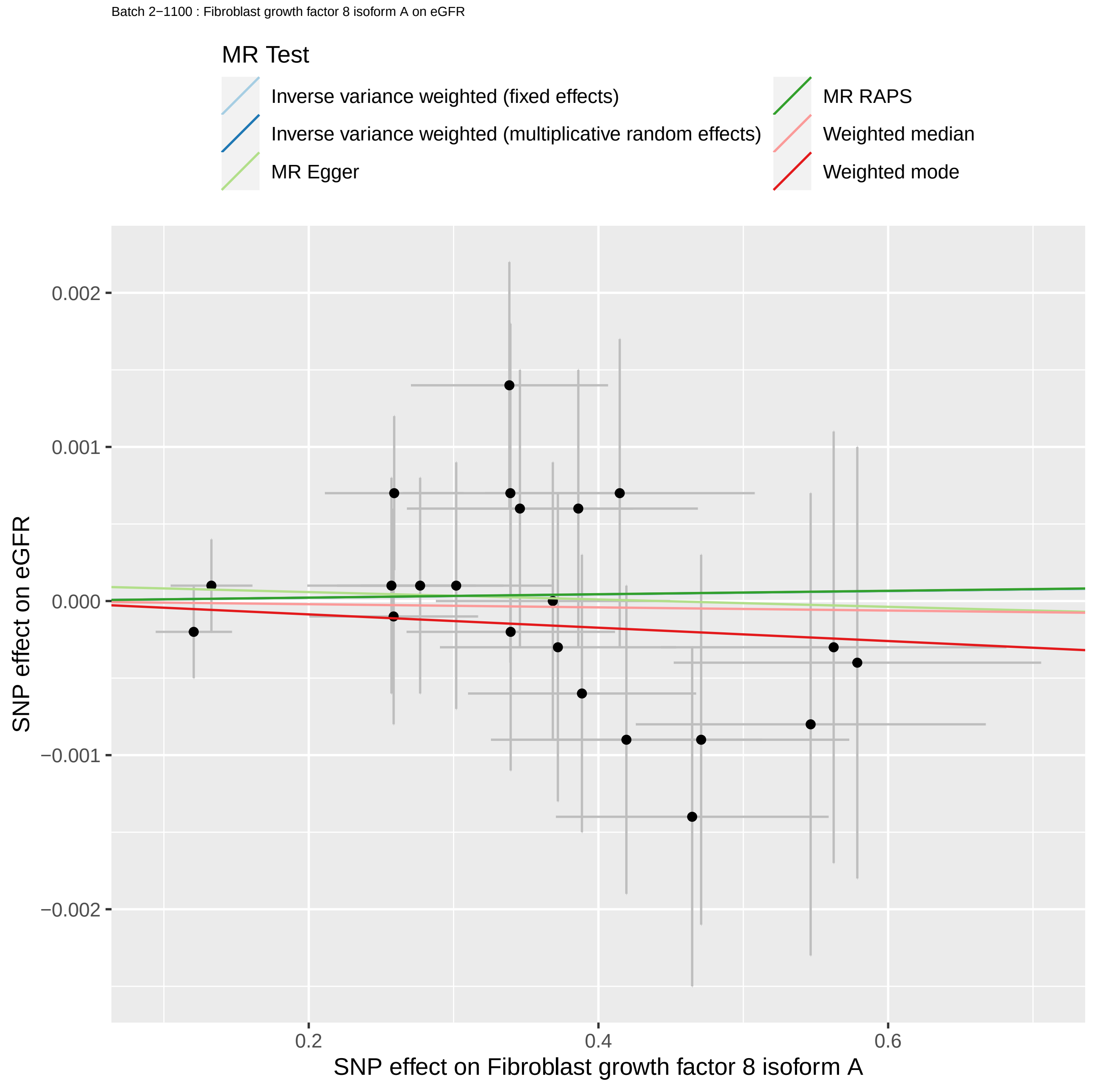
Batch 2−1100 : Fibroblast growth factor 8 isoform A on eGFR
MR Test
Inverse variance weighted (fixed effects)
Inverse variance weighted (multiplicative random effects)
MR Egger
MR RAPS
Weighted median
Weighted mode
0.002
0.001
0.000
−0.001
−0.002
0.2
0.4
0.6
SNP effect on eGFR
SNP effect on Fibroblast growth factor 8 isoform A

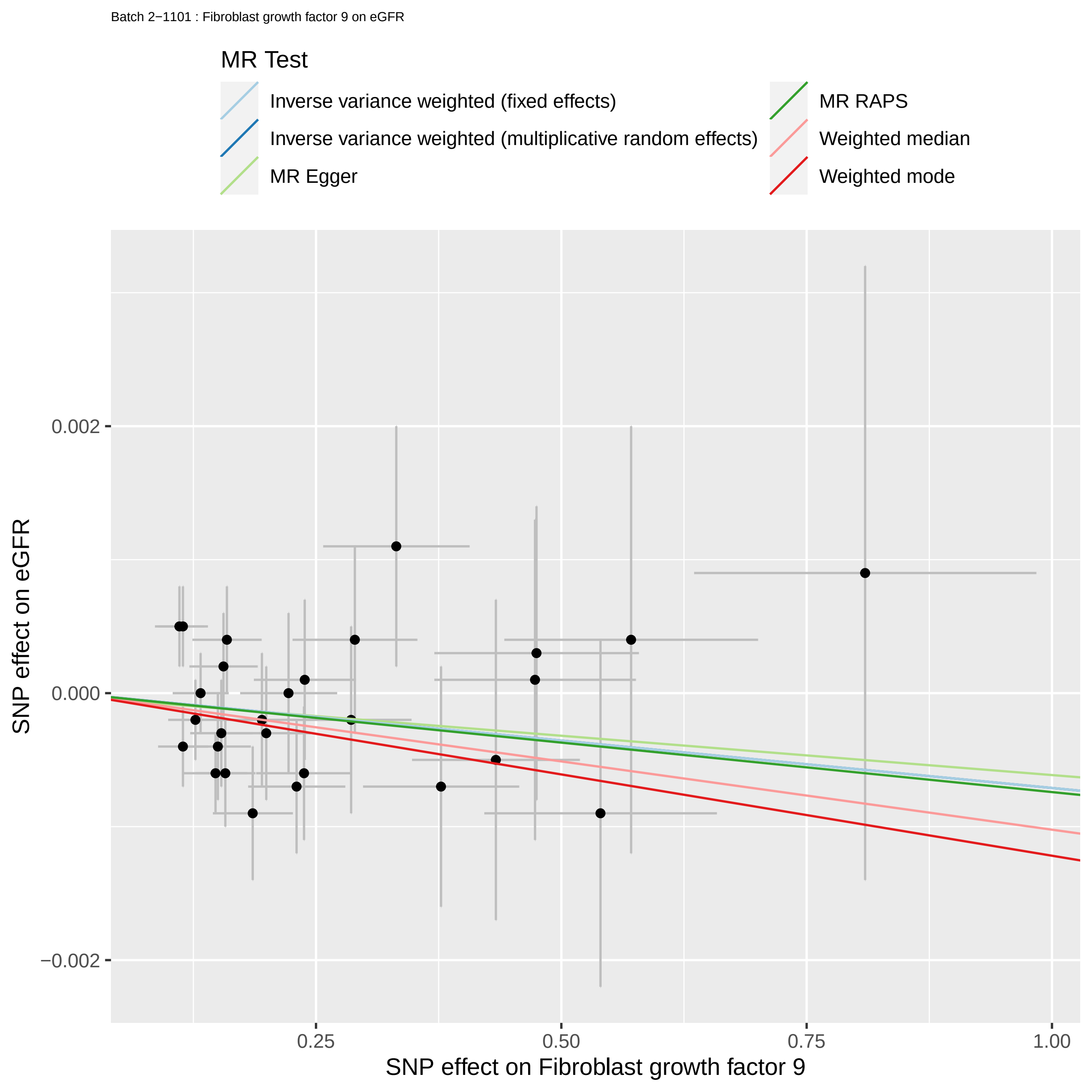
Batch 2−1101 : Fibroblast growth factor 9 on eGFR
MR Test
Inverse variance weighted (fixed effects)
Inverse variance weighted (multiplicative random effects)
MR Egger
MR RAPS
Weighted median
Weighted mode
0.002
0.000
−0.002
0.25
0.50
0.75
1.00
SNP effect on eGFR
SNP effect on Fibroblast growth factor 9

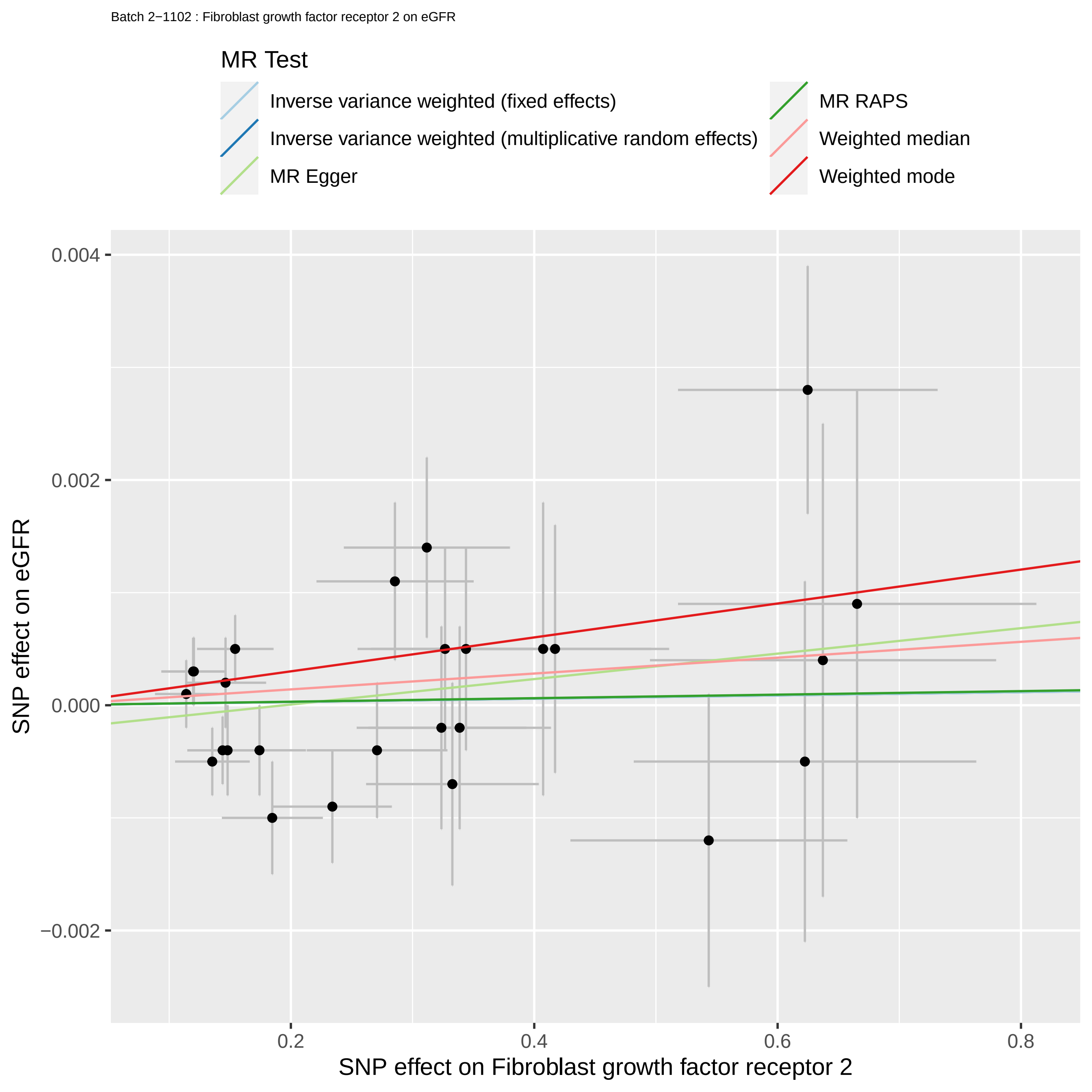

Batch 2−1102 : Fibroblast growth factor receptor 2 on eGFR
MR Test
Inverse variance weighted (fixed effects)
Inverse variance weighted (multiplicative random effects)
MR Egger
MR RAPS
Weighted median
Weighted mode
0.004
0.002
0.000
−0.002
0.2
0.4
0.6
0.8
SNP effect on eGFR
SNP effect on Fibroblast growth factor receptor 2

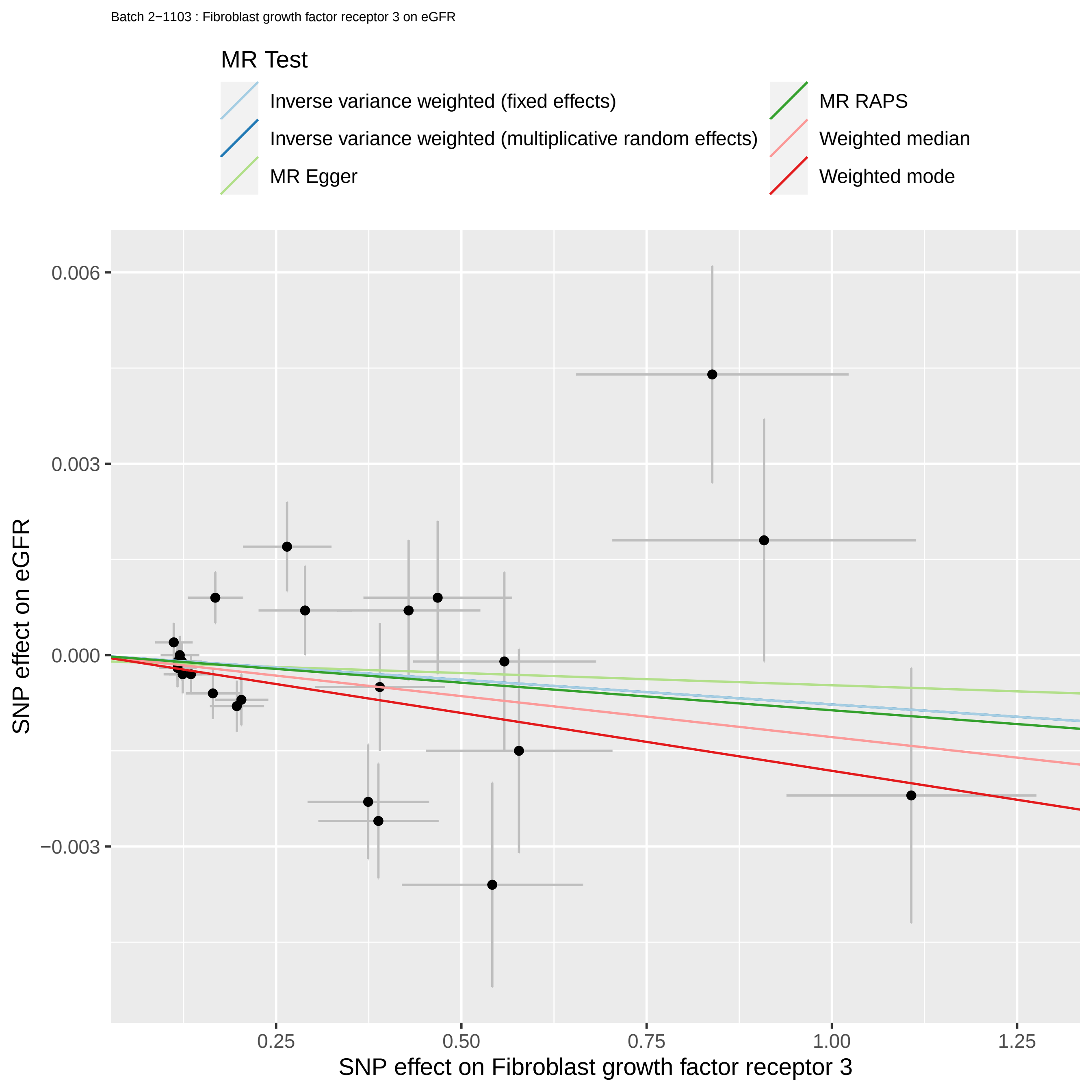

Batch 2−1103 : Fibroblast growth factor receptor 3 on eGFR
MR Test
Inverse variance weighted (fixed effects)
Inverse variance weighted (multiplicative random effects)
MR Egger
MR RAPS
Weighted median
Weighted mode
0.006
0.003
0.000
−0.003
SNP effect on eGFR
0.25
0.50
0.75
1.00
1.25
SNP effect on Fibroblast growth factor receptor 3

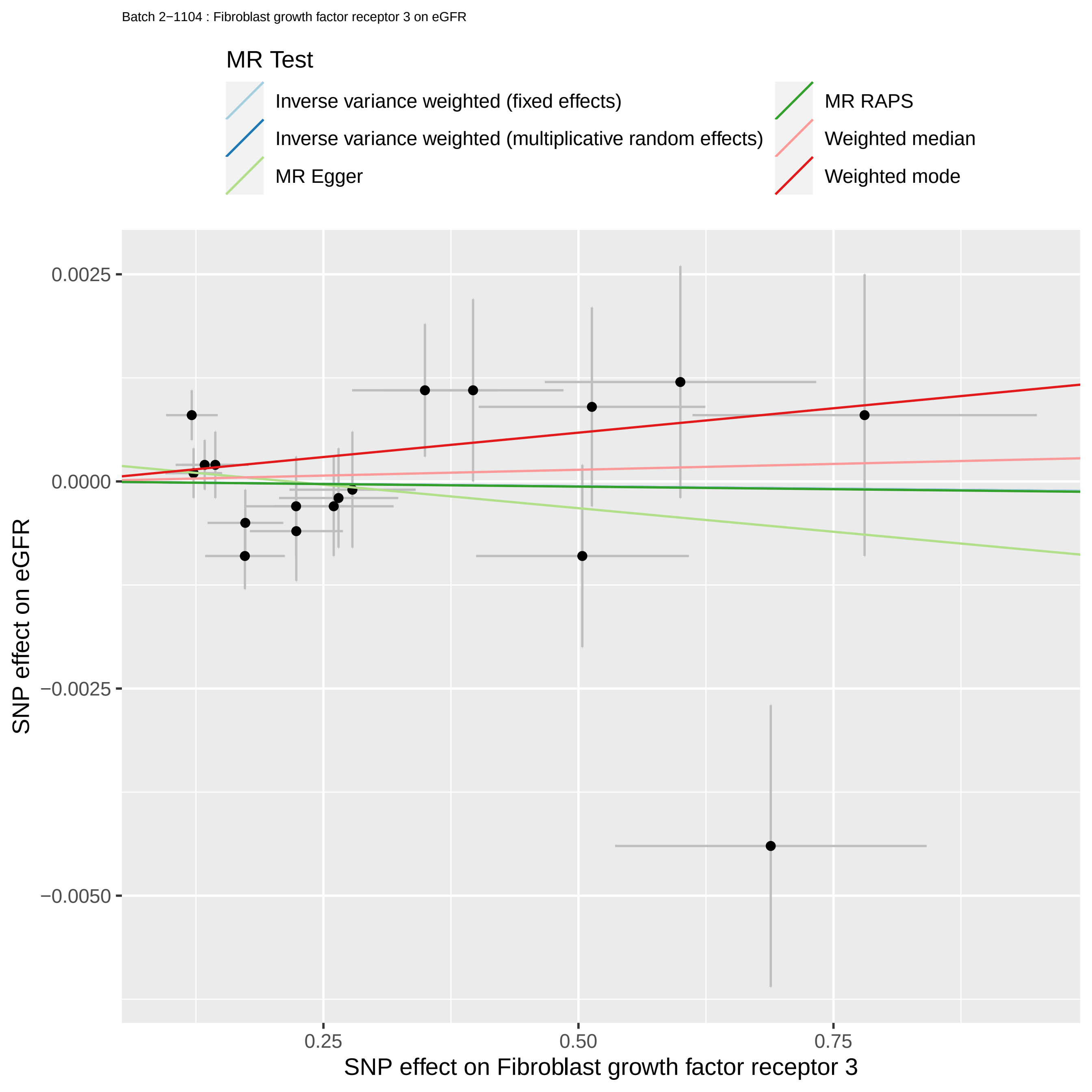
Batch 2−1104 : Fibroblast growth factor receptor 3 on eGFR
MR Test
Inverse variance weighted (fixed effects)
Inverse variance weighted (multiplicative random effects)
MR Egger
MR RAPS
Weighted median
Weighted mode
0.0025
0.0000
−0.0025
−0.0050
0.25
0.50
0.75
SNP effect on eGFR
SNP effect on Fibroblast growth factor receptor 3

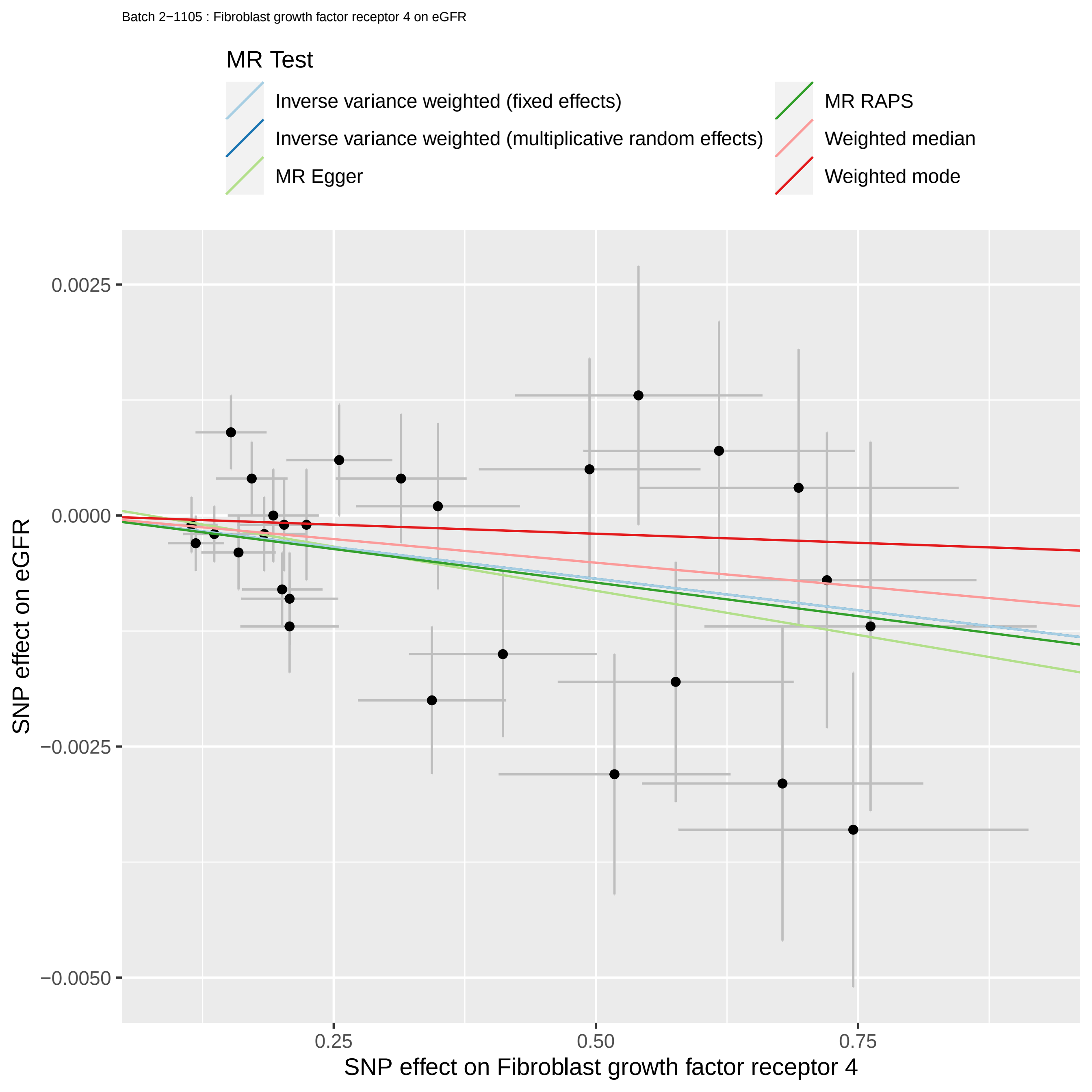
Batch 2−1105 : Fibroblast growth factor receptor 4 on eGFR
MR Test
Inverse variance weighted (fixed effects)
Inverse variance weighted (multiplicative random effects)
MR Egger
MR RAPS
Weighted median
Weighted mode
0.0025
0.0000
−0.0025
−0.0050
0.25
0.50
0.75
SNP effect on eGFR
SNP effect on Fibroblast growth factor receptor 4

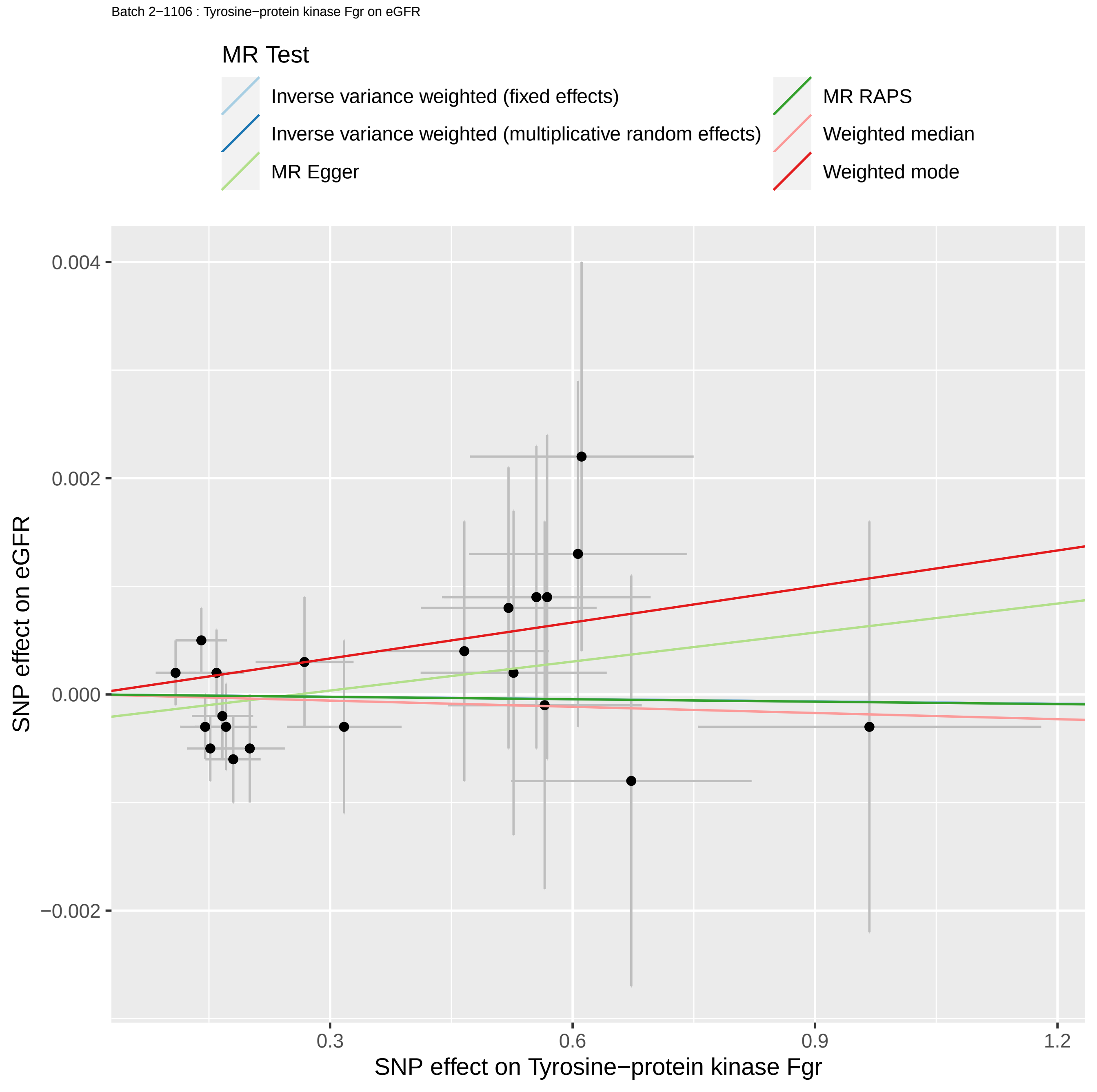
Batch 2−1106 : Tyrosine−protein kinase Fgr on eGFR
MR Test
Inverse variance weighted (fixed effects)
Inverse variance weighted (multiplicative random effects)
MR Egger
MR RAPS
Weighted median
Weighted mode
0.004
0.002
0.000
−0.002
SNP effect on eGFR
0.3
0.6
0.9
1.2
SNP effect on Tyrosine−protein kinase Fgr

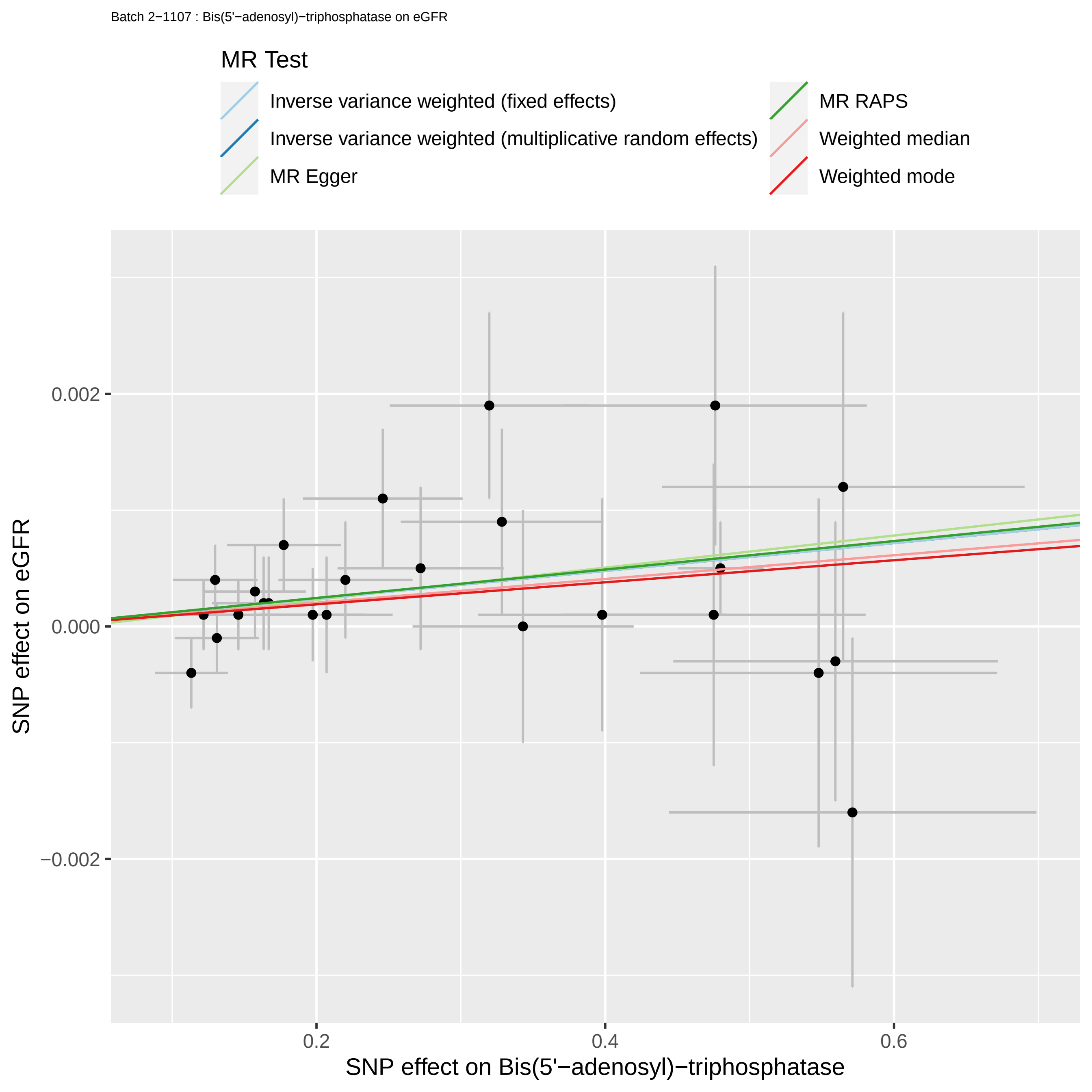
Batch 2−1107 : Bis(5'−adenosyl)−triphosphatase on eGFR
MR Test
Inverse variance weighted (fixed effects)
Inverse variance weighted (multiplicative random effects)
MR Egger
MR RAPS
Weighted median
Weighted mode
0.002
0.000
−0.002
0.2
0.4
0.6
SNP effect on eGFR
SNP effect on Bis(5'−adenosyl)−triphosphatase

Batch 2−1108 : Fibrinogen C domain−containing protein 1 on eGFR

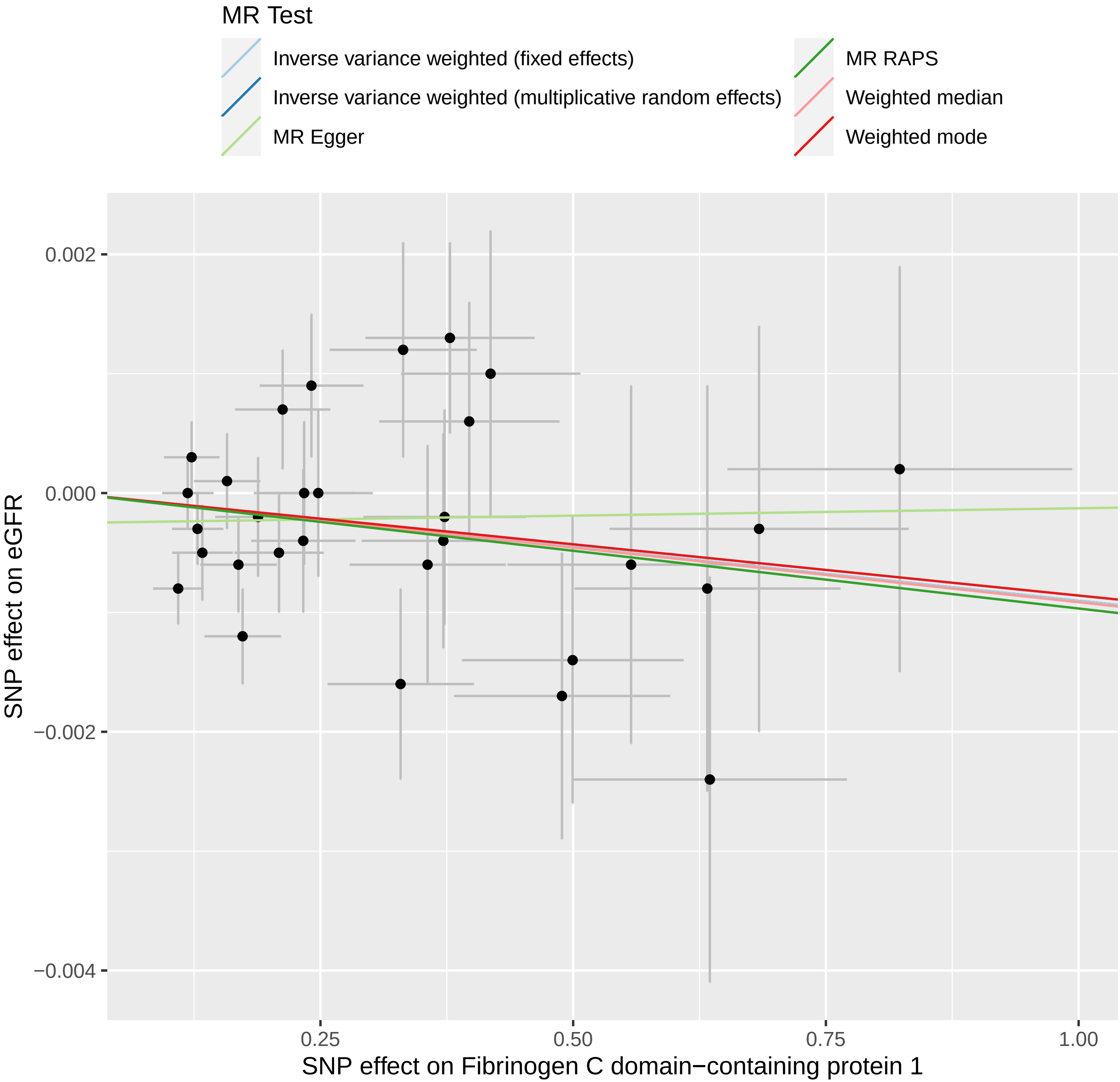

Batch 2−1109 : Polyphosphoinositide phosphatase on eGFR

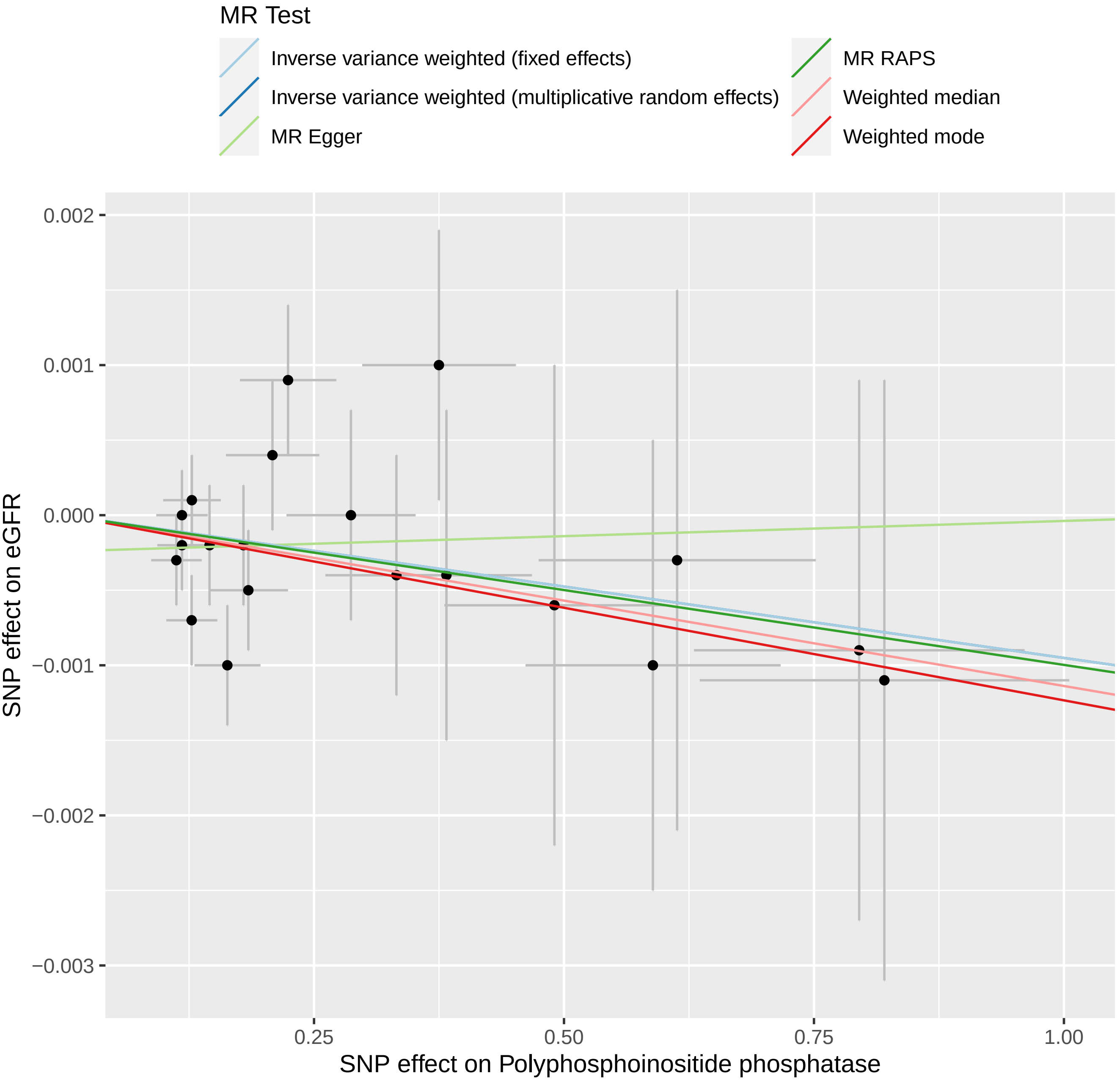

Batch 2−1110 : Vascular endothelial growth factor D on eGFR

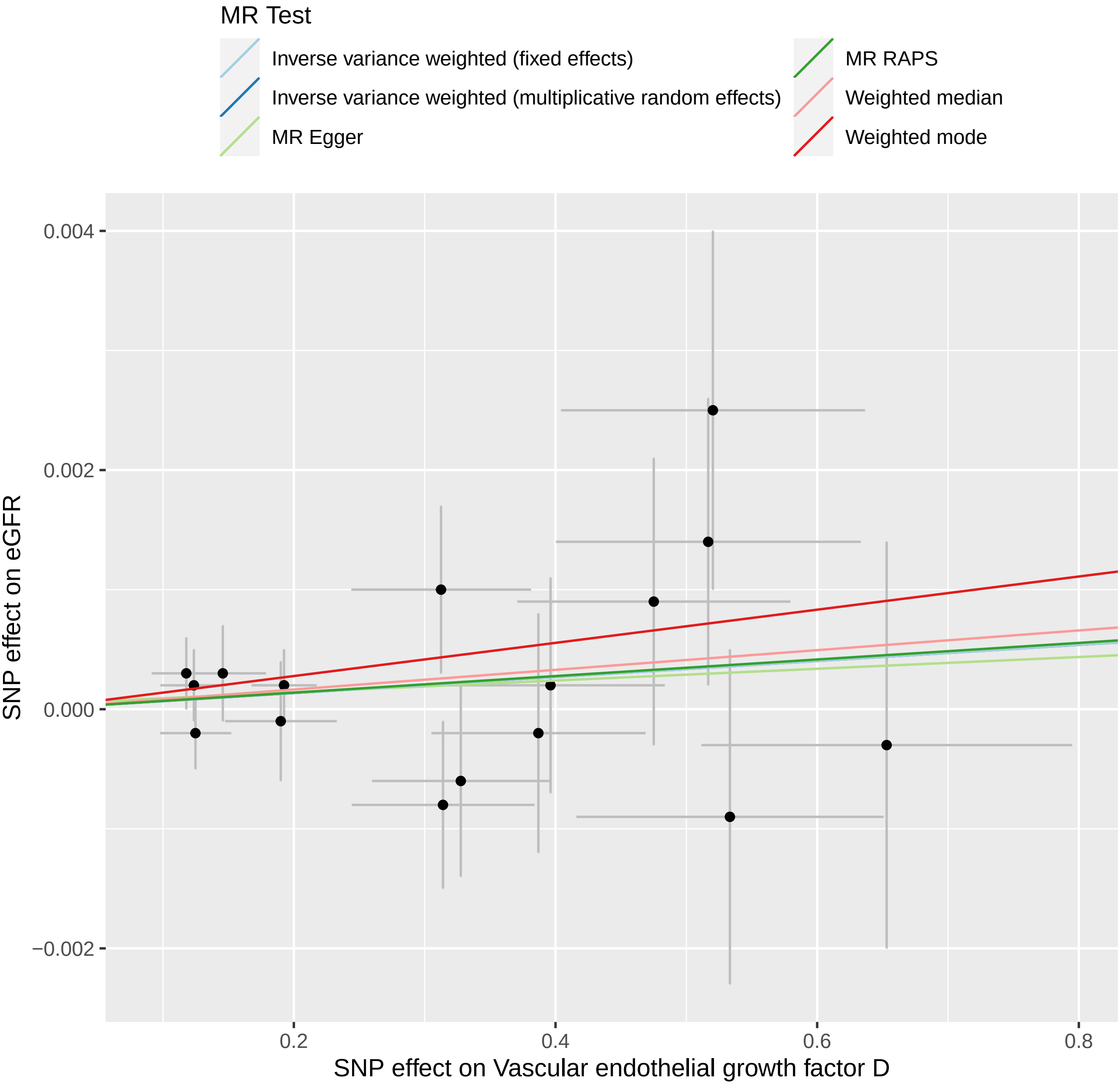

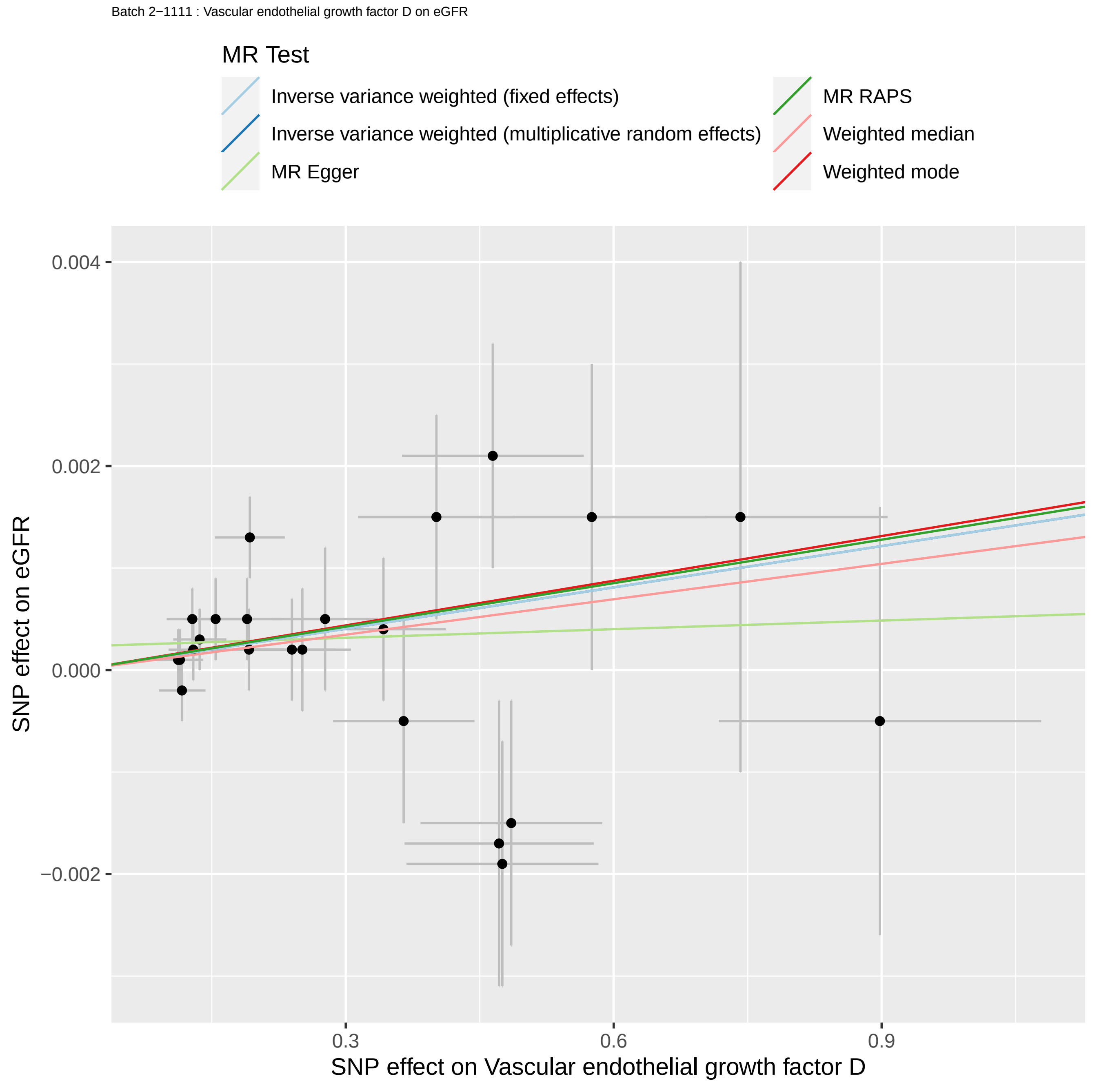

Batch 2−1111 : Vascular endothelial growth factor D on eGFR
MR Test
Inverse variance weighted (fixed effects)
Inverse variance weighted (multiplicative random effects)
MR Egger
MR RAPS
Weighted median
Weighted mode
0.004
0.002
0.000
−0.002
SNP effect on eGFR
0.3
0.6
0.9
SNP effect on Vascular endothelial growth factor D

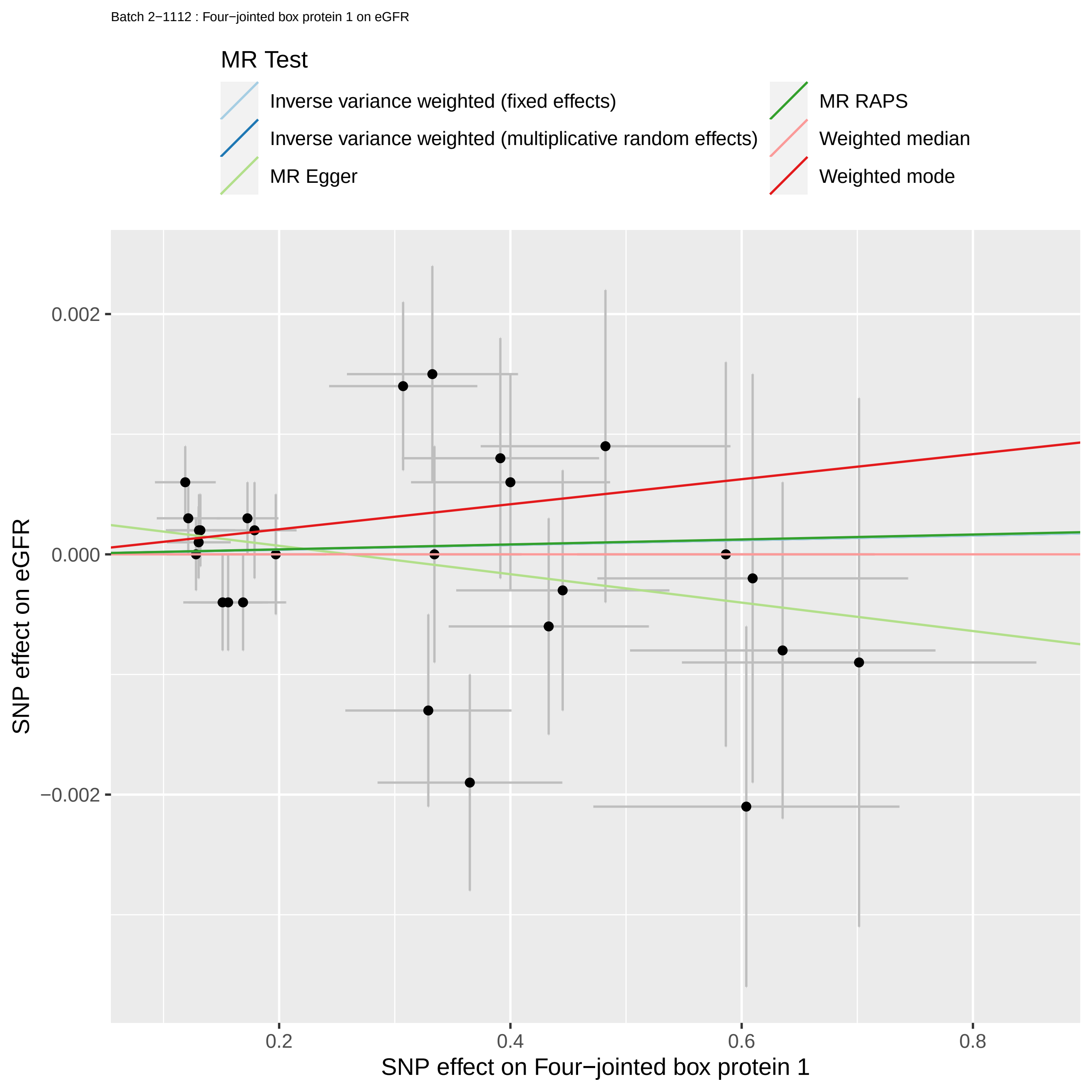

Batch 2−1112 : Four−jointed box protein 1 on eGFR
MR Test
Inverse variance weighted (fixed effects)
Inverse variance weighted (multiplicative random effects)
MR Egger
MR RAPS
Weighted median
Weighted mode
0.002
0.000
−0.002
0.2
0.4
0.6
0.8
SNP effect on eGFR
SNP effect on Four−jointed box protein 1

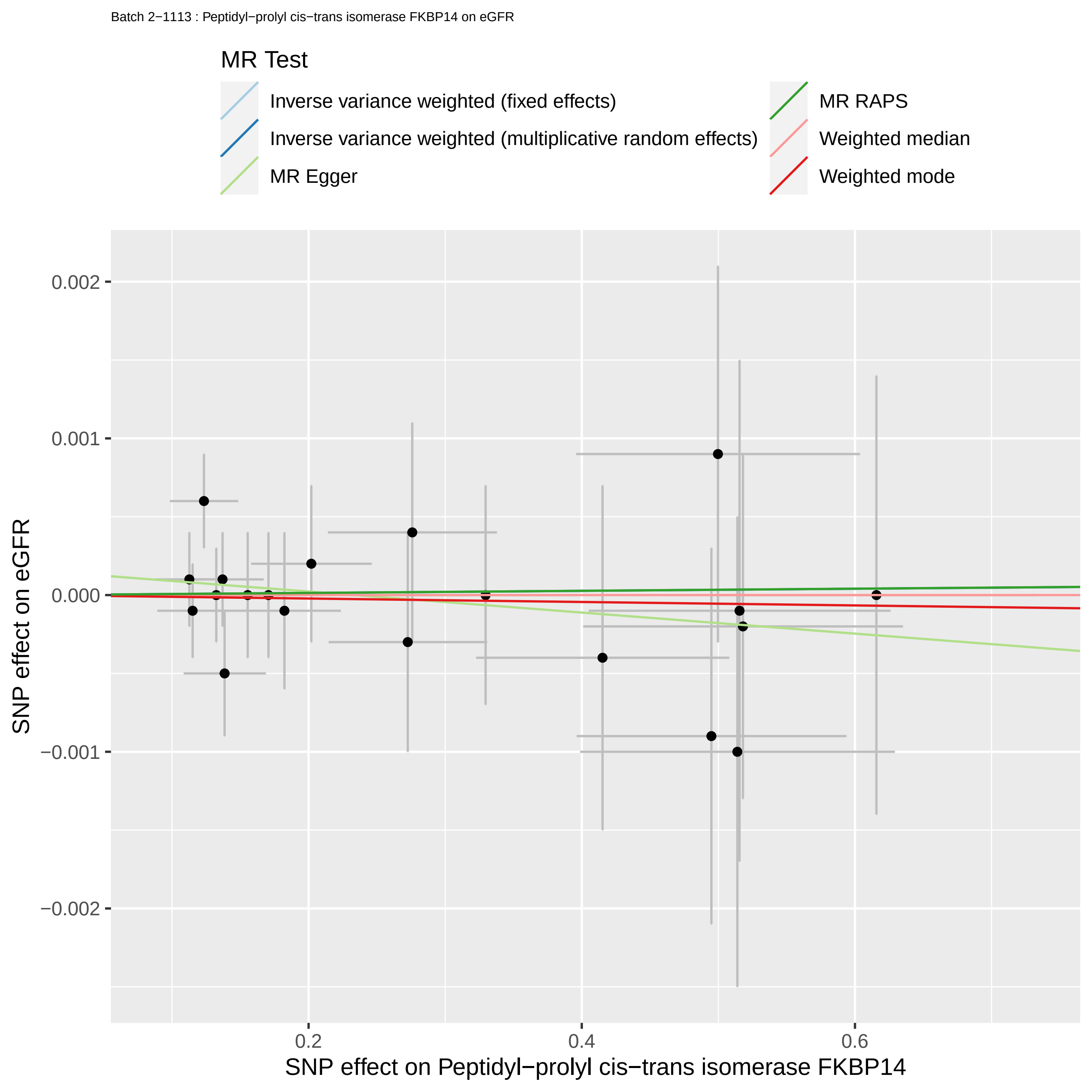

Batch 2−1113 : Peptidyl−prolyl cis−trans isomerase FKBP14 on eGFR
MR Test
Inverse variance weighted (fixed effects)
Inverse variance weighted (multiplicative random effects)
MR Egger
MR RAPS
Weighted median
Weighted mode
0.002
0.001
0.000
−0.001
−0.002
0.2
0.4
0.6
SNP effect on eGFR
SNP effect on Peptidyl−prolyl cis−trans isomerase FKBP14

Batch 2−1114 : Peptidyl−prolyl cis−trans isomerase FKBP2 on eGFR

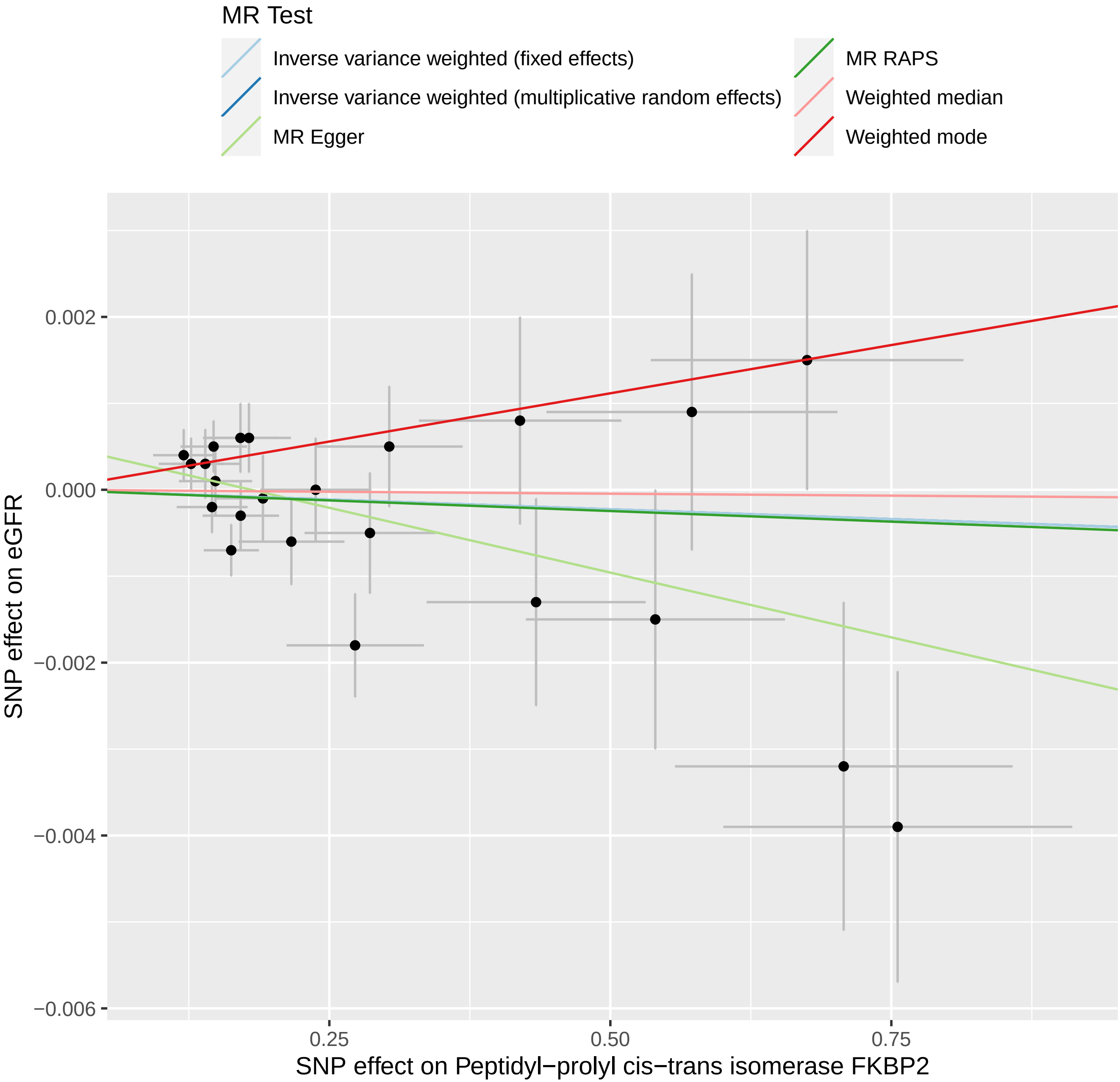

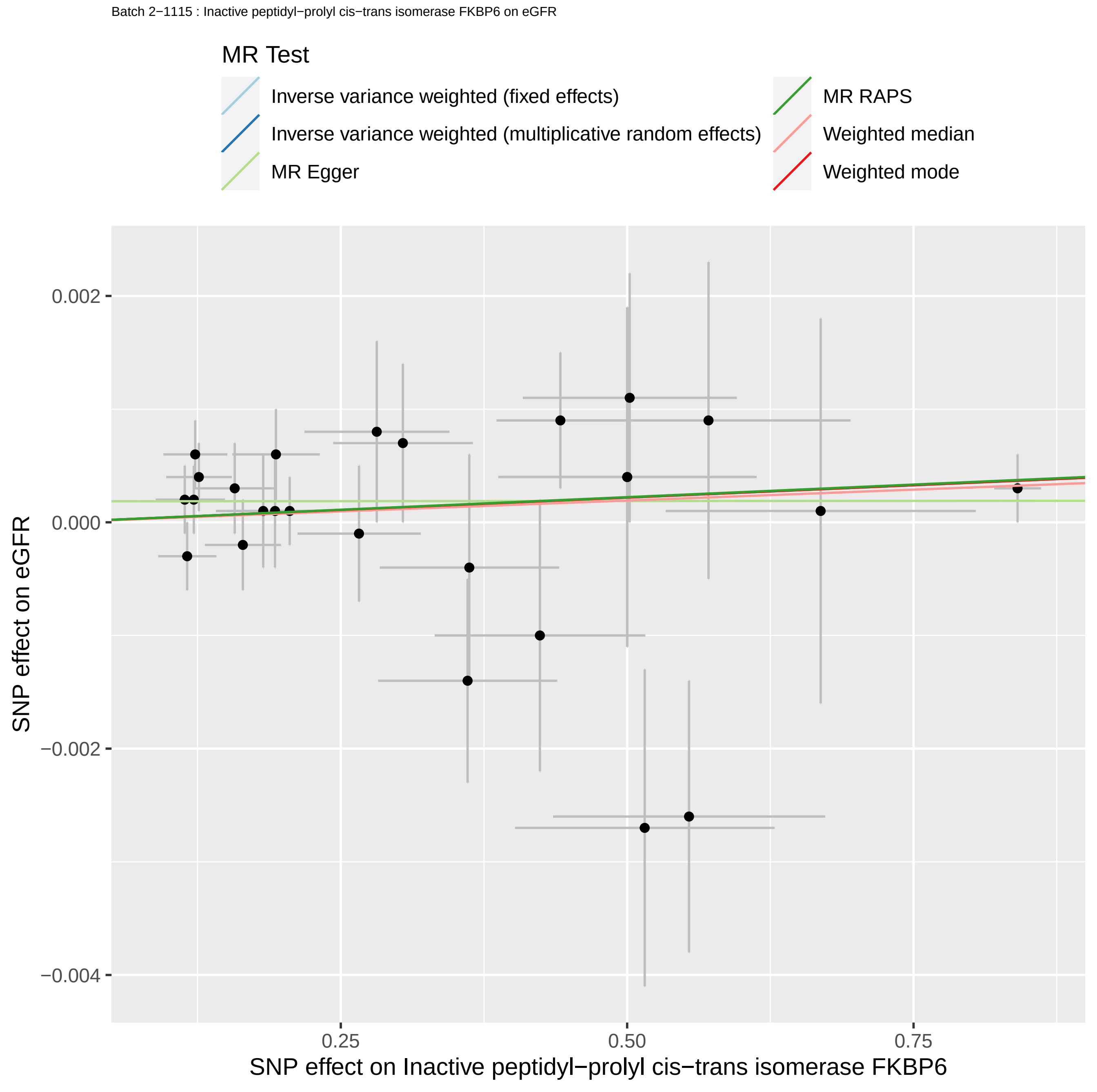

Batch 2−1115 : Inactive peptidyl−prolyl cis−trans isomerase FKBP6 on eGFR
MR Test
Inverse variance weighted (fixed effects)
Inverse variance weighted (multiplicative random effects)
MR Egger
MR RAPS
Weighted median
Weighted mode
SNP effect on eGFR
0.002
0.000
−0.002
−0.004
0.25
0.50
0.75
SNP effect on Inactive peptidyl−prolyl cis−trans isomerase FKBP6

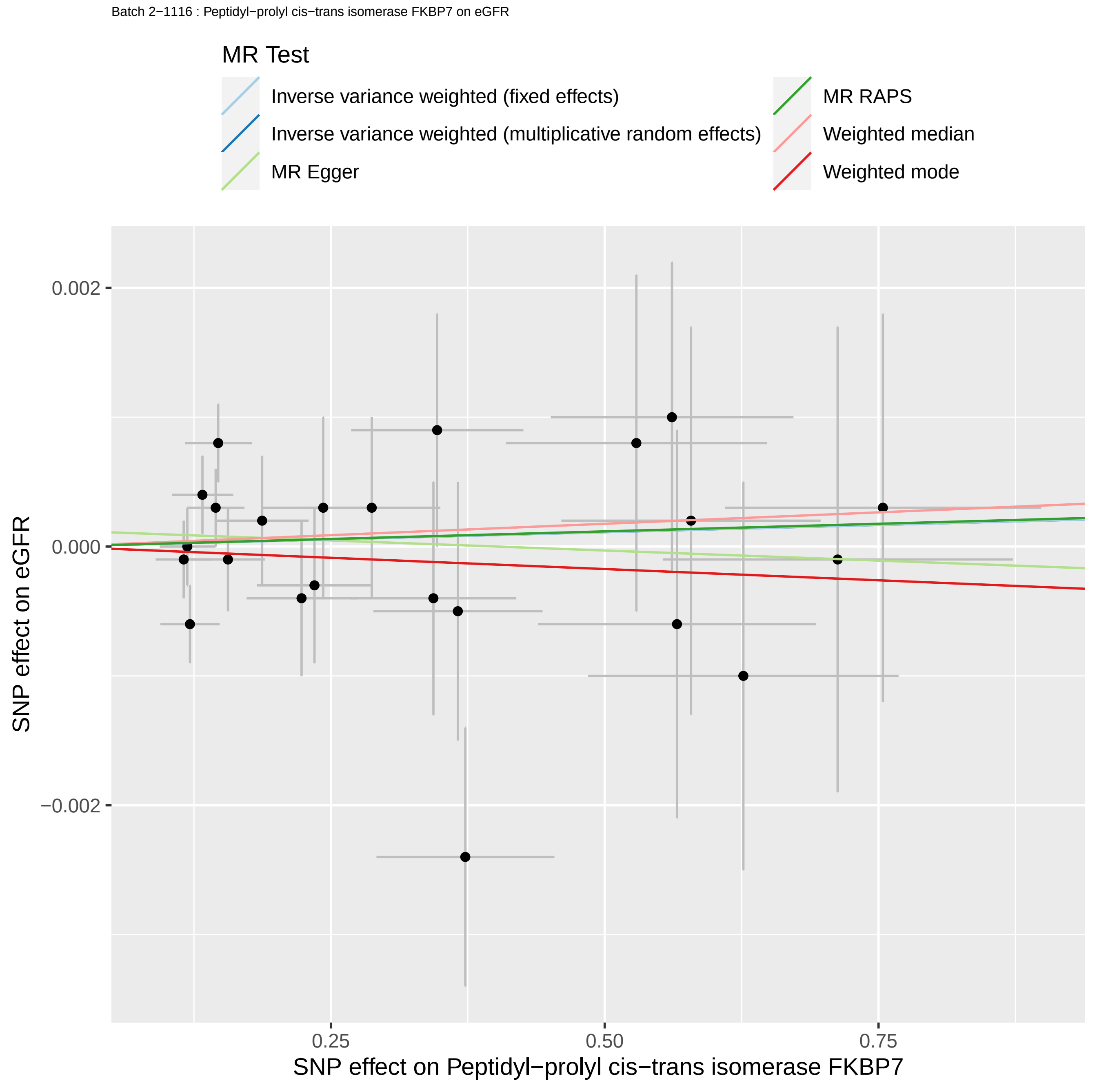
Batch 2−1116 : Peptidyl−prolyl cis−trans isomerase FKBP7 on eGFR
MR Test
Inverse variance weighted (fixed effects)
Inverse variance weighted (multiplicative random effects)
MR Egger
MR RAPS
Weighted median
Weighted mode
0.002
0.000
−0.002
0.25
0.50
0.75
SNP effect on eGFR
SNP effect on Peptidyl−prolyl cis−trans isomerase FKBP7

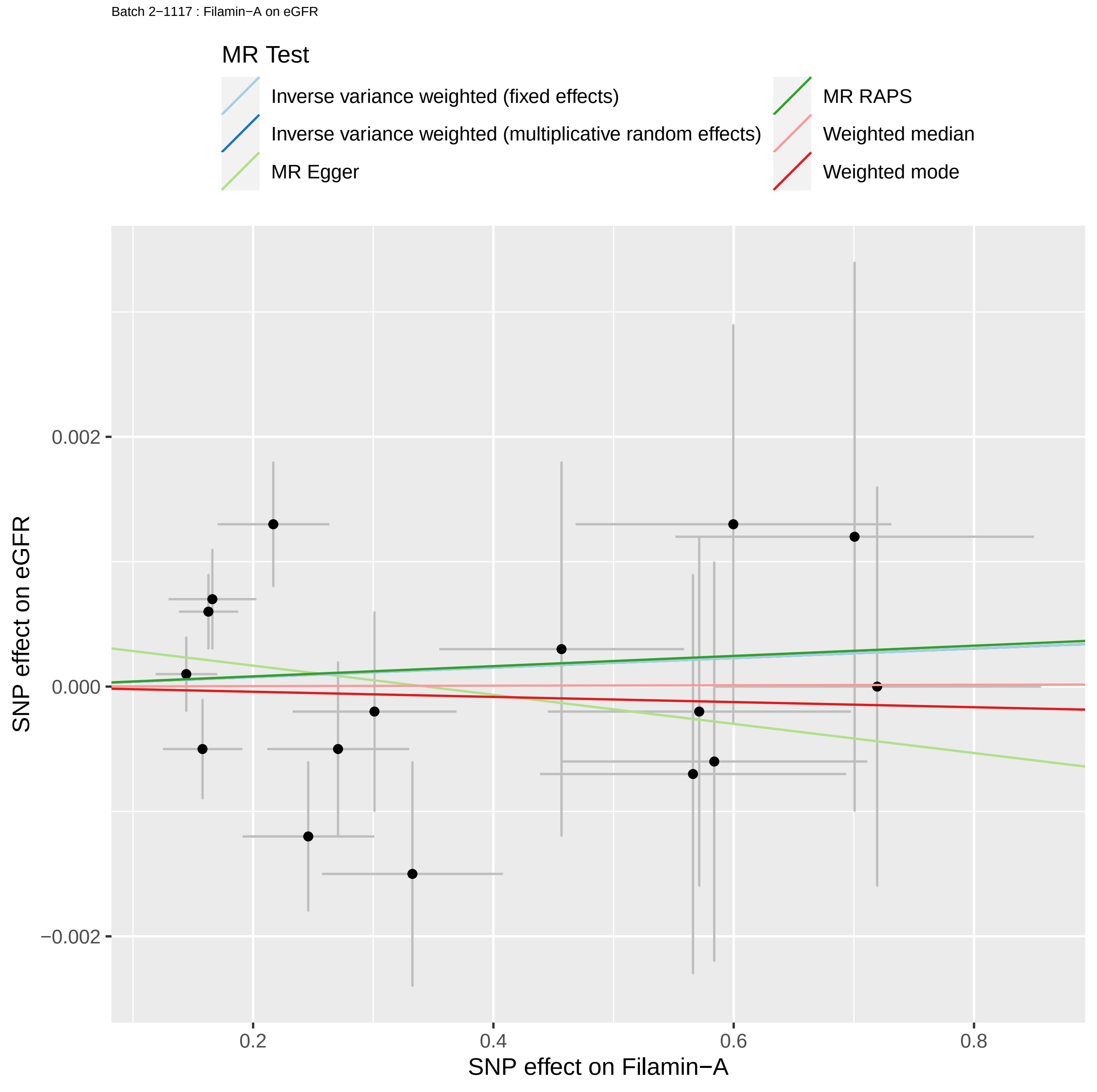
Batch 2−1117 : Filamin−A on eGFR
MR Test
Inverse variance weighted (fixed effects)
Inverse variance weighted (multiplicative random effects)
MR Egger
MR RAPS
Weighted median
Weighted mode
0.002
0.000
−0.002
0.2
0.4
0.6
0.8
SNP effect on eGFR
SNP effect on Filamin−A

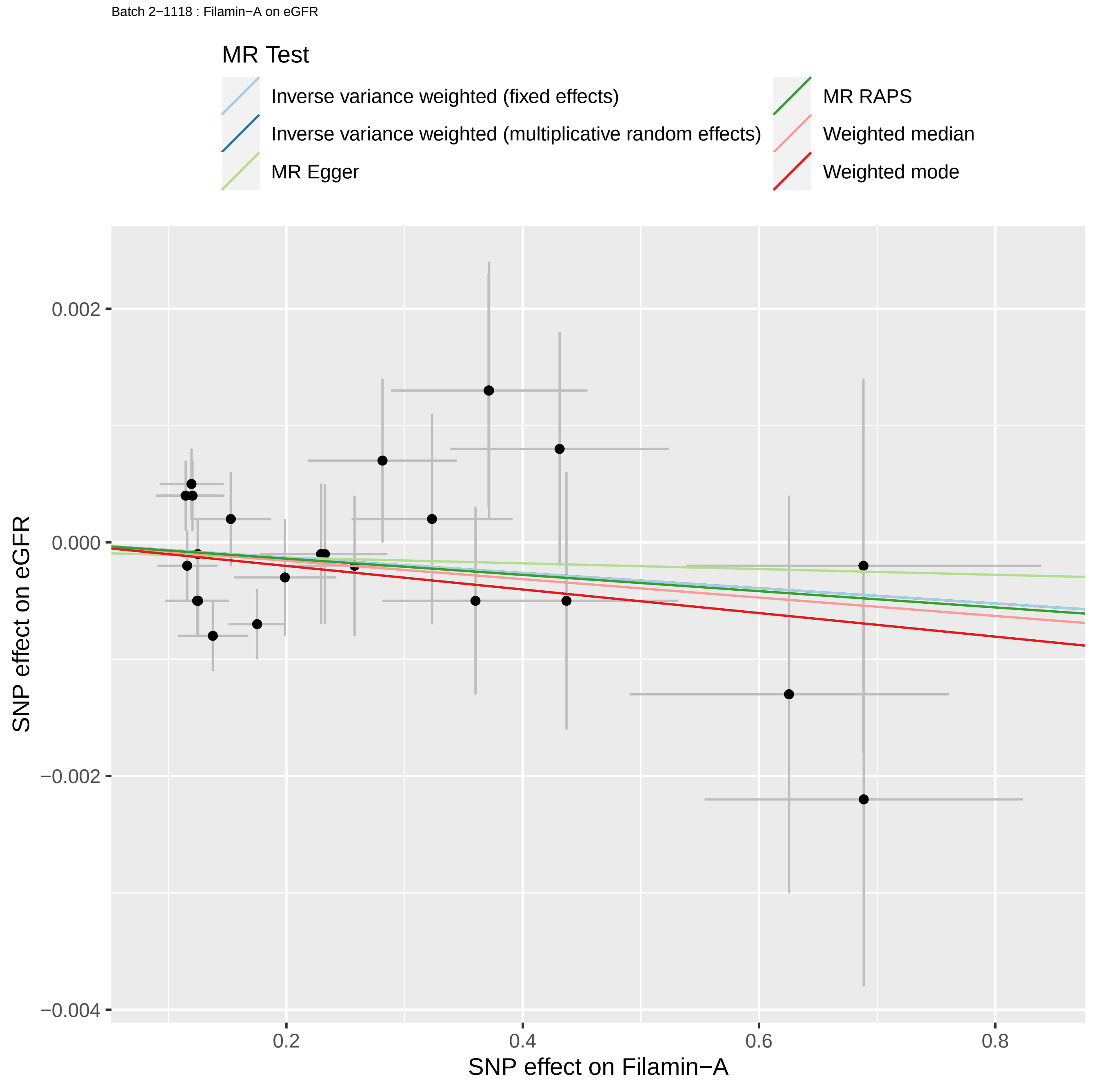

Batch 2−1118 : Filamin−A on eGFR
MR Test
Inverse variance weighted (fixed effects)
Inverse variance weighted (multiplicative random effects)
MR Egger
MR RAPS
Weighted median
Weighted mode
0.002
0.000
−0.002
−0.004
0.2
0.4
0.6
0.8
SNP effect on eGFR
SNP effect on Filamin−A

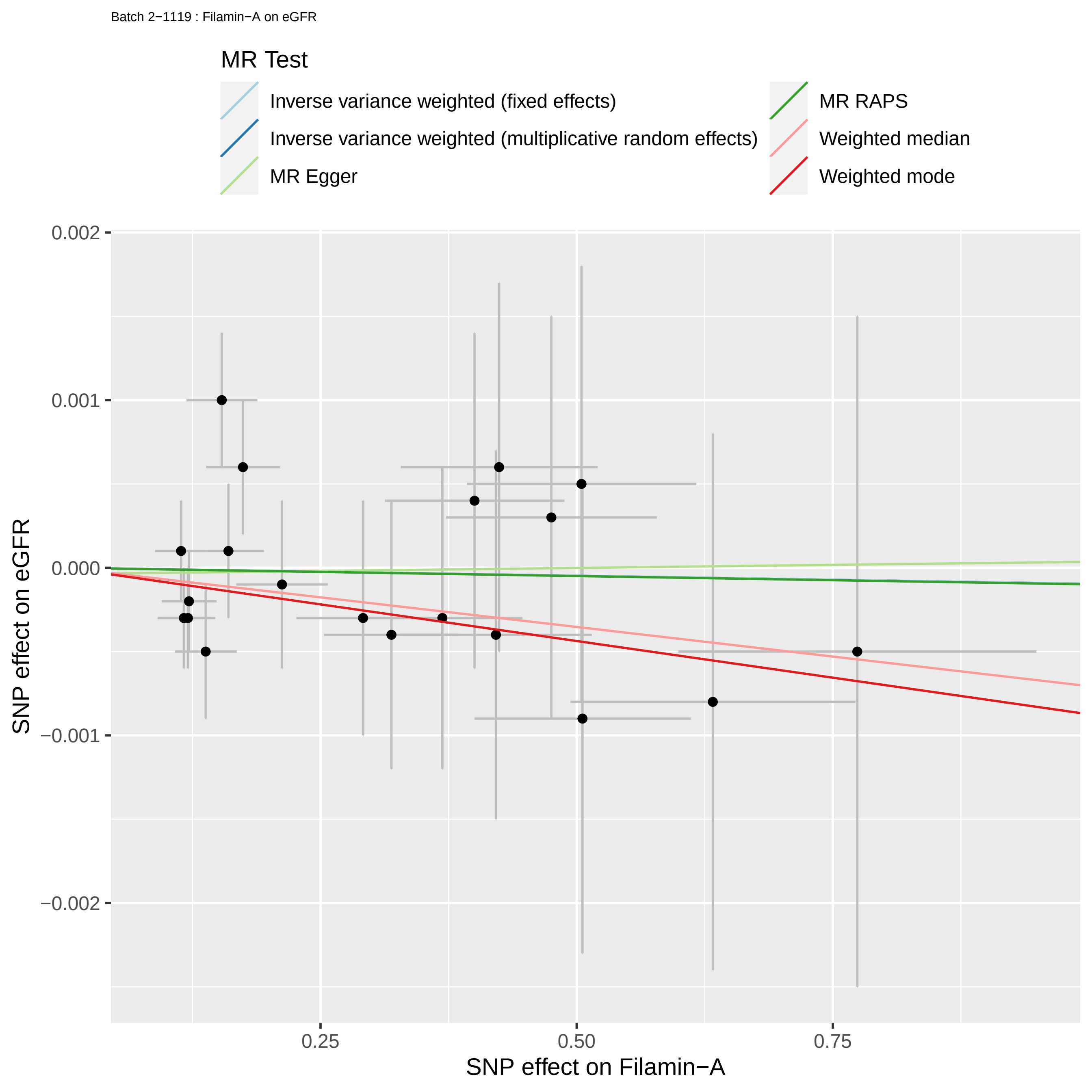

Batch 2−1119 : Filamin−A on eGFR
MR Test
Inverse variance weighted (fixed effects)
Inverse variance weighted (multiplicative random effects)
MR Egger
MR RAPS
Weighted median
Weighted mode
0.002
0.001
0.000
−0.001
−0.002
0.25
0.50
0.75
SNP effect on eGFR
SNP effect on Filamin−A

Batch 2−1120 : Filamin−A on eGFR

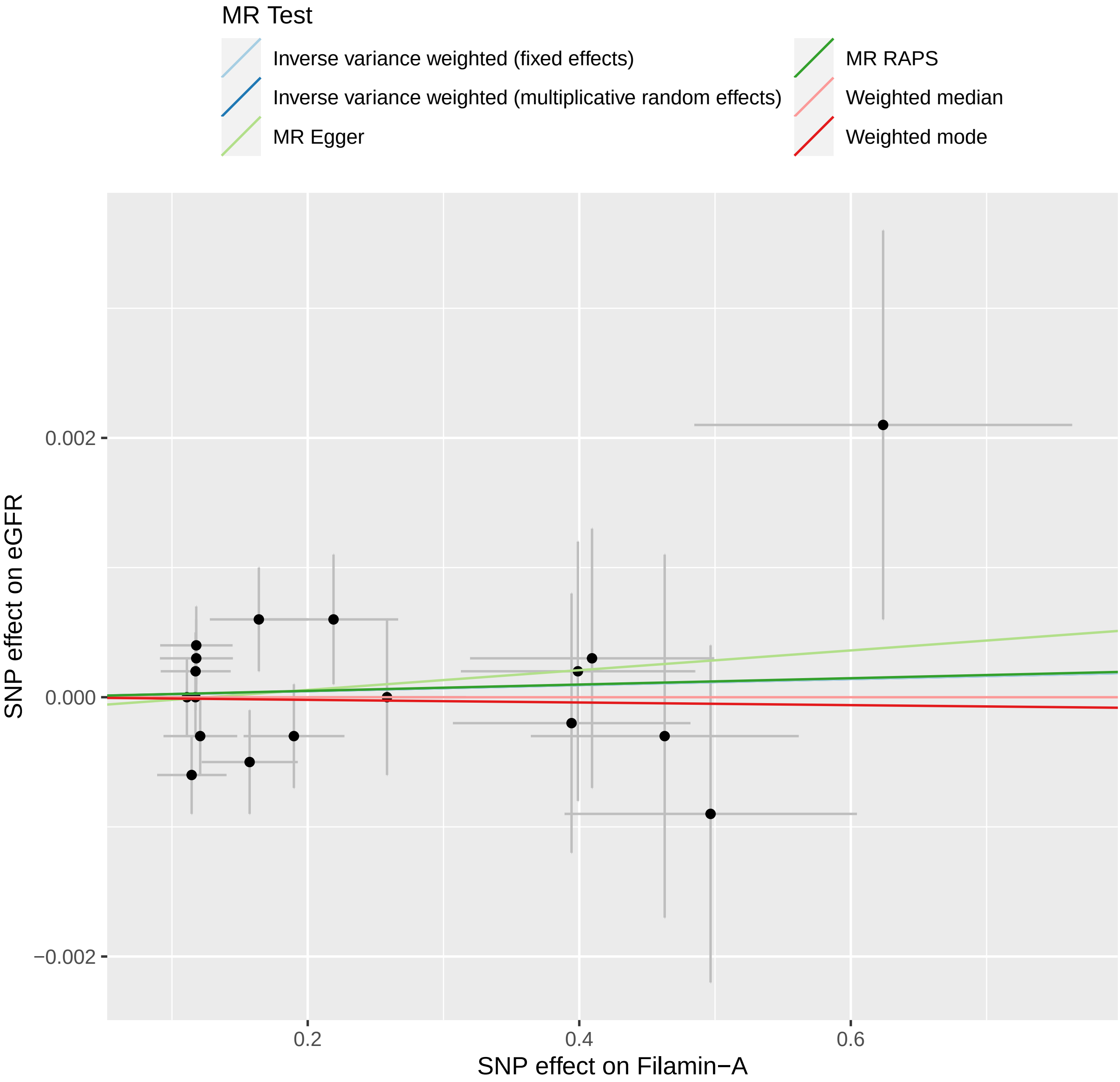

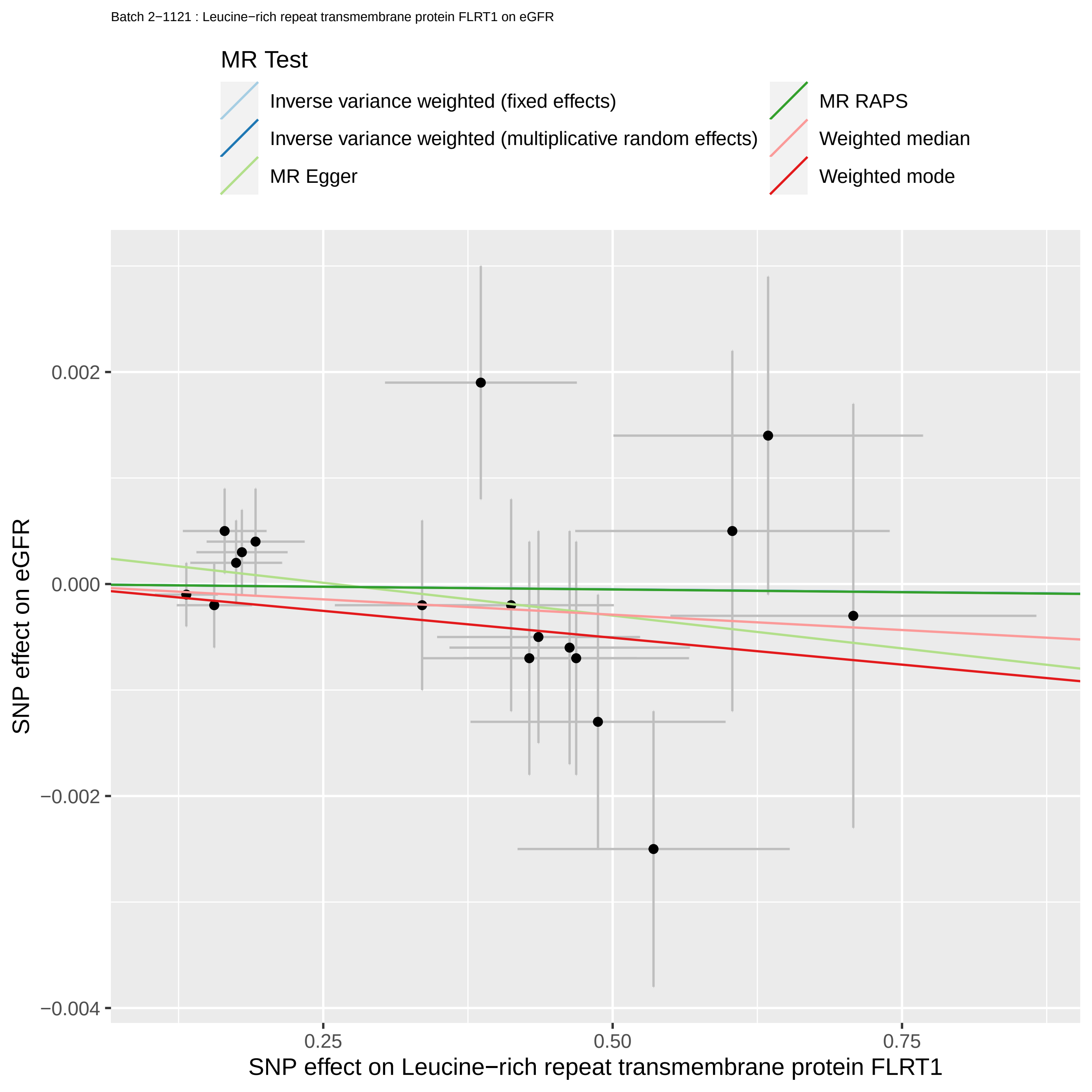

Batch 2−1121 : Leucine−rich repeat transmembrane protein FLRT1 on eGFR
MR Test
Inverse variance weighted (fixed effects)
Inverse variance weighted (multiplicative random effects)
MR Egger
MR RAPS
Weighted median
Weighted mode
SNP effect on eGFR
0.002
0.000
−0.002
−0.004
0.25
0.50
0.75
SNP effect on Leucine−rich repeat transmembrane protein FLRT1

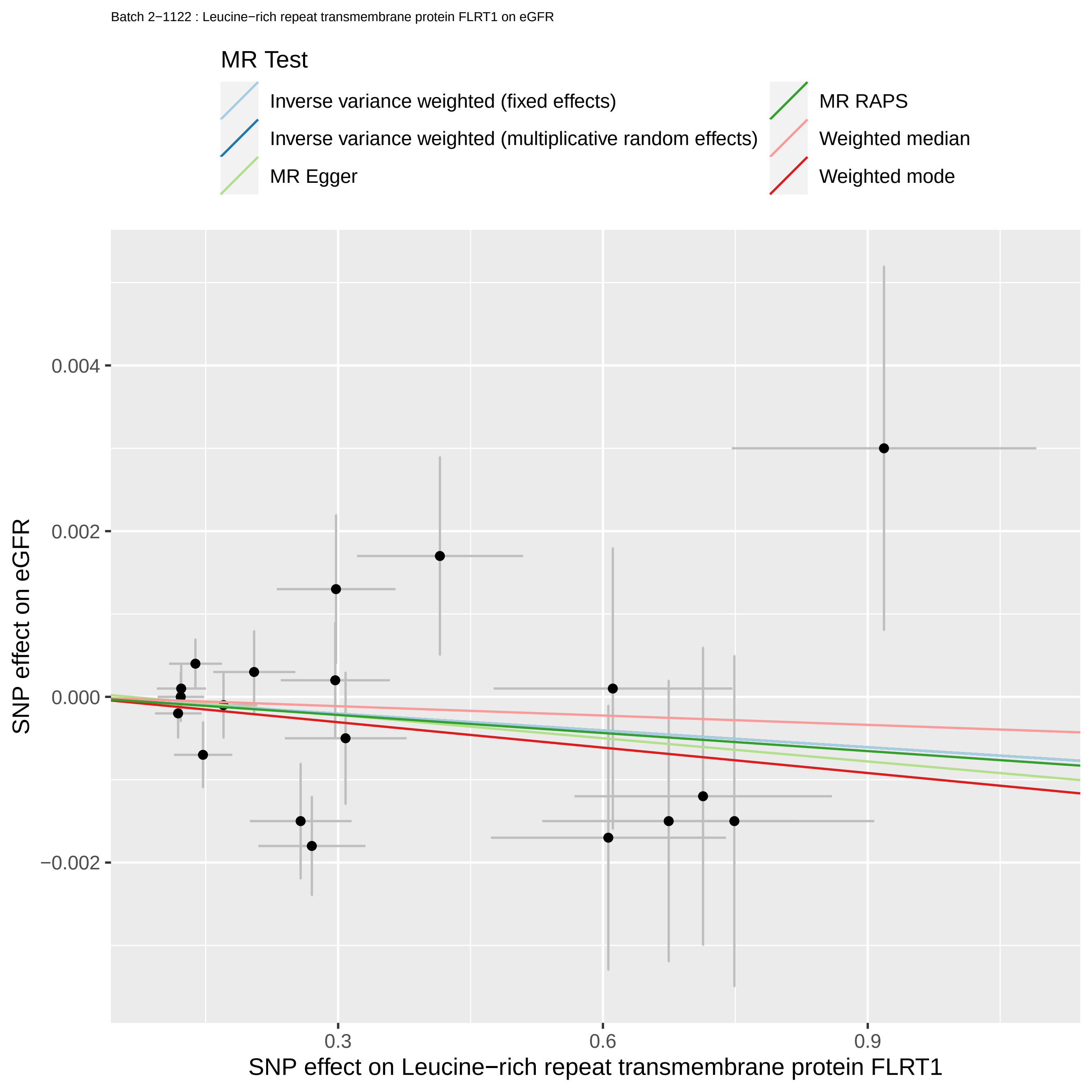
Batch 2−1122 : Leucine−rich repeat transmembrane protein FLRT1 on eGFR
MR Test
Inverse variance weighted (fixed effects)
Inverse variance weighted (multiplicative random effects)
MR Egger
MR RAPS
Weighted median
Weighted mode
0.004
0.002
0.000
−0.002
SNP effect on eGFR
0.3
0.6
0.9
SNP effect on Leucine−rich repeat transmembrane protein FLRT1

Batch 2−1123 : Leucine−rich repeat transmembrane protein FLRT2 on eGFR

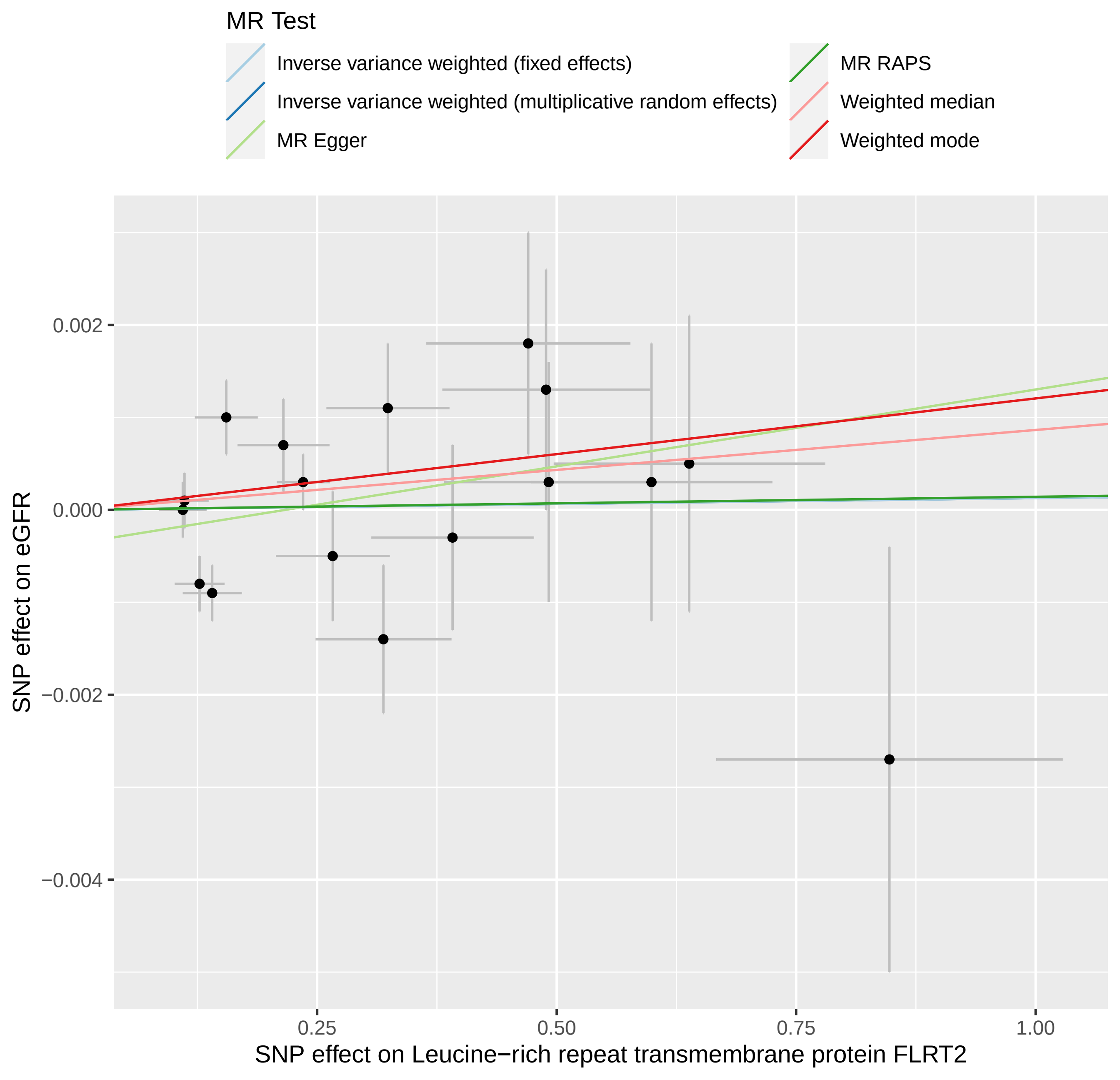

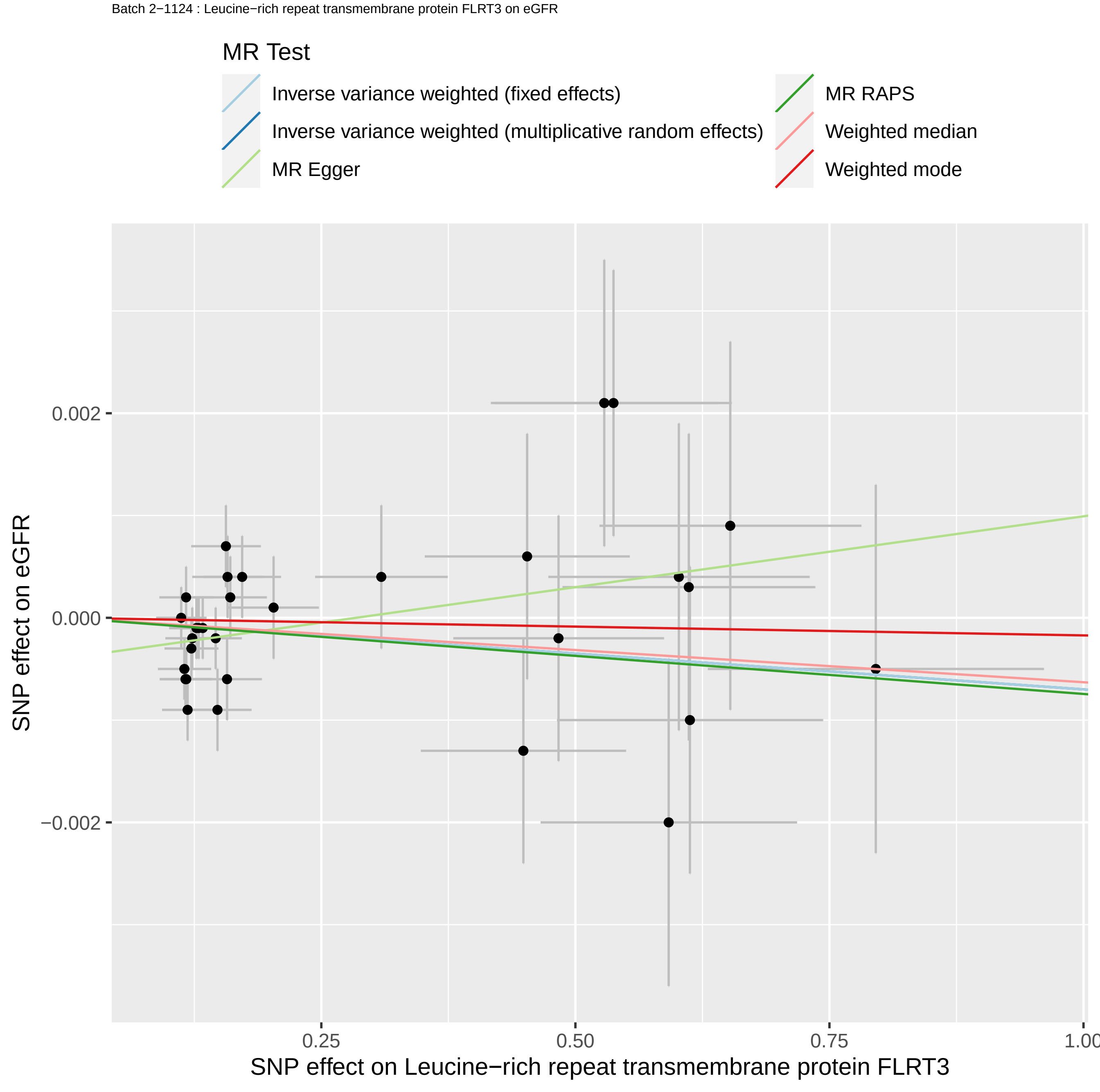

Batch 2−1124 : Leucine−rich repeat transmembrane protein FLRT3 on eGFR
MR Test
Inverse variance weighted (fixed effects)
Inverse variance weighted (multiplicative random effects)
MR Egger
MR RAPS
Weighted median
Weighted mode
0.002
0.000
−0.002
0.25
0.50
0.75
1.00
SNP effect on eGFR
SNP effect on Leucine−rich repeat transmembrane protein FLRT3

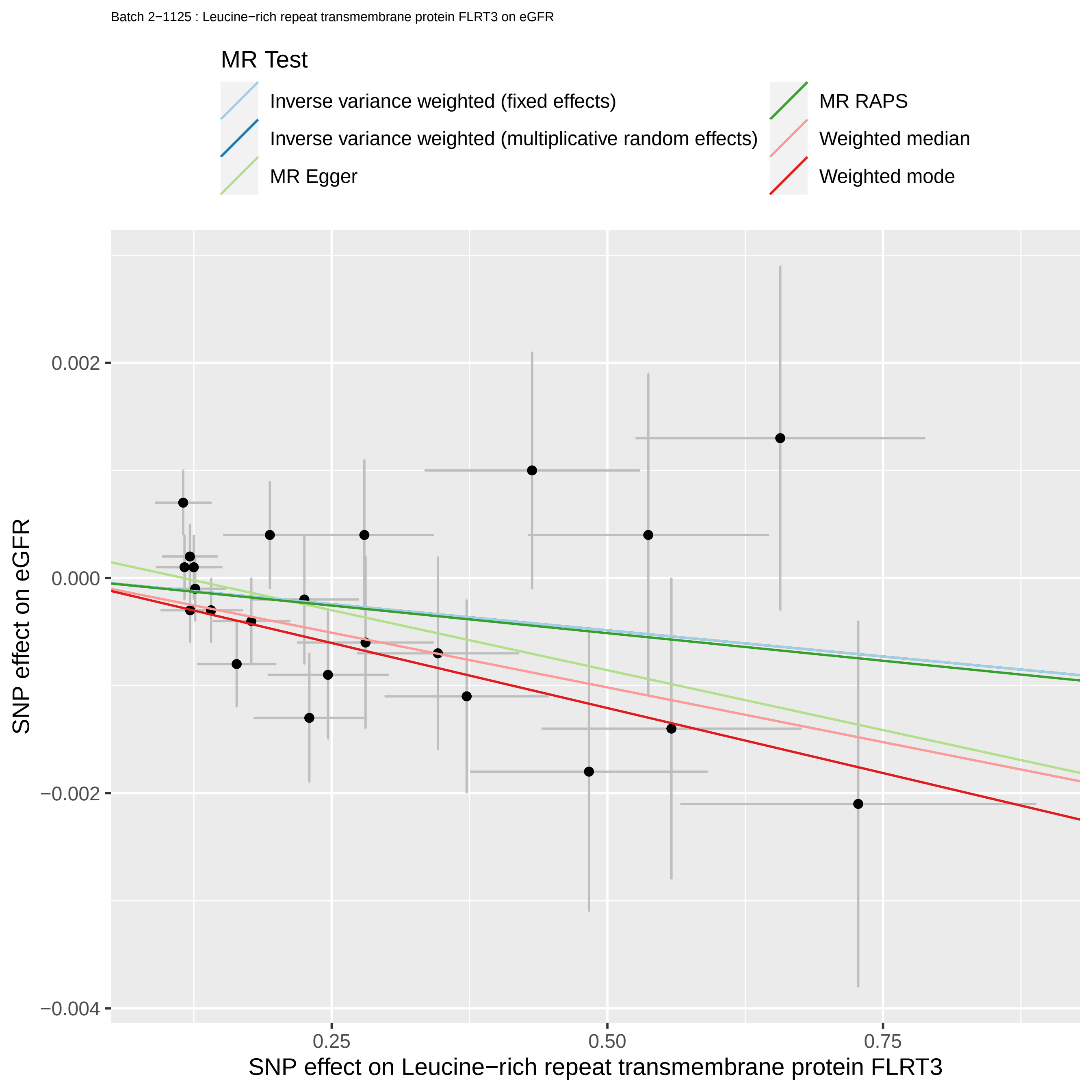

Batch 2−1125 : Leucine−rich repeat transmembrane protein FLRT3 on eGFR
MR Test
Inverse variance weighted (fixed effects)
Inverse variance weighted (multiplicative random effects)
MR Egger
MR RAPS
Weighted median
Weighted mode
0.002
0.000
−0.002
−0.004
0.25
0.50
0.75
SNP effect on eGFR
SNP effect on Leucine−rich repeat transmembrane protein FLRT3

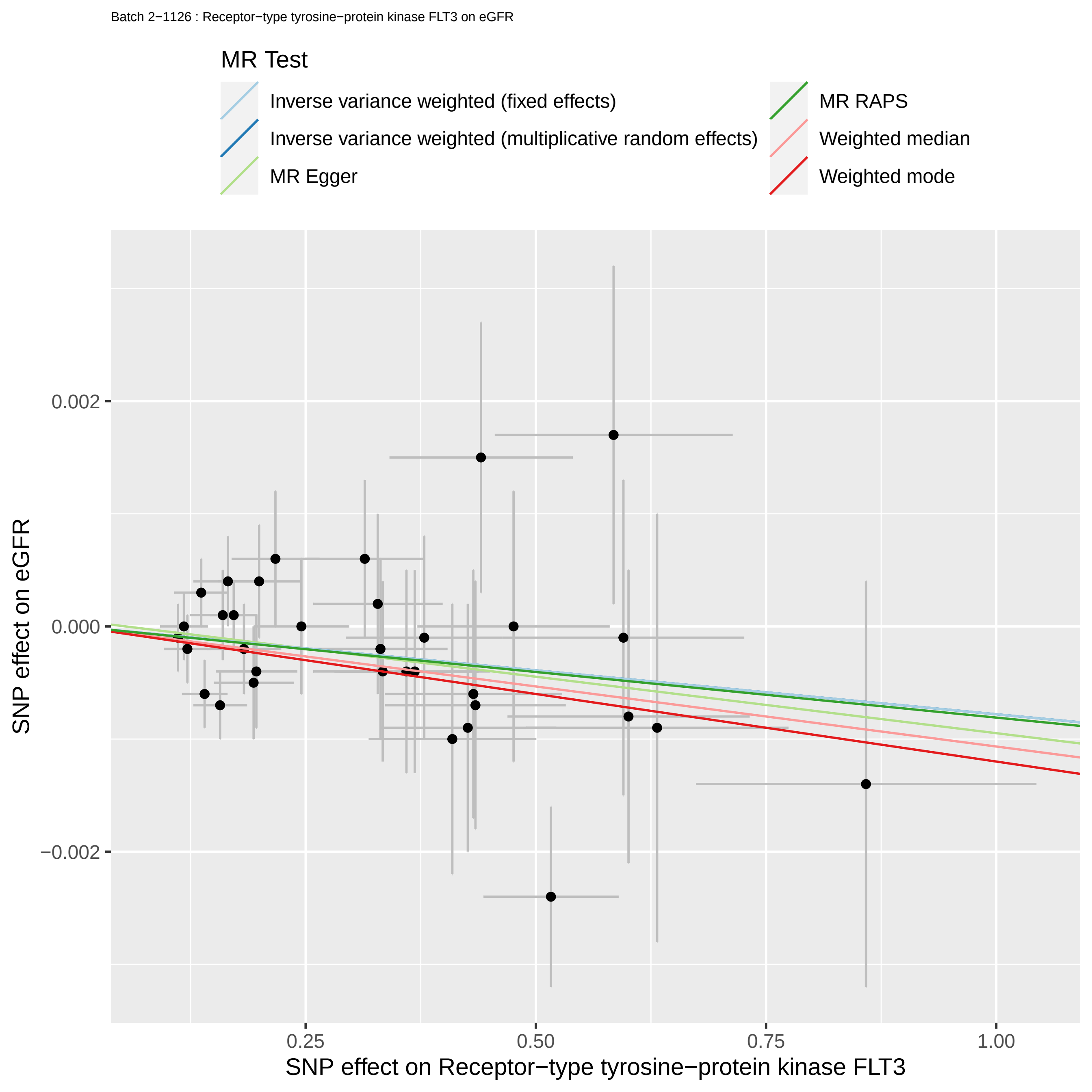

Batch 2−1126 : Receptor−type tyrosine−protein kinase FLT3 on eGFR
MR Test
Inverse variance weighted (fixed effects)
Inverse variance weighted (multiplicative random effects)
MR Egger
MR RAPS
Weighted median
Weighted mode
0.002
0.000
−0.002
SNP effect on eGFR
0.25
0.50
0.75
1.00
SNP effect on Receptor−type tyrosine−protein kinase FLT3

Batch 2−1127 : Fms−related tyrosine kinase 3 ligand on eGFR

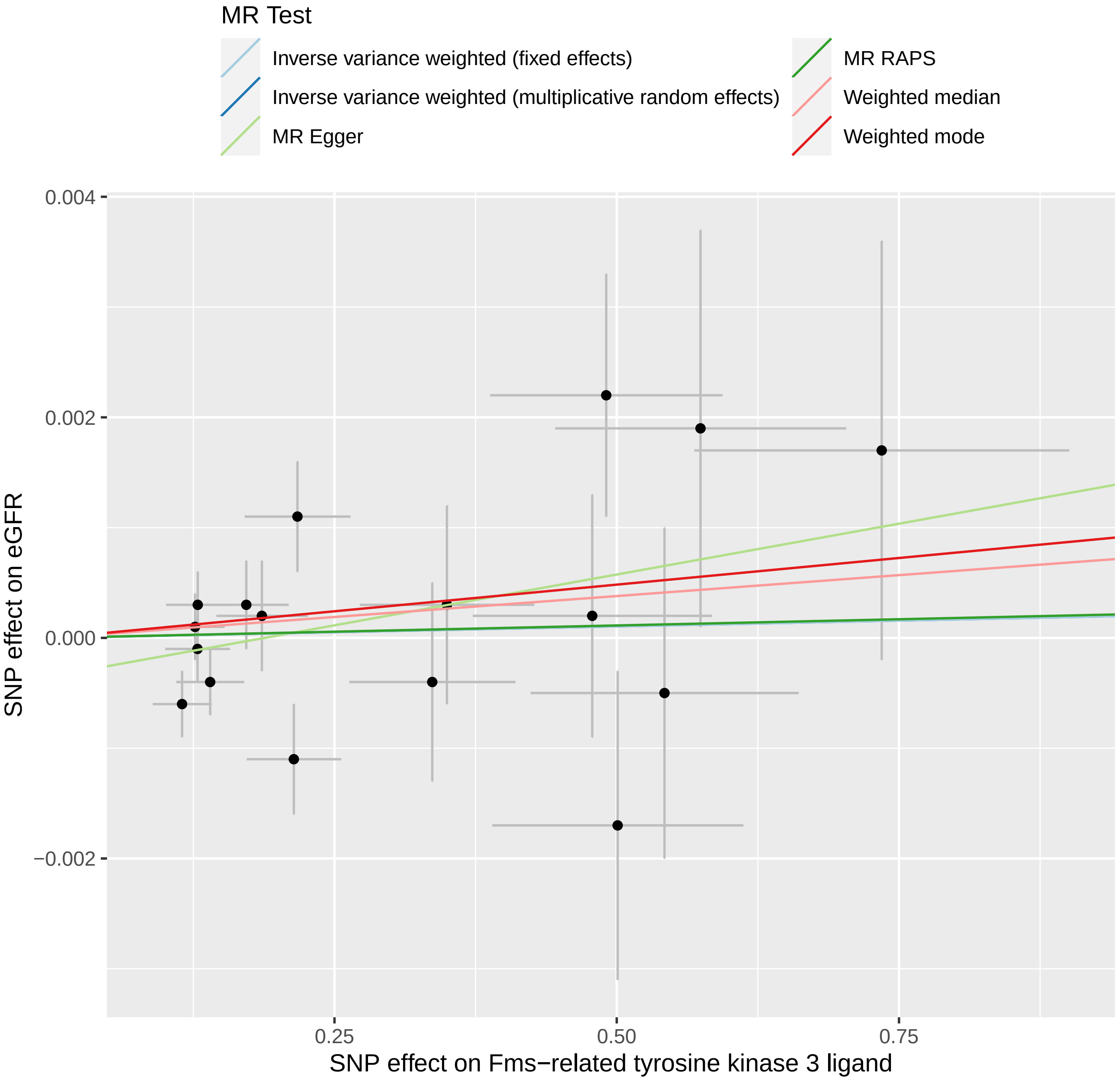

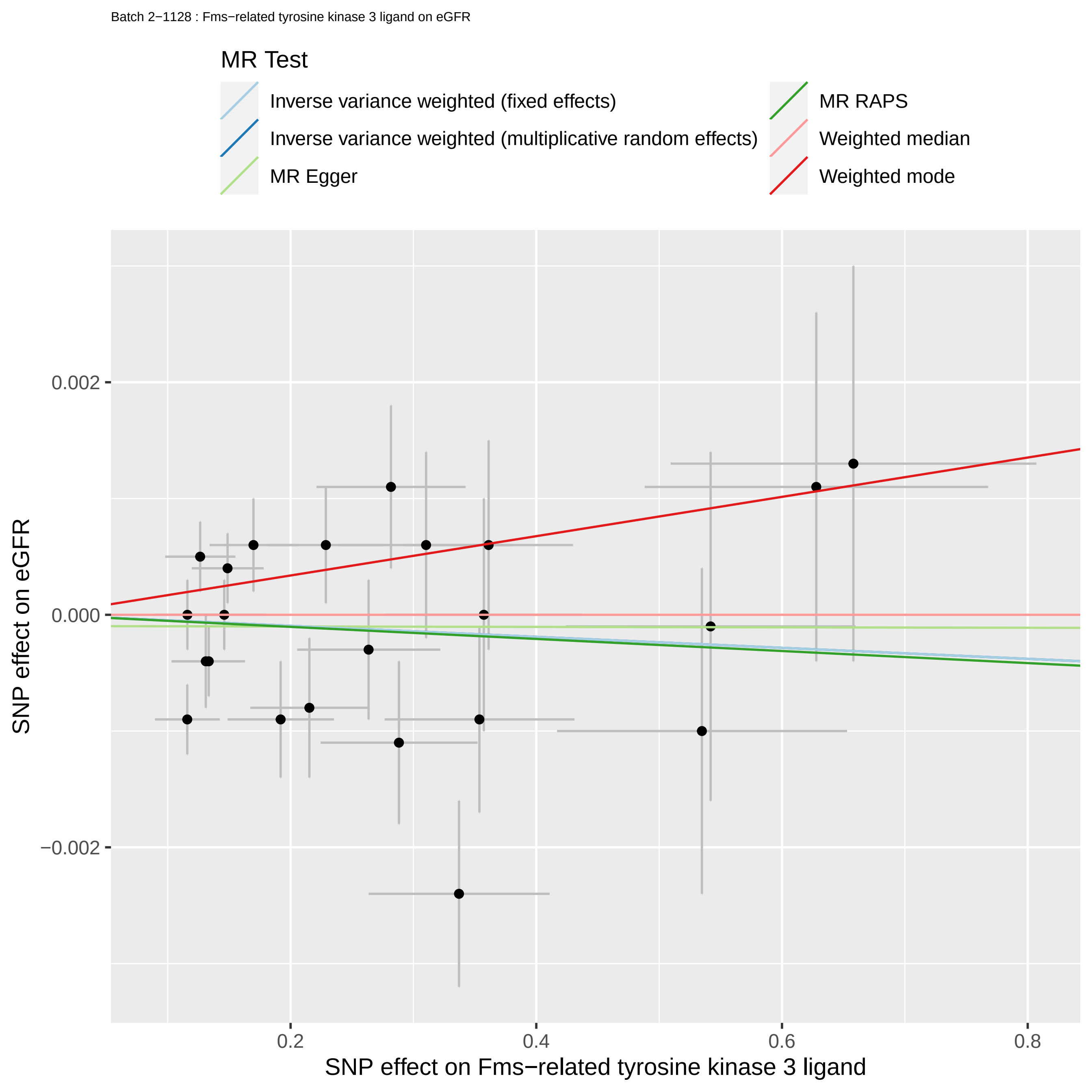

Batch 2−1128 : Fms−related tyrosine kinase 3 ligand on eGFR
MR Test
Inverse variance weighted (fixed effects)
Inverse variance weighted (multiplicative random effects)
MR Egger
MR RAPS
Weighted median
Weighted mode
0.002
0.000
−0.002
0.2
0.4
0.6
0.8
SNP effect on eGFR
SNP effect on Fms−related tyrosine kinase 3 ligand

Batch 2−1129 : Vascular endothelial growth factor receptor 3 on eGFR

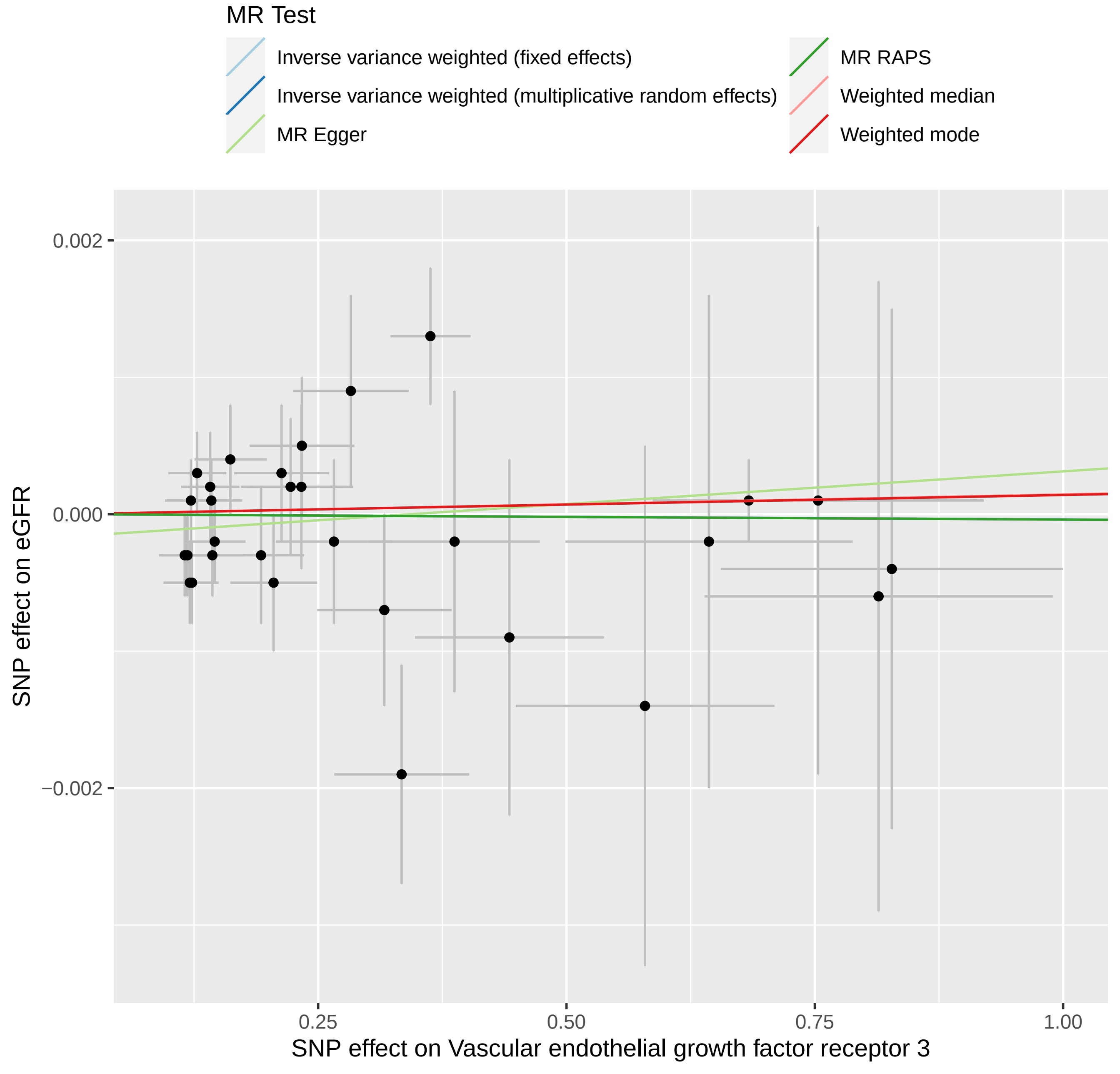

Batch 2−1130 : Feline leukemia virus subgroup C receptor−related protein 1 on eGFR

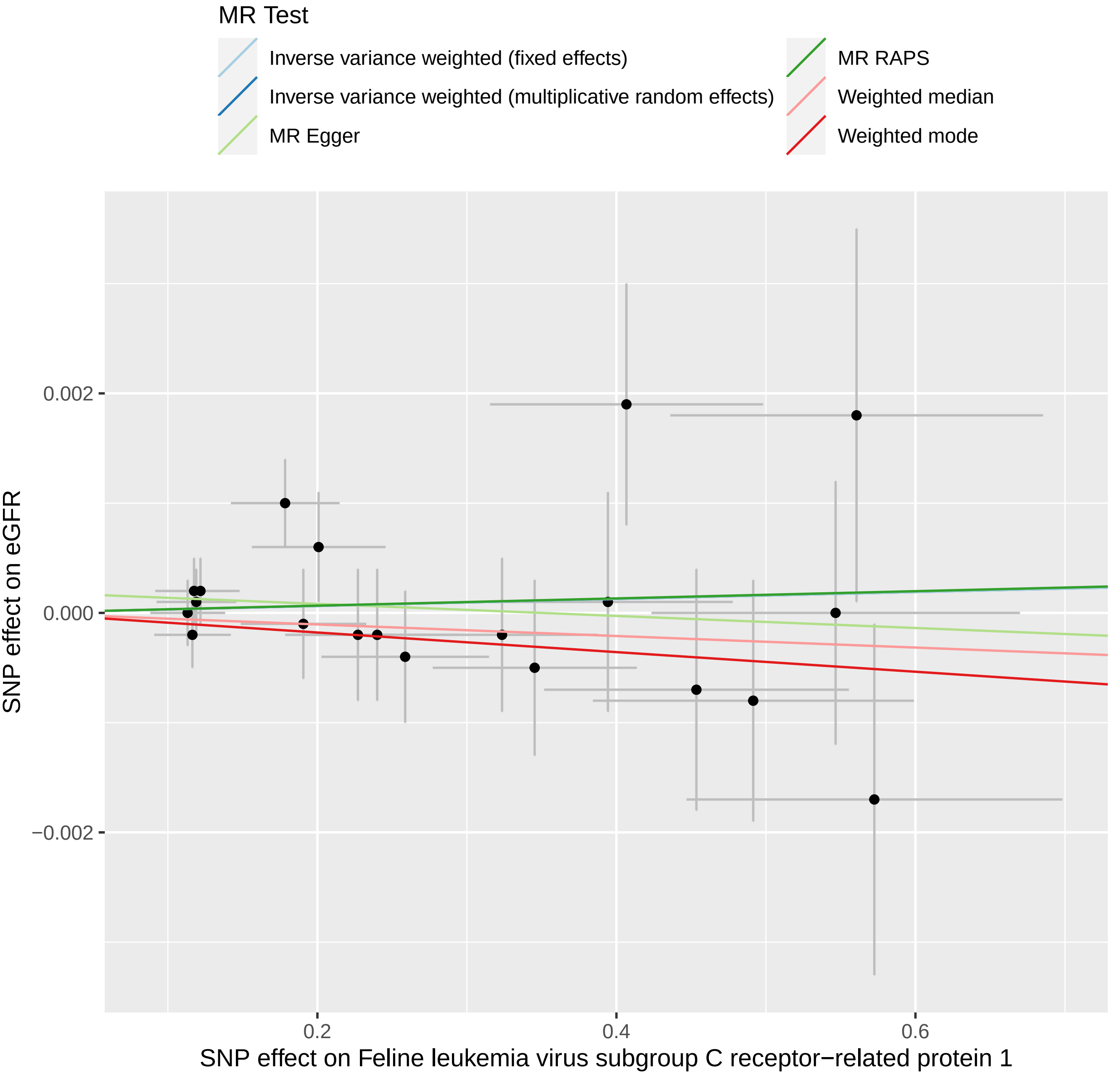

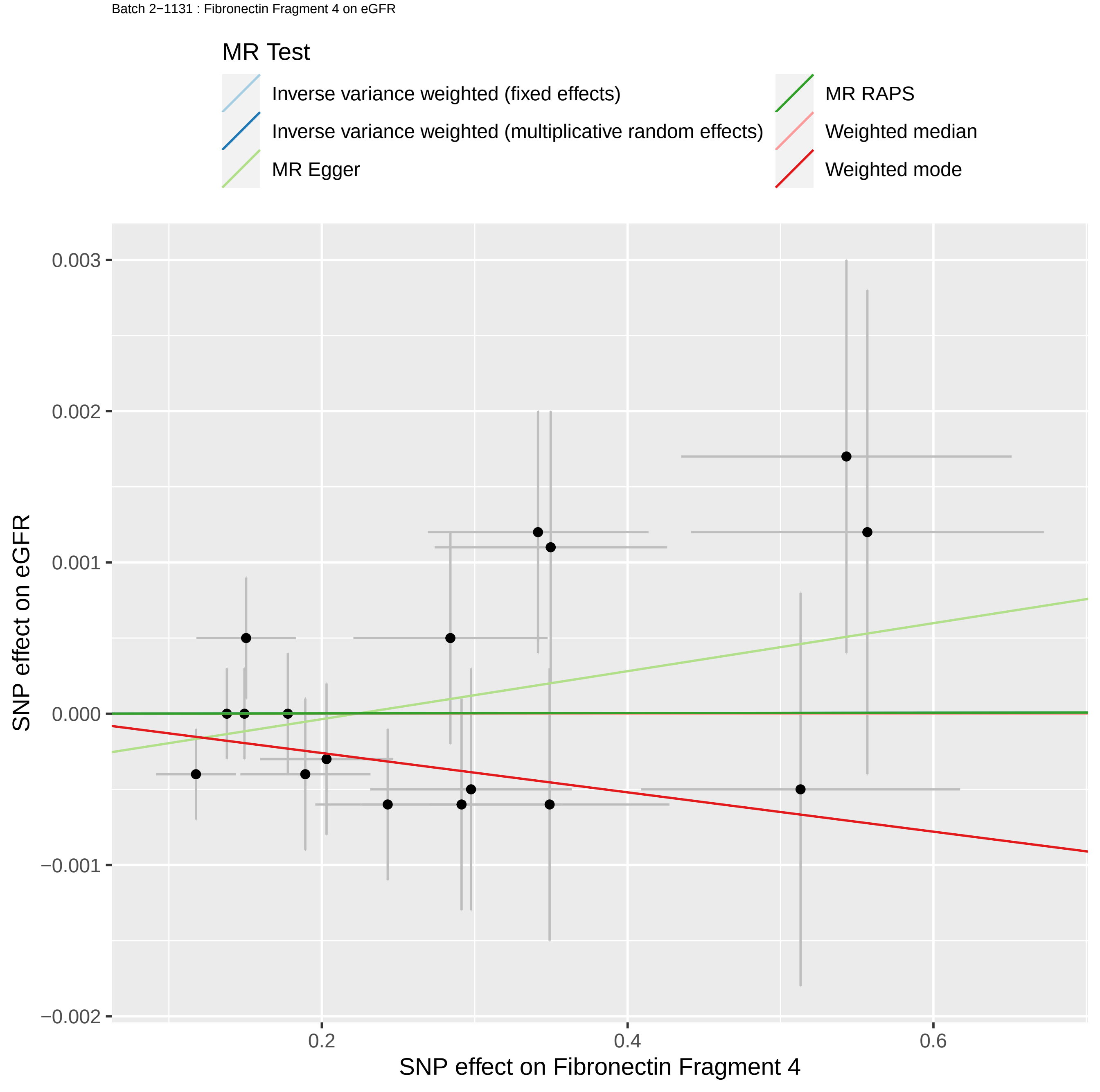

Batch 2−1131 : Fibronectin Fragment 4 on eGFR
MR Test
Inverse variance weighted (fixed effects)
Inverse variance weighted (multiplicative random effects)
MR Egger
MR RAPS
Weighted median
Weighted mode
0.003
0.002
0.001
0.000
−0.001
−0.002
SNP effect on eGFR
0.2
0.4
0.6
SNP effect on Fibronectin Fragment 4

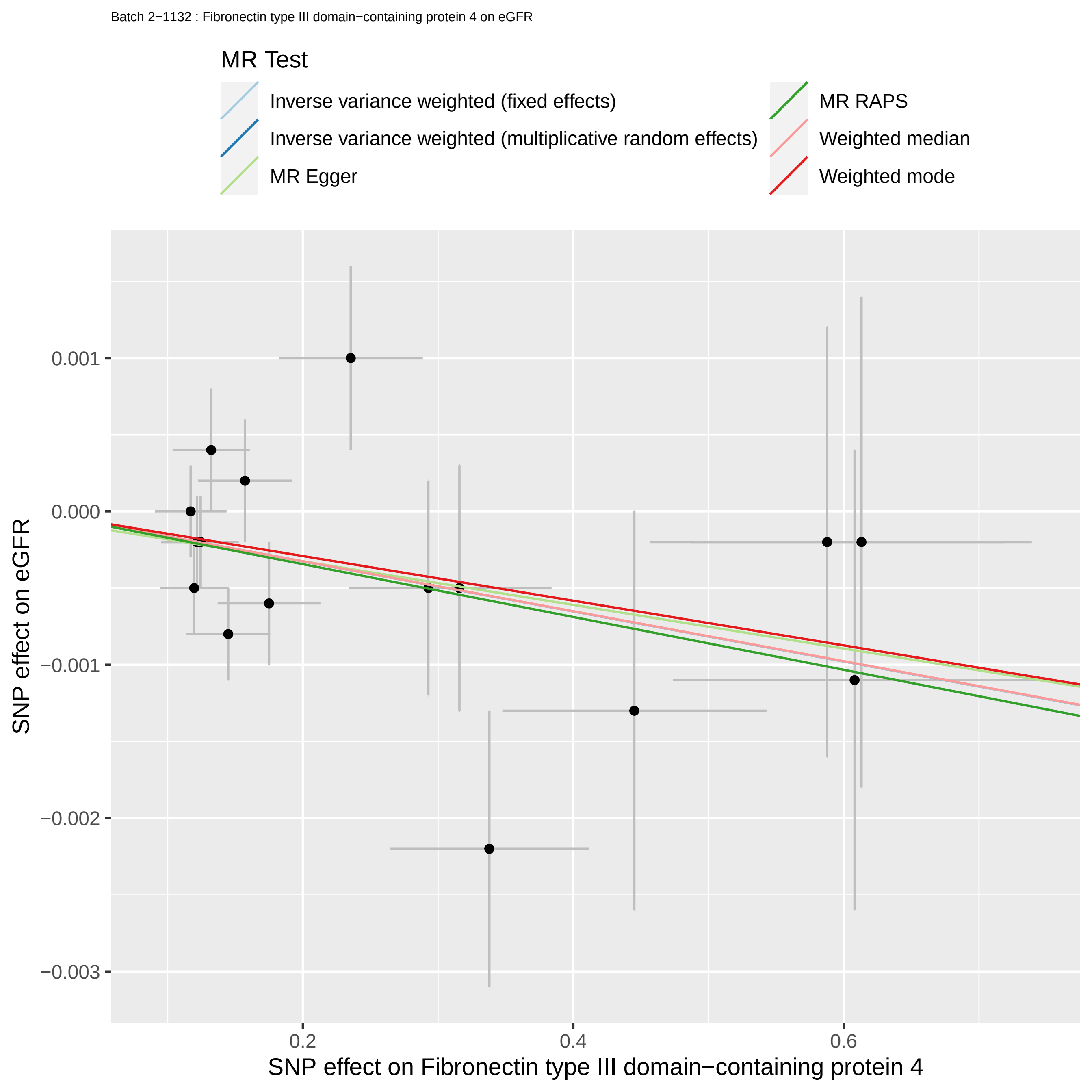
Batch 2−1132 : Fibronectin type III domain−containing protein 4 on eGFR
MR Test
Inverse variance weighted (fixed effects)
Inverse variance weighted (multiplicative random effects)
MR Egger
MR RAPS
Weighted median
Weighted mode
0.001
0.000
−0.001
−0.002
−0.003
0.2
0.4
0.6
SNP effect on eGFR
SNP effect on Fibronectin type III domain−containing protein 4

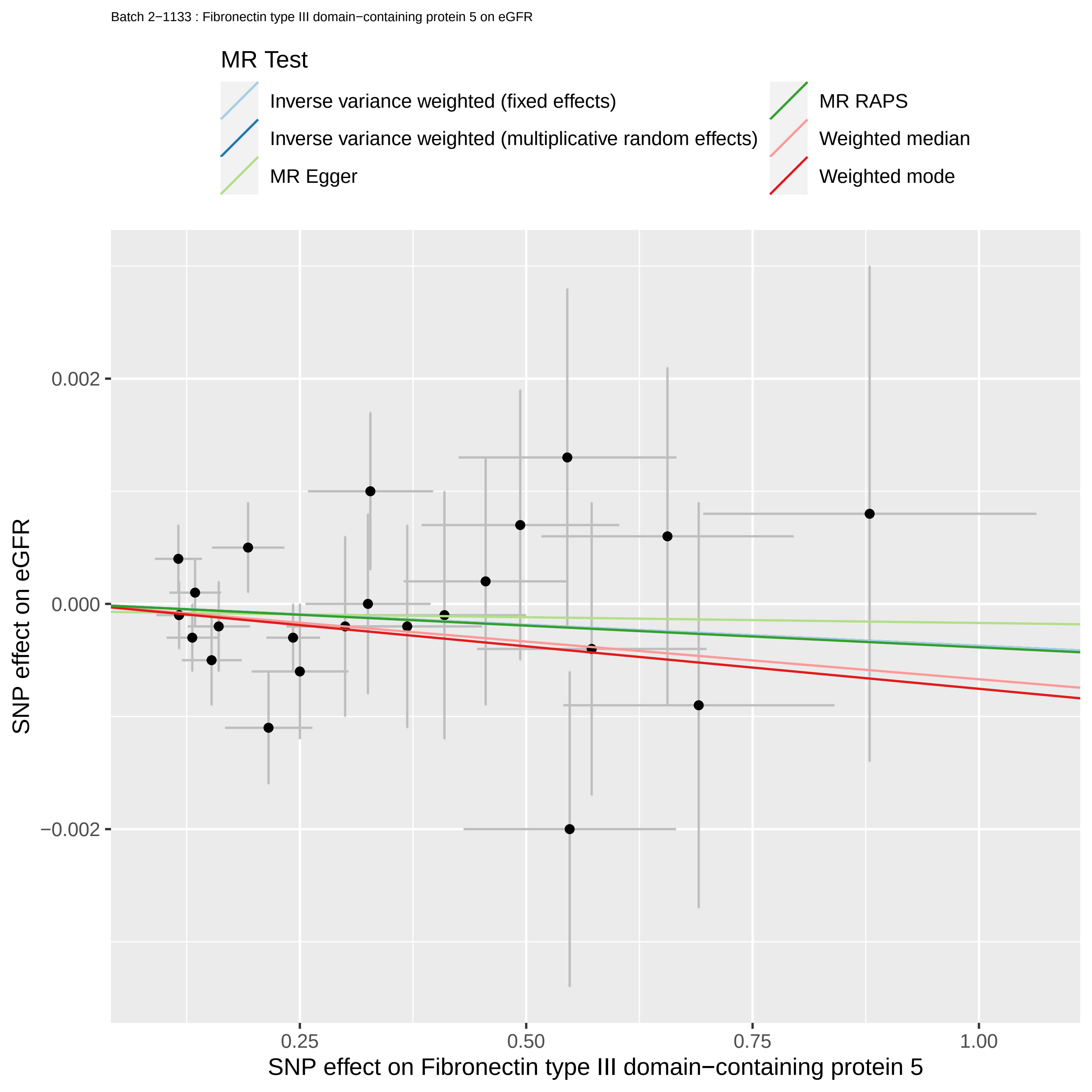
Batch 2−1133 : Fibronectin type III domain−containing protein 5 on eGFR
MR Test
Inverse variance weighted (fixed effects)
Inverse variance weighted (multiplicative random effects)
MR Egger
MR RAPS
Weighted median
Weighted mode
0.002
0.000
−0.002
0.25
0.50
0.75
1.00
SNP effect on eGFR
SNP effect on Fibronectin type III domain−containing protein 5

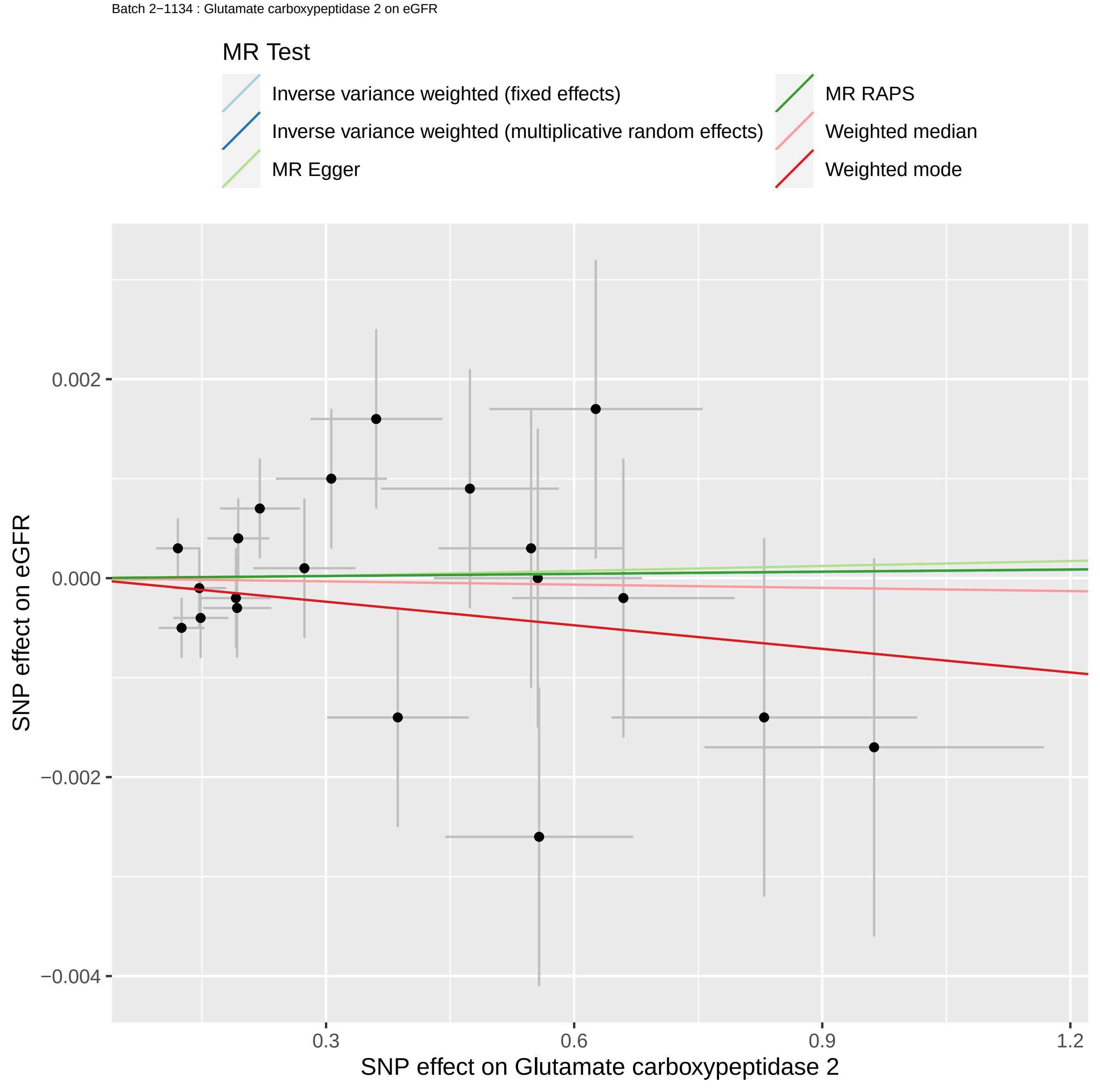

Batch 2−1134 : Glutamate carboxypeptidase 2 on eGFR
MR Test
Inverse variance weighted (fixed effects)
Inverse variance weighted (multiplicative random effects)
MR Egger
MR RAPS
Weighted median
Weighted mode
0.002
0.000
−0.002
−0.004
0.3
0.6
0.9
1.2
SNP effect on eGFR
SNP effect on Glutamate carboxypeptidase 2

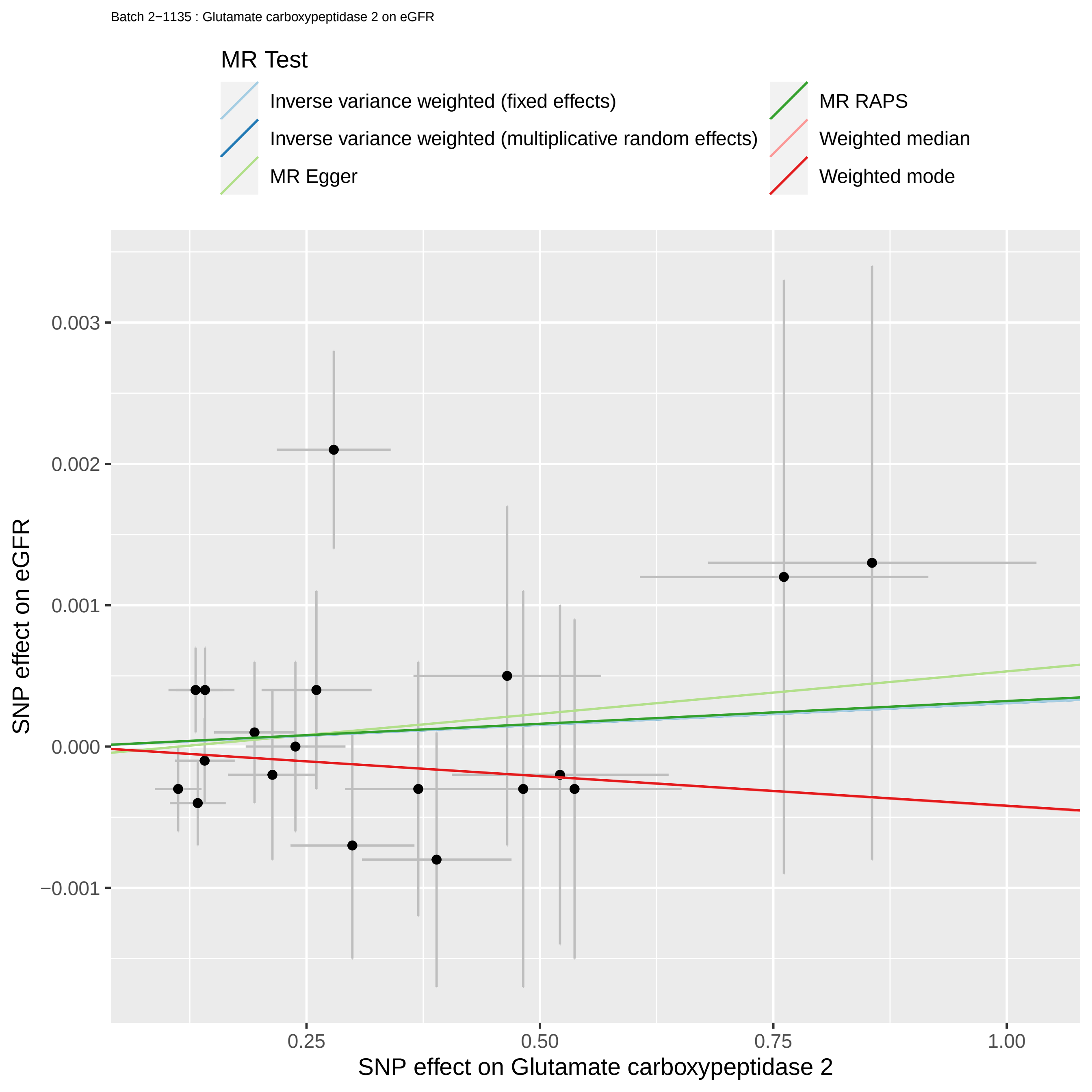
Batch 2−1135 : Glutamate carboxypeptidase 2 on eGFR
MR Test
Inverse variance weighted (fixed effects)
Inverse variance weighted (multiplicative random effects)
MR Egger
MR RAPS
Weighted median
Weighted mode
0.003
0.002
0.001
0.000
−0.001
0.25
0.50
0.75
1.00
SNP effect on eGFR
SNP effect on Glutamate carboxypeptidase 2

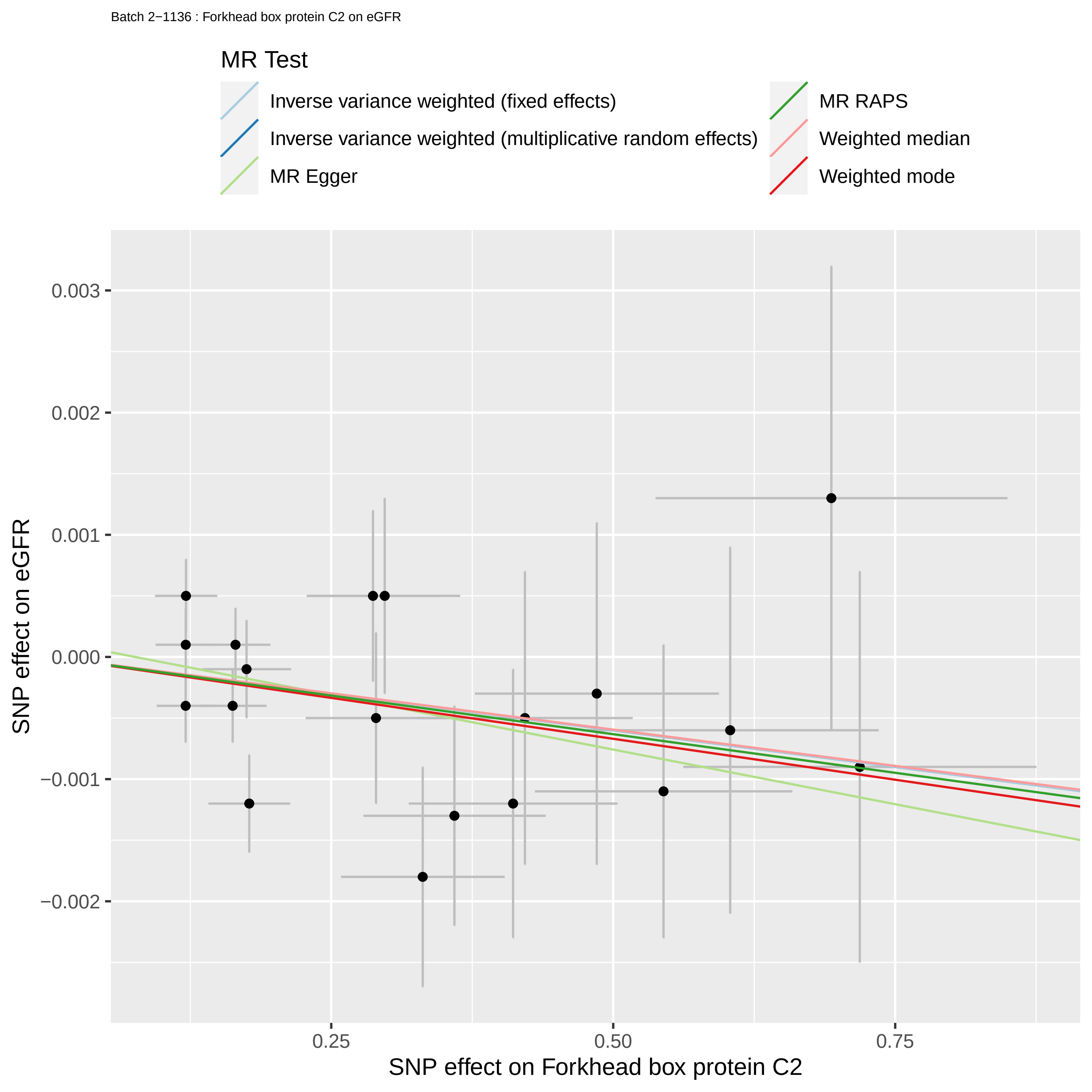
Batch 2−1136 : Forkhead box protein C2 on eGFR
MR Test
Inverse variance weighted (fixed effects)
Inverse variance weighted (multiplicative random effects)
MR Egger
MR RAPS
Weighted median
Weighted mode
0.003
0.002
0.001
0.000
−0.001
−0.002
0.25
0.50
0.75
SNP effect on eGFR
SNP effect on Forkhead box protein C2

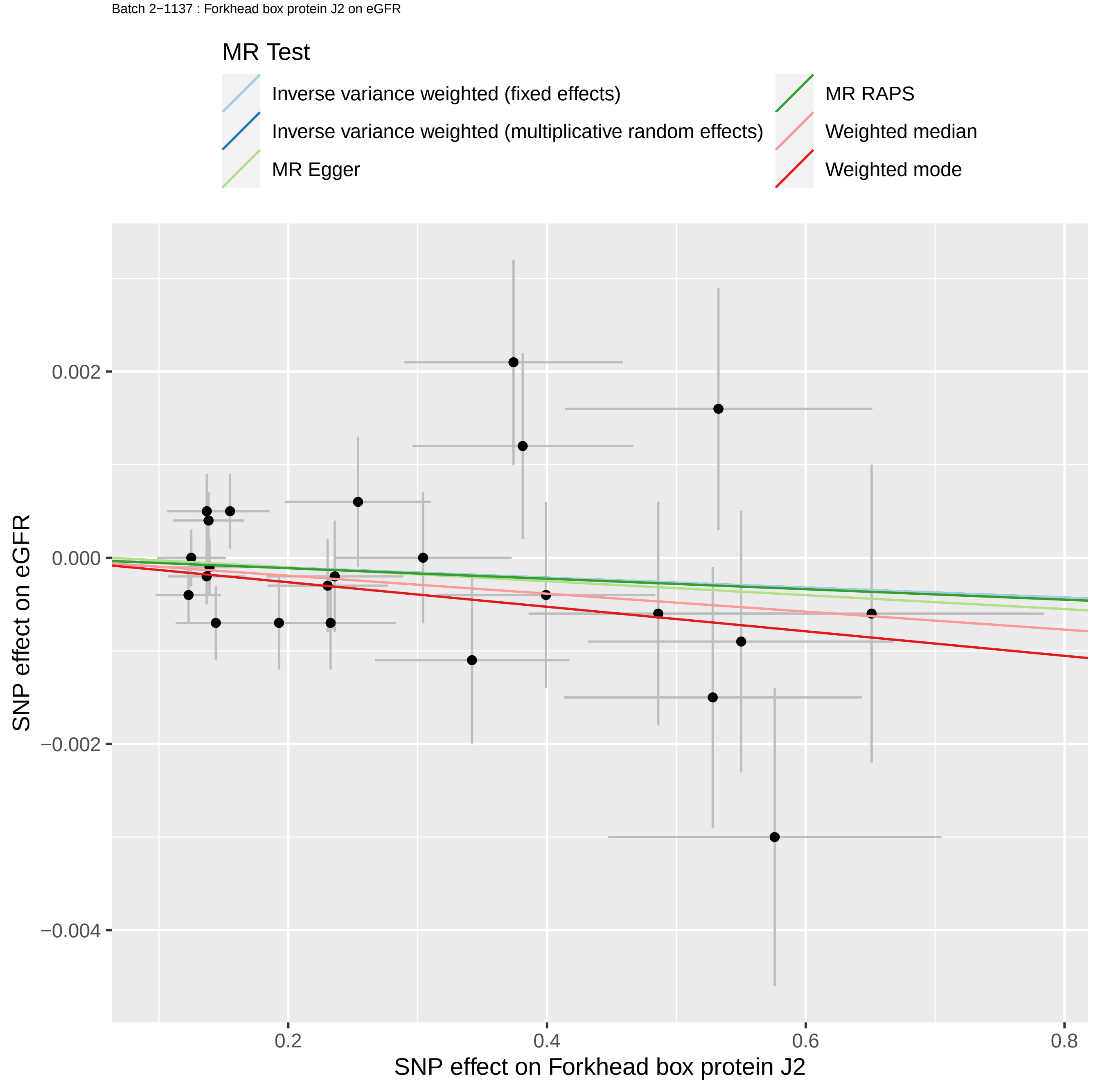
Batch 2−1137 : Forkhead box protein J2 on eGFR
MR Test
Inverse variance weighted (fixed effects)
Inverse variance weighted (multiplicative random effects)
MR Egger
MR RAPS
Weighted median
Weighted mode
SNP effect on eGFR
0.002
0.000
−0.002
−0.004
0.2
0.4
0.6
0.8
SNP effect on Forkhead box protein J2

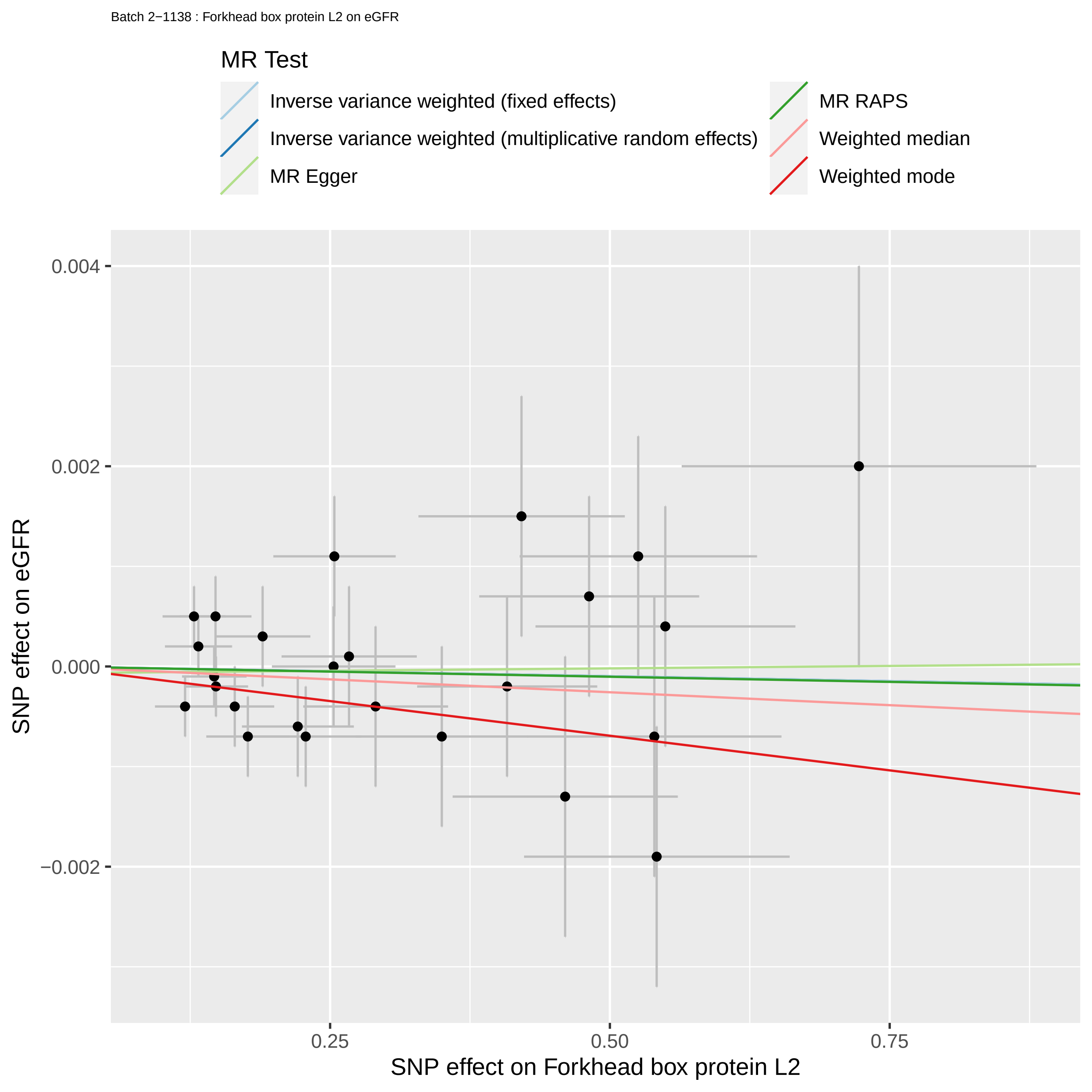
Batch 2−1138 : Forkhead box protein L2 on eGFR
MR Test
Inverse variance weighted (fixed effects)
Inverse variance weighted (multiplicative random effects)
MR Egger
MR RAPS
Weighted median
Weighted mode
0.004
0.002
0.000
−0.002
SNP effect on eGFR
0.25
0.50
0.75
SNP effect on Forkhead box protein L2

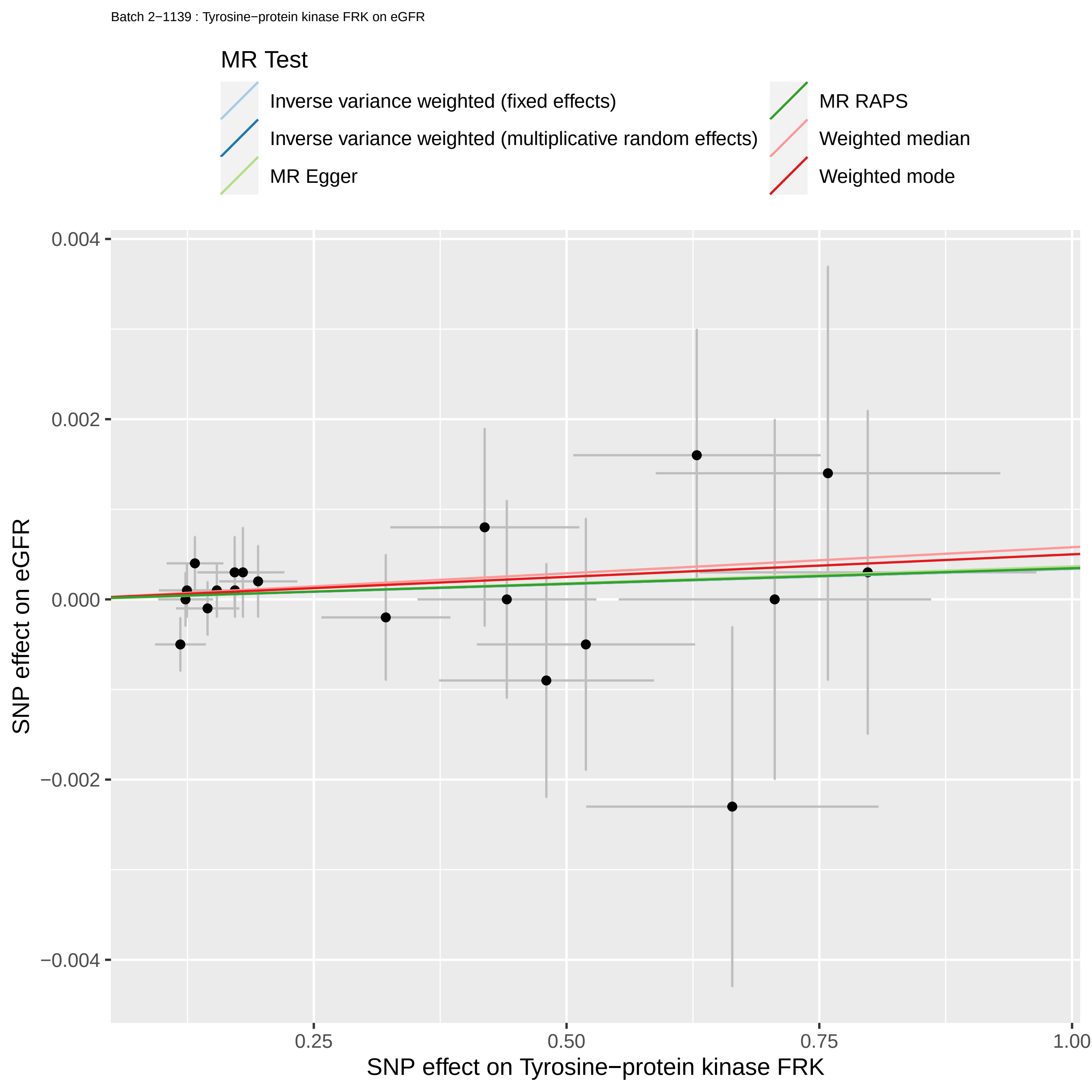

Batch 2−1139 : Tyrosine−protein kinase FRK on eGFR
MR Test
Inverse variance weighted (fixed effects)
Inverse variance weighted (multiplicative random effects)
MR Egger
MR RAPS
Weighted median
Weighted mode
0.004
0.002
0.000
−0.002
−0.004
SNP effect on eGFR
0.25
0.50
0.75
1.00
SNP effect on Tyrosine−protein kinase FRK

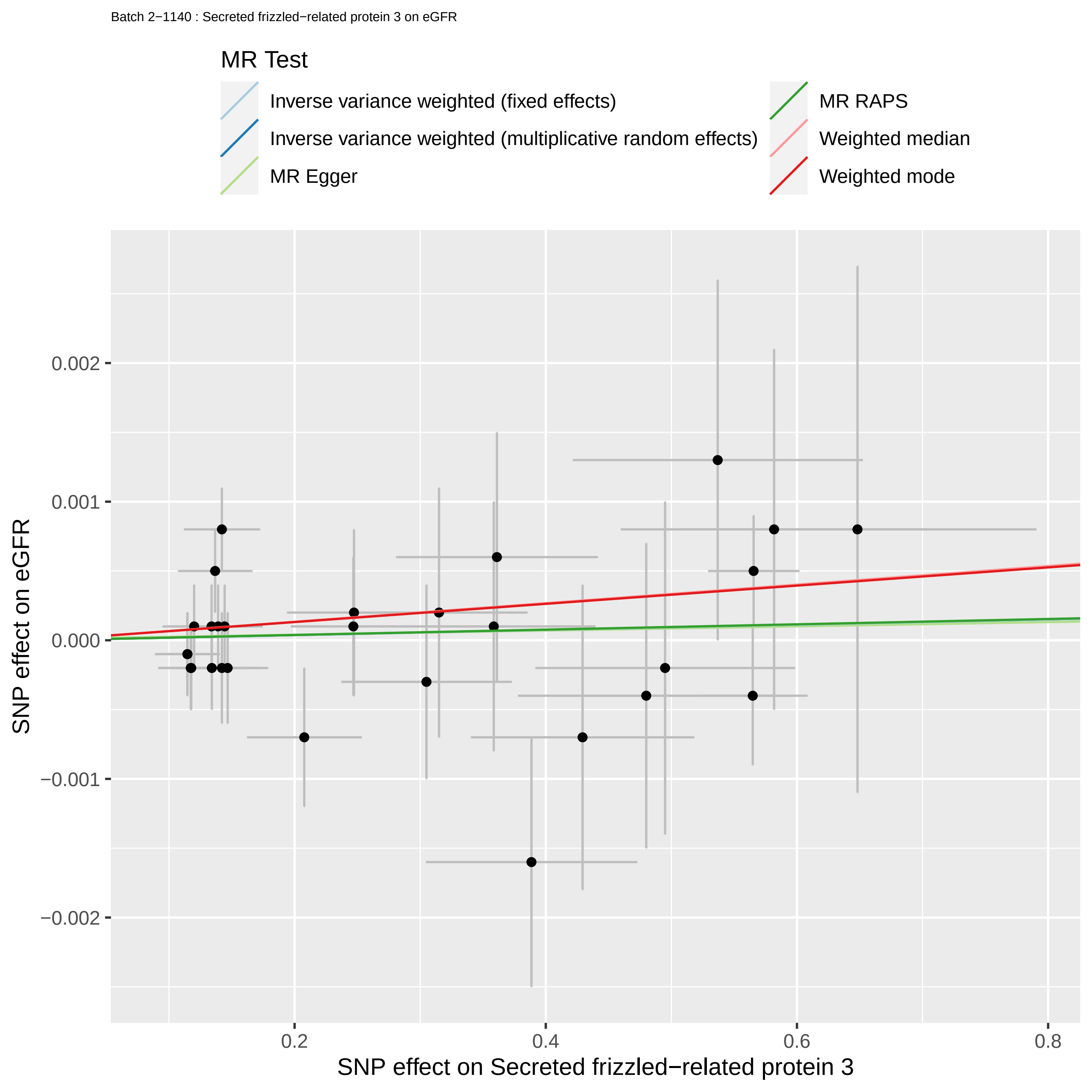

Batch 2−1140 : Secreted frizzled−related protein 3 on eGFR
MR Test
Inverse variance weighted (fixed effects)
Inverse variance weighted (multiplicative random effects)
MR Egger
MR RAPS
Weighted median
Weighted mode
0.002
0.001
0.000
−0.001
−0.002
SNP effect on eGFR
0.2
0.4
0.6
0.8
SNP effect on Secreted frizzled−related protein 3

Batch 2−1141 : Secreted frizzled−related protein 3 on eGFR

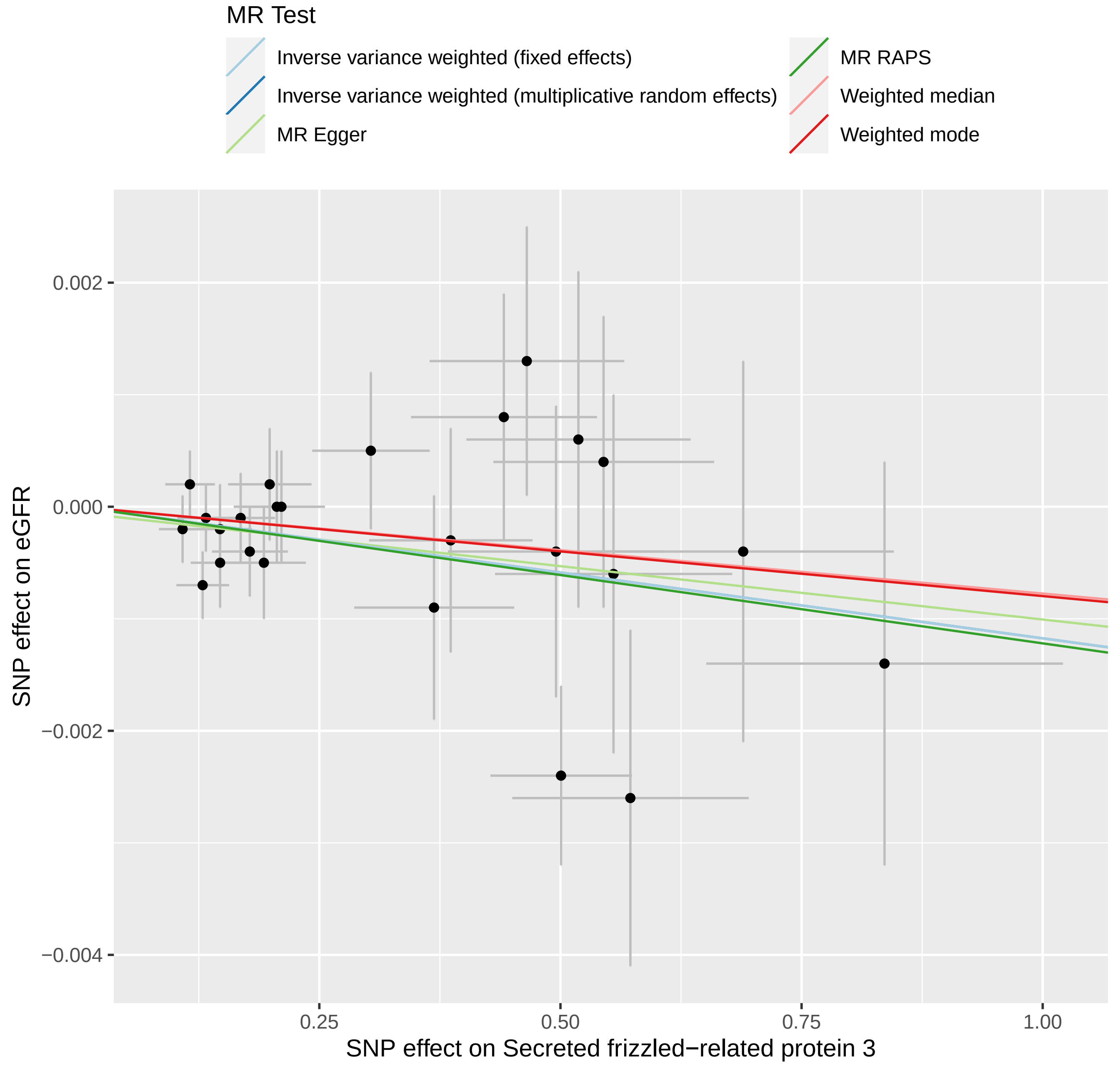

Batch 2−1142 : Follistatin on eGFR

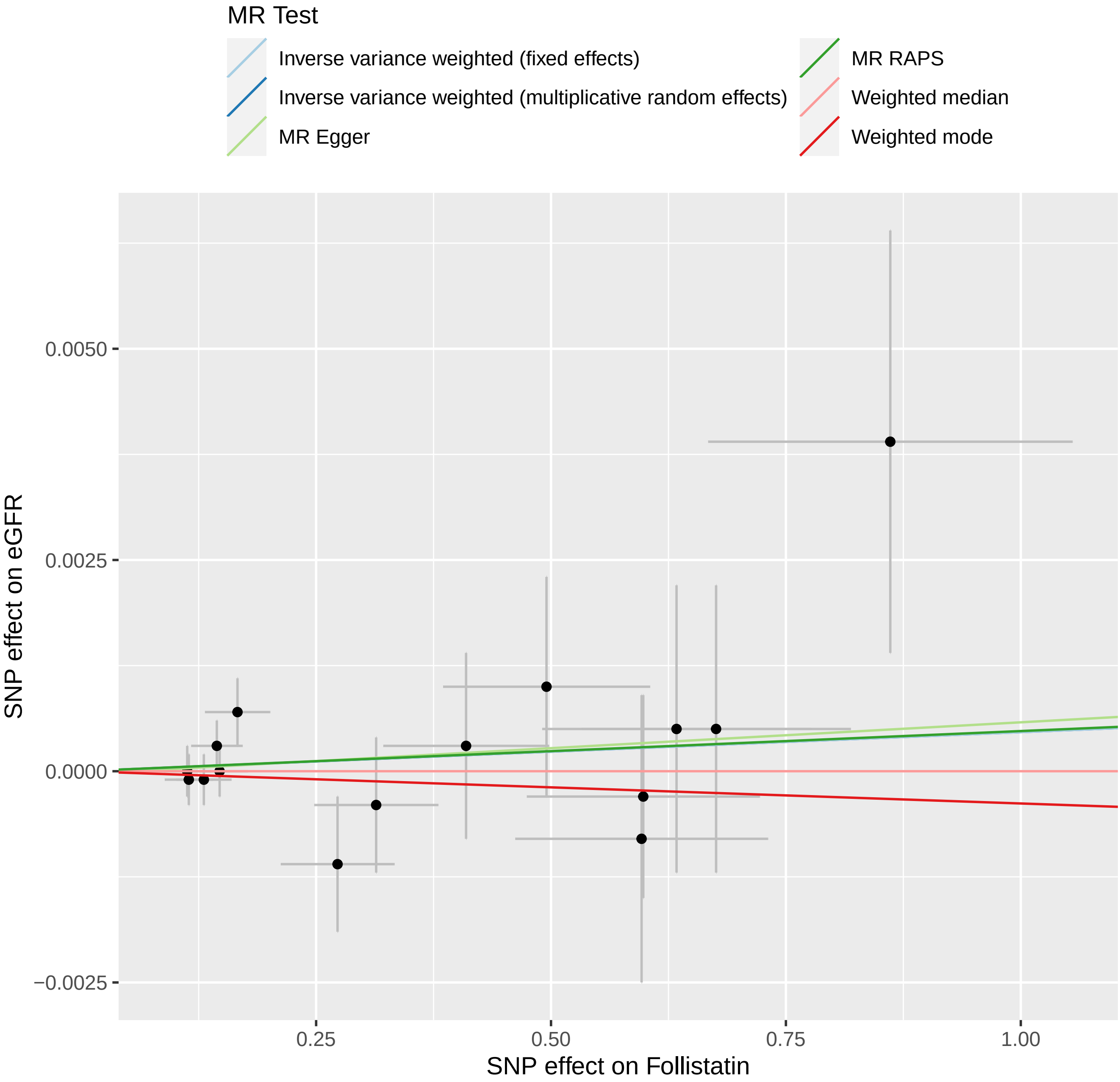

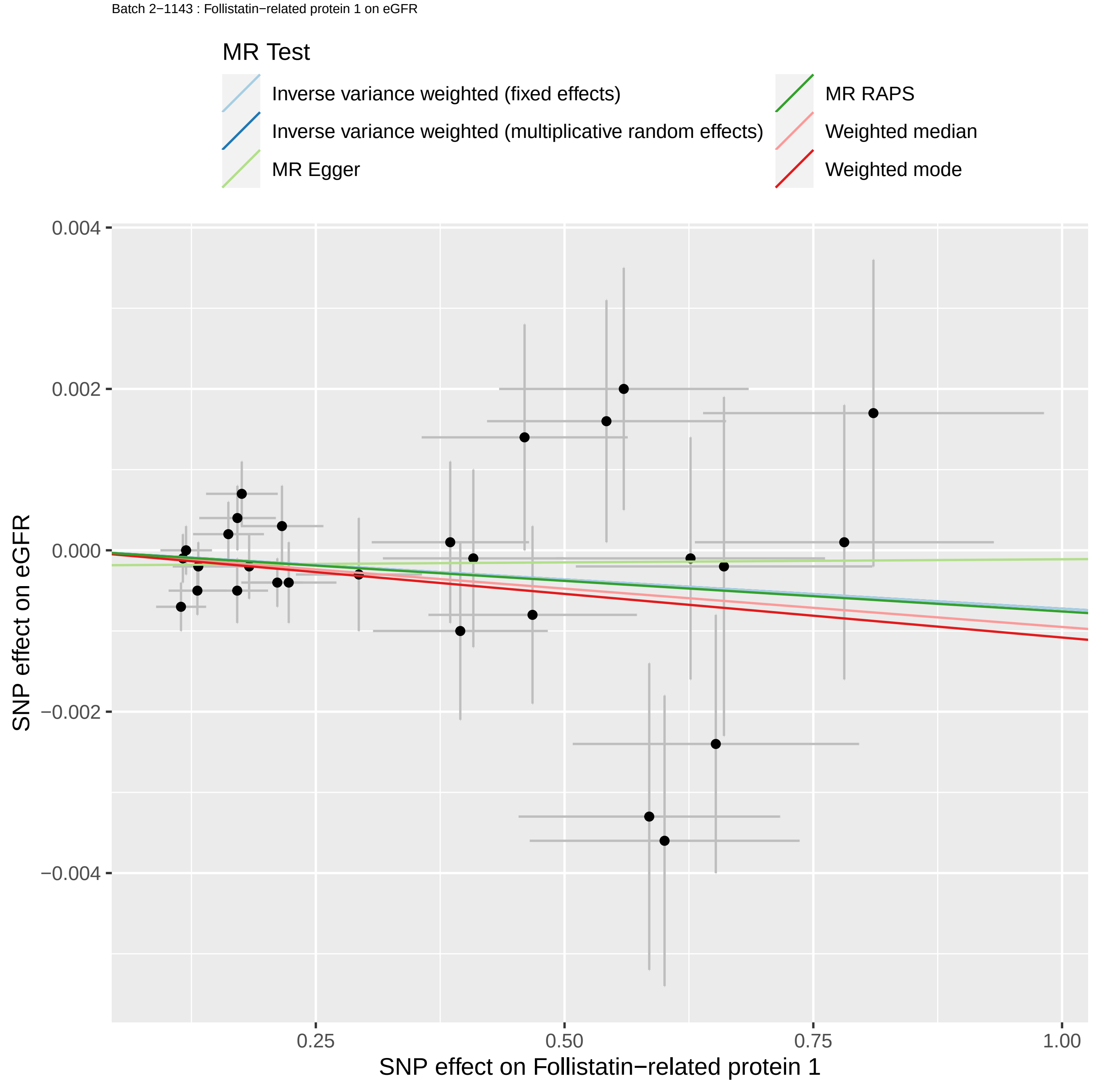

Batch 2−1143 : Follistatin−related protein 1 on eGFR
MR Test
Inverse variance weighted (fixed effects)
Inverse variance weighted (multiplicative random effects)
MR Egger
MR RAPS
Weighted median
Weighted mode
0.004
0.002
0.000
−0.002
−0.004
0.25
0.50
0.75
1.00
SNP effect on eGFR
SNP effect on Follistatin−related protein 1

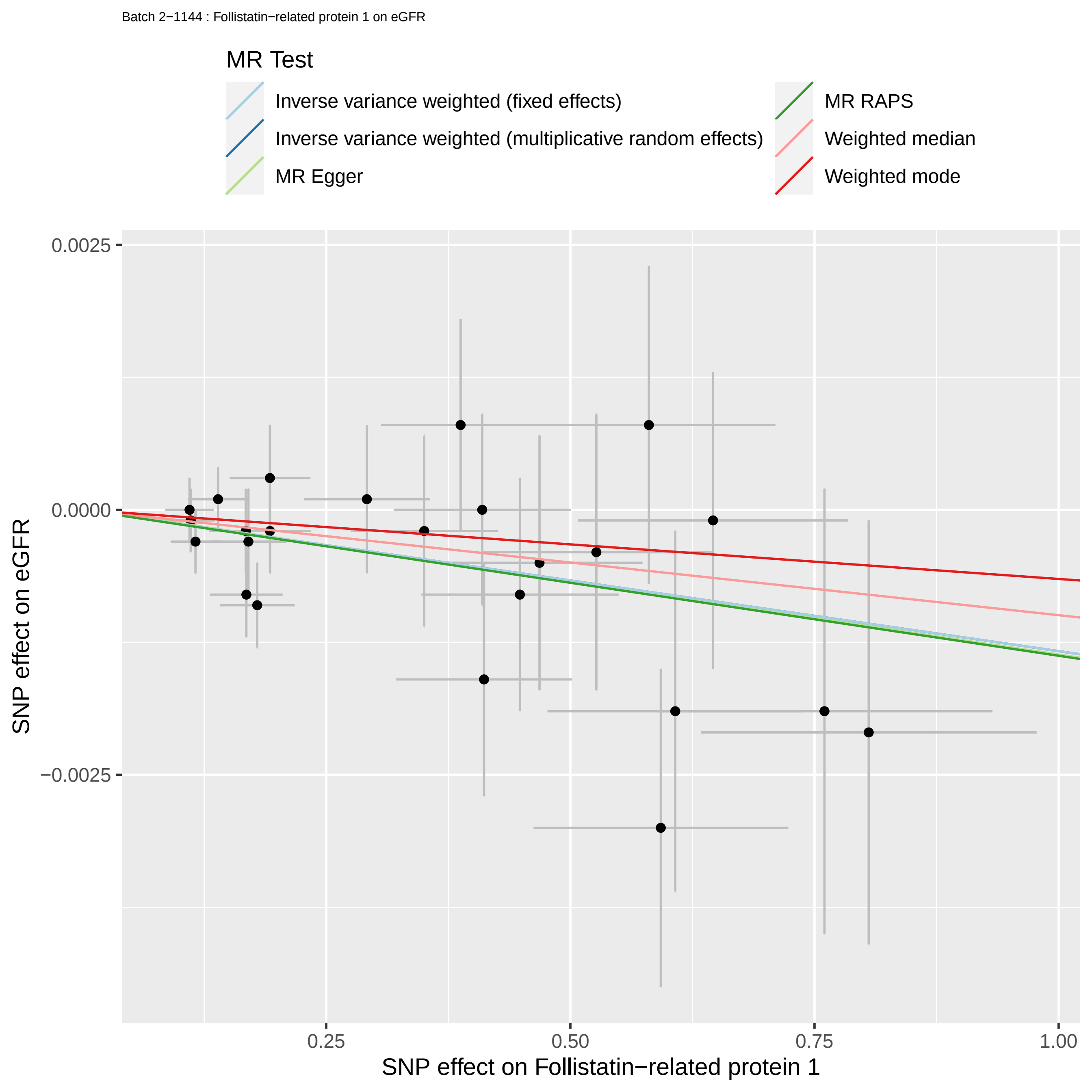

Batch 2−1144 : Follistatin−related protein 1 on eGFR
MR Test
Inverse variance weighted (fixed effects)
Inverse variance weighted (multiplicative random effects)
MR Egger
MR RAPS
Weighted median
Weighted mode
0.0025
0.0000
−0.0025
0.25
0.50
0.75
1.00
SNP effect on eGFR
SNP effect on Follistatin−related protein 1

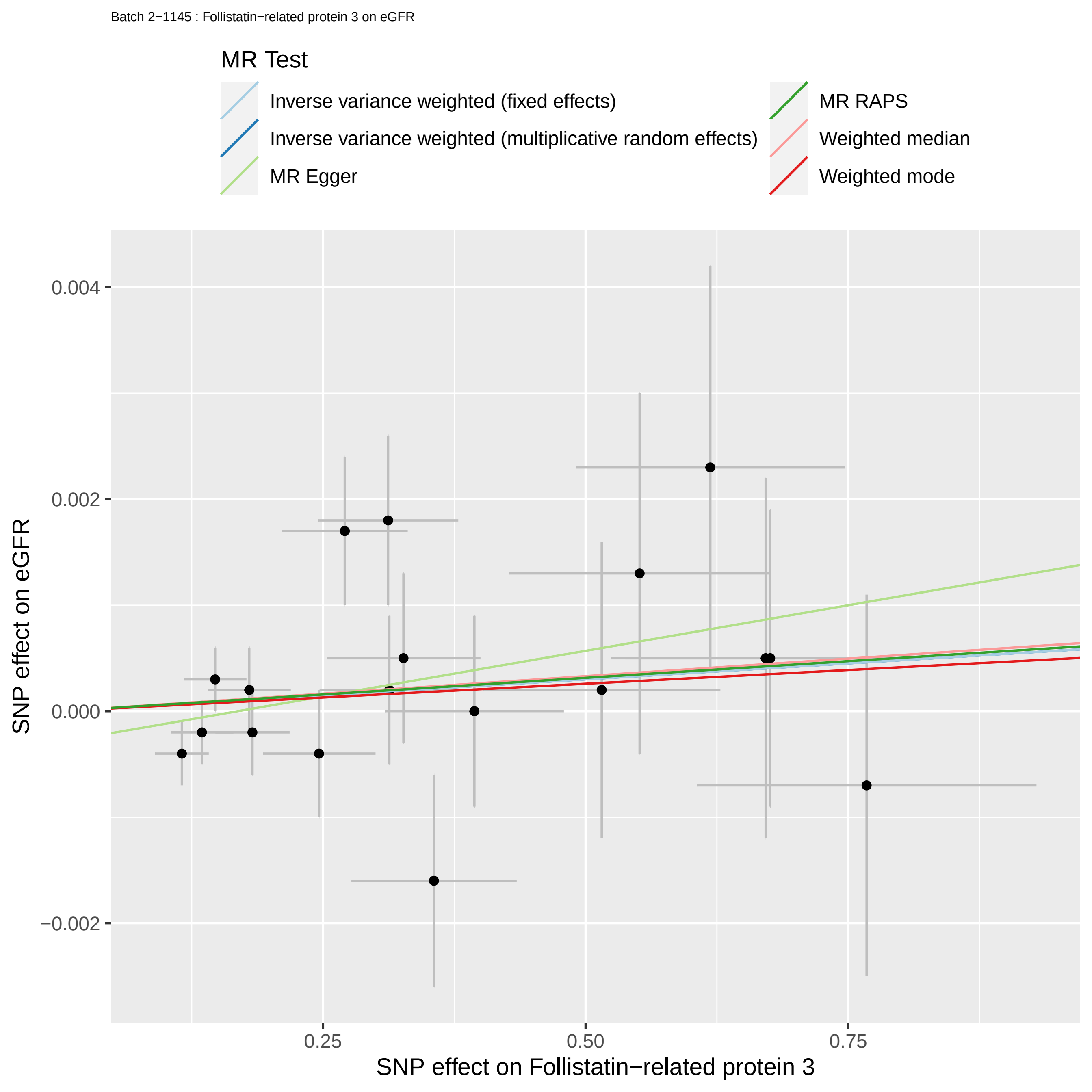
Batch 2−1145 : Follistatin−related protein 3 on eGFR
MR Test
Inverse variance weighted (fixed effects)
Inverse variance weighted (multiplicative random effects)
MR Egger
MR RAPS
Weighted median
Weighted mode
0.004
0.002
0.000
−0.002
0.25
0.50
0.75
SNP effect on eGFR
SNP effect on Follistatin−related protein 3

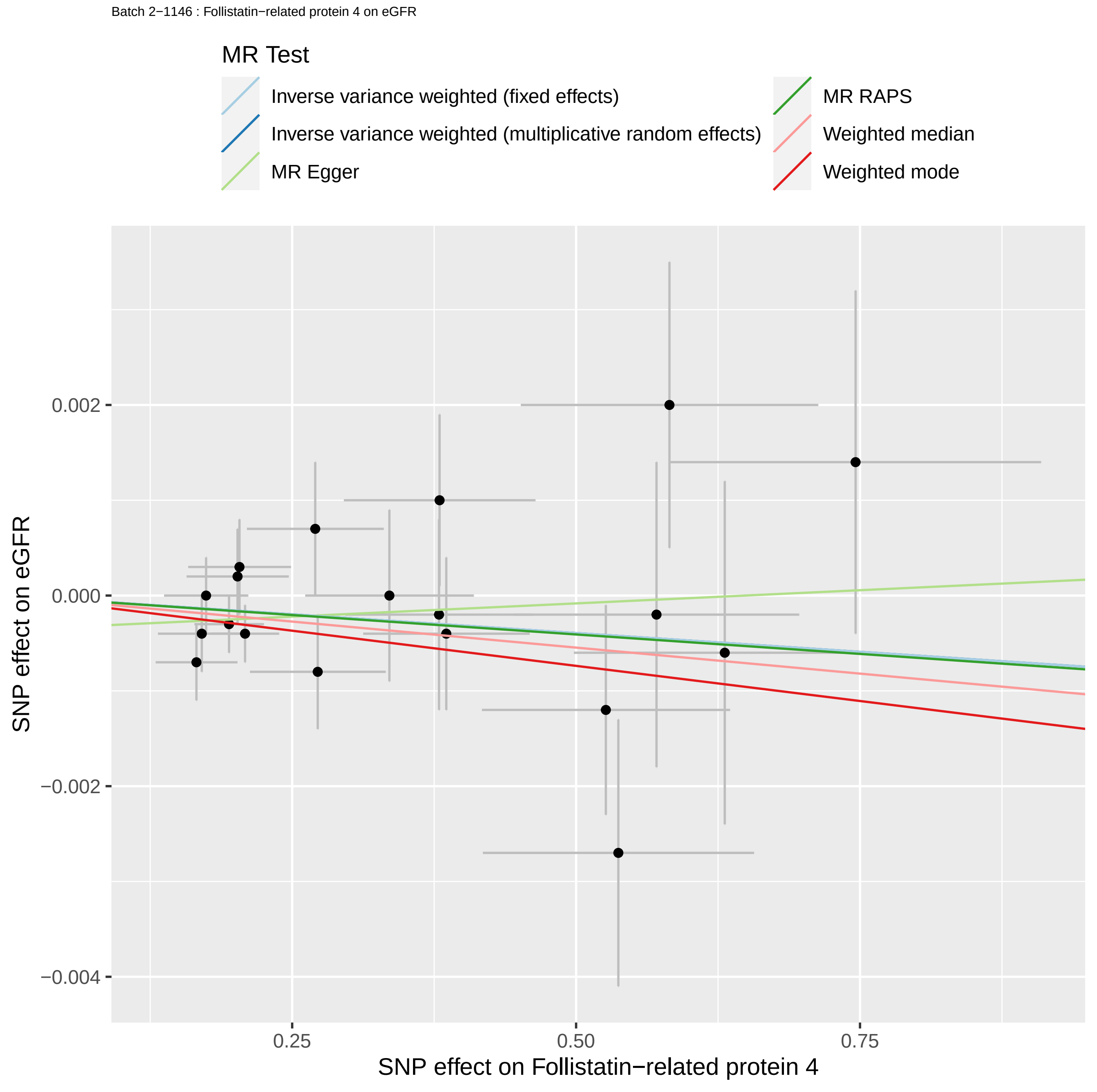

Batch 2−1146 : Follistatin−related protein 4 on eGFR
MR Test
Inverse variance weighted (fixed effects)
Inverse variance weighted (multiplicative random effects)
MR Egger
MR RAPS
Weighted median
Weighted mode
0.002
0.000
−0.002
−0.004
0.25
0.50
0.75
SNP effect on eGFR
SNP effect on Follistatin−related protein 4

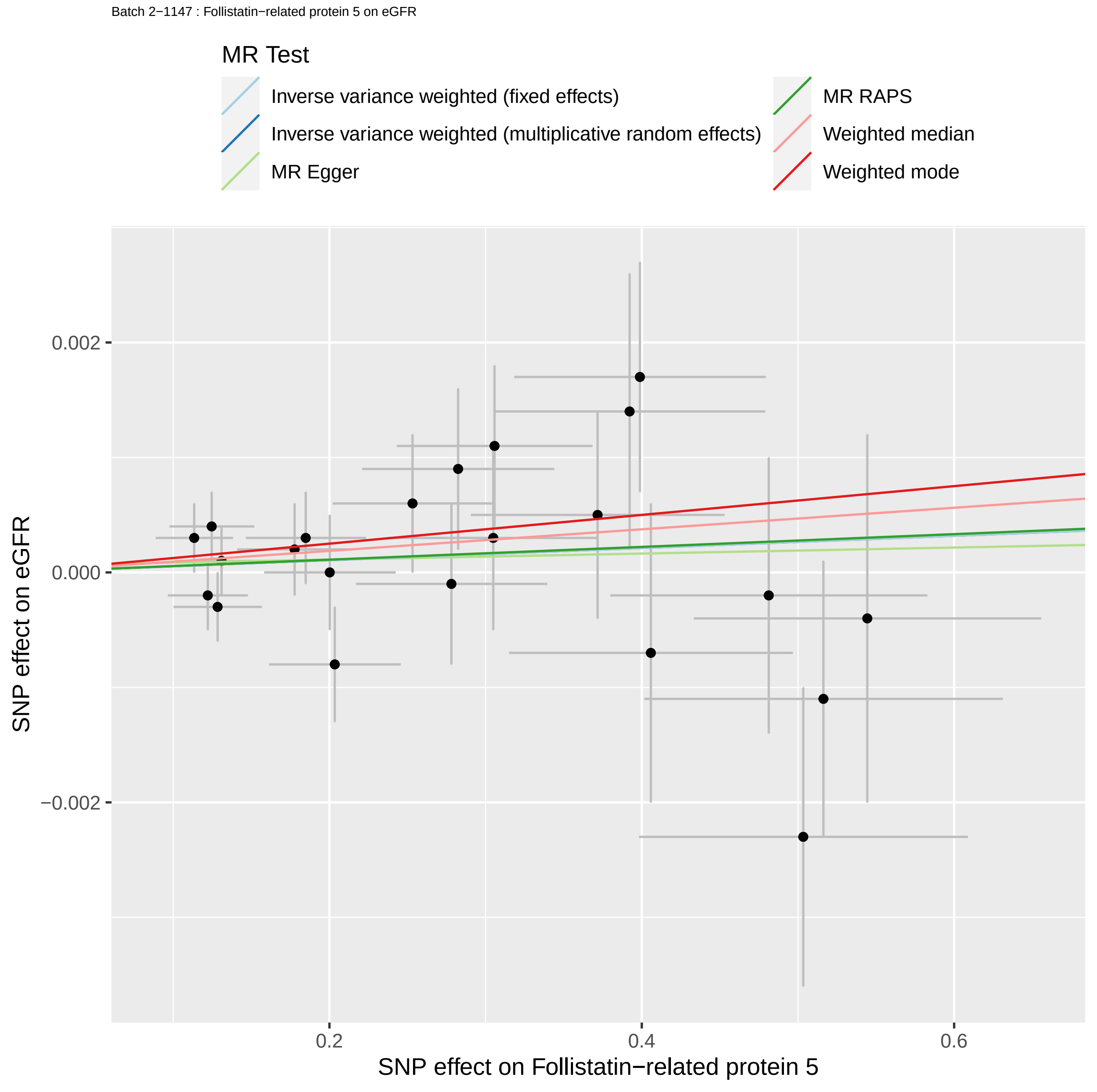
Batch 2−1147 : Follistatin−related protein 5 on eGFR
MR Test
Inverse variance weighted (fixed effects)
Inverse variance weighted (multiplicative random effects)
MR Egger
MR RAPS
Weighted median
Weighted mode
0.002
0.000
−0.002
0.2
0.4
0.6
SNP effect on eGFR
SNP effect on Follistatin−related protein 5

Batch 2−1148 : Ferritin on eGFR

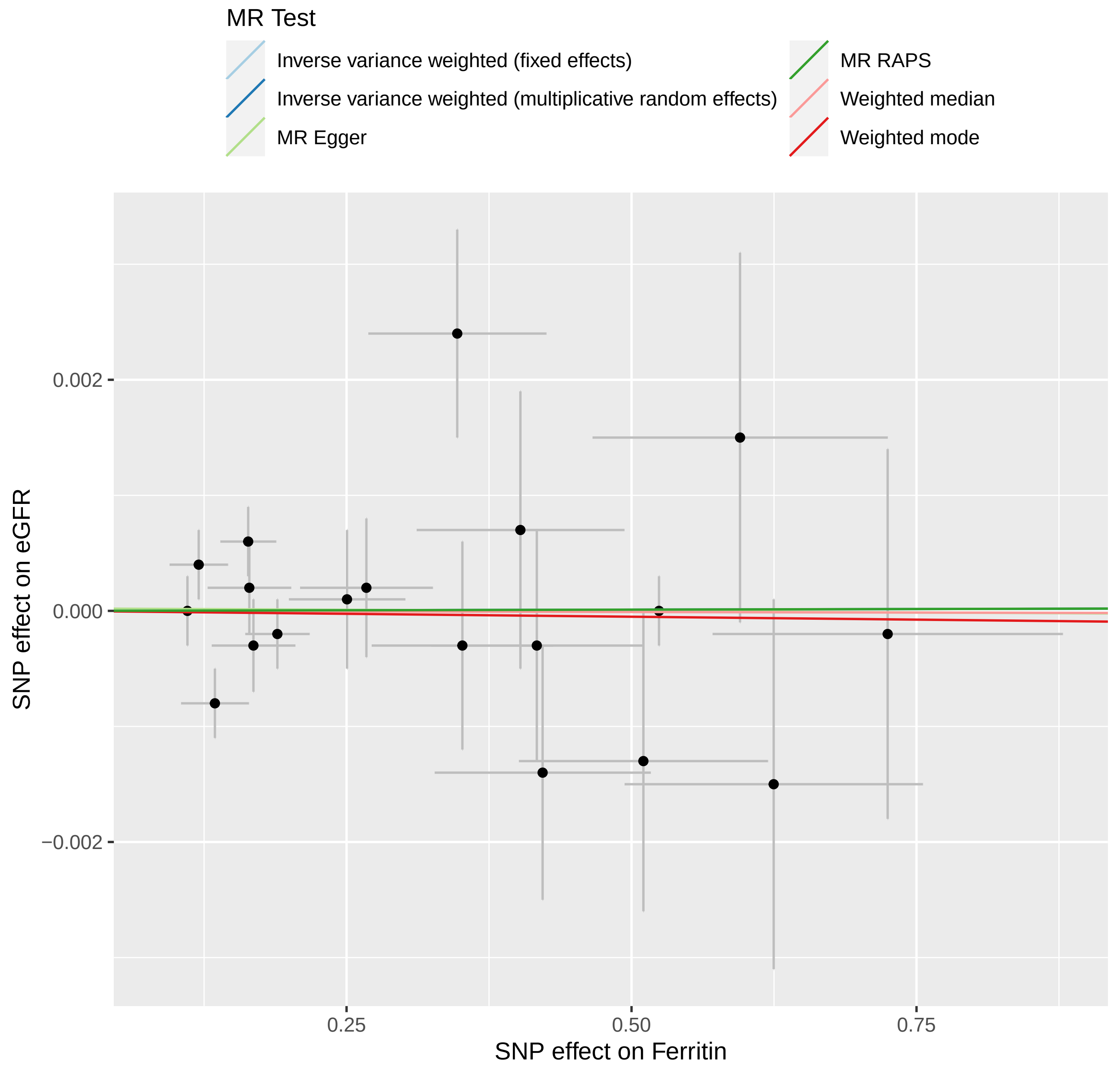

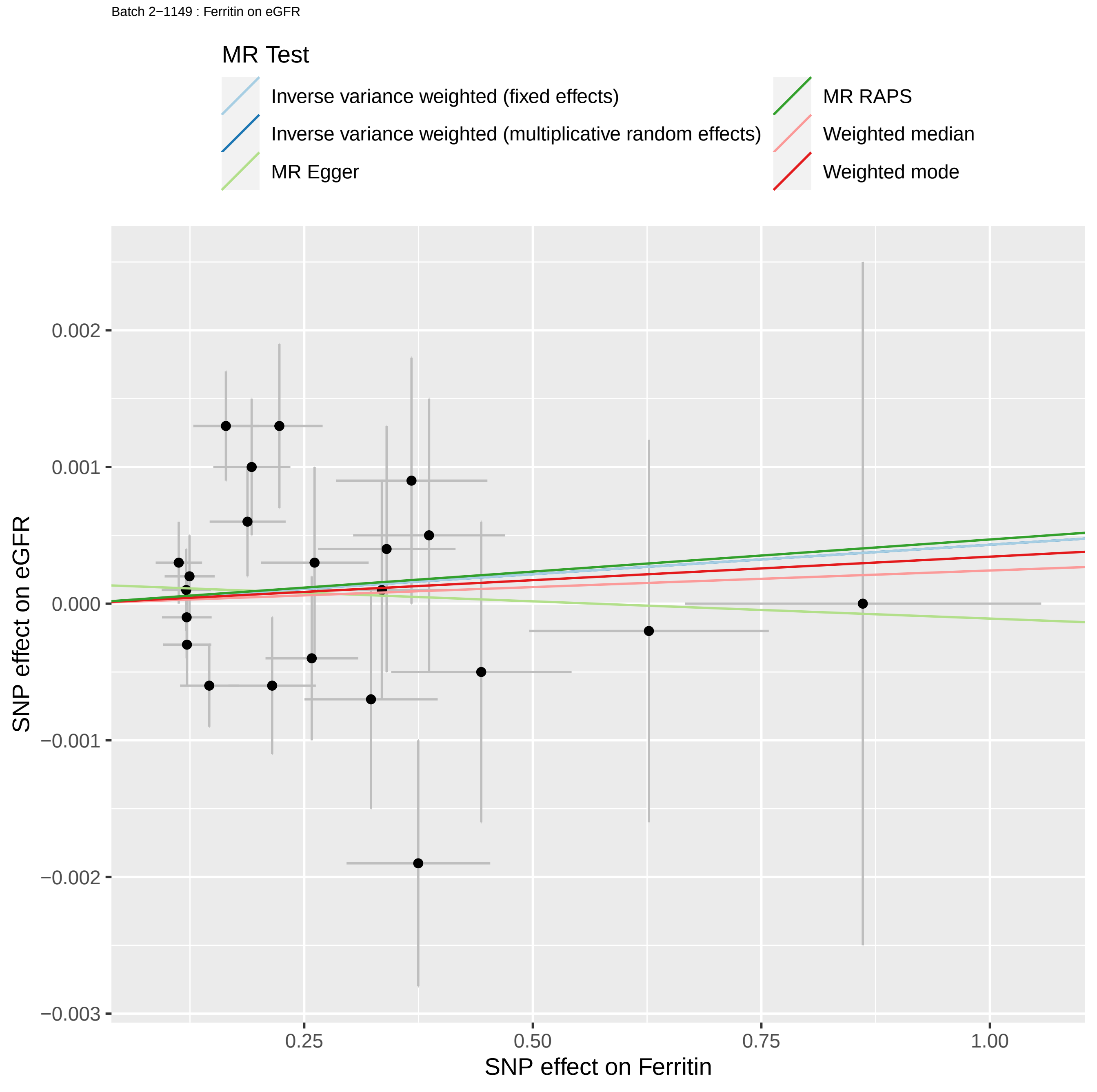
Batch 2−1149 : Ferritin on eGFR
MR Test
Inverse variance weighted (fixed effects)
Inverse variance weighted (multiplicative random effects)
MR Egger
MR RAPS
Weighted median
Weighted mode
0.002
0.001
0.000
−0.001
−0.002
−0.003
0.25
0.50
0.75
1.00
SNP effect on eGFR
SNP effect on Ferritin

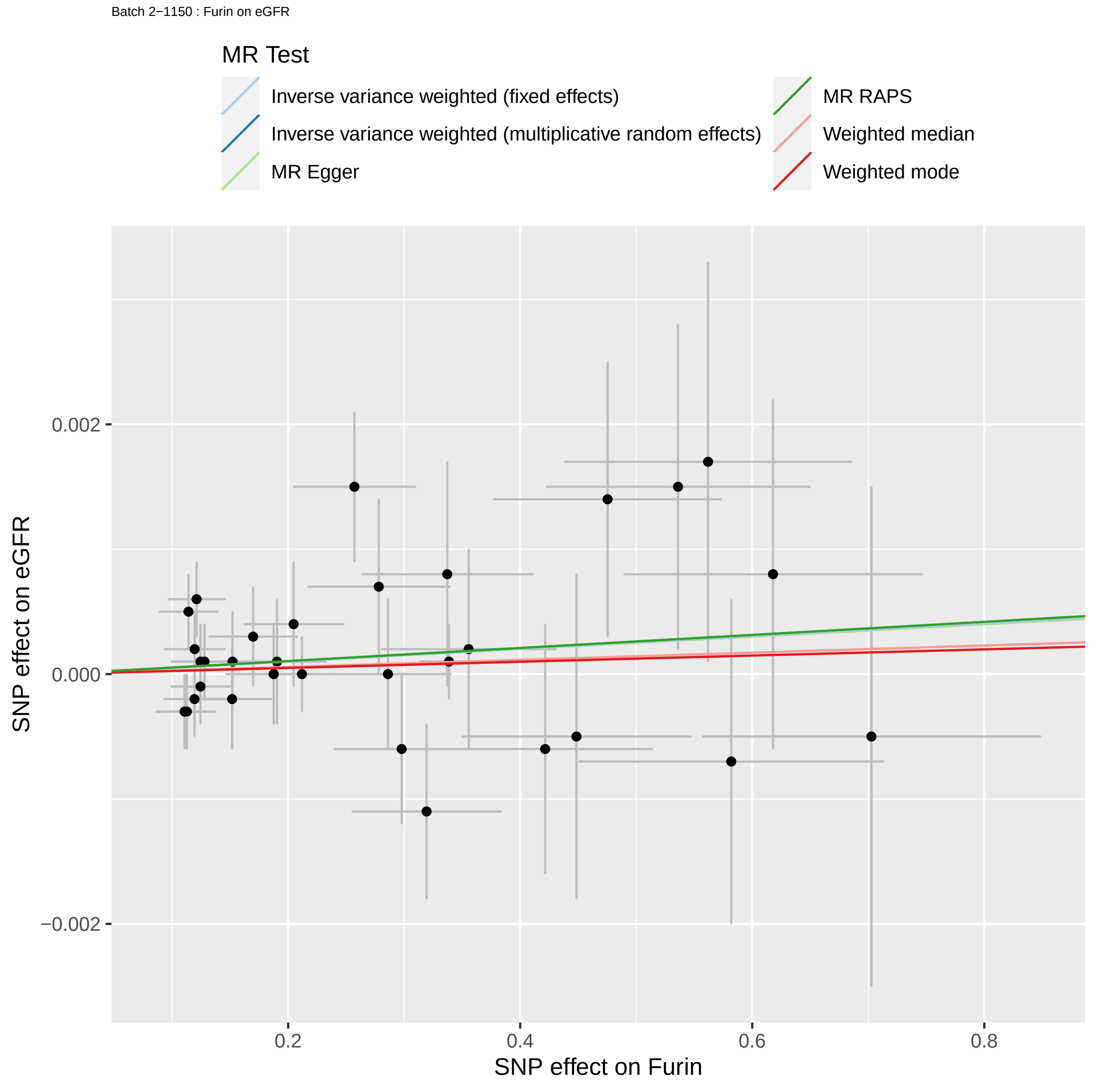

Batch 2−1150 : Furin on eGFR
MR Test
Inverse variance weighted (fixed effects)
Inverse variance weighted (multiplicative random effects)
MR Egger
MR RAPS
Weighted median
Weighted mode
0.002
0.000
−0.002
0.2
0.4
0.6
0.8
SNP effect on eGFR
SNP effect on Furin

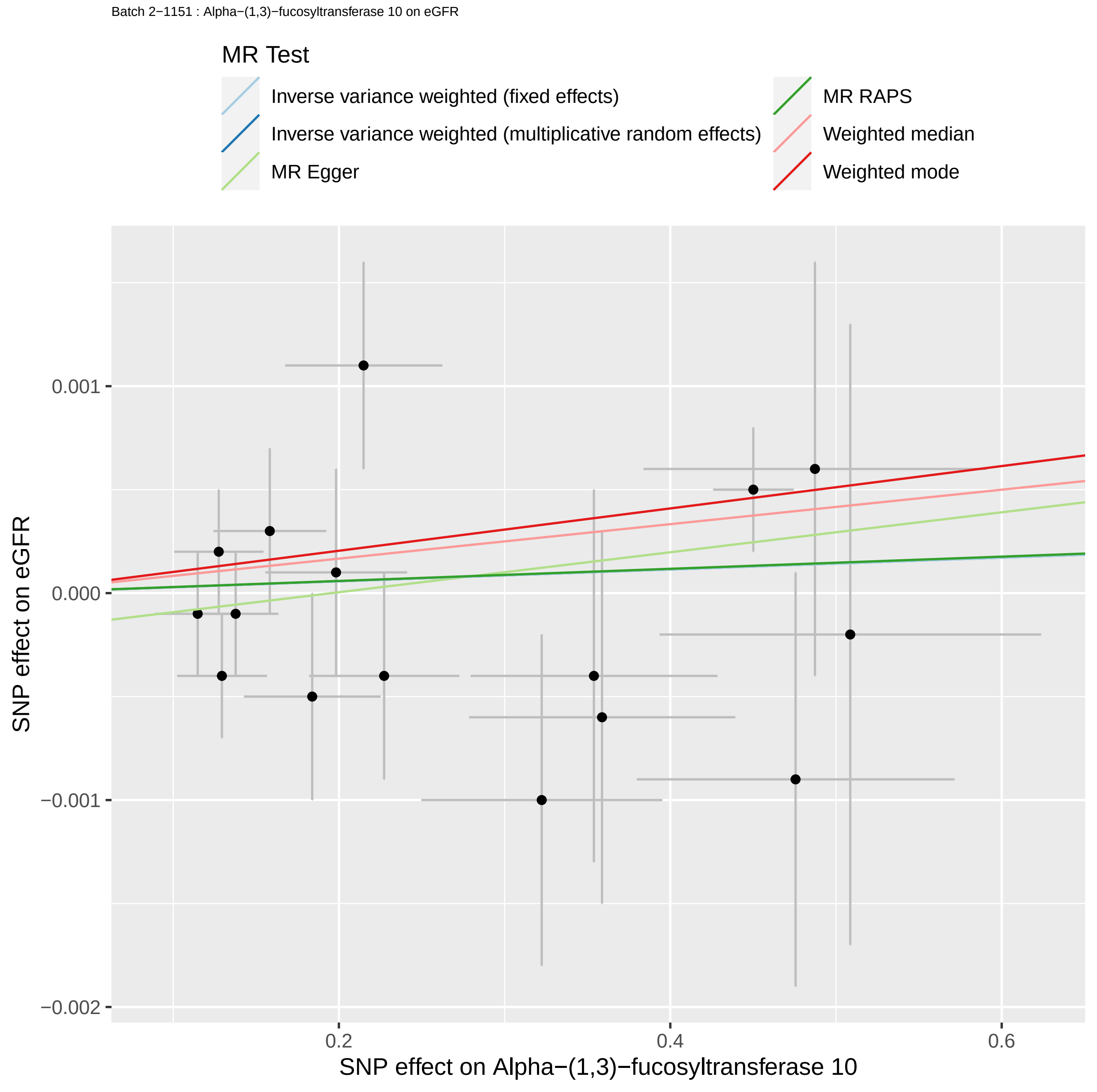
Batch 2−1151 : Alpha−(1,3)−fucosyltransferase 10 on eGFR
MR Test
Inverse variance weighted (fixed effects)
Inverse variance weighted (multiplicative random effects)
MR Egger
MR RAPS
Weighted median
Weighted mode
0.001
0.000
−0.001
−0.002
SNP effect on eGFR
0.2
0.4
0.6
SNP effect on Alpha−(1,3)−fucosyltransferase 10

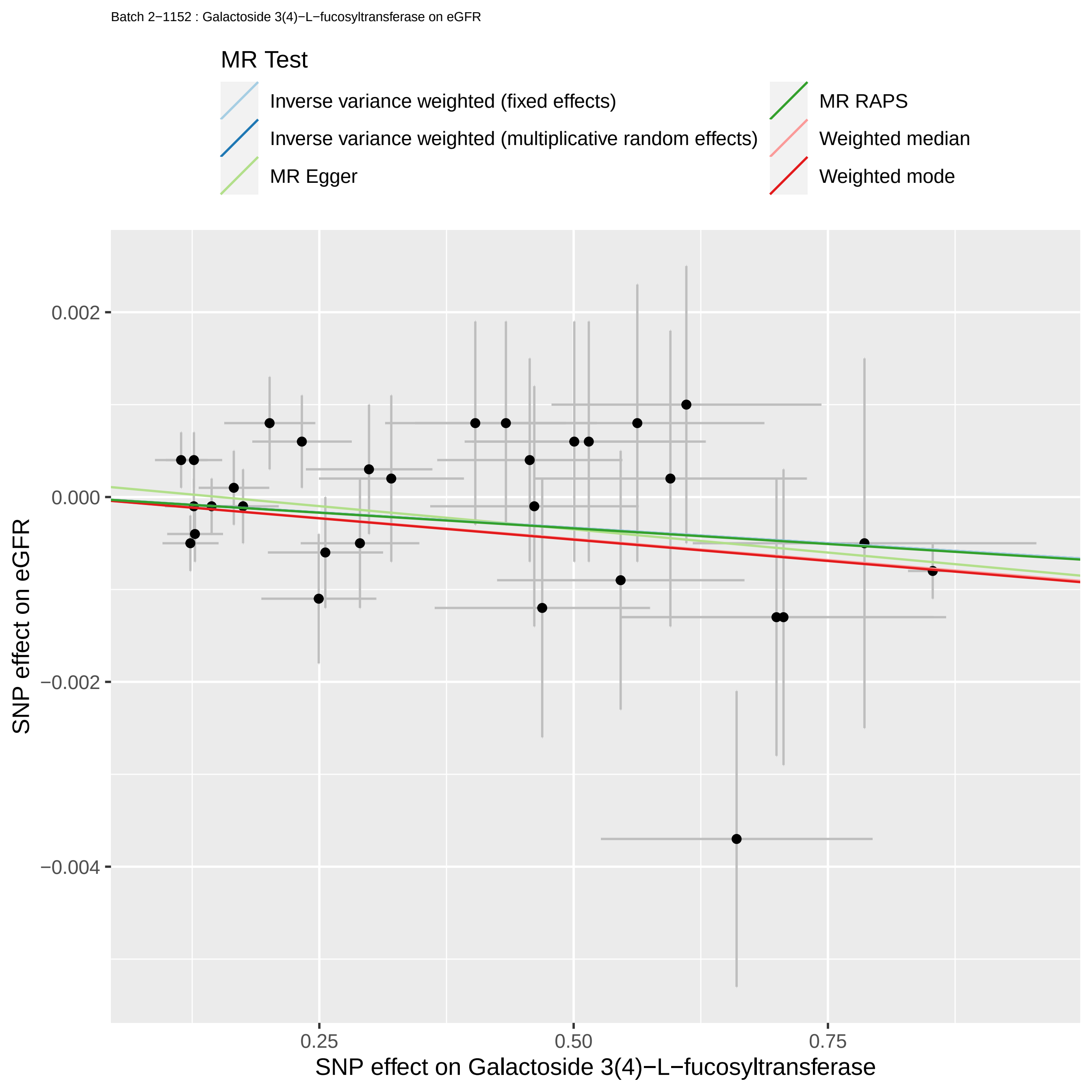
Batch 2−1152 : Galactoside 3(4)−L−fucosyltransferase on eGFR
MR Test
Inverse variance weighted (fixed effects)
Inverse variance weighted (multiplicative random effects)
MR Egger
MR RAPS
Weighted median
Weighted mode
0.002
0.000
−0.002
−0.004
0.25
0.50
0.75
SNP effect on eGFR
SNP effect on Galactoside 3(4)−L−fucosyltransferase

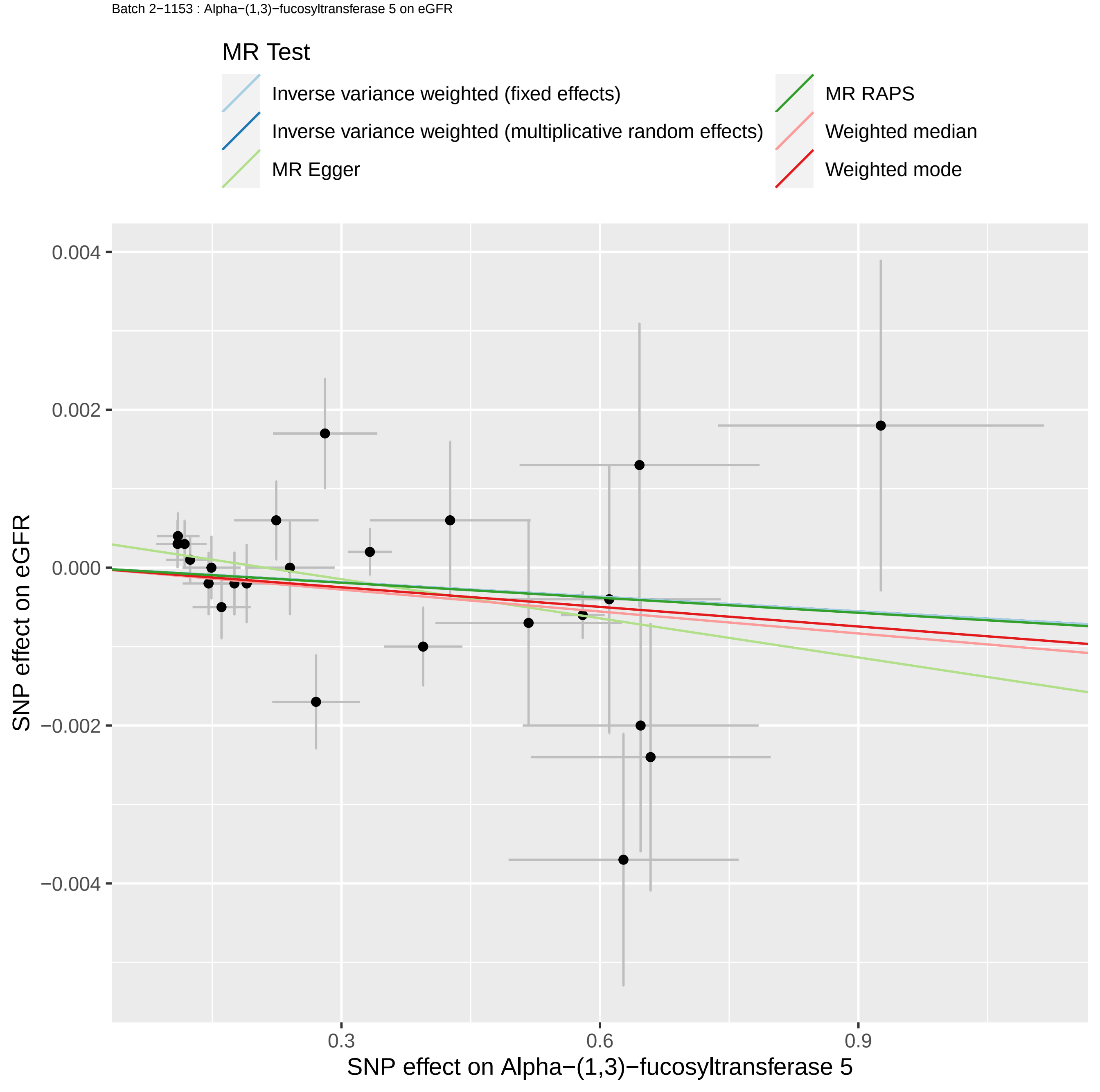
Batch 2−1153 : Alpha−(1,3)−fucosyltransferase 5 on eGFR
MR Test
Inverse variance weighted (fixed effects)
Inverse variance weighted (multiplicative random effects)
MR Egger
MR RAPS
Weighted median
Weighted mode
0.004
0.002
0.000
−0.002
−0.004
0.3
0.6
0.9
SNP effect on eGFR
SNP effect on Alpha−(1,3)−fucosyltransferase 5

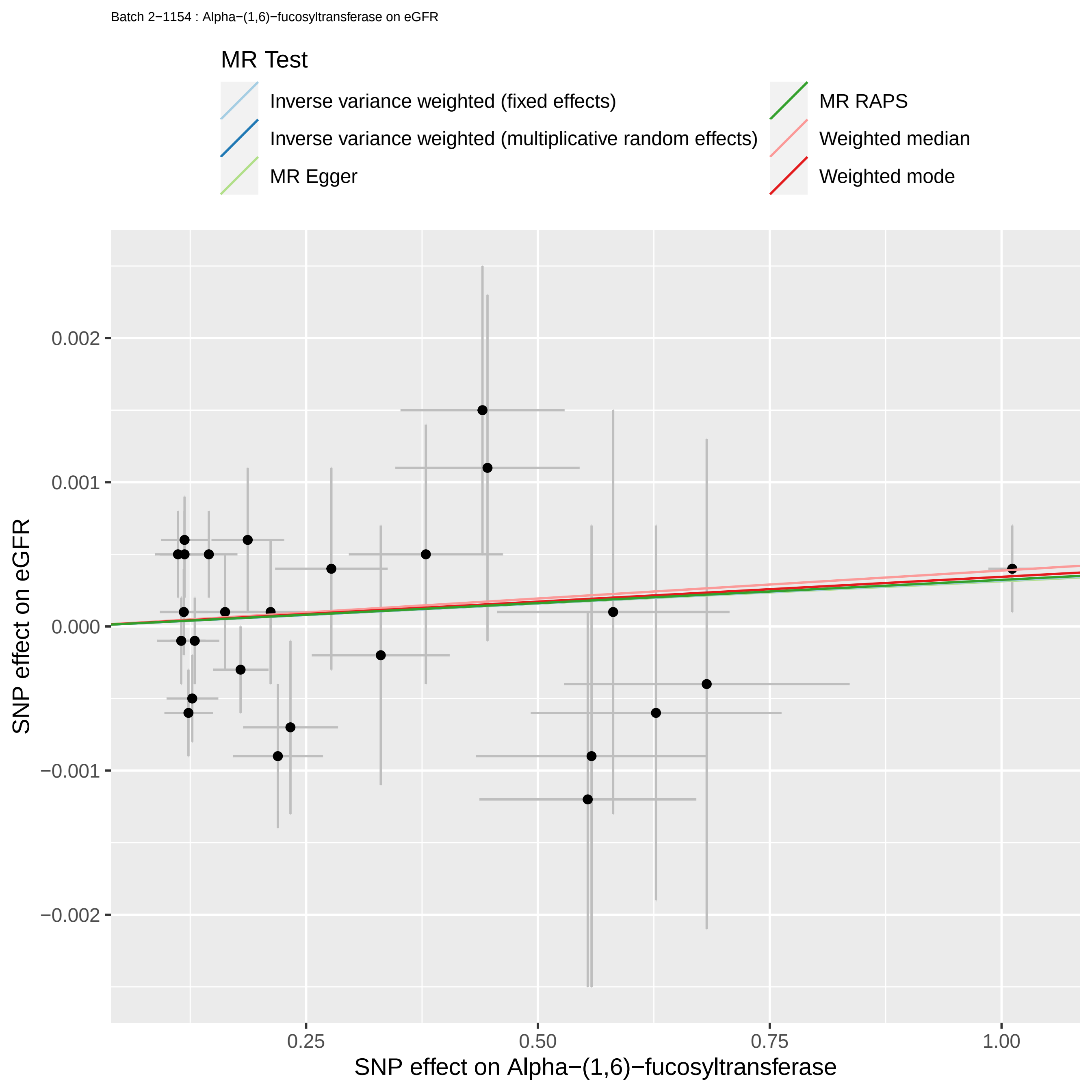

Batch 2−1154 : Alpha−(1,6)−fucosyltransferase on eGFR
MR Test
Inverse variance weighted (fixed effects)
Inverse variance weighted (multiplicative random effects)
MR Egger
MR RAPS
Weighted median
Weighted mode
0.002
0.001
0.000
−0.001
−0.002
0.25
0.50
0.75
1.00
SNP effect on eGFR
SNP effect on Alpha−(1,6)−fucosyltransferase

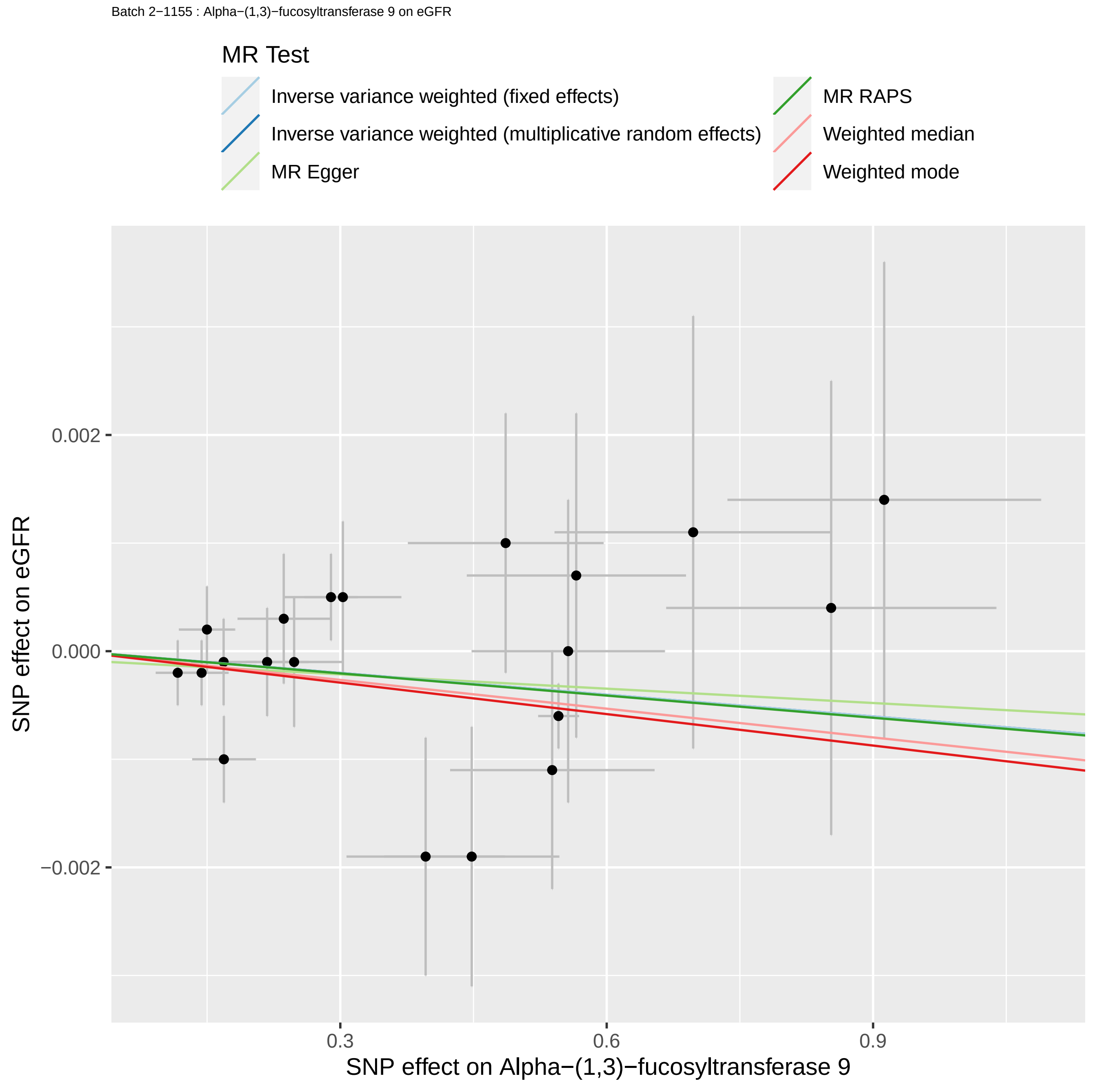
Batch 2−1155 : Alpha−(1,3)−fucosyltransferase 9 on eGFR
MR Test
Inverse variance weighted (fixed effects)
Inverse variance weighted (multiplicative random effects)
MR Egger
MR RAPS
Weighted median
Weighted mode
0.002
0.000
−0.002
0.3
0.6
0.9
SNP effect on eGFR
SNP effect on Alpha−(1,3)−fucosyltransferase 9

Batch 2−1156 : Fragile X mental retardation syndrome−related protein 1 on eGFR

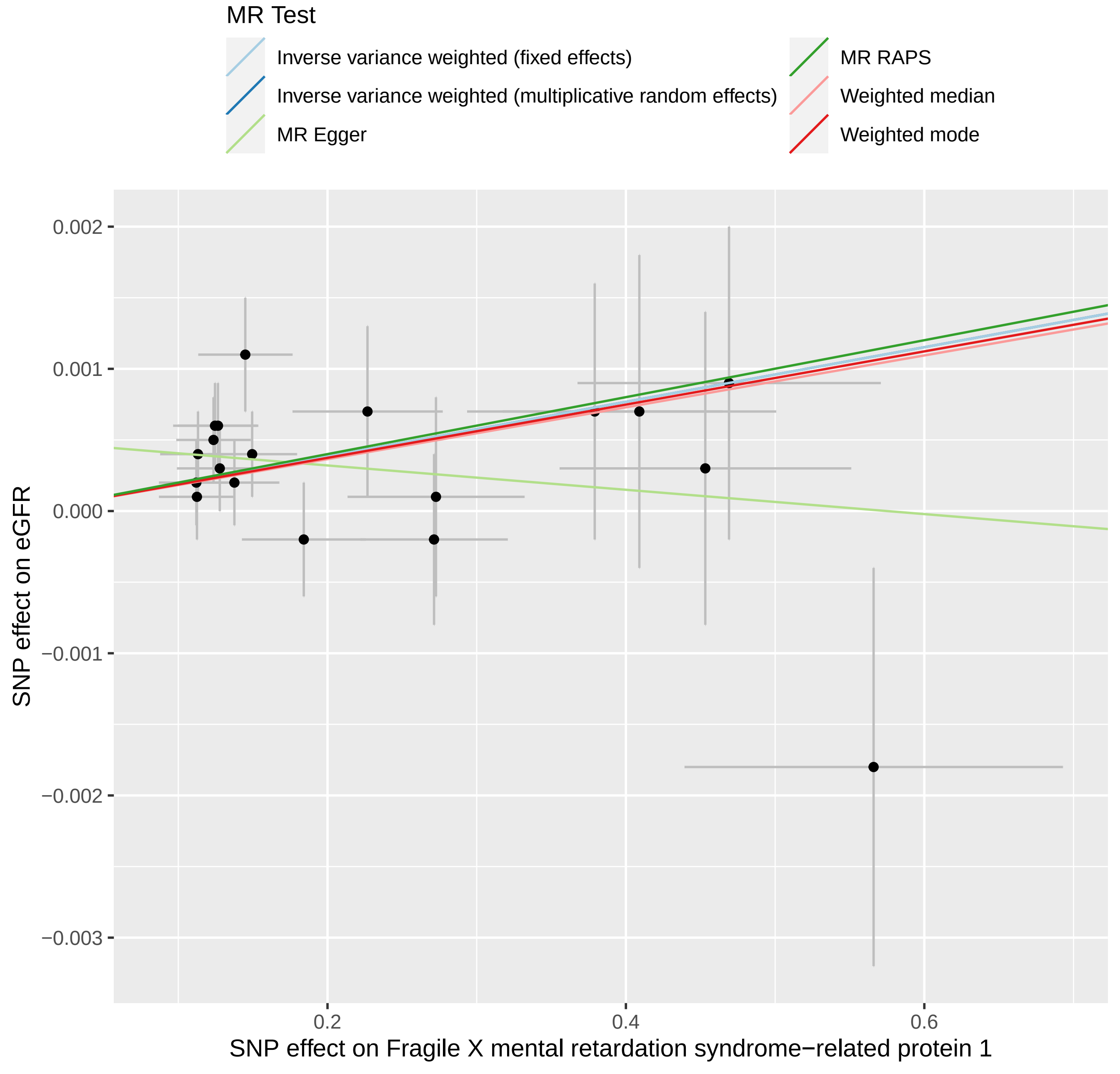

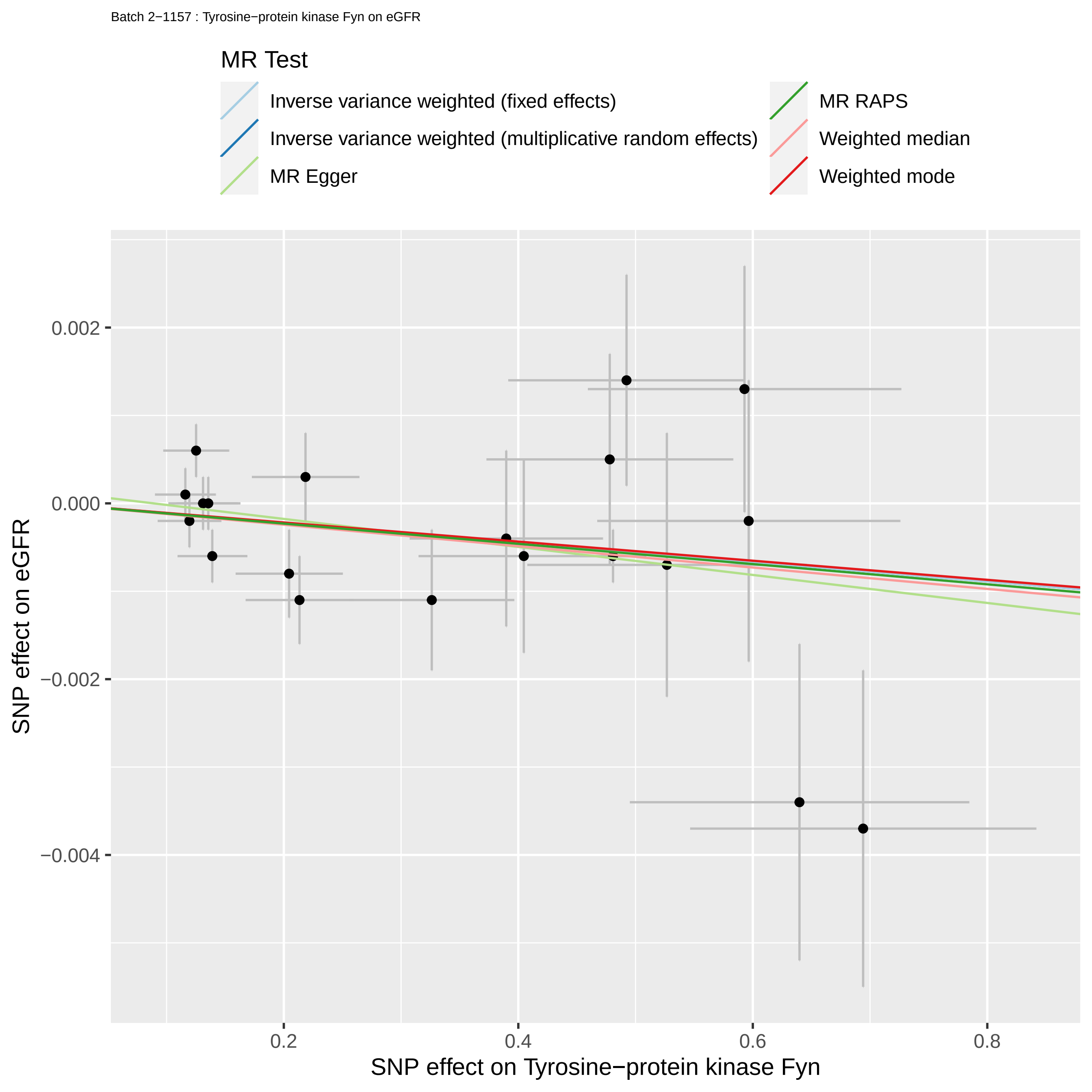
Batch 2−1157 : Tyrosine−protein kinase Fyn on eGFR
MR Test
Inverse variance weighted (fixed effects)
Inverse variance weighted (multiplicative random effects)
MR Egger
MR RAPS
Weighted median
Weighted mode
0.002
0.000
−0.002
−0.004
SNP effect on eGFR
0.2
0.4
0.6
0.8
SNP effect on Tyrosine−protein kinase Fyn

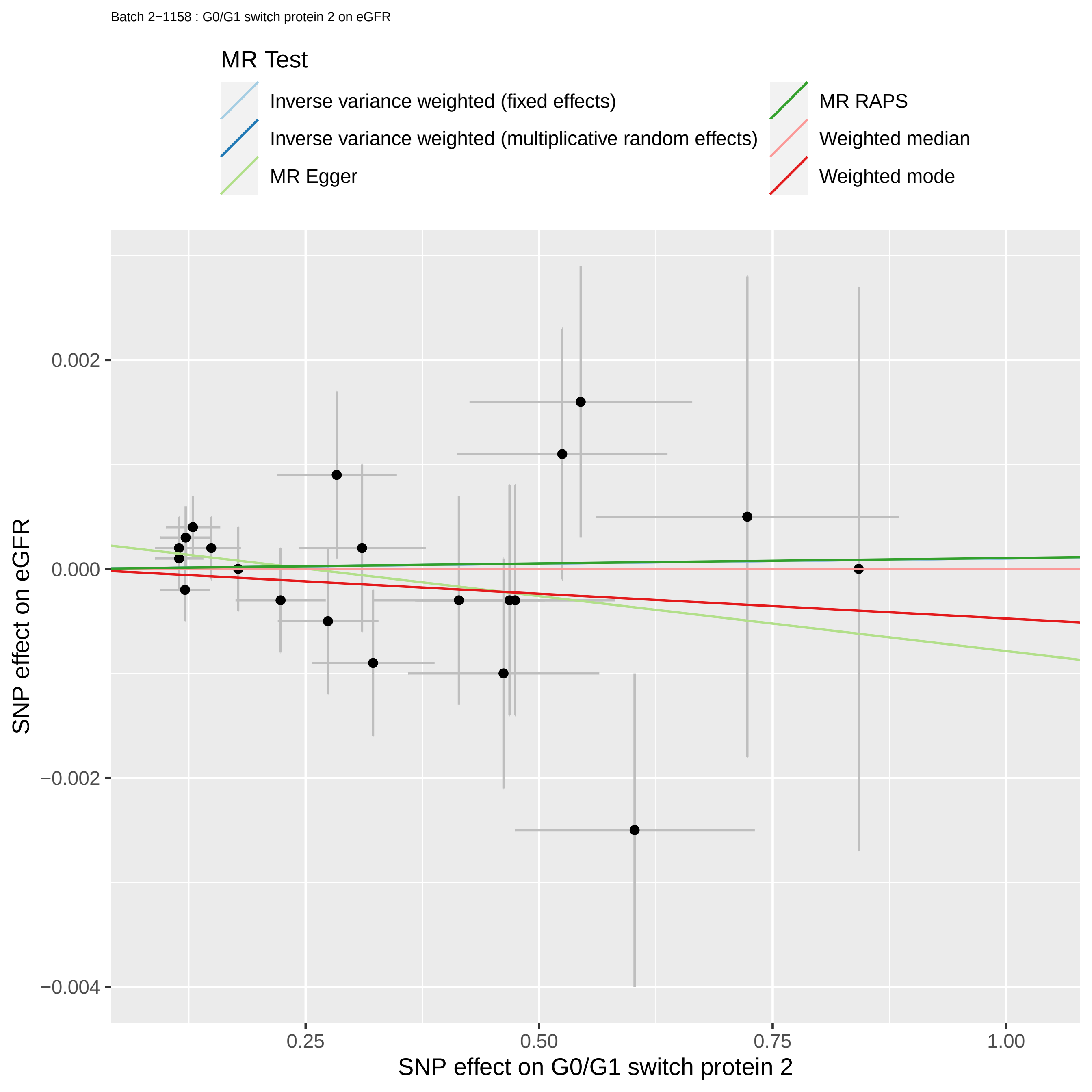

Batch 2−1158 : G0/G1 switch protein 2 on eGFR
MR Test
Inverse variance weighted (fixed effects)
Inverse variance weighted (multiplicative random effects)
MR Egger
MR RAPS
Weighted median
Weighted mode
0.002
0.000
−0.002
−0.004
0.25
0.50
0.75
1.00
SNP effect on eGFR
SNP effect on G0/G1 switch protein 2

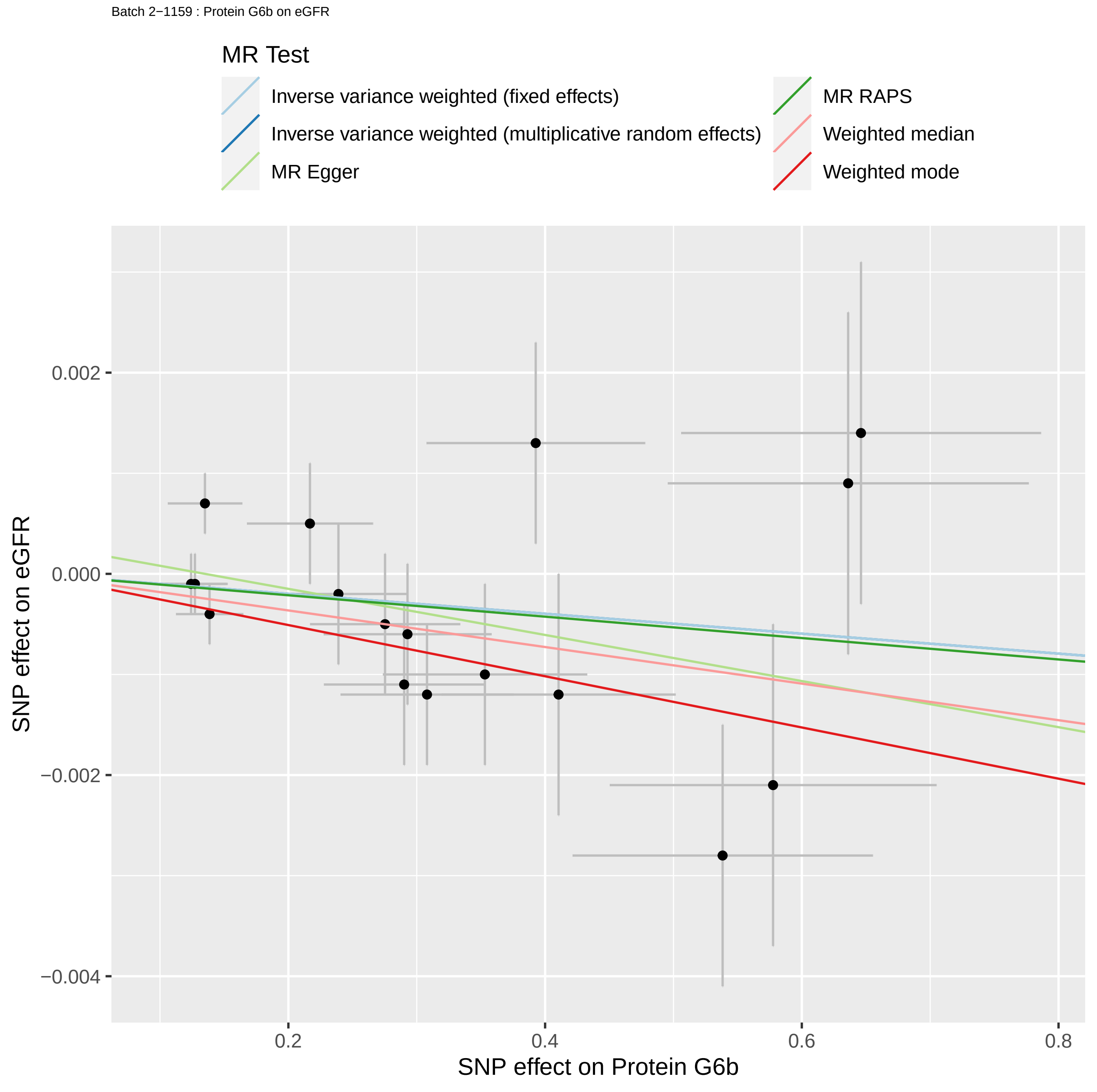
Batch 2−1159 : Protein G6b on eGFR
MR Test
Inverse variance weighted (fixed effects)
Inverse variance weighted (multiplicative random effects)
MR Egger
MR RAPS
Weighted median
Weighted mode
0.002
0.000
−0.002
−0.004
0.2
0.4
0.6
0.8
SNP effect on eGFR
SNP effect on Protein G6b

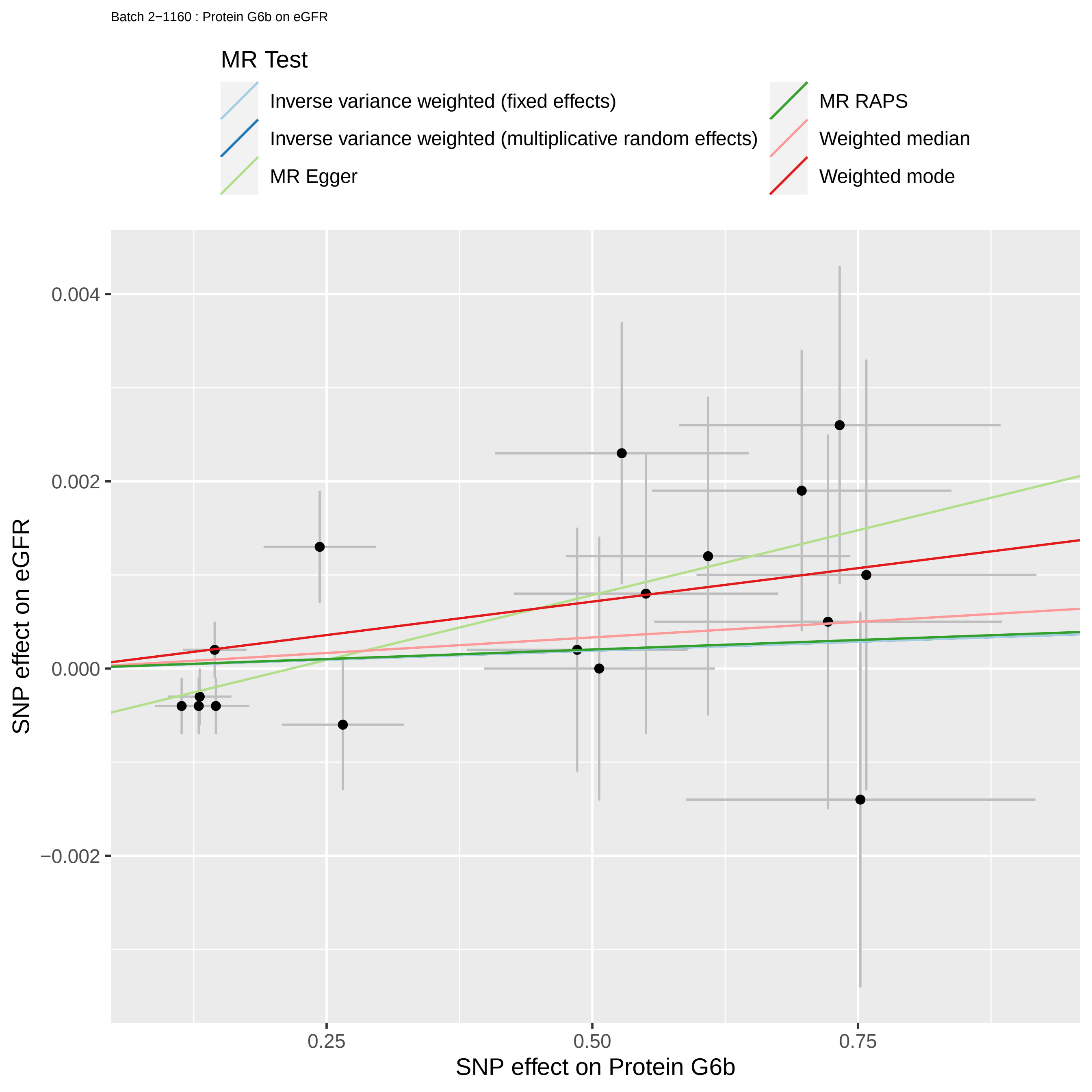
Batch 2−1160 : Protein G6b on eGFR
MR Test
Inverse variance weighted (fixed effects)
Inverse variance weighted (multiplicative random effects)
MR Egger
MR RAPS
Weighted median
Weighted mode
0.004
0.002
0.000
−0.002
SNP effect on eGFR
0.25
0.50
0.75
SNP effect on Protein G6b

Batch 2−1161 : Gamma−aminobutyric acid receptor−associated protein−like 1 on eGFR

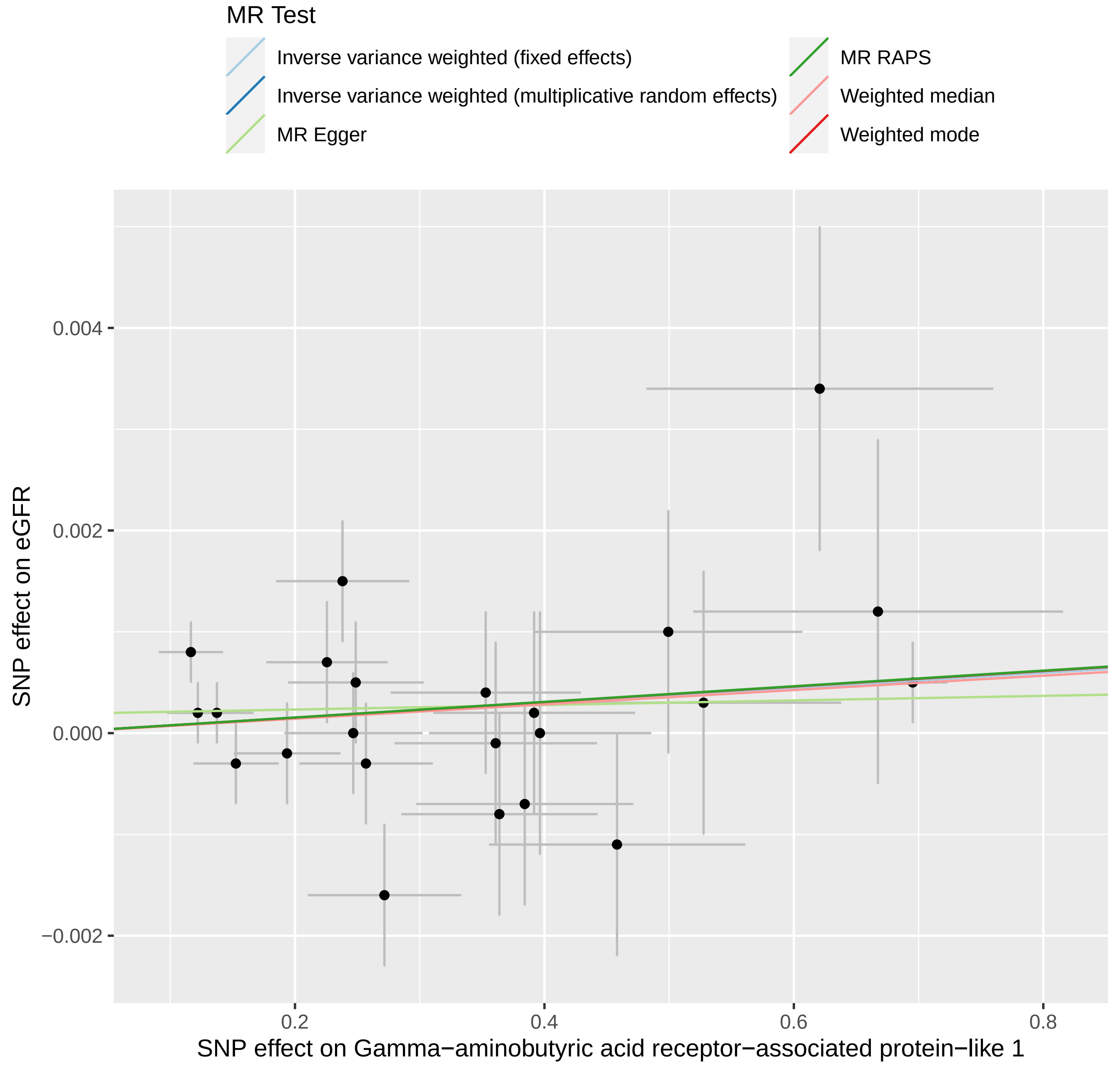

Batch 2−1162 : Gamma−aminobutyric acid receptor−associated protein−like 2 on eGFR

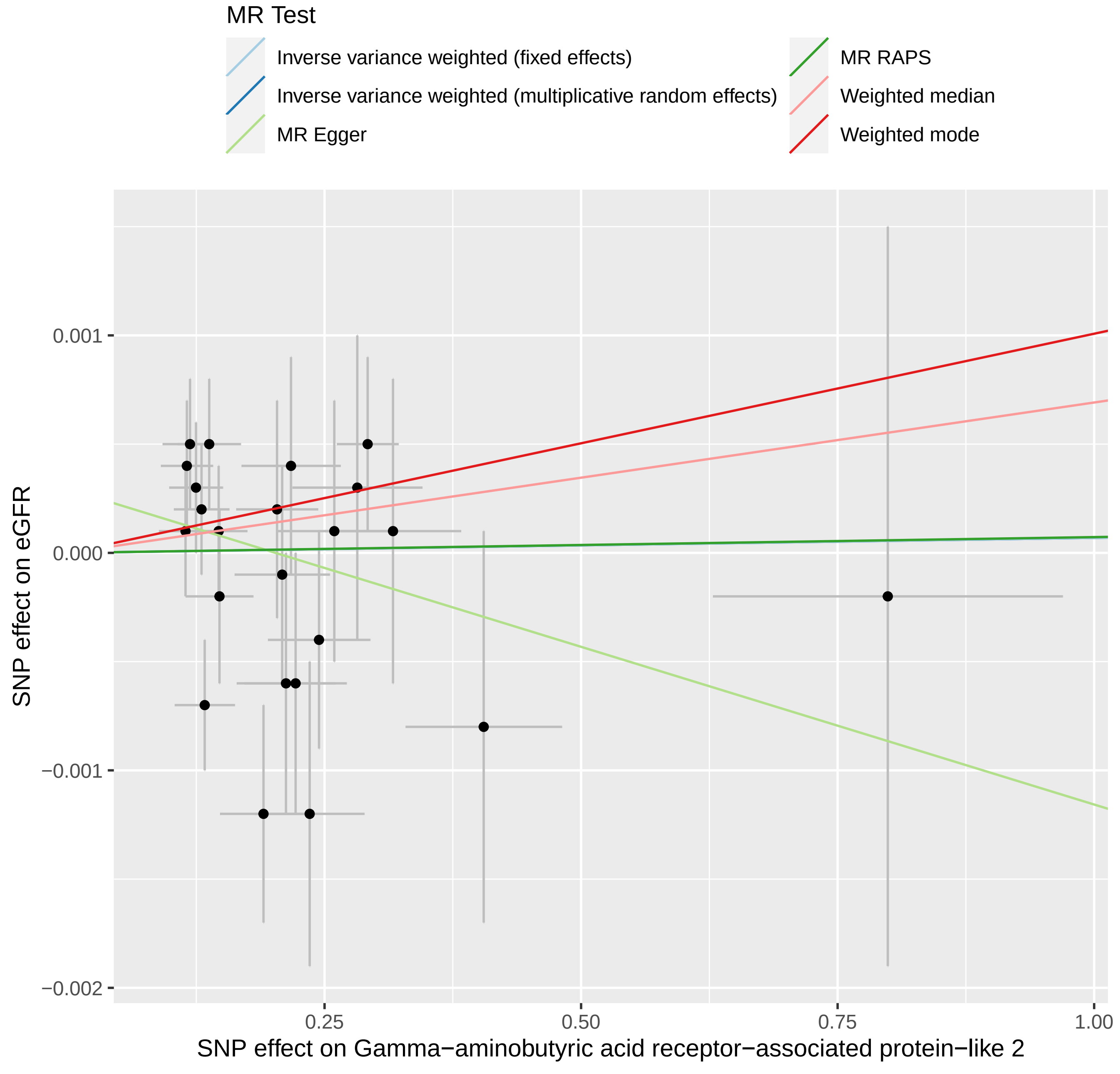

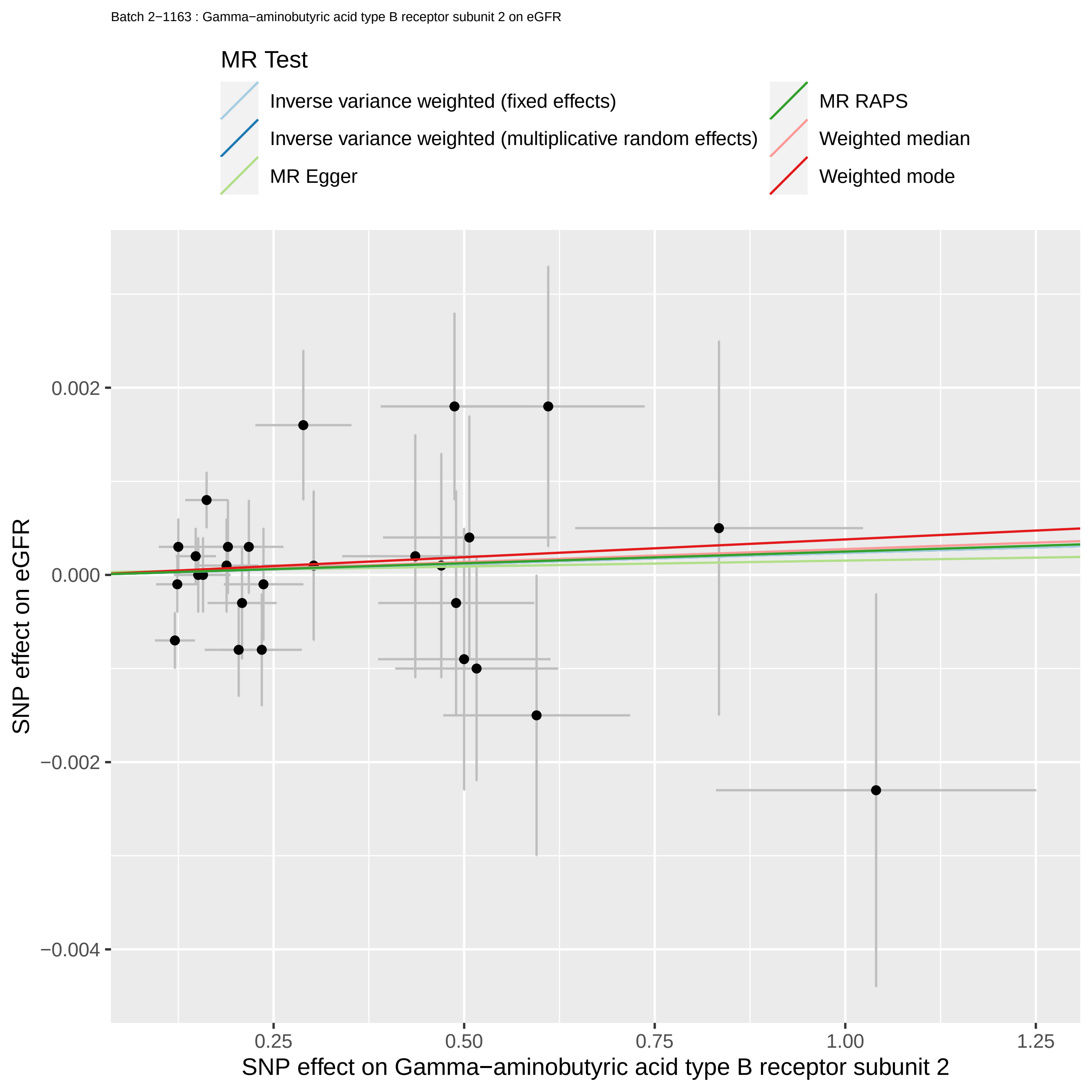
Batch 2−1163 : Gamma−aminobutyric acid type B receptor subunit 2 on eGFR
MR Test
Inverse variance weighted (fixed effects)
Inverse variance weighted (multiplicative random effects)
MR Egger
MR RAPS
Weighted median
Weighted mode
0.002
0.000
−0.002
−0.004
SNP effect on eGFR
0.25
0.50
0.75
1.00
1.25
SNP effect on Gamma−aminobutyric acid type B receptor subunit 2

Batch 2−1164 : Gamma−aminobutyric acid type B receptor subunit 2 on eGFR

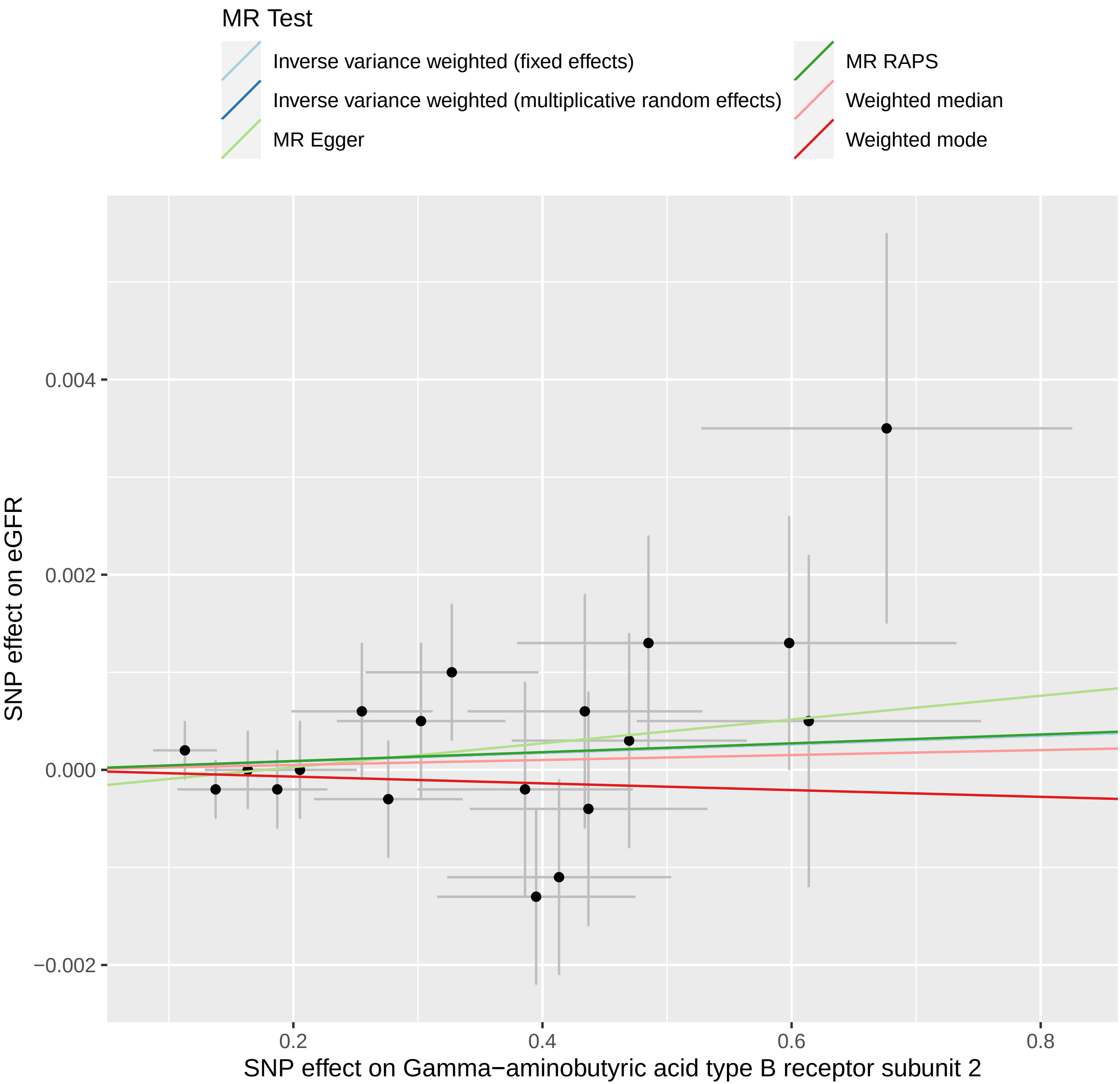

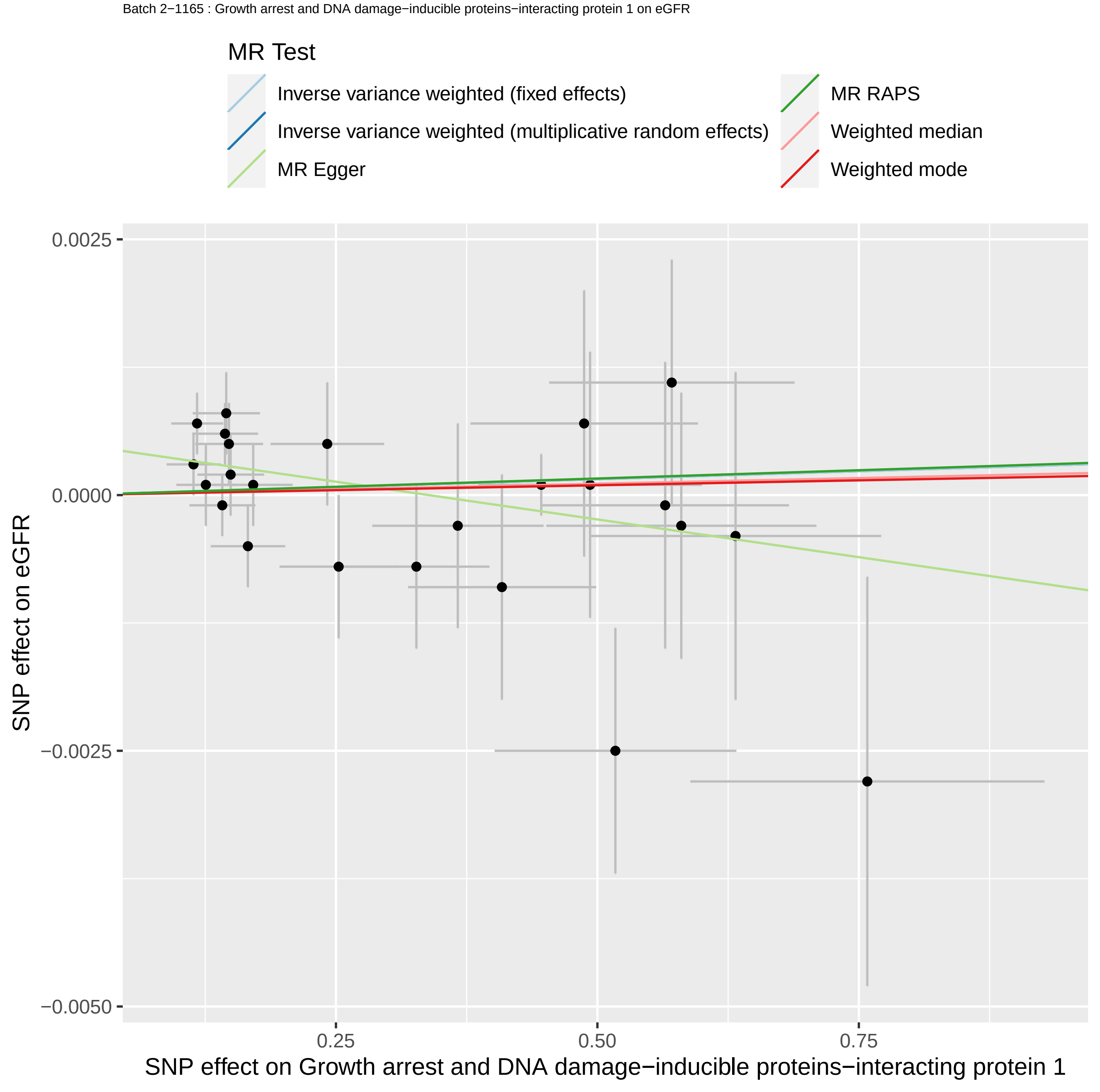

Batch 2−1165 : Growth arrest and DNA damage−inducible proteins−interacting protein 1 on eGFR
MR Test
Inverse variance weighted (fixed effects)
Inverse variance weighted (multiplicative random effects)
MR Egger
MR RAPS
Weighted median
Weighted mode
0.0025
0.0000
−0.0025
−0.0050
0.25
0.50
0.75
SNP effect on eGFR
SNP effect on Growth arrest and DNA damage−inducible proteins−interacting protein 1

Batch 2−1166 : Galanin peptides on eGFR

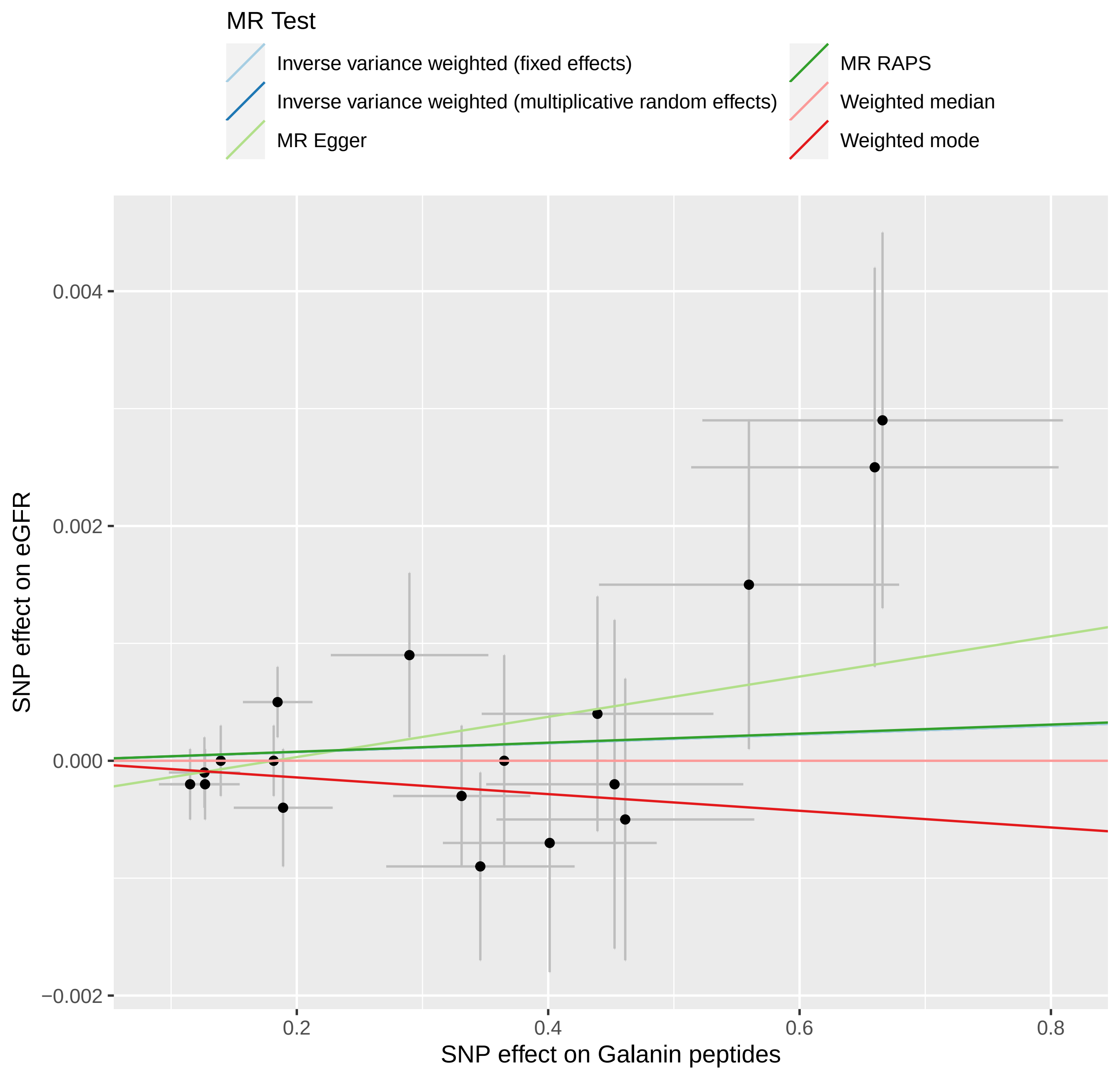

Batch 2−1167 : Polypeptide N−acetylgalactosaminyltransferase 10 on eGFR

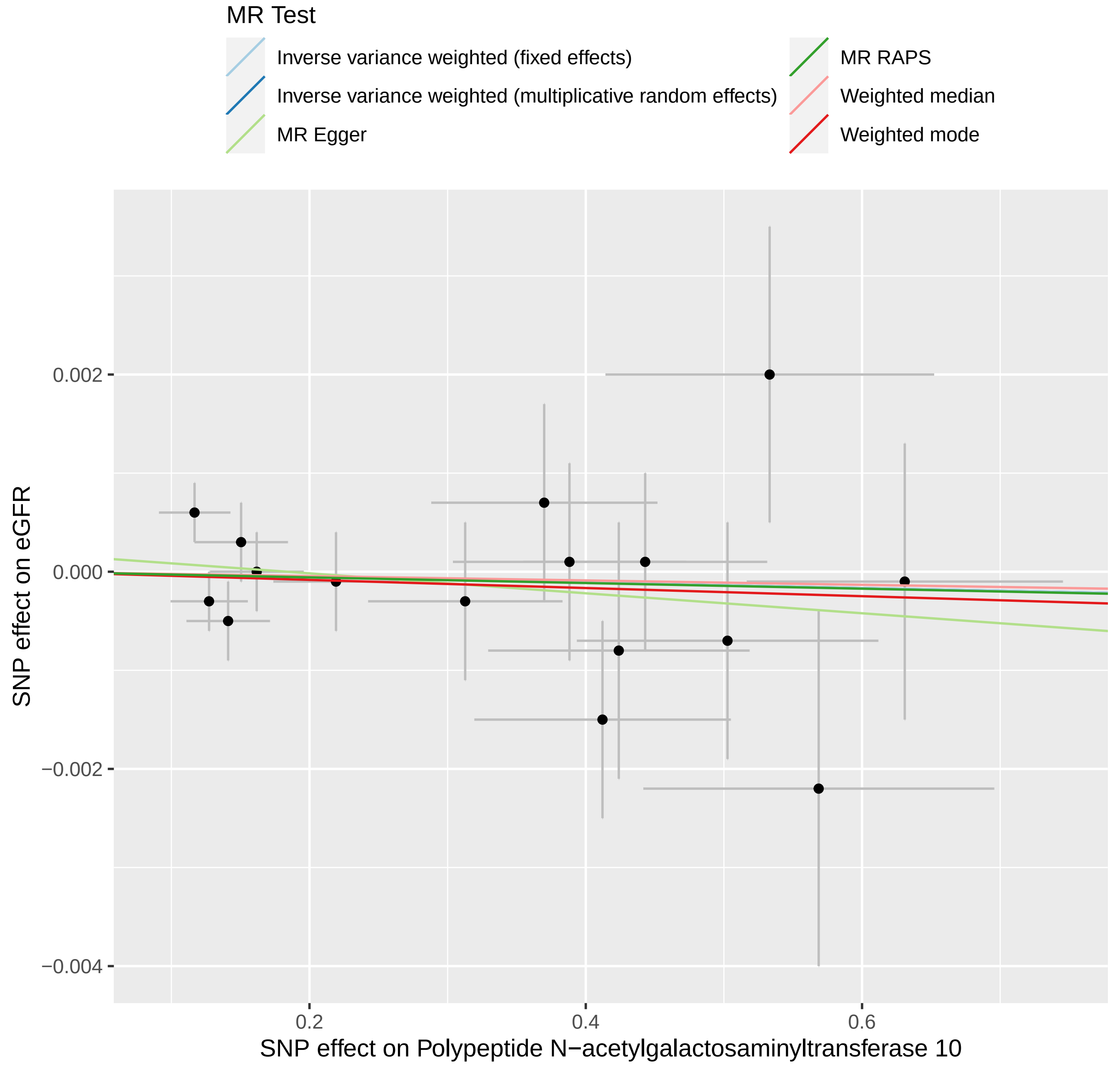

Batch 2−1168 : Polypeptide N−acetylgalactosaminyltransferase 11 on eGFR

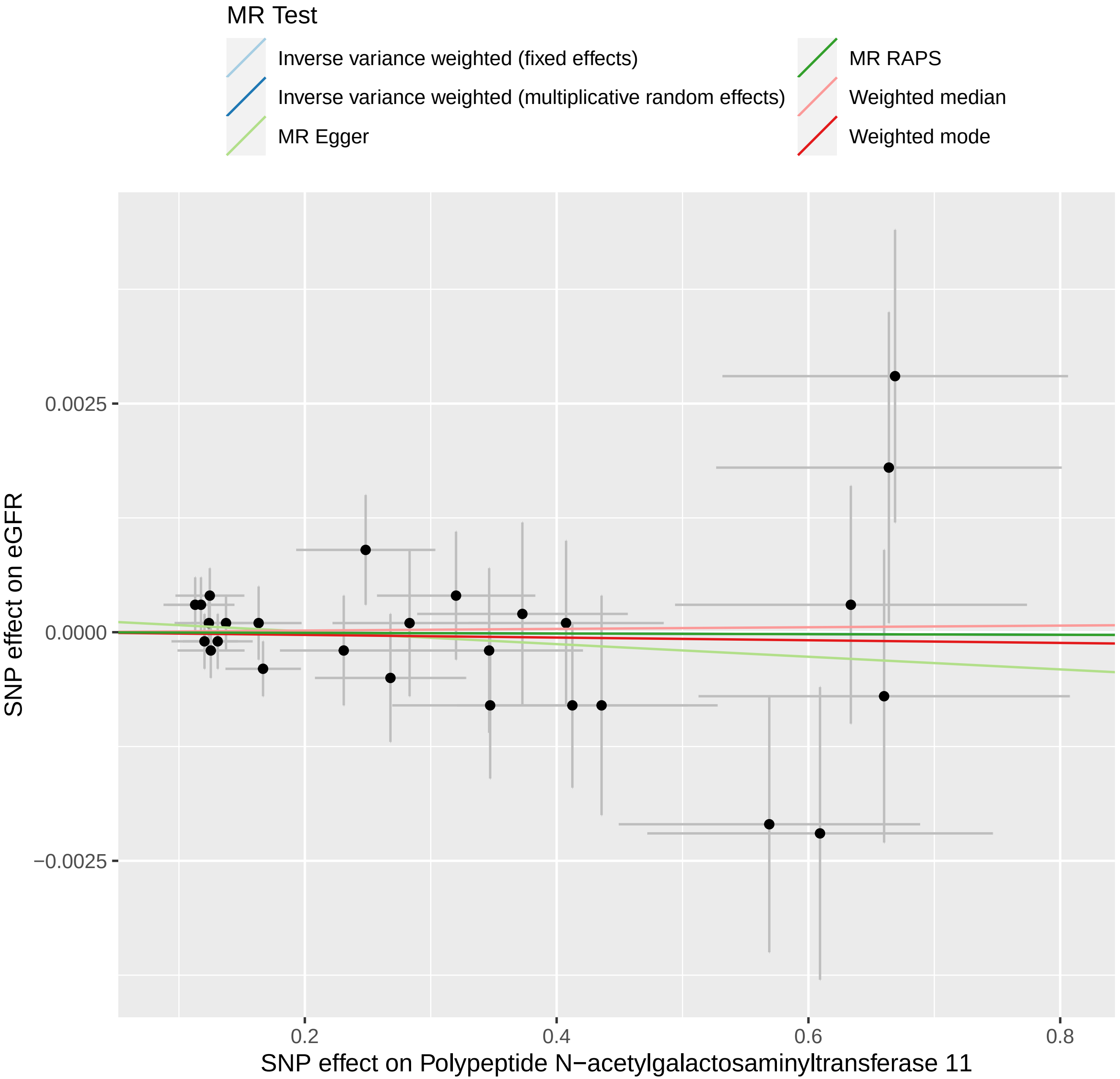

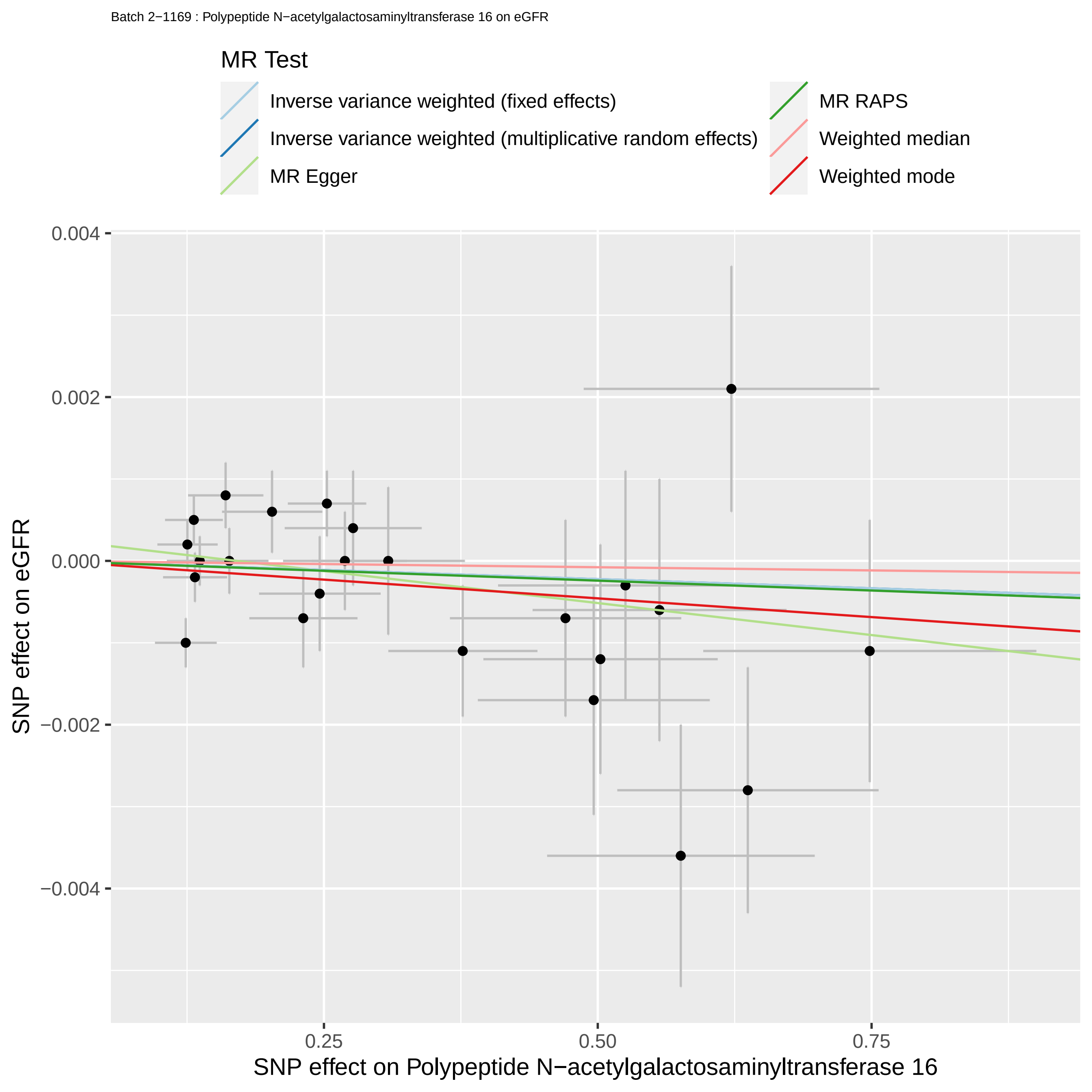

Batch 2−1169 : Polypeptide N−acetylgalactosaminyltransferase 16 on eGFR
MR Test
Inverse variance weighted (fixed effects)
Inverse variance weighted (multiplicative random effects)
MR Egger
MR RAPS
Weighted median
Weighted mode
0.004
0.002
0.000
−0.002
−0.004
0.25
0.50
0.75
SNP effect on eGFR
SNP effect on Polypeptide N−acetylgalactosaminyltransferase 16

Batch 2−1170 : Polypeptide N−acetylgalactosaminyltransferase 1 on eGFR

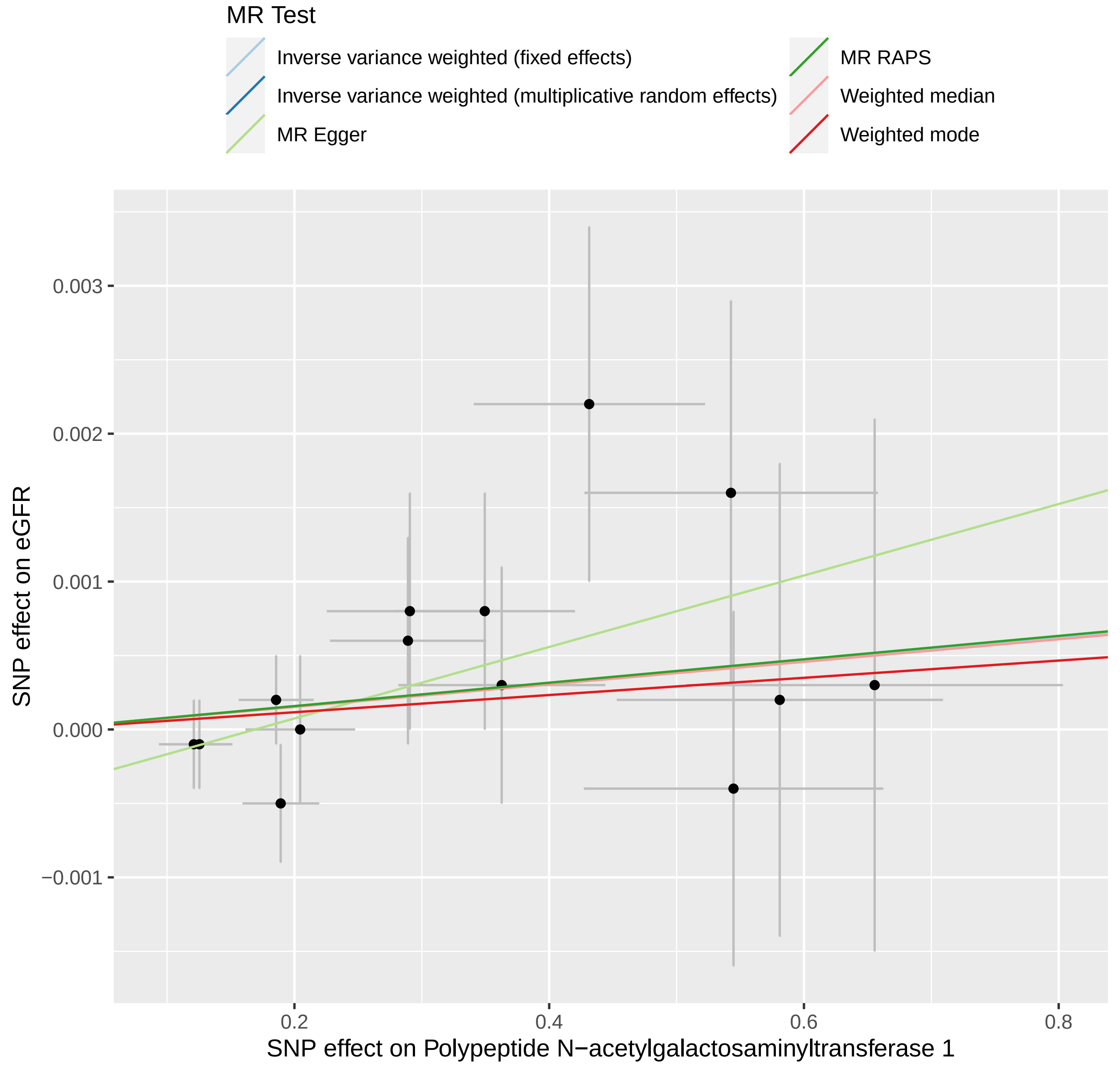

Batch 2−1171 : Polypeptide N−acetylgalactosaminyltransferase 2 on eGFR

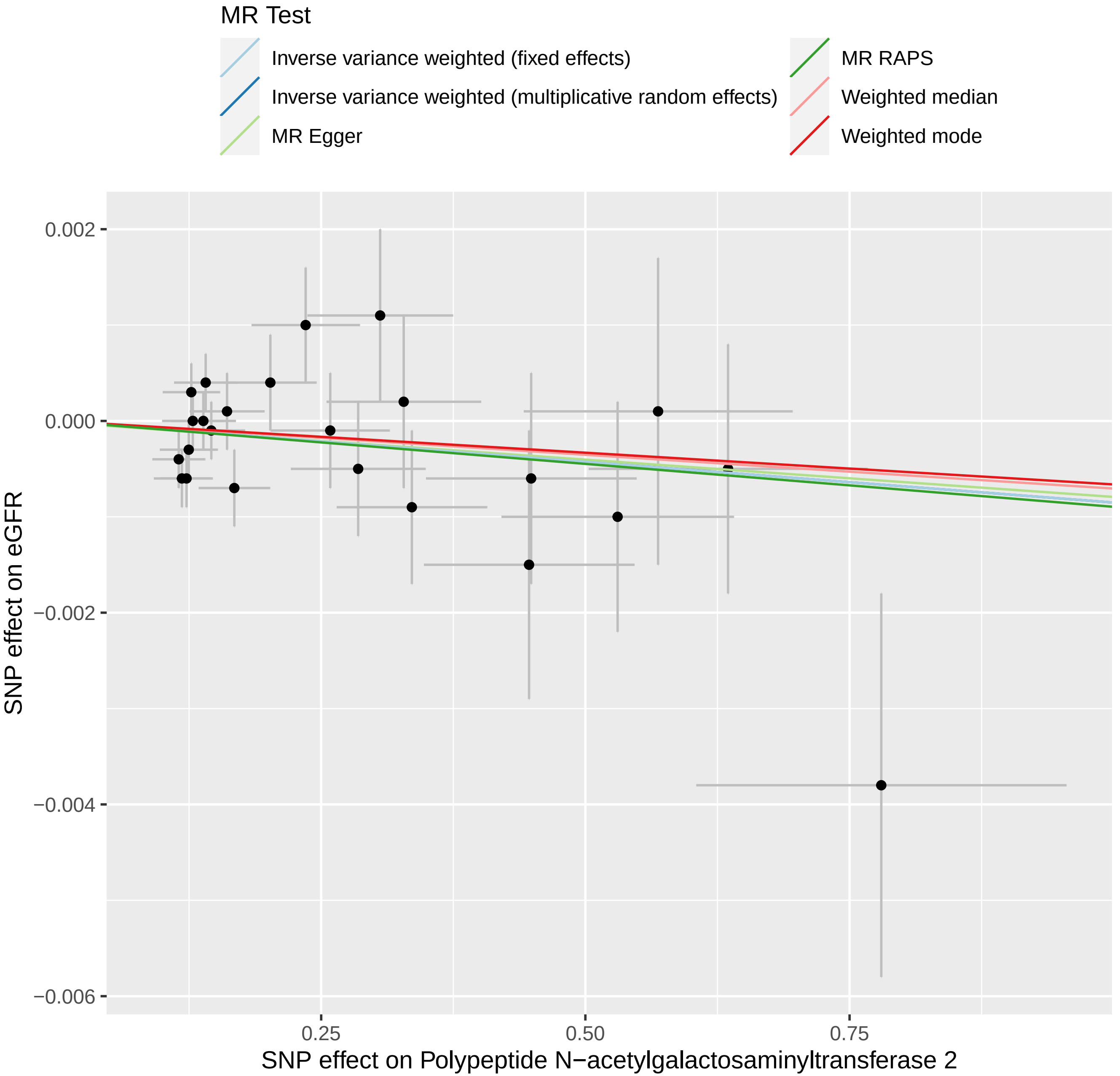

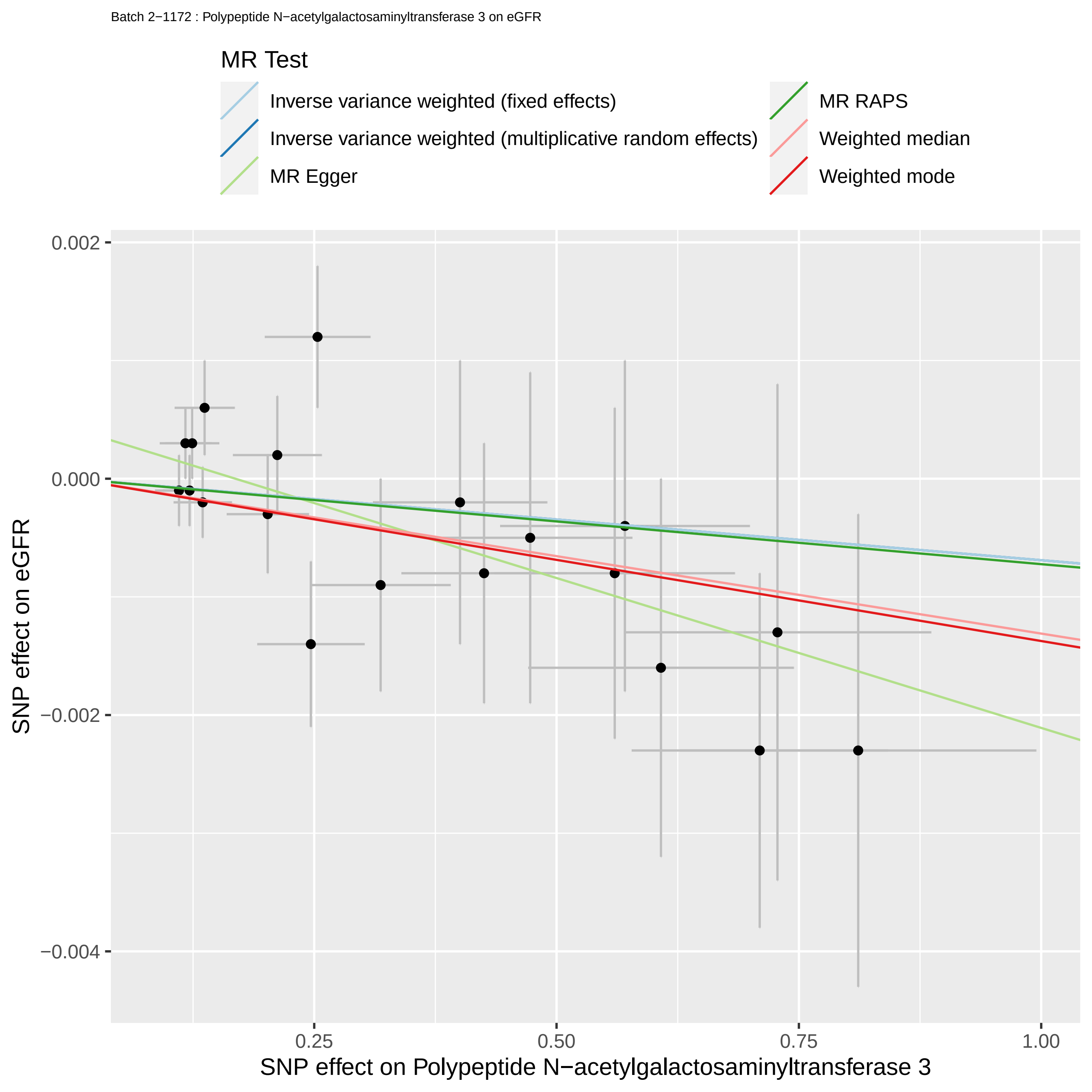
Batch 2−1172 : Polypeptide N−acetylgalactosaminyltransferase 3 on eGFR
MR Test
Inverse variance weighted (fixed effects)
Inverse variance weighted (multiplicative random effects)
MR Egger
MR RAPS
Weighted median
Weighted mode
0.002
0.000
−0.002
−0.004
0.25
0.50
0.75
1.00
SNP effect on eGFR
SNP effect on Polypeptide N−acetylgalactosaminyltransferase 3

Batch 2−1173 : N−acetylgalactosaminyltransferase 7 on eGFR

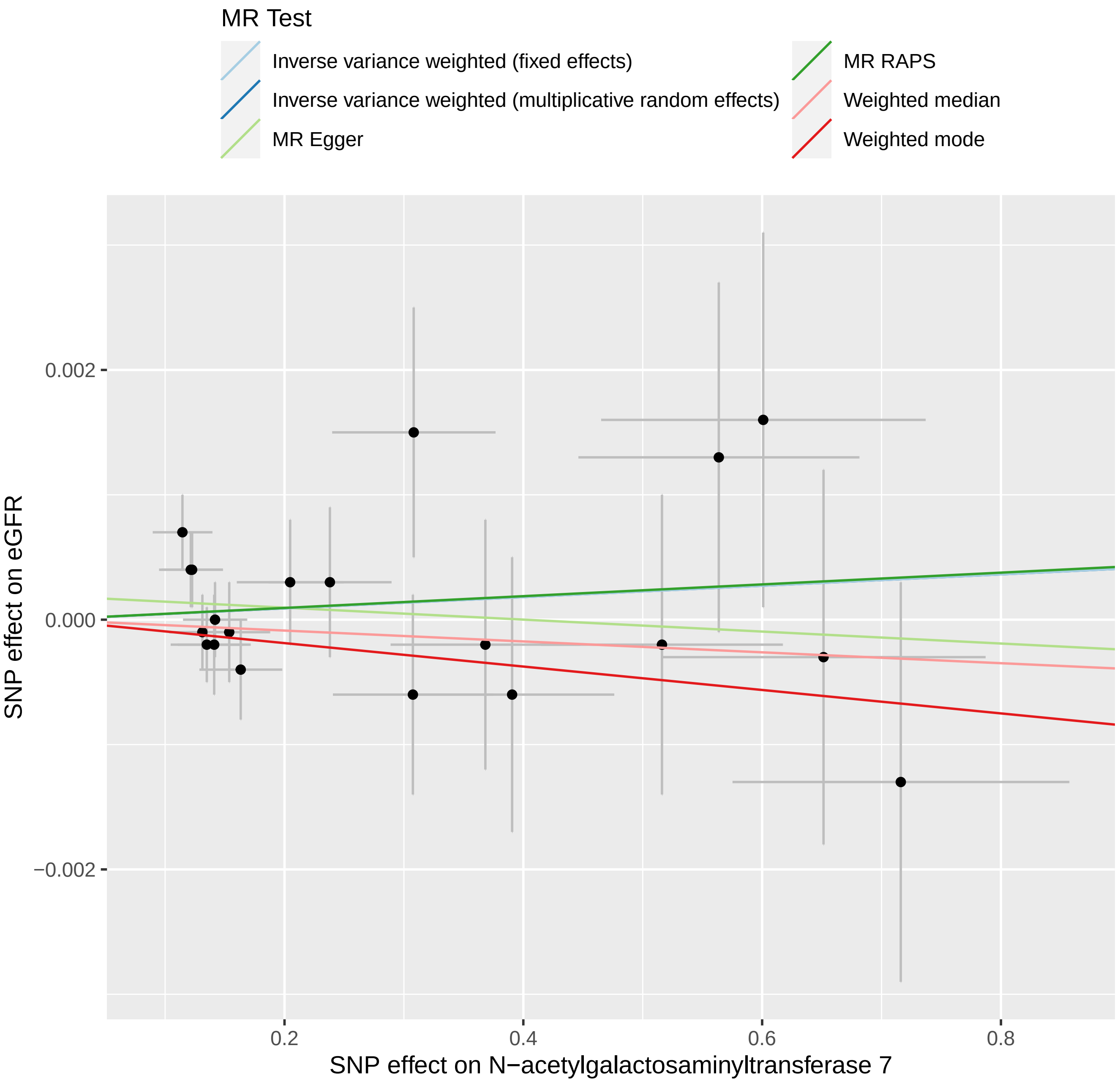

Batch 2−1174 : Galanin−like peptide on eGFR

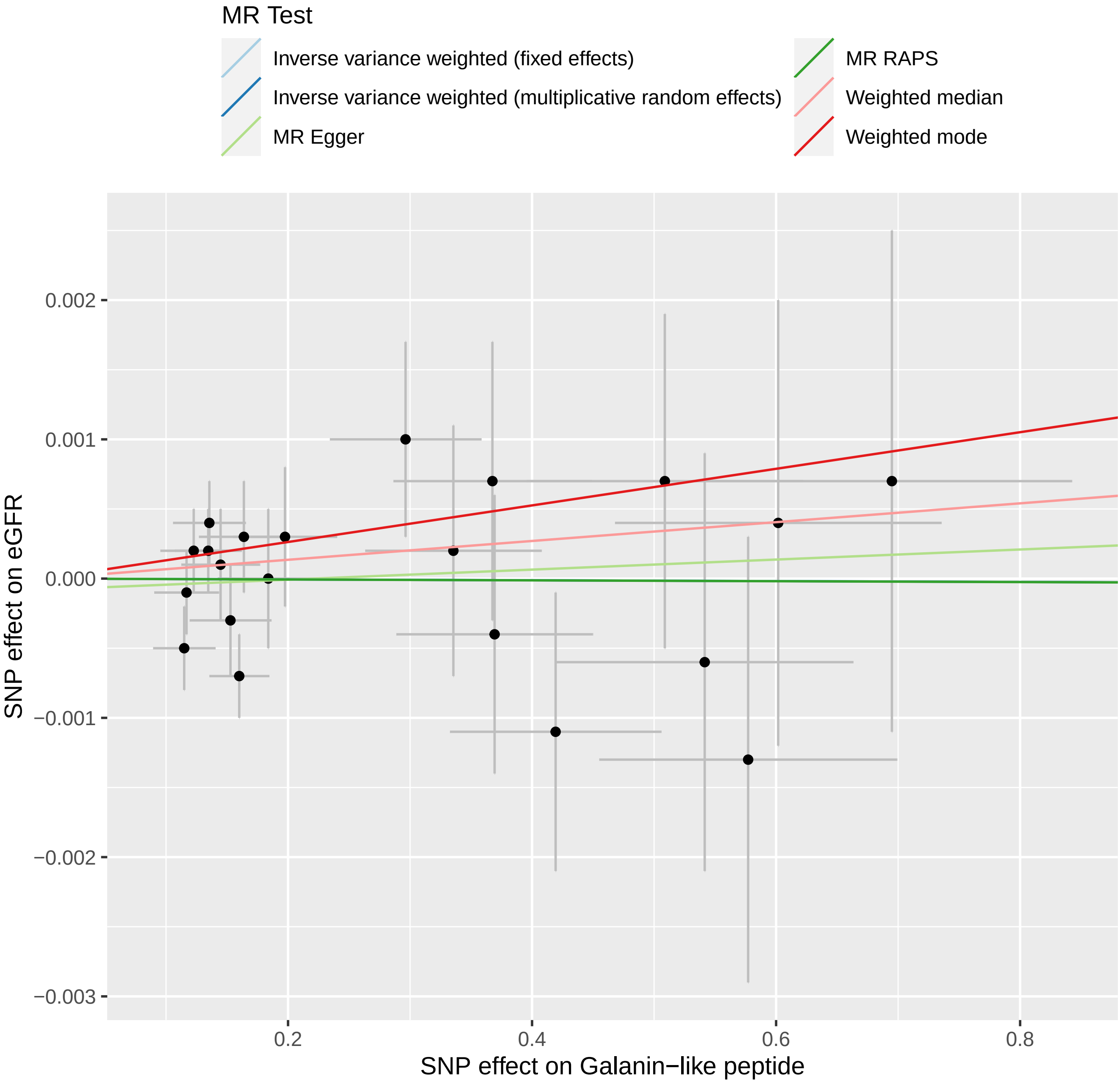

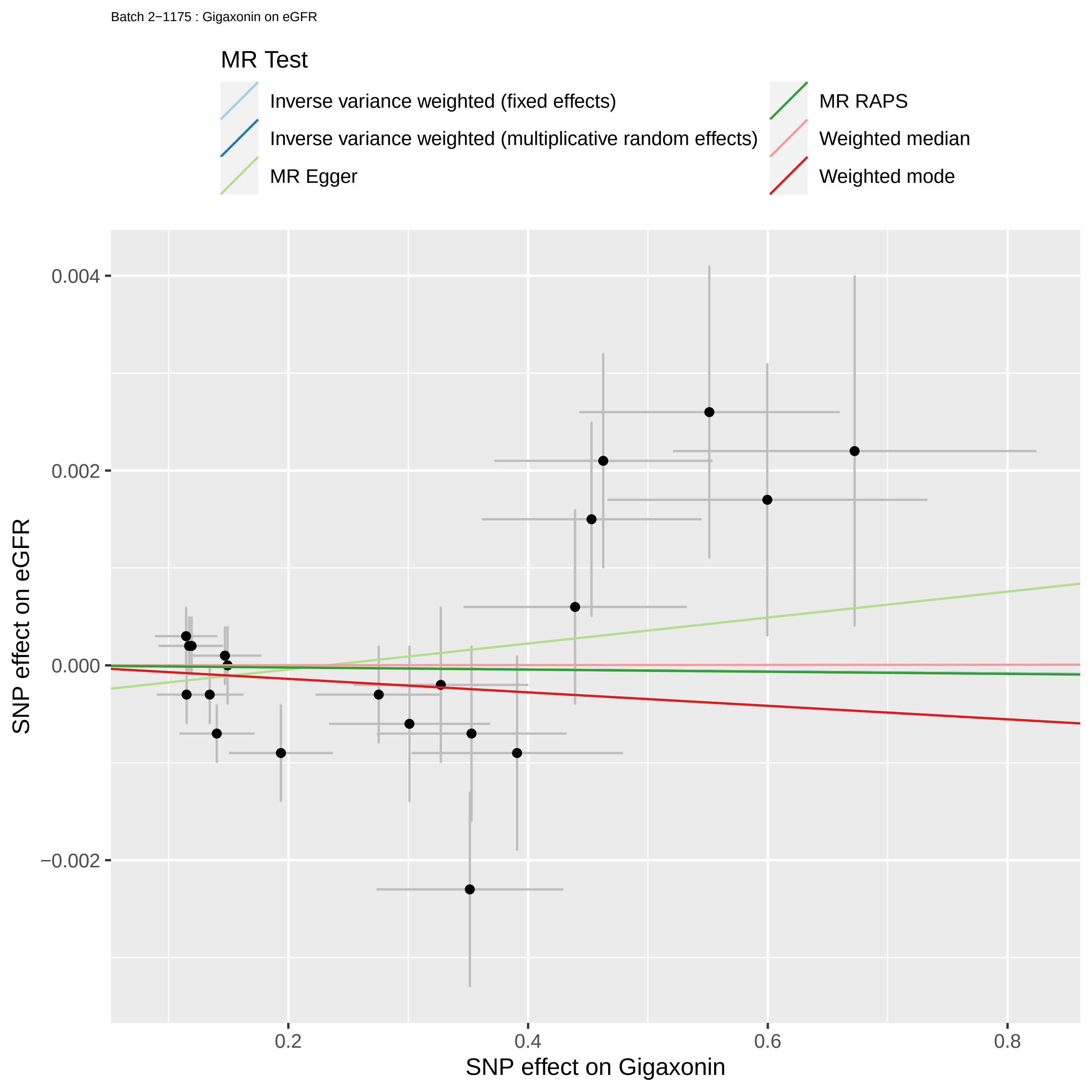
Batch 2−1175 : Gigaxonin on eGFR
MR Test
Inverse variance weighted (fixed effects)
Inverse variance weighted (multiplicative random effects)
MR Egger
MR RAPS
Weighted median
Weighted mode
0.004
0.002
0.000
−0.002
0.2
0.4
0.6
0.8
SNP effect on eGFR
SNP effect on Gigaxonin

Batch 2−1176 : Glyceraldehyde−3−phosphate dehydrogenase, testis−specific on eGFR

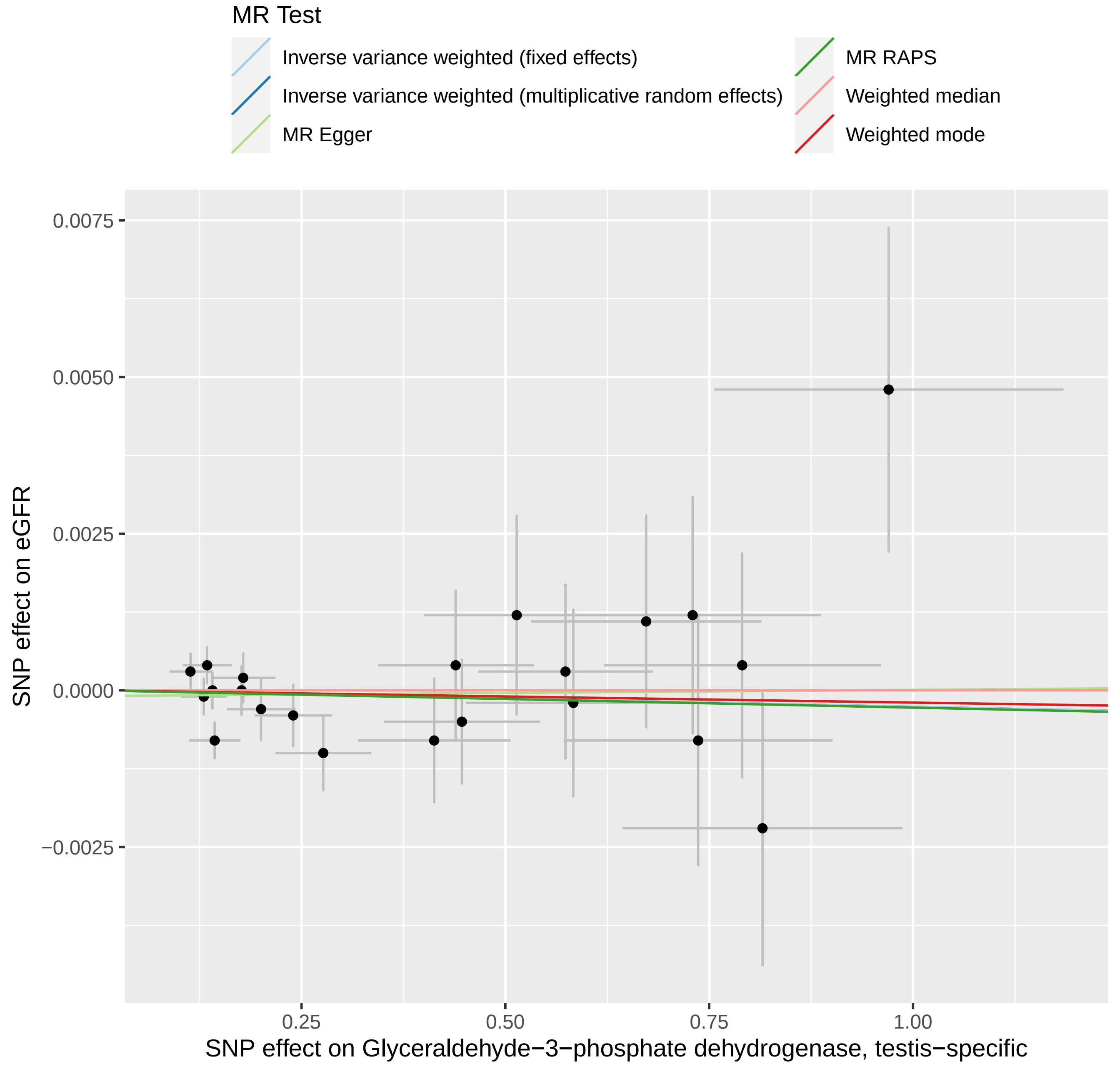

Batch 2−1177 : Growth arrest−specific protein 1 on eGFR

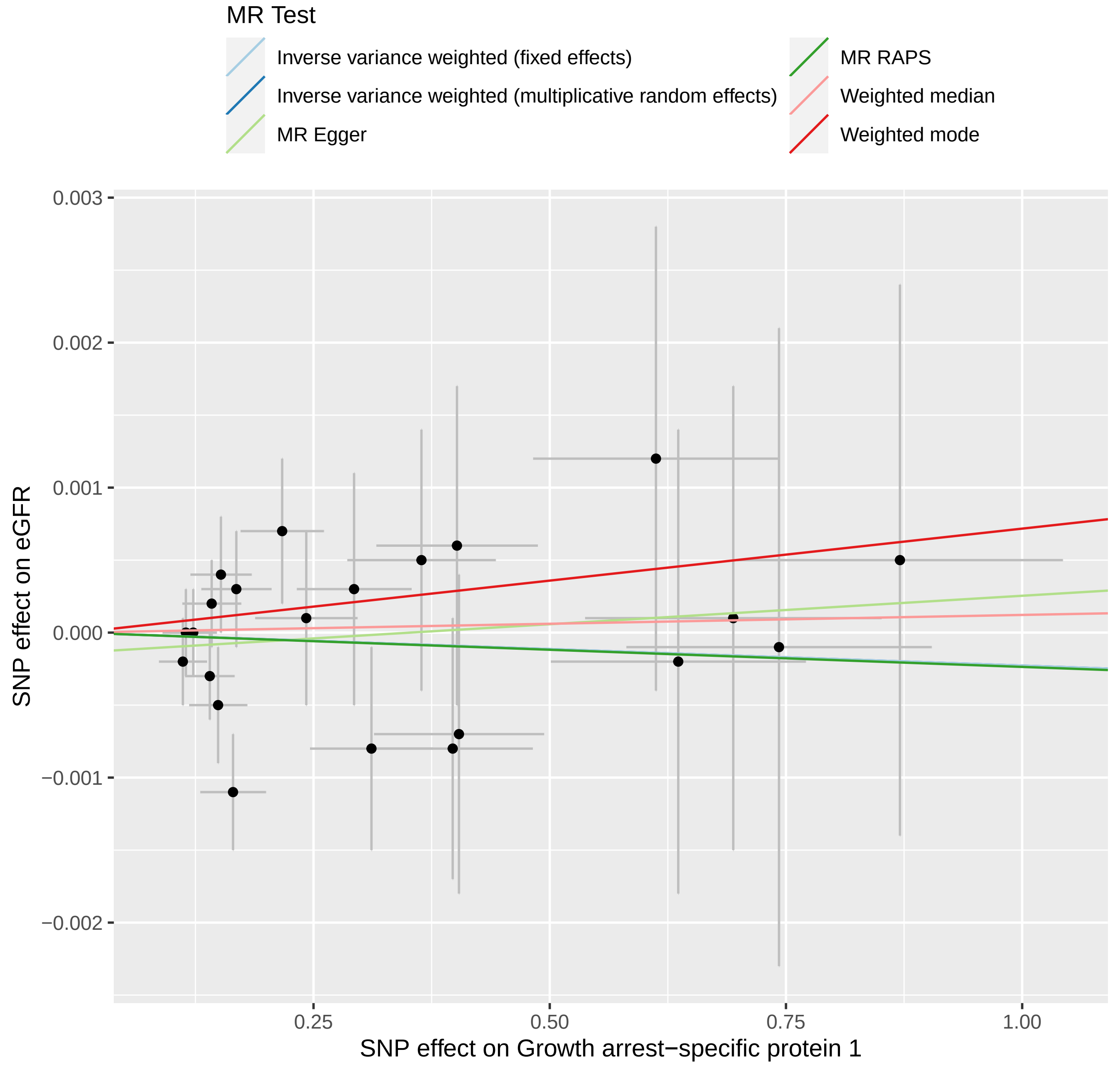

Batch 2−1178 : Guanylate−binding protein 6 on eGFR

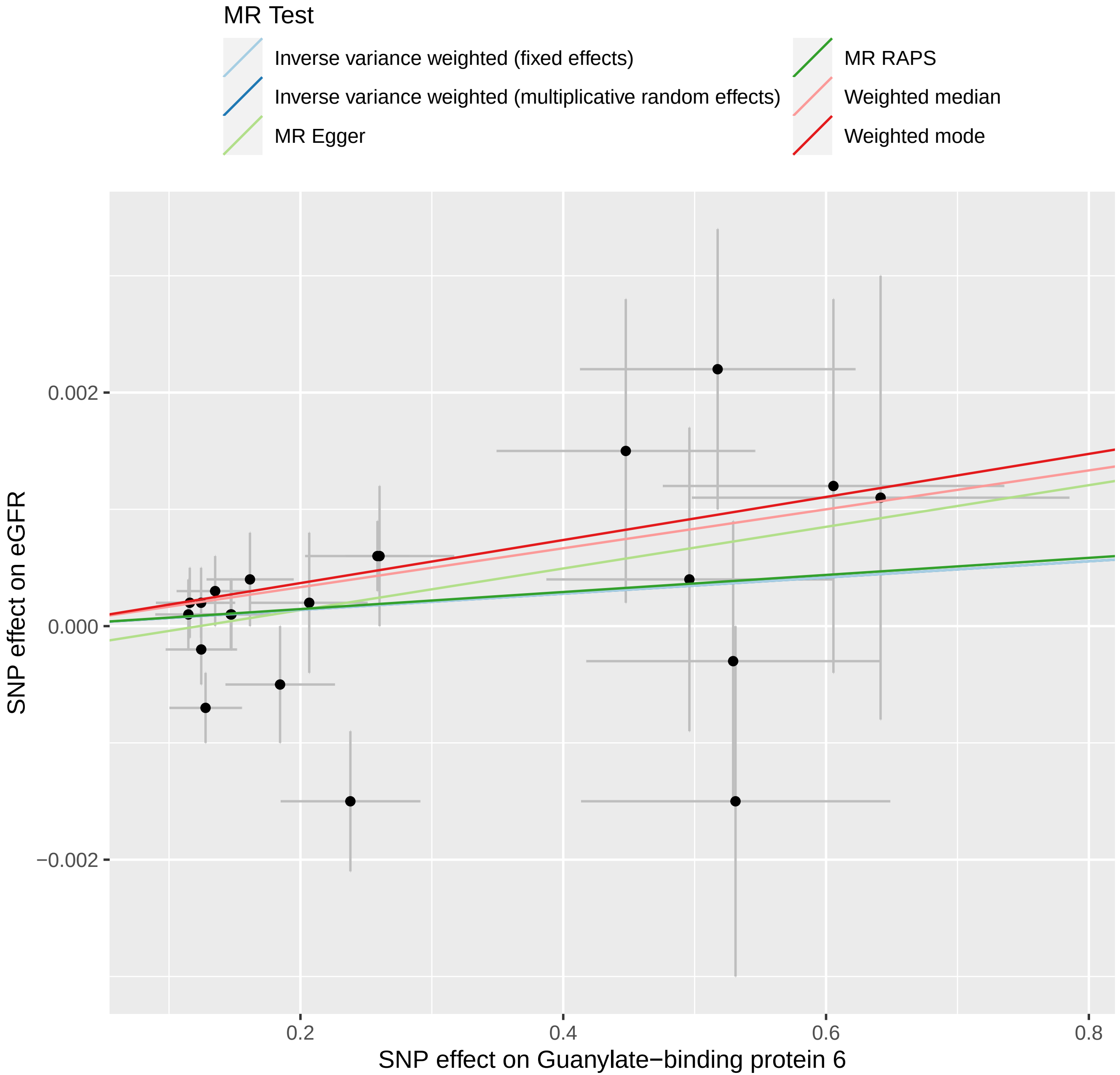

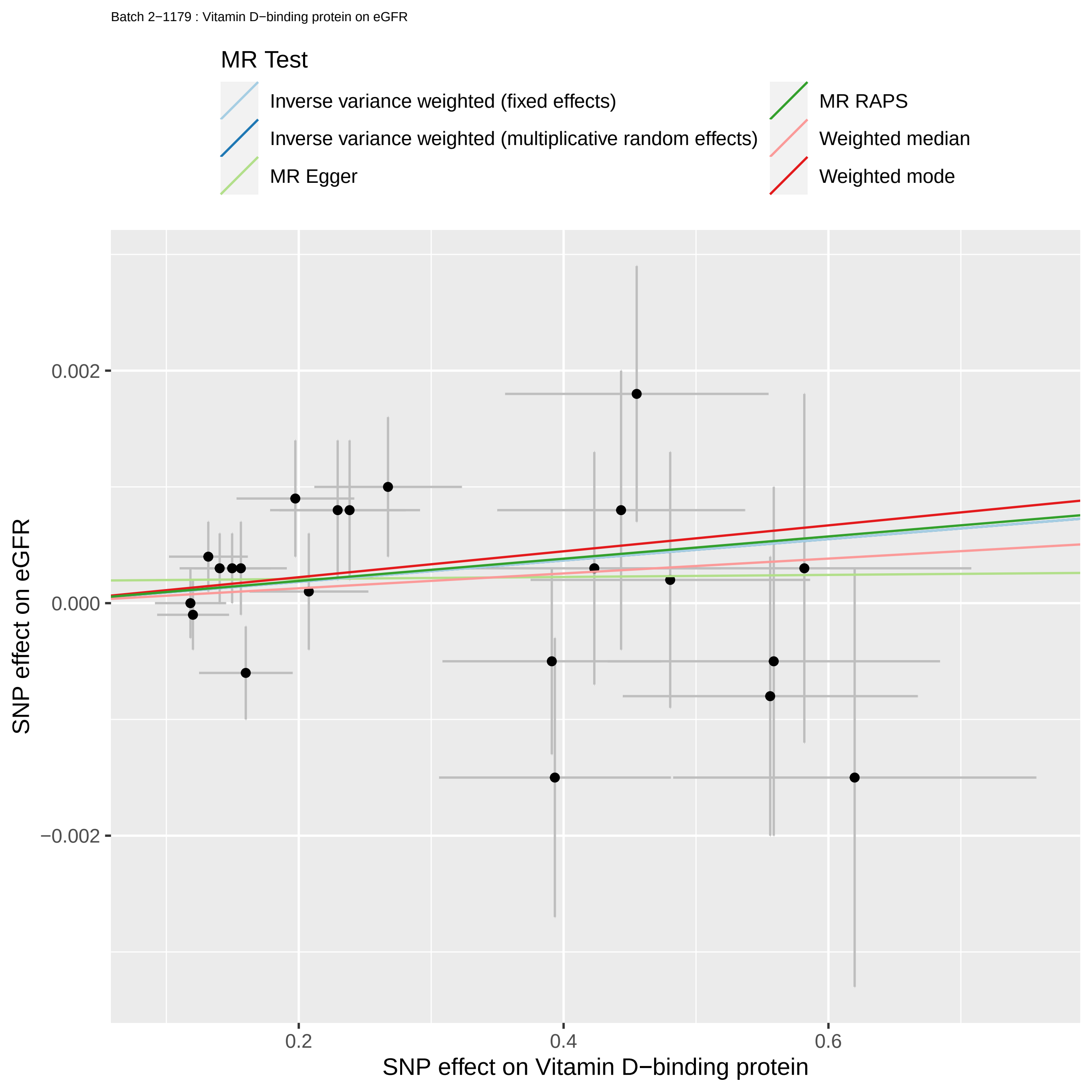
Batch 2−1179 : Vitamin D−binding protein on eGFR
MR Test
Inverse variance weighted (fixed effects)
Inverse variance weighted (multiplicative random effects)
MR Egger
MR RAPS
Weighted median
Weighted mode
0.002
0.000
−0.002
0.2
0.4
0.6
SNP effect on eGFR
SNP effect on Vitamin D−binding protein

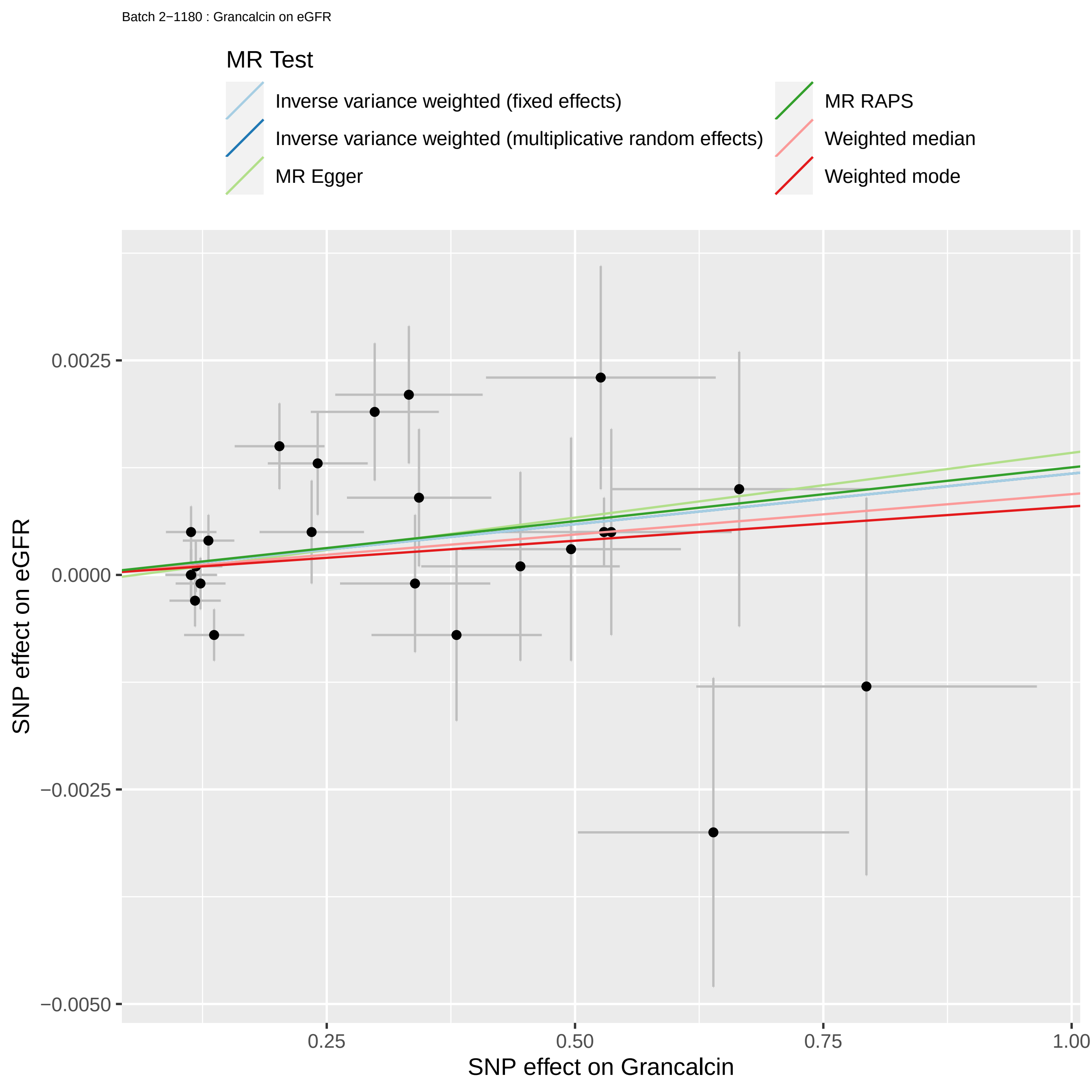
Batch 2−1180 : Grancalcin on eGFR
MR Test
Inverse variance weighted (fixed effects)
Inverse variance weighted (multiplicative random effects)
MR Egger
MR RAPS
Weighted median
Weighted mode
0.0025
0.0000
−0.0025
−0.0050
SNP effect on eGFR
0.25
0.50
0.75
1.00
SNP effect on Grancalcin

Batch 2−1181 : Glucagon on eGFR

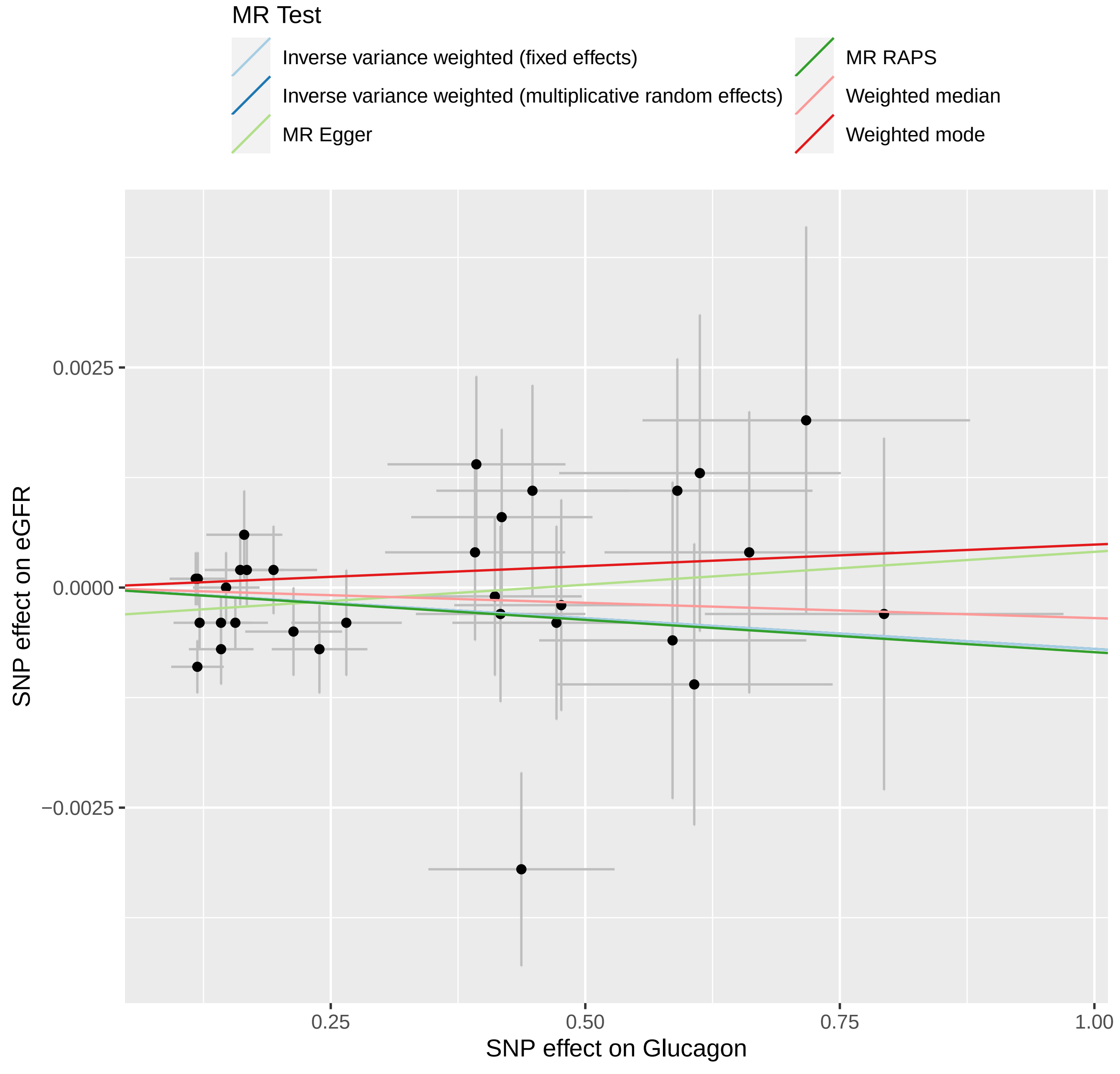

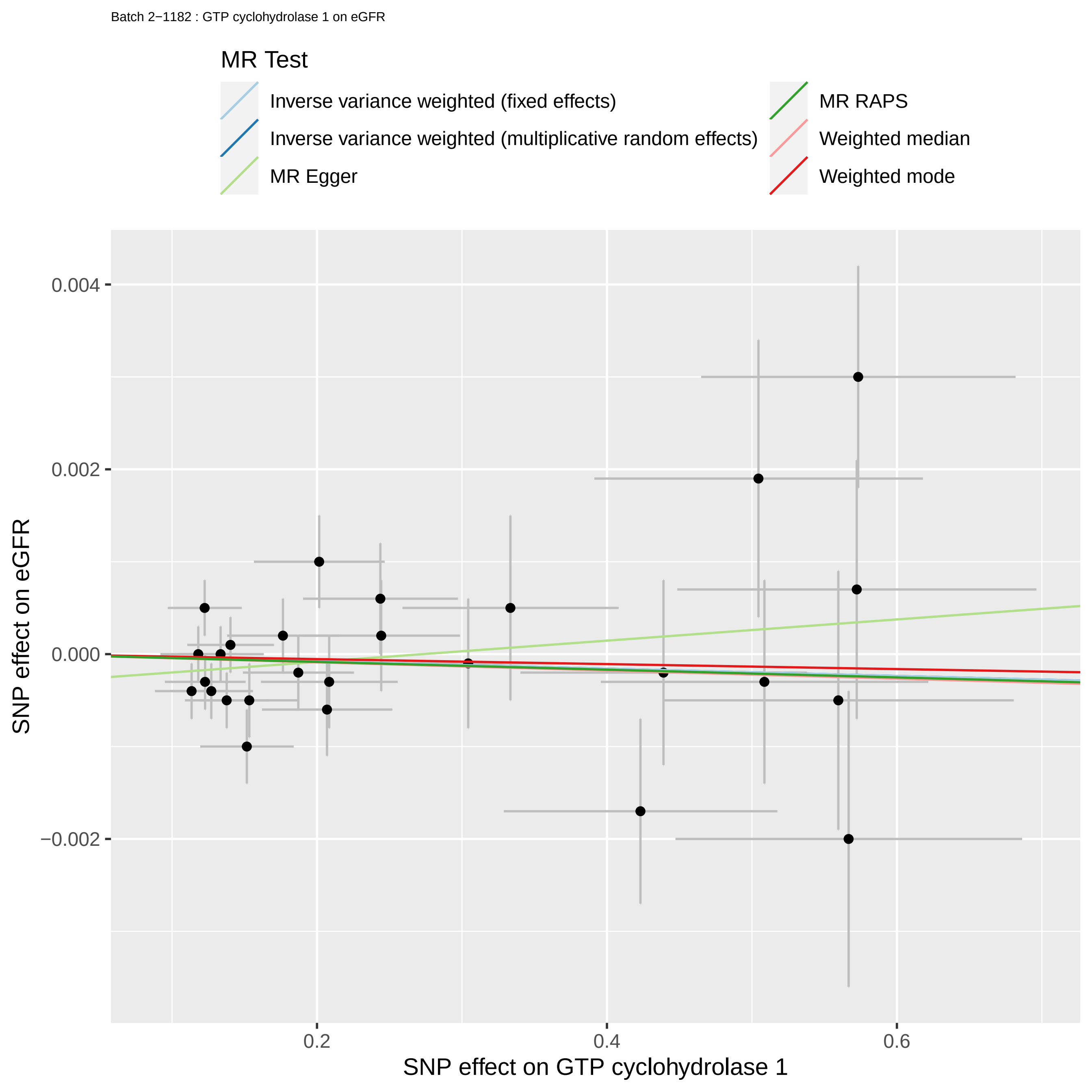

Batch 2−1182 : GTP cyclohydrolase 1 on eGFR
MR Test
Inverse variance weighted (fixed effects)
Inverse variance weighted (multiplicative random effects)
MR Egger
MR RAPS
Weighted median
Weighted mode
0.004
0.002
0.000
−0.002
0.2
0.4
0.6
SNP effect on eGFR
SNP effect on GTP cyclohydrolase 1

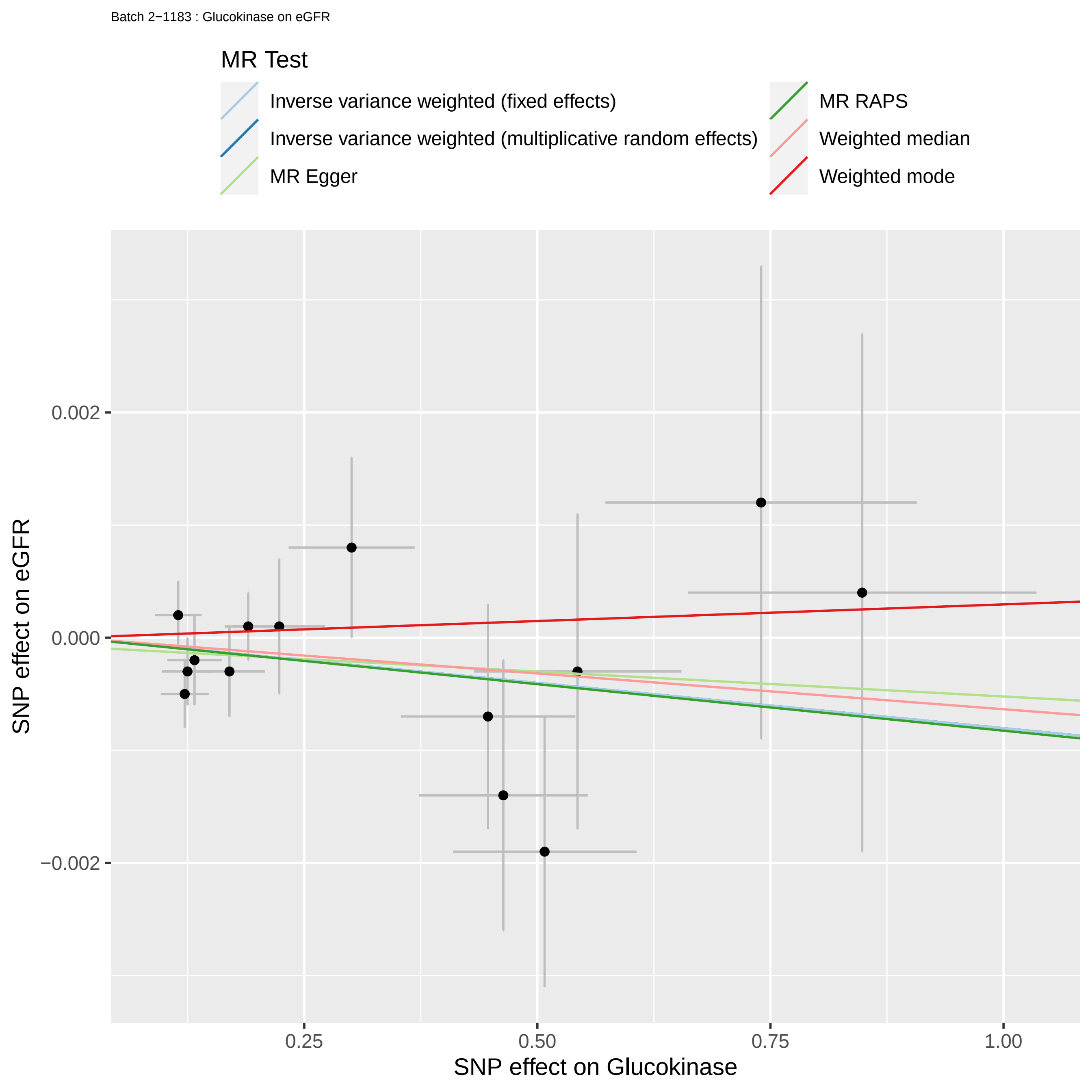

Batch 2−1183 : Glucokinase on eGFR
MR Test
Inverse variance weighted (fixed effects)
Inverse variance weighted (multiplicative random effects)
MR Egger
MR RAPS
Weighted median
Weighted mode
0.002
0.000
−0.002
0.25
0.50
0.75
1.00
SNP effect on Glucokinase
SNP effect on eGFR

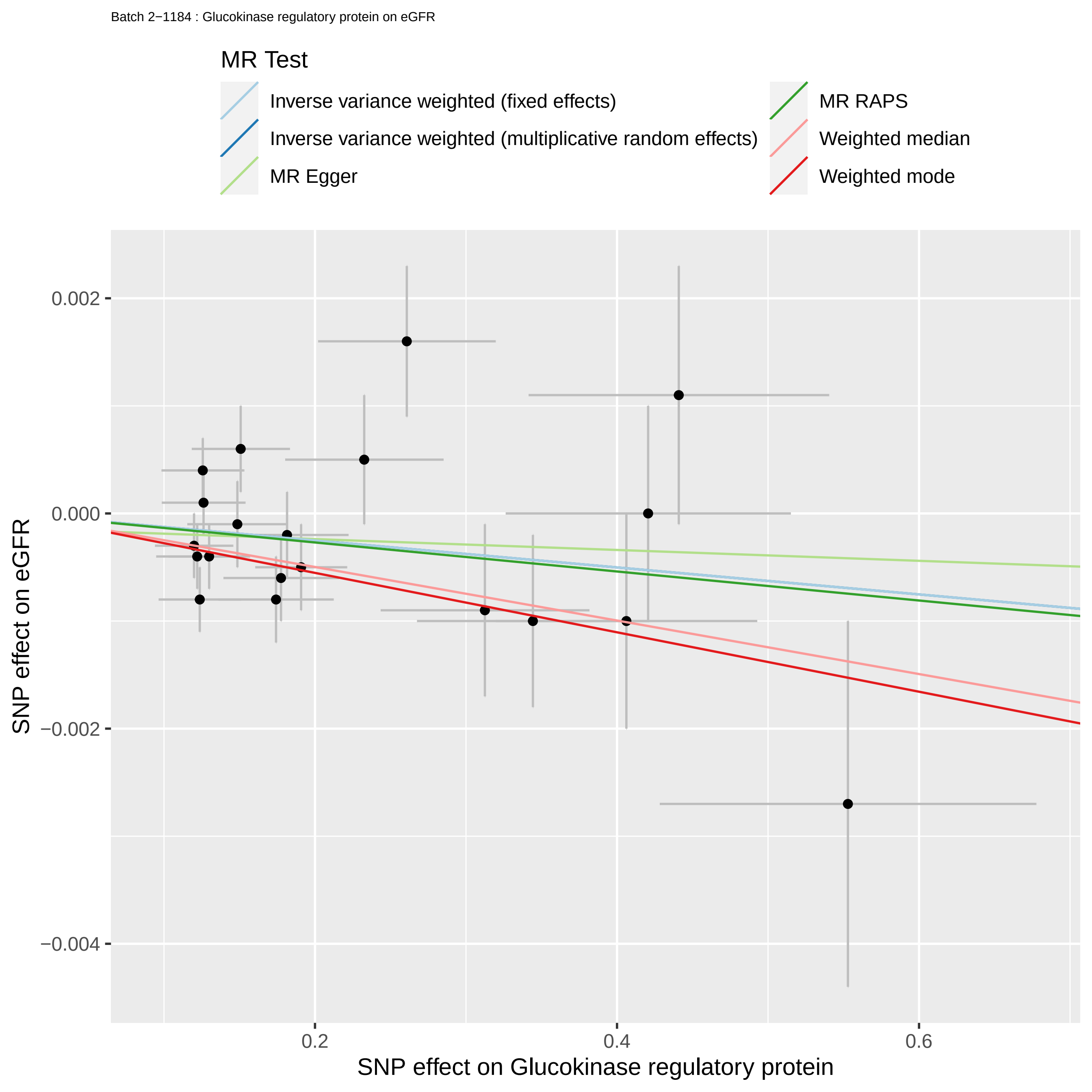

Batch 2−1184 : Glucokinase regulatory protein on eGFR
MR Test
Inverse variance weighted (fixed effects)
Inverse variance weighted (multiplicative random effects)
MR Egger
MR RAPS
Weighted median
Weighted mode
0.002
0.000
−0.002
−0.004
SNP effect on eGFR
0.2
0.4
0.6
SNP effect on Glucokinase regulatory protein

Batch 2−1185 : Beta−1,3−galactosyl−O−glycosyl−glycoprotein beta−1,6−N−acetylglucosaminyltransferase on eGFR

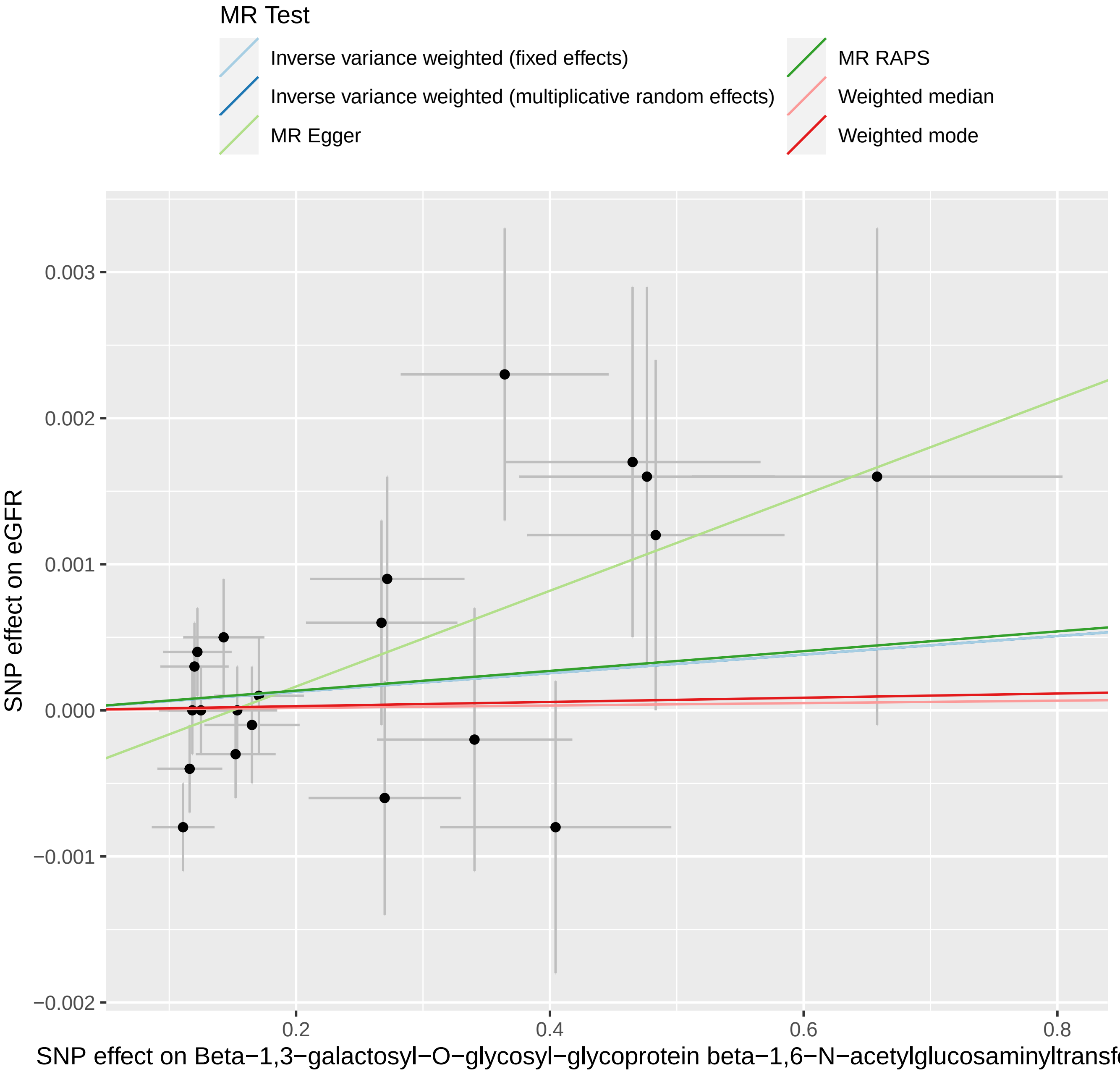

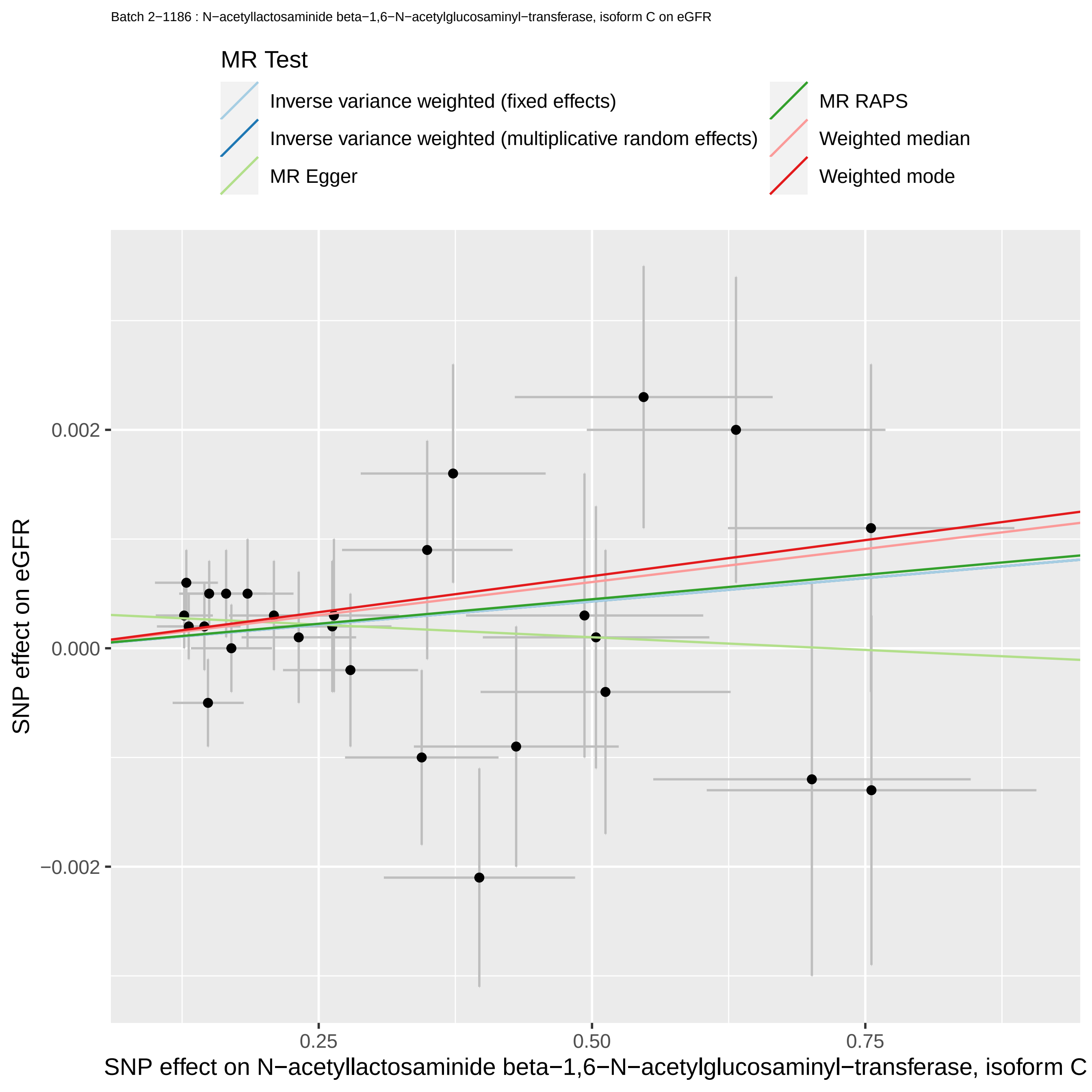

Batch 2−1186 : N−acetyllactosaminide beta−1,6−N−acetylglucosaminyl−transferase, isoform C on eGFR
MR Test
Inverse variance weighted (fixed effects)
Inverse variance weighted (multiplicative random effects)
MR Egger
MR RAPS
Weighted median
Weighted mode
0.002
0.000
−0.002
0.25
0.50
0.75
SNP effect on eGFR
SNP effect on N−acetyllactosaminide beta−1,6−N−acetylglucosaminyl−transferase, isoform C

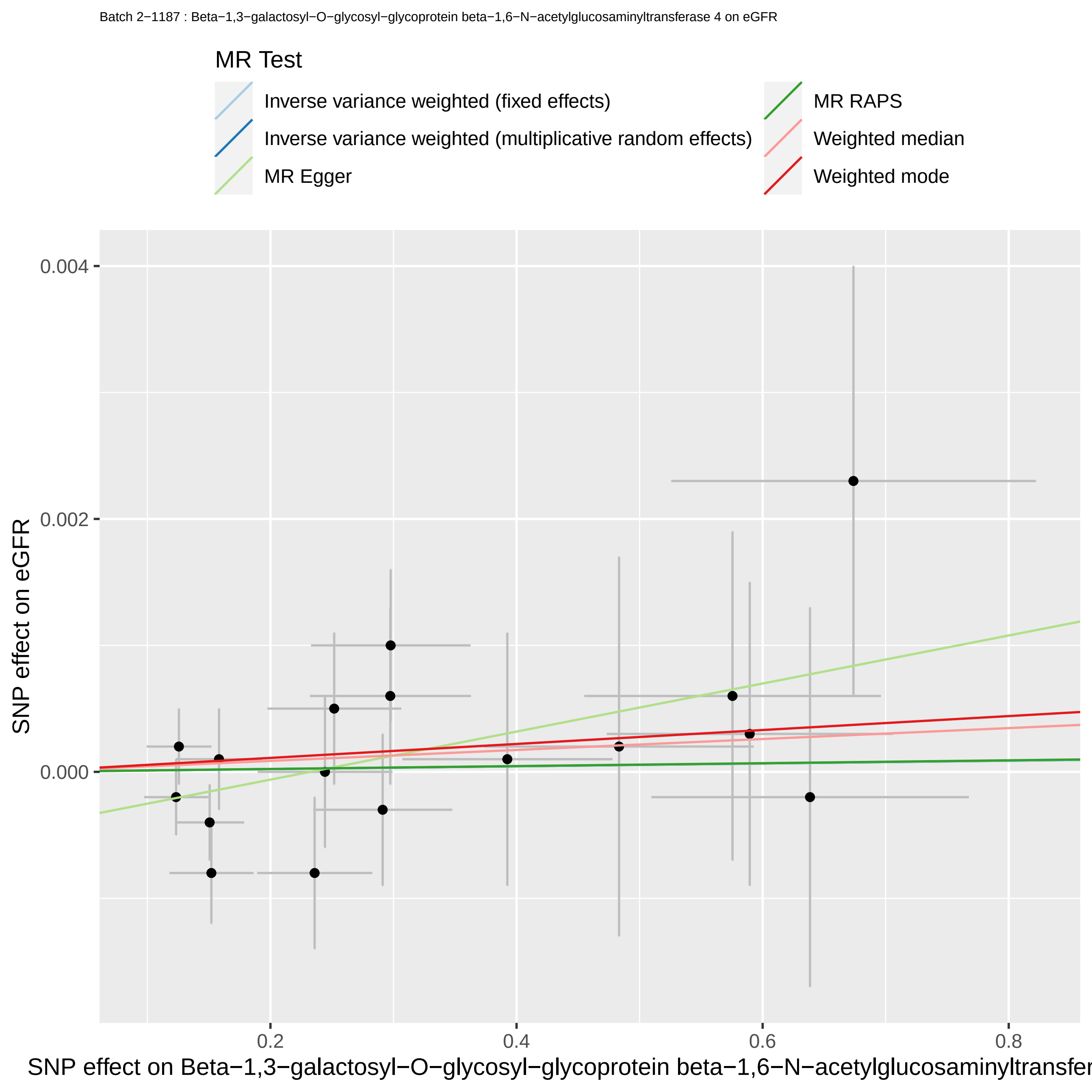
Batch 2−1187 : Beta−1,3−galactosyl−O−glycosyl−glycoprotein beta−1,6−N−acetylglucosaminyltransferase 4 on eGFR
MR Test
Inverse variance weighted (fixed effects)
Inverse variance weighted (multiplicative random effects)
MR Egger
MR RAPS
Weighted median
Weighted mode
0.004
0.002
0.000
0.2
0.4
0.6
0.8
SNP effect on eGFR
SNP effect on Beta−1,3−galactosyl−O−glycosyl−glycoprotein beta−1,6−N−acetylglucosaminyltransfer

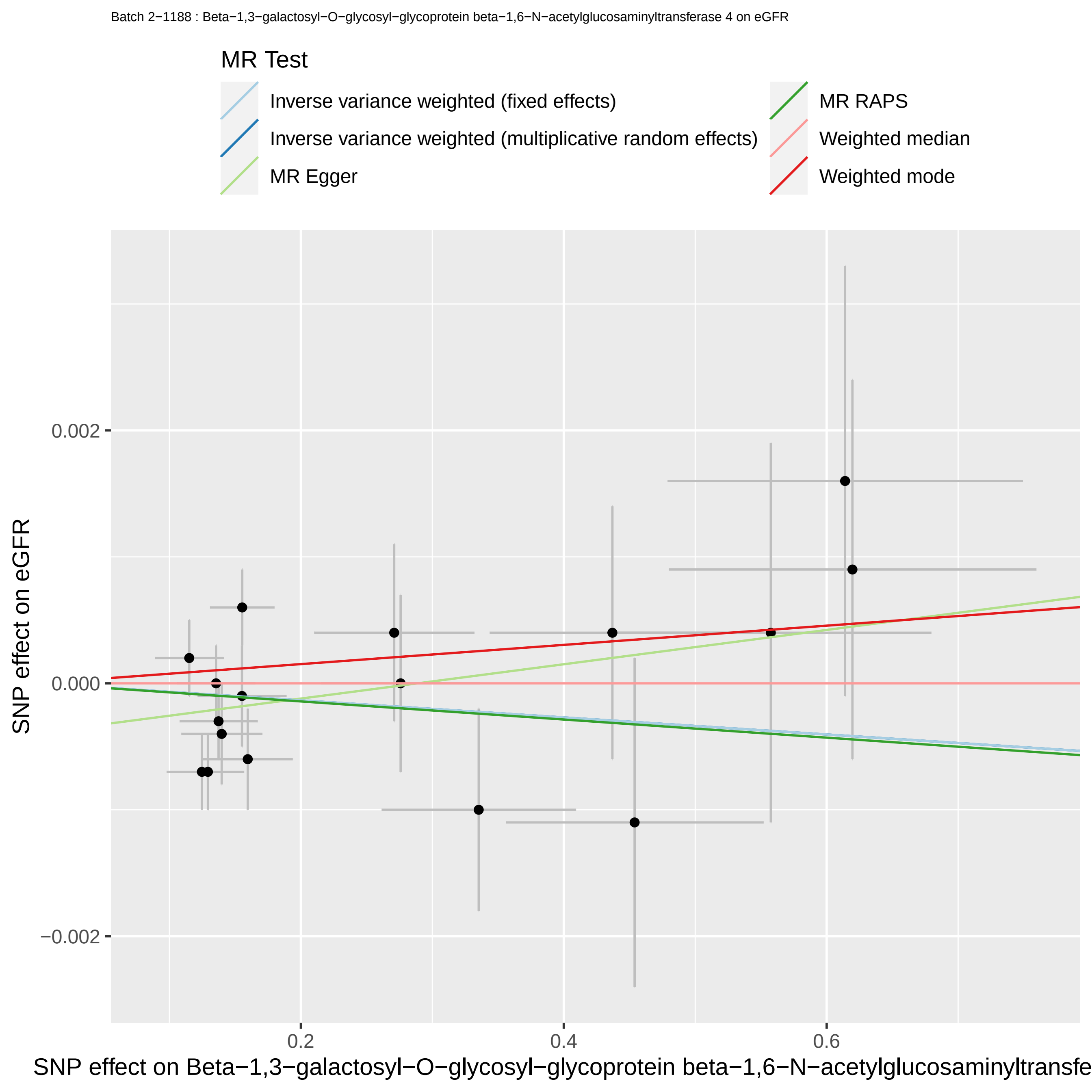

Batch 2−1188 : Beta−1,3−galactosyl−O−glycosyl−glycoprotein beta−1,6−N−acetylglucosaminyltransferase 4 on eGFR
MR Test
Inverse variance weighted (fixed effects)
Inverse variance weighted (multiplicative random effects)
MR Egger
MR RAPS
Weighted median
Weighted mode
0.002
0.000
−0.002
0.2
0.4
0.6
SNP effect on eGFR
SNP effect on Beta−1,3−galactosyl−O−glycosyl−glycoprotein beta−1,6−N−acetylglucosaminyltransfe

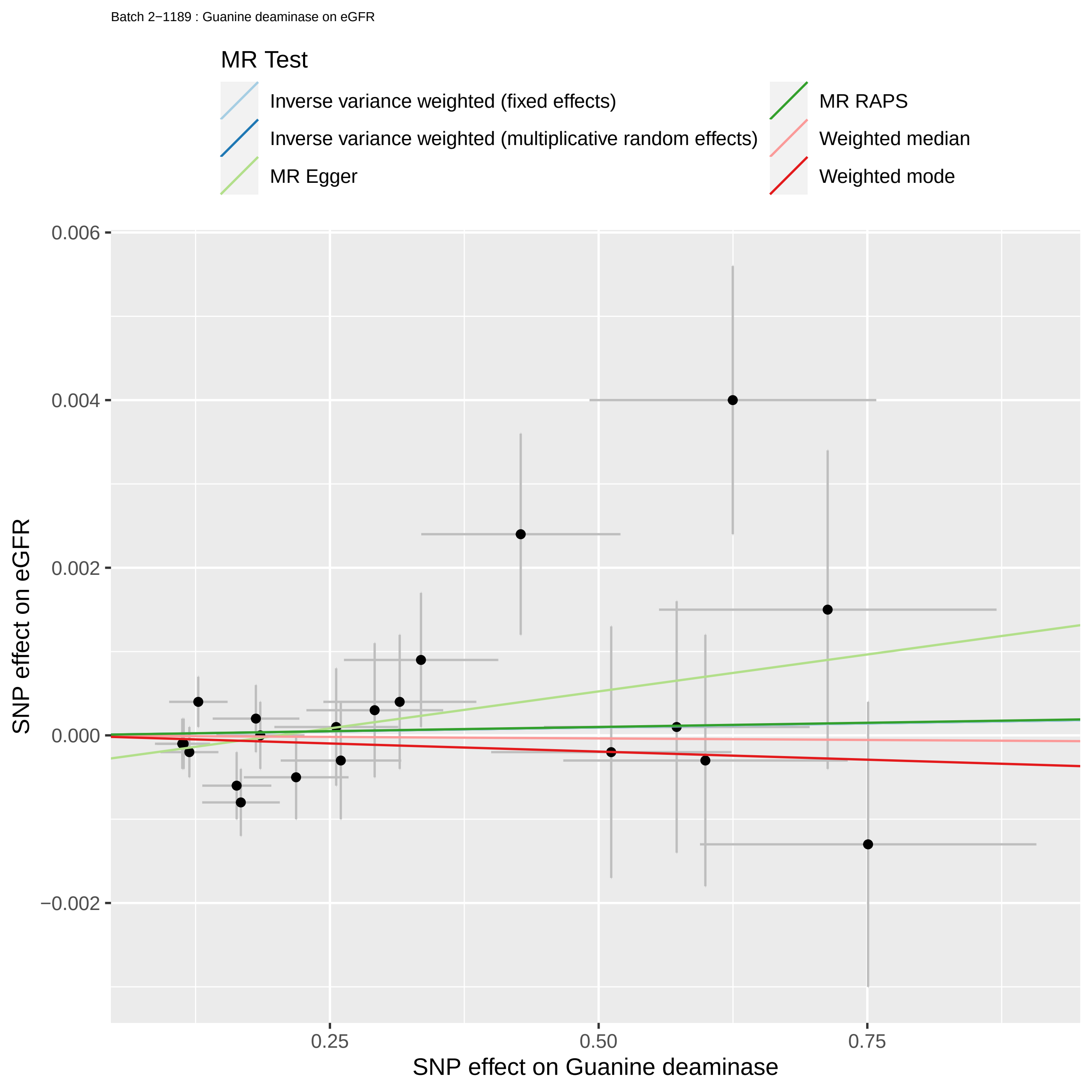
Batch 2−1189 : Guanine deaminase on eGFR
MR Test
Inverse variance weighted (fixed effects)
Inverse variance weighted (multiplicative random effects)
MR Egger
MR RAPS
Weighted median
Weighted mode
0.006
0.004
0.002
0.000
−0.002
0.25
0.50
0.75
SNP effect on eGFR
SNP effect on Guanine deaminase

Batch 2−1190 : Growth/differentiation factor 11 on eGFR

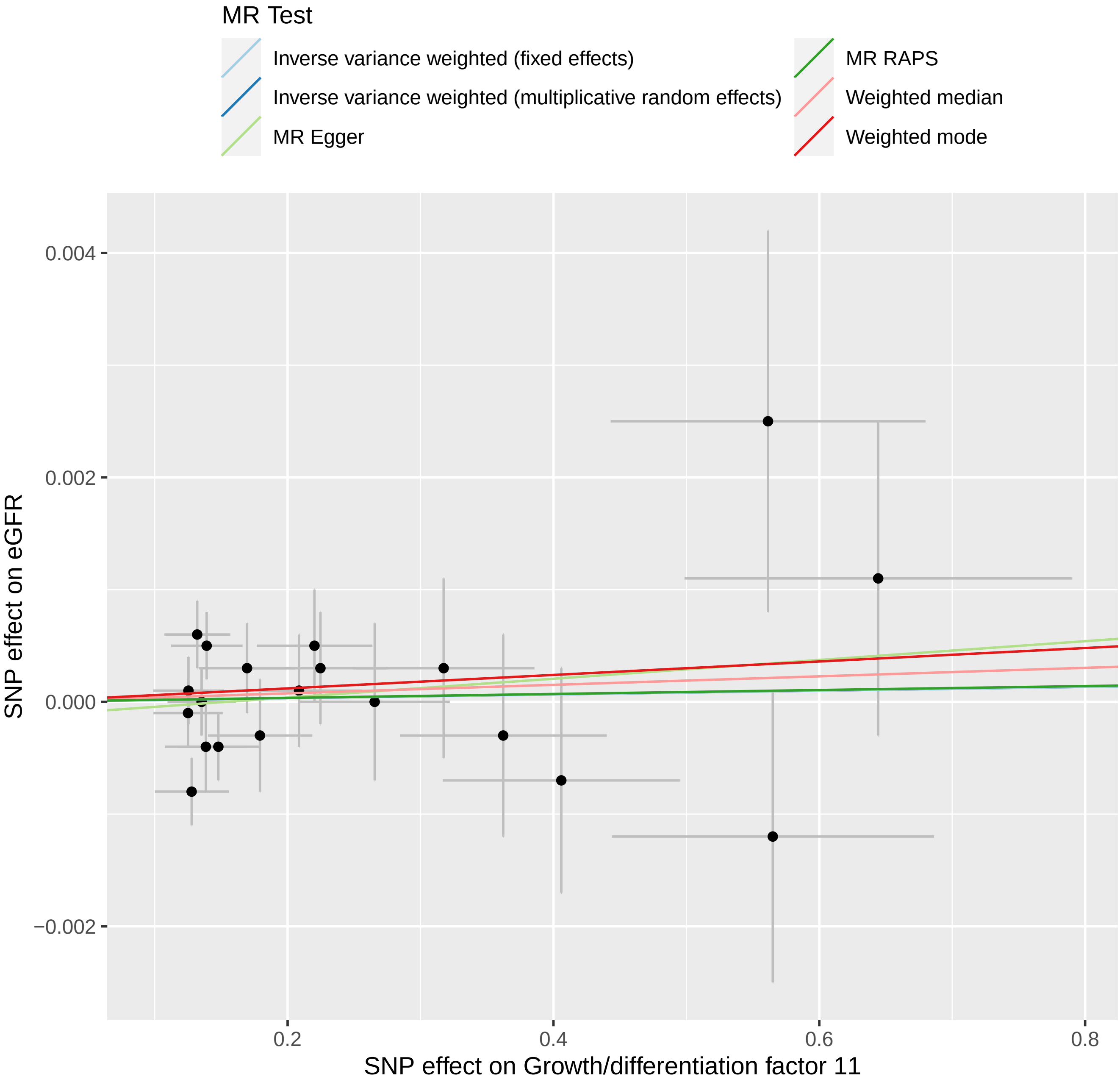

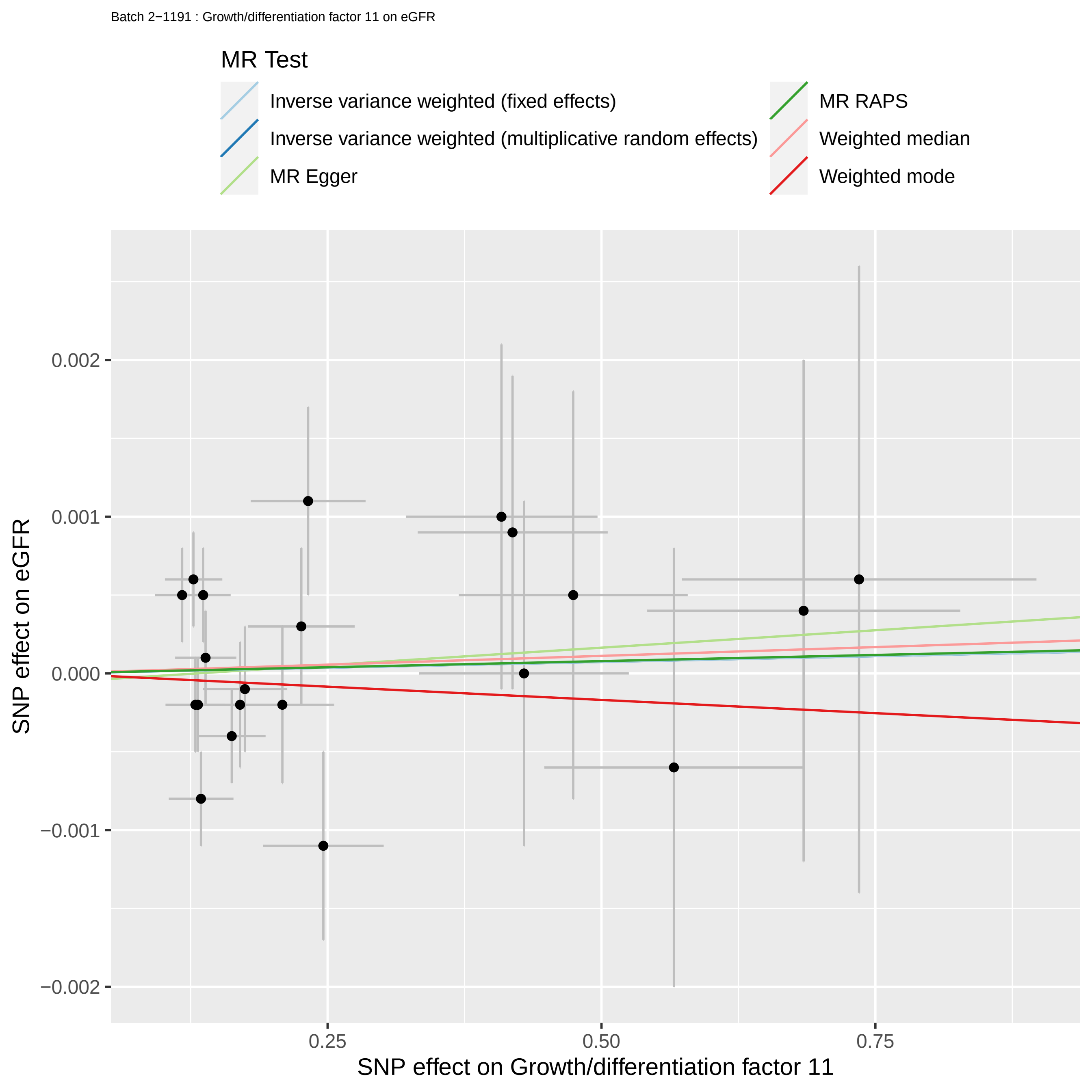
Batch 2−1191 : Growth/differentiation factor 11 on eGFR
MR Test
Inverse variance weighted (fixed effects)
Inverse variance weighted (multiplicative random effects)
MR Egger
MR RAPS
Weighted median
Weighted mode
0.002
0.001
0.000
−0.001
−0.002
0.25
0.50
0.75
SNP effect on eGFR
SNP effect on Growth/differentiation factor 11

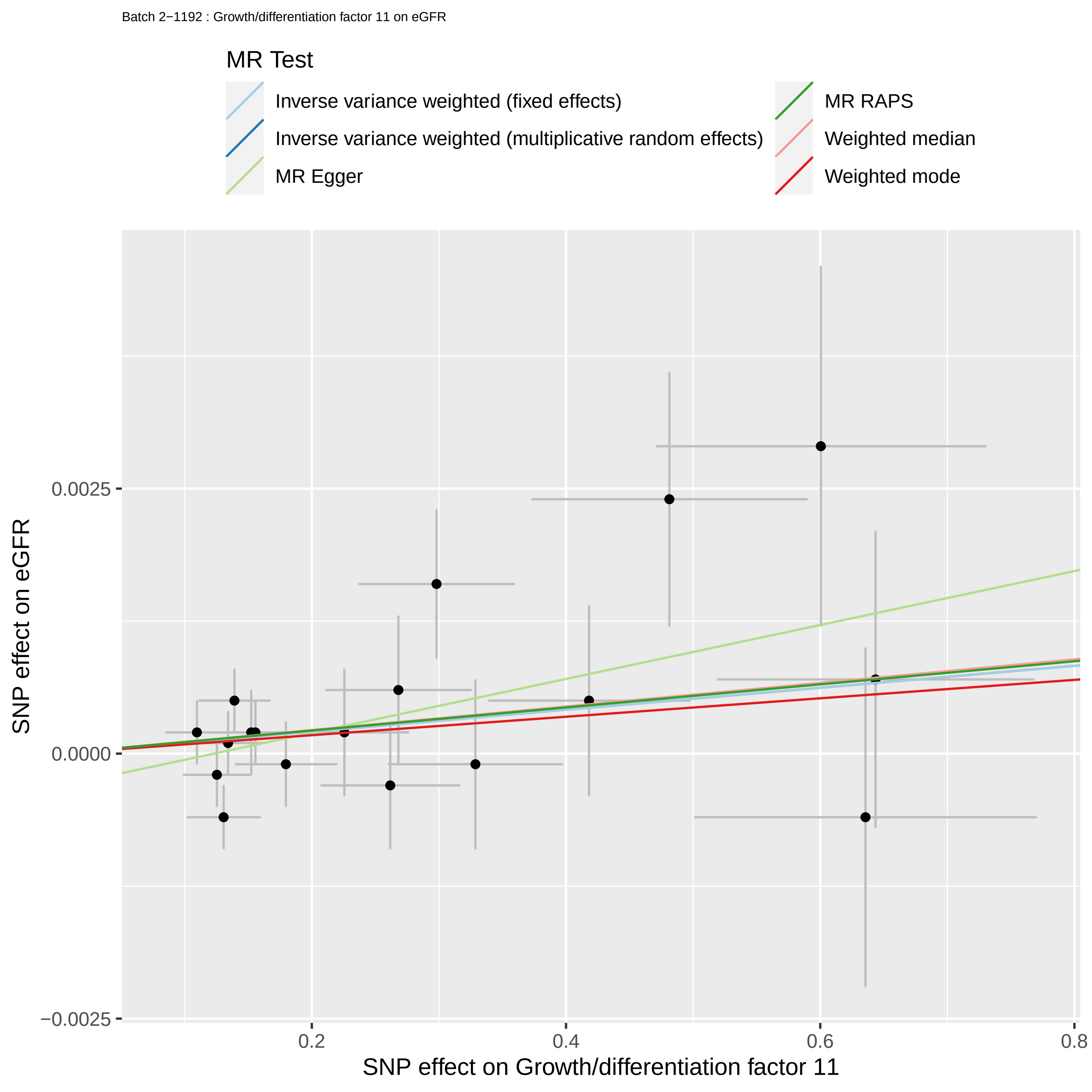

Batch 2−1192 : Growth/differentiation factor 11 on eGFR
MR Test
Inverse variance weighted (fixed effects)
Inverse variance weighted (multiplicative random effects)
MR Egger
MR RAPS
Weighted median
Weighted mode
0.0025
0.0000
−0.0025
0.2
0.4
0.6
0.8
SNP effect on eGFR
SNP effect on Growth/differentiation factor 11

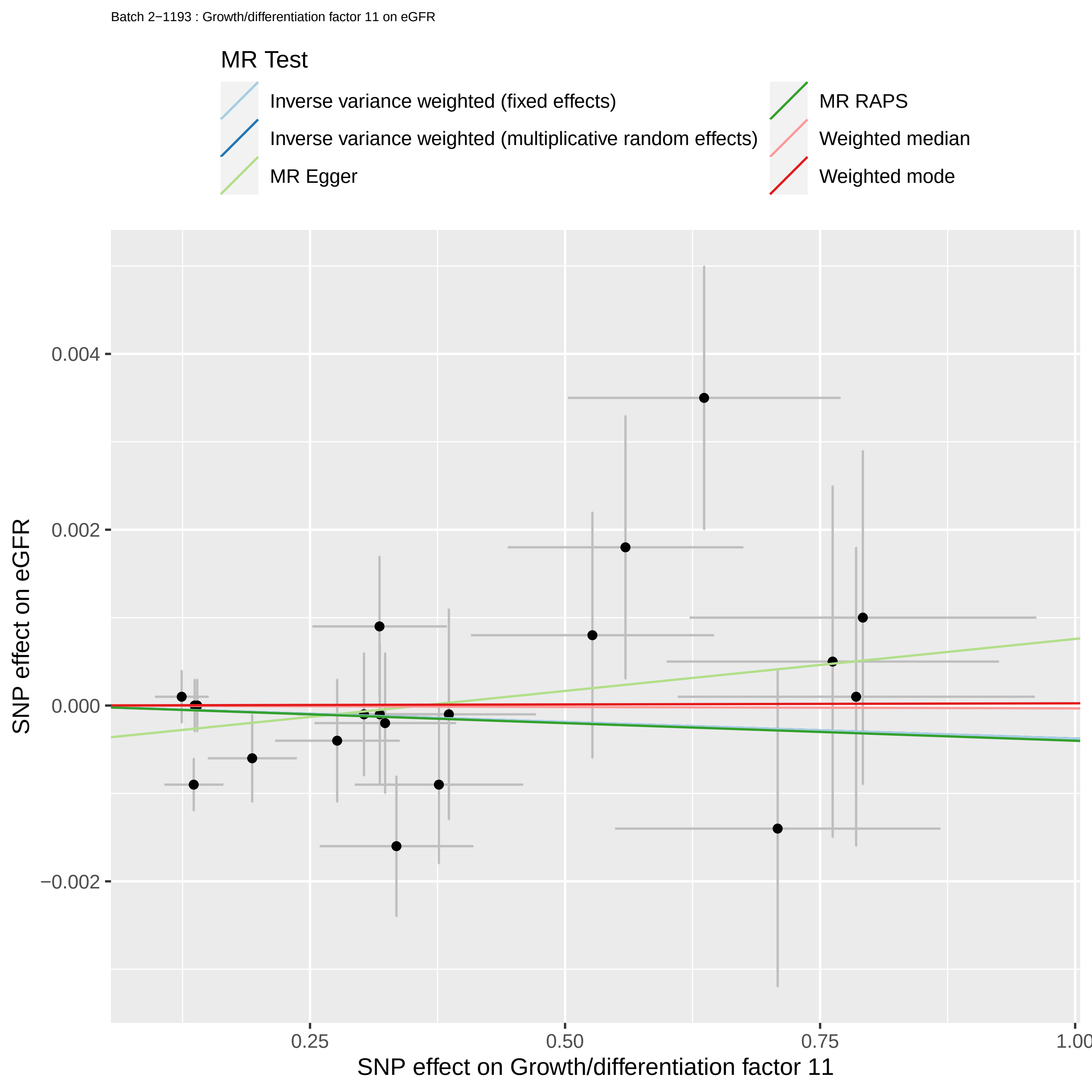
Batch 2−1193 : Growth/differentiation factor 11 on eGFR
MR Test
Inverse variance weighted (fixed effects)
Inverse variance weighted (multiplicative random effects)
MR Egger
MR RAPS
Weighted median
Weighted mode
0.004
0.002
0.000
−0.002
0.25
0.50
0.75
1.00
SNP effect on eGFR
SNP effect on Growth/differentiation factor 11

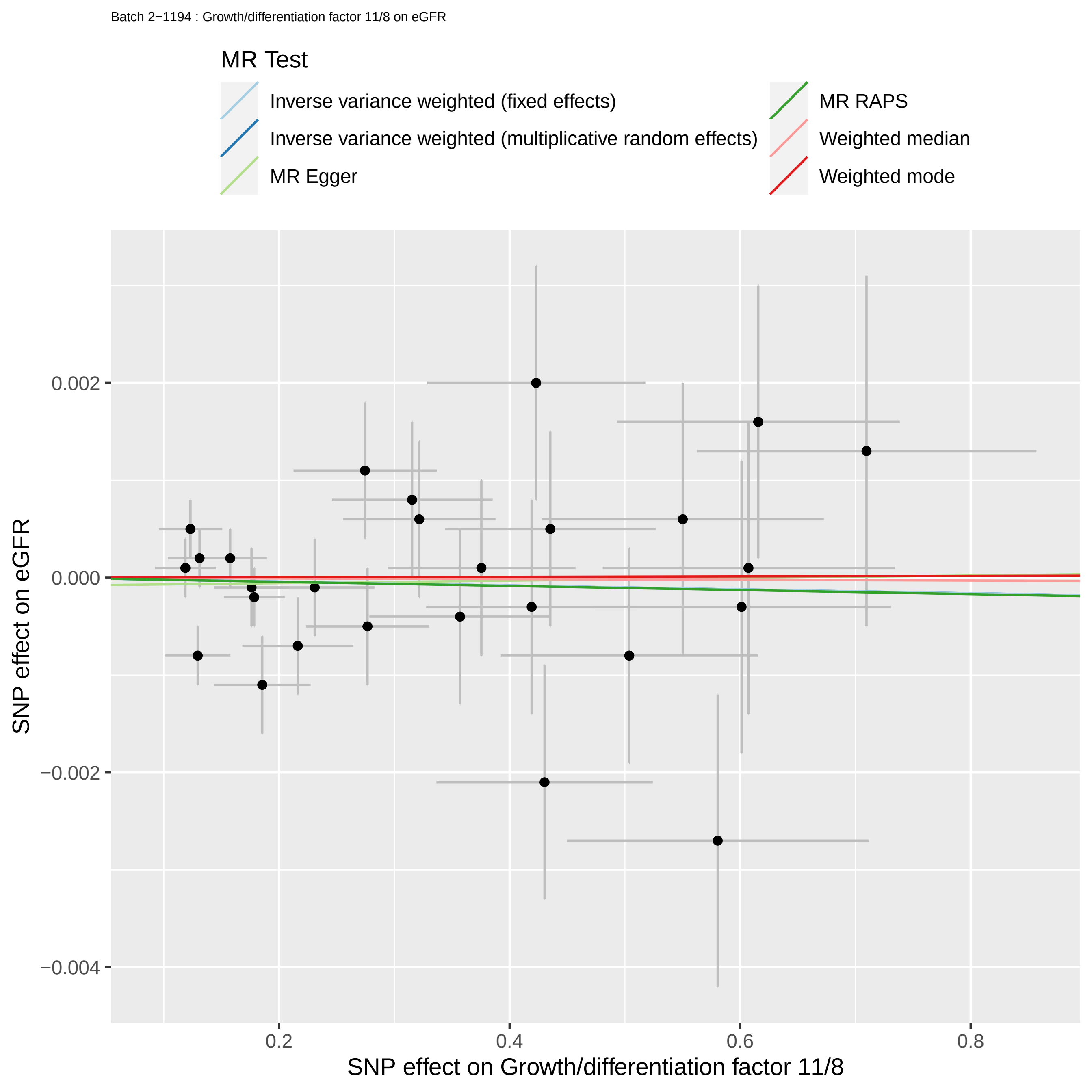

Batch 2−1194 : Growth/differentiation factor 11/8 on eGFR
MR Test
Inverse variance weighted (fixed effects)
Inverse variance weighted (multiplicative random effects)
MR Egger
MR RAPS
Weighted median
Weighted mode
0.002
0.000
−0.002
−0.004
0.2
0.4
0.6
0.8
SNP effect on eGFR
SNP effect on Growth/differentiation factor 11/8

Batch 2−1195 : Growth/differentiation factor 15 on eGFR

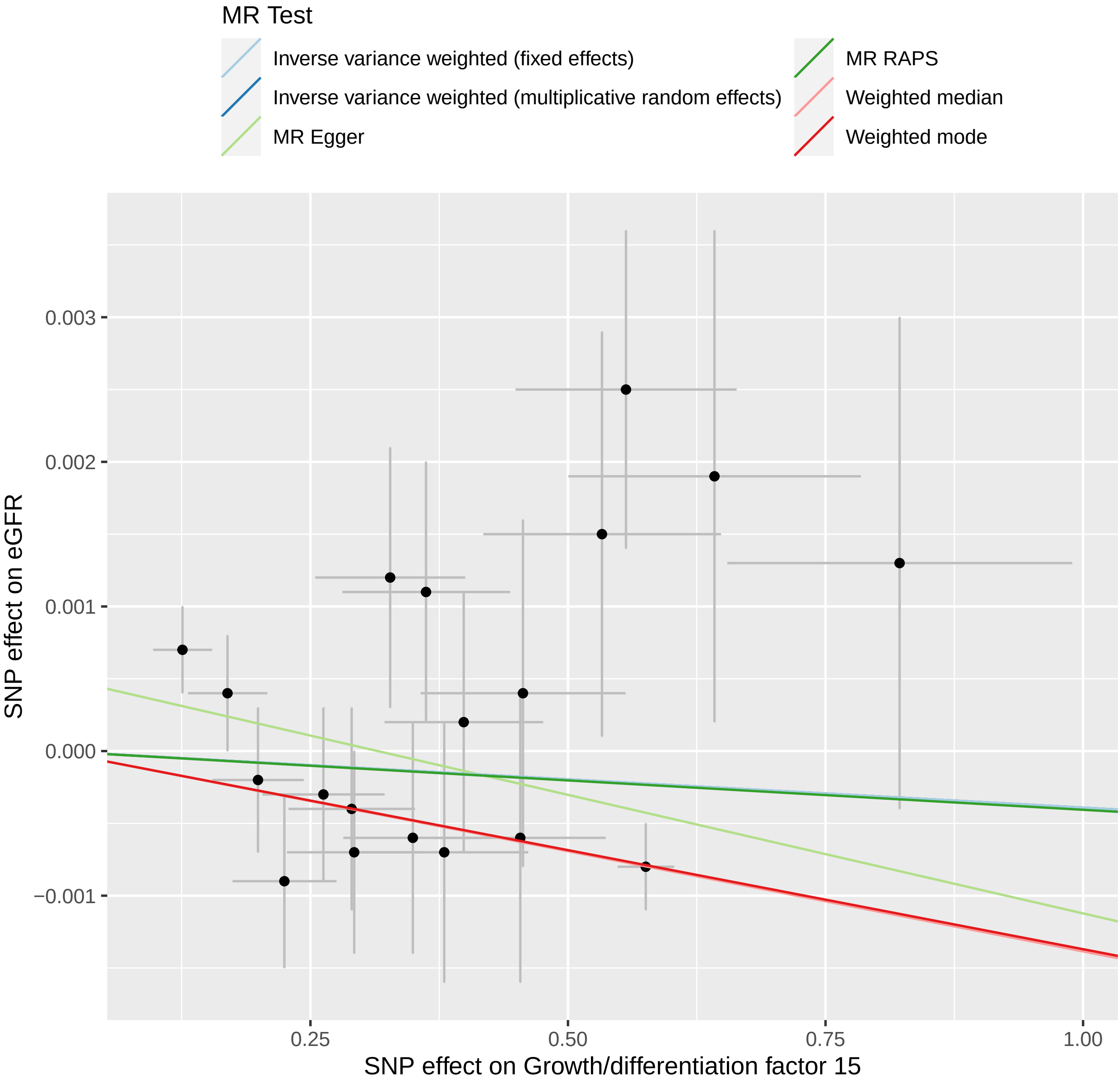

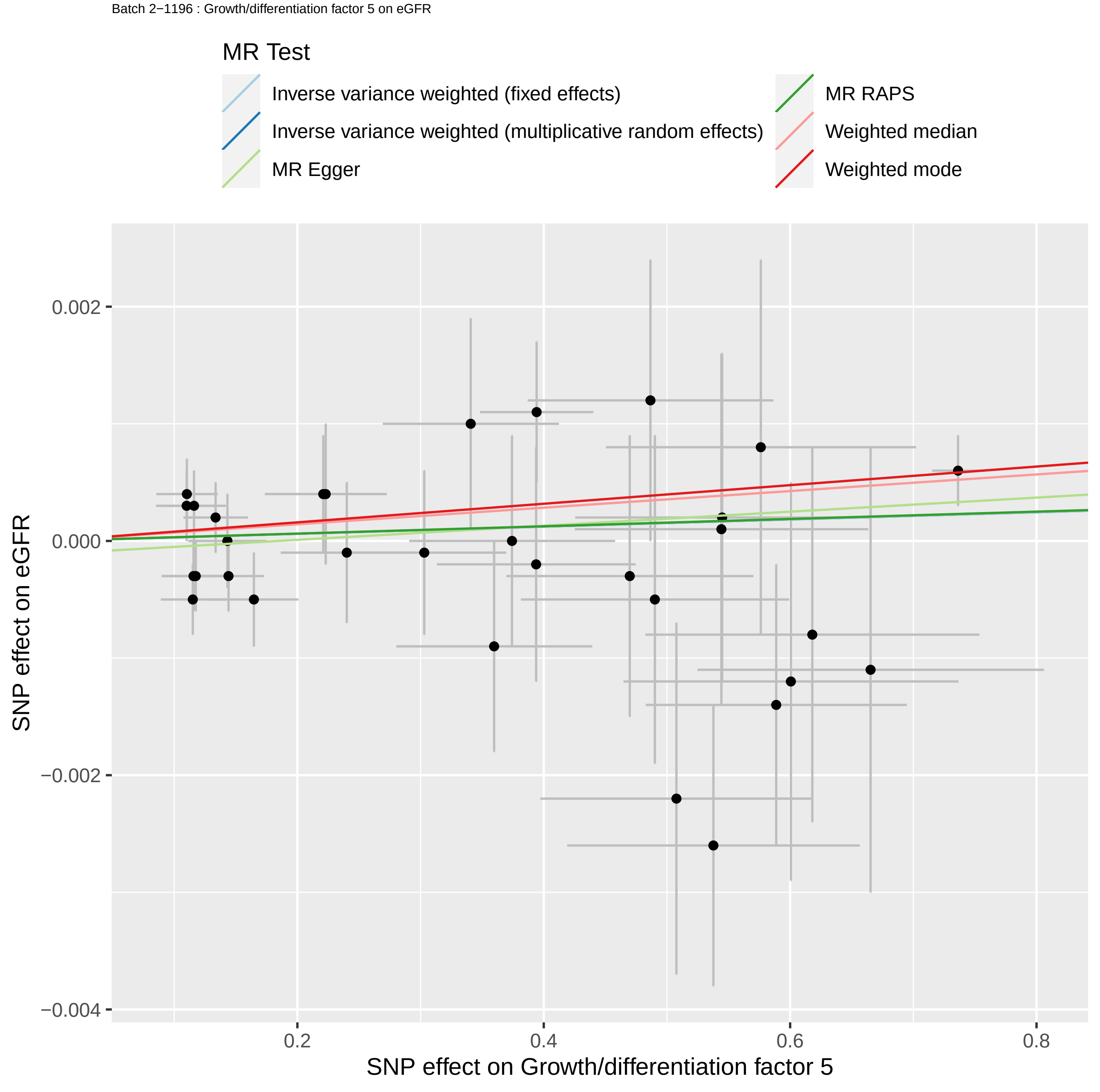

Batch 2−1196 : Growth/differentiation factor 5 on eGFR
MR Test
Inverse variance weighted (fixed effects)
Inverse variance weighted (multiplicative random effects)
MR Egger
MR RAPS
Weighted median
Weighted mode
0.002
0.000
−0.002
−0.004
0.2
0.4
0.6
0.8
SNP effect on eGFR
SNP effect on Growth/differentiation factor 5

Batch 2−1197 : Growth/differentiation factor 9 on eGFR

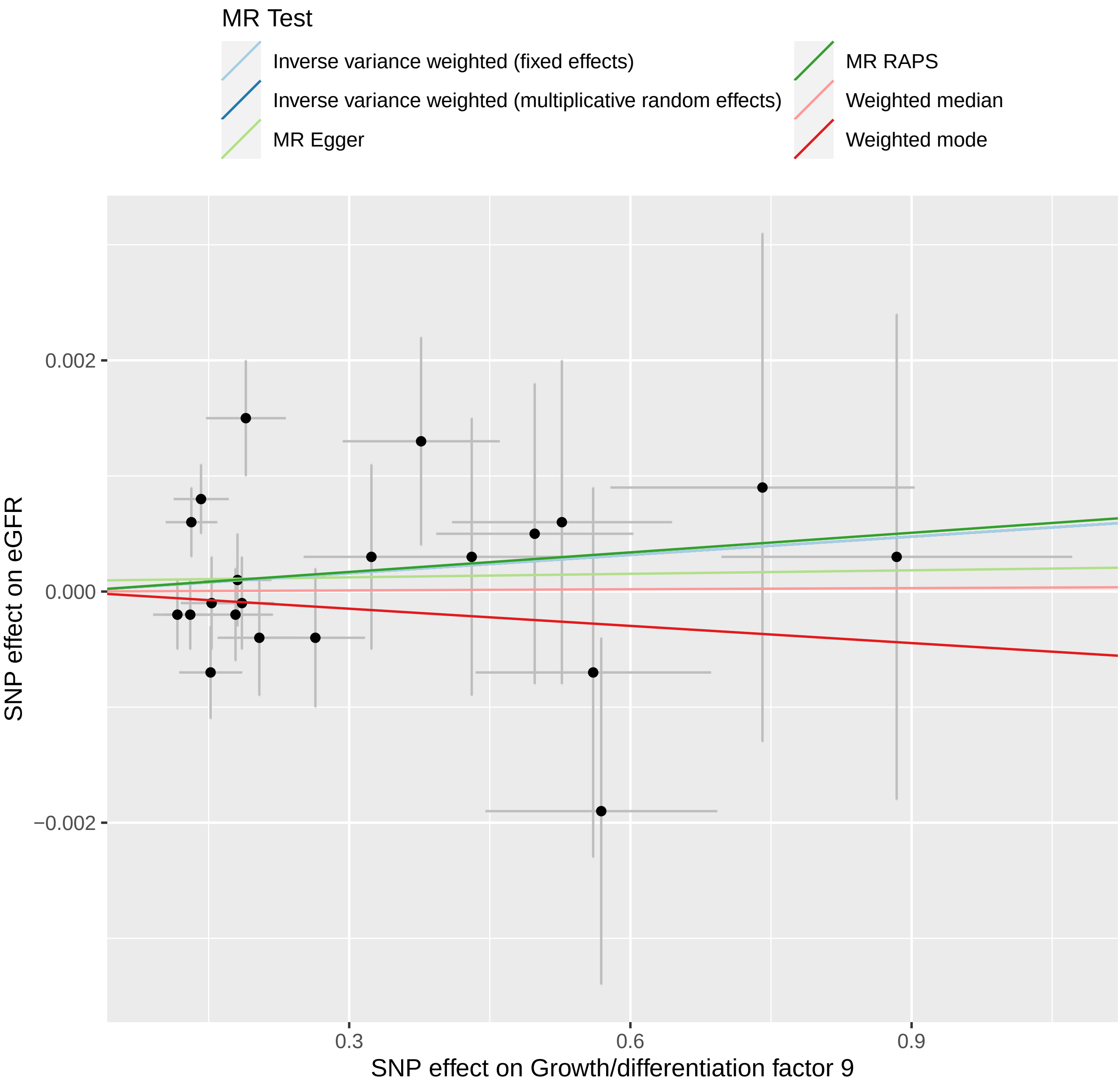

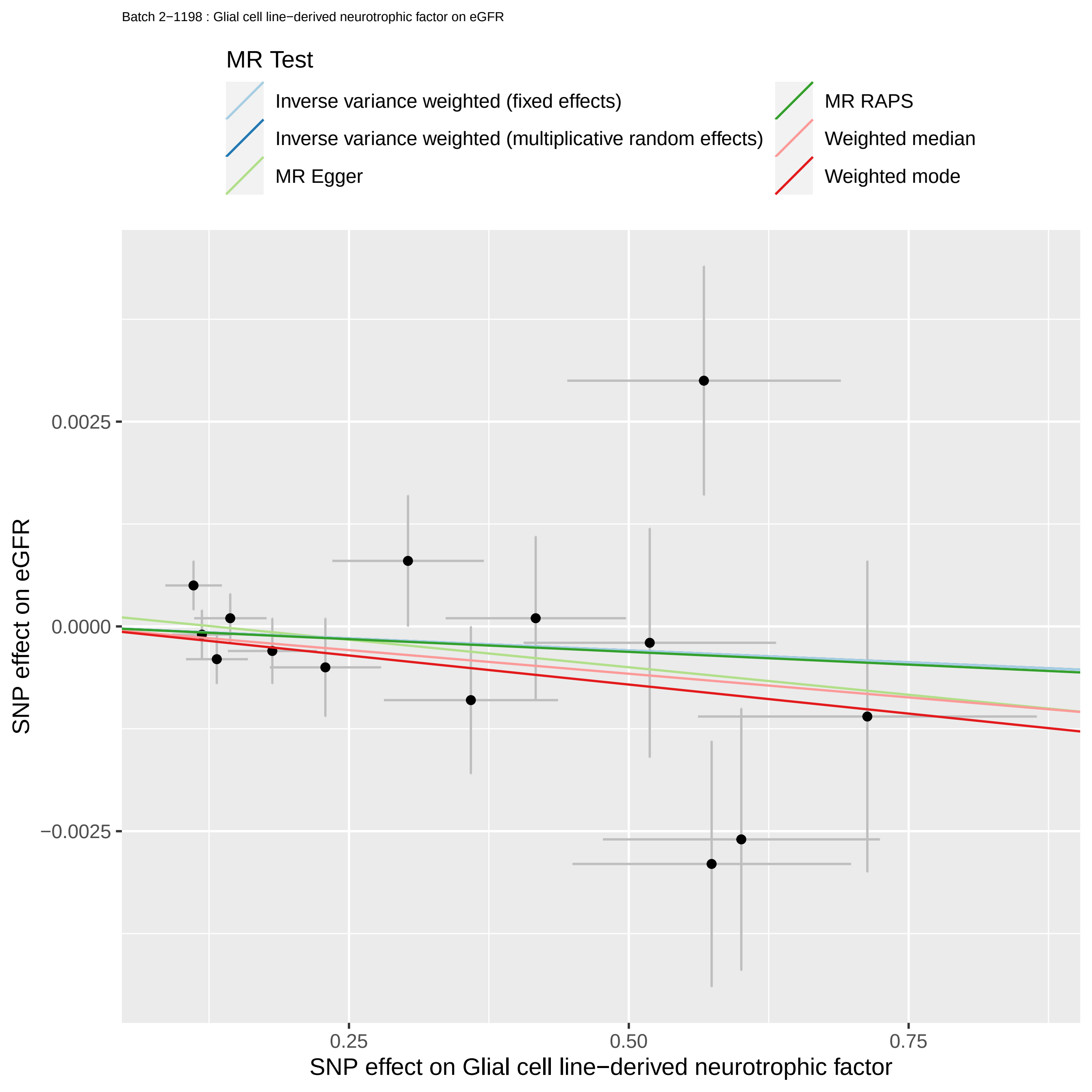

Batch 2−1198 : Glial cell line−derived neurotrophic factor on eGFR
MR Test
Inverse variance weighted (fixed effects)
Inverse variance weighted (multiplicative random effects)
MR Egger
MR RAPS
Weighted median
Weighted mode
0.0025
0.0000
−0.0025
0.25
0.50
0.75
SNP effect on eGFR
SNP effect on Glial cell line−derived neurotrophic factor

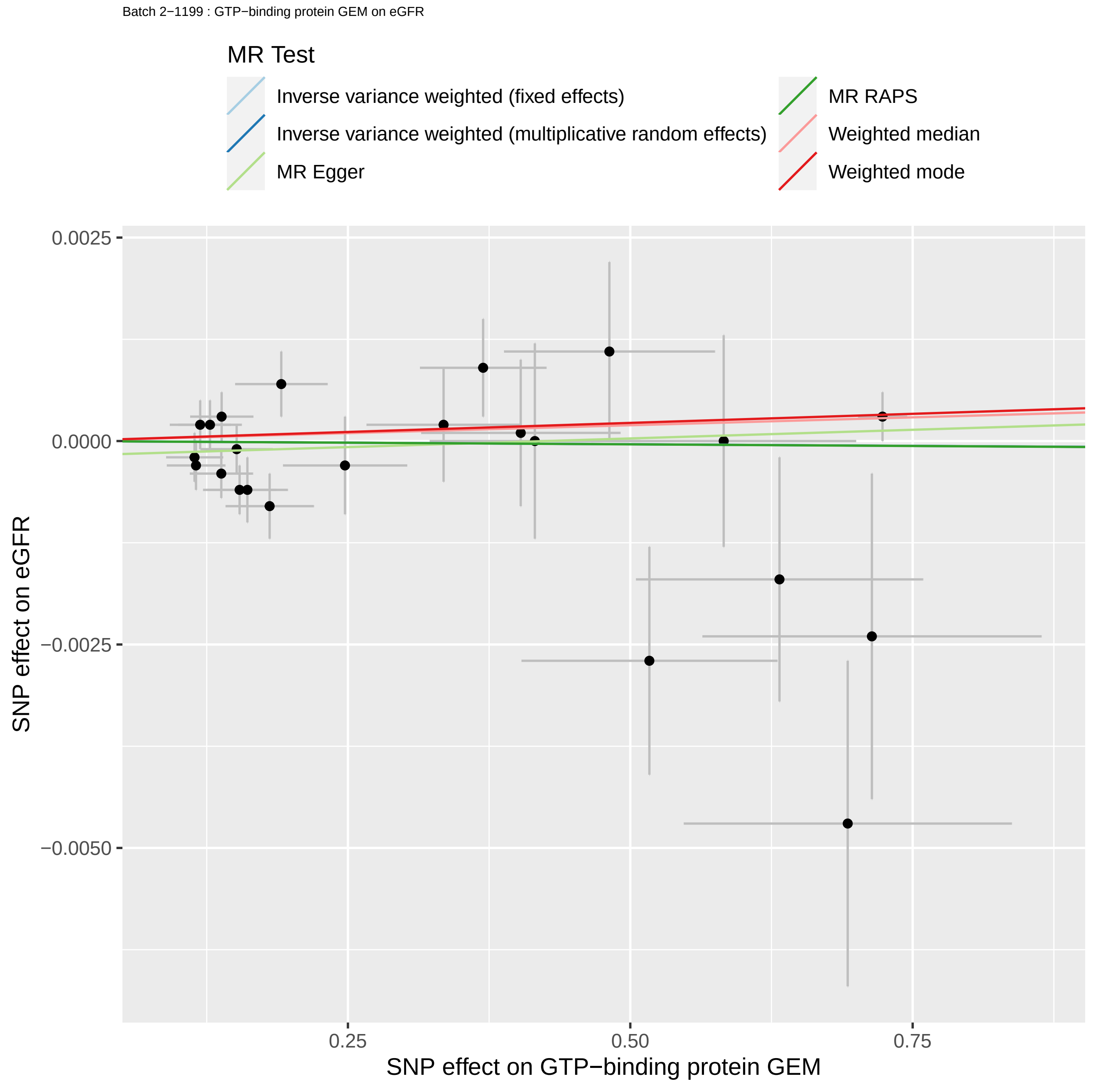
Batch 2−1199 : GTP−binding protein GEM on eGFR
MR Test
Inverse variance weighted (fixed effects)
Inverse variance weighted (multiplicative random effects)
MR Egger
MR RAPS
Weighted median
Weighted mode
0.0025
0.0000
−0.0025
−0.0050
0.25
0.50
0.75
SNP effect on eGFR
SNP effect on GTP−binding protein GEM

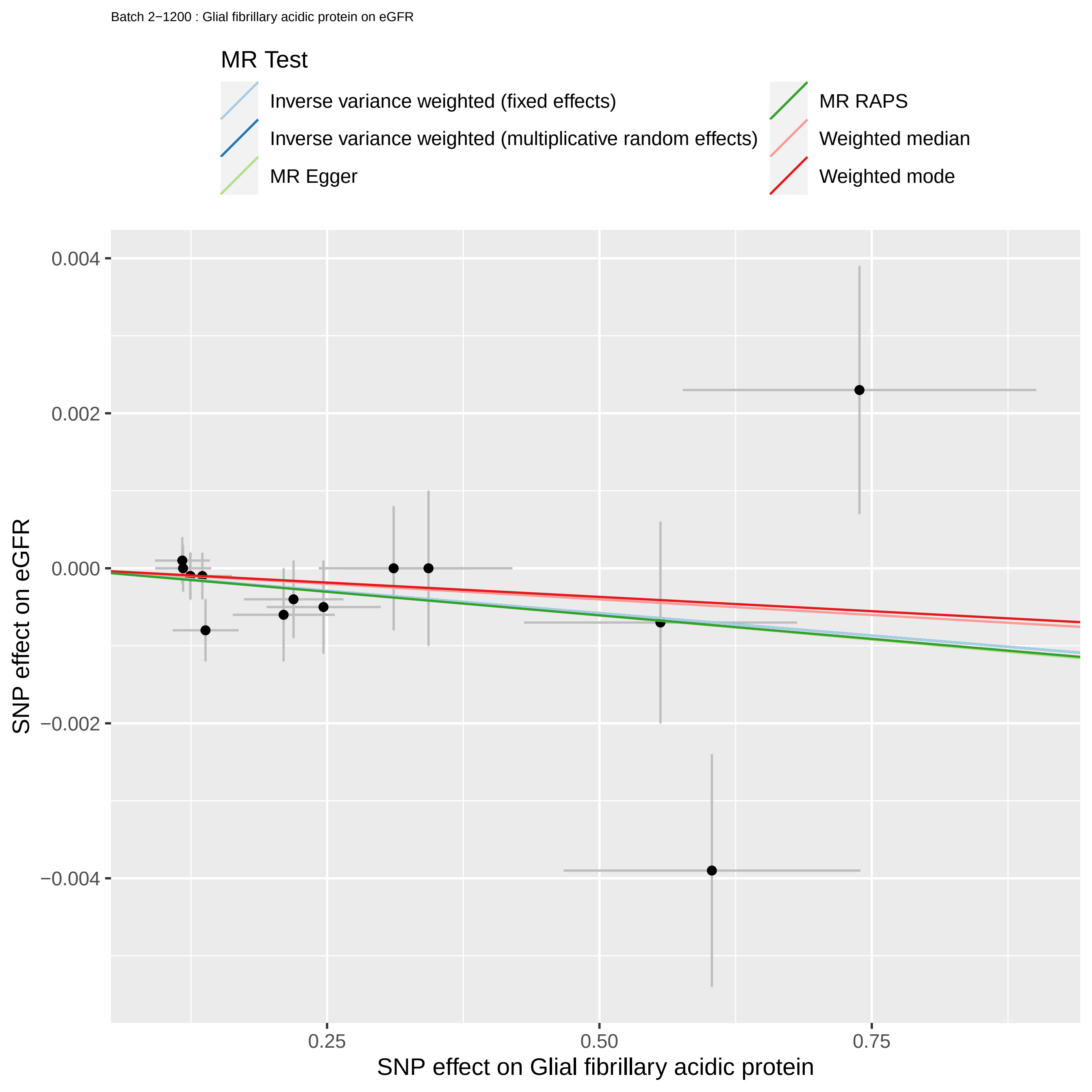

Batch 2−1200 : Glial fibrillary acidic protein on eGFR
MR Test
Inverse variance weighted (fixed effects)
Inverse variance weighted (multiplicative random effects)
MR Egger
MR RAPS
Weighted median
Weighted mode
0.004
0.002
0.000
−0.002
−0.004
0.25
0.50
0.75
SNP effect on eGFR
SNP effect on Glial fibrillary acidic protein

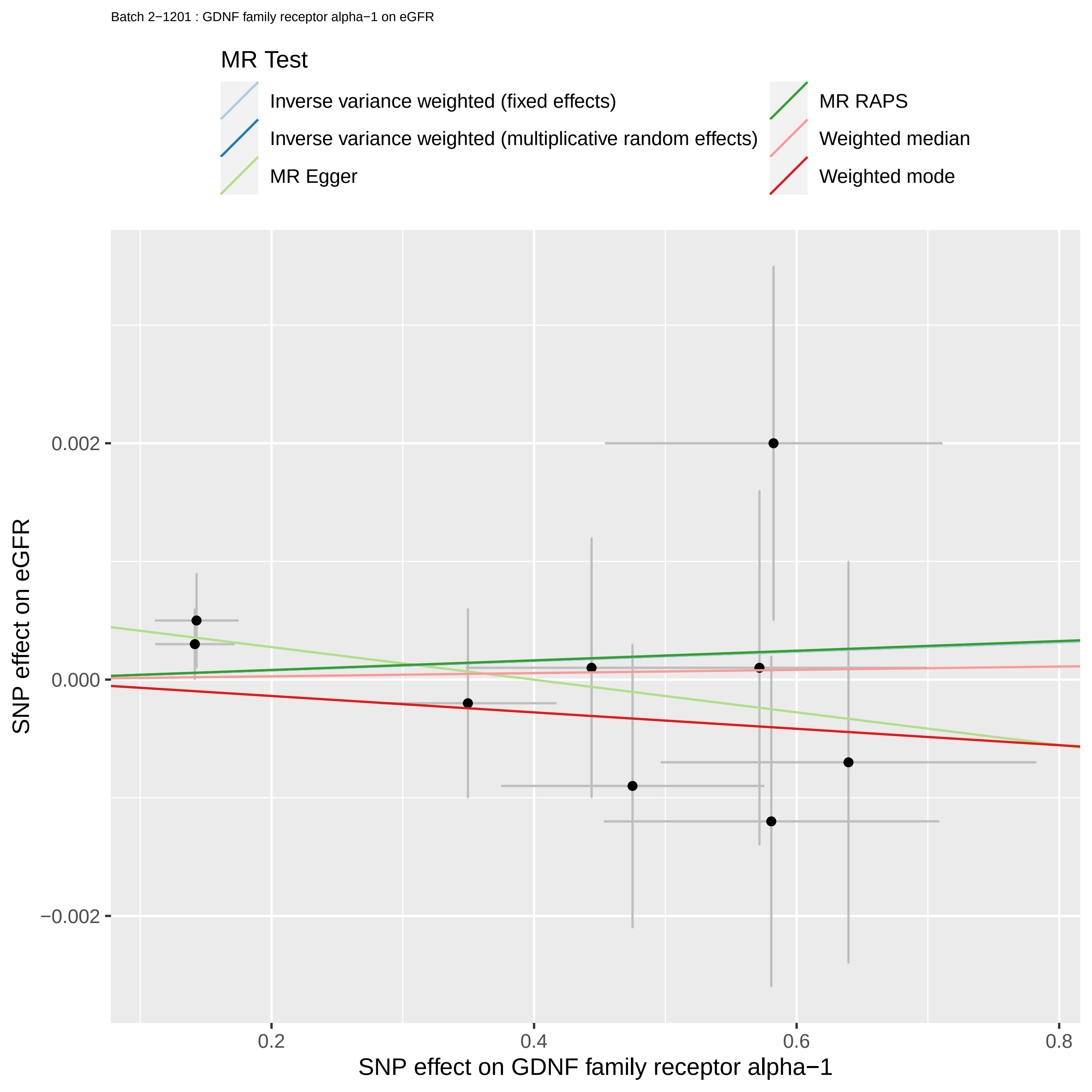
Batch 2−1201 : GDNF family receptor alpha−1 on eGFR
MR Test
Inverse variance weighted (fixed effects)
Inverse variance weighted (multiplicative random effects)
MR Egger
MR RAPS
Weighted median
Weighted mode
0.002
0.000
−0.002
0.2
0.4
0.6
0.8
SNP effect on eGFR
SNP effect on GDNF family receptor alpha−1

Batch 2−1202 : GDNF family receptor alpha−2 on eGFR

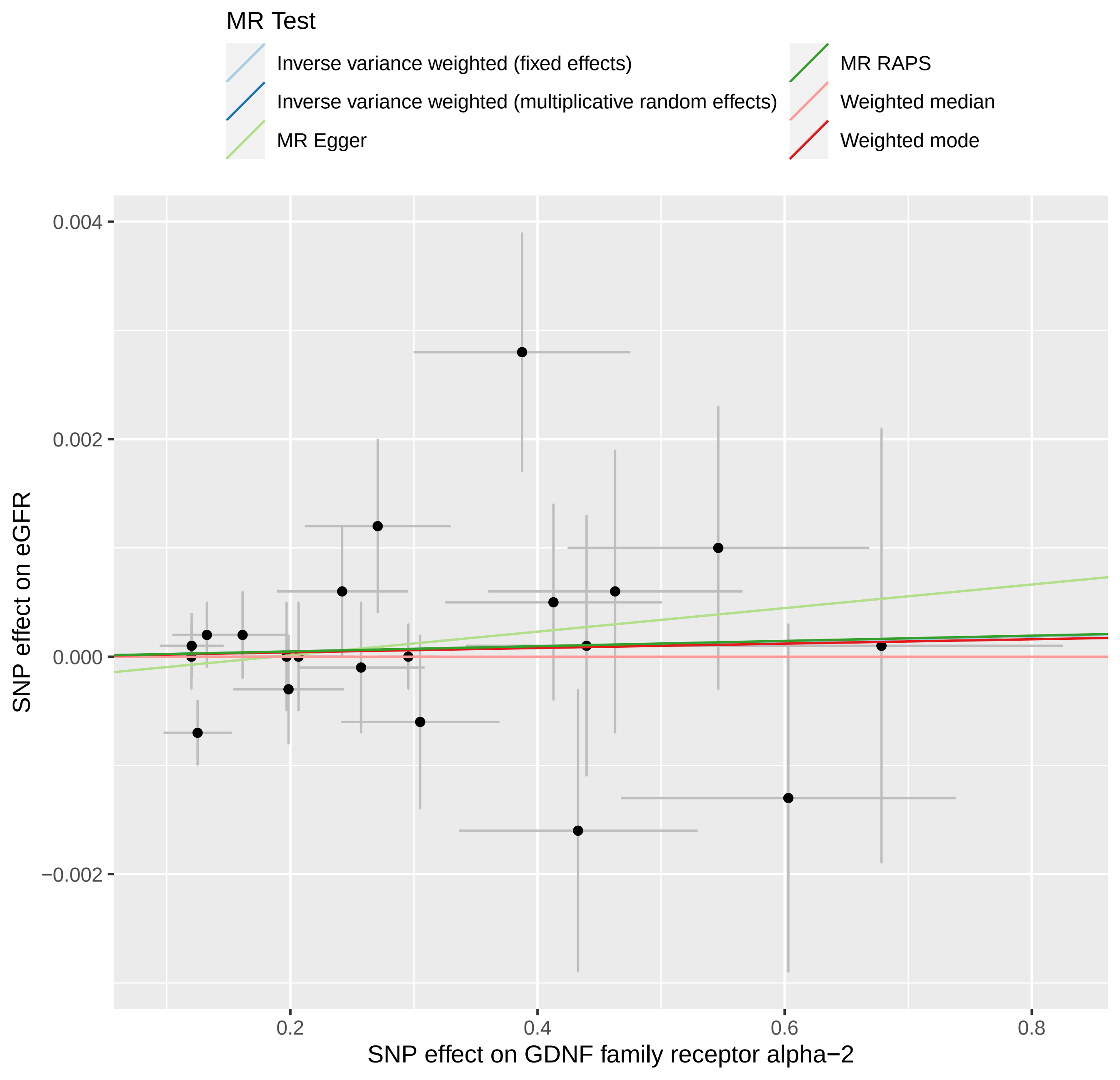

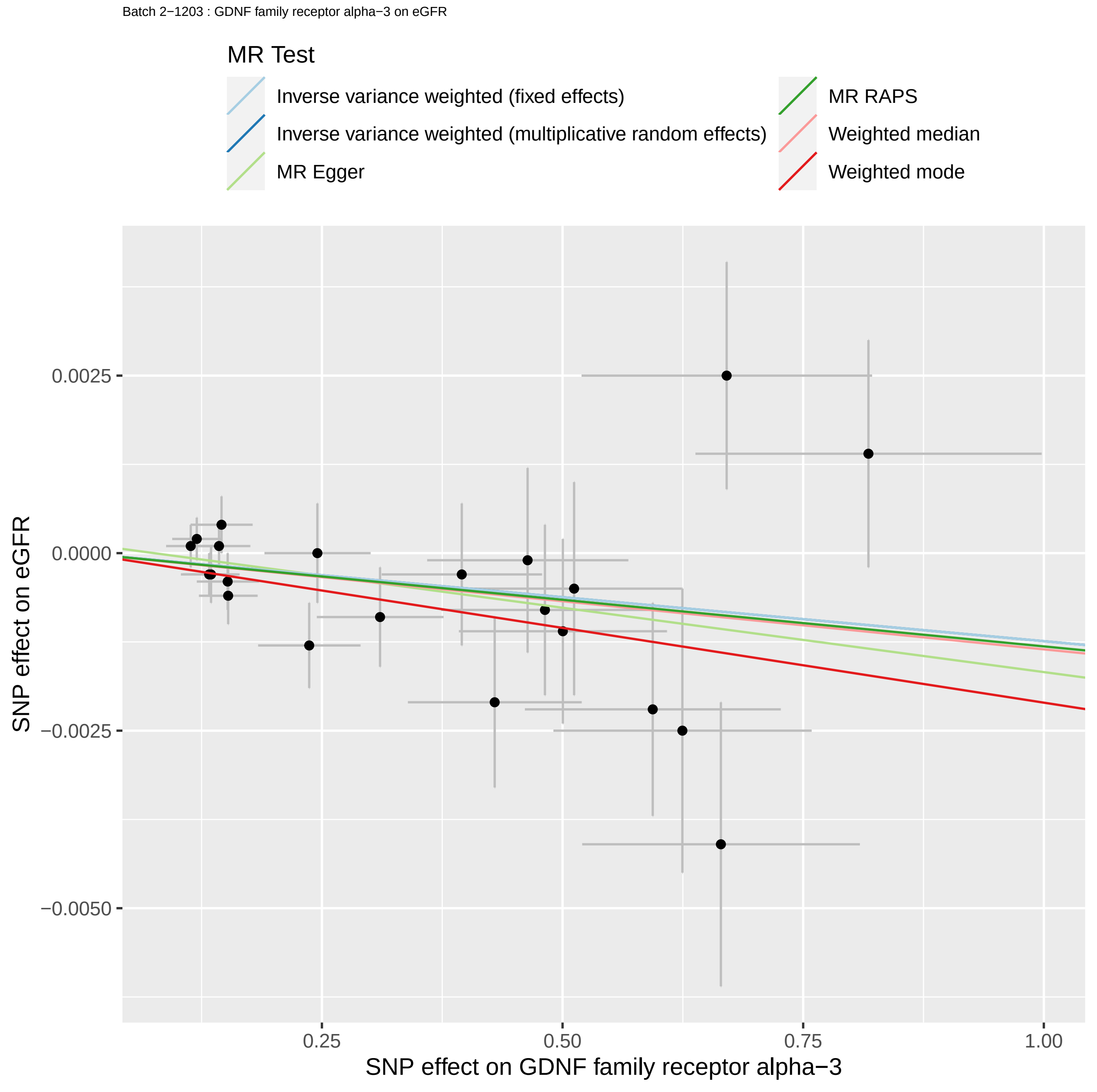

Batch 2−1203 : GDNF family receptor alpha−3 on eGFR
MR Test
Inverse variance weighted (fixed effects)
Inverse variance weighted (multiplicative random effects)
MR Egger
MR RAPS
Weighted median
Weighted mode
0.0025
0.0000
−0.0025
−0.0050
0.25
0.50
0.75
1.00
SNP effect on eGFR
SNP effect on GDNF family receptor alpha−3

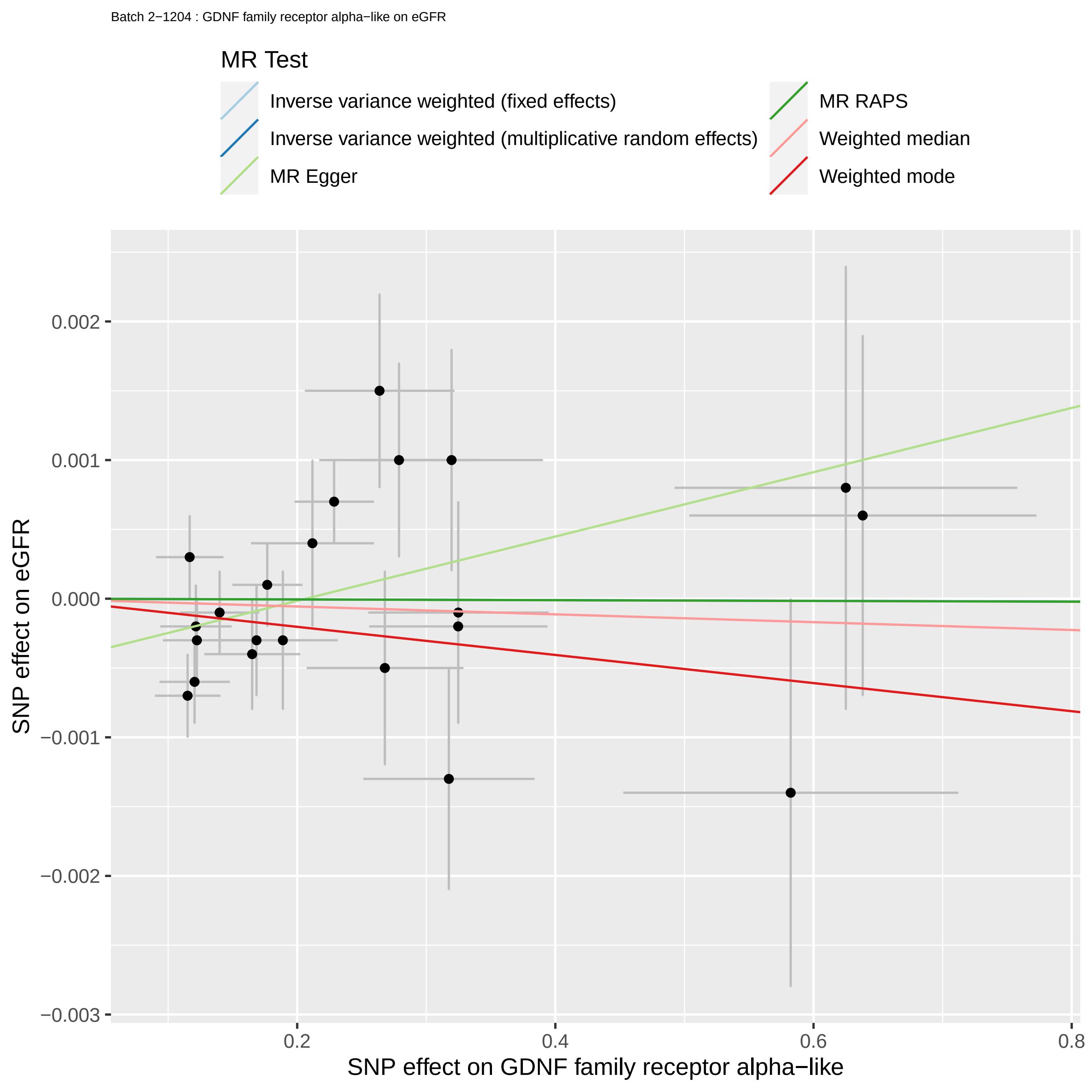
Batch 2−1204 : GDNF family receptor alpha−like on eGFR
MR Test
Inverse variance weighted (fixed effects)
Inverse variance weighted (multiplicative random effects)
MR Egger
MR RAPS
Weighted median
Weighted mode
0.002
0.001
0.000
−0.001
−0.002
−0.003
0.2
0.4
0.6
0.8
SNP effect on eGFR
SNP effect on GDNF family receptor alpha−like

Batch 2−1205 : ADP−ribosylation factor−binding protein GGA1 on eGFR

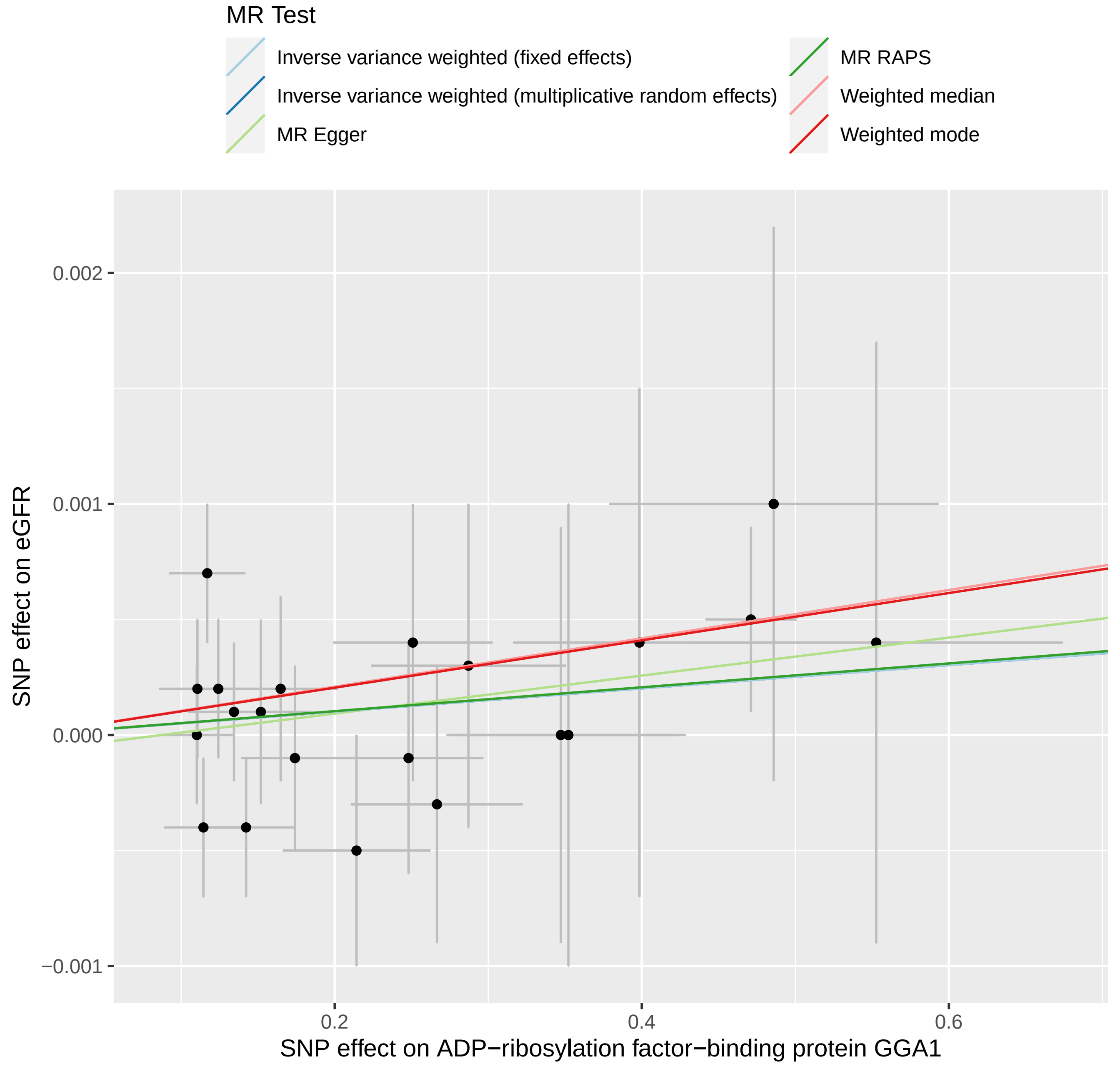

Batch 2−1206 : ADP−ribosylation factor−binding protein GGA3 on eGFR

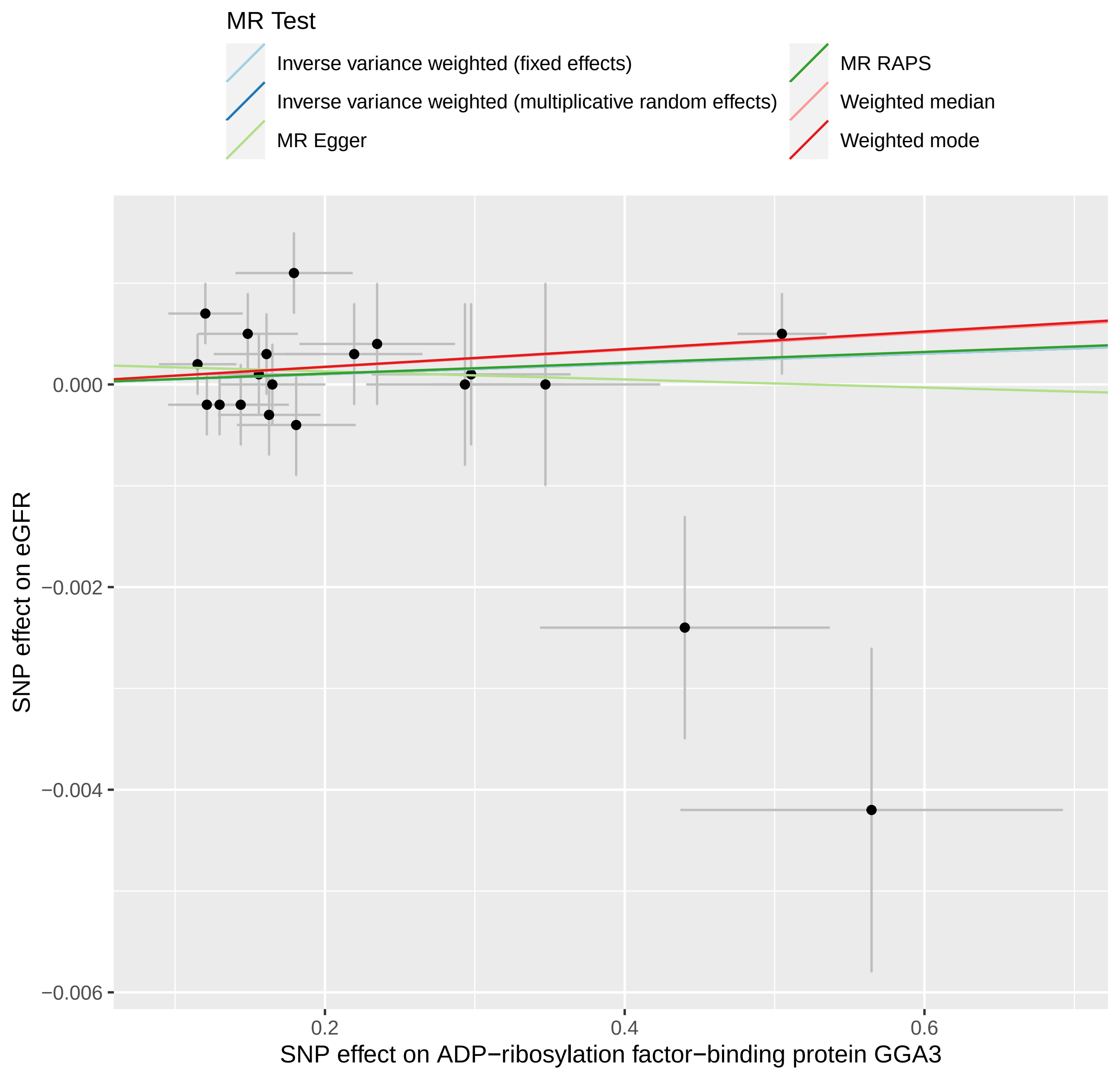

Batch 2−1207 : Gamma−glutamyl hydrolase on eGFR

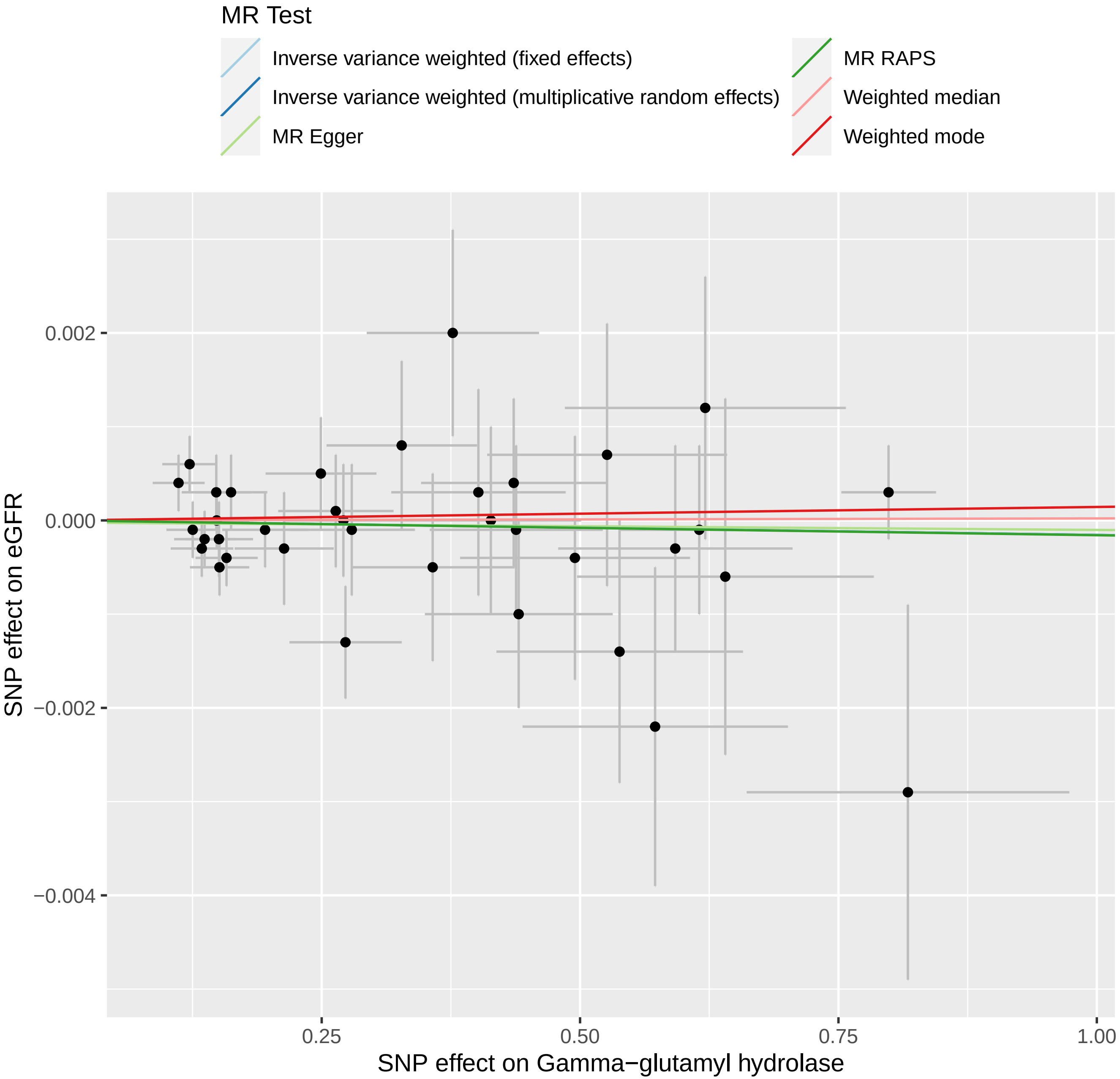

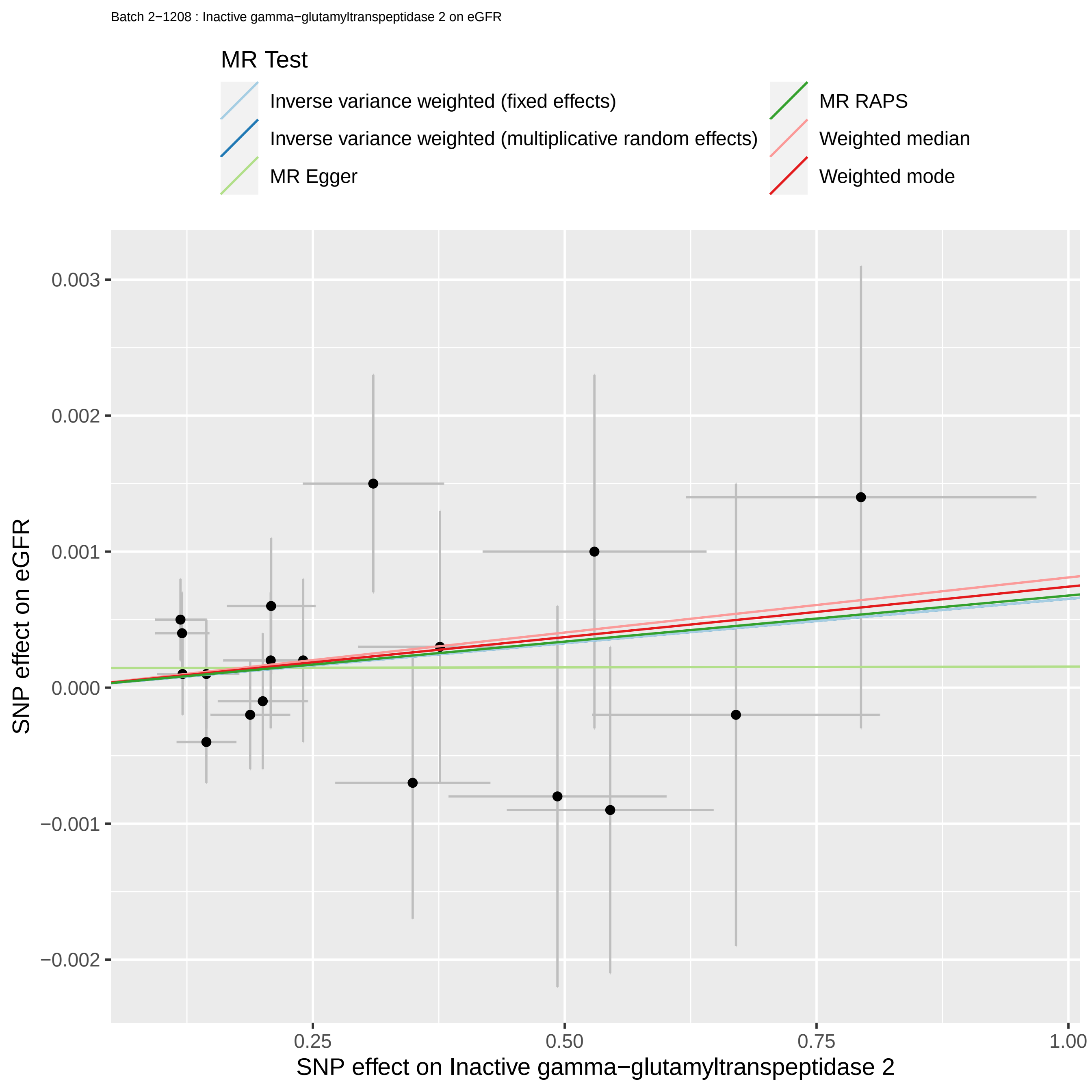
Batch 2−1208 : Inactive gamma−glutamyltranspeptidase 2 on eGFR
MR Test
Inverse variance weighted (fixed effects)
Inverse variance weighted (multiplicative random effects)
MR Egger
MR RAPS
Weighted median
Weighted mode
SNP effect on eGFR
0.003
0.002
0.001
0.000
−0.001
−0.002
0.25
0.50
0.75
1.00
SNP effect on Inactive gamma−glutamyltranspeptidase 2

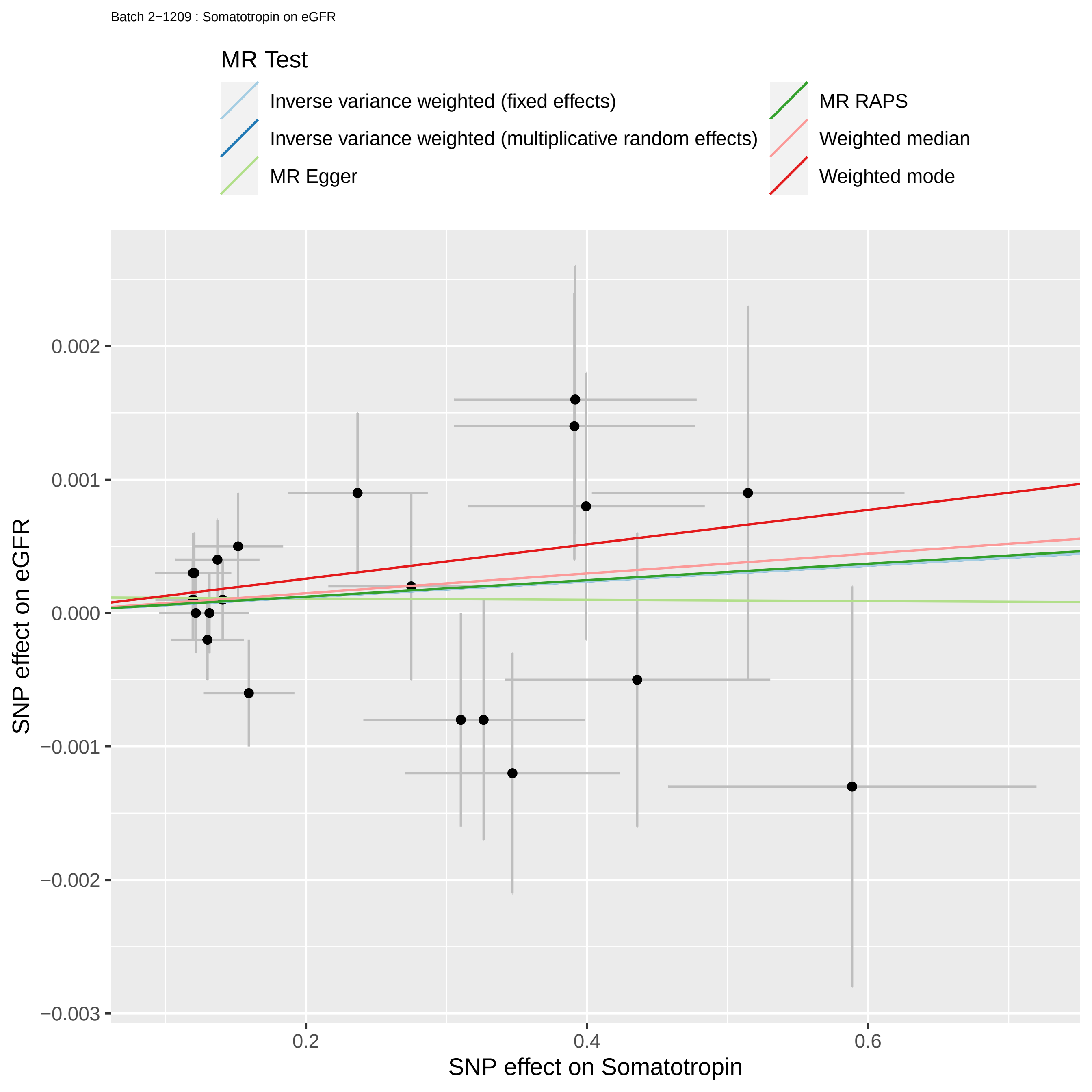

Batch 2−1209 : Somatotropin on eGFR
MR Test
Inverse variance weighted (fixed effects)
Inverse variance weighted (multiplicative random effects)
MR Egger
MR RAPS
Weighted median
Weighted mode
0.002
0.001
0.000
−0.001
−0.002
−0.003
0.2
0.4
0.6
SNP effect on eGFR
SNP effect on Somatotropin

Batch 2−1210 : GH3 domain−containing protein on eGFR

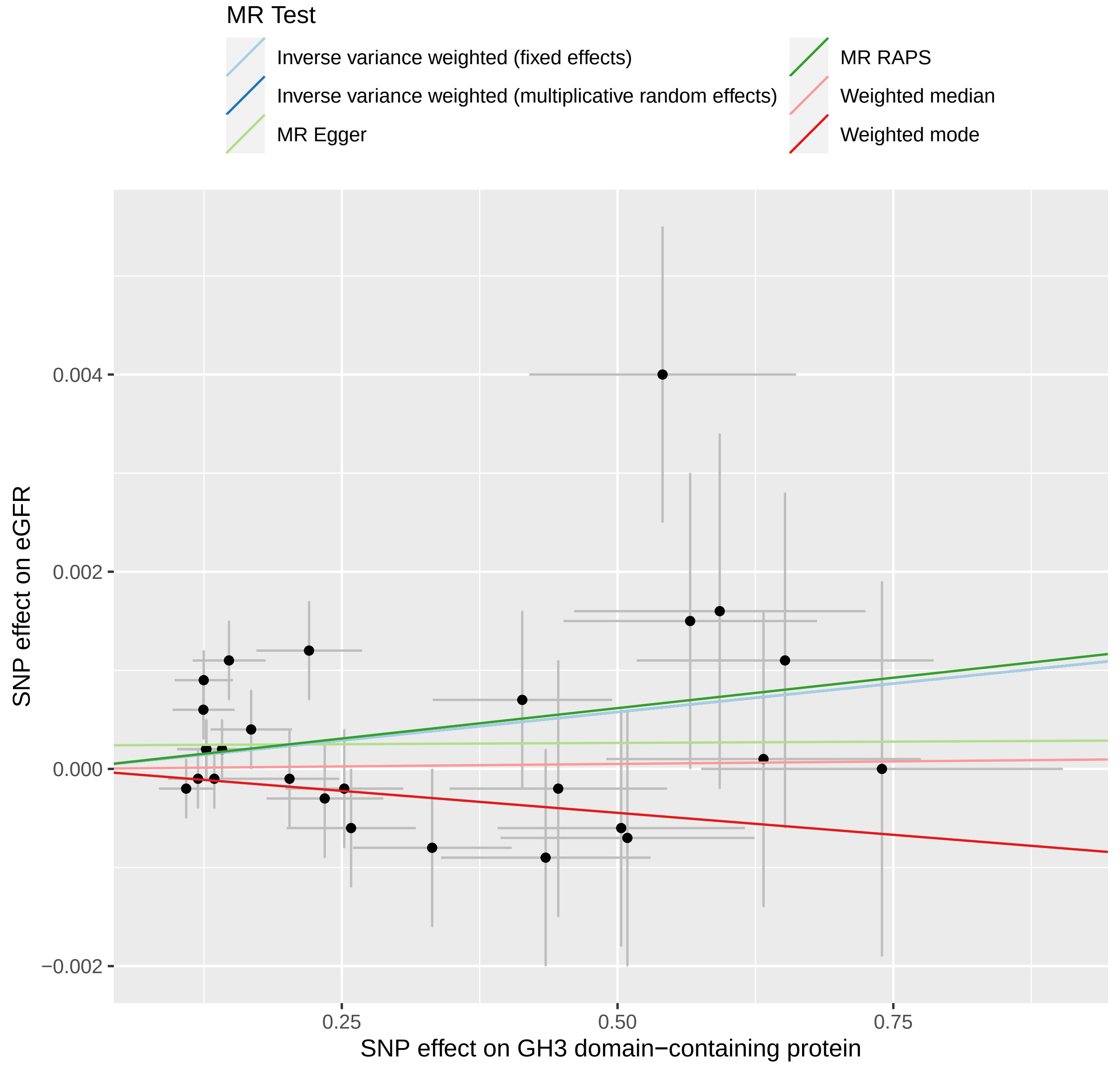

Batch 2−1211 : Growth hormone receptor on eGFR

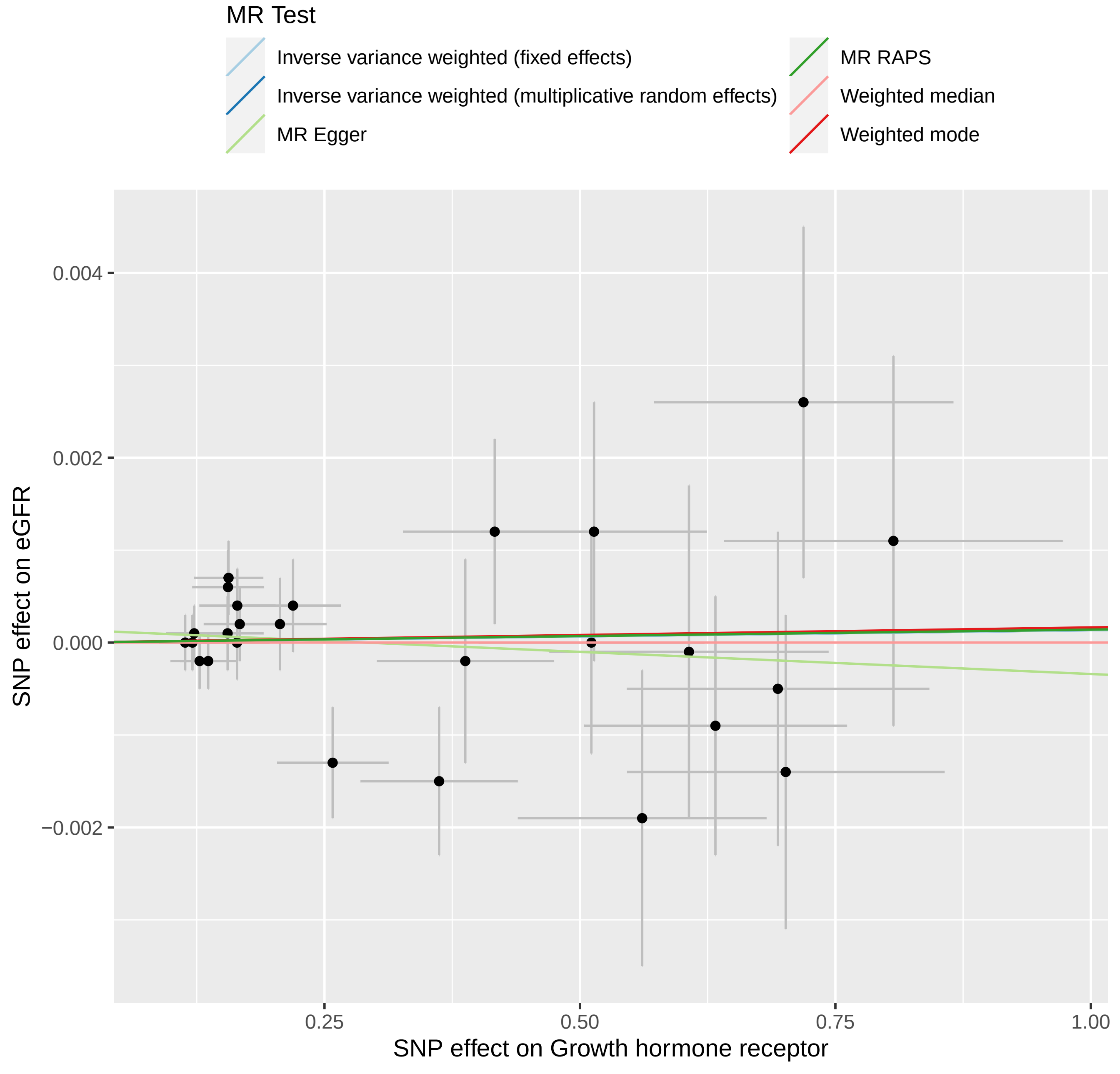

Batch 2−1212 : Appetite−regulating hormone on eGFR

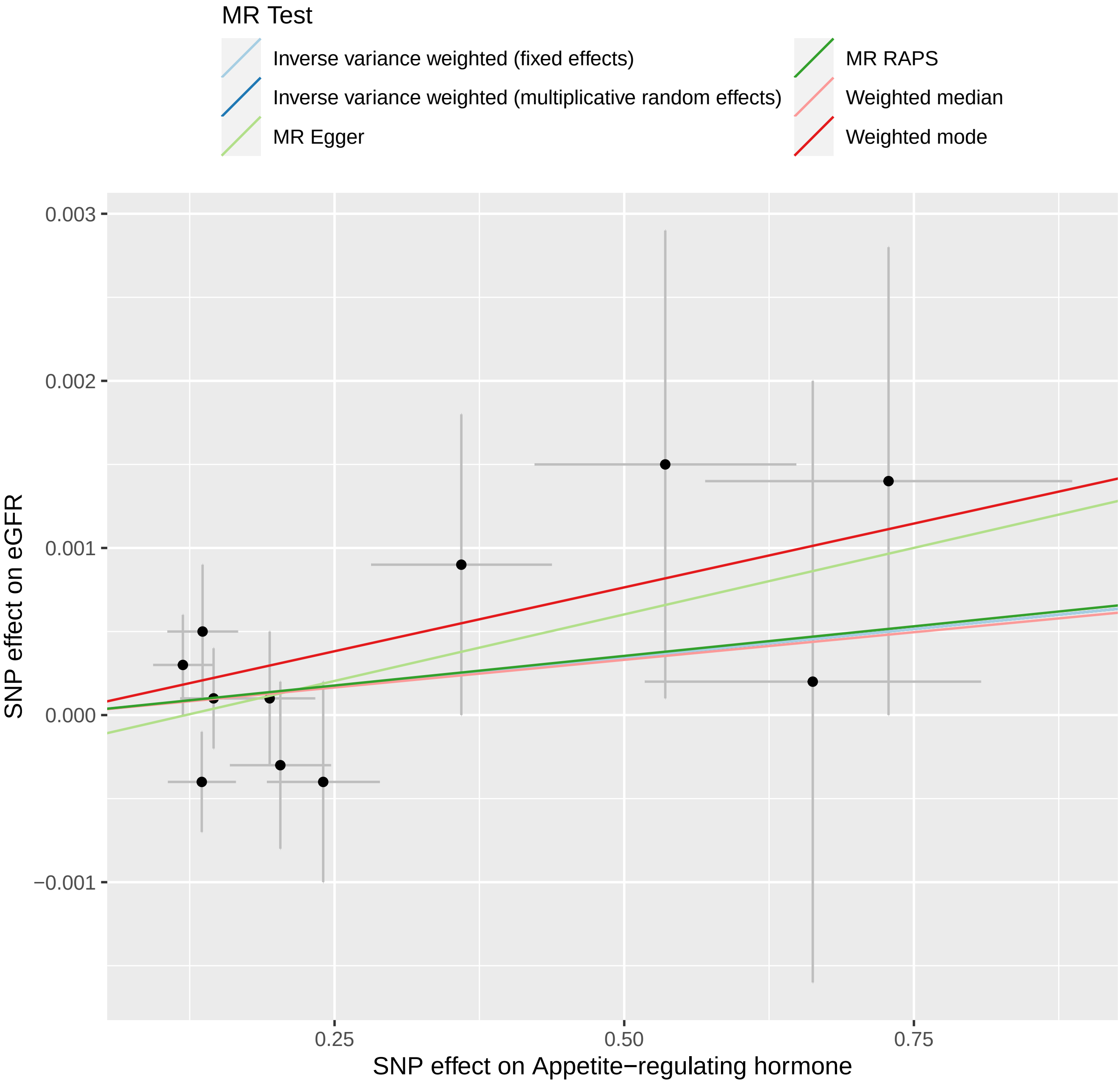

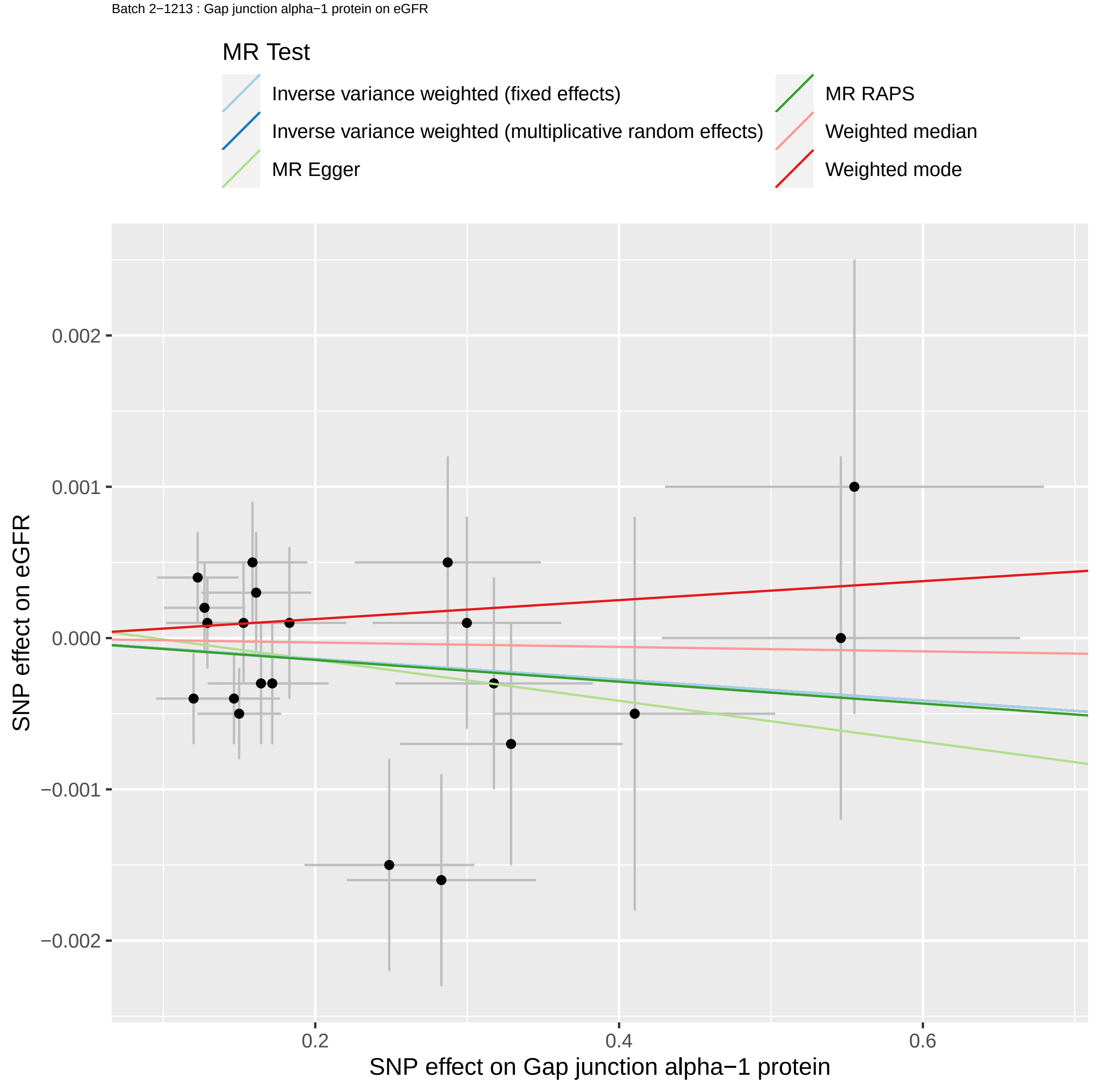
Batch 2−1213 : Gap junction alpha−1 protein on eGFR
MR Test
Inverse variance weighted (fixed effects)
Inverse variance weighted (multiplicative random effects)
MR Egger
MR RAPS
Weighted median
Weighted mode
0.002
0.001
0.000
−0.001
−0.002
SNP effect on eGFR
0.2
0.4
0.6
SNP effect on Gap junction alpha−1 protein

Batch 2−1214 : Gap junction alpha−8 protein on eGFR

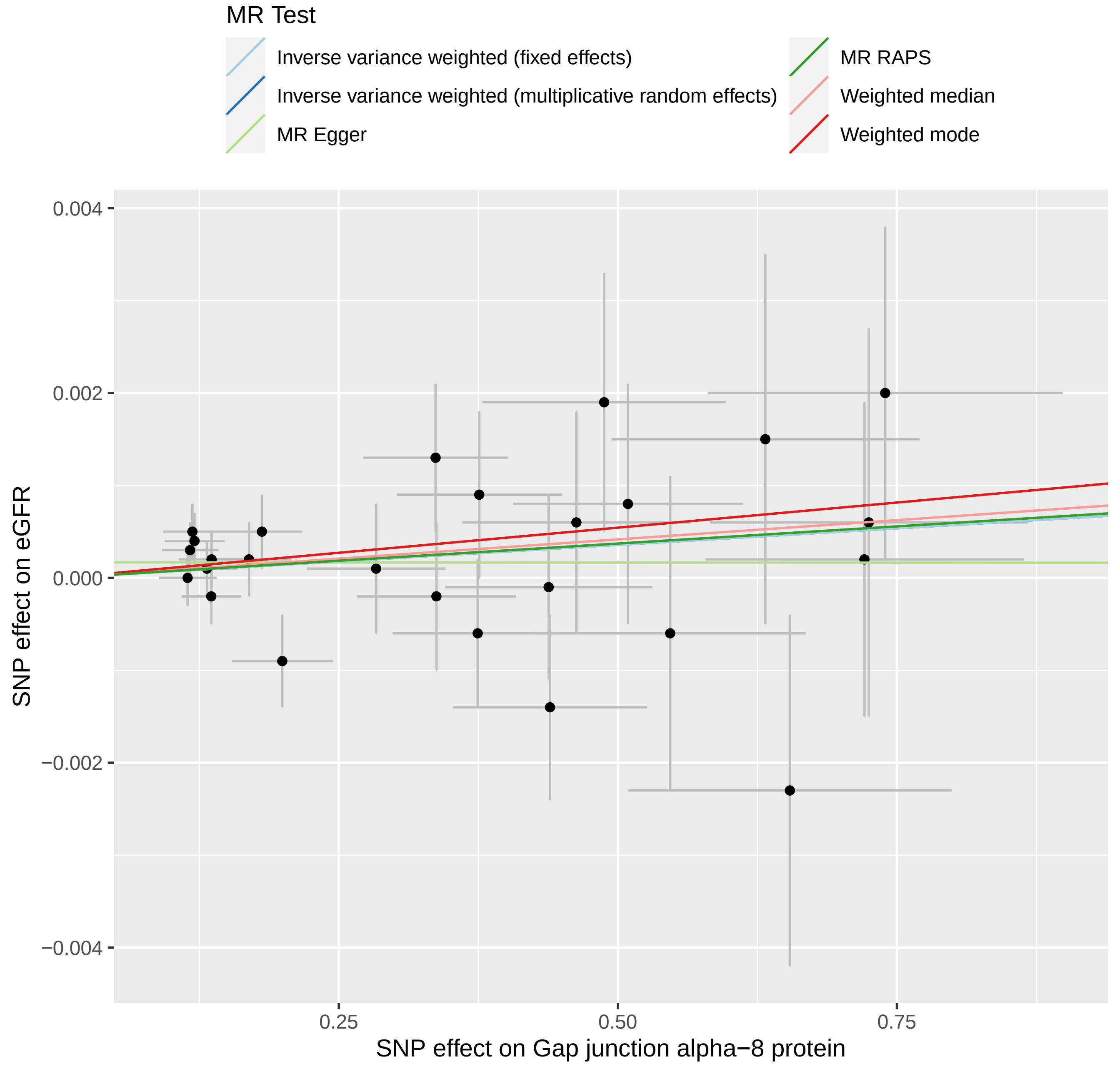

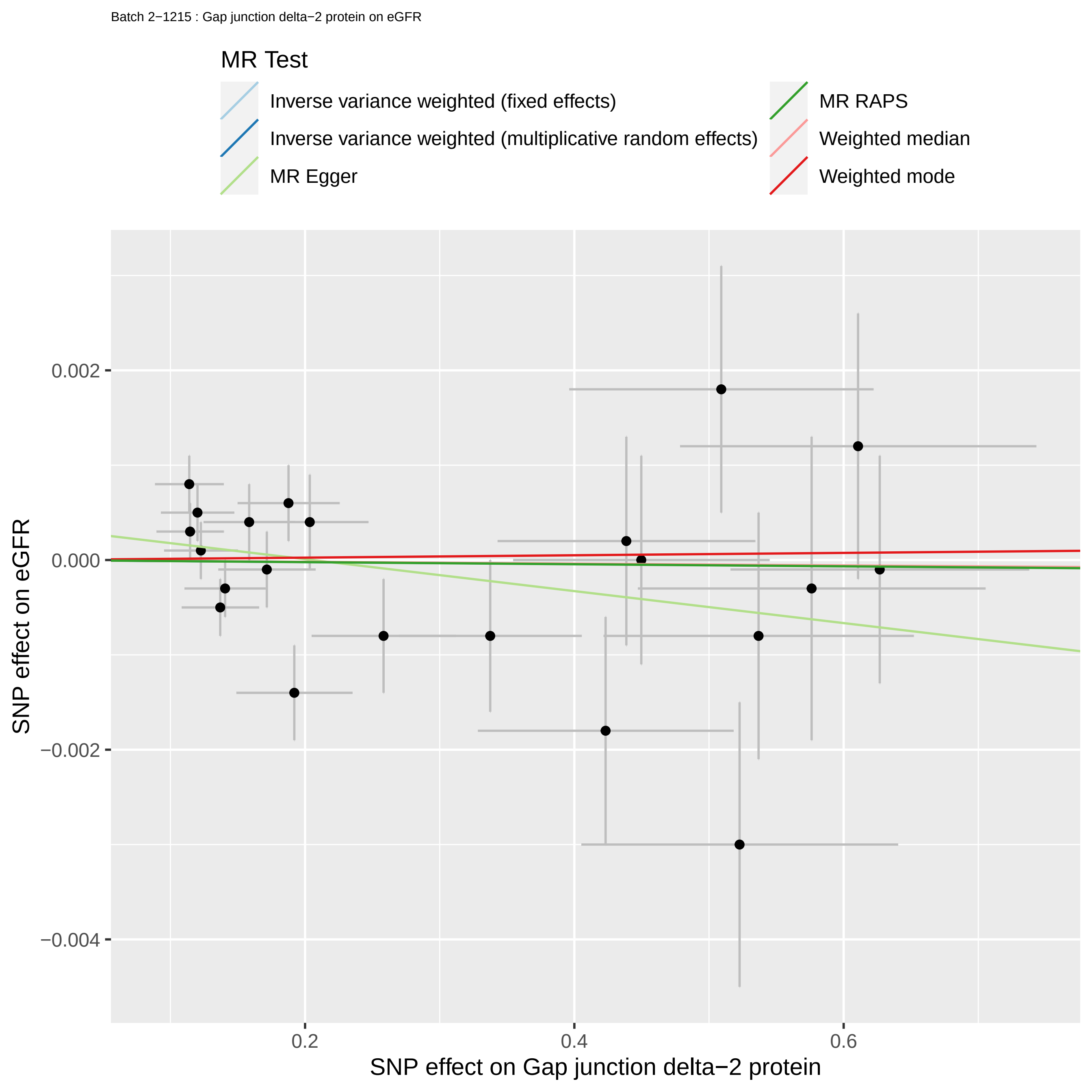

Batch 2−1215 : Gap junction delta−2 protein on eGFR
MR Test
Inverse variance weighted (fixed effects)
Inverse variance weighted (multiplicative random effects)
MR Egger
MR RAPS
Weighted median
Weighted mode
0.002
0.000
−0.002
−0.004
0.2
0.4
0.6
SNP effect on eGFR
SNP effect on Gap junction delta−2 protein

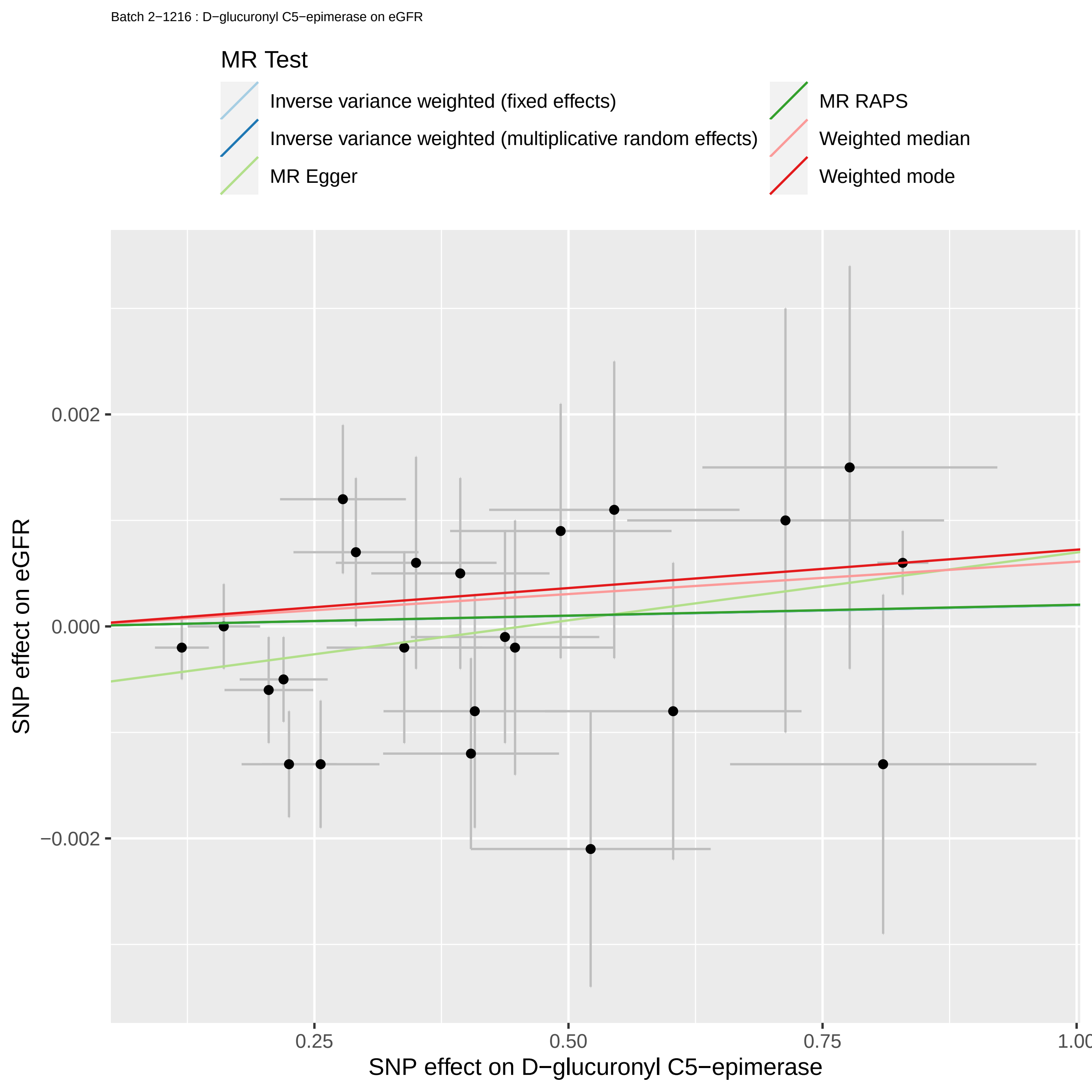
Batch 2−1216 : D−glucuronyl C5−epimerase on eGFR
MR Test
Inverse variance weighted (fixed effects)
Inverse variance weighted (multiplicative random effects)
MR Egger
MR RAPS
Weighted median
Weighted mode
0.002
0.000
−0.002
0.25
0.50
0.75
1.00
SNP effect on eGFR
SNP effect on D−glucuronyl C5−epimerase

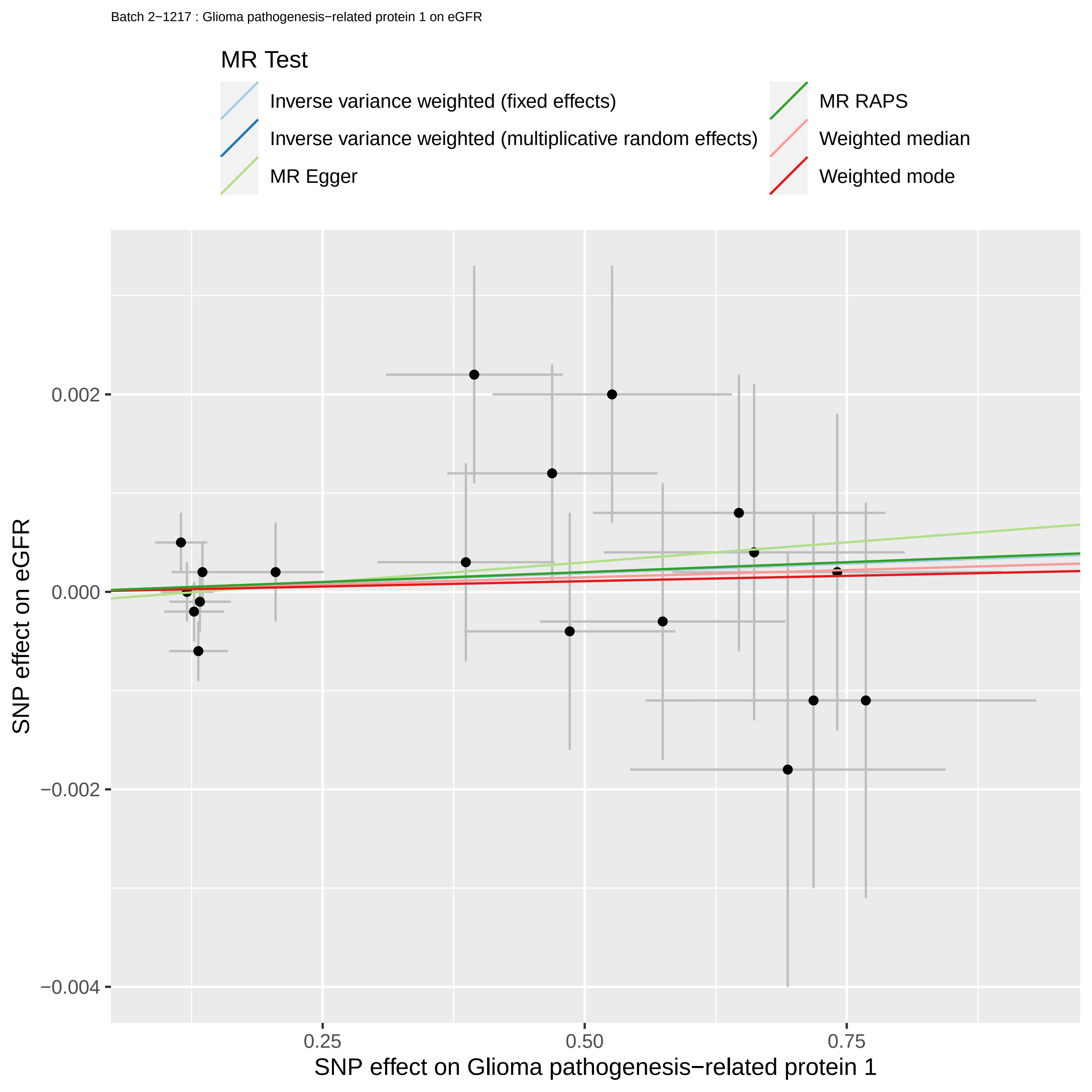

Batch 2−1217 : Glioma pathogenesis−related protein 1 on eGFR
MR Test
Inverse variance weighted (fixed effects)
Inverse variance weighted (multiplicative random effects)
MR Egger
MR RAPS
Weighted median
Weighted mode
0.002
0.000
−0.002
−0.004
0.25
0.50
0.75
SNP effect on eGFR
SNP effect on Glioma pathogenesis−related protein 1

Batch 2−1218 : Lactoylglutathione lyase on eGFR

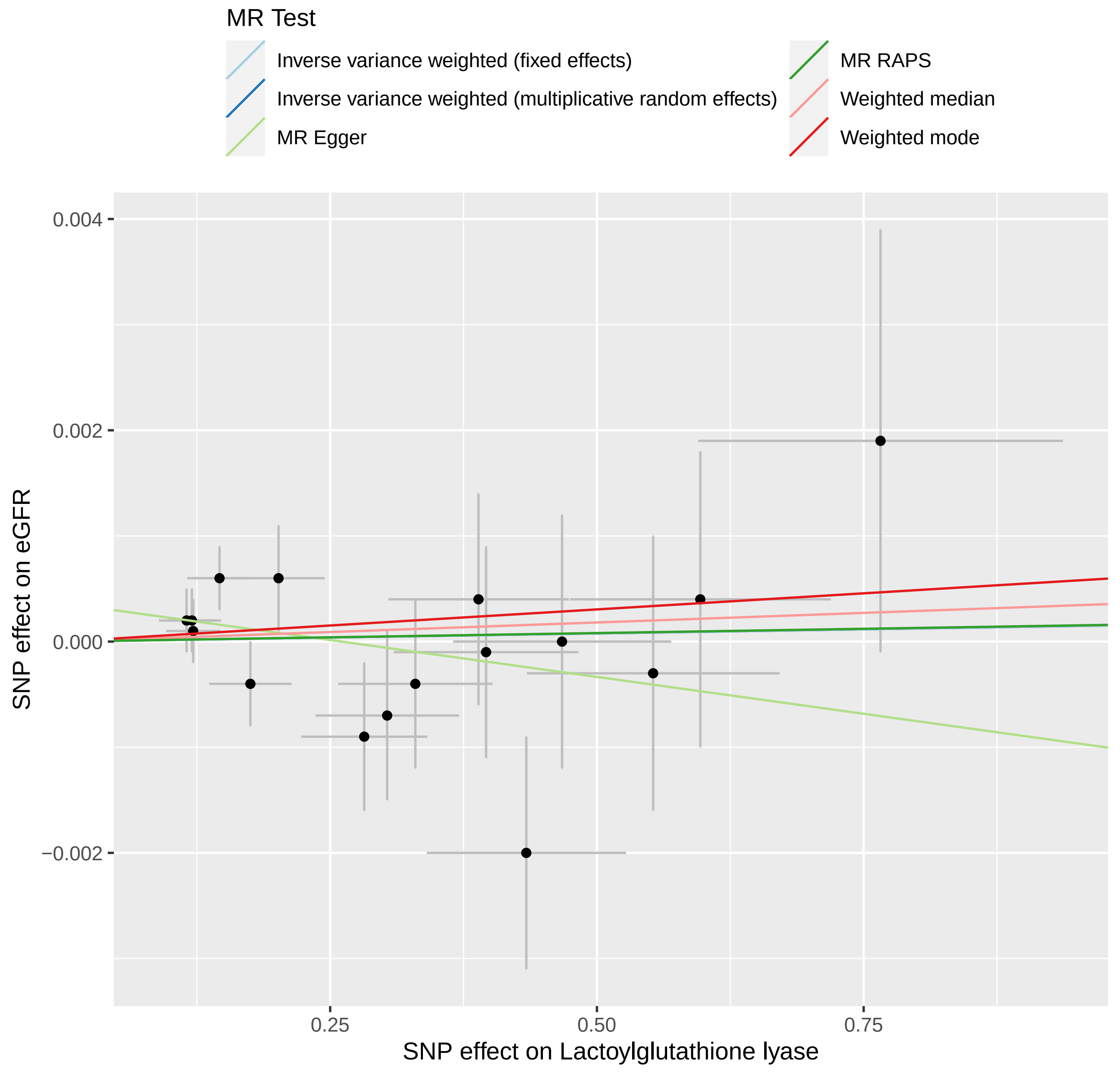

Batch 2−1219 : Glucagon−like peptide 1 receptor on eGFR

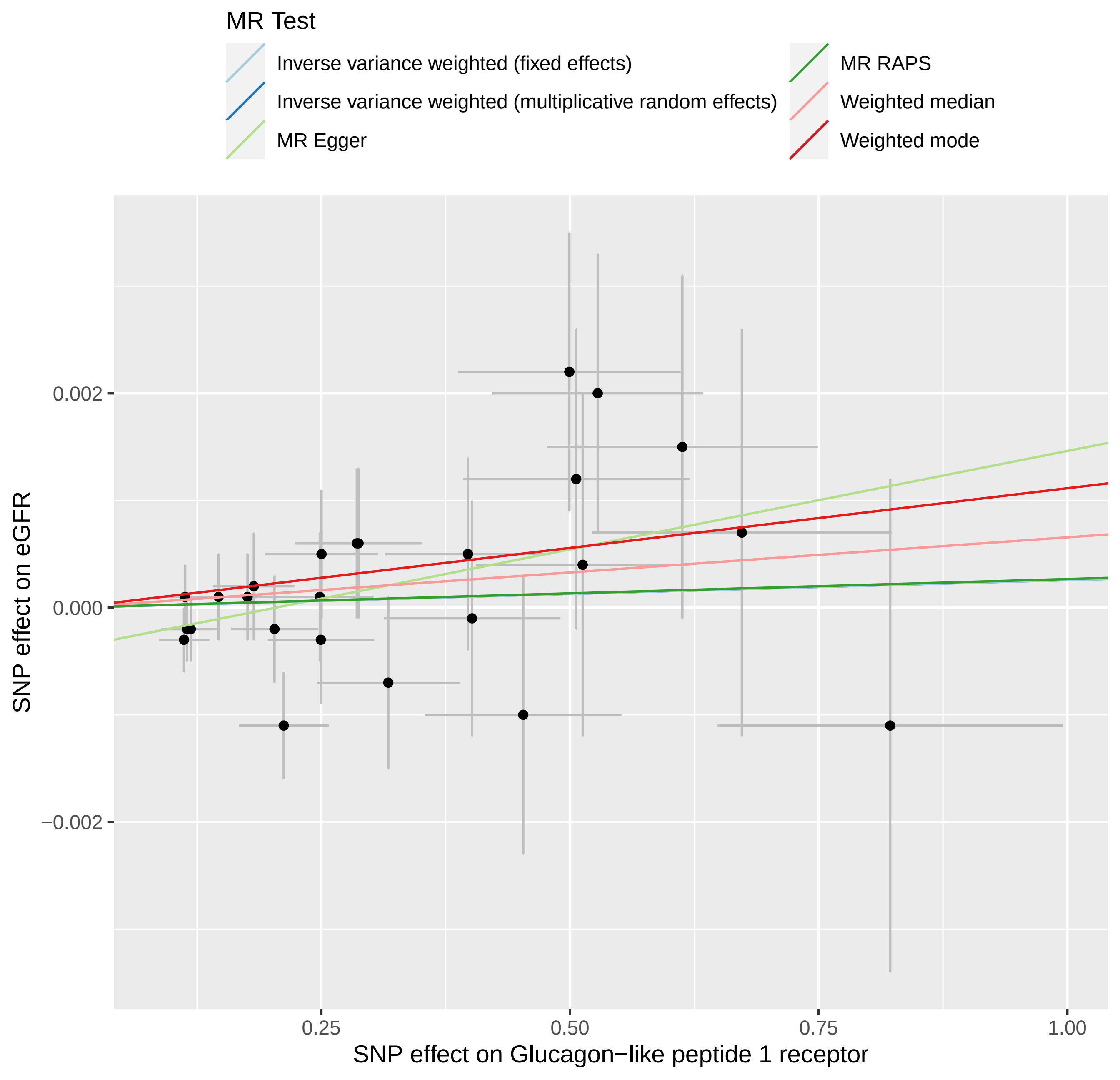

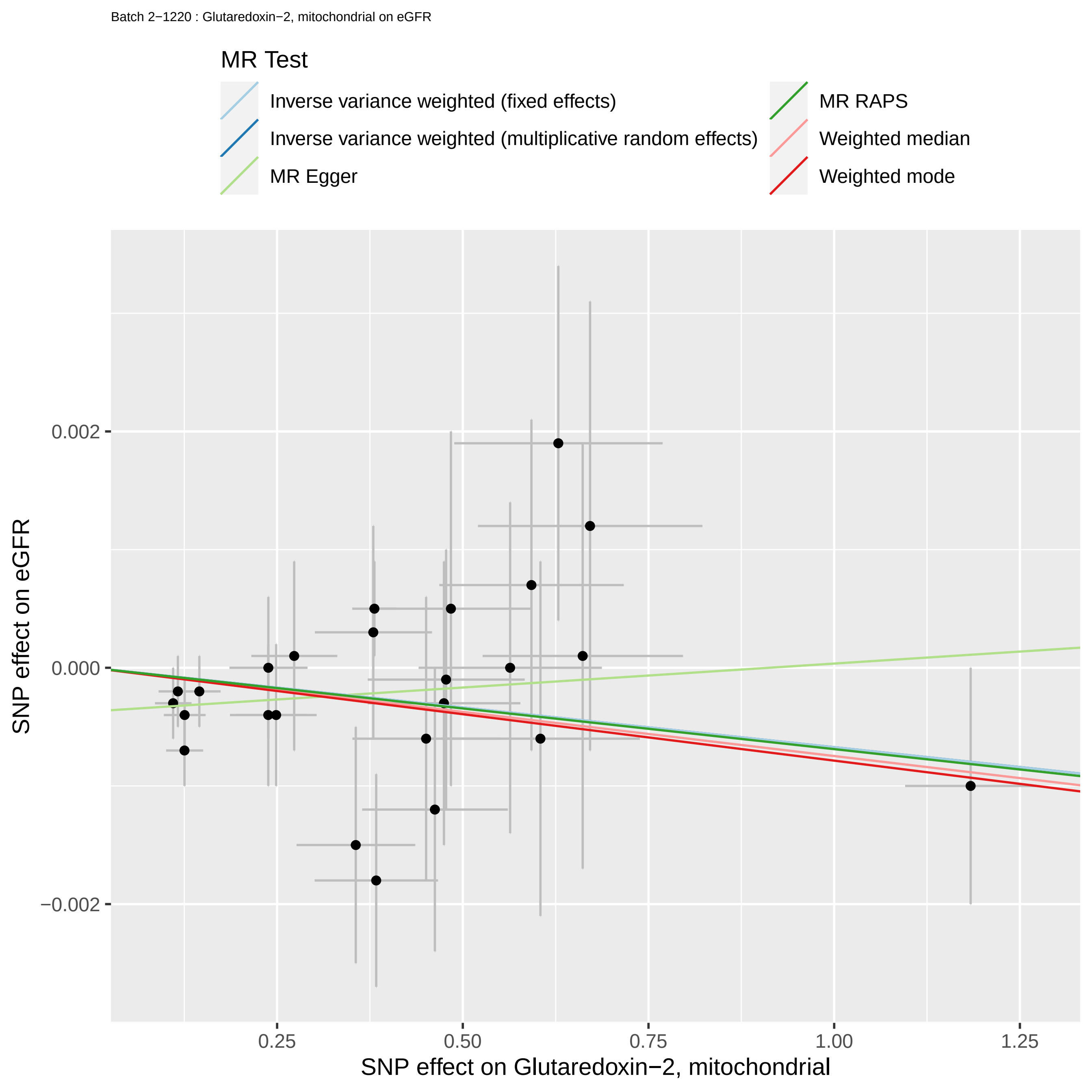
Batch 2−1220 : Glutaredoxin−2, mitochondrial on eGFR
MR Test
Inverse variance weighted (fixed effects)
Inverse variance weighted (multiplicative random effects)
MR Egger
MR RAPS
Weighted median
Weighted mode
0.002
0.000
−0.002
0.25
0.50
0.75
1.00
1.25
SNP effect on eGFR
SNP effect on Glutaredoxin−2, mitochondrial

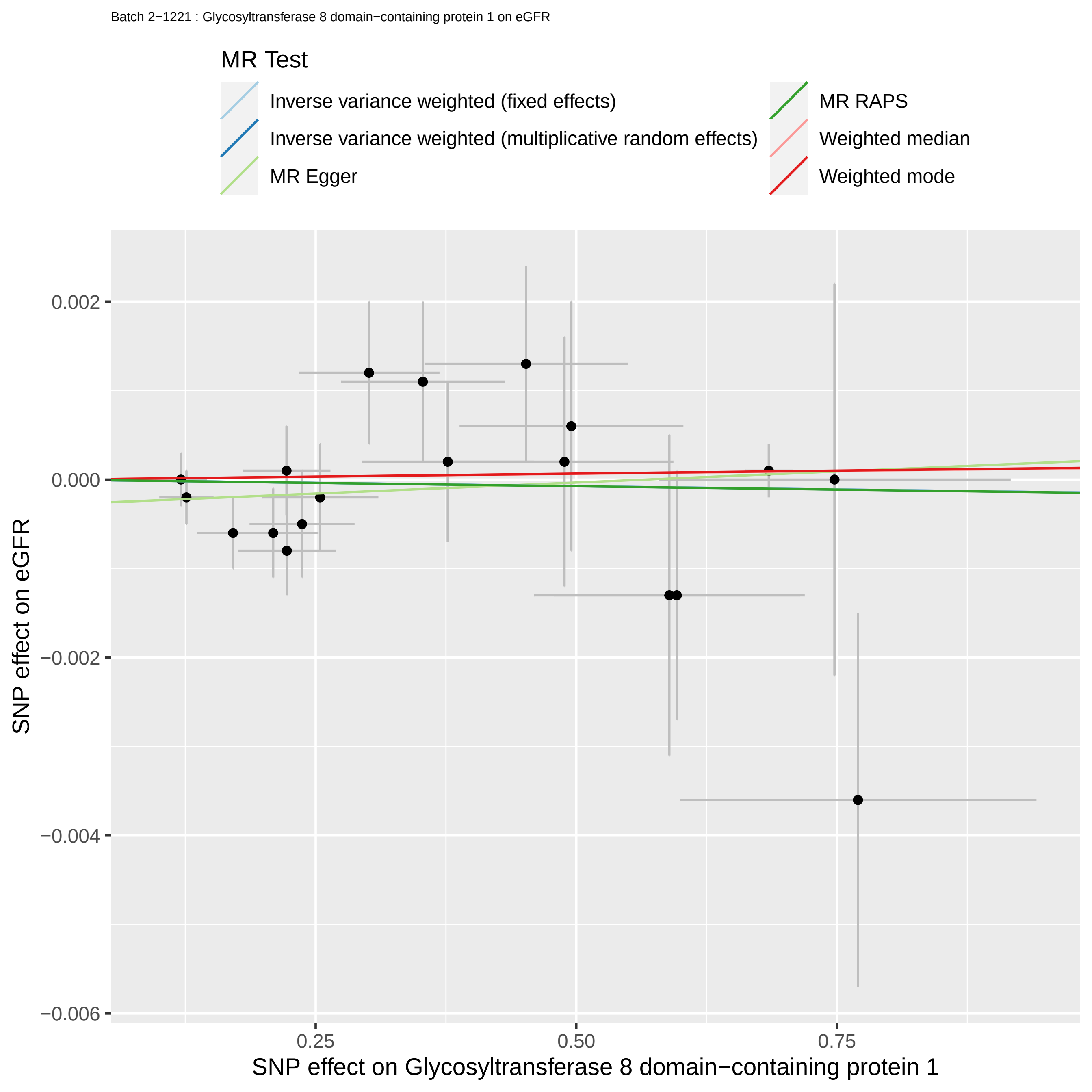

Batch 2−1221 : Glycosyltransferase 8 domain−containing protein 1 on eGFR
MR Test
Inverse variance weighted (fixed effects)
Inverse variance weighted (multiplicative random effects)
MR Egger
MR RAPS
Weighted median
Weighted mode
0.002
0.000
−0.002
−0.004
−0.006
SNP effect on eGFR
0.25
0.50
0.75
SNP effect on Glycosyltransferase 8 domain−containing protein 1

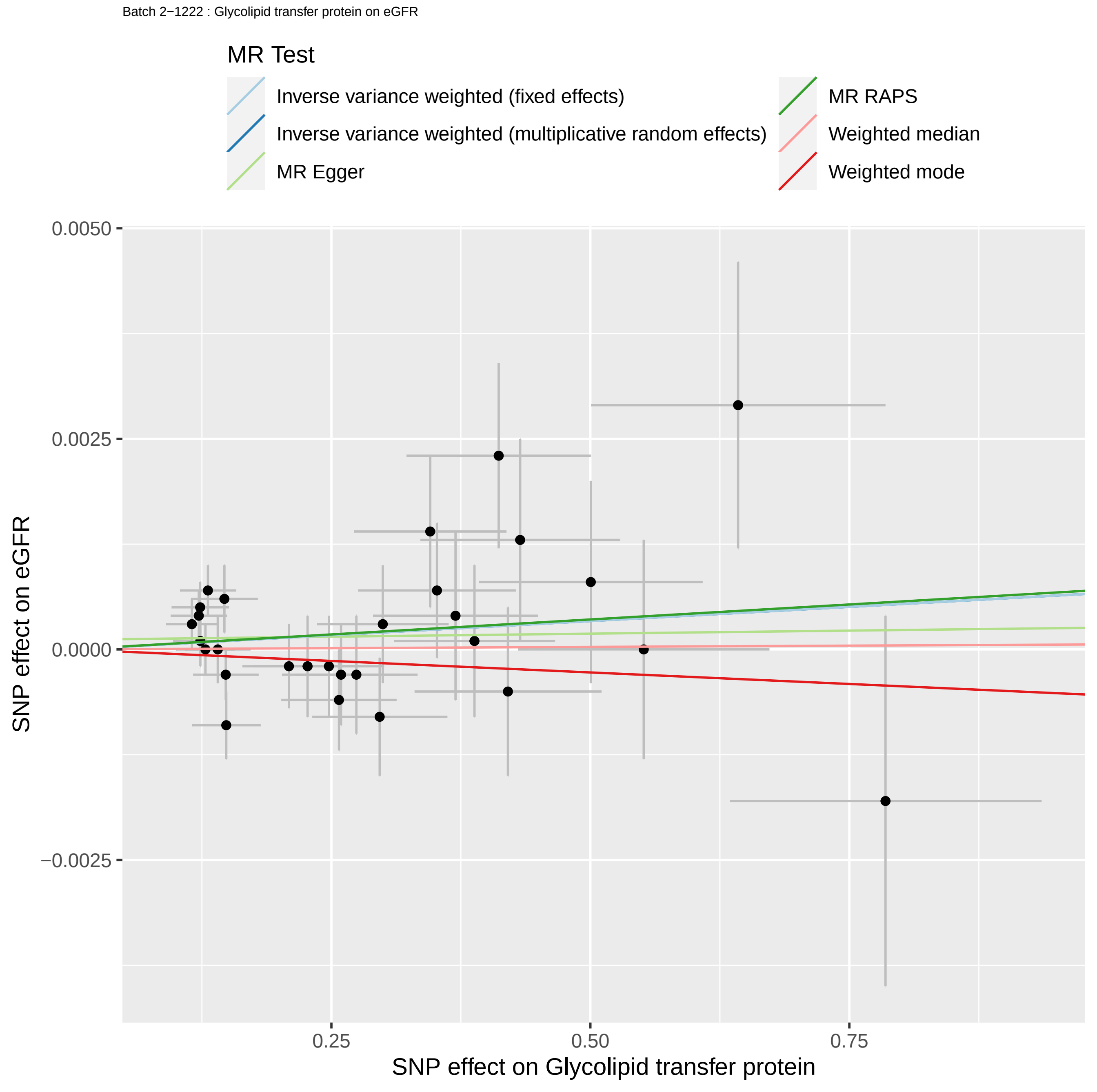

Batch 2−1222 : Glycolipid transfer protein on eGFR
MR Test
Inverse variance weighted (fixed effects)
Inverse variance weighted (multiplicative random effects)
MR Egger
MR RAPS
Weighted median
Weighted mode
0.0050
0.0025
0.0000
−0.0025
0.25
0.50
0.75
SNP effect on eGFR
SNP effect on Glycolipid transfer protein

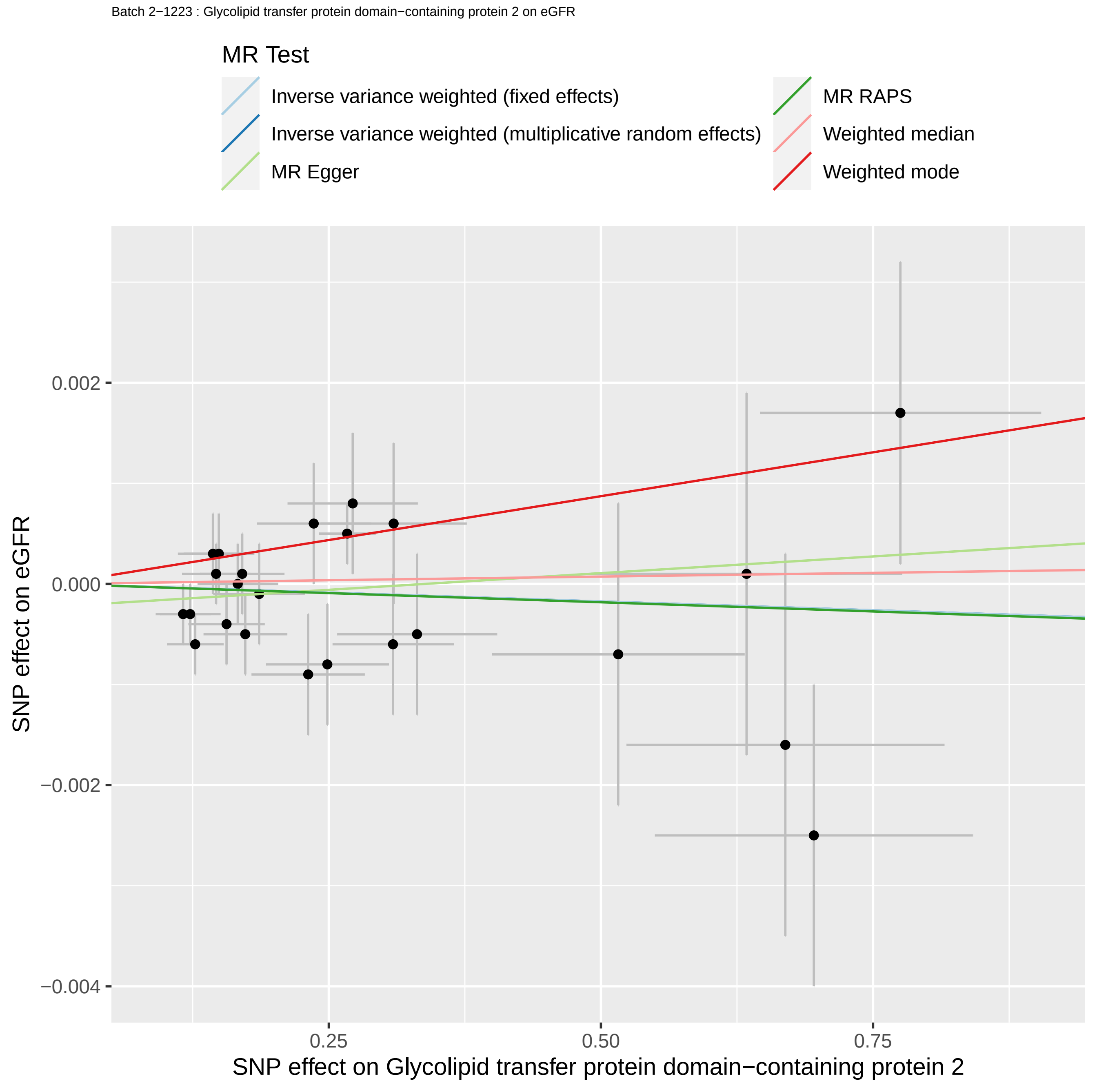
Batch 2−1223 : Glycolipid transfer protein domain−containing protein 2 on eGFR
MR Test
Inverse variance weighted (fixed effects)
Inverse variance weighted (multiplicative random effects)
MR Egger
MR RAPS
Weighted median
Weighted mode
0.002
0.000
−0.002
−0.004
0.25
0.50
0.75
SNP effect on eGFR
SNP effect on Glycolipid transfer protein domain−containing protein 2

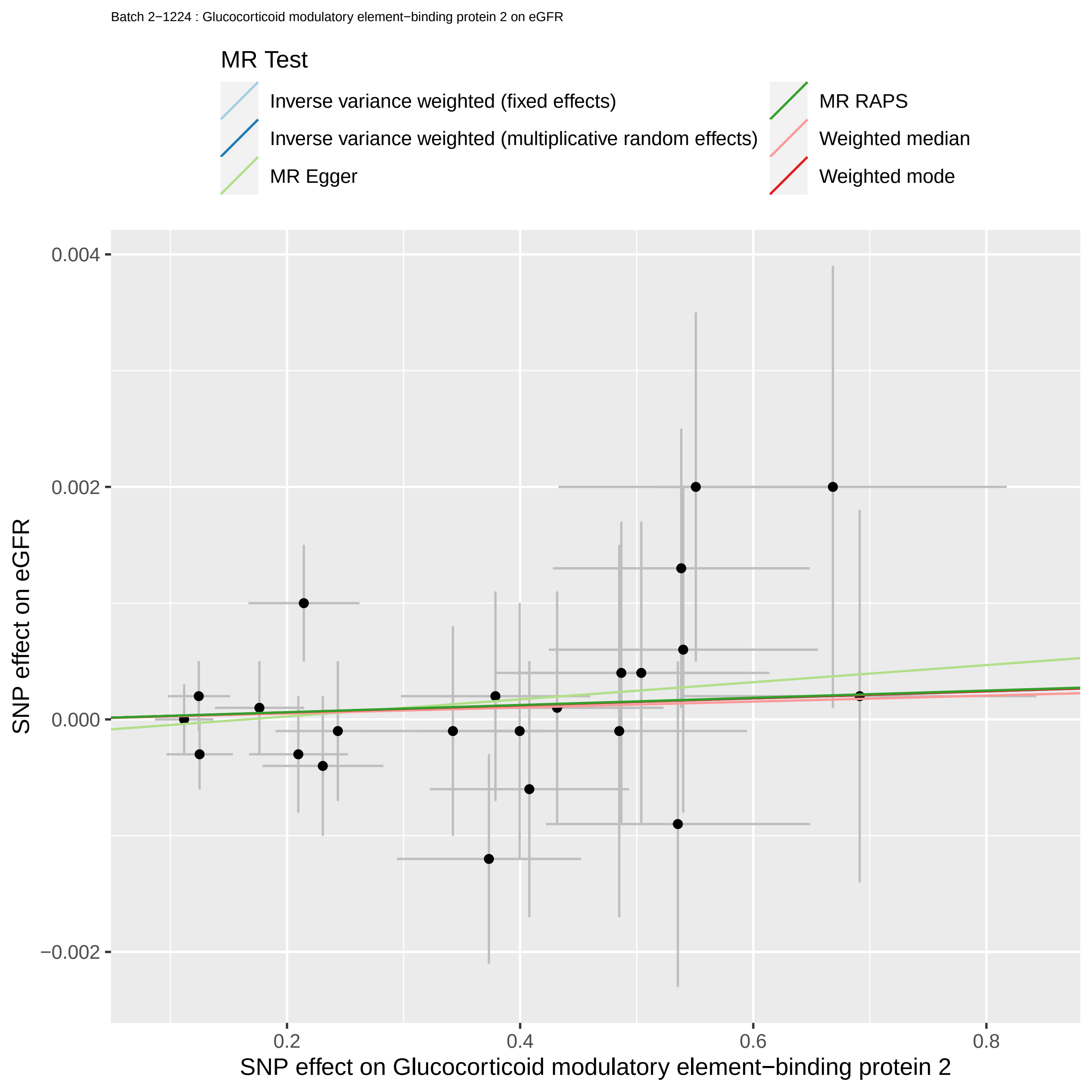

Batch 2−1224 : Glucocorticoid modulatory element−binding protein 2 on eGFR
MR Test
Inverse variance weighted (fixed effects)
Inverse variance weighted (multiplicative random effects)
MR Egger
MR RAPS
Weighted median
Weighted mode
0.004
0.002
0.000
−0.002
0.2
0.4
0.6
0.8
SNP effect on eGFR
SNP effect on Glucocorticoid modulatory element−binding protein 2

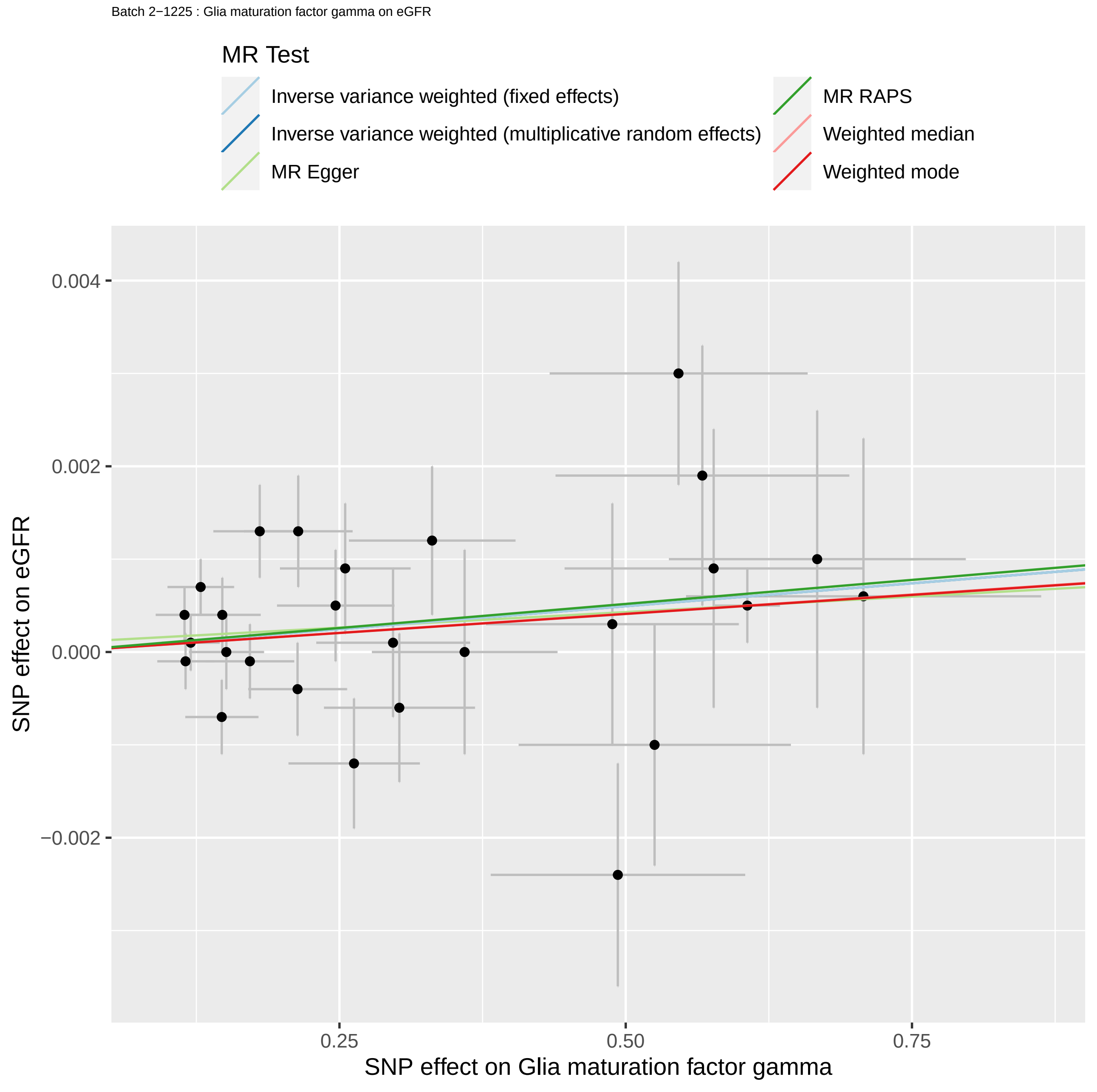

Batch 2−1225 : Glia maturation factor gamma on eGFR
MR Test
Inverse variance weighted (fixed effects)
Inverse variance weighted (multiplicative random effects)
MR Egger
MR RAPS
Weighted median
Weighted mode
0.004
0.002
0.000
−0.002
0.25
0.50
0.75
SNP effect on eGFR
SNP effect on Glia maturation factor gamma

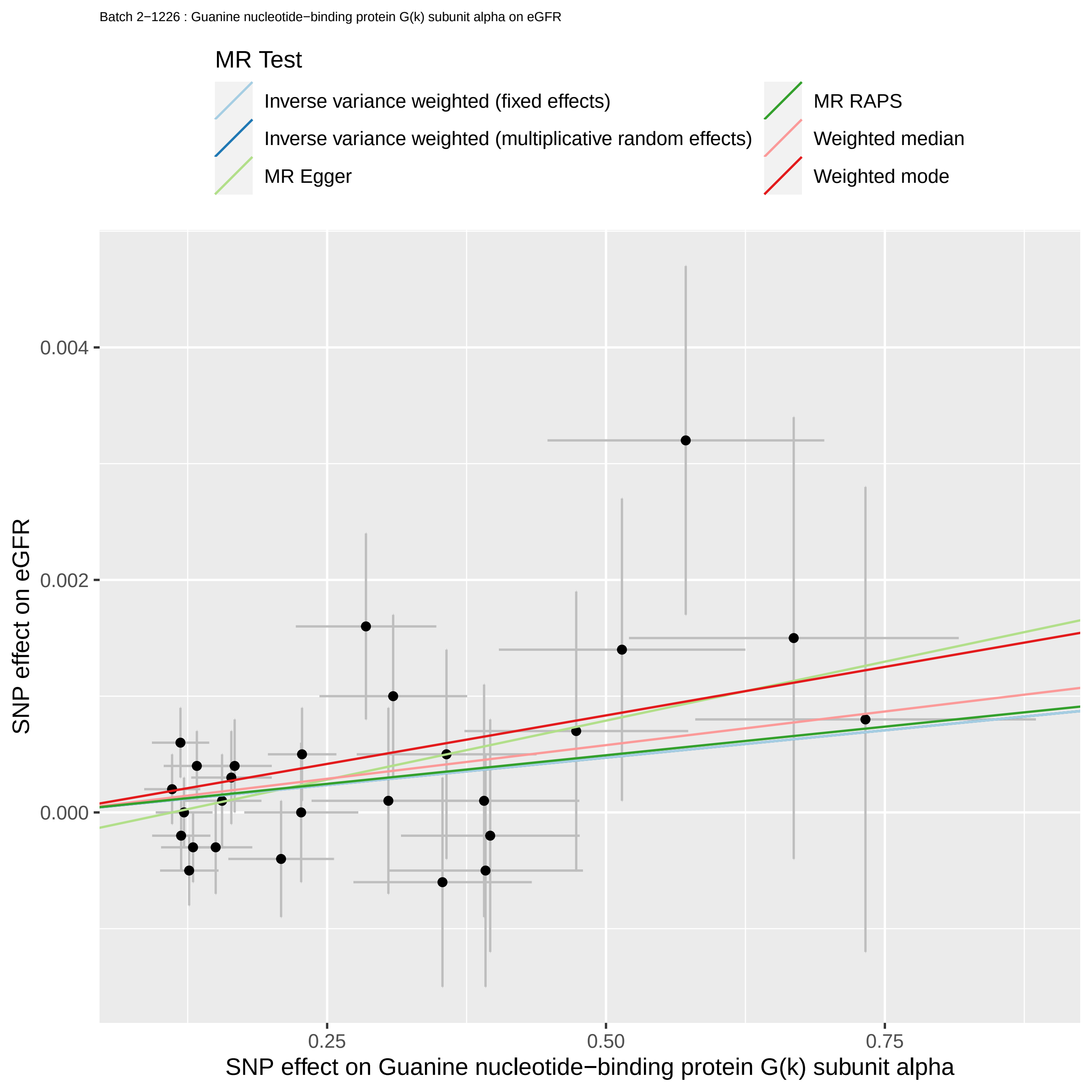

Batch 2−1226 : Guanine nucleotide−binding protein G(k) subunit alpha on eGFR
MR Test
Inverse variance weighted (fixed effects)
Inverse variance weighted (multiplicative random effects)
MR Egger
MR RAPS
Weighted median
Weighted mode
0.004
0.002
0.000
SNP effect on eGFR
0.25
0.50
0.75
SNP effect on Guanine nucleotide−binding protein G(k) subunit alpha

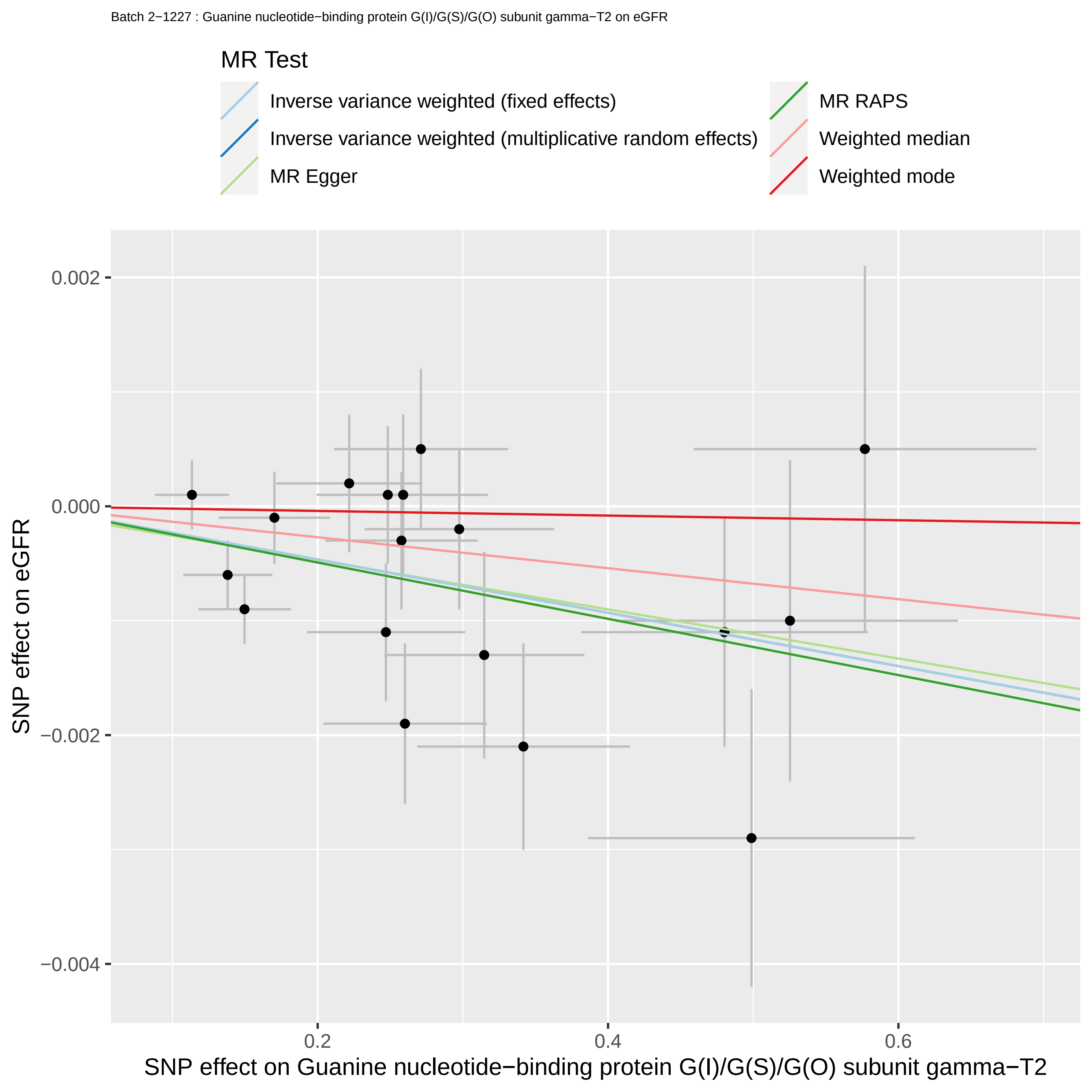
Batch 2−1227 : Guanine nucleotide−binding protein G(I)/G(S)/G(O) subunit gamma−T2 on eGFR
MR Test
Inverse variance weighted (fixed effects)
Inverse variance weighted (multiplicative random effects)
MR Egger
MR RAPS
Weighted median
Weighted mode
0.002
0.000
−0.002
−0.004
0.2
0.4
0.6
SNP effect on eGFR
SNP effect on Guanine nucleotide−binding protein G(I)/G(S)/G(O) subunit gamma−T2

Batch 2−1228 : Granulysin on eGFR

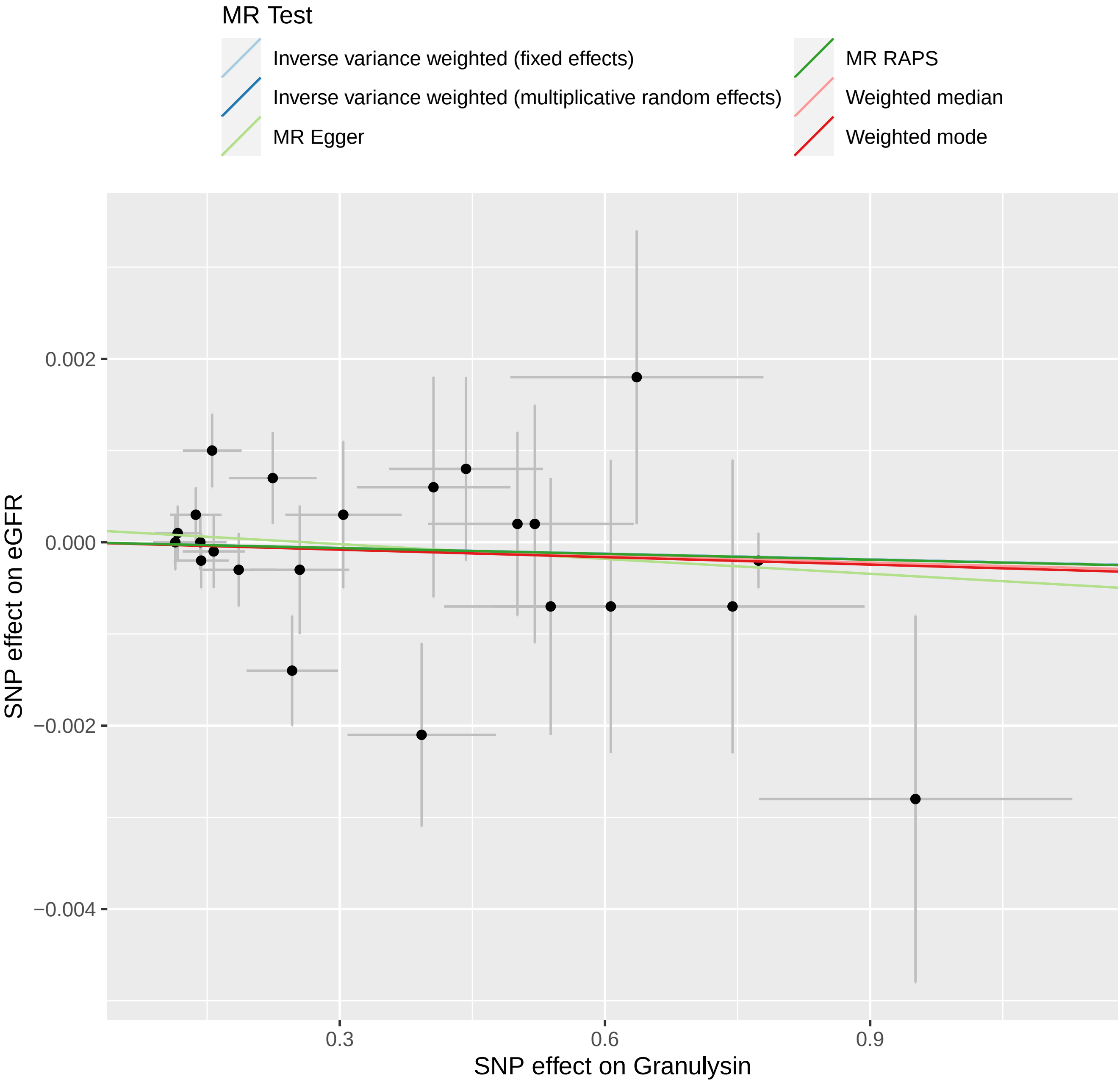

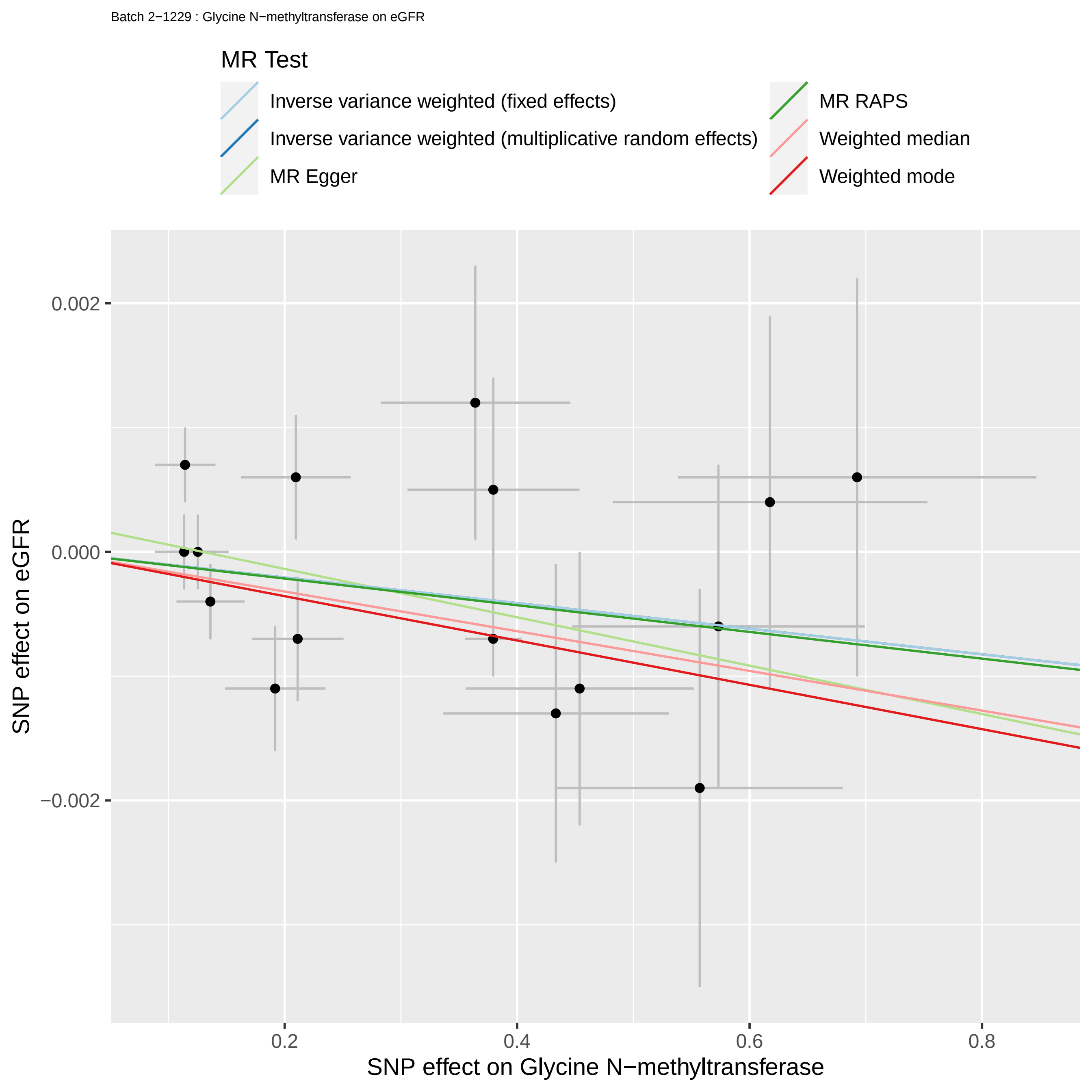

Batch 2−1229 : Glycine N−methyltransferase on eGFR
MR Test
Inverse variance weighted (fixed effects)
Inverse variance weighted (multiplicative random effects)
MR Egger
MR RAPS
Weighted median
Weighted mode
0.002
0.000
−0.002
0.2
0.4
0.6
0.8
SNP effect on eGFR
SNP effect on Glycine N−methyltransferase

Batch 2−1230 : Glucosamine−6−phosphate isomerase 1 on eGFR

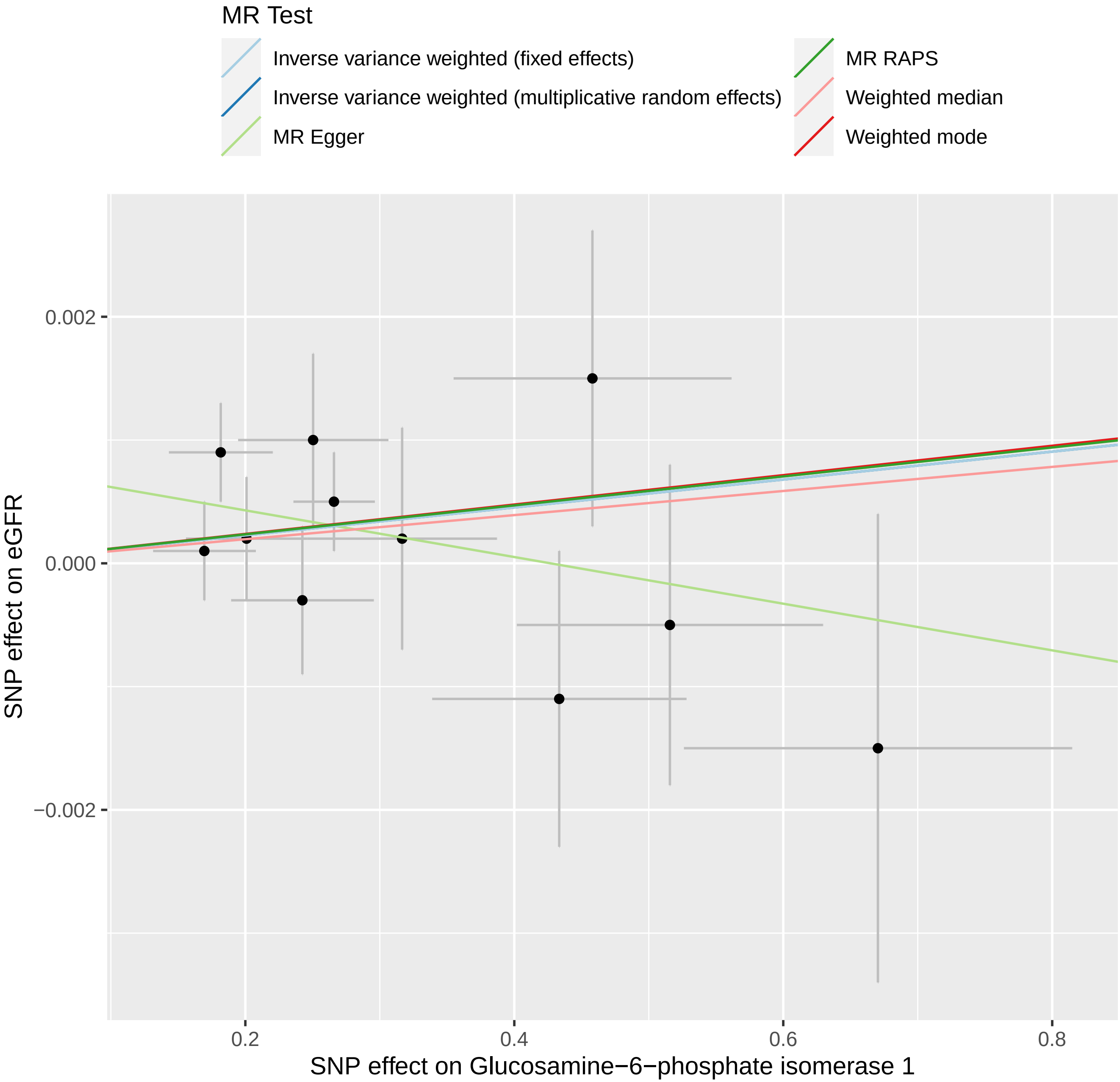

Batch 2−1231 : Glucosamine 6−phosphate N−acetyltransferase on eGFR

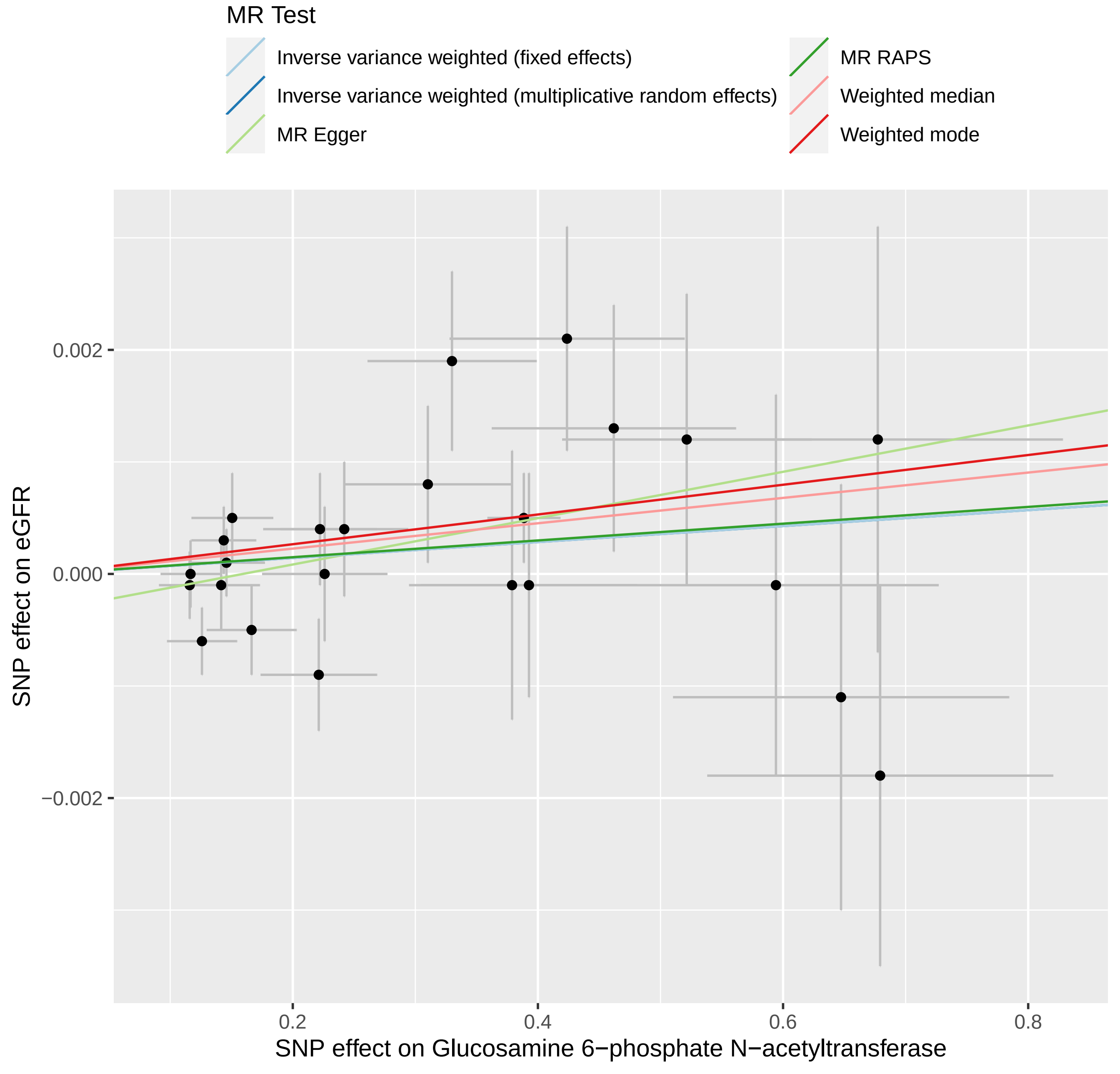

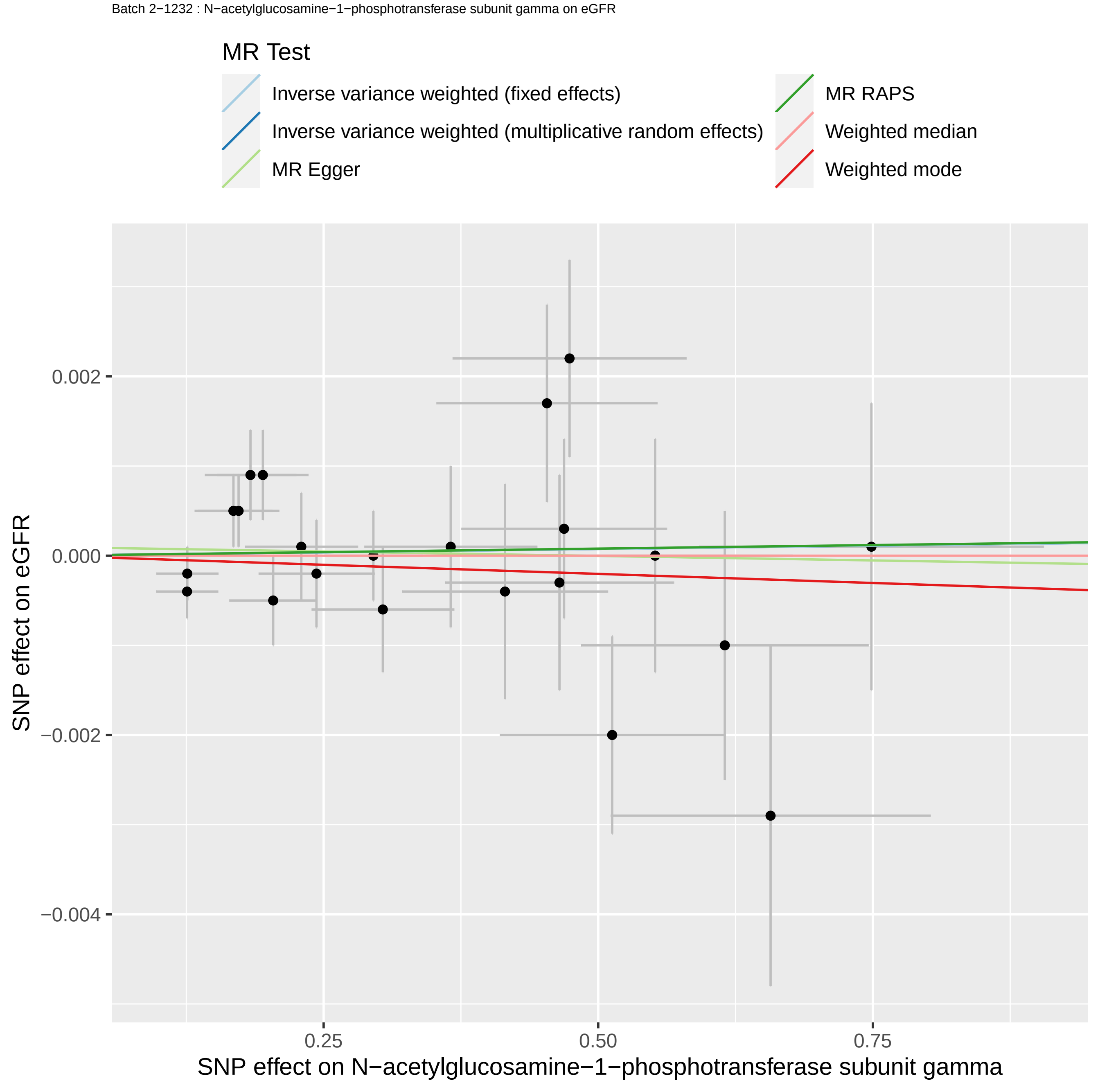
Batch 2−1232 : N−acetylglucosamine−1−phosphotransferase subunit gamma on eGFR
MR Test
Inverse variance weighted (fixed effects)
Inverse variance weighted (multiplicative random effects)
MR Egger
MR RAPS
Weighted median
Weighted mode
0.002
0.000
−0.002
−0.004
0.25
0.50
0.75
SNP effect on eGFR
SNP effect on N−acetylglucosamine−1−phosphotransferase subunit gamma

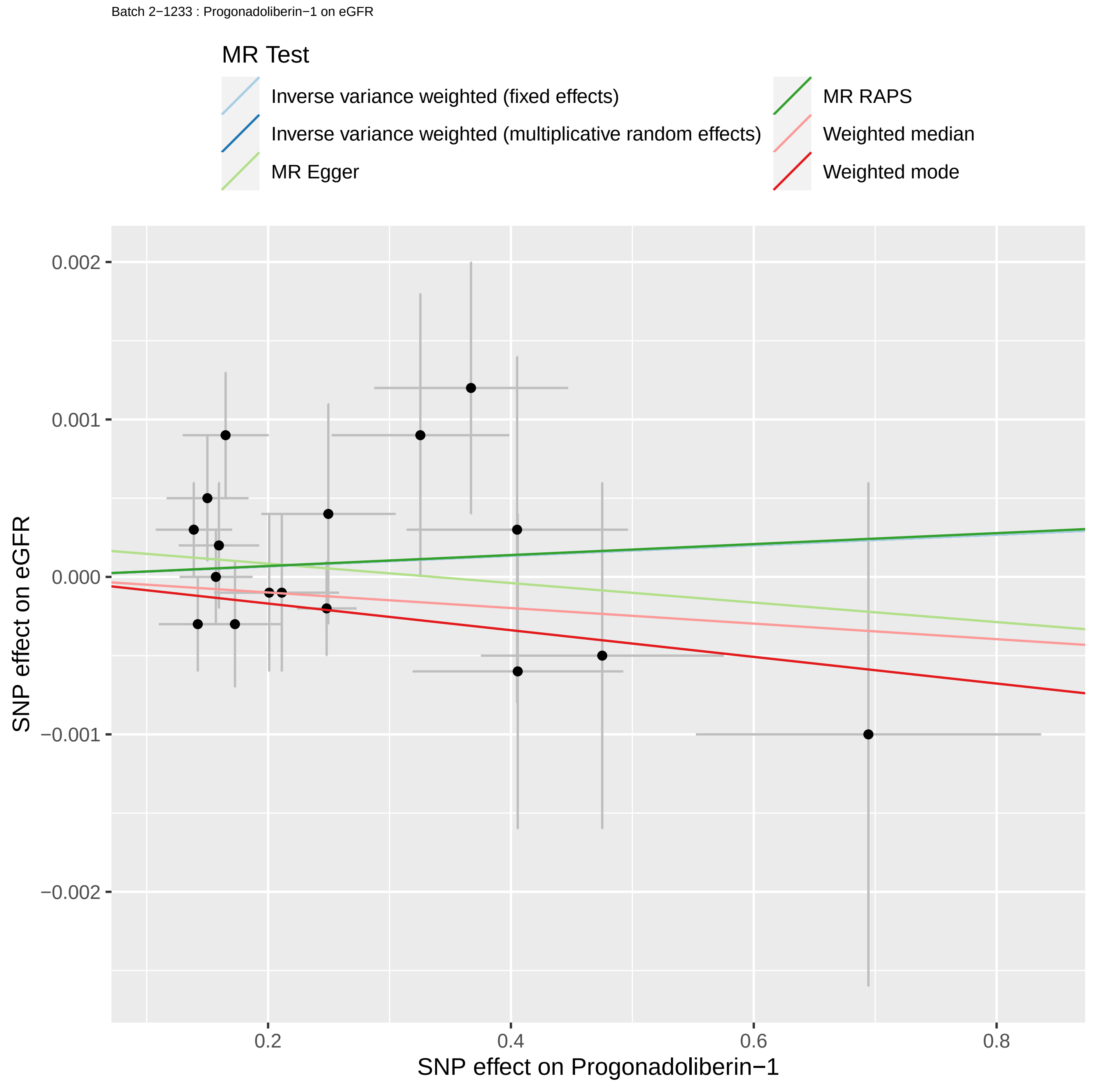
Batch 2−1233 : Progonadoliberin−1 on eGFR
MR Test
Inverse variance weighted (fixed effects)
Inverse variance weighted (multiplicative random effects)
MR Egger
MR RAPS
Weighted median
Weighted mode
0.002
0.001
0.000
−0.001
−0.002
0.2
0.4
0.6
0.8
SNP effect on eGFR
SNP effect on Progonadoliberin−1

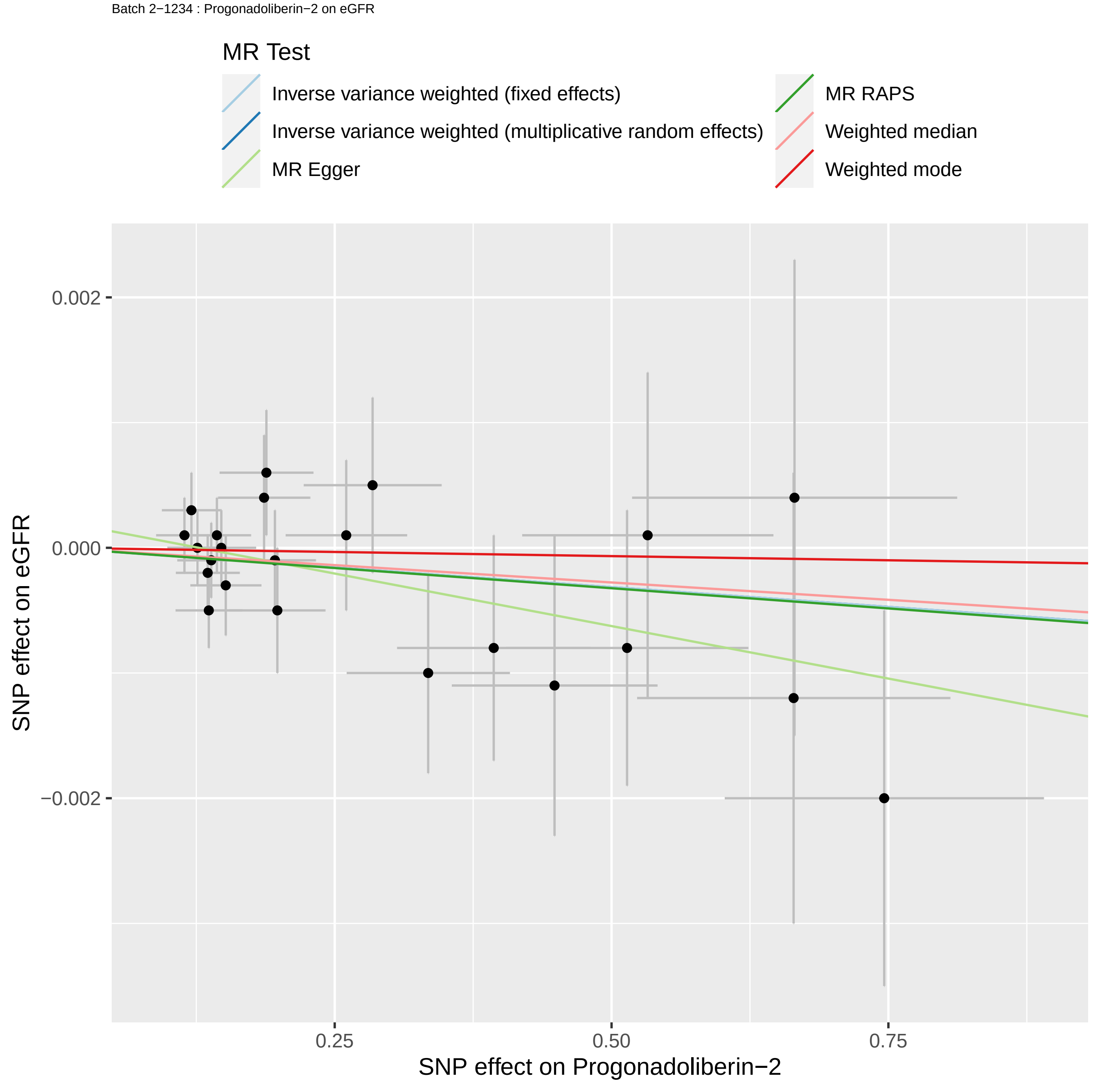

Batch 2−1234 : Progonadoliberin−2 on eGFR
MR Test
Inverse variance weighted (fixed effects)
Inverse variance weighted (multiplicative random effects)
MR Egger
MR RAPS
Weighted median
Weighted mode
0.002
0.000
−0.002
0.25
0.50
0.75
SNP effect on eGFR
SNP effect on Progonadoliberin−2

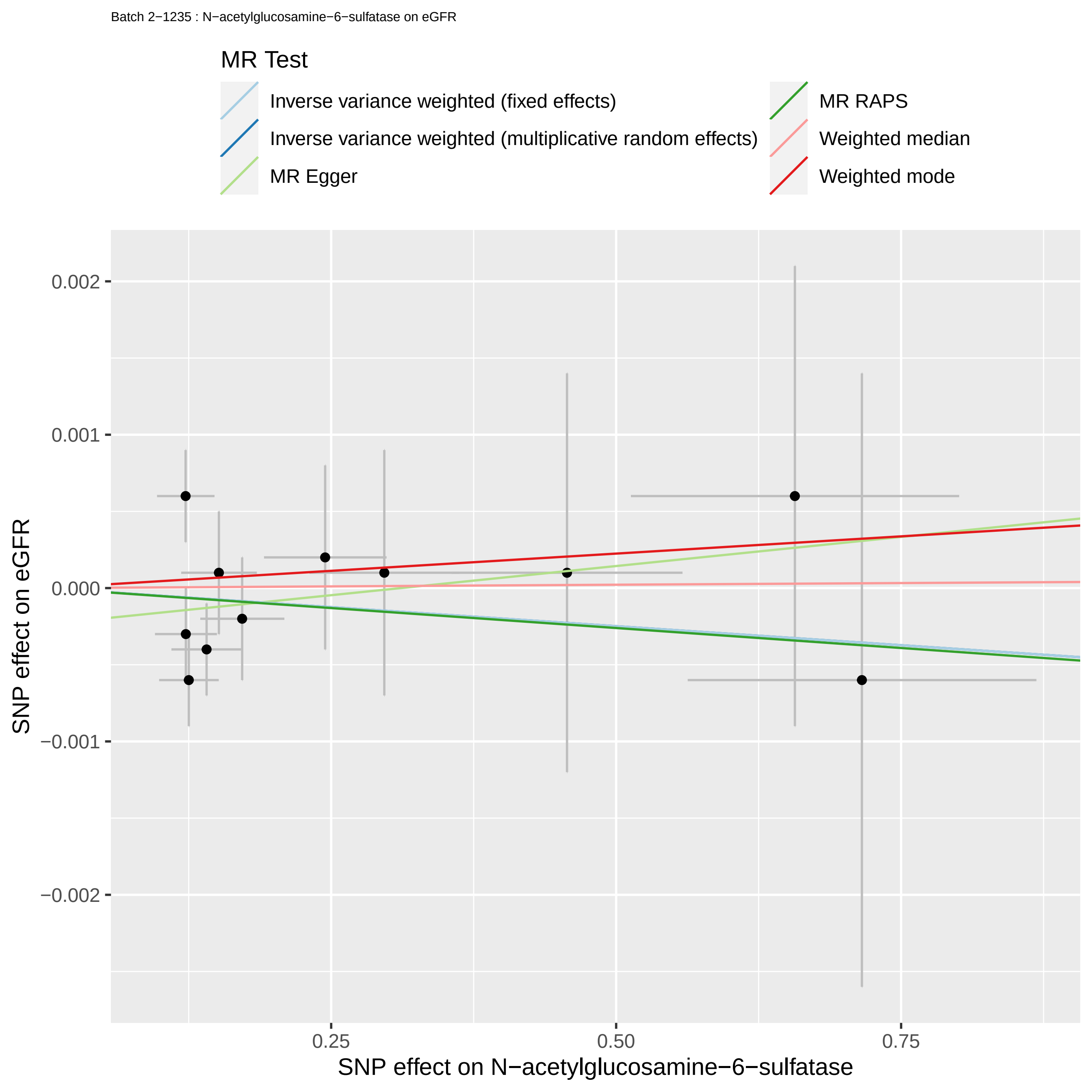
Batch 2−1235 : N−acetylglucosamine−6−sulfatase on eGFR
MR Test
Inverse variance weighted (fixed effects)
Inverse variance weighted (multiplicative random effects)
MR Egger
MR RAPS
Weighted median
Weighted mode
0.002
0.001
0.000
−0.001
−0.002
0.25
0.50
0.75
SNP effect on eGFR
SNP effect on N−acetylglucosamine−6−sulfatase

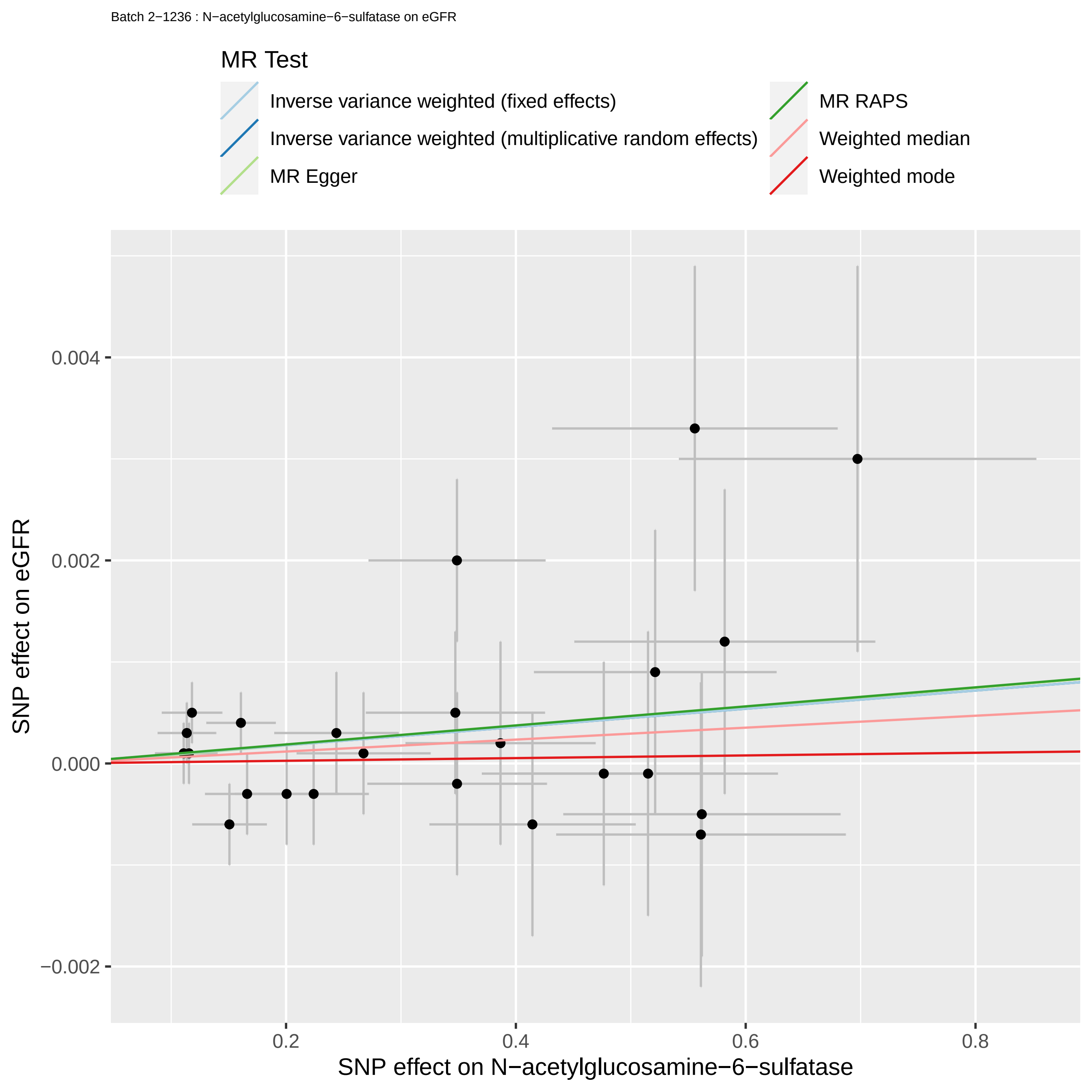

Batch 2−1236 : N−acetylglucosamine−6−sulfatase on eGFR
MR Test
Inverse variance weighted (fixed effects)
Inverse variance weighted (multiplicative random effects)
MR Egger
MR RAPS
Weighted median
Weighted mode
0.004
0.002
0.000
−0.002
0.2
0.4
0.6
0.8
SNP effect on eGFR
SNP effect on N−acetylglucosamine−6−sulfatase

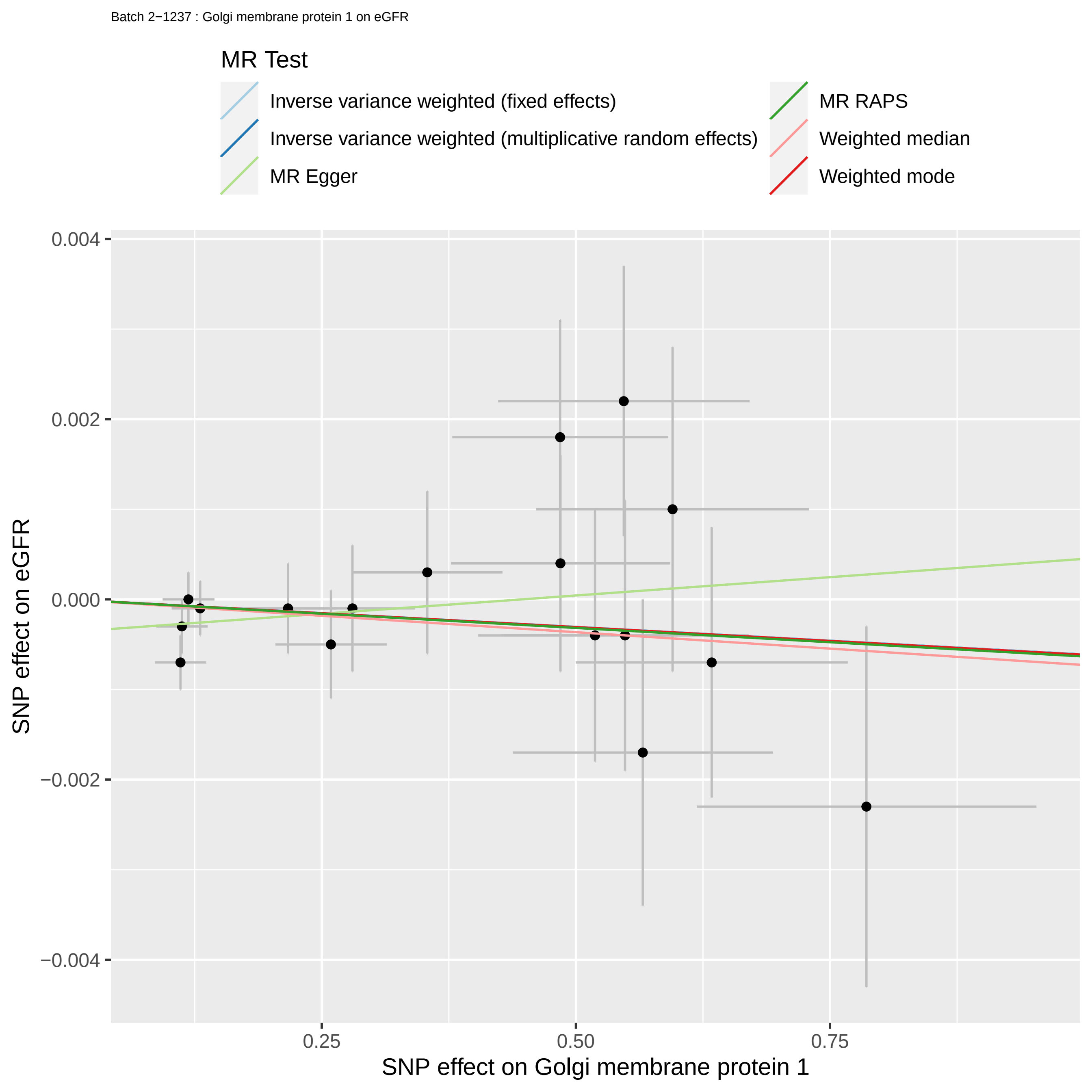

Batch 2−1237 : Golgi membrane protein 1 on eGFR
MR Test
Inverse variance weighted (fixed effects)
Inverse variance weighted (multiplicative random effects)
MR Egger
MR RAPS
Weighted median
Weighted mode
0.004
0.002
0.000
−0.002
−0.004
SNP effect on eGFR
0.25
0.50
0.75
SNP effect on Golgi membrane protein 1

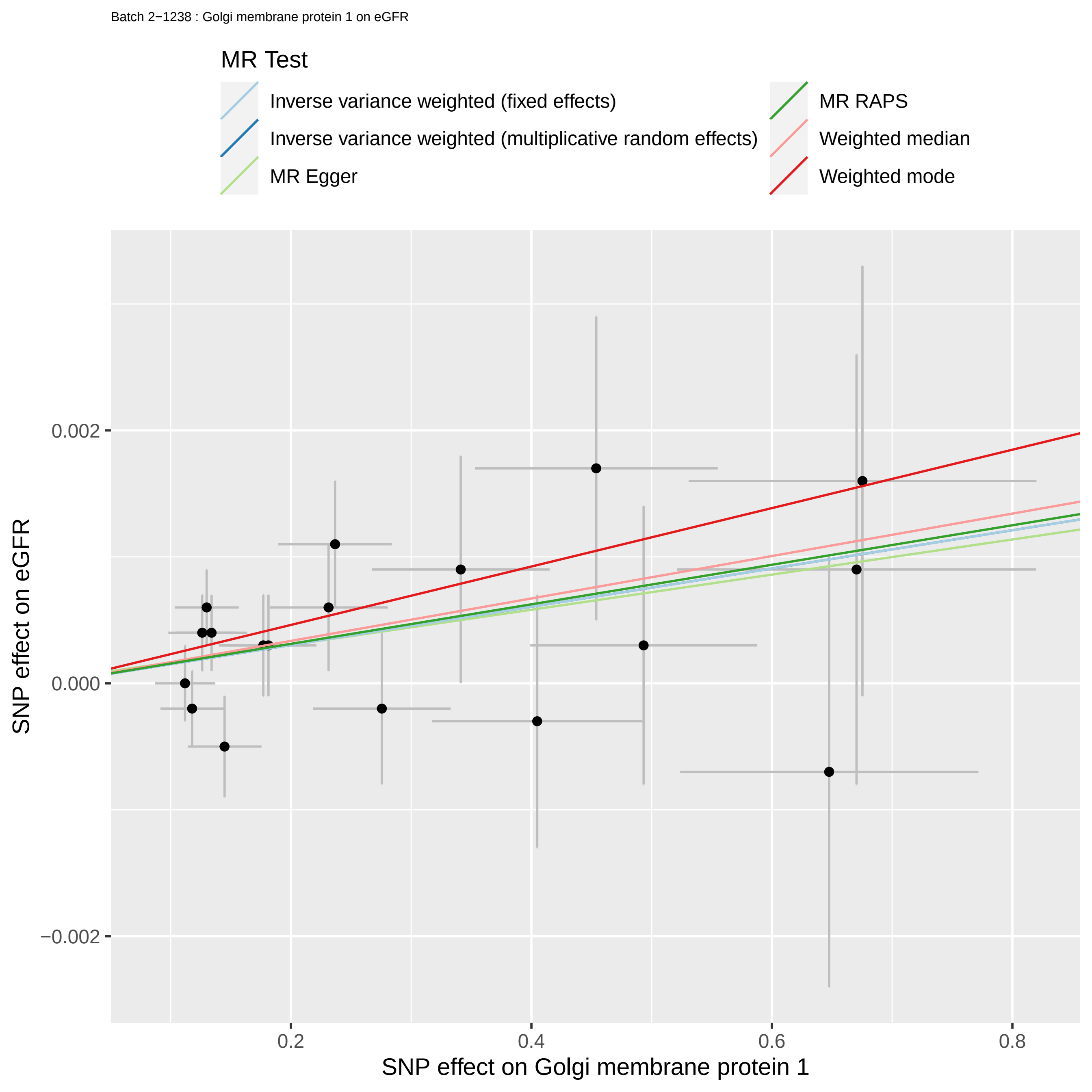

Batch 2−1238 : Golgi membrane protein 1 on eGFR
MR Test
Inverse variance weighted (fixed effects)
Inverse variance weighted (multiplicative random effects)
MR Egger
MR RAPS
Weighted median
Weighted mode
0.002
0.000
−0.002
0.2
0.4
0.6
0.8
SNP effect on eGFR
SNP effect on Golgi membrane protein 1

Batch 2−1239 : RAB6−interacting golgin on eGFR

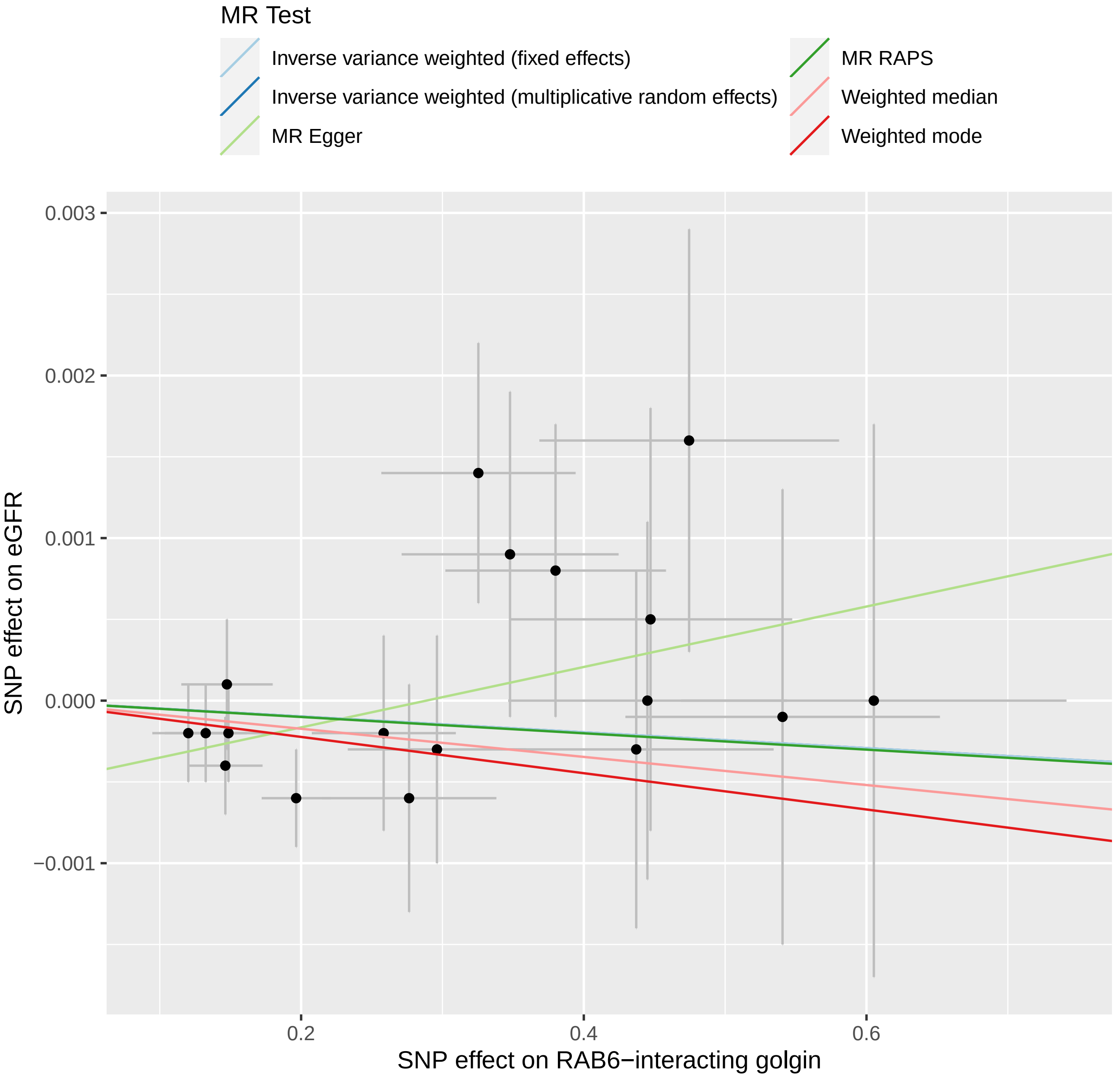

Batch 2−1240 : Golgi SNAP receptor complex member 1 on eGFR

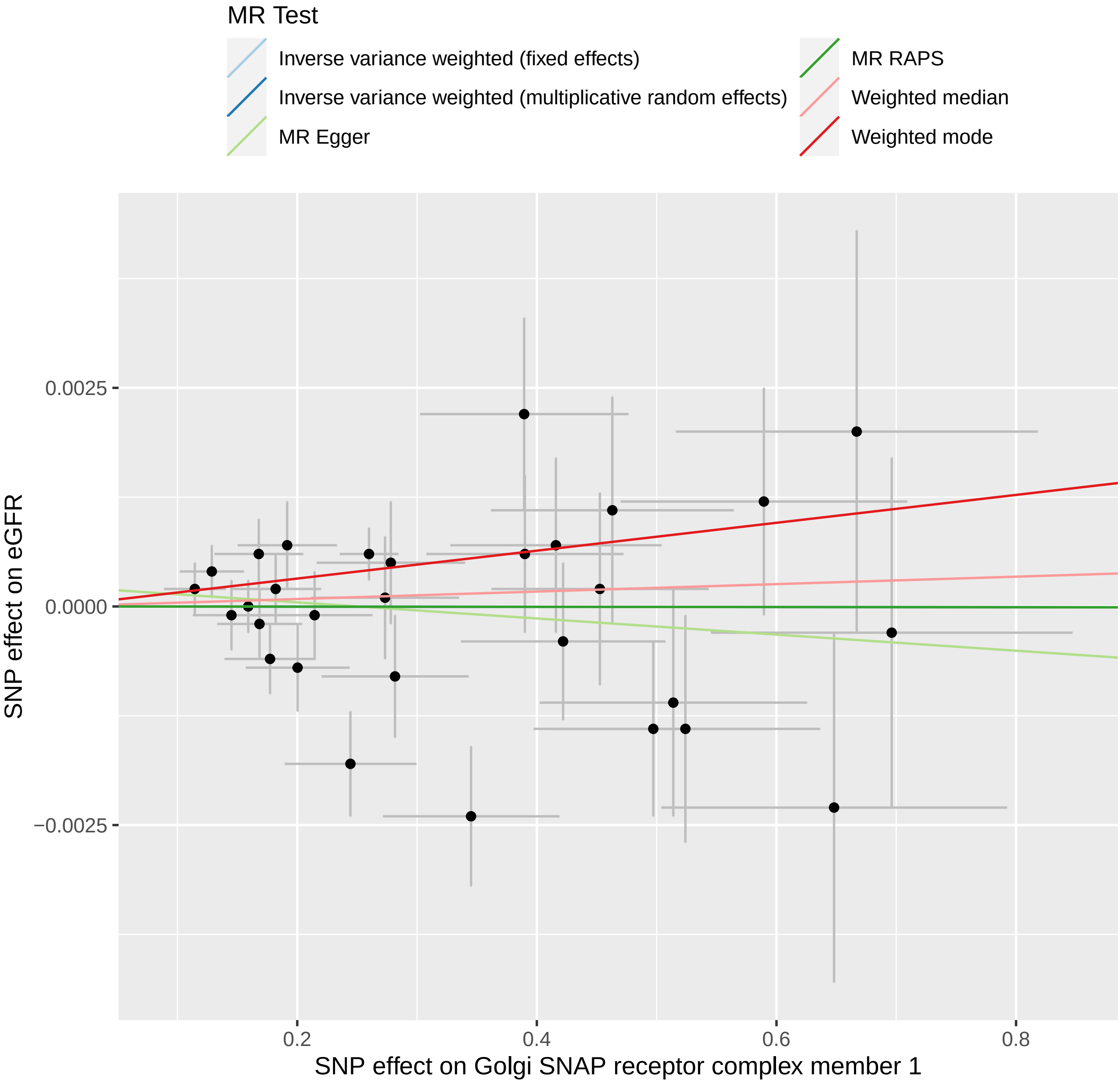

Batch 2−1241 : Aspartate aminotransferase, cytoplasmic on eGFR

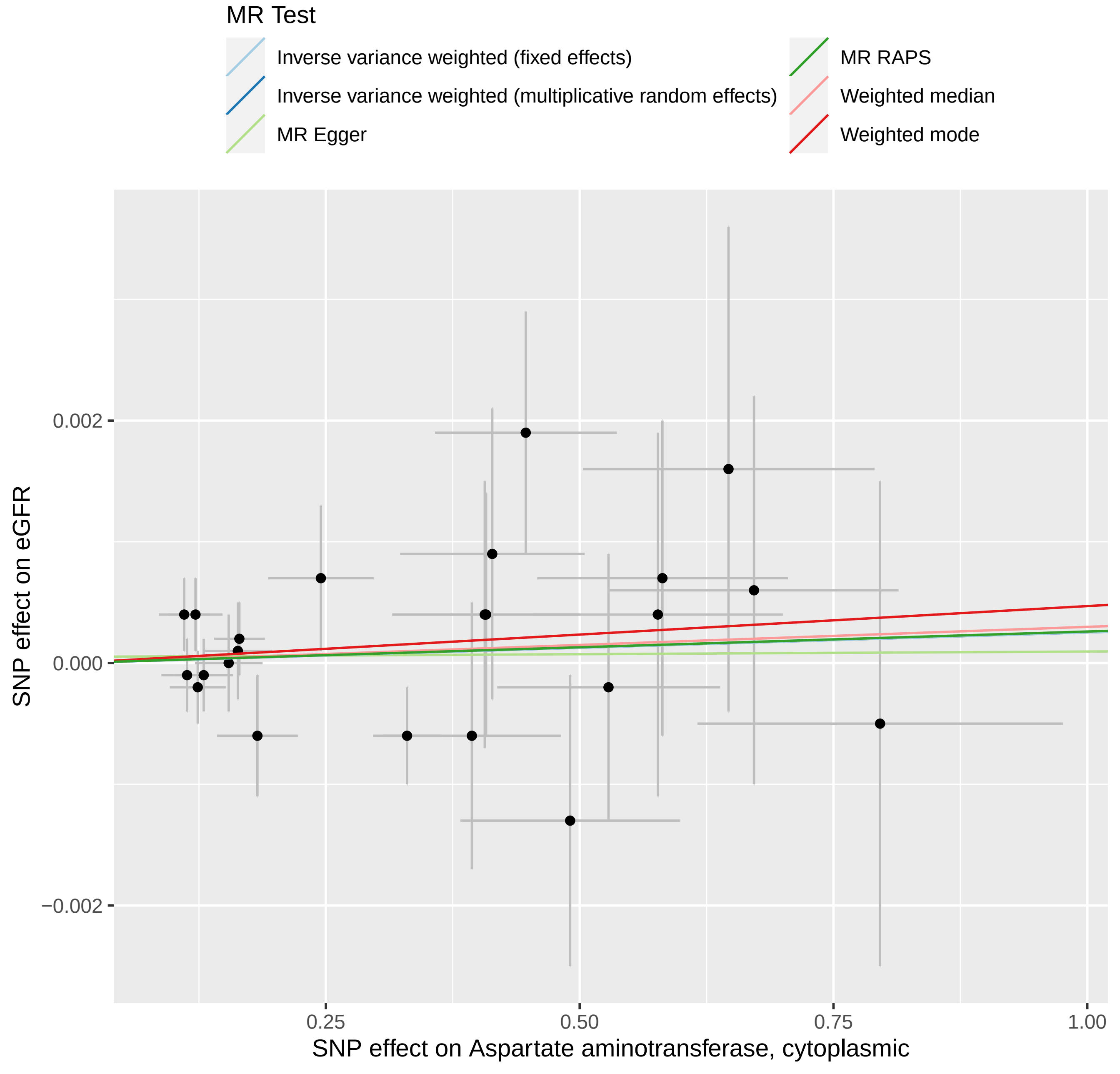

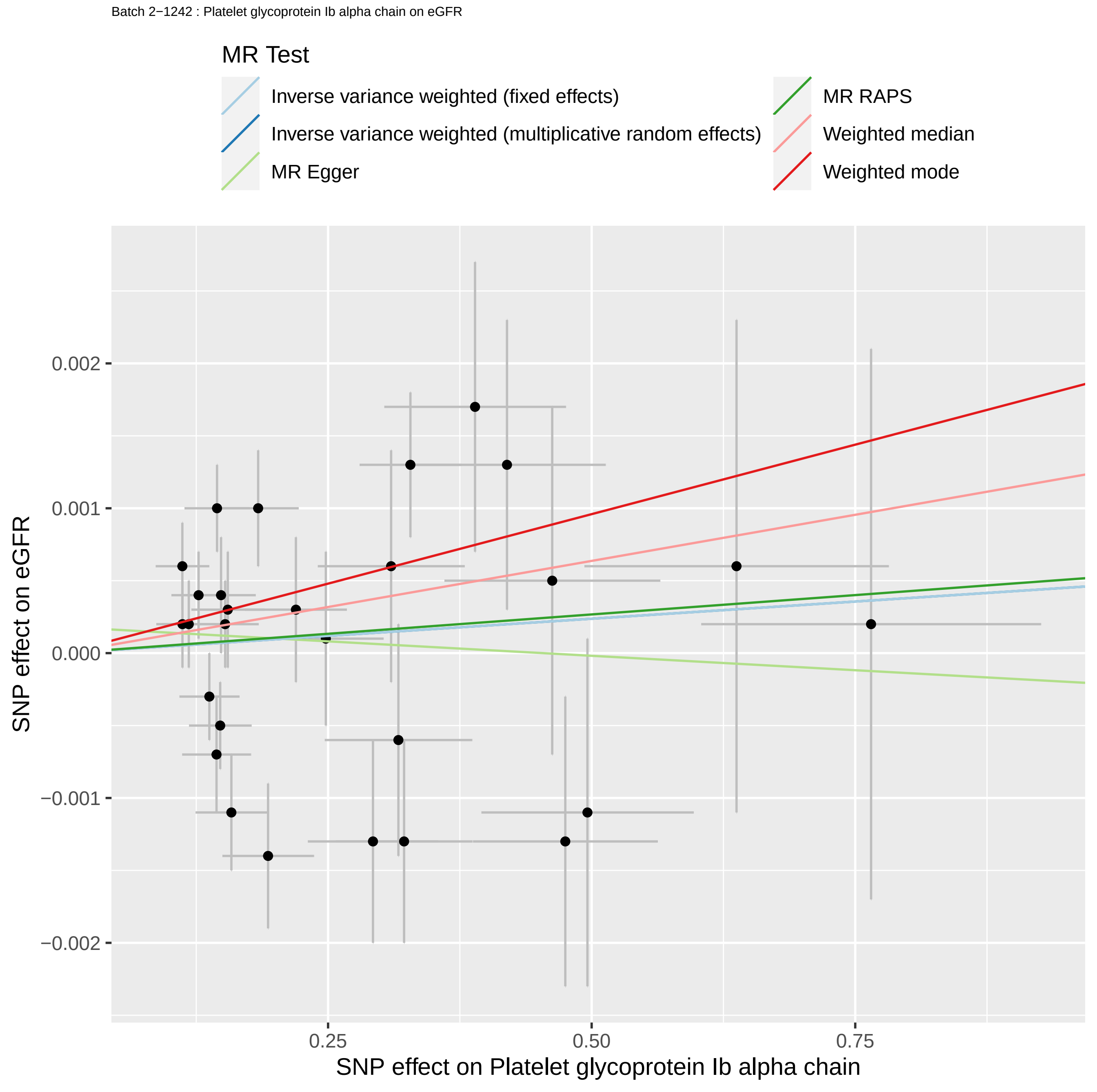
Batch 2−1242 : Platelet glycoprotein Ib alpha chain on eGFR
MR Test
Inverse variance weighted (fixed effects)
Inverse variance weighted (multiplicative random effects)
MR Egger
MR RAPS
Weighted median
Weighted mode
0.002
0.001
0.000
−0.001
−0.002
0.25
0.50
0.75
SNP effect on eGFR
SNP effect on Platelet glycoprotein Ib alpha chain

Batch 2−1243 : Platelet glycoprotein V on eGFR

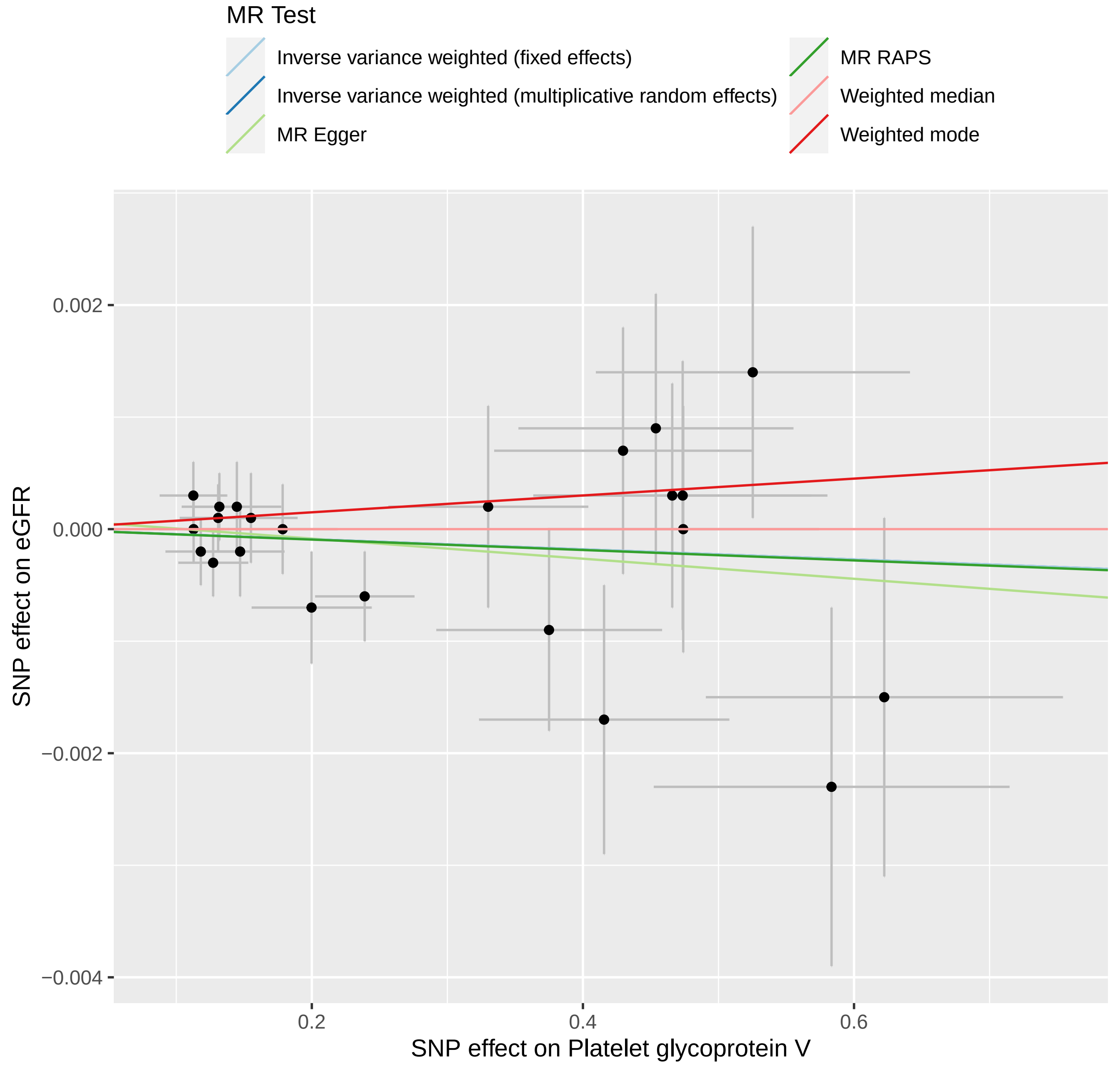

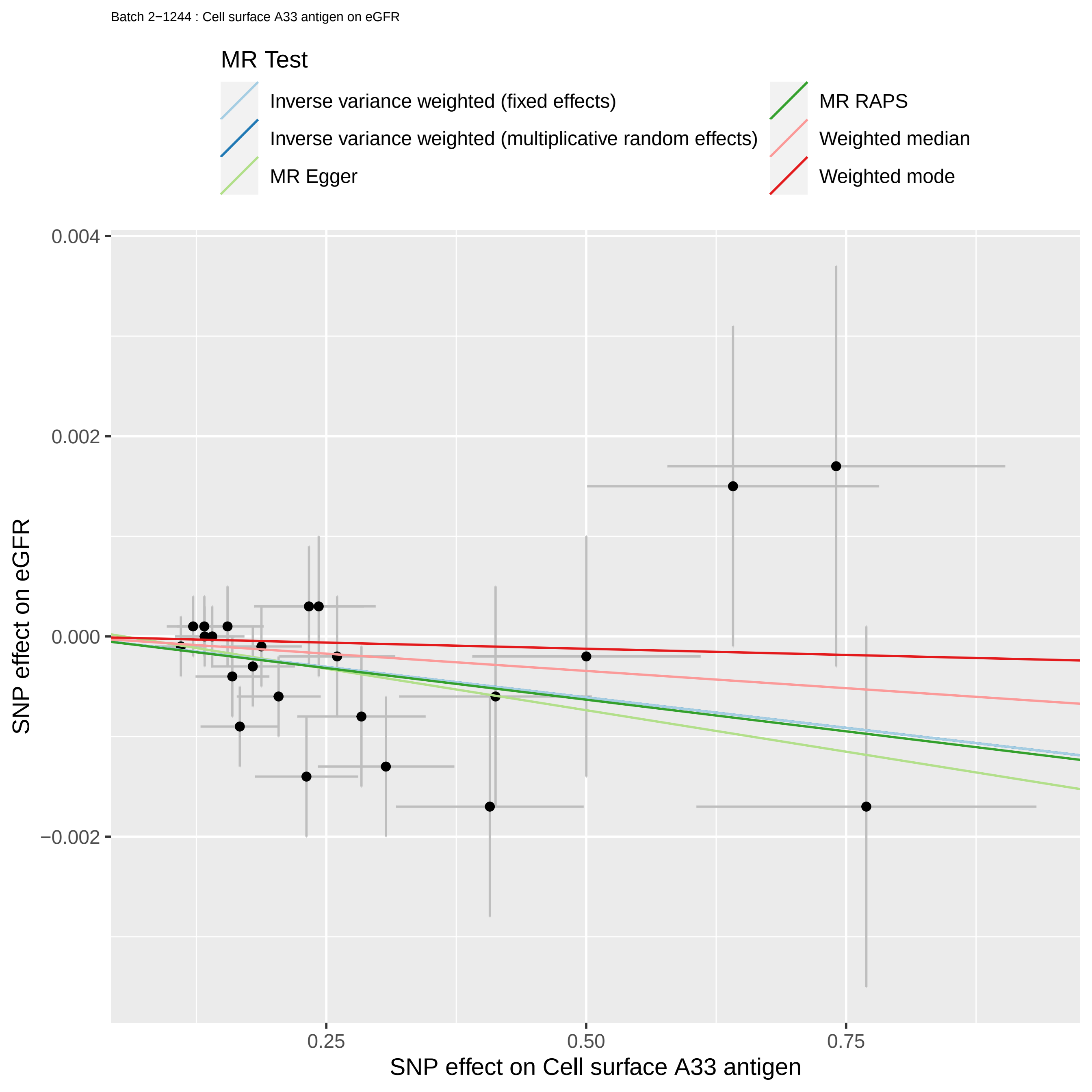
Batch 2−1244 : Cell surface A33 antigen on eGFR
MR Test
Inverse variance weighted (fixed effects)
Inverse variance weighted (multiplicative random effects)
MR Egger
MR RAPS
Weighted median
Weighted mode
0.004
0.002
0.000
−0.002
SNP effect on eGFR
0.25
0.50
0.75
SNP effect on Cell surface A33 antigen

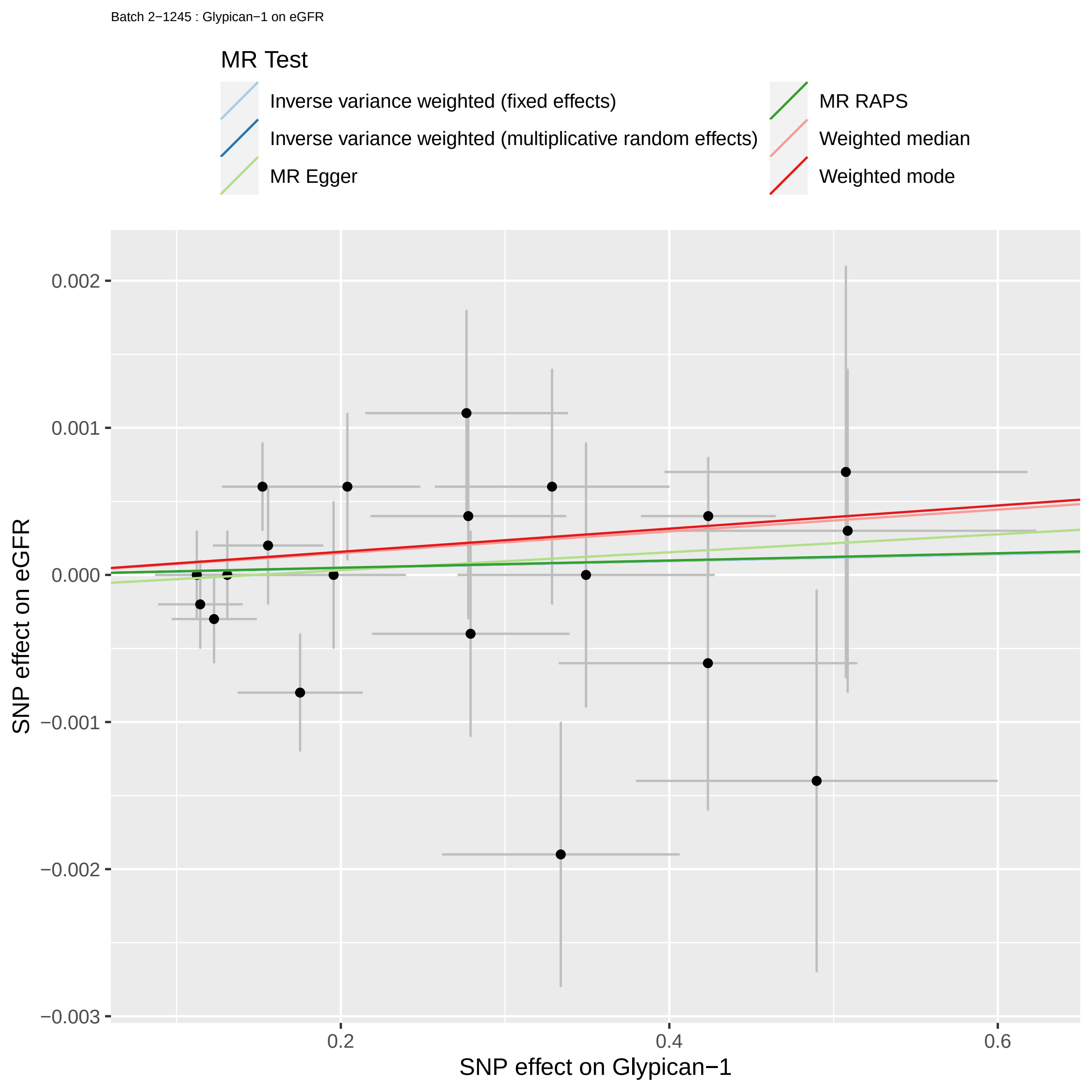

Batch 2−1245 : Glypican−1 on eGFR
MR Test
Inverse variance weighted (fixed effects)
Inverse variance weighted (multiplicative random effects)
MR Egger
MR RAPS
Weighted median
Weighted mode
SNP effect on eGFR
0.002
0.001
0.000
−0.001
−0.002
−0.003
0.2
0.4
0.6
SNP effect on Glypican−1

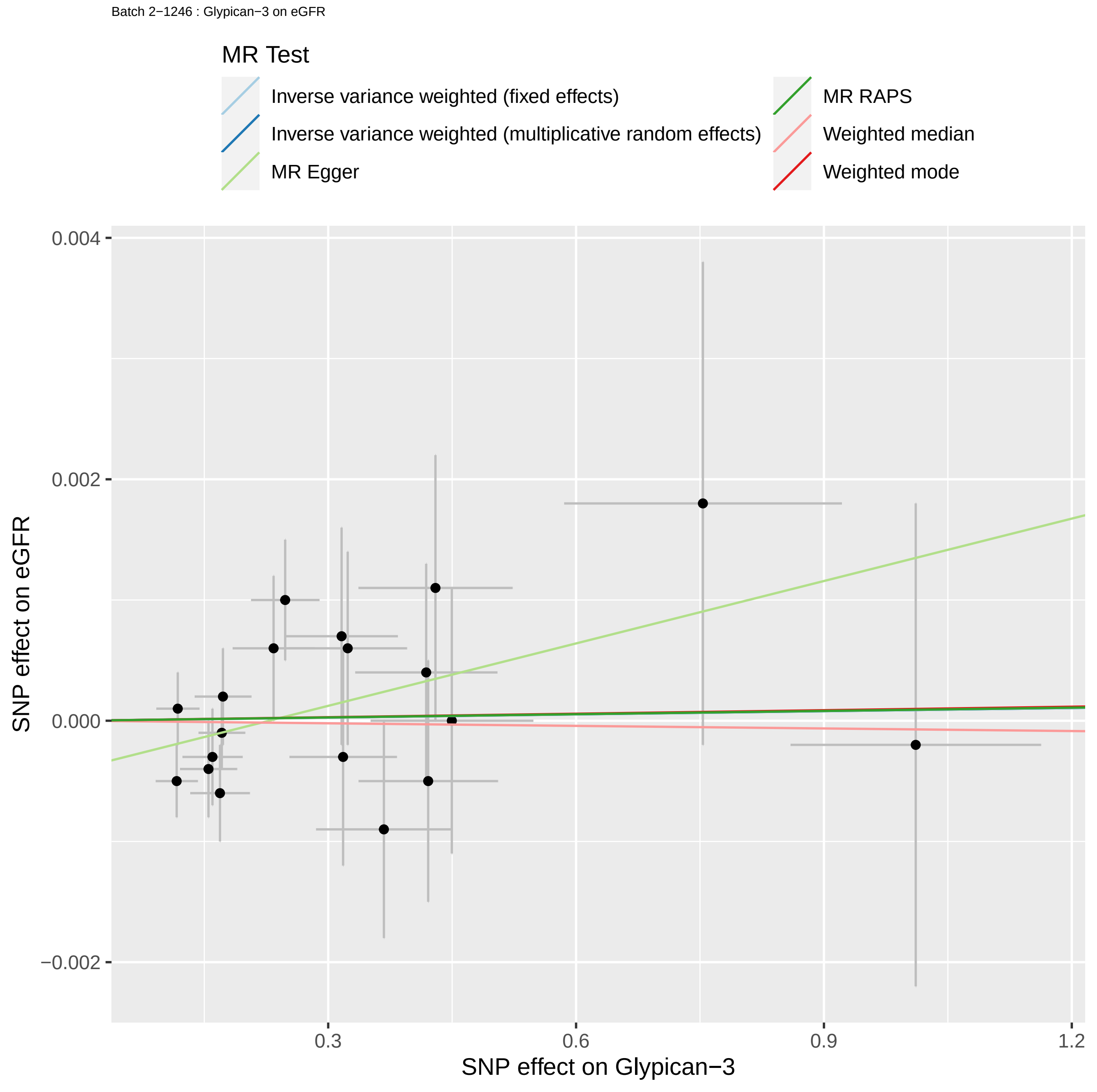

Batch 2−1246 : Glypican−3 on eGFR
MR Test
Inverse variance weighted (fixed effects)
Inverse variance weighted (multiplicative random effects)
MR Egger
MR RAPS
Weighted median
Weighted mode
0.004
0.002
0.000
−0.002
0.3
0.6
0.9
1.2
SNP effect on eGFR
SNP effect on Glypican−3

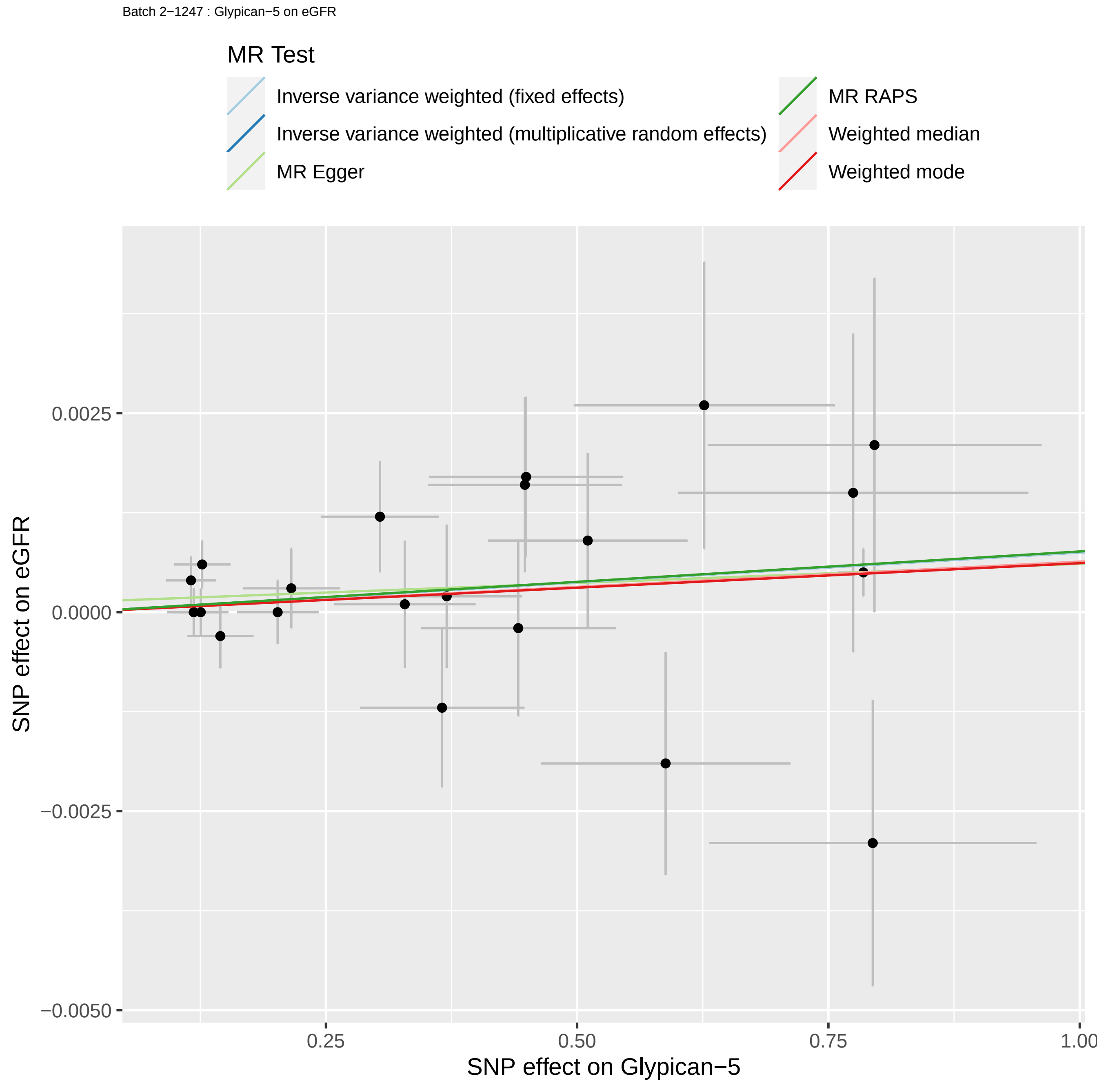

Batch 2−1247 : Glypican−5 on eGFR
MR Test
Inverse variance weighted (fixed effects)
Inverse variance weighted (multiplicative random effects)
MR Egger
MR RAPS
Weighted median
Weighted mode
0.0025
0.0000
−0.0025
−0.0050
0.25
0.50
0.75
1.00
SNP effect on eGFR
SNP effect on Glypican−5

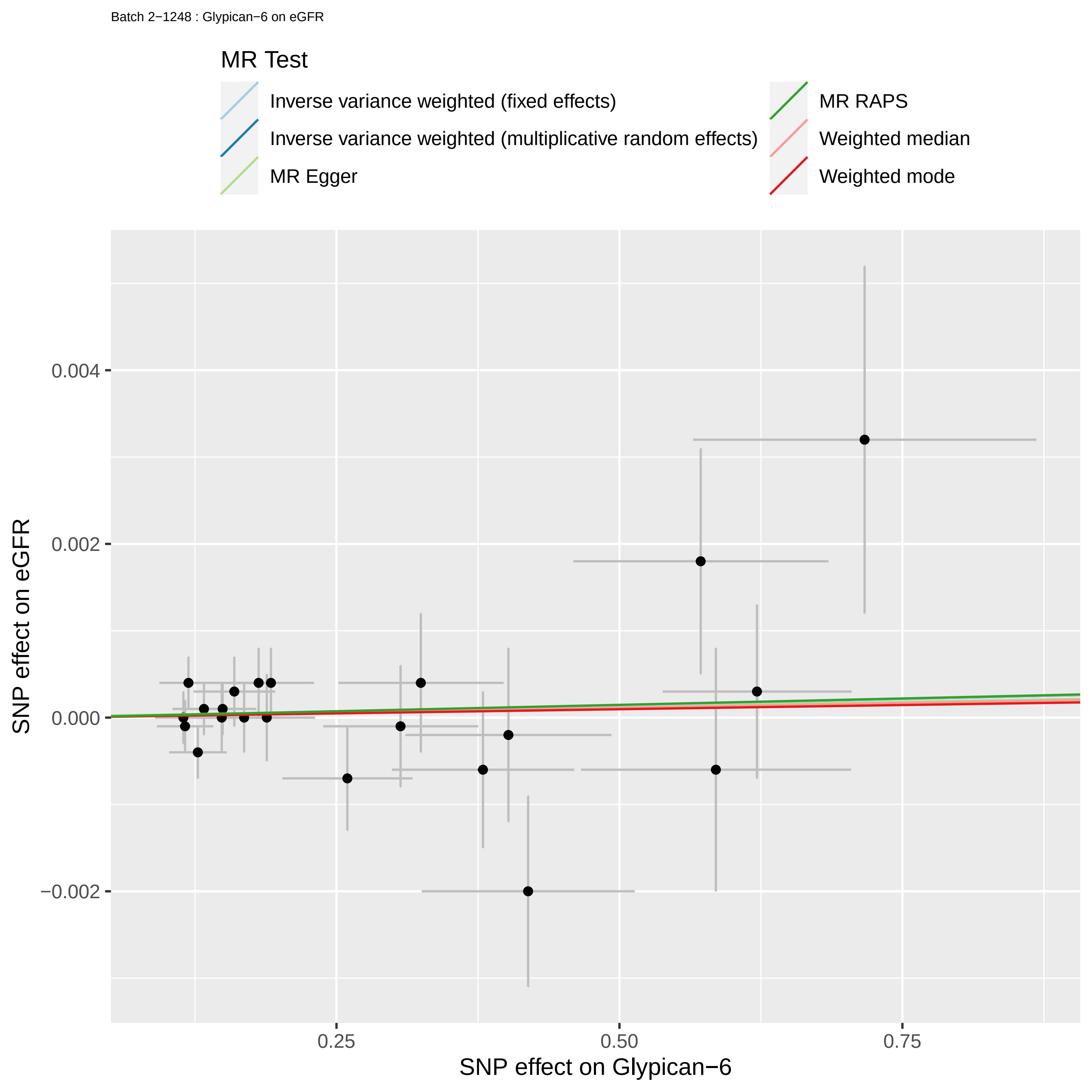

Batch 2−1248 : Glypican−6 on eGFR
MR Test
Inverse variance weighted (fixed effects)
Inverse variance weighted (multiplicative random effects)
MR Egger
MR RAPS
Weighted median
Weighted mode
0.004
0.002
0.000
−0.002
0.25
0.50
0.75
SNP effect on eGFR
SNP effect on Glypican−6

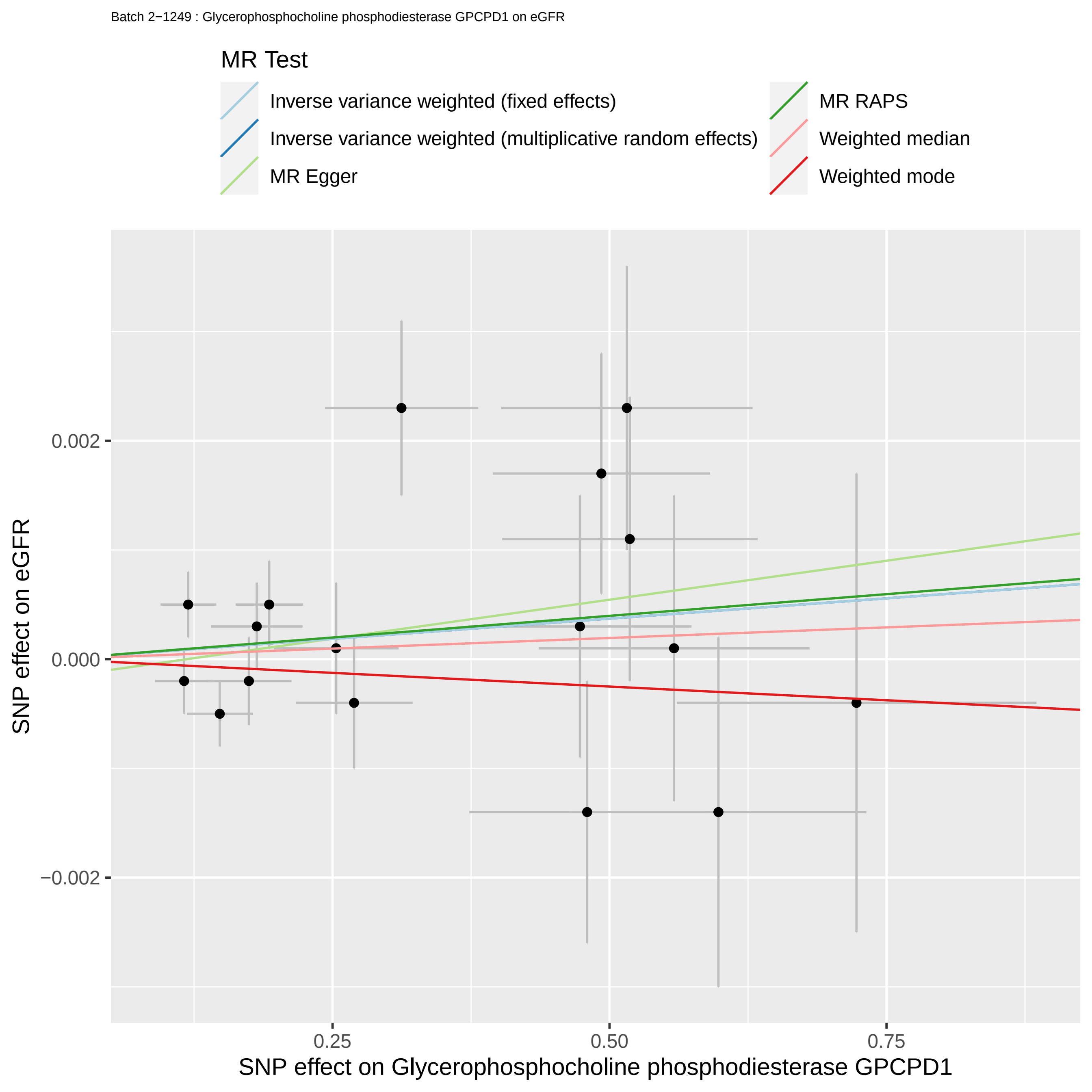

Batch 2−1249 : Glycerophosphocholine phosphodiesterase GPCPD1 on eGFR
MR Test
Inverse variance weighted (fixed effects)
Inverse variance weighted (multiplicative random effects)
MR Egger
MR RAPS
Weighted median
Weighted mode
0.002
0.000
−0.002
0.25
0.50
0.75
SNP effect on eGFR
SNP effect on Glycerophosphocholine phosphodiesterase GPCPD1

Batch 2−1250 : Glycerol−3−phosphate dehydrogenase 1−like protein on eGFR

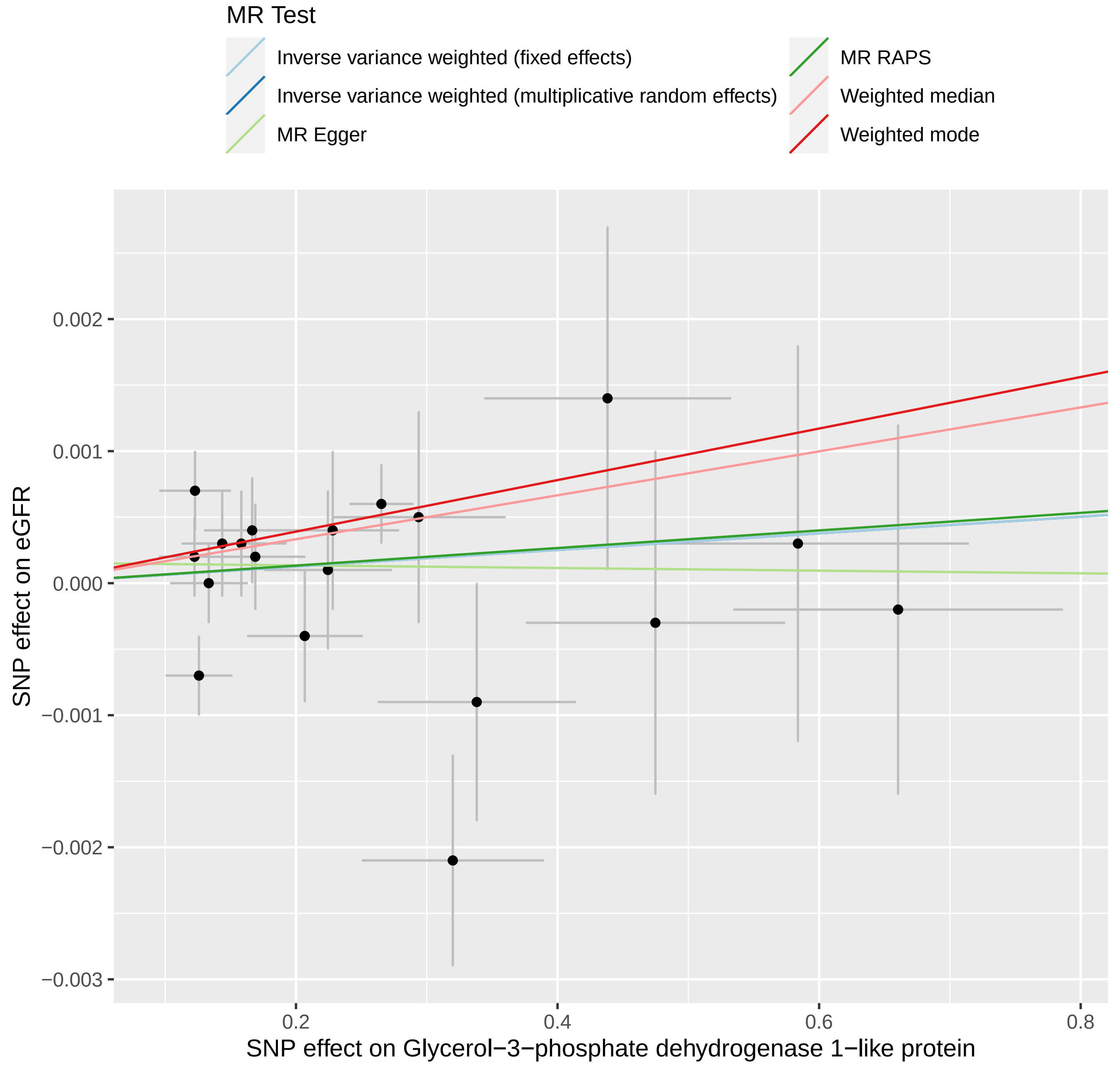

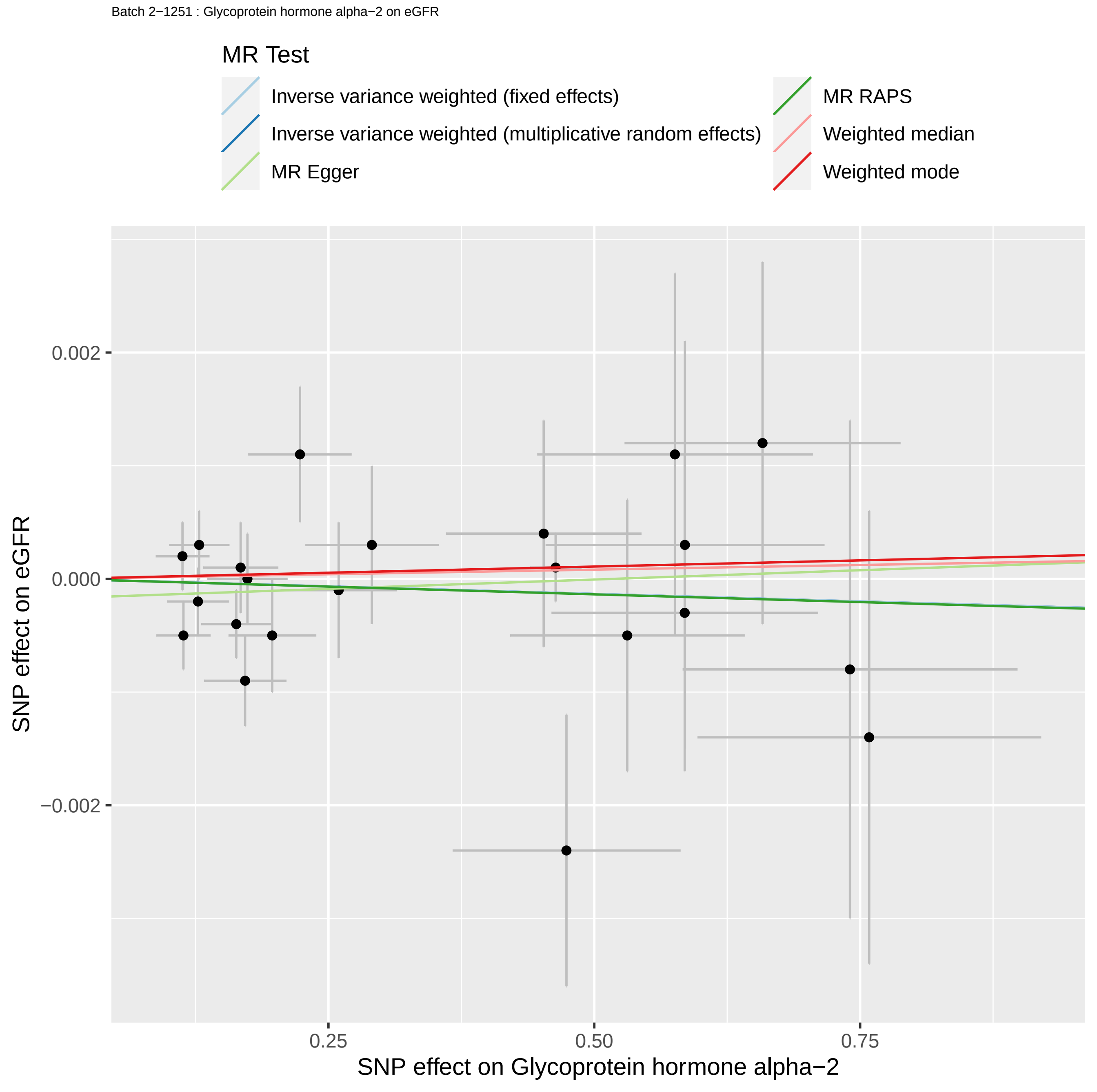
Batch 2−1251 : Glycoprotein hormone alpha−2 on eGFR
MR Test
Inverse variance weighted (fixed effects)
Inverse variance weighted (multiplicative random effects)
MR Egger
MR RAPS
Weighted median
Weighted mode
0.002
0.000
−0.002
0.25
0.50
0.75
SNP effect on eGFR
SNP effect on Glycoprotein hormone alpha−2

Batch 2−1252 : Glucose−6−phosphate isomerase on eGFR

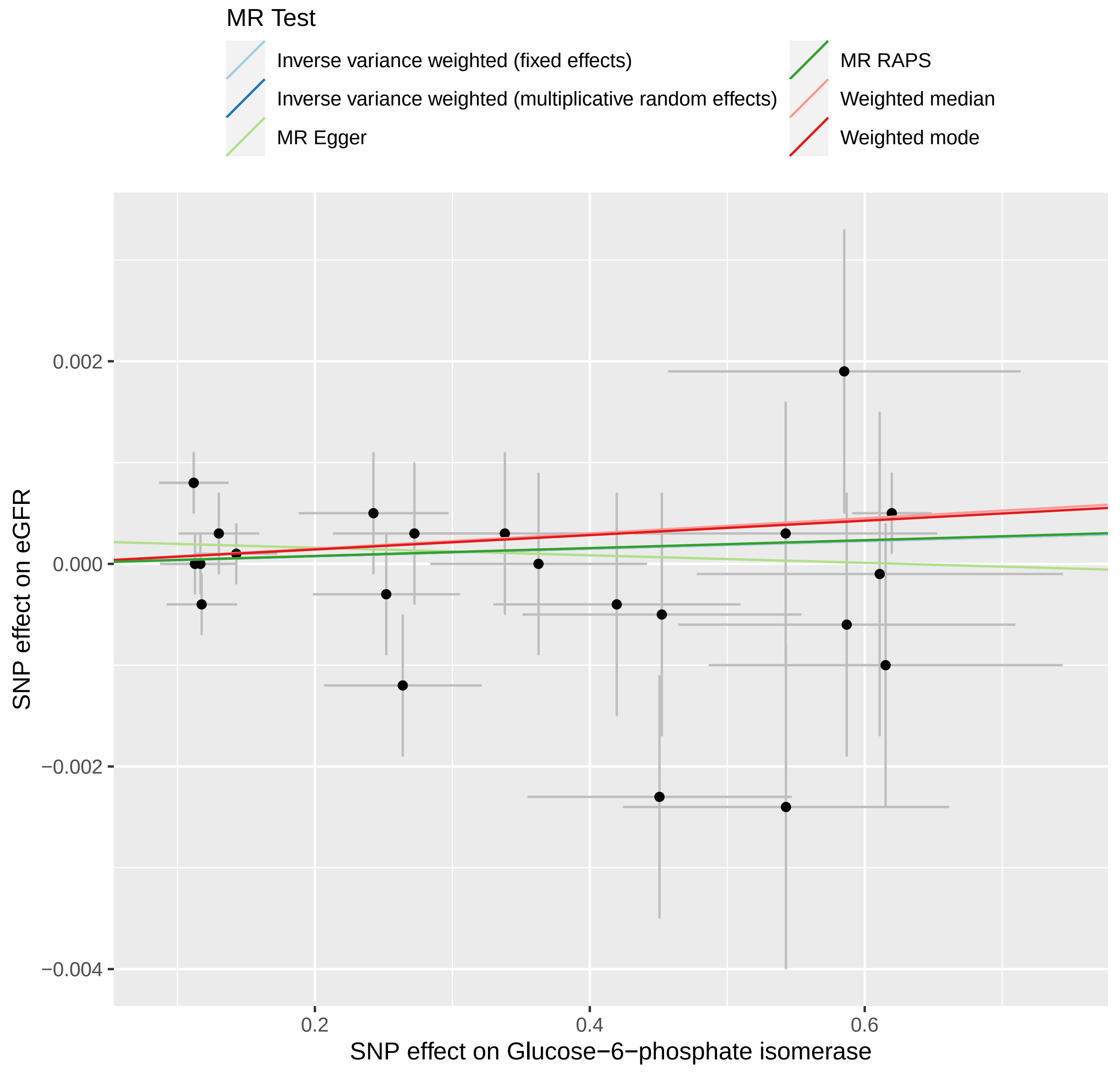

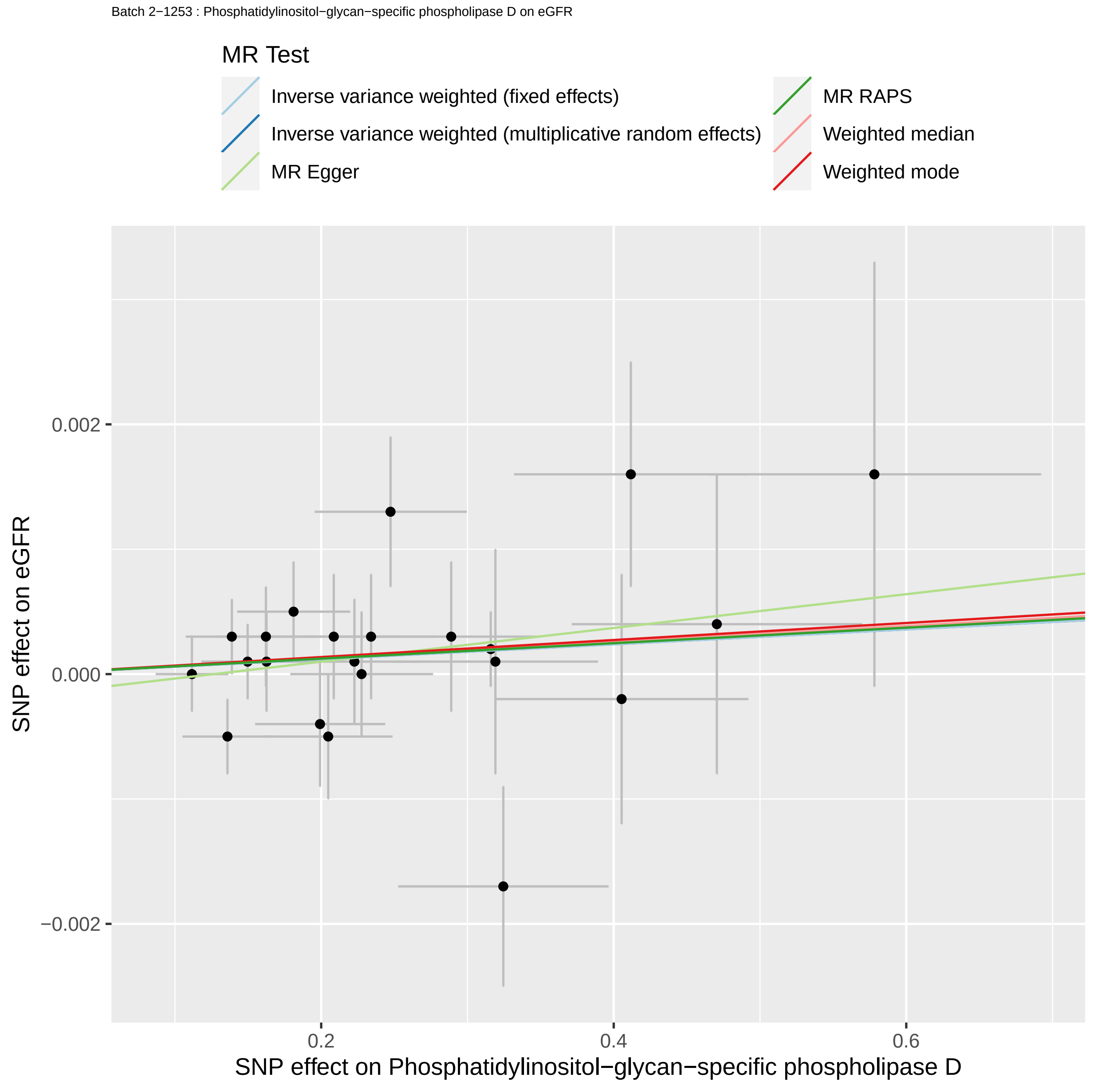
Batch 2−1253 : Phosphatidylinositol−glycan−specific phospholipase D on eGFR
MR Test
Inverse variance weighted (fixed effects)
Inverse variance weighted (multiplicative random effects)
MR Egger
MR RAPS
Weighted median
Weighted mode
0.002
0.000
−0.002
0.2
0.4
0.6
SNP effect on eGFR
SNP effect on Phosphatidylinositol−glycan−specific phospholipase D

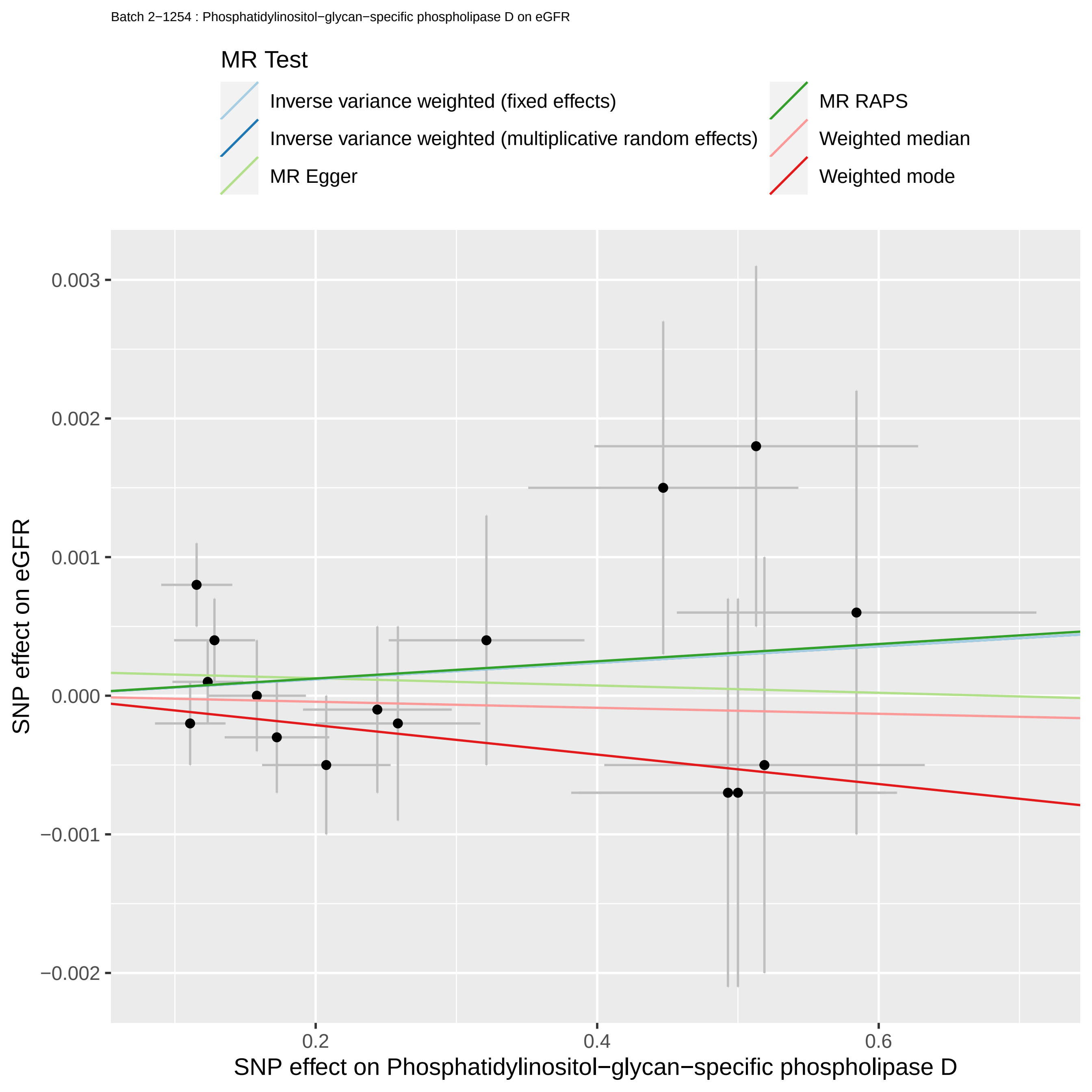

Batch 2−1254 : Phosphatidylinositol−glycan−specific phospholipase D on eGFR
MR Test
Inverse variance weighted (fixed effects)
Inverse variance weighted (multiplicative random effects)
MR Egger
MR RAPS
Weighted median
Weighted mode
0.003
0.002
0.001
0.000
−0.001
−0.002
0.2
0.4
0.6
SNP effect on eGFR
SNP effect on Phosphatidylinositol−glycan−specific phospholipase D

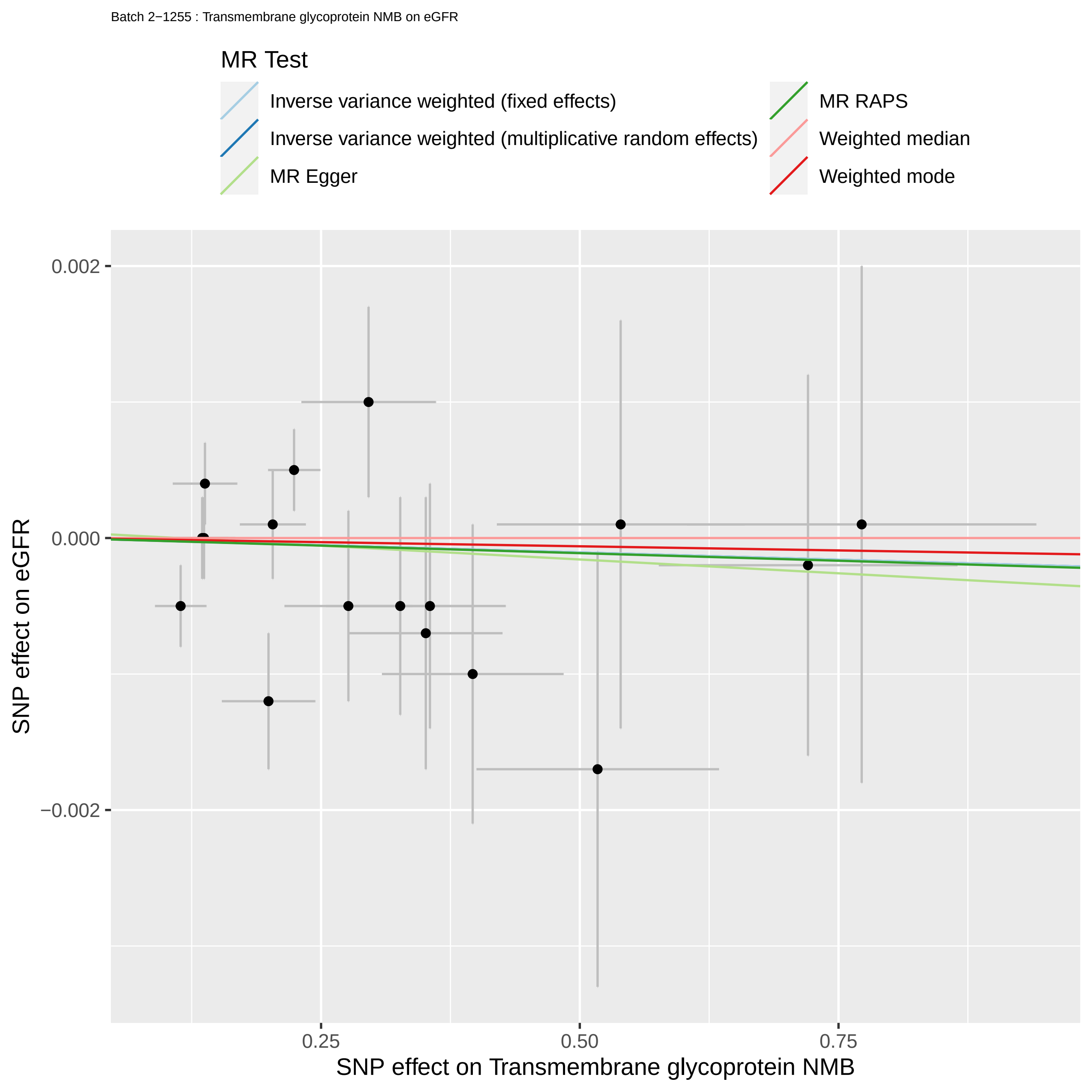

Batch 2−1255 : Transmembrane glycoprotein NMB on eGFR
MR Test
Inverse variance weighted (fixed effects)
Inverse variance weighted (multiplicative random effects)
MR Egger
MR RAPS
Weighted median
Weighted mode
0.002
0.000
−0.002
SNP effect on eGFR
0.25
0.50
0.75
SNP effect on Transmembrane glycoprotein NMB

Batch 2−1256 : Transmembrane glycoprotein NMB on eGFR

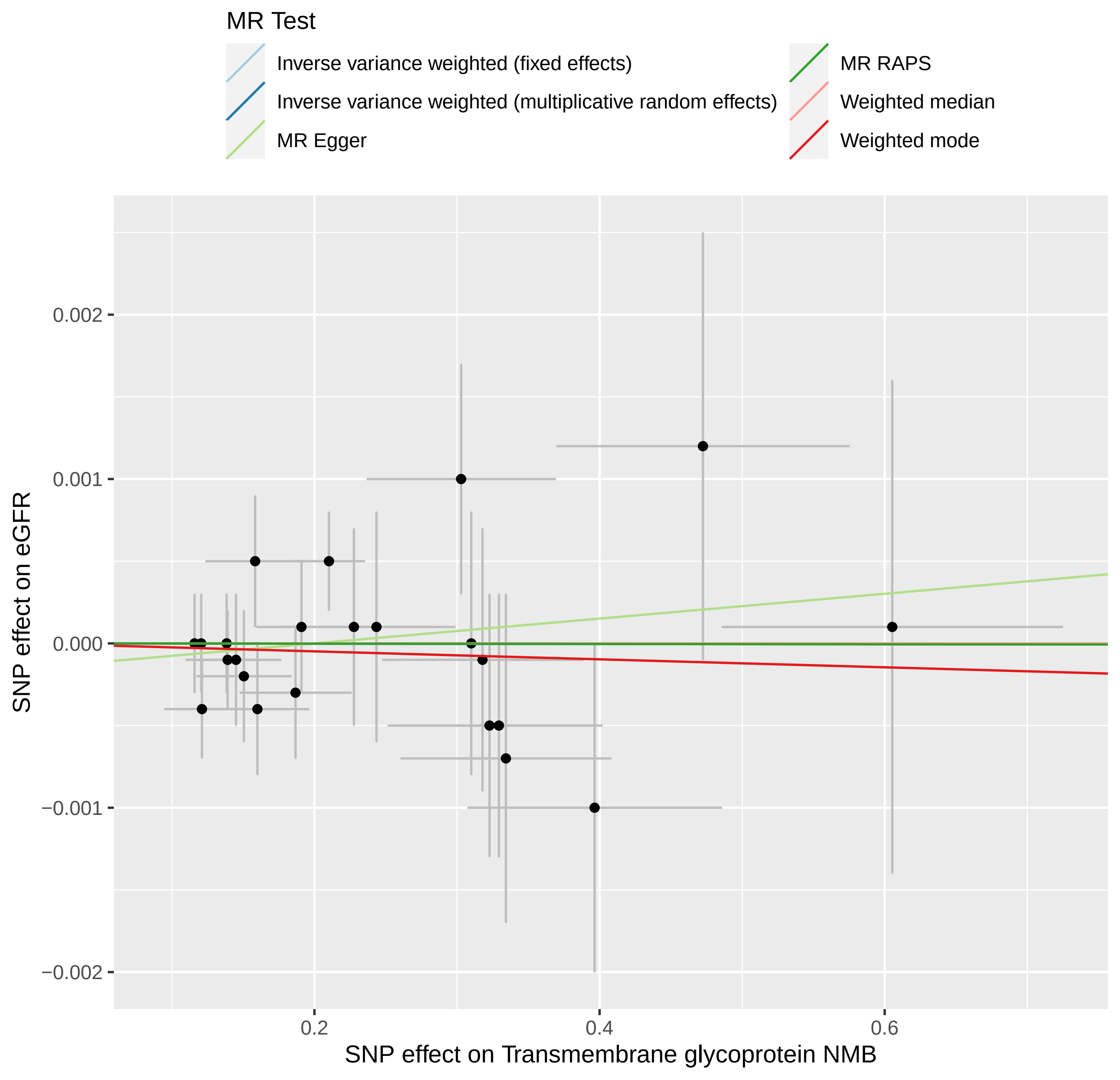

Batch 2−1257 : Transmembrane glycoprotein NMB on eGFR

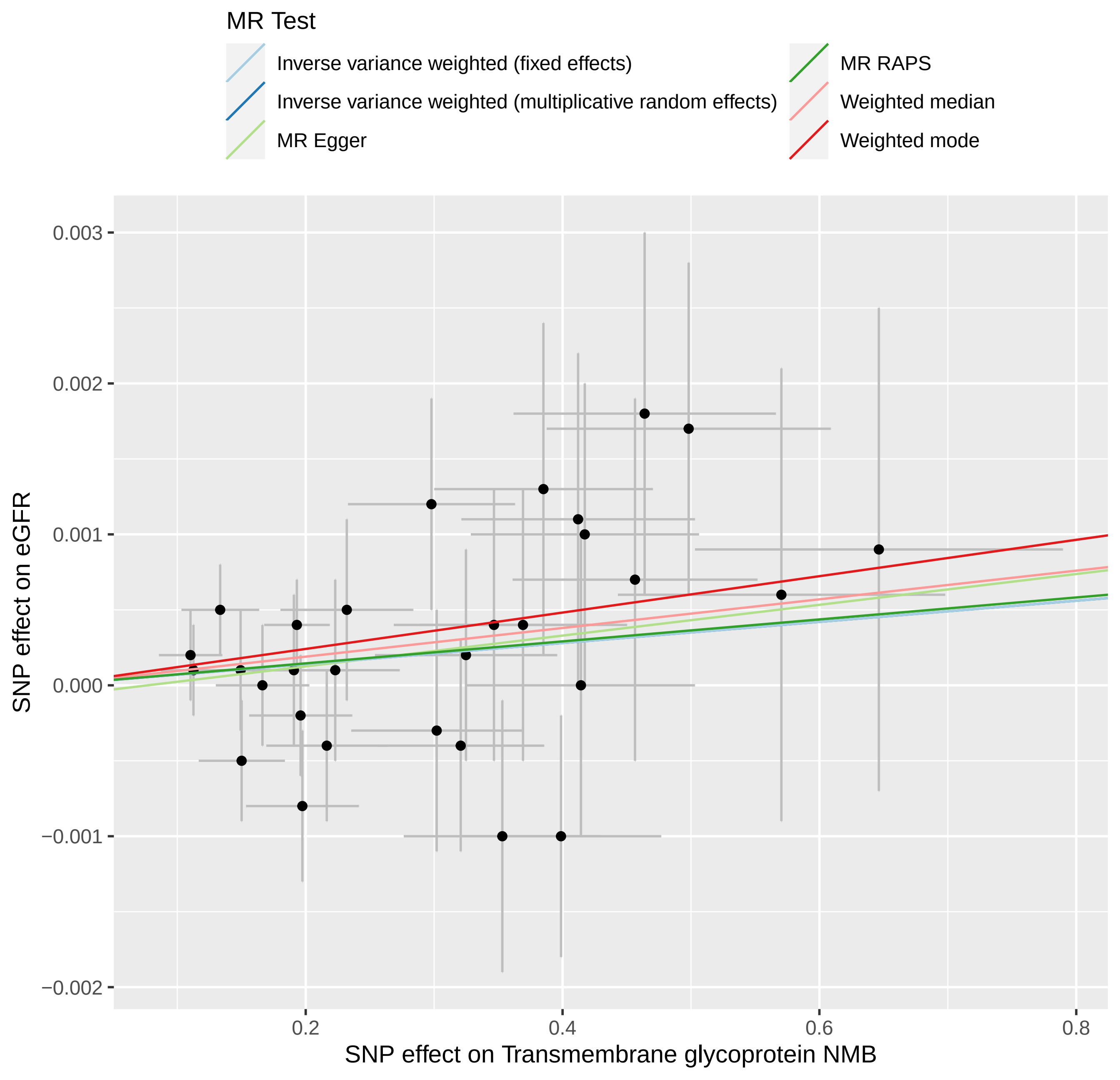

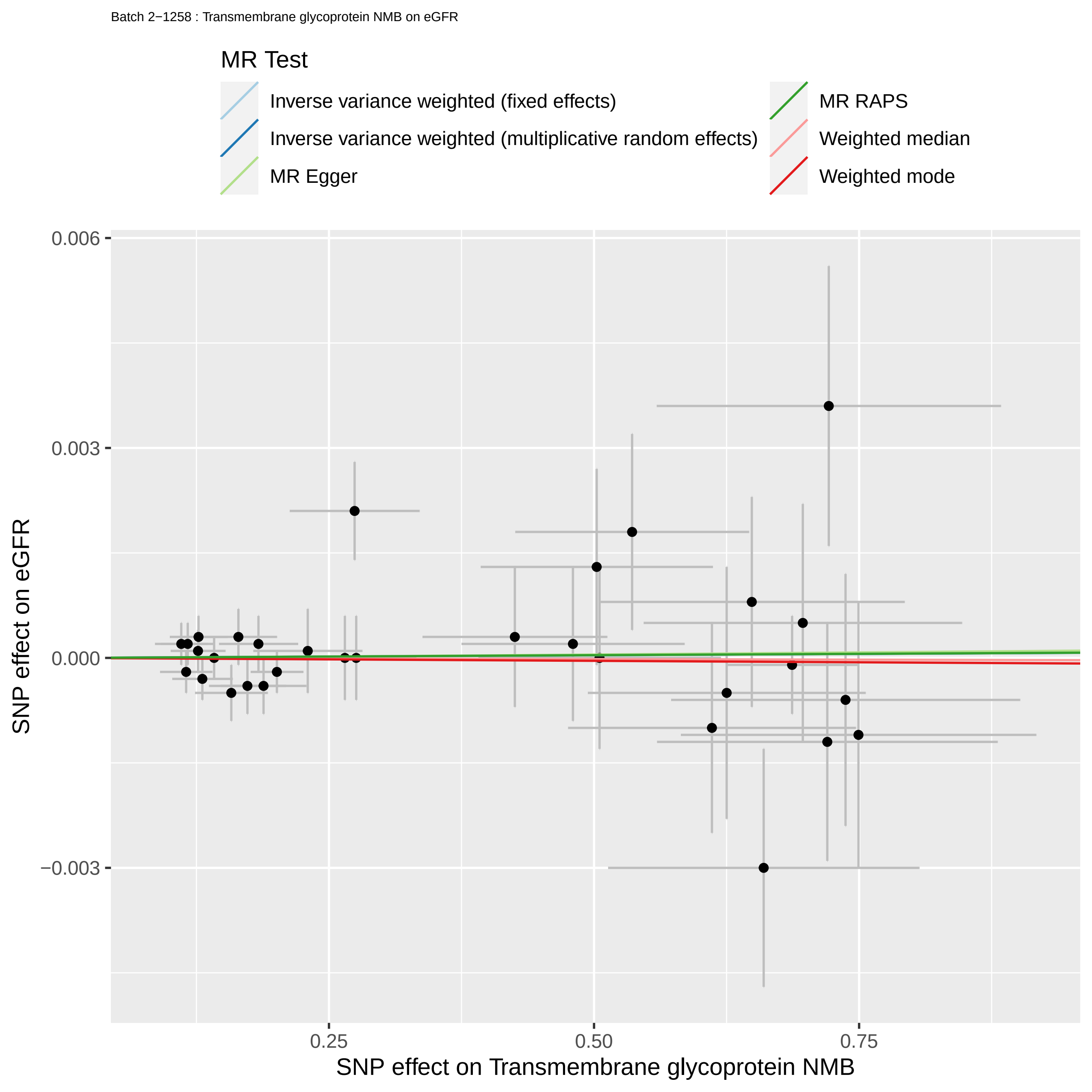

Batch 2−1258 : Transmembrane glycoprotein NMB on eGFR
MR Test
Inverse variance weighted (fixed effects)
Inverse variance weighted (multiplicative random effects)
MR Egger
MR RAPS
Weighted median
Weighted mode
0.006
0.003
0.000
−0.003
SNP effect on eGFR
0.25
0.50
0.75
SNP effect on Transmembrane glycoprotein NMB

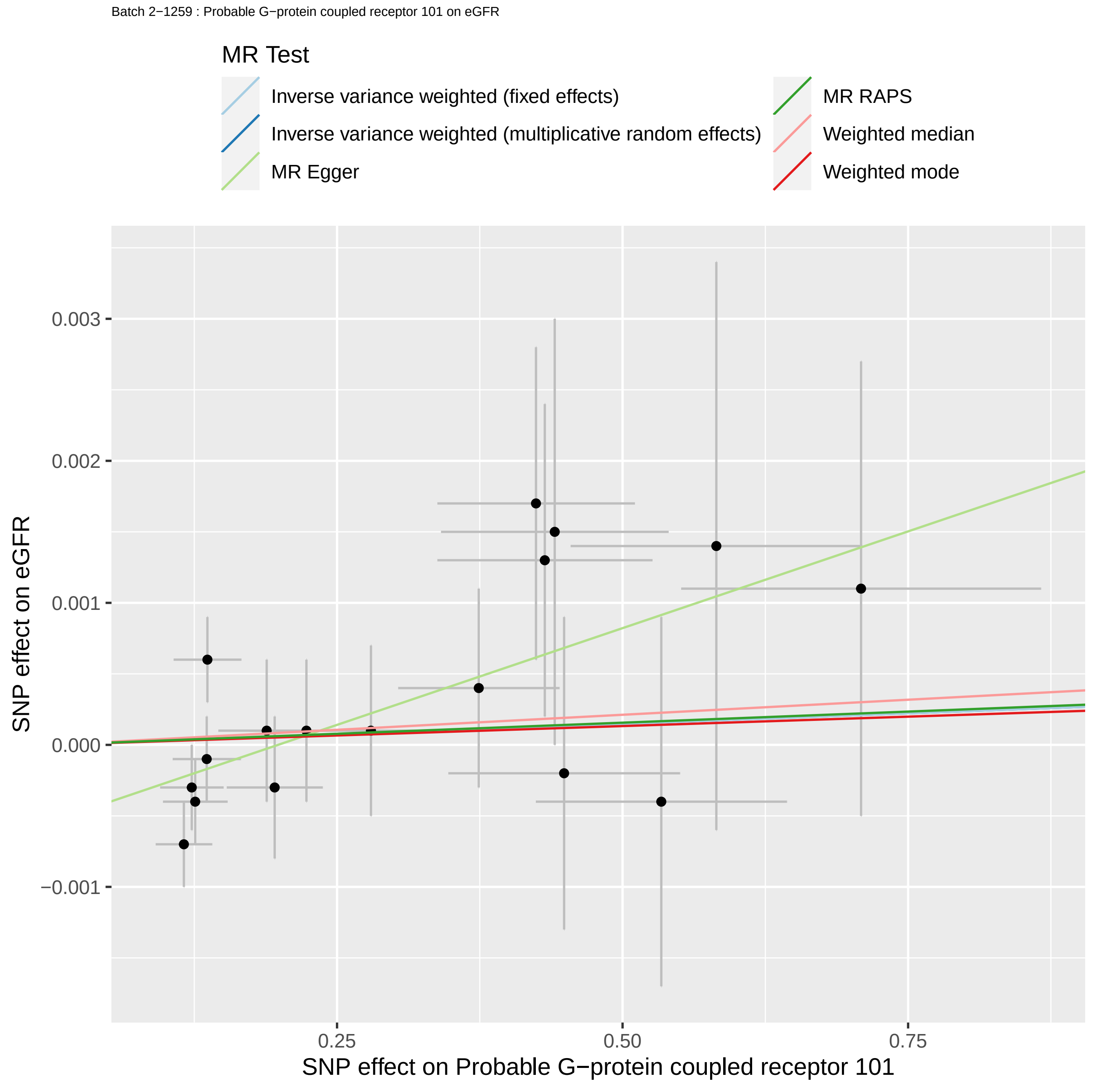
Batch 2−1259 : Probable G−protein coupled receptor 101 on eGFR
MR Test
Inverse variance weighted (fixed effects)
Inverse variance weighted (multiplicative random effects)
MR Egger
MR RAPS
Weighted median
Weighted mode
0.003
0.002
0.001
0.000
−0.001
0.25
0.50
0.75
SNP effect on eGFR
SNP effect on Probable G−protein coupled receptor 101

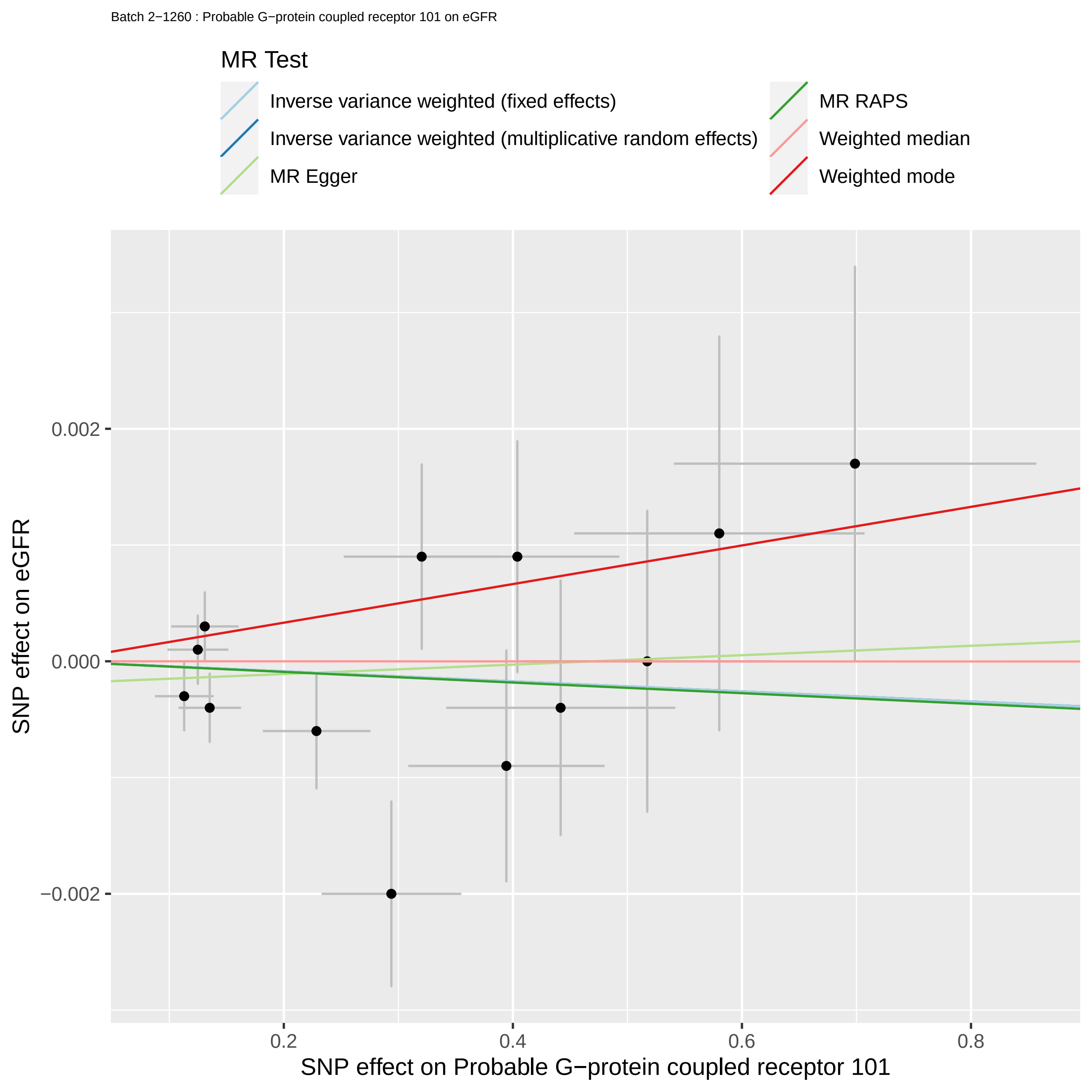
Batch 2−1260 : Probable G−protein coupled receptor 101 on eGFR
MR Test
Inverse variance weighted (fixed effects)
Inverse variance weighted (multiplicative random effects)
MR Egger
MR RAPS
Weighted median
Weighted mode
0.002
0.000
−0.002
0.2
0.4
0.6
0.8
SNP effect on eGFR
SNP effect on Probable G−protein coupled receptor 101

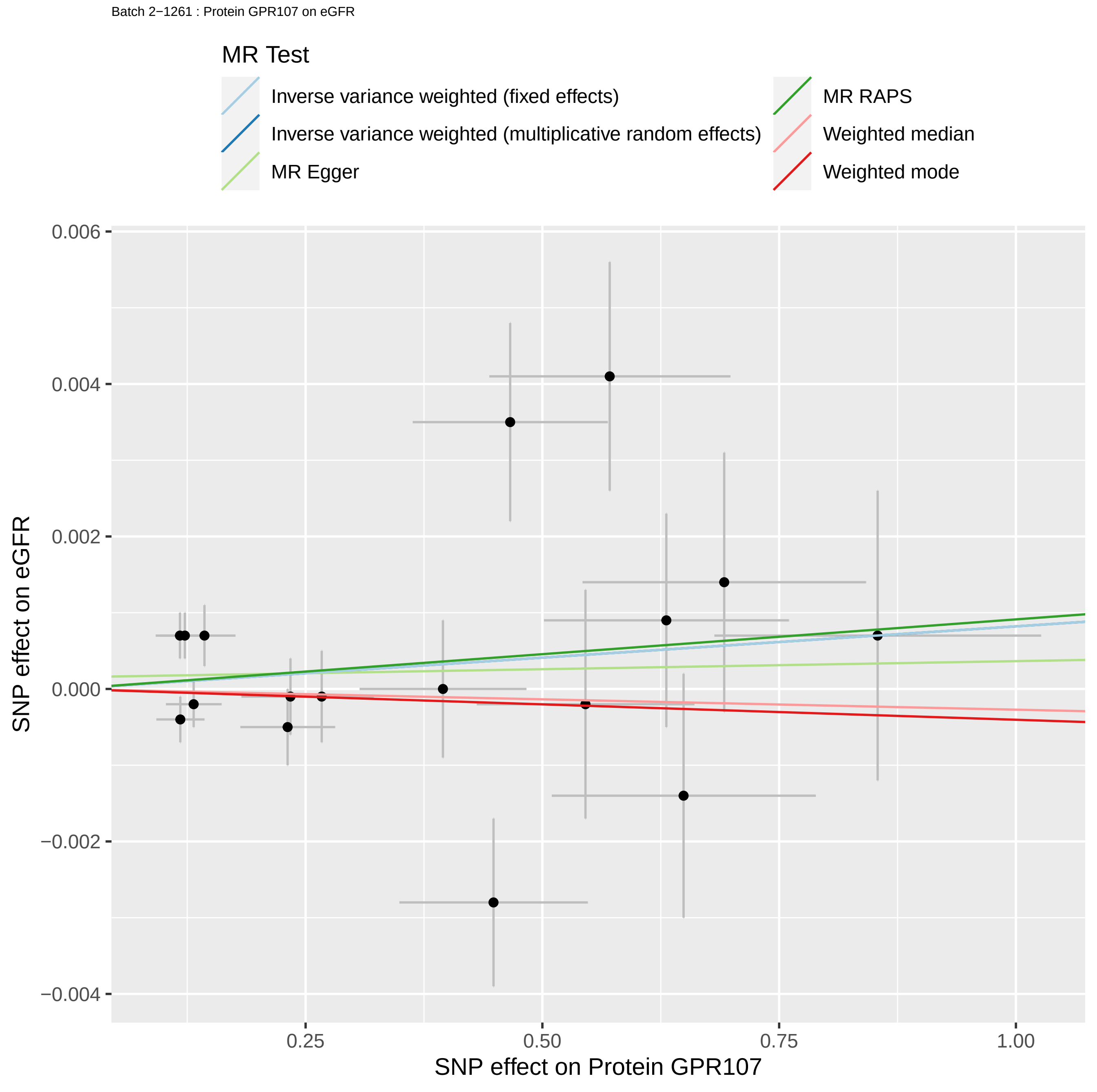

Batch 2−1261 : Protein GPR107 on eGFR
MR Test
Inverse variance weighted (fixed effects)
Inverse variance weighted (multiplicative random effects)
MR Egger
MR RAPS
Weighted median
Weighted mode
0.006
0.004
0.002
0.000
−0.002
−0.004
0.25
0.50
0.75
1.00
SNP effect on eGFR
SNP effect on Protein GPR107

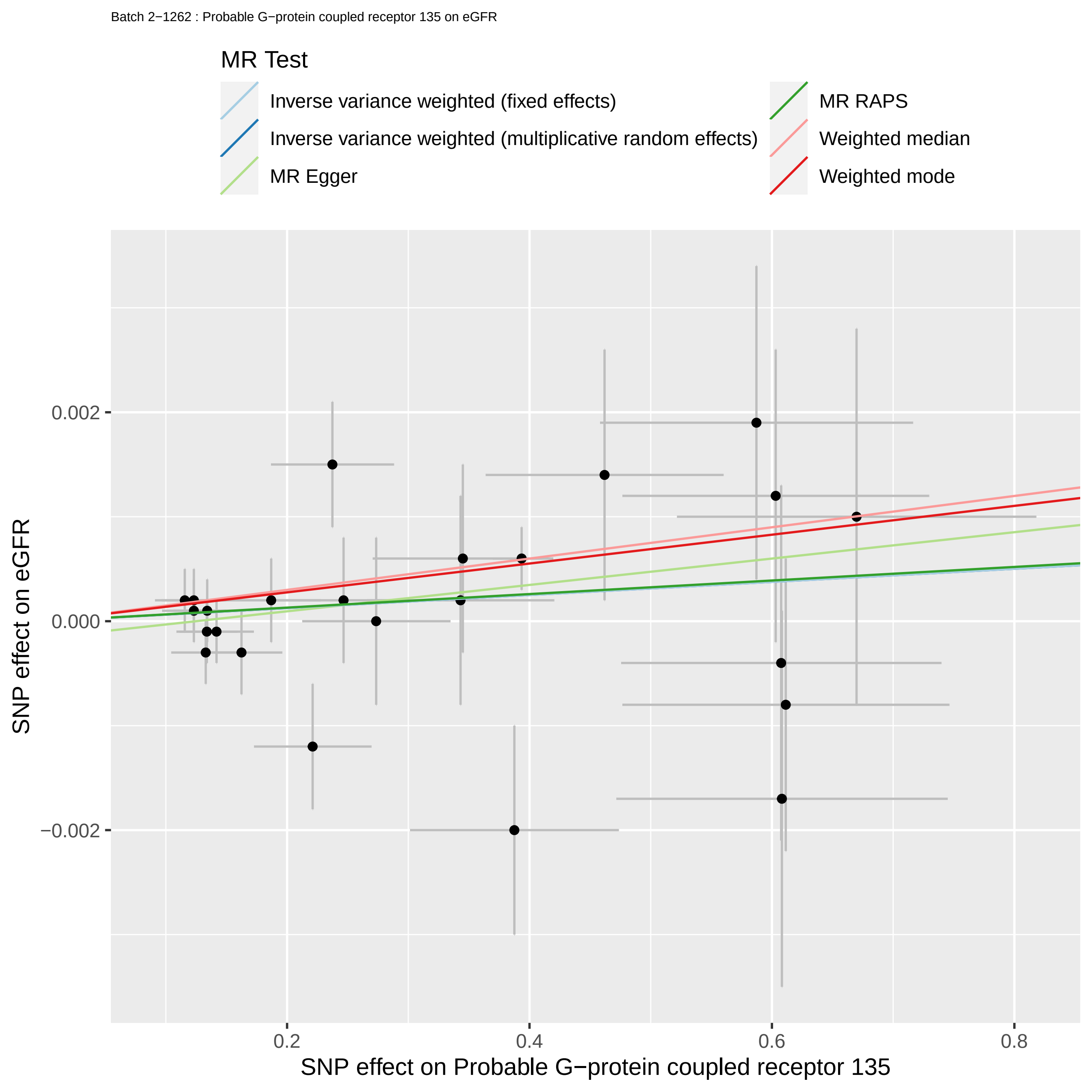

Batch 2−1262 : Probable G−protein coupled receptor 135 on eGFR
MR Test
Inverse variance weighted (fixed effects)
Inverse variance weighted (multiplicative random effects)
MR Egger
MR RAPS
Weighted median
Weighted mode
0.002
0.000
−0.002
0.2
0.4
0.6
0.8
SNP effect on eGFR
SNP effect on Probable G−protein coupled receptor 135

Batch 2−1263 : G−protein coupled receptor 26 on eGFR

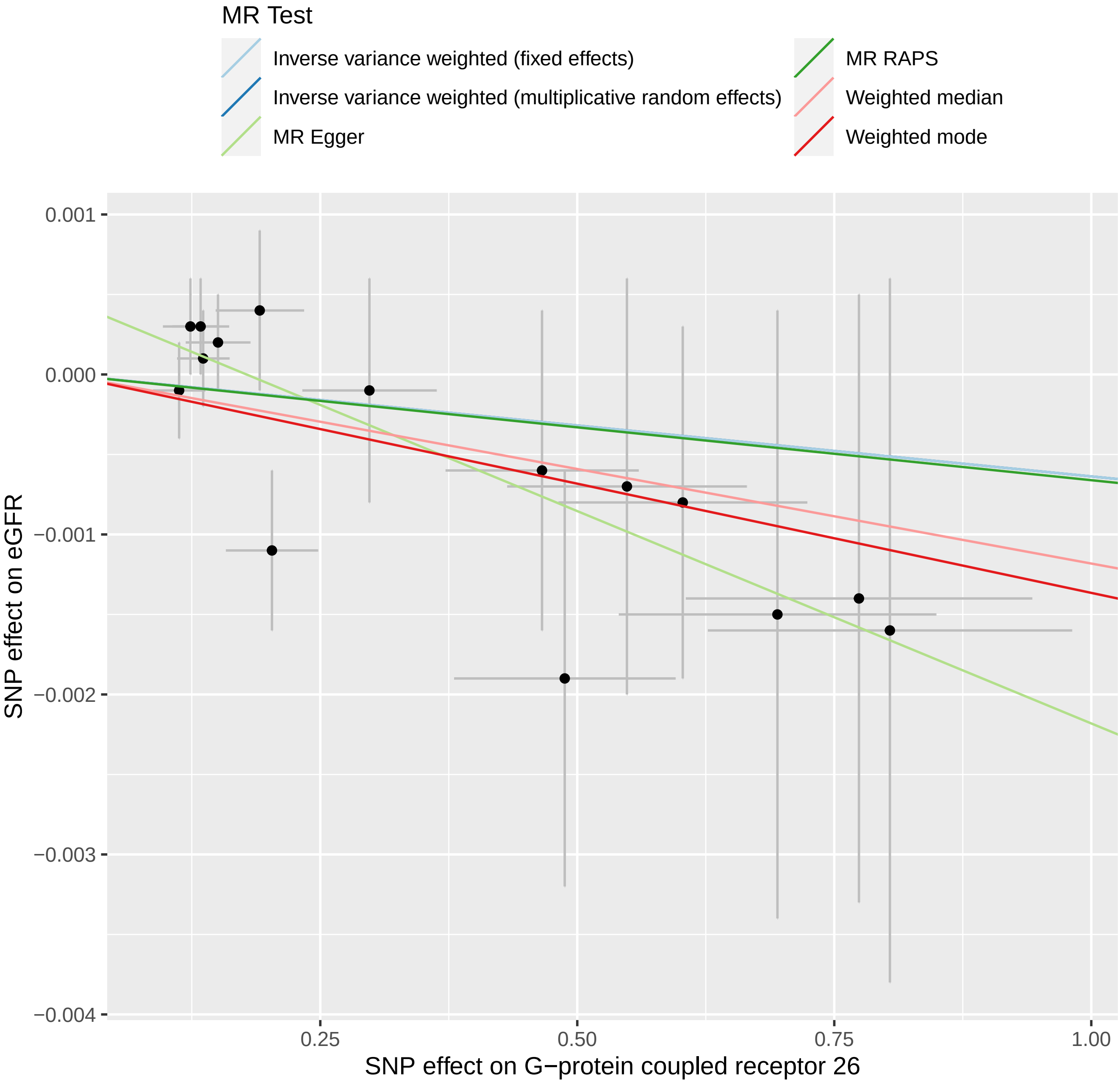

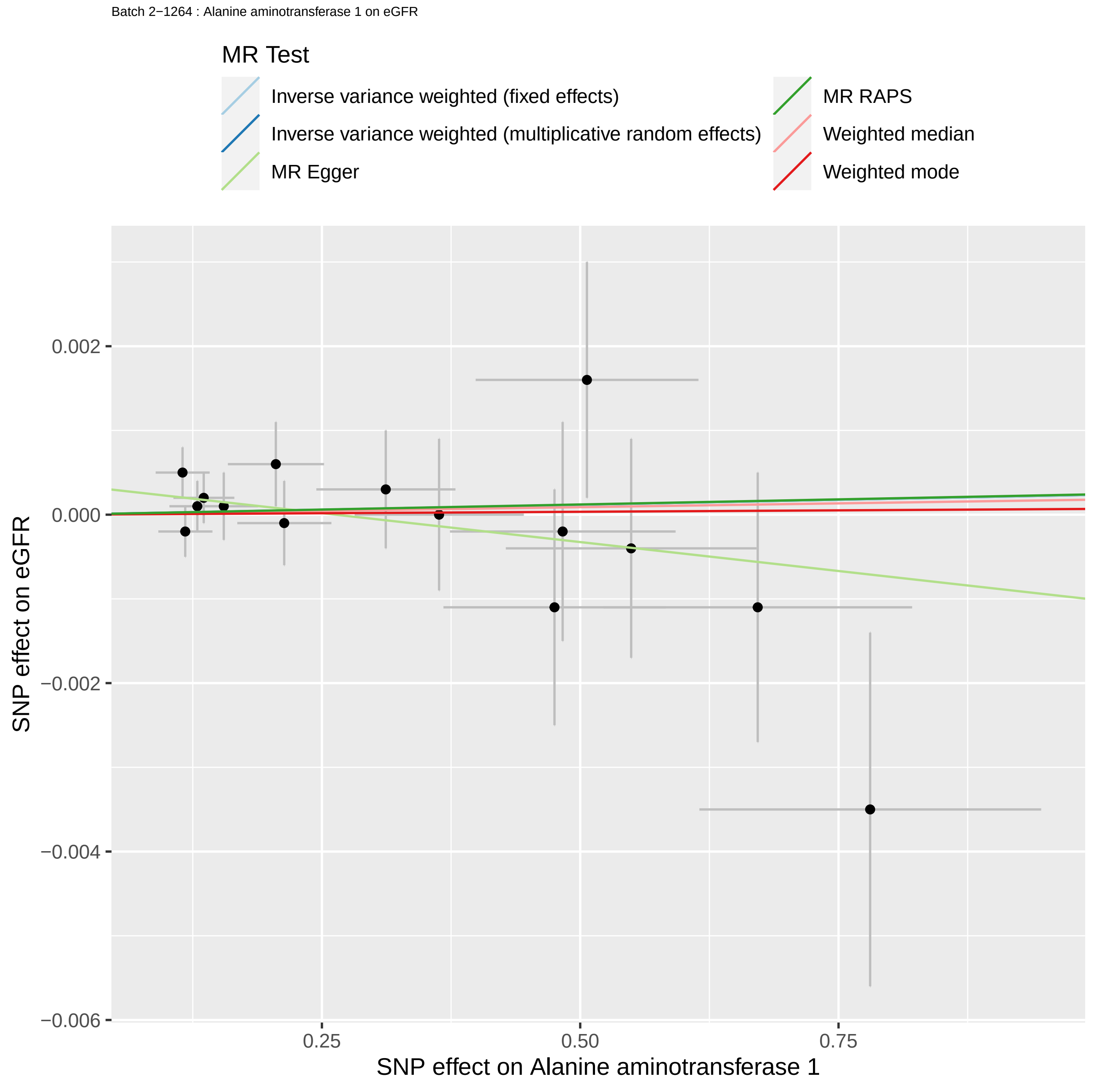
Batch 2−1264 : Alanine aminotransferase 1 on eGFR
MR Test
Inverse variance weighted (fixed effects)
Inverse variance weighted (multiplicative random effects)
MR Egger
MR RAPS
Weighted median
Weighted mode
0.002
0.000
−0.002
−0.004
−0.006
0.25
0.50
0.75
SNP effect on eGFR
SNP effect on Alanine aminotransferase 1

Batch 2−1265 : Glutathione peroxidase 7 on eGFR

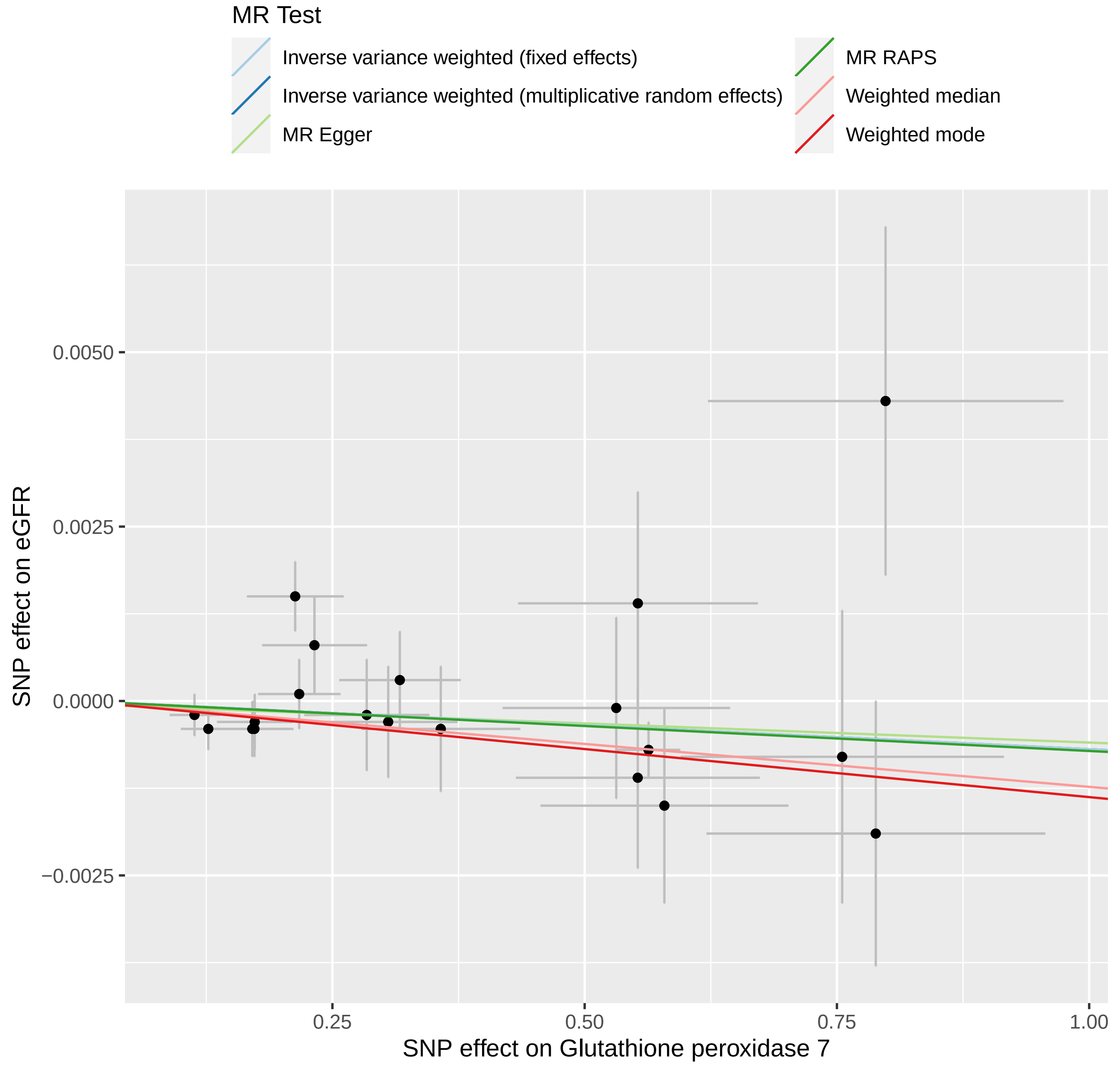

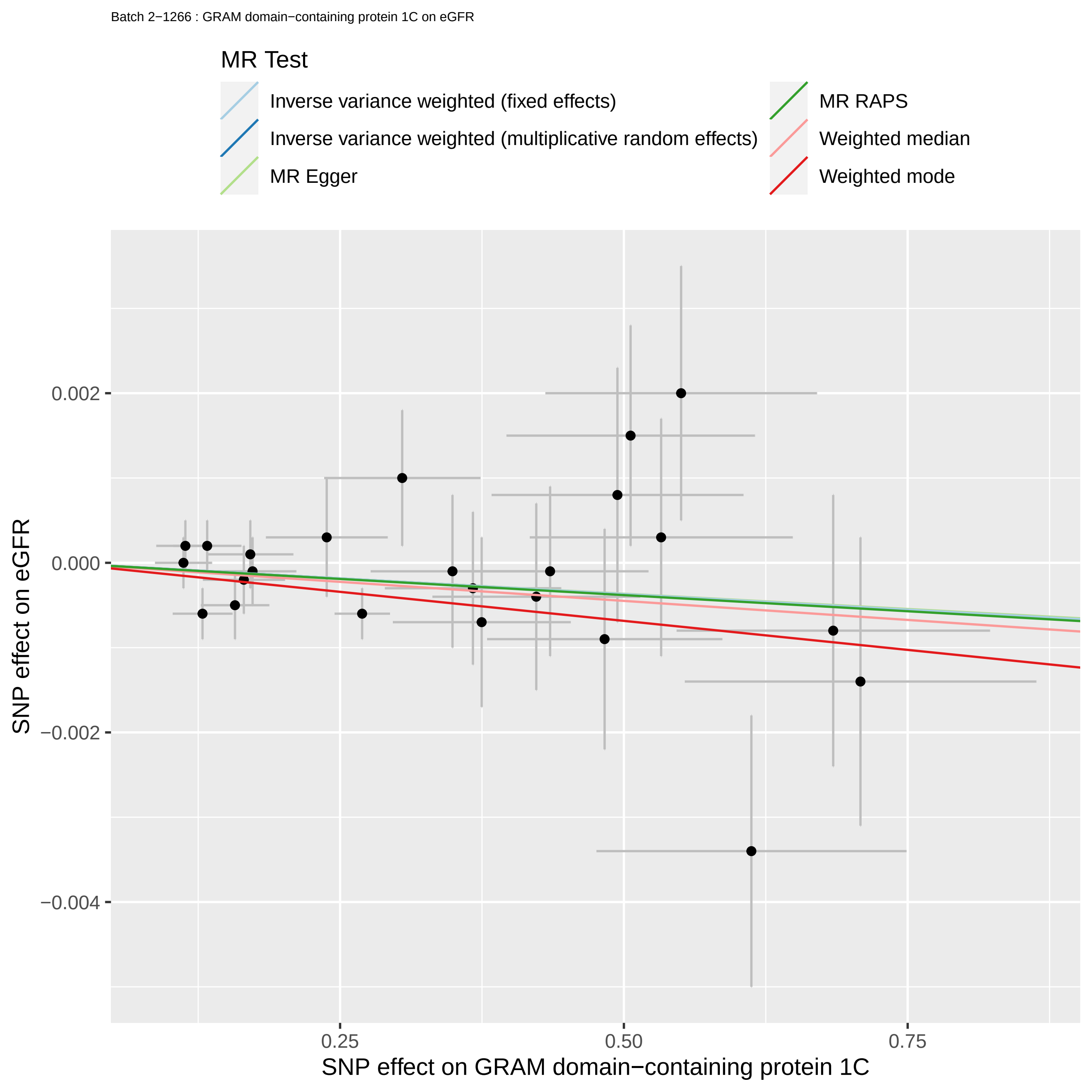

Batch 2−1266 : GRAM domain−containing protein 1C on eGFR
MR Test
Inverse variance weighted (fixed effects)
Inverse variance weighted (multiplicative random effects)
MR Egger
MR RAPS
Weighted median
Weighted mode
0.002
0.000
−0.002
−0.004
SNP effect on eGFR
0.25
0.50
0.75
SNP effect on GRAM domain−containing protein 1C

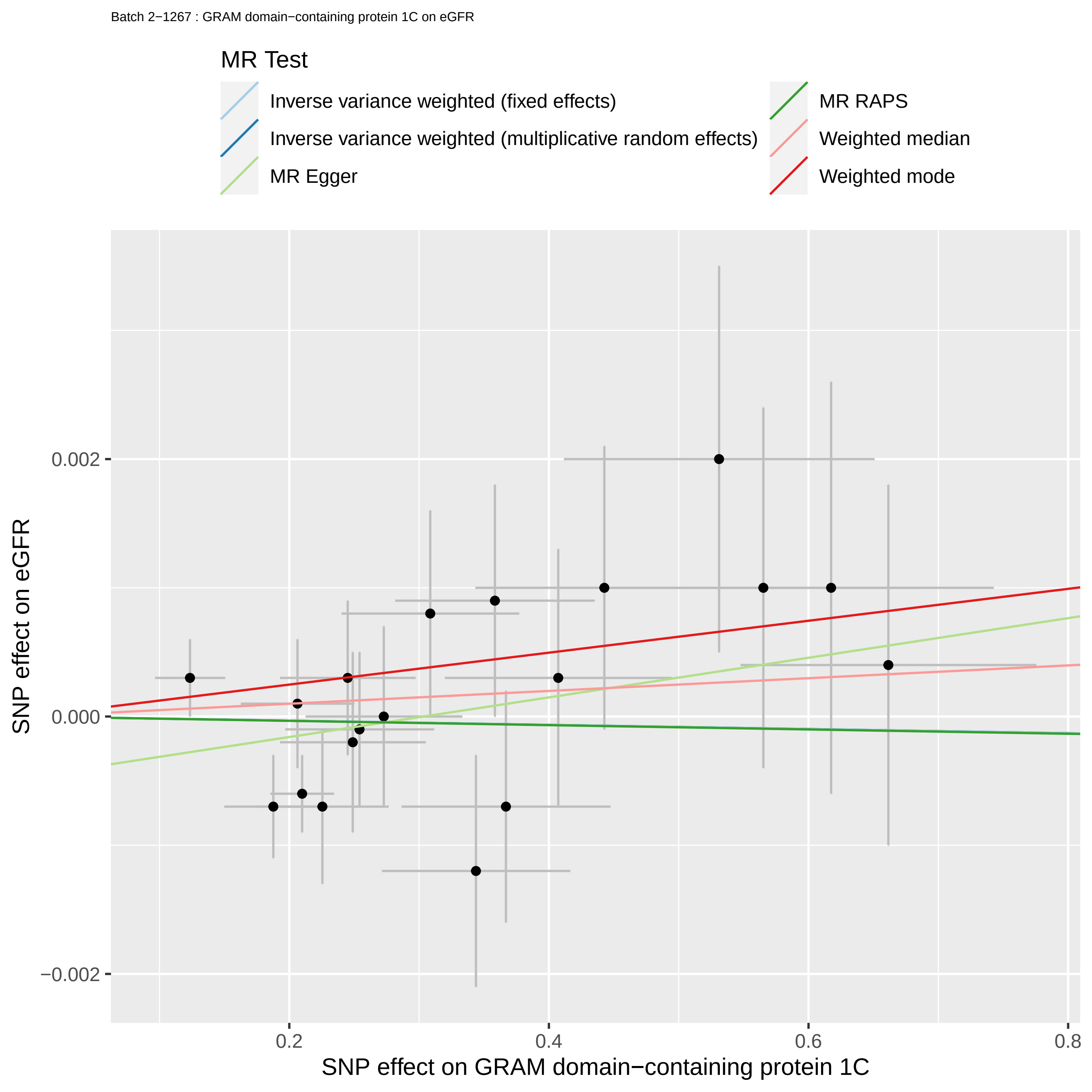
Batch 2−1267 : GRAM domain−containing protein 1C on eGFR
MR Test
Inverse variance weighted (fixed effects)
Inverse variance weighted (multiplicative random effects)
MR Egger
MR RAPS
Weighted median
Weighted mode
0.002
0.000
−0.002
0.2
0.4
0.6
0.8
SNP effect on eGFR
SNP effect on GRAM domain−containing protein 1C

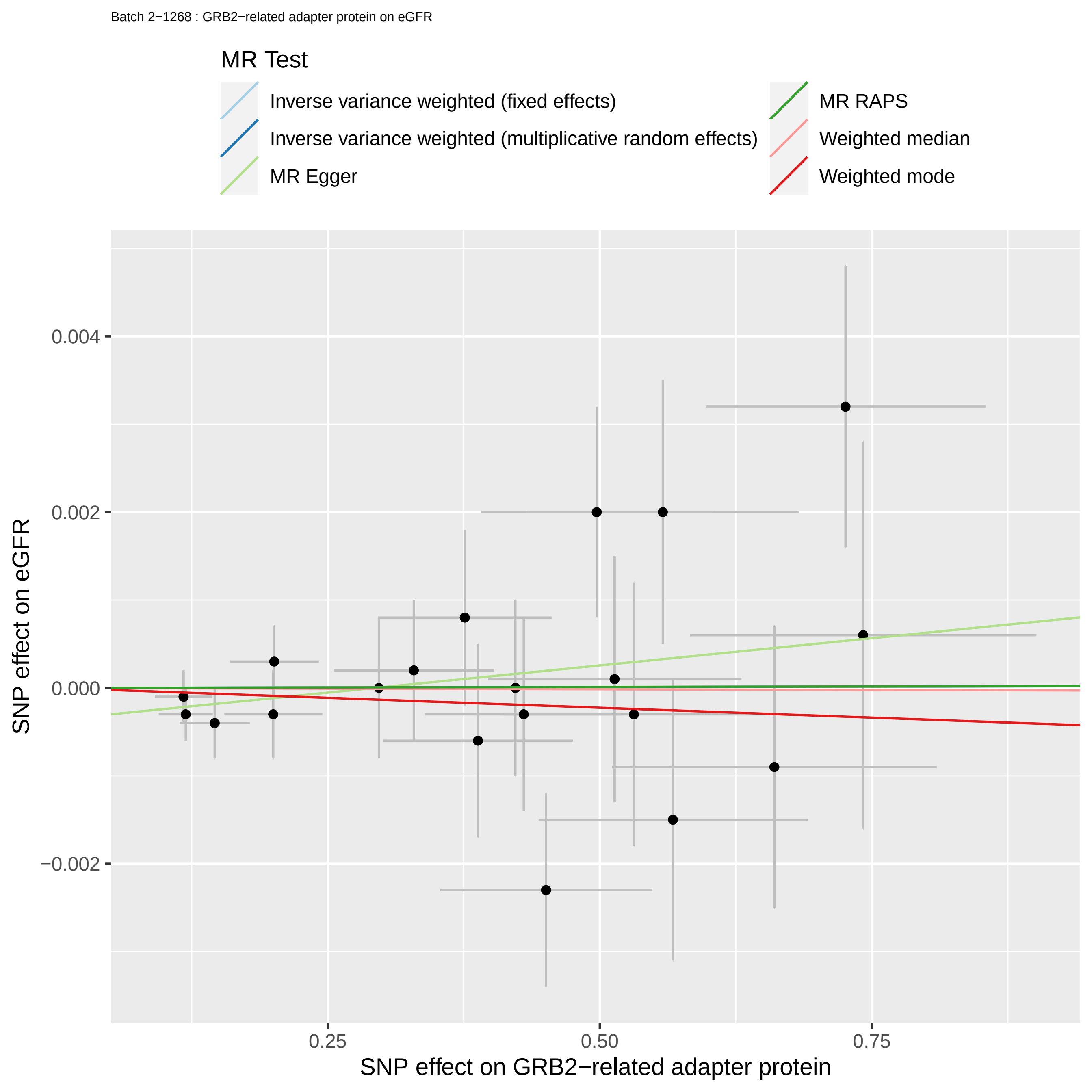

Batch 2−1268 : GRB2−related adapter protein on eGFR
MR Test
Inverse variance weighted (fixed effects)
Inverse variance weighted (multiplicative random effects)
MR Egger
MR RAPS
Weighted median
Weighted mode
0.004
0.002
0.000
−0.002
0.25
0.50
0.75
SNP effect on eGFR
SNP effect on GRB2−related adapter protein

Batch 2−1269 : GRB2−related adapter protein 2 on eGFR

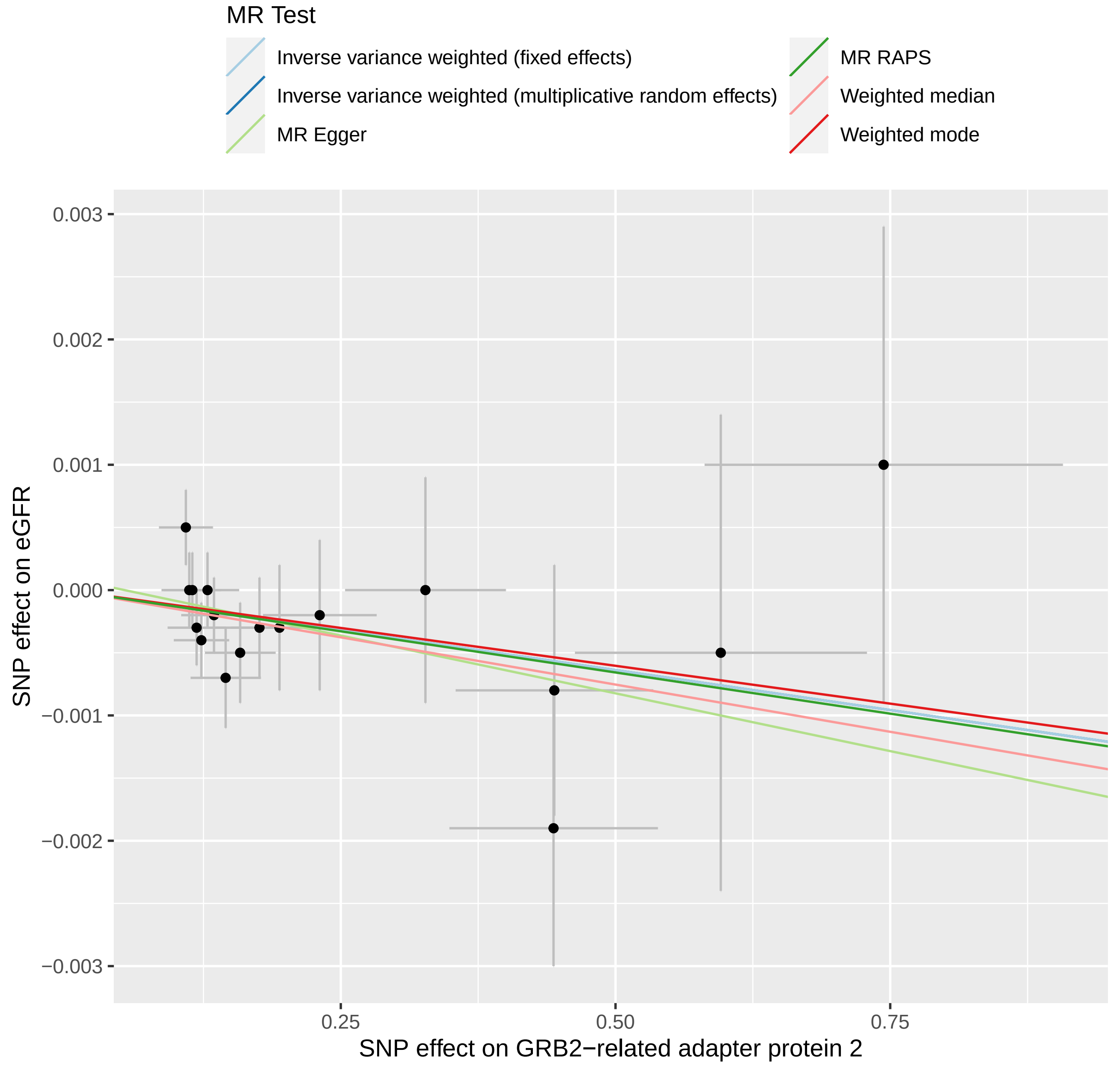

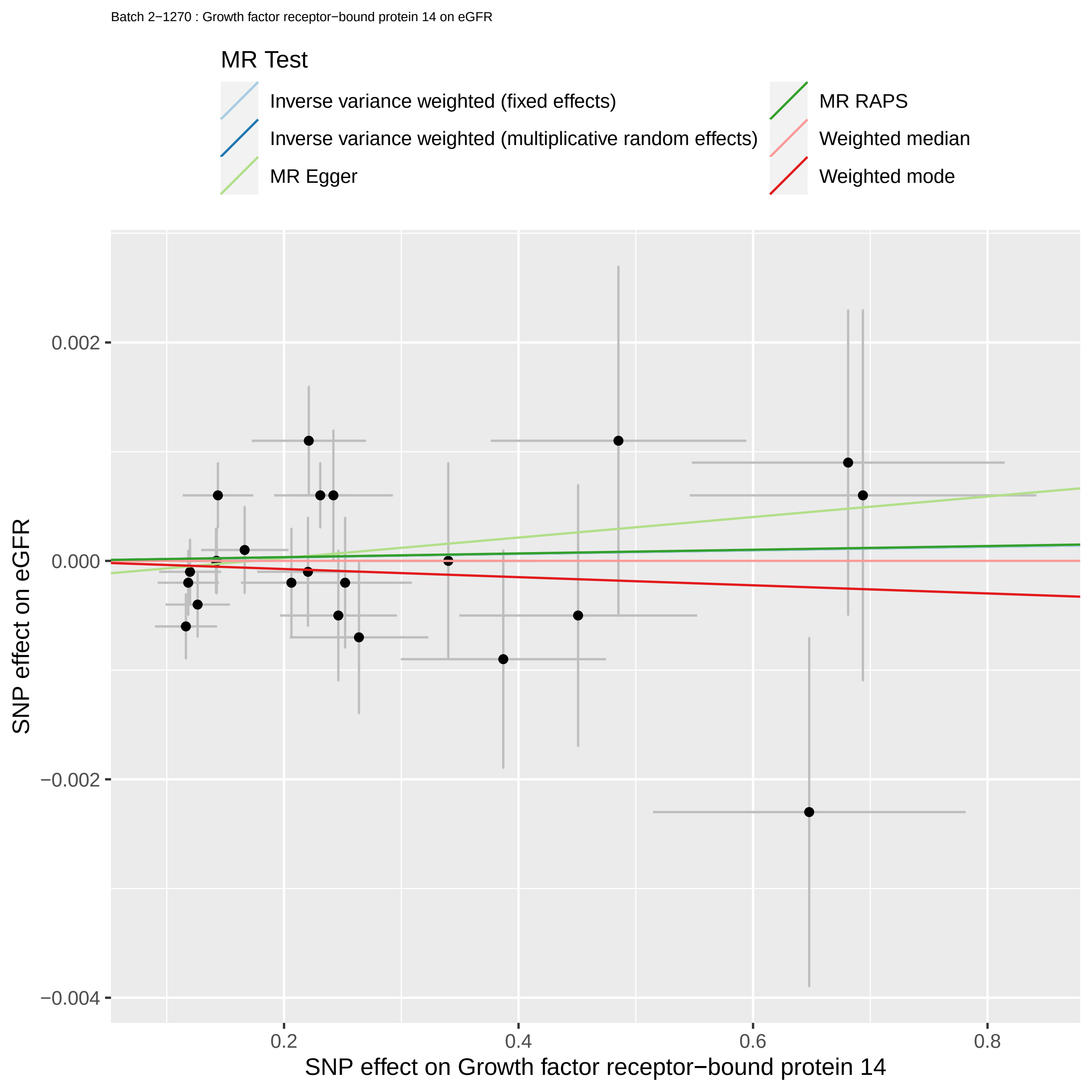

Batch 2−1270 : Growth factor receptor−bound protein 14 on eGFR
MR Test
Inverse variance weighted (fixed effects)
Inverse variance weighted (multiplicative random effects)
MR Egger
MR RAPS
Weighted median
Weighted mode
0.002
0.000
−0.002
−0.004
SNP effect on eGFR
0.2
0.4
0.6
0.8
SNP effect on Growth factor receptor−bound protein 14

Batch 2−1271 : Growth factor receptor−bound protein 2 on eGFR

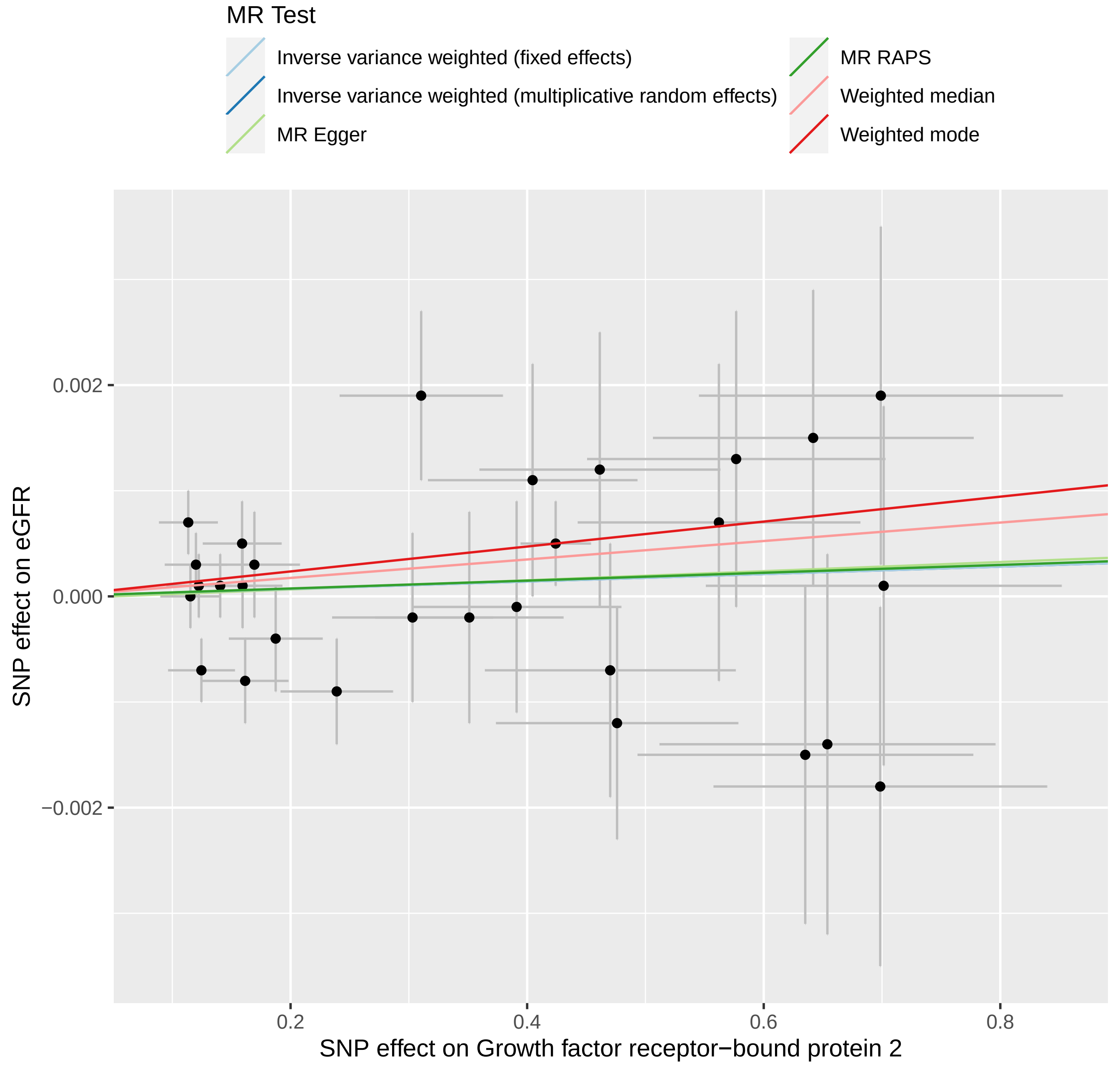

Batch 2−1272 : Growth factor receptor−bound protein 7 on eGFR

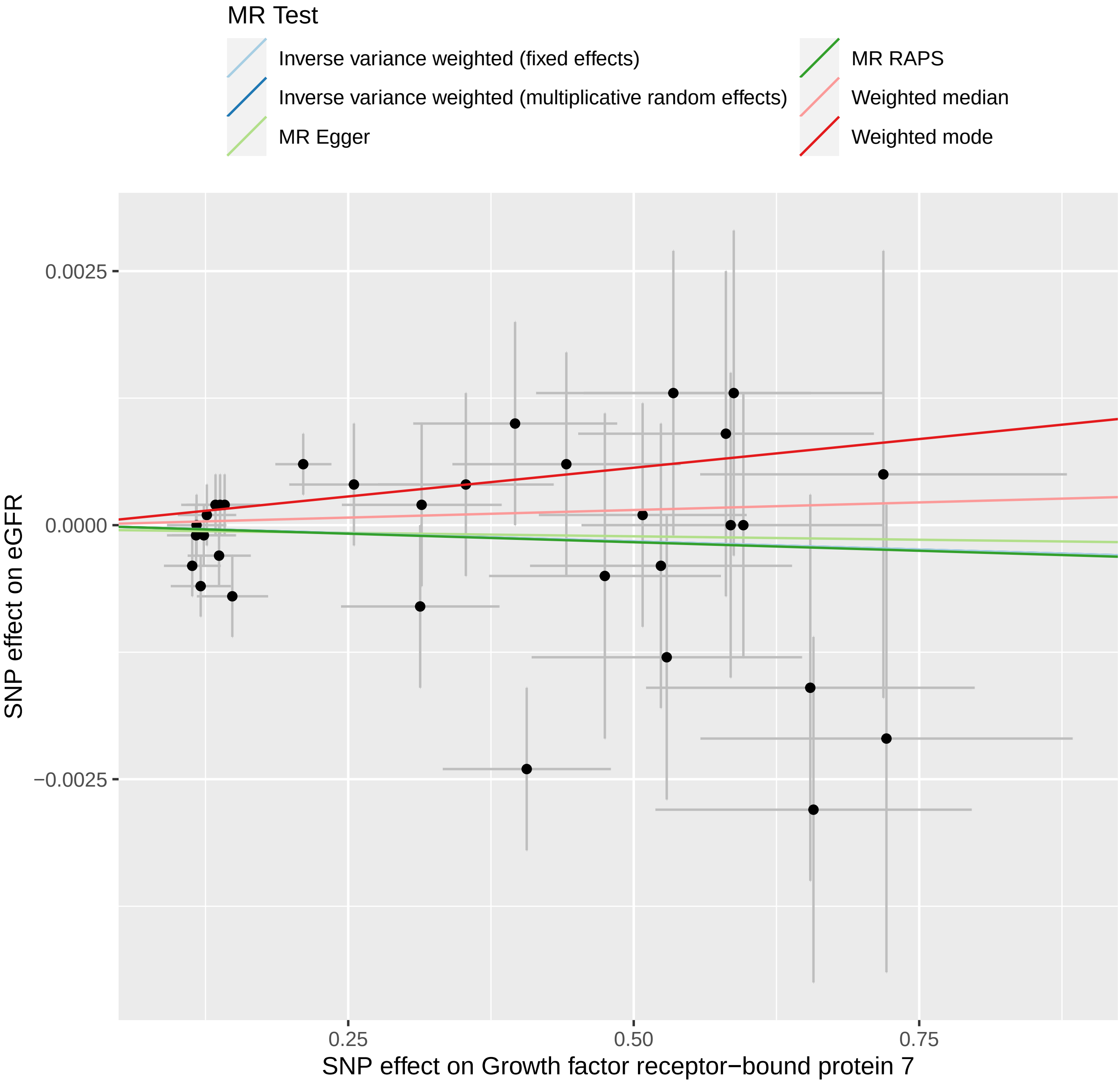

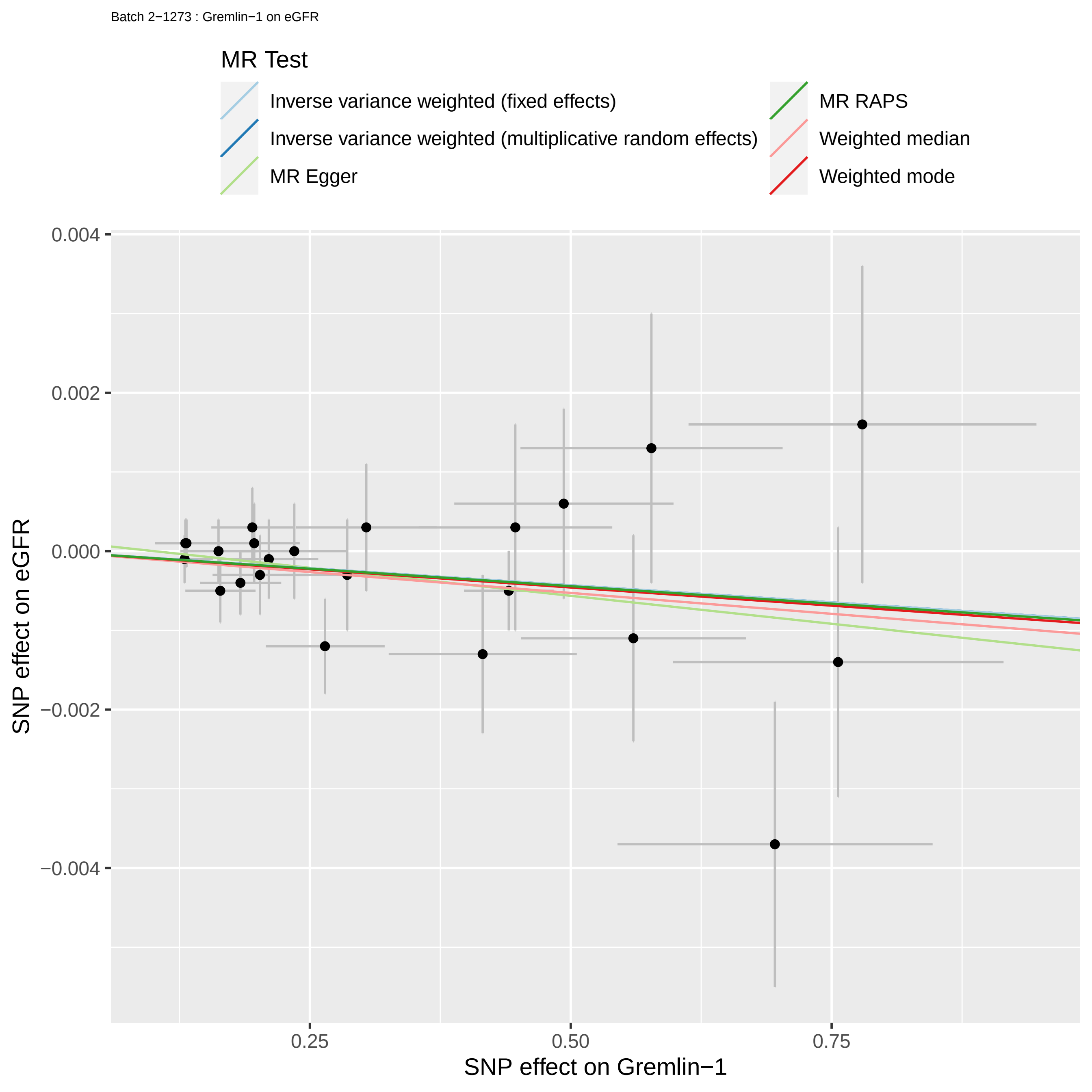
Batch 2−1273 : Gremlin−1 on eGFR
MR Test
Inverse variance weighted (fixed effects)
Inverse variance weighted (multiplicative random effects)
MR Egger
MR RAPS
Weighted median
Weighted mode
0.004
0.002
0.000
−0.002
−0.004
SNP effect on eGFR
0.25
0.50
0.75
SNP effect on Gremlin−1

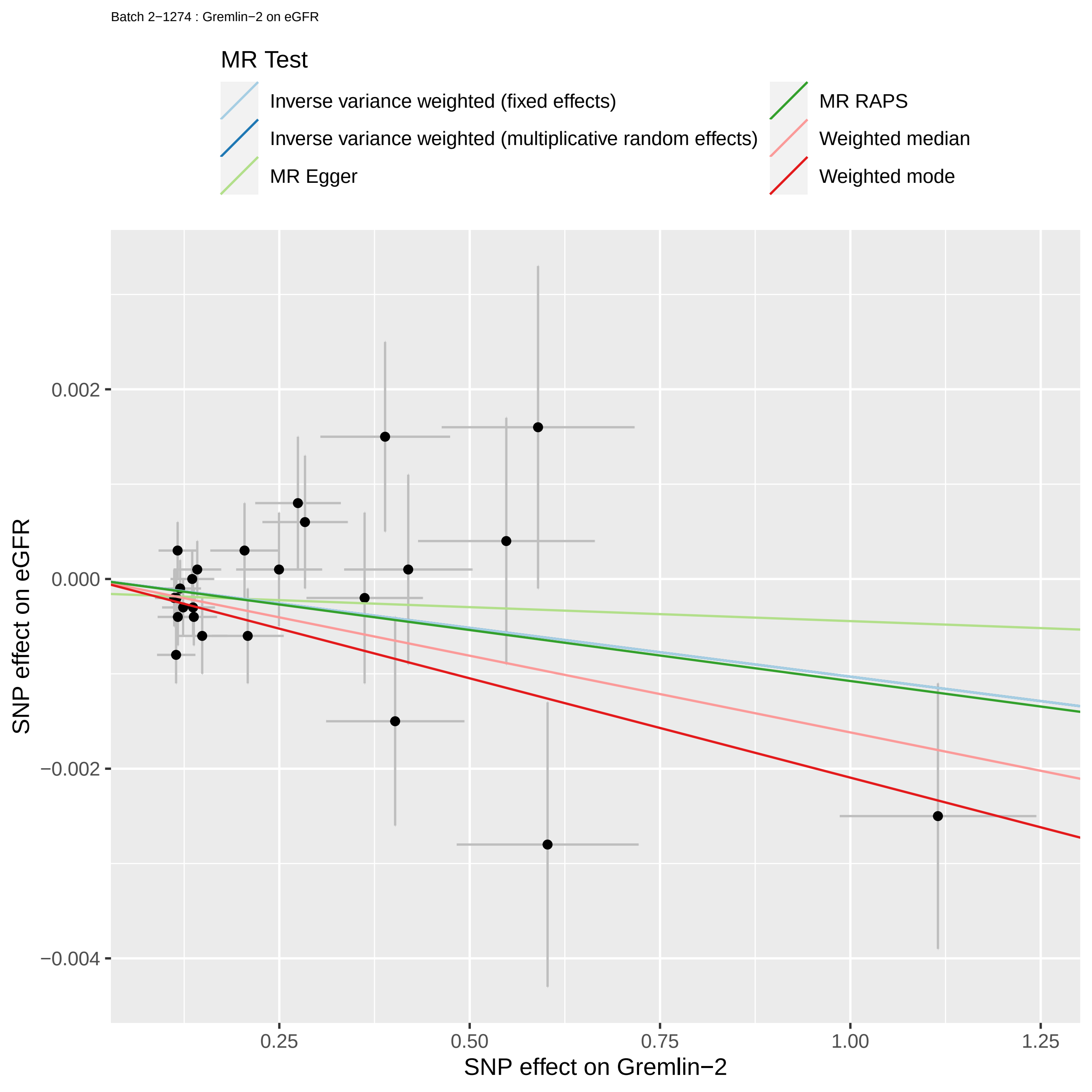
Batch 2−1274 : Gremlin−2 on eGFR
MR Test
Inverse variance weighted (fixed effects)
Inverse variance weighted (multiplicative random effects)
MR Egger
MR RAPS
Weighted median
Weighted mode
0.002
0.000
−0.002
−0.004
SNP effect on eGFR
0.25
0.50
0.75
1.00
1.25
SNP effect on Gremlin−2

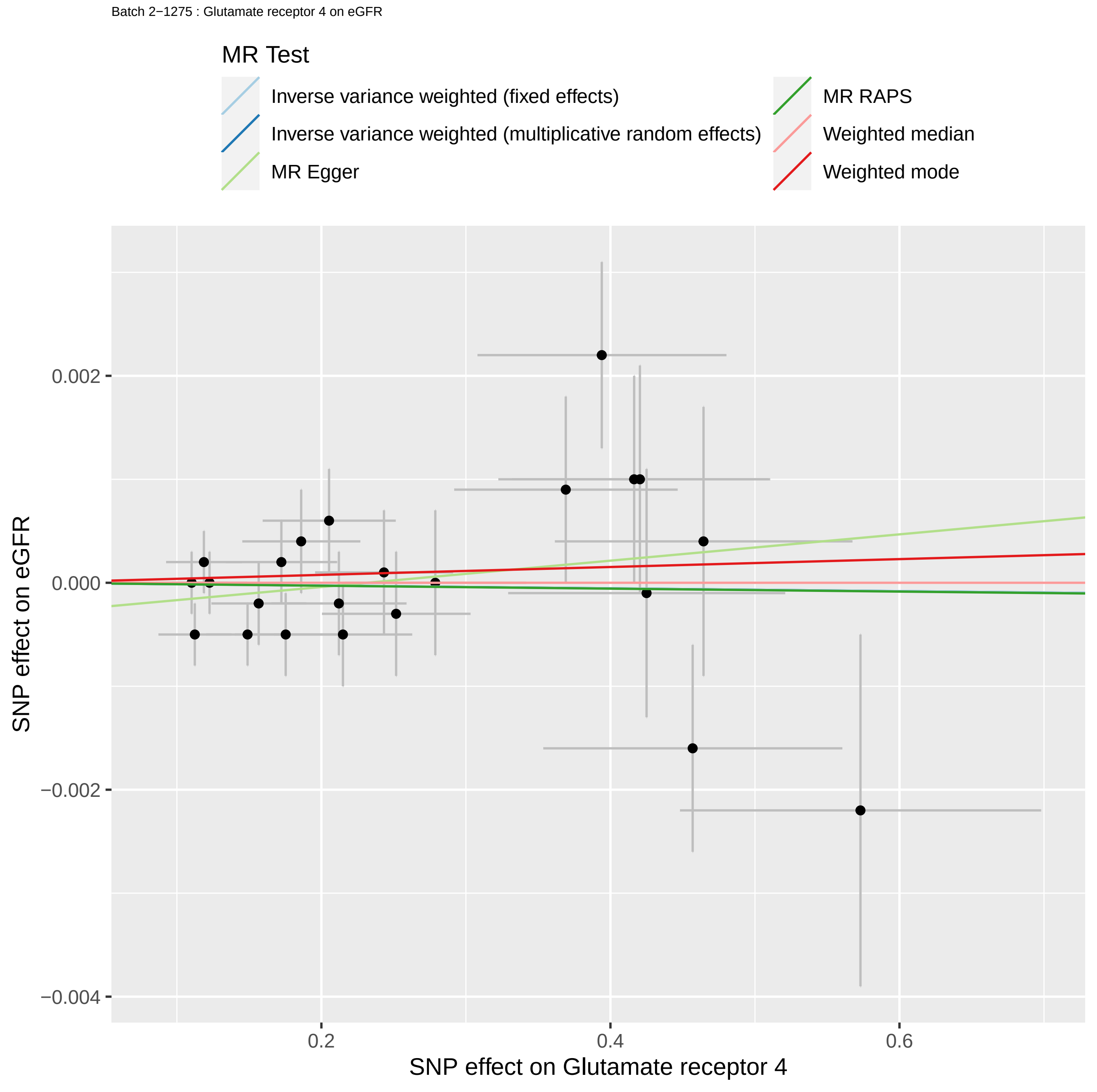
Batch 2−1275 : Glutamate receptor 4 on eGFR
MR Test
Inverse variance weighted (fixed effects)
Inverse variance weighted (multiplicative random effects)
MR Egger
MR RAPS
Weighted median
Weighted mode
0.002
0.000
−0.002
−0.004
0.2
0.4
0.6
SNP effect on eGFR
SNP effect on Glutamate receptor 4

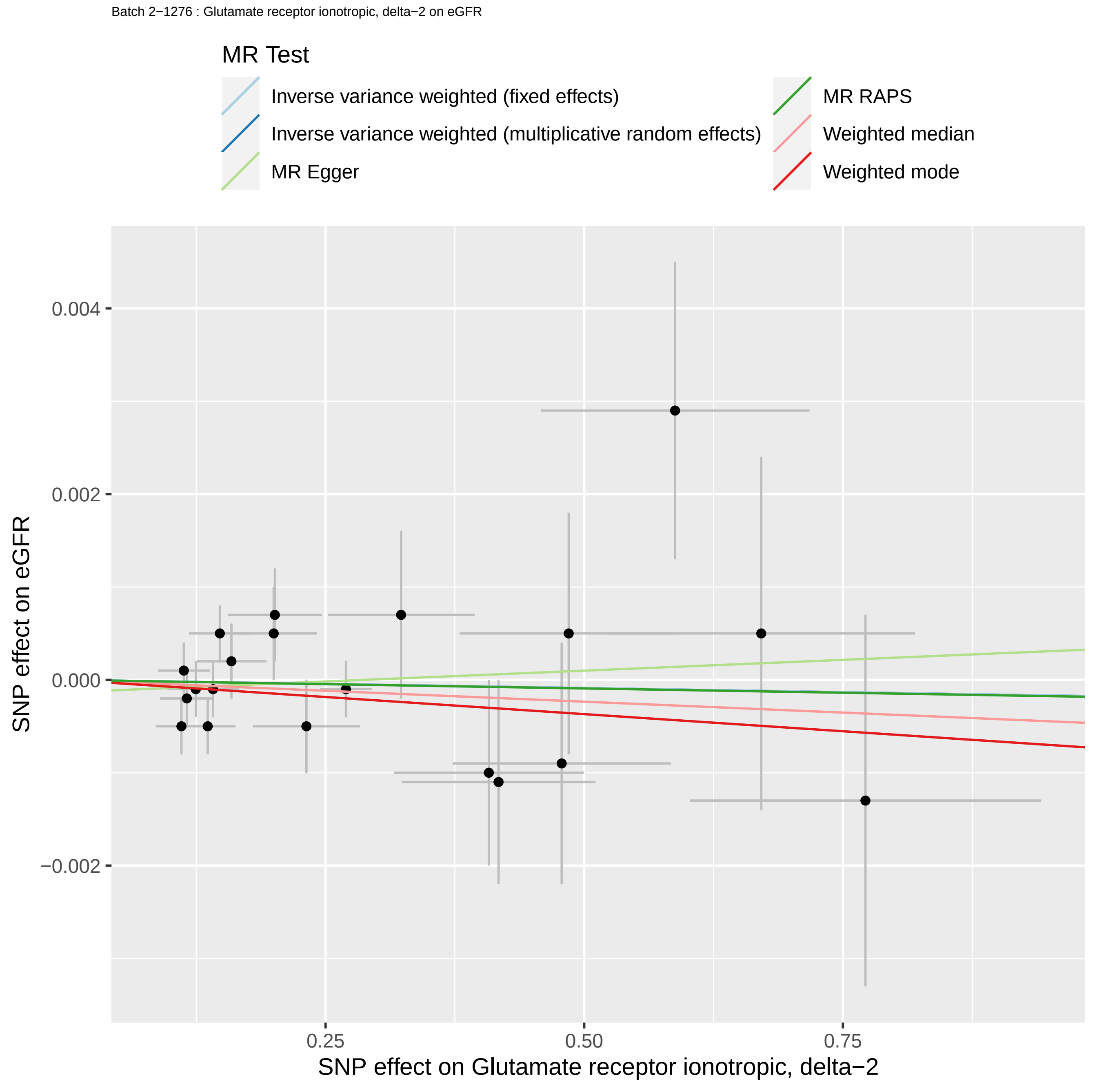
Batch 2−1276 : Glutamate receptor ionotropic, delta−2 on eGFR
MR Test
Inverse variance weighted (fixed effects)
Inverse variance weighted (multiplicative random effects)
MR Egger
MR RAPS
Weighted median
Weighted mode
0.004
0.002
0.000
−0.002
0.25
0.50
0.75
SNP effect on eGFR
SNP effect on Glutamate receptor ionotropic, delta−2

Batch 2−1277 : Granulins on eGFR

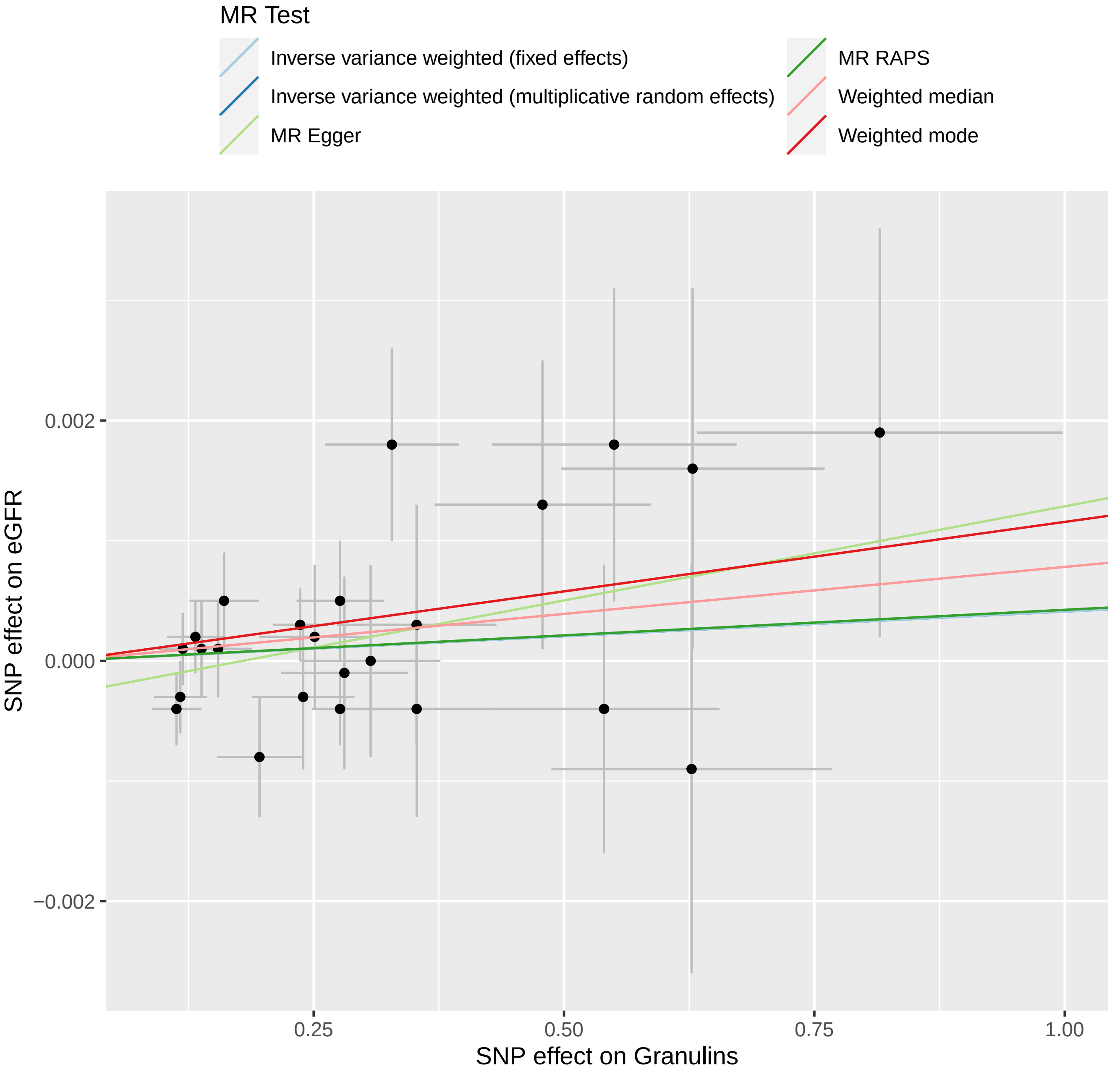

Batch 2−1278 : Gastrin−releasing peptide on eGFR

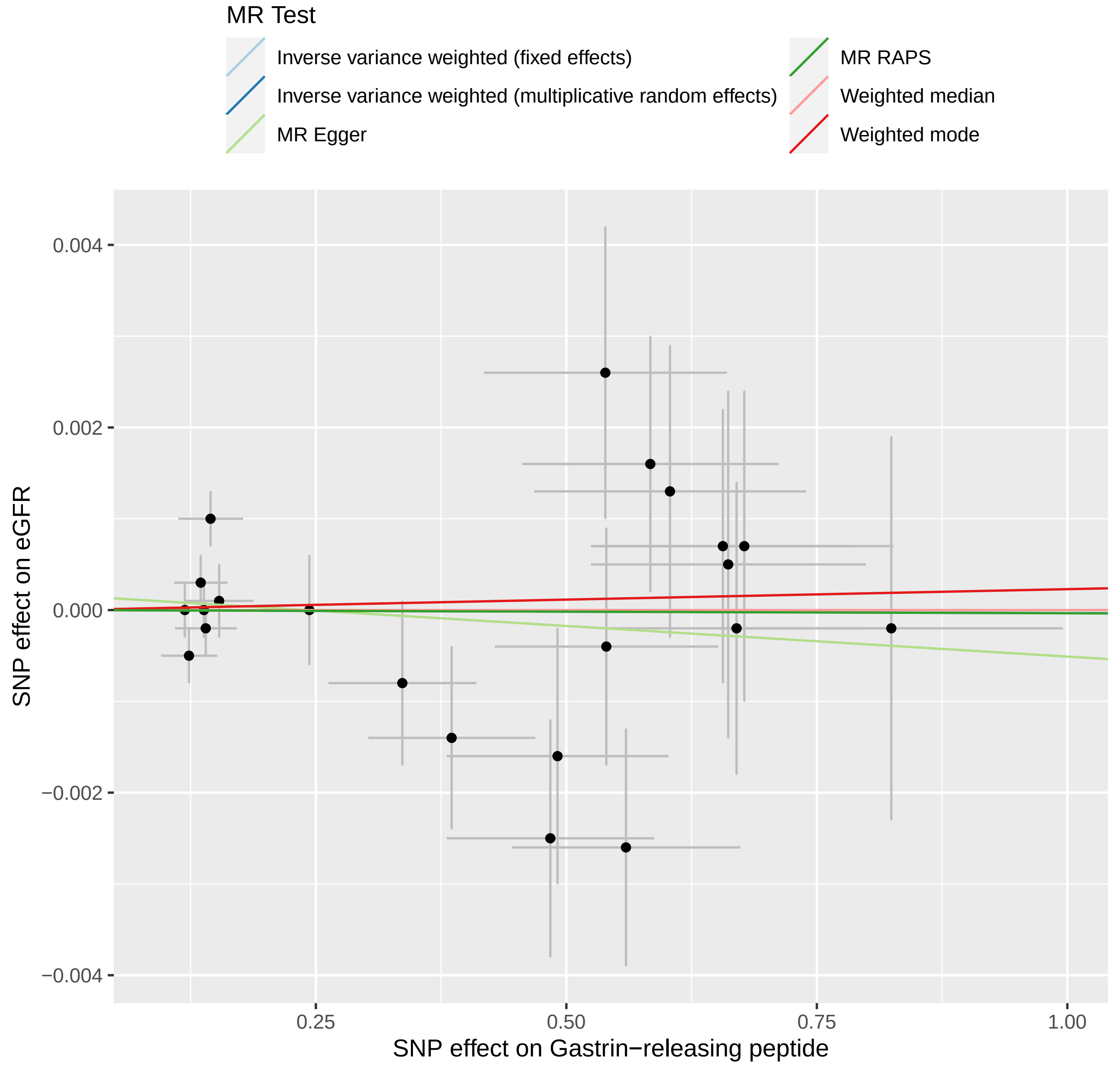

Batch 2−1279 : Gastrin−releasing peptide on eGFR

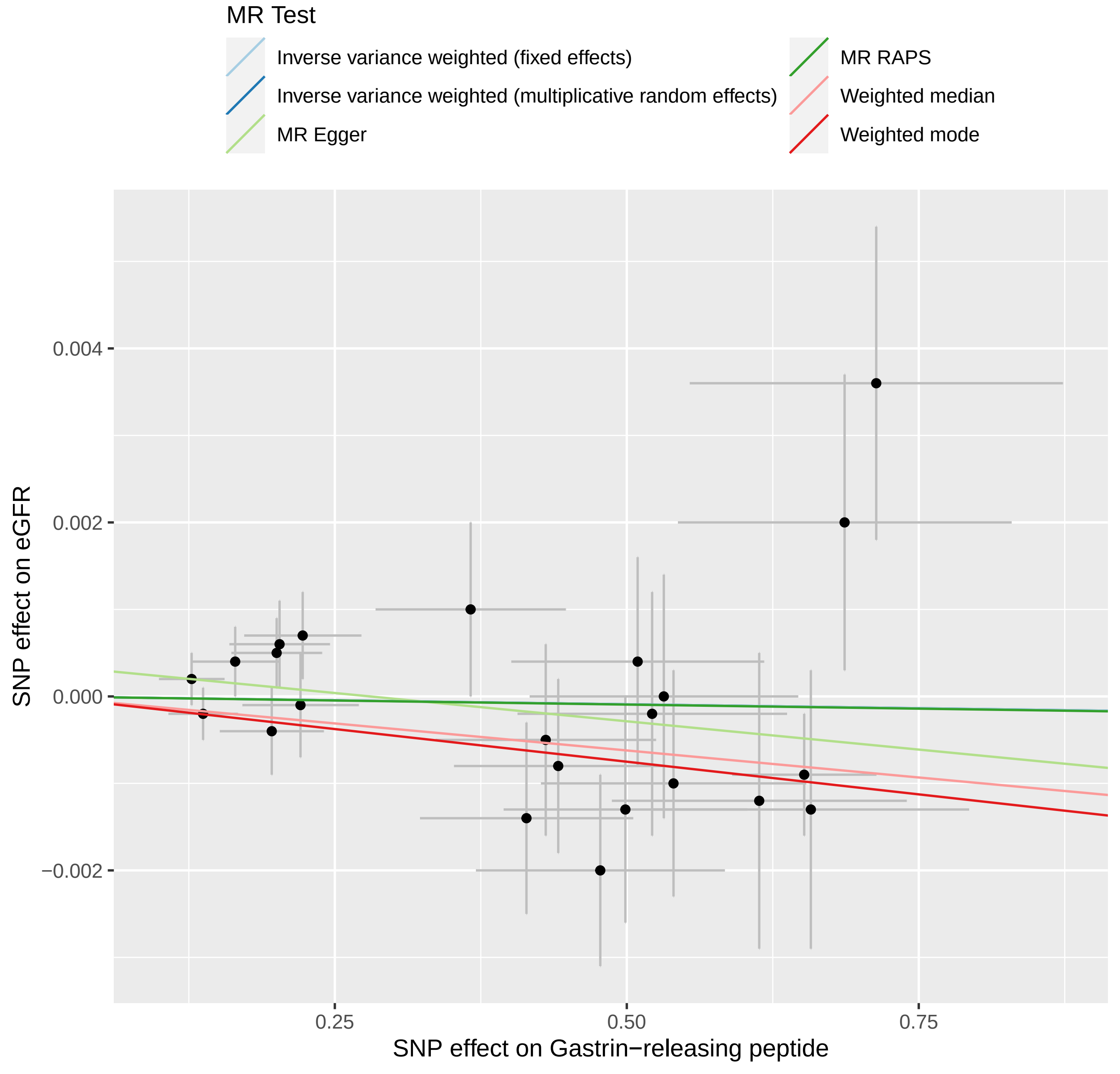

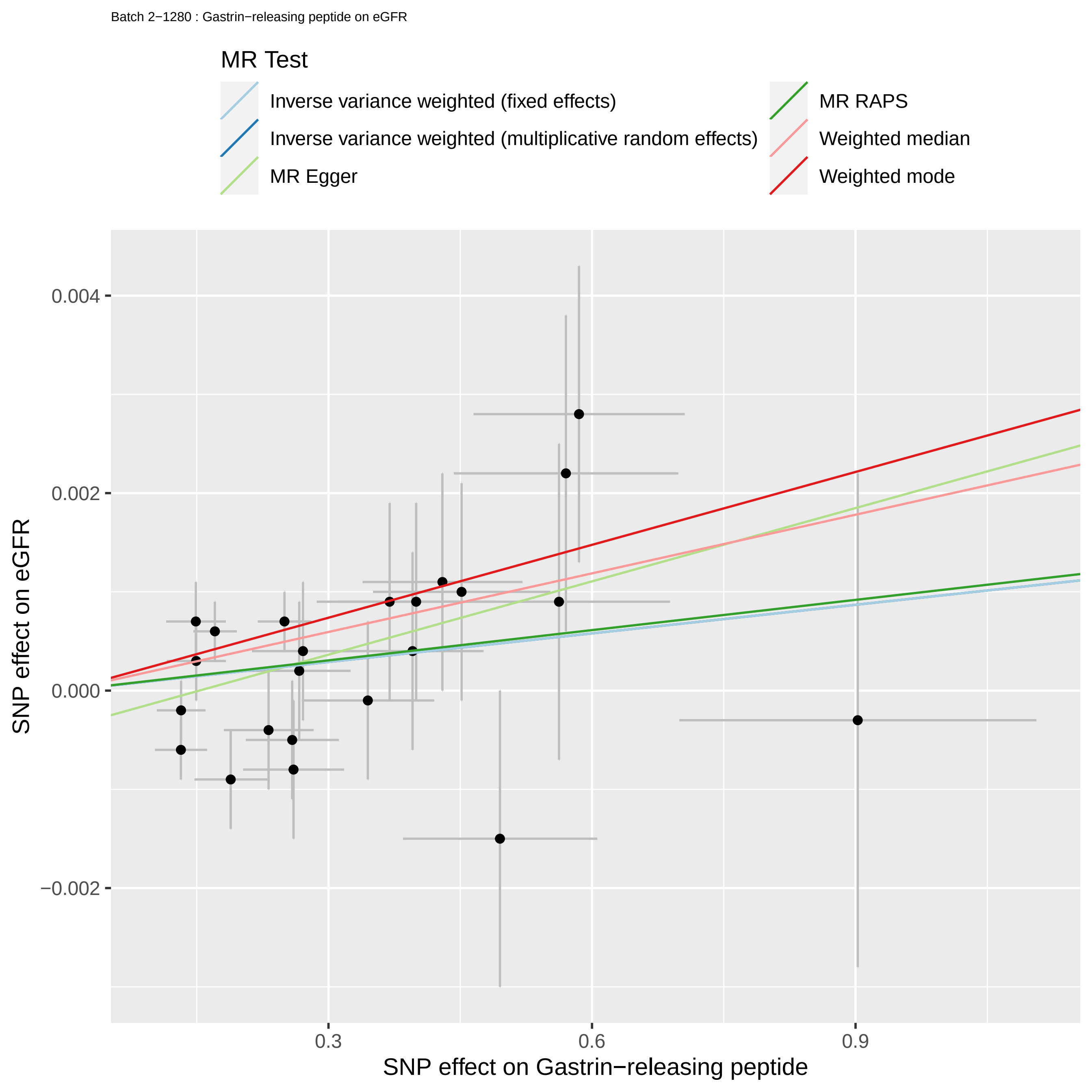
Batch 2−1280 : Gastrin−releasing peptide on eGFR
MR Test
Inverse variance weighted (fixed effects)
Inverse variance weighted (multiplicative random effects)
MR Egger
MR RAPS
Weighted median
Weighted mode
0.004
0.002
0.000
−0.002
0.3
0.6
0.9
SNP effect on eGFR
SNP effect on Gastrin−releasing peptide

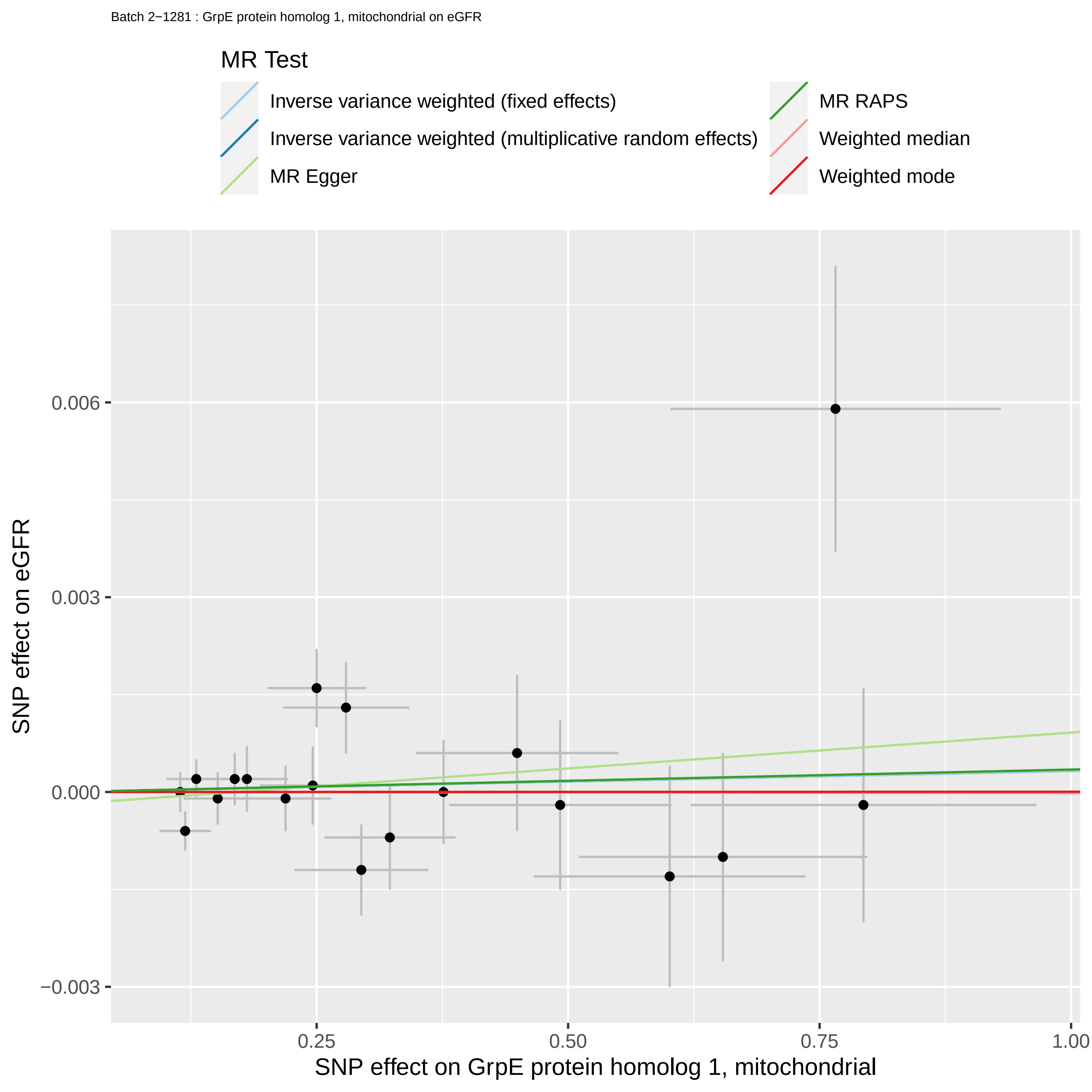
Batch 2−1281 : GrpE protein homolog 1, mitochondrial on eGFR
MR Test
Inverse variance weighted (fixed effects)
Inverse variance weighted (multiplicative random effects)
MR Egger
MR RAPS
Weighted median
Weighted mode
0.006
0.003
0.000
−0.003
SNP effect on eGFR
0.25
0.50
0.75
1.00
SNP effect on GrpE protein homolog 1, mitochondrial

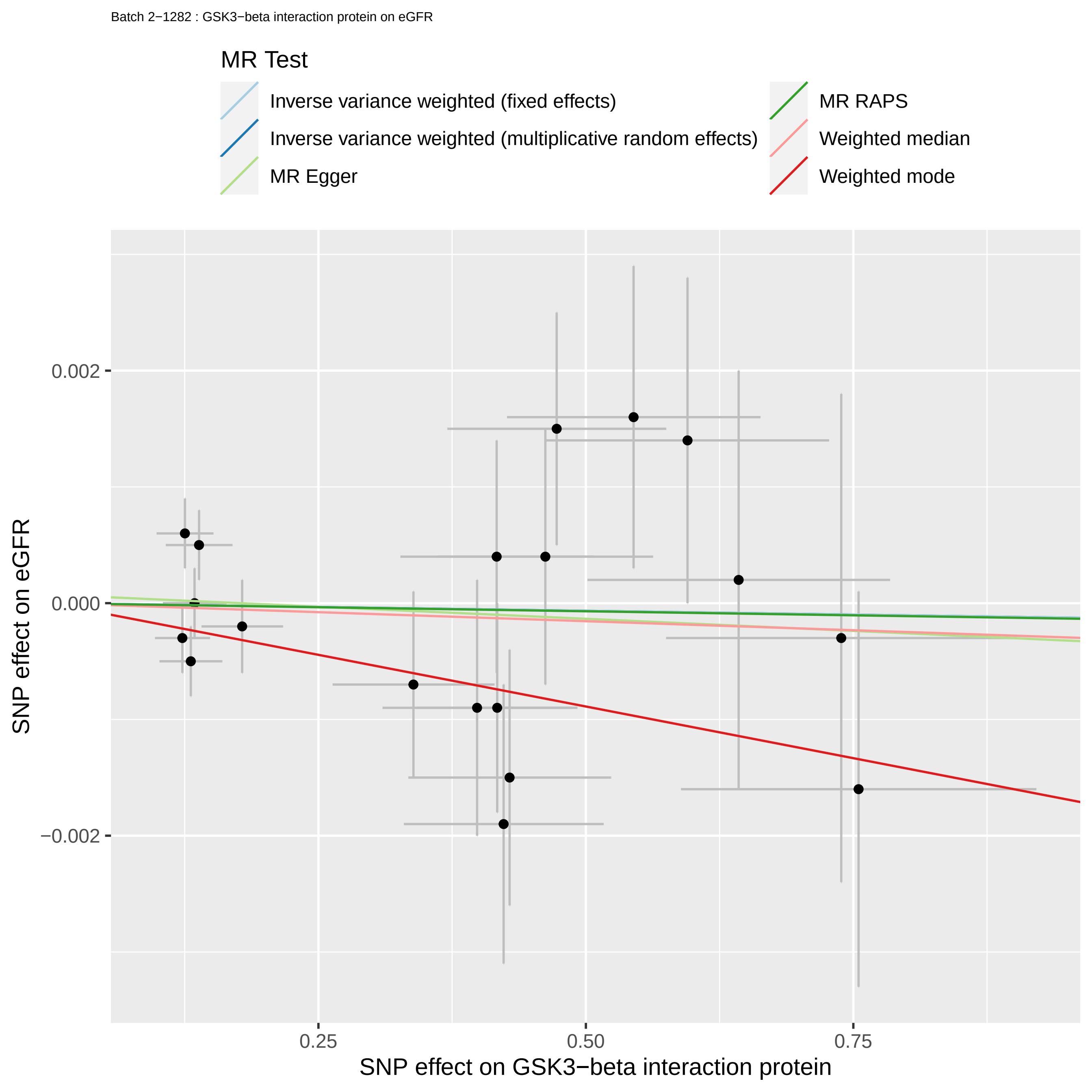
Batch 2−1282 : GSK3−beta interaction protein on eGFR
MR Test
Inverse variance weighted (fixed effects)
Inverse variance weighted (multiplicative random effects)
MR Egger
MR RAPS
Weighted median
Weighted mode
0.002
0.000
−0.002
0.25
0.50
0.75
SNP effect on eGFR
SNP effect on GSK3−beta interaction protein

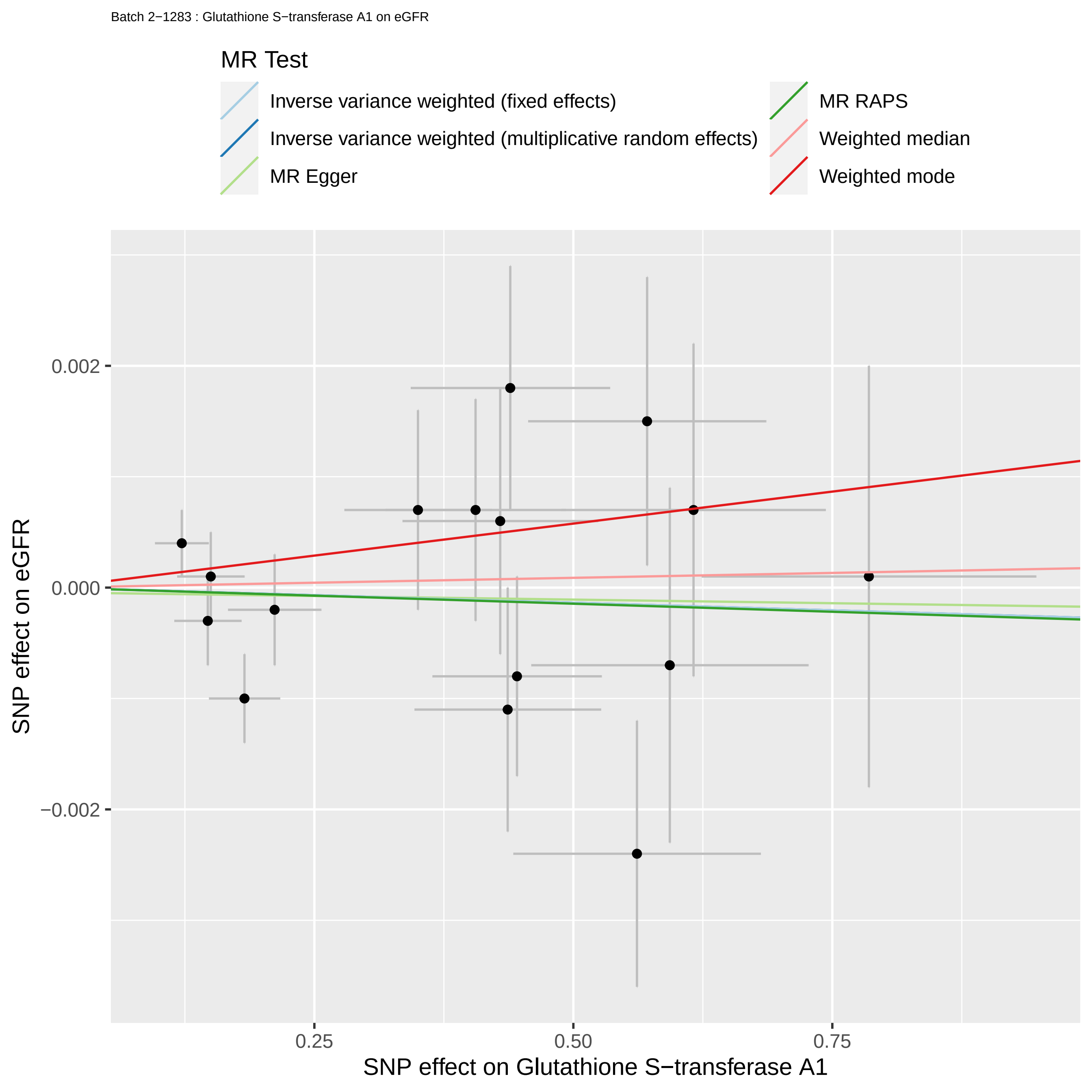

Batch 2−1283 : Glutathione S−transferase A1 on eGFR
MR Test
Inverse variance weighted (fixed effects)
Inverse variance weighted (multiplicative random effects)
MR Egger
MR RAPS
Weighted median
Weighted mode
0.002
0.000
−0.002
0.25
0.50
0.75
SNP effect on eGFR
SNP effect on Glutathione S−transferase A1

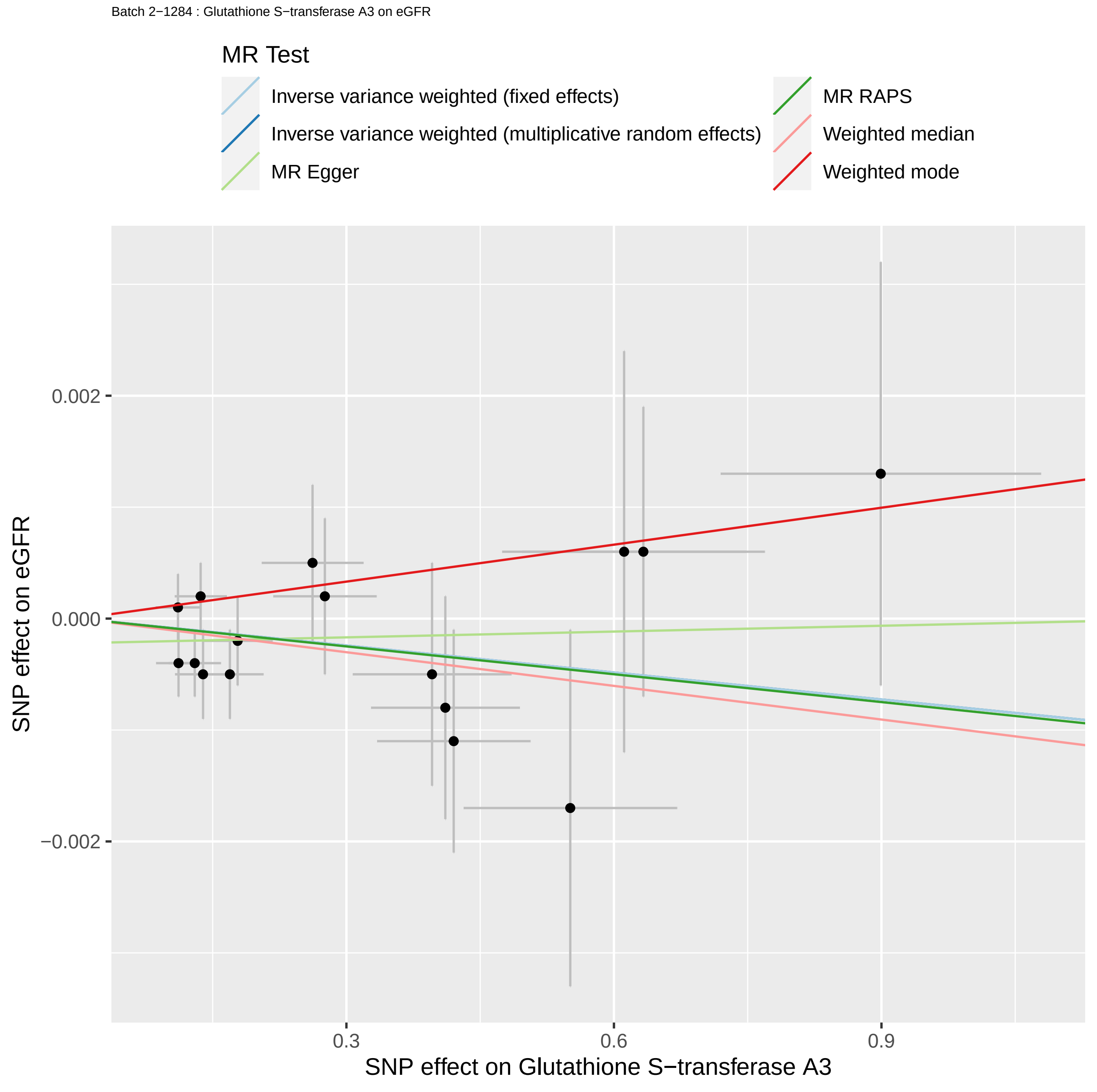

Batch 2−1284 : Glutathione S−transferase A3 on eGFR
MR Test
Inverse variance weighted (fixed effects)
Inverse variance weighted (multiplicative random effects)
MR Egger
MR RAPS
Weighted median
Weighted mode
0.002
0.000
−0.002
SNP effect on eGFR
0.3
0.6
0.9
SNP effect on Glutathione S−transferase A3

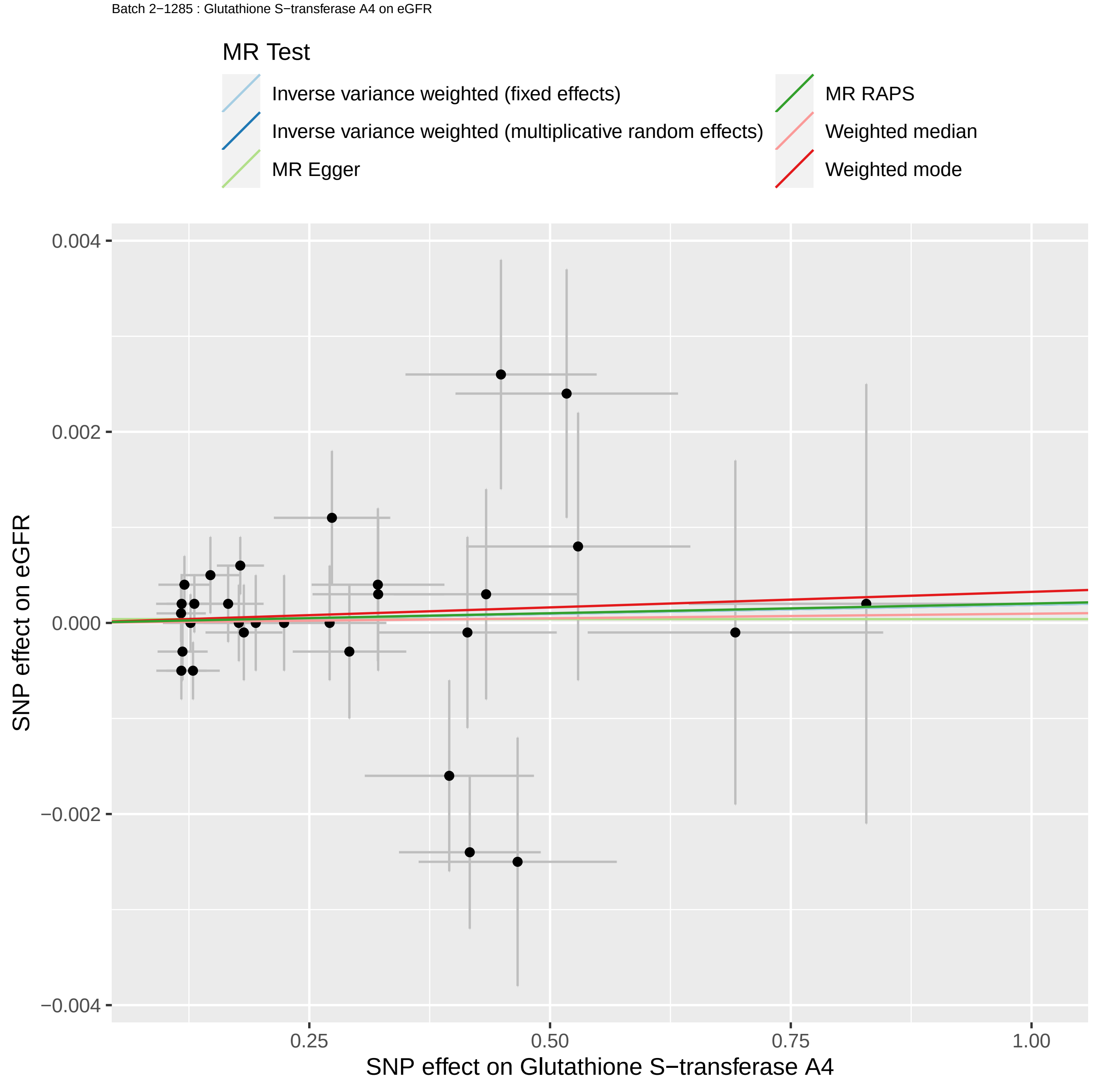
Batch 2−1285 : Glutathione S−transferase A4 on eGFR
MR Test
Inverse variance weighted (fixed effects)
Inverse variance weighted (multiplicative random effects)
MR Egger
MR RAPS
Weighted median
Weighted mode
0.004
0.002
0.000
−0.002
−0.004
0.25
0.50
0.75
1.00
SNP effect on eGFR
SNP effect on Glutathione S−transferase A4

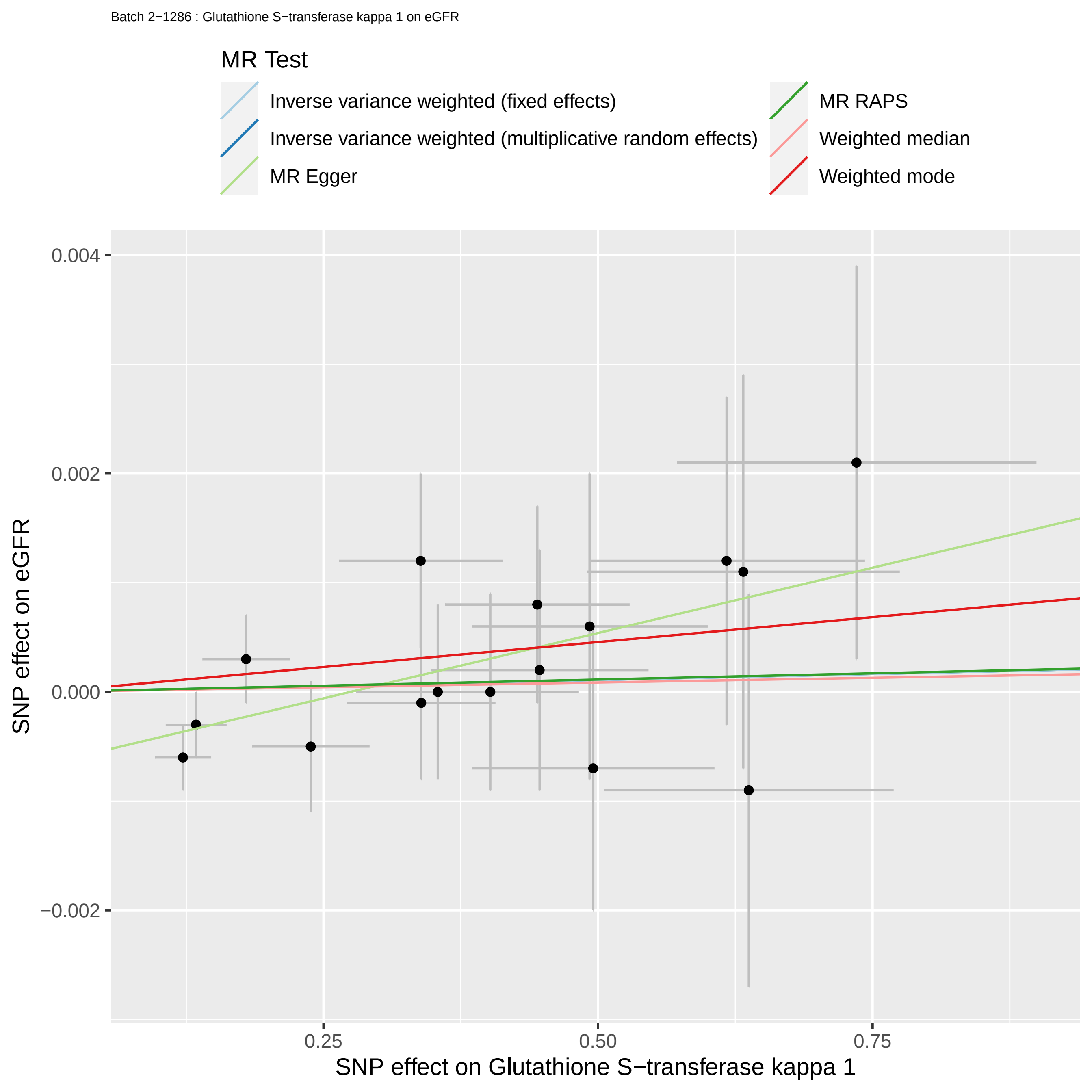

Batch 2−1286 : Glutathione S−transferase kappa 1 on eGFR
MR Test
Inverse variance weighted (fixed effects)
Inverse variance weighted (multiplicative random effects)
MR Egger
MR RAPS
Weighted median
Weighted mode
0.004
0.002
0.000
−0.002
0.25
0.50
0.75
SNP effect on eGFR
SNP effect on Glutathione S−transferase kappa 1

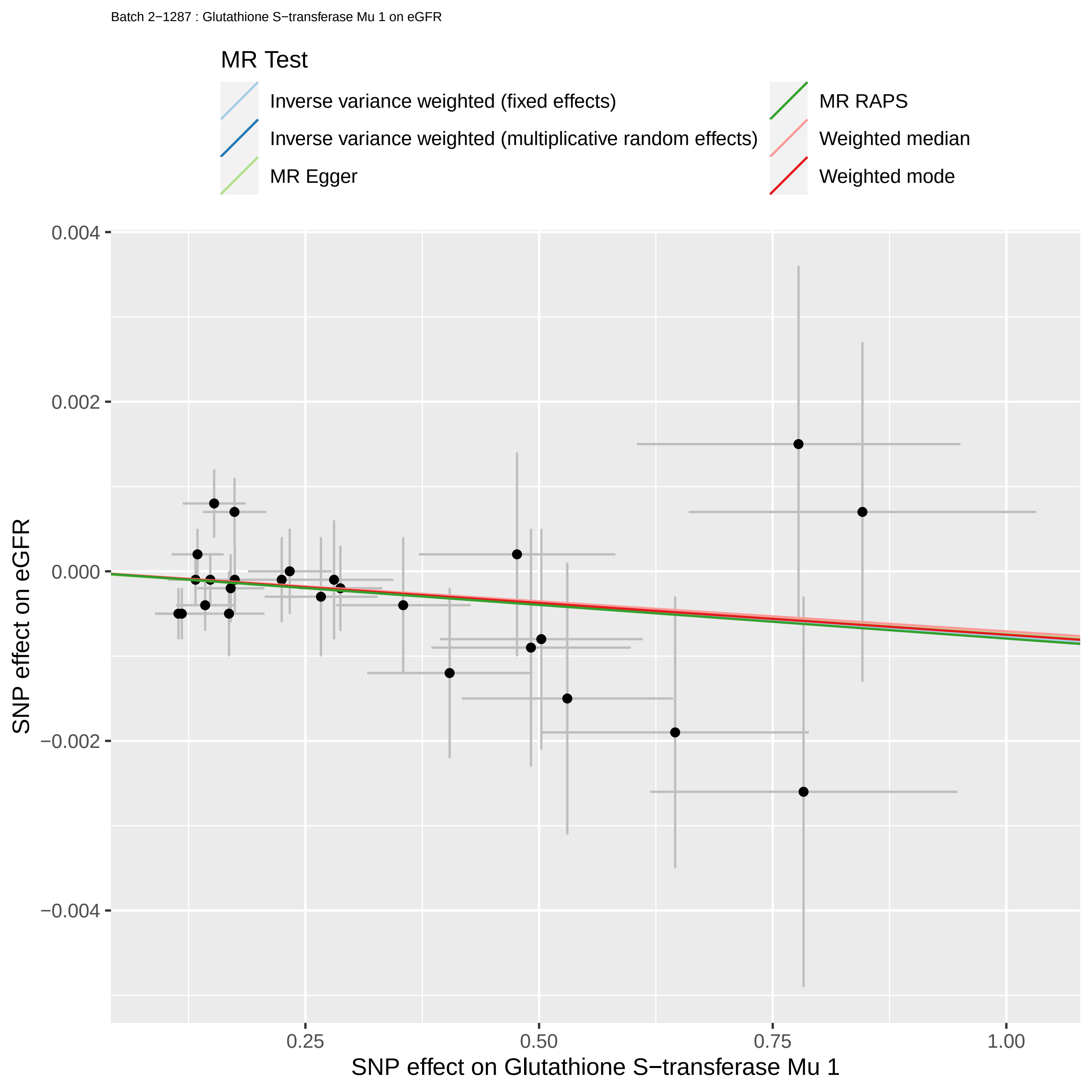

Batch 2−1287 : Glutathione S−transferase Mu 1 on eGFR
MR Test
Inverse variance weighted (fixed effects)
Inverse variance weighted (multiplicative random effects)
MR Egger
MR RAPS
Weighted median
Weighted mode
0.004
0.002
0.000
−0.002
−0.004
SNP effect on eGFR
0.25
0.50
0.75
1.00
SNP effect on Glutathione S−transferase Mu 1

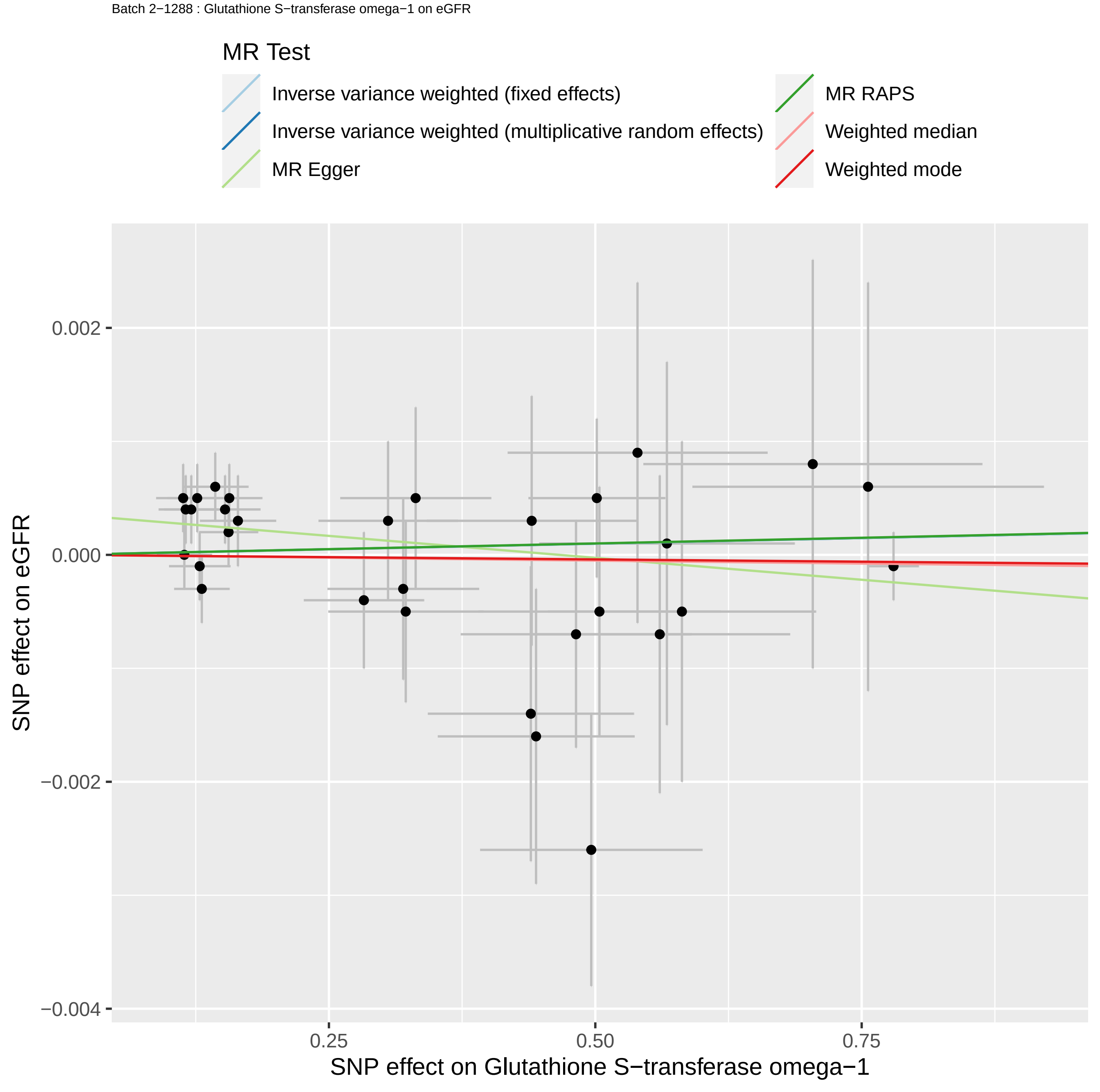

Batch 2−1288 : Glutathione S−transferase omega−1 on eGFR
MR Test
Inverse variance weighted (fixed effects)
Inverse variance weighted (multiplicative random effects)
MR Egger
MR RAPS
Weighted median
Weighted mode
0.002
0.000
−0.002
−0.004
0.25
0.50
0.75
SNP effect on eGFR
SNP effect on Glutathione S−transferase omega−1

Batch 2−1289 : Glutathione S−transferase P on eGFR

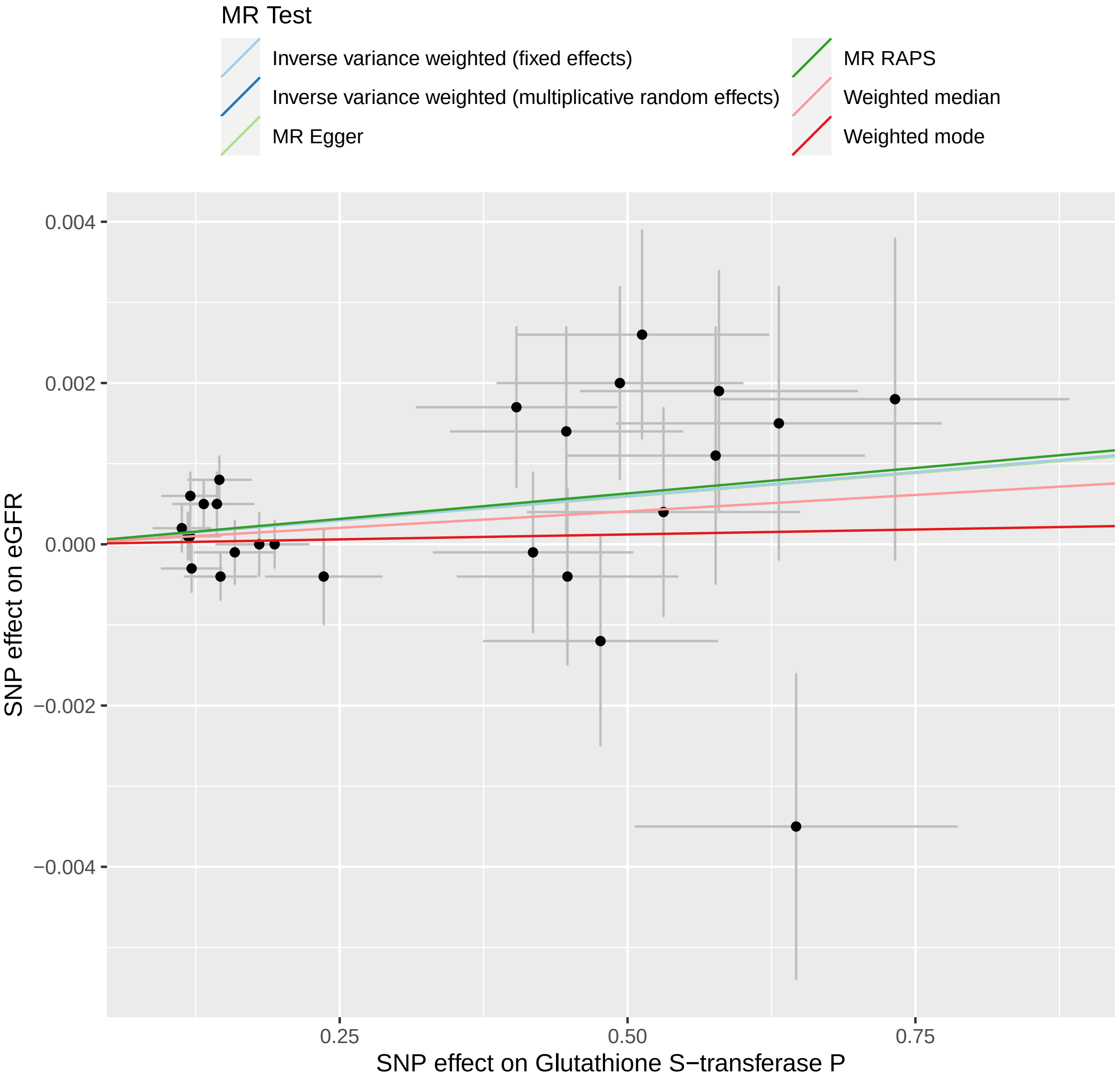

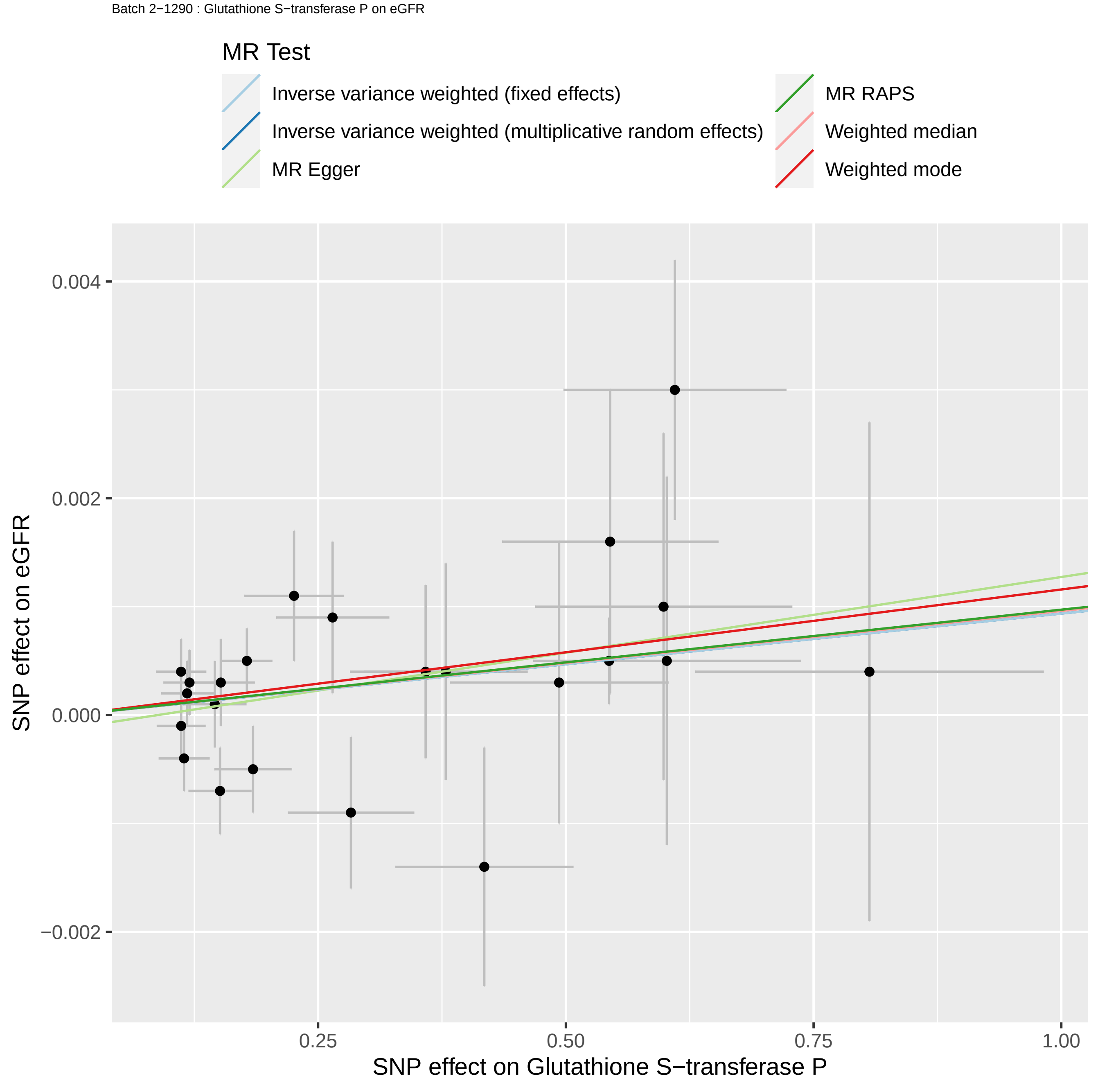

Batch 2−1290 : Glutathione S−transferase P on eGFR
MR Test
Inverse variance weighted (fixed effects)
Inverse variance weighted (multiplicative random effects)
MR Egger
MR RAPS
Weighted median
Weighted mode
0.004
0.002
0.000
−0.002
SNP effect on eGFR
0.25
0.50
0.75
1.00
SNP effect on Glutathione S−transferase P

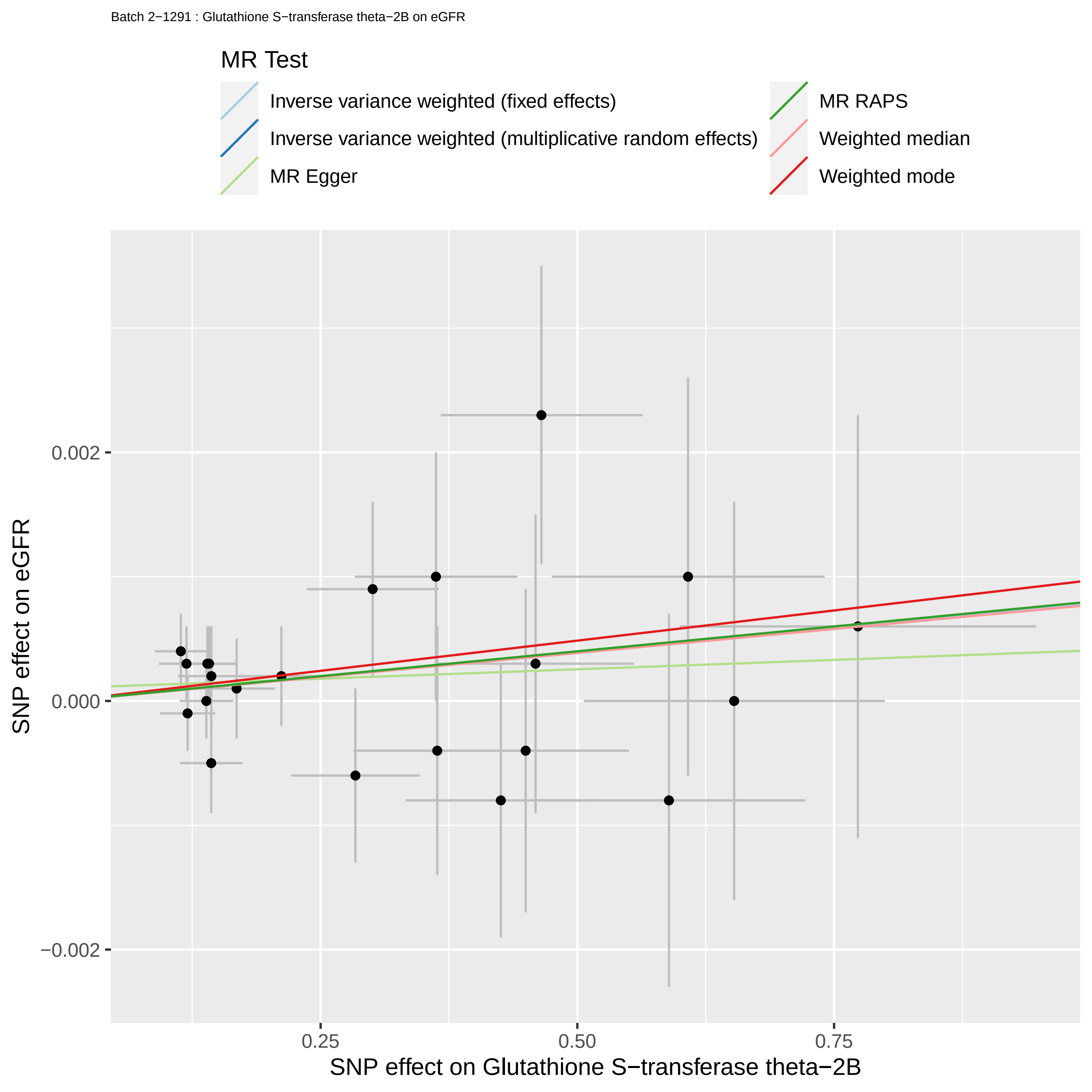

Batch 2−1291 : Glutathione S−transferase theta−2B on eGFR
MR Test
Inverse variance weighted (fixed effects)
Inverse variance weighted (multiplicative random effects)
MR Egger
MR RAPS
Weighted median
Weighted mode
0.002
0.000
−0.002
SNP effect on eGFR
0.25
0.50
0.75
SNP effect on Glutathione S−transferase theta−2B

Batch 2−1292 : General transcription factor II−I on eGFR

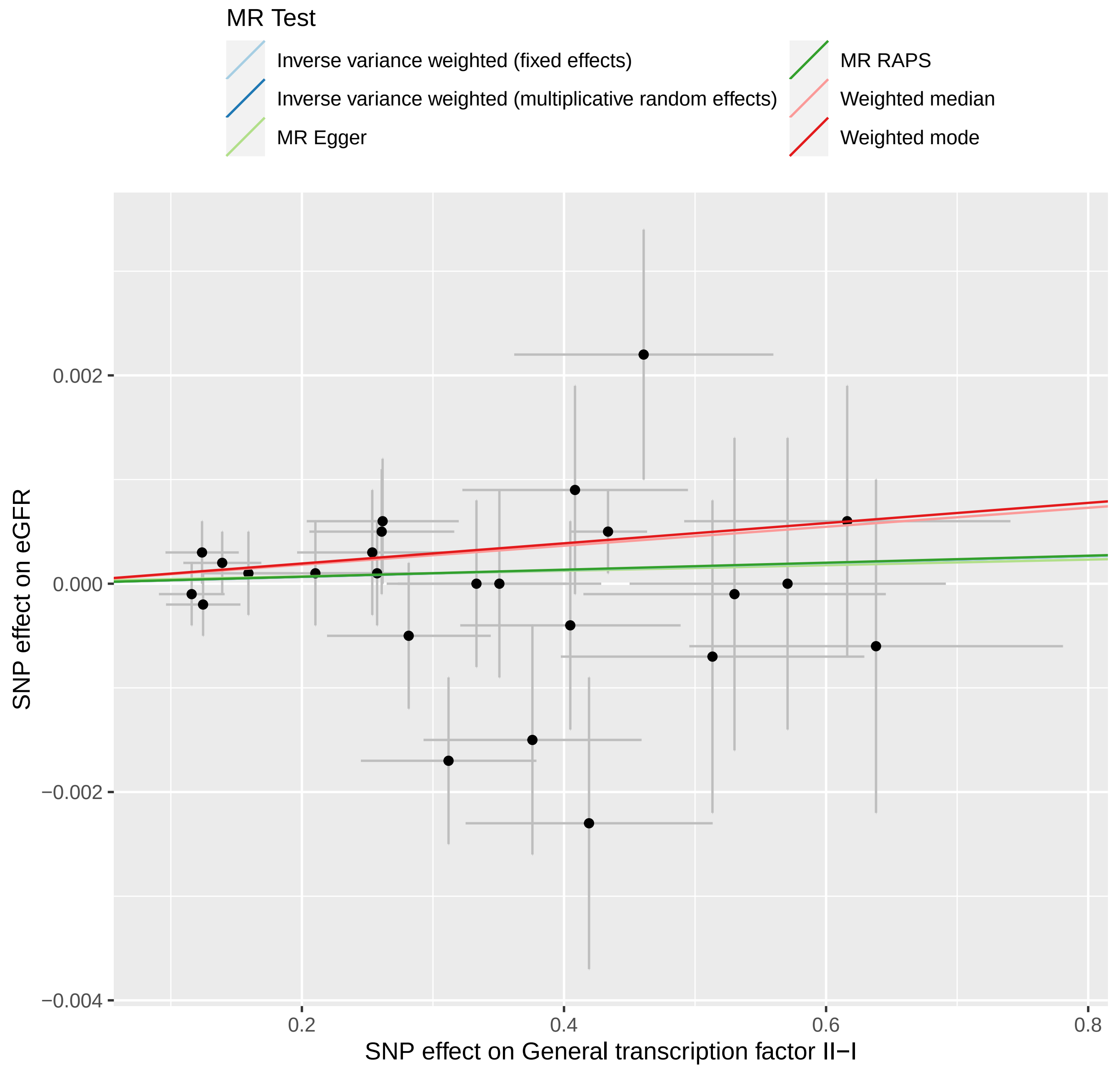

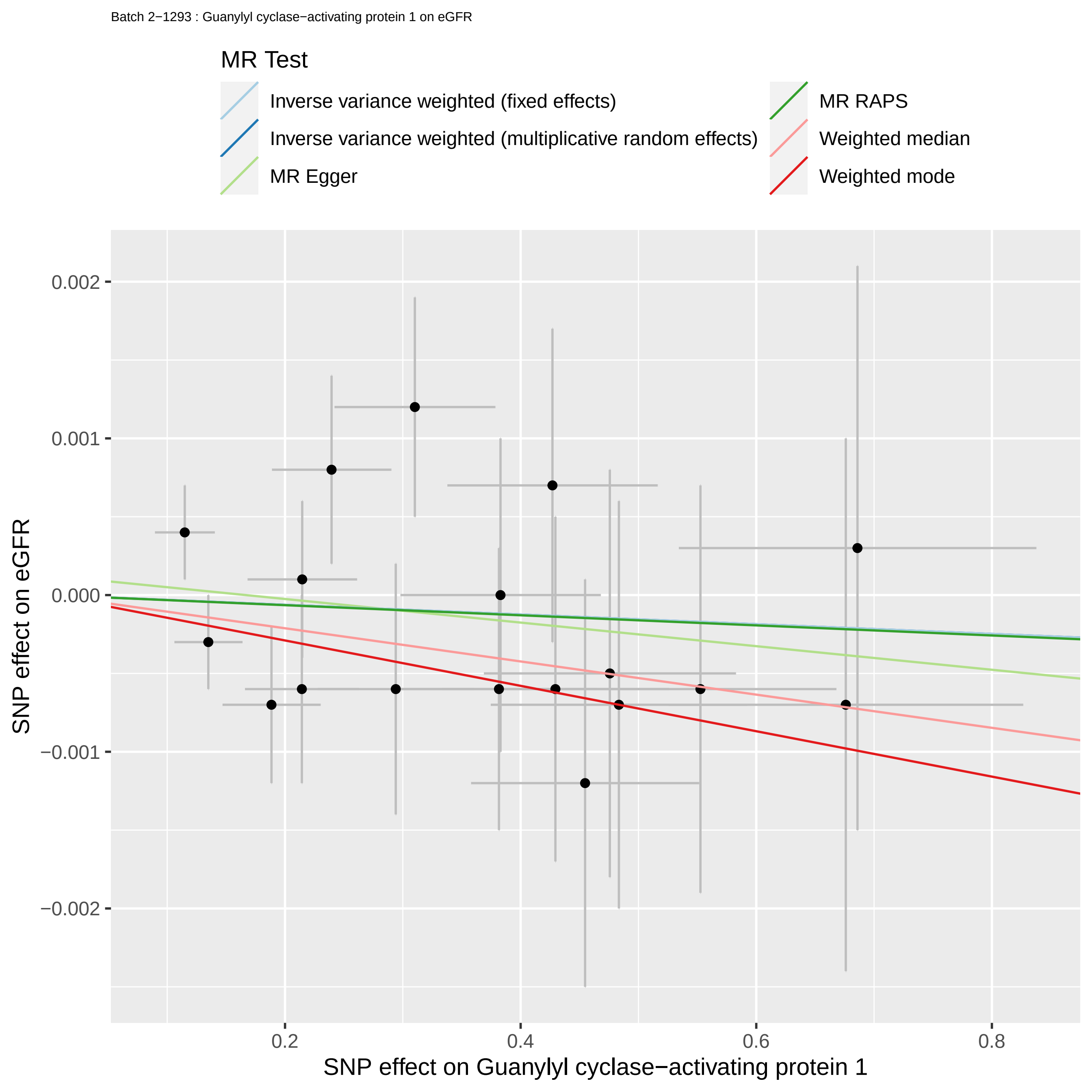
Batch 2−1293 : Guanylyl cyclase−activating protein 1 on eGFR
MR Test
Inverse variance weighted (fixed effects)
Inverse variance weighted (multiplicative random effects)
MR Egger
MR RAPS
Weighted median
Weighted mode
0.002
0.001
0.000
−0.001
−0.002
SNP effect on eGFR
0.2
0.4
0.6
0.8
SNP effect on Guanylyl cyclase−activating protein 1

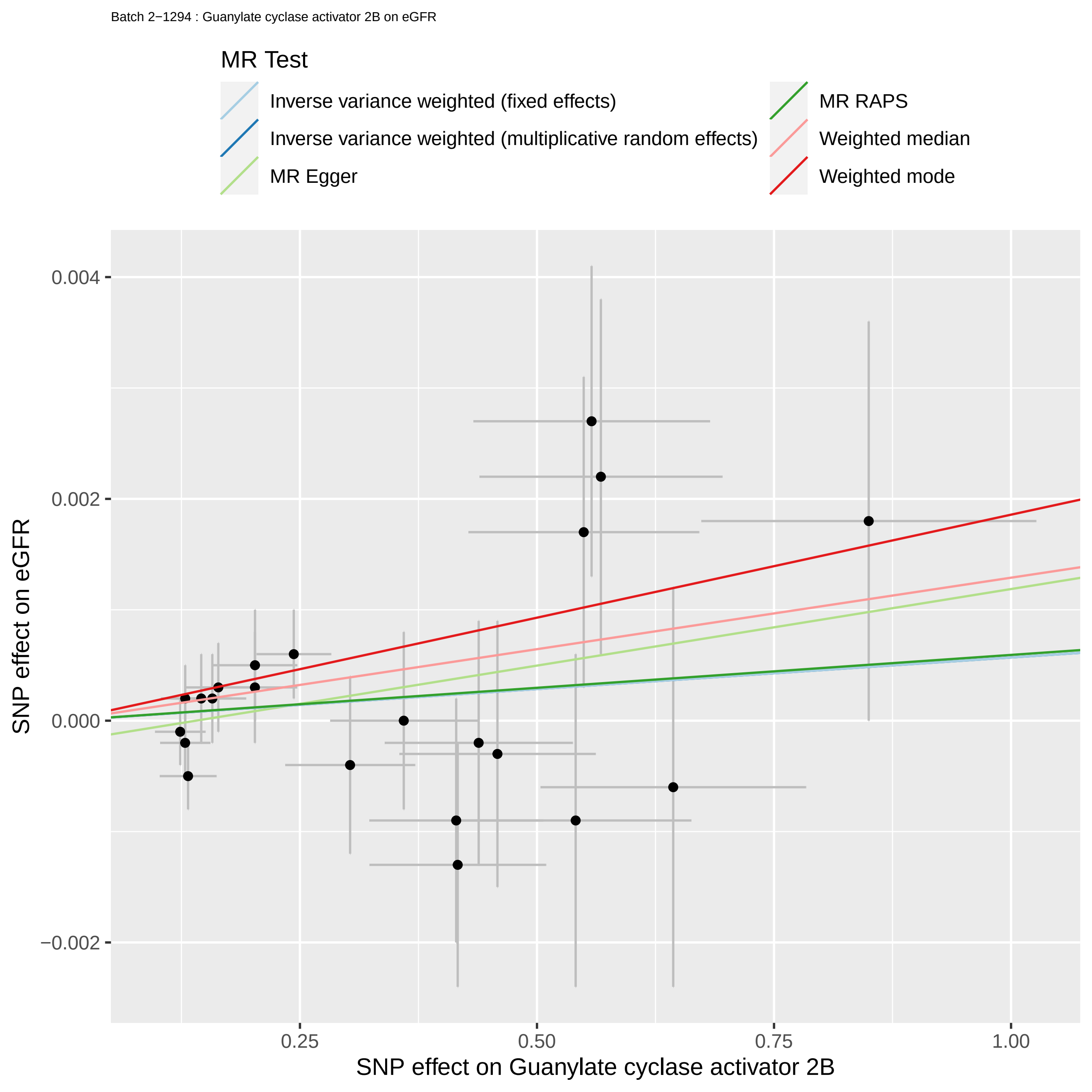

Batch 2−1294 : Guanylate cyclase activator 2B on eGFR
MR Test
Inverse variance weighted (fixed effects)
Inverse variance weighted (multiplicative random effects)
MR Egger
MR RAPS
Weighted median
Weighted mode
0.004
0.002
0.000
−0.002
0.25
0.50
0.75
1.00
SNP effect on eGFR
SNP effect on Guanylate cyclase activator 2B

Batch 2−1295 : Glucoside xylosyltransferase 1 on eGFR

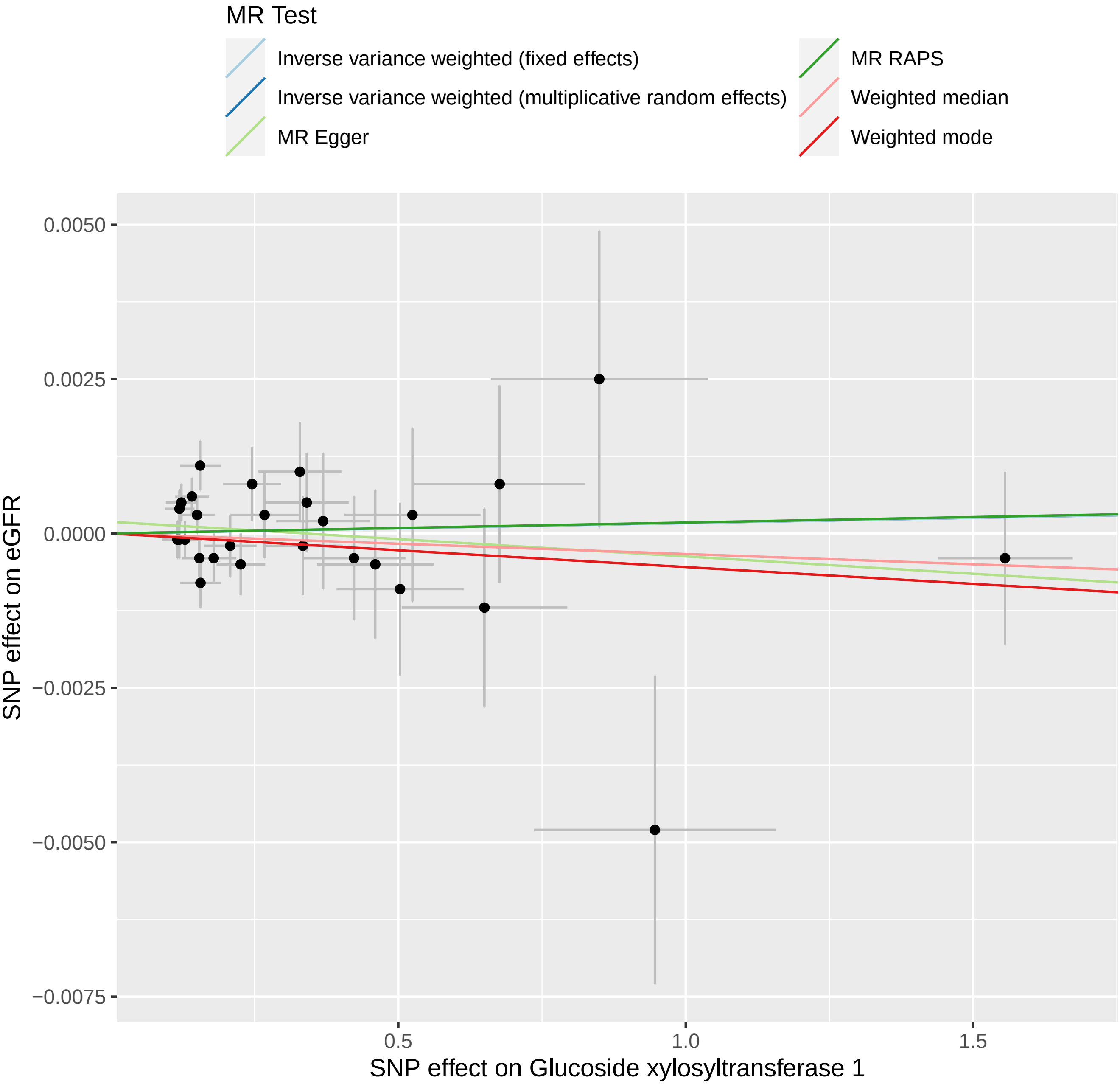

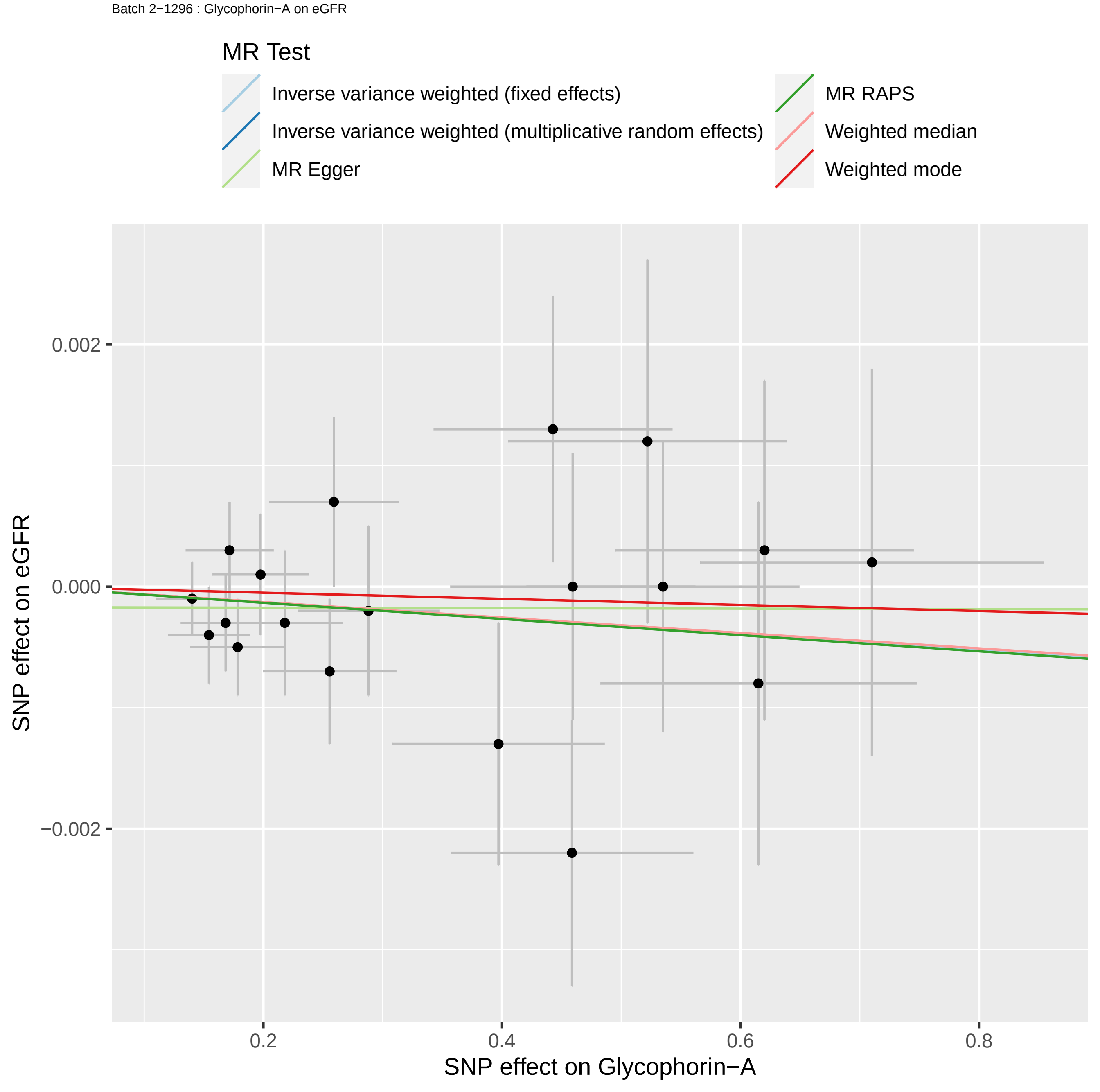

Batch 2−1296 : Glycophorin−A on eGFR
MR Test
Inverse variance weighted (fixed effects)
Inverse variance weighted (multiplicative random effects)
MR Egger
MR RAPS
Weighted median
Weighted mode
0.002
0.000
−0.002
0.2
0.4
0.6
0.8
SNP effect on eGFR
SNP effect on Glycophorin−A

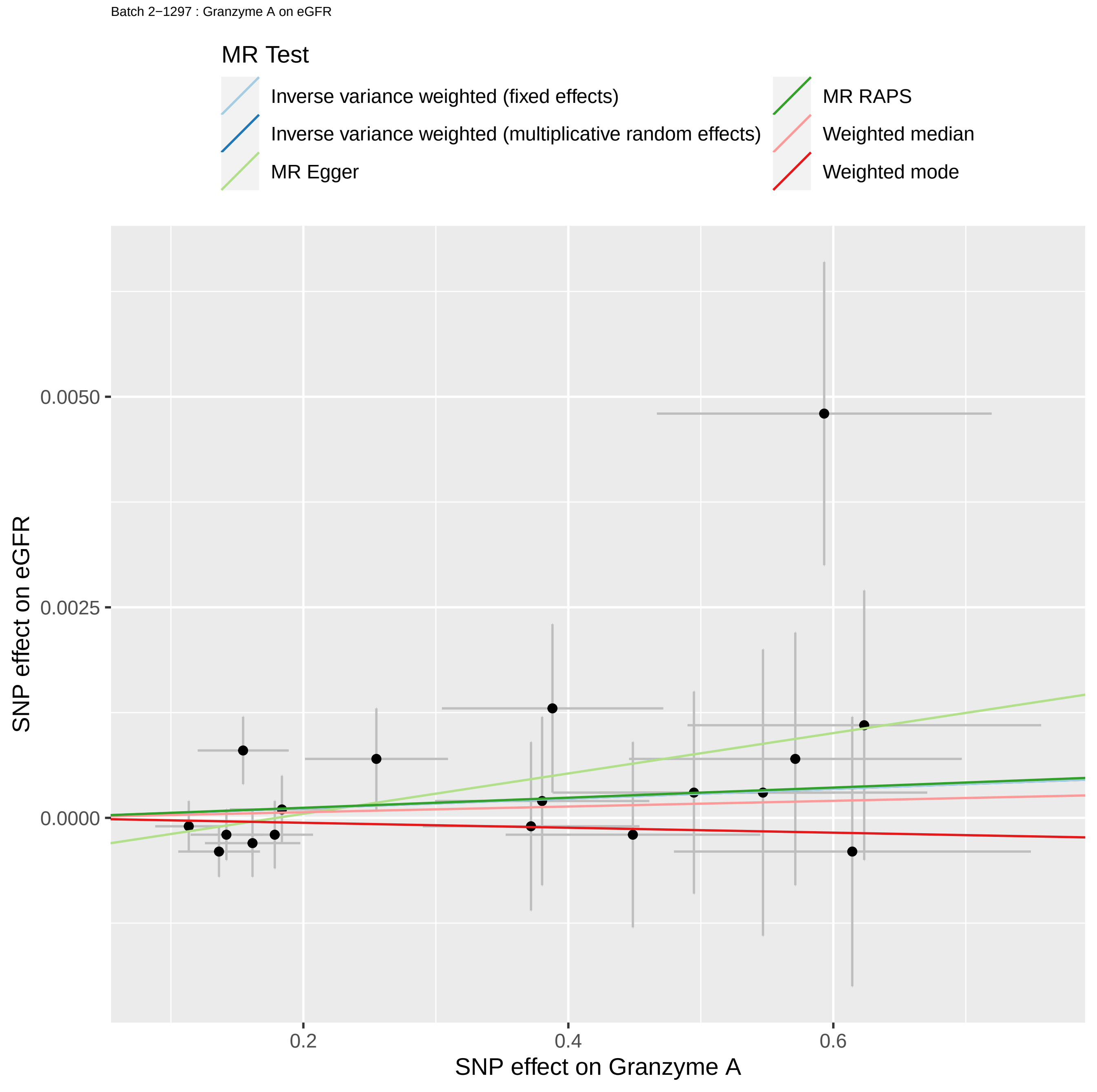
Batch 2−1297 : Granzyme A on eGFR
MR Test
Inverse variance weighted (fixed effects)
Inverse variance weighted (multiplicative random effects)
MR Egger
MR RAPS
Weighted median
Weighted mode
0.0050
0.0025
0.0000
0.2
0.4
0.6
SNP effect on eGFR
SNP effect on Granzyme A

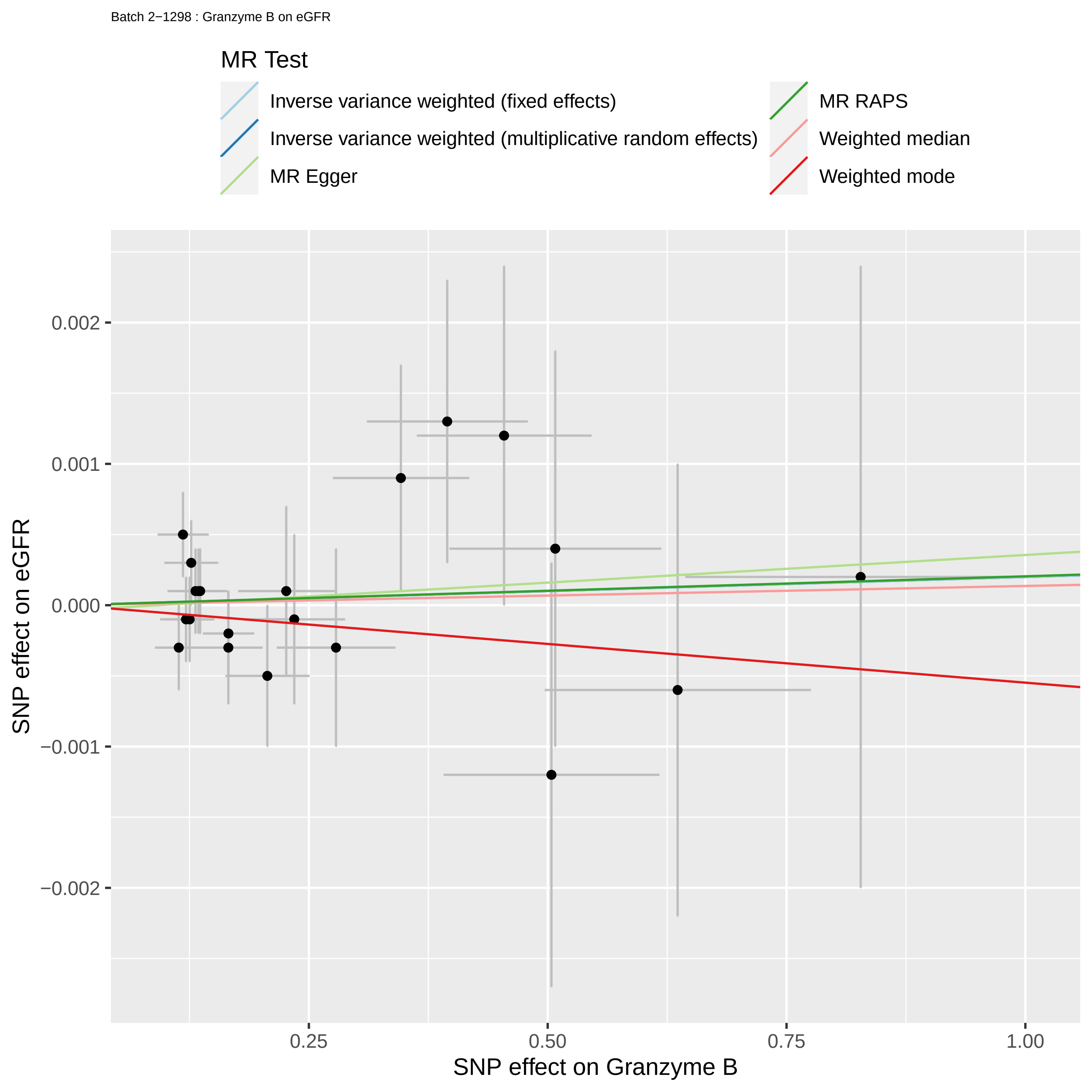

Batch 2−1298 : Granzyme B on eGFR
MR Test
Inverse variance weighted (fixed effects)
Inverse variance weighted (multiplicative random effects)
MR Egger
MR RAPS
Weighted median
Weighted mode
0.002
0.001
0.000
−0.001
−0.002
SNP effect on eGFR
0.25
0.50
0.75
1.00
SNP effect on Granzyme B

Batch 2−1299 : Granzyme B on eGFR

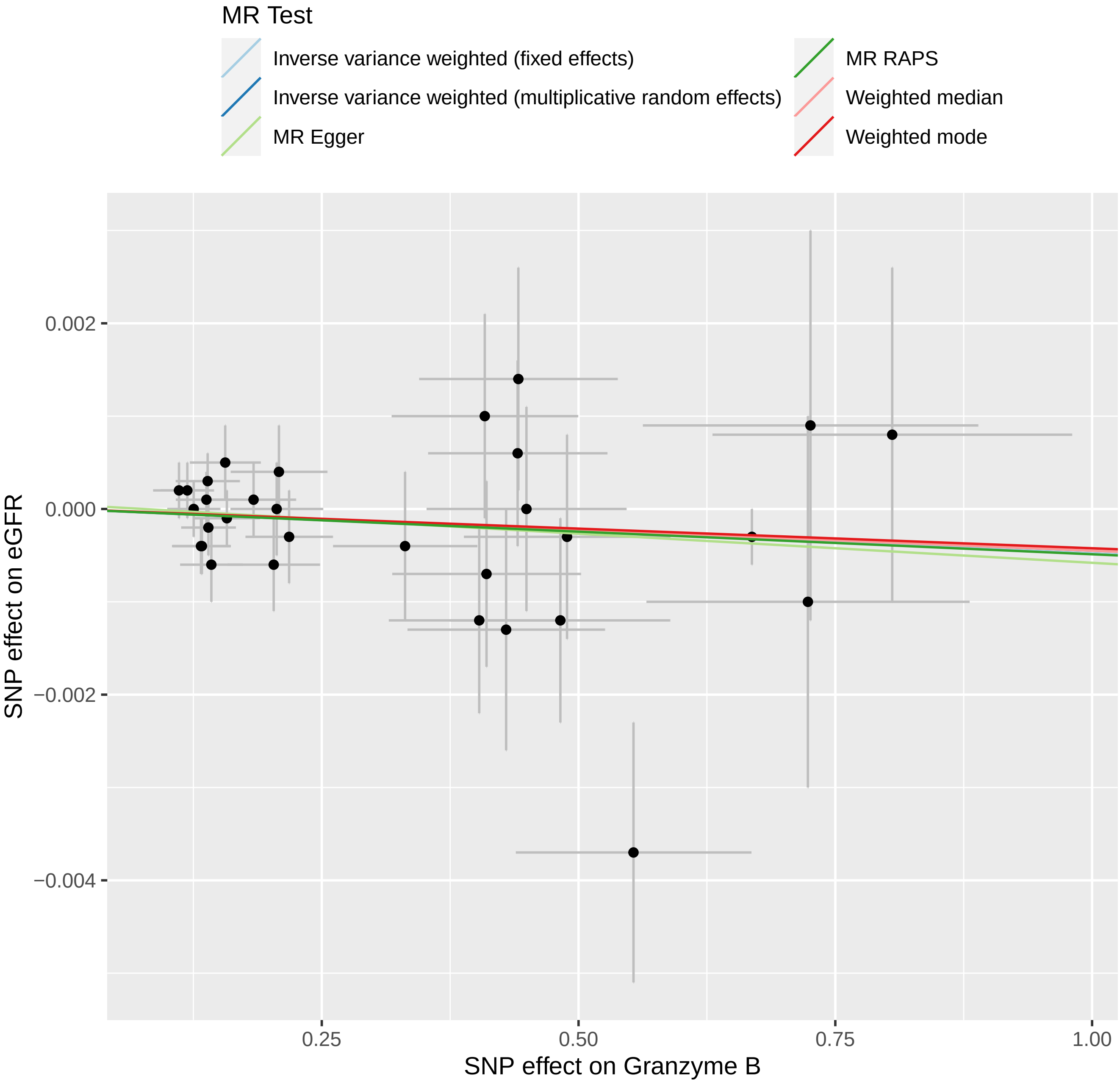

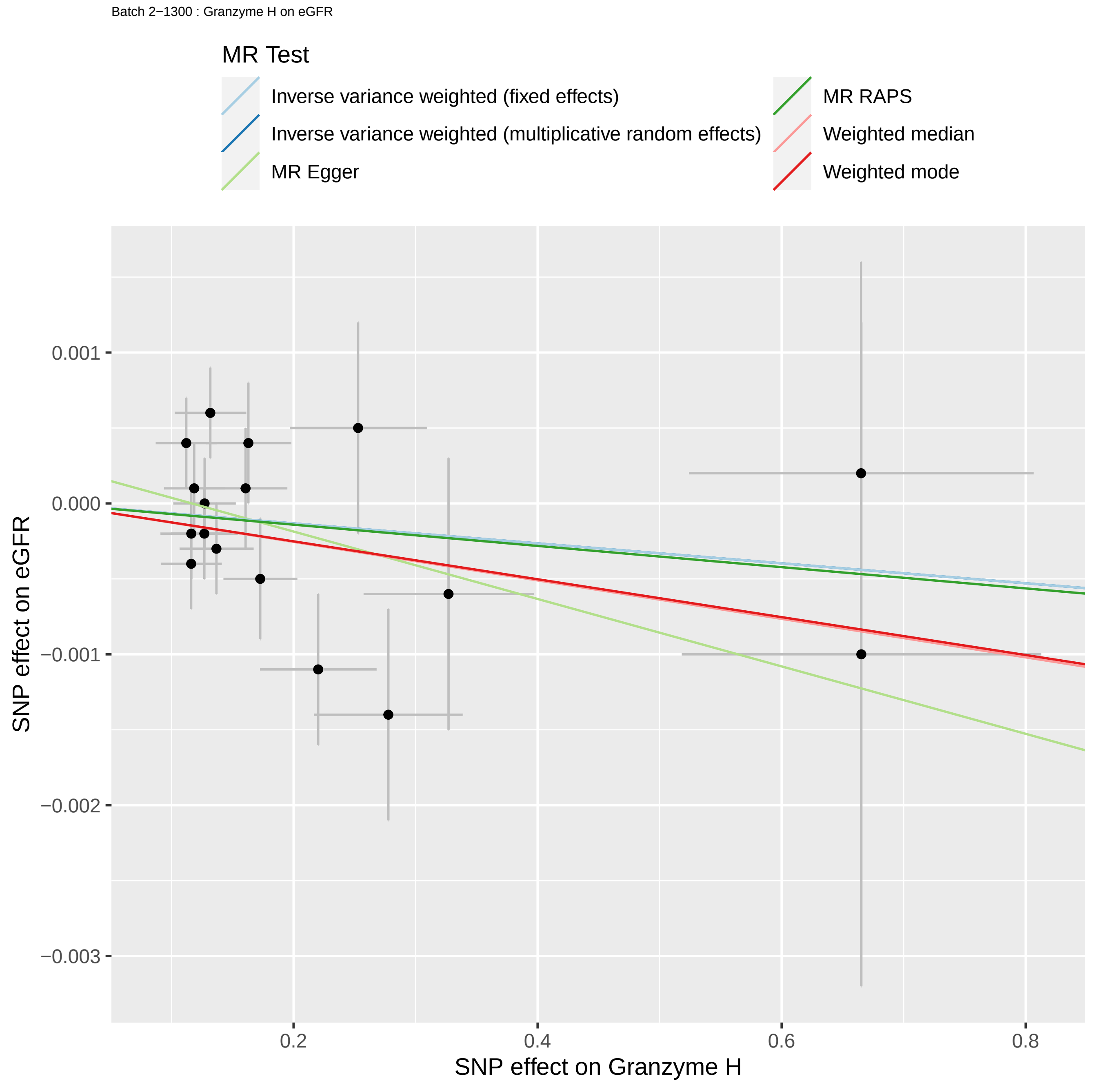

Batch 2−1300 : Granzyme H on eGFR
MR Test
Inverse variance weighted (fixed effects)
Inverse variance weighted (multiplicative random effects)
MR Egger
MR RAPS
Weighted median
Weighted mode
0.001
0.000
−0.001
−0.002
−0.003
SNP effect on eGFR
0.2
0.4
0.6
0.8
SNP effect on Granzyme H

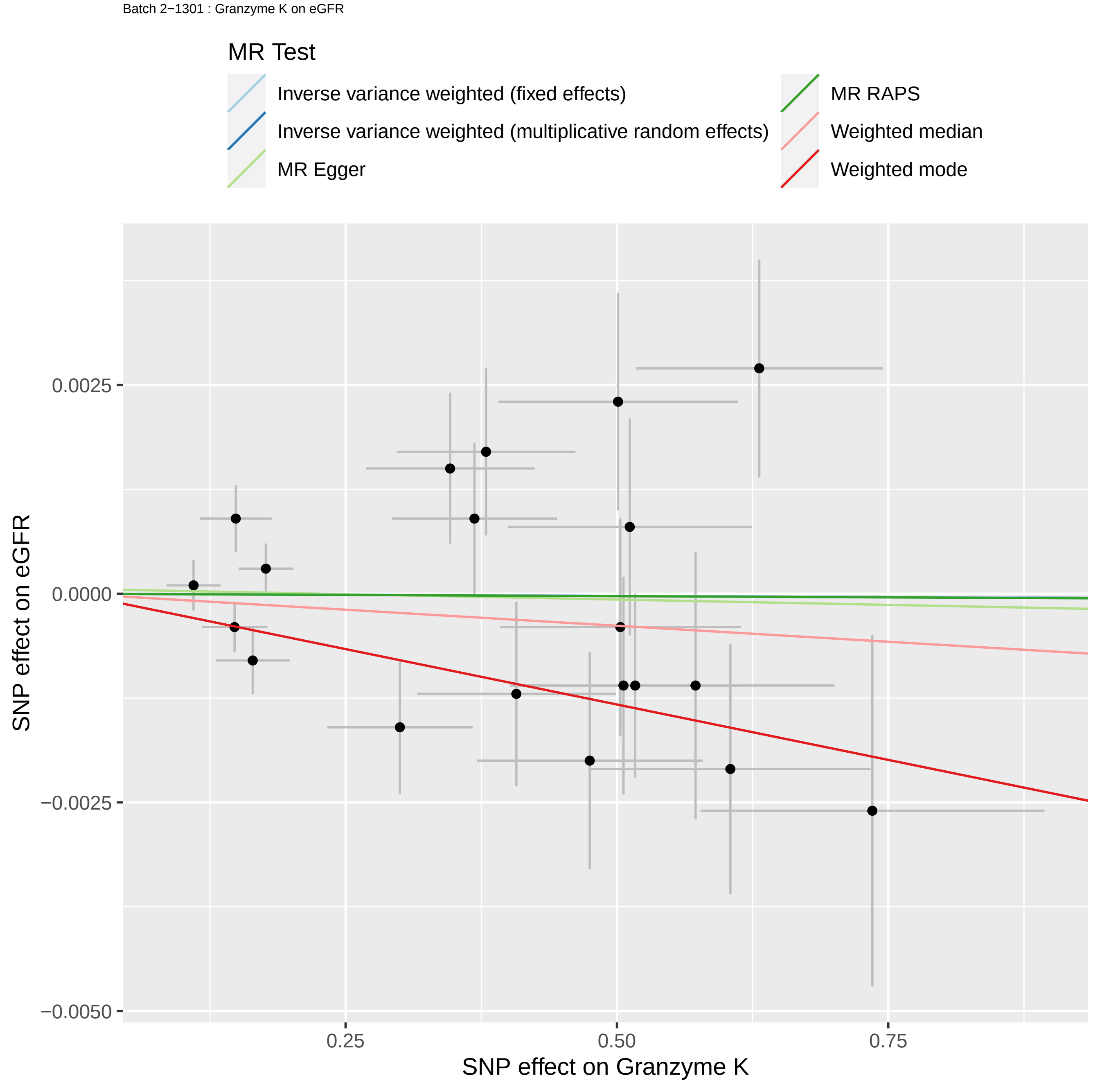

Batch 2−1301 : Granzyme K on eGFR
MR Test
Inverse variance weighted (fixed effects)
Inverse variance weighted (multiplicative random effects)
MR Egger
MR RAPS
Weighted median
Weighted mode
0.0025
0.0000
−0.0025
−0.0050
0.25
0.50
0.75
SNP effect on eGFR
SNP effect on Granzyme K

Batch 2−1302 : Granzyme M on eGFR

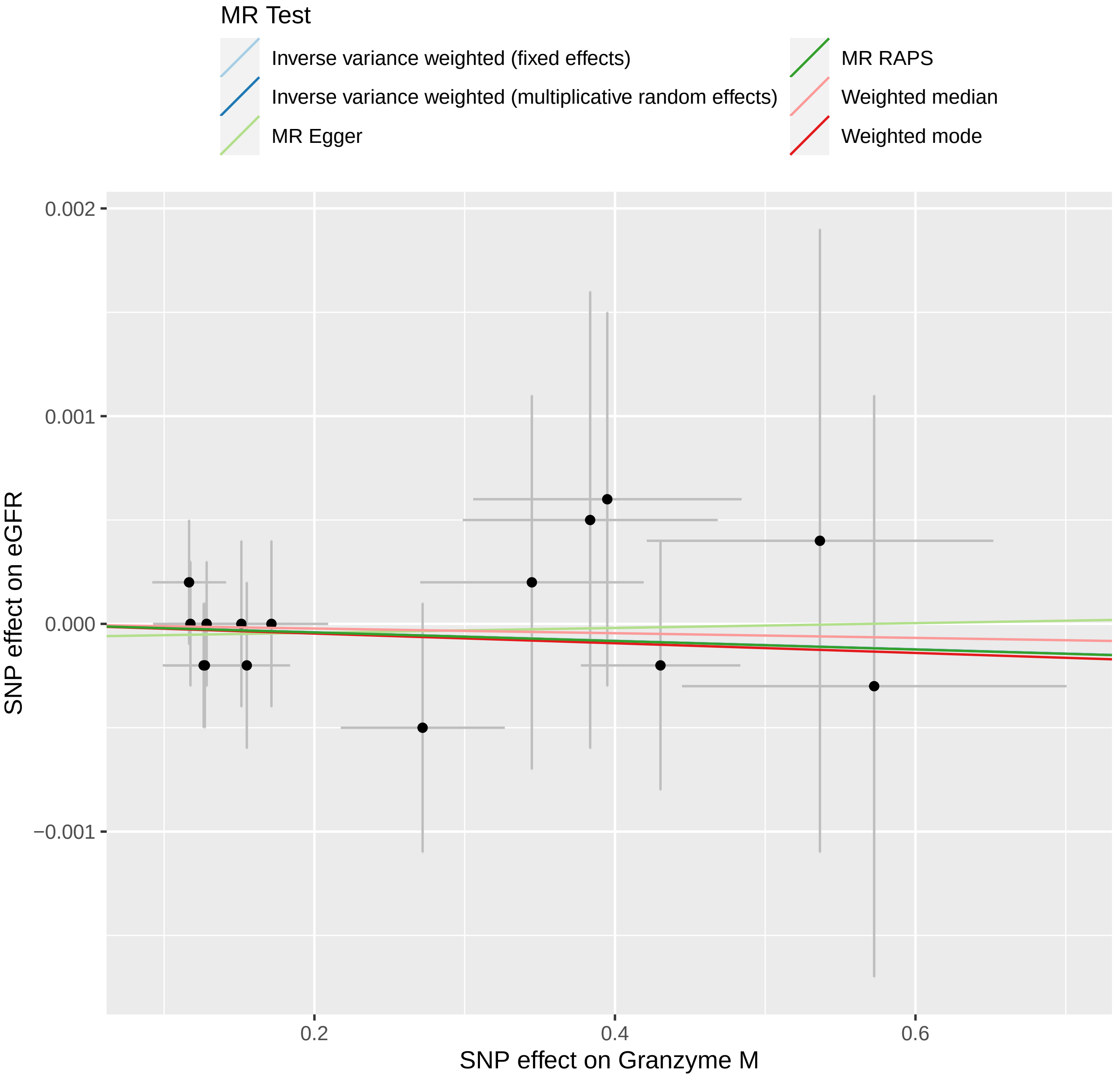

Batch 2−1303 : Histone H1x on eGFR

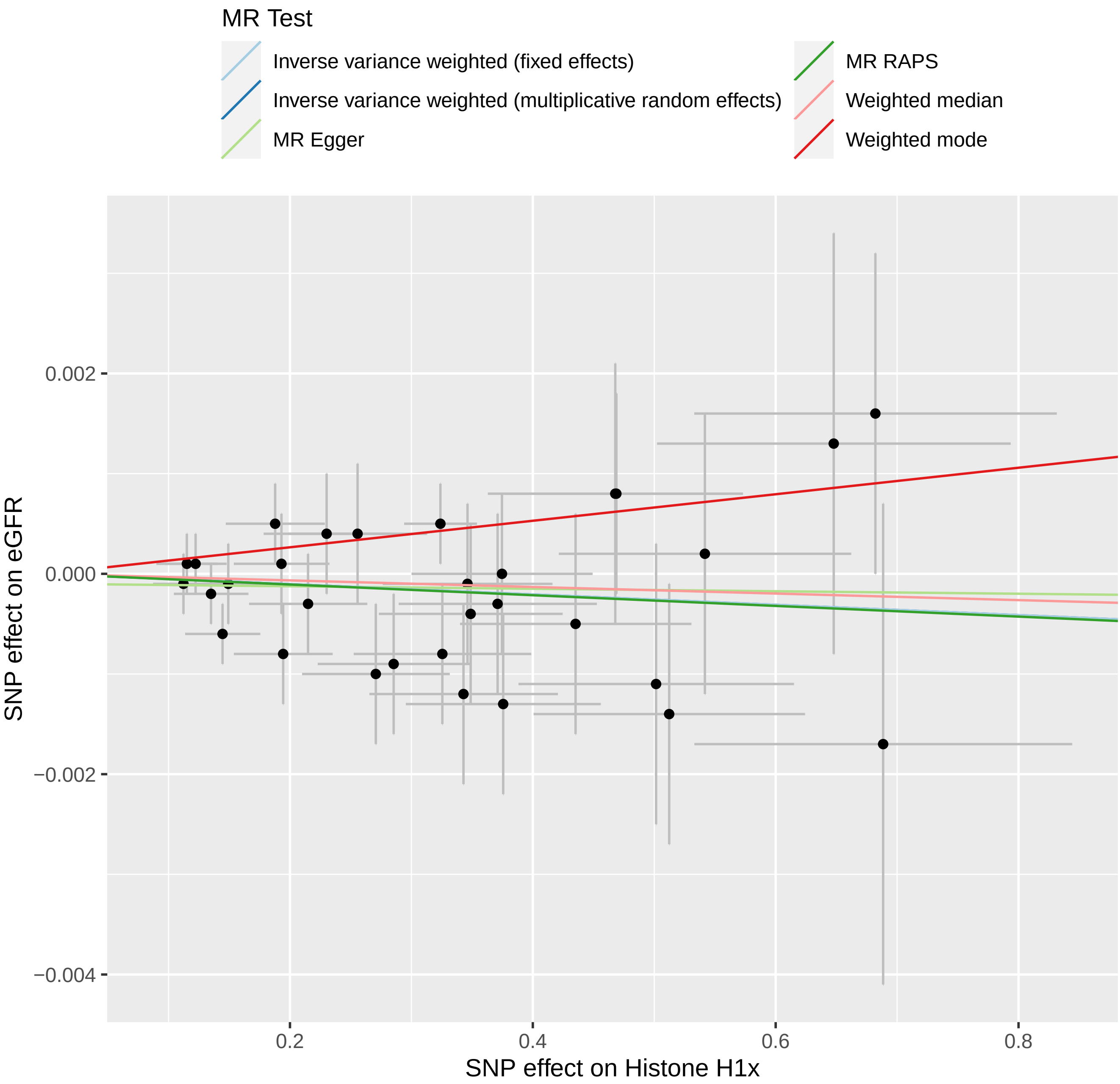

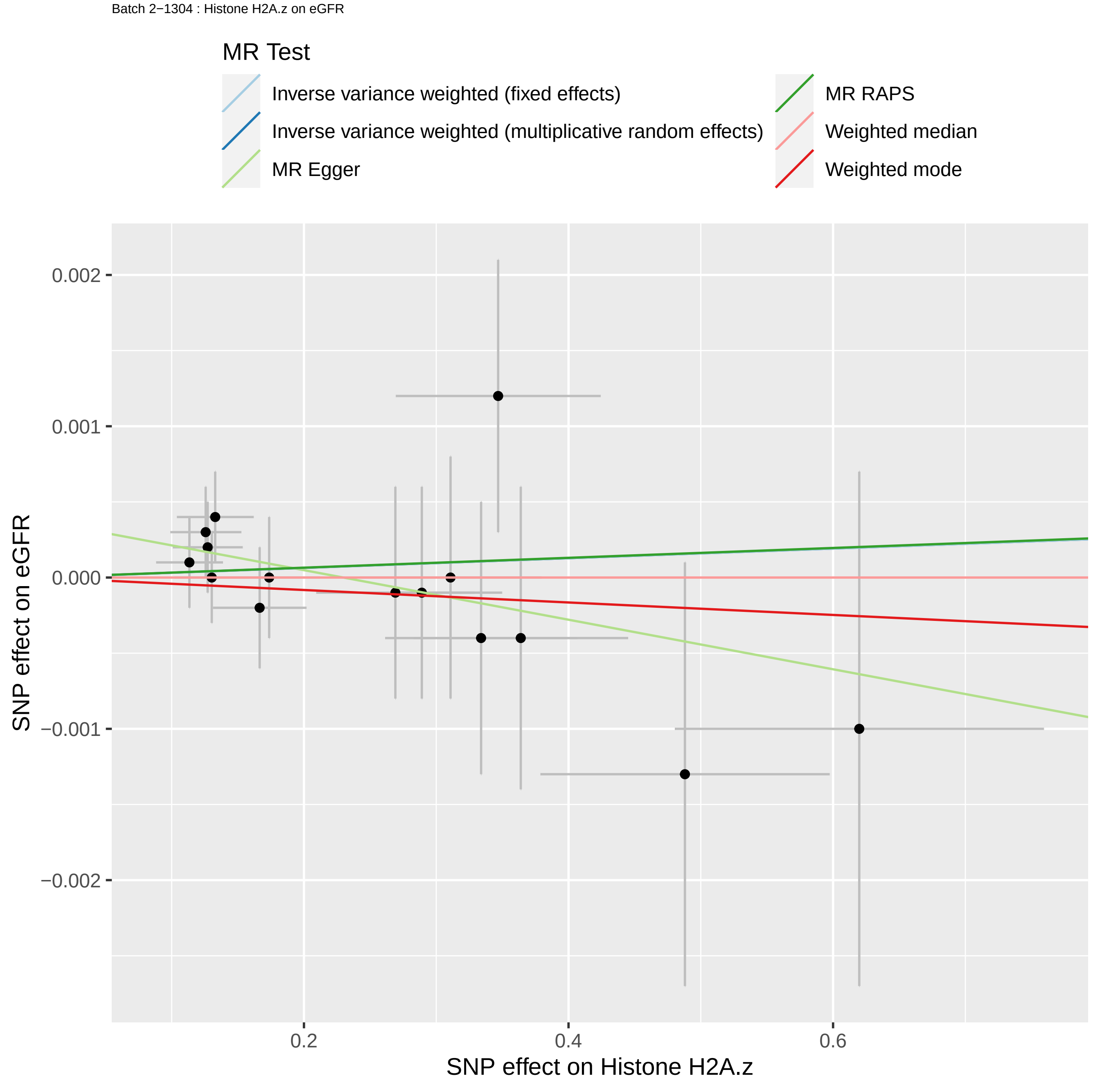

Batch 2−1304 : Histone H2A.z on eGFR
MR Test
Inverse variance weighted (fixed effects)
Inverse variance weighted (multiplicative random effects)
MR Egger
MR RAPS
Weighted median
Weighted mode
0.002
0.001
0.000
−0.001
−0.002
0.2
0.4
0.6
SNP effect on eGFR
SNP effect on Histone H2A.z

Batch 2−1305 : GDH/6PGL endoplasmic bifunctional protein on eGFR

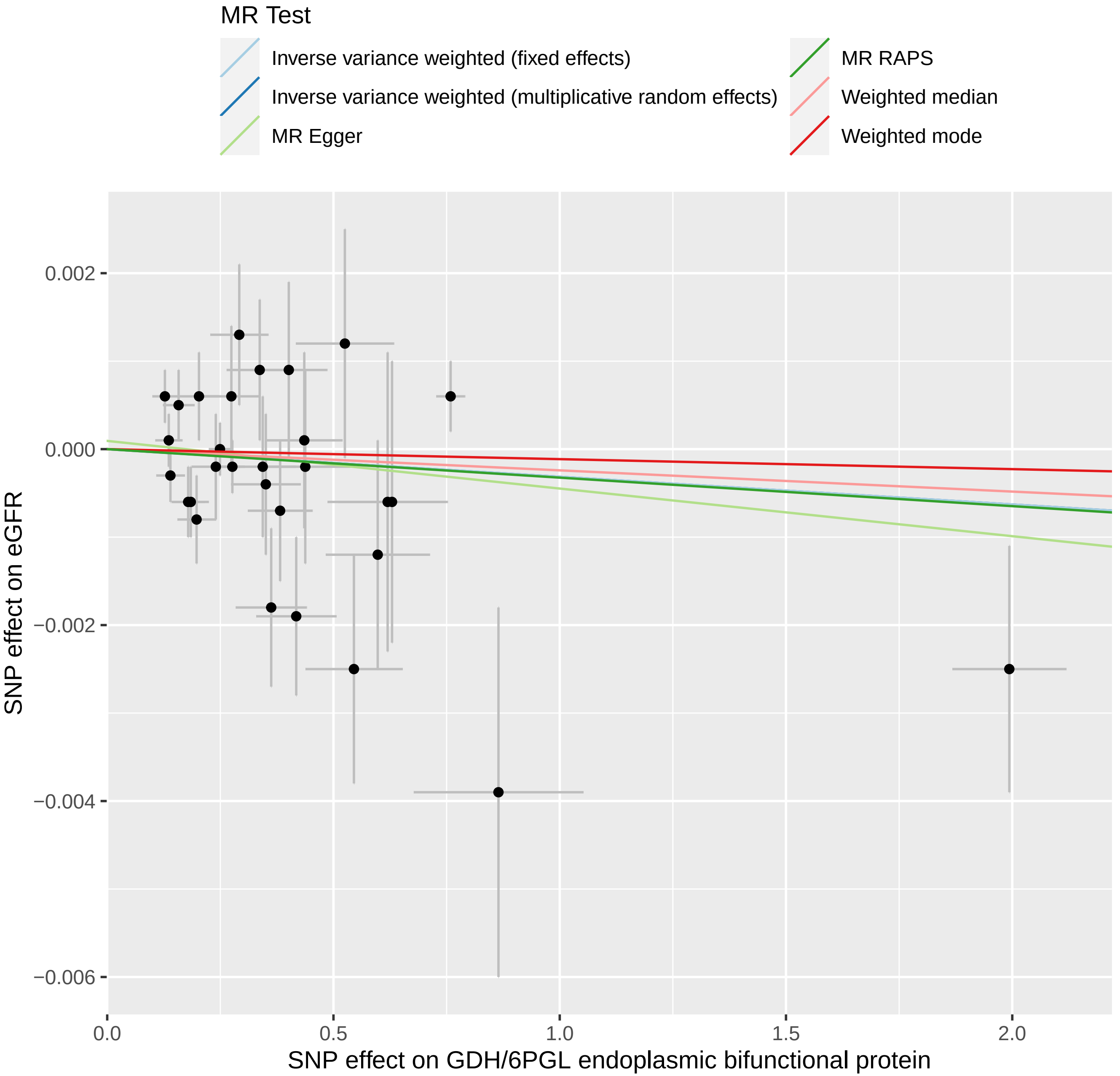

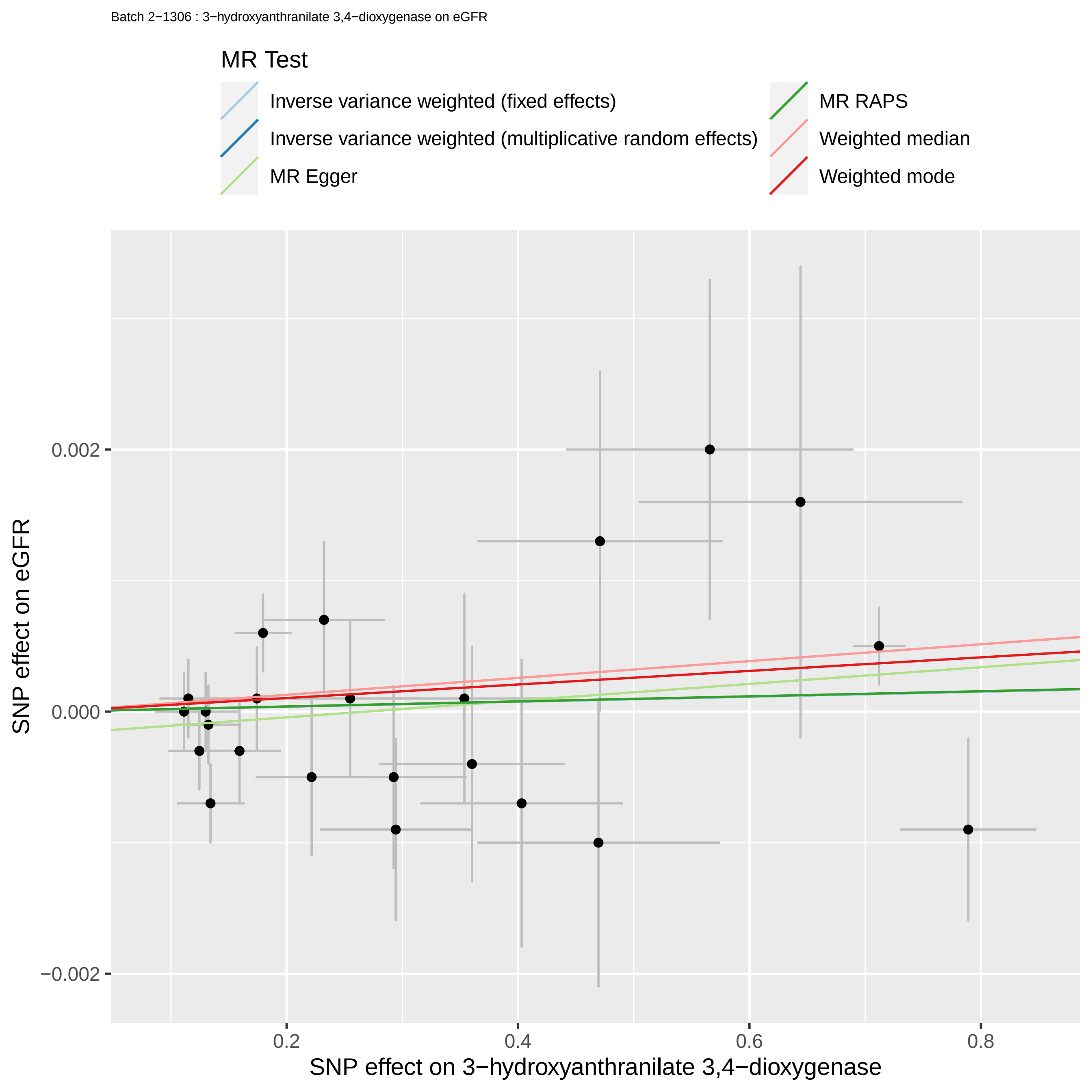

Batch 2−1306 : 3−hydroxyanthranilate 3,4−dioxygenase on eGFR
MR Test
Inverse variance weighted (fixed effects)
Inverse variance weighted (multiplicative random effects)
MR Egger
MR RAPS
Weighted median
Weighted mode
0.002
0.000
−0.002
SNP effect on eGFR
0.2
0.4
0.6
0.8
SNP effect on 3−hydroxyanthranilate 3,4−dioxygenase

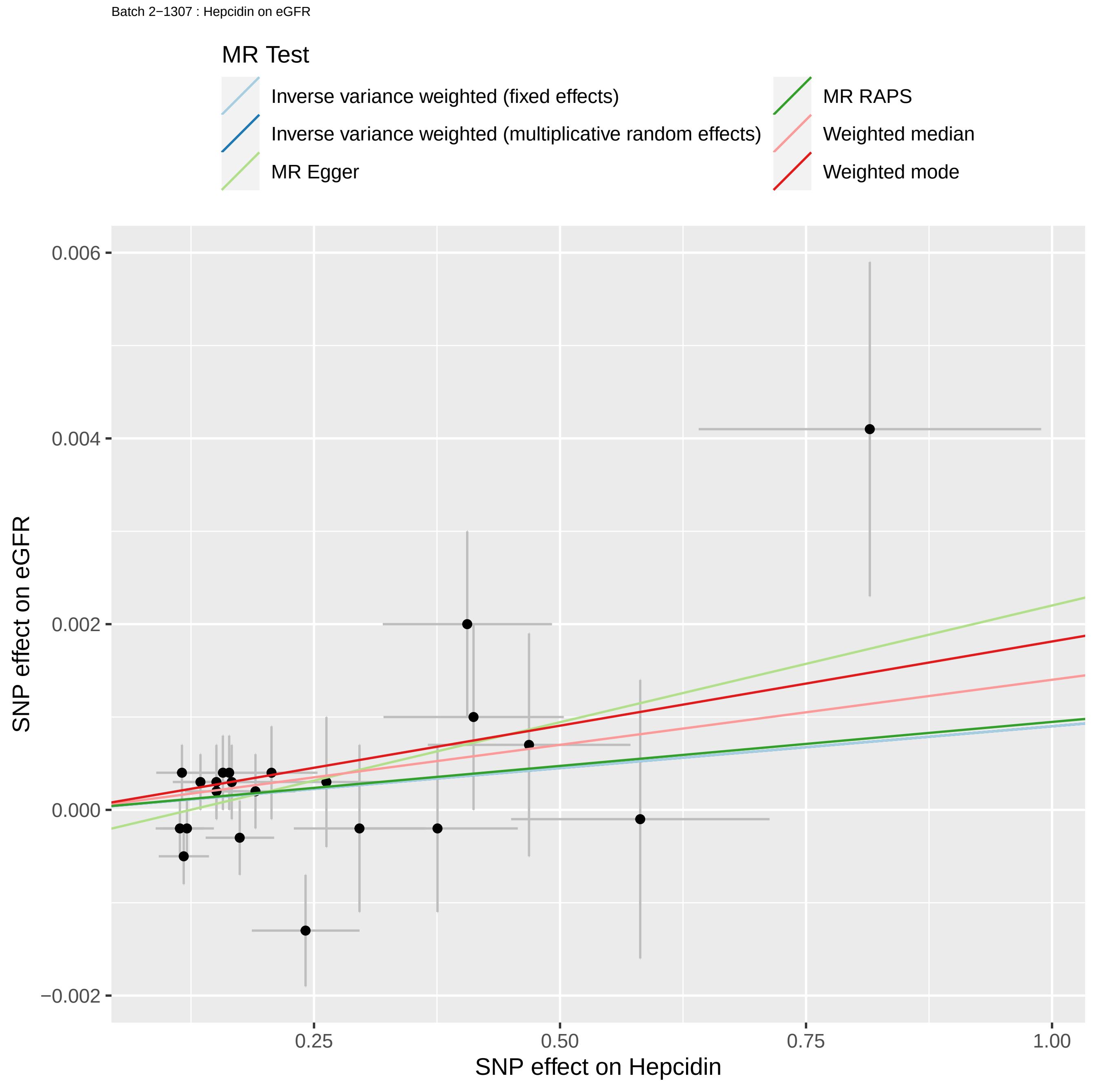

Batch 2−1307 : Hepcidin on eGFR
MR Test
Inverse variance weighted (fixed effects)
Inverse variance weighted (multiplicative random effects)
MR Egger
MR RAPS
Weighted median
Weighted mode
0.006
0.004
0.002
0.000
−0.002
SNP effect on eGFR
0.25
0.50
0.75
1.00
SNP effect on Hepcidin

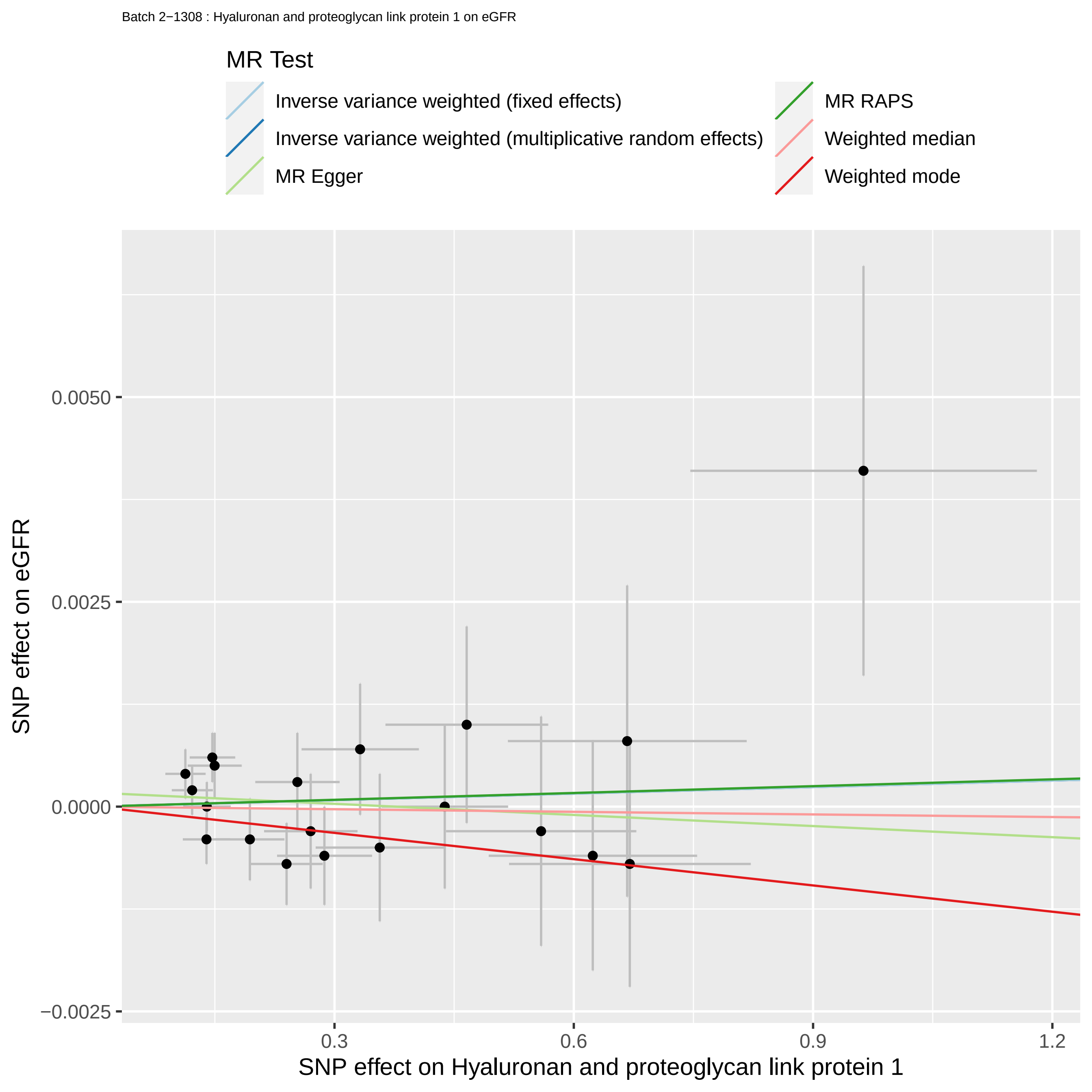

Batch 2−1308 : Hyaluronan and proteoglycan link protein 1 on eGFR
MR Test
Inverse variance weighted (fixed effects)
Inverse variance weighted (multiplicative random effects)
MR Egger
MR RAPS
Weighted median
Weighted mode
0.0050
0.0025
0.0000
−0.0025
0.3
0.6
0.9
1.2
SNP effect on eGFR
SNP effect on Hyaluronan and proteoglycan link protein 1

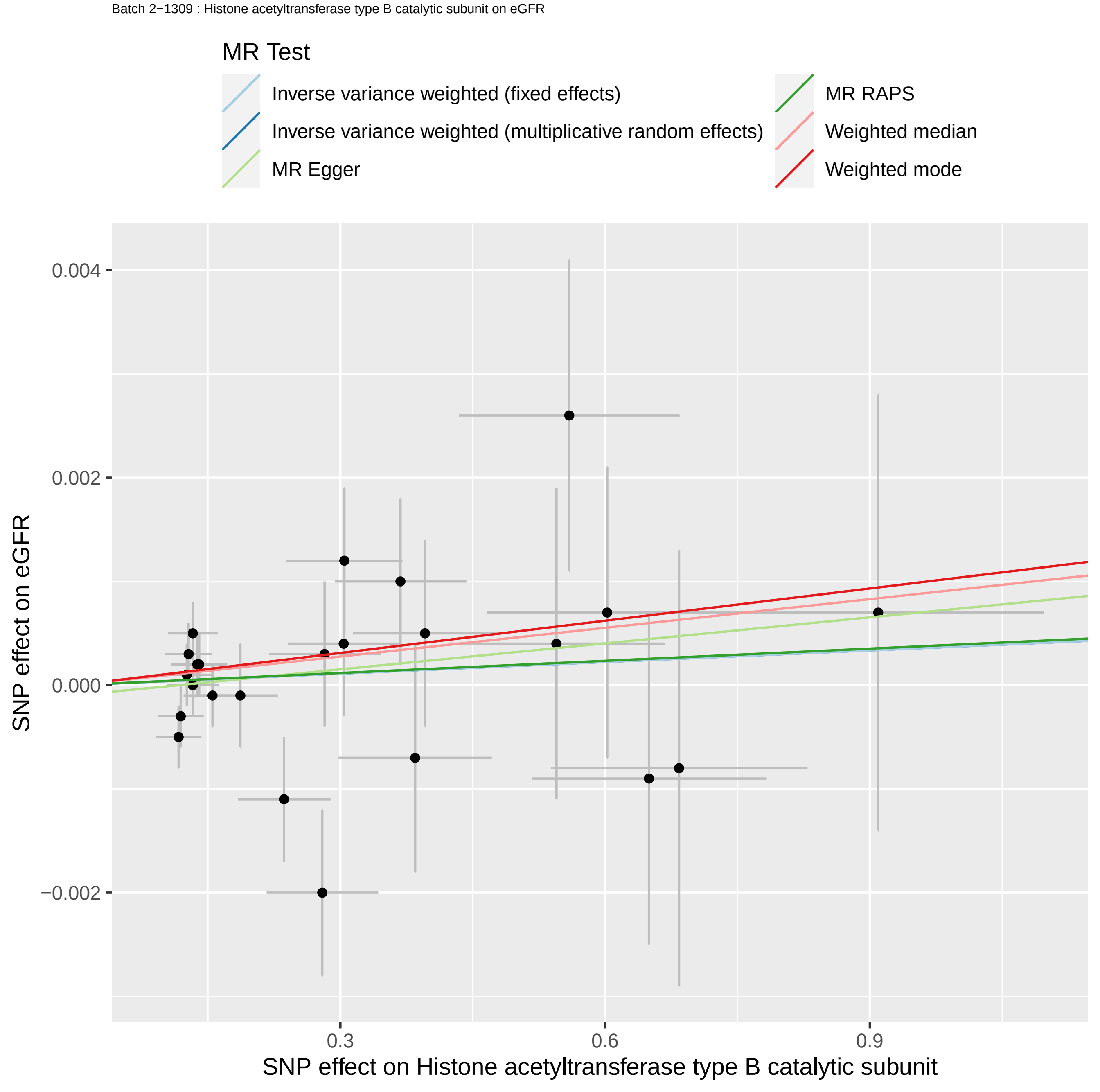
Batch 2−1309 : Histone acetyltransferase type B catalytic subunit on eGFR
MR Test
Inverse variance weighted (fixed effects)
Inverse variance weighted (multiplicative random effects)
MR Egger
MR RAPS
Weighted median
Weighted mode
0.004
0.002
0.000
−0.002
0.3
0.6
0.9
SNP effect on eGFR
SNP effect on Histone acetyltransferase type B catalytic subunit

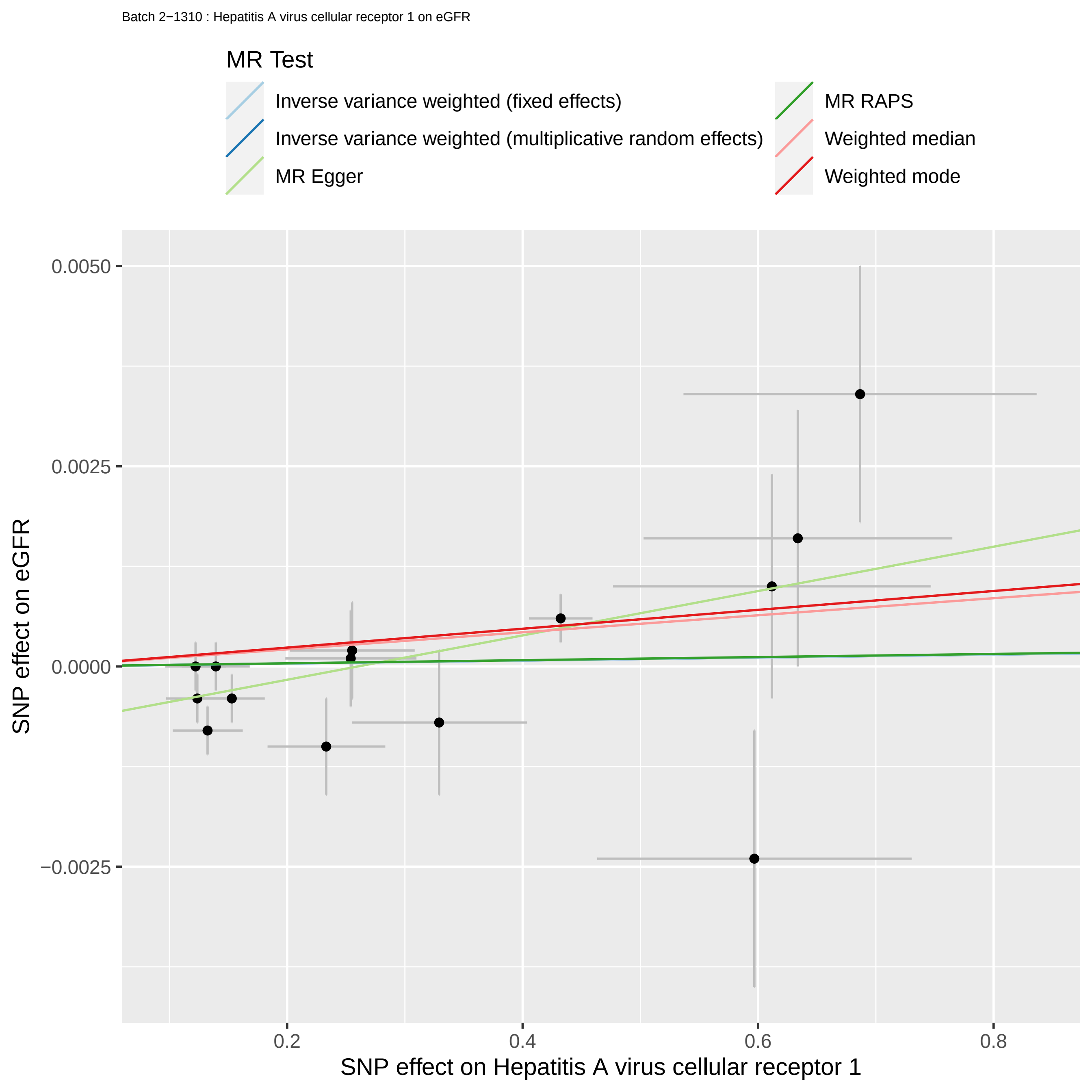
Batch 2−1310 : Hepatitis A virus cellular receptor 1 on eGFR
MR Test
Inverse variance weighted (fixed effects)
Inverse variance weighted (multiplicative random effects)
MR Egger
MR RAPS
Weighted median
Weighted mode
0.0050
0.0025
0.0000
−0.0025
0.2
0.4
0.6
0.8
SNP effect on eGFR
SNP effect on Hepatitis A virus cellular receptor 1

Batch 2−1311 : Hepatitis A virus cellular receptor 2 on eGFR

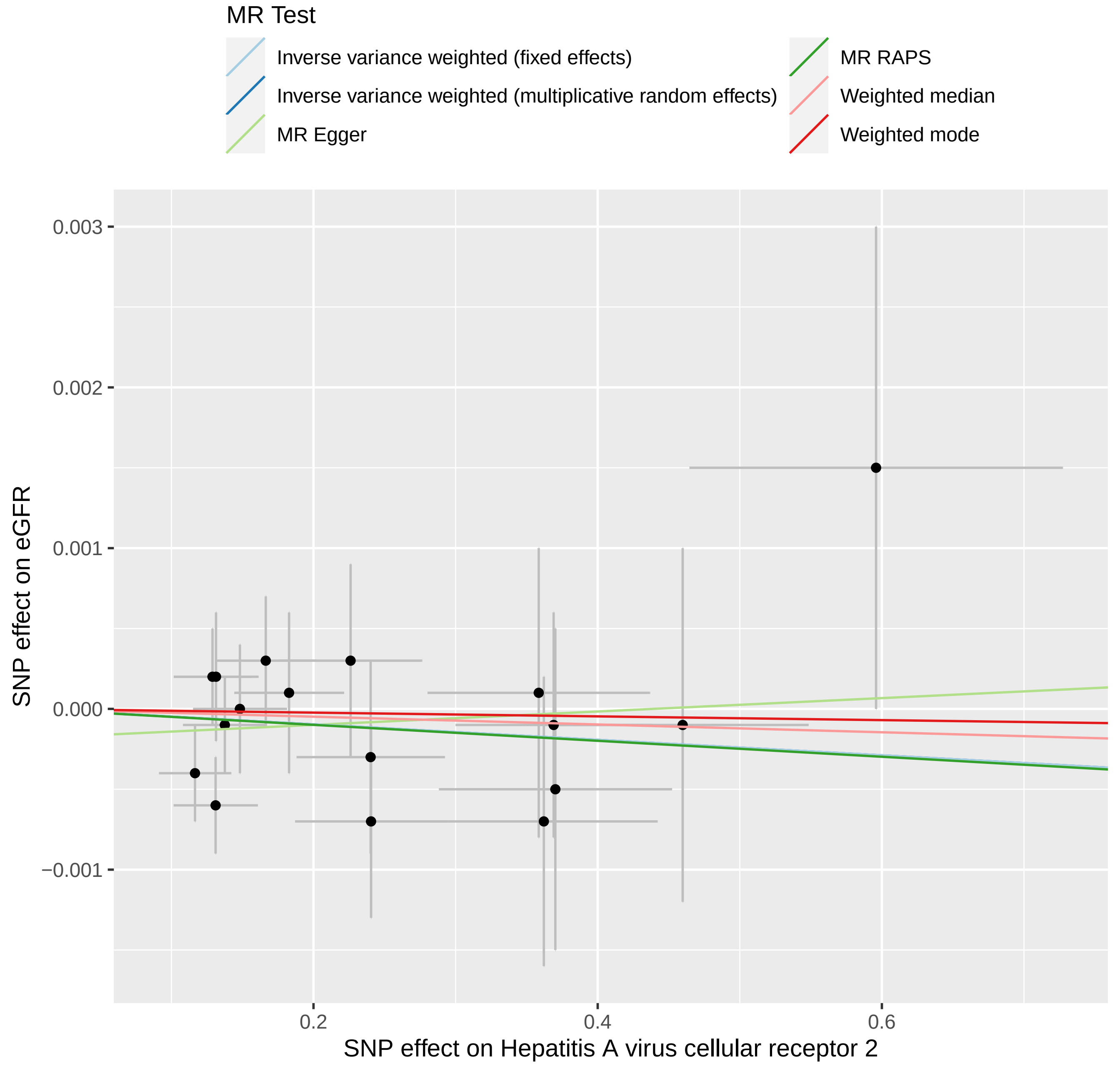

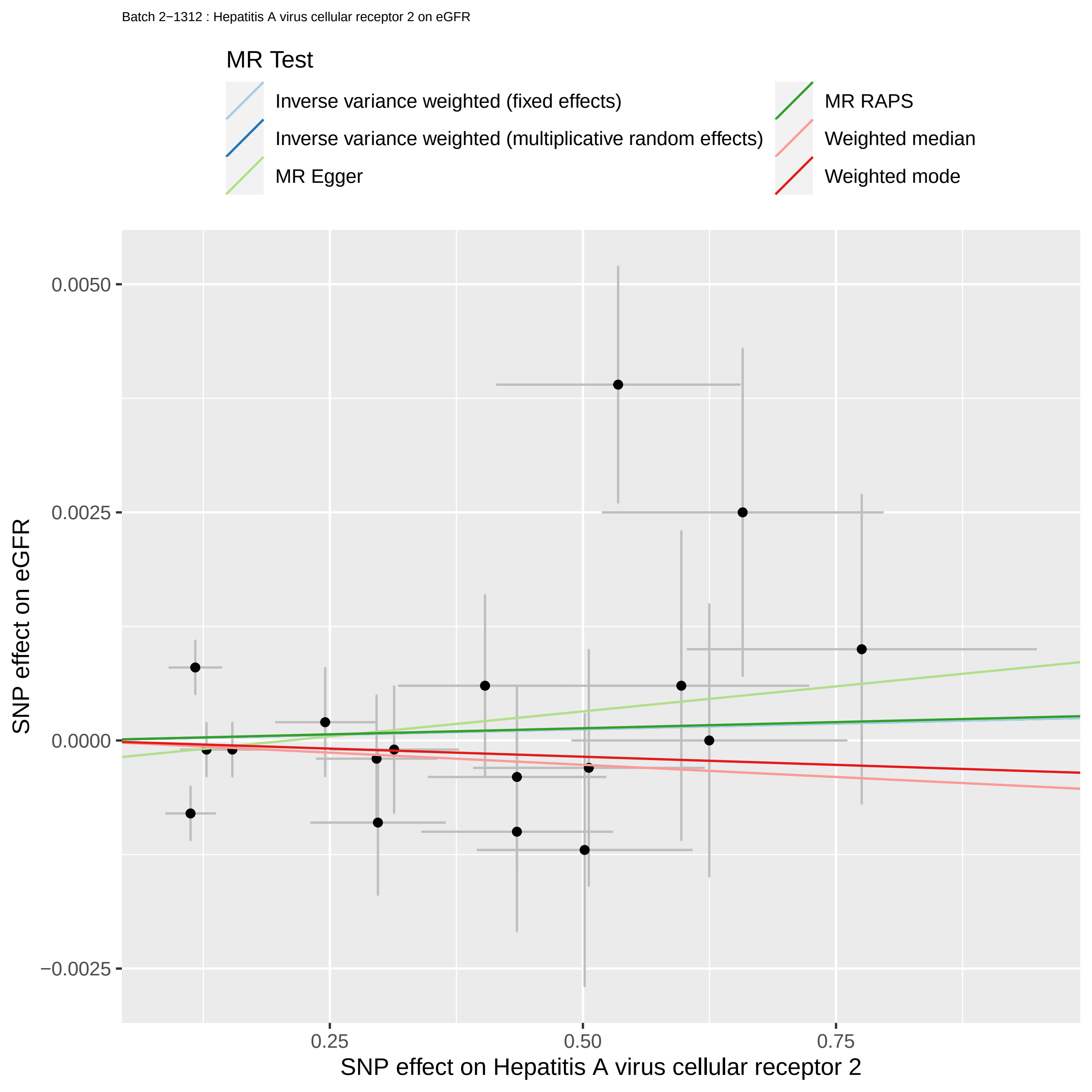

Batch 2−1312 : Hepatitis A virus cellular receptor 2 on eGFR
MR Test
Inverse variance weighted (fixed effects)
Inverse variance weighted (multiplicative random effects)
MR Egger
MR RAPS
Weighted median
Weighted mode
0.0050
0.0025
0.0000
−0.0025
0.25
0.50
0.75
SNP effect on eGFR
SNP effect on Hepatitis A virus cellular receptor 2

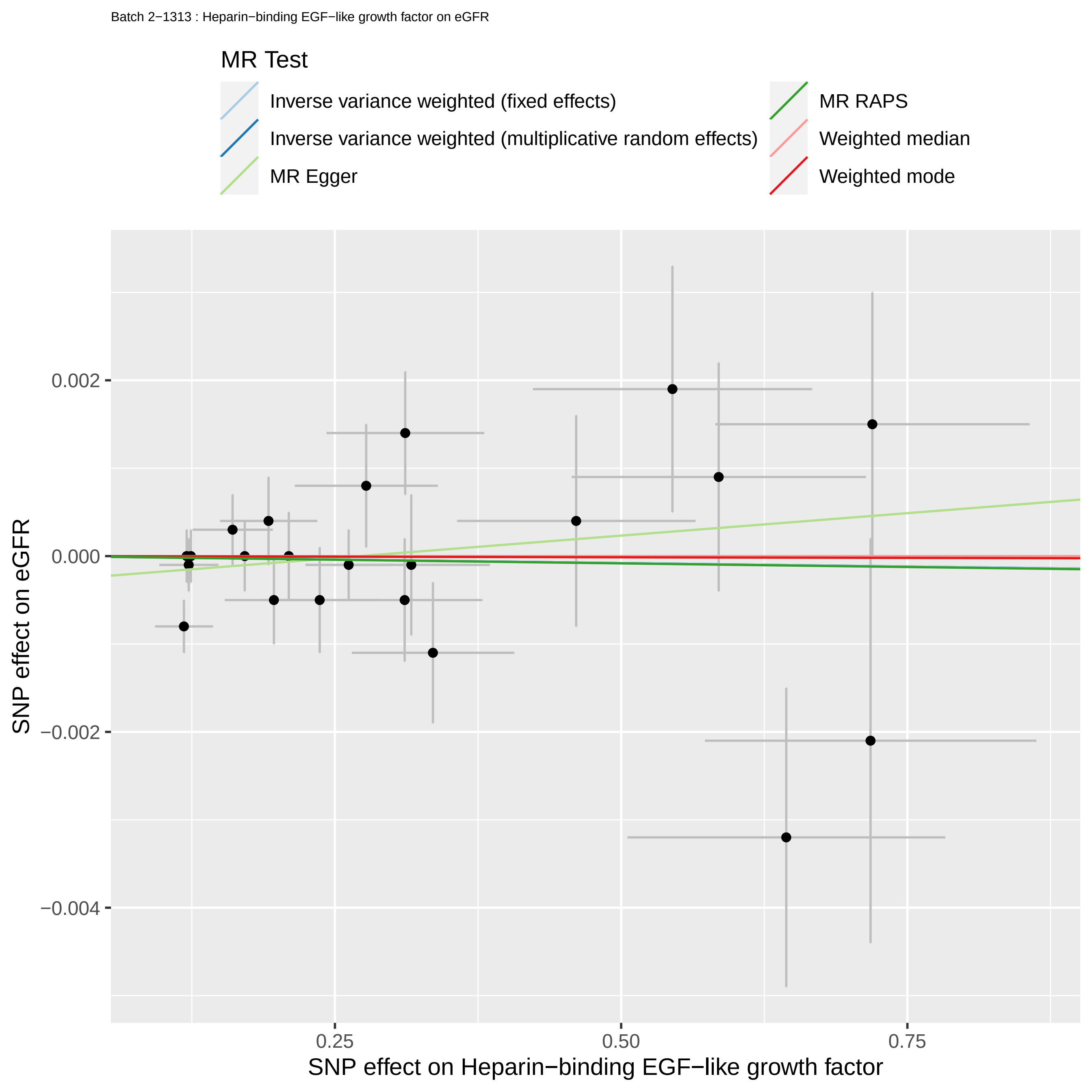

Batch 2−1313 : Heparin−binding EGF−like growth factor on eGFR
MR Test
Inverse variance weighted (fixed effects)
Inverse variance weighted (multiplicative random effects)
MR Egger
MR RAPS
Weighted median
Weighted mode
0.002
0.000
−0.002
−0.004
SNP effect on eGFR
0.25
0.50
0.75
SNP effect on Heparin−binding EGF−like growth factor

Batch 2−1314 : Heparin−binding EGF−like growth factor on eGFR

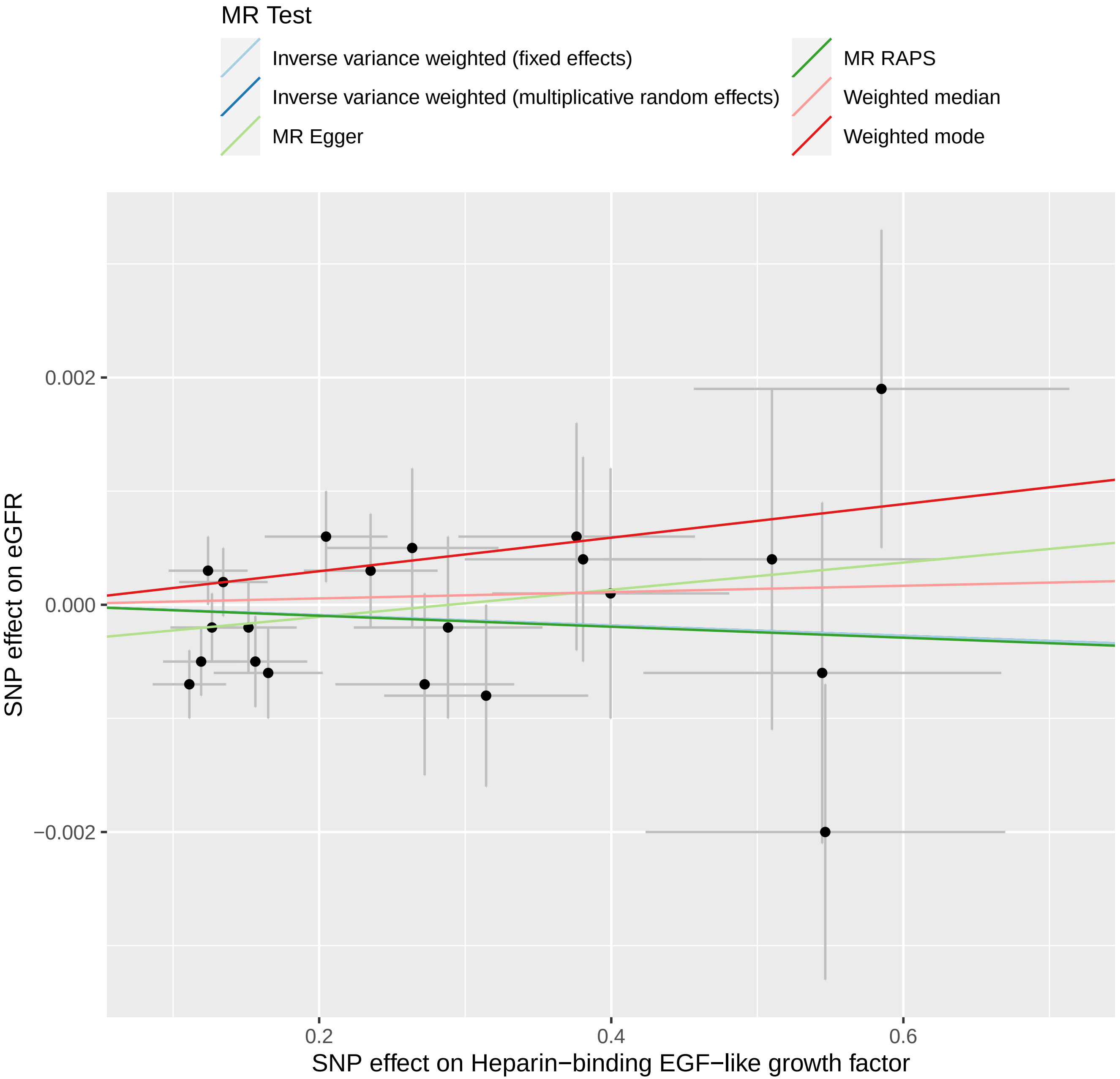

Batch 2−1315 : Hemoglobin subunit gamma−1 on eGFR

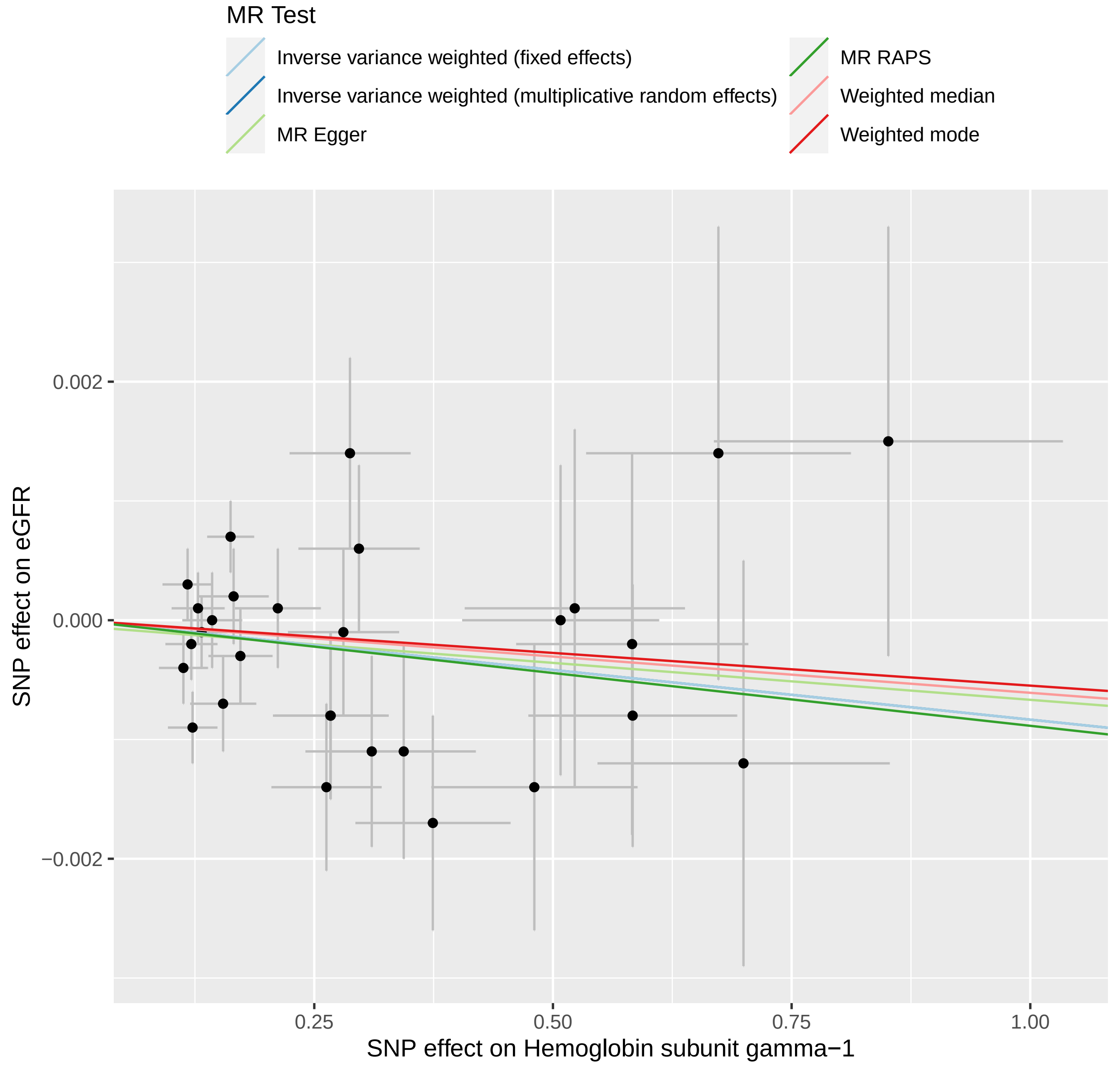

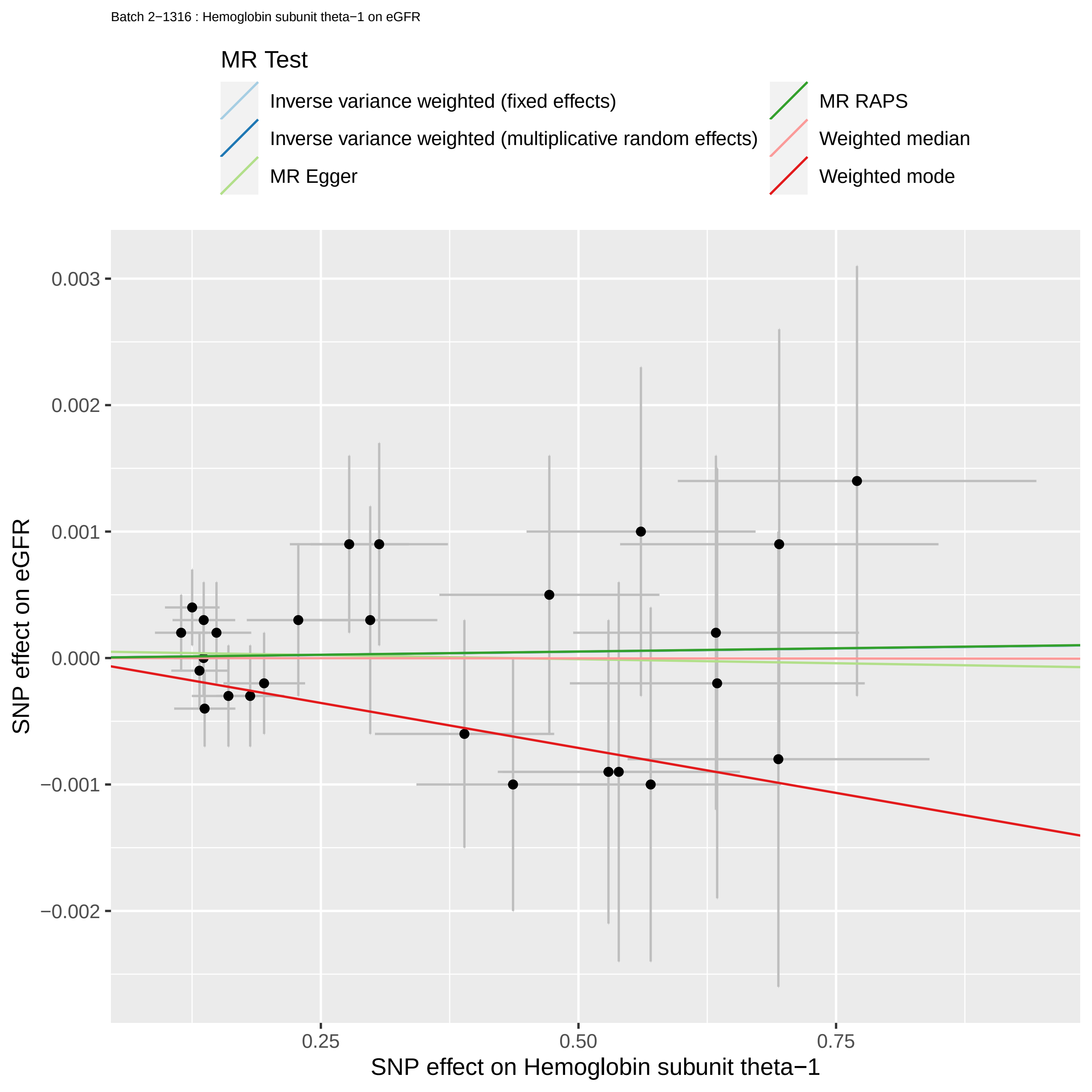

Batch 2−1316 : Hemoglobin subunit theta−1 on eGFR
MR Test
Inverse variance weighted (fixed effects)
Inverse variance weighted (multiplicative random effects)
MR Egger
MR RAPS
Weighted median
Weighted mode
0.003
0.002
0.001
0.000
−0.001
−0.002
SNP effect on eGFR
0.25
0.50
0.75
SNP effect on Hemoglobin subunit theta−1

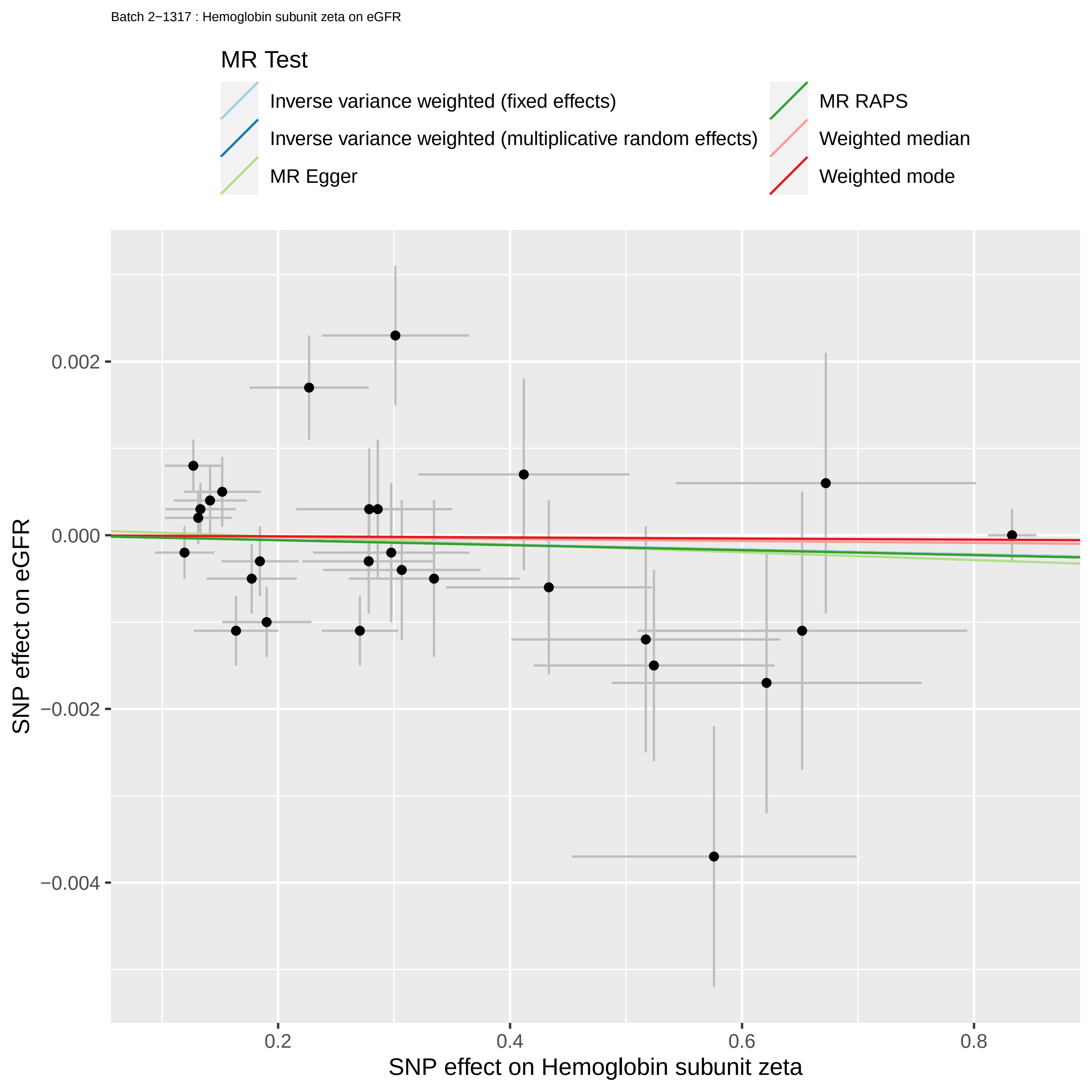

Batch 2−1317 : Hemoglobin subunit zeta on eGFR
MR Test
Inverse variance weighted (fixed effects)
Inverse variance weighted (multiplicative random effects)
MR Egger
MR RAPS
Weighted median
Weighted mode
0.002
0.000
−0.002
−0.004
0.2
0.4
0.6
0.8
SNP effect on eGFR
SNP effect on Hemoglobin subunit zeta

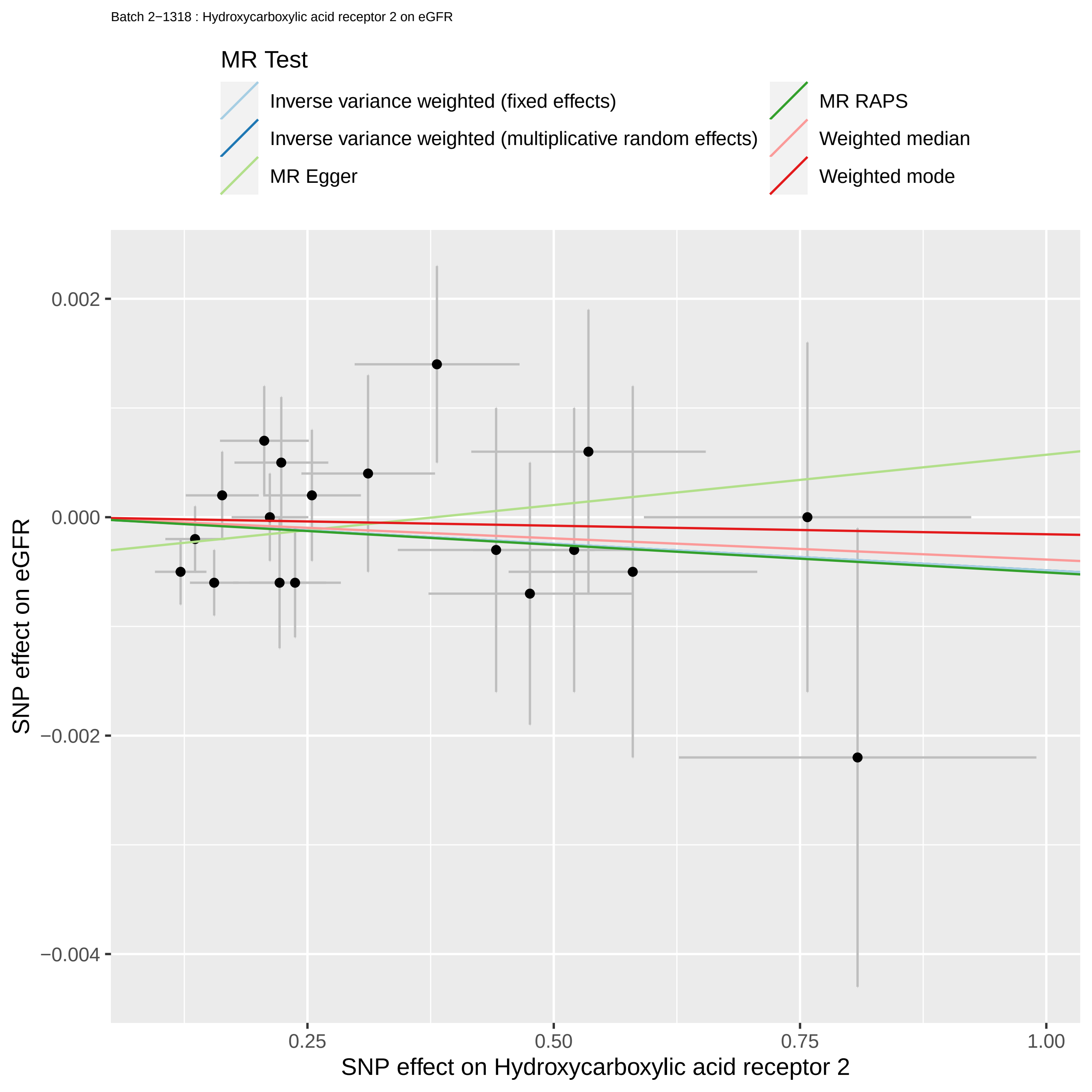

Batch 2−1318 : Hydroxycarboxylic acid receptor 2 on eGFR
MR Test
Inverse variance weighted (fixed effects)
Inverse variance weighted (multiplicative random effects)
MR Egger
MR RAPS
Weighted median
Weighted mode
0.002
0.000
−0.002
−0.004
0.25
0.50
0.75
1.00
SNP effect on eGFR
SNP effect on Hydroxycarboxylic acid receptor 2

Batch 2−1319 : Tyrosine−protein kinase HCK on eGFR

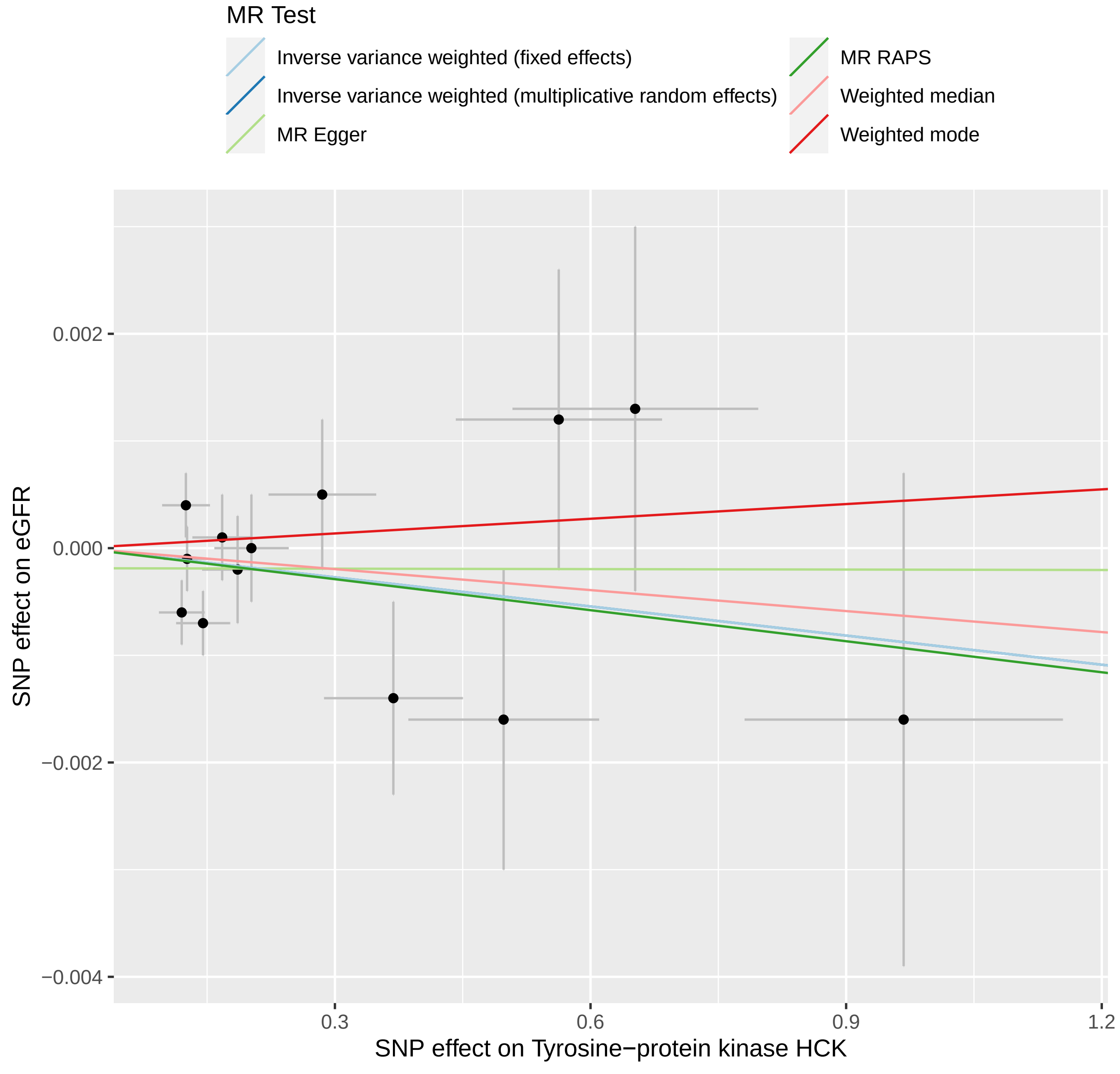

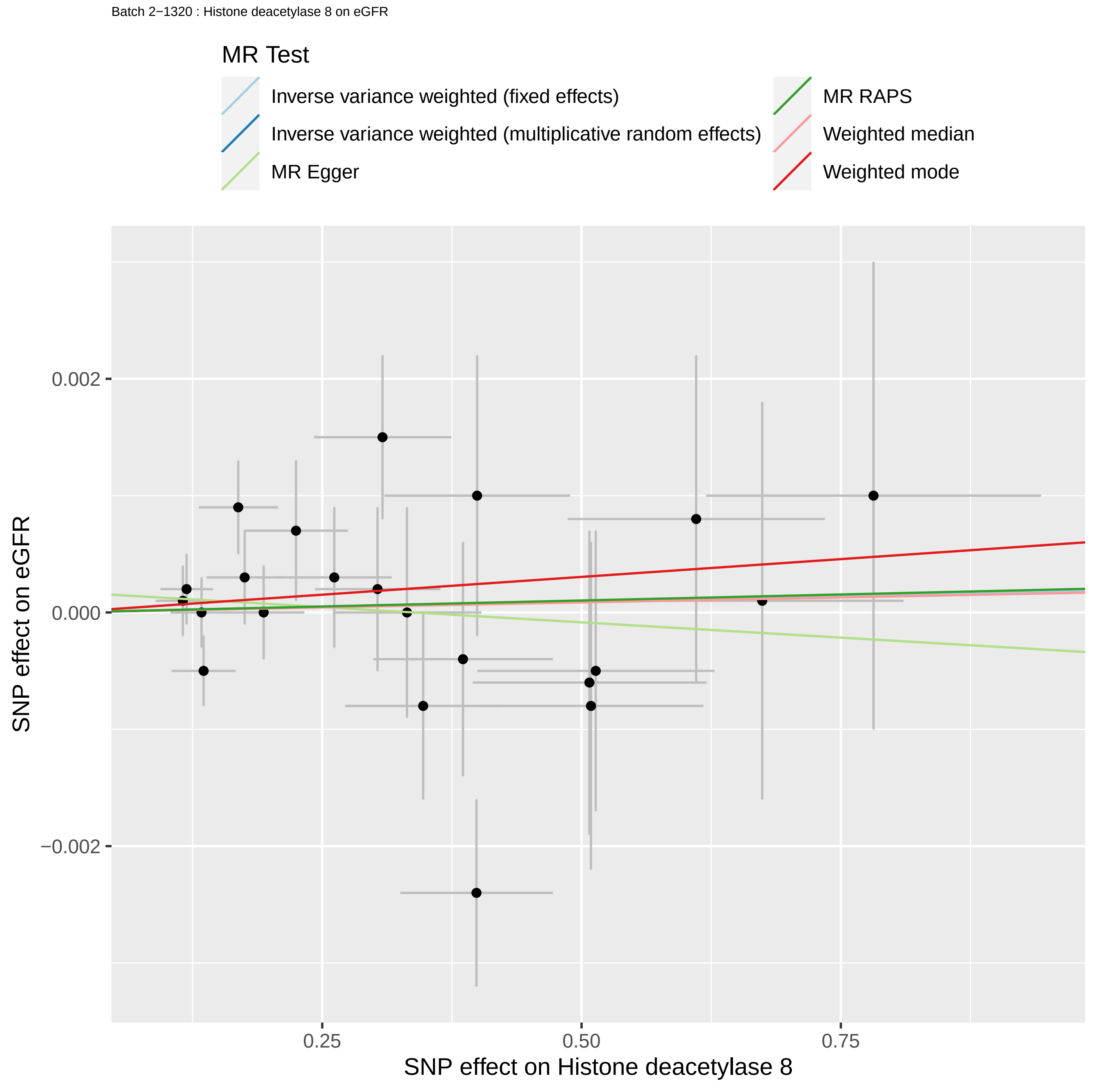

Batch 2−1320 : Histone deacetylase 8 on eGFR
MR Test
Inverse variance weighted (fixed effects)
Inverse variance weighted (multiplicative random effects)
MR Egger
MR RAPS
Weighted median
Weighted mode
0.002
0.000
−0.002
0.25
0.50
0.75
SNP effect on eGFR
SNP effect on Histone deacetylase 8

Batch 2−1321 : Hepatoma−derived growth factor−related protein 2 on eGFR

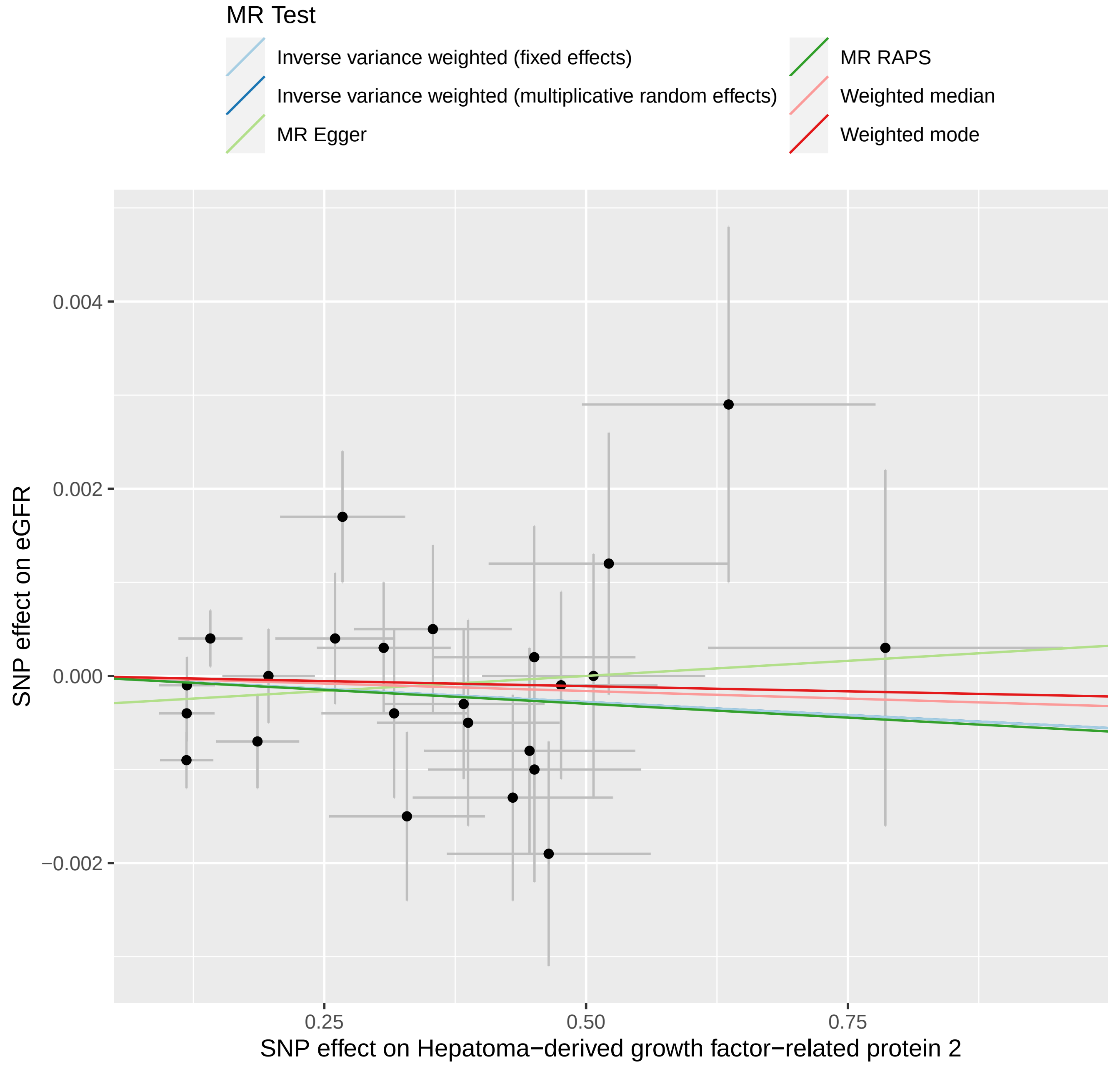

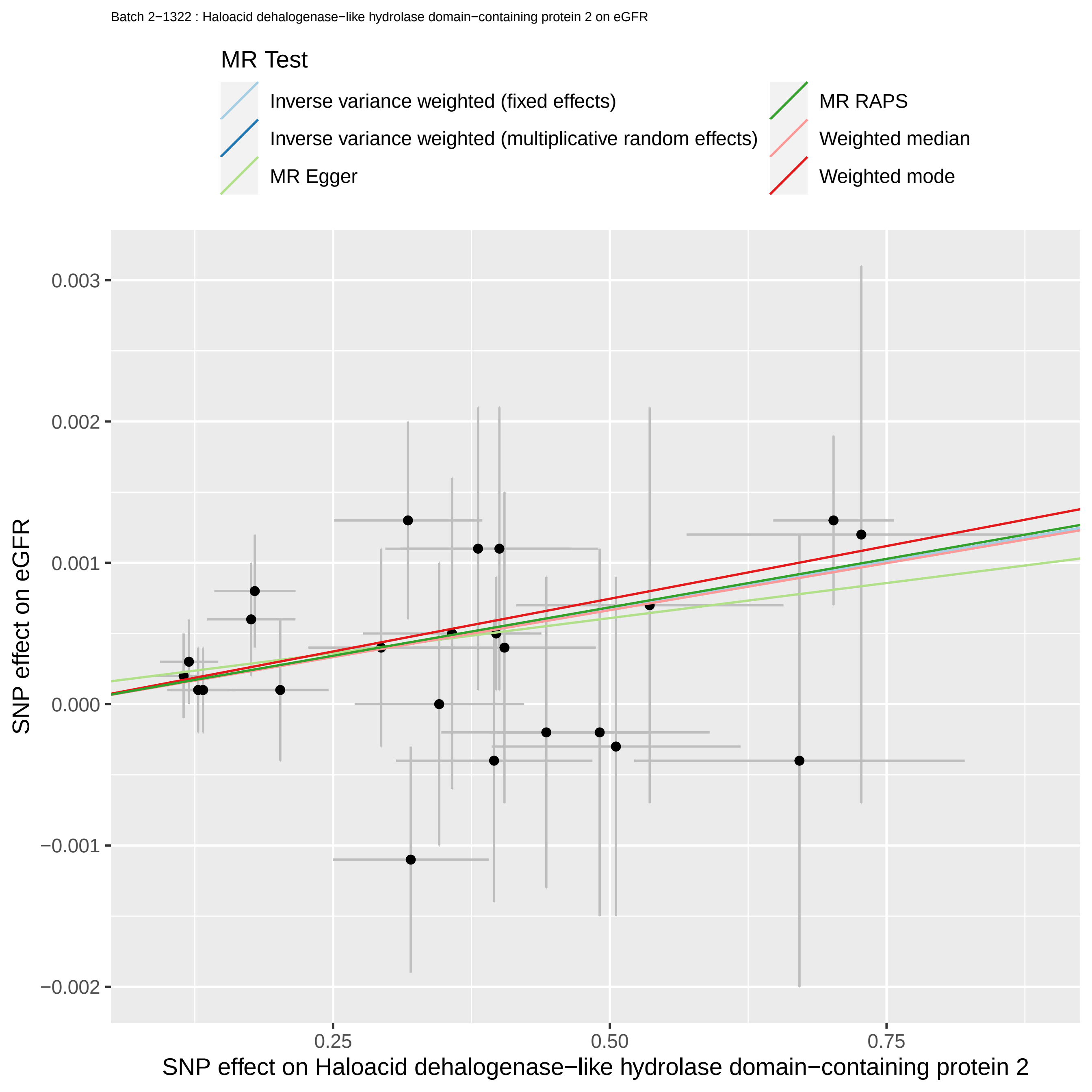
Batch 2−1322 : Haloacid dehalogenase−like hydrolase domain−containing protein 2 on eGFR
MR Test
Inverse variance weighted (fixed effects)
Inverse variance weighted (multiplicative random effects)
MR Egger
MR RAPS
Weighted median
Weighted mode
0.003
0.002
0.001
0.000
−0.001
−0.002
0.25
0.50
0.75
SNP effect on eGFR
SNP effect on Haloacid dehalogenase−like hydrolase domain−containing protein 2

Batch 2−1323 : Vigilin on eGFR

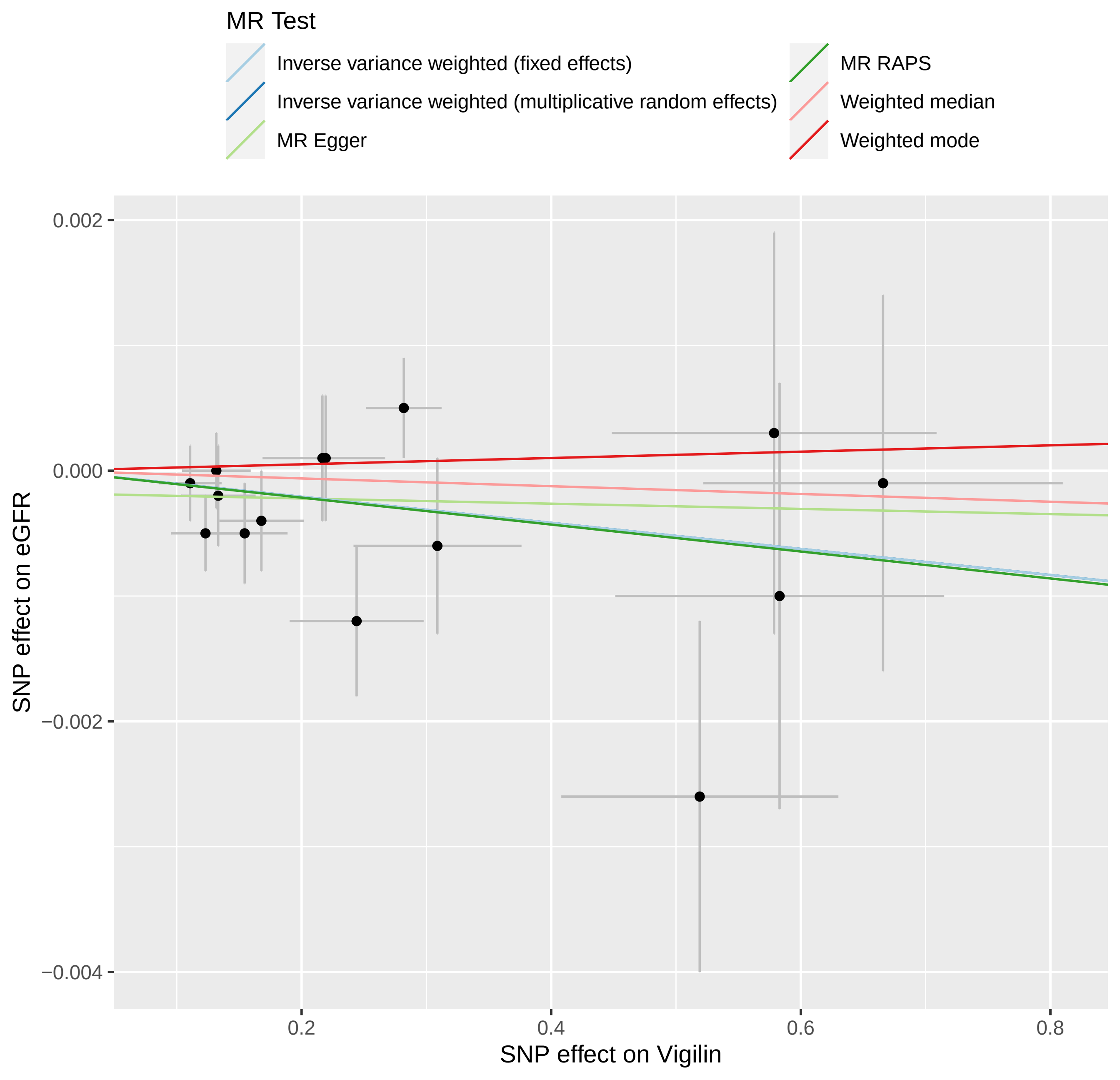

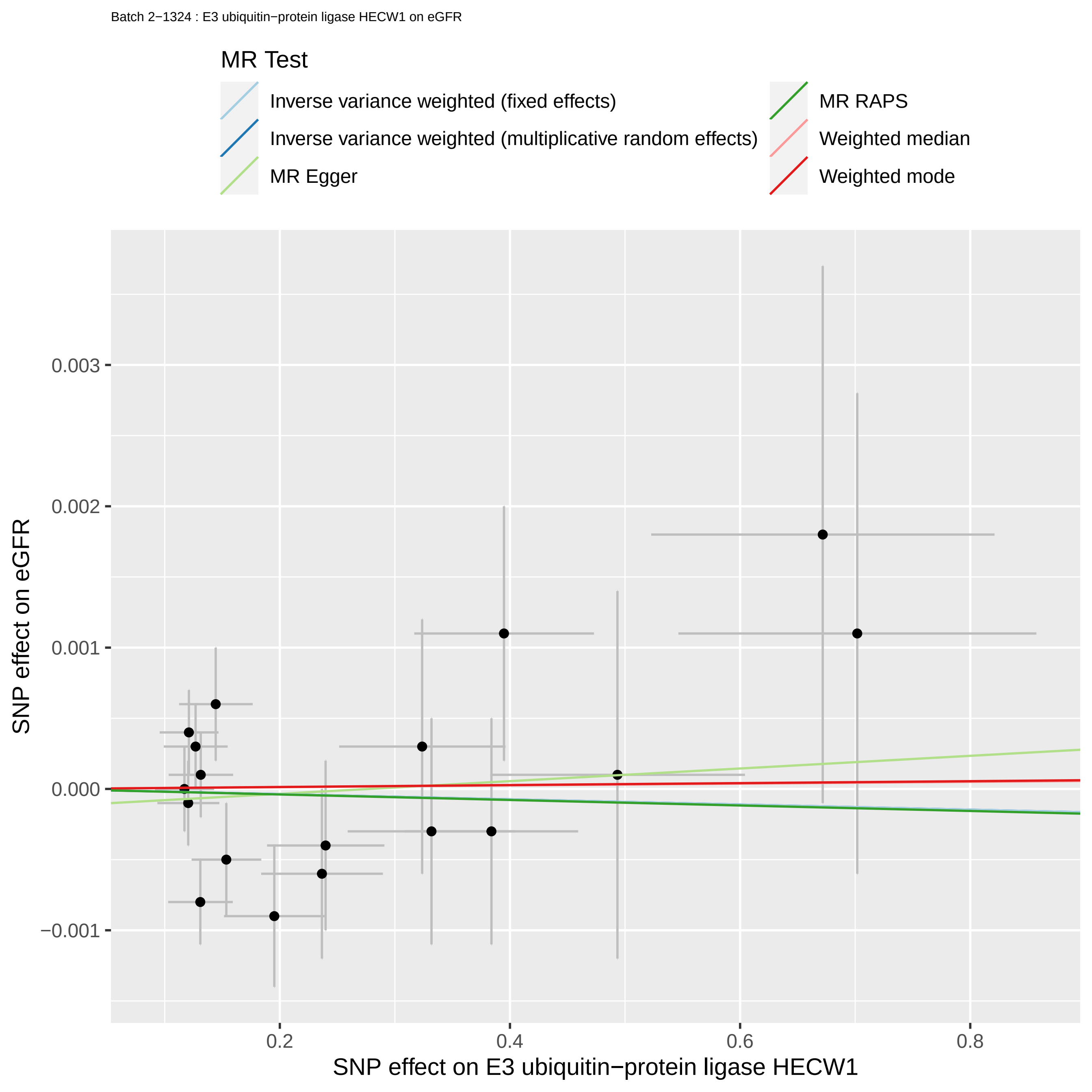

Batch 2−1324 : E3 ubiquitin−protein ligase HECW1 on eGFR
MR Test
Inverse variance weighted (fixed effects)
Inverse variance weighted (multiplicative random effects)
MR Egger
MR RAPS
Weighted median
Weighted mode
0.003
0.002
0.001
0.000
−0.001
SNP effect on eGFR
0.2
0.4
0.6
0.8
SNP effect on E3 ubiquitin−protein ligase HECW1

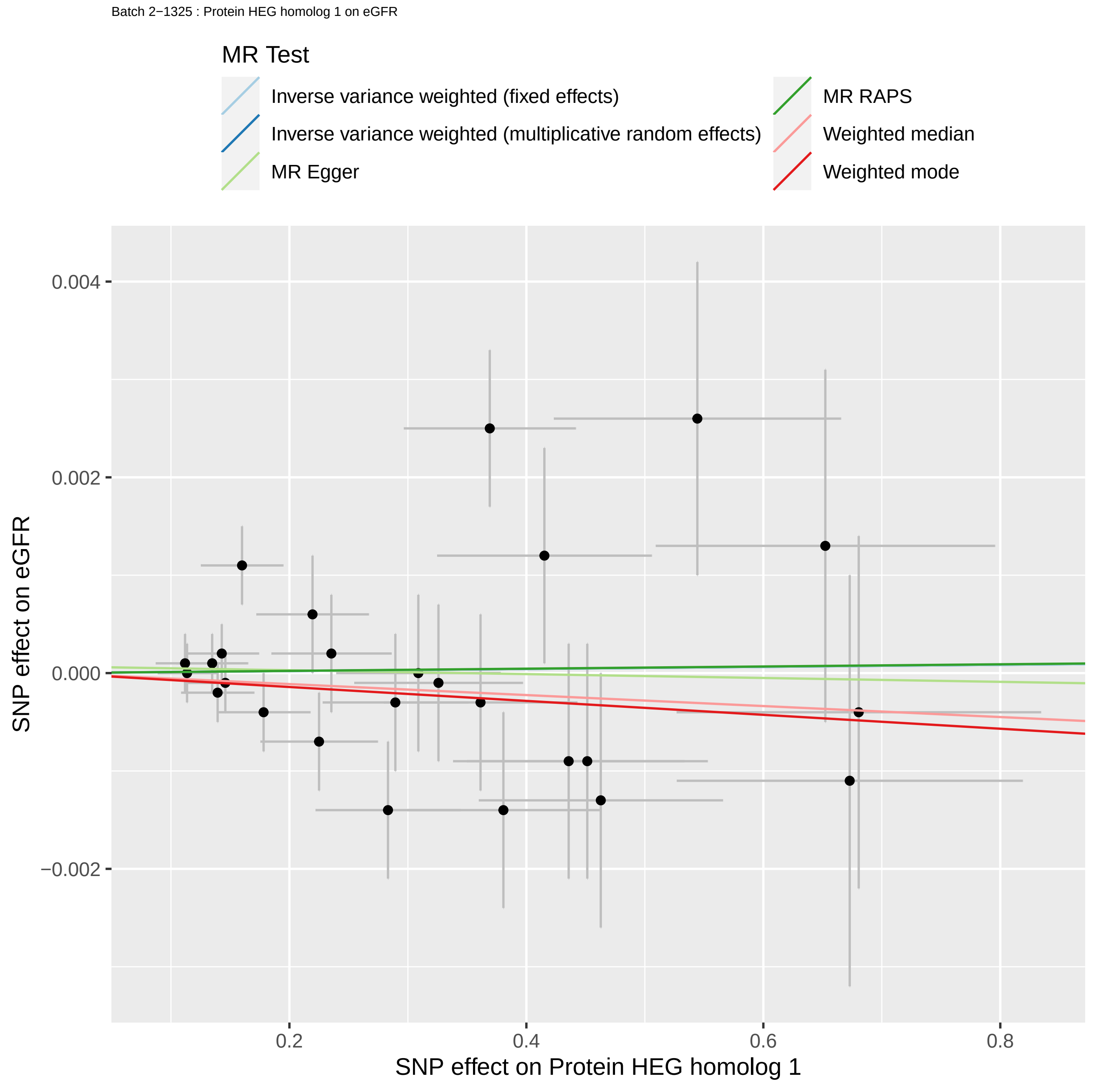

Batch 2−1325 : Protein HEG homolog 1 on eGFR
MR Test
Inverse variance weighted (fixed effects)
Inverse variance weighted (multiplicative random effects)
MR Egger
MR RAPS
Weighted median
Weighted mode
0.004
0.002
0.000
−0.002
0.2
0.4
0.6
0.8
SNP effect on eGFR
SNP effect on Protein HEG homolog 1

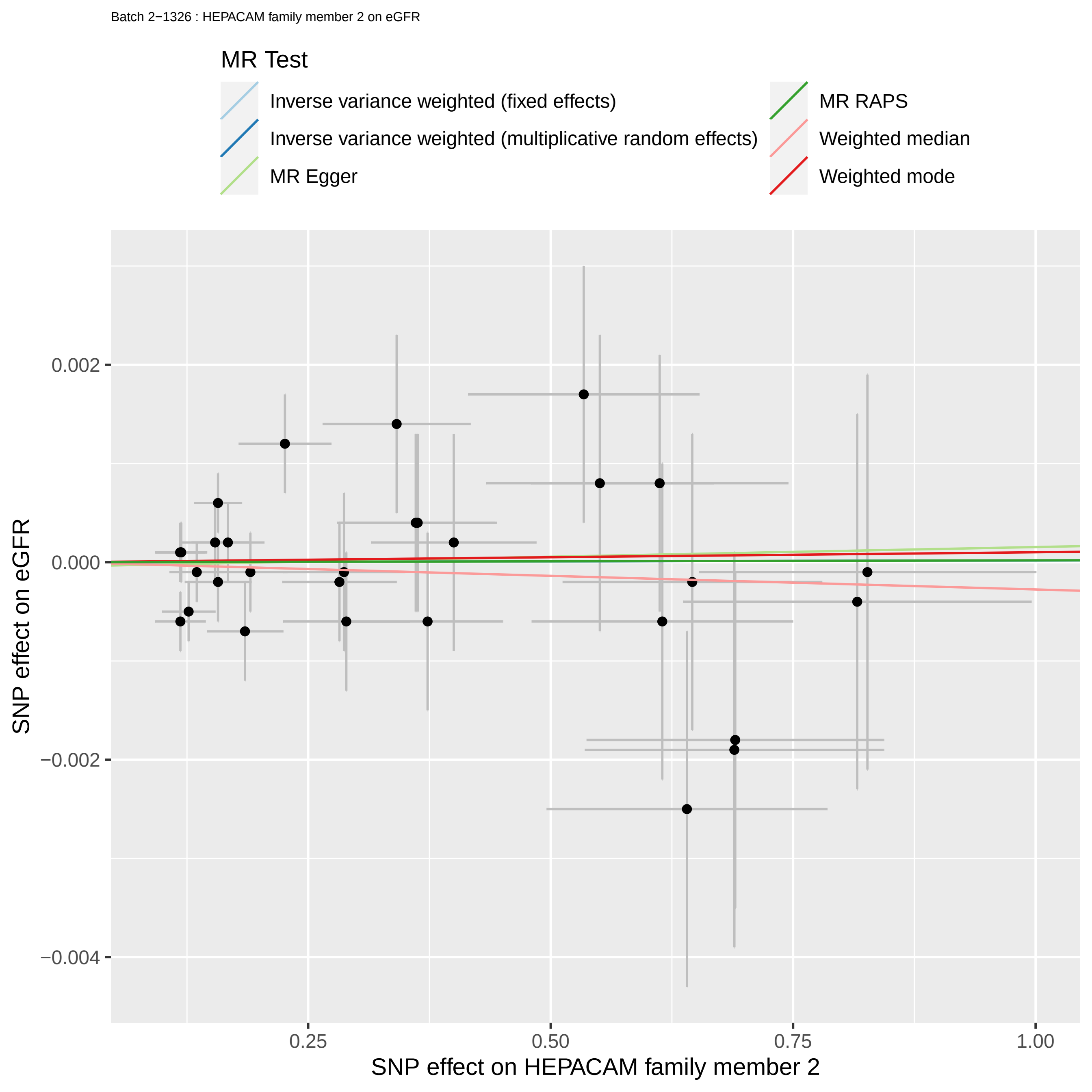
Batch 2−1326 : HEPACAM family member 2 on eGFR
MR Test
Inverse variance weighted (fixed effects)
Inverse variance weighted (multiplicative random effects)
MR Egger
MR RAPS
Weighted median
Weighted mode
0.002
0.000
−0.002
−0.004
SNP effect on eGFR
0.25
0.50
0.75
1.00
SNP effect on HEPACAM family member 2

Batch 2−1327 : HEPACAM family member 2 on eGFR

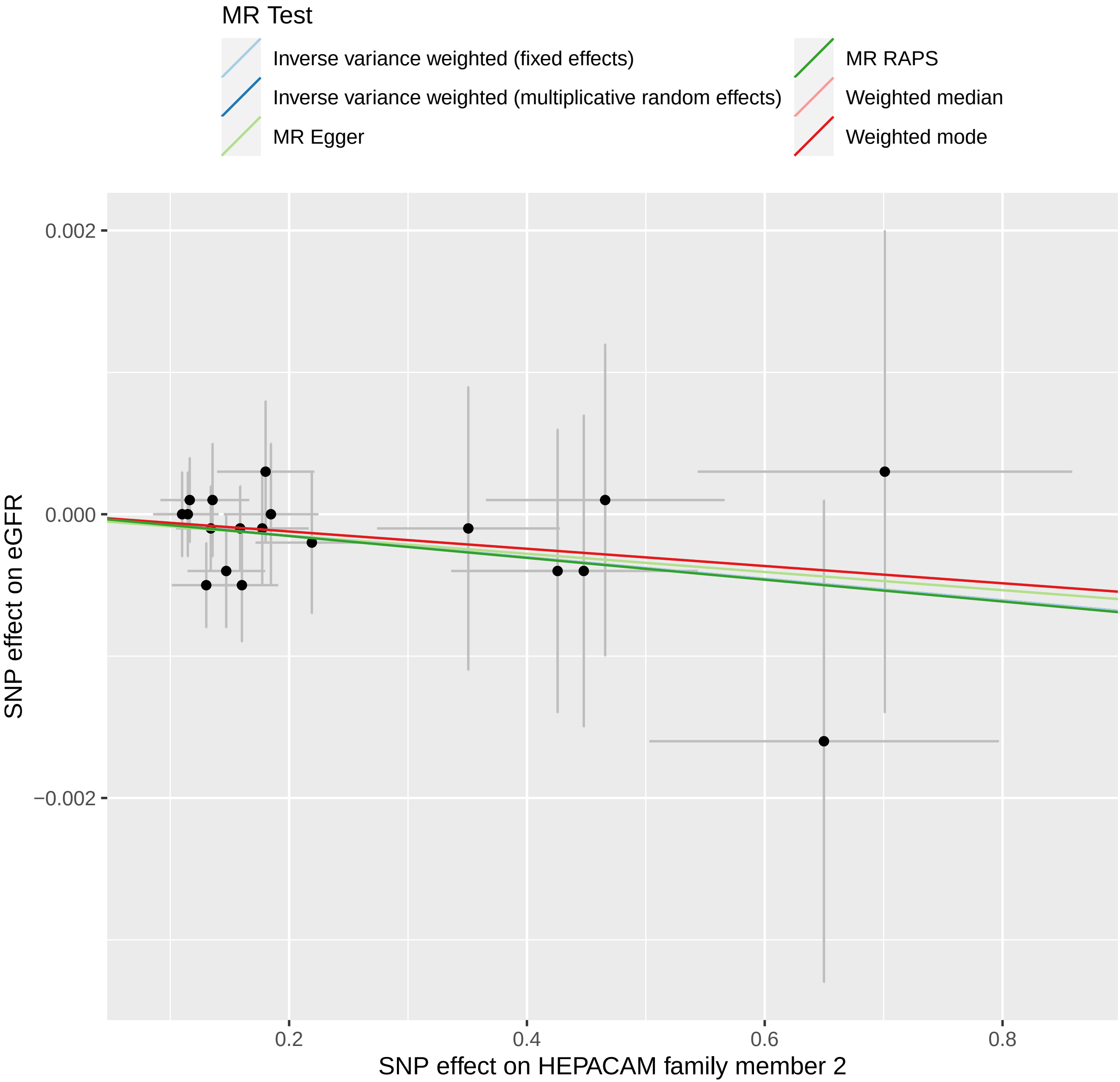

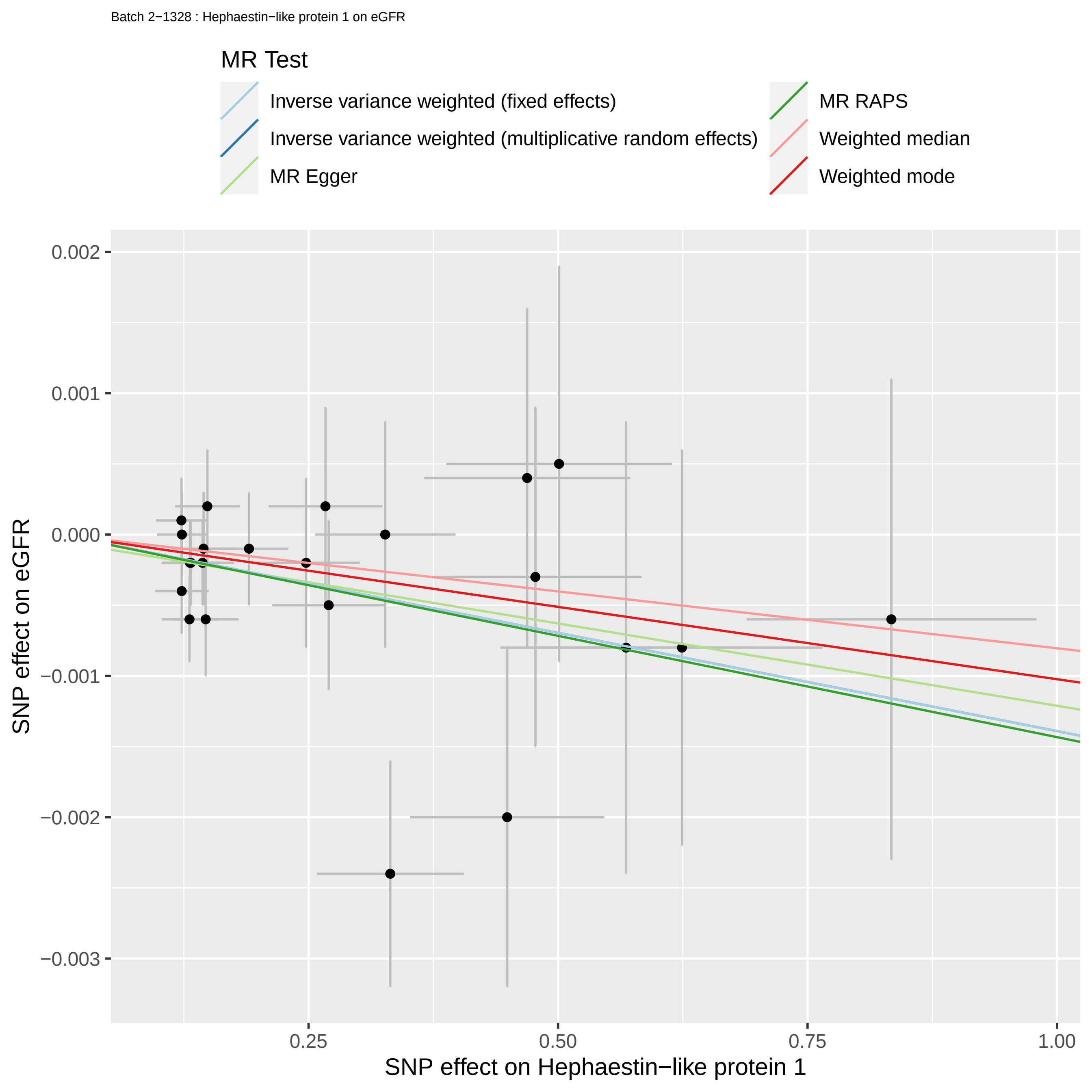

Batch 2−1328 : Hephaestin−like protein 1 on eGFR
MR Test
Inverse variance weighted (fixed effects)
Inverse variance weighted (multiplicative random effects)
MR Egger
MR RAPS
Weighted median
Weighted mode
0.002
0.001
0.000
−0.001
−0.002
−0.003
SNP effect on eGFR
0.25
0.50
0.75
1.00
SNP effect on Hephaestin−like protein 1

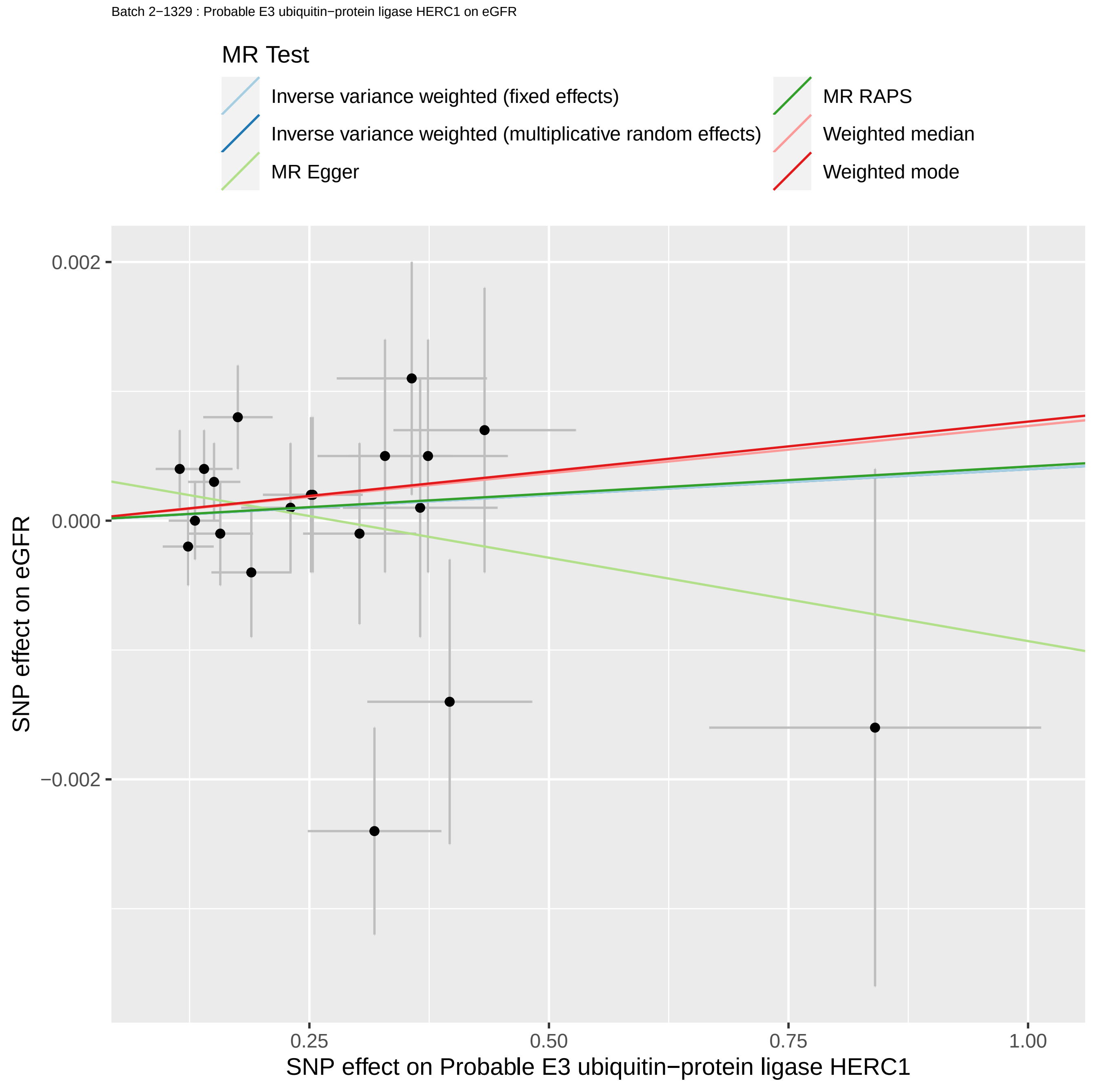
Batch 2−1329 : Probable E3 ubiquitin−protein ligase HERC1 on eGFR
MR Test
Inverse variance weighted (fixed effects)
Inverse variance weighted (multiplicative random effects)
MR Egger
MR RAPS
Weighted median
Weighted mode
0.002
0.000
−0.002
SNP effect on eGFR
0.25
0.50
0.75
1.00
SNP effect on Probable E3 ubiquitin−protein ligase HERC1

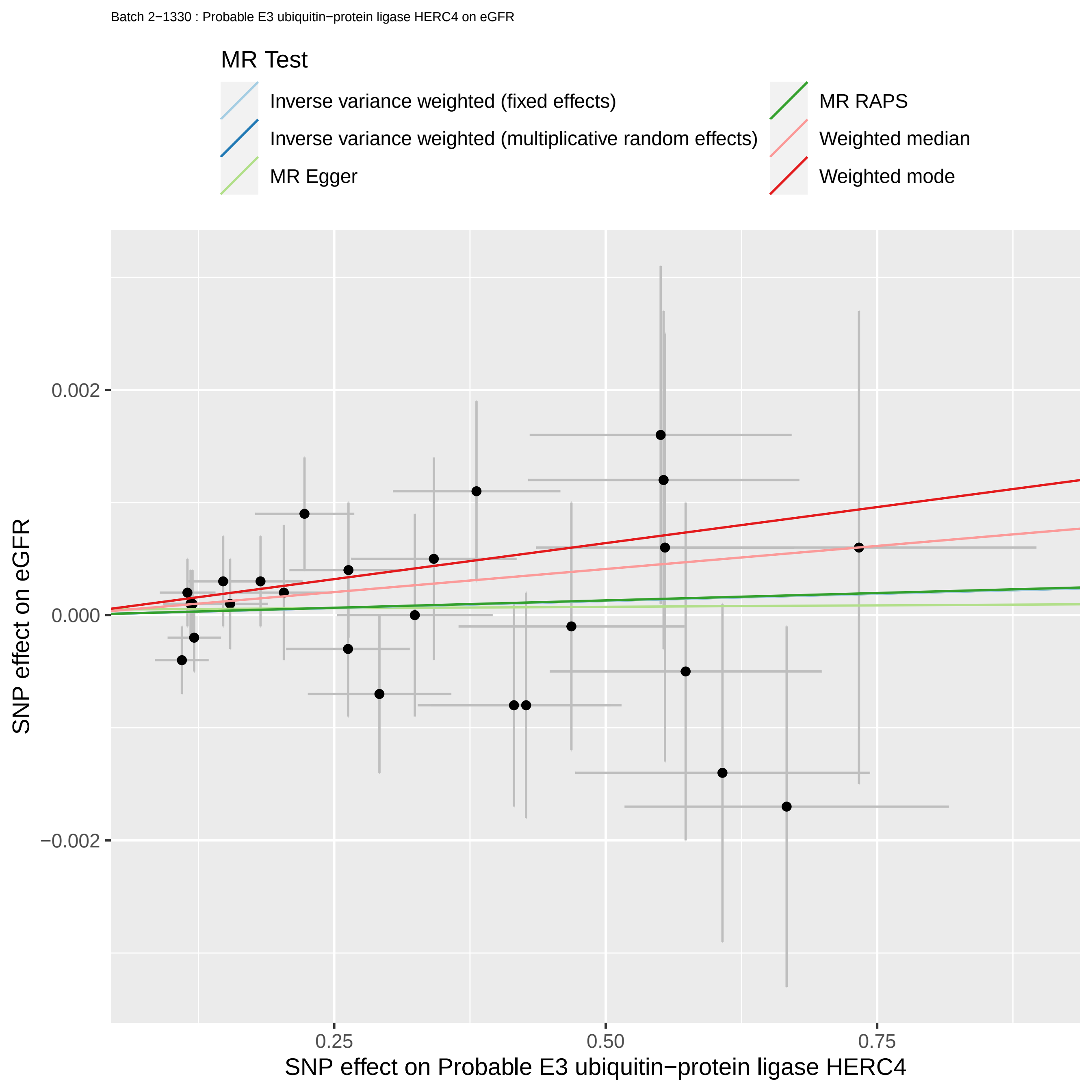

Batch 2−1330 : Probable E3 ubiquitin−protein ligase HERC4 on eGFR
MR Test
Inverse variance weighted (fixed effects)
Inverse variance weighted (multiplicative random effects)
MR Egger
MR RAPS
Weighted median
Weighted mode
0.002
0.000
−0.002
0.25
0.50
0.75
SNP effect on eGFR
SNP effect on Probable E3 ubiquitin−protein ligase HERC4

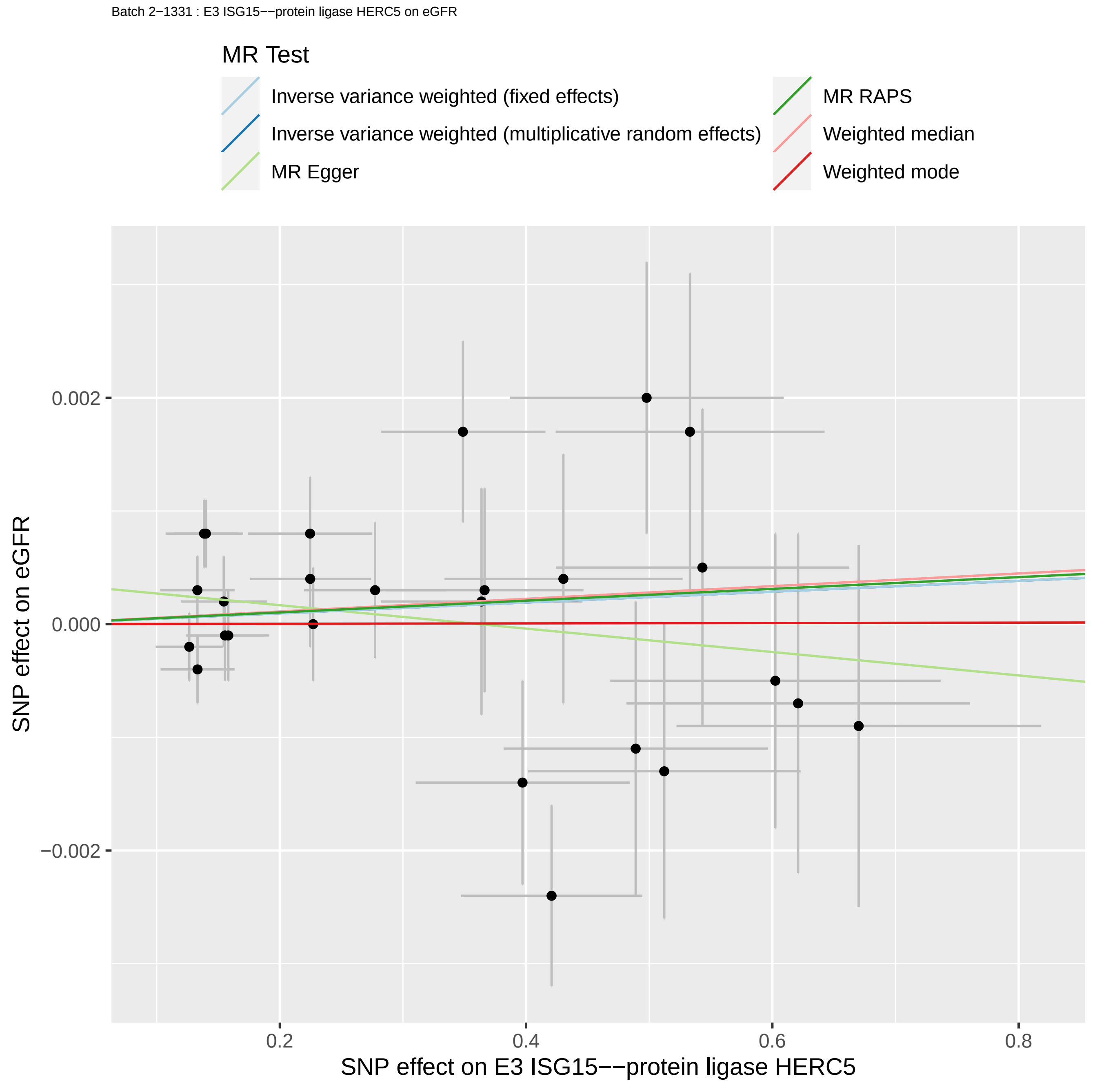
Batch 2−1331 : E3 ISG15−−protein ligase HERC5 on eGFR
MR Test
Inverse variance weighted (fixed effects)
Inverse variance weighted (multiplicative random effects)
MR Egger
MR RAPS
Weighted median
Weighted mode
0.002
0.000
−0.002
0.2
0.4
0.6
0.8
SNP effect on eGFR
SNP effect on E3 ISG15−−protein ligase HERC5

Batch 2−1332 : Hemojuvelin on eGFR

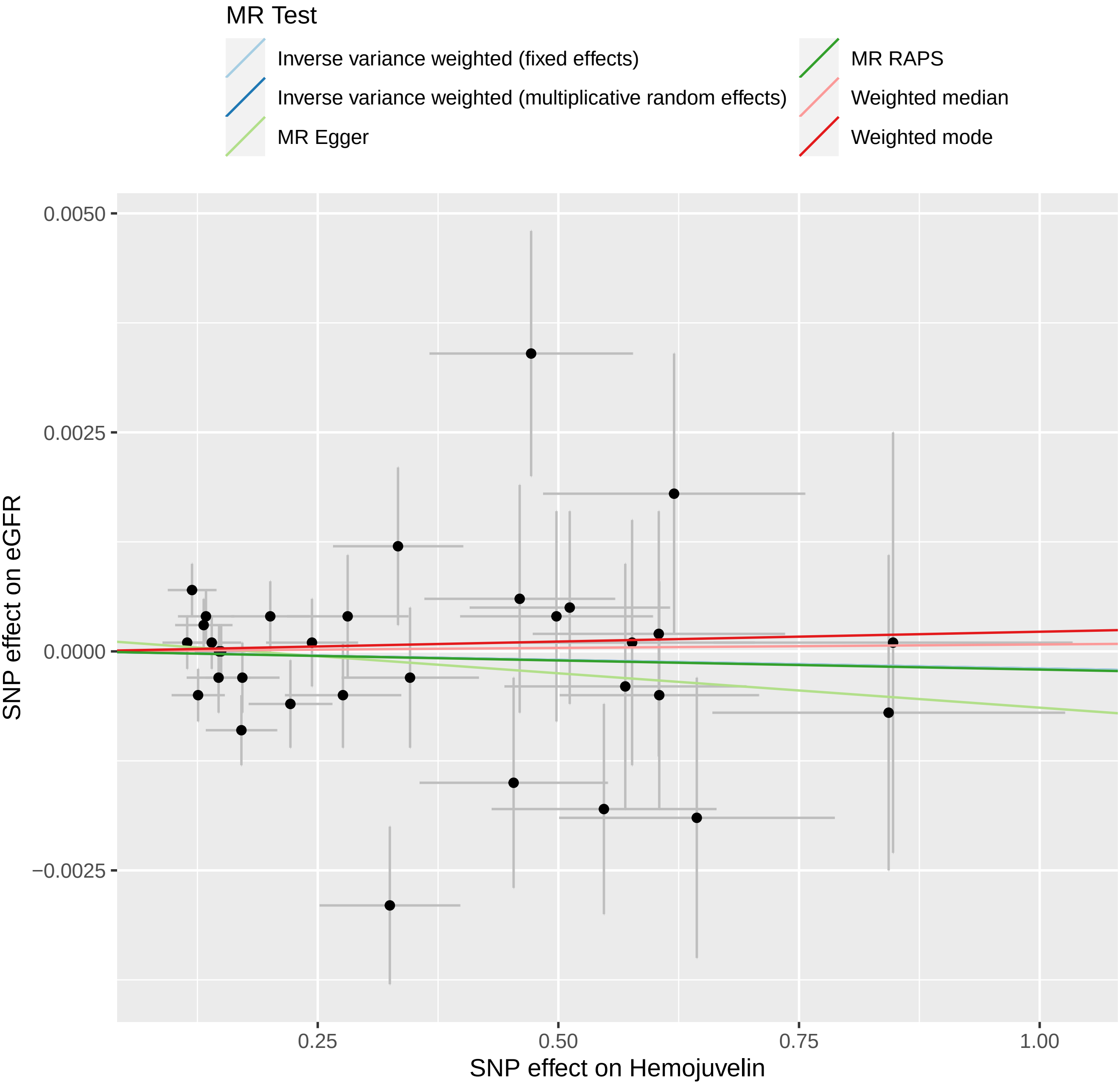

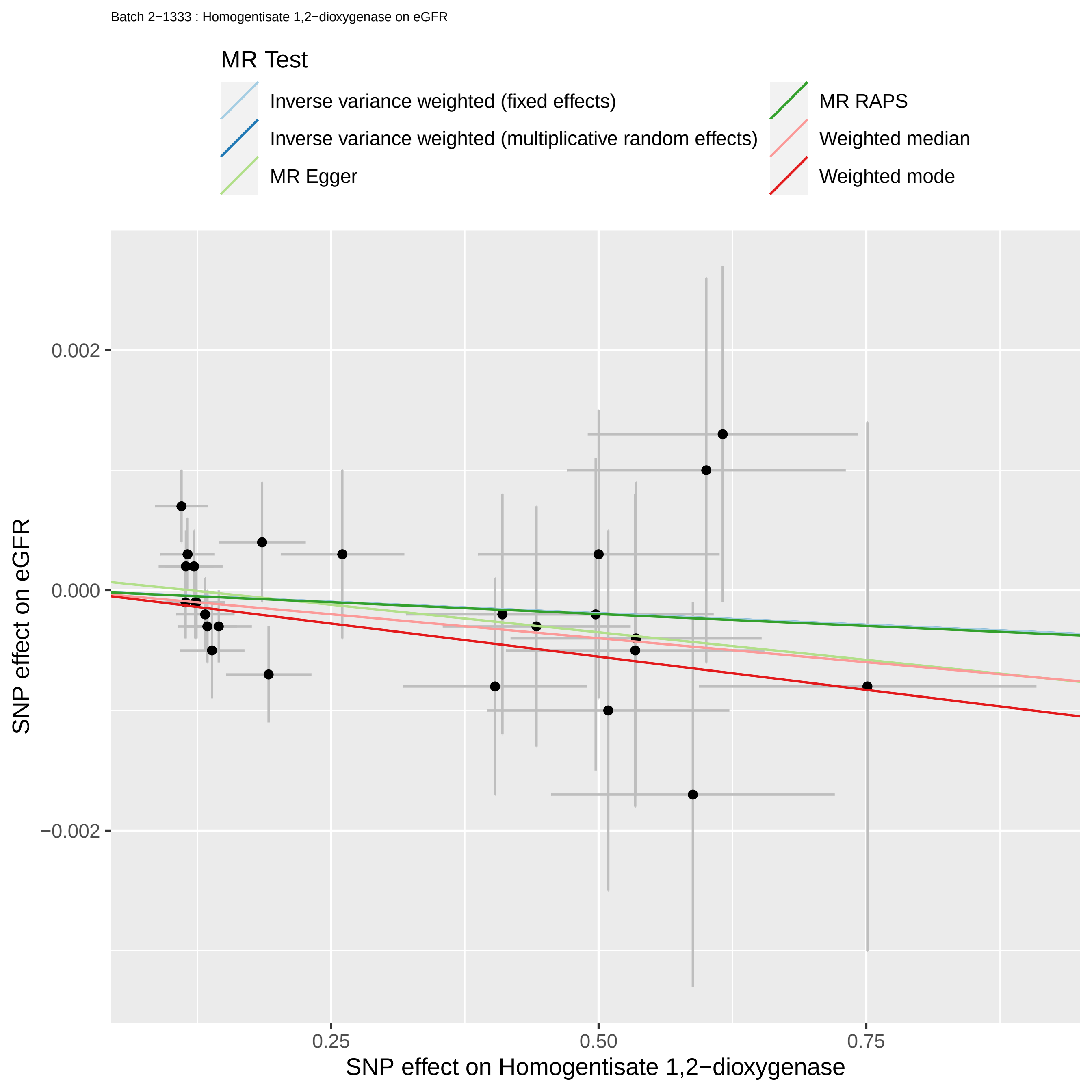

Batch 2−1333 : Homogentisate 1,2−dioxygenase on eGFR
MR Test
Inverse variance weighted (fixed effects)
Inverse variance weighted (multiplicative random effects)
MR Egger
MR RAPS
Weighted median
Weighted mode
0.002
0.000
−0.002
0.25
0.50
0.75
SNP effect on eGFR
SNP effect on Homogentisate 1,2−dioxygenase

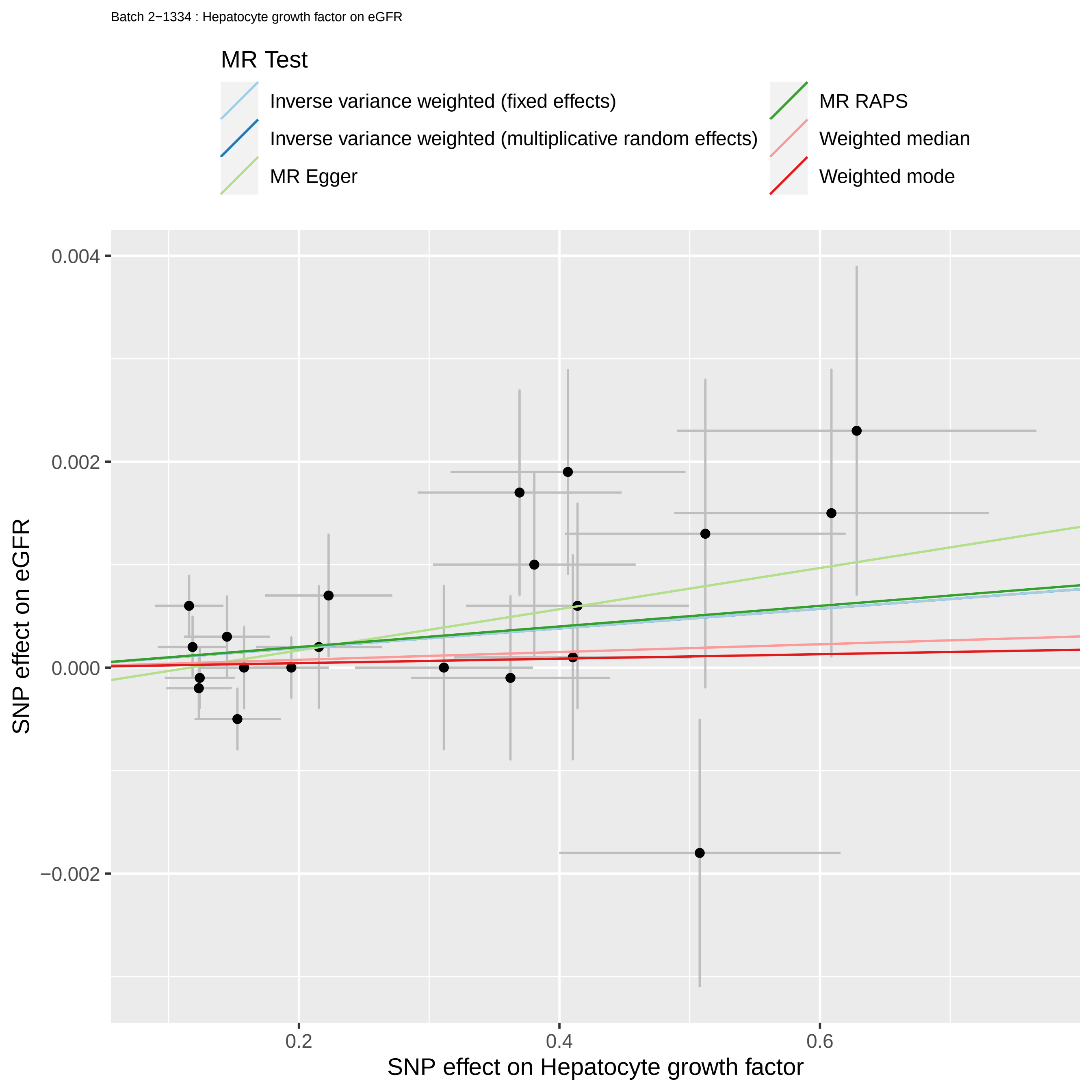

Batch 2−1334 : Hepatocyte growth factor on eGFR
MR Test
Inverse variance weighted (fixed effects)
Inverse variance weighted (multiplicative random effects)
MR Egger
MR RAPS
Weighted median
Weighted mode
0.004
0.002
0.000
−0.002
0.2
0.4
0.6
SNP effect on eGFR
SNP effect on Hepatocyte growth factor

Batch 2−1335 : Hepatocyte growth factor−regulated tyrosine kinase substrate on eGFR

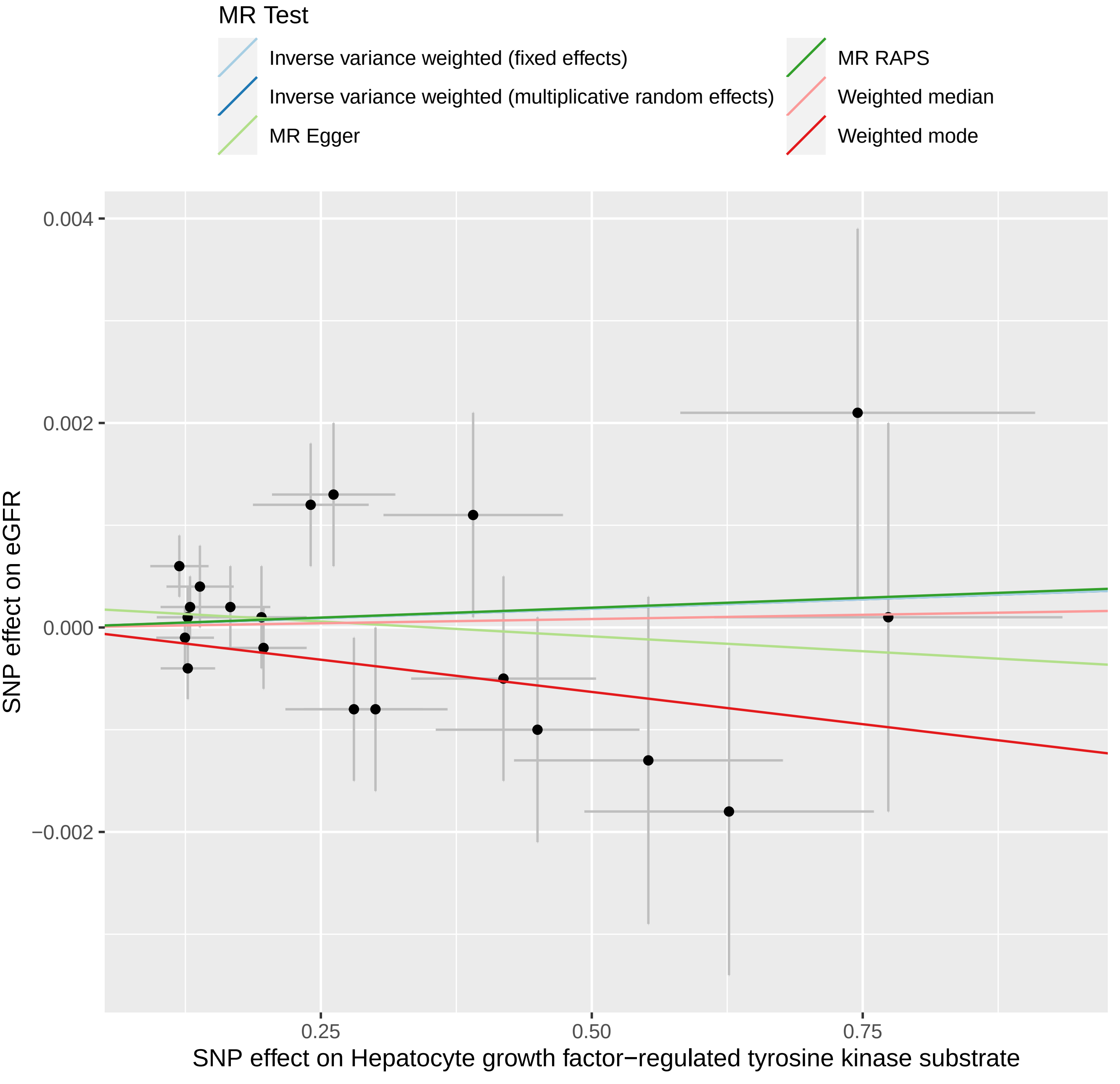

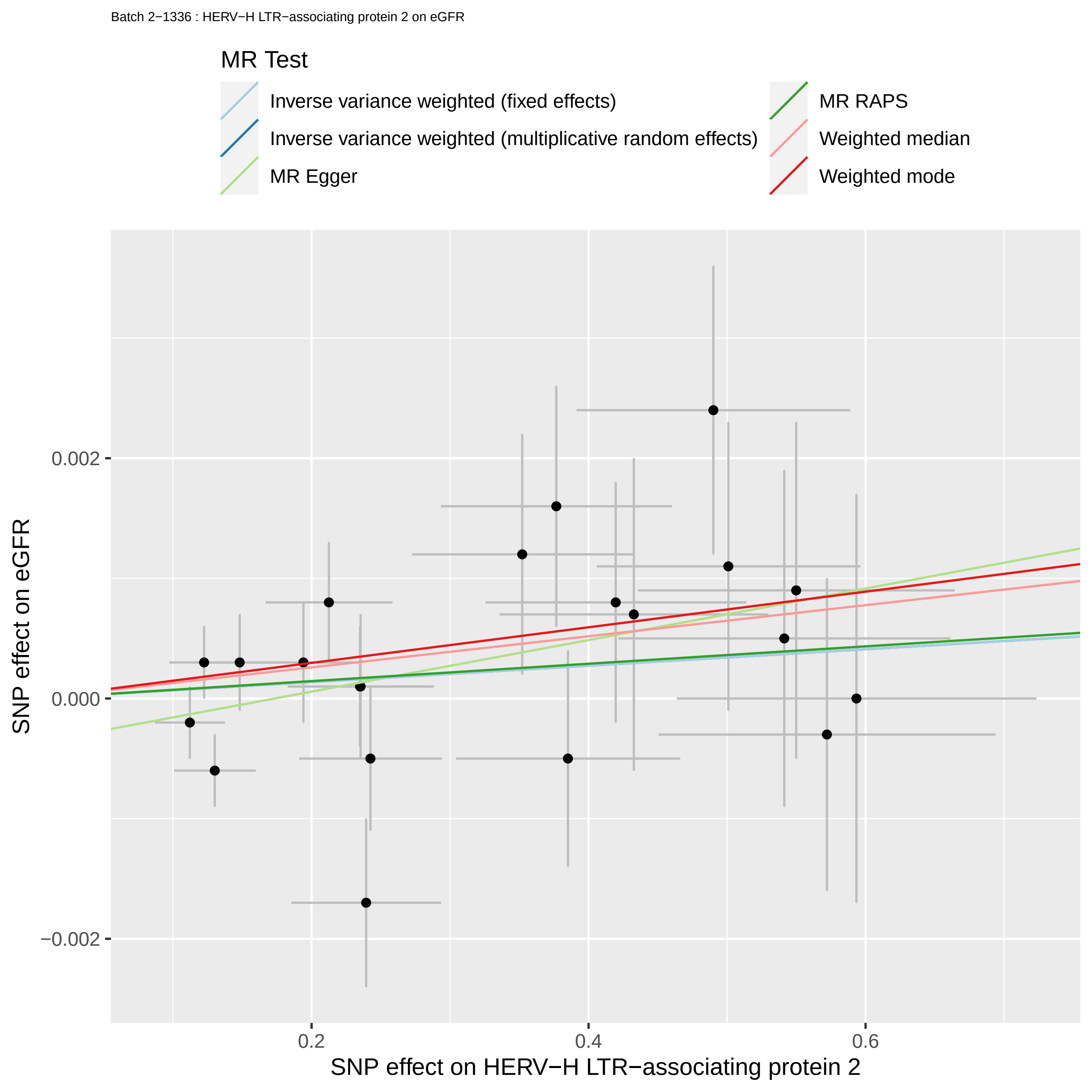

Batch 2−1336 : HERV−H LTR−associating protein 2 on eGFR
MR Test
Inverse variance weighted (fixed effects)
Inverse variance weighted (multiplicative random effects)
MR Egger
MR RAPS
Weighted median
Weighted mode
0.002
0.000
−0.002
0.2
0.4
0.6
SNP effect on eGFR
SNP effect on HERV−H LTR−associating protein 2

Batch 2−1337 : Hypoxia−inducible factor 1−alpha on eGFR

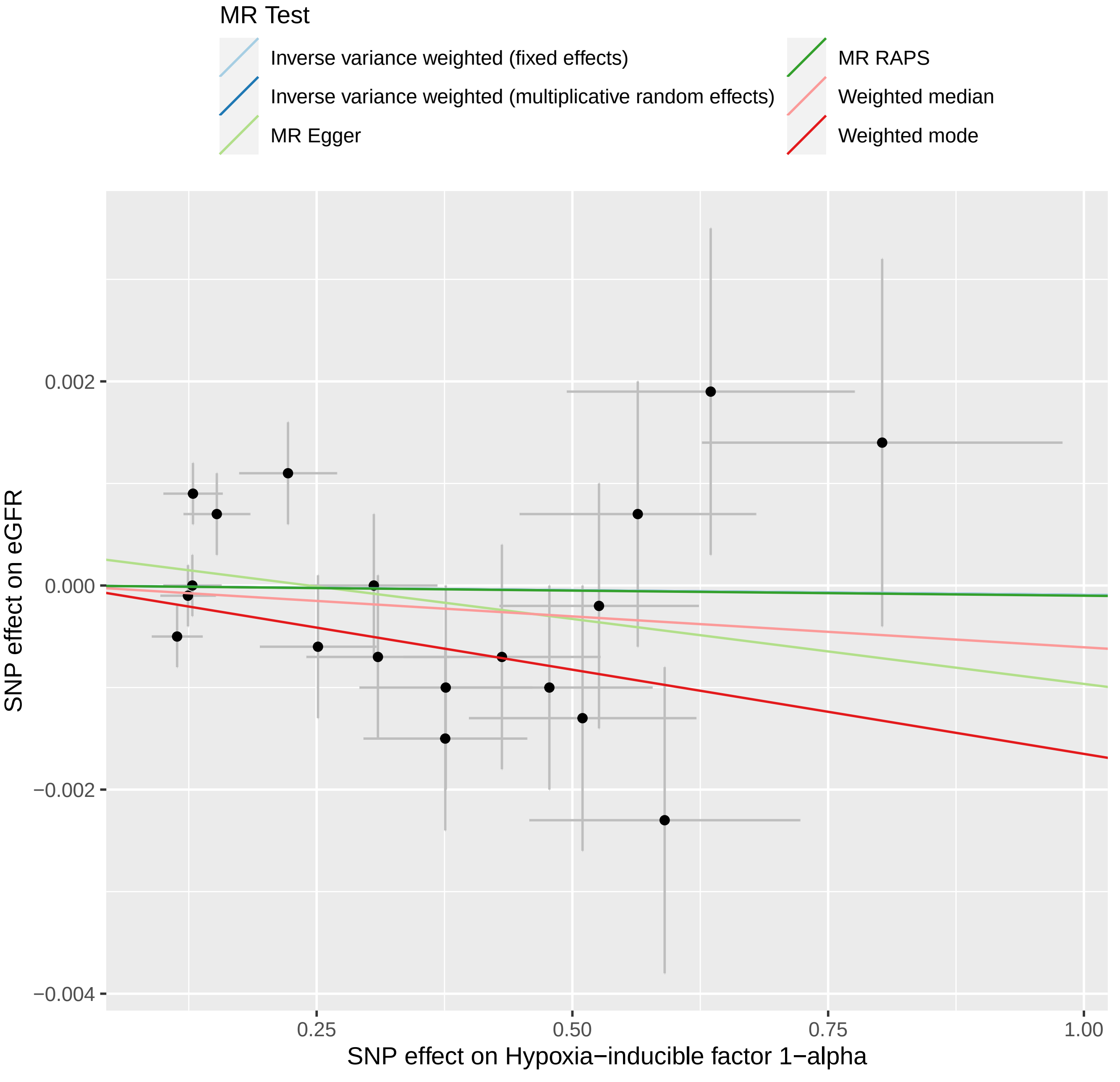

Batch 2−1338 : Histidine triad nucleotide−binding protein 1 on eGFR

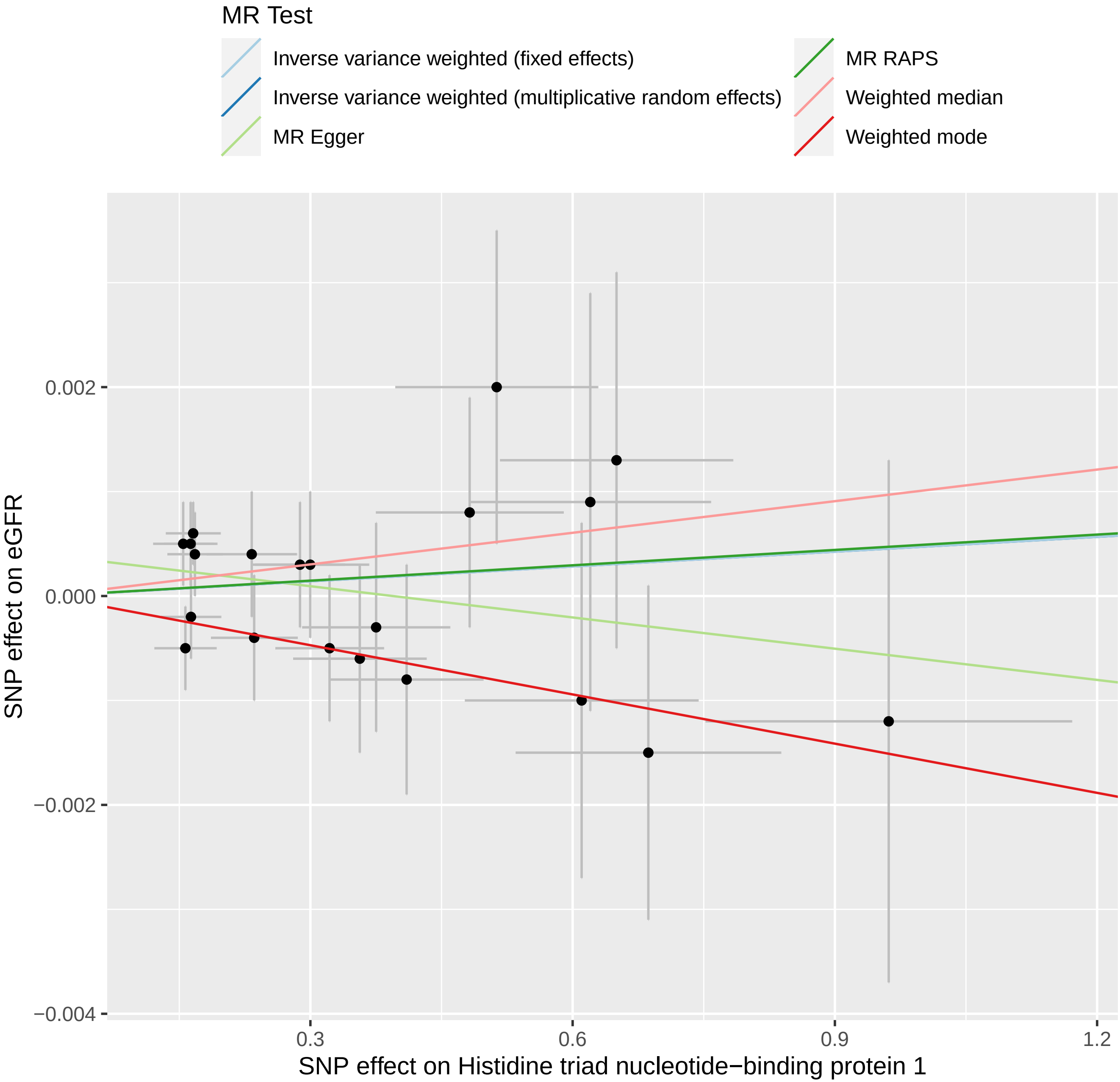

Batch 2−1339 : Histidine triad nucleotide−binding protein 2, mitochondrial on eGFR

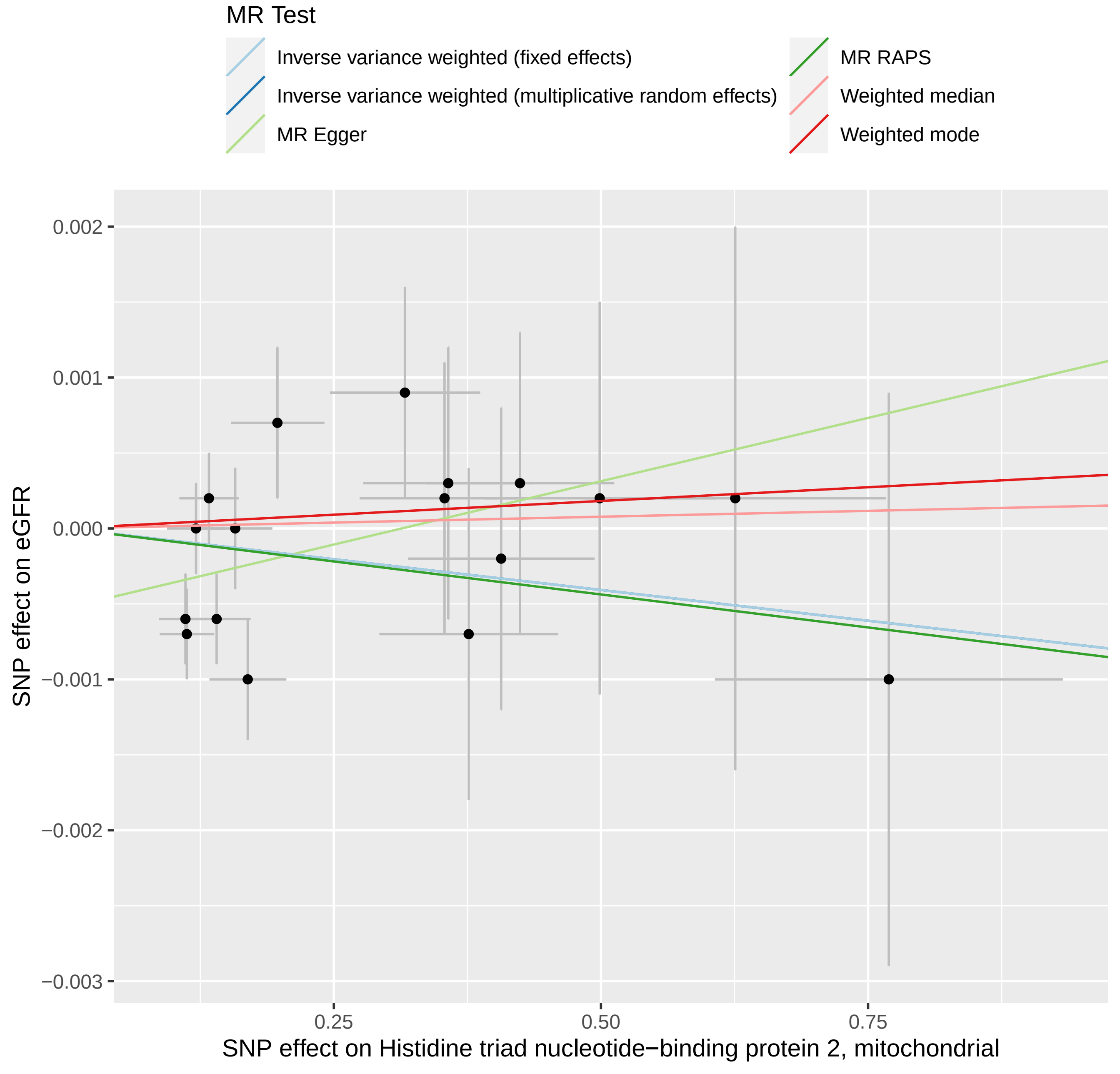

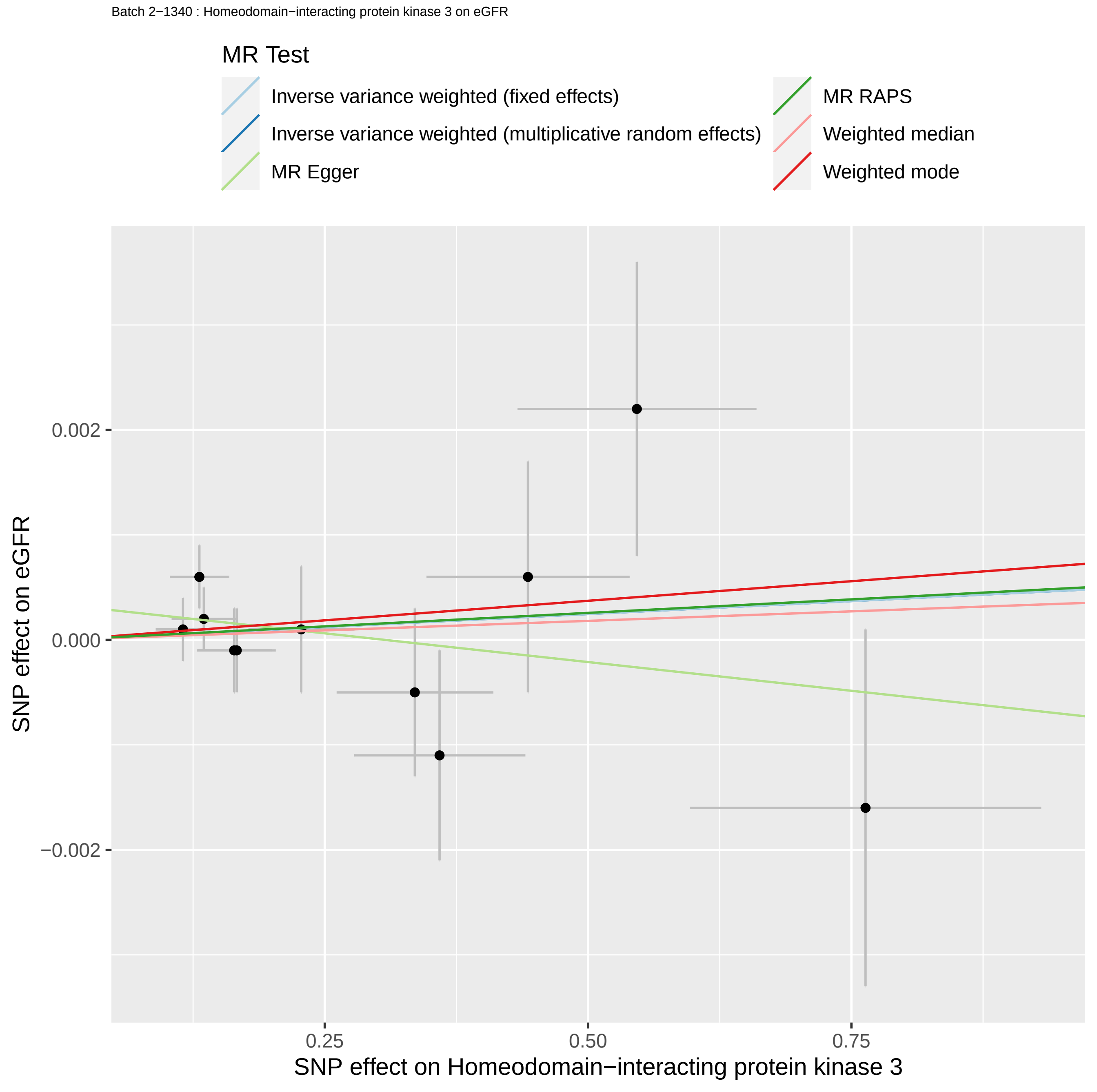

Batch 2−1340 : Homeodomain−interacting protein kinase 3 on eGFR
MR Test
Inverse variance weighted (fixed effects)
Inverse variance weighted (multiplicative random effects)
MR Egger
MR RAPS
Weighted median
Weighted mode
0.002
0.000
−0.002
SNP effect on eGFR
0.25
0.50
0.75
SNP effect on Homeodomain−interacting protein kinase 3

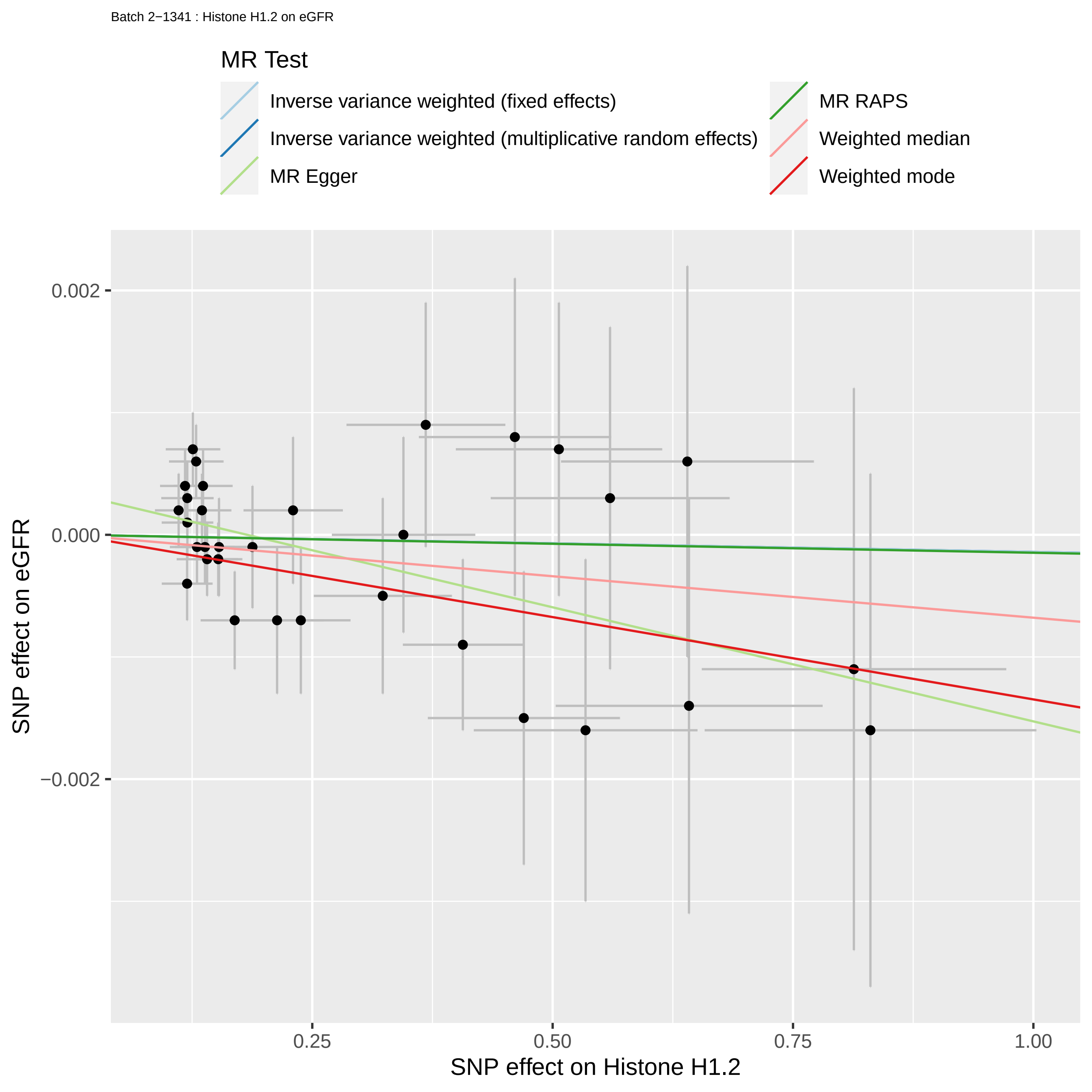

Batch 2−1341 : Histone H1.2 on eGFR
MR Test
Inverse variance weighted (fixed effects)
Inverse variance weighted (multiplicative random effects)
MR Egger
MR RAPS
Weighted median
Weighted mode
0.002
0.000
−0.002
SNP effect on eGFR
0.25
0.50
0.75
1.00
SNP effect on Histone H1.2

Batch 2−1342 : Histone H2A type 3 on eGFR

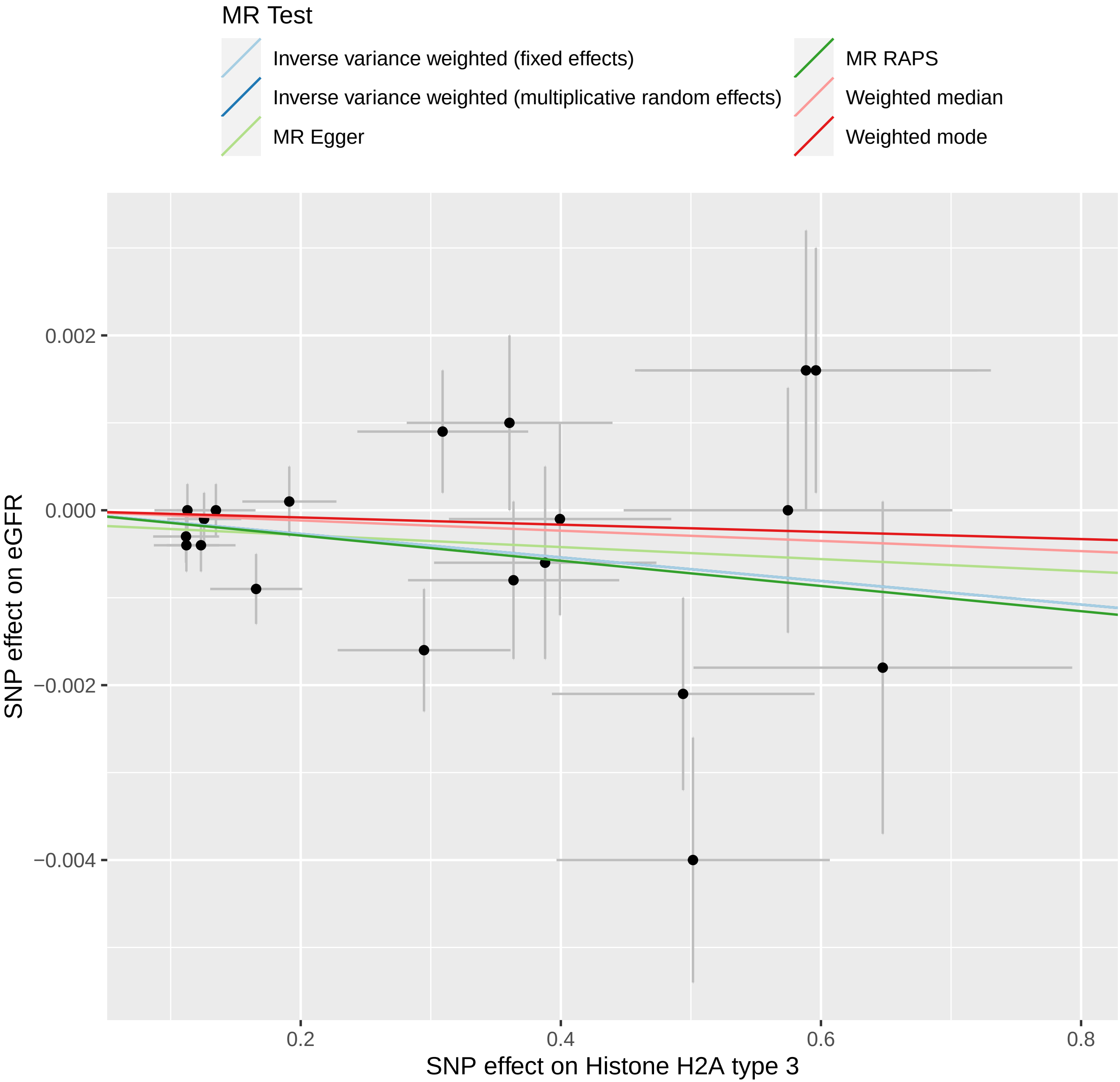

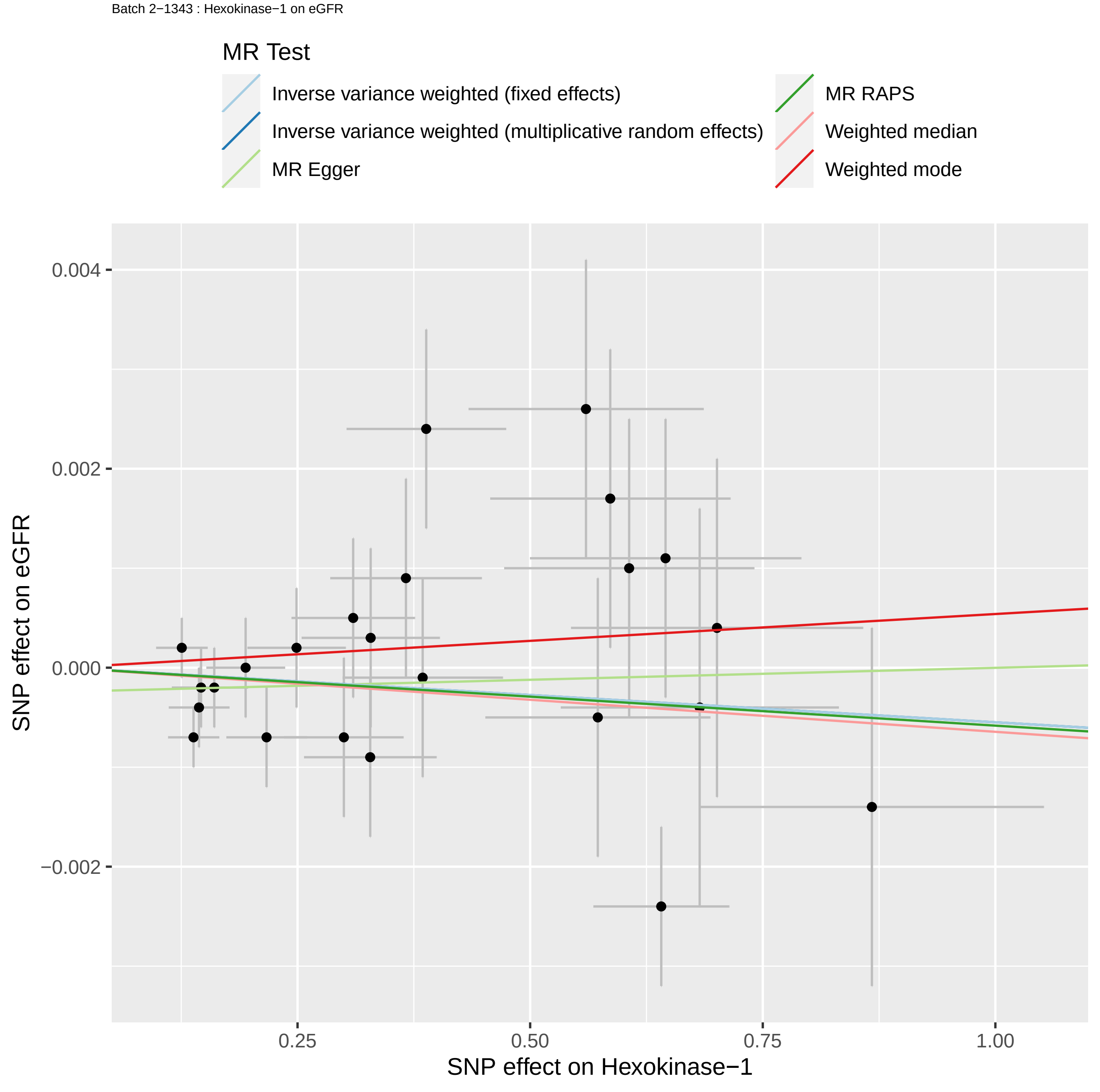

Batch 2−1343 : Hexokinase−1 on eGFR
MR Test
Inverse variance weighted (fixed effects)
Inverse variance weighted (multiplicative random effects)
MR Egger
MR RAPS
Weighted median
Weighted mode
0.004
0.002
0.000
−0.002
0.25
0.50
0.75
1.00
SNP effect on Hexokinase−1
SNP effect on eGFR

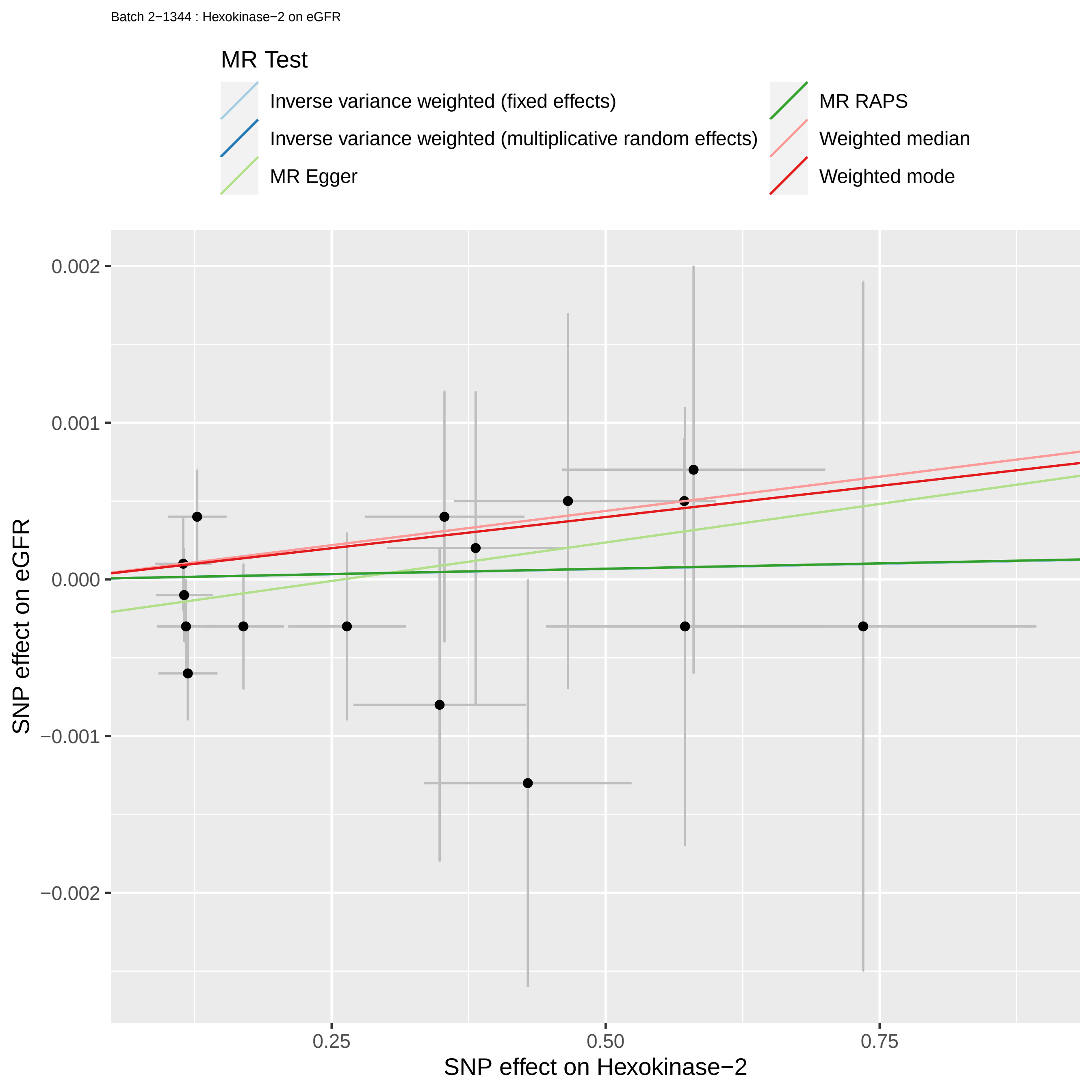
Batch 2−1344 : Hexokinase−2 on eGFR
MR Test
Inverse variance weighted (fixed effects)
Inverse variance weighted (multiplicative random effects)
MR Egger
MR RAPS
Weighted median
Weighted mode
0.002
0.001
0.000
−0.001
−0.002
0.25
0.50
0.75
SNP effect on eGFR
SNP effect on Hexokinase−2

Batch 2−1345 : HLA class II histocompatibility antigen, DM alpha chain on eGFR

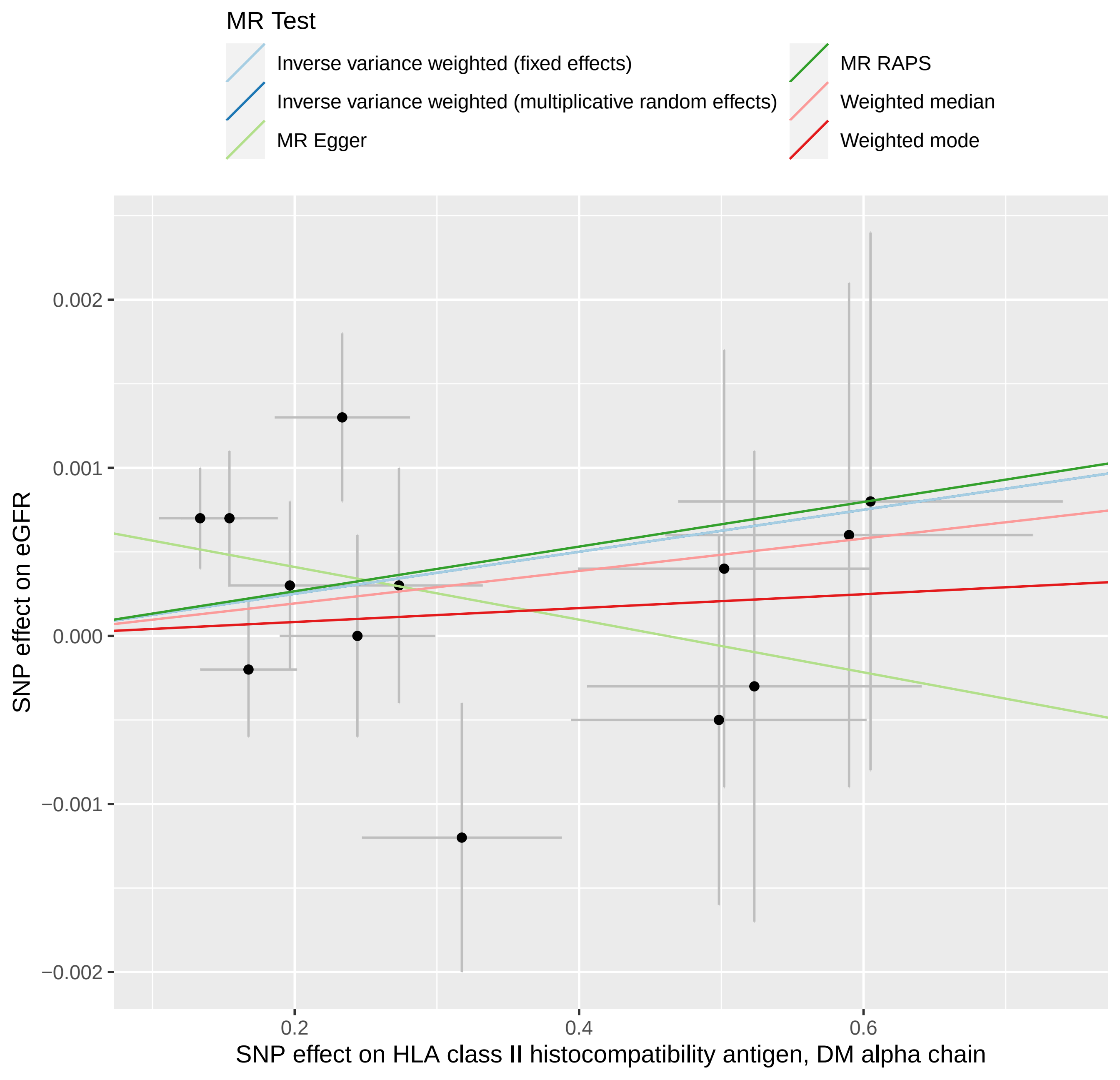

Batch 2−1346 : HLA class II histocompatibility antigen, DP beta 1 chain on eGFR

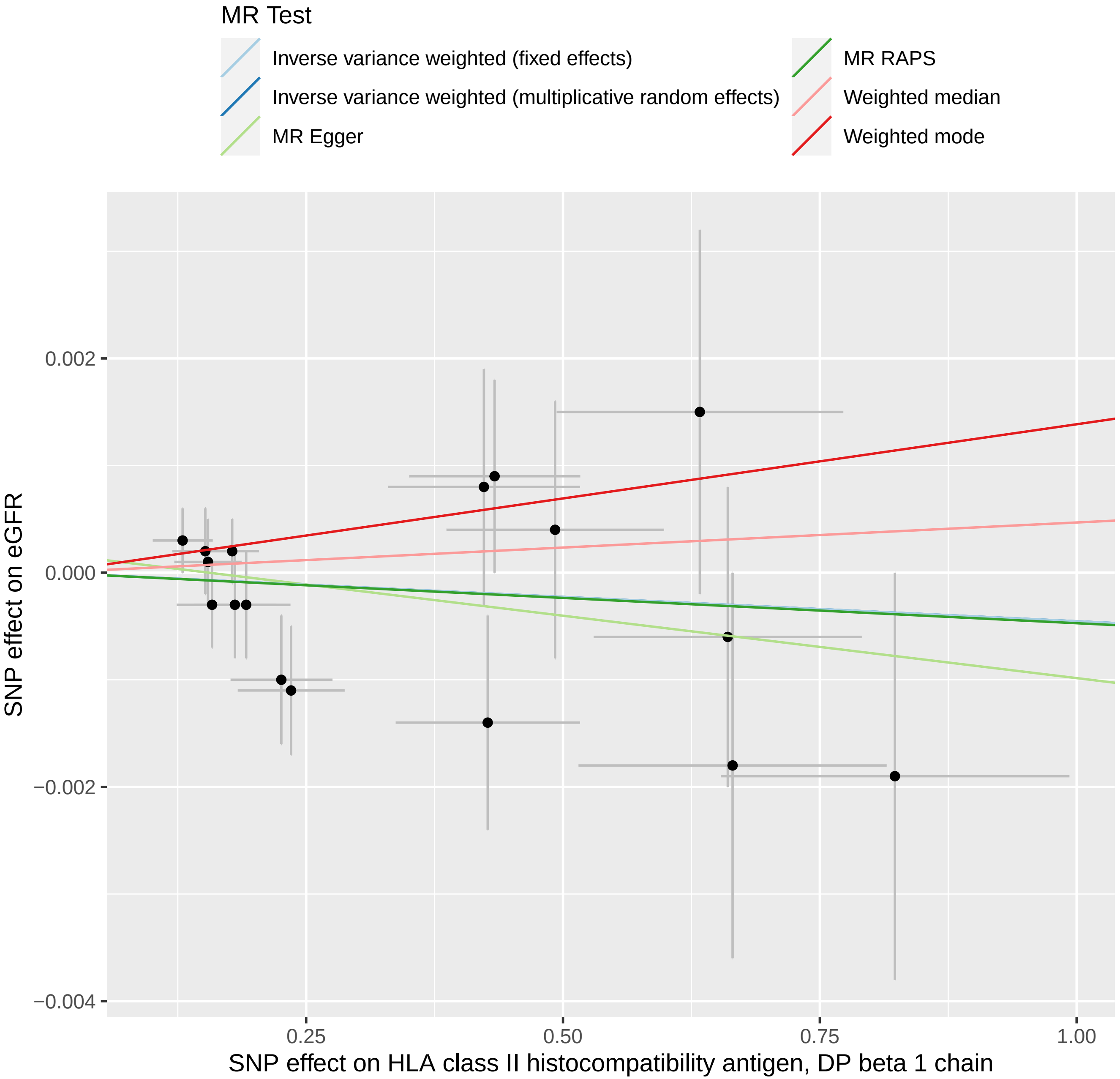

Batch 2−1347 : HLA class II histocompatibility antigen, DQ alpha 2 chain on eGFR

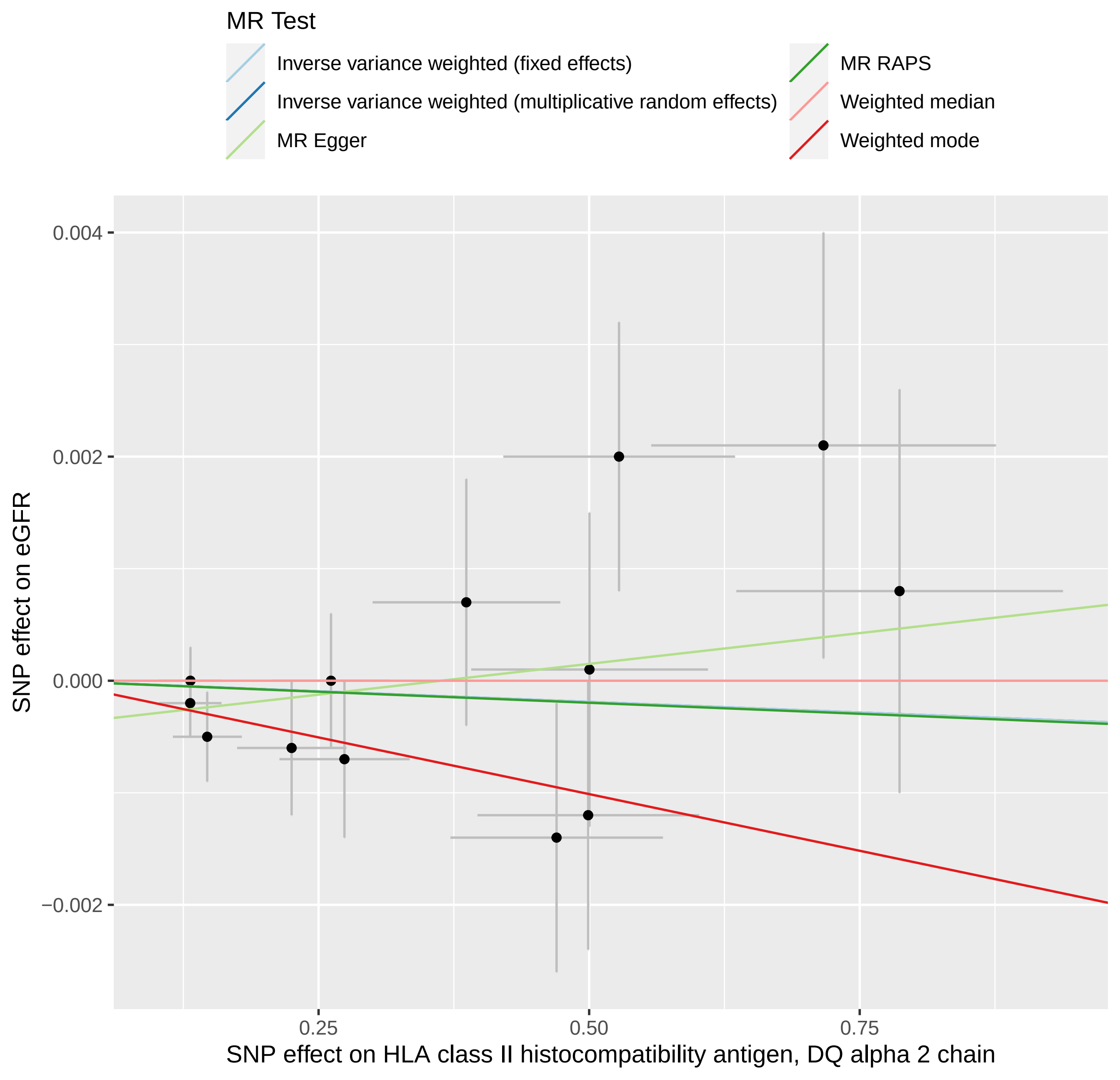

Batch 2−1348 : Porphobilinogen deaminase on eGFR

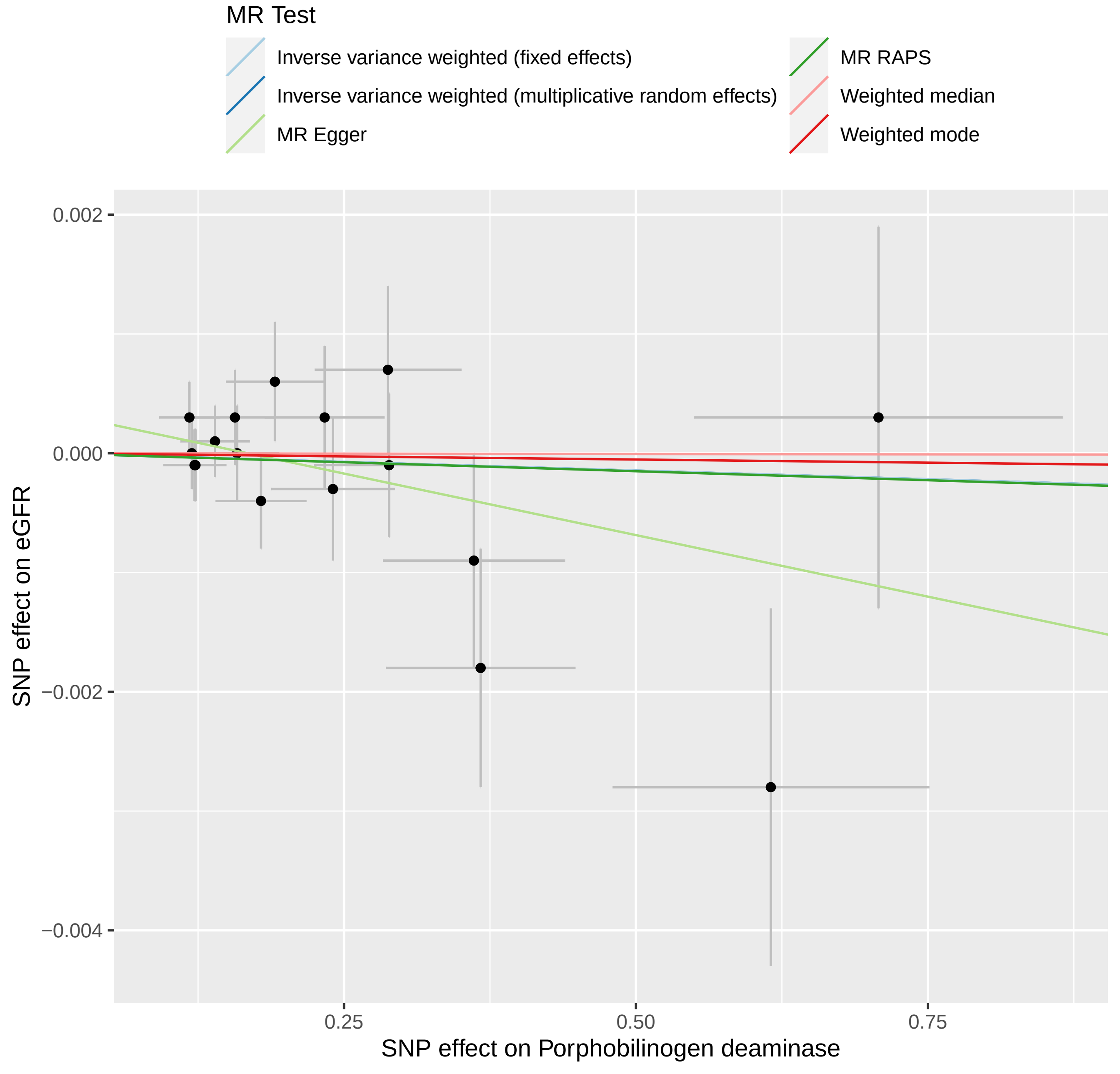

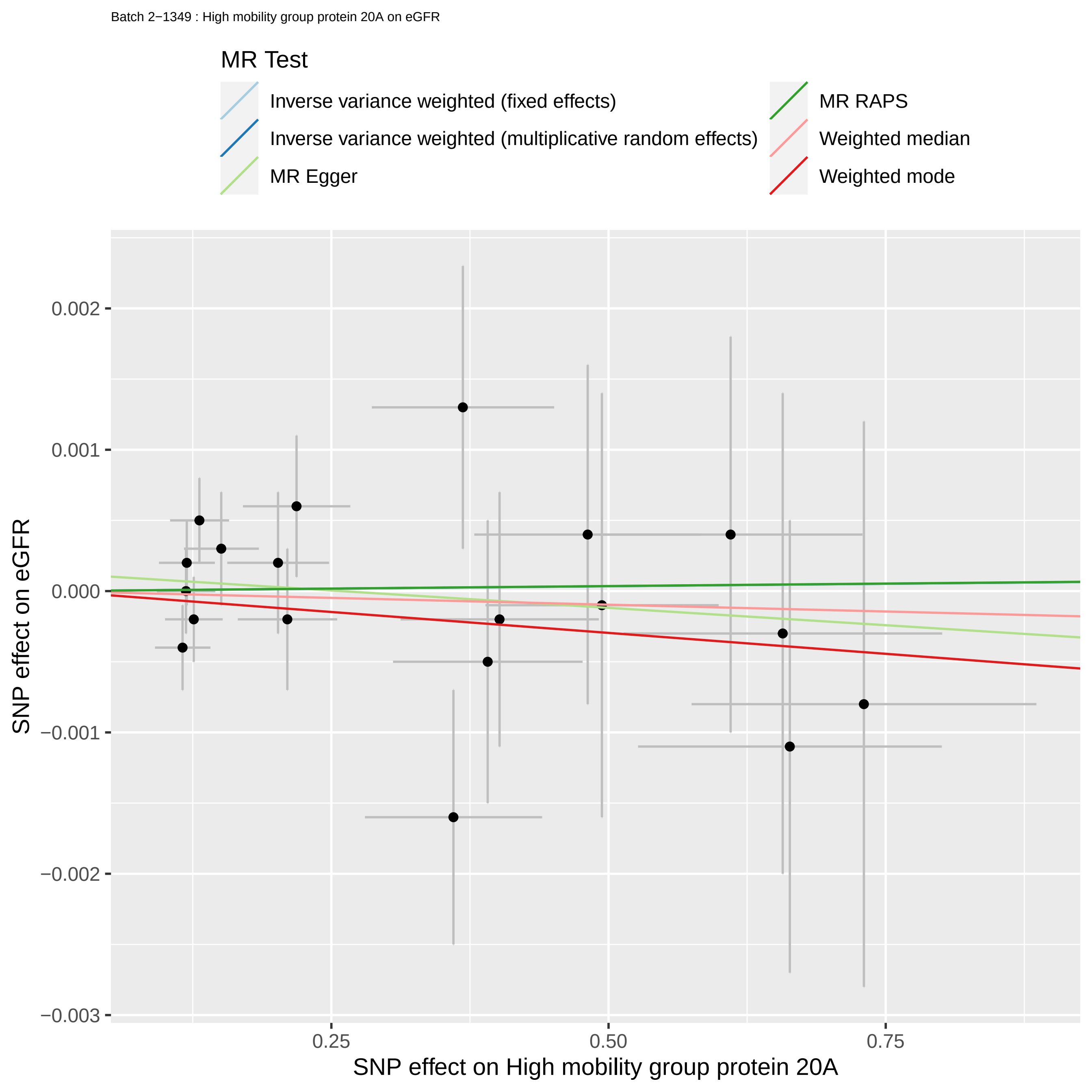

Batch 2−1349 : High mobility group protein 20A on eGFR
MR Test
Inverse variance weighted (fixed effects)
Inverse variance weighted (multiplicative random effects)
MR Egger
MR RAPS
Weighted median
Weighted mode
0.002
0.001
0.000
−0.001
−0.002
−0.003
0.25
0.50
0.75
SNP effect on eGFR
SNP effect on High mobility group protein 20A

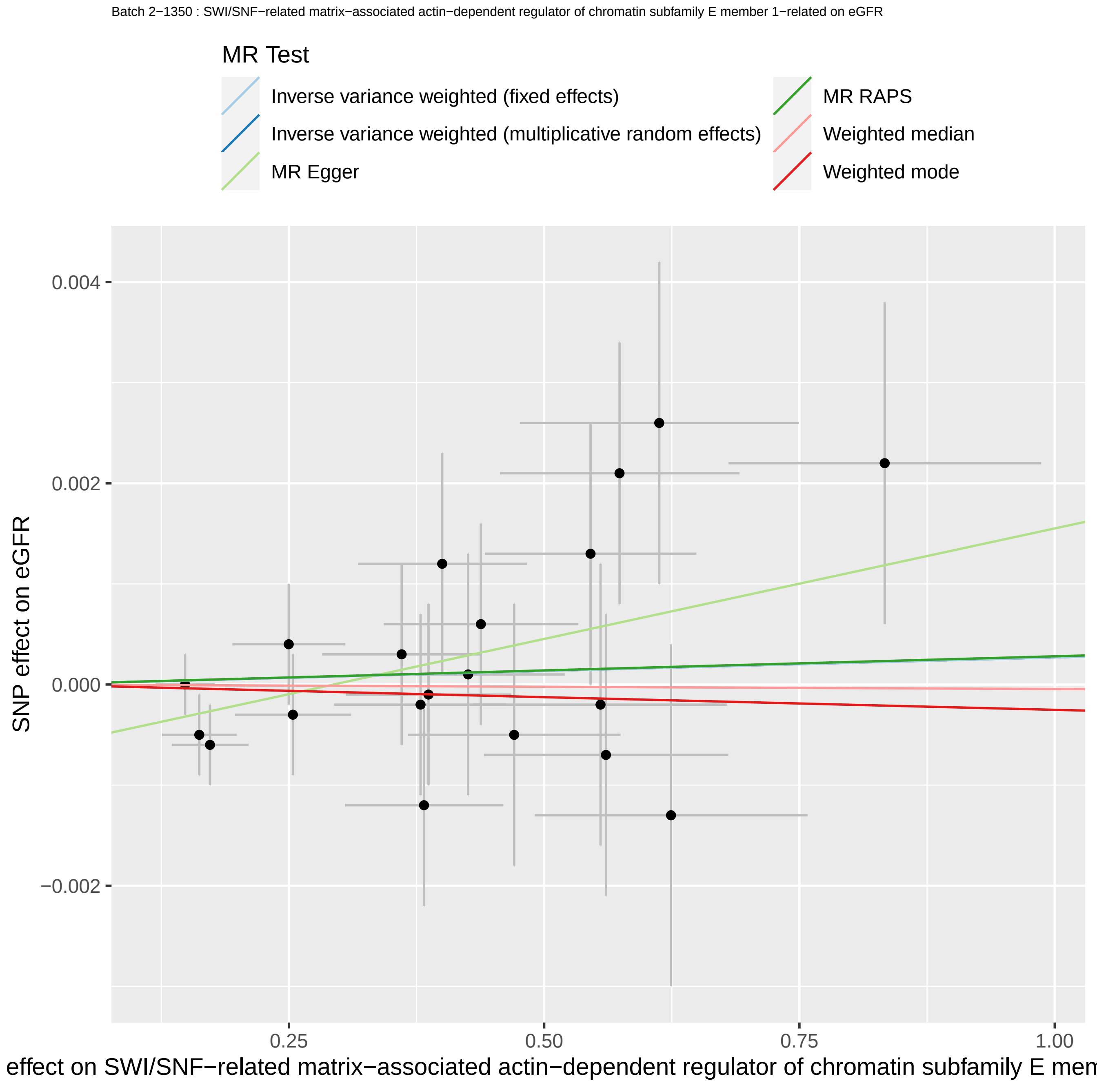

Batch 2−1350 : SWI/SNF−related matrix−associated actin−dependent regulator of chromatin subfamily E member 1−related on eGFR
MR Test
Inverse variance weighted (fixed effects)
Inverse variance weighted (multiplicative random effects)
MR Egger
MR RAPS
Weighted median
Weighted mode
0.004
0.002
0.000
−0.002
SNP effect on eGFR
0.25
0.50
0.75
1.00
effect on SWI/SNF−related matrix−associated actin−dependent regulator of chromatin subfamily E mem

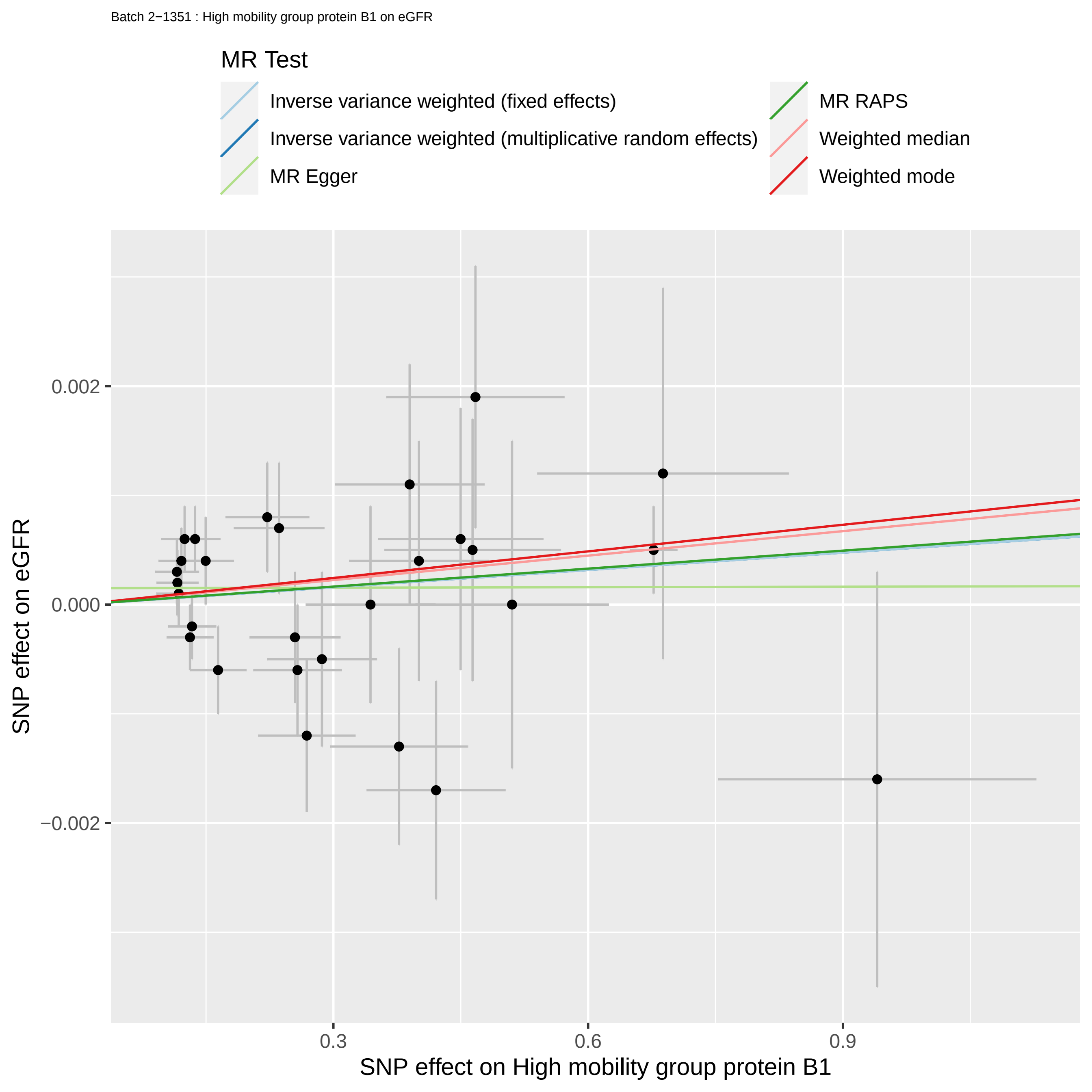

Batch 2−1351 : High mobility group protein B1 on eGFR
MR Test
Inverse variance weighted (fixed effects)
Inverse variance weighted (multiplicative random effects)
MR Egger
MR RAPS
Weighted median
Weighted mode
0.002
0.000
−0.002
0.3
0.6
0.9
SNP effect on eGFR
SNP effect on High mobility group protein B1

Batch 2−1352 : High mobility group protein B2 on eGFR

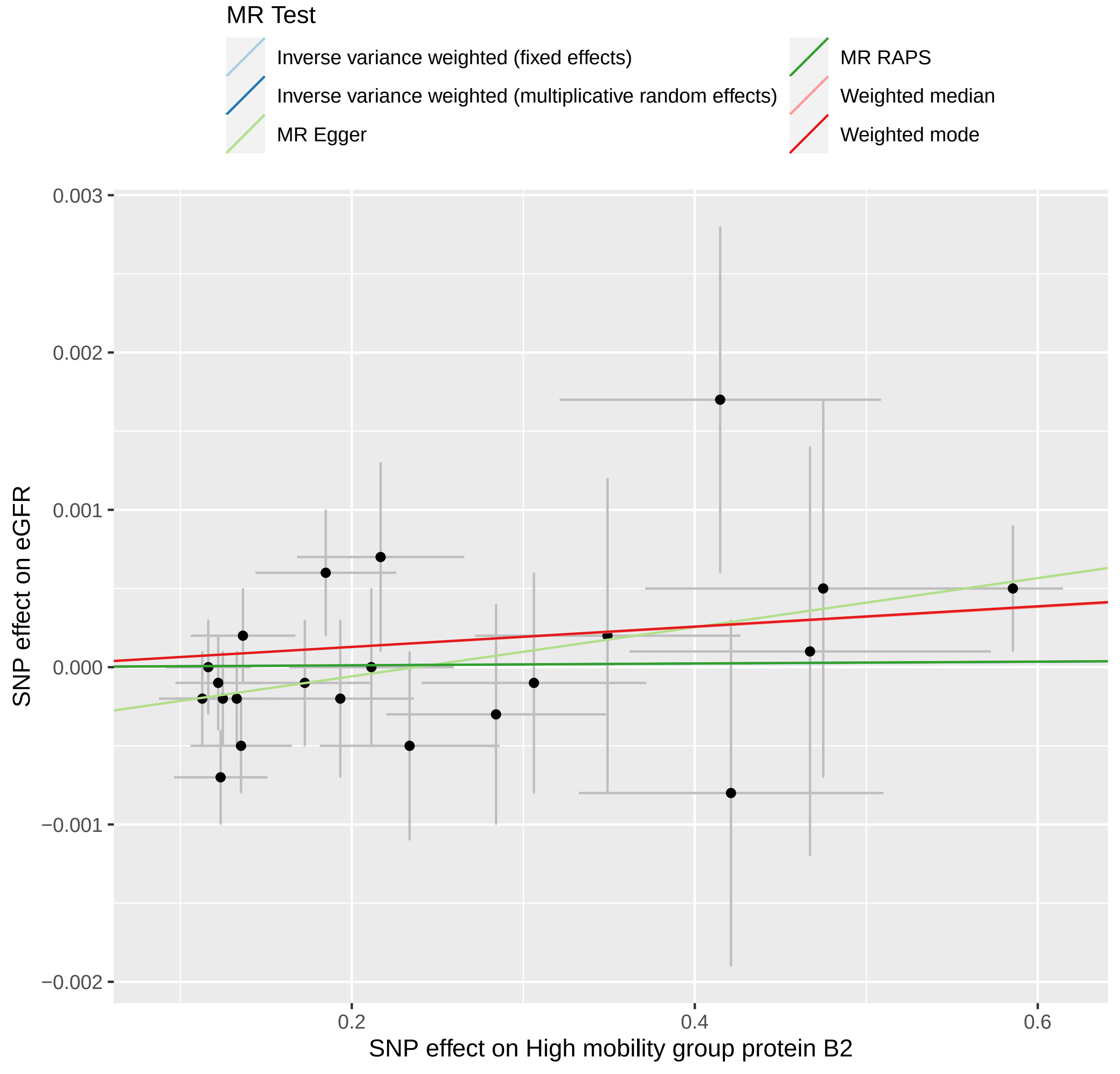

Batch 2−1353 : High mobility group protein B3 on eGFR

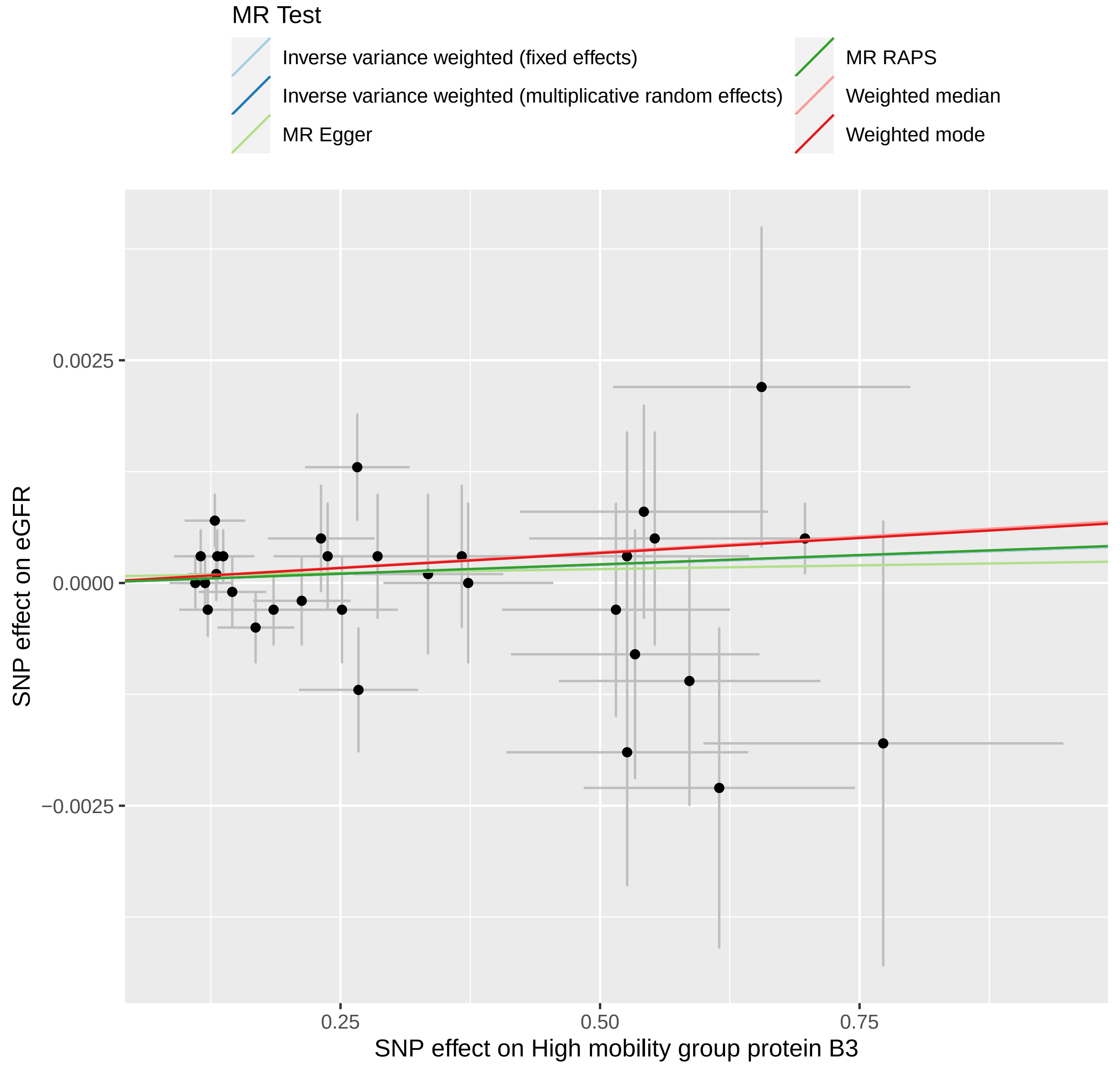

Batch 2−1354 : 3−hydroxy−3−methylglutaryl−coenzyme A reductase on eGFR

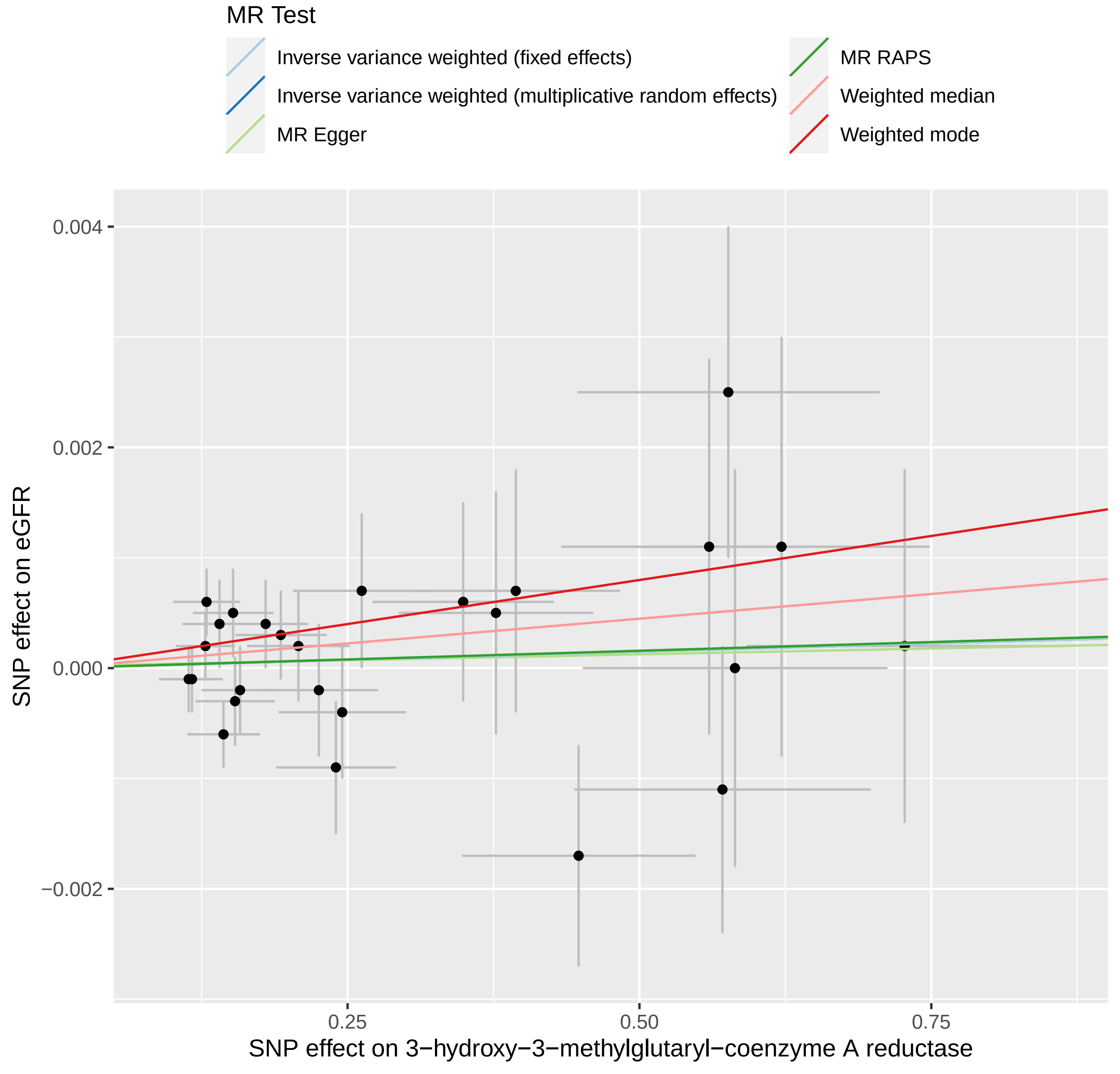

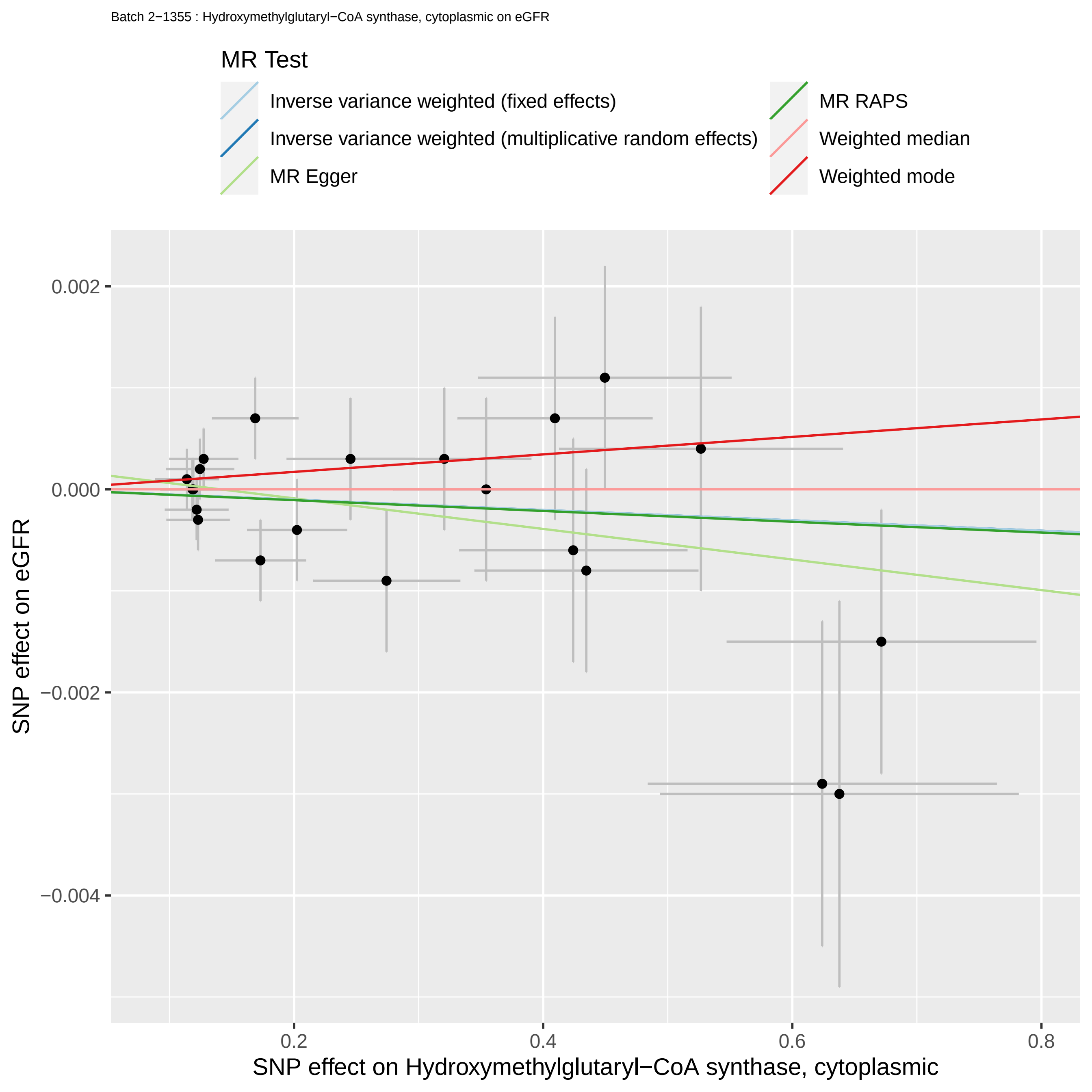

Batch 2−1355 : Hydroxymethylglutaryl−CoA synthase, cytoplasmic on eGFR
MR Test
Inverse variance weighted (fixed effects)
Inverse variance weighted (multiplicative random effects)
MR Egger
MR RAPS
Weighted median
Weighted mode
0.002
0.000
−0.002
−0.004
0.2
0.4
0.6
0.8
SNP effect on eGFR
SNP effect on Hydroxymethylglutaryl−CoA synthase, cytoplasmic

Batch 2−1356 : Hydroxymethylglutaryl−CoA synthase, mitochondrial on eGFR

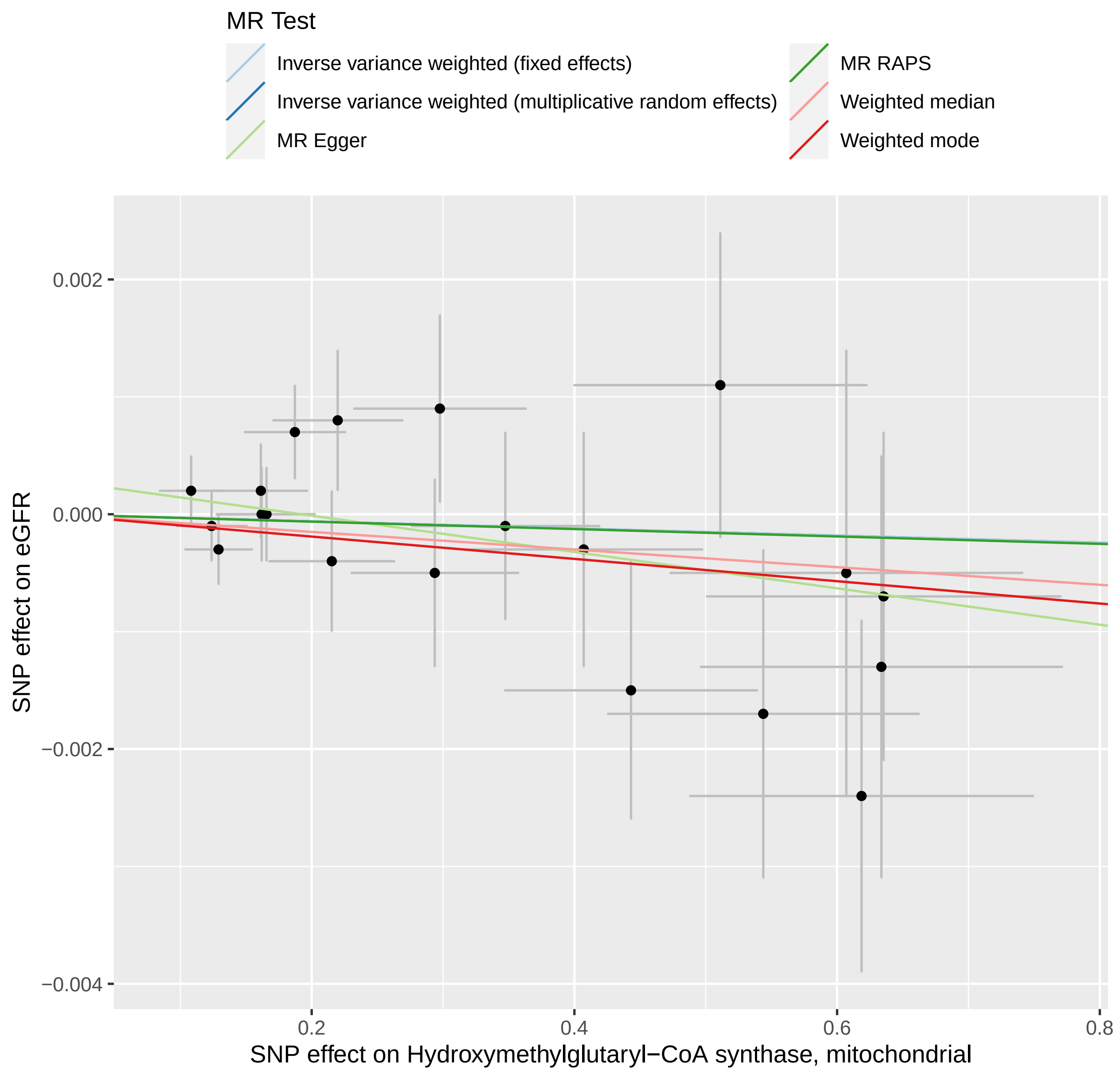

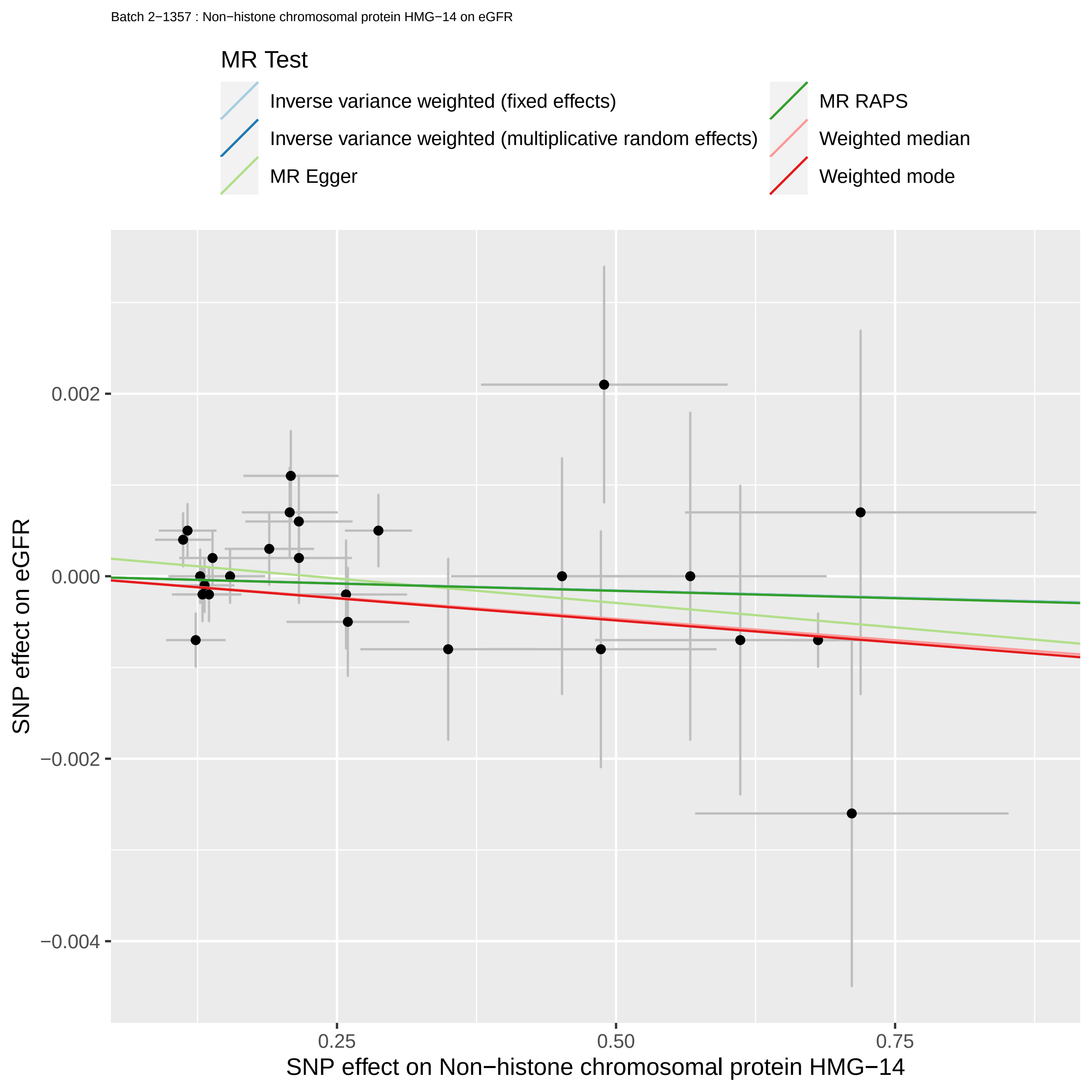

Batch 2−1357 : Non−histone chromosomal protein HMG−14 on eGFR
MR Test
Inverse variance weighted (fixed effects)
Inverse variance weighted (multiplicative random effects)
MR Egger
MR RAPS
Weighted median
Weighted mode
0.002
0.000
−0.002
−0.004
0.25
0.50
0.75
SNP effect on eGFR
SNP effect on Non−histone chromosomal protein HMG−14

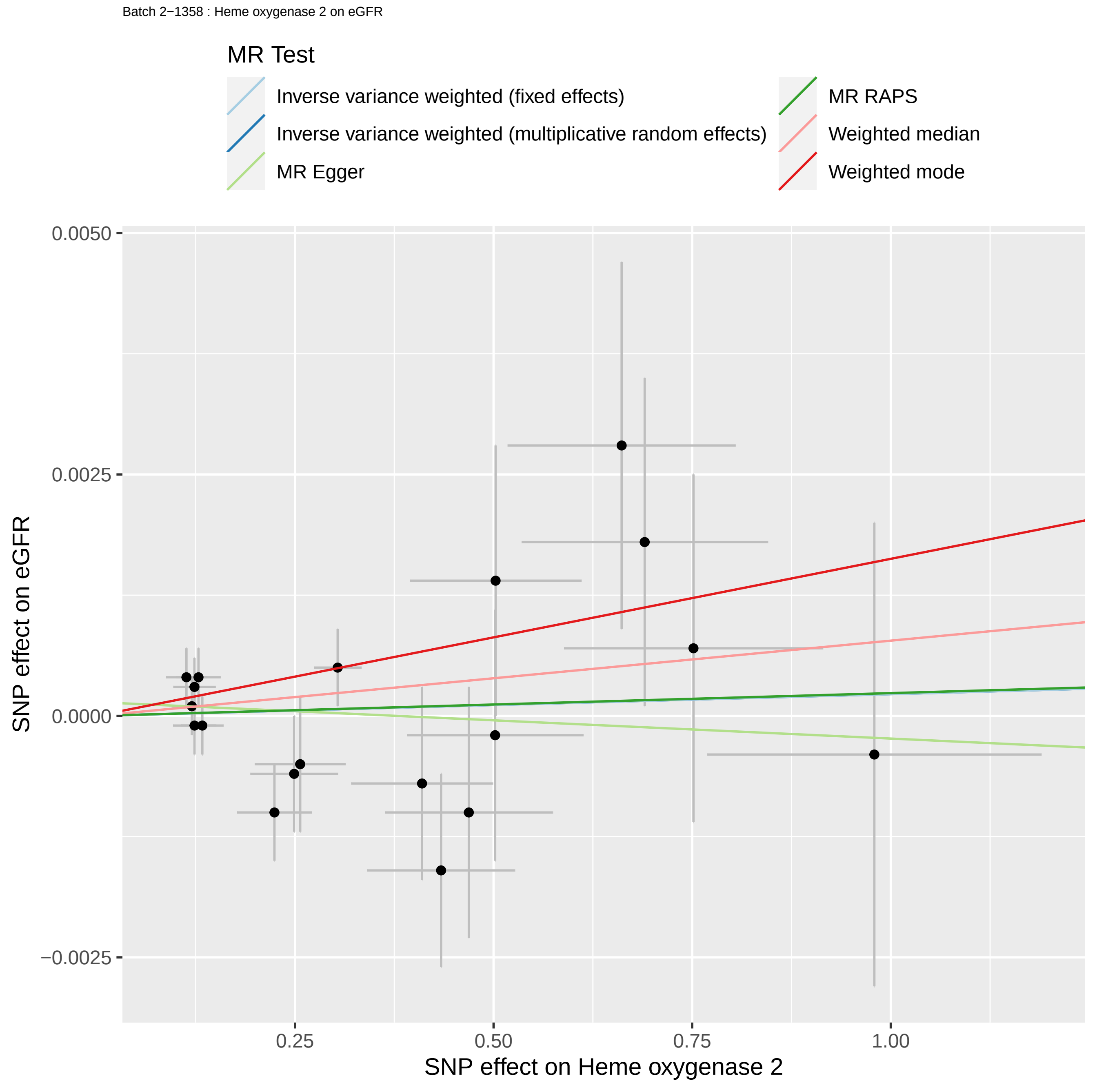

Batch 2−1358 : Heme oxygenase 2 on eGFR
MR Test
Inverse variance weighted (fixed effects)
Inverse variance weighted (multiplicative random effects)
MR Egger
MR RAPS
Weighted median
Weighted mode
0.0050
0.0025
0.0000
−0.0025
0.25
0.50
0.75
1.00
SNP effect on eGFR
SNP effect on Heme oxygenase 2

Batch 2−1359 : Hepatocyte nuclear factor 1−alpha on eGFR

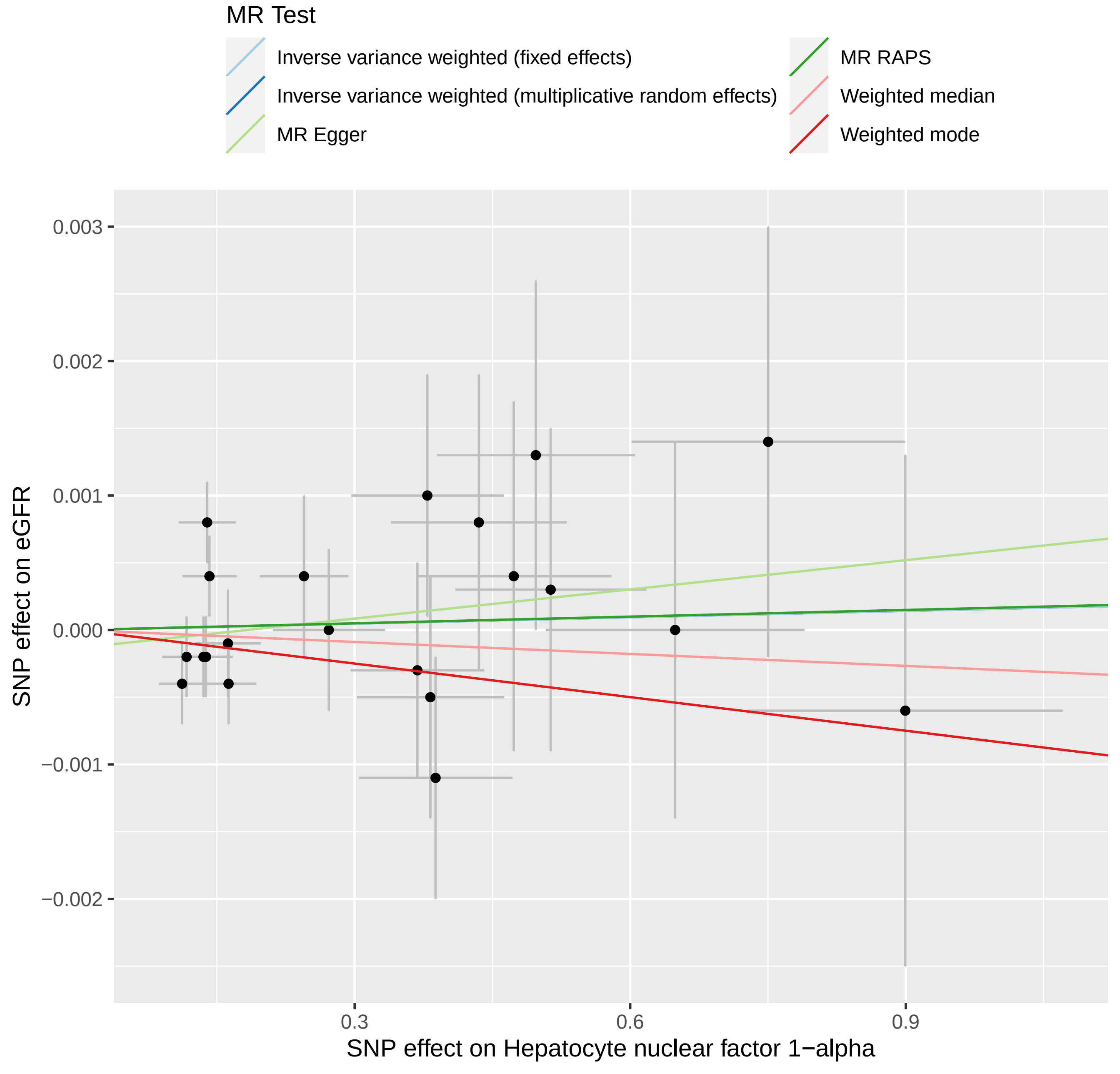

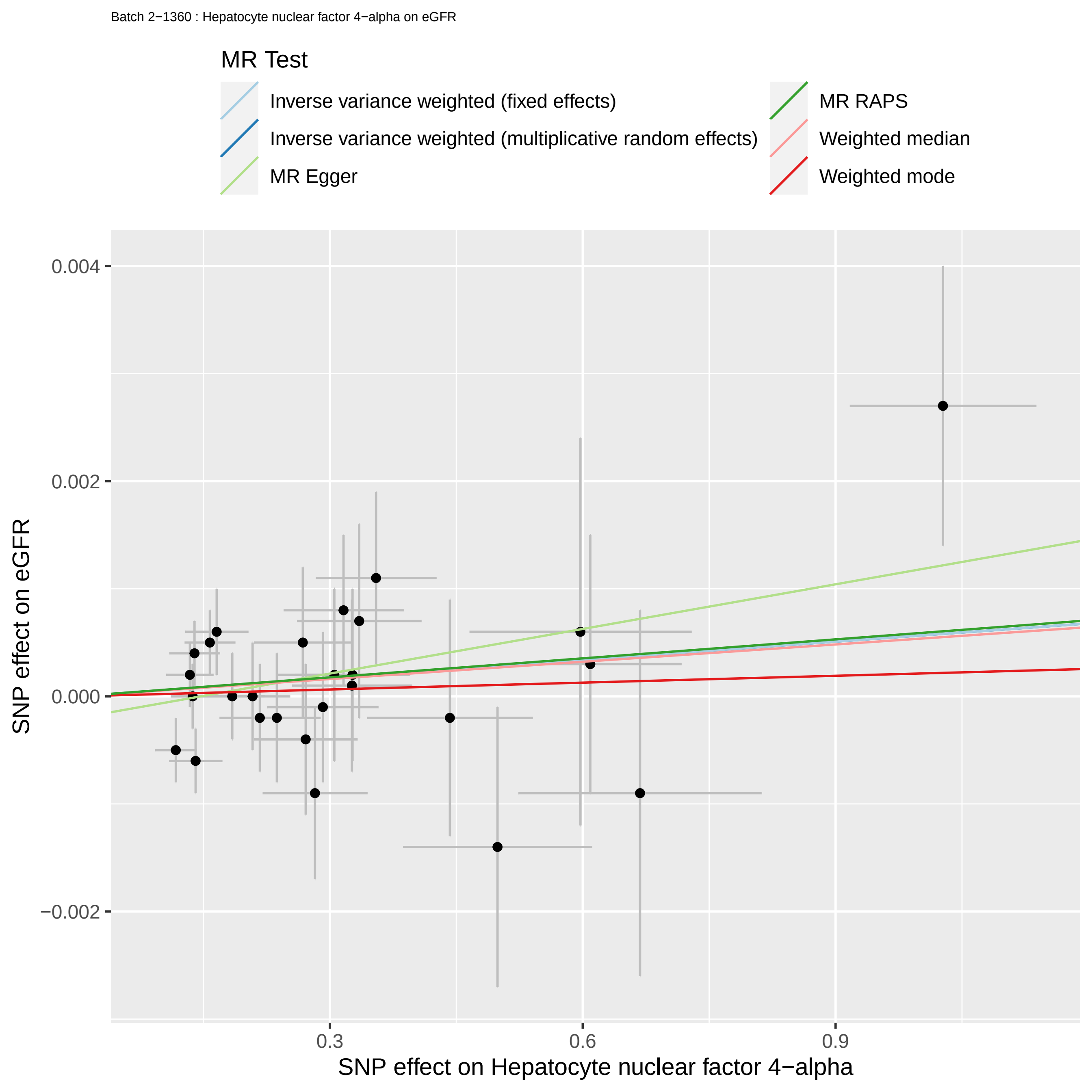
Batch 2−1360 : Hepatocyte nuclear factor 4−alpha on eGFR
MR Test
Inverse variance weighted (fixed effects)
Inverse variance weighted (multiplicative random effects)
MR Egger
MR RAPS
Weighted median
Weighted mode
0.004
0.002
0.000
−0.002
0.3
0.6
0.9
SNP effect on eGFR
SNP effect on Hepatocyte nuclear factor 4−alpha

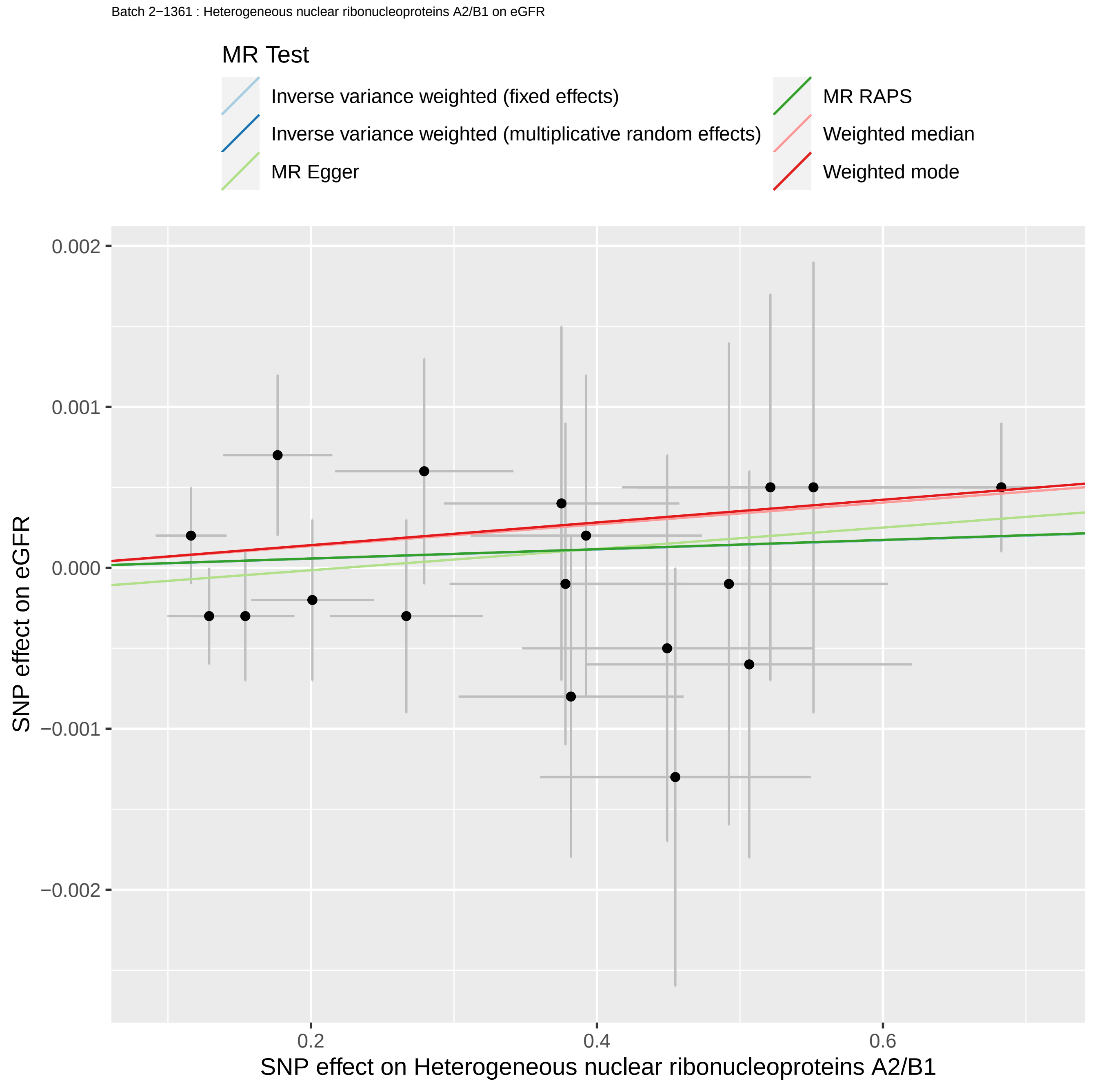

Batch 2−1361 : Heterogeneous nuclear ribonucleoproteins A2/B1 on eGFR
MR Test
Inverse variance weighted (fixed effects)
Inverse variance weighted (multiplicative random effects)
MR Egger
MR RAPS
Weighted median
Weighted mode
0.002
0.001
0.000
−0.001
−0.002
SNP effect on eGFR
0.2
0.4
0.6
SNP effect on Heterogeneous nuclear ribonucleoproteins A2/B1

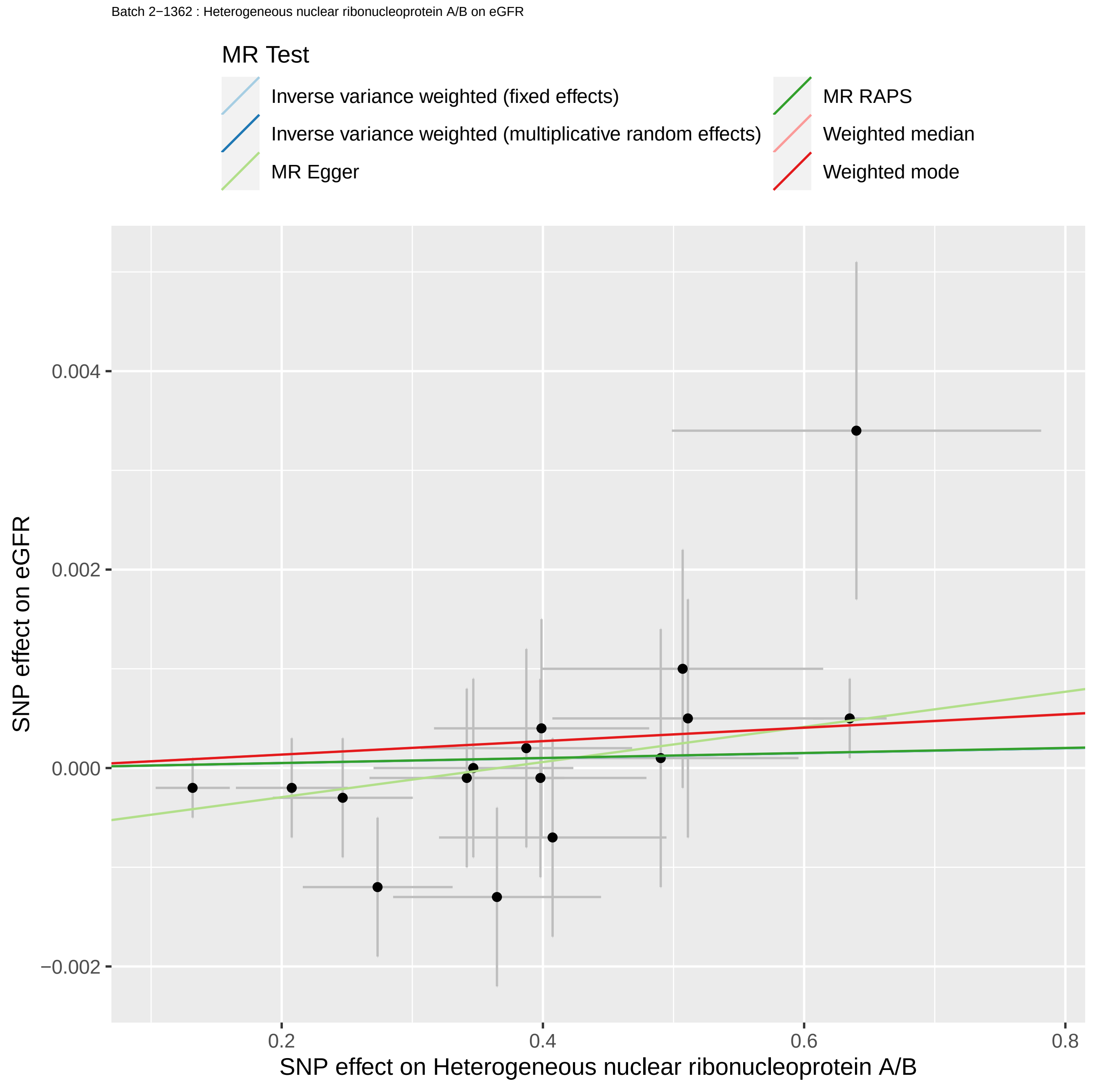

Batch 2−1362 : Heterogeneous nuclear ribonucleoprotein A/B on eGFR
MR Test
Inverse variance weighted (fixed effects)
Inverse variance weighted (multiplicative random effects)
MR Egger
MR RAPS
Weighted median
Weighted mode
0.004
0.002
0.000
−0.002
0.2
0.4
0.6
0.8
SNP effect on eGFR
SNP effect on Heterogeneous nuclear ribonucleoprotein A/B

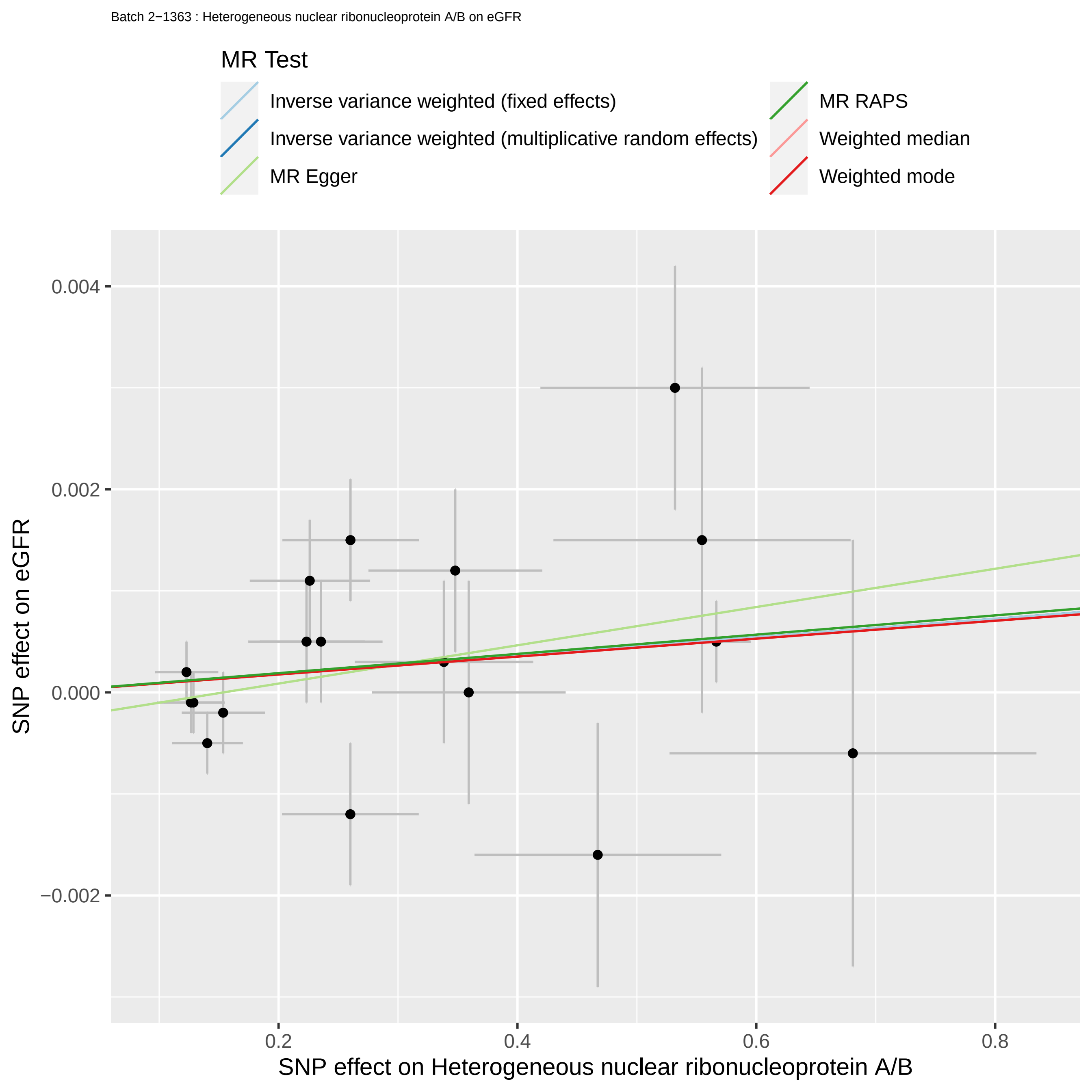

Batch 2−1363 : Heterogeneous nuclear ribonucleoprotein A/B on eGFR
MR Test
Inverse variance weighted (fixed effects)
Inverse variance weighted (multiplicative random effects)
MR Egger
MR RAPS
Weighted median
Weighted mode
0.004
0.002
0.000
−0.002
SNP effect on eGFR
0.2
0.4
0.6
0.8
SNP effect on Heterogeneous nuclear ribonucleoprotein A/B

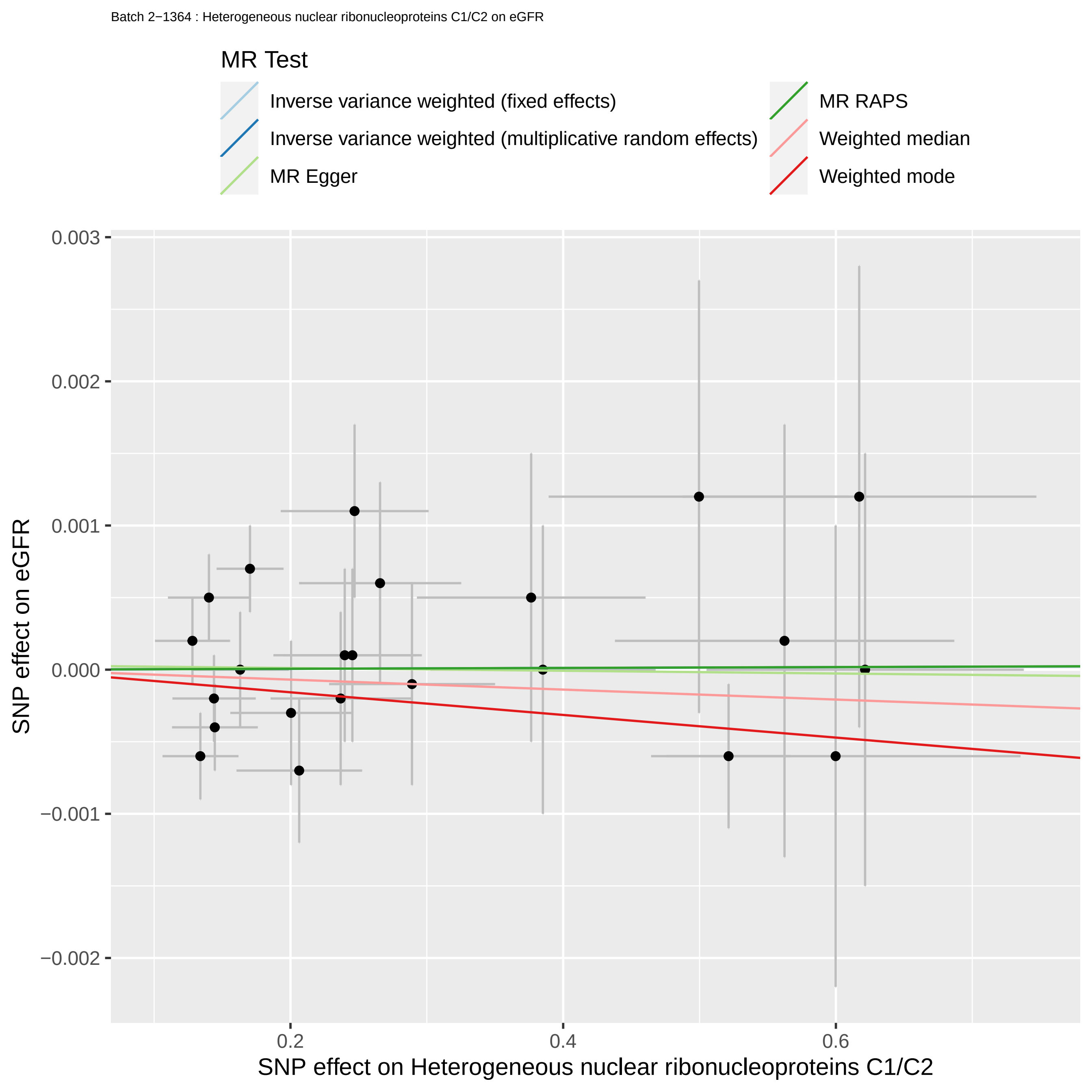

Batch 2−1364 : Heterogeneous nuclear ribonucleoproteins C1/C2 on eGFR
MR Test
Inverse variance weighted (fixed effects)
Inverse variance weighted (multiplicative random effects)
MR Egger
MR RAPS
Weighted median
Weighted mode
0.003
0.002
0.001
0.000
−0.001
−0.002
0.2
0.4
0.6
SNP effect on eGFR
SNP effect on Heterogeneous nuclear ribonucleoproteins C1/C2

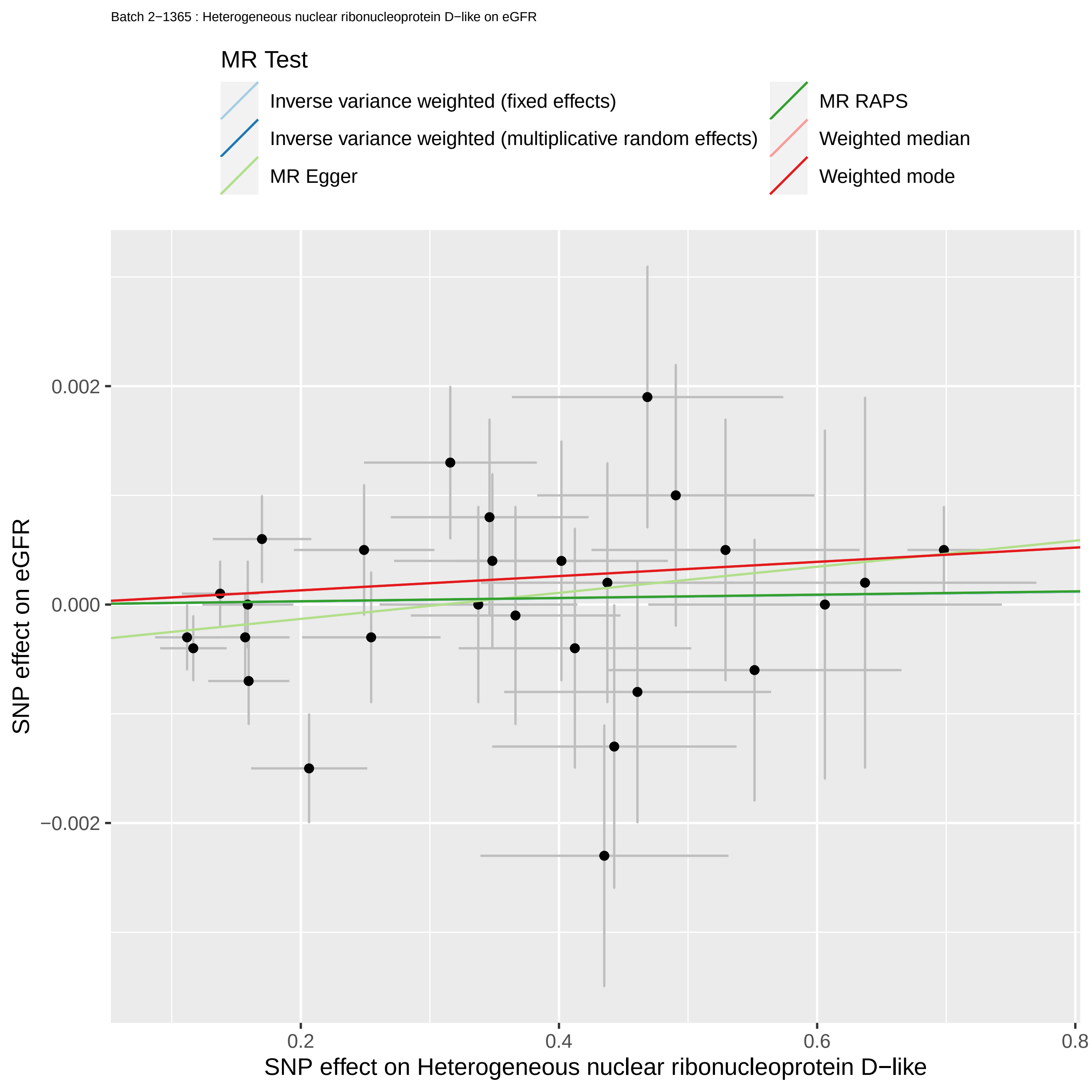
Batch 2−1365 : Heterogeneous nuclear ribonucleoprotein D−like on eGFR
MR Test
Inverse variance weighted (fixed effects)
Inverse variance weighted (multiplicative random effects)
MR Egger
MR RAPS
Weighted median
Weighted mode
0.002
0.000
−0.002
0.2
0.4
0.6
0.8
SNP effect on eGFR
SNP effect on Heterogeneous nuclear ribonucleoprotein D−like

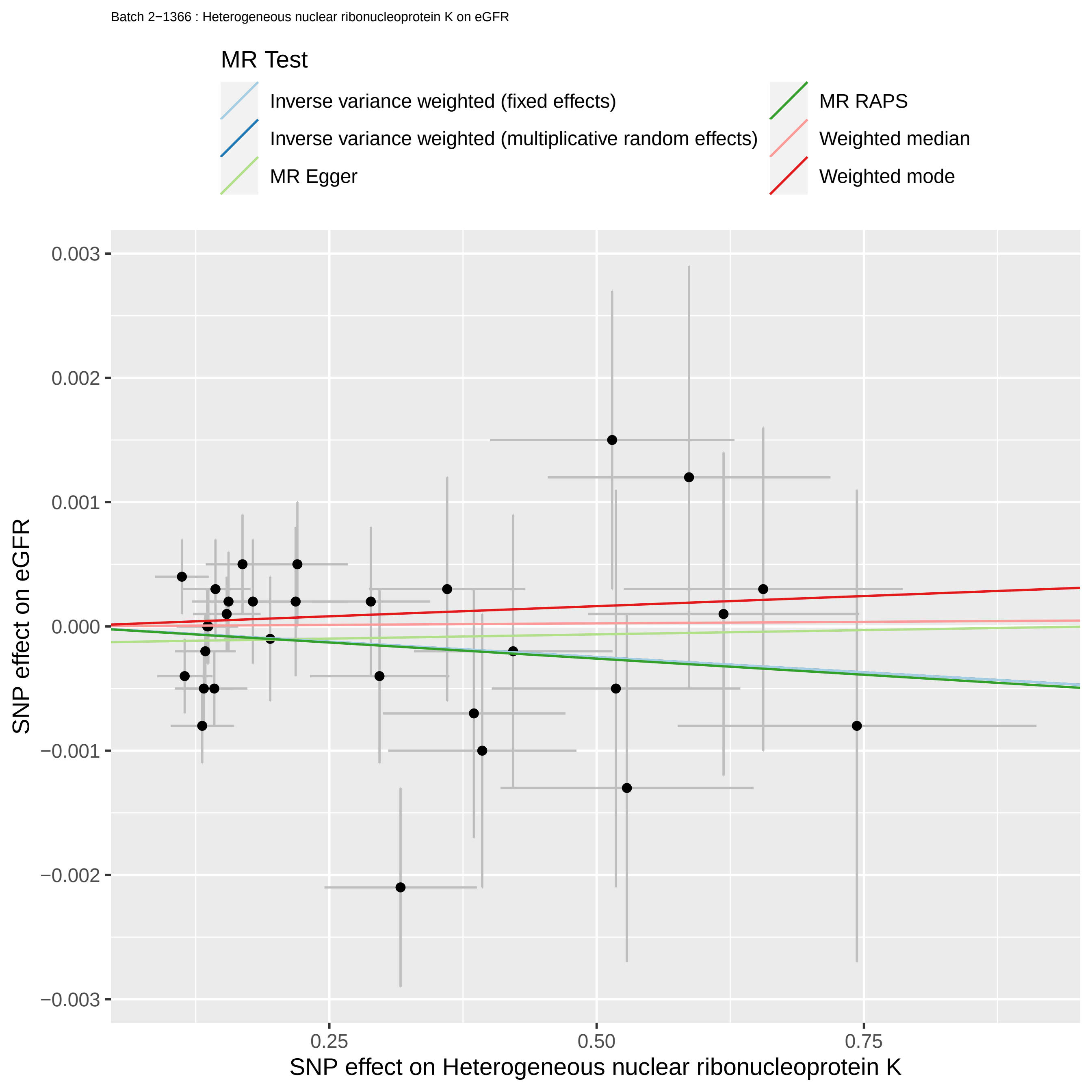
Batch 2−1366 : Heterogeneous nuclear ribonucleoprotein K on eGFR
MR Test
Inverse variance weighted (fixed effects)
Inverse variance weighted (multiplicative random effects)
MR Egger
MR RAPS
Weighted median
Weighted mode
0.003
0.002
0.001
0.000
−0.001
−0.002
−0.003
0.25
0.50
0.75
SNP effect on eGFR
SNP effect on Heterogeneous nuclear ribonucleoprotein K

Batch 2−1367 : Heterogeneous nuclear ribonucleoprotein M on eGFR

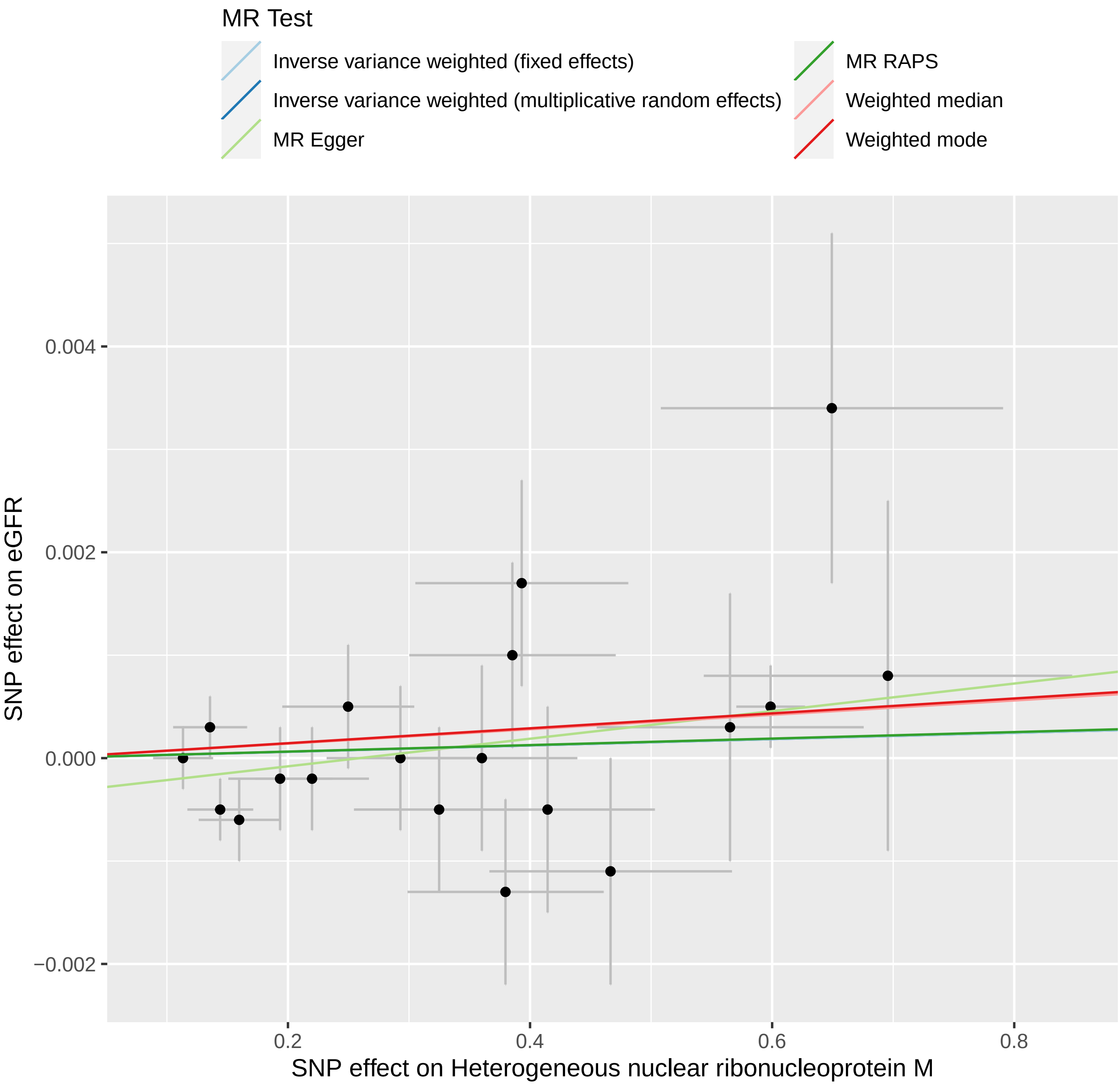

Batch 2−1368 : 4−hydroxy−2−oxoglutarate aldolase, mitochondrial on eGFR

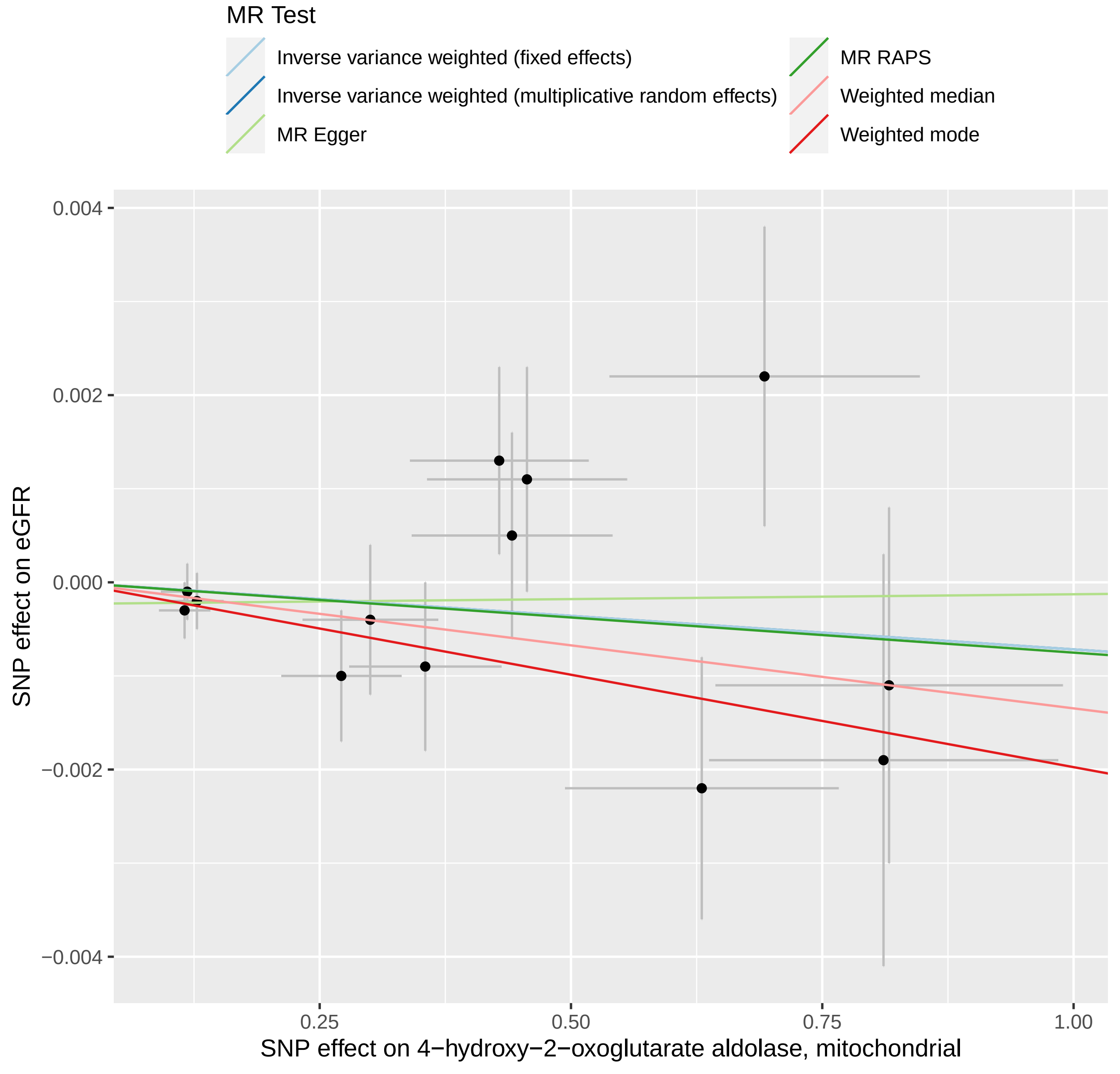

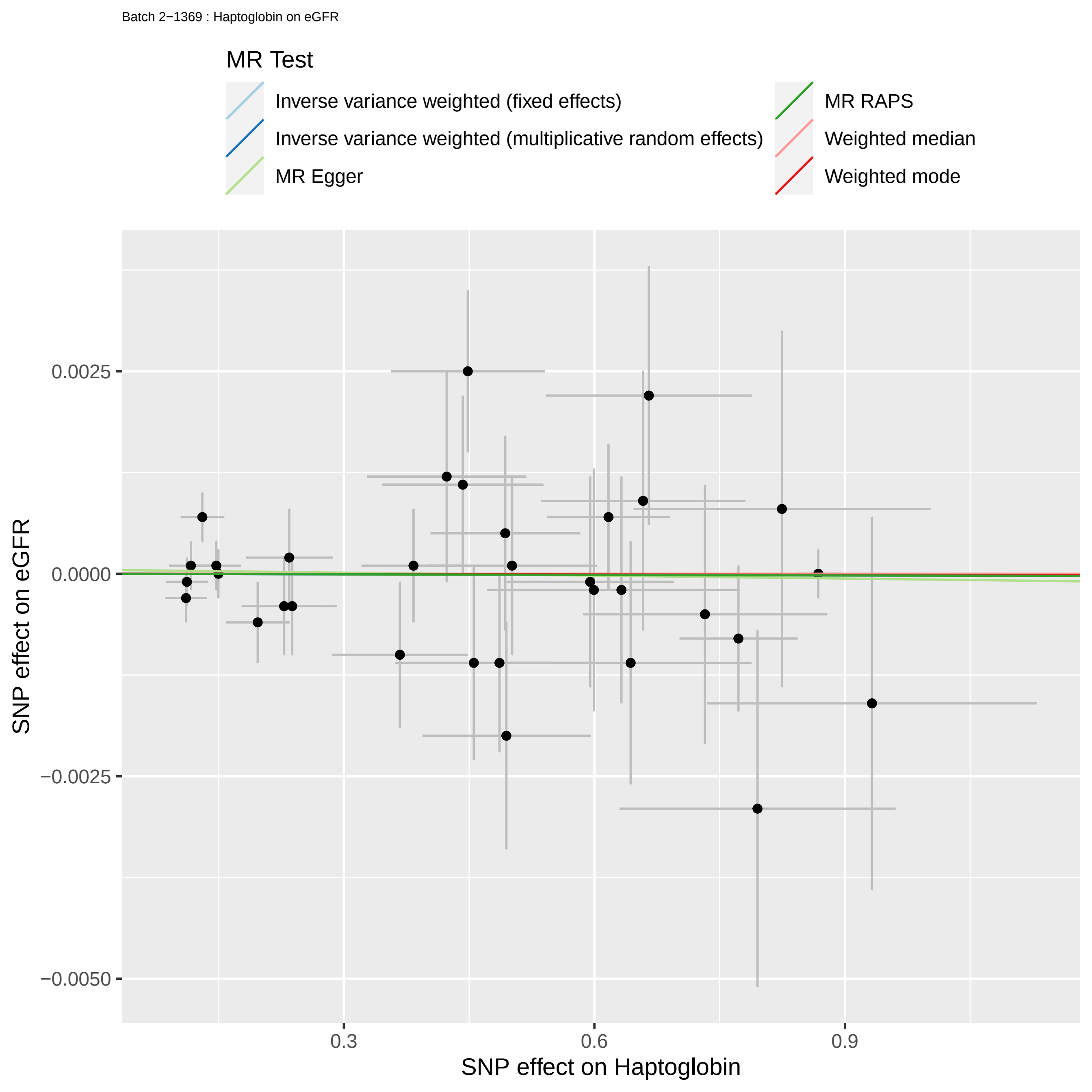

Batch 2−1369 : Haptoglobin on eGFR
MR Test
Inverse variance weighted (fixed effects)
Inverse variance weighted (multiplicative random effects)
MR Egger
MR RAPS
Weighted median
Weighted mode
0.0025
0.0000
−0.0025
−0.0050
SNP effect on eGFR
0.3
0.6
0.9
SNP effect on Haptoglobin

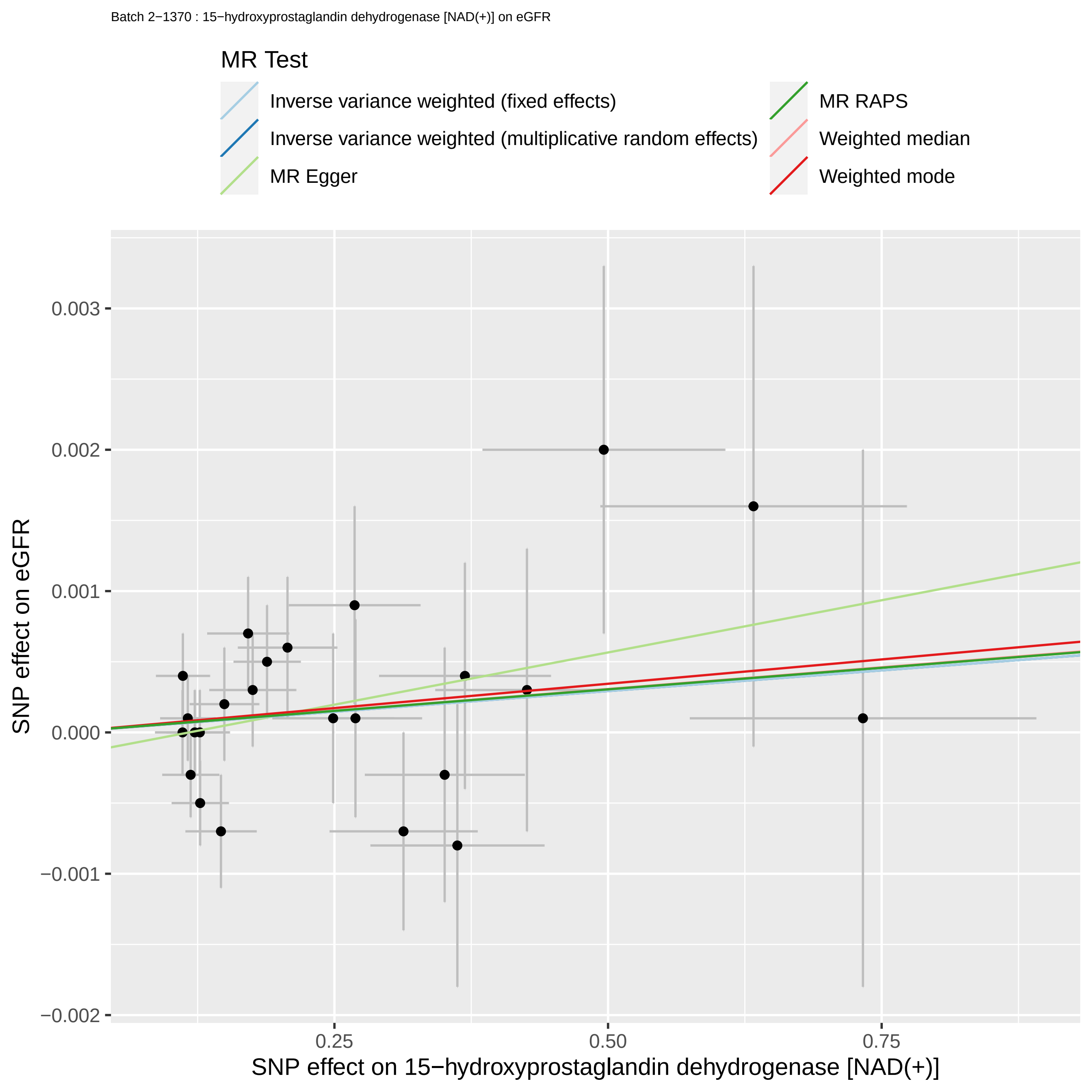

Batch 2−1370 : 15−hydroxyprostaglandin dehydrogenase [NAD(+)] on eGFR
MR Test
Inverse variance weighted (fixed effects)
Inverse variance weighted (multiplicative random effects)
MR Egger
MR RAPS
Weighted median
Weighted mode
0.003
0.002
0.001
0.000
−0.001
−0.002
SNP effect on eGFR
0.25
0.50
0.75
SNP effect on 15−hydroxyprostaglandin dehydrogenase [NAD(+)]

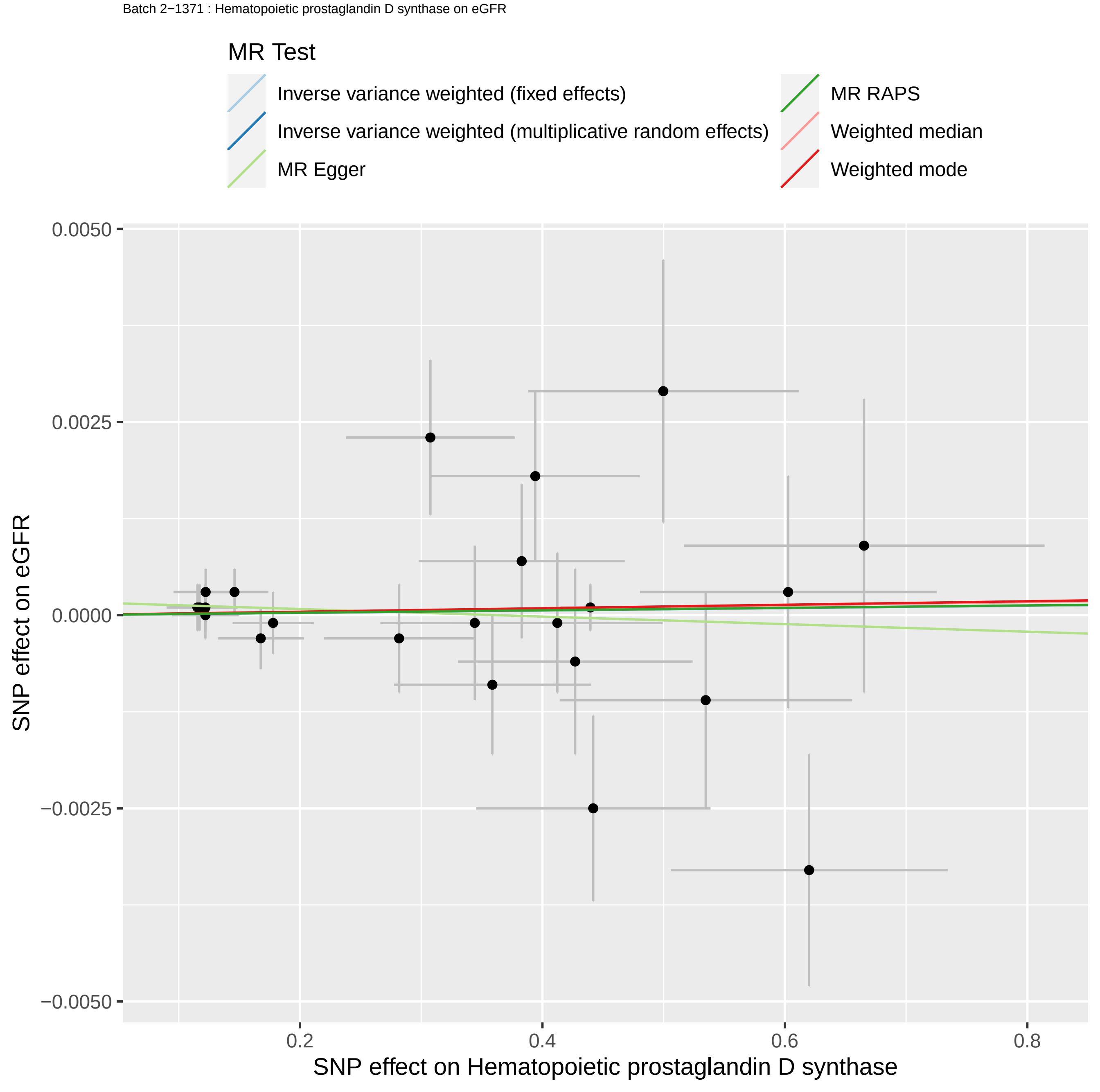

Batch 2−1371 : Hematopoietic prostaglandin D synthase on eGFR
MR Test
Inverse variance weighted (fixed effects)
Inverse variance weighted (multiplicative random effects)
MR Egger
MR RAPS
Weighted median
Weighted mode
0.0050
0.0025
0.0000
−0.0025
−0.0050
SNP effect on eGFR
0.2
0.4
0.6
0.8
SNP effect on Hematopoietic prostaglandin D synthase

Batch 2−1372 : HRAS−like suppressor 2 on eGFR

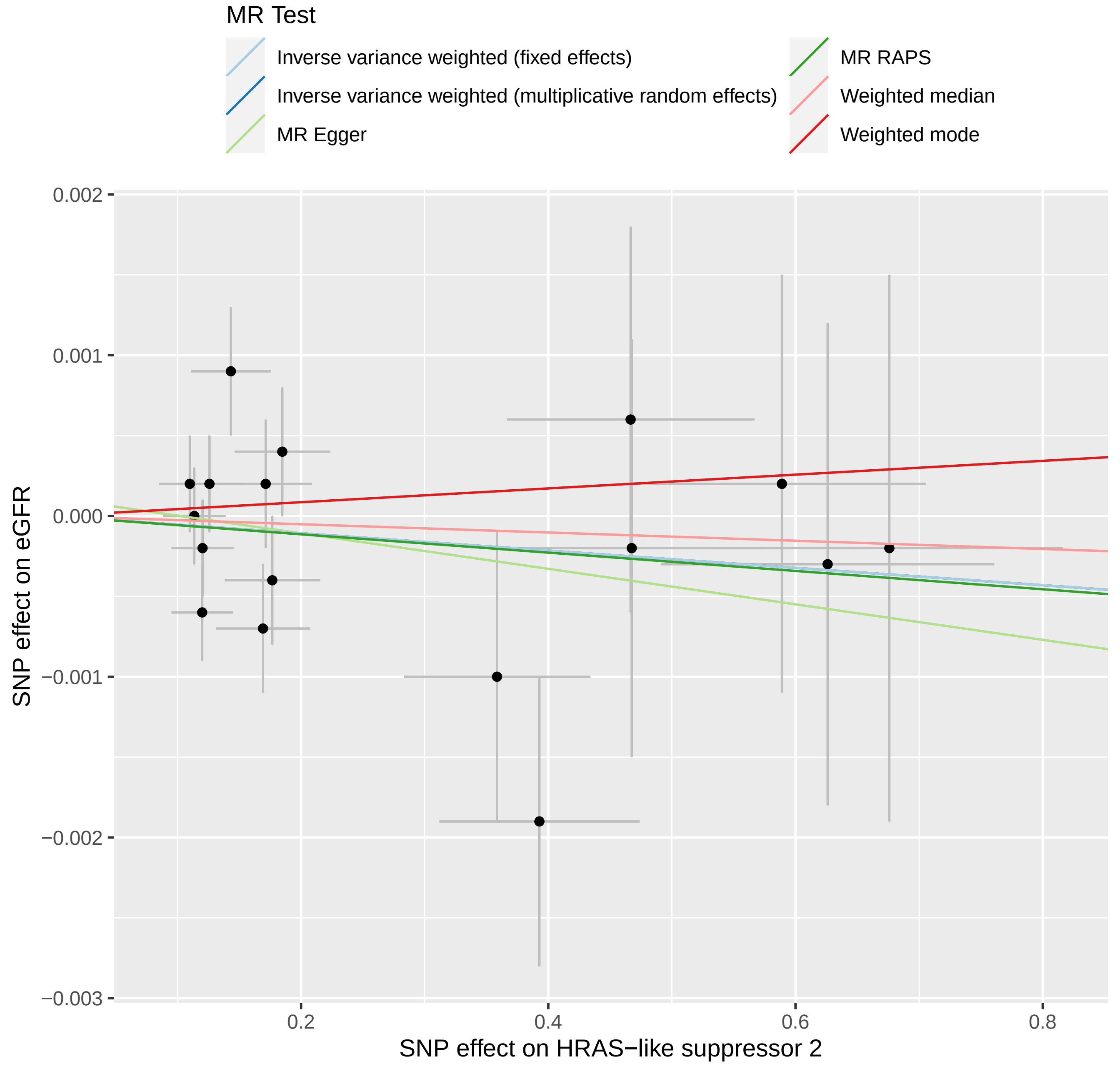

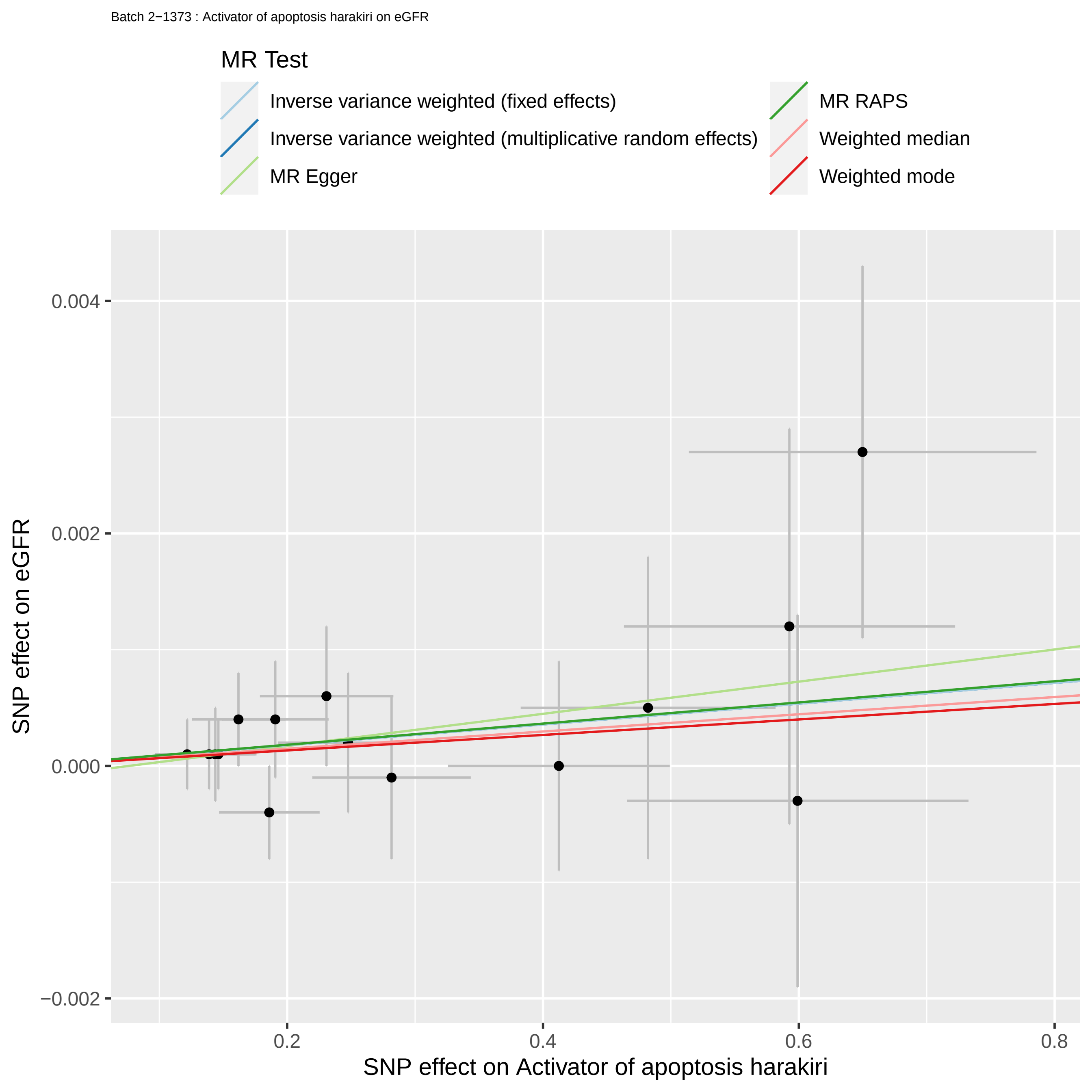

Batch 2−1373 : Activator of apoptosis harakiri on eGFR
MR Test
Inverse variance weighted (fixed effects)
Inverse variance weighted (multiplicative random effects)
MR Egger
MR RAPS
Weighted median
Weighted mode
0.004
0.002
0.000
−0.002
0.2
0.4
0.6
0.8
SNP effect on eGFR
SNP effect on Activator of apoptosis harakiri

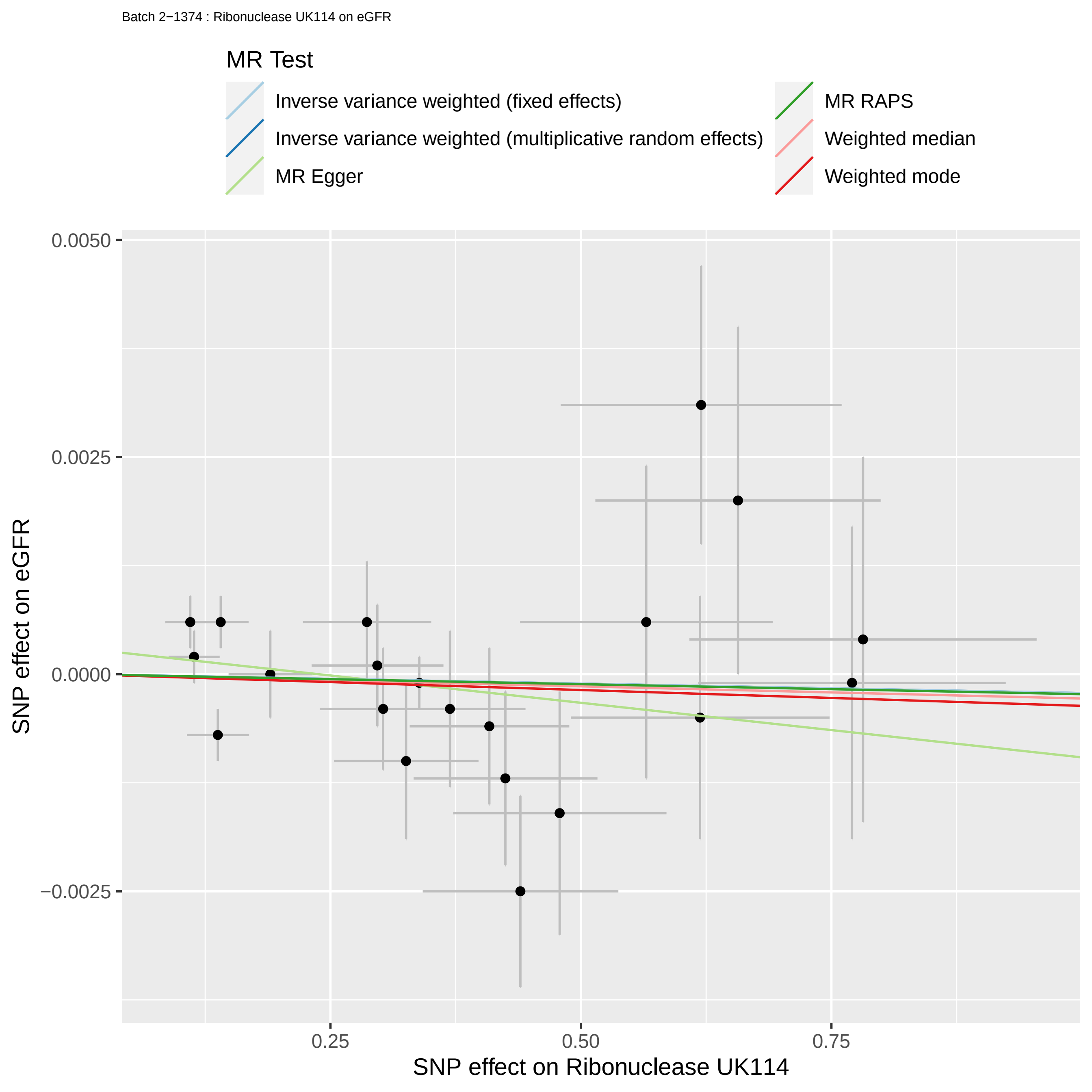

Batch 2−1374 : Ribonuclease UK114 on eGFR
MR Test
Inverse variance weighted (fixed effects)
Inverse variance weighted (multiplicative random effects)
MR Egger
MR RAPS
Weighted median
Weighted mode
0.0050
0.0025
0.0000
−0.0025
0.25
0.50
0.75
SNP effect on eGFR
SNP effect on Ribonuclease UK114

Batch 2−1375 : Heparan sulfate glucosamine 3−O−sulfotransferase 3A1 on eGFR

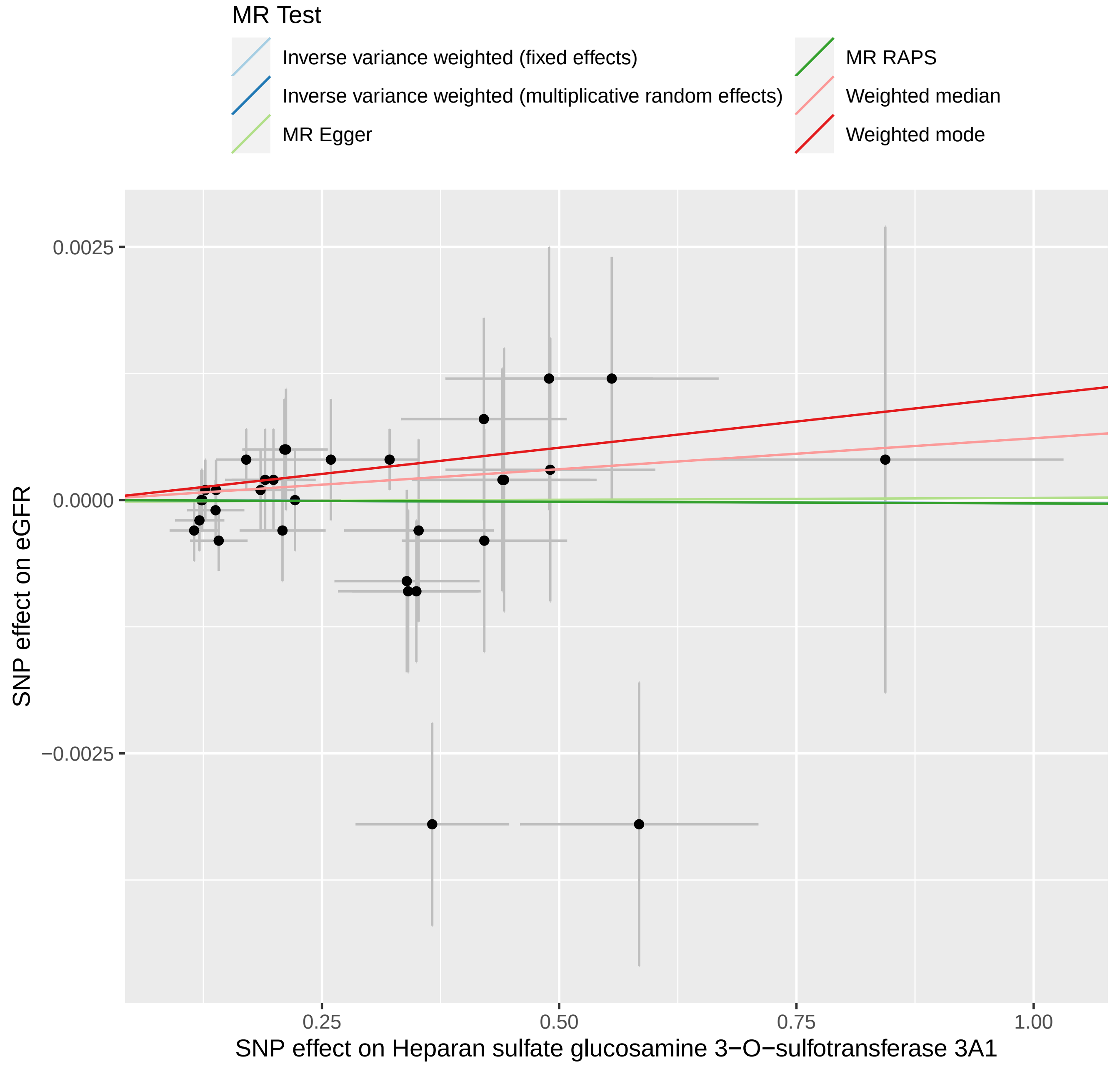

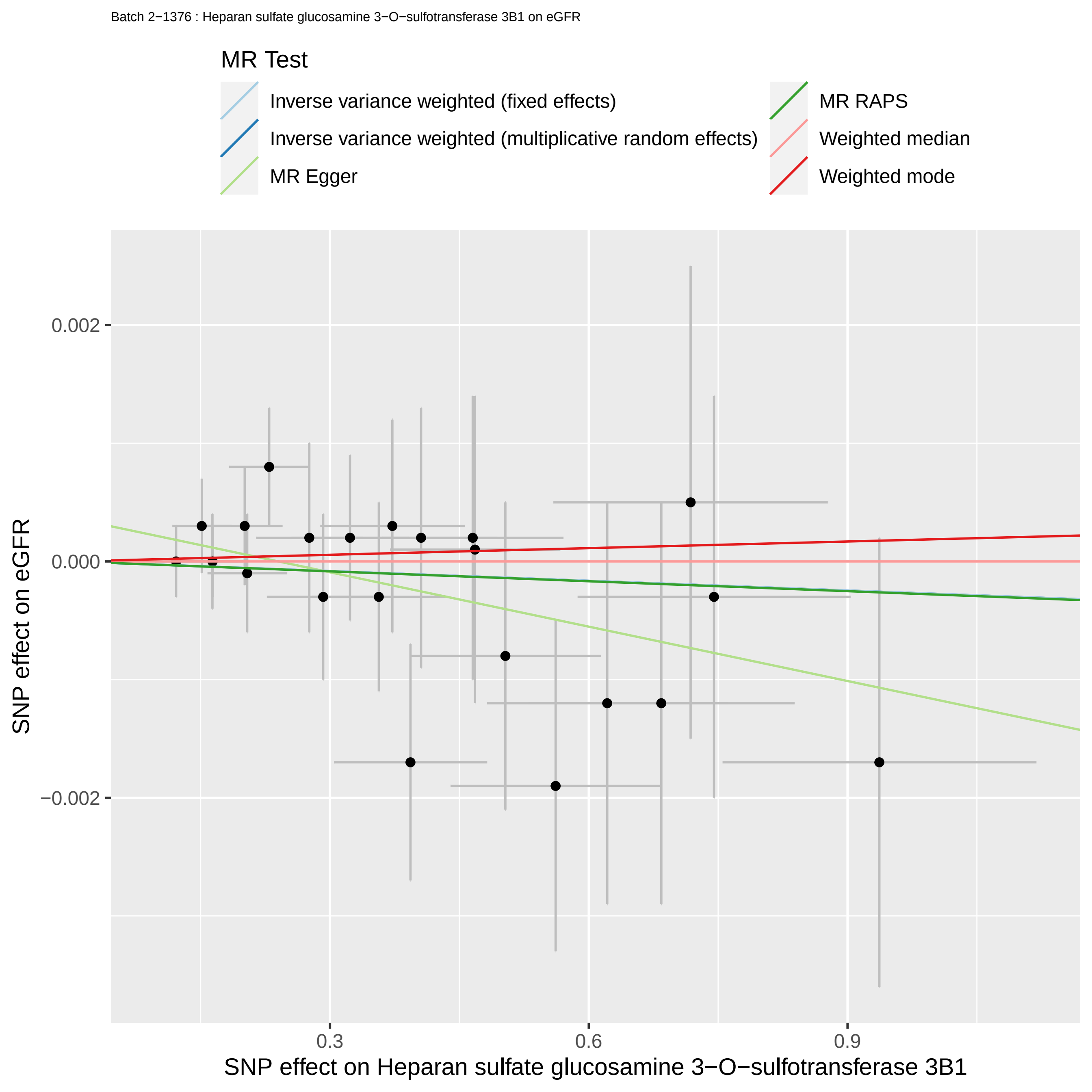
Batch 2−1376 : Heparan sulfate glucosamine 3−O−sulfotransferase 3B1 on eGFR
MR Test
Inverse variance weighted (fixed effects)
Inverse variance weighted (multiplicative random effects)
MR Egger
MR RAPS
Weighted median
Weighted mode
0.002
0.000
−0.002
0.3
0.6
0.9
SNP effect on eGFR
SNP effect on Heparan sulfate glucosamine 3−O−sulfotransferase 3B1

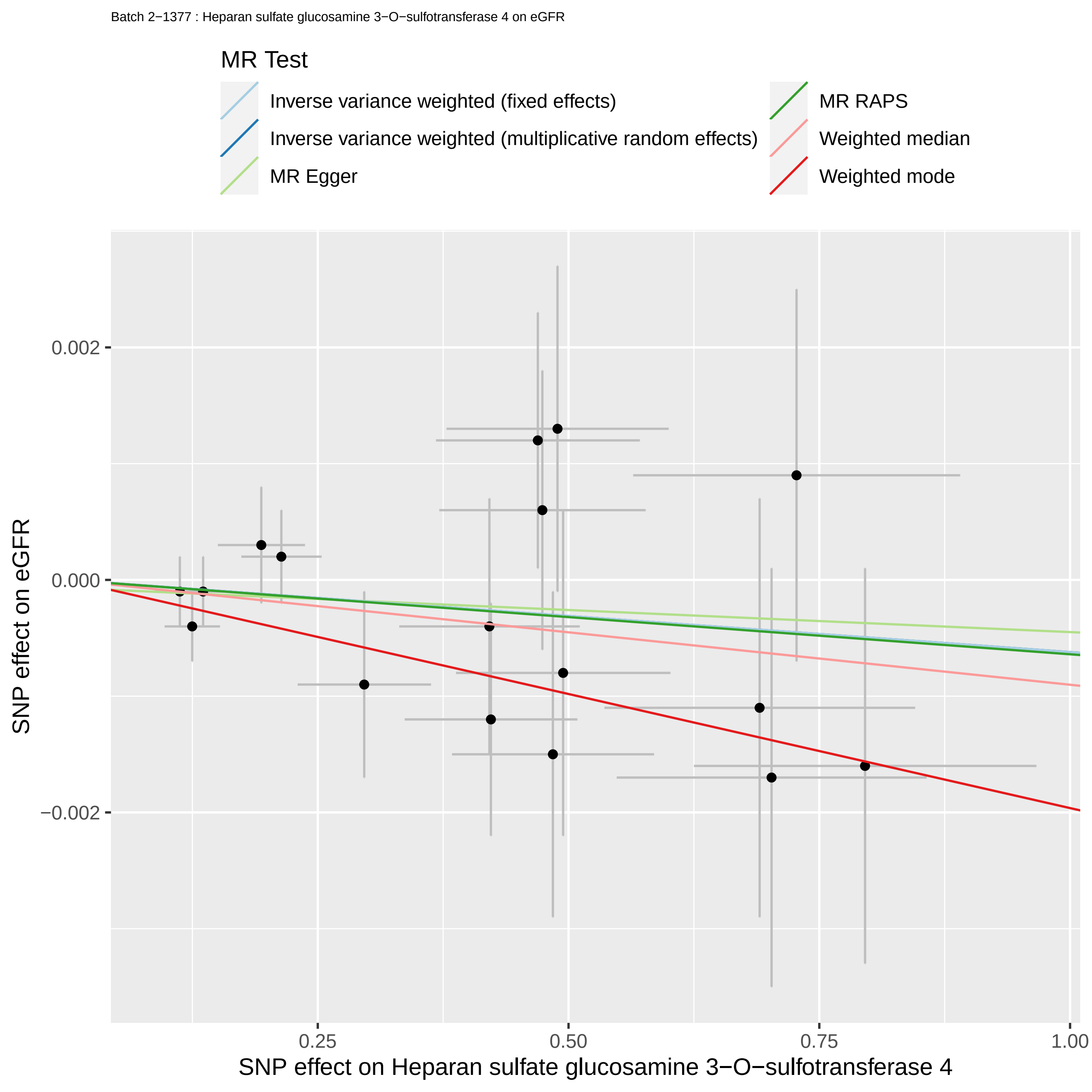
Batch 2−1377 : Heparan sulfate glucosamine 3−O−sulfotransferase 4 on eGFR
MR Test
Inverse variance weighted (fixed effects)
Inverse variance weighted (multiplicative random effects)
MR Egger
MR RAPS
Weighted median
Weighted mode
0.002
0.000
−0.002
0.25
0.50
0.75
1.00
SNP effect on eGFR
SNP effect on Heparan sulfate glucosamine 3−O−sulfotransferase 4

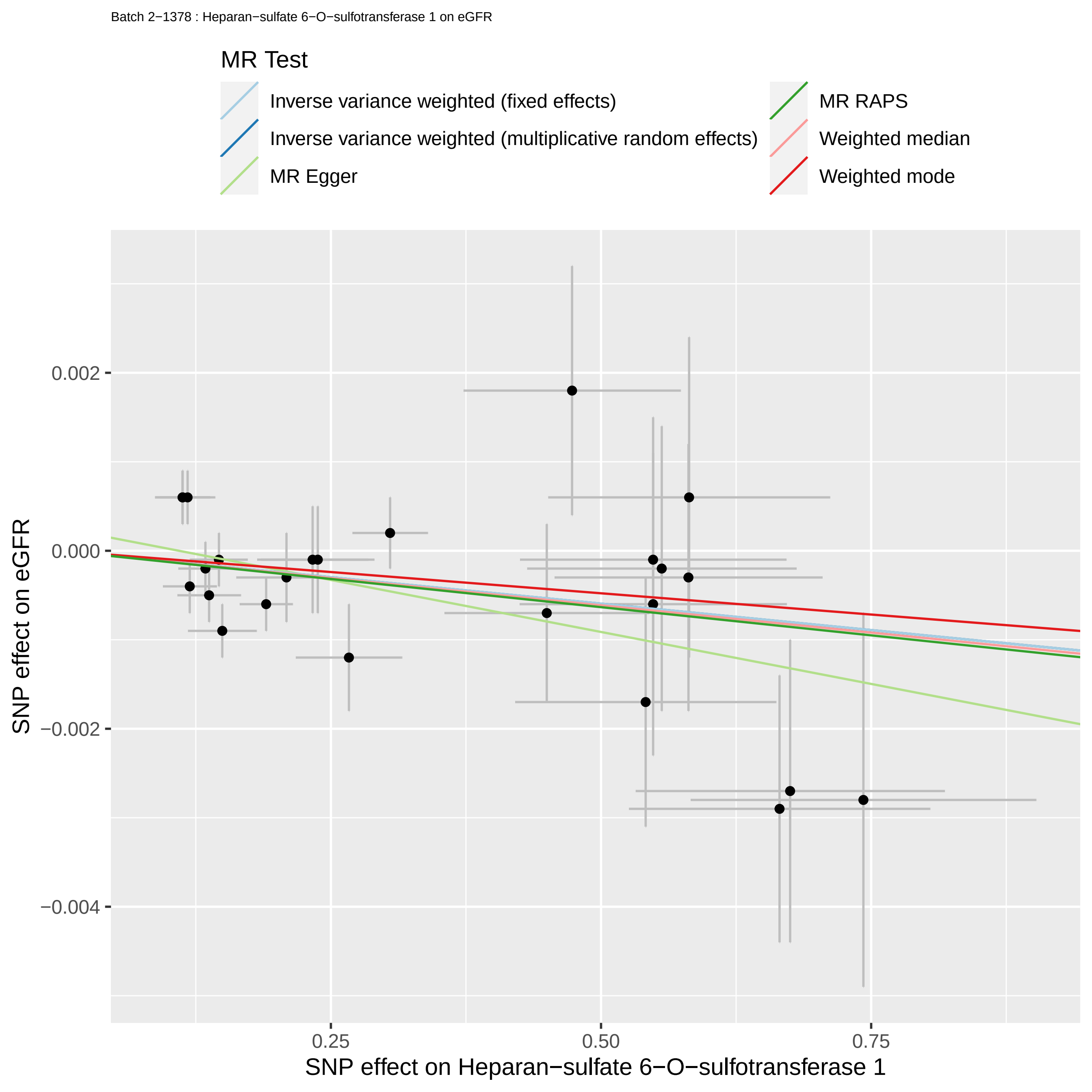

Batch 2−1378 : Heparan−sulfate 6−O−sulfotransferase 1 on eGFR
MR Test
Inverse variance weighted (fixed effects)
Inverse variance weighted (multiplicative random effects)
MR Egger
MR RAPS
Weighted median
Weighted mode
0.002
0.000
−0.002
−0.004
SNP effect on eGFR
0.25
0.50
0.75
SNP effect on Heparan−sulfate 6−O−sulfotransferase 1

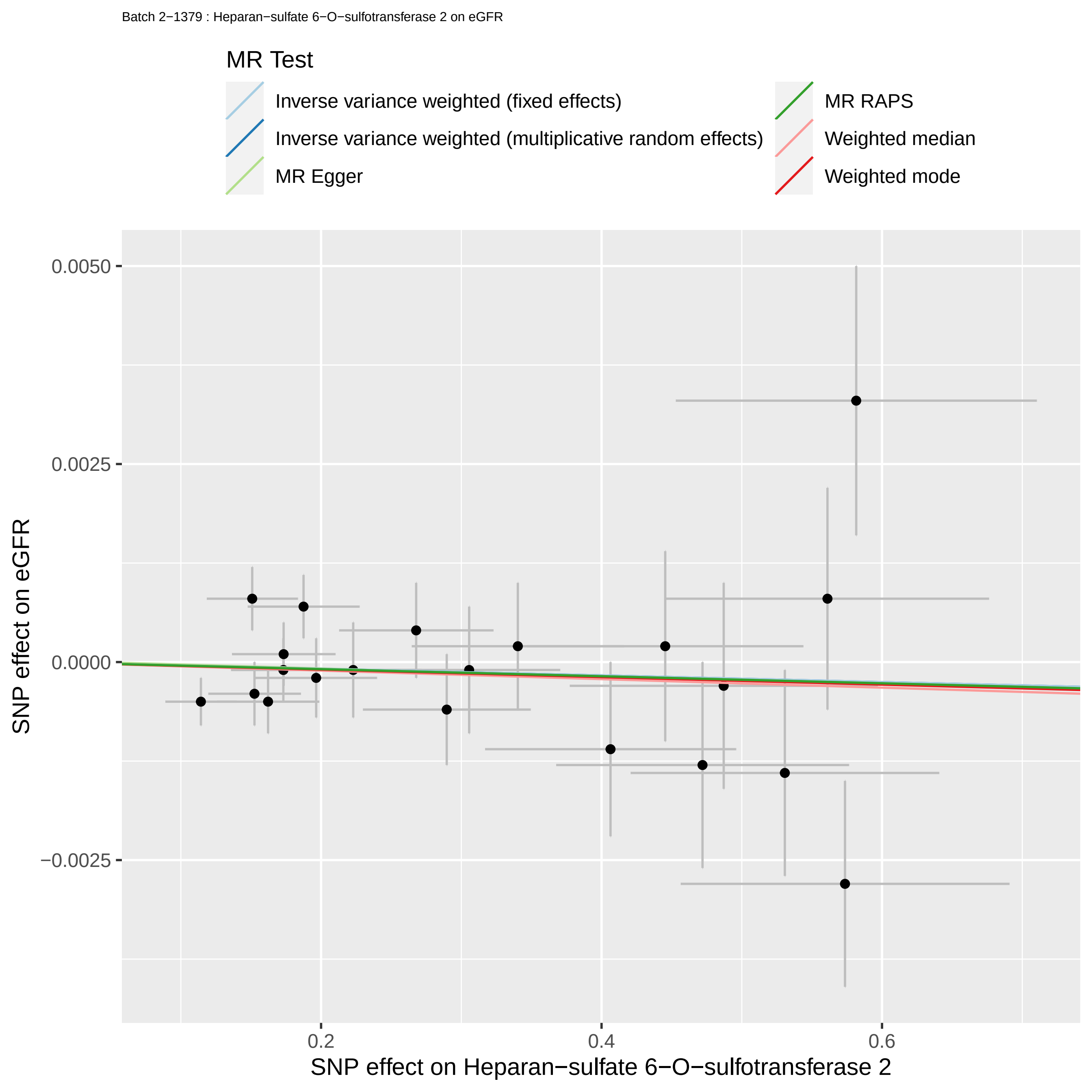

Batch 2−1379 : Heparan−sulfate 6−O−sulfotransferase 2 on eGFR
MR Test
Inverse variance weighted (fixed effects)
Inverse variance weighted (multiplicative random effects)
MR Egger
MR RAPS
Weighted median
Weighted mode
0.0050
0.0025
0.0000
−0.0025
SNP effect on eGFR
0.2
0.4
0.6
SNP effect on Heparan−sulfate 6−O−sulfotransferase 2

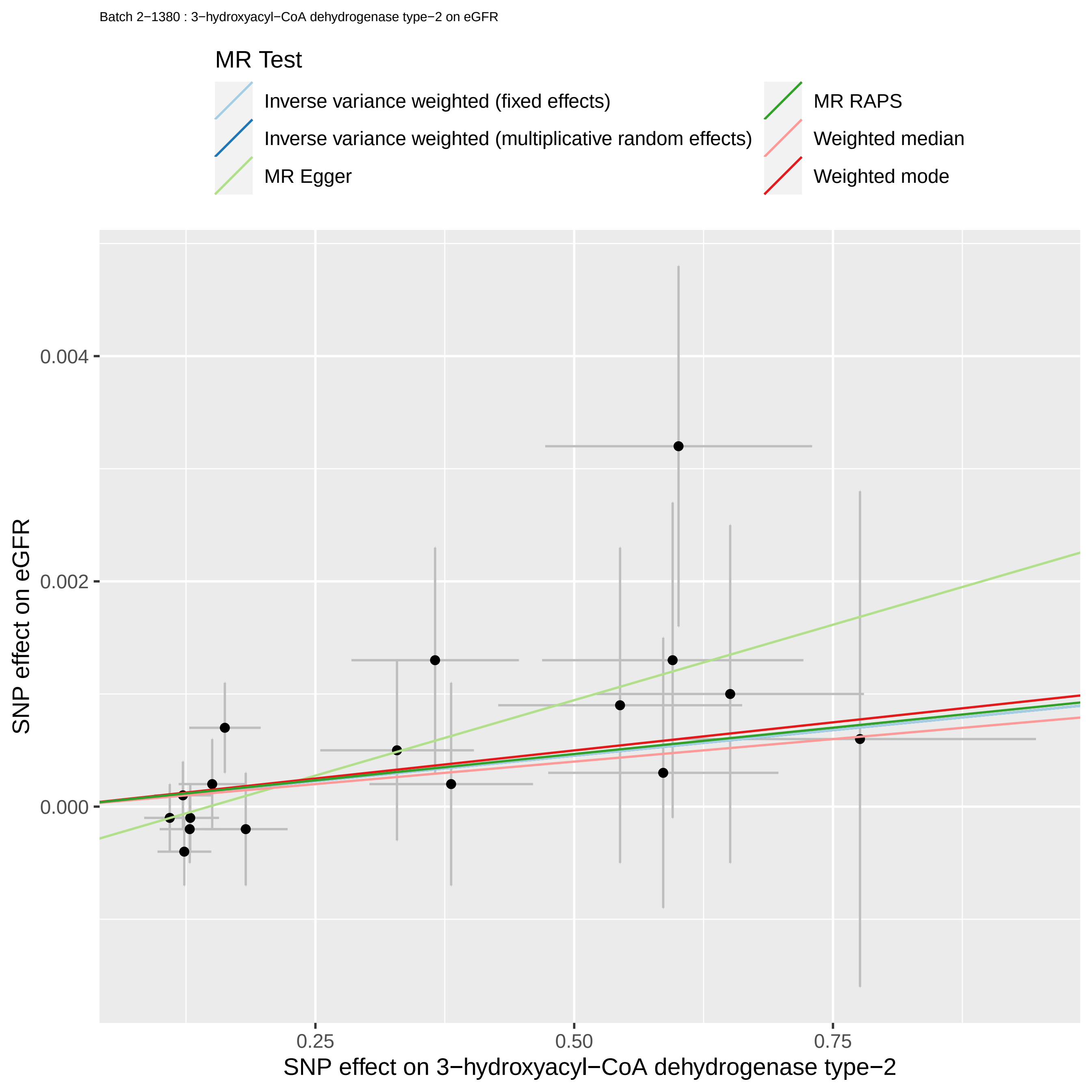
Batch 2−1380 : 3−hydroxyacyl−CoA dehydrogenase type−2 on eGFR
MR Test
Inverse variance weighted (fixed effects)
Inverse variance weighted (multiplicative random effects)
MR Egger
MR RAPS
Weighted median
Weighted mode
0.004
0.002
0.000
SNP effect on eGFR
0.25
0.50
0.75
SNP effect on 3−hydroxyacyl−CoA dehydrogenase type−2

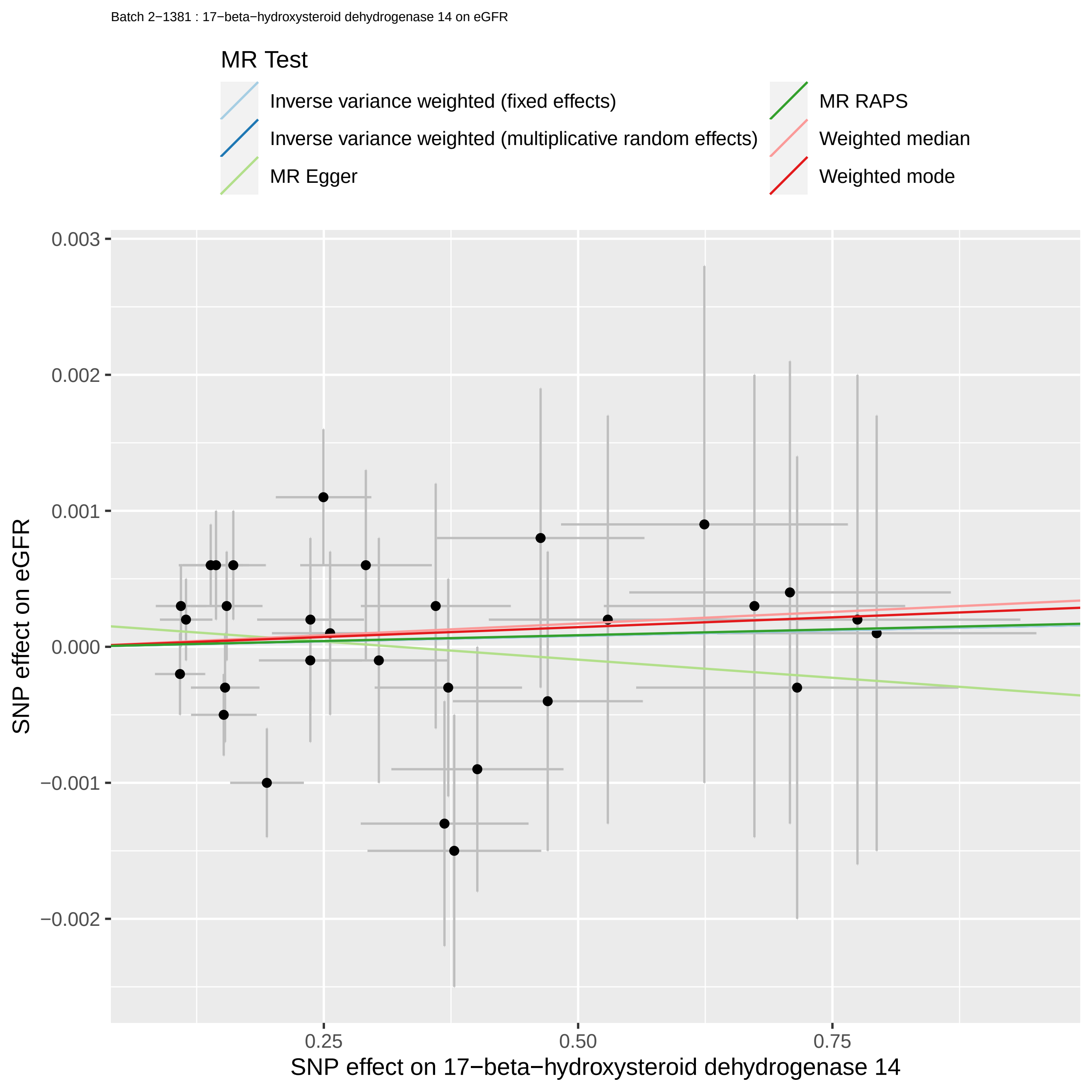
Batch 2−1381 : 17−beta−hydroxysteroid dehydrogenase 14 on eGFR
MR Test
Inverse variance weighted (fixed effects)
Inverse variance weighted (multiplicative random effects)
MR Egger
MR RAPS
Weighted median
Weighted mode
0.003
0.002
0.001
0.000
−0.001
−0.002
SNP effect on eGFR
0.25
0.50
0.75
SNP effect on 17−beta−hydroxysteroid dehydrogenase 14

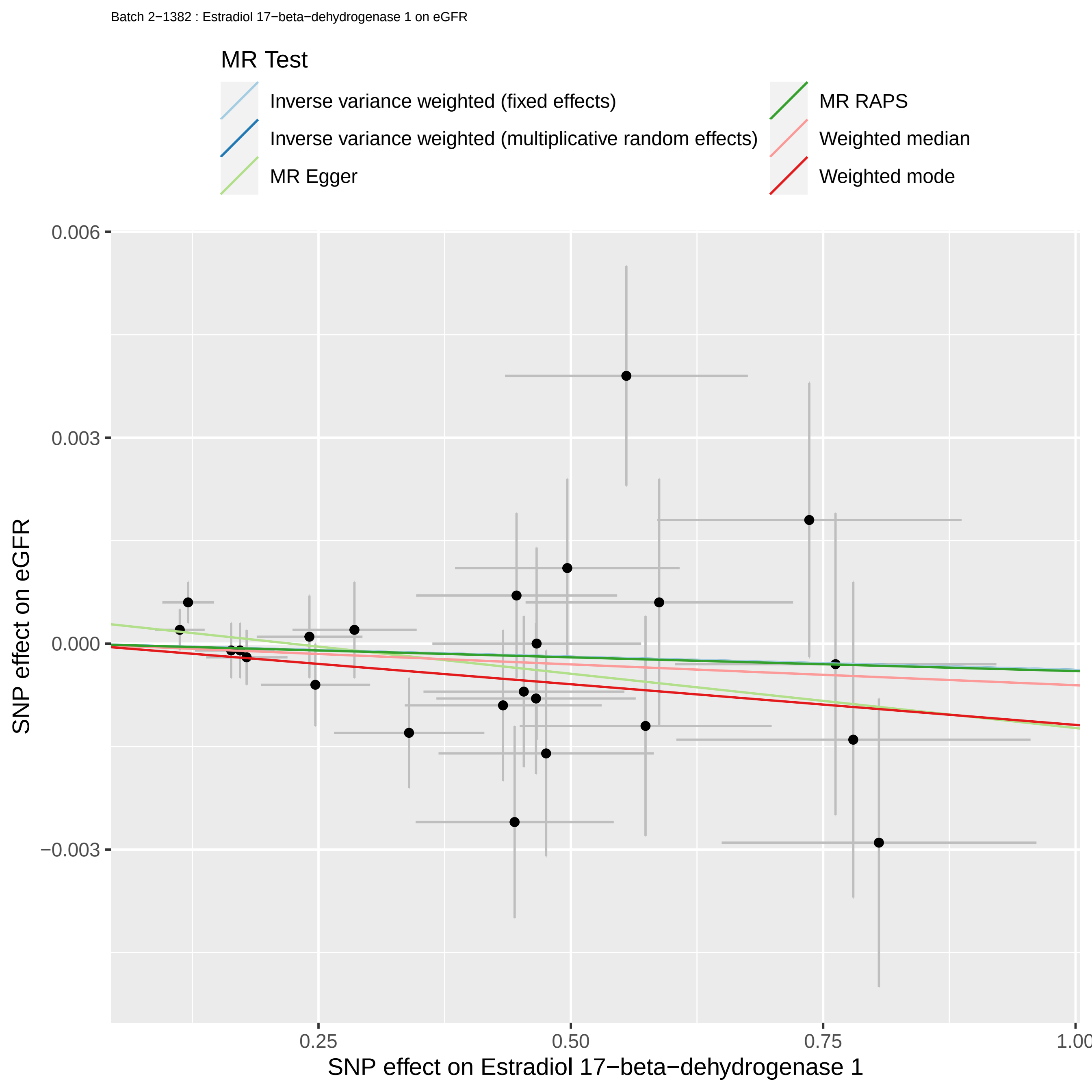

Batch 2−1382 : Estradiol 17−beta−dehydrogenase 1 on eGFR
MR Test
Inverse variance weighted (fixed effects)
Inverse variance weighted (multiplicative random effects)
MR Egger
MR RAPS
Weighted median
Weighted mode
0.006
0.003
0.000
−0.003
0.25
0.50
0.75
1.00
SNP effect on eGFR
SNP effect on Estradiol 17−beta−dehydrogenase 1

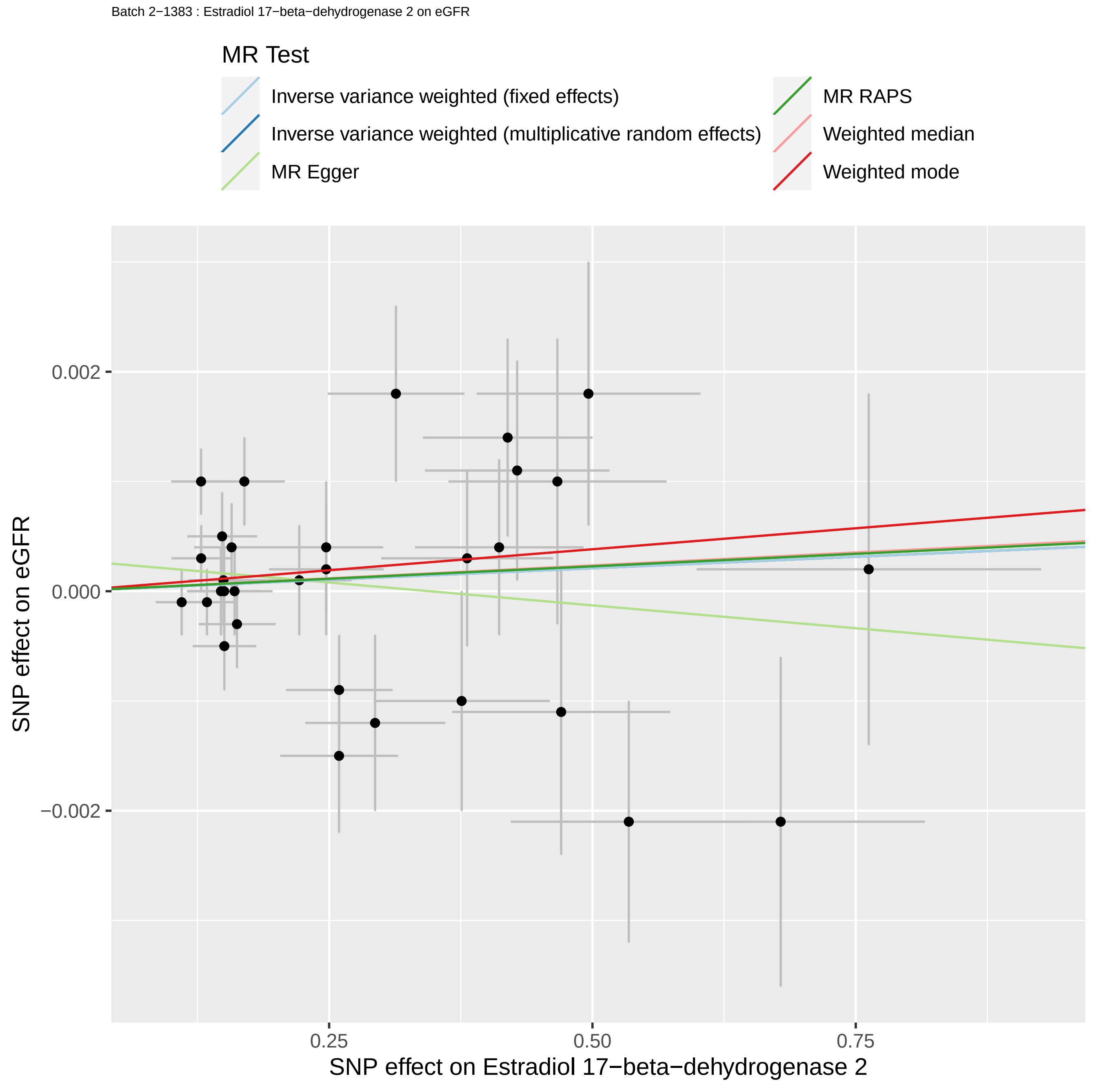

Batch 2−1383 : Estradiol 17−beta−dehydrogenase 2 on eGFR
MR Test
Inverse variance weighted (fixed effects)
Inverse variance weighted (multiplicative random effects)
MR Egger
MR RAPS
Weighted median
Weighted mode
0.002
0.000
−0.002
0.25
0.50
0.75
SNP effect on eGFR
SNP effect on Estradiol 17−beta−dehydrogenase 2

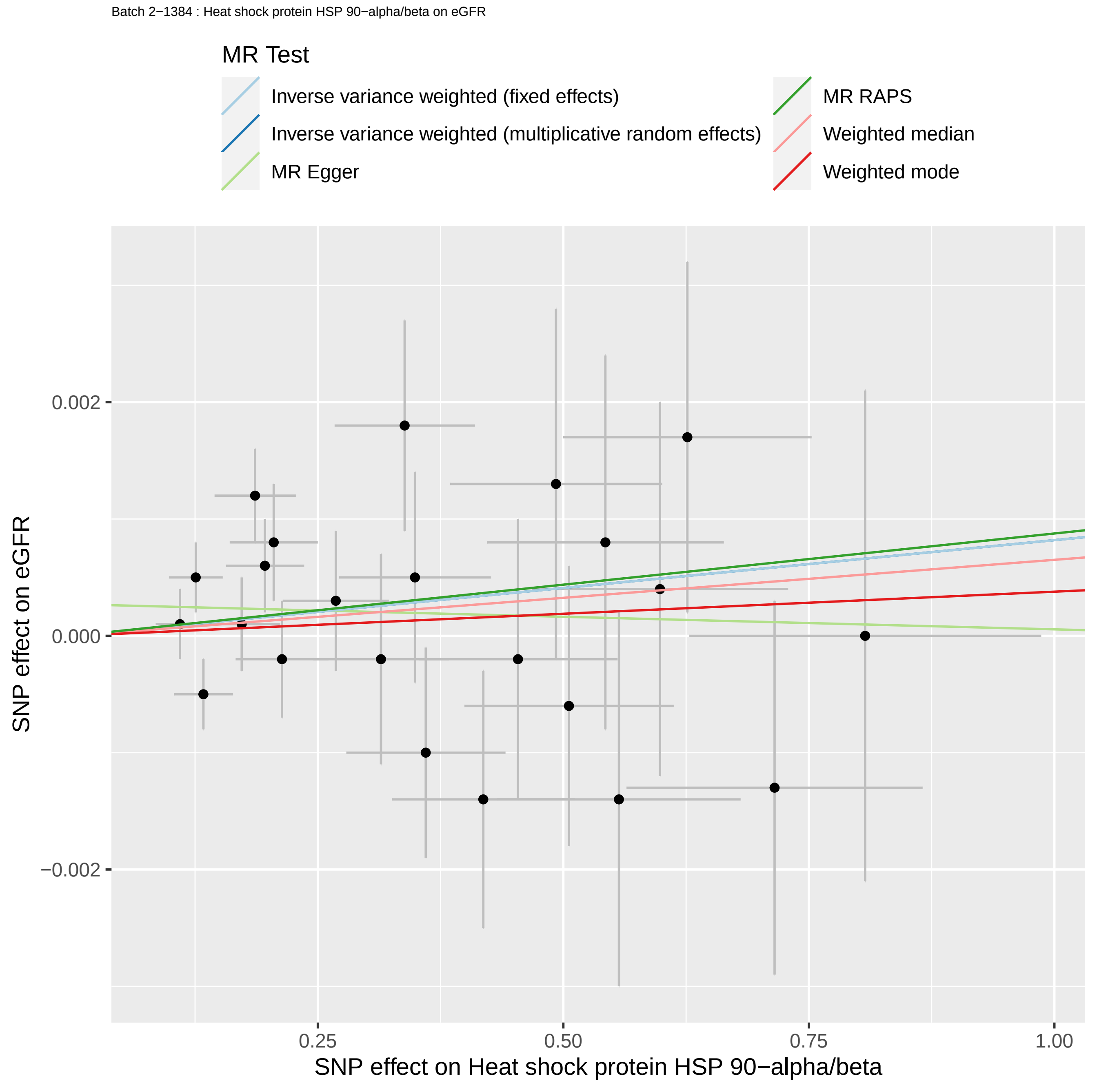

Batch 2−1384 : Heat shock protein HSP 90−alpha/beta on eGFR
MR Test
Inverse variance weighted (fixed effects)
Inverse variance weighted (multiplicative random effects)
MR Egger
MR RAPS
Weighted median
Weighted mode
0.002
0.000
−0.002
0.25
0.50
0.75
1.00
SNP effect on eGFR
SNP effect on Heat shock protein HSP 90−alpha/beta

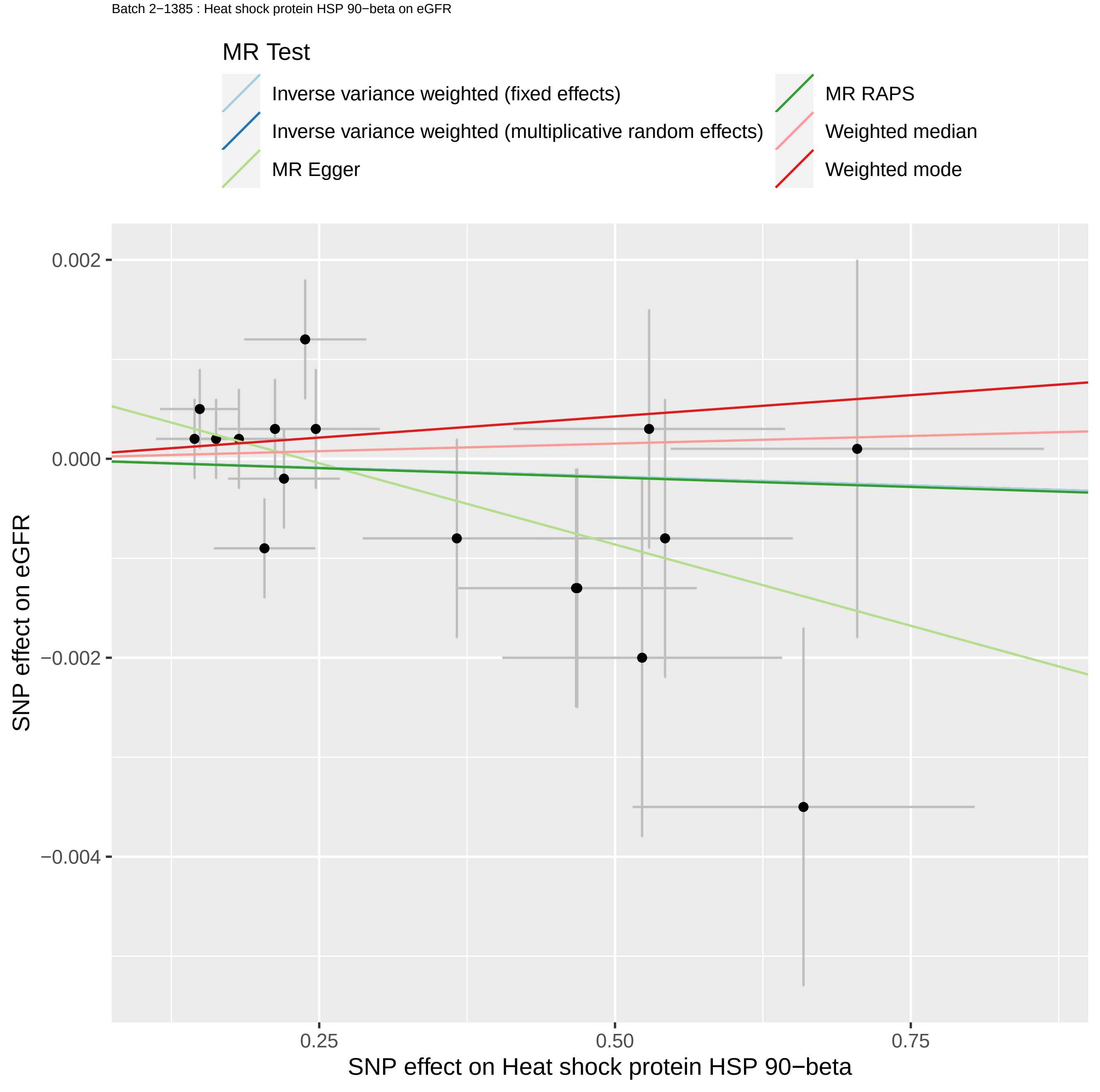
Batch 2−1385 : Heat shock protein HSP 90−beta on eGFR
MR Test
Inverse variance weighted (fixed effects)
Inverse variance weighted (multiplicative random effects)
MR Egger
MR RAPS
Weighted median
Weighted mode
0.002
0.000
−0.002
−0.004
SNP effect on eGFR
0.25
0.50
0.75
SNP effect on Heat shock protein HSP 90−beta

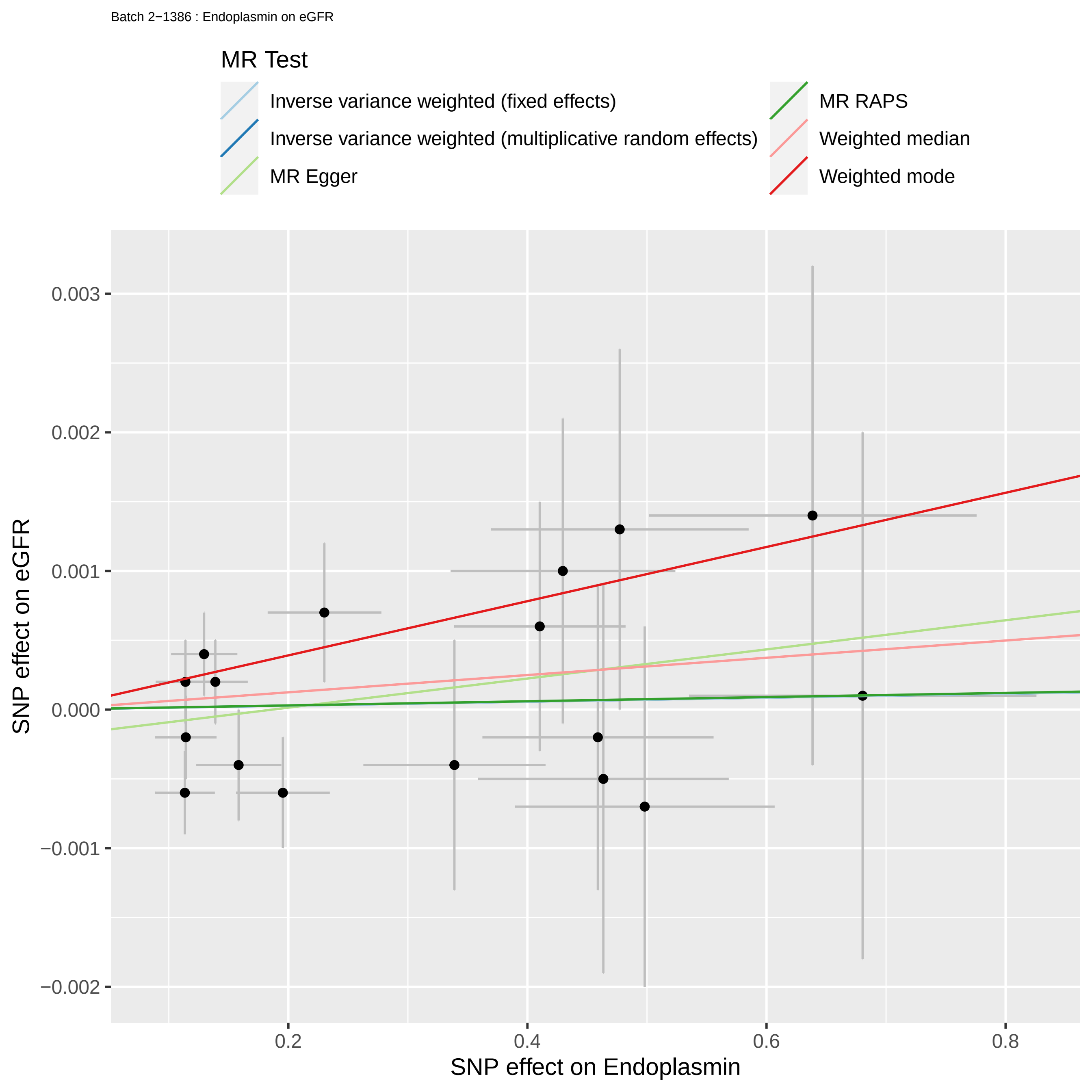

Batch 2−1386 : Endoplasmin on eGFR
MR Test
Inverse variance weighted (fixed effects)
Inverse variance weighted (multiplicative random effects)
MR Egger
MR RAPS
Weighted median
Weighted mode
SNP effect on eGFR
0.003
0.002
0.001
0.000
−0.001
−0.002
0.2
0.4
0.6
0.8
SNP effect on Endoplasmin

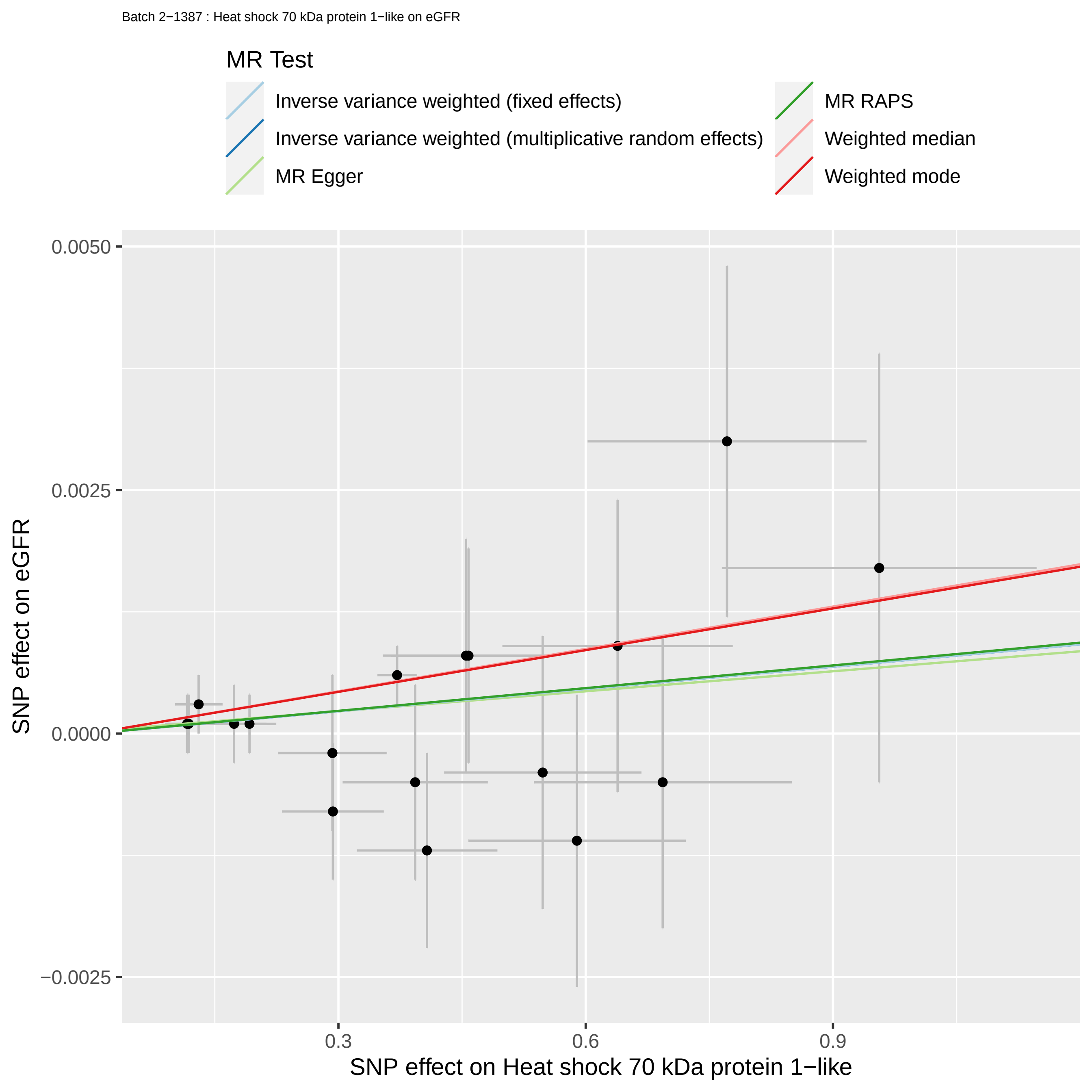

Batch 2−1387 : Heat shock 70 kDa protein 1−like on eGFR
MR Test
Inverse variance weighted (fixed effects)
Inverse variance weighted (multiplicative random effects)
MR Egger
MR RAPS
Weighted median
Weighted mode
0.0050
0.0025
0.0000
−0.0025
0.3
0.6
0.9
SNP effect on eGFR
SNP effect on Heat shock 70 kDa protein 1−like

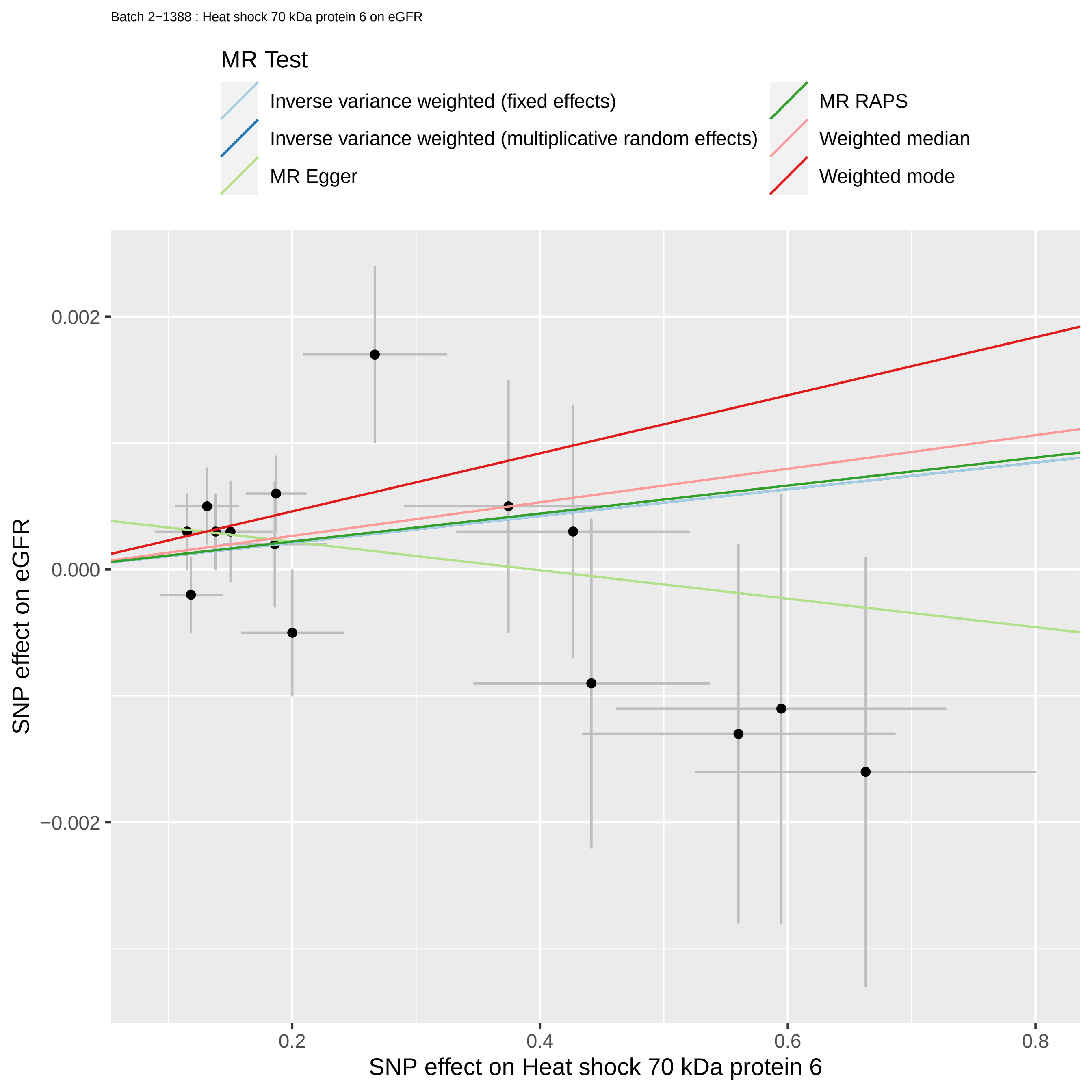
Batch 2−1388 : Heat shock 70 kDa protein 6 on eGFR
MR Test
Inverse variance weighted (fixed effects)
Inverse variance weighted (multiplicative random effects)
MR Egger
MR RAPS
Weighted median
Weighted mode
0.002
0.000
−0.002
0.2
0.4
0.6
0.8
SNP effect on eGFR
SNP effect on Heat shock 70 kDa protein 6

Batch 2−1389 : Oxidoreductase HTATIP2 on eGFR

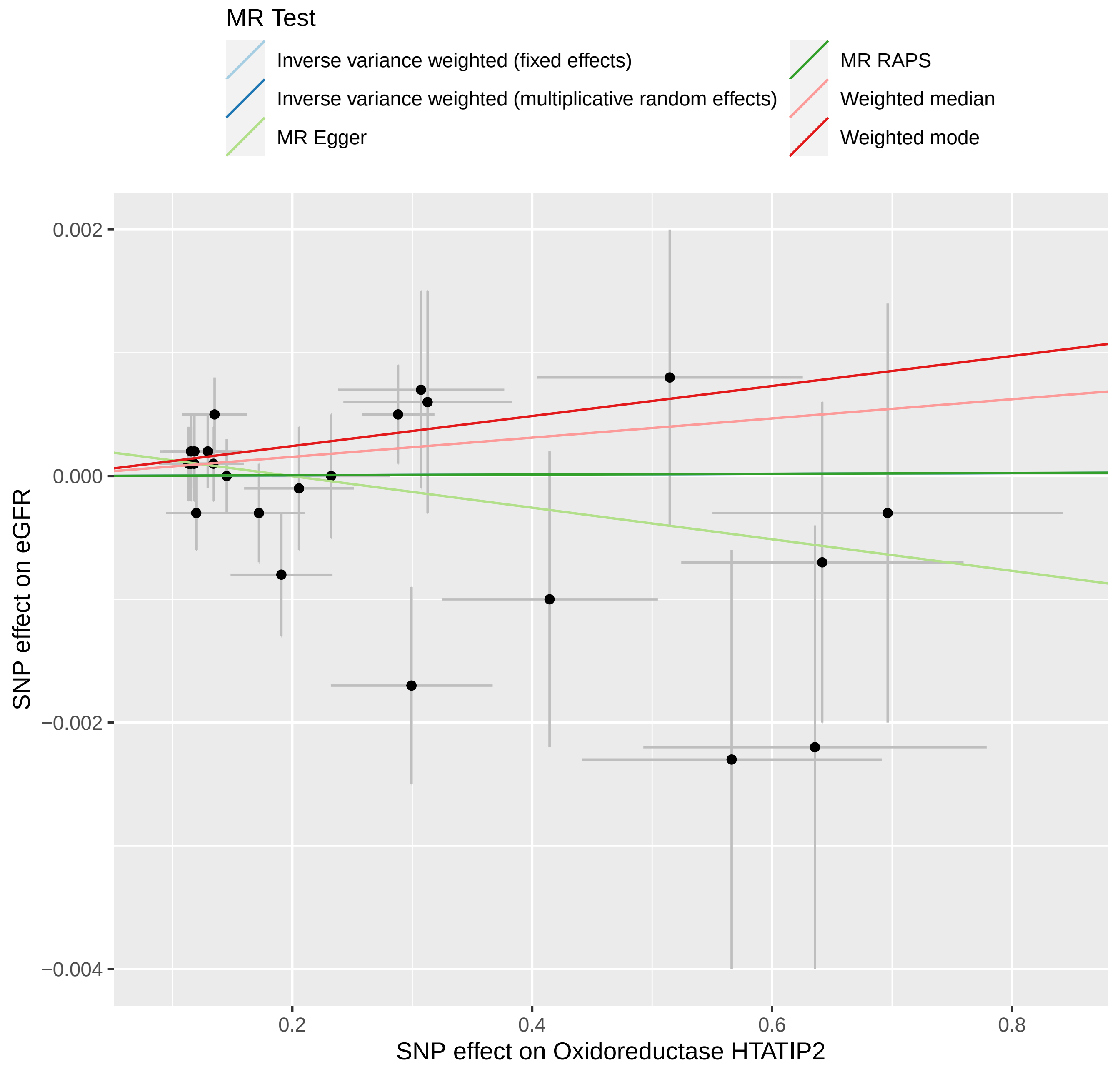

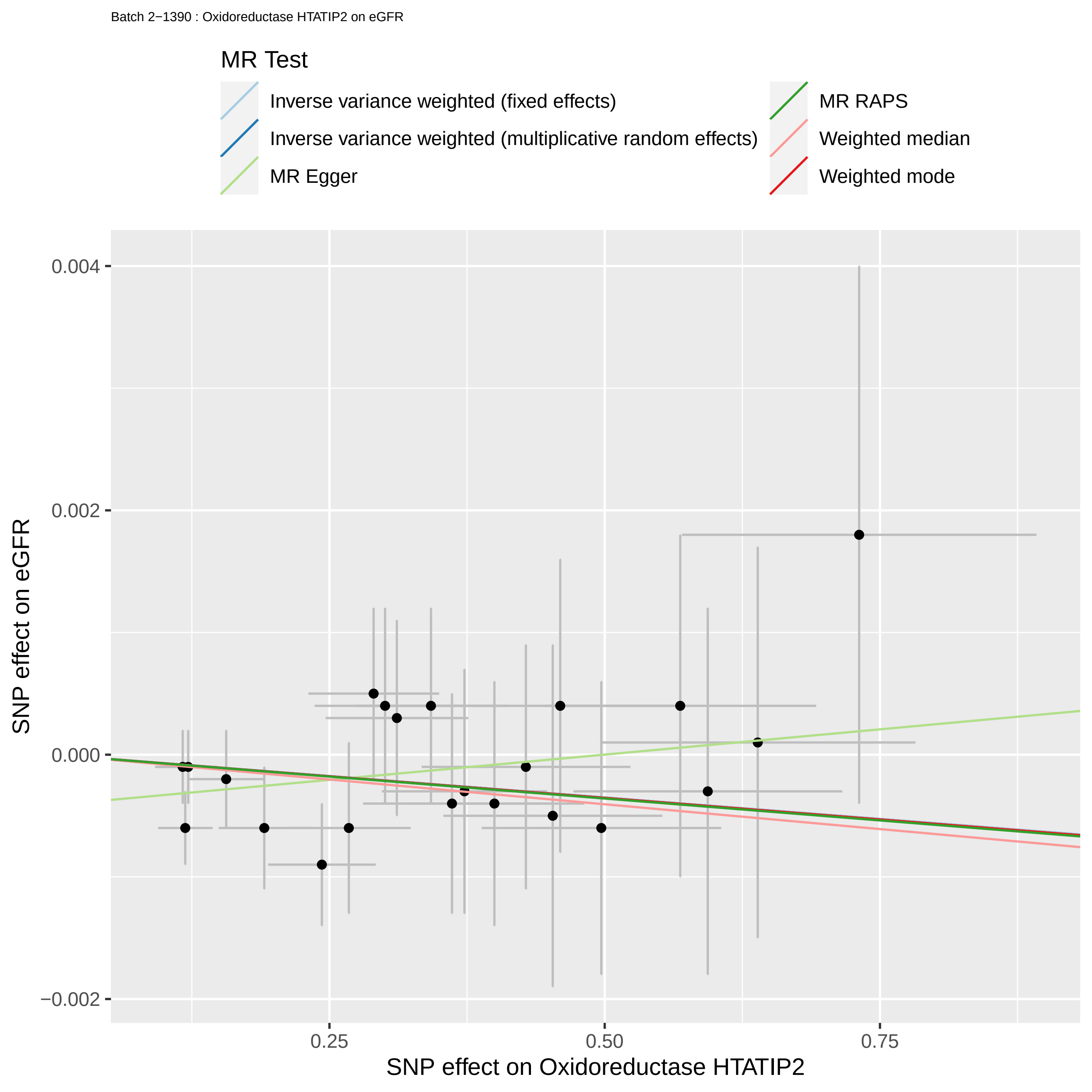

Batch 2−1390 : Oxidoreductase HTATIP2 on eGFR
MR Test
Inverse variance weighted (fixed effects)
Inverse variance weighted (multiplicative random effects)
MR Egger
MR RAPS
Weighted median
Weighted mode
0.004
0.002
0.000
−0.002
0.25
0.50
0.75
SNP effect on eGFR
SNP effect on Oxidoreductase HTATIP2

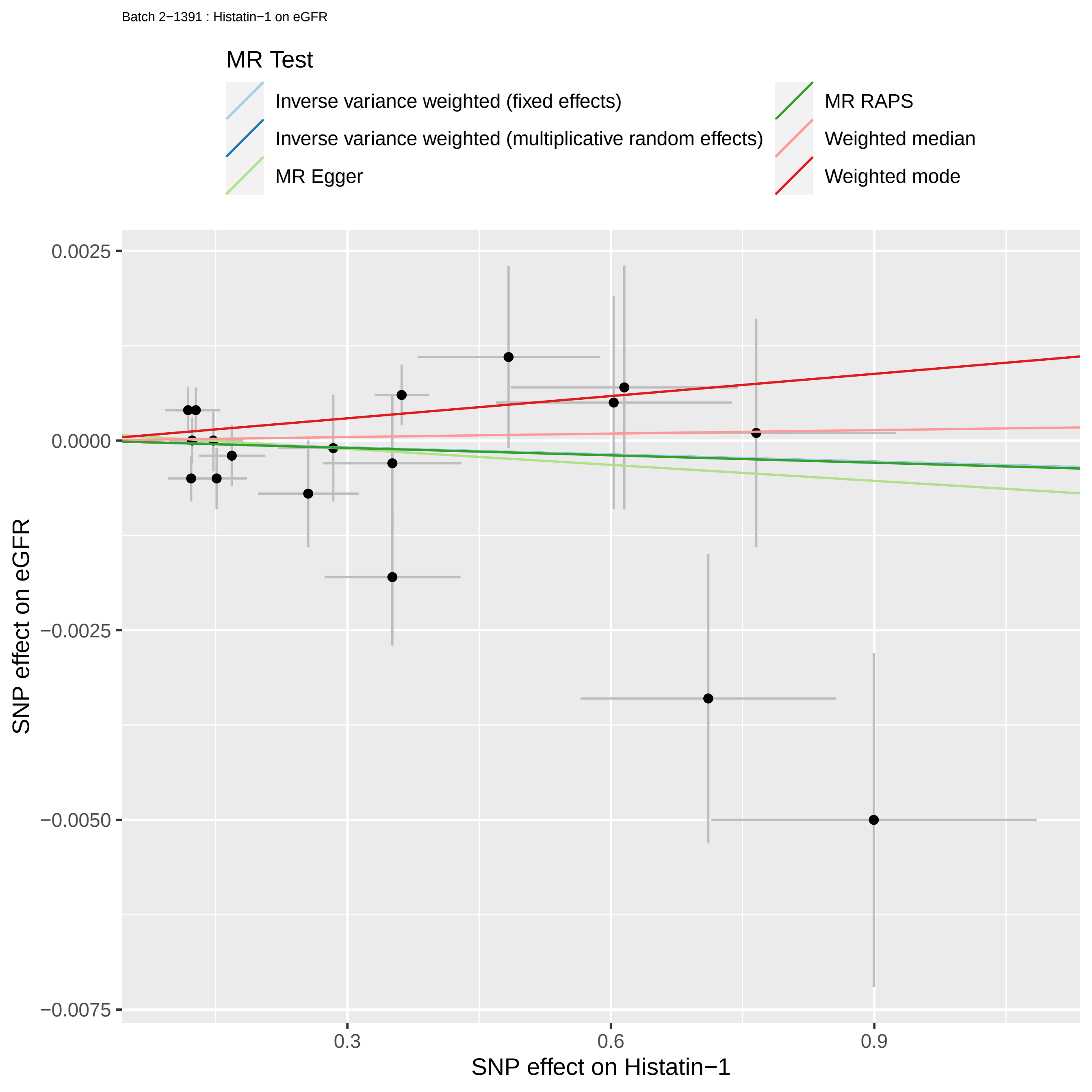

Batch 2−1391 : Histatin−1 on eGFR
MR Test
Inverse variance weighted (fixed effects)
Inverse variance weighted (multiplicative random effects)
MR Egger
MR RAPS
Weighted median
Weighted mode
0.0025
0.0000
−0.0025
−0.0050
−0.0075
SNP effect on eGFR
0.3
0.6
0.9
SNP effect on Histatin−1

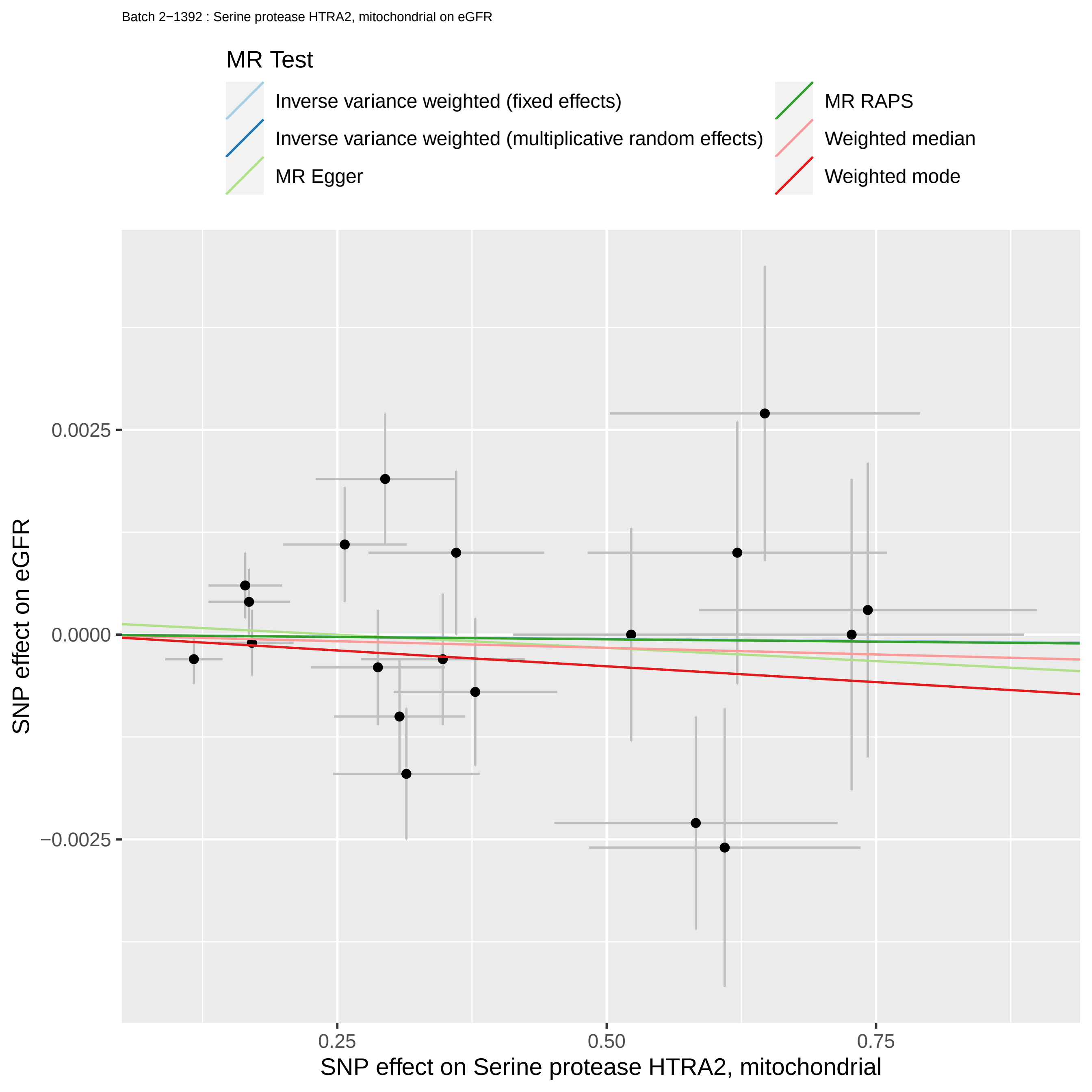

Batch 2−1392 : Serine protease HTRA2, mitochondrial on eGFR
MR Test
Inverse variance weighted (fixed effects)
Inverse variance weighted (multiplicative random effects)
MR Egger
MR RAPS
Weighted median
Weighted mode
0.0025
0.0000
−0.0025
0.25
0.50
0.75
SNP effect on eGFR
SNP effect on Serine protease HTRA2, mitochondrial

Batch 2−1393 : Hyaluronidase−1 on eGFR

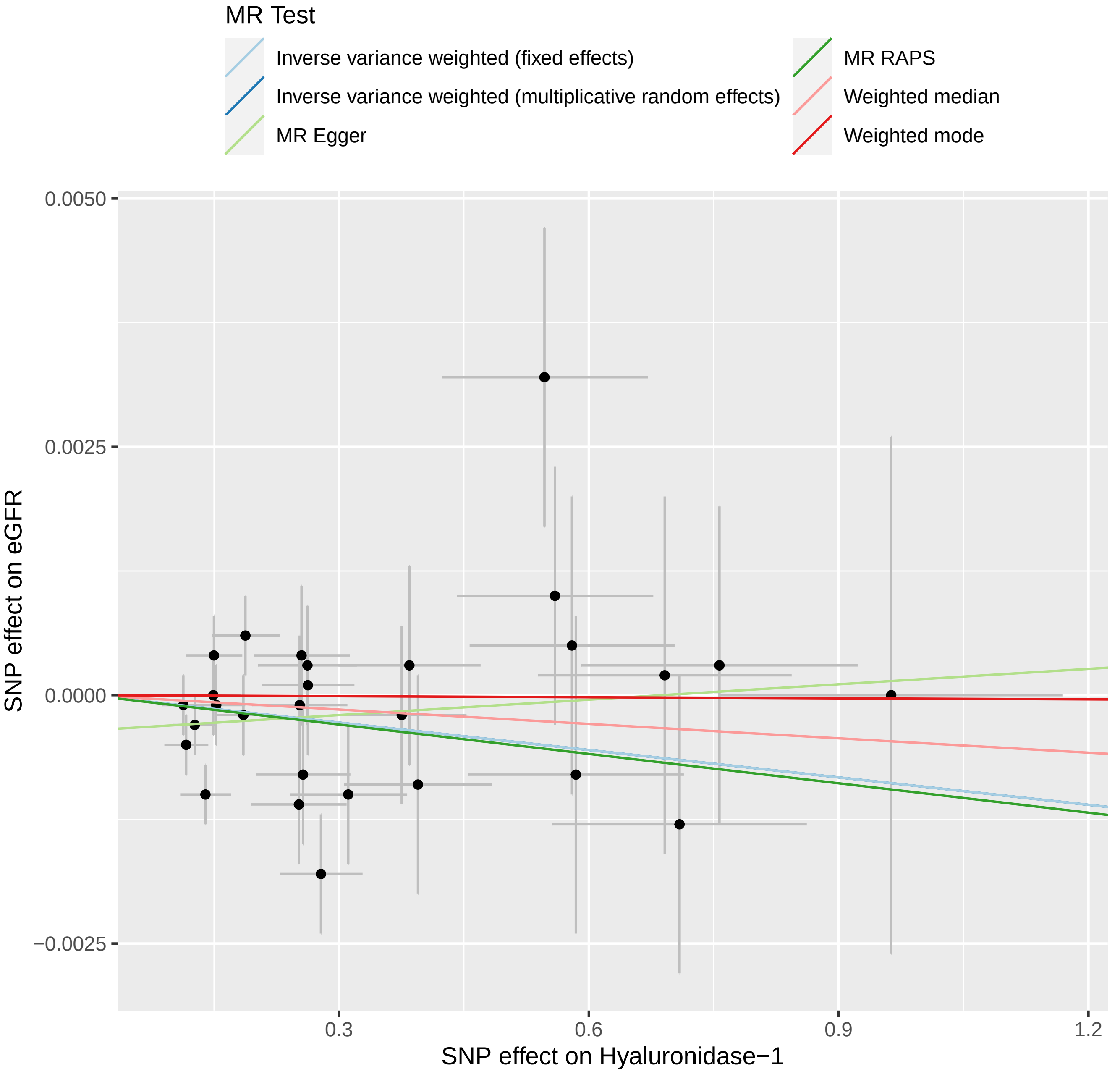

Batch 2−1394 : Isoleucine−−tRNA ligase, cytoplasmic on eGFR

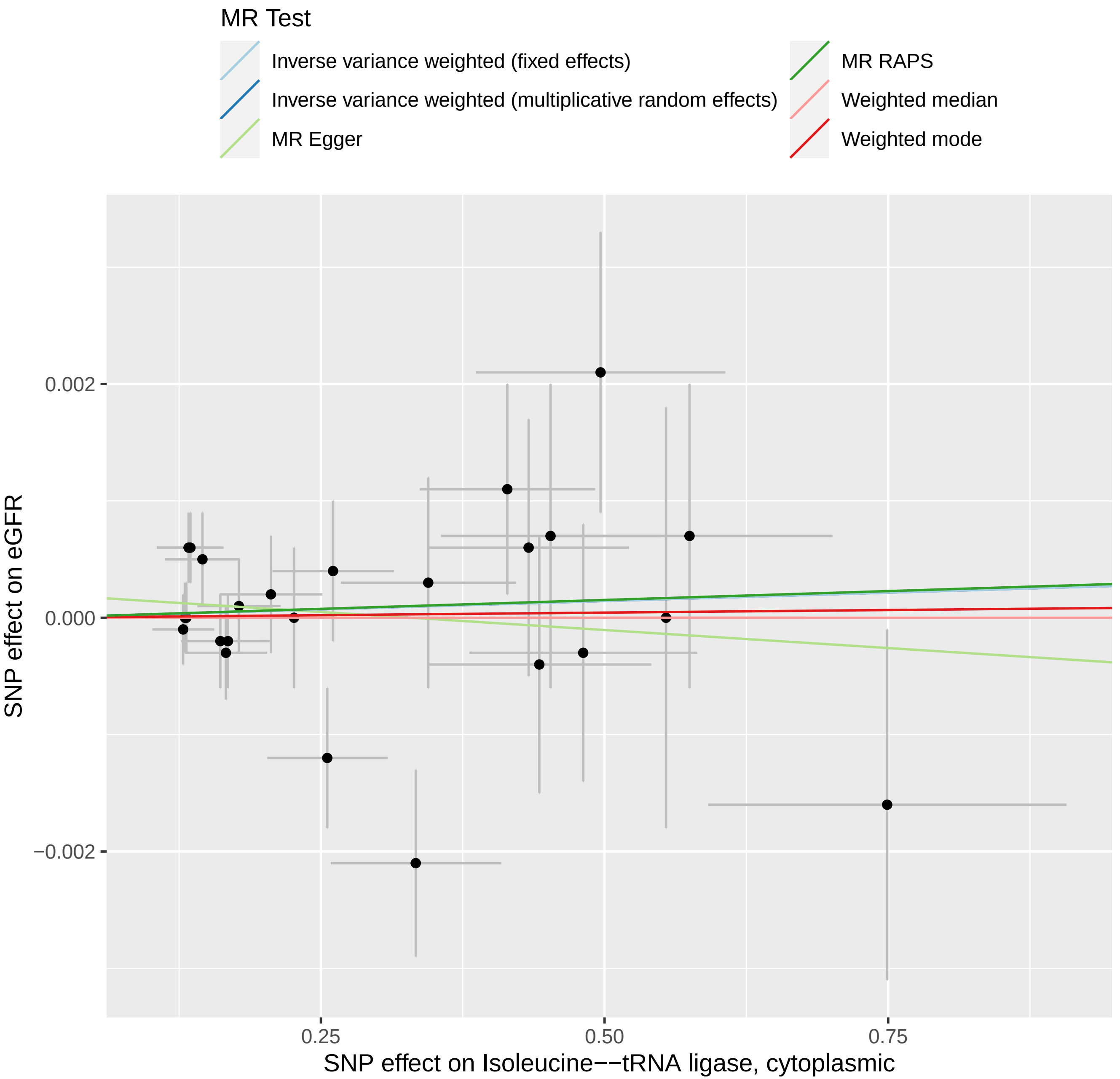

Batch 2−1395 : Bone sialoprotein 2 on eGFR

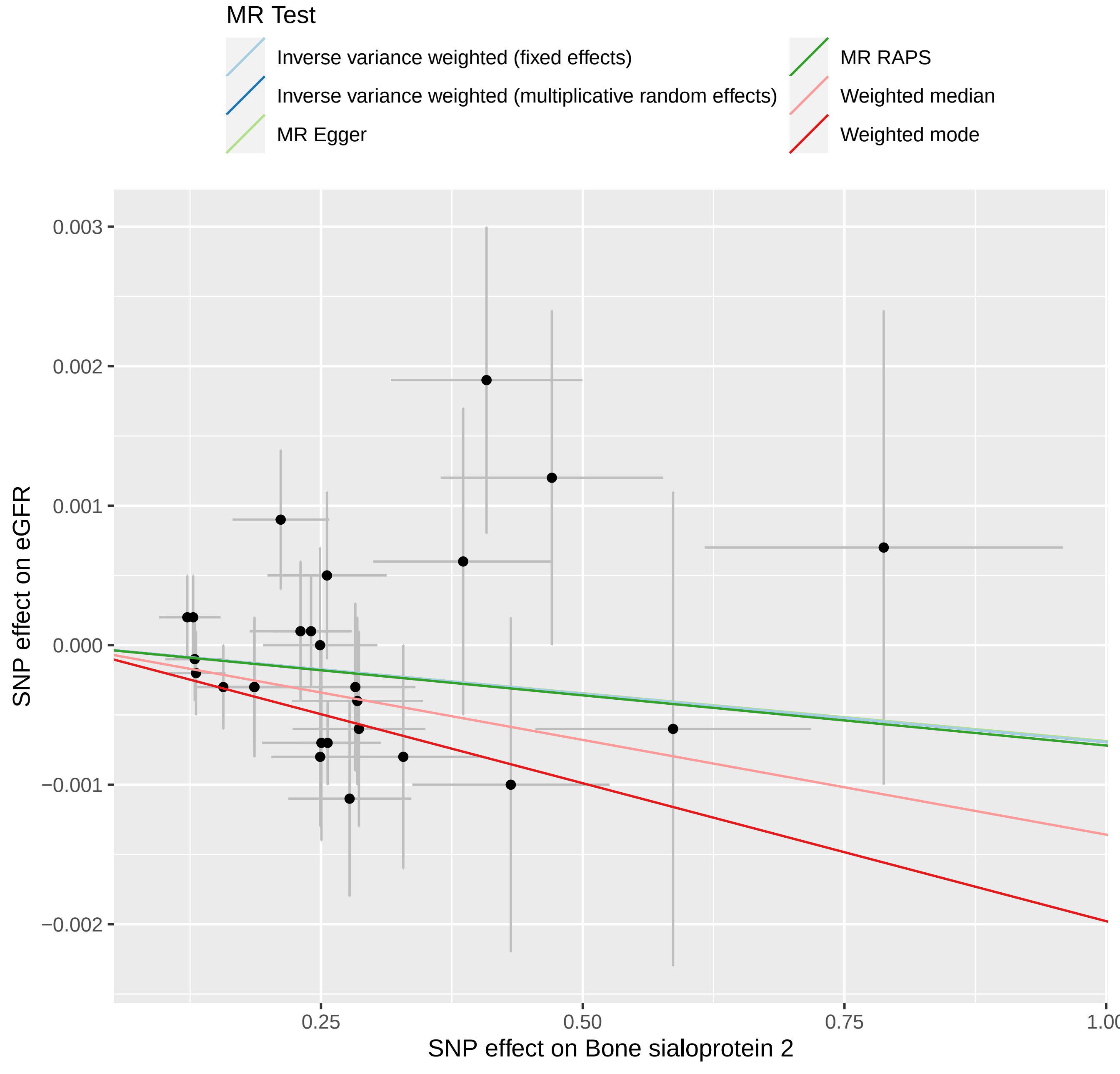

Batch 2−1396 : Bone sialoprotein 2 on eGFR

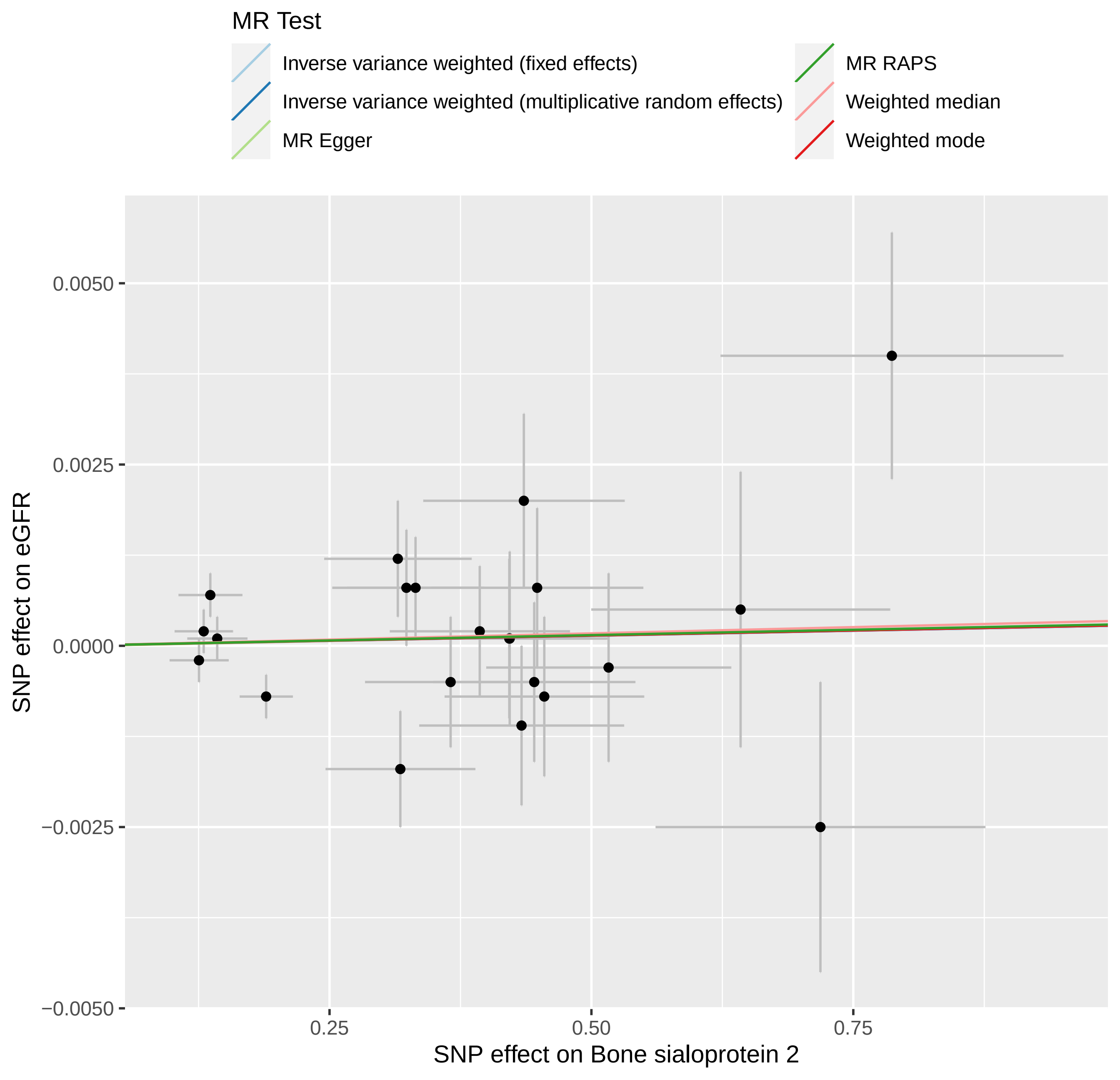

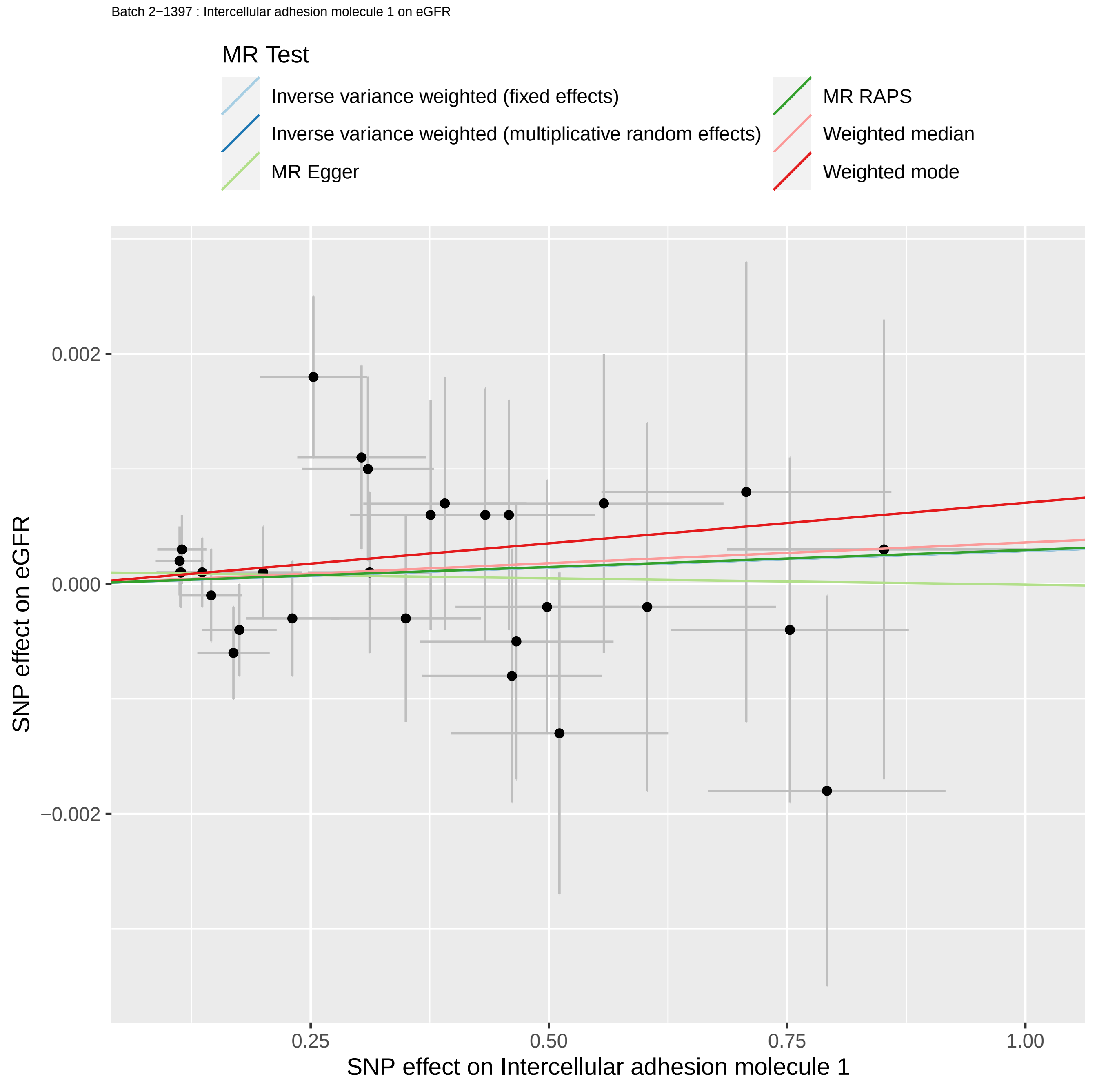
Batch 2−1397 : Intercellular adhesion molecule 1 on eGFR
MR Test
Inverse variance weighted (fixed effects)
Inverse variance weighted (multiplicative random effects)
MR Egger
MR RAPS
Weighted median
Weighted mode
0.002
0.000
−0.002
0.25
0.50
0.75
1.00
SNP effect on eGFR
SNP effect on Intercellular adhesion molecule 1

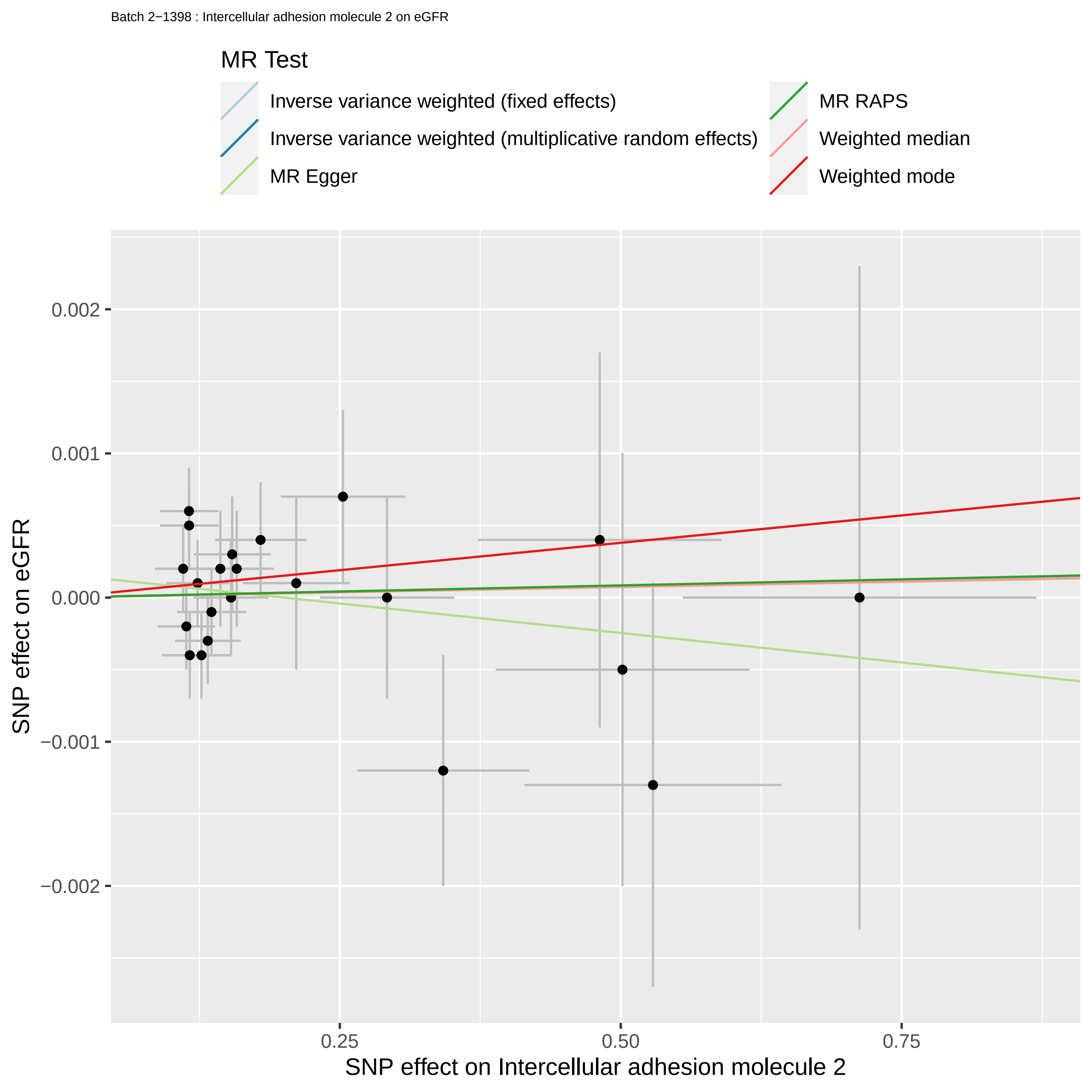
Batch 2−1398 : Intercellular adhesion molecule 2 on eGFR
MR Test
Inverse variance weighted (fixed effects)
Inverse variance weighted (multiplicative random effects)
MR Egger
MR RAPS
Weighted median
Weighted mode
0.002
0.001
0.000
−0.001
−0.002
0.25
0.50
0.75
SNP effect on eGFR
SNP effect on Intercellular adhesion molecule 2

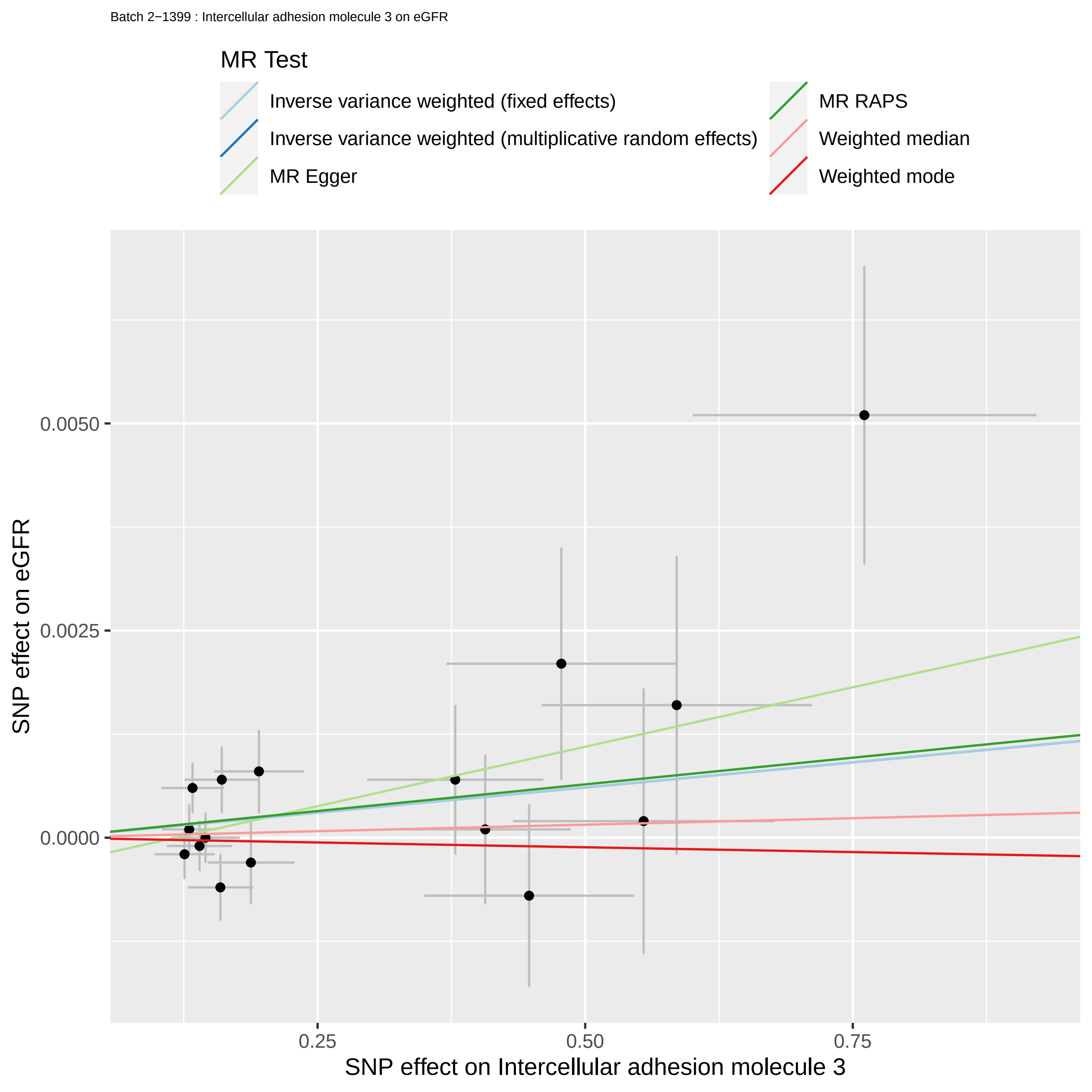

Batch 2−1399 : Intercellular adhesion molecule 3 on eGFR
MR Test
Inverse variance weighted (fixed effects)
Inverse variance weighted (multiplicative random effects)
MR Egger
MR RAPS
Weighted median
Weighted mode
0.0050
0.0025
0.0000
0.25
0.50
0.75
SNP effect on eGFR
SNP effect on Intercellular adhesion molecule 3

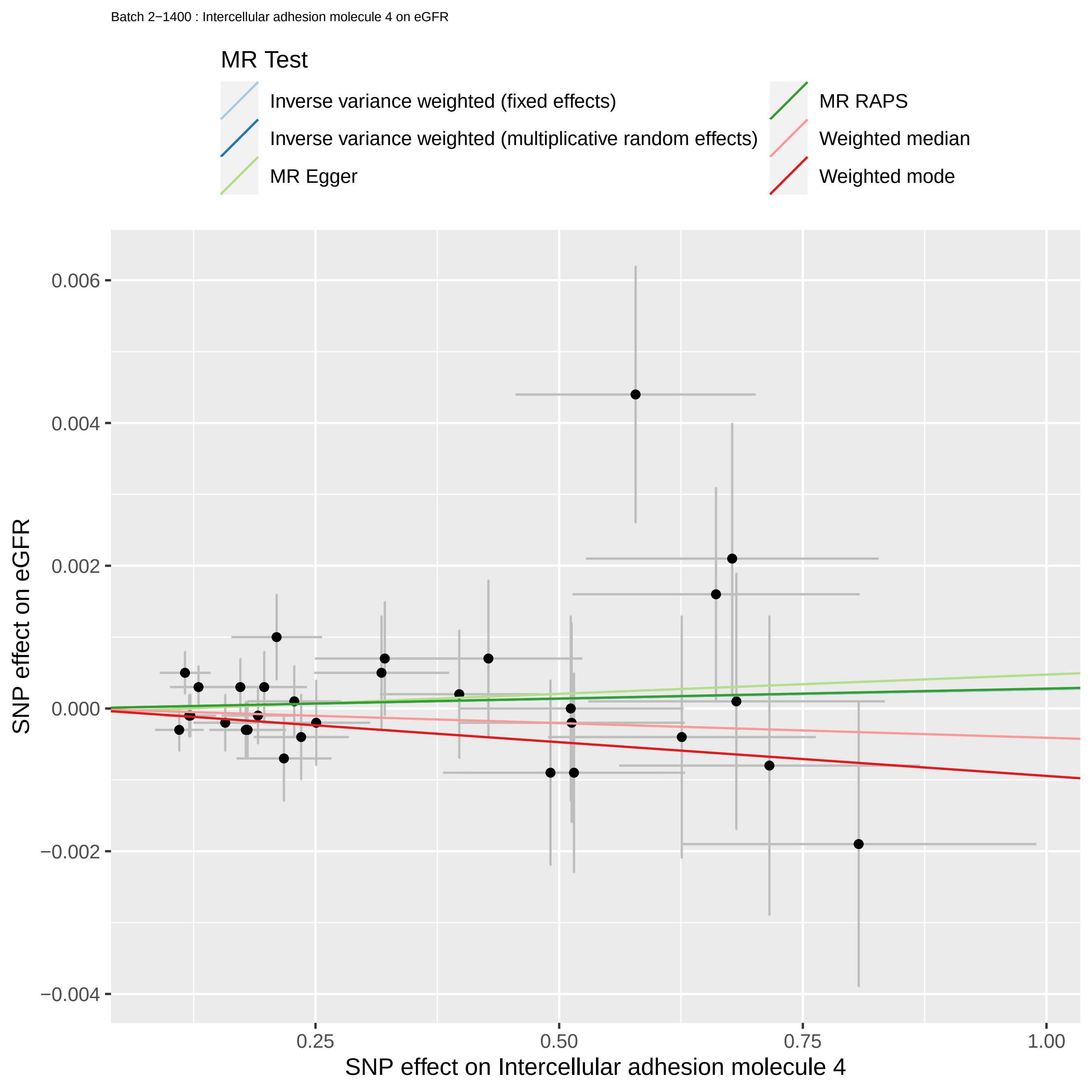
Batch 2−1400 : Intercellular adhesion molecule 4 on eGFR
MR Test
Inverse variance weighted (fixed effects)
Inverse variance weighted (multiplicative random effects)
MR Egger
MR RAPS
Weighted median
Weighted mode
SNP effect on eGFR
0.006
0.004
0.002
0.000
−0.002
−0.004
0.25
0.50
0.75
1.00
SNP effect on Intercellular adhesion molecule 4

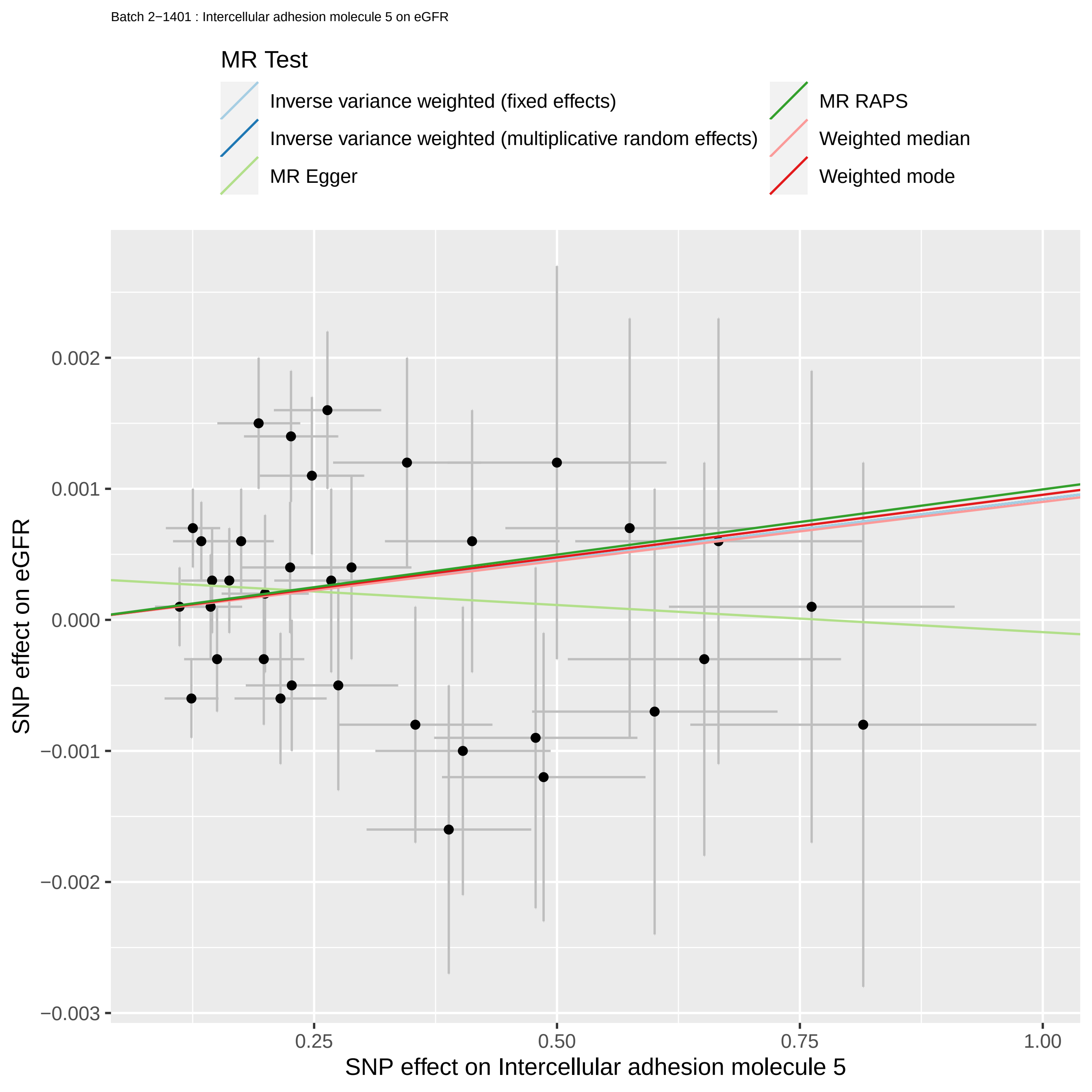
Batch 2−1401 : Intercellular adhesion molecule 5 on eGFR
MR Test
Inverse variance weighted (fixed effects)
Inverse variance weighted (multiplicative random effects)
MR Egger
MR RAPS
Weighted median
Weighted mode
0.002
0.001
0.000
−0.001
−0.002
−0.003
0.25
0.50
0.75
1.00
SNP effect on eGFR
SNP effect on Intercellular adhesion molecule 5

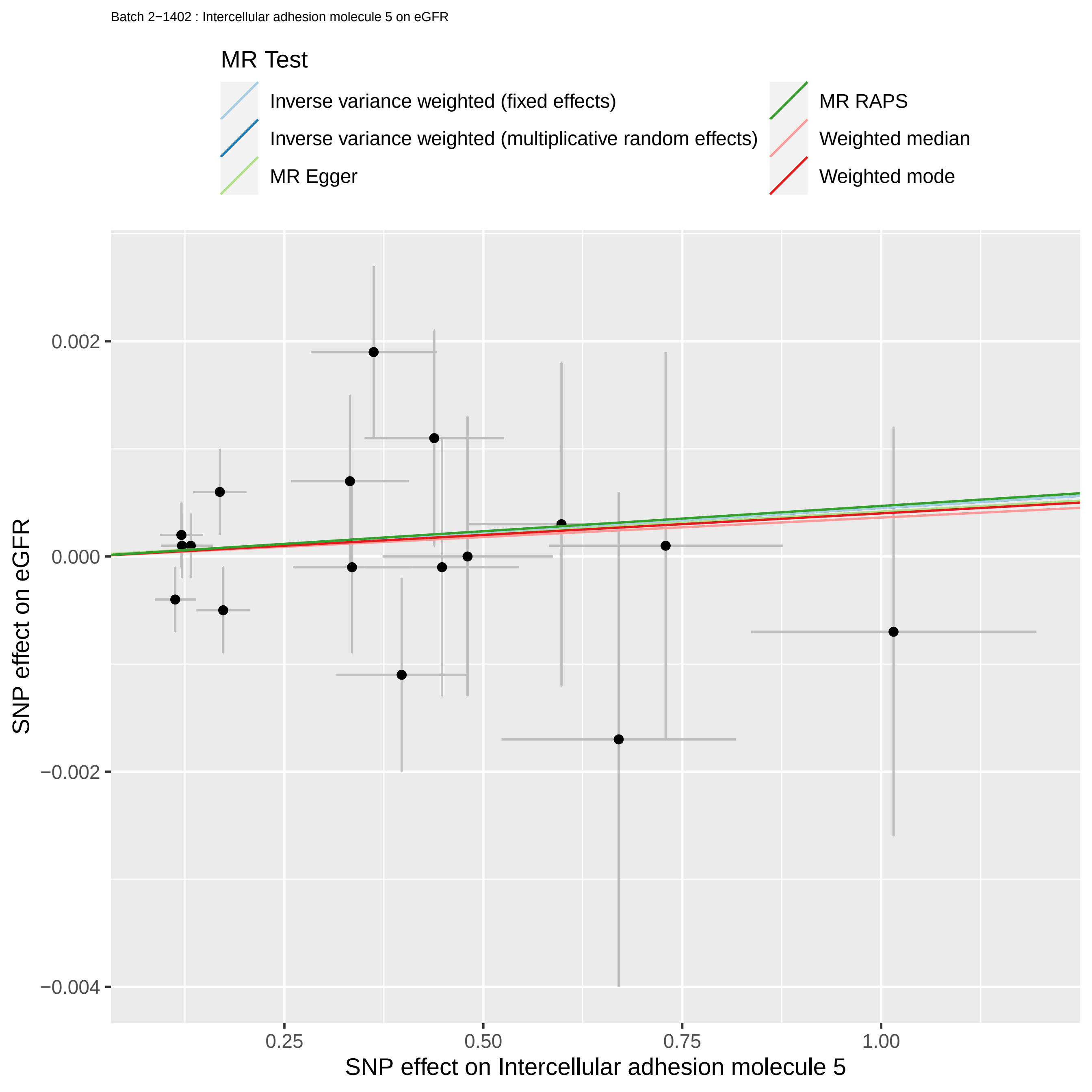

Batch 2−1402 : Intercellular adhesion molecule 5 on eGFR
MR Test
Inverse variance weighted (fixed effects)
Inverse variance weighted (multiplicative random effects)
MR Egger
MR RAPS
Weighted median
Weighted mode
0.002
0.000
−0.002
−0.004
0.25
0.50
0.75
1.00
SNP effect on eGFR
SNP effect on Intercellular adhesion molecule 5

Batch 2−1403 : Inducible T−cell costimulator on eGFR

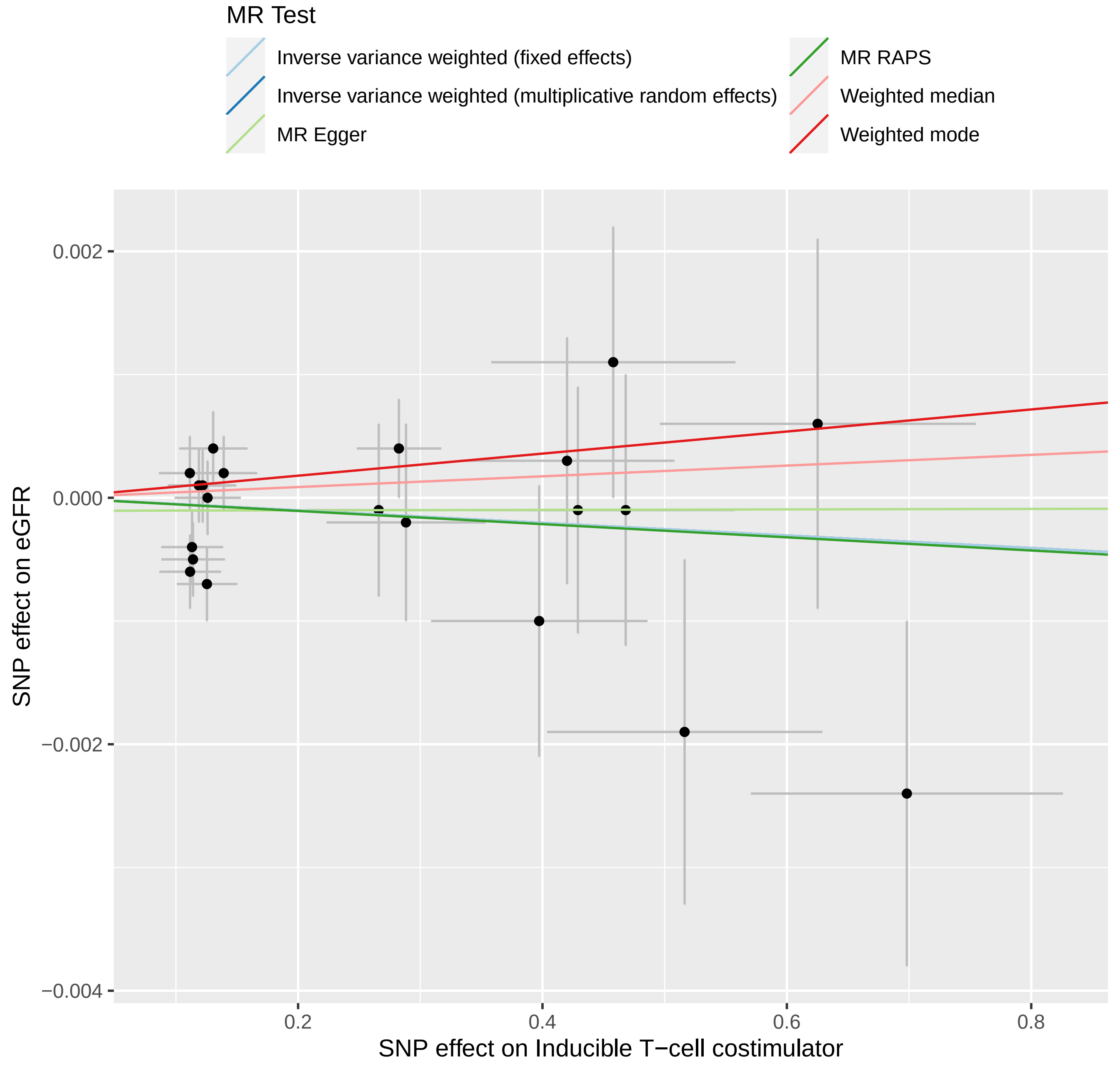

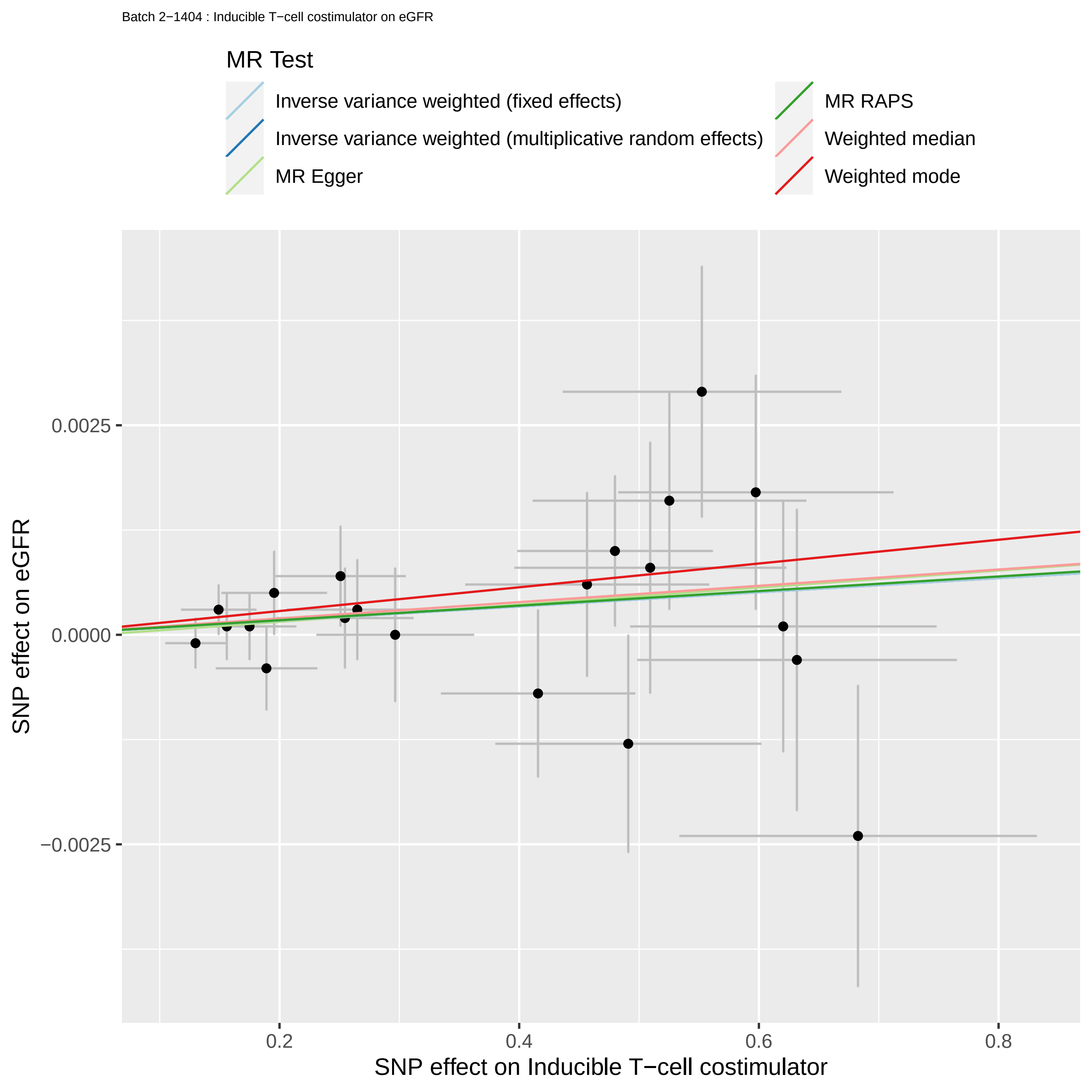

Batch 2−1404 : Inducible T−cell costimulator on eGFR
MR Test
Inverse variance weighted (fixed effects)
Inverse variance weighted (multiplicative random effects)
MR Egger
MR RAPS
Weighted median
Weighted mode
0.0025
0.0000
−0.0025
0.2
0.4
0.6
0.8
SNP effect on eGFR
SNP effect on Inducible T−cell costimulator

Batch 2−1405 : ICOS ligand on eGFR

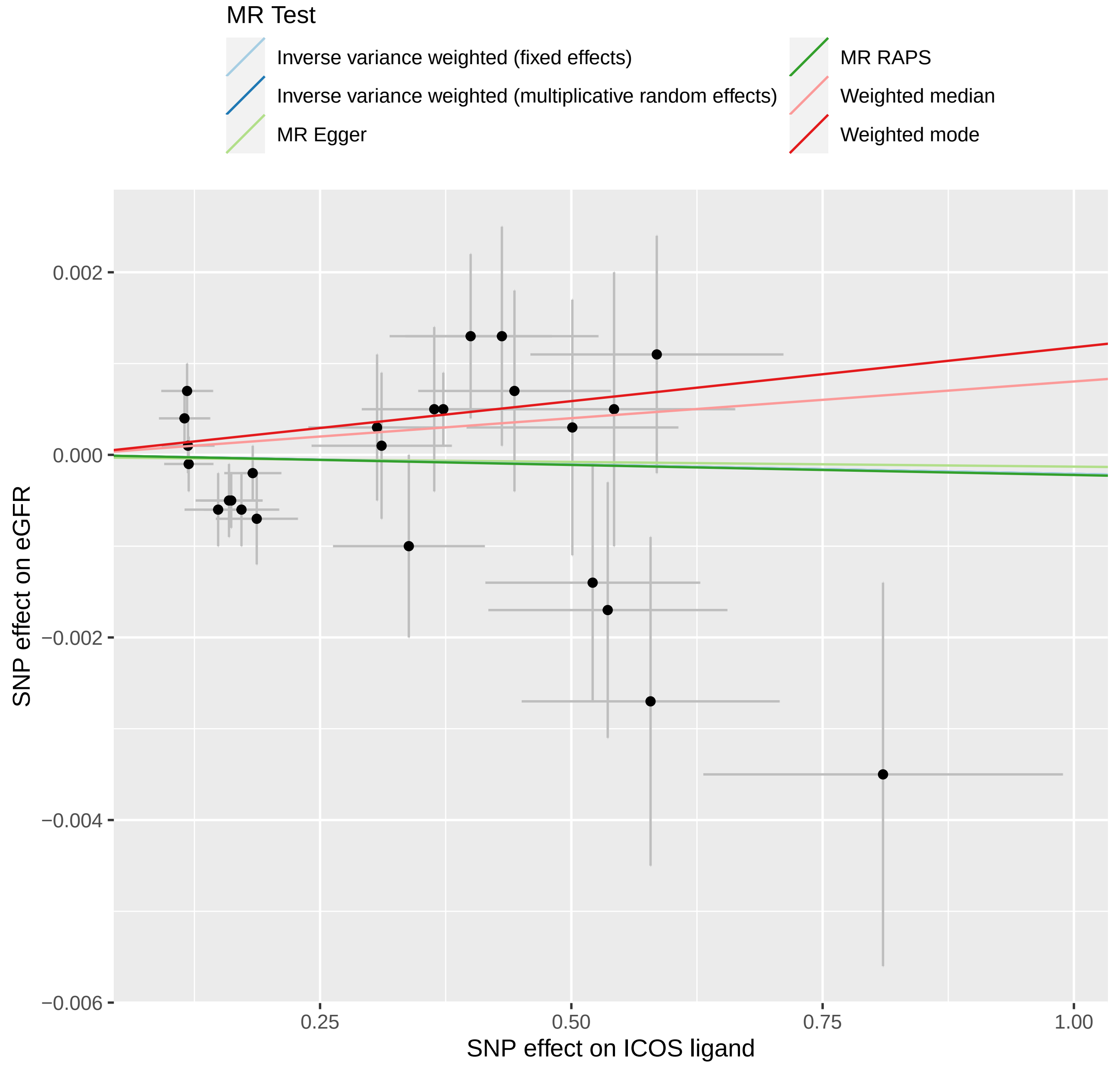

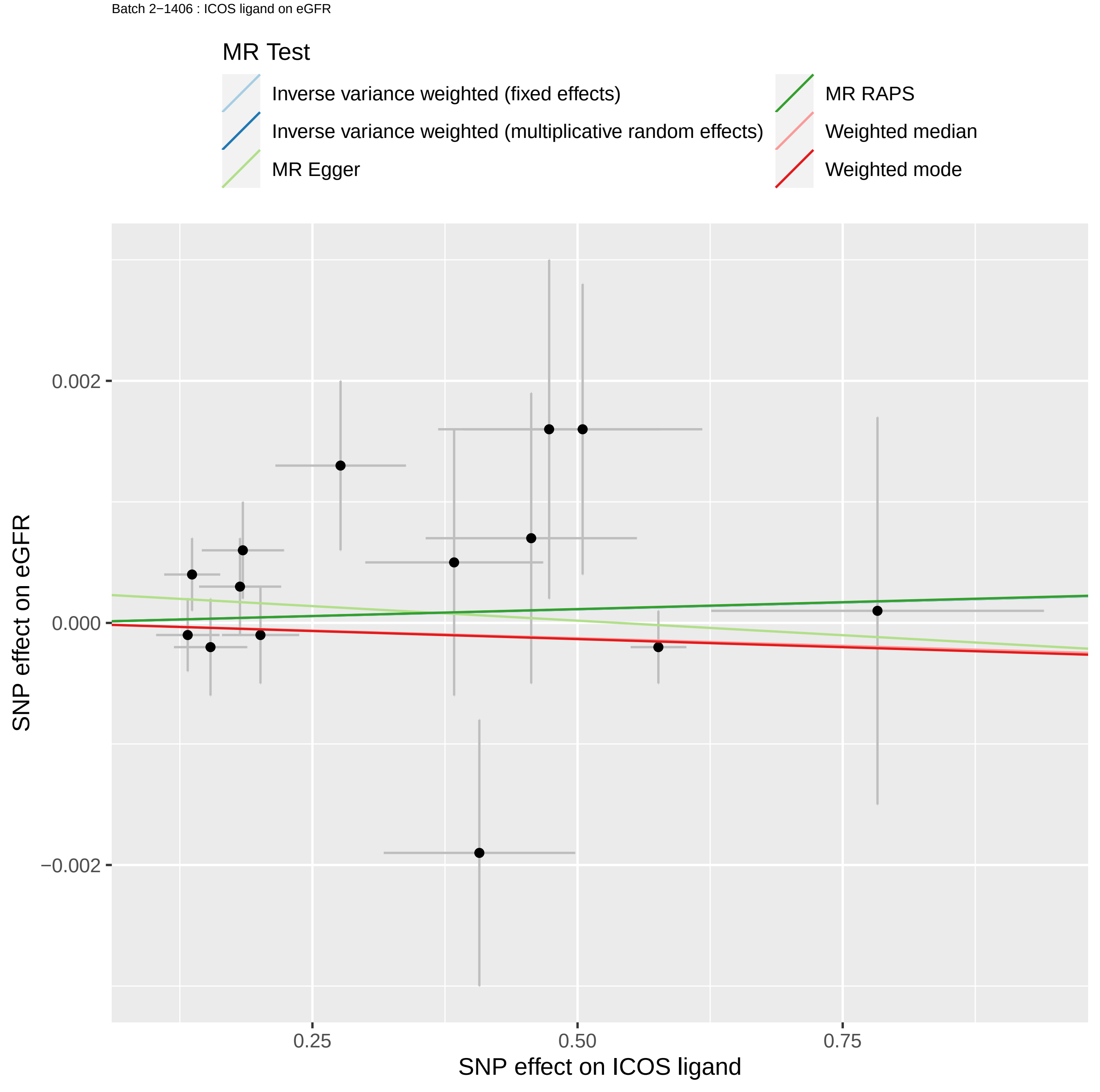

Batch 2−1406 : ICOS ligand on eGFR
MR Test
Inverse variance weighted (fixed effects)
Inverse variance weighted (multiplicative random effects)
MR Egger
MR RAPS
Weighted median
Weighted mode
0.002
0.000
−0.002
0.25
0.50
0.75
SNP effect on ICOS ligand
SNP effect on eGFR

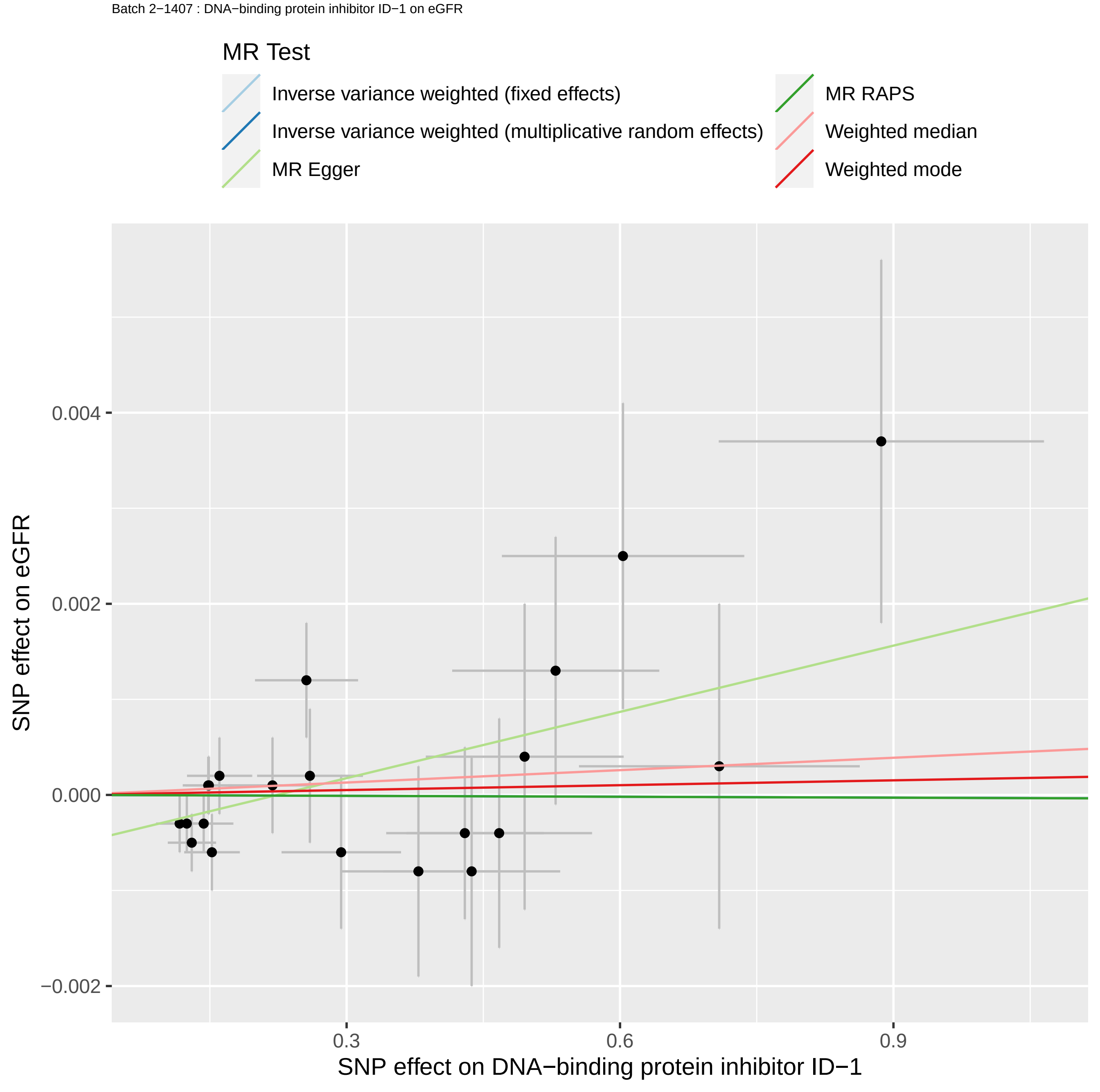

Batch 2−1407 : DNA−binding protein inhibitor ID−1 on eGFR
MR Test
Inverse variance weighted (fixed effects)
Inverse variance weighted (multiplicative random effects)
MR Egger
MR RAPS
Weighted median
Weighted mode
0.004
0.002
0.000
−0.002
0.3
0.6
0.9
SNP effect on eGFR
SNP effect on DNA−binding protein inhibitor ID−1

Batch 2−1408 : DNA−binding protein inhibitor ID−2 on eGFR

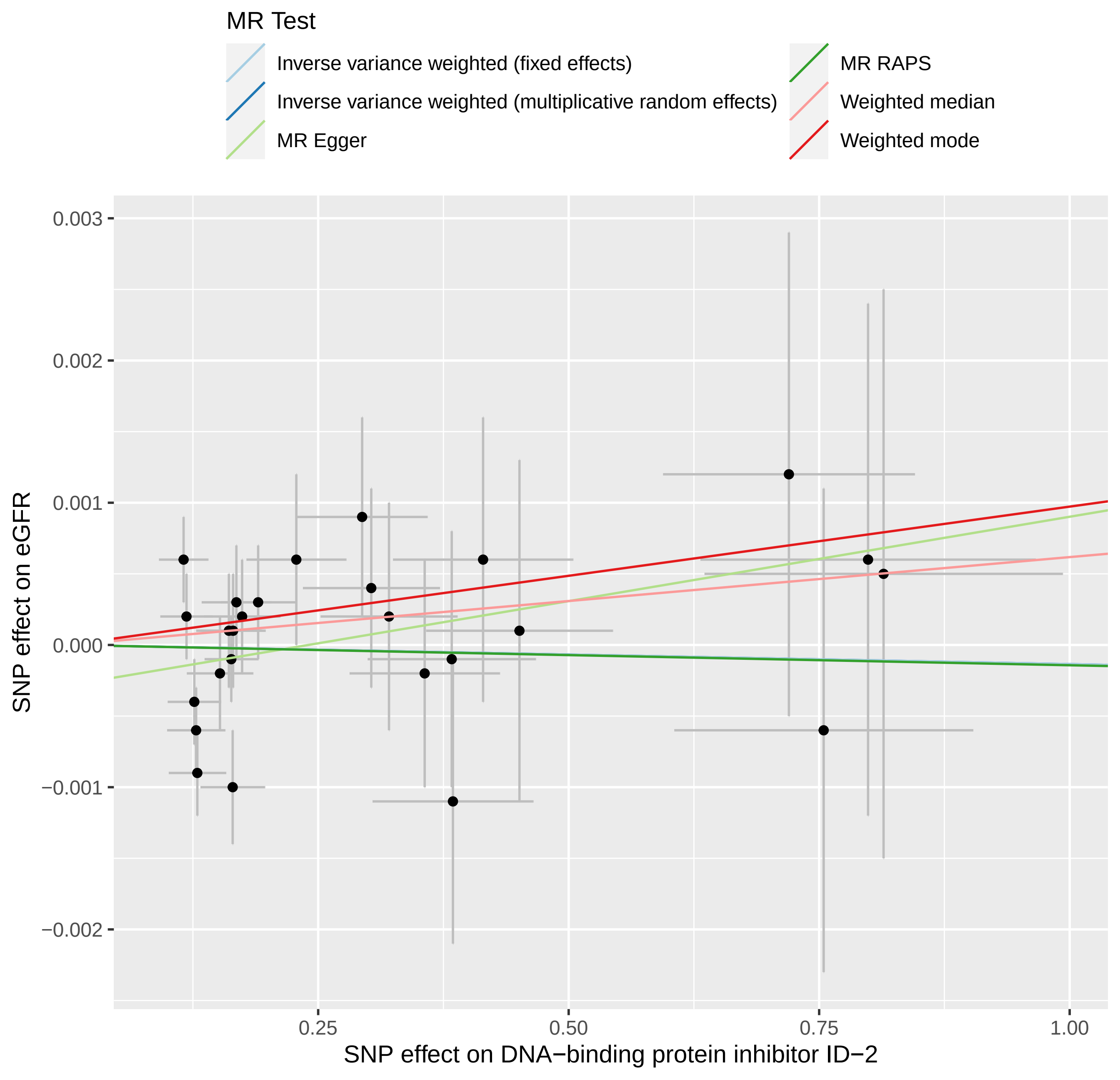

Batch 2−1409 : Insulin−degrading enzyme on eGFR

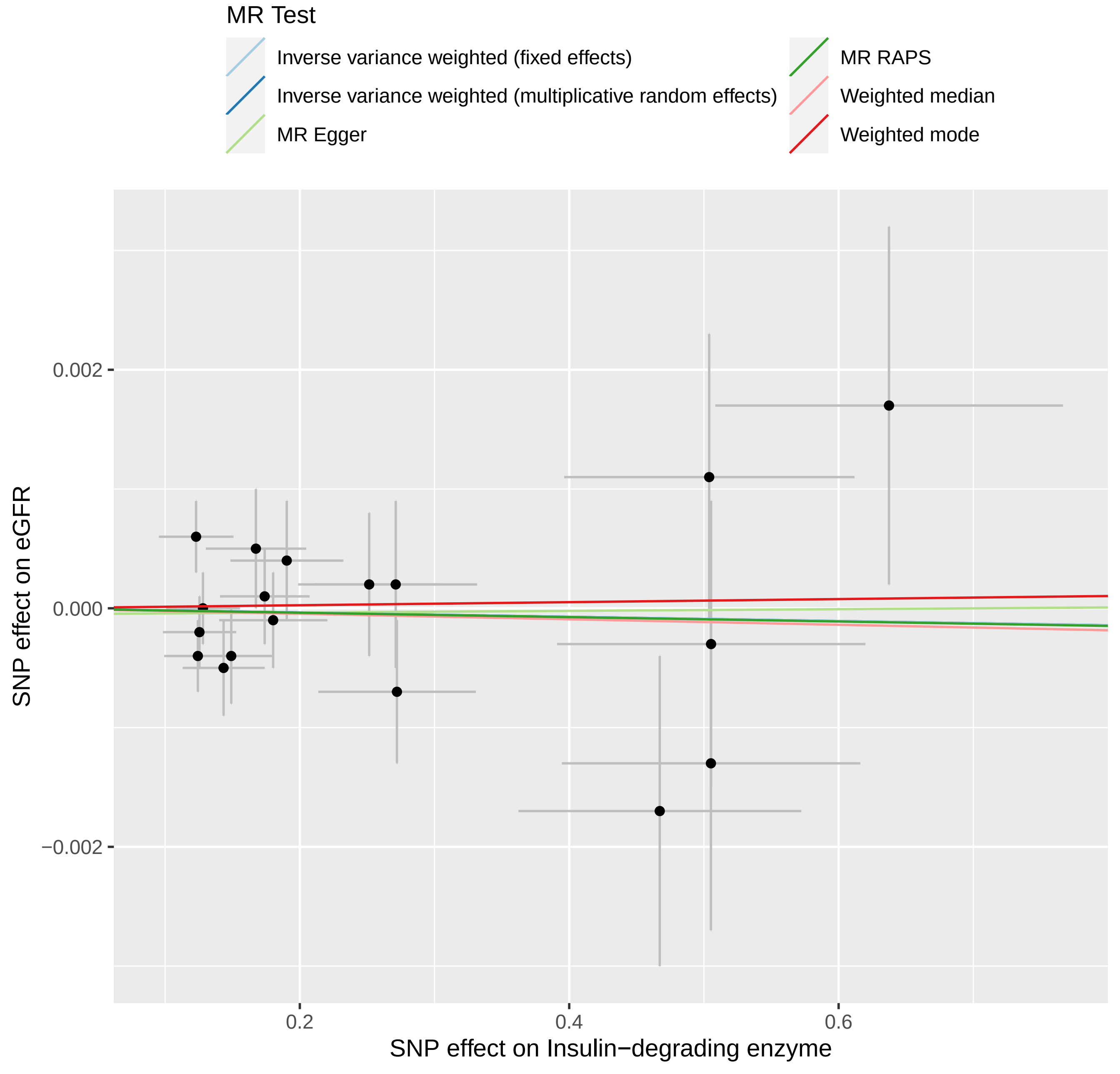

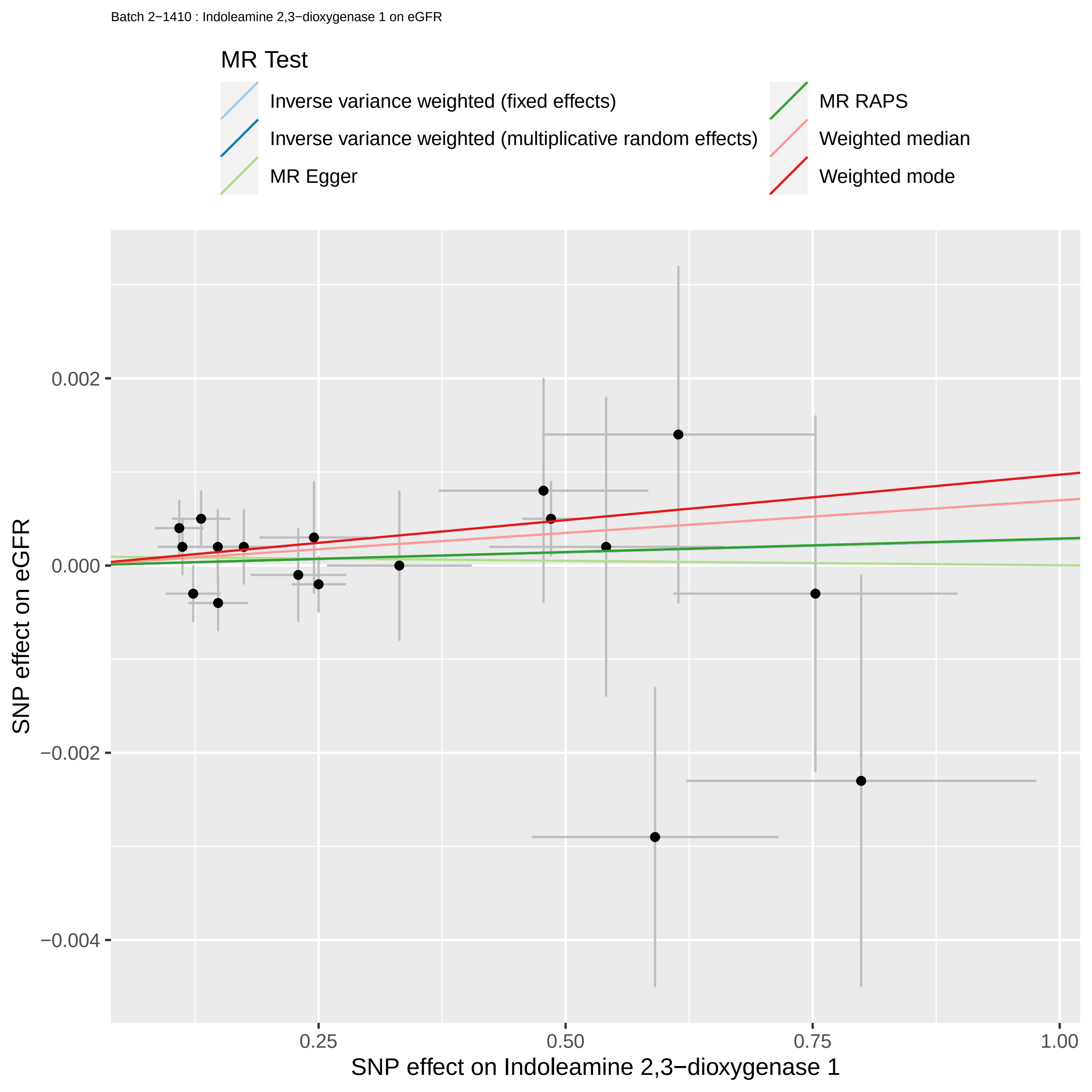

Batch 2−1410 : Indoleamine 2,3−dioxygenase 1 on eGFR
MR Test
Inverse variance weighted (fixed effects)
Inverse variance weighted (multiplicative random effects)
MR Egger
MR RAPS
Weighted median
Weighted mode
0.002
0.000
−0.002
−0.004
SNP effect on eGFR
0.25
0.50
0.75
1.00
SNP effect on Indoleamine 2,3−dioxygenase 1

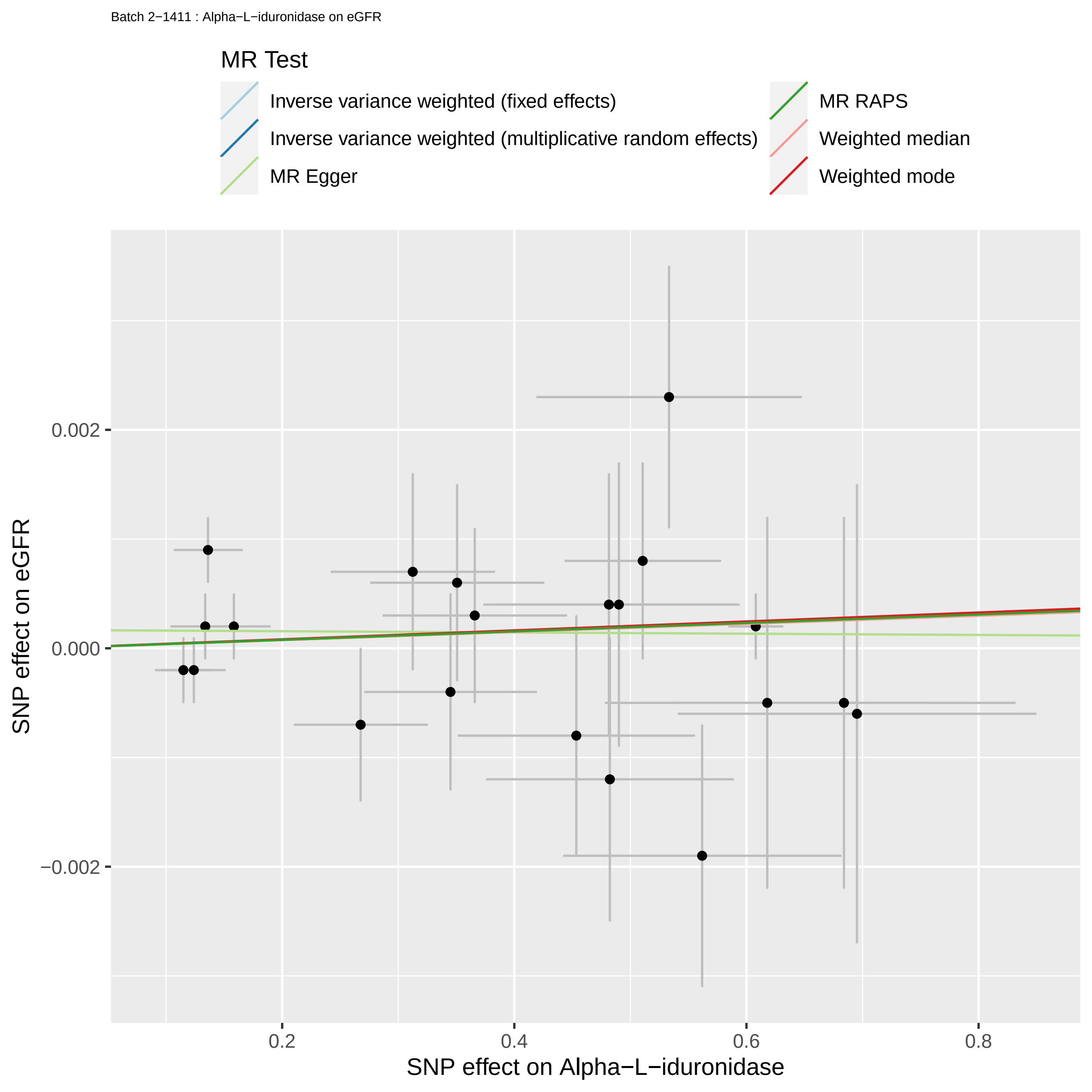

Batch 2−1411 : Alpha−L−iduronidase on eGFR
MR Test
Inverse variance weighted (fixed effects)
Inverse variance weighted (multiplicative random effects)
MR Egger
MR RAPS
Weighted median
Weighted mode
0.002
0.000
−0.002
0.2
0.4
0.6
0.8
SNP effect on eGFR
SNP effect on Alpha−L−iduronidase

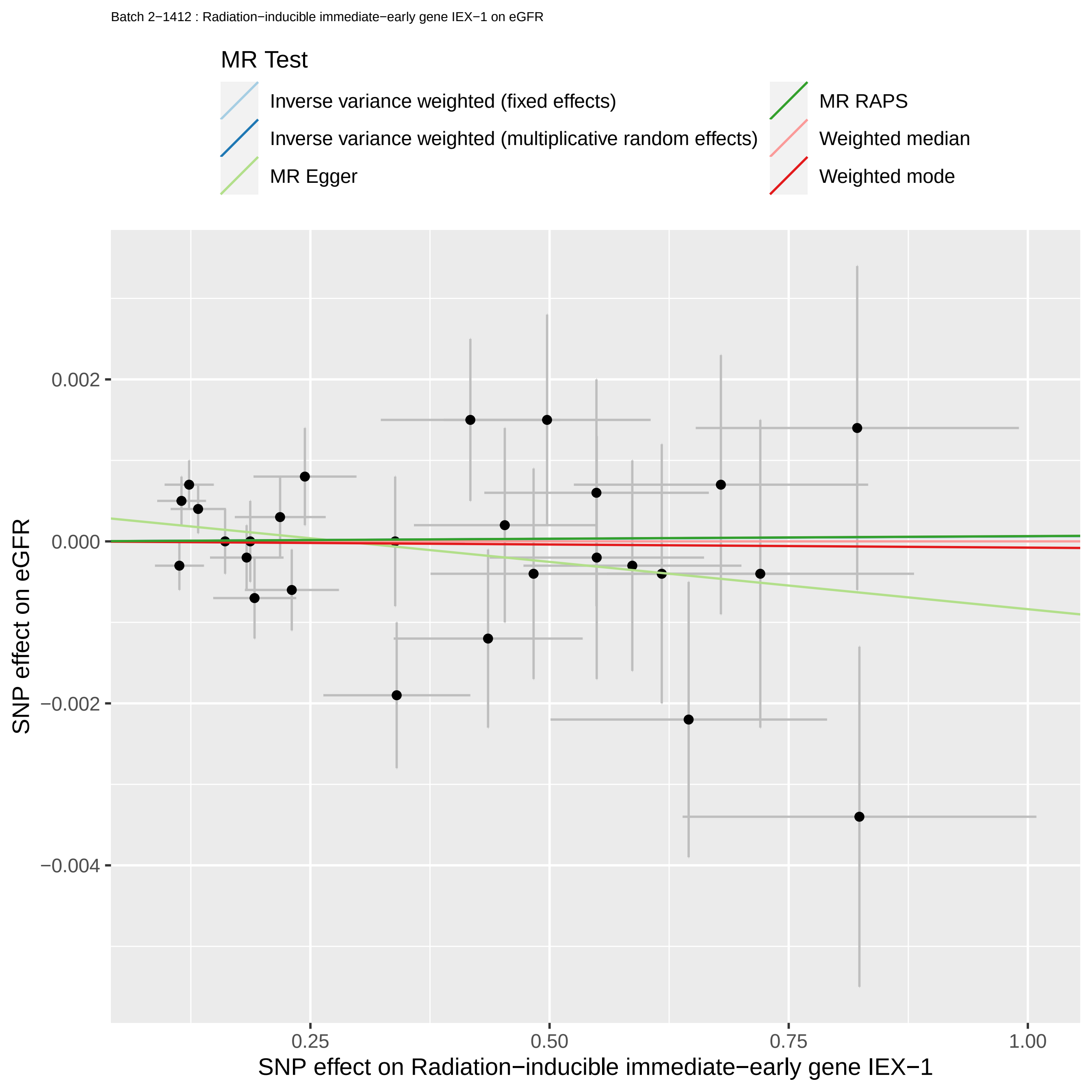

Batch 2−1412 : Radiation−inducible immediate−early gene IEX−1 on eGFR
MR Test
Inverse variance weighted (fixed effects)
Inverse variance weighted (multiplicative random effects)
MR Egger
MR RAPS
Weighted median
Weighted mode
0.002
0.000
−0.002
−0.004
SNP effect on eGFR
0.25
0.50
0.75
1.00
SNP effect on Radiation−inducible immediate−early gene IEX−1

Batch 2−1413 : Immediate early response 3−interacting protein 1 on eGFR

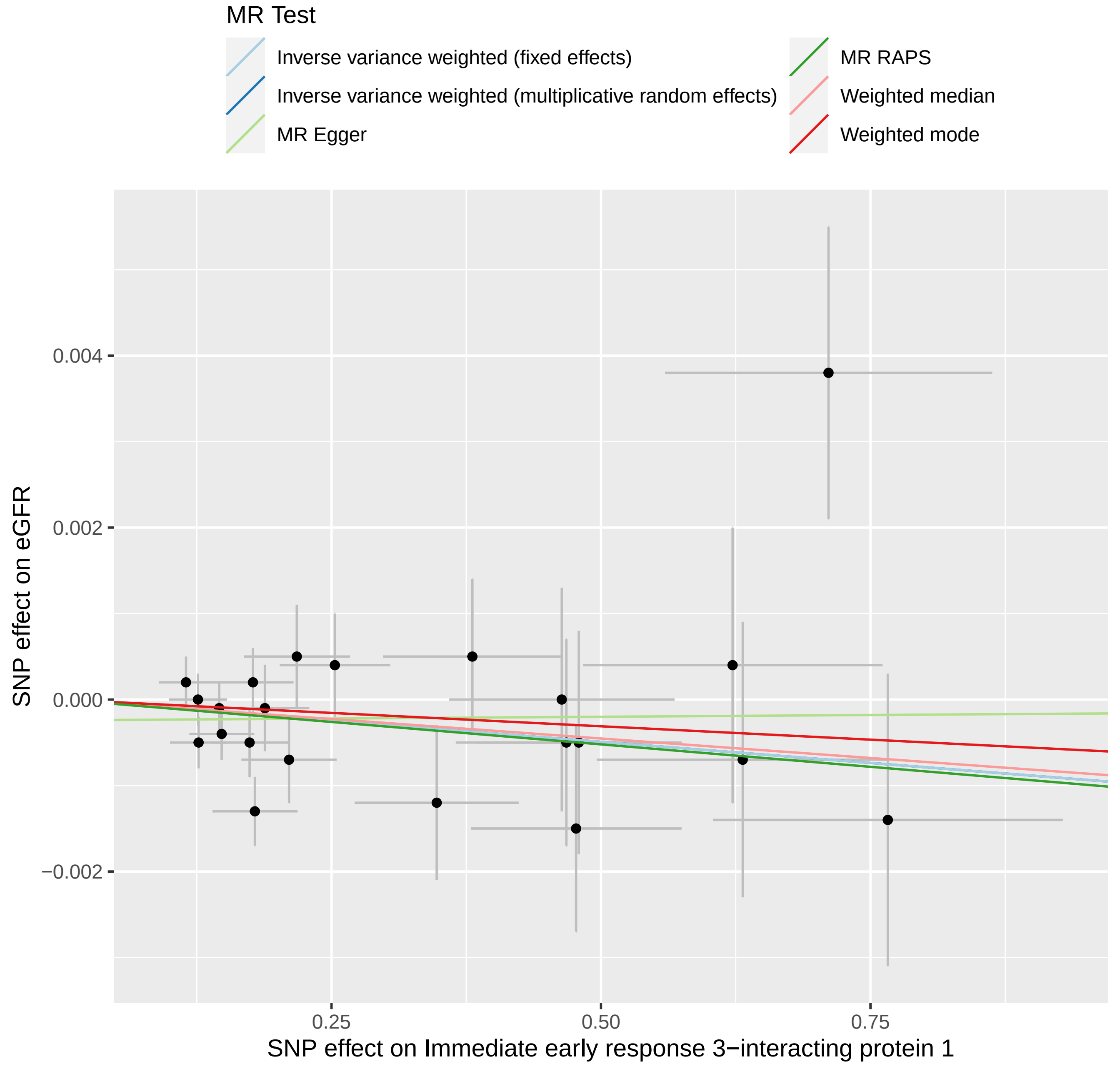

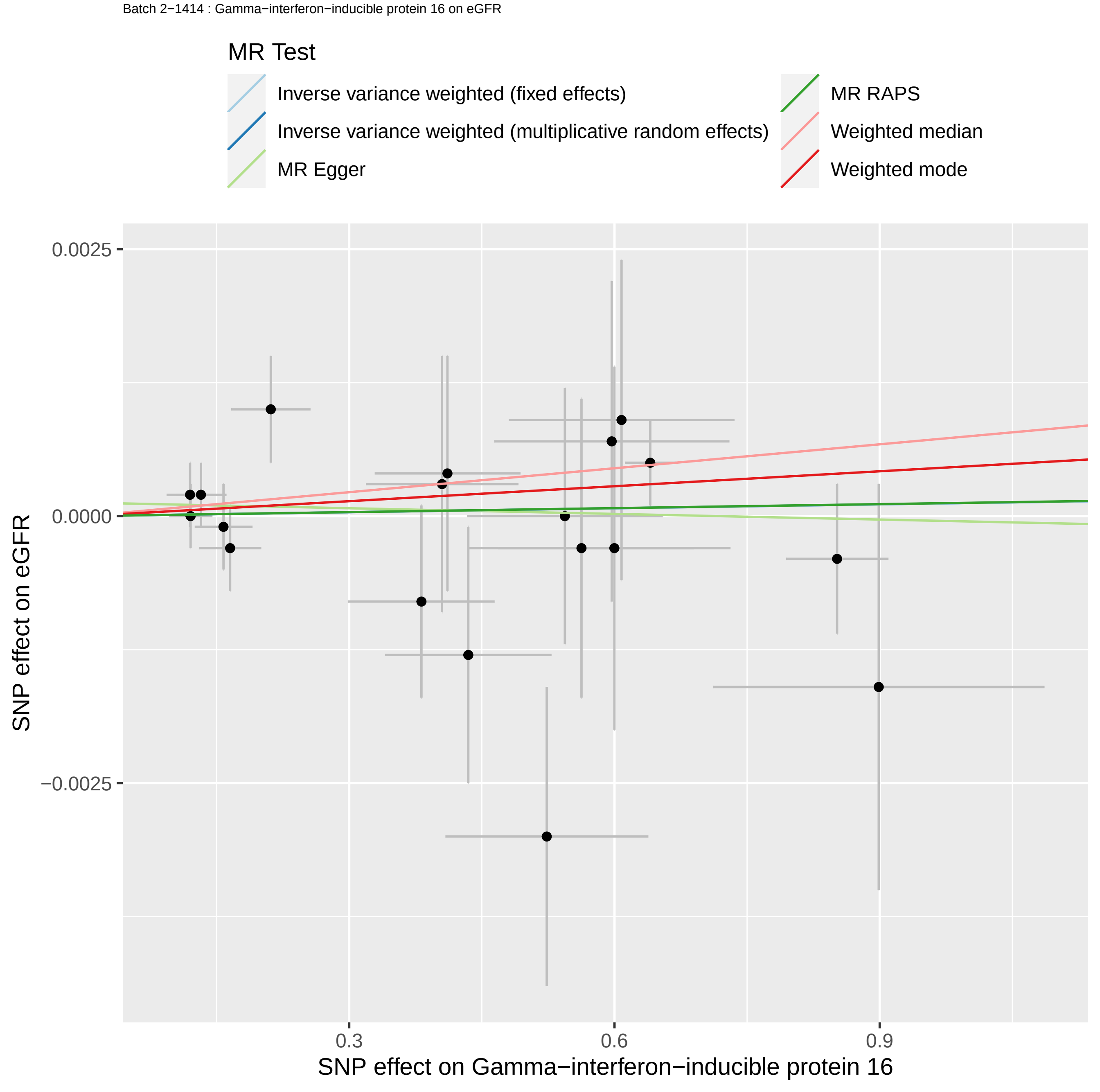

Batch 2−1414 : Gamma−interferon−inducible protein 16 on eGFR
MR Test
Inverse variance weighted (fixed effects)
Inverse variance weighted (multiplicative random effects)
MR Egger
MR RAPS
Weighted median
Weighted mode
0.0025
0.0000
−0.0025
0.3
0.6
0.9
SNP effect on eGFR
SNP effect on Gamma−interferon−inducible protein 16

Batch 2−1415 : Gamma−interferon−inducible protein 16 on eGFR

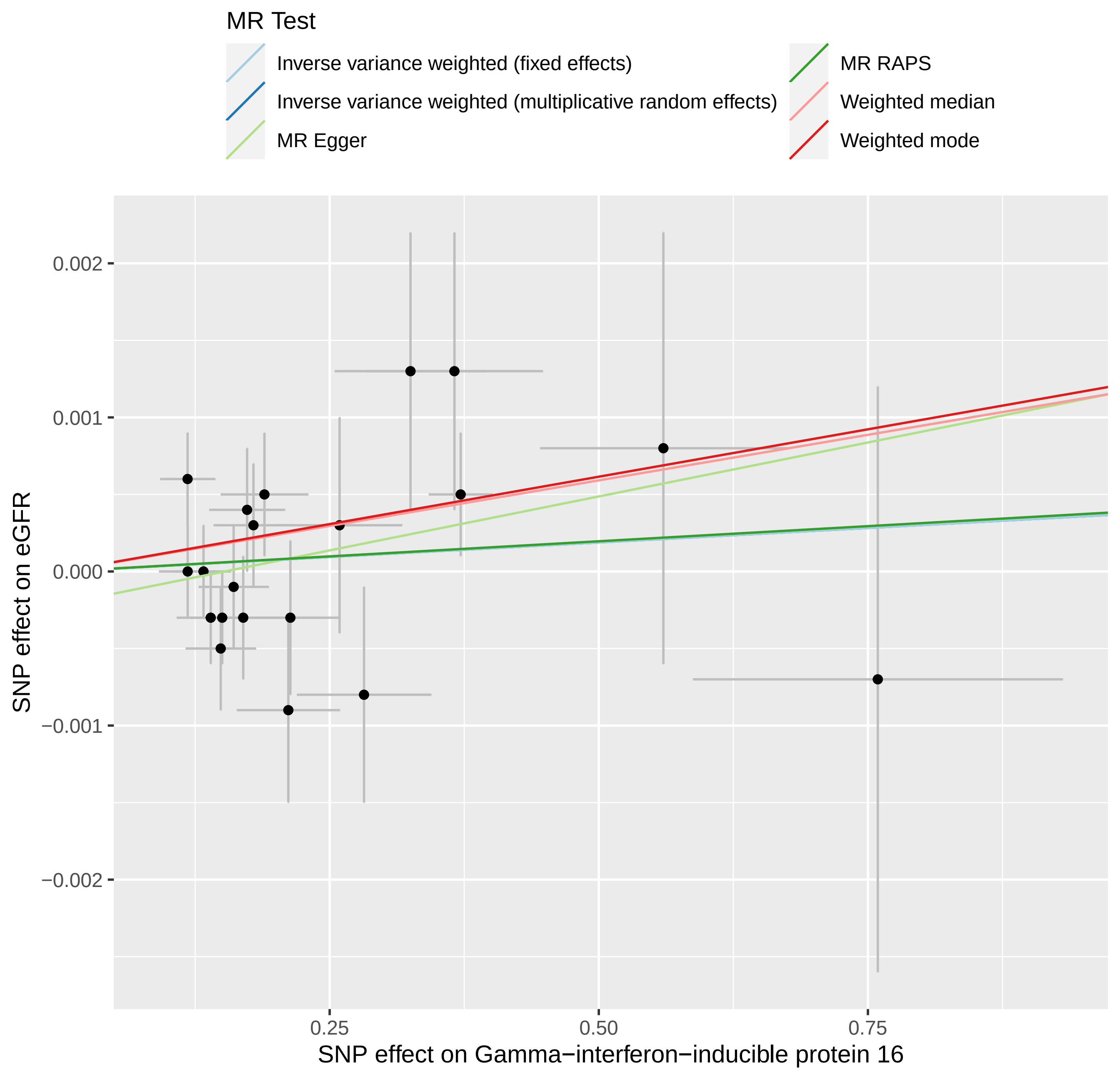

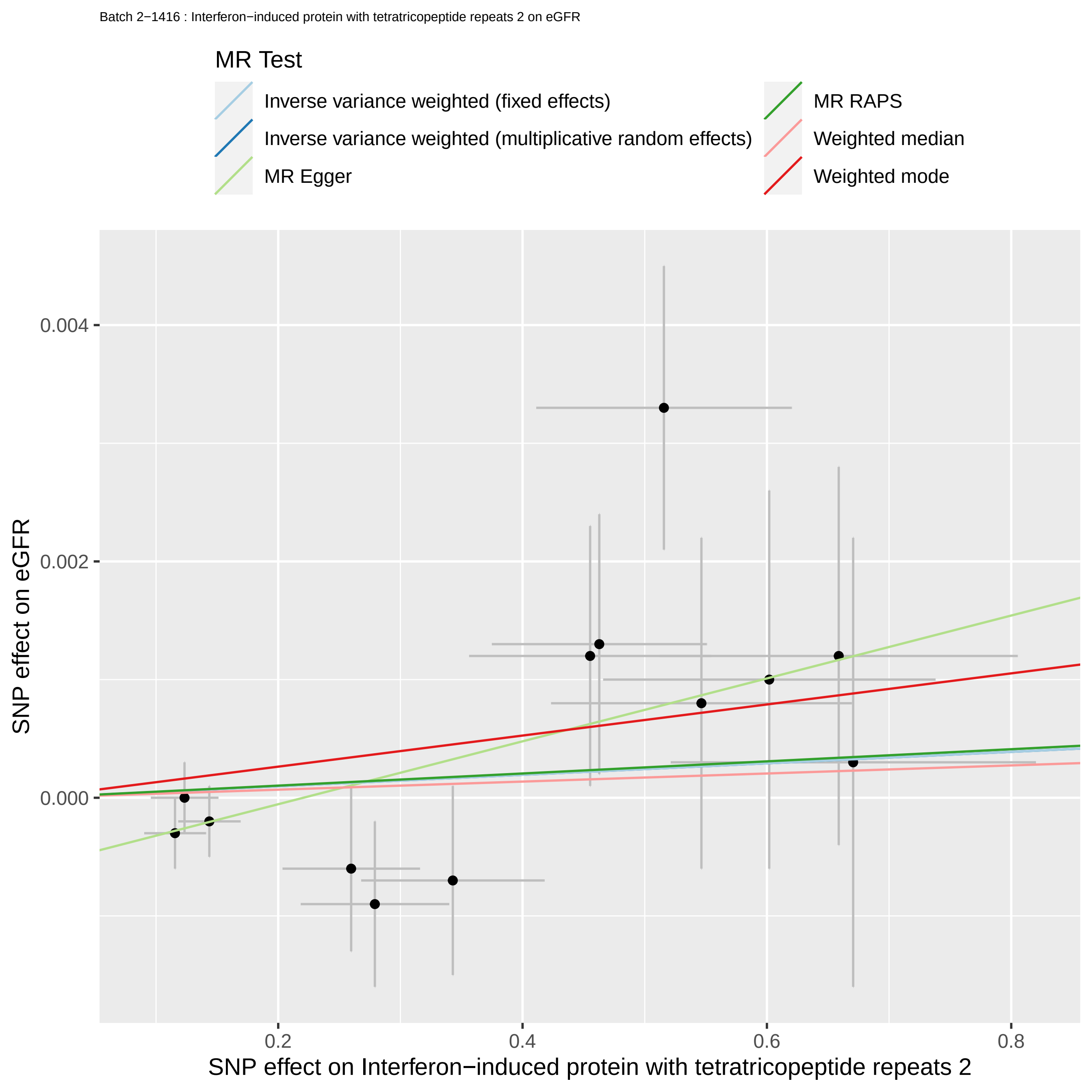
Batch 2−1416 : Interferon−induced protein with tetratricopeptide repeats 2 on eGFR
MR Test
Inverse variance weighted (fixed effects)
Inverse variance weighted (multiplicative random effects)
MR Egger
MR RAPS
Weighted median
Weighted mode
0.004
0.002
0.000
0.2
0.4
0.6
0.8
SNP effect on eGFR
SNP effect on Interferon−induced protein with tetratricopeptide repeats 2

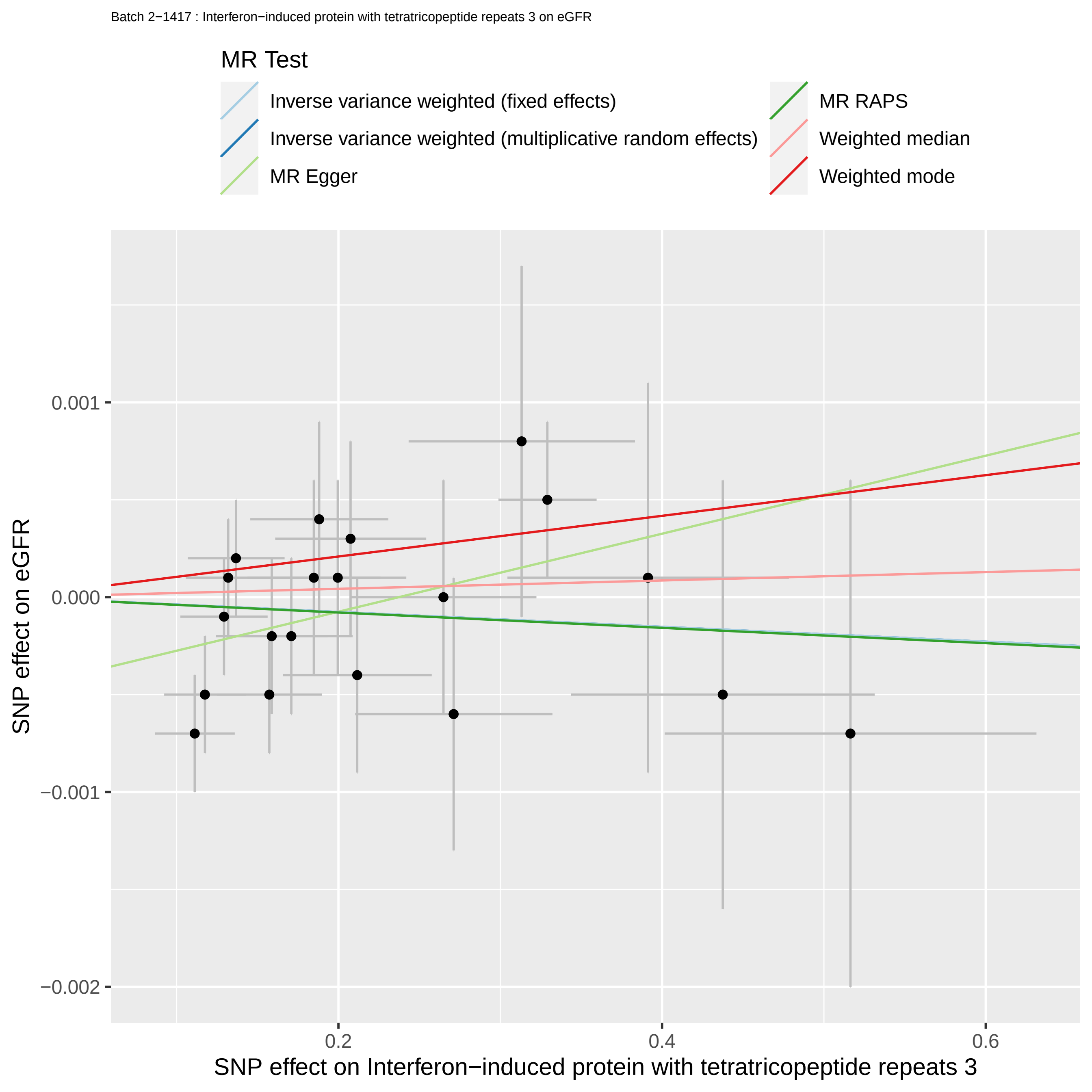

Batch 2−1417 : Interferon−induced protein with tetratricopeptide repeats 3 on eGFR
MR Test
Inverse variance weighted (fixed effects)
Inverse variance weighted (multiplicative random effects)
MR Egger
MR RAPS
Weighted median
Weighted mode
0.001
0.000
−0.001
−0.002
SNP effect on eGFR
0.2
0.4
0.6
SNP effect on Interferon−induced protein with tetratricopeptide repeats 3

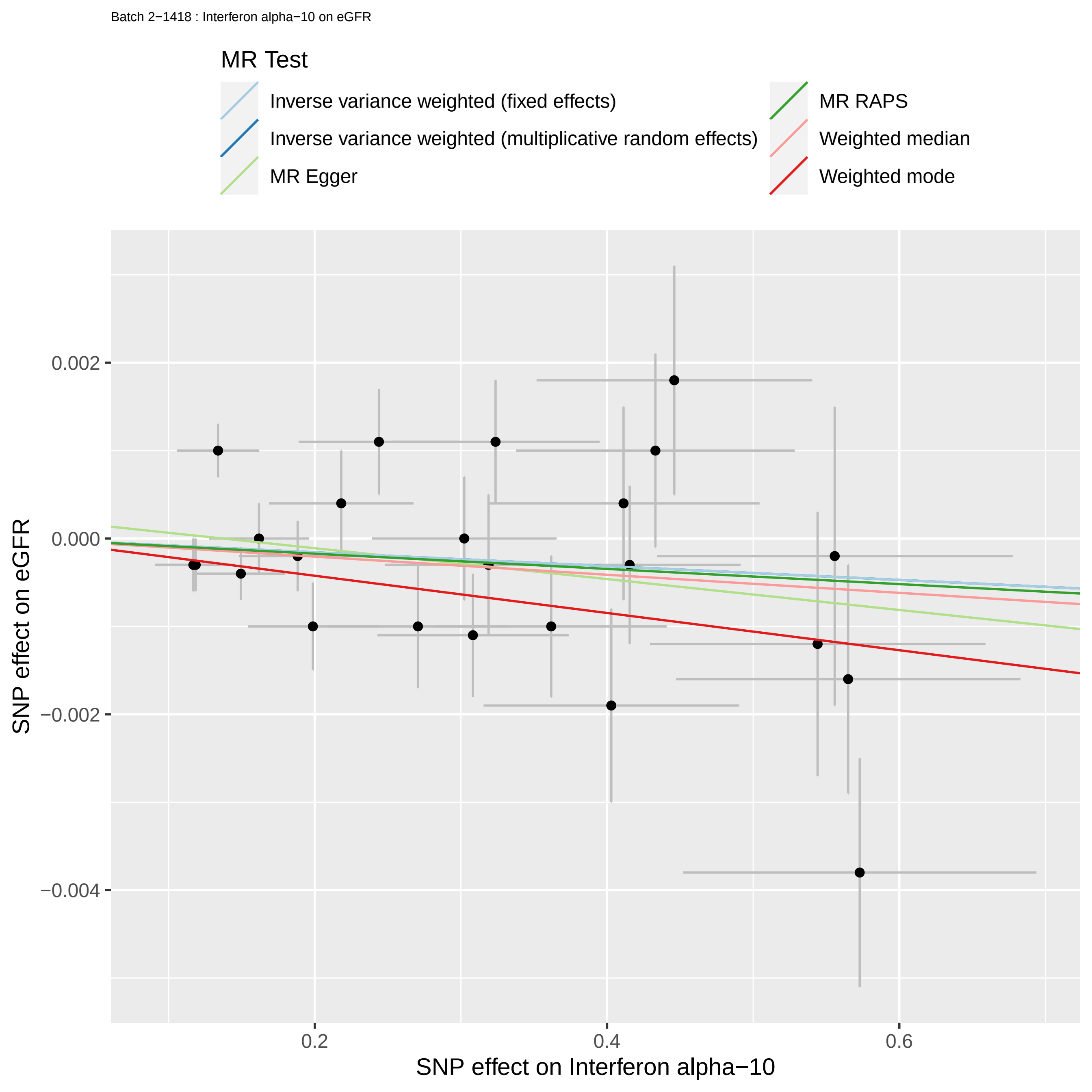

Batch 2−1418 : Interferon alpha−10 on eGFR
MR Test
Inverse variance weighted (fixed effects)
Inverse variance weighted (multiplicative random effects)
MR Egger
MR RAPS
Weighted median
Weighted mode
0.002
0.000
−0.002
−0.004
0.2
0.4
0.6
SNP effect on eGFR
SNP effect on Interferon alpha−10

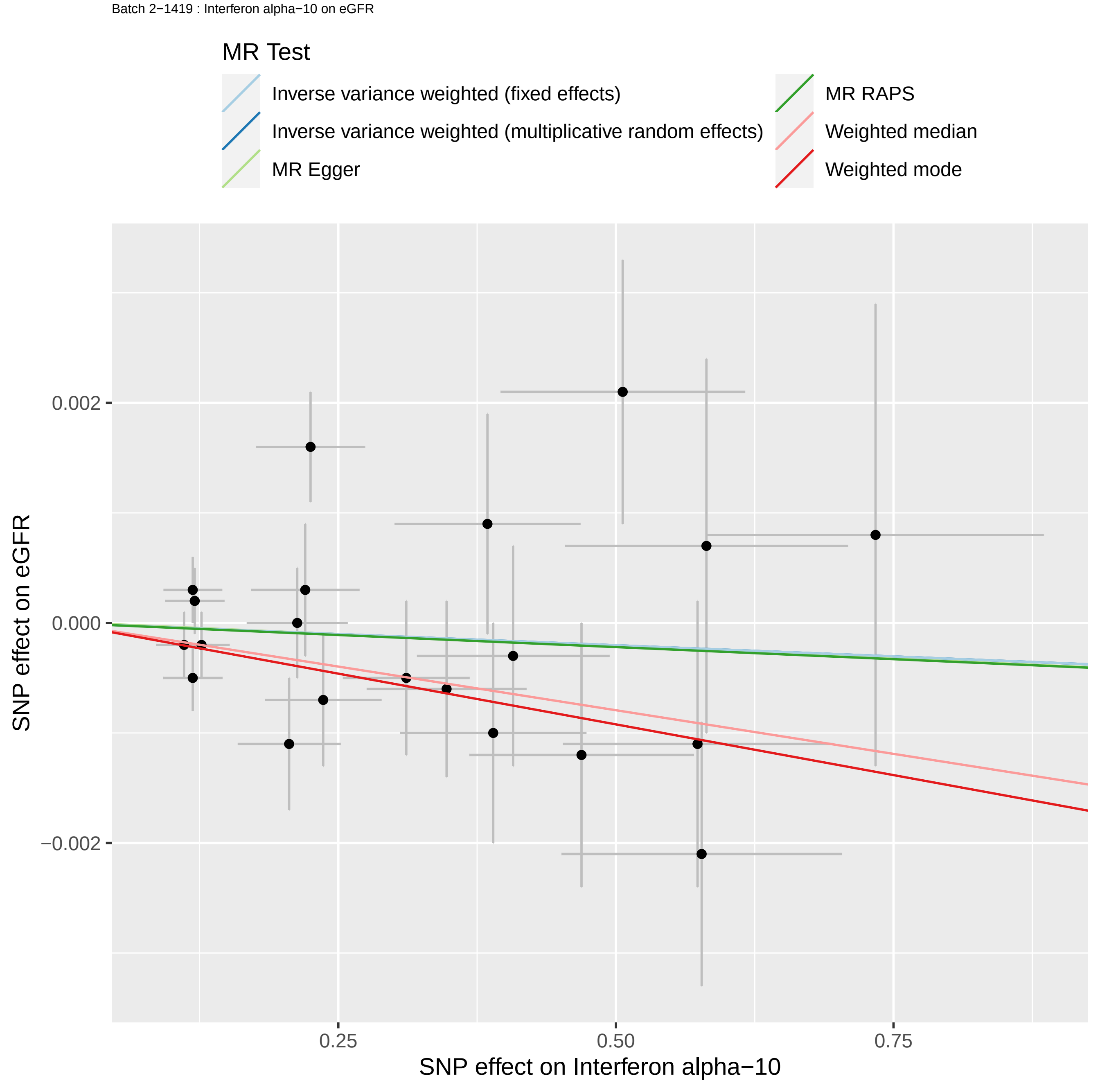
Batch 2−1419 : Interferon alpha−10 on eGFR
MR Test
Inverse variance weighted (fixed effects)
Inverse variance weighted (multiplicative random effects)
MR Egger
MR RAPS
Weighted median
Weighted mode
0.002
0.000
−0.002
SNP effect on eGFR
0.25
0.50
0.75
SNP effect on Interferon alpha−10

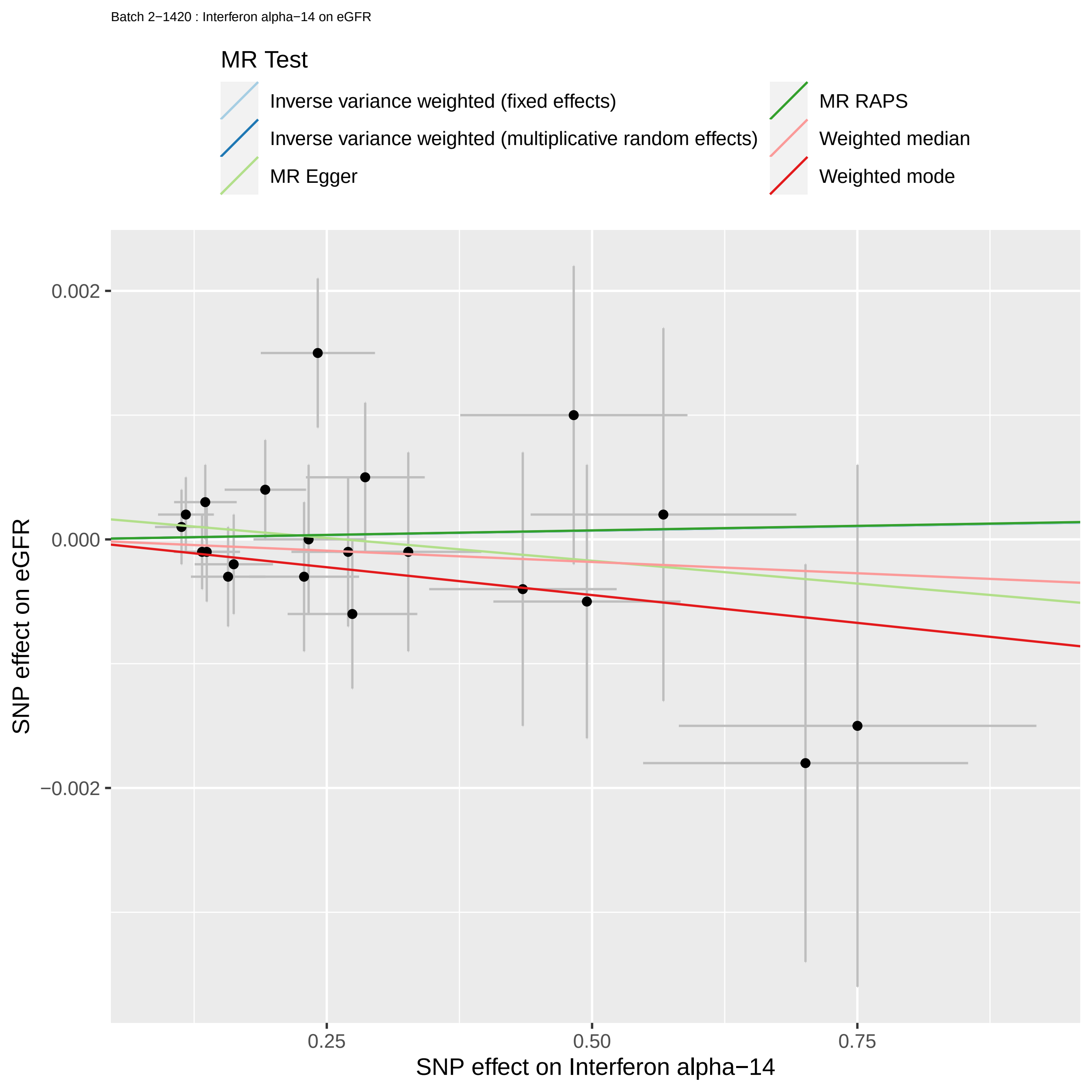
Batch 2−1420 : Interferon alpha−14 on eGFR
MR Test
Inverse variance weighted (fixed effects)
Inverse variance weighted (multiplicative random effects)
MR Egger
MR RAPS
Weighted median
Weighted mode
0.002
0.000
−0.002
0.25
0.50
0.75
SNP effect on eGFR
SNP effect on Interferon alpha−14

Batch 2−1421 : Interferon alpha−4 on eGFR

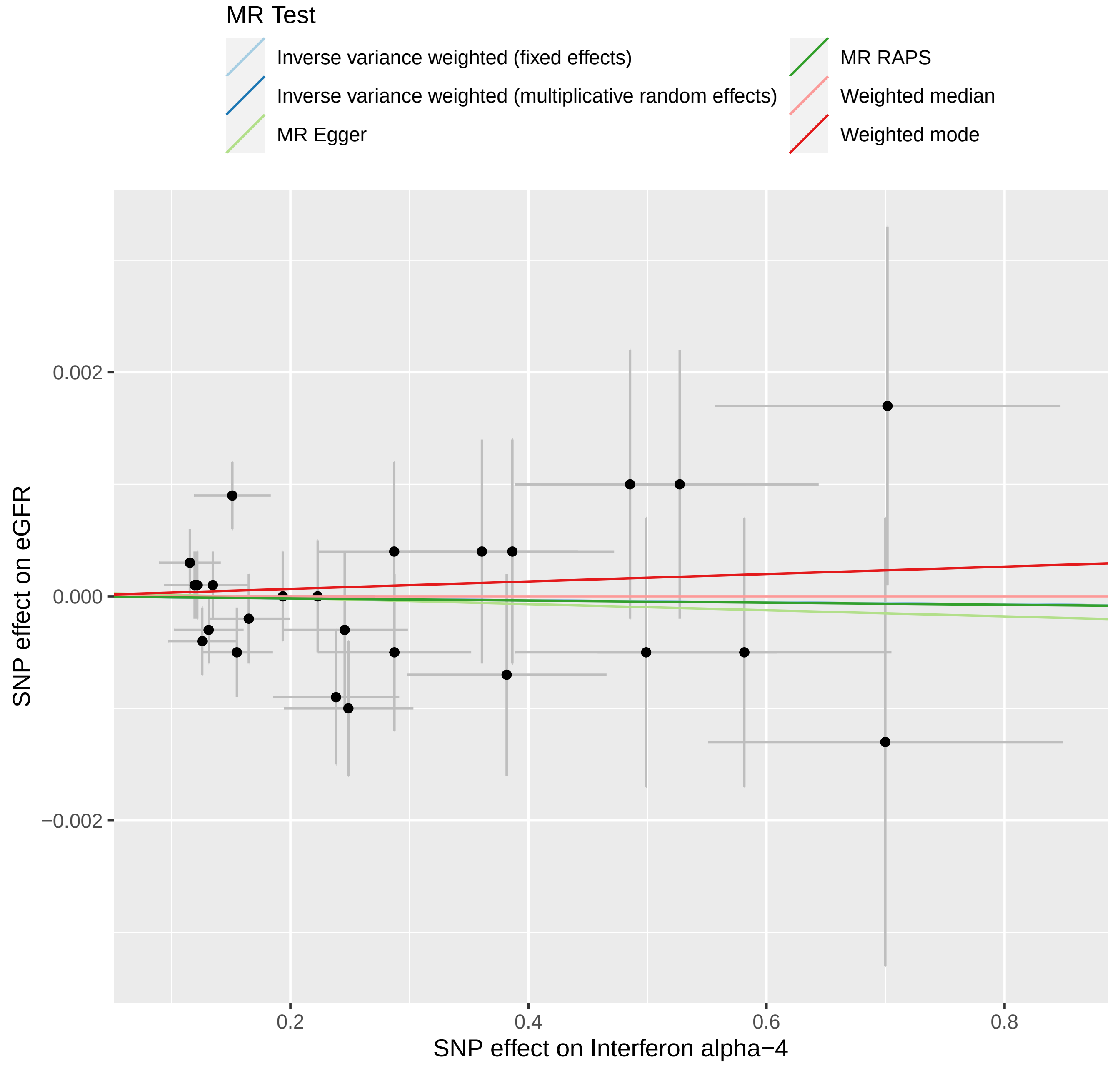

Batch 2−1422 : Interferon alpha−5 on eGFR

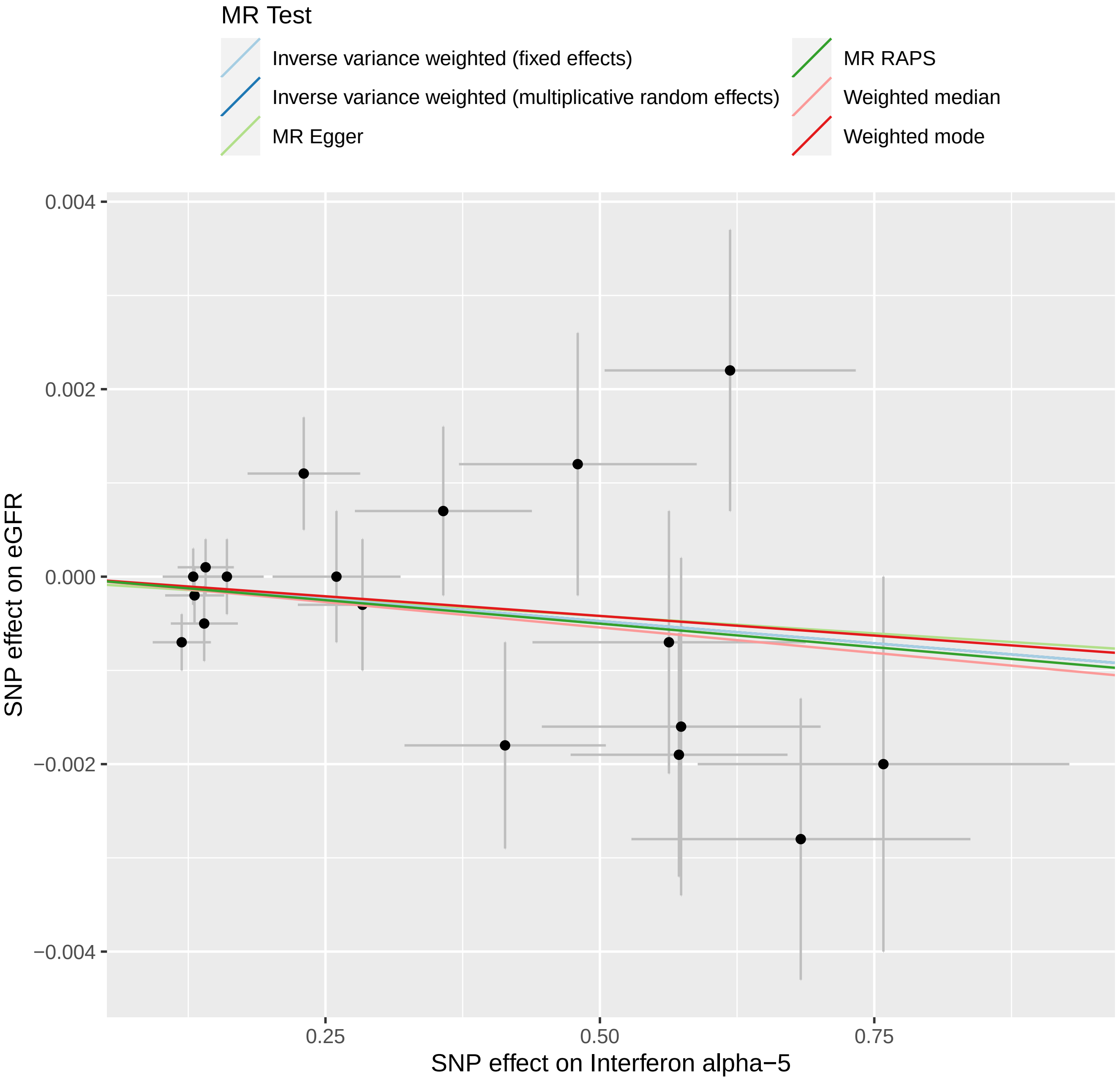

Batch 2−1423 : Interferon alpha−6 on eGFR

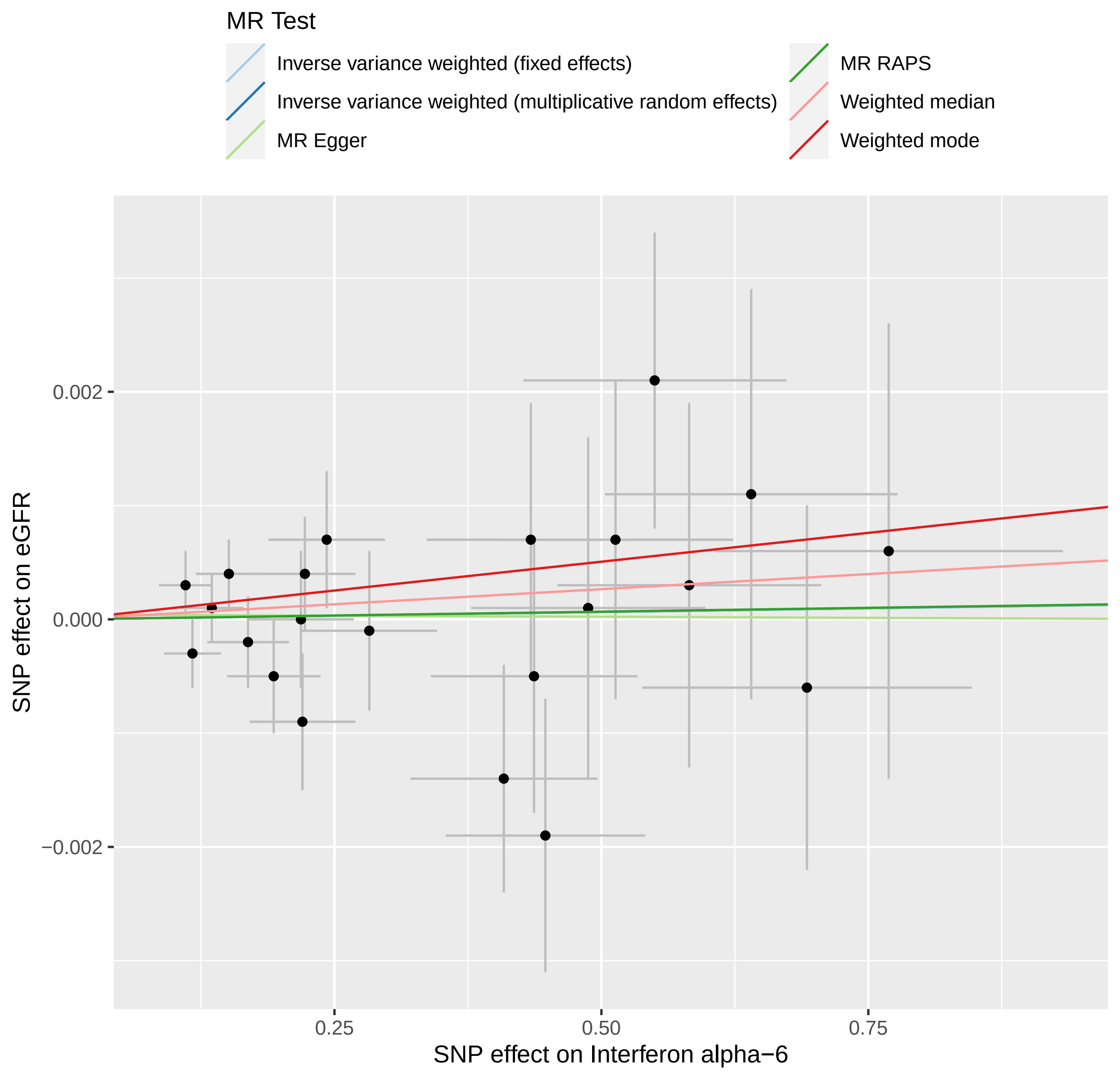

Batch 2−1424 : Interferon alpha−7 on eGFR

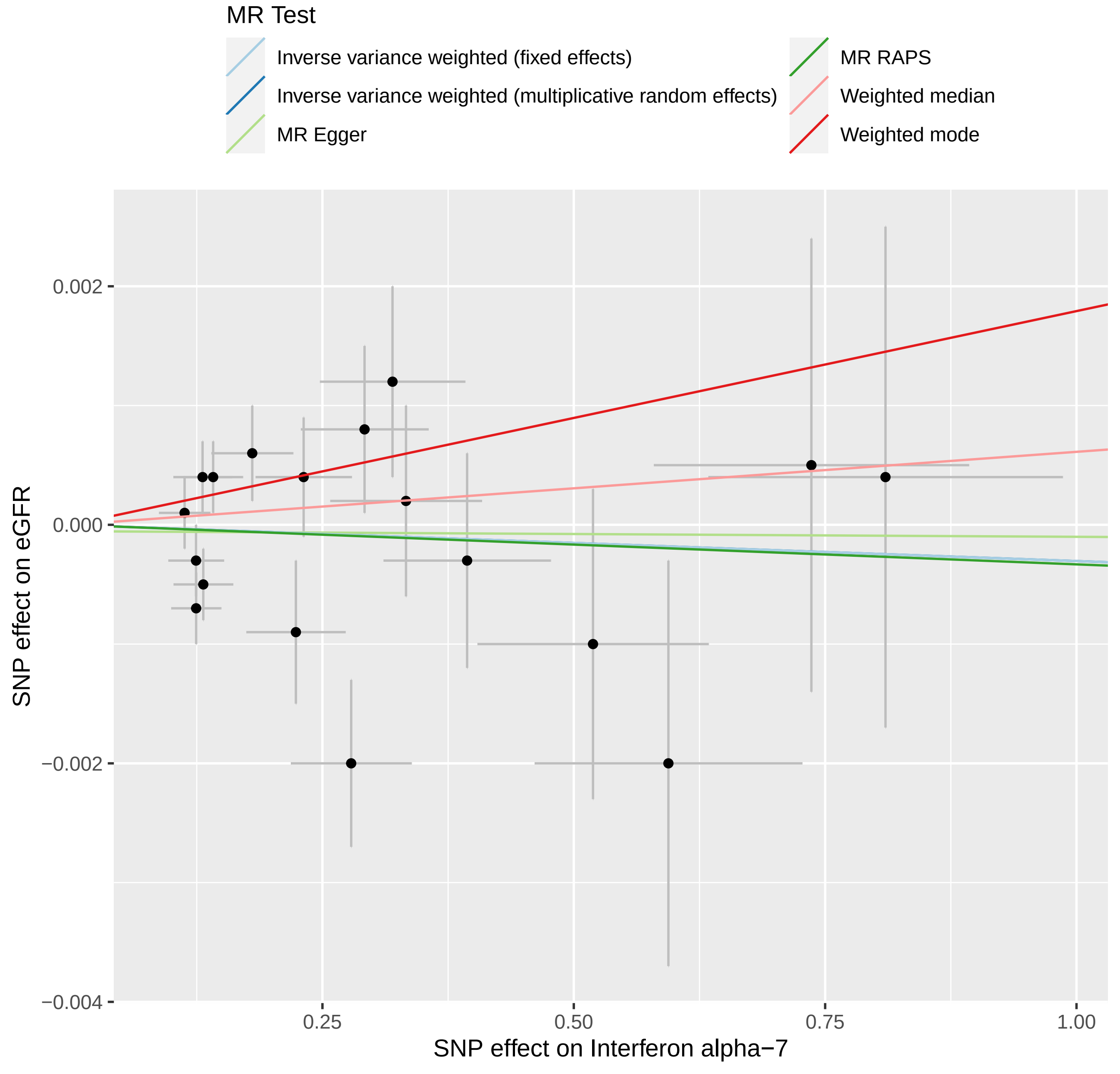

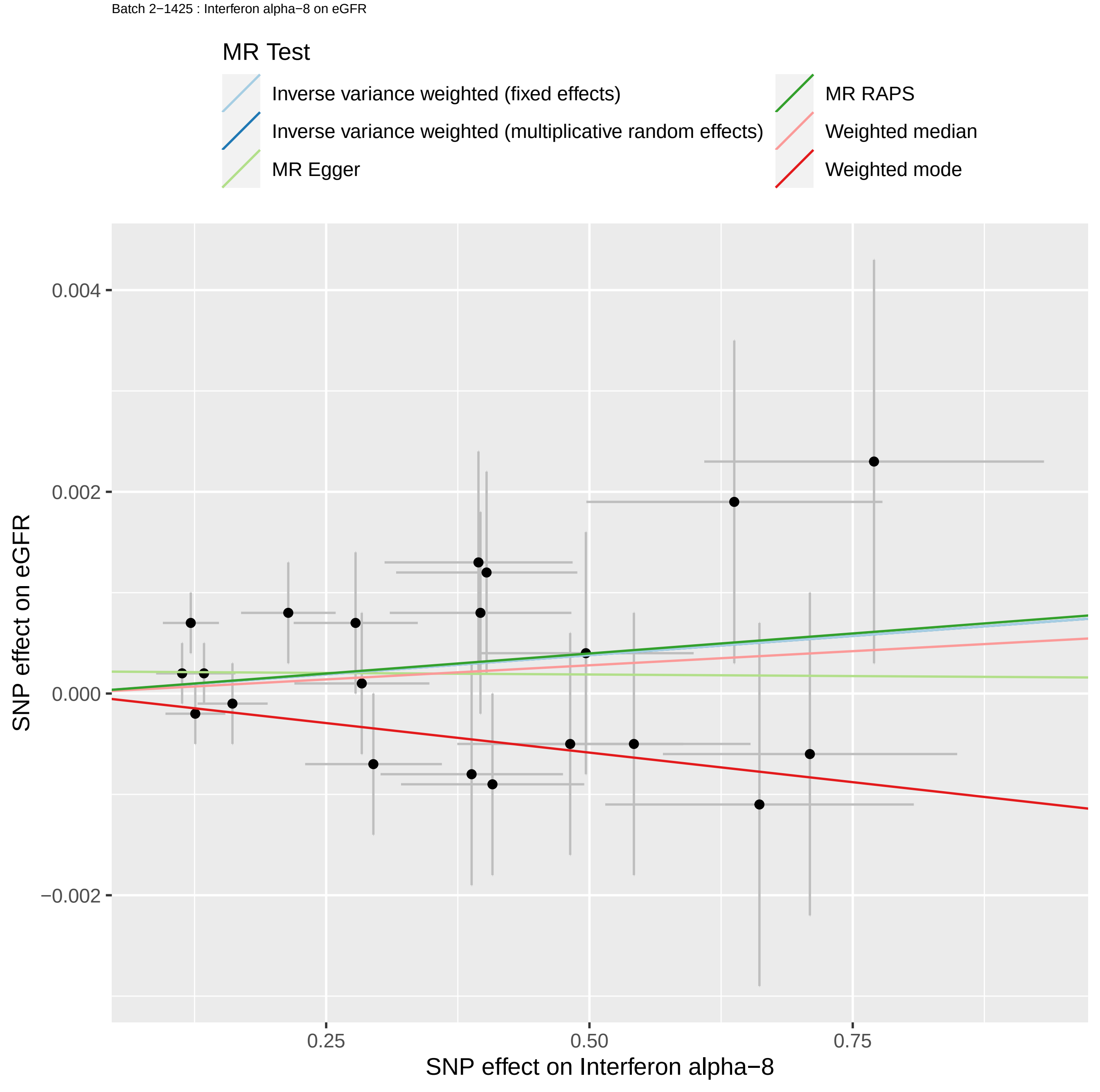

Batch 2−1425 : Interferon alpha−8 on eGFR
MR Test
Inverse variance weighted (fixed effects)
Inverse variance weighted (multiplicative random effects)
MR Egger
MR RAPS
Weighted median
Weighted mode
0.004
0.002
0.000
−0.002
0.25
0.50
0.75
SNP effect on eGFR
SNP effect on Interferon alpha−8

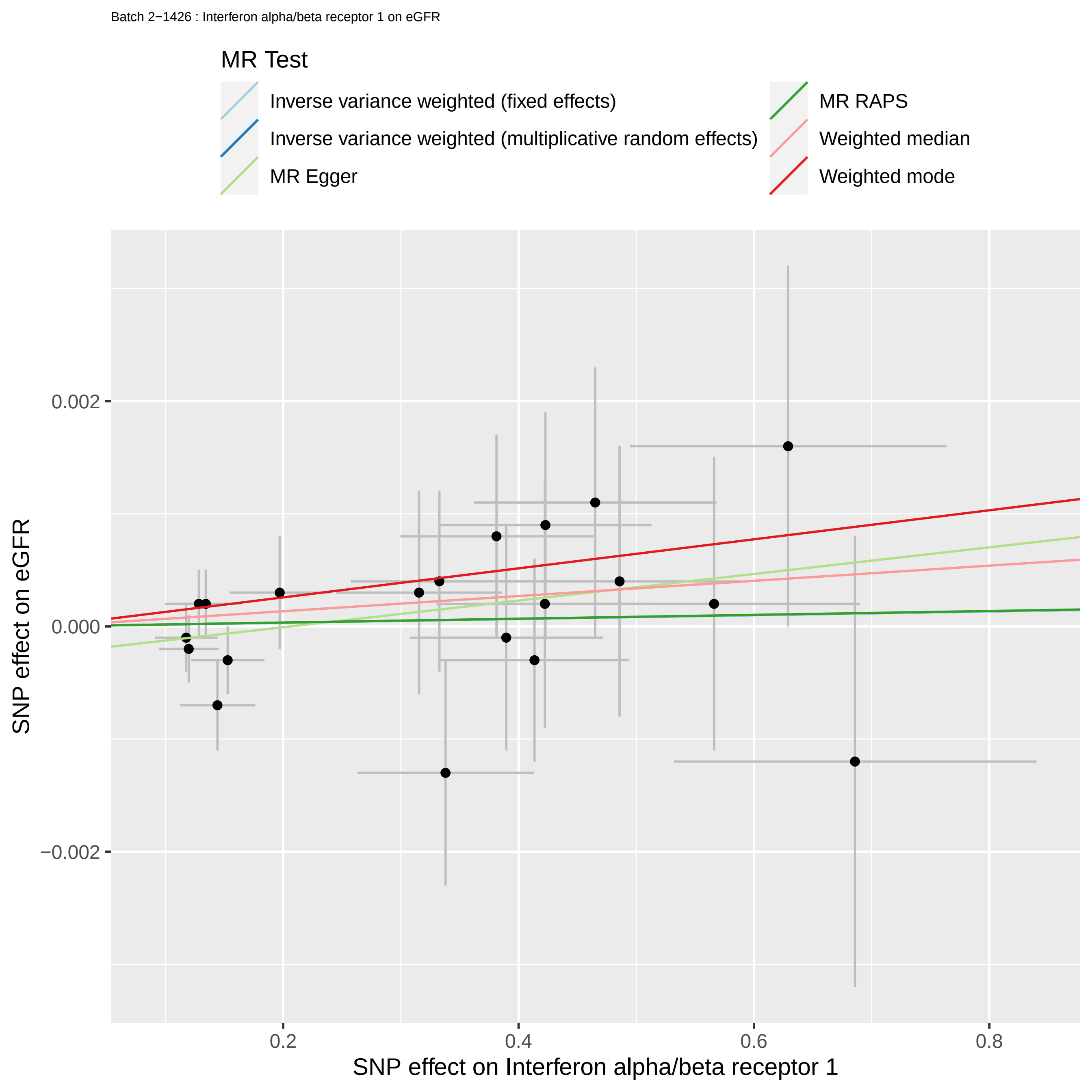

Batch 2−1426 : Interferon alpha/beta receptor 1 on eGFR
MR Test
Inverse variance weighted (fixed effects)
Inverse variance weighted (multiplicative random effects)
MR Egger
MR RAPS
Weighted median
Weighted mode
0.002
0.000
−0.002
0.2
0.4
0.6
0.8
SNP effect on eGFR
SNP effect on Interferon alpha/beta receptor 1

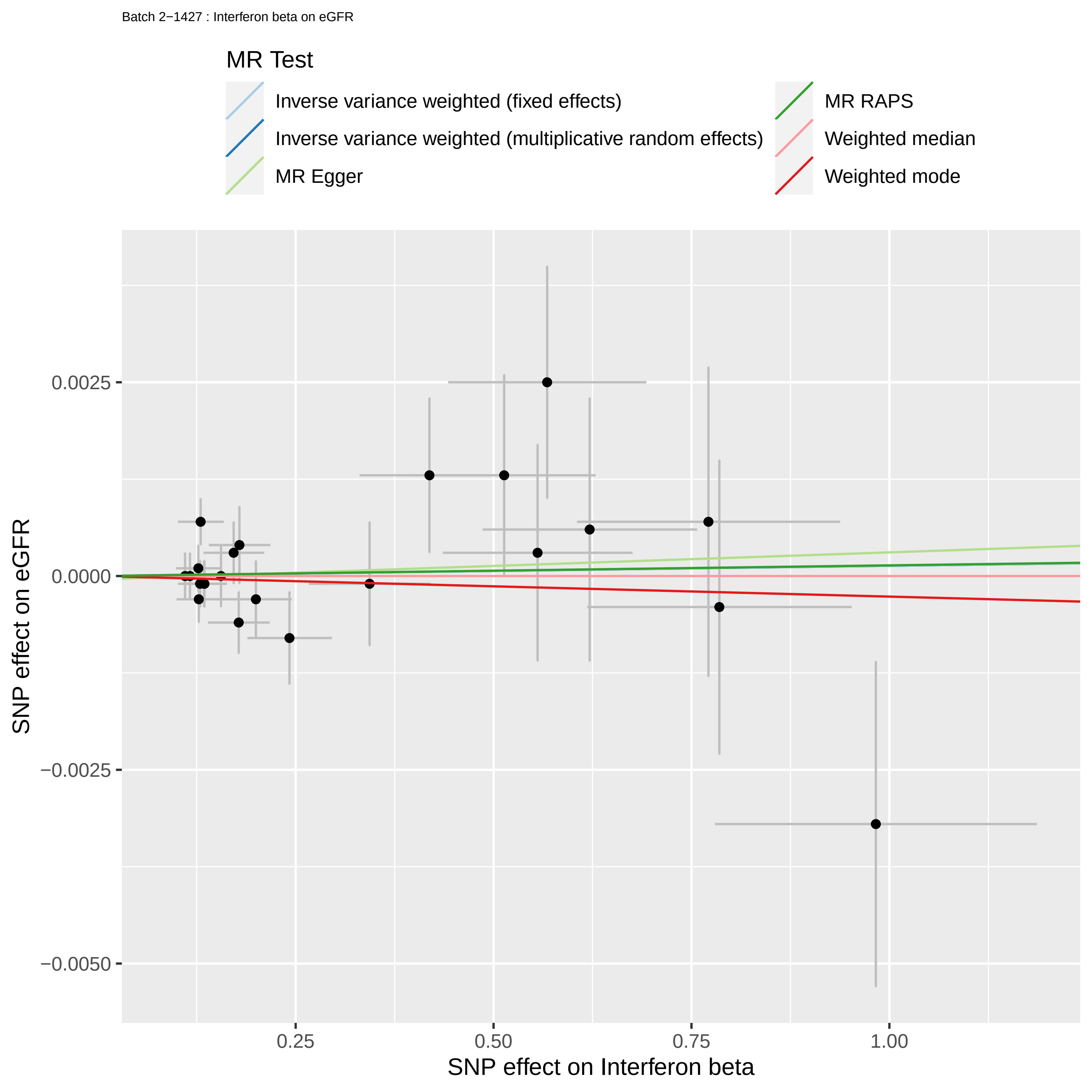
Batch 2−1427 : Interferon beta on eGFR
MR Test
Inverse variance weighted (fixed effects)
Inverse variance weighted (multiplicative random effects)
MR Egger
MR RAPS
Weighted median
Weighted mode
0.0025
0.0000
−0.0025
−0.0050
0.25
0.50
0.75
1.00
SNP effect on Interferon beta
SNP effect on eGFR

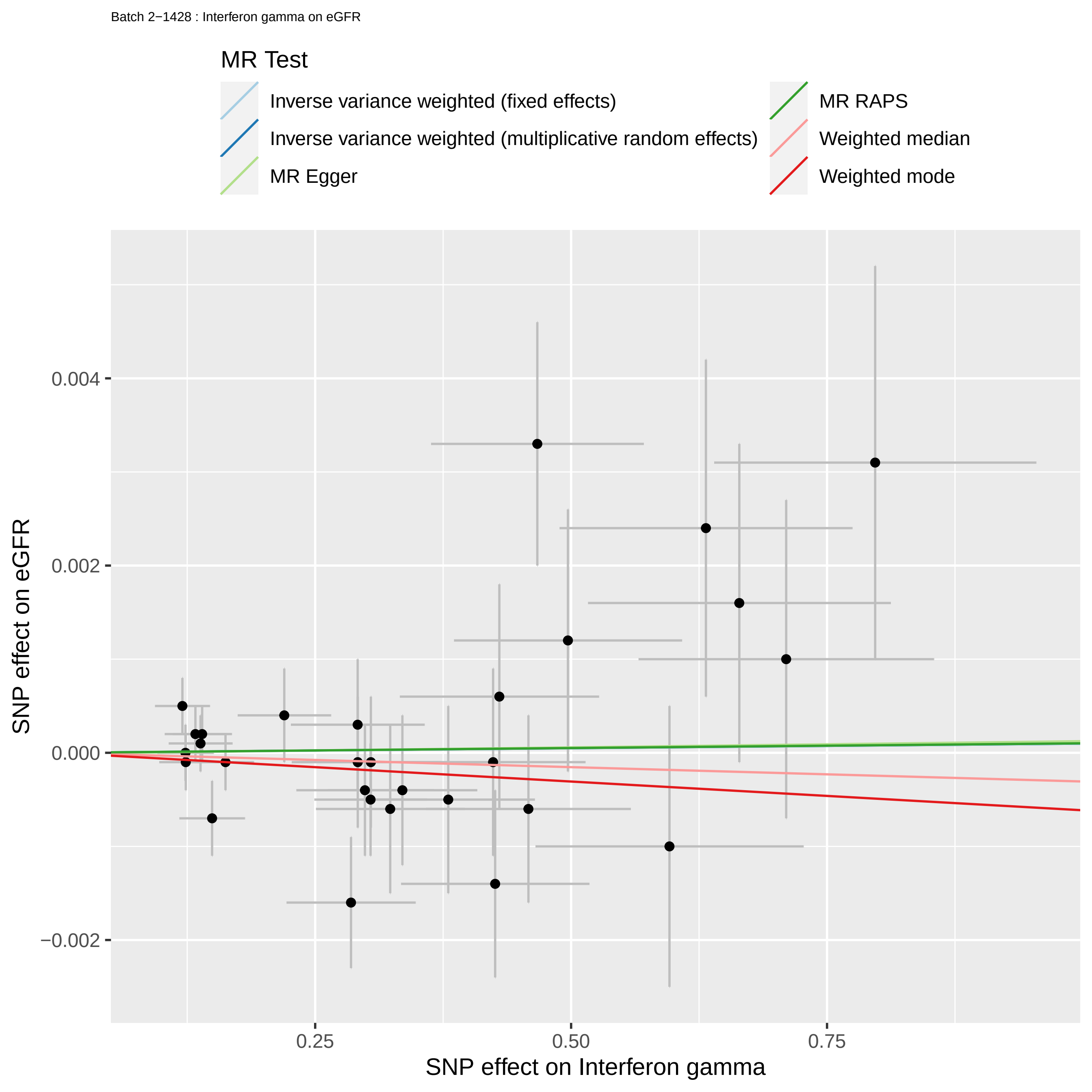

Batch 2−1428 : Interferon gamma on eGFR
MR Test
Inverse variance weighted (fixed effects)
Inverse variance weighted (multiplicative random effects)
MR Egger
MR RAPS
Weighted median
Weighted mode
0.004
0.002
0.000
−0.002
SNP effect on eGFR
0.25
0.50
0.75
SNP effect on Interferon gamma

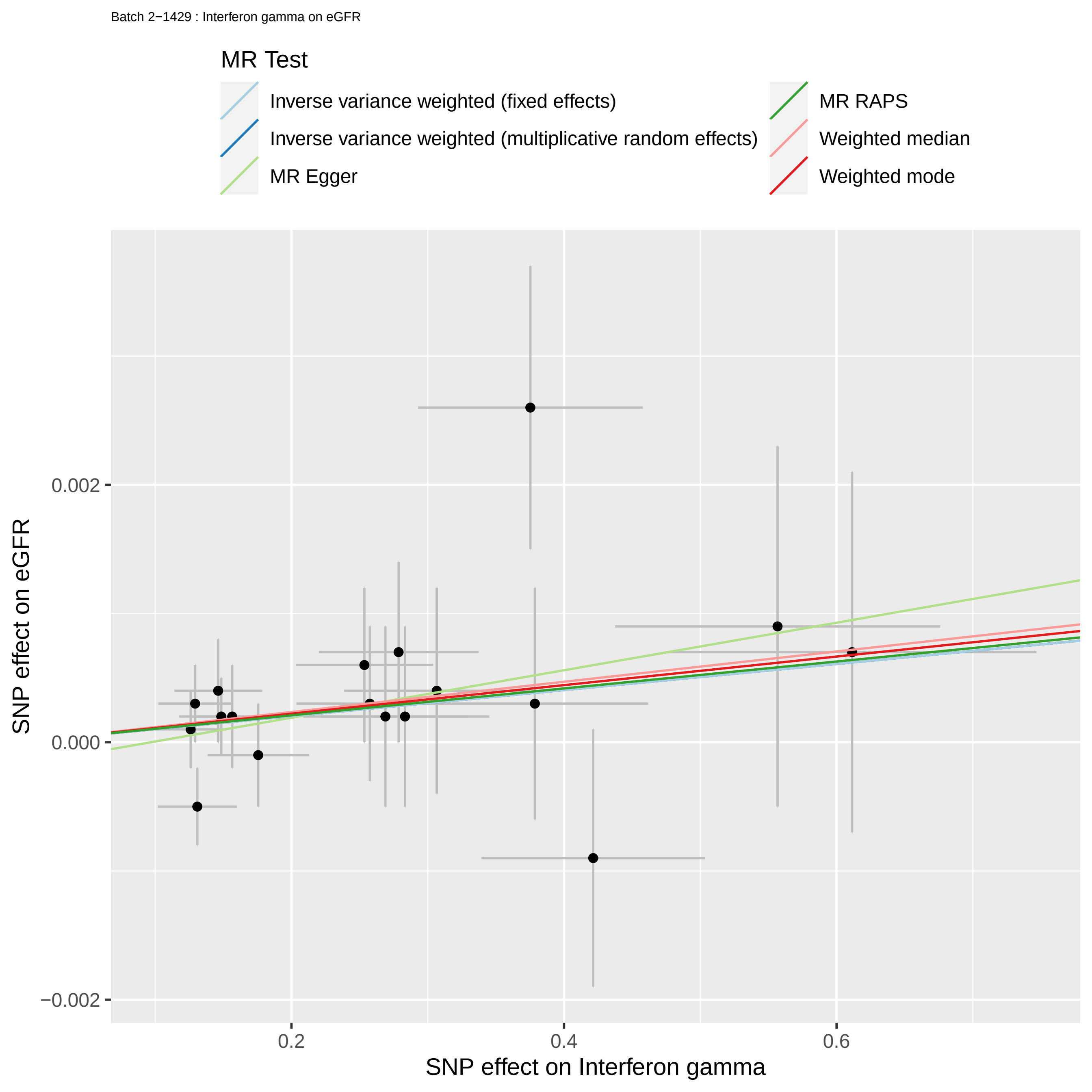
Batch 2−1429 : Interferon gamma on eGFR
MR Test
Inverse variance weighted (fixed effects)
Inverse variance weighted (multiplicative random effects)
MR Egger
MR RAPS
Weighted median
Weighted mode
0.002
0.000
−0.002
SNP effect on eGFR
0.2
0.4
0.6
SNP effect on Interferon gamma

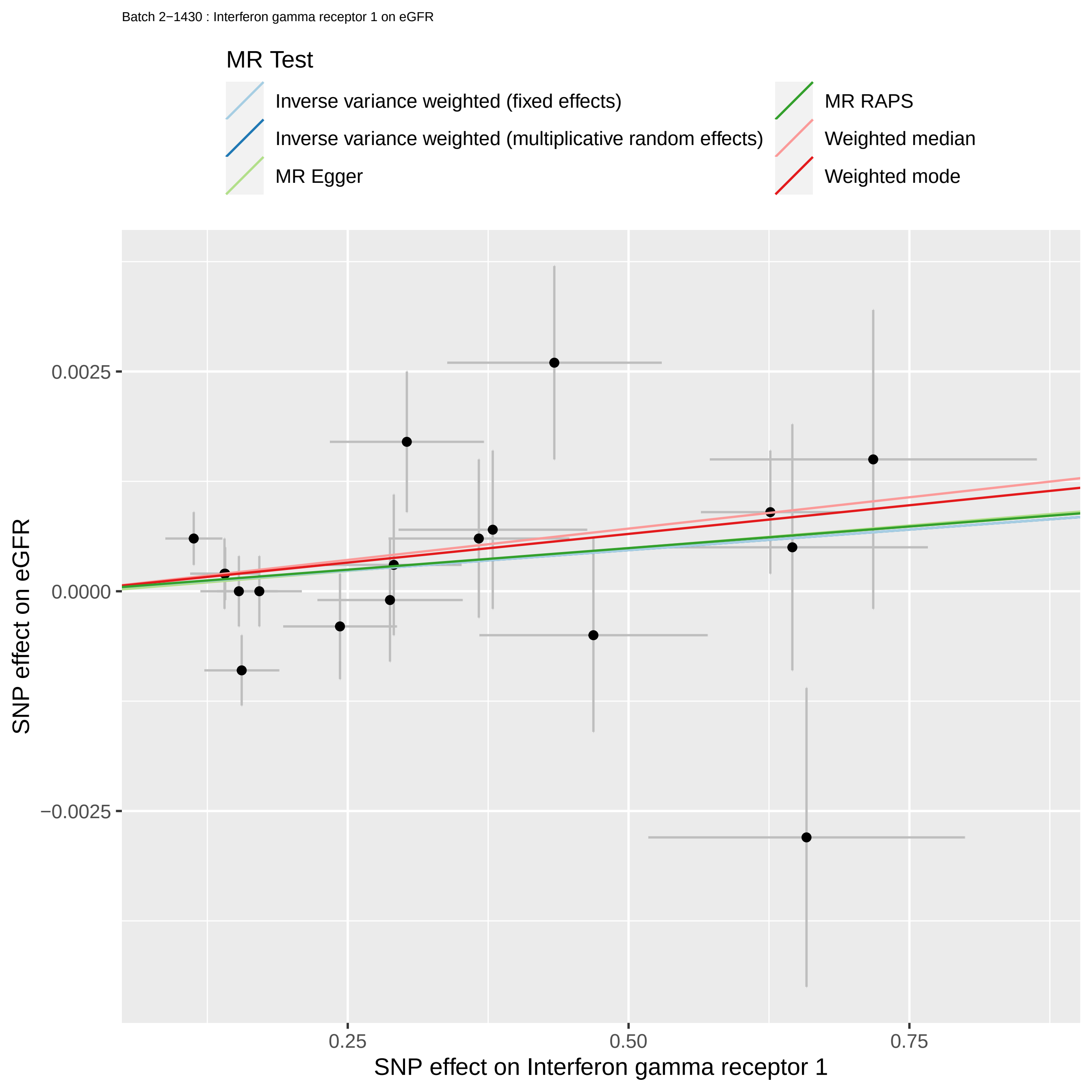

Batch 2−1430 : Interferon gamma receptor 1 on eGFR
MR Test
Inverse variance weighted (fixed effects)
Inverse variance weighted (multiplicative random effects)
MR Egger
MR RAPS
Weighted median
Weighted mode
0.0025
0.0000
−0.0025
0.25
0.50
0.75
SNP effect on eGFR
SNP effect on Interferon gamma receptor 1

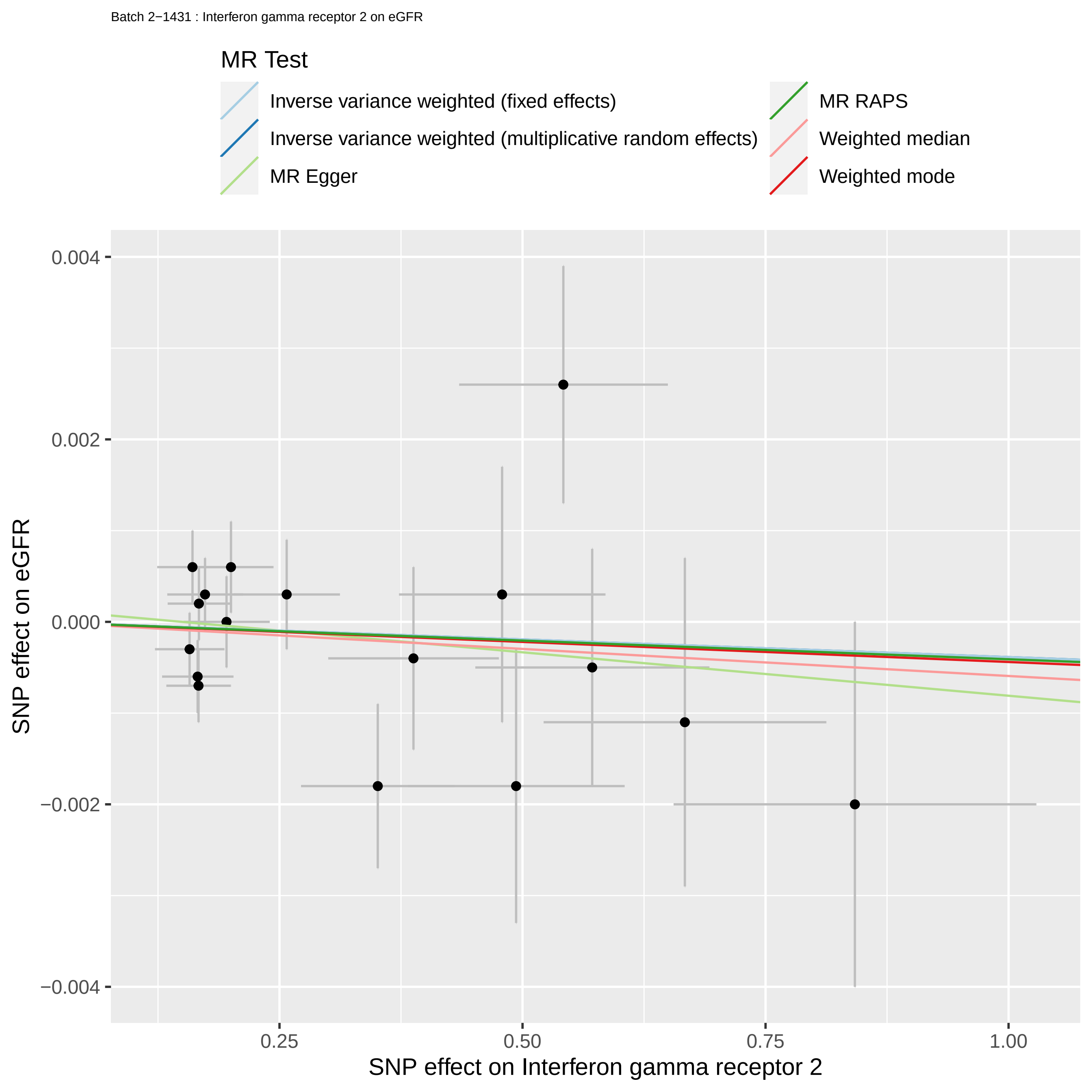

Batch 2−1431 : Interferon gamma receptor 2 on eGFR
MR Test
Inverse variance weighted (fixed effects)
Inverse variance weighted (multiplicative random effects)
MR Egger
MR RAPS
Weighted median
Weighted mode
0.004
0.002
0.000
−0.002
−0.004
SNP effect on eGFR
0.25
0.50
0.75
1.00
SNP effect on Interferon gamma receptor 2

Batch 2−1432 : Interferon gamma receptor 2 on eGFR

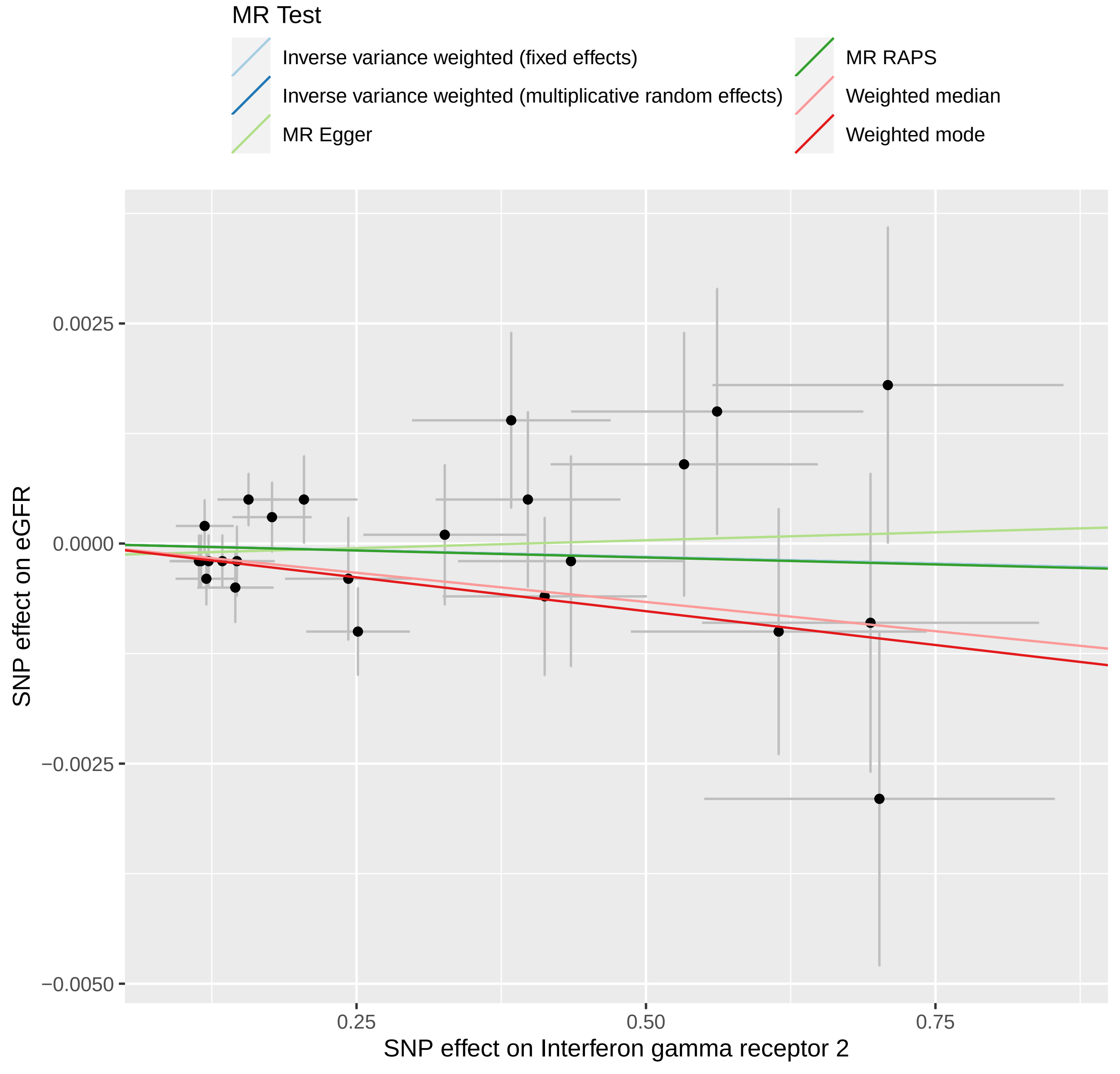

Batch 2−1433 : Interferon gamma receptor 2 on eGFR

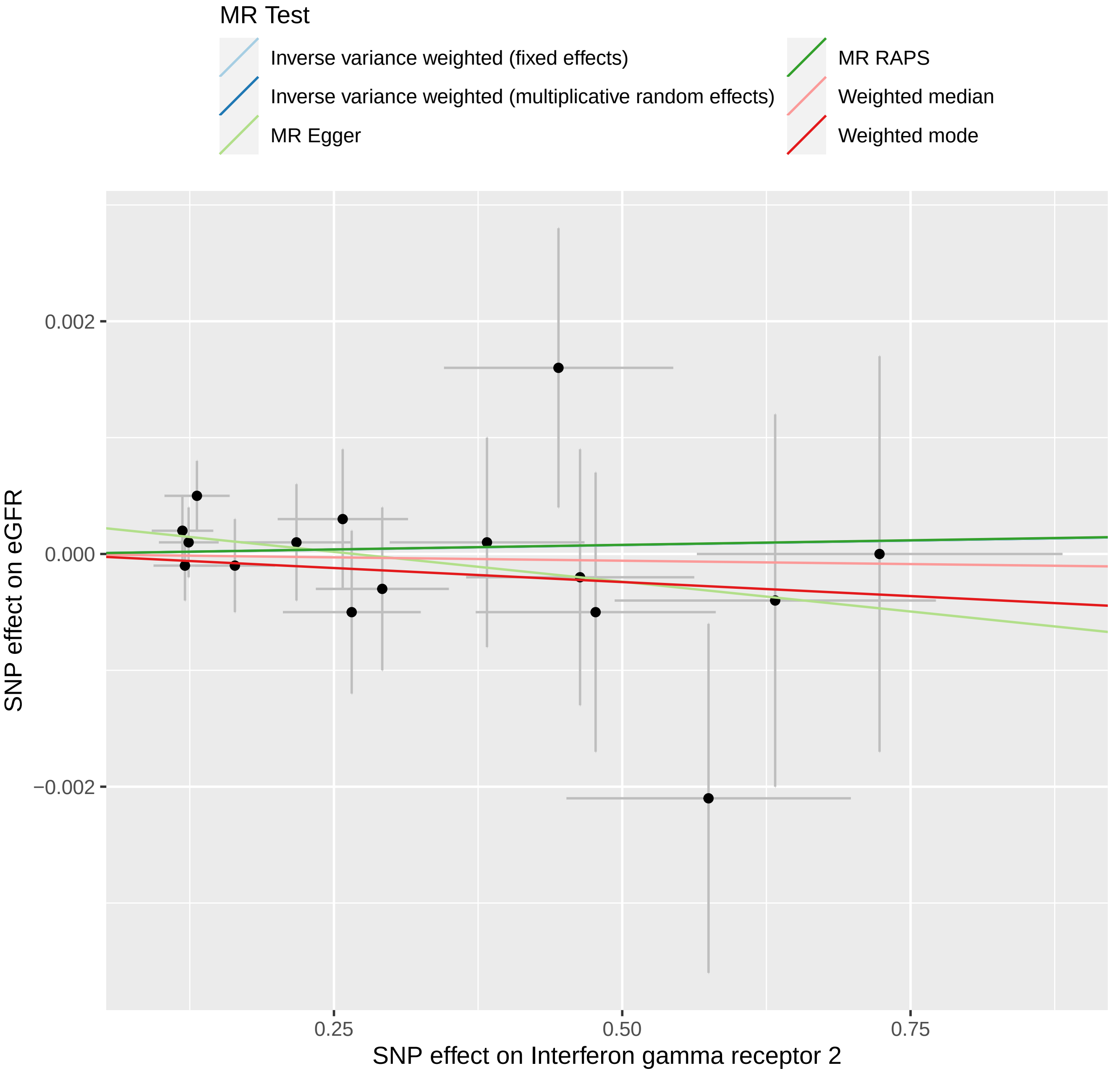

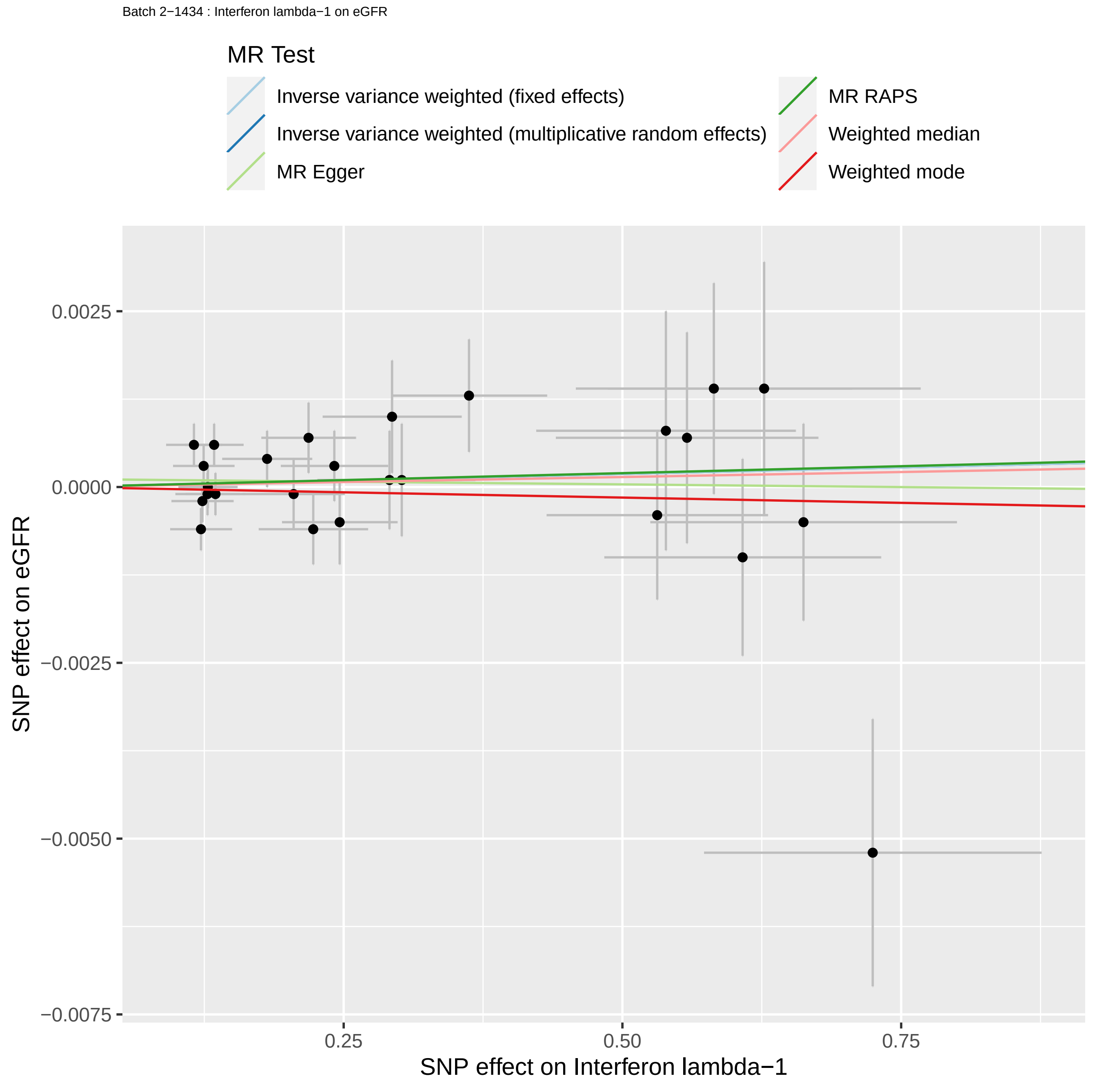
Batch 2−1434 : Interferon lambda−1 on eGFR
MR Test
Inverse variance weighted (fixed effects)
Inverse variance weighted (multiplicative random effects)
MR Egger
MR RAPS
Weighted median
Weighted mode
0.0025
0.0000
−0.0025
−0.0050
−0.0075
0.25
0.50
0.75
SNP effect on eGFR
SNP effect on Interferon lambda−1

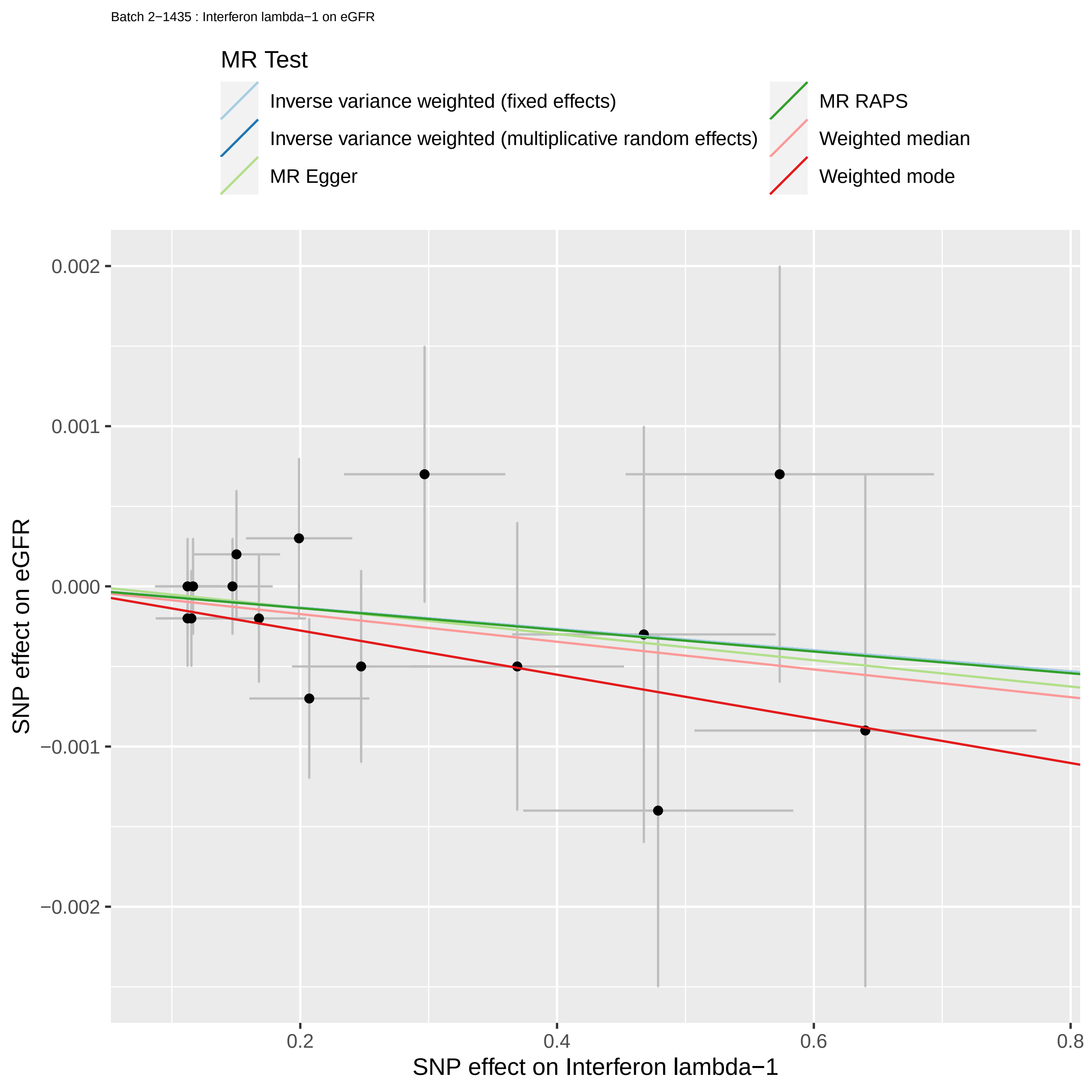

Batch 2−1435 : Interferon lambda−1 on eGFR
MR Test
Inverse variance weighted (fixed effects)
Inverse variance weighted (multiplicative random effects)
MR Egger
MR RAPS
Weighted median
Weighted mode
0.002
0.001
0.000
−0.001
−0.002
SNP effect on eGFR
0.2
0.4
0.6
0.8
SNP effect on Interferon lambda−1

Batch 2−1436 : Interferon lambda−2 on eGFR

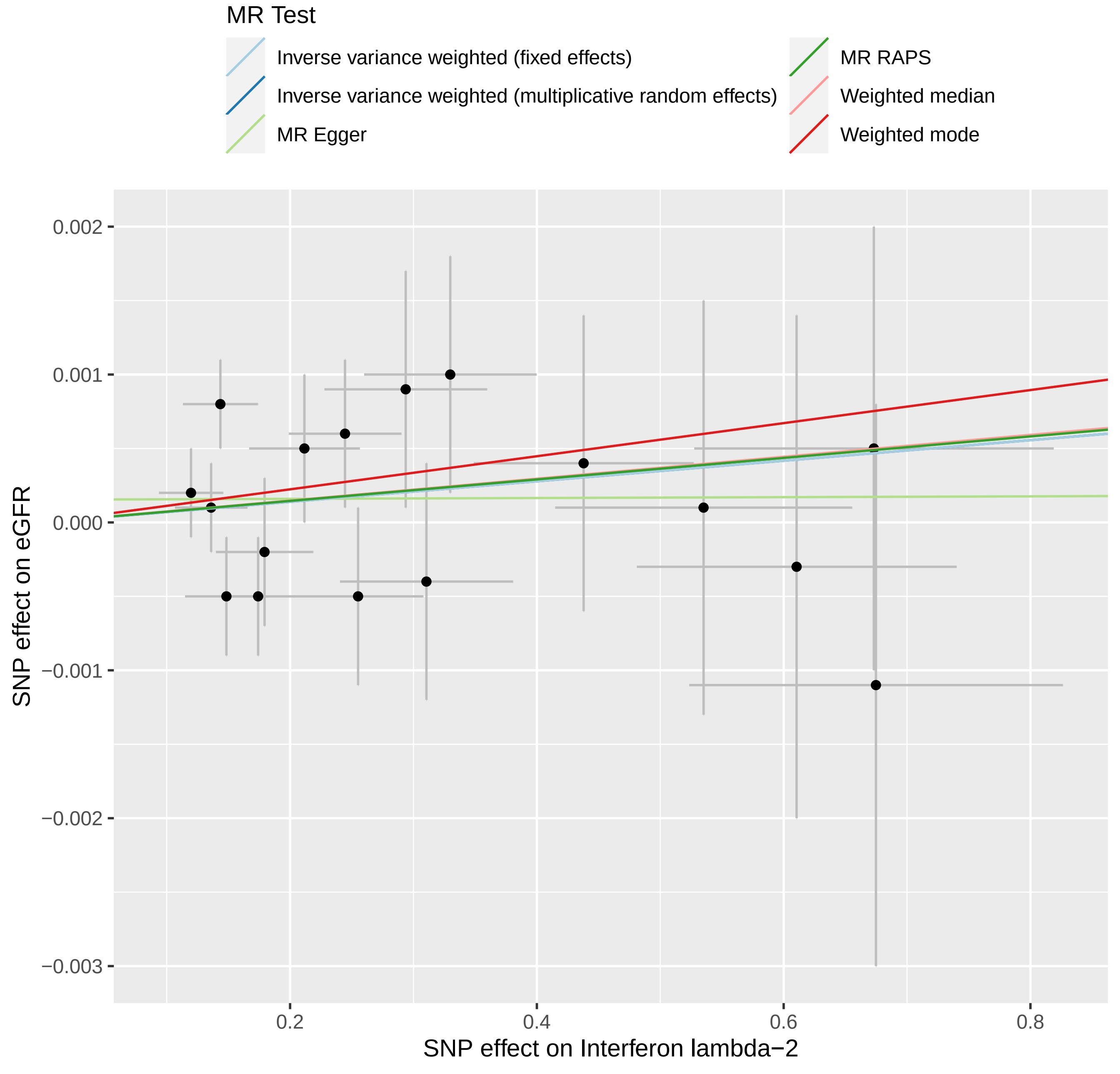

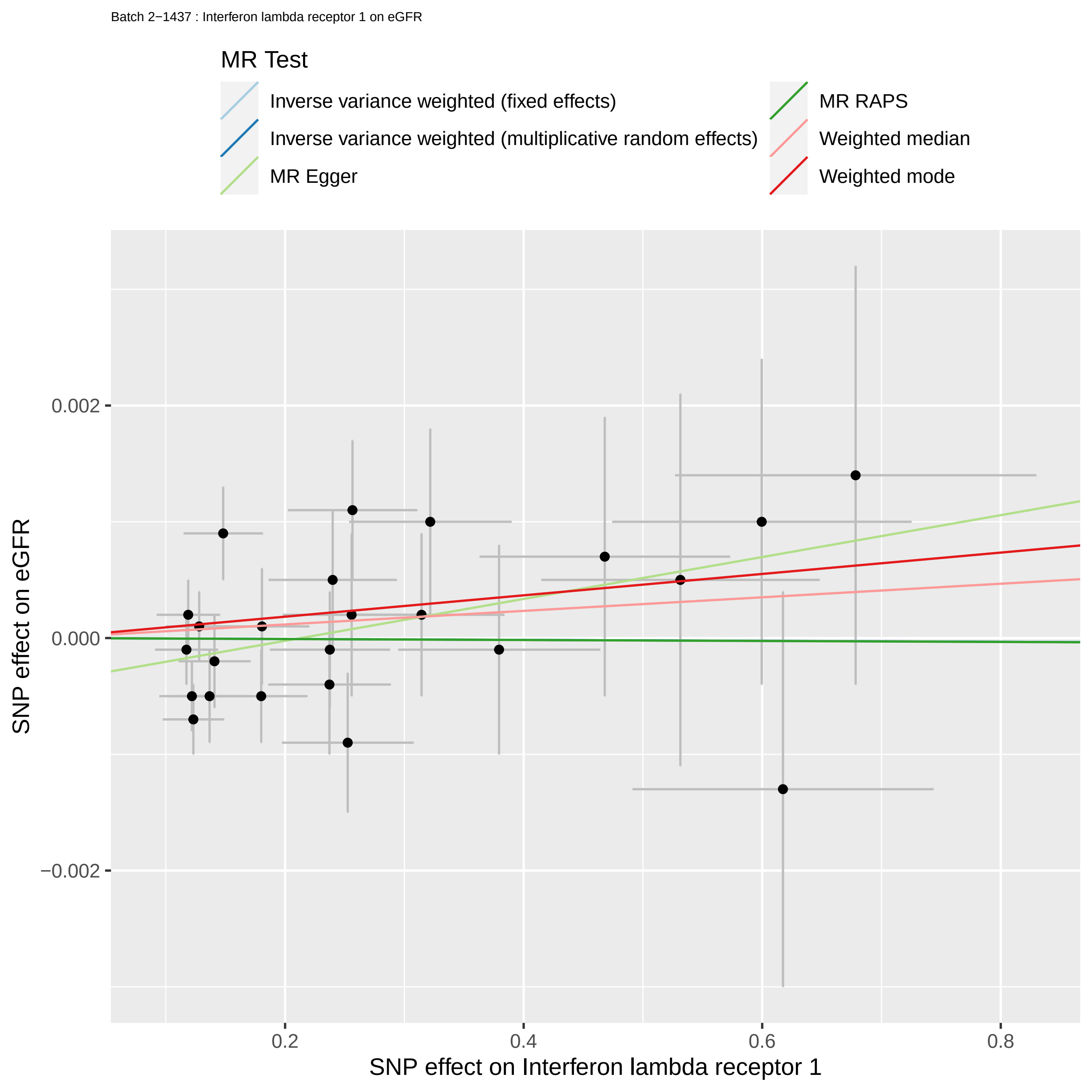

Batch 2−1437 : Interferon lambda receptor 1 on eGFR
MR Test
Inverse variance weighted (fixed effects)
Inverse variance weighted (multiplicative random effects)
MR Egger
MR RAPS
Weighted median
Weighted mode
0.002
0.000
−0.002
SNP effect on eGFR
0.2
0.4
0.6
0.8
SNP effect on Interferon lambda receptor 1

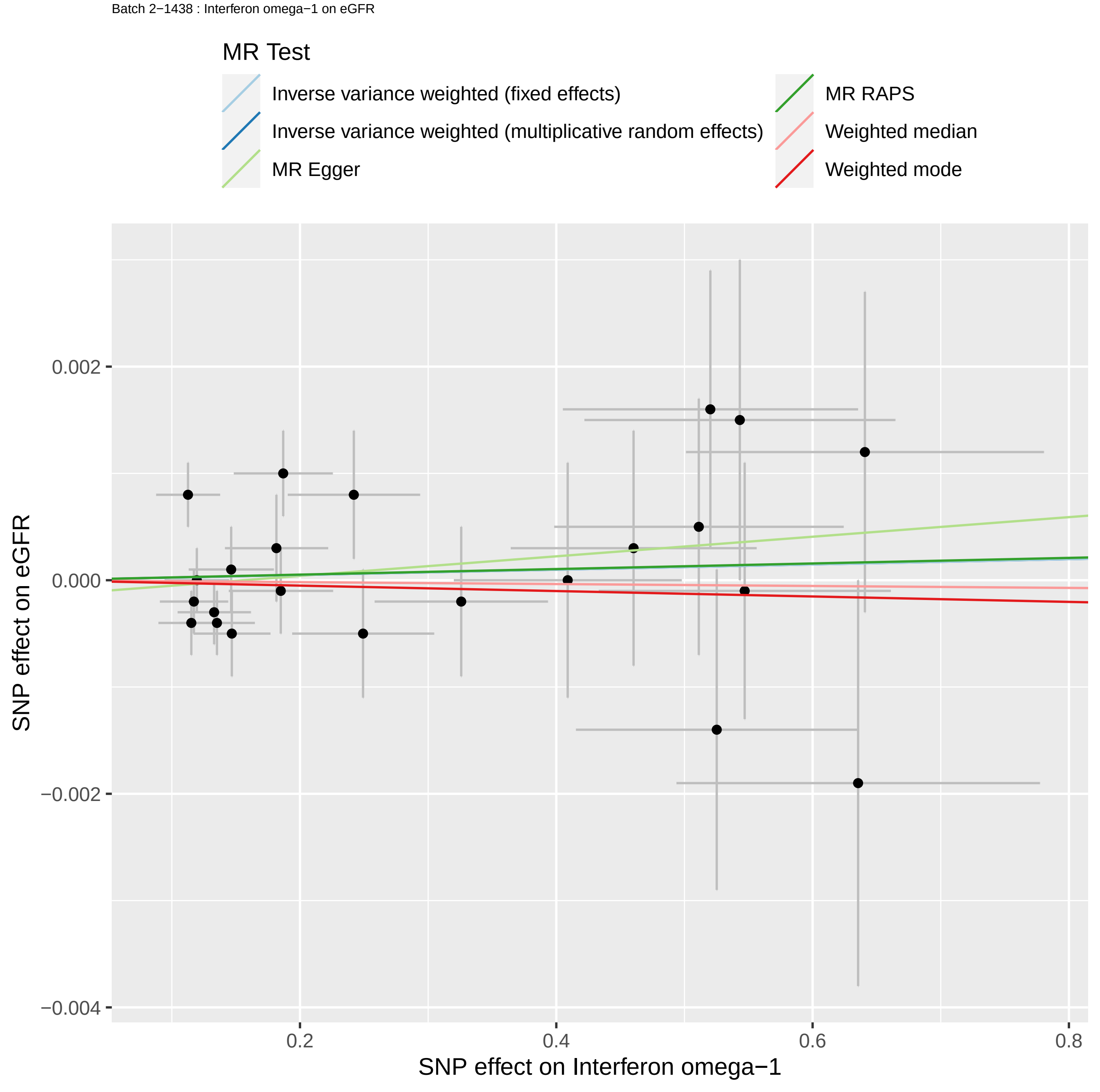
Batch 2−1438 : Interferon omega−1 on eGFR
MR Test
Inverse variance weighted (fixed effects)
Inverse variance weighted (multiplicative random effects)
MR Egger
MR RAPS
Weighted median
Weighted mode
0.002
0.000
−0.002
−0.004
0.2
0.4
0.6
0.8
SNP effect on eGFR
SNP effect on Interferon omega−1

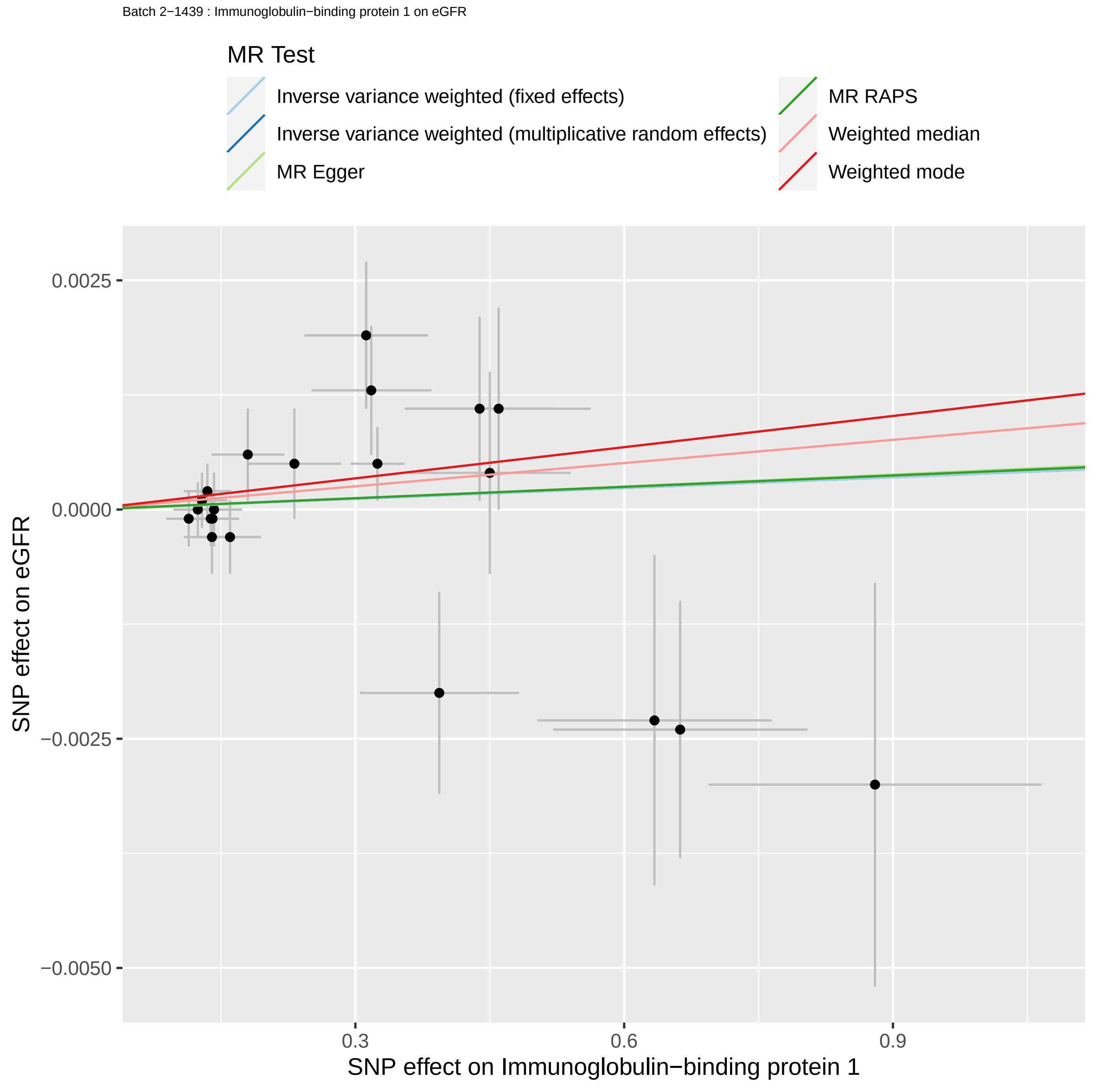

Batch 2−1439 : Immunoglobulin−binding protein 1 on eGFR
MR Test
Inverse variance weighted (fixed effects)
Inverse variance weighted (multiplicative random effects)
MR Egger
MR RAPS
Weighted median
Weighted mode
0.0025
0.0000
−0.0025
−0.0050
0.3
0.6
0.9
SNP effect on eGFR
SNP effect on Immunoglobulin−binding protein 1

Batch 2−1440 : Immunoglobulin superfamily DCC subclass member 3 on eGFR

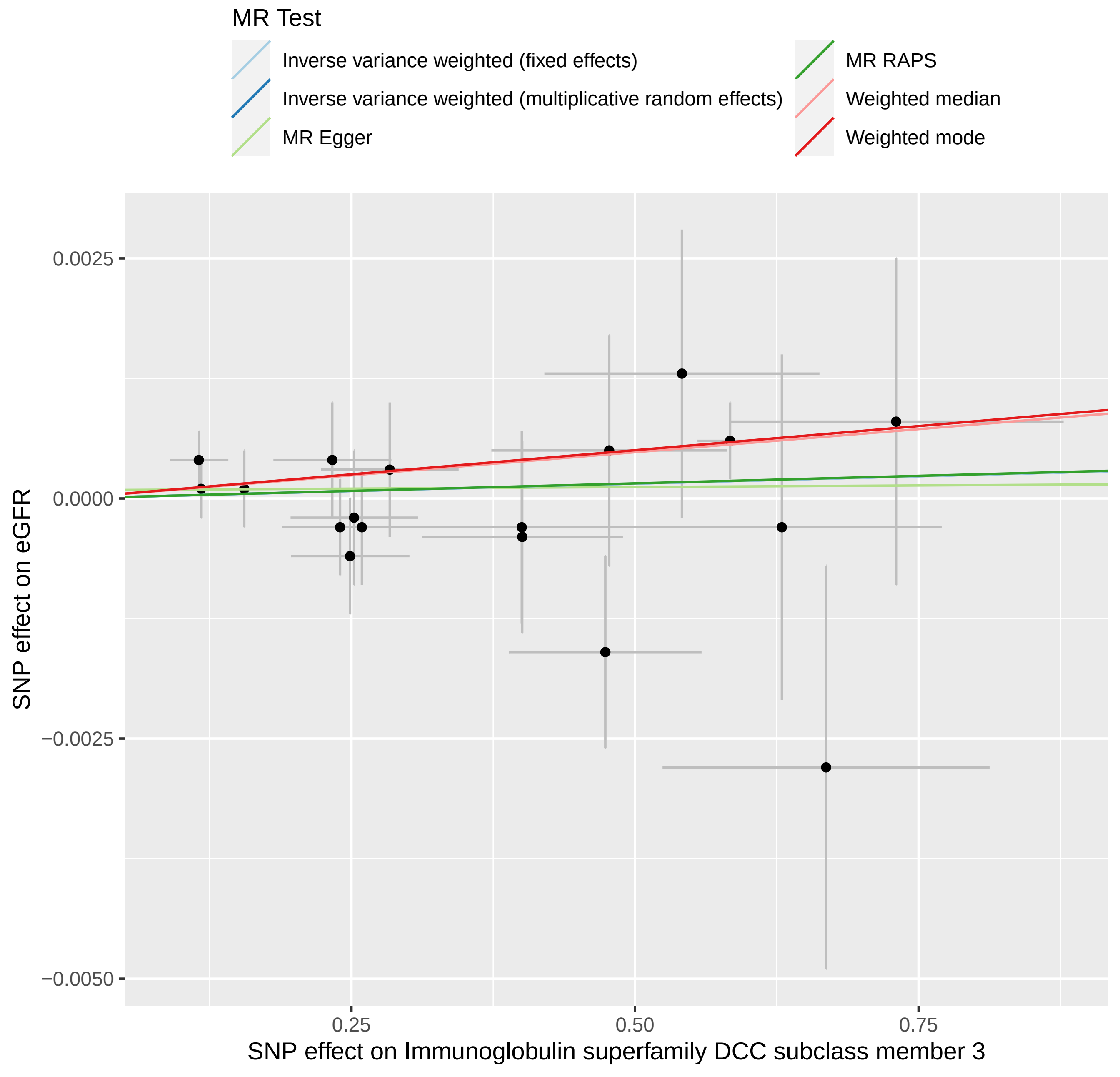

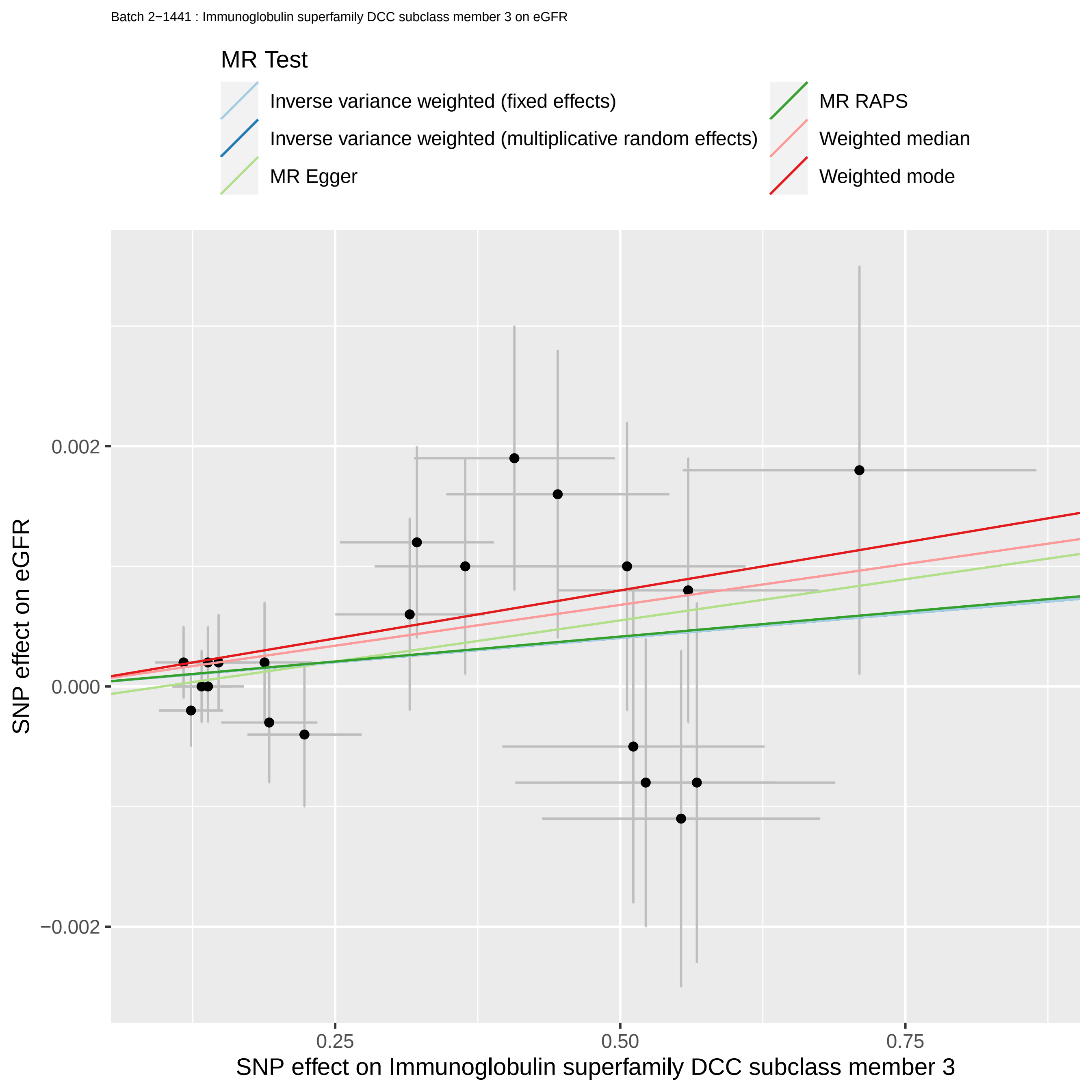

Batch 2−1441 : Immunoglobulin superfamily DCC subclass member 3 on eGFR
MR Test
Inverse variance weighted (fixed effects)
Inverse variance weighted (multiplicative random effects)
MR Egger
MR RAPS
Weighted median
Weighted mode
SNP effect on eGFR
0.002
0.000
−0.002
0.25
0.50
0.75
SNP effect on Immunoglobulin superfamily DCC subclass member 3

Batch 2−1442 : Immunoglobulin superfamily DCC subclass member 4 on eGFR

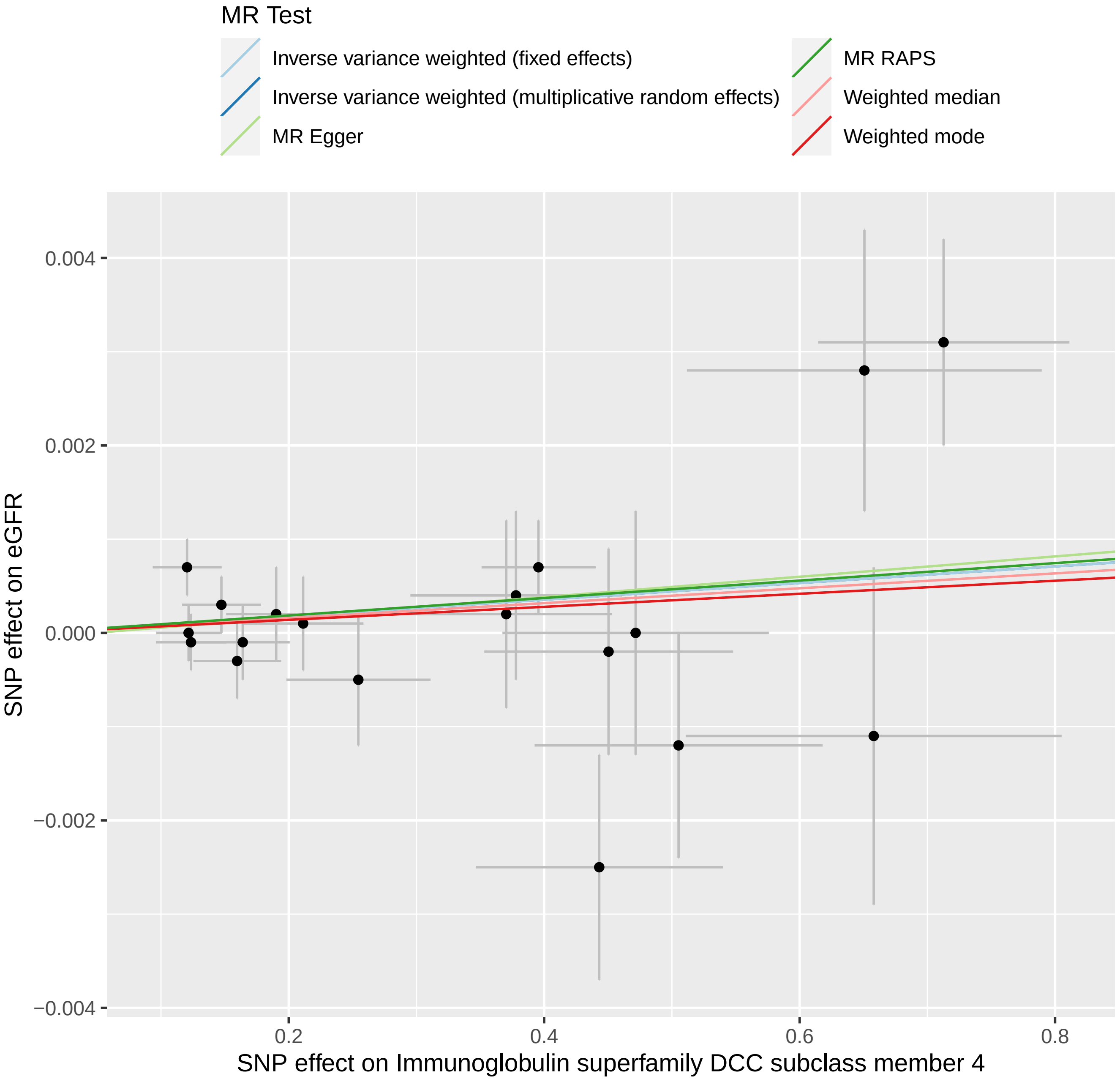

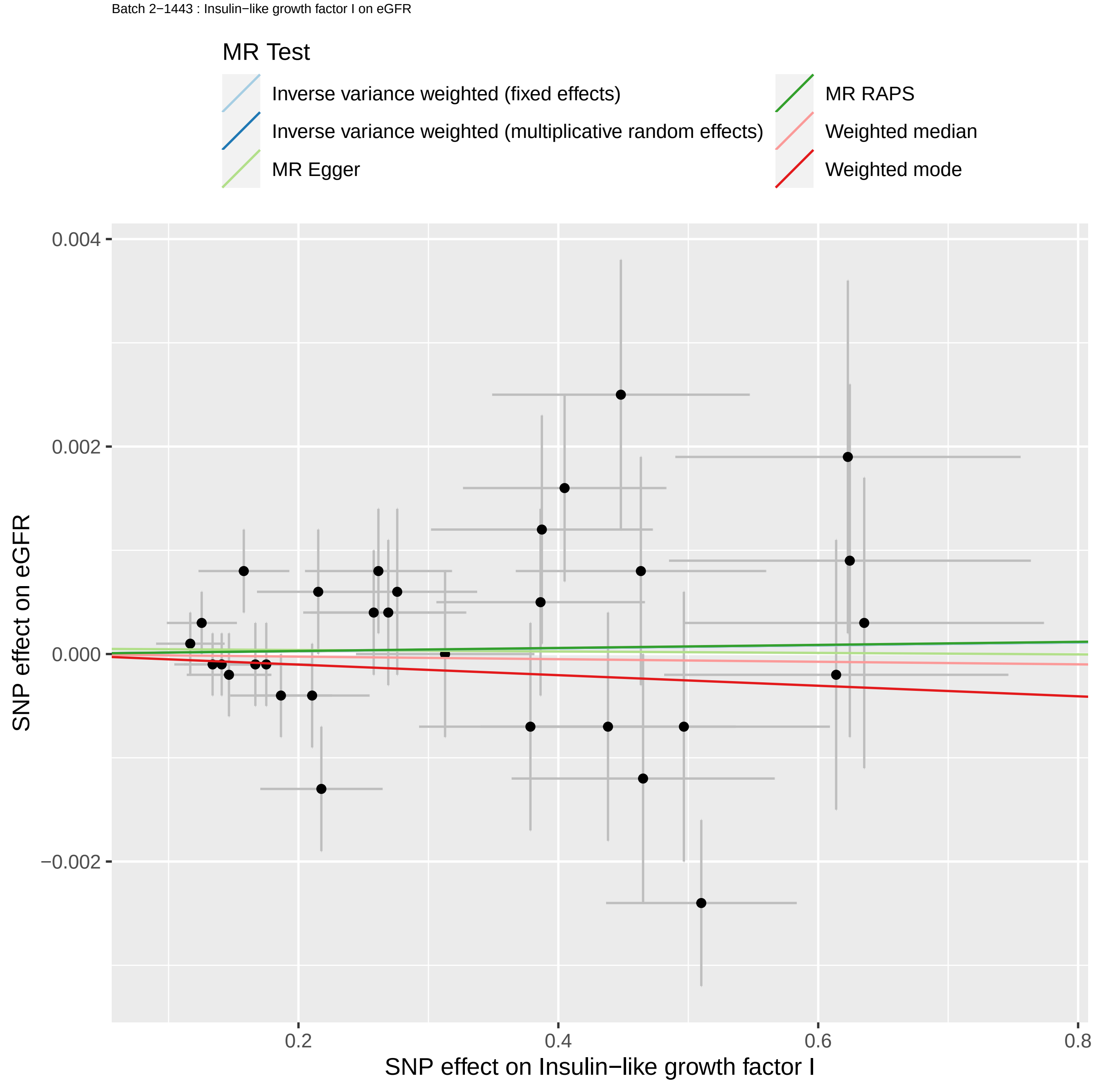

Batch 2−1443 : Insulin−like growth factor I on eGFR
MR Test
Inverse variance weighted (fixed effects)
Inverse variance weighted (multiplicative random effects)
MR Egger
MR RAPS
Weighted median
Weighted mode
0.004
0.002
0.000
−0.002
0.2
0.4
0.6
0.8
SNP effect on eGFR
SNP effect on Insulin−like growth factor I

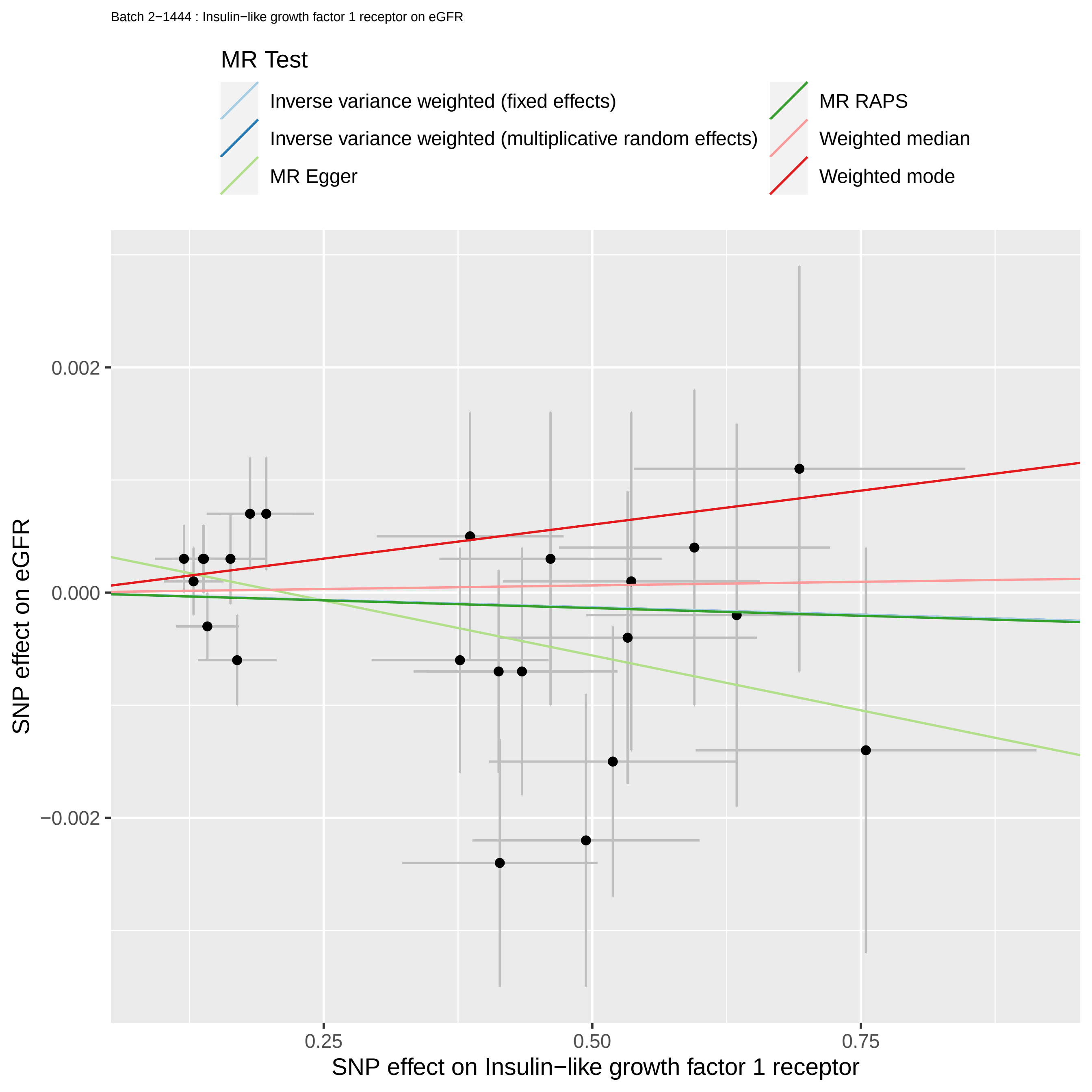

Batch 2−1444 : Insulin−like growth factor 1 receptor on eGFR
MR Test
Inverse variance weighted (fixed effects)
Inverse variance weighted (multiplicative random effects)
MR Egger
MR RAPS
Weighted median
Weighted mode
0.002
0.000
−0.002
0.25
0.50
0.75
SNP effect on eGFR
SNP effect on Insulin−like growth factor 1 receptor

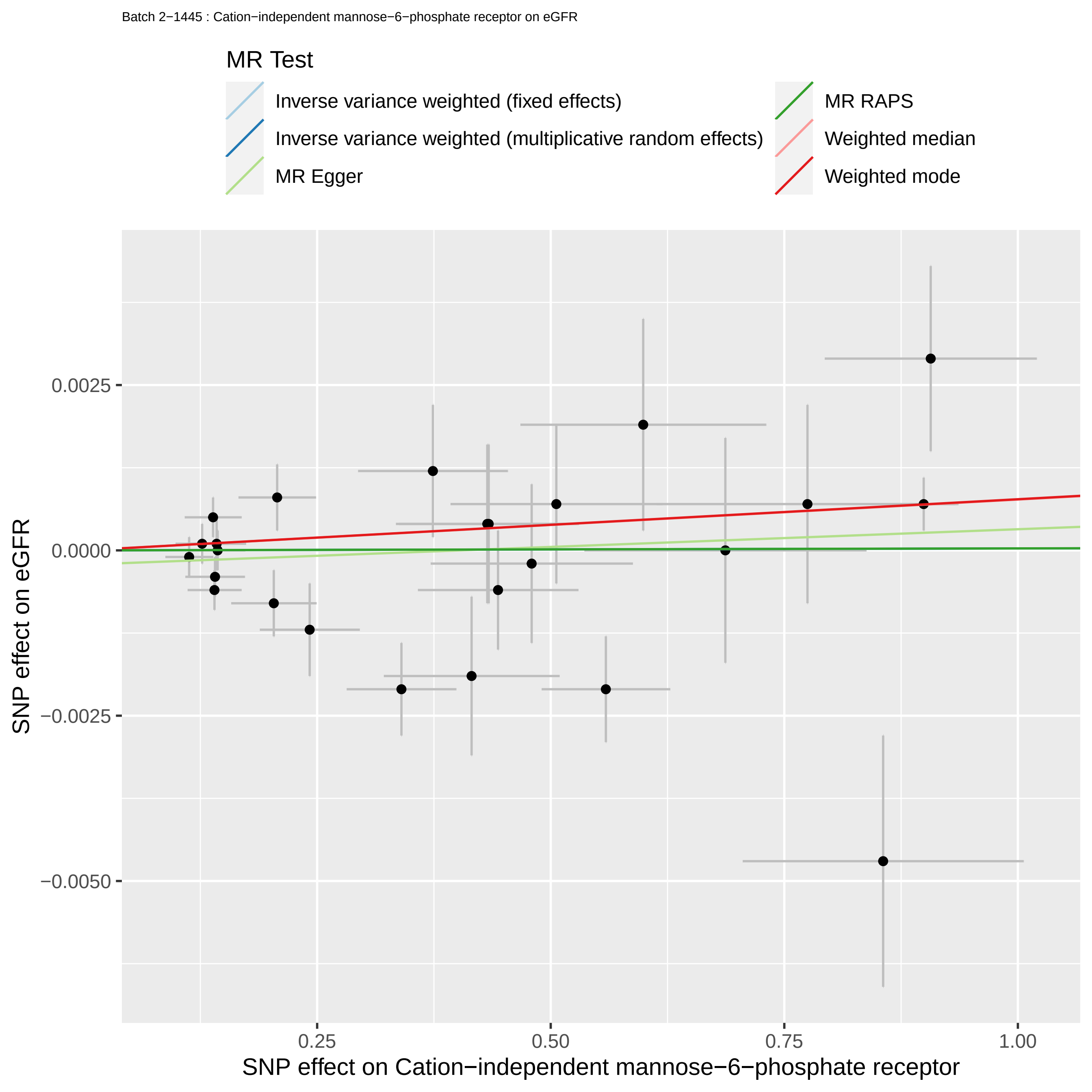

Batch 2−1445 : Cation−independent mannose−6−phosphate receptor on eGFR
MR Test
Inverse variance weighted (fixed effects)
Inverse variance weighted (multiplicative random effects)
MR Egger
MR RAPS
Weighted median
Weighted mode
0.0025
0.0000
−0.0025
−0.0050
SNP effect on eGFR
0.25
0.50
0.75
1.00
SNP effect on Cation−independent mannose−6−phosphate receptor

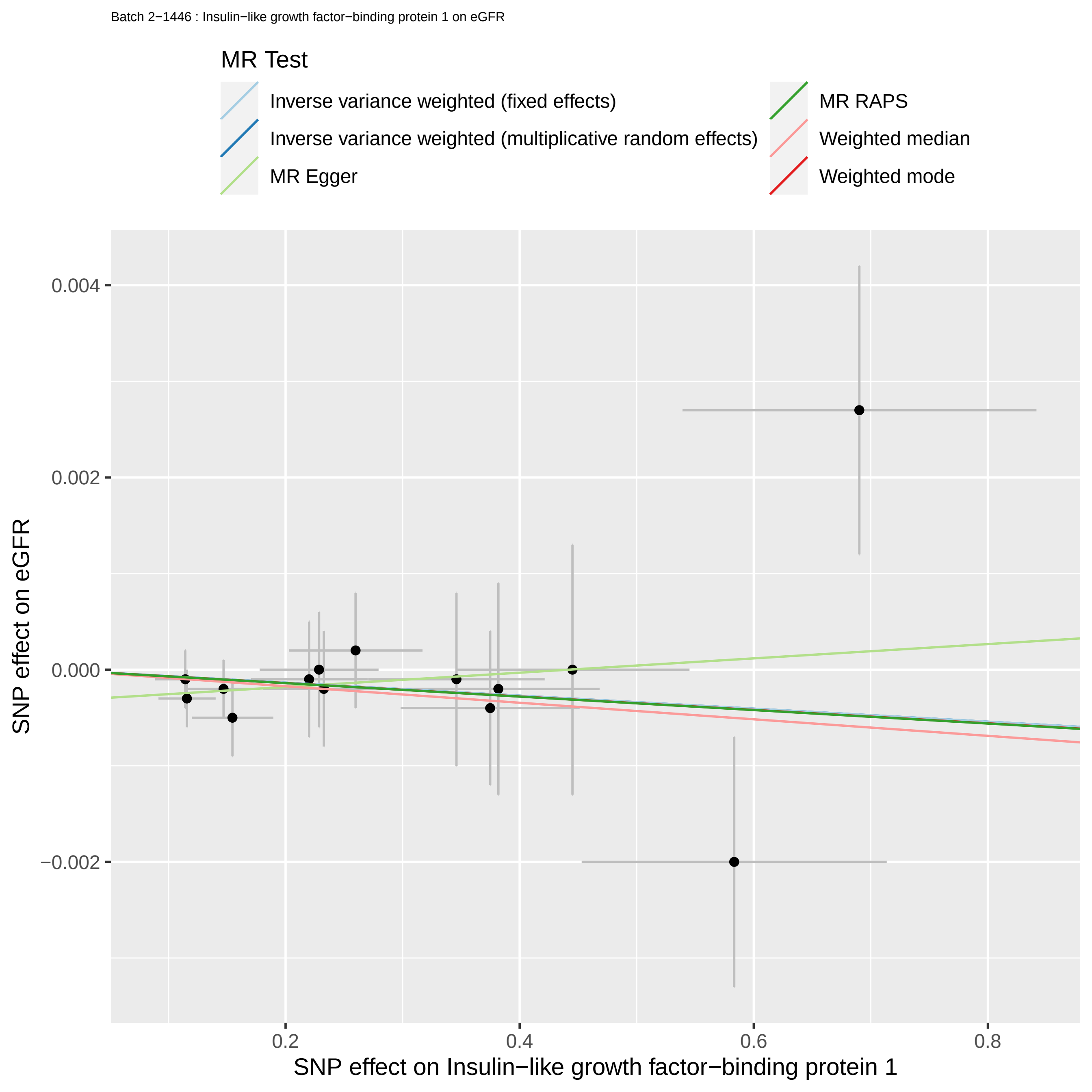

Batch 2−1446 : Insulin−like growth factor−binding protein 1 on eGFR
MR Test
Inverse variance weighted (fixed effects)
Inverse variance weighted (multiplicative random effects)
MR Egger
MR RAPS
Weighted median
Weighted mode
0.004
0.002
0.000
−0.002
SNP effect on eGFR
0.2
0.4
0.6
0.8
SNP effect on Insulin−like growth factor−binding protein 1

Batch 2−1447 : Insulin−like growth factor−binding protein 1 on eGFR

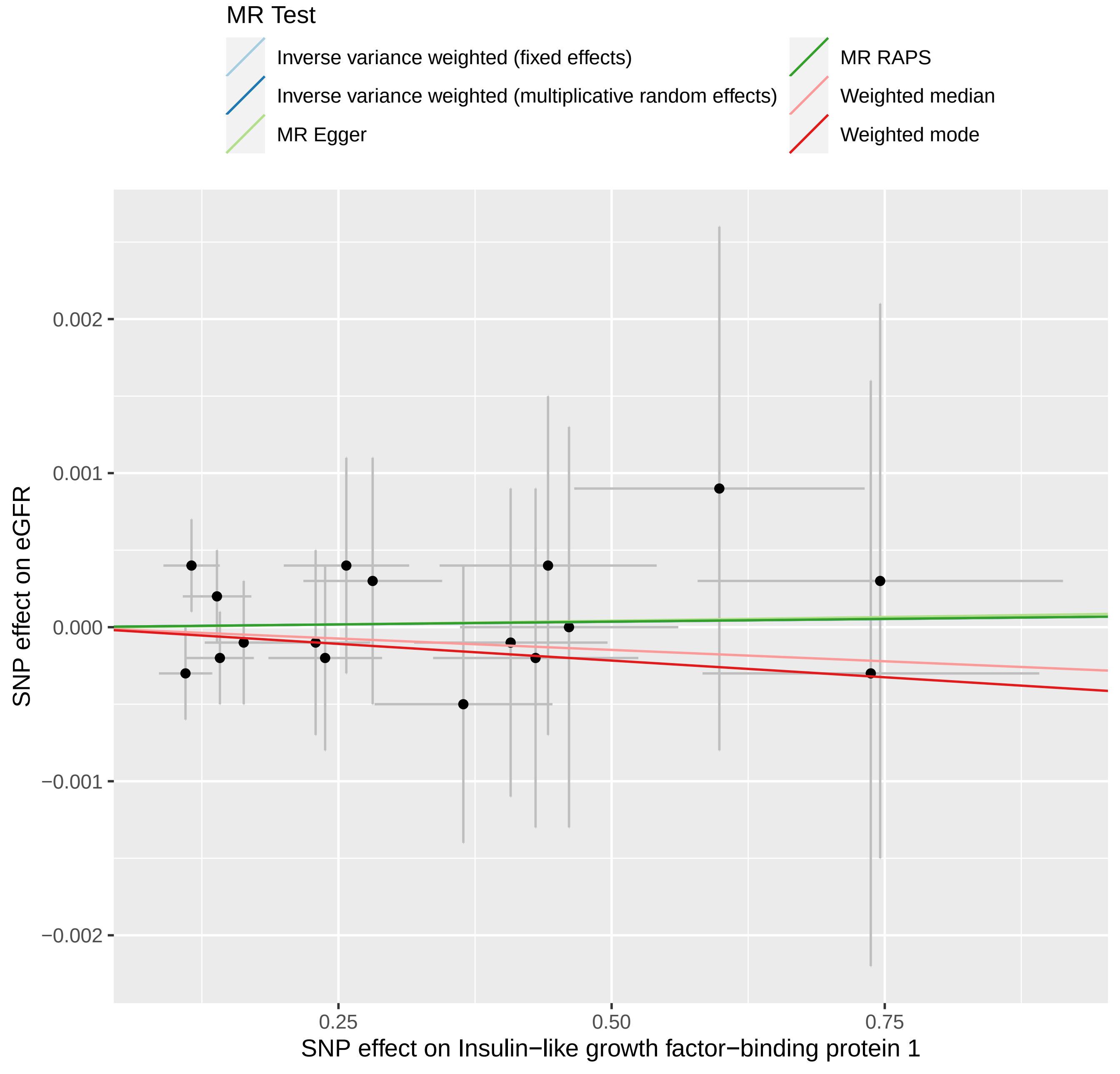

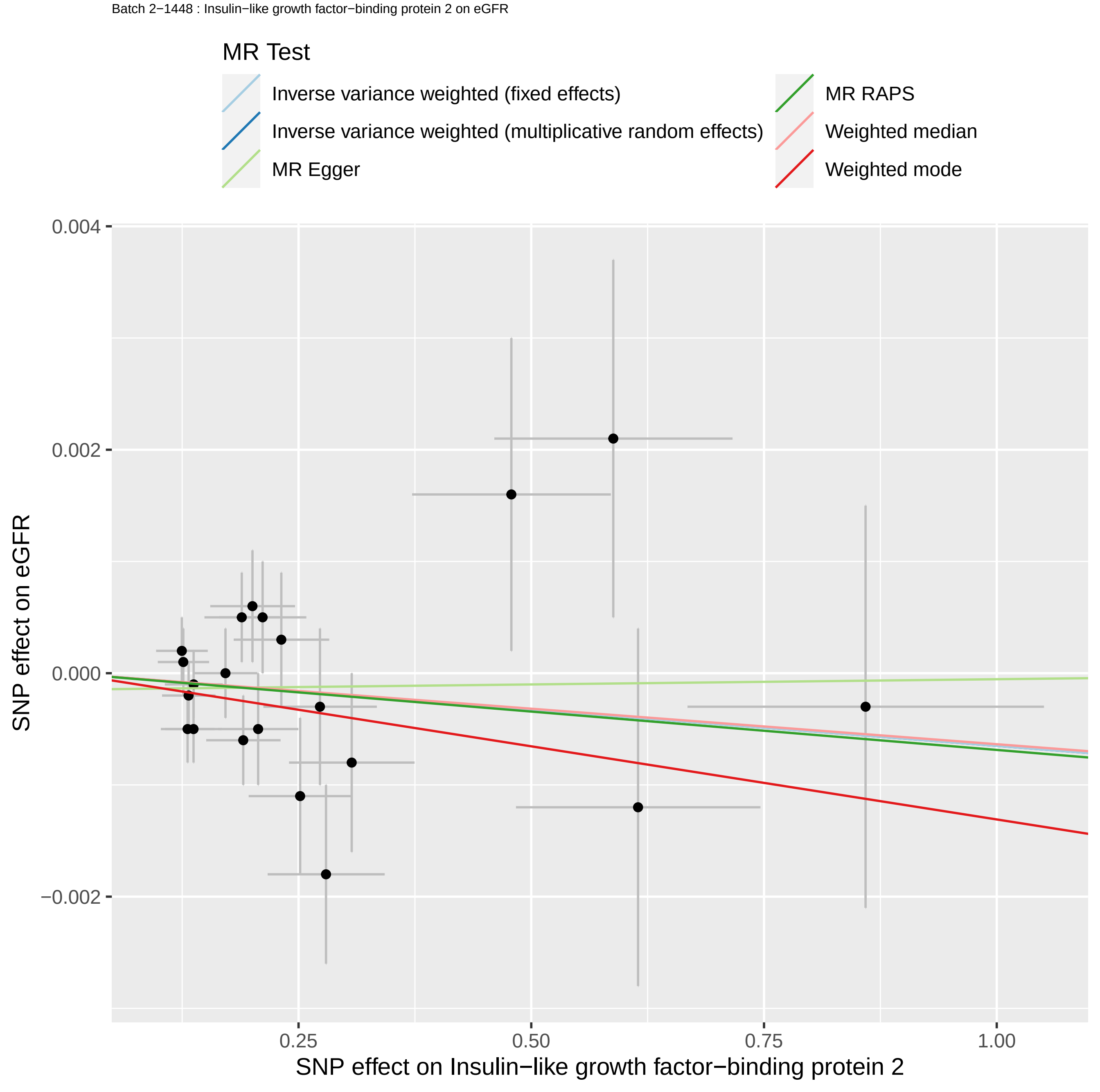

Batch 2−1448 : Insulin−like growth factor−binding protein 2 on eGFR
MR Test
Inverse variance weighted (fixed effects)
Inverse variance weighted (multiplicative random effects)
MR Egger
MR RAPS
Weighted median
Weighted mode
0.004
0.002
0.000
−0.002
SNP effect on eGFR
0.25
0.50
0.75
1.00
SNP effect on Insulin−like growth factor−binding protein 2

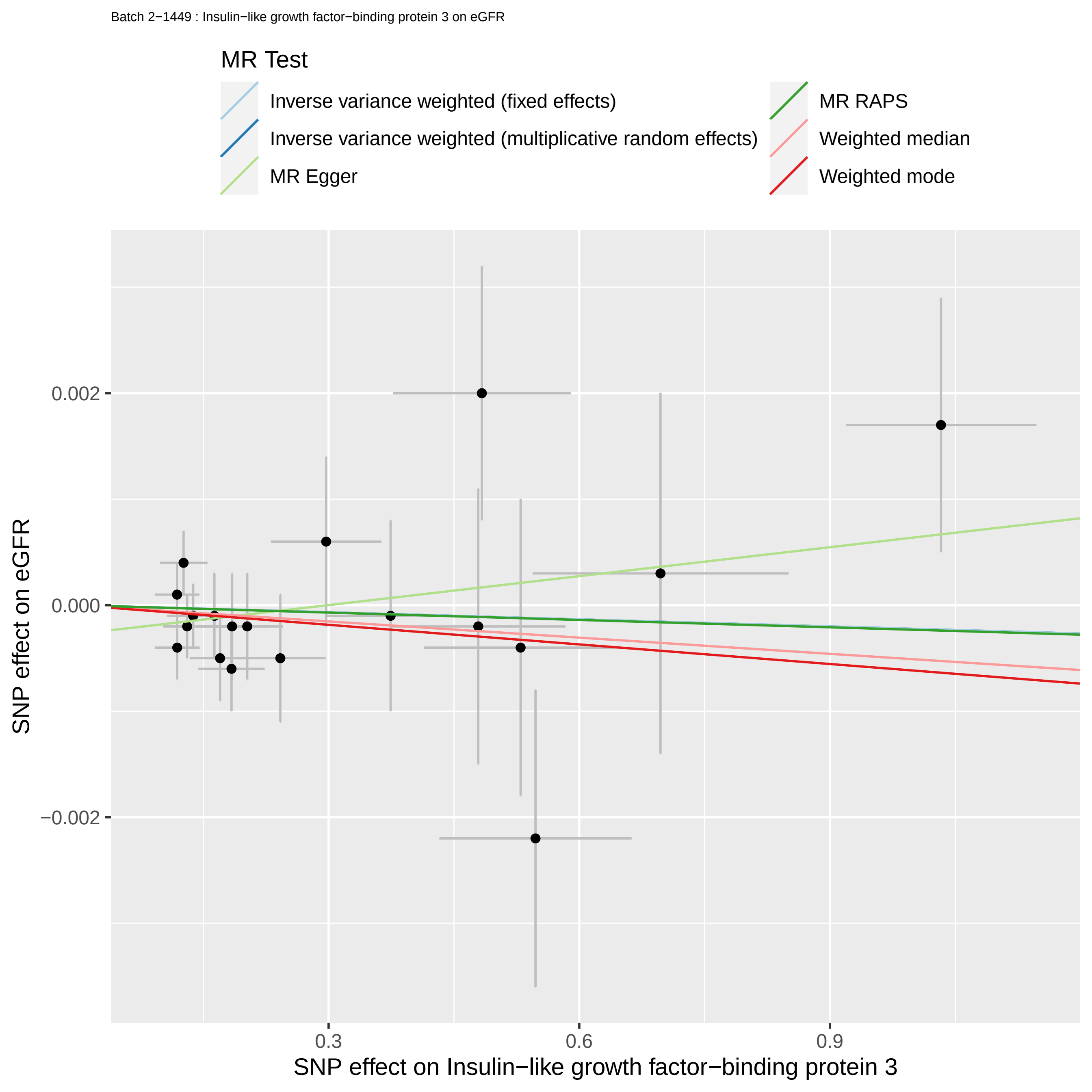
Batch 2−1449 : Insulin−like growth factor−binding protein 3 on eGFR
MR Test
Inverse variance weighted (fixed effects)
Inverse variance weighted (multiplicative random effects)
MR Egger
MR RAPS
Weighted median
Weighted mode
0.002
0.000
−0.002
SNP effect on eGFR
0.3
0.6
0.9
SNP effect on Insulin−like growth factor−binding protein 3

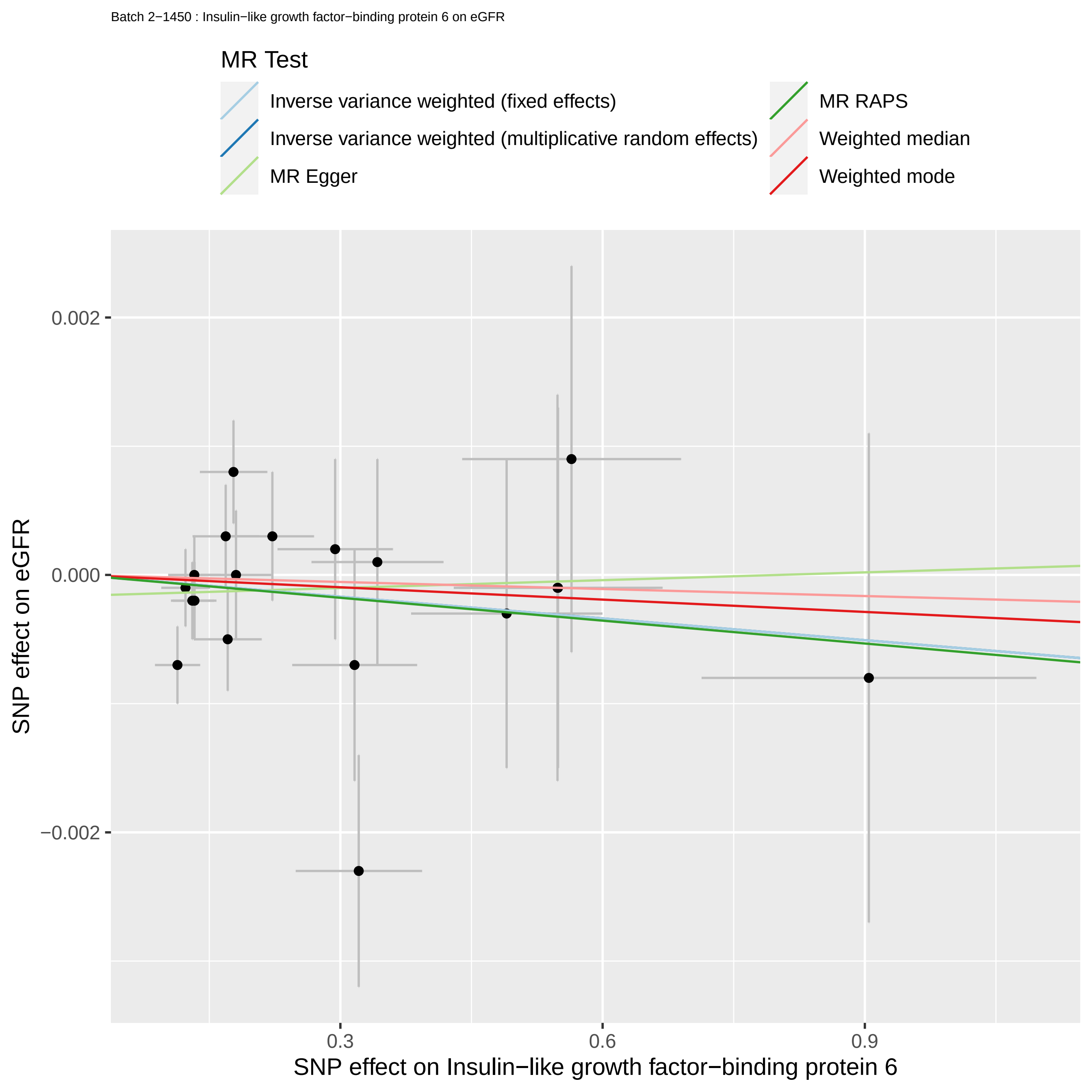
Batch 2−1450 : Insulin−like growth factor−binding protein 6 on eGFR
MR Test
Inverse variance weighted (fixed effects)
Inverse variance weighted (multiplicative random effects)
MR Egger
MR RAPS
Weighted median
Weighted mode
0.002
0.000
−0.002
0.3
0.6
0.9
SNP effect on eGFR
SNP effect on Insulin−like growth factor−binding protein 6

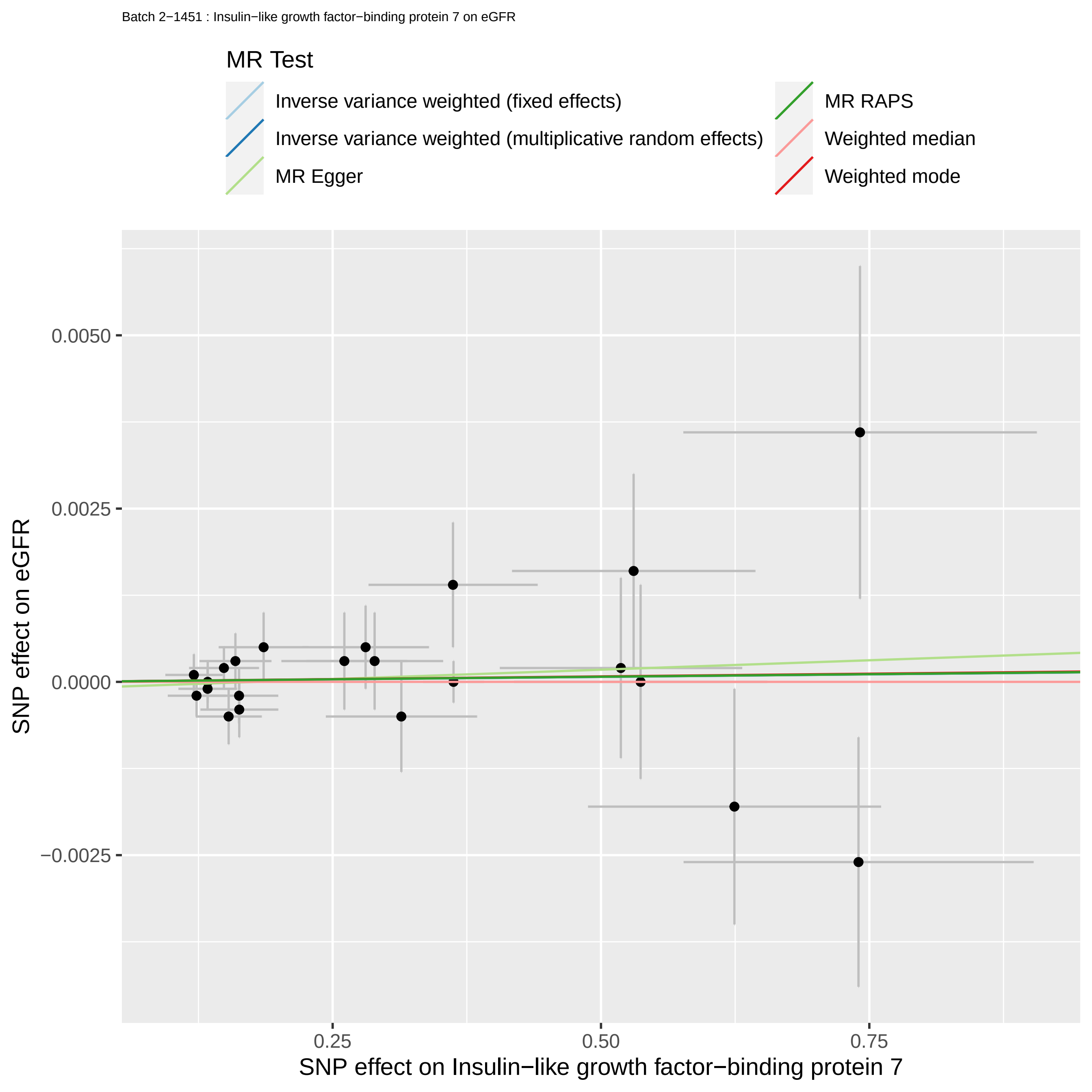

Batch 2−1451 : Insulin−like growth factor−binding protein 7 on eGFR
MR Test
Inverse variance weighted (fixed effects)
Inverse variance weighted (multiplicative random effects)
MR Egger
MR RAPS
Weighted median
Weighted mode
0.0050
0.0025
0.0000
−0.0025
SNP effect on eGFR
0.25
0.50
0.75
SNP effect on Insulin−like growth factor−binding protein 7

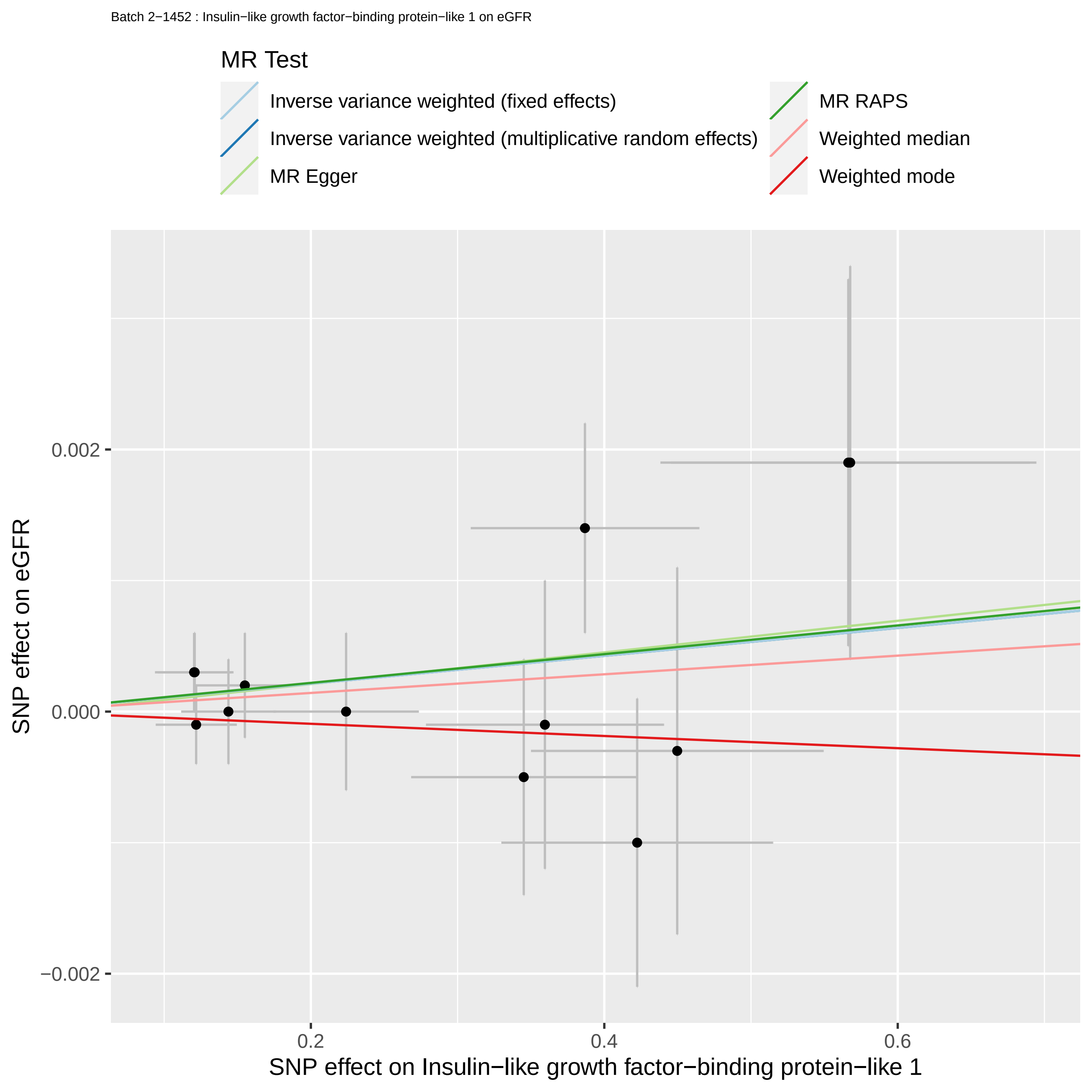
Batch 2−1452 : Insulin−like growth factor−binding protein−like 1 on eGFR
MR Test
Inverse variance weighted (fixed effects)
Inverse variance weighted (multiplicative random effects)
MR Egger
MR RAPS
Weighted median
Weighted mode
0.002
0.000
−0.002
0.2
0.4
0.6
SNP effect on eGFR
SNP effect on Insulin−like growth factor−binding protein−like 1

Batch 2−1453 : Insulin growth factor−like family member 3 on eGFR

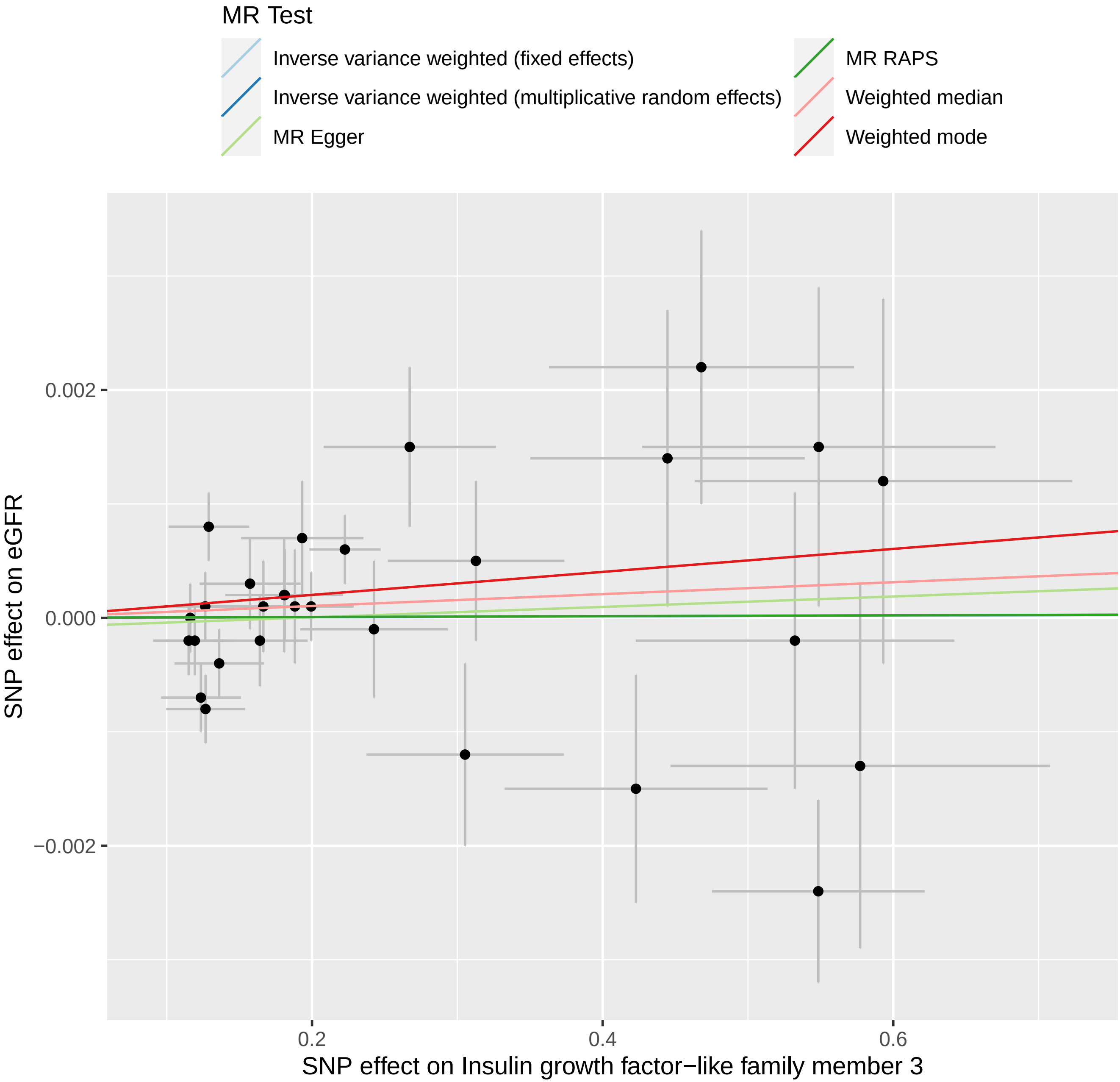

Batch 2−1454 : Insulin growth factor−like family member 4 on eGFR

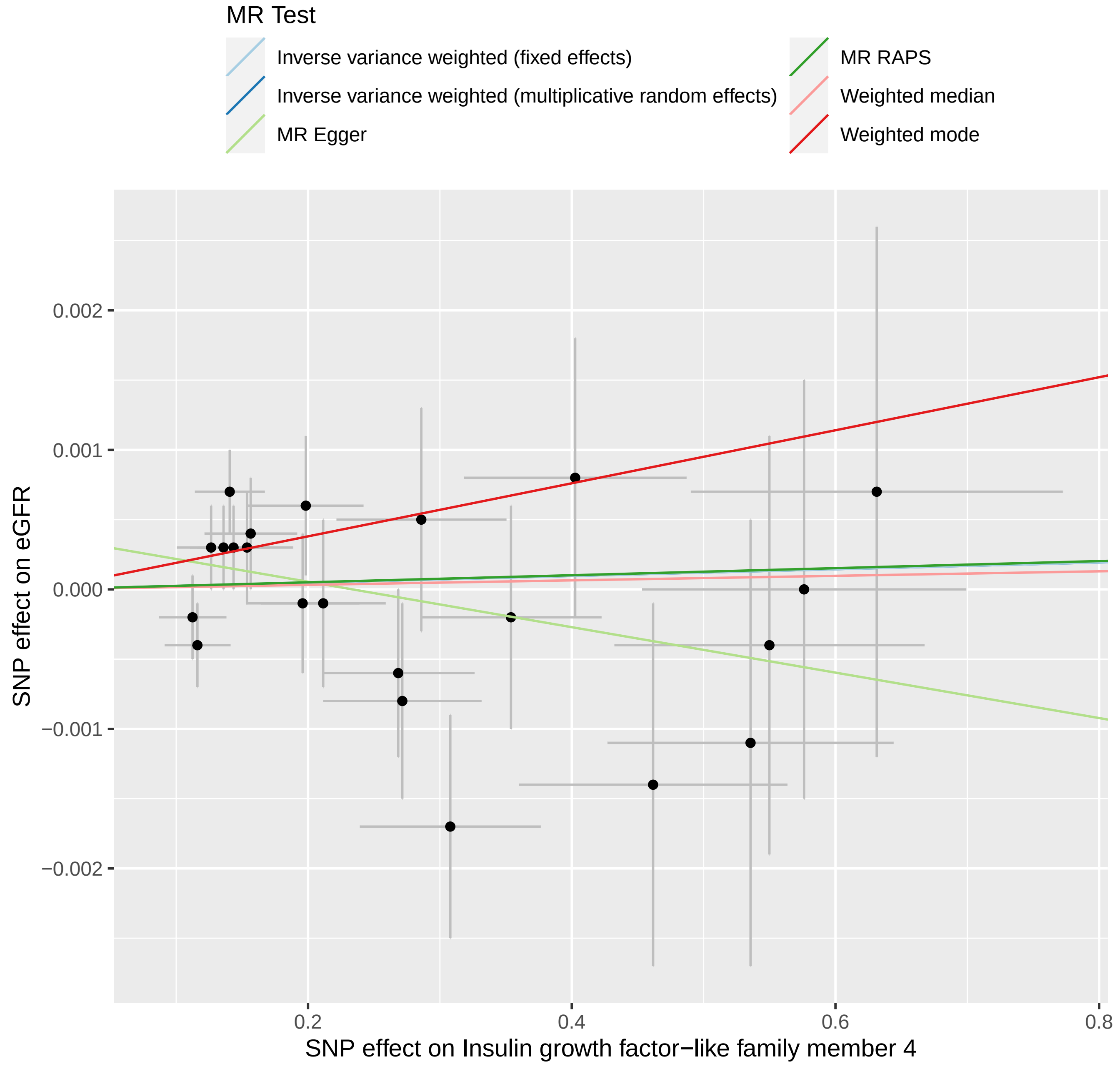

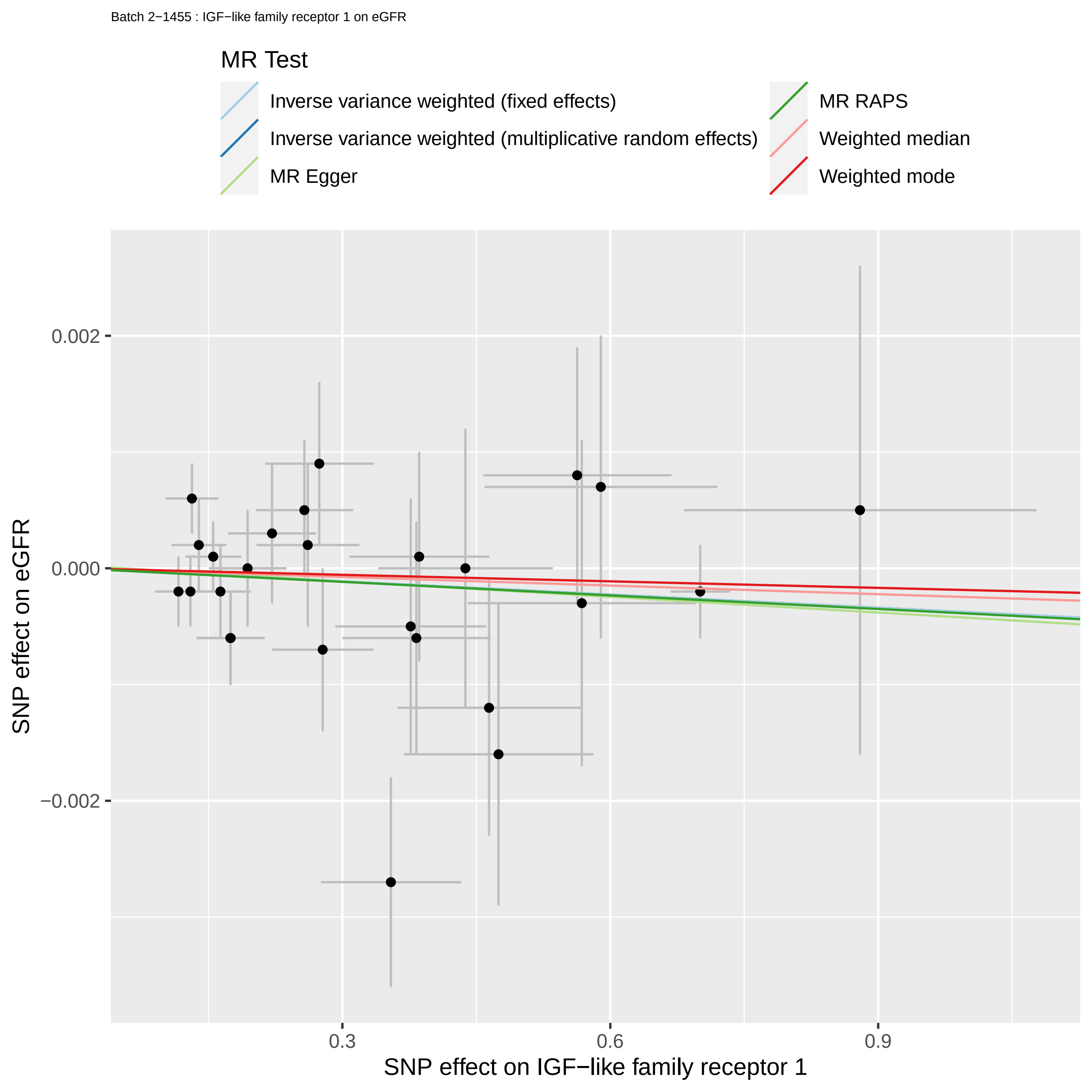

Batch 2−1455 : IGF−like family receptor 1 on eGFR
MR Test
Inverse variance weighted (fixed effects)
Inverse variance weighted (multiplicative random effects)
MR Egger
MR RAPS
Weighted median
Weighted mode
0.002
0.000
−0.002
0.3
0.6
0.9
SNP effect on eGFR
SNP effect on IGF−like family receptor 1

Batch 2−1456 : Immunoglobulin E on eGFR

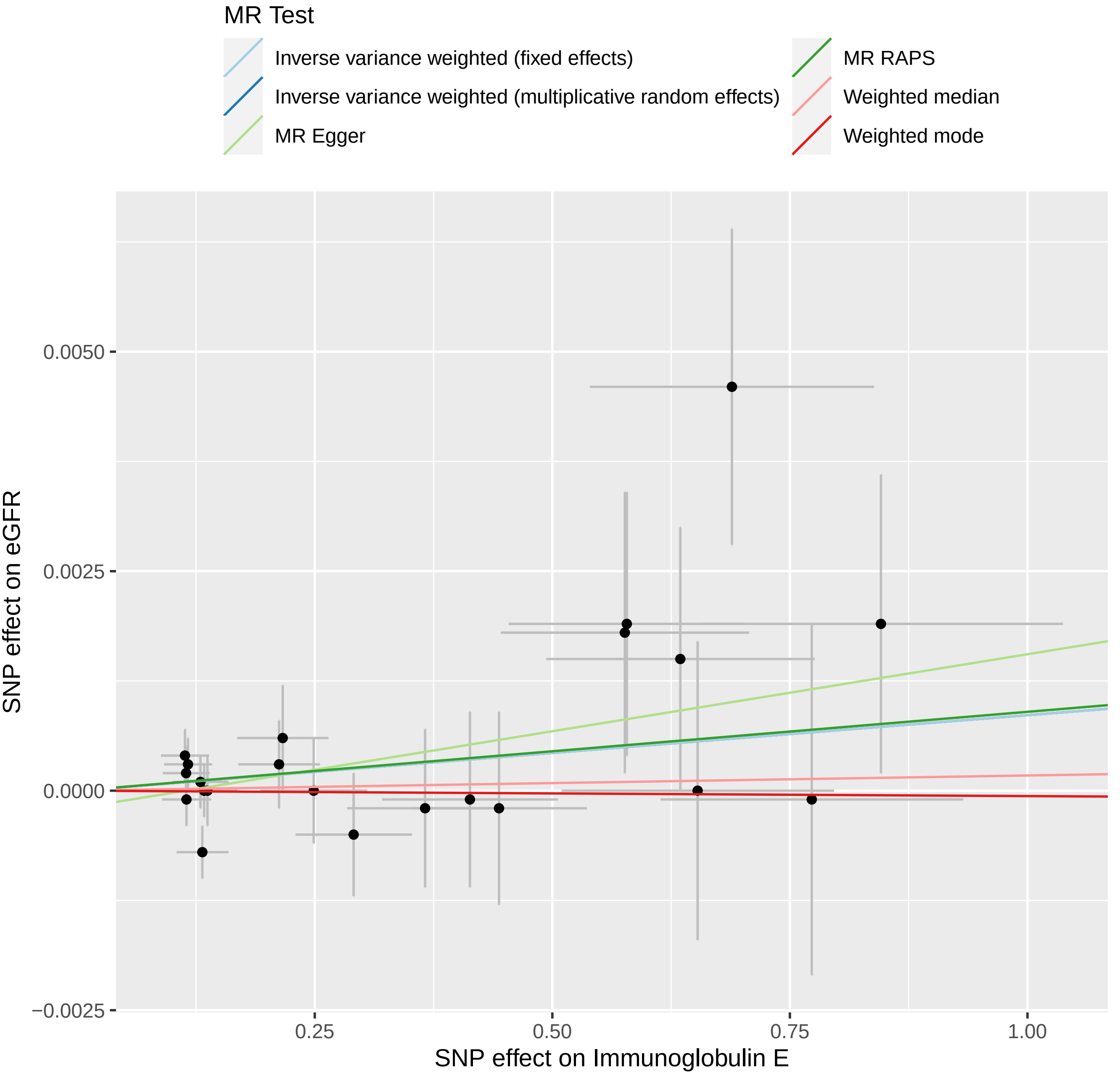

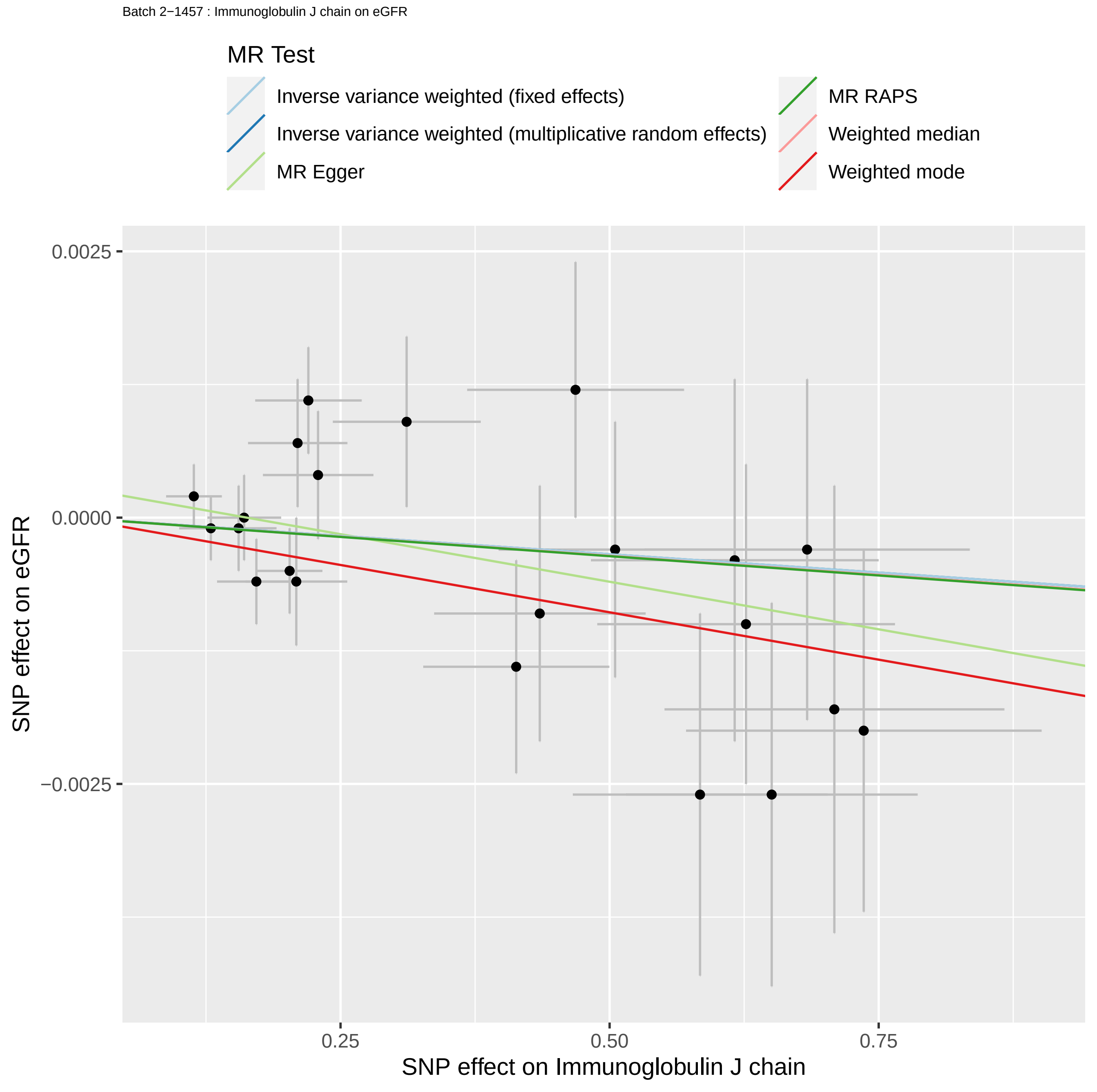

Batch 2−1457 : Immunoglobulin J chain on eGFR
MR Test
Inverse variance weighted (fixed effects)
Inverse variance weighted (multiplicative random effects)
MR Egger
MR RAPS
Weighted median
Weighted mode
0.0025
0.0000
−0.0025
0.25
0.50
0.75
SNP effect on eGFR
SNP effect on Immunoglobulin J chain

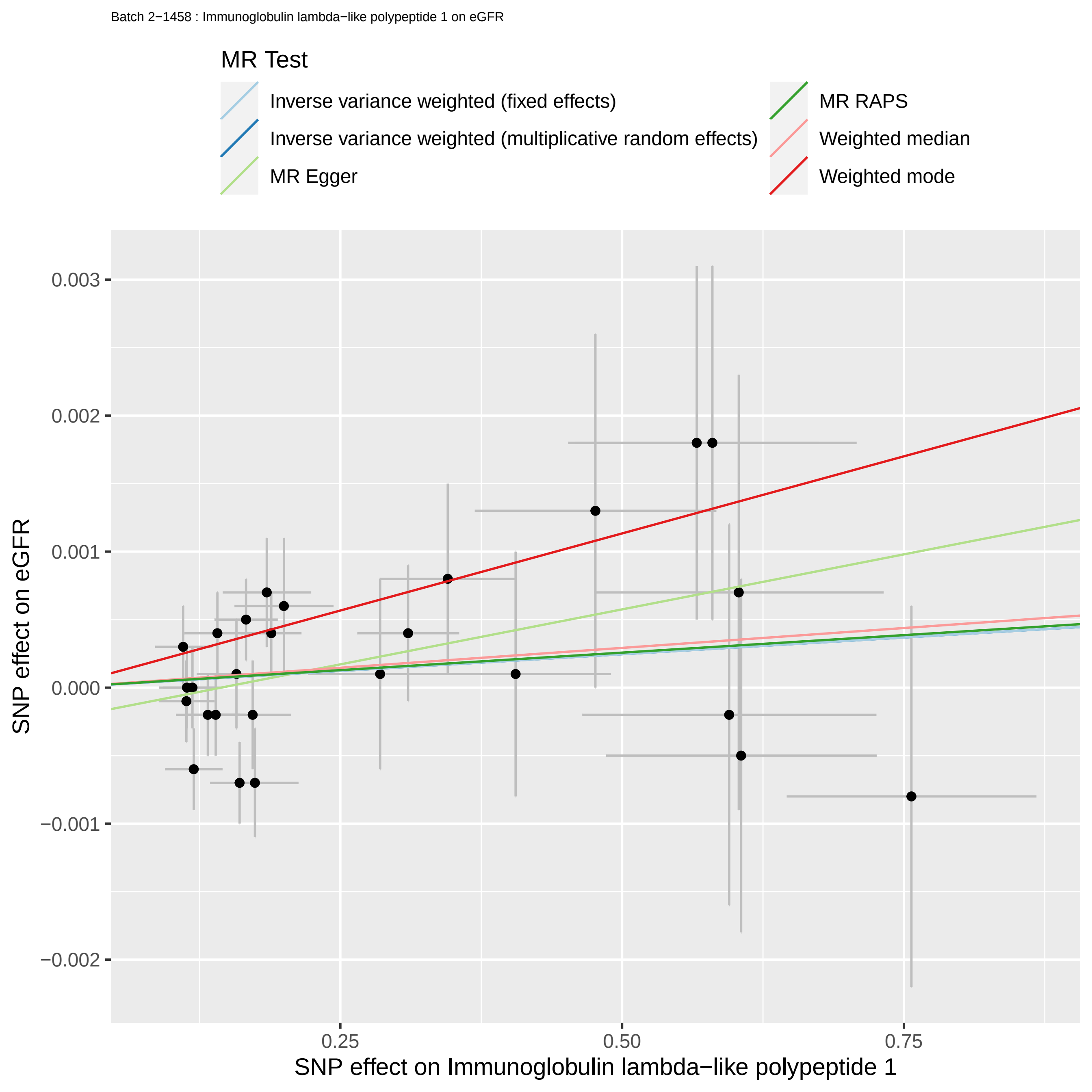

Batch 2−1458 : Immunoglobulin lambda−like polypeptide 1 on eGFR
MR Test
Inverse variance weighted (fixed effects)
Inverse variance weighted (multiplicative random effects)
MR Egger
MR RAPS
Weighted median
Weighted mode
0.003
0.002
0.001
0.000
−0.001
−0.002
0.25
0.50
0.75
SNP effect on eGFR
SNP effect on Immunoglobulin lambda−like polypeptide 1

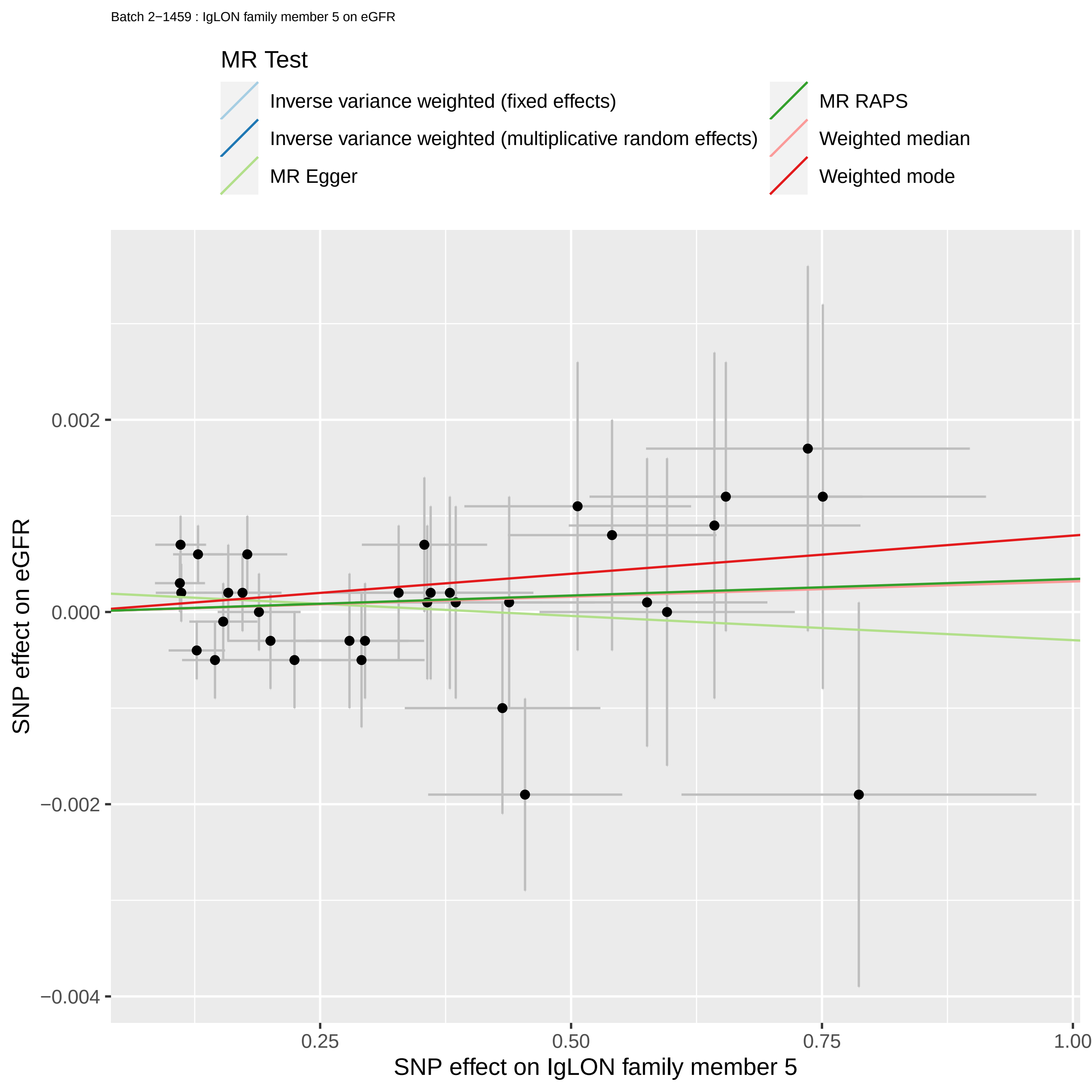
Batch 2−1459 : IgLON family member 5 on eGFR
MR Test
Inverse variance weighted (fixed effects)
Inverse variance weighted (multiplicative random effects)
MR Egger
MR RAPS
Weighted median
Weighted mode
0.002
0.000
−0.002
−0.004
SNP effect on eGFR
0.25
0.50
0.75
1.00
SNP effect on IgLON family member 5

Batch 2−1460 : Immunoglobulin superfamily member 11 on eGFR

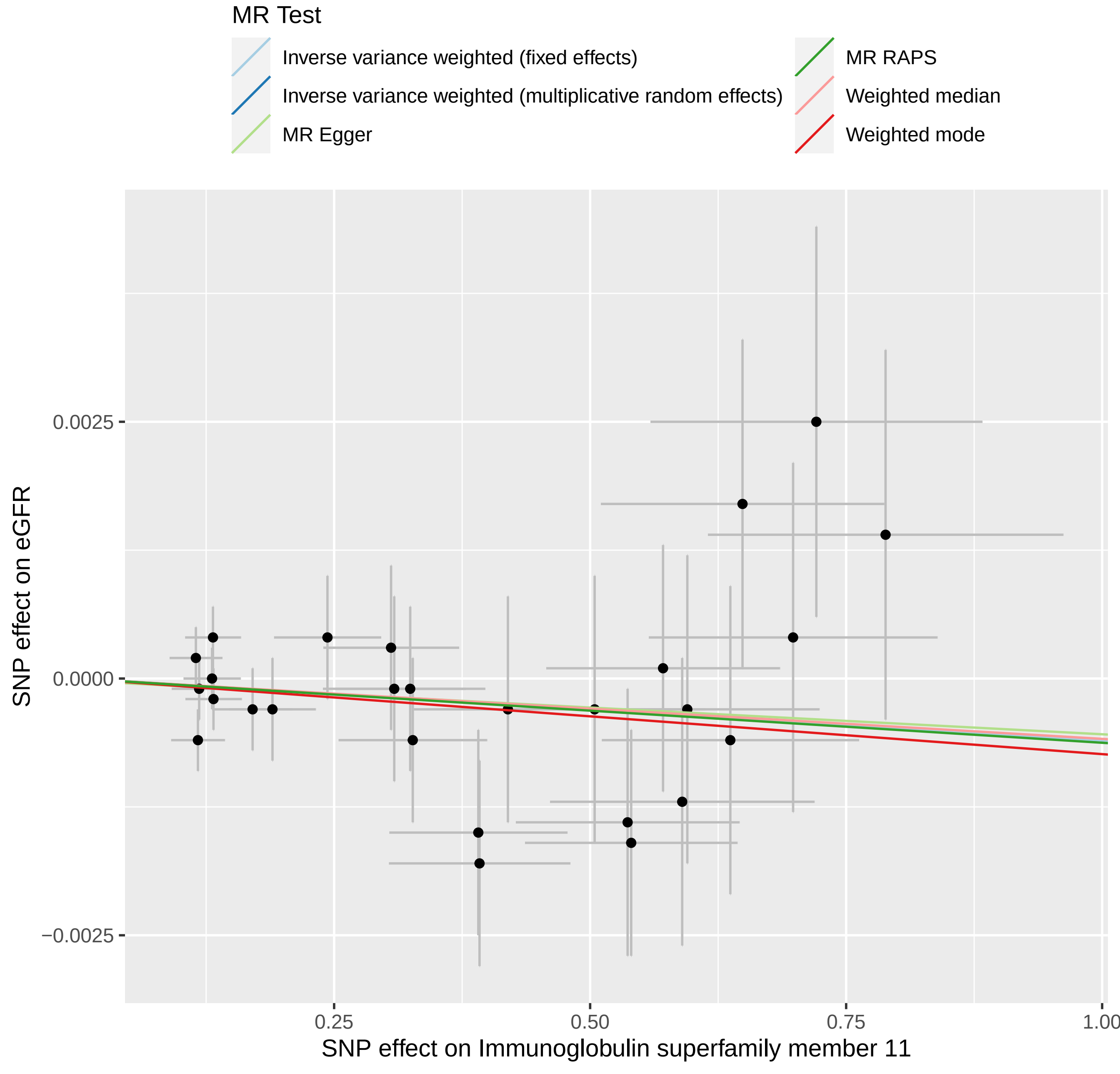

Batch 2−1461 : Immunoglobulin superfamily member 11 on eGFR

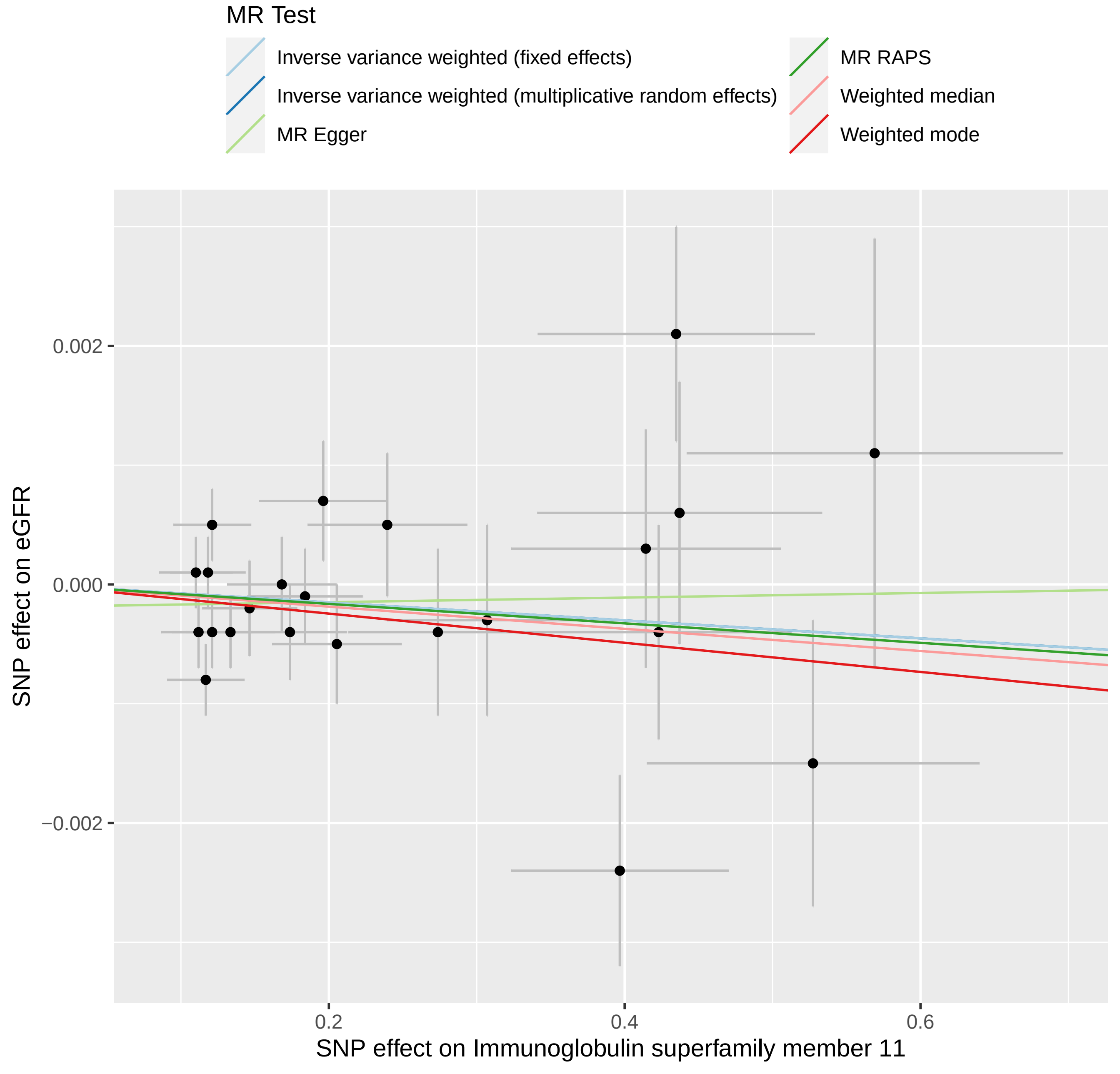

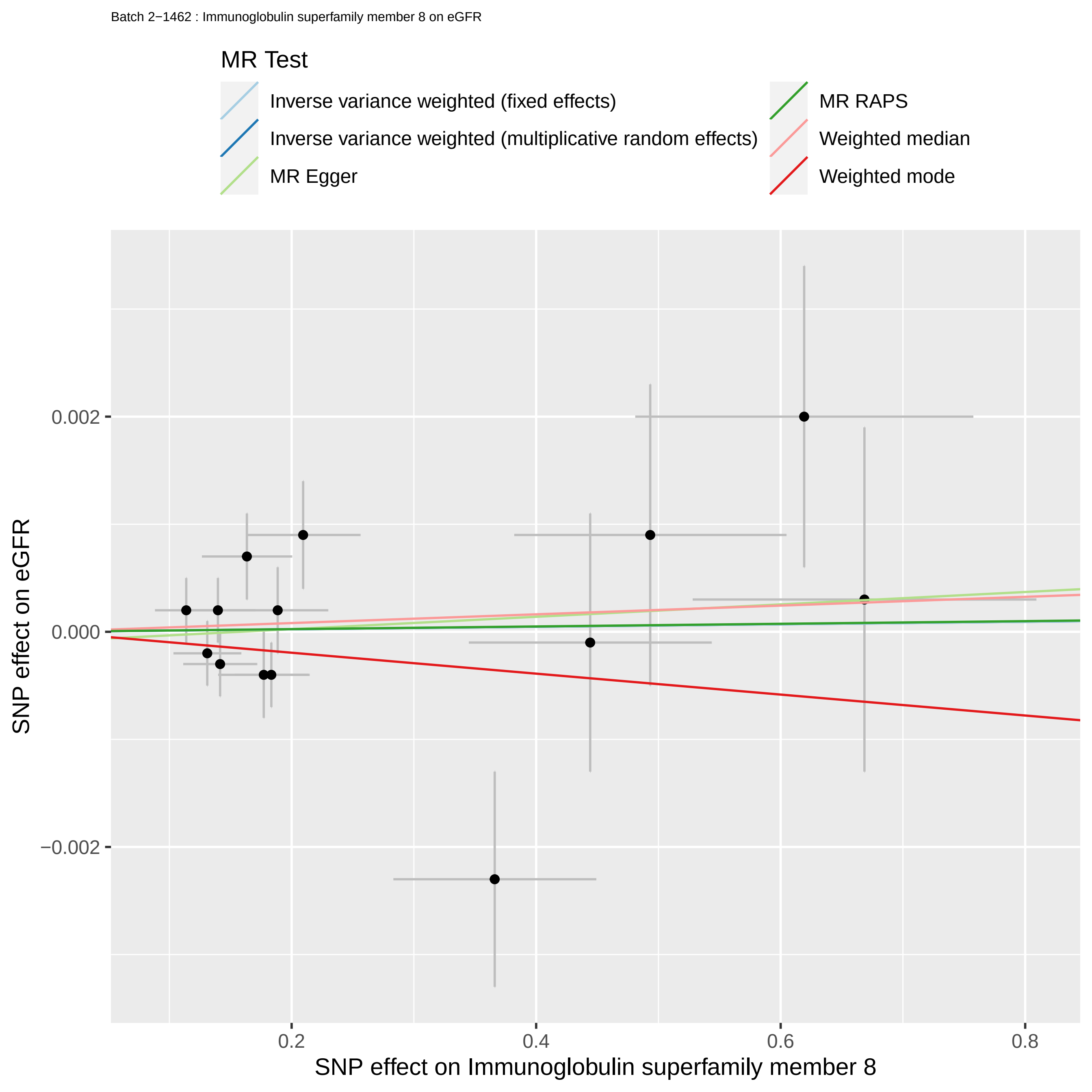
Batch 2−1462 : Immunoglobulin superfamily member 8 on eGFR
MR Test
Inverse variance weighted (fixed effects)
Inverse variance weighted (multiplicative random effects)
MR Egger
MR RAPS
Weighted median
Weighted mode
0.002
0.000
−0.002
0.2
0.4
0.6
0.8
SNP effect on eGFR
SNP effect on Immunoglobulin superfamily member 8

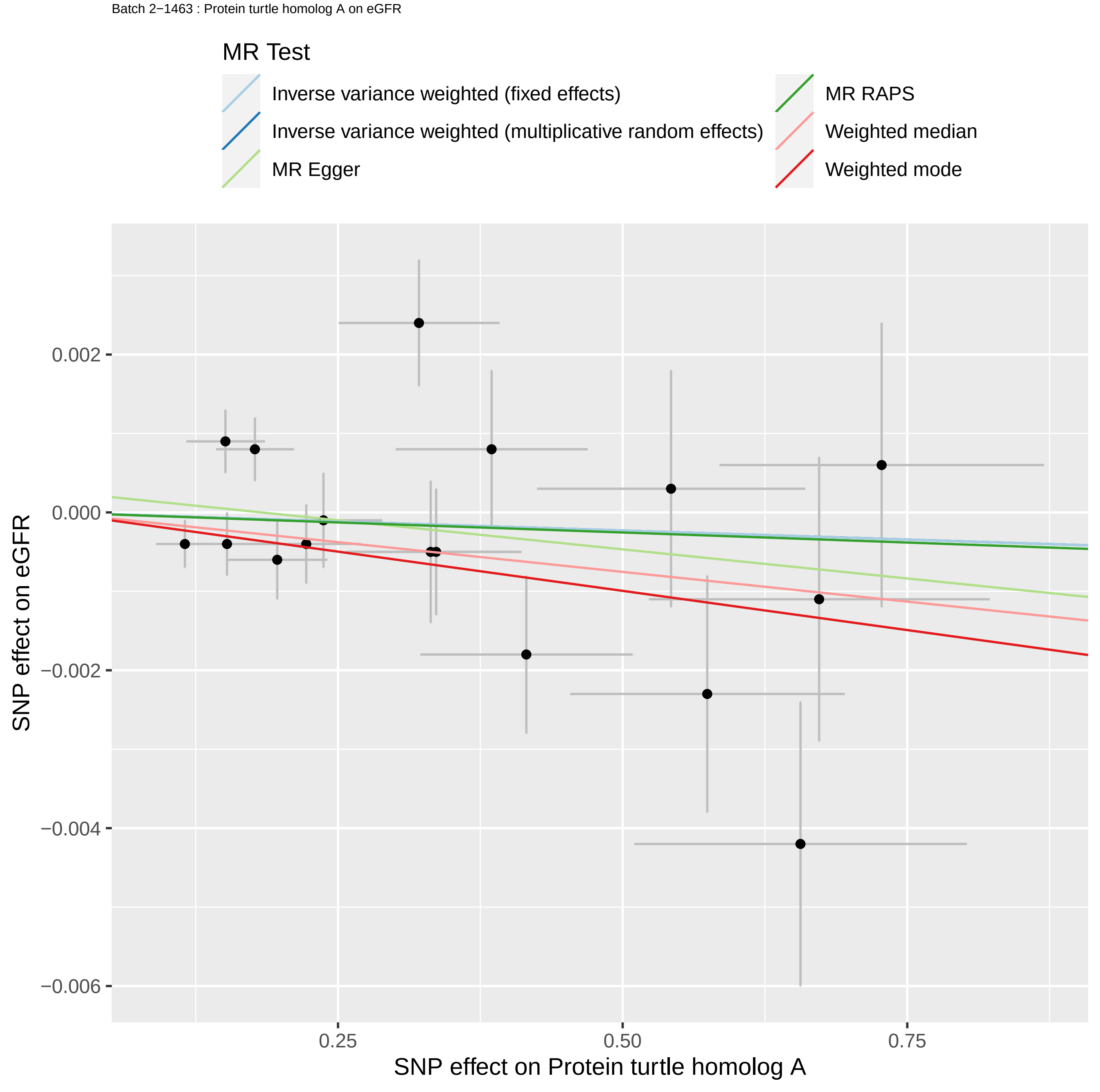

Batch 2−1463 : Protein turtle homolog A on eGFR
MR Test
Inverse variance weighted (fixed effects)
Inverse variance weighted (multiplicative random effects)
MR Egger
MR RAPS
Weighted median
Weighted mode
0.002
0.000
−0.002
−0.004
−0.006
0.25
0.50
0.75
SNP effect on eGFR
SNP effect on Protein turtle homolog A

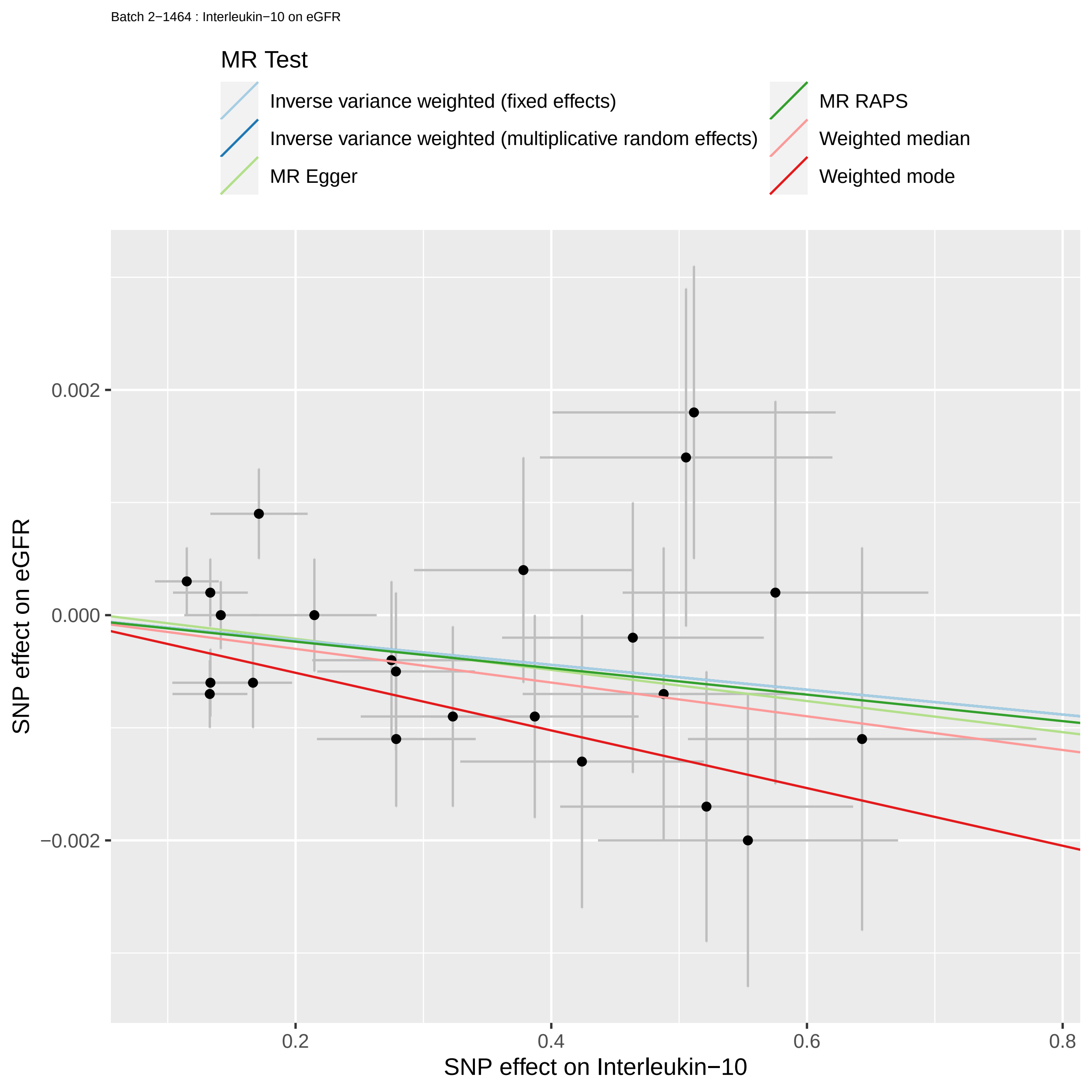

Batch 2−1464 : Interleukin−10 on eGFR
MR Test
Inverse variance weighted (fixed effects)
Inverse variance weighted (multiplicative random effects)
MR Egger
MR RAPS
Weighted median
Weighted mode
0.002
0.000
−0.002
0.2
0.4
0.6
0.8
SNP effect on eGFR
SNP effect on Interleukin−10

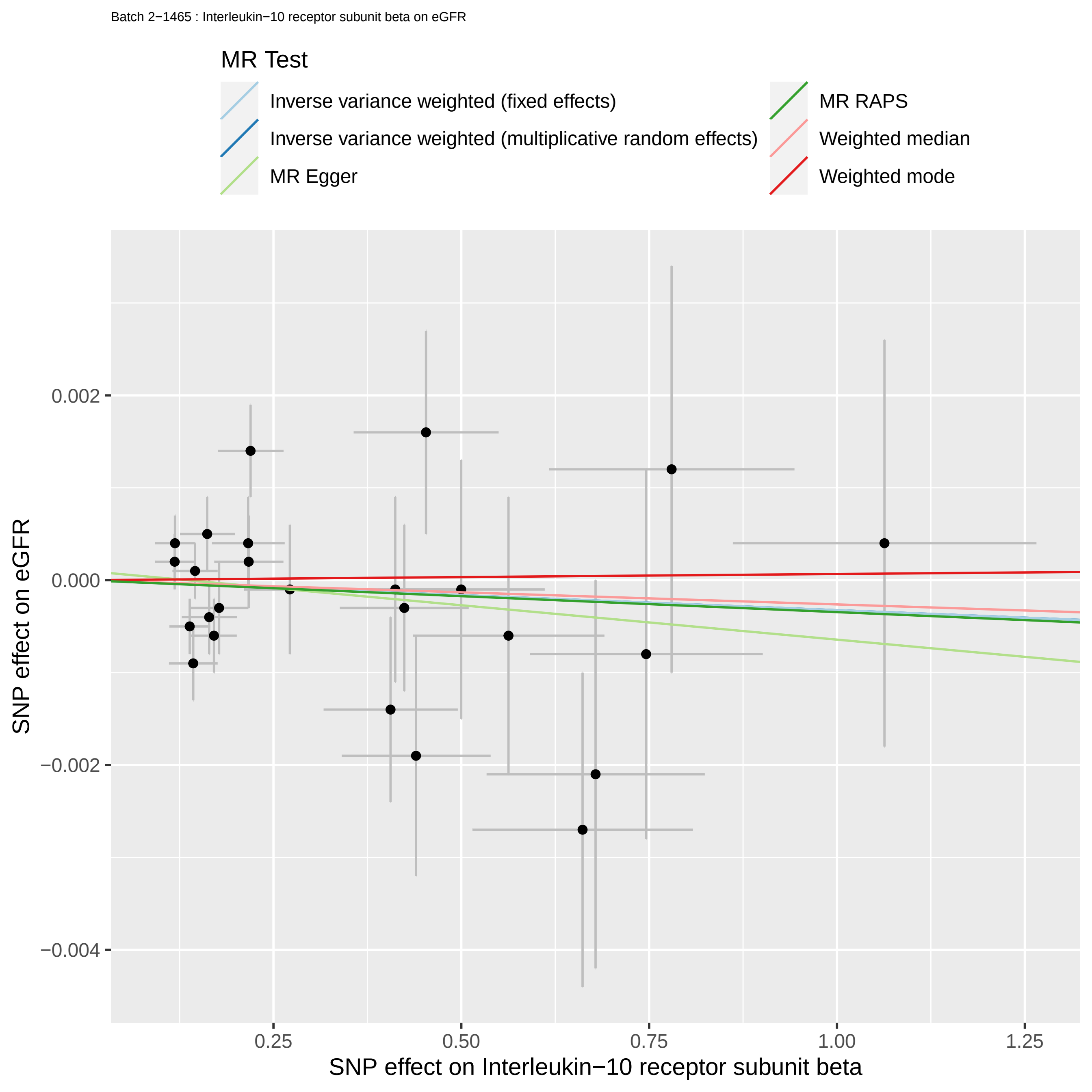

Batch 2−1465 : Interleukin−10 receptor subunit beta on eGFR
MR Test
Inverse variance weighted (fixed effects)
Inverse variance weighted (multiplicative random effects)
MR Egger
MR RAPS
Weighted median
Weighted mode
0.002
0.000
−0.002
−0.004
0.25
0.50
0.75
1.00
1.25
SNP effect on eGFR
SNP effect on Interleukin−10 receptor subunit beta

Batch 2−1466 : Interleukin−11 on eGFR

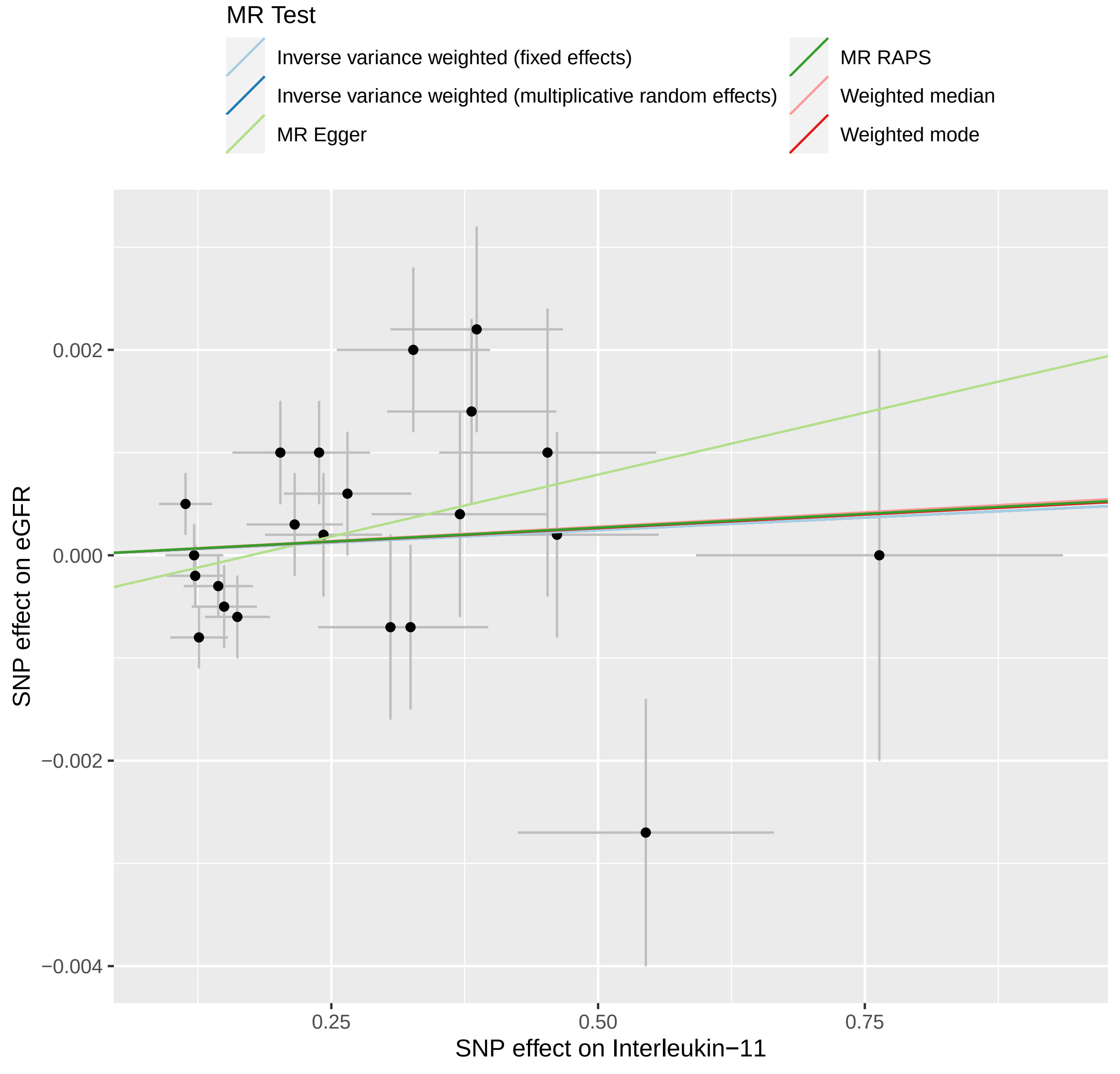

Batch 2−1467 : Interleukin−11 receptor subunit alpha on eGFR

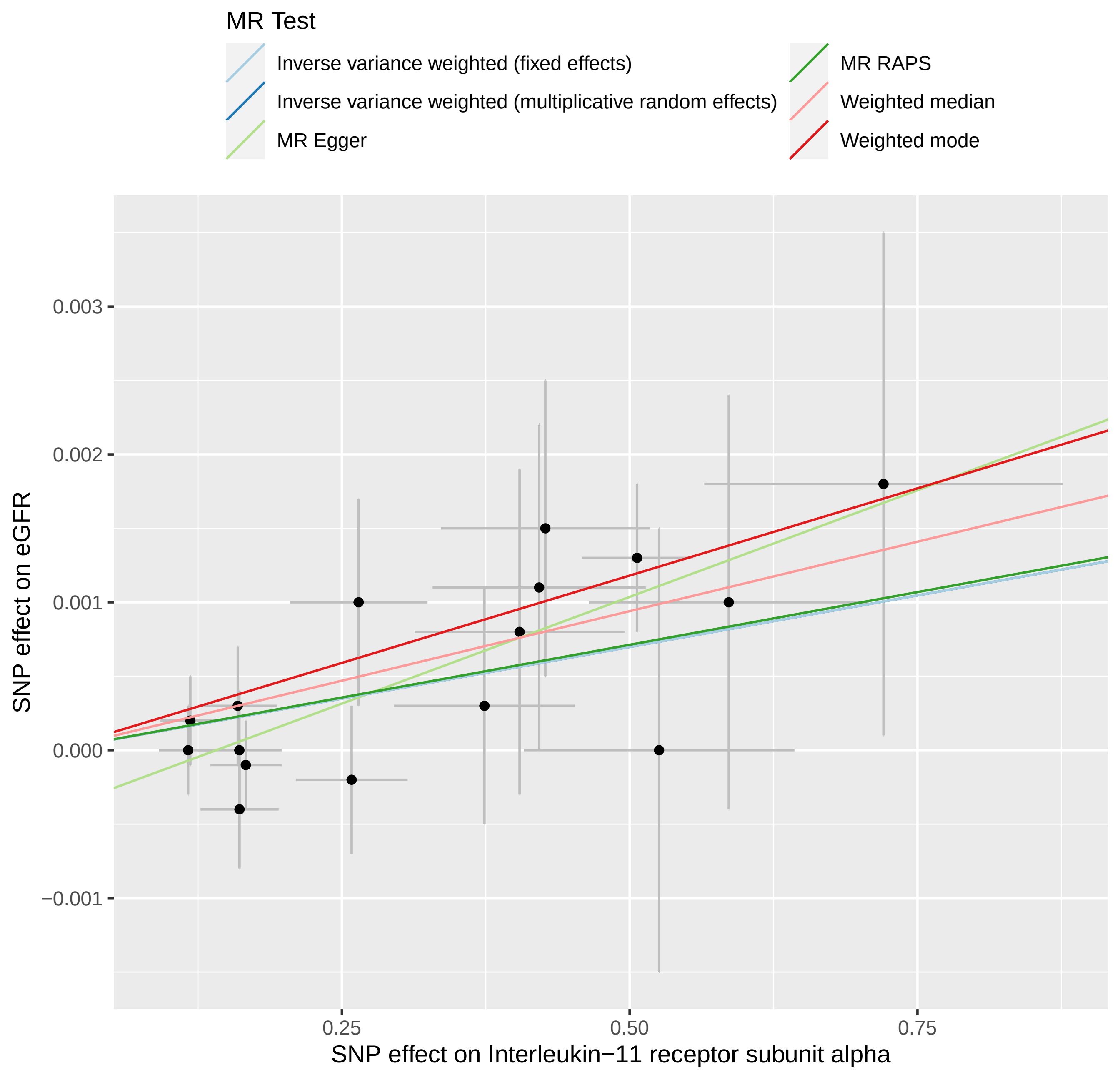

Batch 2−1468 : Interleukin−12 on eGFR

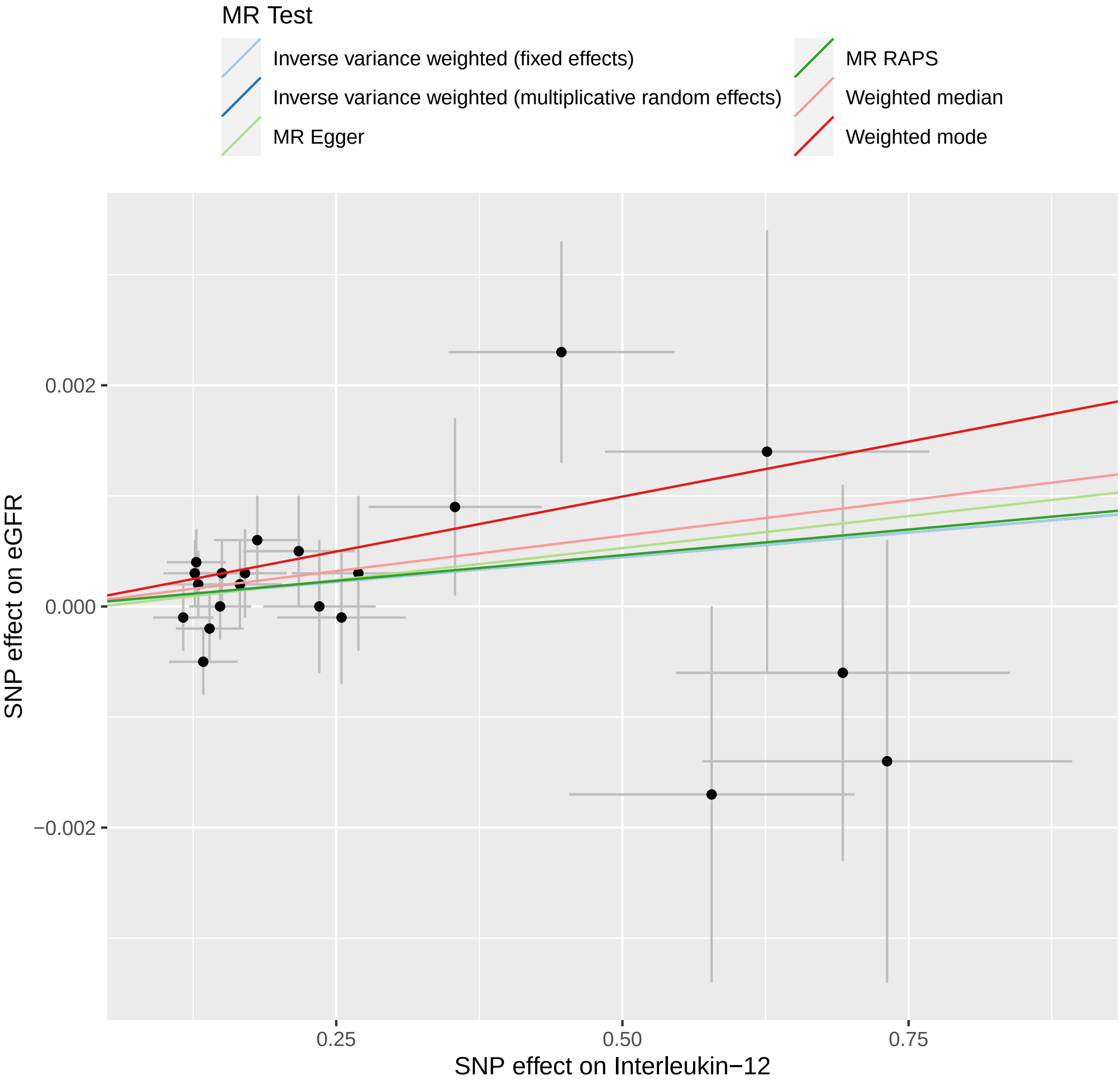

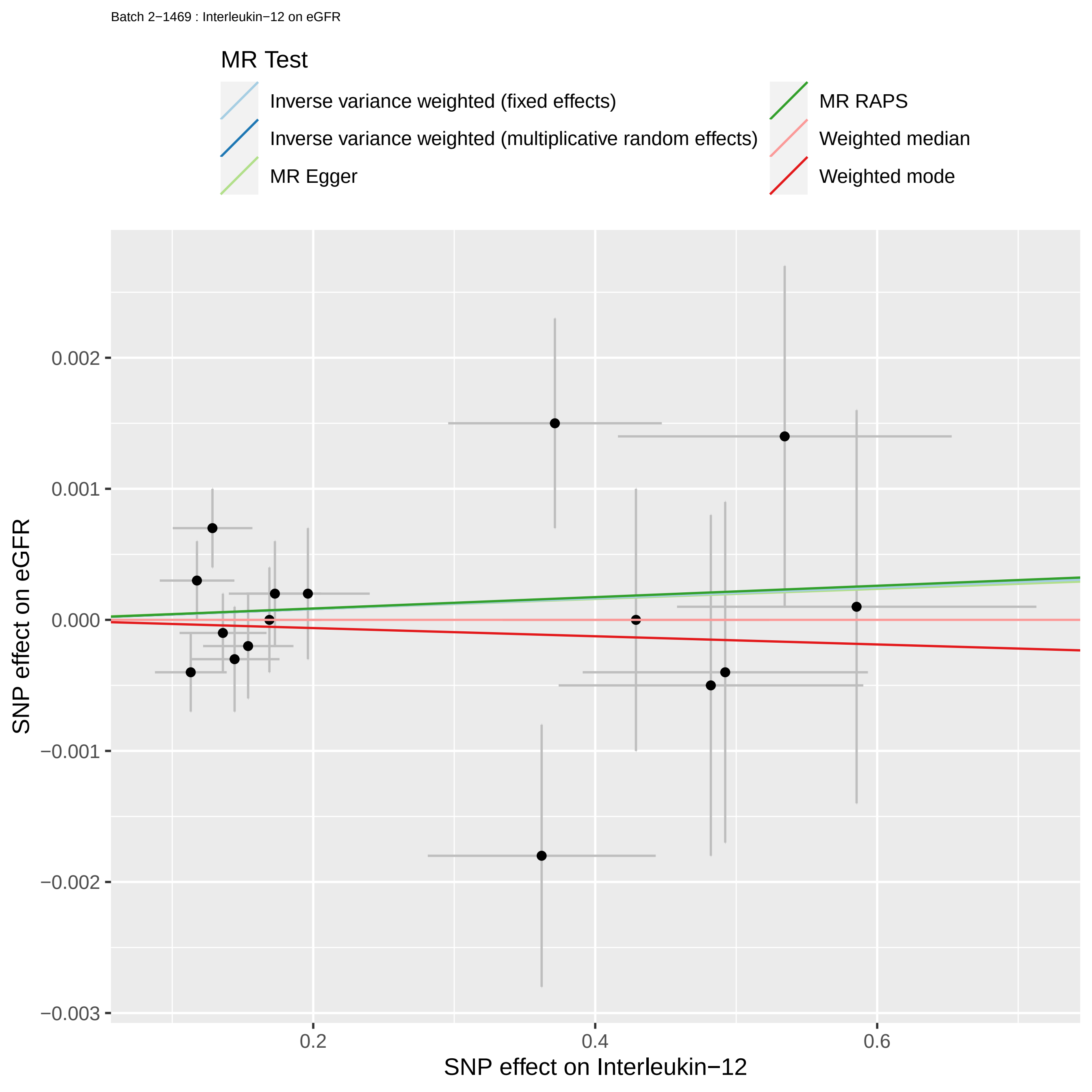

Batch 2−1469 : Interleukin−12 on eGFR
MR Test
Inverse variance weighted (fixed effects)
Inverse variance weighted (multiplicative random effects)
MR Egger
MR RAPS
Weighted median
Weighted mode
0.002
0.001
0.000
−0.001
−0.002
−0.003
SNP effect on eGFR
0.2
0.4
0.6
SNP effect on Interleukin−12

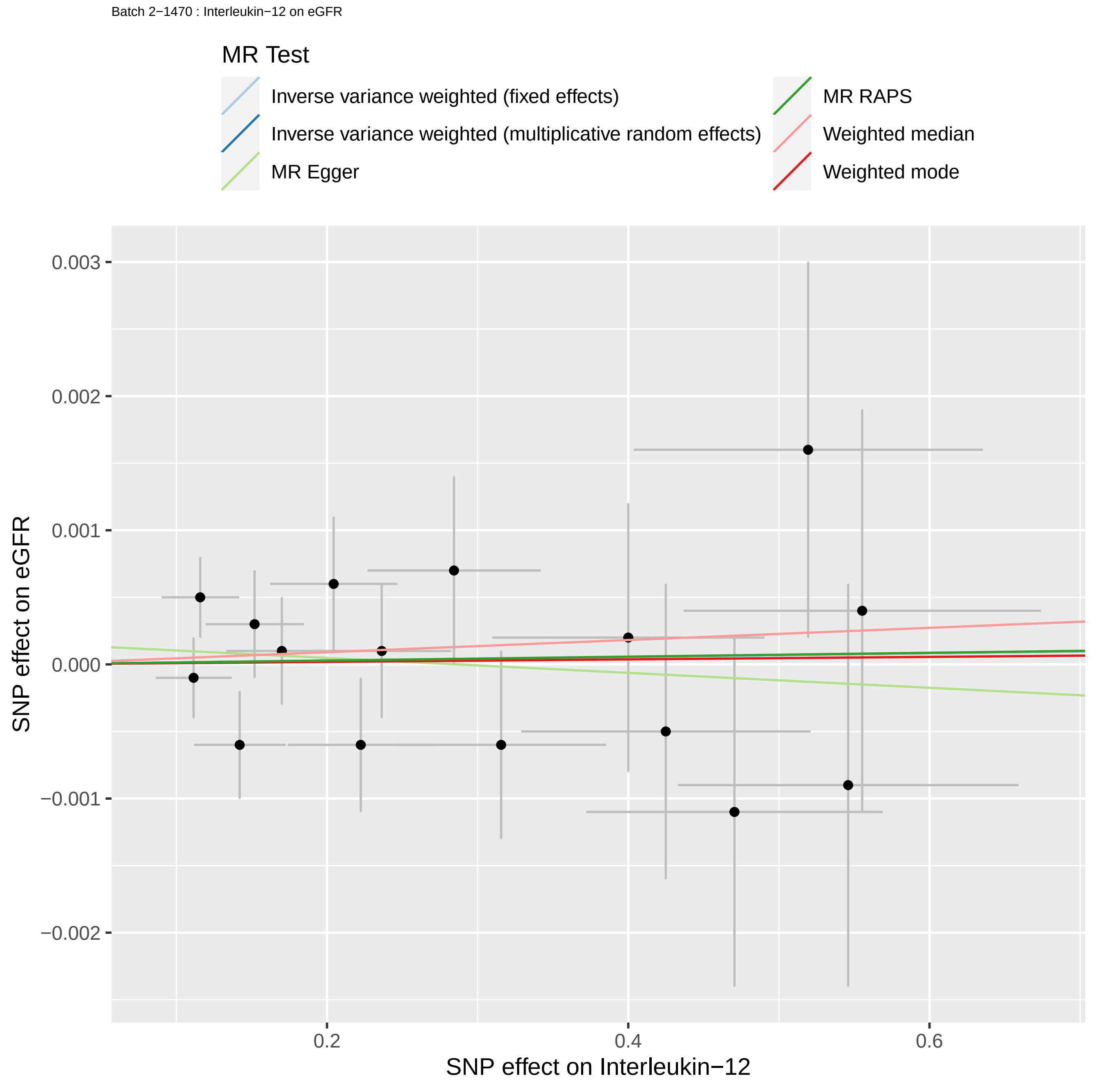
Batch 2−1470 : Interleukin−12 on eGFR
MR Test
Inverse variance weighted (fixed effects)
Inverse variance weighted (multiplicative random effects)
MR Egger
MR RAPS
Weighted median
Weighted mode
0.003
0.002
0.001
0.000
−0.001
−0.002
SNP effect on eGFR
0.2
0.4
0.6
SNP effect on Interleukin−12

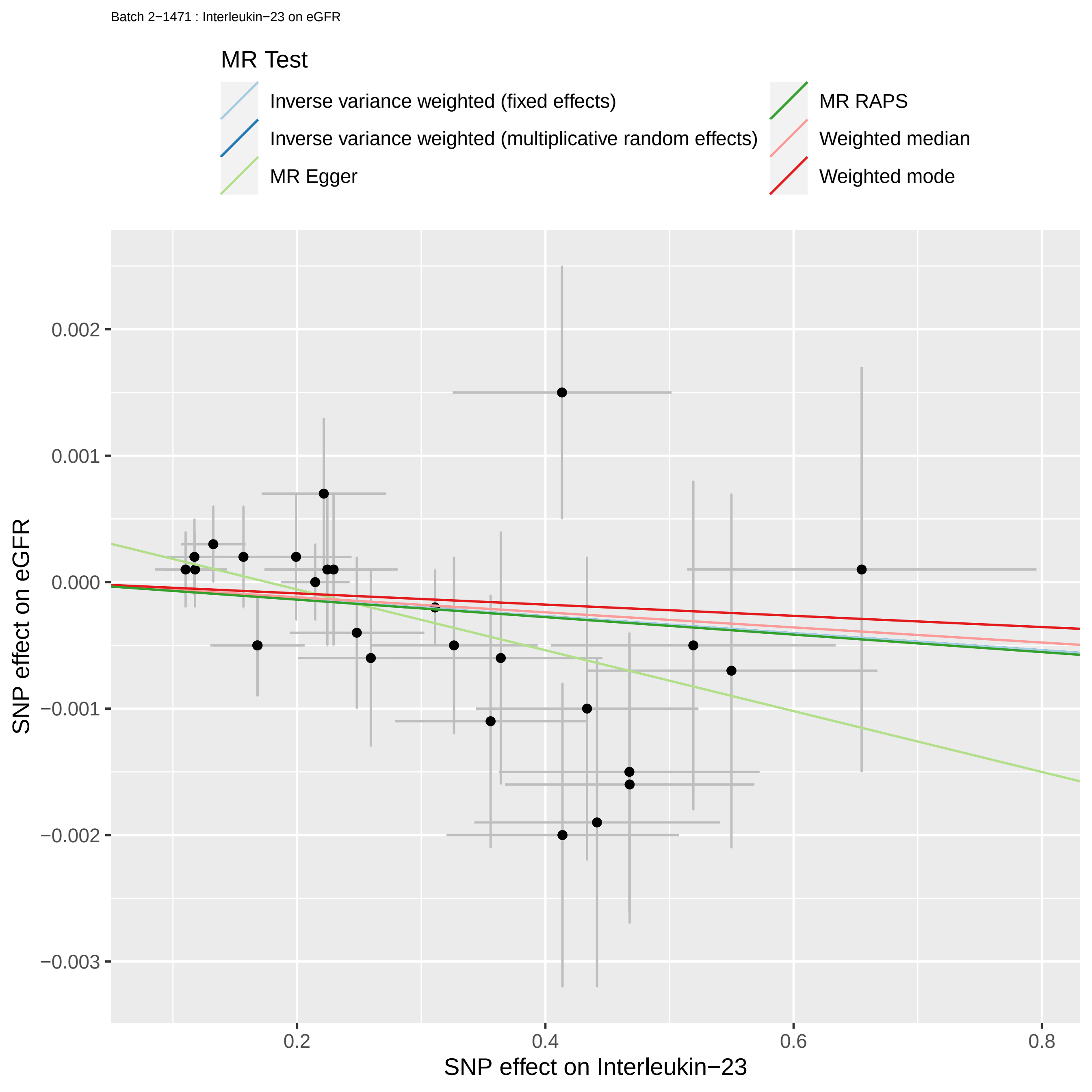

Batch 2−1471 : Interleukin−23 on eGFR
MR Test
Inverse variance weighted (fixed effects)
Inverse variance weighted (multiplicative random effects)
MR Egger
MR RAPS
Weighted median
Weighted mode
0.002
0.001
0.000
−0.001
−0.002
−0.003
SNP effect on eGFR
0.2
0.4
0.6
0.8
SNP effect on Interleukin−23

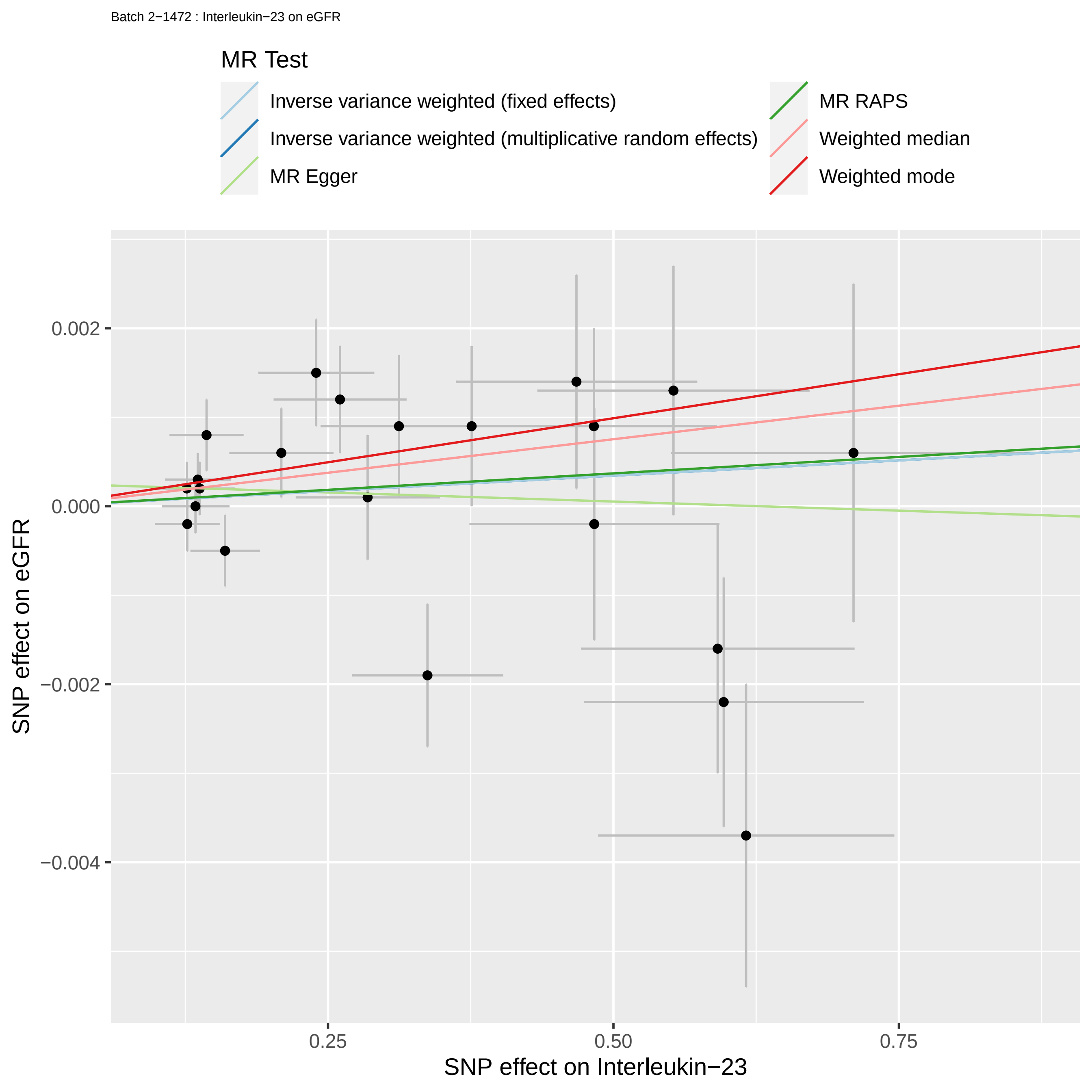
Batch 2−1472 : Interleukin−23 on eGFR
MR Test
Inverse variance weighted (fixed effects)
Inverse variance weighted (multiplicative random effects)
MR Egger
MR RAPS
Weighted median
Weighted mode
0.002
0.000
−0.002
−0.004
0.25
0.50
0.75
SNP effect on eGFR
SNP effect on Interleukin−23

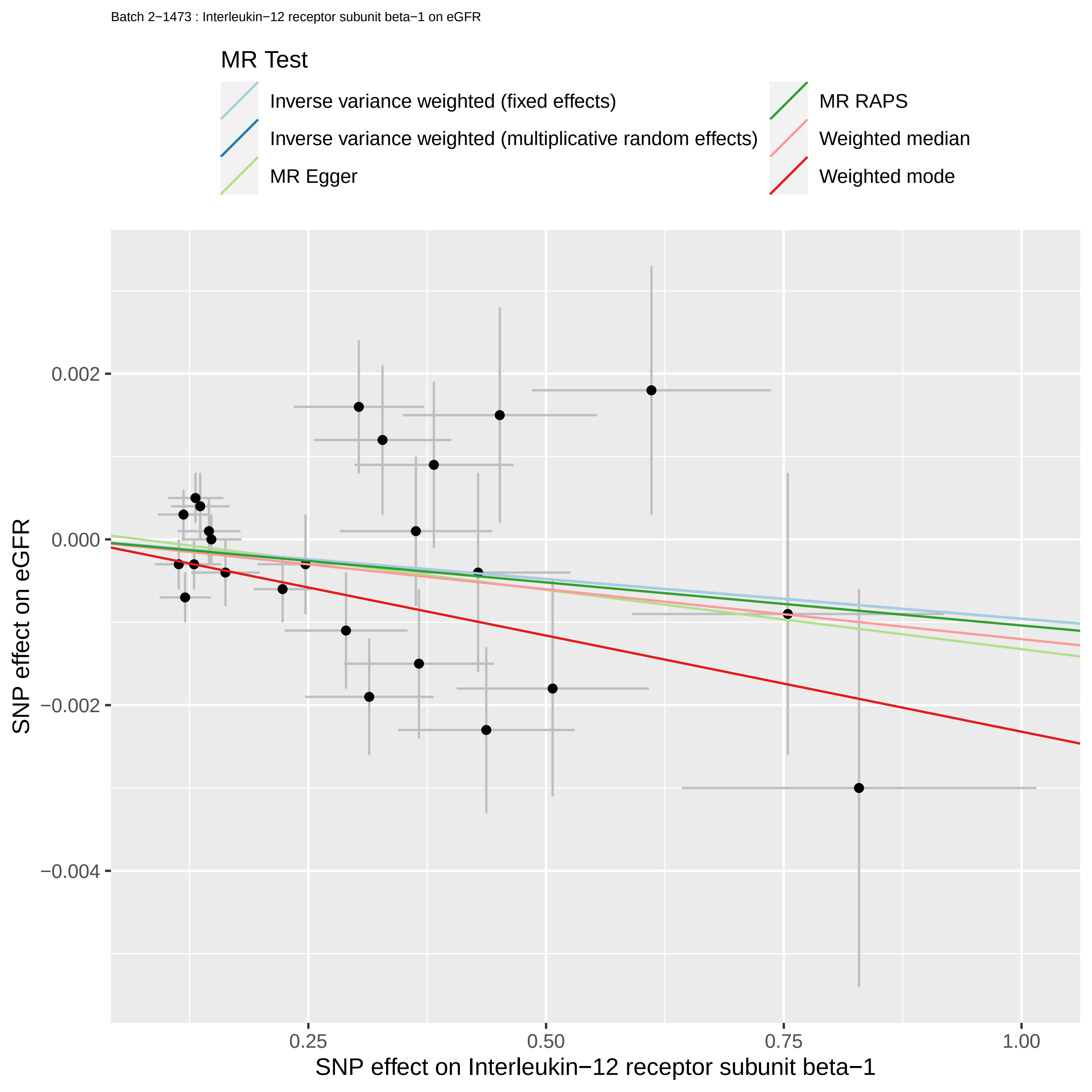
Batch 2−1473 : Interleukin−12 receptor subunit beta−1 on eGFR
MR Test
Inverse variance weighted (fixed effects)
Inverse variance weighted (multiplicative random effects)
MR Egger
MR RAPS
Weighted median
Weighted mode
0.002
0.000
−0.002
−0.004
SNP effect on eGFR
0.25
0.50
0.75
1.00
SNP effect on Interleukin−12 receptor subunit beta−1

Batch 2−1474 : Interleukin−12 receptor subunit beta−2 on eGFR

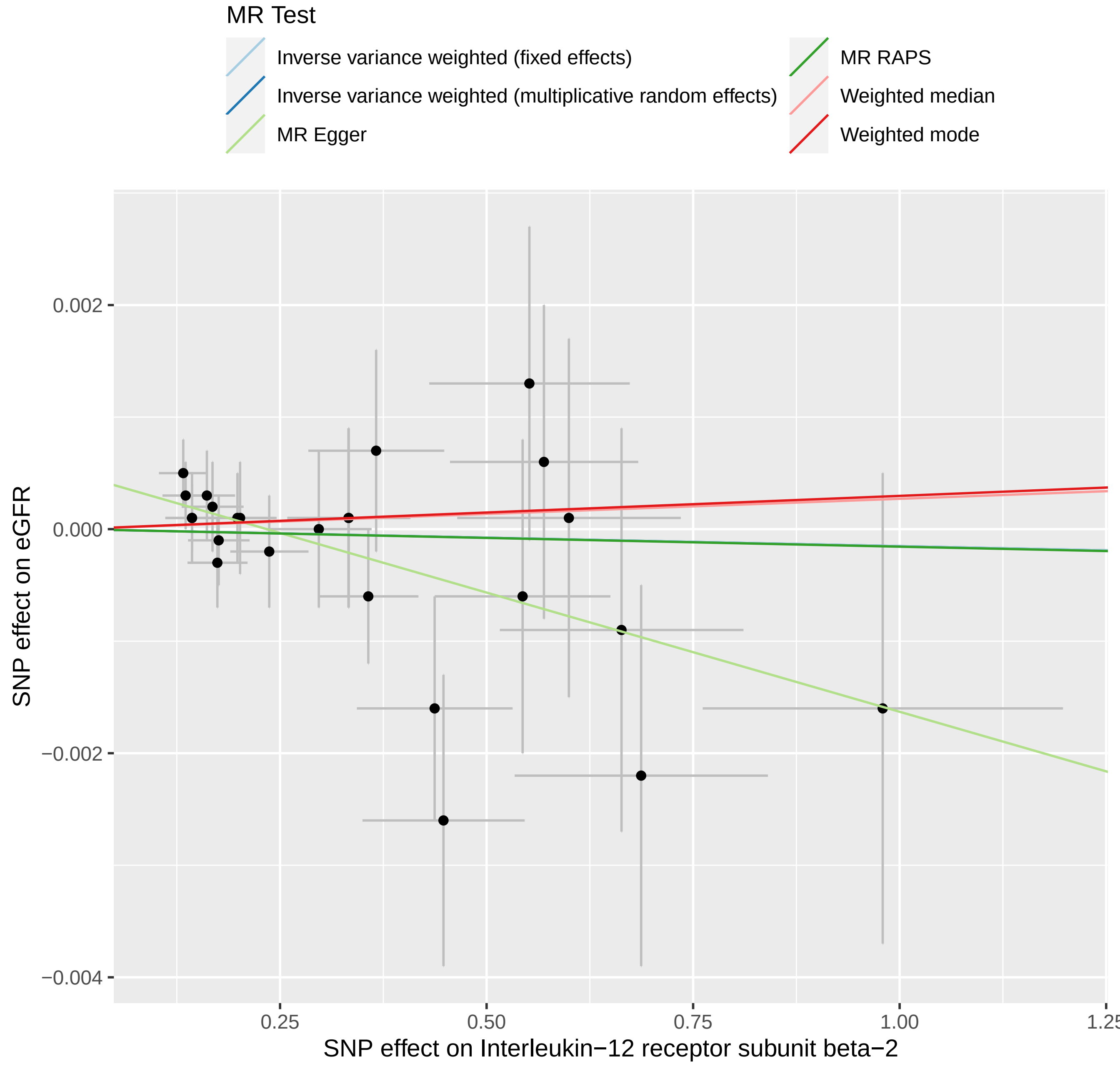

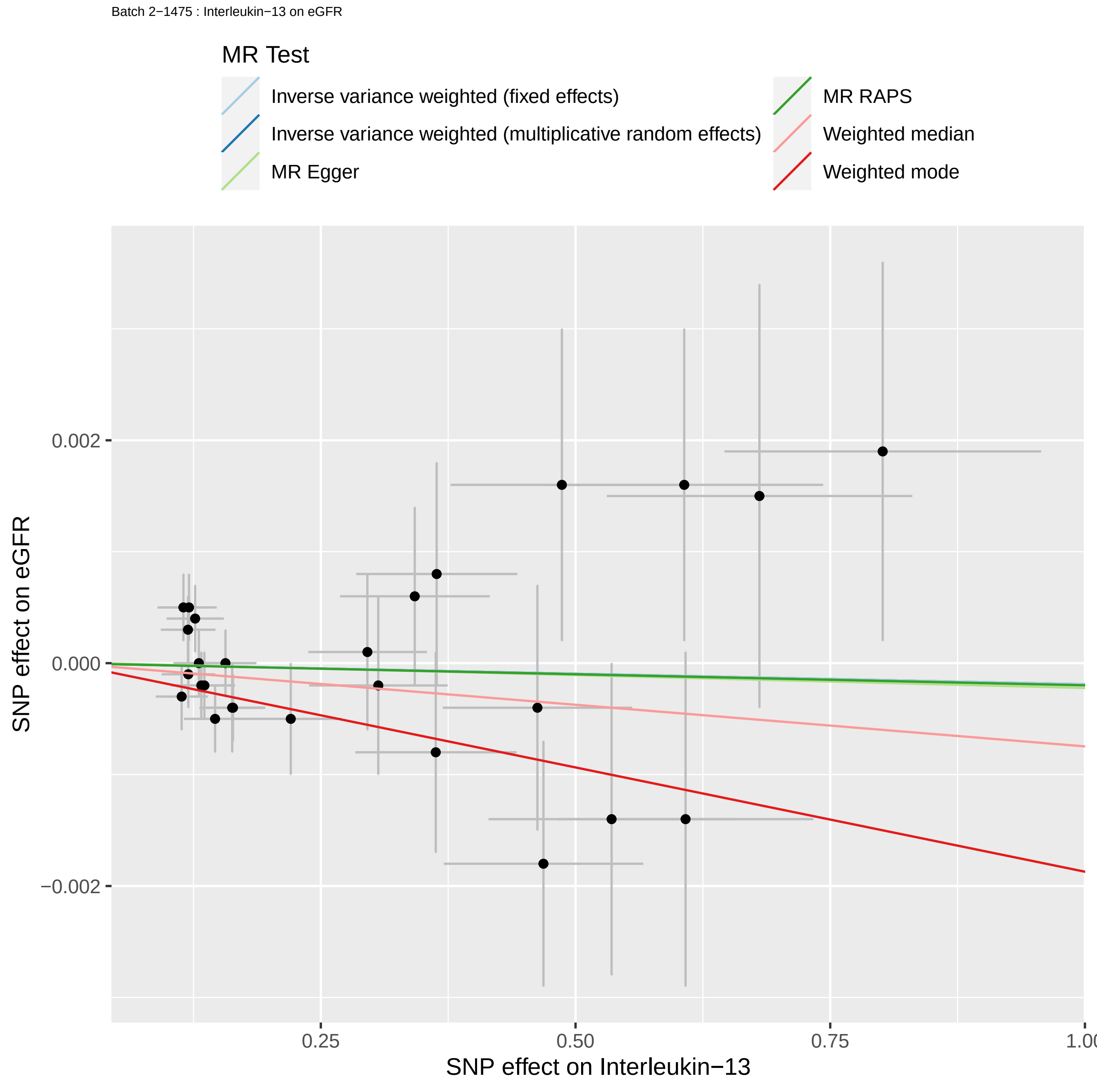

Batch 2−1475 : Interleukin−13 on eGFR
MR Test
Inverse variance weighted (fixed effects)
Inverse variance weighted (multiplicative random effects)
MR Egger
MR RAPS
Weighted median
Weighted mode
0.002
0.000
−0.002
0.25
0.50
0.75
1.00
SNP effect on eGFR
SNP effect on Interleukin−13

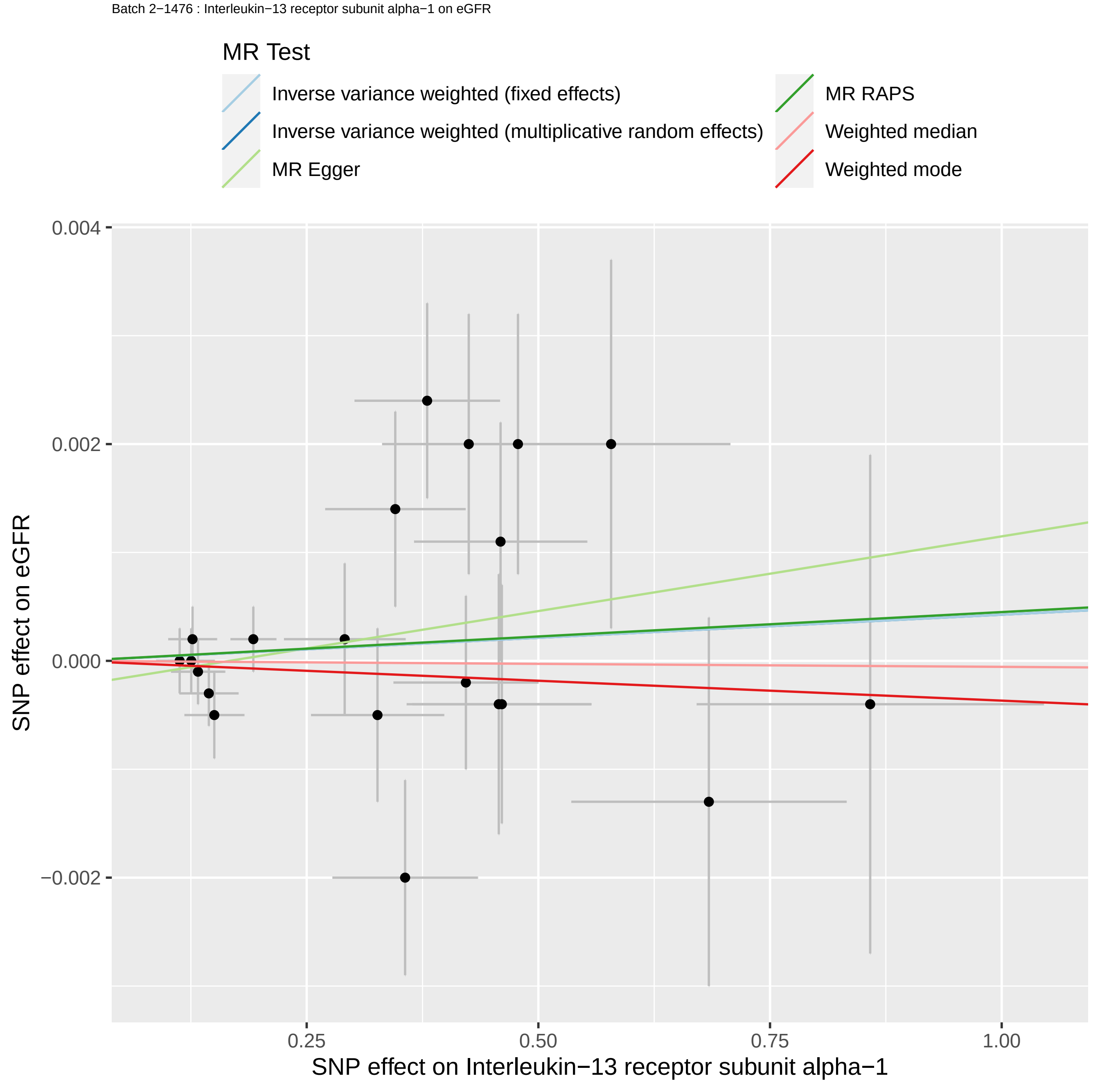

Batch 2−1476 : Interleukin−13 receptor subunit alpha−1 on eGFR
MR Test
Inverse variance weighted (fixed effects)
Inverse variance weighted (multiplicative random effects)
MR Egger
MR RAPS
Weighted median
Weighted mode
0.004
0.002
0.000
−0.002
0.25
0.50
0.75
1.00
SNP effect on eGFR
SNP effect on Interleukin−13 receptor subunit alpha−1

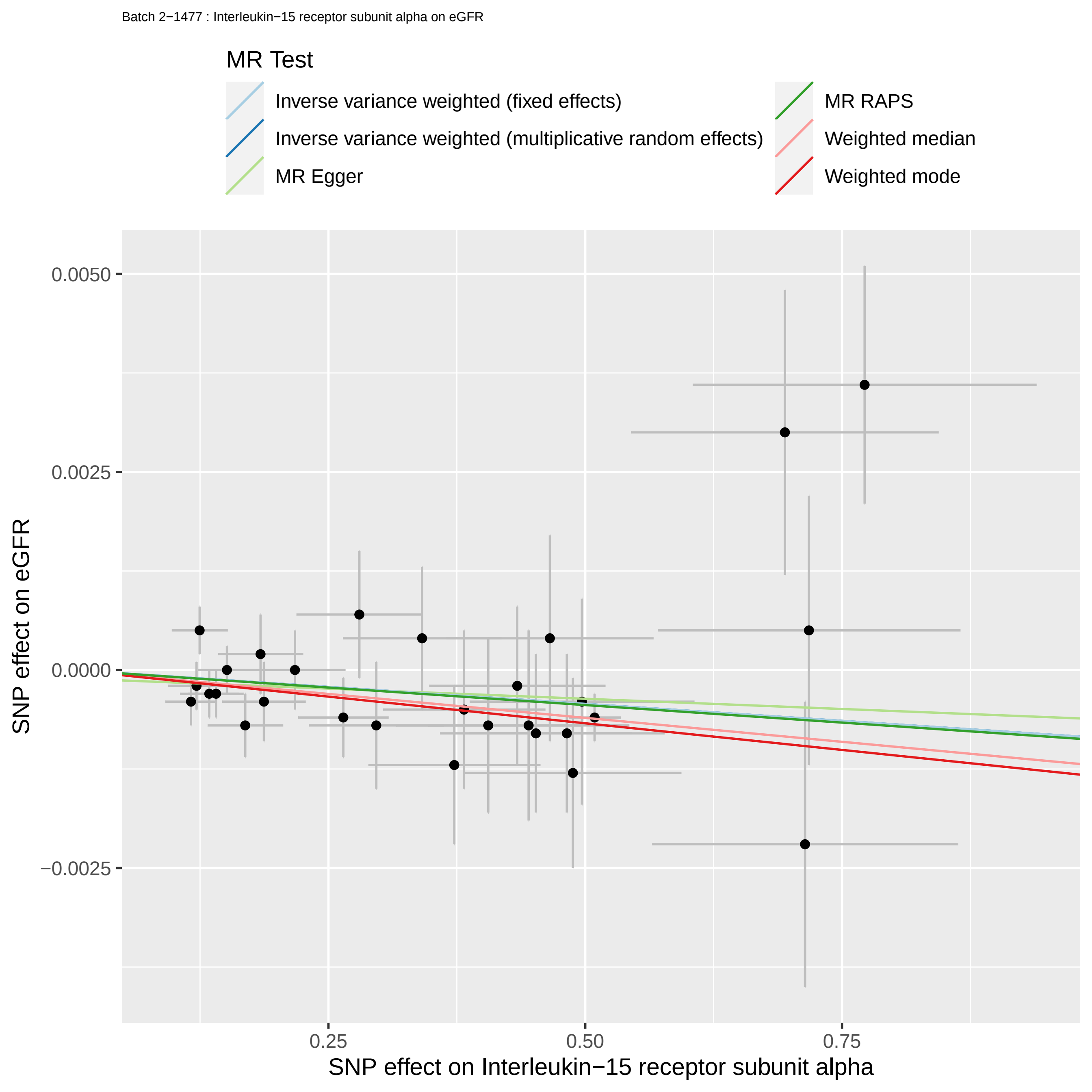

Batch 2−1477 : Interleukin−15 receptor subunit alpha on eGFR
MR Test
Inverse variance weighted (fixed effects)
Inverse variance weighted (multiplicative random effects)
MR Egger
MR RAPS
Weighted median
Weighted mode
0.0050
0.0025
0.0000
−0.0025
0.25
0.50
0.75
SNP effect on eGFR
SNP effect on Interleukin−15 receptor subunit alpha

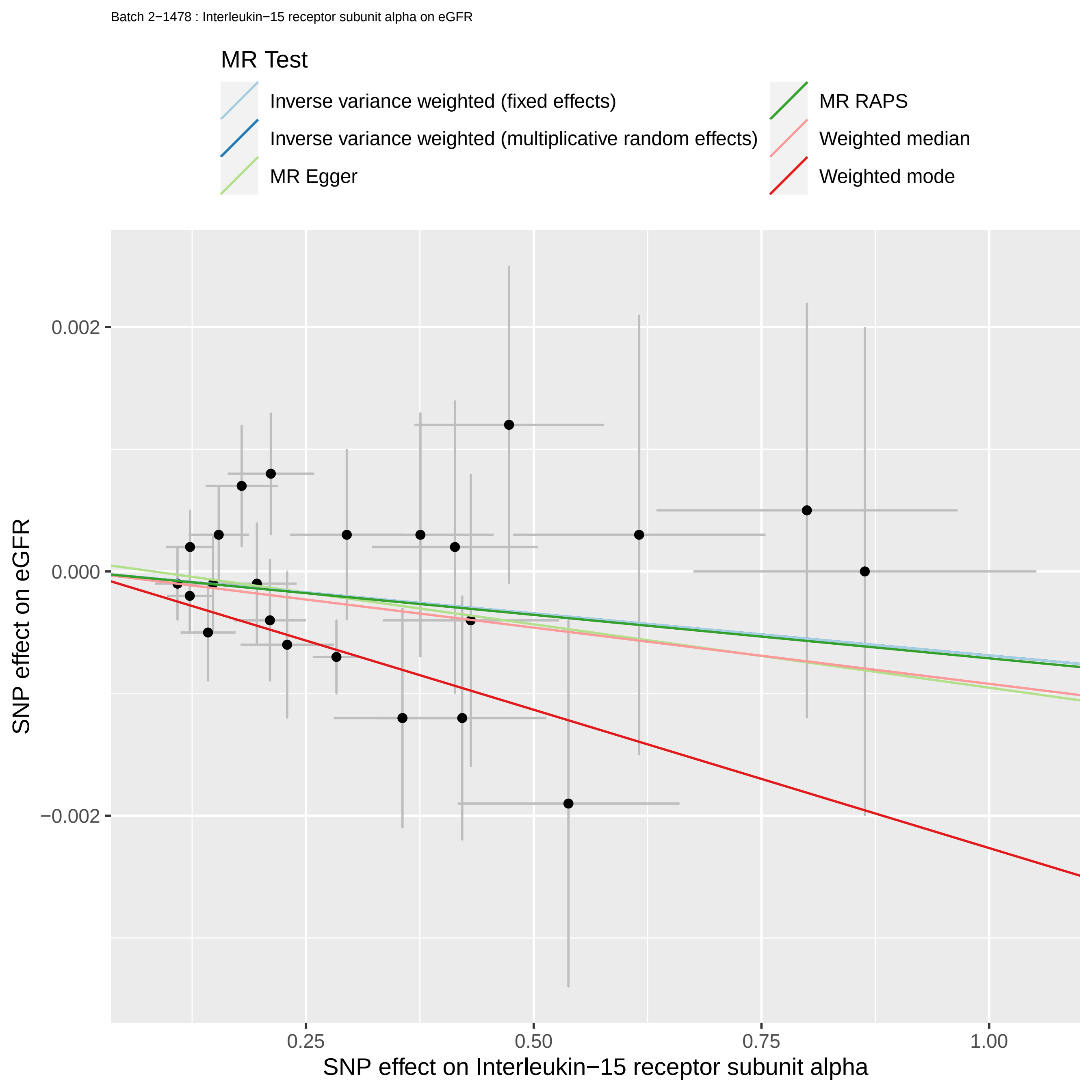
Batch 2−1478 : Interleukin−15 receptor subunit alpha on eGFR
MR Test
Inverse variance weighted (fixed effects)
Inverse variance weighted (multiplicative random effects)
MR Egger
MR RAPS
Weighted median
Weighted mode
0.002
0.000
−0.002
SNP effect on eGFR
0.25
0.50
0.75
1.00
SNP effect on Interleukin−15 receptor subunit alpha

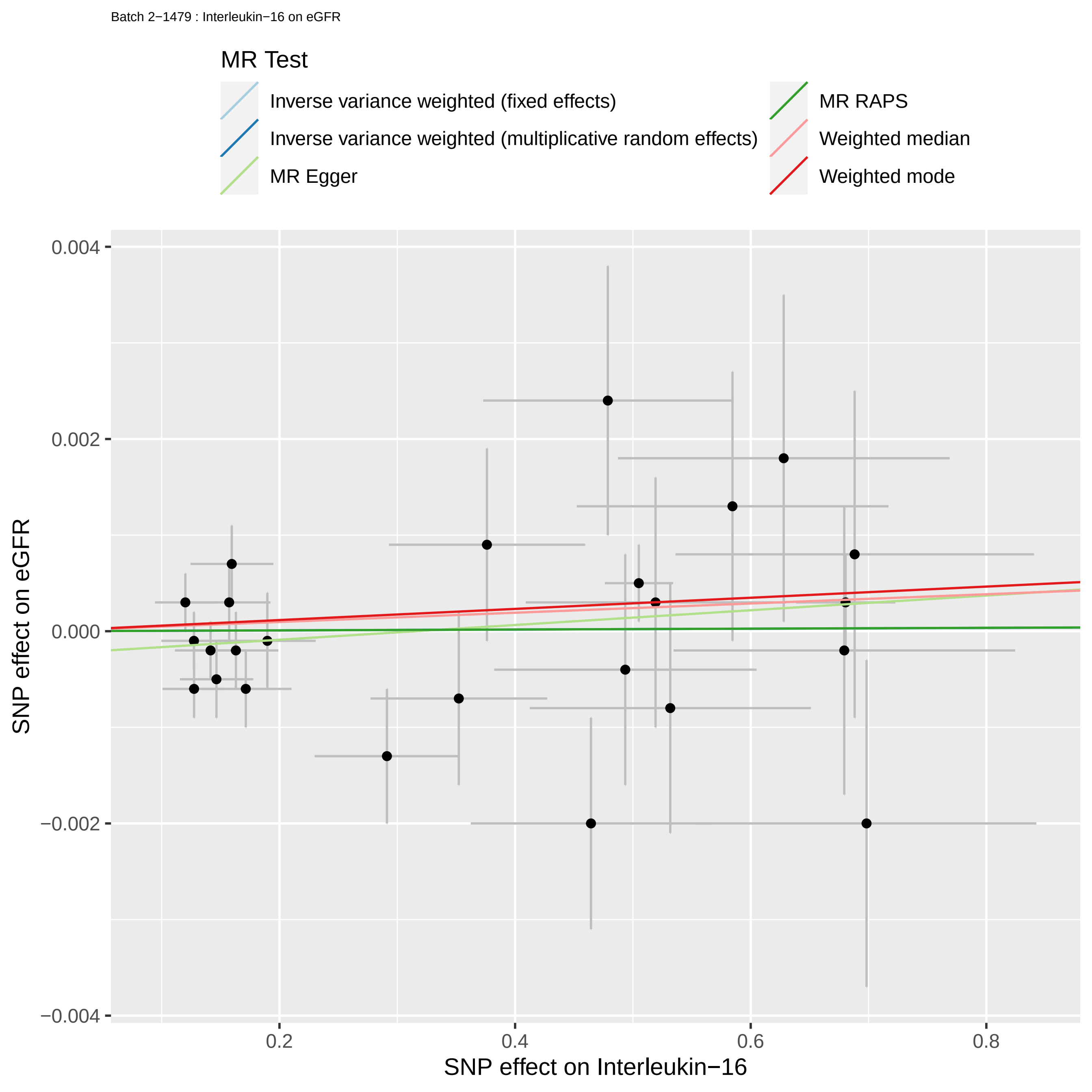

Batch 2−1479 : Interleukin−16 on eGFR
MR Test
Inverse variance weighted (fixed effects)
Inverse variance weighted (multiplicative random effects)
MR Egger
MR RAPS
Weighted median
Weighted mode
0.004
0.002
0.000
−0.002
−0.004
SNP effect on eGFR
0.2
0.4
0.6
0.8
SNP effect on Interleukin−16

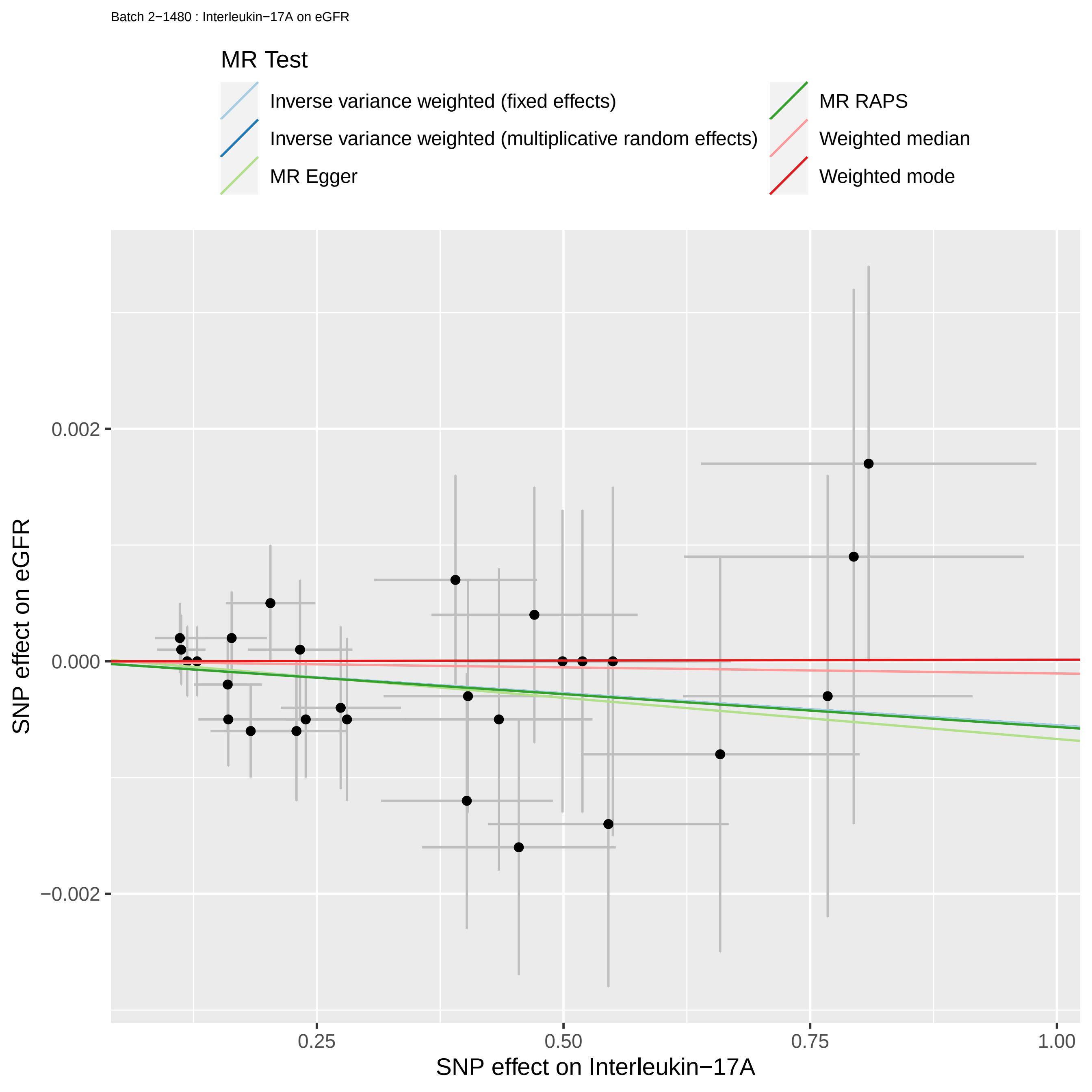

Batch 2−1480 : Interleukin−17A on eGFR
MR Test
Inverse variance weighted (fixed effects)
Inverse variance weighted (multiplicative random effects)
MR Egger
MR RAPS
Weighted median
Weighted mode
0.002
0.000
−0.002
0.25
0.50
0.75
1.00
SNP effect on eGFR
SNP effect on Interleukin−17A

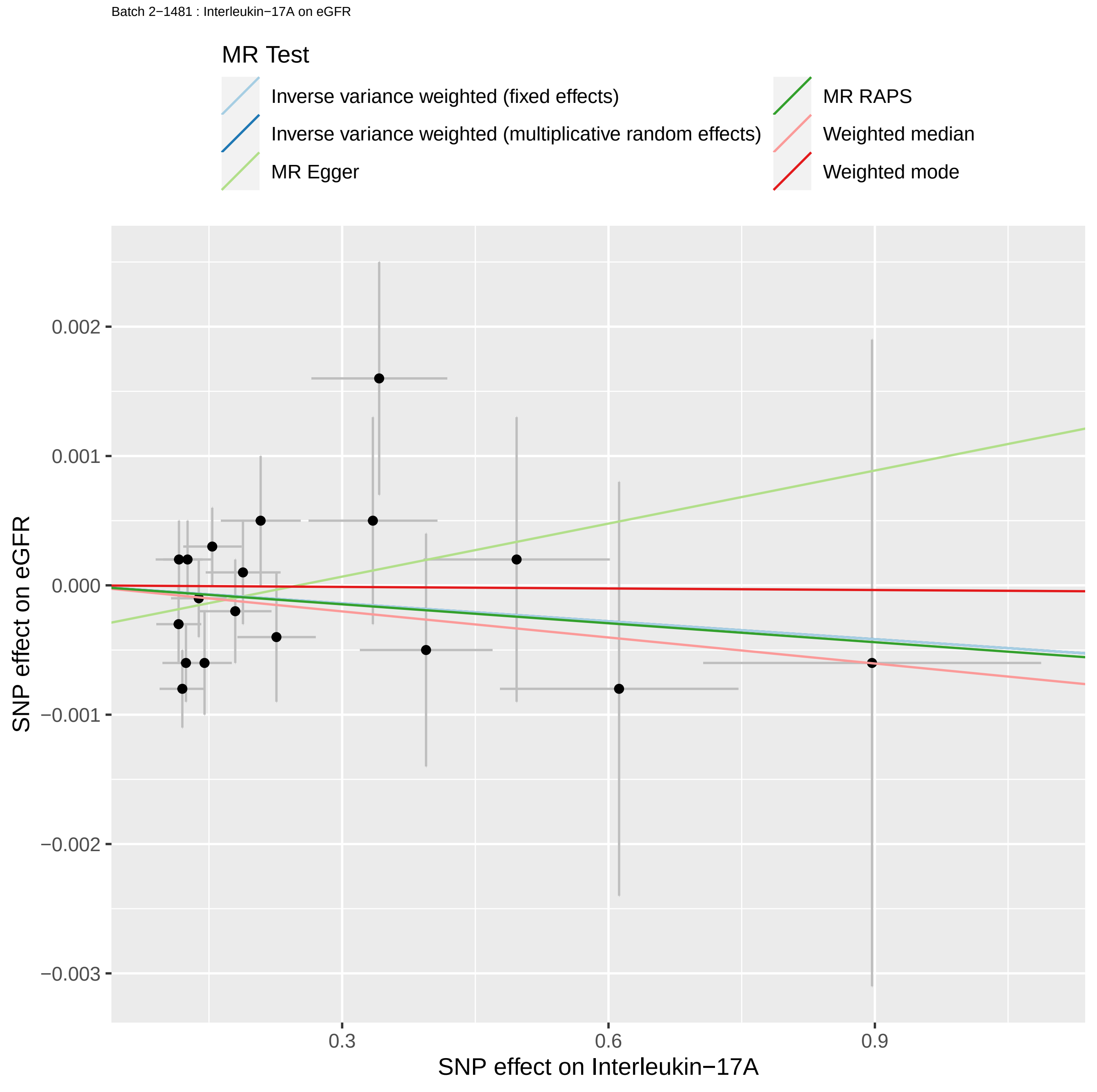

Batch 2−1481 : Interleukin−17A on eGFR
MR Test
Inverse variance weighted (fixed effects)
Inverse variance weighted (multiplicative random effects)
MR Egger
MR RAPS
Weighted median
Weighted mode
0.002
0.001
0.000
−0.001
−0.002
−0.003
SNP effect on eGFR
0.3
0.6
0.9
SNP effect on Interleukin−17A

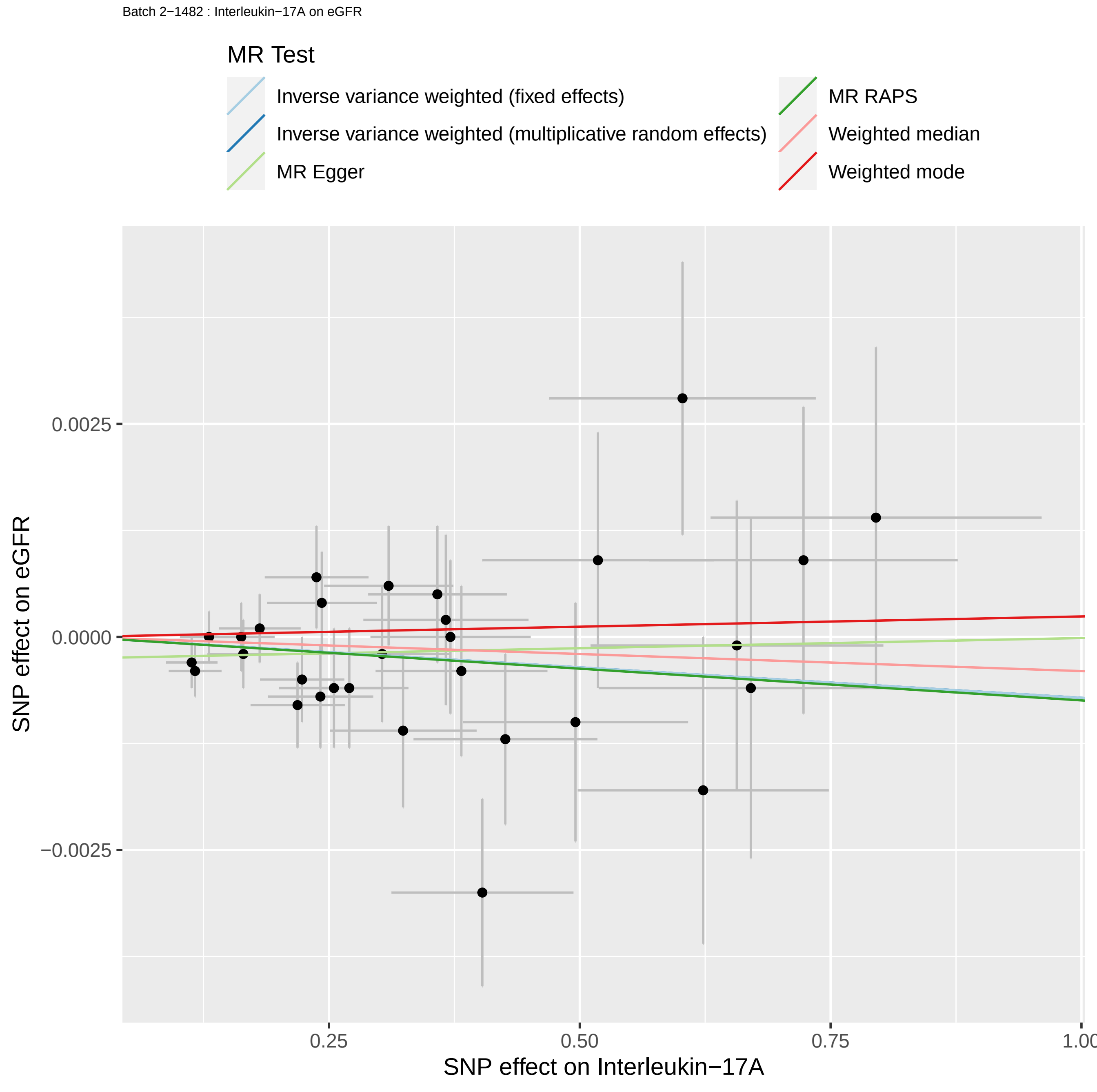
Batch 2−1482 : Interleukin−17A on eGFR
MR Test
Inverse variance weighted (fixed effects)
Inverse variance weighted (multiplicative random effects)
MR Egger
MR RAPS
Weighted median
Weighted mode
0.0025
0.0000
−0.0025
SNP effect on eGFR
0.25
0.50
0.75
1.00
SNP effect on Interleukin−17A

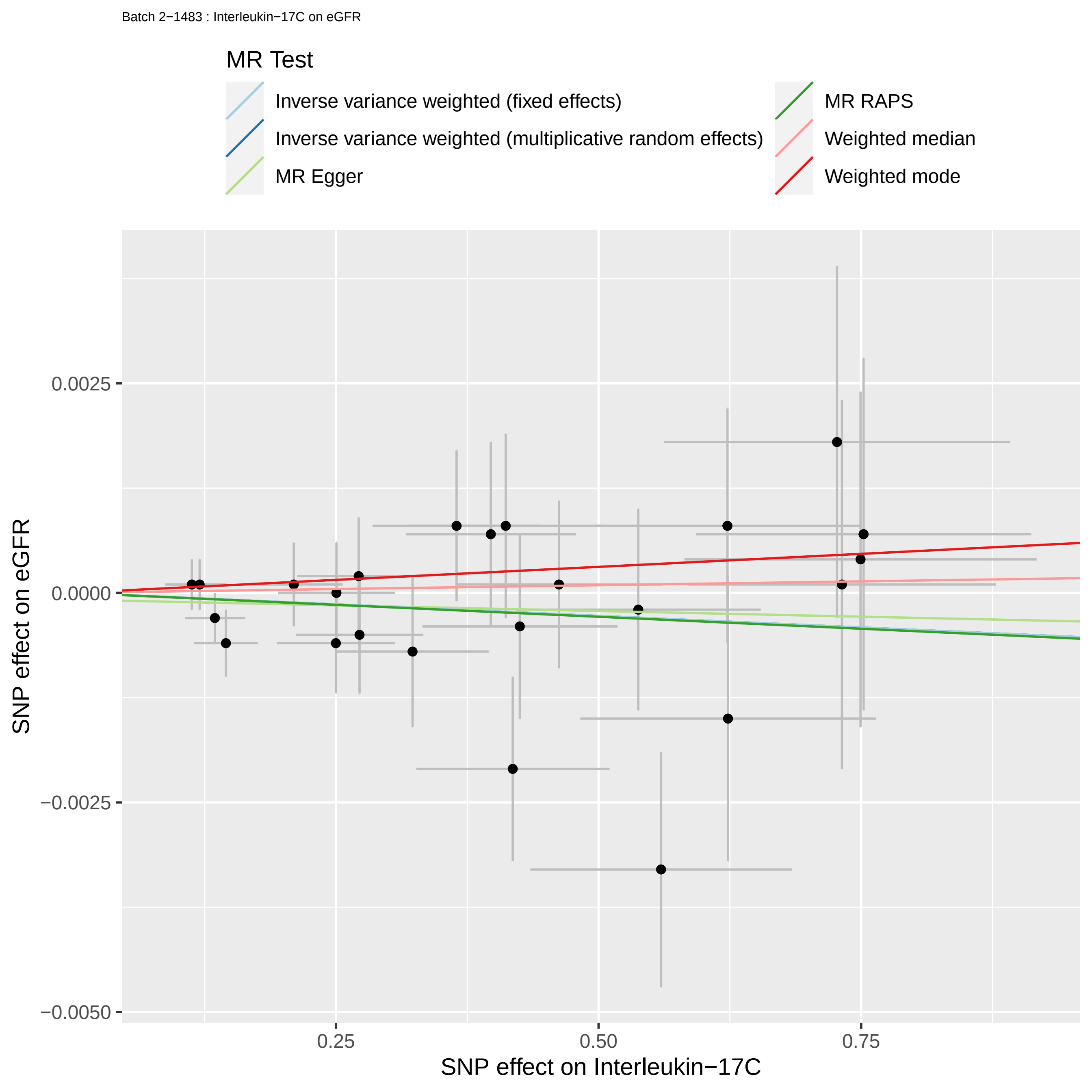

Batch 2−1483 : Interleukin−17C on eGFR
MR Test
Inverse variance weighted (fixed effects)
Inverse variance weighted (multiplicative random effects)
MR Egger
MR RAPS
Weighted median
Weighted mode
0.0025
0.0000
−0.0025
−0.0050
0.25
0.50
0.75
SNP effect on eGFR
SNP effect on Interleukin−17C

Batch 2−1484 : Interleukin−17D on eGFR

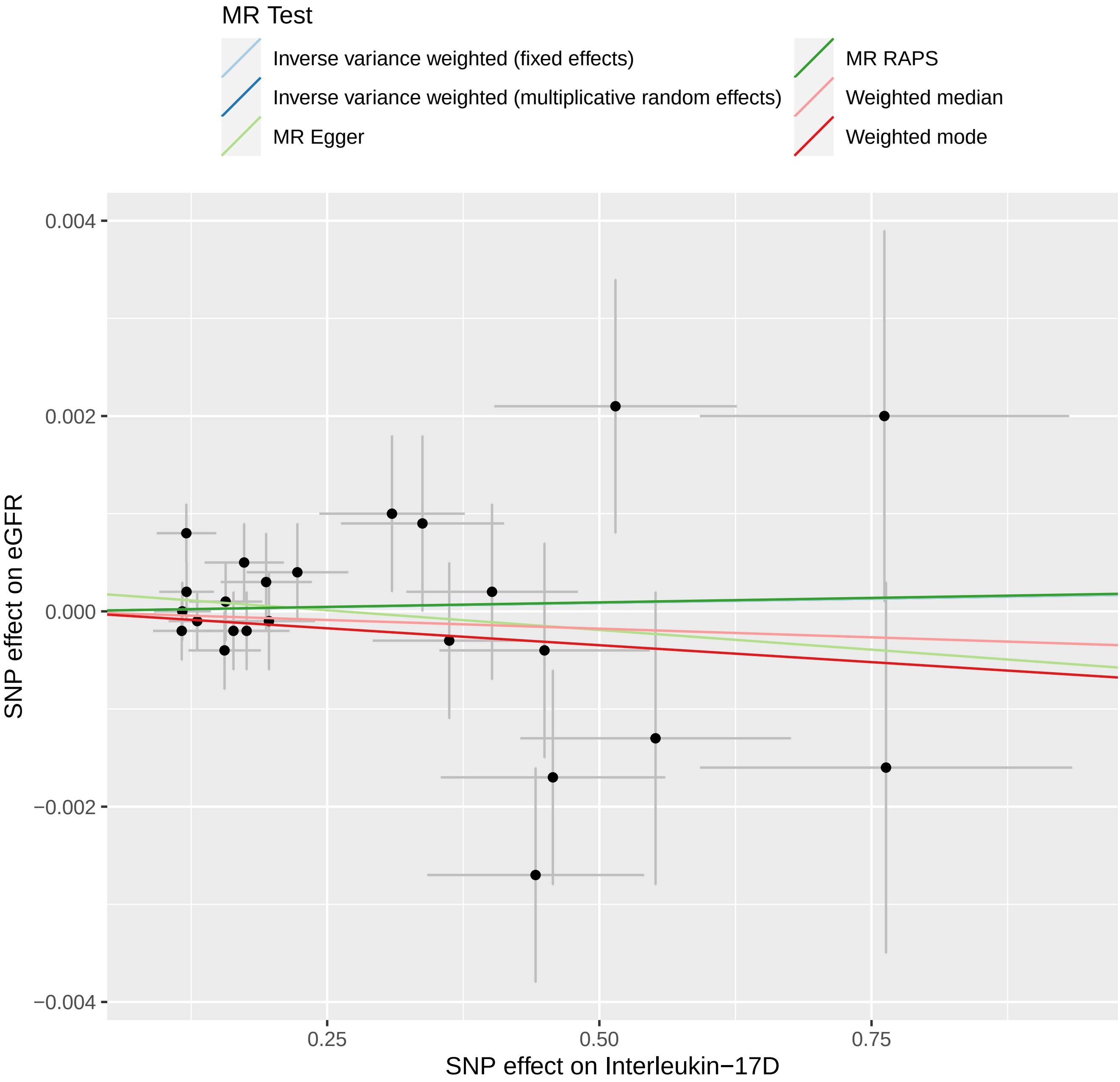

Batch 2−1485 : Interleukin−17F on eGFR

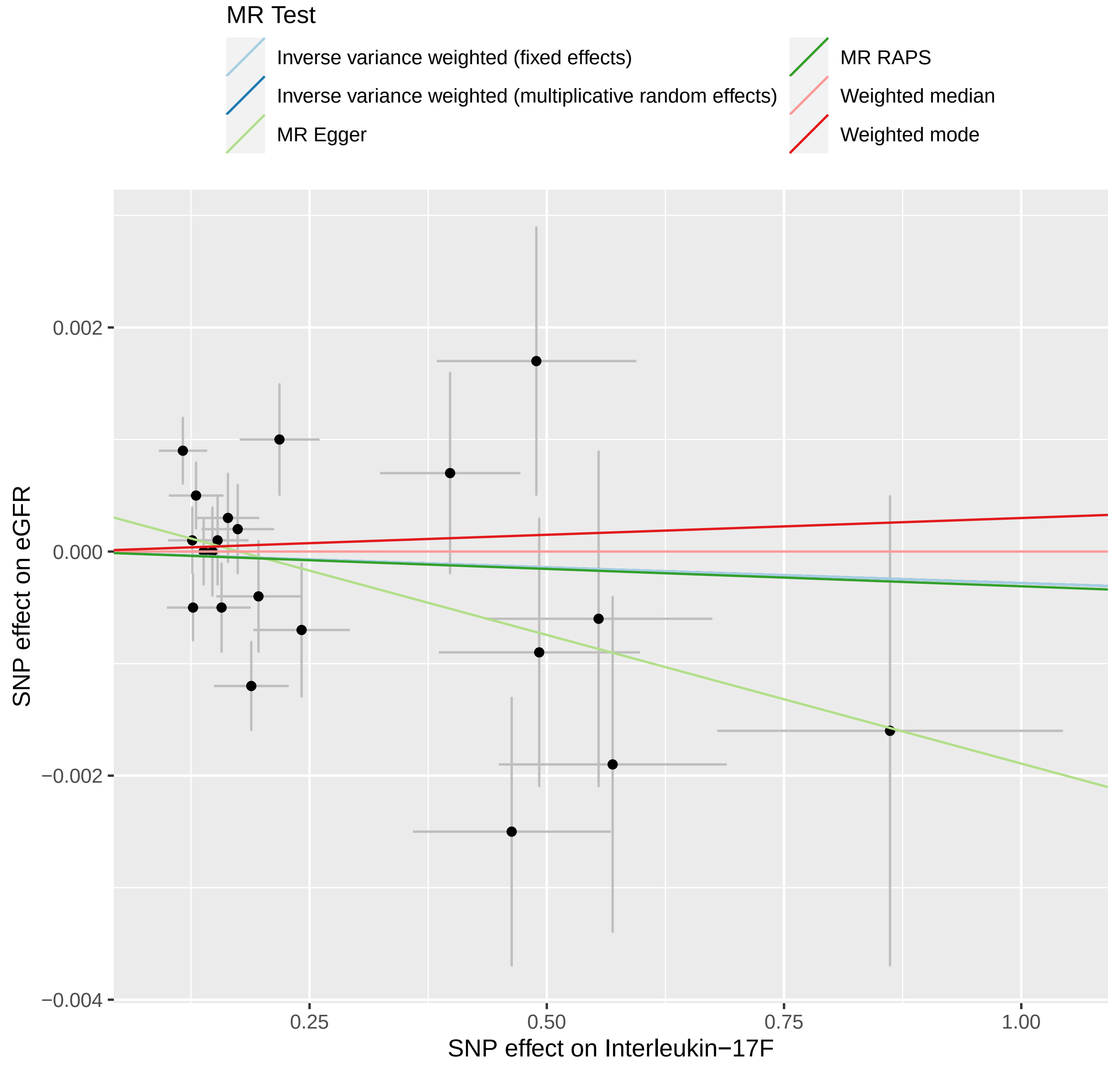

Batch 2−1486 : Interleukin−17 receptor A on eGFR

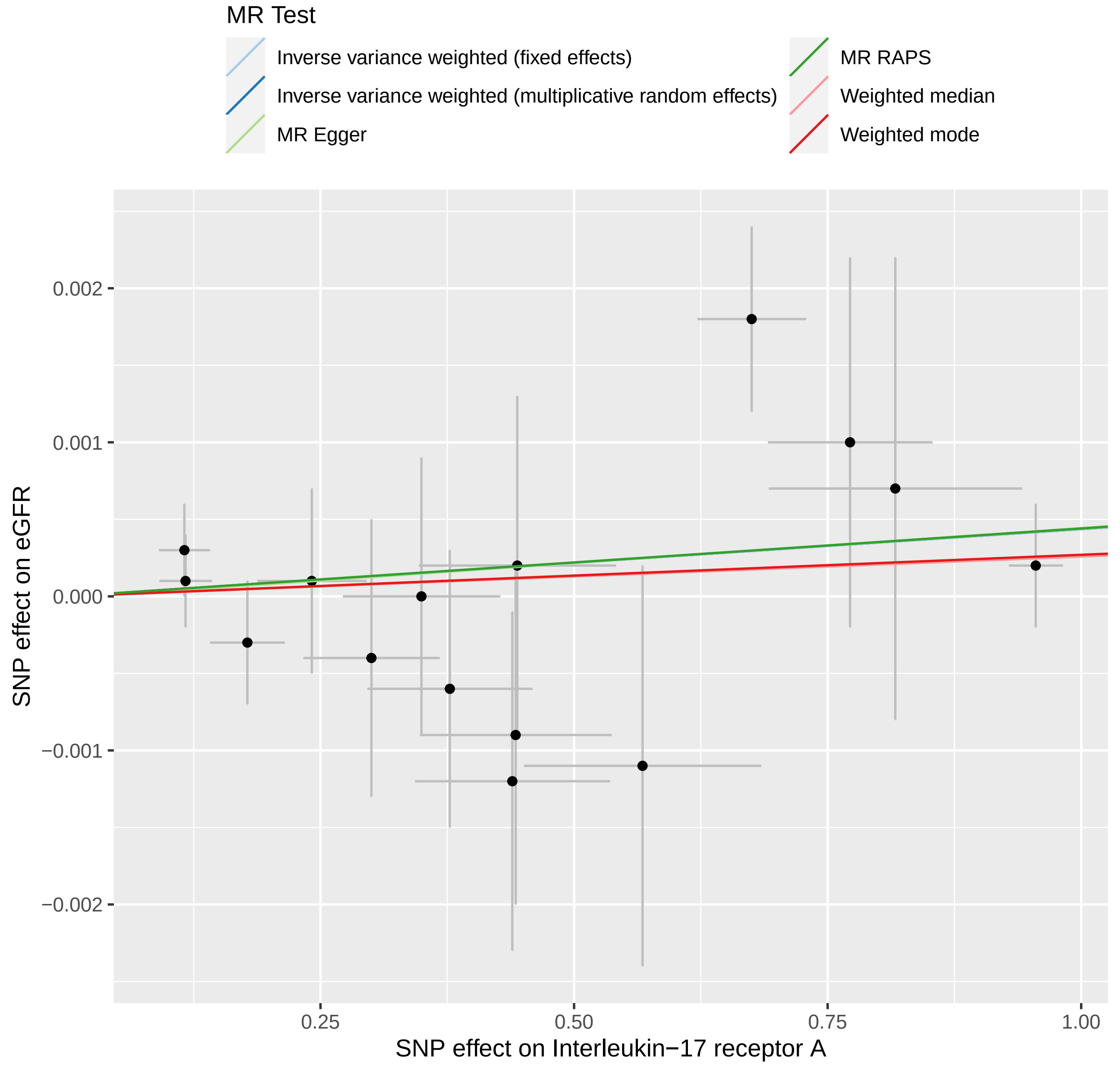

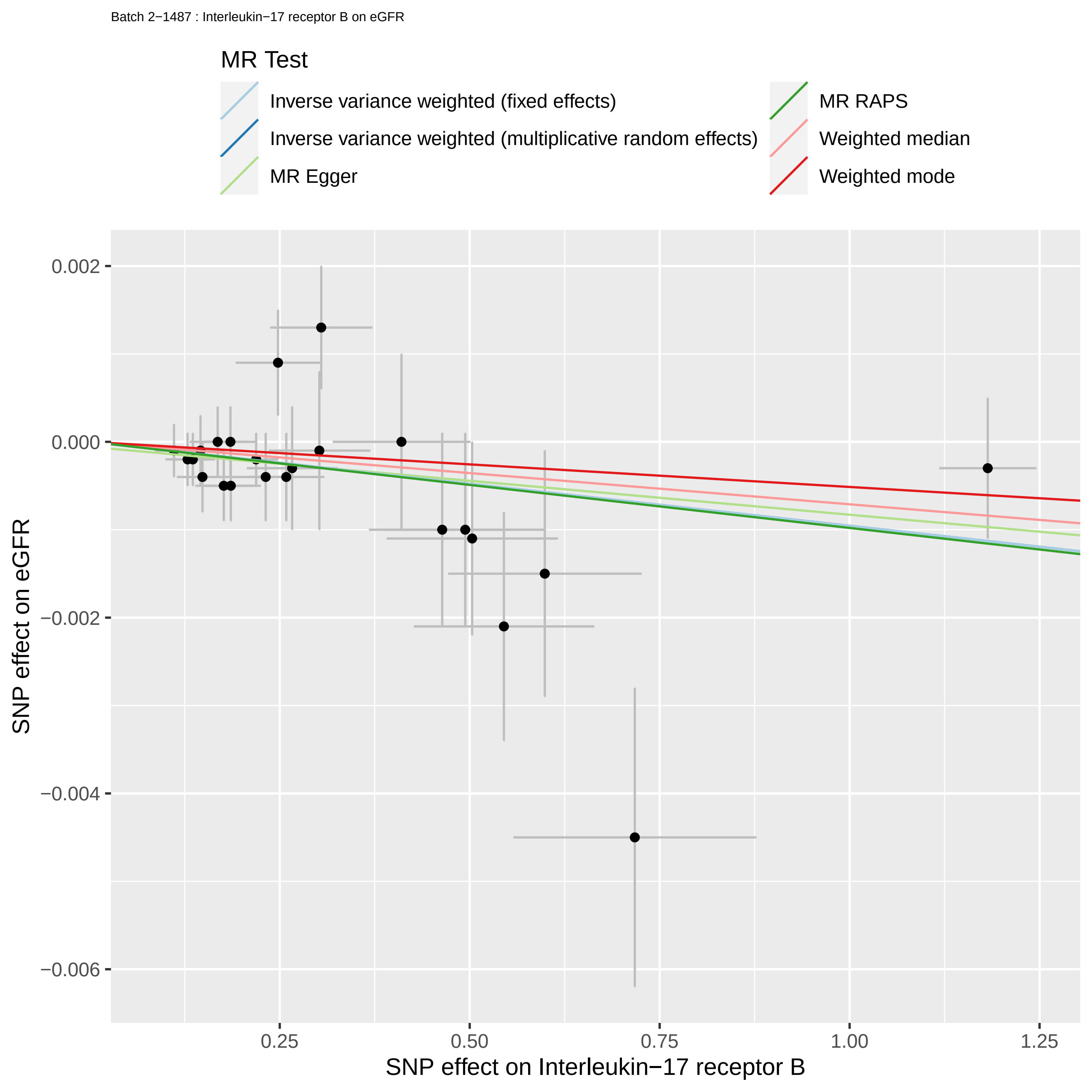

Batch 2−1487 : Interleukin−17 receptor B on eGFR
MR Test
Inverse variance weighted (fixed effects)
Inverse variance weighted (multiplicative random effects)
MR Egger
MR RAPS
Weighted median
Weighted mode
0.002
0.000
−0.002
−0.004
−0.006
0.25
0.50
0.75
1.00
1.25
SNP effect on eGFR
SNP effect on Interleukin−17 receptor B

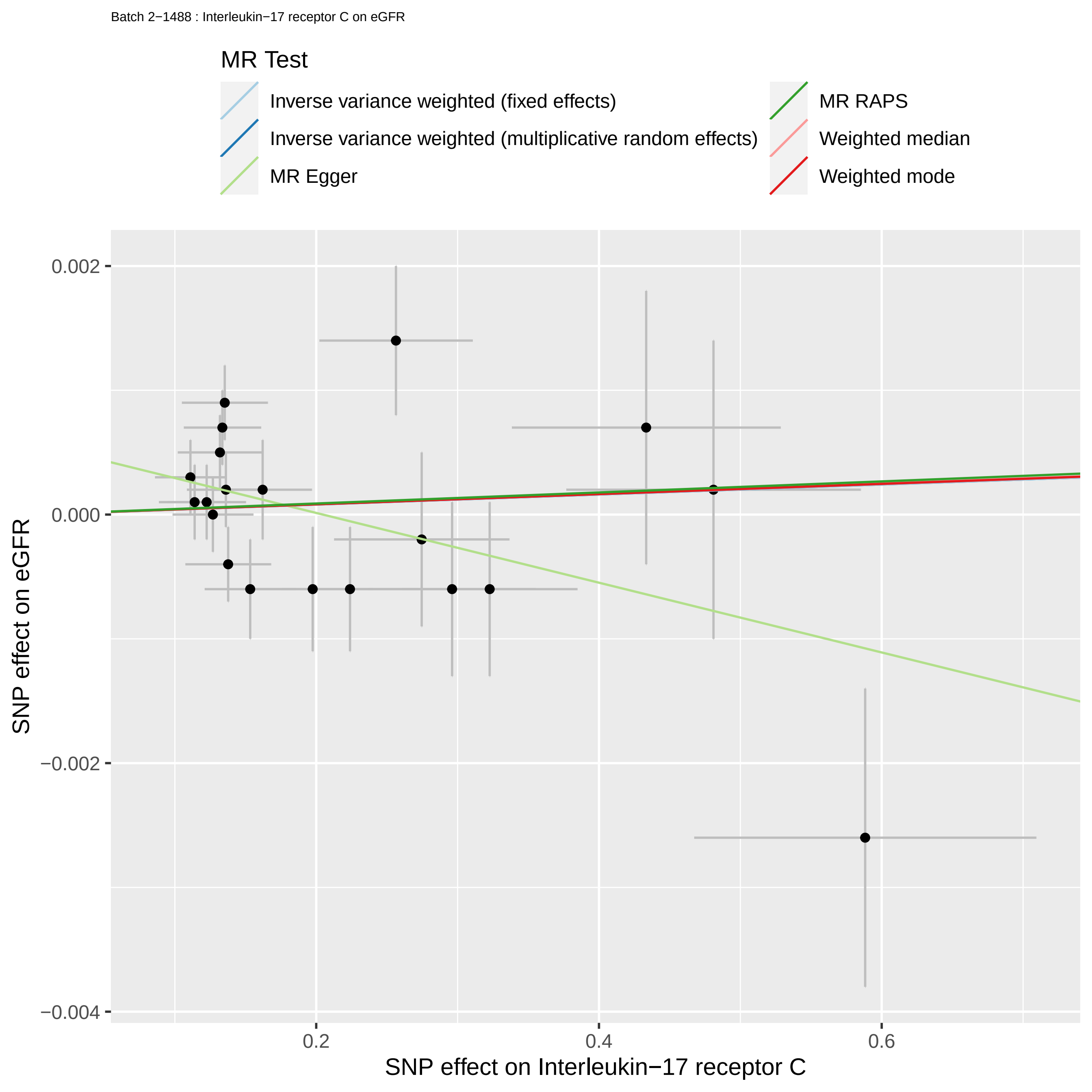

Batch 2−1488 : Interleukin−17 receptor C on eGFR
MR Test
Inverse variance weighted (fixed effects)
Inverse variance weighted (multiplicative random effects)
MR Egger
MR RAPS
Weighted median
Weighted mode
0.002
0.000
−0.002
−0.004
SNP effect on eGFR
0.2
0.4
0.6
SNP effect on Interleukin−17 receptor C

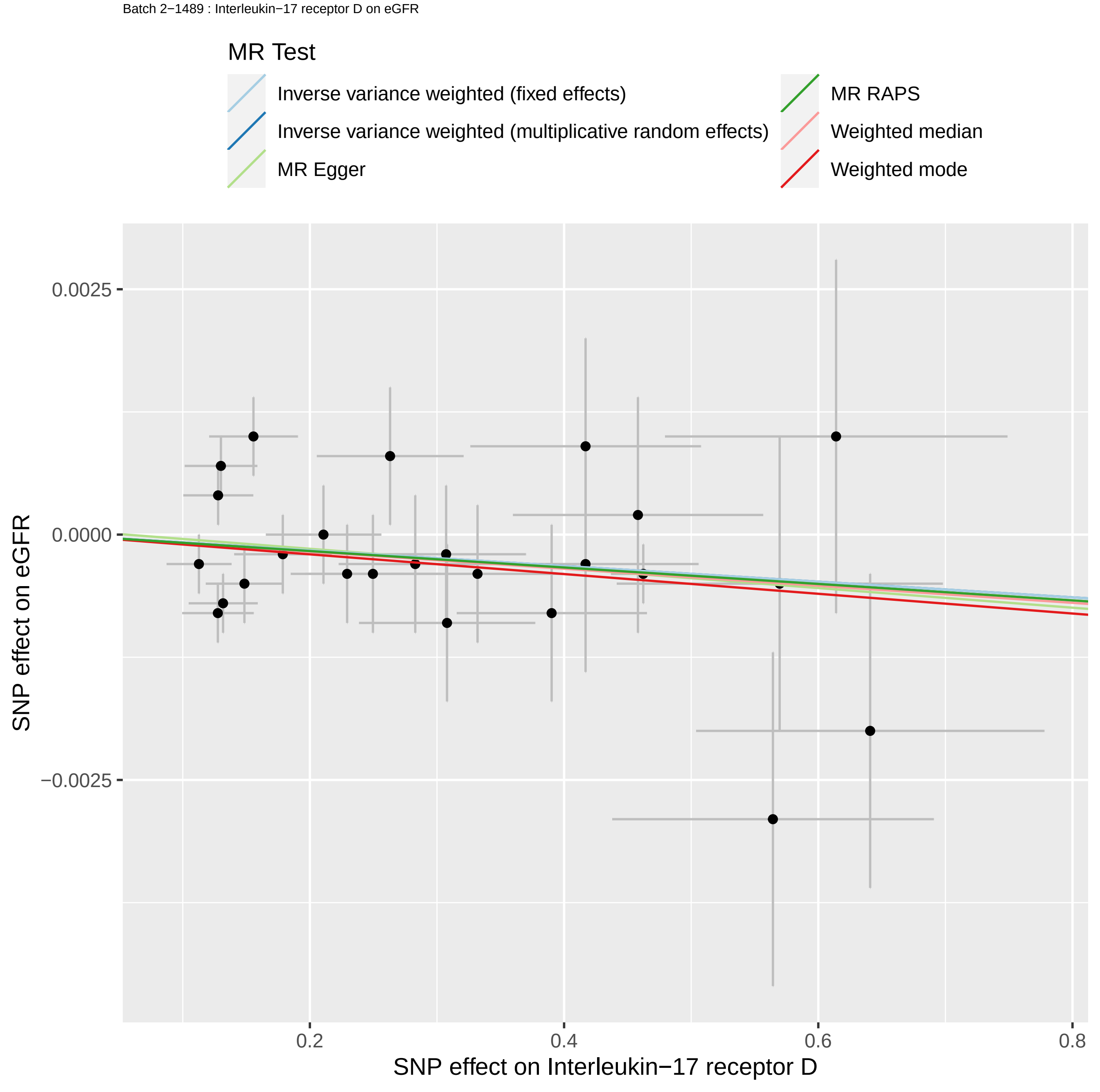
Batch 2−1489 : Interleukin−17 receptor D on eGFR
MR Test
Inverse variance weighted (fixed effects)
Inverse variance weighted (multiplicative random effects)
MR Egger
MR RAPS
Weighted median
Weighted mode
0.0025
0.0000
−0.0025
SNP effect on eGFR
0.2
0.4
0.6
0.8
SNP effect on Interleukin−17 receptor D

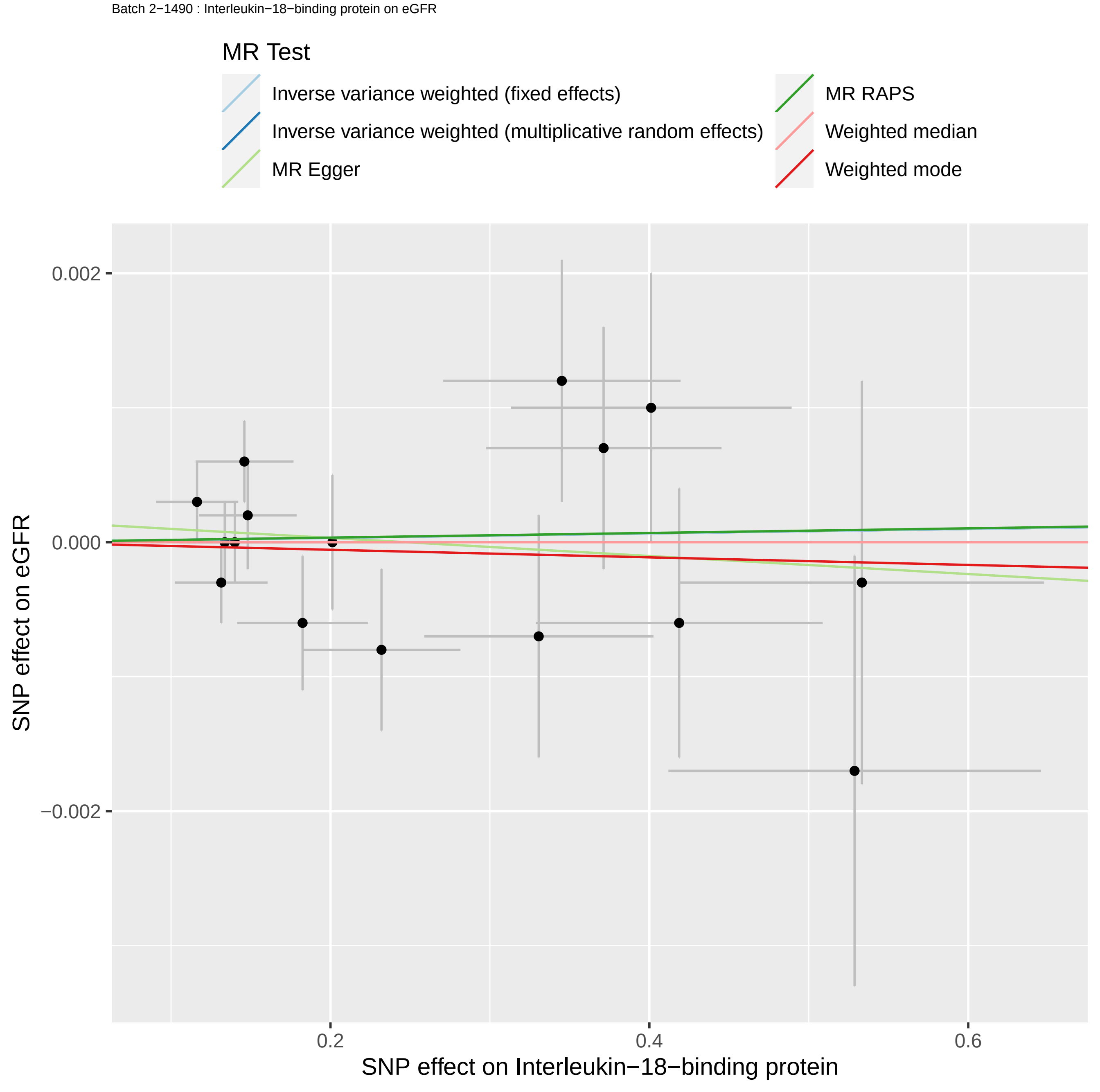
Batch 2−1490 : Interleukin−18−binding protein on eGFR
MR Test
Inverse variance weighted (fixed effects)
Inverse variance weighted (multiplicative random effects)
MR Egger
MR RAPS
Weighted median
Weighted mode
0.002
0.000
−0.002
0.2
0.4
0.6
SNP effect on eGFR
SNP effect on Interleukin−18−binding protein

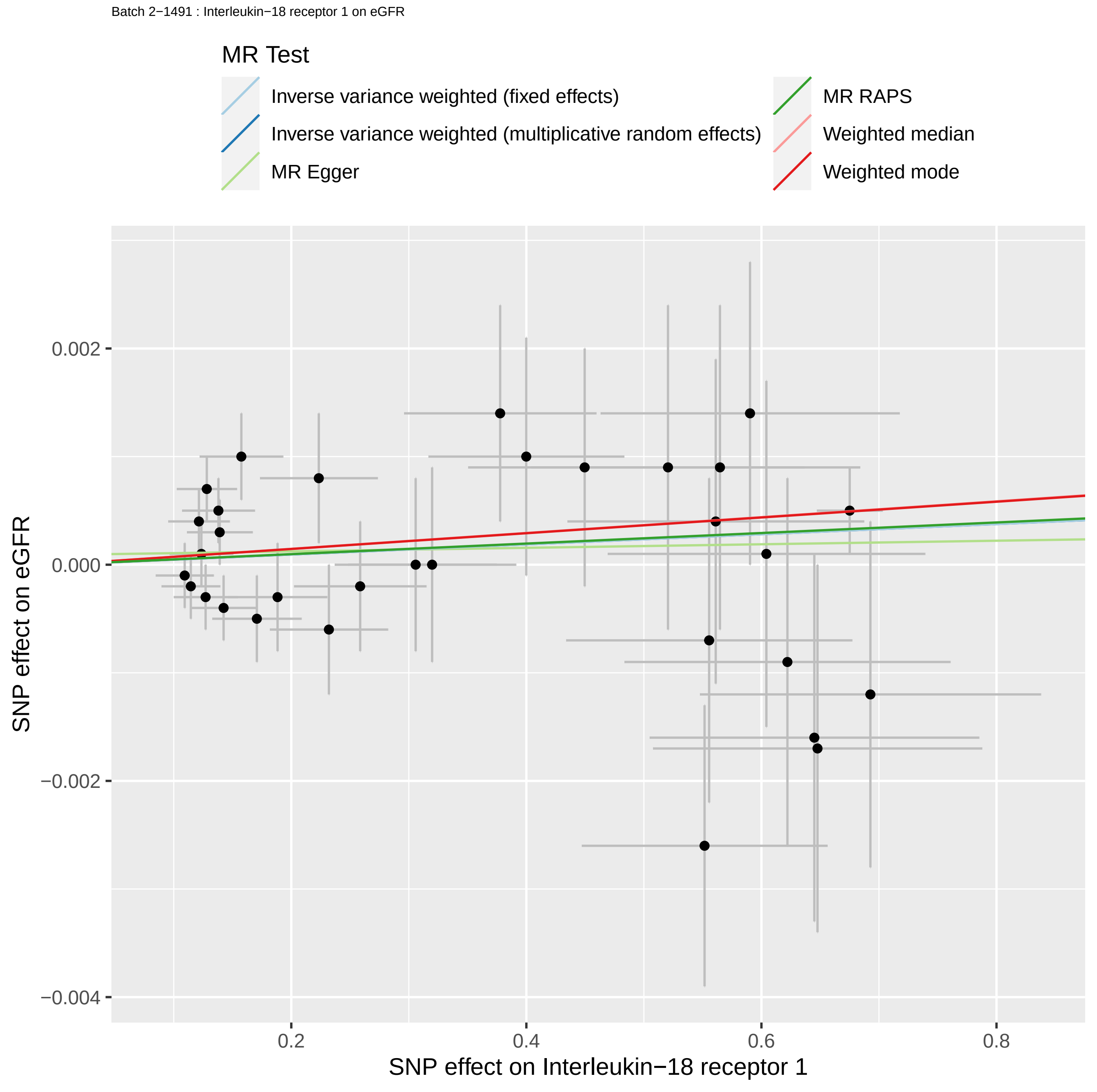

Batch 2−1491 : Interleukin−18 receptor 1 on eGFR
MR Test
Inverse variance weighted (fixed effects)
Inverse variance weighted (multiplicative random effects)
MR Egger
MR RAPS
Weighted median
Weighted mode
SNP effect on eGFR
0.002
0.000
−0.002
−0.004
0.2
0.4
0.6
0.8
SNP effect on Interleukin−18 receptor 1

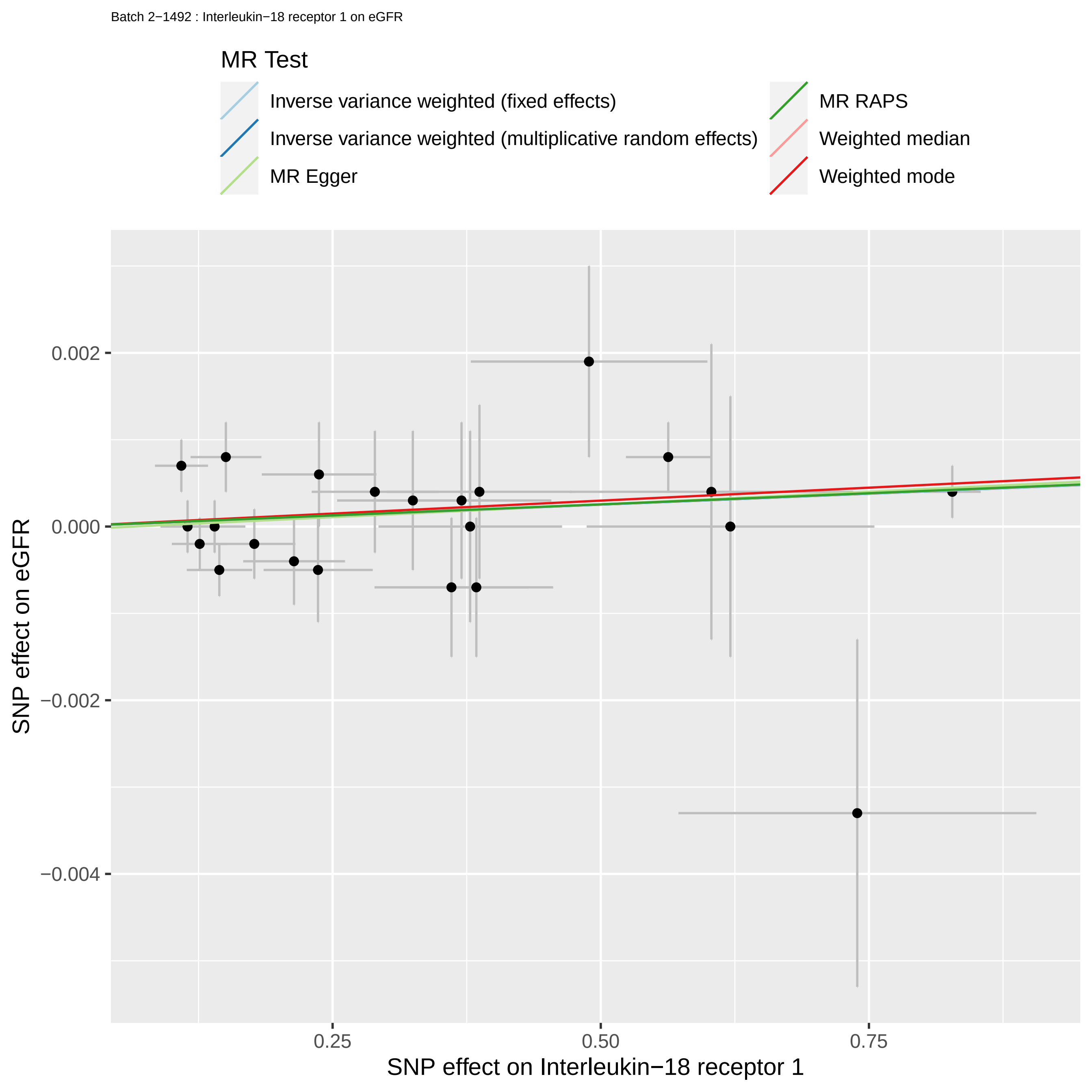

Batch 2−1492 : Interleukin−18 receptor 1 on eGFR
MR Test
Inverse variance weighted (fixed effects)
Inverse variance weighted (multiplicative random effects)
MR Egger
MR RAPS
Weighted median
Weighted mode
0.002
0.000
−0.002
−0.004
SNP effect on eGFR
0.25
0.50
0.75
SNP effect on Interleukin−18 receptor 1

Batch 2−1493 : Interleukin−19 on eGFR

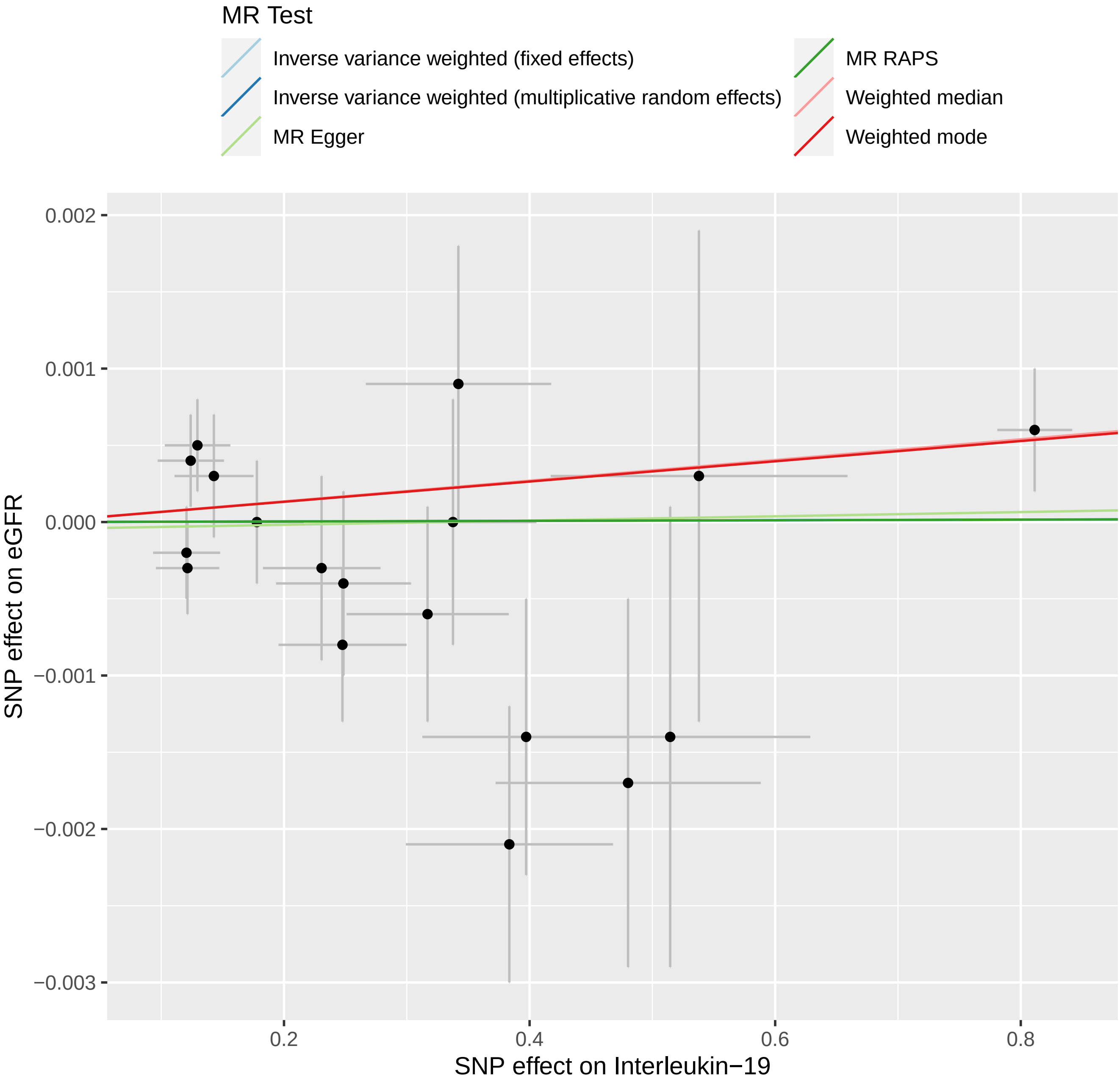

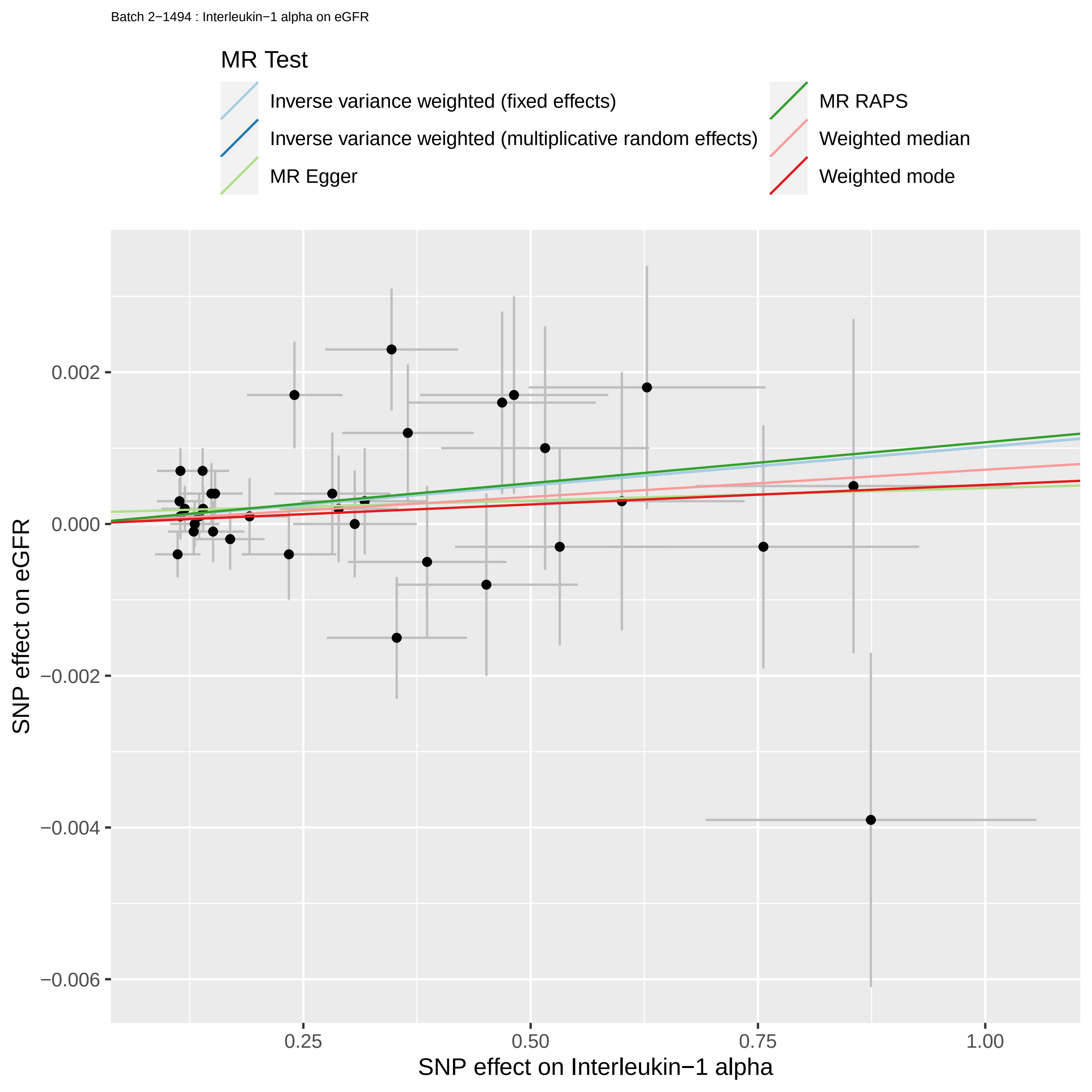

Batch 2−1494 : Interleukin−1 alpha on eGFR
MR Test
Inverse variance weighted (fixed effects)
Inverse variance weighted (multiplicative random effects)
MR Egger
MR RAPS
Weighted median
Weighted mode
0.002
0.000
−0.002
−0.004
−0.006
0.25
0.50
0.75
1.00
SNP effect on Interleukin−1 alpha
SNP effect on eGFR

Batch 2−1495 : Interleukin−1 beta on eGFR

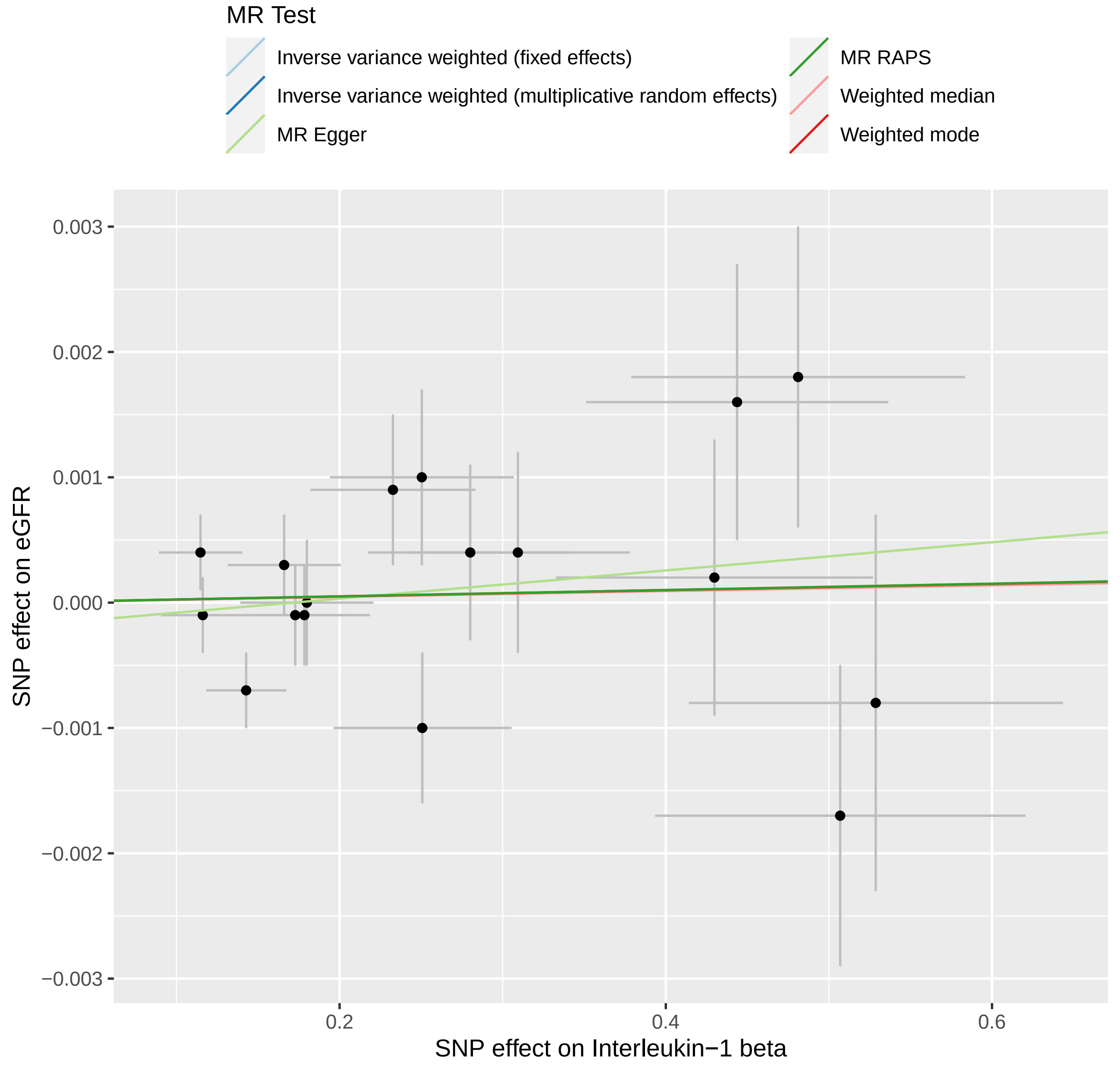

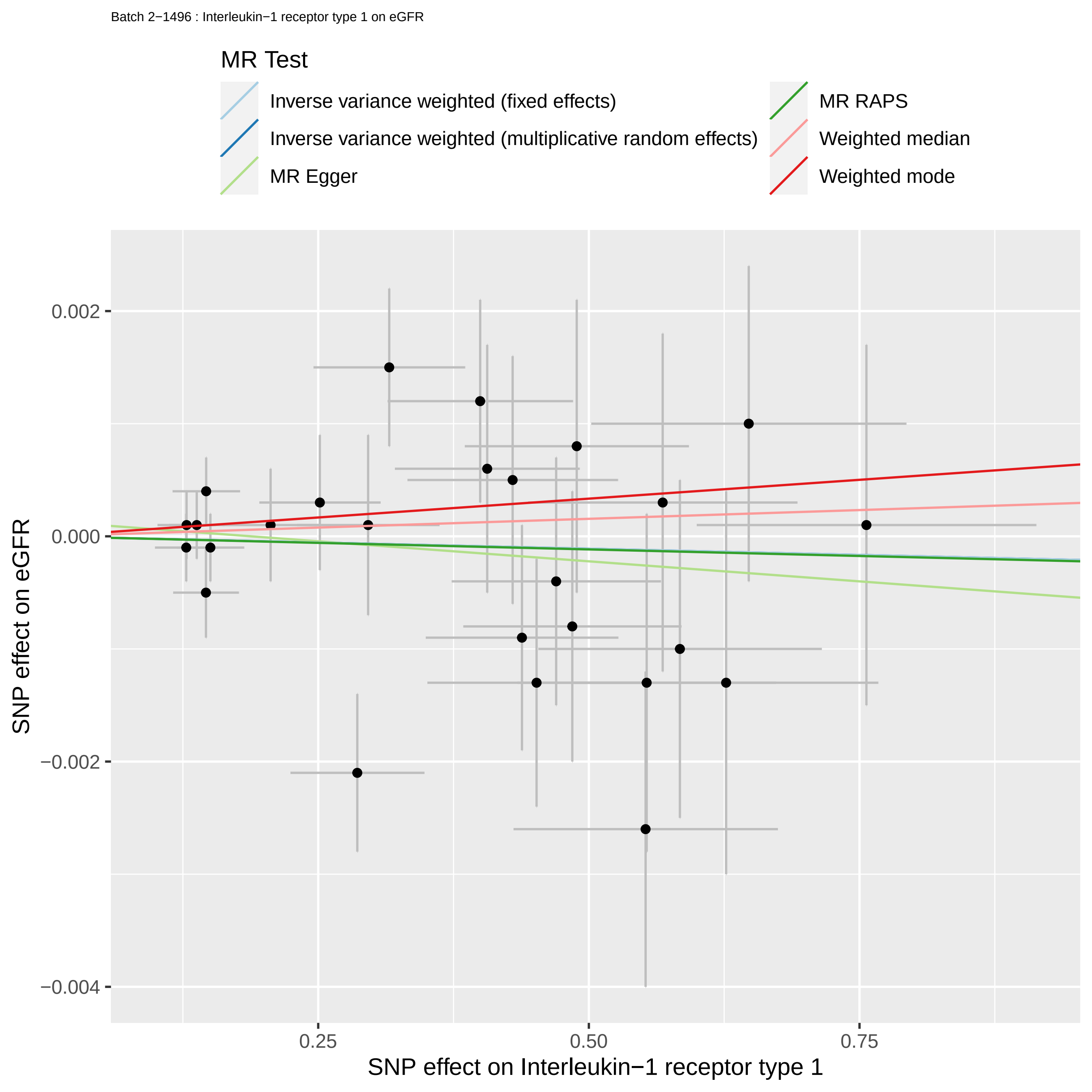

Batch 2−1496 : Interleukin−1 receptor type 1 on eGFR
MR Test
Inverse variance weighted (fixed effects)
Inverse variance weighted (multiplicative random effects)
MR Egger
MR RAPS
Weighted median
Weighted mode
0.002
0.000
−0.002
−0.004
SNP effect on eGFR
0.25
0.50
0.75
SNP effect on Interleukin−1 receptor type 1

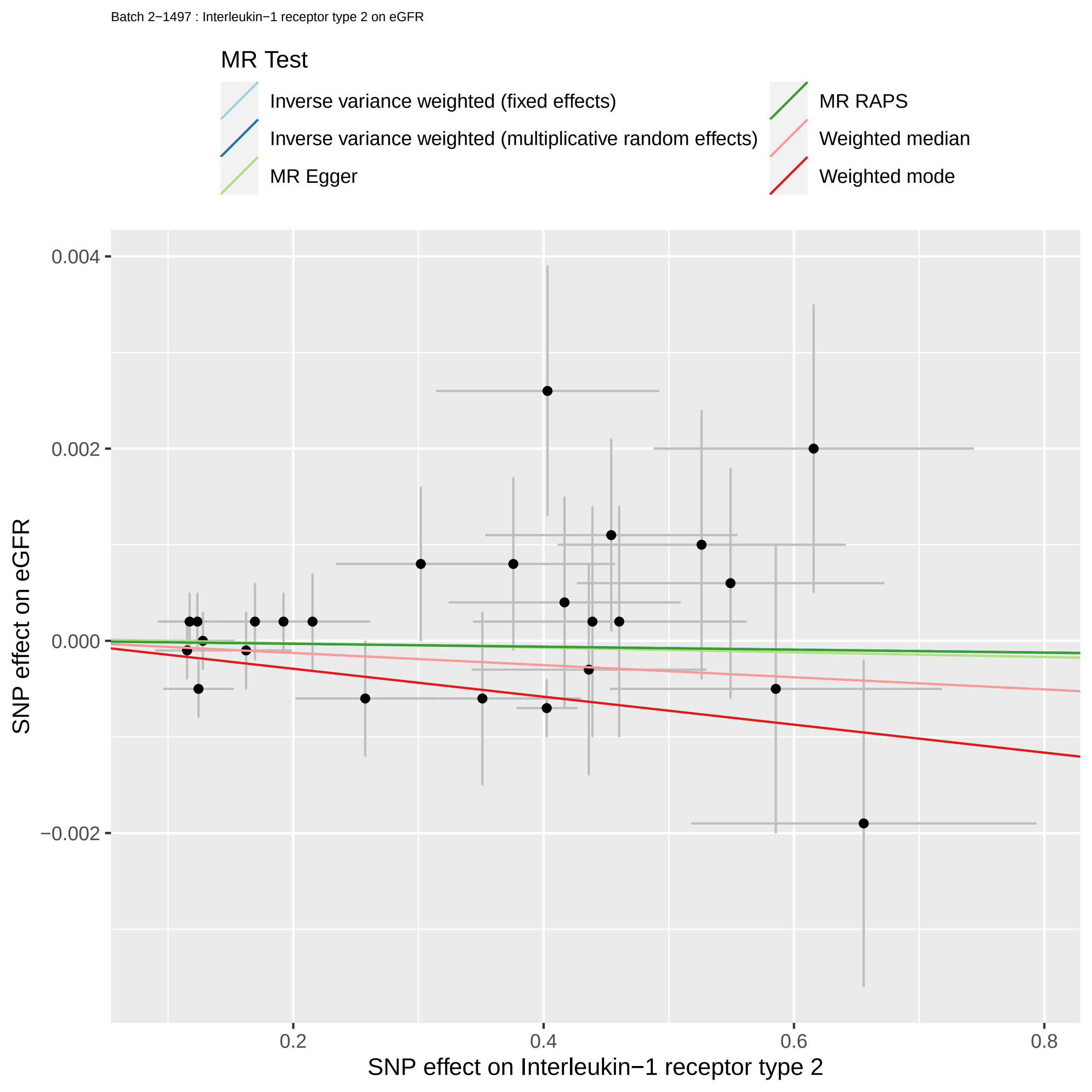

Batch 2−1497 : Interleukin−1 receptor type 2 on eGFR
MR Test
Inverse variance weighted (fixed effects)
Inverse variance weighted (multiplicative random effects)
MR Egger
MR RAPS
Weighted median
Weighted mode
0.004
0.002
0.000
−0.002
SNP effect on eGFR
0.2
0.4
0.6
0.8
SNP effect on Interleukin−1 receptor type 2

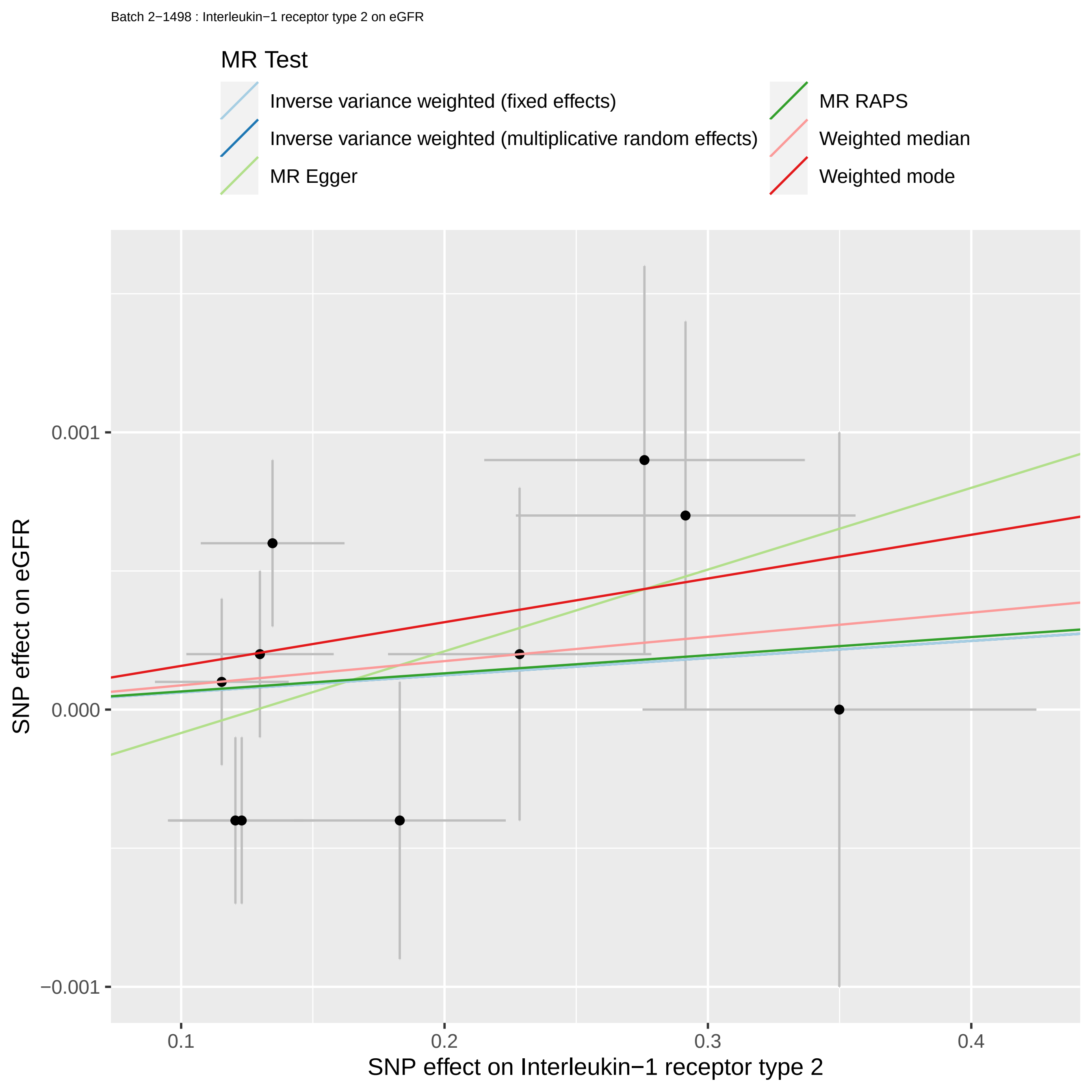

Batch 2−1498 : Interleukin−1 receptor type 2 on eGFR
MR Test
Inverse variance weighted (fixed effects)
Inverse variance weighted (multiplicative random effects)
MR Egger
MR RAPS
Weighted median
Weighted mode
SNP effect on eGFR
0.001
0.000
−0.001
0.1
0.2
0.3
0.4
SNP effect on Interleukin−1 receptor type 2

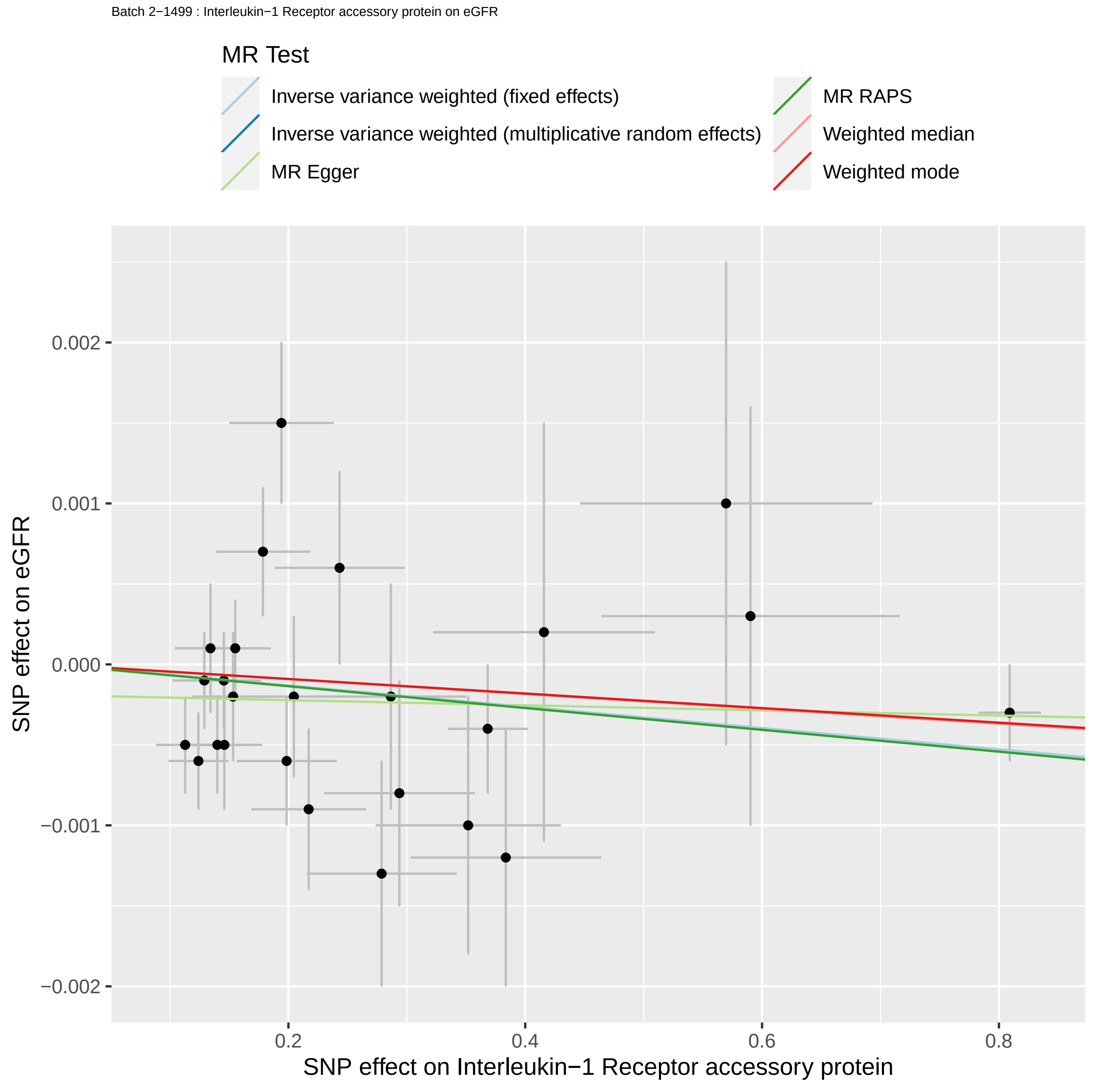

Batch 2−1499 : Interleukin−1 Receptor accessory protein on eGFR
MR Test
Inverse variance weighted (fixed effects)
Inverse variance weighted (multiplicative random effects)
MR Egger
MR RAPS
Weighted median
Weighted mode
0.002
0.001
0.000
−0.001
−0.002
0.2
0.4
0.6
0.8
SNP effect on eGFR
SNP effect on Interleukin−1 Receptor accessory protein

Batch 2−1500 : Interleukin−1 Receptor accessory protein on eGFR

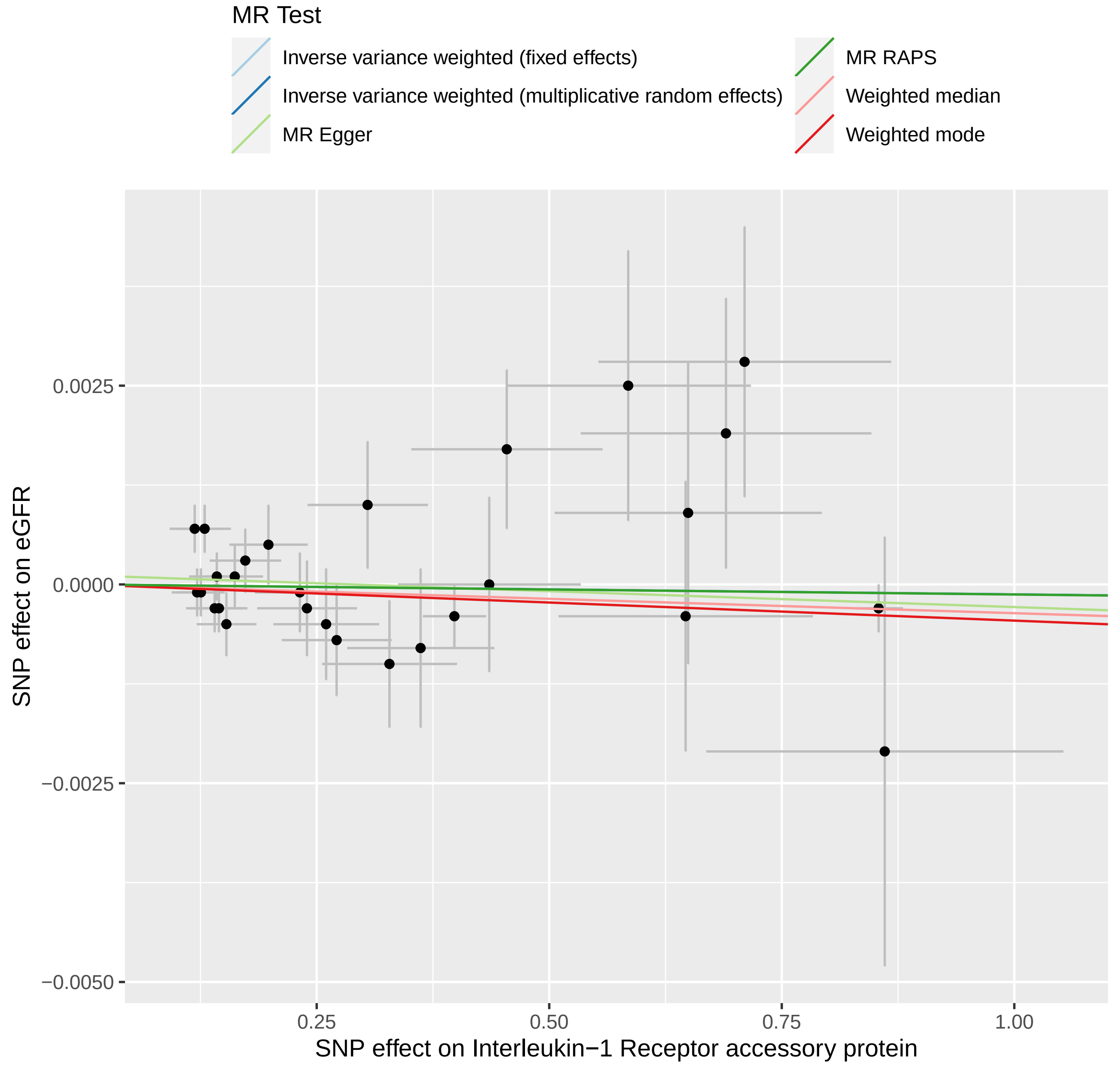

Batch 2−1501 : X−linked interleukin−1 receptor accessory protein−like 2 on eGFR

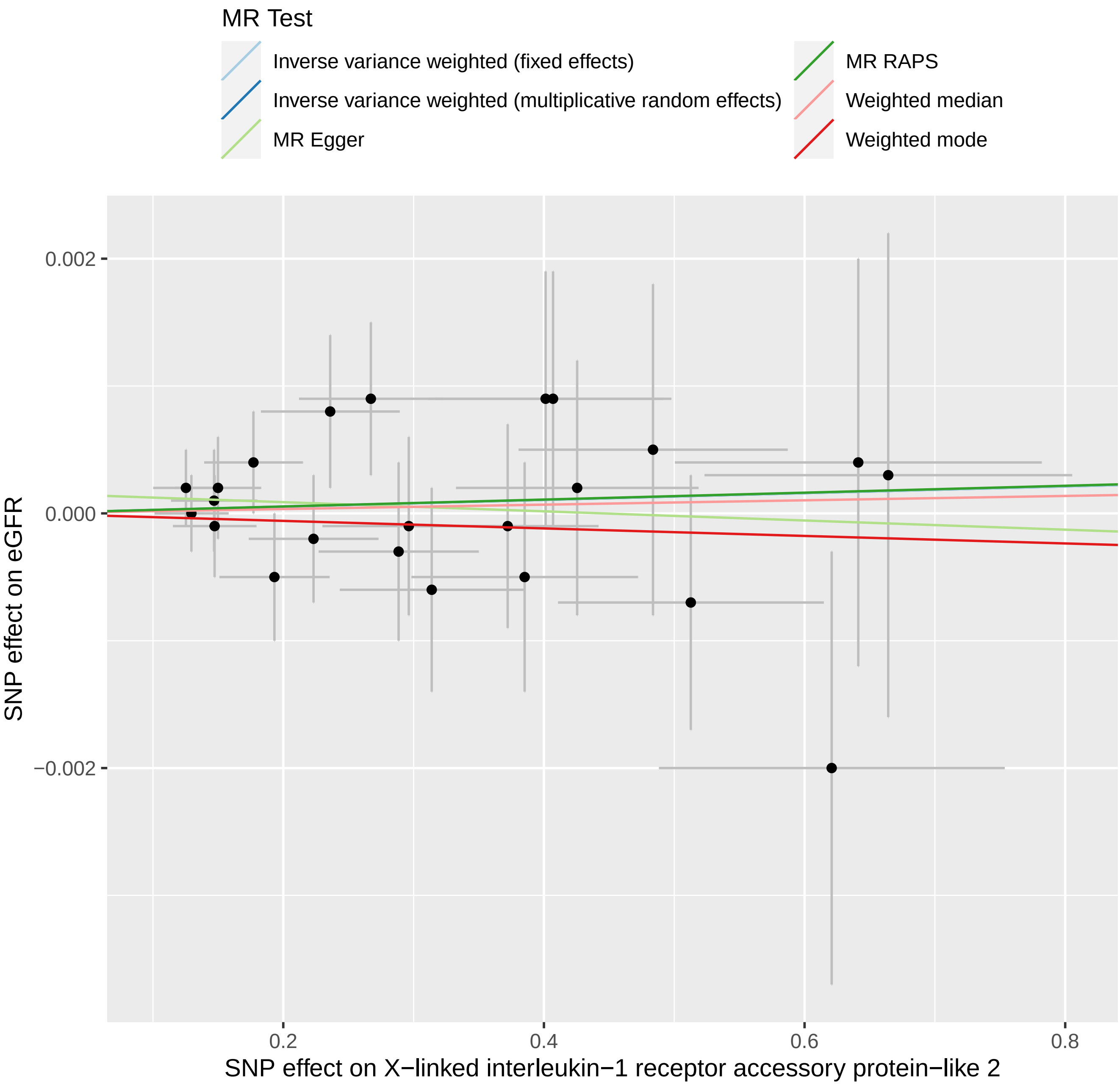

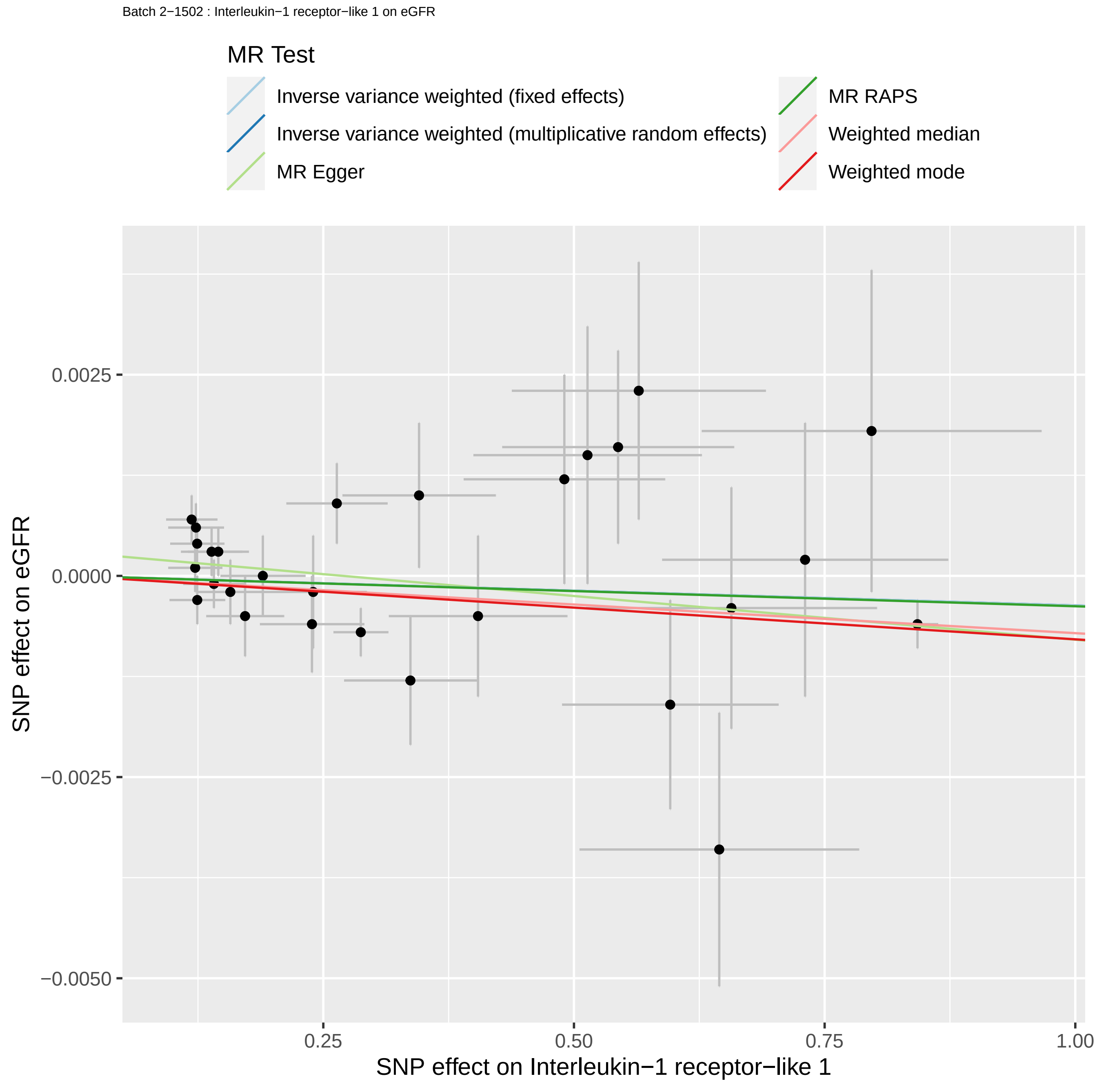
Batch 2−1502 : Interleukin−1 receptor−like 1 on eGFR
MR Test
Inverse variance weighted (fixed effects)
Inverse variance weighted (multiplicative random effects)
MR Egger
MR RAPS
Weighted median
Weighted mode
0.0025
0.0000
−0.0025
−0.0050
0.25
0.50
0.75
1.00
SNP effect on eGFR
SNP effect on Interleukin−1 receptor−like 1

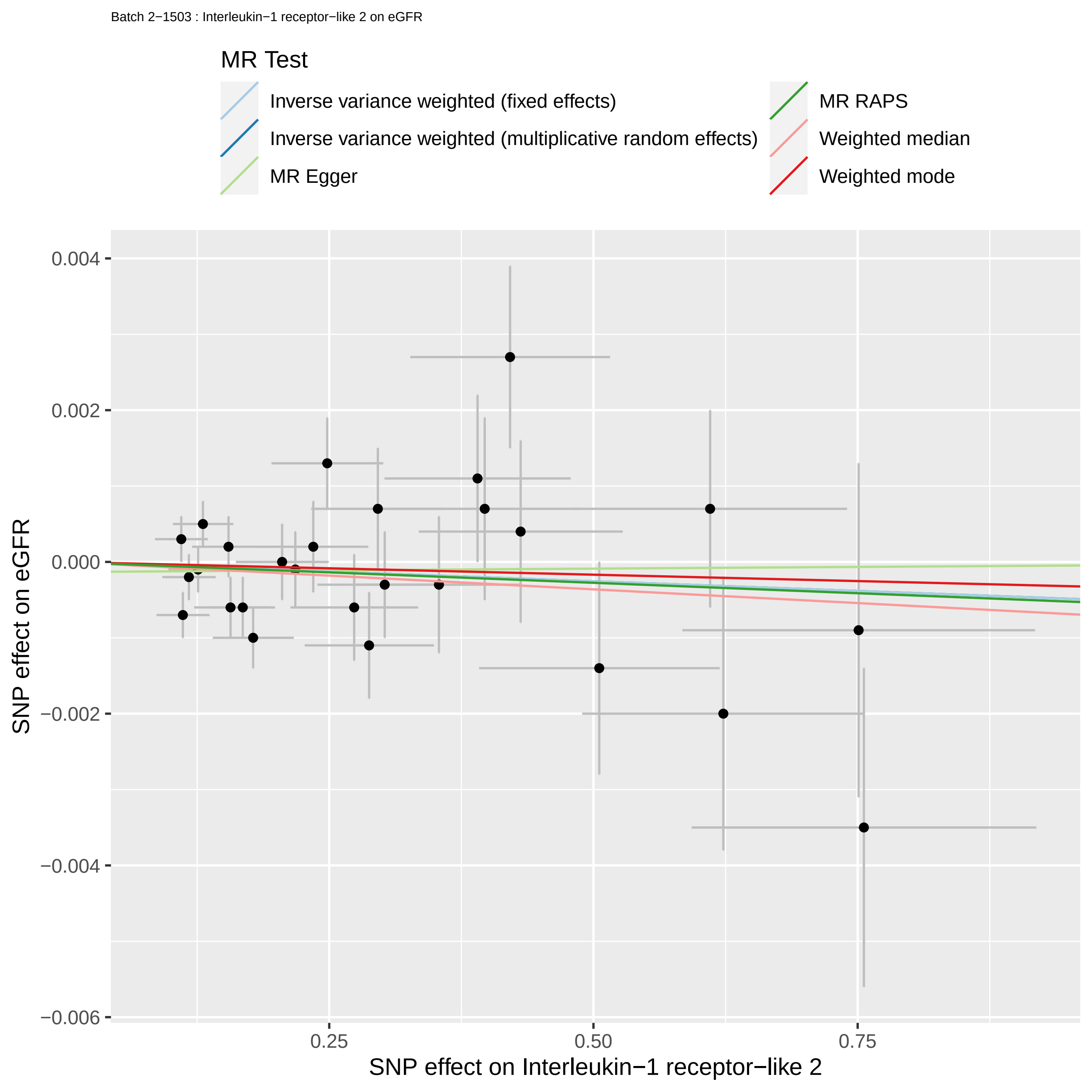
Batch 2−1503 : Interleukin−1 receptor−like 2 on eGFR
MR Test
Inverse variance weighted (fixed effects)
Inverse variance weighted (multiplicative random effects)
MR Egger
MR RAPS
Weighted median
Weighted mode
0.004
0.002
0.000
−0.002
−0.004
−0.006
0.25
0.50
0.75
SNP effect on eGFR
SNP effect on Interleukin−1 receptor−like 2

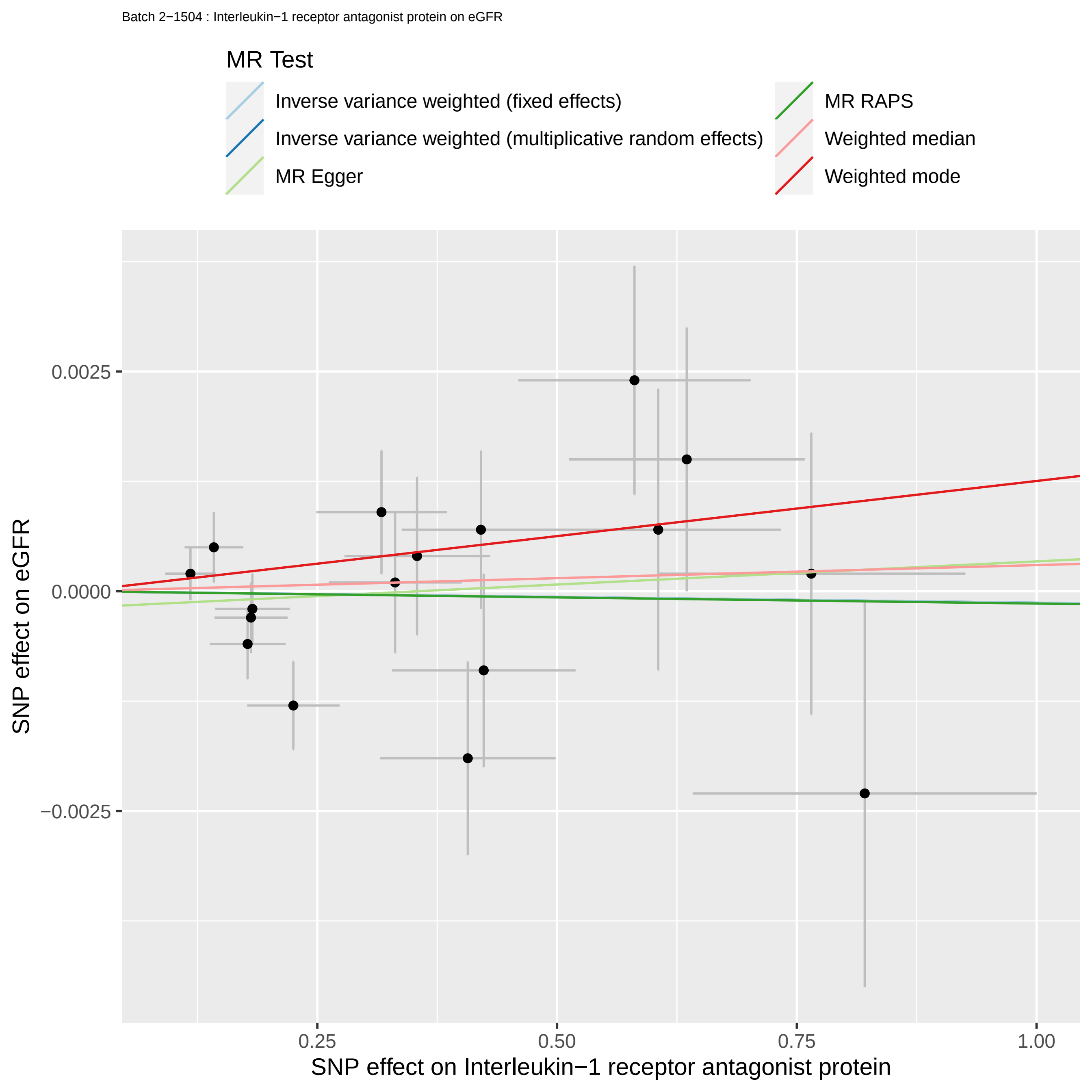

Batch 2−1504 : Interleukin−1 receptor antagonist protein on eGFR
MR Test
Inverse variance weighted (fixed effects)
Inverse variance weighted (multiplicative random effects)
MR Egger
MR RAPS
Weighted median
Weighted mode
0.0025
0.0000
−0.0025
0.25
0.50
0.75
1.00
SNP effect on eGFR
SNP effect on Interleukin−1 receptor antagonist protein

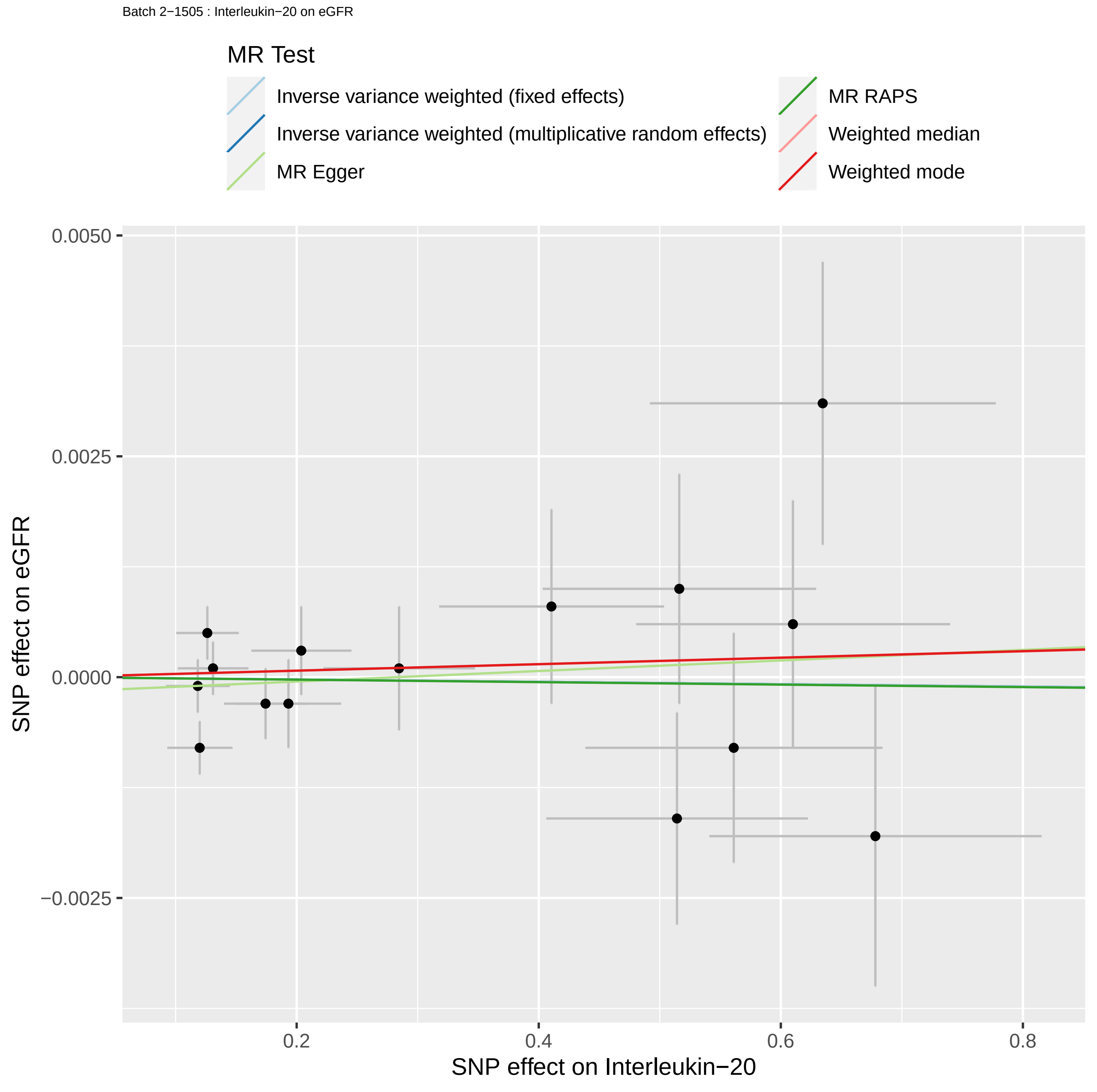
Batch 2−1505 : Interleukin−20 on eGFR
MR Test
Inverse variance weighted (fixed effects)
Inverse variance weighted (multiplicative random effects)
MR Egger
MR RAPS
Weighted median
Weighted mode
0.0050
0.0025
0.0000
−0.0025
0.2
0.4
0.6
0.8
SNP effect on eGFR
SNP effect on Interleukin−20

Batch 2−1506 : Interleukin−21 on eGFR

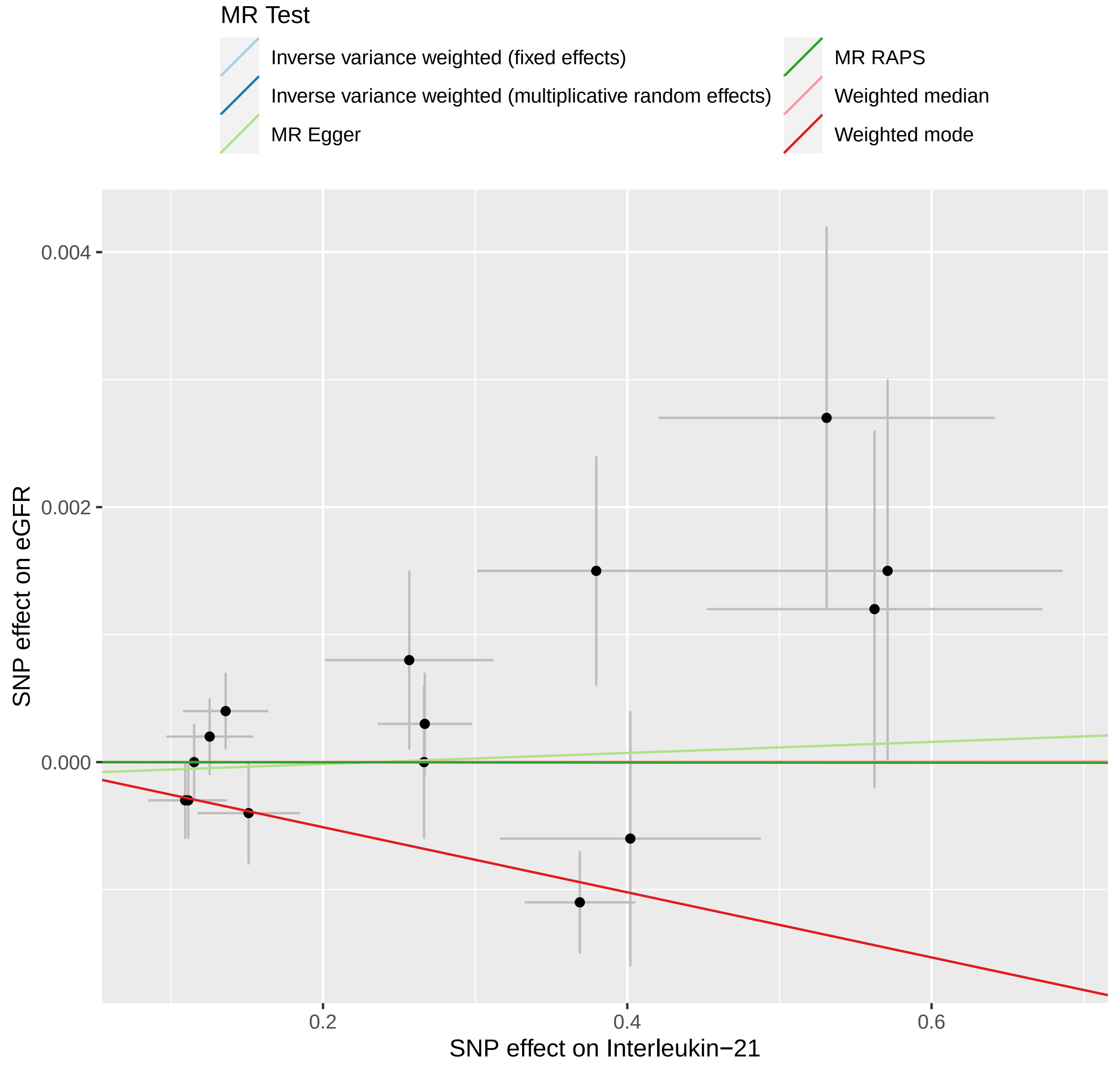

Batch 2−1507 : Interleukin−22 on eGFR

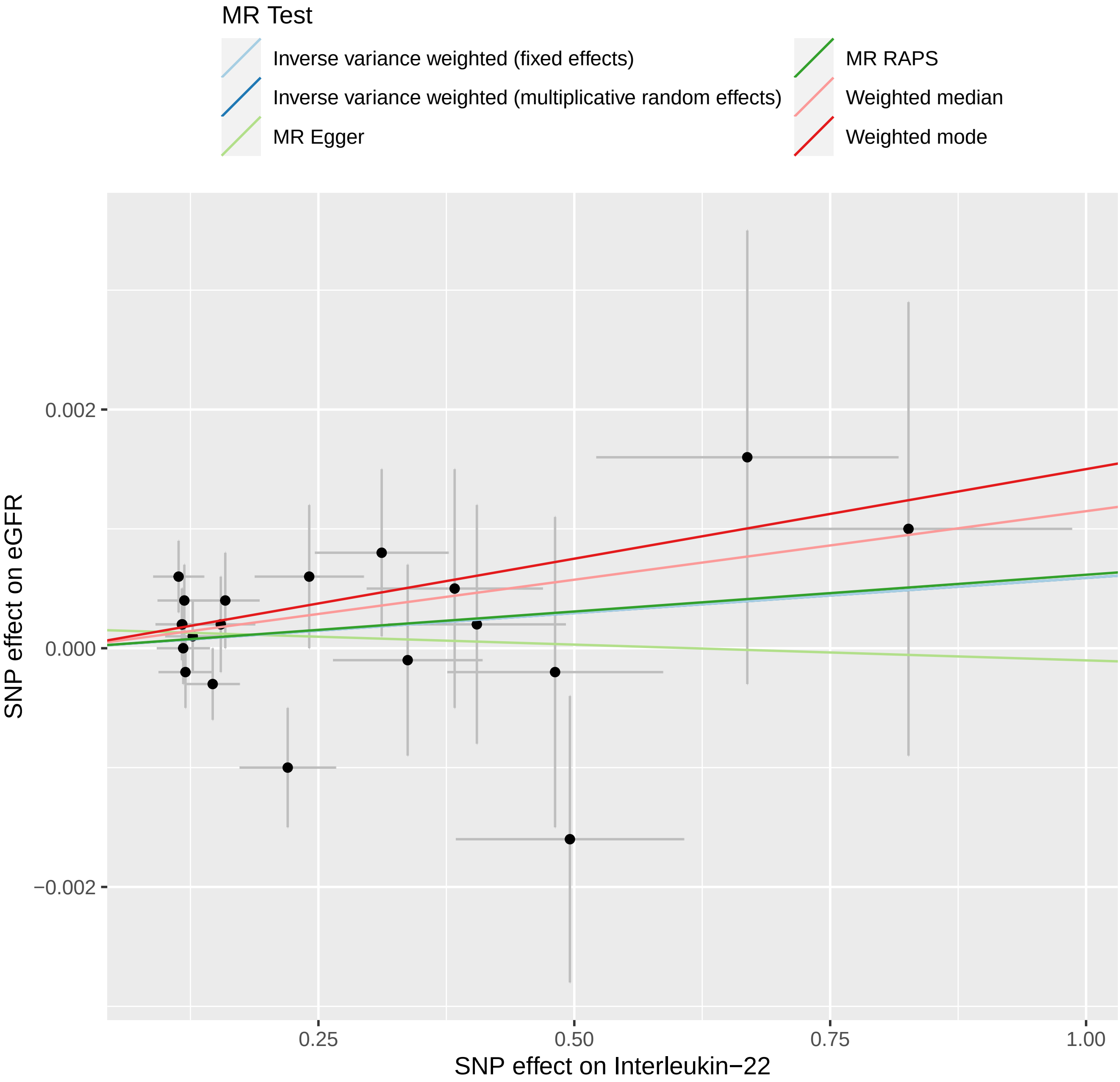

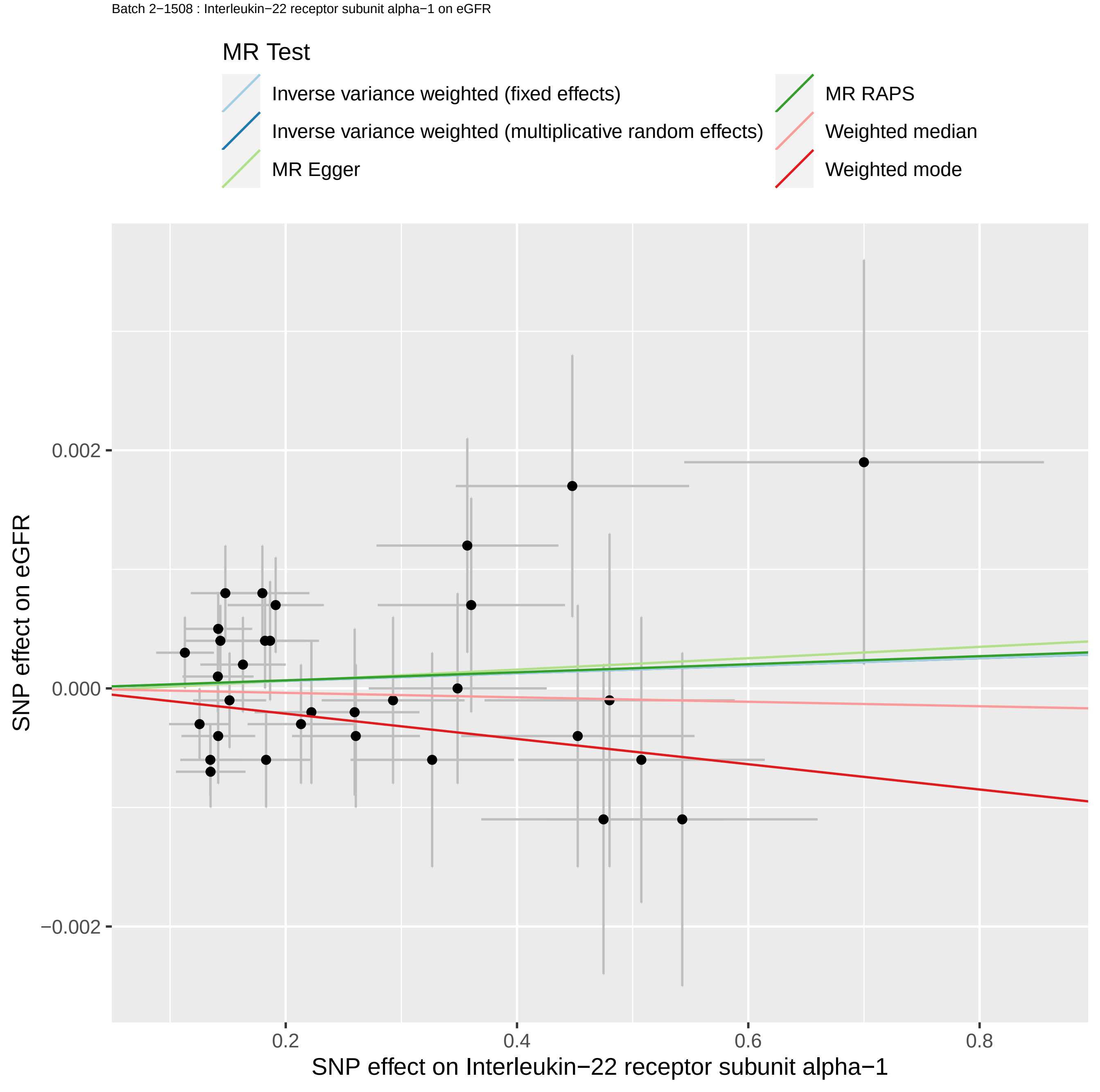

Batch 2−1508 : Interleukin−22 receptor subunit alpha−1 on eGFR
MR Test
Inverse variance weighted (fixed effects)
Inverse variance weighted (multiplicative random effects)
MR Egger
MR RAPS
Weighted median
Weighted mode
0.002
0.000
−0.002
SNP effect on eGFR
0.2
0.4
0.6
0.8
SNP effect on Interleukin−22 receptor subunit alpha−1

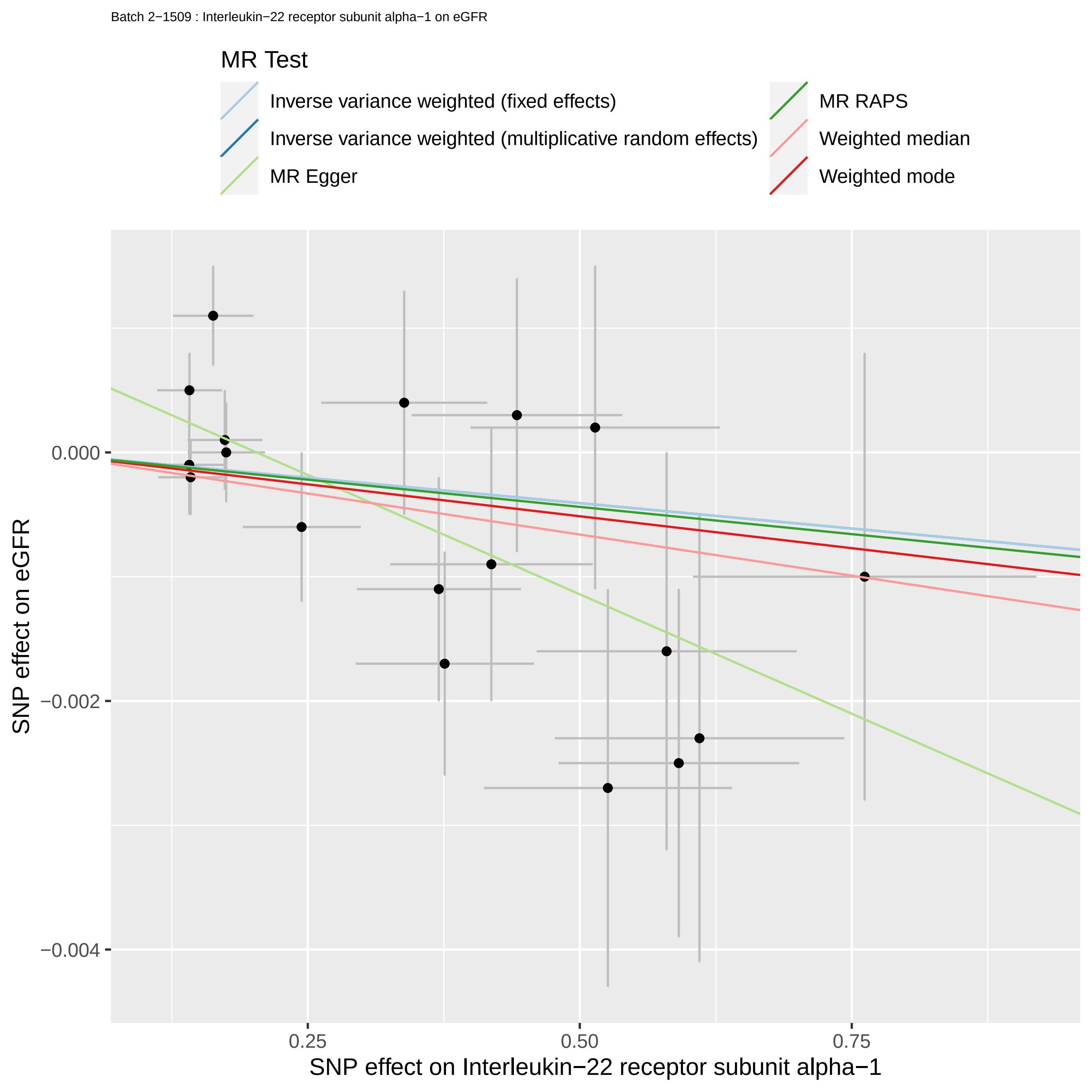

Batch 2−1509 : Interleukin−22 receptor subunit alpha−1 on eGFR
MR Test
Inverse variance weighted (fixed effects)
Inverse variance weighted (multiplicative random effects)
MR Egger
MR RAPS
Weighted median
Weighted mode
0.000
−0.002
−0.004
0.25
0.50
0.75
SNP effect on eGFR
SNP effect on Interleukin−22 receptor subunit alpha−1

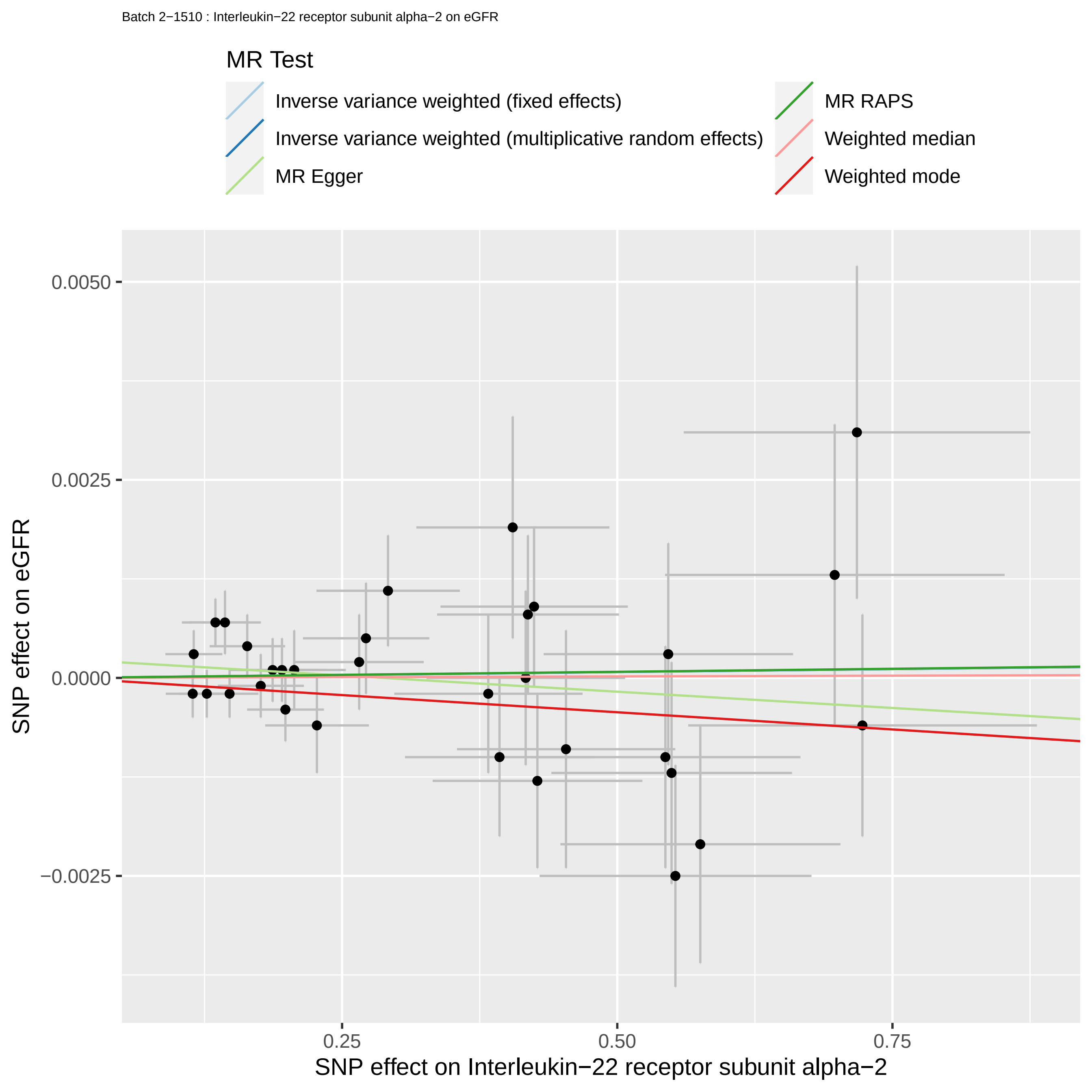

Batch 2−1510 : Interleukin−22 receptor subunit alpha−2 on eGFR
MR Test
Inverse variance weighted (fixed effects)
Inverse variance weighted (multiplicative random effects)
MR Egger
MR RAPS
Weighted median
Weighted mode
0.0050
0.0025
0.0000
−0.0025
SNP effect on eGFR
0.25
0.50
0.75
SNP effect on Interleukin−22 receptor subunit alpha−2

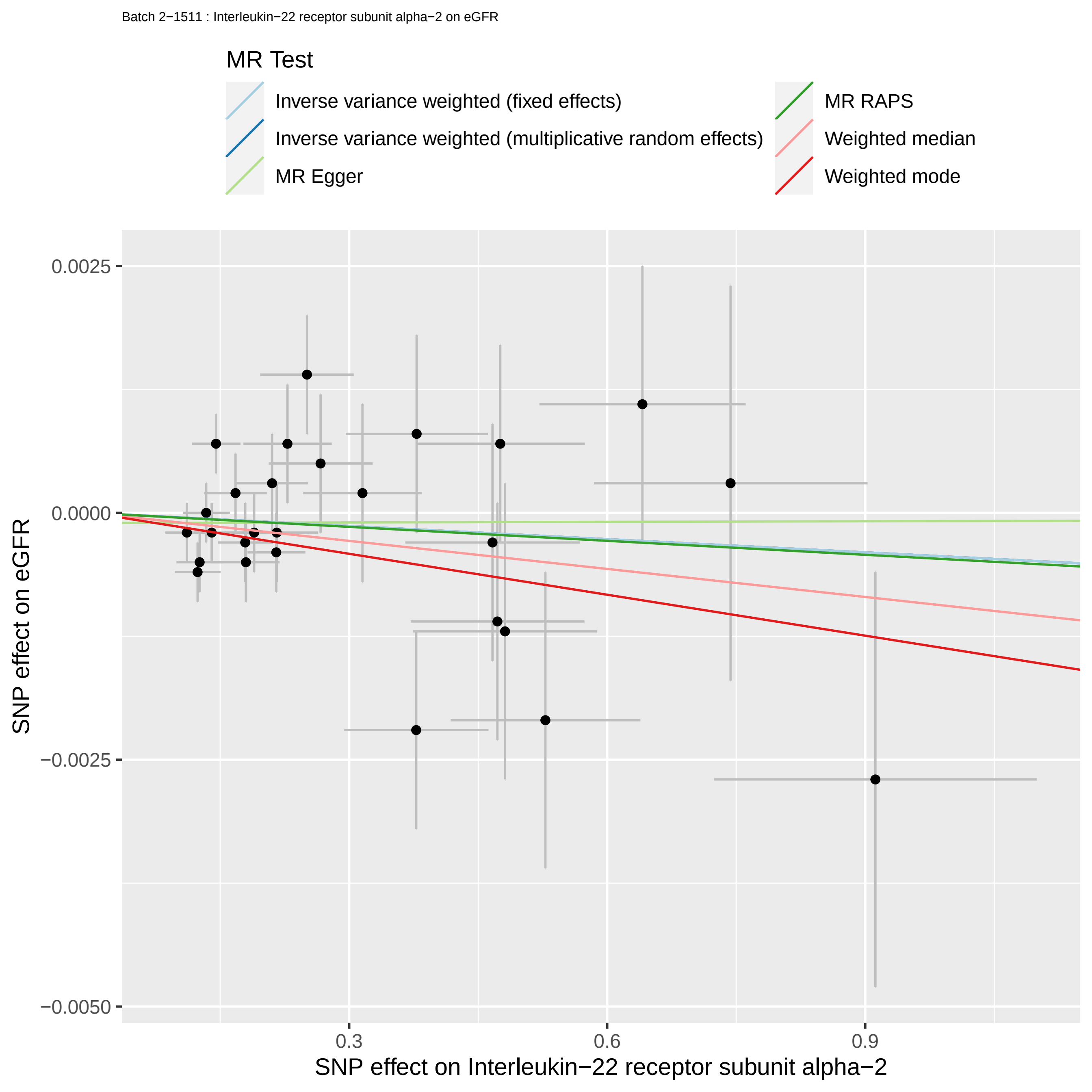

Batch 2−1511 : Interleukin−22 receptor subunit alpha−2 on eGFR
MR Test
Inverse variance weighted (fixed effects)
Inverse variance weighted (multiplicative random effects)
MR Egger
MR RAPS
Weighted median
Weighted mode
0.0025
0.0000
−0.0025
−0.0050
SNP effect on eGFR
0.3
0.6
0.9
SNP effect on Interleukin−22 receptor subunit alpha−2

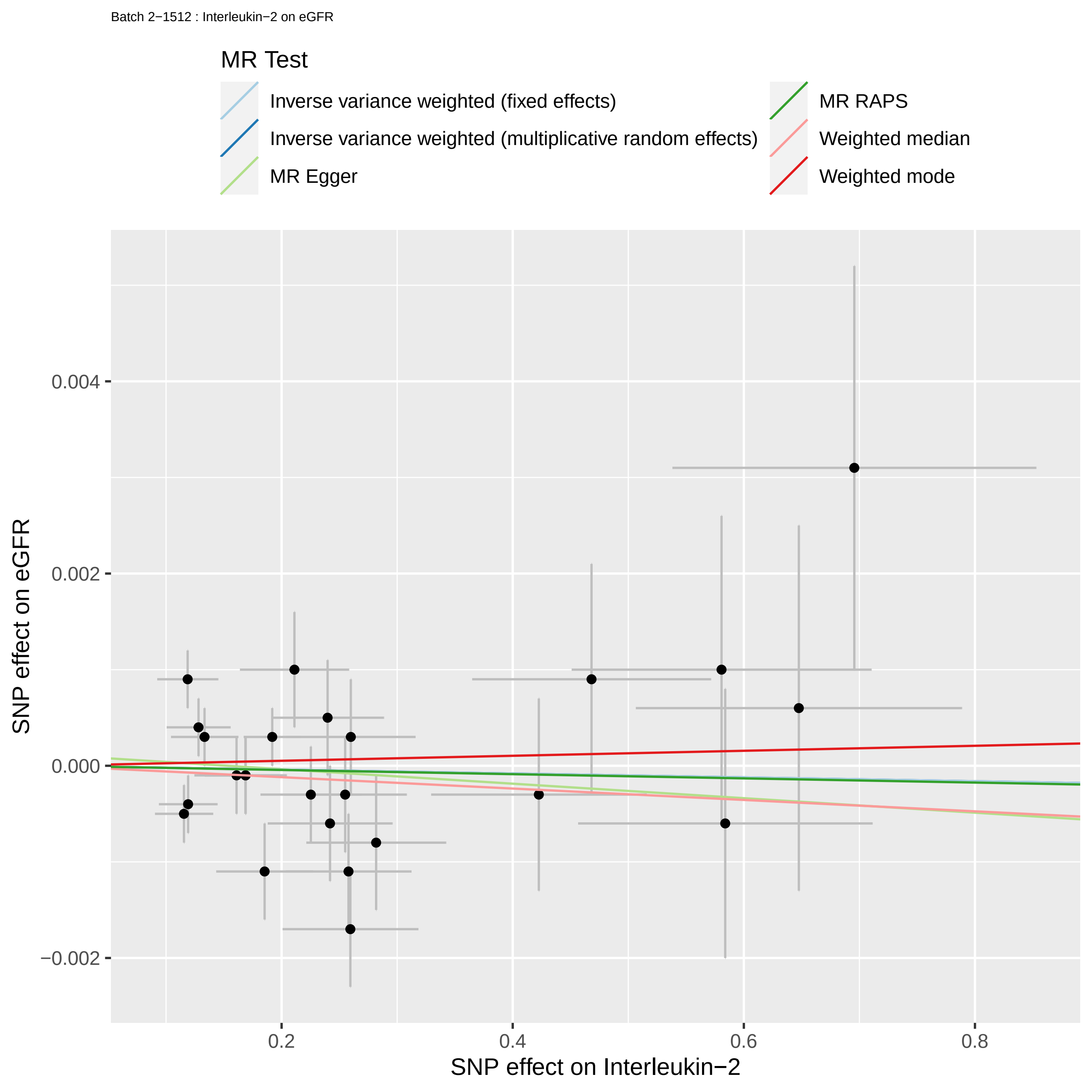

Batch 2−1512 : Interleukin−2 on eGFR
MR Test
Inverse variance weighted (fixed effects)
Inverse variance weighted (multiplicative random effects)
MR Egger
MR RAPS
Weighted median
Weighted mode
0.004
0.002
0.000
−0.002
0.2
0.4
0.6
0.8
SNP effect on eGFR
SNP effect on Interleukin−2

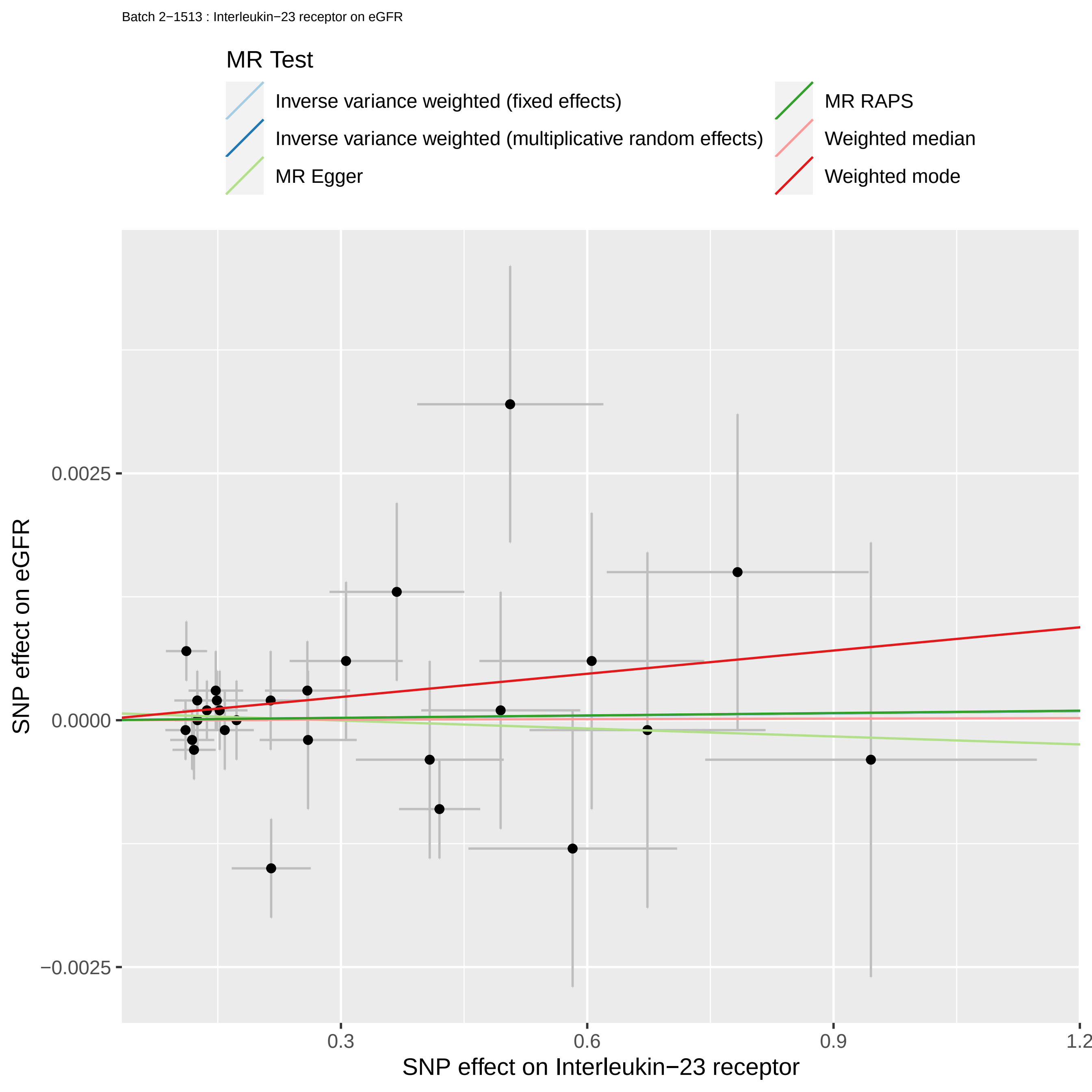
Batch 2−1513 : Interleukin−23 receptor on eGFR
MR Test
Inverse variance weighted (fixed effects)
Inverse variance weighted (multiplicative random effects)
MR Egger
MR RAPS
Weighted median
Weighted mode
0.0025
0.0000
−0.0025
0.3
0.6
0.9
1.2
SNP effect on eGFR
SNP effect on Interleukin−23 receptor

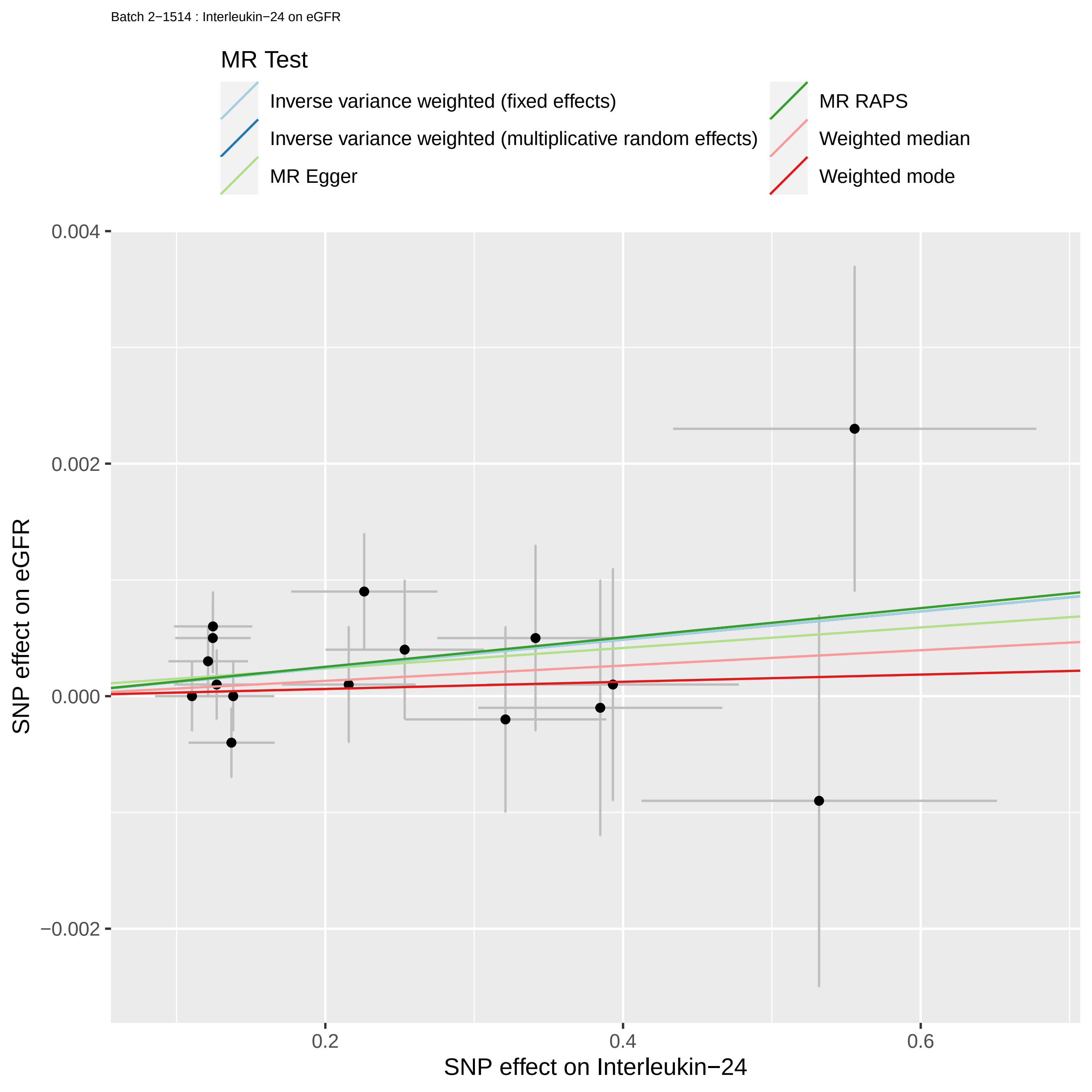

Batch 2−1514 : Interleukin−24 on eGFR
MR Test
Inverse variance weighted (fixed effects)
Inverse variance weighted (multiplicative random effects)
MR Egger
MR RAPS
Weighted median
Weighted mode
0.004
0.002
0.000
−0.002
SNP effect on eGFR
0.2
0.4
0.6
SNP effect on Interleukin−24

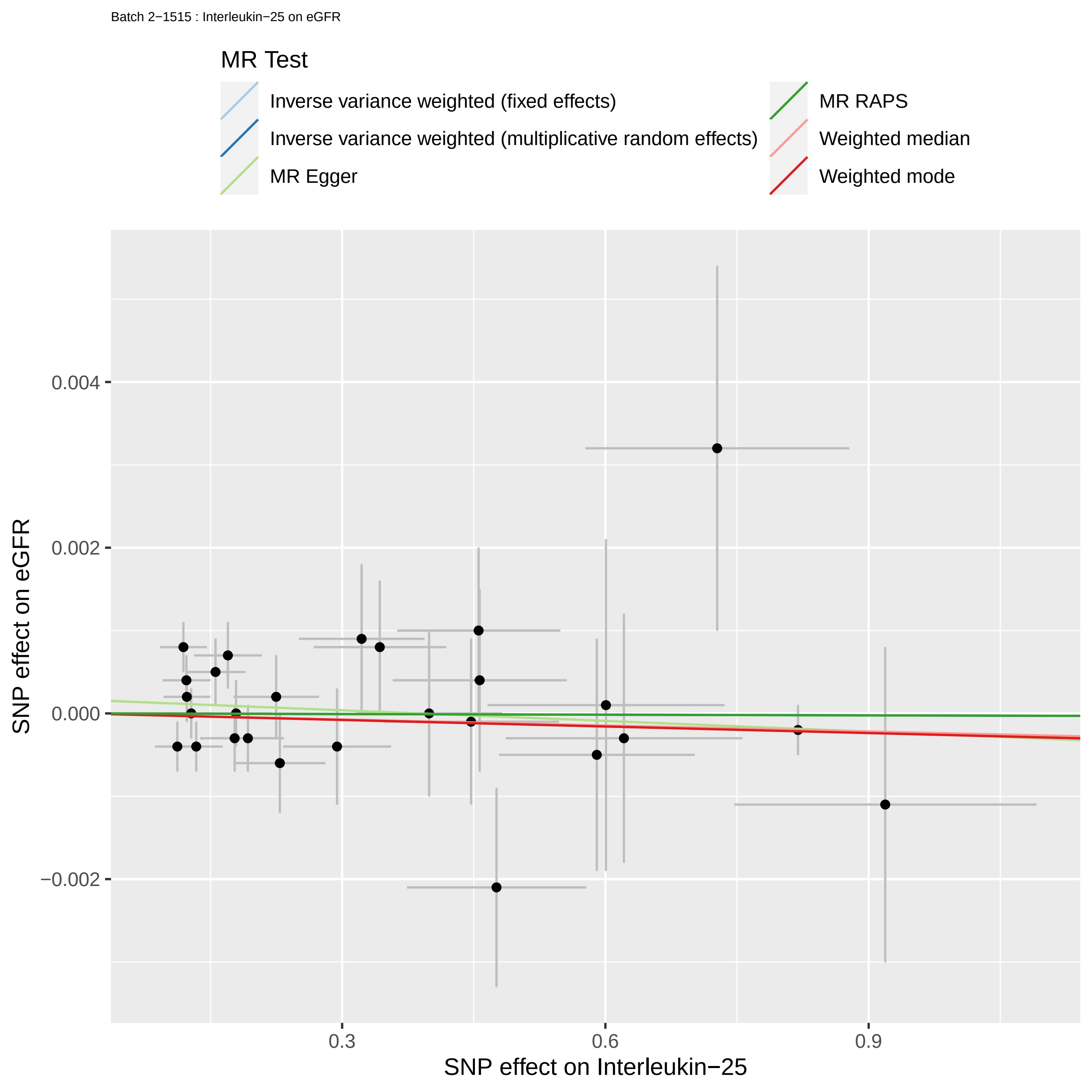

Batch 2−1515 : Interleukin−25 on eGFR
MR Test
Inverse variance weighted (fixed effects)
Inverse variance weighted (multiplicative random effects)
MR Egger
MR RAPS
Weighted median
Weighted mode
0.004
0.002
0.000
−0.002
0.3
0.6
0.9
SNP effect on eGFR
SNP effect on Interleukin−25

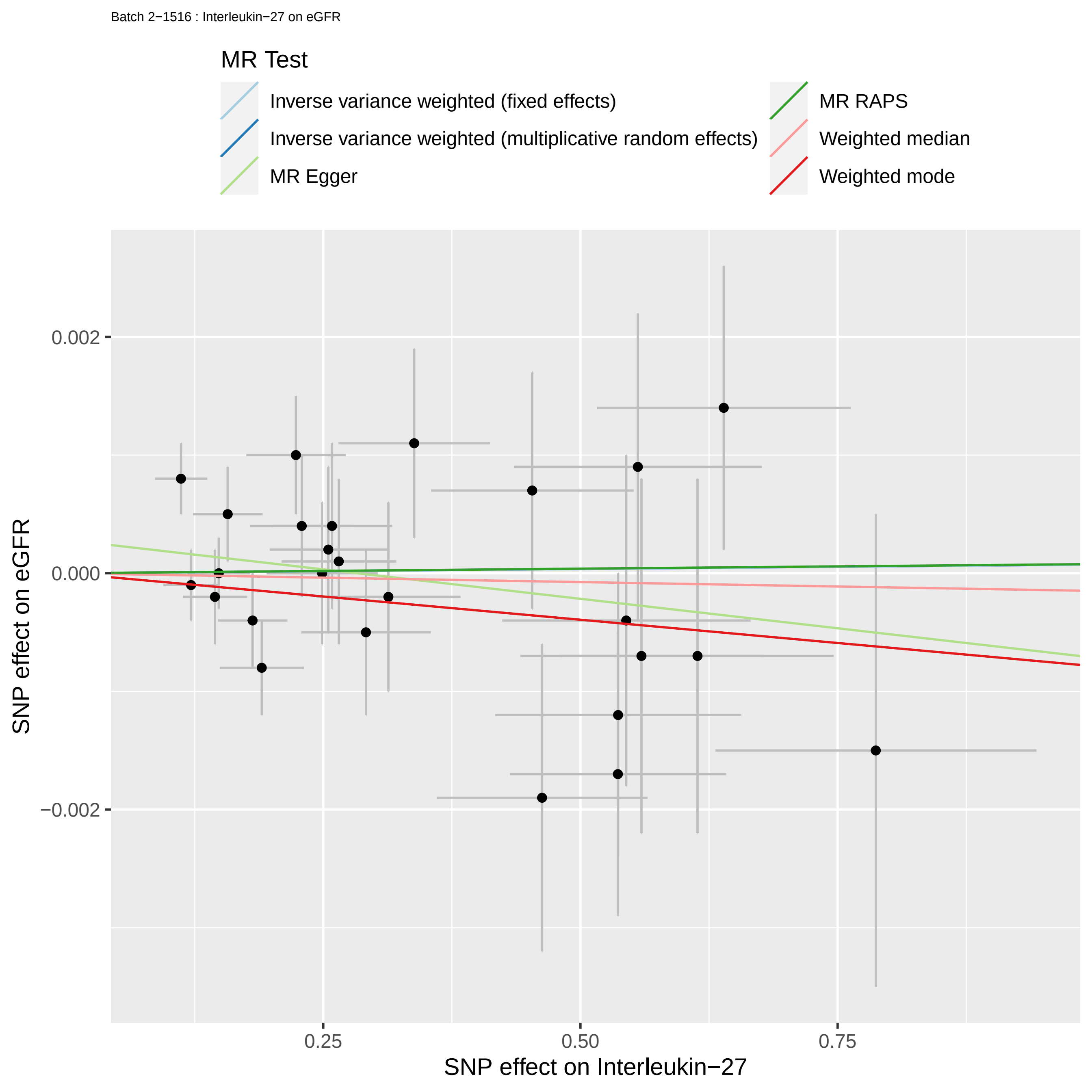

Batch 2−1516 : Interleukin−27 on eGFR
MR Test
Inverse variance weighted (fixed effects)
Inverse variance weighted (multiplicative random effects)
MR Egger
MR RAPS
Weighted median
Weighted mode
0.002
0.000
−0.002
0.25
0.50
0.75
SNP effect on eGFR
SNP effect on Interleukin−27

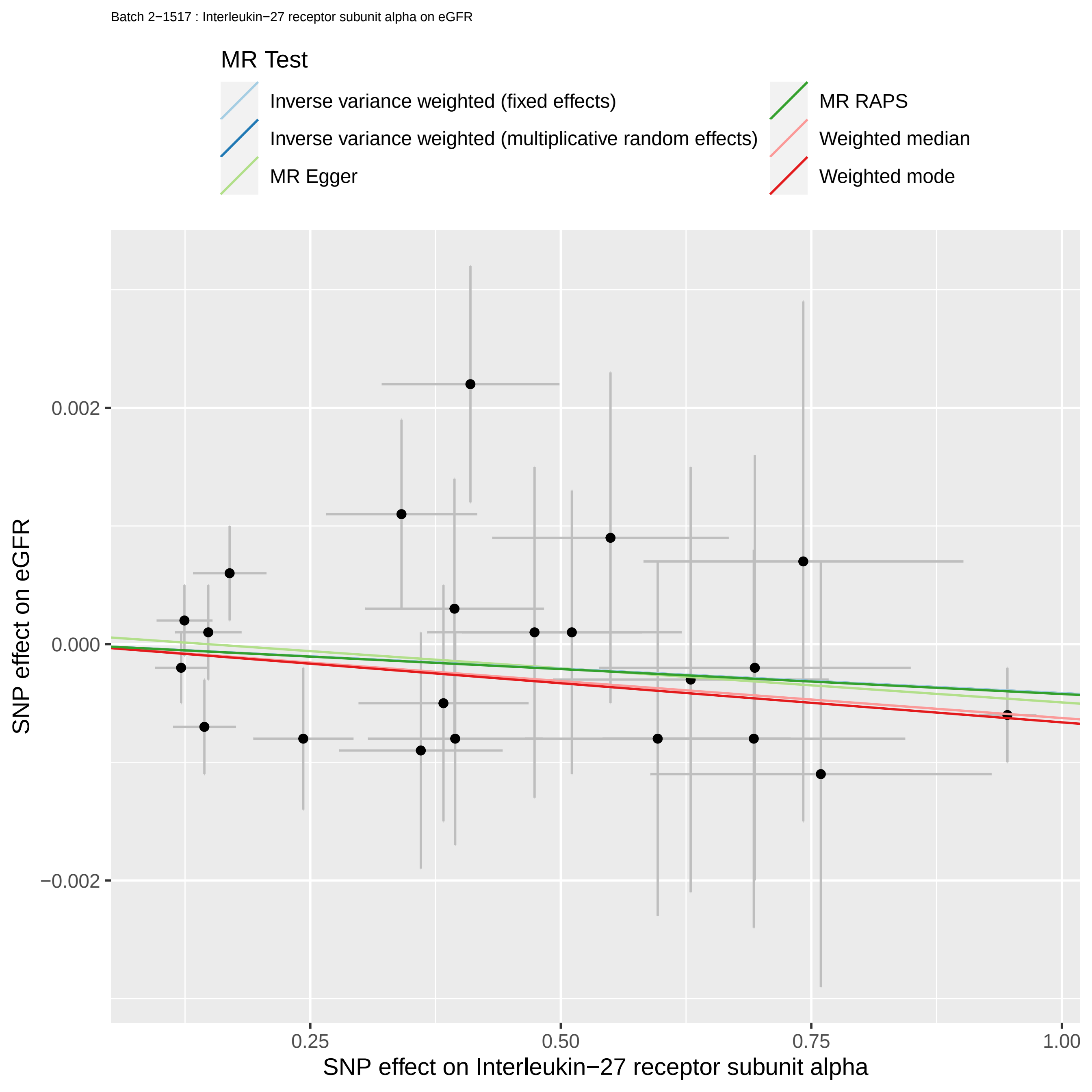

Batch 2−1517 : Interleukin−27 receptor subunit alpha on eGFR
MR Test
Inverse variance weighted (fixed effects)
Inverse variance weighted (multiplicative random effects)
MR Egger
MR RAPS
Weighted median
Weighted mode
0.002
0.000
−0.002
0.25
0.50
0.75
1.00
SNP effect on eGFR
SNP effect on Interleukin−27 receptor subunit alpha

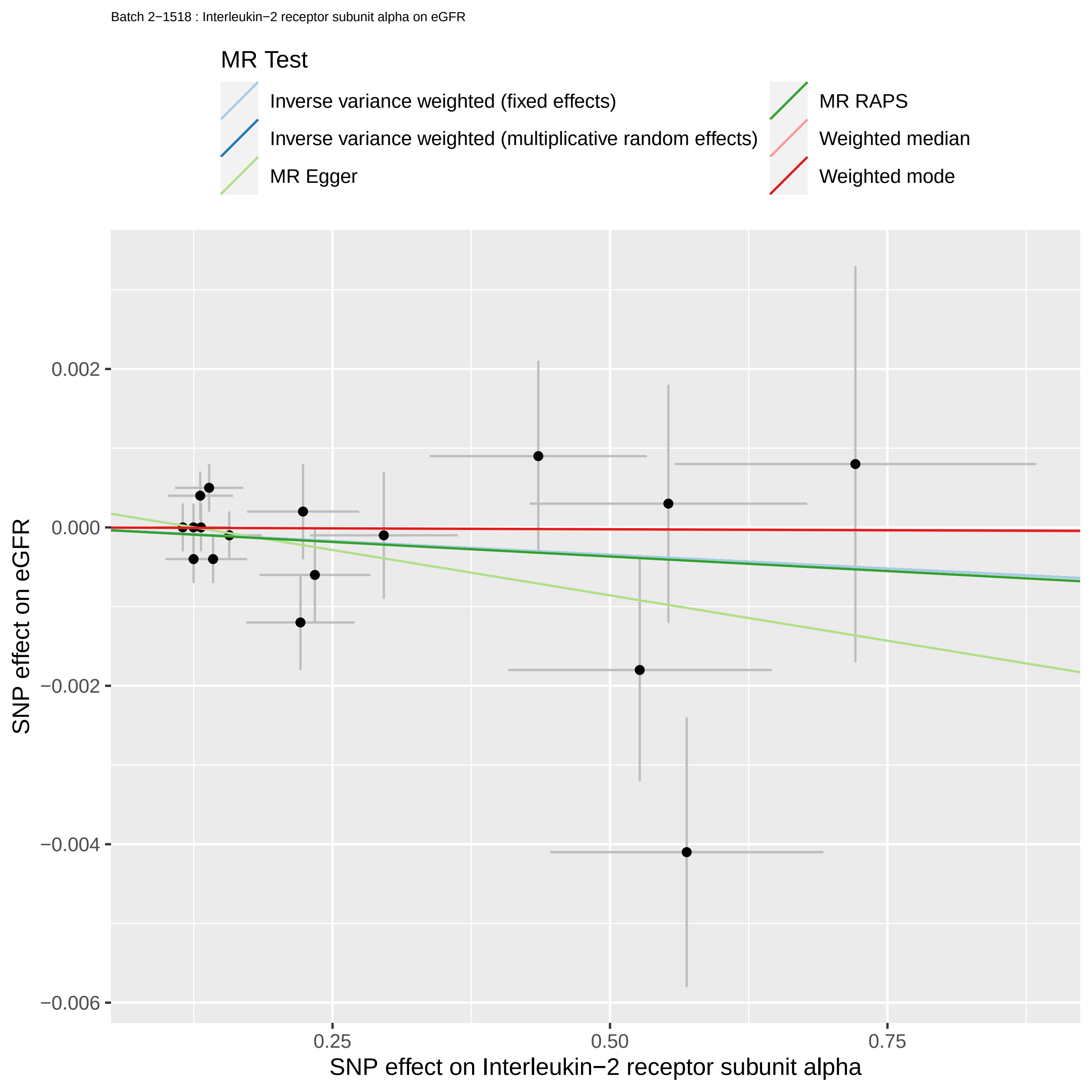
Batch 2−1518 : Interleukin−2 receptor subunit alpha on eGFR
MR Test
Inverse variance weighted (fixed effects)
Inverse variance weighted (multiplicative random effects)
MR Egger
MR RAPS
Weighted median
Weighted mode
0.002
0.000
−0.002
−0.004
−0.006
0.25
0.50
0.75
SNP effect on eGFR
SNP effect on Interleukin−2 receptor subunit alpha

Batch 2−1519 : Interleukin−2 receptor subunit beta on eGFR

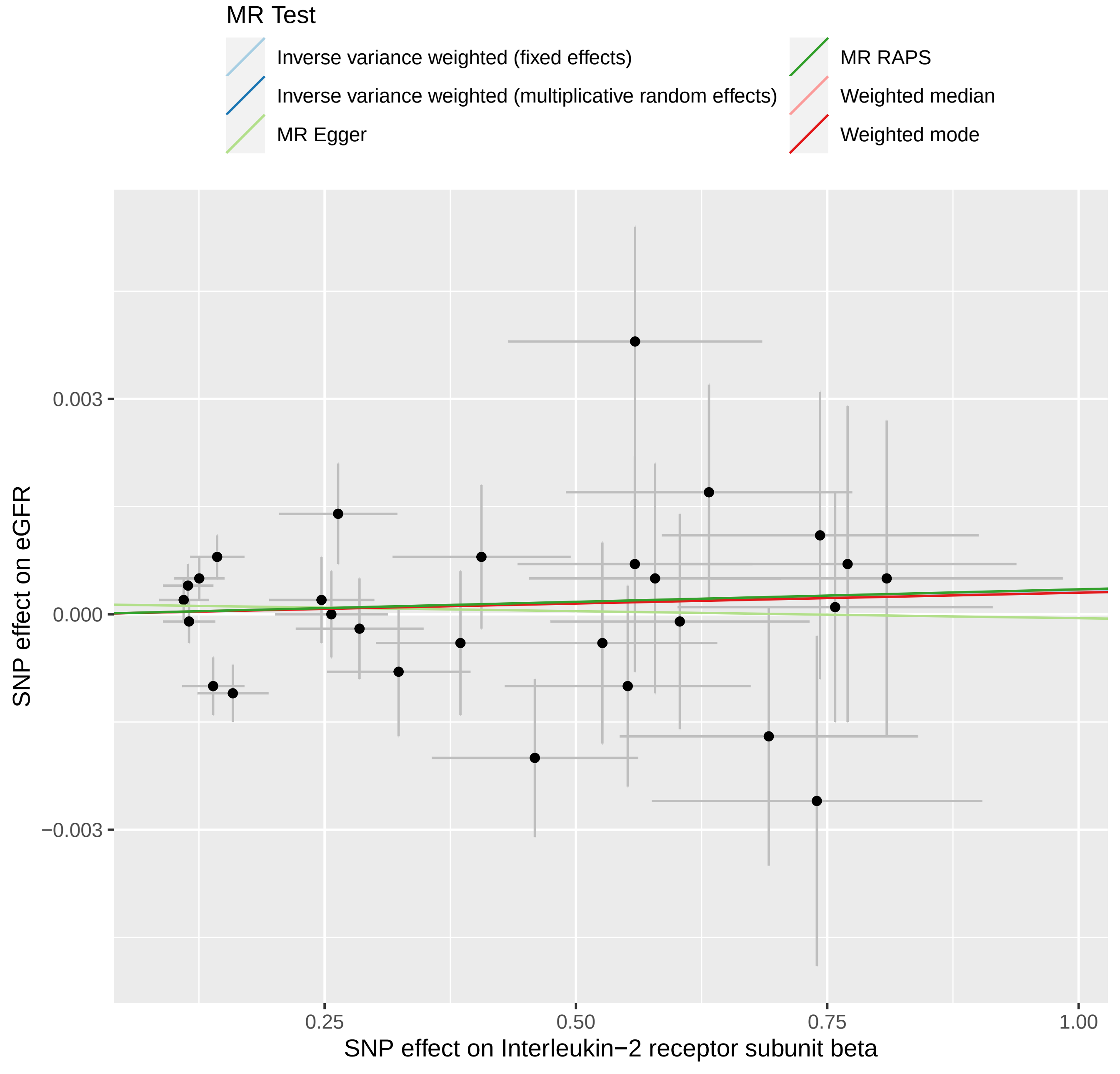

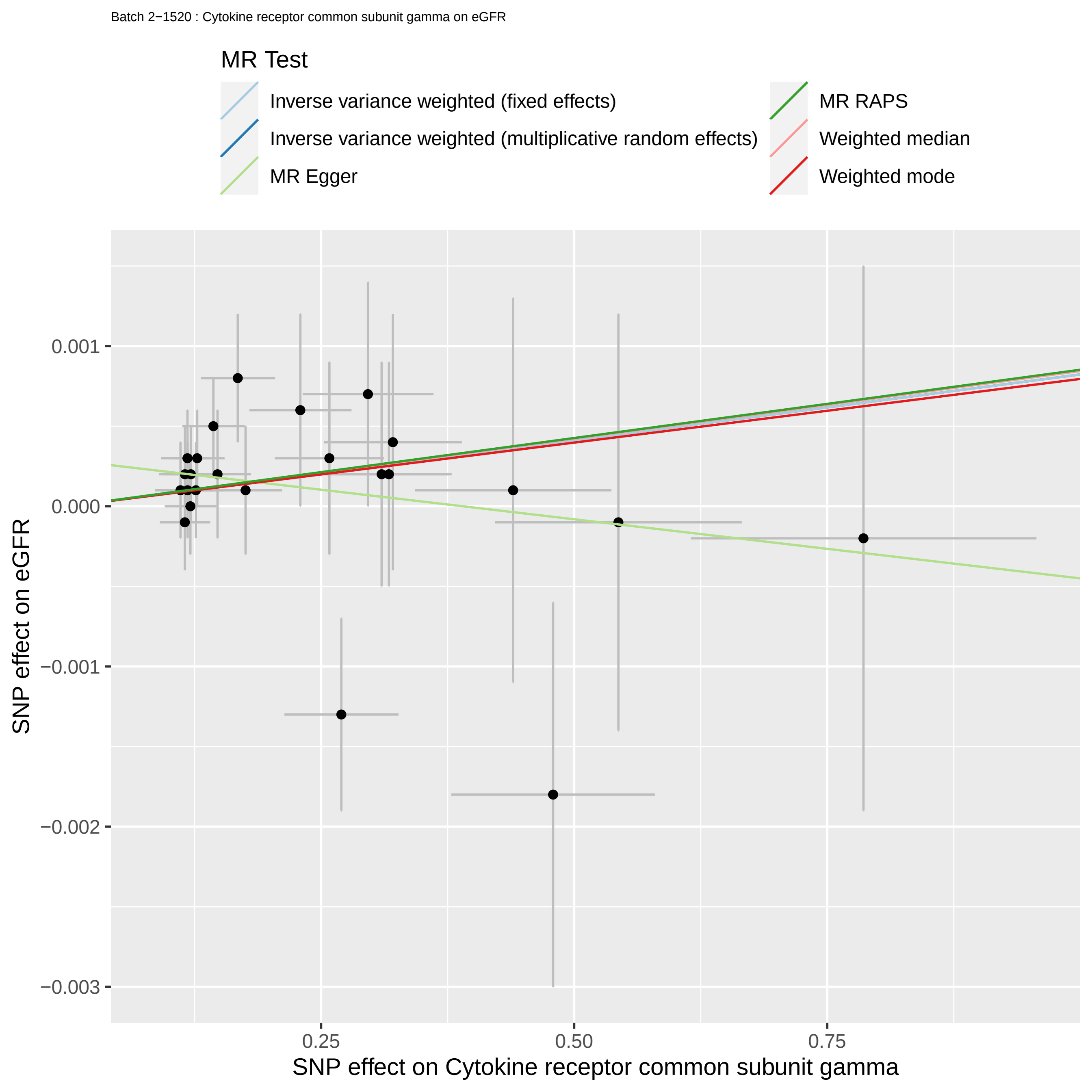

Batch 2−1520 : Cytokine receptor common subunit gamma on eGFR
MR Test
Inverse variance weighted (fixed effects)
Inverse variance weighted (multiplicative random effects)
MR Egger
MR RAPS
Weighted median
Weighted mode
0.001
0.000
−0.001
−0.002
−0.003
0.25
0.50
0.75
SNP effect on eGFR
SNP effect on Cytokine receptor common subunit gamma

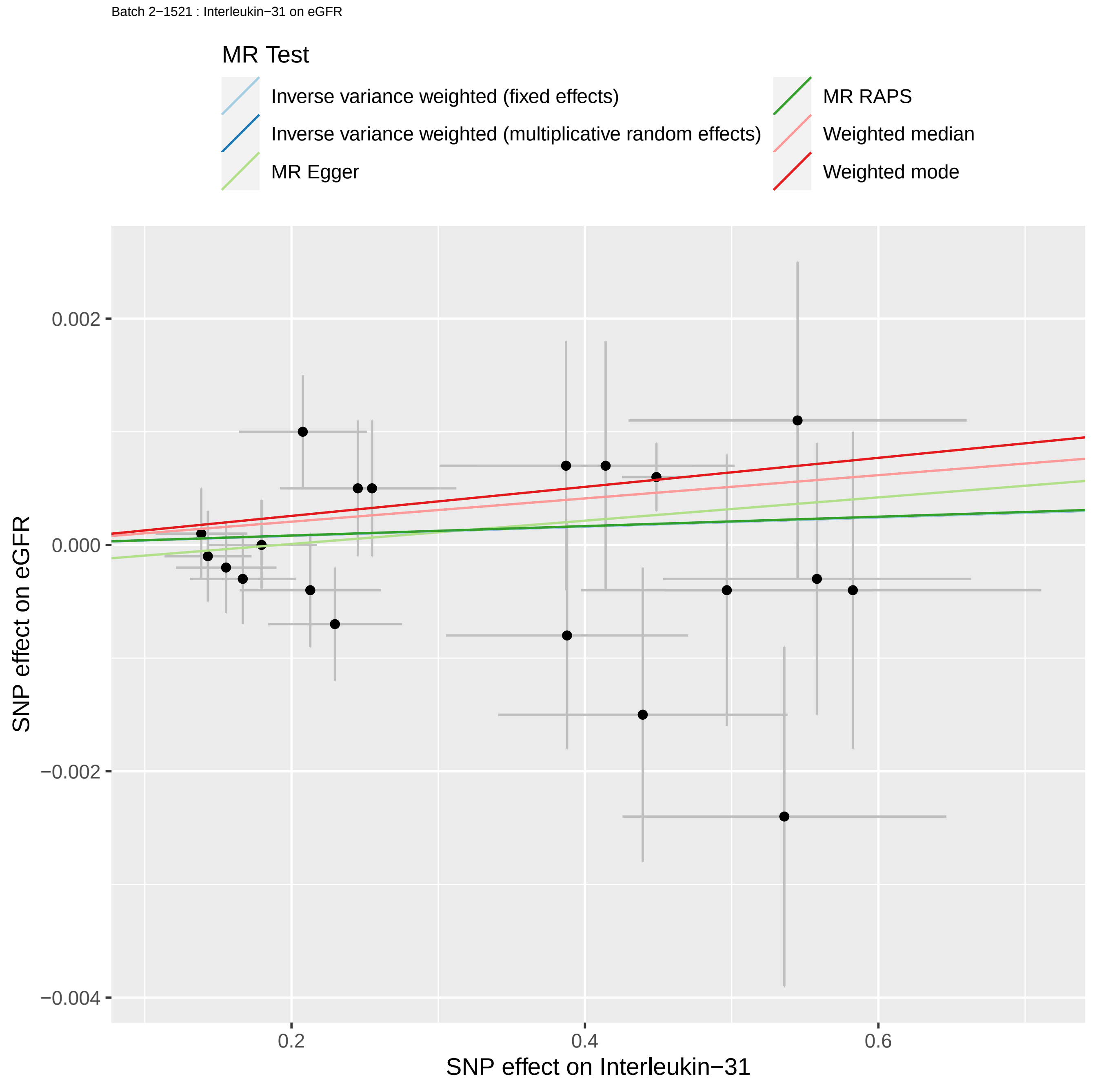

Batch 2−1521 : Interleukin−31 on eGFR
MR Test
Inverse variance weighted (fixed effects)
Inverse variance weighted (multiplicative random effects)
MR Egger
MR RAPS
Weighted median
Weighted mode
0.002
0.000
−0.002
−0.004
0.2
0.4
0.6
SNP effect on eGFR
SNP effect on Interleukin−31

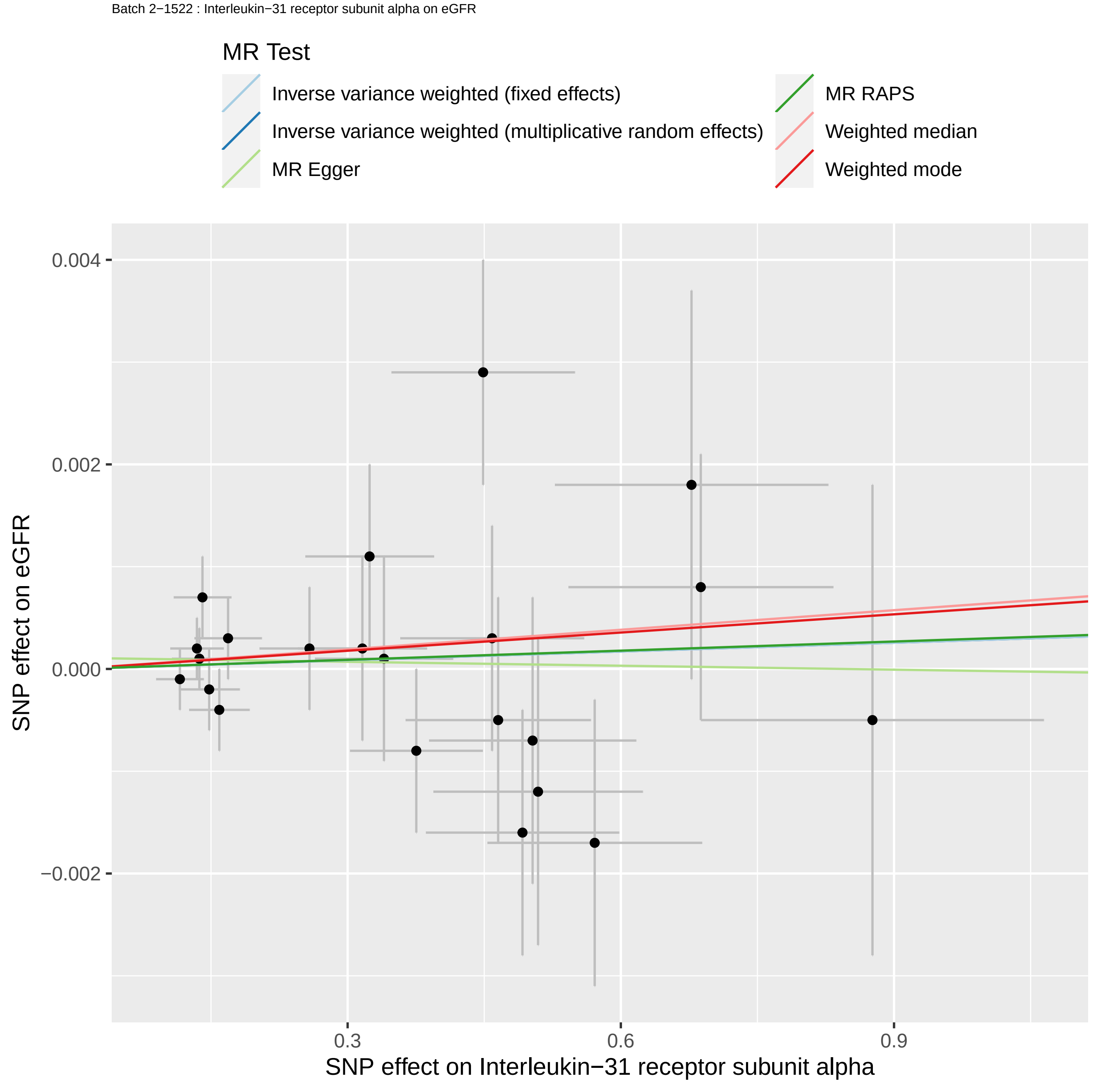
Batch 2−1522 : Interleukin−31 receptor subunit alpha on eGFR
MR Test
Inverse variance weighted (fixed effects)
Inverse variance weighted (multiplicative random effects)
MR Egger
MR RAPS
Weighted median
Weighted mode
0.004
0.002
0.000
−0.002
0.3
0.6
0.9
SNP effect on eGFR
SNP effect on Interleukin−31 receptor subunit alpha

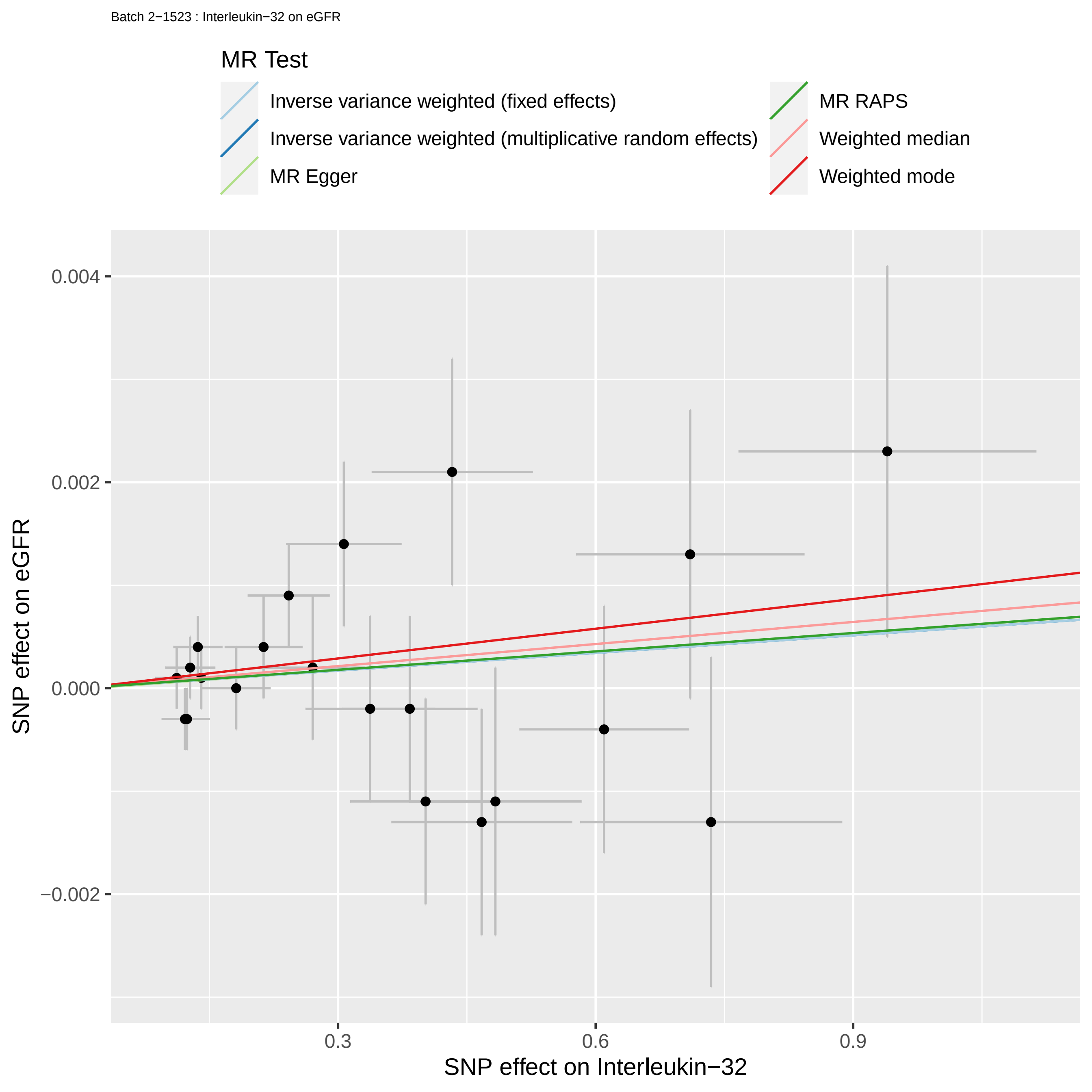

Batch 2−1523 : Interleukin−32 on eGFR
MR Test
Inverse variance weighted (fixed effects)
Inverse variance weighted (multiplicative random effects)
MR Egger
MR RAPS
Weighted median
Weighted mode
SNP effect on eGFR
0.004
0.002
0.000
−0.002
0.3
0.6
0.9
SNP effect on Interleukin−32

Batch 2−1524 : Interleukin−34 on eGFR

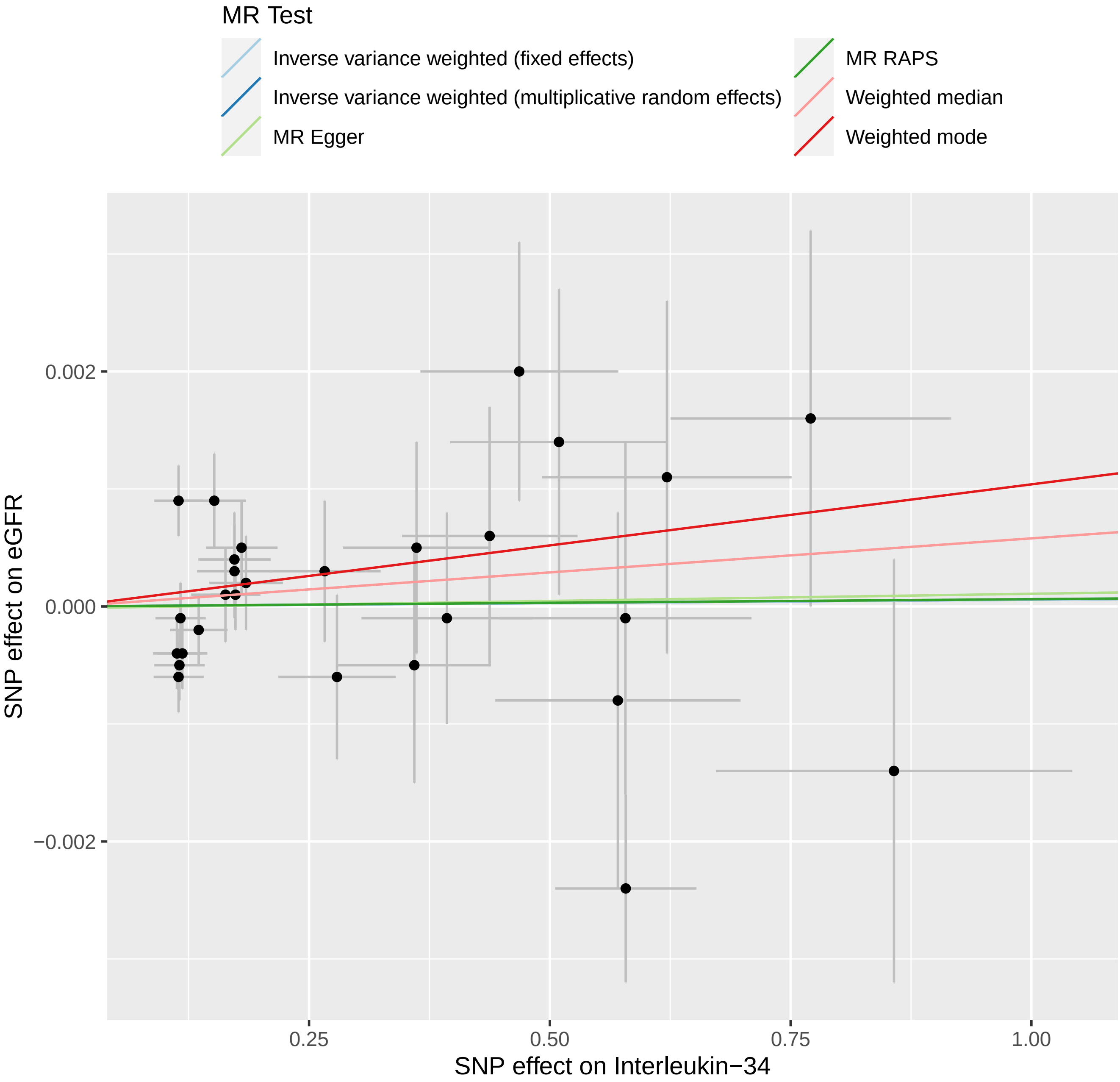

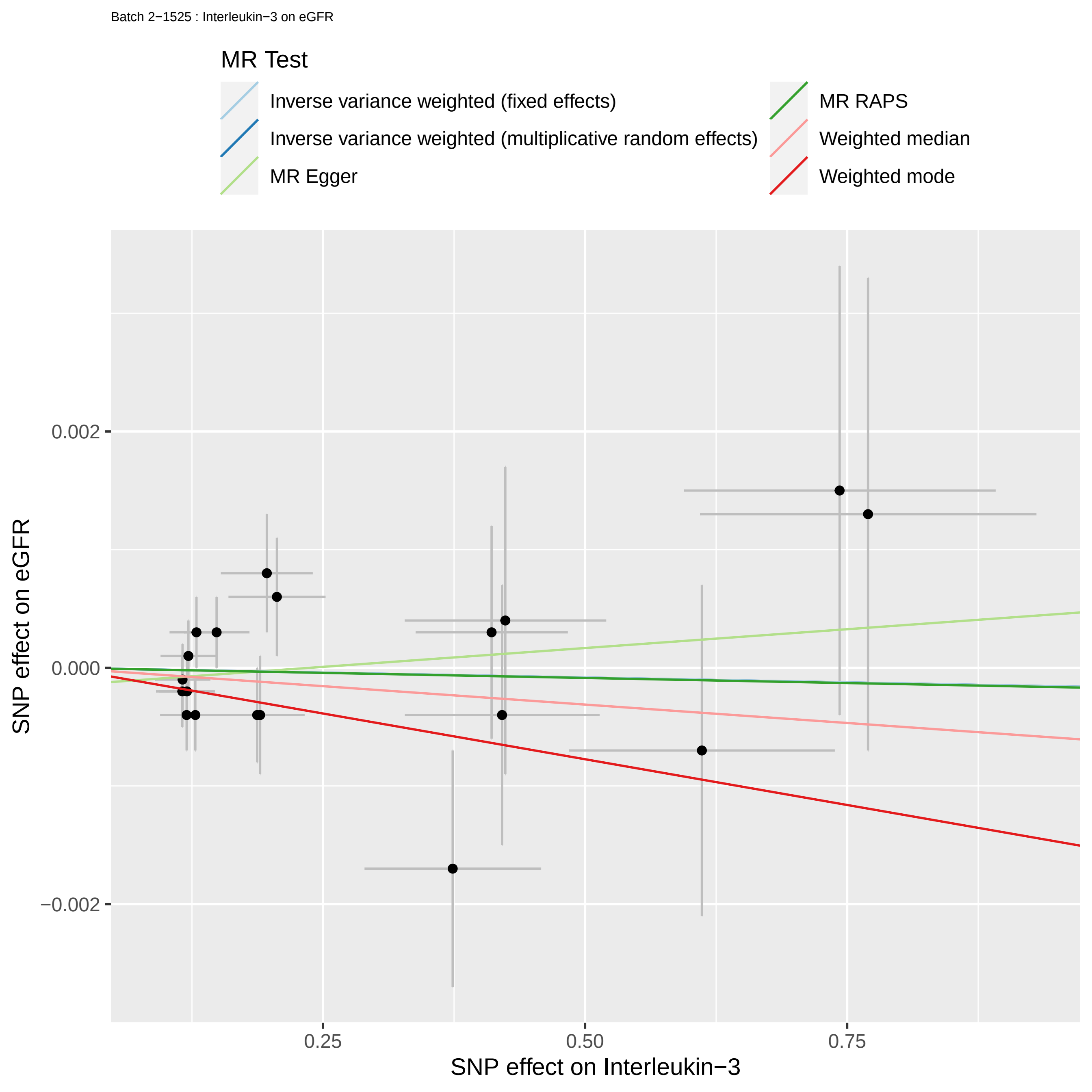

Batch 2−1525 : Interleukin−3 on eGFR
MR Test
Inverse variance weighted (fixed effects)
Inverse variance weighted (multiplicative random effects)
MR Egger
MR RAPS
Weighted median
Weighted mode
0.002
0.000
−0.002
SNP effect on eGFR
0.25
0.50
0.75
SNP effect on Interleukin−3

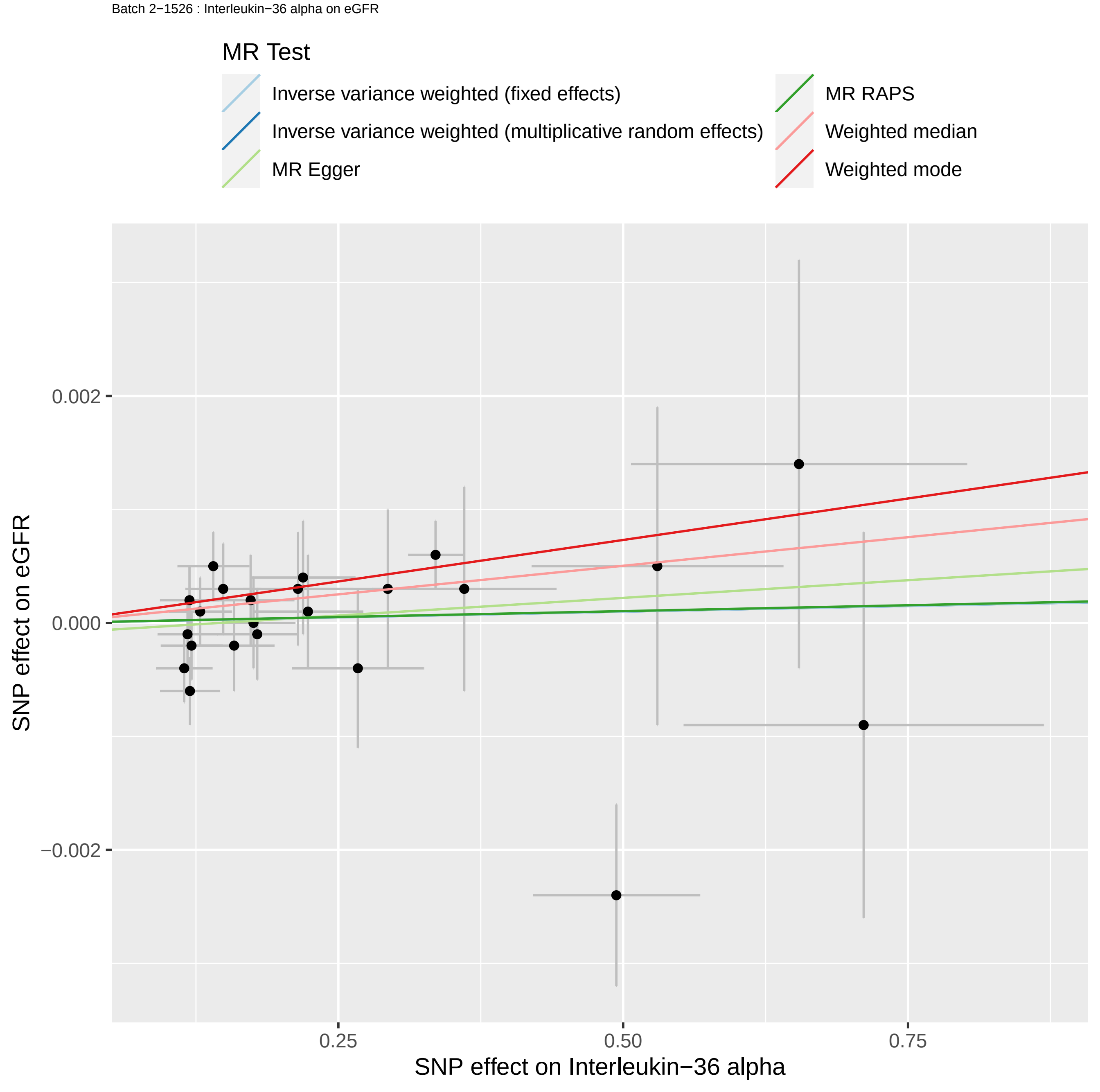
Batch 2−1526 : Interleukin−36 alpha on eGFR
MR Test
Inverse variance weighted (fixed effects)
Inverse variance weighted (multiplicative random effects)
MR Egger
MR RAPS
Weighted median
Weighted mode
0.002
0.000
−0.002
0.25
0.50
0.75
SNP effect on eGFR
SNP effect on Interleukin−36 alpha

Batch 2−1527 : Interleukin−36 beta on eGFR

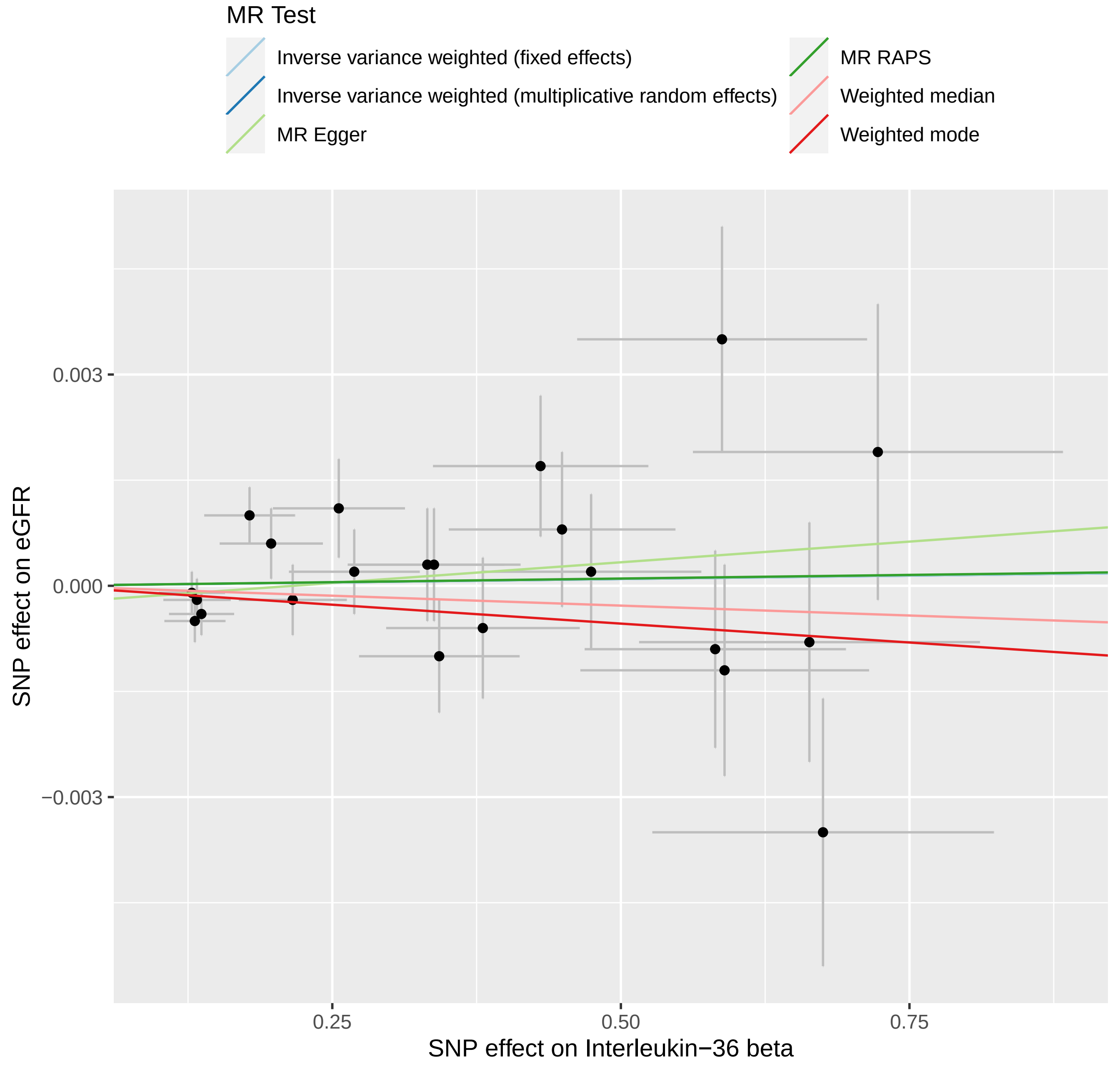

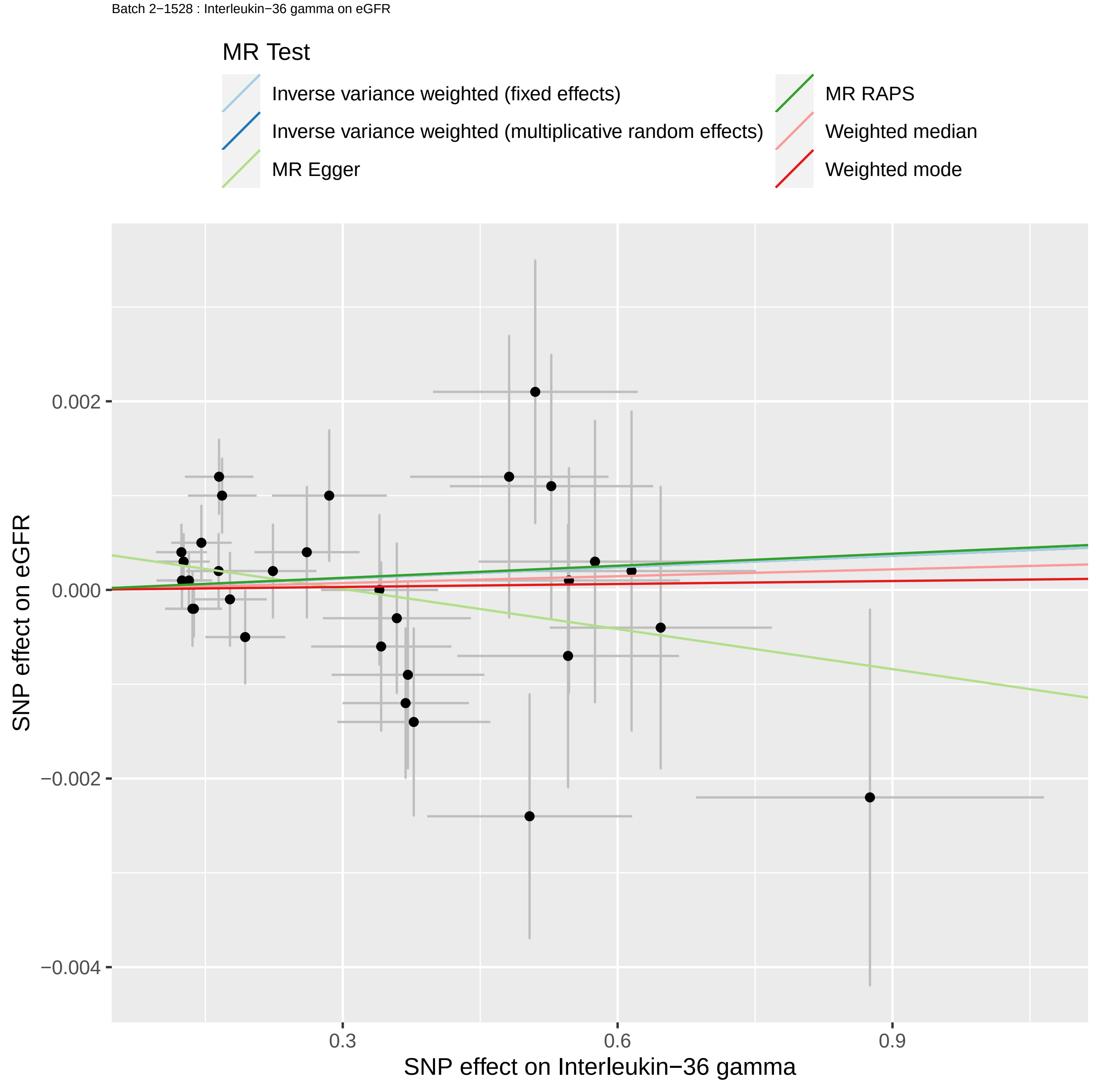

Batch 2−1528 : Interleukin−36 gamma on eGFR
MR Test
Inverse variance weighted (fixed effects)
Inverse variance weighted (multiplicative random effects)
MR Egger
MR RAPS
Weighted median
Weighted mode
0.002
0.000
−0.002
−0.004
0.3
0.6
0.9
SNP effect on eGFR
SNP effect on Interleukin−36 gamma

Batch 2−1529 : Interleukin−37 on eGFR

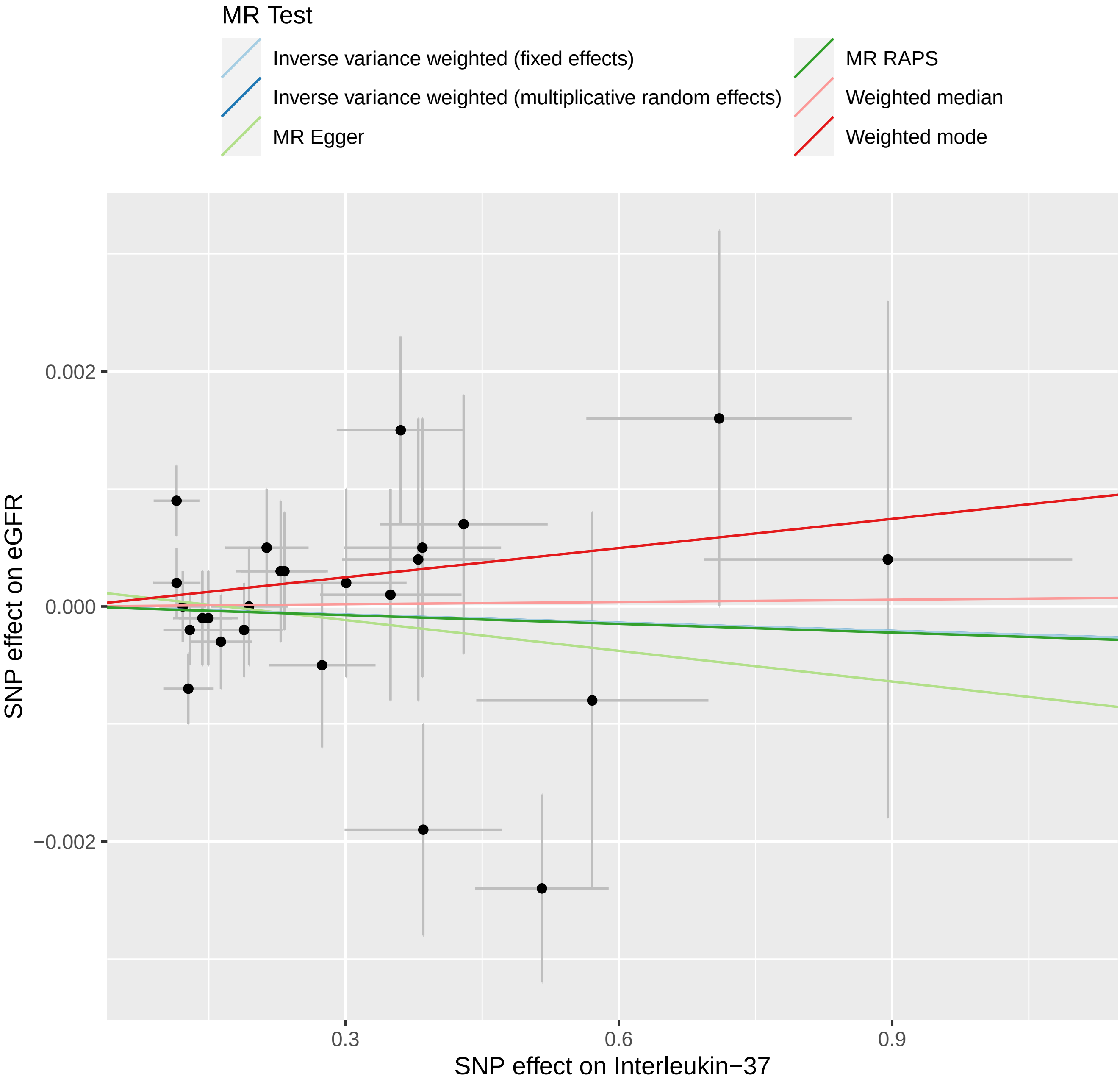

Batch 2−1530 : Interleukin−3 receptor subunit alpha on eGFR

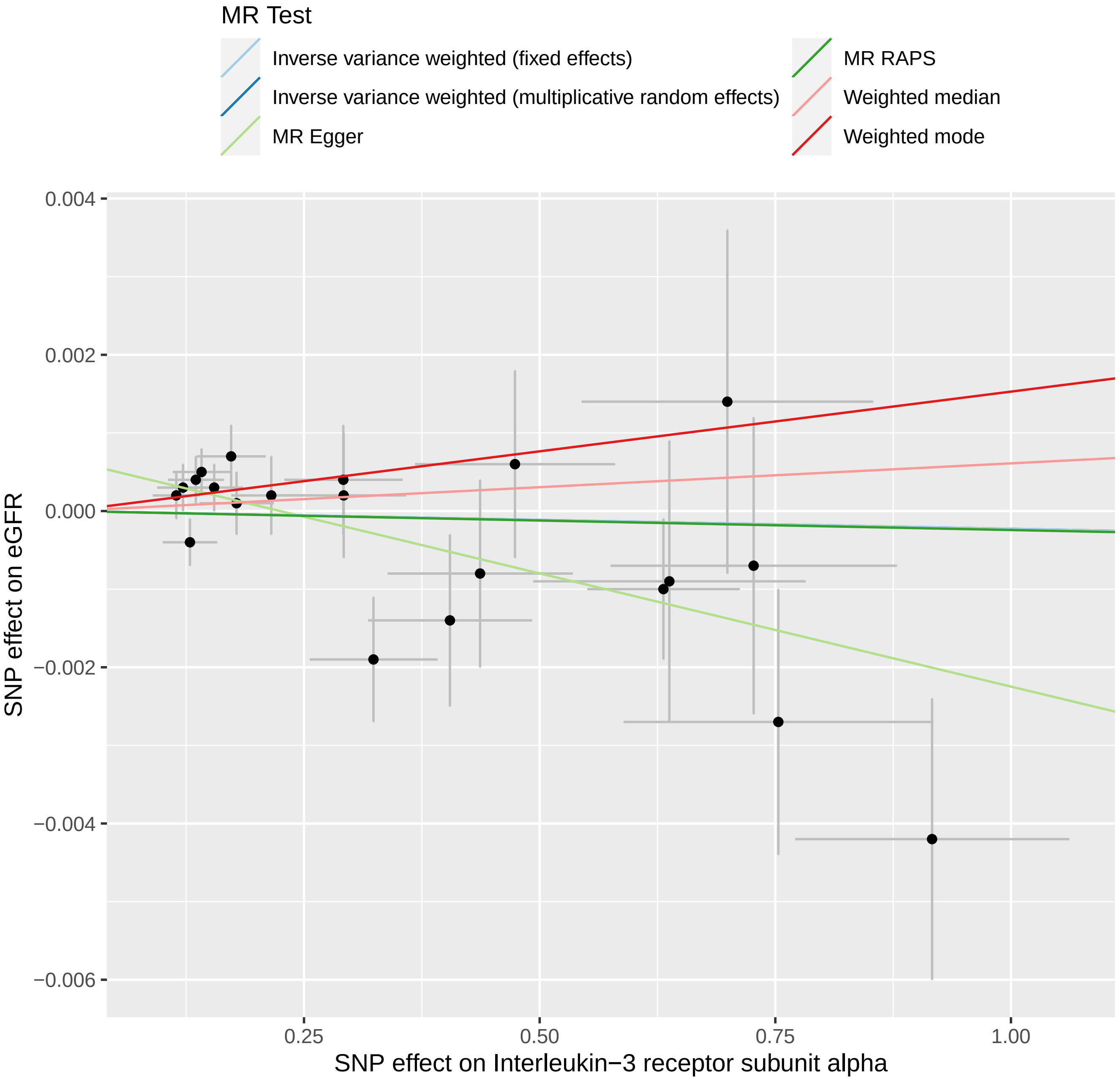

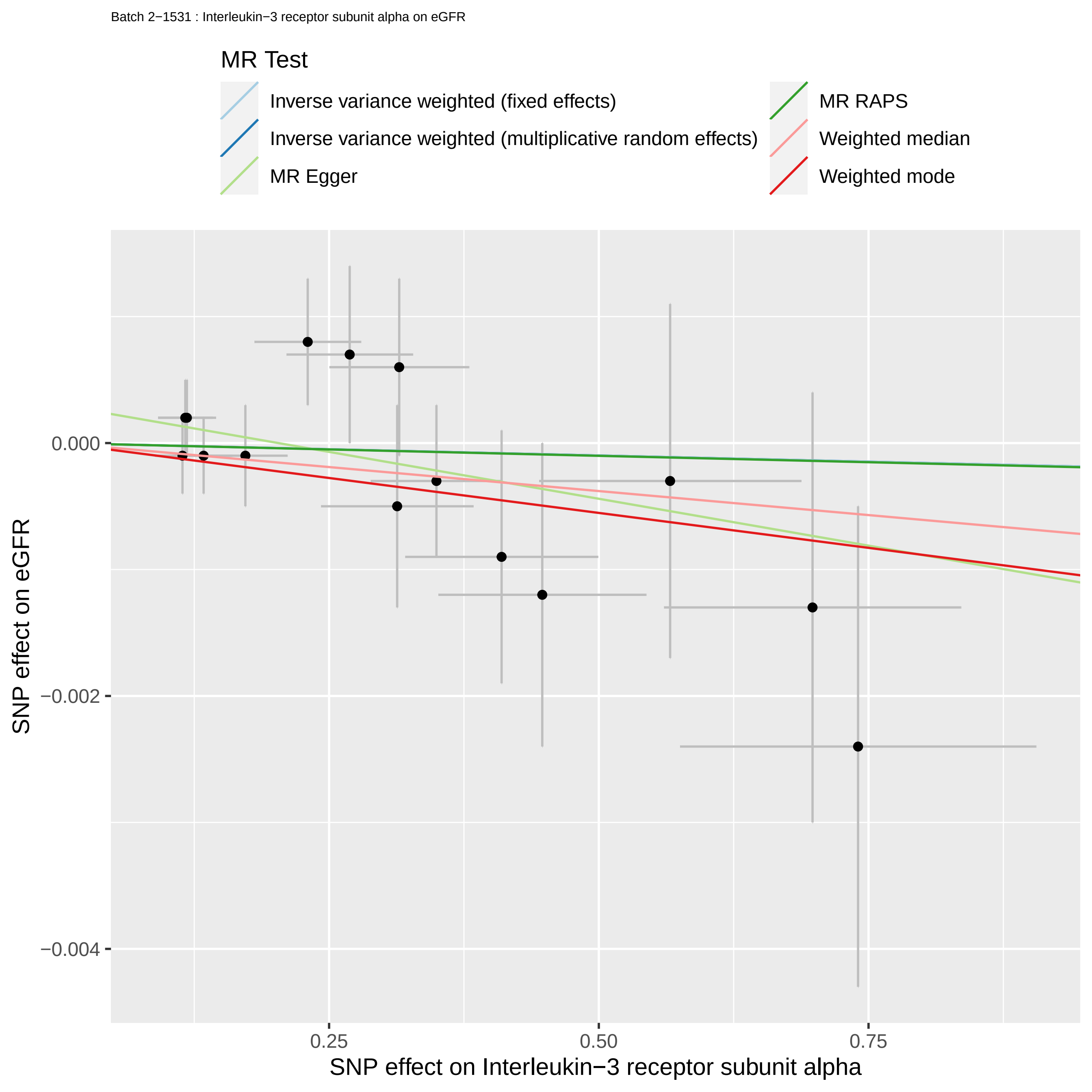
Batch 2−1531 : Interleukin−3 receptor subunit alpha on eGFR
MR Test
Inverse variance weighted (fixed effects)
Inverse variance weighted (multiplicative random effects)
MR Egger
MR RAPS
Weighted median
Weighted mode
0.000
−0.002
−0.004
0.25
0.50
0.75
SNP effect on eGFR
SNP effect on Interleukin−3 receptor subunit alpha

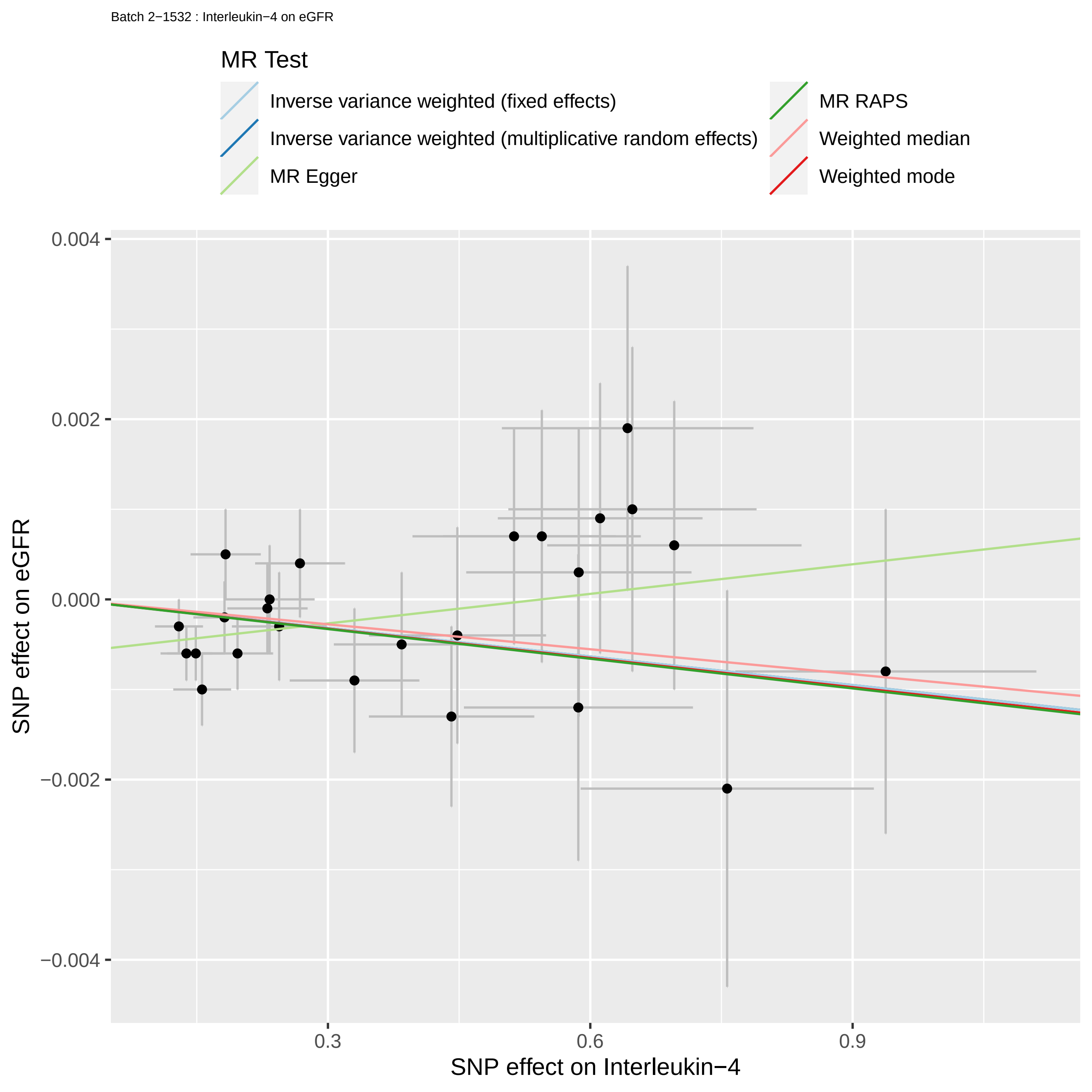

Batch 2−1532 : Interleukin−4 on eGFR
MR Test
Inverse variance weighted (fixed effects)
Inverse variance weighted (multiplicative random effects)
MR Egger
MR RAPS
Weighted median
Weighted mode
0.004
0.002
0.000
−0.002
−0.004
SNP effect on eGFR
0.3
0.6
0.9
SNP effect on Interleukin−4

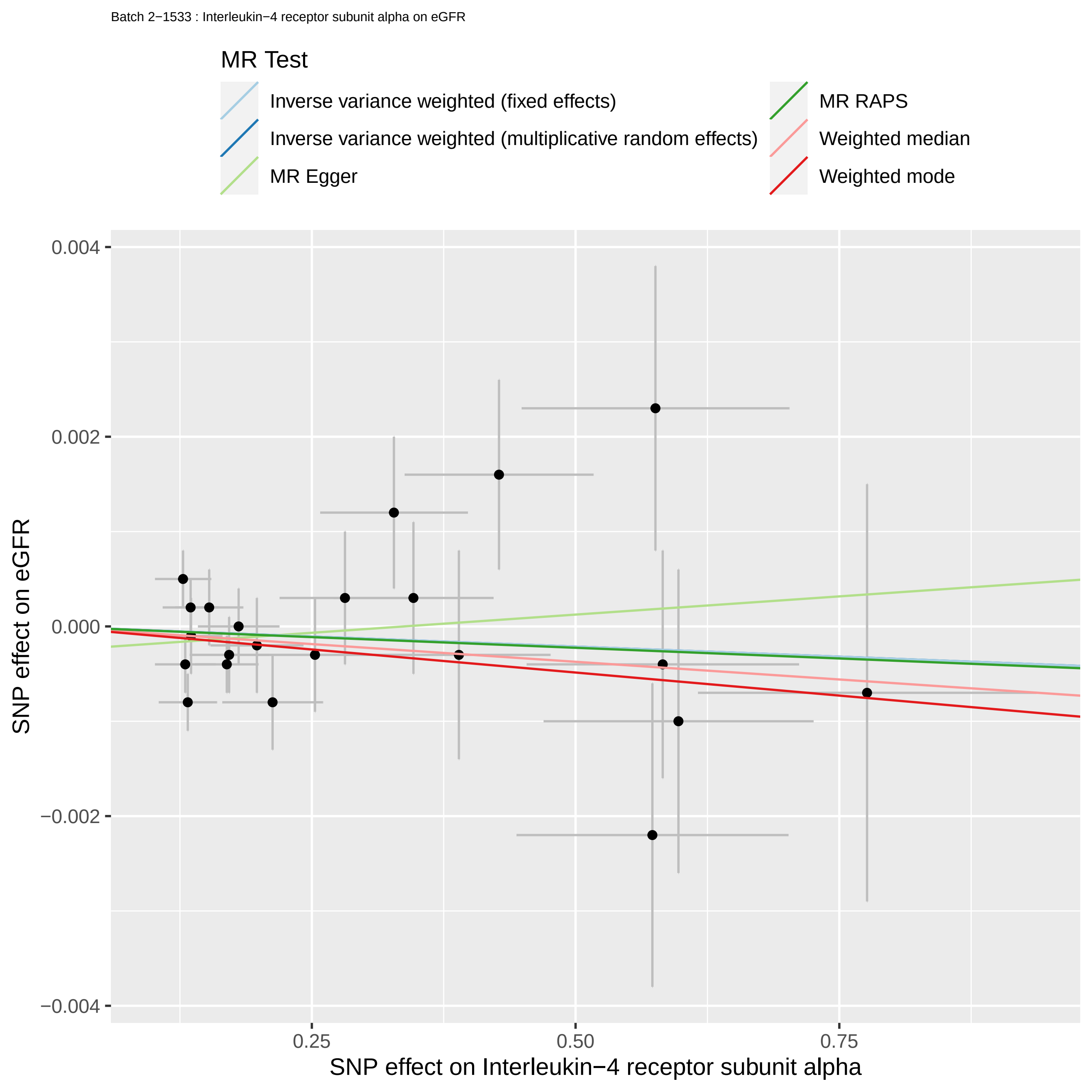

Batch 2−1533 : Interleukin−4 receptor subunit alpha on eGFR
MR Test
Inverse variance weighted (fixed effects)
Inverse variance weighted (multiplicative random effects)
MR Egger
MR RAPS
Weighted median
Weighted mode
0.004
0.002
0.000
−0.002
−0.004
SNP effect on eGFR
0.25
0.50
0.75
SNP effect on Interleukin−4 receptor subunit alpha

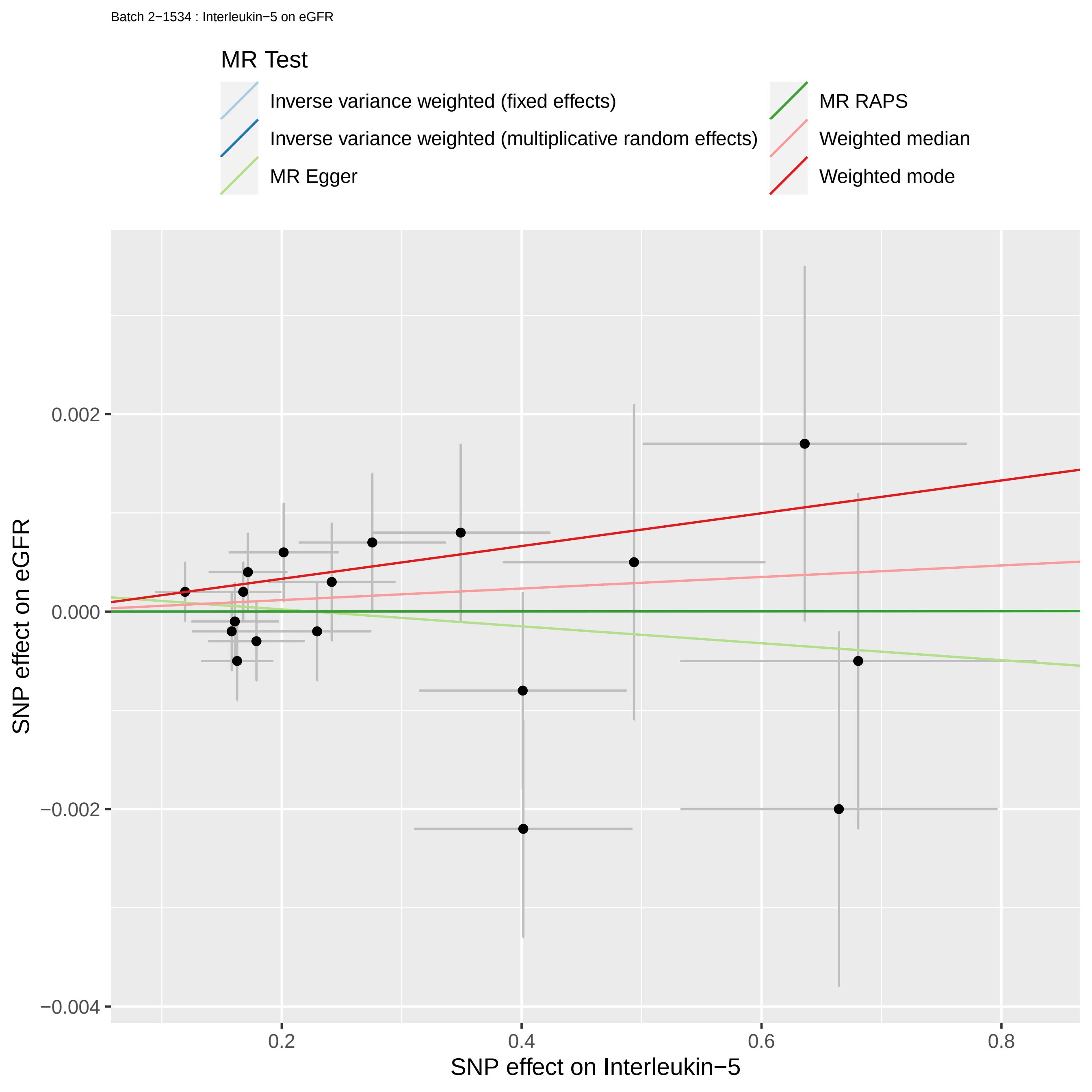

Batch 2−1534 : Interleukin−5 on eGFR
MR Test
Inverse variance weighted (fixed effects)
Inverse variance weighted (multiplicative random effects)
MR Egger
MR RAPS
Weighted median
Weighted mode
0.002
0.000
−0.002
−0.004
SNP effect on eGFR
0.2
0.4
0.6
0.8
SNP effect on Interleukin−5

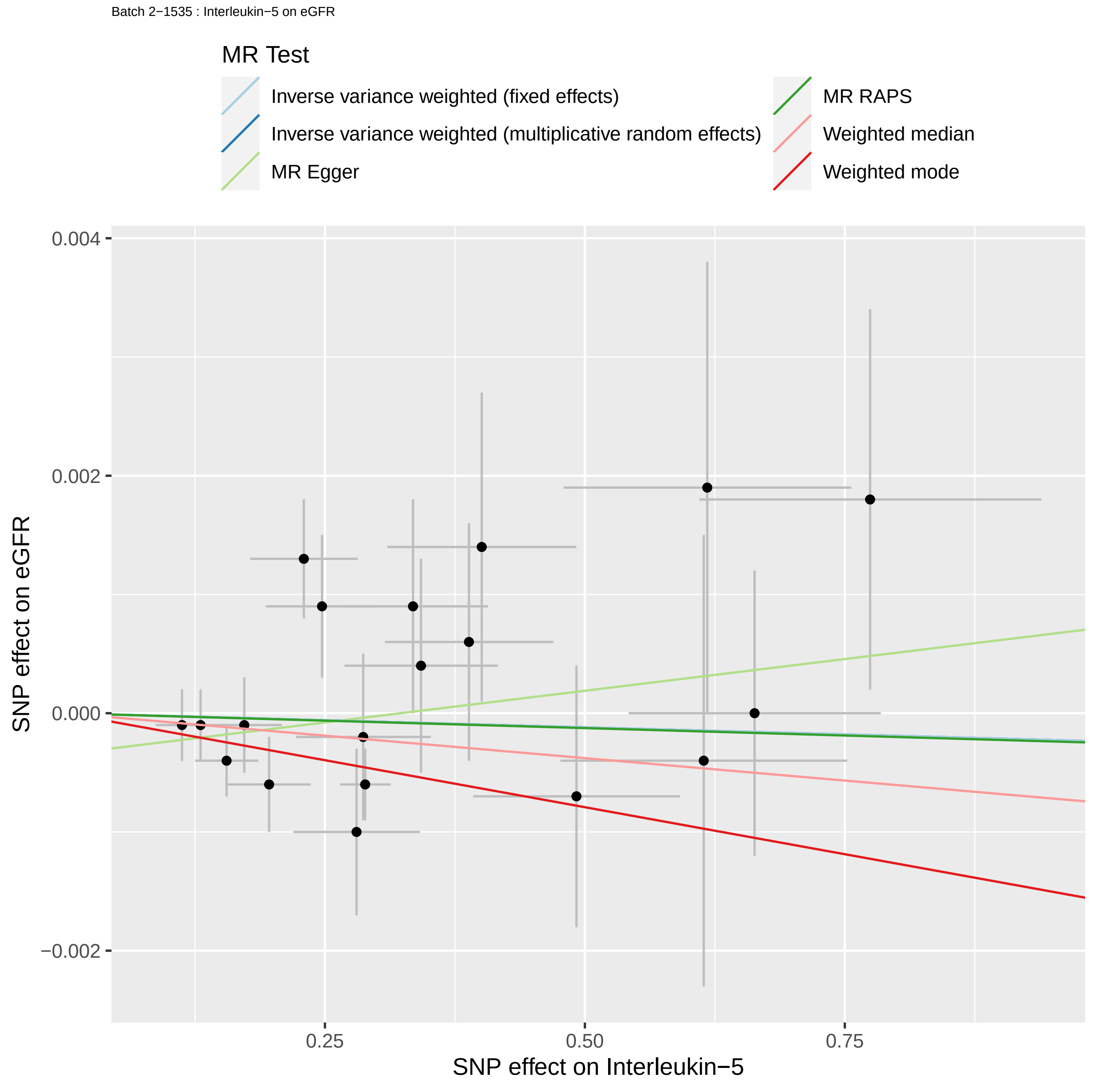

Batch 2−1535 : Interleukin−5 on eGFR
MR Test
Inverse variance weighted (fixed effects)
Inverse variance weighted (multiplicative random effects)
MR Egger
MR RAPS
Weighted median
Weighted mode
0.004
0.002
0.000
−0.002
SNP effect on eGFR
0.25
0.50
0.75
SNP effect on Interleukin−5

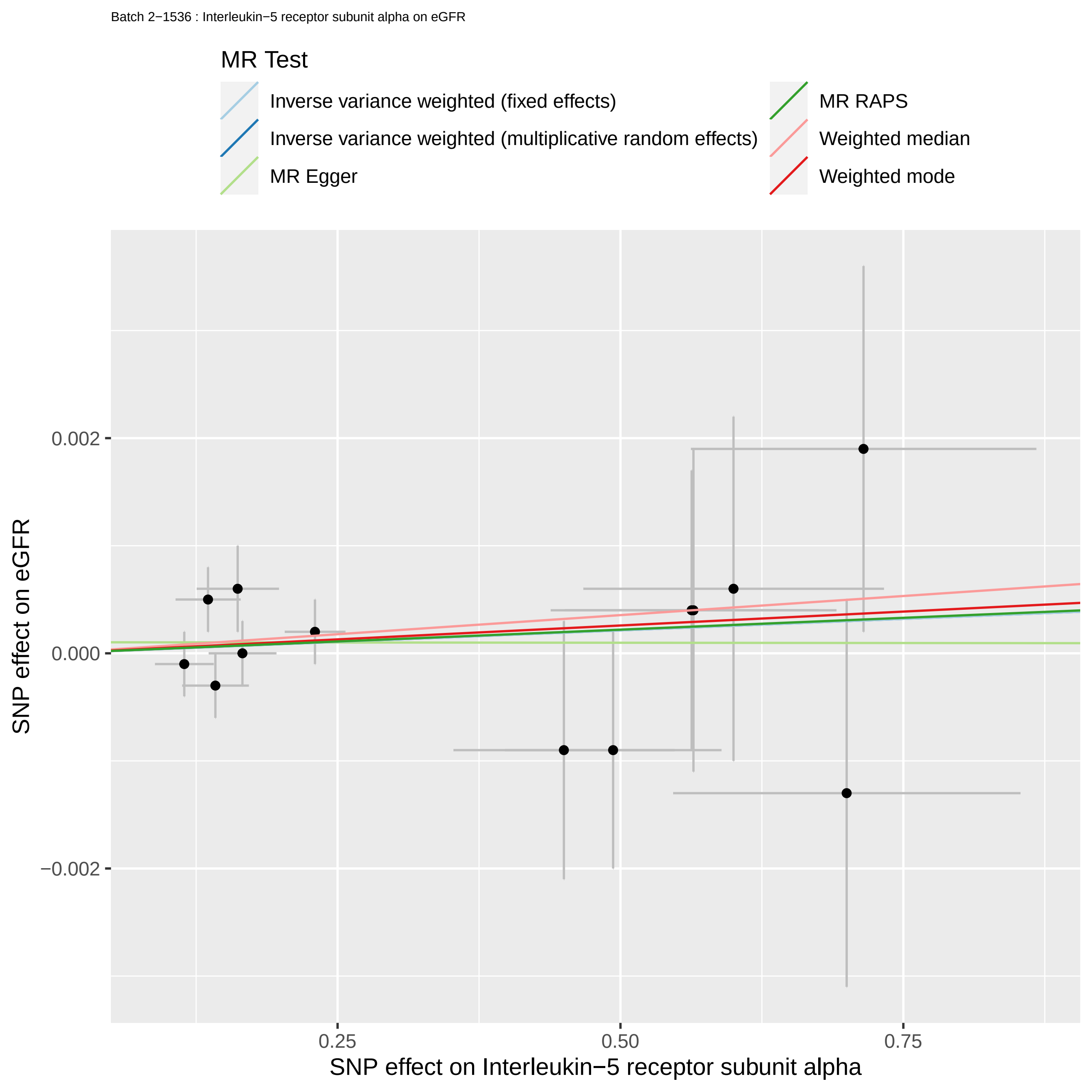
Batch 2−1536 : Interleukin−5 receptor subunit alpha on eGFR
MR Test
Inverse variance weighted (fixed effects)
Inverse variance weighted (multiplicative random effects)
MR Egger
MR RAPS
Weighted median
Weighted mode
0.002
0.000
−0.002
0.25
0.50
0.75
SNP effect on eGFR
SNP effect on Interleukin−5 receptor subunit alpha

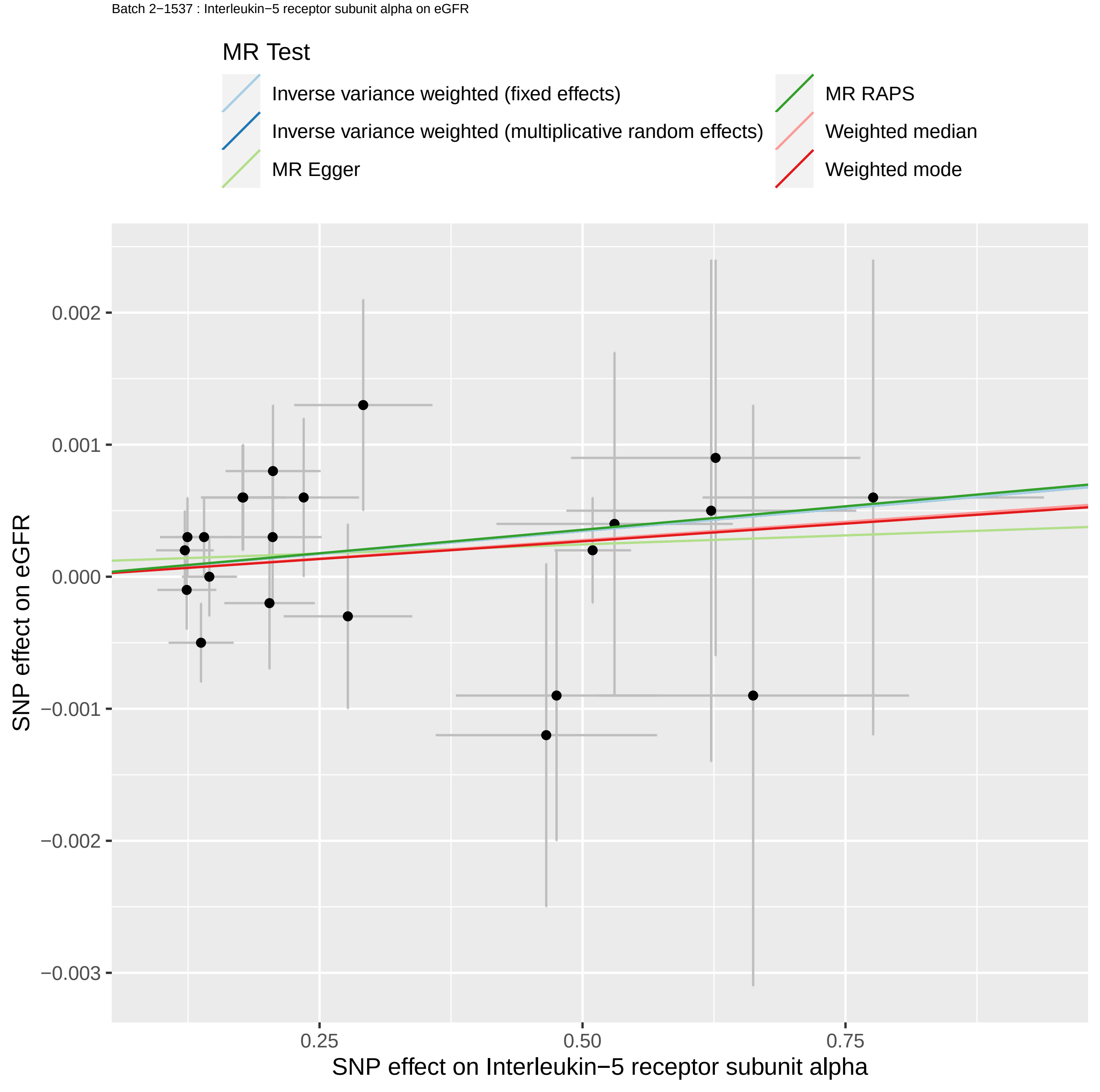

Batch 2−1537 : Interleukin−5 receptor subunit alpha on eGFR
MR Test
Inverse variance weighted (fixed effects)
Inverse variance weighted (multiplicative random effects)
MR Egger
MR RAPS
Weighted median
Weighted mode
0.002
0.001
0.000
−0.001
−0.002
−0.003
0.25
0.50
0.75
SNP effect on eGFR
SNP effect on Interleukin−5 receptor subunit alpha

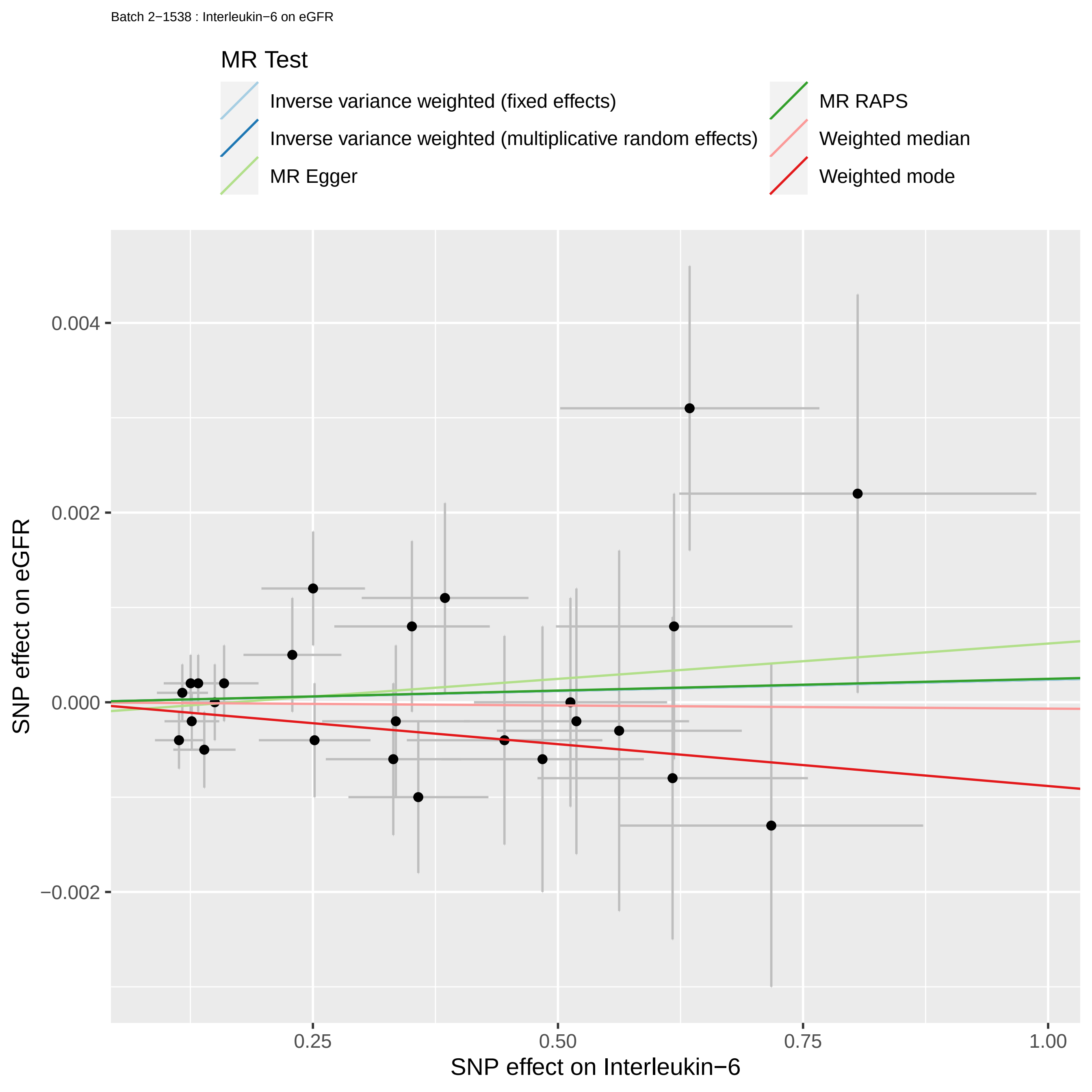

Batch 2−1538 : Interleukin−6 on eGFR
MR Test
Inverse variance weighted (fixed effects)
Inverse variance weighted (multiplicative random effects)
MR Egger
MR RAPS
Weighted median
Weighted mode
0.004
0.002
0.000
−0.002
SNP effect on eGFR
0.25
0.50
0.75
1.00
SNP effect on Interleukin−6

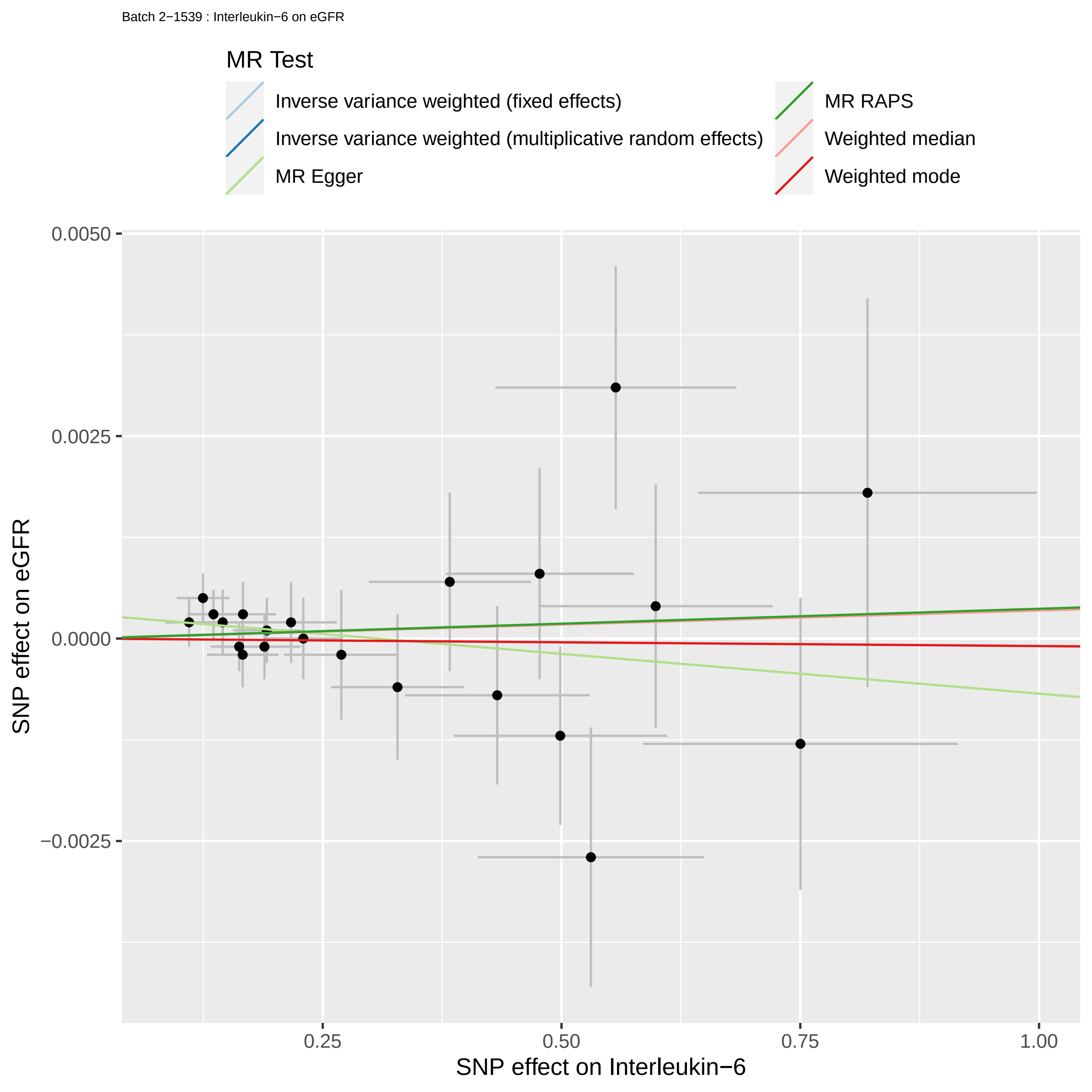
Batch 2−1539 : Interleukin−6 on eGFR
MR Test
Inverse variance weighted (fixed effects)
Inverse variance weighted (multiplicative random effects)
MR Egger
MR RAPS
Weighted median
Weighted mode
0.0050
0.0025
0.0000
−0.0025
SNP effect on eGFR
0.25
0.50
0.75
1.00
SNP effect on Interleukin−6

Batch 2−1540 : Interleukin−6 receptor subunit alpha on eGFR

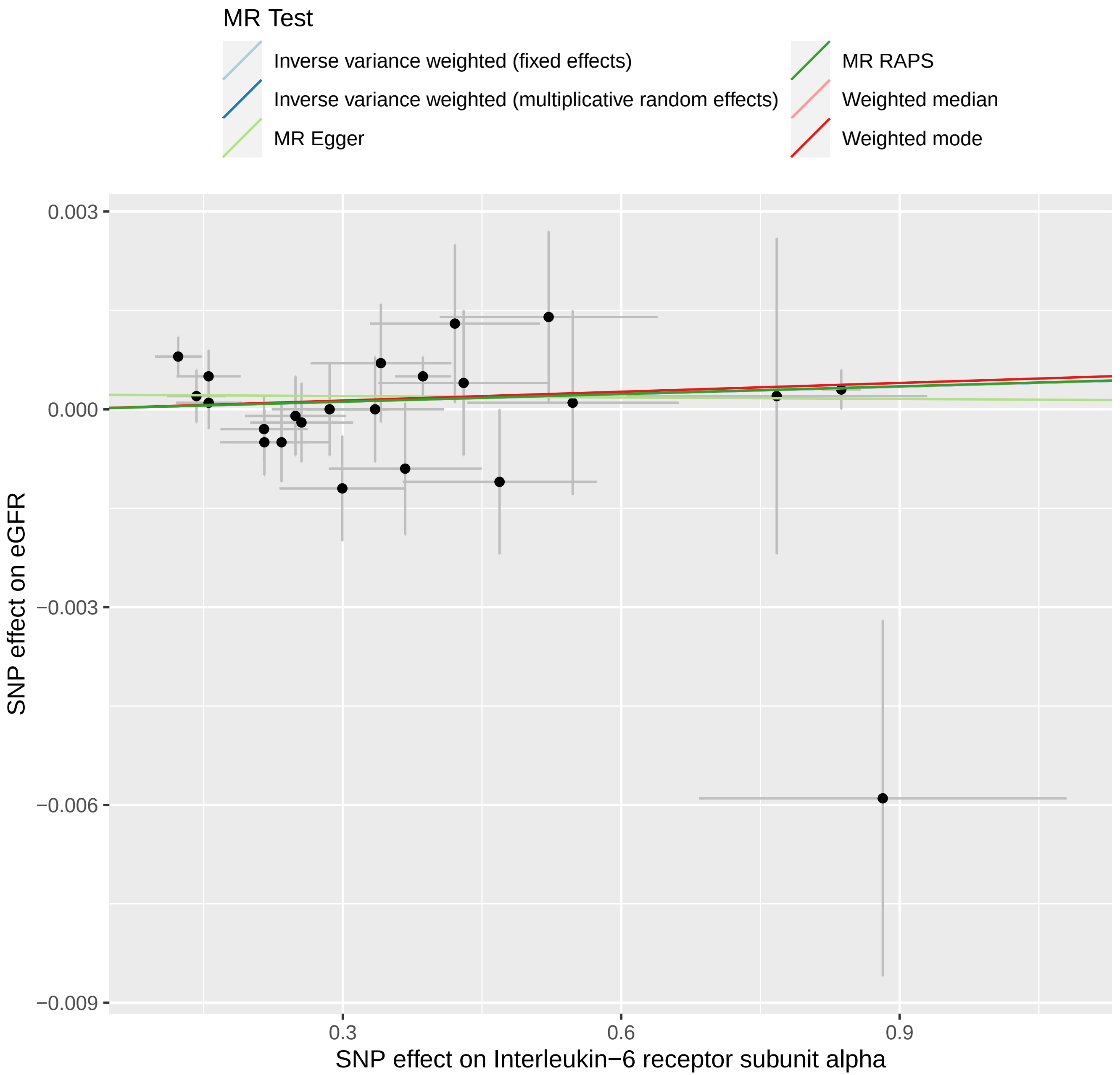

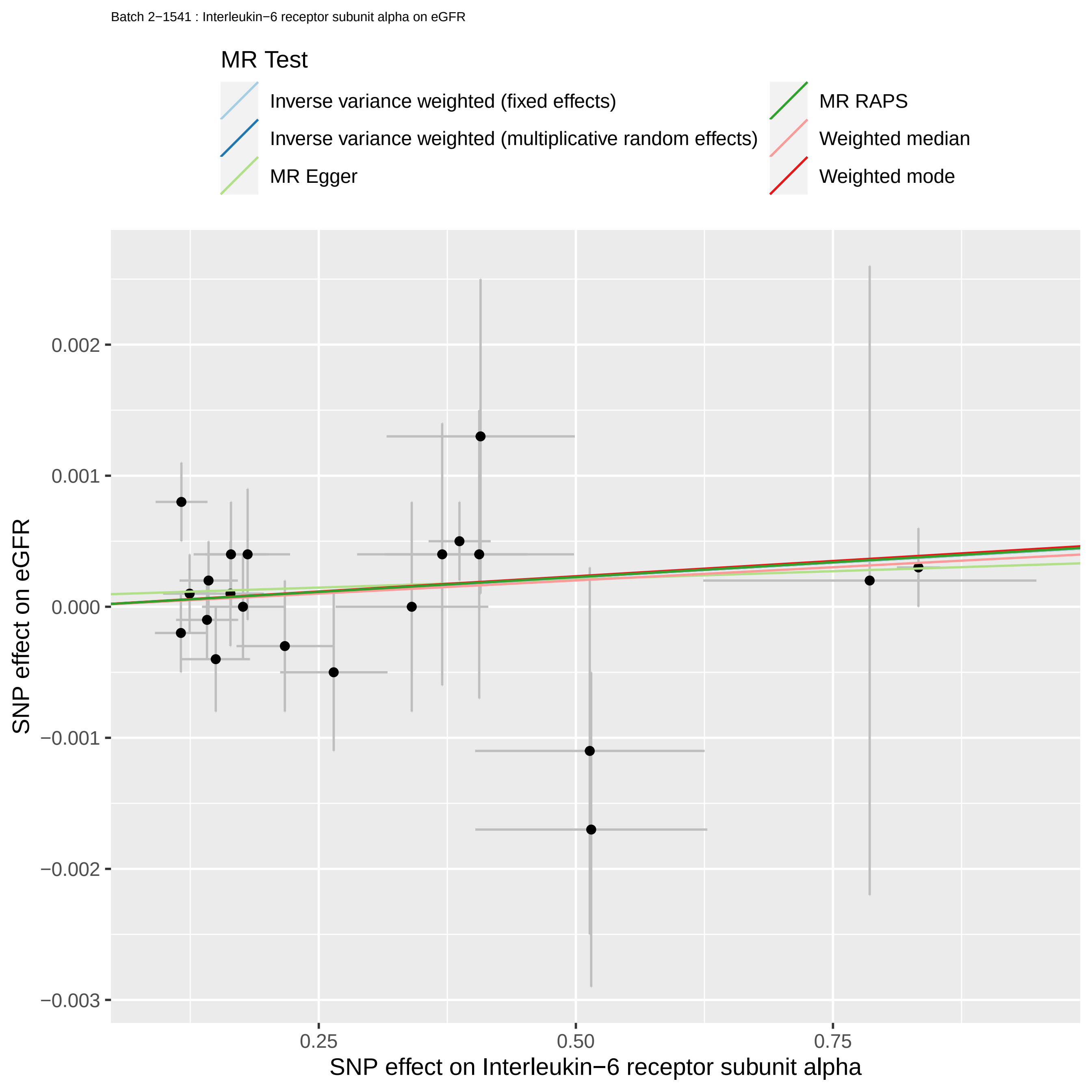

Batch 2−1541 : Interleukin−6 receptor subunit alpha on eGFR
MR Test
Inverse variance weighted (fixed effects)
Inverse variance weighted (multiplicative random effects)
MR Egger
MR RAPS
Weighted median
Weighted mode
0.002
0.001
0.000
−0.001
−0.002
−0.003
SNP effect on eGFR
0.25
0.50
0.75
SNP effect on Interleukin−6 receptor subunit alpha

Batch 2−1542 : Interleukin−6 receptor subunit beta on eGFR

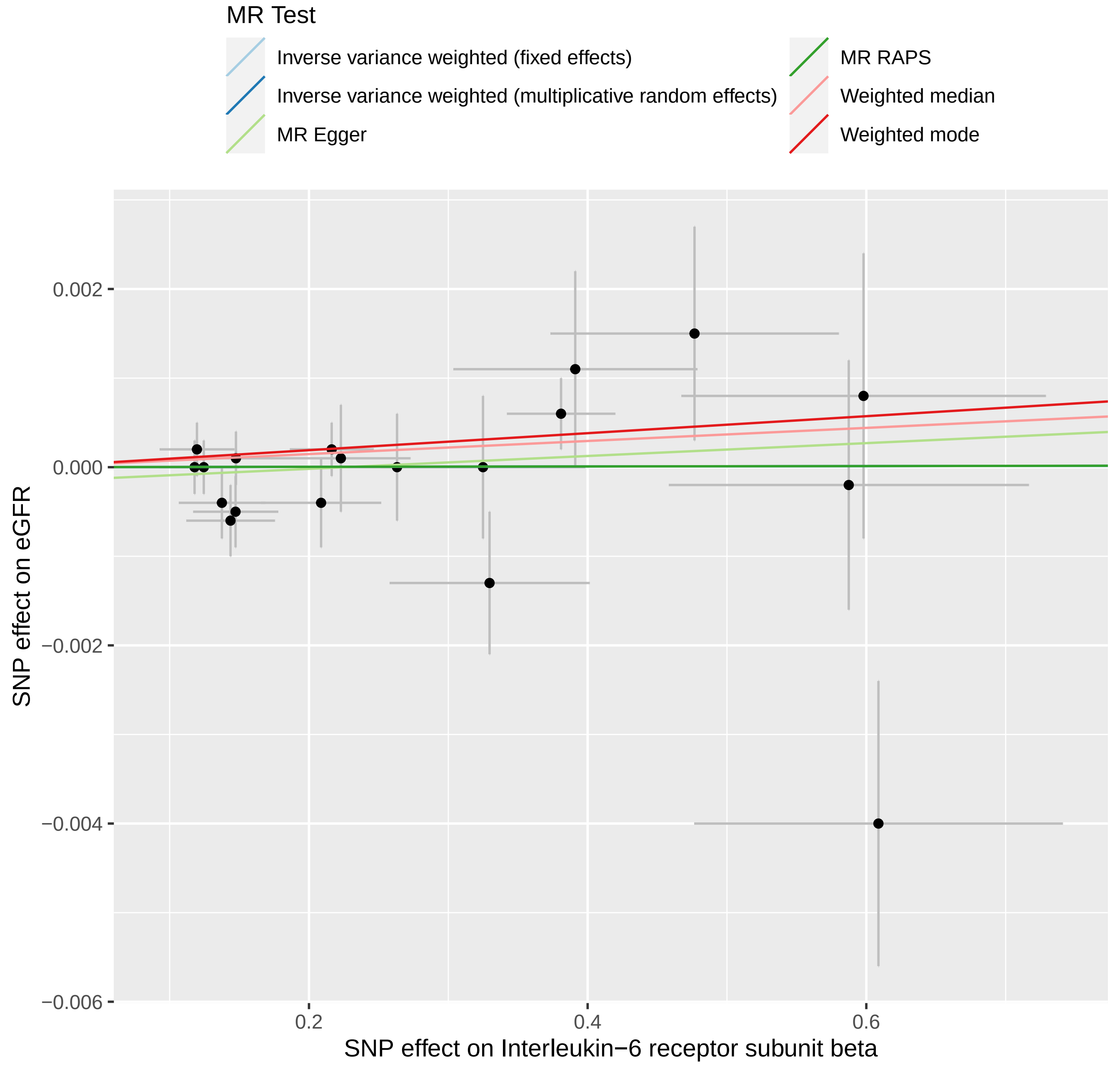

Batch 2−1543 : Interleukin−7 on eGFR

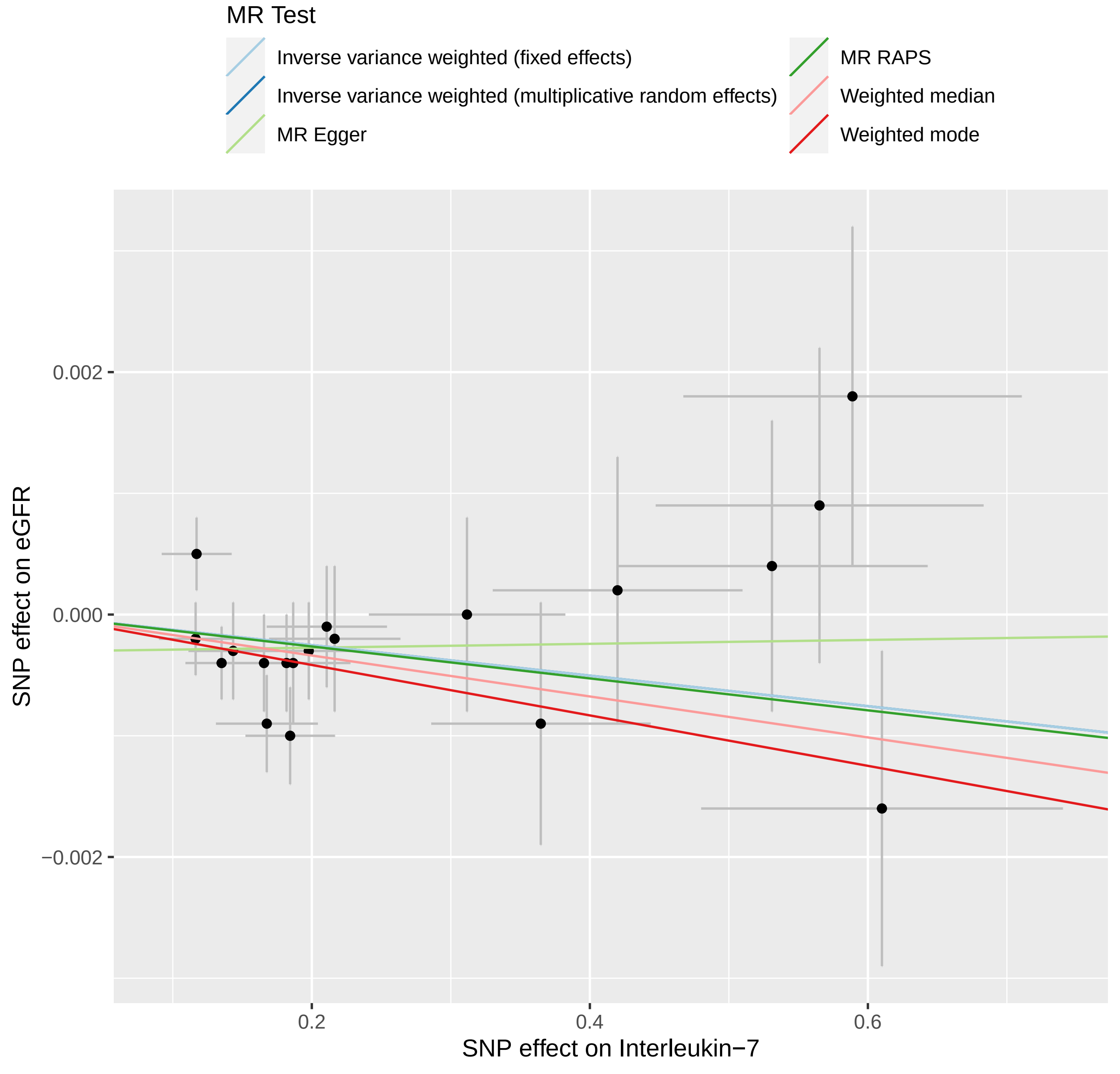

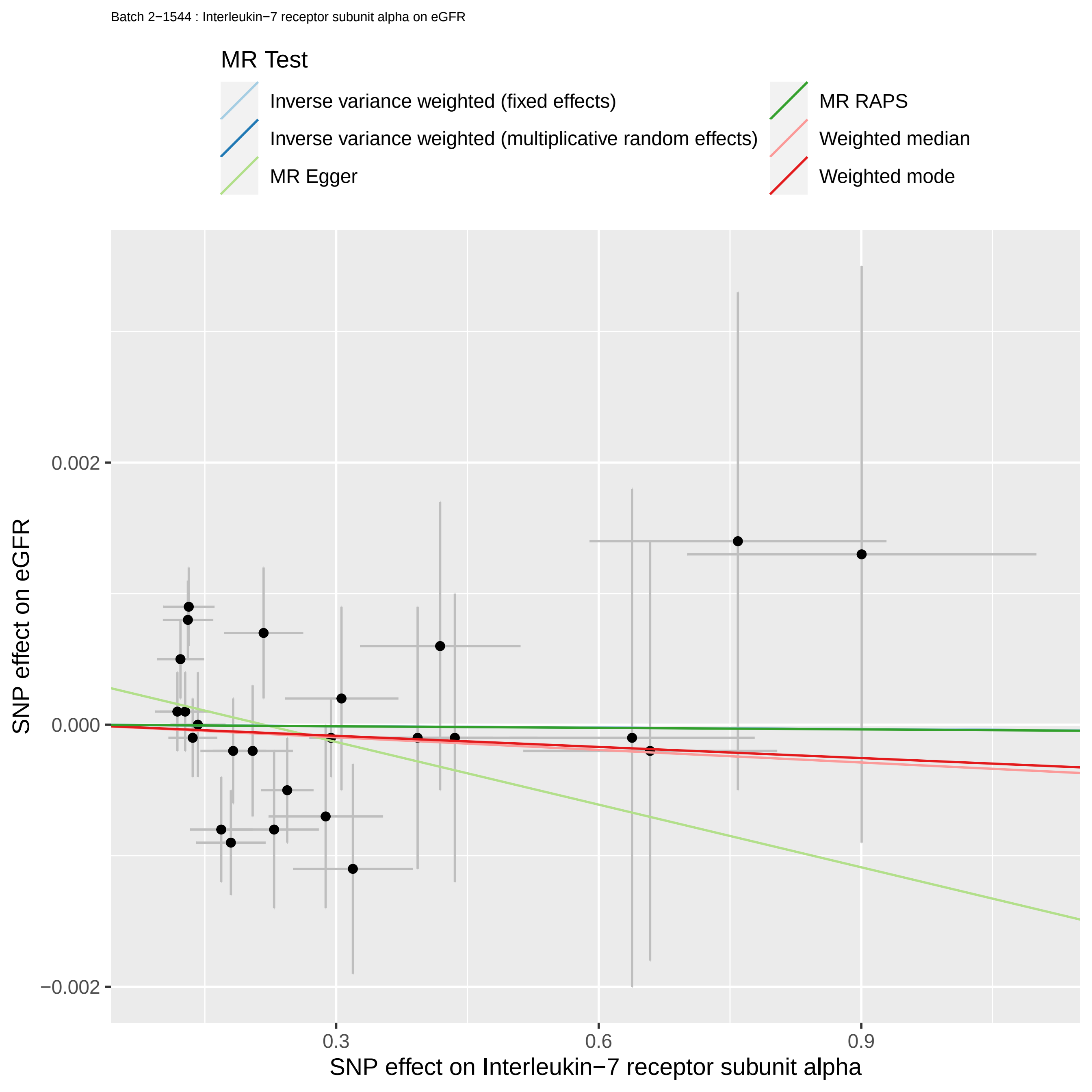
Batch 2−1544 : Interleukin−7 receptor subunit alpha on eGFR
MR Test
Inverse variance weighted (fixed effects)
Inverse variance weighted (multiplicative random effects)
MR Egger
MR RAPS
Weighted median
Weighted mode
0.002
0.000
−0.002
0.3
0.6
0.9
SNP effect on eGFR
SNP effect on Interleukin−7 receptor subunit alpha

Batch 2−1545 : Interleukin−9 on eGFR

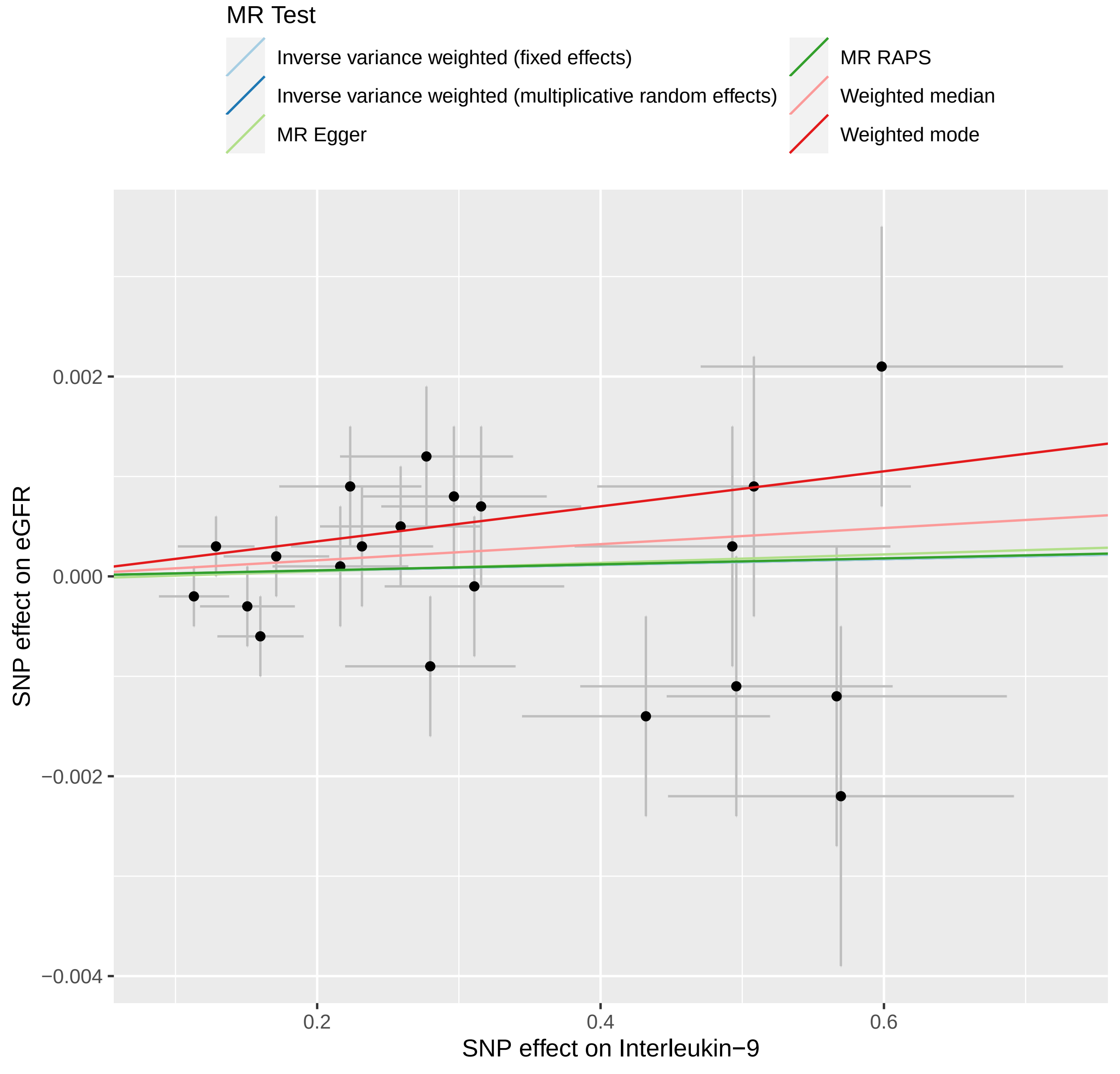

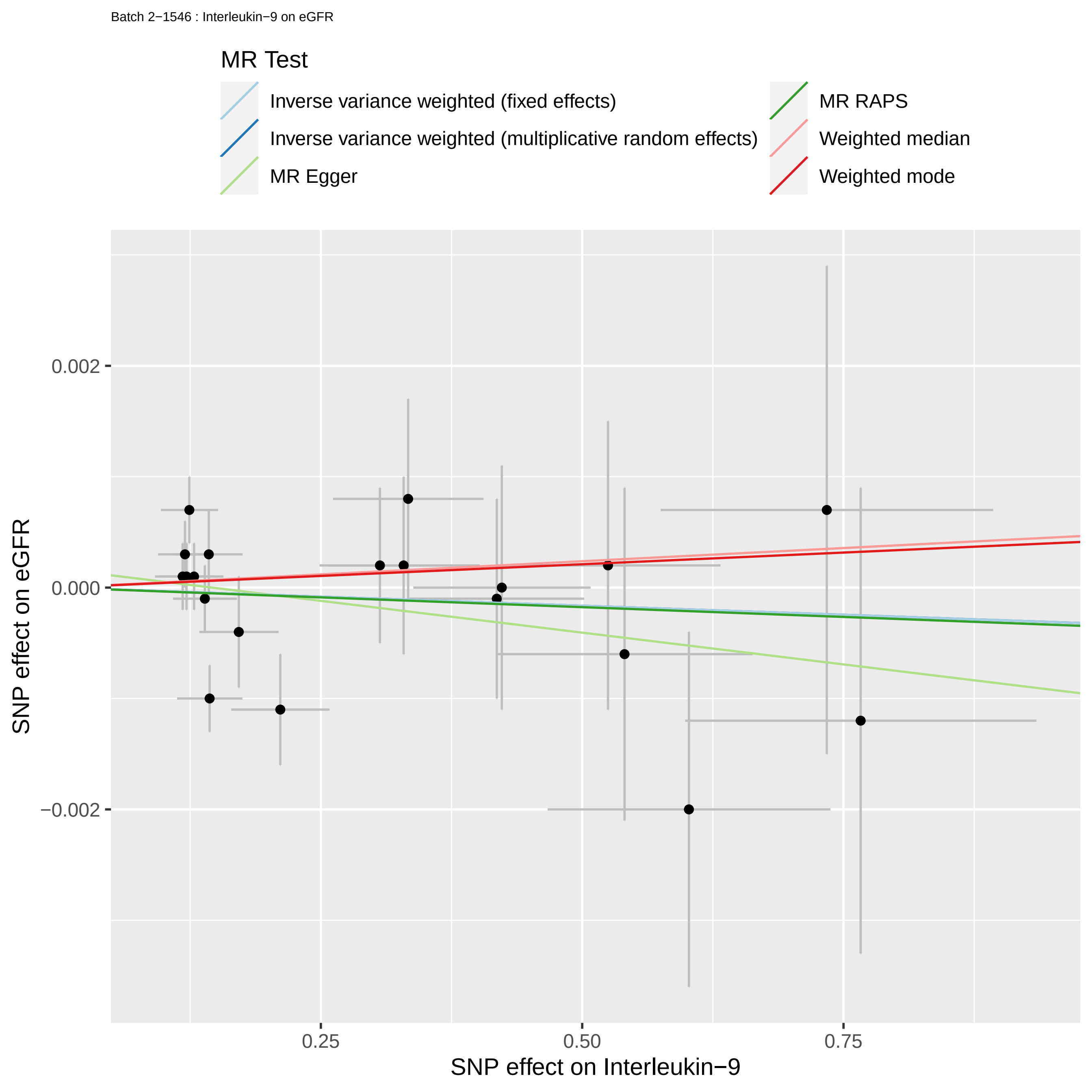

Batch 2−1546 : Interleukin−9 on eGFR
MR Test
Inverse variance weighted (fixed effects)
Inverse variance weighted (multiplicative random effects)
MR Egger
MR RAPS
Weighted median
Weighted mode
0.002
0.000
−0.002
0.25
0.50
0.75
SNP effect on eGFR
SNP effect on Interleukin−9

Batch 2−1547 : Interleukin enhancer−binding factor 3 on eGFR

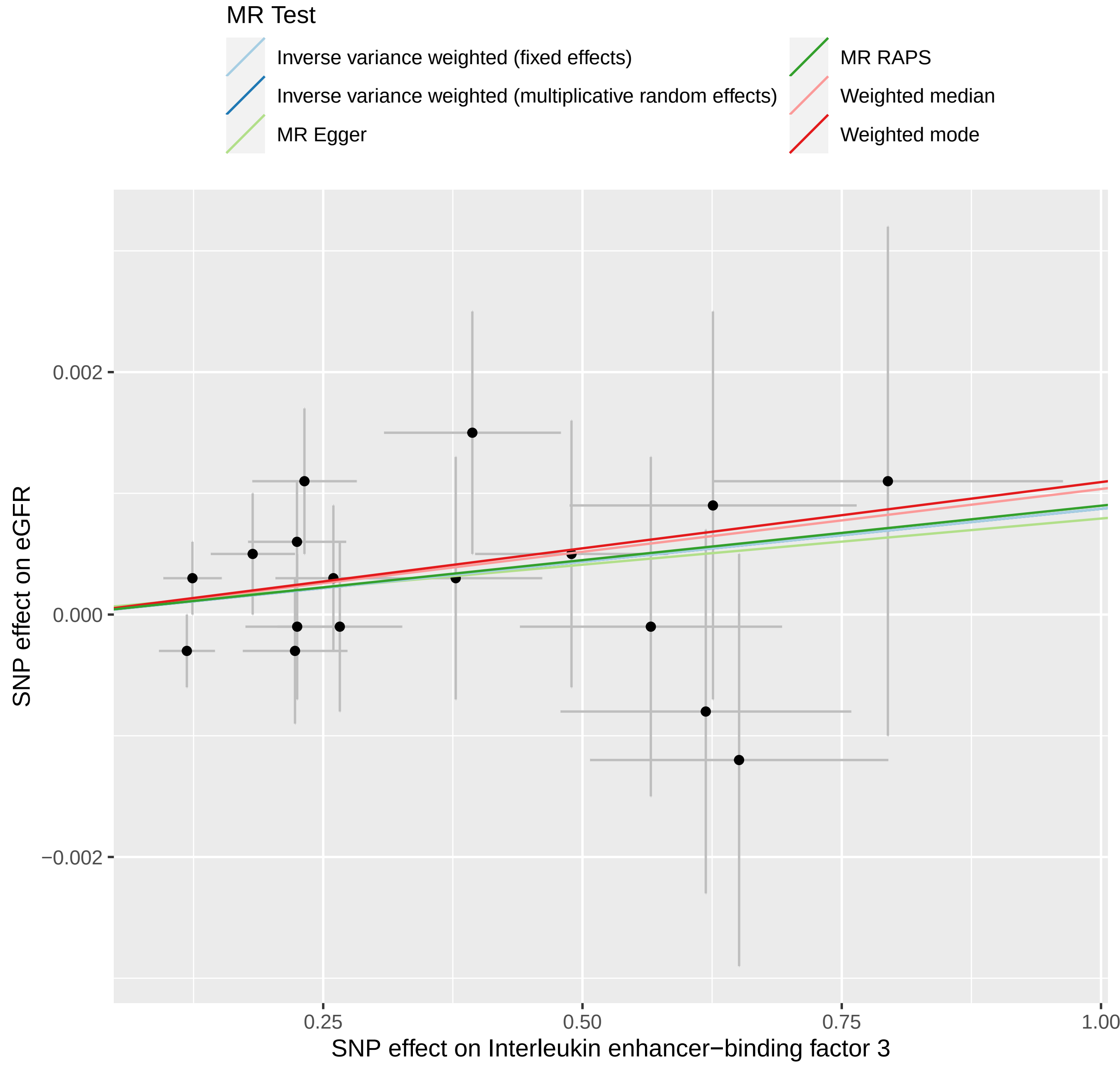

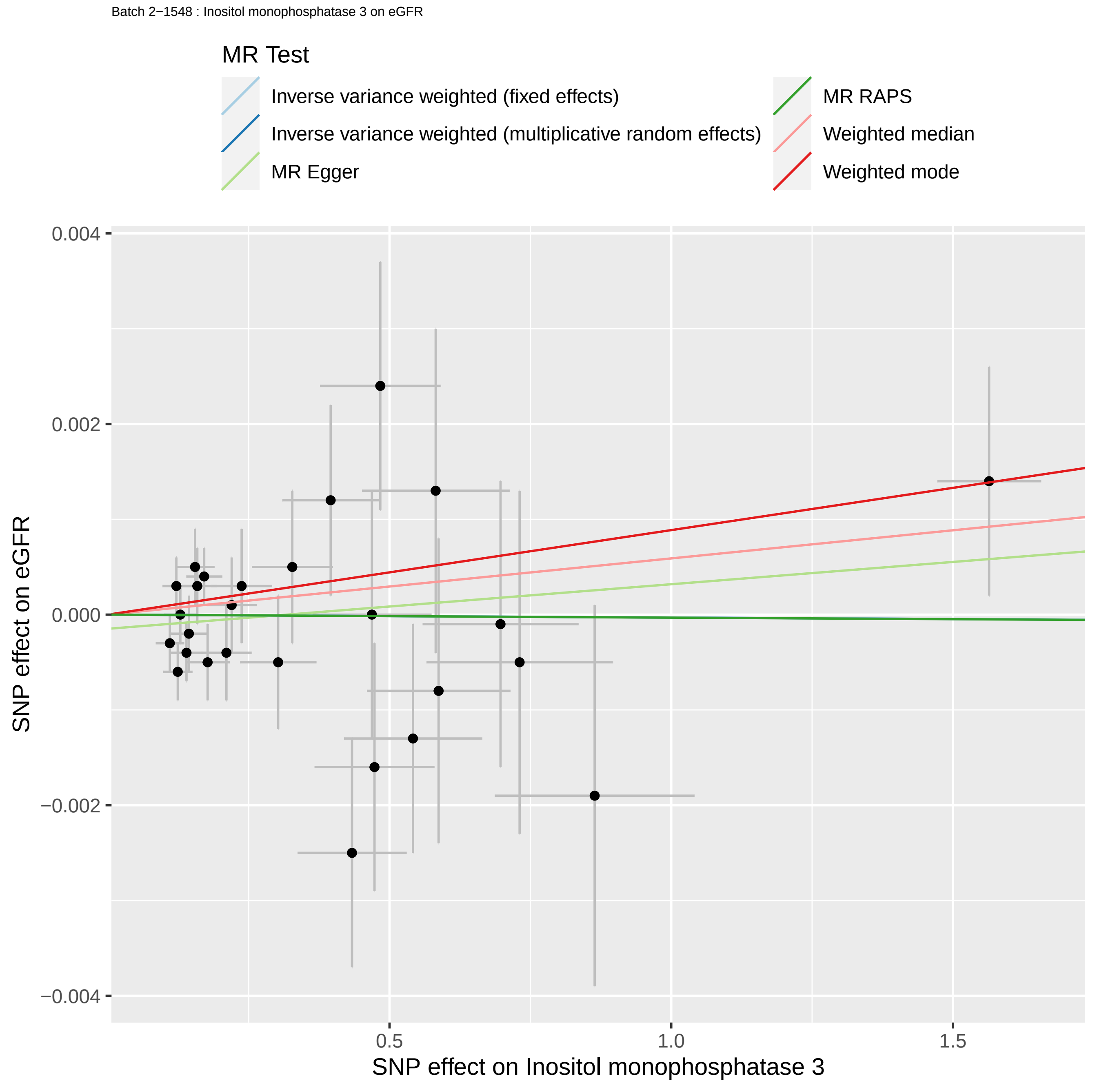

Batch 2−1548 : Inositol monophosphatase 3 on eGFR
MR Test
Inverse variance weighted (fixed effects)
Inverse variance weighted (multiplicative random effects)
MR Egger
MR RAPS
Weighted median
Weighted mode
0.004
0.002
0.000
−0.002
−0.004
0.5
1.0
1.5
SNP effect on eGFR
SNP effect on Inositol monophosphatase 3

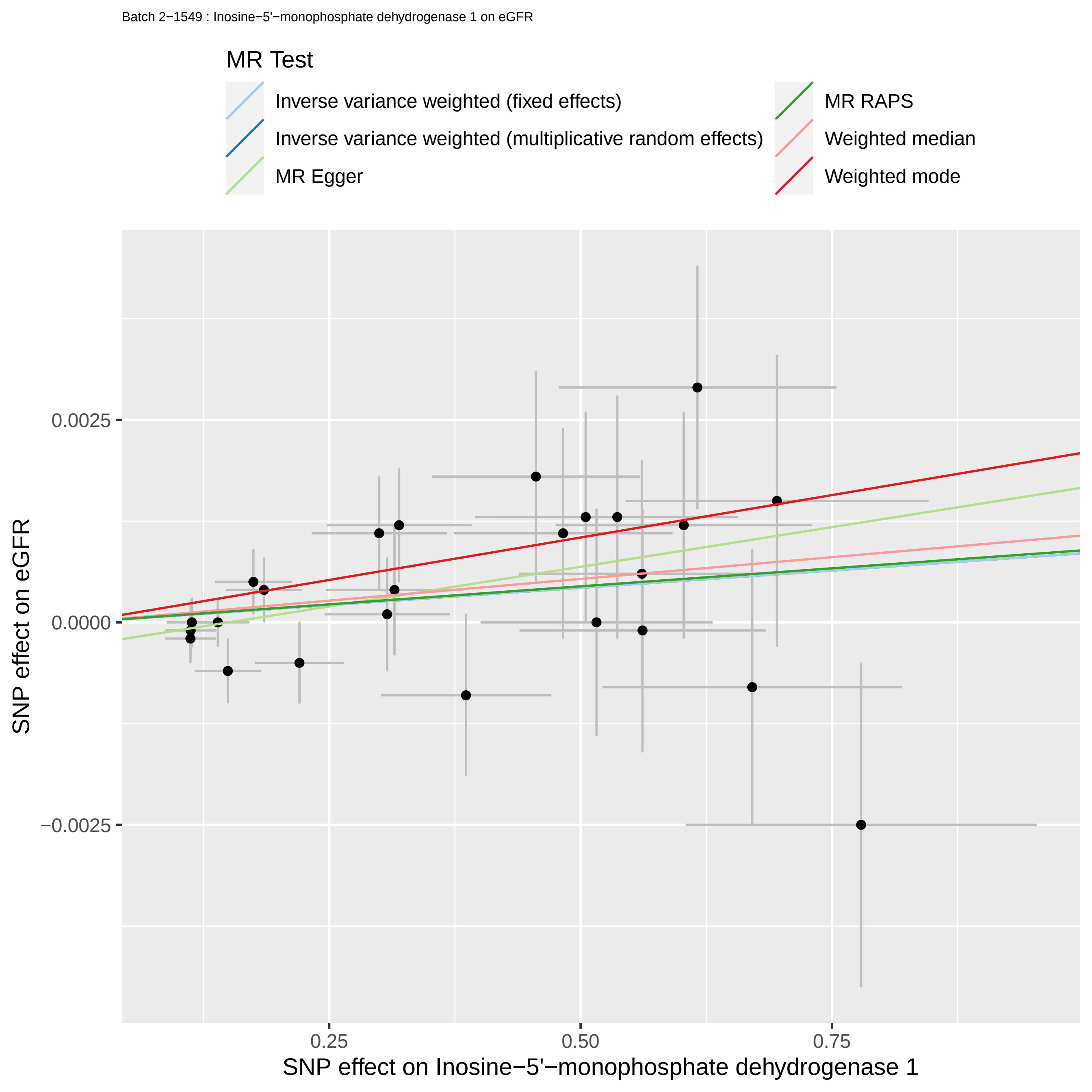
Batch 2−1549 : Inosine−5'−monophosphate dehydrogenase 1 on eGFR
MR Test
Inverse variance weighted (fixed effects)
Inverse variance weighted (multiplicative random effects)
MR Egger
MR RAPS
Weighted median
Weighted mode
0.0025
0.0000
−0.0025
0.25
0.50
0.75
SNP effect on eGFR
SNP effect on Inosine−5'−monophosphate dehydrogenase 1

Batch 2−1550 : Inosine−5'−monophosphate dehydrogenase 2 on eGFR

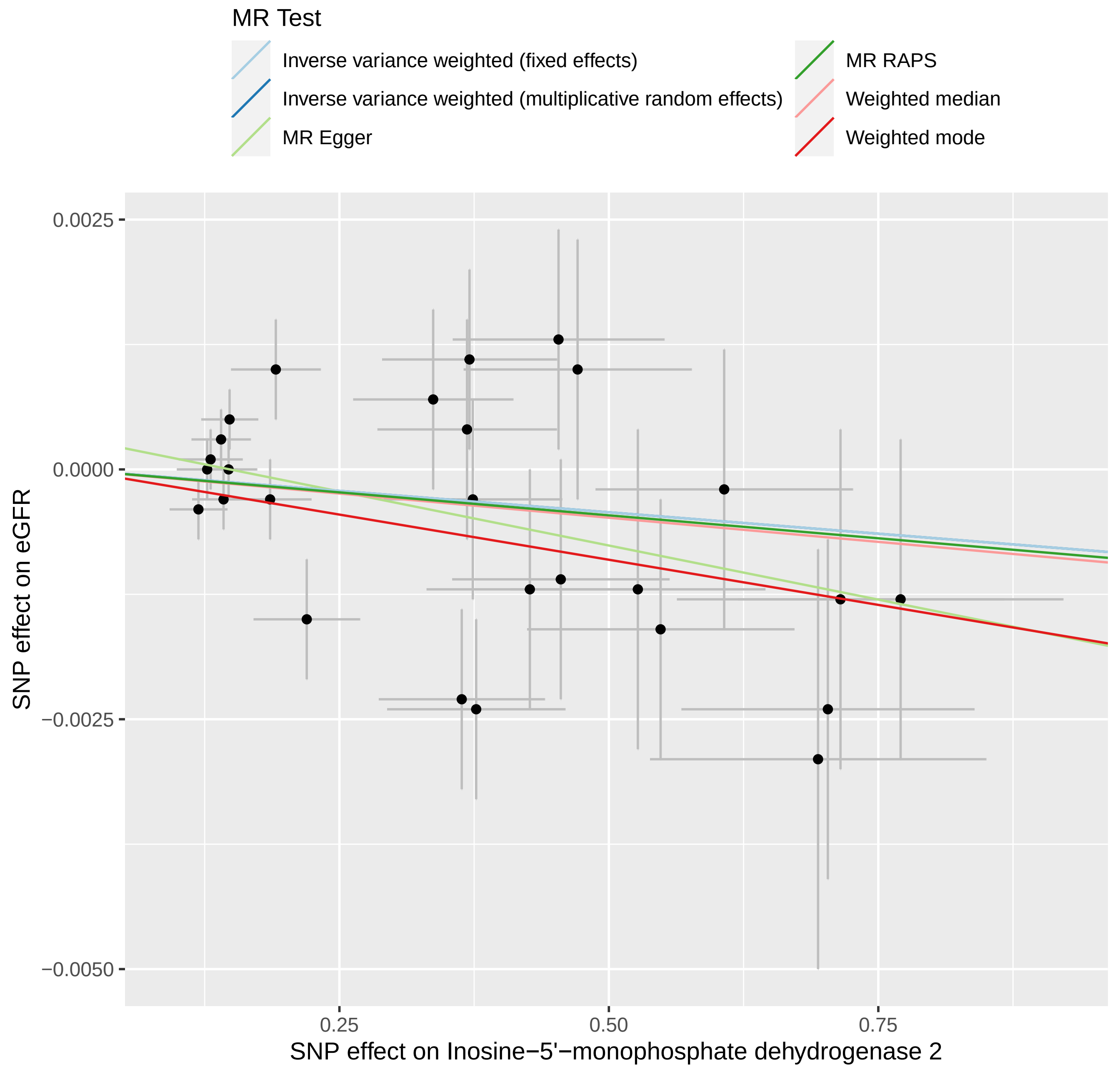

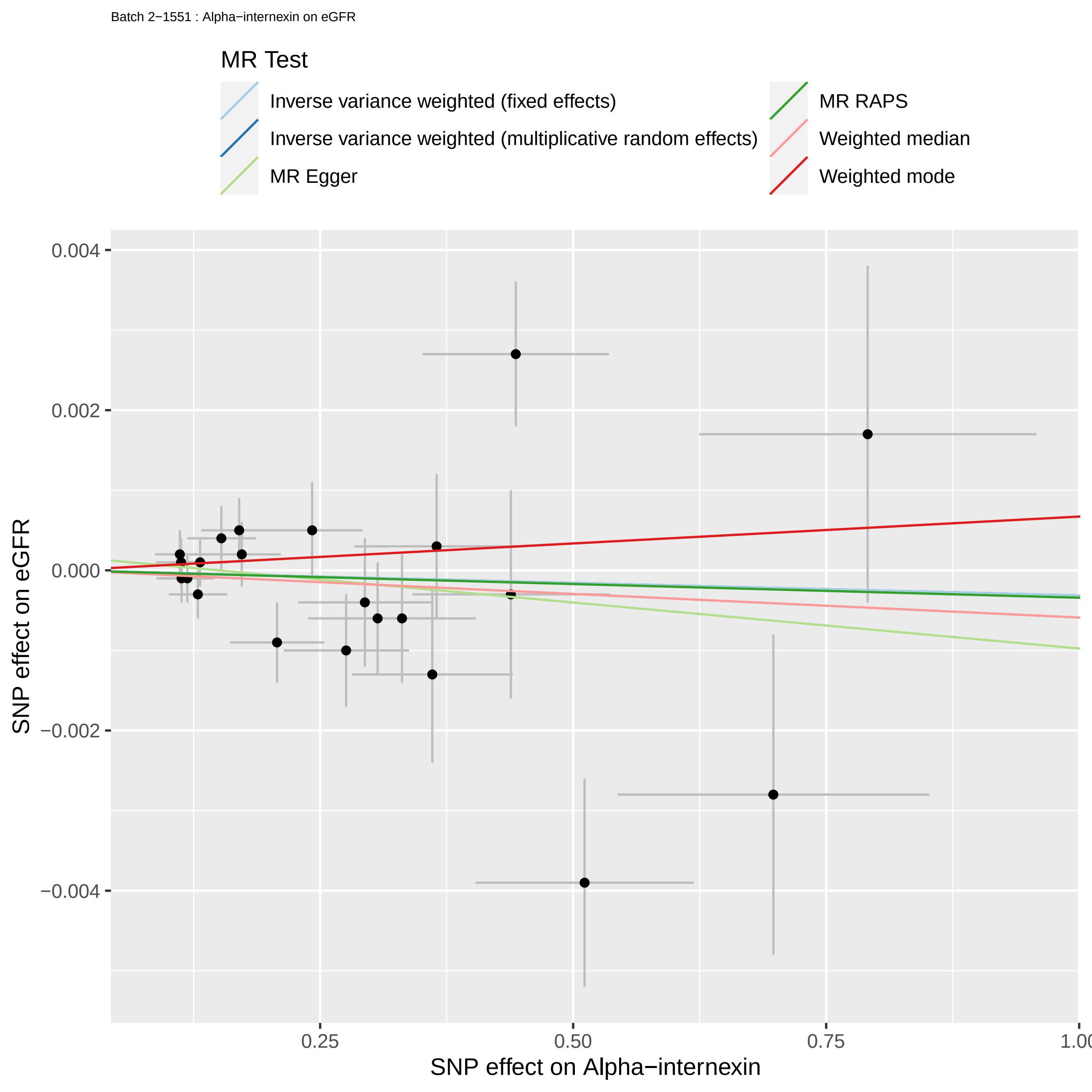
Batch 2−1551 : Alpha−internexin on eGFR
MR Test
Inverse variance weighted (fixed effects)
Inverse variance weighted (multiplicative random effects)
MR Egger
MR RAPS
Weighted median
Weighted mode
0.004
0.002
0.000
−0.002
−0.004
SNP effect on eGFR
0.25
0.50
0.75
1.00
SNP effect on Alpha−internexin

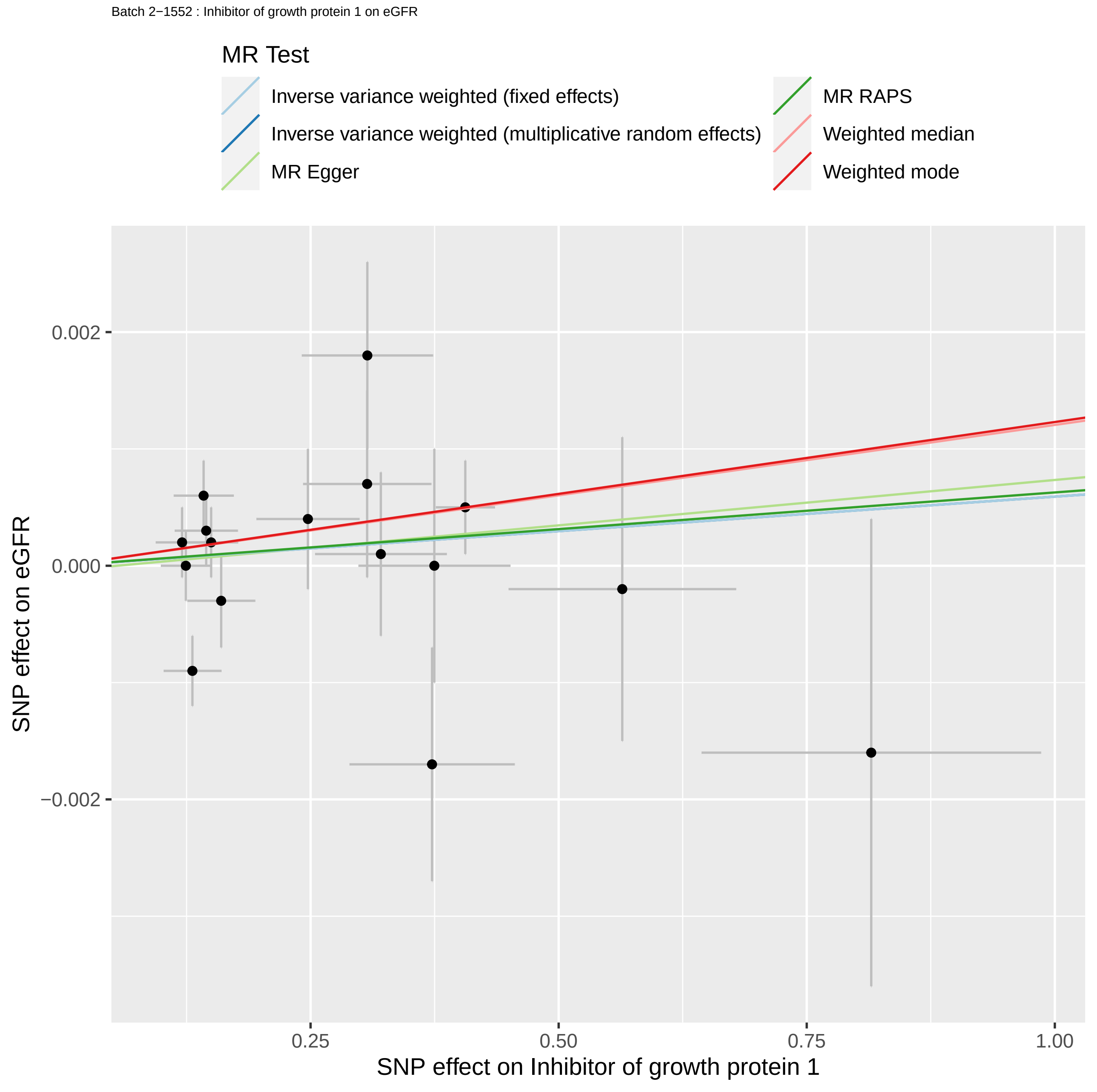
Batch 2−1552 : Inhibitor of growth protein 1 on eGFR
MR Test
Inverse variance weighted (fixed effects)
Inverse variance weighted (multiplicative random effects)
MR Egger
MR RAPS
Weighted median
Weighted mode
0.002
0.000
−0.002
0.25
0.50
0.75
1.00
SNP effect on eGFR
SNP effect on Inhibitor of growth protein 1

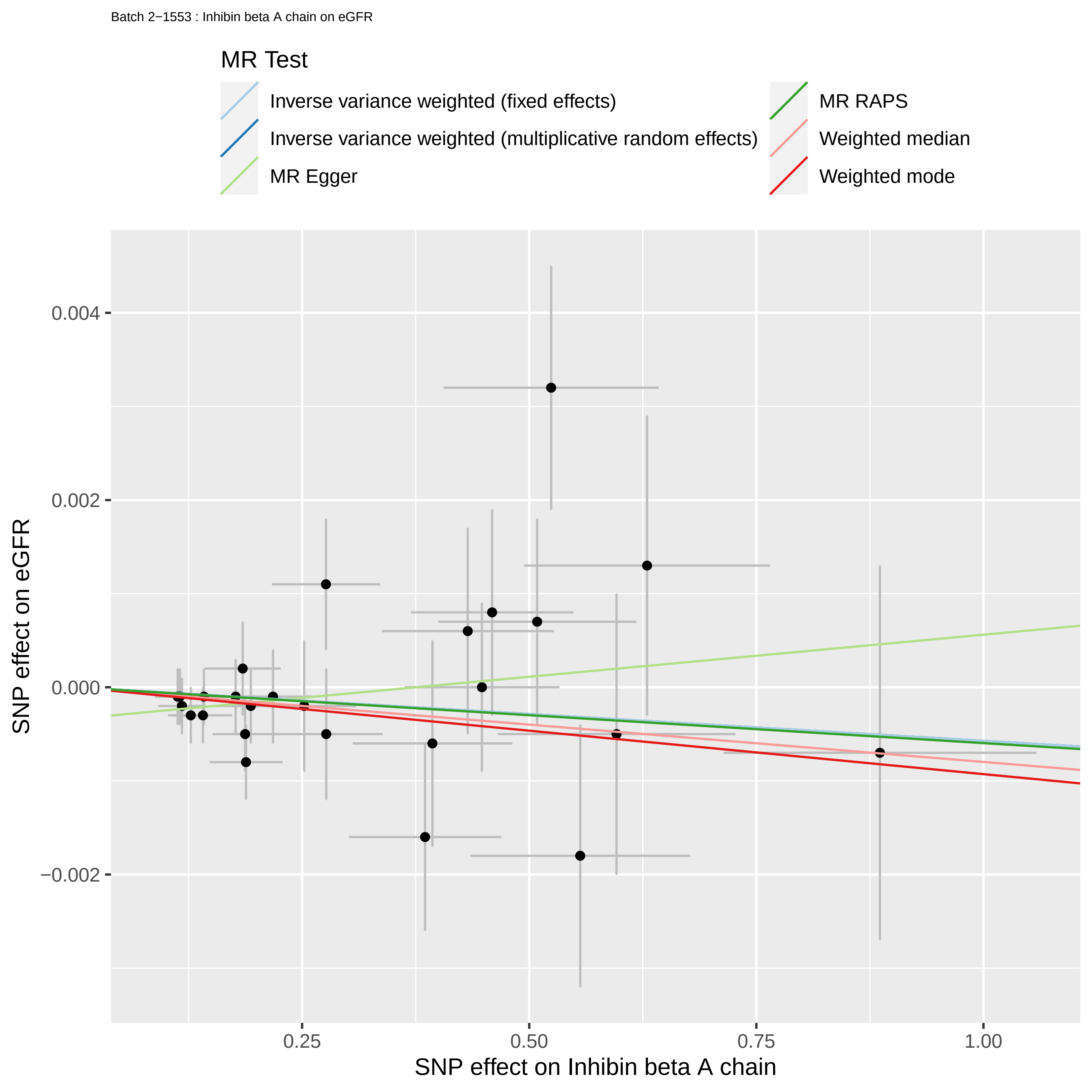

Batch 2−1553 : Inhibin beta A chain on eGFR
MR Test
Inverse variance weighted (fixed effects)
Inverse variance weighted (multiplicative random effects)
MR Egger
MR RAPS
Weighted median
Weighted mode
0.004
0.002
0.000
−0.002
0.25
0.50
0.75
1.00
SNP effect on eGFR
SNP effect on Inhibin beta A chain

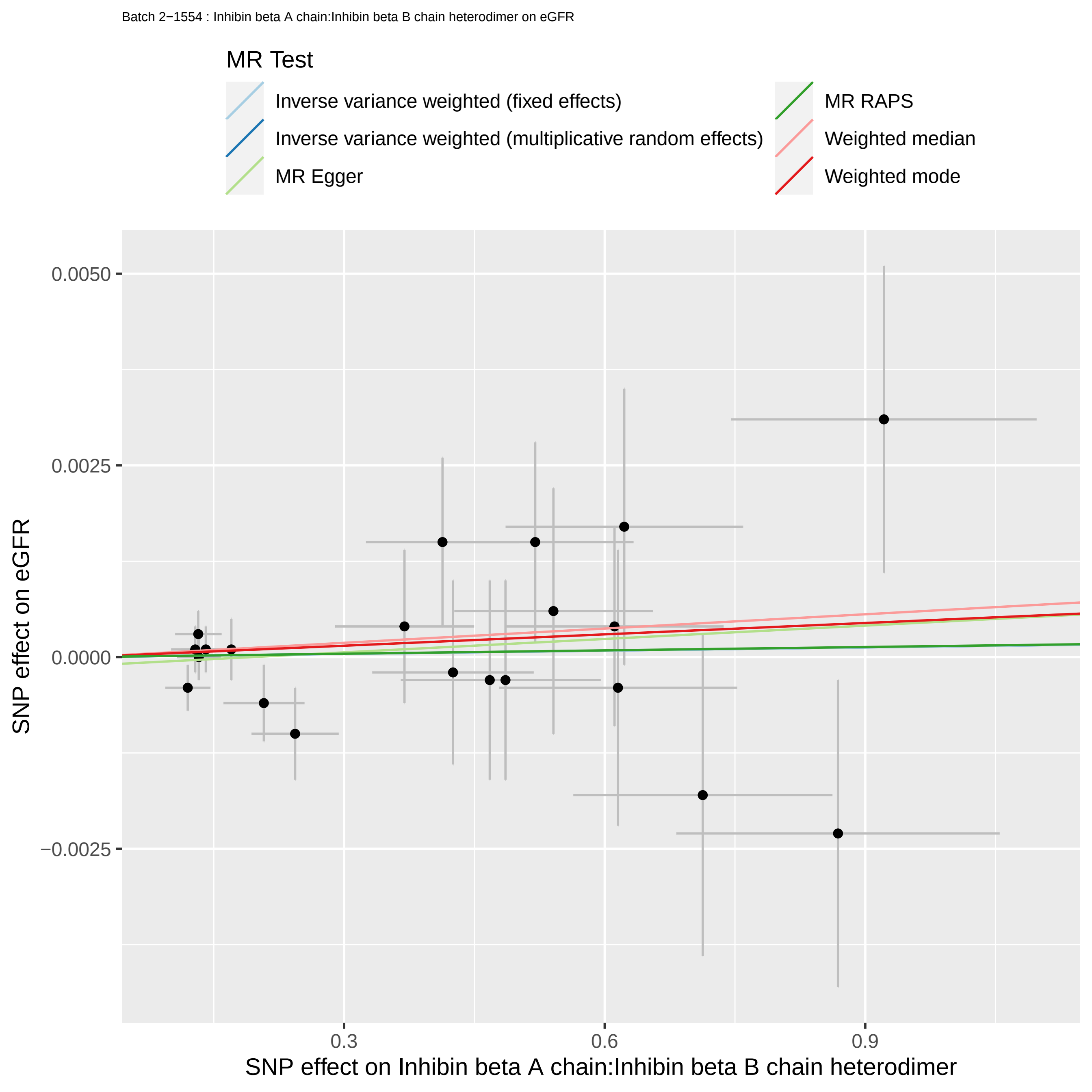

Batch 2−1554 : Inhibin beta A chain:Inhibin beta B chain heterodimer on eGFR
MR Test
Inverse variance weighted (fixed effects)
Inverse variance weighted (multiplicative random effects)
MR Egger
MR RAPS
Weighted median
Weighted mode
0.0050
0.0025
0.0000
−0.0025
0.3
0.6
0.9
SNP effect on eGFR
SNP effect on Inhibin beta A chain:Inhibin beta B chain heterodimer

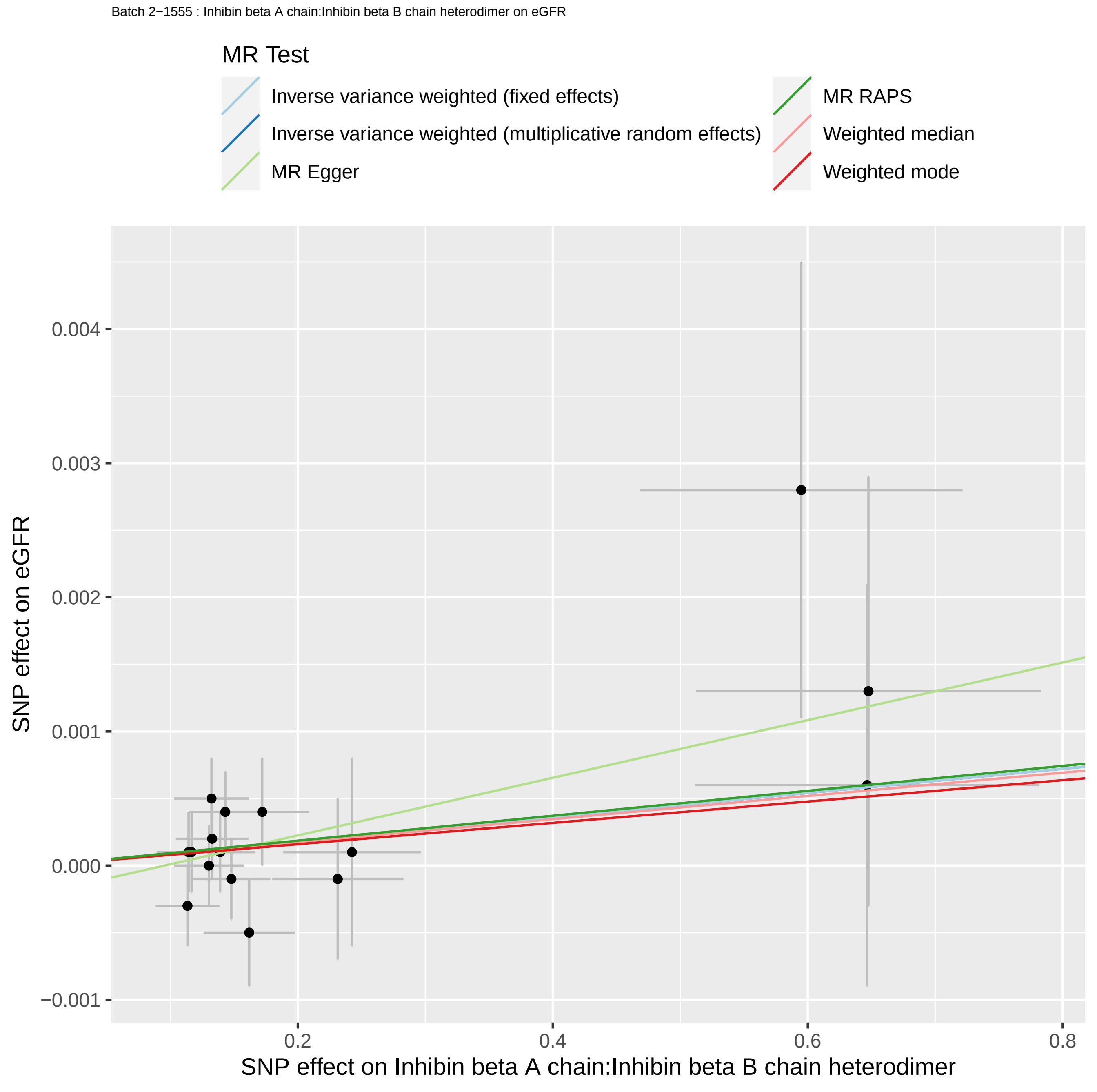

Batch 2−1555 : Inhibin beta A chain:Inhibin beta B chain heterodimer on eGFR
MR Test
Inverse variance weighted (fixed effects)
Inverse variance weighted (multiplicative random effects)
MR Egger
MR RAPS
Weighted median
Weighted mode
0.004
0.003
0.002
0.001
0.000
−0.001
0.2
0.4
0.6
0.8
SNP effect on eGFR
SNP effect on Inhibin beta A chain:Inhibin beta B chain heterodimer

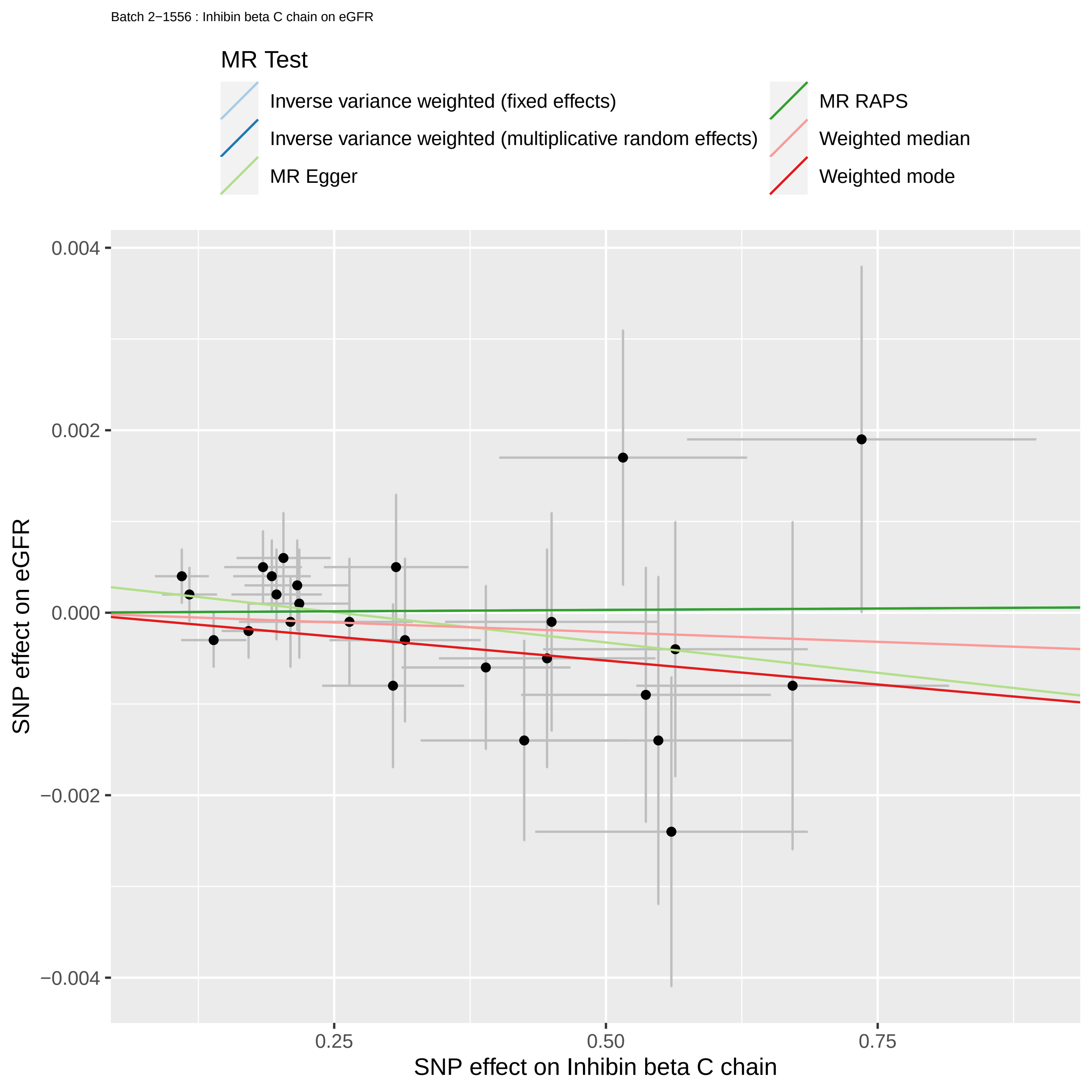

Batch 2−1556 : Inhibin beta C chain on eGFR
MR Test
Inverse variance weighted (fixed effects)
Inverse variance weighted (multiplicative random effects)
MR Egger
MR RAPS
Weighted median
Weighted mode
0.004
0.002
0.000
−0.002
−0.004
SNP effect on eGFR
0.25
0.50
0.75
SNP effect on Inhibin beta C chain

Batch 2−1557 : 72 kDa inositol polyphosphate 5−phosphatase on eGFR

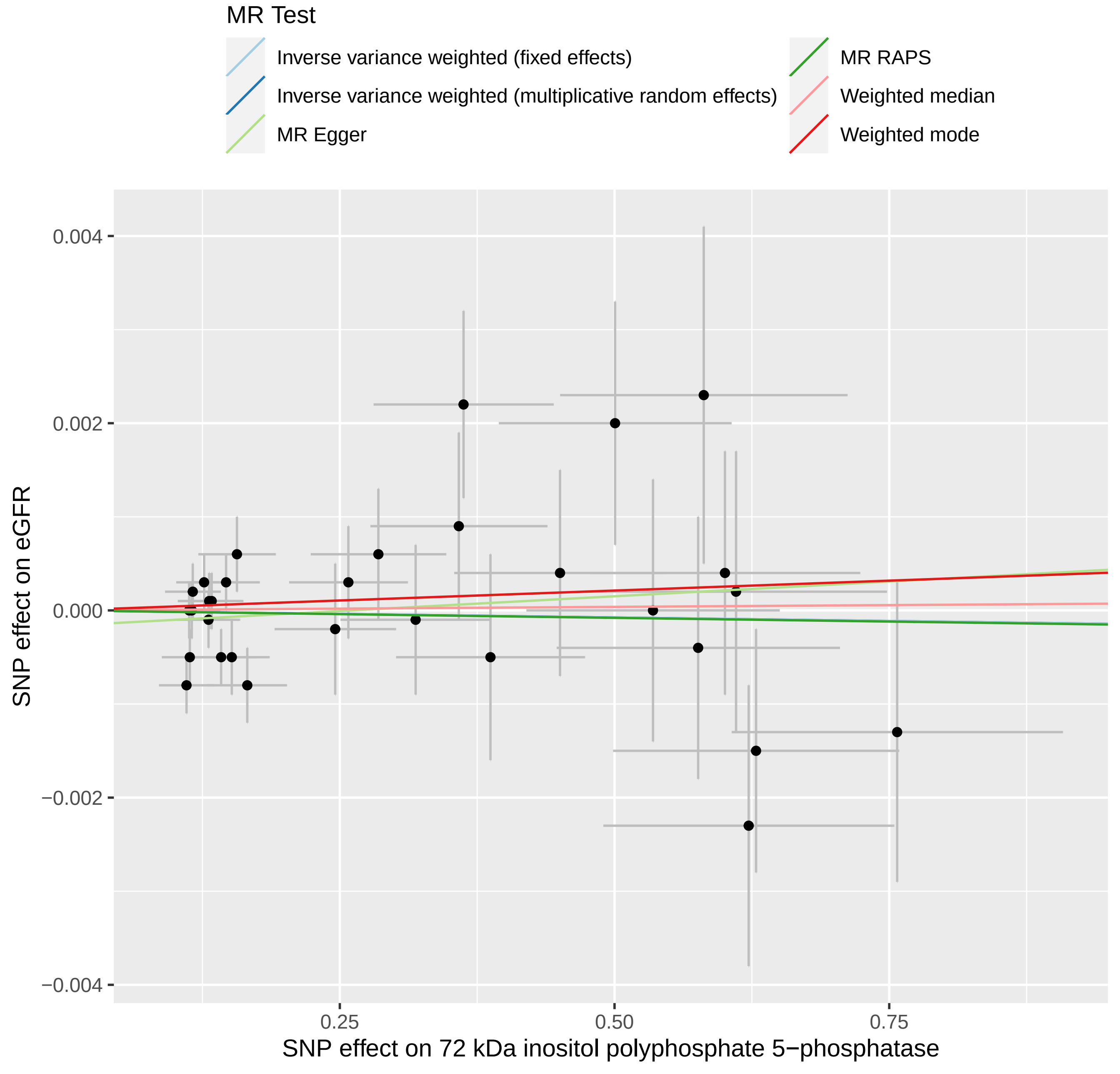

Batch 2−1558 : Insulin on eGFR

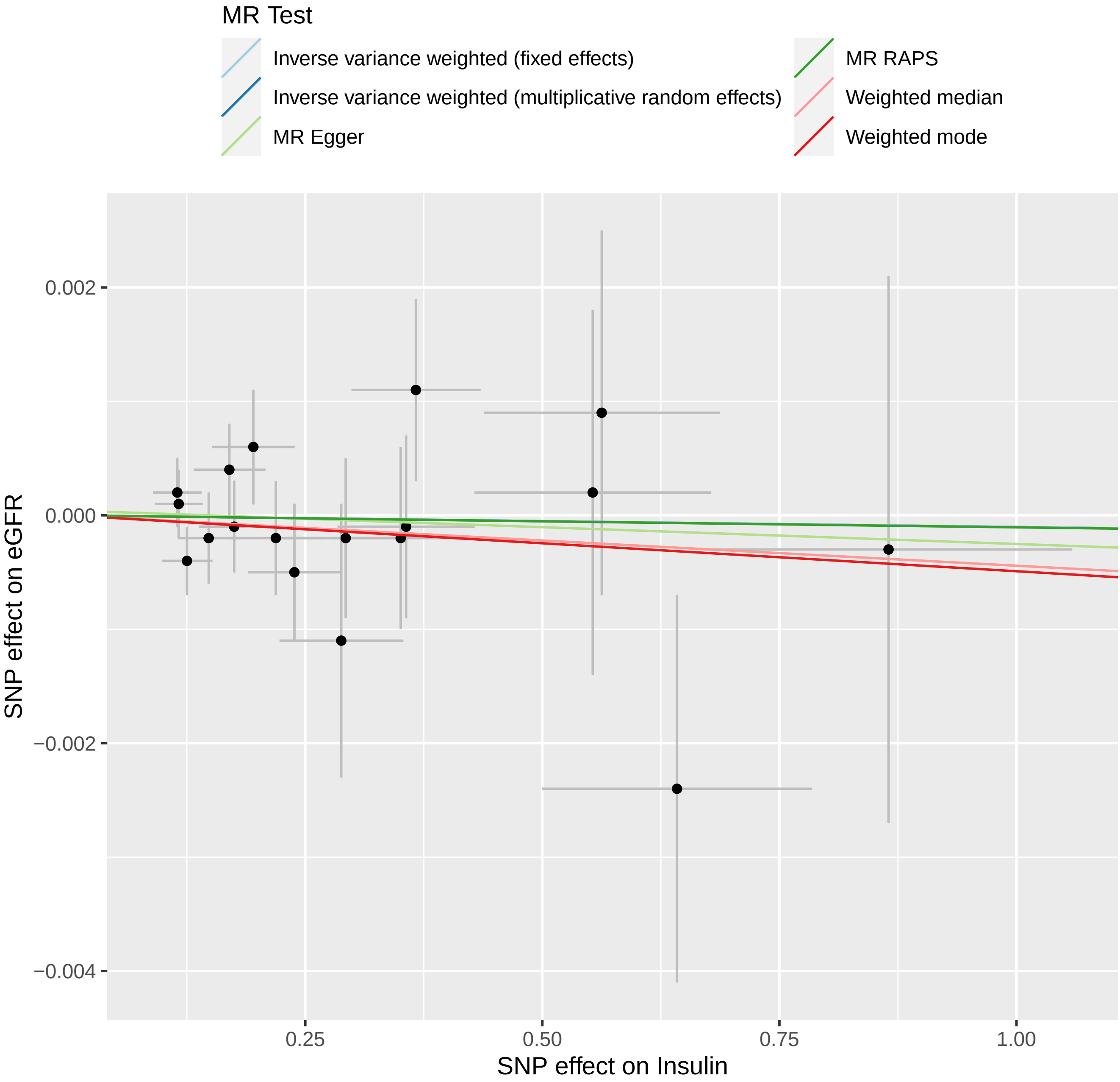

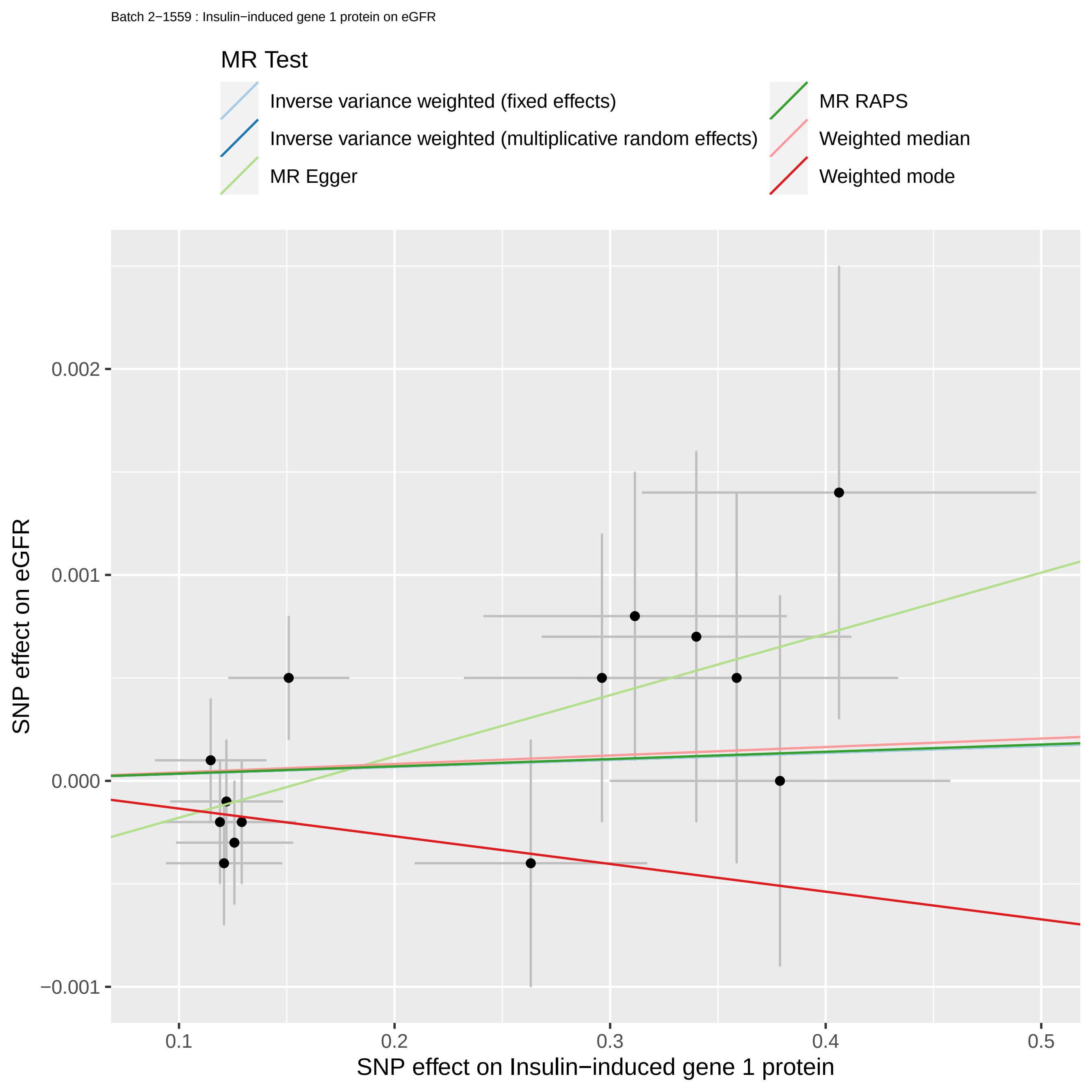
Batch 2−1559 : Insulin−induced gene 1 protein on eGFR
MR Test
Inverse variance weighted (fixed effects)
Inverse variance weighted (multiplicative random effects)
MR Egger
MR RAPS
Weighted median
Weighted mode
0.002
0.001
0.000
−0.001
0.1
0.2
0.3
0.4
0.5
SNP effect on eGFR
SNP effect on Insulin−induced gene 1 protein

Batch 2−1560 : Insulin−like 3 on eGFR

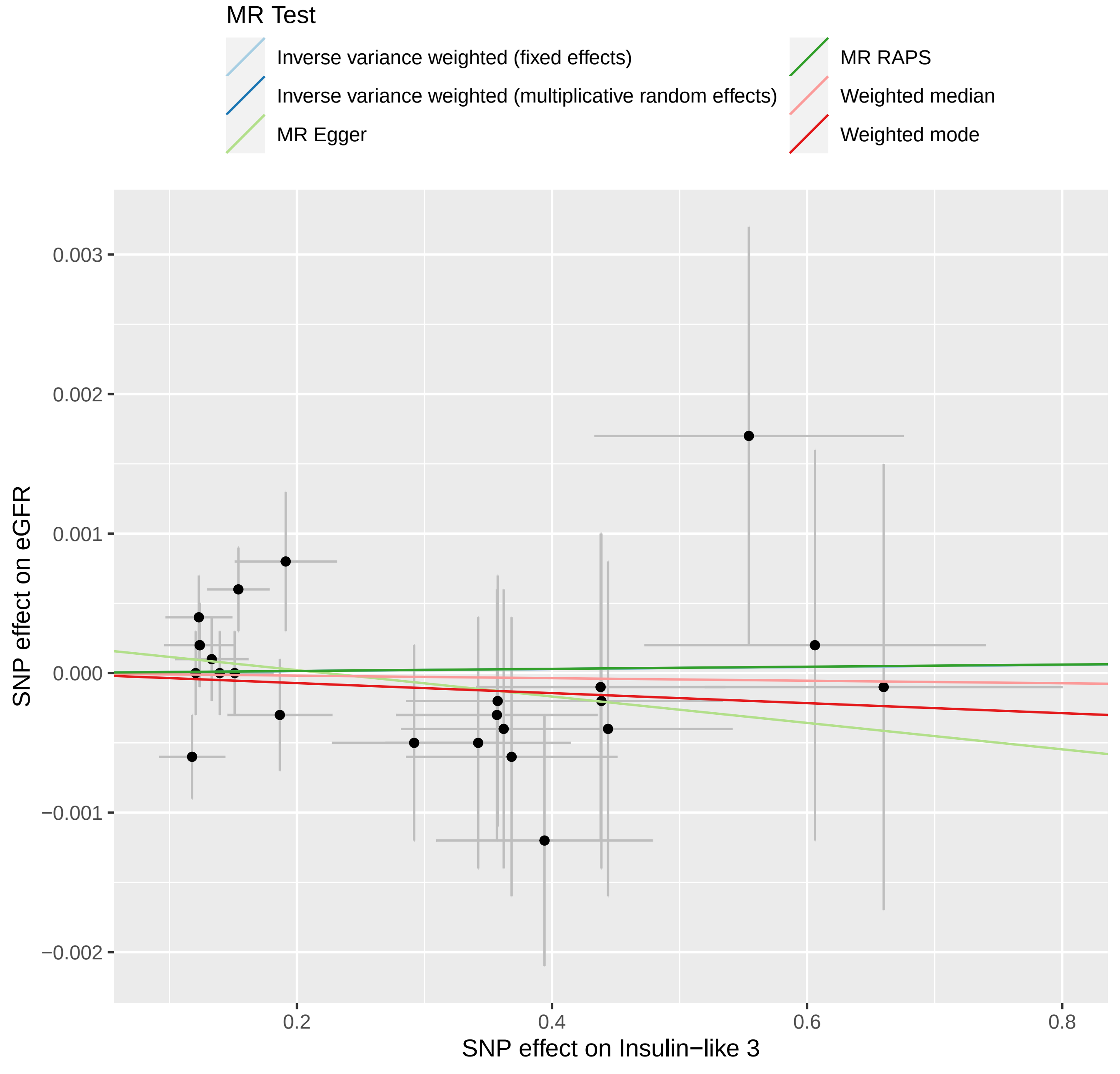

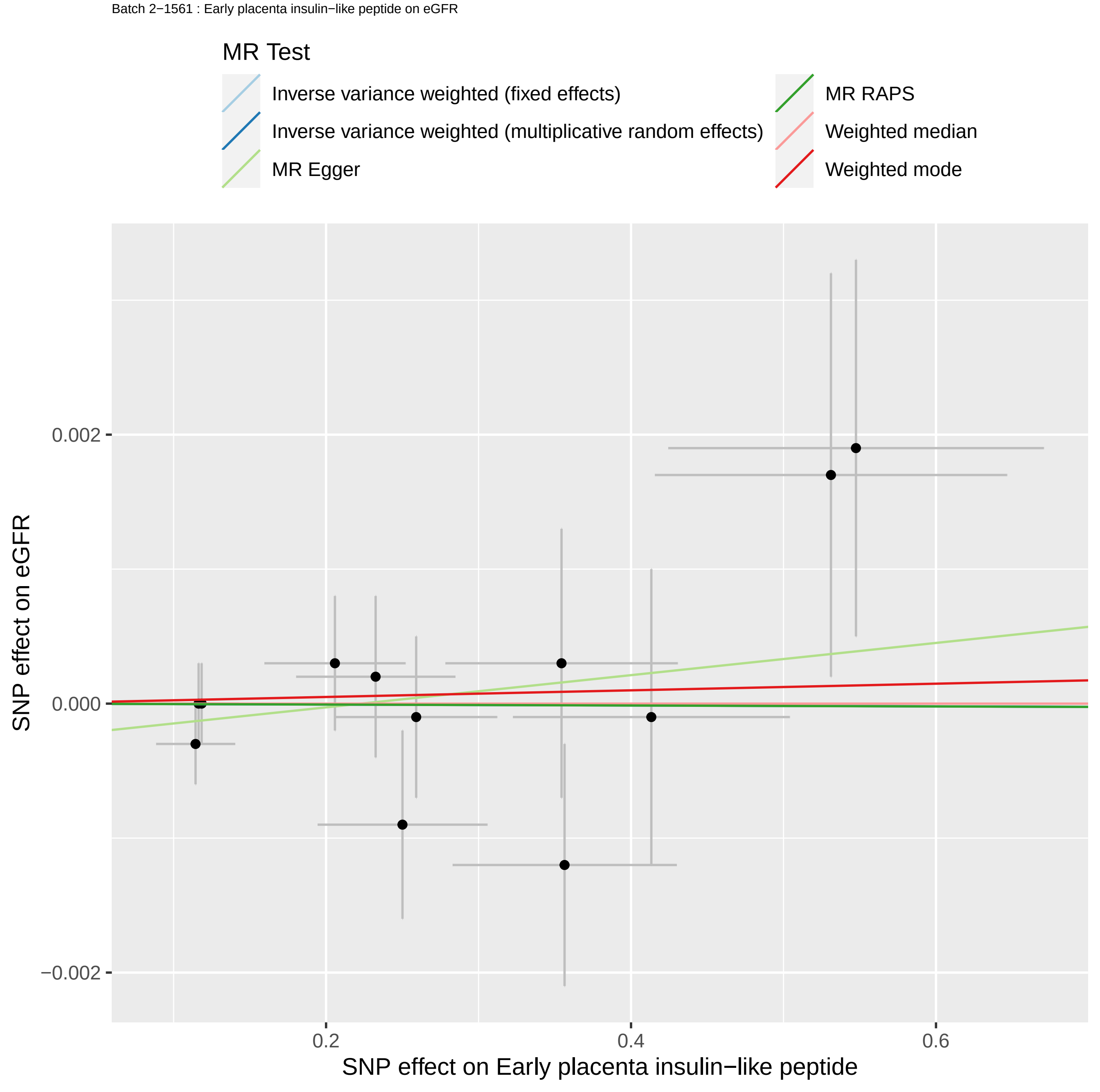
Batch 2−1561 : Early placenta insulin−like peptide on eGFR
MR Test
Inverse variance weighted (fixed effects)
Inverse variance weighted (multiplicative random effects)
MR Egger
MR RAPS
Weighted median
Weighted mode
0.002
0.000
−0.002
0.2
0.4
0.6
SNP effect on eGFR
SNP effect on Early placenta insulin−like peptide

Batch 2−1562 : Insulin−like peptide INSL5 on eGFR

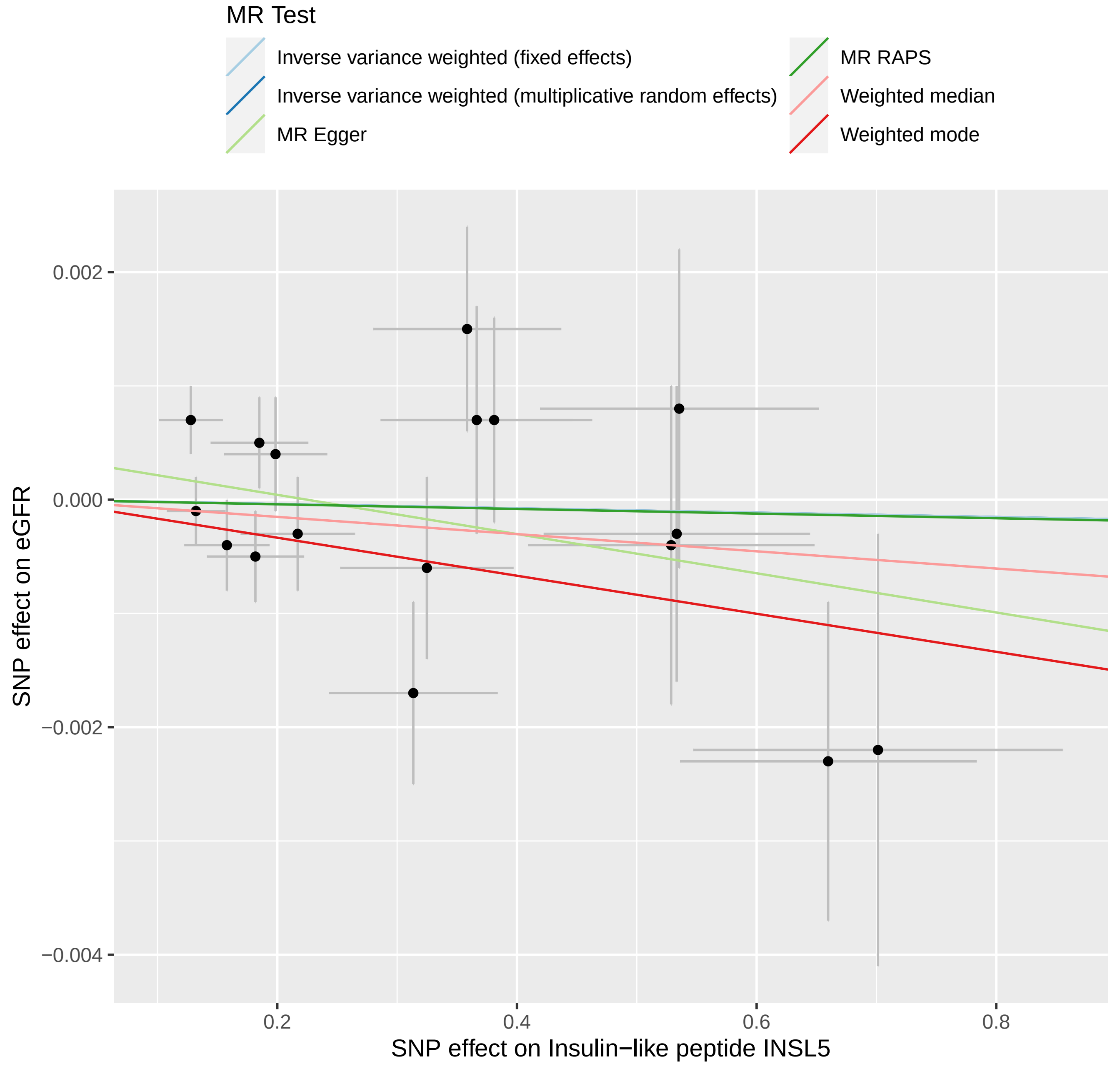

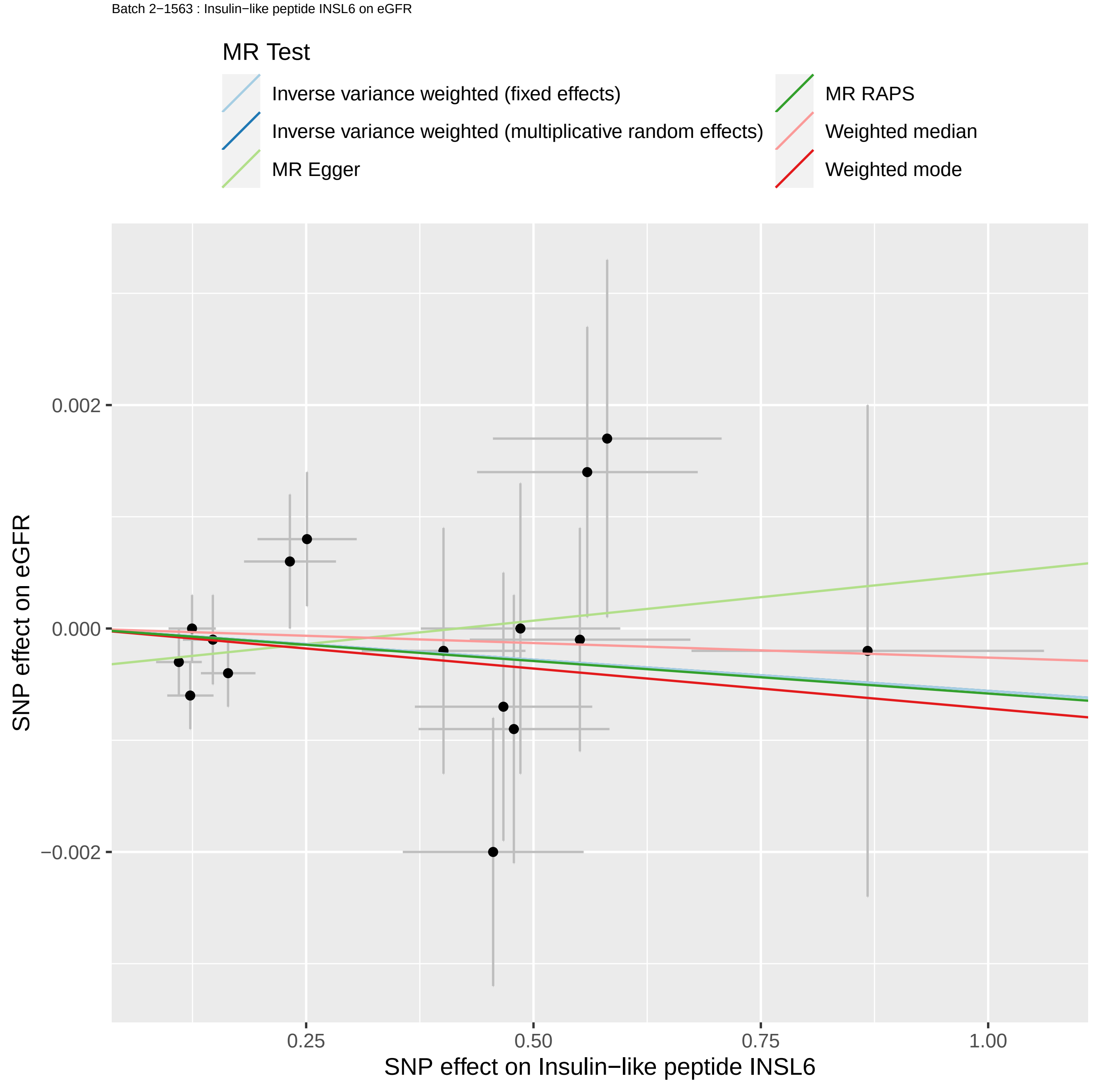

Batch 2−1563 : Insulin−like peptide INSL6 on eGFR
MR Test
Inverse variance weighted (fixed effects)
Inverse variance weighted (multiplicative random effects)
MR Egger
MR RAPS
Weighted median
Weighted mode
0.002
0.000
−0.002
0.25
0.50
0.75
1.00
SNP effect on eGFR
SNP effect on Insulin−like peptide INSL6

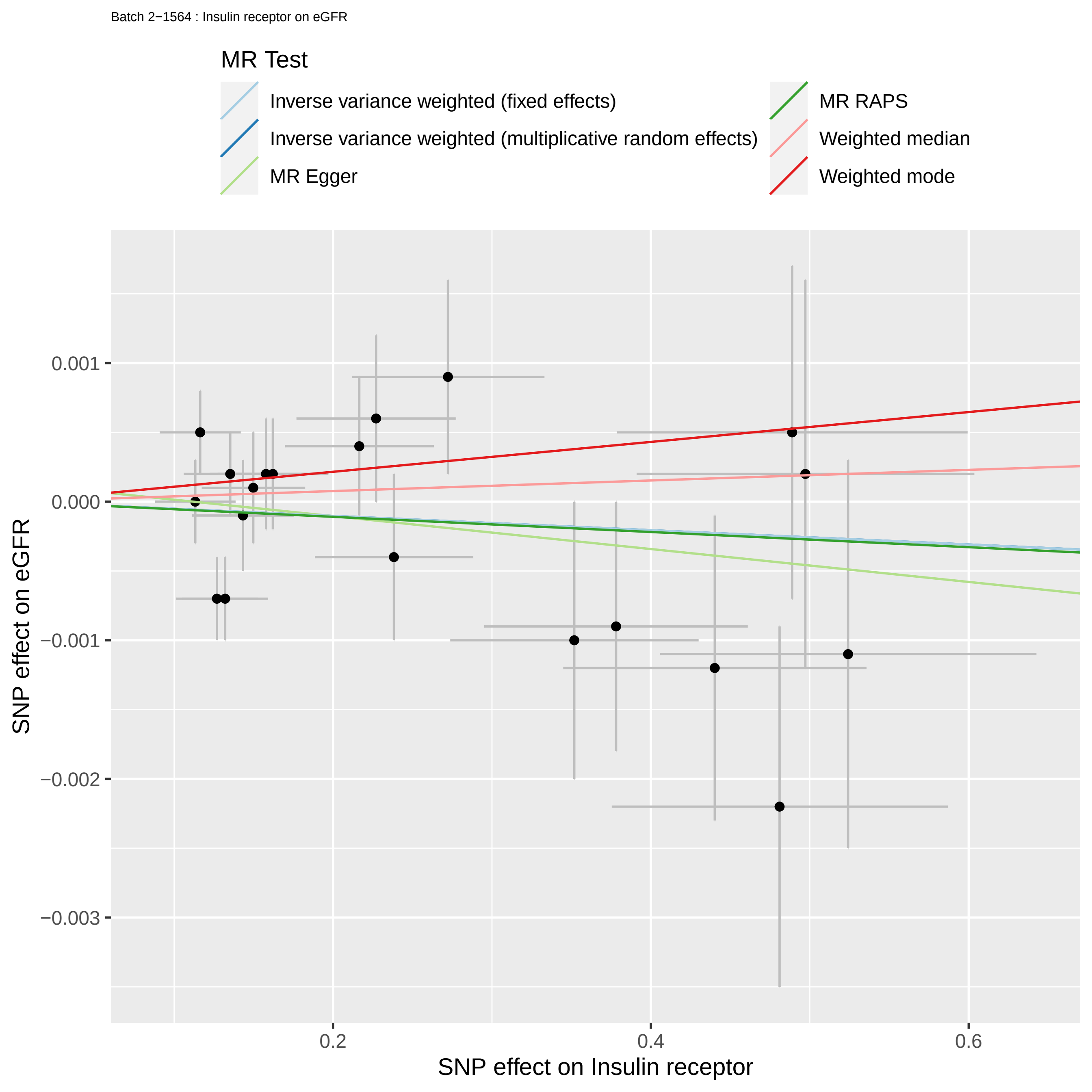
Batch 2−1564 : Insulin receptor on eGFR
MR Test
Inverse variance weighted (fixed effects)
Inverse variance weighted (multiplicative random effects)
MR Egger
MR RAPS
Weighted median
Weighted mode
SNP effect on eGFR
0.001
0.000
−0.001
−0.002
−0.003
SNP effect on Insulin receptor
0.2
0.4
0.6

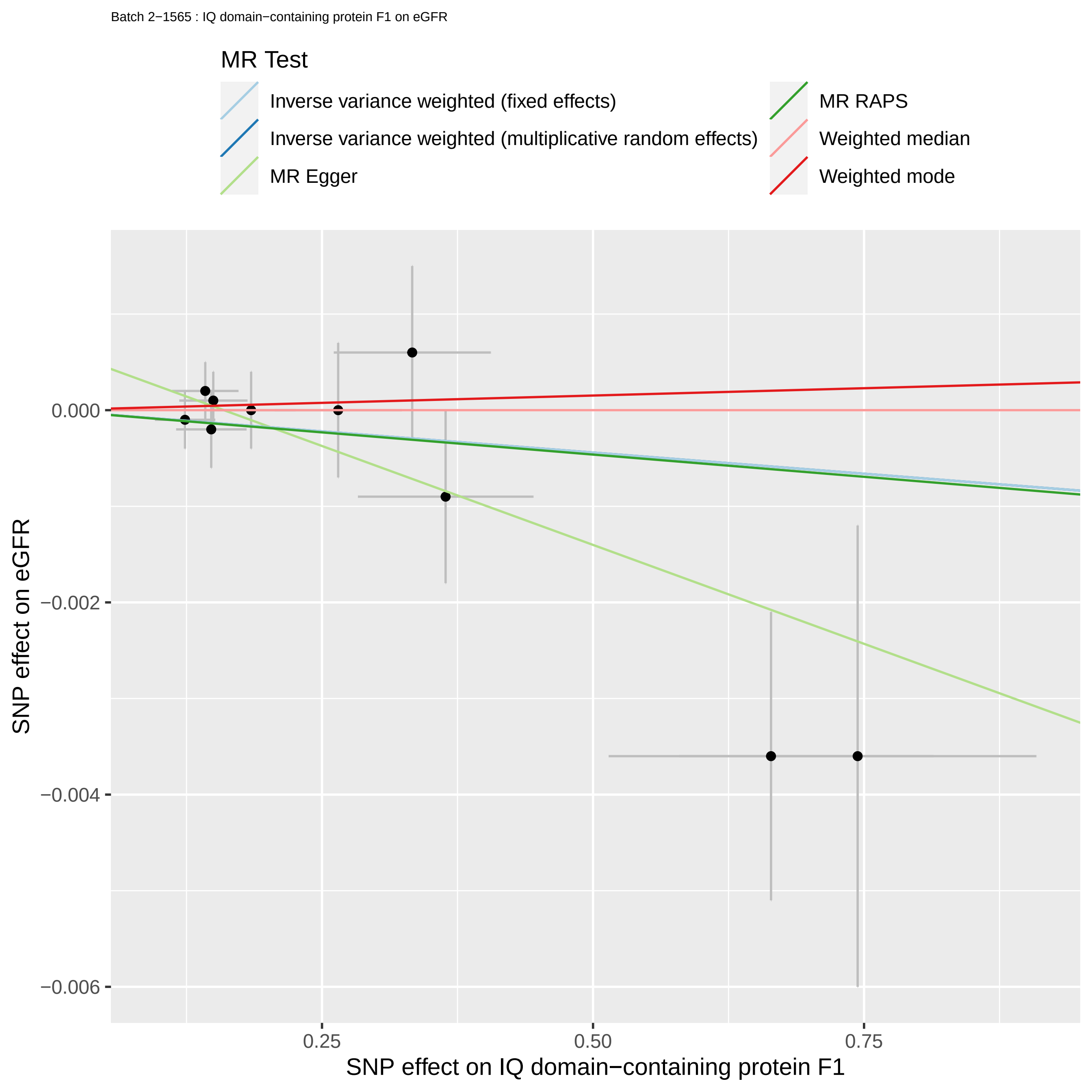

Batch 2−1565 : IQ domain−containing protein F1 on eGFR
MR Test
Inverse variance weighted (fixed effects)
Inverse variance weighted (multiplicative random effects)
MR Egger
MR RAPS
Weighted median
Weighted mode
0.000
−0.002
−0.004
−0.006
0.25
0.50
0.75
SNP effect on eGFR
SNP effect on IQ domain−containing protein F1

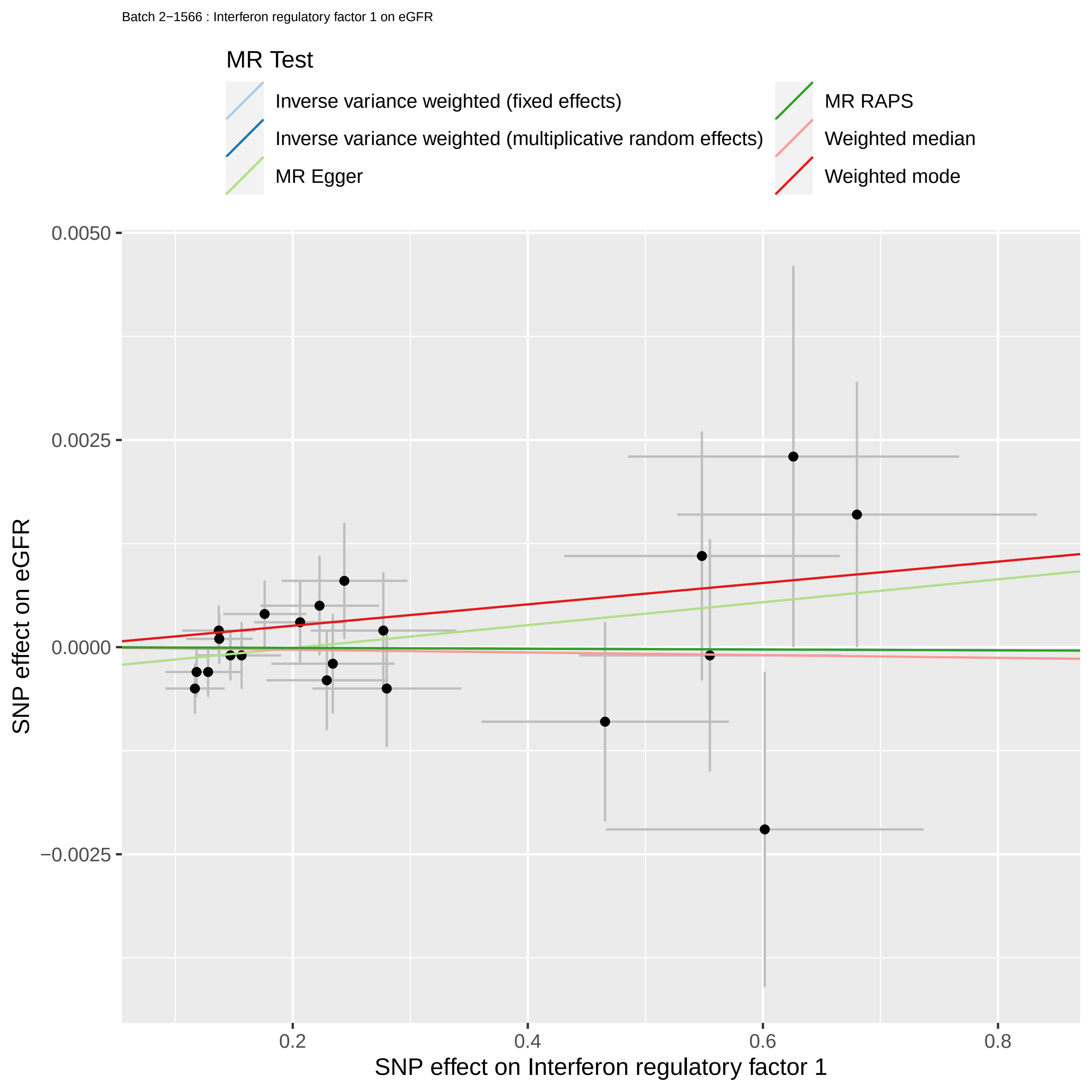

Batch 2−1566 : Interferon regulatory factor 1 on eGFR
MR Test
Inverse variance weighted (fixed effects)
Inverse variance weighted (multiplicative random effects)
MR Egger
MR RAPS
Weighted median
Weighted mode
0.0050
0.0025
0.0000
−0.0025
SNP effect on eGFR
0.2
0.4
0.6
0.8
SNP effect on Interferon regulatory factor 1

Batch 2−1567 : Interferon regulatory factor 1 on eGFR

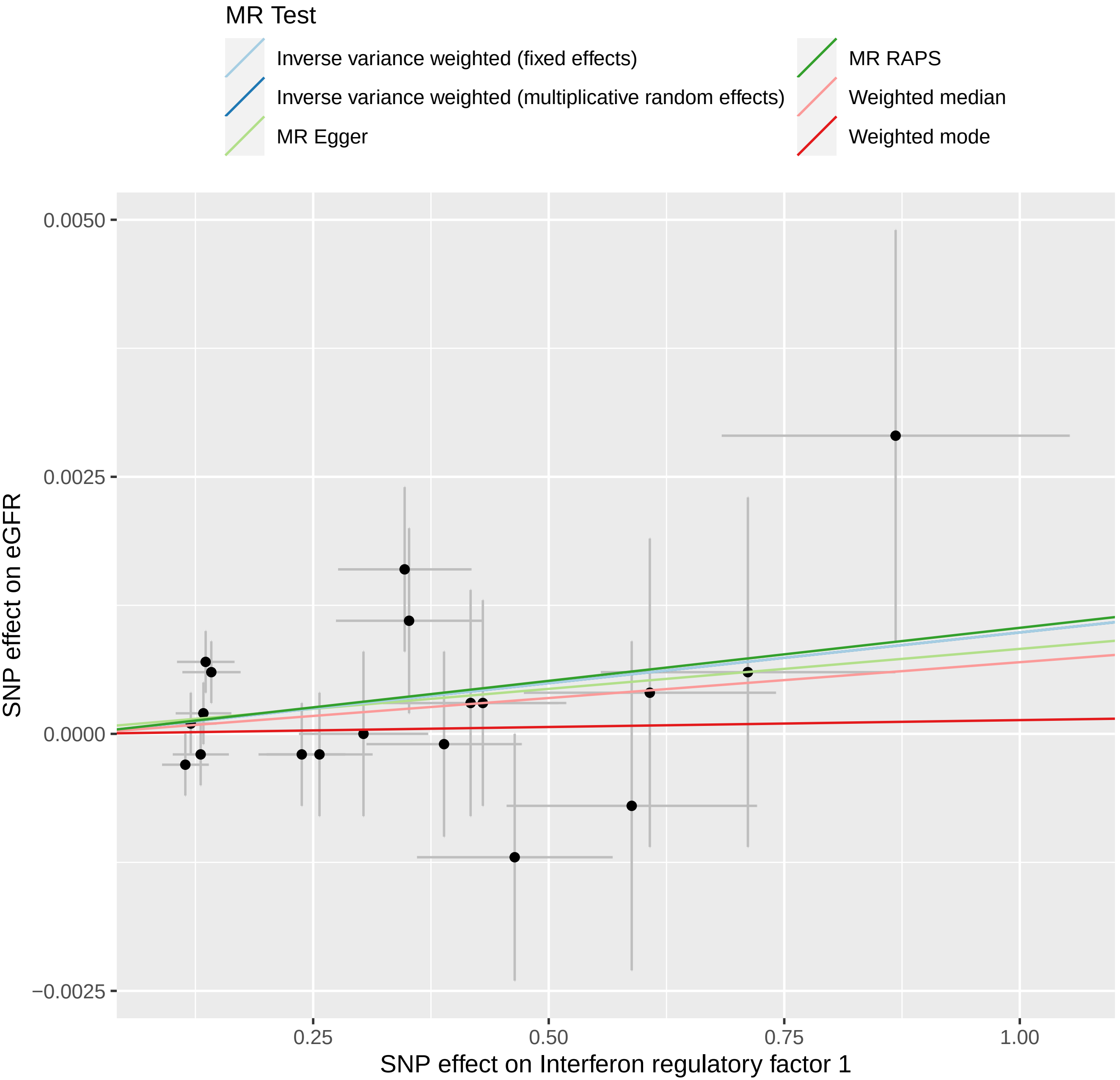

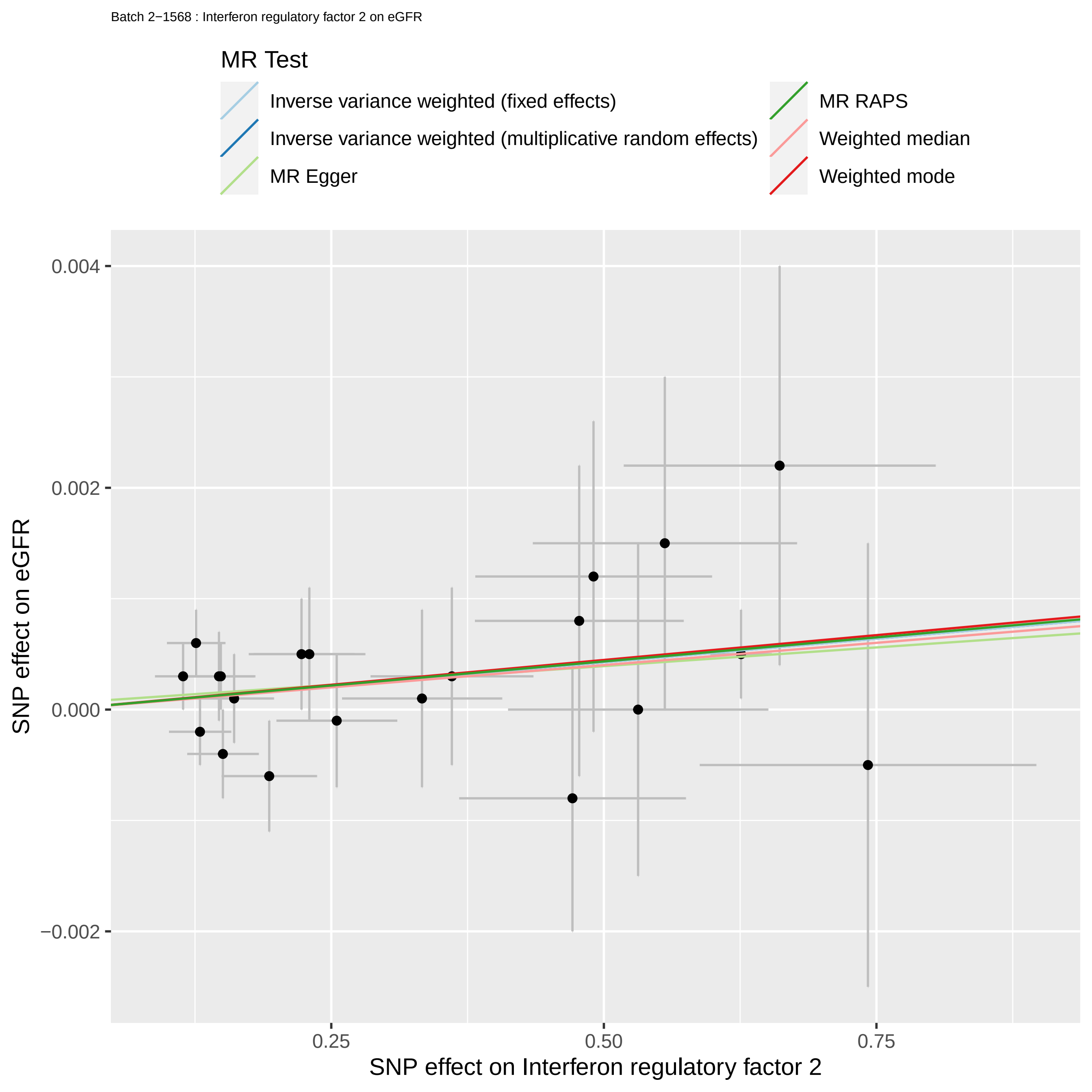
Batch 2−1568 : Interferon regulatory factor 2 on eGFR
MR Test
Inverse variance weighted (fixed effects)
Inverse variance weighted (multiplicative random effects)
MR Egger
MR RAPS
Weighted median
Weighted mode
0.004
0.002
0.000
−0.002
SNP effect on eGFR
0.25
0.50
0.75
SNP effect on Interferon regulatory factor 2

Batch 2−1569 : Interferon regulatory factor 4 on eGFR

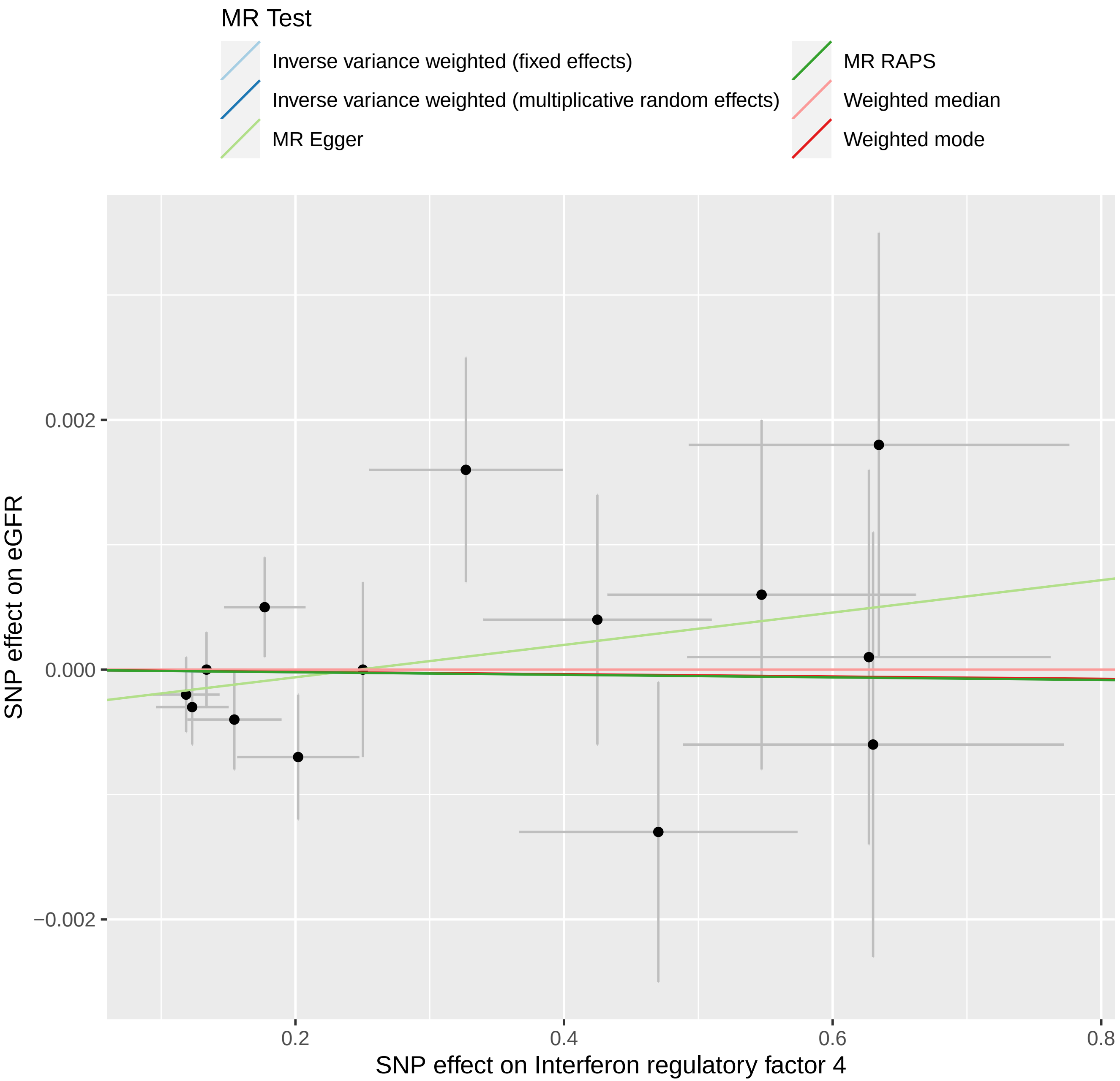

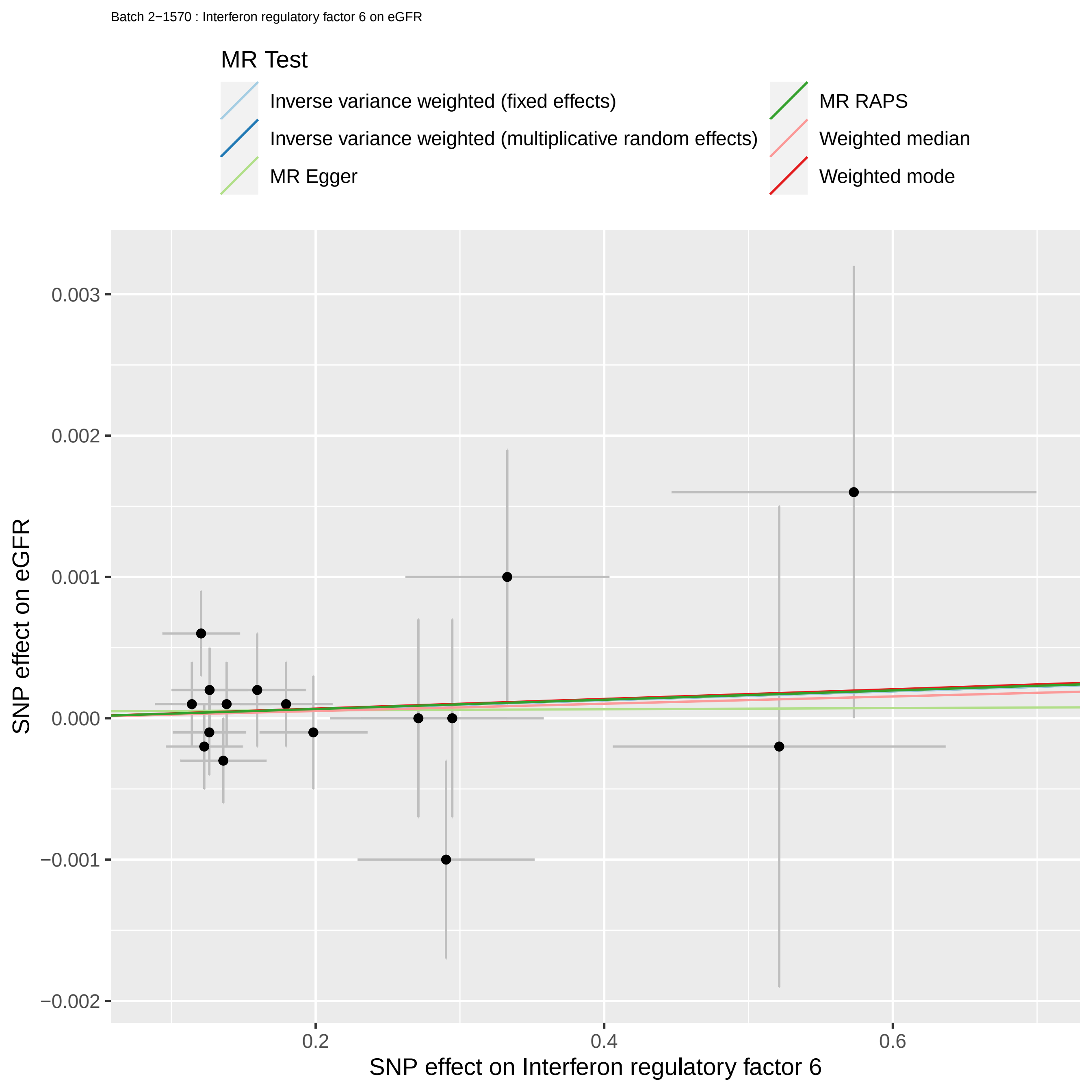
Batch 2−1570 : Interferon regulatory factor 6 on eGFR
MR Test
Inverse variance weighted (fixed effects)
Inverse variance weighted (multiplicative random effects)
MR Egger
MR RAPS
Weighted median
Weighted mode
0.003
0.002
0.001
0.000
−0.001
−0.002
0.2
0.4
0.6
SNP effect on eGFR
SNP effect on Interferon regulatory factor 6

Batch 2−1571 : Interferon regulatory factor 9 on eGFR

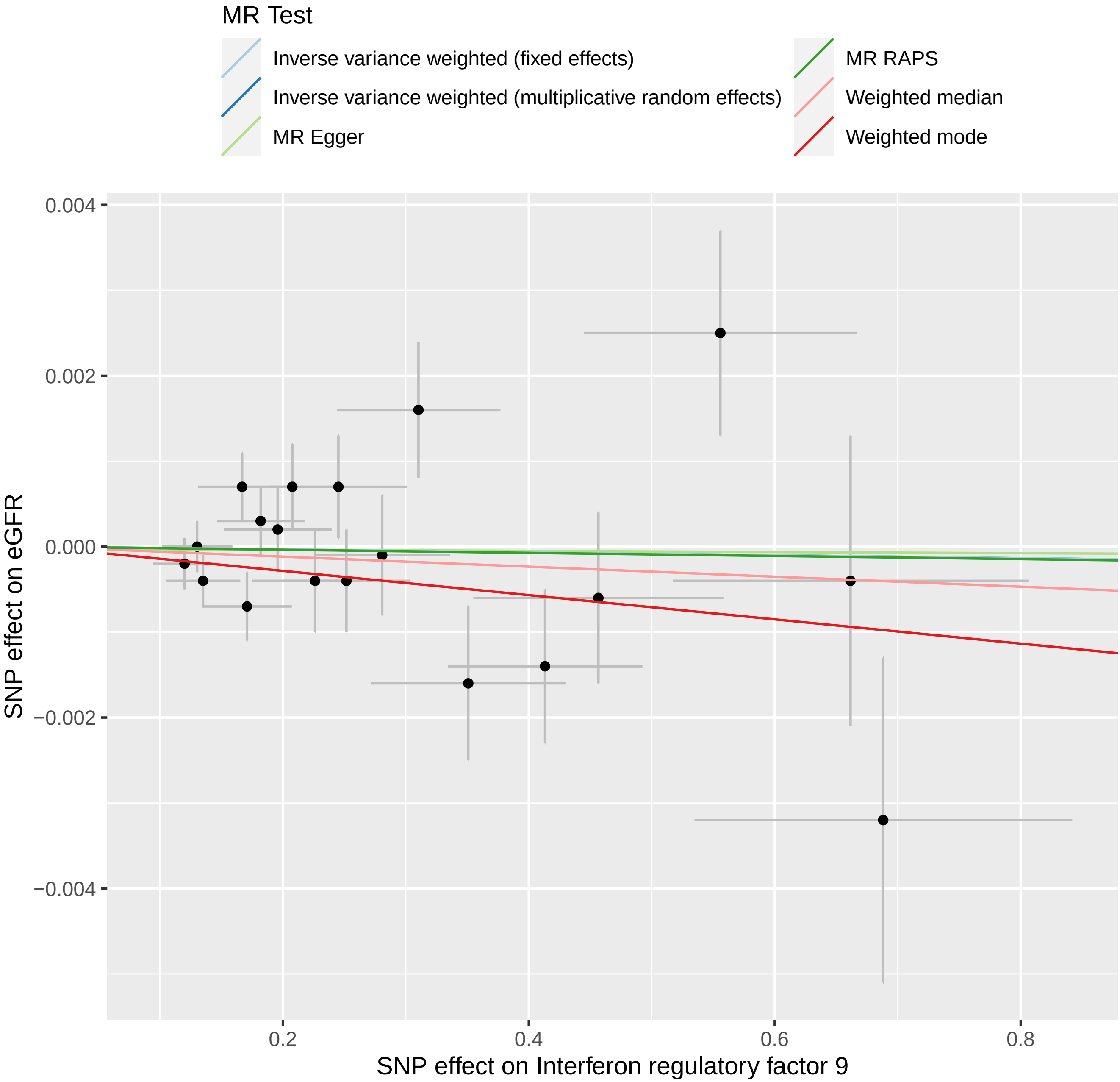

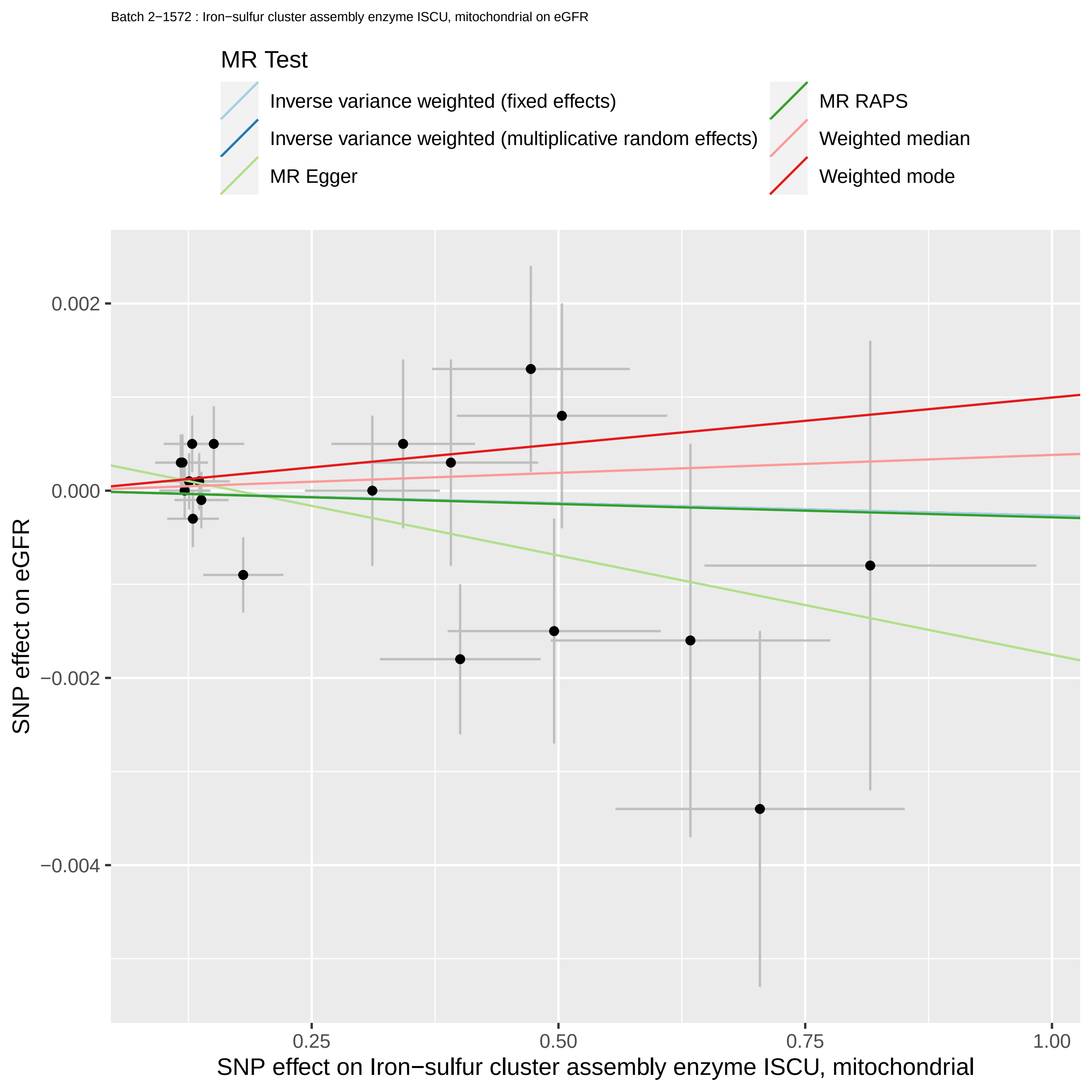

Batch 2−1572 : Iron−sulfur cluster assembly enzyme ISCU, mitochondrial on eGFR
MR Test
Inverse variance weighted (fixed effects)
Inverse variance weighted (multiplicative random effects)
MR Egger
MR RAPS
Weighted median
Weighted mode
0.002
0.000
−0.002
−0.004
0.25
0.50
0.75
1.00
SNP effect on eGFR
SNP effect on Iron−sulfur cluster assembly enzyme ISCU, mitochondrial

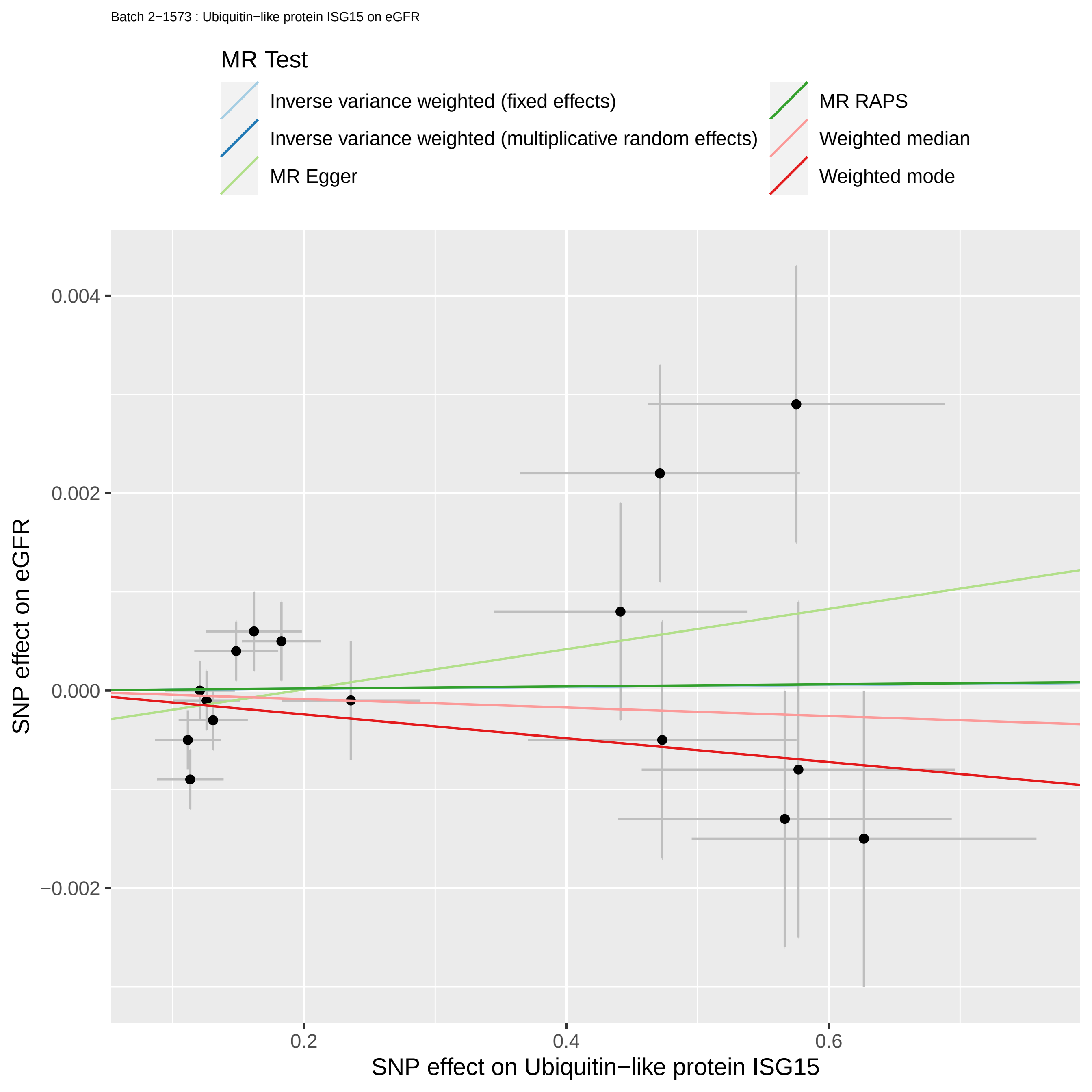
Batch 2−1573 : Ubiquitin−like protein ISG15 on eGFR
MR Test
Inverse variance weighted (fixed effects)
Inverse variance weighted (multiplicative random effects)
MR Egger
MR RAPS
Weighted median
Weighted mode
0.004
0.002
0.000
−0.002
0.2
0.4
0.6
SNP effect on eGFR
SNP effect on Ubiquitin−like protein ISG15

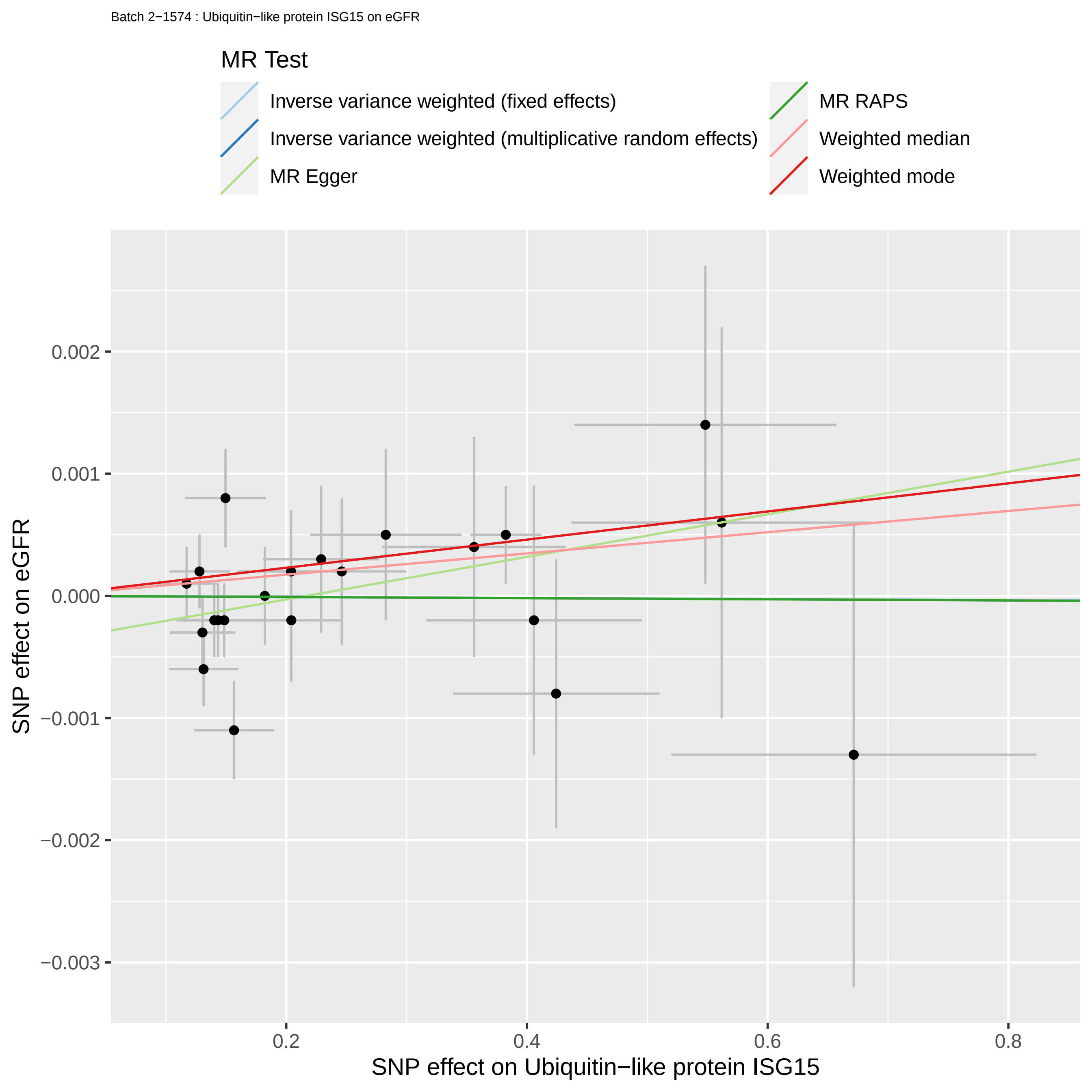

Batch 2−1574 : Ubiquitin−like protein ISG15 on eGFR
MR Test
Inverse variance weighted (fixed effects)
Inverse variance weighted (multiplicative random effects)
MR Egger
MR RAPS
Weighted median
Weighted mode
0.002
0.001
0.000
−0.001
−0.002
−0.003
SNP effect on eGFR
0.2
0.4
0.6
0.8
SNP effect on Ubiquitin−like protein ISG15

Batch 2−1575 : Insulin gene enhancer protein ISL−1 on eGFR

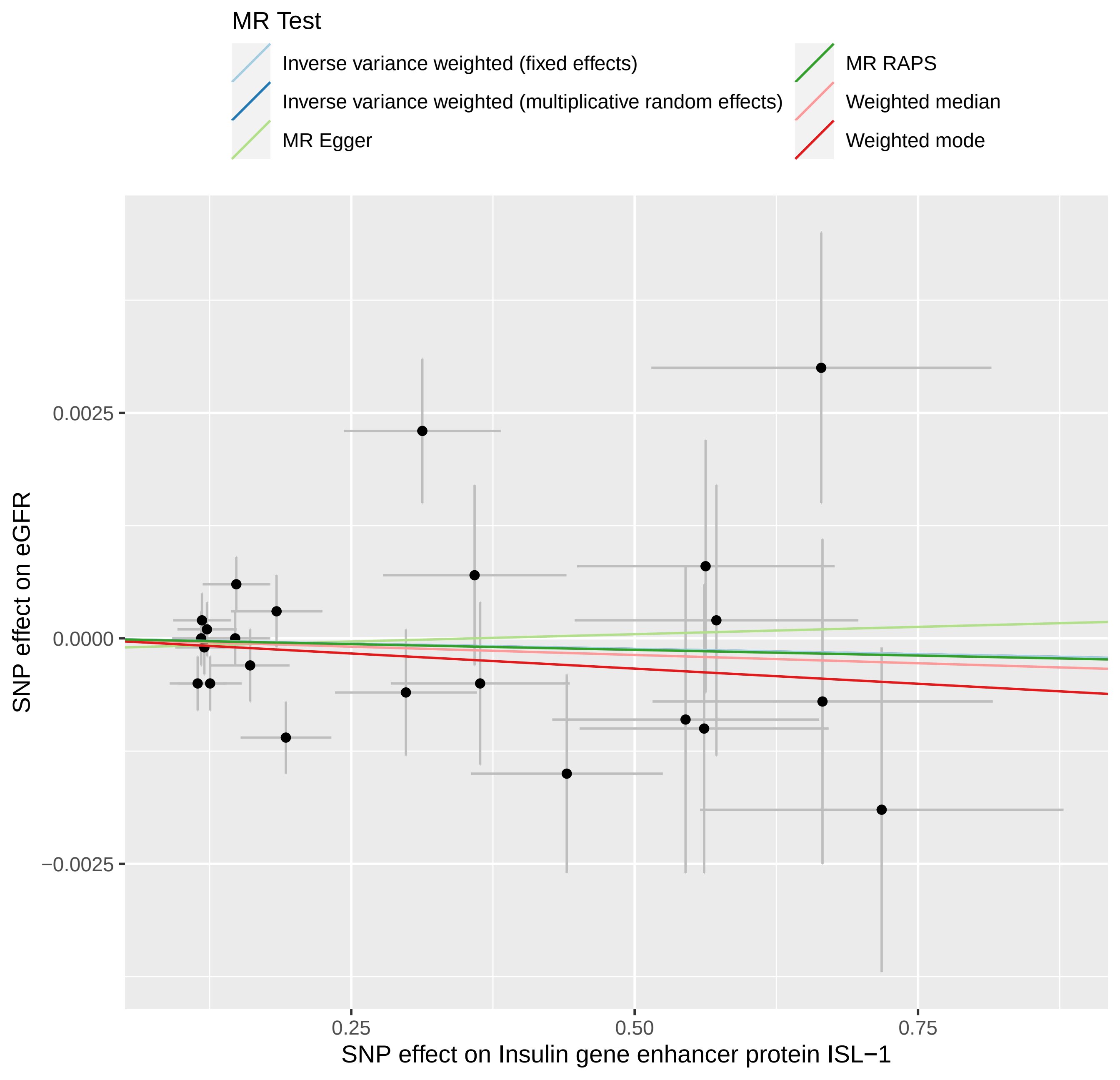

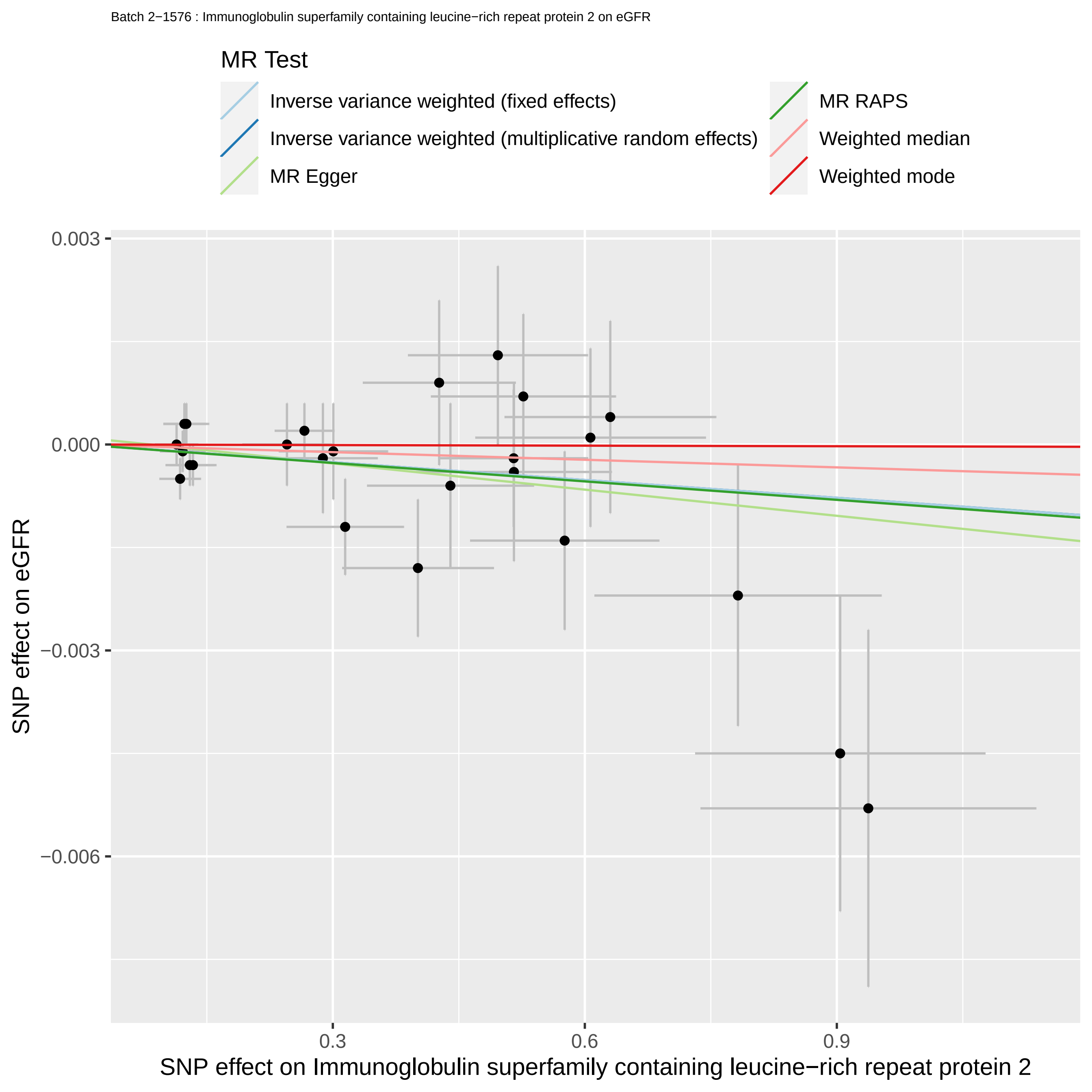

Batch 2−1576 : Immunoglobulin superfamily containing leucine−rich repeat protein 2 on eGFR
MR Test
Inverse variance weighted (fixed effects)
Inverse variance weighted (multiplicative random effects)
MR Egger
MR RAPS
Weighted median
Weighted mode
0.003
0.000
−0.003
−0.006
SNP effect on eGFR
0.3
0.6
0.9
SNP effect on Immunoglobulin superfamily containing leucine−rich repeat protein 2

Batch 2−1577 : Isthmin−1 on eGFR

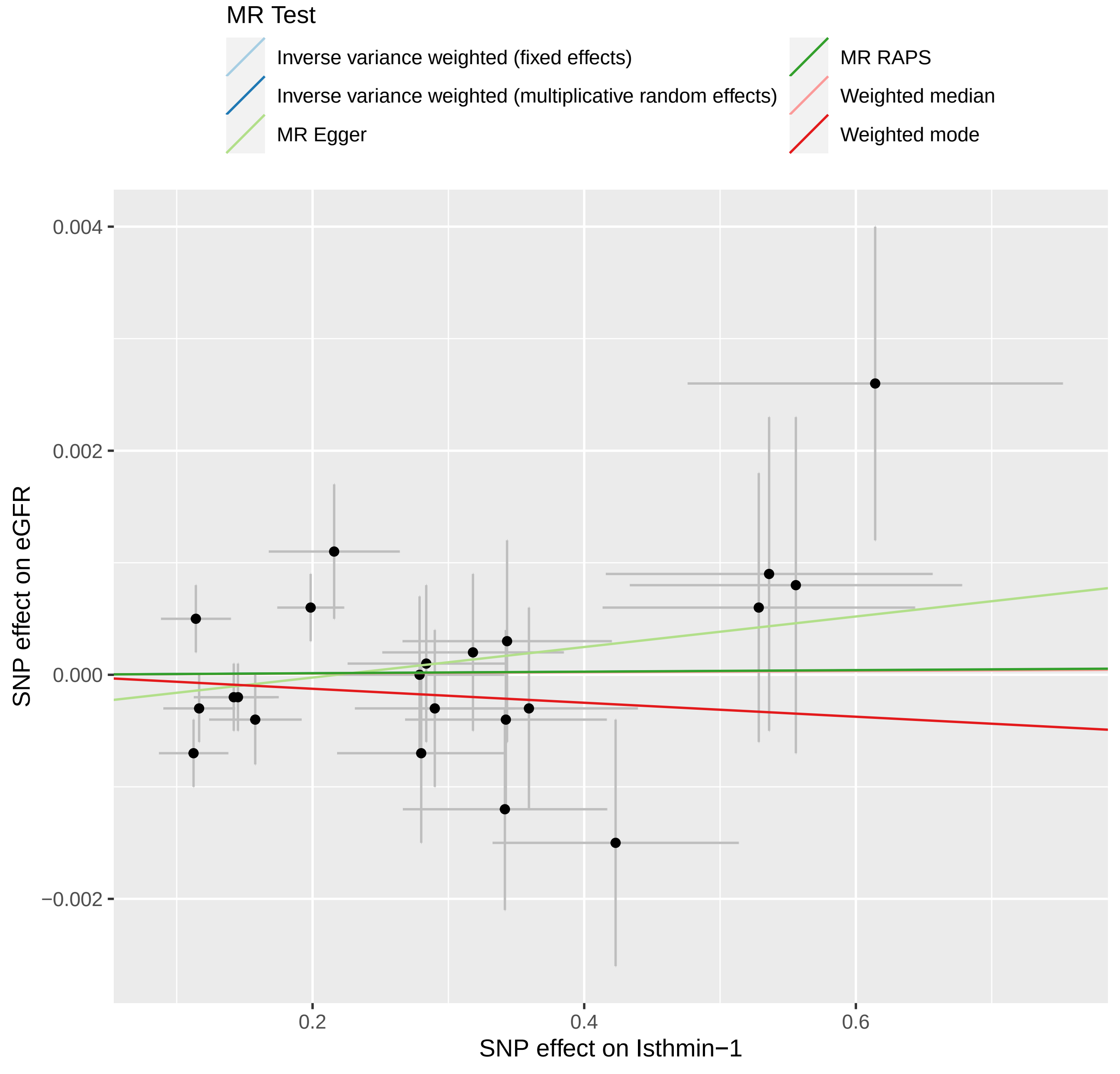

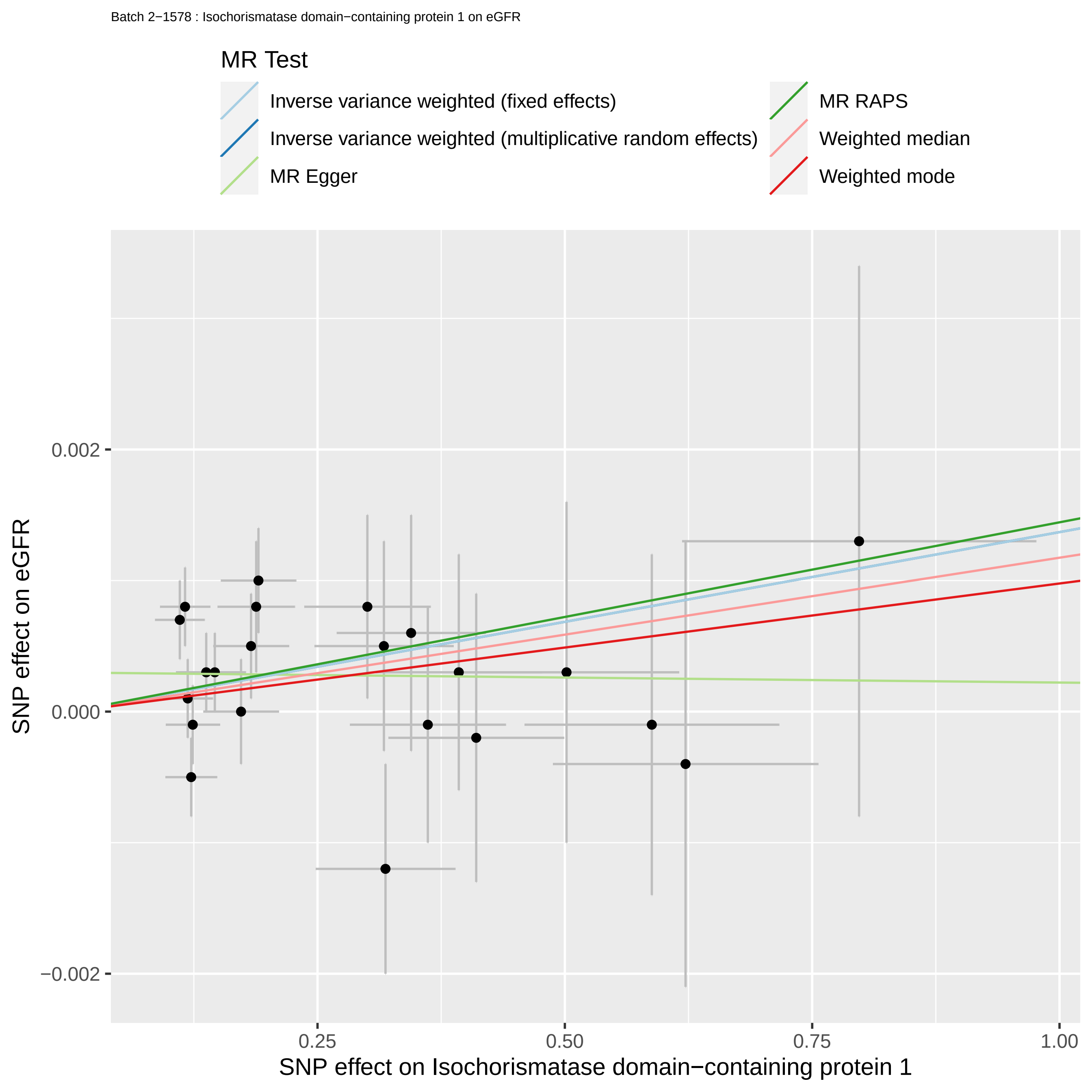

Batch 2−1578 : Isochorismatase domain−containing protein 1 on eGFR
MR Test
Inverse variance weighted (fixed effects)
Inverse variance weighted (multiplicative random effects)
MR Egger
MR RAPS
Weighted median
Weighted mode
0.002
0.000
−0.002
0.25
0.50
0.75
1.00
SNP effect on eGFR
SNP effect on Isochorismatase domain−containing protein 1

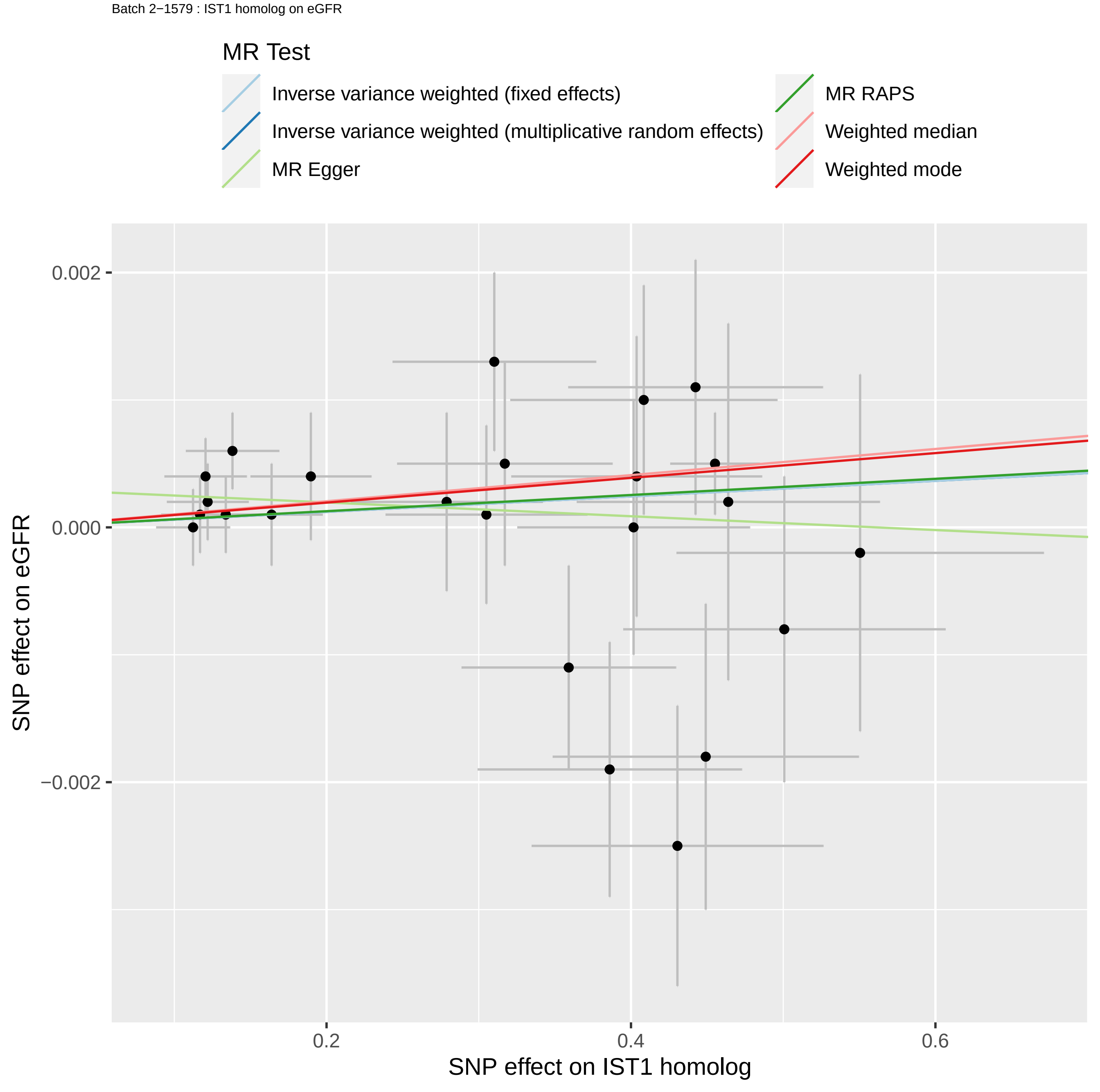
Batch 2−1579 : IST1 homolog on eGFR
MR Test
Inverse variance weighted (fixed effects)
Inverse variance weighted (multiplicative random effects)
MR Egger
MR RAPS
Weighted median
Weighted mode
0.002
0.000
−0.002
0.2
0.4
0.6
SNP effect on eGFR
SNP effect on IST1 homolog

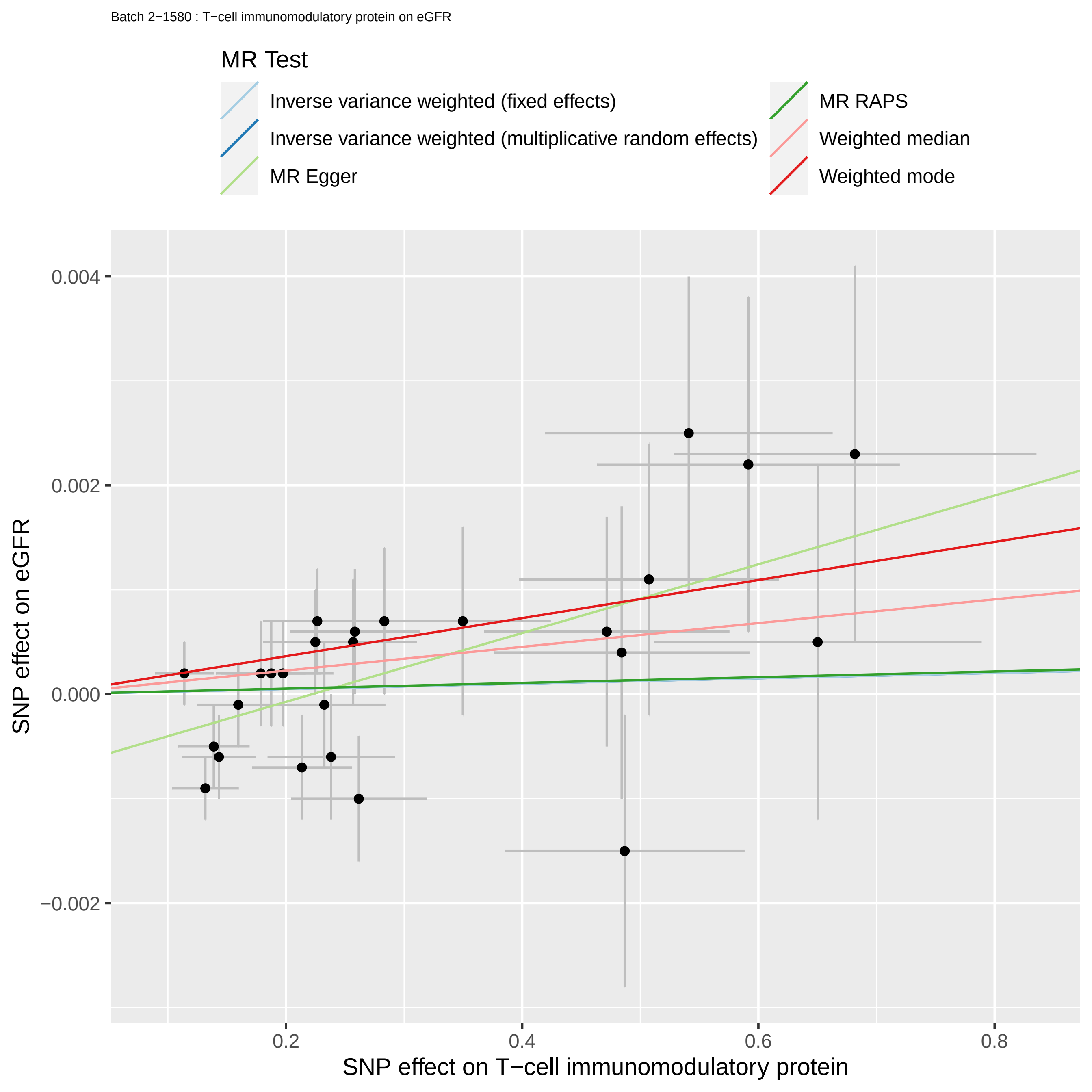

Batch 2−1580 : T−cell immunomodulatory protein on eGFR
MR Test
Inverse variance weighted (fixed effects)
Inverse variance weighted (multiplicative random effects)
MR Egger
MR RAPS
Weighted median
Weighted mode
0.004
0.002
0.000
−0.002
0.2
0.4
0.6
0.8
SNP effect on eGFR
SNP effect on T−cell immunomodulatory protein

Batch 2−1581 : Integrin alpha−I: beta−1 complex on eGFR

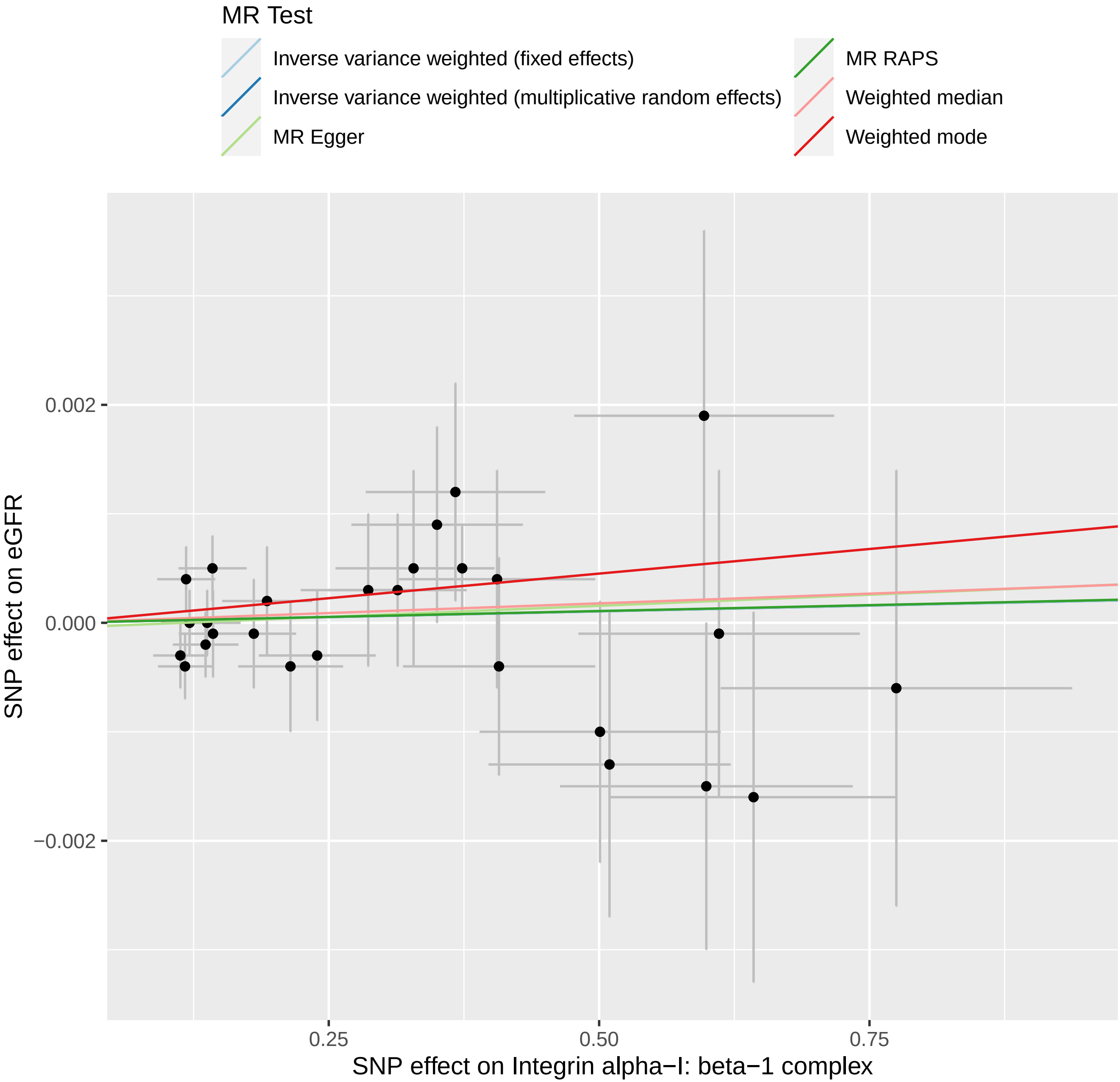

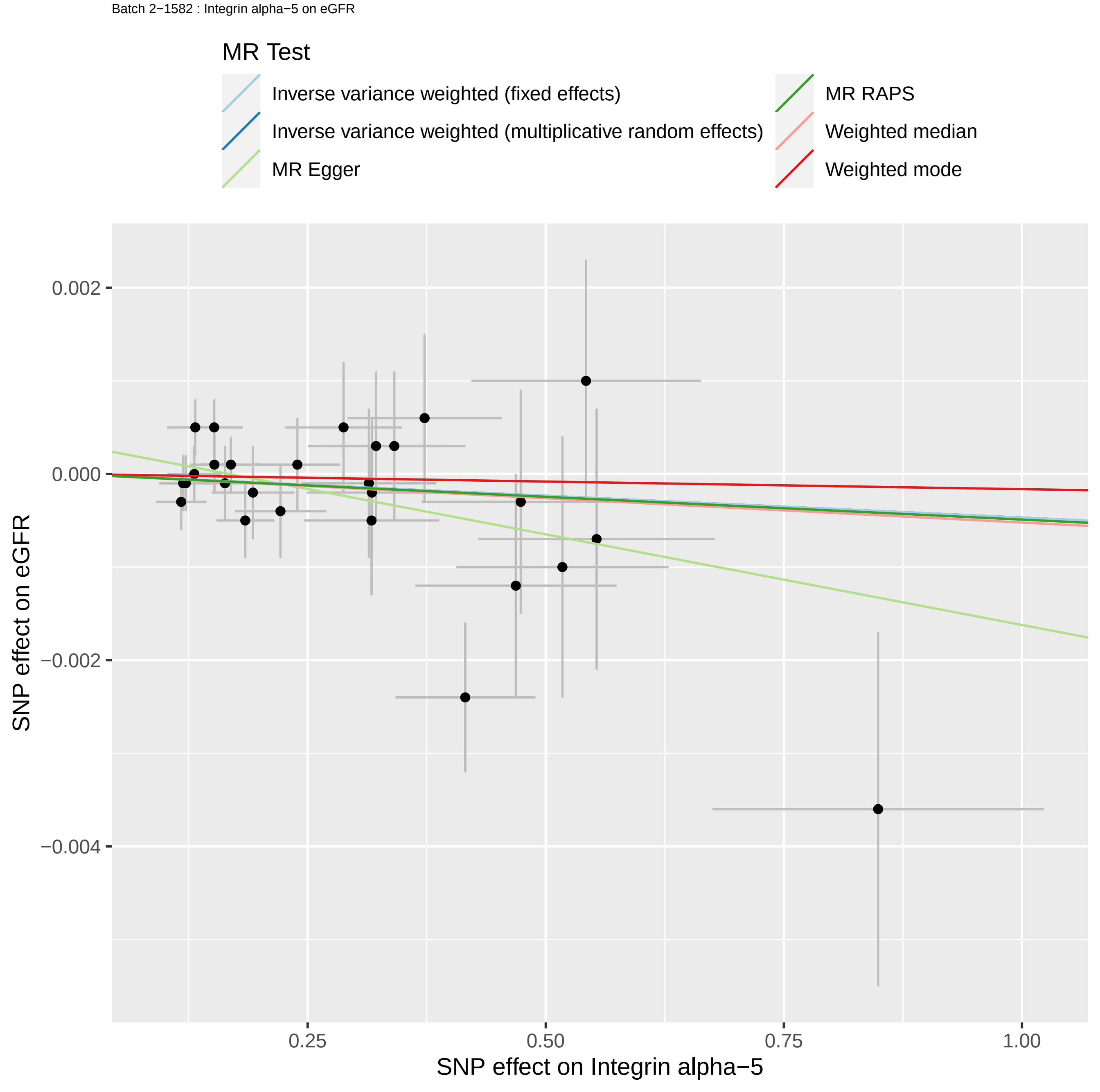
Batch 2−1582 : Integrin alpha−5 on eGFR
MR Test
Inverse variance weighted (fixed effects)
Inverse variance weighted (multiplicative random effects)
MR Egger
MR RAPS
Weighted median
Weighted mode
0.002
0.000
−0.002
−0.004
0.25
0.50
0.75
1.00
SNP effect on eGFR
SNP effect on Integrin alpha−5

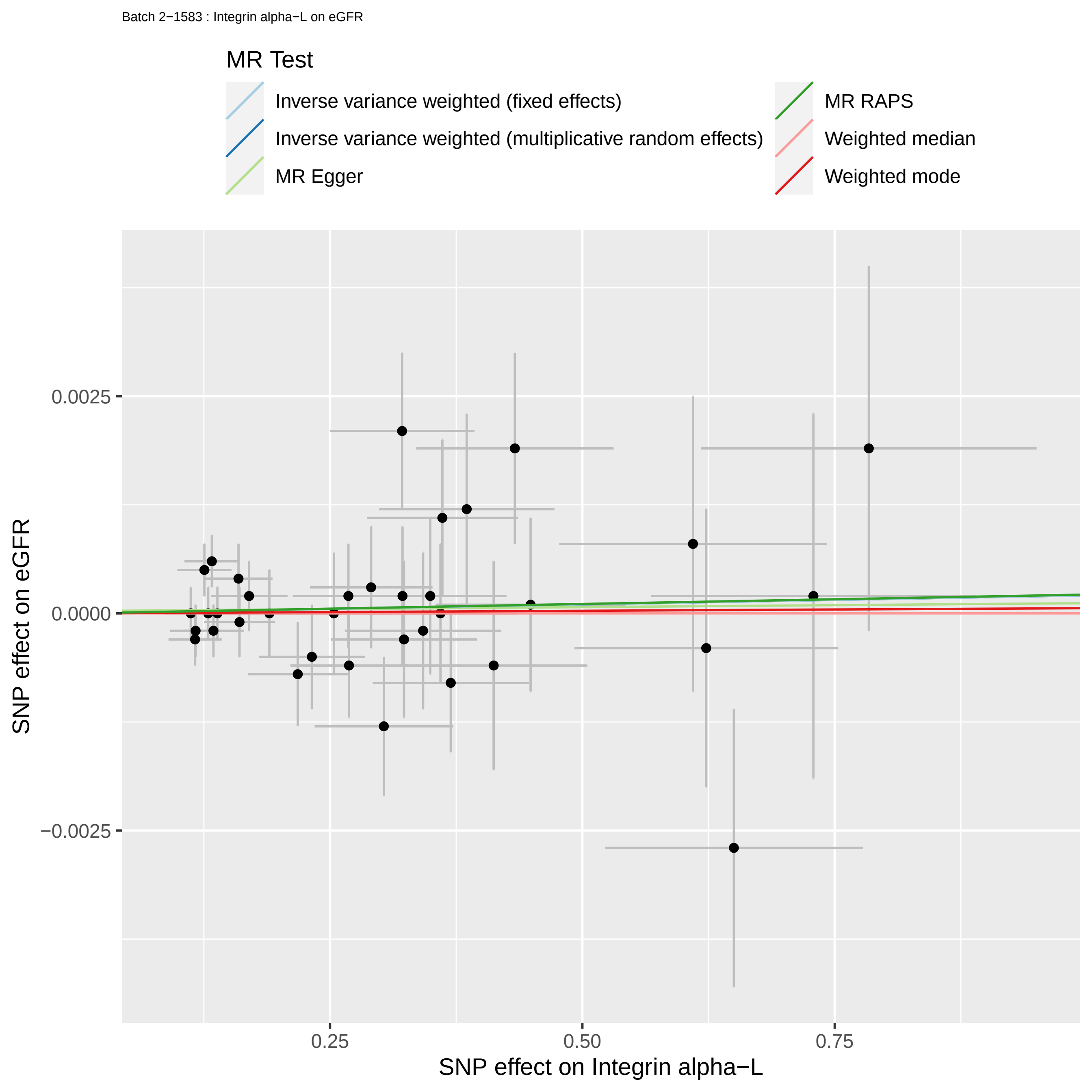

Batch 2−1583 : Integrin alpha−L on eGFR
MR Test
Inverse variance weighted (fixed effects)
Inverse variance weighted (multiplicative random effects)
MR Egger
MR RAPS
Weighted median
Weighted mode
0.0025
0.0000
−0.0025
0.25
0.50
0.75
SNP effect on eGFR
SNP effect on Integrin alpha−L

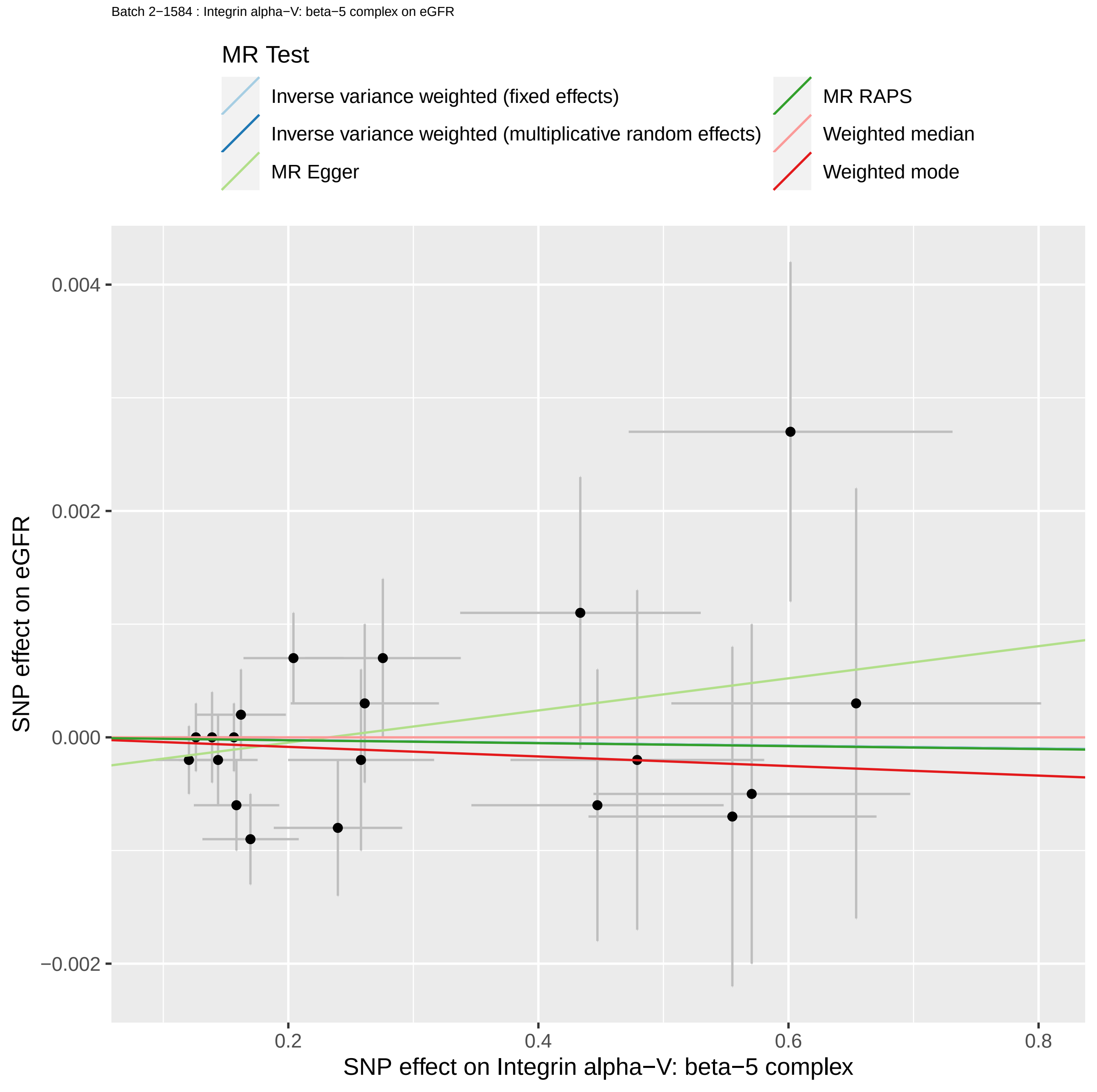
Batch 2−1584 : Integrin alpha−V: beta−5 complex on eGFR
MR Test
Inverse variance weighted (fixed effects)
Inverse variance weighted (multiplicative random effects)
MR Egger
MR RAPS
Weighted median
Weighted mode
SNP effect on eGFR
0.004
0.002
0.000
−0.002
0.2
0.4
0.6
0.8
SNP effect on Integrin alpha−V: beta−5 complex

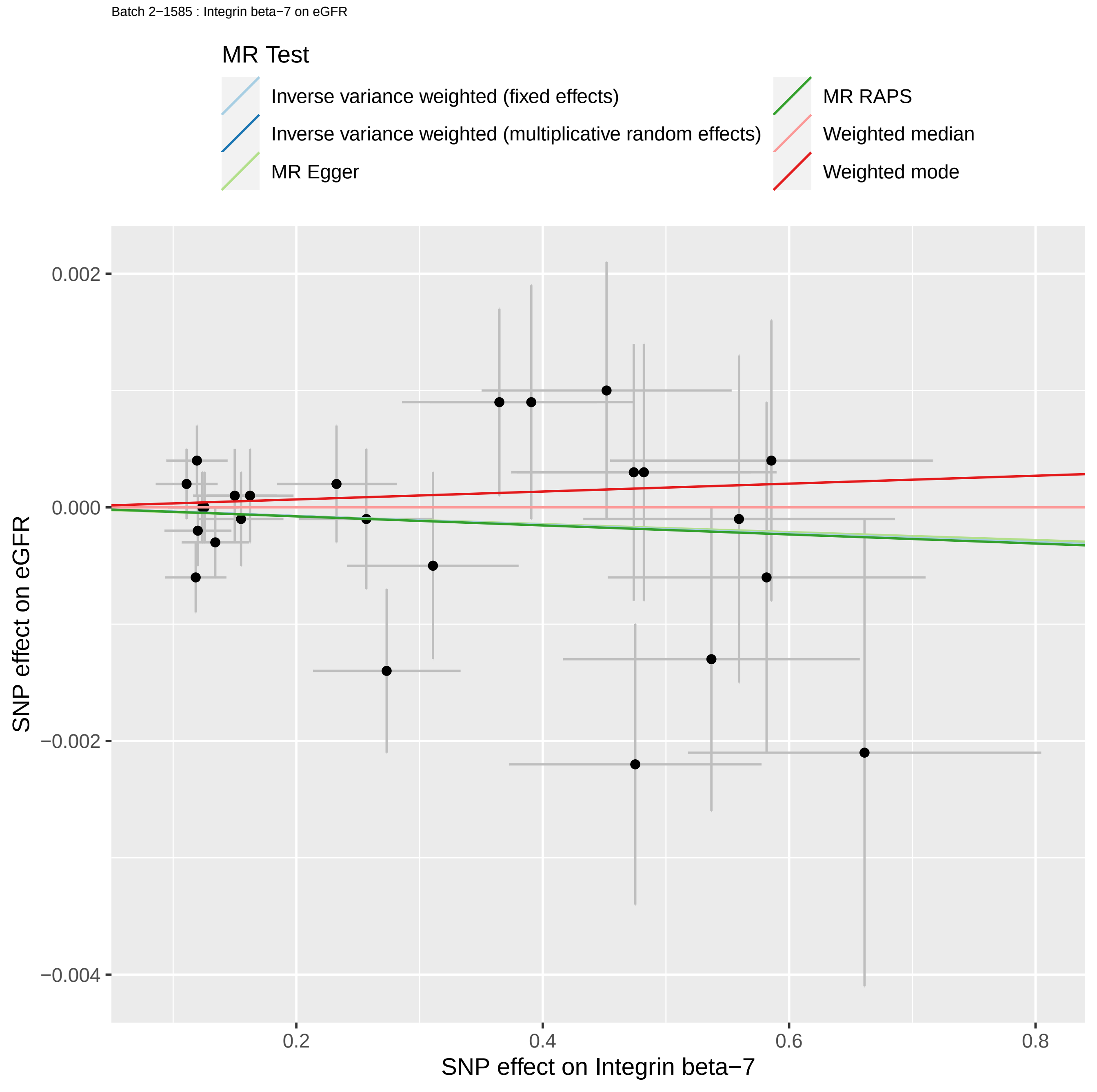
Batch 2−1585 : Integrin beta−7 on eGFR
MR Test
Inverse variance weighted (fixed effects)
Inverse variance weighted (multiplicative random effects)
MR Egger
MR RAPS
Weighted median
Weighted mode
0.002
0.000
−0.002
−0.004
0.2
0.4
0.6
0.8
SNP effect on eGFR
SNP effect on Integrin beta−7

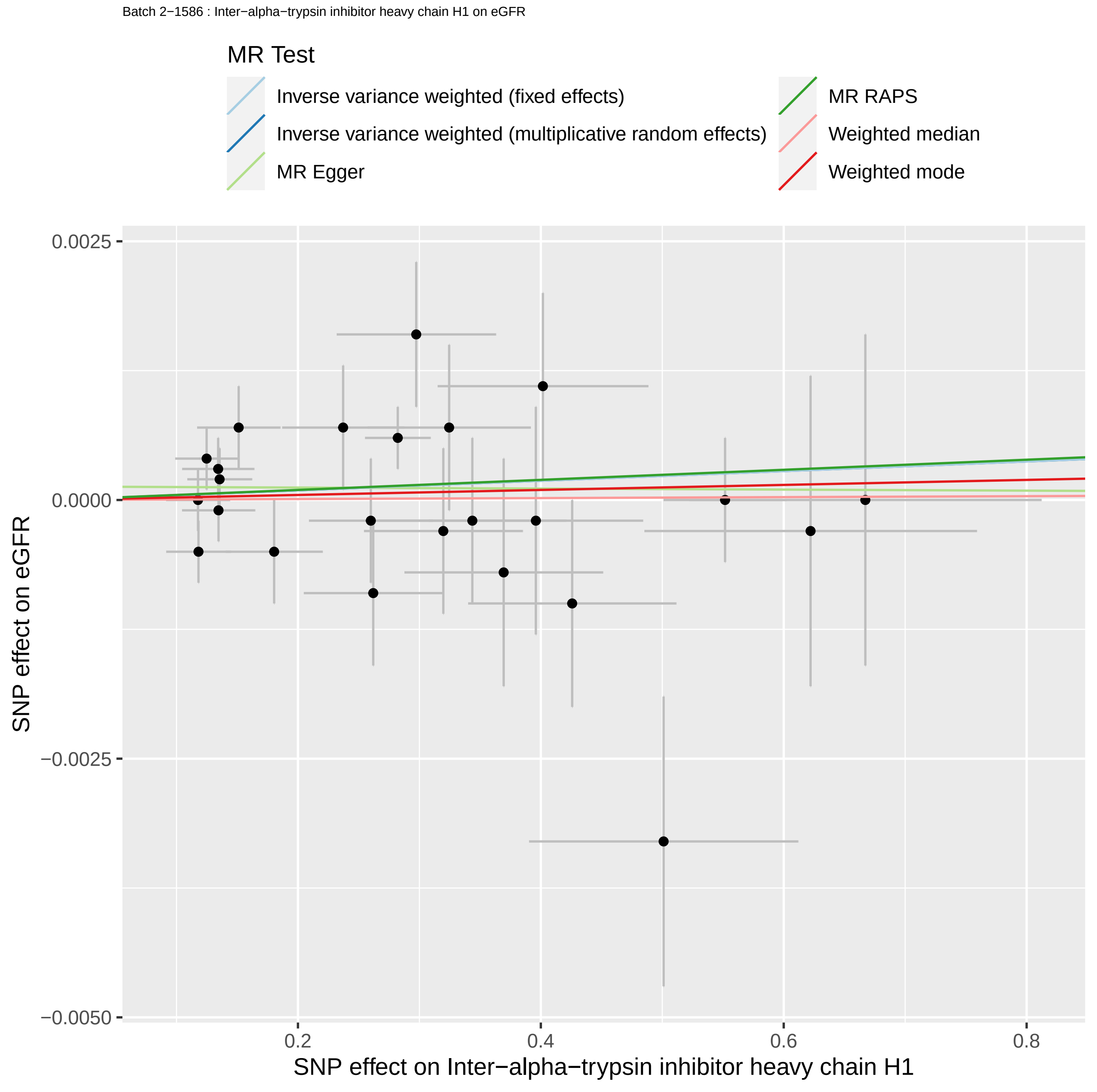

Batch 2−1586 : Inter−alpha−trypsin inhibitor heavy chain H1 on eGFR
MR Test
Inverse variance weighted (fixed effects)
Inverse variance weighted (multiplicative random effects)
MR Egger
MR RAPS
Weighted median
Weighted mode
0.0025
0.0000
−0.0025
−0.0050
0.2
0.4
0.6
0.8
SNP effect on eGFR
SNP effect on Inter−alpha−trypsin inhibitor heavy chain H1

Batch 2−1587 : Inter−alpha−trypsin inhibitor heavy chain H5 on eGFR

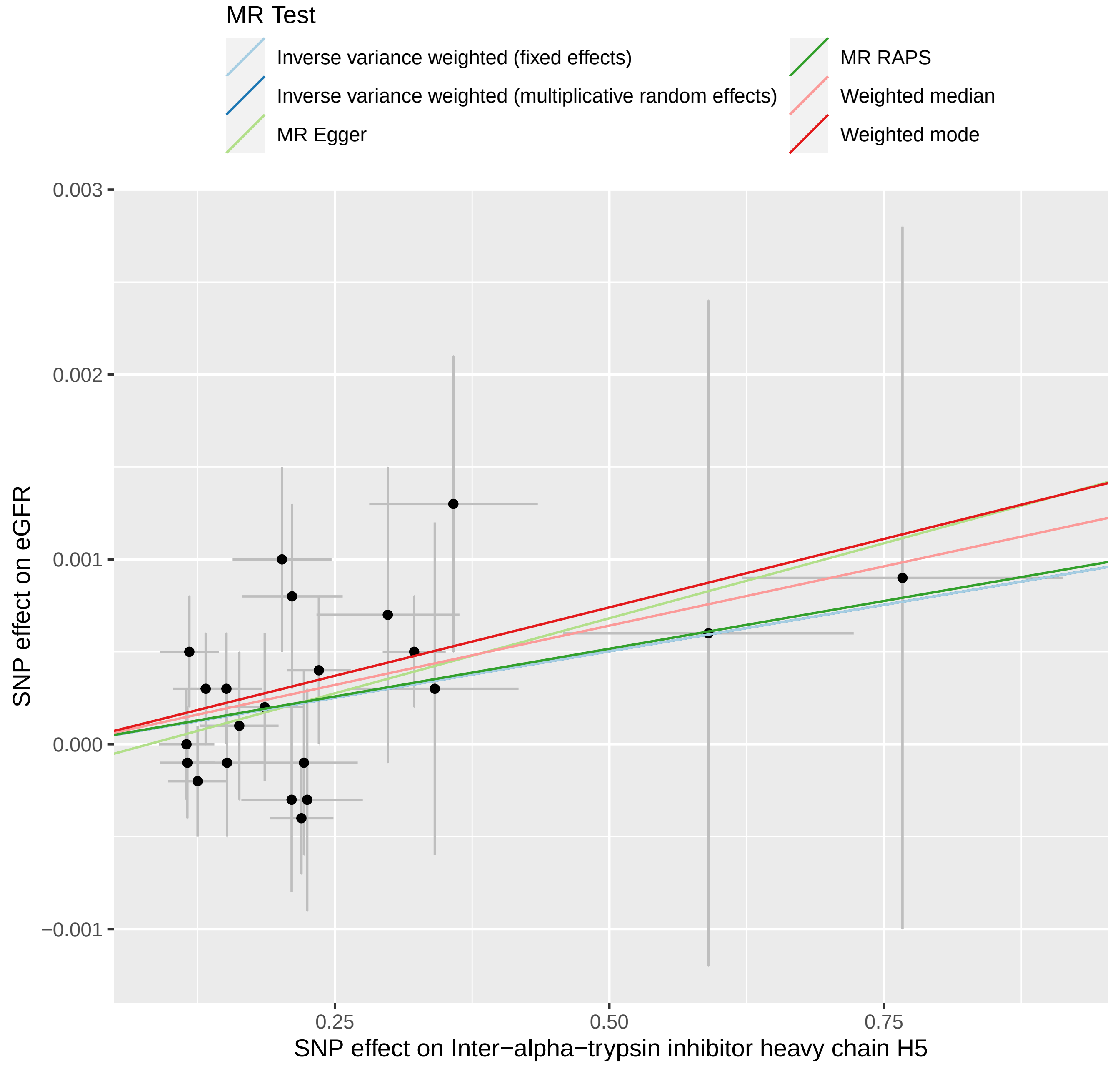

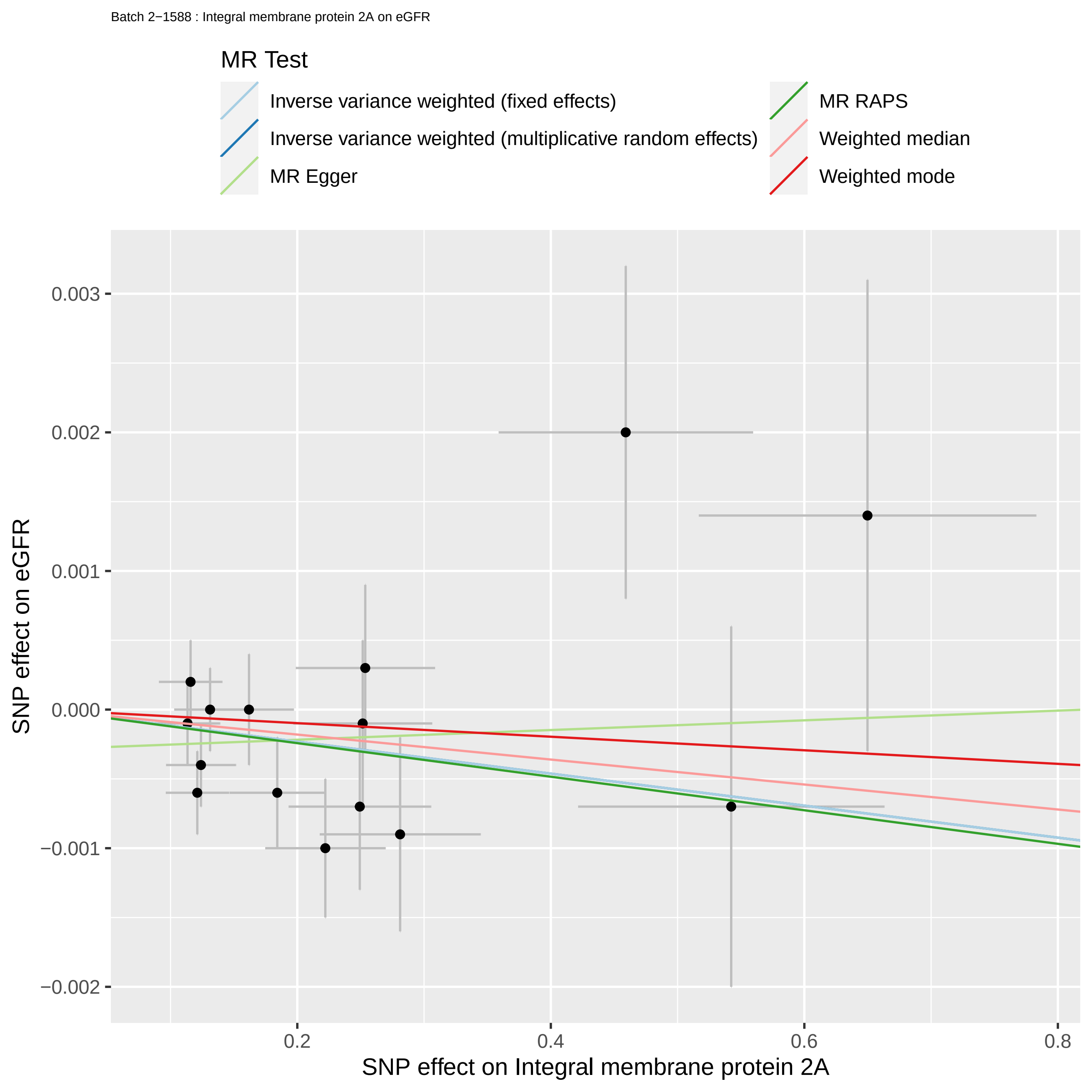

Batch 2−1588 : Integral membrane protein 2A on eGFR
MR Test
Inverse variance weighted (fixed effects)
Inverse variance weighted (multiplicative random effects)
MR Egger
MR RAPS
Weighted median
Weighted mode
0.003
0.002
0.001
0.000
−0.001
−0.002
0.2
0.4
0.6
0.8
SNP effect on eGFR
SNP effect on Integral membrane protein 2A

Batch 2−1589 : Integral membrane protein 2A on eGFR

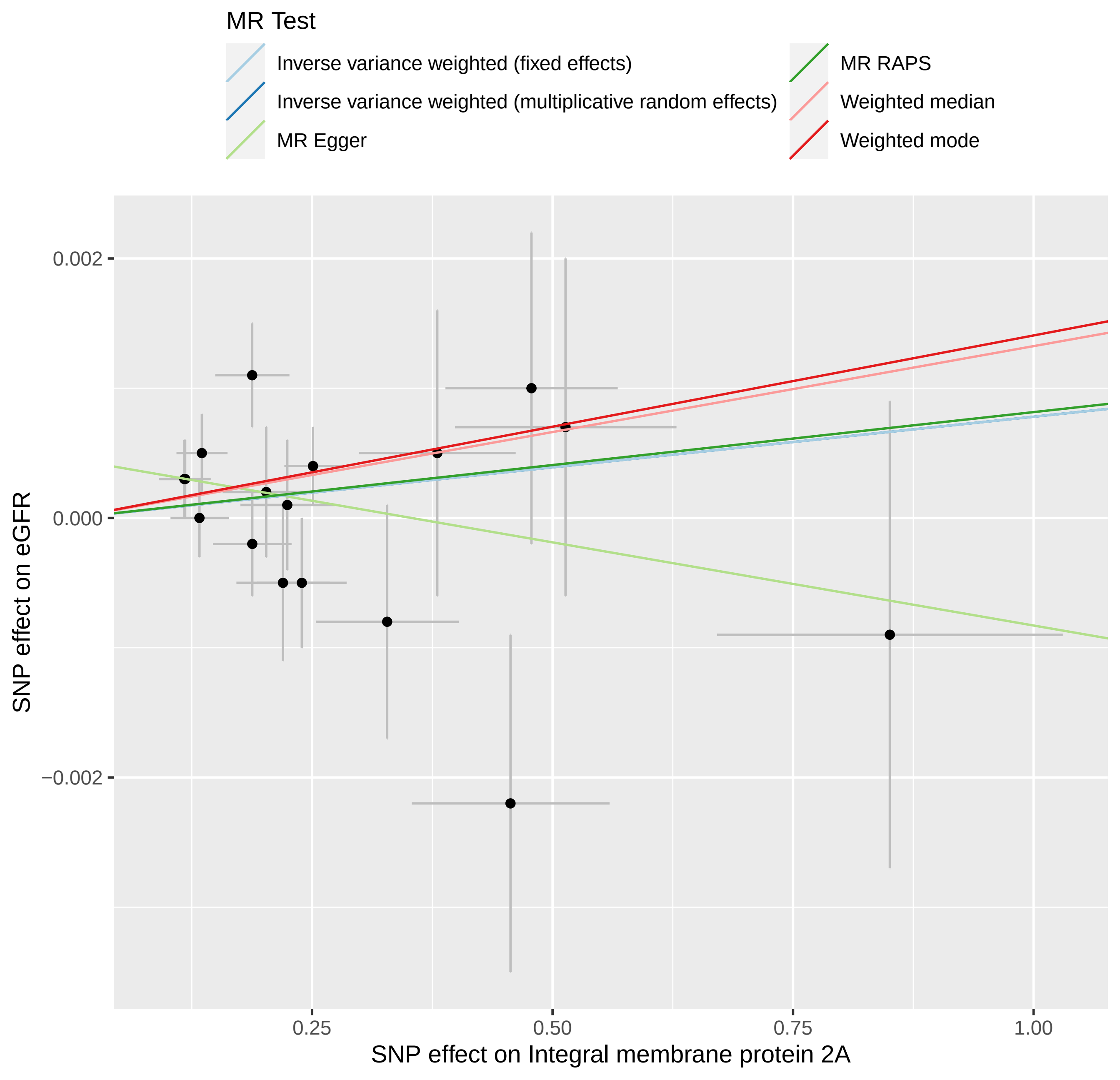

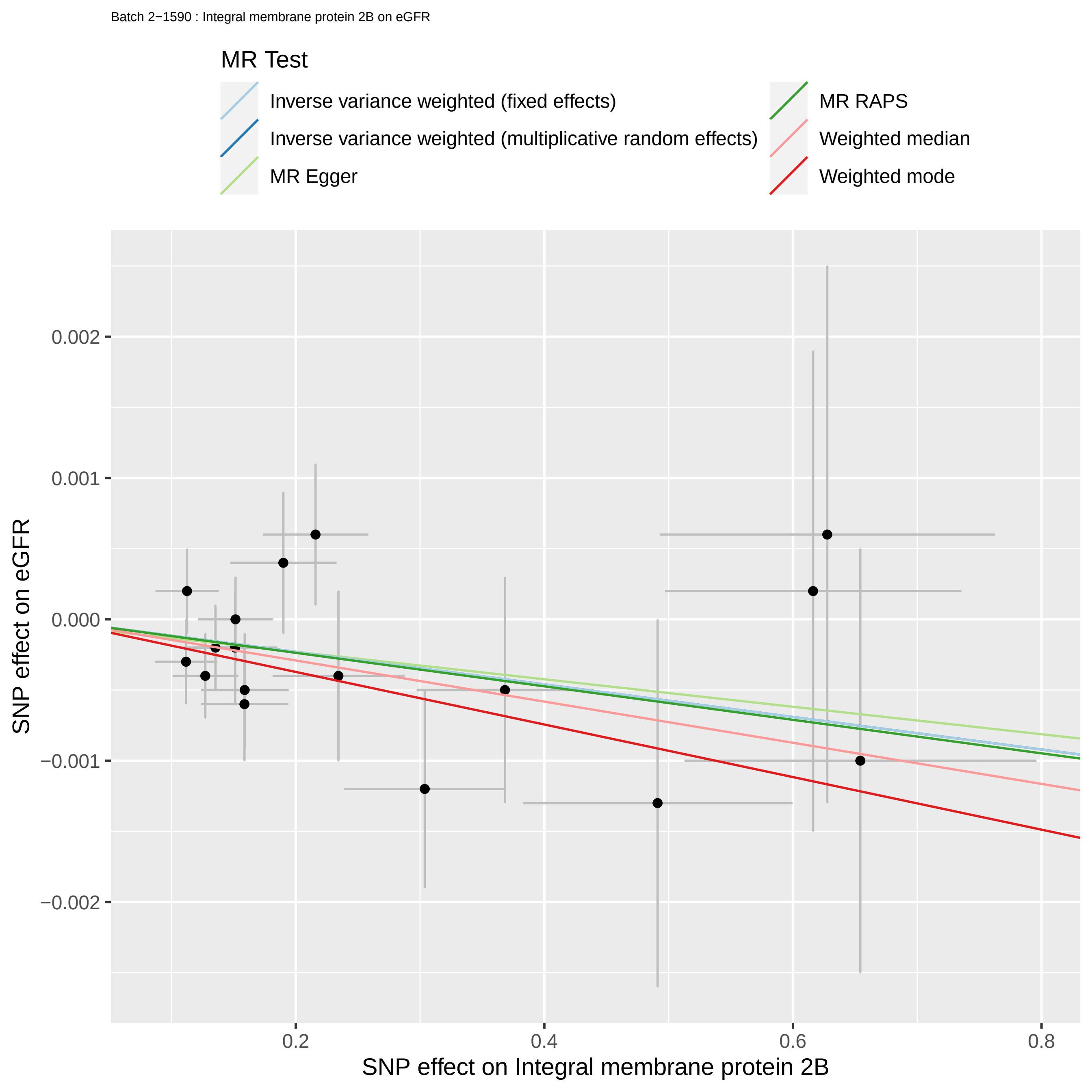
Batch 2−1590 : Integral membrane protein 2B on eGFR
MR Test
Inverse variance weighted (fixed effects)
Inverse variance weighted (multiplicative random effects)
MR Egger
MR RAPS
Weighted median
Weighted mode
0.002
0.001
0.000
−0.001
−0.002
SNP effect on eGFR
0.2
0.4
0.6
0.8
SNP effect on Integral membrane protein 2B

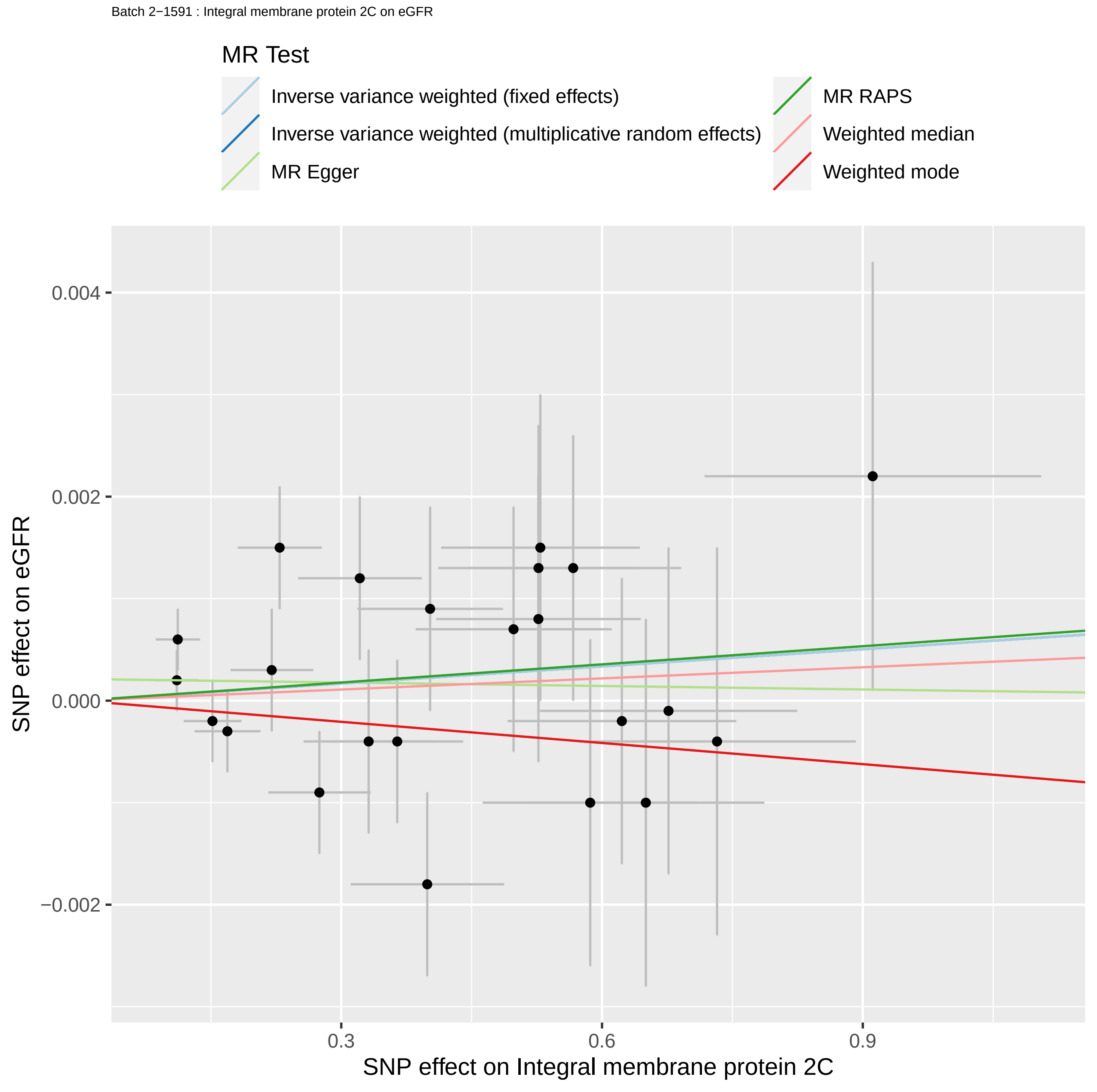

Batch 2−1591 : Integral membrane protein 2C on eGFR
MR Test
Inverse variance weighted (fixed effects)
Inverse variance weighted (multiplicative random effects)
MR Egger
MR RAPS
Weighted median
Weighted mode
0.004
0.002
0.000
−0.002
0.3
0.6
0.9
SNP effect on eGFR
SNP effect on Integral membrane protein 2C

Batch 2−1592 : Integral membrane protein 2C on eGFR

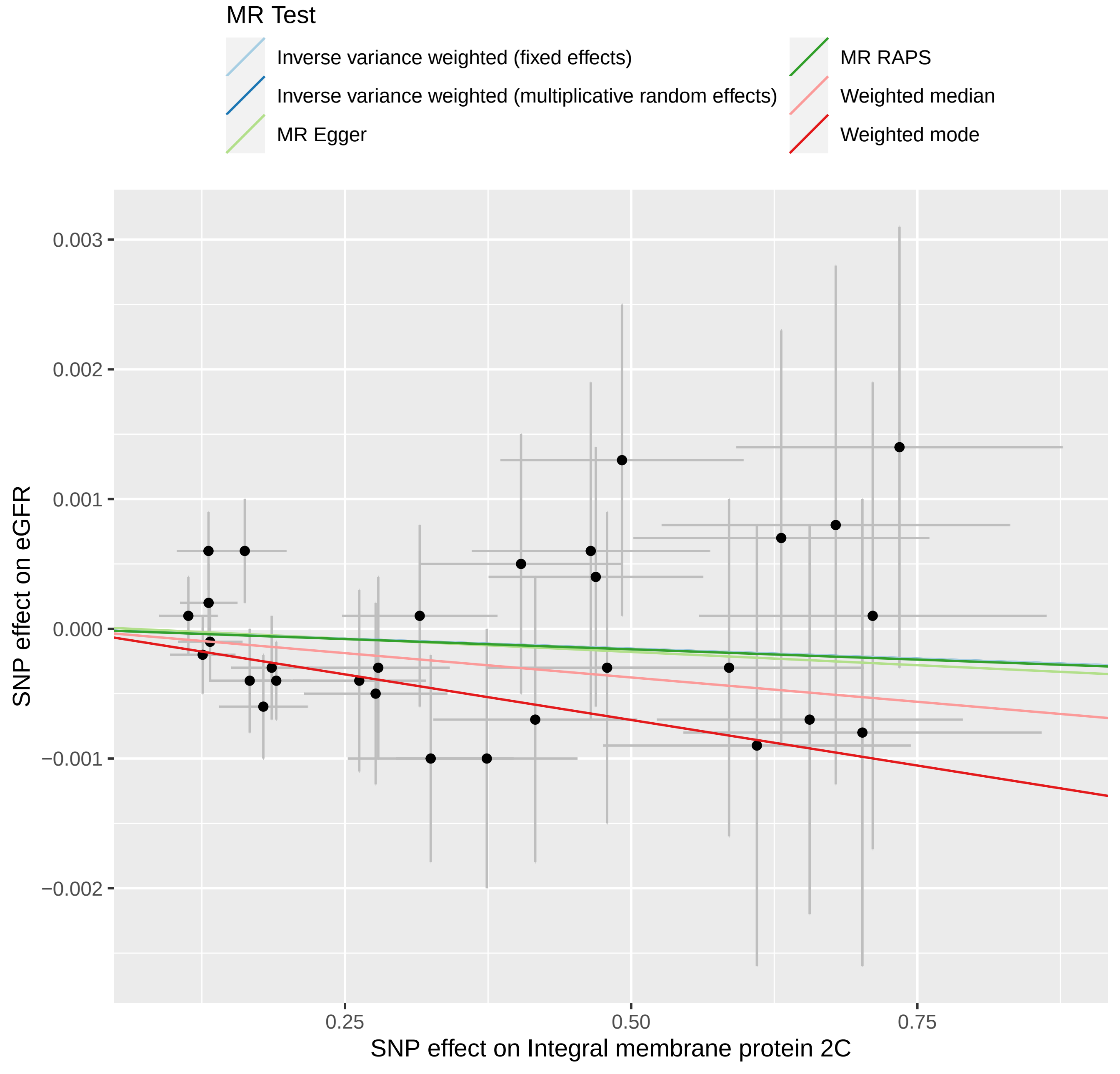

Batch 2−1593 : Inositol−trisphosphate 3−kinase A on eGFR

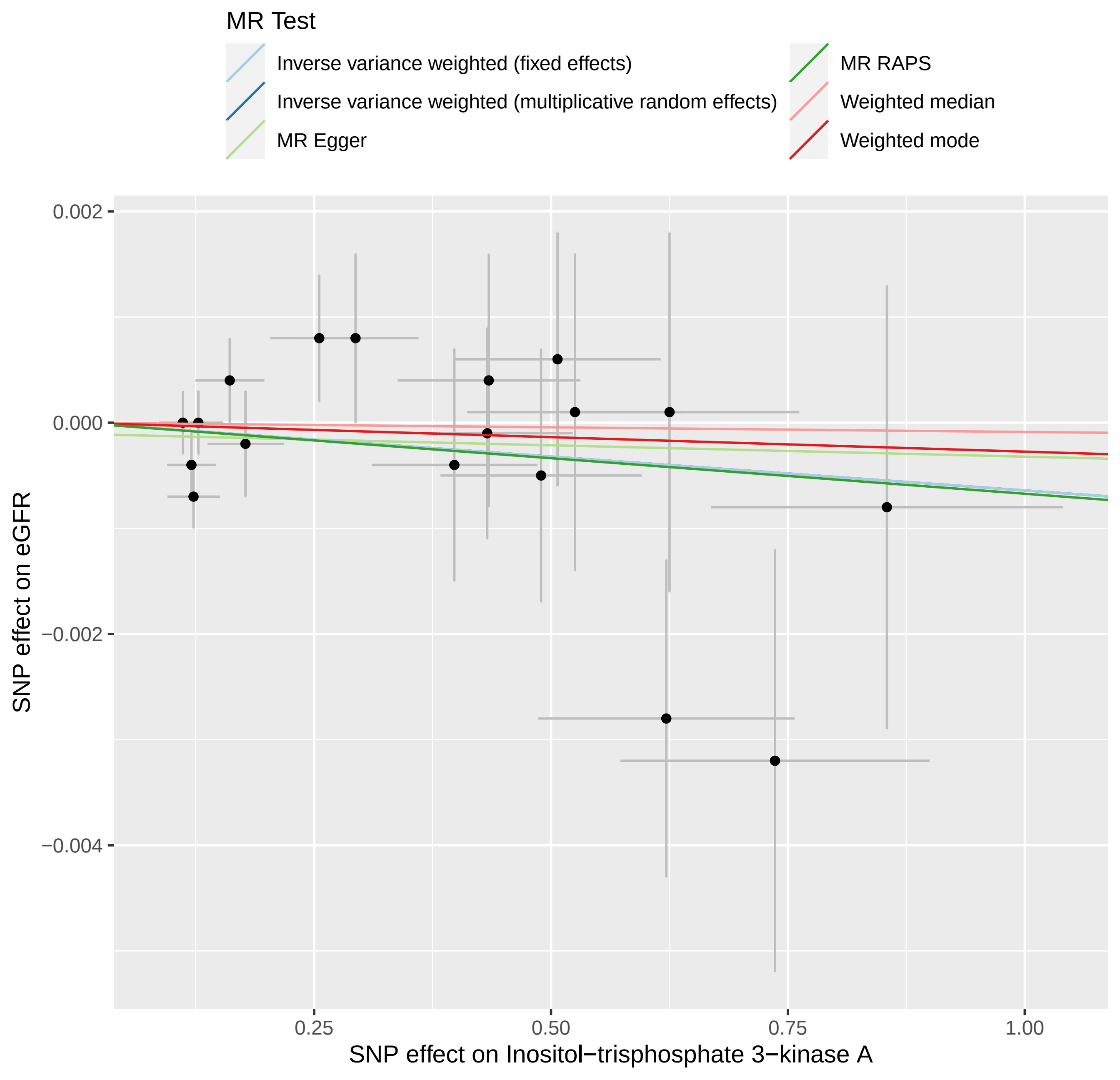

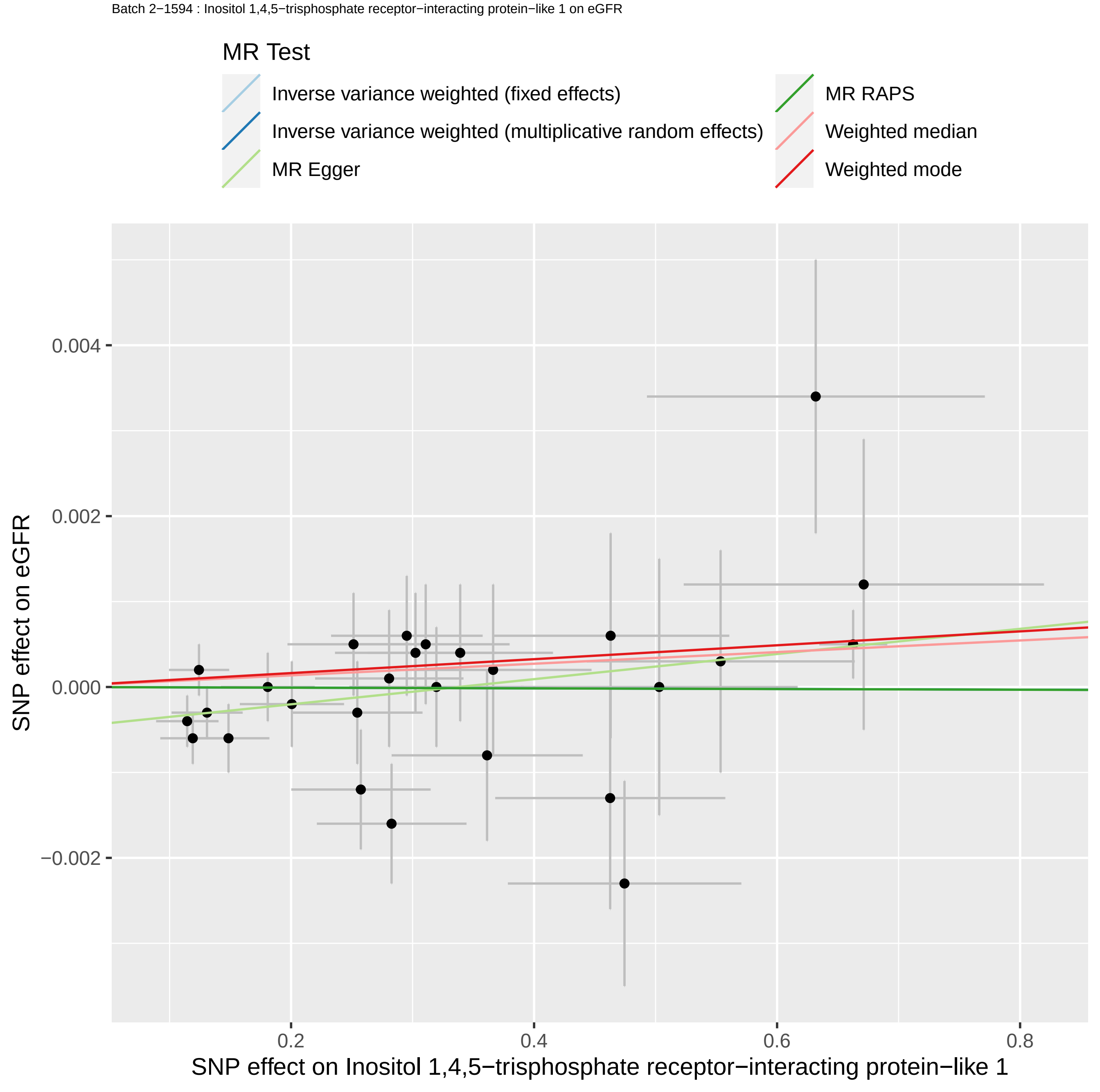

Batch 2−1594 : Inositol 1,4,5−trisphosphate receptor−interacting protein−like 1 on eGFR
MR Test
Inverse variance weighted (fixed effects)
Inverse variance weighted (multiplicative random effects)
MR Egger
MR RAPS
Weighted median
Weighted mode
0.004
0.002
0.000
−0.002
SNP effect on eGFR
0.2
0.4
0.6
0.8
SNP effect on Inositol 1,4,5−trisphosphate receptor−interacting protein−like 1

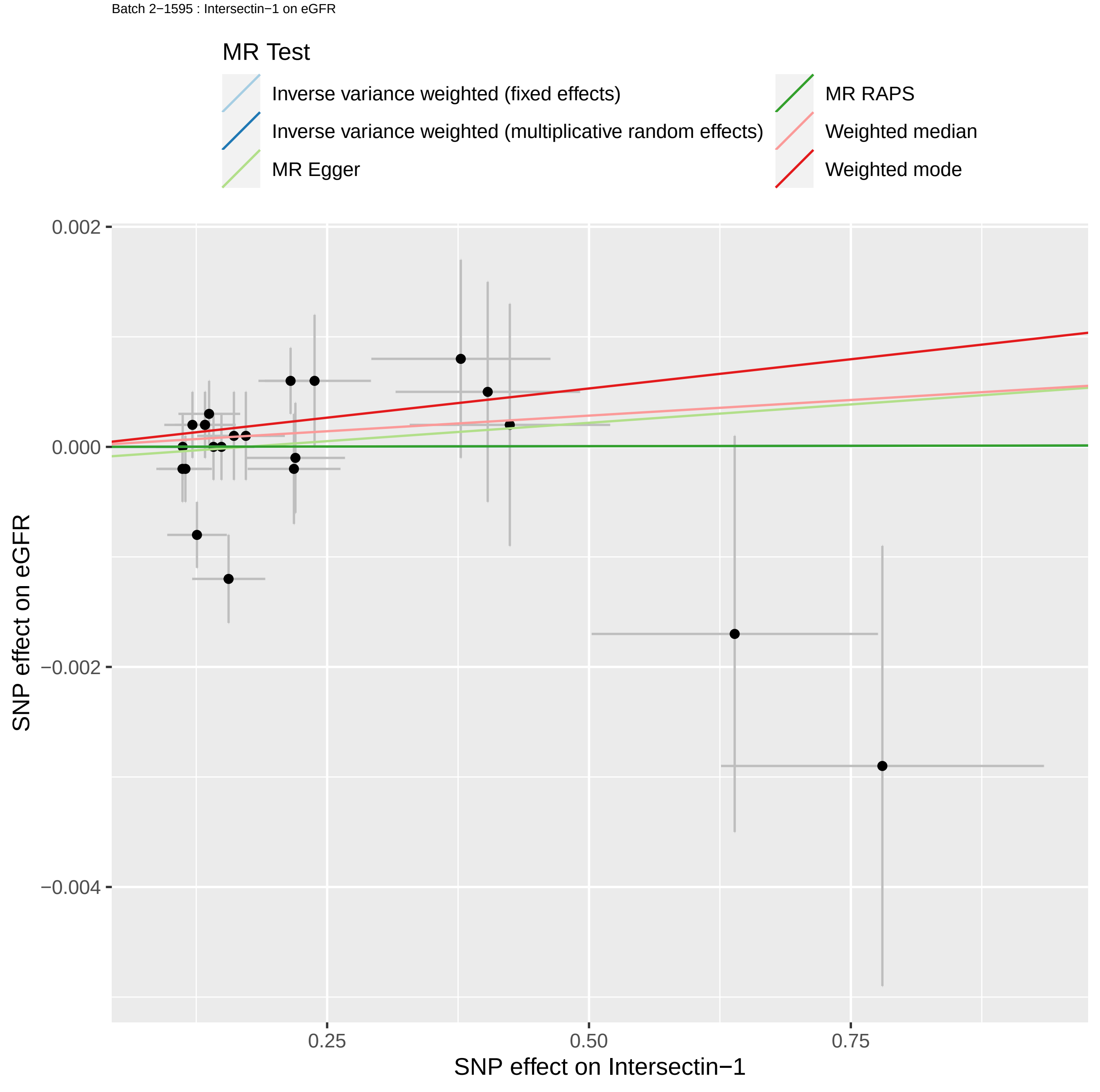

Batch 2−1595 : Intersectin−1 on eGFR
MR Test
Inverse variance weighted (fixed effects)
Inverse variance weighted (multiplicative random effects)
MR Egger
MR RAPS
Weighted median
Weighted mode
0.002
0.000
−0.002
−0.004
0.25
0.50
0.75
SNP effect on eGFR
SNP effect on Intersectin−1

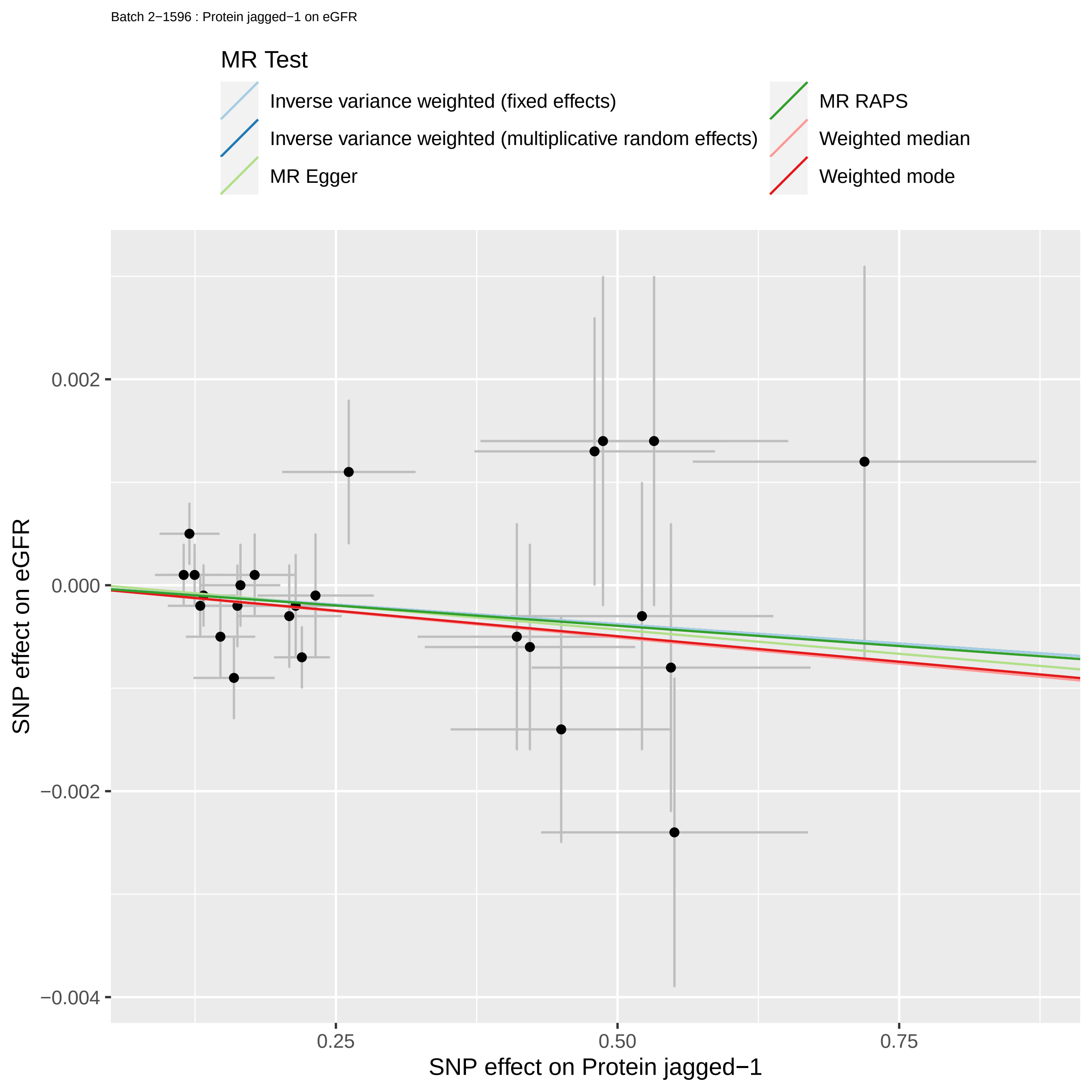
Batch 2−1596 : Protein jagged−1 on eGFR
MR Test
Inverse variance weighted (fixed effects)
Inverse variance weighted (multiplicative random effects)
MR Egger
MR RAPS
Weighted median
Weighted mode
0.002
0.000
−0.002
−0.004
SNP effect on eGFR
0.25
0.50
0.75
SNP effect on Protein jagged−1

Batch 2−1597 : Protein jagged−2 on eGFR

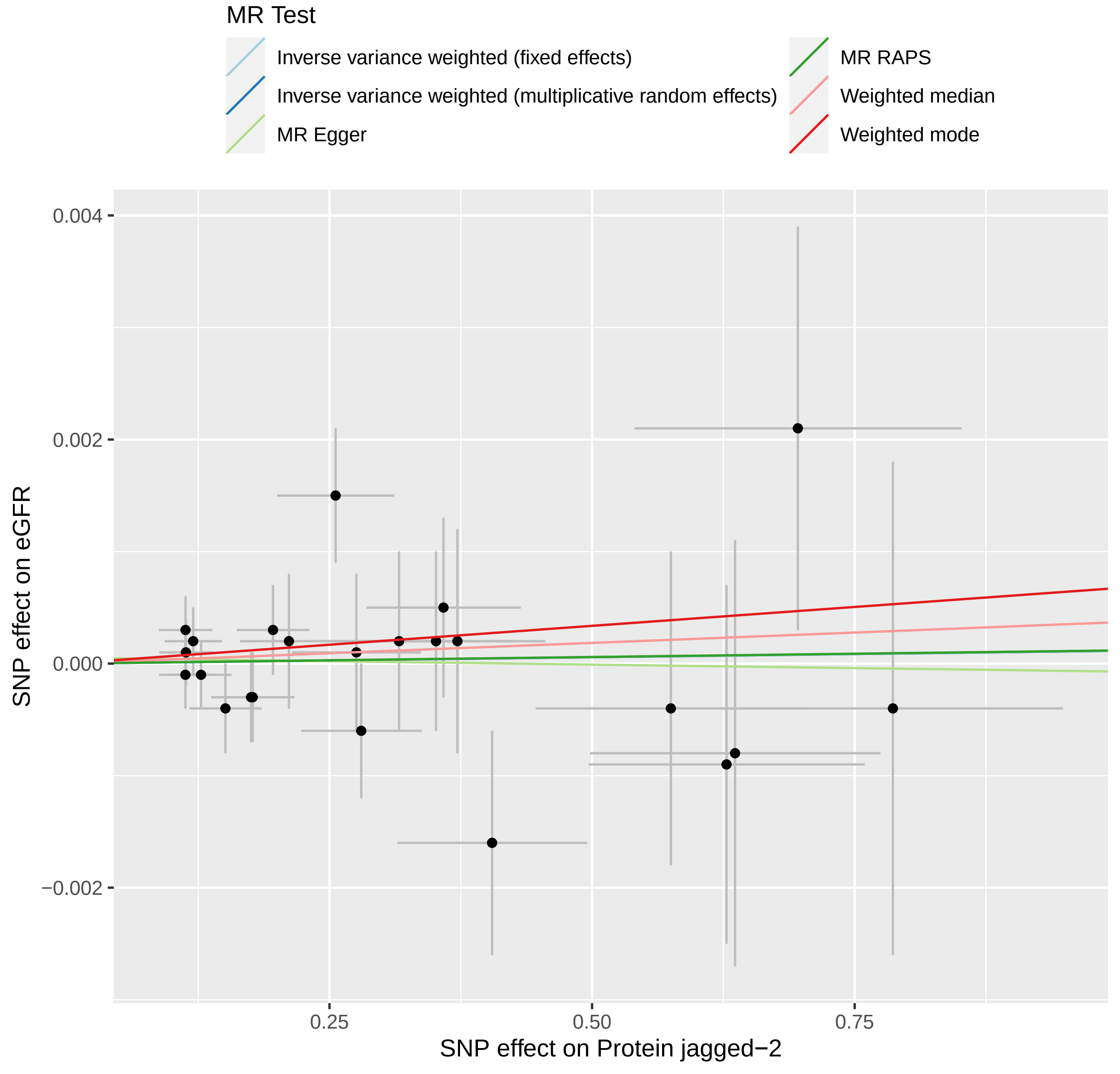

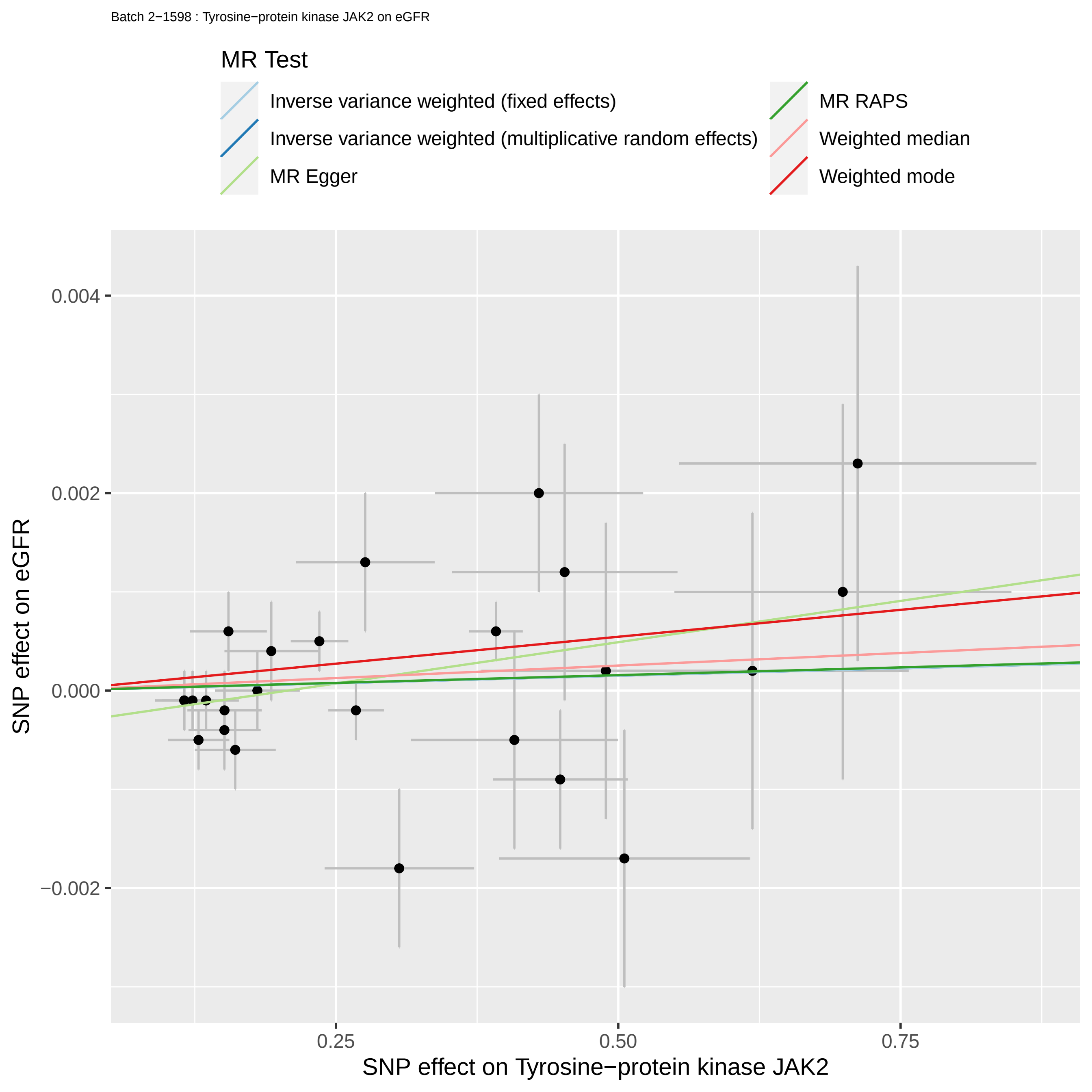
Batch 2−1598 : Tyrosine−protein kinase JAK2 on eGFR
MR Test
Inverse variance weighted (fixed effects)
Inverse variance weighted (multiplicative random effects)
MR Egger
MR RAPS
Weighted median
Weighted mode
0.004
0.002
0.000
−0.002
SNP effect on eGFR
0.25
0.50
0.75
SNP effect on Tyrosine−protein kinase JAK2

Batch 2−1599 : Janus kinase and microtubule−interacting protein 3 on eGFR

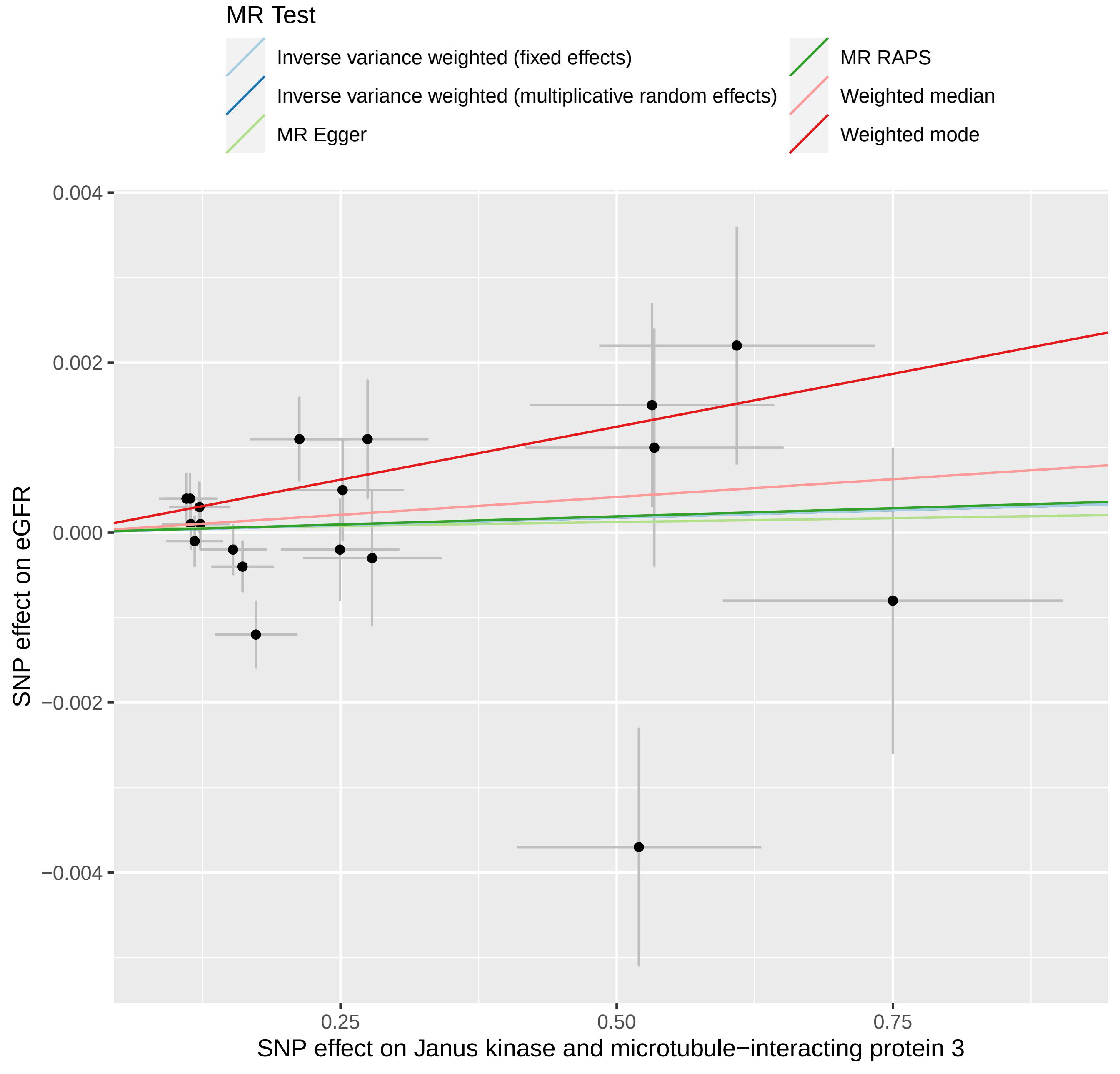

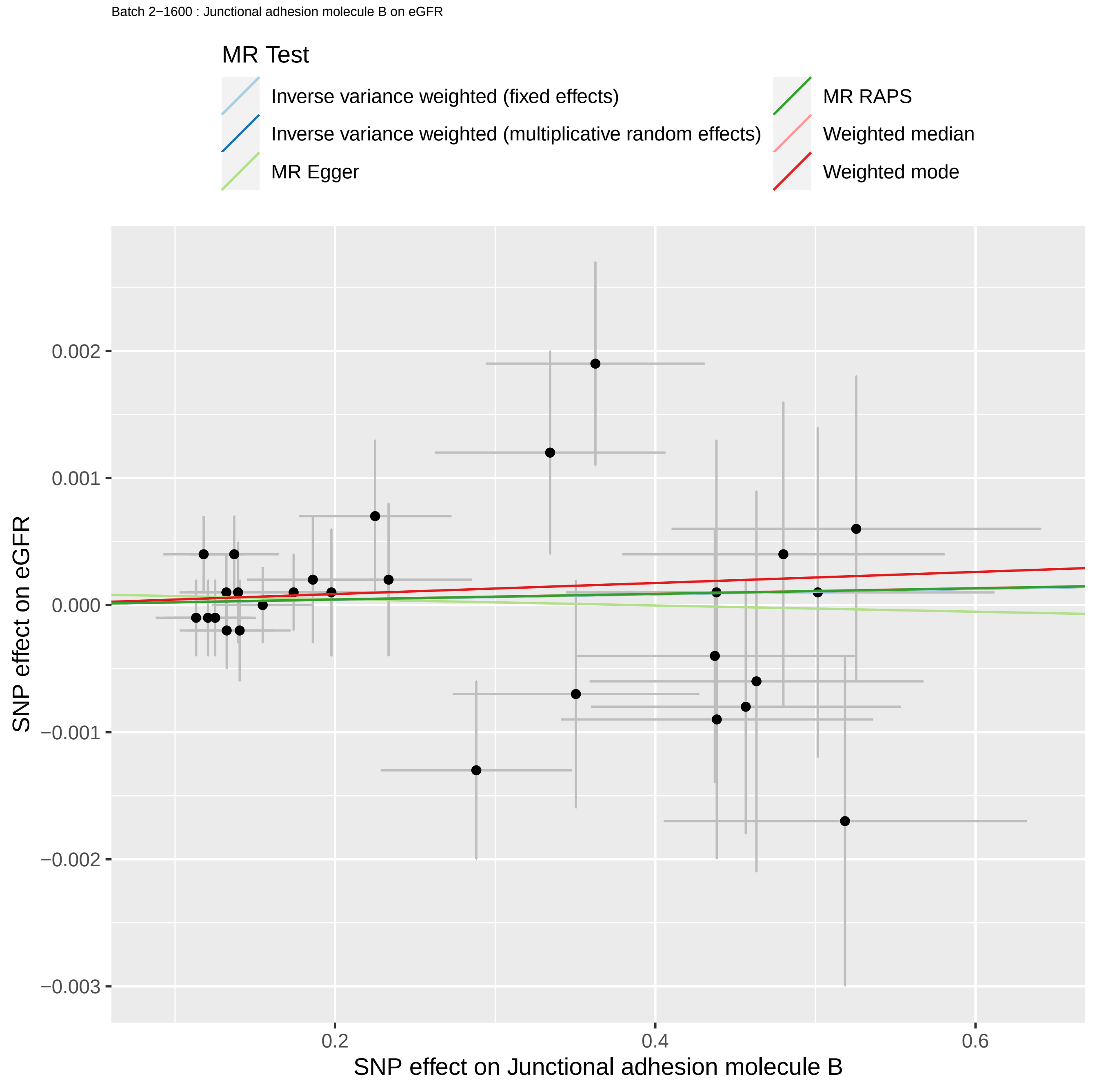

Batch 2−1600 : Junctional adhesion molecule B on eGFR
MR Test
Inverse variance weighted (fixed effects)
Inverse variance weighted (multiplicative random effects)
MR Egger
MR RAPS
Weighted median
Weighted mode
0.002
0.001
0.000
−0.001
−0.002
−0.003
SNP effect on eGFR
0.2
0.4
0.6
SNP effect on Junctional adhesion molecule B

Batch 2−1601 : Junctional adhesion molecule C on eGFR

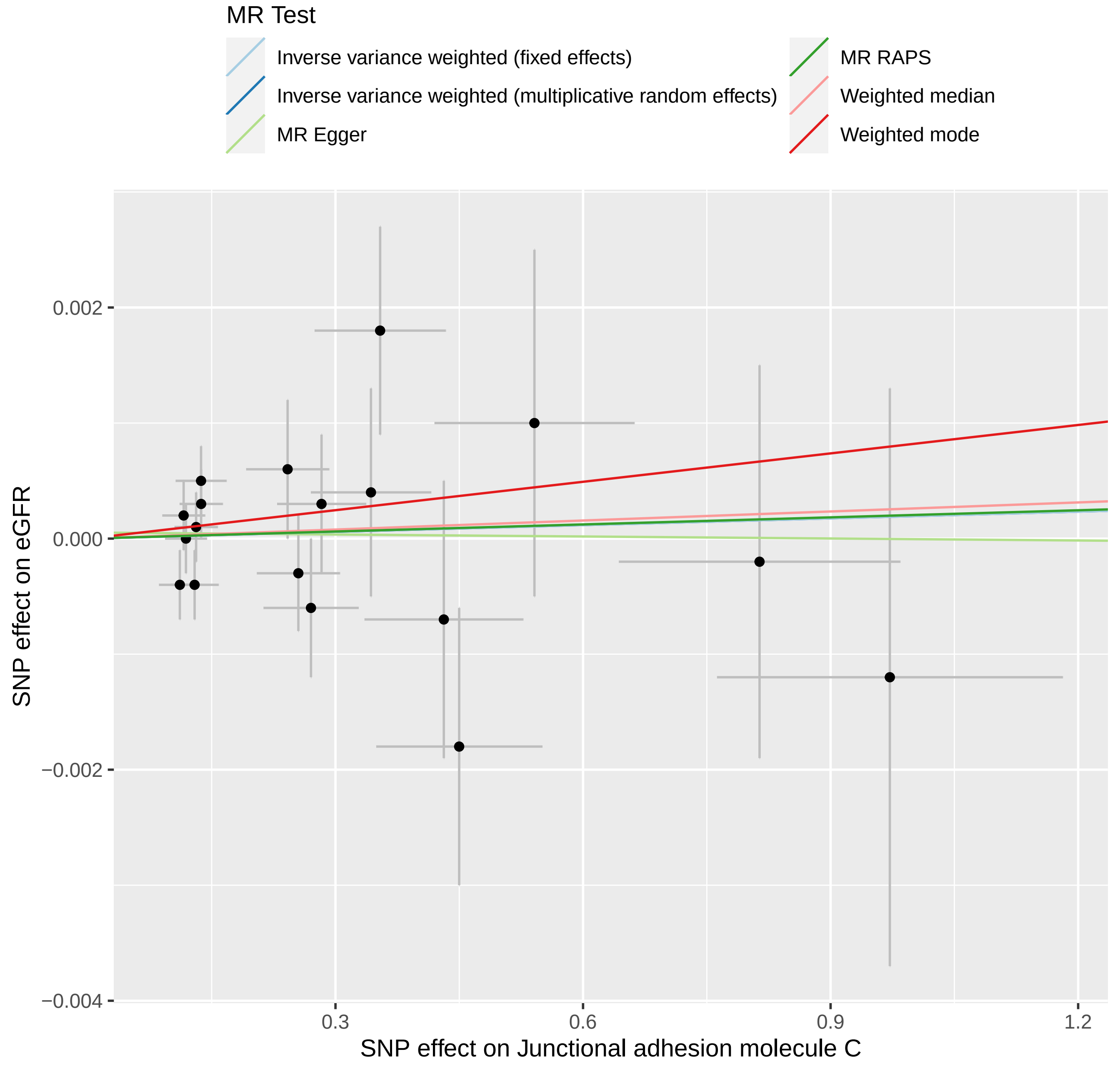

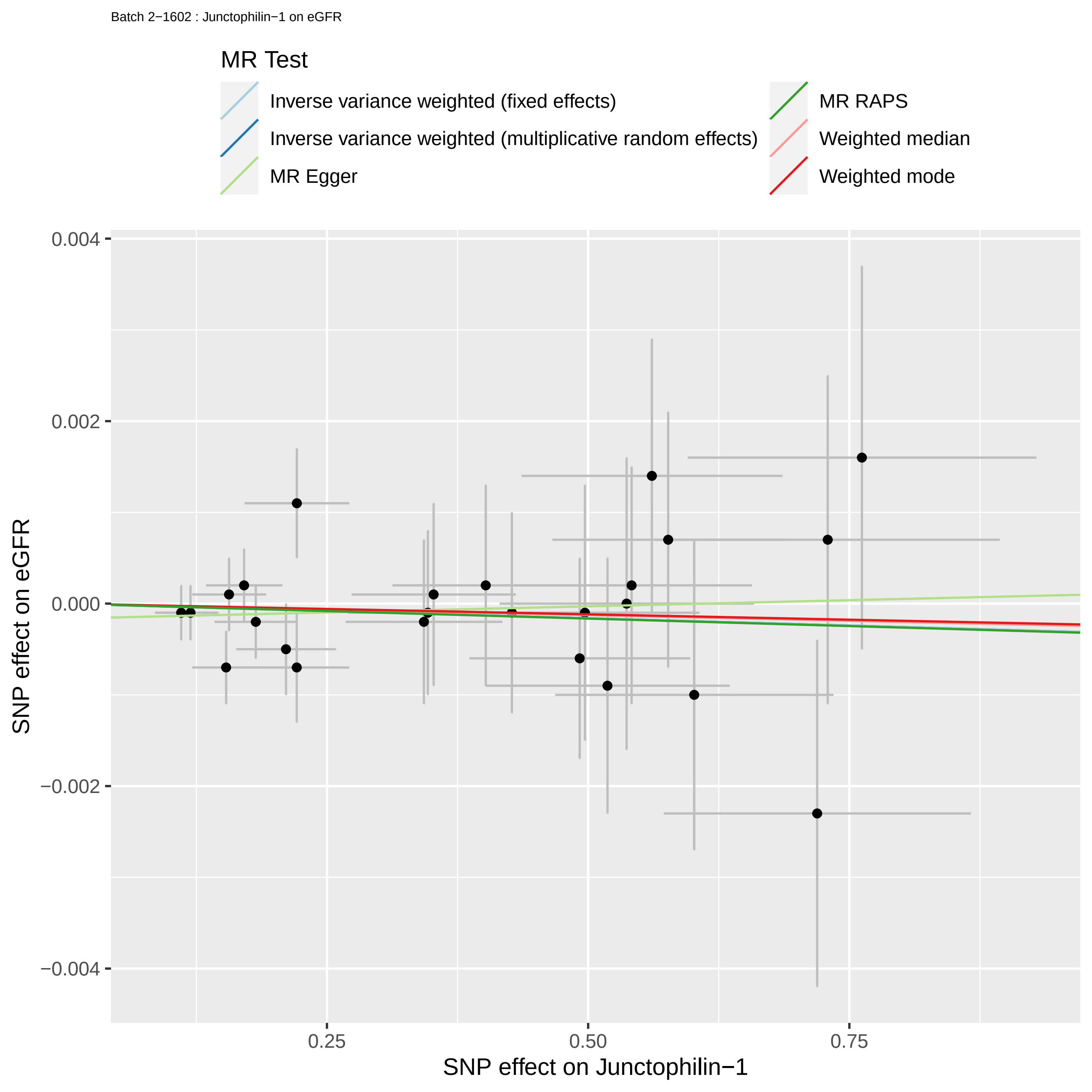

Batch 2−1602 : Junctophilin−1 on eGFR
MR Test
Inverse variance weighted (fixed effects)
Inverse variance weighted (multiplicative random effects)
MR Egger
MR RAPS
Weighted median
Weighted mode
0.004
0.002
0.000
−0.002
−0.004
0.25
0.50
0.75
SNP effect on eGFR
SNP effect on Junctophilin−1

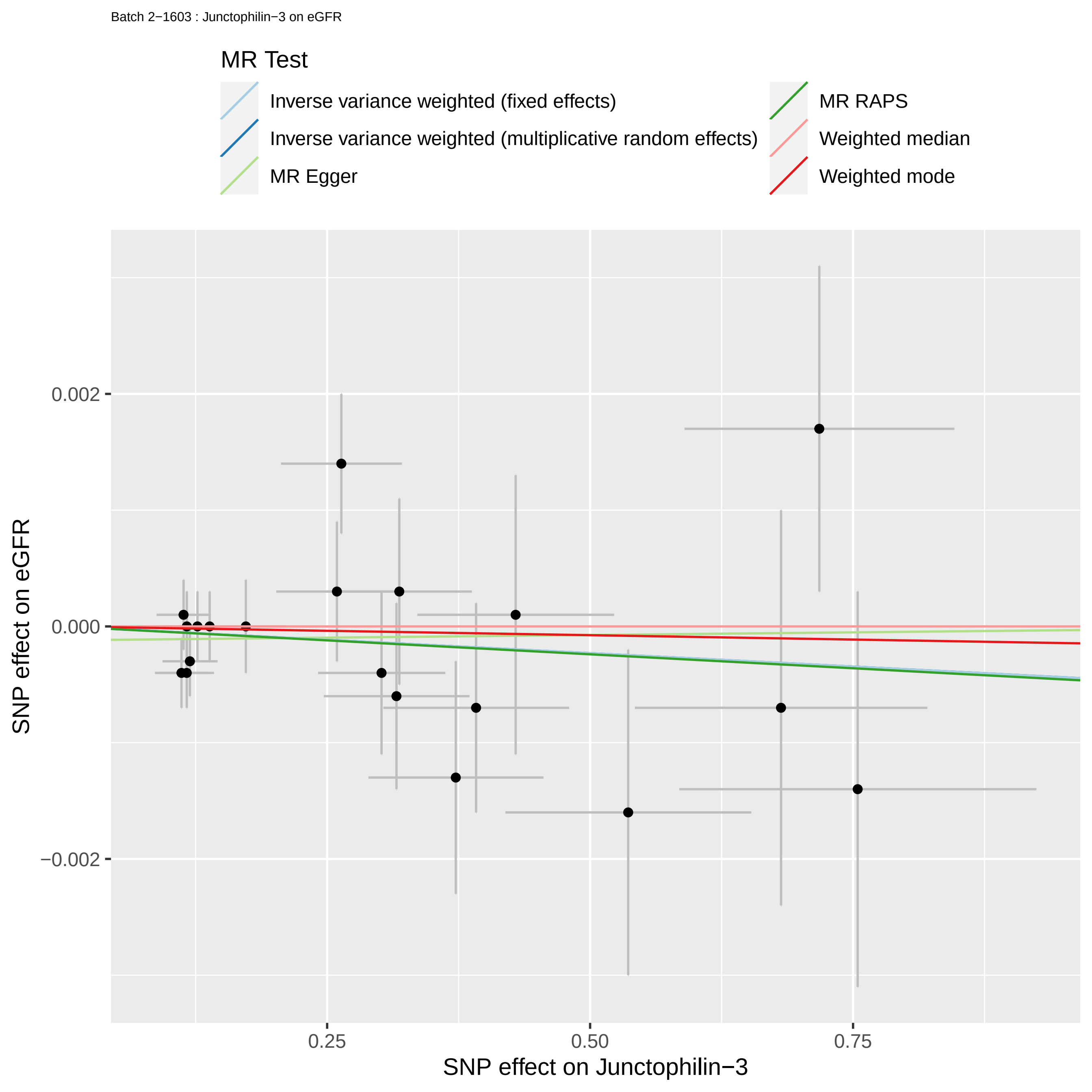
Batch 2−1603 : Junctophilin−3 on eGFR
MR Test
Inverse variance weighted (fixed effects)
Inverse variance weighted (multiplicative random effects)
MR Egger
MR RAPS
Weighted median
Weighted mode
0.002
0.000
−0.002
0.25
0.50
0.75
SNP effect on eGFR
SNP effect on Junctophilin−3

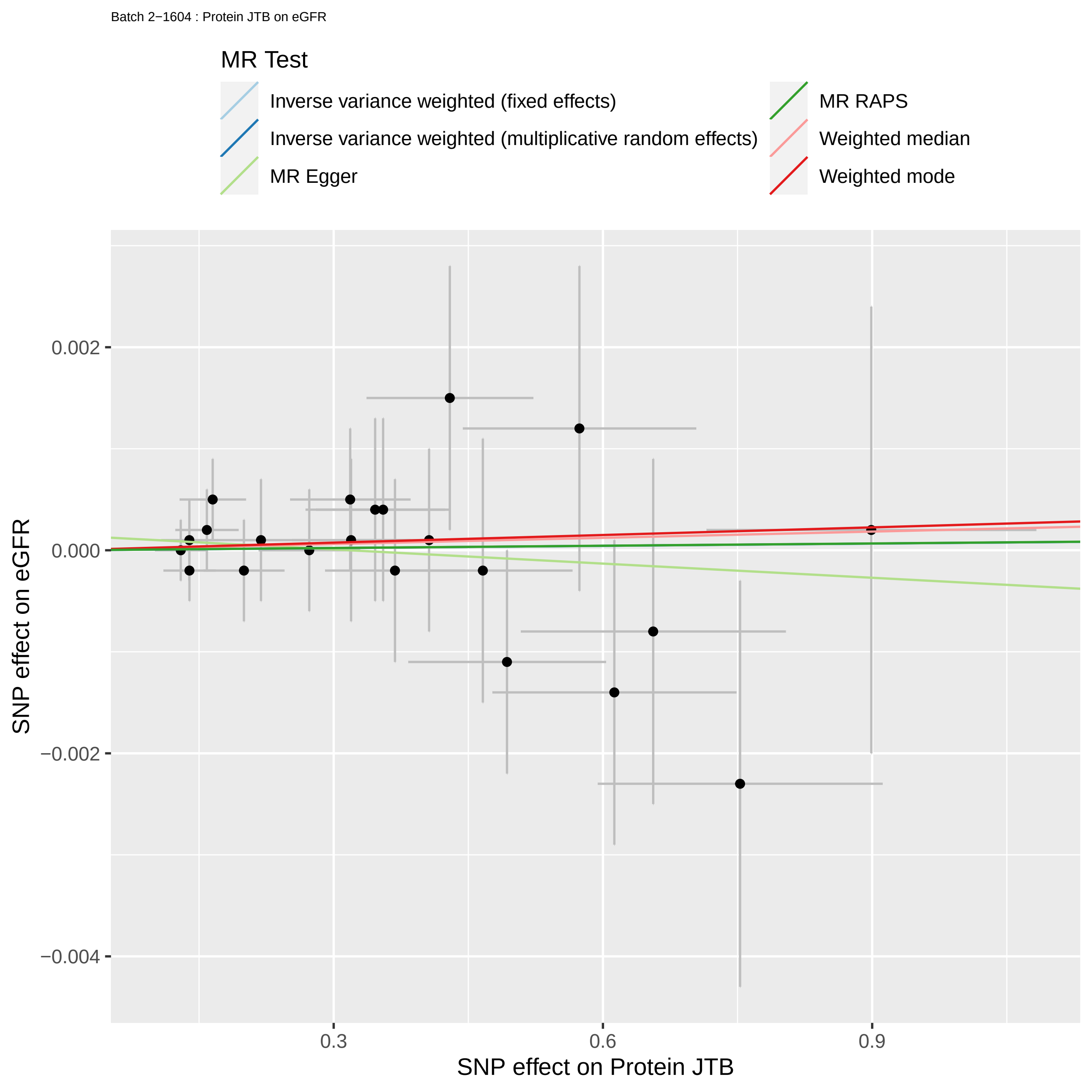
Batch 2−1604 : Protein JTB on eGFR
MR Test
Inverse variance weighted (fixed effects)
Inverse variance weighted (multiplicative random effects)
MR Egger
MR RAPS
Weighted median
Weighted mode
0.002
0.000
−0.002
−0.004
0.3
0.6
0.9
SNP effect on eGFR
SNP effect on Protein JTB

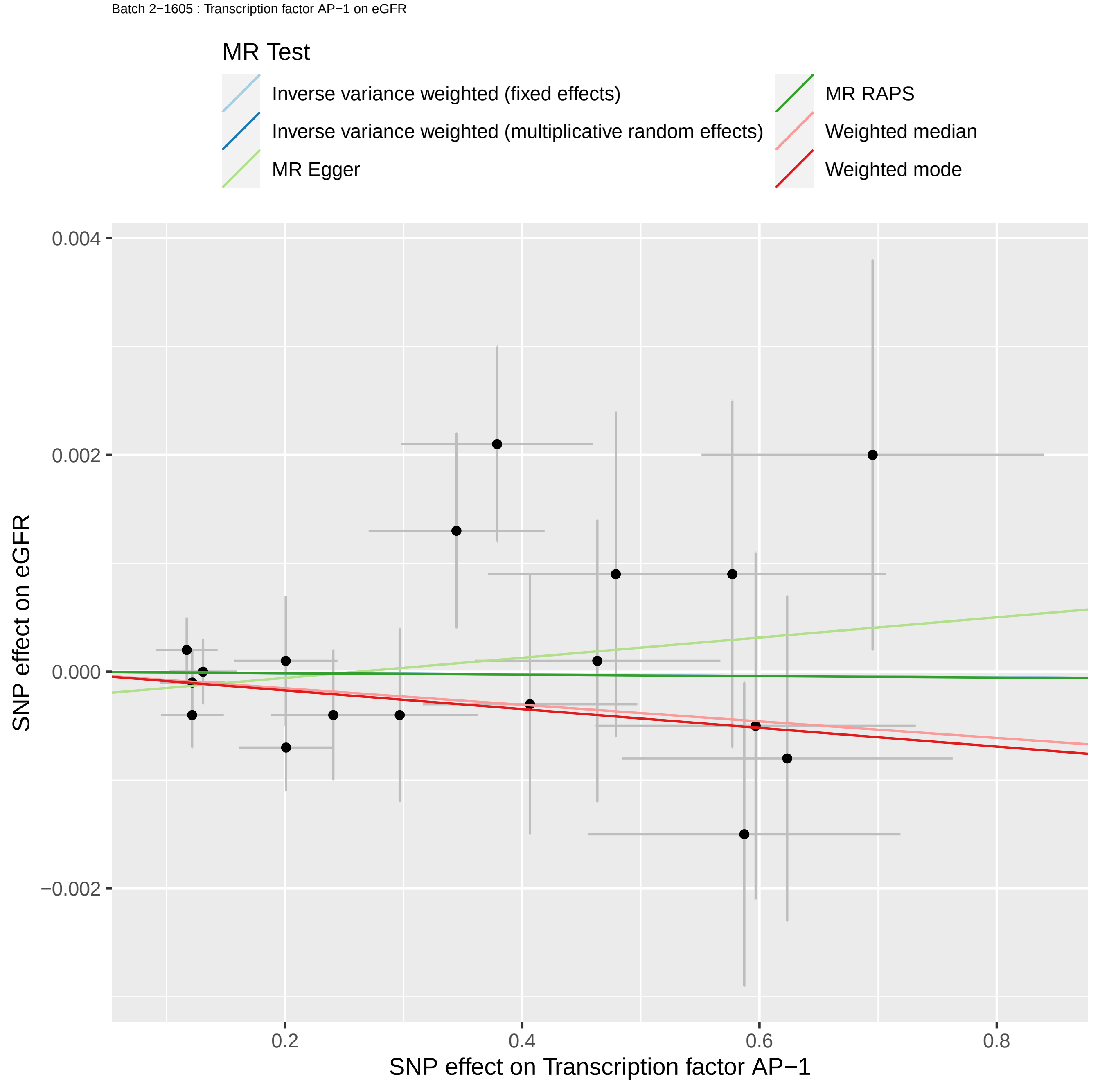

Batch 2−1605 : Transcription factor AP−1 on eGFR
MR Test
Inverse variance weighted (fixed effects)
Inverse variance weighted (multiplicative random effects)
MR Egger
MR RAPS
Weighted median
Weighted mode
0.004
0.002
0.000
−0.002
0.2
0.4
0.6
0.8
SNP effect on eGFR
SNP effect on Transcription factor AP−1

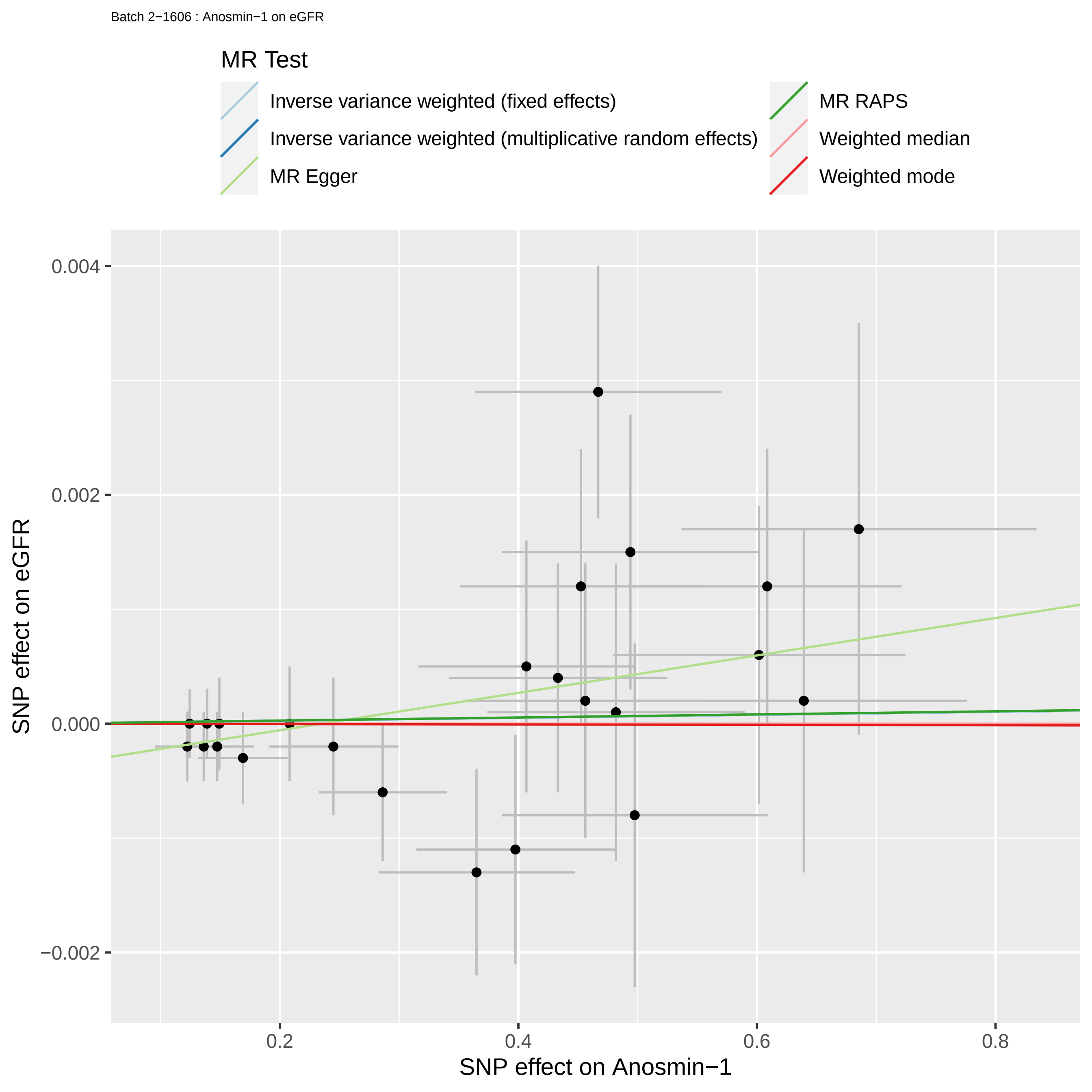
Batch 2−1606 : Anosmin−1 on eGFR
MR Test
Inverse variance weighted (fixed effects)
Inverse variance weighted (multiplicative random effects)
MR Egger
MR RAPS
Weighted median
Weighted mode
0.004
0.002
0.000
−0.002
0.2
0.4
0.6
0.8
SNP effect on eGFR
SNP effect on Anosmin−1

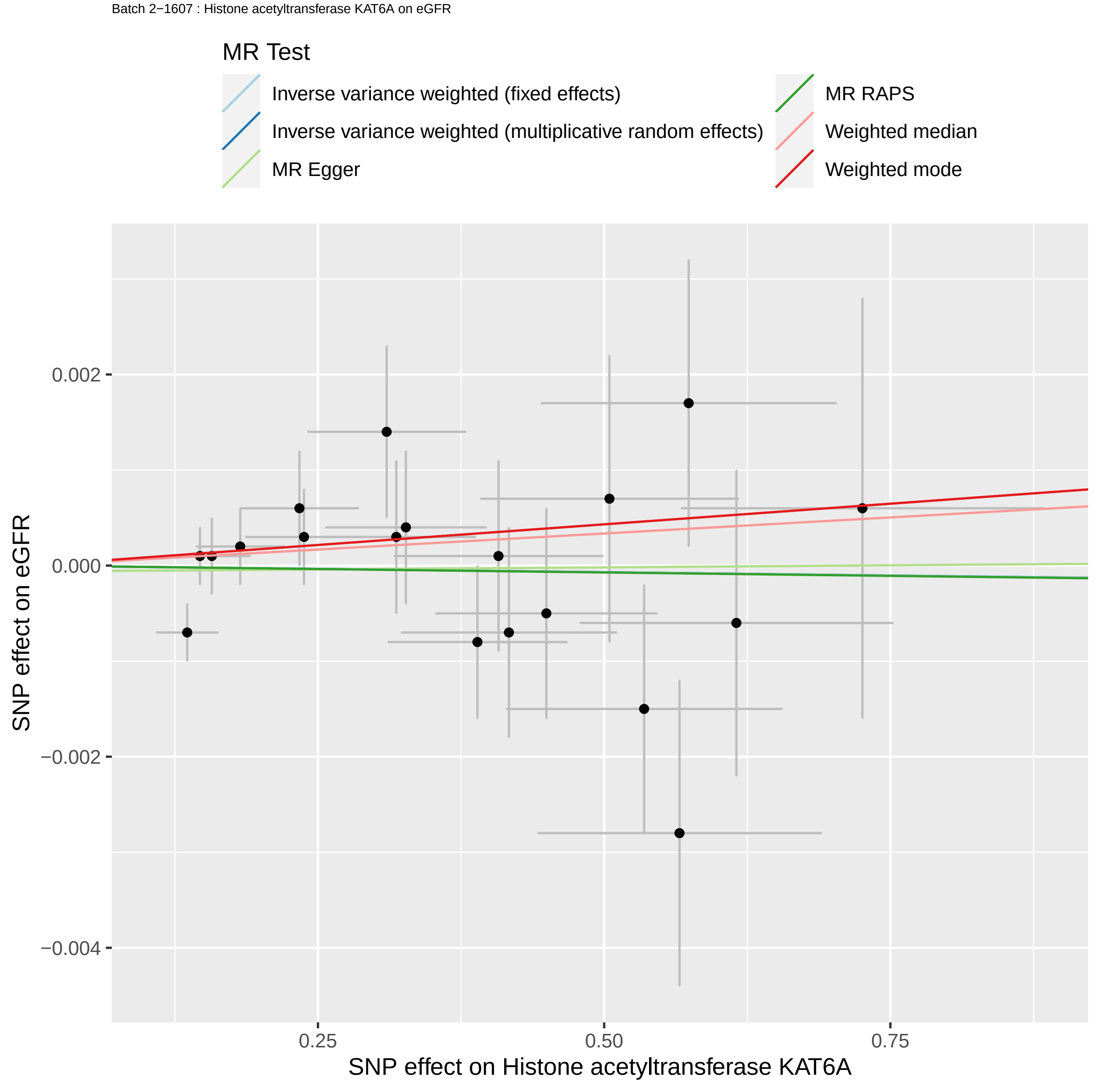

Batch 2−1607 : Histone acetyltransferase KAT6A on eGFR
MR Test
Inverse variance weighted (fixed effects)
Inverse variance weighted (multiplicative random effects)
MR Egger
MR RAPS
Weighted median
Weighted mode
0.002
0.000
−0.002
−0.004
0.25
0.50
0.75
SNP effect on eGFR
SNP effect on Histone acetyltransferase KAT6A

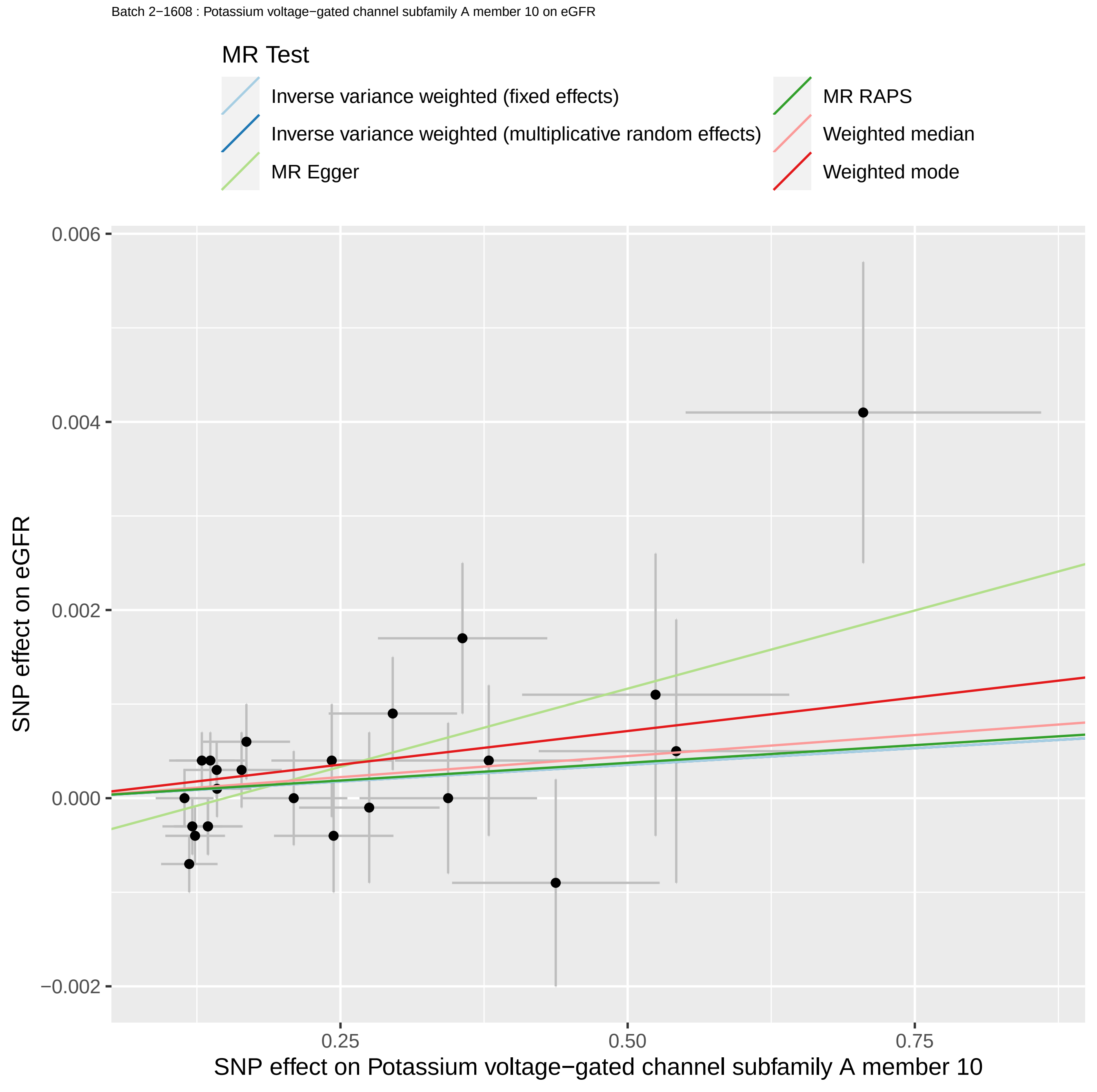

Batch 2−1608 : Potassium voltage−gated channel subfamily A member 10 on eGFR
MR Test
Inverse variance weighted (fixed effects)
Inverse variance weighted (multiplicative random effects)
MR Egger
MR RAPS
Weighted median
Weighted mode
0.006
0.004
0.002
0.000
−0.002
0.25
0.50
0.75
SNP effect on eGFR
SNP effect on Potassium voltage−gated channel subfamily A member 10

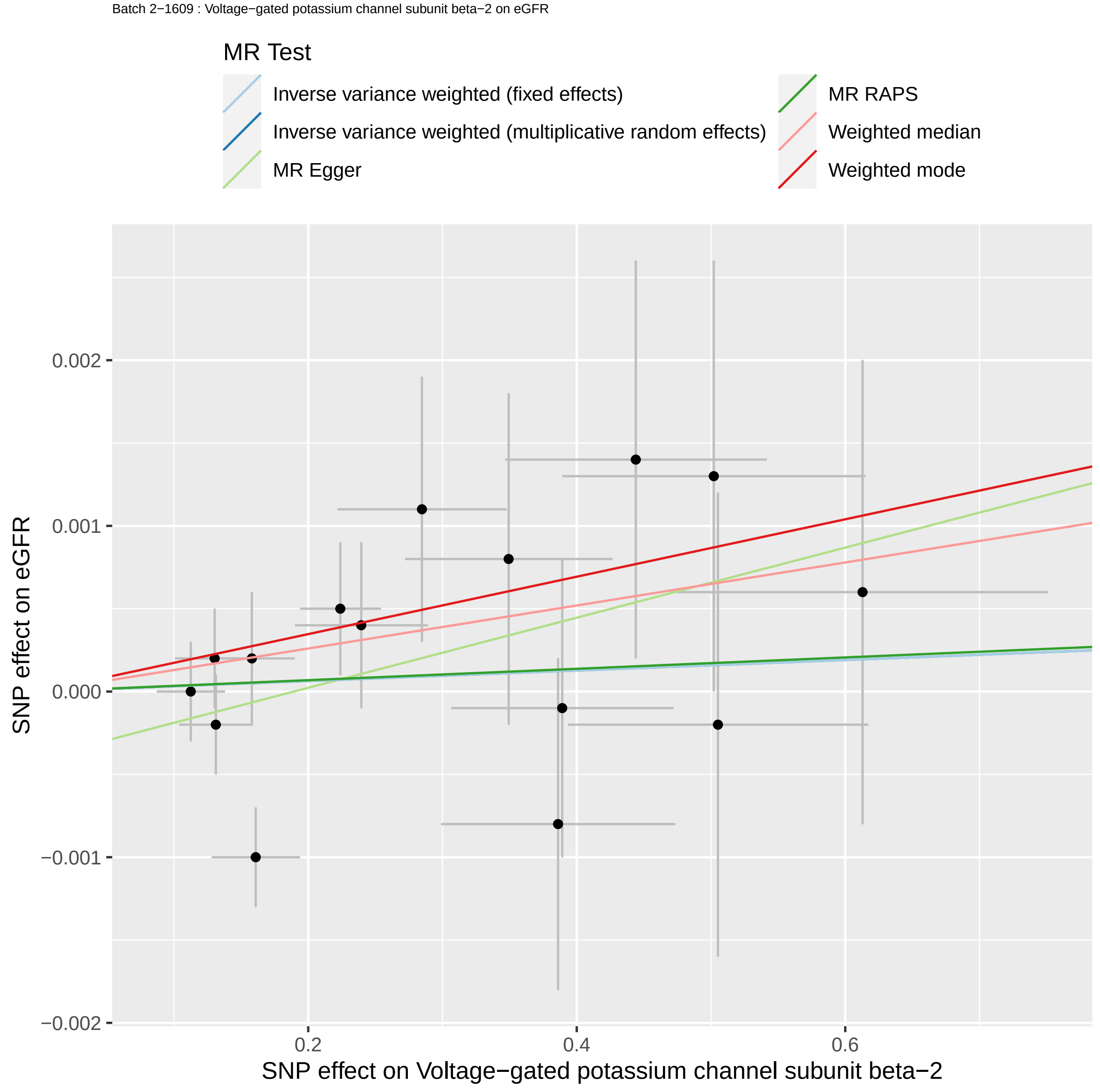
Batch 2−1609 : Voltage−gated potassium channel subunit beta−2 on eGFR
MR Test
Inverse variance weighted (fixed effects)
Inverse variance weighted (multiplicative random effects)
MR Egger
MR RAPS
Weighted median
Weighted mode
0.002
0.001
0.000
−0.001
−0.002
0.2
0.4
0.6
SNP effect on eGFR
SNP effect on Voltage−gated potassium channel subunit beta−2

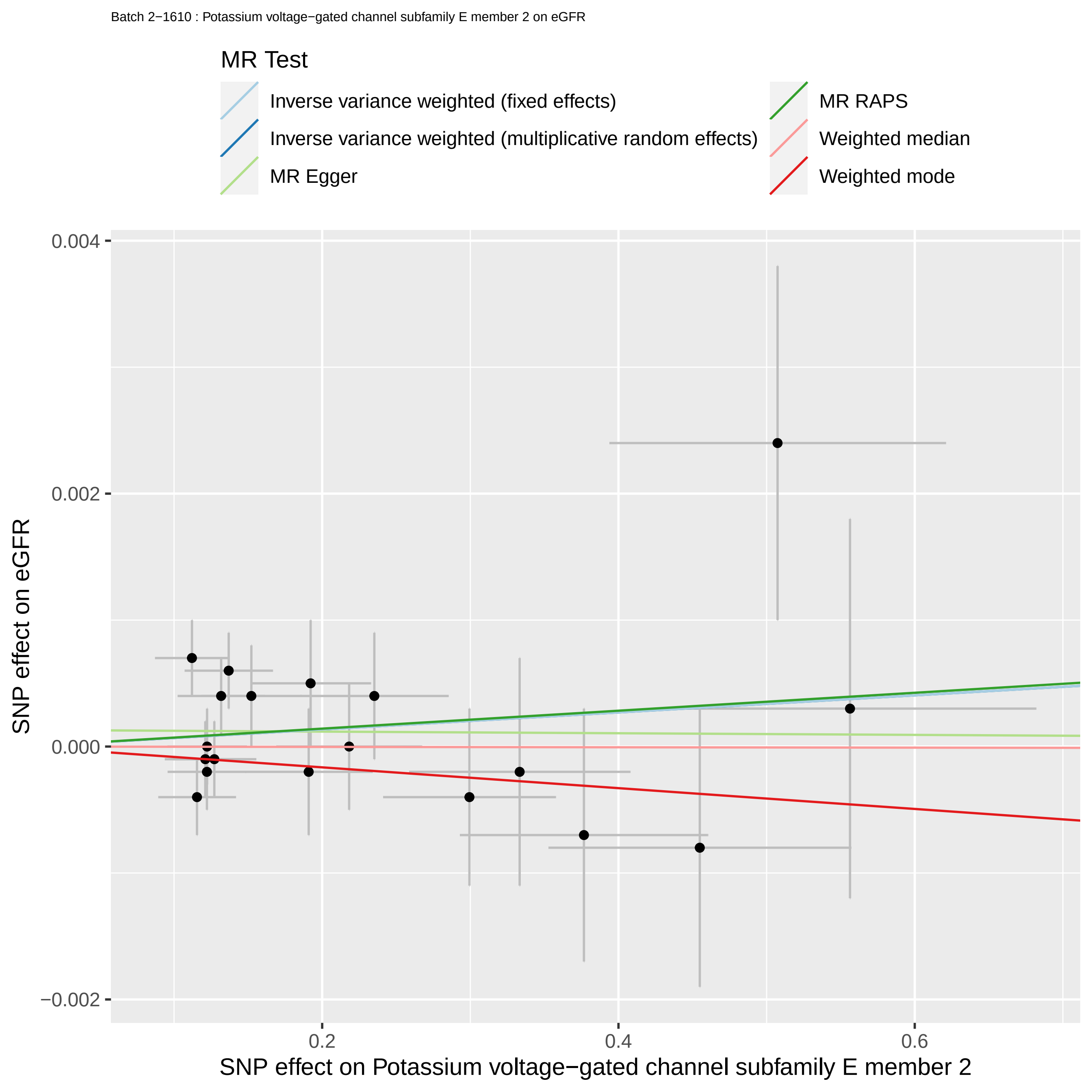

Batch 2−1610 : Potassium voltage−gated channel subfamily E member 2 on eGFR
MR Test
Inverse variance weighted (fixed effects)
Inverse variance weighted (multiplicative random effects)
MR Egger
MR RAPS
Weighted median
Weighted mode
0.004
0.002
0.000
−0.002
SNP effect on eGFR
0.2
0.4
0.6
SNP effect on Potassium voltage−gated channel subfamily E member 2

Batch 2−1611 : Potassium voltage−gated channel subfamily E member 3 on eGFR

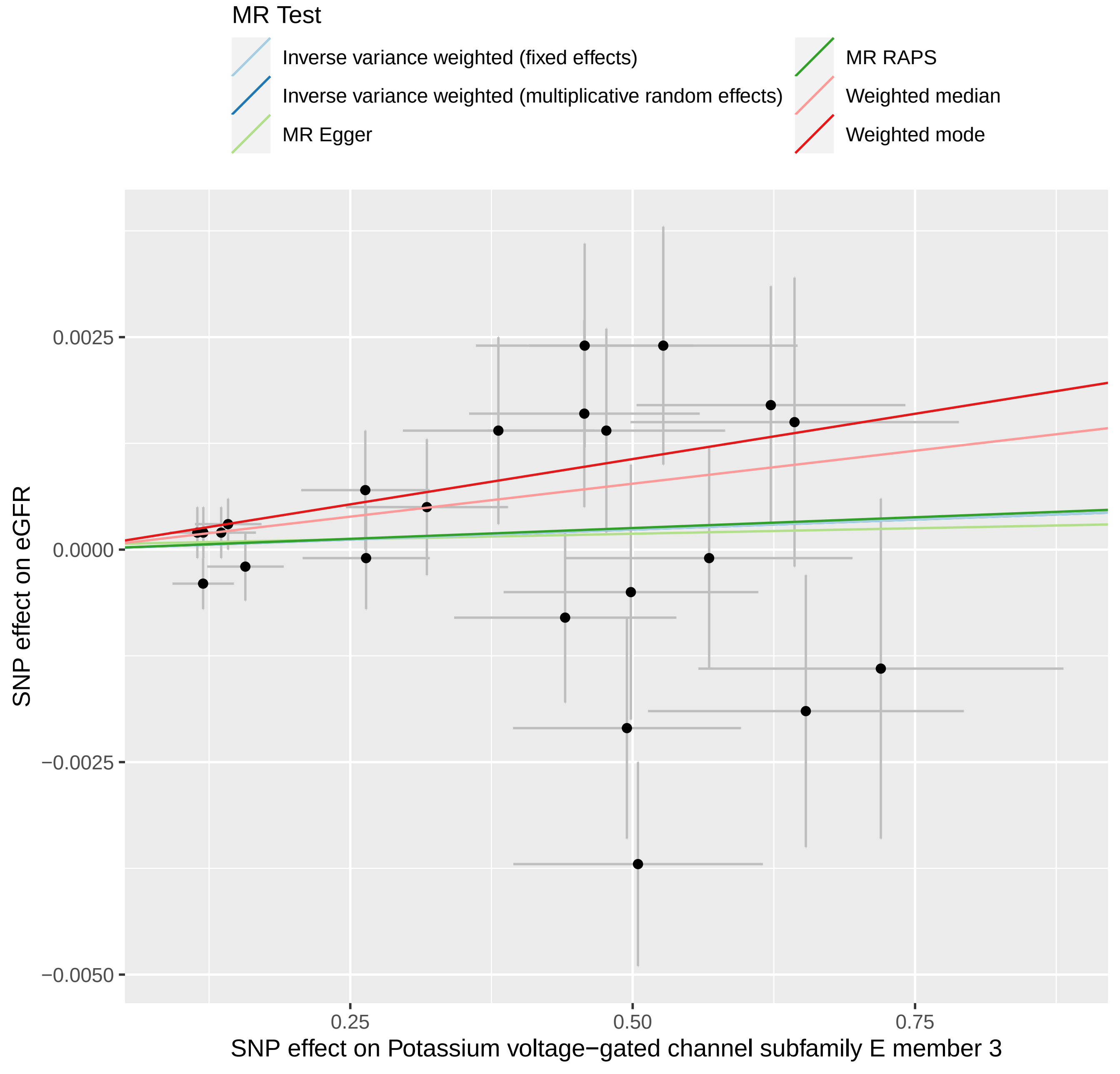

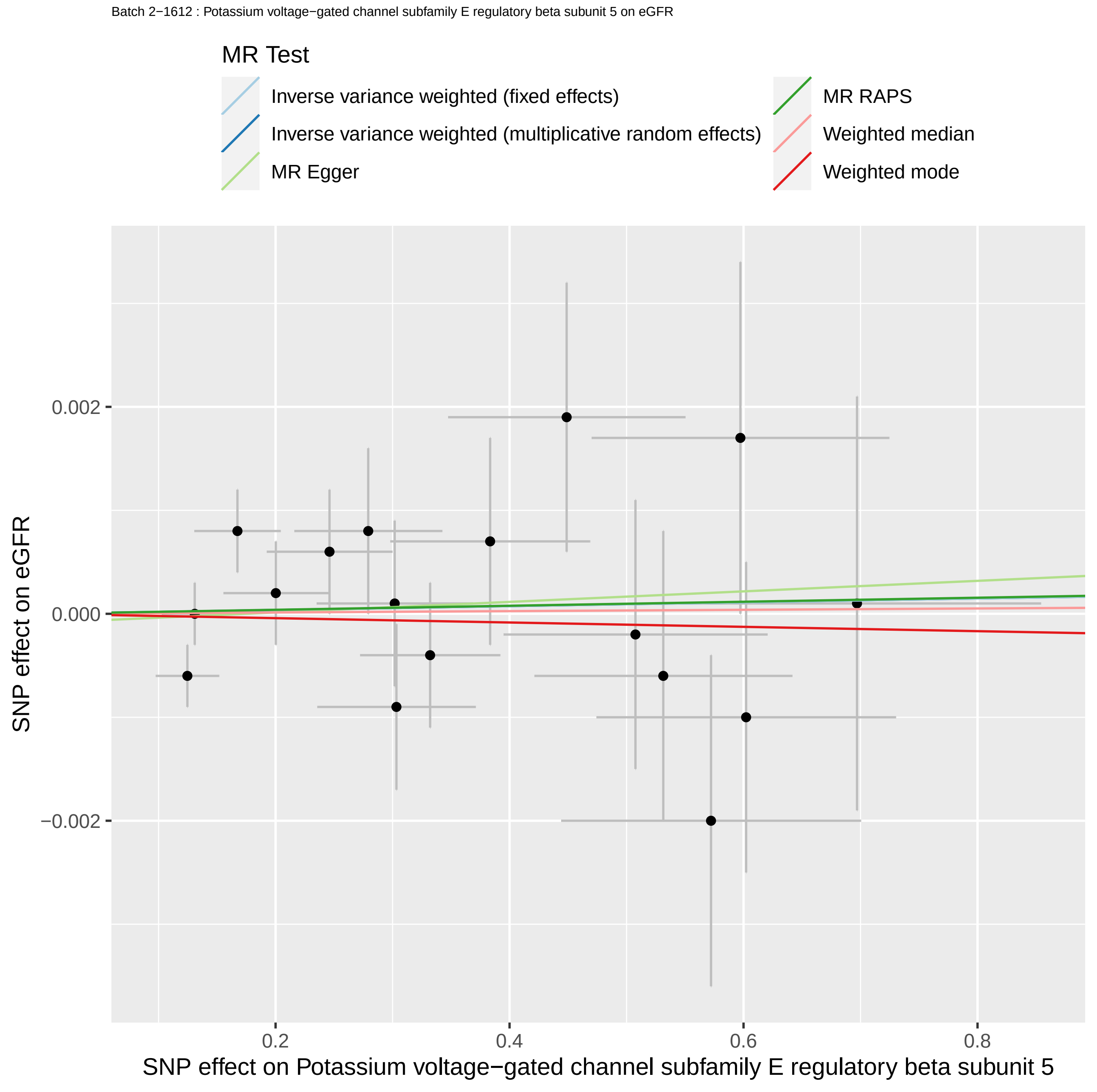

Batch 2−1612 : Potassium voltage−gated channel subfamily E regulatory beta subunit 5 on eGFR
MR Test
Inverse variance weighted (fixed effects)
Inverse variance weighted (multiplicative random effects)
MR Egger
MR RAPS
Weighted median
Weighted mode
0.002
0.000
−0.002
0.2
0.4
0.6
0.8
SNP effect on eGFR
SNP effect on Potassium voltage−gated channel subfamily E regulatory beta subunit 5

Batch 2−1613 : Potassium voltage−gated channel subfamily E regulatory beta subunit 5 on eGFR

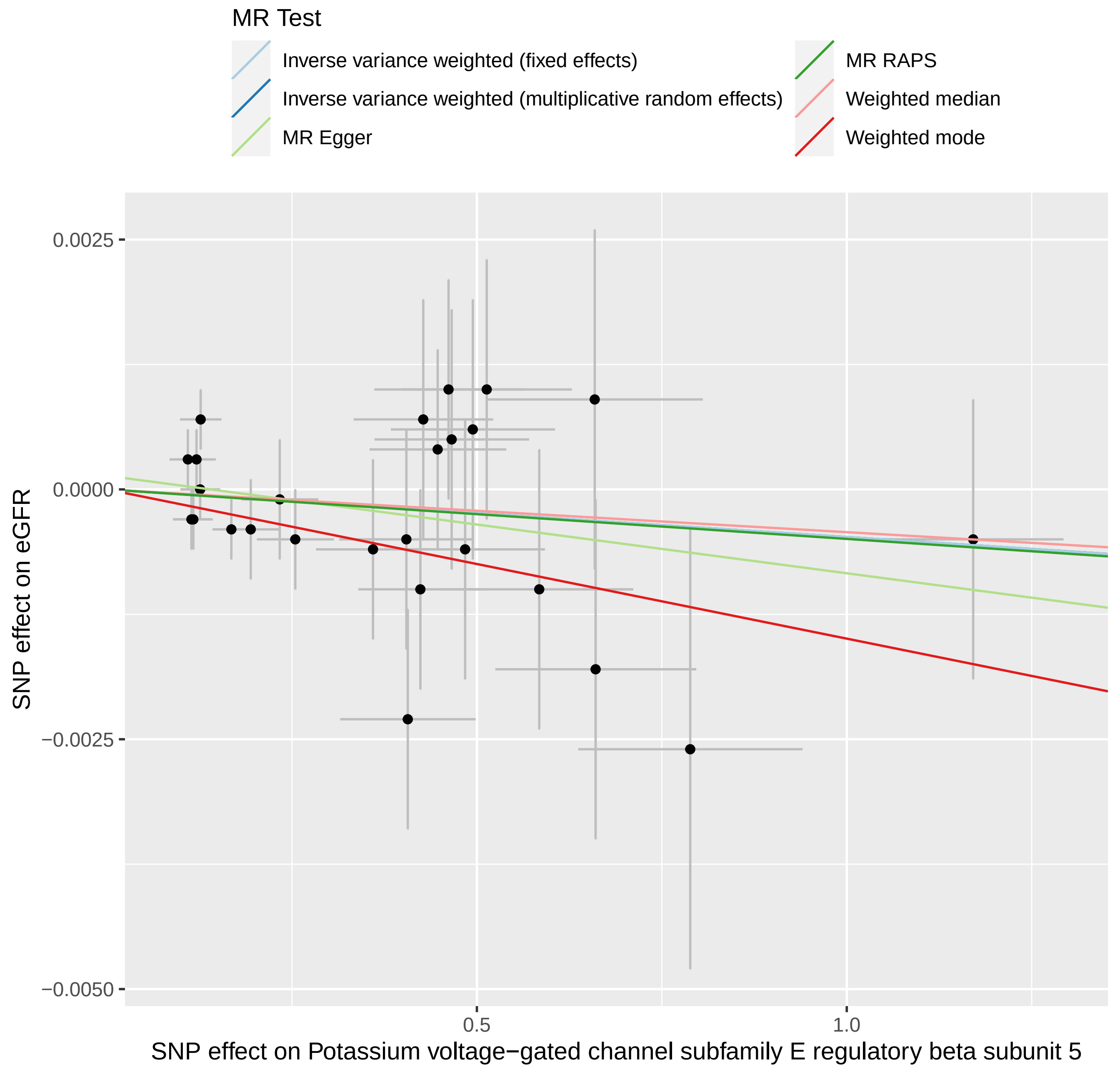

Batch 2−1614 : Potassium voltage−gated channel subfamily F member 1 on eGFR

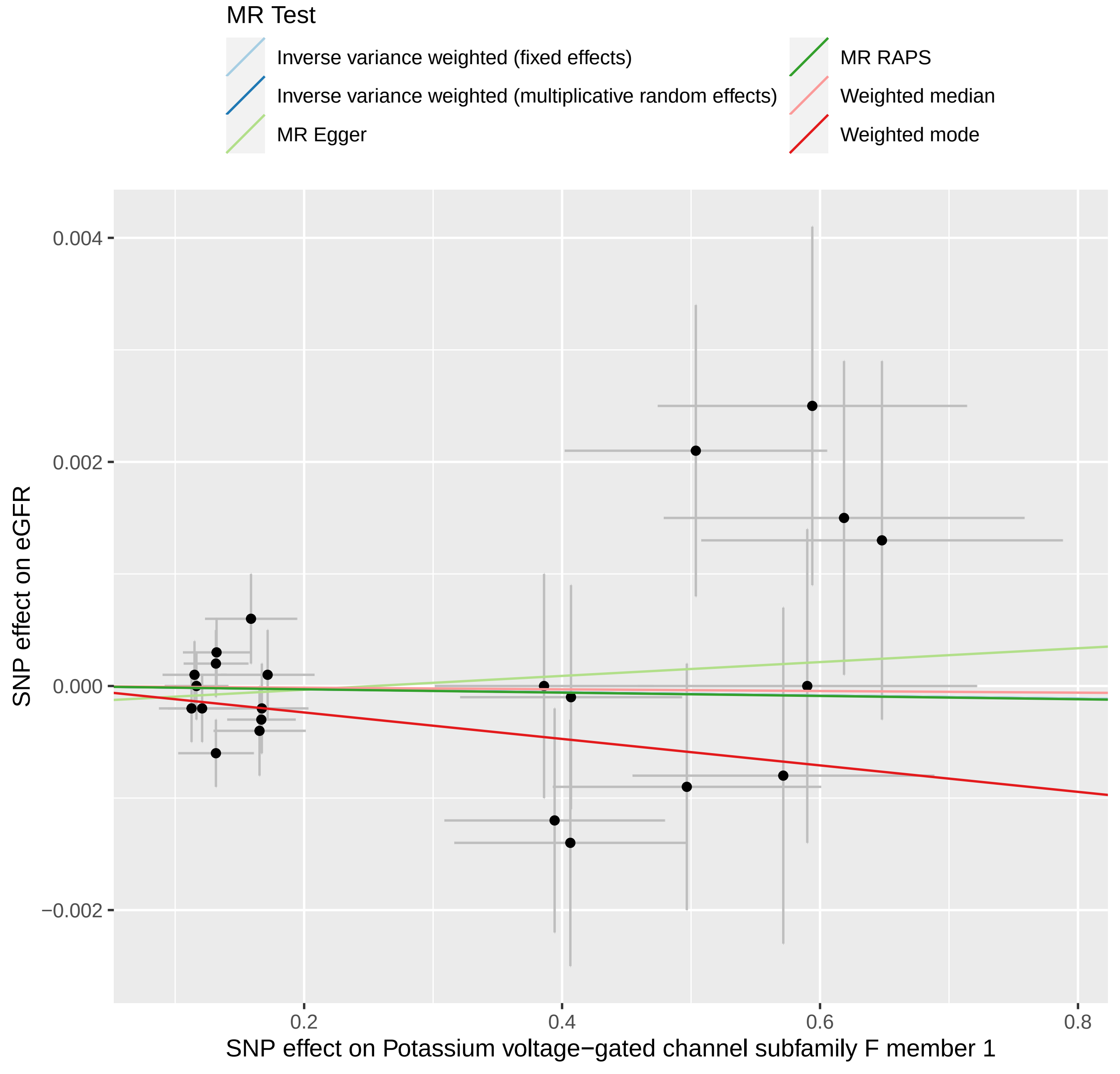

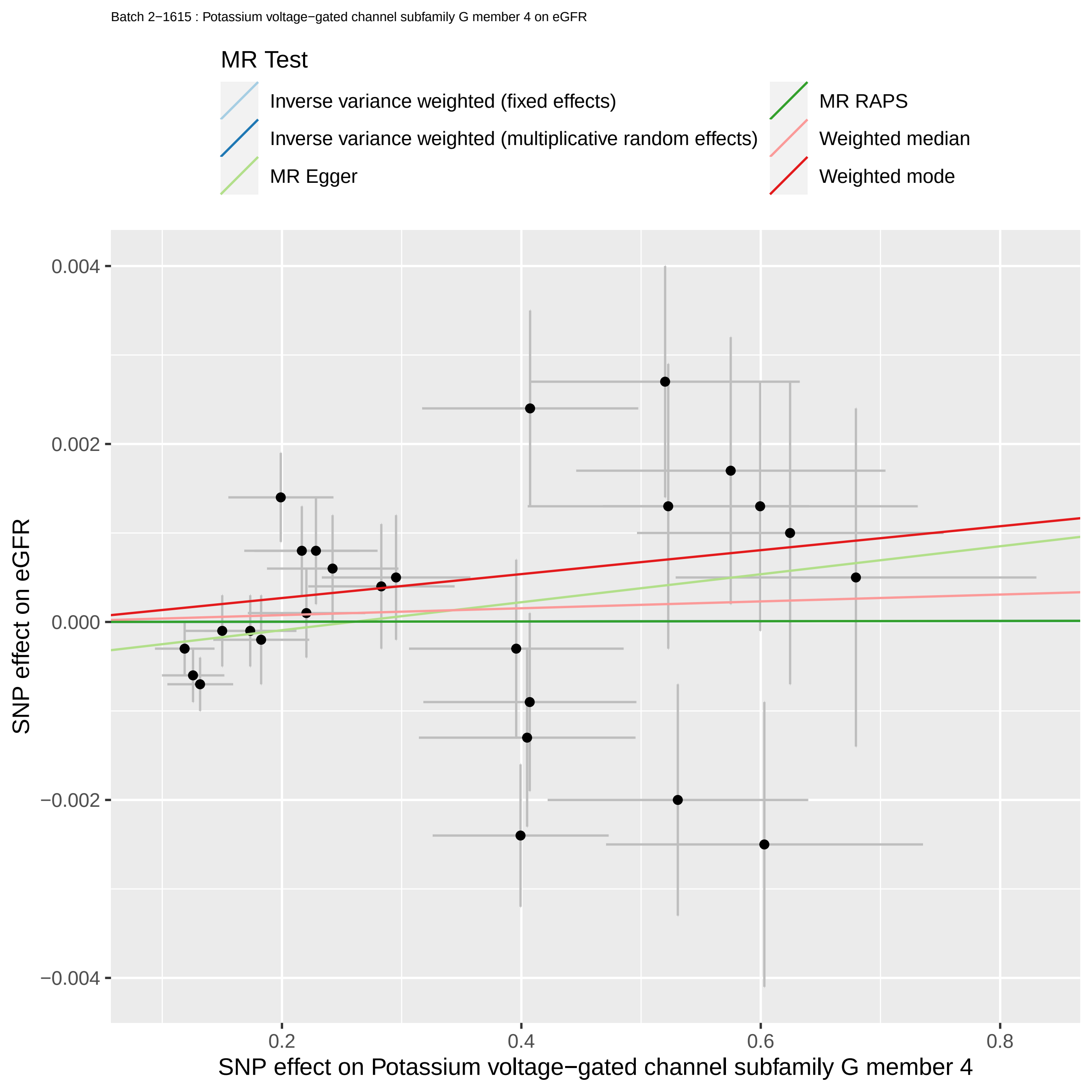
Batch 2−1615 : Potassium voltage−gated channel subfamily G member 4 on eGFR
MR Test
Inverse variance weighted (fixed effects)
Inverse variance weighted (multiplicative random effects)
MR Egger
MR RAPS
Weighted median
Weighted mode
0.004
0.002
0.000
−0.002
−0.004
0.2
0.4
0.6
0.8
SNP effect on eGFR
SNP effect on Potassium voltage−gated channel subfamily G member 4

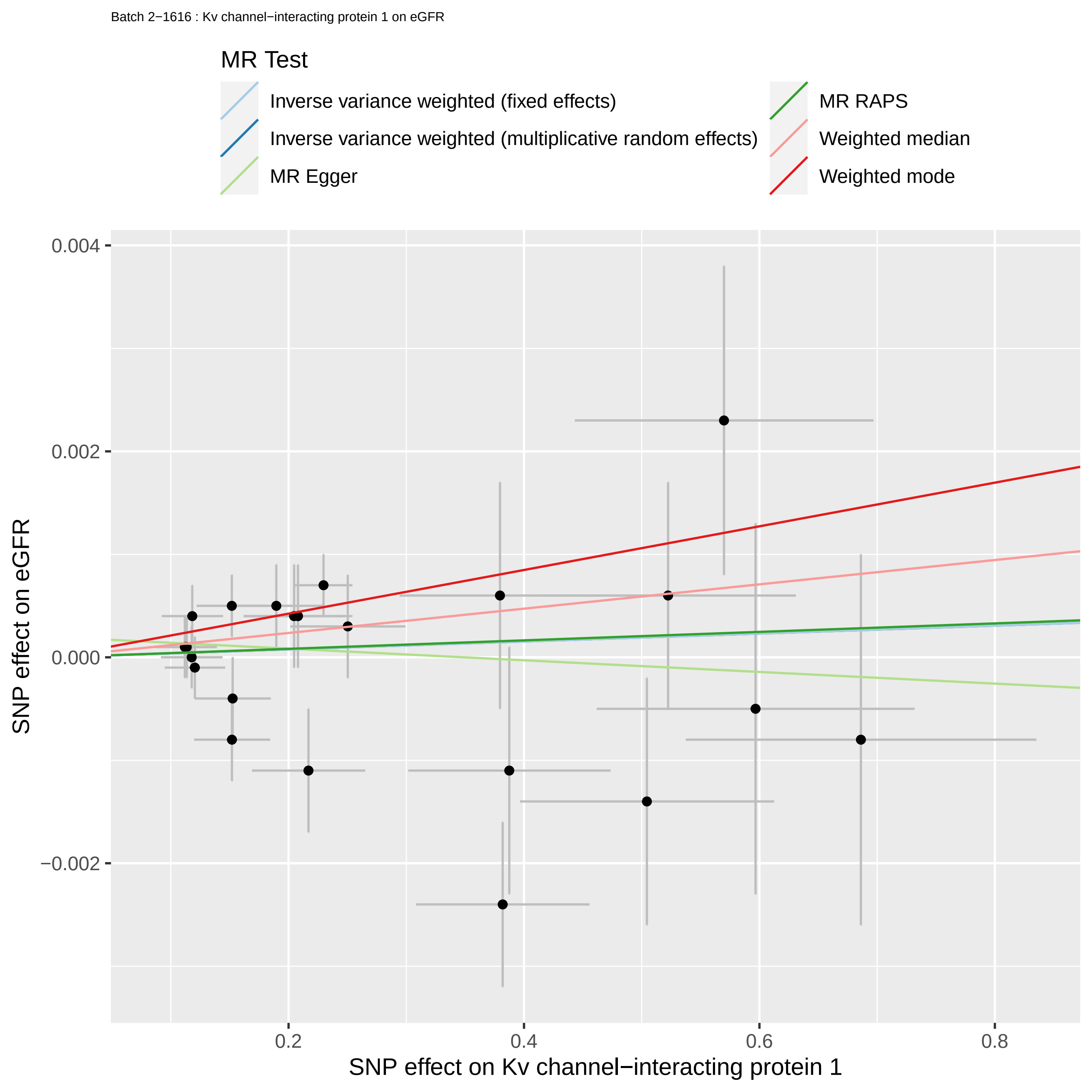
Batch 2−1616 : Kv channel−interacting protein 1 on eGFR
MR Test
Inverse variance weighted (fixed effects)
Inverse variance weighted (multiplicative random effects)
MR Egger
MR RAPS
Weighted median
Weighted mode
0.004
0.002
0.000
−0.002
SNP effect on eGFR
0.2
0.4
0.6
0.8
SNP effect on Kv channel−interacting protein 1

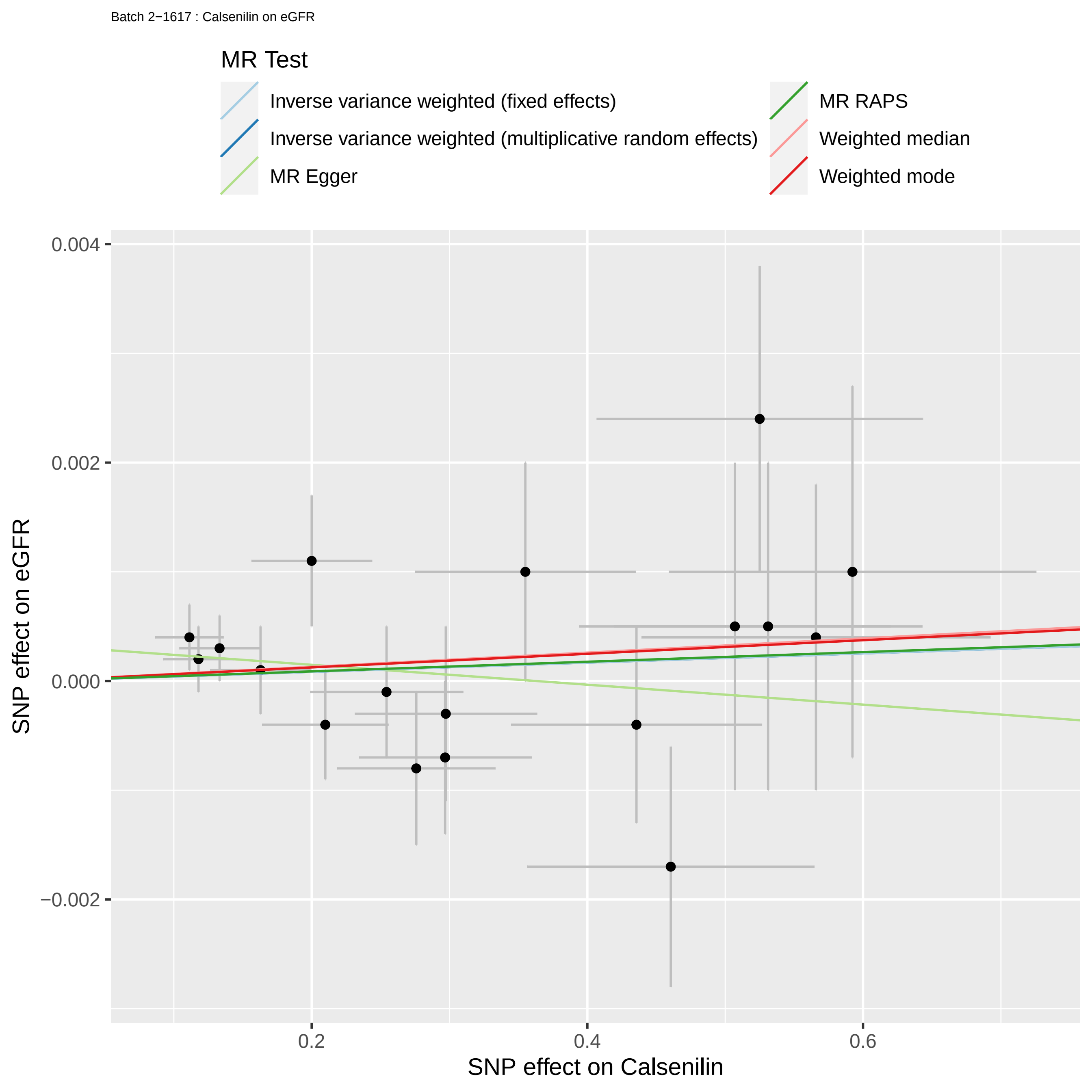
Batch 2−1617 : Calsenilin on eGFR
MR Test
Inverse variance weighted (fixed effects)
Inverse variance weighted (multiplicative random effects)
MR Egger
MR RAPS
Weighted median
Weighted mode
0.004
0.002
0.000
−0.002
0.2
0.4
0.6
SNP effect on eGFR
SNP effect on Calsenilin

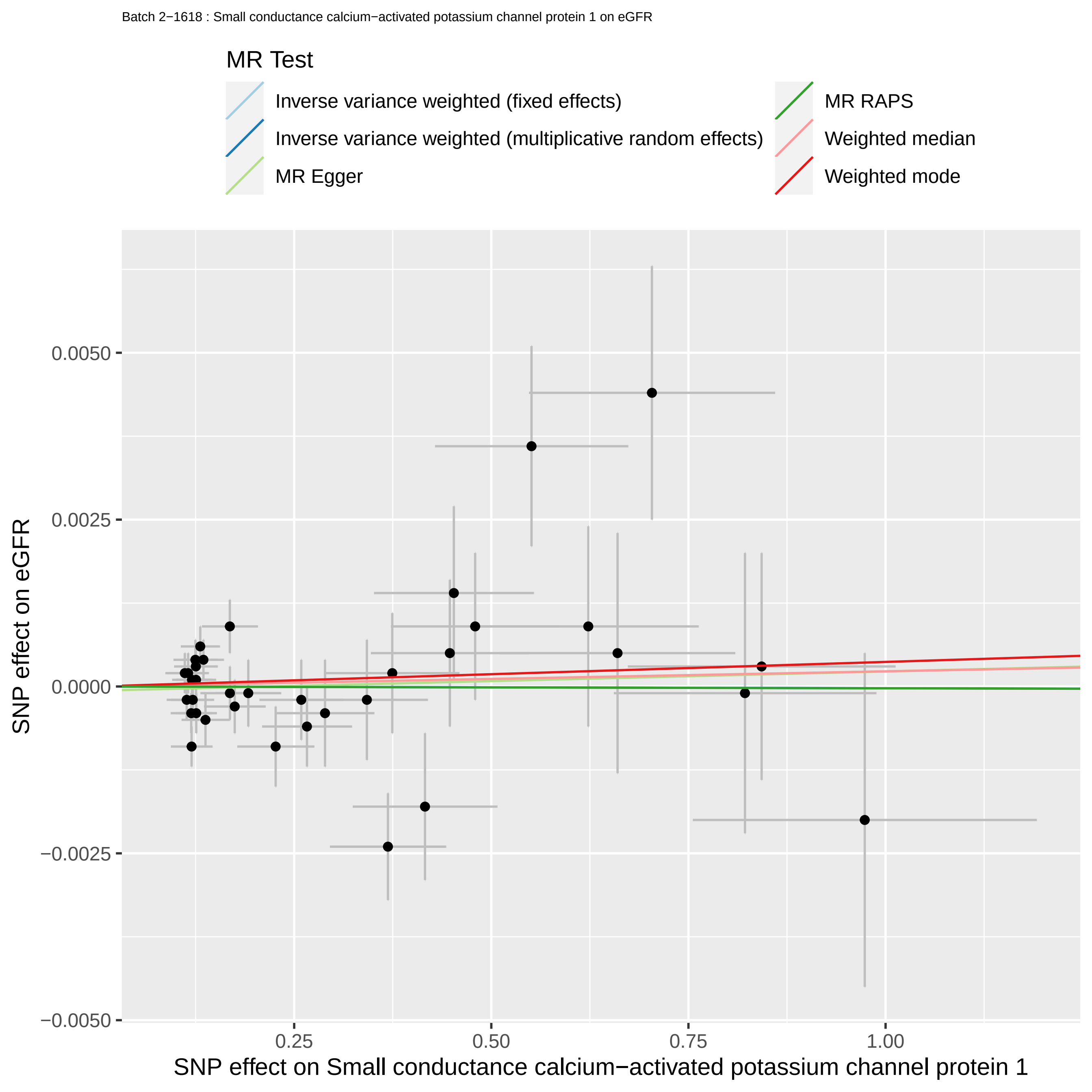

Batch 2−1618 : Small conductance calcium−activated potassium channel protein 1 on eGFR
MR Test
Inverse variance weighted (fixed effects)
Inverse variance weighted (multiplicative random effects)
MR Egger
MR RAPS
Weighted median
Weighted mode
0.0050
0.0025
0.0000
−0.0025
−0.0050
0.25
0.50
0.75
1.00
SNP effect on eGFR
SNP effect on Small conductance calcium−activated potassium channel protein 1

Batch 2−1619 : Keratinocyte−associated transmembrane protein 2 on eGFR

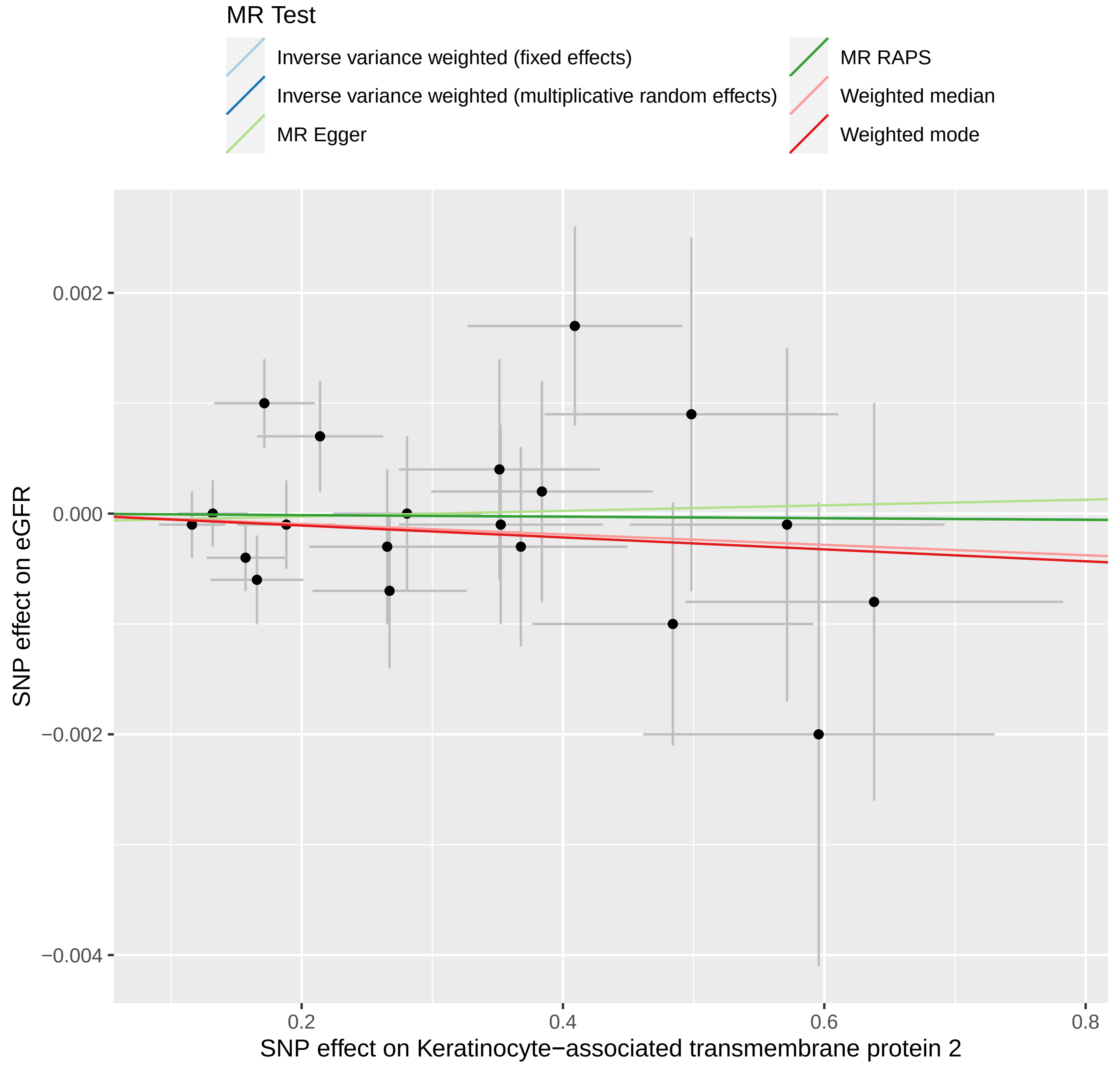

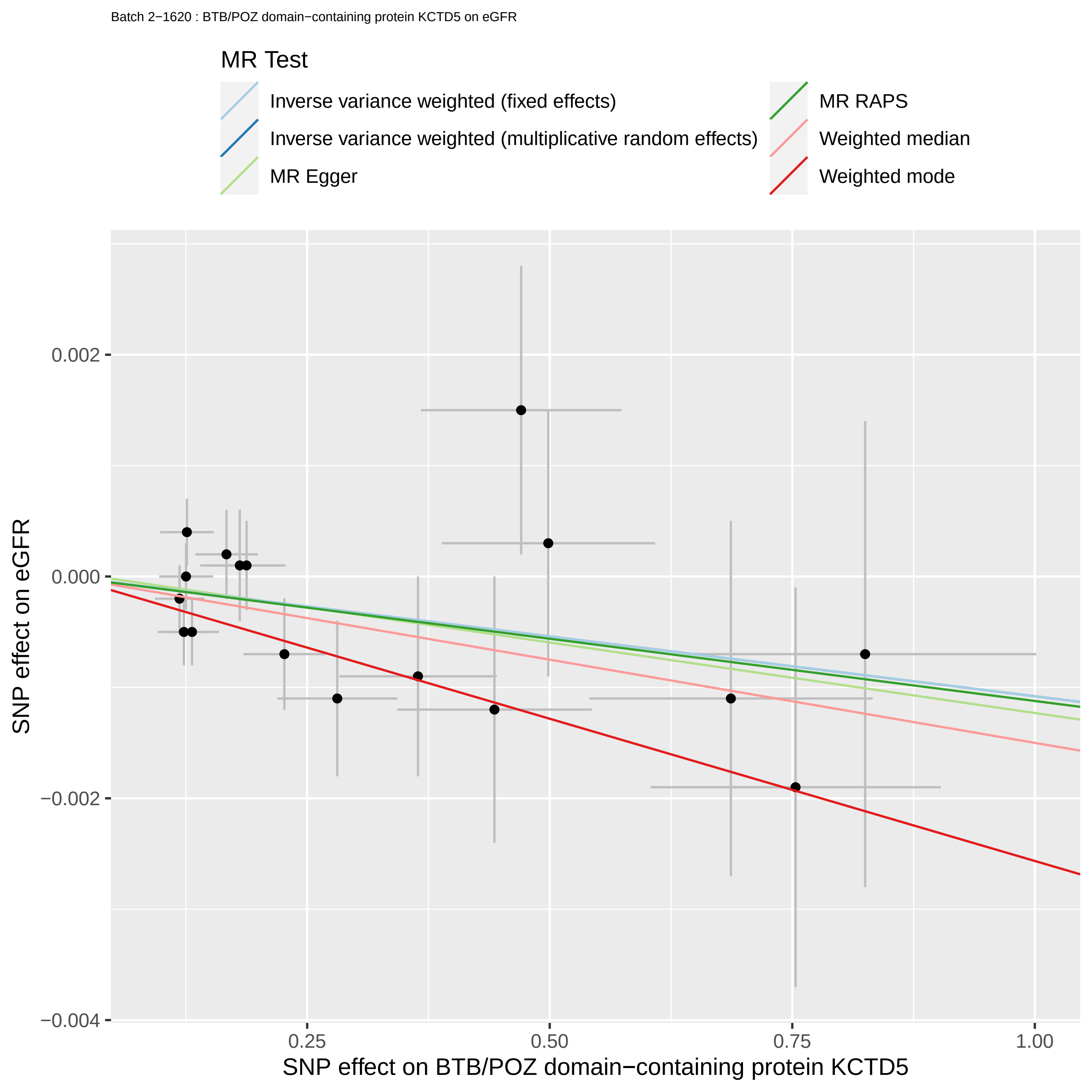

Batch 2−1620 : BTB/POZ domain−containing protein KCTD5 on eGFR
MR Test
Inverse variance weighted (fixed effects)
Inverse variance weighted (multiplicative random effects)
MR Egger
MR RAPS
Weighted median
Weighted mode
0.002
0.000
−0.002
−0.004
SNP effect on eGFR
0.25
0.50
0.75
1.00
SNP effect on BTB/POZ domain−containing protein KCTD5

Batch 2−1621 : KDEL motif−containing protein 2 on eGFR

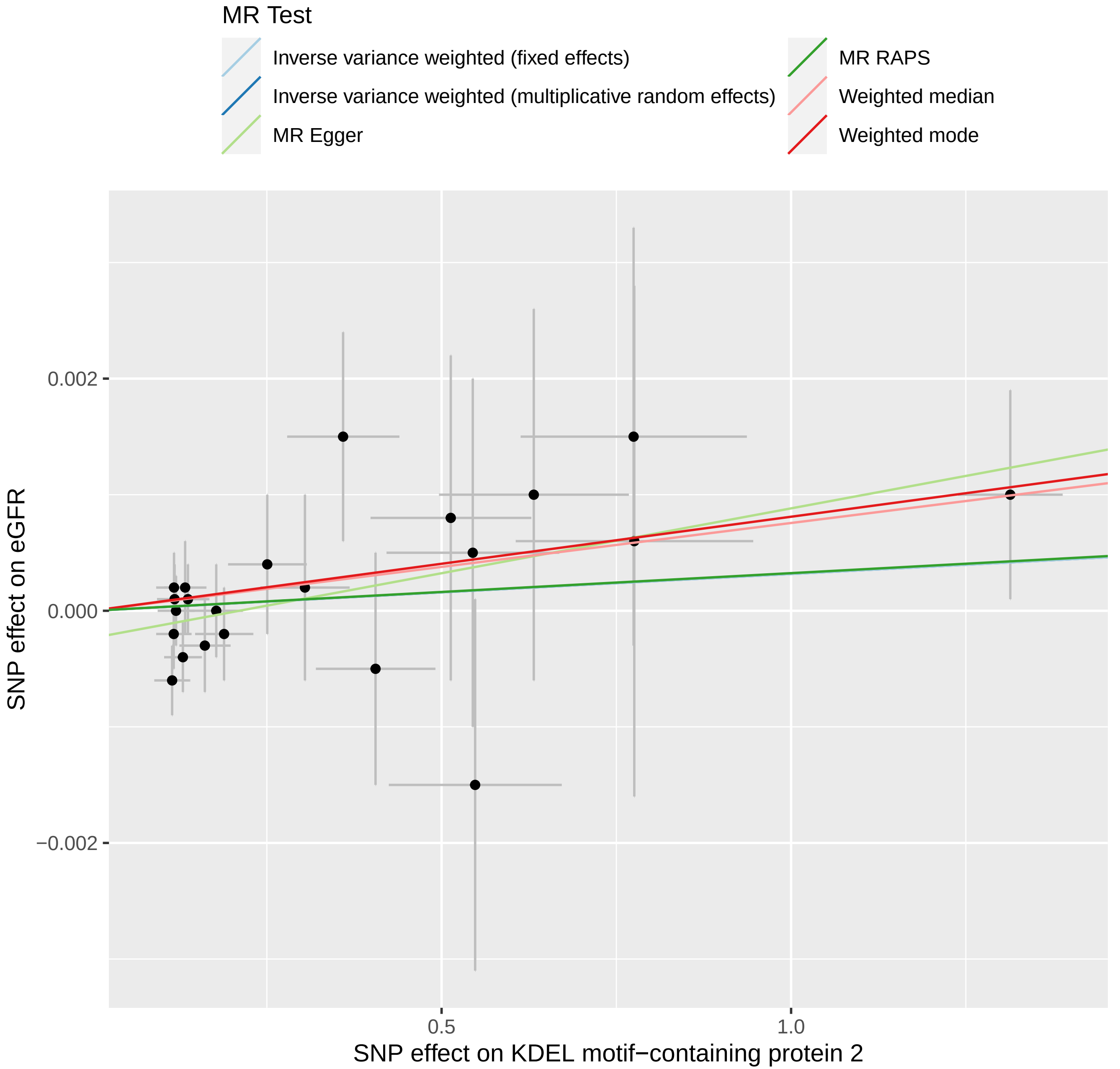

Batch 2−1622 : Vascular endothelial growth factor receptor 2 on eGFR

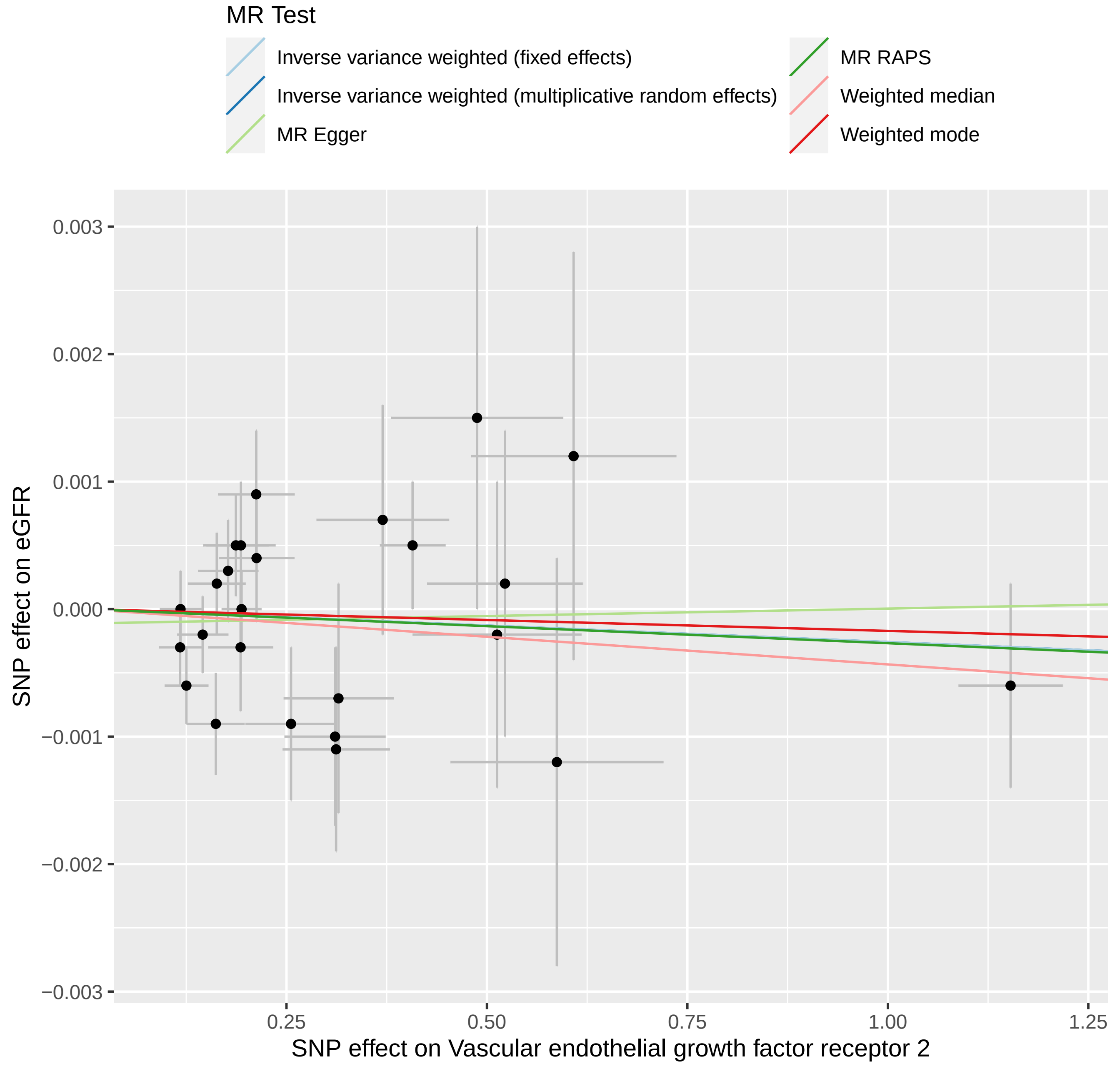

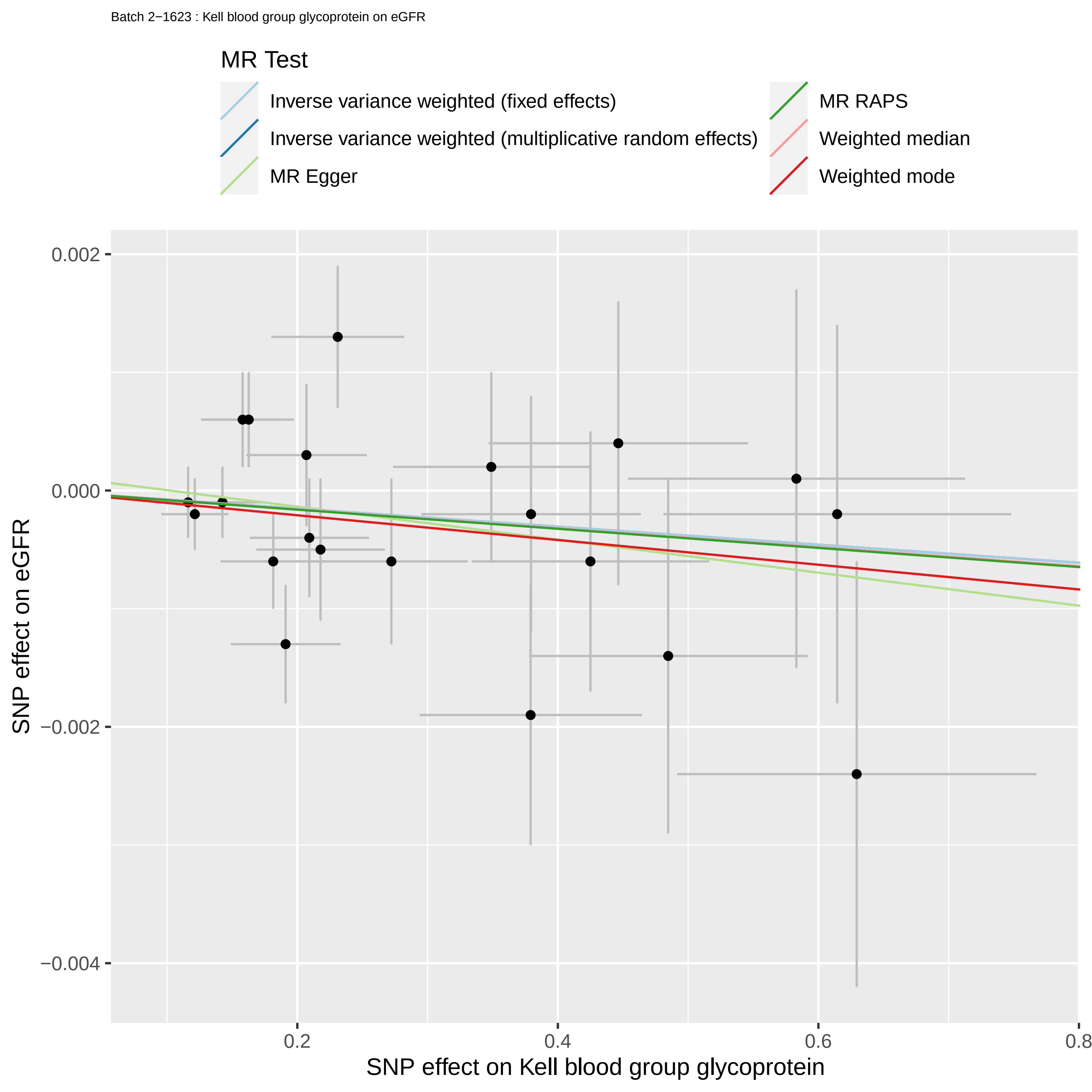

Batch 2−1623 : Kell blood group glycoprotein on eGFR
MR Test
Inverse variance weighted (fixed effects)
Inverse variance weighted (multiplicative random effects)
MR Egger
MR RAPS
Weighted median
Weighted mode
0.002
0.000
−0.002
−0.004
SNP effect on eGFR
0.2
0.4
0.6
0.8
SNP effect on Kell blood group glycoprotein

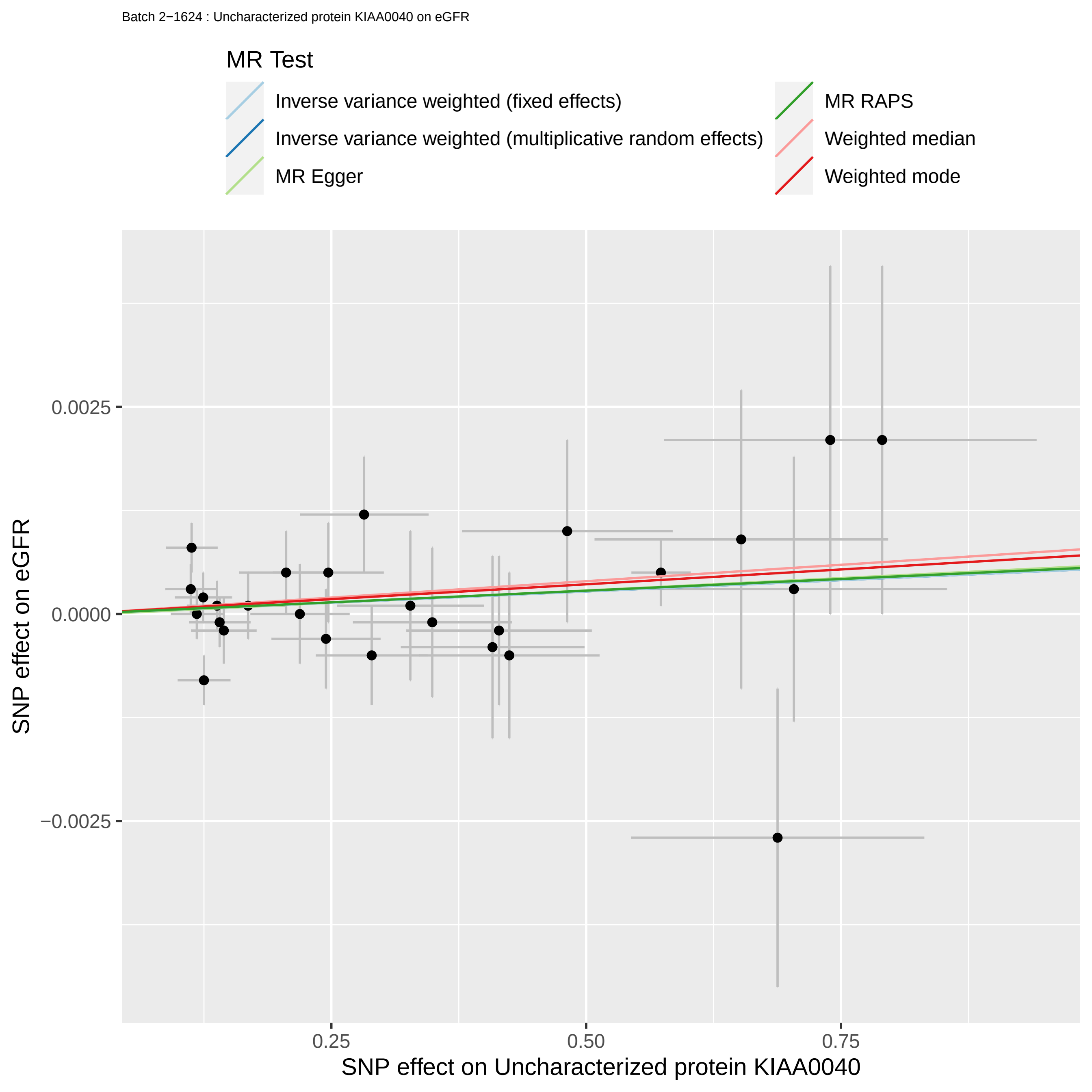

Batch 2−1624 : Uncharacterized protein KIAA0040 on eGFR
MR Test
Inverse variance weighted (fixed effects)
Inverse variance weighted (multiplicative random effects)
MR Egger
MR RAPS
Weighted median
Weighted mode
SNP effect on eGFR
0.0025
0.0000
−0.0025
0.25
0.50
0.75
SNP effect on Uncharacterized protein KIAA0040

Batch 2−1625 : Dyslexia−associated protein KIAA0319 on eGFR

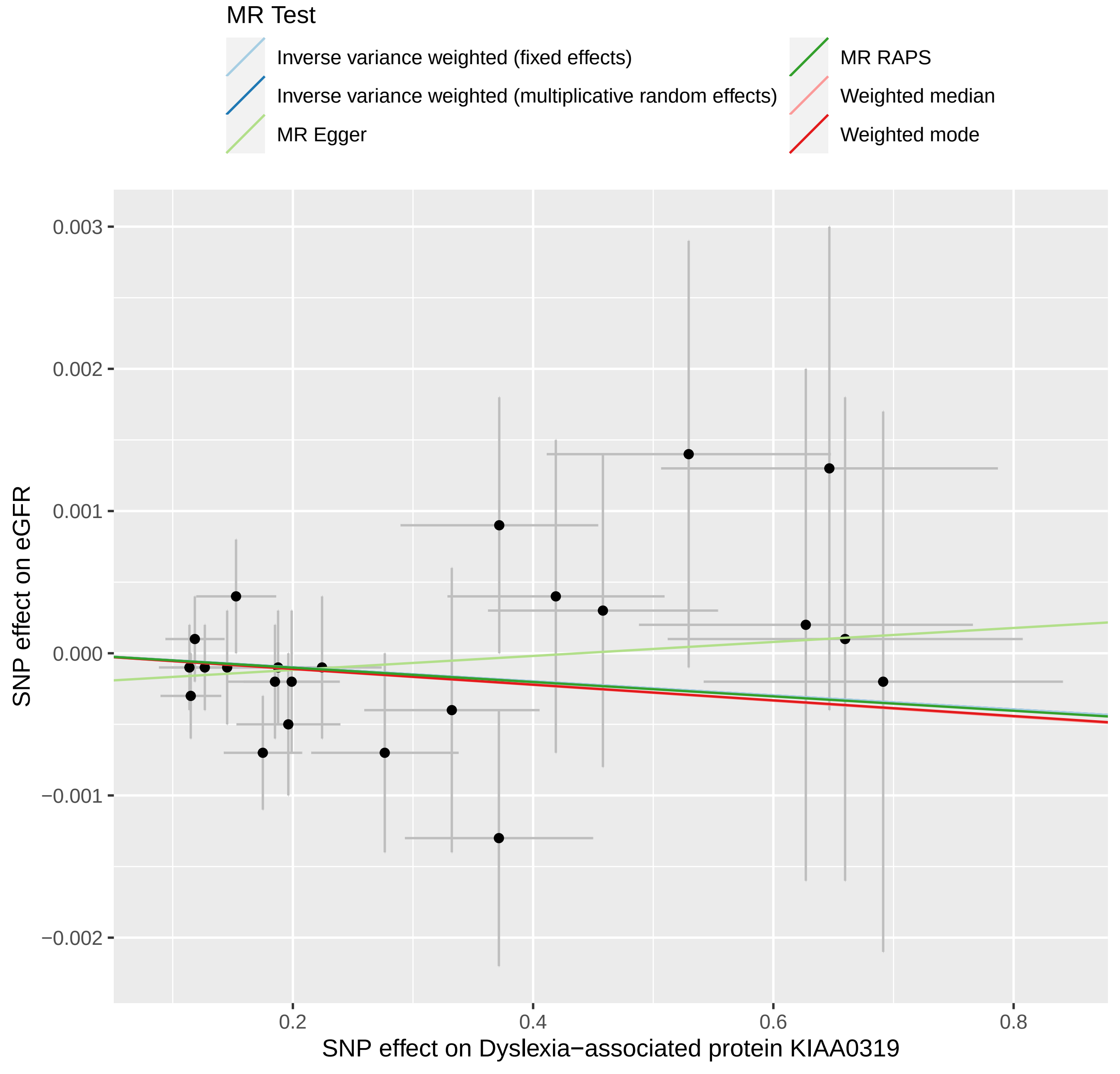

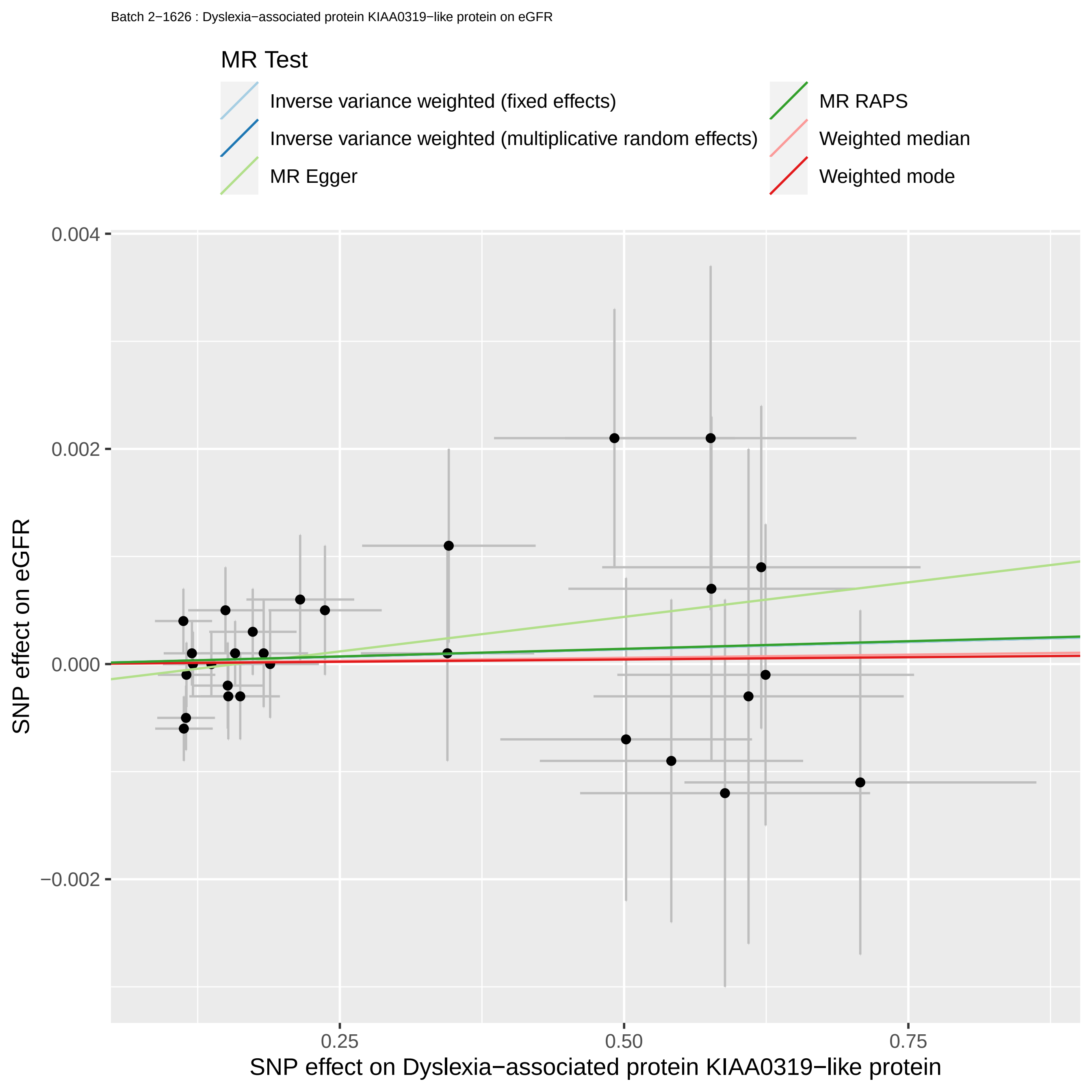
Batch 2−1626 : Dyslexia−associated protein KIAA0319−like protein on eGFR
MR Test
Inverse variance weighted (fixed effects)
Inverse variance weighted (multiplicative random effects)
MR Egger
MR RAPS
Weighted median
Weighted mode
0.004
0.002
0.000
−0.002
0.25
0.50
0.75
SNP effect on eGFR
SNP effect on Dyslexia−associated protein KIAA0319−like protein

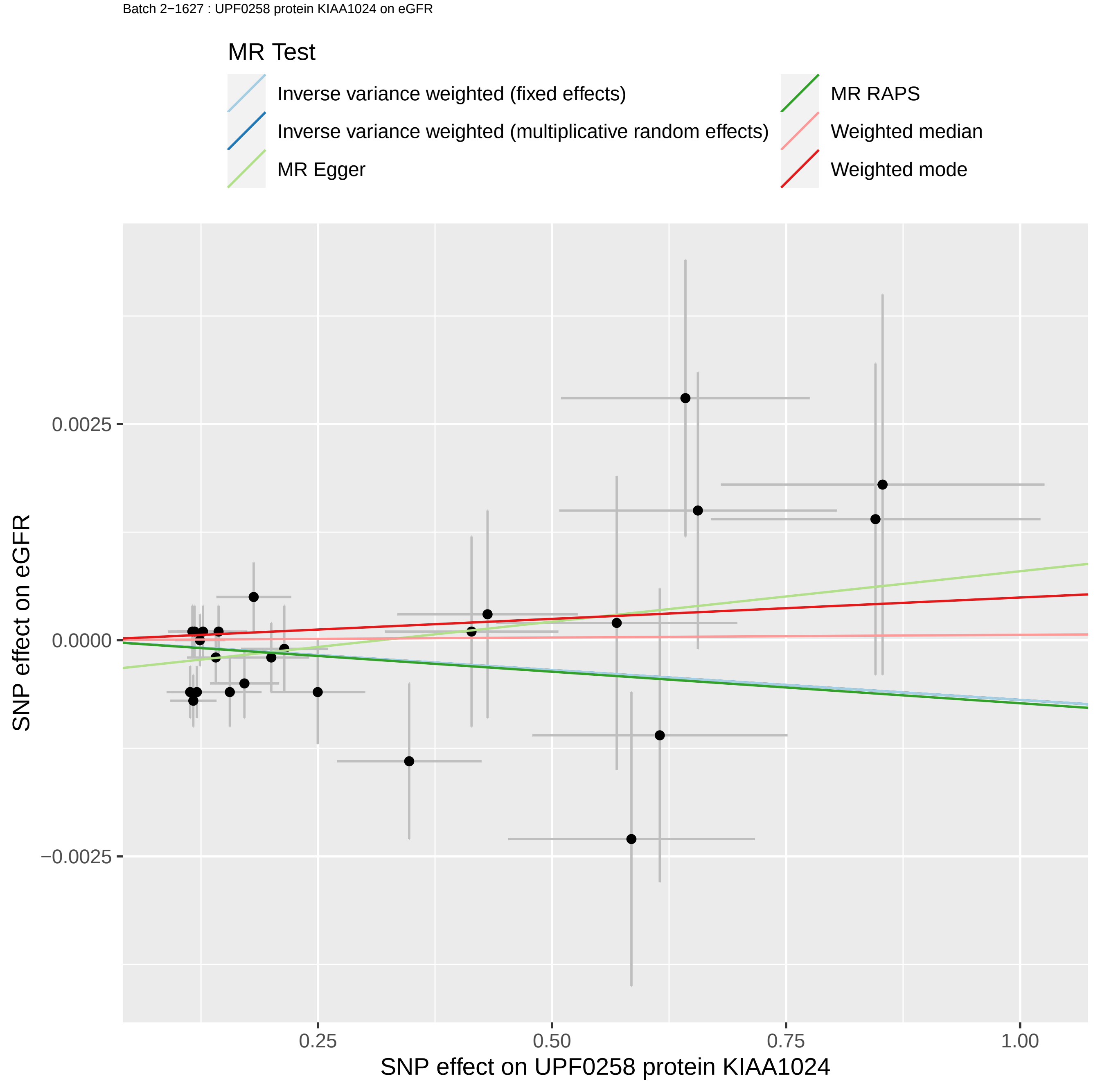

Batch 2−1627 : UPF0258 protein KIAA1024 on eGFR
MR Test
Inverse variance weighted (fixed effects)
Inverse variance weighted (multiplicative random effects)
MR Egger
MR RAPS
Weighted median
Weighted mode
0.0025
0.0000
−0.0025
0.25
0.50
0.75
1.00
SNP effect on eGFR
SNP effect on UPF0258 protein KIAA1024

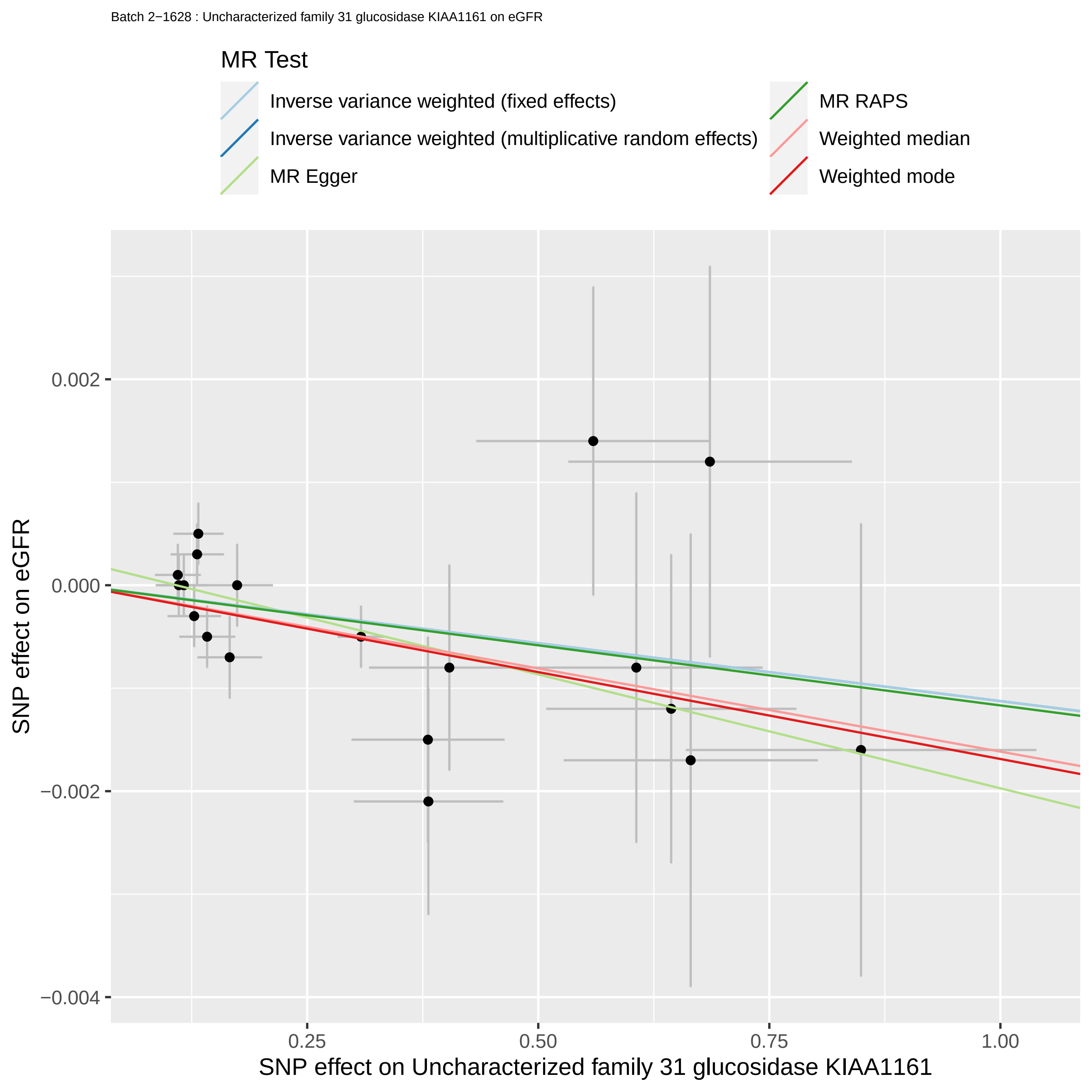

Batch 2−1628 : Uncharacterized family 31 glucosidase KIAA1161 on eGFR
MR Test
Inverse variance weighted (fixed effects)
Inverse variance weighted (multiplicative random effects)
MR Egger
MR RAPS
Weighted median
Weighted mode
0.002
0.000
−0.002
−0.004
0.25
0.50
0.75
1.00
SNP effect on eGFR
SNP effect on Uncharacterized family 31 glucosidase KIAA1161

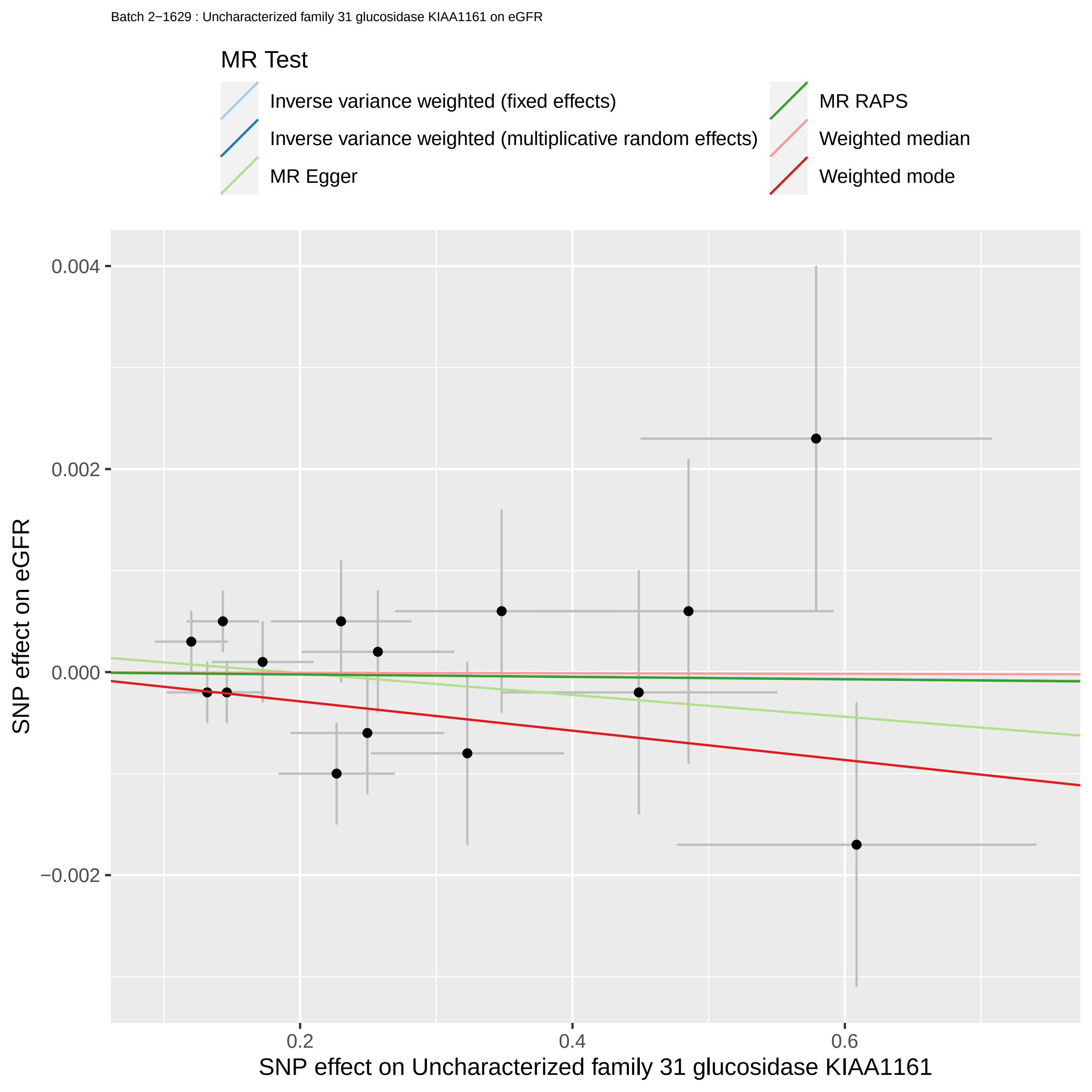

Batch 2−1629 : Uncharacterized family 31 glucosidase KIAA1161 on eGFR
MR Test
Inverse variance weighted (fixed effects)
Inverse variance weighted (multiplicative random effects)
MR Egger
MR RAPS
Weighted median
Weighted mode
0.004
0.002
0.000
−0.002
SNP effect on eGFR
0.2
0.4
0.6
SNP effect on Uncharacterized family 31 glucosidase KIAA1161

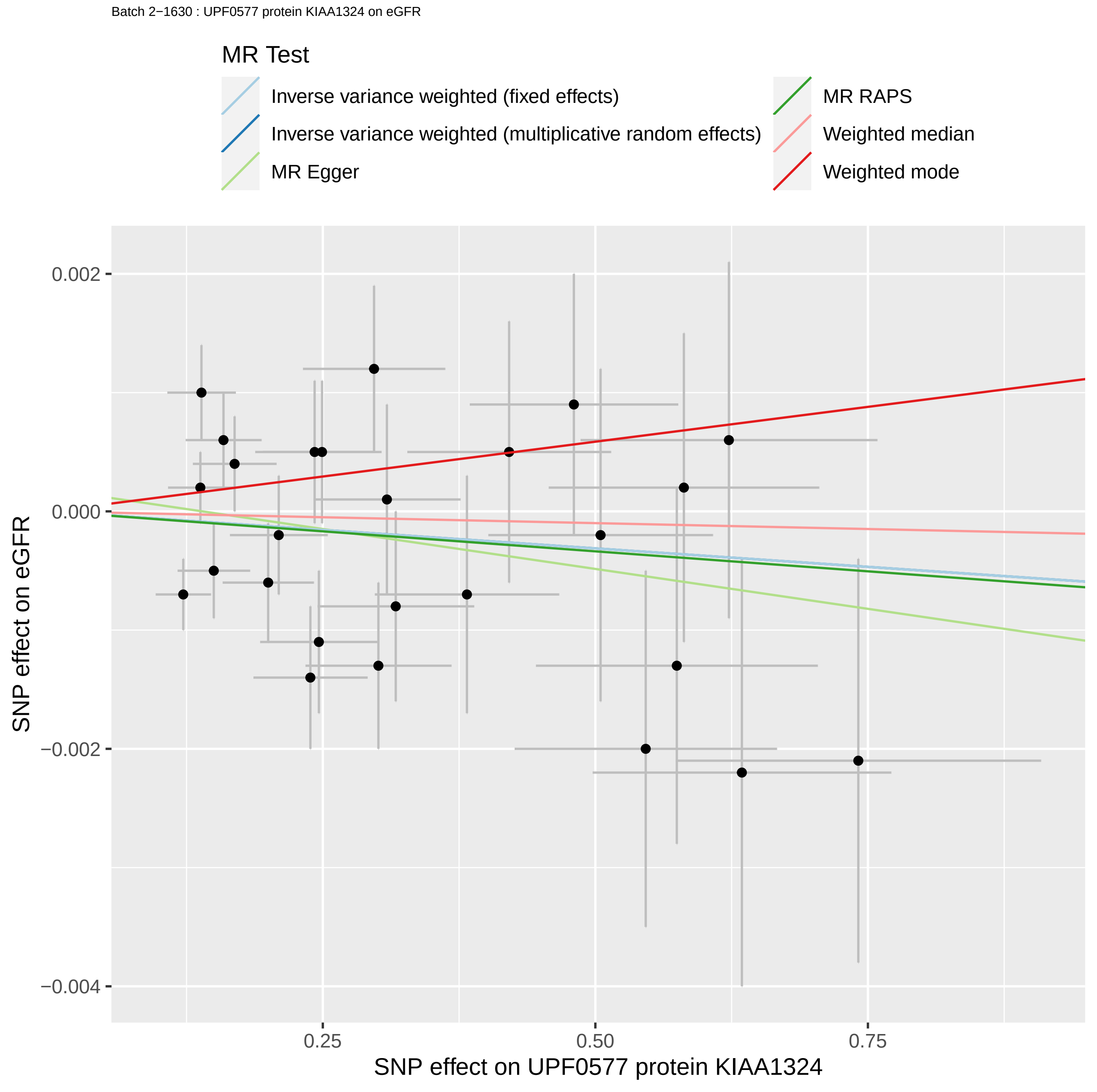
Batch 2−1630 : UPF0577 protein KIAA1324 on eGFR
MR Test
Inverse variance weighted (fixed effects)
Inverse variance weighted (multiplicative random effects)
MR Egger
MR RAPS
Weighted median
Weighted mode
0.002
0.000
−0.002
−0.004
0.25
0.50
0.75
SNP effect on eGFR
SNP effect on UPF0577 protein KIAA1324

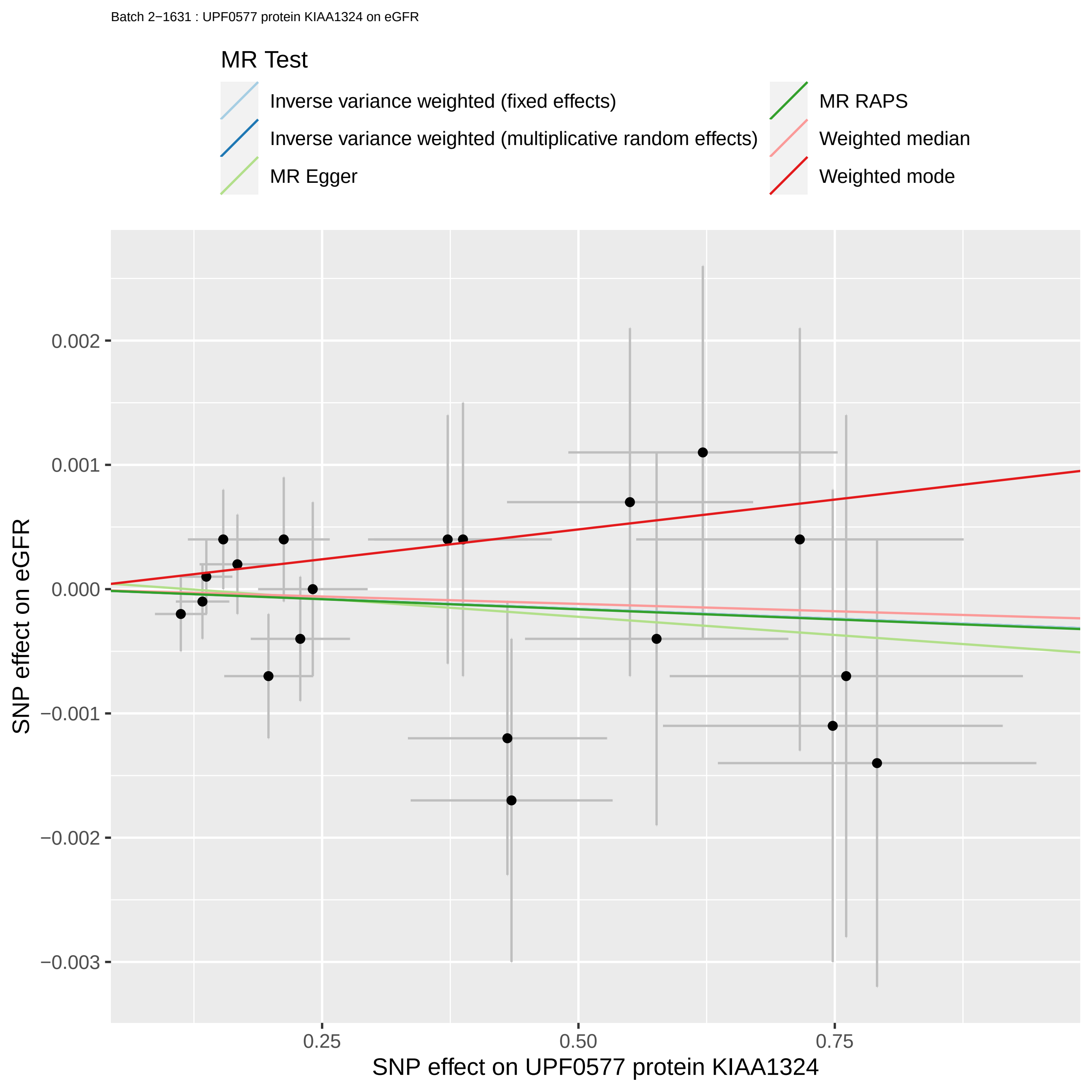
Batch 2−1631 : UPF0577 protein KIAA1324 on eGFR
MR Test
Inverse variance weighted (fixed effects)
Inverse variance weighted (multiplicative random effects)
MR Egger
MR RAPS
Weighted median
Weighted mode
0.002
0.001
0.000
−0.001
−0.002
−0.003
0.25
0.50
0.75
SNP effect on eGFR
SNP effect on UPF0577 protein KIAA1324

Batch 2−1632 : UPF0577 protein KIAA1324−like on eGFR

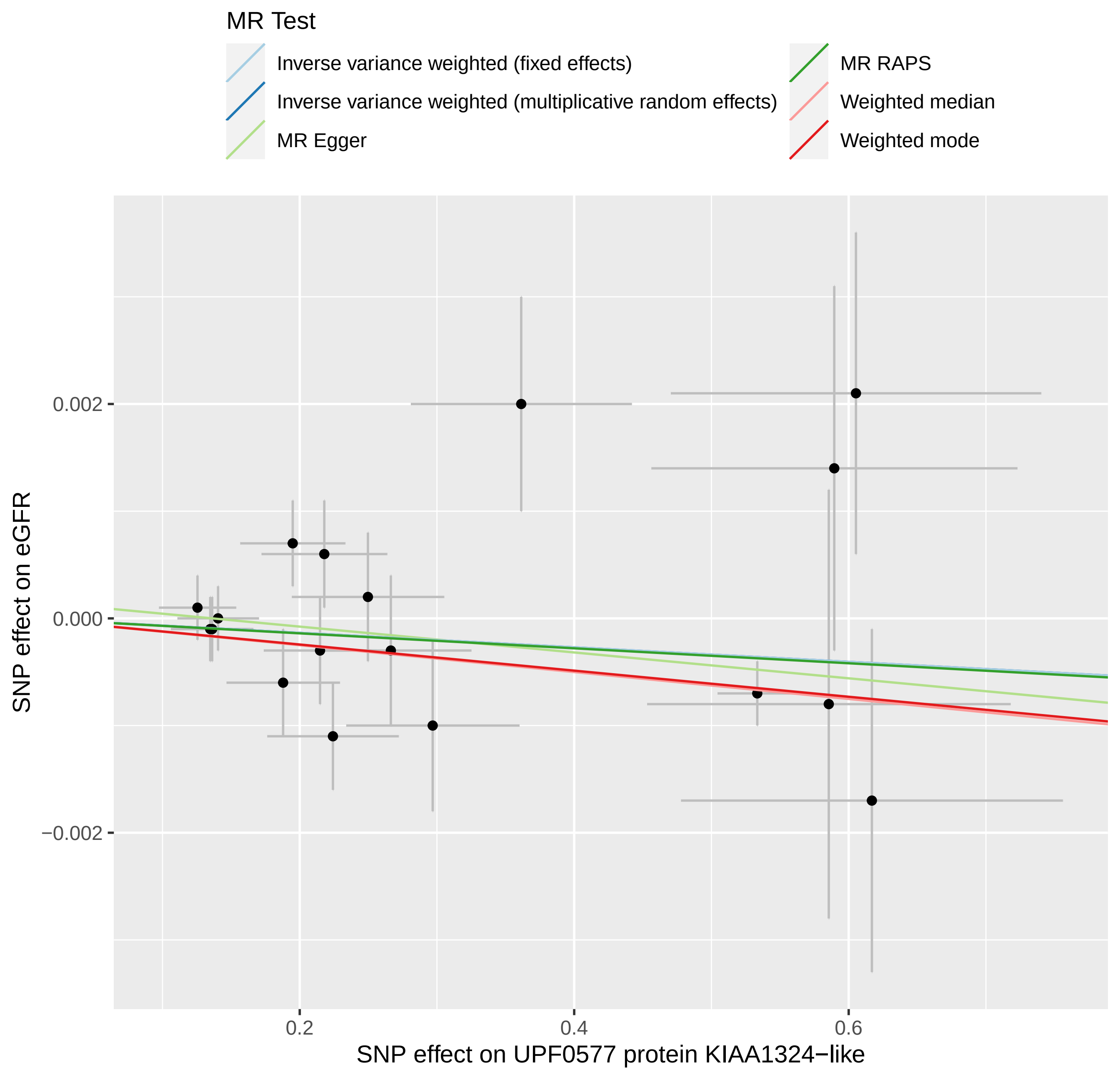

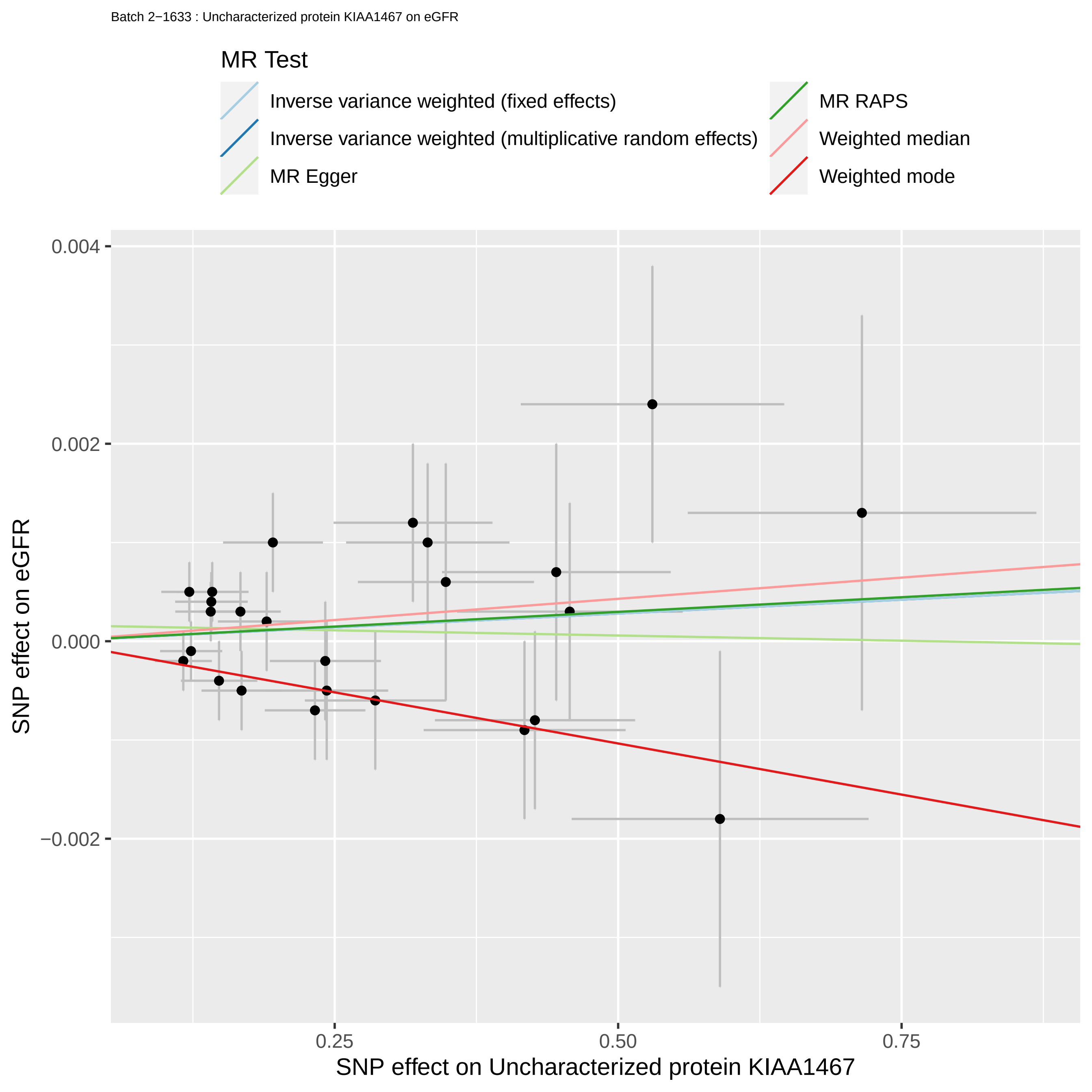
Batch 2−1633 : Uncharacterized protein KIAA1467 on eGFR
MR Test
Inverse variance weighted (fixed effects)
Inverse variance weighted (multiplicative random effects)
MR Egger
MR RAPS
Weighted median
Weighted mode
0.004
0.002
0.000
−0.002
0.25
0.50
0.75
SNP effect on eGFR
SNP effect on Uncharacterized protein KIAA1467

Batch 2−1634 : Uncharacterized protein KIAA2013 on eGFR

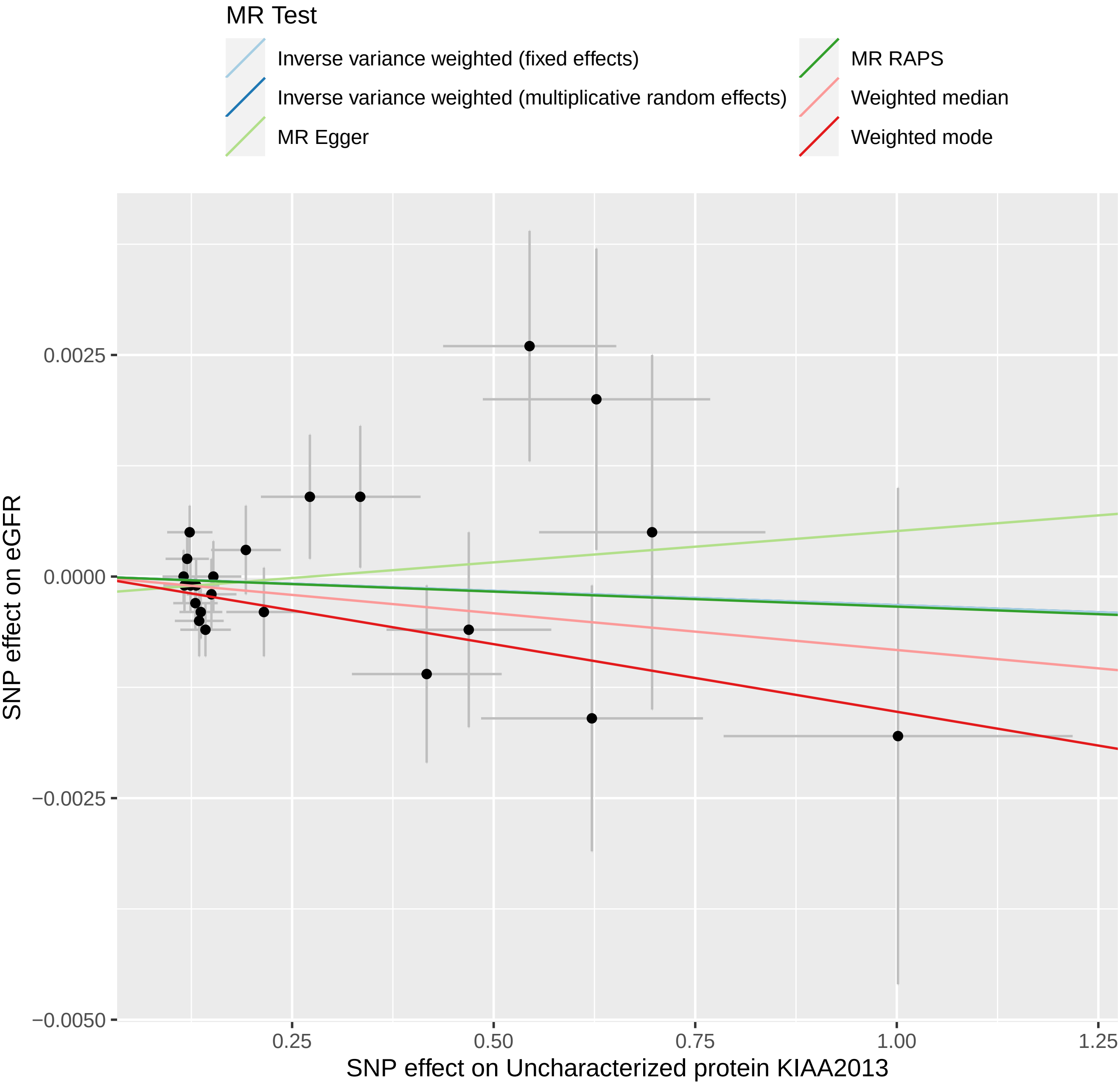

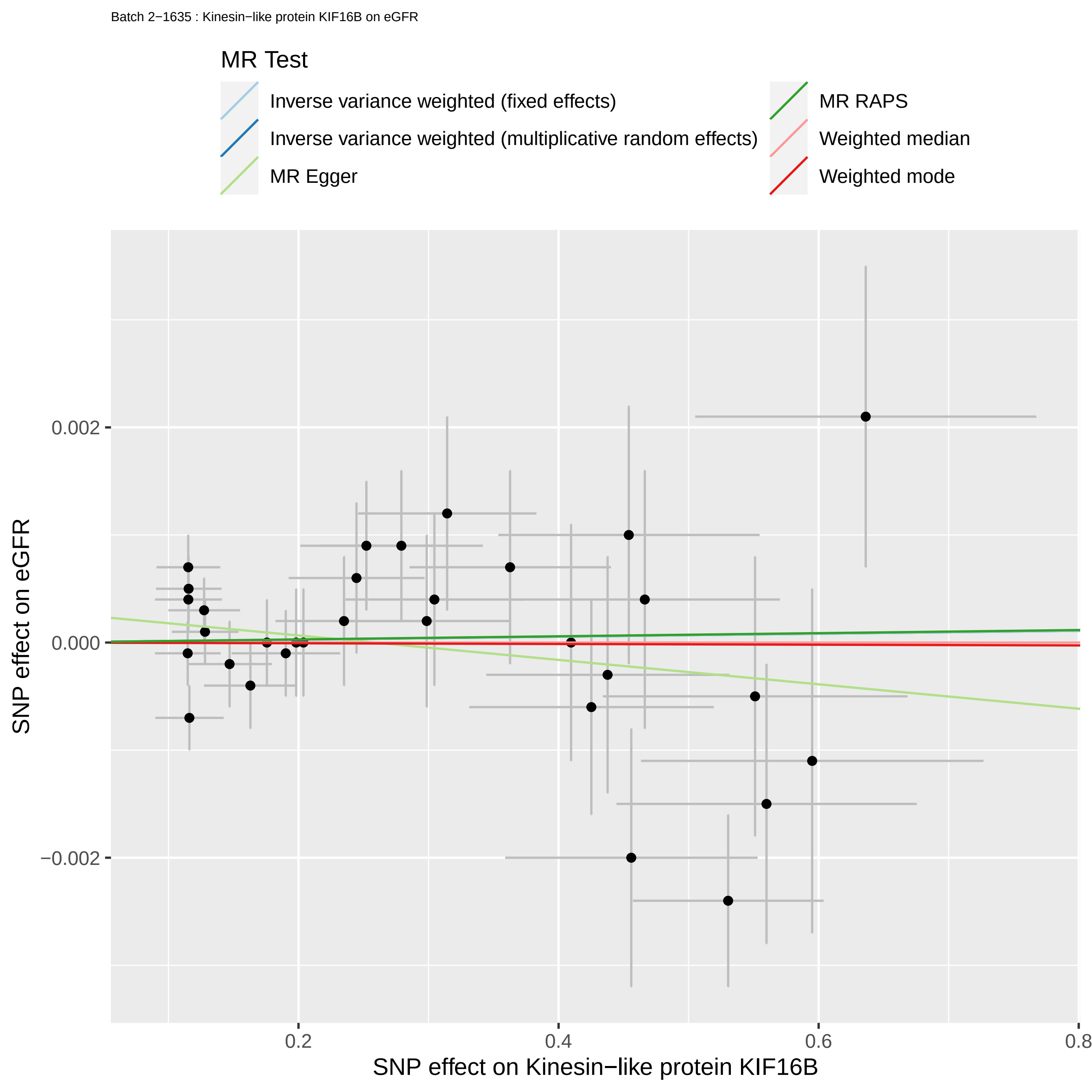

Batch 2−1635 : Kinesin−like protein KIF16B on eGFR
MR Test
Inverse variance weighted (fixed effects)
Inverse variance weighted (multiplicative random effects)
MR Egger
MR RAPS
Weighted median
Weighted mode
0.002
0.000
−0.002
0.2
0.4
0.6
0.8
SNP effect on eGFR
SNP effect on Kinesin−like protein KIF16B

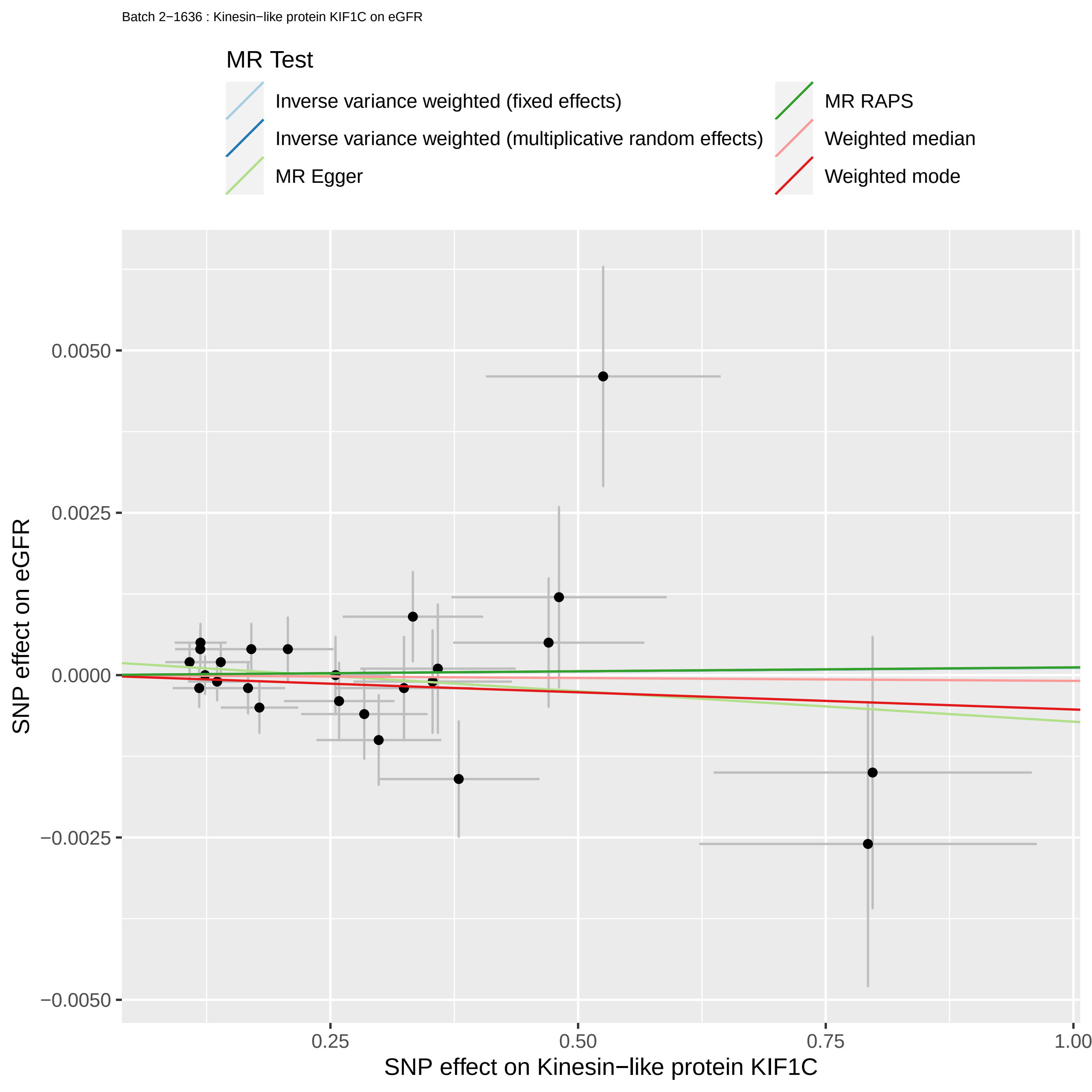
Batch 2−1636 : Kinesin−like protein KIF1C on eGFR
MR Test
Inverse variance weighted (fixed effects)
Inverse variance weighted (multiplicative random effects)
MR Egger
MR RAPS
Weighted median
Weighted mode
0.0050
0.0025
0.0000
−0.0025
−0.0050
SNP effect on eGFR
0.25
0.50
0.75
1.00
SNP effect on Kinesin−like protein KIF1C

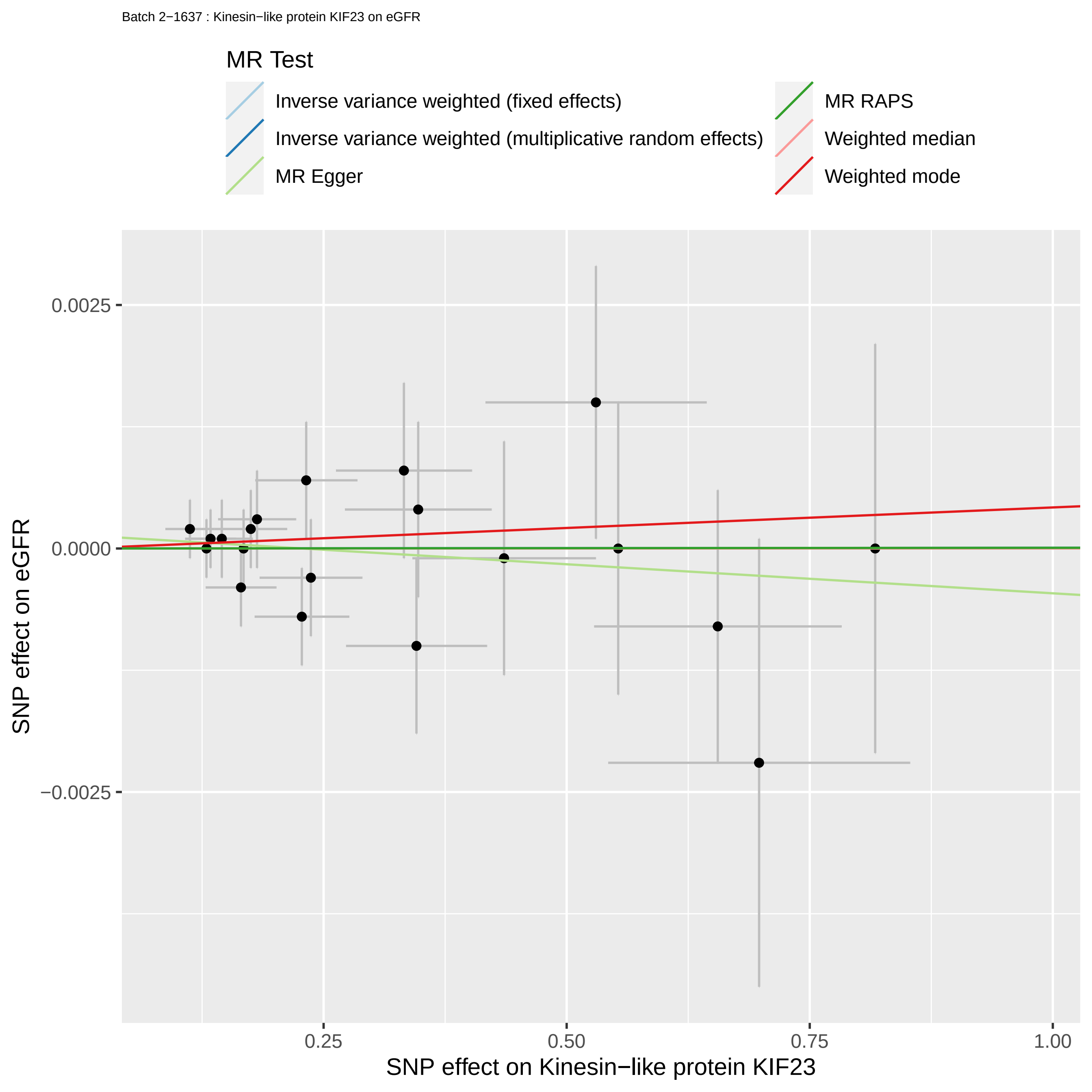

Batch 2−1637 : Kinesin−like protein KIF23 on eGFR
MR Test
Inverse variance weighted (fixed effects)
Inverse variance weighted (multiplicative random effects)
MR Egger
MR RAPS
Weighted median
Weighted mode
0.0025
0.0000
−0.0025
0.25
0.50
0.75
1.00
SNP effect on eGFR
SNP effect on Kinesin−like protein KIF23

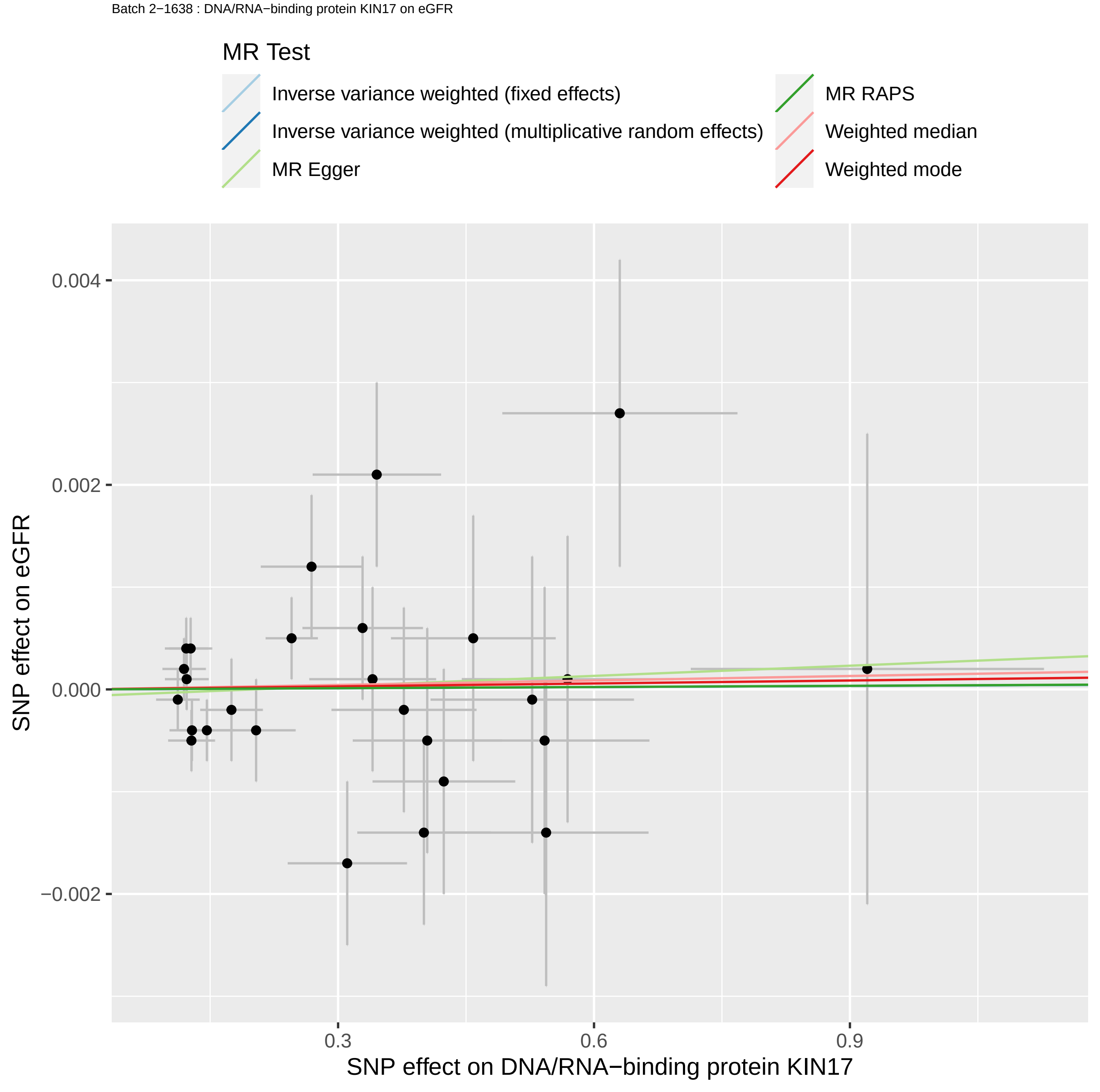

Batch 2−1638 : DNA/RNA−binding protein KIN17 on eGFR
MR Test
Inverse variance weighted (fixed effects)
Inverse variance weighted (multiplicative random effects)
MR Egger
MR RAPS
Weighted median
Weighted mode
0.004
0.002
0.000
−0.002
SNP effect on eGFR
0.3
0.6
0.9
SNP effect on DNA/RNA−binding protein KIN17

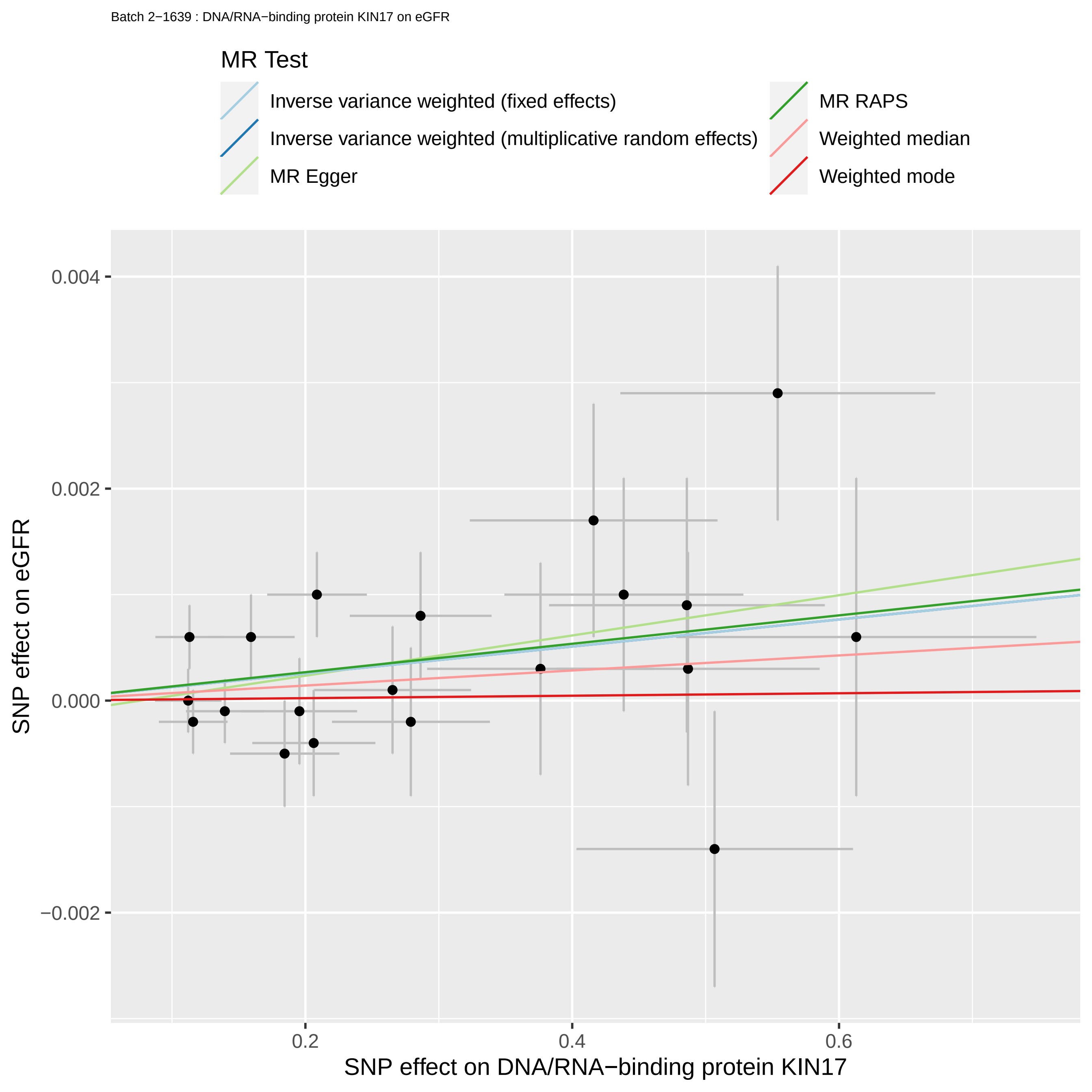

Batch 2−1639 : DNA/RNA−binding protein KIN17 on eGFR
MR Test
Inverse variance weighted (fixed effects)
Inverse variance weighted (multiplicative random effects)
MR Egger
MR RAPS
Weighted median
Weighted mode
0.004
0.002
0.000
−0.002
0.2
0.4
0.6
SNP effect on eGFR
SNP effect on DNA/RNA−binding protein KIN17

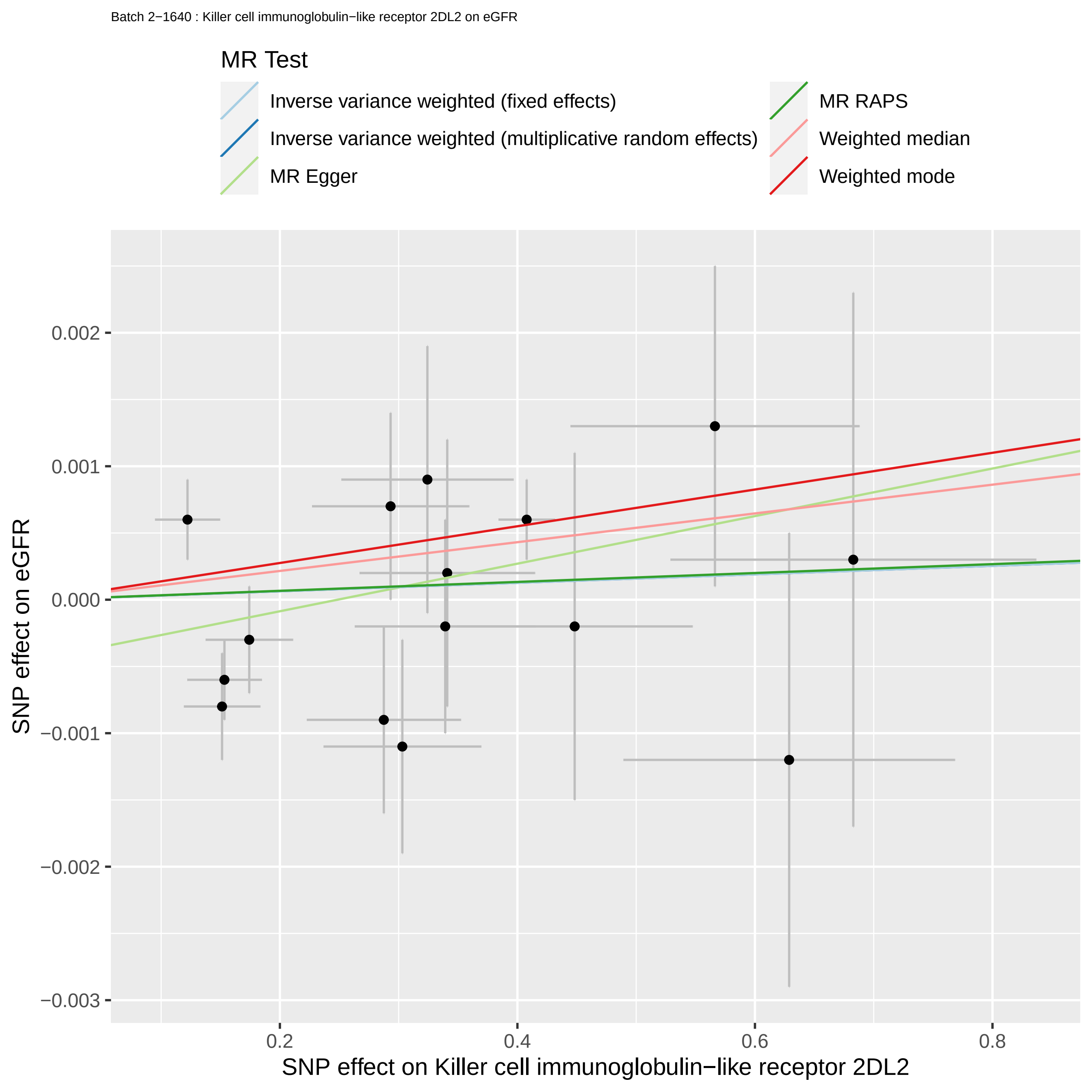
Batch 2−1640 : Killer cell immunoglobulin−like receptor 2DL2 on eGFR
MR Test
Inverse variance weighted (fixed effects)
Inverse variance weighted (multiplicative random effects)
MR Egger
MR RAPS
Weighted median
Weighted mode
0.002
0.001
0.000
−0.001
−0.002
−0.003
0.2
0.4
0.6
0.8
SNP effect on eGFR
SNP effect on Killer cell immunoglobulin−like receptor 2DL2

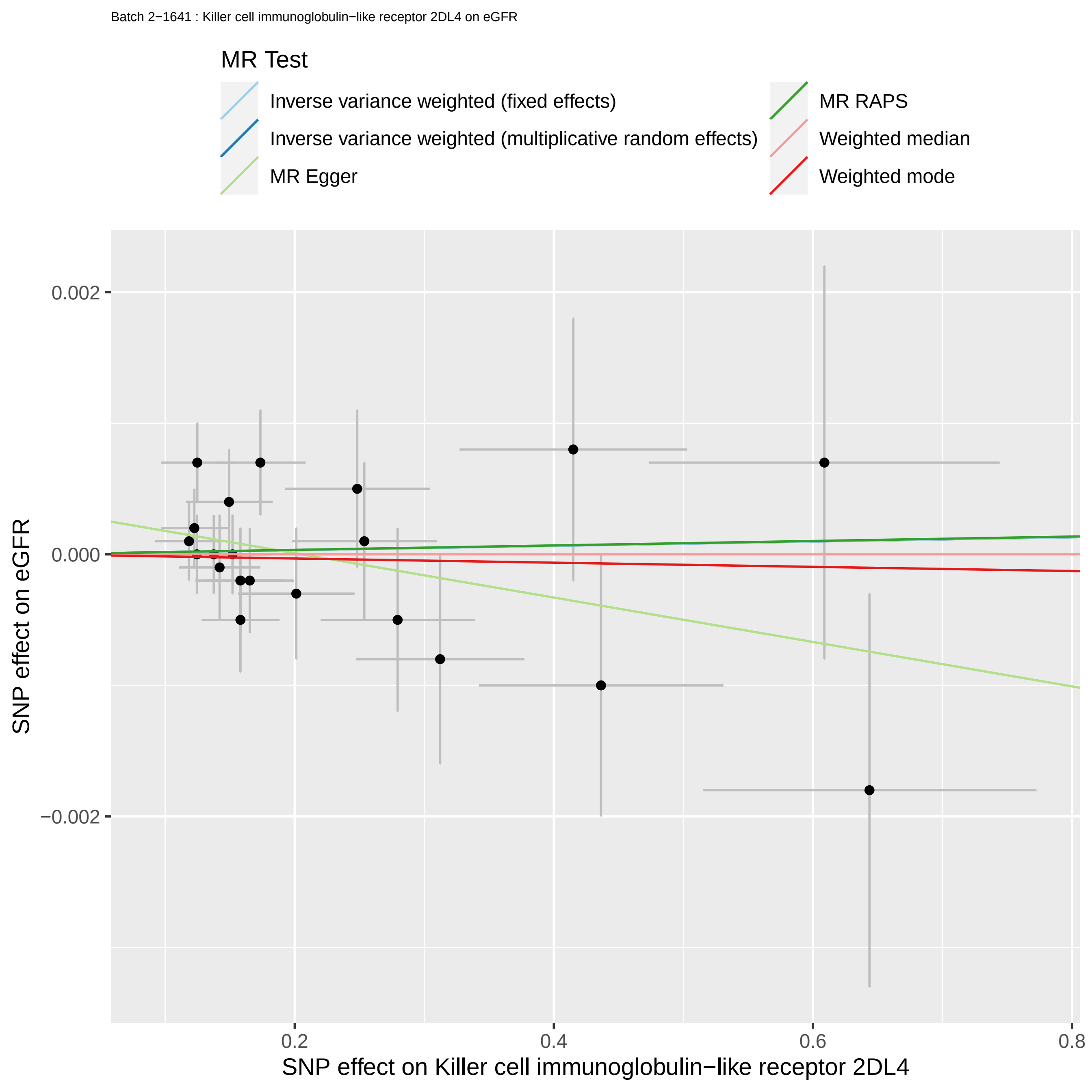

Batch 2−1641 : Killer cell immunoglobulin−like receptor 2DL4 on eGFR
MR Test
Inverse variance weighted (fixed effects)
Inverse variance weighted (multiplicative random effects)
MR Egger
MR RAPS
Weighted median
Weighted mode
0.002
0.000
−0.002
0.2
0.4
0.6
0.8
SNP effect on eGFR
SNP effect on Killer cell immunoglobulin−like receptor 2DL4

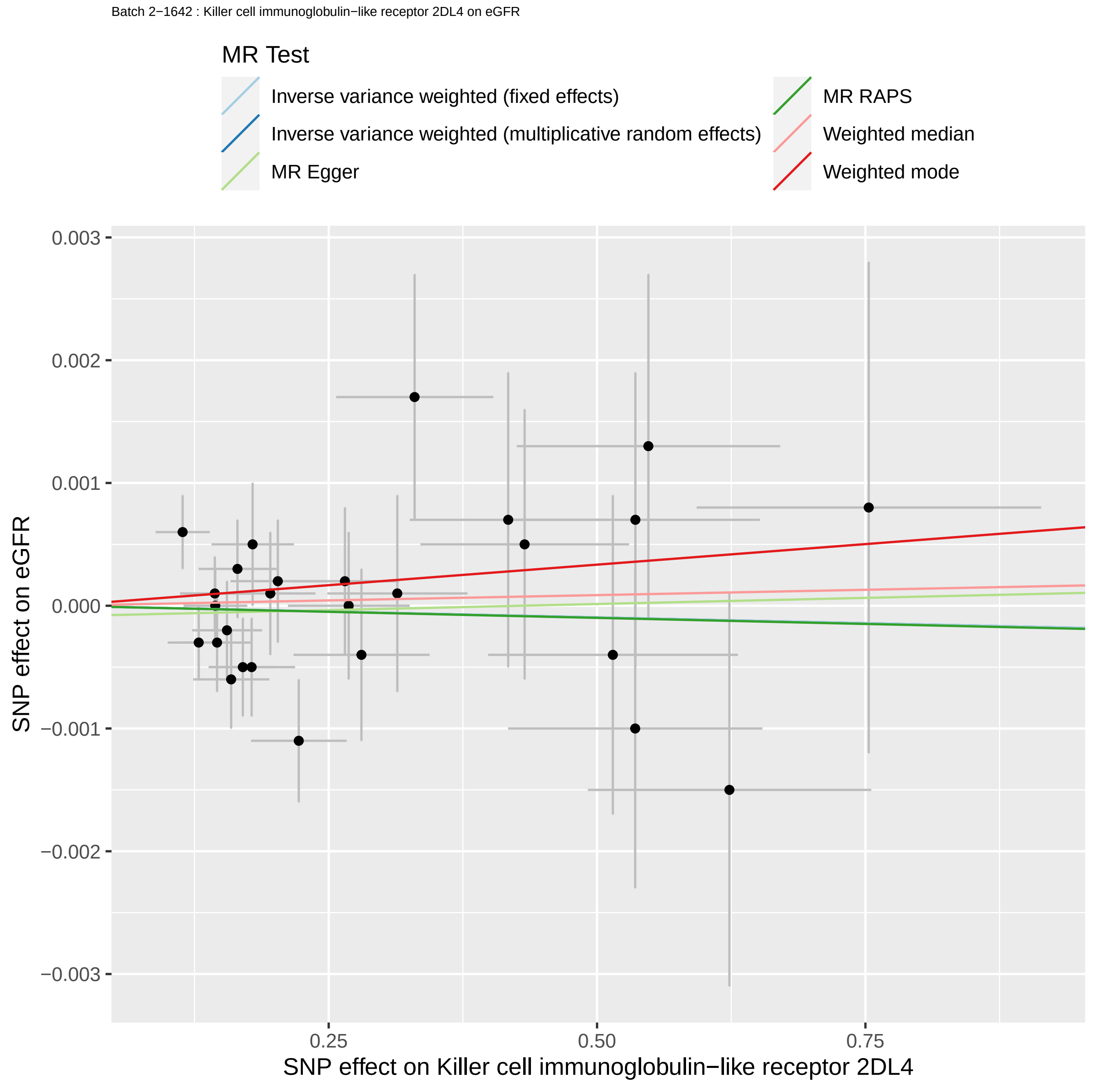

Batch 2−1642 : Killer cell immunoglobulin−like receptor 2DL4 on eGFR
MR Test
Inverse variance weighted (fixed effects)
Inverse variance weighted (multiplicative random effects)
MR Egger
MR RAPS
Weighted median
Weighted mode
0.003
0.002
0.001
0.000
−0.001
−0.002
−0.003
0.25
0.50
0.75
SNP effect on eGFR
SNP effect on Killer cell immunoglobulin−like receptor 2DL4

Batch 2−1643 : Killer cell immunoglobulin−like receptor 2DL5A on eGFR

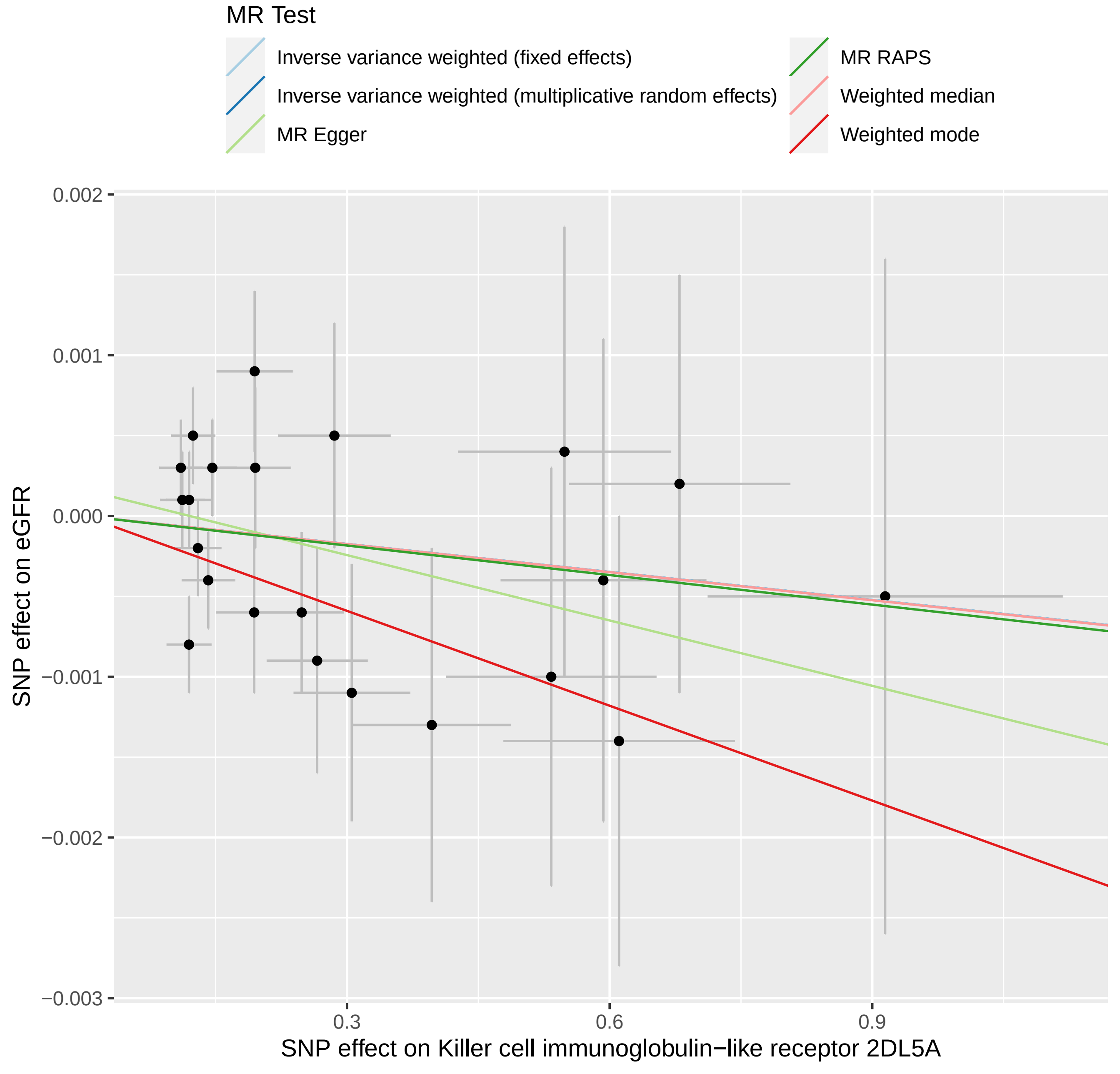

Batch 2−1644 : Killer cell immunoglobulin−like receptor 2DL5A on eGFR

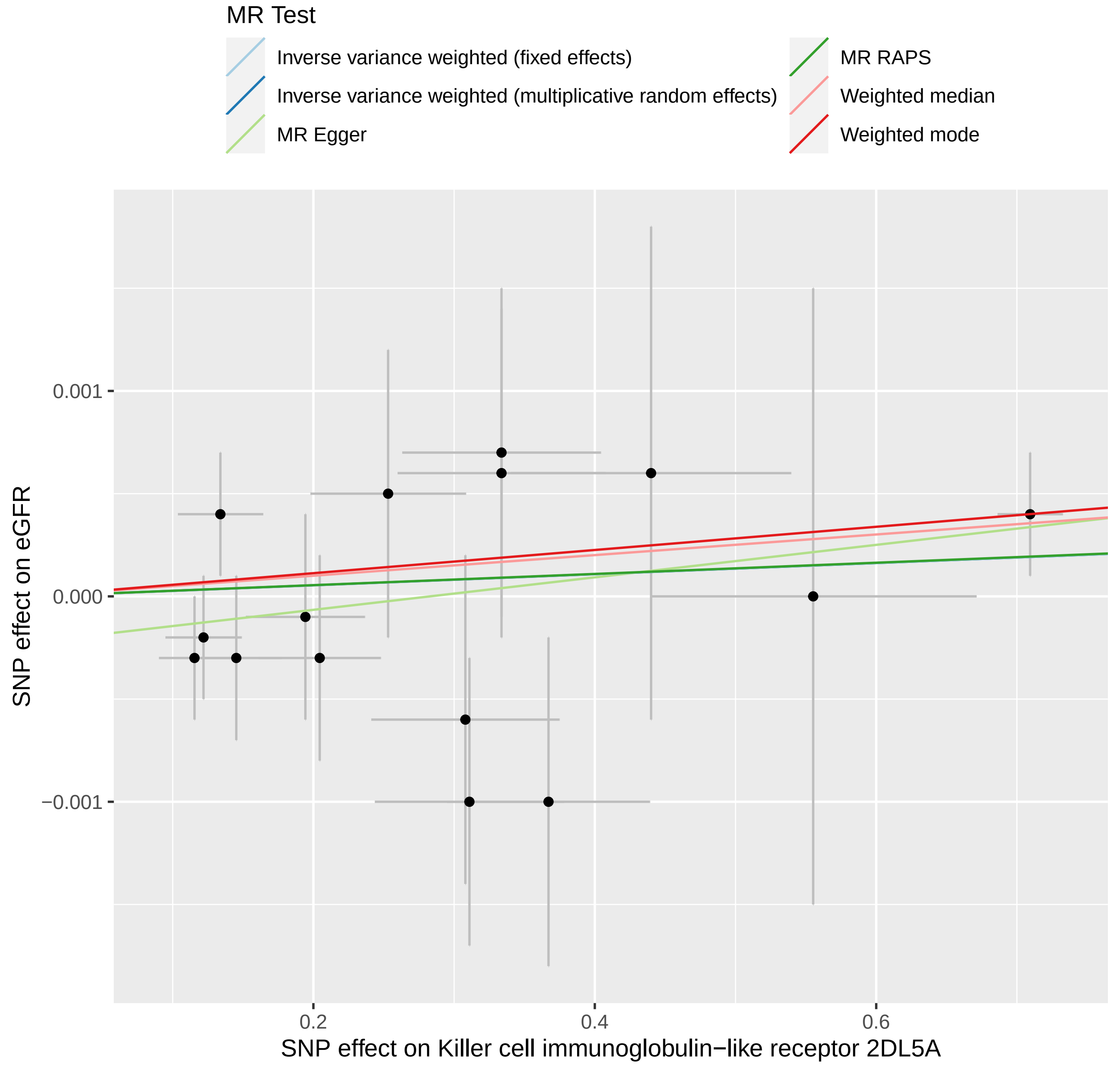

Batch 2−1645 : Killer cell immunoglobulin−like receptor 2DS2 on eGFR

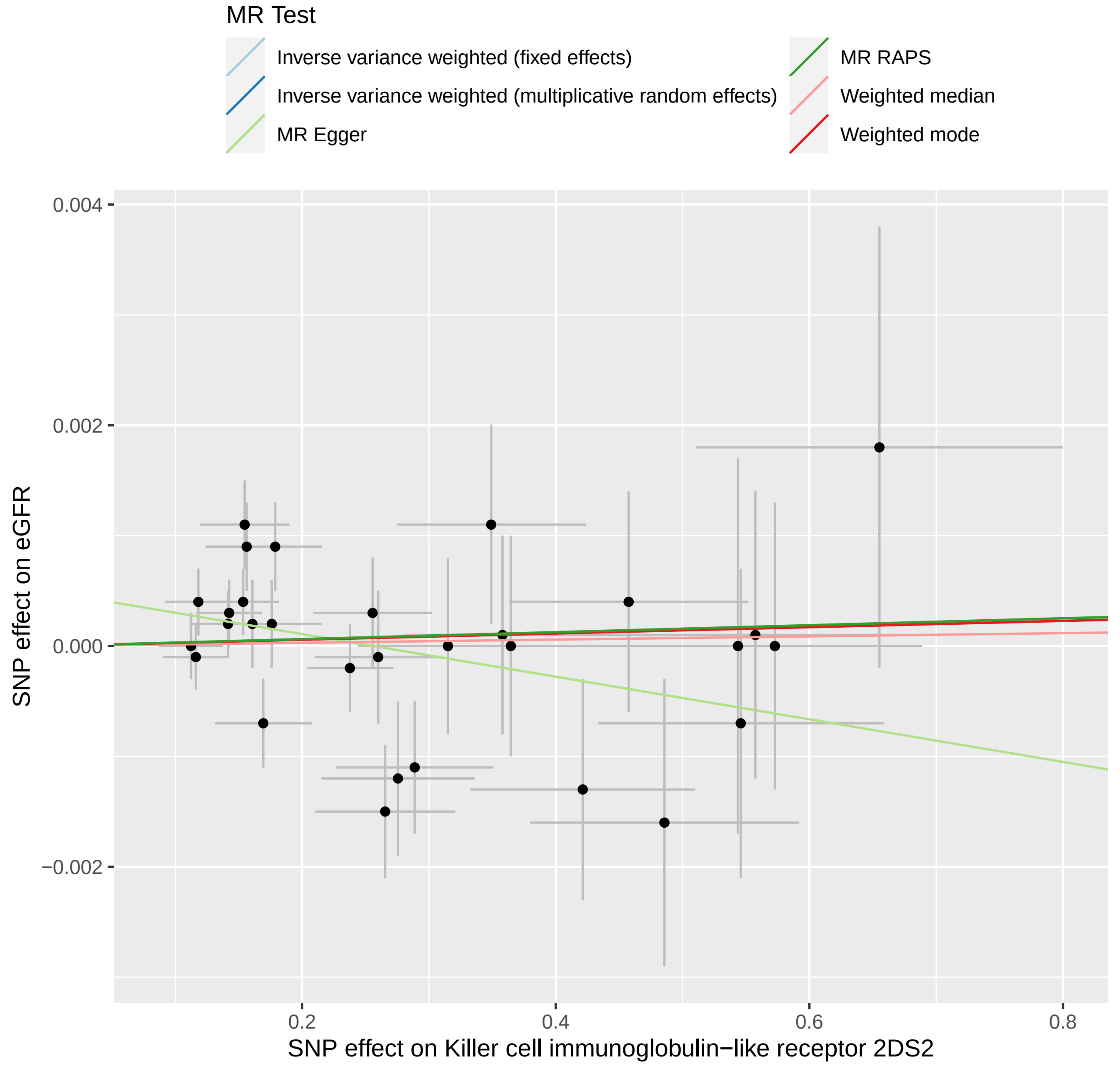

Batch 2−1646 : Killer cell immunoglobulin−like receptor 3DL2 on eGFR

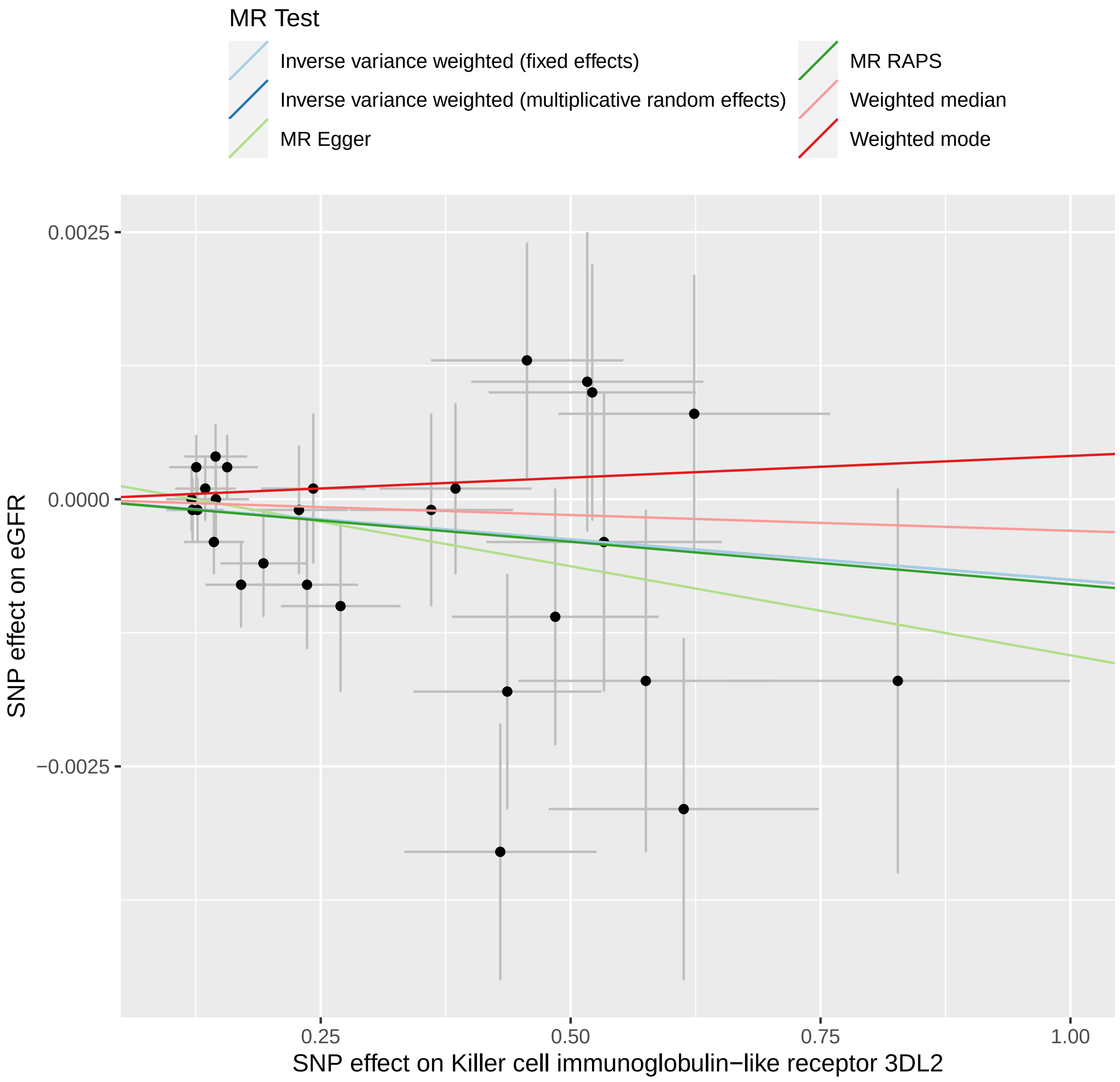

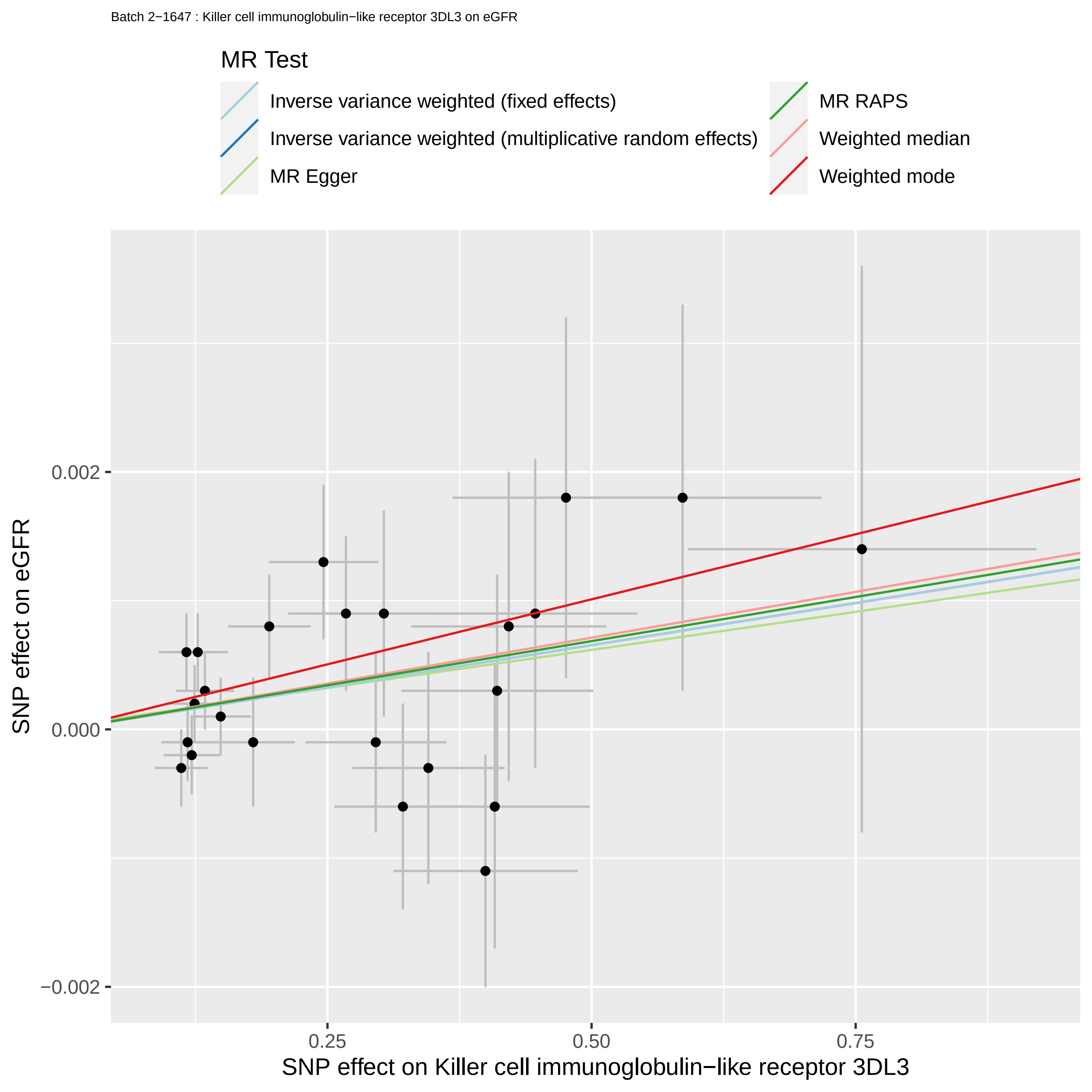

Batch 2−1647 : Killer cell immunoglobulin−like receptor 3DL3 on eGFR
MR Test
Inverse variance weighted (fixed effects)
Inverse variance weighted (multiplicative random effects)
MR Egger
MR RAPS
Weighted median
Weighted mode
0.002
0.000
−0.002
0.25
0.50
0.75
SNP effect on eGFR
SNP effect on Killer cell immunoglobulin−like receptor 3DL3

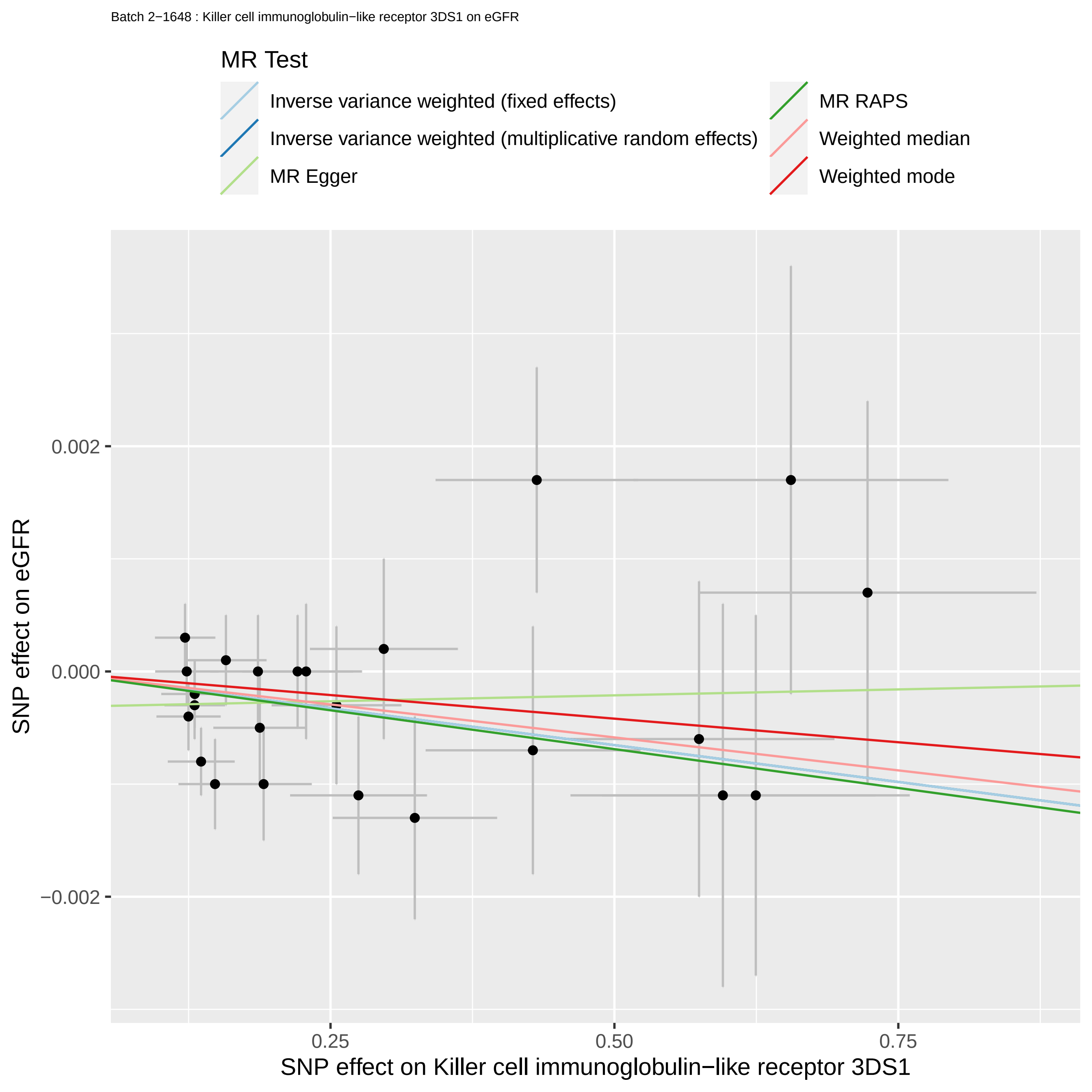

Batch 2−1648 : Killer cell immunoglobulin−like receptor 3DS1 on eGFR
MR Test
Inverse variance weighted (fixed effects)
Inverse variance weighted (multiplicative random effects)
MR Egger
MR RAPS
Weighted median
Weighted mode
0.002
0.000
−0.002
0.25
0.50
0.75
SNP effect on eGFR
SNP effect on Killer cell immunoglobulin−like receptor 3DS1

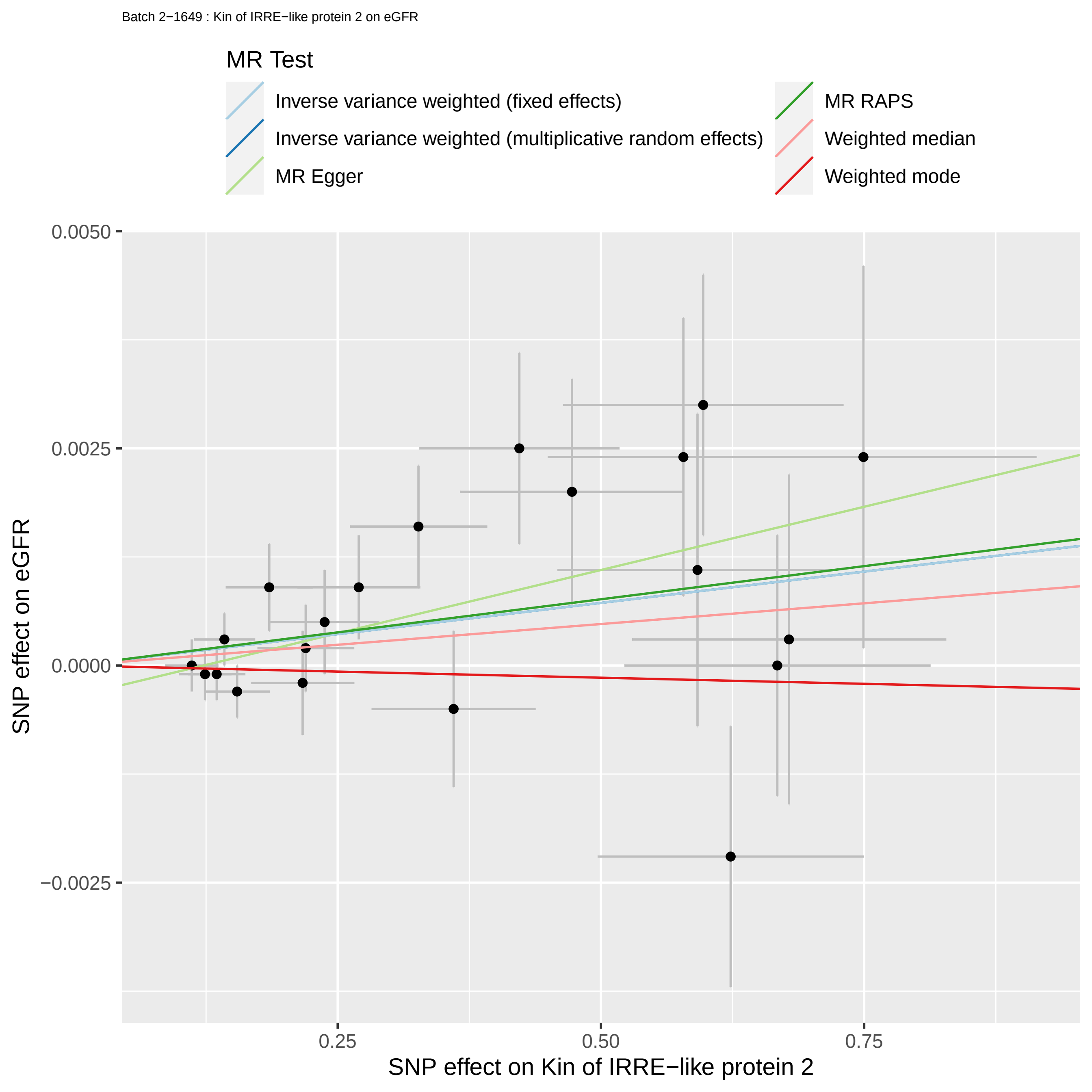

Batch 2−1649 : Kin of IRRE−like protein 2 on eGFR
MR Test
Inverse variance weighted (fixed effects)
Inverse variance weighted (multiplicative random effects)
MR Egger
MR RAPS
Weighted median
Weighted mode
0.0050
0.0025
0.0000
−0.0025
SNP effect on eGFR
0.25
0.50
0.75
SNP effect on Kin of IRRE−like protein 2

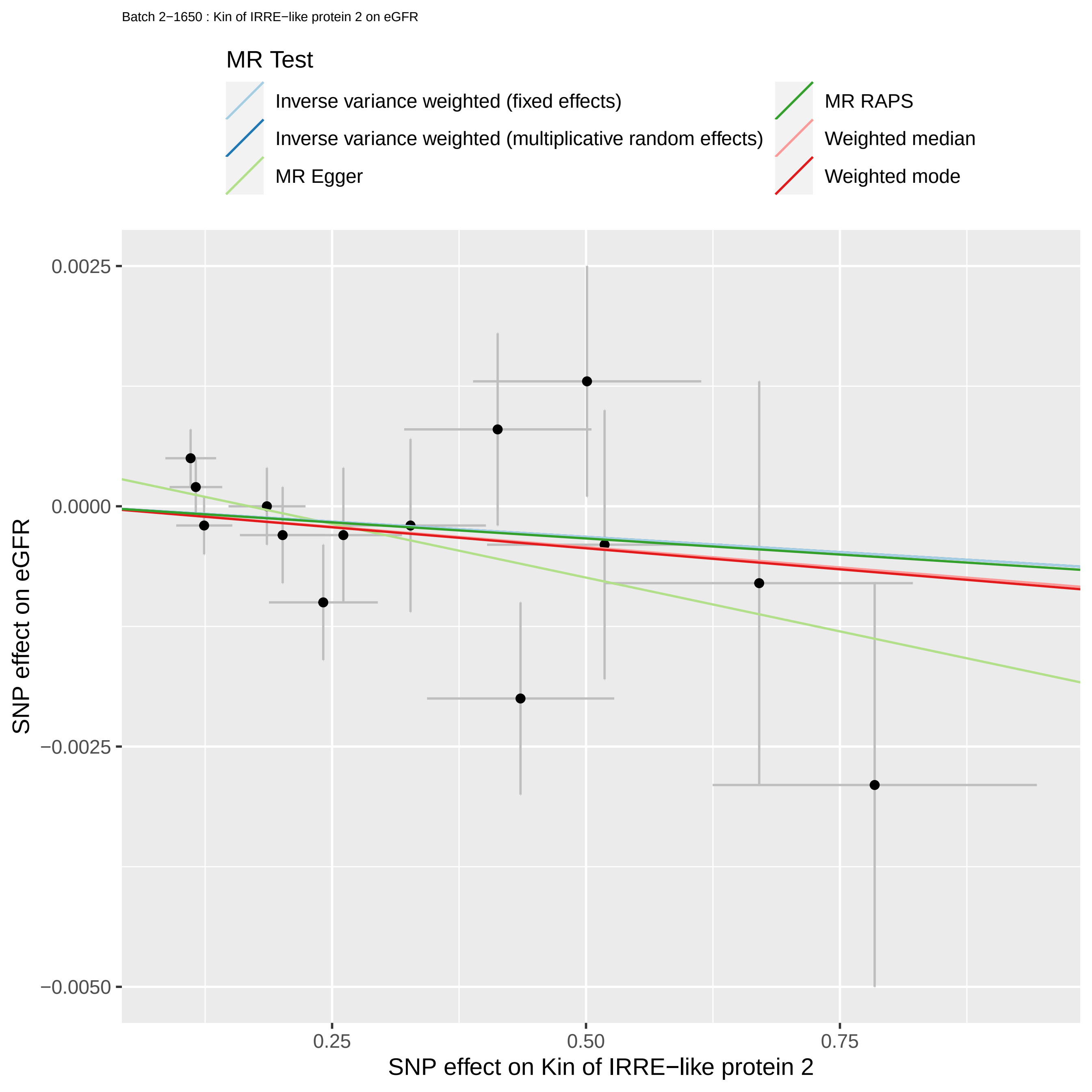

Batch 2−1650 : Kin of IRRE−like protein 2 on eGFR
MR Test
Inverse variance weighted (fixed effects)
Inverse variance weighted (multiplicative random effects)
MR Egger
MR RAPS
Weighted median
Weighted mode
0.0025
0.0000
−0.0025
−0.0050
0.25
0.50
0.75
SNP effect on eGFR
SNP effect on Kin of IRRE−like protein 2

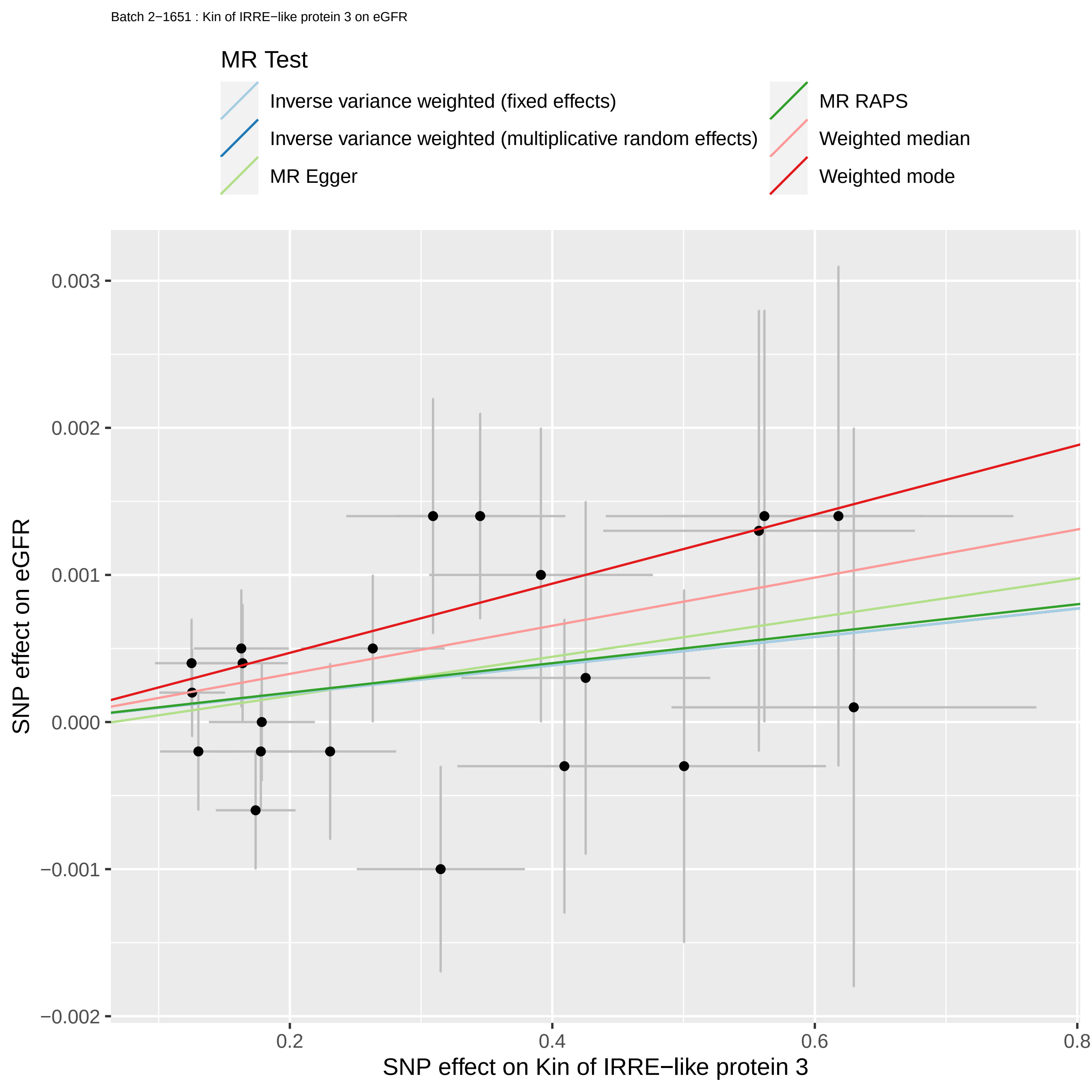

Batch 2−1651 : Kin of IRRE−like protein 3 on eGFR
MR Test
Inverse variance weighted (fixed effects)
Inverse variance weighted (multiplicative random effects)
MR Egger
MR RAPS
Weighted median
Weighted mode
0.003
0.002
0.001
0.000
−0.001
−0.002
0.2
0.4
0.6
0.8
SNP effect on eGFR
SNP effect on Kin of IRRE−like protein 3

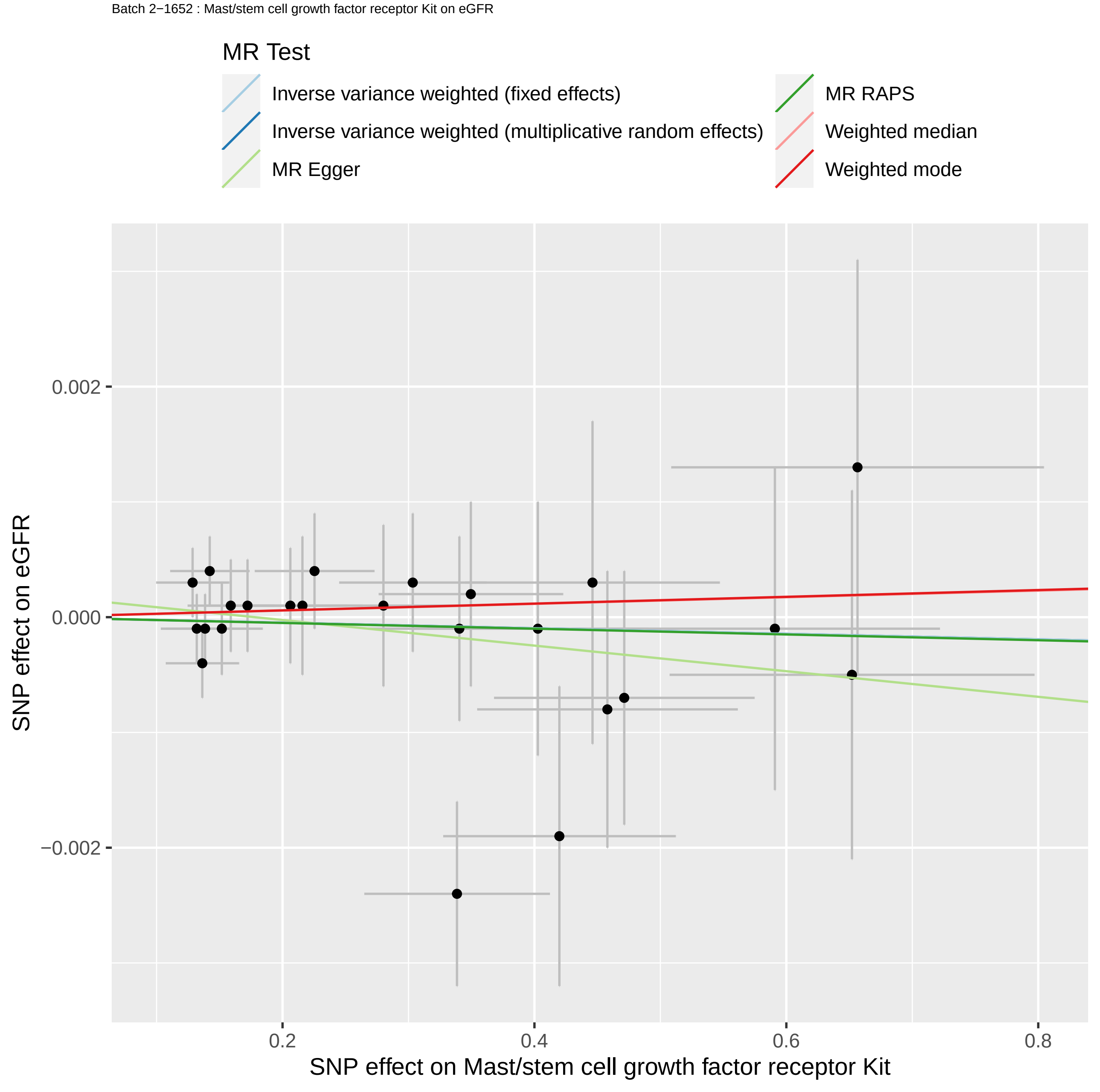
Batch 2−1652 : Mast/stem cell growth factor receptor Kit on eGFR
MR Test
Inverse variance weighted (fixed effects)
Inverse variance weighted (multiplicative random effects)
MR Egger
MR RAPS
Weighted median
Weighted mode
0.002
0.000
−0.002
0.2
0.4
0.6
0.8
SNP effect on eGFR
SNP effect on Mast/stem cell growth factor receptor Kit

Batch 2−1653 : Kit ligand on eGFR

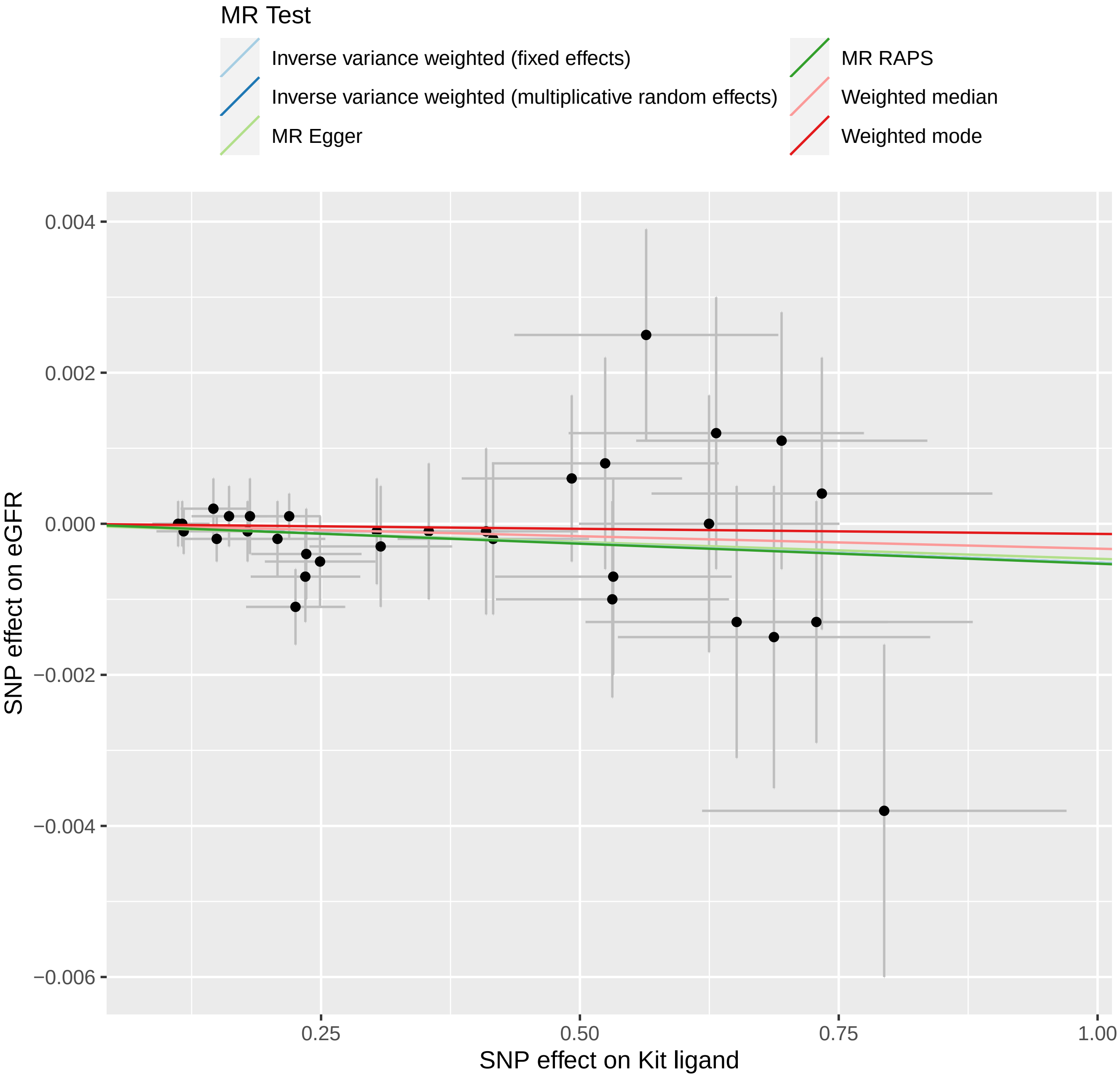

Batch 2−1654 : Kelch−like protein 12 on eGFR

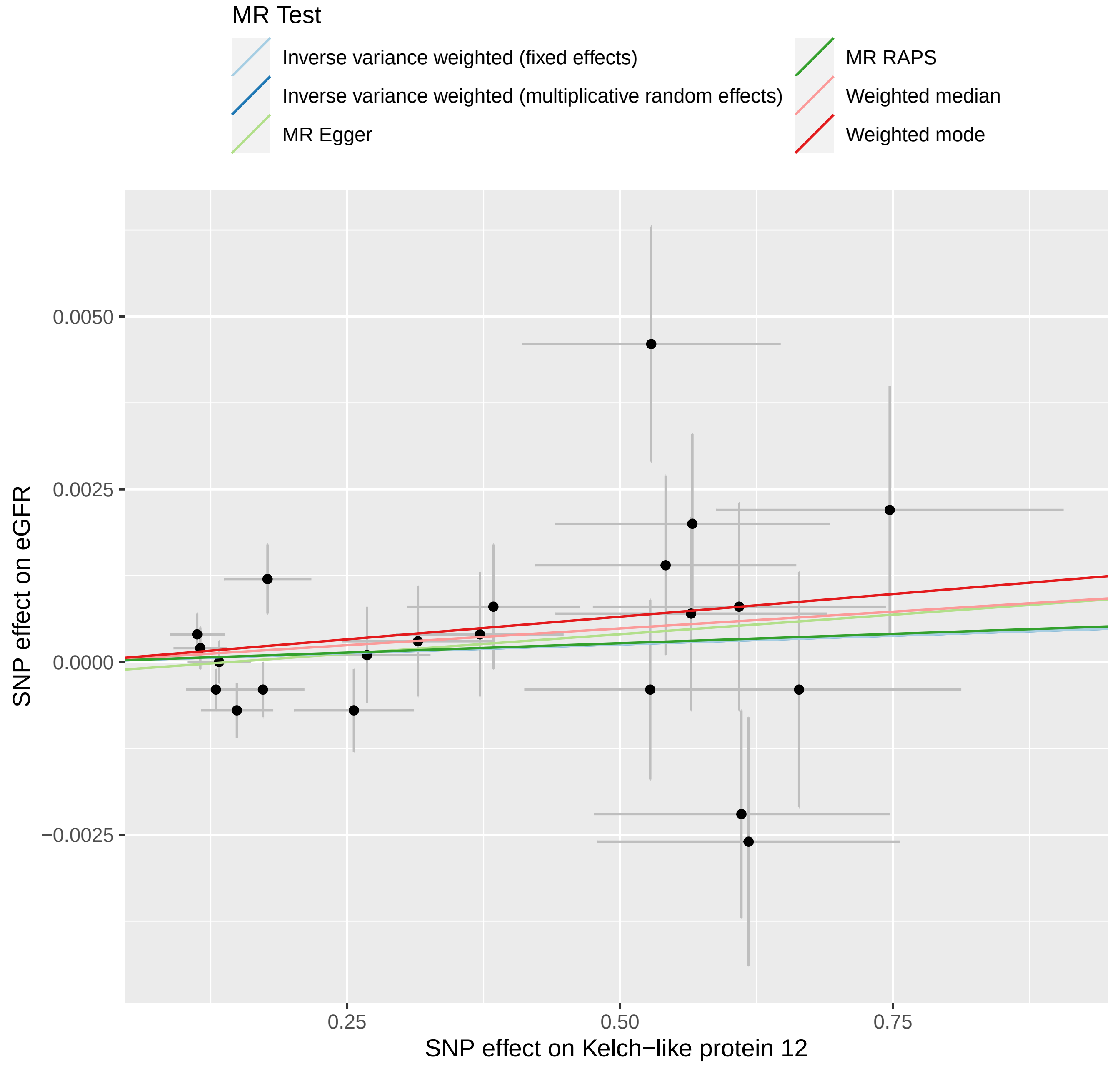

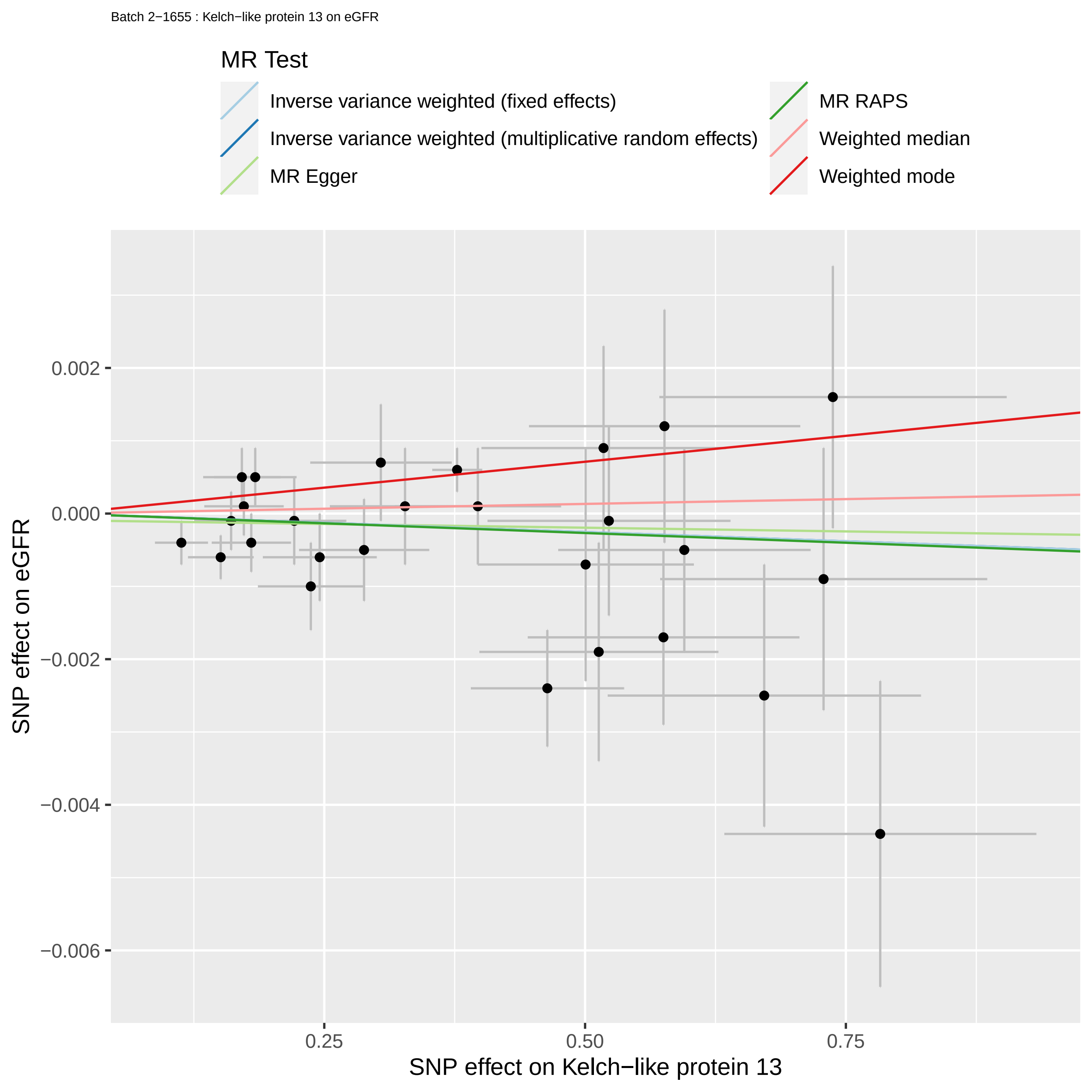

Batch 2−1655 : Kelch−like protein 13 on eGFR
MR Test
Inverse variance weighted (fixed effects)
Inverse variance weighted (multiplicative random effects)
MR Egger
MR RAPS
Weighted median
Weighted mode
0.002
0.000
−0.002
−0.004
−0.006
0.25
0.50
0.75
SNP effect on eGFR
SNP effect on Kelch−like protein 13

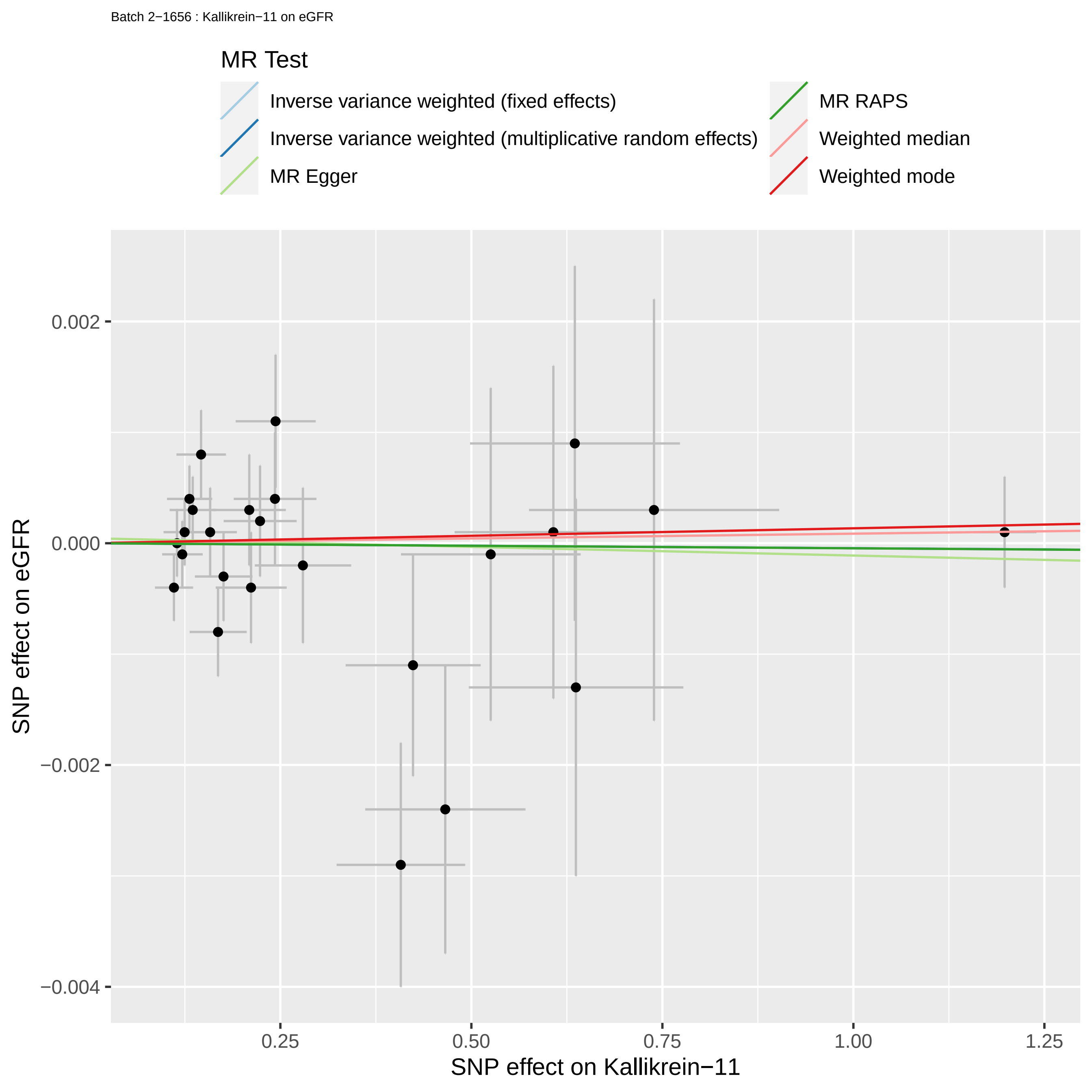
Batch 2−1656 : Kallikrein−11 on eGFR
MR Test
Inverse variance weighted (fixed effects)
Inverse variance weighted (multiplicative random effects)
MR Egger
MR RAPS
Weighted median
Weighted mode
0.002
0.000
−0.002
−0.004
SNP effect on eGFR
0.25
0.50
0.75
1.00
1.25
SNP effect on Kallikrein−11

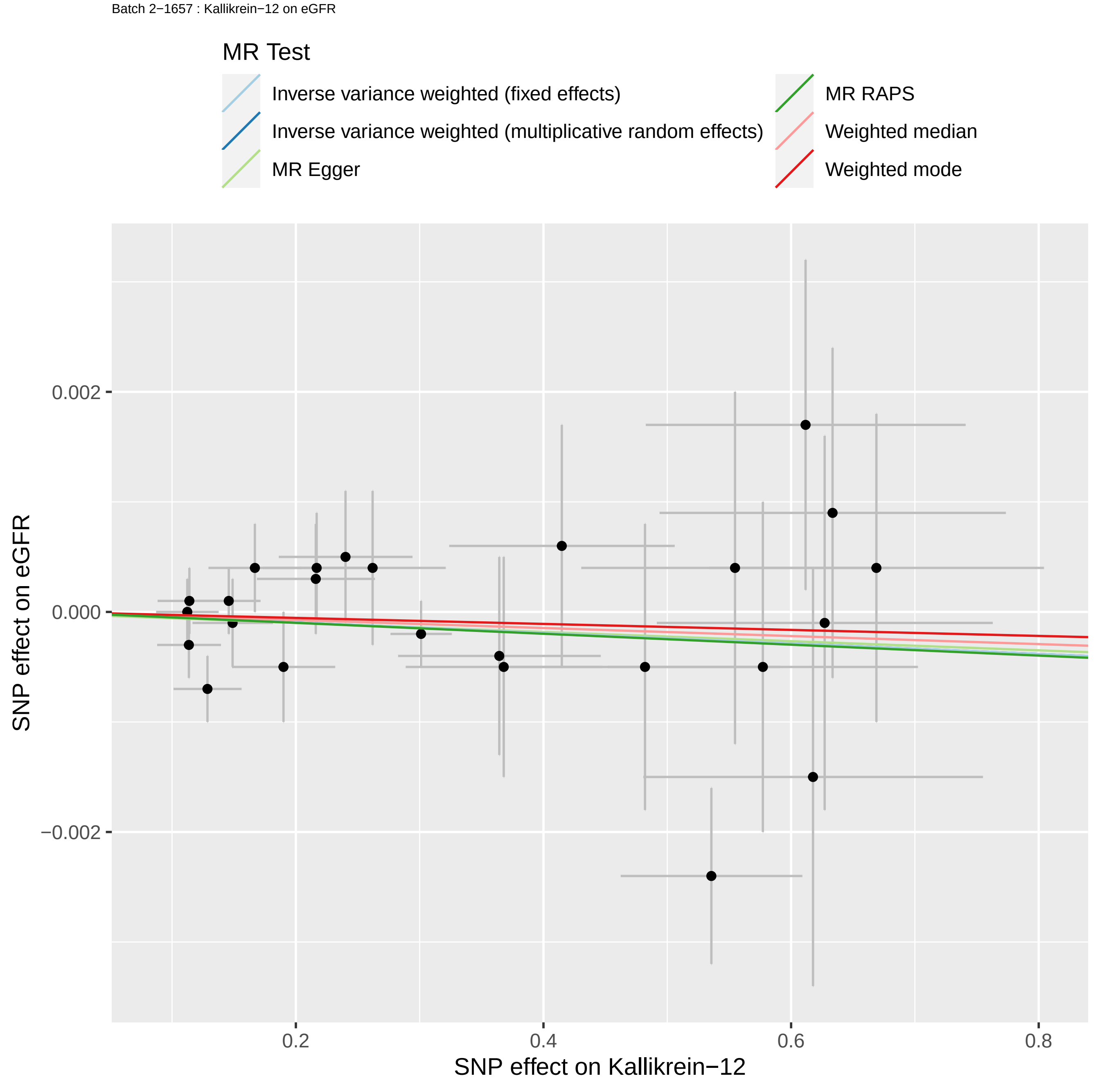

Batch 2−1657 : Kallikrein−12 on eGFR
MR Test
Inverse variance weighted (fixed effects)
Inverse variance weighted (multiplicative random effects)
MR Egger
MR RAPS
Weighted median
Weighted mode
0.002
0.000
−0.002
0.2
0.4
0.6
0.8
SNP effect on eGFR
SNP effect on Kallikrein−12

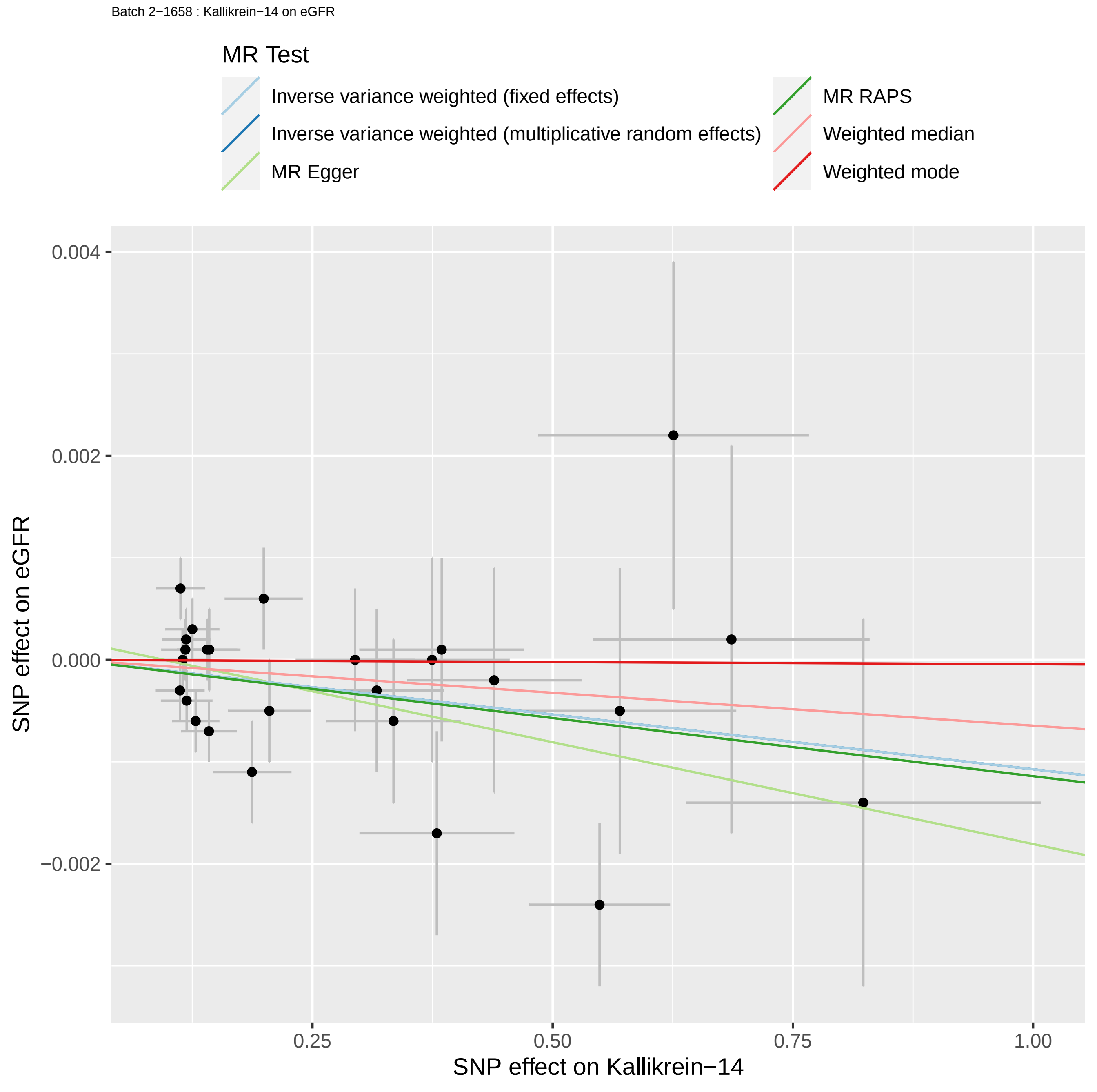

Batch 2−1658 : Kallikrein−14 on eGFR
MR Test
Inverse variance weighted (fixed effects)
Inverse variance weighted (multiplicative random effects)
MR Egger
MR RAPS
Weighted median
Weighted mode
0.004
0.002
0.000
−0.002
SNP effect on eGFR
0.25
0.50
0.75
1.00
SNP effect on Kallikrein−14

Batch 2−1659 : Kallikrein−14 on eGFR

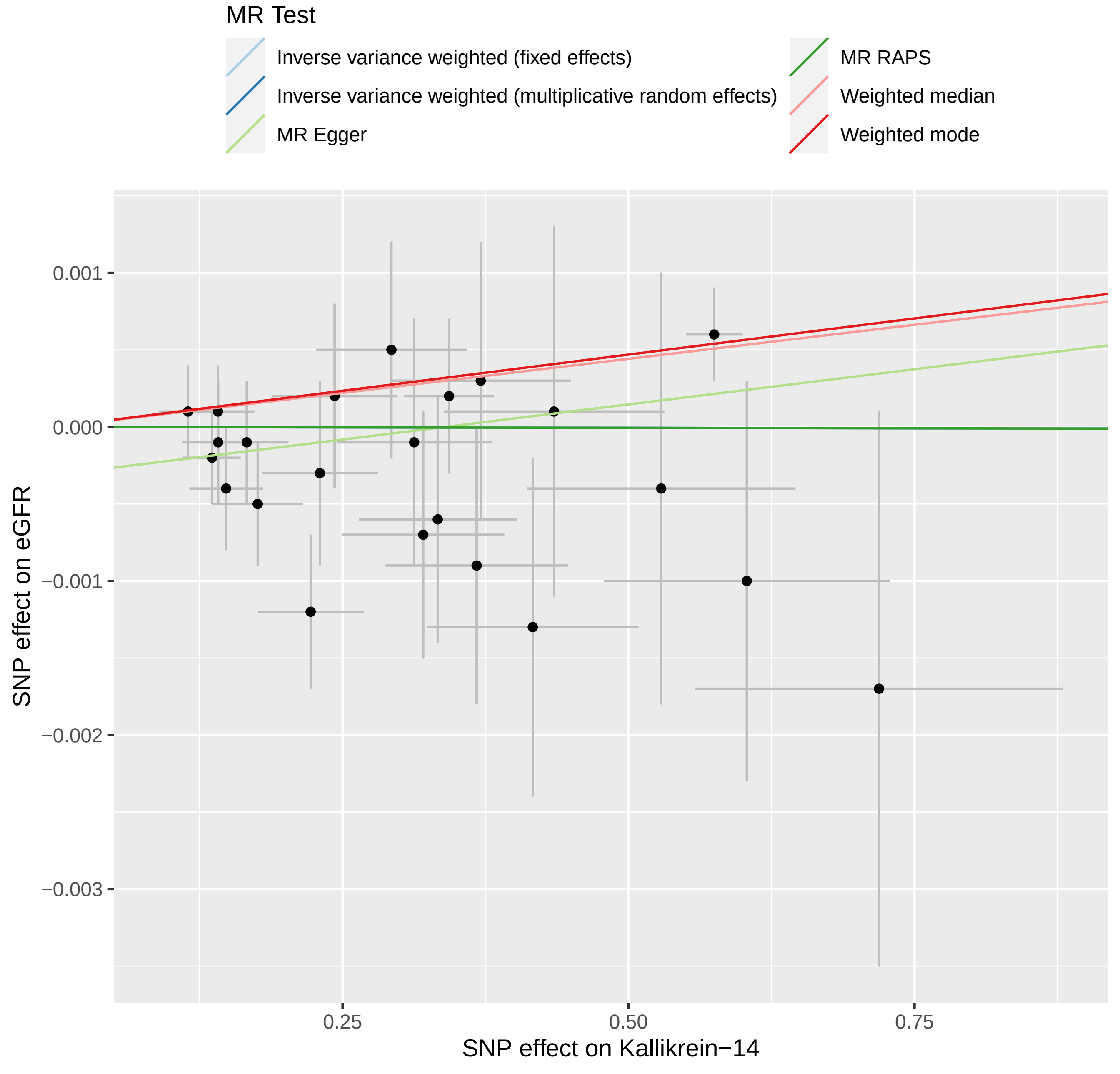

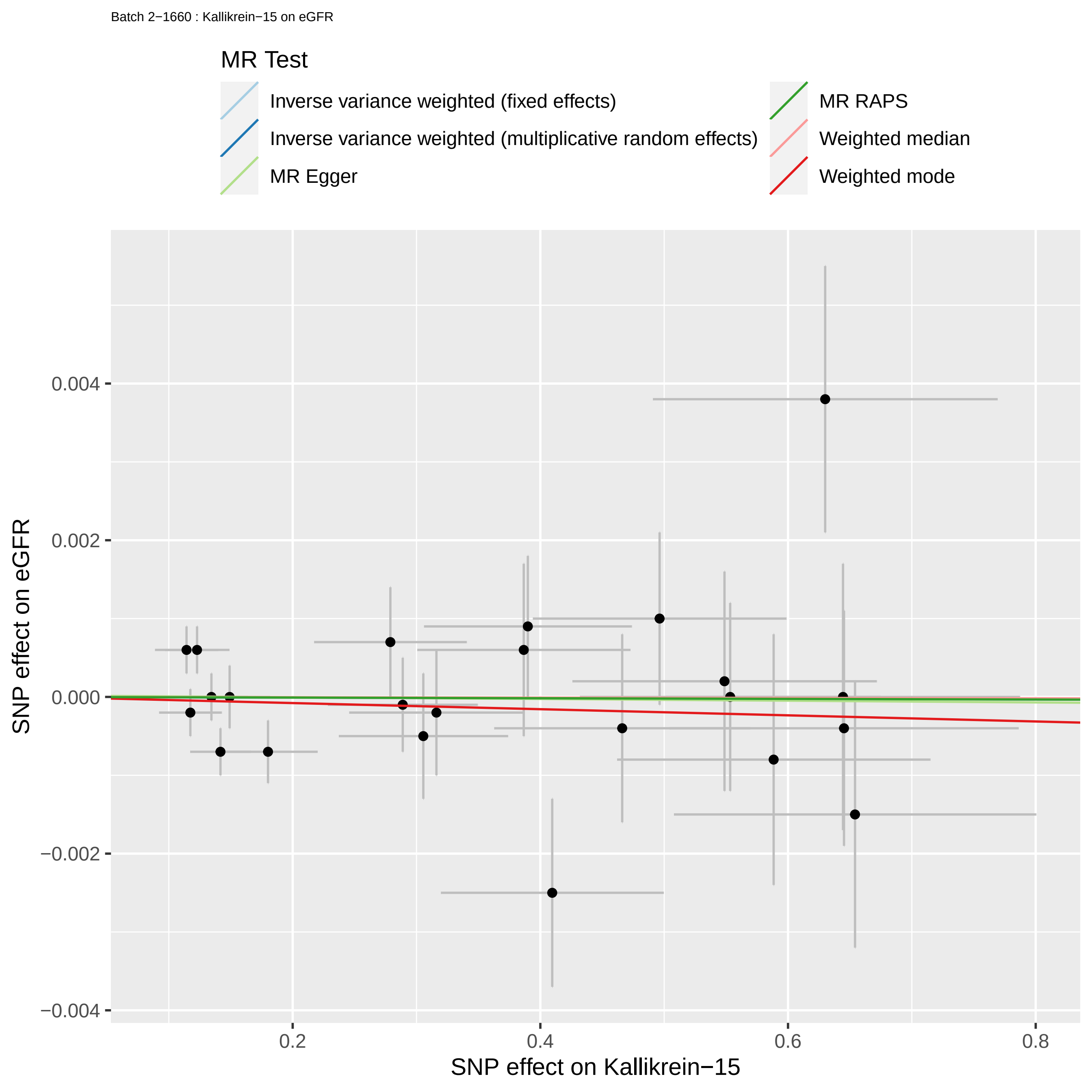
Batch 2−1660 : Kallikrein−15 on eGFR
MR Test
Inverse variance weighted (fixed effects)
Inverse variance weighted (multiplicative random effects)
MR Egger
MR RAPS
Weighted median
Weighted mode
SNP effect on eGFR
0.004
0.002
0.000
−0.002
−0.004
0.2
0.4
0.6
0.8
SNP effect on Kallikrein−15

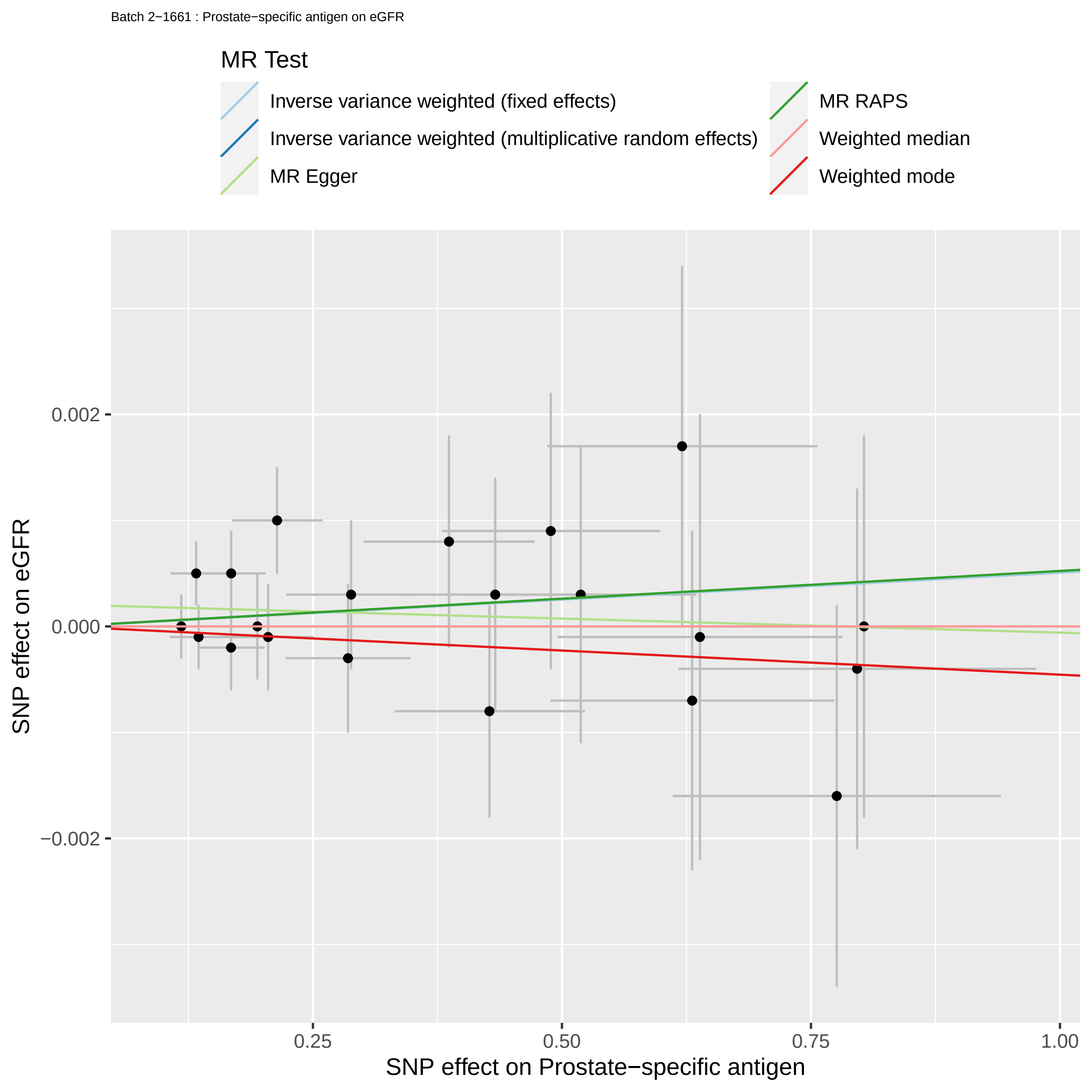
Batch 2−1661 : Prostate−specific antigen on eGFR
MR Test
Inverse variance weighted (fixed effects)
Inverse variance weighted (multiplicative random effects)
MR Egger
MR RAPS
Weighted median
Weighted mode
0.002
0.000
−0.002
0.25
0.50
0.75
1.00
SNP effect on eGFR
SNP effect on Prostate−specific antigen

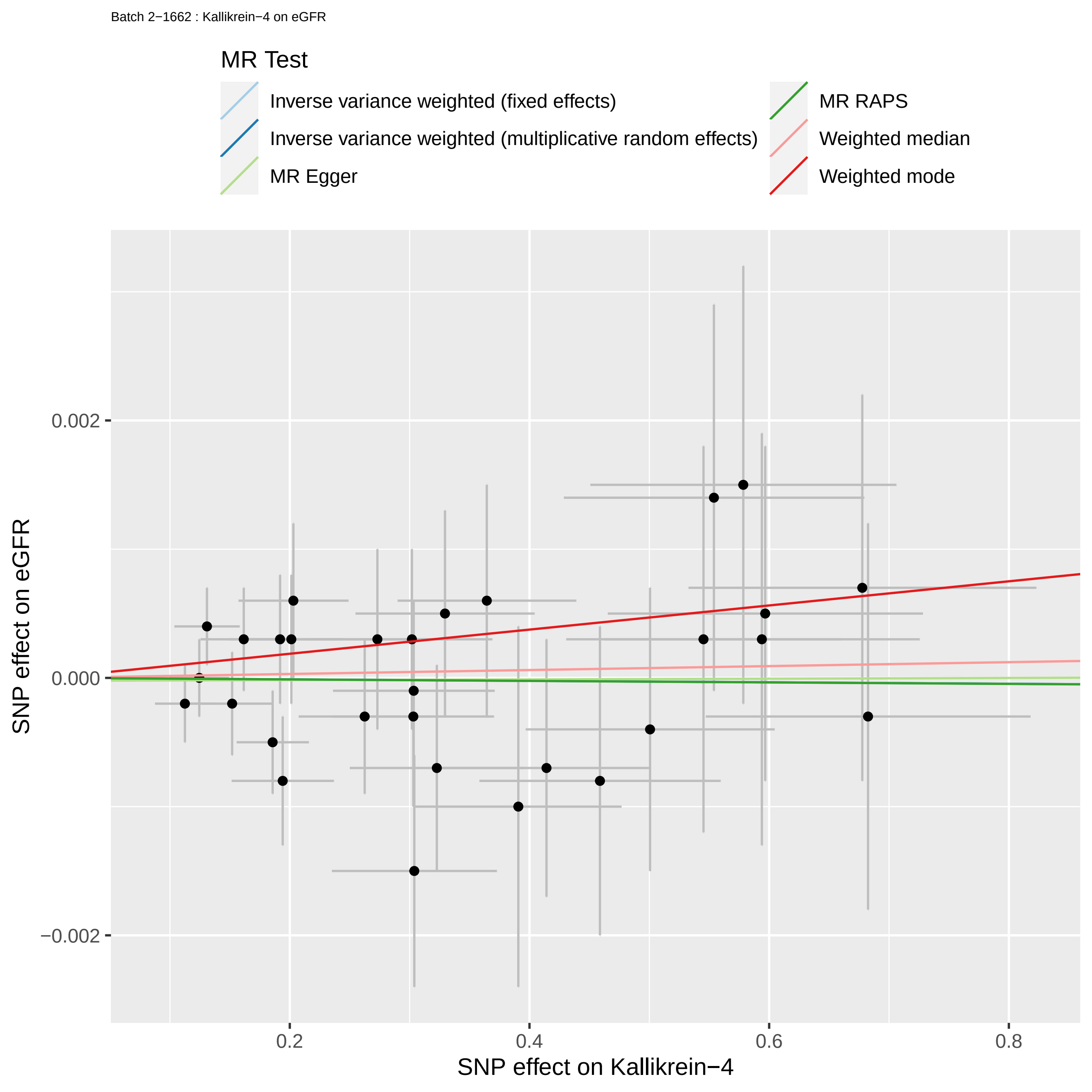

Batch 2−1662 : Kallikrein−4 on eGFR
MR Test
Inverse variance weighted (fixed effects)
Inverse variance weighted (multiplicative random effects)
MR Egger
MR RAPS
Weighted median
Weighted mode
0.002
0.000
−0.002
0.2
0.4
0.6
0.8
SNP effect on Kallikrein−4
SNP effect on eGFR

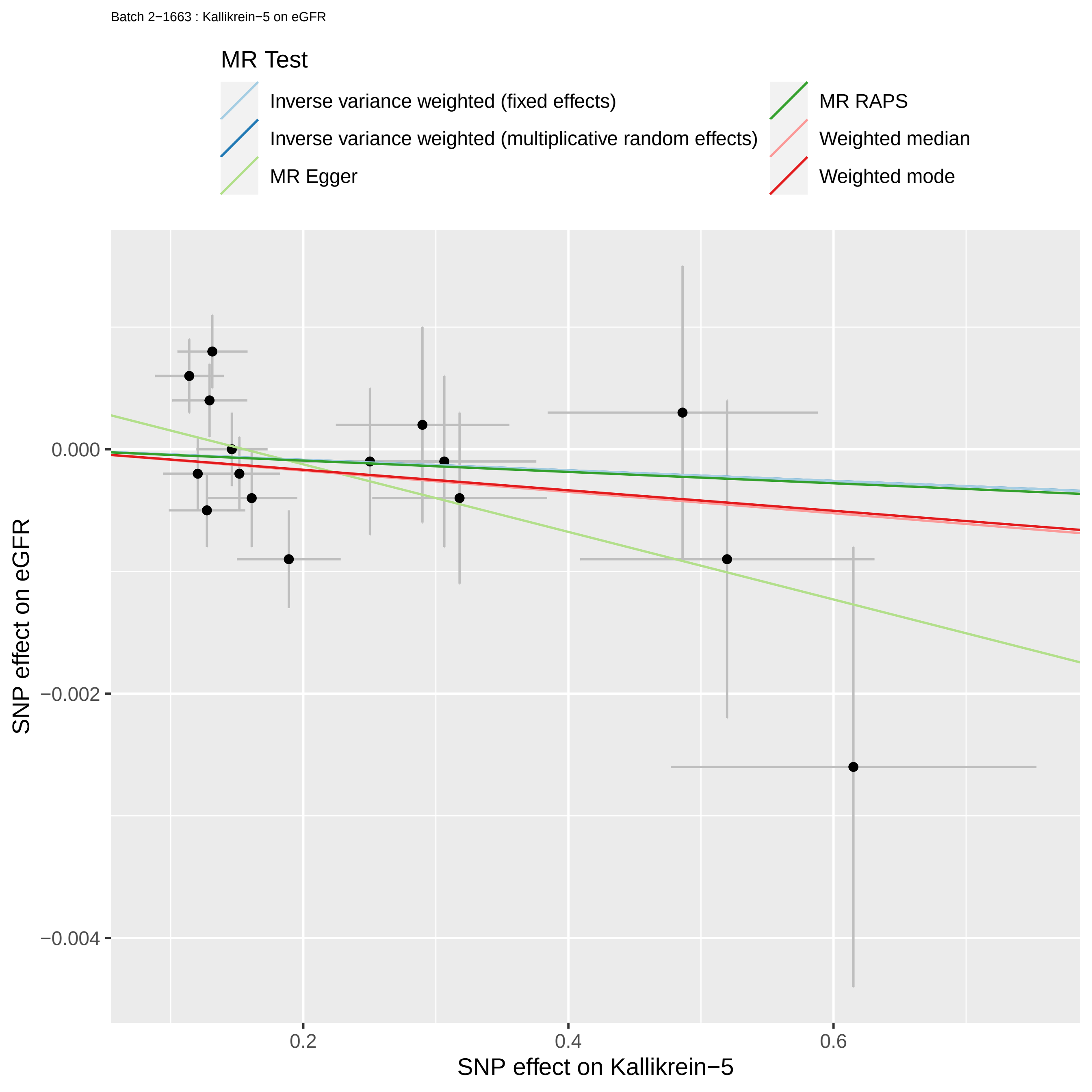

Batch 2−1663 : Kallikrein−5 on eGFR
MR Test
Inverse variance weighted (fixed effects)
Inverse variance weighted (multiplicative random effects)
MR Egger
MR RAPS
Weighted median
Weighted mode
0.000
−0.002
−0.004
0.2
0.4
0.6
SNP effect on eGFR
SNP effect on Kallikrein−5

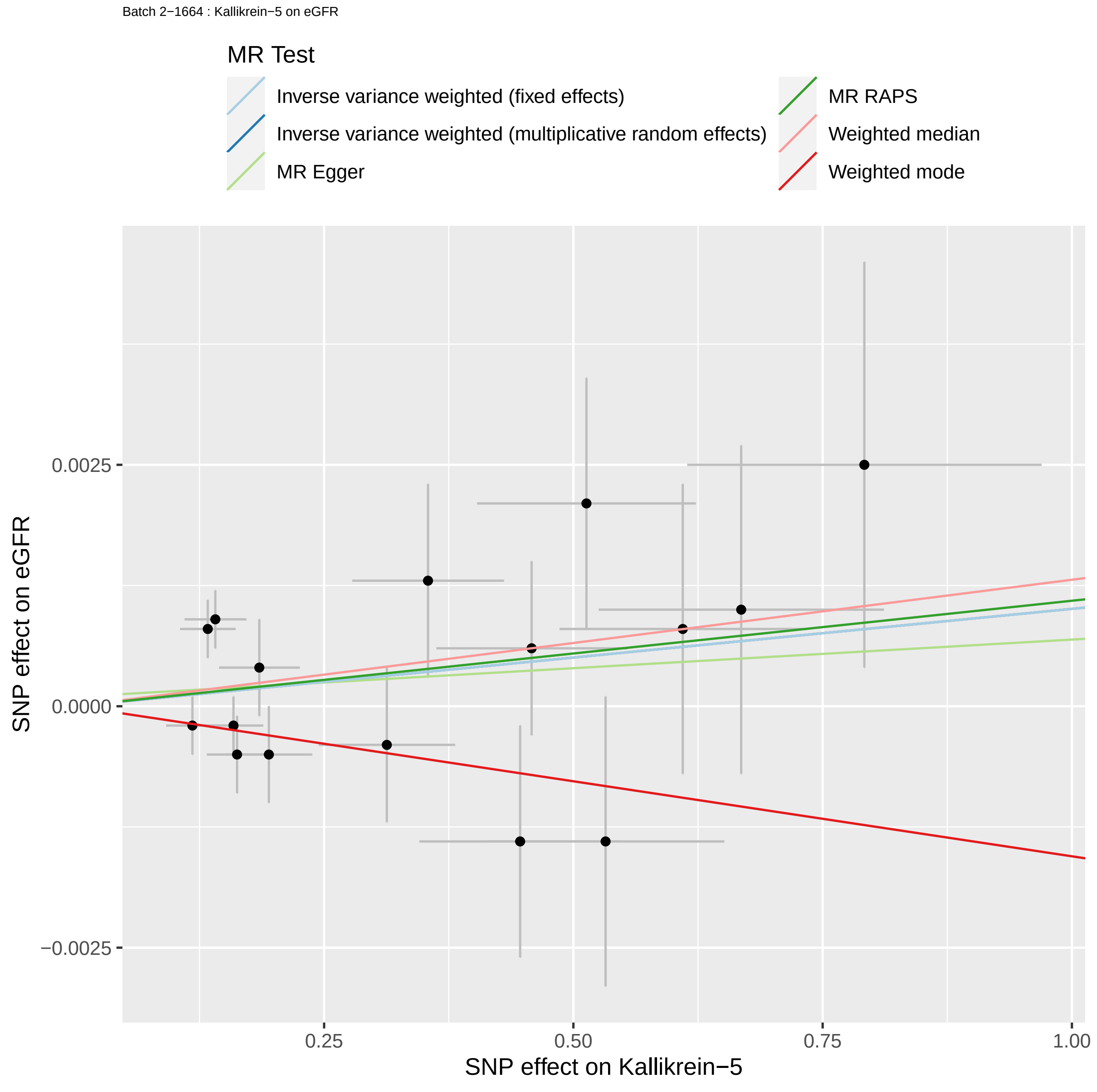

Batch 2−1664 : Kallikrein−5 on eGFR
MR Test
Inverse variance weighted (fixed effects)
Inverse variance weighted (multiplicative random effects)
MR Egger
MR RAPS
Weighted median
Weighted mode
0.0025
0.0000
−0.0025
0.25
0.50
0.75
1.00
SNP effect on eGFR
SNP effect on Kallikrein−5

Batch 2−1665 : Kallikrein−6 on eGFR

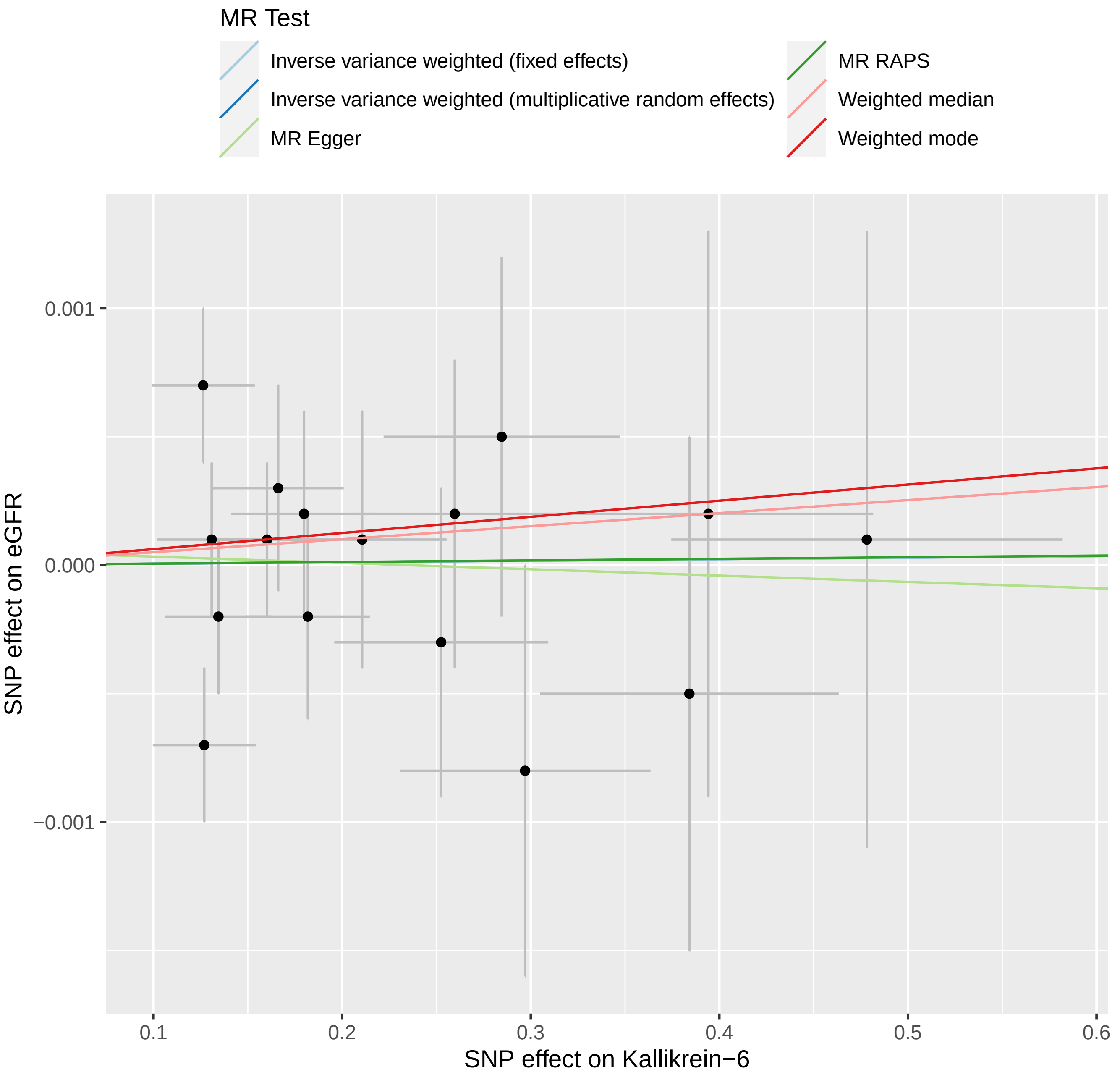

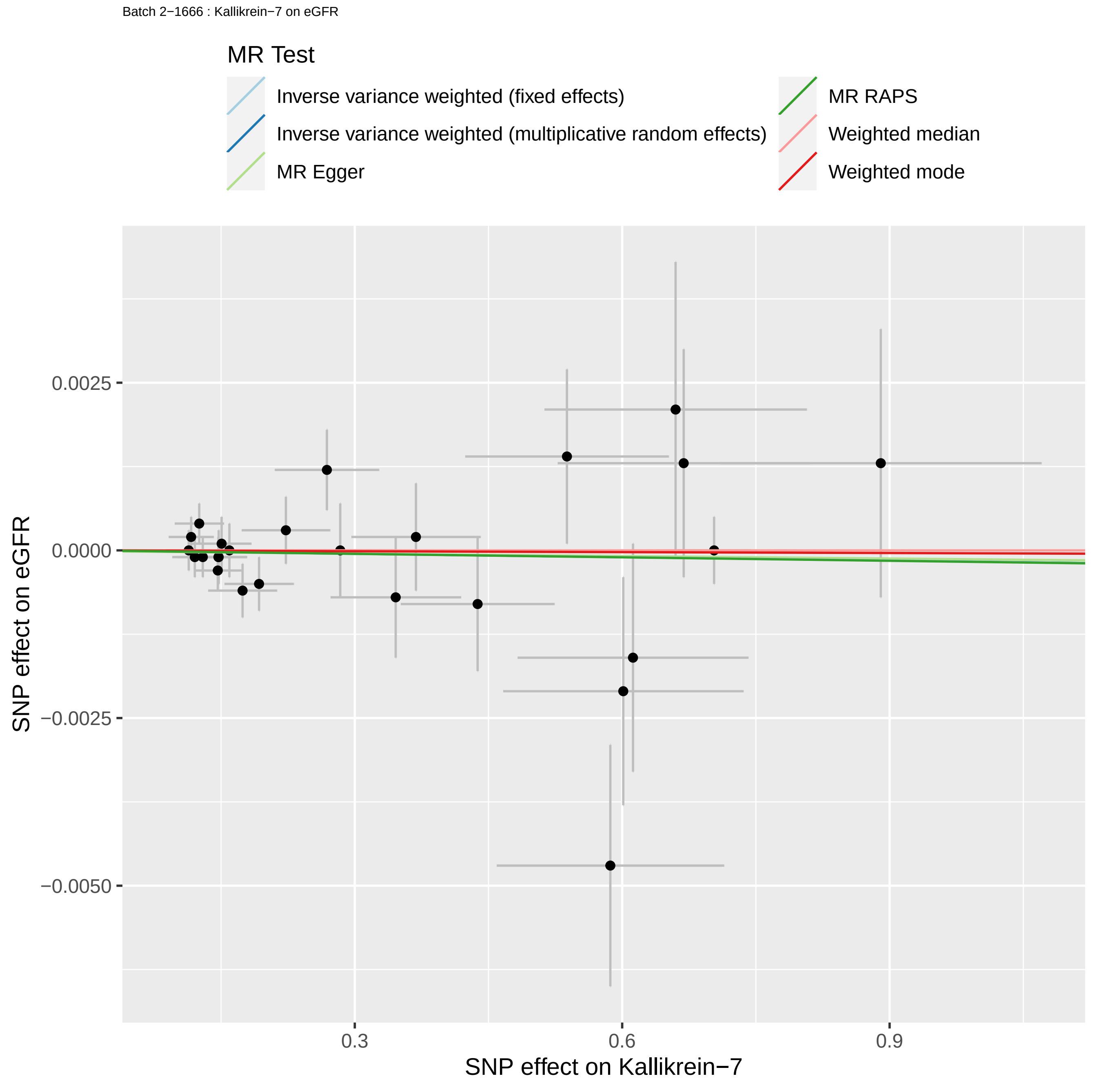

Batch 2−1666 : Kallikrein−7 on eGFR
MR Test
Inverse variance weighted (fixed effects)
Inverse variance weighted (multiplicative random effects)
MR Egger
MR RAPS
Weighted median
Weighted mode
0.0025
0.0000
−0.0025
−0.0050
0.3
0.6
0.9
SNP effect on eGFR
SNP effect on Kallikrein−7

Batch 2−1667 : Kallikrein−8 on eGFR

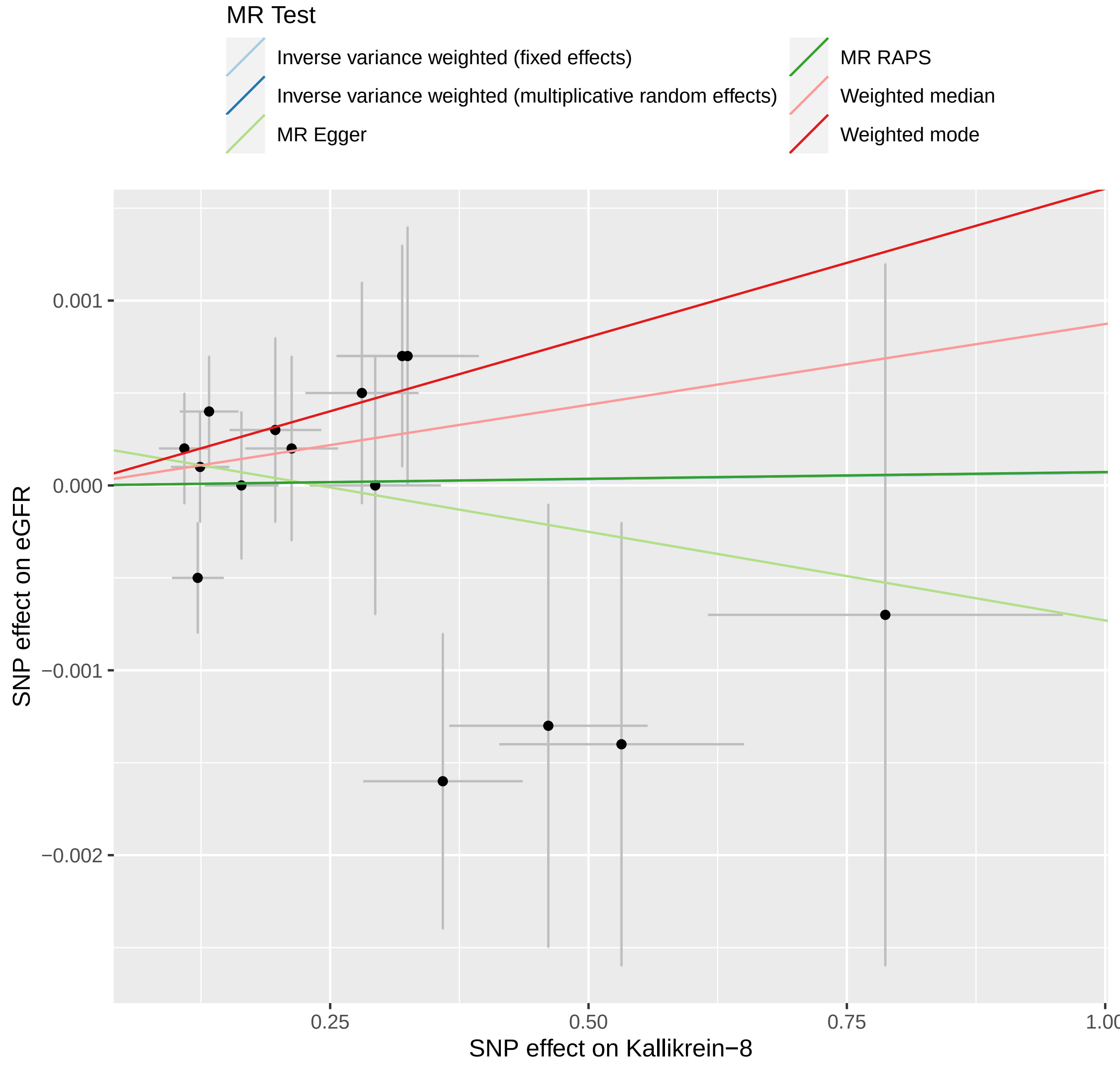

Batch 2−1668 : Kallikrein−8 on eGFR

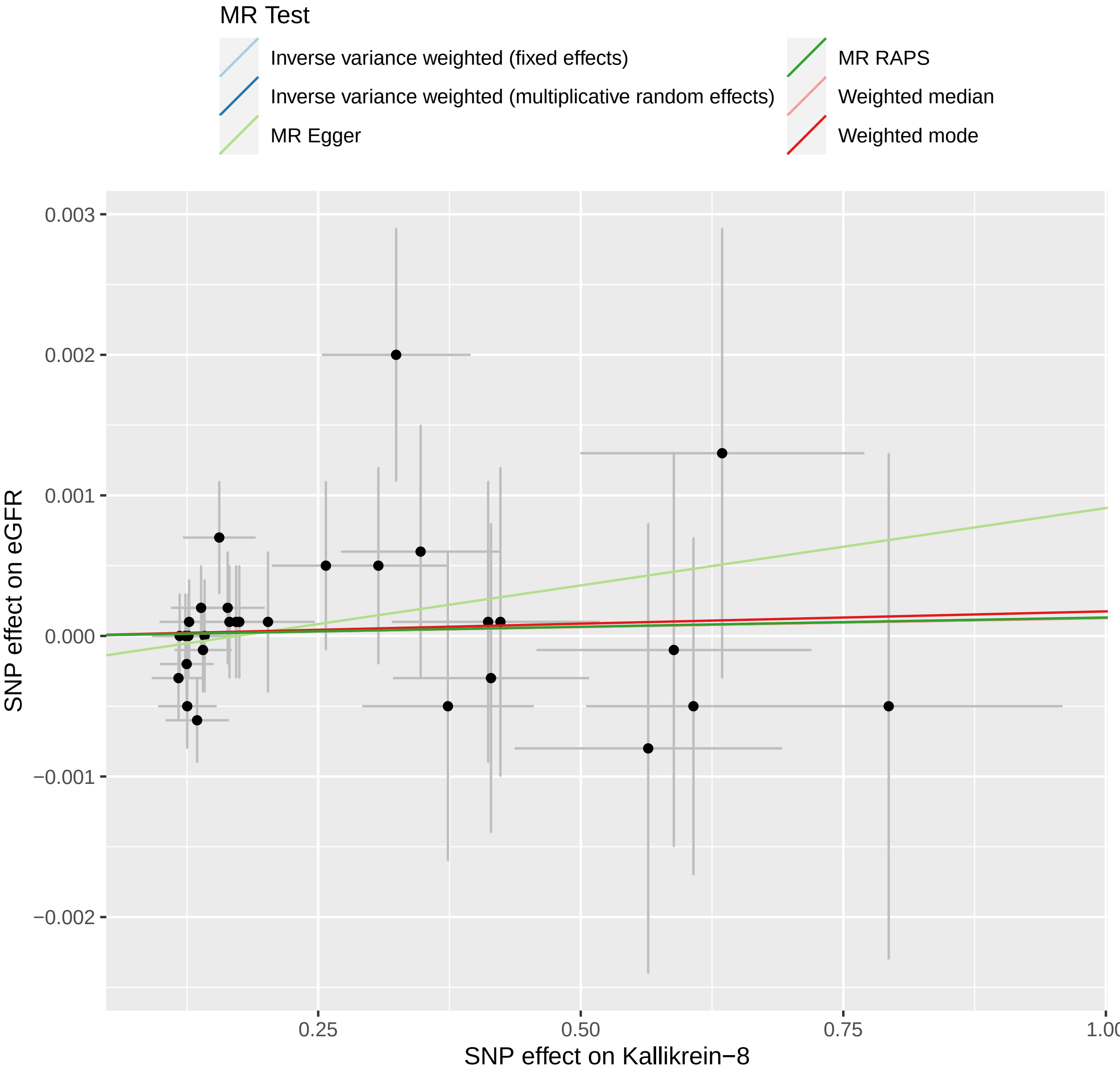

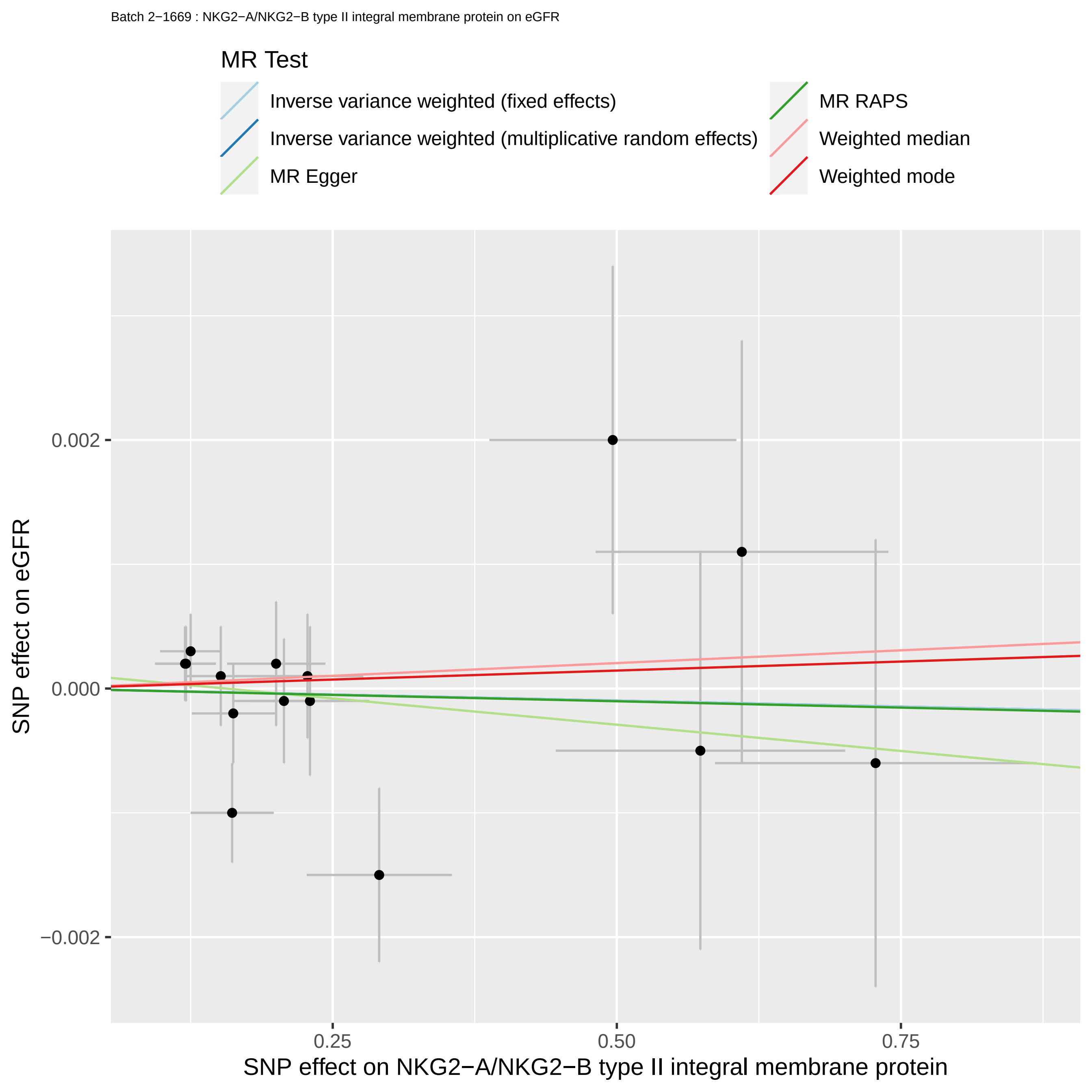

Batch 2−1669 : NKG2−A/NKG2−B type II integral membrane protein on eGFR
MR Test
Inverse variance weighted (fixed effects)
Inverse variance weighted (multiplicative random effects)
MR Egger
MR RAPS
Weighted median
Weighted mode
0.002
0.000
−0.002
0.25
0.50
0.75
SNP effect on eGFR
SNP effect on NKG2−A/NKG2−B type II integral membrane protein

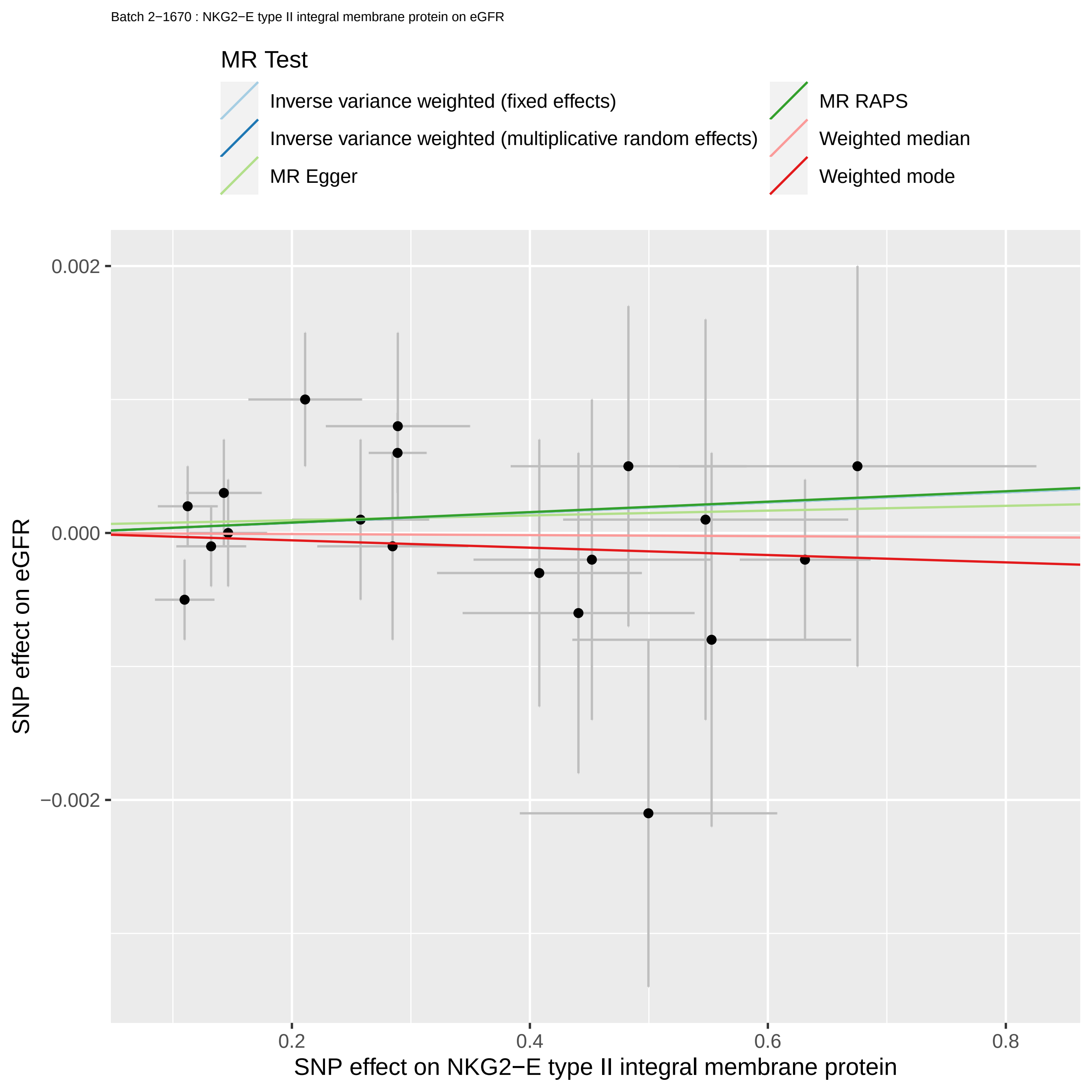
Batch 2−1670 : NKG2−E type II integral membrane protein on eGFR
MR Test
Inverse variance weighted (fixed effects)
Inverse variance weighted (multiplicative random effects)
MR Egger
MR RAPS
Weighted median
Weighted mode
0.002
0.000
−0.002
0.2
0.4
0.6
0.8
SNP effect on eGFR
SNP effect on NKG2−E type II integral membrane protein

Batch 2−1671 : NKG2−E type II integral membrane protein on eGFR

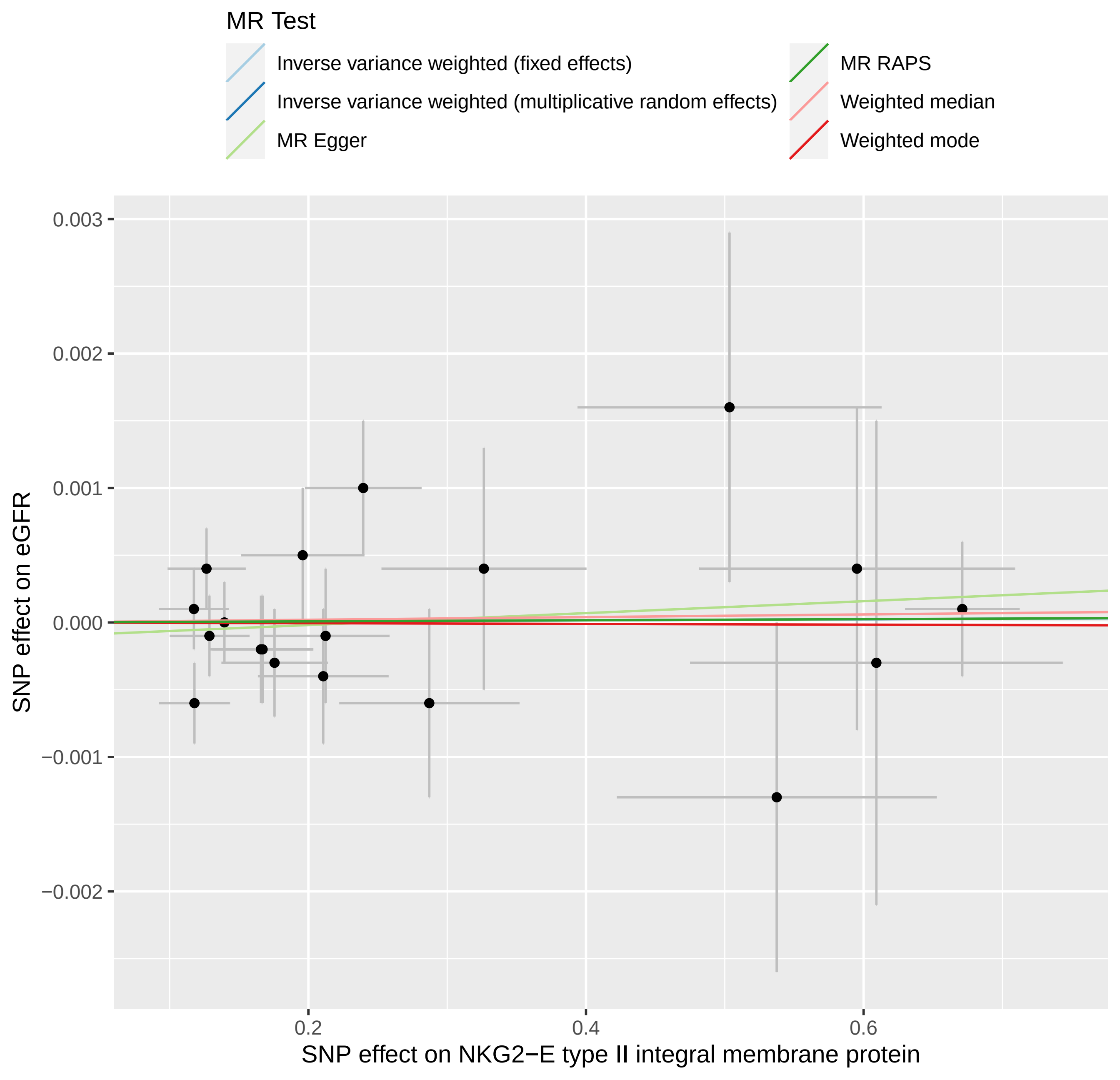

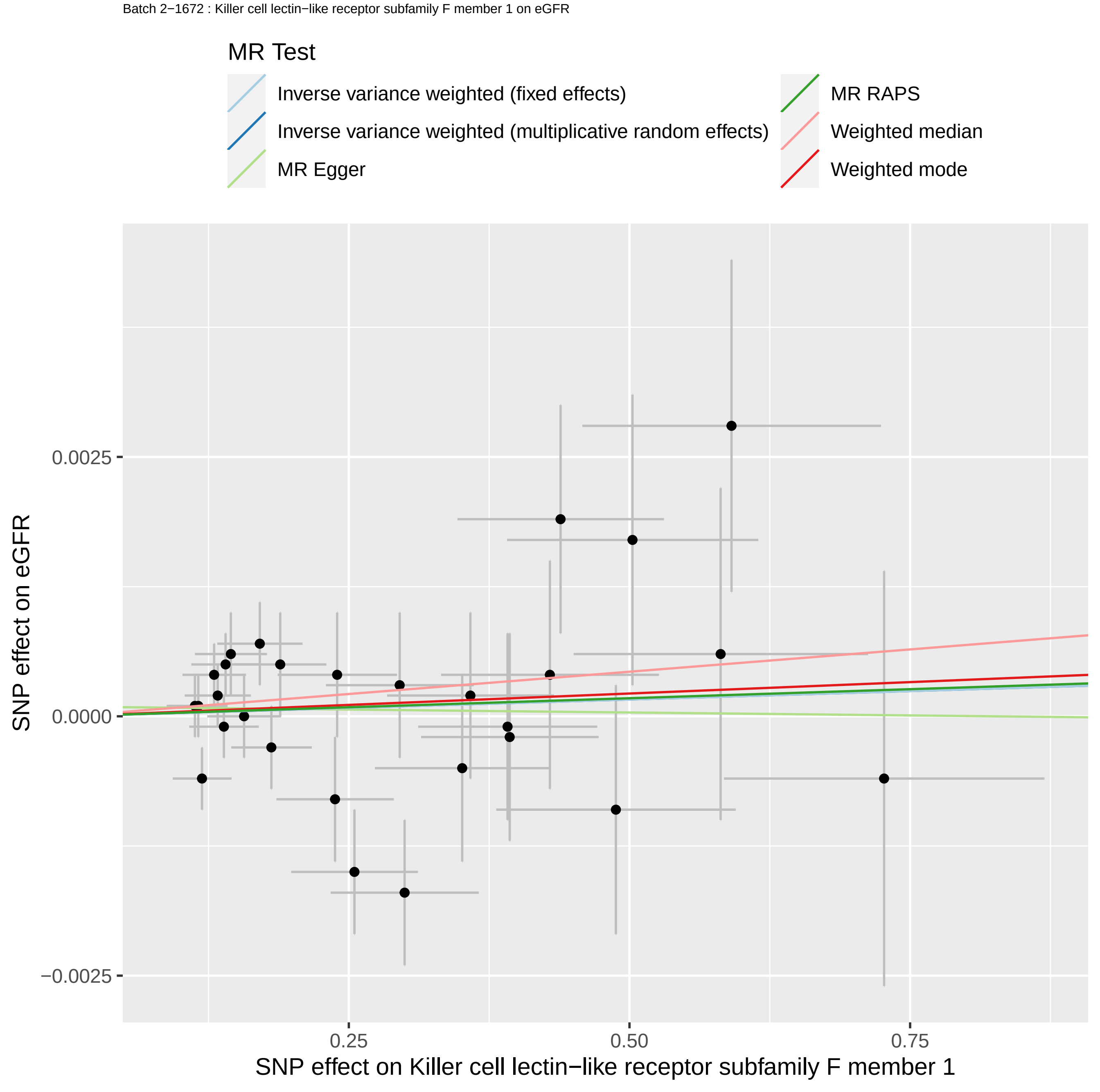
Batch 2−1672 : Killer cell lectin−like receptor subfamily F member 1 on eGFR
MR Test
Inverse variance weighted (fixed effects)
Inverse variance weighted (multiplicative random effects)
MR Egger
MR RAPS
Weighted median
Weighted mode
SNP effect on eGFR
0.0025
0.0000
−0.0025
0.25
0.50
0.75
SNP effect on Killer cell lectin−like receptor subfamily F member 1

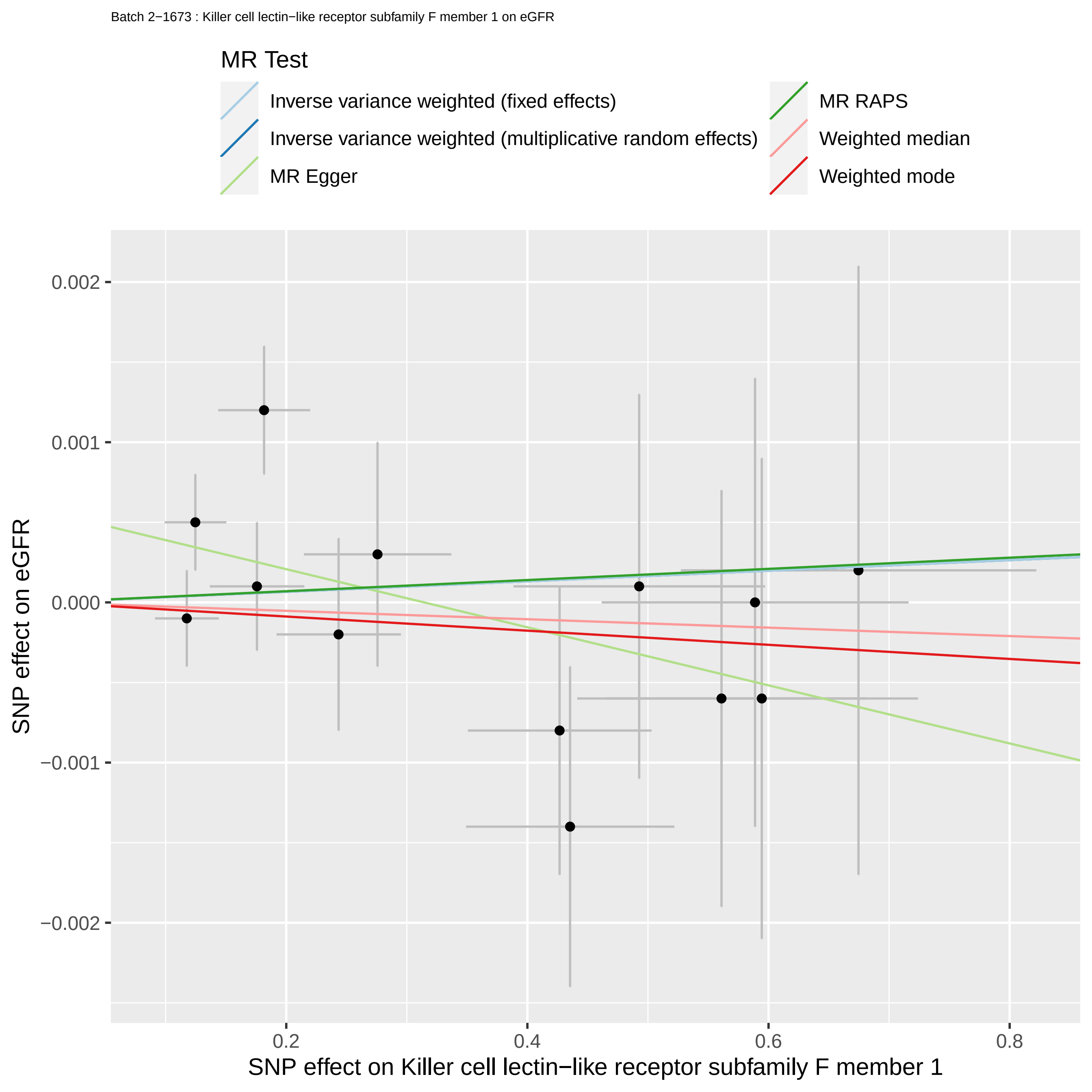

Batch 2−1673 : Killer cell lectin−like receptor subfamily F member 1 on eGFR
MR Test
Inverse variance weighted (fixed effects)
Inverse variance weighted (multiplicative random effects)
MR Egger
MR RAPS
Weighted median
Weighted mode
0.002
0.001
0.000
−0.001
−0.002
SNP effect on eGFR
0.2
0.4
0.6
0.8
SNP effect on Killer cell lectin−like receptor subfamily F member 1

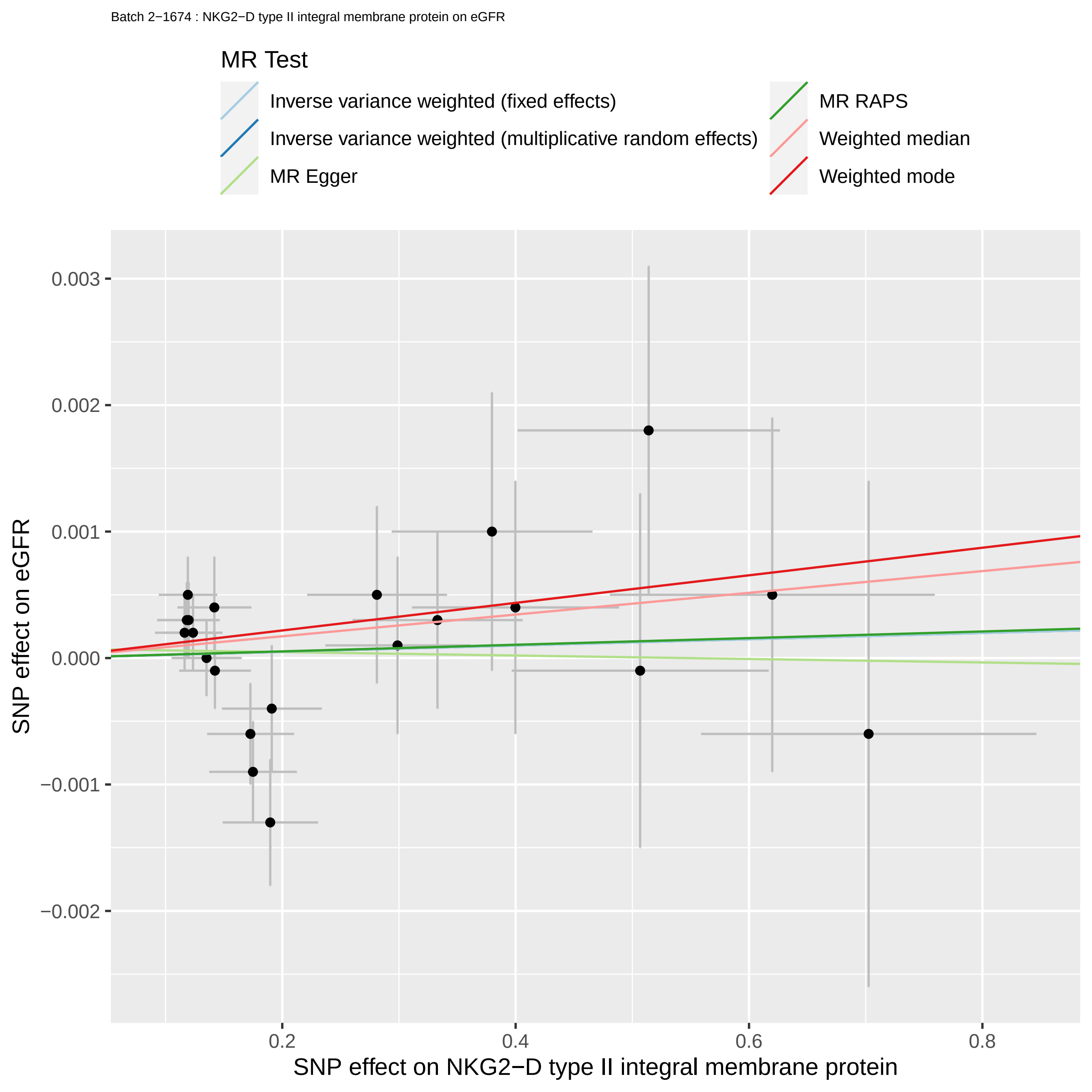

Batch 2−1674 : NKG2−D type II integral membrane protein on eGFR
MR Test
Inverse variance weighted (fixed effects)
Inverse variance weighted (multiplicative random effects)
MR Egger
MR RAPS
Weighted median
Weighted mode
0.003
0.002
0.001
0.000
−0.001
−0.002
0.2
0.4
0.6
0.8
SNP effect on eGFR
SNP effect on NKG2−D type II integral membrane protein

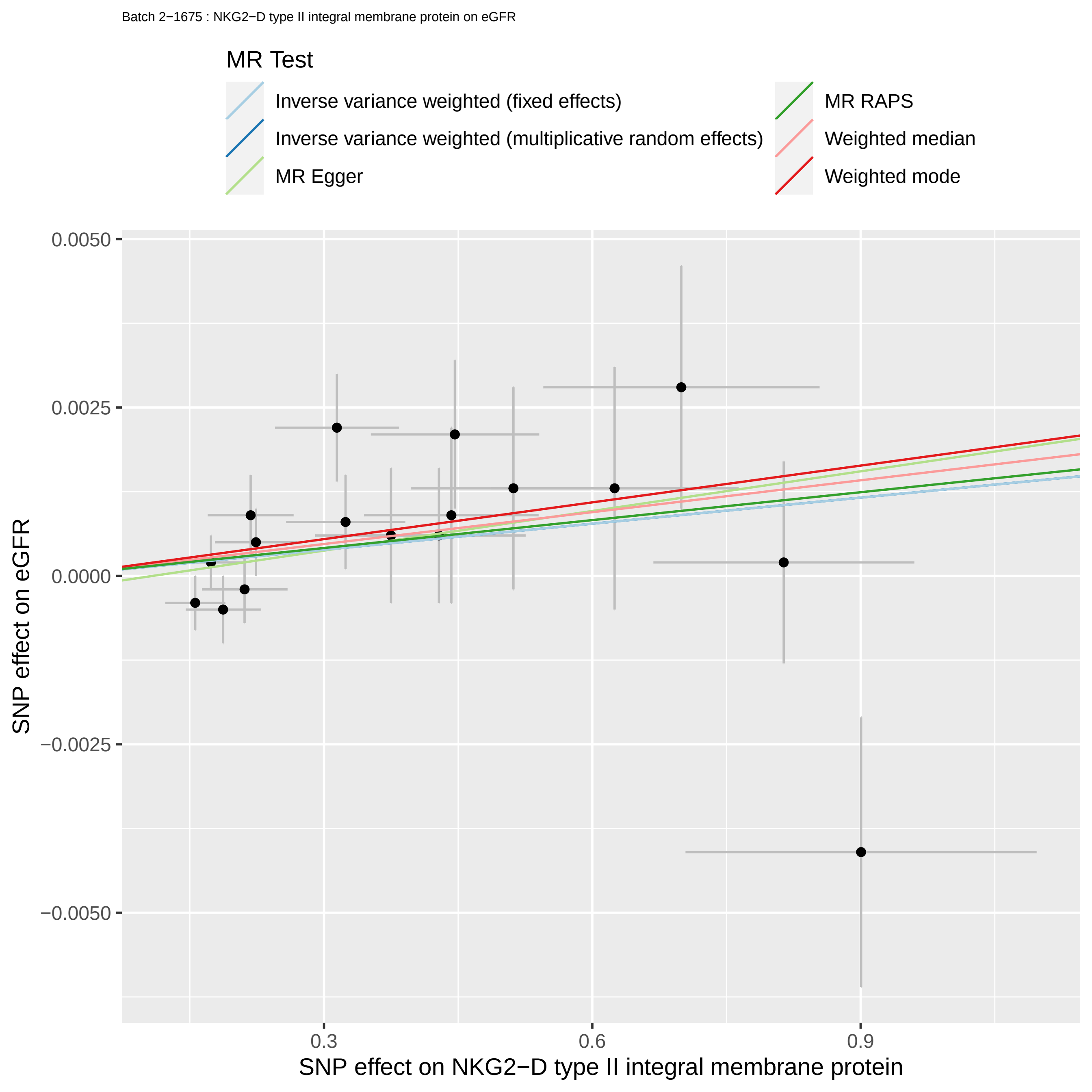

Batch 2−1675 : NKG2−D type II integral membrane protein on eGFR
MR Test
Inverse variance weighted (fixed effects)
Inverse variance weighted (multiplicative random effects)
MR Egger
MR RAPS
Weighted median
Weighted mode
0.0050
0.0025
0.0000
−0.0025
−0.0050
SNP effect on eGFR
0.3
0.6
0.9
SNP effect on NKG2−D type II integral membrane protein

Batch 2−1676 : Histone−lysine N−methyltransferase 2C on eGFR

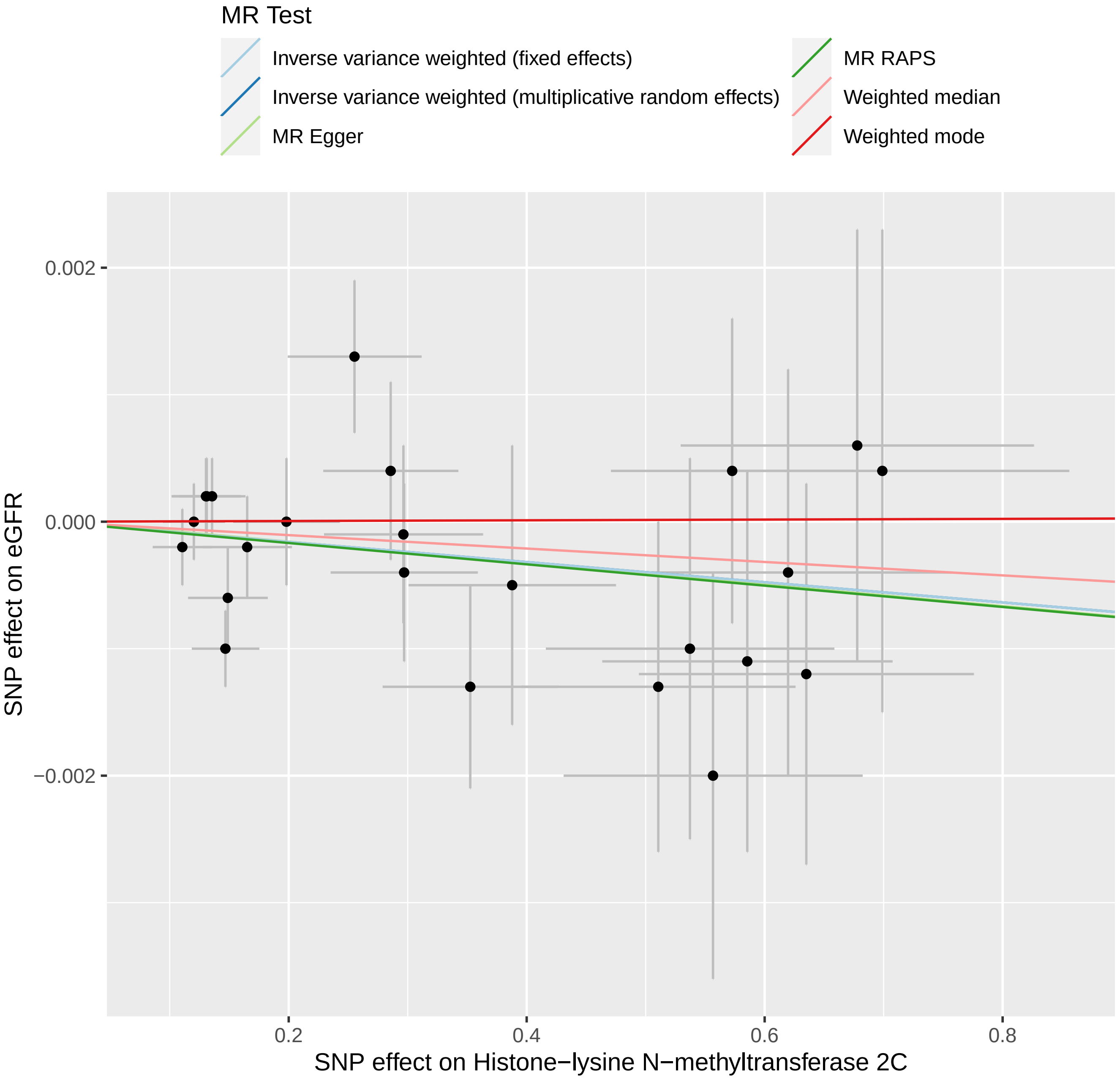

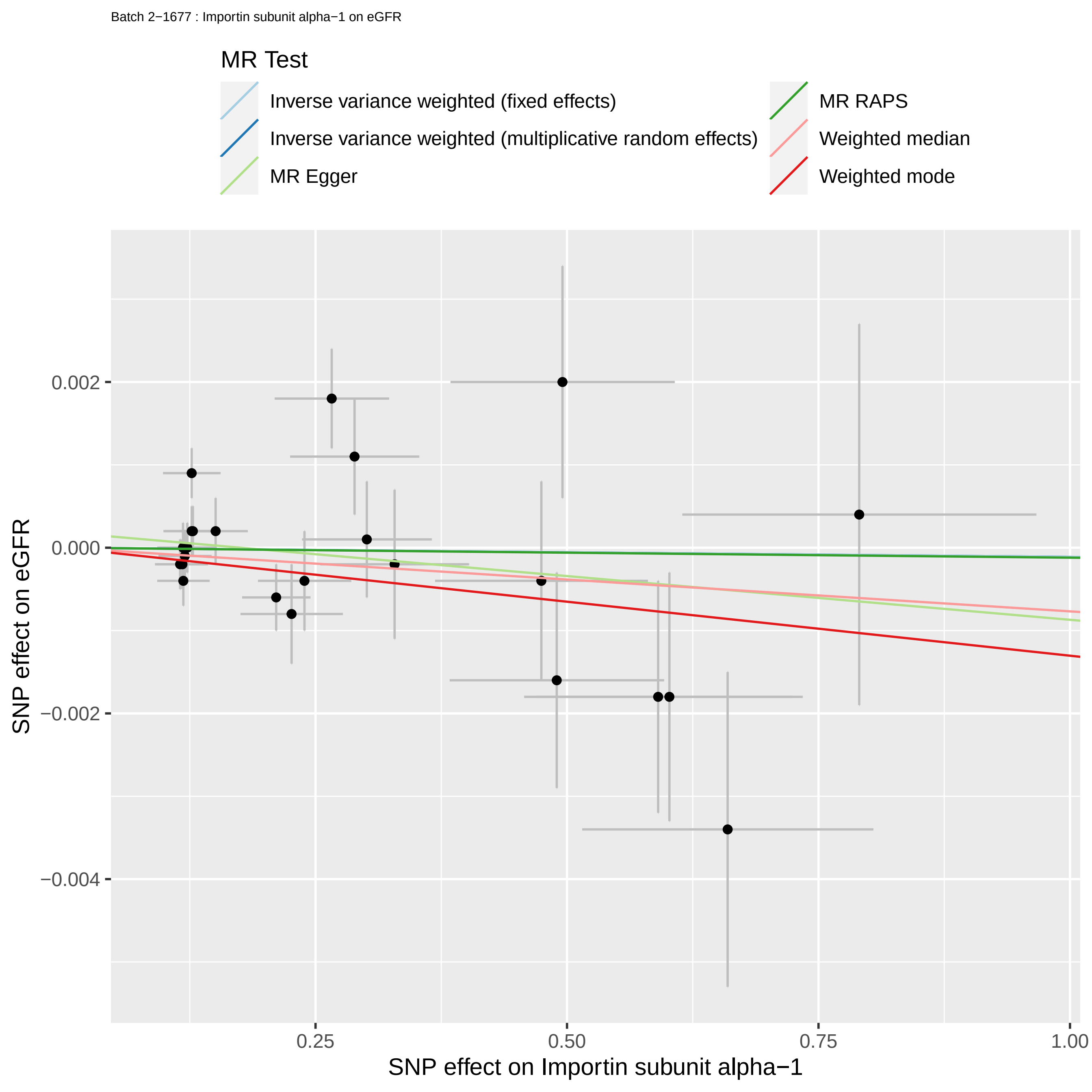

Batch 2−1677 : Importin subunit alpha−1 on eGFR
MR Test
Inverse variance weighted (fixed effects)
Inverse variance weighted (multiplicative random effects)
MR Egger
MR RAPS
Weighted median
Weighted mode
0.002
0.000
−0.002
−0.004
SNP effect on eGFR
0.25
0.50
0.75
1.00
SNP effect on Importin subunit alpha−1

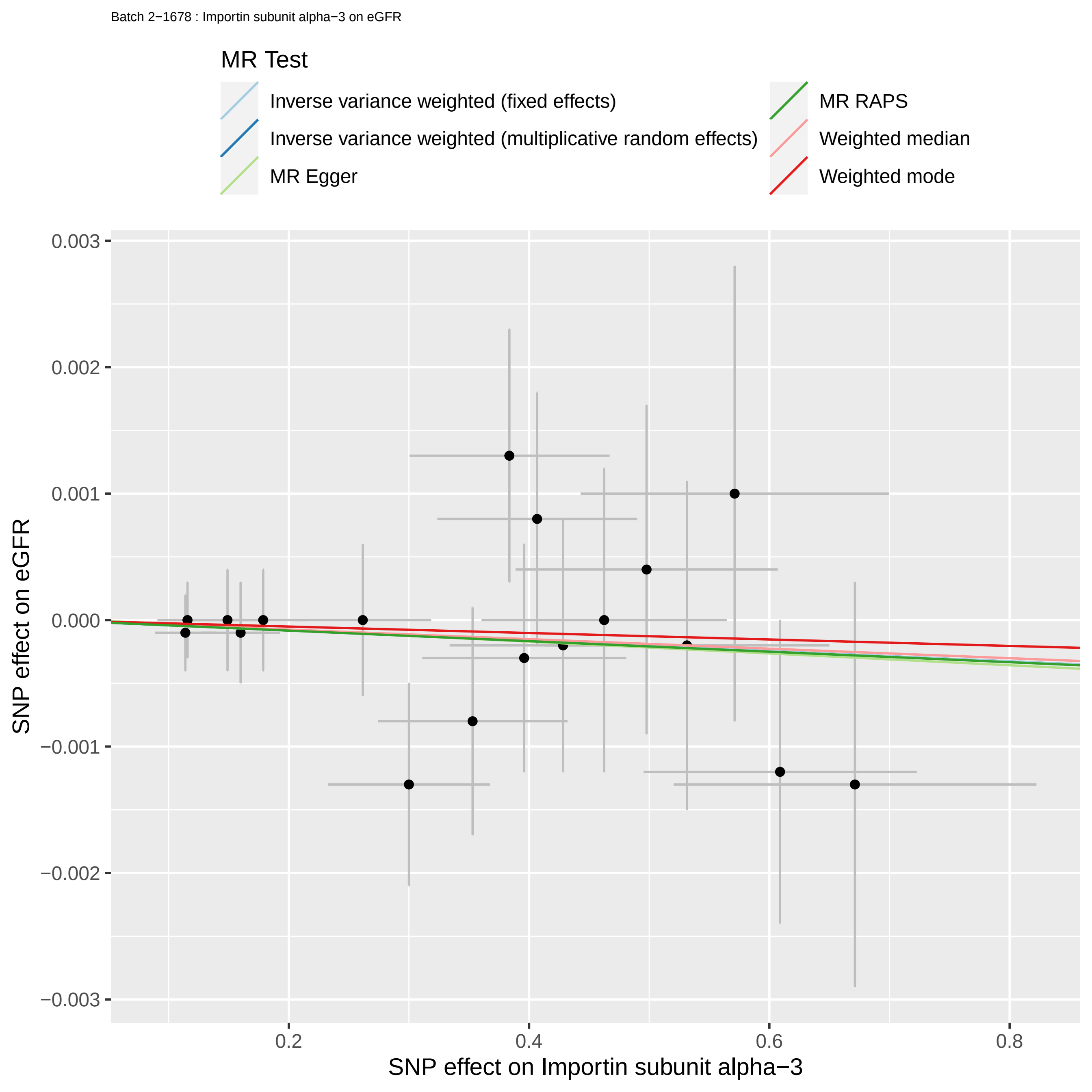
Batch 2−1678 : Importin subunit alpha−3 on eGFR
MR Test
Inverse variance weighted (fixed effects)
Inverse variance weighted (multiplicative random effects)
MR Egger
MR RAPS
Weighted median
Weighted mode
0.003
0.002
0.001
0.000
−0.001
−0.002
−0.003
SNP effect on eGFR
0.2
0.4
0.6
0.8
SNP effect on Importin subunit alpha−3

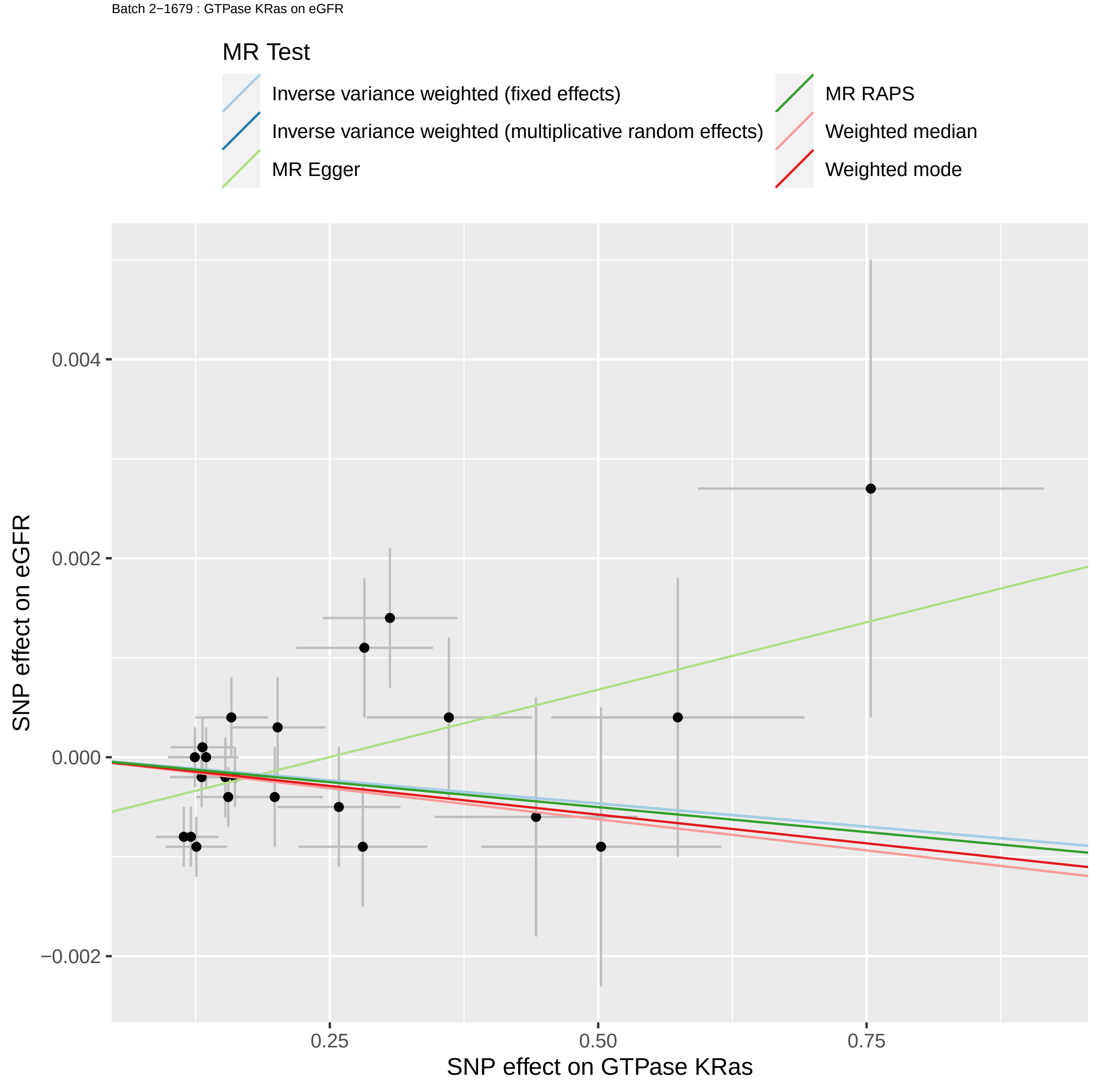

Batch 2−1679 : GTPase KRas on eGFR
MR Test
Inverse variance weighted (fixed effects)
Inverse variance weighted (multiplicative random effects)
MR Egger
MR RAPS
Weighted median
Weighted mode
0.004
0.002
0.000
−0.002
0.25
0.50
0.75
SNP effect on eGFR
SNP effect on GTPase KRas

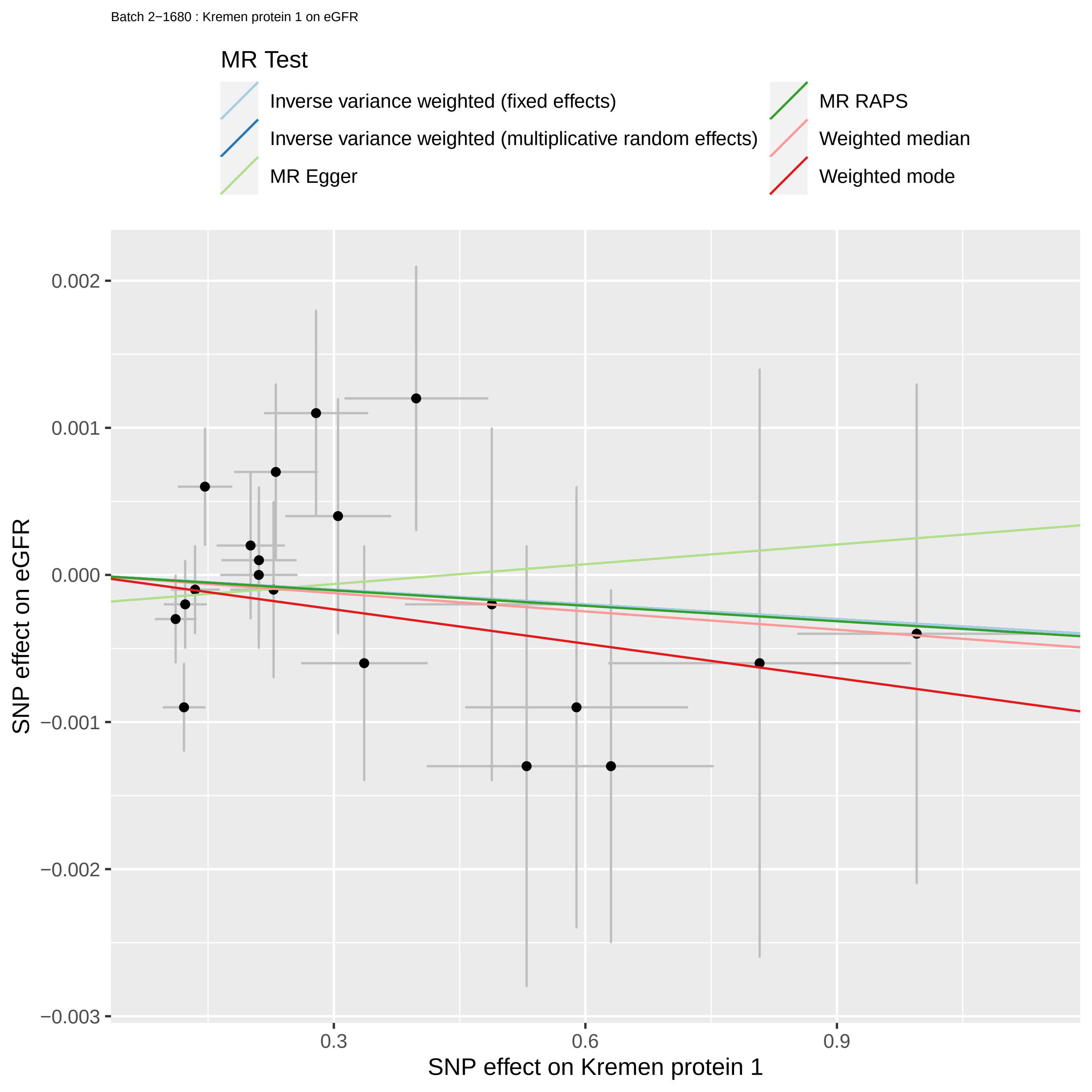

Batch 2−1680 : Kremen protein 1 on eGFR
MR Test
Inverse variance weighted (fixed effects)
Inverse variance weighted (multiplicative random effects)
MR Egger
MR RAPS
Weighted median
Weighted mode
SNP effect on eGFR
0.002
0.001
0.000
−0.001
−0.002
−0.003
0.3
0.6
0.9
SNP effect on Kremen protein 1

Batch 2−1681 : Kremen protein 2 on eGFR

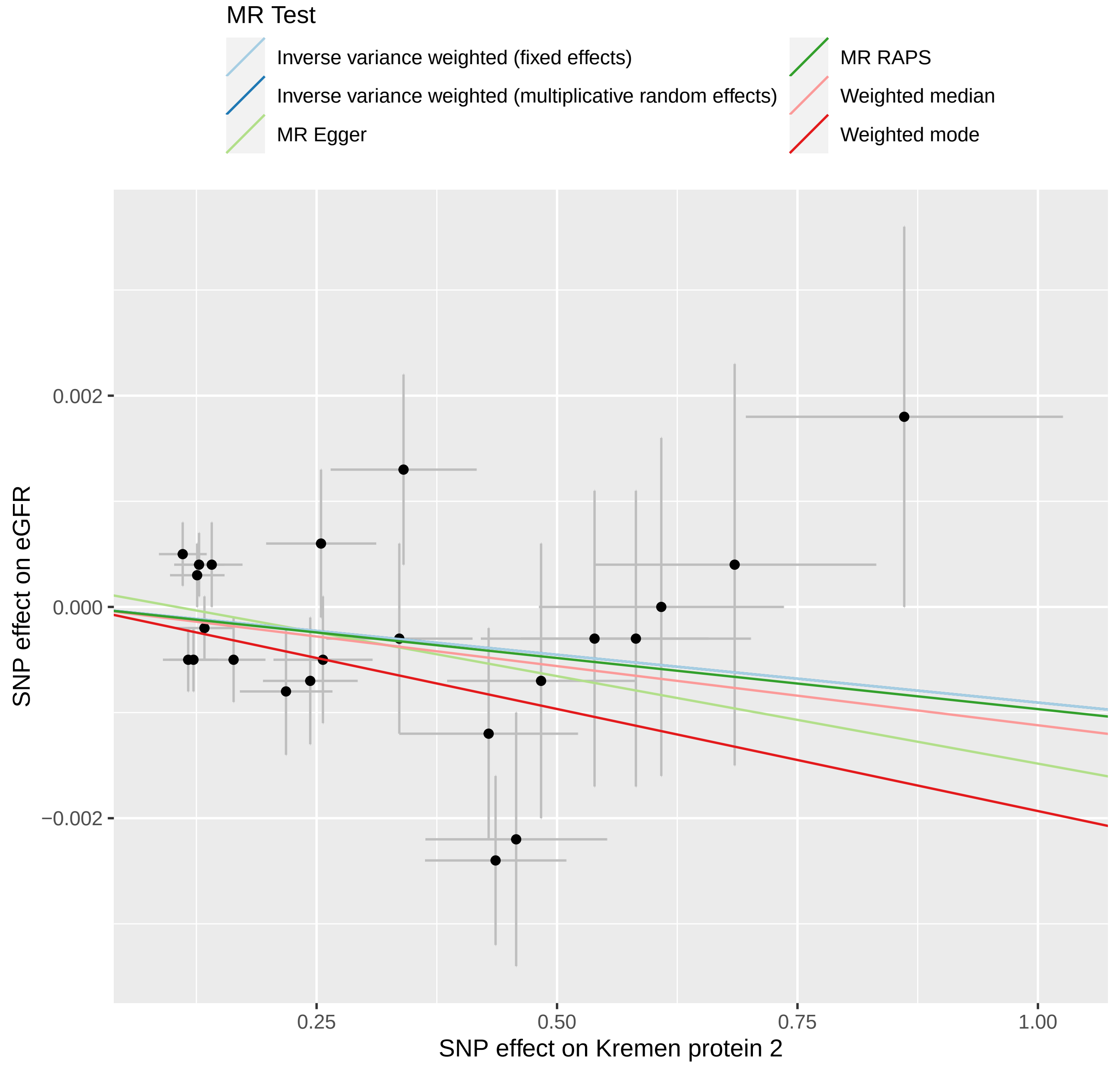

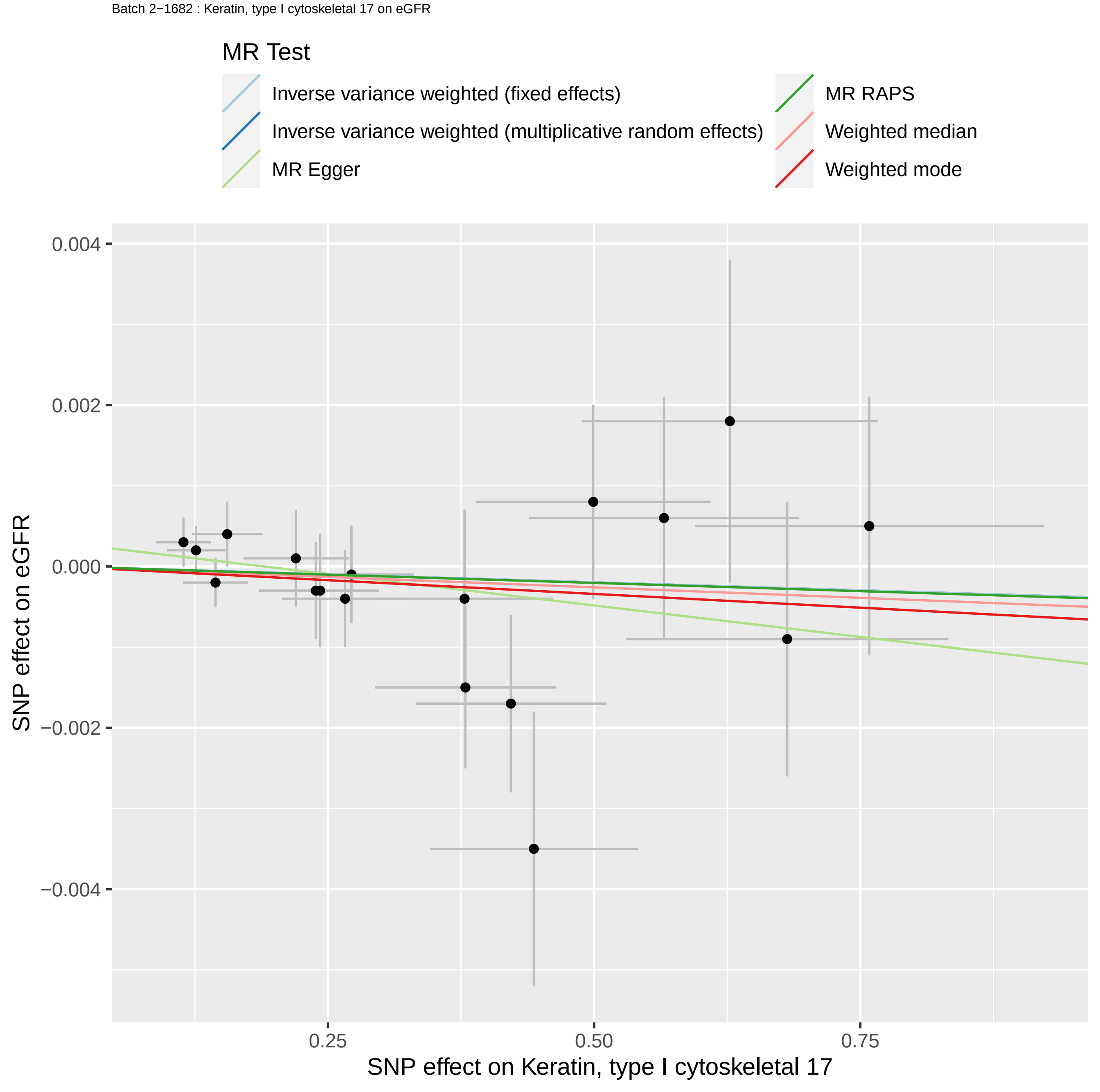
Batch 2−1682 : Keratin, type I cytoskeletal 17 on eGFR
MR Test
Inverse variance weighted (fixed effects)
Inverse variance weighted (multiplicative random effects)
MR Egger
MR RAPS
Weighted median
Weighted mode
0.004
0.002
0.000
−0.002
−0.004
SNP effect on eGFR
0.25
0.50
0.75
SNP effect on Keratin, type I cytoskeletal 17

Batch 2−1683 : Keratin, type I cytoskeletal 18 on eGFR

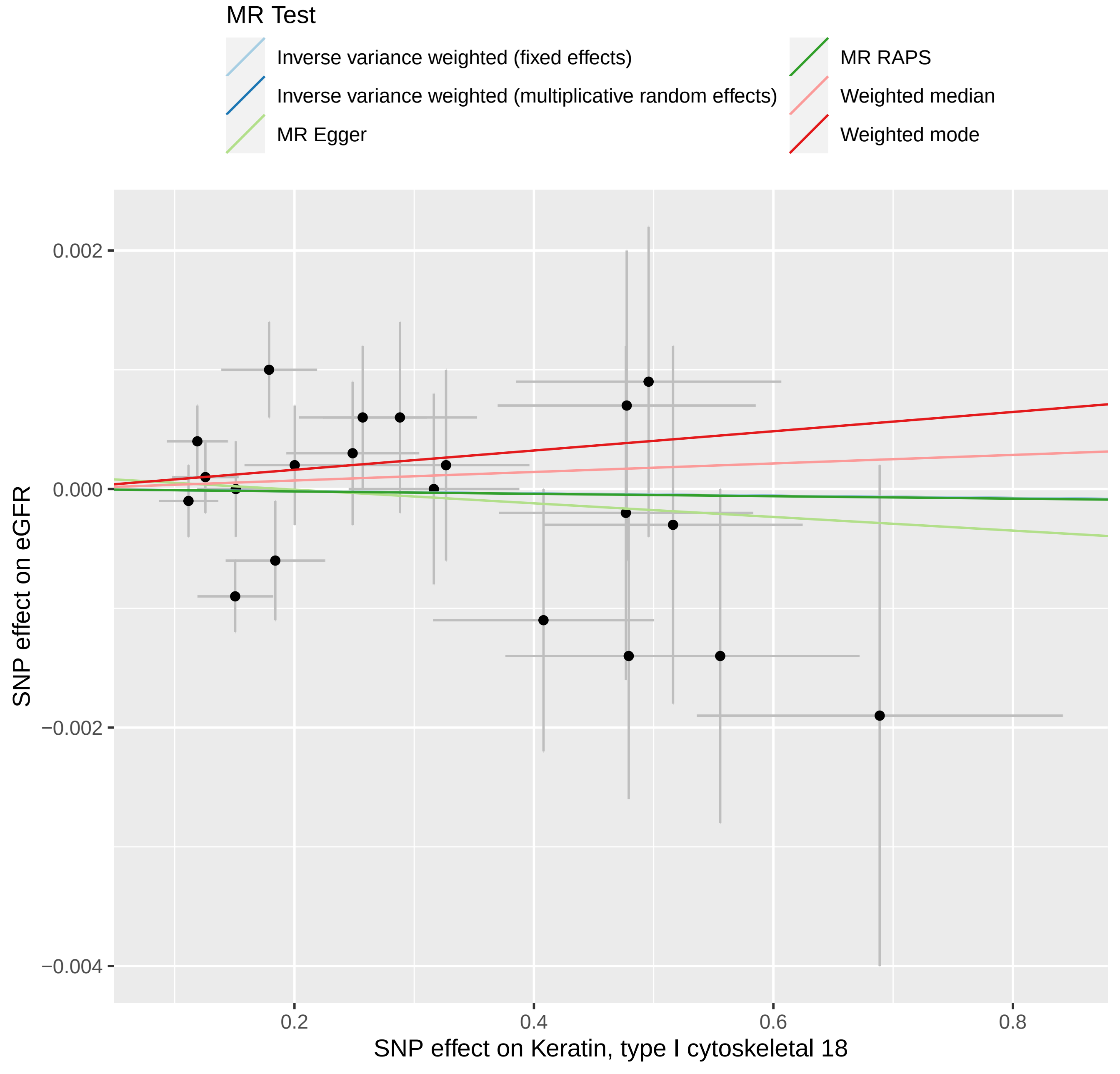

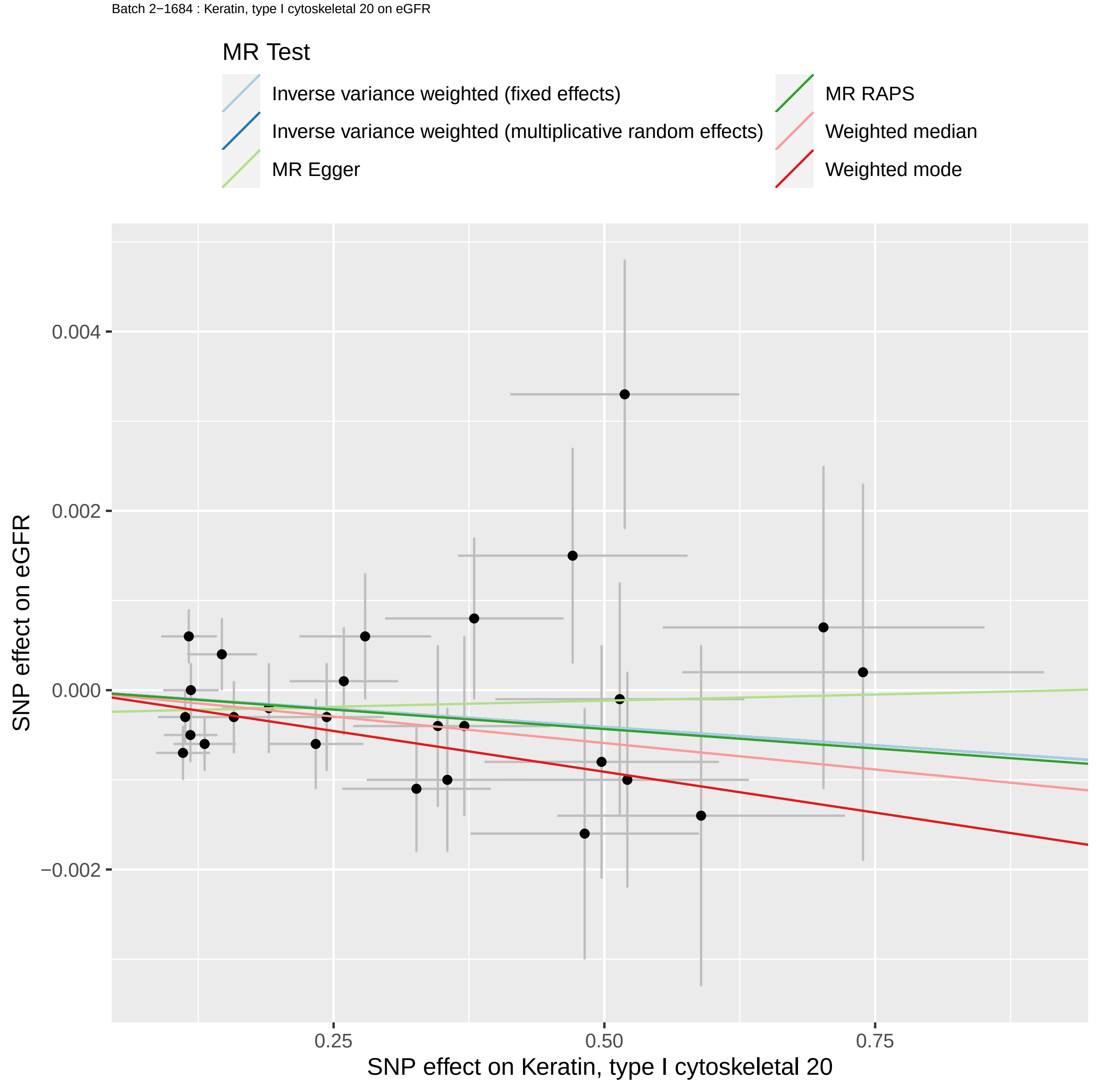
Batch 2−1684 : Keratin, type I cytoskeletal 20 on eGFR
MR Test
Inverse variance weighted (fixed effects)
Inverse variance weighted (multiplicative random effects)
MR Egger
MR RAPS
Weighted median
Weighted mode
0.004
0.002
0.000
−0.002
SNP effect on eGFR
0.25
0.50
0.75
SNP effect on Keratin, type I cytoskeletal 20

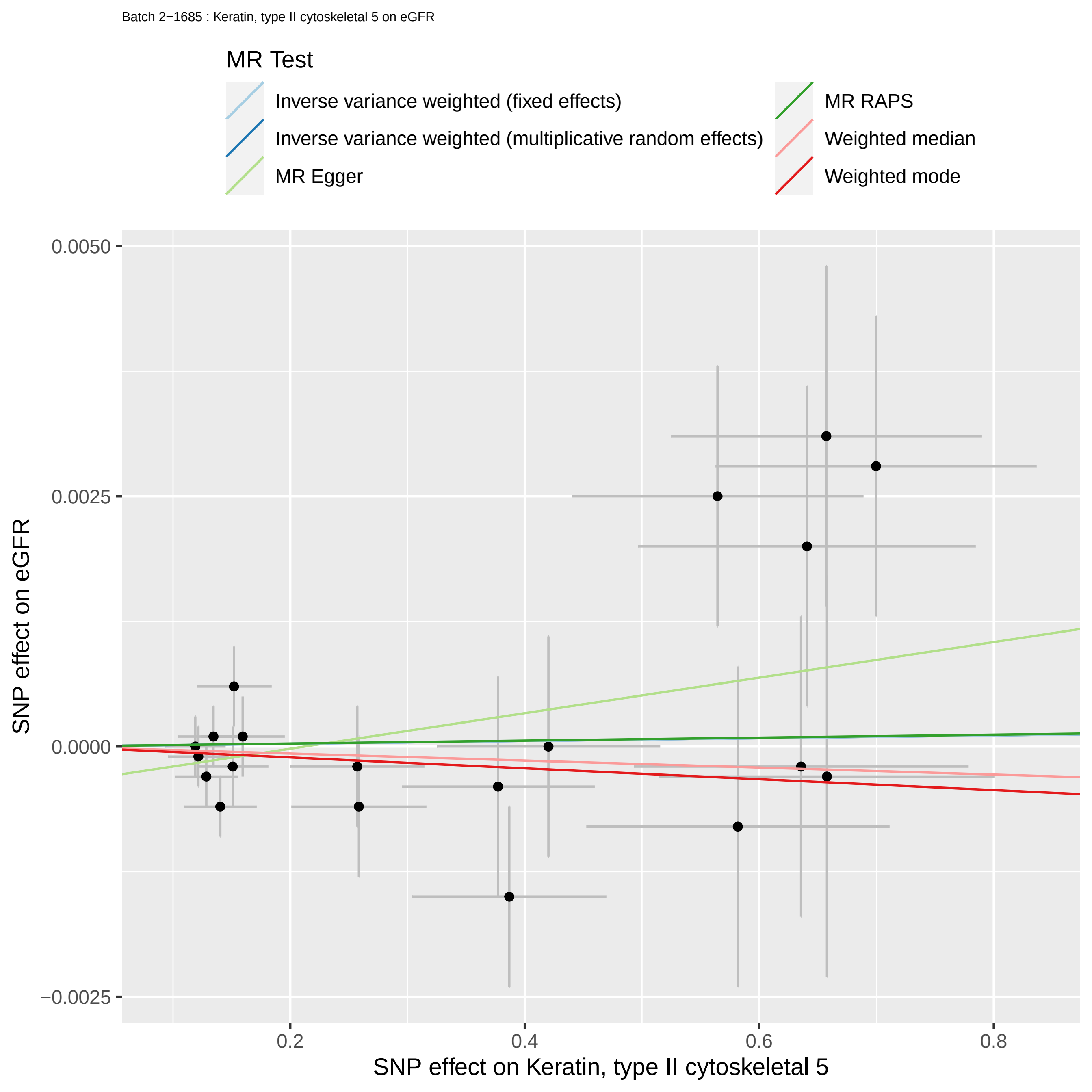

Batch 2−1685 : Keratin, type II cytoskeletal 5 on eGFR
MR Test
Inverse variance weighted (fixed effects)
Inverse variance weighted (multiplicative random effects)
MR Egger
MR RAPS
Weighted median
Weighted mode
0.0050
0.0025
0.0000
−0.0025
SNP effect on eGFR
0.2
0.4
0.6
0.8
SNP effect on Keratin, type II cytoskeletal 5

Batch 2−1686 : Keratin, type II cytoskeletal 7 on eGFR

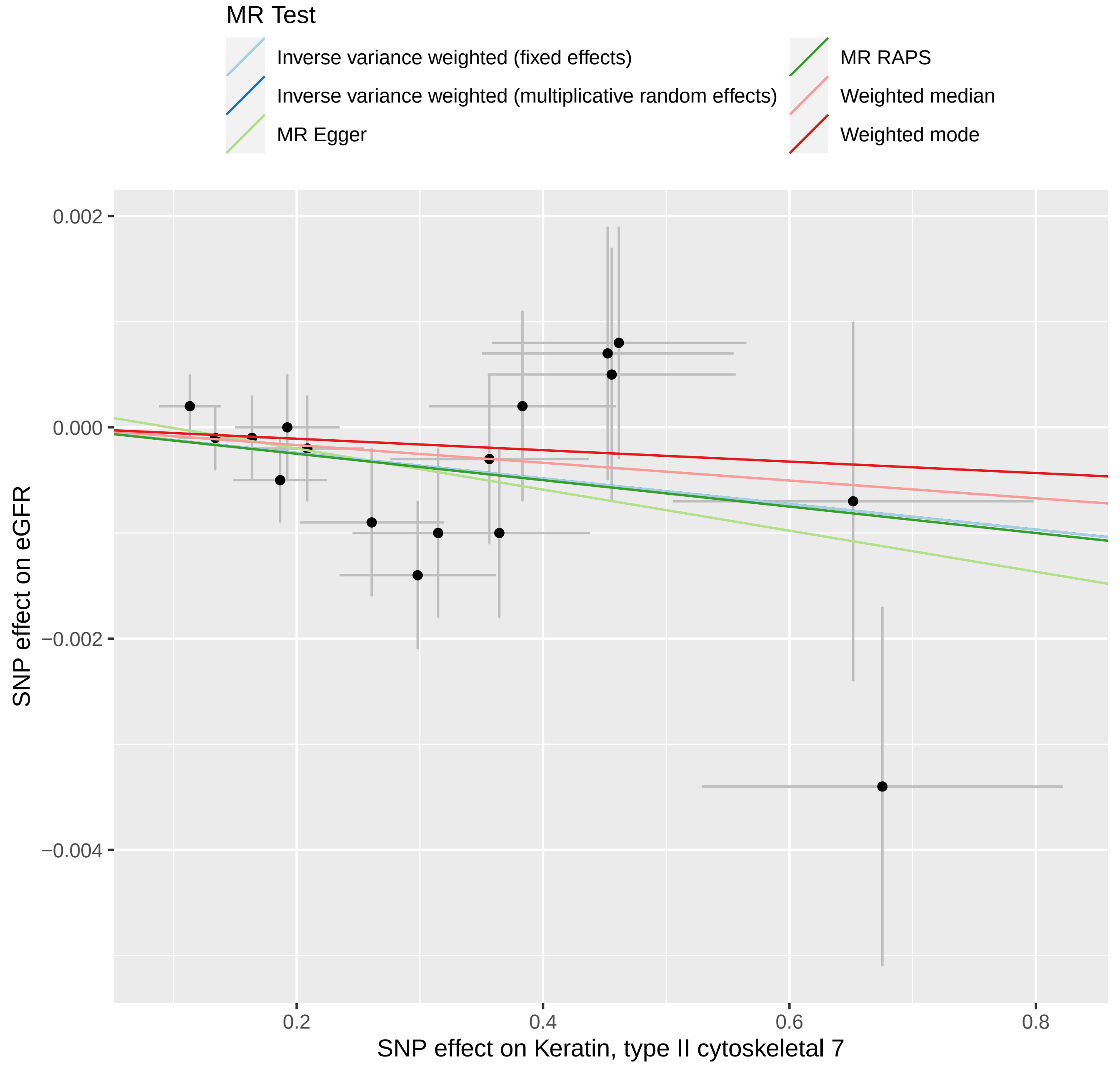

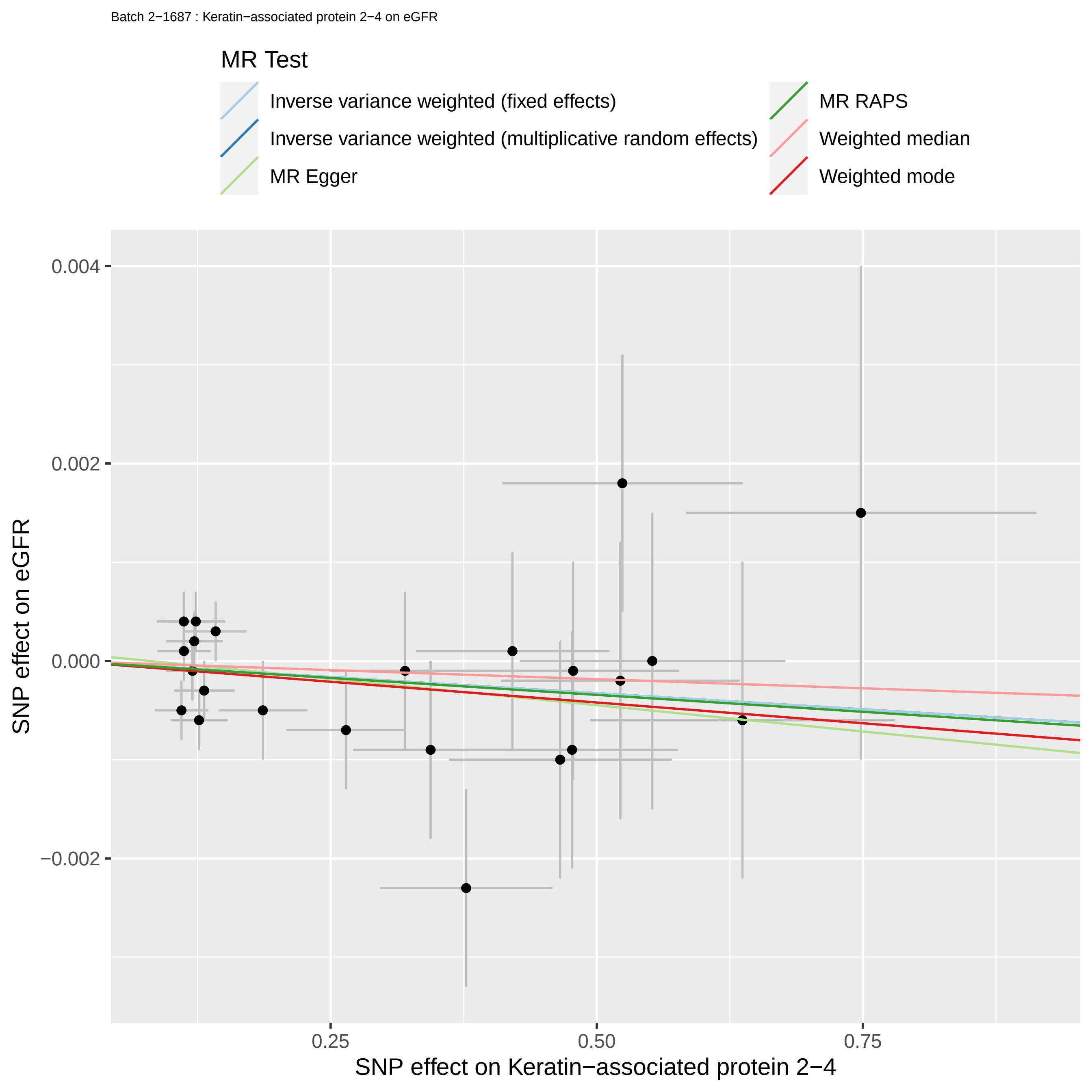

Batch 2−1687 : Keratin−associated protein 2−4 on eGFR
MR Test
Inverse variance weighted (fixed effects)
Inverse variance weighted (multiplicative random effects)
MR Egger
MR RAPS
Weighted median
Weighted mode
0.004
0.002
0.000
−0.002
0.25
0.50
0.75
SNP effect on eGFR
SNP effect on Keratin−associated protein 2−4

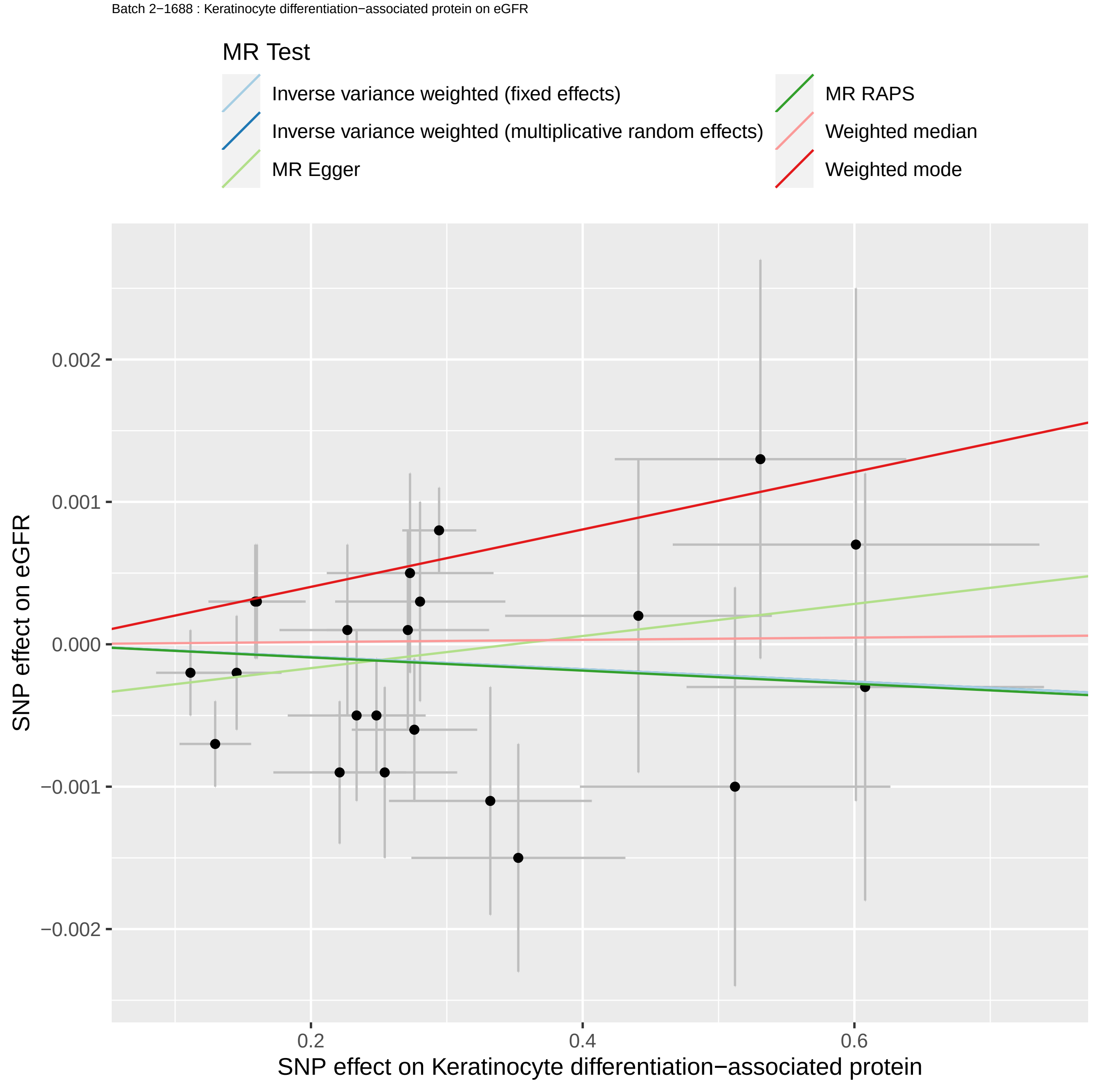
Batch 2−1688 : Keratinocyte differentiation−associated protein on eGFR
MR Test
Inverse variance weighted (fixed effects)
Inverse variance weighted (multiplicative random effects)
MR Egger
MR RAPS
Weighted median
Weighted mode
0.002
0.001
0.000
−0.001
−0.002
0.2
0.4
0.6
SNP effect on eGFR
SNP effect on Keratinocyte differentiation−associated protein

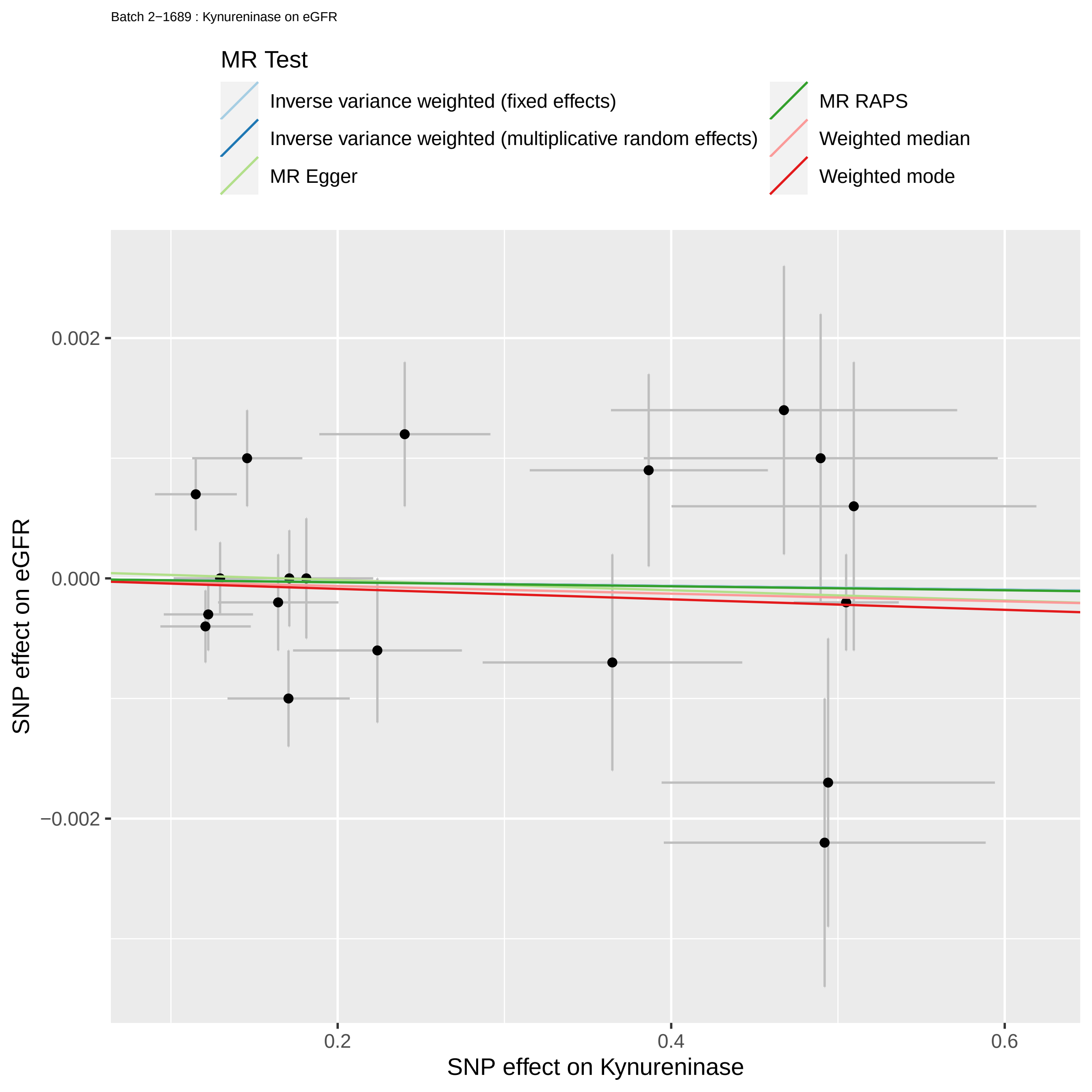

Batch 2−1689 : Kynureninase on eGFR
MR Test
Inverse variance weighted (fixed effects)
Inverse variance weighted (multiplicative random effects)
MR Egger
MR RAPS
Weighted median
Weighted mode
0.002
0.000
−0.002
0.2
0.4
0.6
SNP effect on eGFR
SNP effect on Kynureninase

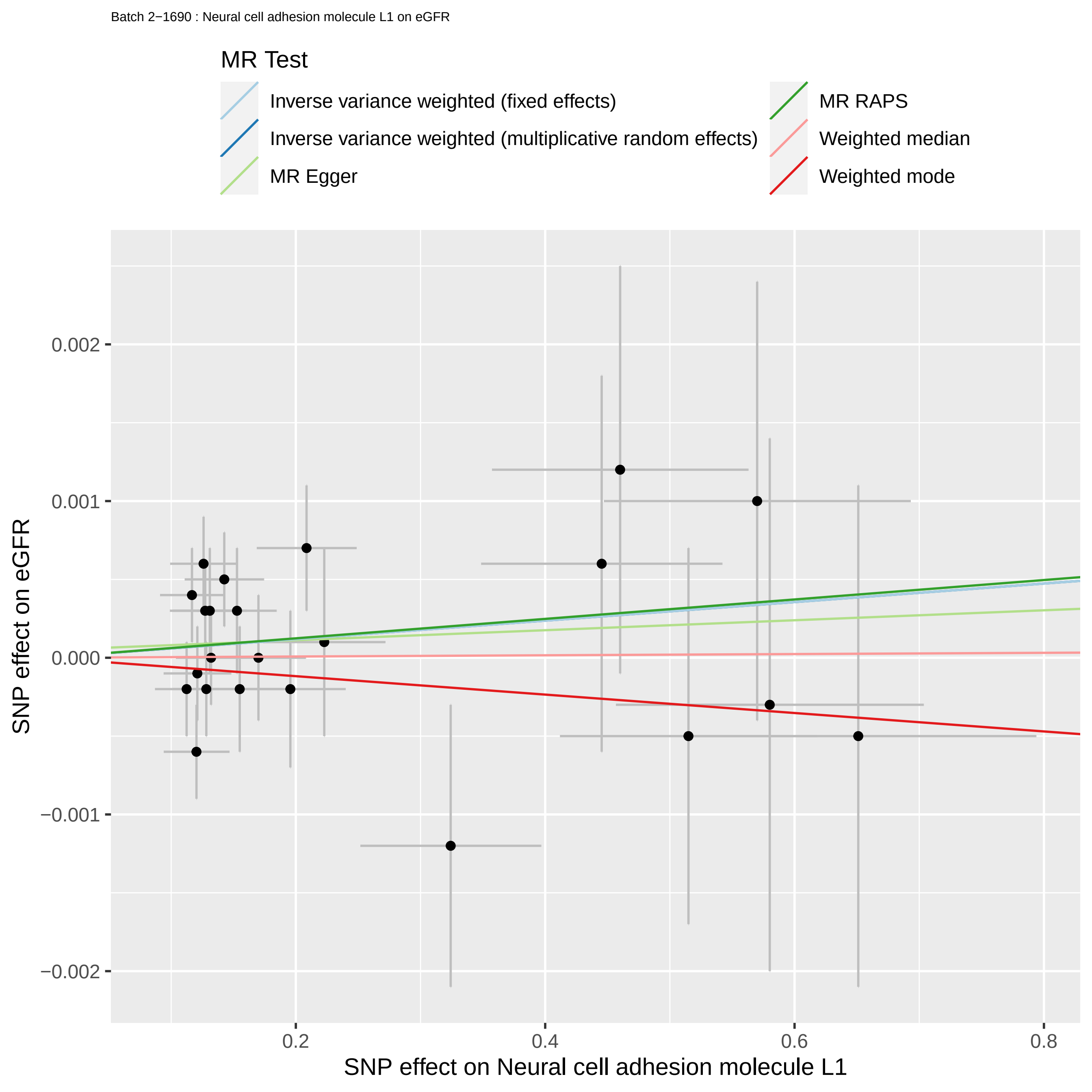

Batch 2−1690 : Neural cell adhesion molecule L1 on eGFR
MR Test
Inverse variance weighted (fixed effects)
Inverse variance weighted (multiplicative random effects)
MR Egger
MR RAPS
Weighted median
Weighted mode
0.002
0.001
0.000
−0.001
−0.002
SNP effect on eGFR
0.2
0.4
0.6
0.8
SNP effect on Neural cell adhesion molecule L1

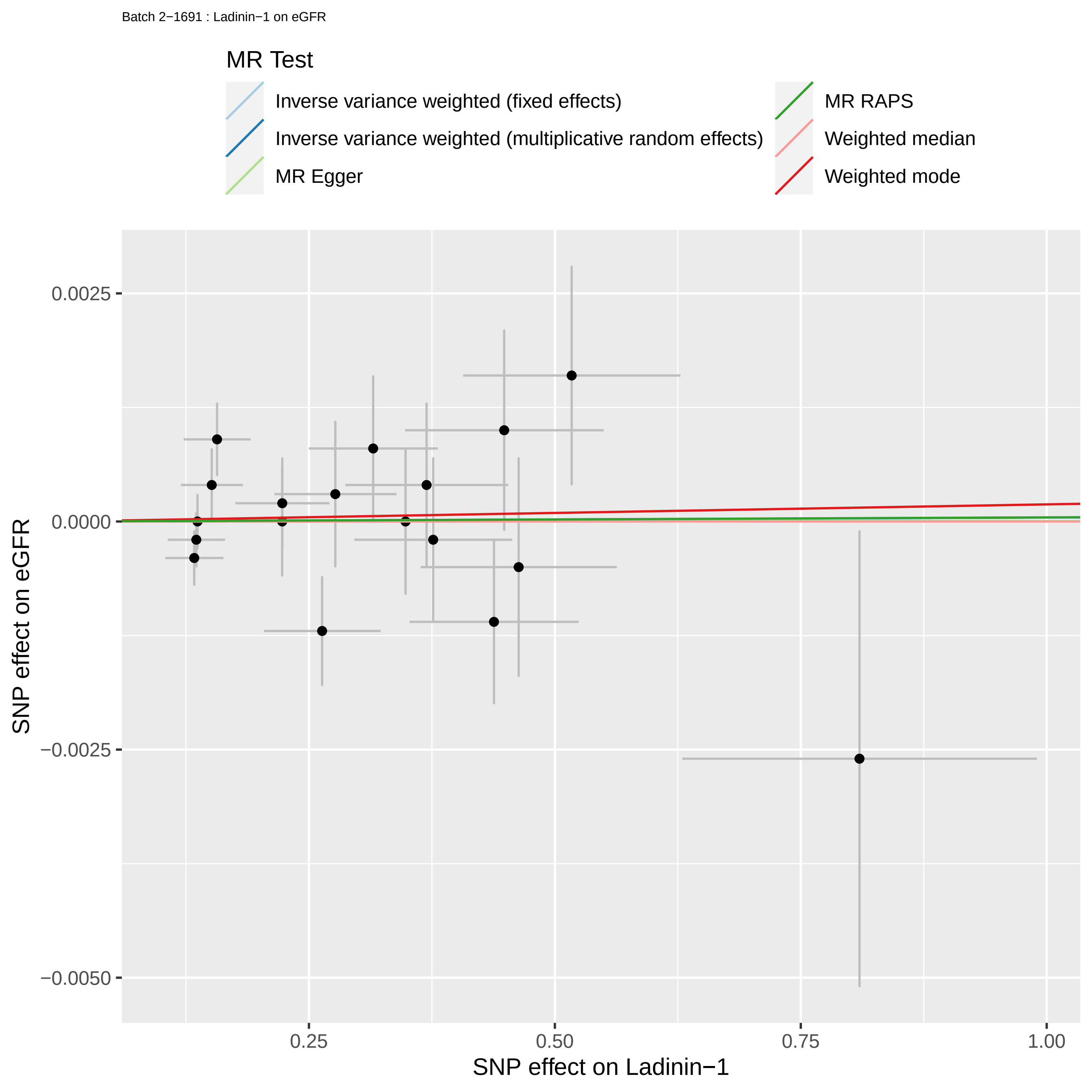

Batch 2−1691 : Ladinin−1 on eGFR
MR Test
Inverse variance weighted (fixed effects)
Inverse variance weighted (multiplicative random effects)
MR Egger
MR RAPS
Weighted median
Weighted mode
0.0025
0.0000
−0.0025
−0.0050
SNP effect on eGFR
0.25
0.50
0.75
1.00
SNP effect on Ladinin−1

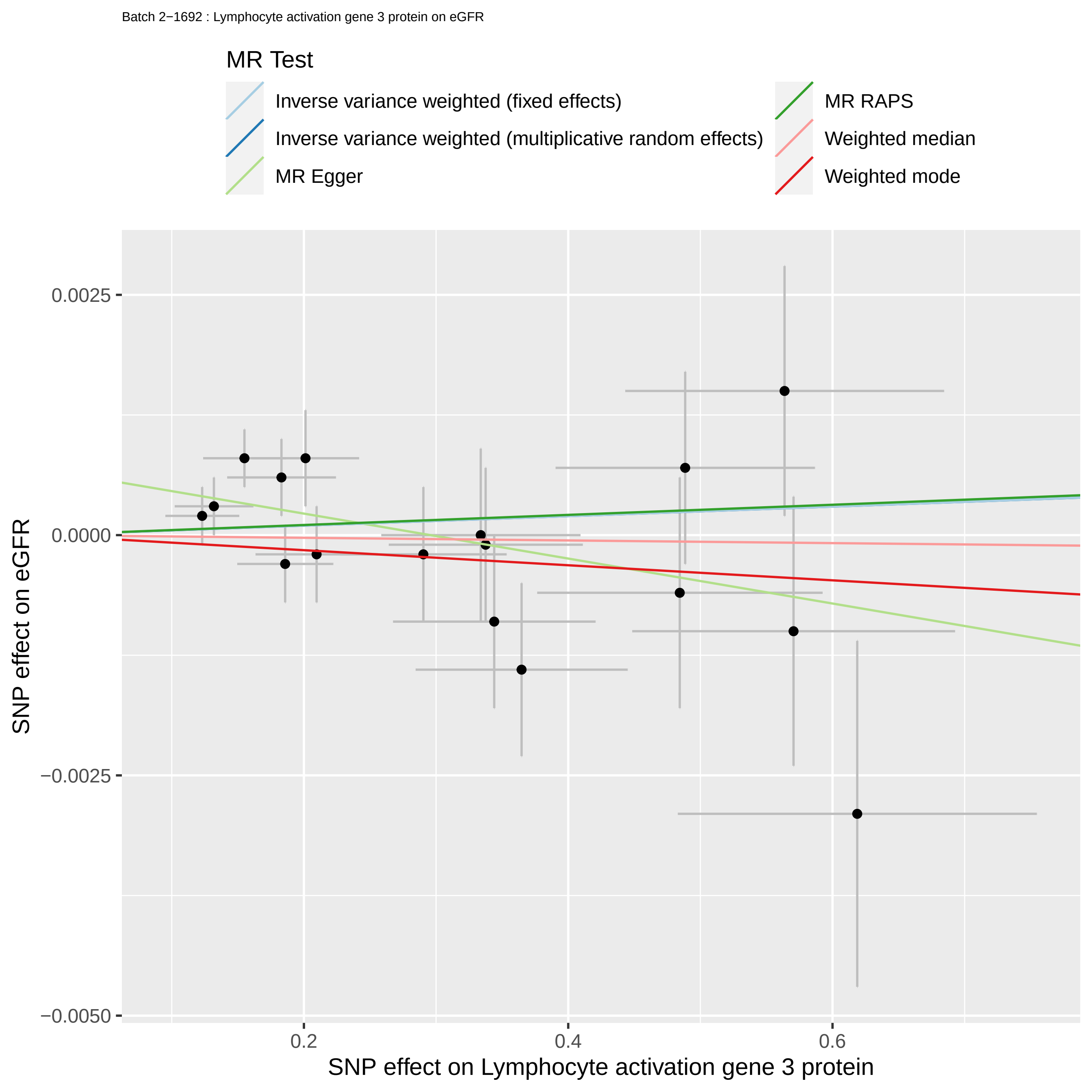

Batch 2−1692 : Lymphocyte activation gene 3 protein on eGFR
MR Test
Inverse variance weighted (fixed effects)
Inverse variance weighted (multiplicative random effects)
MR Egger
MR RAPS
Weighted median
Weighted mode
0.0025
0.0000
−0.0025
−0.0050
0.2
0.4
0.6
SNP effect on eGFR
SNP effect on Lymphocyte activation gene 3 protein

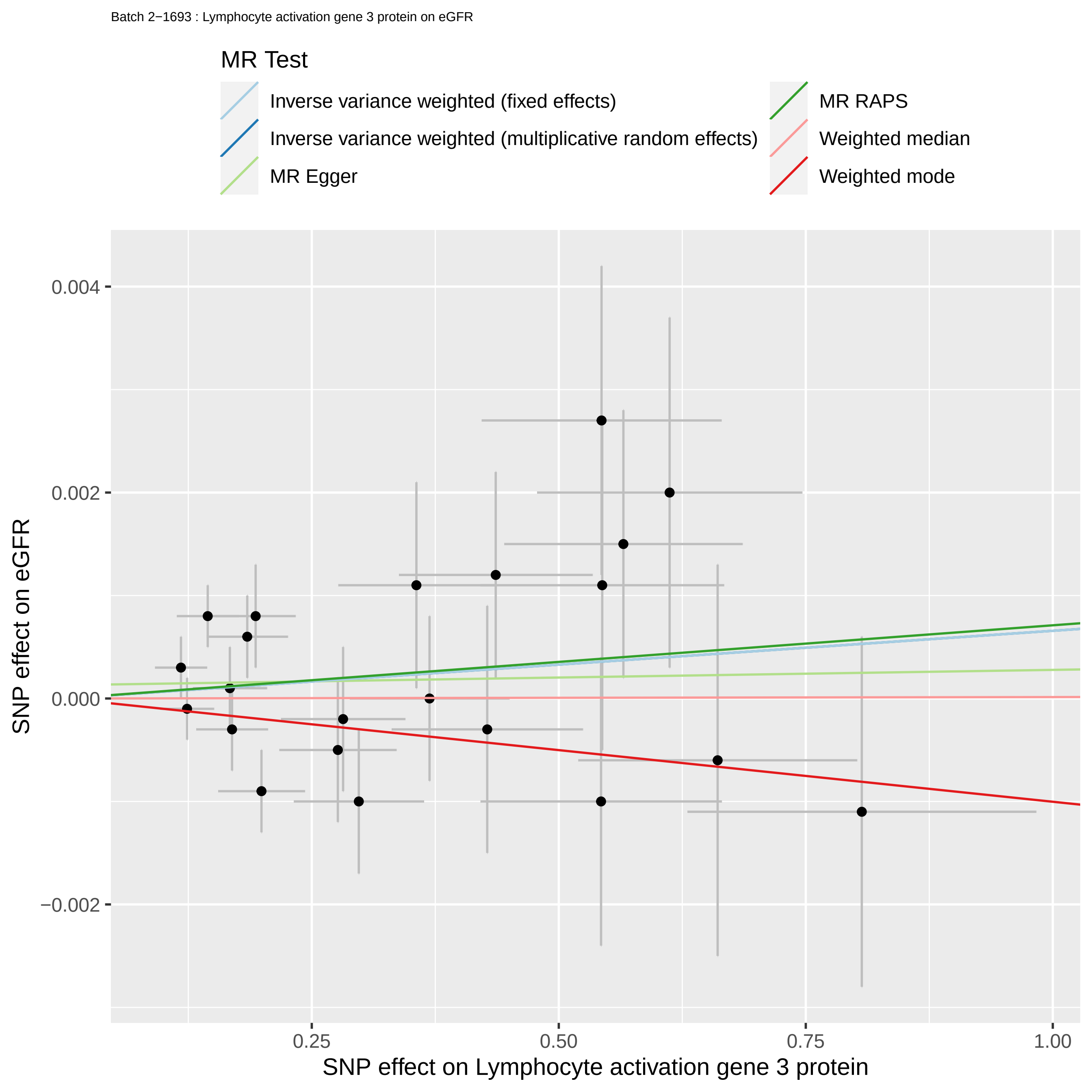
Batch 2−1693 : Lymphocyte activation gene 3 protein on eGFR
MR Test
Inverse variance weighted (fixed effects)
Inverse variance weighted (multiplicative random effects)
MR Egger
MR RAPS
Weighted median
Weighted mode
0.004
0.002
0.000
−0.002
SNP effect on eGFR
0.25
0.50
0.75
1.00
SNP effect on Lymphocyte activation gene 3 protein

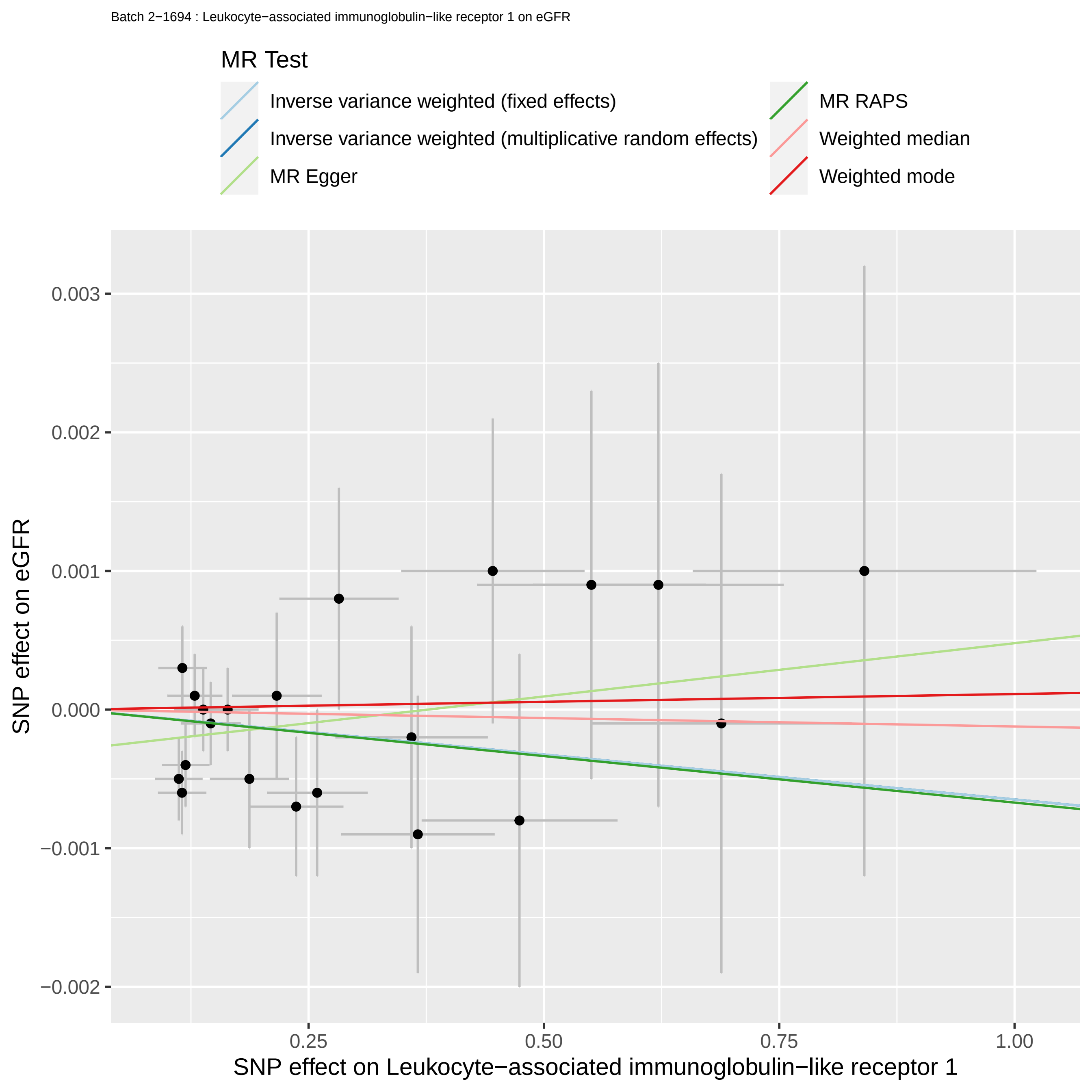
Batch 2−1694 : Leukocyte−associated immunoglobulin−like receptor 1 on eGFR
MR Test
Inverse variance weighted (fixed effects)
Inverse variance weighted (multiplicative random effects)
MR Egger
MR RAPS
Weighted median
Weighted mode
SNP effect on eGFR
0.003
0.002
0.001
0.000
−0.001
−0.002
0.25
0.50
0.75
1.00
SNP effect on Leukocyte−associated immunoglobulin−like receptor 1

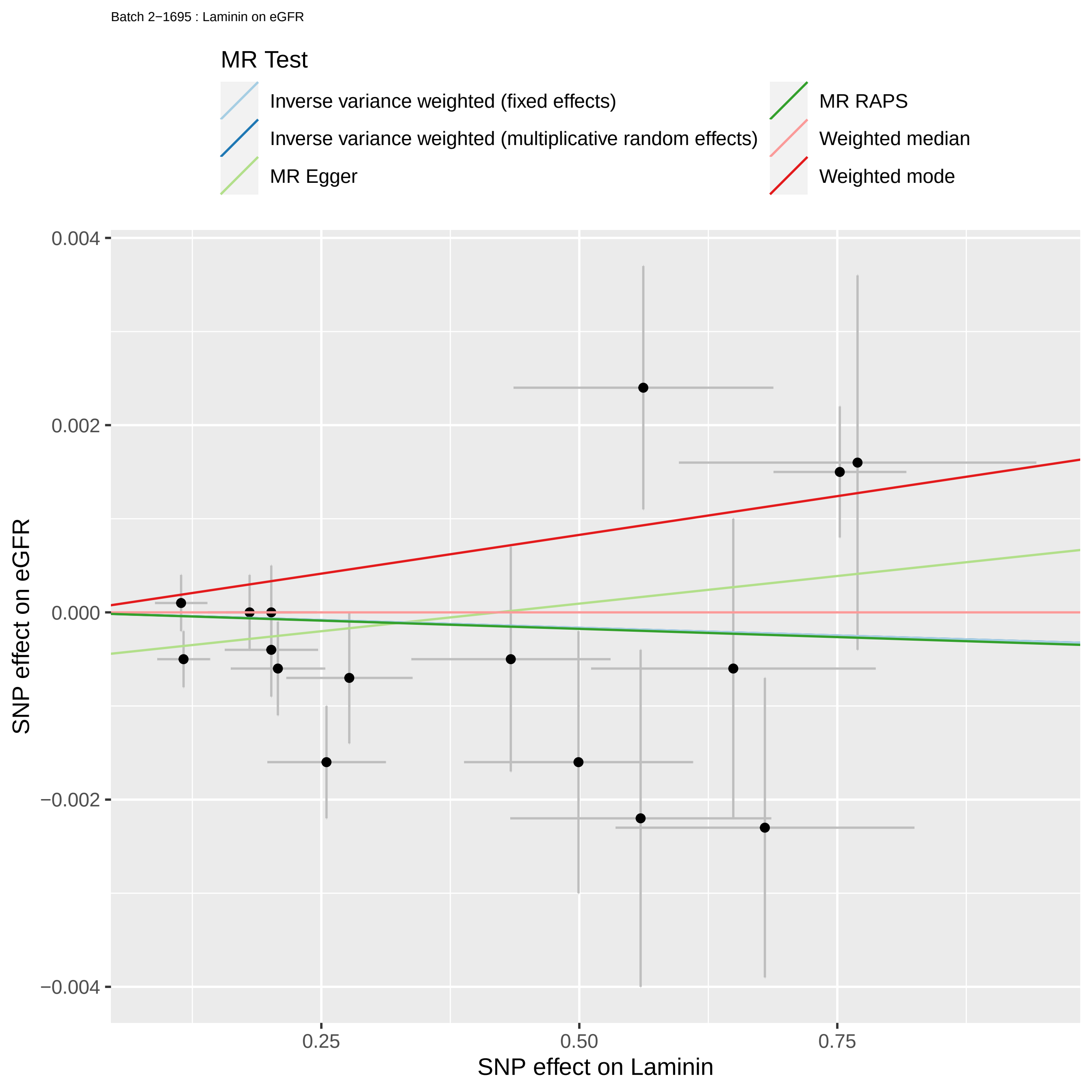

Batch 2−1695 : Laminin on eGFR
MR Test
Inverse variance weighted (fixed effects)
Inverse variance weighted (multiplicative random effects)
MR Egger
MR RAPS
Weighted median
Weighted mode
0.004
0.002
0.000
−0.002
−0.004
SNP effect on eGFR
0.25
0.50
0.75
SNP effect on Laminin

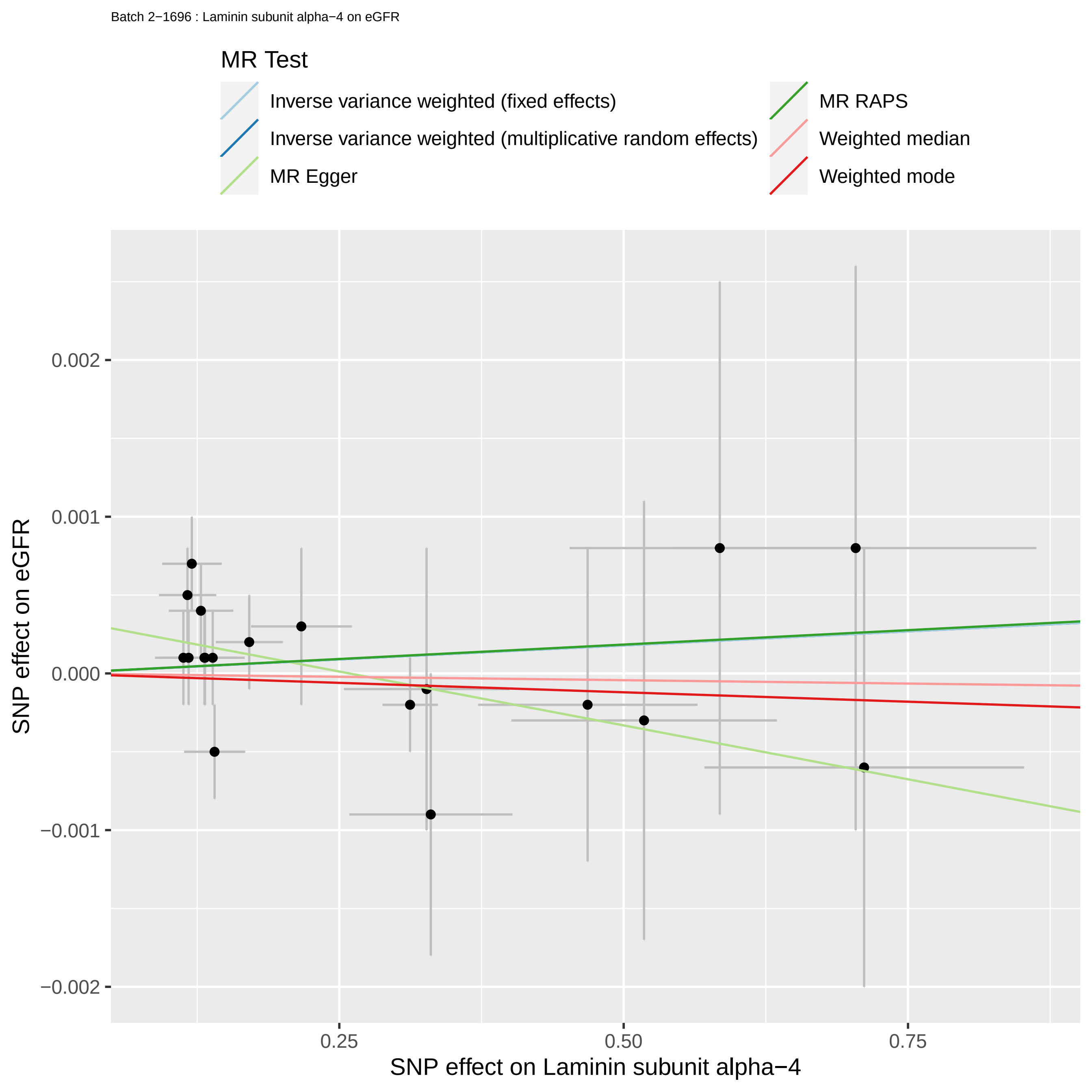

Batch 2−1696 : Laminin subunit alpha−4 on eGFR
MR Test
Inverse variance weighted (fixed effects)
Inverse variance weighted (multiplicative random effects)
MR Egger
MR RAPS
Weighted median
Weighted mode
0.002
0.001
0.000
−0.001
−0.002
SNP effect on eGFR
0.25
0.50
0.75
SNP effect on Laminin subunit alpha−4

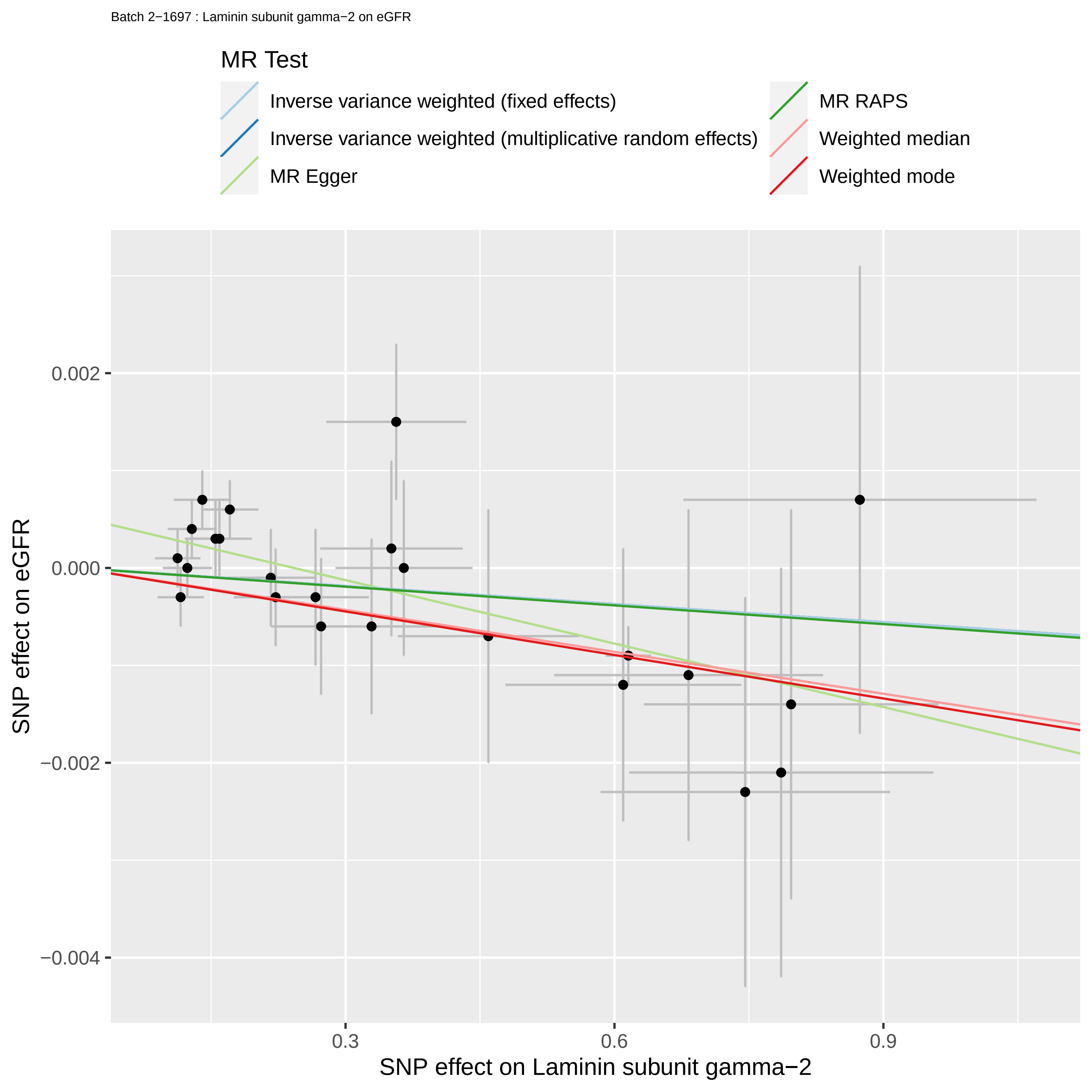
Batch 2−1697 : Laminin subunit gamma−2 on eGFR
MR Test
Inverse variance weighted (fixed effects)
Inverse variance weighted (multiplicative random effects)
MR Egger
MR RAPS
Weighted median
Weighted mode
0.002
0.000
−0.002
−0.004
0.3
0.6
0.9
SNP effect on eGFR
SNP effect on Laminin subunit gamma−2

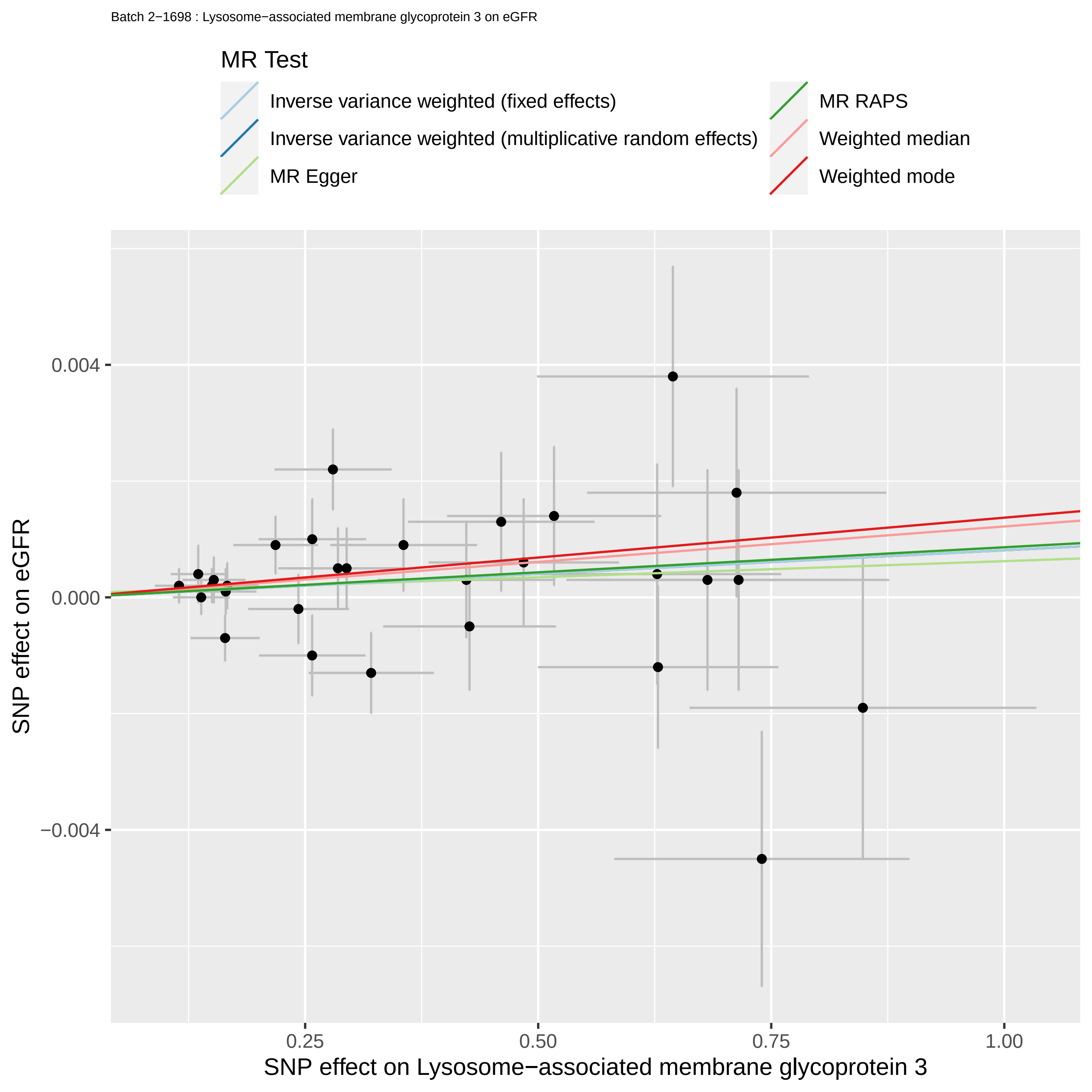

Batch 2−1698 : Lysosome−associated membrane glycoprotein 3 on eGFR
MR Test
Inverse variance weighted (fixed effects)
Inverse variance weighted (multiplicative random effects)
MR Egger
MR RAPS
Weighted median
Weighted mode
0.004
0.000
−0.004
0.25
0.50
0.75
1.00
SNP effect on eGFR
SNP effect on Lysosome−associated membrane glycoprotein 3

Batch 2−1699 : Ragulator complex protein LAMTOR3 on eGFR

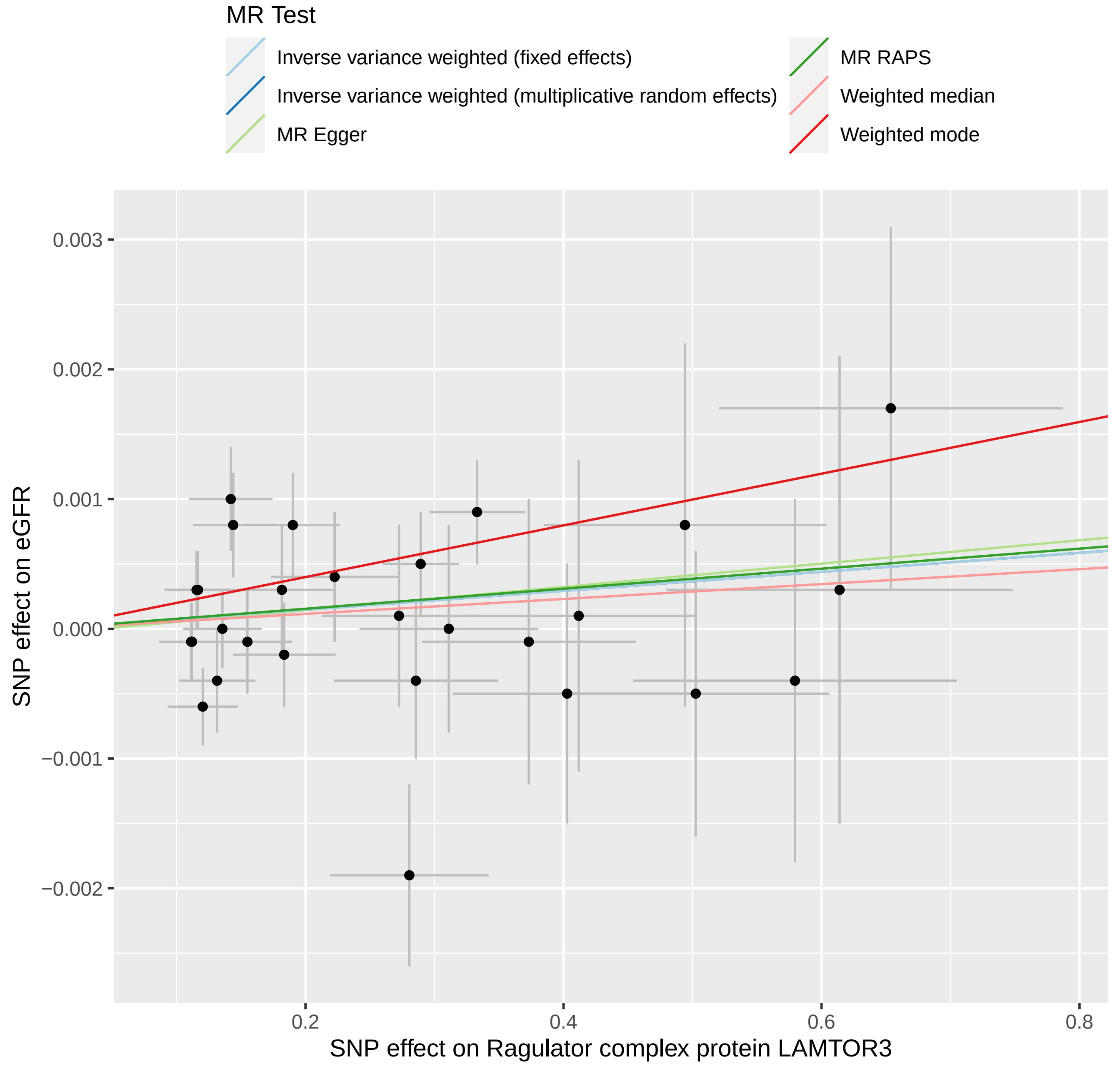

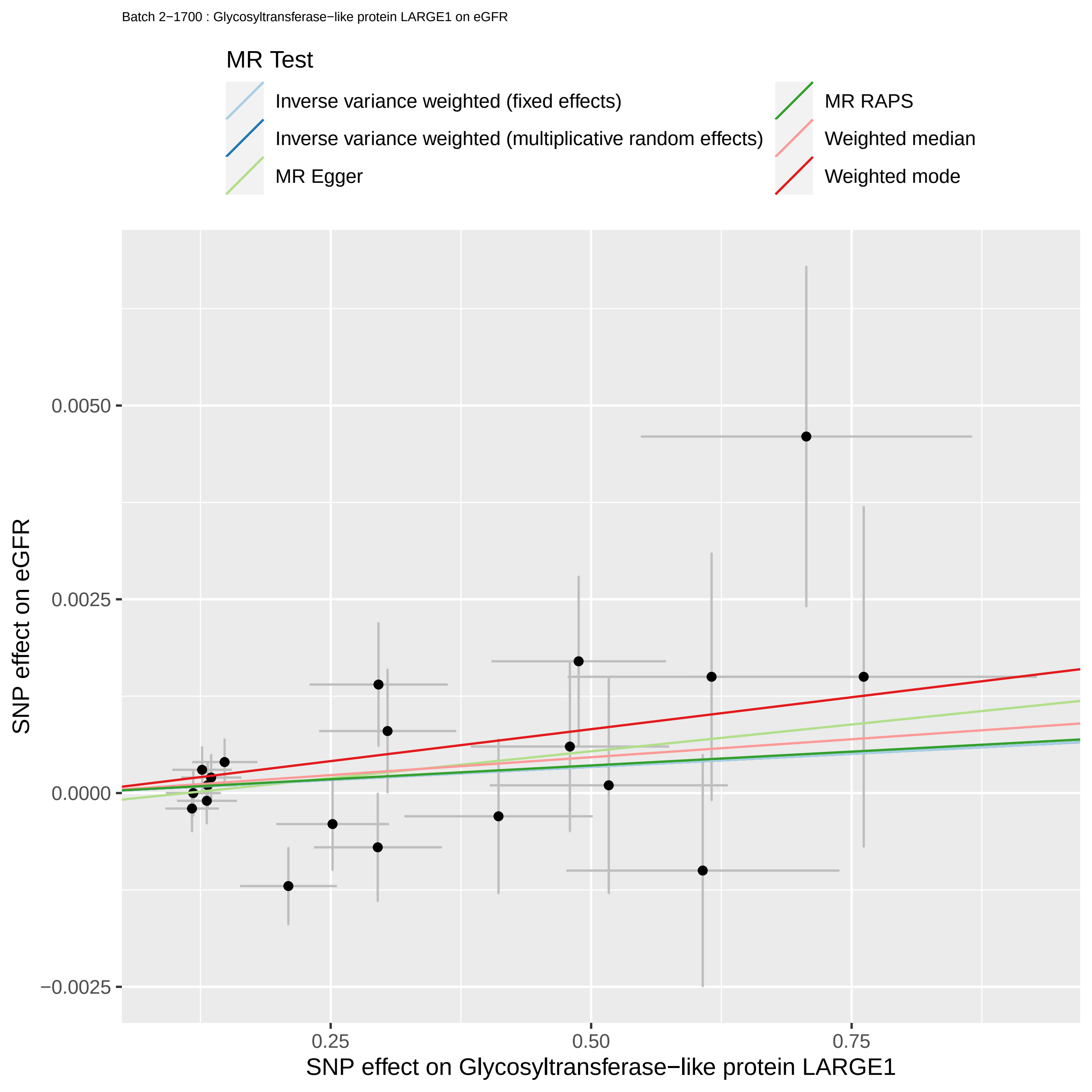

Batch 2−1700 : Glycosyltransferase−like protein LARGE1 on eGFR
MR Test
Inverse variance weighted (fixed effects)
Inverse variance weighted (multiplicative random effects)
MR Egger
MR RAPS
Weighted median
Weighted mode
0.0050
0.0025
0.0000
−0.0025
SNP effect on eGFR
0.25
0.50
0.75
SNP effect on Glycosyltransferase−like protein LARGE1

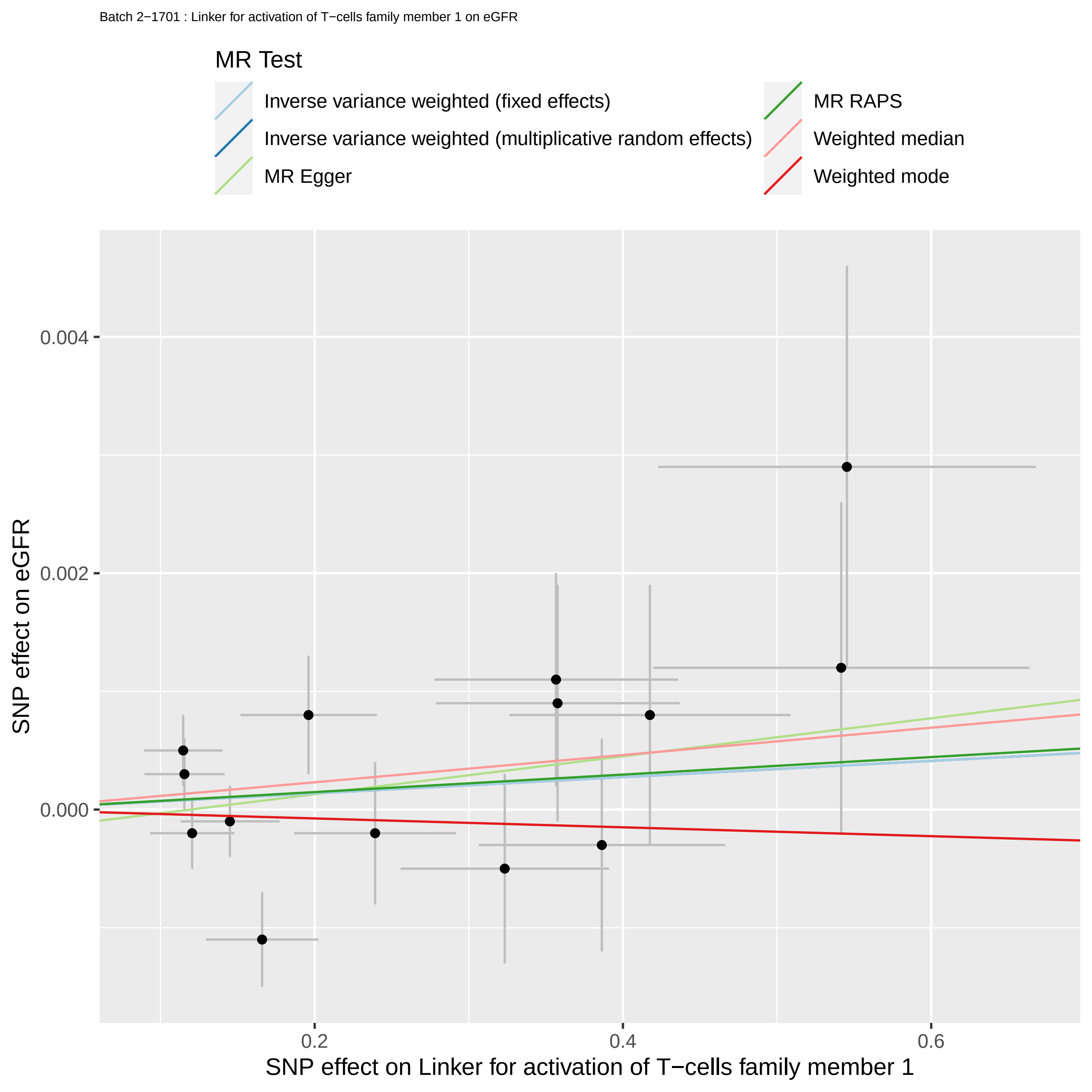
Batch 2−1701 : Linker for activation of T−cells family member 1 on eGFR
MR Test
Inverse variance weighted (fixed effects)
Inverse variance weighted (multiplicative random effects)
MR Egger
MR RAPS
Weighted median
Weighted mode
0.004
0.002
0.000
SNP effect on eGFR
0.2
0.4
0.6
SNP effect on Linker for activation of T−cells family member 1

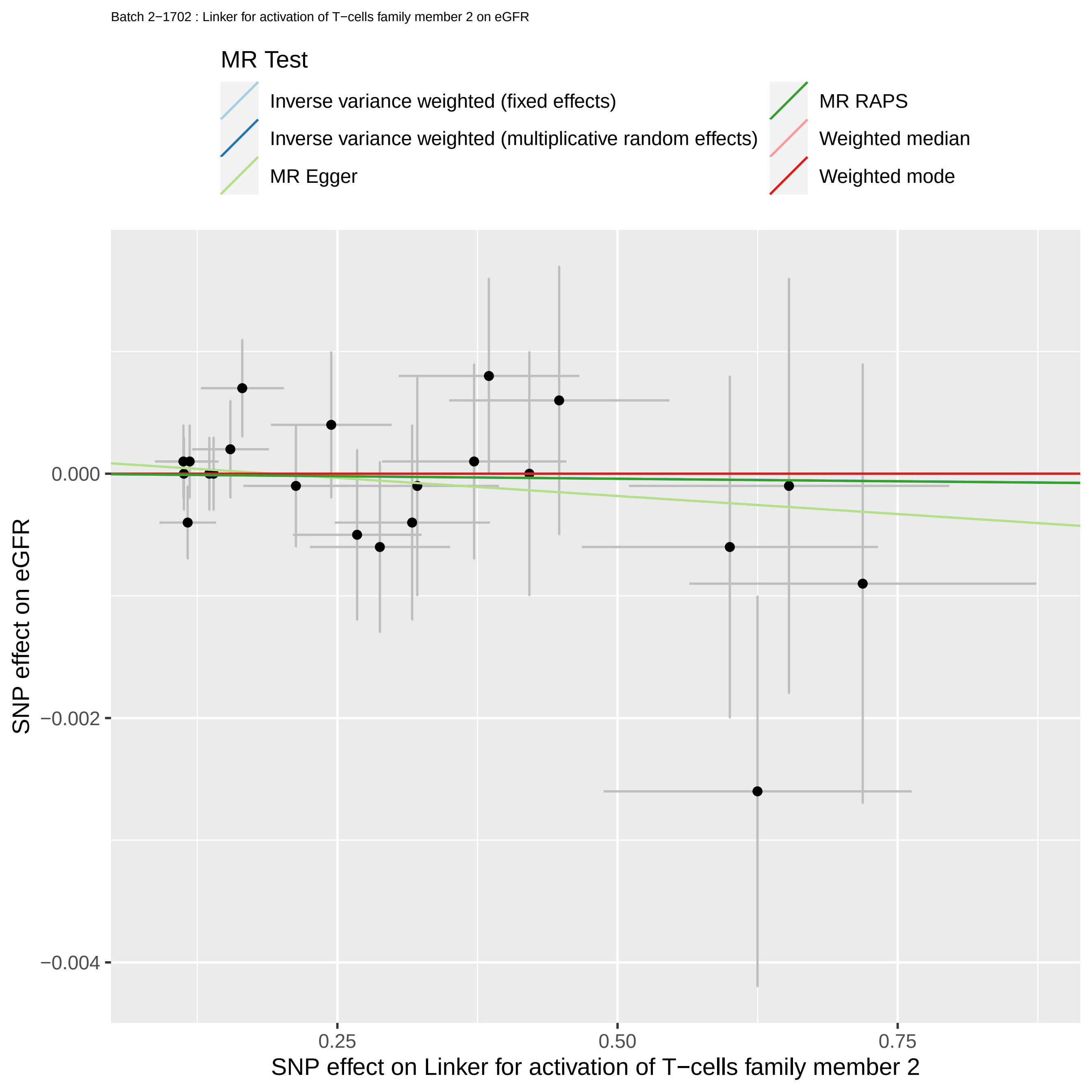

Batch 2−1702 : Linker for activation of T−cells family member 2 on eGFR
MR Test
Inverse variance weighted (fixed effects)
Inverse variance weighted (multiplicative random effects)
MR Egger
MR RAPS
Weighted median
Weighted mode
SNP effect on eGFR
0.000
−0.002
−0.004
0.25
0.50
0.75
SNP effect on Linker for activation of T−cells family member 2

Batch 2−1703 : Layilin on eGFR

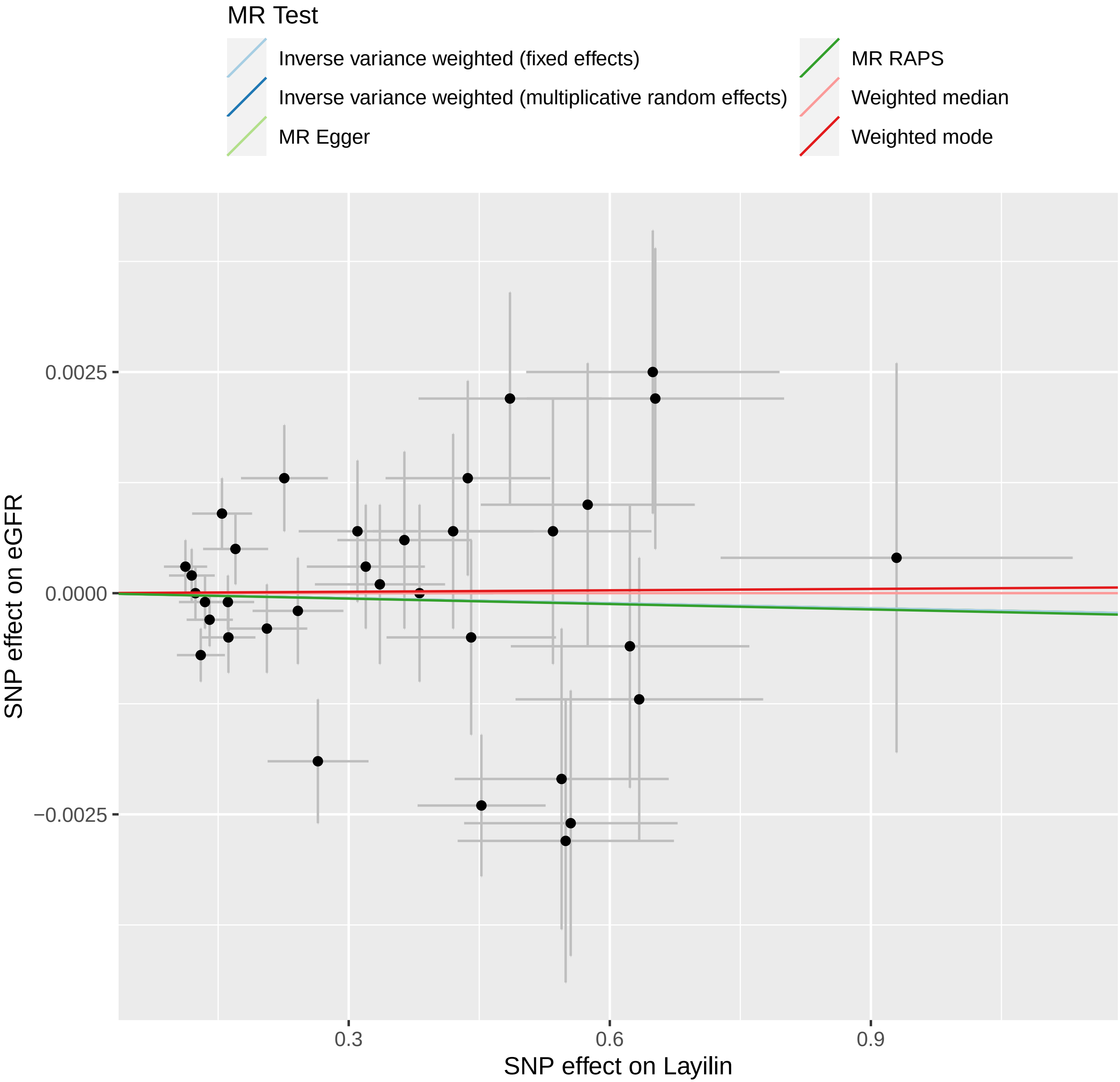

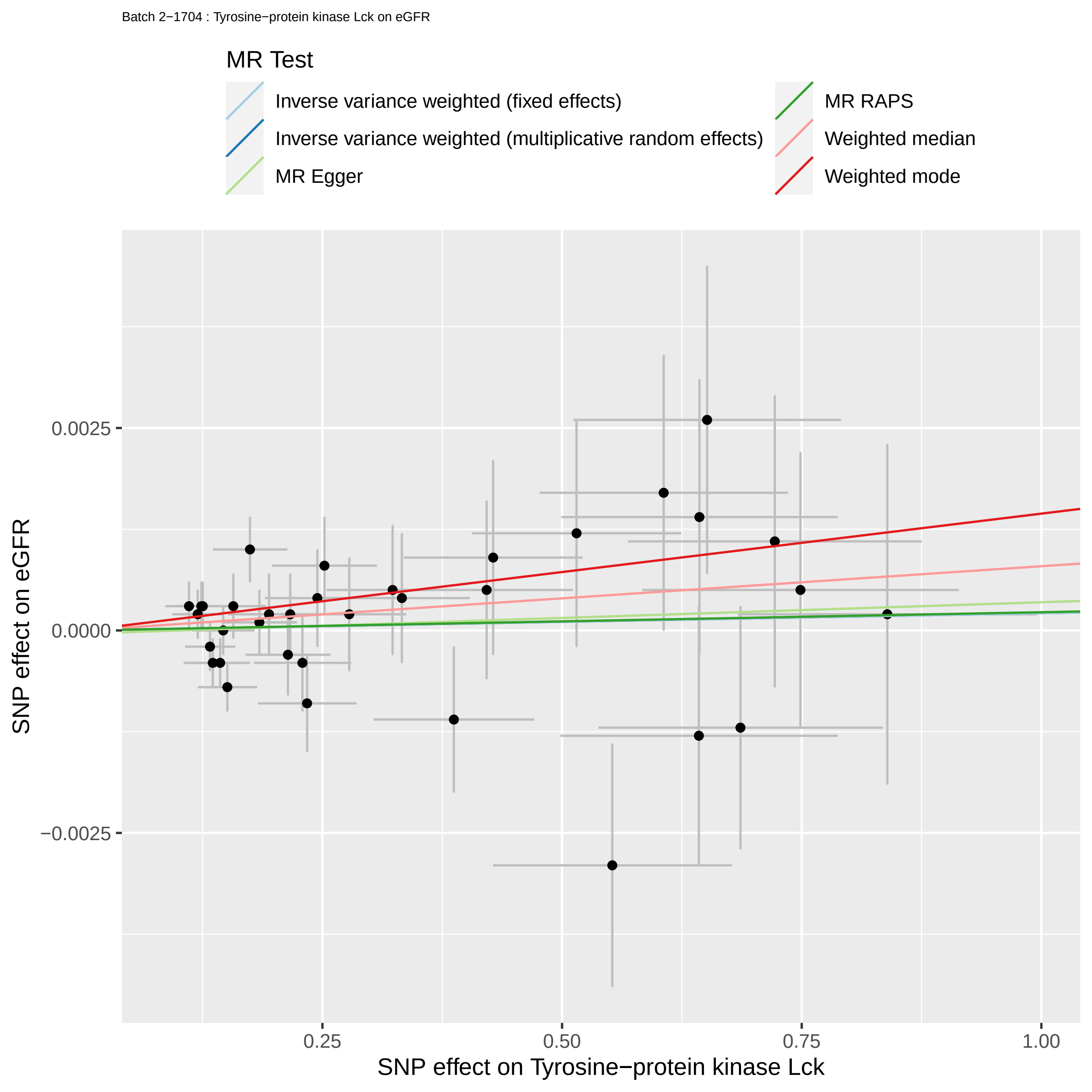
Batch 2−1704 : Tyrosine−protein kinase Lck on eGFR
MR Test
Inverse variance weighted (fixed effects)
Inverse variance weighted (multiplicative random effects)
MR Egger
MR RAPS
Weighted median
Weighted mode
0.0025
0.0000
−0.0025
0.25
0.50
0.75
1.00
SNP effect on eGFR
SNP effect on Tyrosine−protein kinase Lck

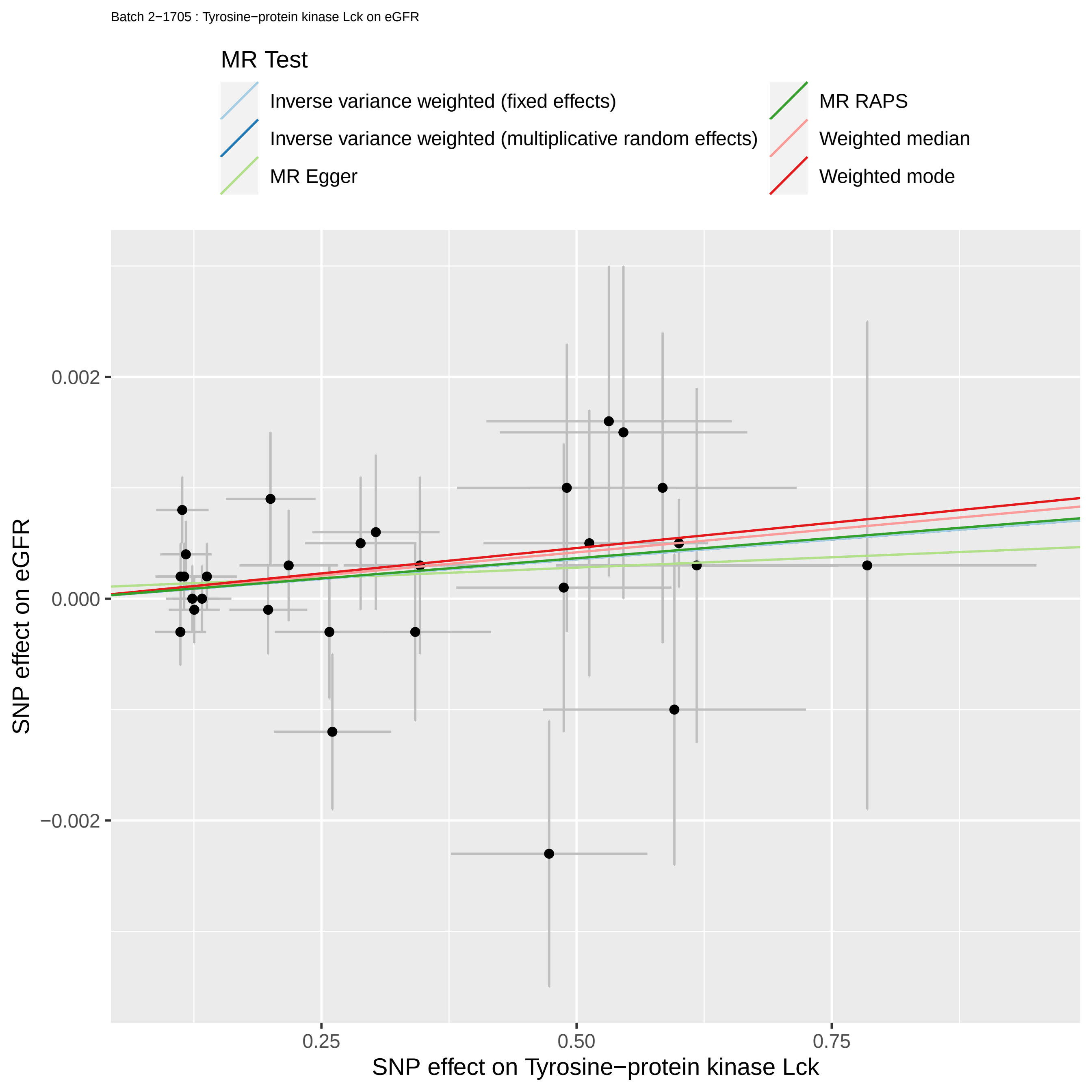

Batch 2−1705 : Tyrosine−protein kinase Lck on eGFR
MR Test
Inverse variance weighted (fixed effects)
Inverse variance weighted (multiplicative random effects)
MR Egger
MR RAPS
Weighted median
Weighted mode
0.002
0.000
−0.002
0.25
0.50
0.75
SNP effect on eGFR
SNP effect on Tyrosine−protein kinase Lck

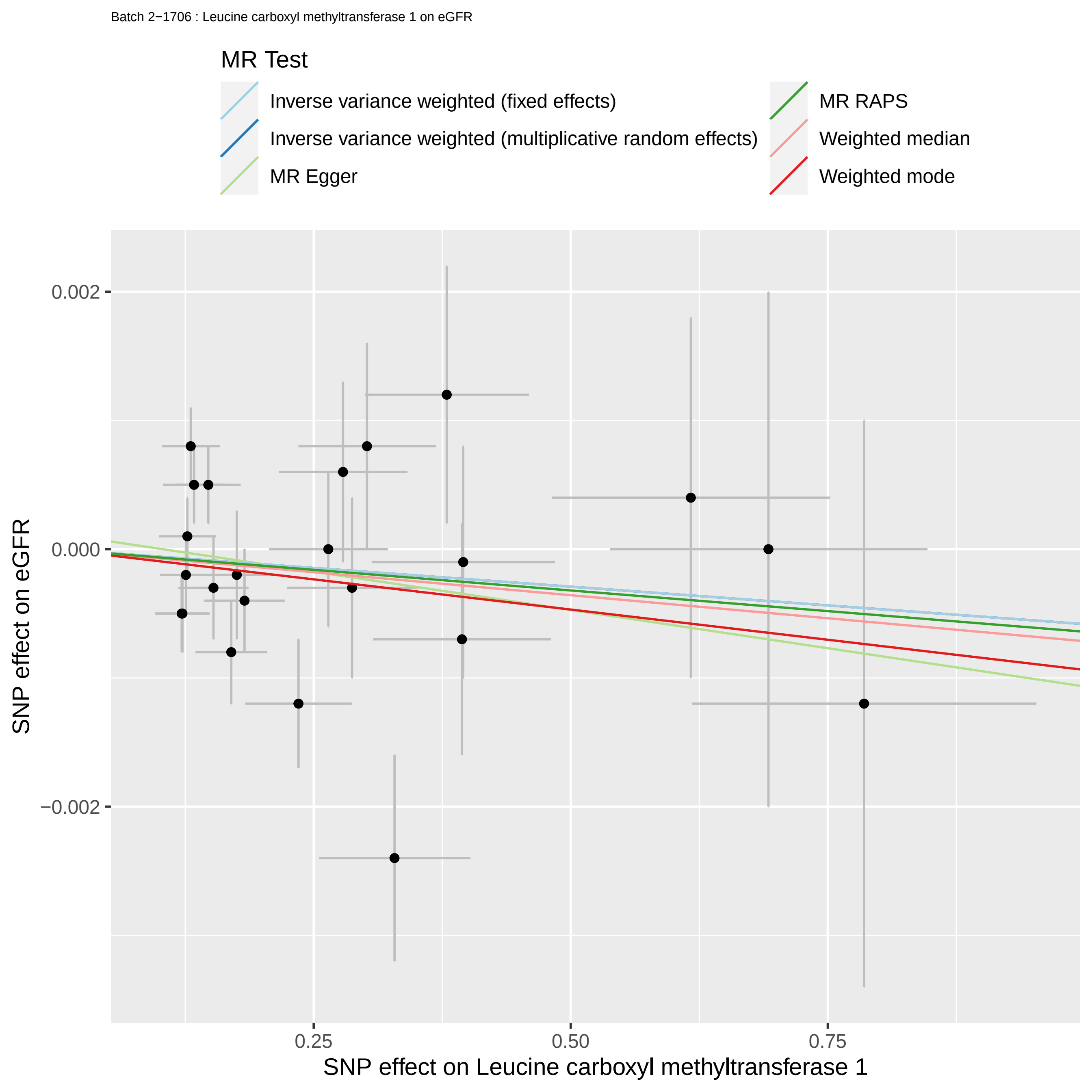
Batch 2−1706 : Leucine carboxyl methyltransferase 1 on eGFR
MR Test
Inverse variance weighted (fixed effects)
Inverse variance weighted (multiplicative random effects)
MR Egger
MR RAPS
Weighted median
Weighted mode
0.002
0.000
−0.002
SNP effect on eGFR
0.25
0.50
0.75
SNP effect on Leucine carboxyl methyltransferase 1

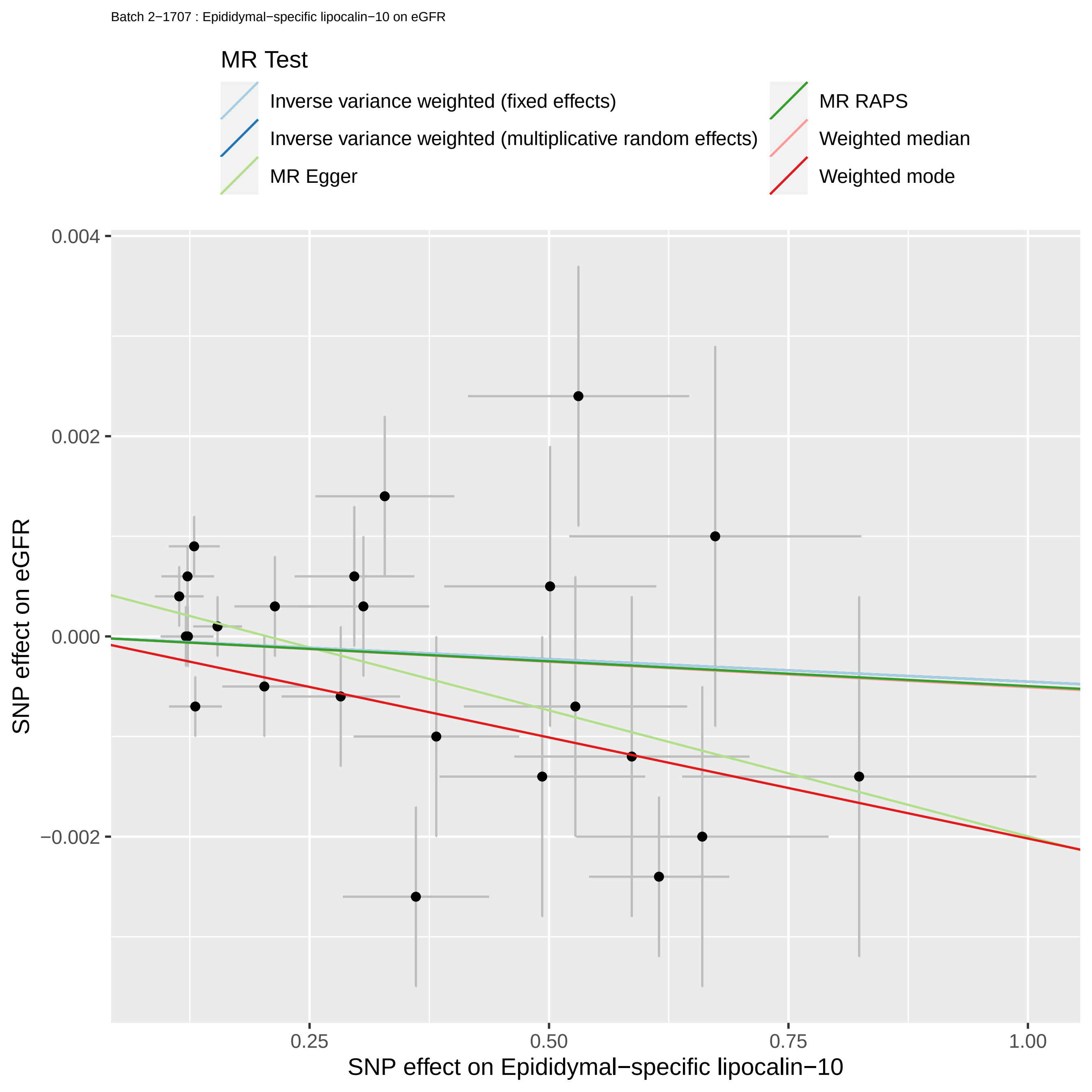

Batch 2−1707 : Epididymal−specific lipocalin−10 on eGFR
MR Test
Inverse variance weighted (fixed effects)
Inverse variance weighted (multiplicative random effects)
MR Egger
MR RAPS
Weighted median
Weighted mode
0.004
0.002
0.000
−0.002
SNP effect on eGFR
0.25
0.50
0.75
1.00
SNP effect on Epididymal−specific lipocalin−10

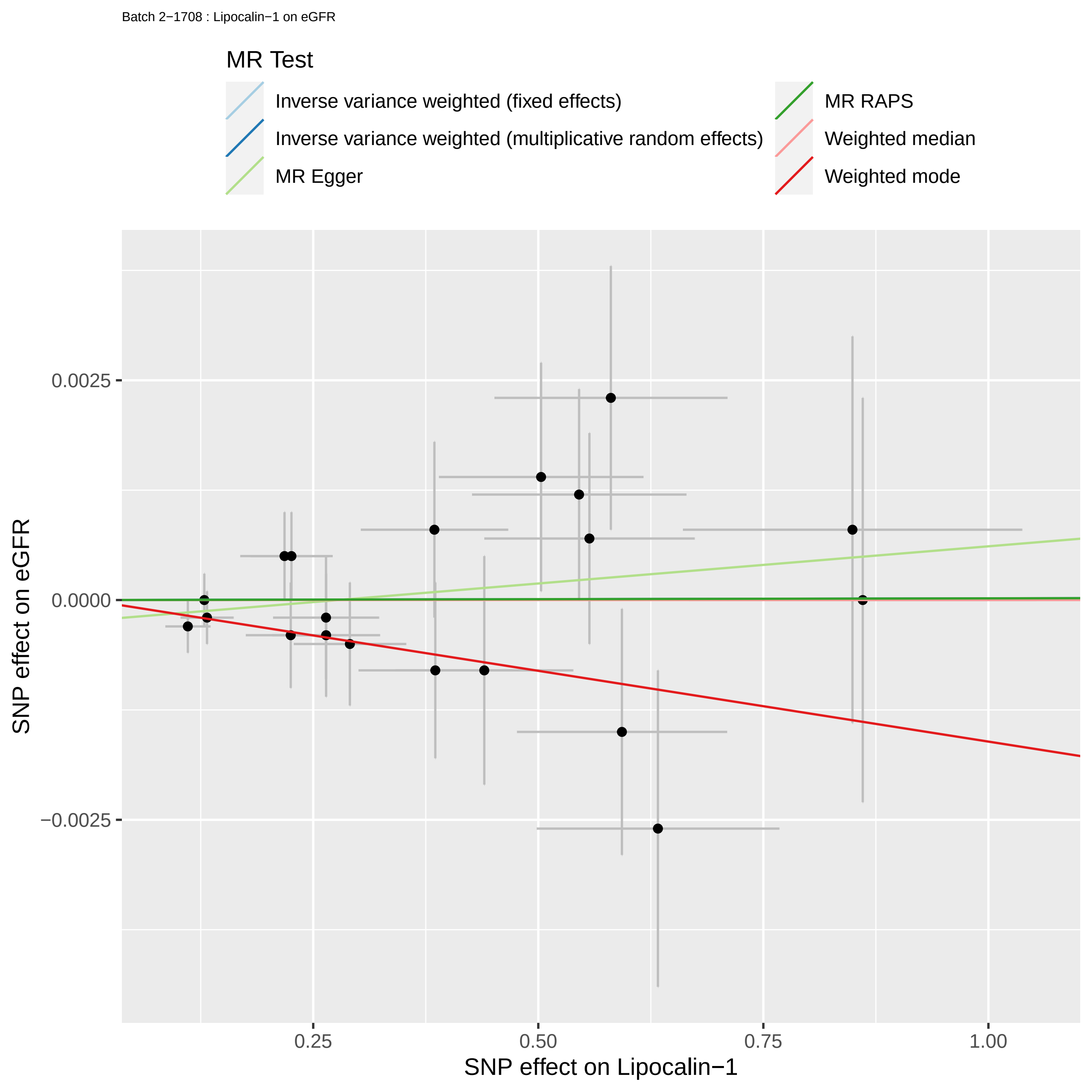

Batch 2−1708 : Lipocalin−1 on eGFR
MR Test
Inverse variance weighted (fixed effects)
Inverse variance weighted (multiplicative random effects)
MR Egger
MR RAPS
Weighted median
Weighted mode
0.0025
0.0000
−0.0025
0.25
0.50
0.75
1.00
SNP effect on Lipocalin−1
SNP effect on eGFR

Batch 2−1709 : Neutrophil gelatinase−associated lipocalin on eGFR

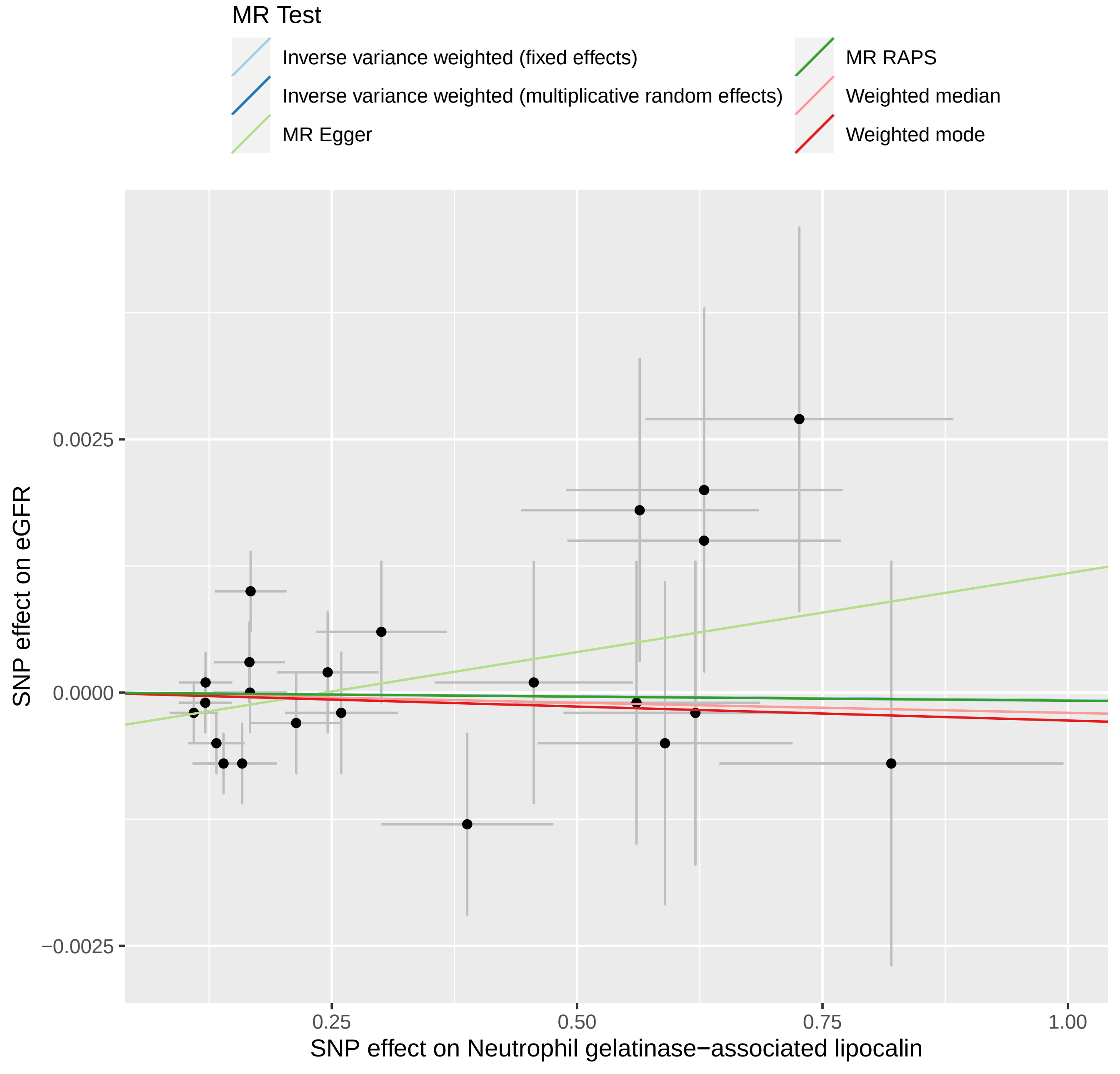

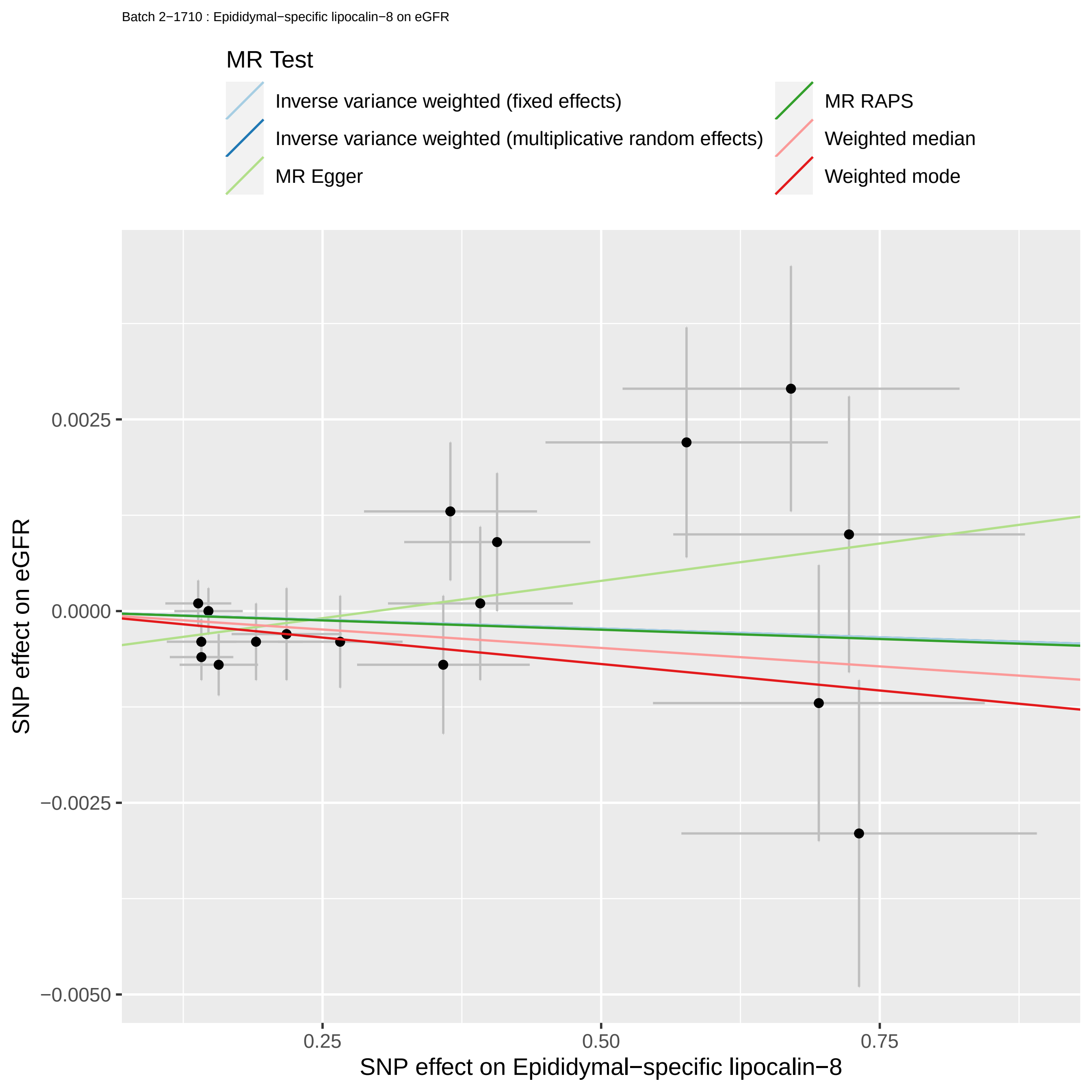
Batch 2−1710 : Epididymal−specific lipocalin−8 on eGFR
MR Test
Inverse variance weighted (fixed effects)
Inverse variance weighted (multiplicative random effects)
MR Egger
MR RAPS
Weighted median
Weighted mode
0.0025
0.0000
−0.0025
−0.0050
0.25
0.50
0.75
SNP effect on eGFR
SNP effect on Epididymal−specific lipocalin−8

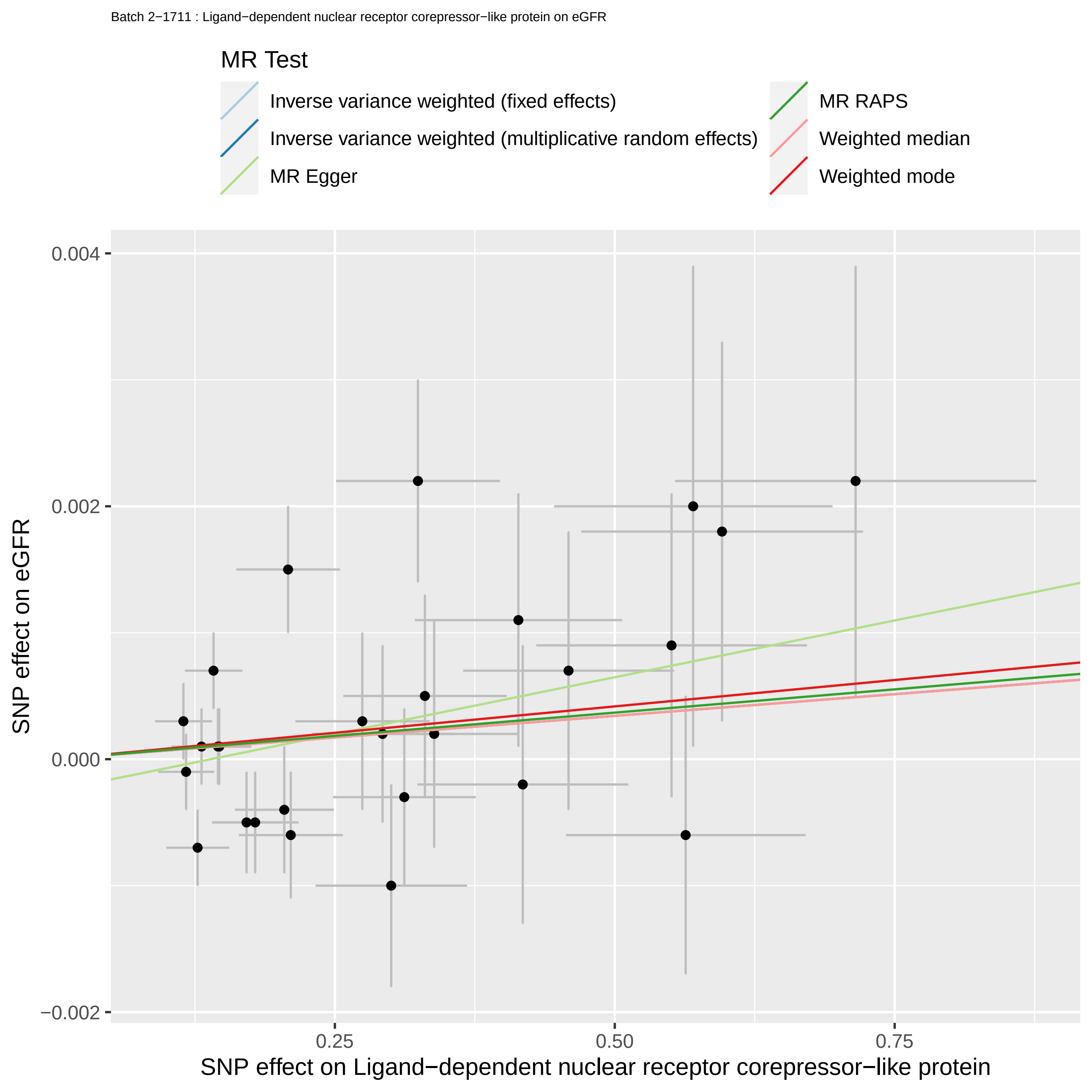

Batch 2−1711 : Ligand−dependent nuclear receptor corepressor−like protein on eGFR
MR Test
Inverse variance weighted (fixed effects)
Inverse variance weighted (multiplicative random effects)
MR Egger
MR RAPS
Weighted median
Weighted mode
0.004
0.002
0.000
−0.002
0.25
0.50
0.75
SNP effect on eGFR
SNP effect on Ligand−dependent nuclear receptor corepressor−like protein

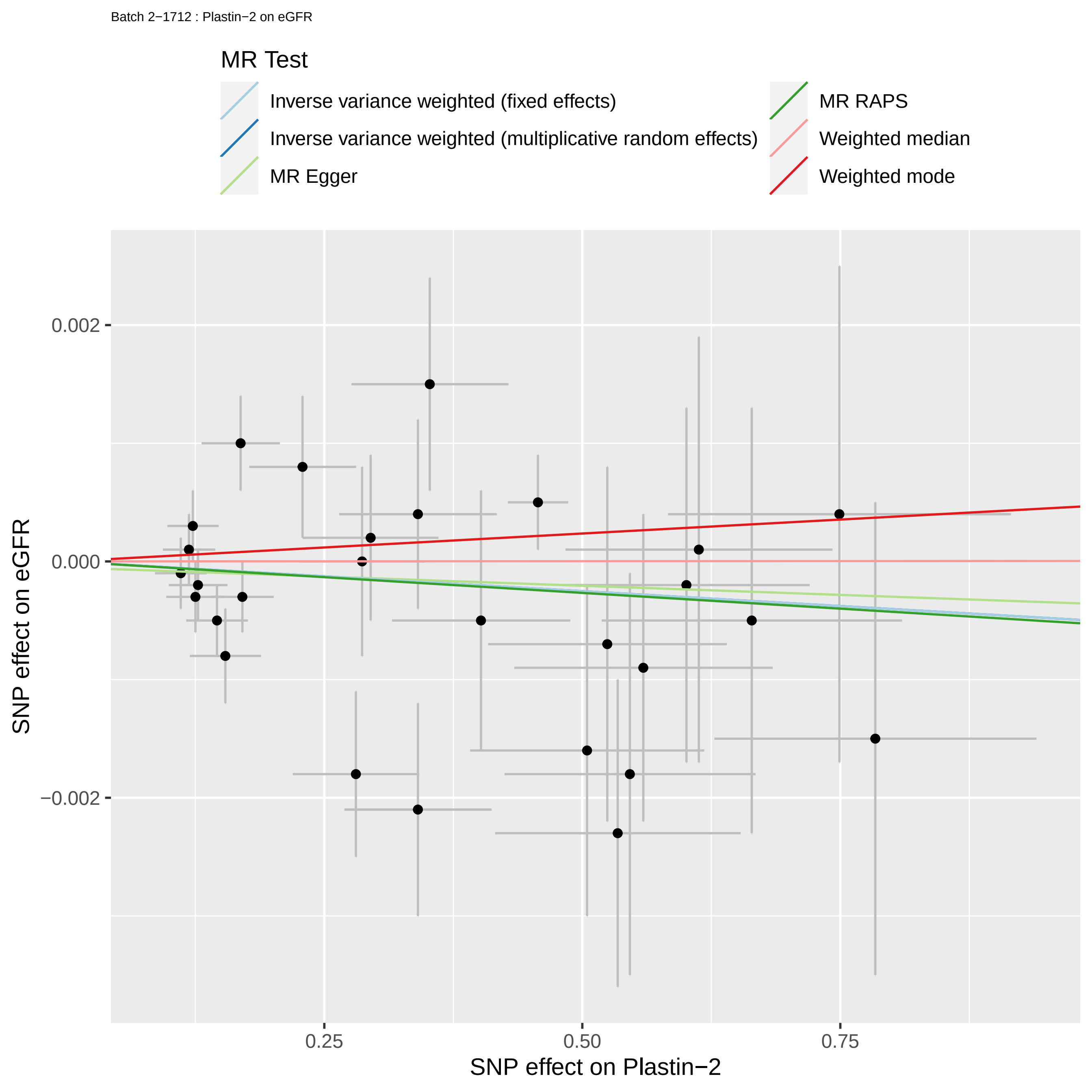
Batch 2−1712 : Plastin−2 on eGFR
MR Test
Inverse variance weighted (fixed effects)
Inverse variance weighted (multiplicative random effects)
MR Egger
MR RAPS
Weighted median
Weighted mode
0.002
0.000
−0.002
0.25
0.50
0.75
SNP effect on eGFR
SNP effect on Plastin−2

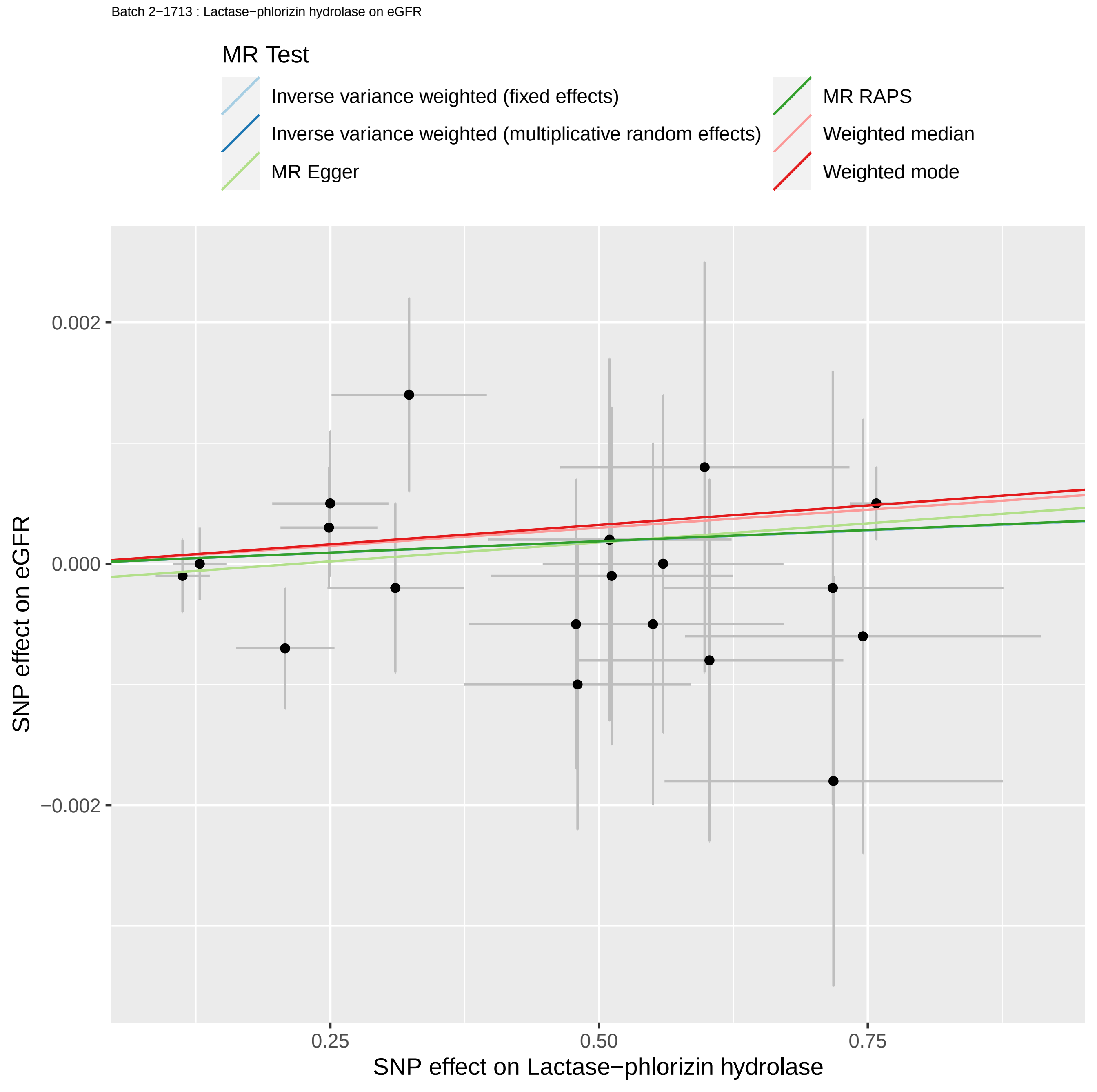

Batch 2−1713 : Lactase−phlorizin hydrolase on eGFR
MR Test
Inverse variance weighted (fixed effects)
Inverse variance weighted (multiplicative random effects)
MR Egger
MR RAPS
Weighted median
Weighted mode
0.002
0.000
−0.002
0.25
0.50
0.75
SNP effect on eGFR
SNP effect on Lactase−phlorizin hydrolase

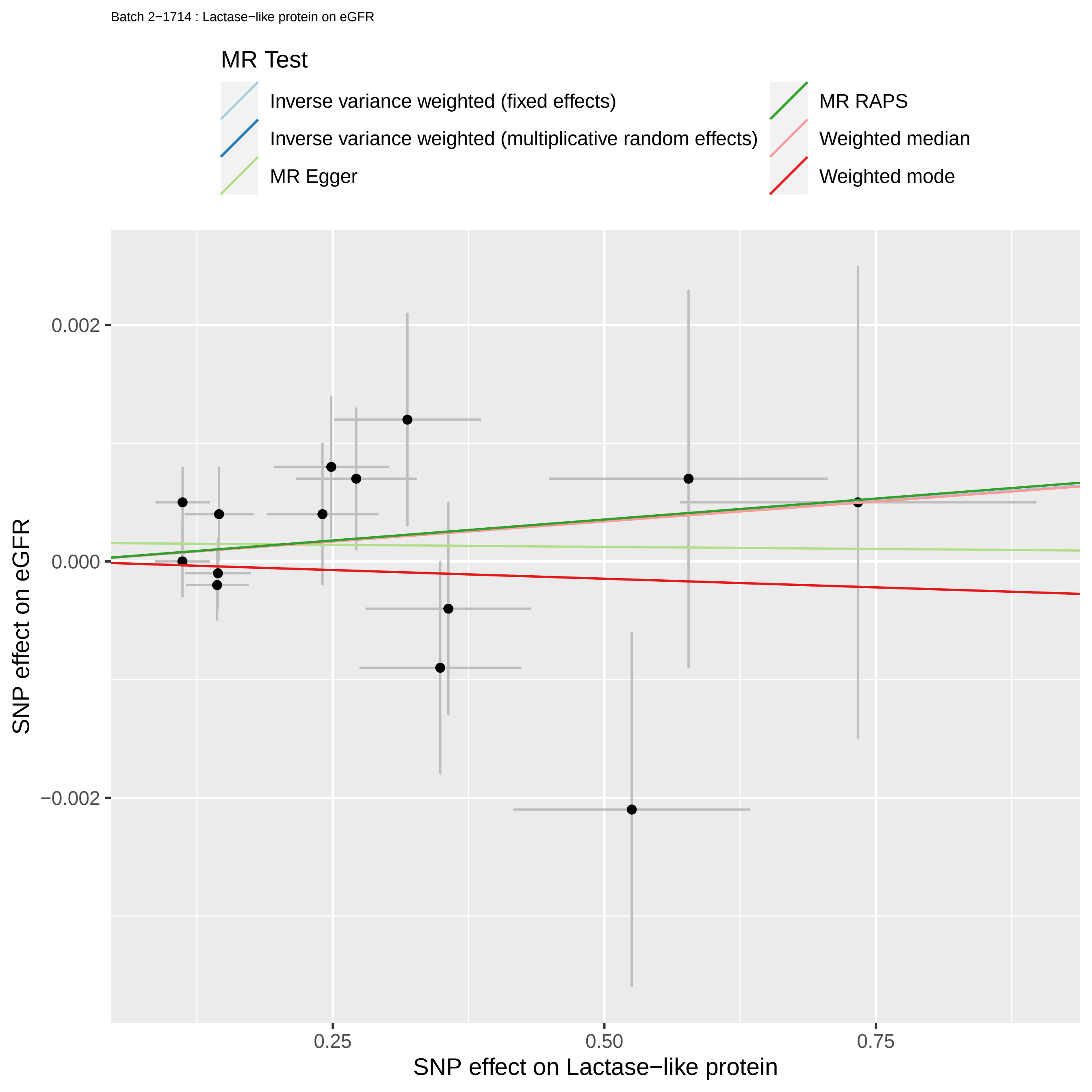
Batch 2−1714 : Lactase−like protein on eGFR
MR Test
Inverse variance weighted (fixed effects)
Inverse variance weighted (multiplicative random effects)
MR Egger
MR RAPS
Weighted median
Weighted mode
0.002
0.000
−0.002
0.25
0.50
0.75
SNP effect on eGFR
SNP effect on Lactase−like protein

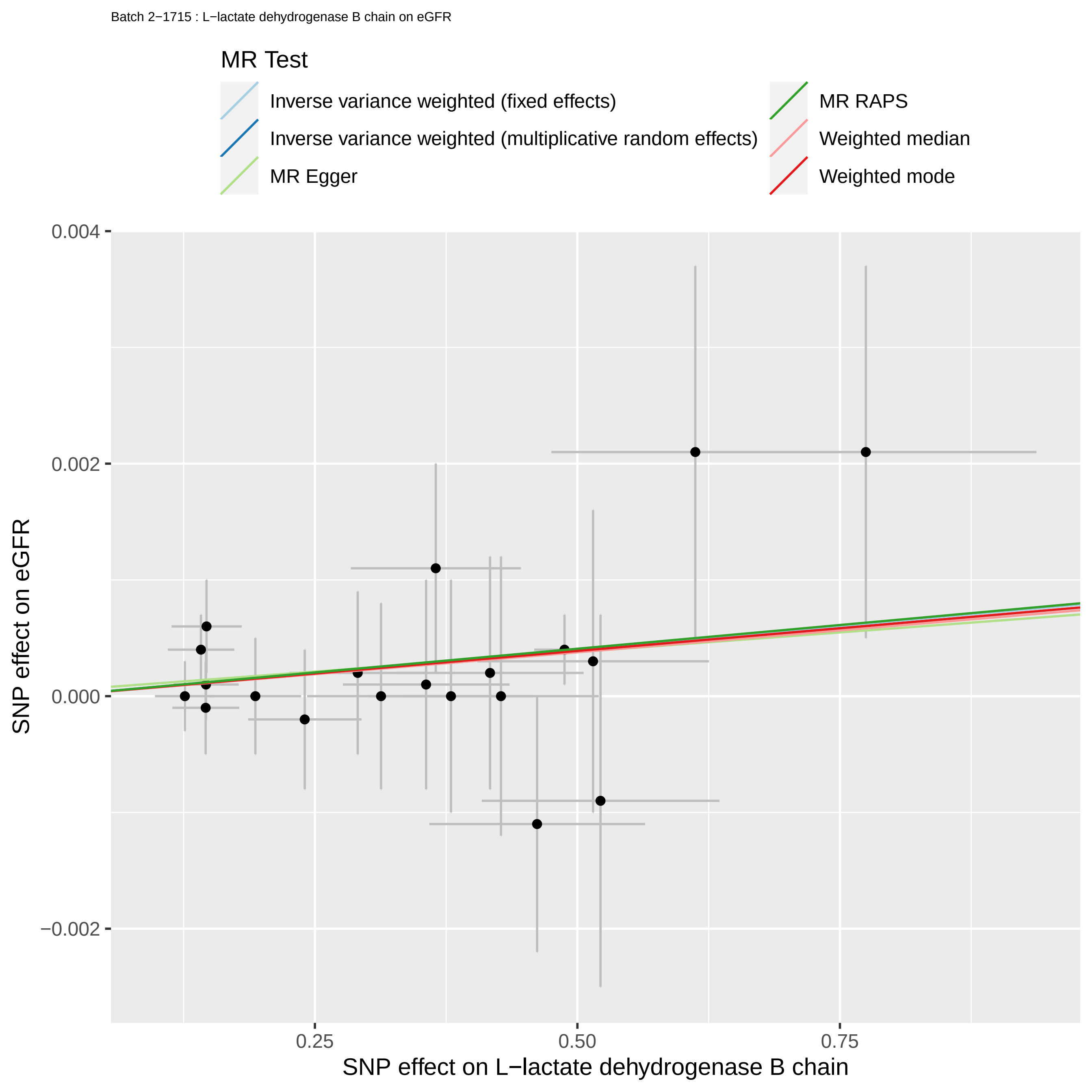

Batch 2−1715 : L−lactate dehydrogenase B chain on eGFR
MR Test
Inverse variance weighted (fixed effects)
Inverse variance weighted (multiplicative random effects)
MR Egger
MR RAPS
Weighted median
Weighted mode
0.004
0.002
0.000
−0.002
0.25
0.50
0.75
SNP effect on eGFR
SNP effect on L−lactate dehydrogenase B chain

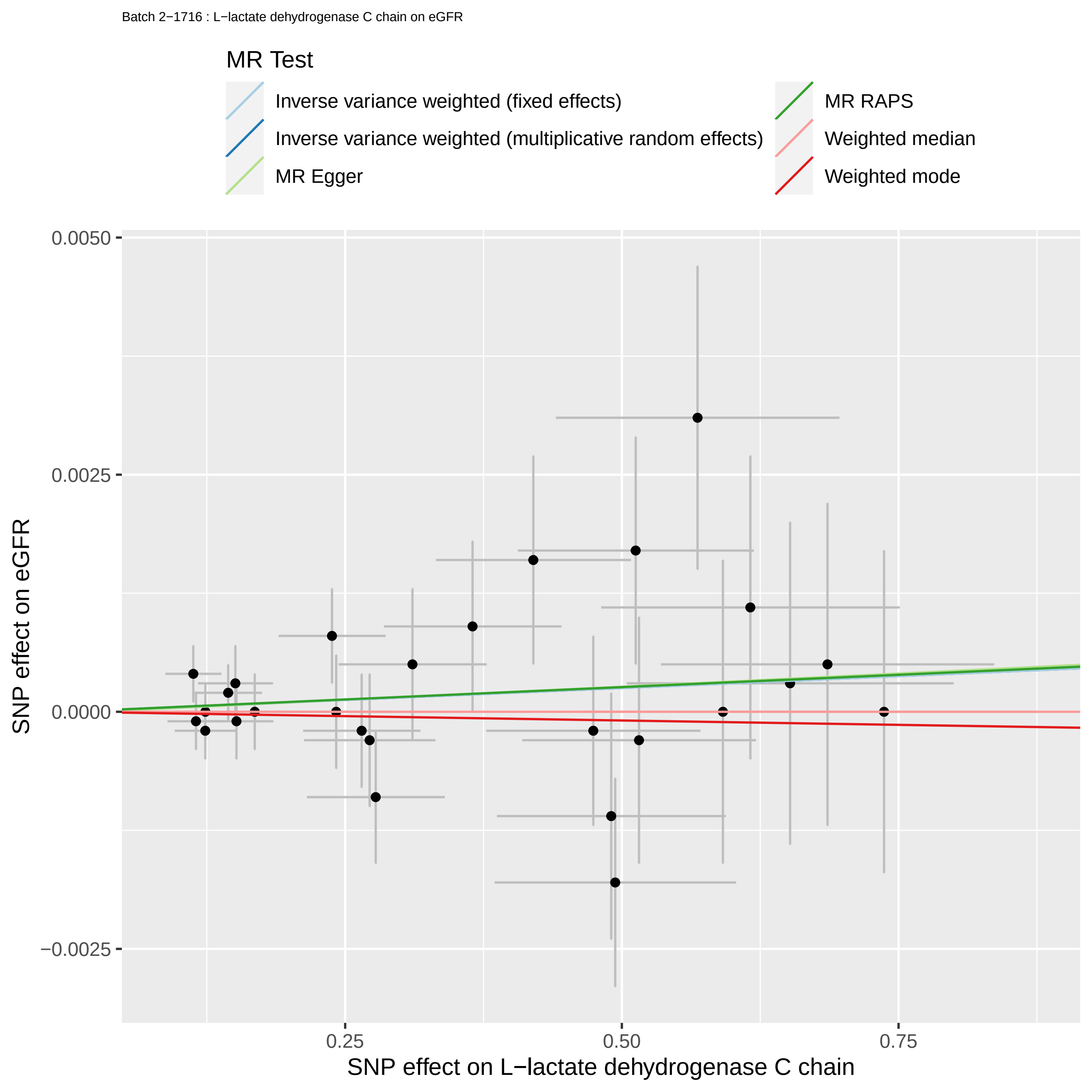

Batch 2−1716 : L−lactate dehydrogenase C chain on eGFR
MR Test
Inverse variance weighted (fixed effects)
Inverse variance weighted (multiplicative random effects)
MR Egger
MR RAPS
Weighted median
Weighted mode
0.0050
0.0025
0.0000
−0.0025
SNP effect on eGFR
0.25
0.50
0.75
SNP effect on L−lactate dehydrogenase C chain

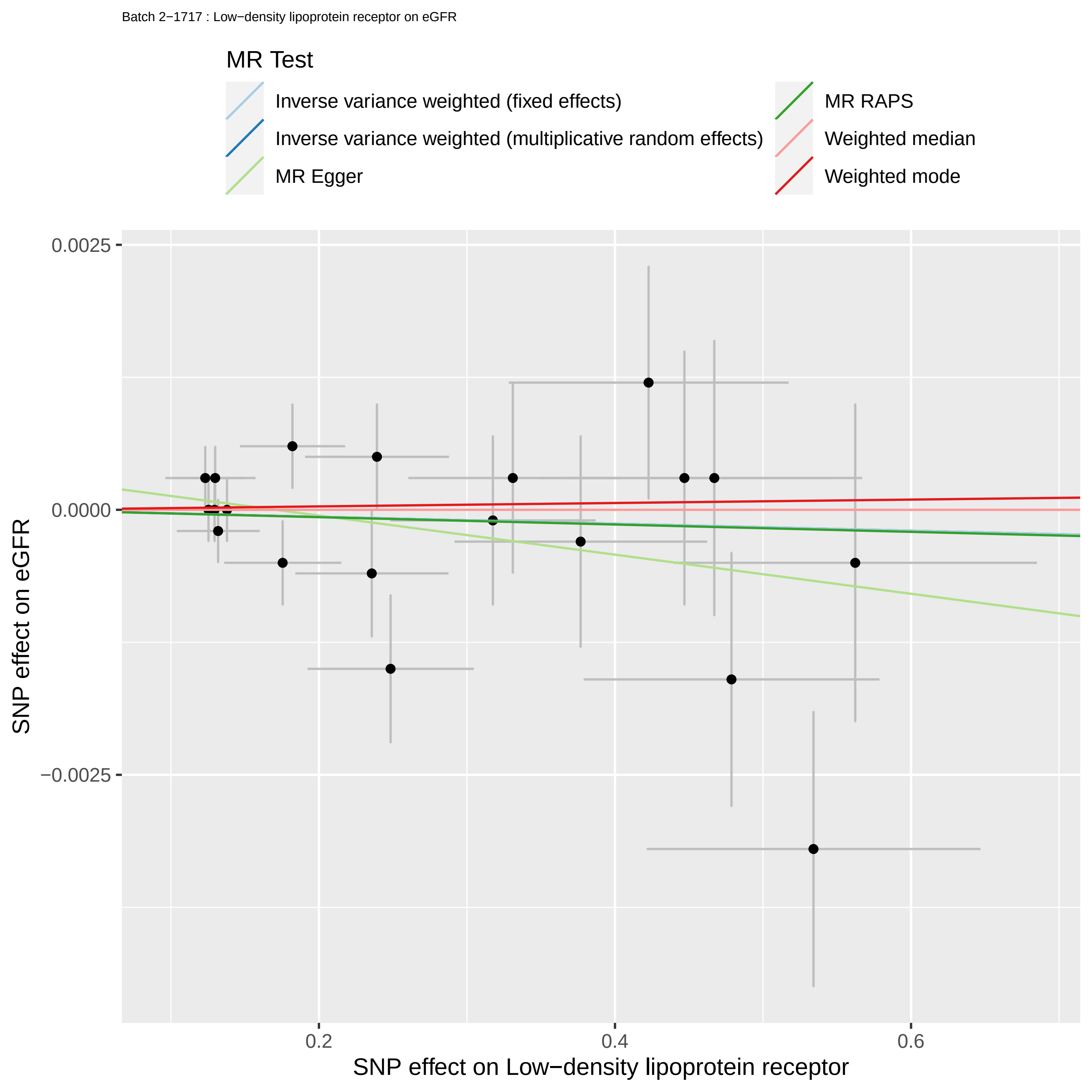

Batch 2−1717 : Low−density lipoprotein receptor on eGFR
MR Test
Inverse variance weighted (fixed effects)
Inverse variance weighted (multiplicative random effects)
MR Egger
MR RAPS
Weighted median
Weighted mode
0.0025
0.0000
−0.0025
0.2
0.4
0.6
SNP effect on eGFR
SNP effect on Low−density lipoprotein receptor

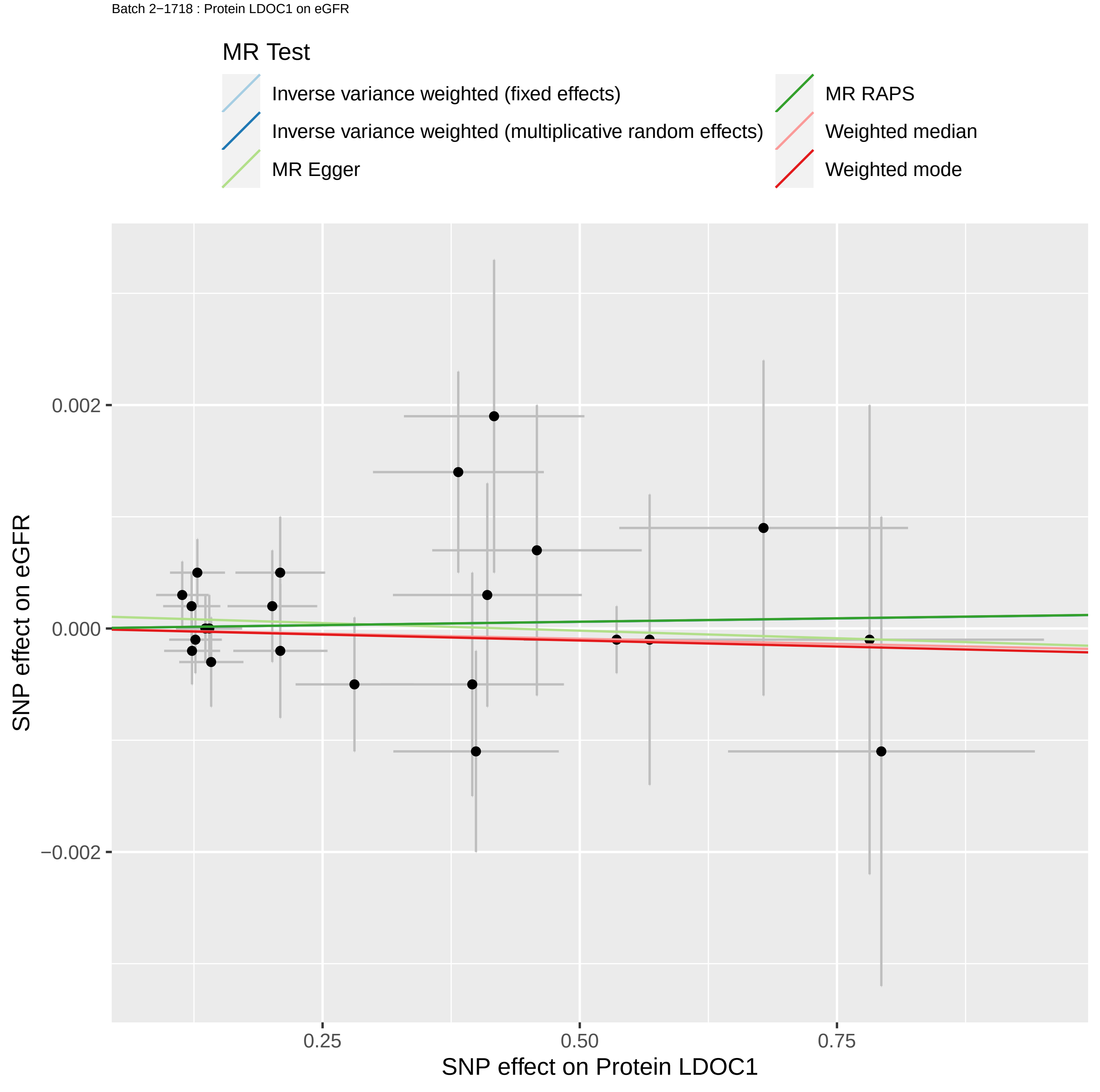

Batch 2−1718 : Protein LDOC1 on eGFR
MR Test
Inverse variance weighted (fixed effects)
Inverse variance weighted (multiplicative random effects)
MR Egger
MR RAPS
Weighted median
Weighted mode
0.002
0.000
−0.002
SNP effect on eGFR
0.25
0.50
0.75
SNP effect on Protein LDOC1

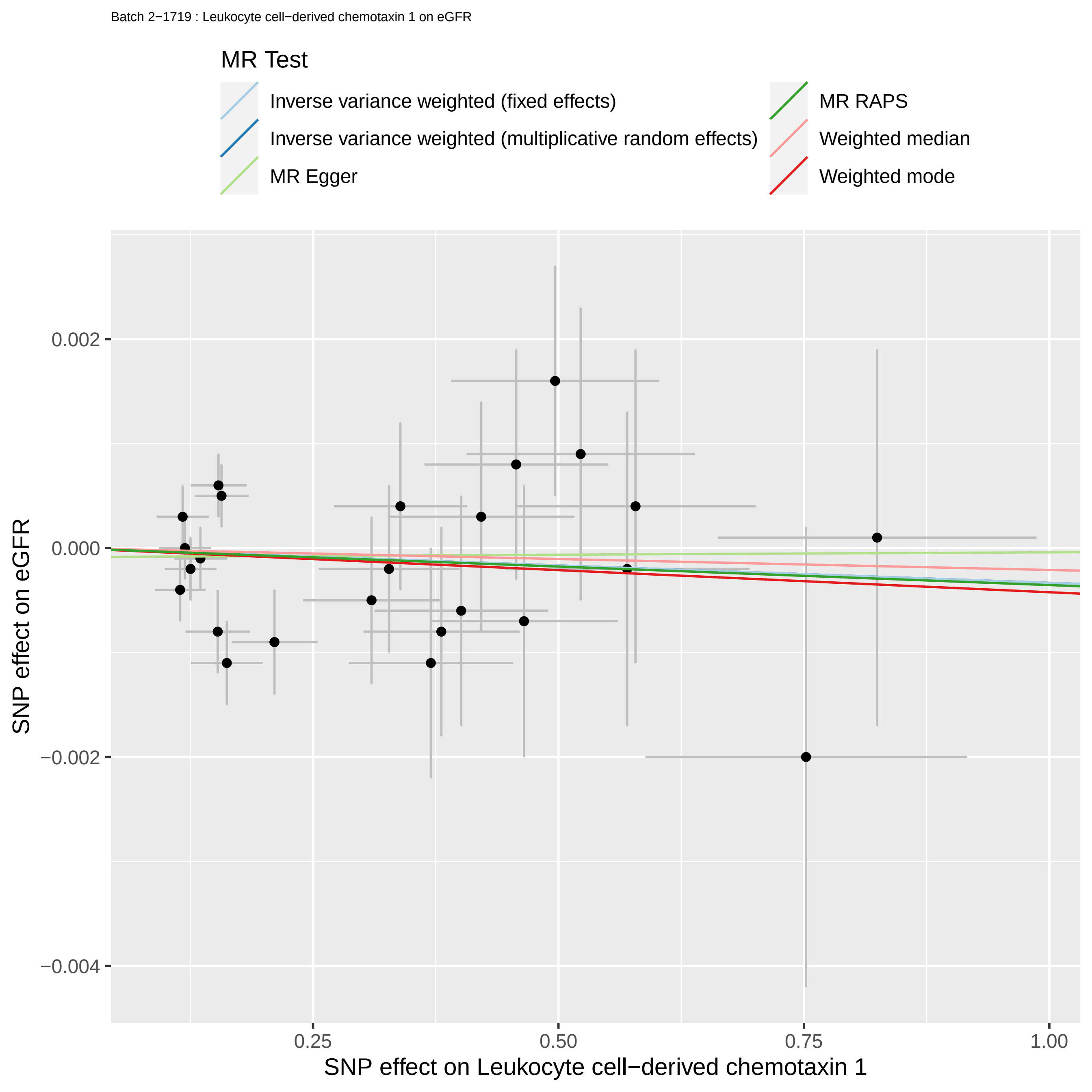
Batch 2−1719 : Leukocyte cell−derived chemotaxin 1 on eGFR
MR Test
Inverse variance weighted (fixed effects)
Inverse variance weighted (multiplicative random effects)
MR Egger
MR RAPS
Weighted median
Weighted mode
0.002
0.000
−0.002
−0.004
SNP effect on eGFR
0.25
0.50
0.75
1.00
SNP effect on Leukocyte cell−derived chemotaxin 1

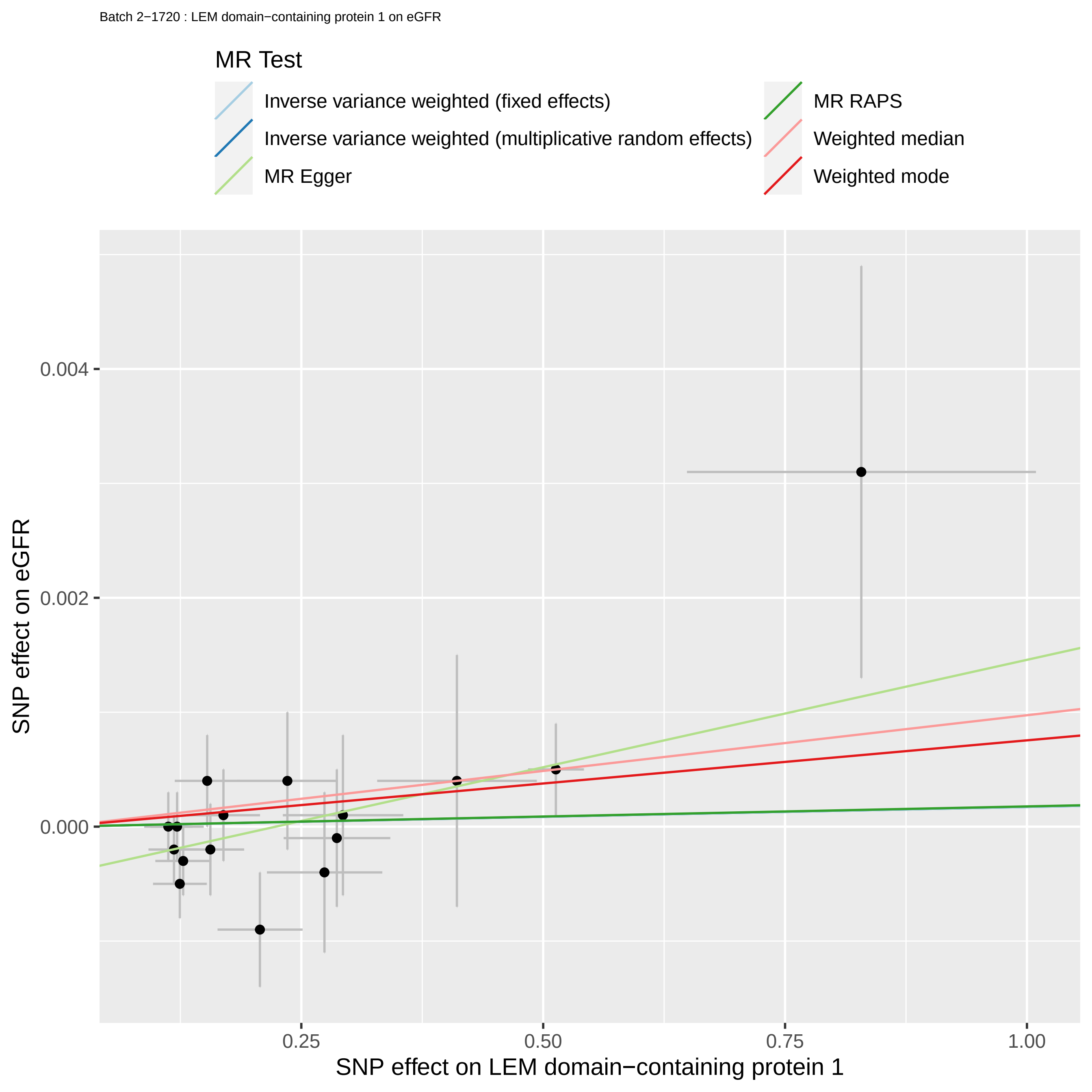

Batch 2−1720 : LEM domain−containing protein 1 on eGFR
MR Test
Inverse variance weighted (fixed effects)
Inverse variance weighted (multiplicative random effects)
MR Egger
MR RAPS
Weighted median
Weighted mode
0.004
0.002
0.000
0.25
0.50
0.75
1.00
SNP effect on eGFR
SNP effect on LEM domain−containing protein 1

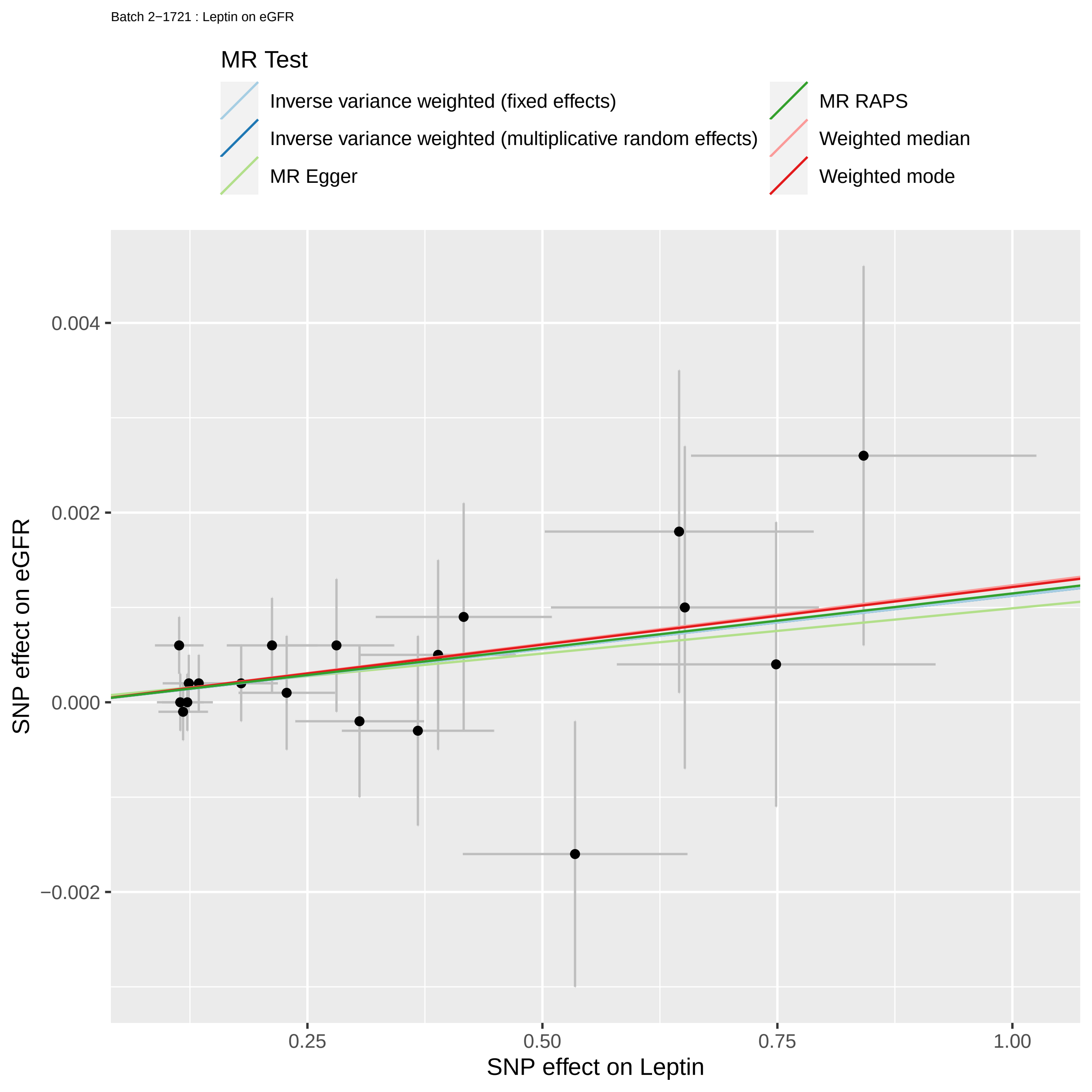
Batch 2−1721 : Leptin on eGFR
MR Test
Inverse variance weighted (fixed effects)
Inverse variance weighted (multiplicative random effects)
MR Egger
MR RAPS
Weighted median
Weighted mode
0.004
0.002
0.000
−0.002
SNP effect on eGFR
0.25
0.50
0.75
1.00
SNP effect on Leptin

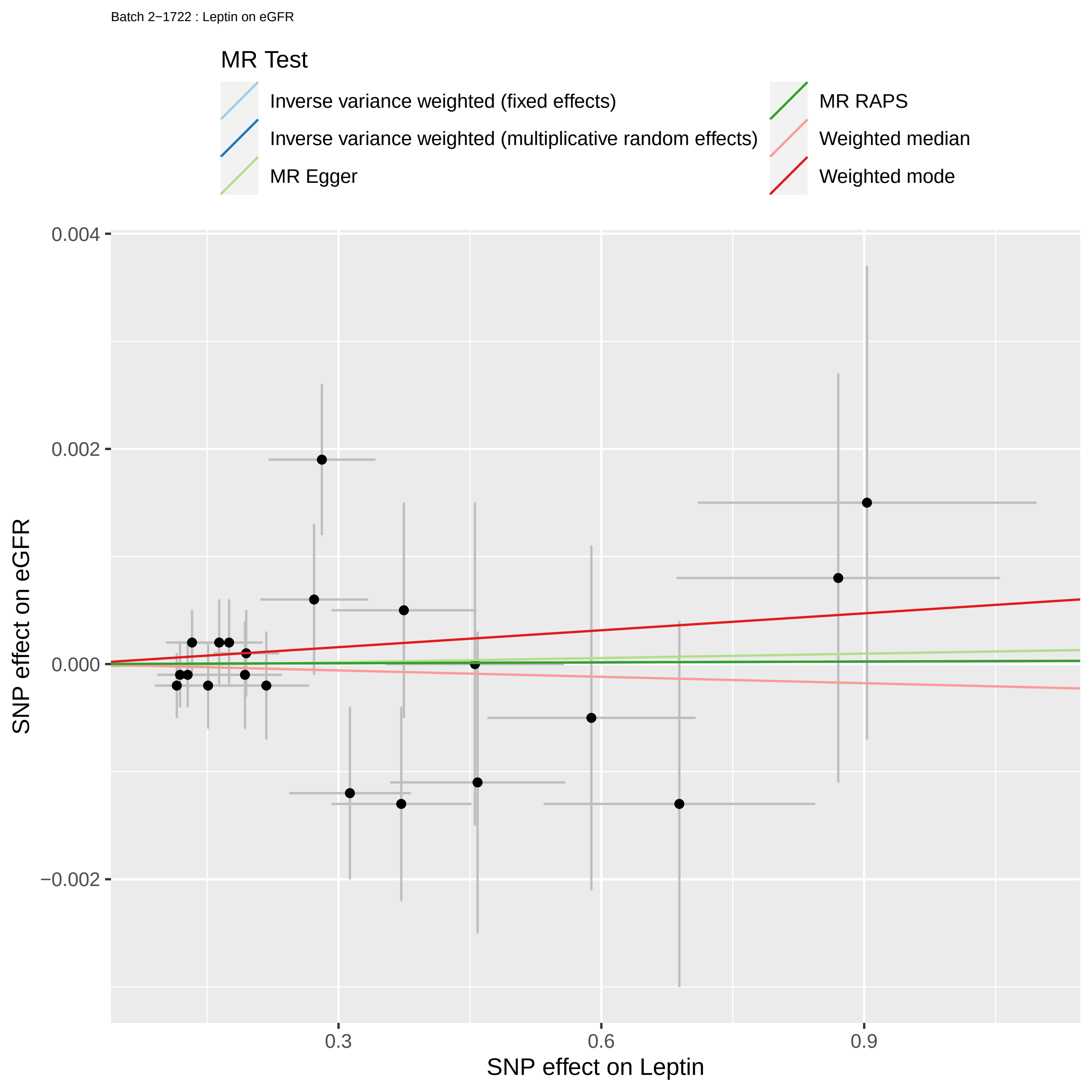

Batch 2−1722 : Leptin on eGFR
MR Test
Inverse variance weighted (fixed effects)
Inverse variance weighted (multiplicative random effects)
MR Egger
MR RAPS
Weighted median
Weighted mode
0.004
0.002
0.000
−0.002
0.3
0.6
0.9
SNP effect on eGFR
SNP effect on Leptin

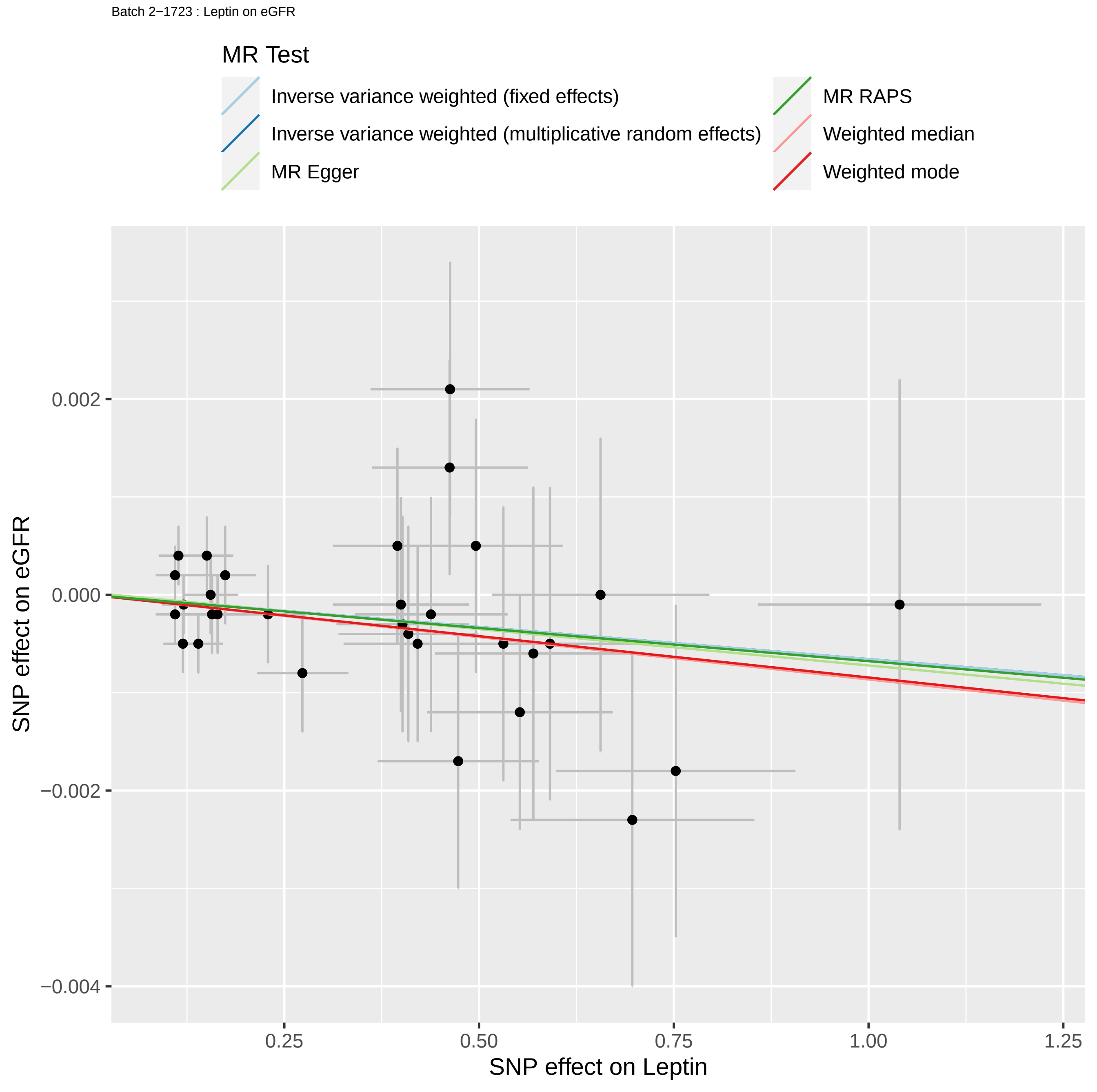

Batch 2−1723 : Leptin on eGFR
MR Test
Inverse variance weighted (fixed effects)
Inverse variance weighted (multiplicative random effects)
MR Egger
MR RAPS
Weighted median
Weighted mode
0.002
0.000
−0.002
−0.004
0.25
0.50
0.75
1.00
1.25
SNP effect on Leptin
SNP effect on eGFR

Batch 2−1724 : Leptin receptor on eGFR

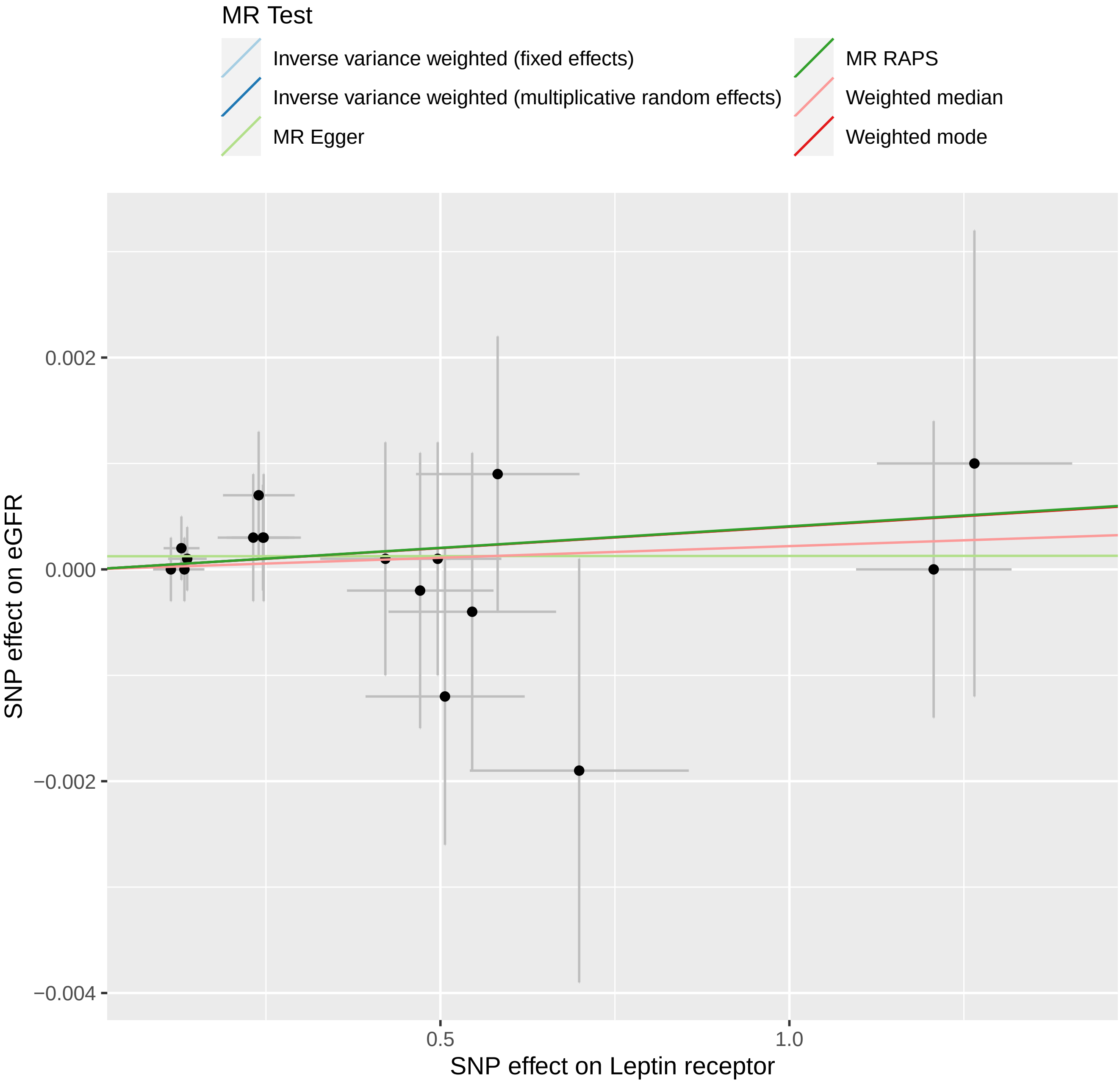

Batch 2−1725 : Leptin receptor on eGFR

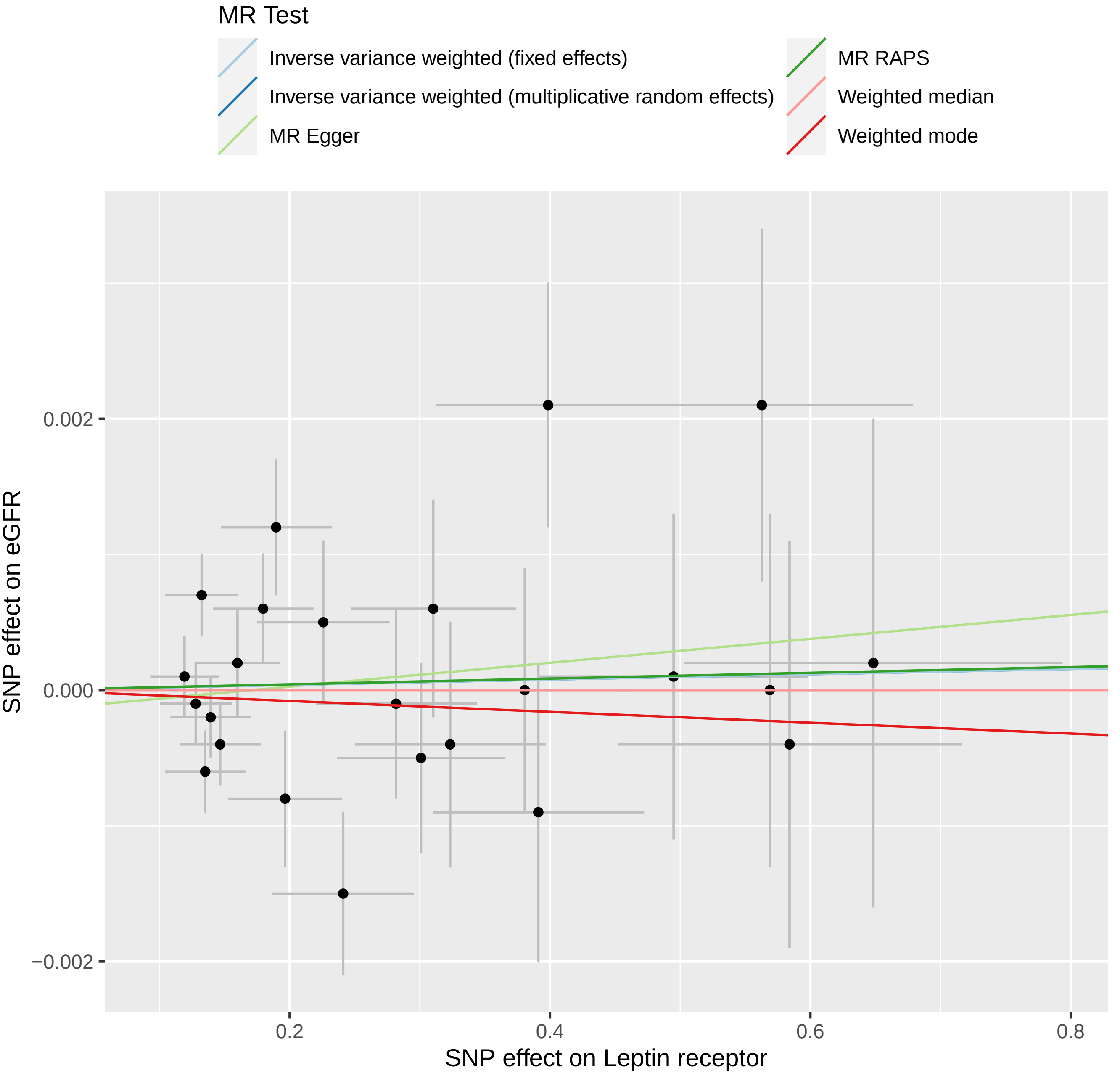

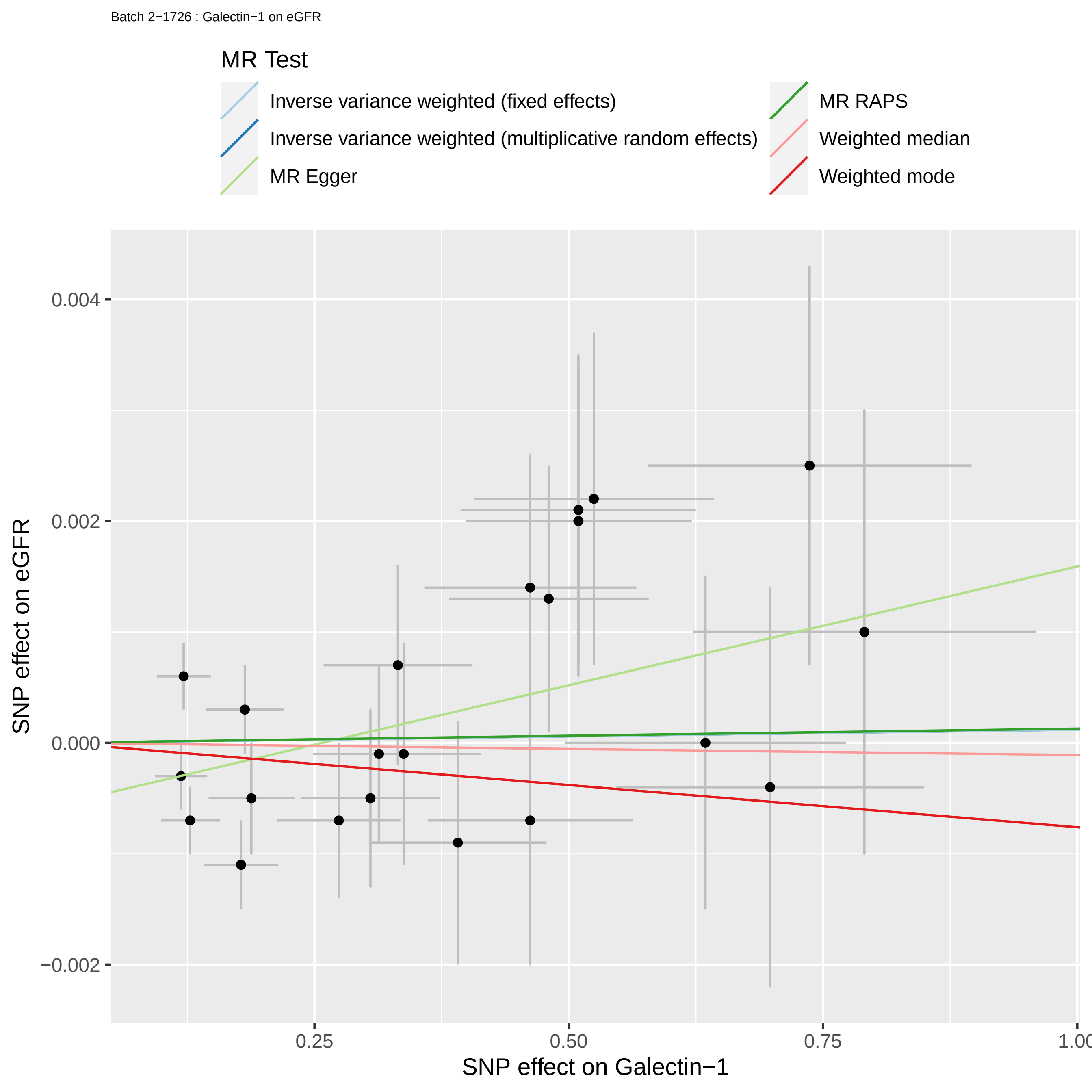
Batch 2−1726 : Galectin−1 on eGFR
MR Test
Inverse variance weighted (fixed effects)
Inverse variance weighted (multiplicative random effects)
MR Egger
MR RAPS
Weighted median
Weighted mode
0.004
0.002
0.000
−0.002
SNP effect on eGFR
0.25
0.50
0.75
1.00
SNP effect on Galectin−1

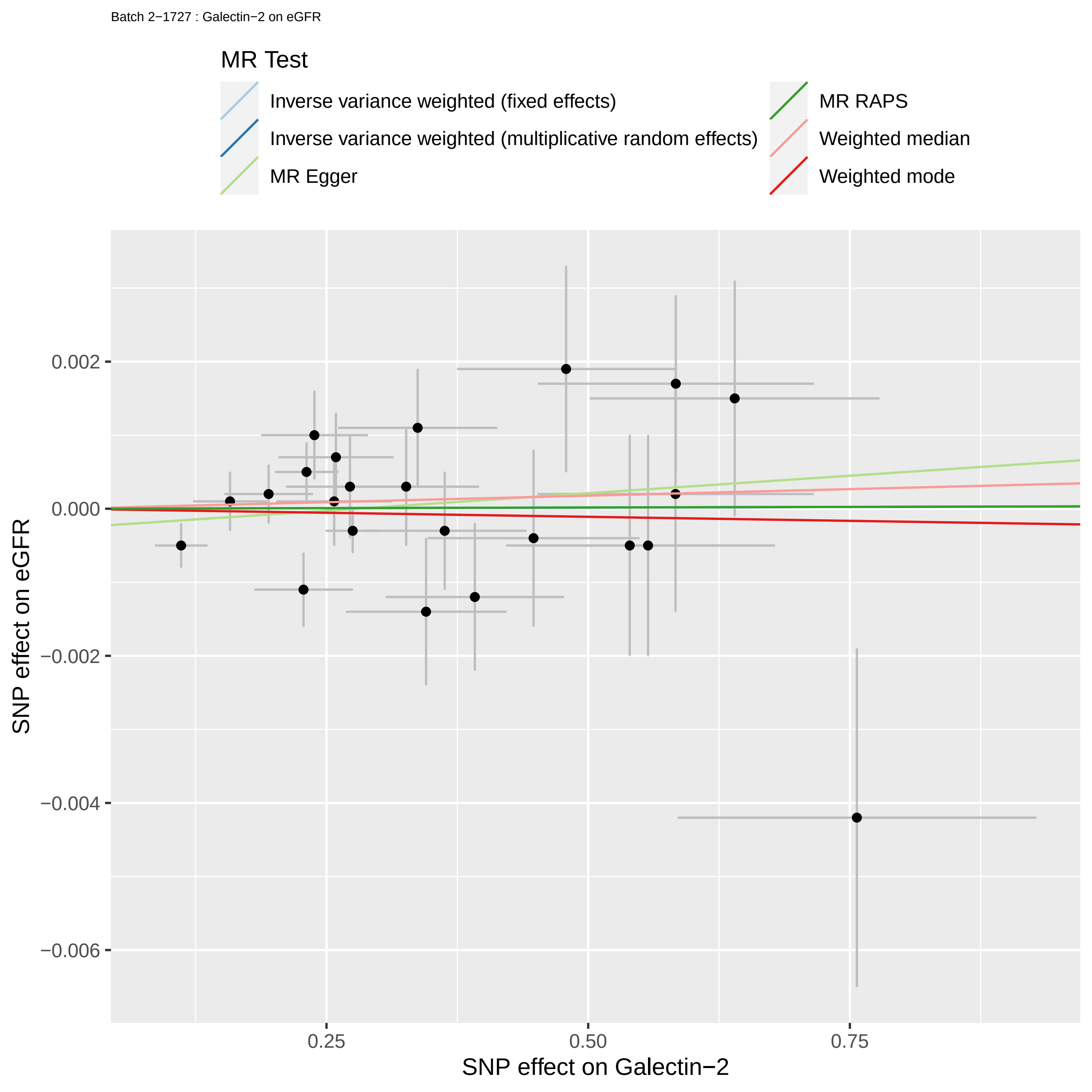

Batch 2−1727 : Galectin−2 on eGFR
MR Test
Inverse variance weighted (fixed effects)
Inverse variance weighted (multiplicative random effects)
MR Egger
MR RAPS
Weighted median
Weighted mode
0.002
0.000
−0.002
−0.004
−0.006
0.25
0.50
0.75
SNP effect on Galectin−2
SNP effect on eGFR

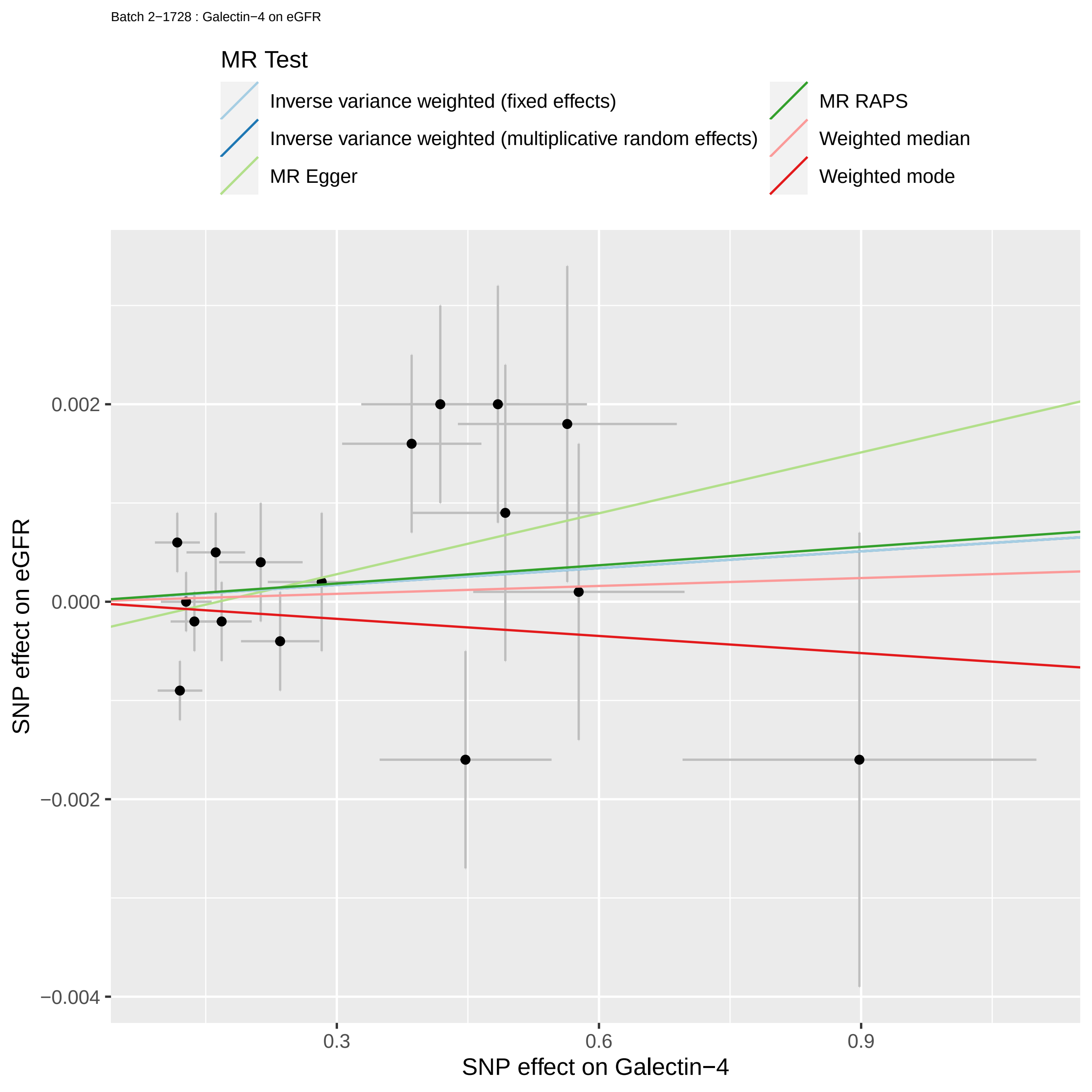

Batch 2−1728 : Galectin−4 on eGFR
MR Test
Inverse variance weighted (fixed effects)
Inverse variance weighted (multiplicative random effects)
MR Egger
MR RAPS
Weighted median
Weighted mode
0.002
0.000
−0.002
−0.004
0.3
0.6
0.9
SNP effect on Galectin−4
SNP effect on eGFR

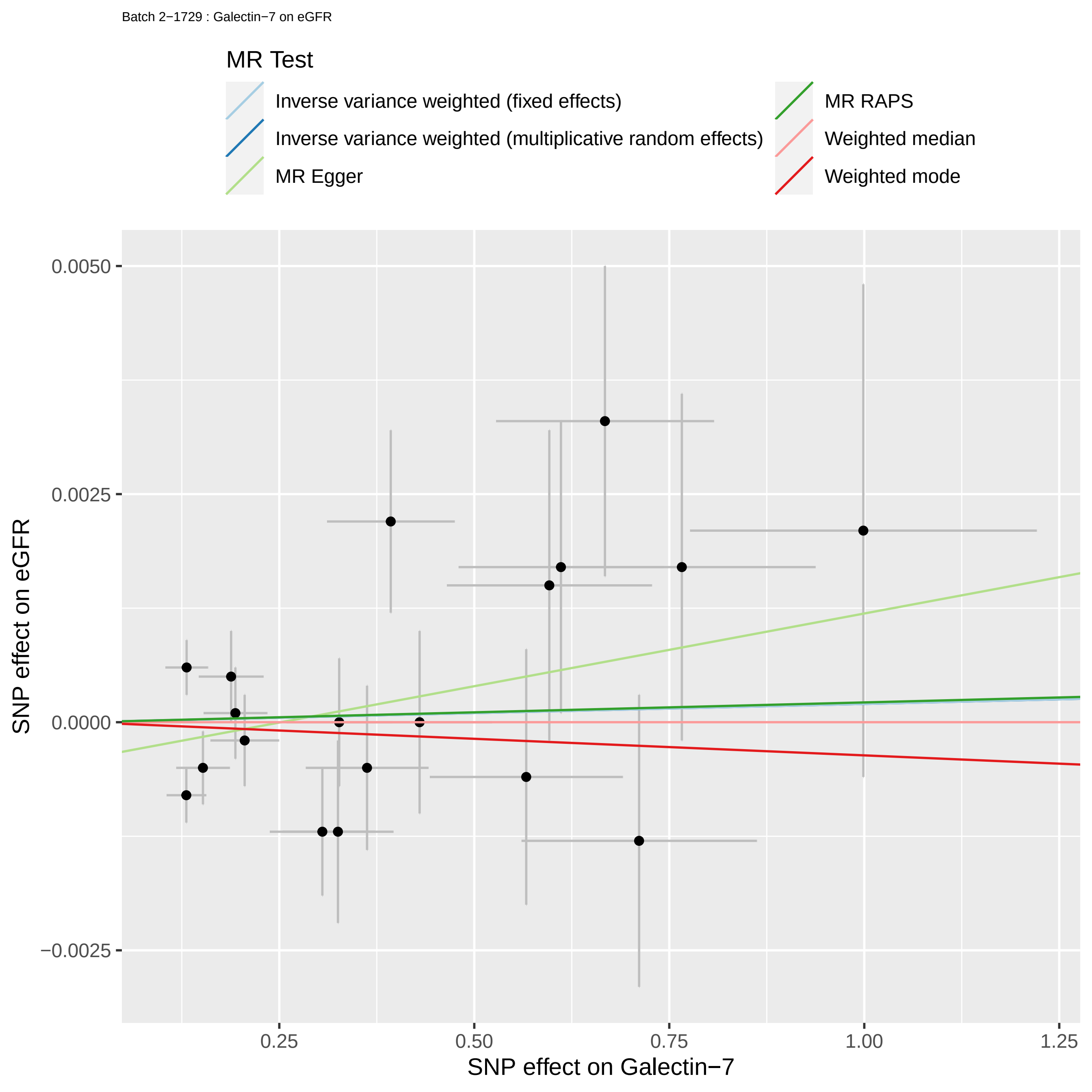

Batch 2−1729 : Galectin−7 on eGFR
MR Test
Inverse variance weighted (fixed effects)
Inverse variance weighted (multiplicative random effects)
MR Egger
MR RAPS
Weighted median
Weighted mode
SNP effect on eGFR
0.0050
0.0025
0.0000
−0.0025
0.25
0.50
0.75
1.00
1.25
SNP effect on Galectin−7

Batch 2−1730 : Galectin−7 on eGFR

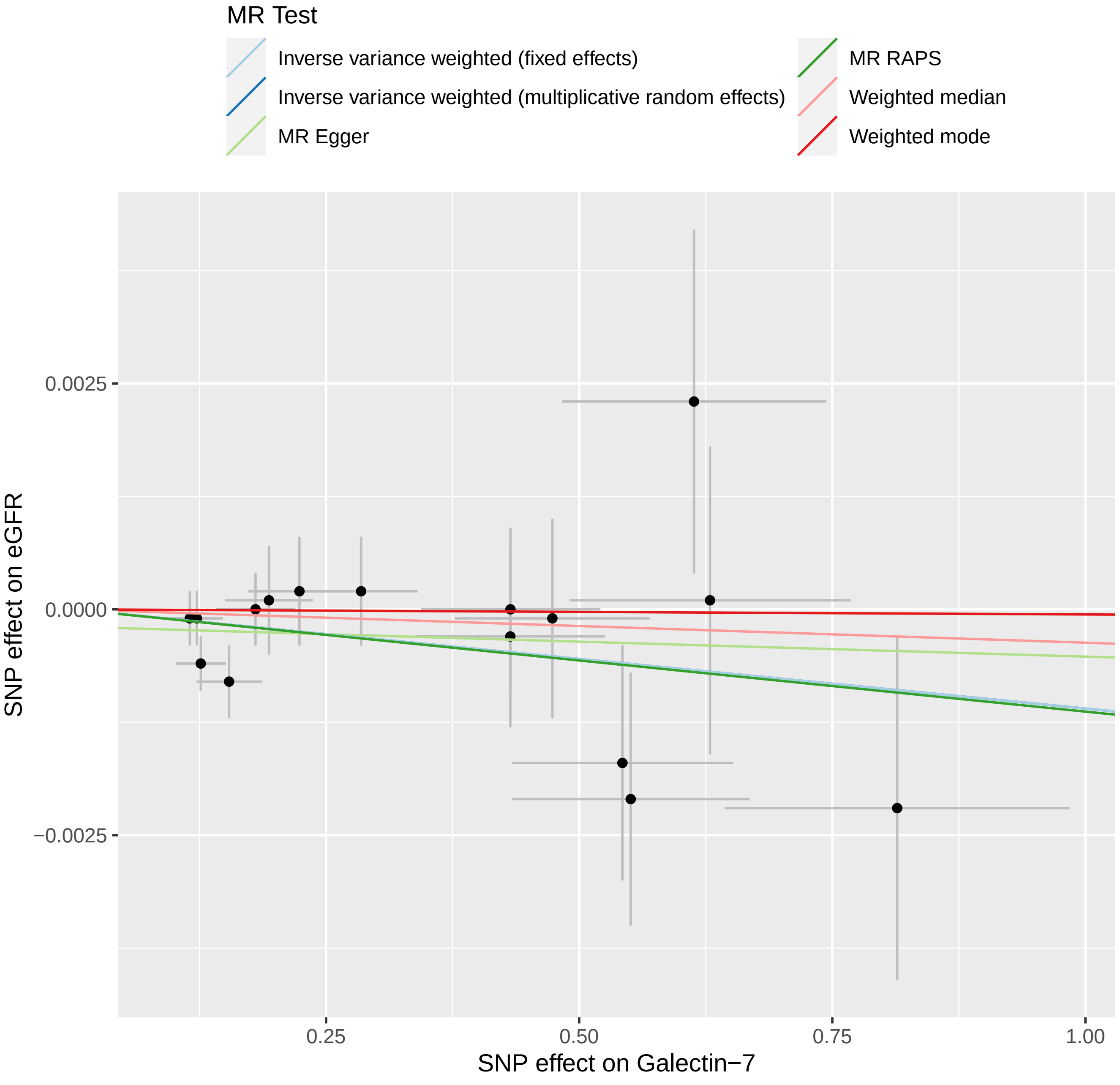

Batch 2−1731 : Galectin−8 on eGFR

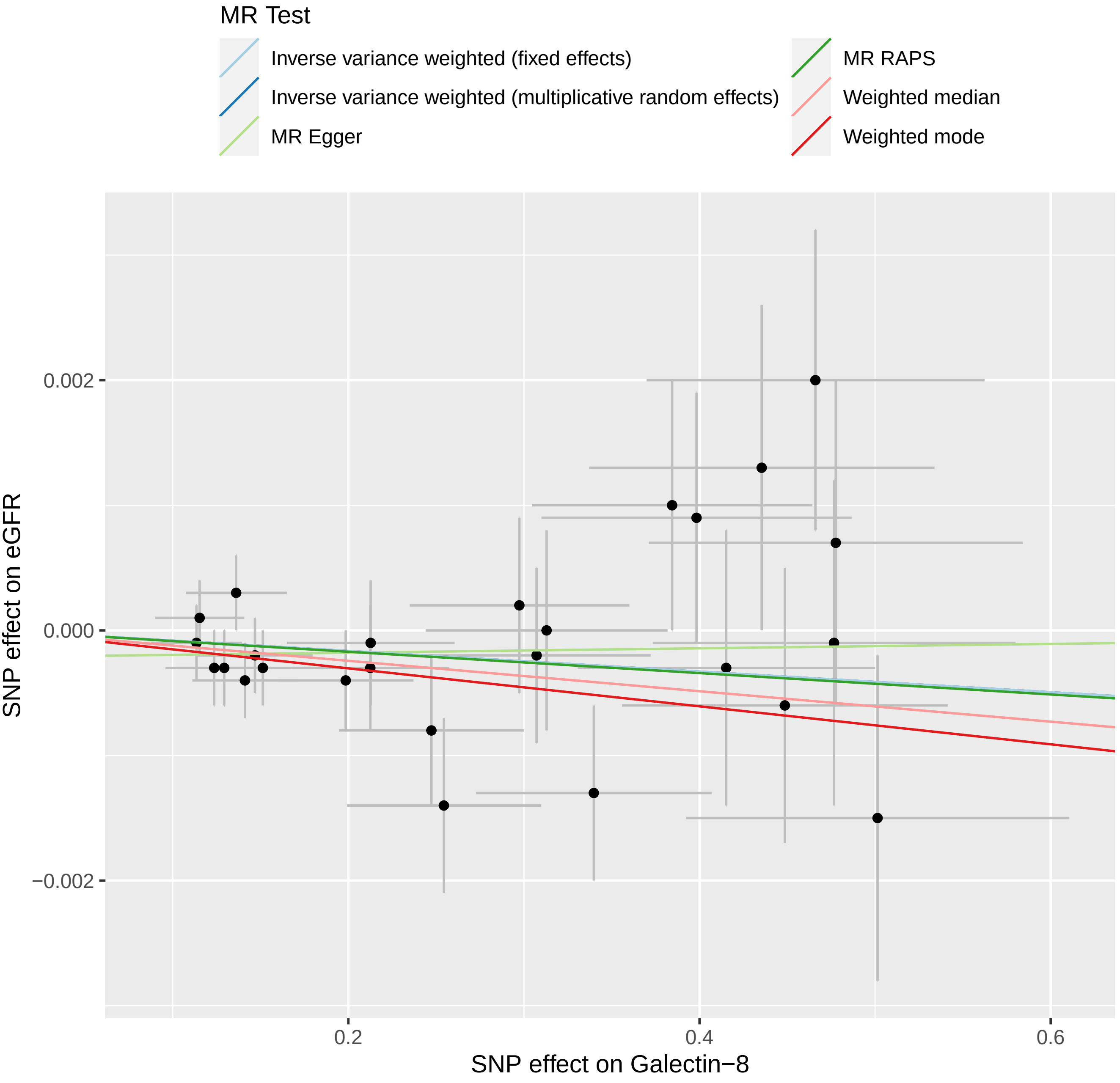

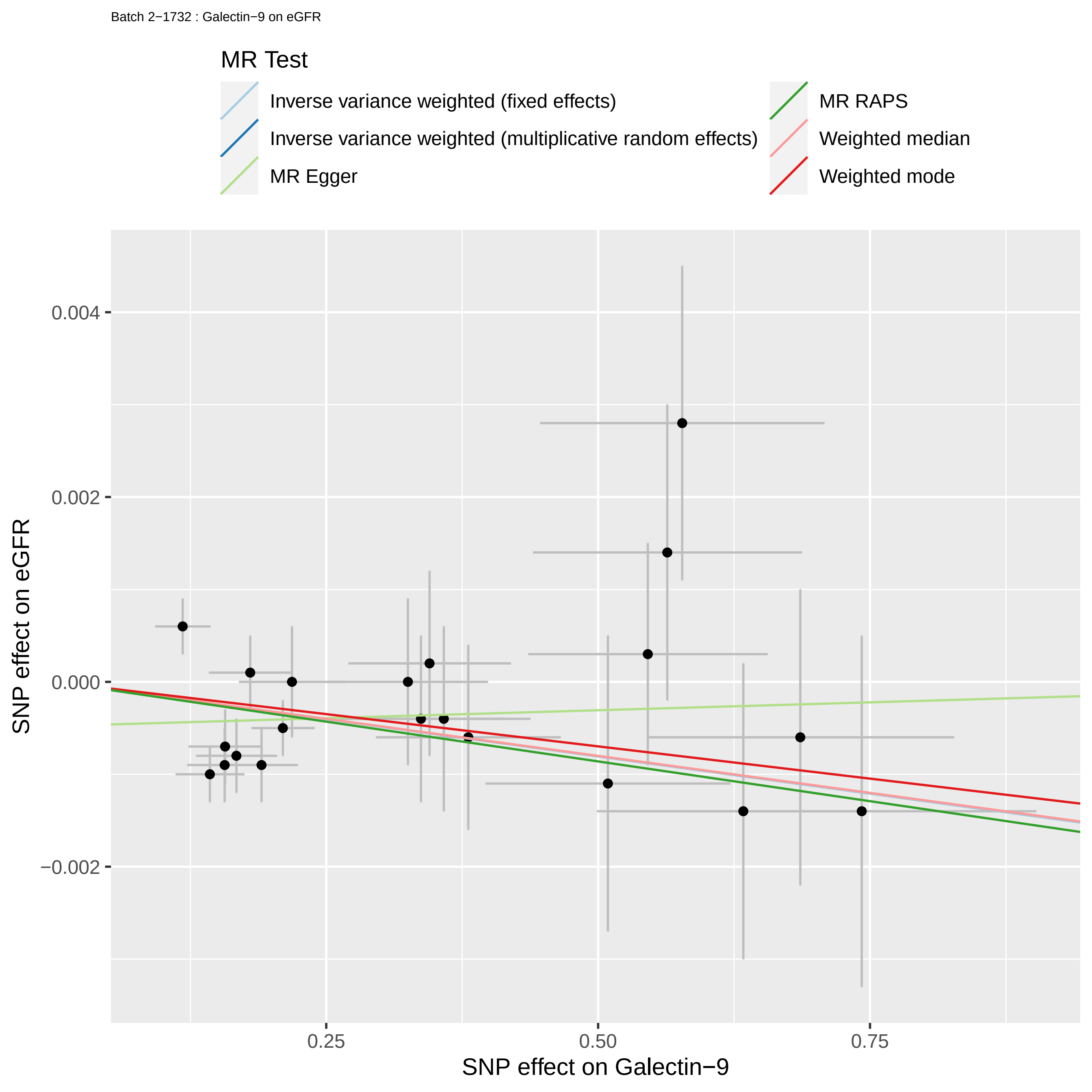
Batch 2−1732 : Galectin−9 on eGFR
MR Test
Inverse variance weighted (fixed effects)
Inverse variance weighted (multiplicative random effects)
MR Egger
MR RAPS
Weighted median
Weighted mode
0.004
0.002
0.000
−0.002
0.25
0.50
0.75
SNP effect on eGFR
SNP effect on Galectin−9

Batch 2−1733 : Leucine−rich repeat LGI family member 3 on eGFR

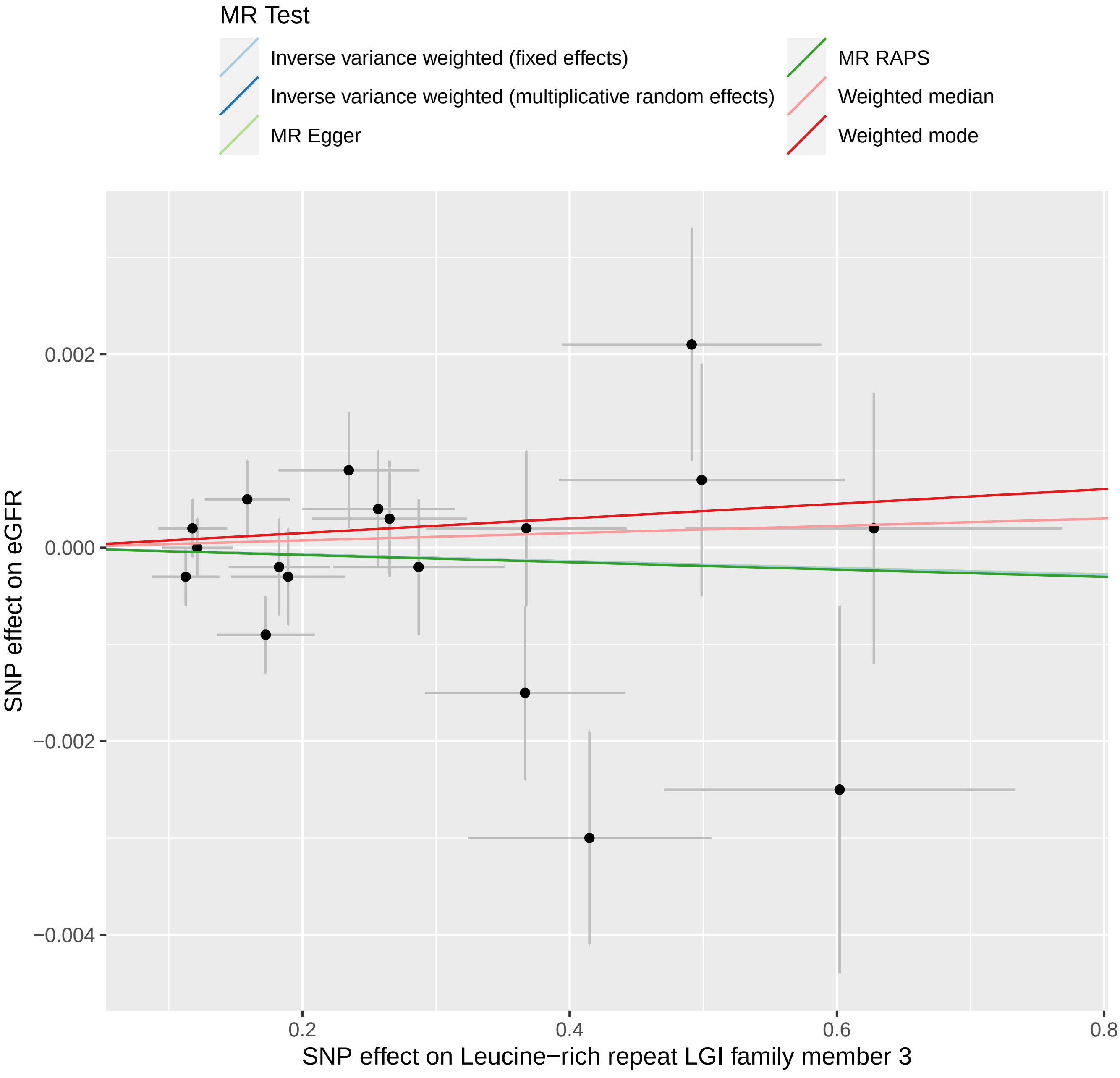

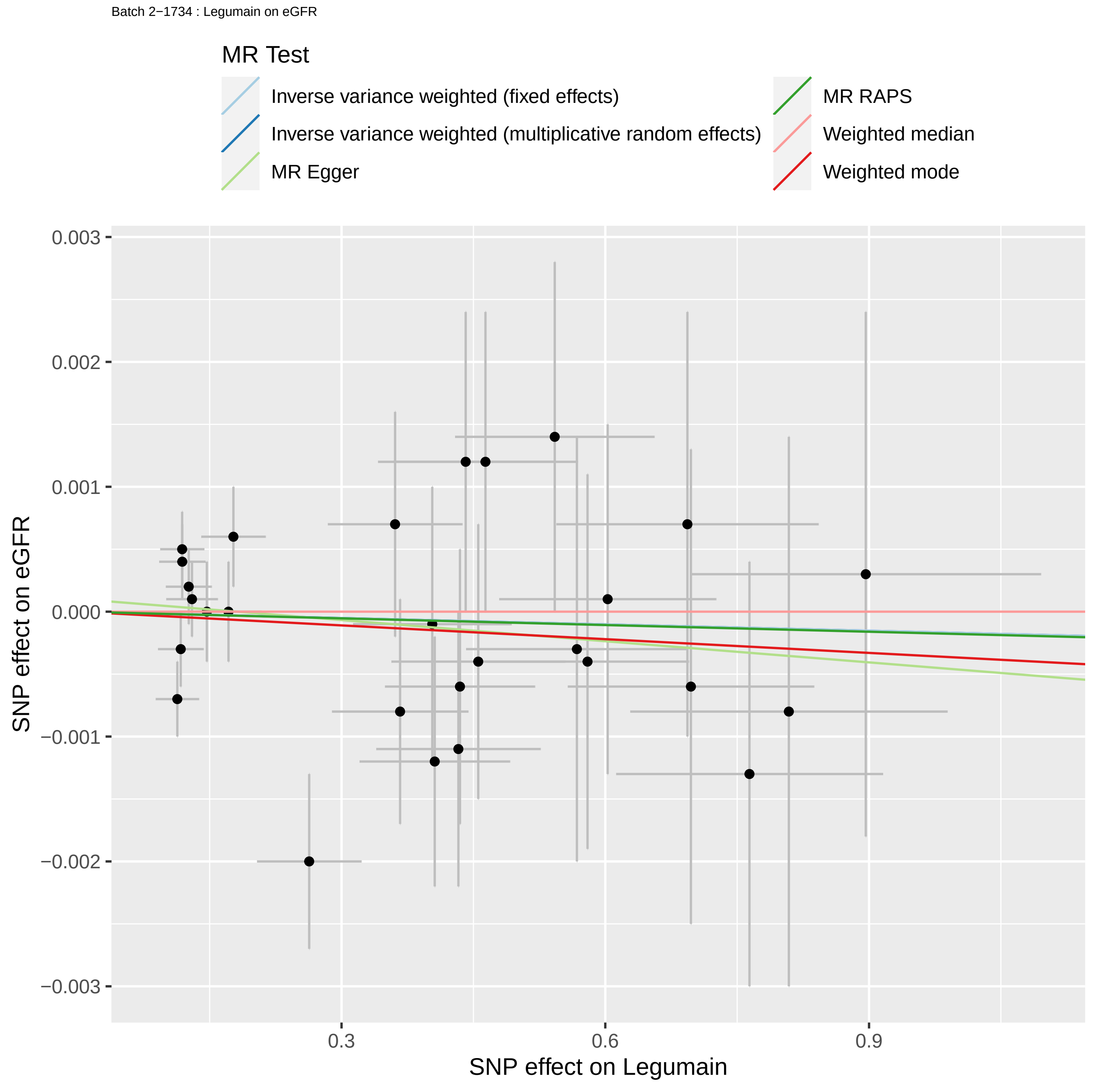
Batch 2−1734 : Legumain on eGFR
MR Test
Inverse variance weighted (fixed effects)
Inverse variance weighted (multiplicative random effects)
MR Egger
MR RAPS
Weighted median
Weighted mode
0.003
0.002
0.001
0.000
−0.001
−0.002
−0.003
SNP effect on eGFR
0.3
0.6
0.9
SNP effect on Legumain

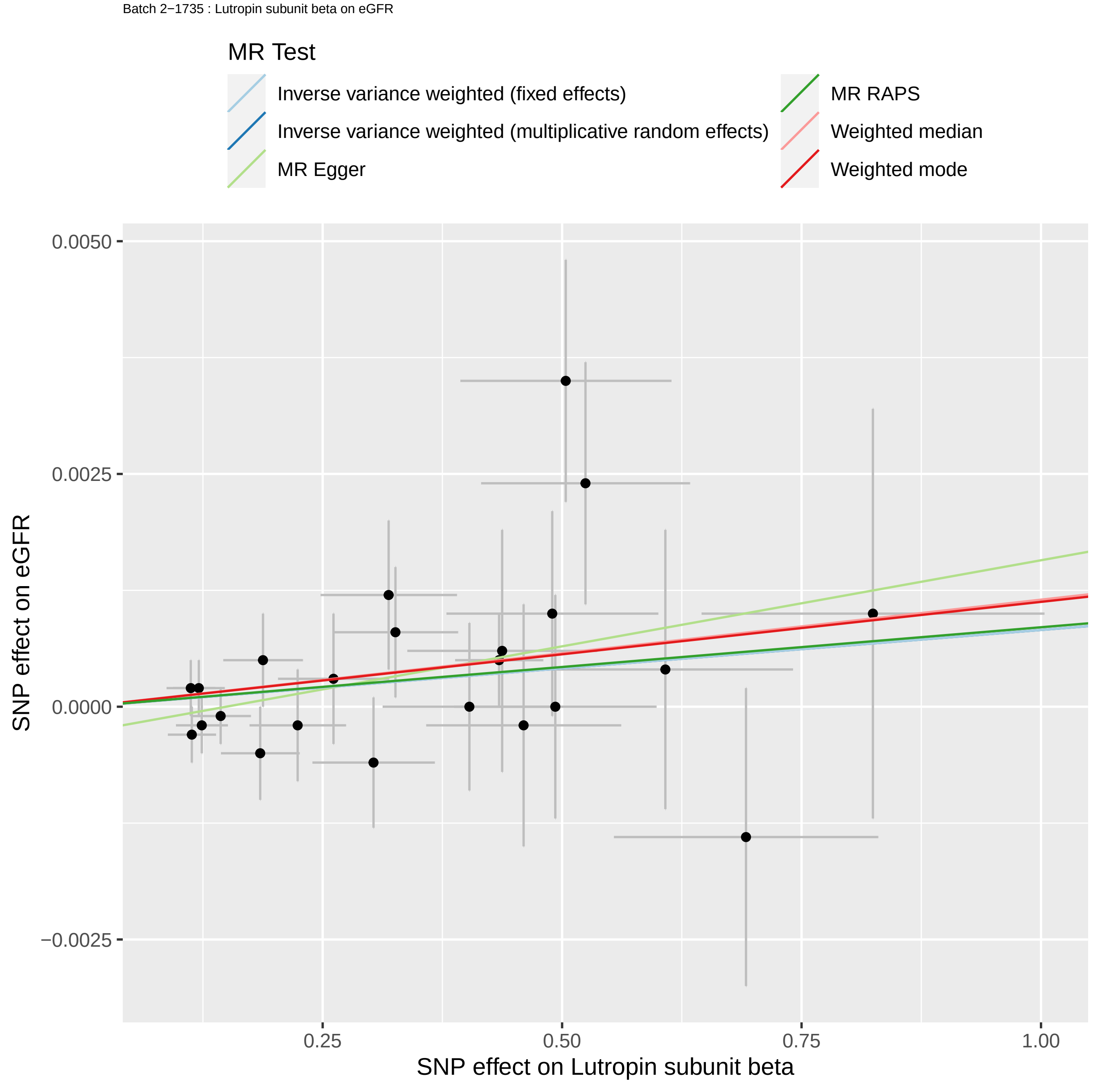

Batch 2−1735 : Lutropin subunit beta on eGFR
MR Test
Inverse variance weighted (fixed effects)
Inverse variance weighted (multiplicative random effects)
MR Egger
MR RAPS
Weighted median
Weighted mode
0.0050
0.0025
0.0000
−0.0025
0.25
0.50
0.75
1.00
SNP effect on eGFR
SNP effect on Lutropin subunit beta

Batch 2−1736 : Leukemia inhibitory factor on eGFR

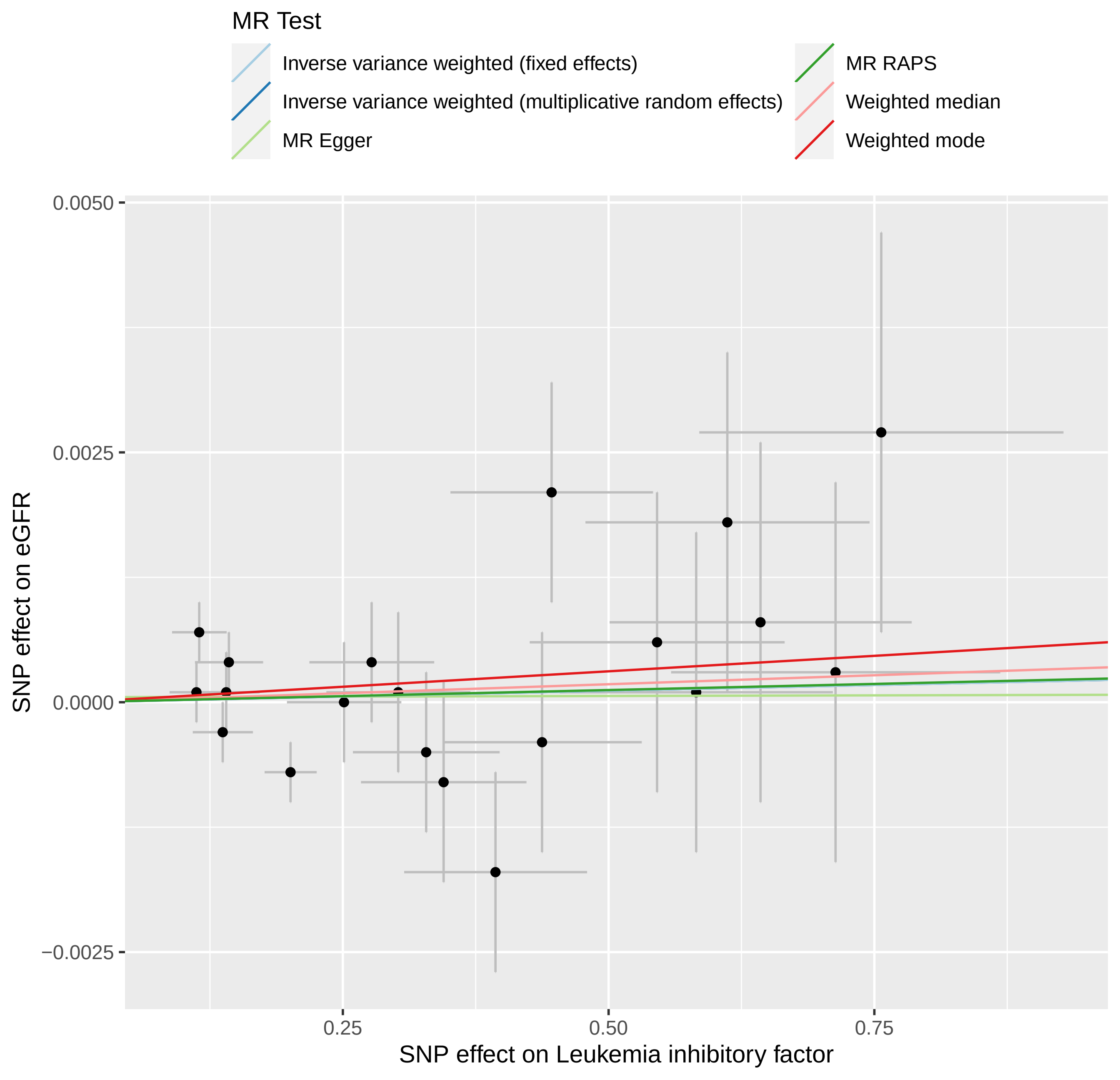

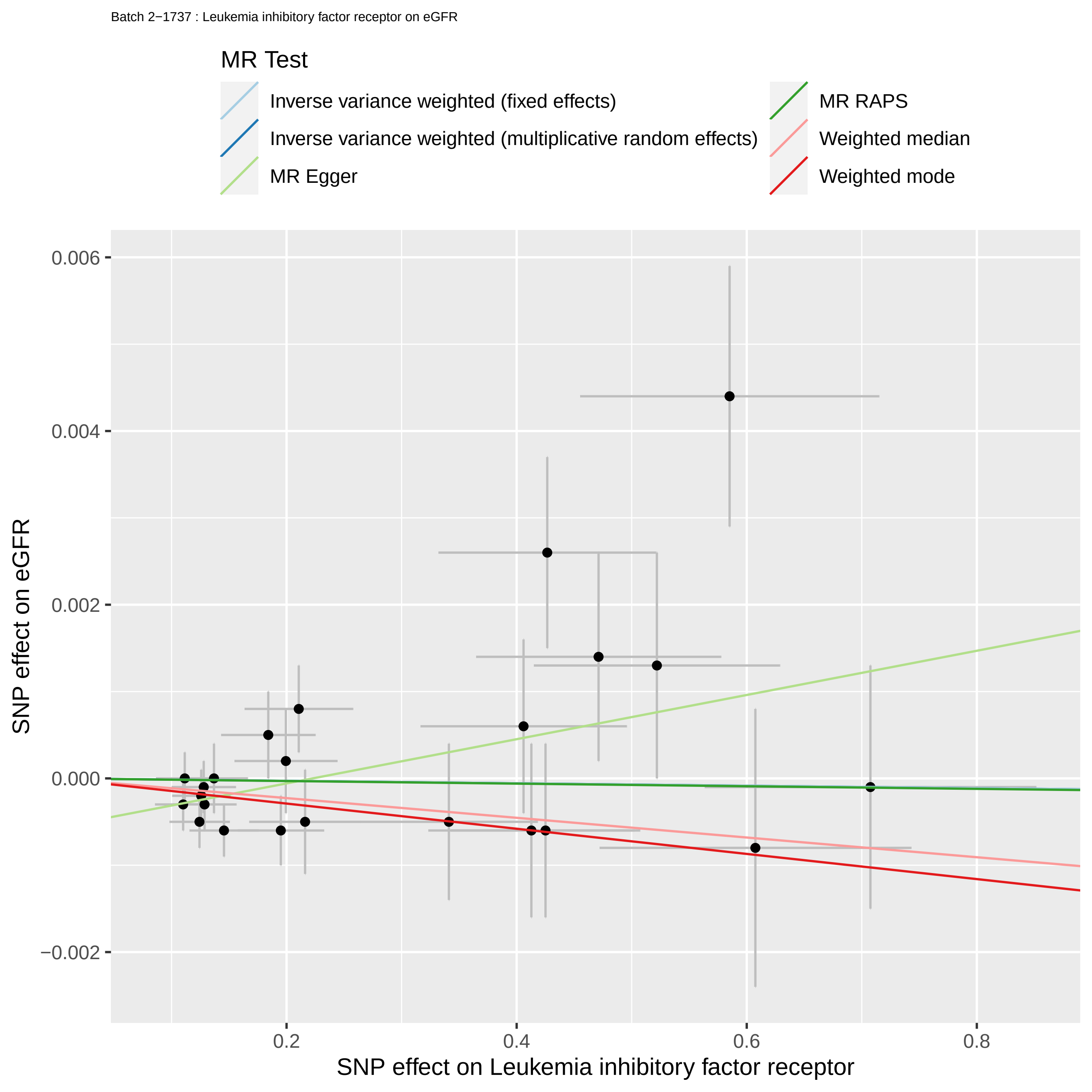

Batch 2−1737 : Leukemia inhibitory factor receptor on eGFR
MR Test
Inverse variance weighted (fixed effects)
Inverse variance weighted (multiplicative random effects)
MR Egger
MR RAPS
Weighted median
Weighted mode
0.006
0.004
0.002
0.000
−0.002
0.2
0.4
0.6
0.8
SNP effect on eGFR
SNP effect on Leukemia inhibitory factor receptor

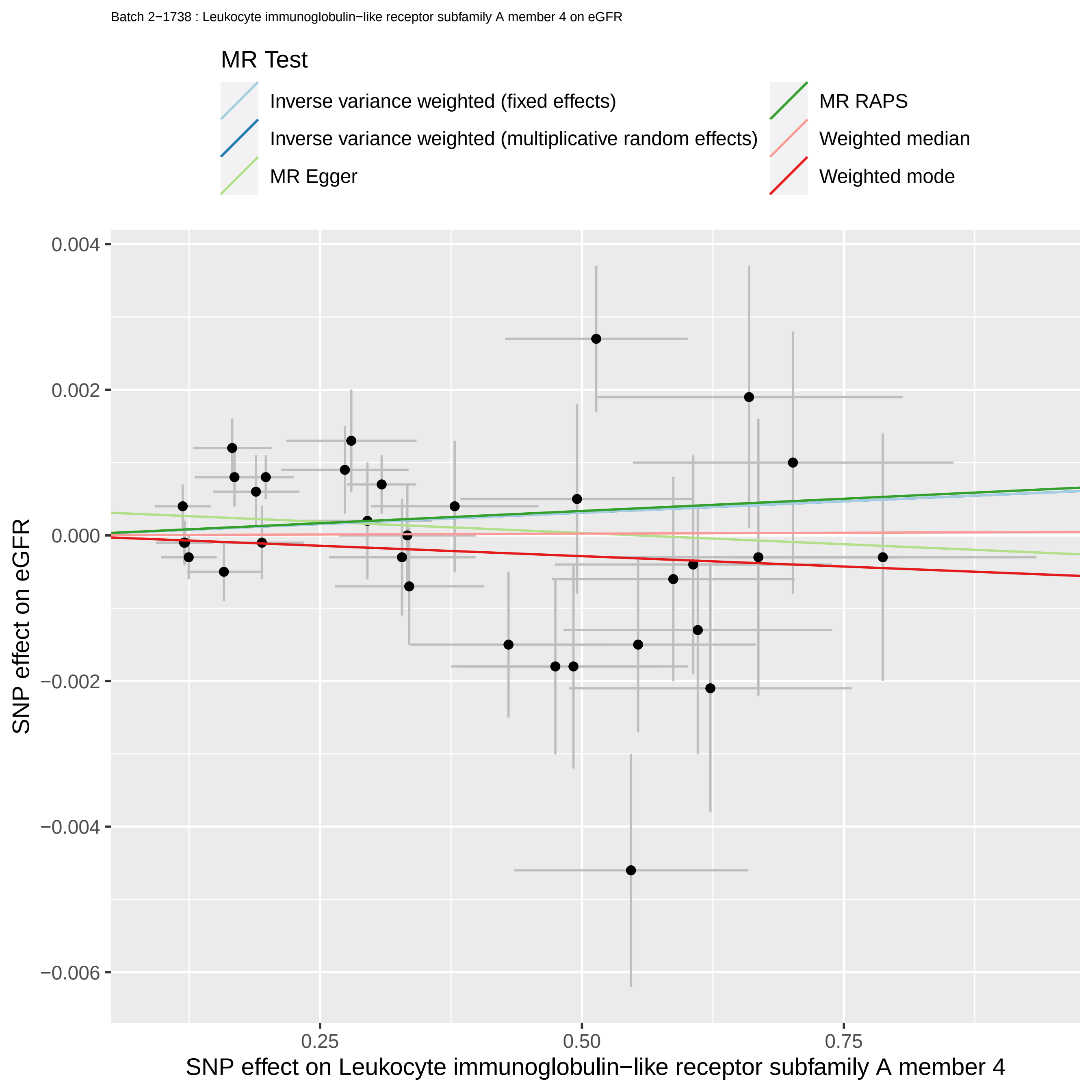

Batch 2−1738 : Leukocyte immunoglobulin−like receptor subfamily A member 4 on eGFR
MR Test
Inverse variance weighted (fixed effects)
Inverse variance weighted (multiplicative random effects)
MR Egger
MR RAPS
Weighted median
Weighted mode
0.004
0.002
0.000
−0.002
−0.004
−0.006
SNP effect on eGFR
0.25
0.50
0.75
SNP effect on Leukocyte immunoglobulin−like receptor subfamily A member 4

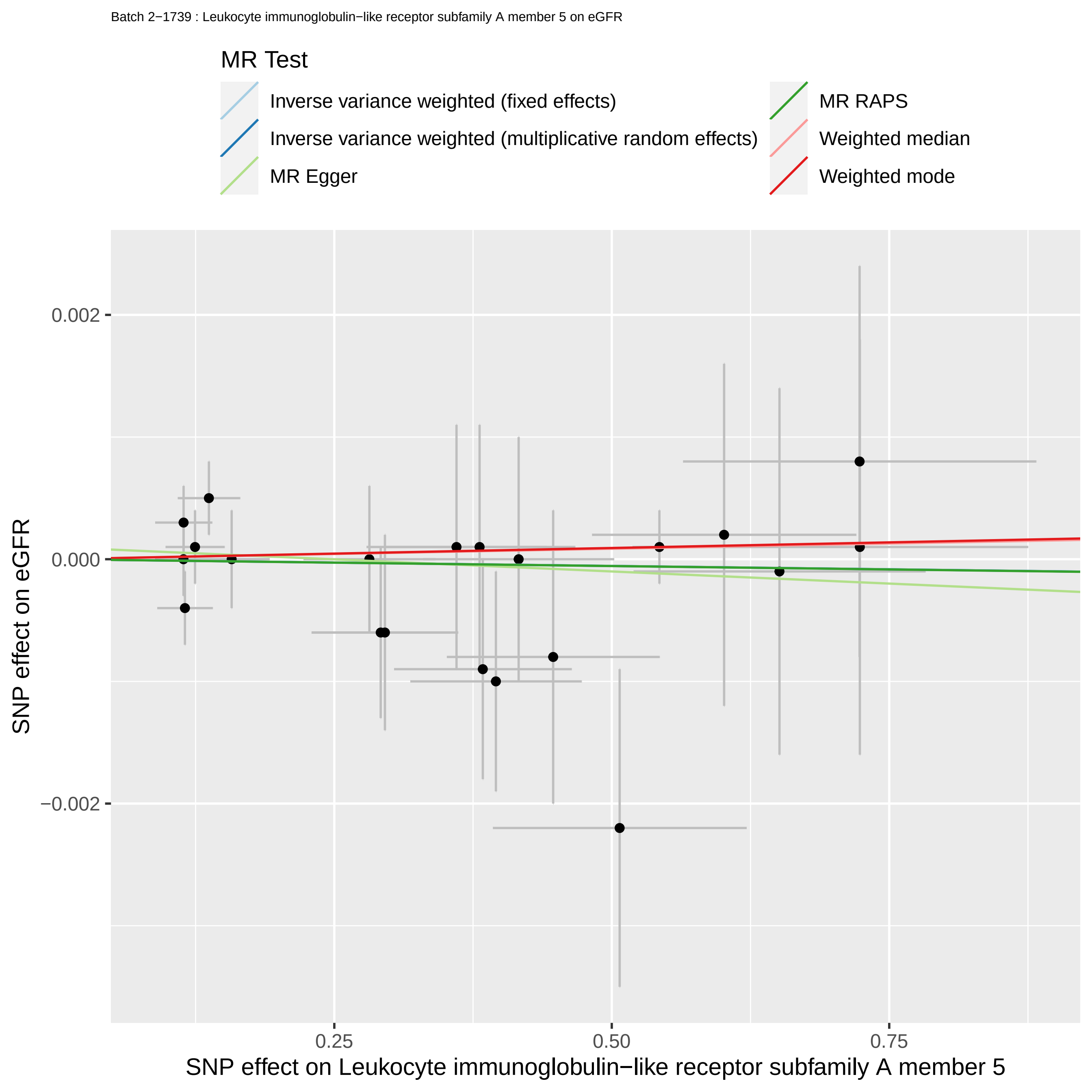

Batch 2−1739 : Leukocyte immunoglobulin−like receptor subfamily A member 5 on eGFR
MR Test
Inverse variance weighted (fixed effects)
Inverse variance weighted (multiplicative random effects)
MR Egger
MR RAPS
Weighted median
Weighted mode
0.002
0.000
−0.002
0.25
0.50
0.75
SNP effect on eGFR
SNP effect on Leukocyte immunoglobulin−like receptor subfamily A member 5

Batch 2−1740 : Leukocyte immunoglobulin−like receptor subfamily A member 5 on eGFR

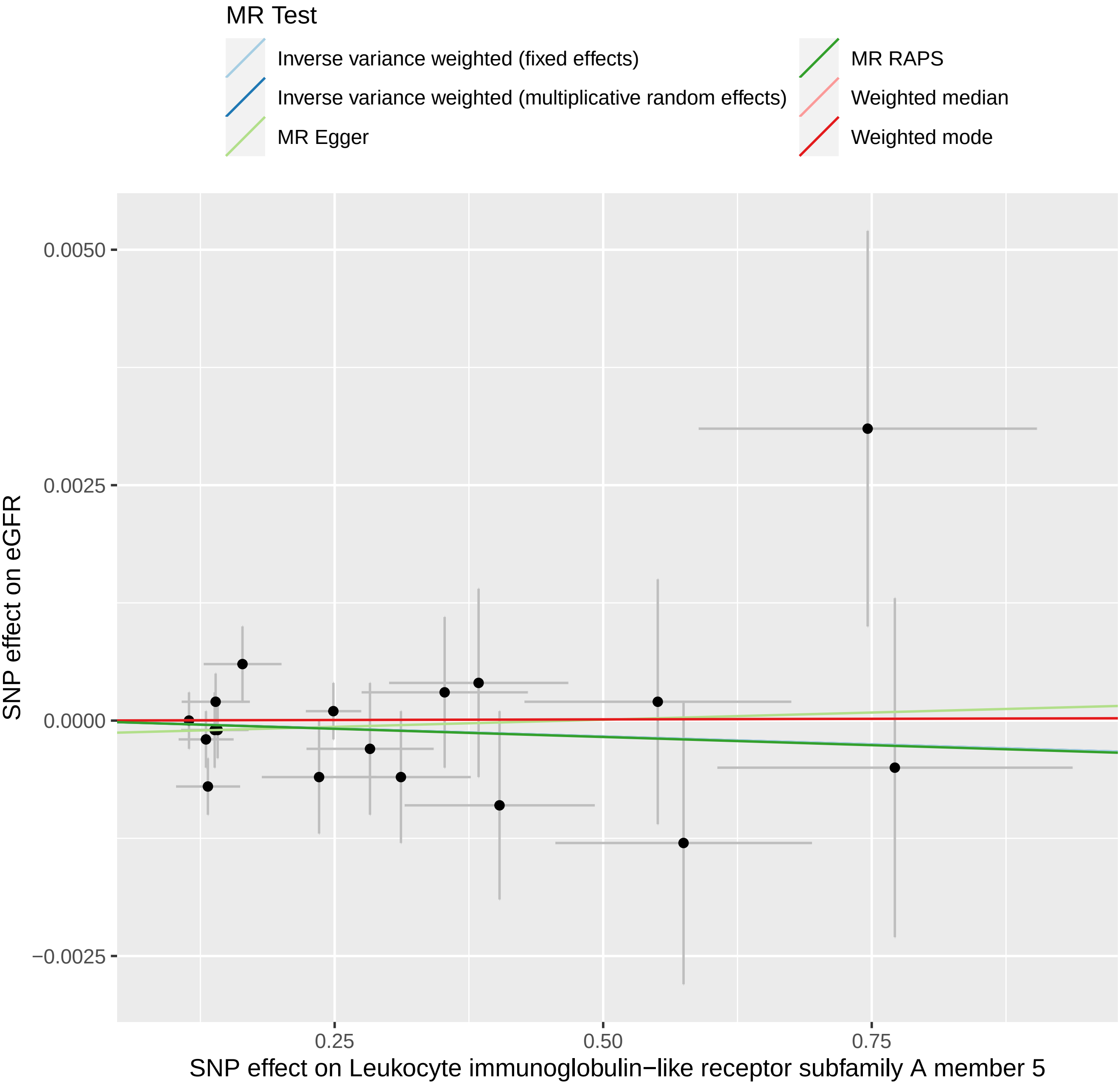

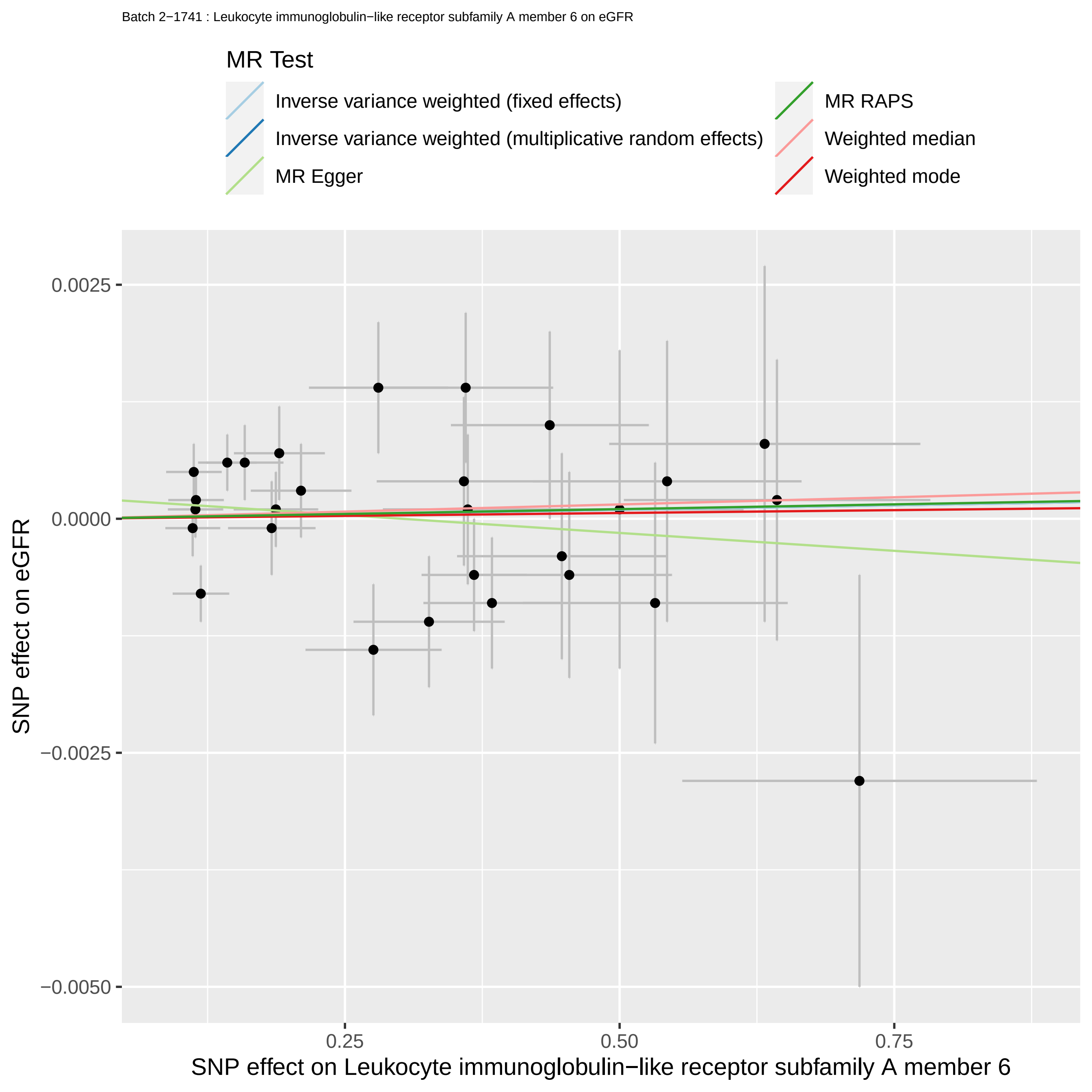

Batch 2−1741 : Leukocyte immunoglobulin−like receptor subfamily A member 6 on eGFR
MR Test
Inverse variance weighted (fixed effects)
Inverse variance weighted (multiplicative random effects)
MR Egger
MR RAPS
Weighted median
Weighted mode
0.0025
0.0000
−0.0025
−0.0050
0.25
0.50
0.75
SNP effect on eGFR
SNP effect on Leukocyte immunoglobulin−like receptor subfamily A member 6

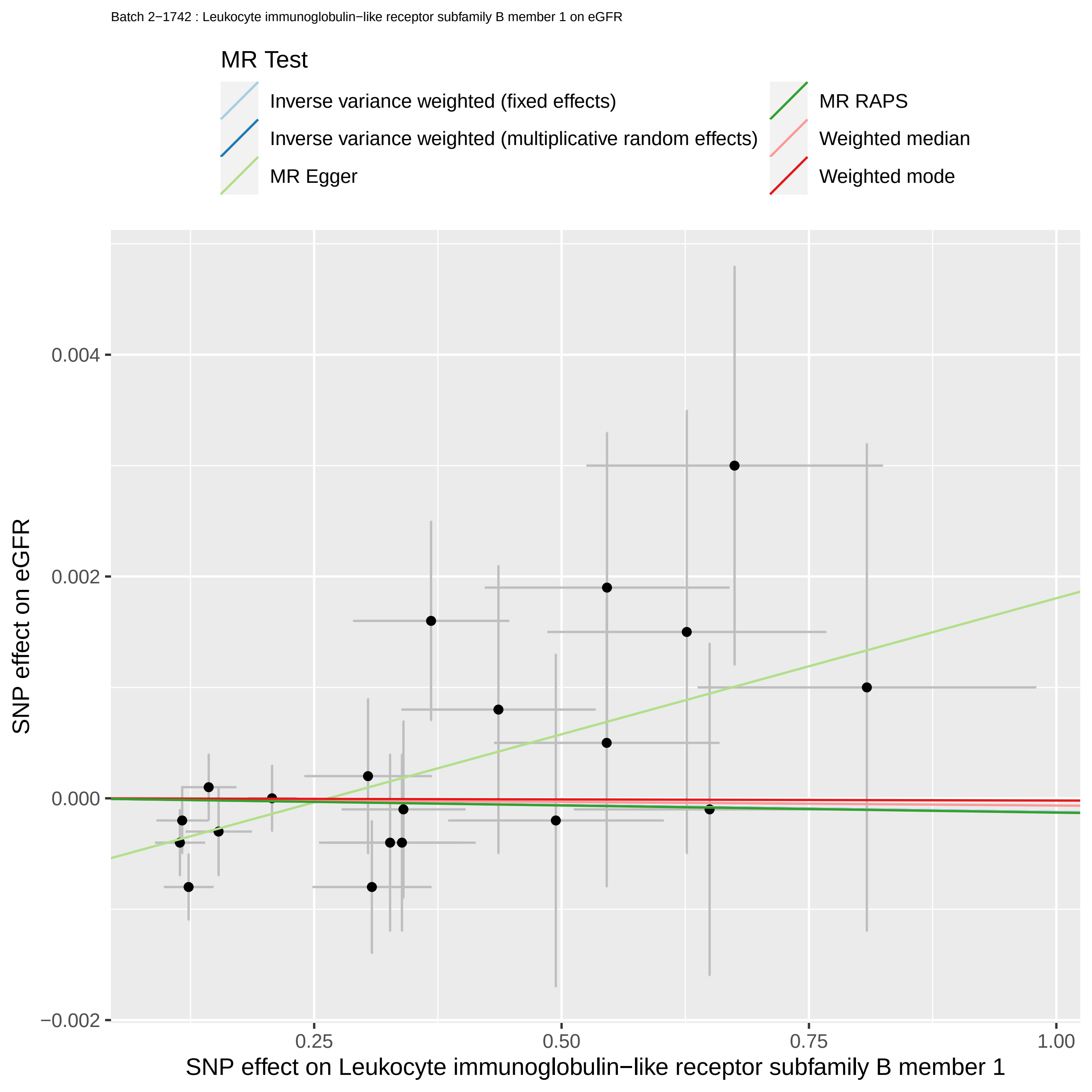

Batch 2−1742 : Leukocyte immunoglobulin−like receptor subfamily B member 1 on eGFR
MR Test
Inverse variance weighted (fixed effects)
Inverse variance weighted (multiplicative random effects)
MR Egger
MR RAPS
Weighted median
Weighted mode
0.004
0.002
0.000
−0.002
0.25
0.50
0.75
1.00
SNP effect on eGFR
SNP effect on Leukocyte immunoglobulin−like receptor subfamily B member 1

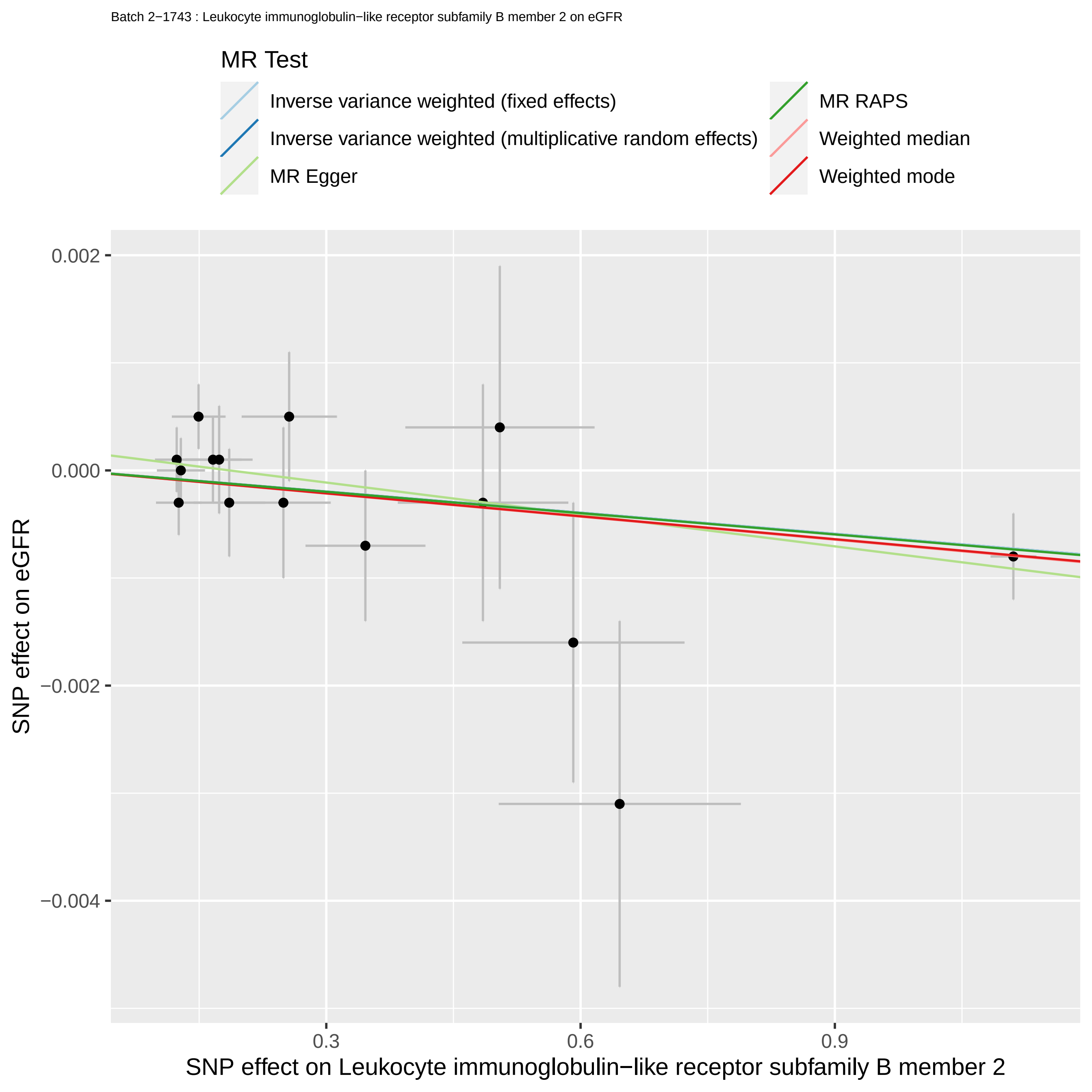
Batch 2−1743 : Leukocyte immunoglobulin−like receptor subfamily B member 2 on eGFR
MR Test
Inverse variance weighted (fixed effects)
Inverse variance weighted (multiplicative random effects)
MR Egger
MR RAPS
Weighted median
Weighted mode
0.002
0.000
−0.002
−0.004
0.3
0.6
0.9
SNP effect on eGFR
SNP effect on Leukocyte immunoglobulin−like receptor subfamily B member 2

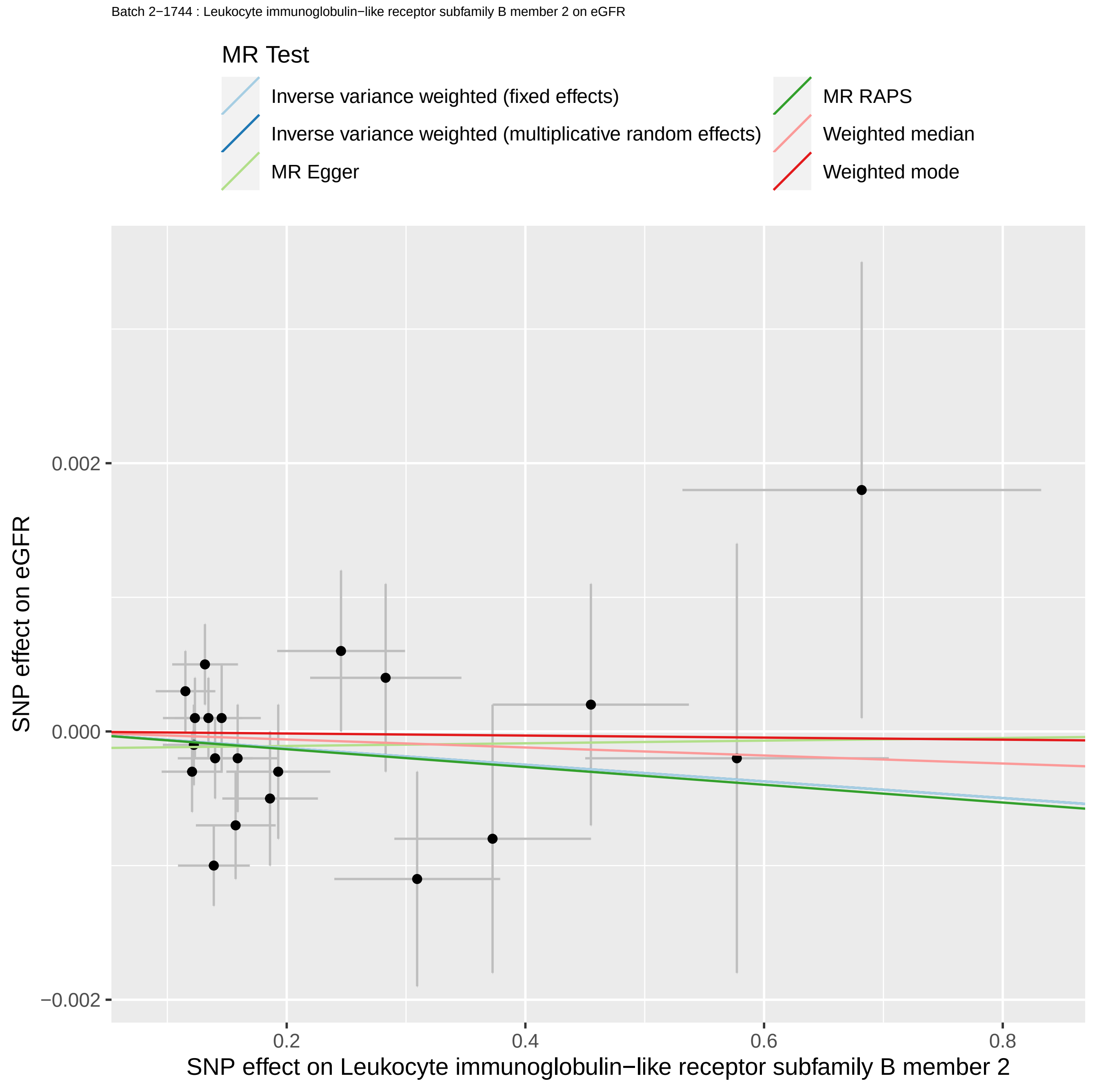

Batch 2−1744 : Leukocyte immunoglobulin−like receptor subfamily B member 2 on eGFR
MR Test
Inverse variance weighted (fixed effects)
Inverse variance weighted (multiplicative random effects)
MR Egger
MR RAPS
Weighted median
Weighted mode
SNP effect on eGFR
0.002
0.000
−0.002
0.2
0.4
0.6
0.8
SNP effect on Leukocyte immunoglobulin−like receptor subfamily B member 2

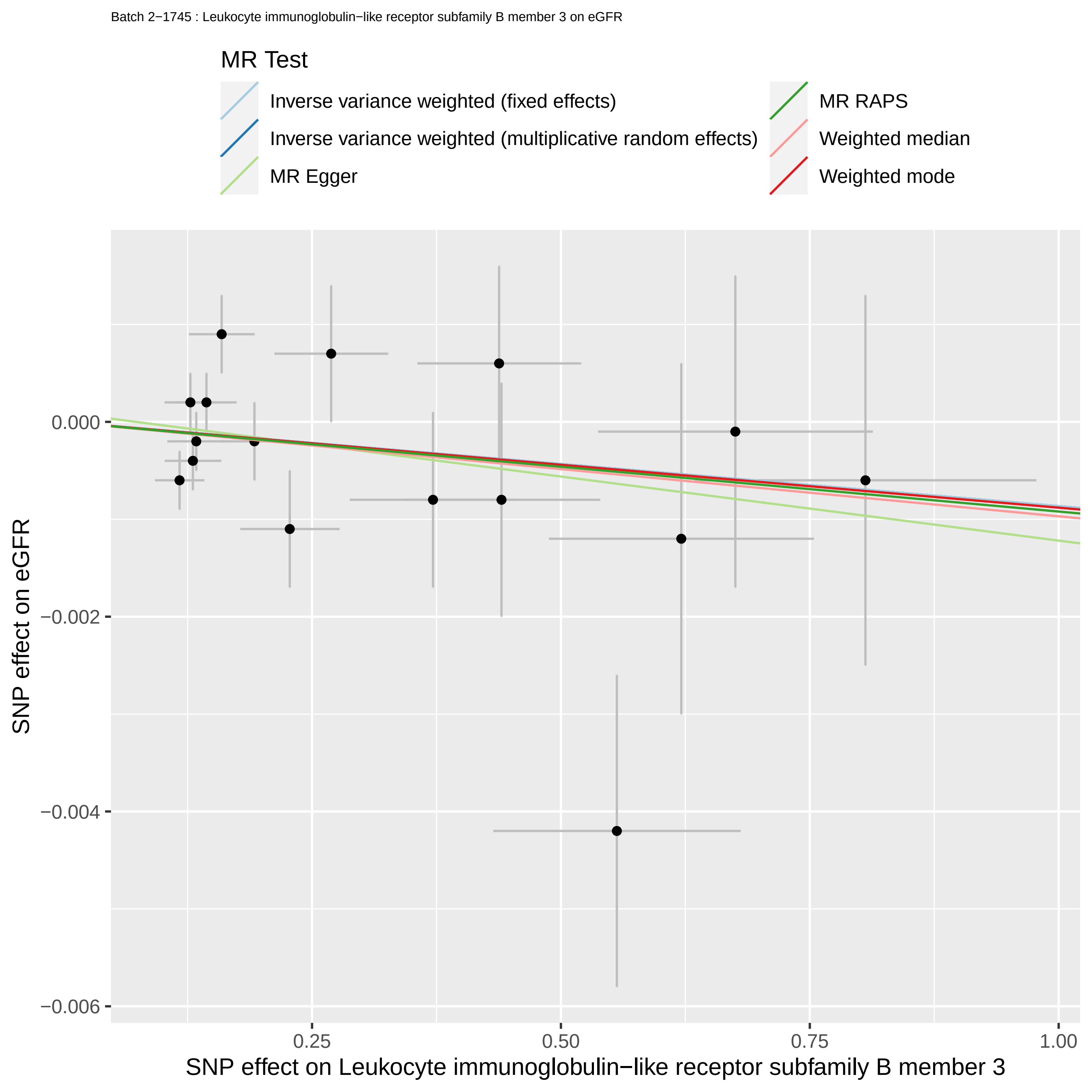

Batch 2−1745 : Leukocyte immunoglobulin−like receptor subfamily B member 3 on eGFR
MR Test
Inverse variance weighted (fixed effects)
Inverse variance weighted (multiplicative random effects)
MR Egger
MR RAPS
Weighted median
Weighted mode
0.000
−0.002
−0.004
−0.006
SNP effect on eGFR
0.25
0.50
0.75
1.00
SNP effect on Leukocyte immunoglobulin−like receptor subfamily B member 3

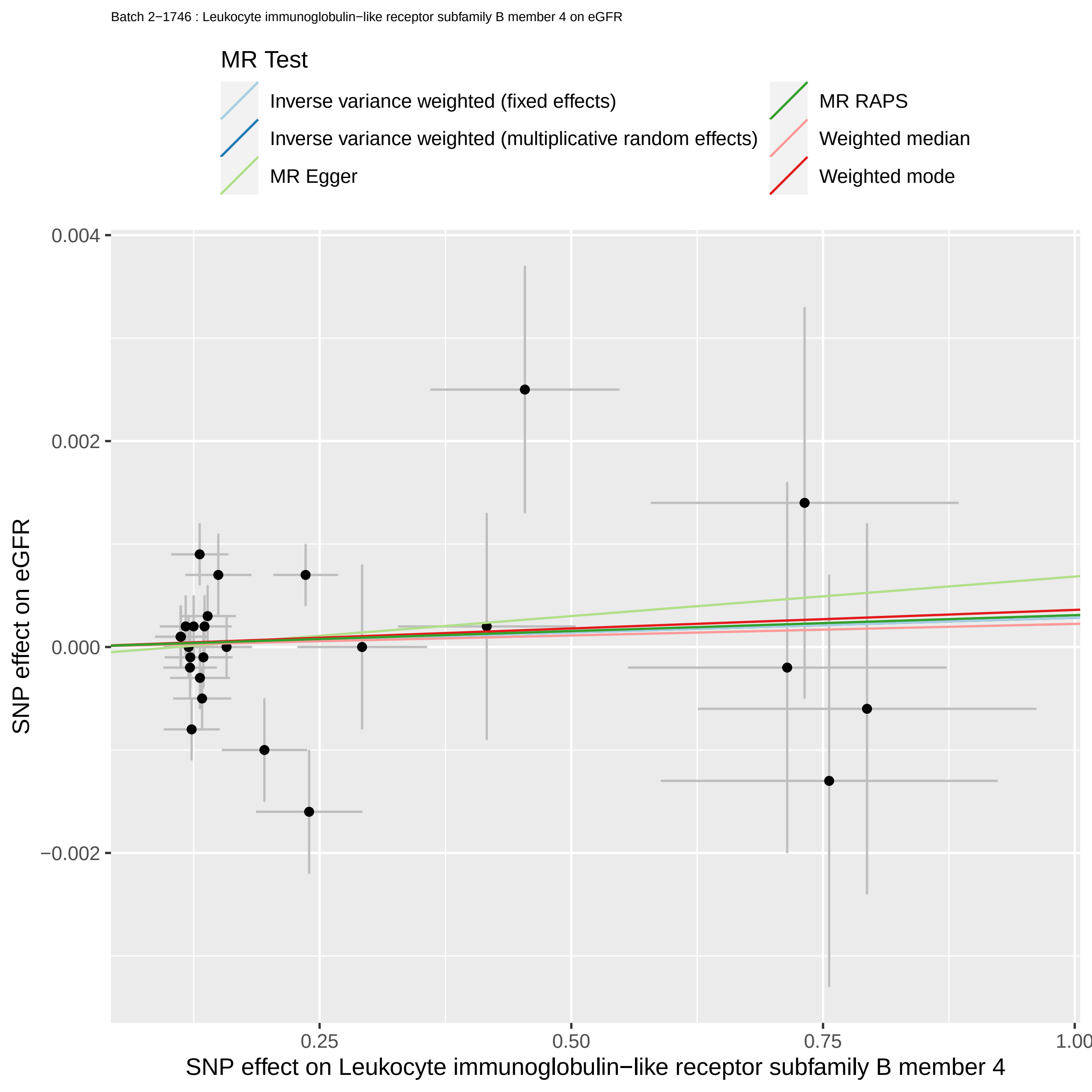

Batch 2−1746 : Leukocyte immunoglobulin−like receptor subfamily B member 4 on eGFR
MR Test
Inverse variance weighted (fixed effects)
Inverse variance weighted (multiplicative random effects)
MR Egger
MR RAPS
Weighted median
Weighted mode
0.004
0.002
0.000
−0.002
0.25
0.50
0.75
1.00
SNP effect on eGFR
SNP effect on Leukocyte immunoglobulin−like receptor subfamily B member 4

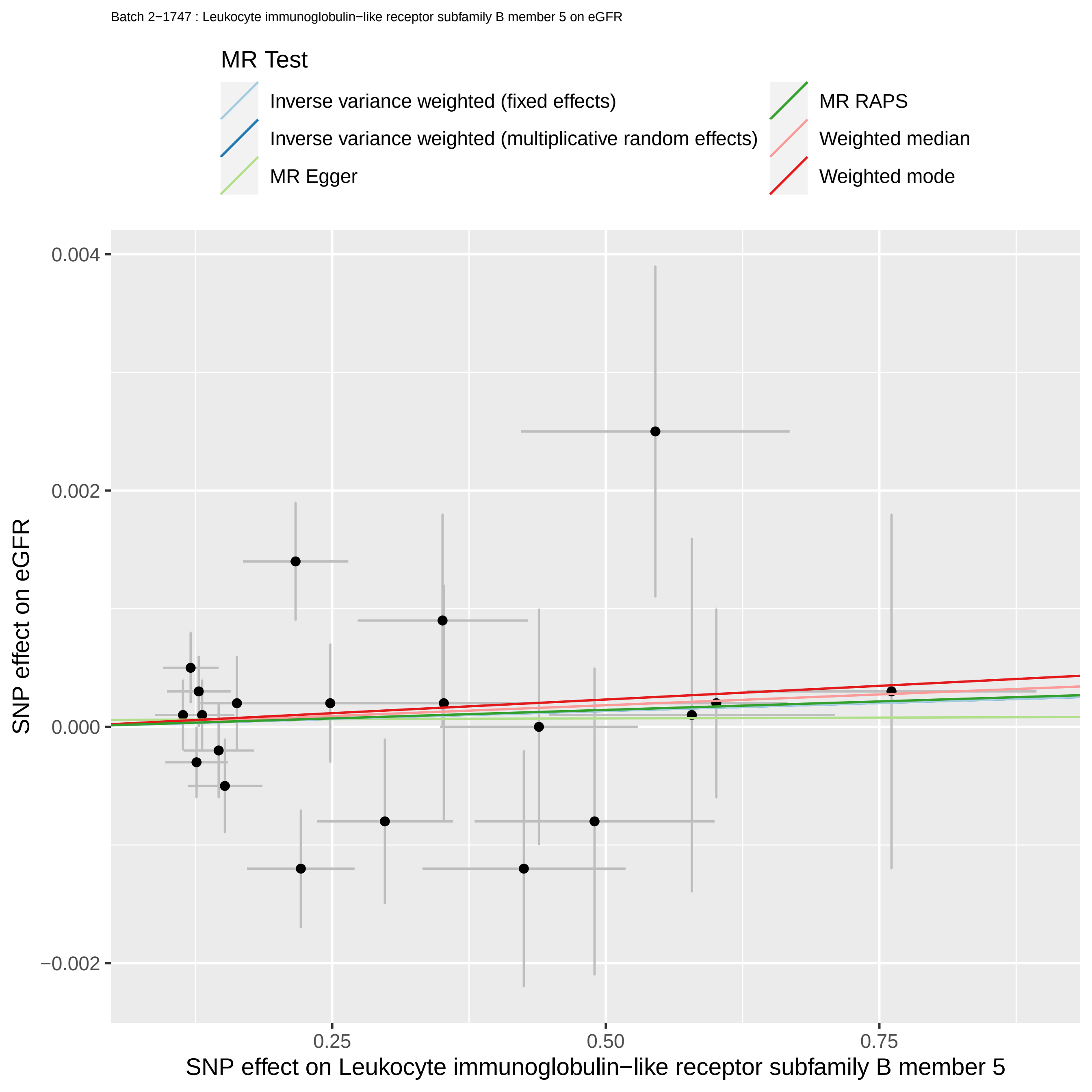
Batch 2−1747 : Leukocyte immunoglobulin−like receptor subfamily B member 5 on eGFR
MR Test
Inverse variance weighted (fixed effects)
Inverse variance weighted (multiplicative random effects)
MR Egger
MR RAPS
Weighted median
Weighted mode
0.004
0.002
0.000
−0.002
0.25
0.50
0.75
SNP effect on eGFR
SNP effect on Leukocyte immunoglobulin−like receptor subfamily B member 5

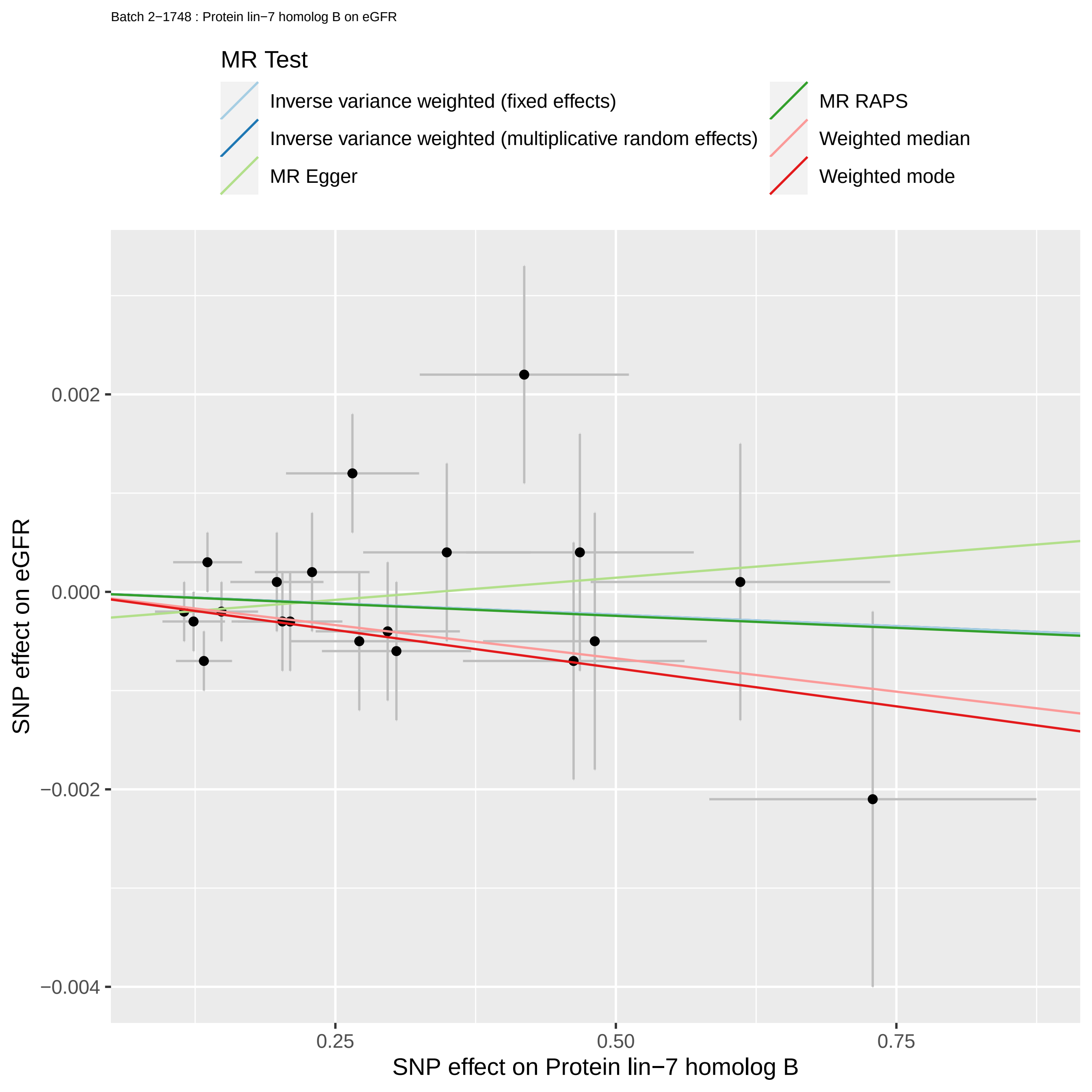

Batch 2−1748 : Protein lin−7 homolog B on eGFR
MR Test
Inverse variance weighted (fixed effects)
Inverse variance weighted (multiplicative random effects)
MR Egger
MR RAPS
Weighted median
Weighted mode
0.002
0.000
−0.002
−0.004
SNP effect on eGFR
0.25
0.50
0.75
SNP effect on Protein lin−7 homolog B

Batch 2−1749 : Leucine−rich repeat and immunoglobulin−like domain−containing nogo receptor−interacting protein 1 on eGFR

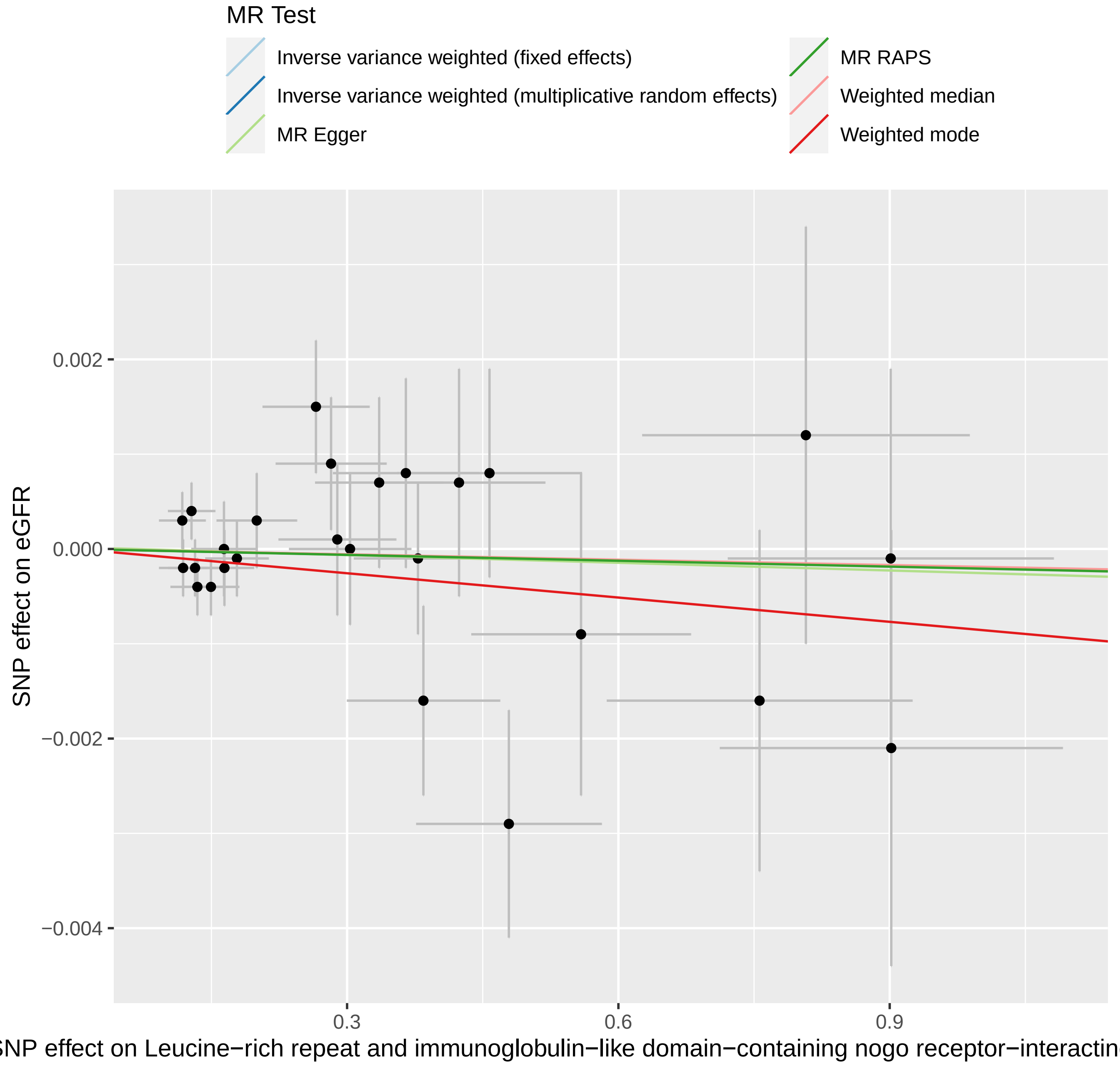

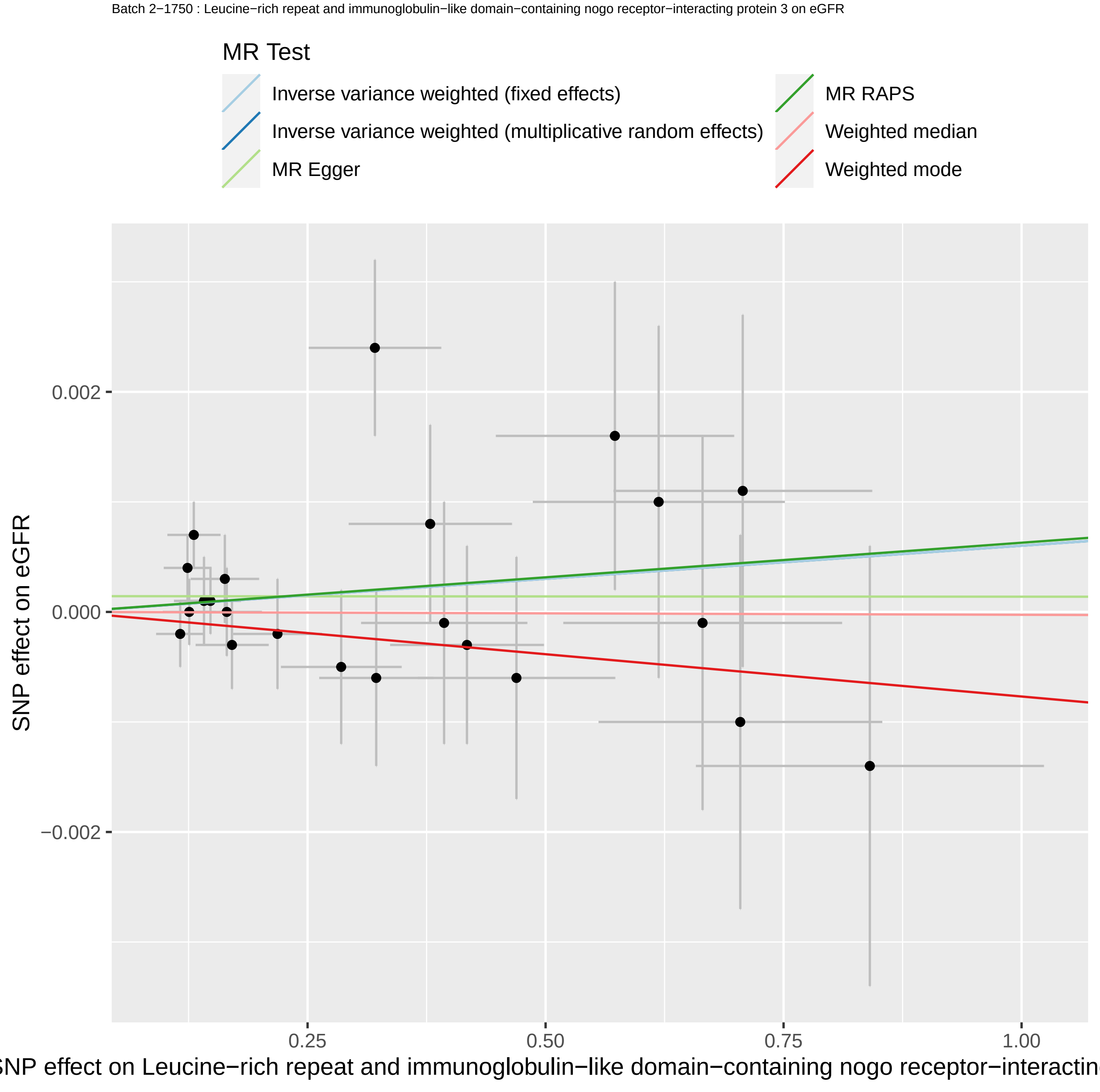
Batch 2−1750 : Leucine−rich repeat and immunoglobulin−like domain−containing nogo receptor−interacting protein 3 on eGFR
MR Test
Inverse variance weighted (fixed effects)
Inverse variance weighted (multiplicative random effects)
MR Egger
MR RAPS
Weighted median
Weighted mode
SNP effect on eGFR
0.002
0.000
−0.002
0.25
0.50
0.75
1.00
SNP effect on Leucine−rich repeat and immunoglobulin−like domain−containing nogo receptor−interactin

Batch 2−1751 : Gastric triacylglycerol lipase on eGFR

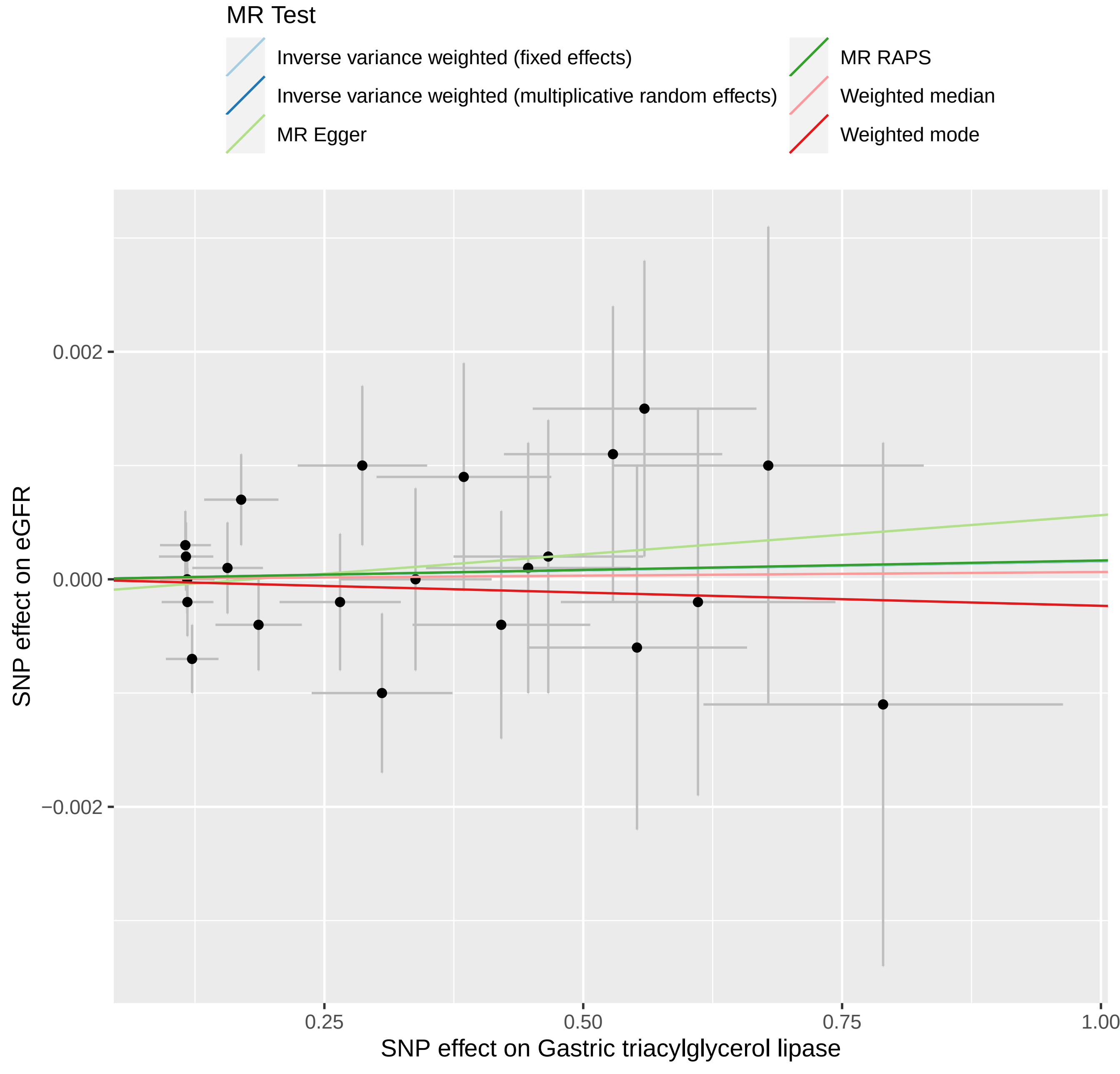

Batch 2−1752 : Lipase member K on eGFR

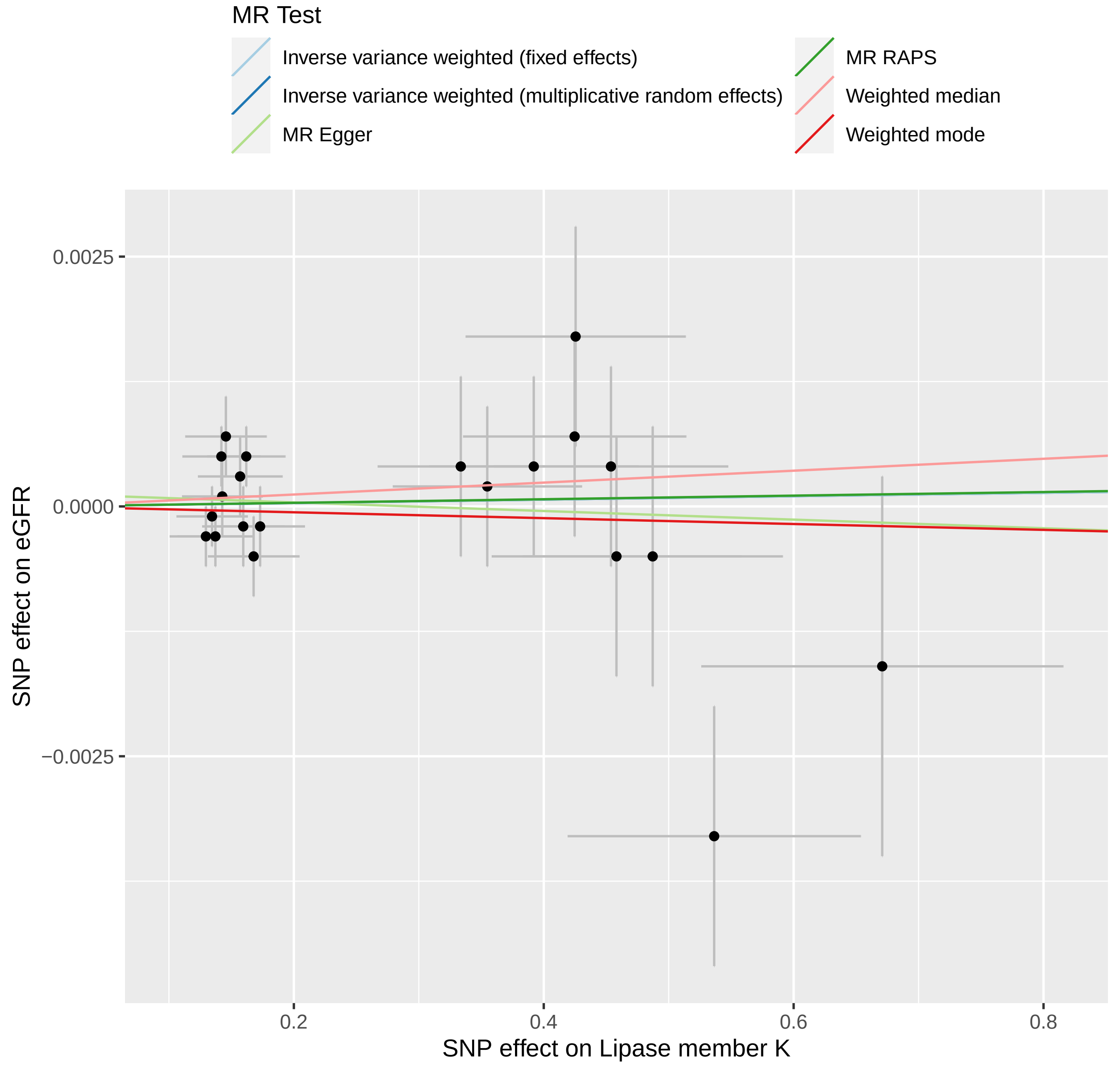

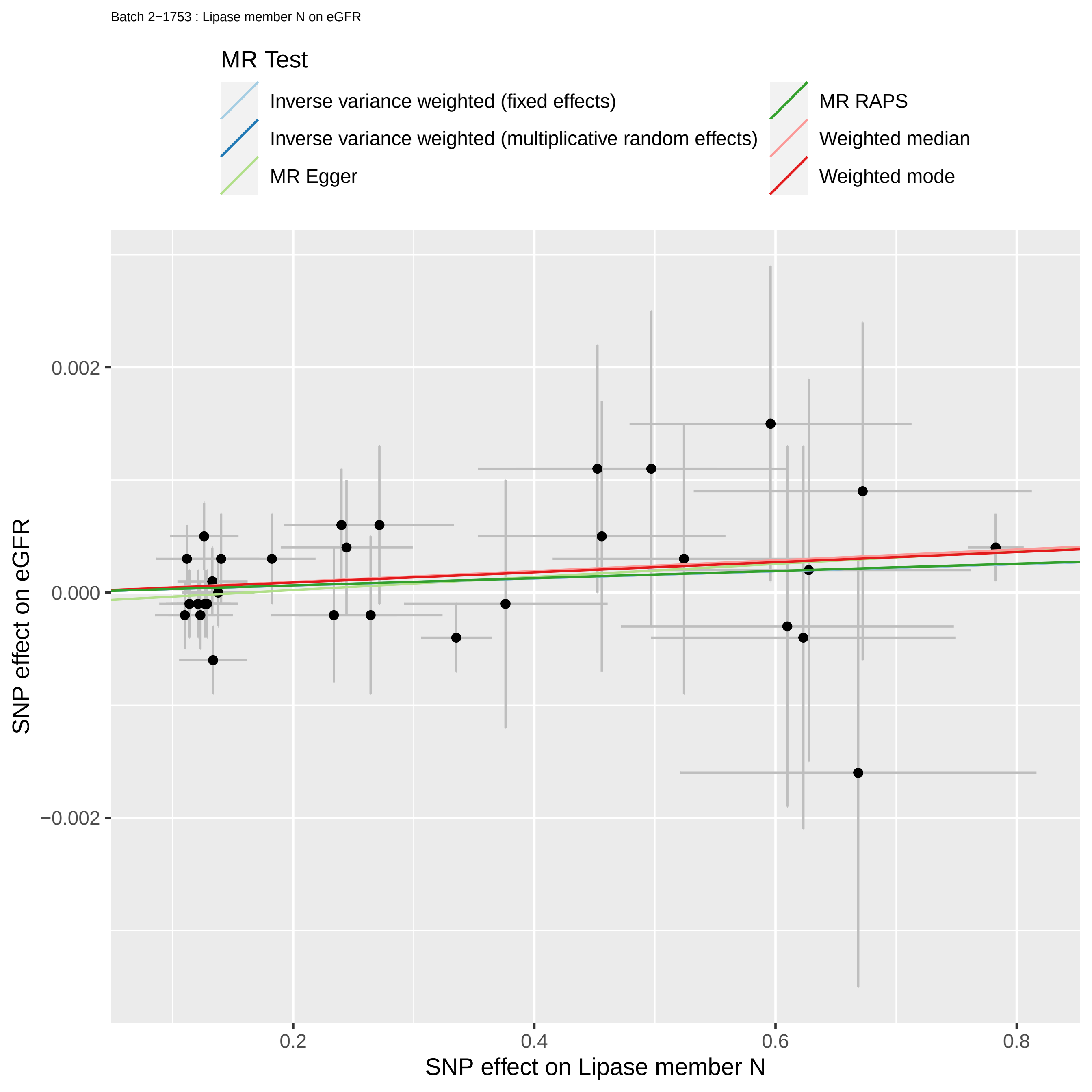

Batch 2−1753 : Lipase member N on eGFR
MR Test
Inverse variance weighted (fixed effects)
Inverse variance weighted (multiplicative random effects)
MR Egger
MR RAPS
Weighted median
Weighted mode
0.002
0.000
−0.002
0.2
0.4
0.6
0.8
SNP effect on eGFR
SNP effect on Lipase member N

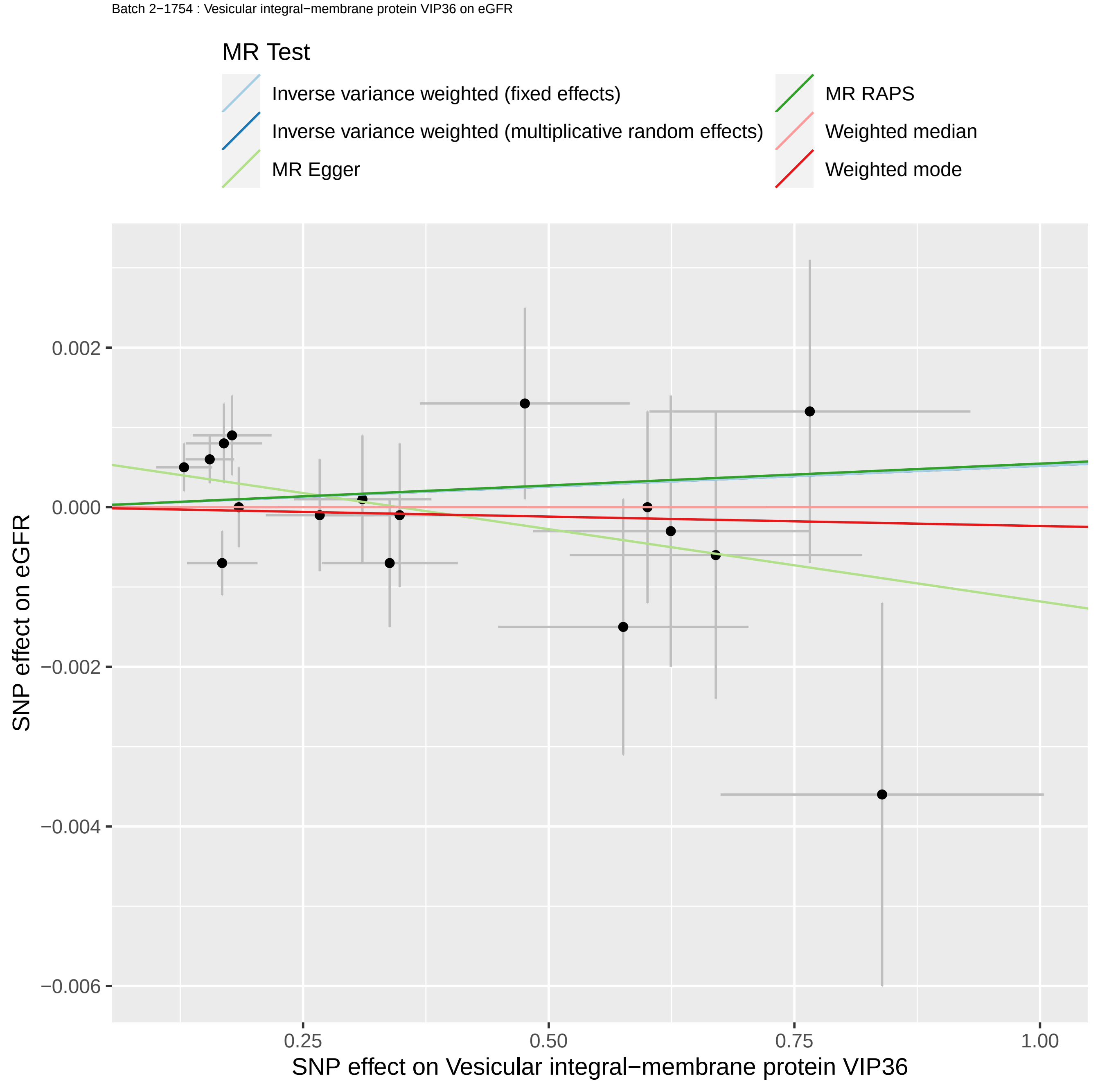

Batch 2−1754 : Vesicular integral−membrane protein VIP36 on eGFR
MR Test
Inverse variance weighted (fixed effects)
Inverse variance weighted (multiplicative random effects)
MR Egger
MR RAPS
Weighted median
Weighted mode
0.002
0.000
−0.002
−0.004
−0.006
SNP effect on eGFR
0.25
0.50
0.75
1.00
SNP effect on Vesicular integral−membrane protein VIP36

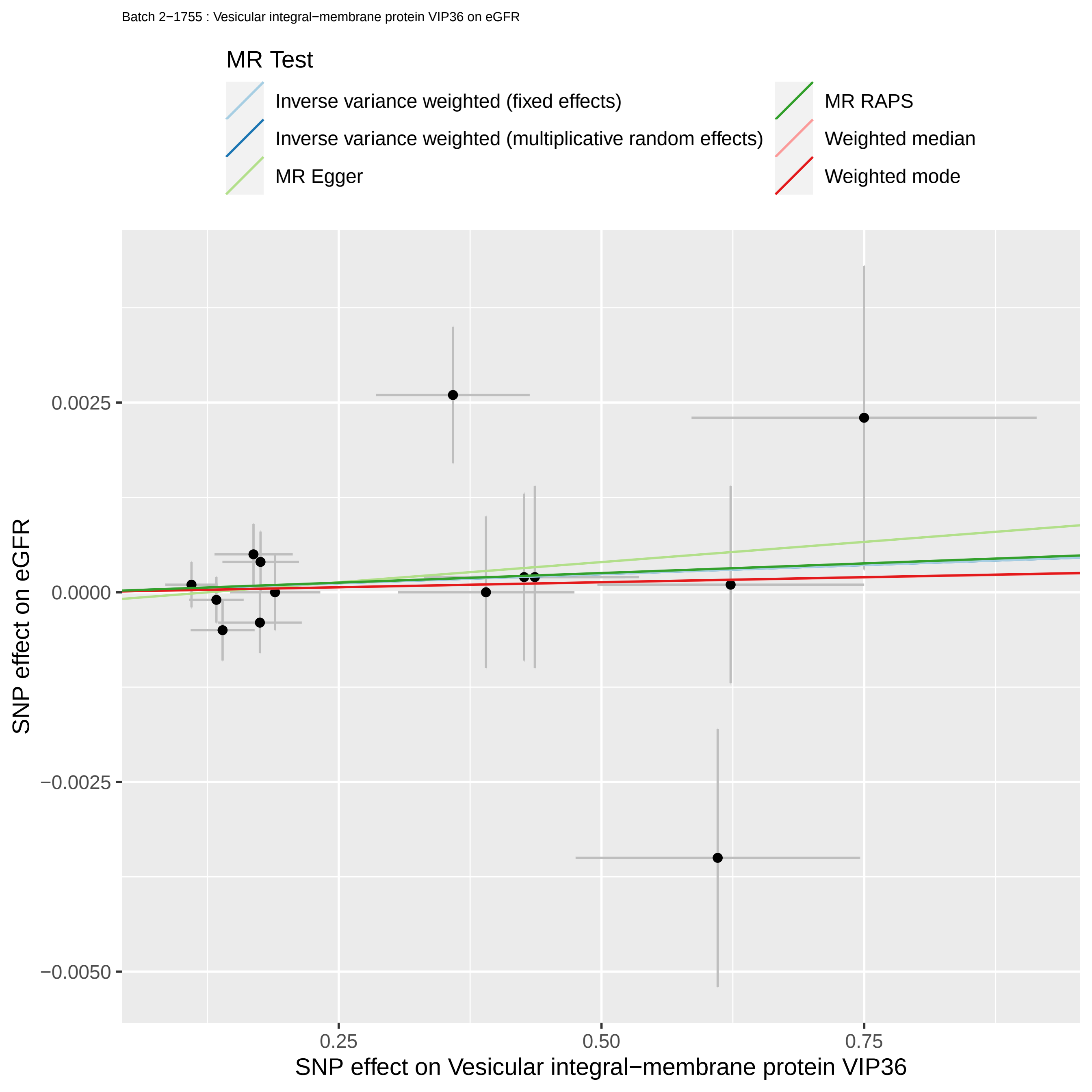

Batch 2−1755 : Vesicular integral−membrane protein VIP36 on eGFR
MR Test
Inverse variance weighted (fixed effects)
Inverse variance weighted (multiplicative random effects)
MR Egger
MR RAPS
Weighted median
Weighted mode
0.0025
0.0000
−0.0025
−0.0050
0.25
0.50
0.75
SNP effect on eGFR
SNP effect on Vesicular integral−membrane protein VIP36

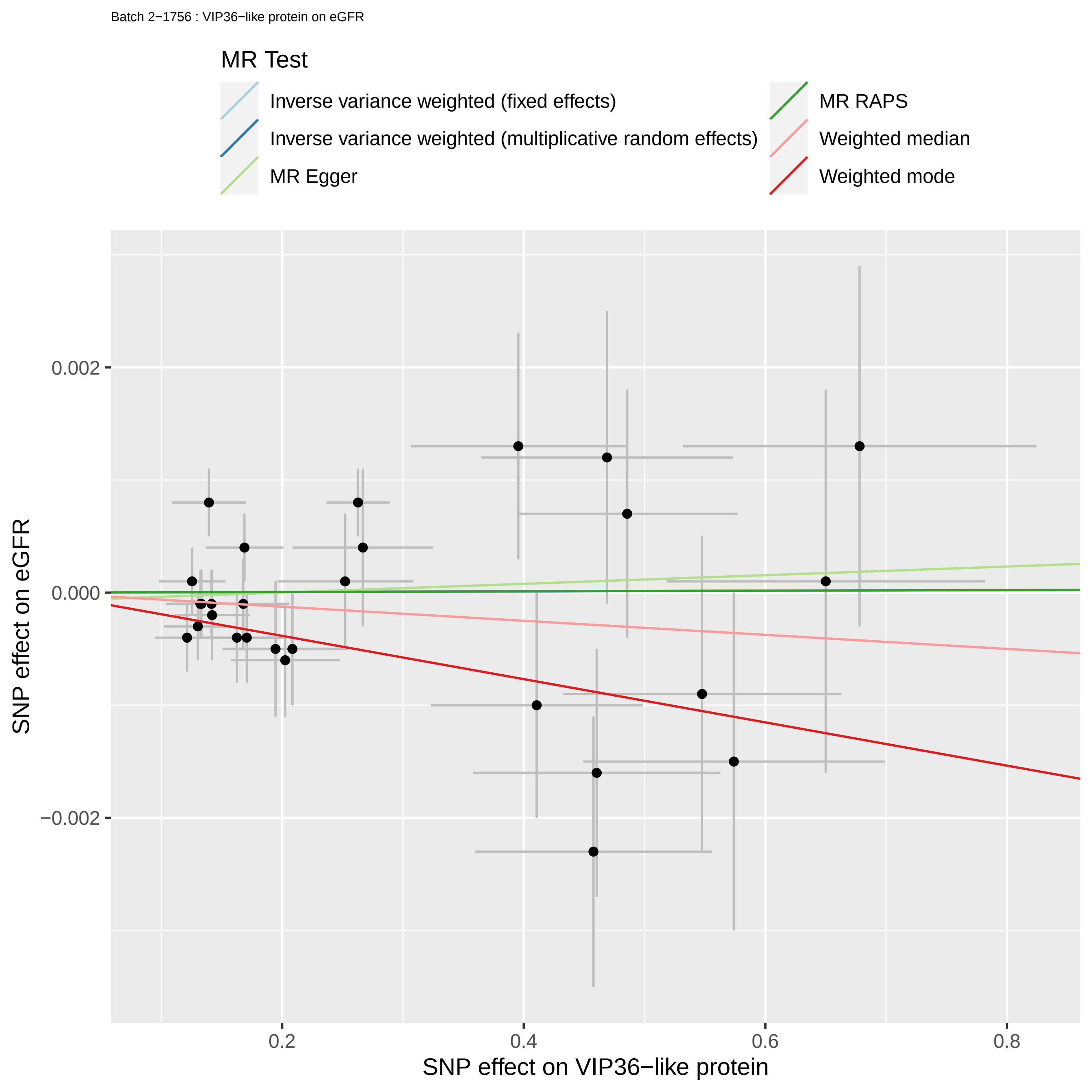

Batch 2−1756 : VIP36−like protein on eGFR
MR Test
Inverse variance weighted (fixed effects)
Inverse variance weighted (multiplicative random effects)
MR Egger
MR RAPS
Weighted median
Weighted mode
0.002
0.000
−0.002
0.2
0.4
0.6
0.8
SNP effect on eGFR
SNP effect on VIP36−like protein

Batch 2−1757 : LIM and cysteine−rich domains protein 1 on eGFR

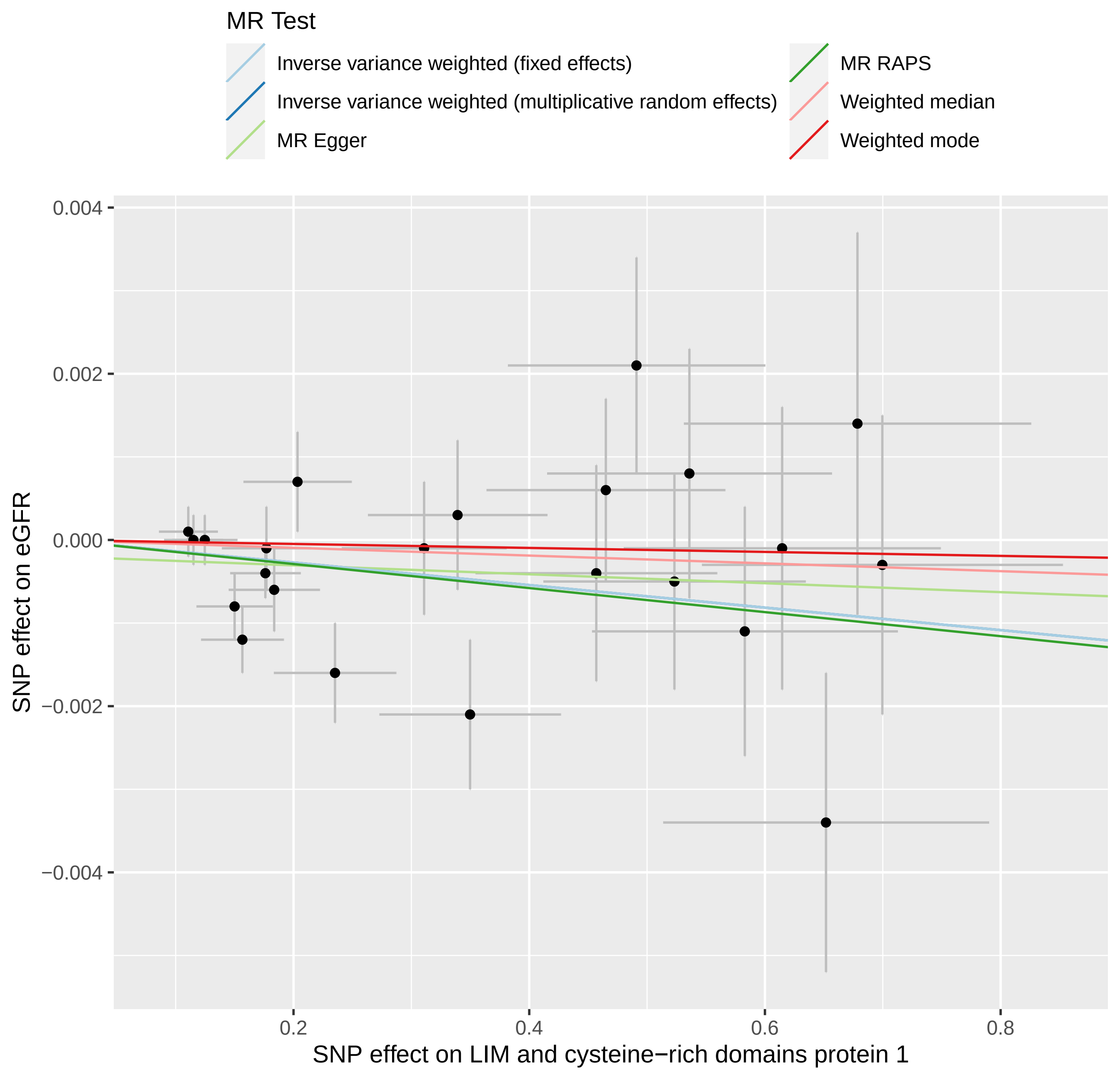

Batch 2−1758 : Lamin−B1 on eGFR

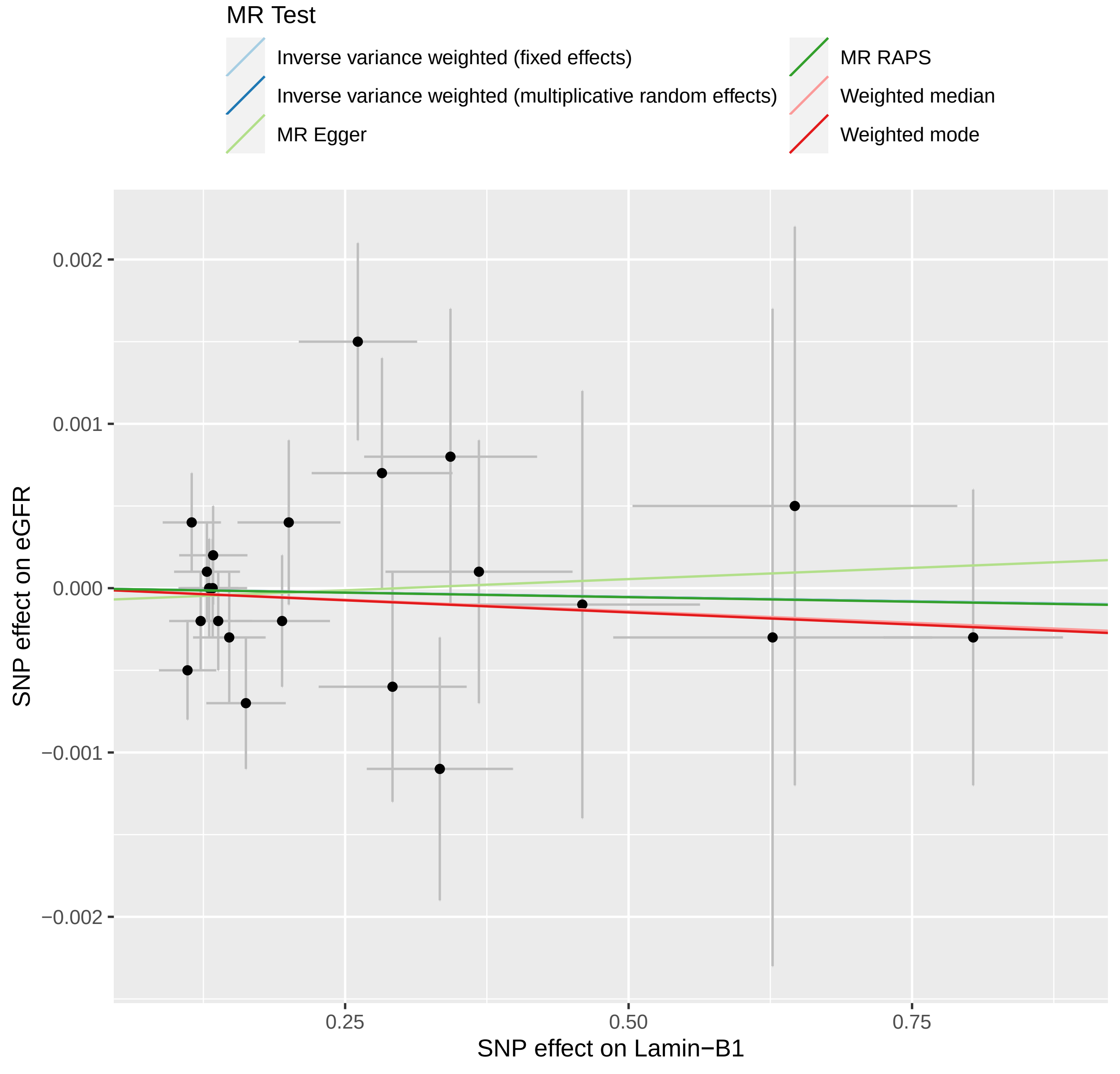

Batch 2−1759 : Leiomodin−1 on eGFR

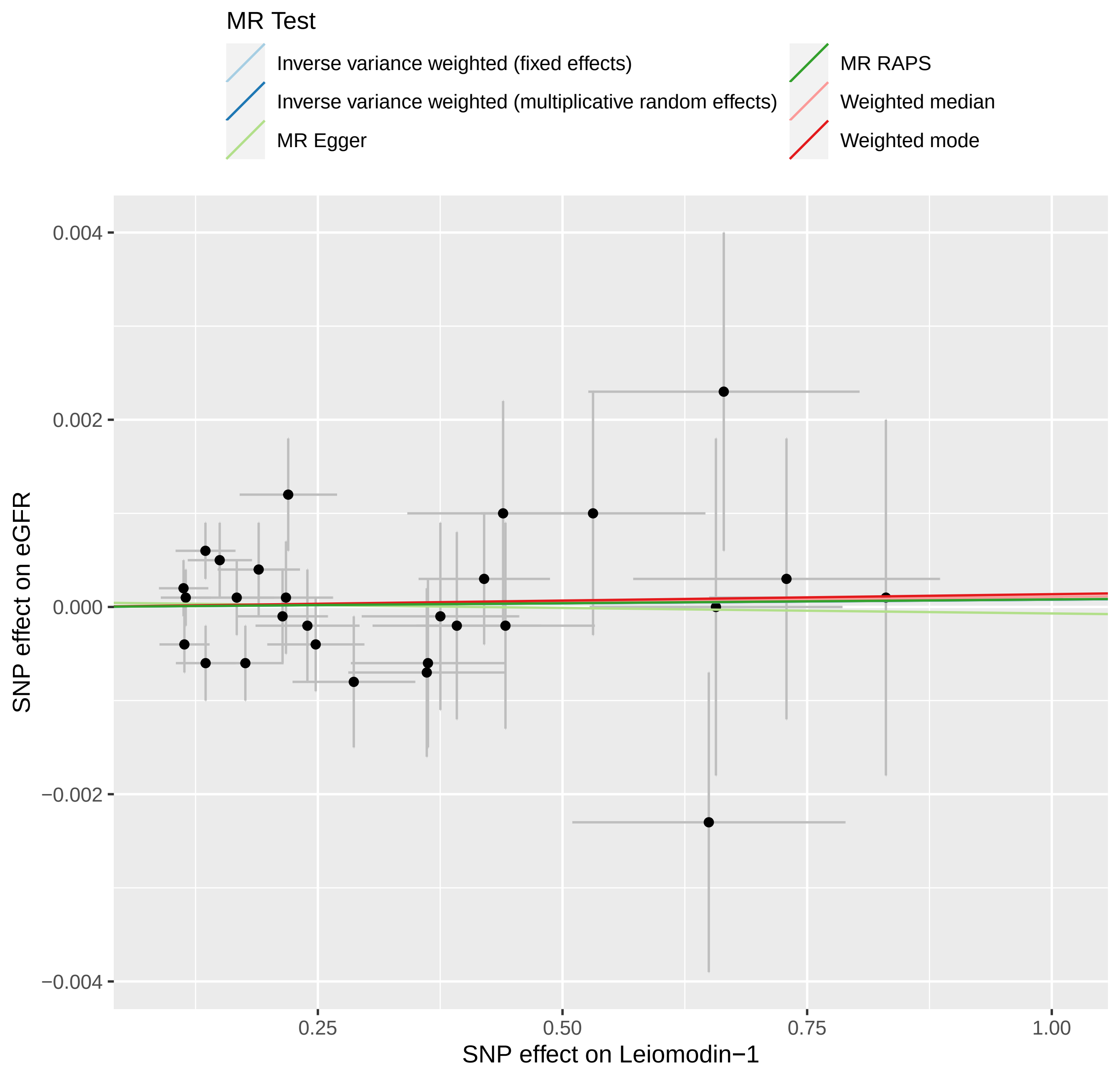

Batch 2−1760 : Ig Kappa chain V−I region HK102− like on eGFR

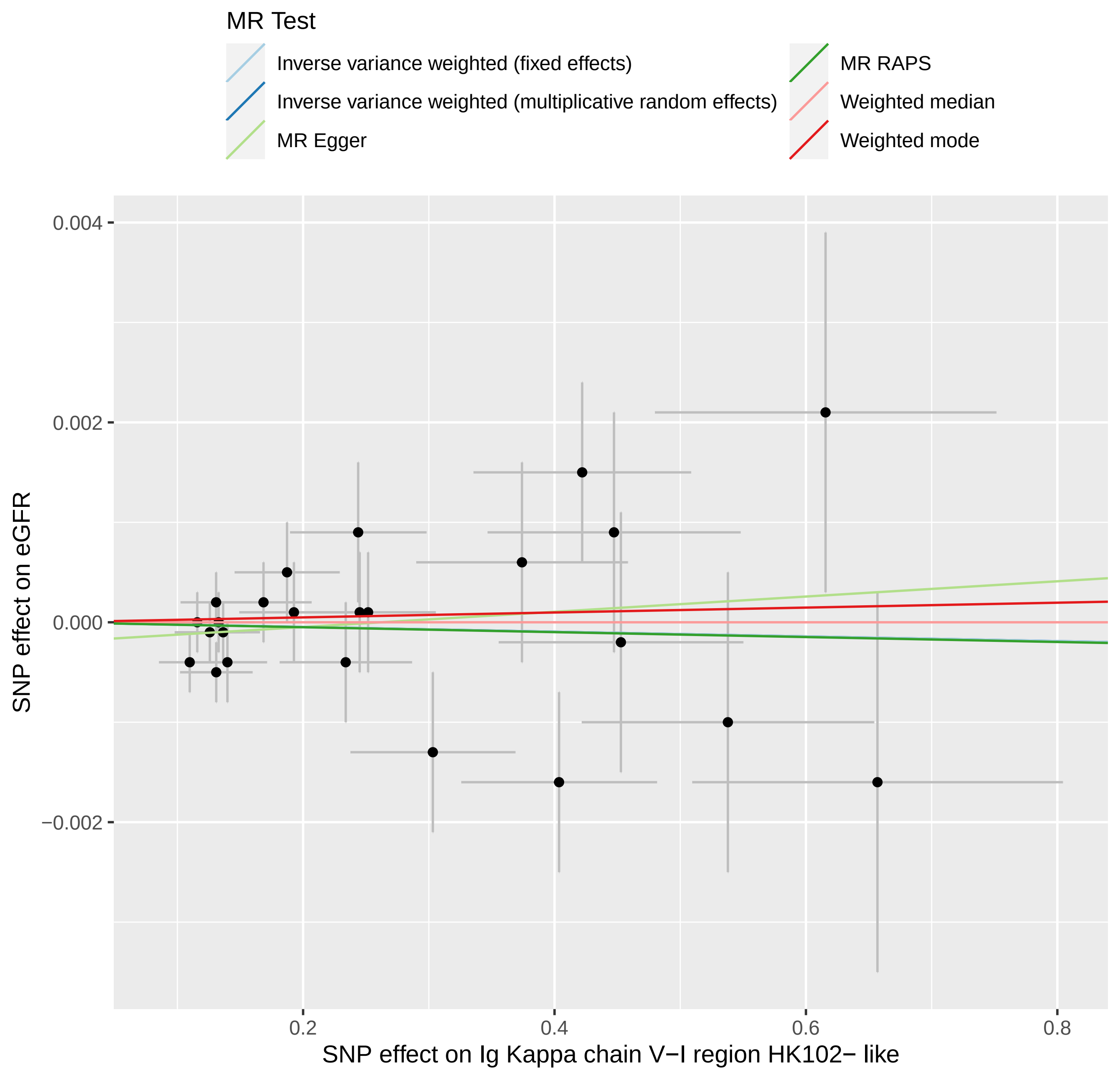

Batch 2−1761 : Lon protease homolog, mitochondrial on eGFR

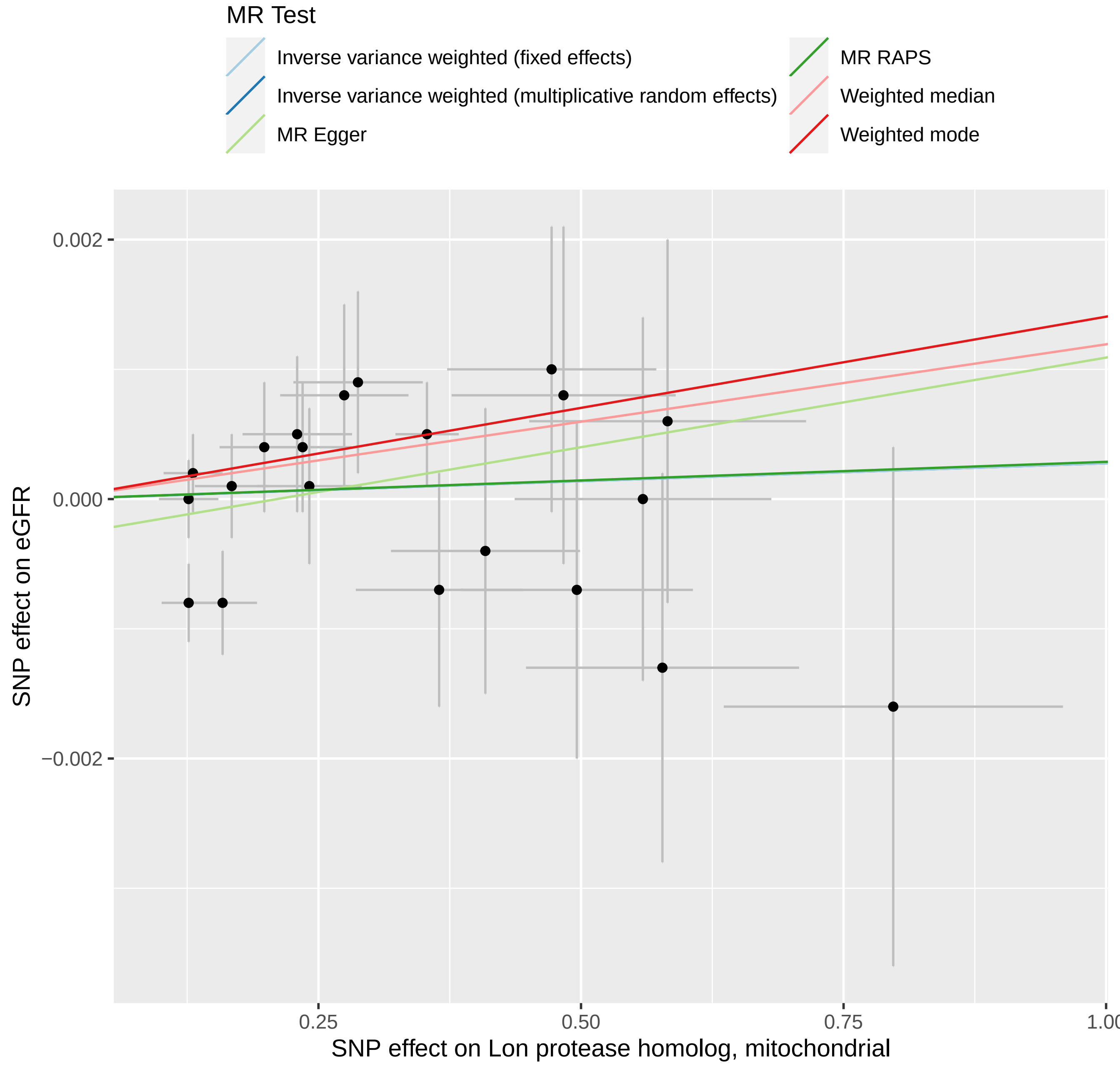

Batch 2−1762 : Lysyl oxidase homolog 2 on eGFR

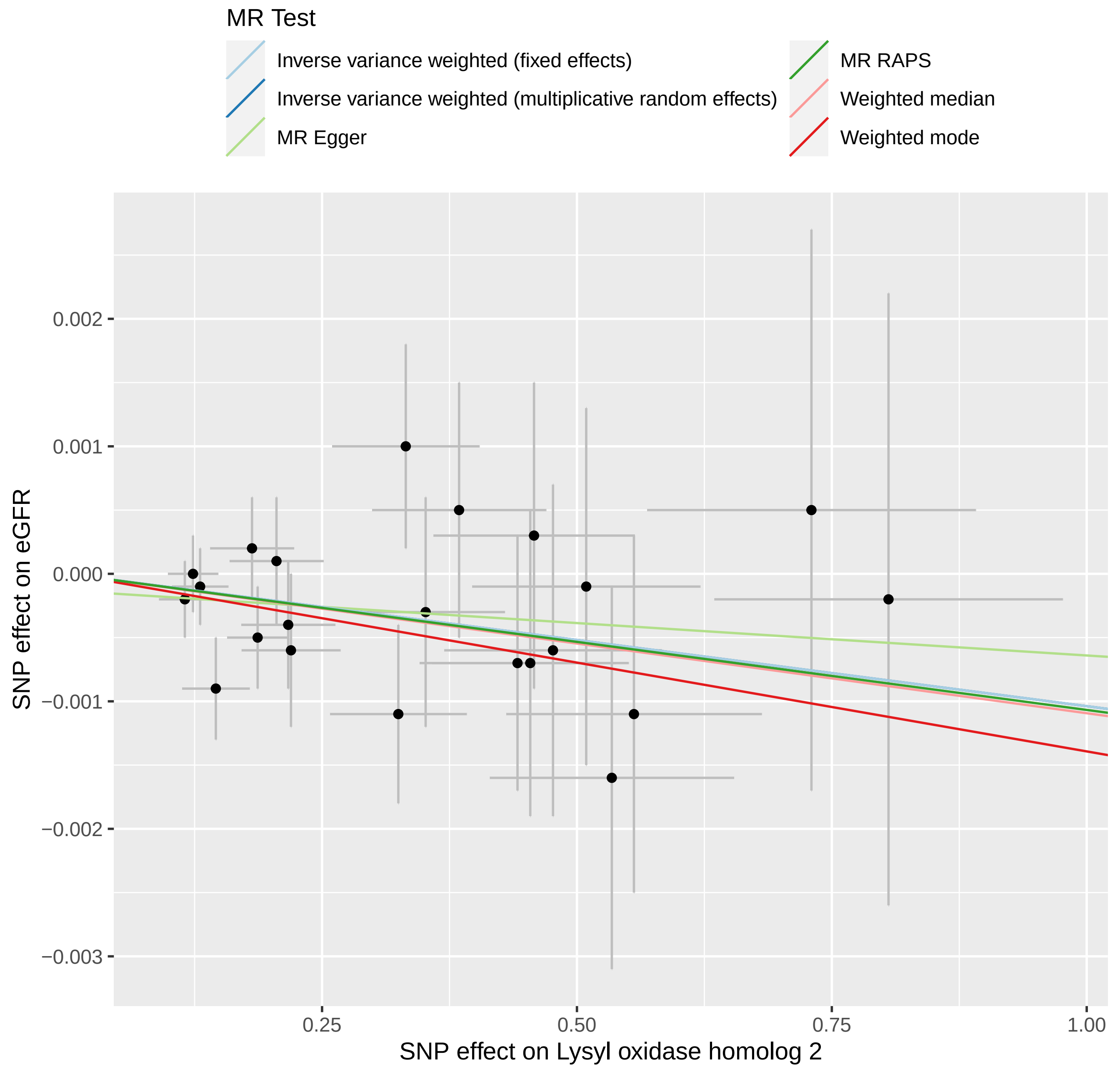

Batch 2−1763 : Putative apolipoprotein(a)−like protein 2 on eGFR

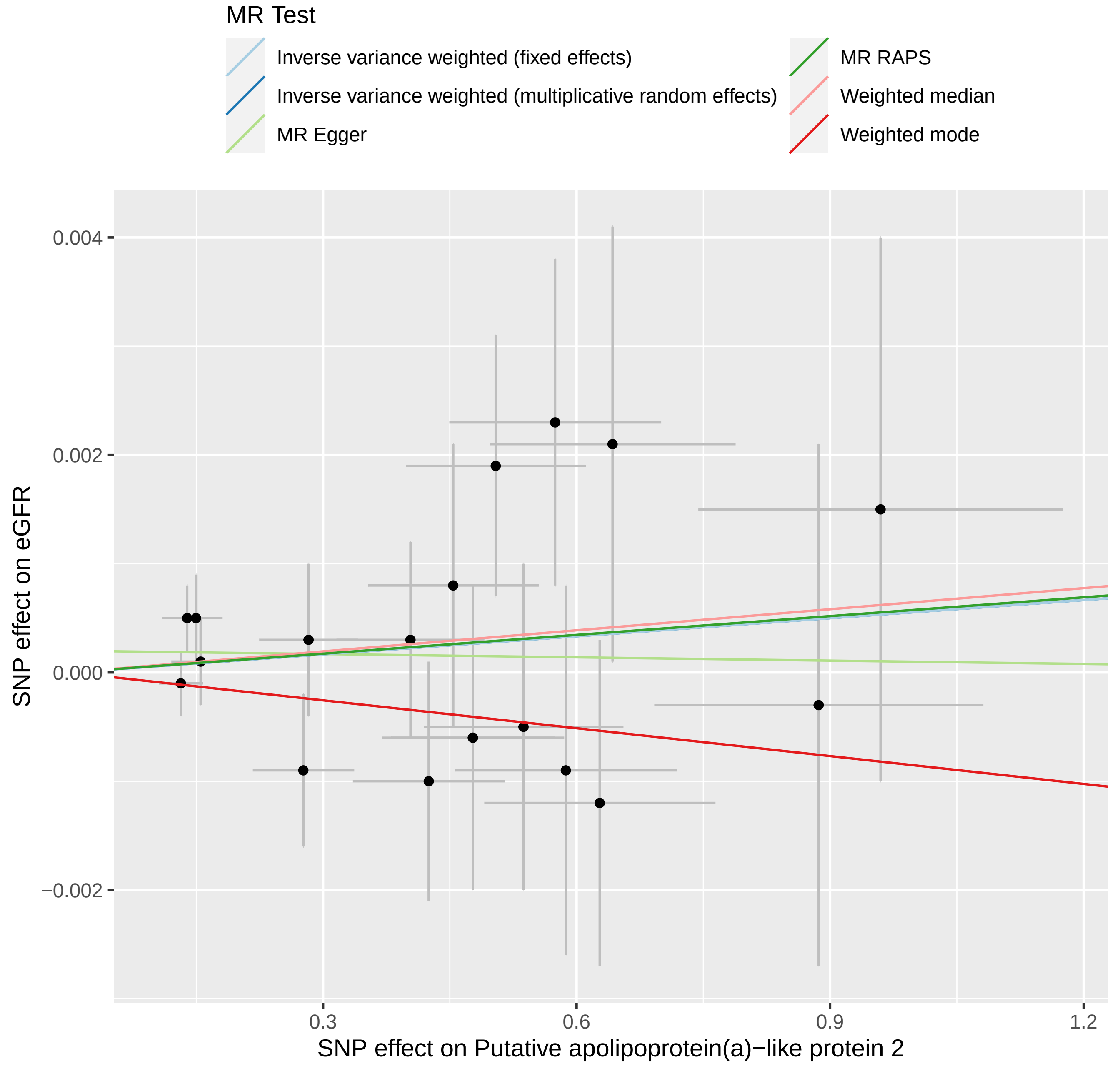

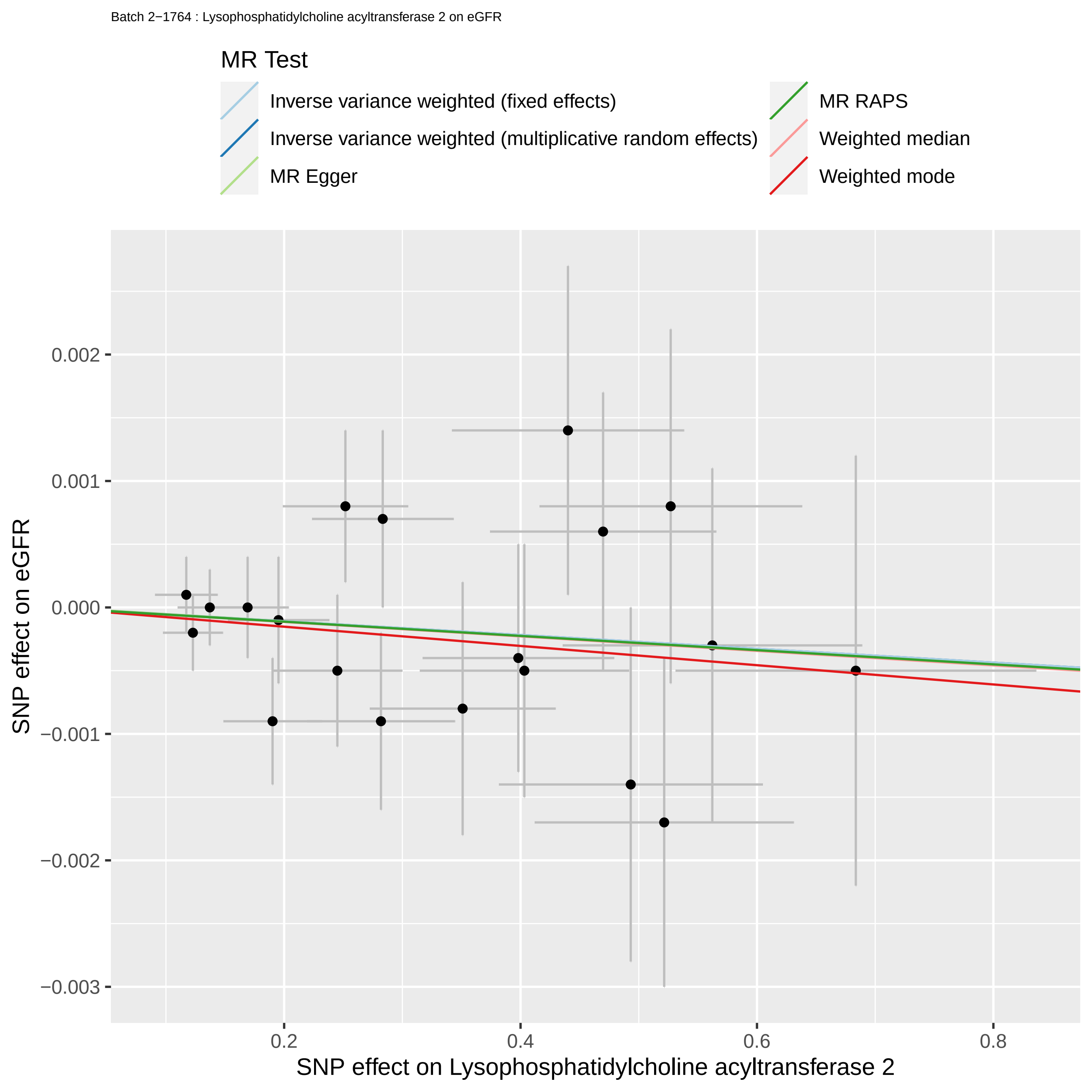

Batch 2−1764 : Lysophosphatidylcholine acyltransferase 2 on eGFR
MR Test
Inverse variance weighted (fixed effects)
Inverse variance weighted (multiplicative random effects)
MR Egger
MR RAPS
Weighted median
Weighted mode
0.002
0.001
0.000
−0.001
−0.002
−0.003
0.2
0.4
0.6
0.8
SNP effect on eGFR
SNP effect on Lysophosphatidylcholine acyltransferase 2

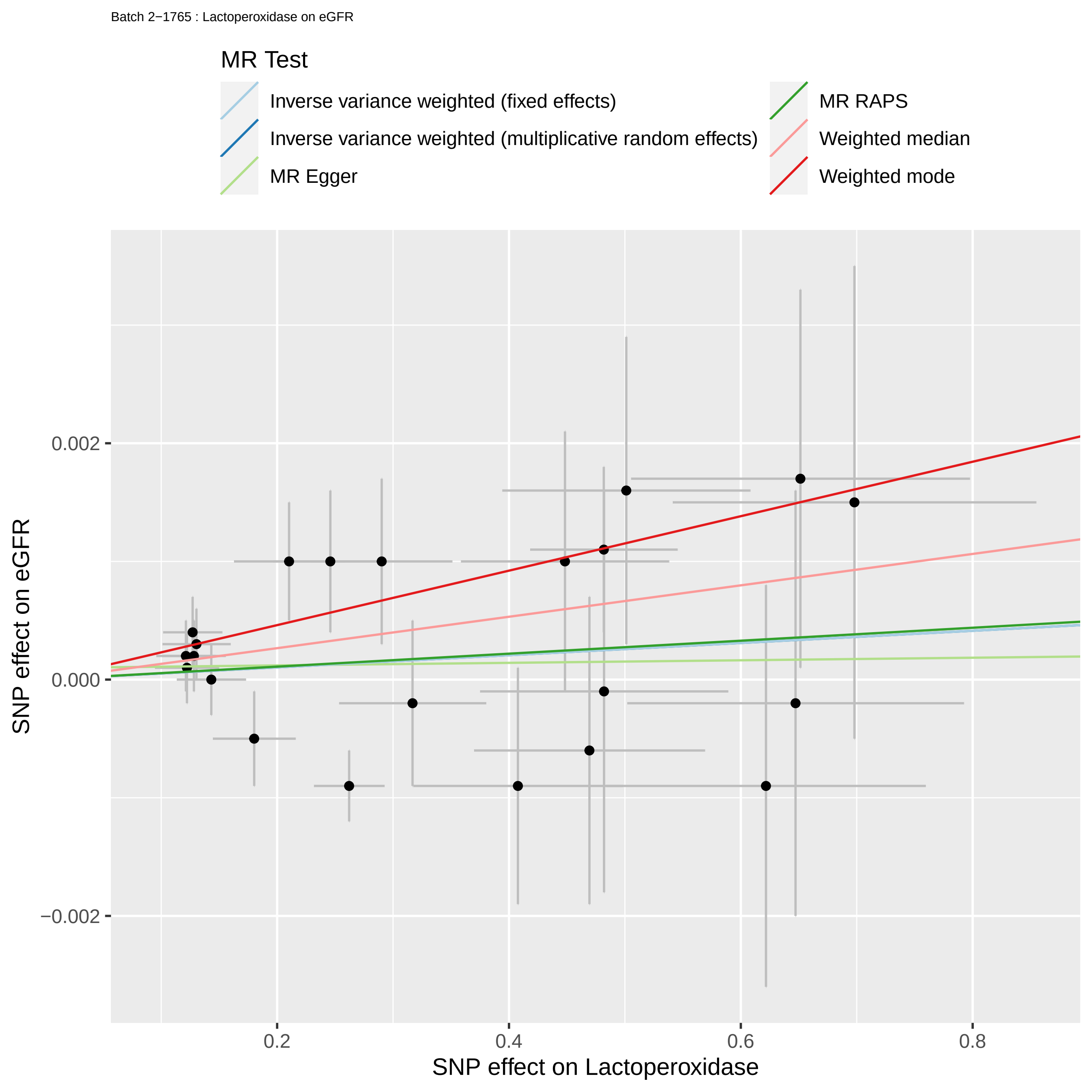
Batch 2−1765 : Lactoperoxidase on eGFR
MR Test
Inverse variance weighted (fixed effects)
Inverse variance weighted (multiplicative random effects)
MR Egger
MR RAPS
Weighted median
Weighted mode
0.002
0.000
−0.002
0.2
0.4
0.6
0.8
SNP effect on eGFR
SNP effect on Lactoperoxidase

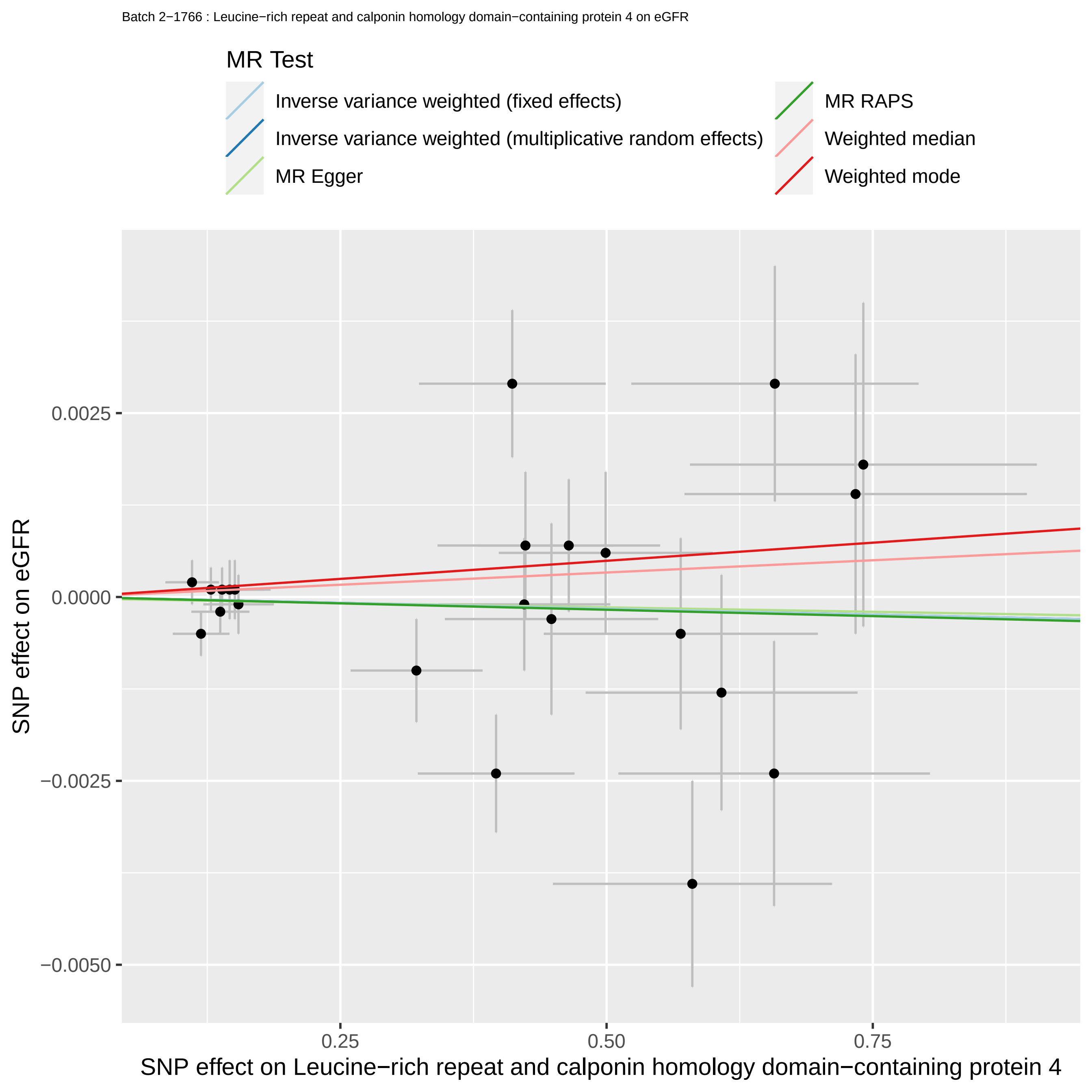

Batch 2−1766 : Leucine−rich repeat and calponin homology domain−containing protein 4 on eGFR
MR Test
Inverse variance weighted (fixed effects)
Inverse variance weighted (multiplicative random effects)
MR Egger
MR RAPS
Weighted median
Weighted mode
SNP effect on eGFR
0.0025
0.0000
−0.0025
−0.0050
0.25
0.50
0.75
SNP effect on Leucine−rich repeat and calponin homology domain−containing protein 4

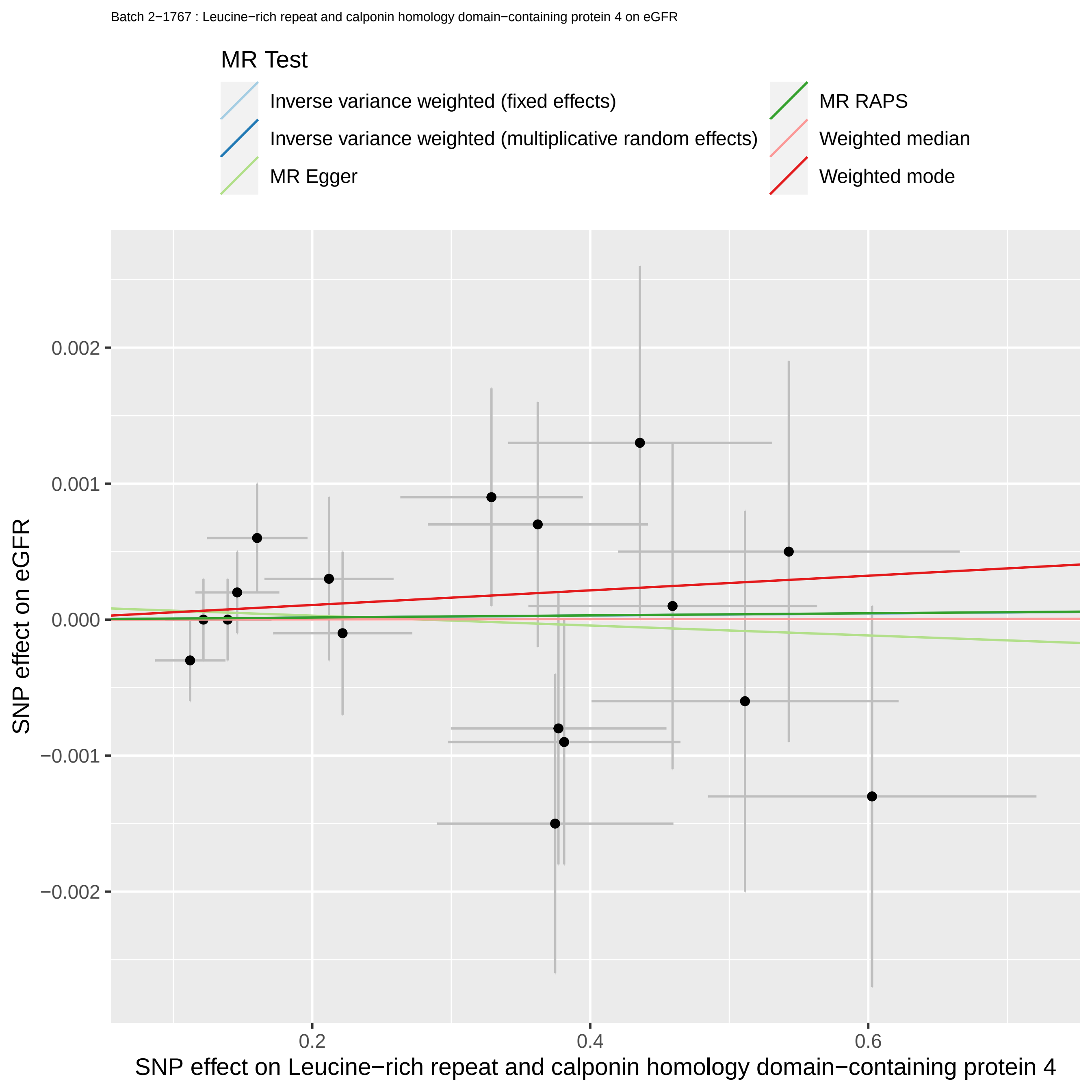
Batch 2−1767 : Leucine−rich repeat and calponin homology domain−containing protein 4 on eGFR
MR Test
Inverse variance weighted (fixed effects)
Inverse variance weighted (multiplicative random effects)
MR Egger
MR RAPS
Weighted median
Weighted mode
0.002
0.001
0.000
−0.001
−0.002
0.2
0.4
0.6
SNP effect on eGFR
SNP effect on Leucine−rich repeat and calponin homology domain−containing protein 4

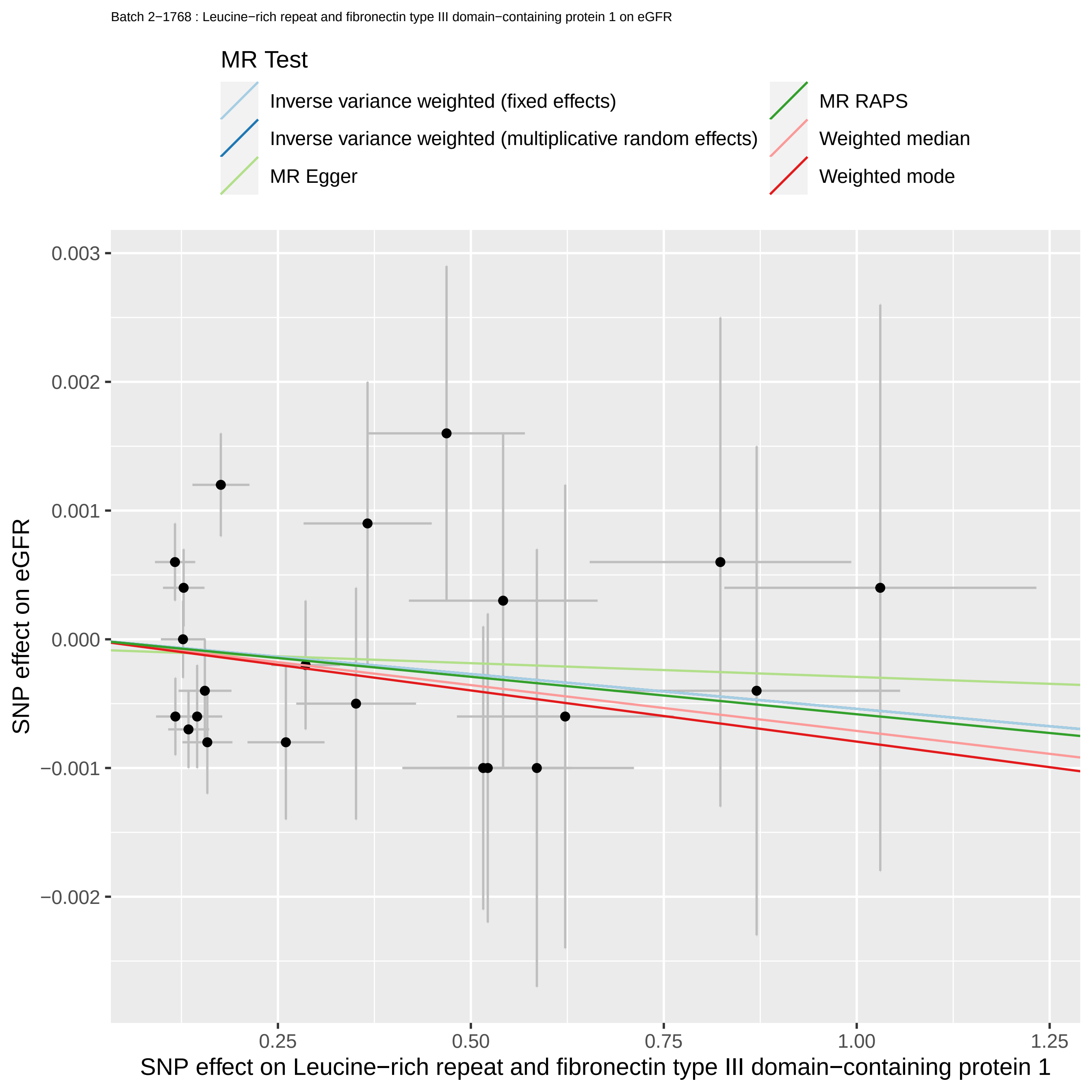

Batch 2−1768 : Leucine−rich repeat and fibronectin type III domain−containing protein 1 on eGFR
MR Test
Inverse variance weighted (fixed effects)
Inverse variance weighted (multiplicative random effects)
MR Egger
MR RAPS
Weighted median
Weighted mode
0.003
0.002
0.001
0.000
−0.001
−0.002
SNP effect on eGFR
0.25
0.50
0.75
1.00
1.25
SNP effect on Leucine−rich repeat and fibronectin type III domain−containing protein 1

Batch 2−1769 : Leucine−rich repeat and fibronectin type−III domain−containing protein 2 on eGFR

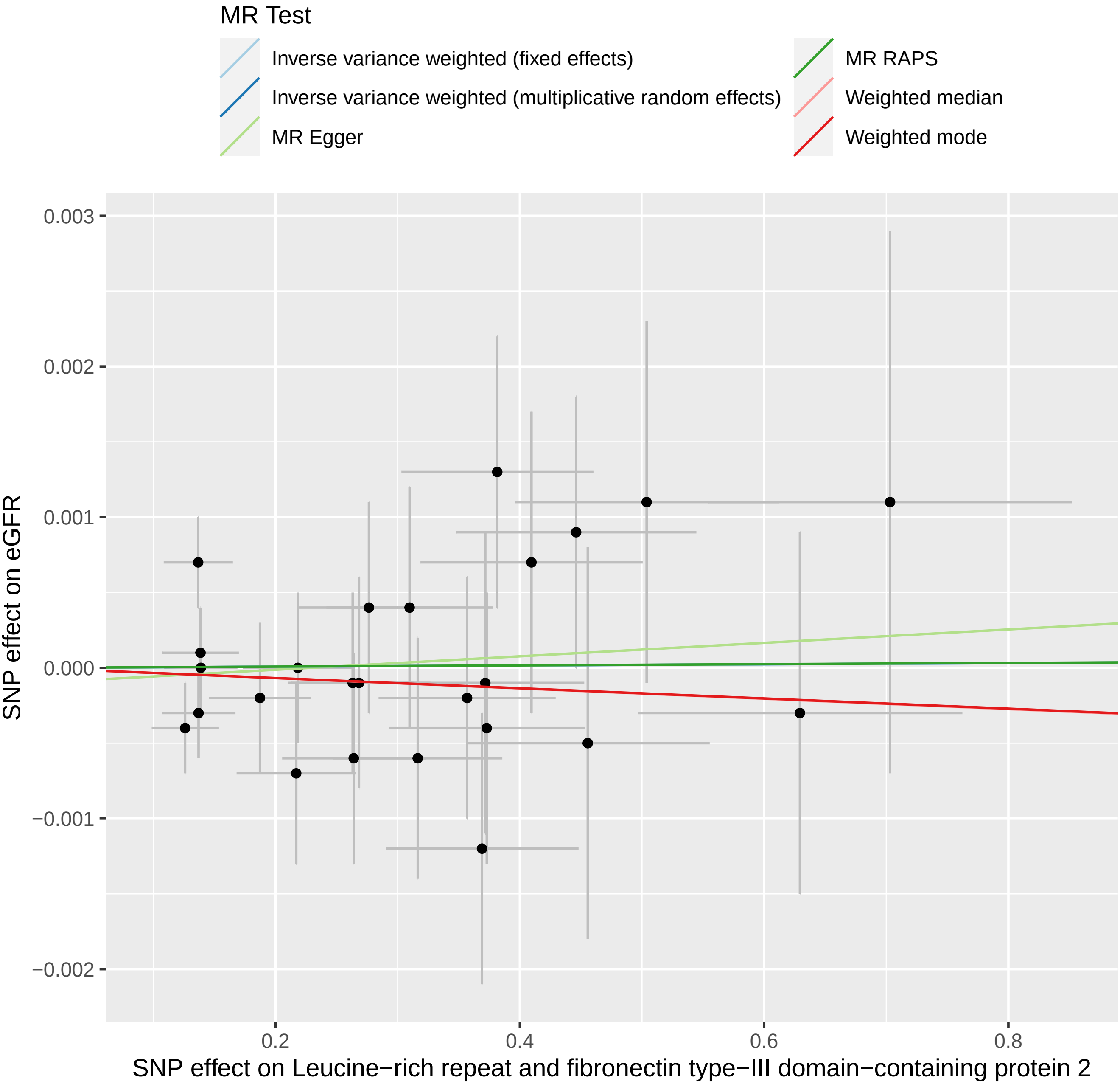

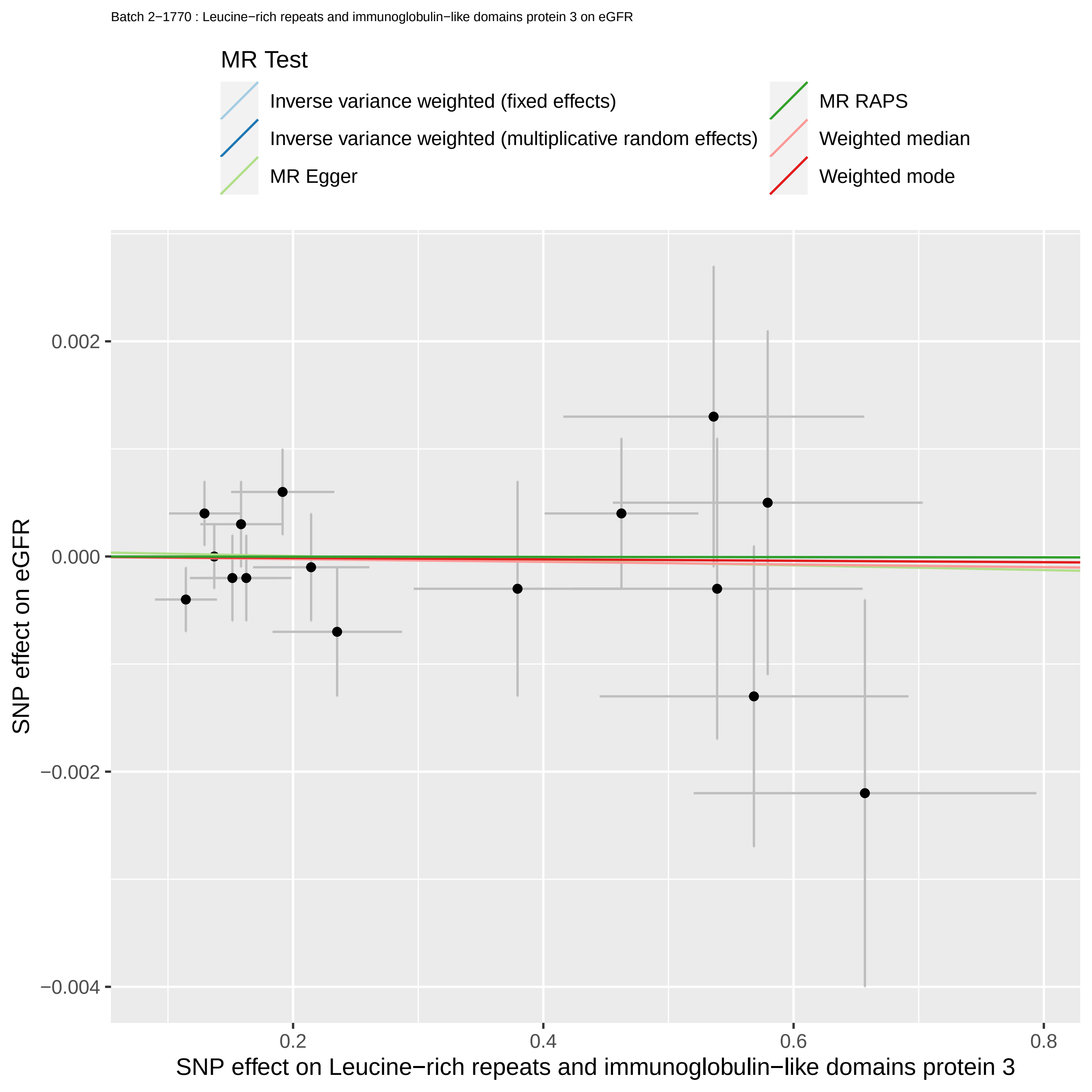

Batch 2−1770 : Leucine−rich repeats and immunoglobulin−like domains protein 3 on eGFR
MR Test
Inverse variance weighted (fixed effects)
Inverse variance weighted (multiplicative random effects)
MR Egger
MR RAPS
Weighted median
Weighted mode
0.002
0.000
−0.002
−0.004
SNP effect on eGFR
0.2
0.4
0.6
0.8
SNP effect on Leucine−rich repeats and immunoglobulin−like domains protein 3

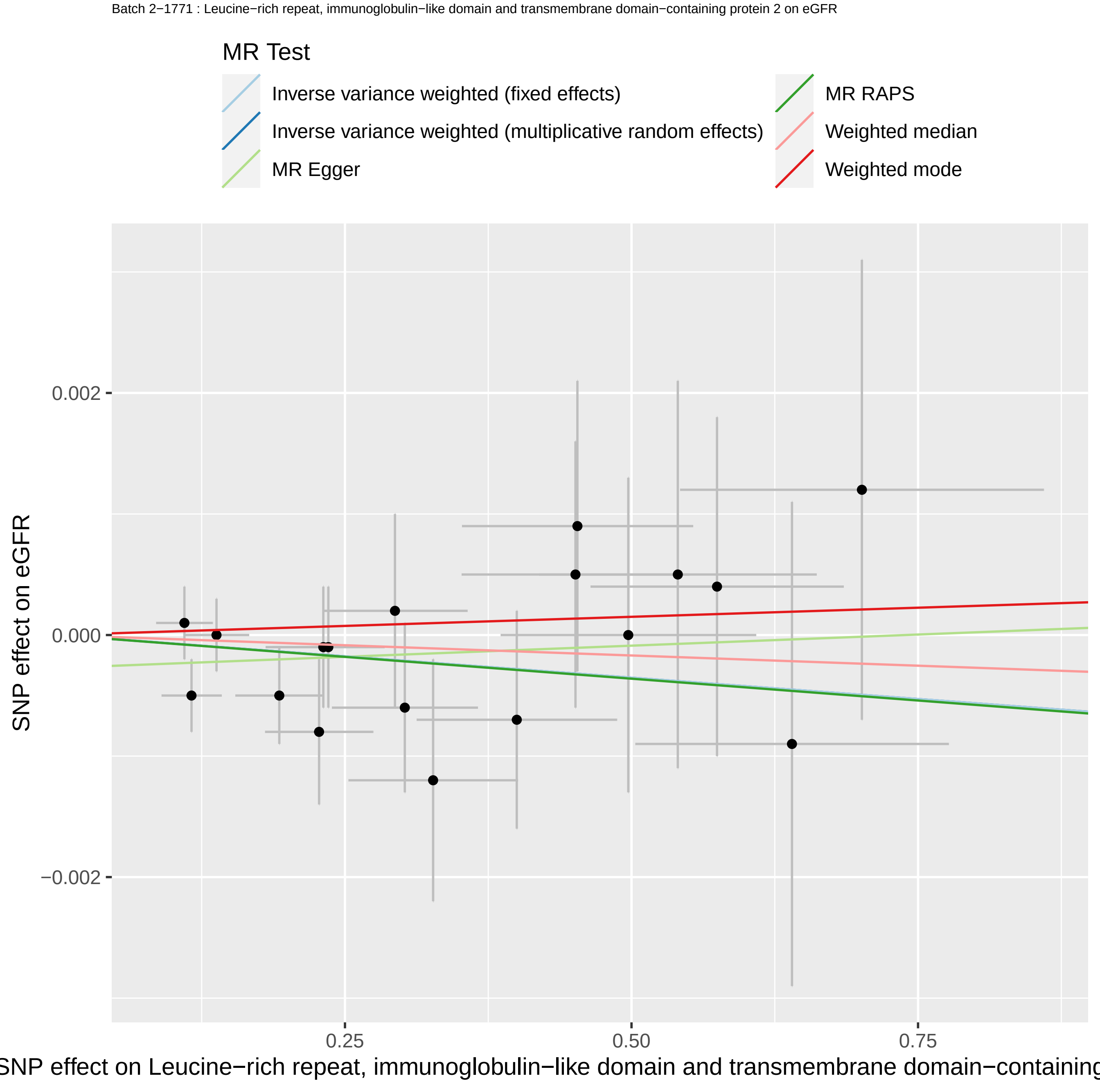

Batch 2−1771 : Leucine−rich repeat, immunoglobulin−like domain and transmembrane domain−containing protein 2 on eGFR
MR Test
Inverse variance weighted (fixed effects)
Inverse variance weighted (multiplicative random effects)
MR Egger
MR RAPS
Weighted median
Weighted mode
0.002
0.000
−0.002
0.25
0.50
0.75
SNP effect on eGFR
SNP effect on Leucine−rich repeat, immunoglobulin−like domain and transmembrane domain−containing

Batch 2−1772 : Leucine−rich repeat, immunoglobulin−like domain and transmembrane domain−containing protein 3 on eGFR

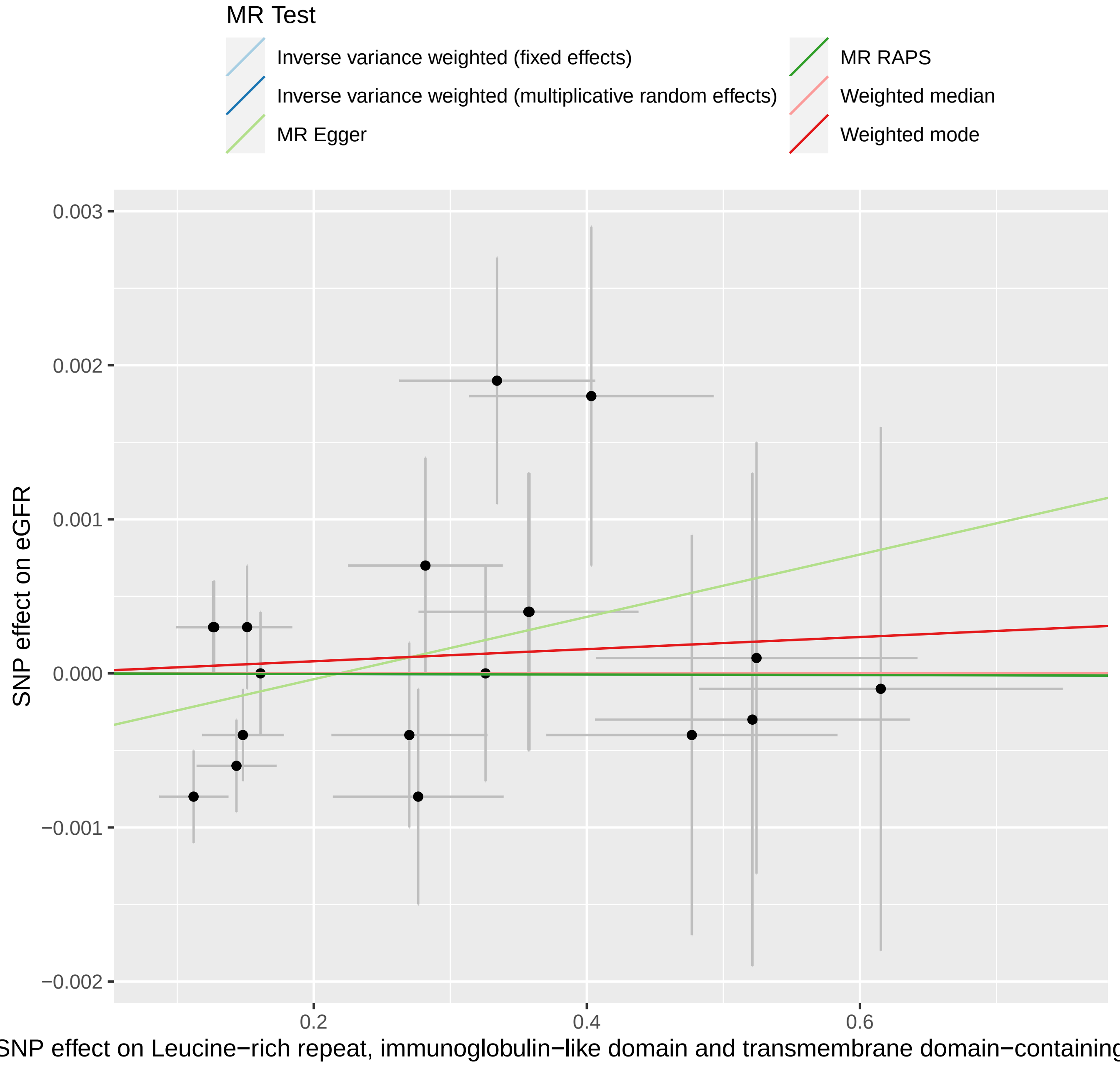

Batch 2−1773 : Leucine−rich repeat, immunoglobulin−like domain and transmembrane domain−containing protein 3 on eGFR

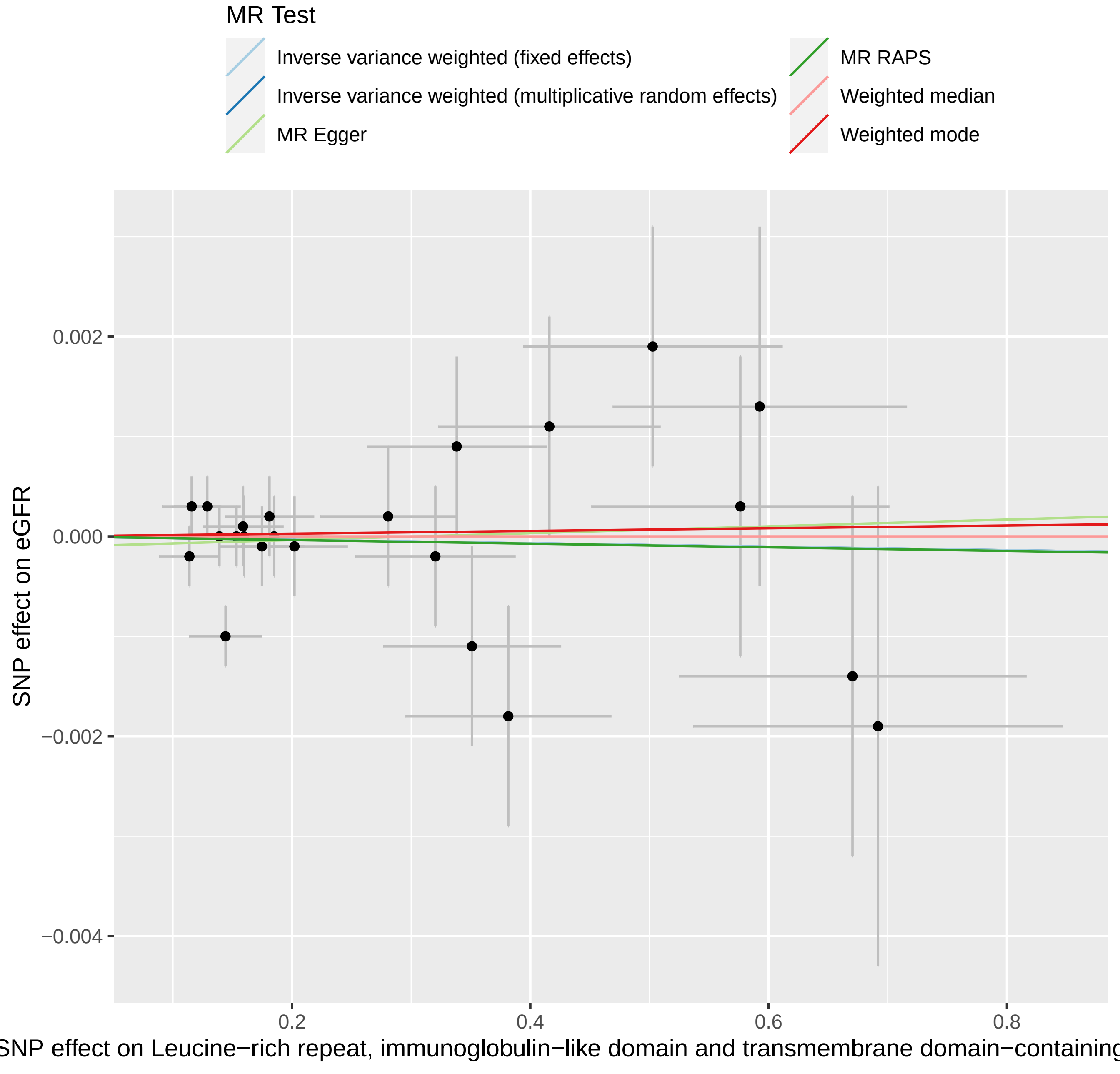

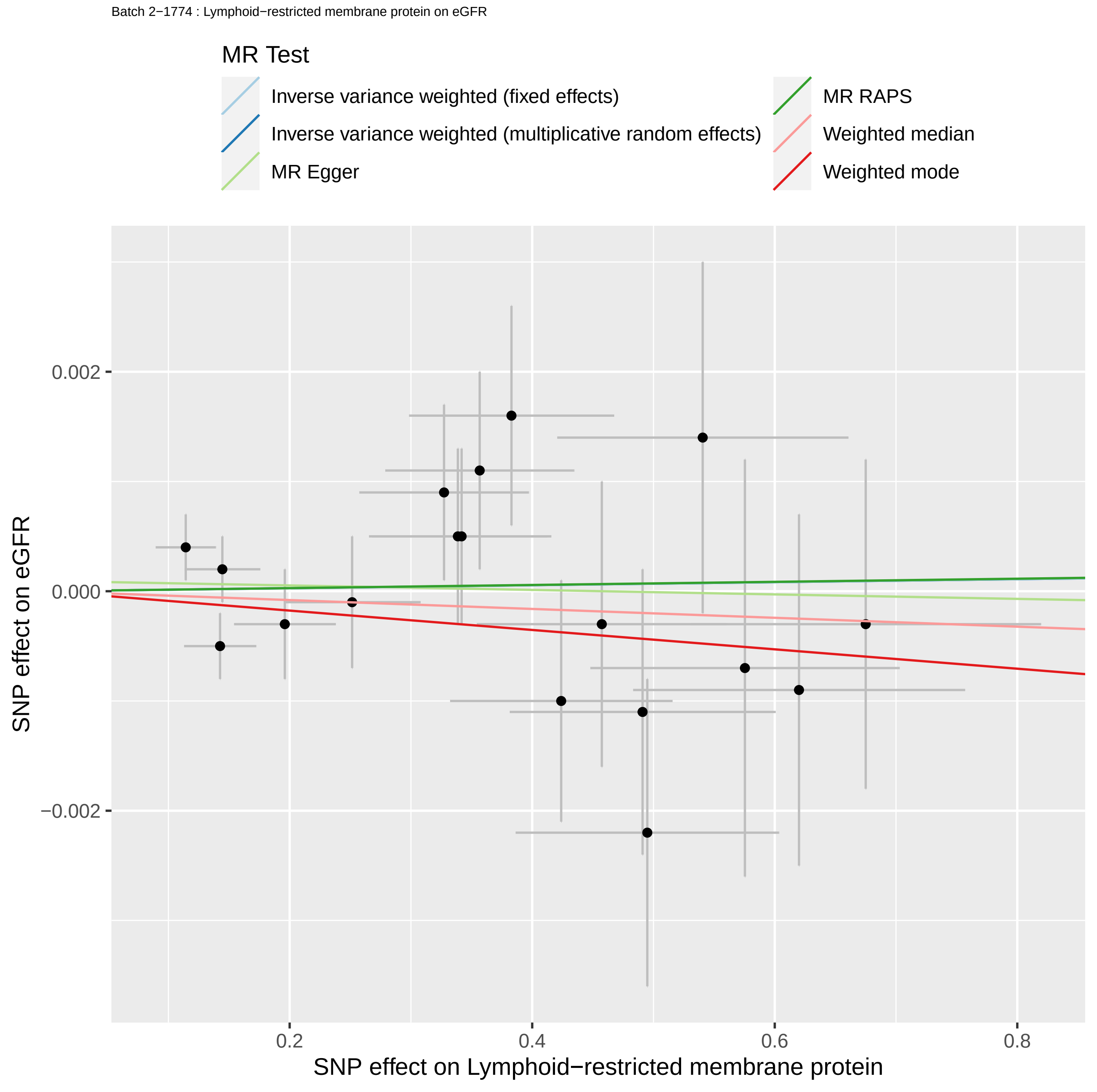

Batch 2−1774 : Lymphoid−restricted membrane protein on eGFR
MR Test
Inverse variance weighted (fixed effects)
Inverse variance weighted (multiplicative random effects)
MR Egger
MR RAPS
Weighted median
Weighted mode
0.002
0.000
−0.002
SNP effect on eGFR
0.2
0.4
0.6
0.8
SNP effect on Lymphoid−restricted membrane protein

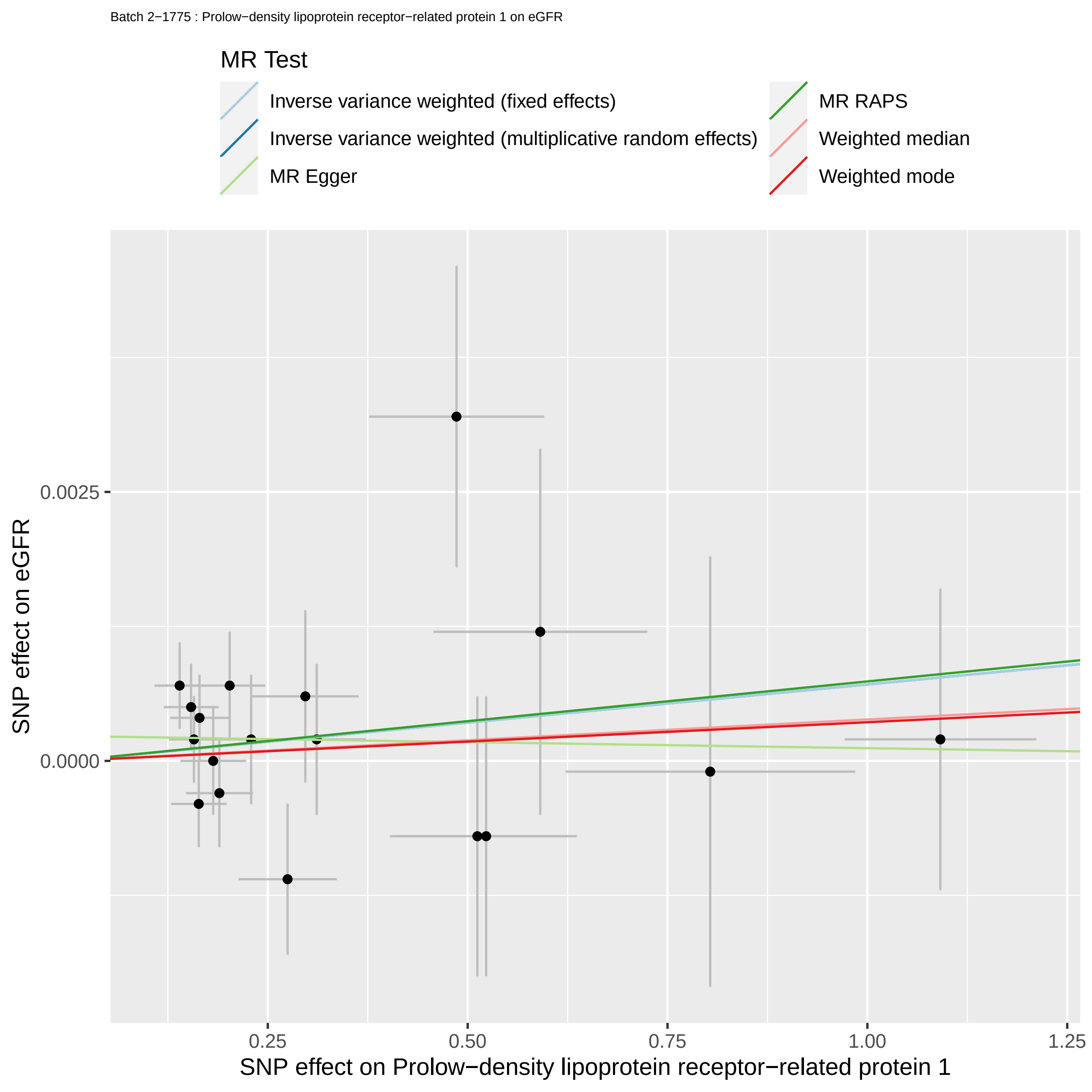

Batch 2−1775 : Prolow−density lipoprotein receptor−related protein 1 on eGFR
MR Test
Inverse variance weighted (fixed effects)
Inverse variance weighted (multiplicative random effects)
MR Egger
MR RAPS
Weighted median
Weighted mode
0.0025
0.0000
0.25
0.50
0.75
1.00
1.25
SNP effect on eGFR
SNP effect on Prolow−density lipoprotein receptor−related protein 1

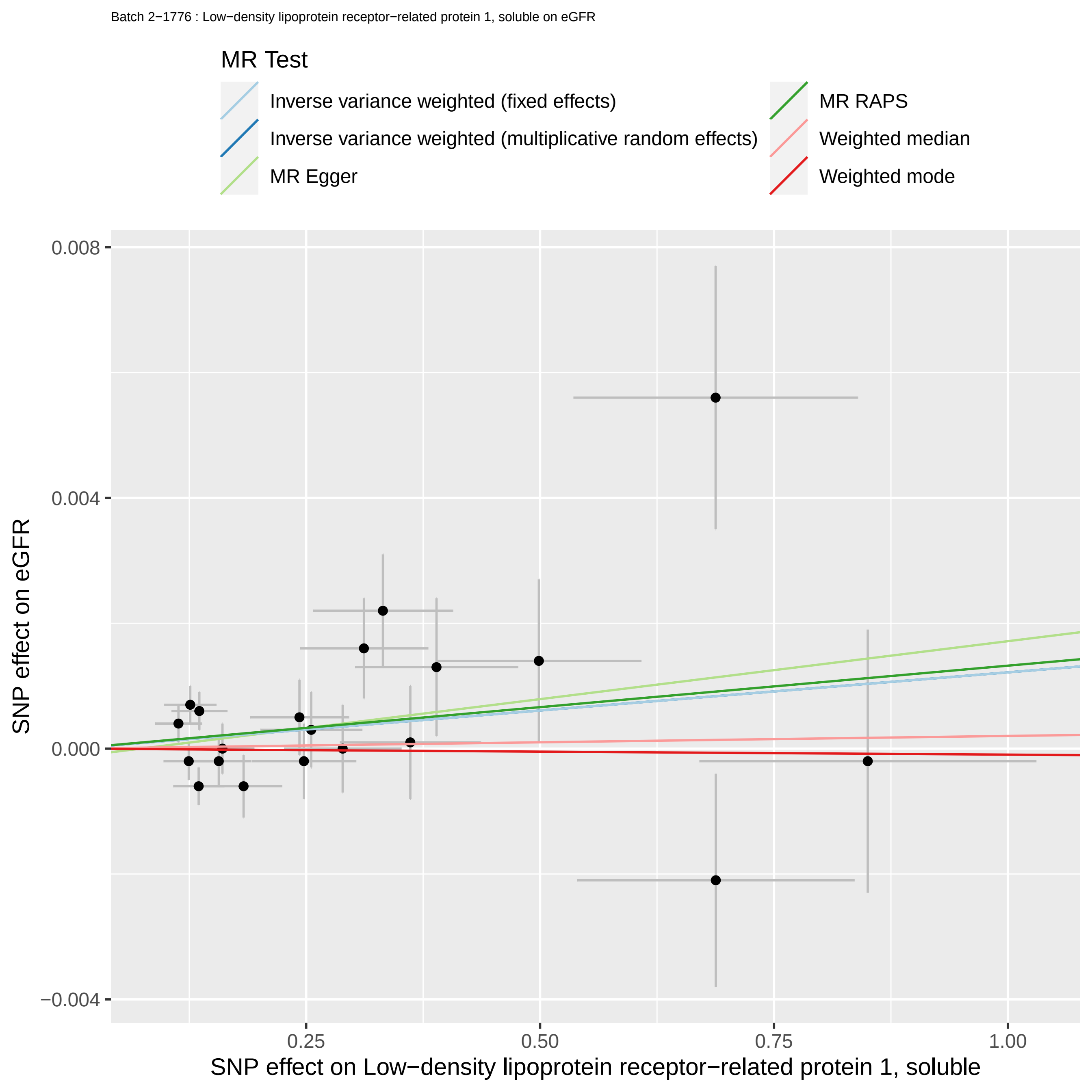

Batch 2−1776 : Low−density lipoprotein receptor−related protein 1, soluble on eGFR
MR Test
Inverse variance weighted (fixed effects)
Inverse variance weighted (multiplicative random effects)
MR Egger
MR RAPS
Weighted median
Weighted mode
0.008
0.004
0.000
−0.004
0.25
0.50
0.75
1.00
SNP effect on eGFR
SNP effect on Low−density lipoprotein receptor−related protein 1, soluble

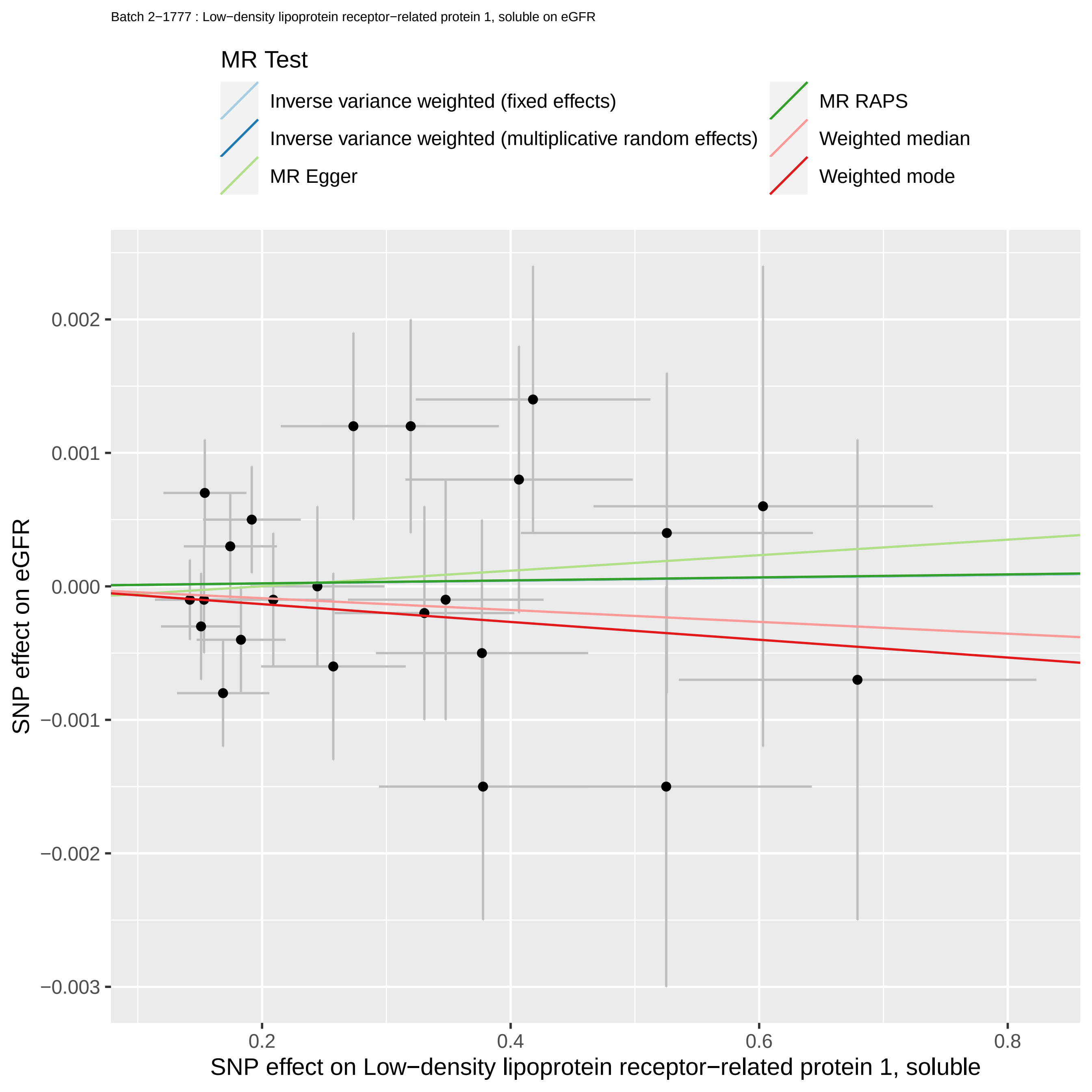

Batch 2−1777 : Low−density lipoprotein receptor−related protein 1, soluble on eGFR
MR Test
Inverse variance weighted (fixed effects)
Inverse variance weighted (multiplicative random effects)
MR Egger
MR RAPS
Weighted median
Weighted mode
SNP effect on eGFR
0.002
0.001
0.000
−0.001
−0.002
−0.003
0.2
0.4
0.6
0.8
SNP effect on Low−density lipoprotein receptor−related protein 1, soluble

Batch 2−1778 : Low−density lipoprotein receptor−related protein 1B on eGFR

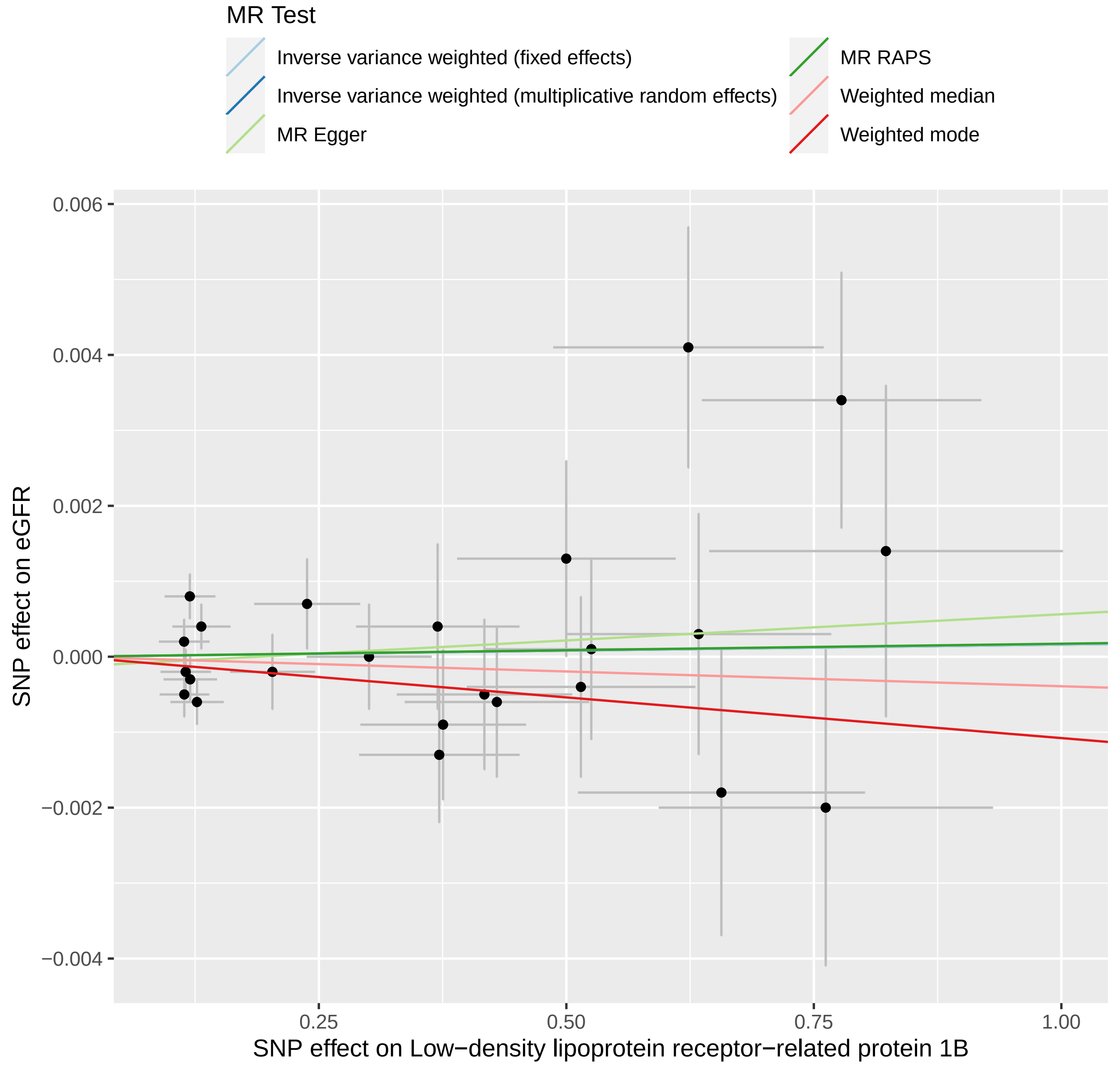

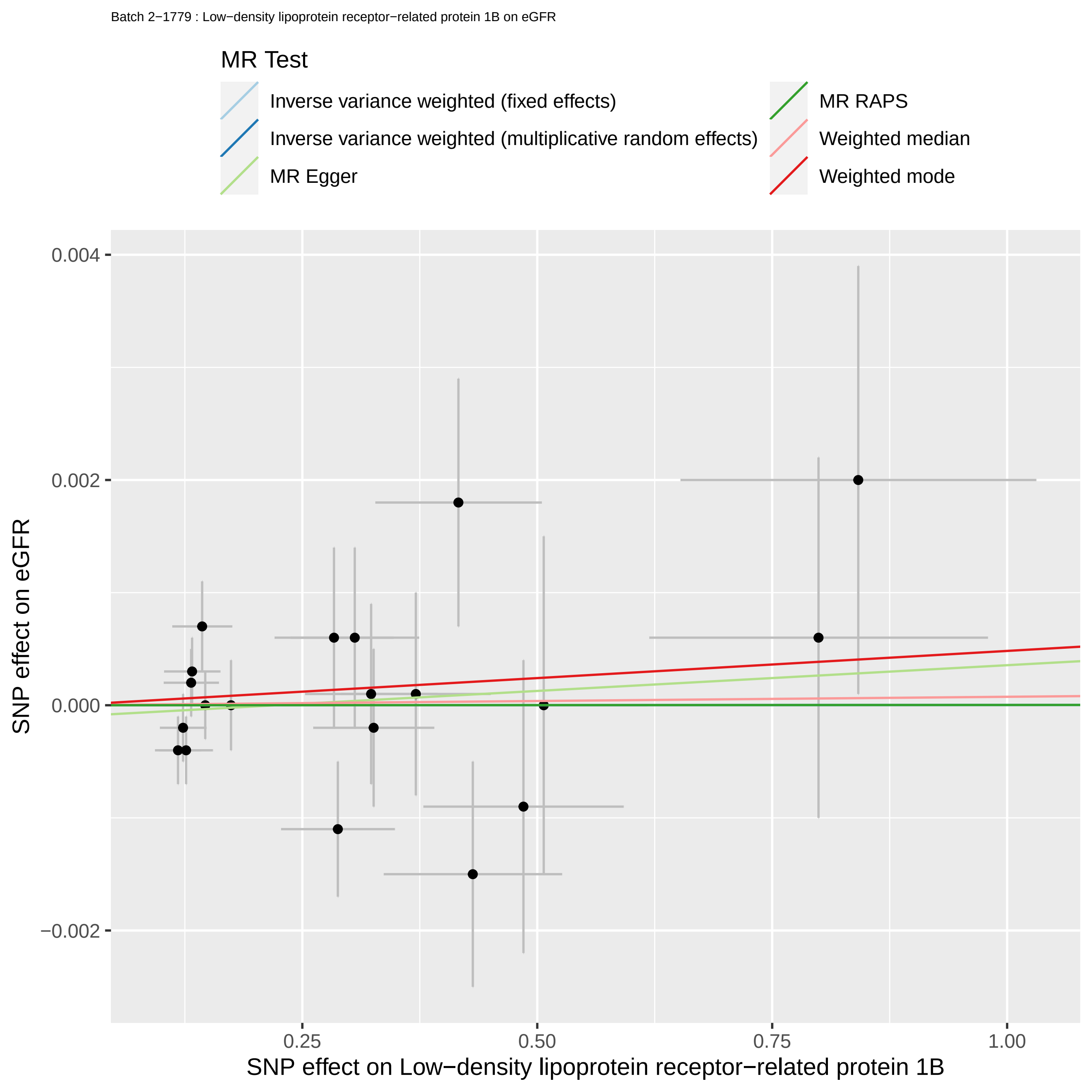

Batch 2−1779 : Low−density lipoprotein receptor−related protein 1B on eGFR
MR Test
Inverse variance weighted (fixed effects)
Inverse variance weighted (multiplicative random effects)
MR Egger
MR RAPS
Weighted median
Weighted mode
0.004
0.002
0.000
−0.002
0.25
0.50
0.75
1.00
SNP effect on eGFR
SNP effect on Low−density lipoprotein receptor−related protein 1B

Batch 2−1780 : Low−density lipoprotein receptor−related protein 8 on eGFR

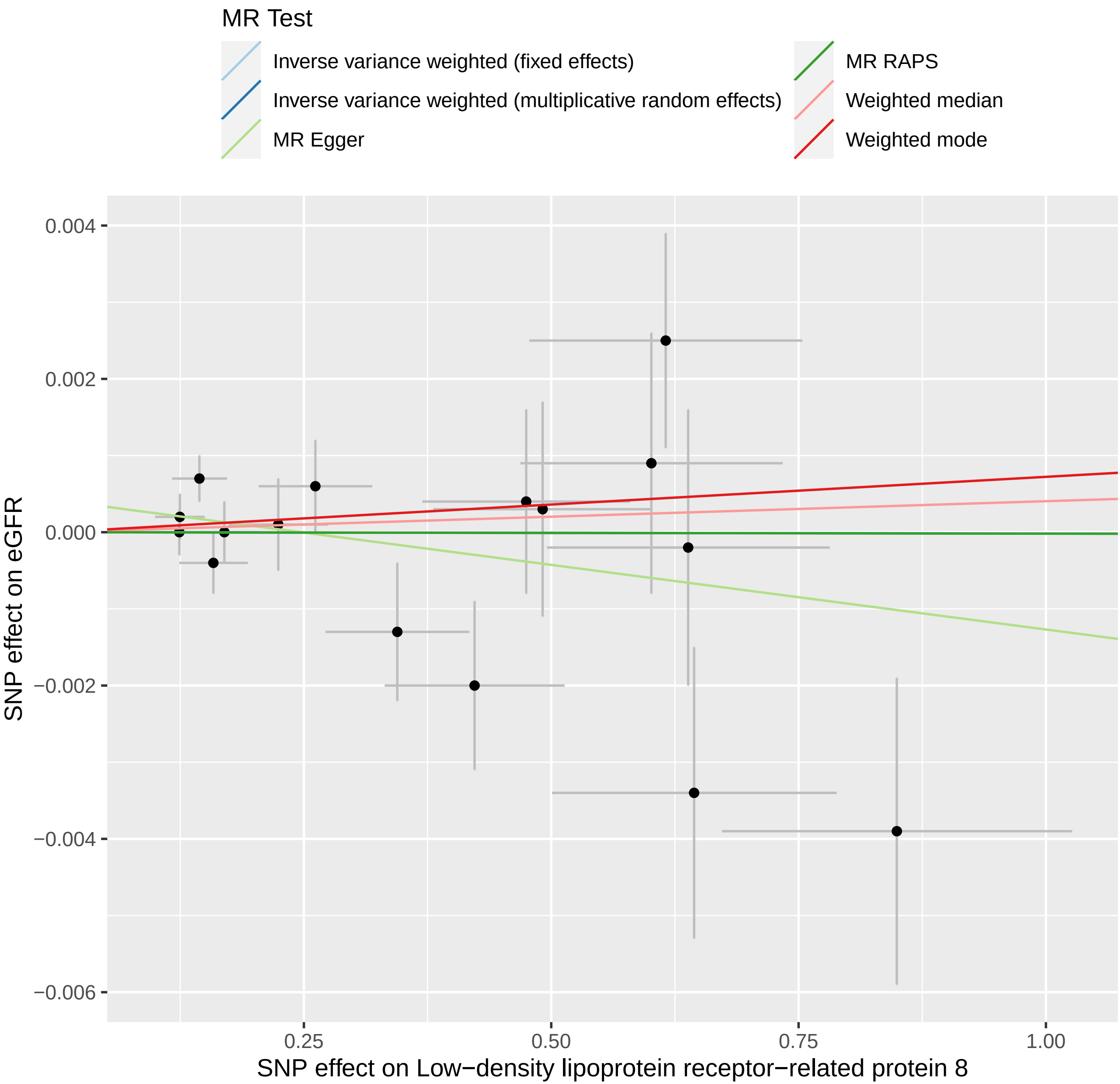

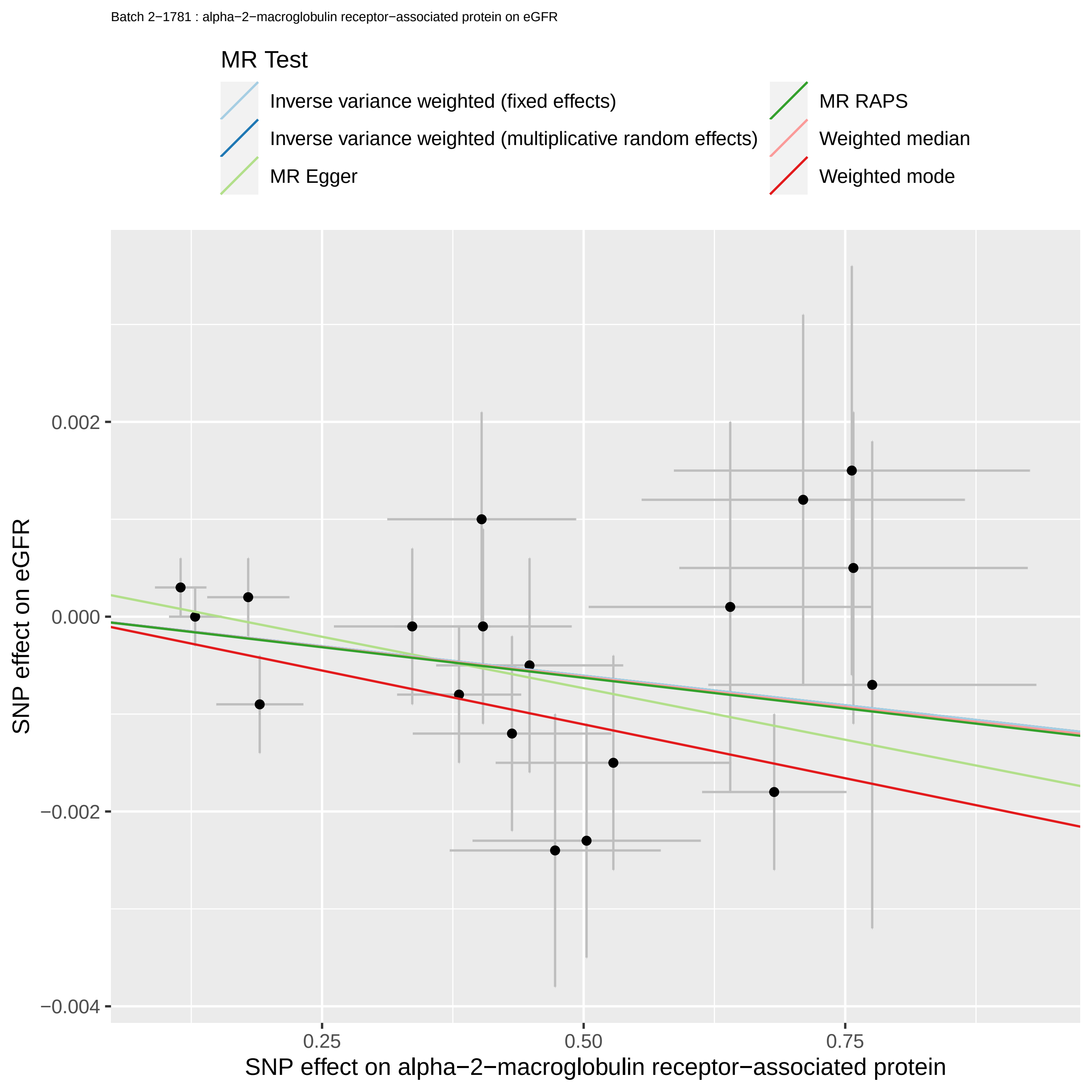
Batch 2−1781 : alpha−2−macroglobulin receptor−associated protein on eGFR
MR Test
Inverse variance weighted (fixed effects)
Inverse variance weighted (multiplicative random effects)
MR Egger
MR RAPS
Weighted median
Weighted mode
0.002
0.000
−0.002
−0.004
0.25
0.50
0.75
SNP effect on eGFR
SNP effect on alpha−2−macroglobulin receptor−associated protein

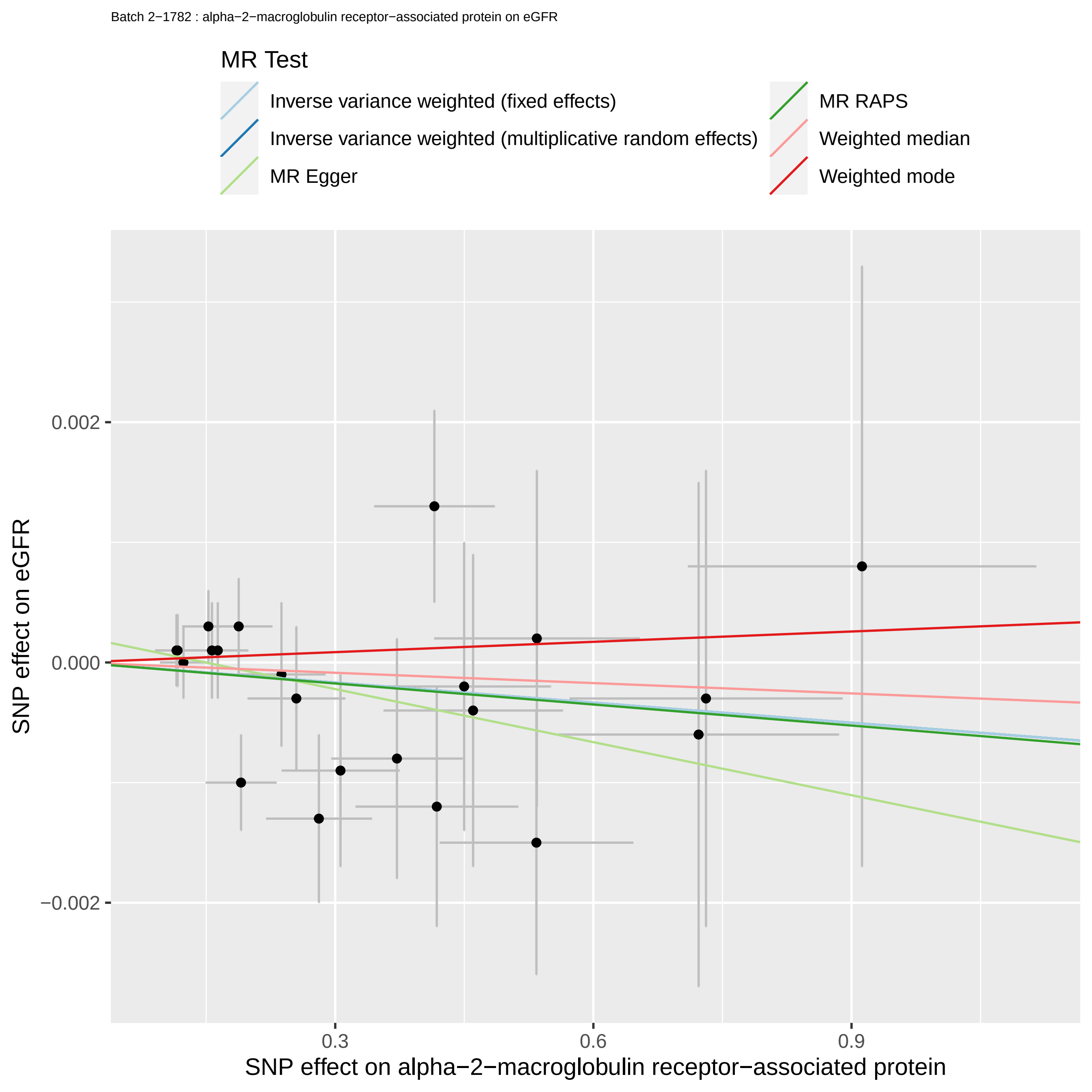

Batch 2−1782 : alpha−2−macroglobulin receptor−associated protein on eGFR
MR Test
Inverse variance weighted (fixed effects)
Inverse variance weighted (multiplicative random effects)
MR Egger
MR RAPS
Weighted median
Weighted mode
0.002
0.000
−0.002
0.3
0.6
0.9
SNP effect on eGFR
SNP effect on alpha−2−macroglobulin receptor−associated protein

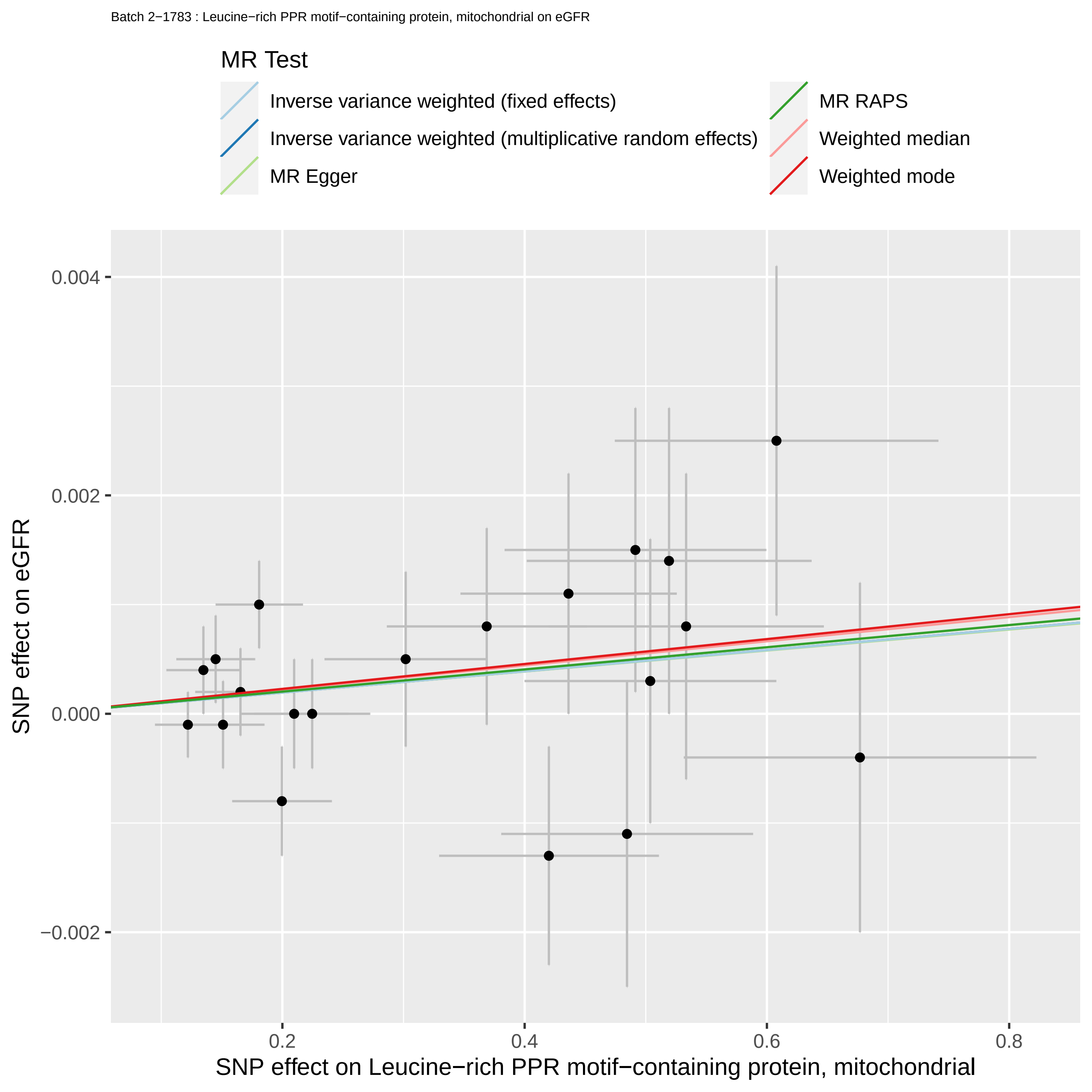

Batch 2−1783 : Leucine−rich PPR motif−containing protein, mitochondrial on eGFR
MR Test
Inverse variance weighted (fixed effects)
Inverse variance weighted (multiplicative random effects)
MR Egger
MR RAPS
Weighted median
Weighted mode
0.004
0.002
0.000
−0.002
0.2
0.4
0.6
0.8
SNP effect on eGFR
SNP effect on Leucine−rich PPR motif−containing protein, mitochondrial

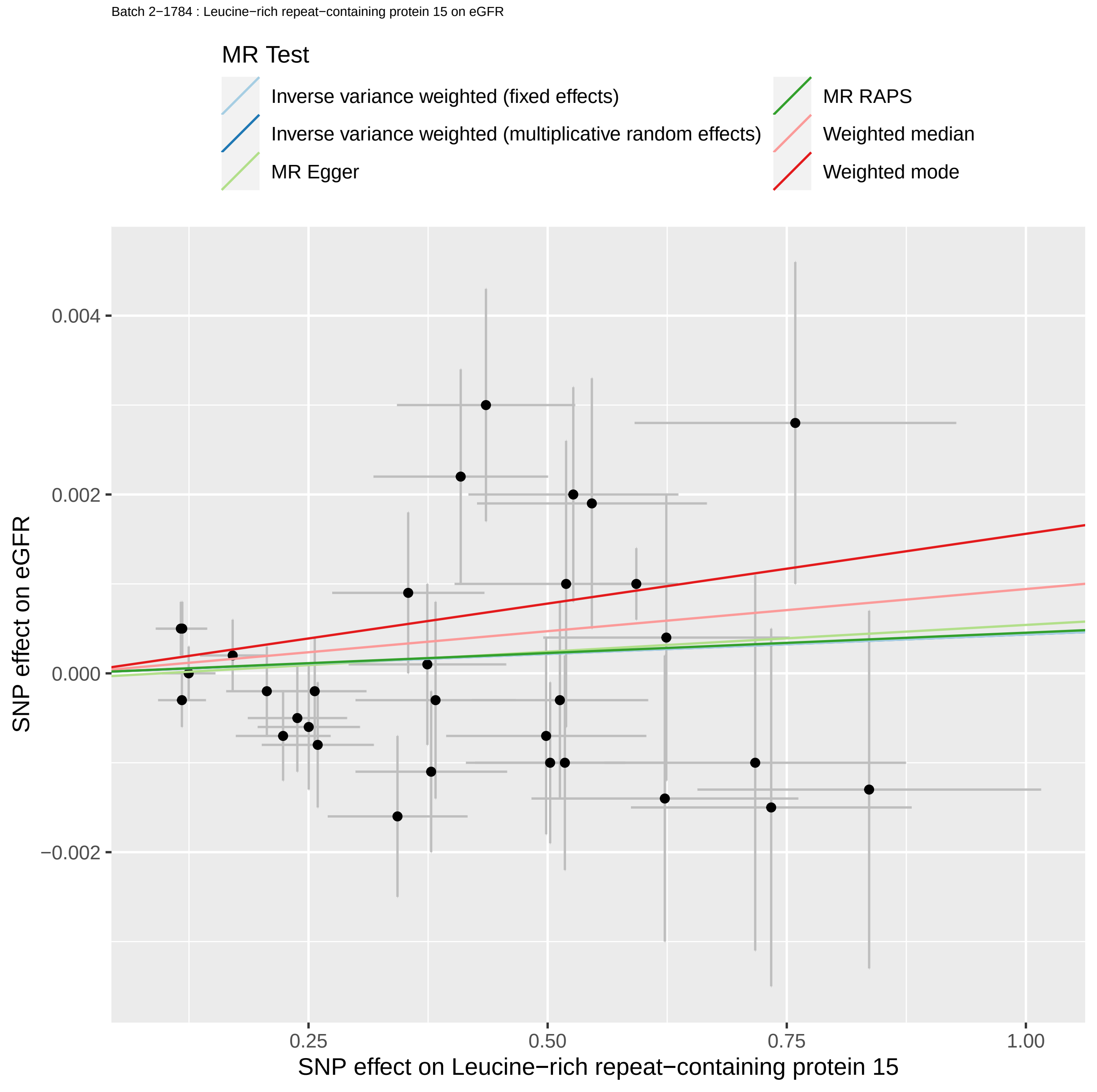
Batch 2−1784 : Leucine−rich repeat−containing protein 15 on eGFR
MR Test
Inverse variance weighted (fixed effects)
Inverse variance weighted (multiplicative random effects)
MR Egger
MR RAPS
Weighted median
Weighted mode
0.004
0.002
0.000
−0.002
0.25
0.50
0.75
1.00
SNP effect on eGFR
SNP effect on Leucine−rich repeat−containing protein 15

Batch 2−1785 : Leucine−rich repeat−containing protein 19 on eGFR

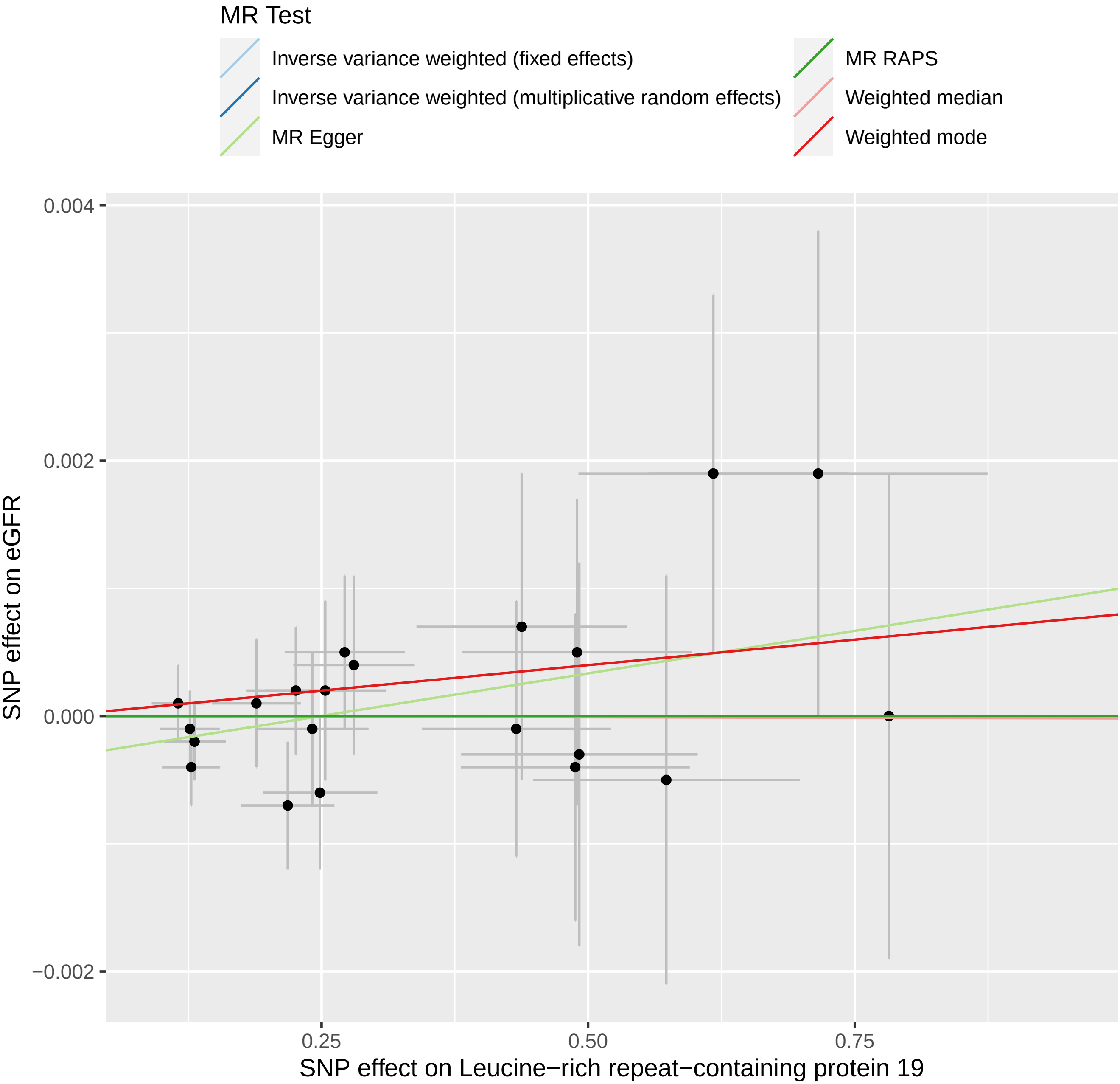

Batch 2−1786 : Leucine−rich repeat−containing protein 3 on eGFR

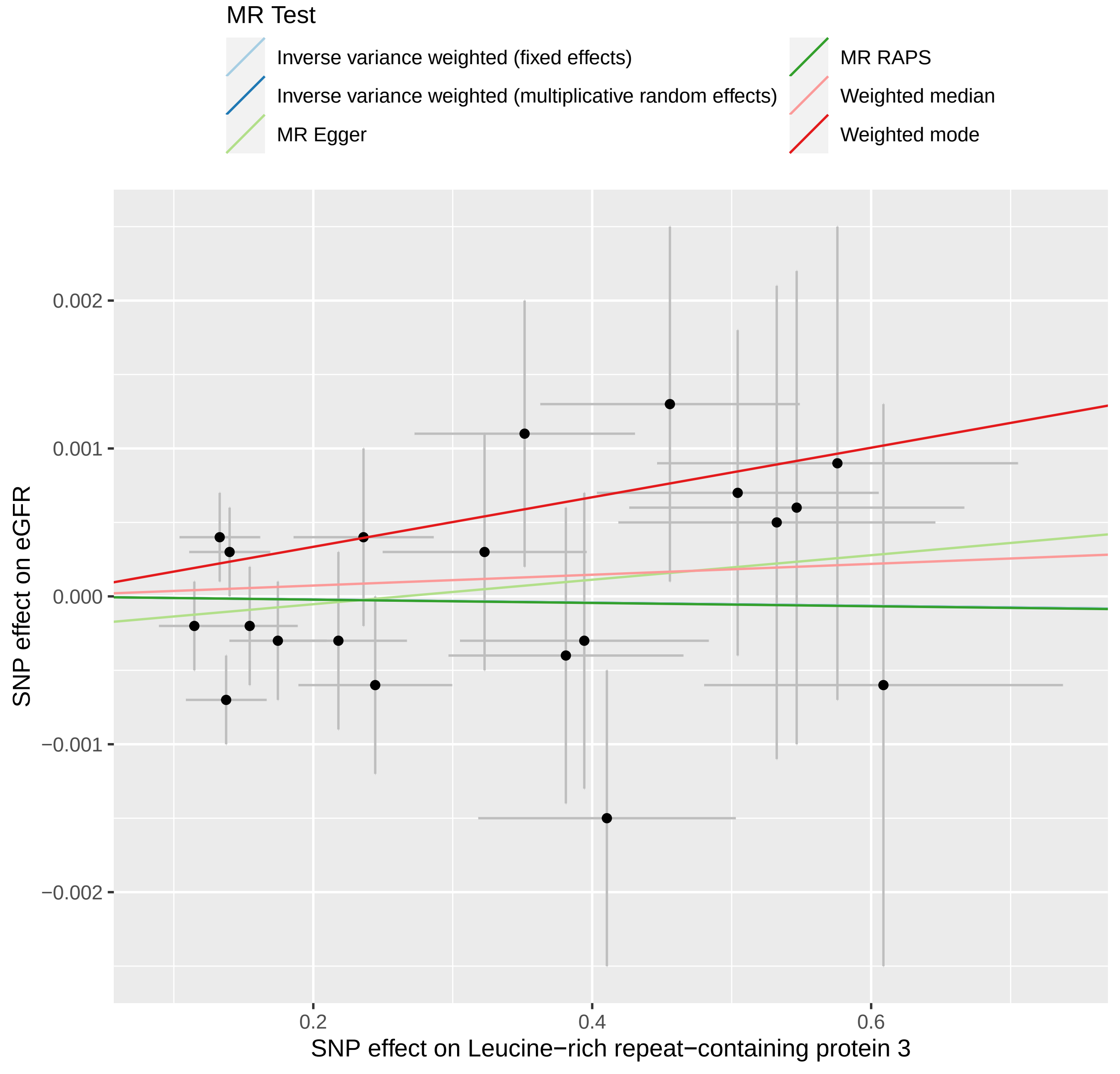

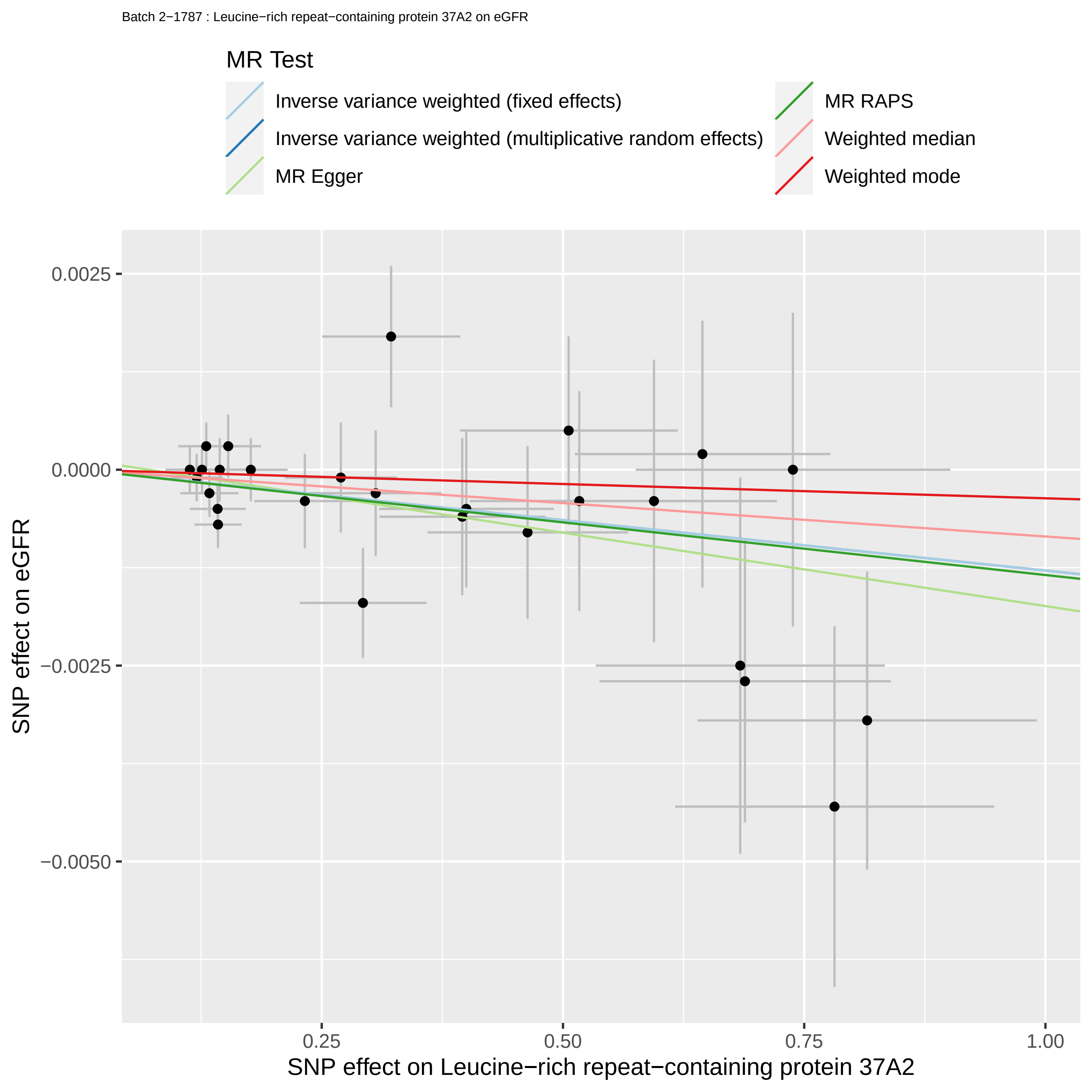
Batch 2−1787 : Leucine−rich repeat−containing protein 37A2 on eGFR
MR Test
Inverse variance weighted (fixed effects)
Inverse variance weighted (multiplicative random effects)
MR Egger
MR RAPS
Weighted median
Weighted mode
0.0025
0.0000
−0.0025
−0.0050
SNP effect on eGFR
0.25
0.50
0.75
1.00
SNP effect on Leucine−rich repeat−containing protein 37A2

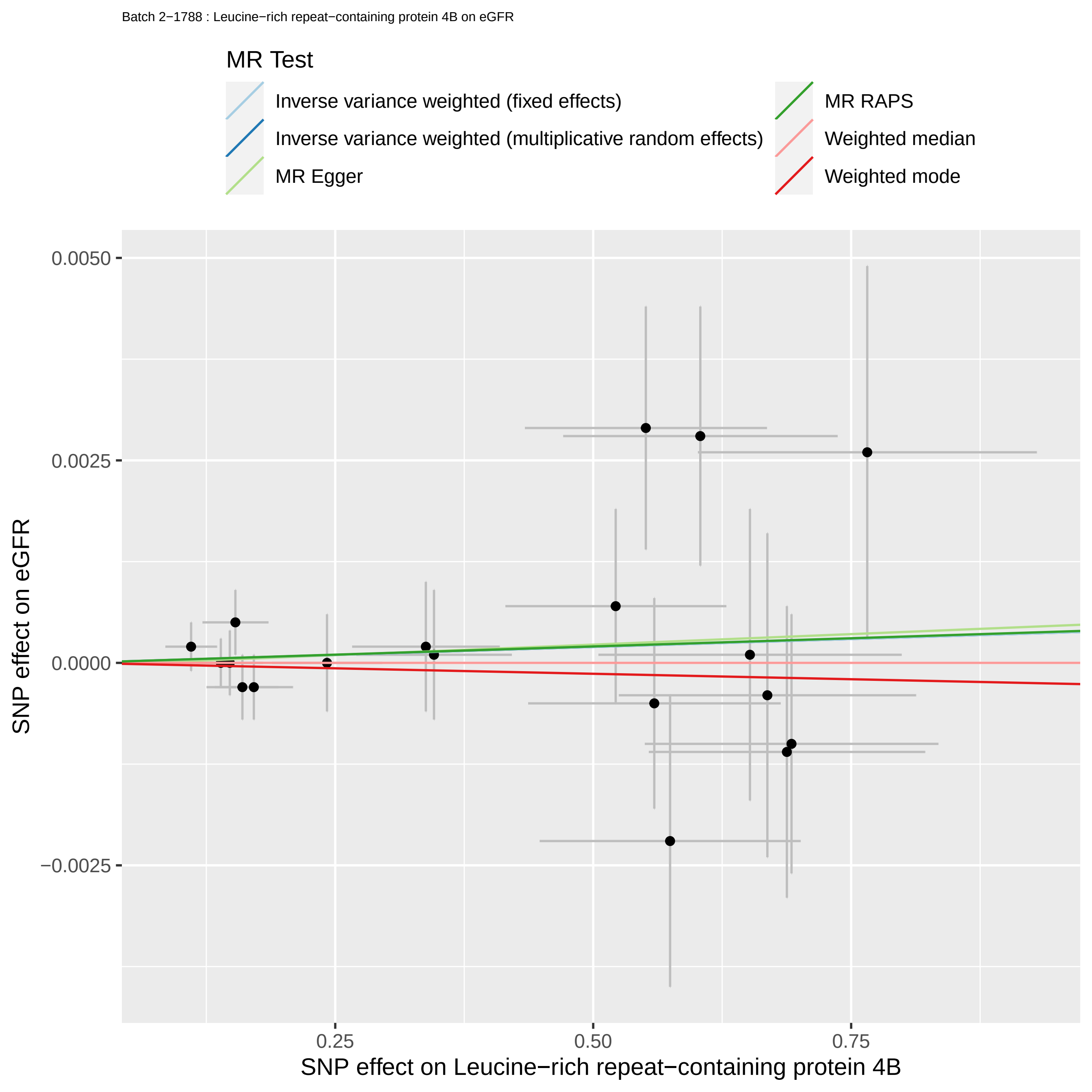
Batch 2−1788 : Leucine−rich repeat−containing protein 4B on eGFR
MR Test
Inverse variance weighted (fixed effects)
Inverse variance weighted (multiplicative random effects)
MR Egger
MR RAPS
Weighted median
Weighted mode
0.0050
0.0025
0.0000
−0.0025
0.25
0.50
0.75
SNP effect on eGFR
SNP effect on Leucine−rich repeat−containing protein 4B

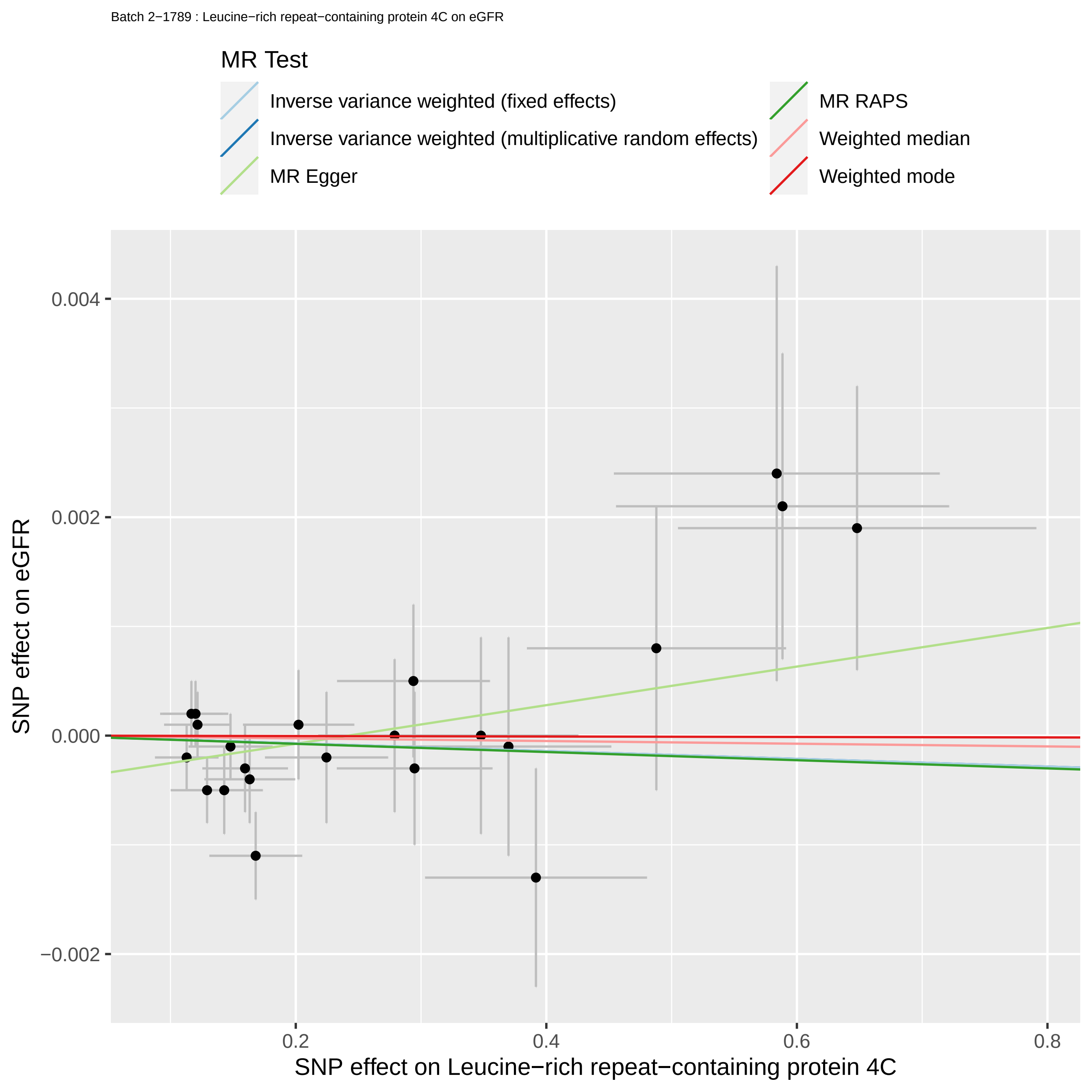

Batch 2−1789 : Leucine−rich repeat−containing protein 4C on eGFR
MR Test
Inverse variance weighted (fixed effects)
Inverse variance weighted (multiplicative random effects)
MR Egger
MR RAPS
Weighted median
Weighted mode
0.004
0.002
0.000
−0.002
0.2
0.4
0.6
0.8
SNP effect on eGFR
SNP effect on Leucine−rich repeat−containing protein 4C

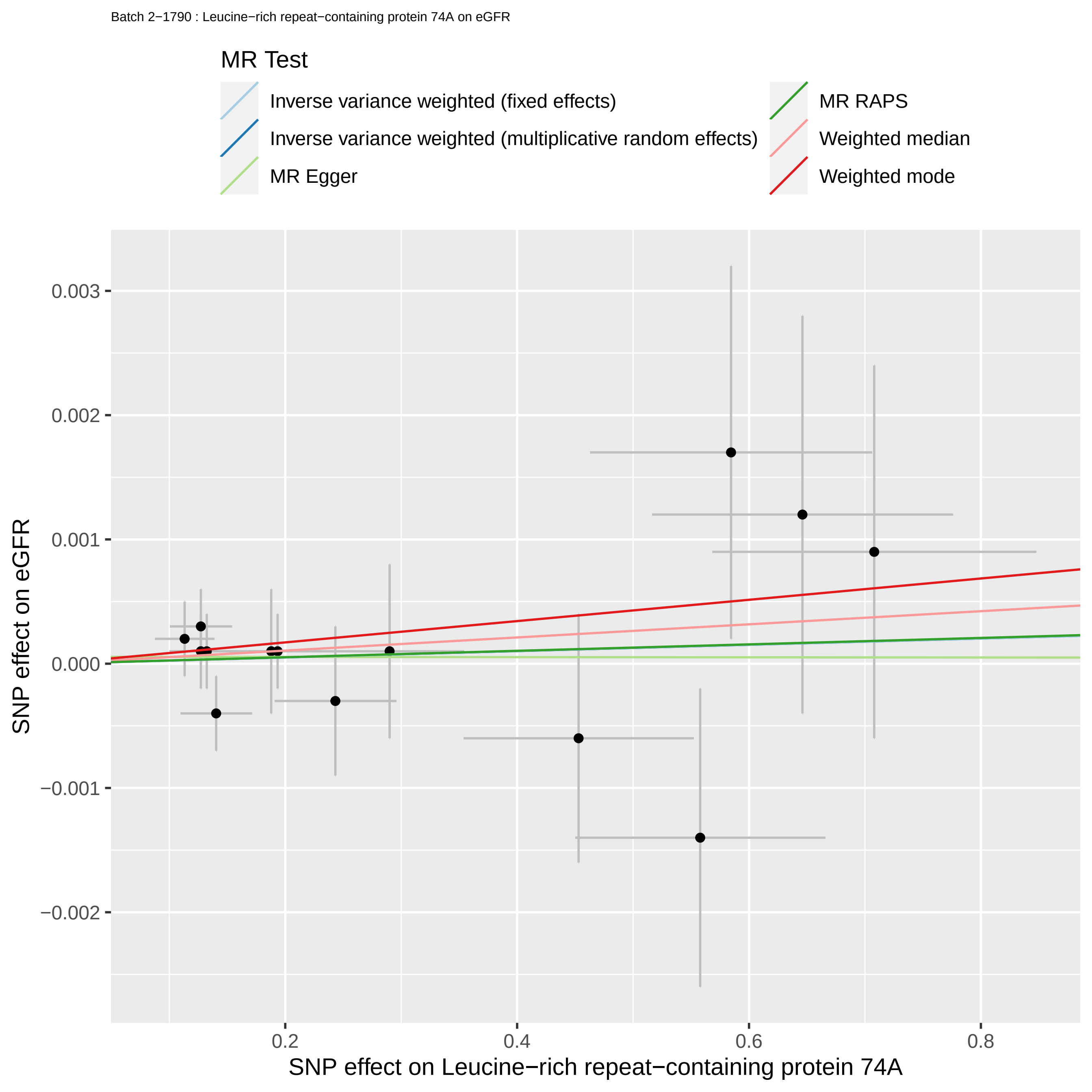

Batch 2−1790 : Leucine−rich repeat−containing protein 74A on eGFR
MR Test
Inverse variance weighted (fixed effects)
Inverse variance weighted (multiplicative random effects)
MR Egger
MR RAPS
Weighted median
Weighted mode
0.003
0.002
0.001
0.000
−0.001
−0.002
SNP effect on eGFR
0.2
0.4
0.6
0.8
SNP effect on Leucine−rich repeat−containing protein 74A

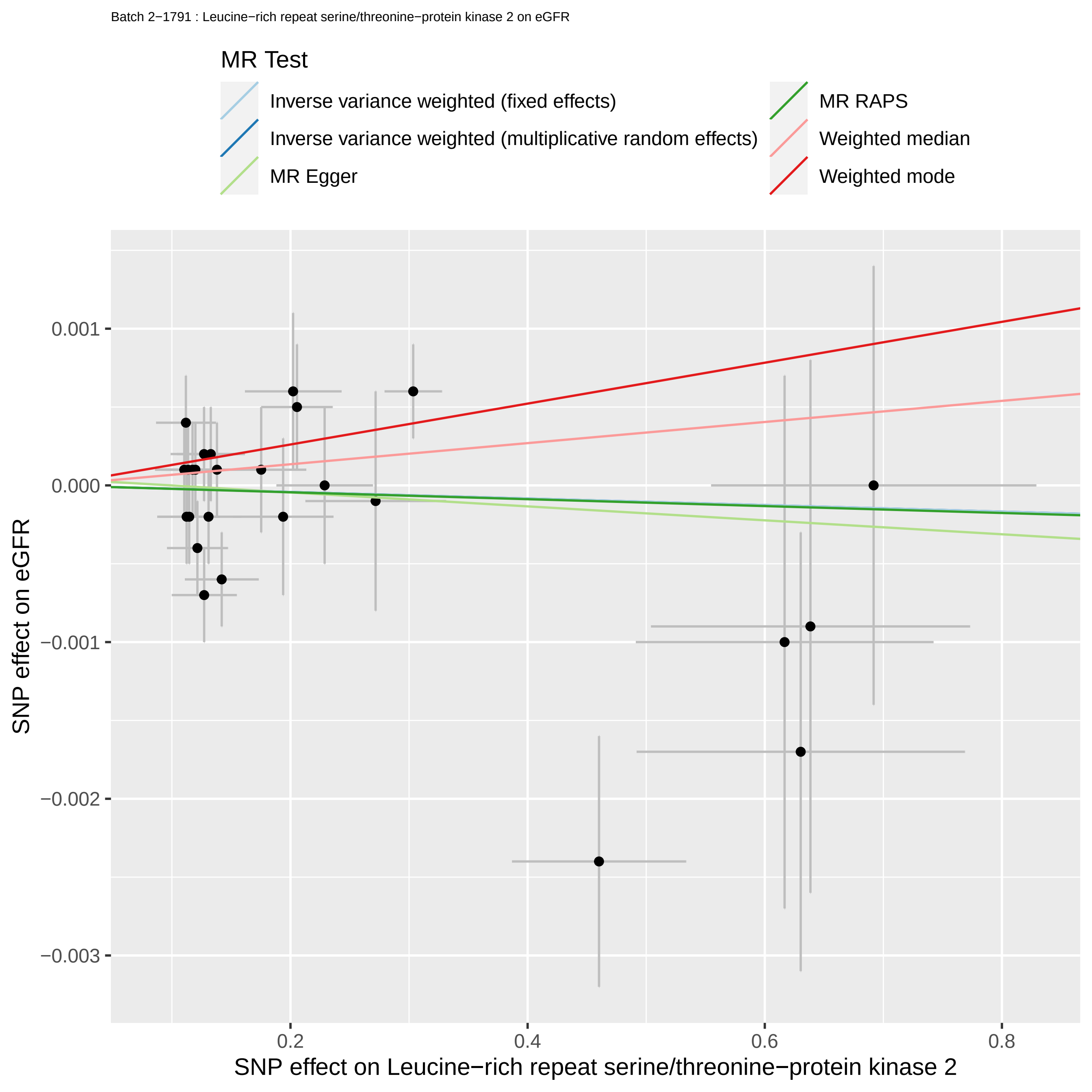

Batch 2−1791 : Leucine−rich repeat serine/threonine−protein kinase 2 on eGFR
MR Test
Inverse variance weighted (fixed effects)
Inverse variance weighted (multiplicative random effects)
MR Egger
MR RAPS
Weighted median
Weighted mode
0.001
0.000
−0.001
−0.002
−0.003
0.2
0.4
0.6
0.8
SNP effect on eGFR
SNP effect on Leucine−rich repeat serine/threonine−protein kinase 2

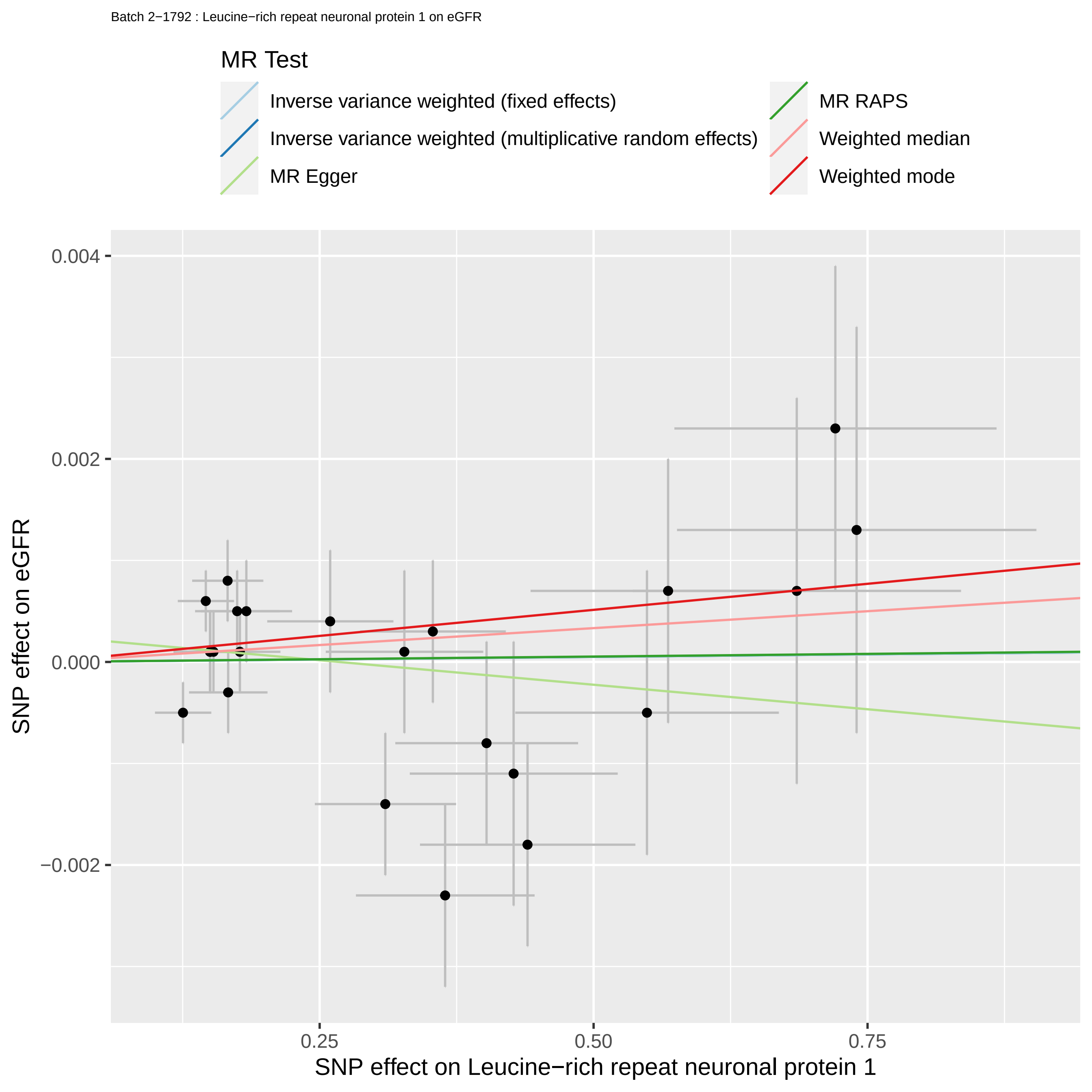

Batch 2−1792 : Leucine−rich repeat neuronal protein 1 on eGFR
MR Test
Inverse variance weighted (fixed effects)
Inverse variance weighted (multiplicative random effects)
MR Egger
MR RAPS
Weighted median
Weighted mode
0.004
0.002
0.000
−0.002
0.25
0.50
0.75
SNP effect on eGFR
SNP effect on Leucine−rich repeat neuronal protein 1

Batch 2−1793 : Leucine−rich repeat neuronal protein 1 on eGFR

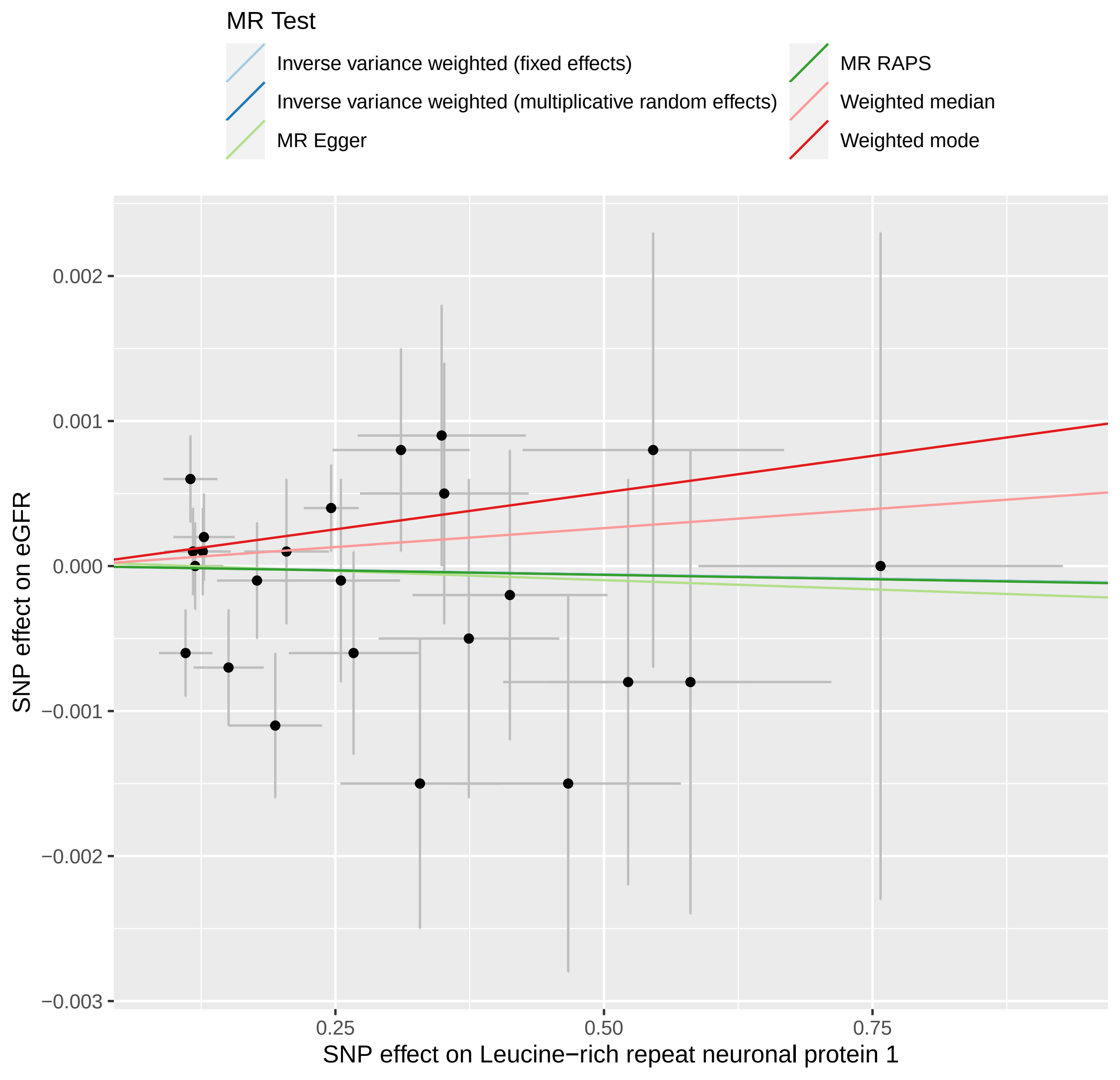

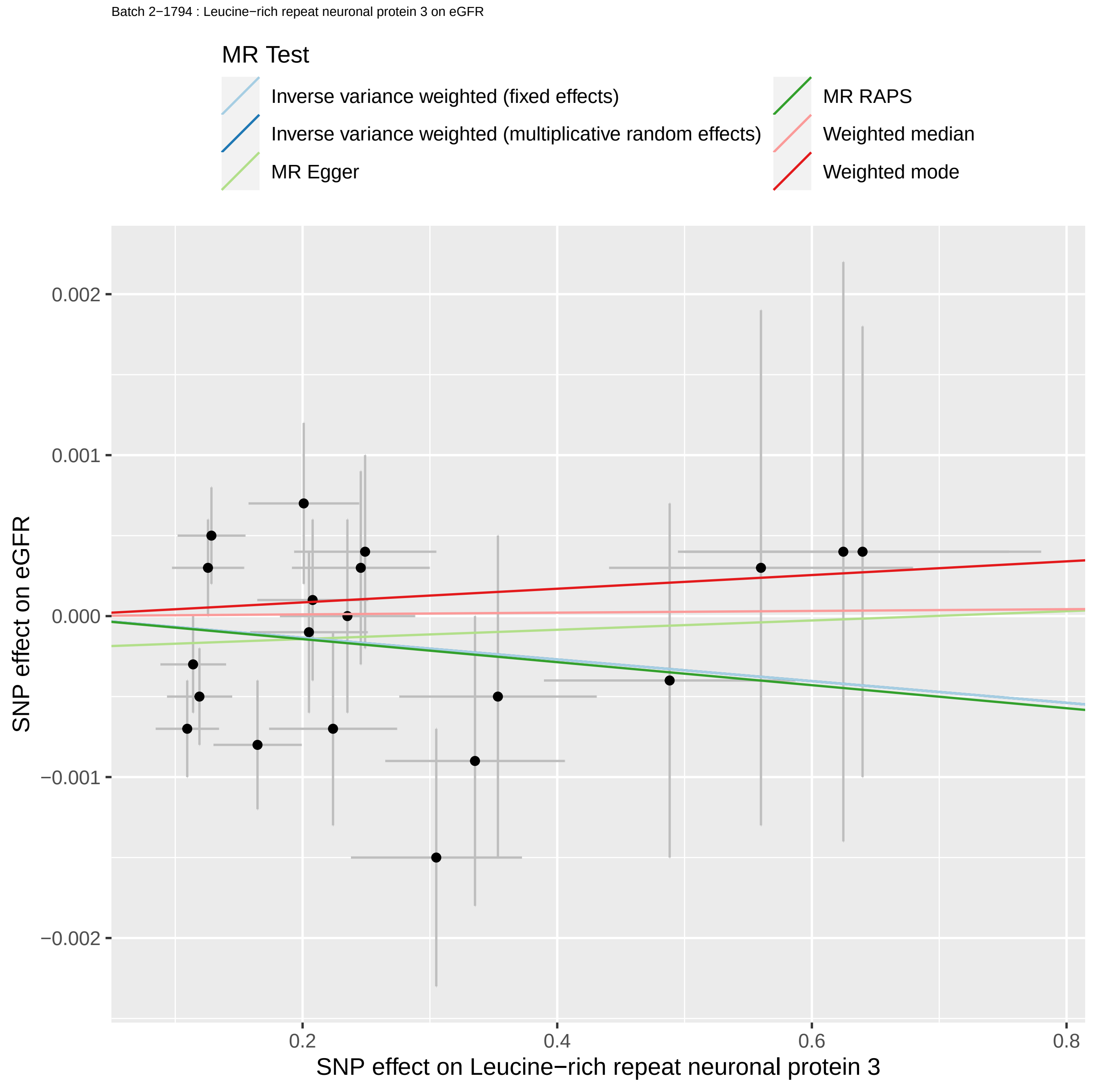

Batch 2−1794 : Leucine−rich repeat neuronal protein 3 on eGFR
MR Test
Inverse variance weighted (fixed effects)
Inverse variance weighted (multiplicative random effects)
MR Egger
MR RAPS
Weighted median
Weighted mode
0.002
0.001
0.000
−0.001
−0.002
0.2
0.4
0.6
0.8
SNP effect on eGFR
SNP effect on Leucine−rich repeat neuronal protein 3

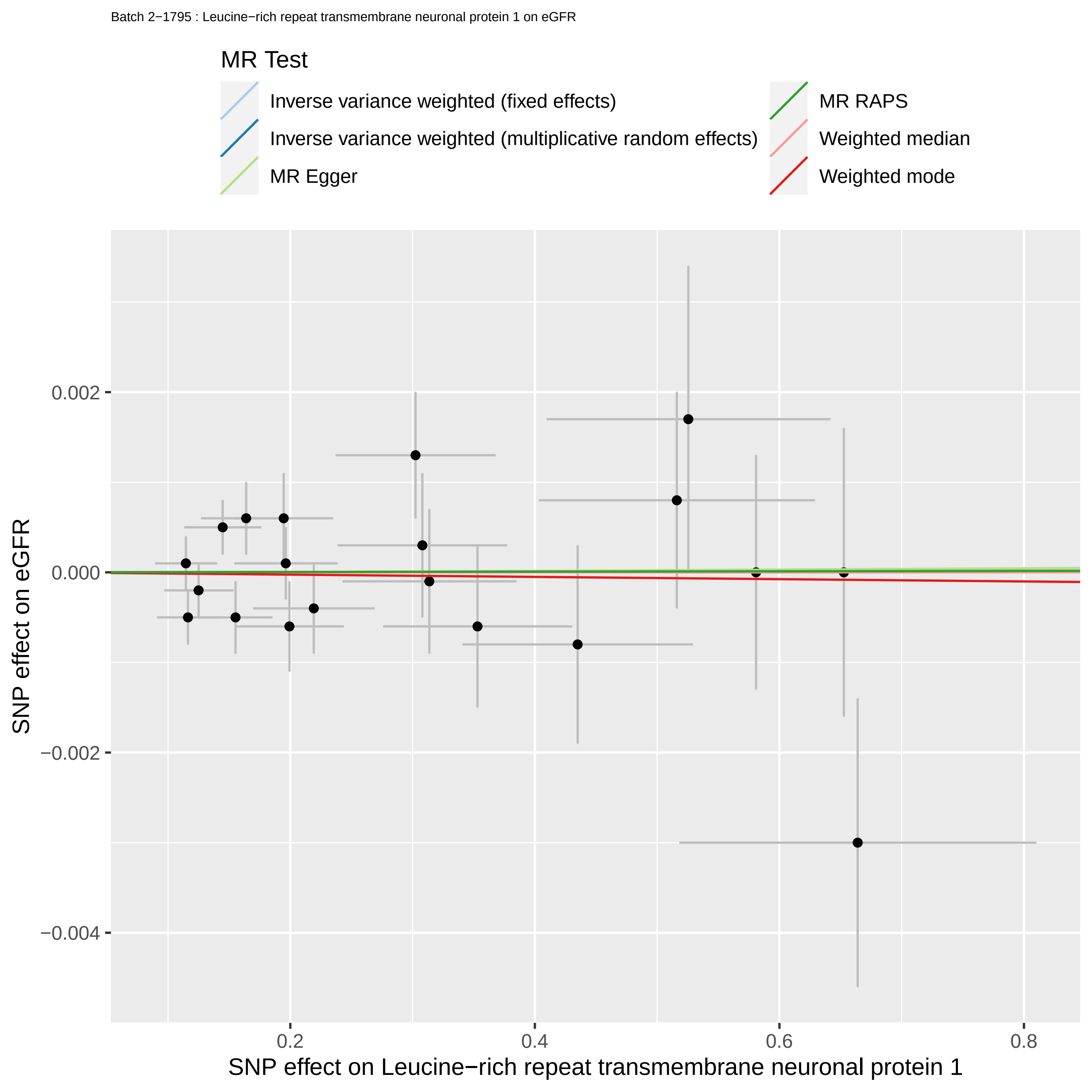
Batch 2−1795 : Leucine−rich repeat transmembrane neuronal protein 1 on eGFR
MR Test
Inverse variance weighted (fixed effects)
Inverse variance weighted (multiplicative random effects)
MR Egger
MR RAPS
Weighted median
Weighted mode
SNP effect on eGFR
0.002
0.000
−0.002
−0.004
0.2
0.4
0.6
0.8
SNP effect on Leucine−rich repeat transmembrane neuronal protein 1

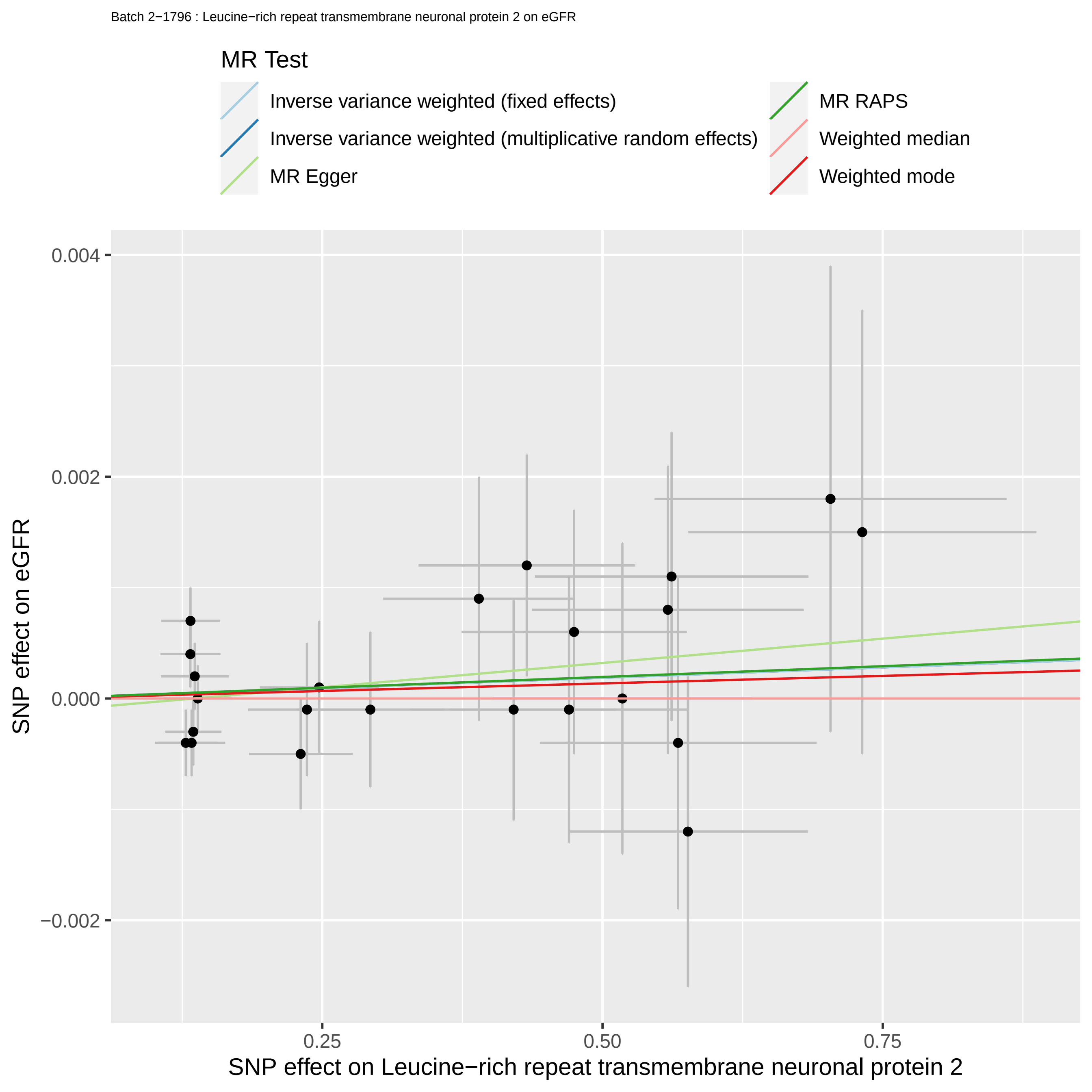
Batch 2−1796 : Leucine−rich repeat transmembrane neuronal protein 2 on eGFR
MR Test
Inverse variance weighted (fixed effects)
Inverse variance weighted (multiplicative random effects)
MR Egger
MR RAPS
Weighted median
Weighted mode
0.004
0.002
0.000
−0.002
0.25
0.50
0.75
SNP effect on eGFR
SNP effect on Leucine−rich repeat transmembrane neuronal protein 2

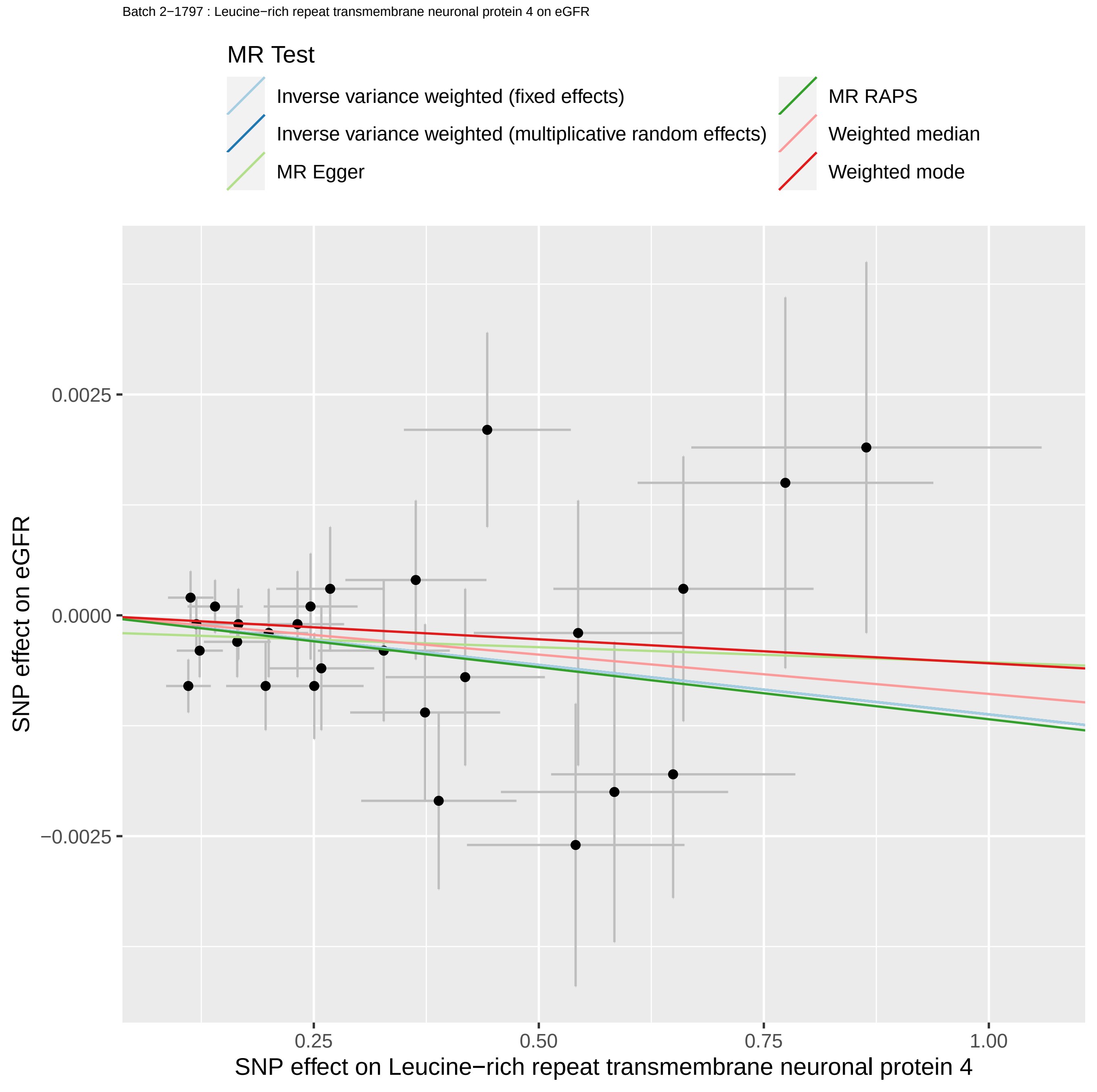
Batch 2−1797 : Leucine−rich repeat transmembrane neuronal protein 4 on eGFR
MR Test
Inverse variance weighted (fixed effects)
Inverse variance weighted (multiplicative random effects)
MR Egger
MR RAPS
Weighted median
Weighted mode
0.0025
0.0000
−0.0025
0.25
0.50
0.75
1.00
SNP effect on eGFR
SNP effect on Leucine−rich repeat transmembrane neuronal protein 4

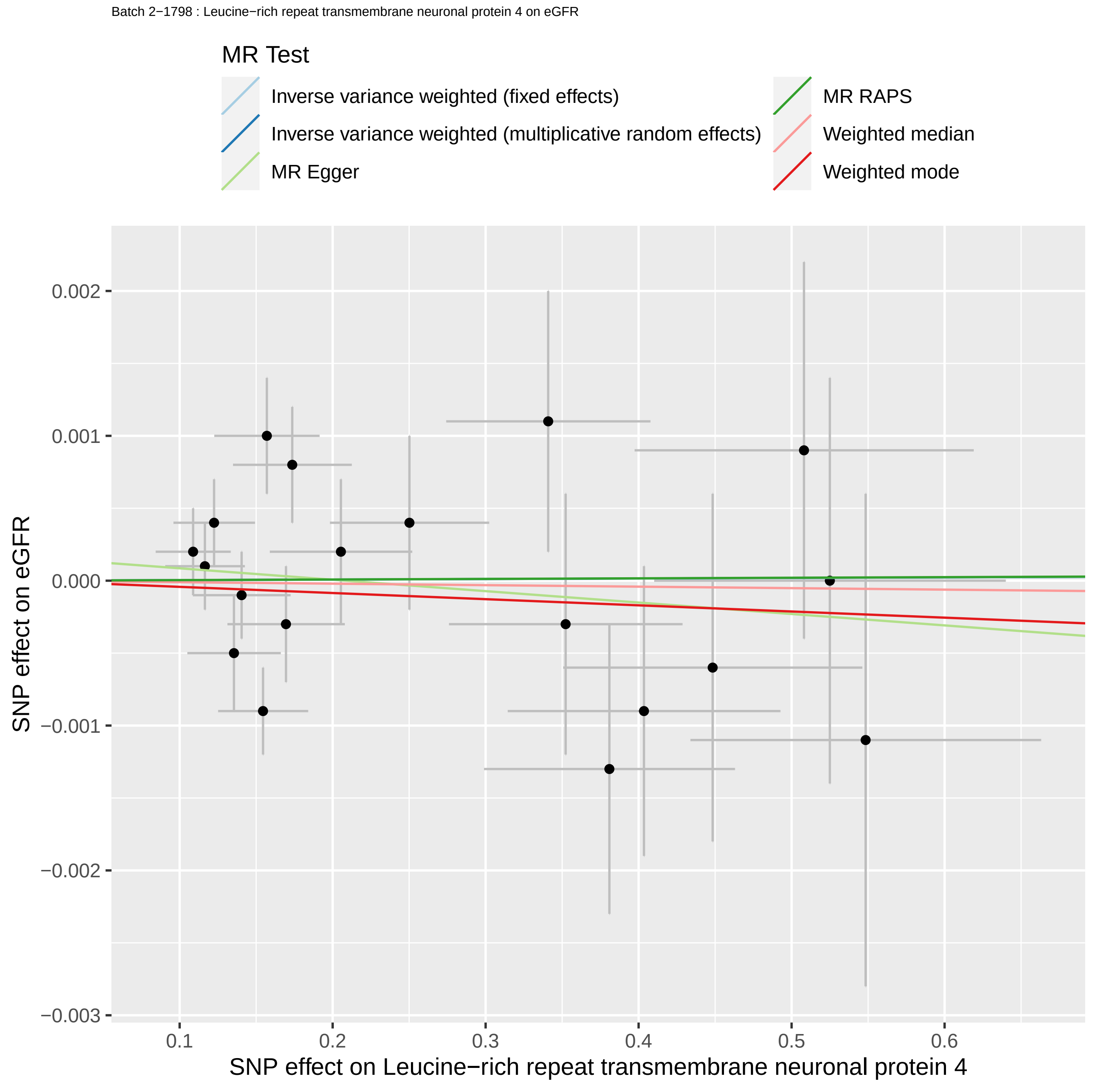

Batch 2−1798 : Leucine−rich repeat transmembrane neuronal protein 4 on eGFR
MR Test
Inverse variance weighted (fixed effects)
Inverse variance weighted (multiplicative random effects)
MR Egger
MR RAPS
Weighted median
Weighted mode
0.002
0.001
0.000
−0.001
−0.002
−0.003
0.1
0.2
0.3
0.4
0.5
0.6
SNP effect on eGFR
SNP effect on Leucine−rich repeat transmembrane neuronal protein 4

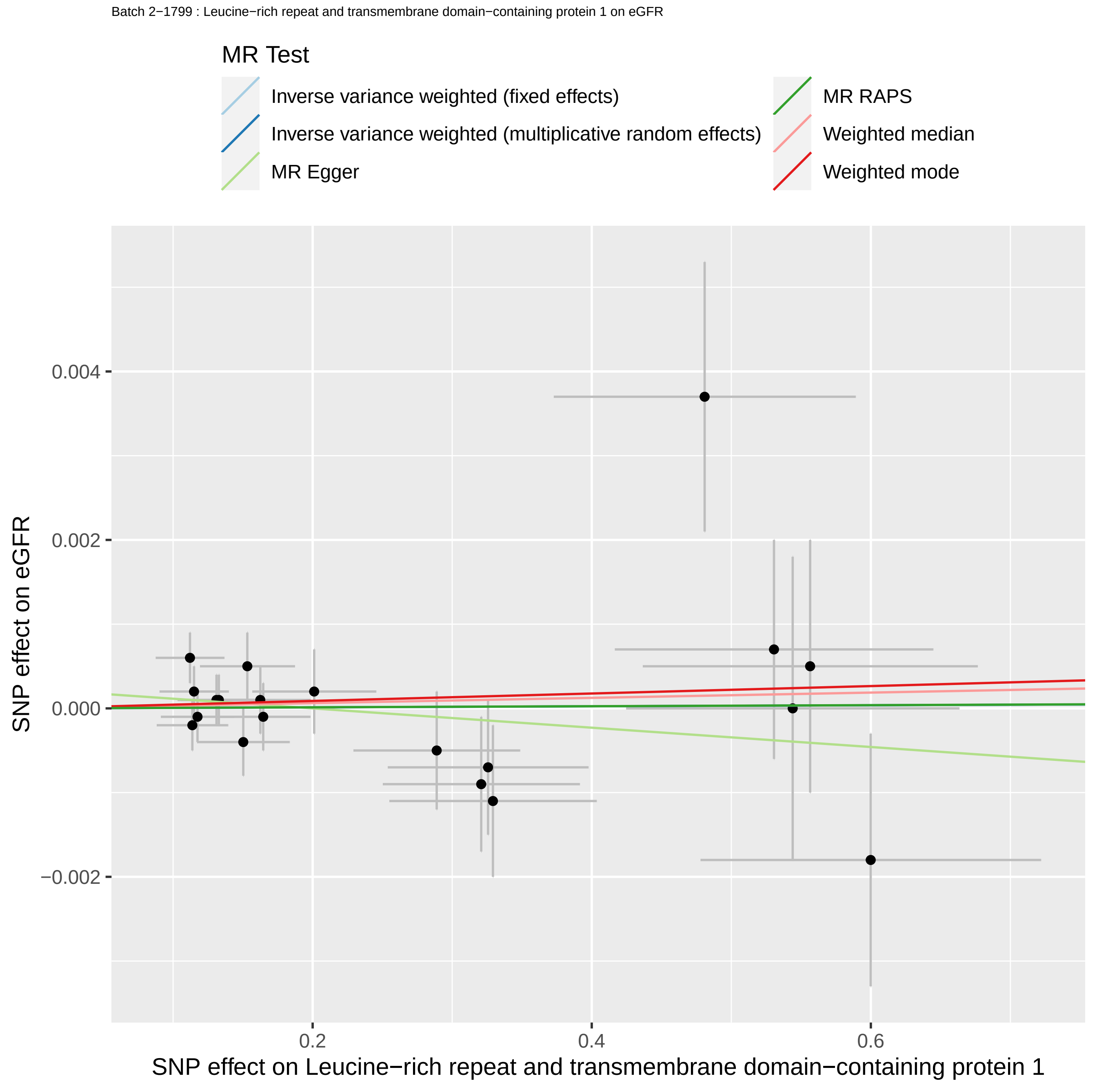
Batch 2−1799 : Leucine−rich repeat and transmembrane domain−containing protein 1 on eGFR
MR Test
Inverse variance weighted (fixed effects)
Inverse variance weighted (multiplicative random effects)
MR Egger
MR RAPS
Weighted median
Weighted mode
0.004
0.002
0.000
−0.002
0.2
0.4
0.6
SNP effect on eGFR
SNP effect on Leucine−rich repeat and transmembrane domain−containing protein 1

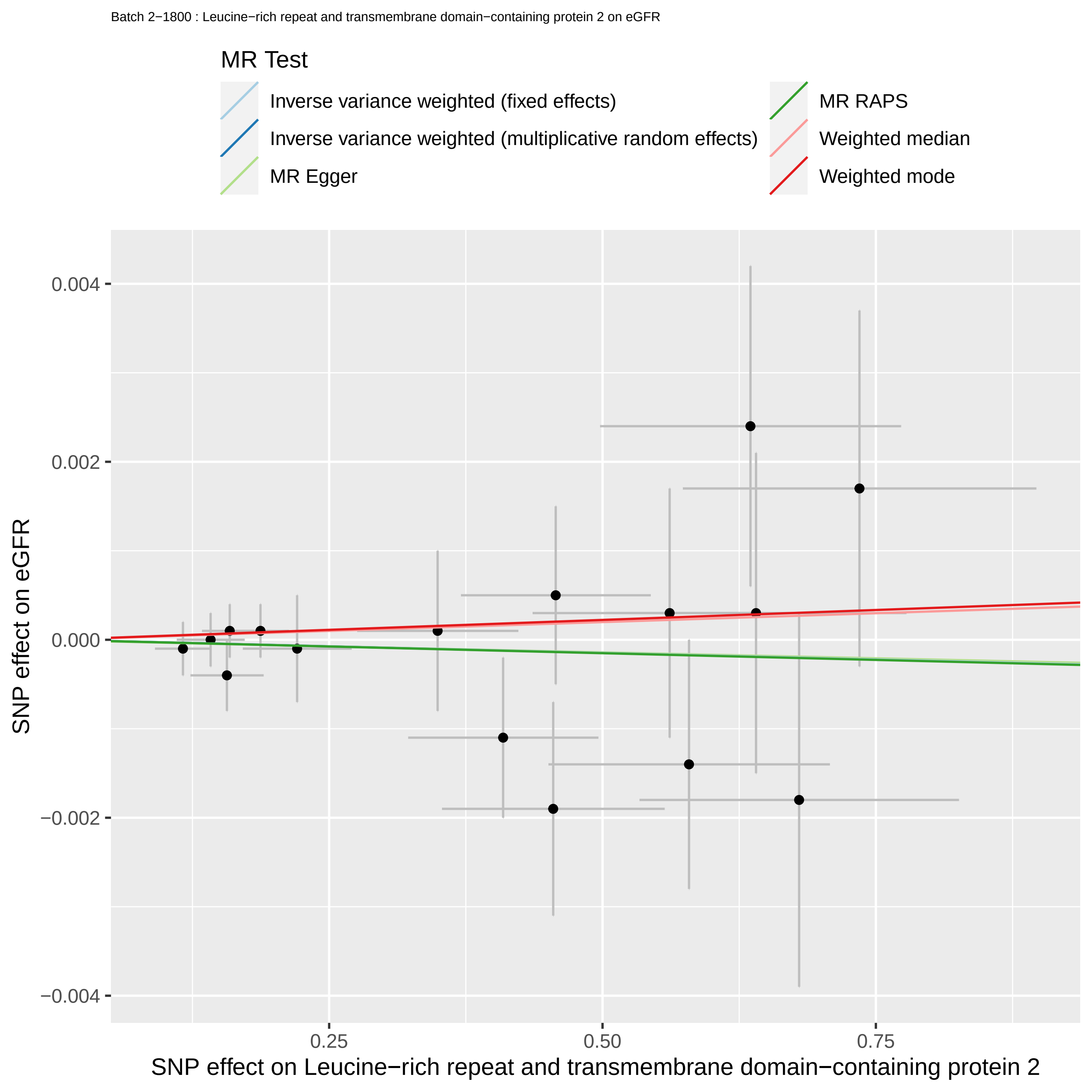

Batch 2−1800 : Leucine−rich repeat and transmembrane domain−containing protein 2 on eGFR
MR Test
Inverse variance weighted (fixed effects)
Inverse variance weighted (multiplicative random effects)
MR Egger
MR RAPS
Weighted median
Weighted mode
0.004
0.002
0.000
−0.002
−0.004
0.25
0.50
0.75
SNP effect on eGFR
SNP effect on Leucine−rich repeat and transmembrane domain−containing protein 2

Batch 2−1801 : Limbic system−associated membrane protein on eGFR

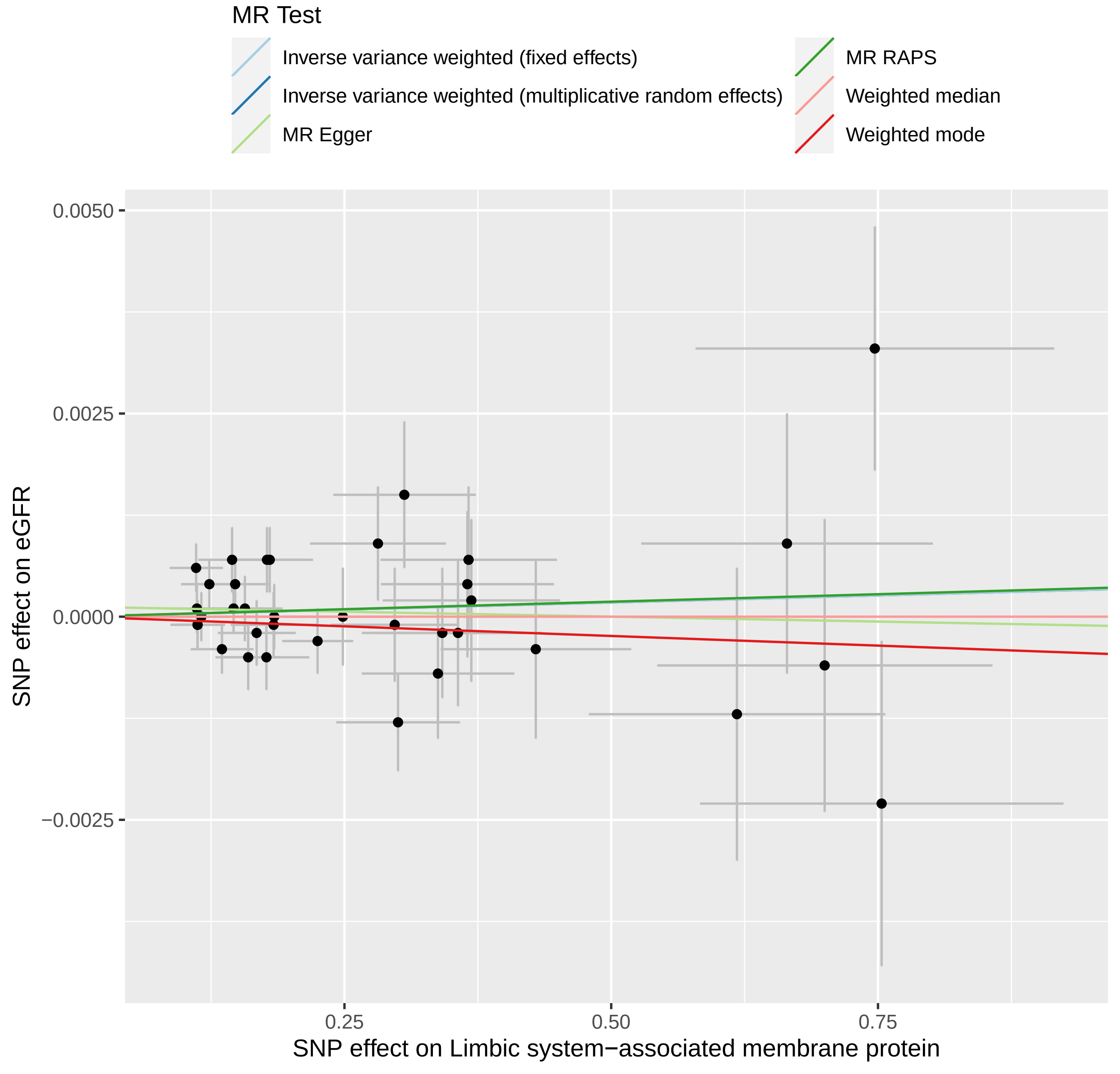

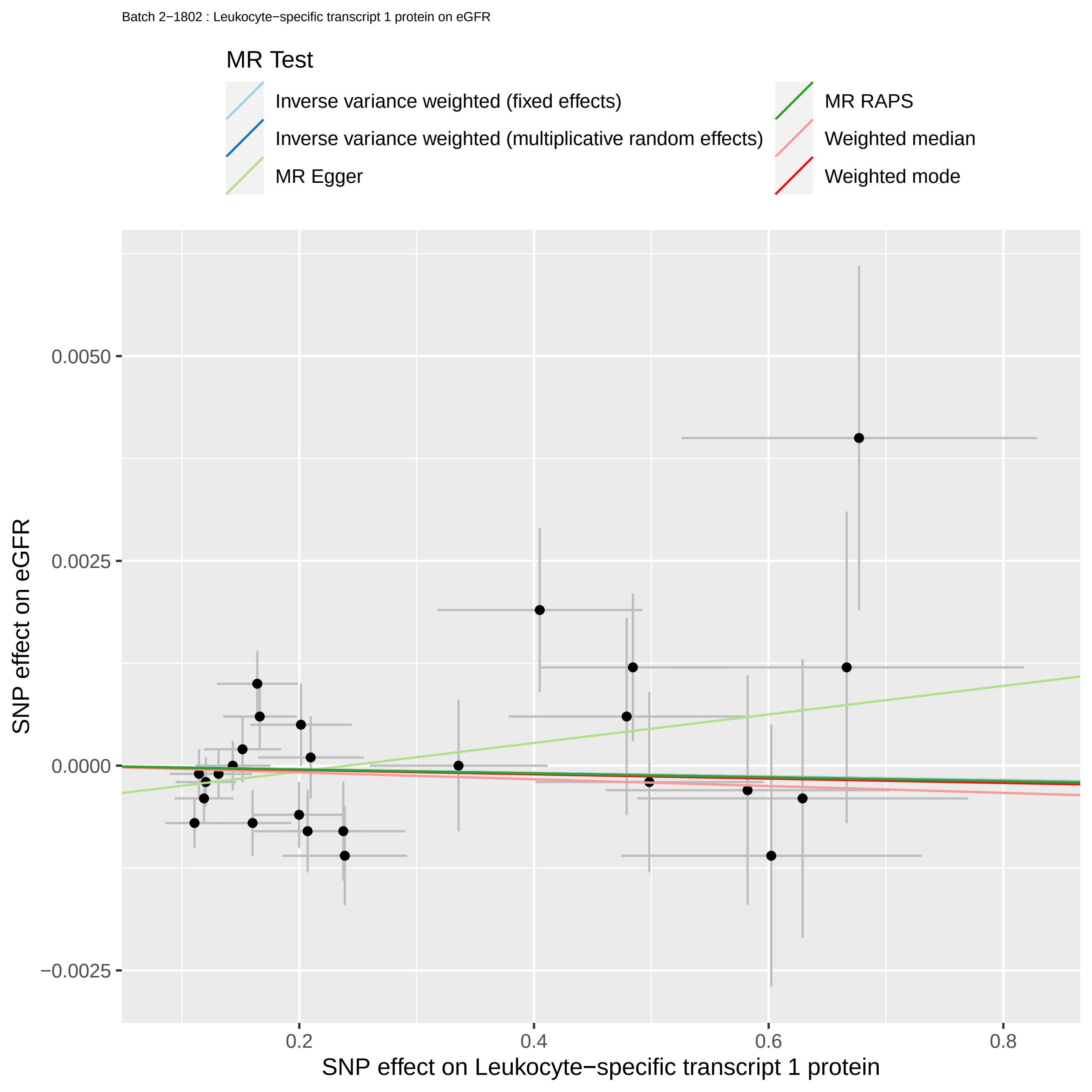
Batch 2−1802 : Leukocyte−specific transcript 1 protein on eGFR
MR Test
Inverse variance weighted (fixed effects)
Inverse variance weighted (multiplicative random effects)
MR Egger
MR RAPS
Weighted median
Weighted mode
0.0050
0.0025
0.0000
−0.0025
SNP effect on eGFR
0.2
0.4
0.6
0.8
SNP effect on Leukocyte−specific transcript 1 protein

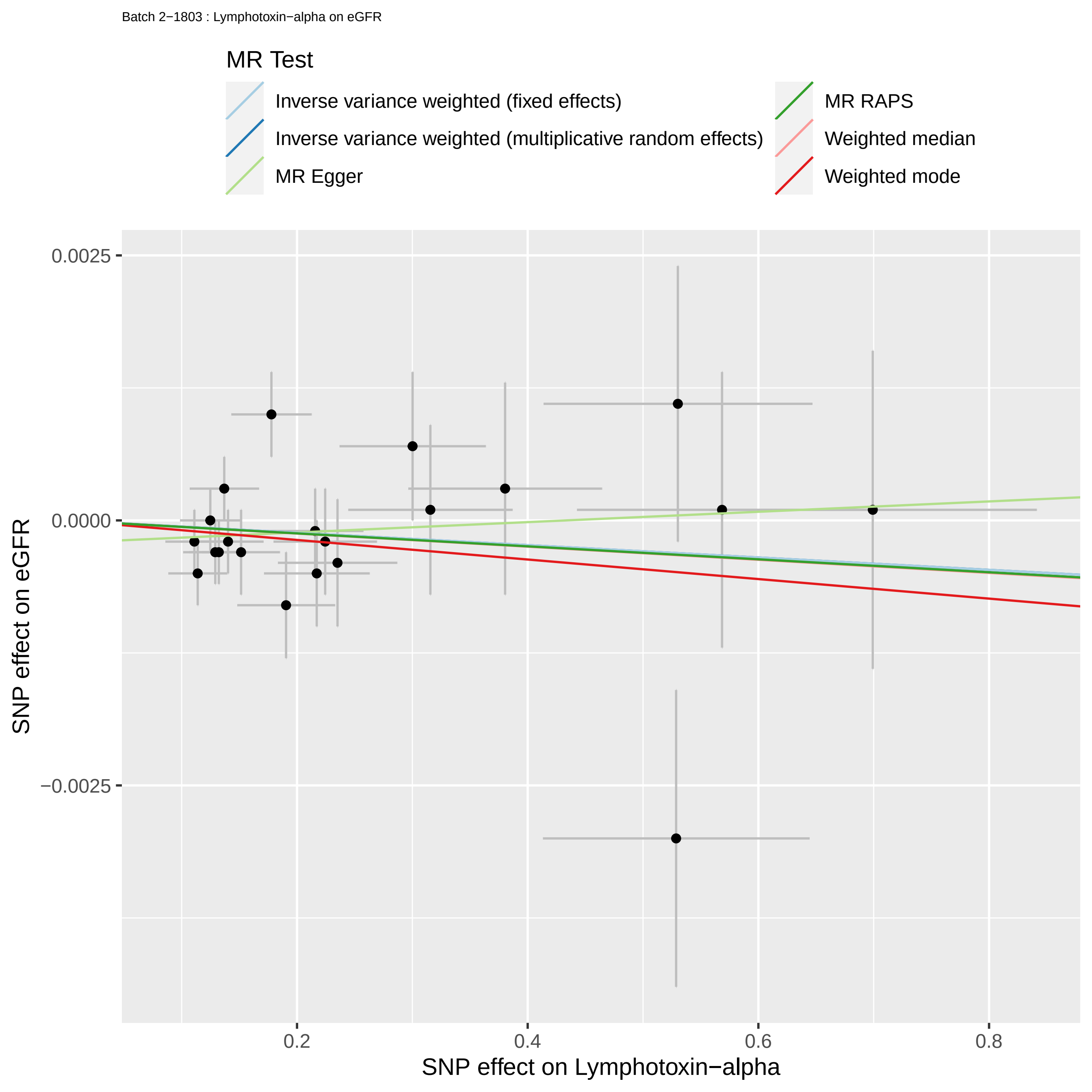

Batch 2−1803 : Lymphotoxin−alpha on eGFR
MR Test
Inverse variance weighted (fixed effects)
Inverse variance weighted (multiplicative random effects)
MR Egger
MR RAPS
Weighted median
Weighted mode
0.0025
0.0000
−0.0025
0.2
0.4
0.6
0.8
SNP effect on Lymphotoxin−alpha
SNP effect on eGFR

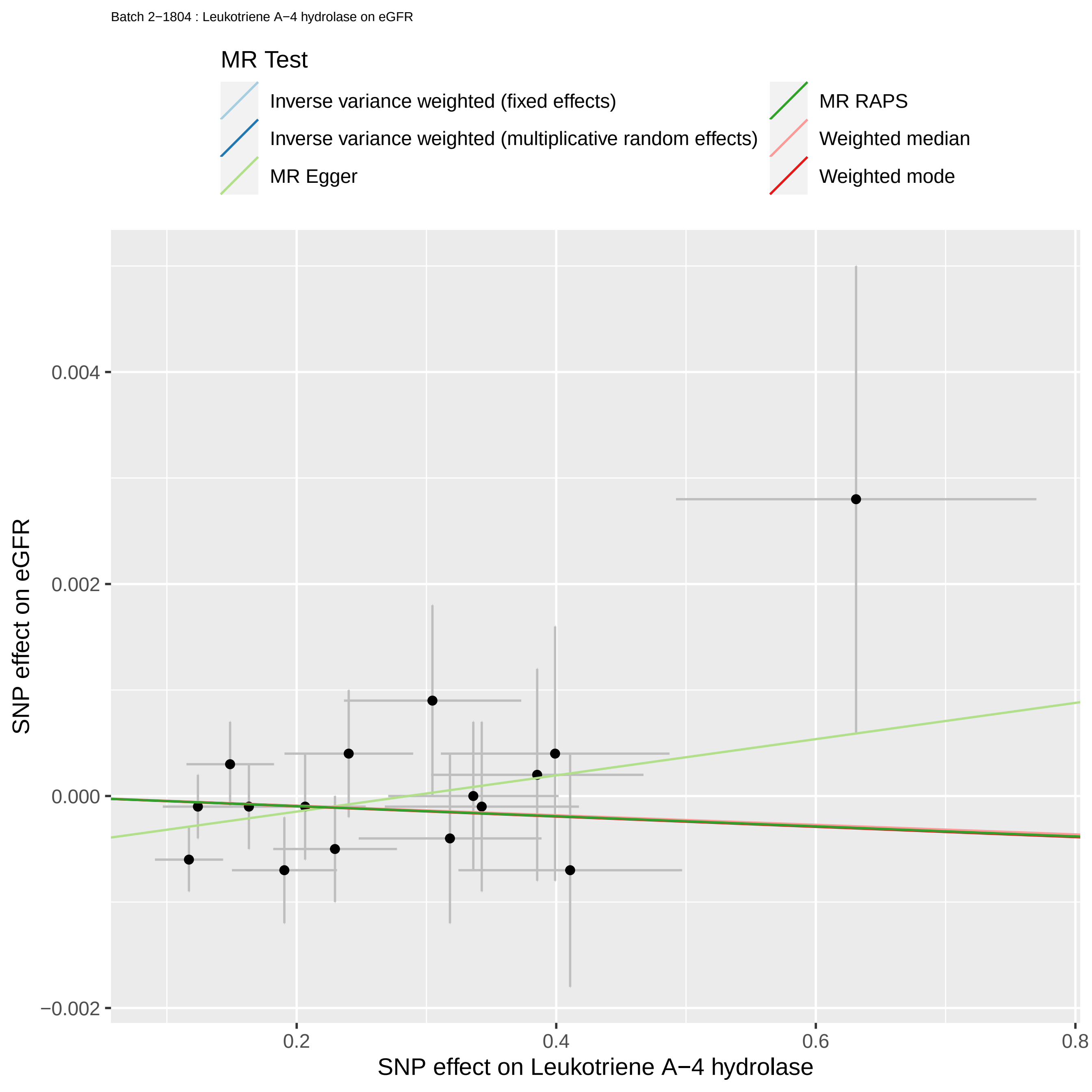
Batch 2−1804 : Leukotriene A−4 hydrolase on eGFR
MR Test
Inverse variance weighted (fixed effects)
Inverse variance weighted (multiplicative random effects)
MR Egger
MR RAPS
Weighted median
Weighted mode
0.004
0.002
0.000
−0.002
SNP effect on eGFR
0.2
0.4
0.6
0.8
SNP effect on Leukotriene A−4 hydrolase

Batch 2−1805 : Lymphotoxin alpha2:beta1 on eGFR

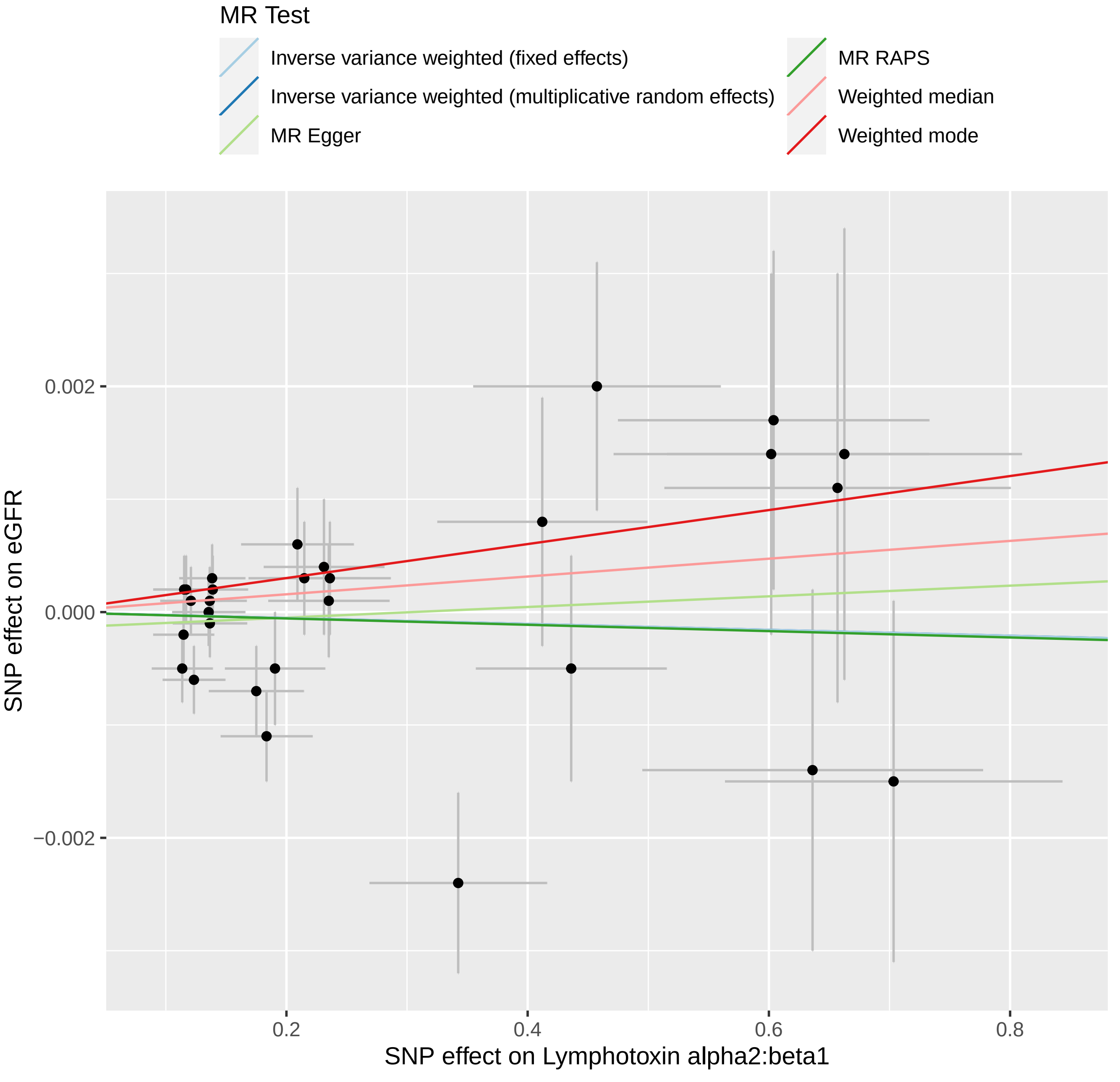

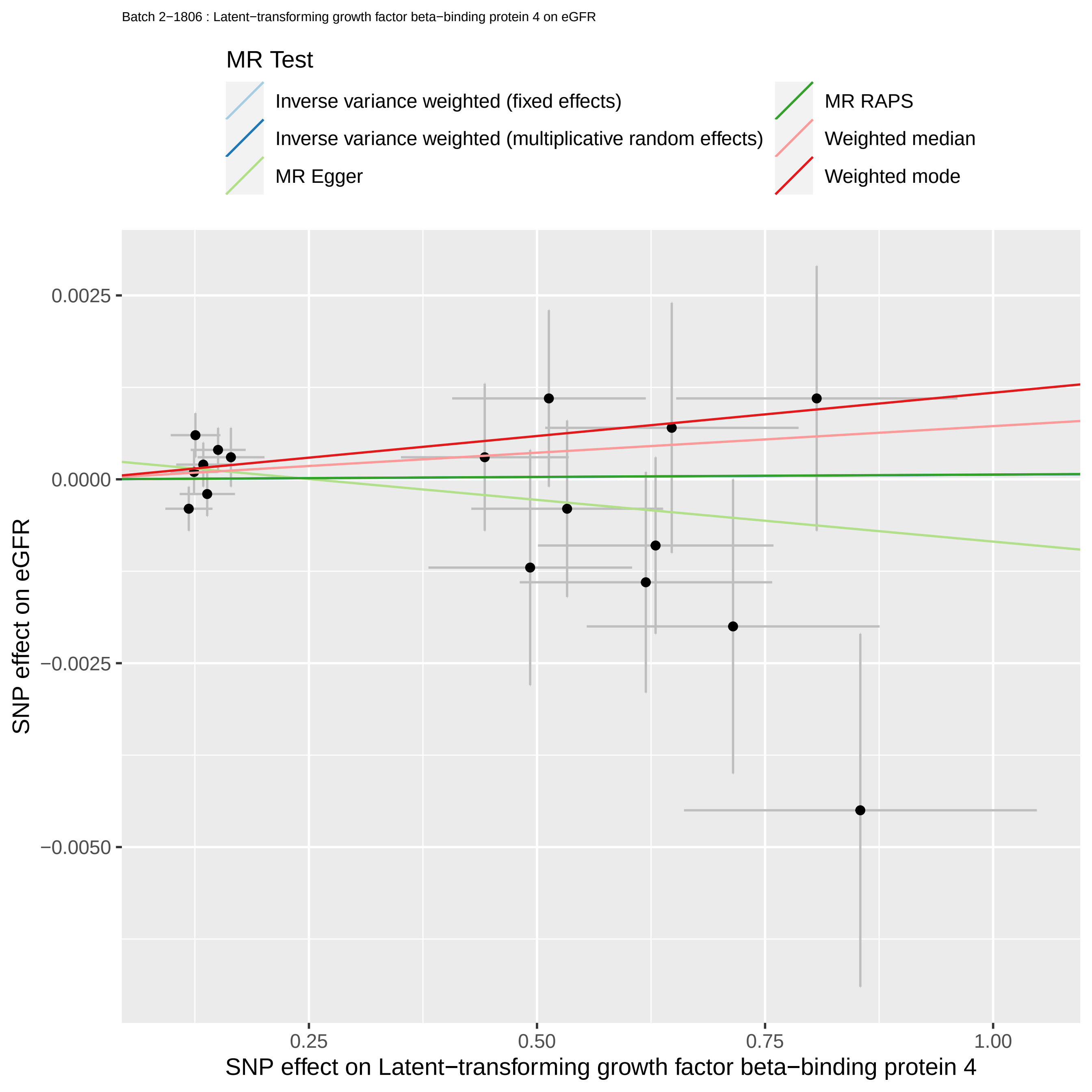
Batch 2−1806 : Latent−transforming growth factor beta−binding protein 4 on eGFR
MR Test
Inverse variance weighted (fixed effects)
Inverse variance weighted (multiplicative random effects)
MR Egger
MR RAPS
Weighted median
Weighted mode
0.0025
0.0000
−0.0025
−0.0050
0.25
0.50
0.75
1.00
SNP effect on eGFR
SNP effect on Latent−transforming growth factor beta−binding protein 4

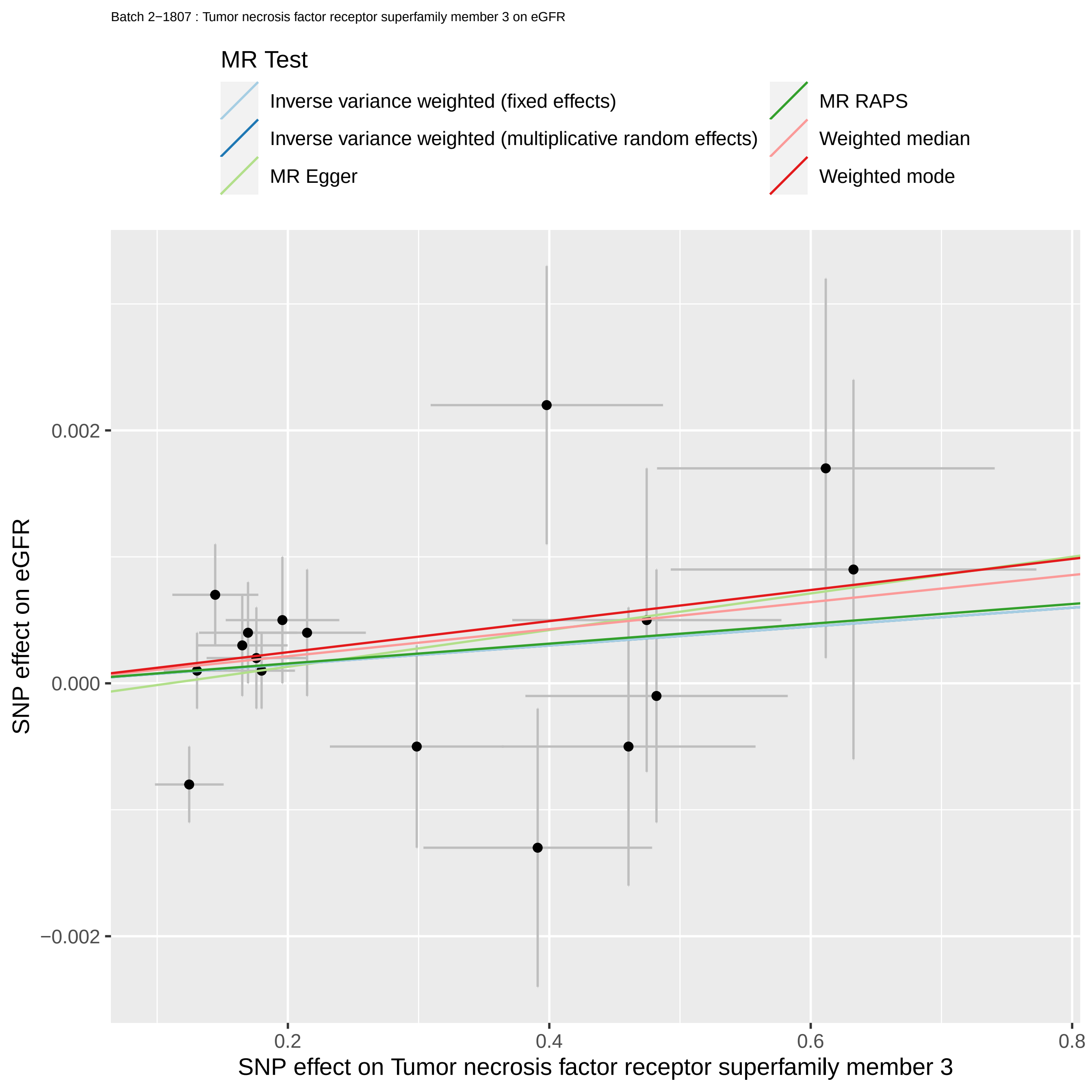
Batch 2−1807 : Tumor necrosis factor receptor superfamily member 3 on eGFR
MR Test
Inverse variance weighted (fixed effects)
Inverse variance weighted (multiplicative random effects)
MR Egger
MR RAPS
Weighted median
Weighted mode
0.002
0.000
−0.002
SNP effect on eGFR
0.2
0.4
0.6
0.8
SNP effect on Tumor necrosis factor receptor superfamily member 3

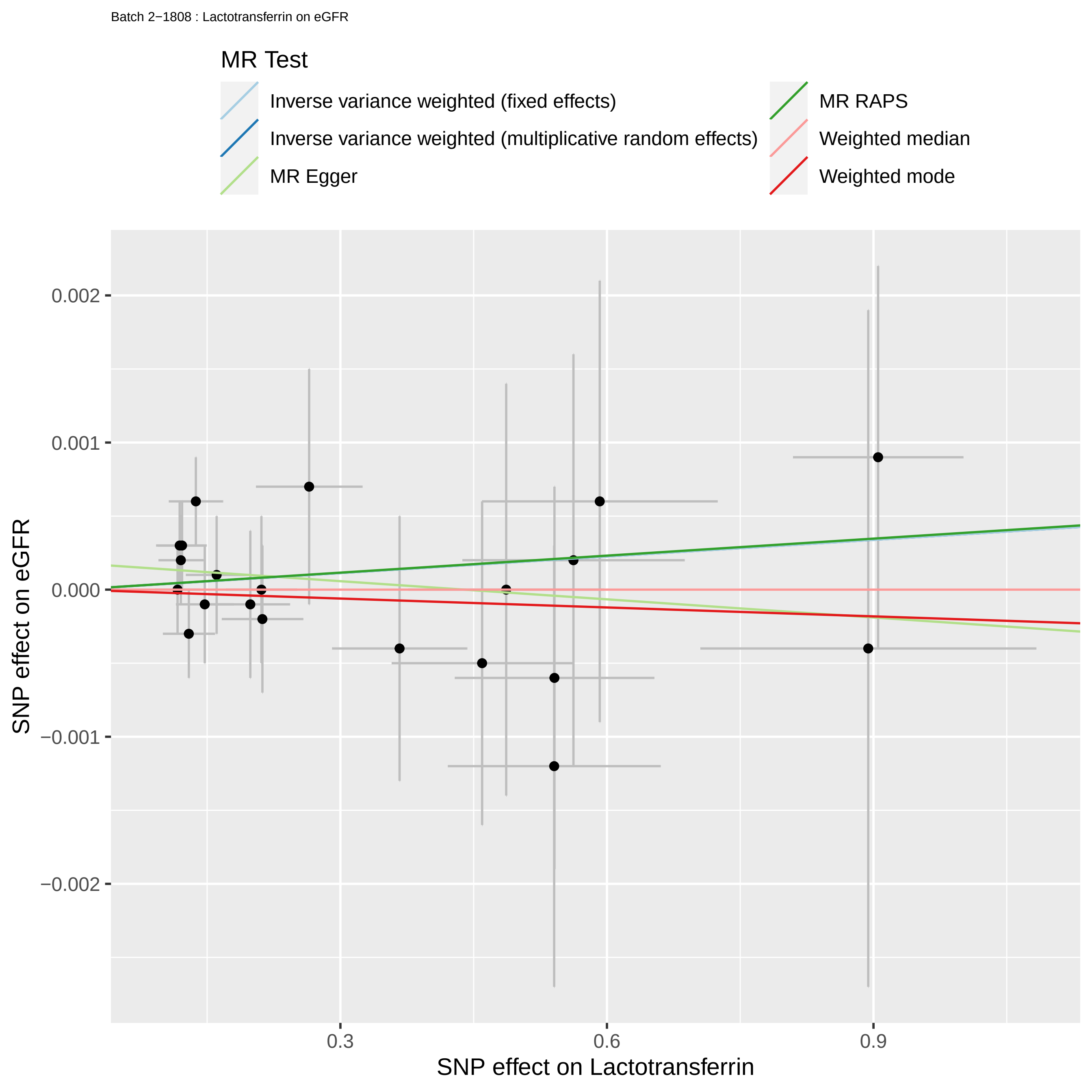

Batch 2−1808 : Lactotransferrin on eGFR
MR Test
Inverse variance weighted (fixed effects)
Inverse variance weighted (multiplicative random effects)
MR Egger
MR RAPS
Weighted median
Weighted mode
0.002
0.001
0.000
−0.001
−0.002
0.3
0.6
0.9
SNP effect on Lactotransferrin
SNP effect on eGFR

Batch 2−1809 : Lactotransferrin on eGFR

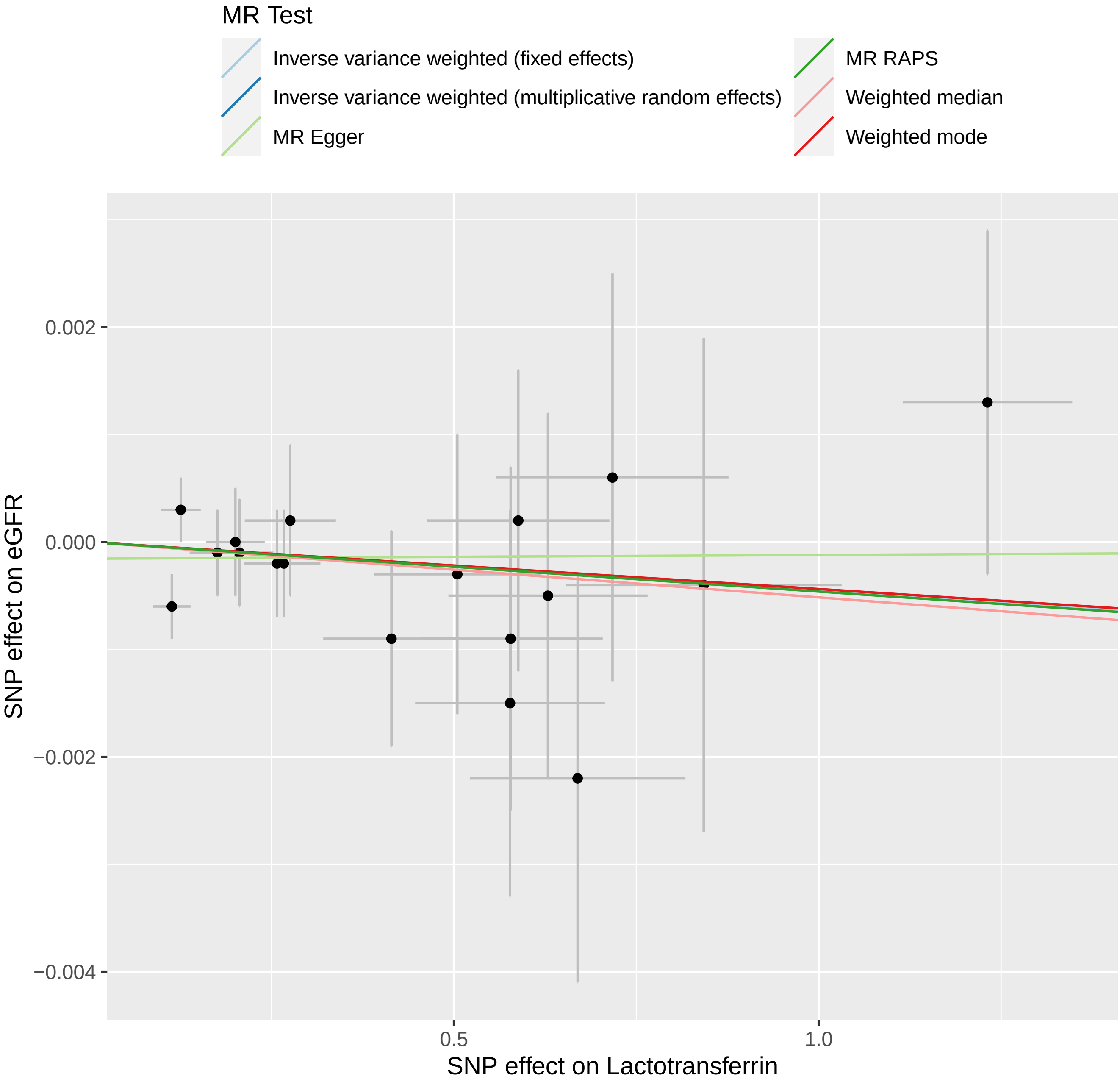

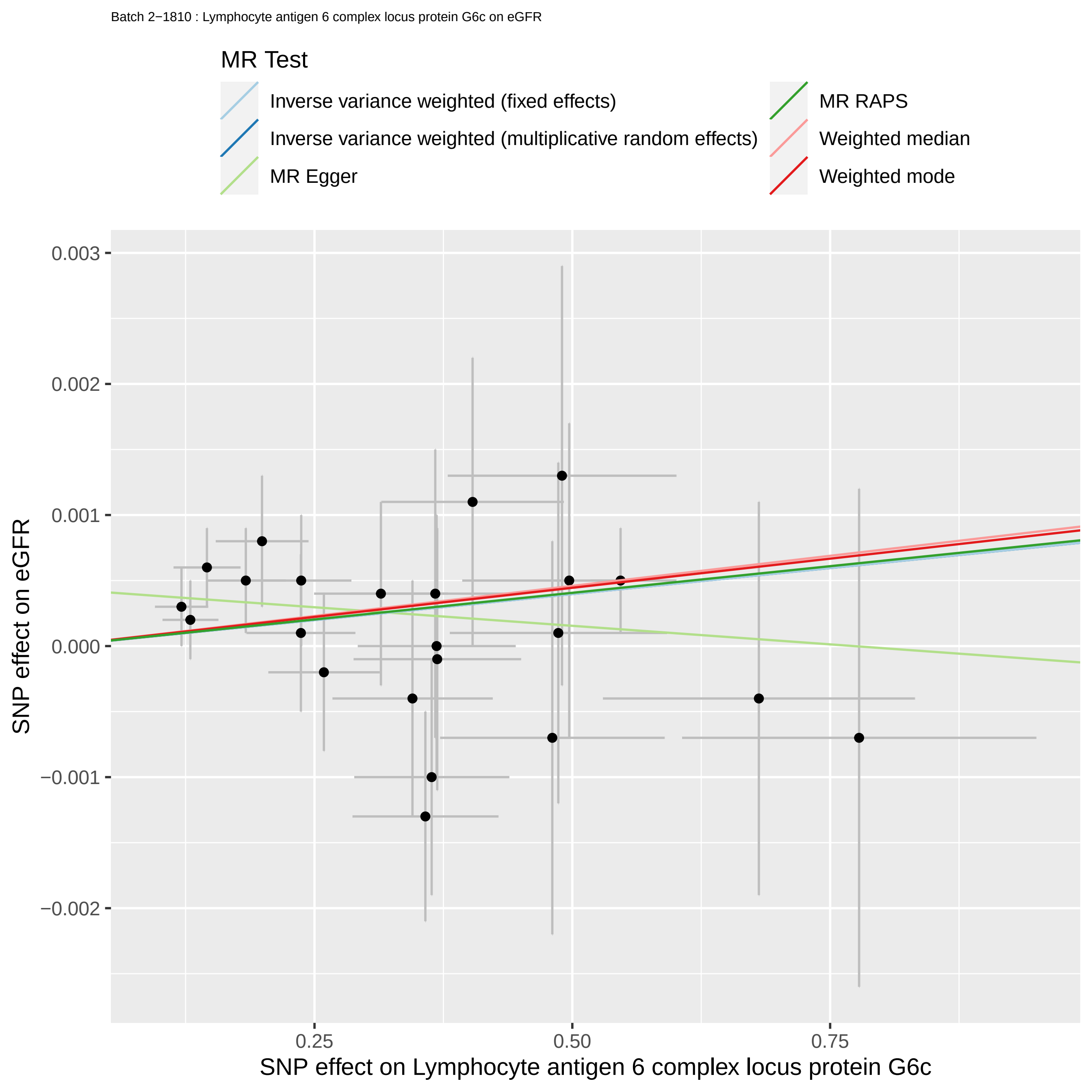

Batch 2−1810 : Lymphocyte antigen 6 complex locus protein G6c on eGFR
MR Test
Inverse variance weighted (fixed effects)
Inverse variance weighted (multiplicative random effects)
MR Egger
MR RAPS
Weighted median
Weighted mode
0.003
0.002
0.001
0.000
−0.001
−0.002
0.25
0.50
0.75
SNP effect on eGFR
SNP effect on Lymphocyte antigen 6 complex locus protein G6c

Batch 2−1811 : Lymphocyte antigen 6 complex locus protein G6d on eGFR

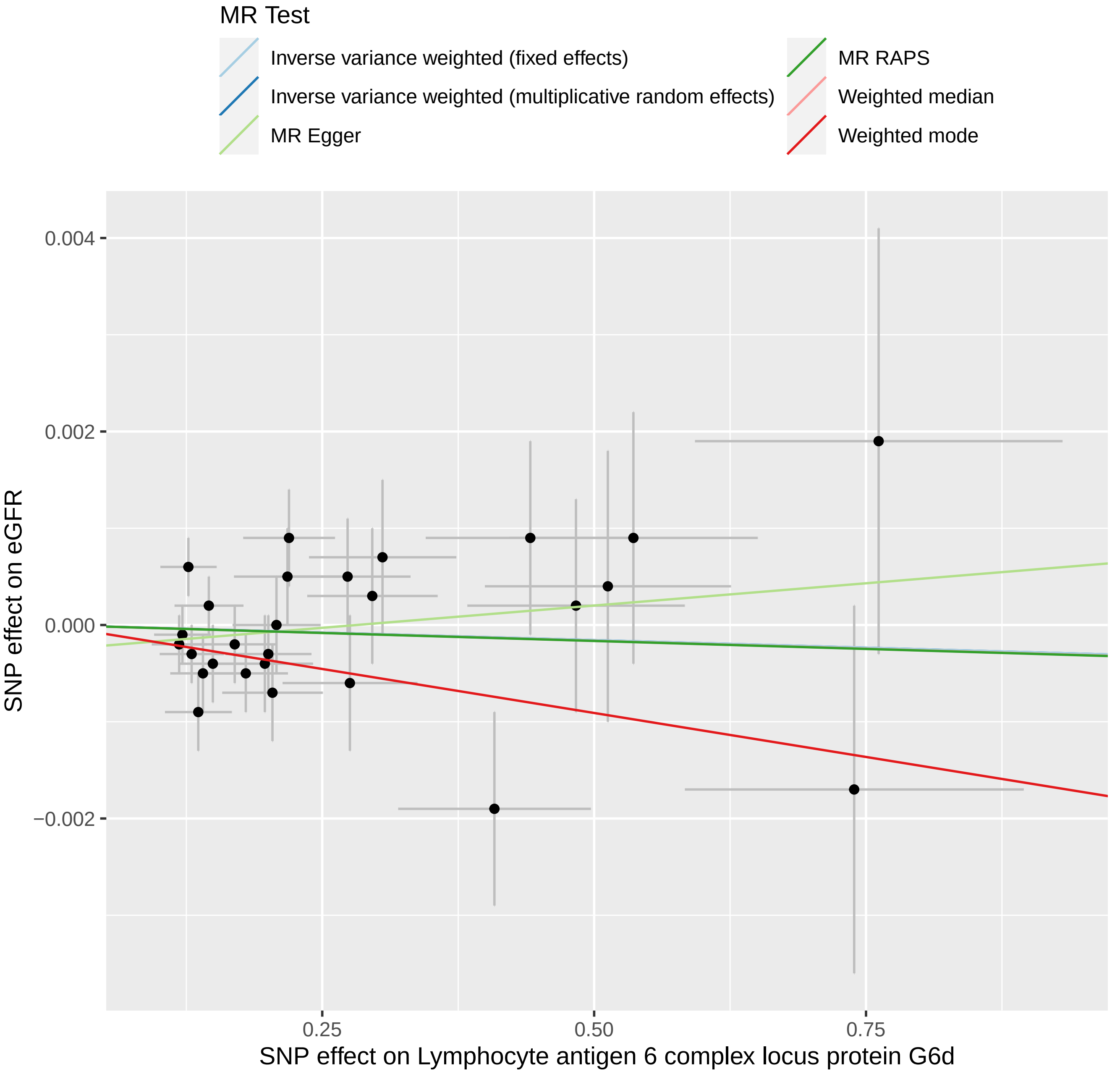

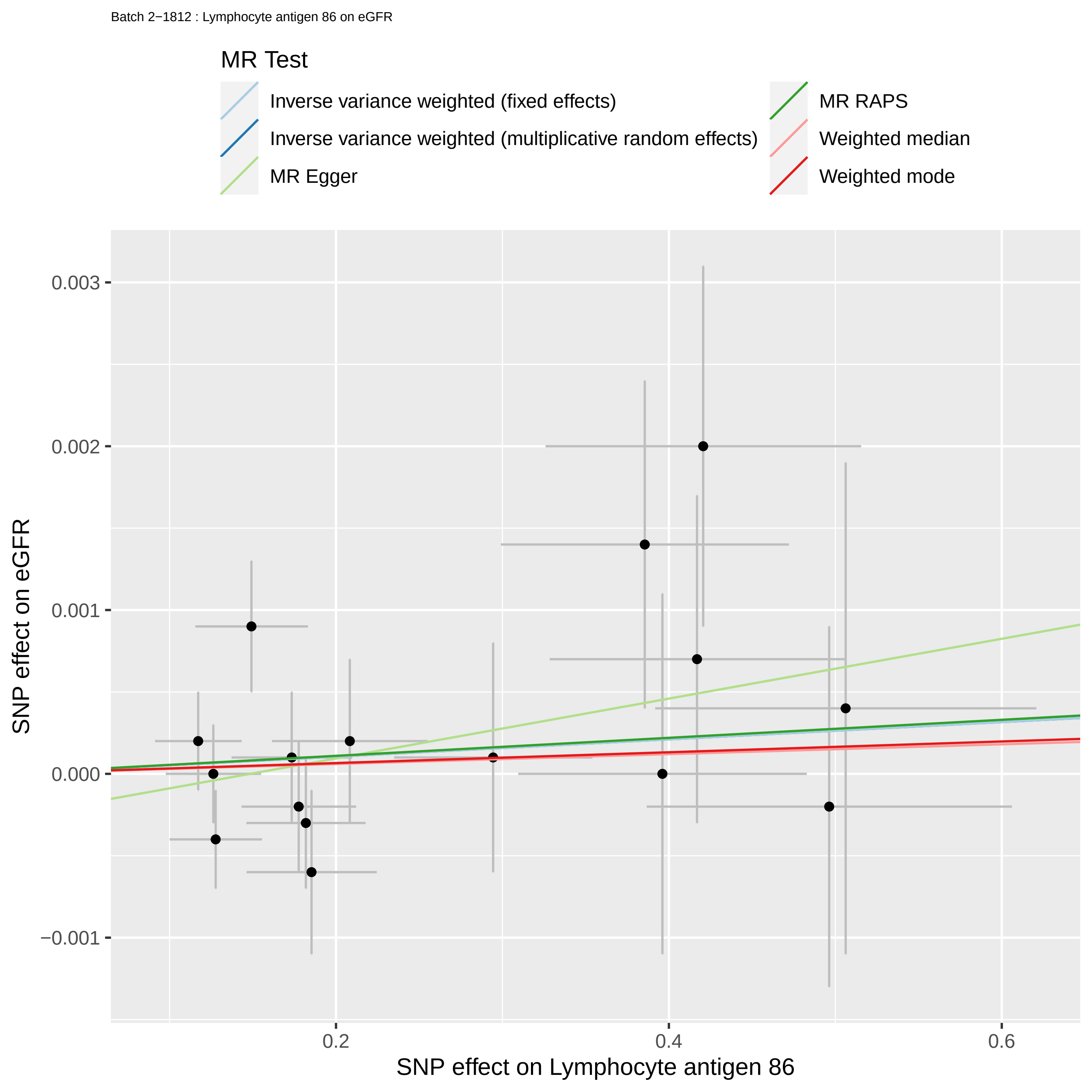
Batch 2−1812 : Lymphocyte antigen 86 on eGFR
MR Test
Inverse variance weighted (fixed effects)
Inverse variance weighted (multiplicative random effects)
MR Egger
MR RAPS
Weighted median
Weighted mode
0.003
0.002
0.001
0.000
−0.001
SNP effect on eGFR
0.2
0.4
0.6
SNP effect on Lymphocyte antigen 86

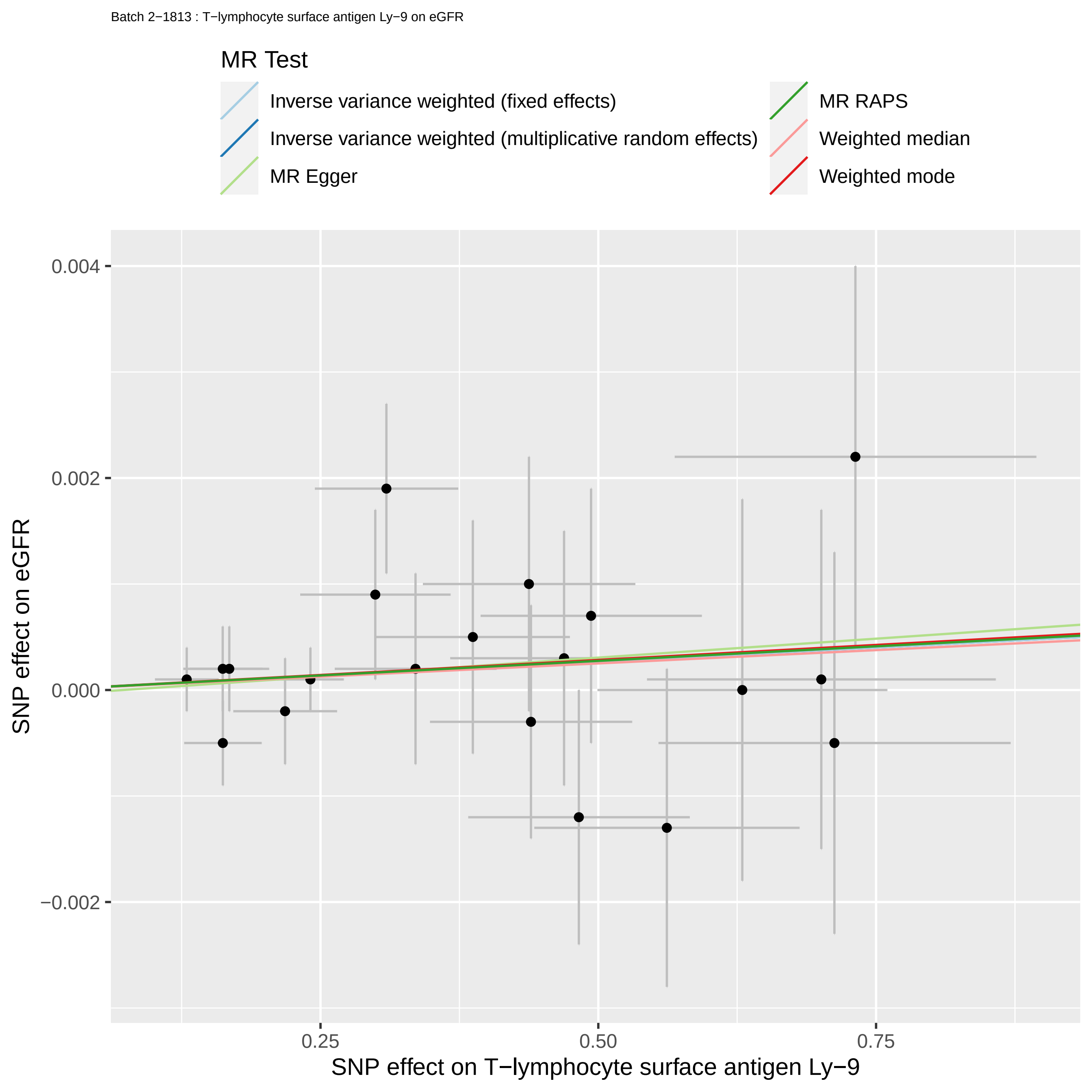
Batch 2−1813 : T−lymphocyte surface antigen Ly−9 on eGFR
MR Test
Inverse variance weighted (fixed effects)
Inverse variance weighted (multiplicative random effects)
MR Egger
MR RAPS
Weighted median
Weighted mode
0.004
0.002
0.000
−0.002
0.25
0.50
0.75
SNP effect on eGFR
SNP effect on T−lymphocyte surface antigen Ly−9

Batch 2−1814 : Lysozyme g−like protein 1 on eGFR

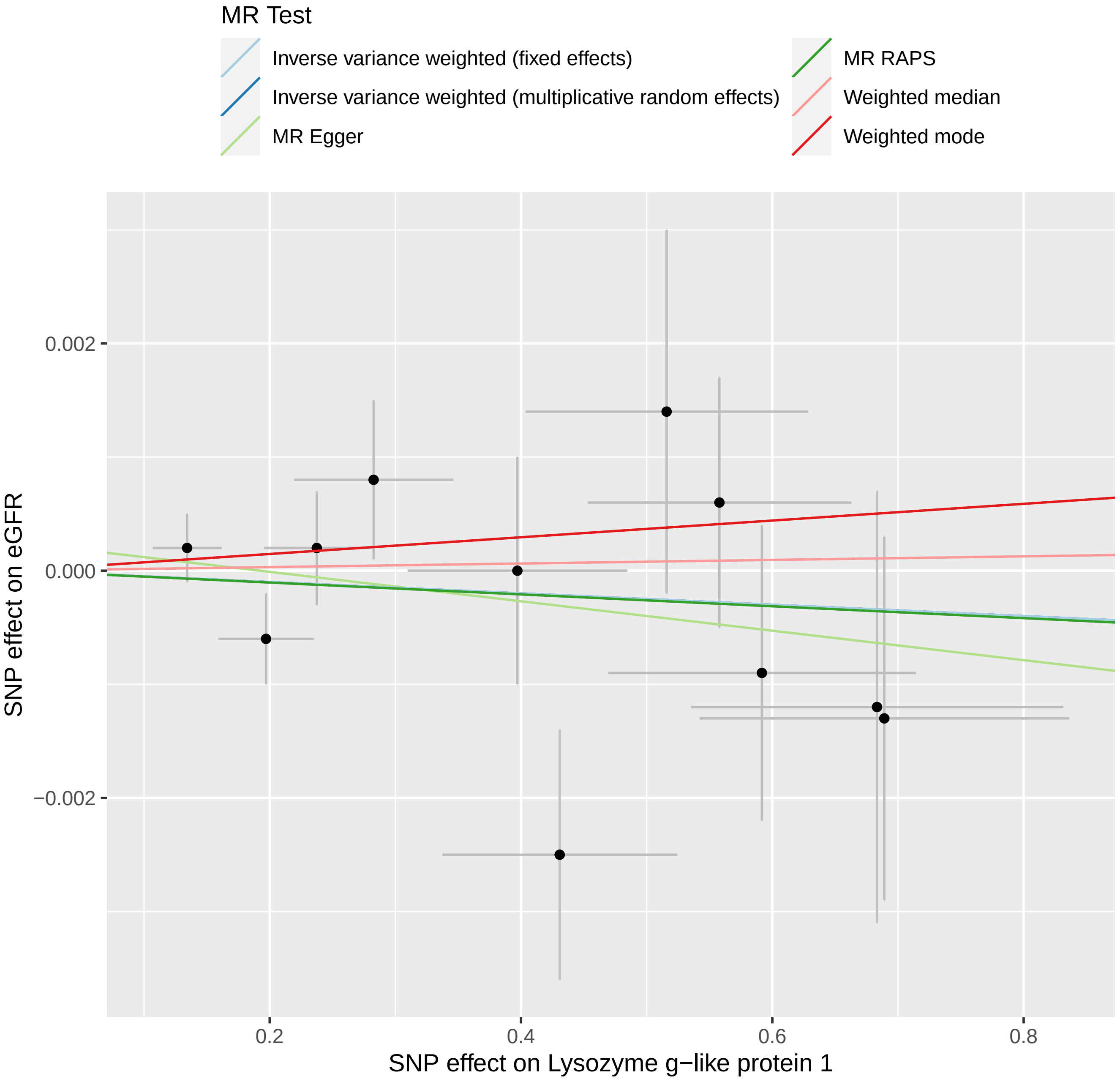

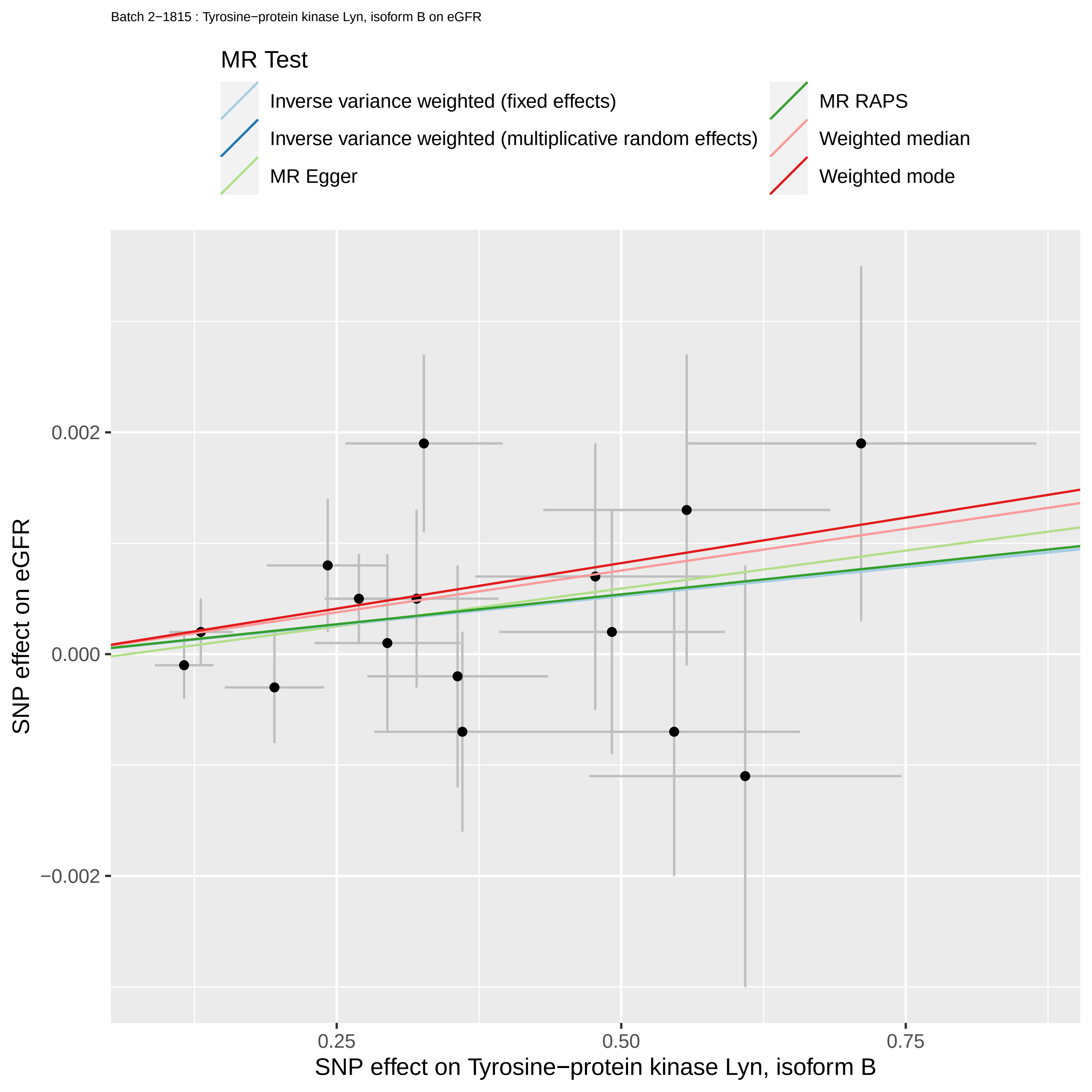
Batch 2−1815 : Tyrosine−protein kinase Lyn, isoform B on eGFR
MR Test
Inverse variance weighted (fixed effects)
Inverse variance weighted (multiplicative random effects)
MR Egger
MR RAPS
Weighted median
Weighted mode
0.002
0.000
−0.002
0.25
0.50
0.75
SNP effect on eGFR
SNP effect on Tyrosine−protein kinase Lyn, isoform B

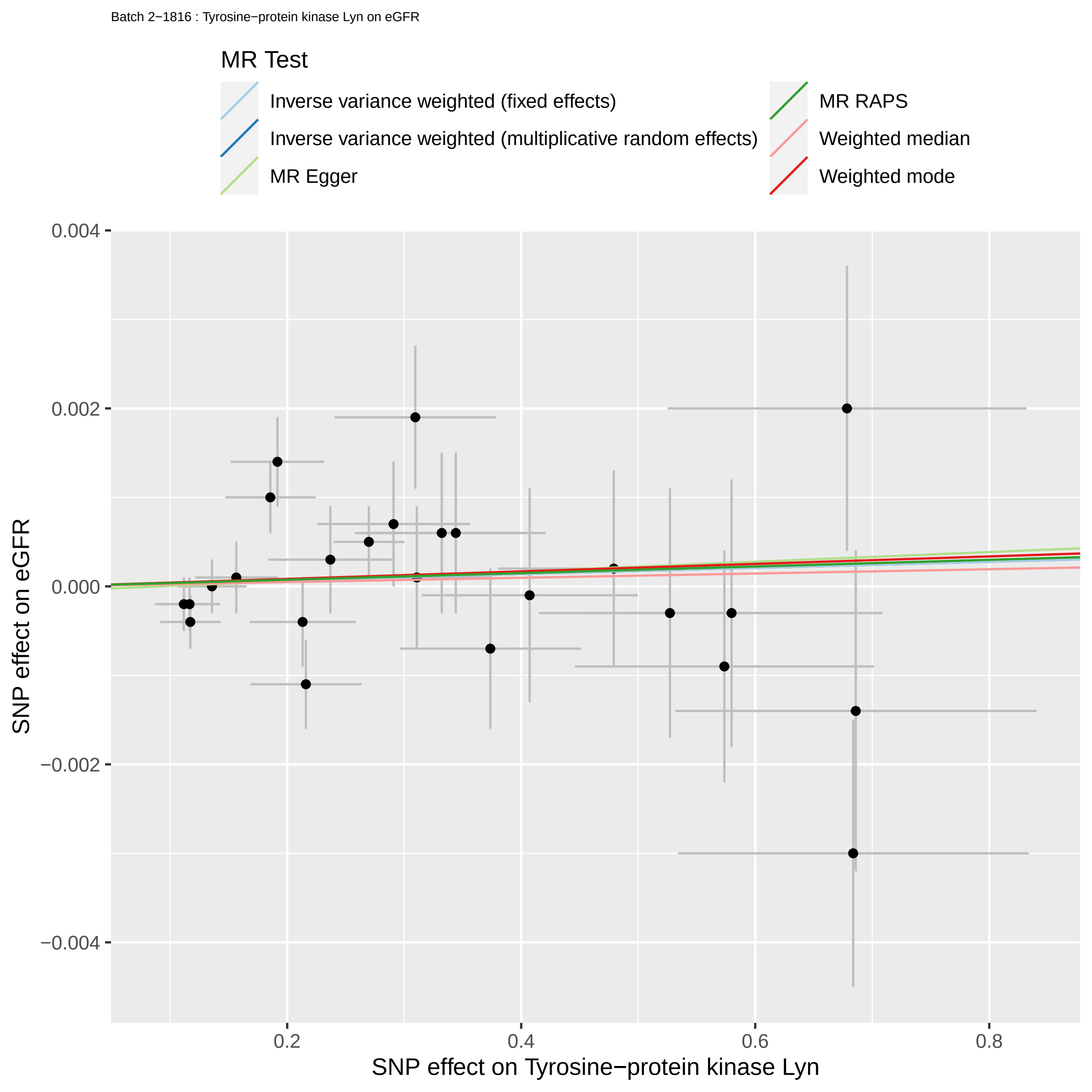
Batch 2−1816 : Tyrosine−protein kinase Lyn on eGFR
MR Test
Inverse variance weighted (fixed effects)
Inverse variance weighted (multiplicative random effects)
MR Egger
MR RAPS
Weighted median
Weighted mode
0.004
0.002
0.000
−0.002
−0.004
0.2
0.4
0.6
0.8
SNP effect on eGFR
SNP effect on Tyrosine−protein kinase Lyn

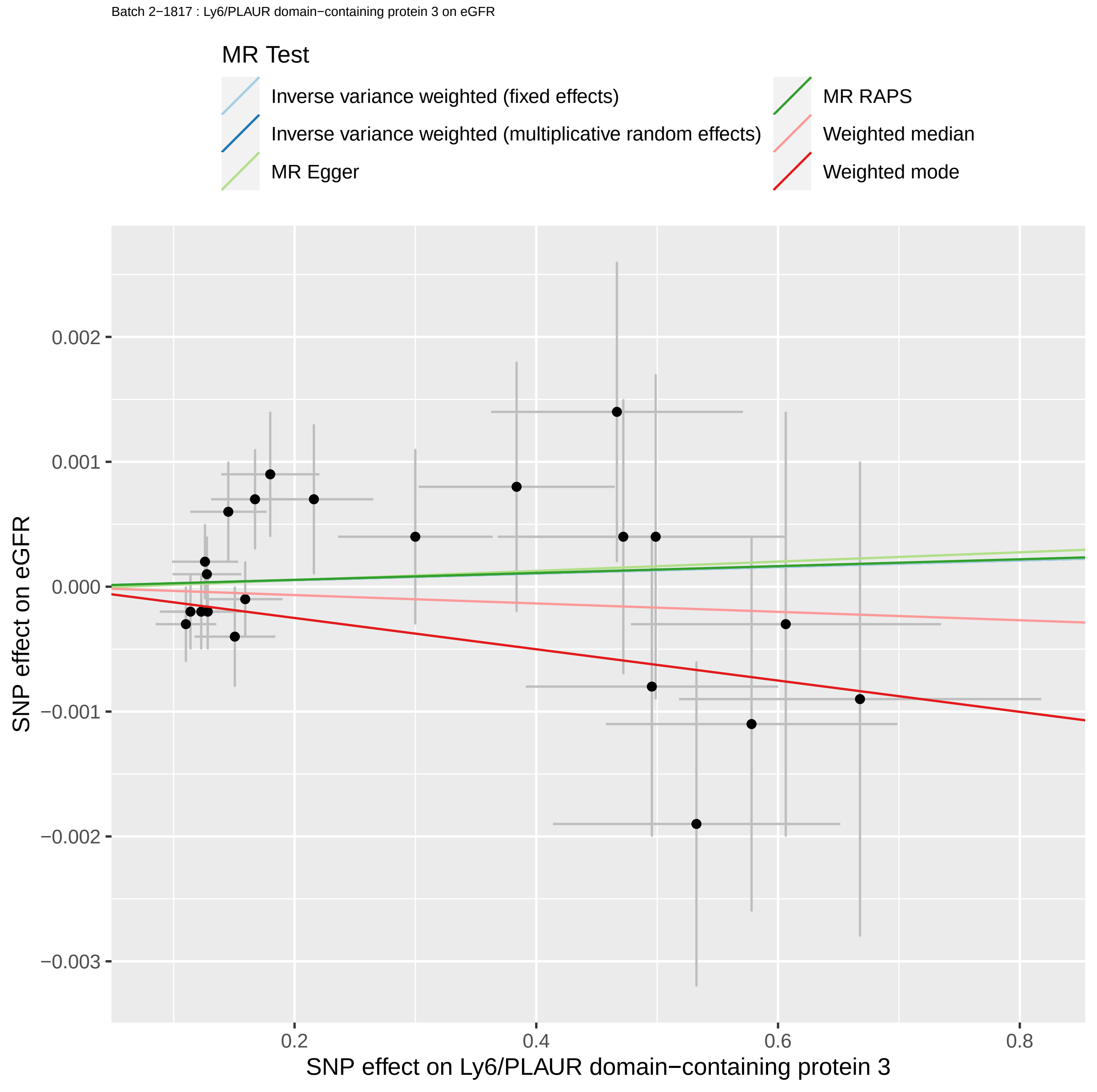
Batch 2−1817 : Ly6/PLAUR domain−containing protein 3 on eGFR
MR Test
Inverse variance weighted (fixed effects)
Inverse variance weighted (multiplicative random effects)
MR Egger
MR RAPS
Weighted median
Weighted mode
0.002
0.001
0.000
−0.001
−0.002
−0.003
SNP effect on eGFR
0.2
0.4
0.6
0.8
SNP effect on Ly6/PLAUR domain−containing protein 3

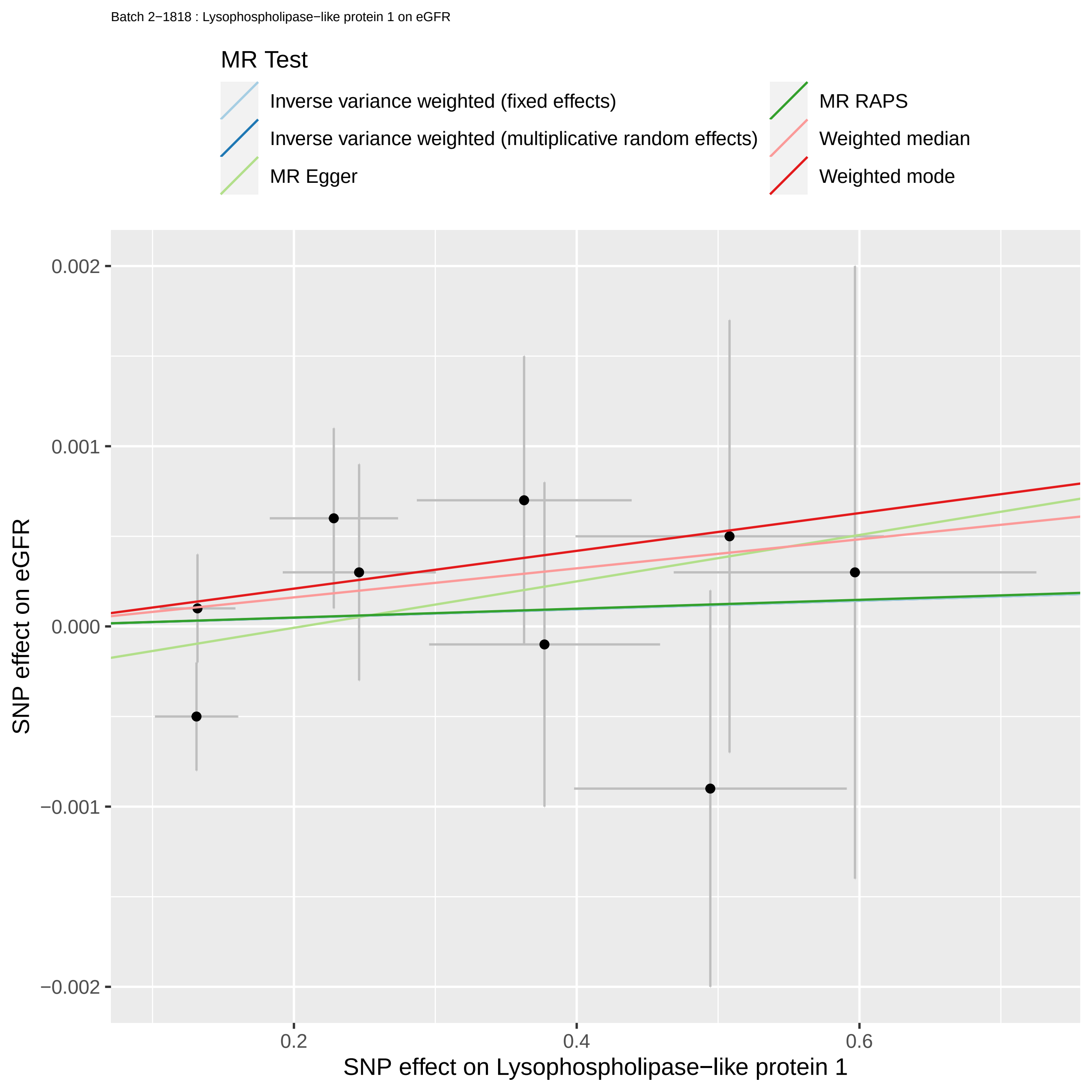

Batch 2−1818 : Lysophospholipase−like protein 1 on eGFR
MR Test
Inverse variance weighted (fixed effects)
Inverse variance weighted (multiplicative random effects)
MR Egger
MR RAPS
Weighted median
Weighted mode
0.002
0.001
0.000
−0.001
−0.002
0.2
0.4
0.6
SNP effect on eGFR
SNP effect on Lysophospholipase−like protein 1

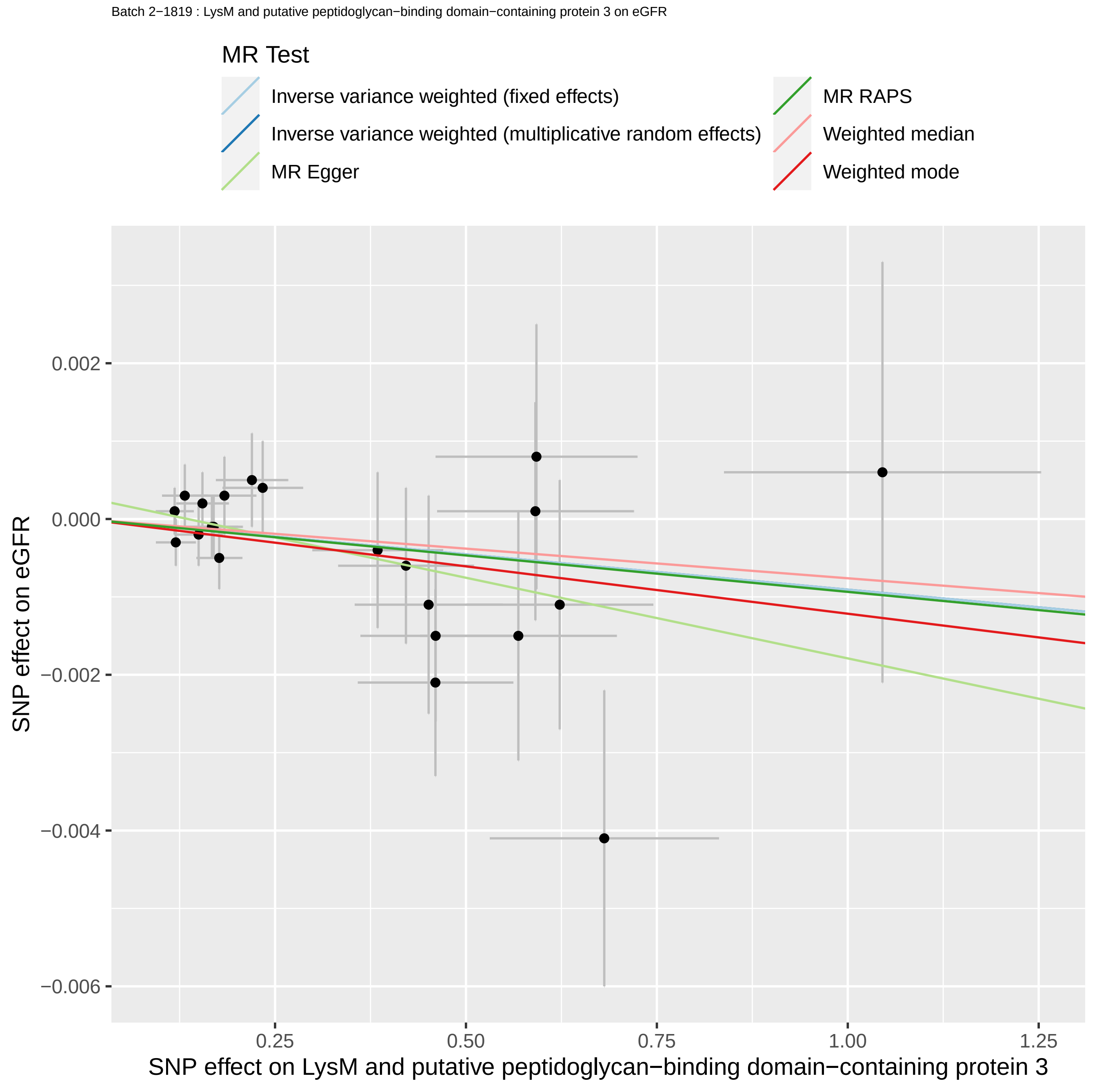
Batch 2−1819 : LysM and putative peptidoglycan−binding domain−containing protein 3 on eGFR
MR Test
Inverse variance weighted (fixed effects)
Inverse variance weighted (multiplicative random effects)
MR Egger
MR RAPS
Weighted median
Weighted mode
0.002
0.000
−0.002
−0.004
−0.006
0.25
0.50
0.75
1.00
1.25
SNP effect on eGFR
SNP effect on LysM and putative peptidoglycan−binding domain−containing protein 3

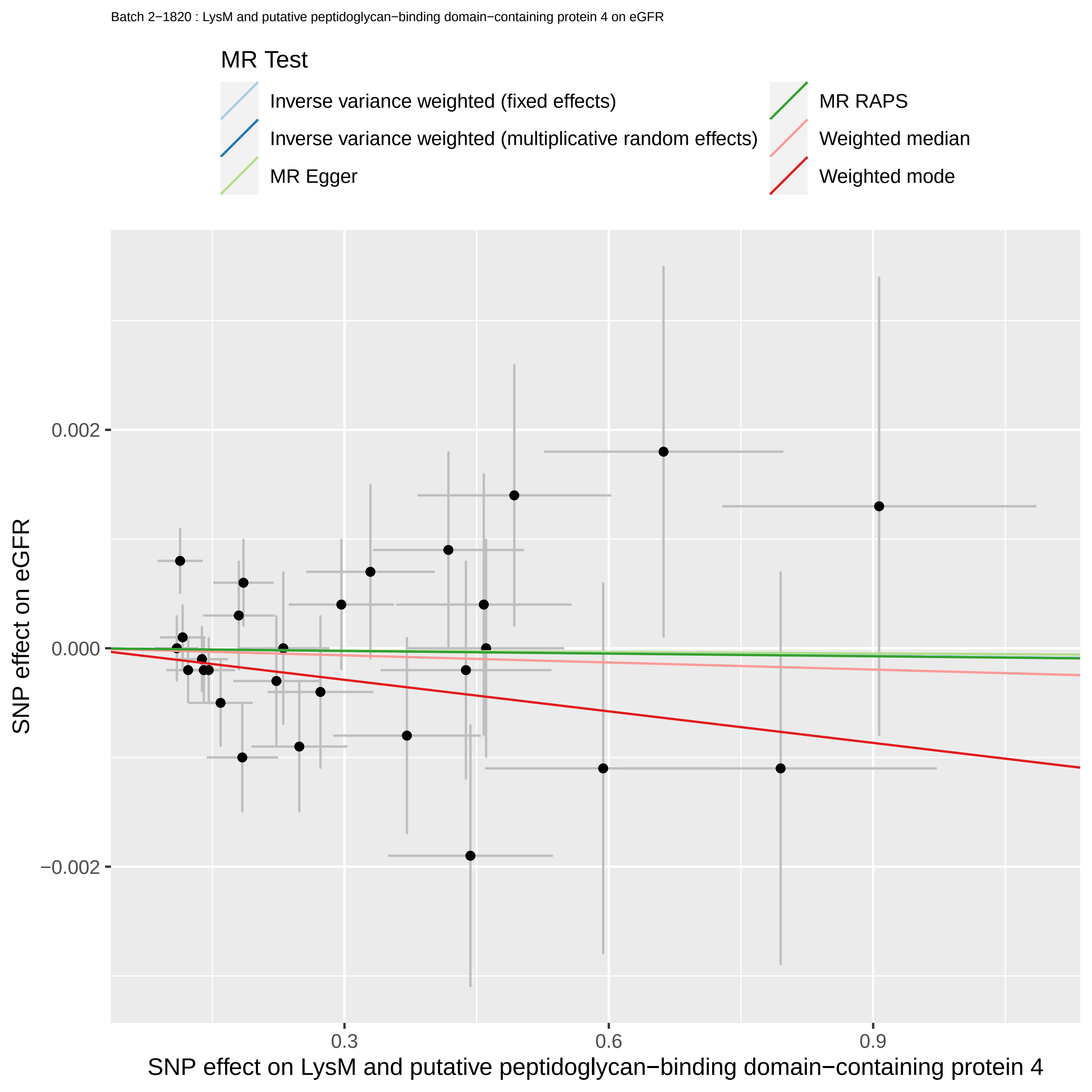

Batch 2−1820 : LysM and putative peptidoglycan−binding domain−containing protein 4 on eGFR
MR Test
Inverse variance weighted (fixed effects)
Inverse variance weighted (multiplicative random effects)
MR Egger
MR RAPS
Weighted median
Weighted mode
0.002
0.000
−0.002
0.3
0.6
0.9
SNP effect on eGFR
SNP effect on LysM and putative peptidoglycan−binding domain−containing protein 4

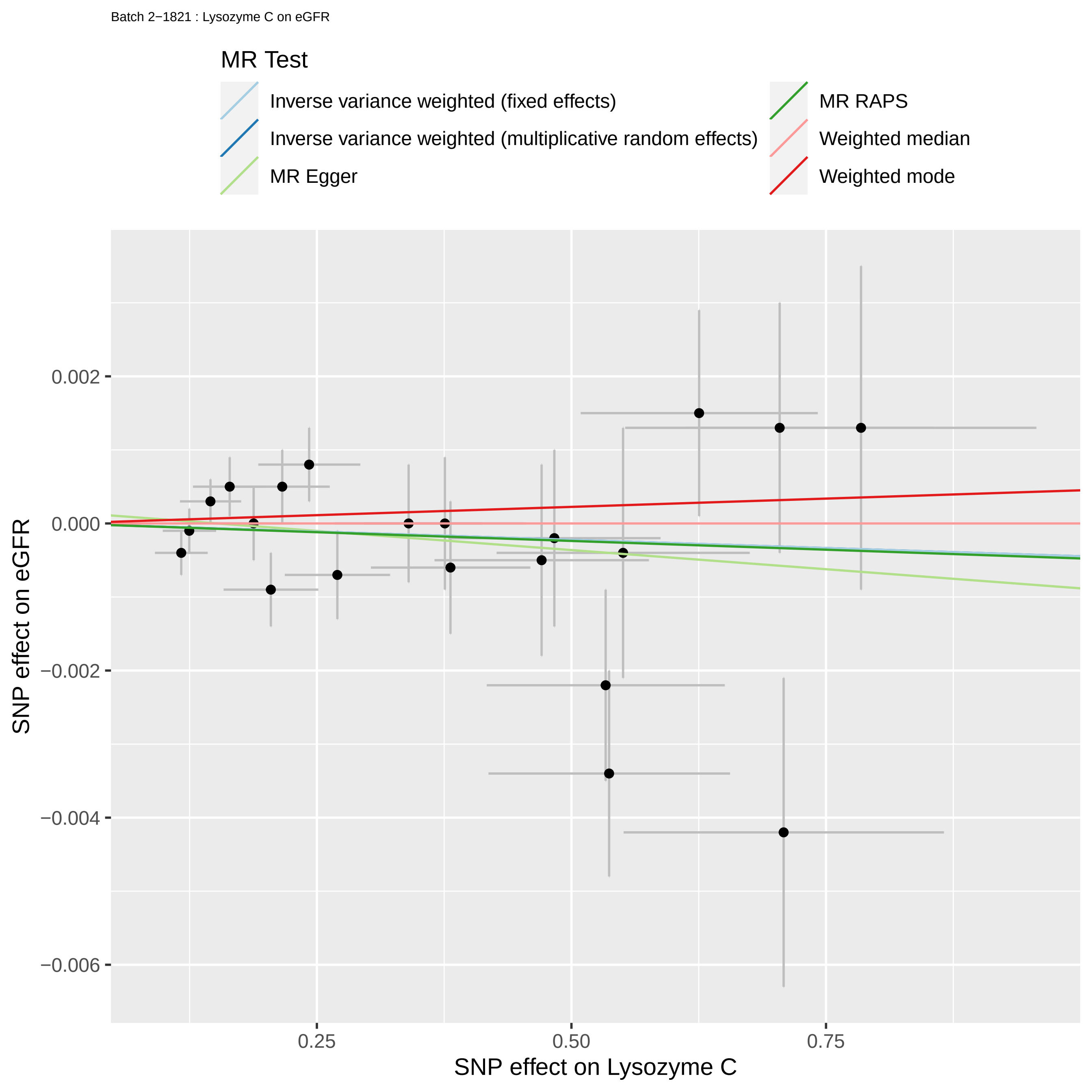
Batch 2−1821 : Lysozyme C on eGFR
MR Test
Inverse variance weighted (fixed effects)
Inverse variance weighted (multiplicative random effects)
MR Egger
MR RAPS
Weighted median
Weighted mode
0.002
0.000
−0.002
−0.004
−0.006
0.25
0.50
0.75
SNP effect on eGFR
SNP effect on Lysozyme C

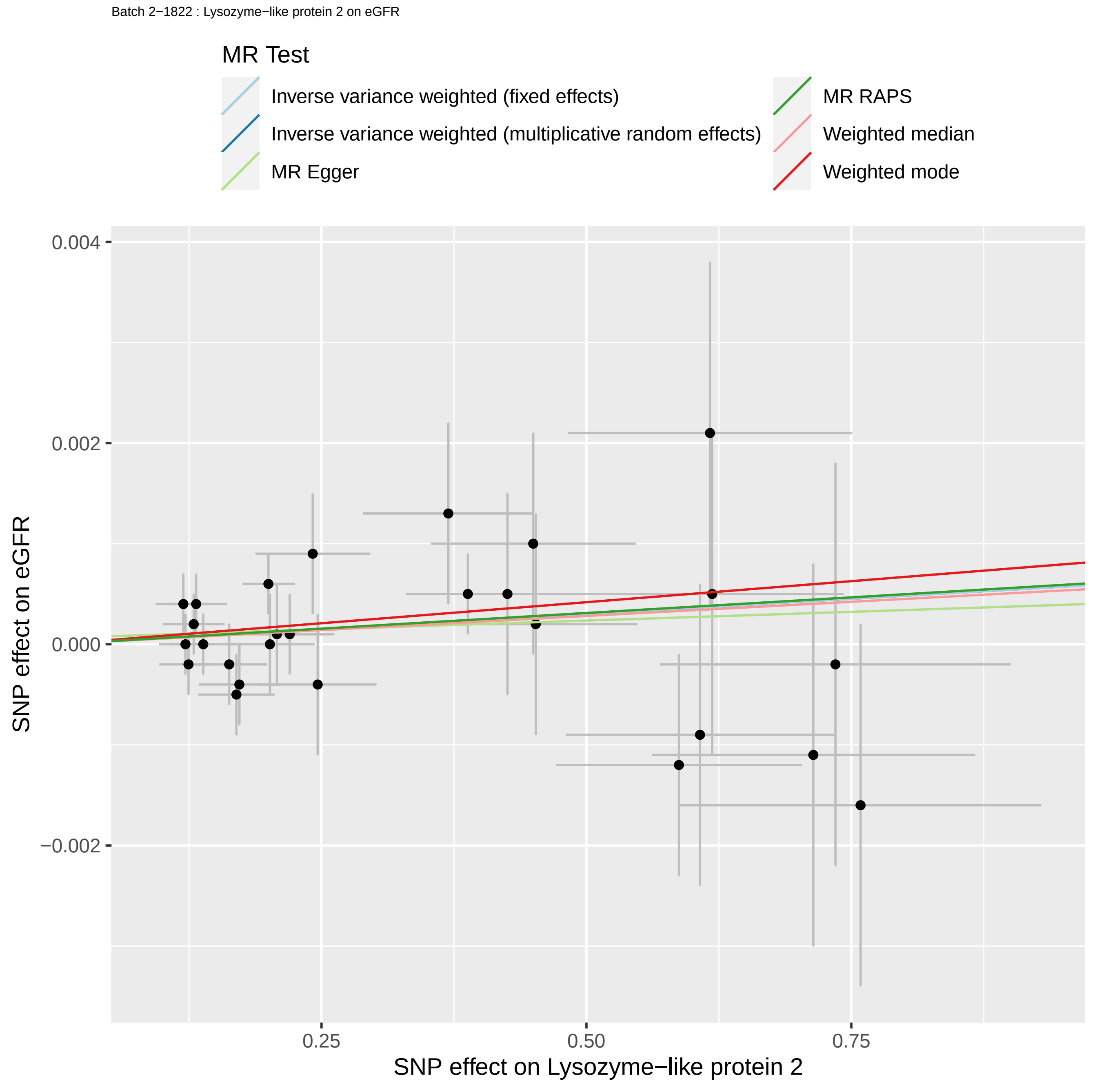
Batch 2−1822 : Lysozyme−like protein 2 on eGFR
MR Test
Inverse variance weighted (fixed effects)
Inverse variance weighted (multiplicative random effects)
MR Egger
MR RAPS
Weighted median
Weighted mode
0.004
0.002
0.000
−0.002
0.25
0.50
0.75
SNP effect on eGFR
SNP effect on Lysozyme−like protein 2

Batch 2−1823 : Cation−dependent mannose−6−phosphate receptor on eGFR

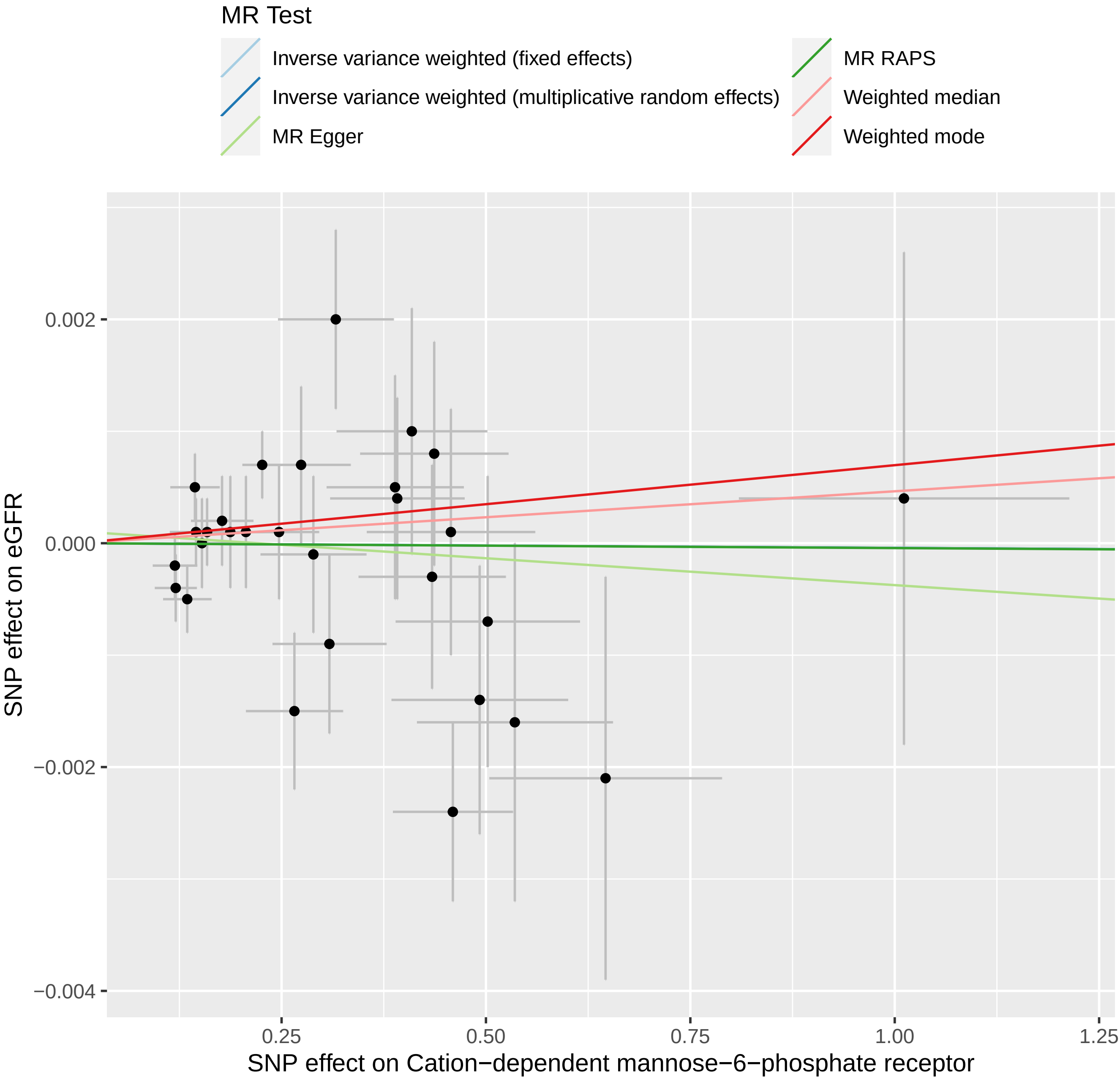

Batch 2−1824 : O−acetyl−ADP−ribose deacetylase MACROD1 on eGFR

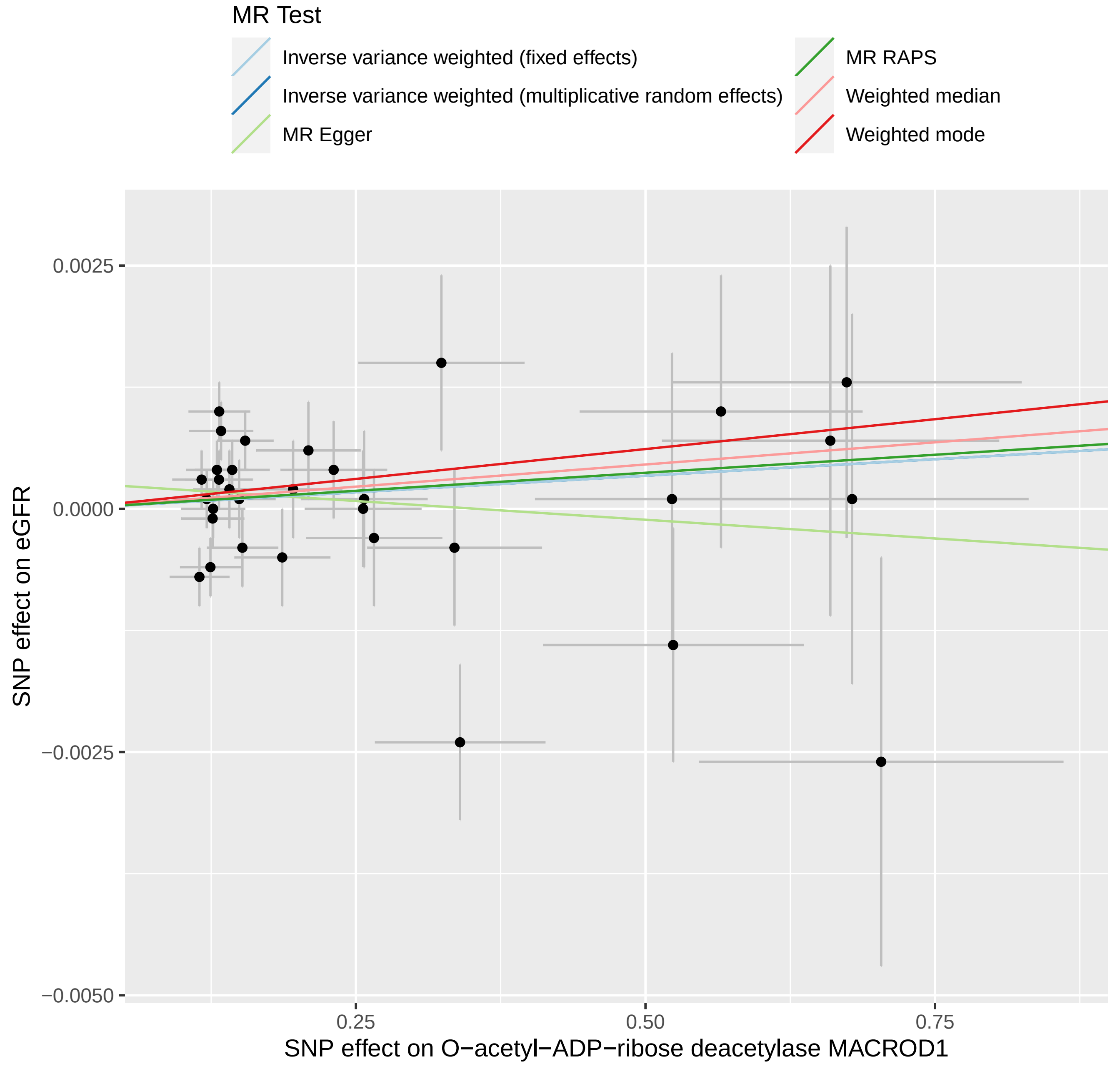

Batch 2−1825 : Mitotic spindle assembly checkpoint protein MAD1 on eGFR

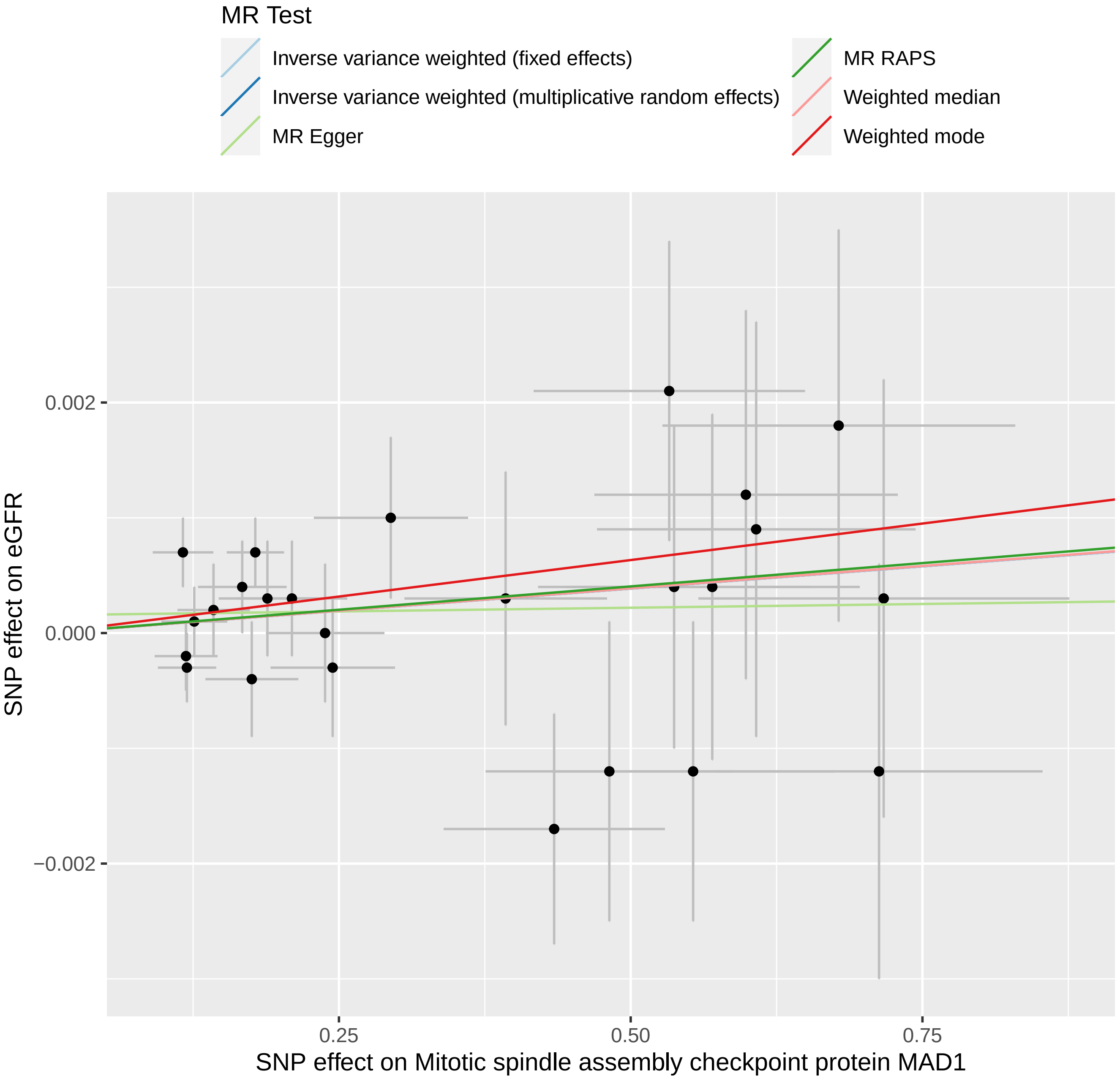

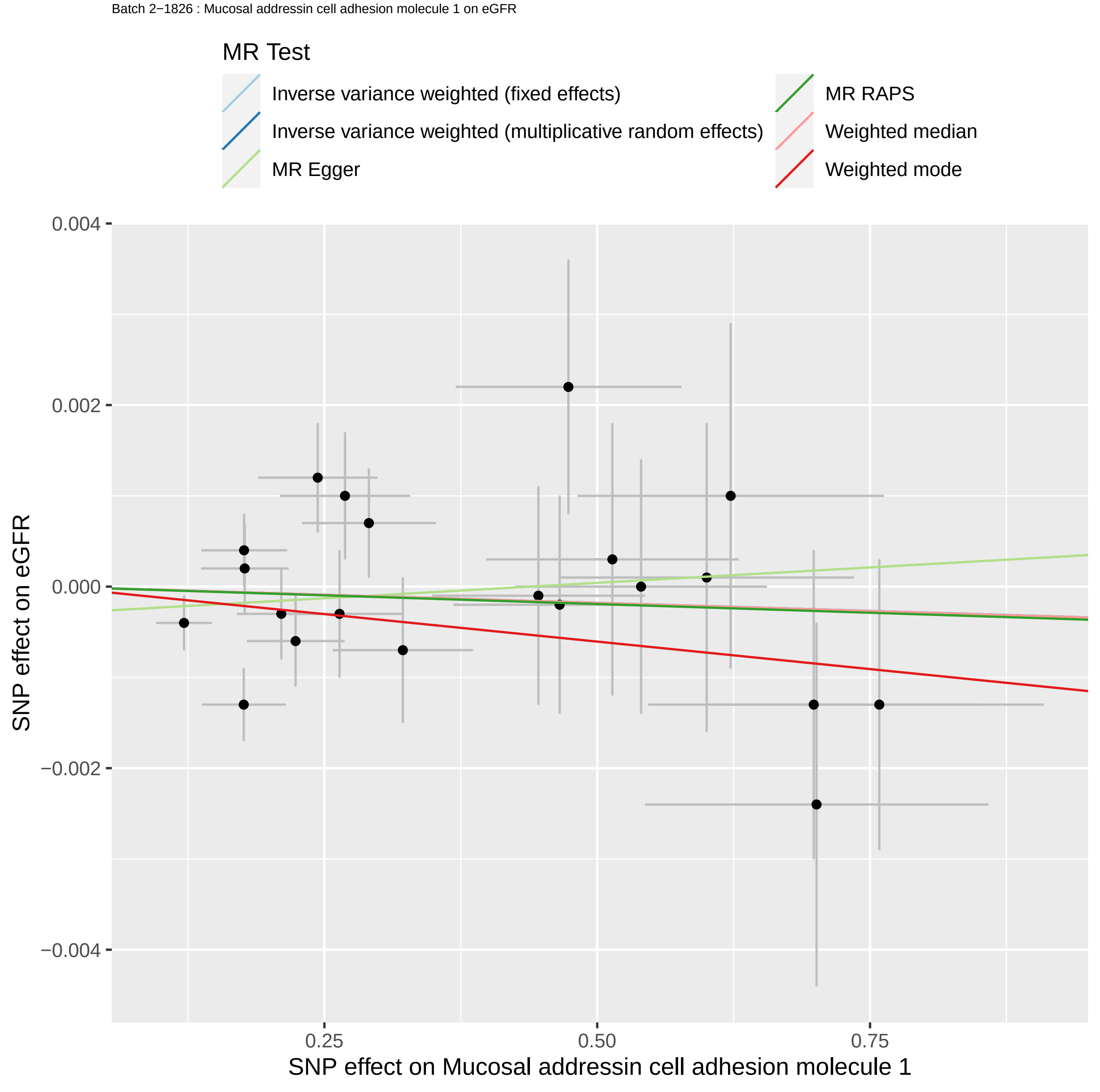

Batch 2−1826 : Mucosal addressin cell adhesion molecule 1 on eGFR
MR Test
Inverse variance weighted (fixed effects)
Inverse variance weighted (multiplicative random effects)
MR Egger
MR RAPS
Weighted median
Weighted mode
0.004
0.002
0.000
−0.002
−0.004
0.25
0.50
0.75
SNP effect on eGFR
SNP effect on Mucosal addressin cell adhesion molecule 1

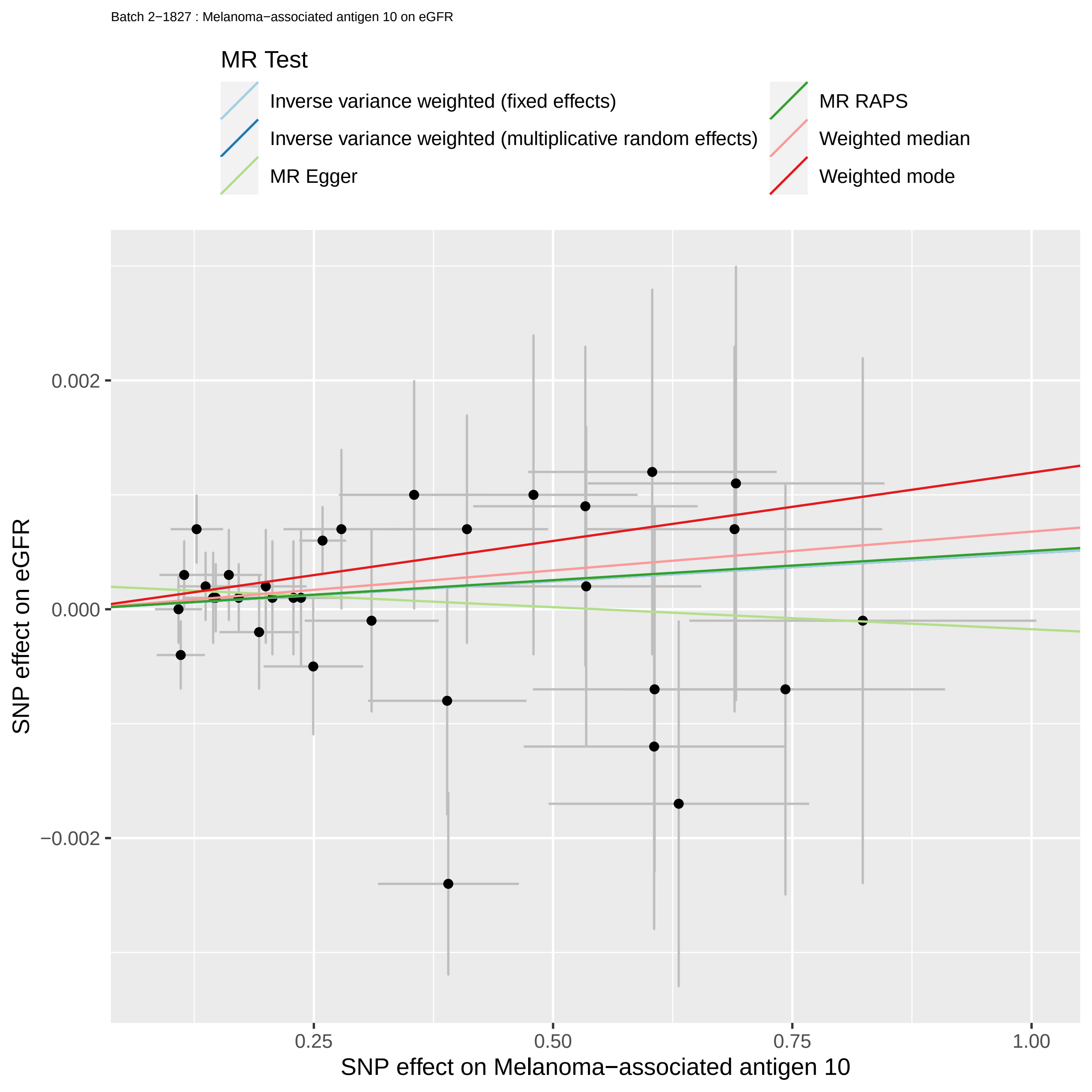

Batch 2−1827 : Melanoma−associated antigen 10 on eGFR
MR Test
Inverse variance weighted (fixed effects)
Inverse variance weighted (multiplicative random effects)
MR Egger
MR RAPS
Weighted median
Weighted mode
0.002
0.000
−0.002
0.25
0.50
0.75
1.00
SNP effect on eGFR
SNP effect on Melanoma−associated antigen 10

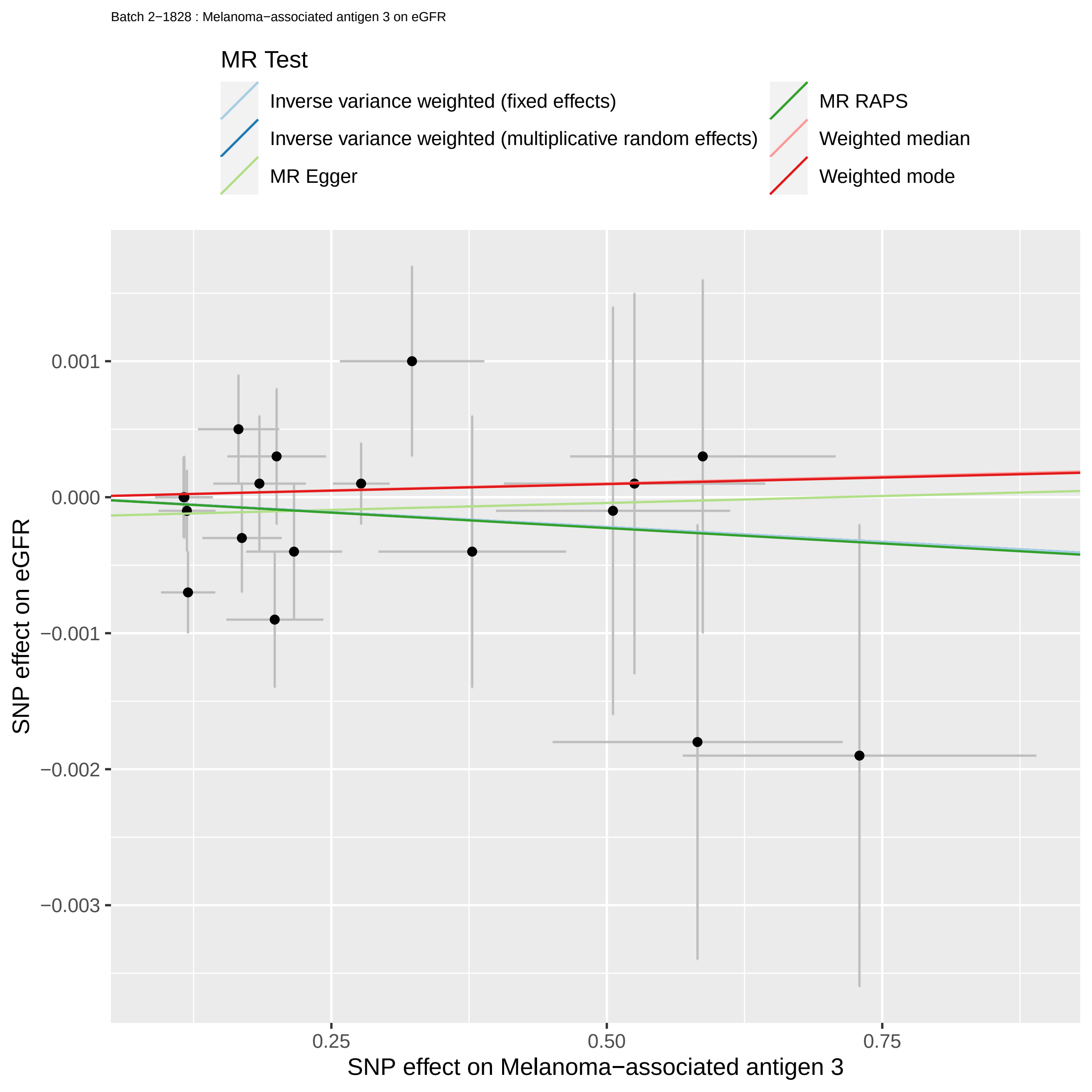
Batch 2−1828 : Melanoma−associated antigen 3 on eGFR
MR Test
Inverse variance weighted (fixed effects)
Inverse variance weighted (multiplicative random effects)
MR Egger
MR RAPS
Weighted median
Weighted mode
0.001
0.000
−0.001
−0.002
−0.003
SNP effect on eGFR
0.25
0.50
0.75
SNP effect on Melanoma−associated antigen 3

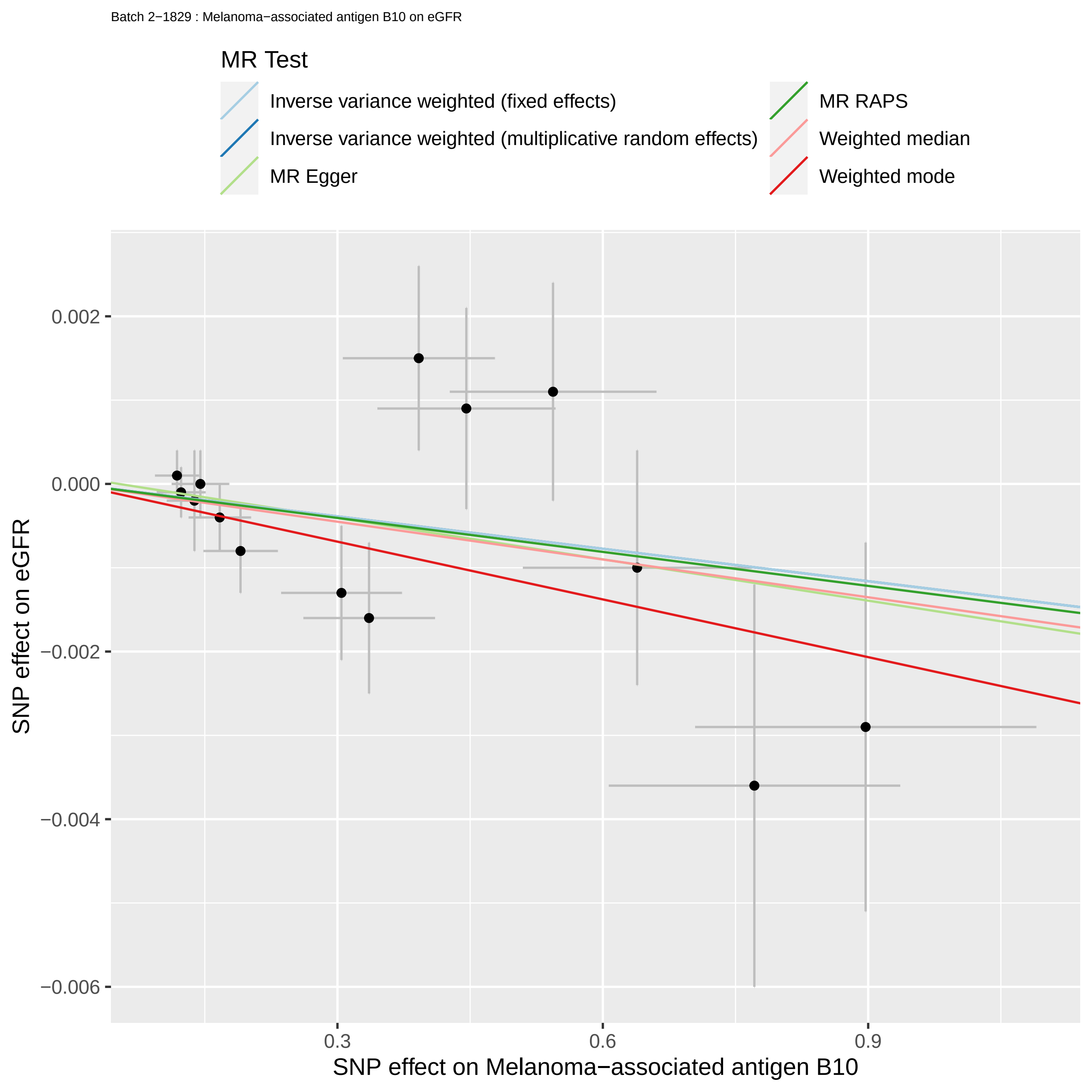
Batch 2−1829 : Melanoma−associated antigen B10 on eGFR
MR Test
Inverse variance weighted (fixed effects)
Inverse variance weighted (multiplicative random effects)
MR Egger
MR RAPS
Weighted median
Weighted mode
0.002
0.000
−0.002
−0.004
−0.006
0.3
0.6
0.9
SNP effect on eGFR
SNP effect on Melanoma−associated antigen B10

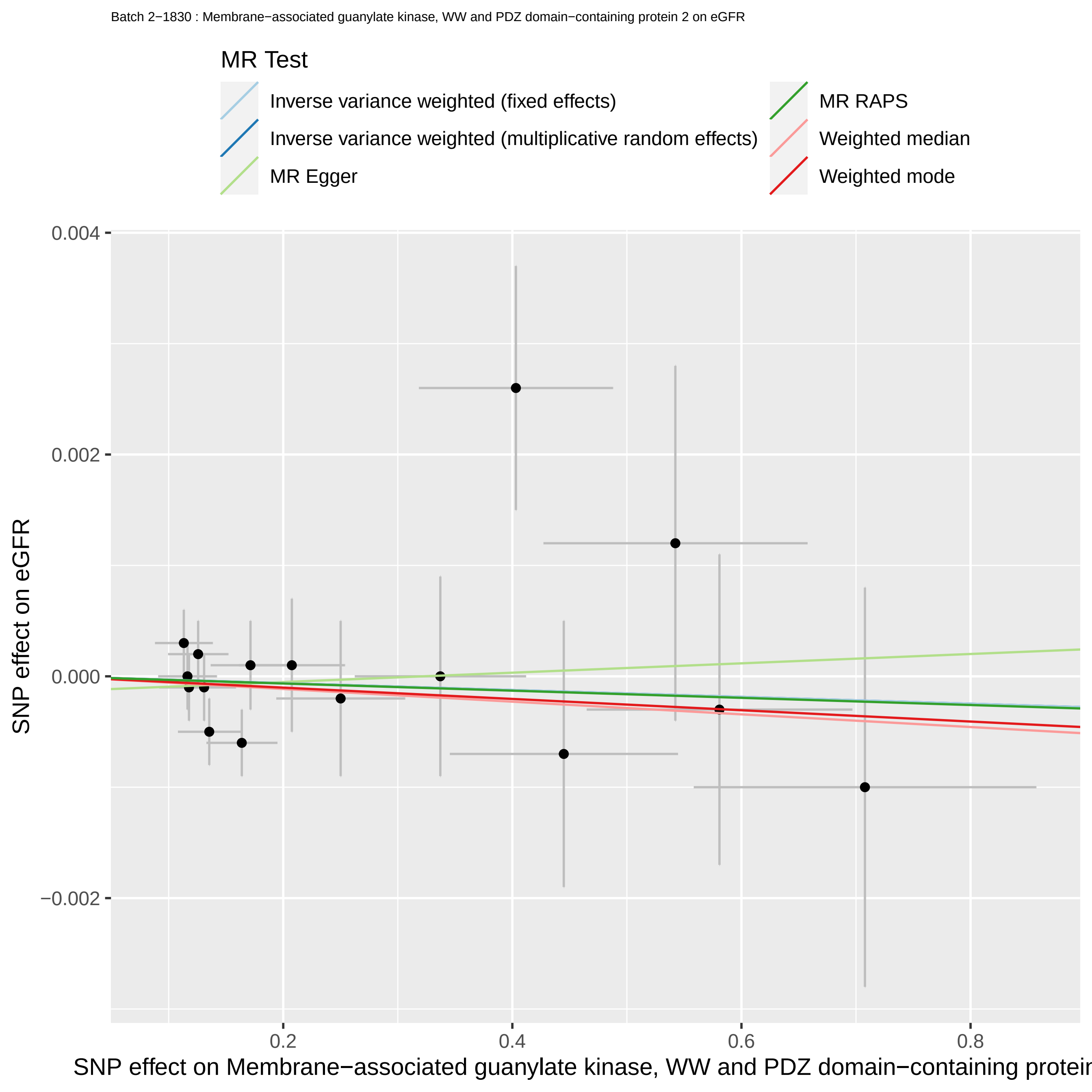

Batch 2−1830 : Membrane−associated guanylate kinase, WW and PDZ domain−containing protein 2 on eGFR
MR Test
Inverse variance weighted (fixed effects)
Inverse variance weighted (multiplicative random effects)
MR Egger
MR RAPS
Weighted median
Weighted mode
0.004
0.002
0.000
−0.002
0.2
0.4
0.6
0.8
SNP effect on eGFR
SNP effect on Membrane−associated guanylate kinase, WW and PDZ domain−containing protein

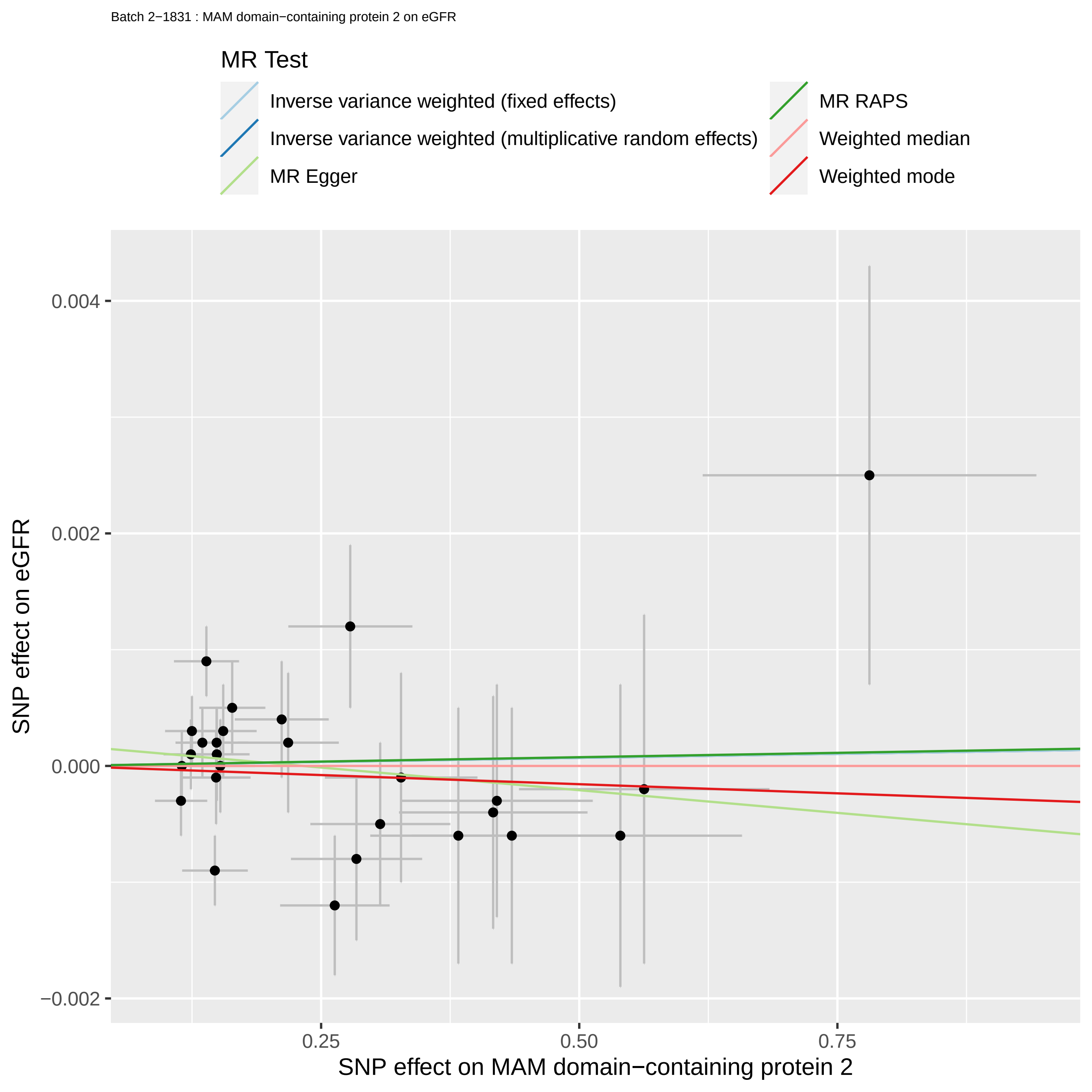

Batch 2−1831 : MAM domain−containing protein 2 on eGFR
MR Test
Inverse variance weighted (fixed effects)
Inverse variance weighted (multiplicative random effects)
MR Egger
MR RAPS
Weighted median
Weighted mode
SNP effect on eGFR
0.004
0.002
0.000
−0.002
0.25
0.50
0.75
SNP effect on MAM domain−containing protein 2

Batch 2−1832 : Mannosyl−oligosaccharide 1,2−alpha−mannosidase IA on eGFR

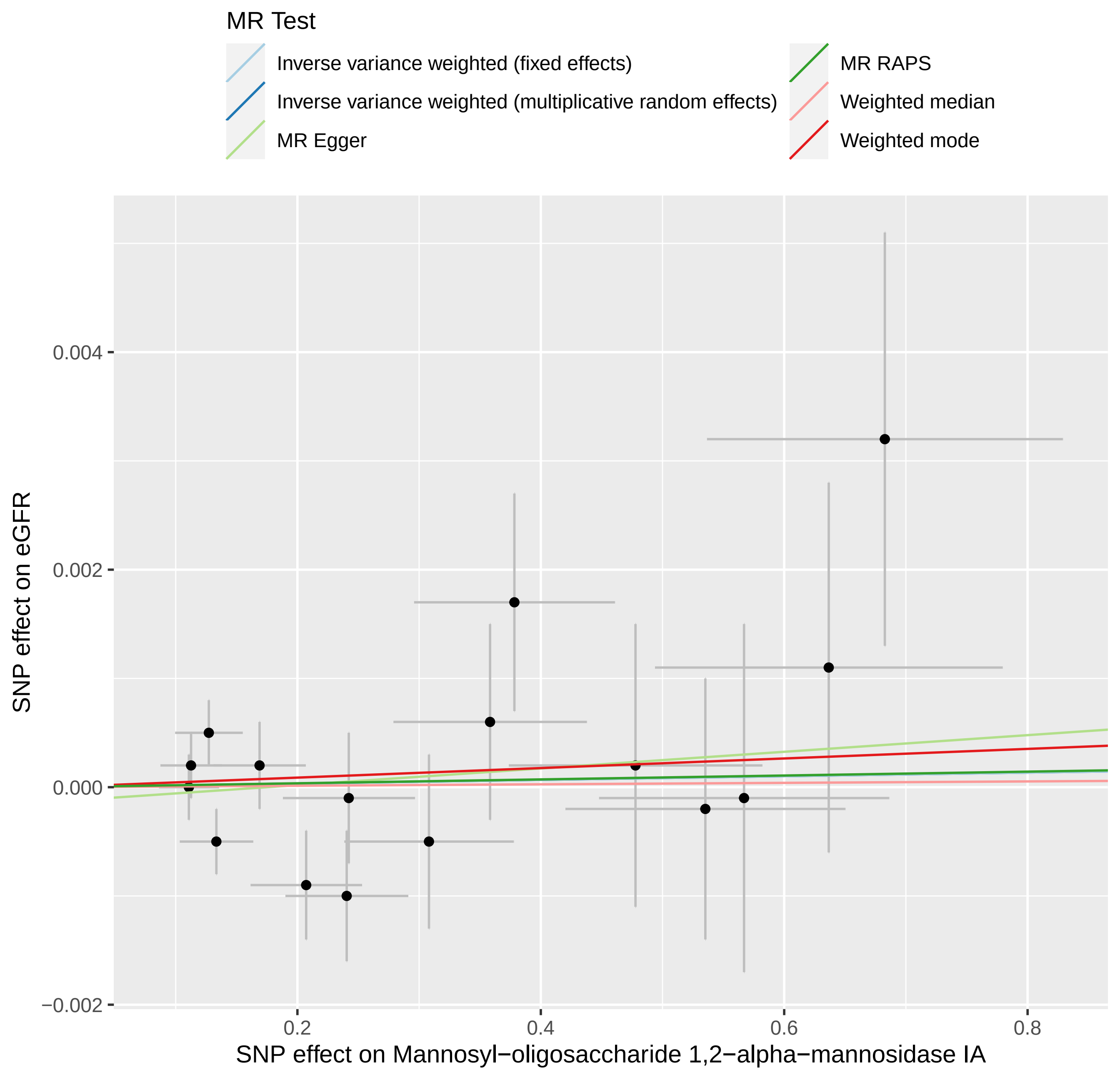

Batch 2−1833 : Mannosyl−oligosaccharide 1,2−alpha−mannosidase IB on eGFR

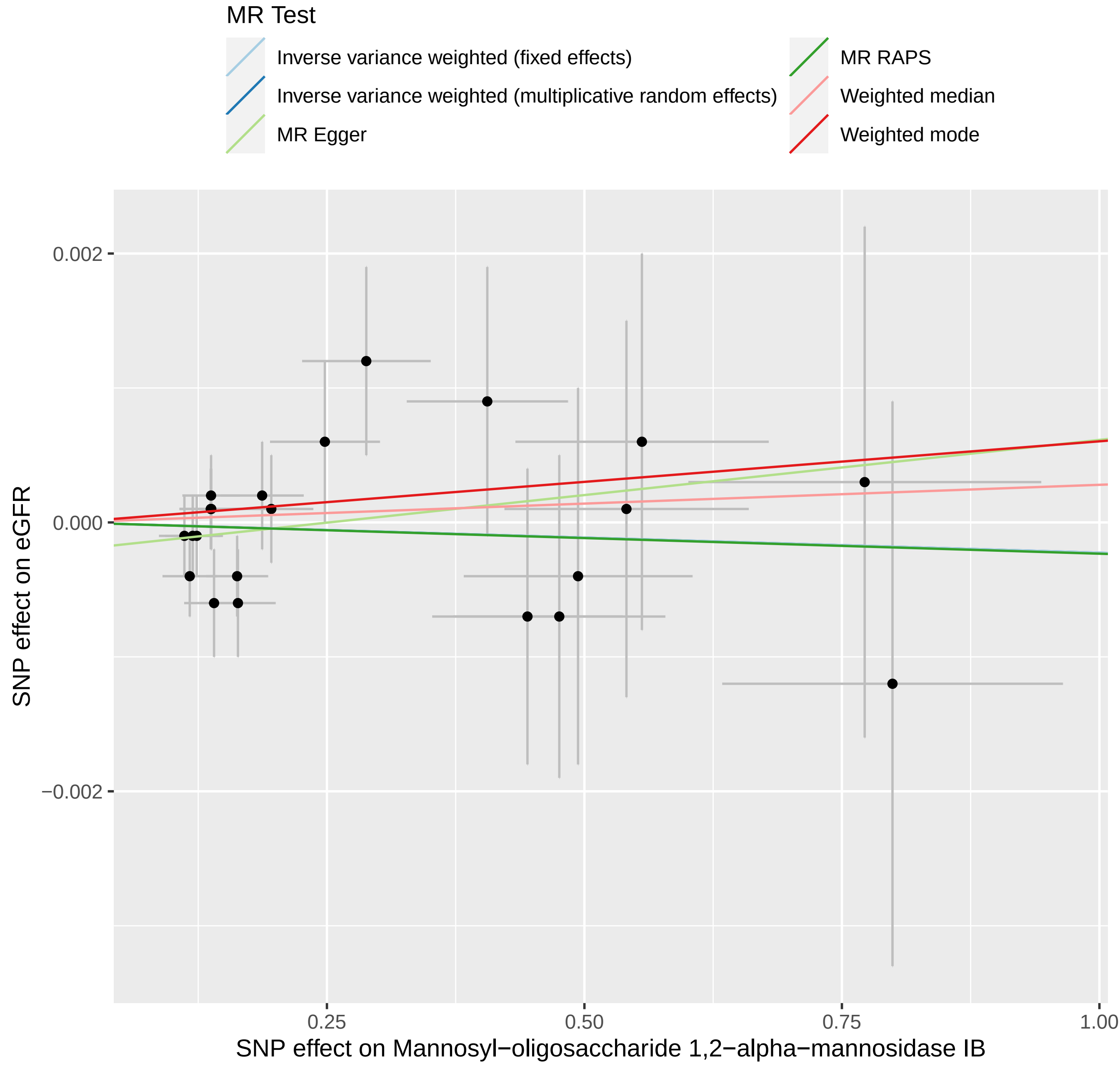

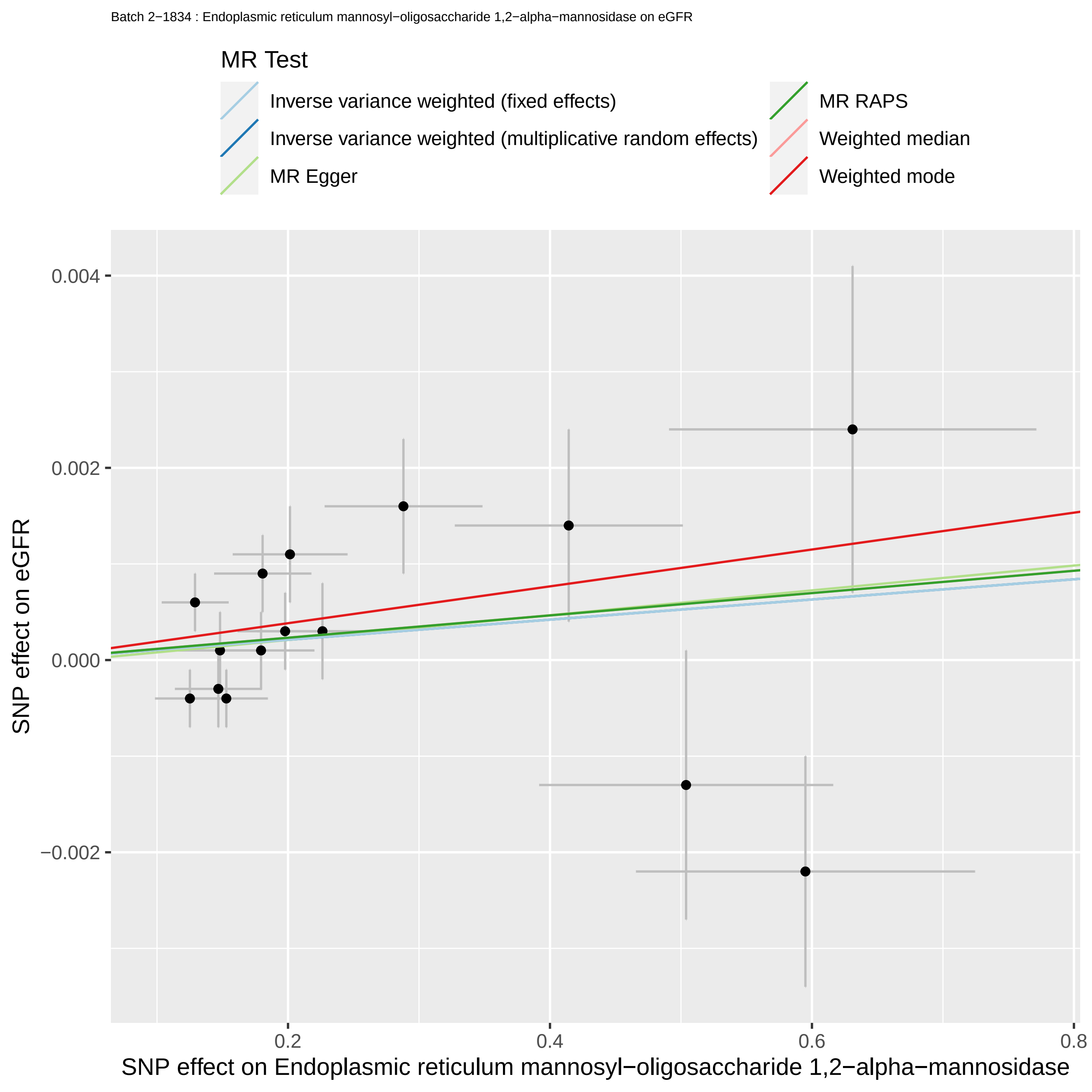

Batch 2−1834 : Endoplasmic reticulum mannosyl−oligosaccharide 1,2−alpha−mannosidase on eGFR
MR Test
Inverse variance weighted (fixed effects)
Inverse variance weighted (multiplicative random effects)
MR Egger
MR RAPS
Weighted median
Weighted mode
SNP effect on eGFR
0.004
0.002
0.000
−0.002
0.2
0.4
0.6
0.8
SNP effect on Endoplasmic reticulum mannosyl−oligosaccharide 1,2−alpha−mannosidase

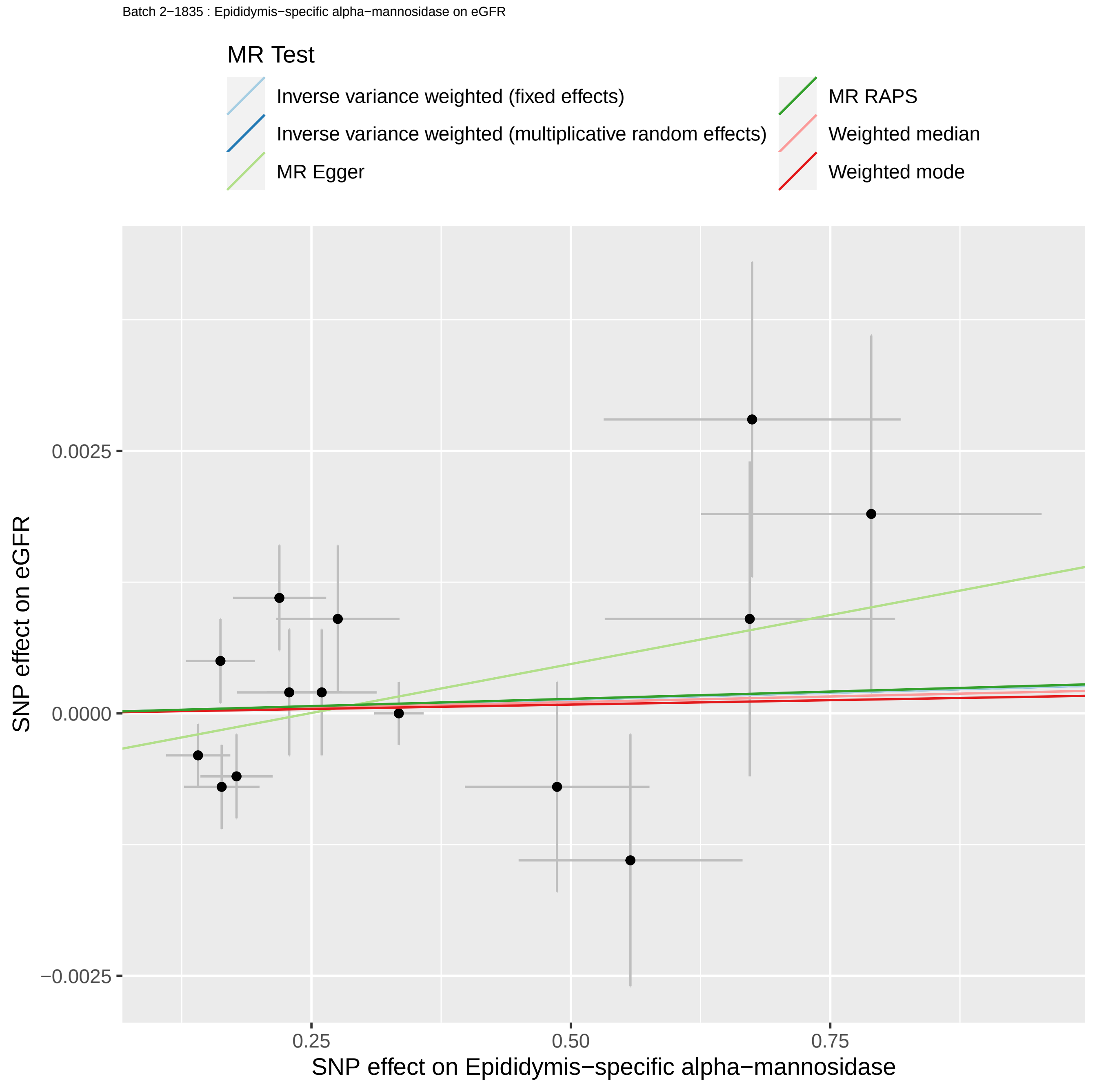
Batch 2−1835 : Epididymis−specific alpha−mannosidase on eGFR
MR Test
Inverse variance weighted (fixed effects)
Inverse variance weighted (multiplicative random effects)
MR Egger
MR RAPS
Weighted median
Weighted mode
SNP effect on eGFR
0.0025
0.0000
−0.0025
0.25
0.50
0.75
SNP effect on Epididymis−specific alpha−mannosidase

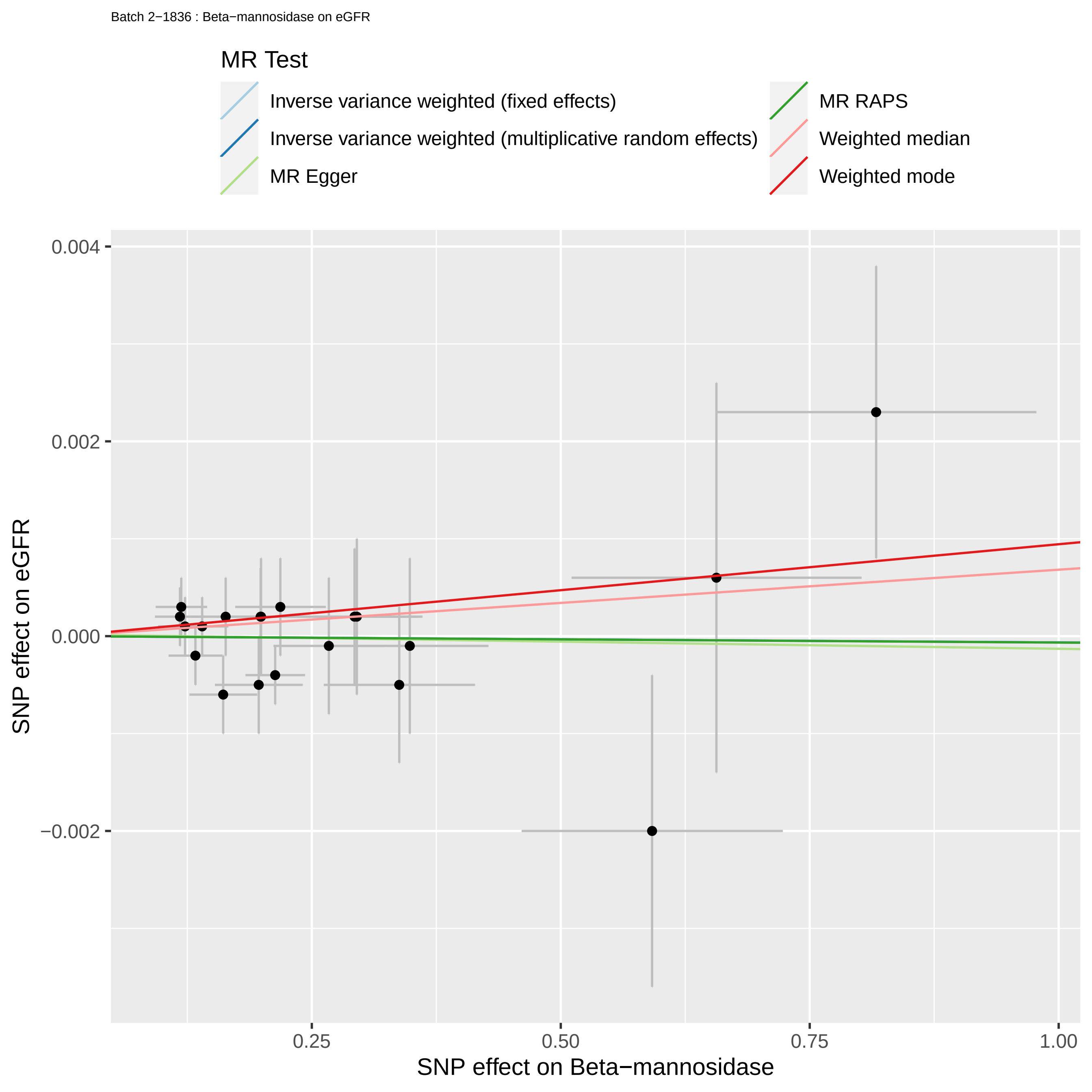
Batch 2−1836 : Beta−mannosidase on eGFR
MR Test
Inverse variance weighted (fixed effects)
Inverse variance weighted (multiplicative random effects)
MR Egger
MR RAPS
Weighted median
Weighted mode
0.004
0.002
0.000
−0.002
SNP effect on eGFR
0.25
0.50
0.75
1.00
SNP effect on Beta−mannosidase

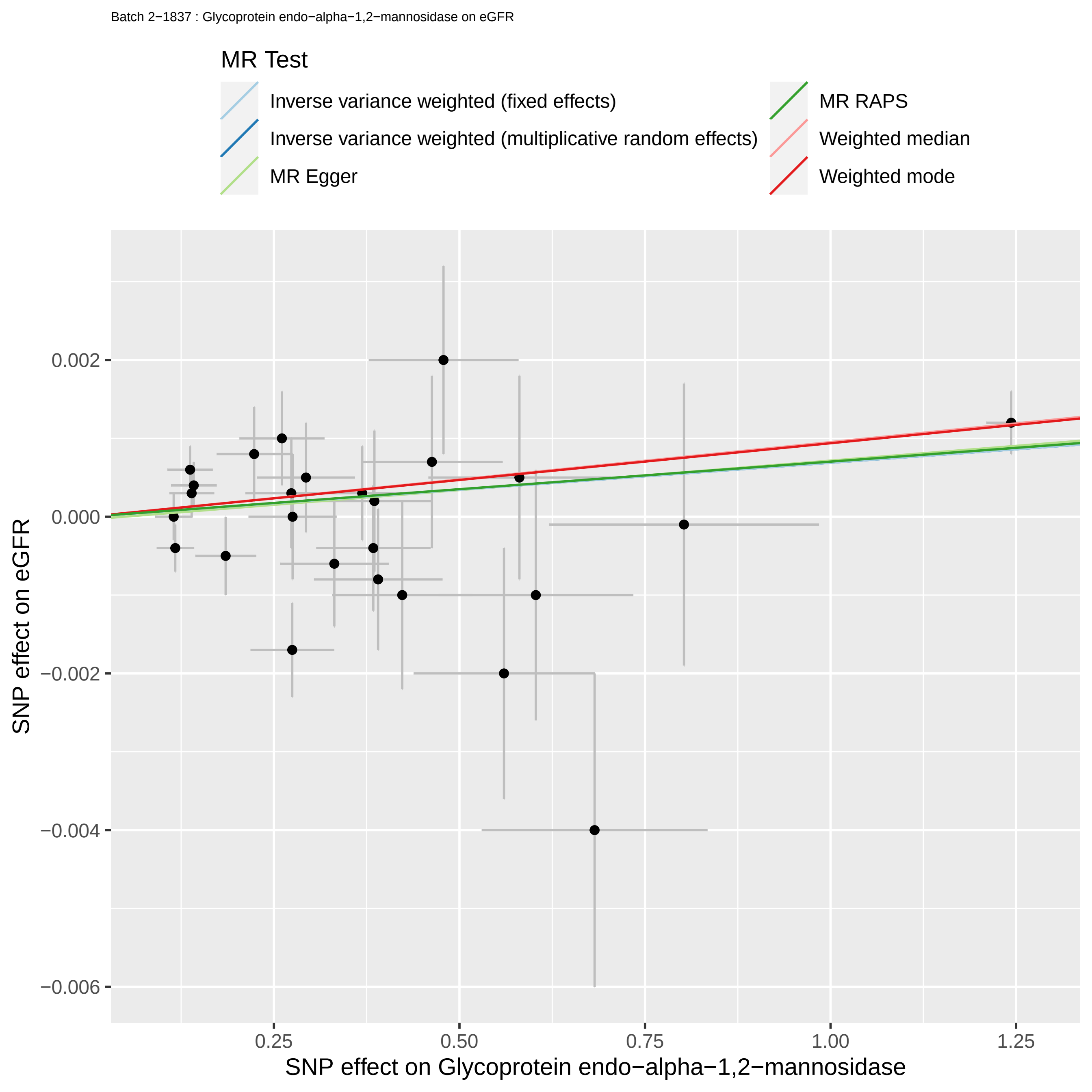

Batch 2−1837 : Glycoprotein endo−alpha−1,2−mannosidase on eGFR
MR Test
Inverse variance weighted (fixed effects)
Inverse variance weighted (multiplicative random effects)
MR Egger
MR RAPS
Weighted median
Weighted mode
0.002
0.000
−0.002
−0.004
−0.006
SNP effect on eGFR
0.25
0.50
0.75
1.00
1.25
SNP effect on Glycoprotein endo−alpha−1,2−mannosidase

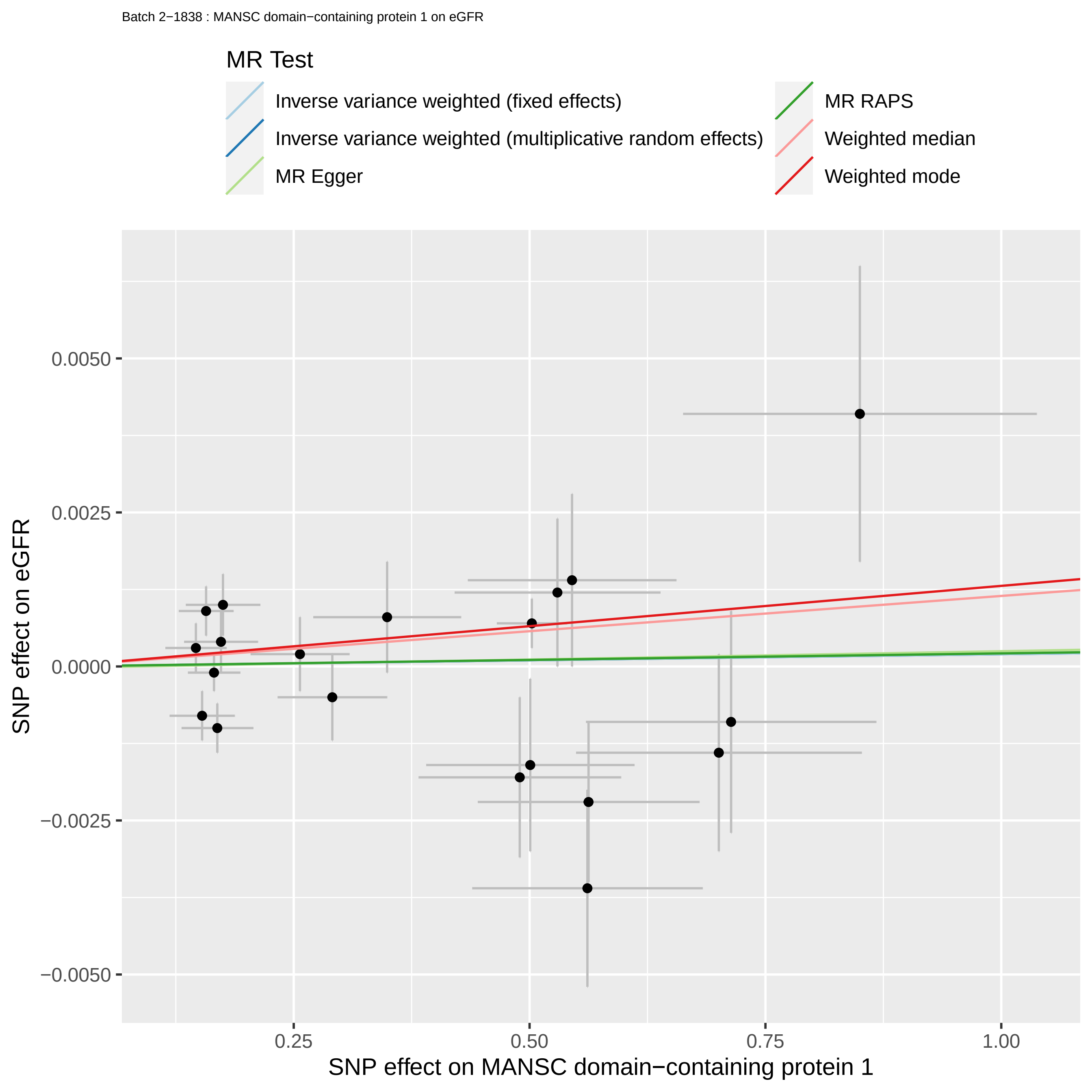

Batch 2−1838 : MANSC domain−containing protein 1 on eGFR
MR Test
Inverse variance weighted (fixed effects)
Inverse variance weighted (multiplicative random effects)
MR Egger
MR RAPS
Weighted median
Weighted mode
0.0050
0.0025
0.0000
−0.0025
−0.0050
0.25
0.50
0.75
1.00
SNP effect on eGFR
SNP effect on MANSC domain−containing protein 1

Batch 2−1839 : MANSC domain−containing protein 4 on eGFR

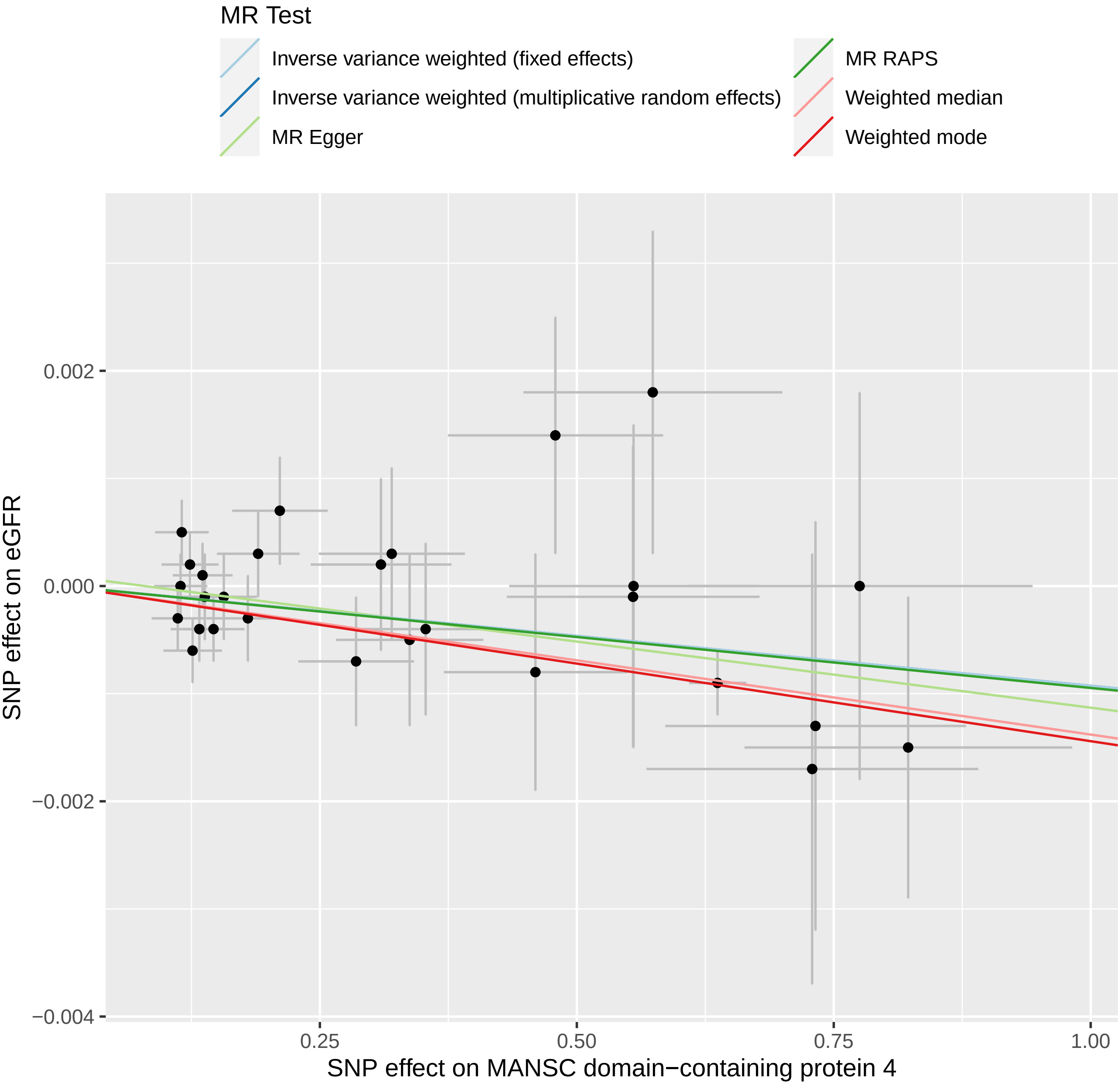

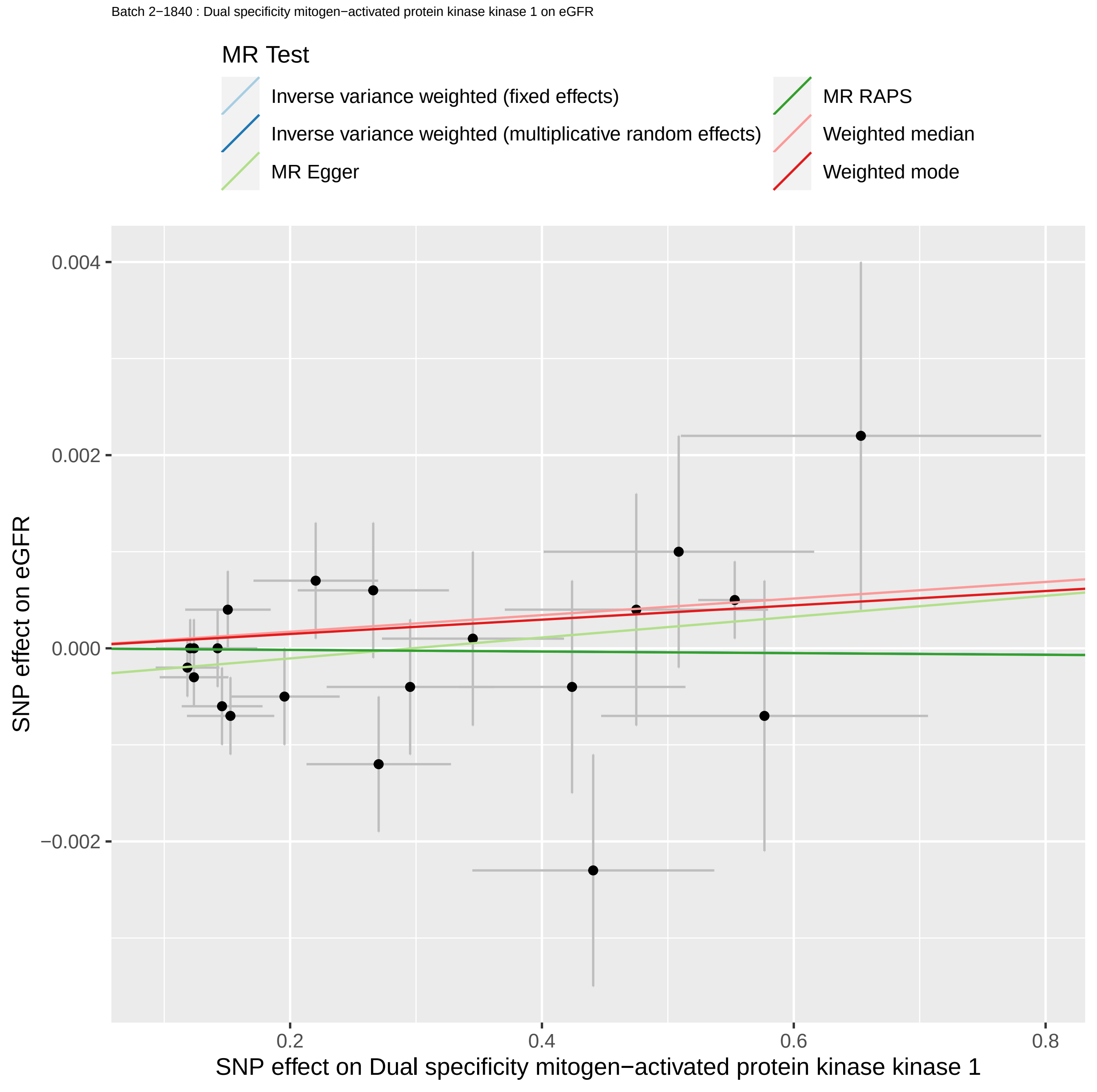

Batch 2−1840 : Dual specificity mitogen−activated protein kinase kinase 1 on eGFR
MR Test
Inverse variance weighted (fixed effects)
Inverse variance weighted (multiplicative random effects)
MR Egger
MR RAPS
Weighted median
Weighted mode
0.004
0.002
0.000
−0.002
0.2
0.4
0.6
0.8
SNP effect on eGFR
SNP effect on Dual specificity mitogen−activated protein kinase kinase 1

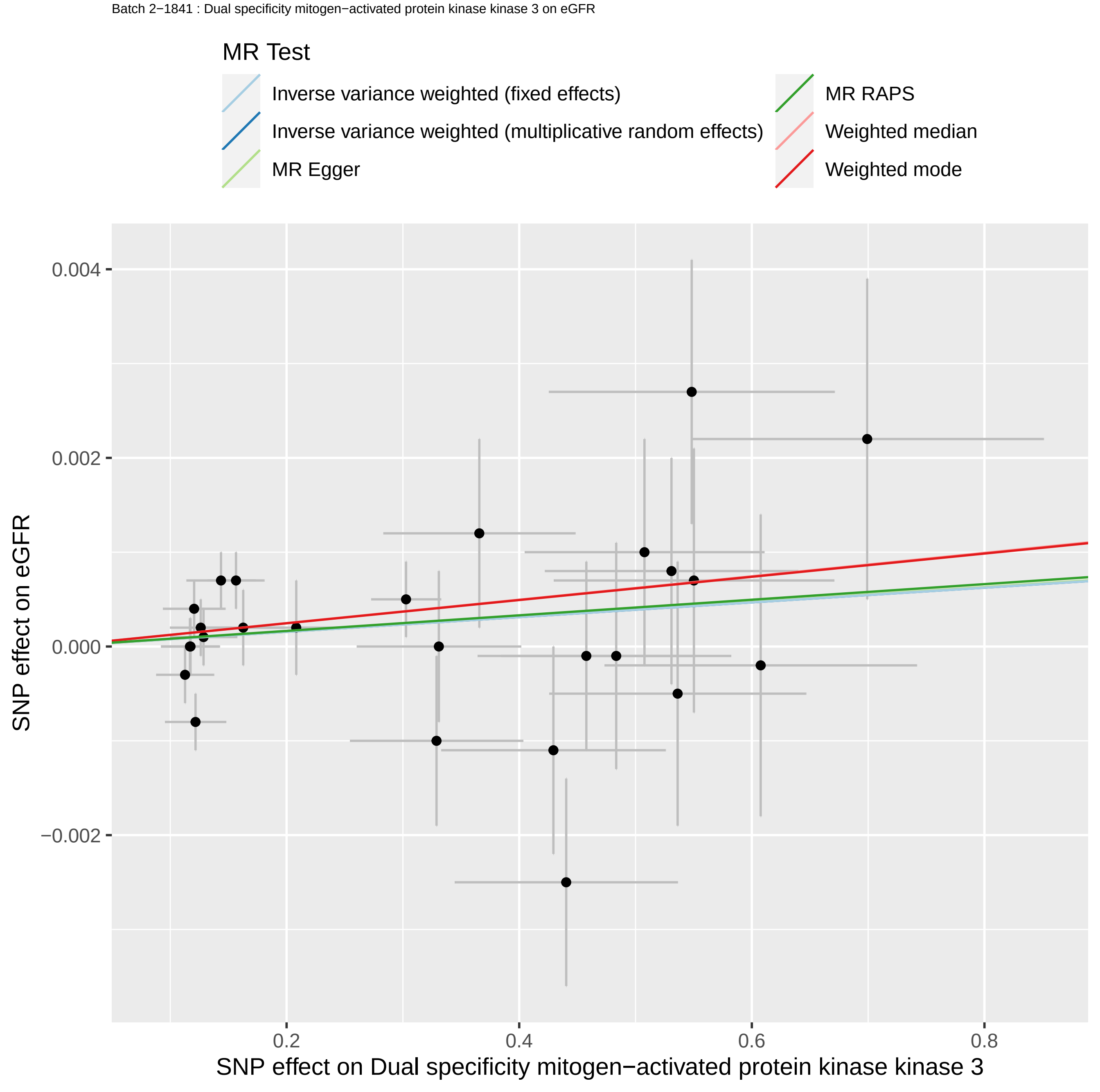

Batch 2−1841 : Dual specificity mitogen−activated protein kinase kinase 3 on eGFR
MR Test
Inverse variance weighted (fixed effects)
Inverse variance weighted (multiplicative random effects)
MR Egger
MR RAPS
Weighted median
Weighted mode
0.004
0.002
0.000
−0.002
0.2
0.4
0.6
0.8
SNP effect on eGFR
SNP effect on Dual specificity mitogen−activated protein kinase kinase 3

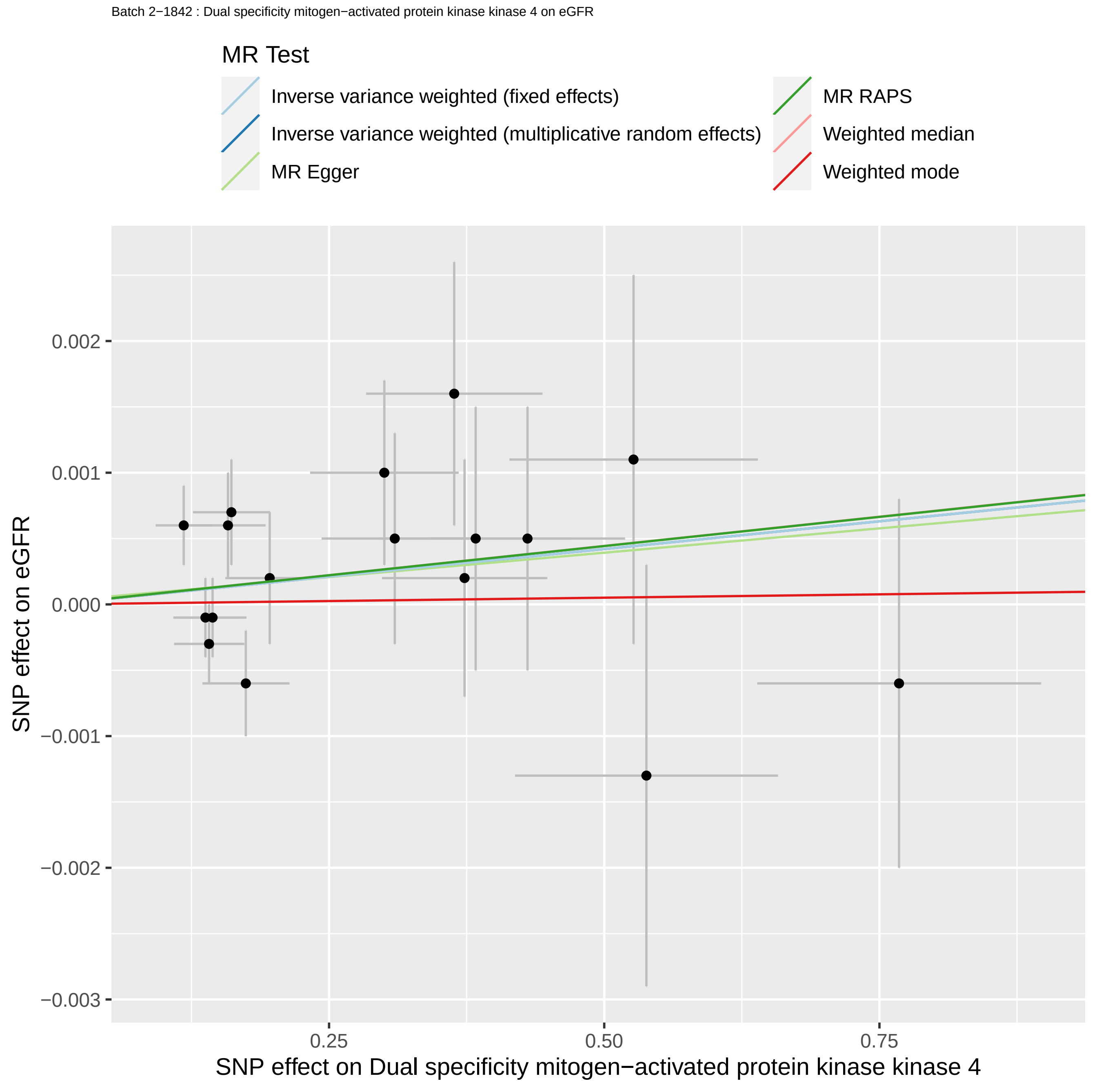
Batch 2−1842 : Dual specificity mitogen−activated protein kinase kinase 4 on eGFR
MR Test
Inverse variance weighted (fixed effects)
Inverse variance weighted (multiplicative random effects)
MR Egger
MR RAPS
Weighted median
Weighted mode
0.002
0.001
0.000
−0.001
−0.002
−0.003
0.25
0.50
0.75
SNP effect on eGFR
SNP effect on Dual specificity mitogen−activated protein kinase kinase 4

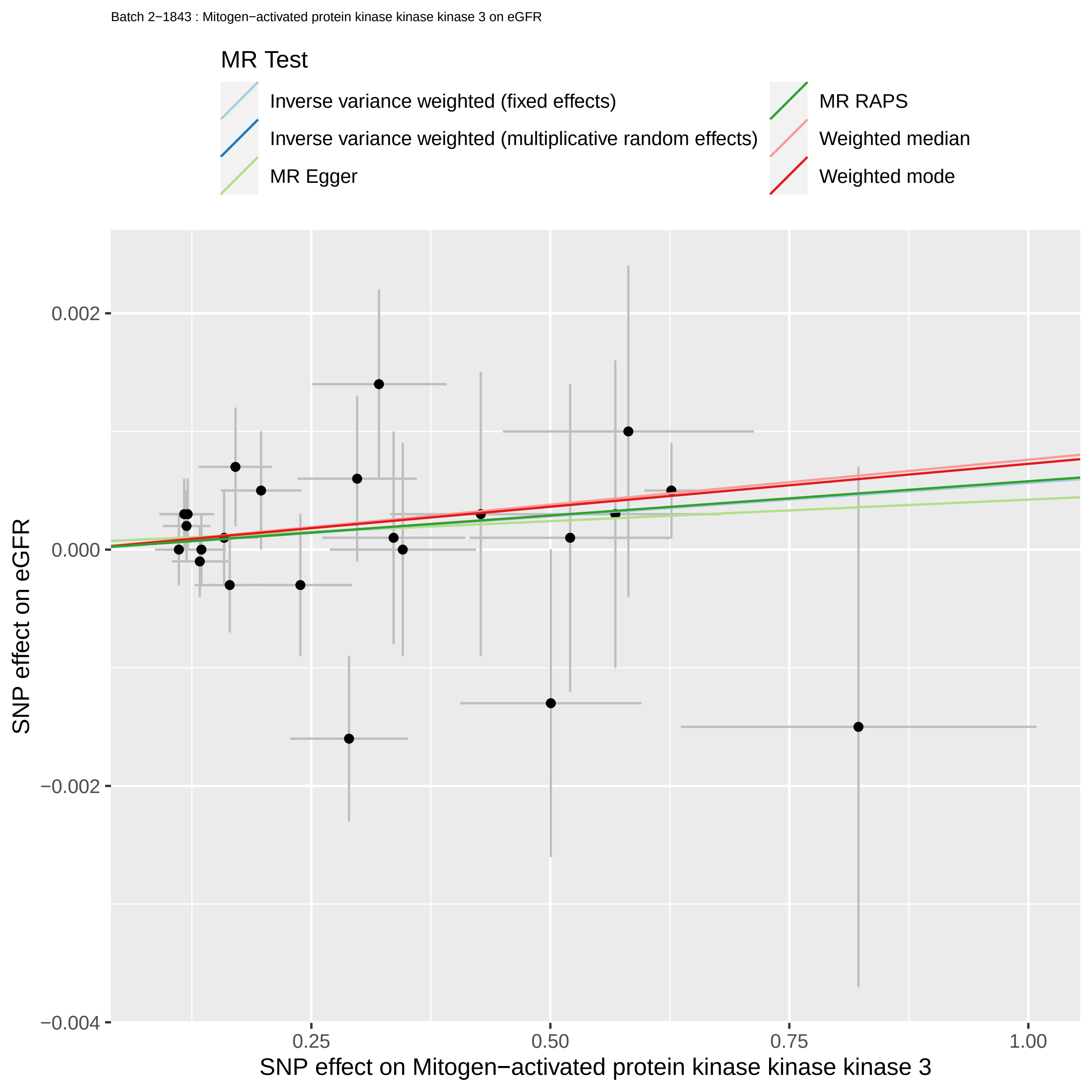

Batch 2−1843 : Mitogen−activated protein kinase kinase kinase 3 on eGFR
MR Test
Inverse variance weighted (fixed effects)
Inverse variance weighted (multiplicative random effects)
MR Egger
MR RAPS
Weighted median
Weighted mode
0.002
0.000
−0.002
−0.004
SNP effect on eGFR
0.25
0.50
0.75
1.00
SNP effect on Mitogen−activated protein kinase kinase kinase 3

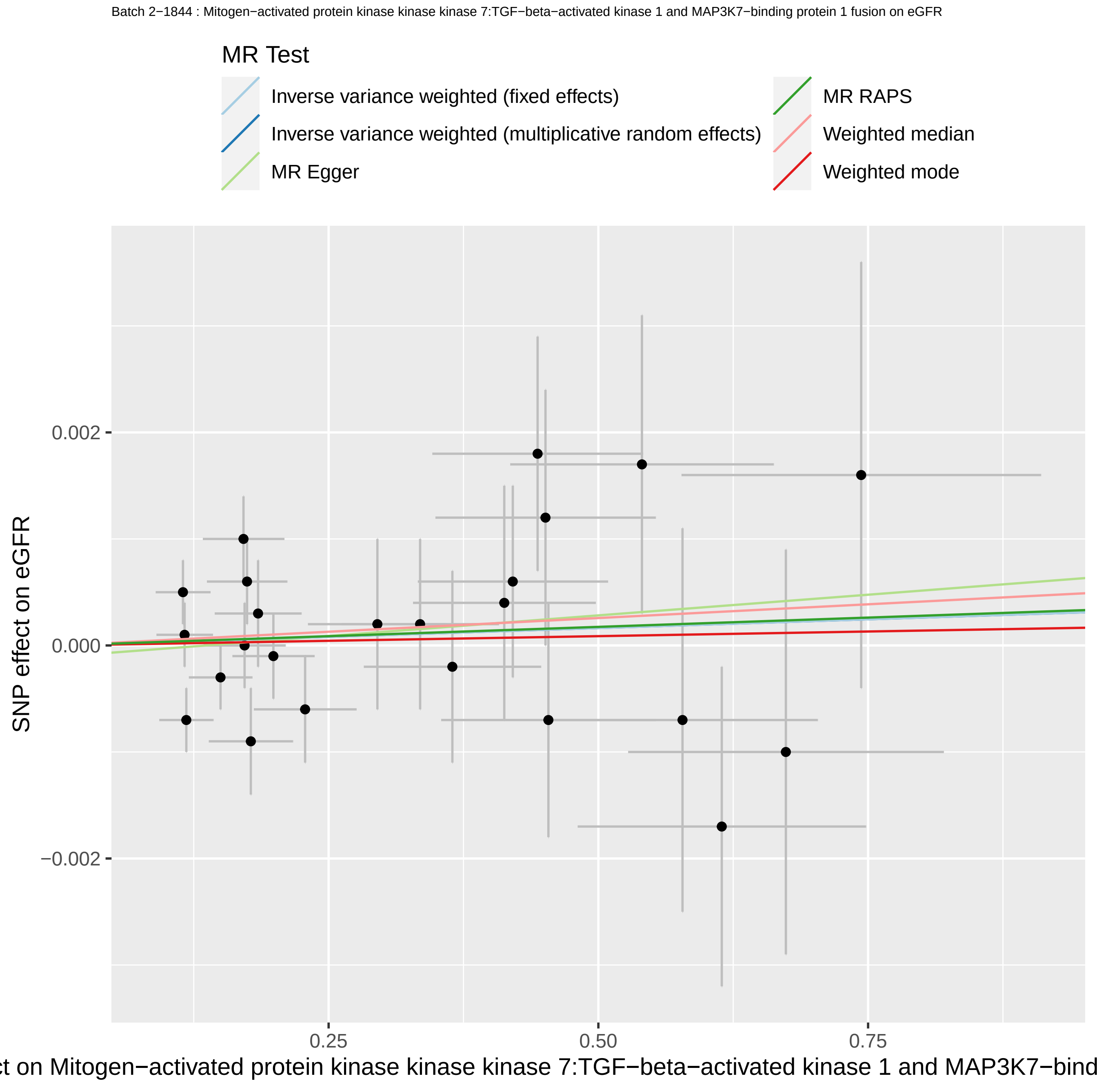

Batch 2−1844 : Mitogen−activated protein kinase kinase kinase 7:TGF−beta−activated kinase 1 and MAP3K7−binding protein 1 fusion on eGFR
MR Test
Inverse variance weighted (fixed effects)
Inverse variance weighted (multiplicative random effects)
MR Egger
MR RAPS
Weighted median
Weighted mode
0.002
0.000
−0.002
SNP effect on eGFR
0.25
0.50
0.75
ct on Mitogen−activated protein kinase kinase kinase 7:TGF−beta−activated kinase 1 and MAP3K7−bind

Batch 2−1845 : Mitogen−activated protein kinase 1 on eGFR

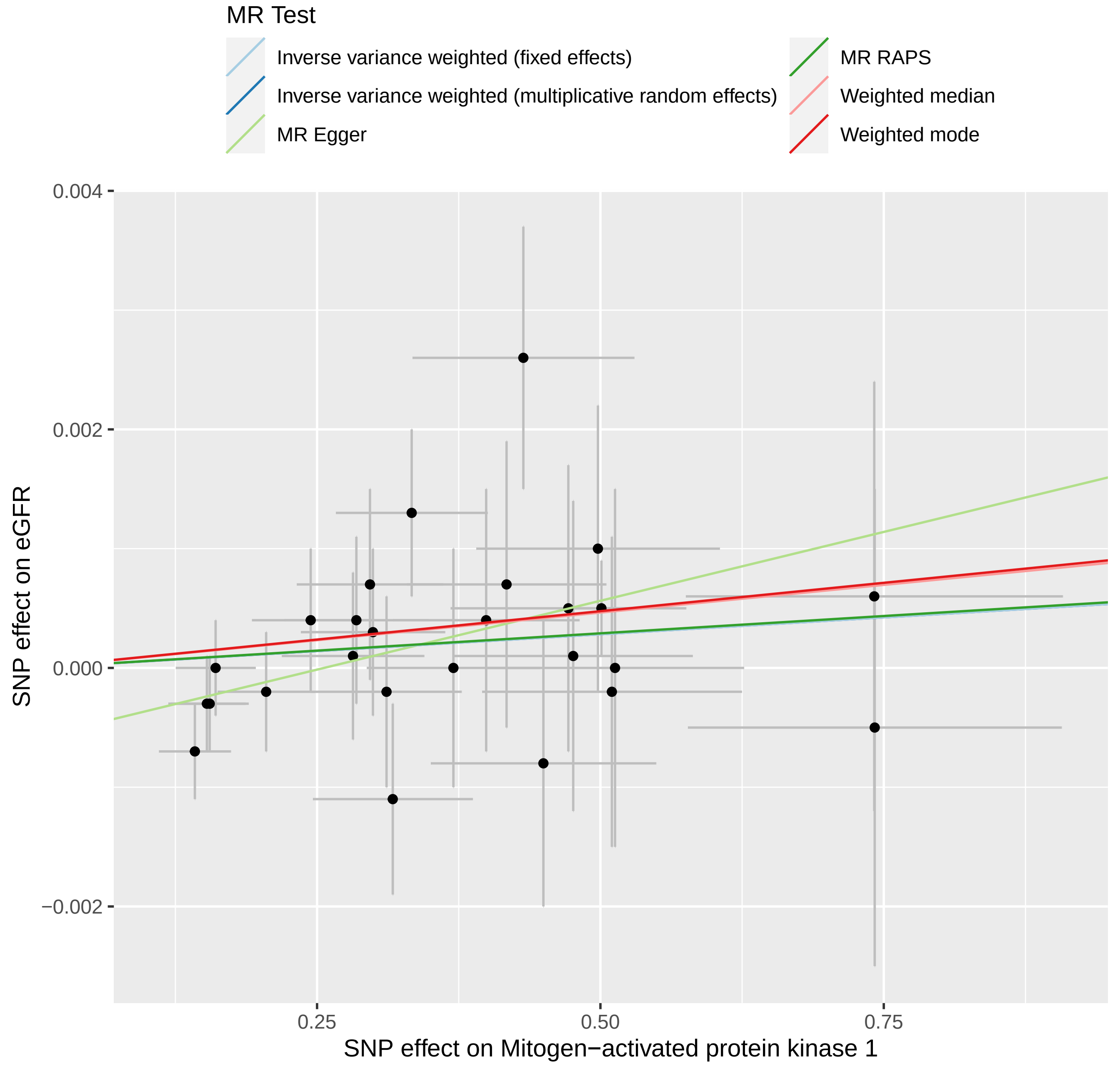

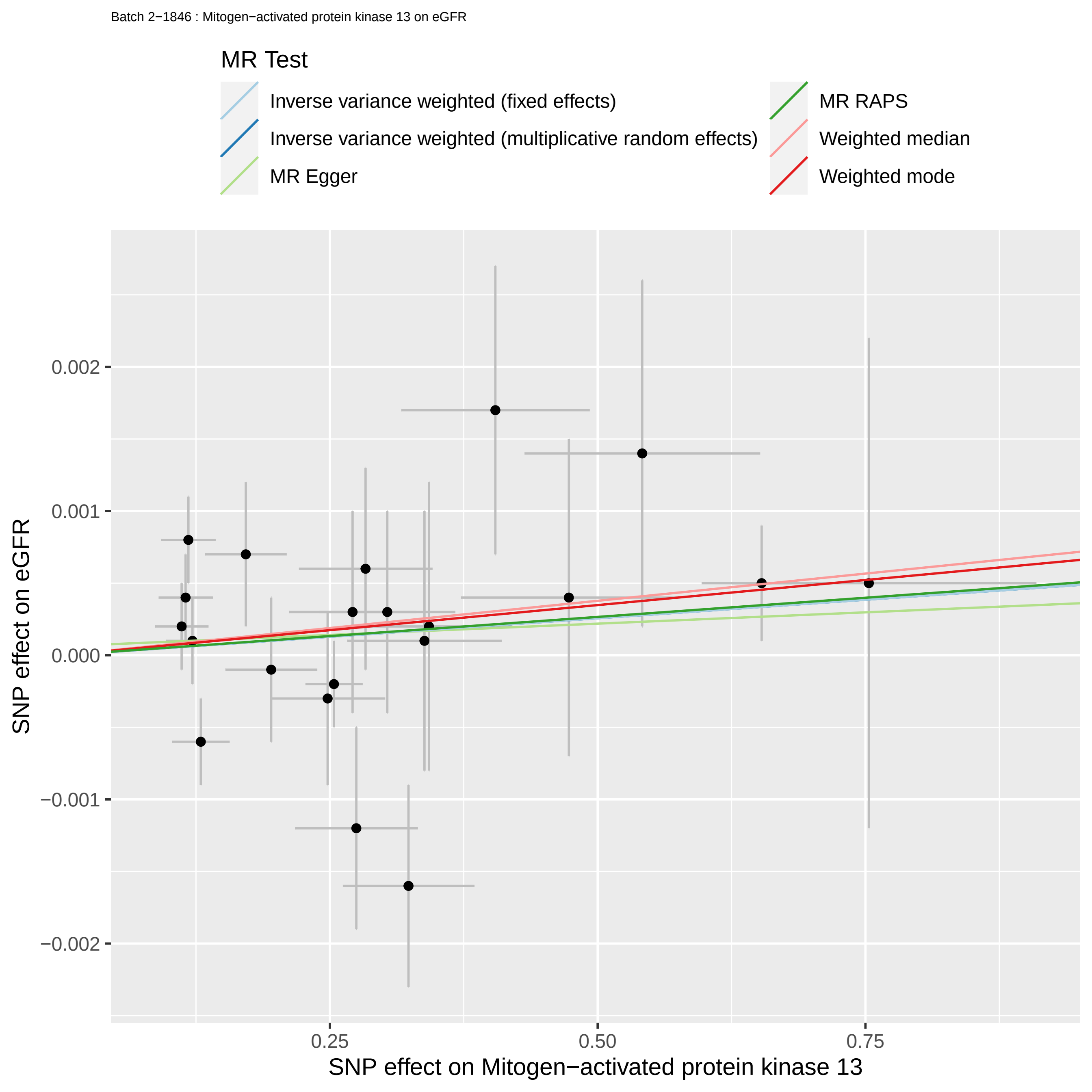
Batch 2−1846 : Mitogen−activated protein kinase 13 on eGFR
MR Test
Inverse variance weighted (fixed effects)
Inverse variance weighted (multiplicative random effects)
MR Egger
MR RAPS
Weighted median
Weighted mode
0.002
0.001
0.000
−0.001
−0.002
SNP effect on eGFR
0.25
0.50
0.75
SNP effect on Mitogen−activated protein kinase 13

Batch 2−1847 : Mitogen−activated protein kinase 14 on eGFR

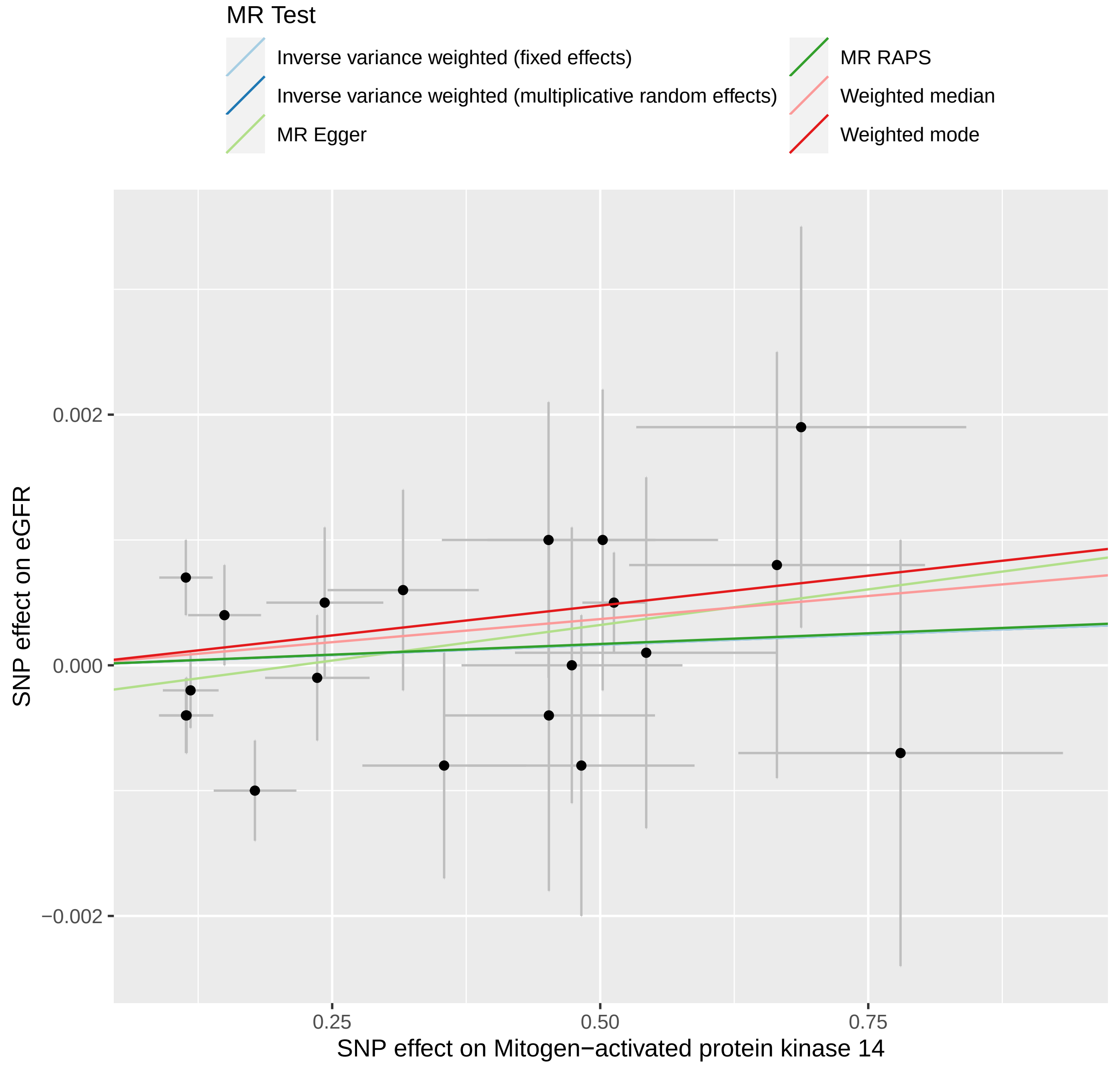

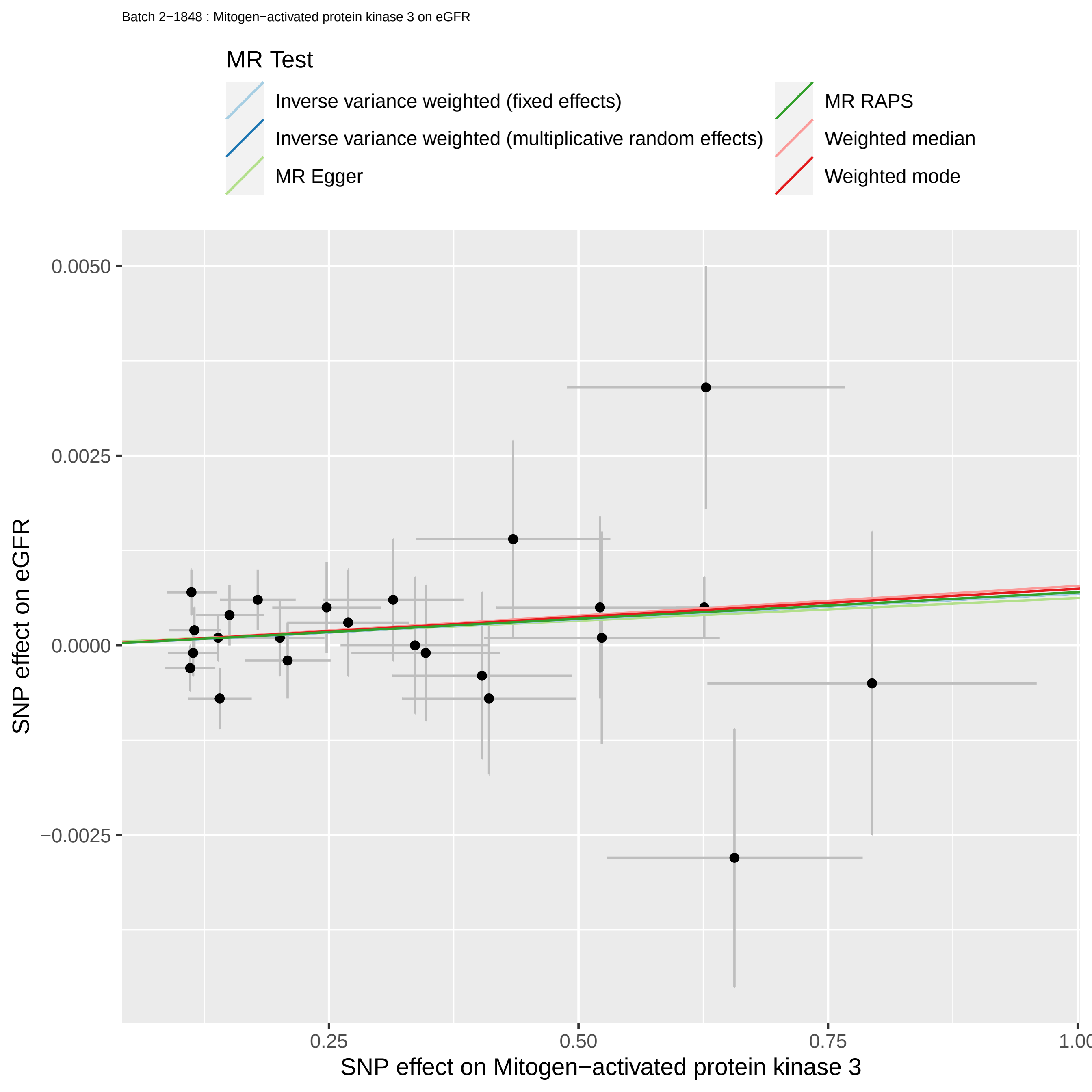

Batch 2−1848 : Mitogen−activated protein kinase 3 on eGFR
MR Test
Inverse variance weighted (fixed effects)
Inverse variance weighted (multiplicative random effects)
MR Egger
MR RAPS
Weighted median
Weighted mode
0.0050
0.0025
0.0000
−0.0025
SNP effect on eGFR
0.25
0.50
0.75
1.00
SNP effect on Mitogen−activated protein kinase 3

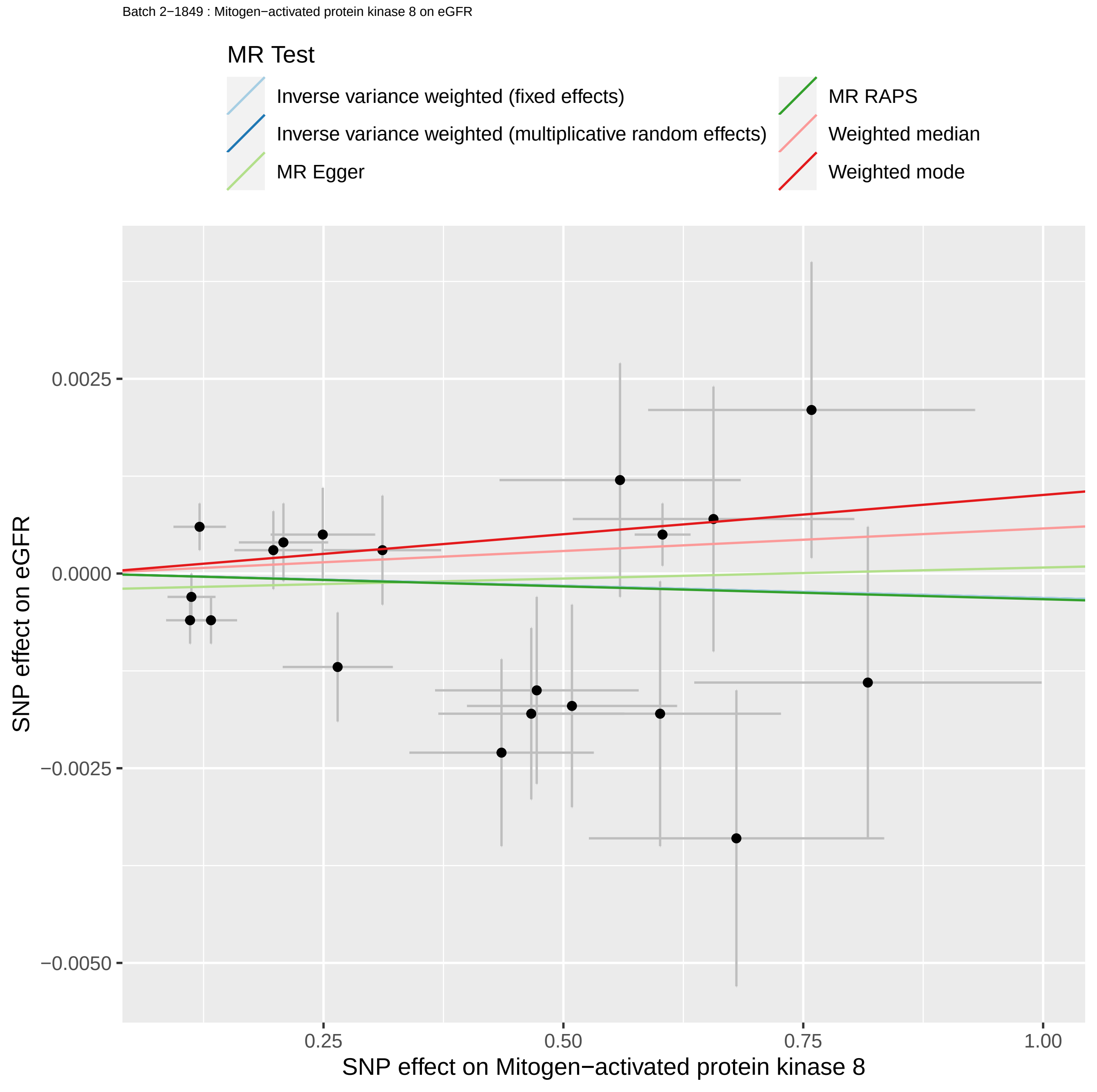

Batch 2−1849 : Mitogen−activated protein kinase 8 on eGFR
MR Test
Inverse variance weighted (fixed effects)
Inverse variance weighted (multiplicative random effects)
MR Egger
MR RAPS
Weighted median
Weighted mode
0.0025
0.0000
−0.0025
−0.0050
0.25
0.50
0.75
1.00
SNP effect on Mitogen−activated protein kinase 8
SNP effect on eGFR

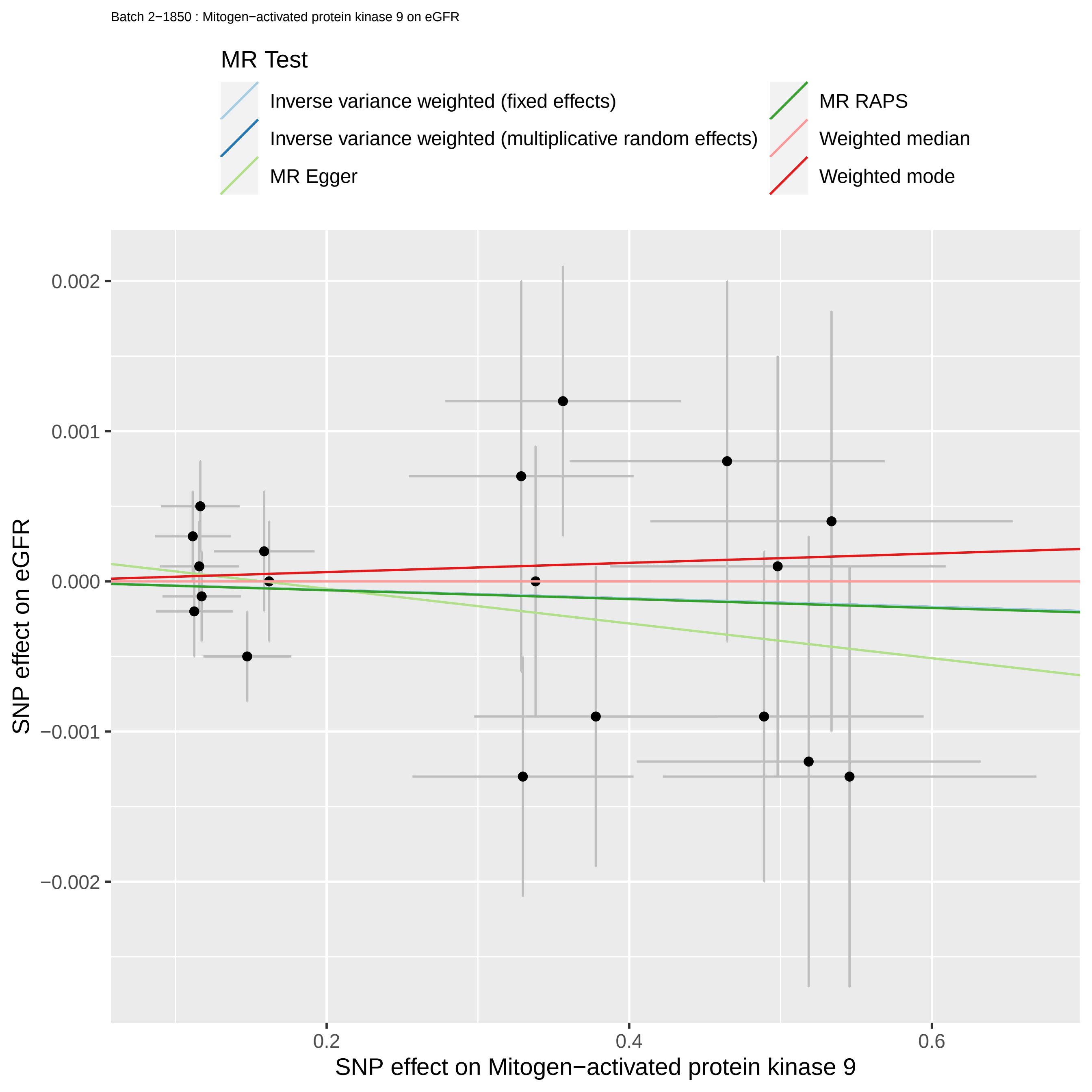
Batch 2−1850 : Mitogen−activated protein kinase 9 on eGFR
MR Test
Inverse variance weighted (fixed effects)
Inverse variance weighted (multiplicative random effects)
MR Egger
MR RAPS
Weighted median
Weighted mode
0.002
0.001
0.000
−0.001
−0.002
0.2
0.4
0.6
SNP effect on eGFR
SNP effect on Mitogen−activated protein kinase 9

Batch 2−1851 : MAP kinase−activated protein kinase 2 on eGFR

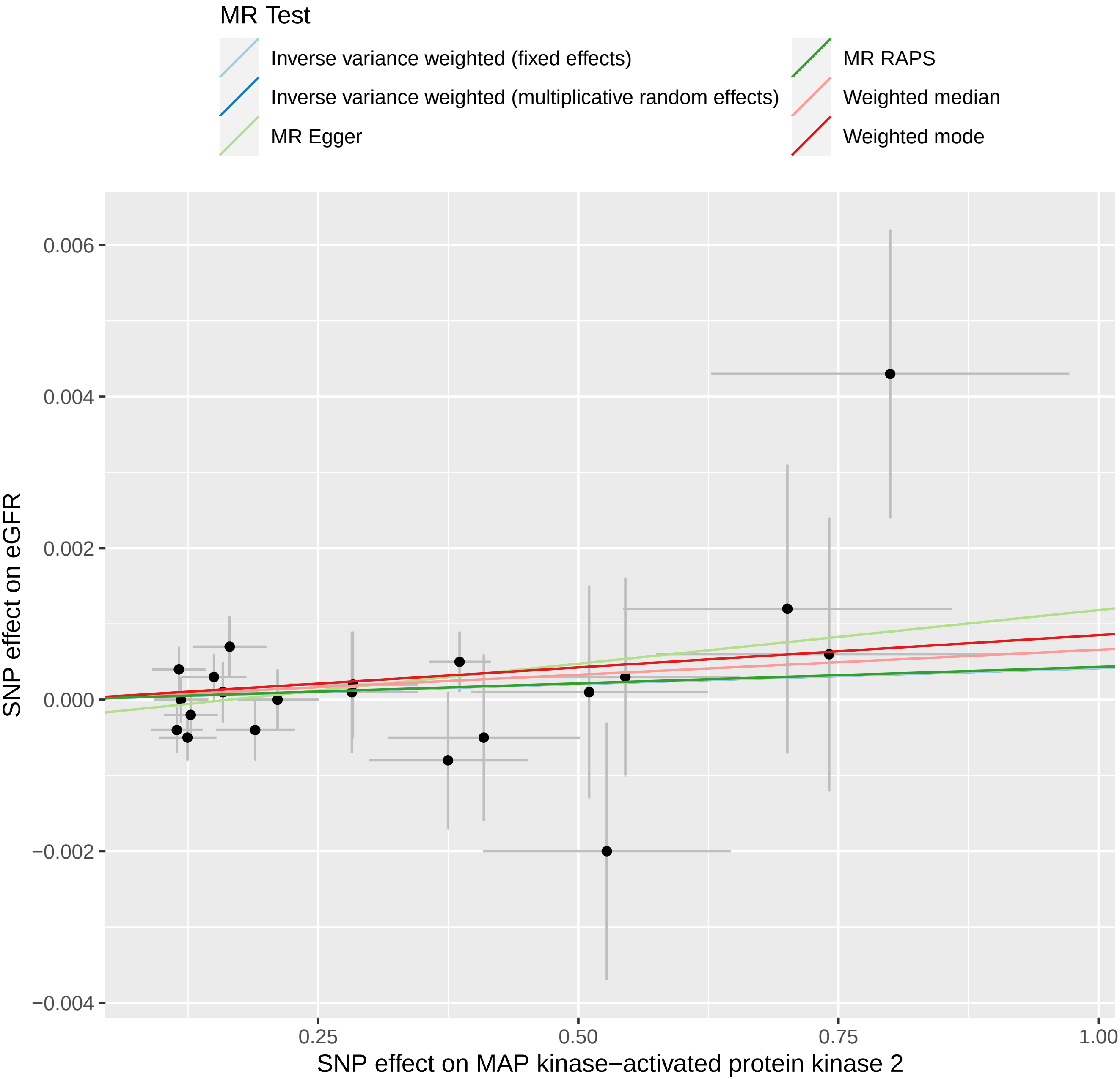

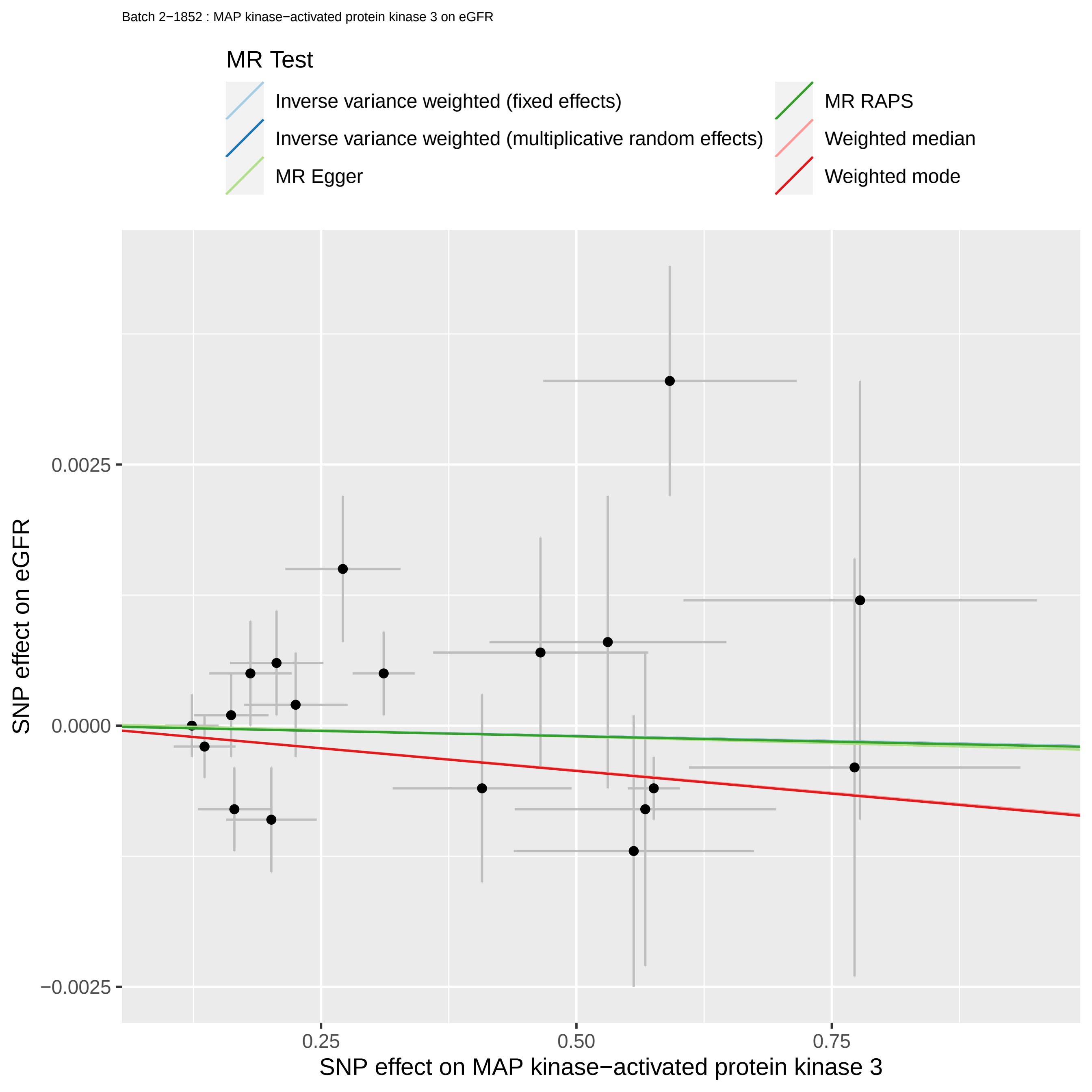

Batch 2−1852 : MAP kinase−activated protein kinase 3 on eGFR
MR Test
Inverse variance weighted (fixed effects)
Inverse variance weighted (multiplicative random effects)
MR Egger
MR RAPS
Weighted median
Weighted mode
0.0025
0.0000
−0.0025
0.25
0.50
0.75
SNP effect on eGFR
SNP effect on MAP kinase−activated protein kinase 3

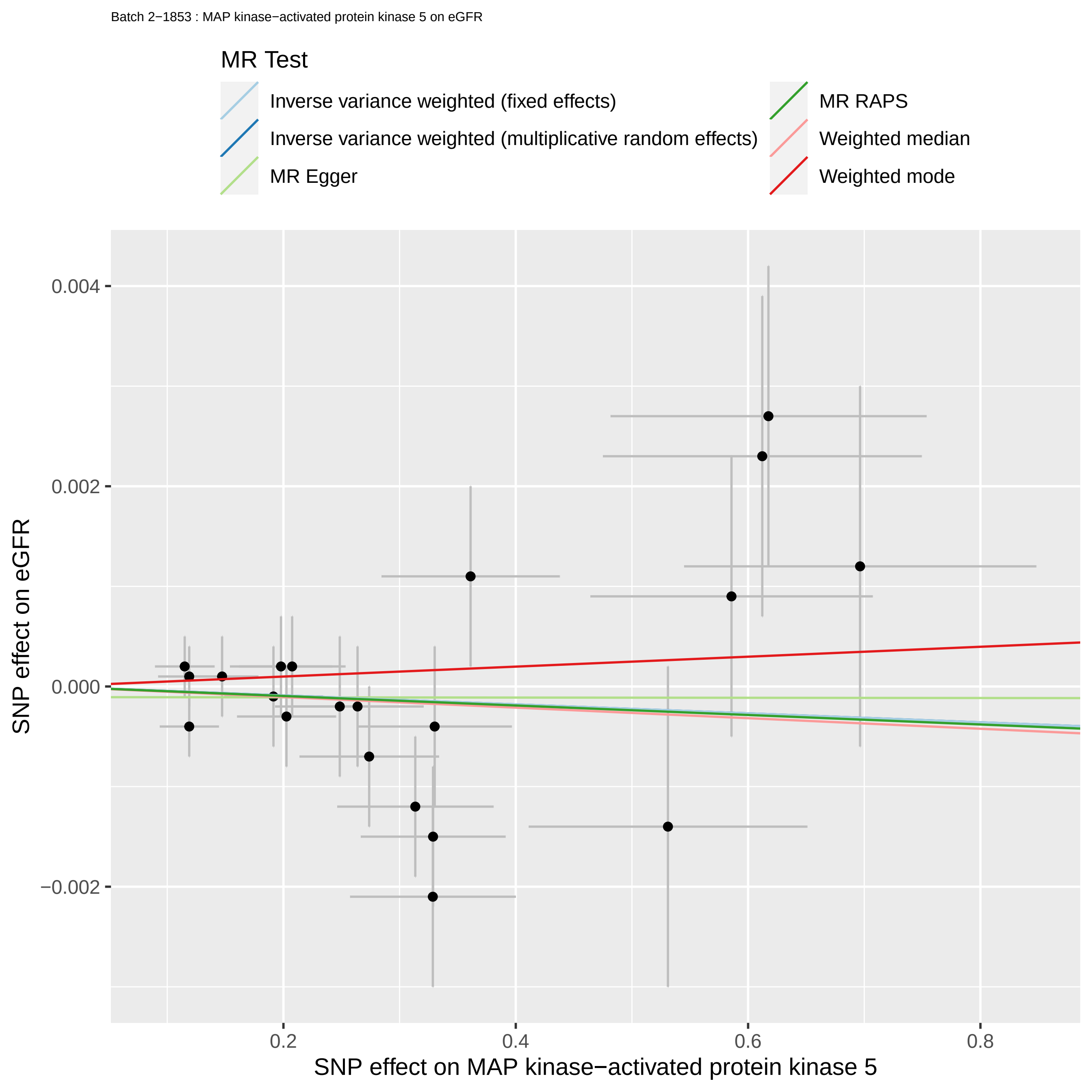

Batch 2−1853 : MAP kinase−activated protein kinase 5 on eGFR
MR Test
Inverse variance weighted (fixed effects)
Inverse variance weighted (multiplicative random effects)
MR Egger
MR RAPS
Weighted median
Weighted mode
0.004
0.002
0.000
−0.002
SNP effect on eGFR
0.2
0.4
0.6
0.8
SNP effect on MAP kinase−activated protein kinase 5

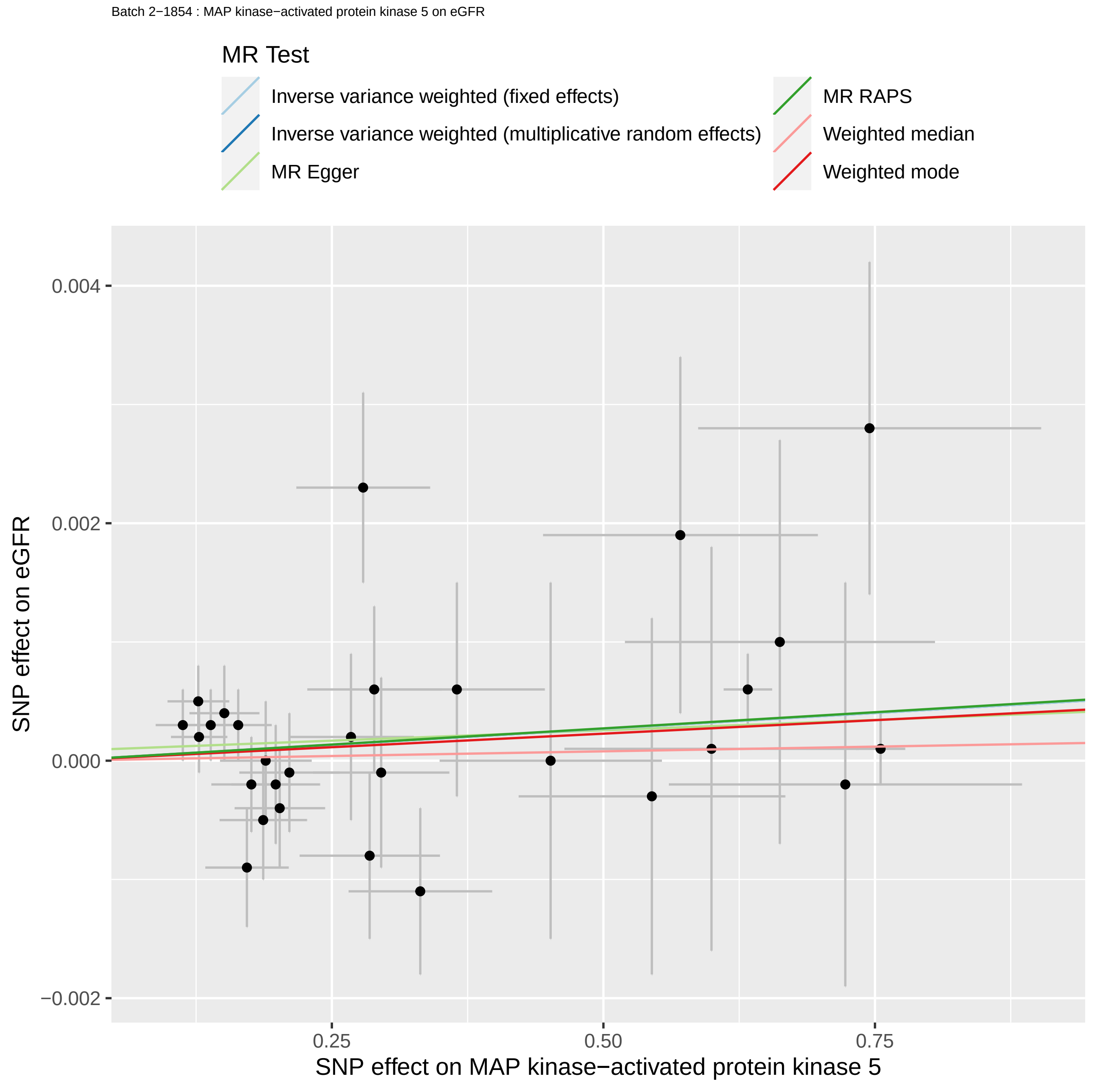
Batch 2−1854 : MAP kinase−activated protein kinase 5 on eGFR
MR Test
Inverse variance weighted (fixed effects)
Inverse variance weighted (multiplicative random effects)
MR Egger
MR RAPS
Weighted median
Weighted mode
0.004
0.002
0.000
−0.002
0.25
0.50
0.75
SNP effect on eGFR
SNP effect on MAP kinase−activated protein kinase 5

Batch 2−1855 : Mannan−binding lectin serine protease 1 on eGFR

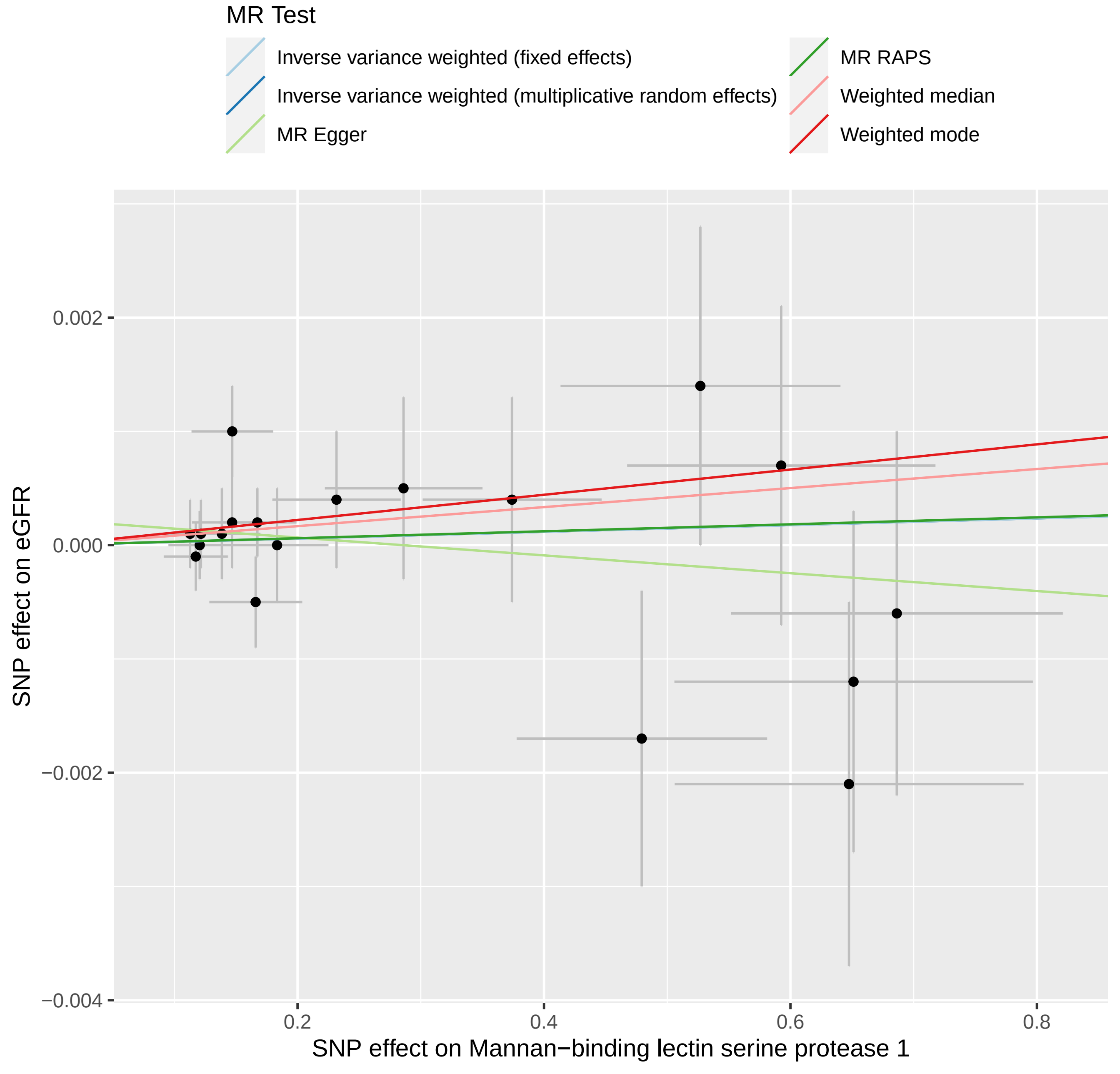

Batch 2−1856 : Megakaryocyte−associated tyrosine−protein kinase on eGFR

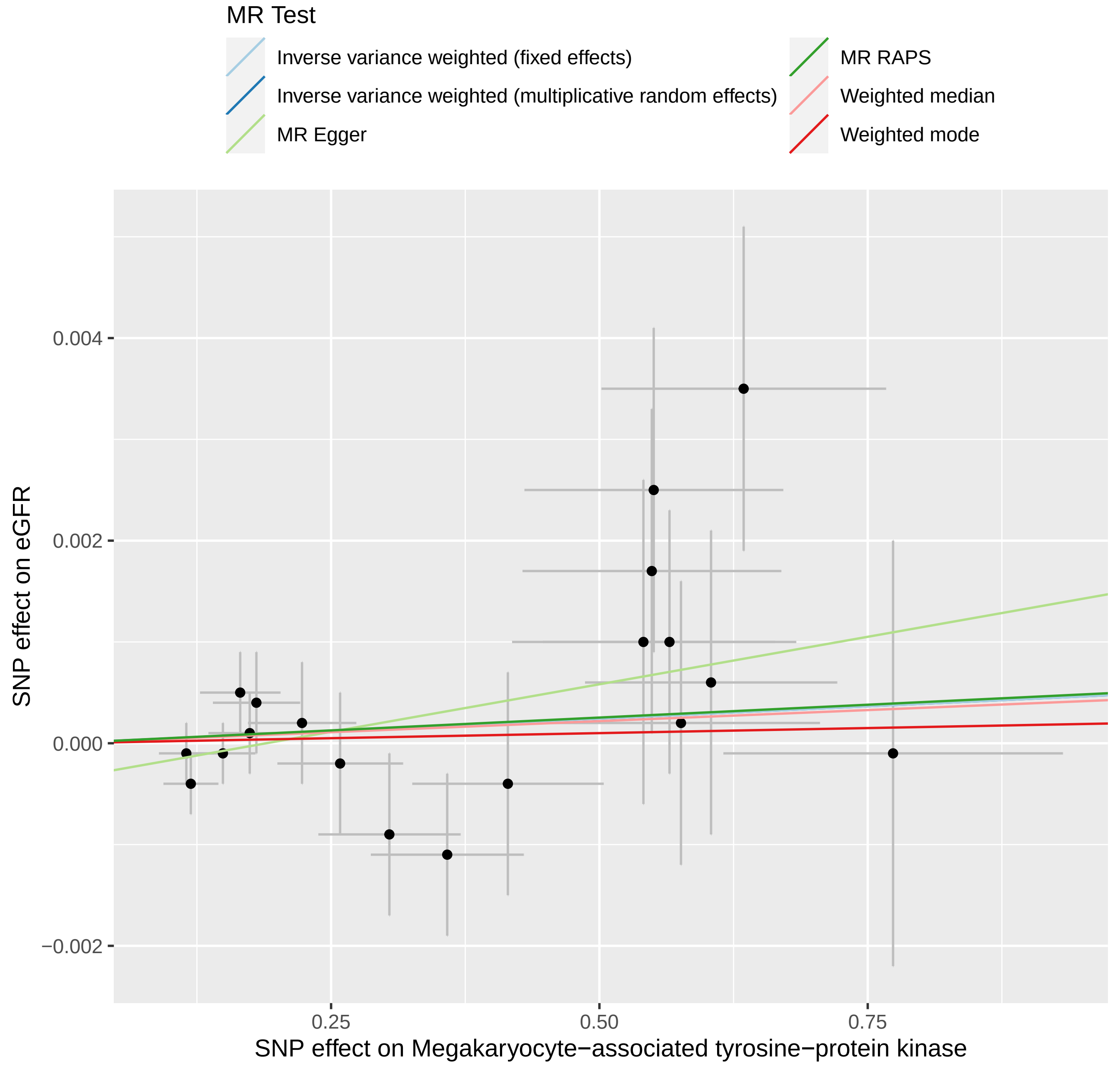

Batch 2−1857 : Matrilin−2 on eGFR

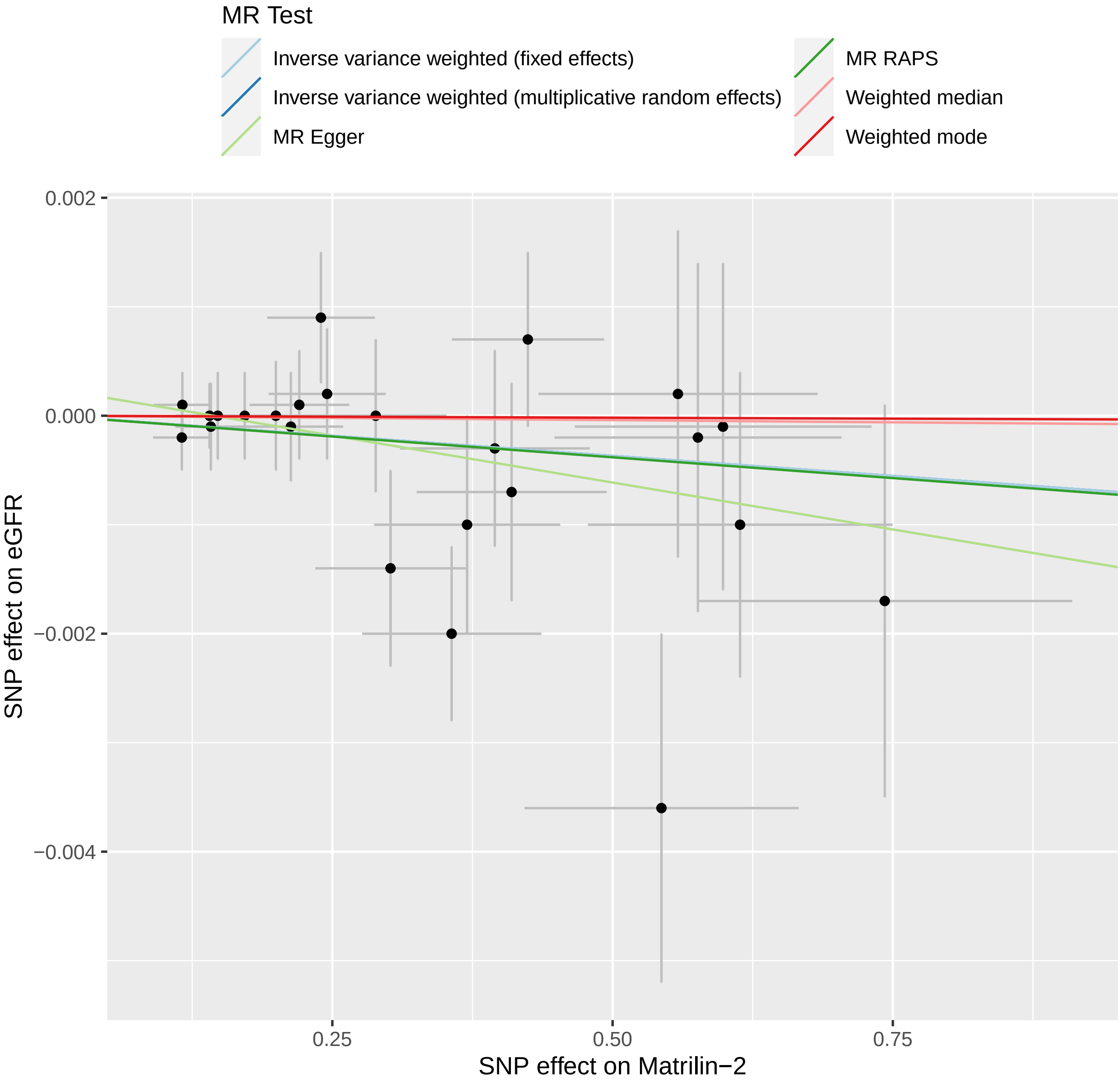

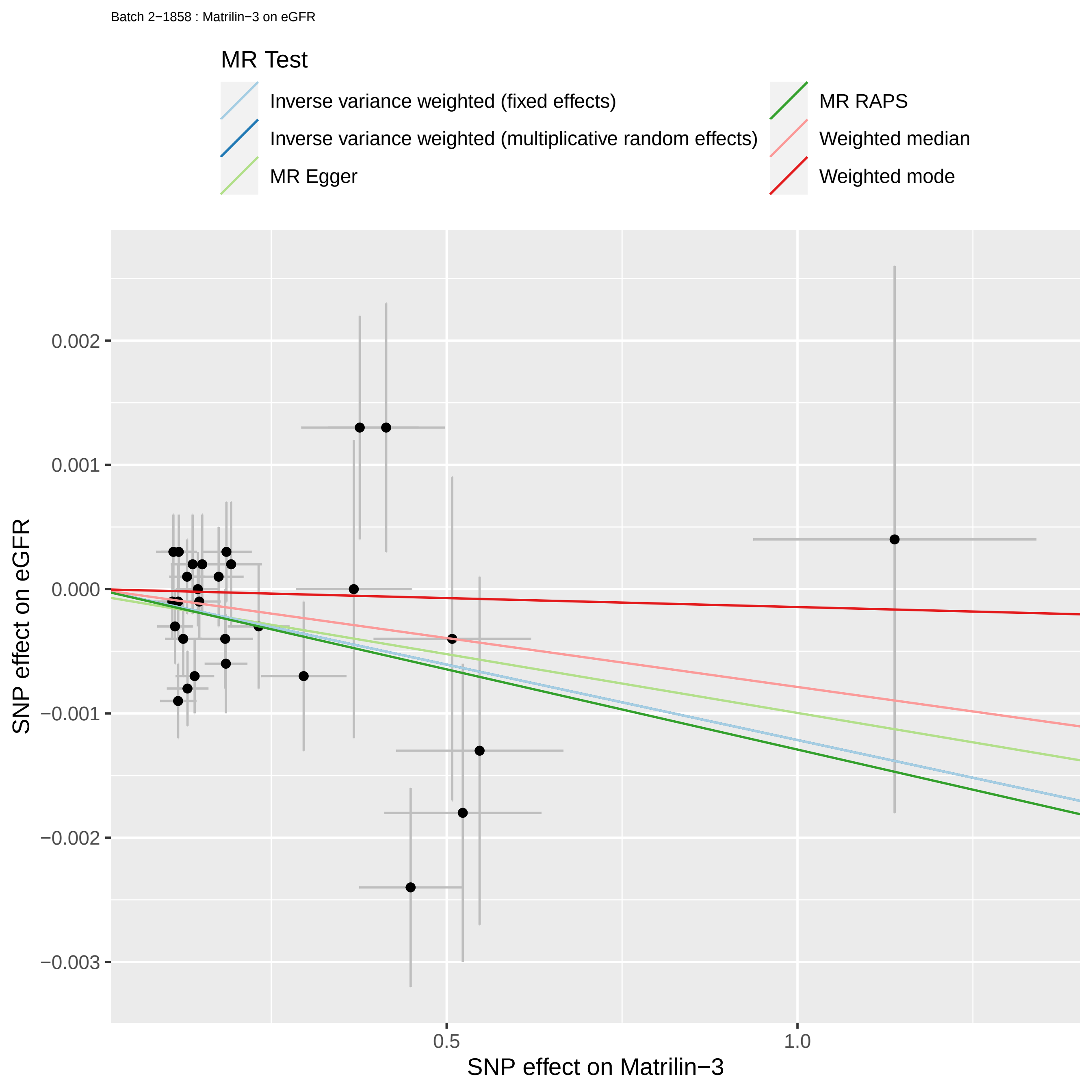
Batch 2−1858 : Matrilin−3 on eGFR
MR Test
Inverse variance weighted (fixed effects)
Inverse variance weighted (multiplicative random effects)
MR Egger
MR RAPS
Weighted median
Weighted mode
0.002
0.001
0.000
−0.001
−0.002
−0.003
SNP effect on eGFR
0.5
1.0
SNP effect on Matrilin−3

Batch 2−1859 : Matrilin−4 on eGFR

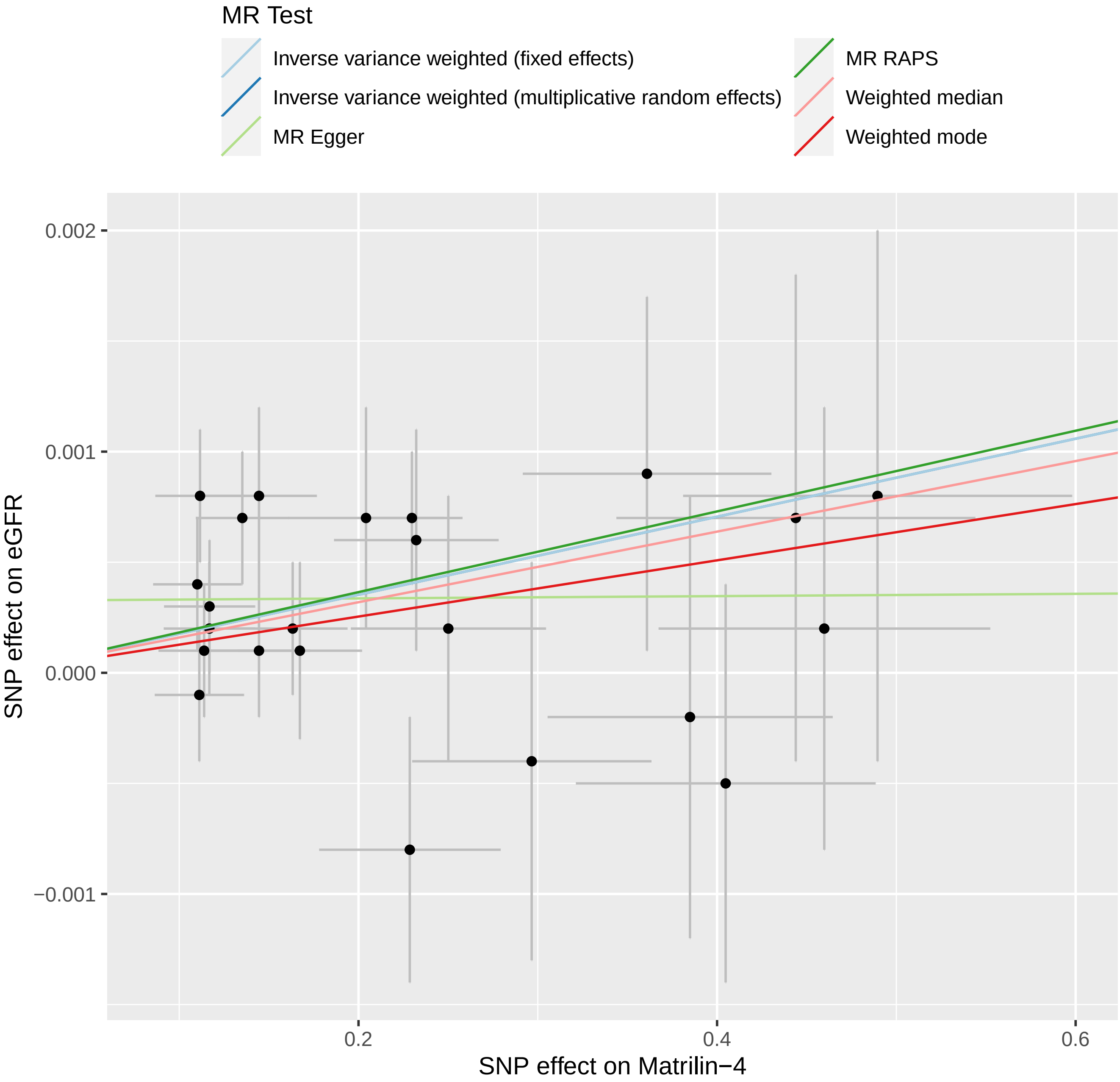

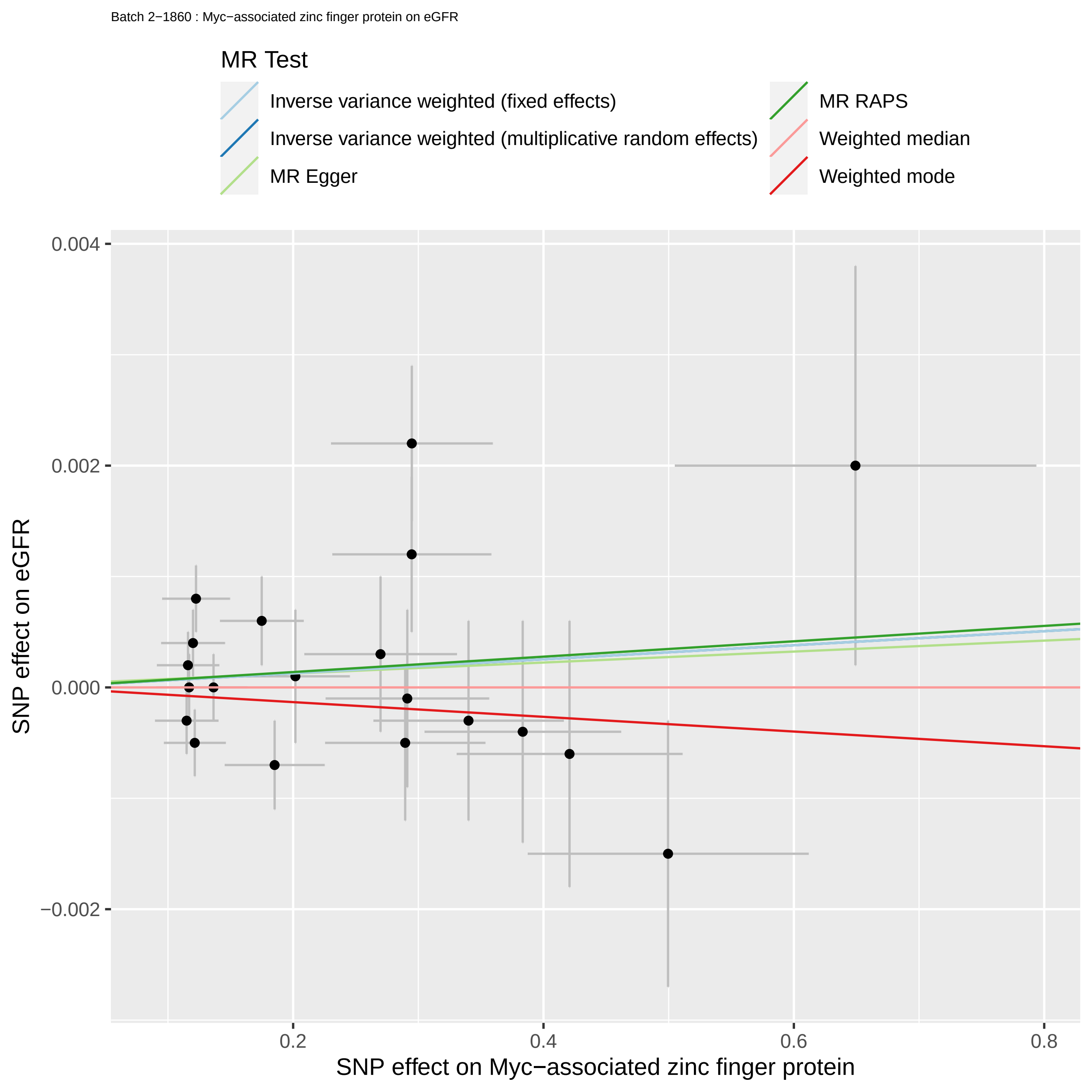

Batch 2−1860 : Myc−associated zinc finger protein on eGFR
MR Test
Inverse variance weighted (fixed effects)
Inverse variance weighted (multiplicative random effects)
MR Egger
MR RAPS
Weighted median
Weighted mode
0.004
0.002
0.000
−0.002
SNP effect on eGFR
0.2
0.4
0.6
0.8
SNP effect on Myc−associated zinc finger protein

Batch 2−1861 : Myoglobin on eGFR

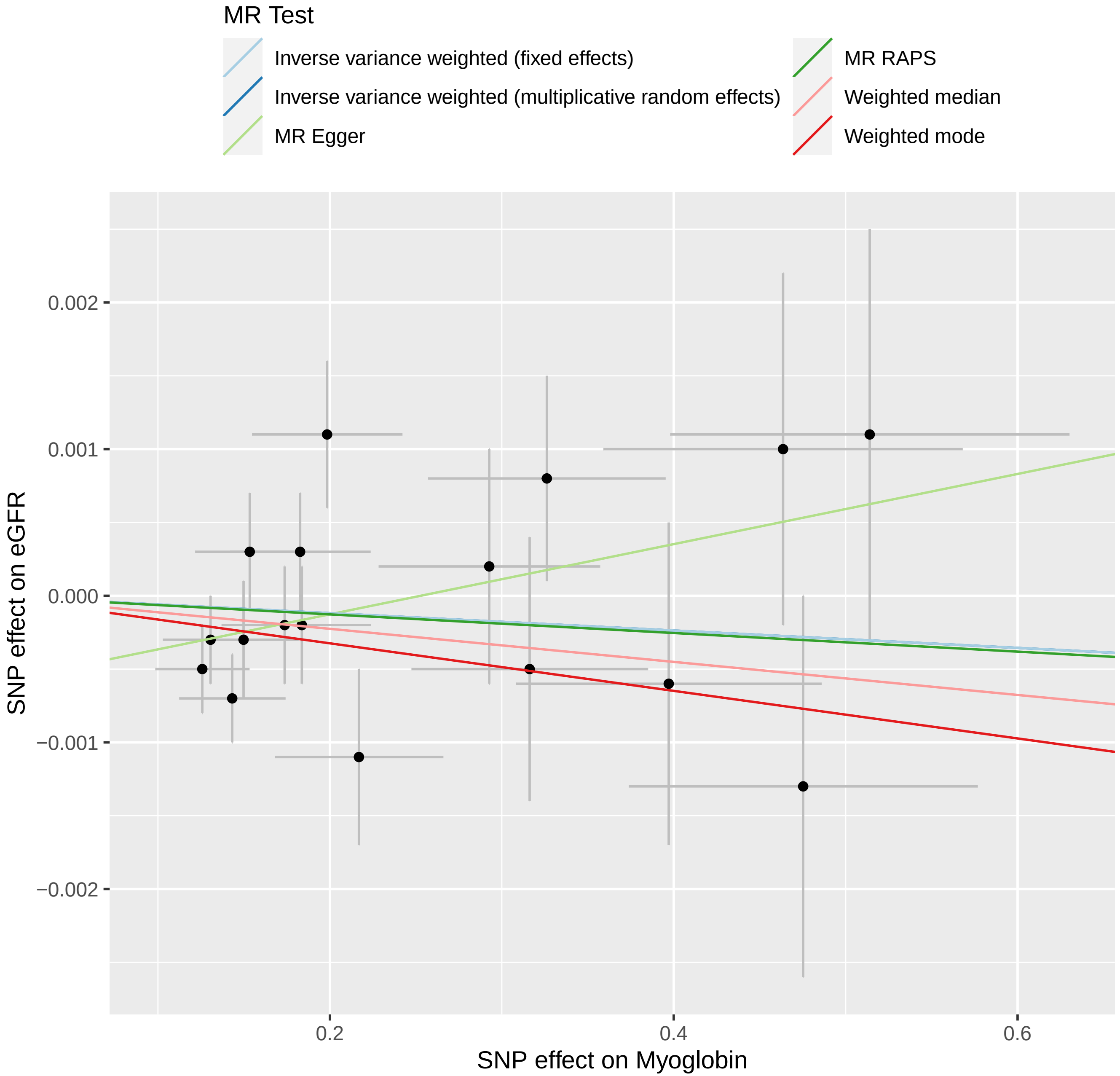

Batch 2−1862 : Methyl−CpG−binding domain protein 4 on eGFR

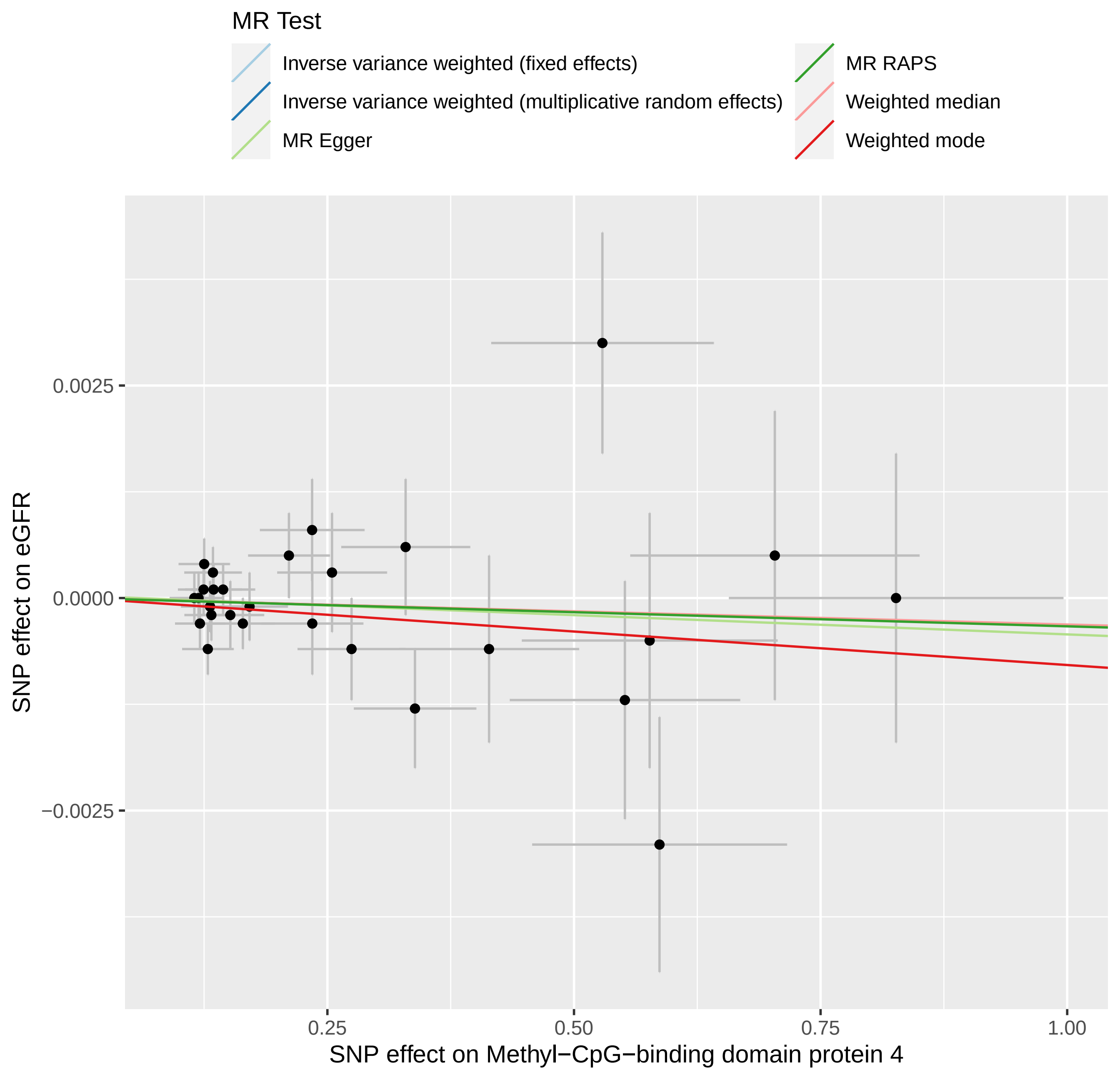

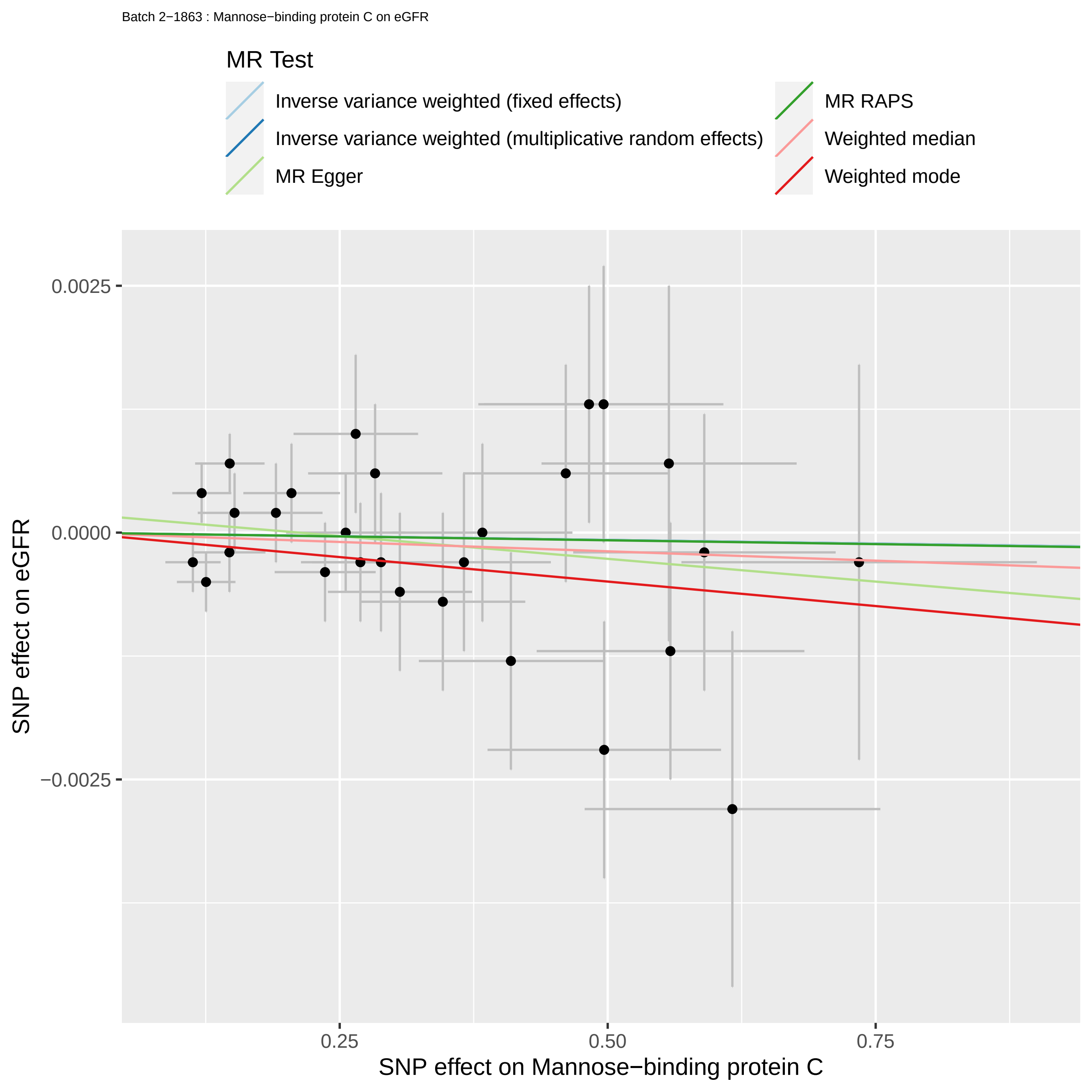

Batch 2−1863 : Mannose−binding protein C on eGFR
MR Test
Inverse variance weighted (fixed effects)
Inverse variance weighted (multiplicative random effects)
MR Egger
MR RAPS
Weighted median
Weighted mode
0.0025
0.0000
−0.0025
0.25
0.50
0.75
SNP effect on eGFR
SNP effect on Mannose−binding protein C

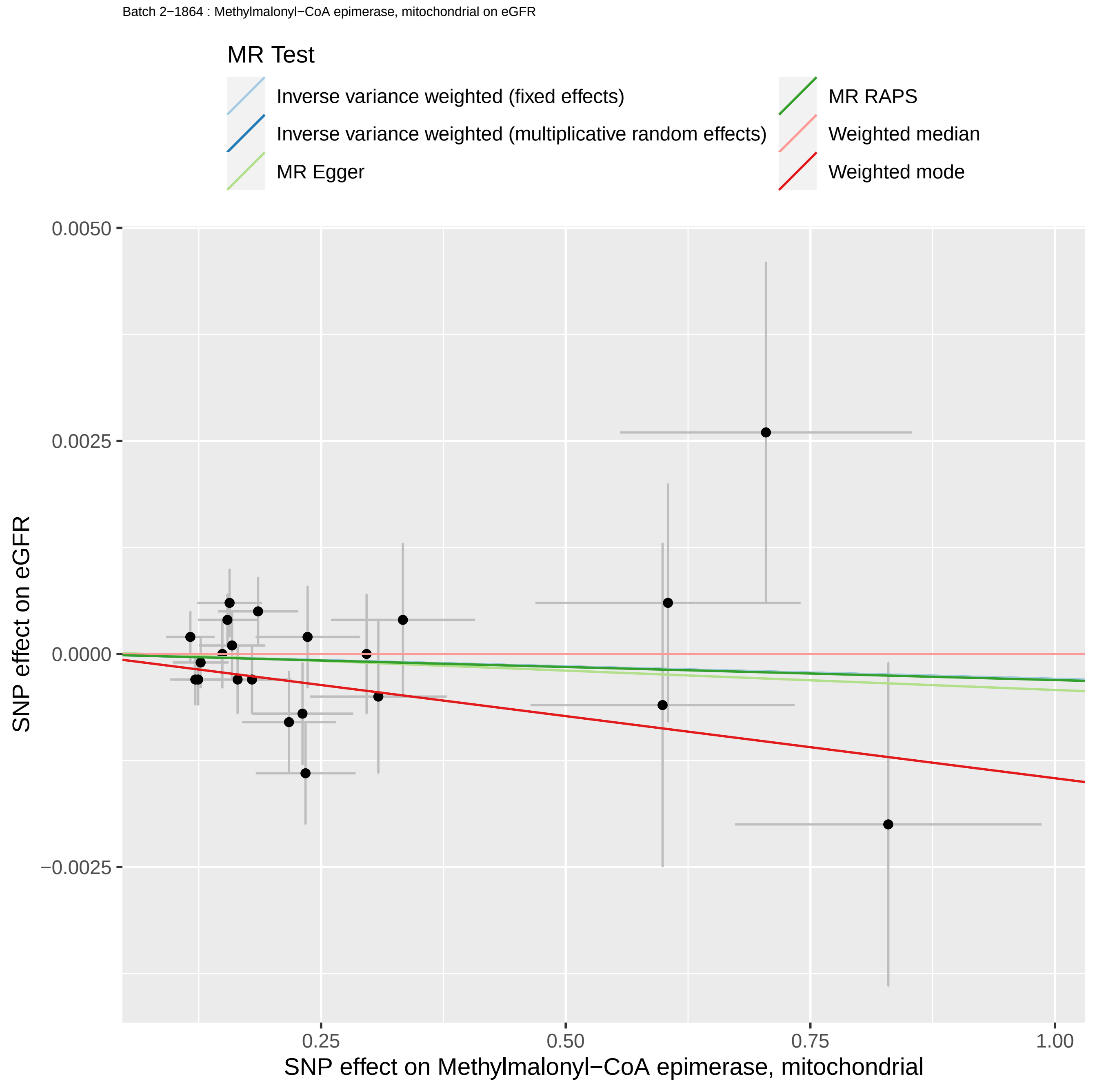
Batch 2−1864 : Methylmalonyl−CoA epimerase, mitochondrial on eGFR
MR Test
Inverse variance weighted (fixed effects)
Inverse variance weighted (multiplicative random effects)
MR Egger
MR RAPS
Weighted median
Weighted mode
0.0050
0.0025
0.0000
−0.0025
SNP effect on eGFR
0.25
0.50
0.75
1.00
SNP effect on Methylmalonyl−CoA epimerase, mitochondrial

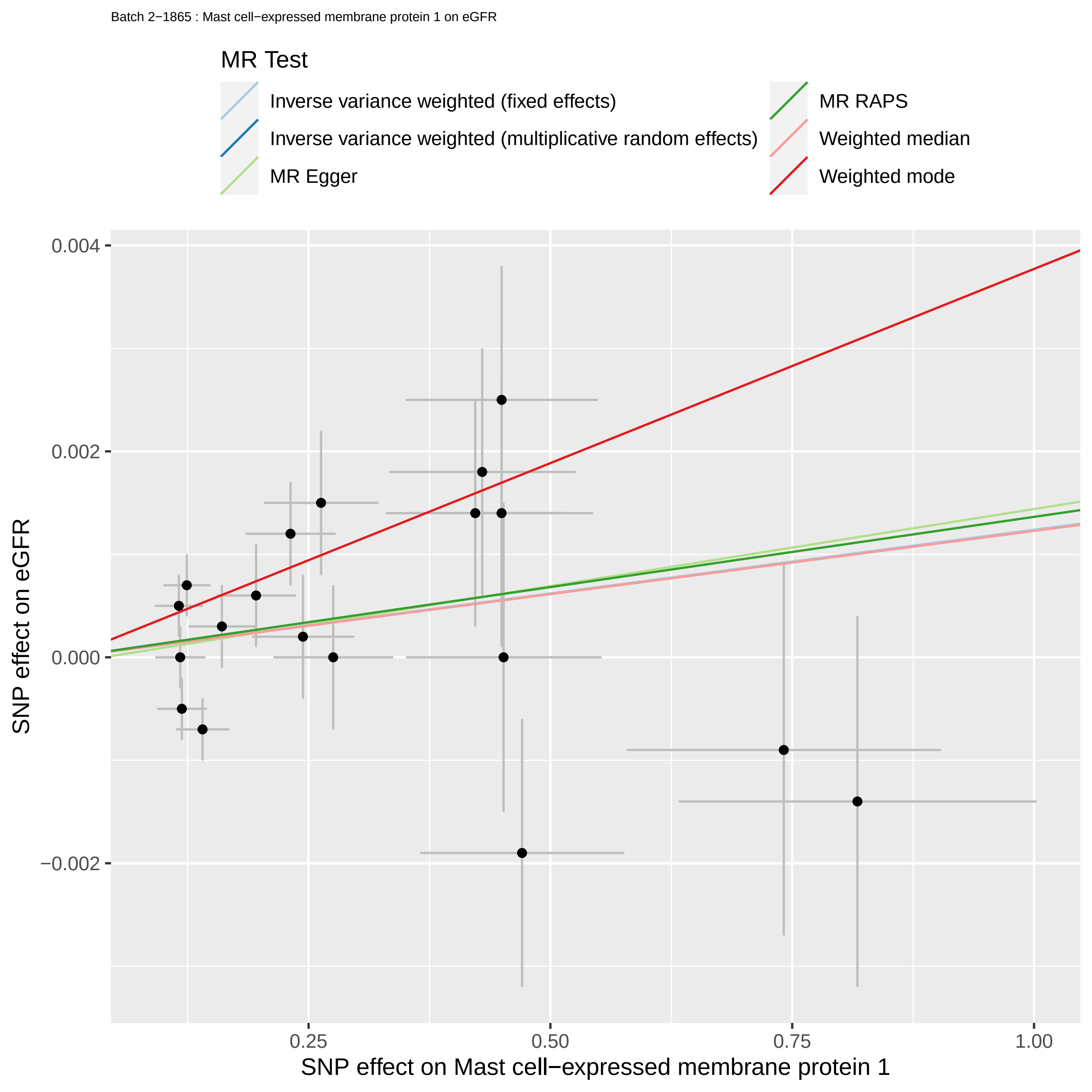

Batch 2−1865 : Mast cell−expressed membrane protein 1 on eGFR
MR Test
Inverse variance weighted (fixed effects)
Inverse variance weighted (multiplicative random effects)
MR Egger
MR RAPS
Weighted median
Weighted mode
0.004
0.002
0.000
−0.002
SNP effect on eGFR
0.25
0.50
0.75
1.00
SNP effect on Mast cell−expressed membrane protein 1

Batch 2−1866 : Guanine nucleotide exchange factor DBS on eGFR

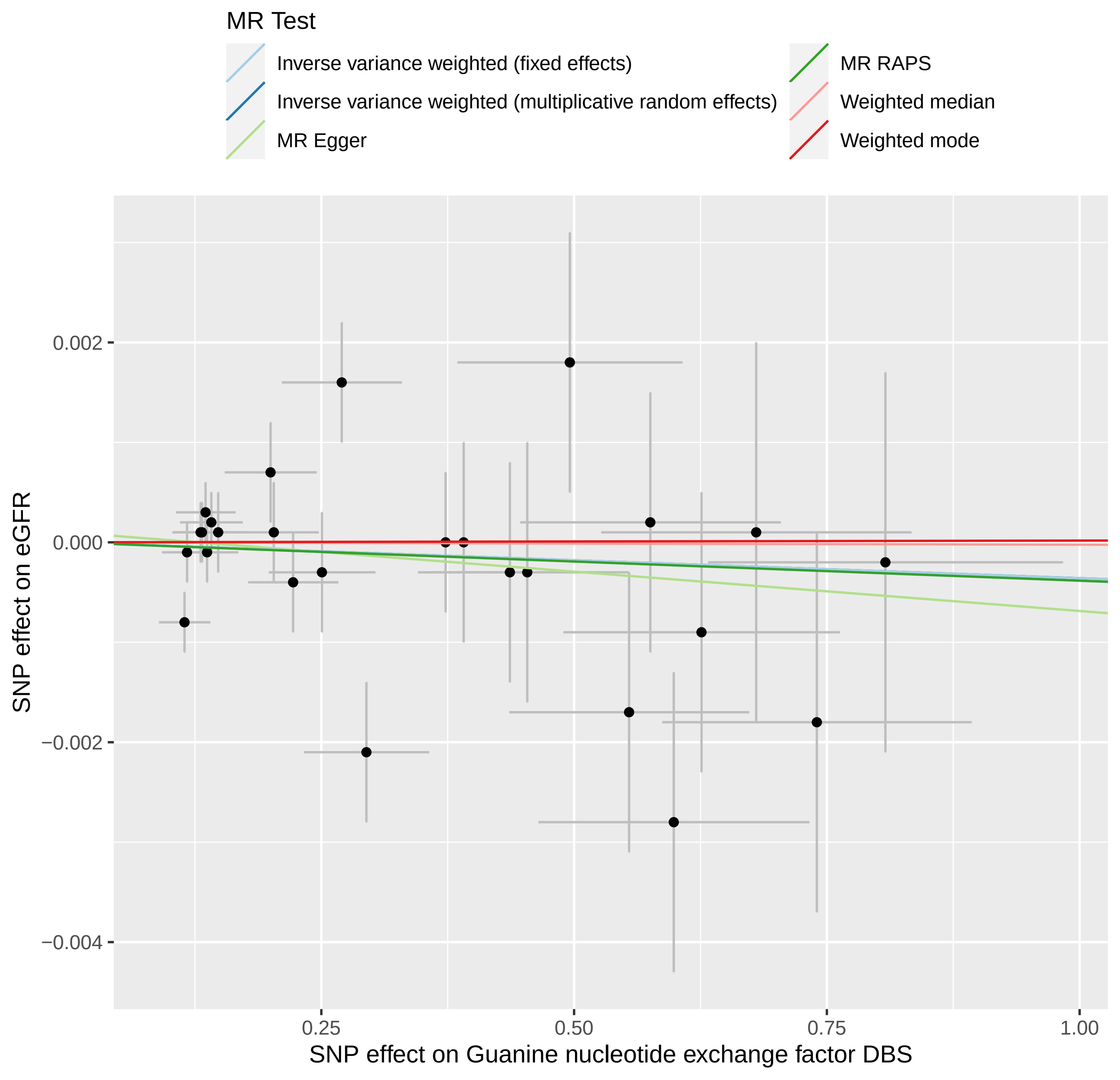

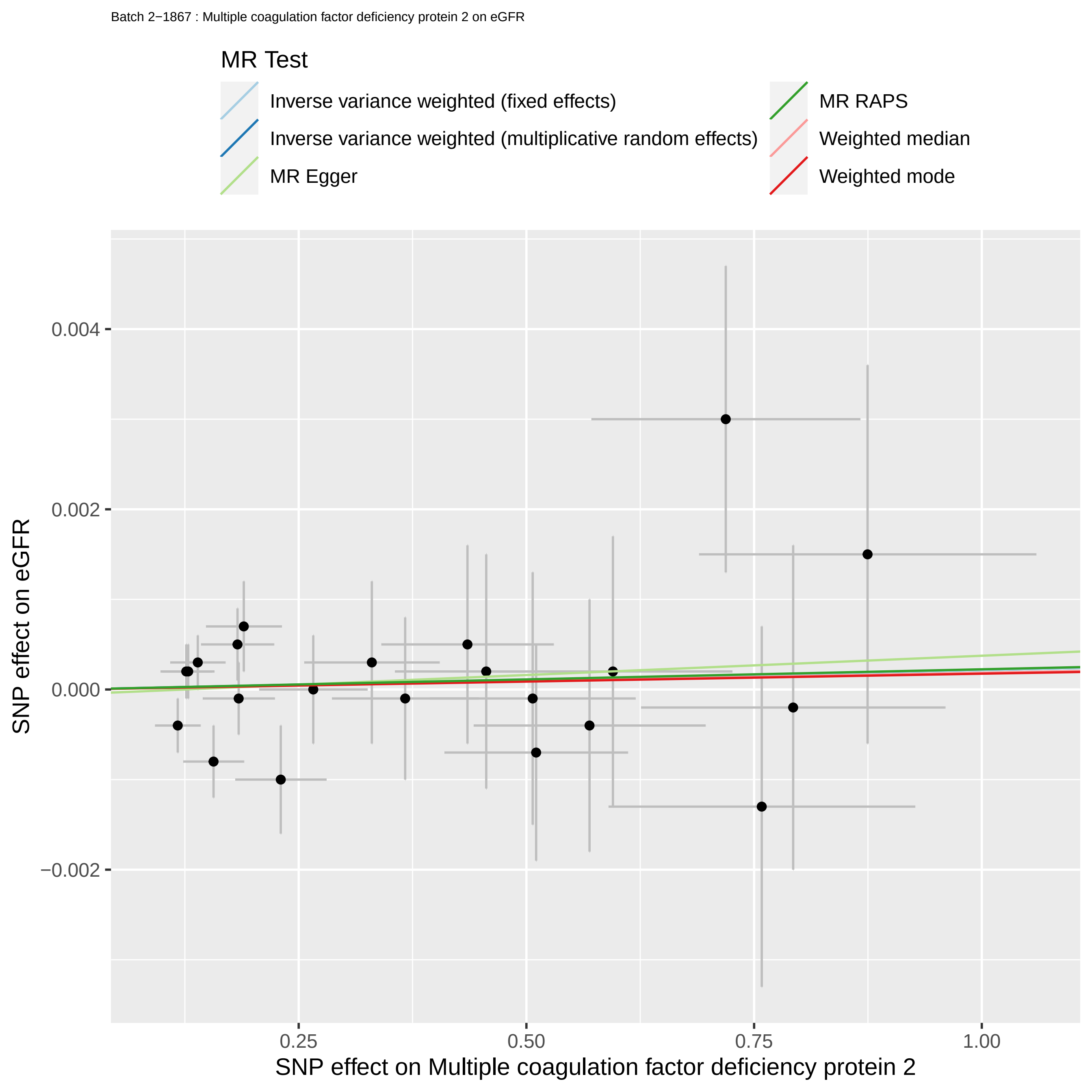
Batch 2−1867 : Multiple coagulation factor deficiency protein 2 on eGFR
MR Test
Inverse variance weighted (fixed effects)
Inverse variance weighted (multiplicative random effects)
MR Egger
MR RAPS
Weighted median
Weighted mode
0.004
0.002
0.000
−0.002
SNP effect on eGFR
0.25
0.50
0.75
1.00
SNP effect on Multiple coagulation factor deficiency protein 2

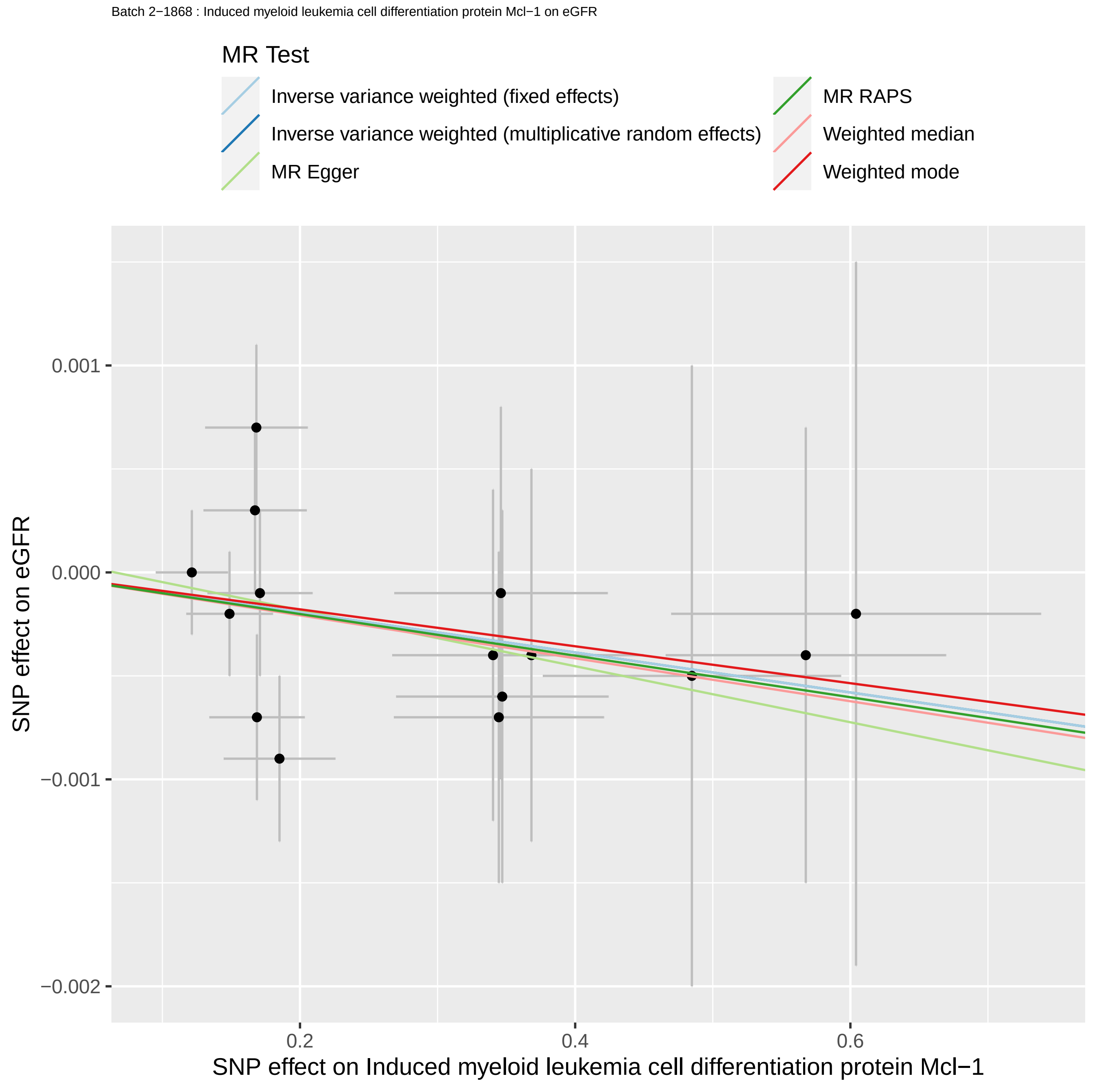

Batch 2−1868 : Induced myeloid leukemia cell differentiation protein Mcl−1 on eGFR
MR Test
Inverse variance weighted (fixed effects)
Inverse variance weighted (multiplicative random effects)
MR Egger
MR RAPS
Weighted median
Weighted mode
SNP effect on eGFR
0.001
0.000
−0.001
−0.002
0.2
0.4
0.6
SNP effect on Induced myeloid leukemia cell differentiation protein Mcl−1

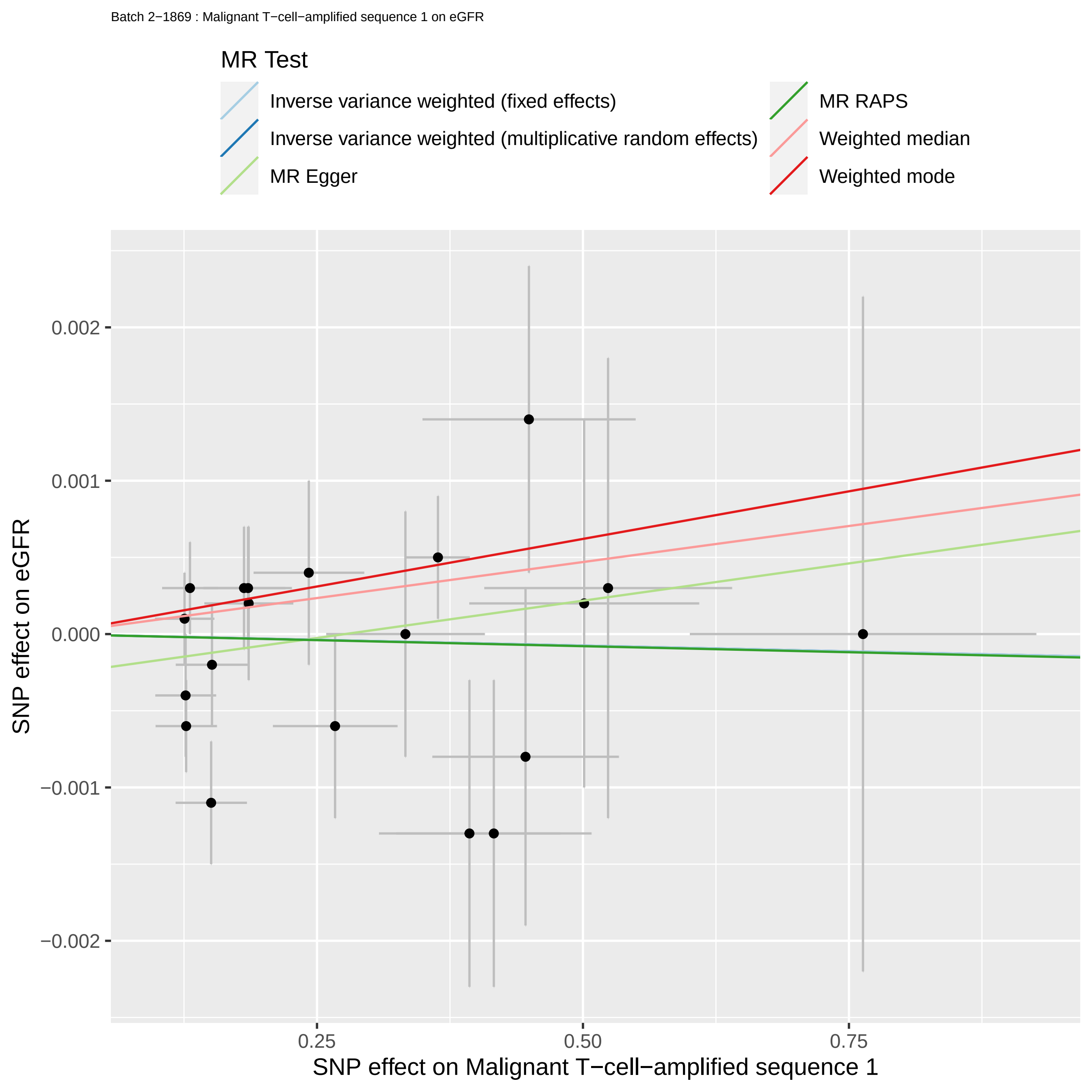

Batch 2−1869 : Malignant T−cell−amplified sequence 1 on eGFR
MR Test
Inverse variance weighted (fixed effects)
Inverse variance weighted (multiplicative random effects)
MR Egger
MR RAPS
Weighted median
Weighted mode
0.002
0.001
0.000
−0.001
−0.002
0.25
0.50
0.75
SNP effect on eGFR
SNP effect on Malignant T−cell−amplified sequence 1

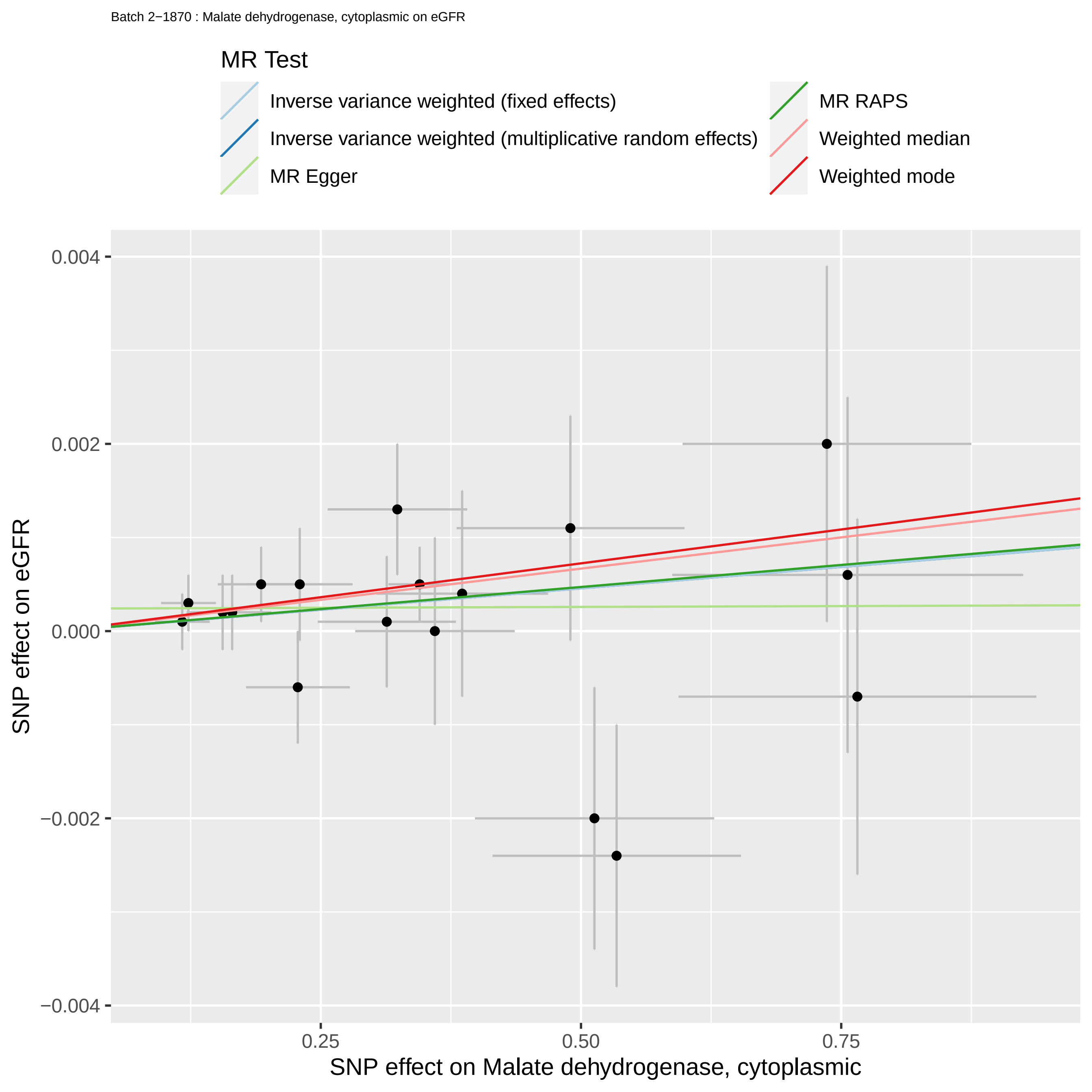

Batch 2−1870 : Malate dehydrogenase, cytoplasmic on eGFR
MR Test
Inverse variance weighted (fixed effects)
Inverse variance weighted (multiplicative random effects)
MR Egger
MR RAPS
Weighted median
Weighted mode
SNP effect on eGFR
0.004
0.002
0.000
−0.002
−0.004
0.25
0.50
0.75
SNP effect on Malate dehydrogenase, cytoplasmic

Batch 2−1871 : Midkine on eGFR

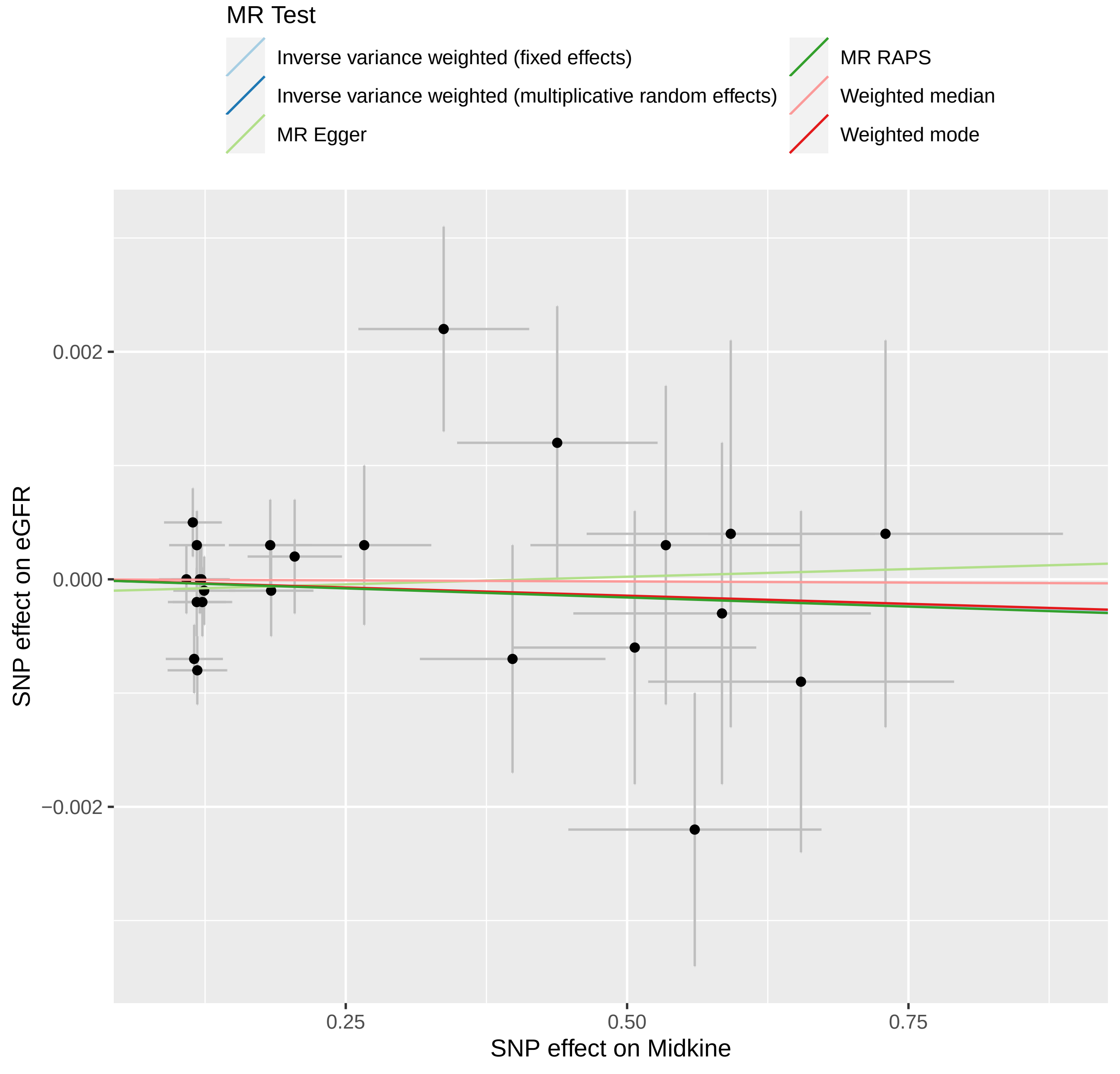

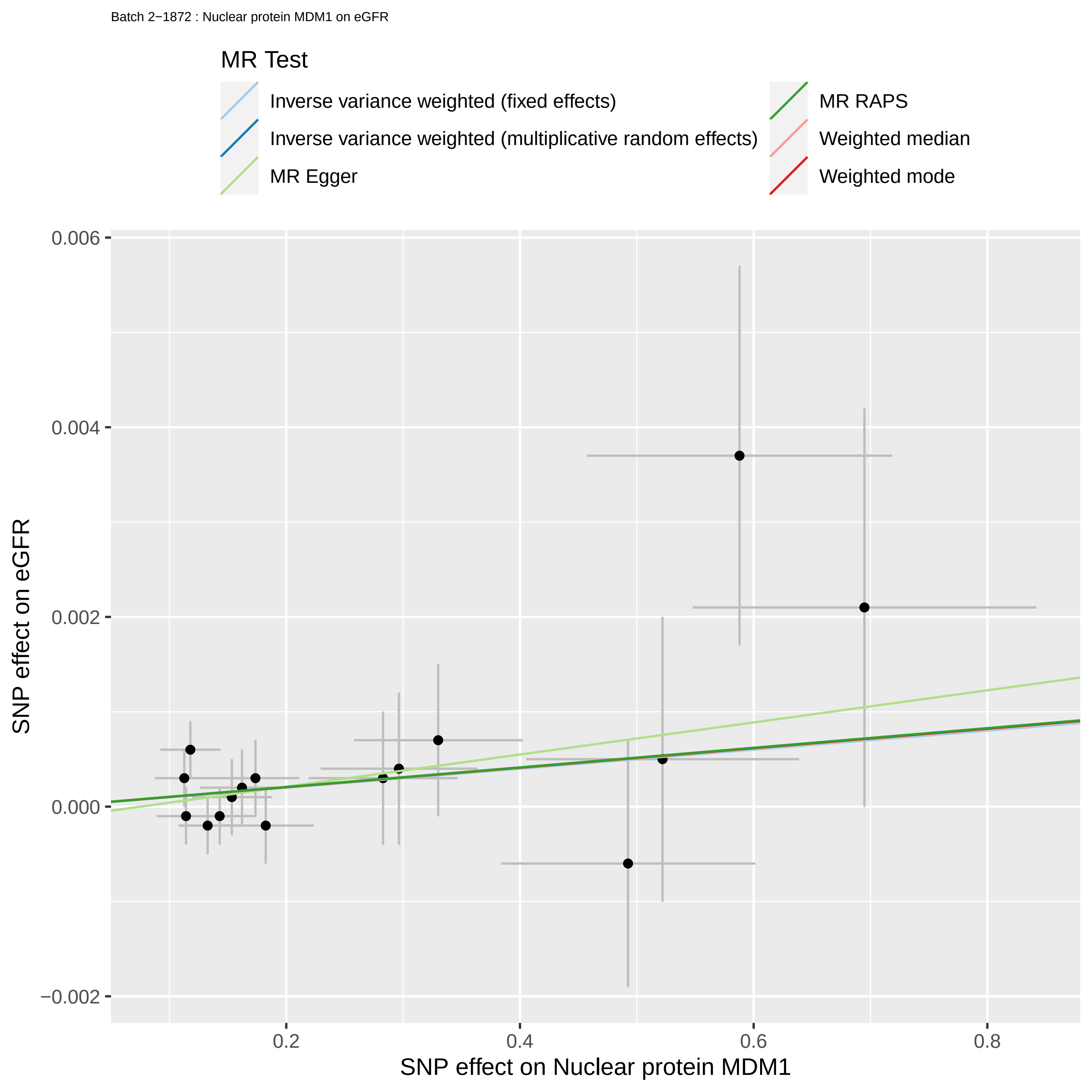

Batch 2−1872 : Nuclear protein MDM1 on eGFR
MR Test
Inverse variance weighted (fixed effects)
Inverse variance weighted (multiplicative random effects)
MR Egger
MR RAPS
Weighted median
Weighted mode
0.006
0.004
0.002
0.000
−0.002
0.2
0.4
0.6
0.8
SNP effect on eGFR
SNP effect on Nuclear protein MDM1

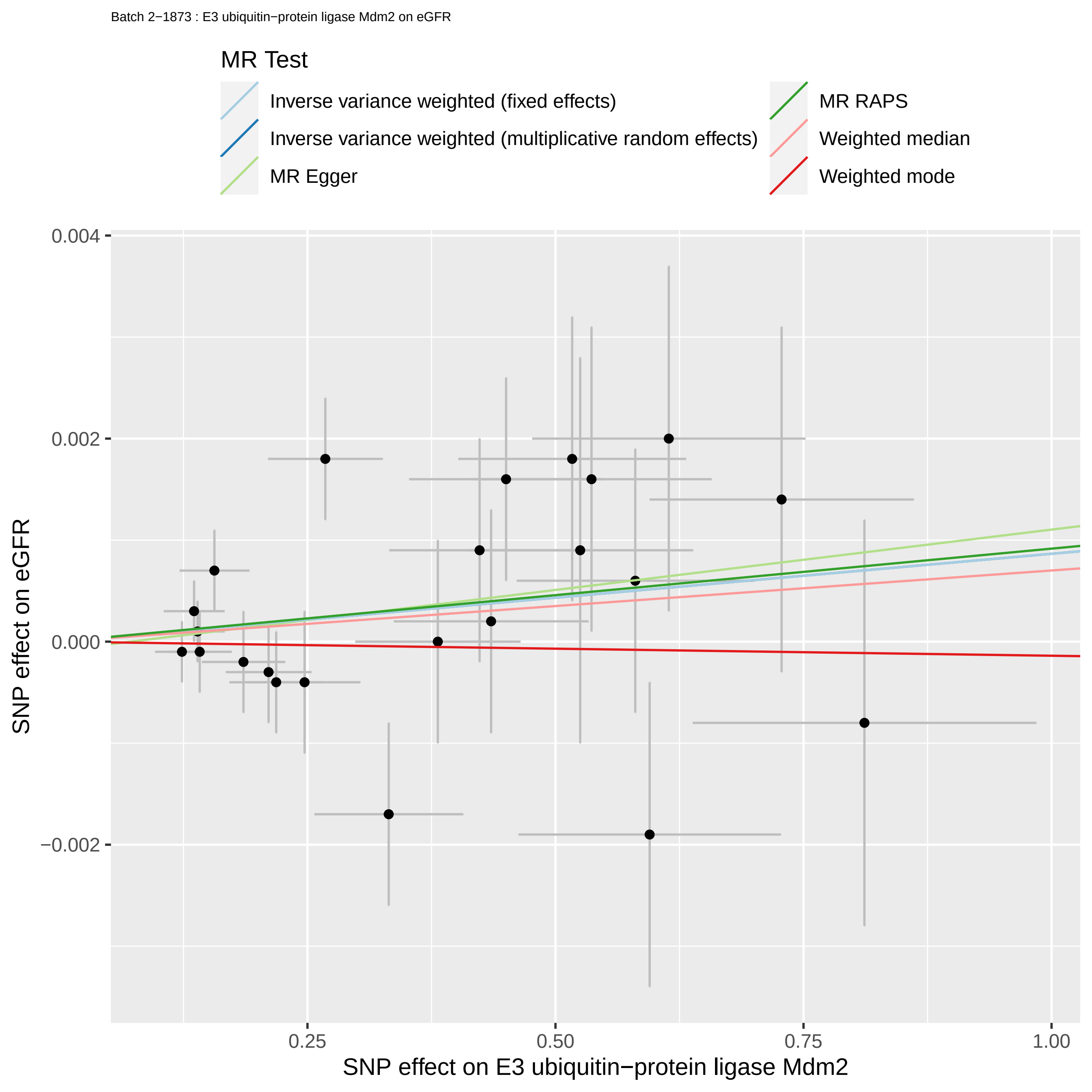

Batch 2−1873 : E3 ubiquitin−protein ligase Mdm2 on eGFR
MR Test
Inverse variance weighted (fixed effects)
Inverse variance weighted (multiplicative random effects)
MR Egger
MR RAPS
Weighted median
Weighted mode
0.004
0.002
0.000
−0.002
0.25
0.50
0.75
1.00
SNP effect on eGFR
SNP effect on E3 ubiquitin−protein ligase Mdm2

Batch 2−1874 : Mediator of RNA polymerase II transcription subunit 1 on eGFR

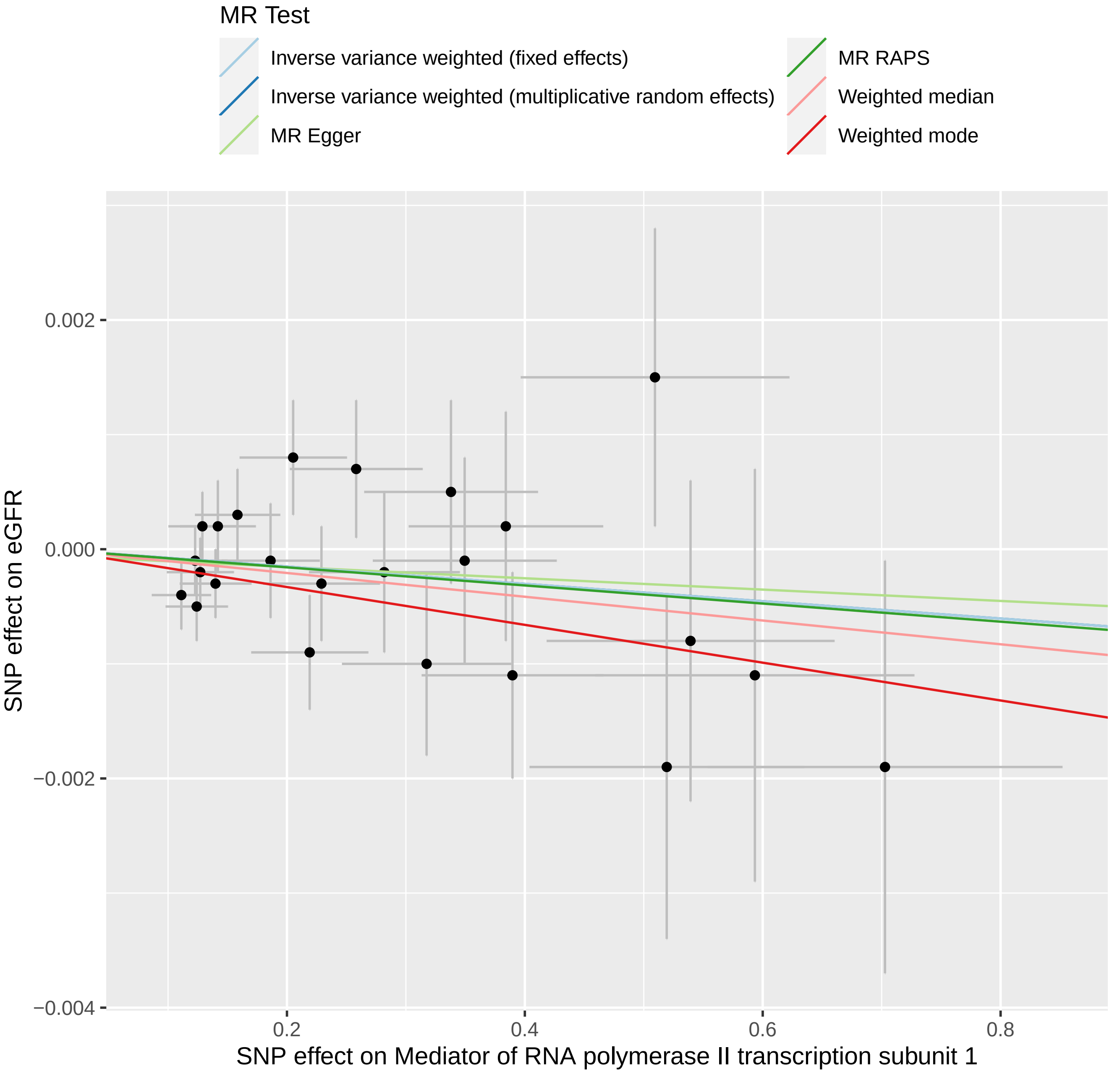

Batch 2−1875 : Mediator of RNA polymerase II transcription subunit 1 on eGFR

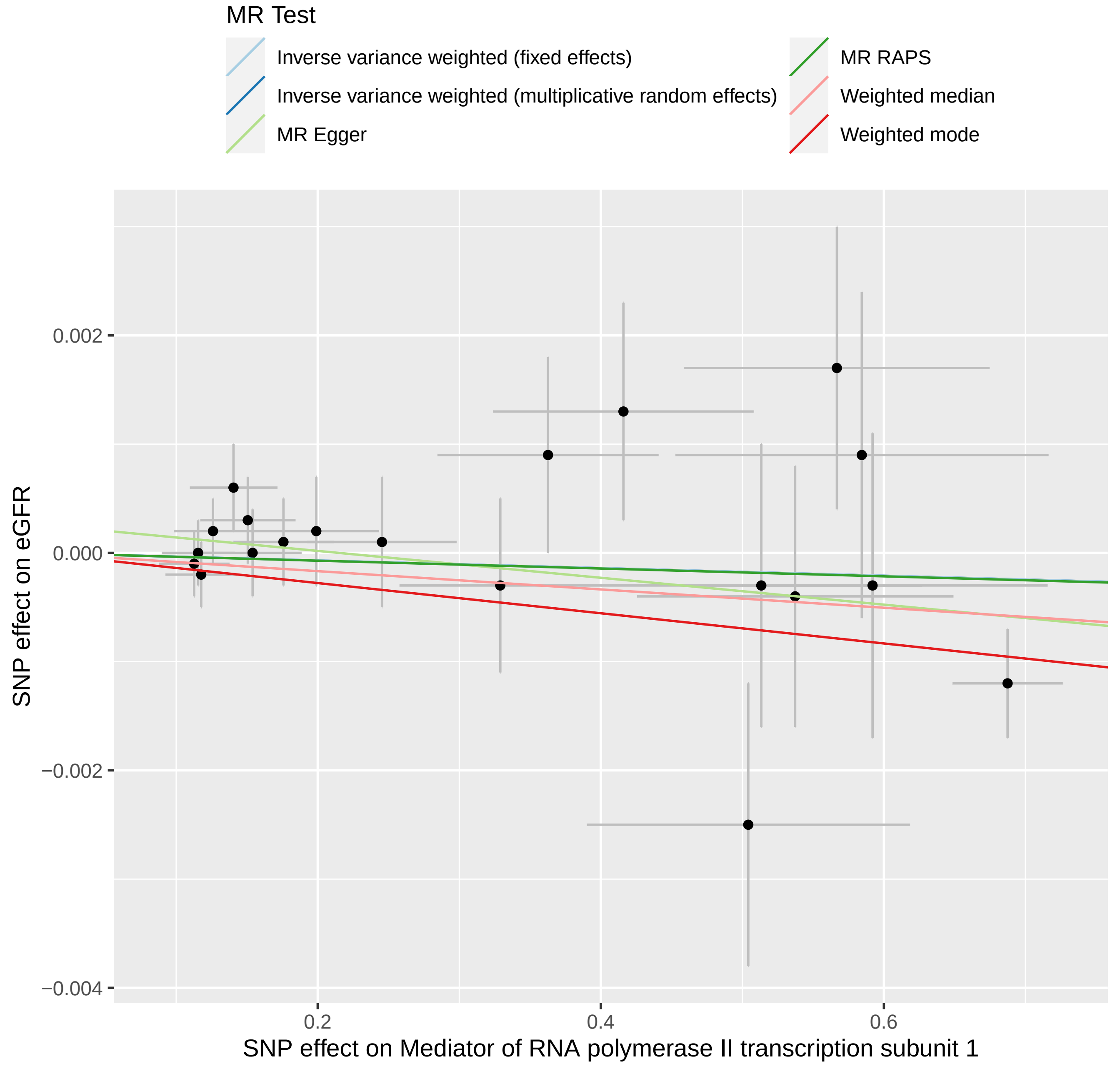

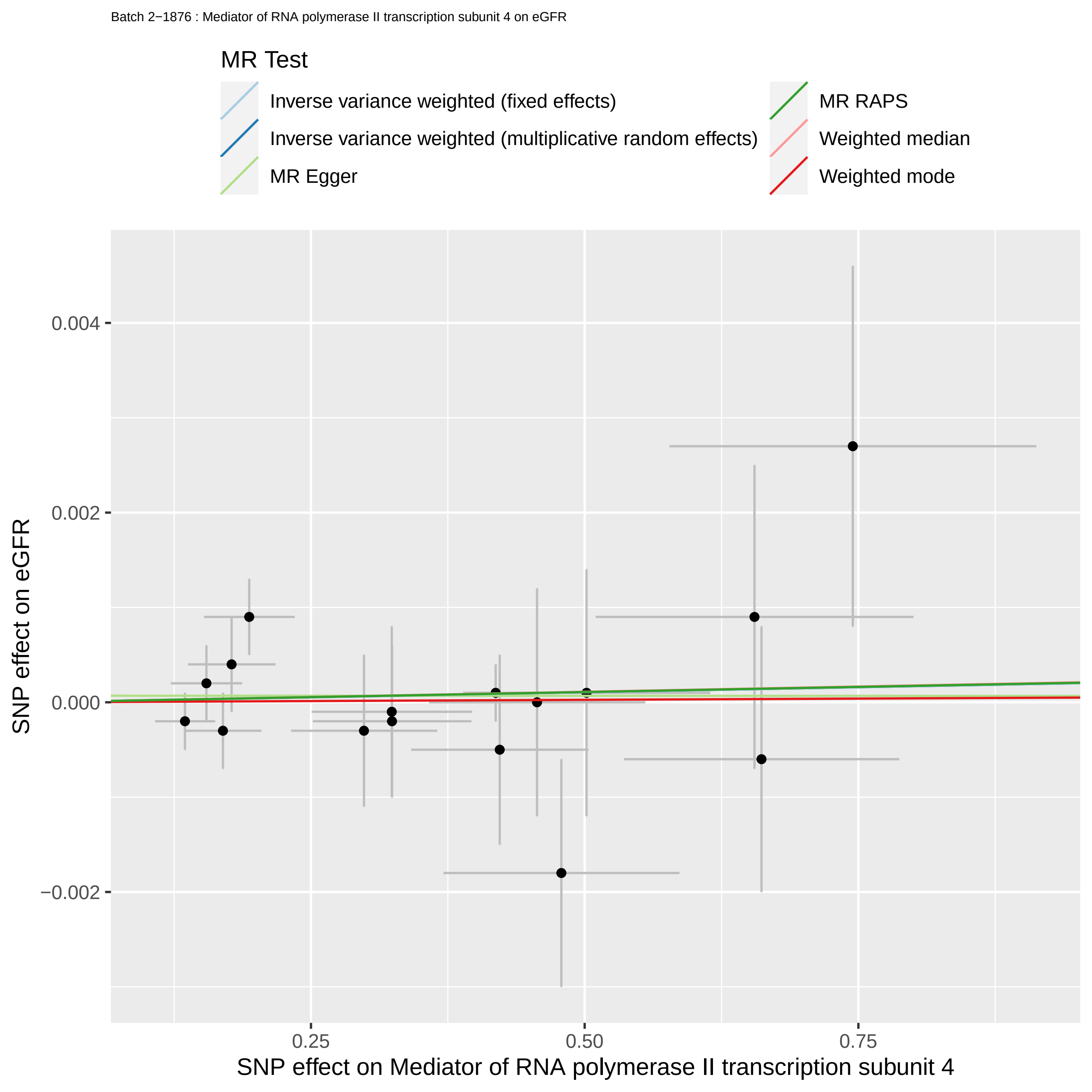

Batch 2−1876 : Mediator of RNA polymerase II transcription subunit 4 on eGFR
MR Test
Inverse variance weighted (fixed effects)
Inverse variance weighted (multiplicative random effects)
MR Egger
MR RAPS
Weighted median
Weighted mode
SNP effect on eGFR
0.004
0.002
0.000
−0.002
0.25
0.50
0.75
SNP effect on Mediator of RNA polymerase II transcription subunit 4

Batch 2−1877 : Myocyte−specific enhancer factor 2C on eGFR

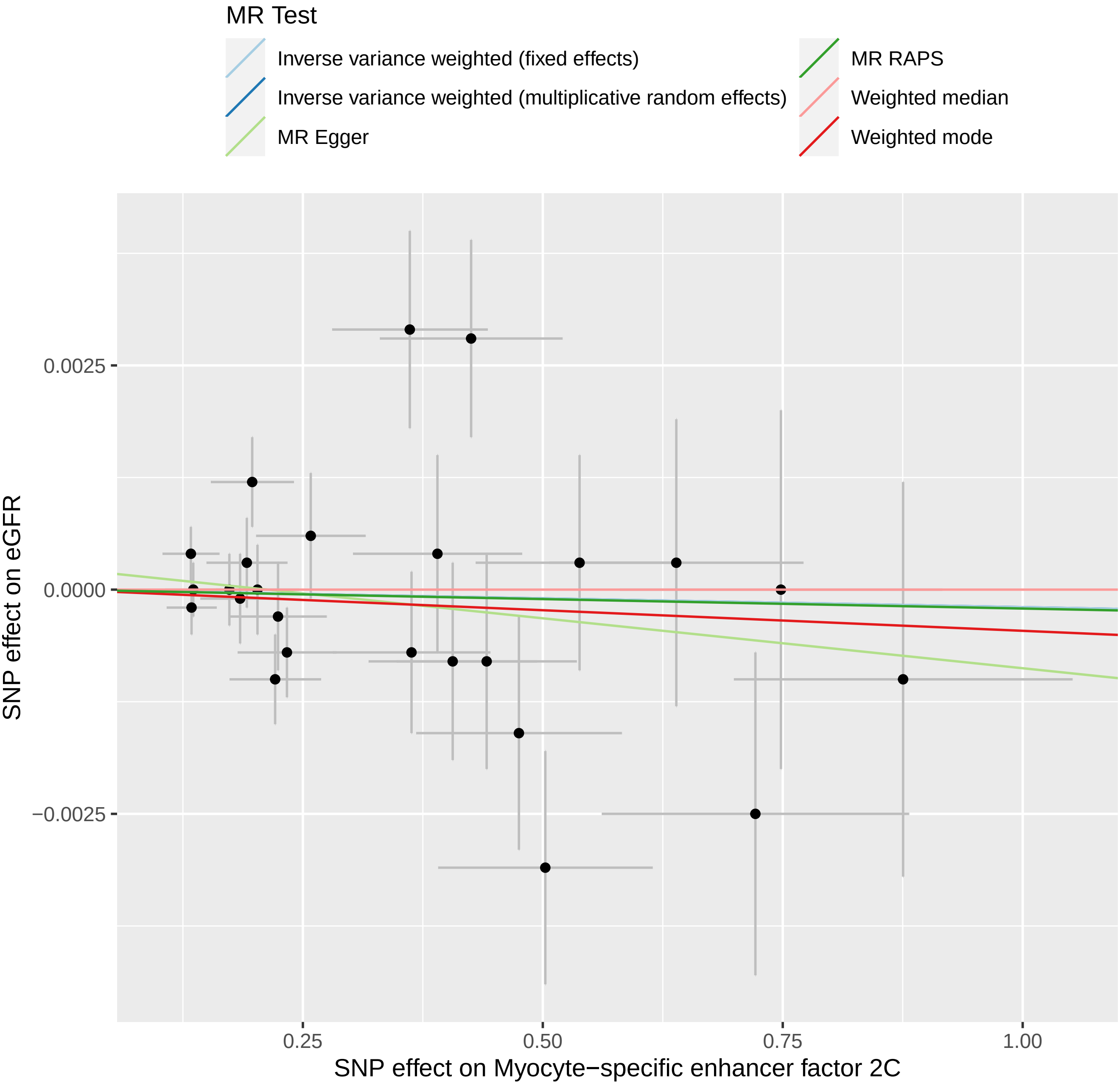

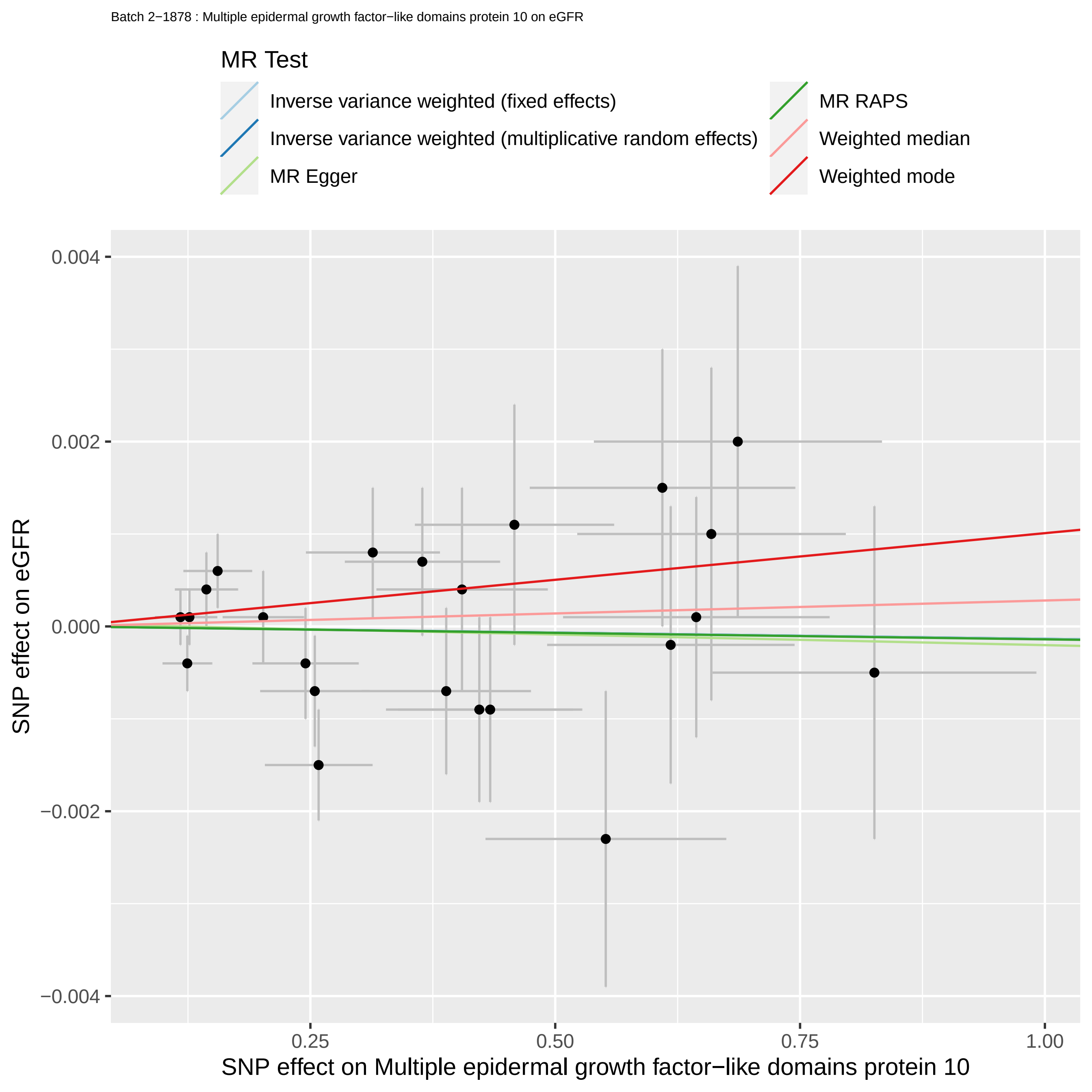

Batch 2−1878 : Multiple epidermal growth factor−like domains protein 10 on eGFR
MR Test
Inverse variance weighted (fixed effects)
Inverse variance weighted (multiplicative random effects)
MR Egger
MR RAPS
Weighted median
Weighted mode
0.004
0.002
0.000
−0.002
−0.004
SNP effect on eGFR
0.25
0.50
0.75
1.00
SNP effect on Multiple epidermal growth factor−like domains protein 10

Batch 2−1879 : Protein MENT on eGFR

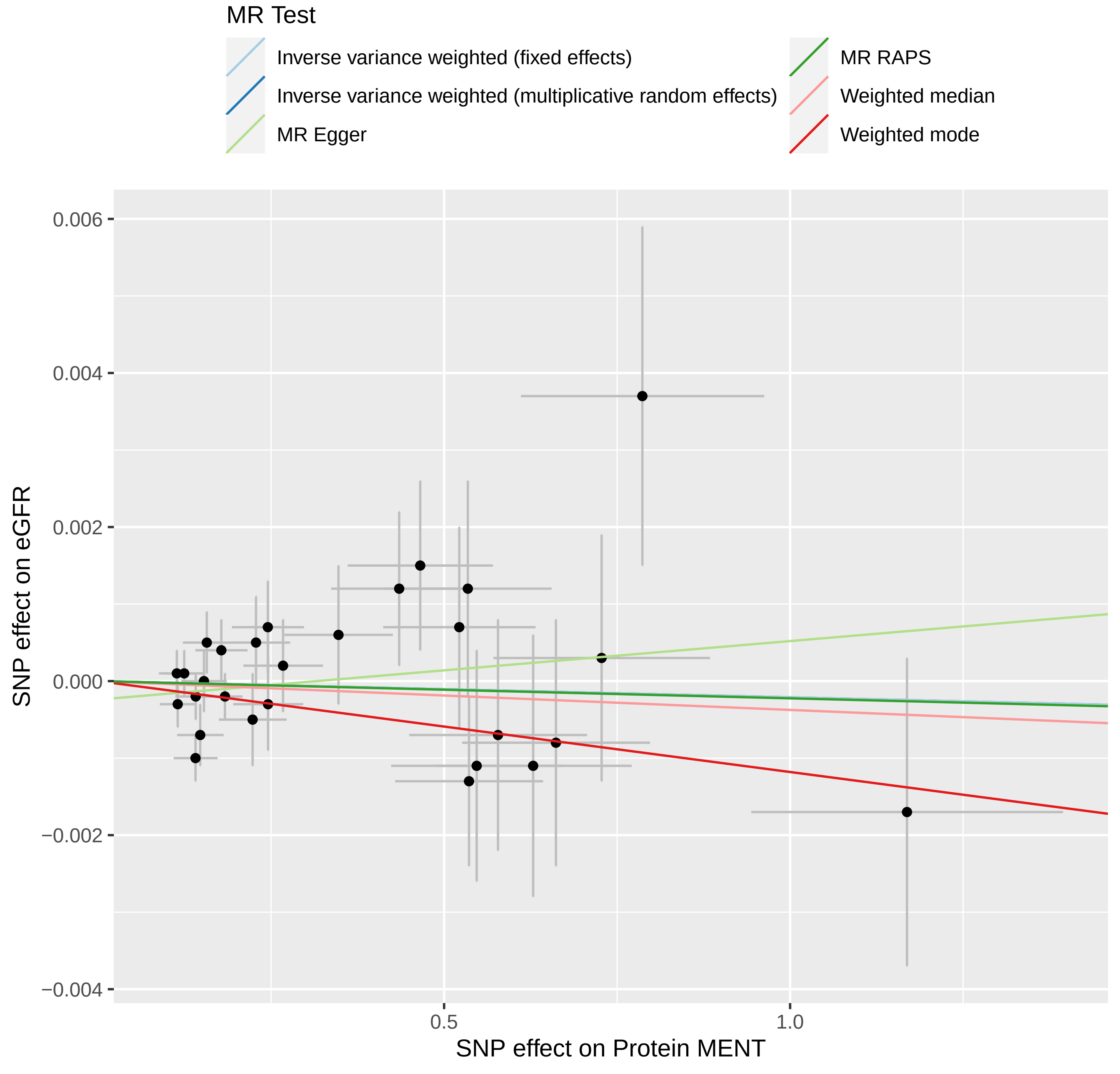

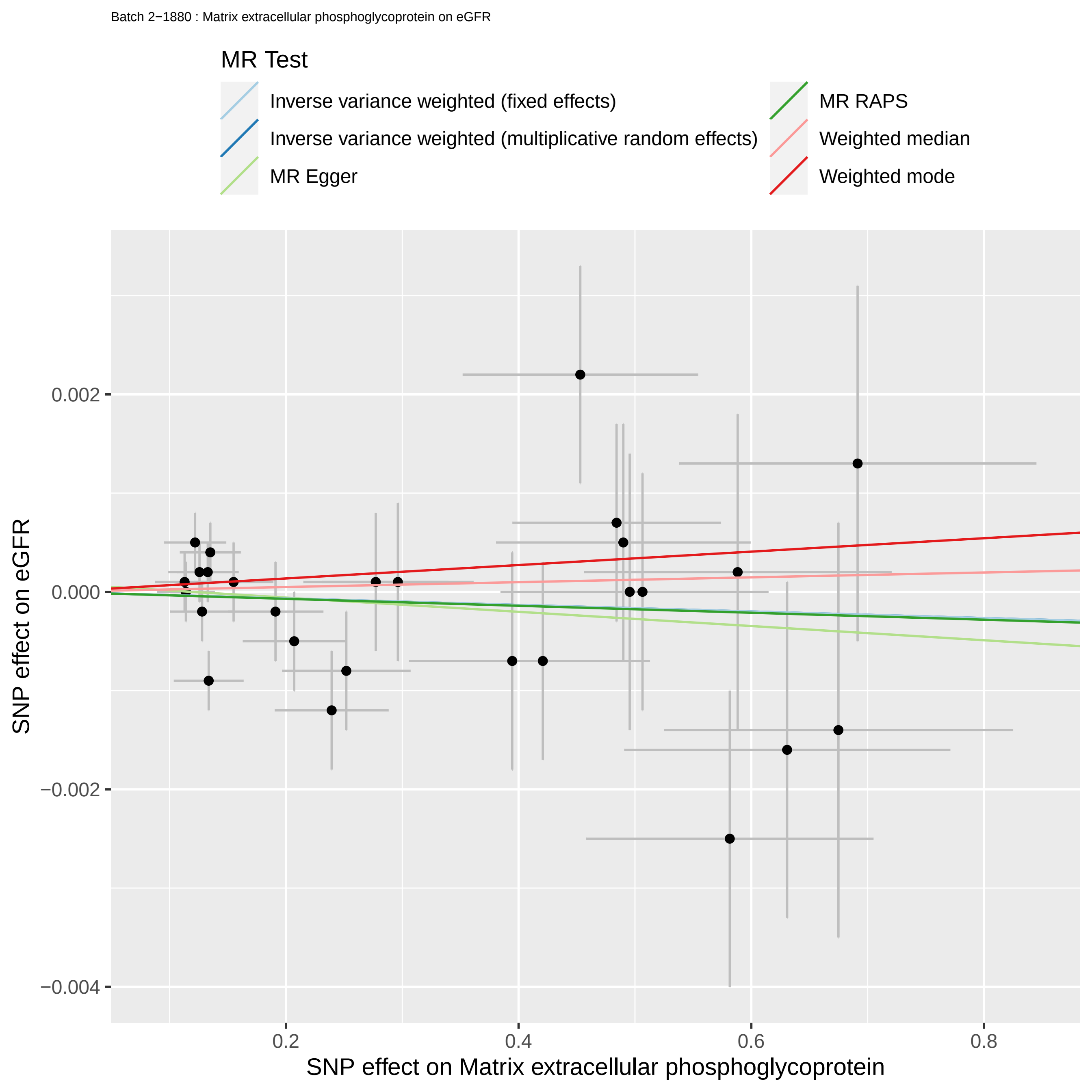
Batch 2−1880 : Matrix extracellular phosphoglycoprotein on eGFR
MR Test
Inverse variance weighted (fixed effects)
Inverse variance weighted (multiplicative random effects)
MR Egger
MR RAPS
Weighted median
Weighted mode
0.002
0.000
−0.002
−0.004
0.2
0.4
0.6
0.8
SNP effect on eGFR
SNP effect on Matrix extracellular phosphoglycoprotein

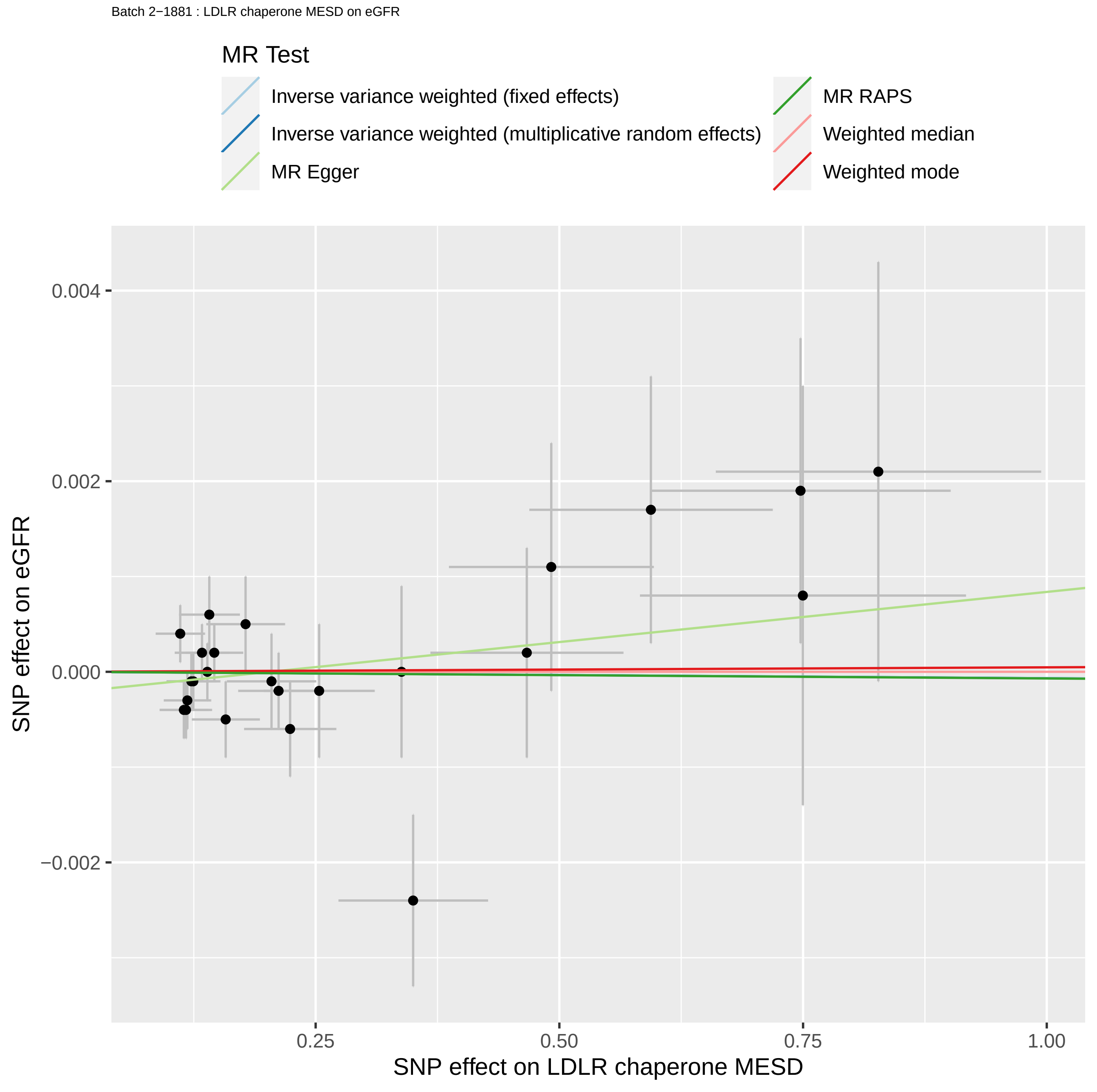
Batch 2−1881 : LDLR chaperone MESD on eGFR
MR Test
Inverse variance weighted (fixed effects)
Inverse variance weighted (multiplicative random effects)
MR Egger
MR RAPS
Weighted median
Weighted mode
0.004
0.002
0.000
−0.002
SNP effect on eGFR
0.25
0.50
0.75
1.00
SNP effect on LDLR chaperone MESD

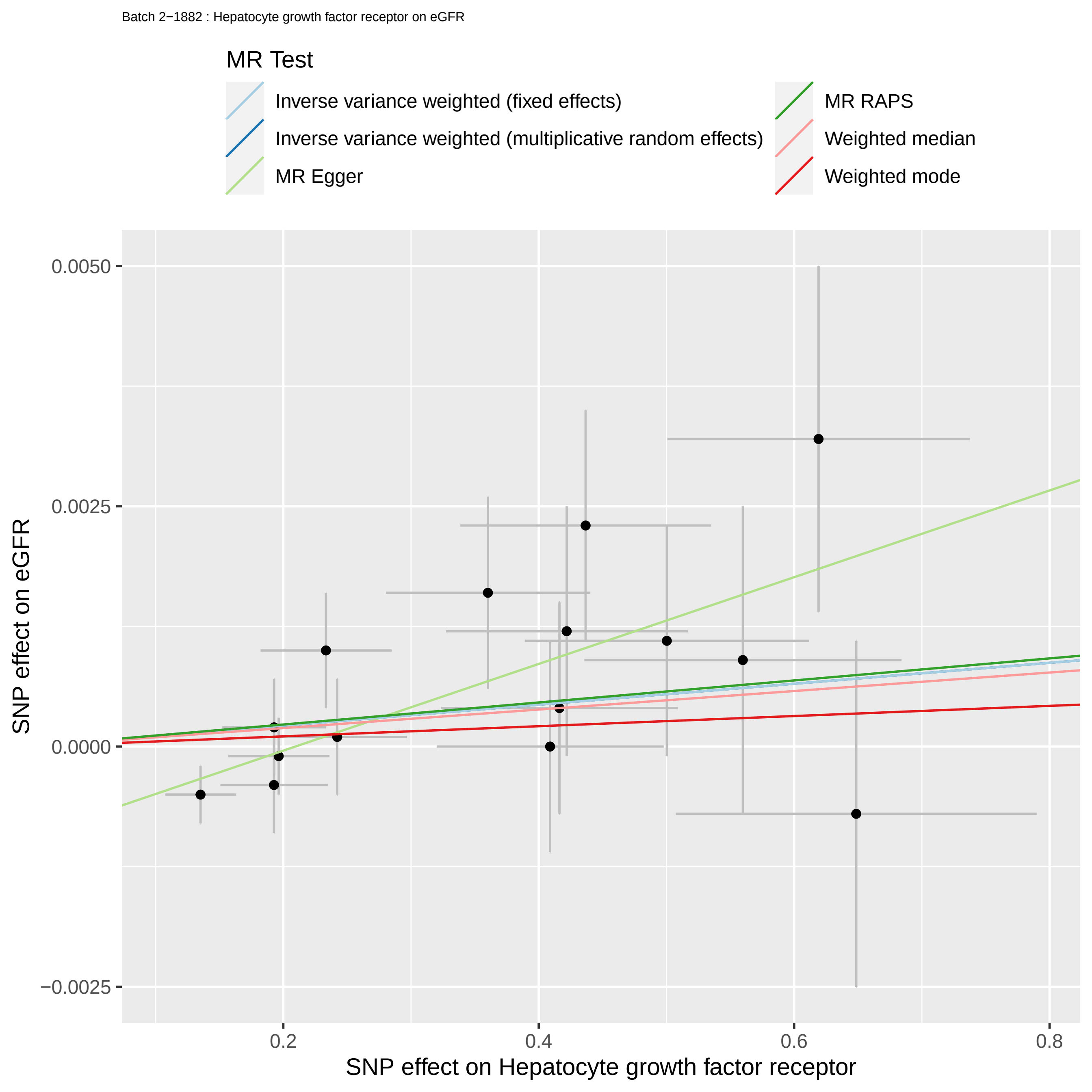
Batch 2−1882 : Hepatocyte growth factor receptor on eGFR
MR Test
Inverse variance weighted (fixed effects)
Inverse variance weighted (multiplicative random effects)
MR Egger
MR RAPS
Weighted median
Weighted mode
0.0050
0.0025
0.0000
−0.0025
0.2
0.4
0.6
0.8
SNP effect on eGFR
SNP effect on Hepatocyte growth factor receptor

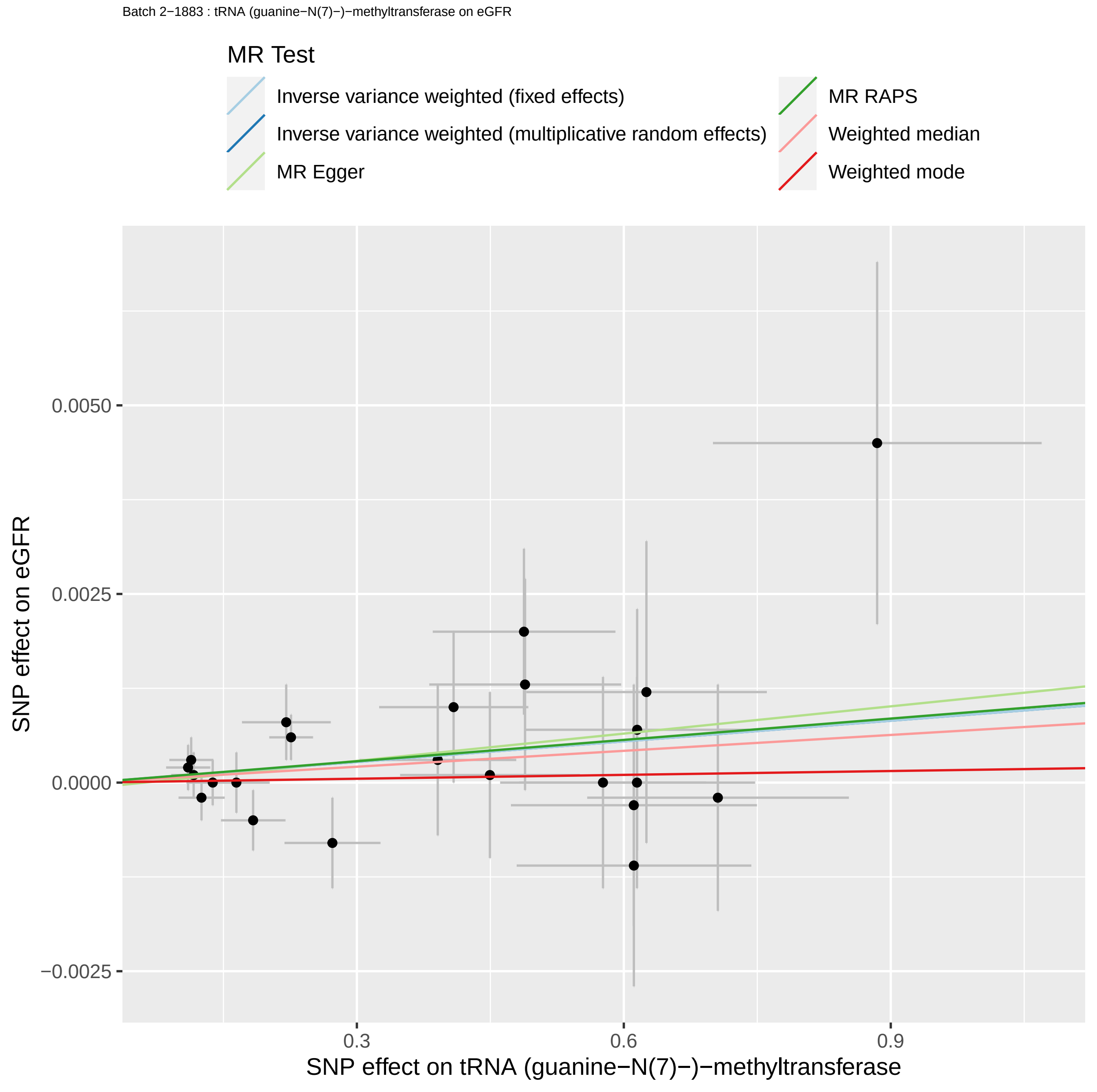
Batch 2−1883 : tRNA (guanine−N(7)−)−methyltransferase on eGFR
MR Test
Inverse variance weighted (fixed effects)
Inverse variance weighted (multiplicative random effects)
MR Egger
MR RAPS
Weighted median
Weighted mode
0.0050
0.0025
0.0000
−0.0025
0.3
0.6
0.9
SNP effect on eGFR
SNP effect on tRNA (guanine−N(7)−)−methyltransferase

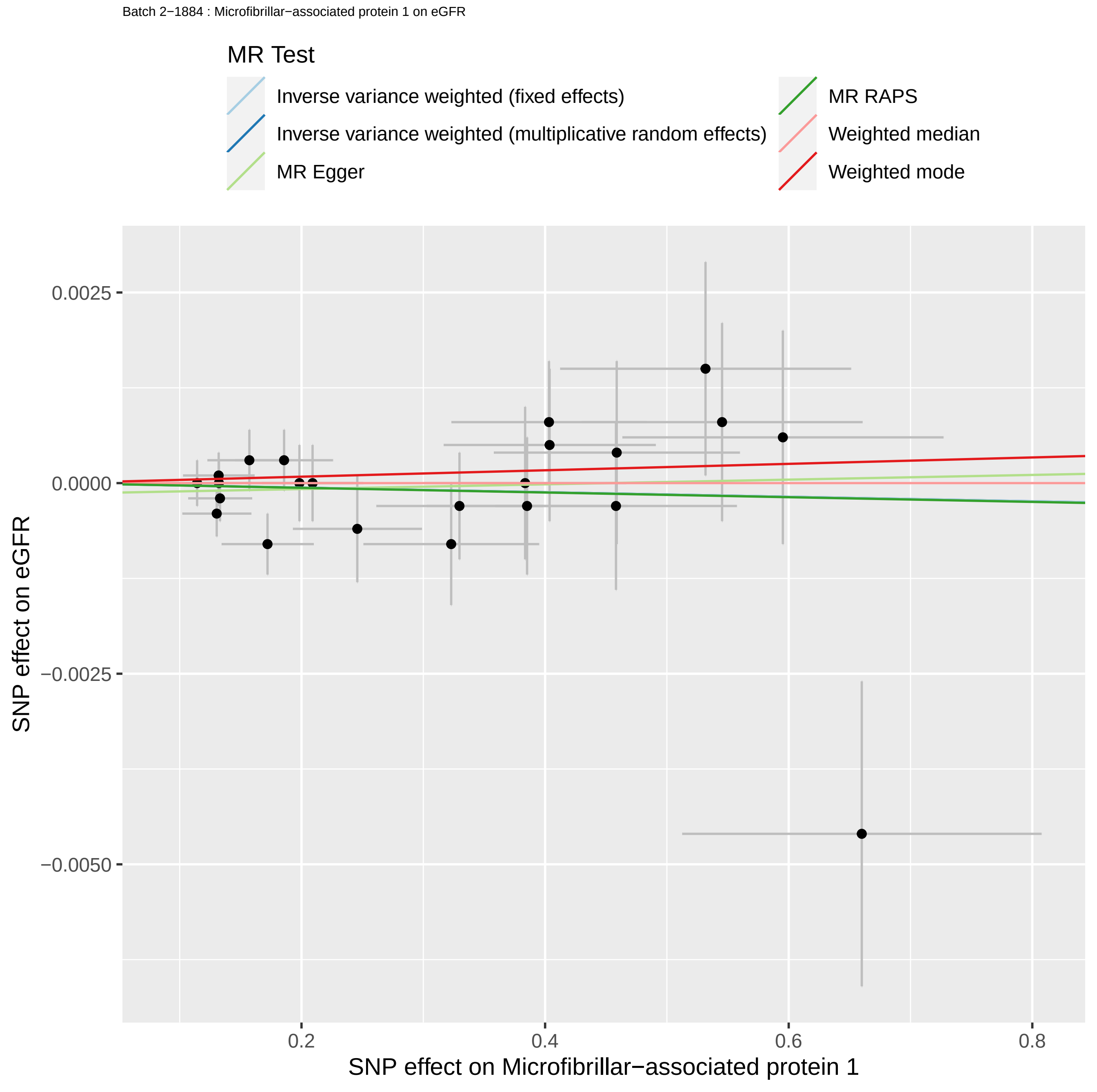
Batch 2−1884 : Microfibrillar−associated protein 1 on eGFR
MR Test
Inverse variance weighted (fixed effects)
Inverse variance weighted (multiplicative random effects)
MR Egger
MR RAPS
Weighted median
Weighted mode
0.0025
0.0000
−0.0025
−0.0050
0.2
0.4
0.6
0.8
SNP effect on eGFR
SNP effect on Microfibrillar−associated protein 1

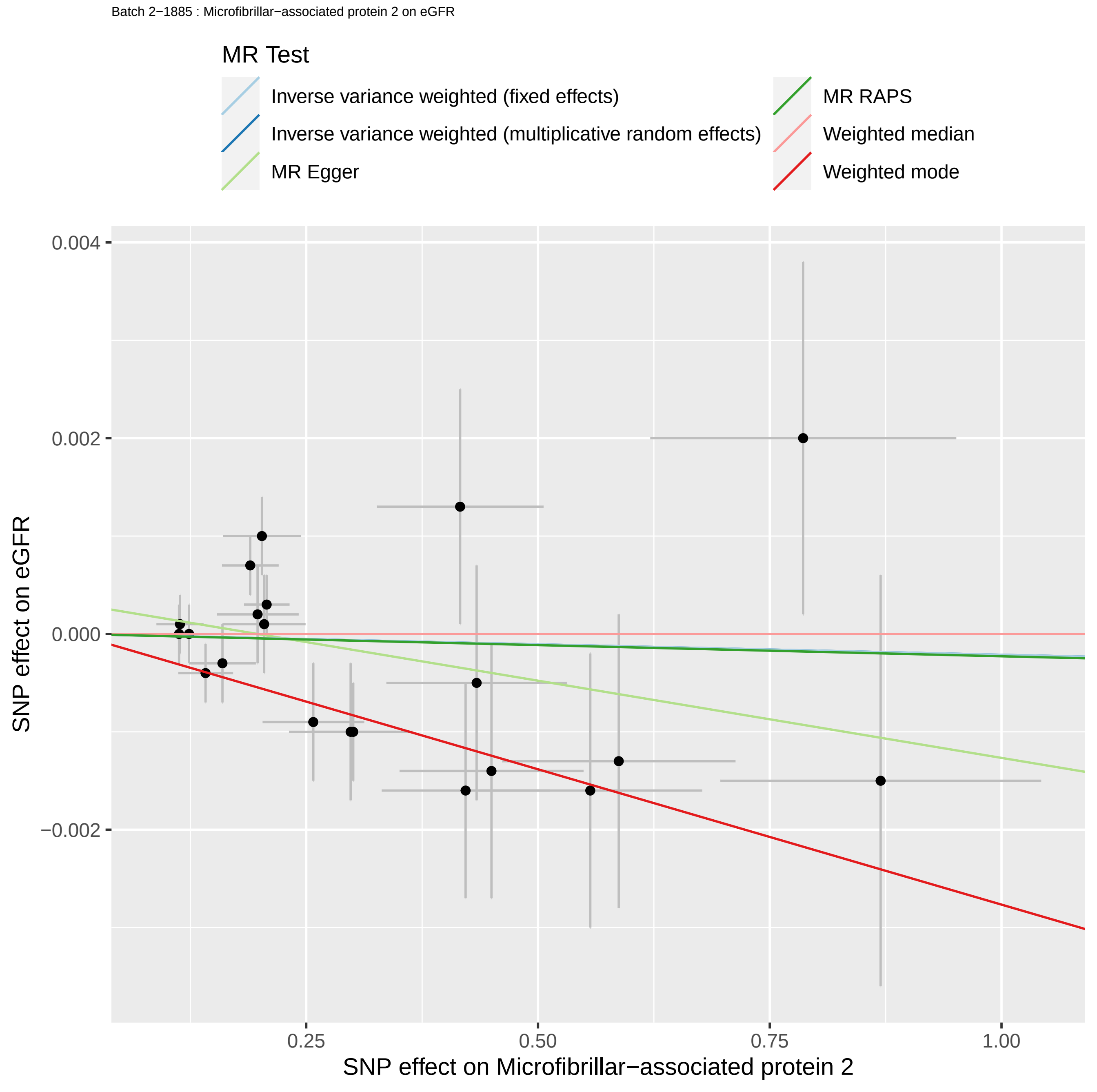
Batch 2−1885 : Microfibrillar−associated protein 2 on eGFR
MR Test
Inverse variance weighted (fixed effects)
Inverse variance weighted (multiplicative random effects)
MR Egger
MR RAPS
Weighted median
Weighted mode
0.004
0.002
0.000
−0.002
0.25
0.50
0.75
1.00
SNP effect on eGFR
SNP effect on Microfibrillar−associated protein 2

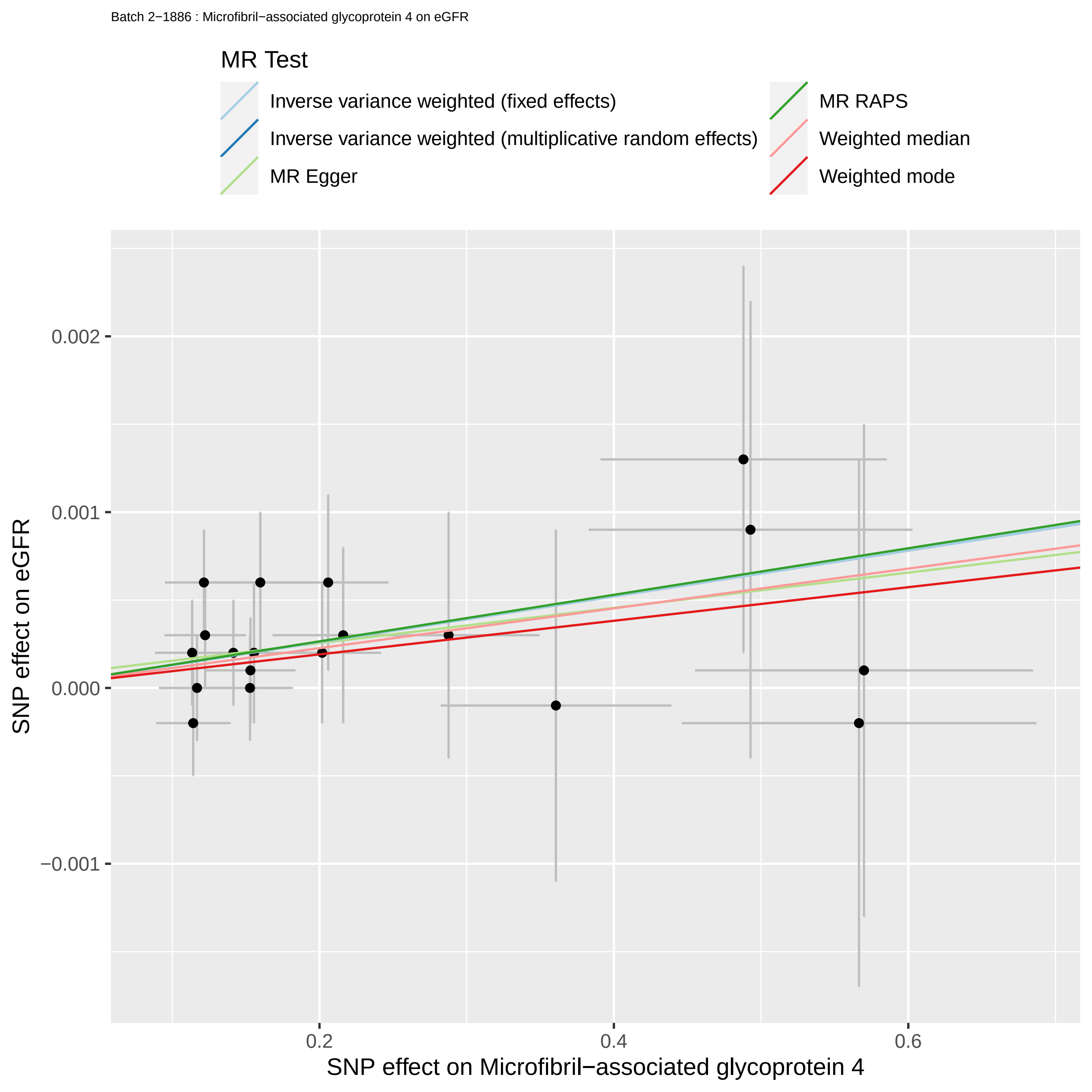
Batch 2−1886 : Microfibril−associated glycoprotein 4 on eGFR
MR Test
Inverse variance weighted (fixed effects)
Inverse variance weighted (multiplicative random effects)
MR Egger
MR RAPS
Weighted median
Weighted mode
0.002
0.001
0.000
−0.001
0.2
0.4
0.6
SNP effect on eGFR
SNP effect on Microfibril−associated glycoprotein 4

Batch 2−1887 : Microfibrillar−associated protein 5 on eGFR

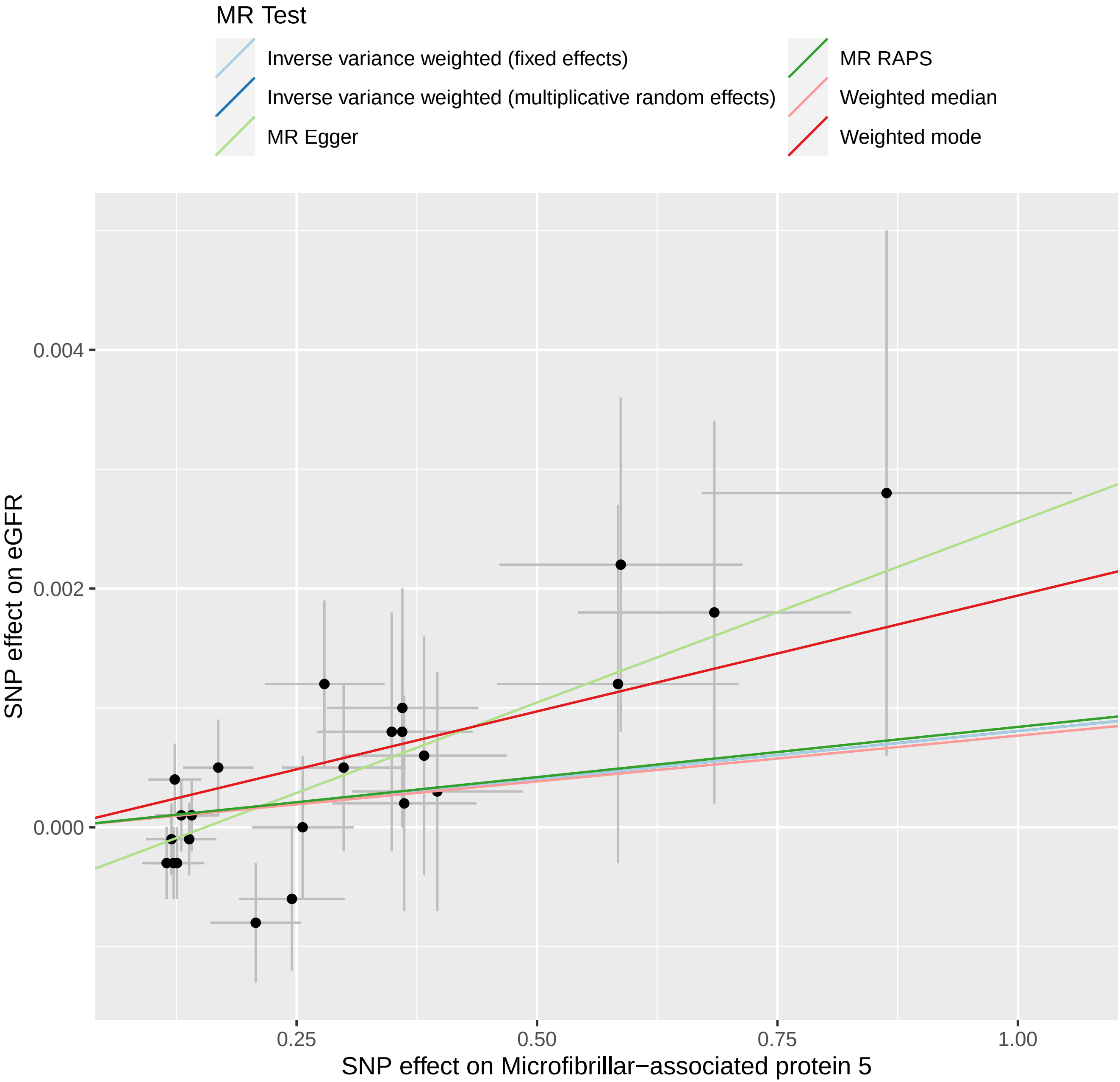

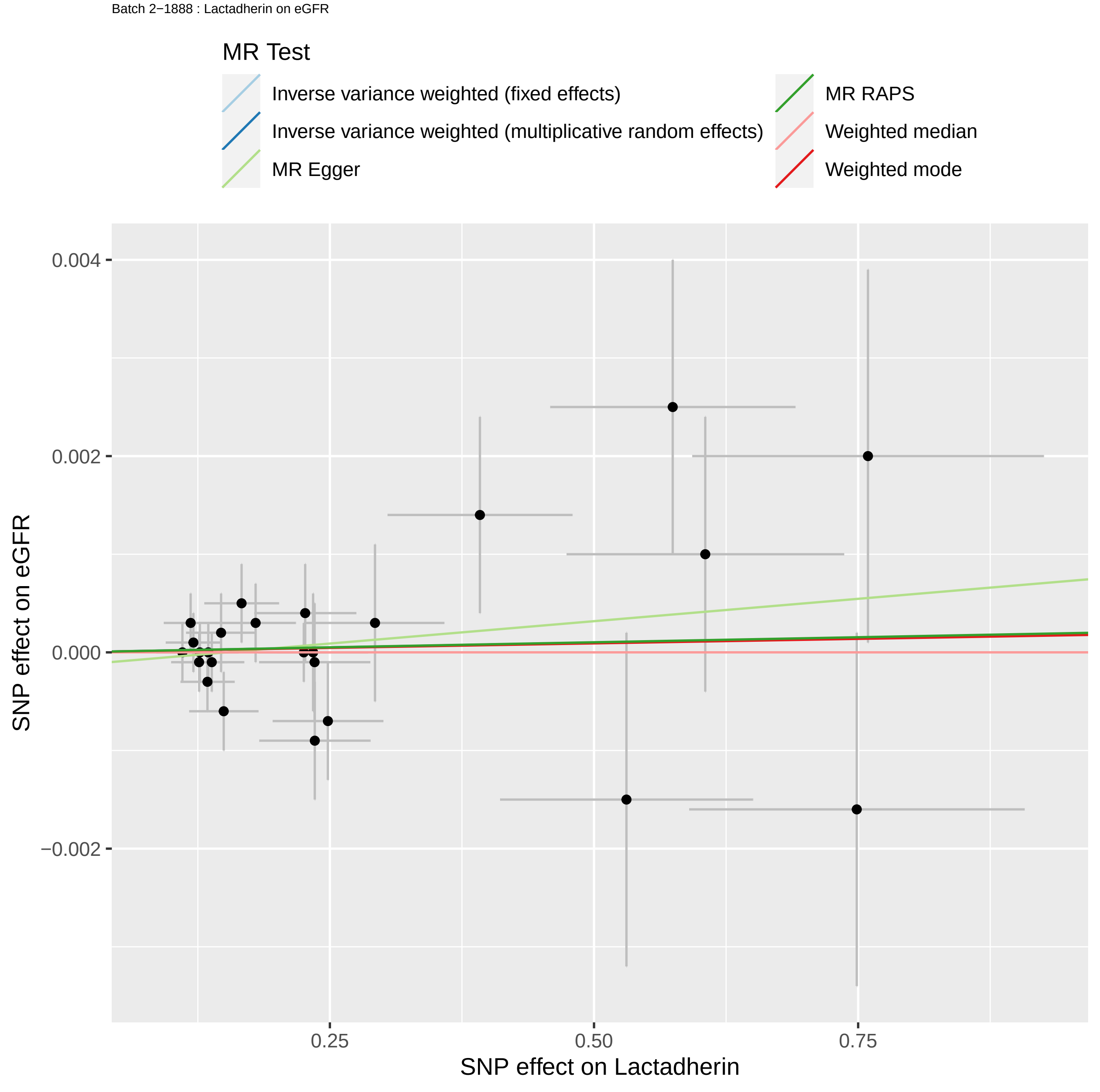

Batch 2−1888 : Lactadherin on eGFR
MR Test
Inverse variance weighted (fixed effects)
Inverse variance weighted (multiplicative random effects)
MR Egger
MR RAPS
Weighted median
Weighted mode
0.004
0.002
0.000
−0.002
SNP effect on eGFR
0.25
0.50
0.75
SNP effect on Lactadherin

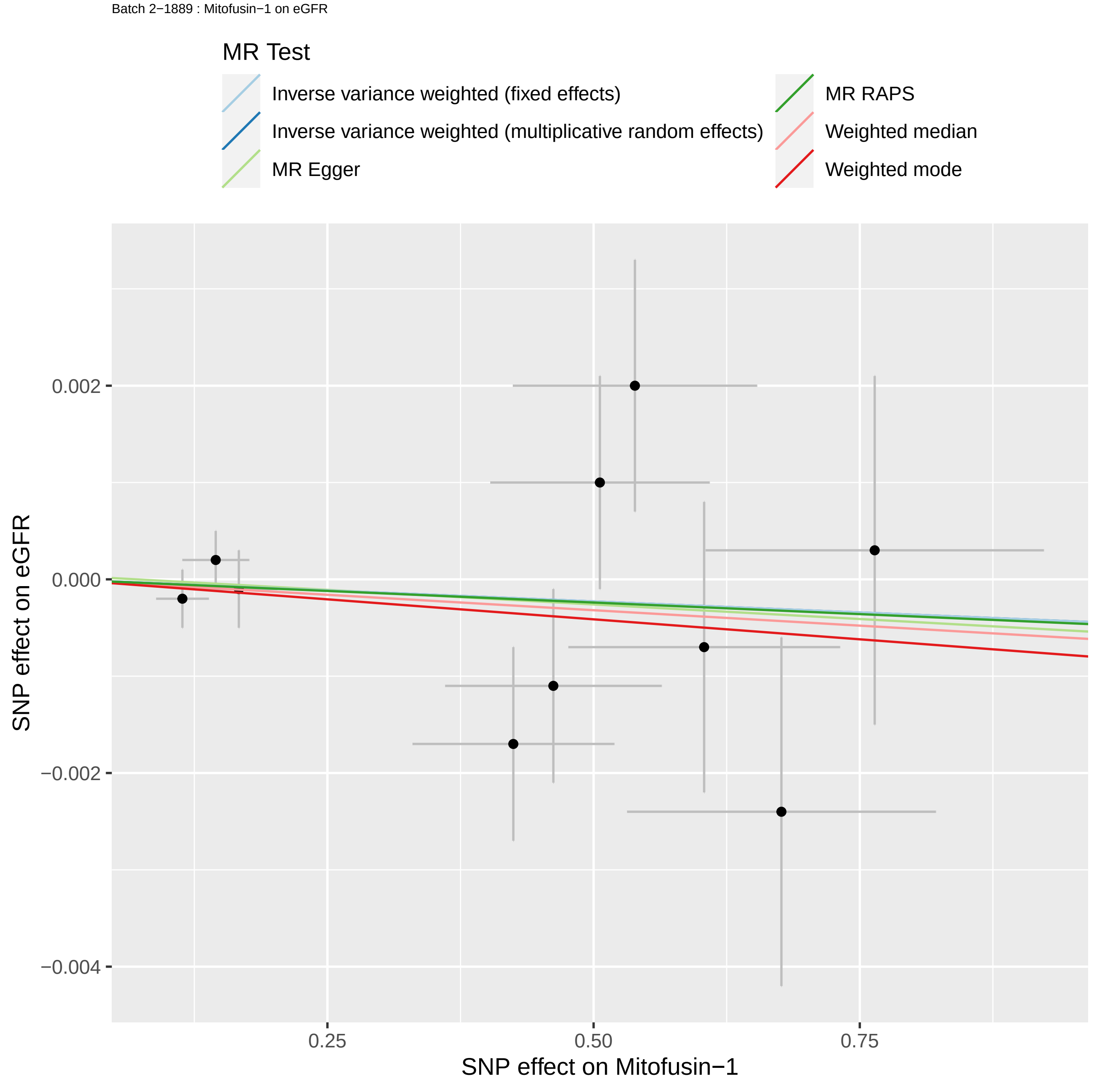

Batch 2−1889 : Mitofusin−1 on eGFR
MR Test
Inverse variance weighted (fixed effects)
Inverse variance weighted (multiplicative random effects)
MR Egger
MR RAPS
Weighted median
Weighted mode
0.002
0.000
−0.002
−0.004
0.25
0.50
0.75
SNP effect on eGFR
SNP effect on Mitofusin−1

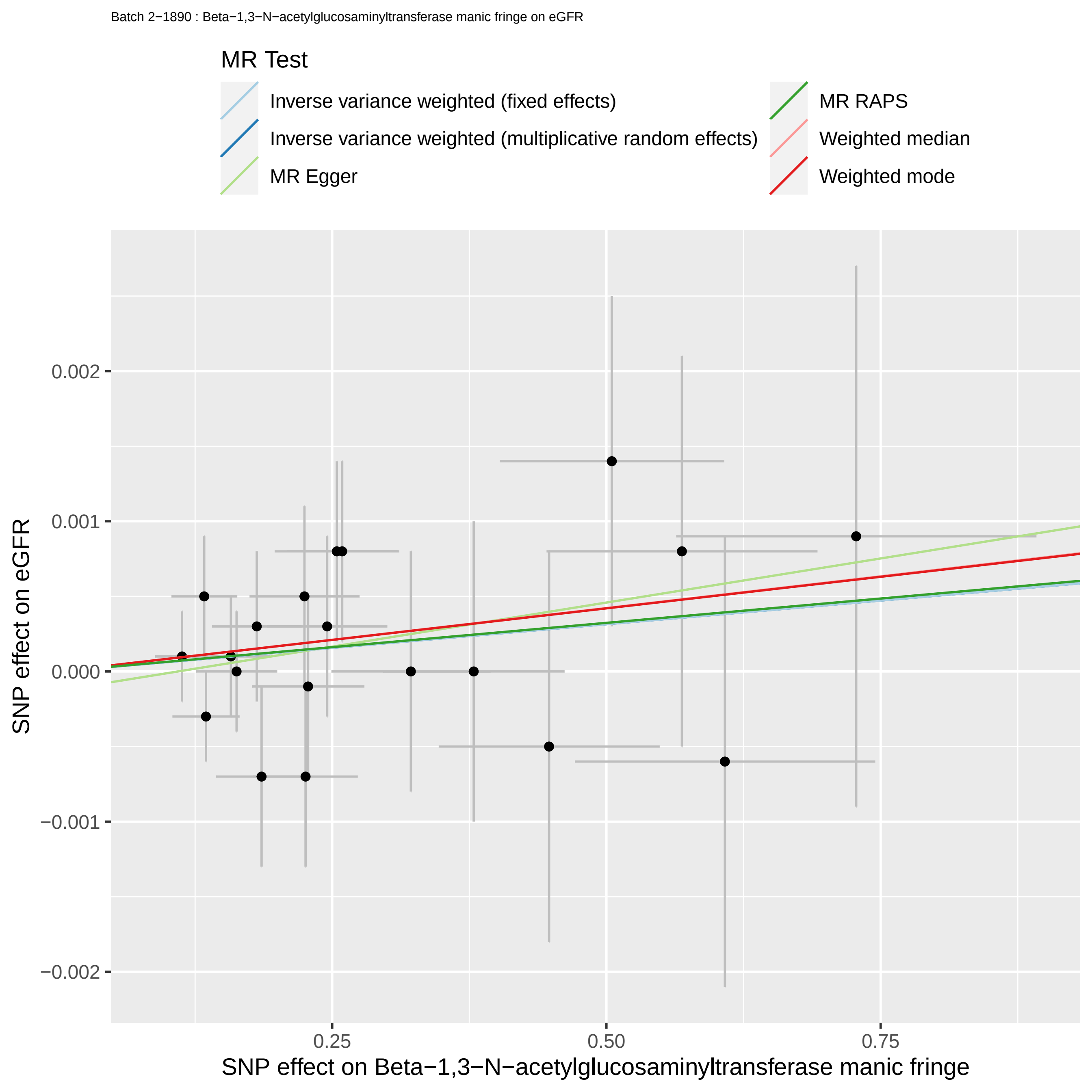

Batch 2−1890 : Beta−1,3−N−acetylglucosaminyltransferase manic fringe on eGFR
MR Test
Inverse variance weighted (fixed effects)
Inverse variance weighted (multiplicative random effects)
MR Egger
MR RAPS
Weighted median
Weighted mode
0.002
0.001
0.000
−0.001
−0.002
SNP effect on eGFR
0.25
0.50
0.75
SNP effect on Beta−1,3−N−acetylglucosaminyltransferase manic fringe

Batch 2−1891 : MAX gene−associated protein on eGFR

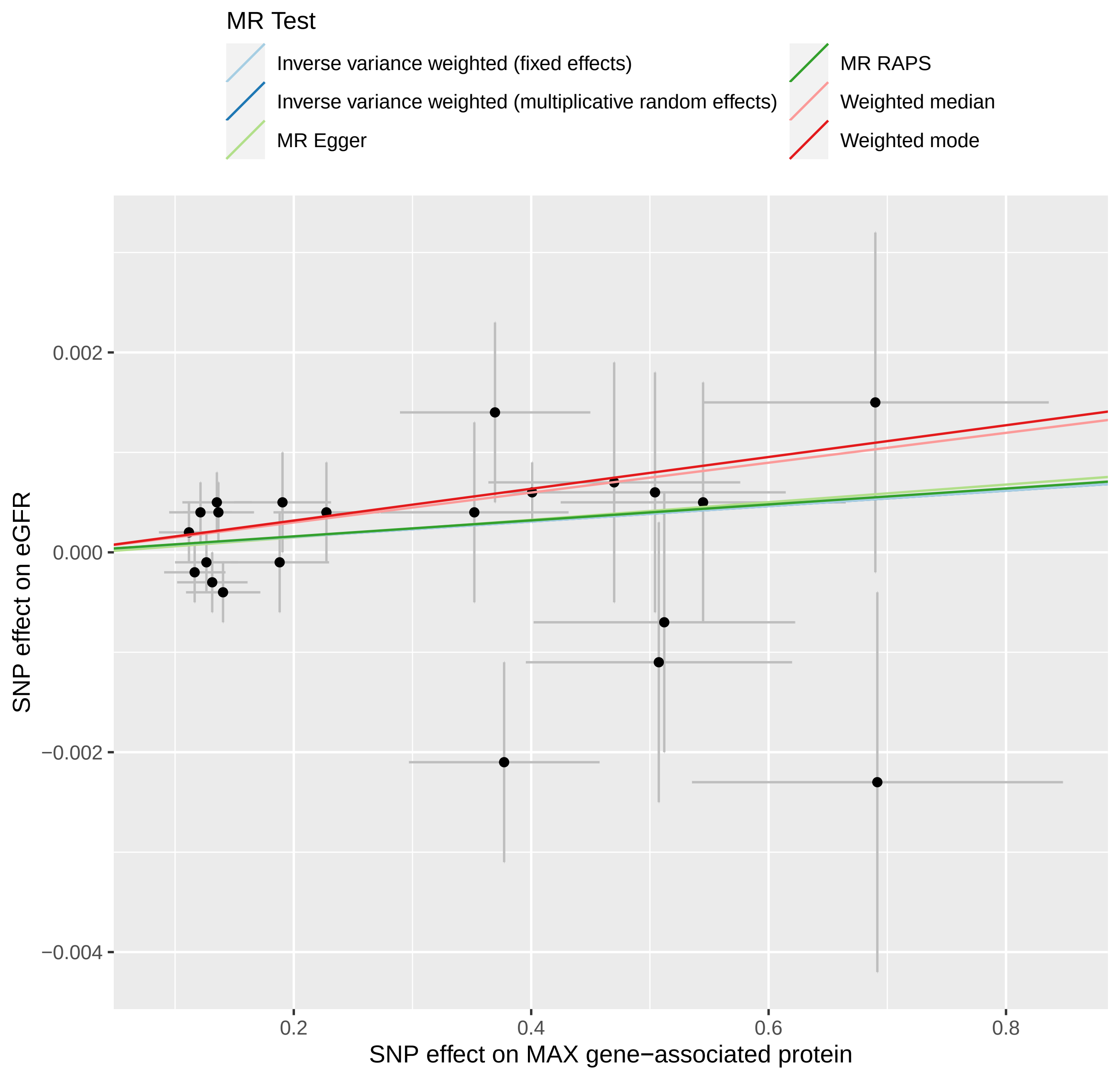

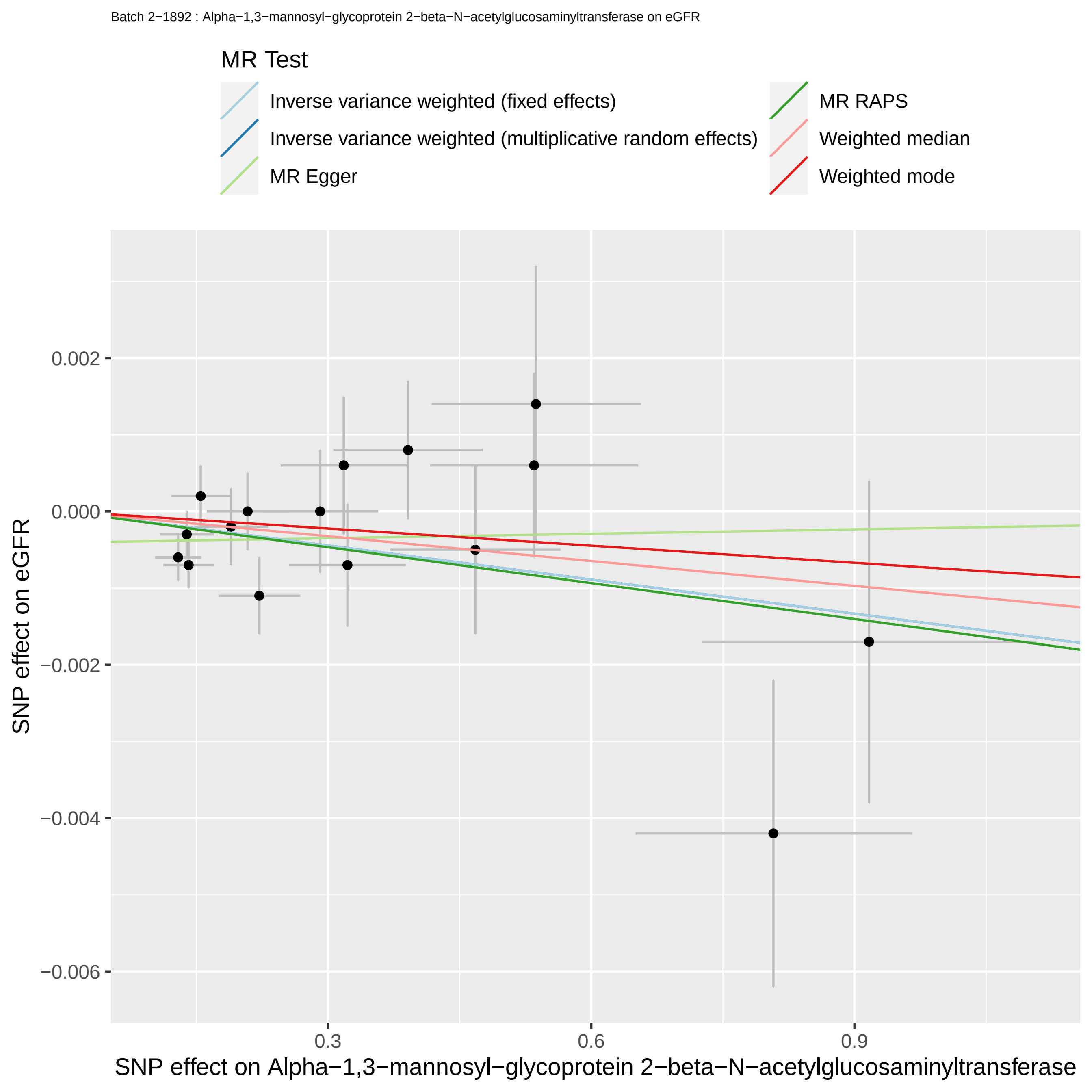

Batch 2−1892 : Alpha−1,3−mannosyl−glycoprotein 2−beta−N−acetylglucosaminyltransferase on eGFR
MR Test
Inverse variance weighted (fixed effects)
Inverse variance weighted (multiplicative random effects)
MR Egger
MR RAPS
Weighted median
Weighted mode
0.002
0.000
−0.002
−0.004
−0.006
0.3
0.6
0.9
SNP effect on eGFR
SNP effect on Alpha−1,3−mannosyl−glycoprotein 2−beta−N−acetylglucosaminyltransferase

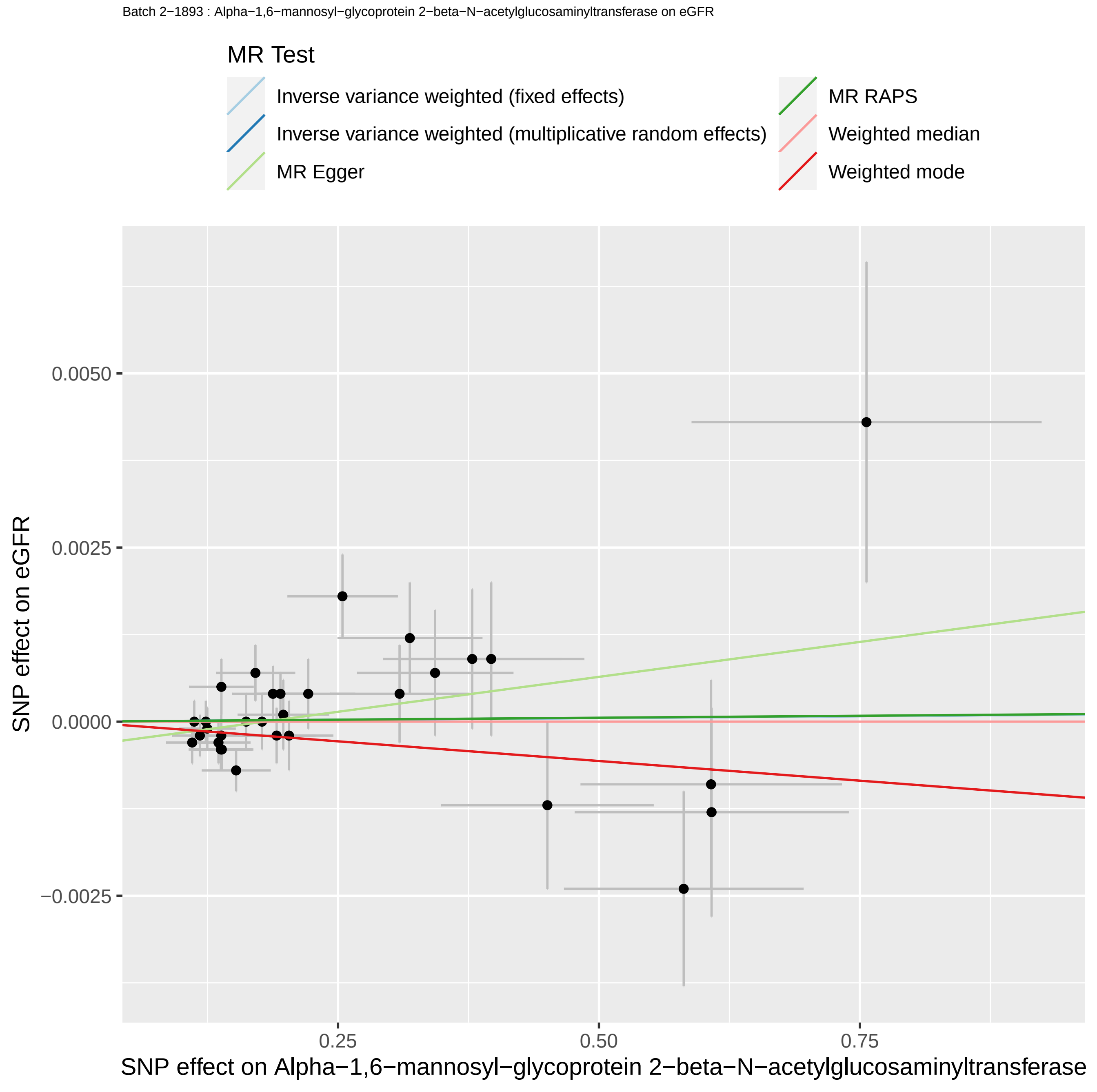
Batch 2−1893 : Alpha−1,6−mannosyl−glycoprotein 2−beta−N−acetylglucosaminyltransferase on eGFR
MR Test
Inverse variance weighted (fixed effects)
Inverse variance weighted (multiplicative random effects)
MR Egger
MR RAPS
Weighted median
Weighted mode
0.0050
0.0025
0.0000
−0.0025
0.25
0.50
0.75
SNP effect on eGFR
SNP effect on Alpha−1,6−mannosyl−glycoprotein 2−beta−N−acetylglucosaminyltransferase

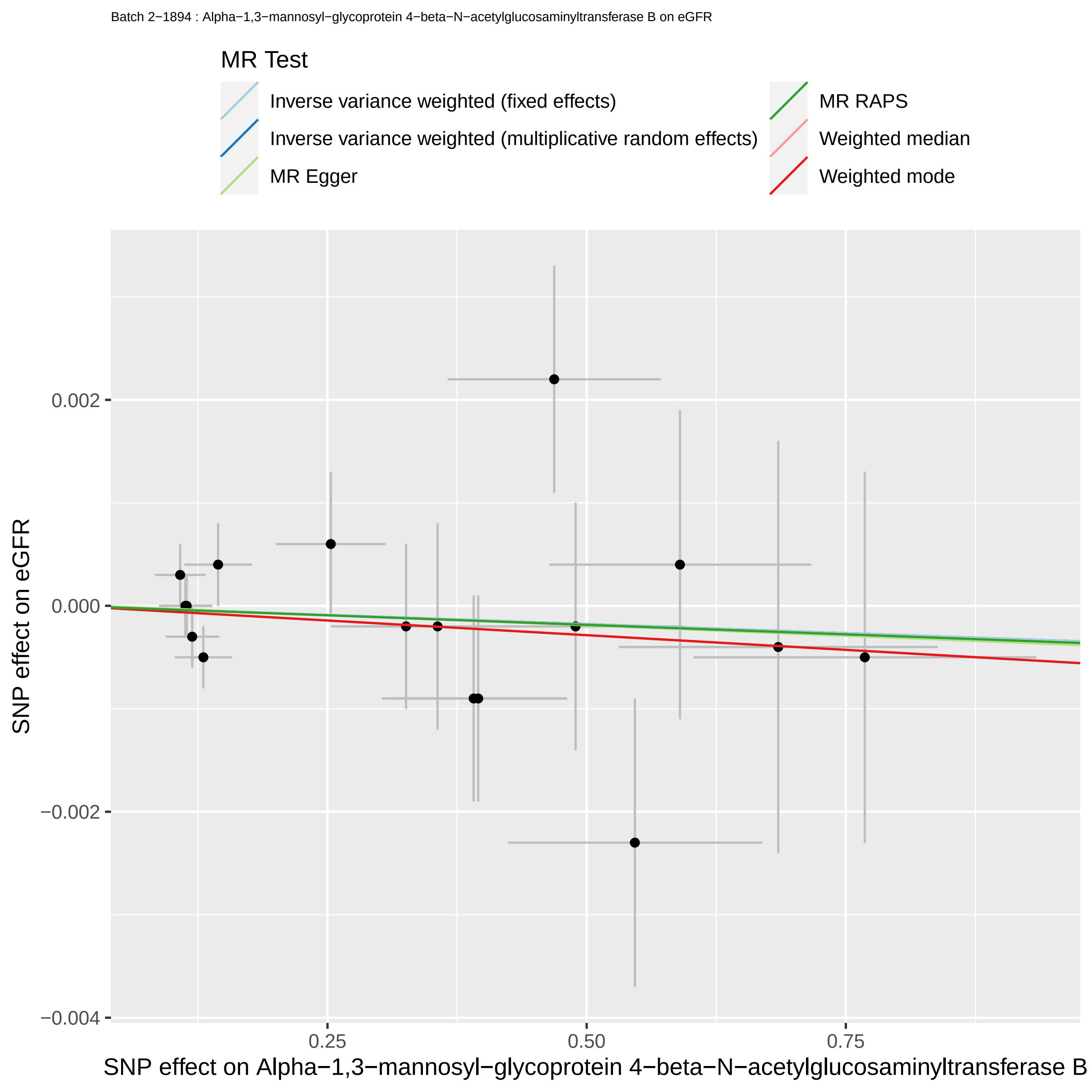
Batch 2−1894 : Alpha−1,3−mannosyl−glycoprotein 4−beta−N−acetylglucosaminyltransferase B on eGFR
MR Test
Inverse variance weighted (fixed effects)
Inverse variance weighted (multiplicative random effects)
MR Egger
MR RAPS
Weighted median
Weighted mode
0.002
0.000
−0.002
−0.004
0.25
0.50
0.75
SNP effect on eGFR
SNP effect on Alpha−1,3−mannosyl−glycoprotein 4−beta−N−acetylglucosaminyltransferase B

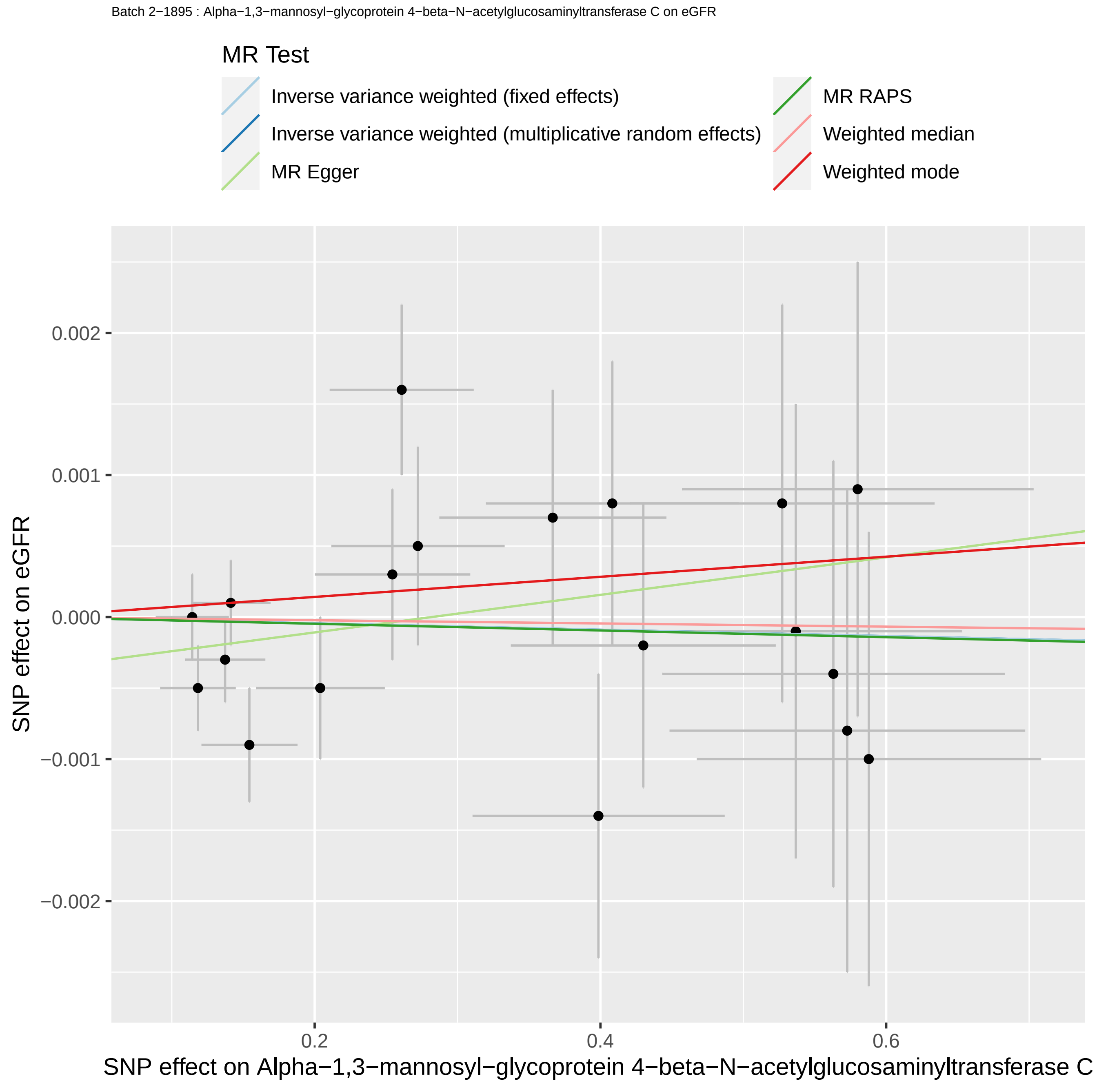

Batch 2−1895 : Alpha−1,3−mannosyl−glycoprotein 4−beta−N−acetylglucosaminyltransferase C on eGFR
MR Test
Inverse variance weighted (fixed effects)
Inverse variance weighted (multiplicative random effects)
MR Egger
MR RAPS
Weighted median
Weighted mode
0.002
0.001
0.000
−0.001
−0.002
SNP effect on eGFR
0.2
0.4
0.6
SNP effect on Alpha−1,3−mannosyl−glycoprotein 4−beta−N−acetylglucosaminyltransferase C

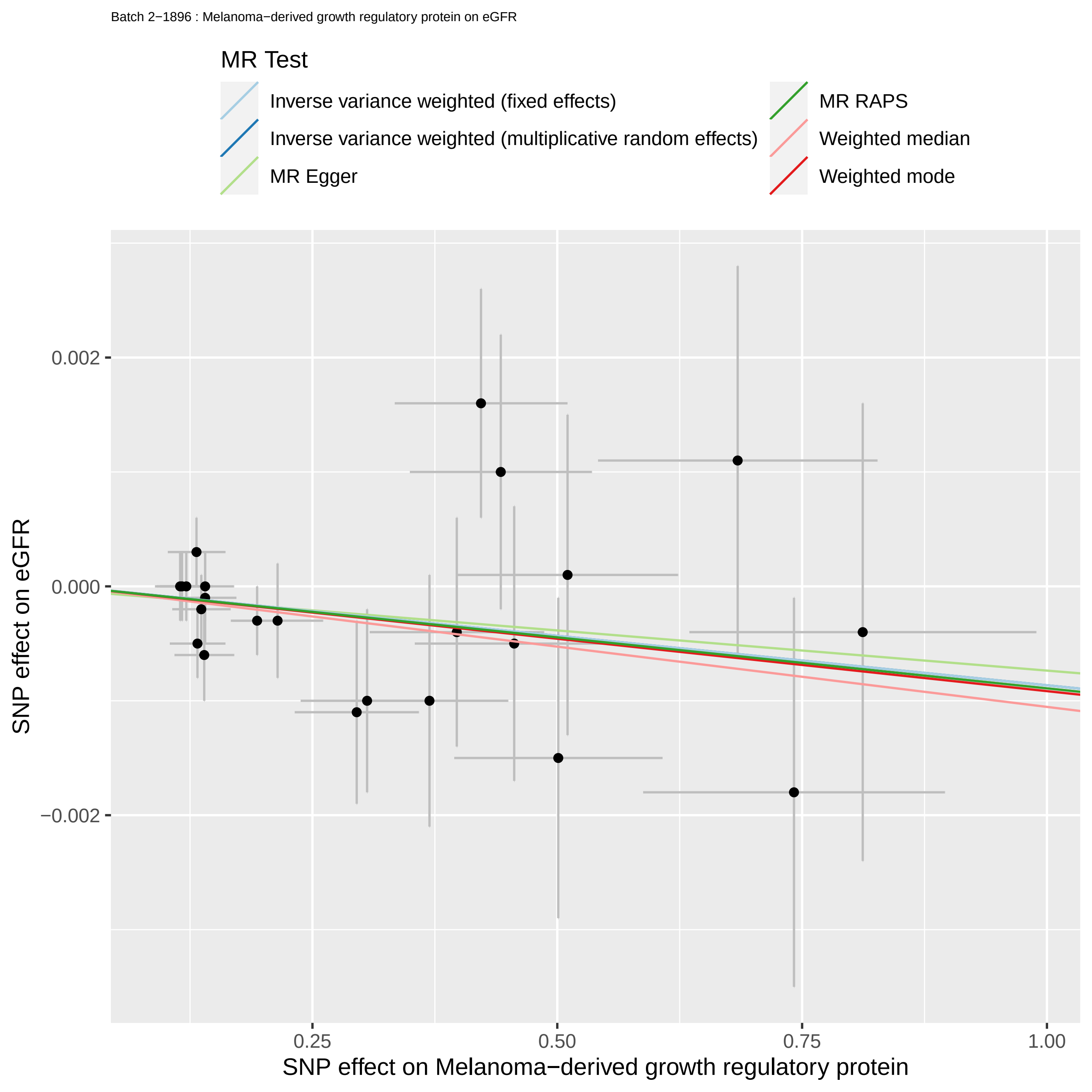
Batch 2−1896 : Melanoma−derived growth regulatory protein on eGFR
MR Test
Inverse variance weighted (fixed effects)
Inverse variance weighted (multiplicative random effects)
MR Egger
MR RAPS
Weighted median
Weighted mode
0.002
0.000
−0.002
SNP effect on eGFR
0.25
0.50
0.75
1.00
SNP effect on Melanoma−derived growth regulatory protein

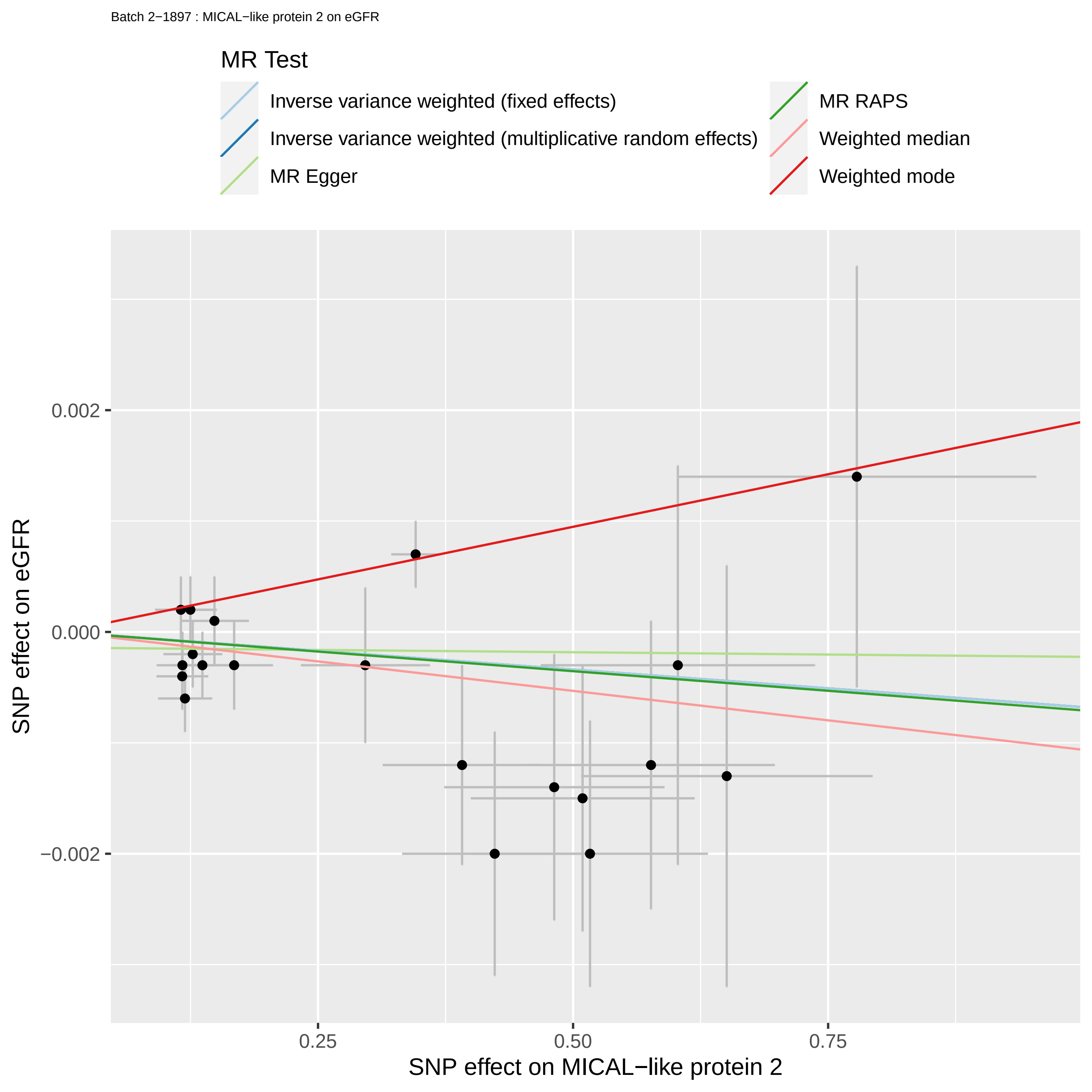
Batch 2−1897 : MICAL−like protein 2 on eGFR
MR Test
Inverse variance weighted (fixed effects)
Inverse variance weighted (multiplicative random effects)
MR Egger
MR RAPS
Weighted median
Weighted mode
0.002
0.000
−0.002
0.25
0.50
0.75
SNP effect on eGFR
SNP effect on MICAL−like protein 2

Batch 2−1898 : MHC class I polypeptide−related sequence B on eGFR

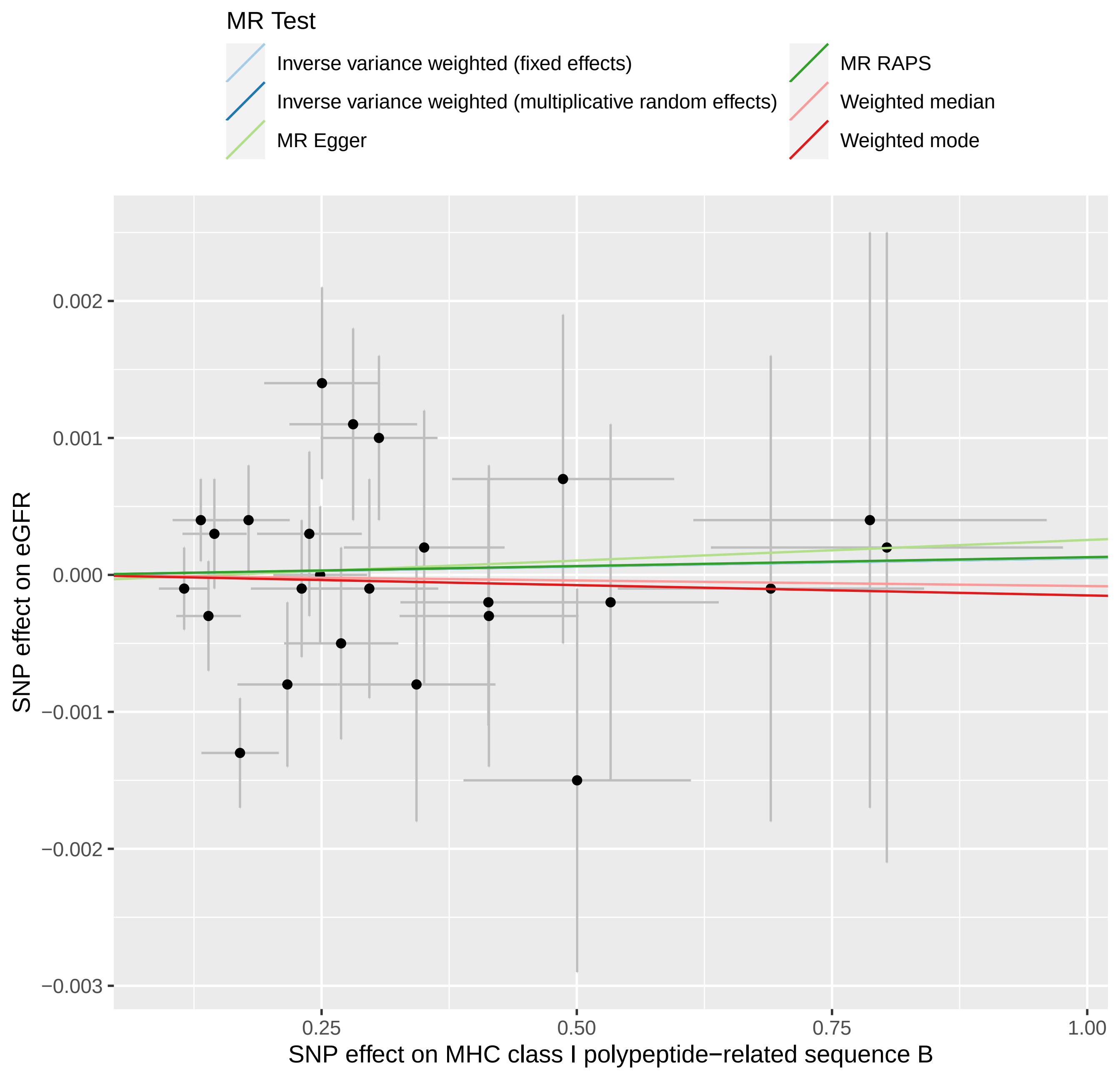

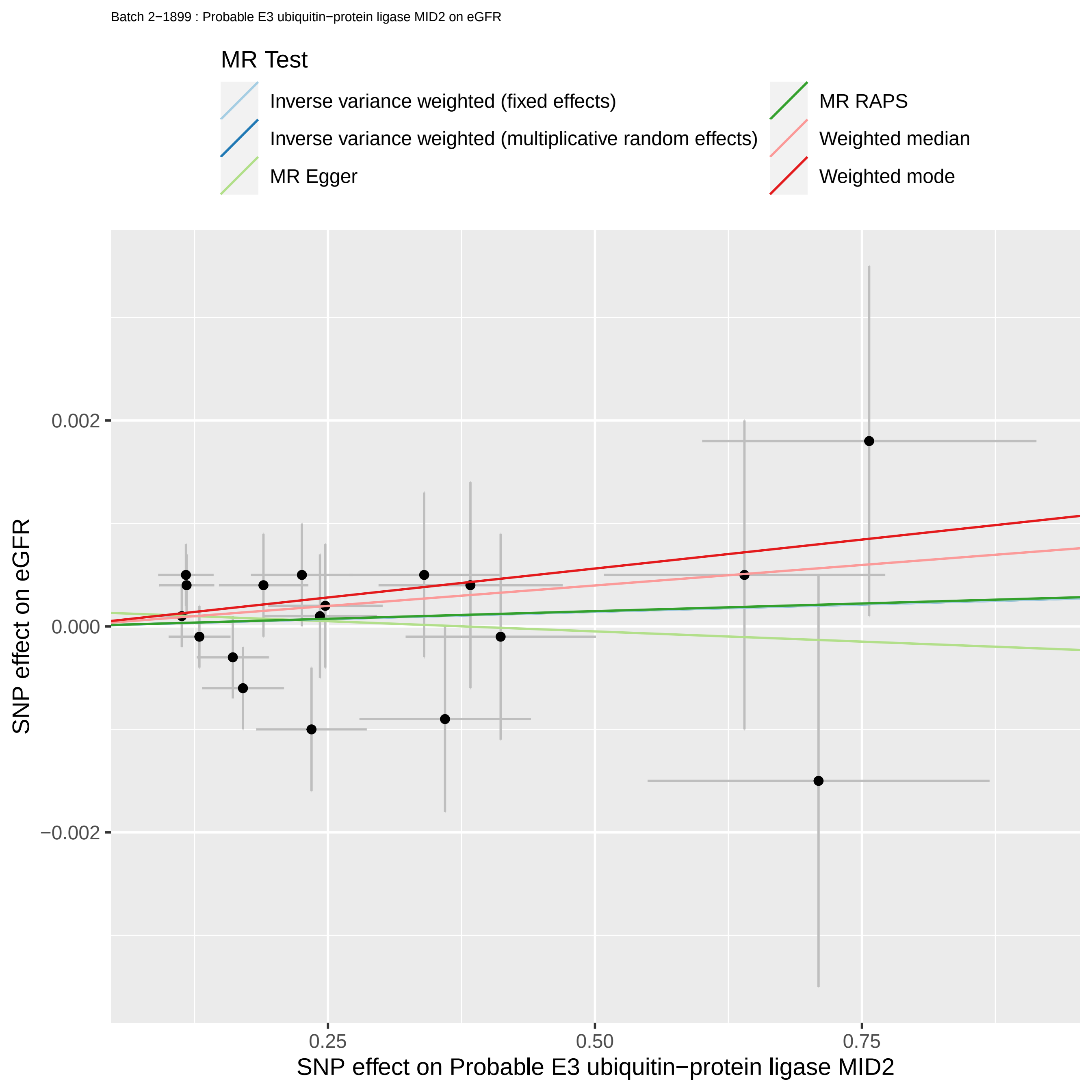
Batch 2−1899 : Probable E3 ubiquitin−protein ligase MID2 on eGFR
MR Test
Inverse variance weighted (fixed effects)
Inverse variance weighted (multiplicative random effects)
MR Egger
MR RAPS
Weighted median
Weighted mode
0.002
0.000
−0.002
0.25
0.50
0.75
SNP effect on eGFR
SNP effect on Probable E3 ubiquitin−protein ligase MID2

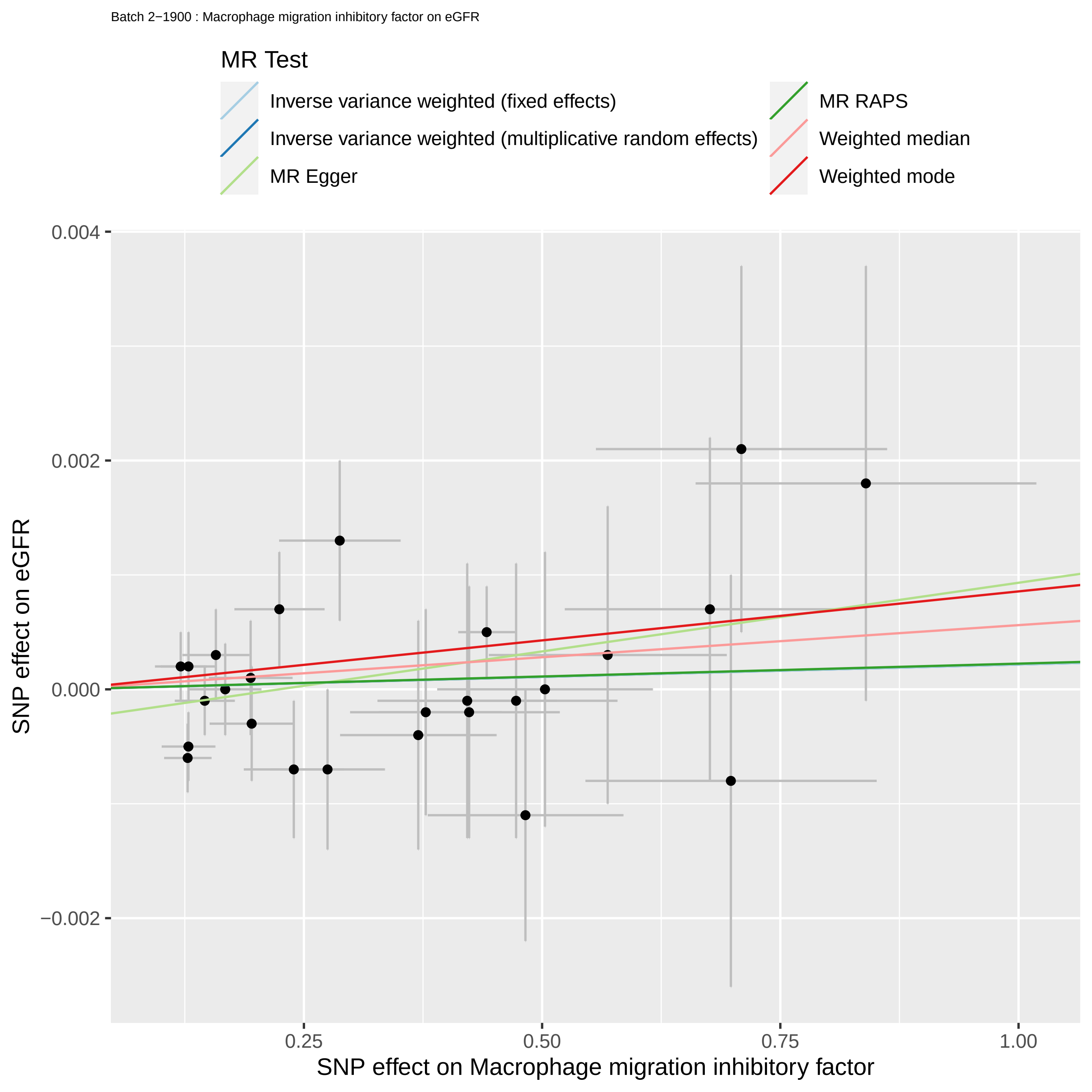
Batch 2−1900 : Macrophage migration inhibitory factor on eGFR
MR Test
Inverse variance weighted (fixed effects)
Inverse variance weighted (multiplicative random effects)
MR Egger
MR RAPS
Weighted median
Weighted mode
0.004
0.002
0.000
−0.002
SNP effect on eGFR
0.25
0.50
0.75
1.00
SNP effect on Macrophage migration inhibitory factor

Batch 2−1901 : Allergin−1 on eGFR

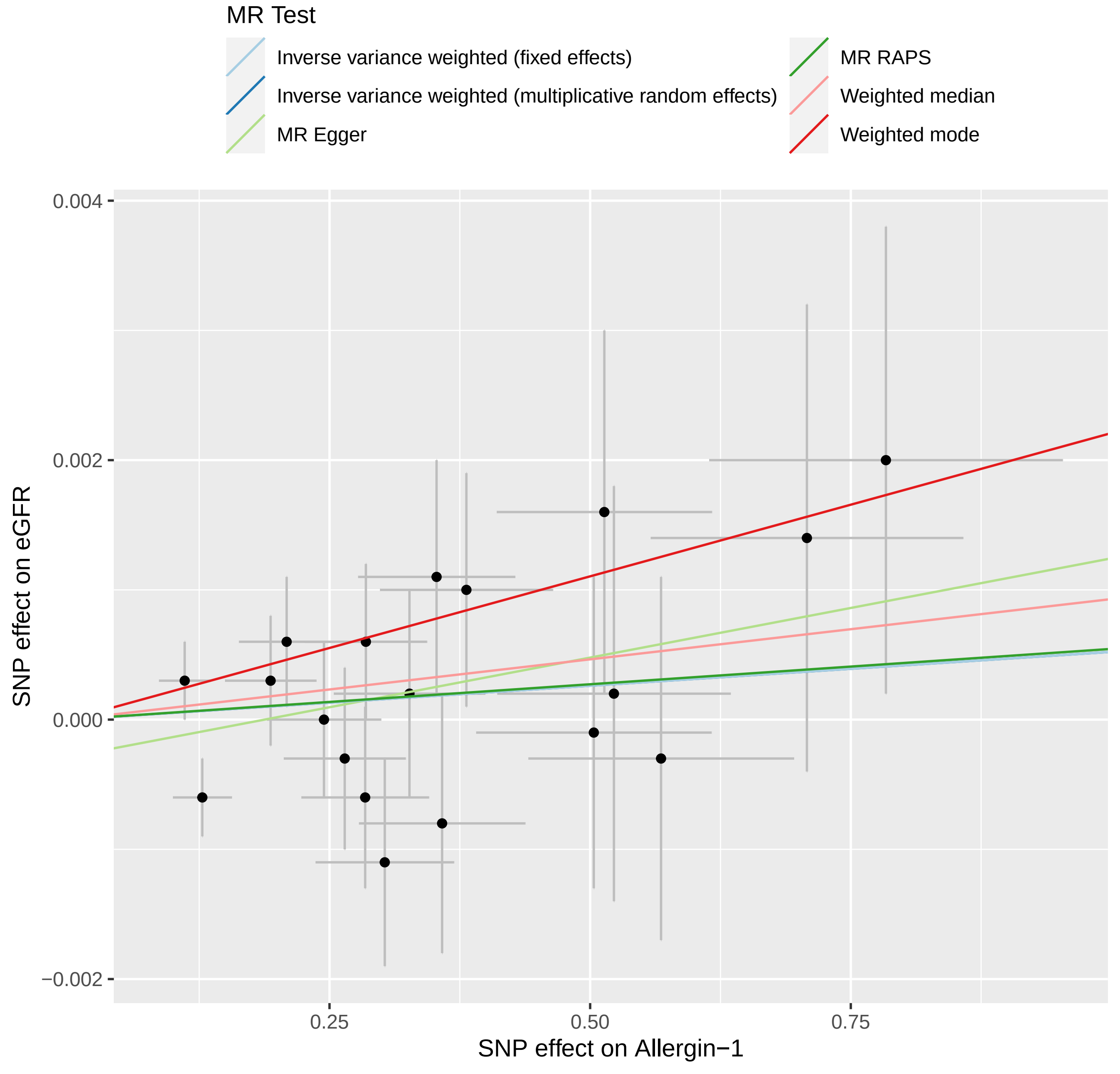

Batch 2−1902 : MICOS complex subunit MIC10 on eGFR

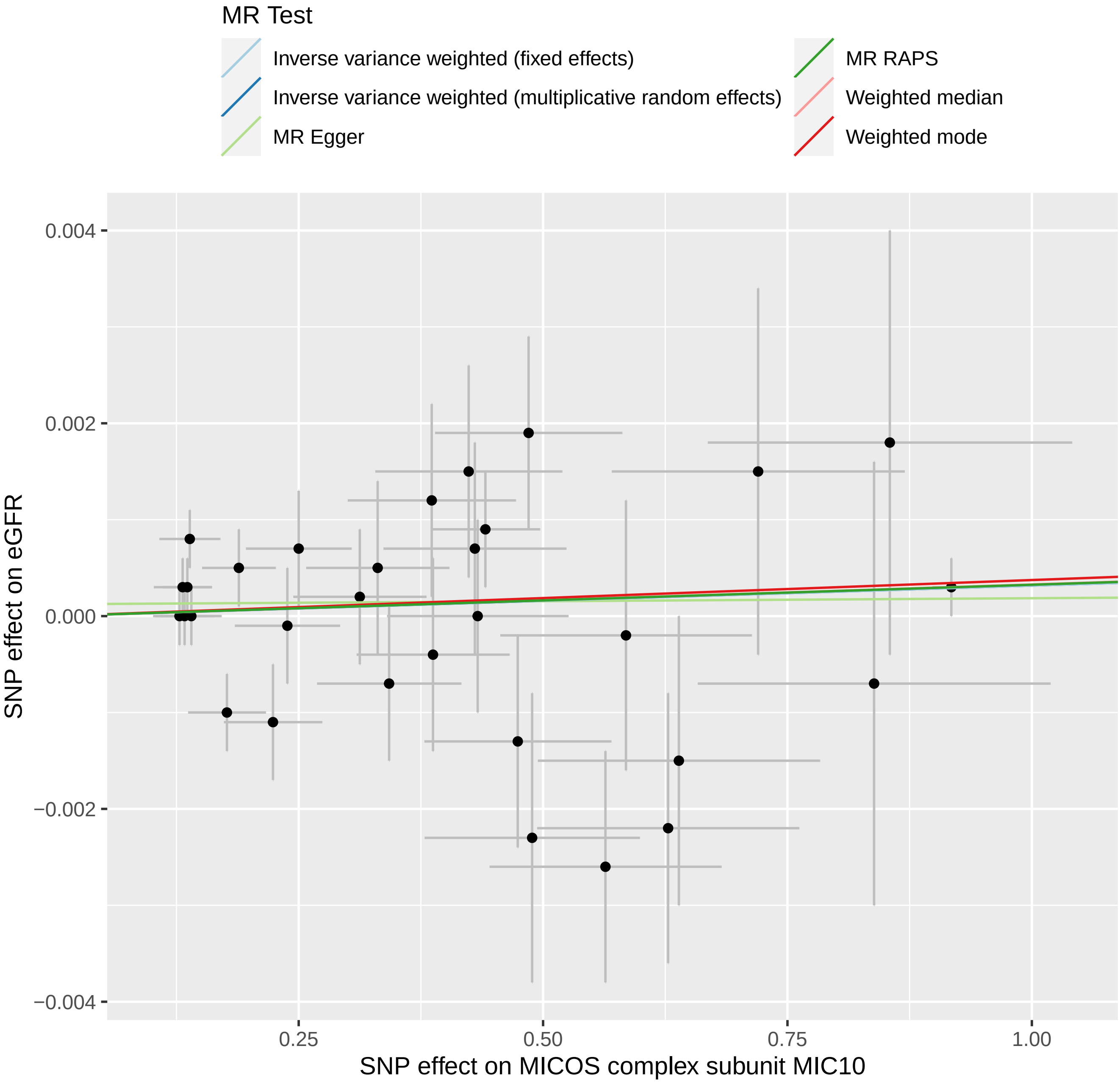

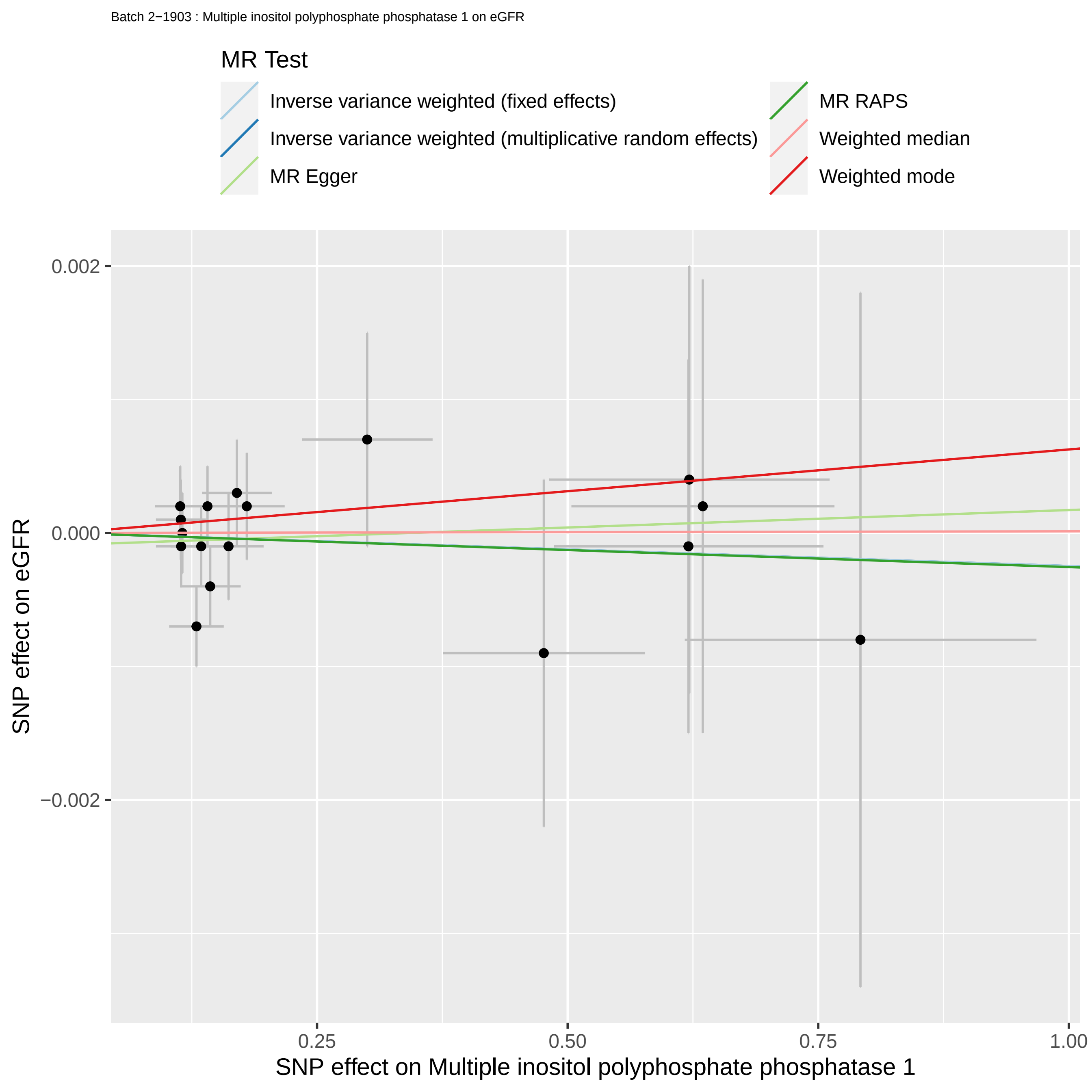
Batch 2−1903 : Multiple inositol polyphosphate phosphatase 1 on eGFR
MR Test
Inverse variance weighted (fixed effects)
Inverse variance weighted (multiplicative random effects)
MR Egger
MR RAPS
Weighted median
Weighted mode
0.002
0.000
−0.002
SNP effect on eGFR
0.25
0.50
0.75
1.00
SNP effect on Multiple inositol polyphosphate phosphatase 1

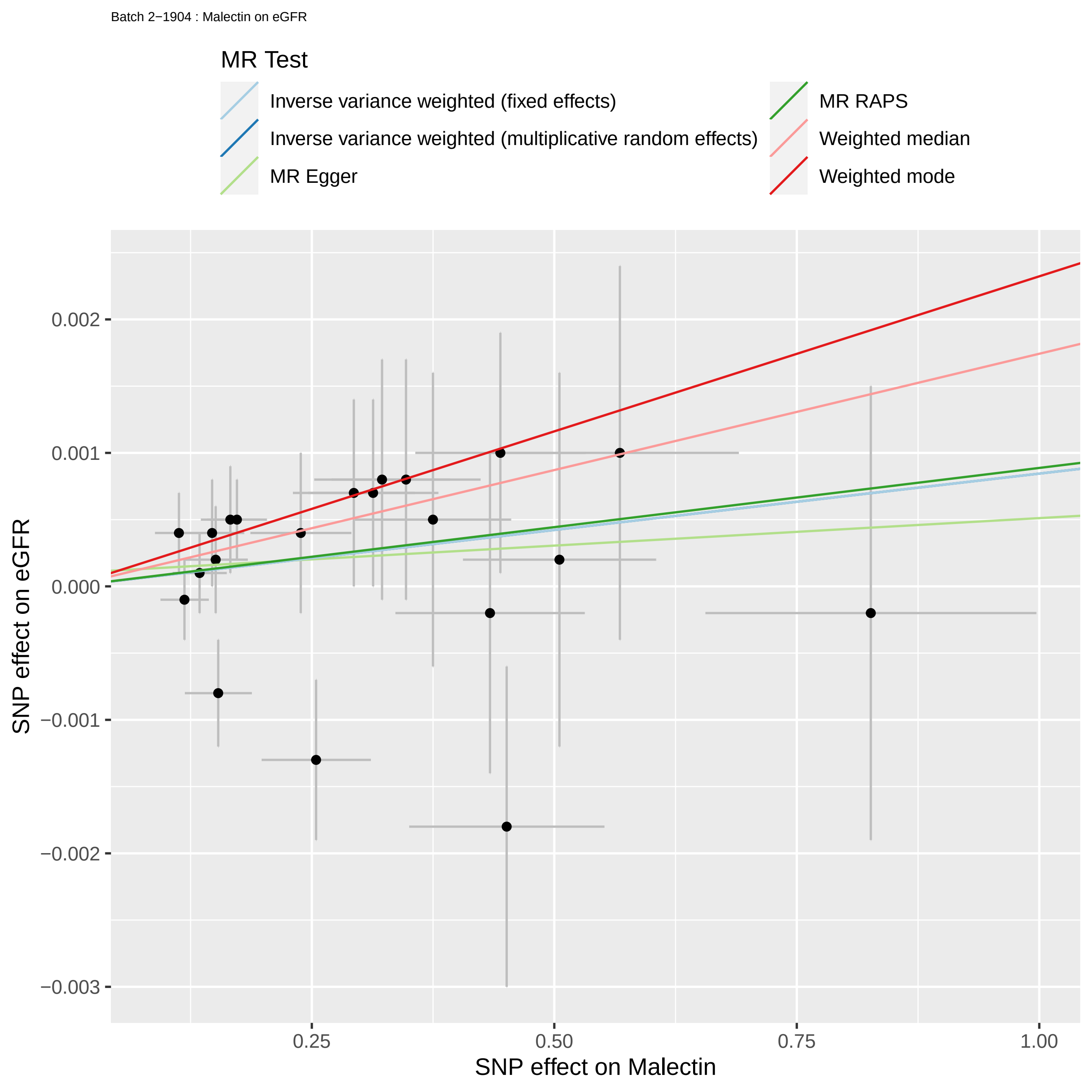

Batch 2−1904 : Malectin on eGFR
MR Test
Inverse variance weighted (fixed effects)
Inverse variance weighted (multiplicative random effects)
MR Egger
MR RAPS
Weighted median
Weighted mode
0.002
0.001
0.000
−0.001
−0.002
−0.003
0.25
0.50
0.75
1.00
SNP effect on Malectin
SNP effect on eGFR

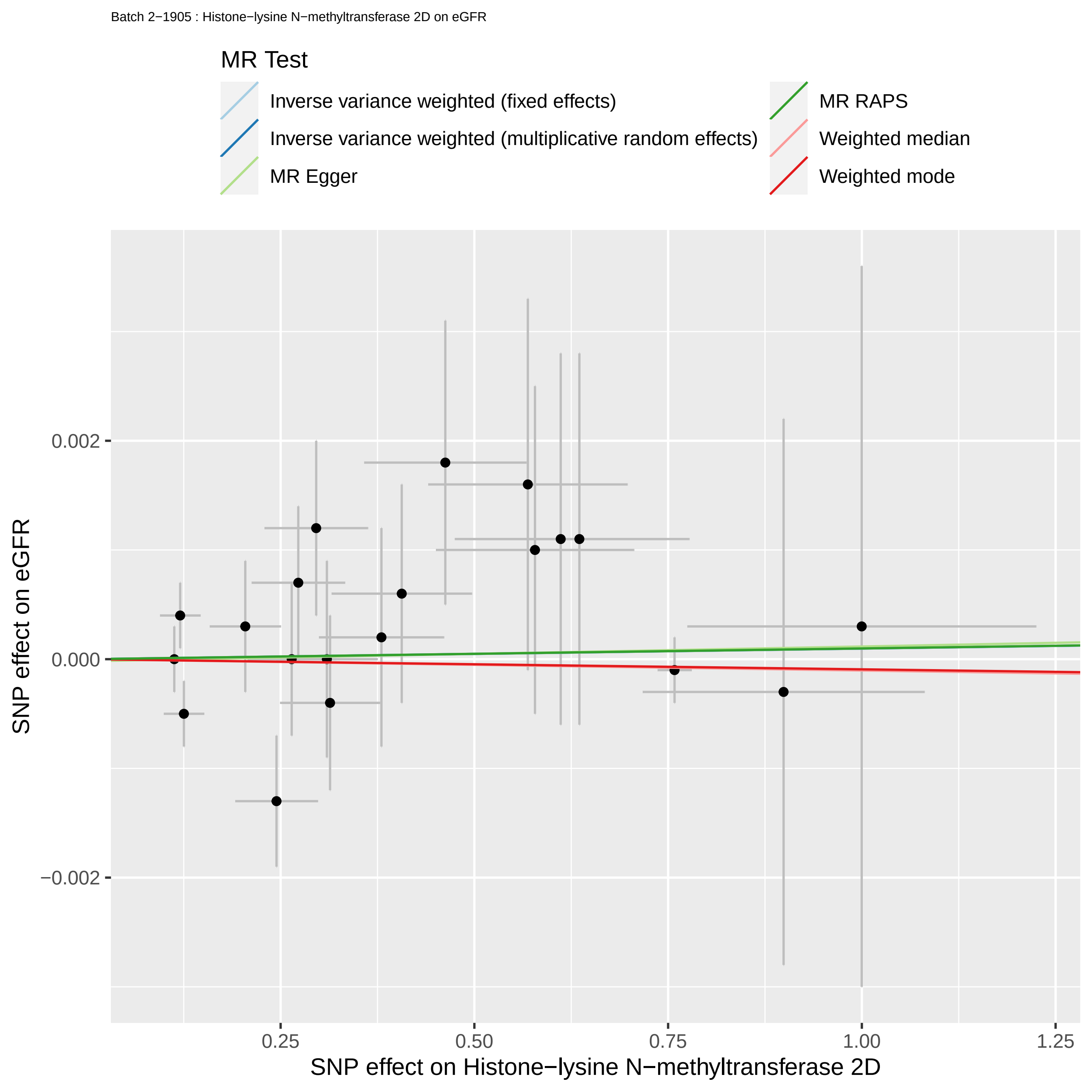

Batch 2−1905 : Histone−lysine N−methyltransferase 2D on eGFR
MR Test
Inverse variance weighted (fixed effects)
Inverse variance weighted (multiplicative random effects)
MR Egger
MR RAPS
Weighted median
Weighted mode
0.002
0.000
−0.002
SNP effect on eGFR
0.25
0.50
0.75
1.00
1.25
SNP effect on Histone−lysine N−methyltransferase 2D

Batch 2−1906 : Promotilin on eGFR

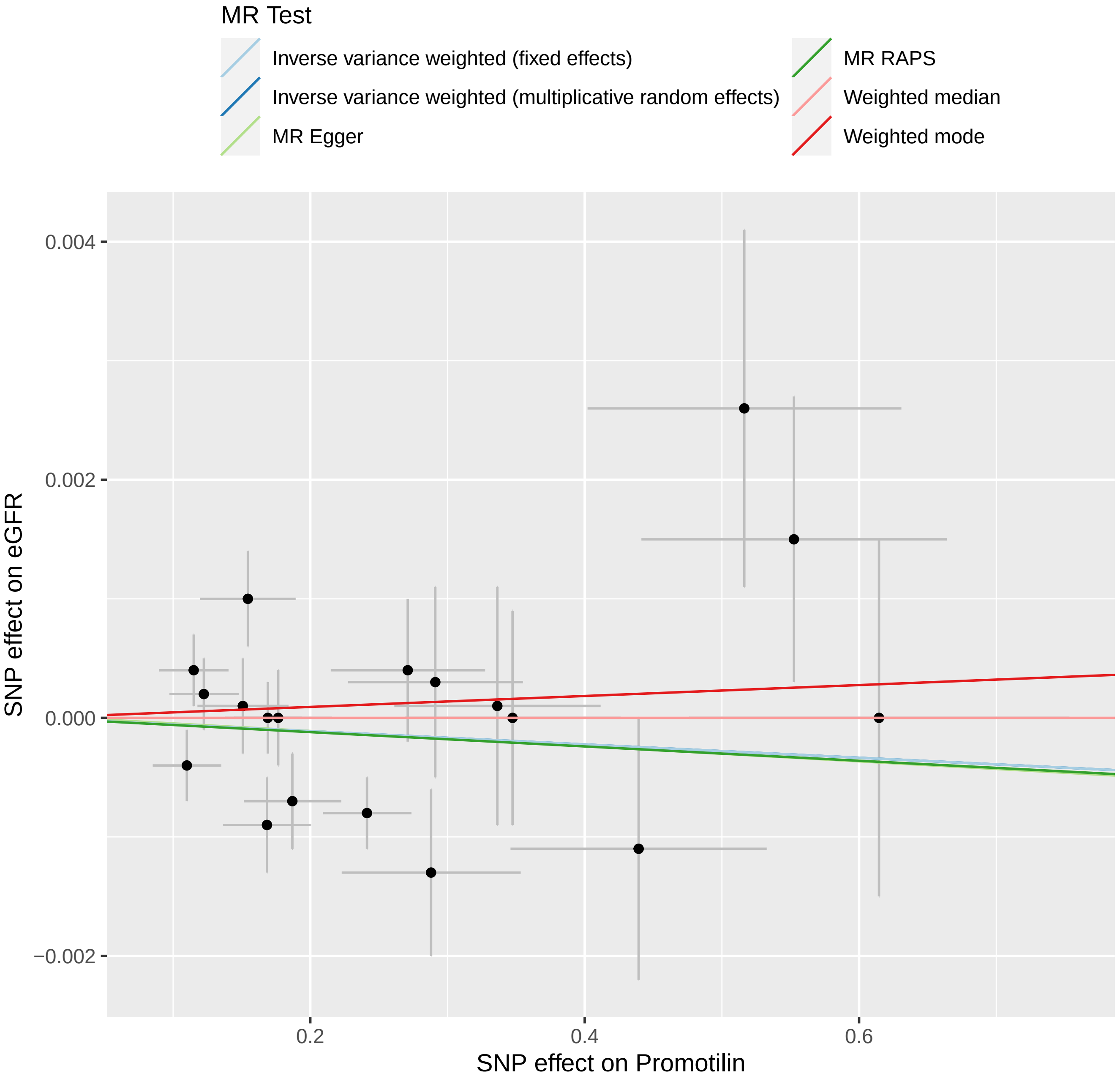

Batch 2−1907 : Malonyl−CoA decarboxylase, mitochondrial on eGFR

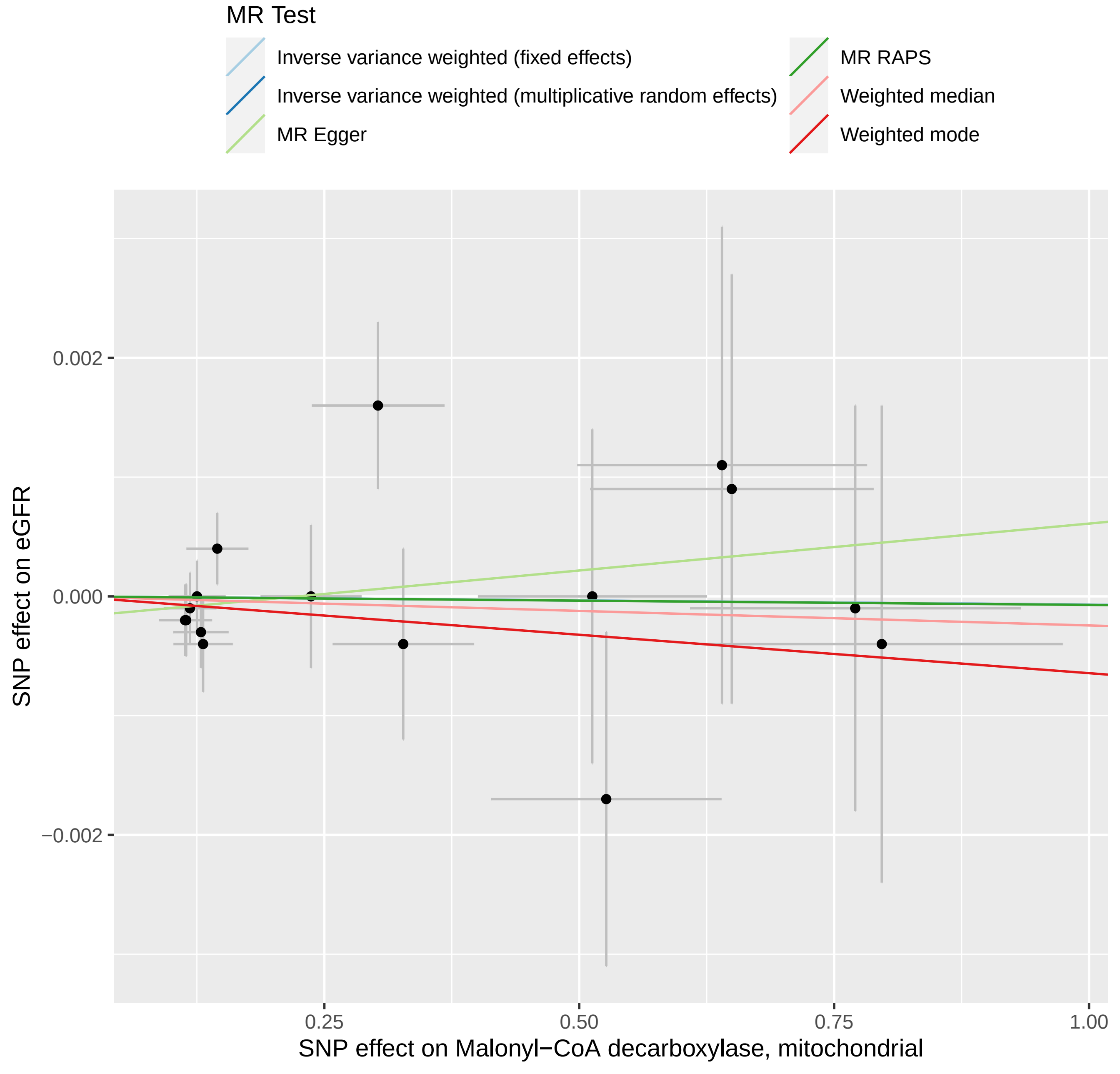

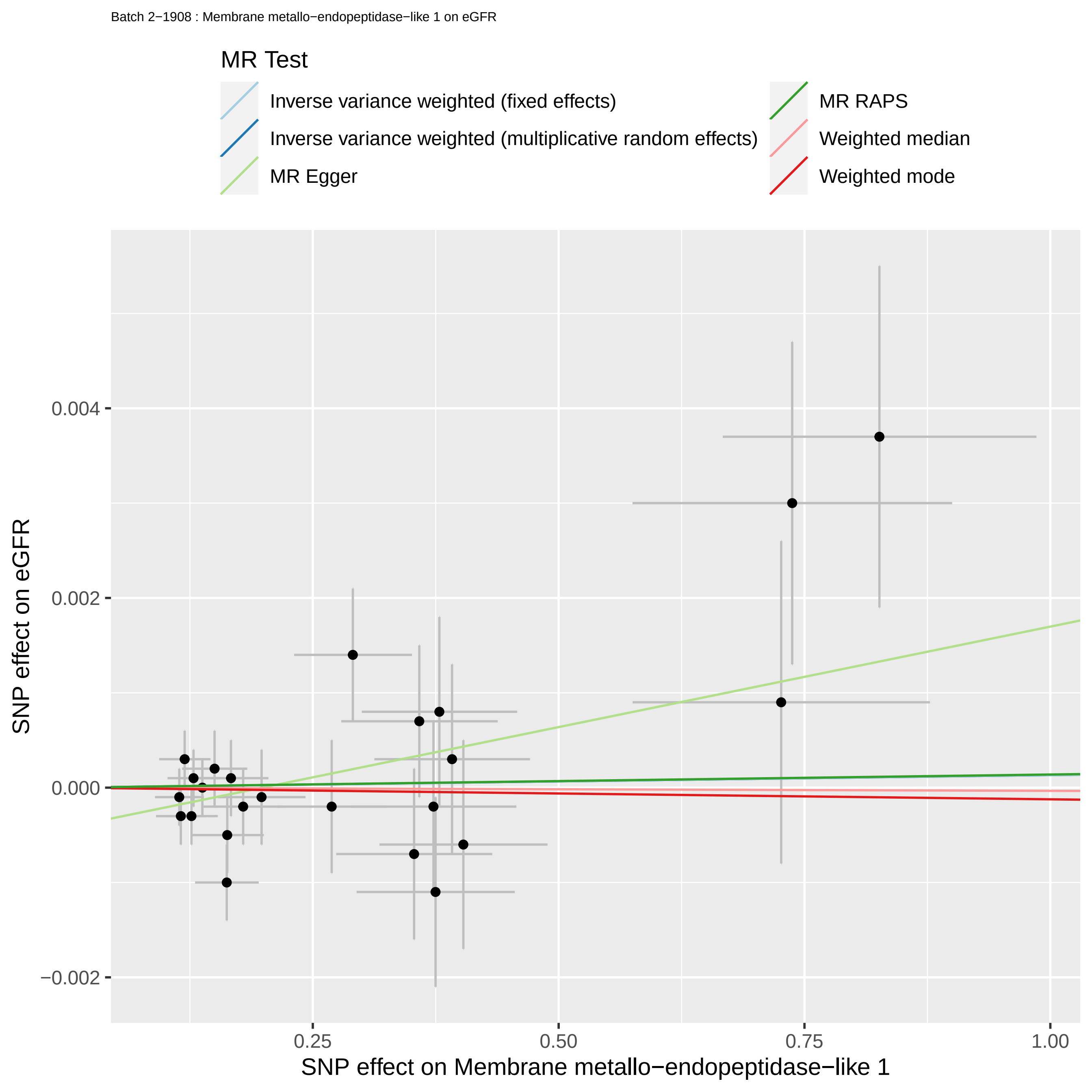

Batch 2−1908 : Membrane metallo−endopeptidase−like 1 on eGFR
MR Test
Inverse variance weighted (fixed effects)
Inverse variance weighted (multiplicative random effects)
MR Egger
MR RAPS
Weighted median
Weighted mode
0.004
0.002
0.000
−0.002
SNP effect on eGFR
0.25
0.50
0.75
1.00
SNP effect on Membrane metallo−endopeptidase−like 1

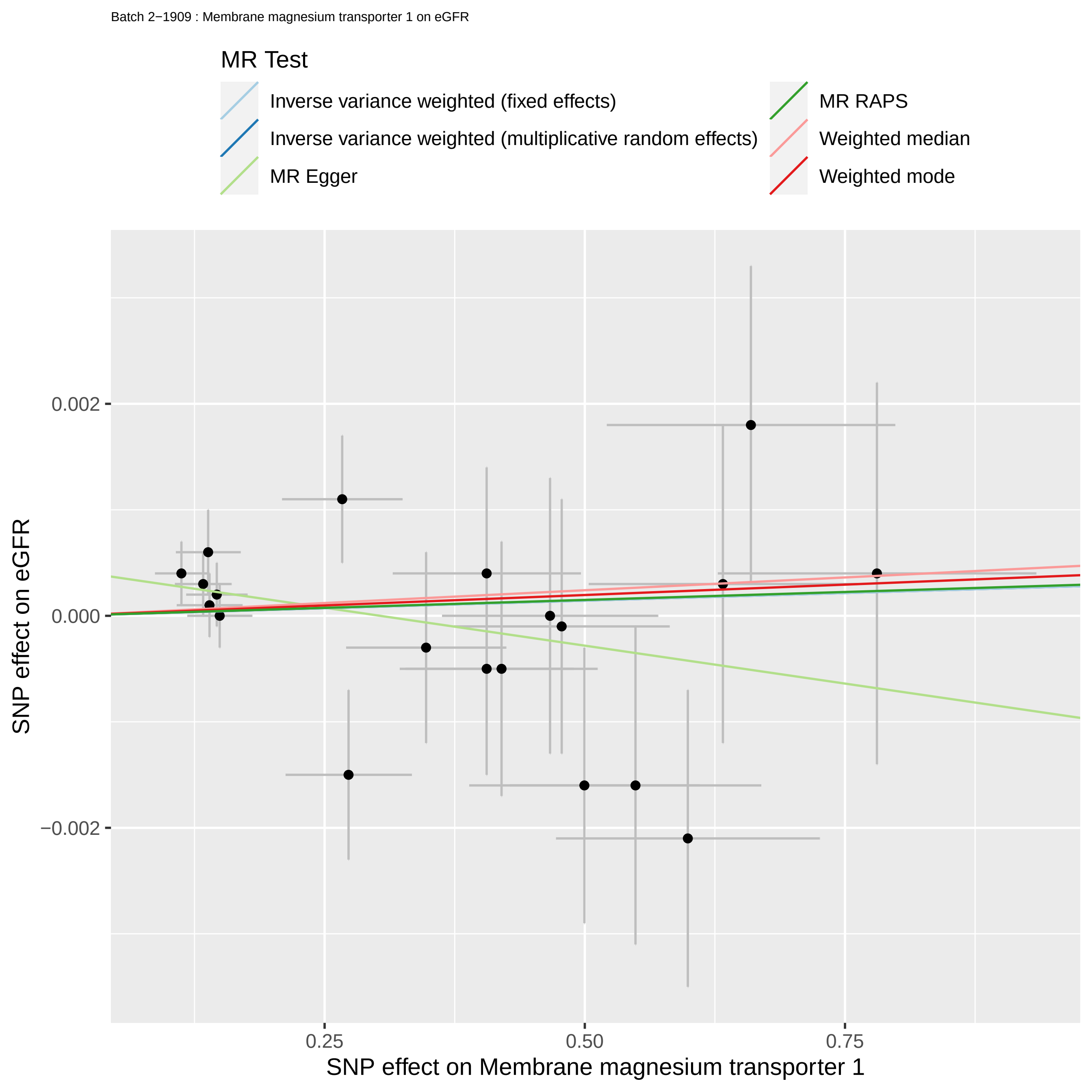

Batch 2−1909 : Membrane magnesium transporter 1 on eGFR
MR Test
Inverse variance weighted (fixed effects)
Inverse variance weighted (multiplicative random effects)
MR Egger
MR RAPS
Weighted median
Weighted mode
0.002
0.000
−0.002
0.25
0.50
0.75
SNP effect on eGFR
SNP effect on Membrane magnesium transporter 1

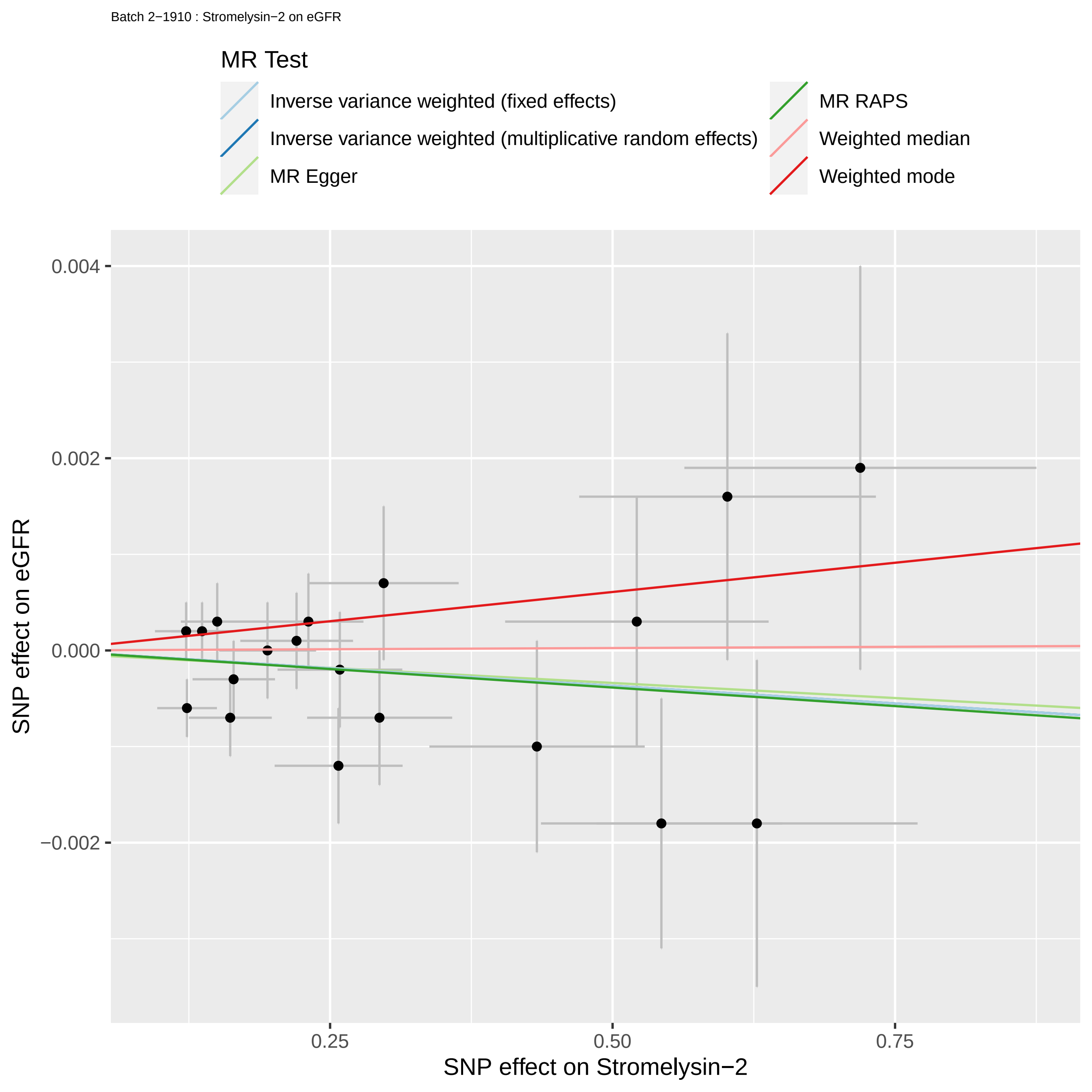

Batch 2−1910 : Stromelysin−2 on eGFR
MR Test
Inverse variance weighted (fixed effects)
Inverse variance weighted (multiplicative random effects)
MR Egger
MR RAPS
Weighted median
Weighted mode
0.004
0.002
0.000
−0.002
SNP effect on eGFR
0.25
0.50
0.75
SNP effect on Stromelysin−2

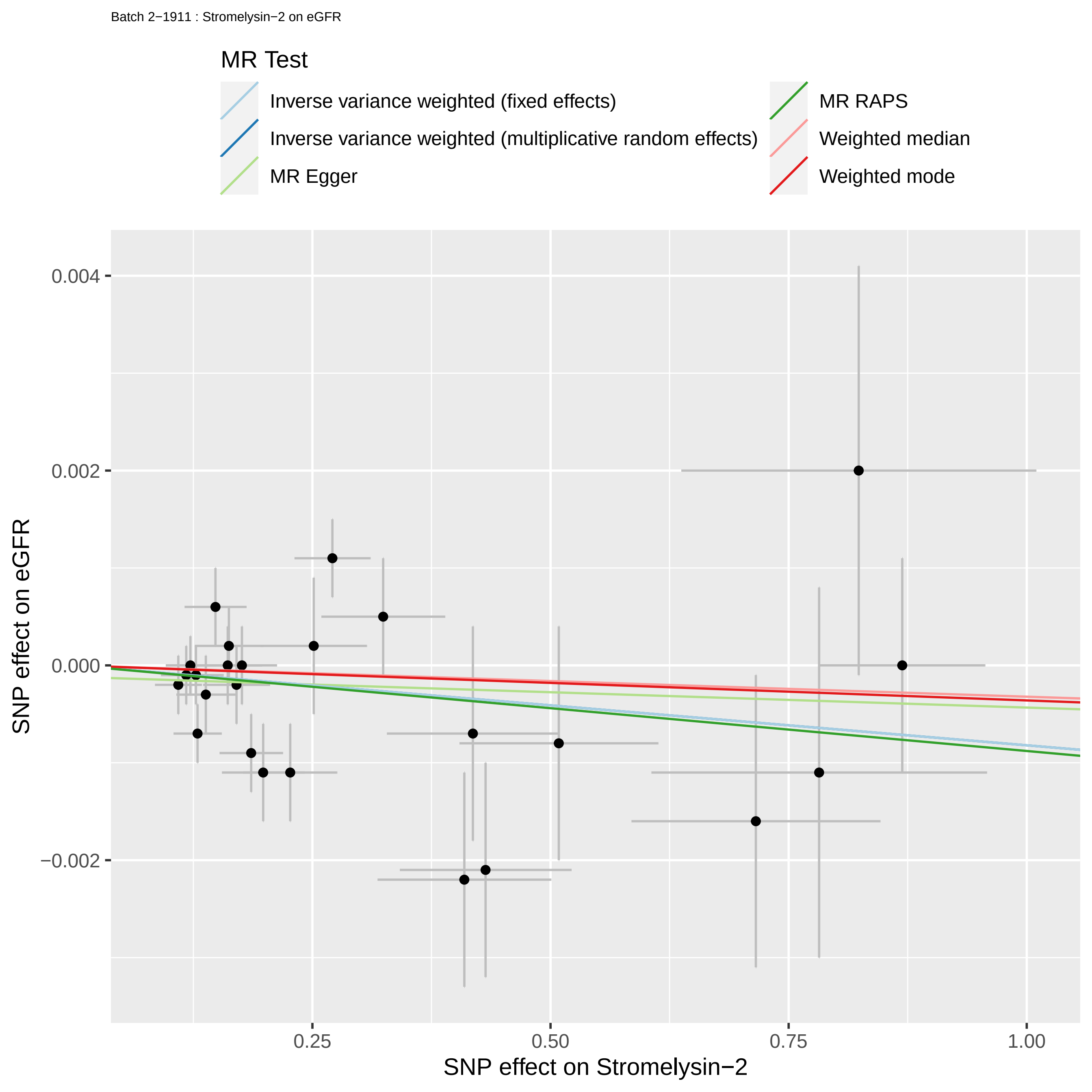
Batch 2−1911 : Stromelysin−2 on eGFR
MR Test
Inverse variance weighted (fixed effects)
Inverse variance weighted (multiplicative random effects)
MR Egger
MR RAPS
Weighted median
Weighted mode
0.004
0.002
0.000
−0.002
SNP effect on eGFR
0.25
0.50
0.75
1.00
SNP effect on Stromelysin−2

Batch 2−1912 : Macrophage metalloelastase on eGFR

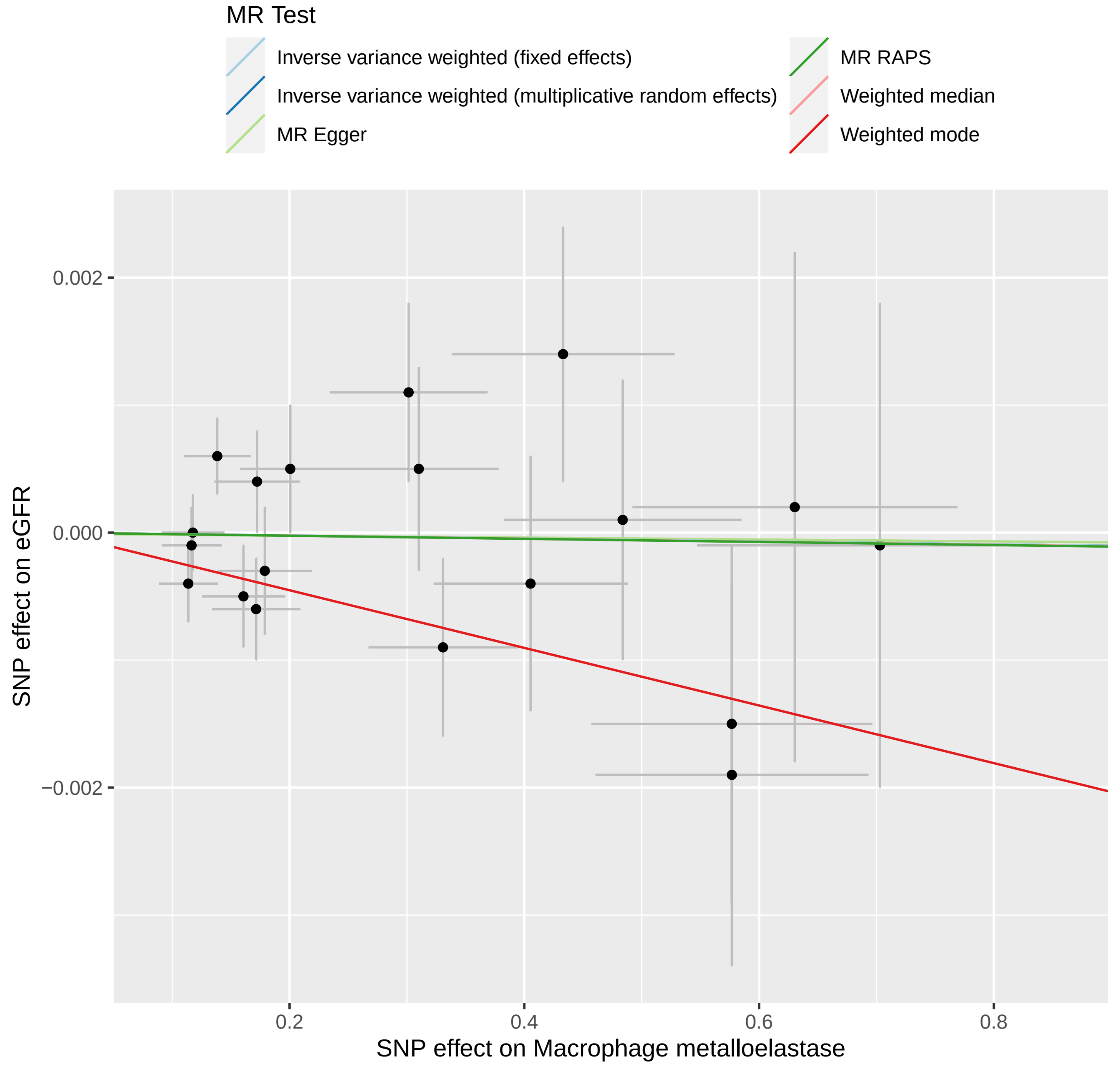

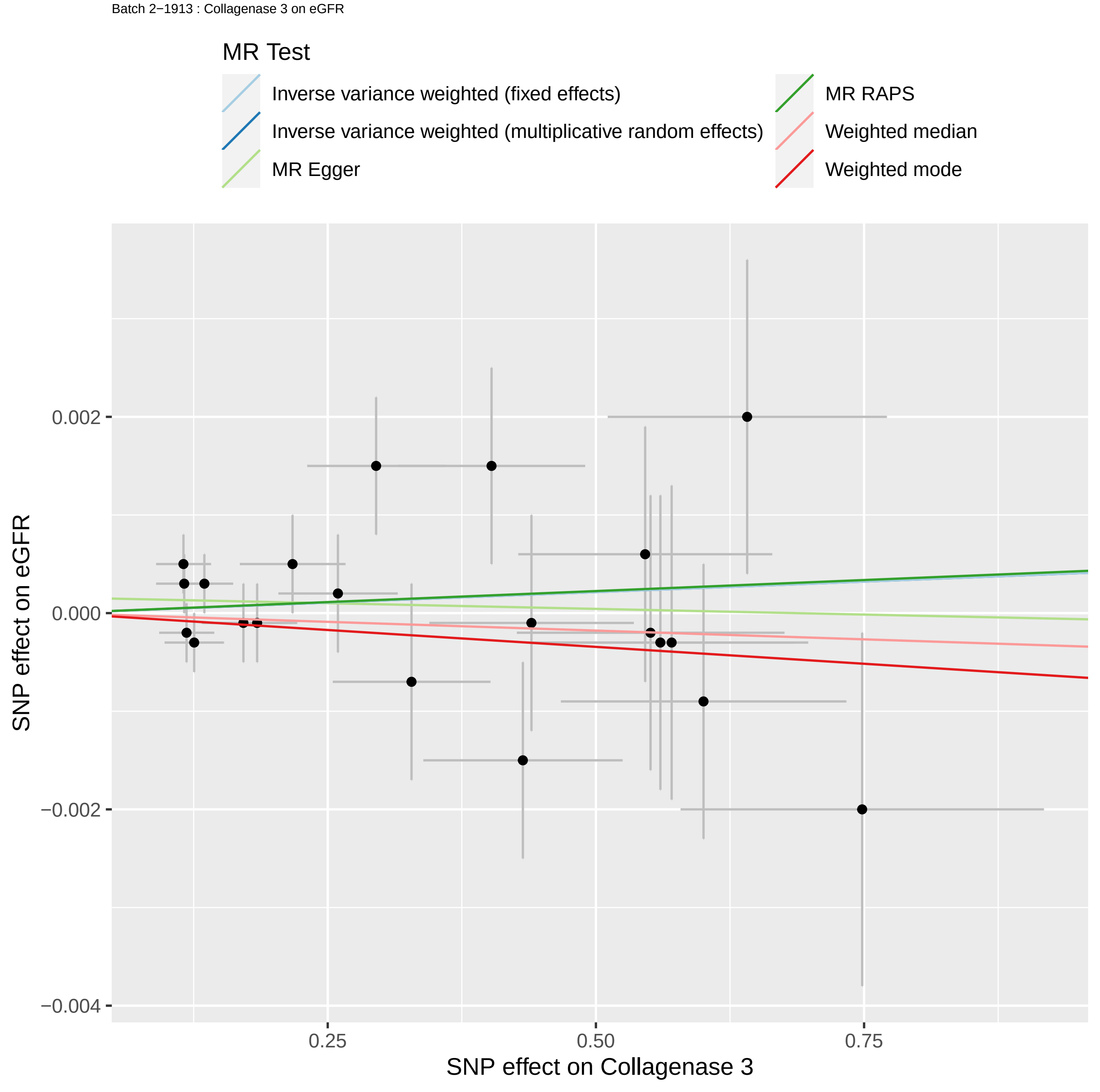

Batch 2−1913 : Collagenase 3 on eGFR
MR Test
Inverse variance weighted (fixed effects)
Inverse variance weighted (multiplicative random effects)
MR Egger
MR RAPS
Weighted median
Weighted mode
0.002
0.000
−0.002
−0.004
0.25
0.50
0.75
SNP effect on eGFR
SNP effect on Collagenase 3

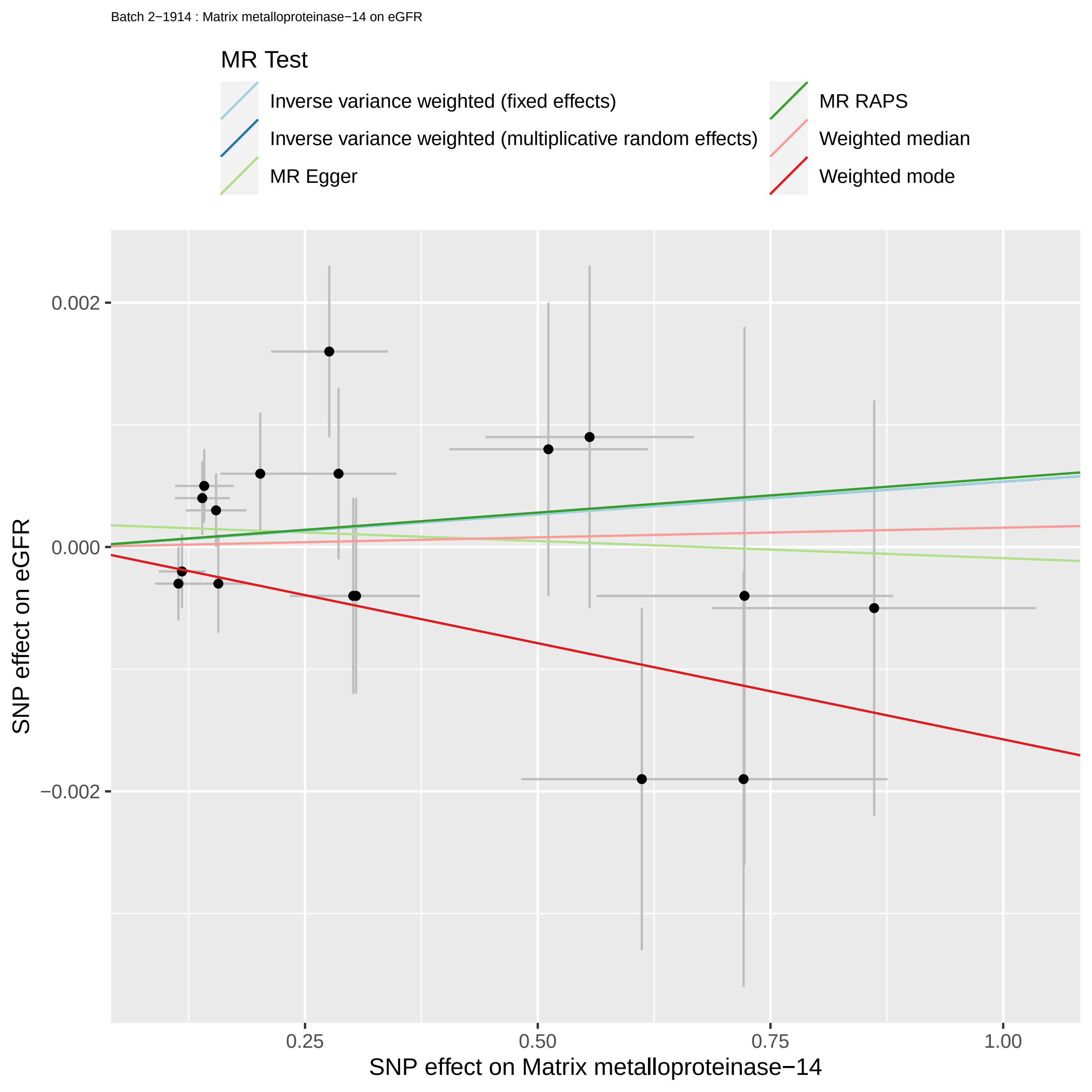
Batch 2−1914 : Matrix metalloproteinase−14 on eGFR
MR Test
Inverse variance weighted (fixed effects)
Inverse variance weighted (multiplicative random effects)
MR Egger
MR RAPS
Weighted median
Weighted mode
0.002
0.000
−0.002
0.25
0.50
0.75
1.00
SNP effect on eGFR
SNP effect on Matrix metalloproteinase−14

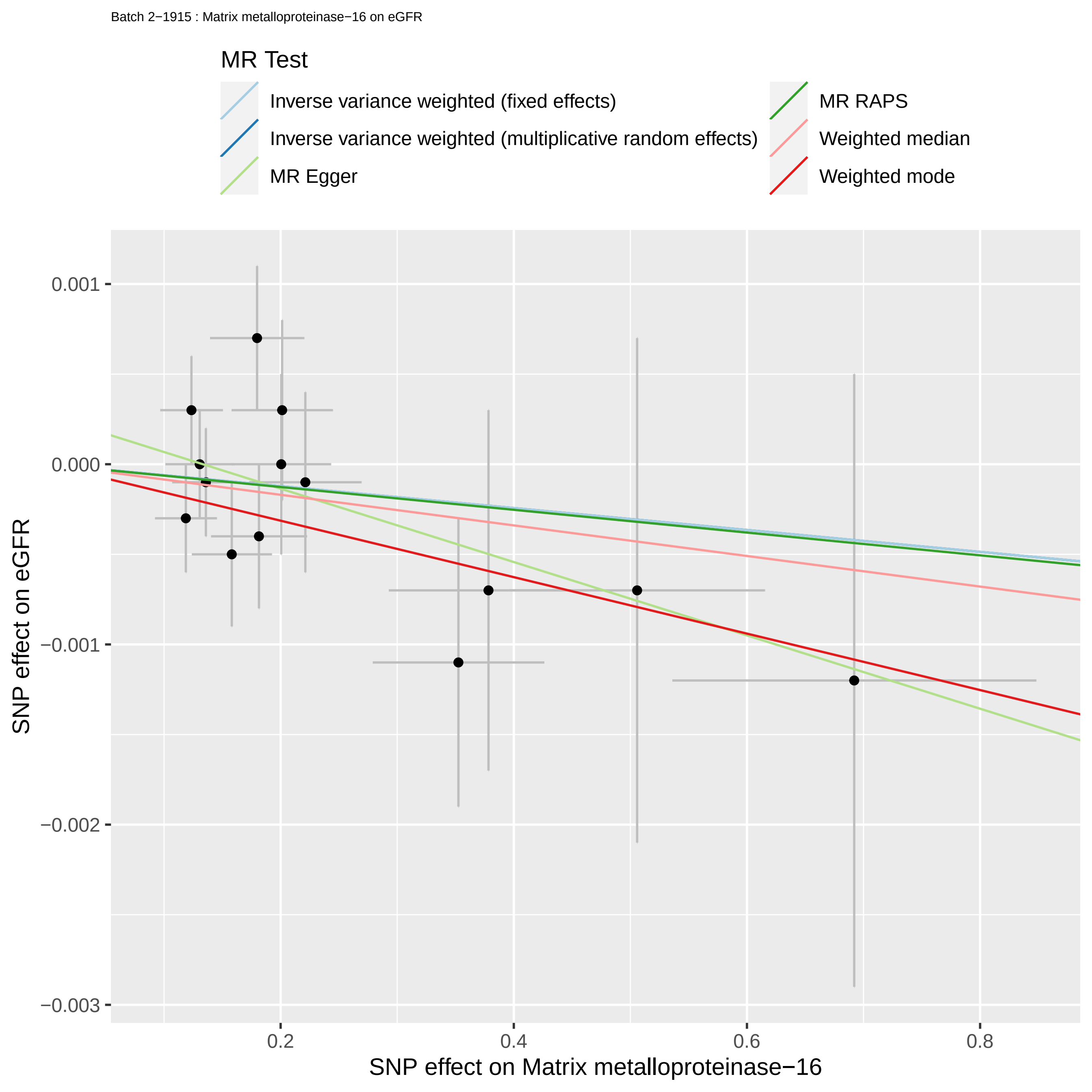

Batch 2−1915 : Matrix metalloproteinase−16 on eGFR
MR Test
Inverse variance weighted (fixed effects)
Inverse variance weighted (multiplicative random effects)
MR Egger
MR RAPS
Weighted median
Weighted mode
0.001
0.000
−0.001
−0.002
−0.003
0.2
0.4
0.6
0.8
SNP effect on eGFR
SNP effect on Matrix metalloproteinase−16

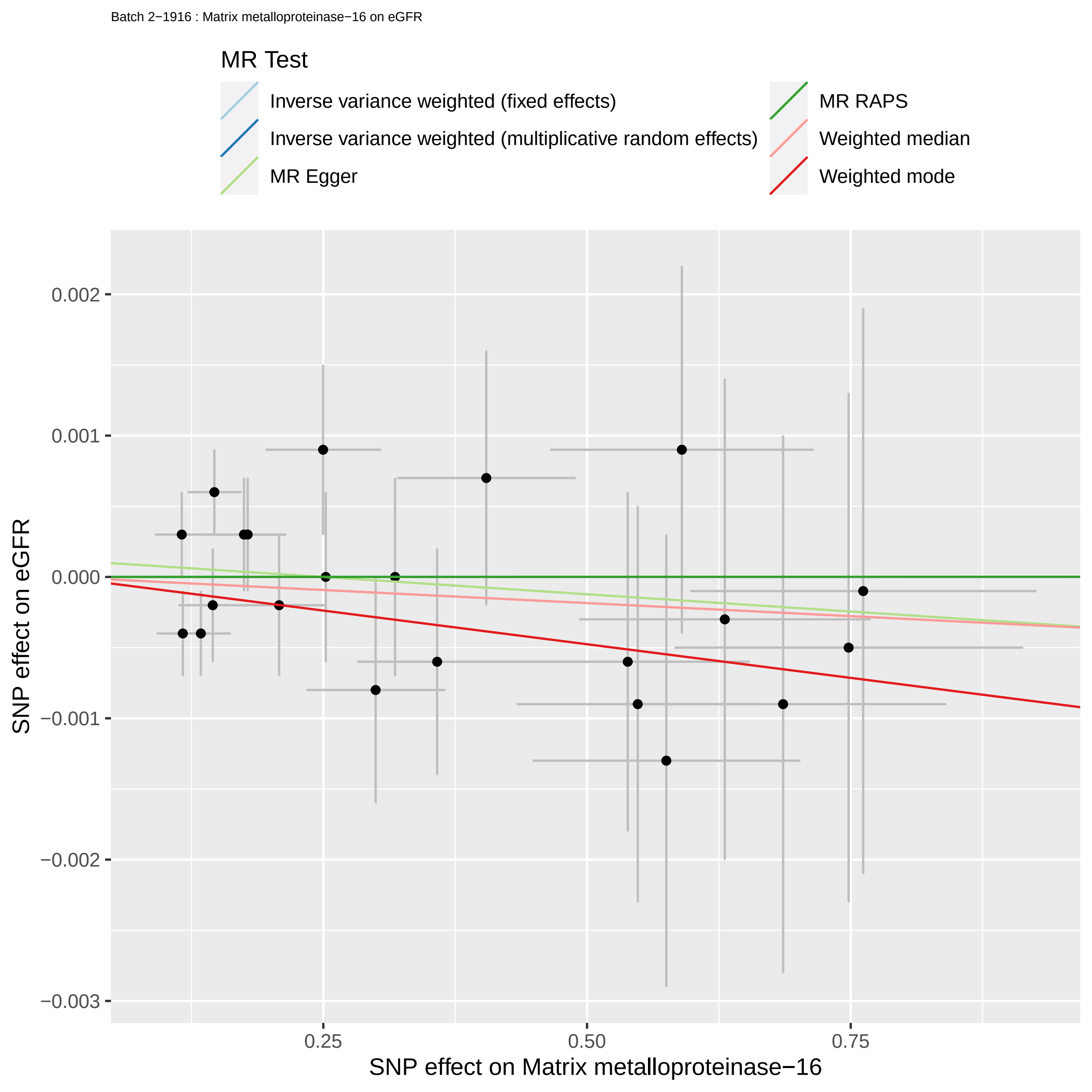

Batch 2−1916 : Matrix metalloproteinase−16 on eGFR
MR Test
Inverse variance weighted (fixed effects)
Inverse variance weighted (multiplicative random effects)
MR Egger
MR RAPS
Weighted median
Weighted mode
0.002
0.001
0.000
−0.001
−0.002
−0.003
SNP effect on eGFR
0.25
0.50
0.75
SNP effect on Matrix metalloproteinase−16

Batch 2−1917 : Matrix metalloproteinase−17 on eGFR

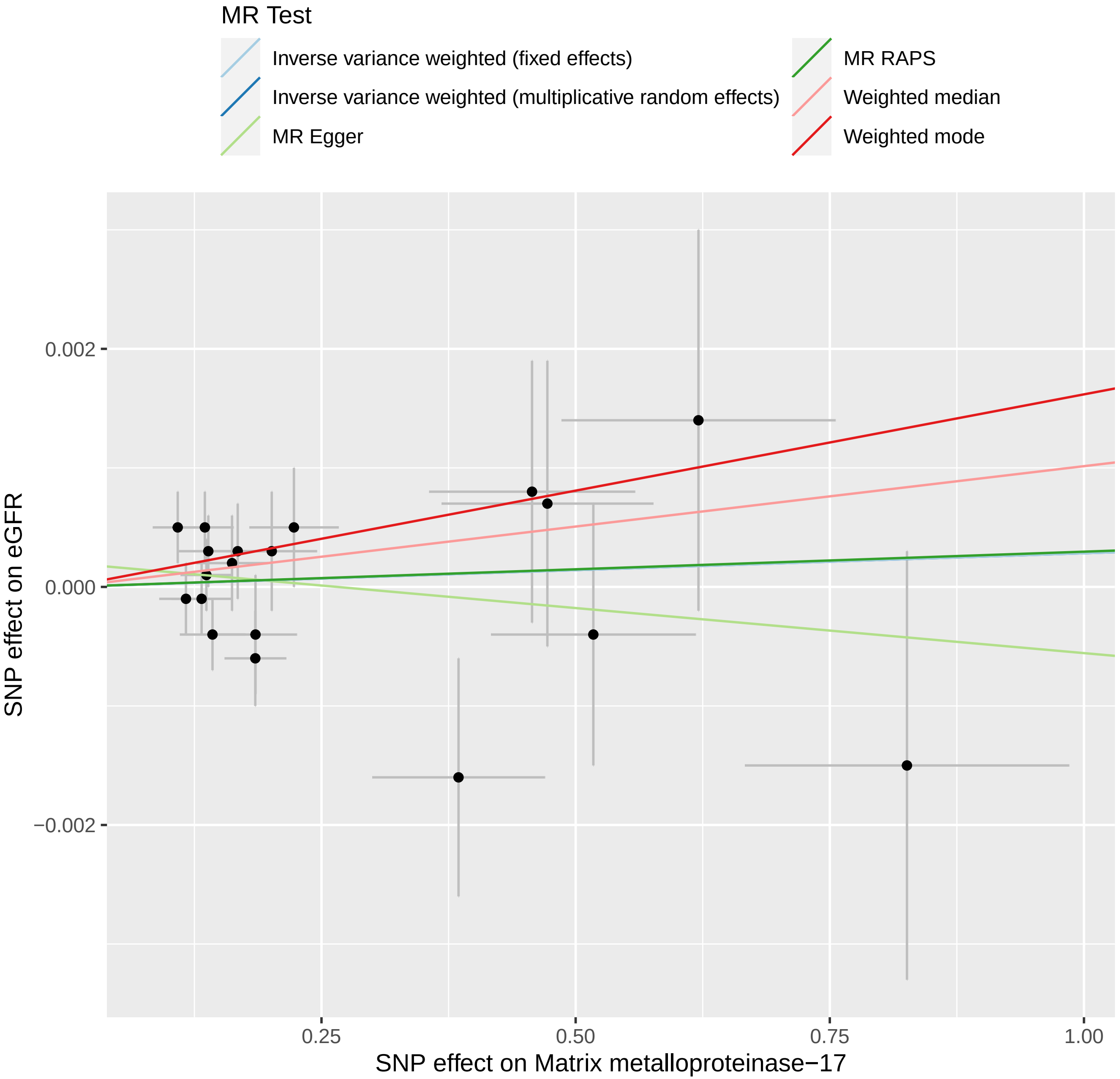

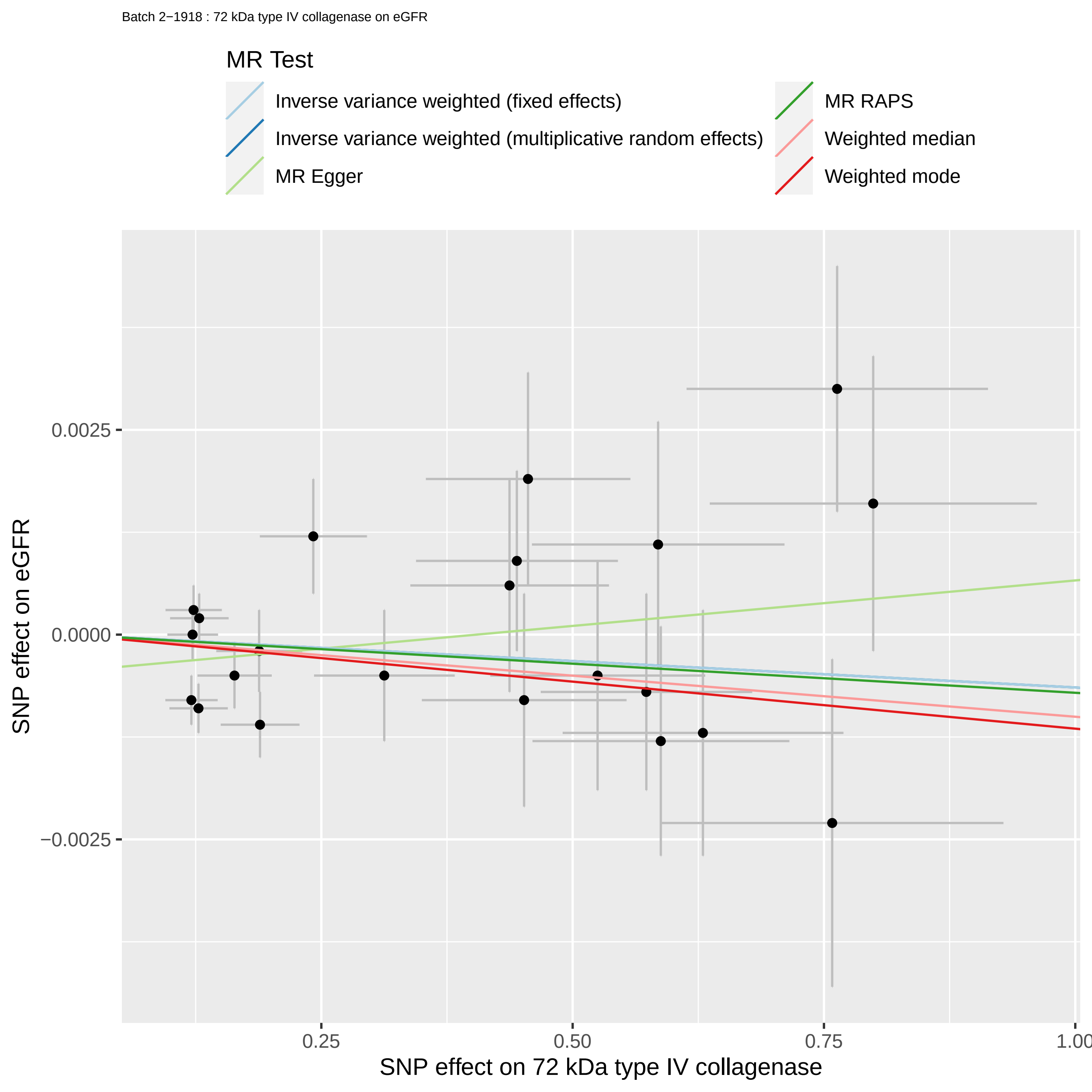

Batch 2−1918 : 72 kDa type IV collagenase on eGFR
MR Test
Inverse variance weighted (fixed effects)
Inverse variance weighted (multiplicative random effects)
MR Egger
MR RAPS
Weighted median
Weighted mode
0.0025
0.0000
−0.0025
0.25
0.50
0.75
1.00
SNP effect on eGFR
SNP effect on 72 kDa type IV collagenase

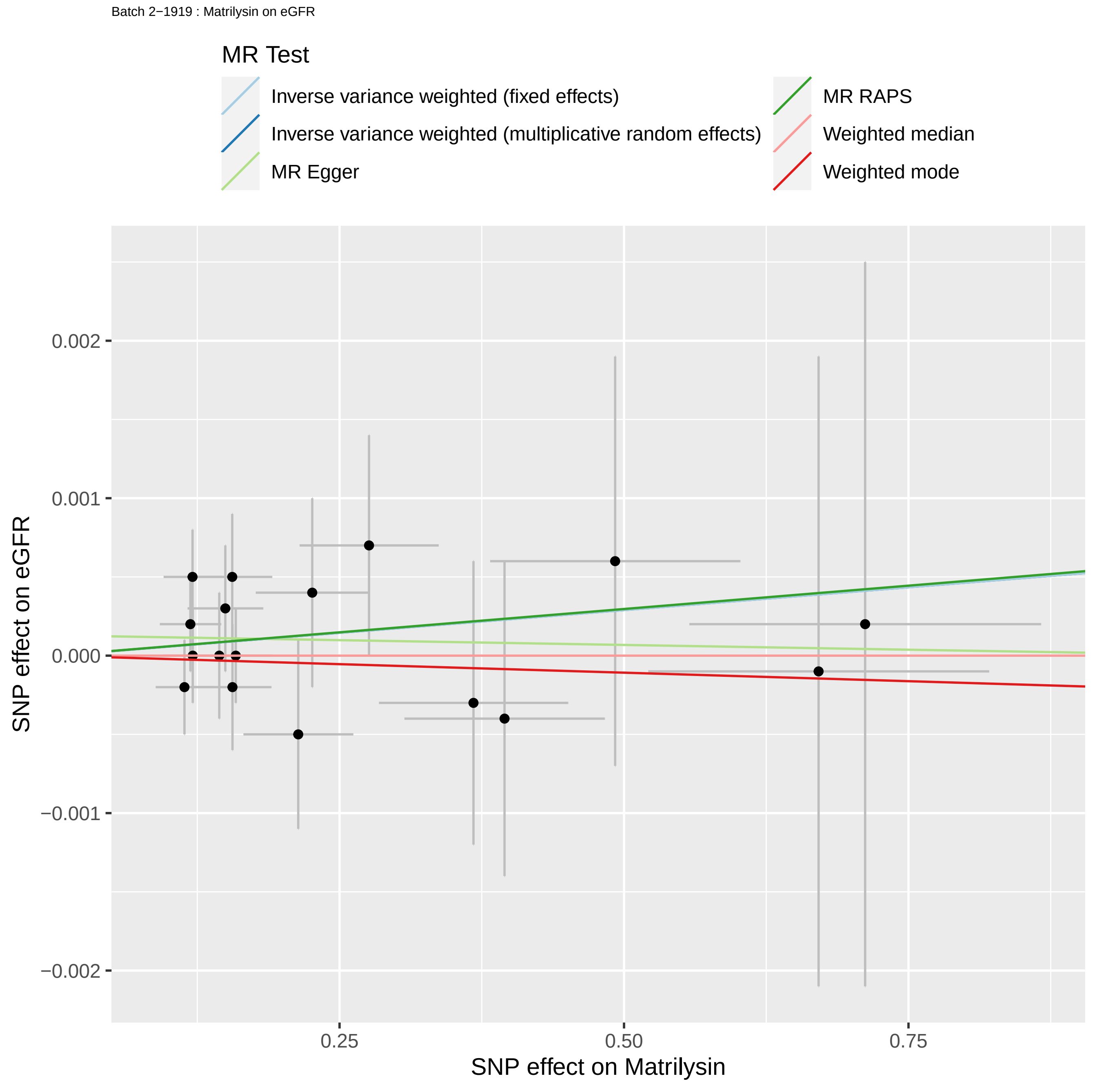

Batch 2−1919 : Matrilysin on eGFR
MR Test
Inverse variance weighted (fixed effects)
Inverse variance weighted (multiplicative random effects)
MR Egger
MR RAPS
Weighted median
Weighted mode
0.002
0.001
0.000
−0.001
−0.002
SNP effect on eGFR
0.25
0.50
0.75
SNP effect on Matrilysin

Batch 2−1920 : Neutrophil collagenase on eGFR

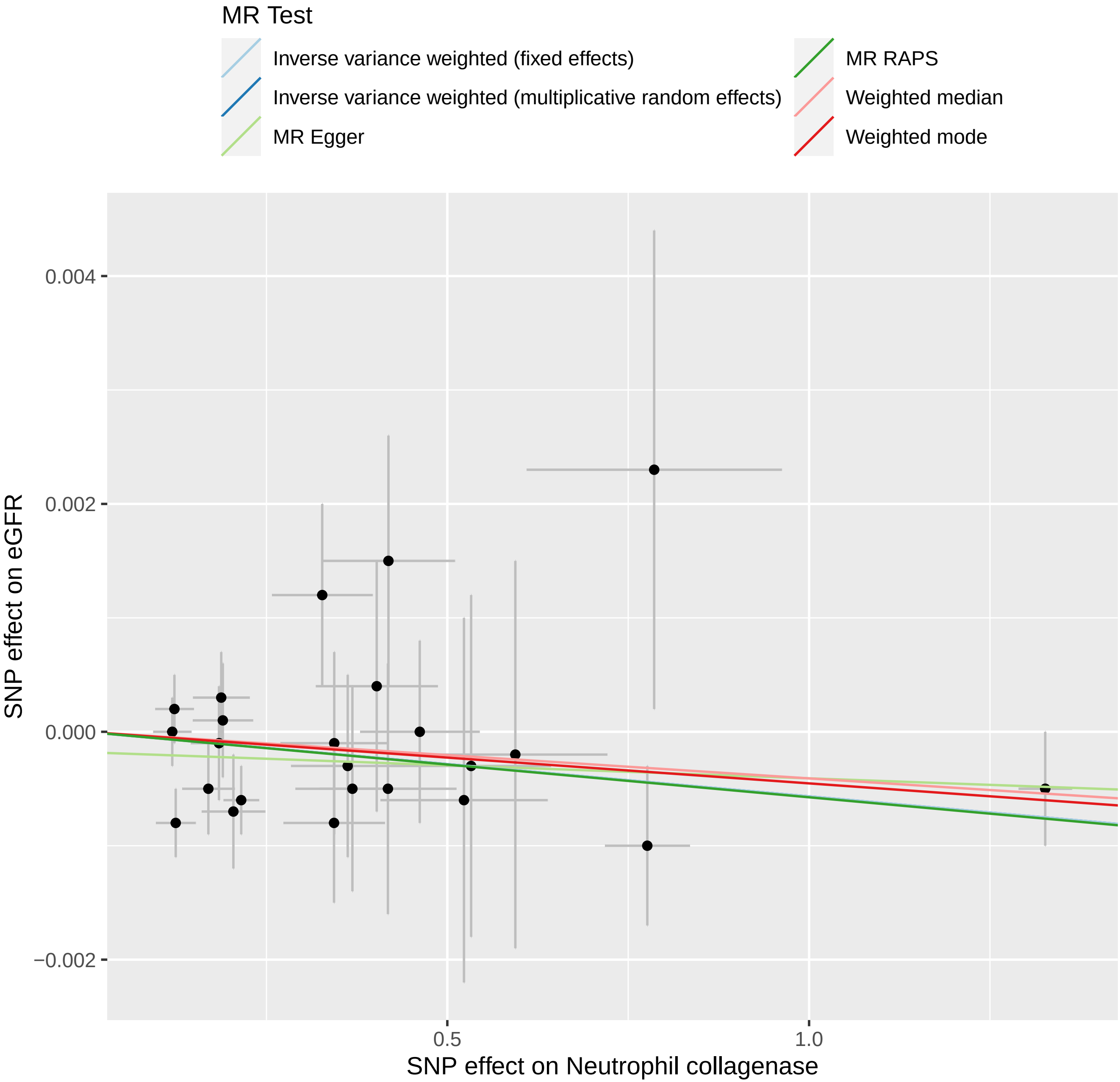

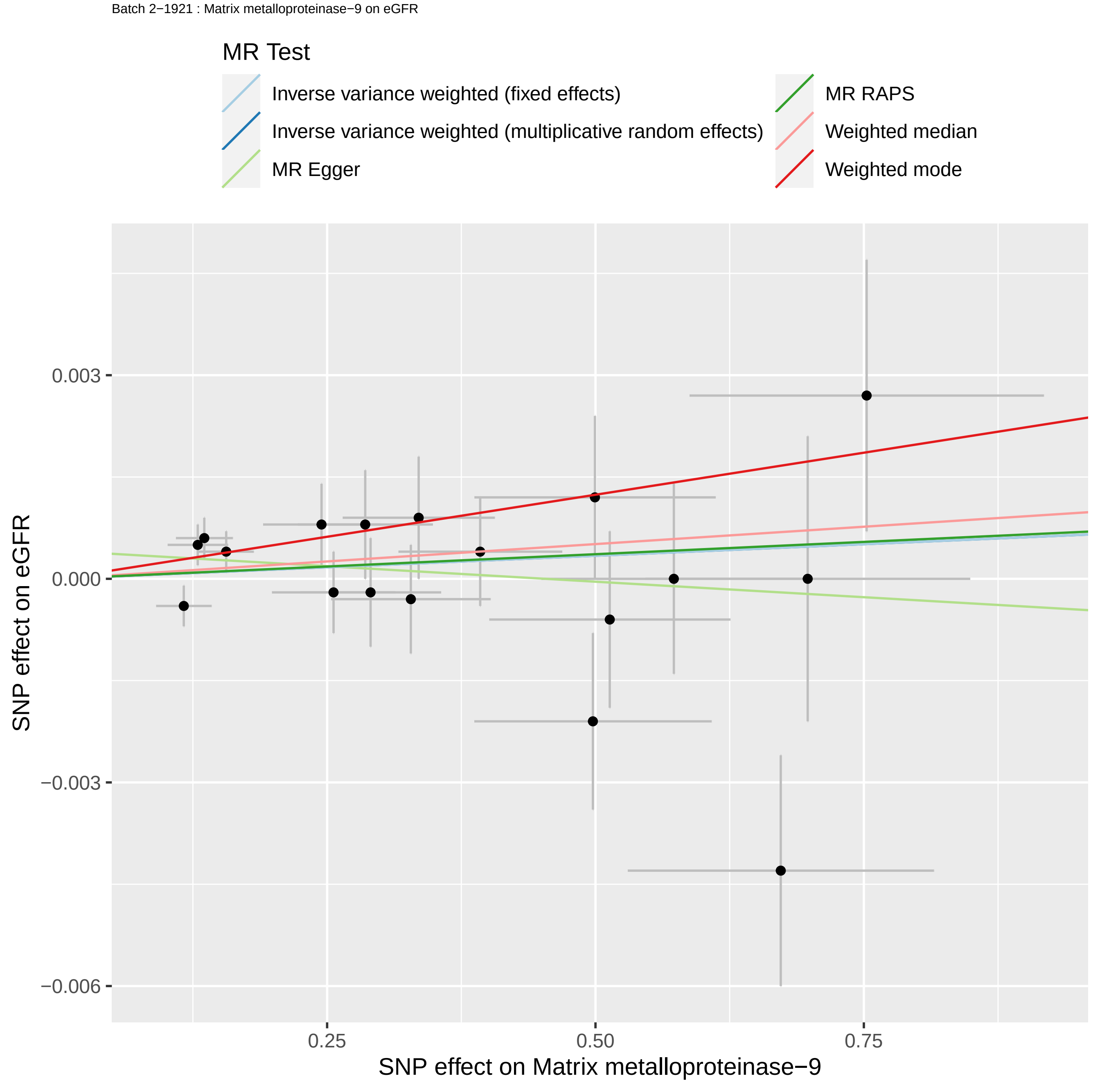

Batch 2−1921 : Matrix metalloproteinase−9 on eGFR
MR Test
Inverse variance weighted (fixed effects)
Inverse variance weighted (multiplicative random effects)
MR Egger
MR RAPS
Weighted median
Weighted mode
0.003
0.000
−0.003
−0.006
0.25
0.50
0.75
SNP effect on eGFR
SNP effect on Matrix metalloproteinase−9

Batch 2−1922 : Multimerin−2 on eGFR

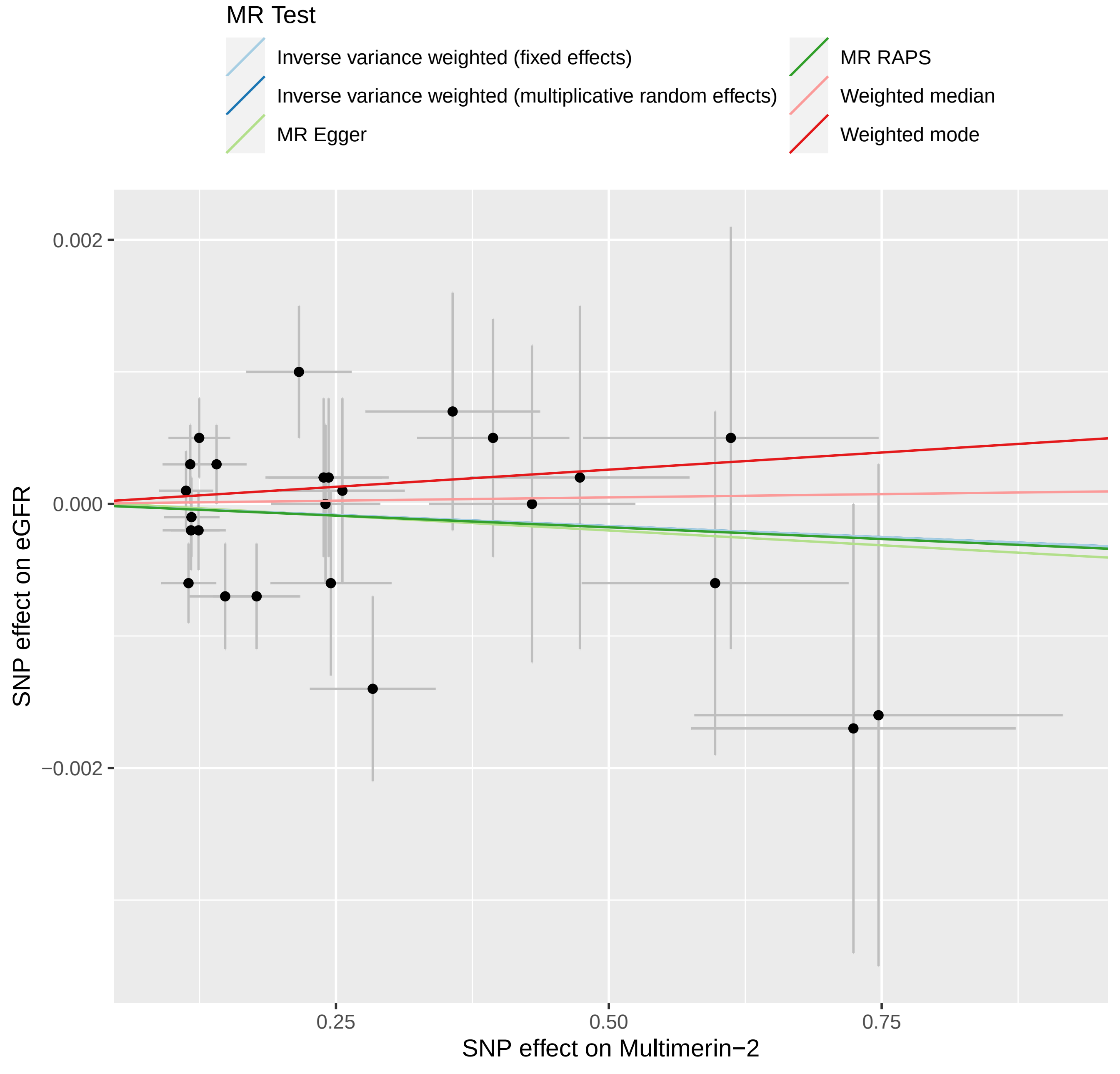

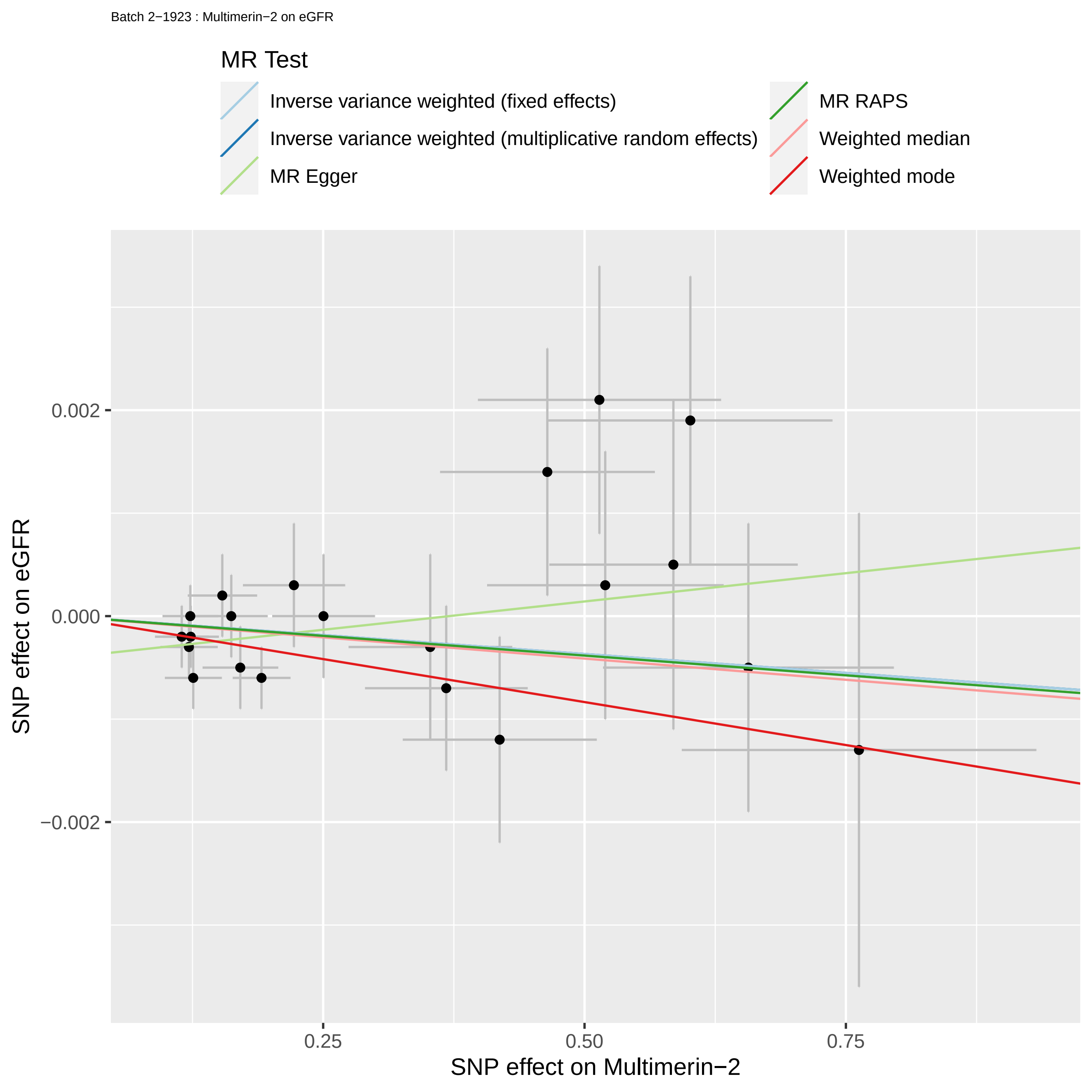
Batch 2−1923 : Multimerin−2 on eGFR
MR Test
Inverse variance weighted (fixed effects)
Inverse variance weighted (multiplicative random effects)
MR Egger
MR RAPS
Weighted median
Weighted mode
0.002
0.000
−0.002
0.25
0.50
0.75
SNP effect on eGFR
SNP effect on Multimerin−2

Batch 2−1924 : Motor neuron and pancreas homeobox protein 1 on eGFR

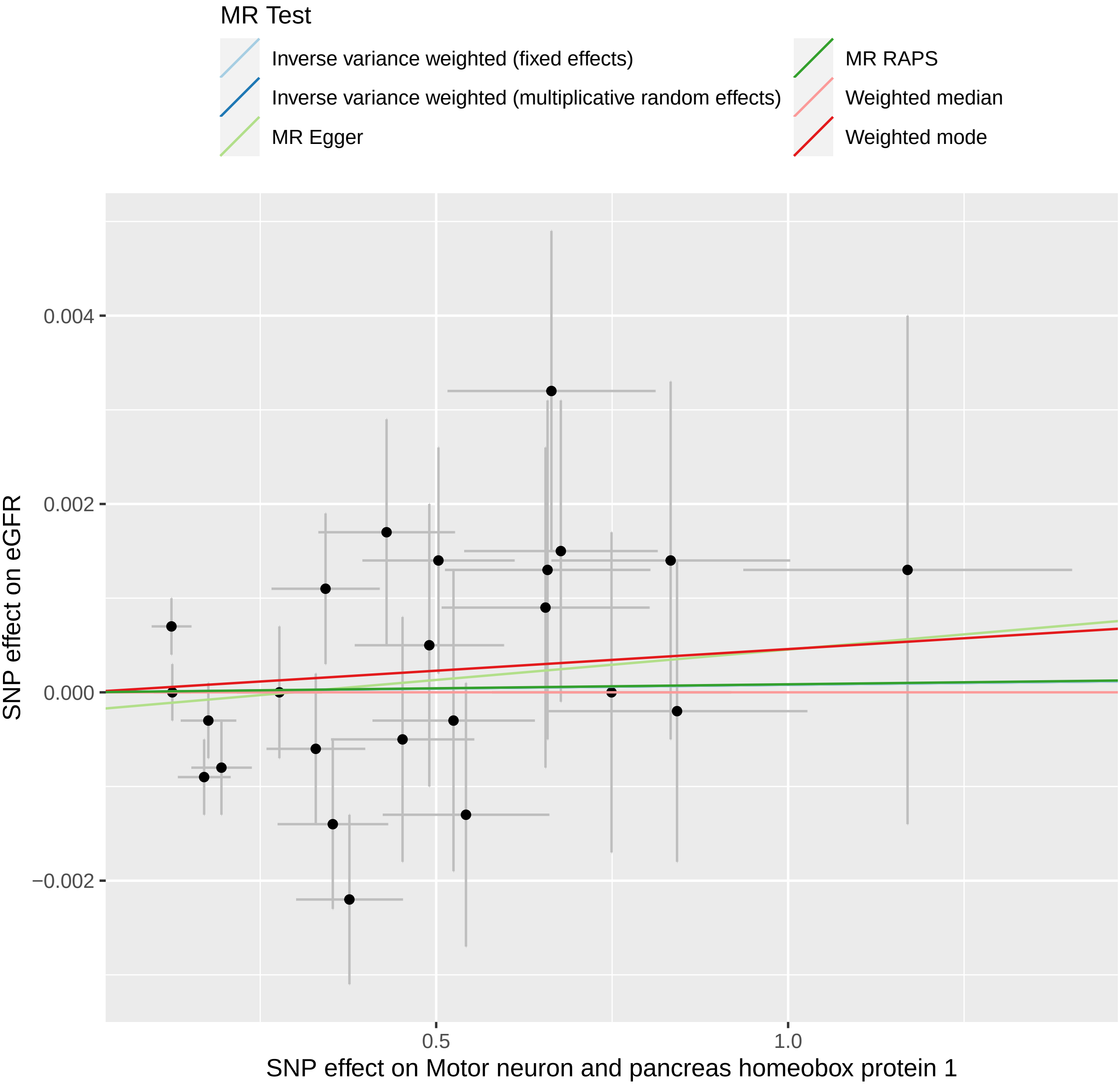

Batch 2−1925 : MOB kinase activator 1A on eGFR

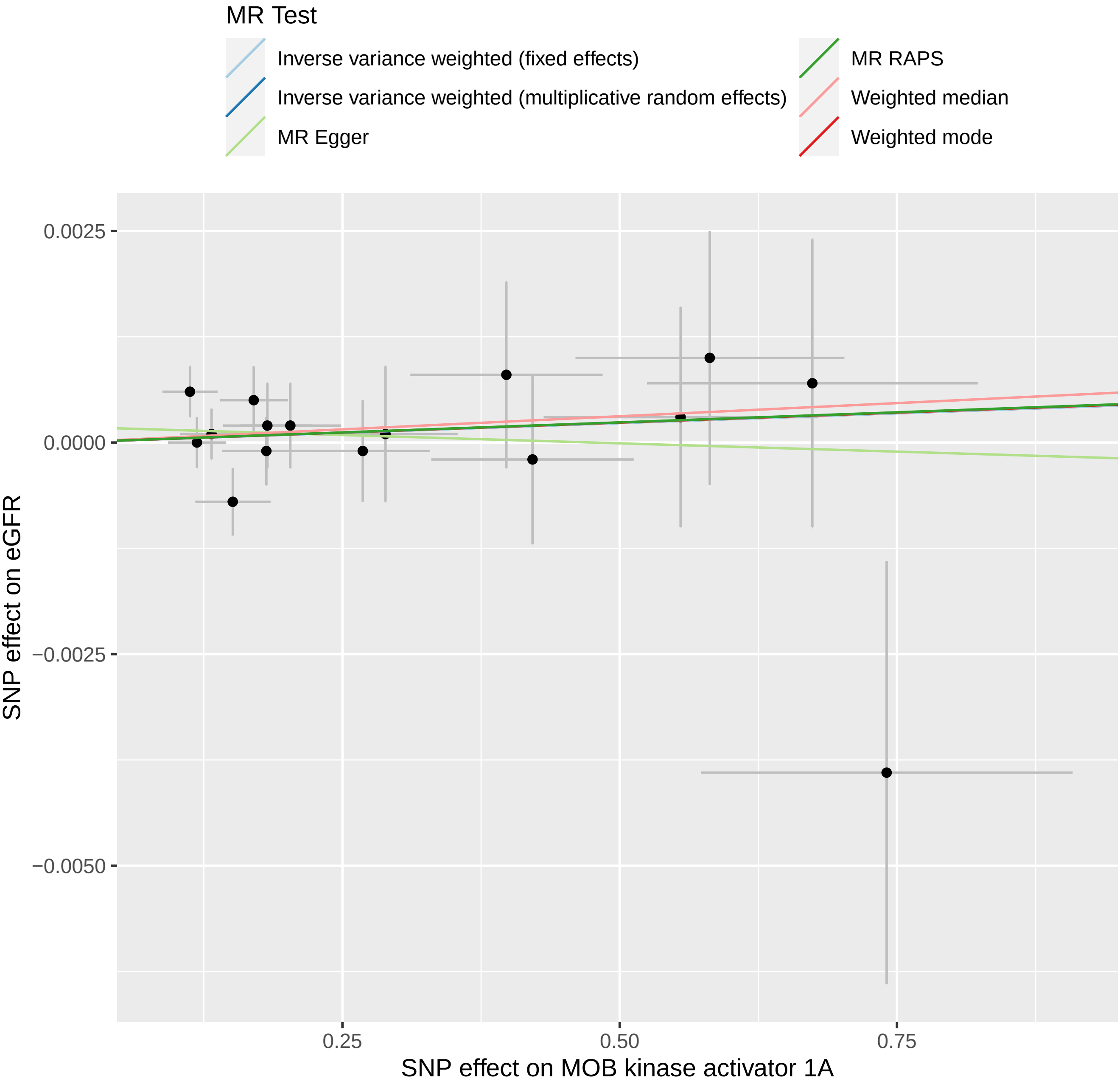

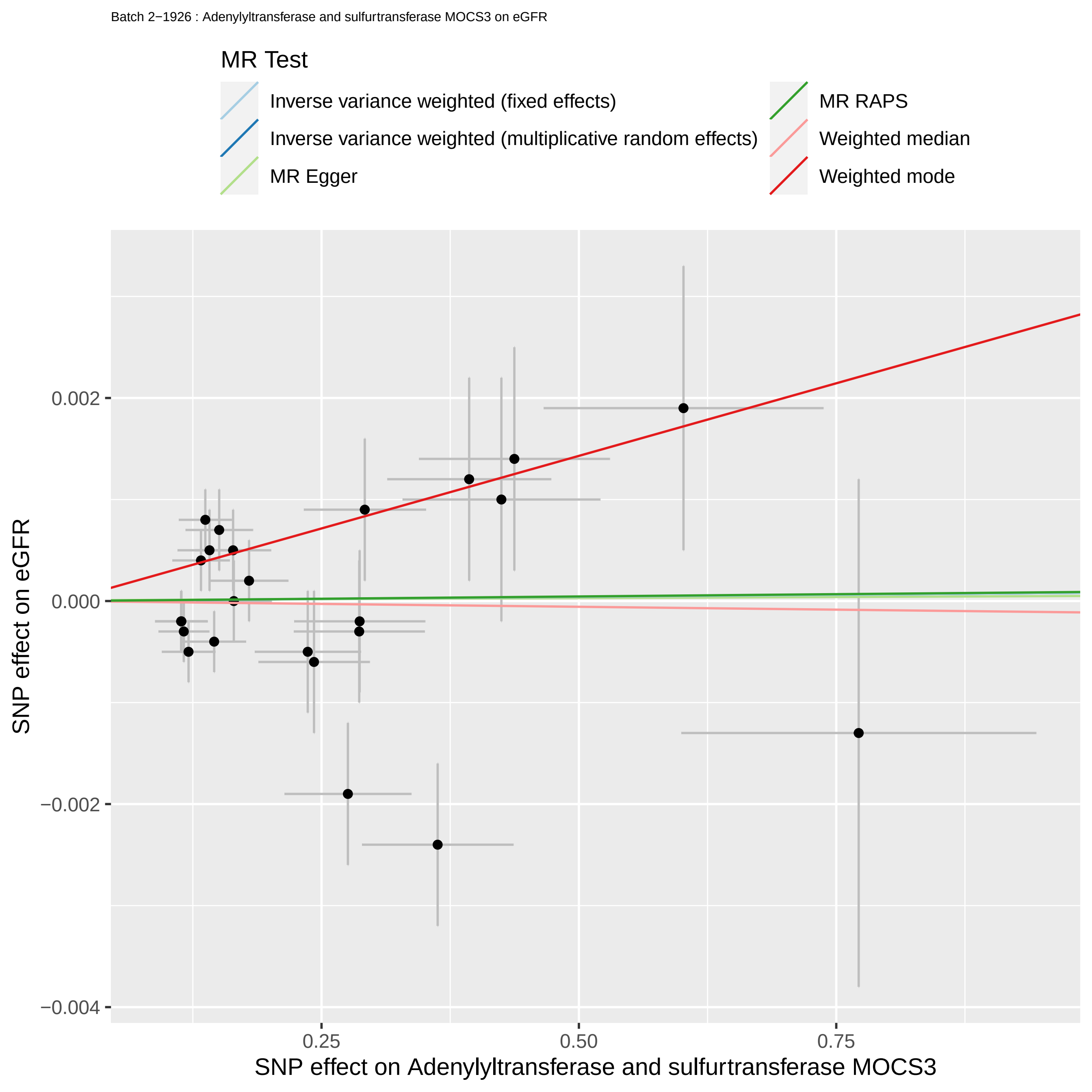

Batch 2−1926 : Adenylyltransferase and sulfurtransferase MOCS3 on eGFR
MR Test
Inverse variance weighted (fixed effects)
Inverse variance weighted (multiplicative random effects)
MR Egger
MR RAPS
Weighted median
Weighted mode
0.002
0.000
−0.002
−0.004
SNP effect on eGFR
0.25
0.50
0.75
SNP effect on Adenylyltransferase and sulfurtransferase MOCS3

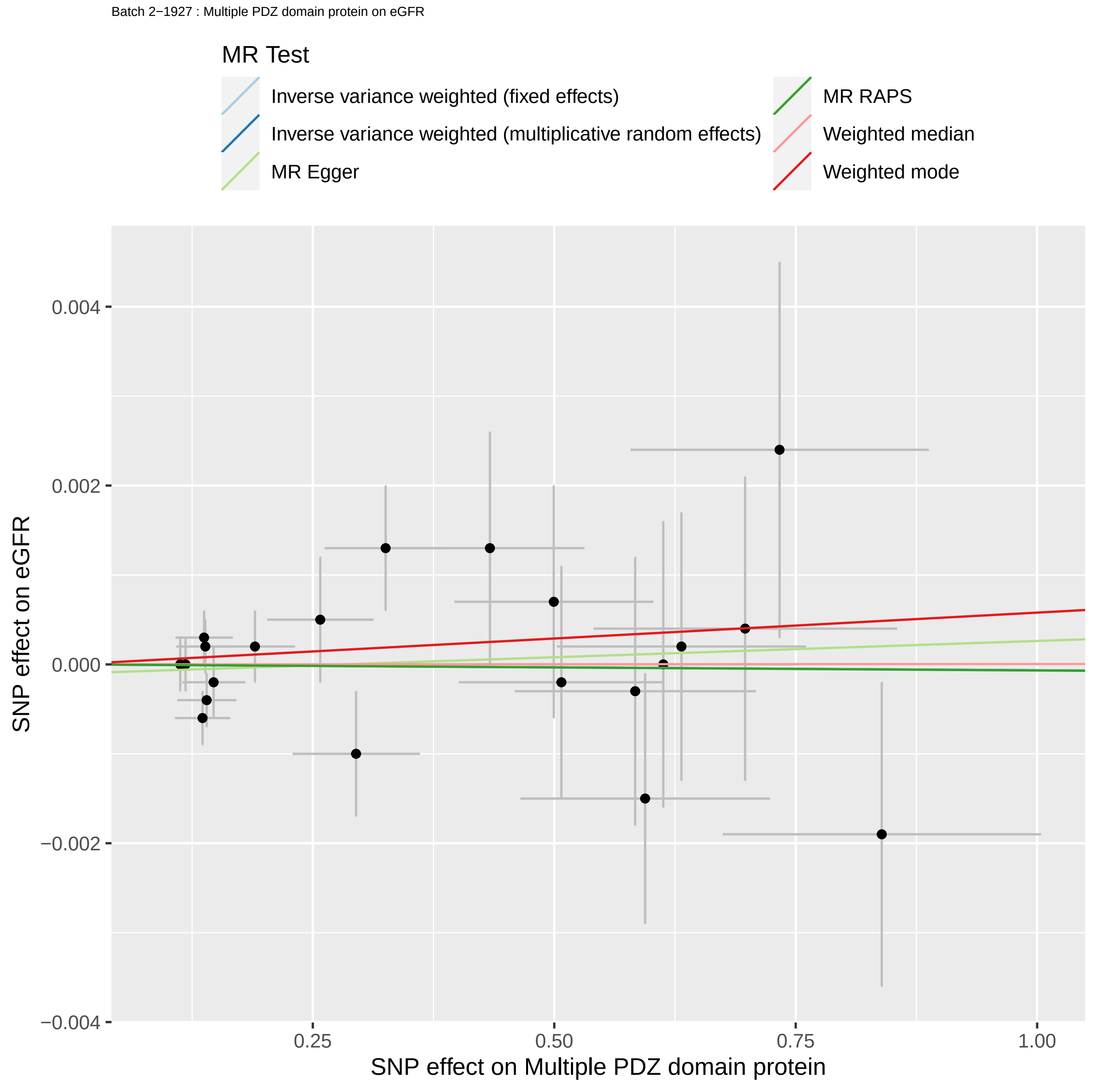

Batch 2−1927 : Multiple PDZ domain protein on eGFR
MR Test
Inverse variance weighted (fixed effects)
Inverse variance weighted (multiplicative random effects)
MR Egger
MR RAPS
Weighted median
Weighted mode
0.004
0.002
0.000
−0.002
−0.004
0.25
0.50
0.75
1.00
SNP effect on eGFR
SNP effect on Multiple PDZ domain protein

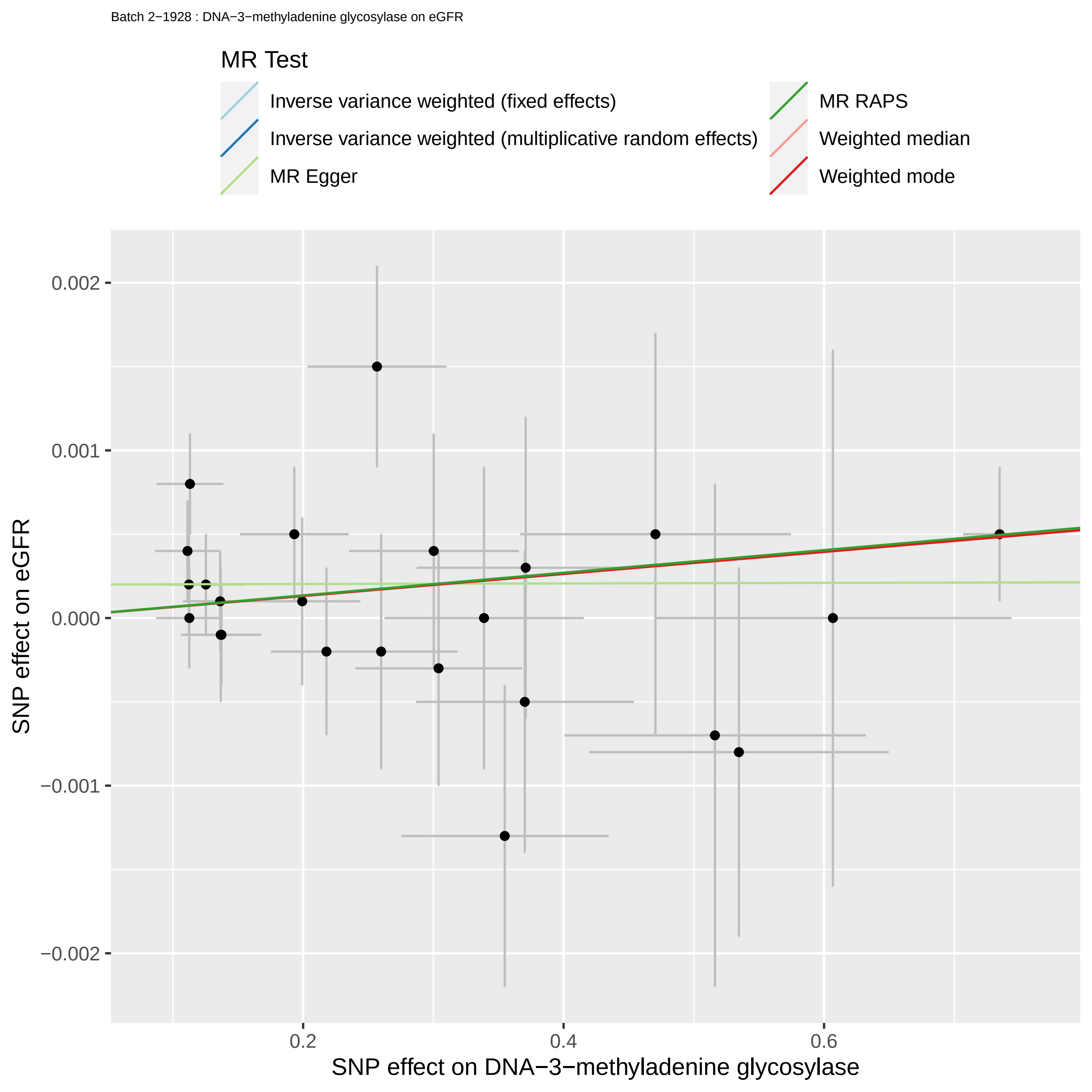

Batch 2−1928 : DNA−3−methyladenine glycosylase on eGFR
MR Test
Inverse variance weighted (fixed effects)
Inverse variance weighted (multiplicative random effects)
MR Egger
MR RAPS
Weighted median
Weighted mode
0.002
0.001
0.000
−0.001
−0.002
SNP effect on eGFR
0.2
0.4
0.6
SNP effect on DNA−3−methyladenine glycosylase

Batch 2−1929 : Thrombopoietin Receptor on eGFR

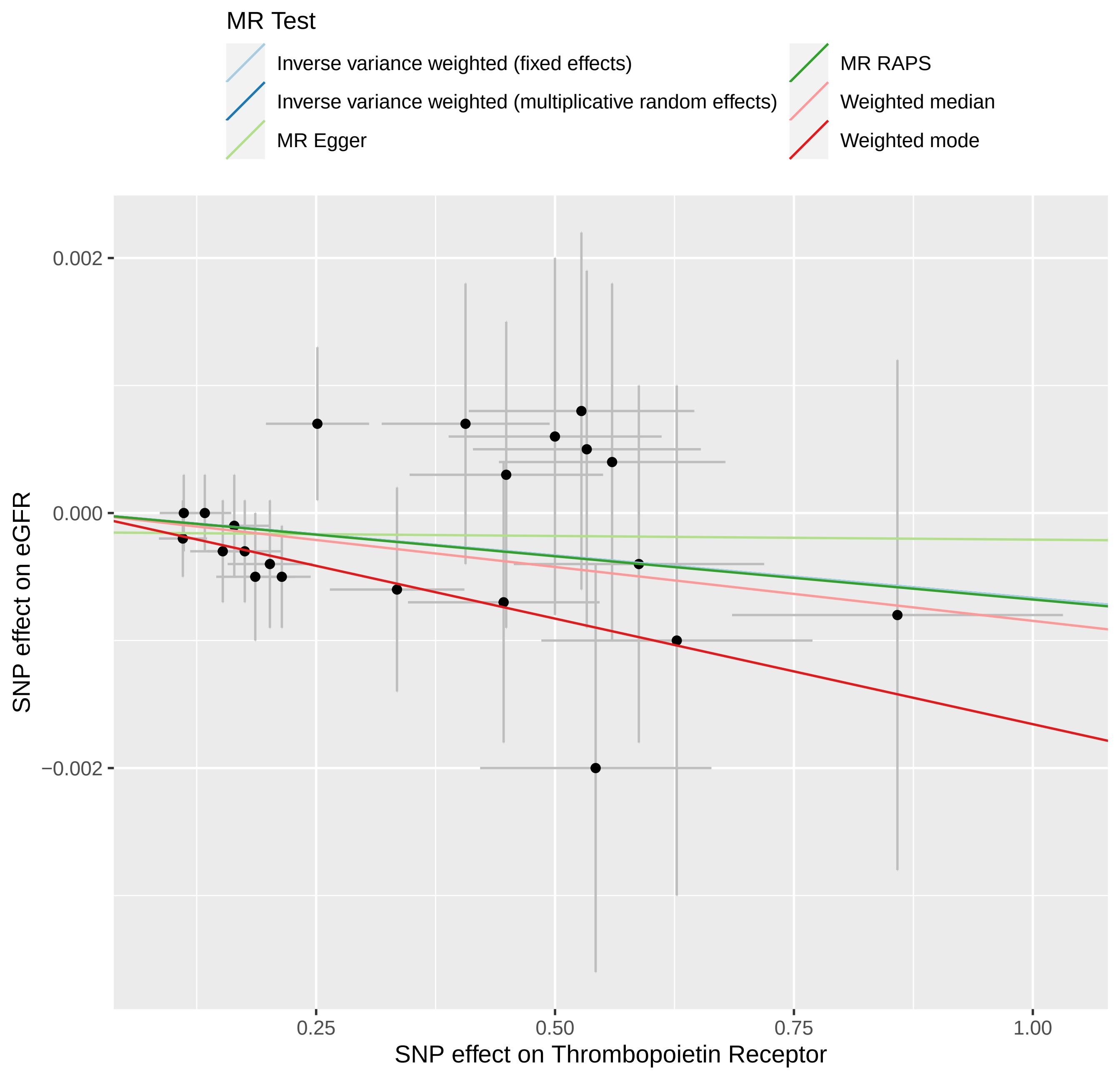

Batch 2−1930 : Myeloperoxidase on eGFR

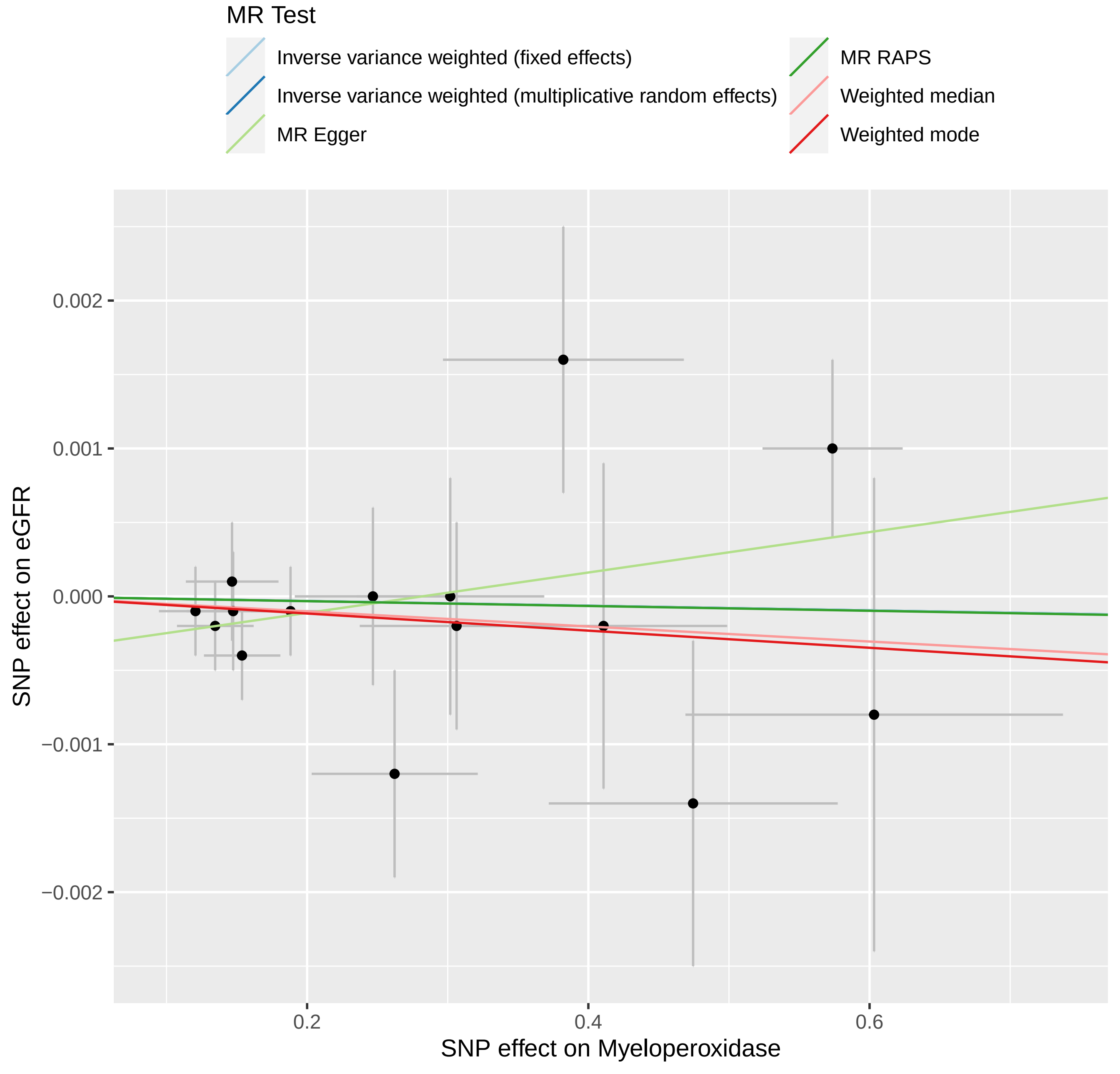

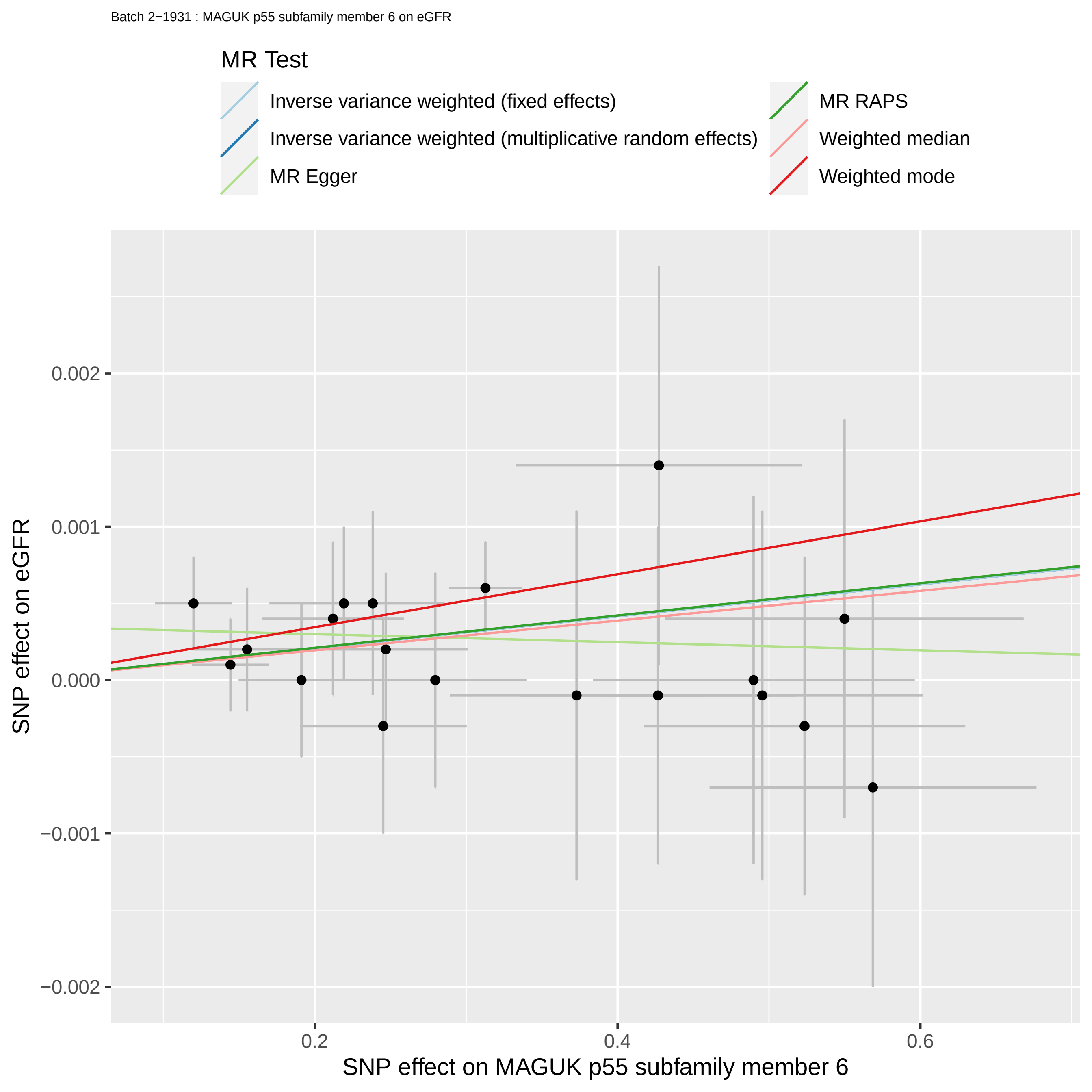

Batch 2−1931 : MAGUK p55 subfamily member 6 on eGFR
MR Test
Inverse variance weighted (fixed effects)
Inverse variance weighted (multiplicative random effects)
MR Egger
MR RAPS
Weighted median
Weighted mode
0.002
0.001
0.000
−0.001
−0.002
SNP effect on eGFR
0.2
0.4
0.6
SNP effect on MAGUK p55 subfamily member 6

Batch 2−1932 : MAGUK p55 subfamily member 7 on eGFR

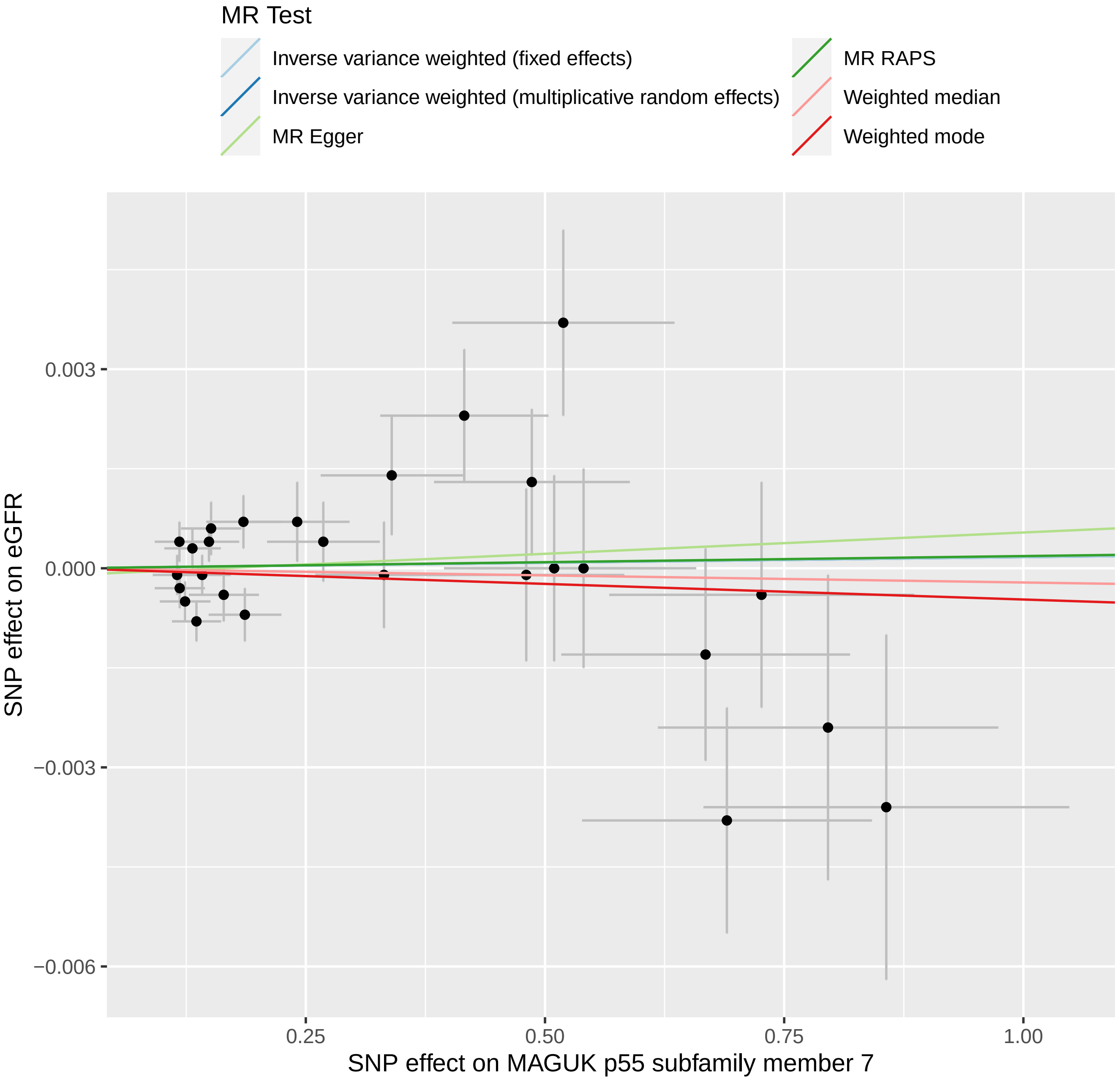

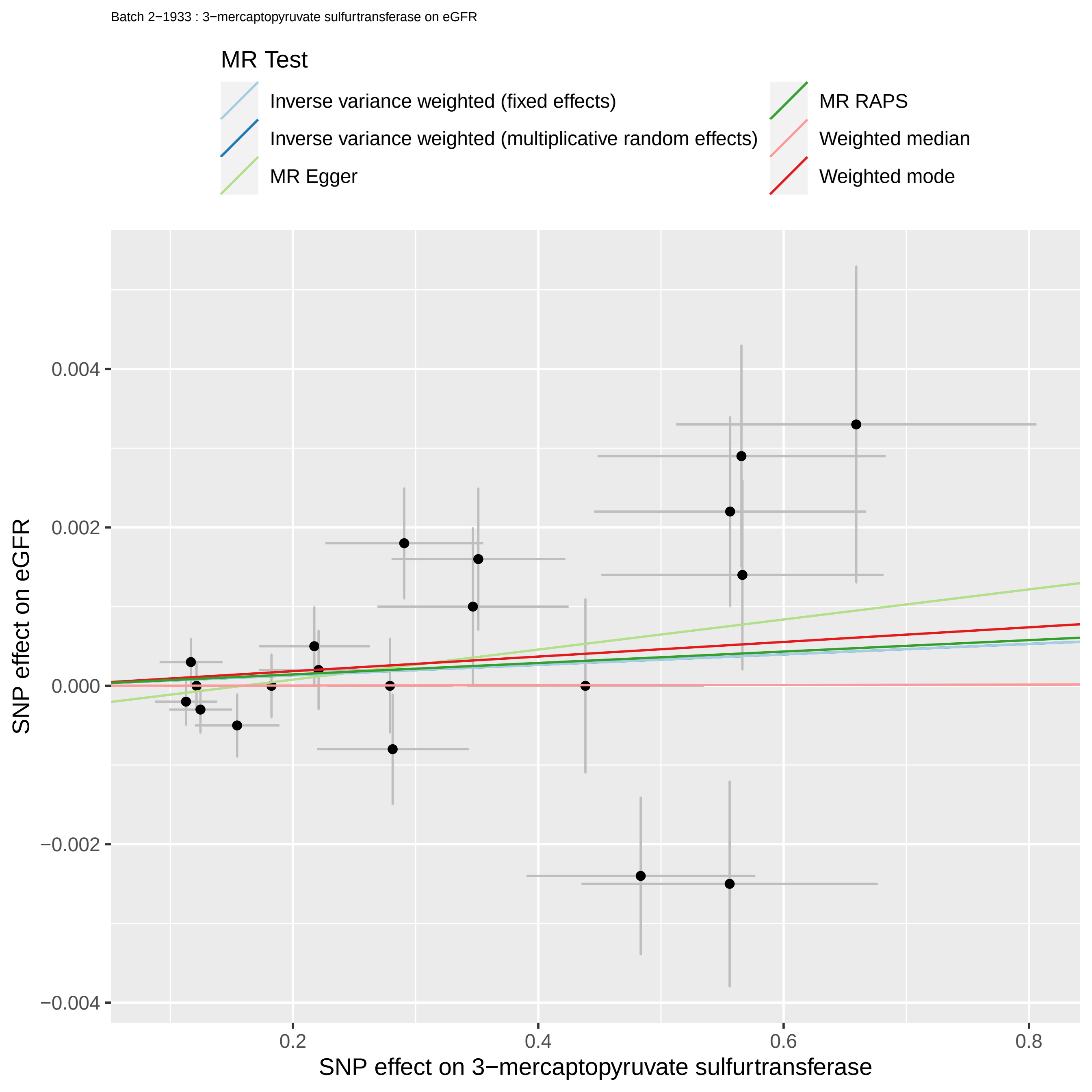
Batch 2−1933 : 3−mercaptopyruvate sulfurtransferase on eGFR
MR Test
Inverse variance weighted (fixed effects)
Inverse variance weighted (multiplicative random effects)
MR Egger
MR RAPS
Weighted median
Weighted mode
0.004
0.002
0.000
−0.002
−0.004
SNP effect on eGFR
0.2
0.4
0.6
0.8
SNP effect on 3−mercaptopyruvate sulfurtransferase

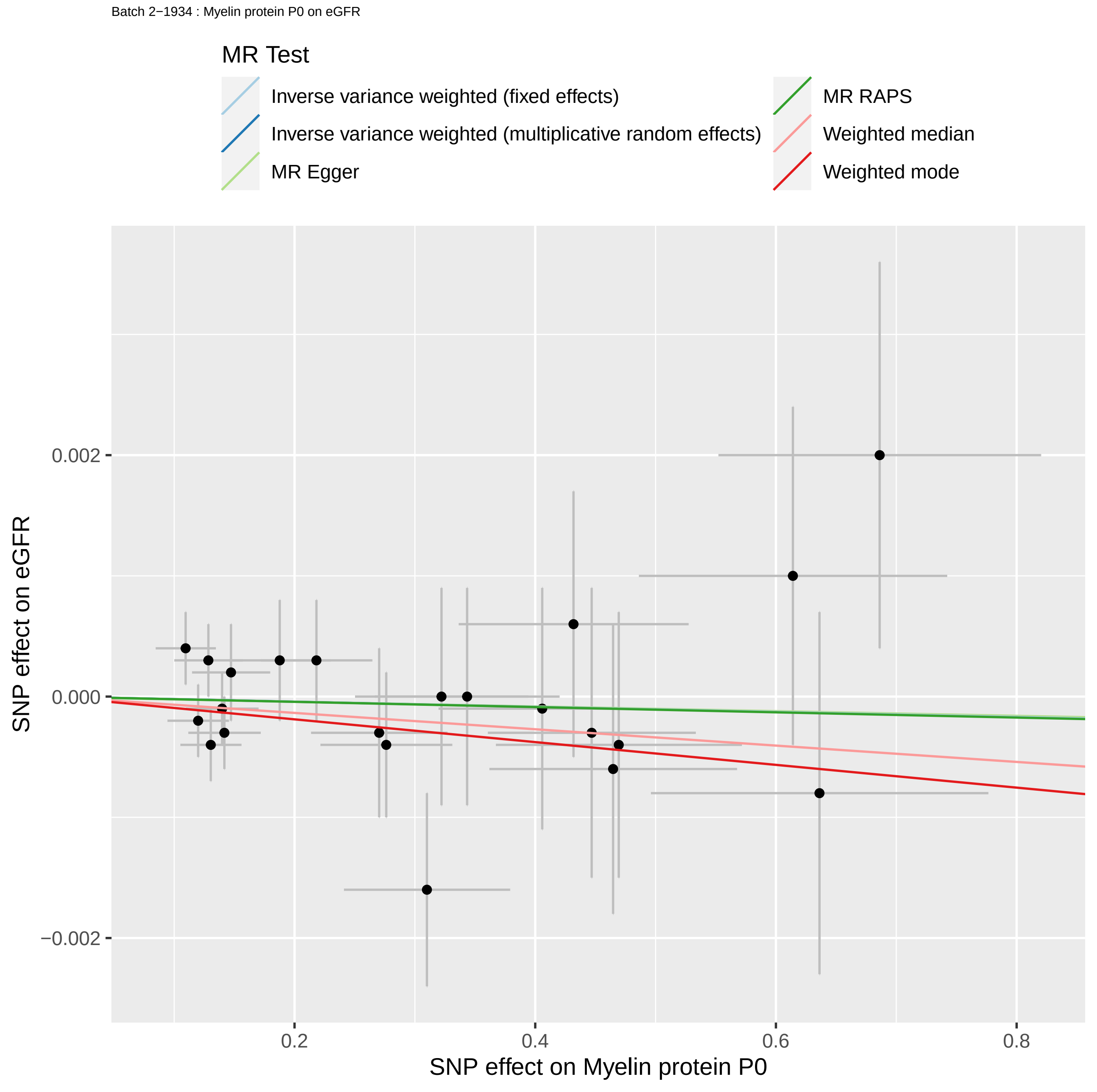

Batch 2−1934 : Myelin protein P0 on eGFR
MR Test
Inverse variance weighted (fixed effects)
Inverse variance weighted (multiplicative random effects)
MR Egger
MR RAPS
Weighted median
Weighted mode
0.002
0.000
−0.002
SNP effect on eGFR
0.2
0.4
0.6
0.8
SNP effect on Myelin protein P0

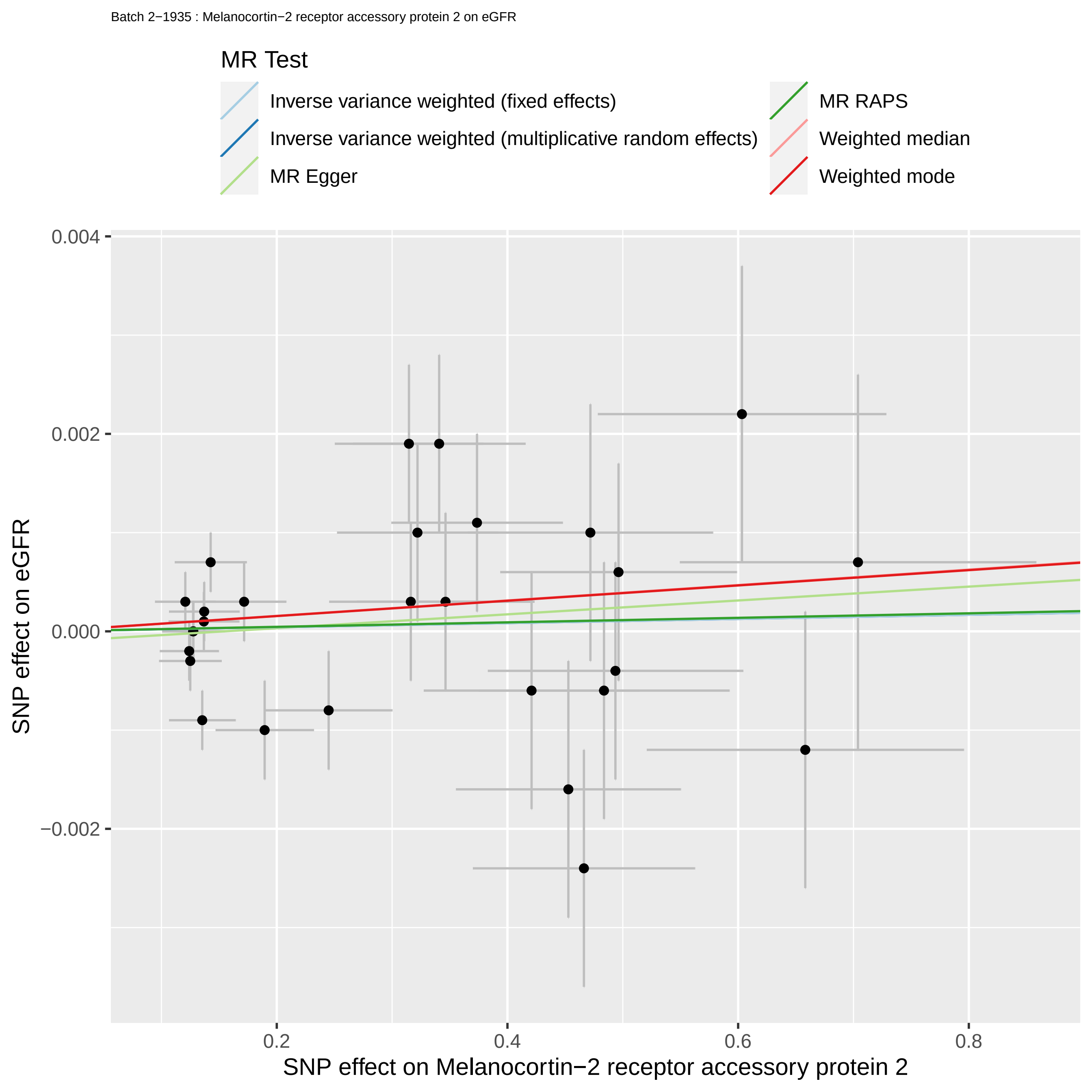
Batch 2−1935 : Melanocortin−2 receptor accessory protein 2 on eGFR
MR Test
Inverse variance weighted (fixed effects)
Inverse variance weighted (multiplicative random effects)
MR Egger
MR RAPS
Weighted median
Weighted mode
0.004
0.002
0.000
−0.002
0.2
0.4
0.6
0.8
SNP effect on eGFR
SNP effect on Melanocortin−2 receptor accessory protein 2

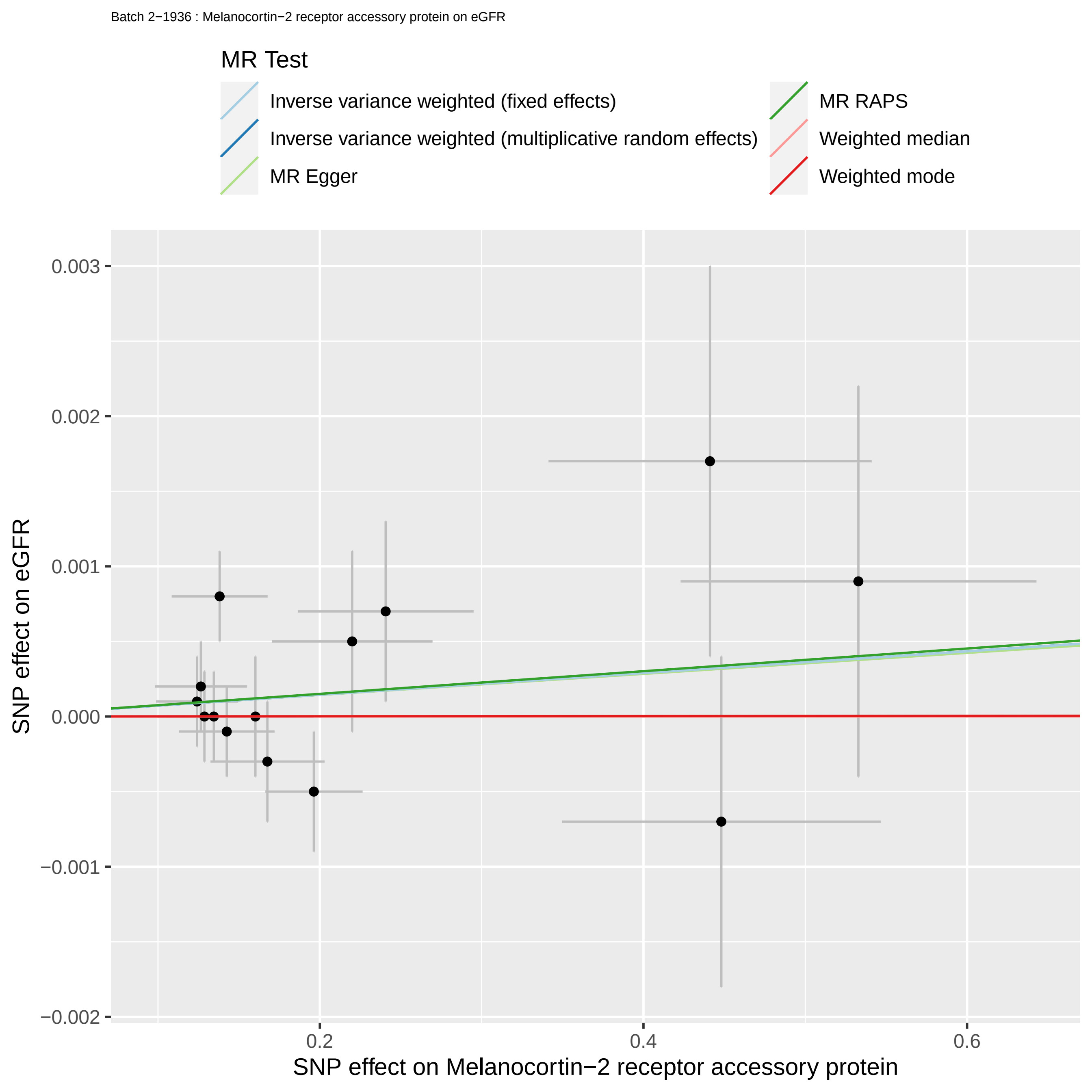
Batch 2−1936 : Melanocortin−2 receptor accessory protein on eGFR
MR Test
Inverse variance weighted (fixed effects)
Inverse variance weighted (multiplicative random effects)
MR Egger
MR RAPS
Weighted median
Weighted mode
0.003
0.002
0.001
0.000
−0.001
−0.002
0.2
0.4
0.6
SNP effect on eGFR
SNP effect on Melanocortin−2 receptor accessory protein

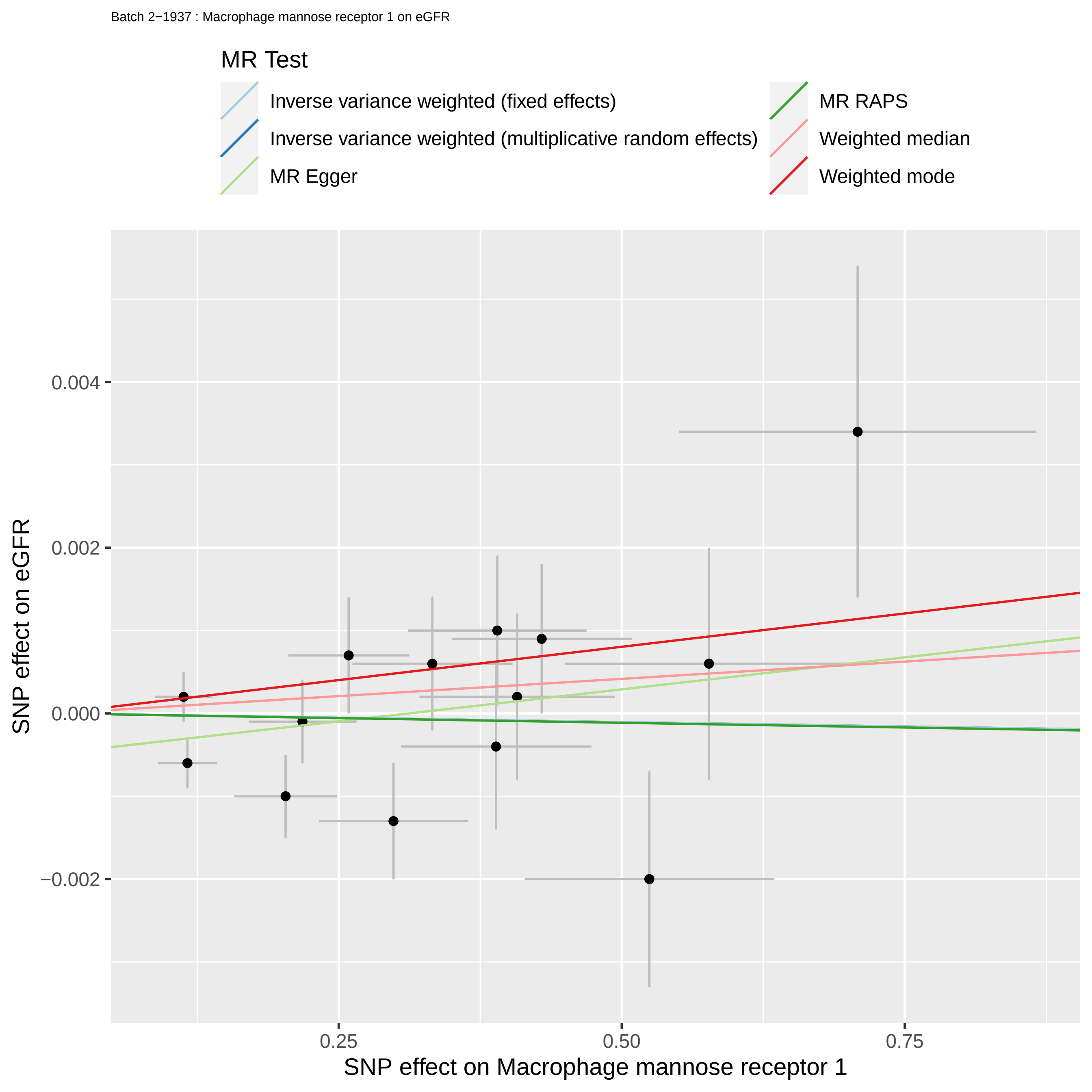
Batch 2−1937 : Macrophage mannose receptor 1 on eGFR
MR Test
Inverse variance weighted (fixed effects)
Inverse variance weighted (multiplicative random effects)
MR Egger
MR RAPS
Weighted median
Weighted mode
0.004
0.002
0.000
−0.002
0.25
0.50
0.75
SNP effect on eGFR
SNP effect on Macrophage mannose receptor 1

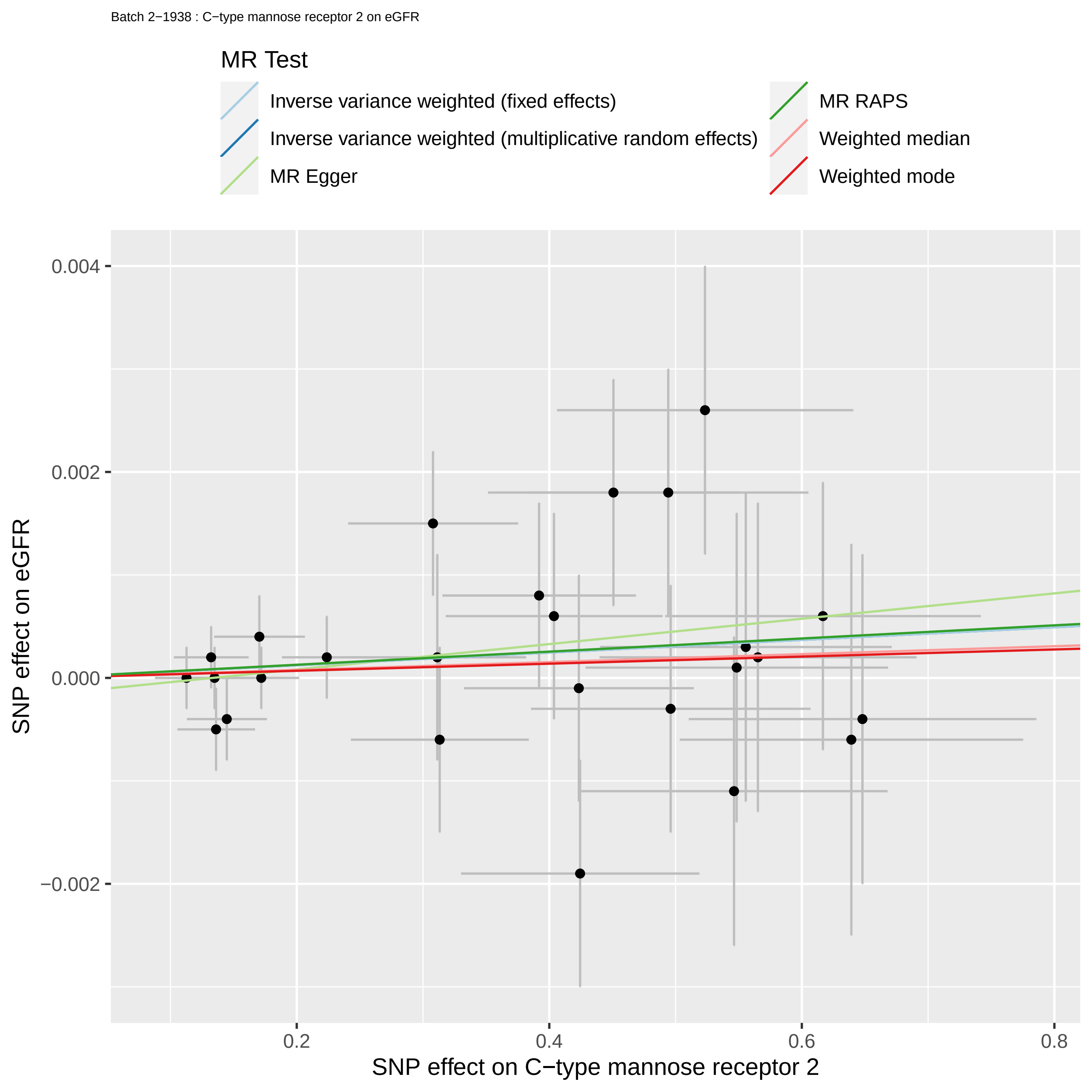

Batch 2−1938 : C−type mannose receptor 2 on eGFR
MR Test
Inverse variance weighted (fixed effects)
Inverse variance weighted (multiplicative random effects)
MR Egger
MR RAPS
Weighted median
Weighted mode
0.004
0.002
0.000
−0.002
SNP effect on eGFR
0.2
0.4
0.6
0.8
SNP effect on C−type mannose receptor 2

Batch 2−1939 : Double−strand break repair protein MRE11A on eGFR

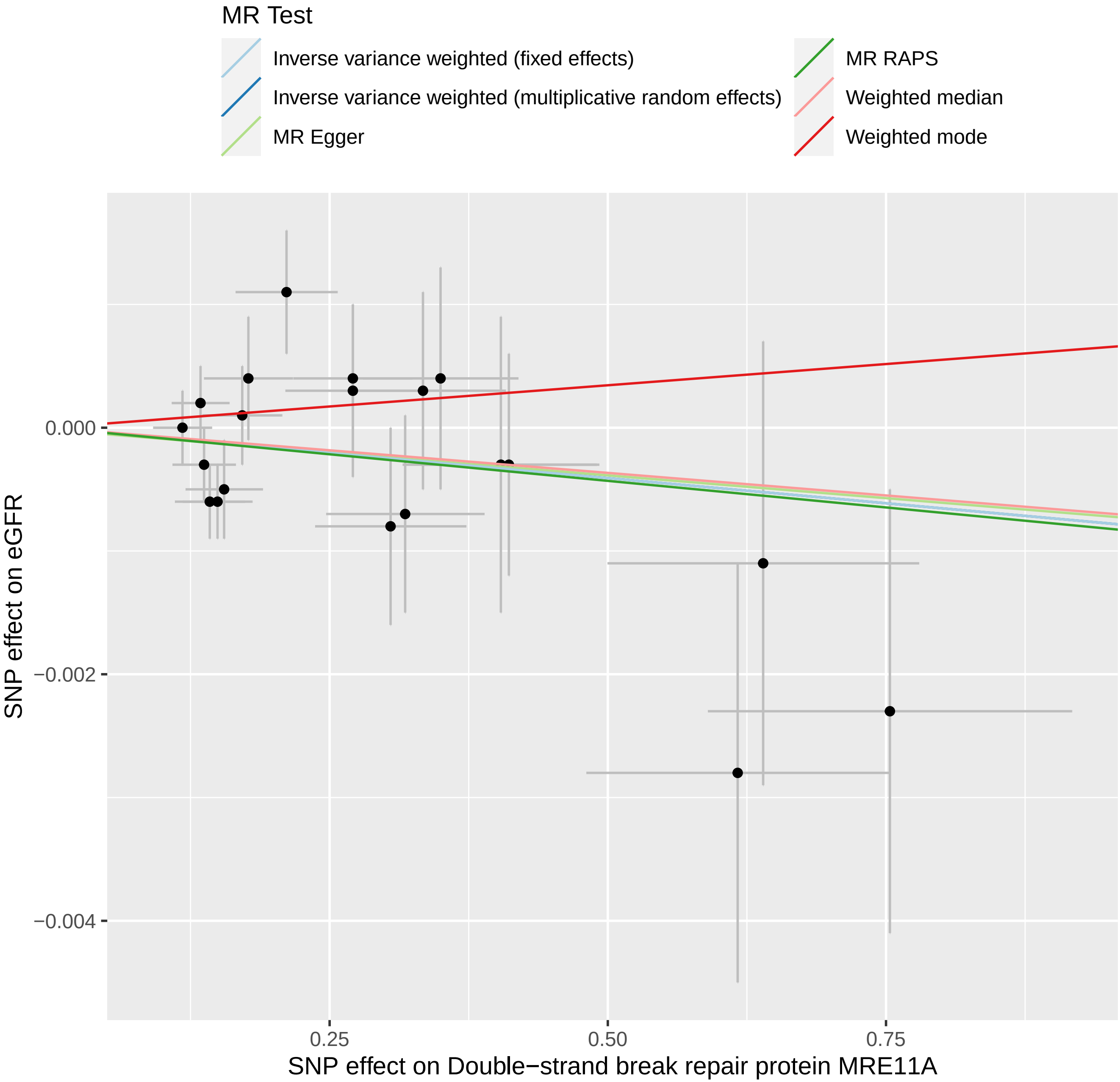

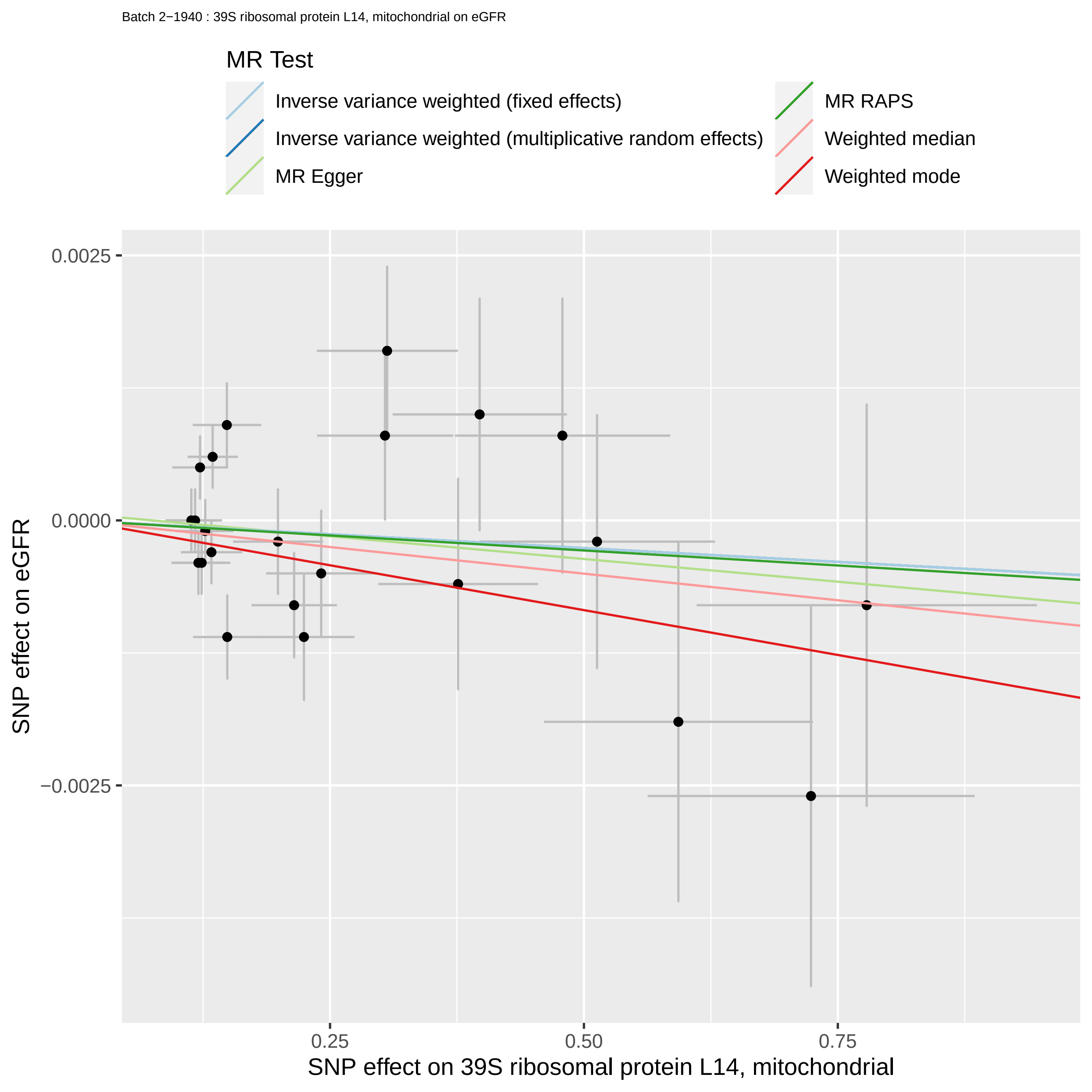

Batch 2−1940 : 39S ribosomal protein L14, mitochondrial on eGFR
MR Test
Inverse variance weighted (fixed effects)
Inverse variance weighted (multiplicative random effects)
MR Egger
MR RAPS
Weighted median
Weighted mode
0.0025
0.0000
−0.0025
0.25
0.50
0.75
SNP effect on eGFR
SNP effect on 39S ribosomal protein L14, mitochondrial

Batch 2−1941 : 39S ribosomal protein L32, mitochondrial on eGFR

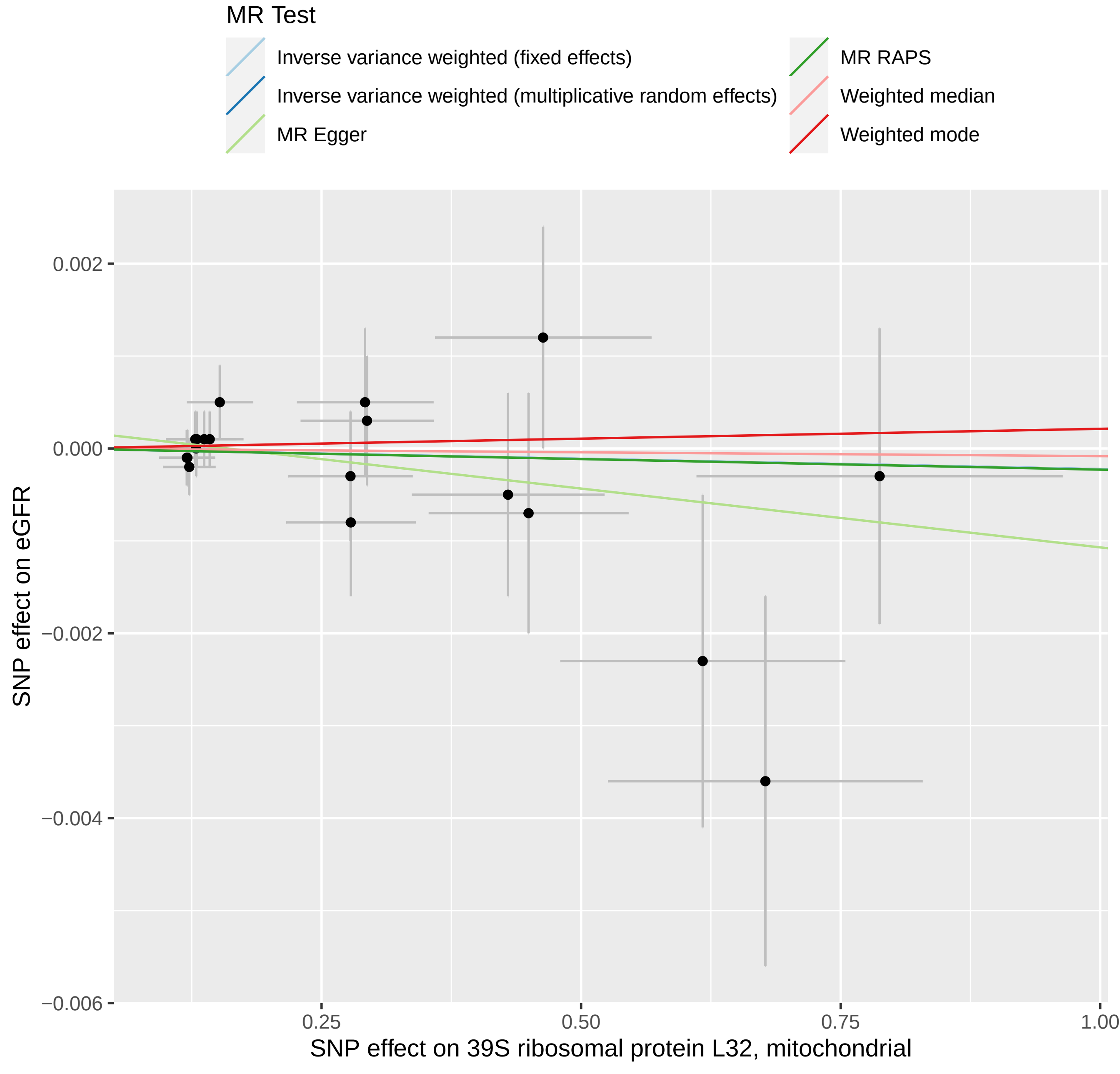

Batch 2−1942 : 39S ribosomal protein L33, mitochondrial on eGFR

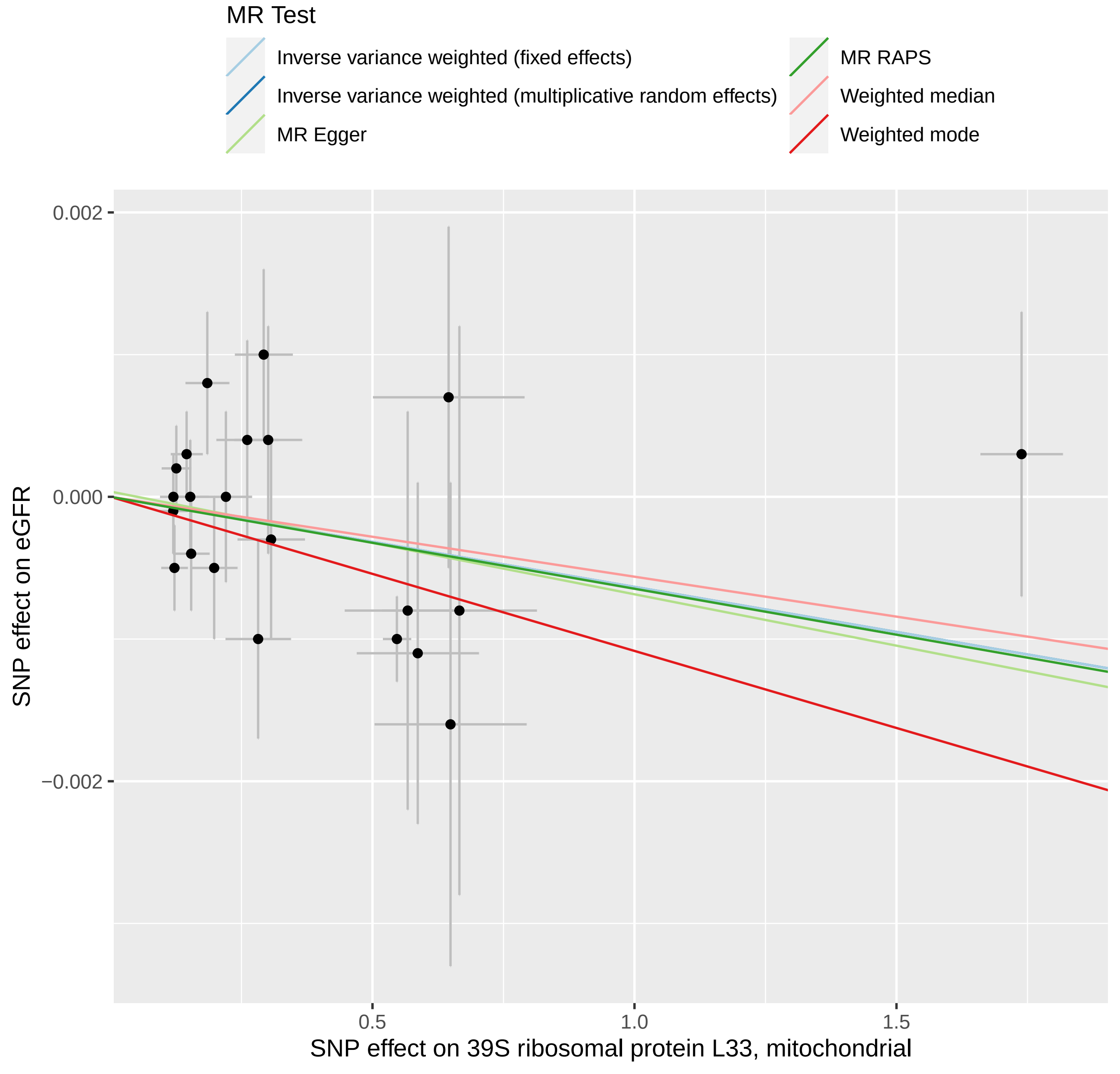

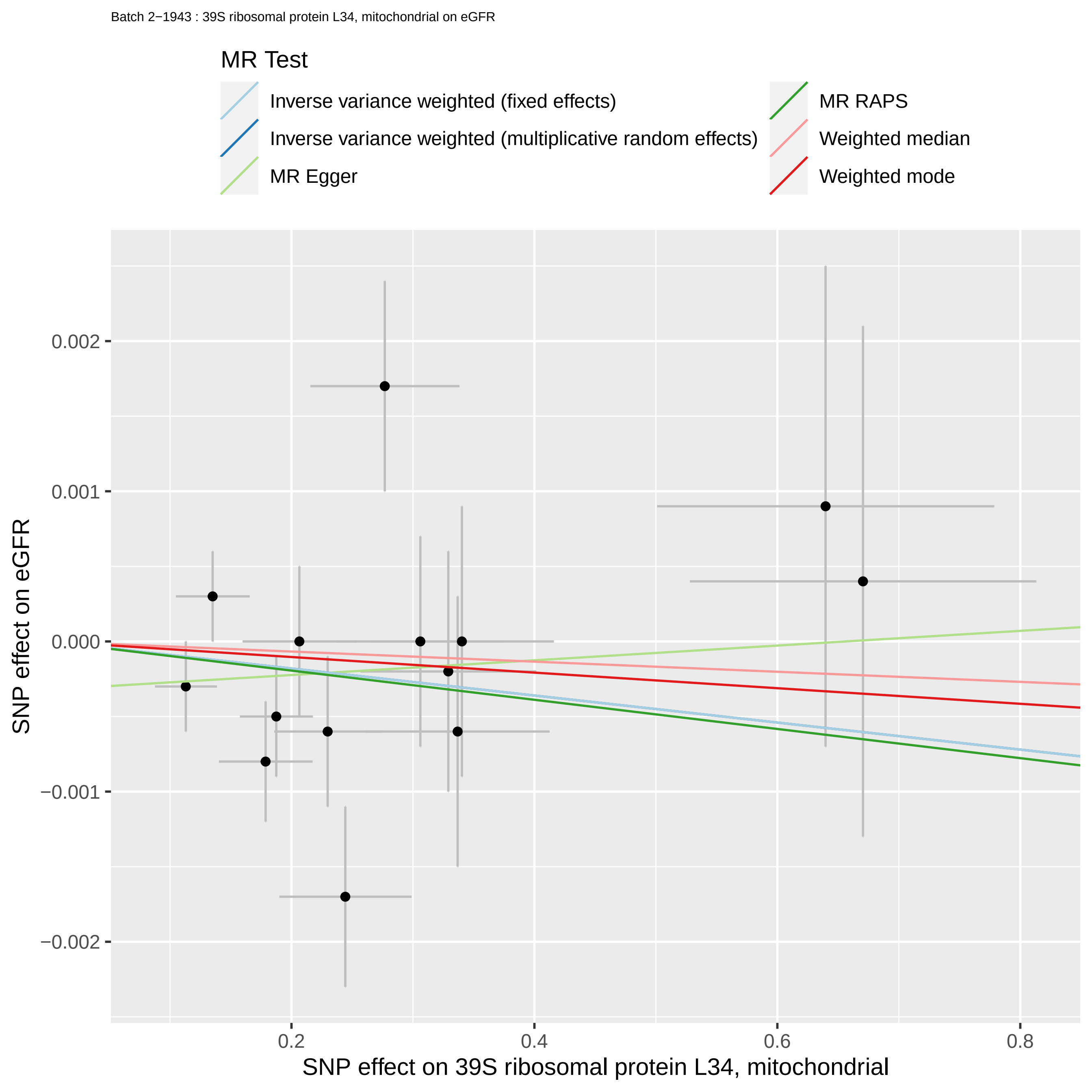
Batch 2−1943 : 39S ribosomal protein L34, mitochondrial on eGFR
MR Test
Inverse variance weighted (fixed effects)
Inverse variance weighted (multiplicative random effects)
MR Egger
MR RAPS
Weighted median
Weighted mode
0.002
0.001
0.000
−0.001
−0.002
0.2
0.4
0.6
0.8
SNP effect on eGFR
SNP effect on 39S ribosomal protein L34, mitochondrial

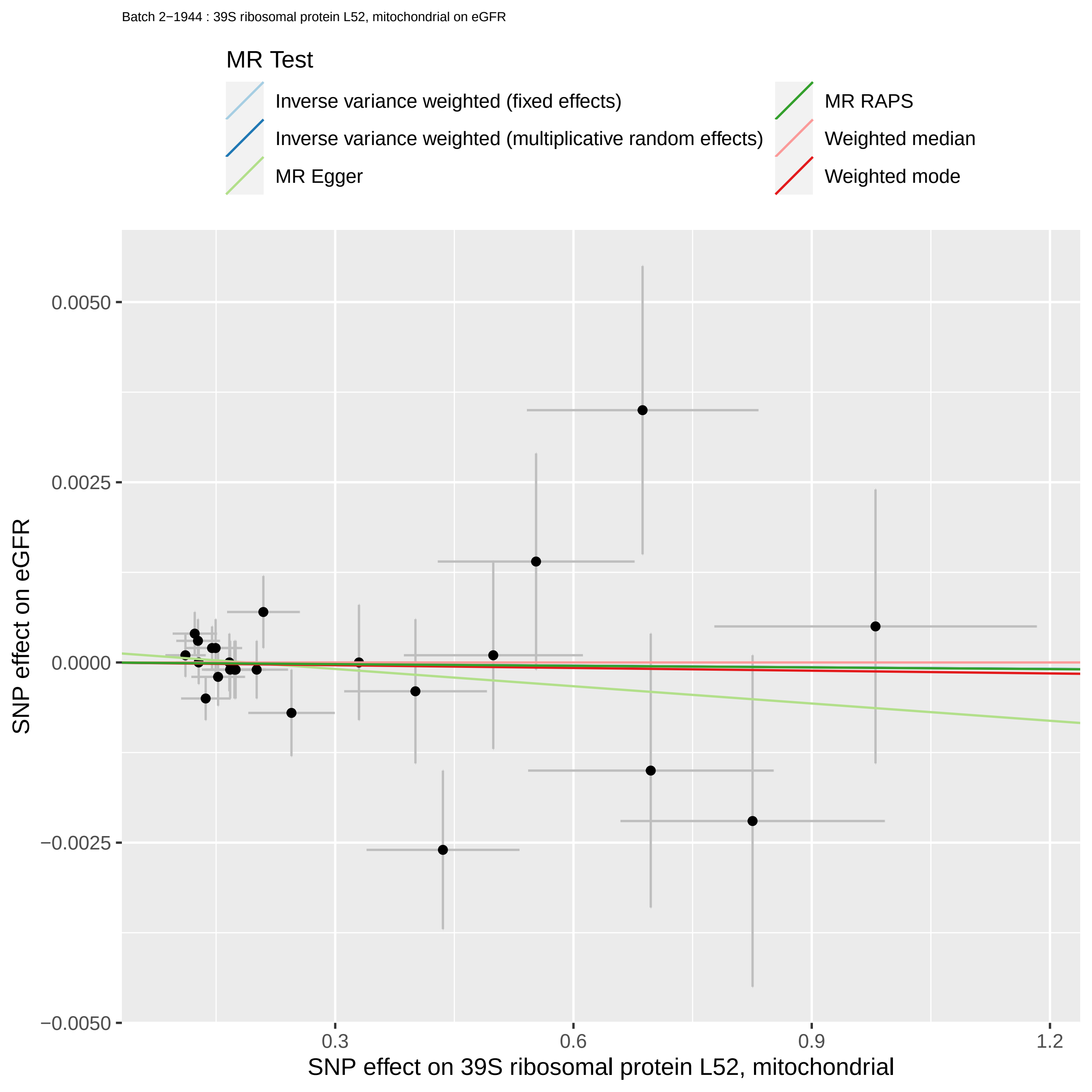

Batch 2−1944 : 39S ribosomal protein L52, mitochondrial on eGFR
MR Test
Inverse variance weighted (fixed effects)
Inverse variance weighted (multiplicative random effects)
MR Egger
MR RAPS
Weighted median
Weighted mode
0.0050
0.0025
0.0000
−0.0025
−0.0050
SNP effect on eGFR
0.3
0.6
0.9
1.2
SNP effect on 39S ribosomal protein L52, mitochondrial

Batch 2−1945 : Ribosome−recycling factor, mitochondrial on eGFR

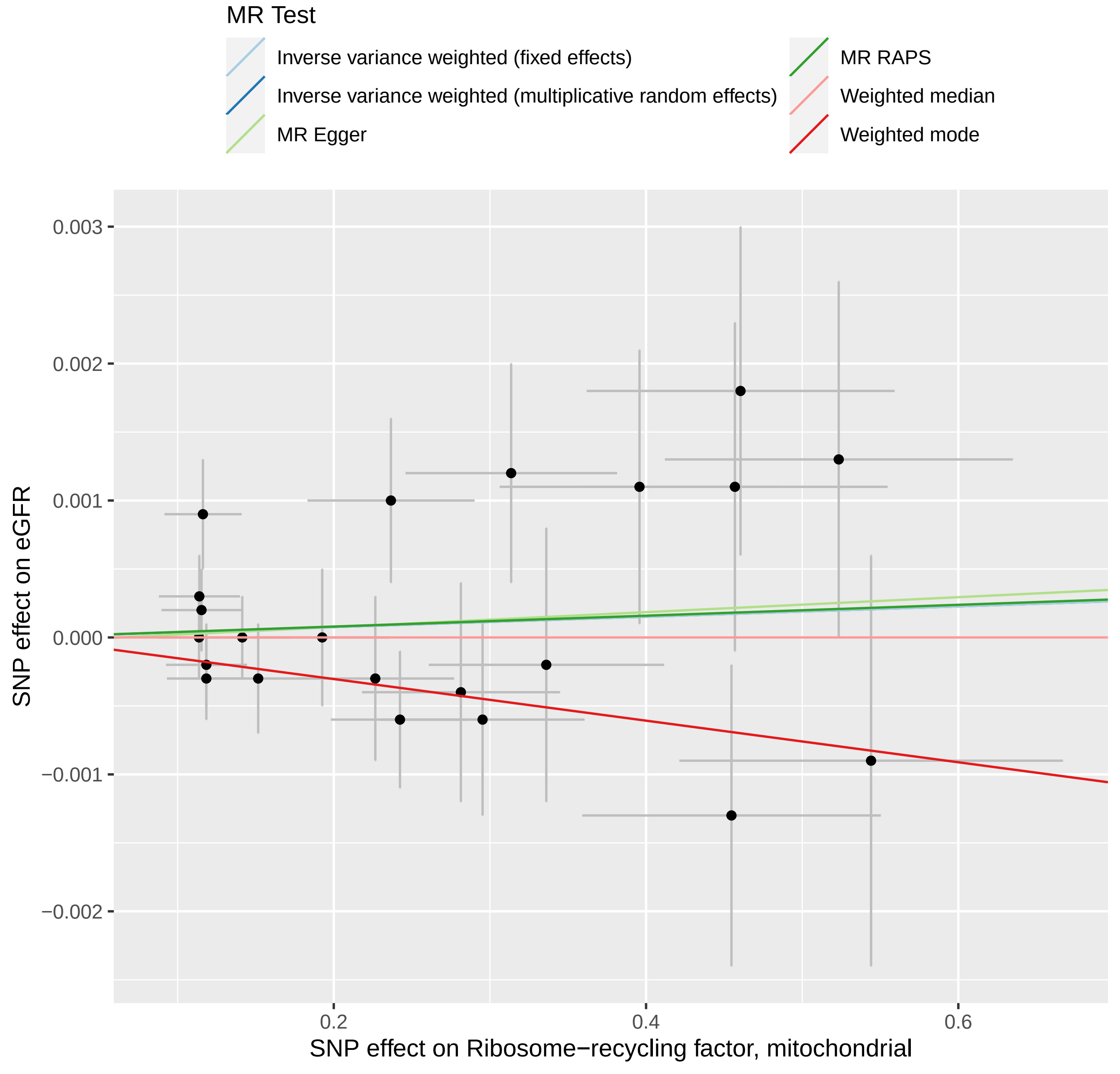

Batch 2−1946 : Protein MRVI1 on eGFR

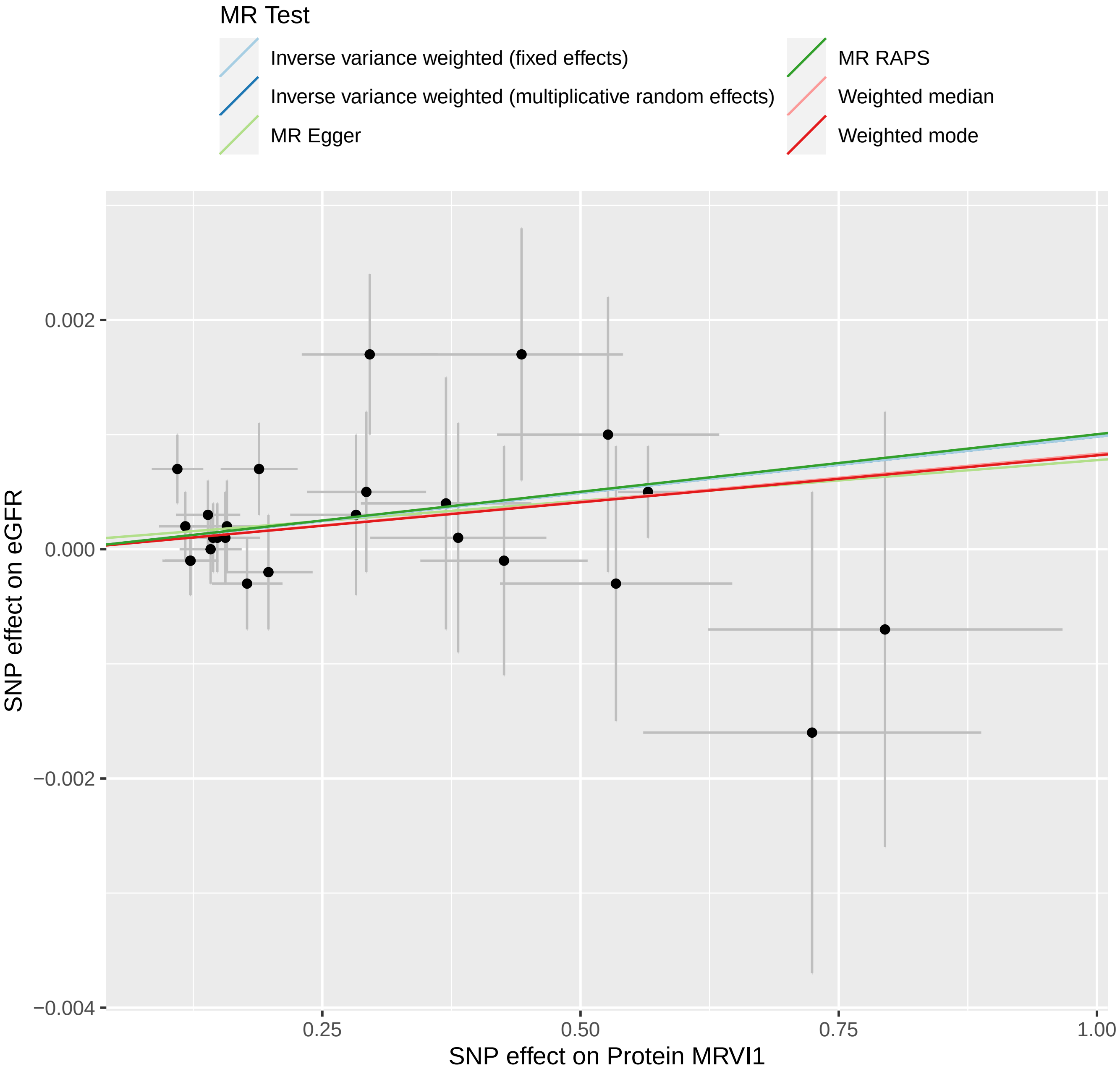

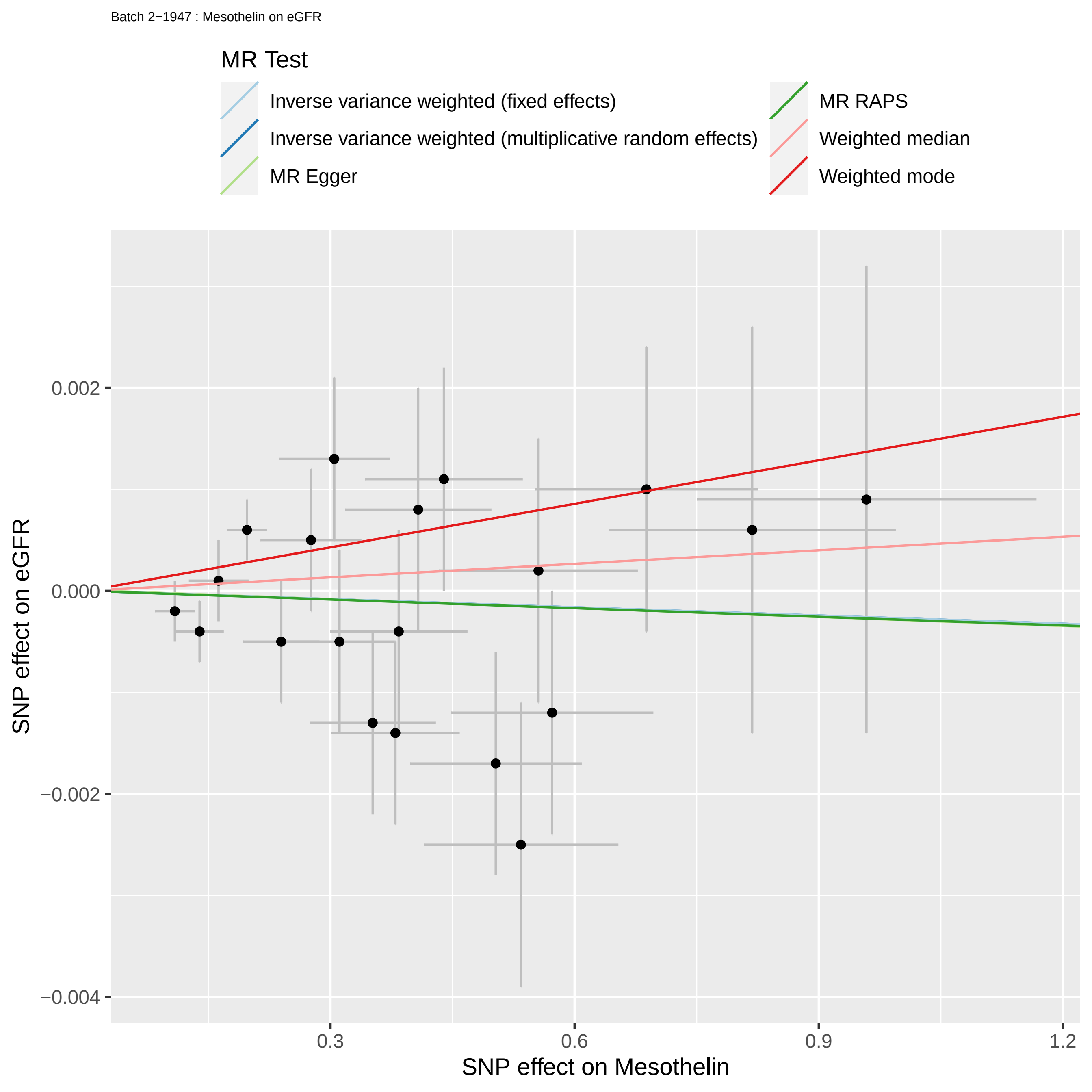

Batch 2−1947 : Mesothelin on eGFR
MR Test
Inverse variance weighted (fixed effects)
Inverse variance weighted (multiplicative random effects)
MR Egger
MR RAPS
Weighted median
Weighted mode
0.002
0.000
−0.002
−0.004
0.3
0.6
0.9
1.2
SNP effect on Mesothelin
SNP effect on eGFR

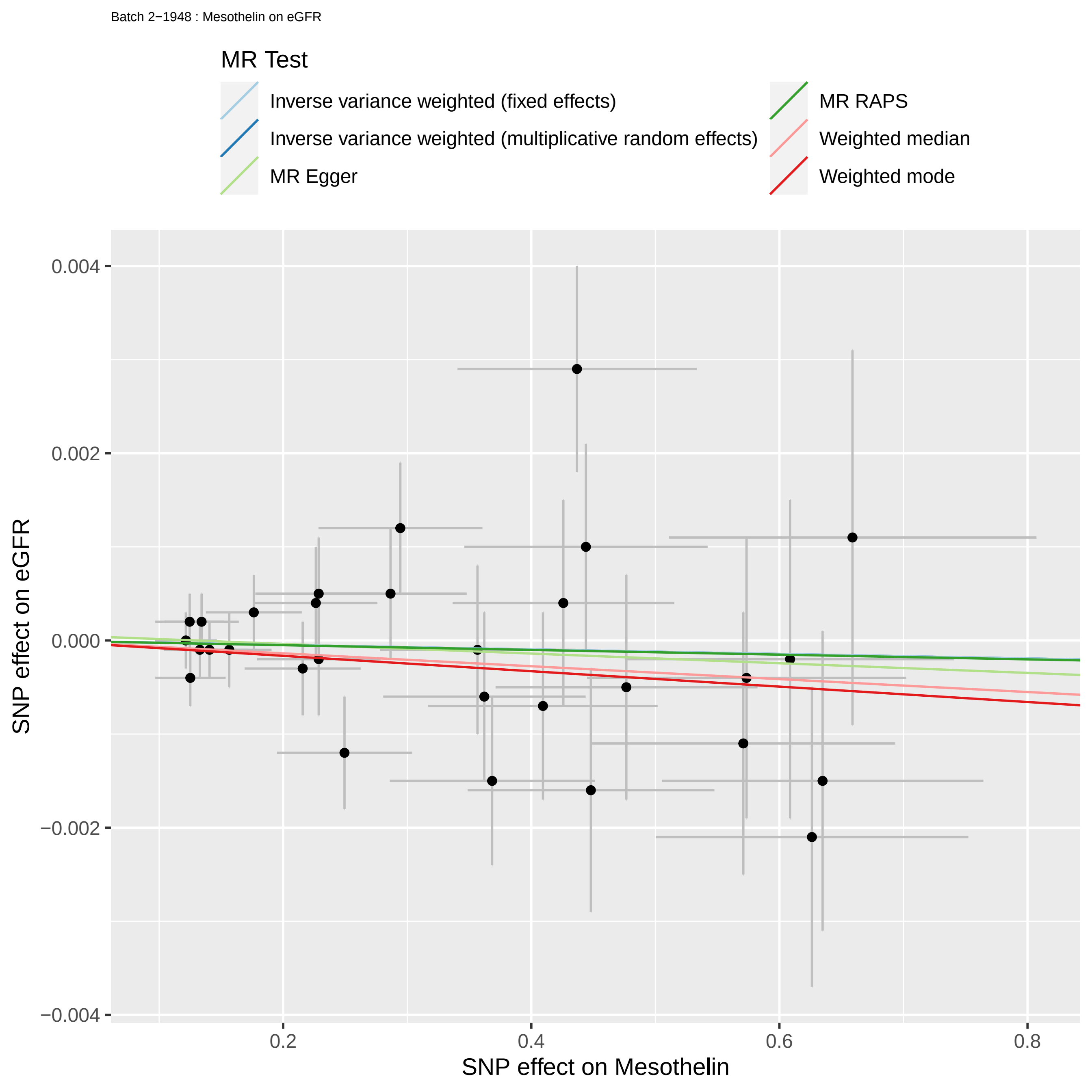

Batch 2−1948 : Mesothelin on eGFR
MR Test
Inverse variance weighted (fixed effects)
Inverse variance weighted (multiplicative random effects)
MR Egger
MR RAPS
Weighted median
Weighted mode
0.004
0.002
0.000
−0.002
−0.004
0.2
0.4
0.6
0.8
SNP effect on eGFR
SNP effect on Mesothelin

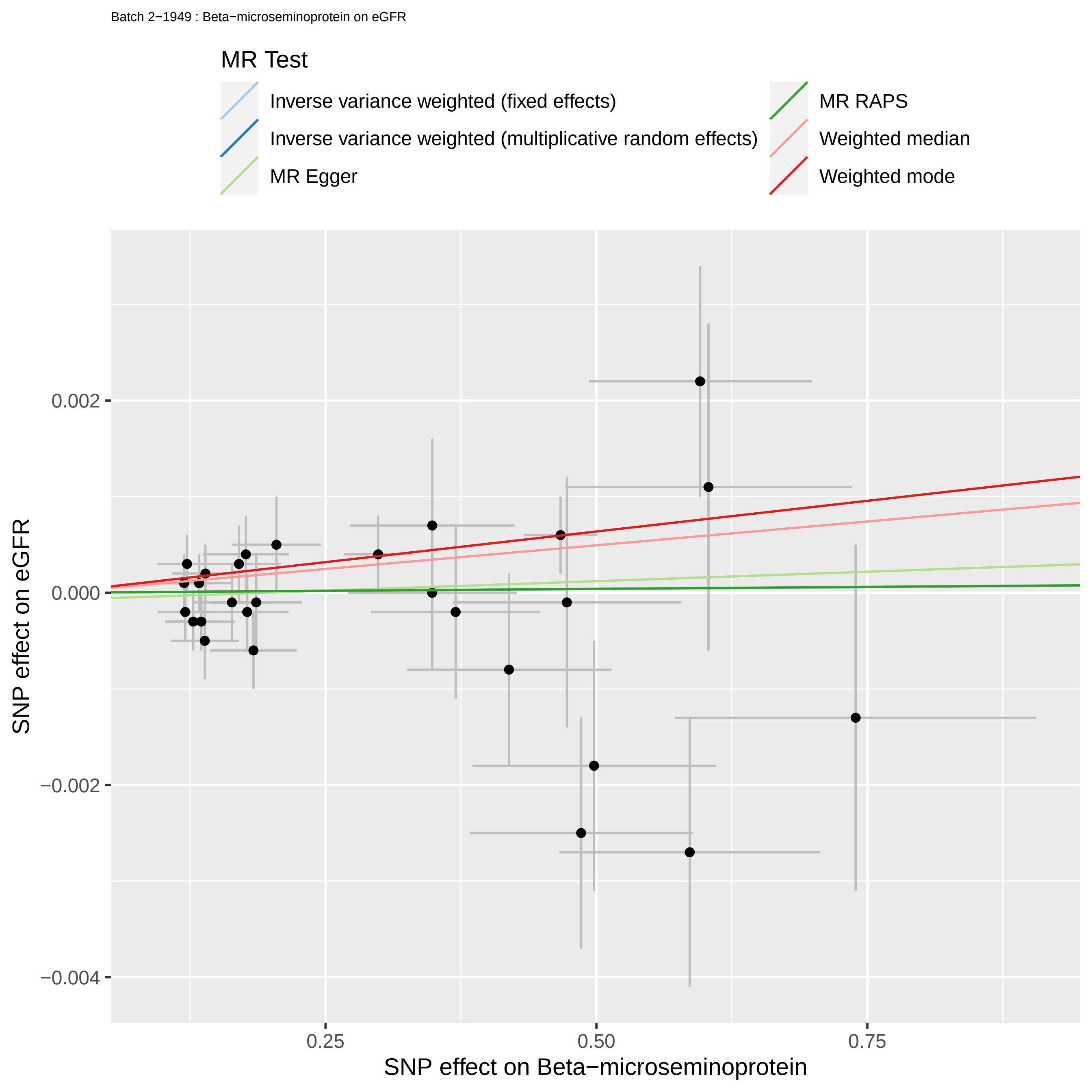
Batch 2−1949 : Beta−microseminoprotein on eGFR
MR Test
Inverse variance weighted (fixed effects)
Inverse variance weighted (multiplicative random effects)
MR Egger
MR RAPS
Weighted median
Weighted mode
0.002
0.000
−0.002
−0.004
0.25
0.50
0.75
SNP effect on eGFR
SNP effect on Beta−microseminoprotein

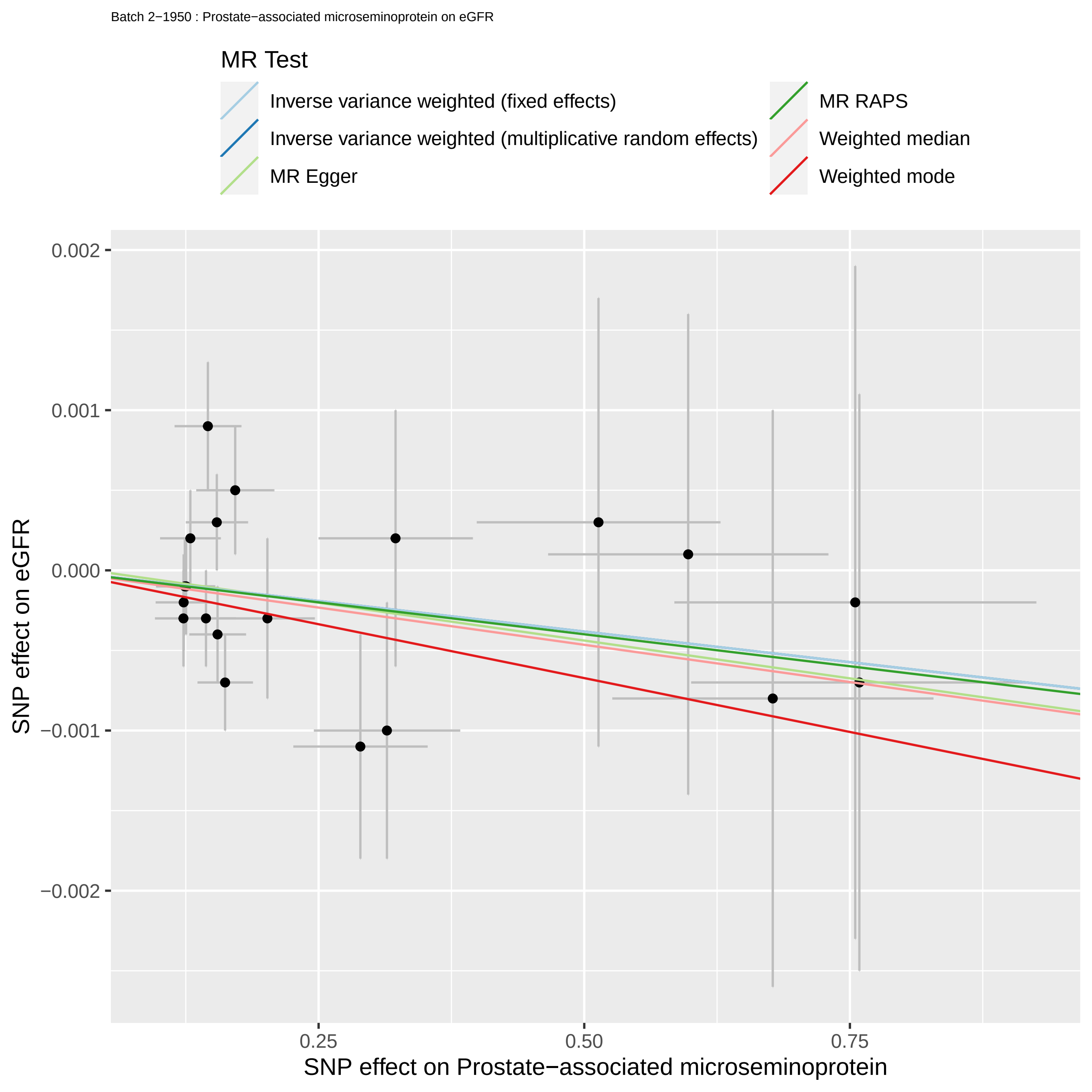

Batch 2−1950 : Prostate−associated microseminoprotein on eGFR
MR Test
Inverse variance weighted (fixed effects)
Inverse variance weighted (multiplicative random effects)
MR Egger
MR RAPS
Weighted median
Weighted mode
0.002
0.001
0.000
−0.001
−0.002
SNP effect on eGFR
0.25
0.50
0.75
SNP effect on Prostate−associated microseminoprotein

Batch 2−1951 : Macrophage scavenger receptor types I and II on eGFR

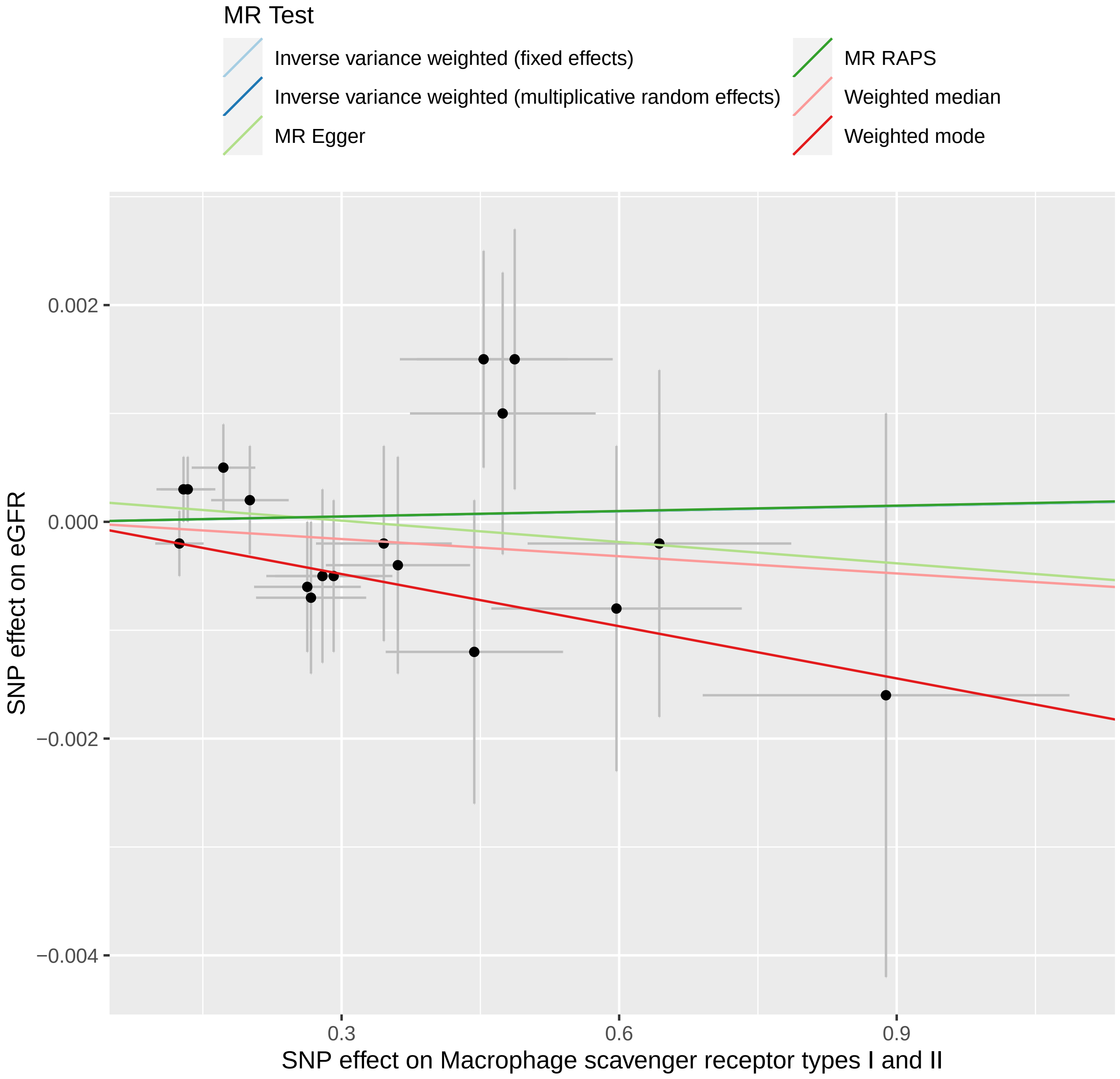

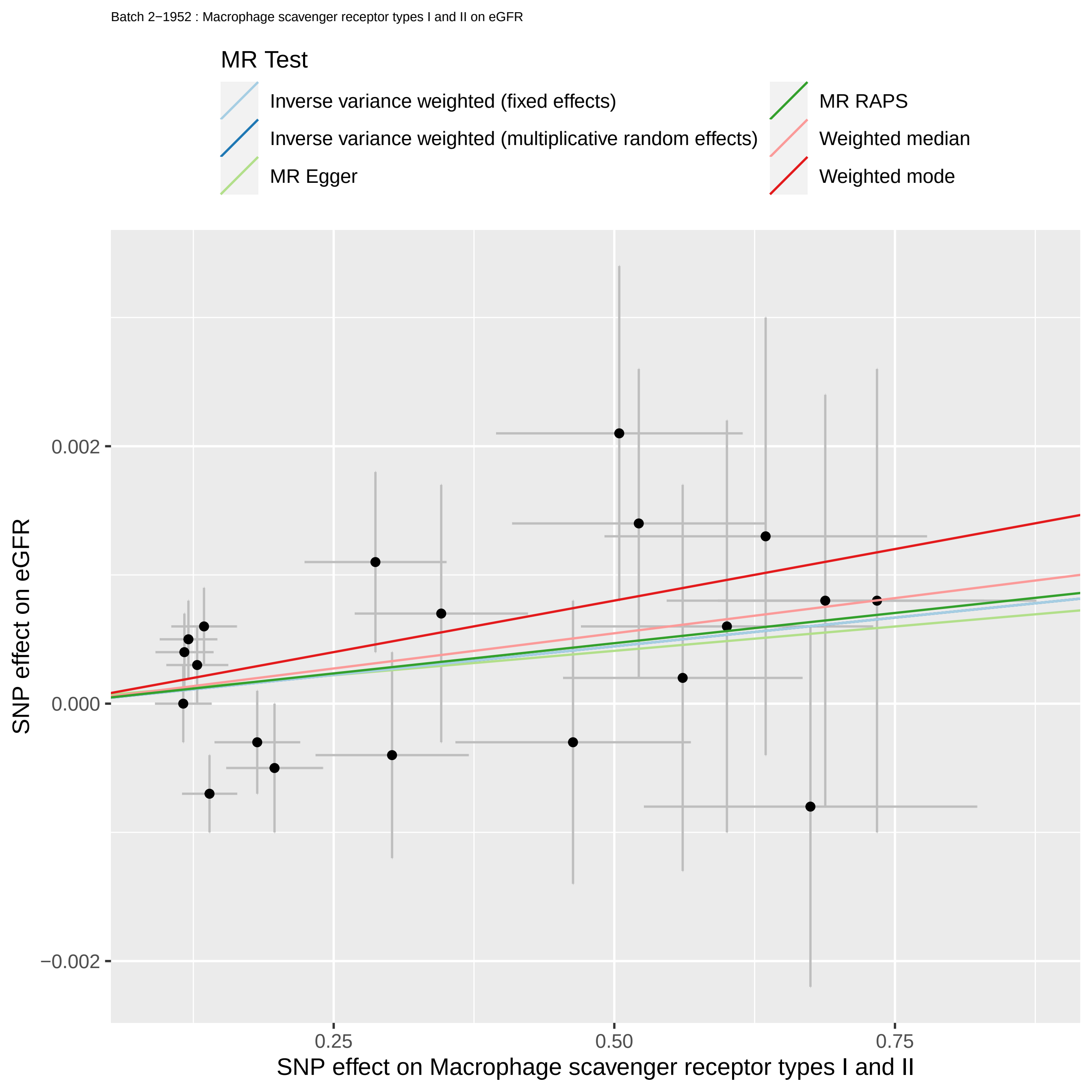
Batch 2−1952 : Macrophage scavenger receptor types I and II on eGFR
MR Test
Inverse variance weighted (fixed effects)
Inverse variance weighted (multiplicative random effects)
MR Egger
MR RAPS
Weighted median
Weighted mode
0.002
0.000
−0.002
0.25
0.50
0.75
SNP effect on eGFR
SNP effect on Macrophage scavenger receptor types I and II

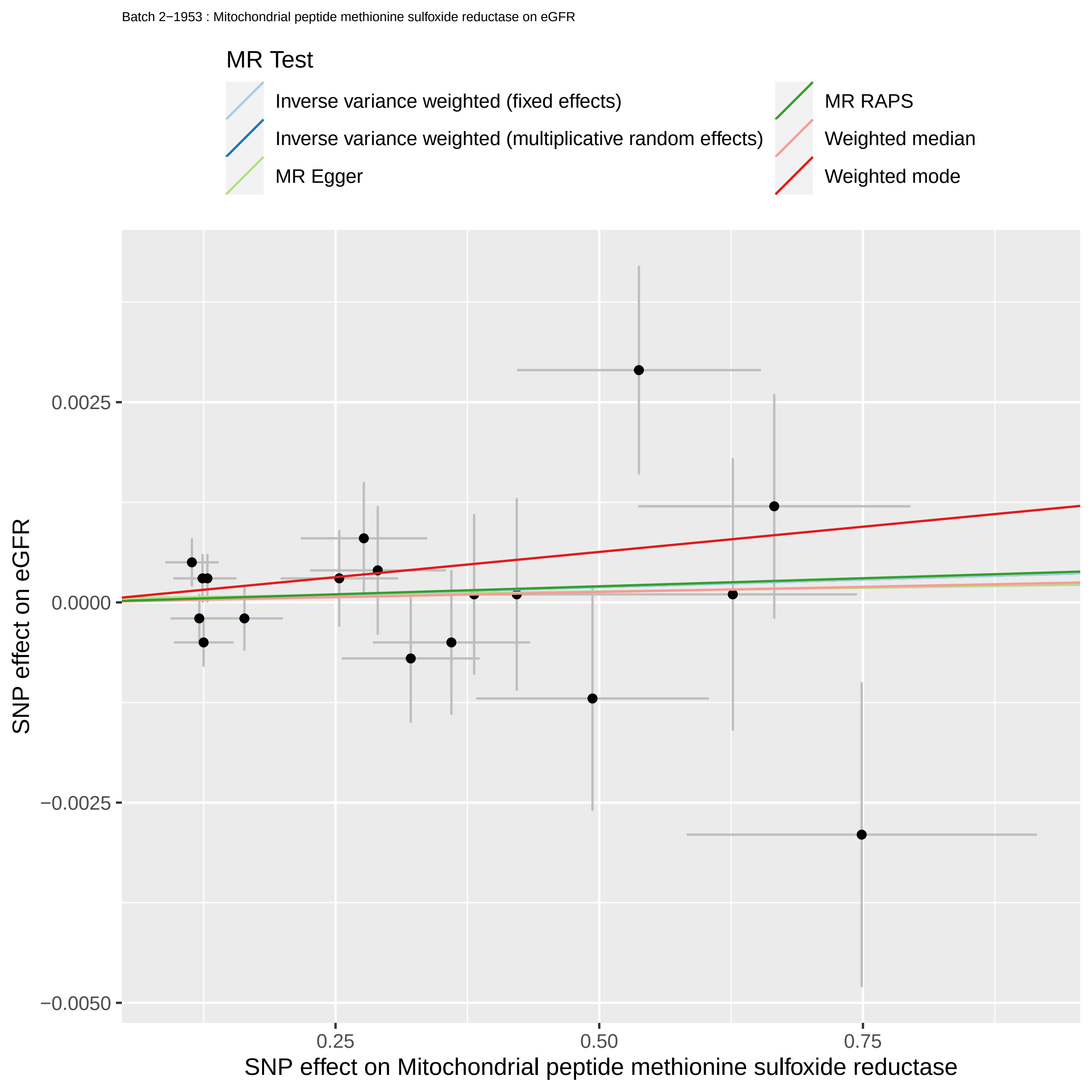
Batch 2−1953 : Mitochondrial peptide methionine sulfoxide reductase on eGFR
MR Test
Inverse variance weighted (fixed effects)
Inverse variance weighted (multiplicative random effects)
MR Egger
MR RAPS
Weighted median
Weighted mode
0.0025
0.0000
−0.0025
−0.0050
SNP effect on eGFR
0.25
0.50
0.75
SNP effect on Mitochondrial peptide methionine sulfoxide reductase

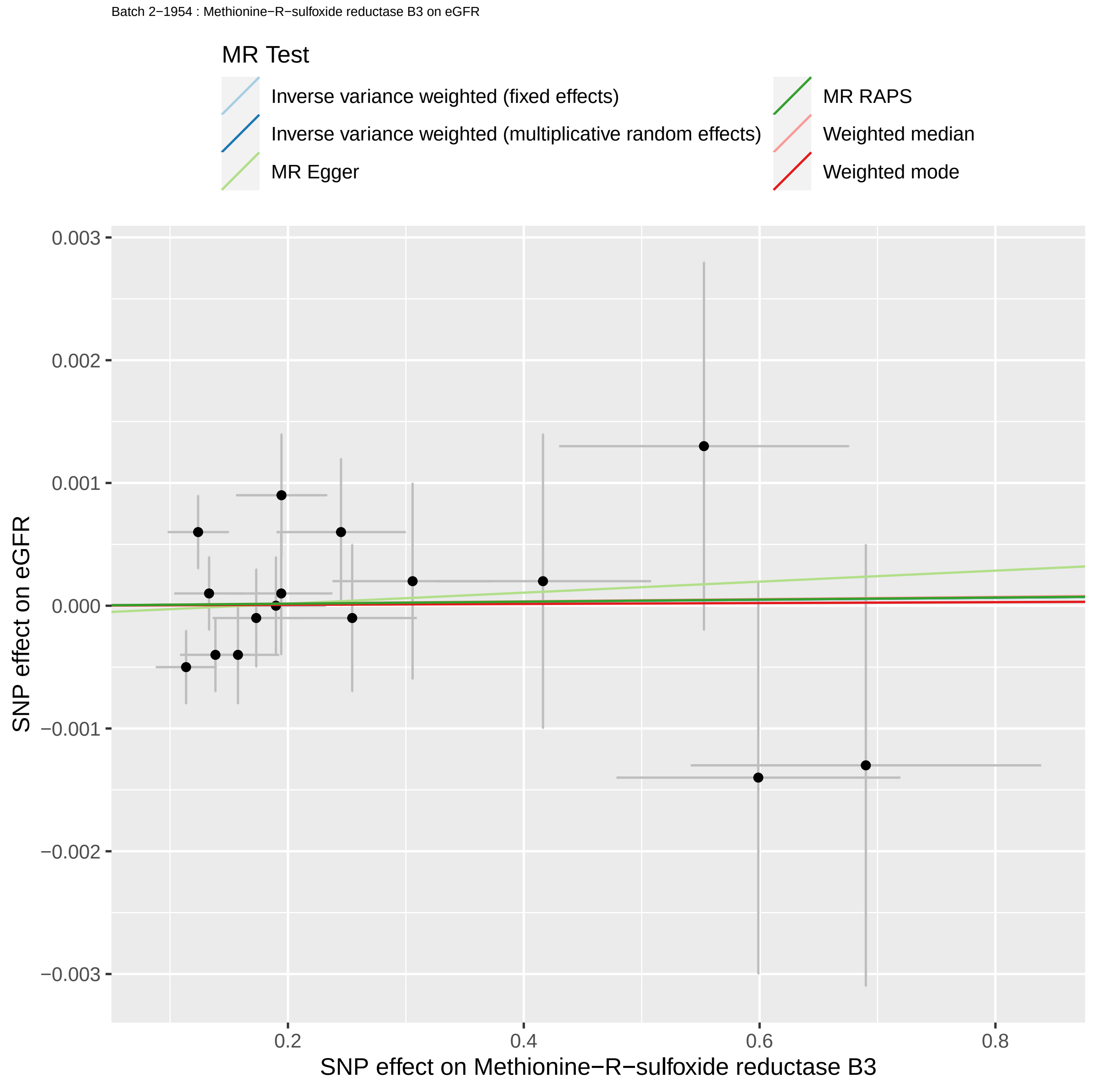

Batch 2−1954 : Methionine−R−sulfoxide reductase B3 on eGFR
MR Test
Inverse variance weighted (fixed effects)
Inverse variance weighted (multiplicative random effects)
MR Egger
MR RAPS
Weighted median
Weighted mode
0.003
0.002
0.001
0.000
−0.001
−0.002
−0.003
SNP effect on eGFR
0.2
0.4
0.6
0.8
SNP effect on Methionine−R−sulfoxide reductase B3

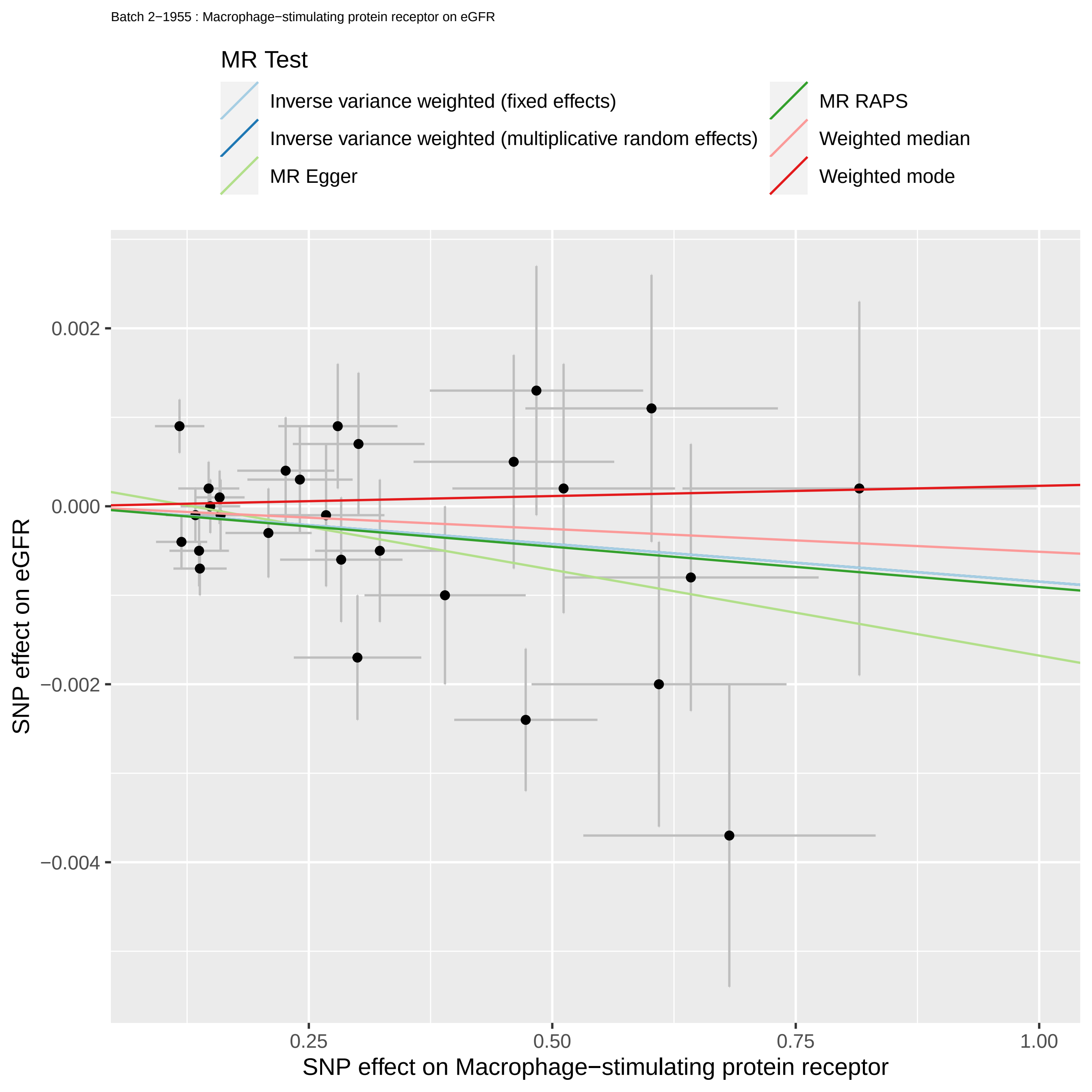

Batch 2−1955 : Macrophage−stimulating protein receptor on eGFR
MR Test
Inverse variance weighted (fixed effects)
Inverse variance weighted (multiplicative random effects)
MR Egger
MR RAPS
Weighted median
Weighted mode
0.002
0.000
−0.002
−0.004
SNP effect on eGFR
0.25
0.50
0.75
1.00
SNP effect on Macrophage−stimulating protein receptor

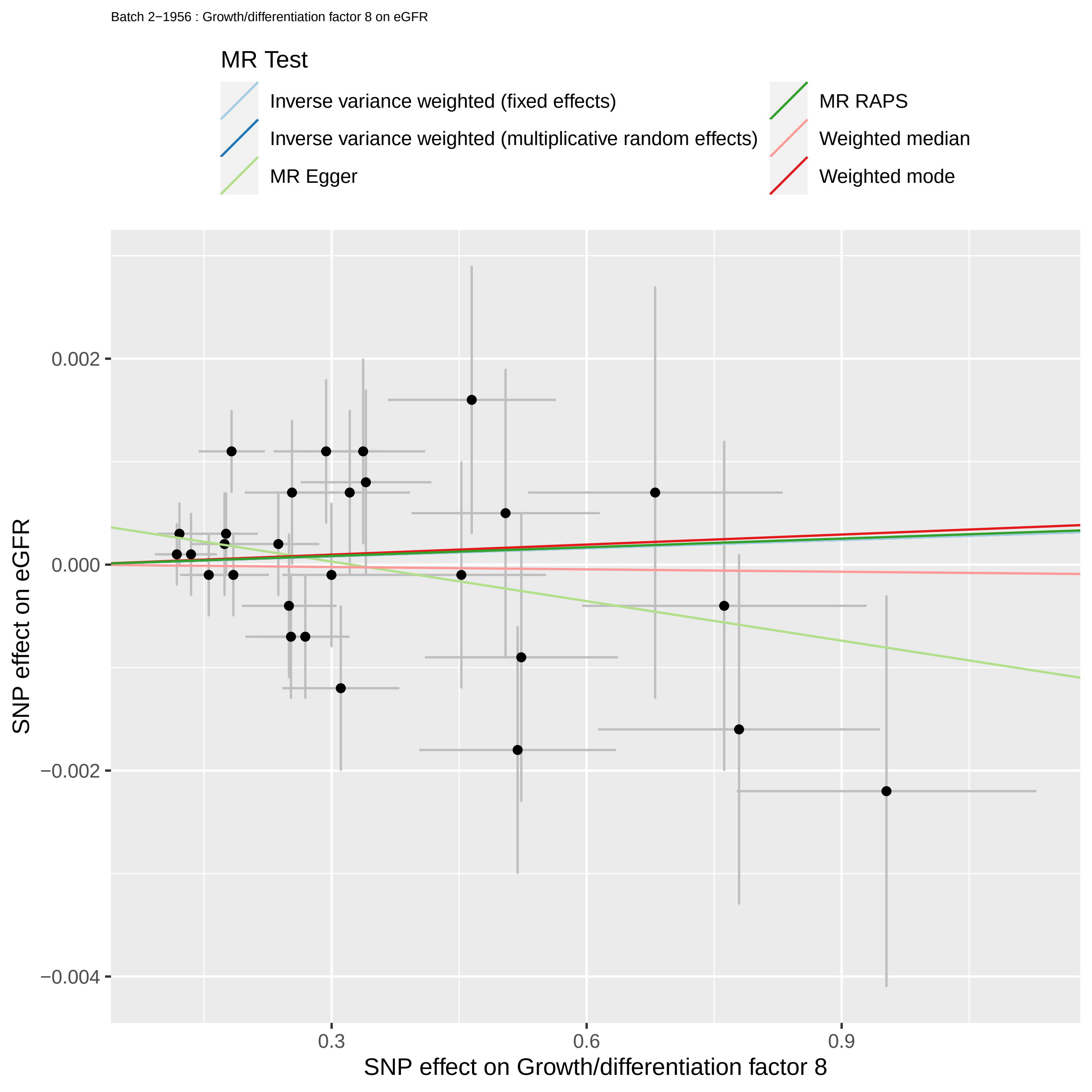

Batch 2−1956 : Growth/differentiation factor 8 on eGFR
MR Test
Inverse variance weighted (fixed effects)
Inverse variance weighted (multiplicative random effects)
MR Egger
MR RAPS
Weighted median
Weighted mode
0.002
0.000
−0.002
−0.004
SNP effect on eGFR
0.3
0.6
0.9
SNP effect on Growth/differentiation factor 8

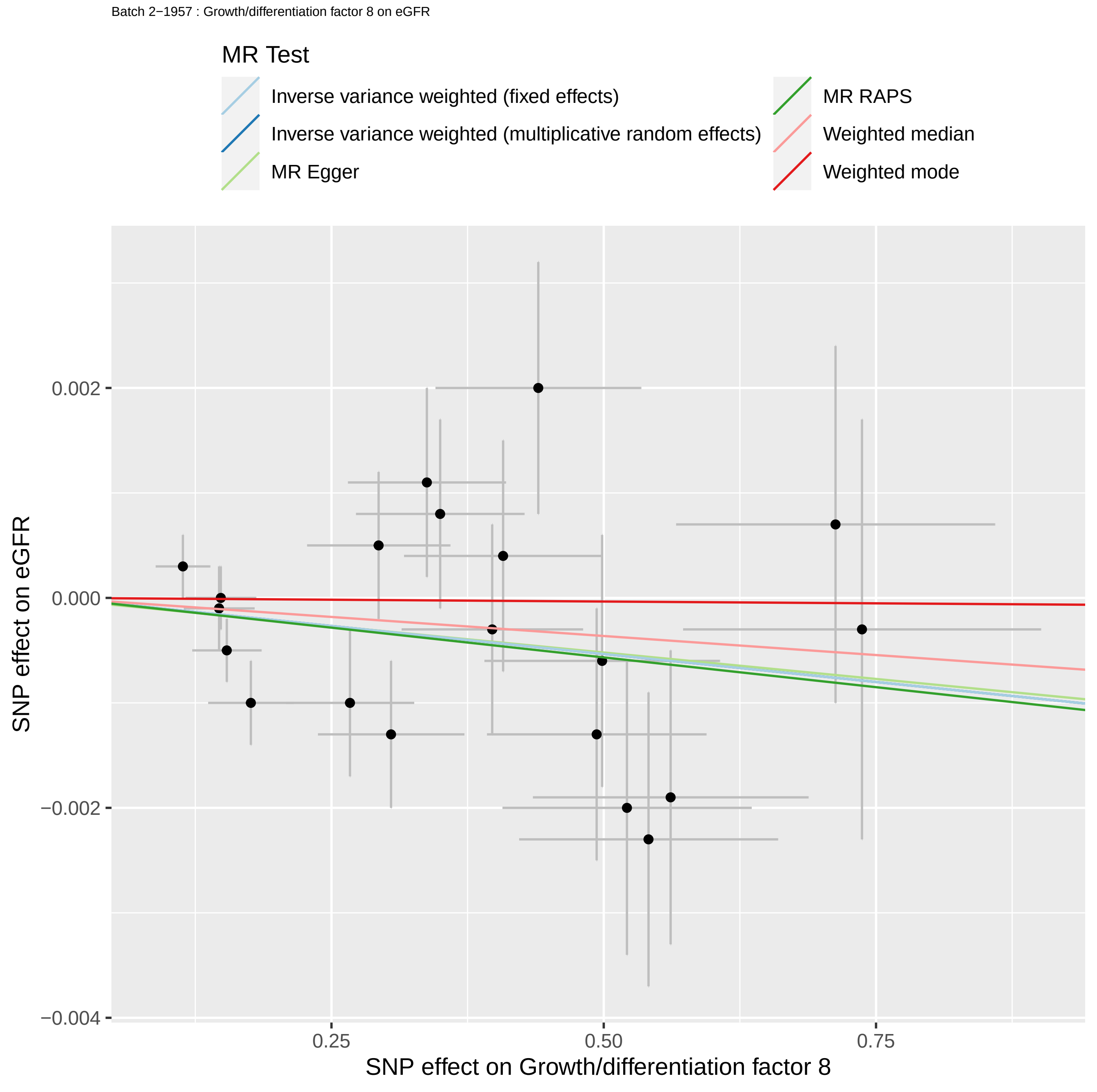
Batch 2−1957 : Growth/differentiation factor 8 on eGFR
MR Test
Inverse variance weighted (fixed effects)
Inverse variance weighted (multiplicative random effects)
MR Egger
MR RAPS
Weighted median
Weighted mode
0.002
0.000
−0.002
−0.004
0.25
0.50
0.75
SNP effect on eGFR
SNP effect on Growth/differentiation factor 8

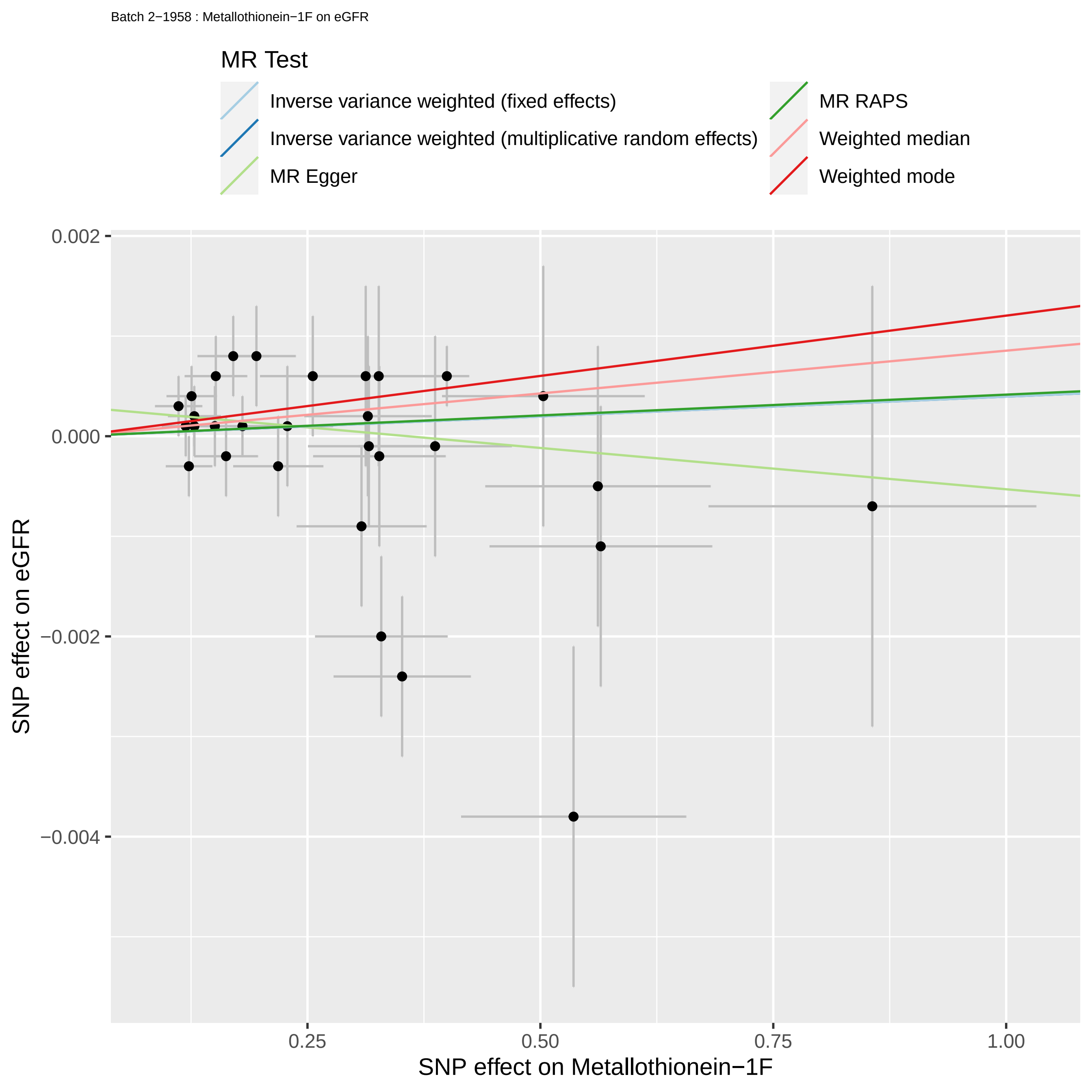

Batch 2−1958 : Metallothionein−1F on eGFR
MR Test
Inverse variance weighted (fixed effects)
Inverse variance weighted (multiplicative random effects)
MR Egger
MR RAPS
Weighted median
Weighted mode
0.002
0.000
−0.002
−0.004
0.25
0.50
0.75
1.00
SNP effect on eGFR
SNP effect on Metallothionein−1F

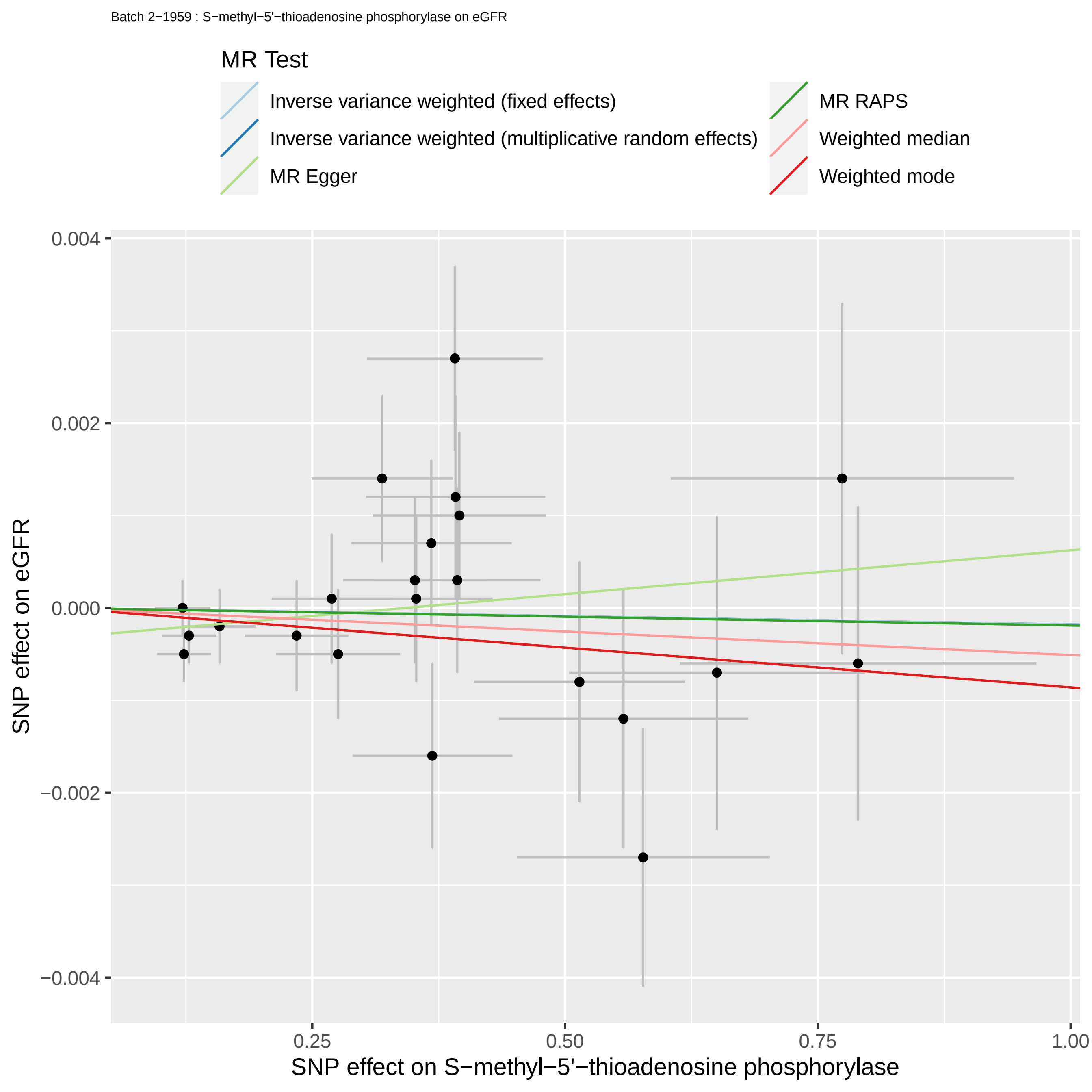
Batch 2−1959 : S−methyl−5'−thioadenosine phosphorylase on eGFR
MR Test
Inverse variance weighted (fixed effects)
Inverse variance weighted (multiplicative random effects)
MR Egger
MR RAPS
Weighted median
Weighted mode
0.004
0.002
0.000
−0.002
−0.004
0.25
0.50
0.75
1.00
SNP effect on eGFR
SNP effect on S−methyl−5'−thioadenosine phosphorylase

Batch 2−1960 : Protein p13 MTCP−1 on eGFR

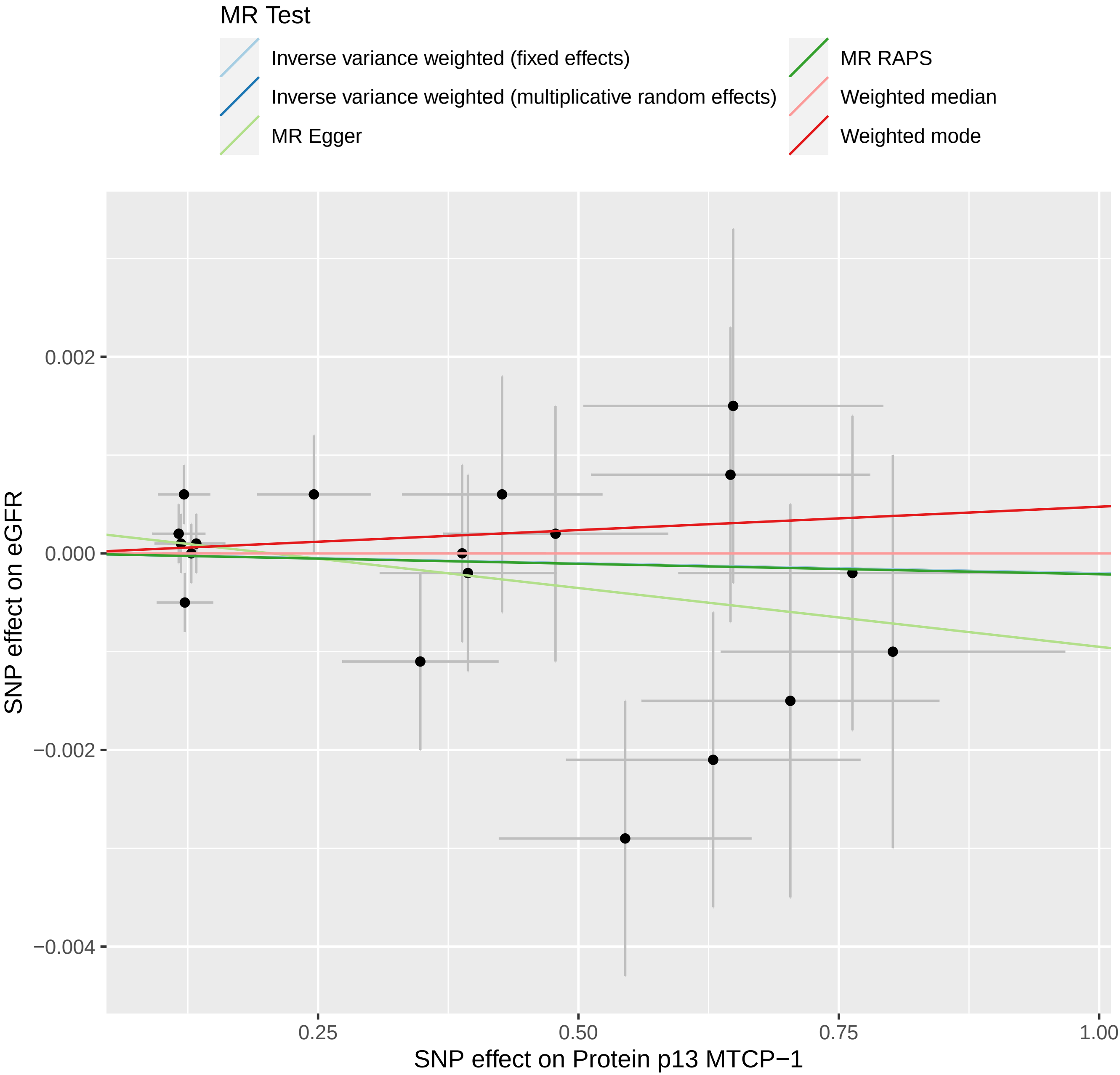

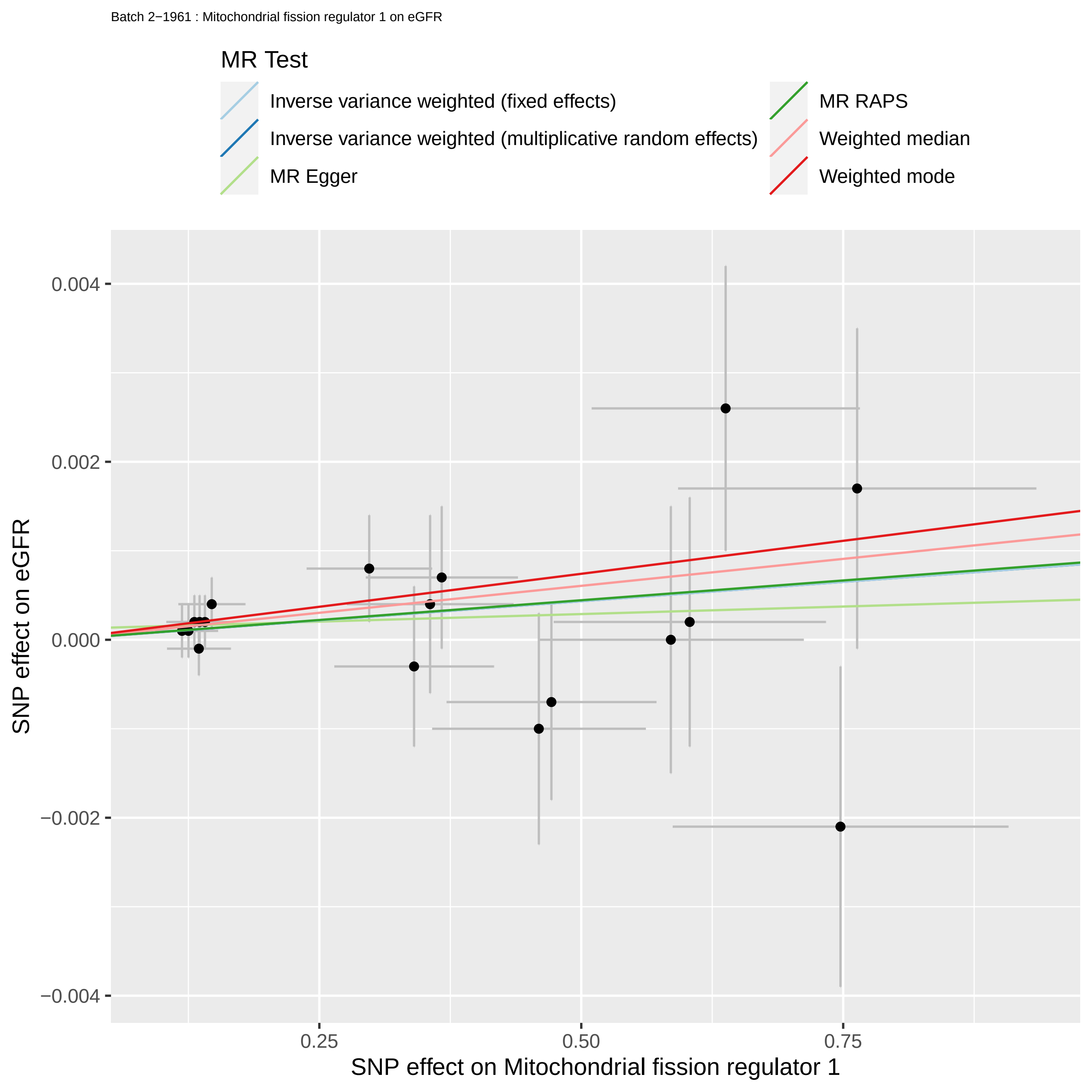

Batch 2−1961 : Mitochondrial fission regulator 1 on eGFR
MR Test
Inverse variance weighted (fixed effects)
Inverse variance weighted (multiplicative random effects)
MR Egger
MR RAPS
Weighted median
Weighted mode
0.004
0.002
0.000
−0.002
−0.004
0.25
0.50
0.75
SNP effect on eGFR
SNP effect on Mitochondrial fission regulator 1

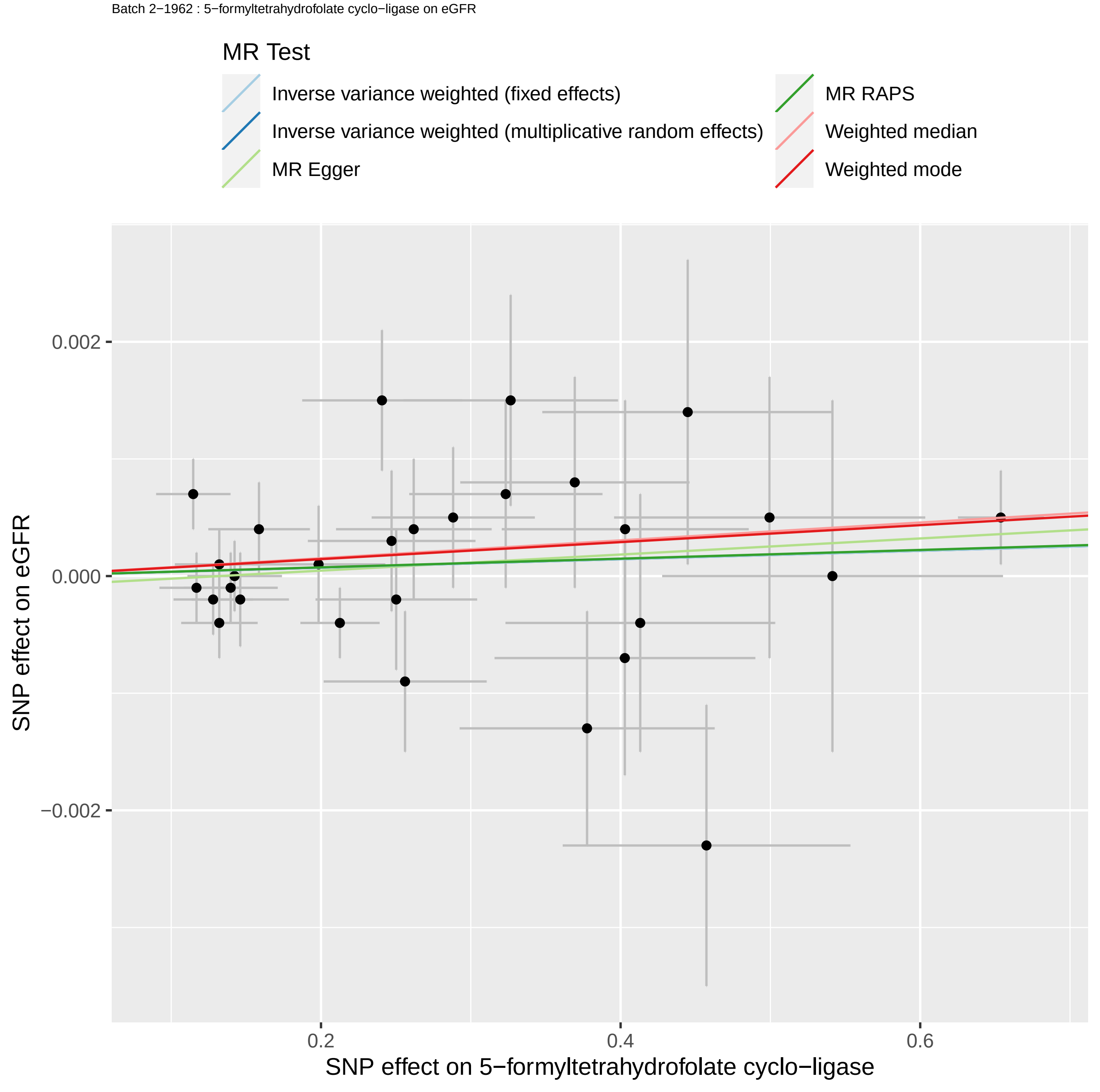

Batch 2−1962 : 5−formyltetrahydrofolate cyclo−ligase on eGFR
MR Test
Inverse variance weighted (fixed effects)
Inverse variance weighted (multiplicative random effects)
MR Egger
MR RAPS
Weighted median
Weighted mode
0.002
0.000
−0.002
0.2
0.4
0.6
SNP effect on eGFR
SNP effect on 5−formyltetrahydrofolate cyclo−ligase

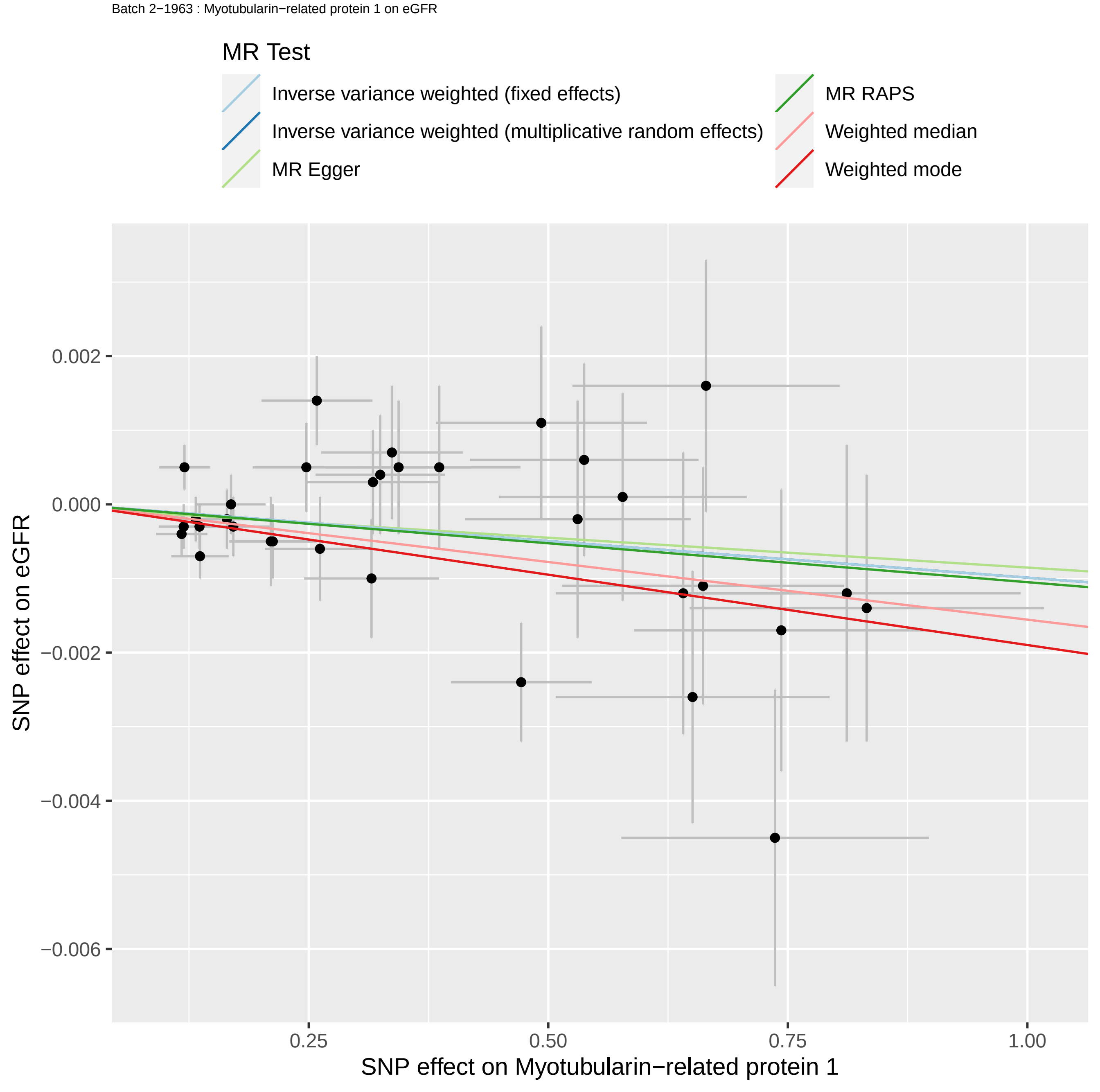
Batch 2−1963 : Myotubularin−related protein 1 on eGFR
MR Test
Inverse variance weighted (fixed effects)
Inverse variance weighted (multiplicative random effects)
MR Egger
MR RAPS
Weighted median
Weighted mode
0.002
0.000
−0.002
−0.004
−0.006
0.25
0.50
0.75
1.00
SNP effect on eGFR
SNP effect on Myotubularin−related protein 1

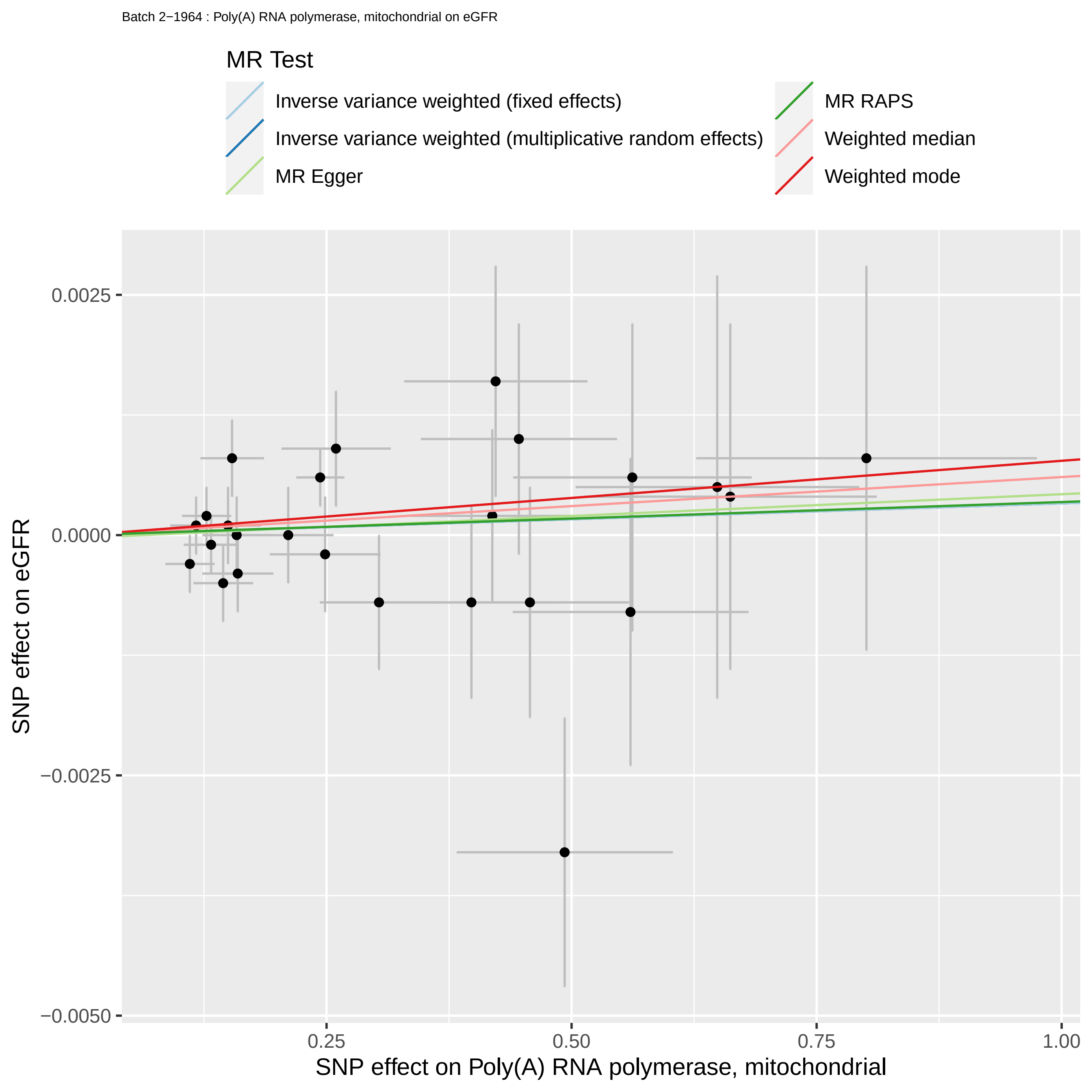
Batch 2−1964 : Poly(A) RNA polymerase, mitochondrial on eGFR
MR Test
Inverse variance weighted (fixed effects)
Inverse variance weighted (multiplicative random effects)
MR Egger
MR RAPS
Weighted median
Weighted mode
0.0025
0.0000
−0.0025
−0.0050
0.25
0.50
0.75
1.00
SNP effect on eGFR
SNP effect on Poly(A) RNA polymerase, mitochondrial

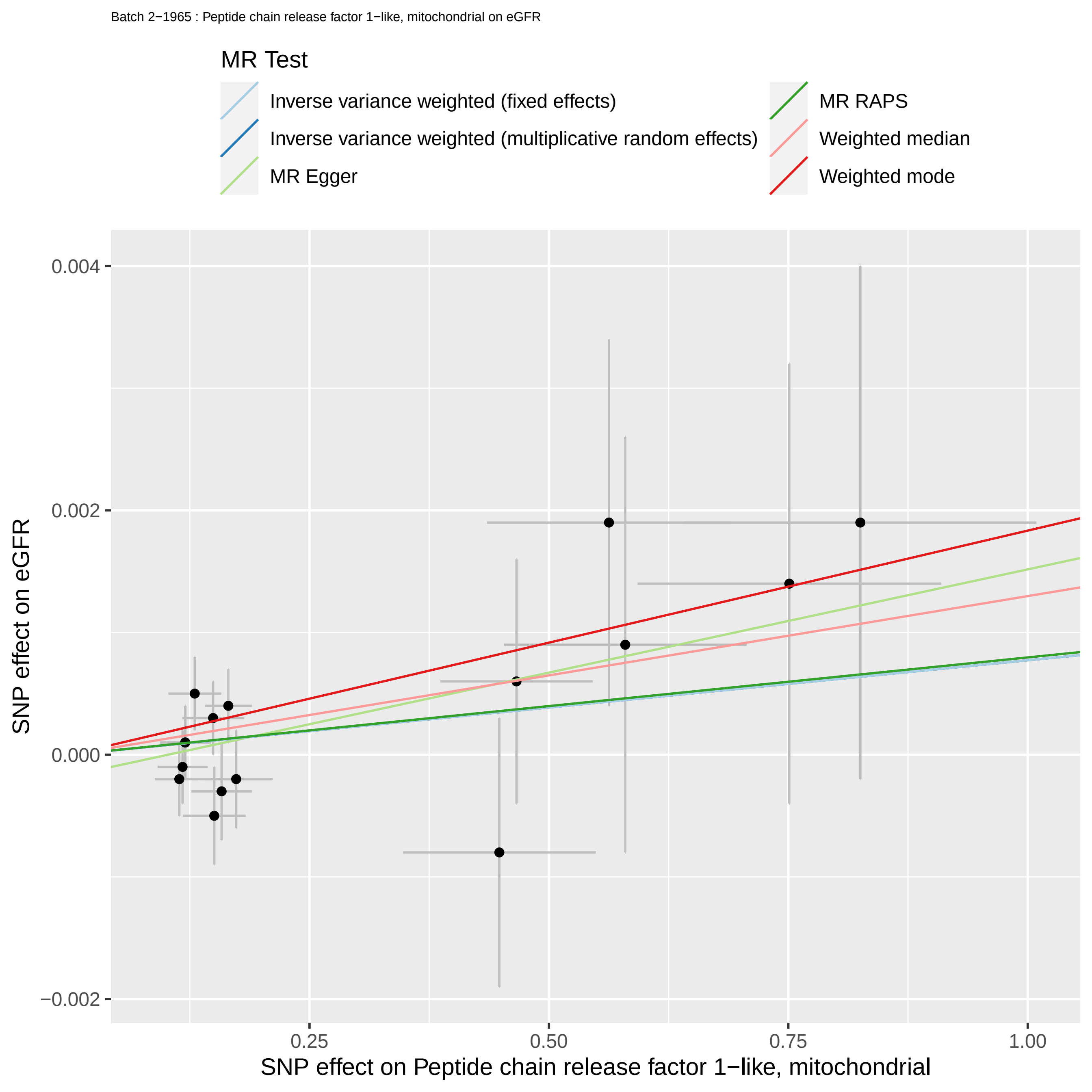

Batch 2−1965 : Peptide chain release factor 1−like, mitochondrial on eGFR
MR Test
Inverse variance weighted (fixed effects)
Inverse variance weighted (multiplicative random effects)
MR Egger
MR RAPS
Weighted median
Weighted mode
0.004
0.002
0.000
−0.002
0.25
0.50
0.75
1.00
SNP effect on eGFR
SNP effect on Peptide chain release factor 1−like, mitochondrial

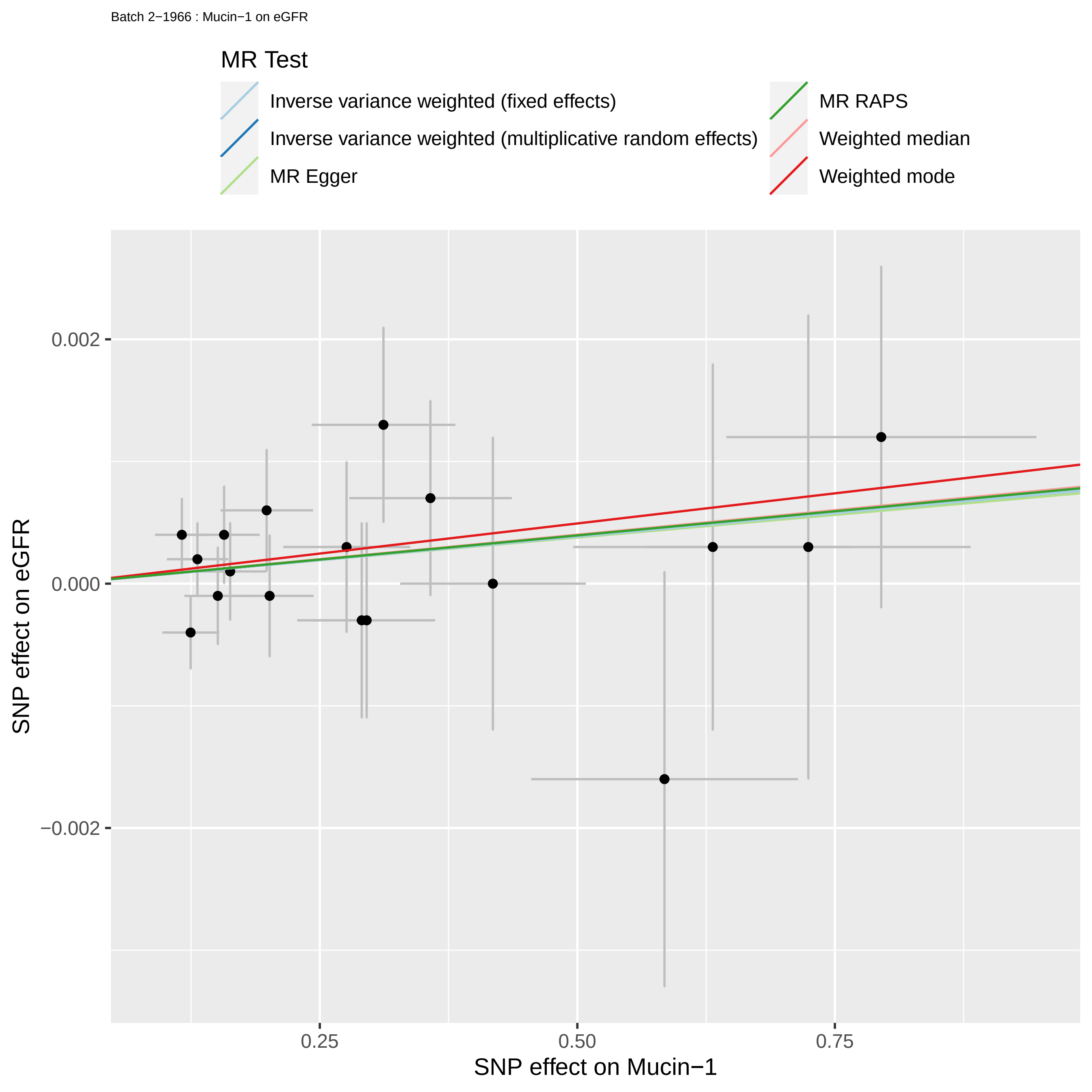
Batch 2−1966 : Mucin−1 on eGFR
MR Test
Inverse variance weighted (fixed effects)
Inverse variance weighted (multiplicative random effects)
MR Egger
MR RAPS
Weighted median
Weighted mode
SNP effect on eGFR
0.002
0.000
−0.002
0.25
0.50
0.75
SNP effect on Mucin−1

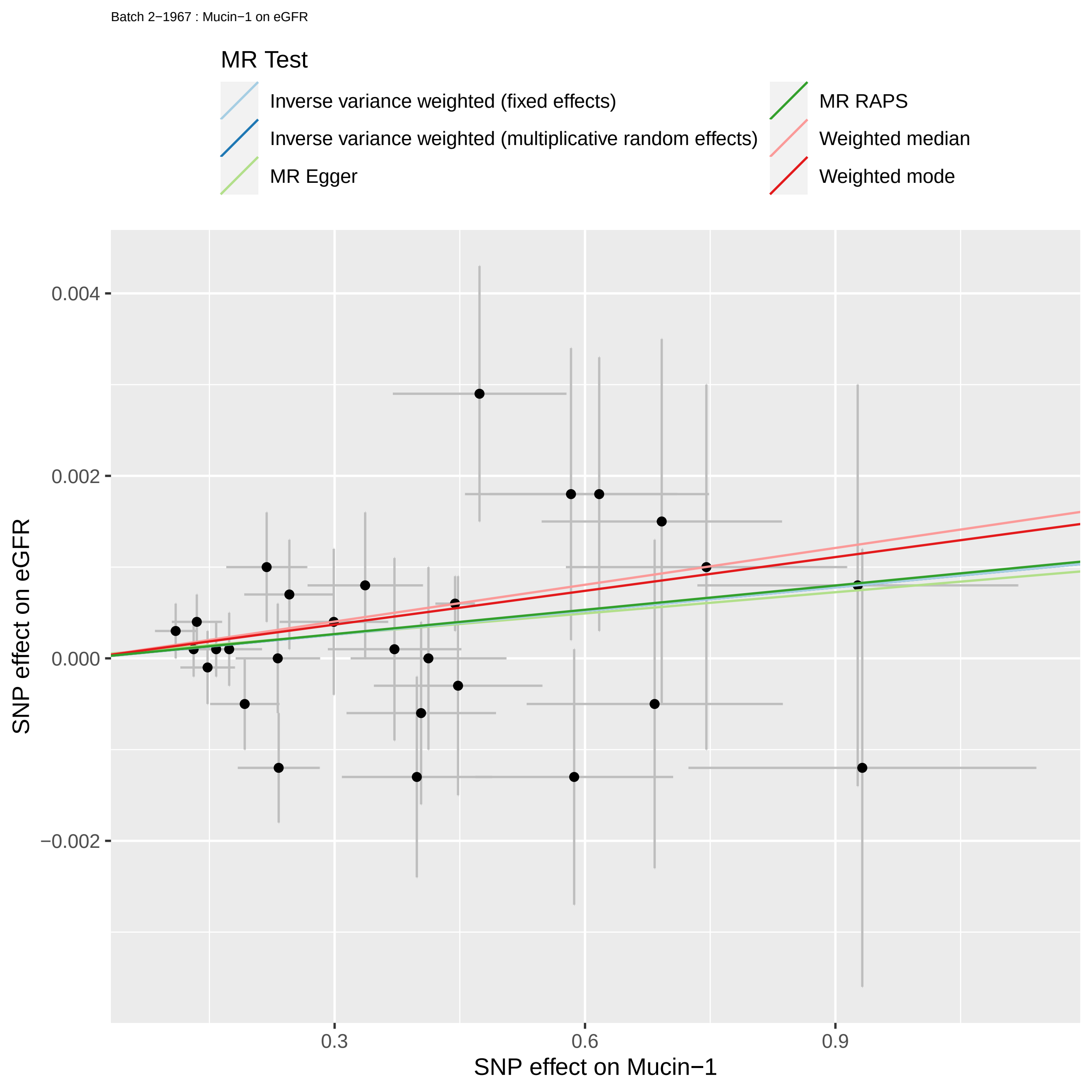
Batch 2−1967 : Mucin−1 on eGFR
MR Test
Inverse variance weighted (fixed effects)
Inverse variance weighted (multiplicative random effects)
MR Egger
MR RAPS
Weighted median
Weighted mode
0.004
0.002
0.000
−0.002
SNP effect on eGFR
0.3
0.6
0.9
SNP effect on Mucin−1

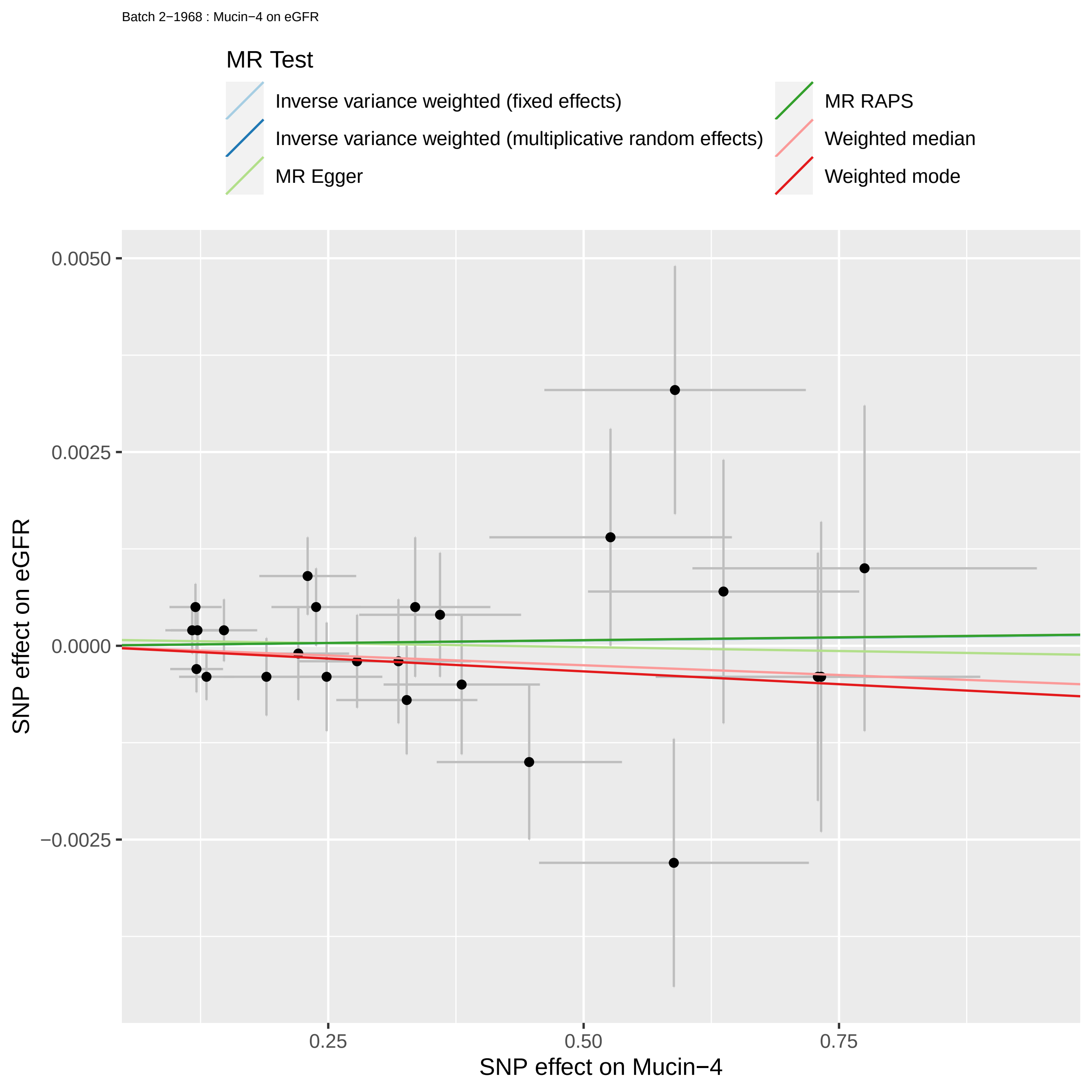
Batch 2−1968 : Mucin−4 on eGFR
MR Test
Inverse variance weighted (fixed effects)
Inverse variance weighted (multiplicative random effects)
MR Egger
MR RAPS
Weighted median
Weighted mode
0.0050
0.0025
0.0000
−0.0025
0.25
0.50
0.75
SNP effect on eGFR
SNP effect on Mucin−4

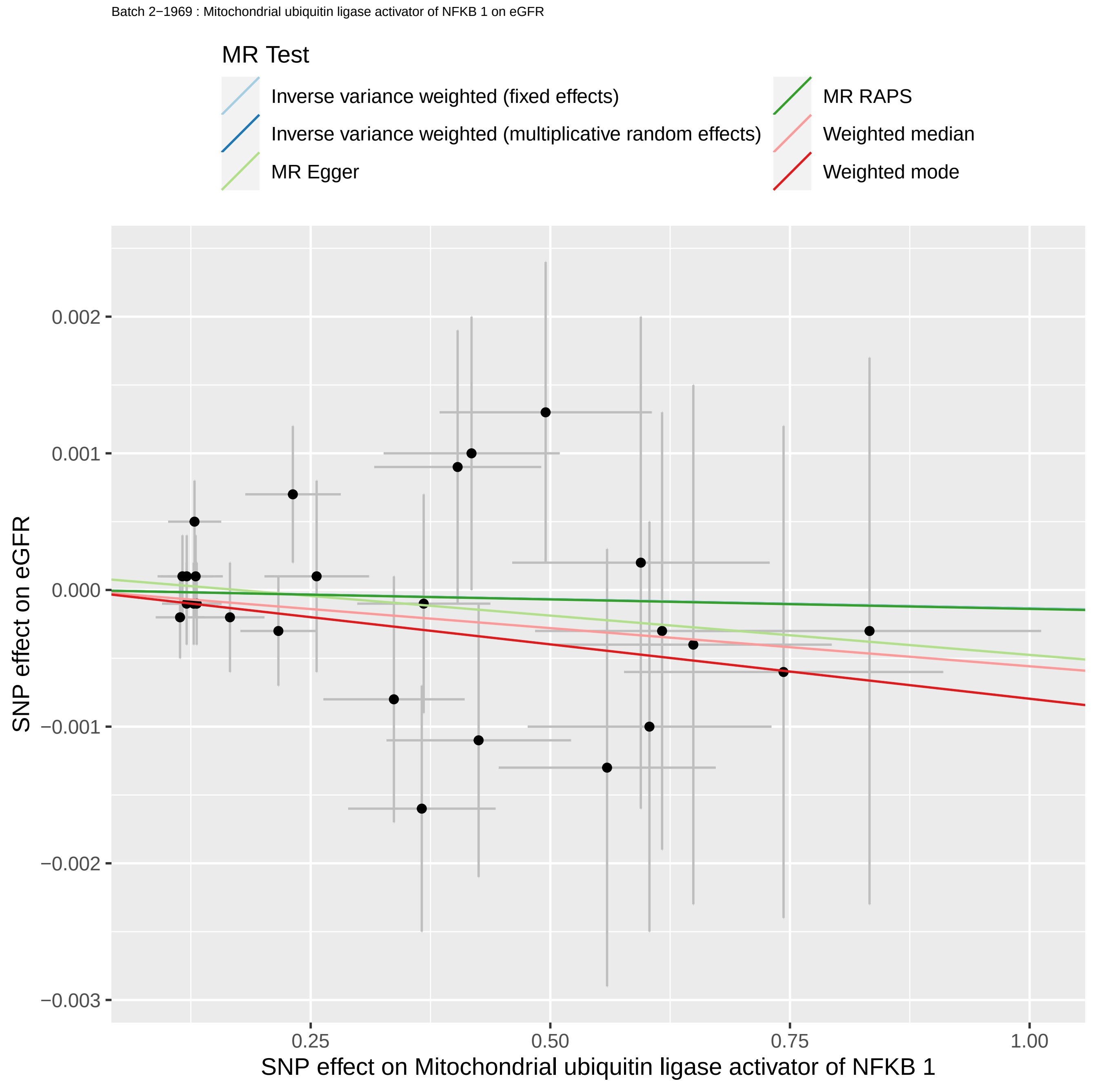

Batch 2−1969 : Mitochondrial ubiquitin ligase activator of NFKB 1 on eGFR
MR Test
Inverse variance weighted (fixed effects)
Inverse variance weighted (multiplicative random effects)
MR Egger
MR RAPS
Weighted median
Weighted mode
0.002
0.001
0.000
−0.001
−0.002
−0.003
0.25
0.50
0.75
1.00
SNP effect on eGFR
SNP effect on Mitochondrial ubiquitin ligase activator of NFKB 1

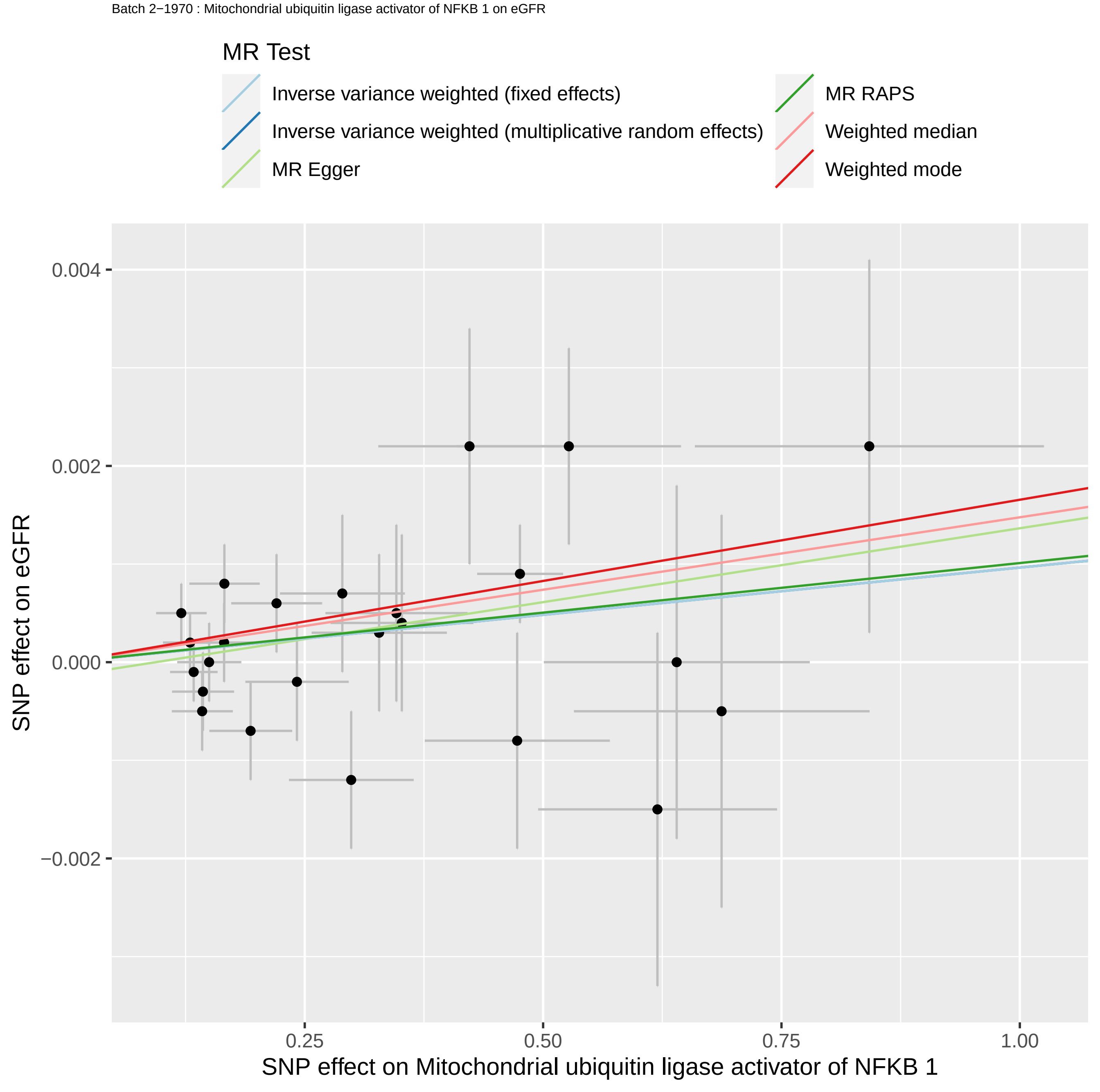

Batch 2−1970 : Mitochondrial ubiquitin ligase activator of NFKB 1 on eGFR
MR Test
Inverse variance weighted (fixed effects)
Inverse variance weighted (multiplicative random effects)
MR Egger
MR RAPS
Weighted median
Weighted mode
0.004
0.002
0.000
−0.002
0.25
0.50
0.75
1.00
SNP effect on eGFR
SNP effect on Mitochondrial ubiquitin ligase activator of NFKB 1

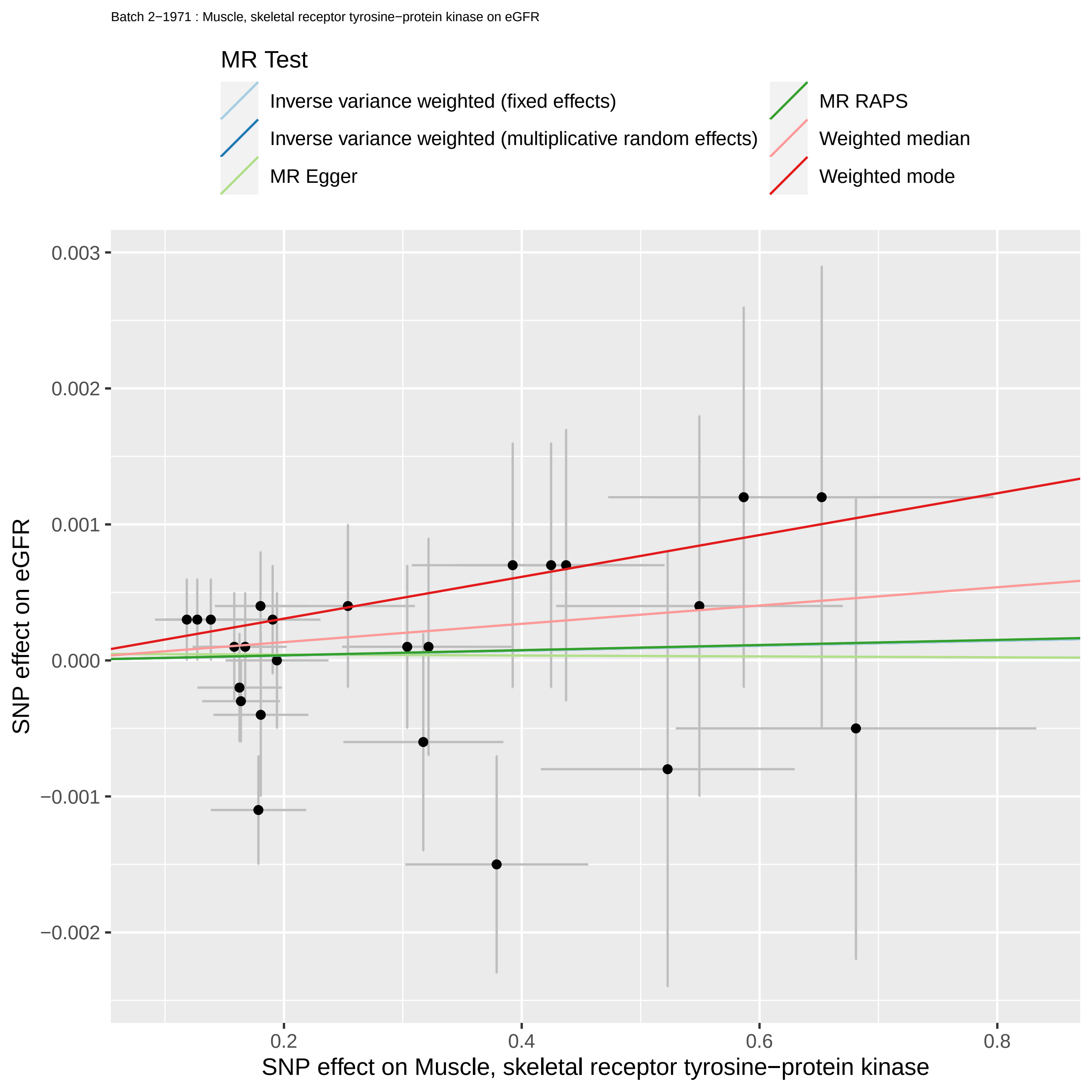
Batch 2−1971 : Muscle, skeletal receptor tyrosine−protein kinase on eGFR
MR Test
Inverse variance weighted (fixed effects)
Inverse variance weighted (multiplicative random effects)
MR Egger
MR RAPS
Weighted median
Weighted mode
0.003
0.002
0.001
0.000
−0.001
−0.002
0.2
0.4
0.6
0.8
SNP effect on eGFR
SNP effect on Muscle, skeletal receptor tyrosine−protein kinase

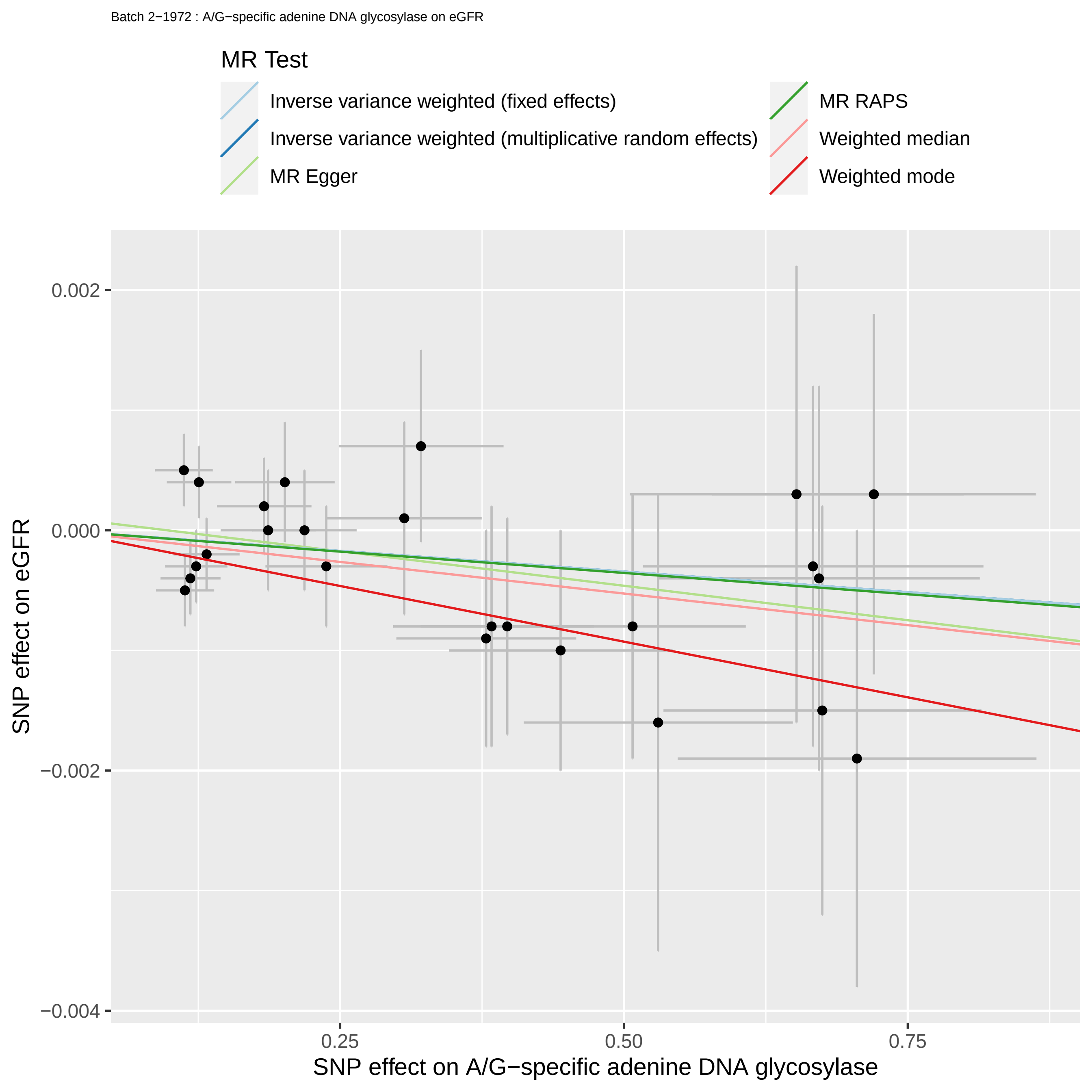

Batch 2−1972 : A/G−specific adenine DNA glycosylase on eGFR
MR Test
Inverse variance weighted (fixed effects)
Inverse variance weighted (multiplicative random effects)
MR Egger
MR RAPS
Weighted median
Weighted mode
0.002
0.000
−0.002
−0.004
0.25
0.50
0.75
SNP effect on eGFR
SNP effect on A/G−specific adenine DNA glycosylase

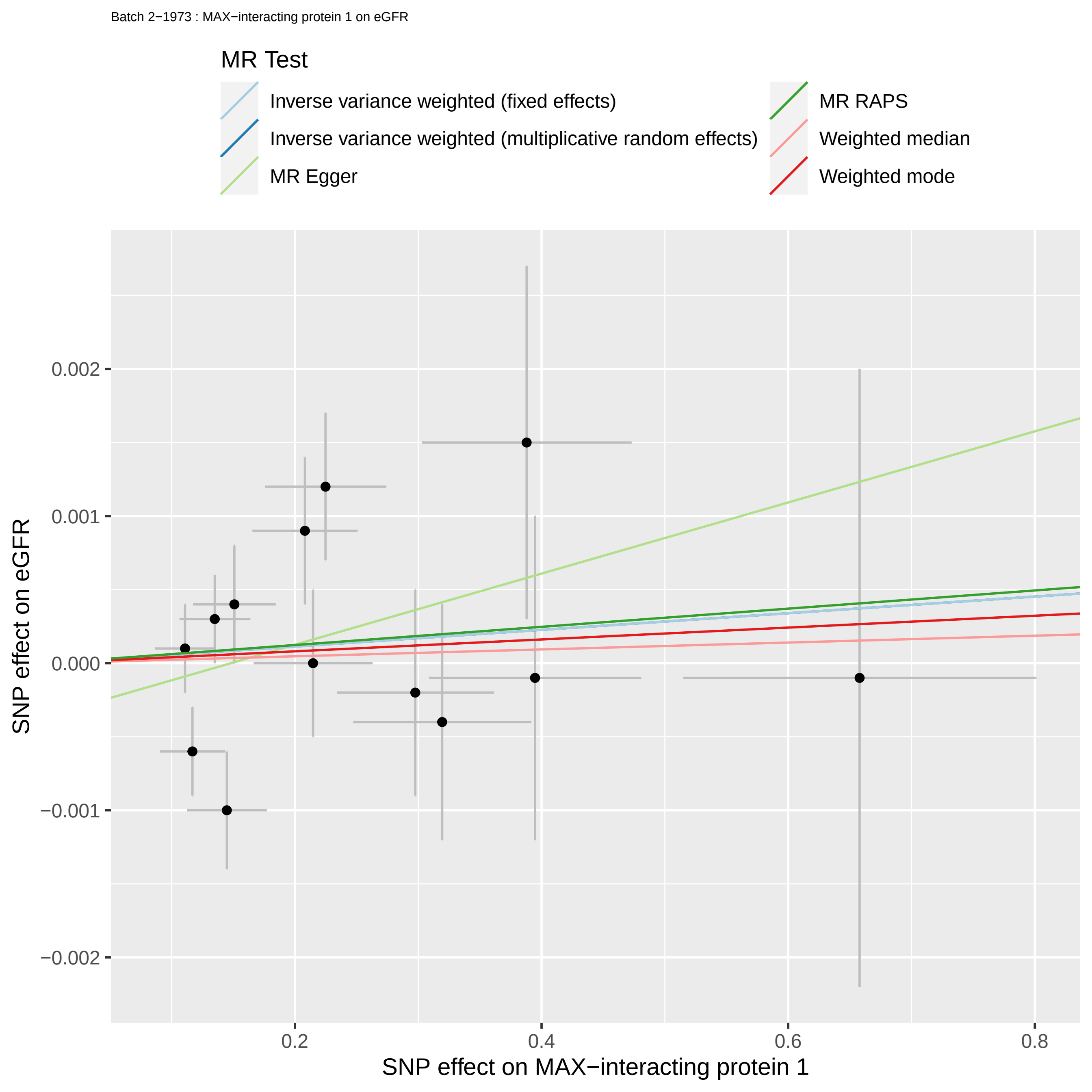

Batch 2−1973 : MAX−interacting protein 1 on eGFR
MR Test
Inverse variance weighted (fixed effects)
Inverse variance weighted (multiplicative random effects)
MR Egger
MR RAPS
Weighted median
Weighted mode
0.002
0.001
0.000
−0.001
−0.002
0.2
0.4
0.6
0.8
SNP effect on MAX−interacting protein 1
SNP effect on eGFR

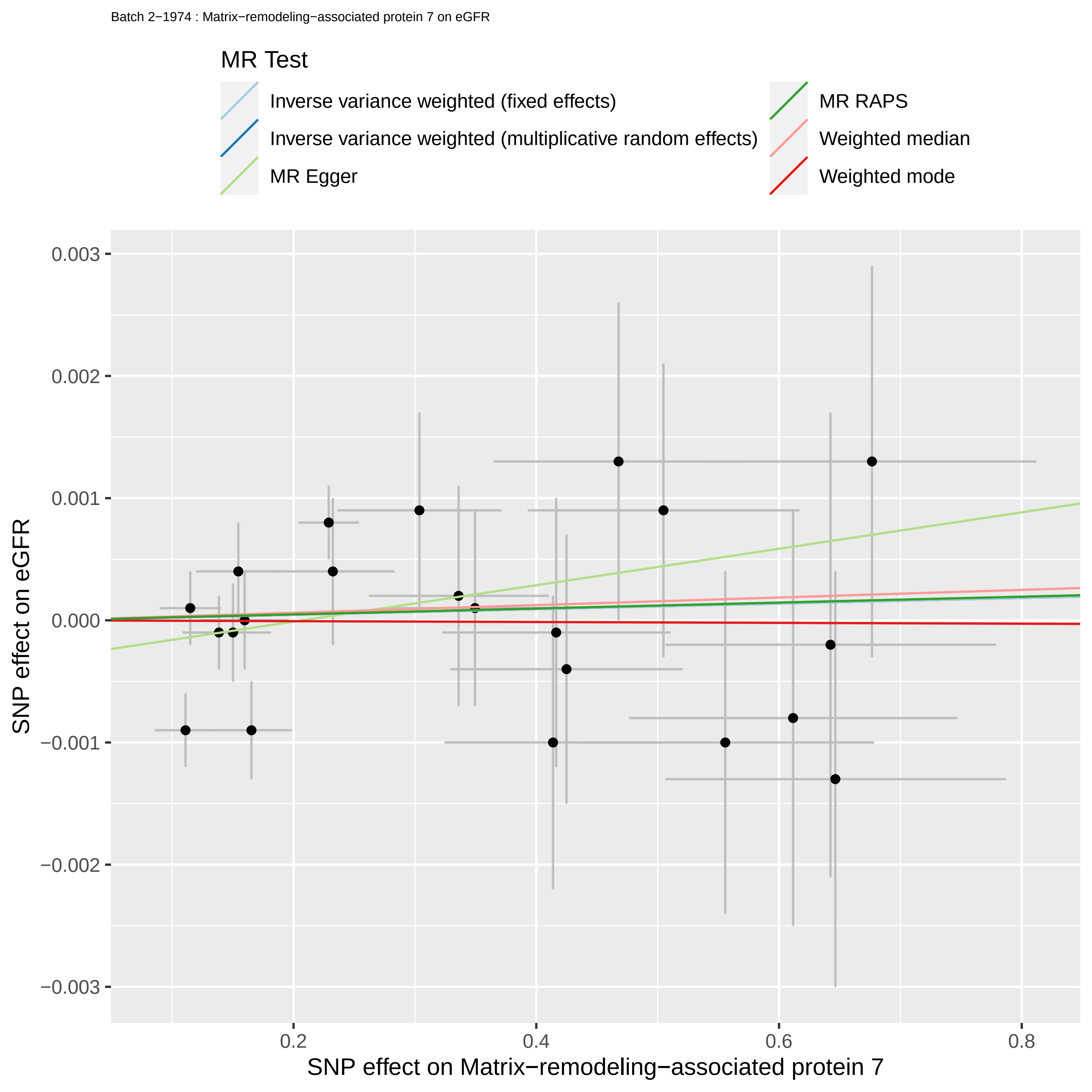
Batch 2−1974 : Matrix−remodeling−associated protein 7 on eGFR
MR Test
Inverse variance weighted (fixed effects)
Inverse variance weighted (multiplicative random effects)
MR Egger
MR RAPS
Weighted median
Weighted mode
SNP effect on eGFR
0.003
0.002
0.001
0.000
−0.001
−0.002
−0.003
0.2
0.4
0.6
0.8
SNP effect on Matrix−remodeling−associated protein 7

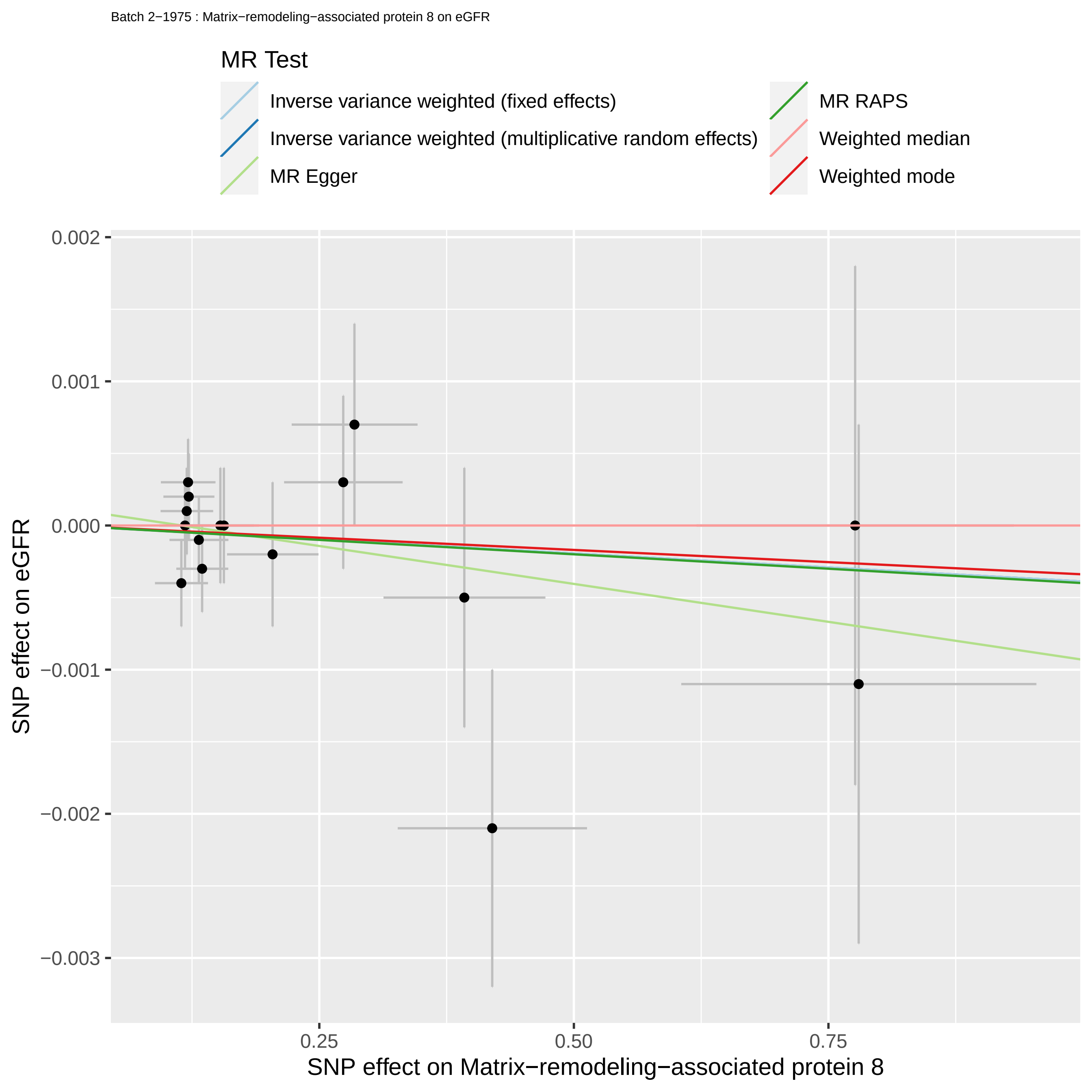

Batch 2−1975 : Matrix−remodeling−associated protein 8 on eGFR
MR Test
Inverse variance weighted (fixed effects)
Inverse variance weighted (multiplicative random effects)
MR Egger
MR RAPS
Weighted median
Weighted mode
0.002
0.001
0.000
−0.001
−0.002
−0.003
SNP effect on eGFR
0.25
0.50
0.75
SNP effect on Matrix−remodeling−associated protein 8

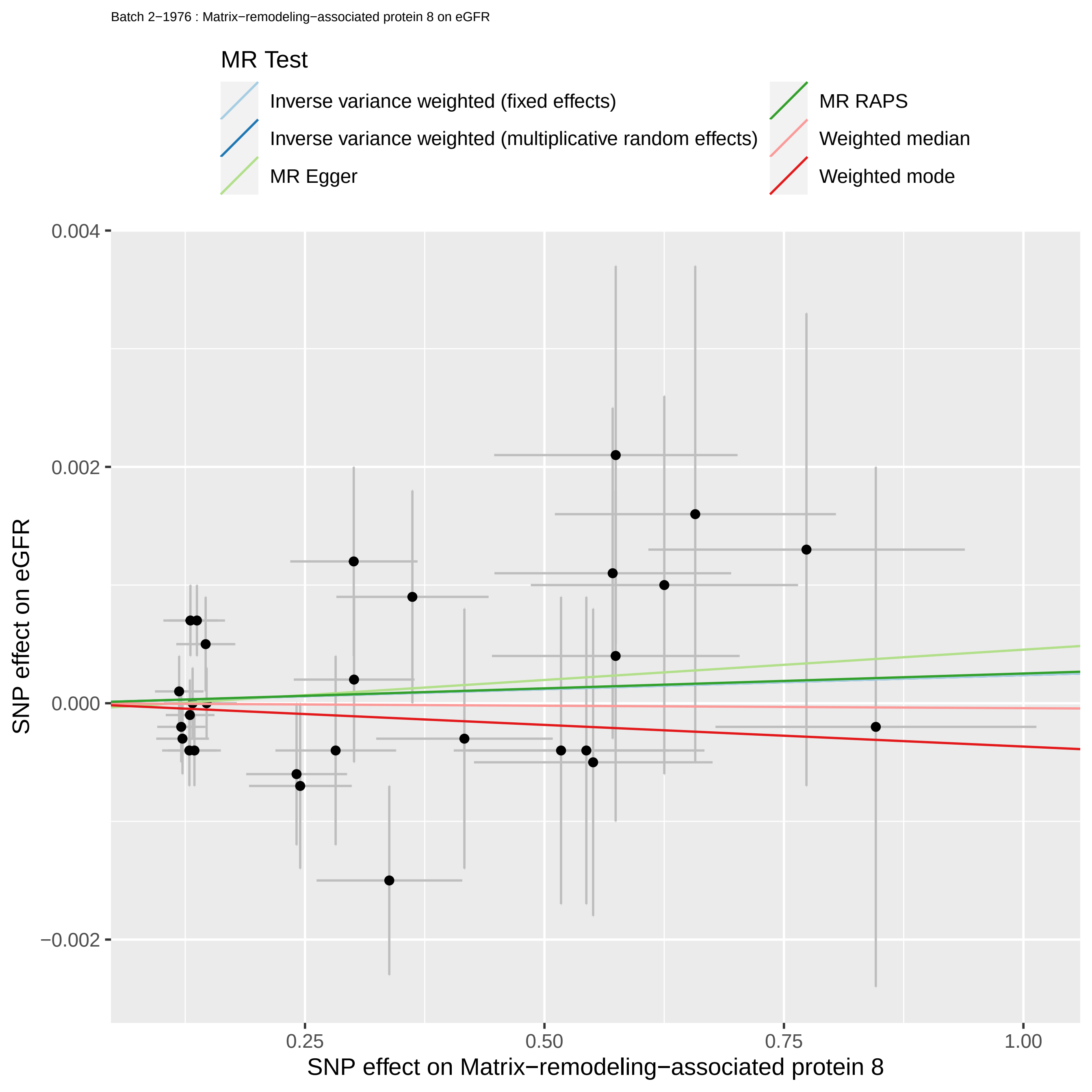
Batch 2−1976 : Matrix−remodeling−associated protein 8 on eGFR
MR Test
Inverse variance weighted (fixed effects)
Inverse variance weighted (multiplicative random effects)
MR Egger
MR RAPS
Weighted median
Weighted mode
0.004
0.002
0.000
−0.002
0.25
0.50
0.75
1.00
SNP effect on eGFR
SNP effect on Matrix−remodeling−associated protein 8

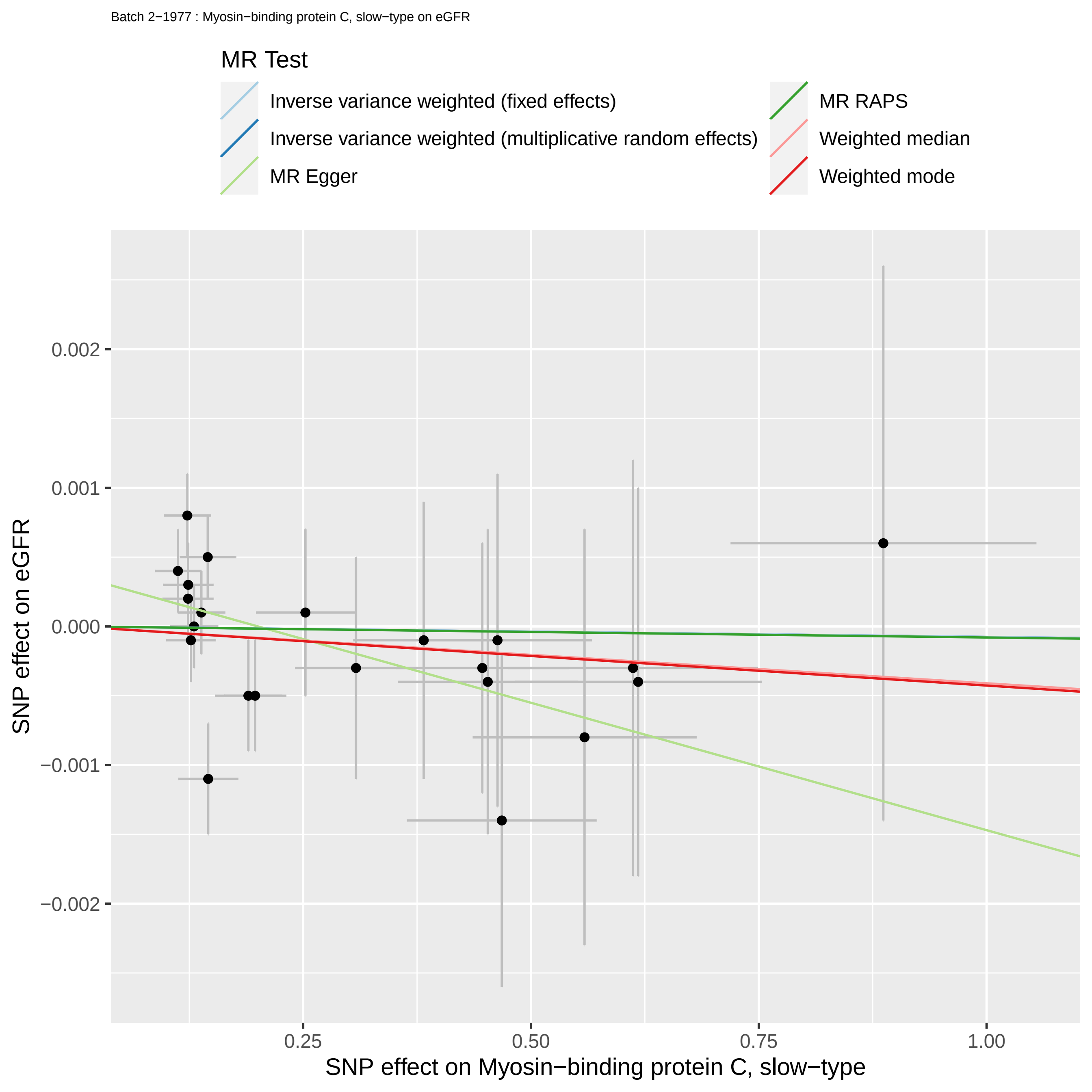

Batch 2−1977 : Myosin−binding protein C, slow−type on eGFR
MR Test
Inverse variance weighted (fixed effects)
Inverse variance weighted (multiplicative random effects)
MR Egger
MR RAPS
Weighted median
Weighted mode
0.002
0.001
0.000
−0.001
−0.002
0.25
0.50
0.75
1.00
SNP effect on eGFR
SNP effect on Myosin−binding protein C, slow−type

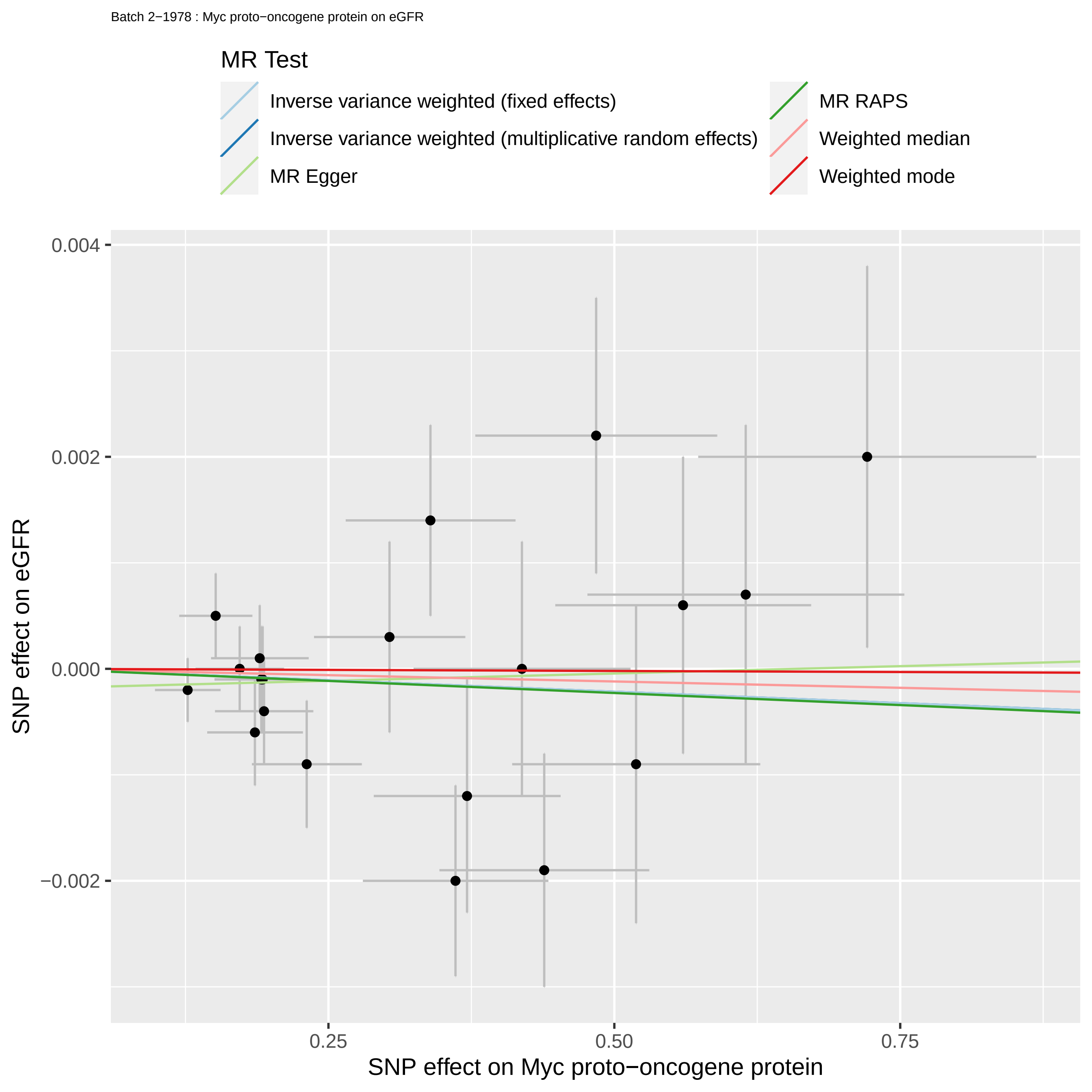

Batch 2−1978 : Myc proto−oncogene protein on eGFR
MR Test
Inverse variance weighted (fixed effects)
Inverse variance weighted (multiplicative random effects)
MR Egger
MR RAPS
Weighted median
Weighted mode
0.004
0.002
0.000
−0.002
0.25
0.50
0.75
SNP effect on eGFR
SNP effect on Myc proto−oncogene protein

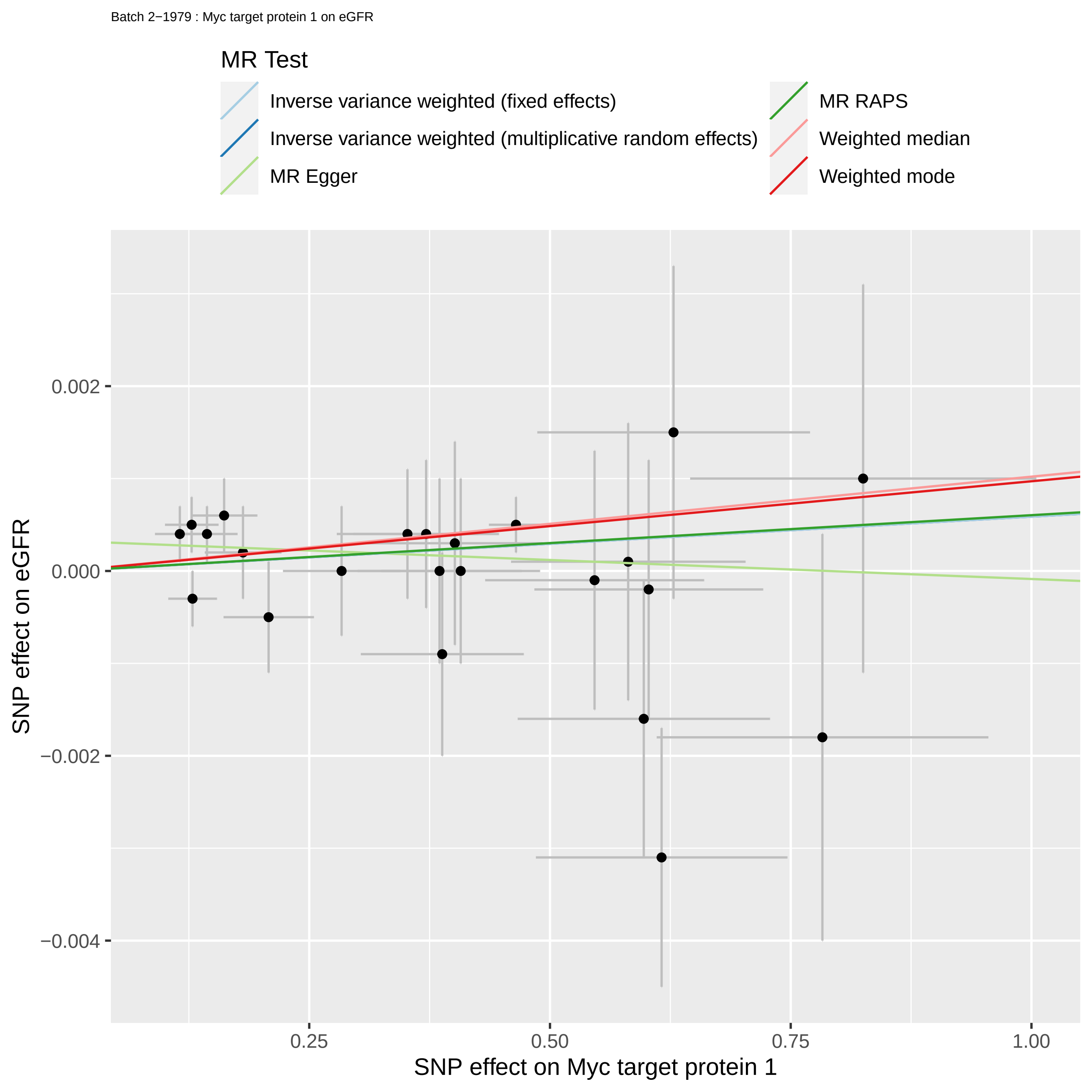

Batch 2−1979 : Myc target protein 1 on eGFR
MR Test
Inverse variance weighted (fixed effects)
Inverse variance weighted (multiplicative random effects)
MR Egger
MR RAPS
Weighted median
Weighted mode
0.002
0.000
−0.002
−0.004
0.25
0.50
0.75
1.00
SNP effect on eGFR
SNP effect on Myc target protein 1

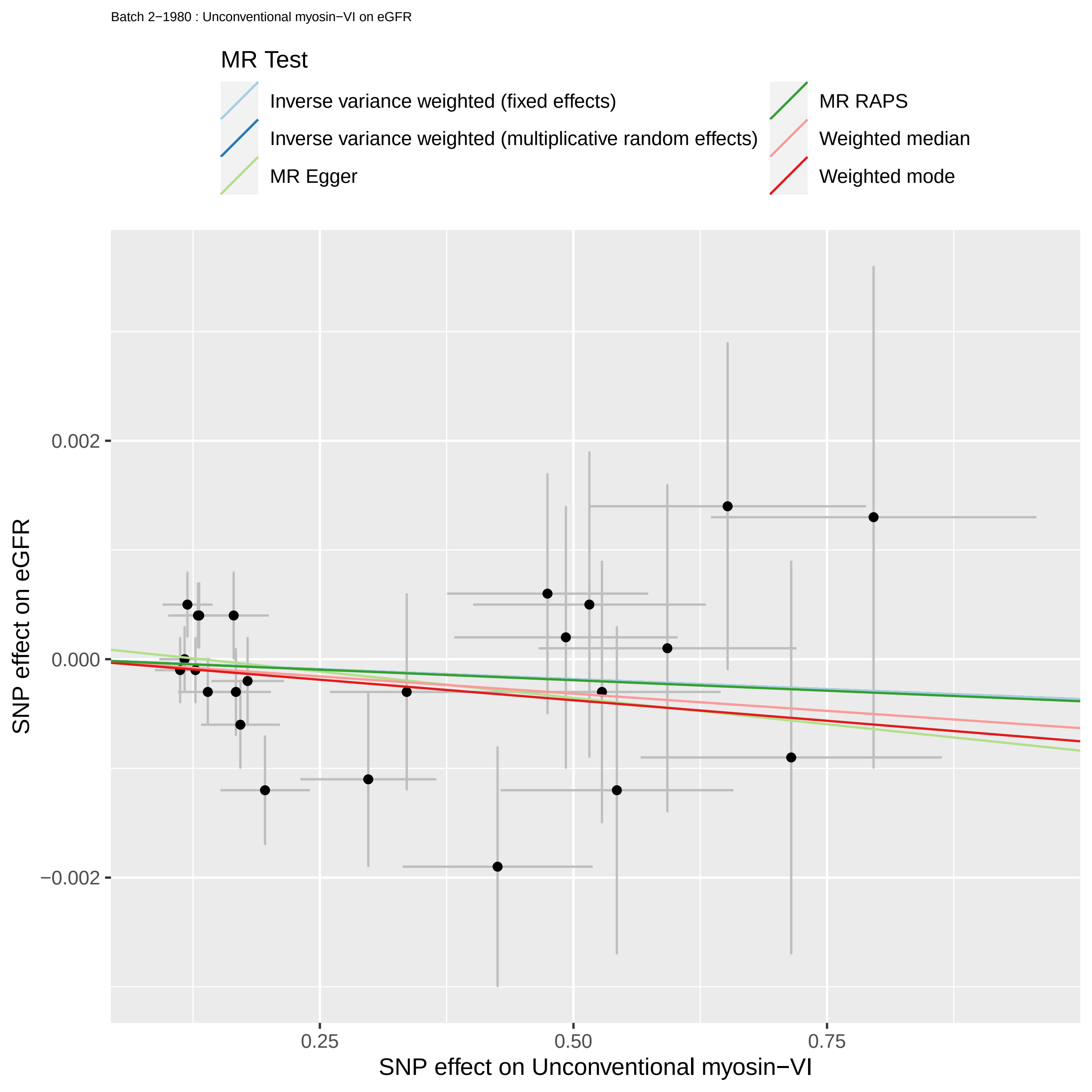

Batch 2−1980 : Unconventional myosin−VI on eGFR
MR Test
Inverse variance weighted (fixed effects)
Inverse variance weighted (multiplicative random effects)
MR Egger
MR RAPS
Weighted median
Weighted mode
0.002
0.000
−0.002
0.25
0.50
0.75
SNP effect on eGFR
SNP effect on Unconventional myosin−VI

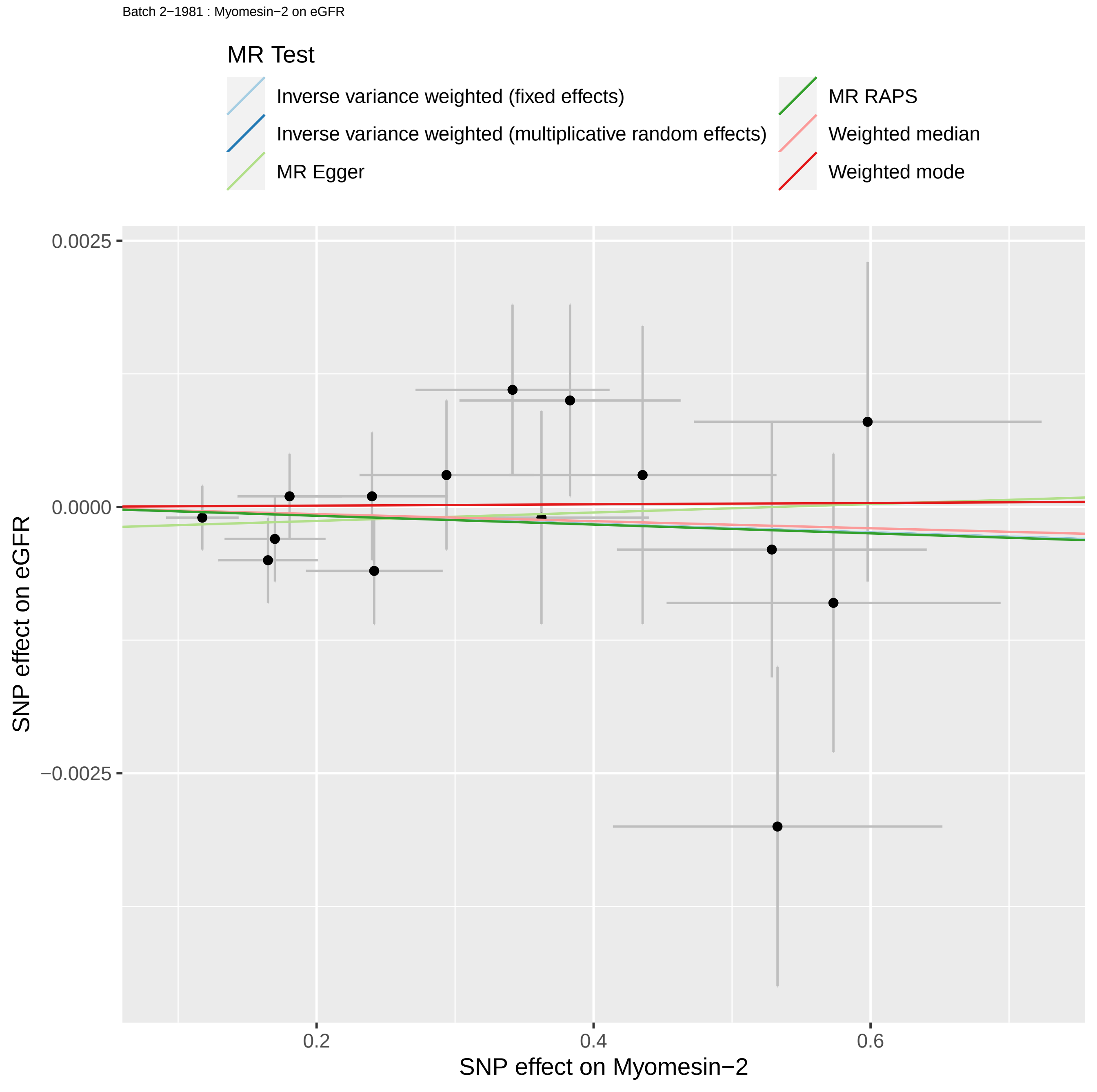

Batch 2−1981 : Myomesin−2 on eGFR
MR Test
Inverse variance weighted (fixed effects)
Inverse variance weighted (multiplicative random effects)
MR Egger
MR RAPS
Weighted median
Weighted mode
0.0025
0.0000
−0.0025
0.2
0.4
0.6
SNP effect on eGFR
SNP effect on Myomesin−2

Batch 2−1982 : Myelin regulatory factor on eGFR

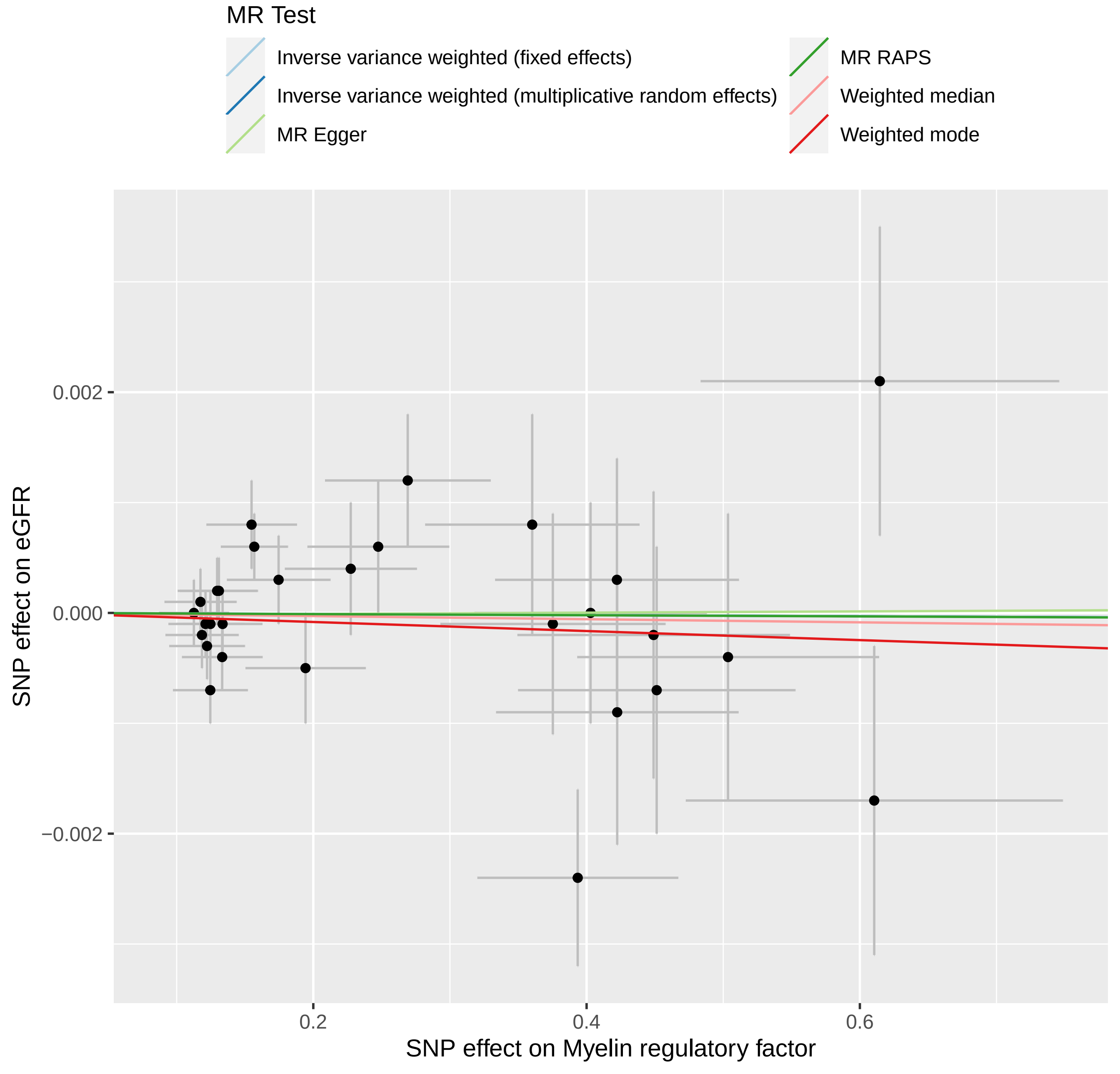

Batch 2−1983 : Histone H2A deubiquitinase MYSM1 on eGFR

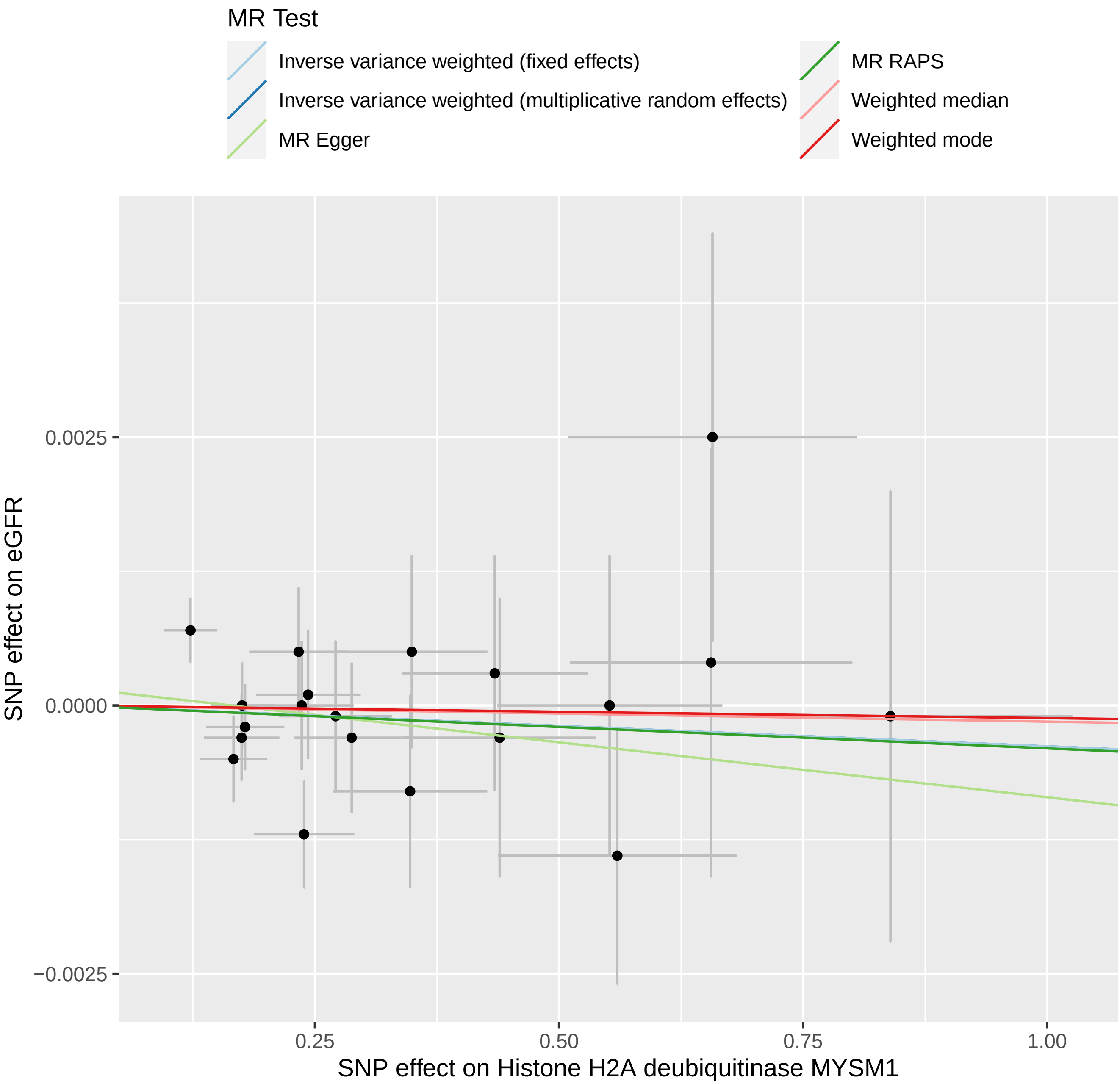

Batch 2−1984 : Myocardial zonula adherens protein on eGFR

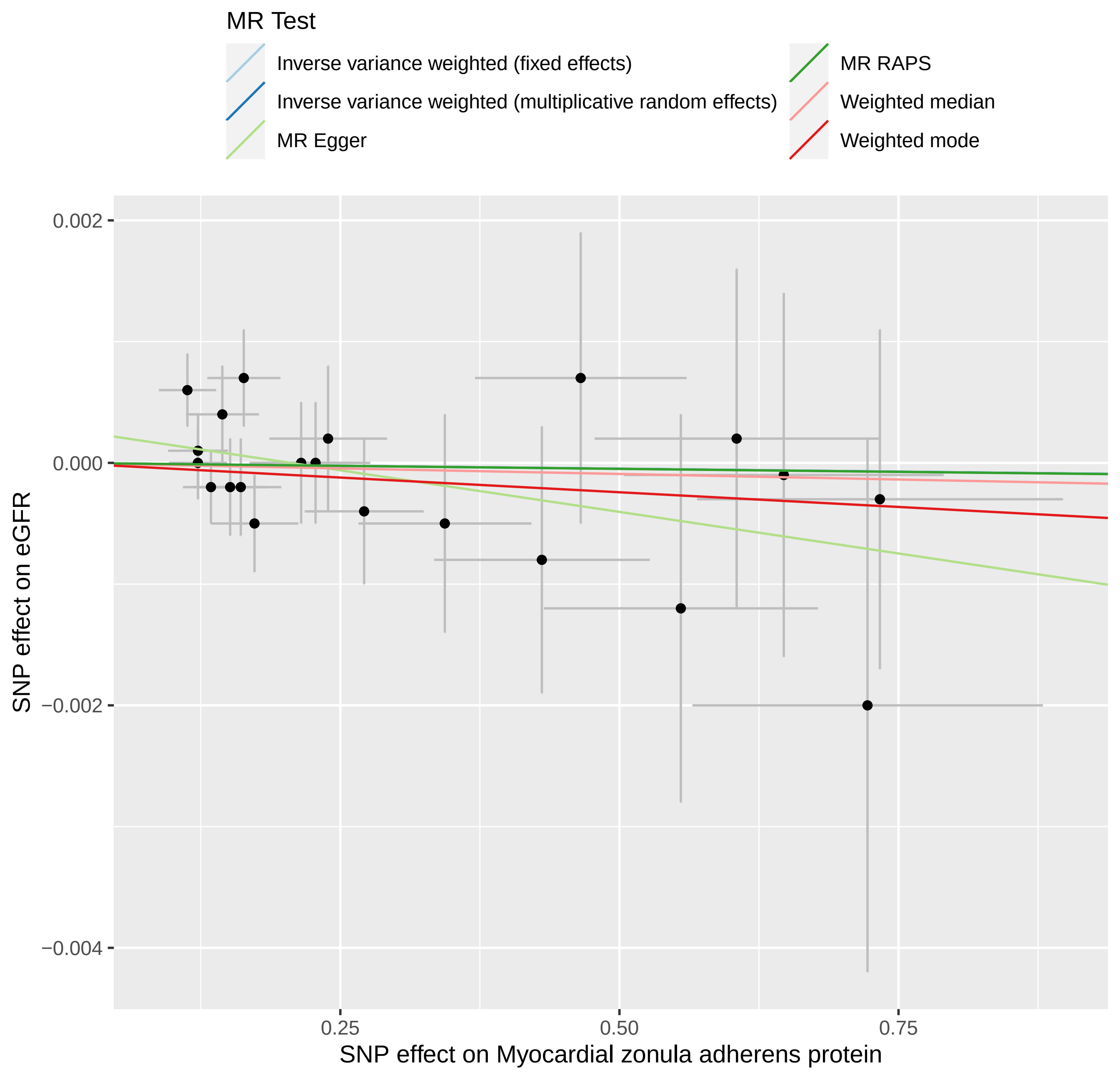

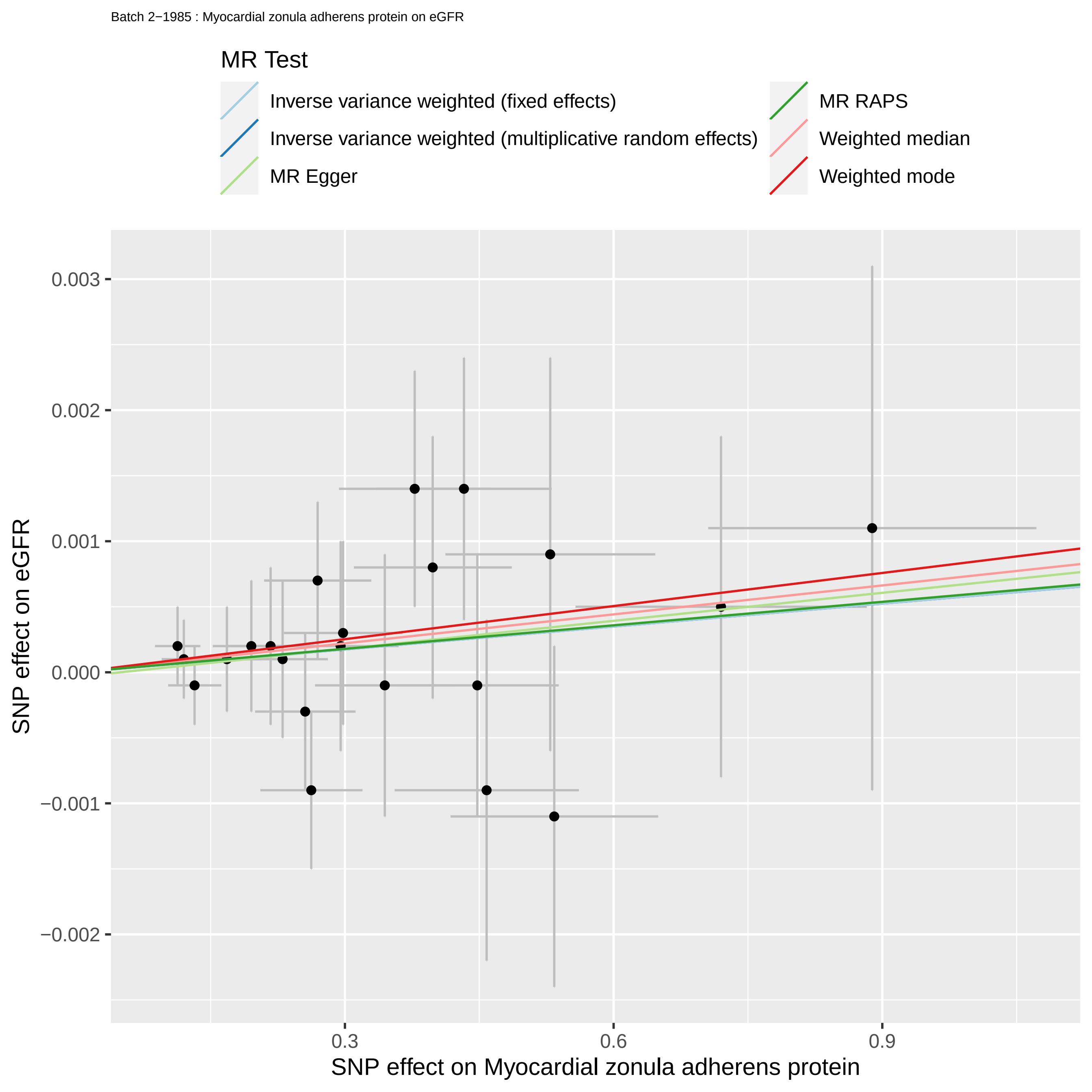

Batch 2−1985 : Myocardial zonula adherens protein on eGFR
MR Test
Inverse variance weighted (fixed effects)
Inverse variance weighted (multiplicative random effects)
MR Egger
MR RAPS
Weighted median
Weighted mode
0.003
0.002
0.001
0.000
−0.001
−0.002
0.3
0.6
0.9
SNP effect on eGFR
SNP effect on Myocardial zonula adherens protein

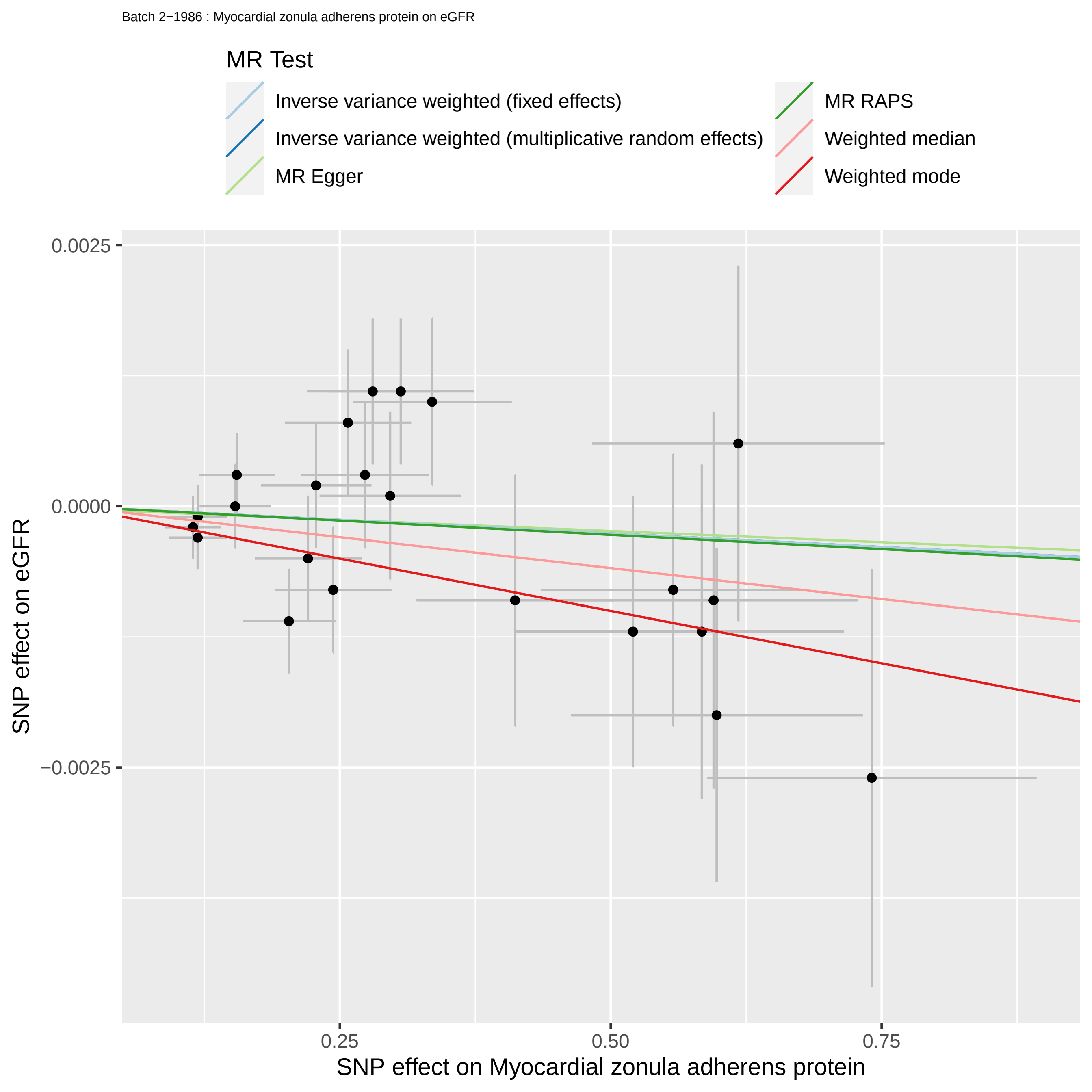
Batch 2−1986 : Myocardial zonula adherens protein on eGFR
MR Test
Inverse variance weighted (fixed effects)
Inverse variance weighted (multiplicative random effects)
MR Egger
MR RAPS
Weighted median
Weighted mode
0.0025
0.0000
−0.0025
0.25
0.50
0.75
SNP effect on eGFR
SNP effect on Myocardial zonula adherens protein

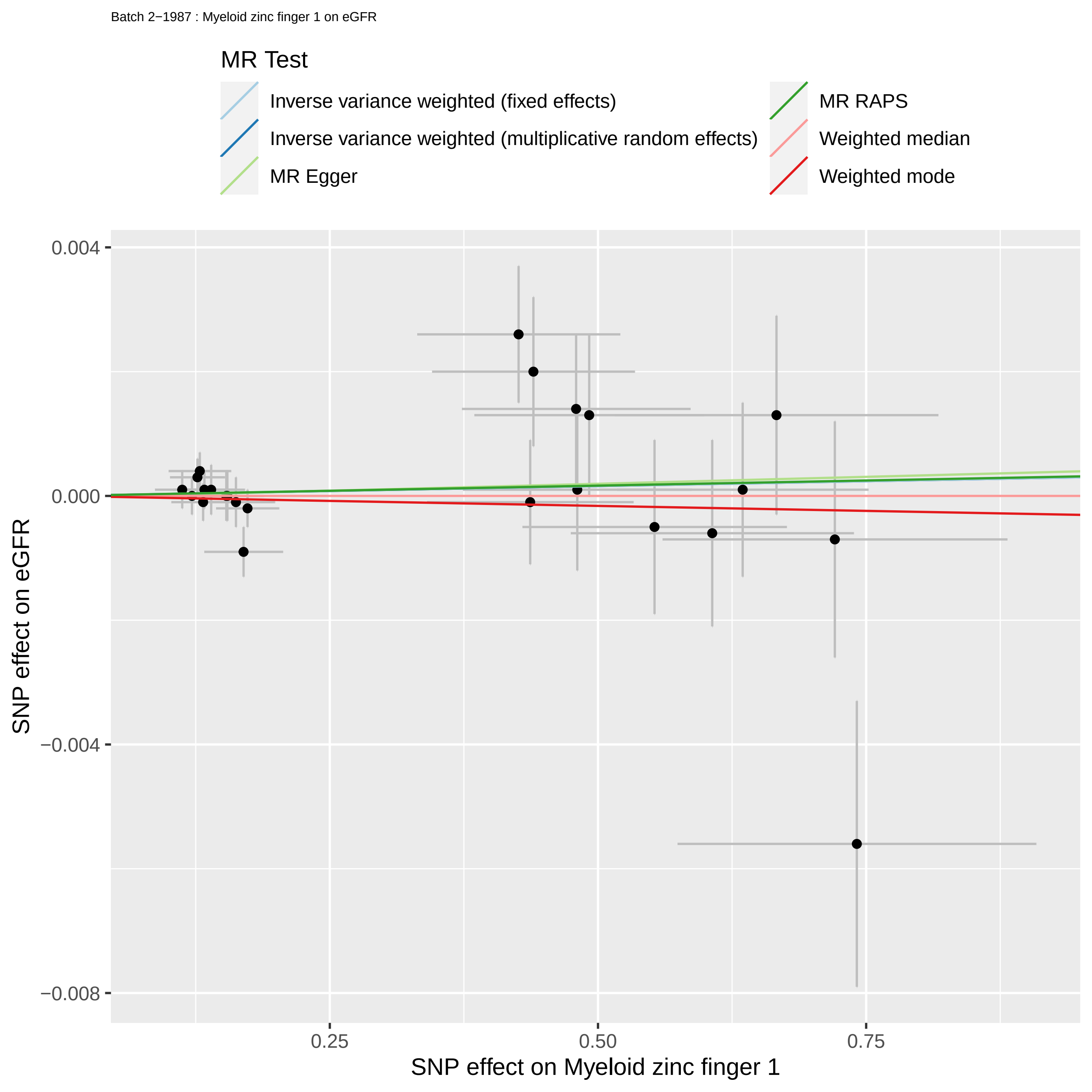

Batch 2−1987 : Myeloid zinc finger 1 on eGFR
MR Test
Inverse variance weighted (fixed effects)
Inverse variance weighted (multiplicative random effects)
MR Egger
MR RAPS
Weighted median
Weighted mode
0.004
0.000
−0.004
−0.008
SNP effect on eGFR
0.25
0.50
0.75
SNP effect on Myeloid zinc finger 1

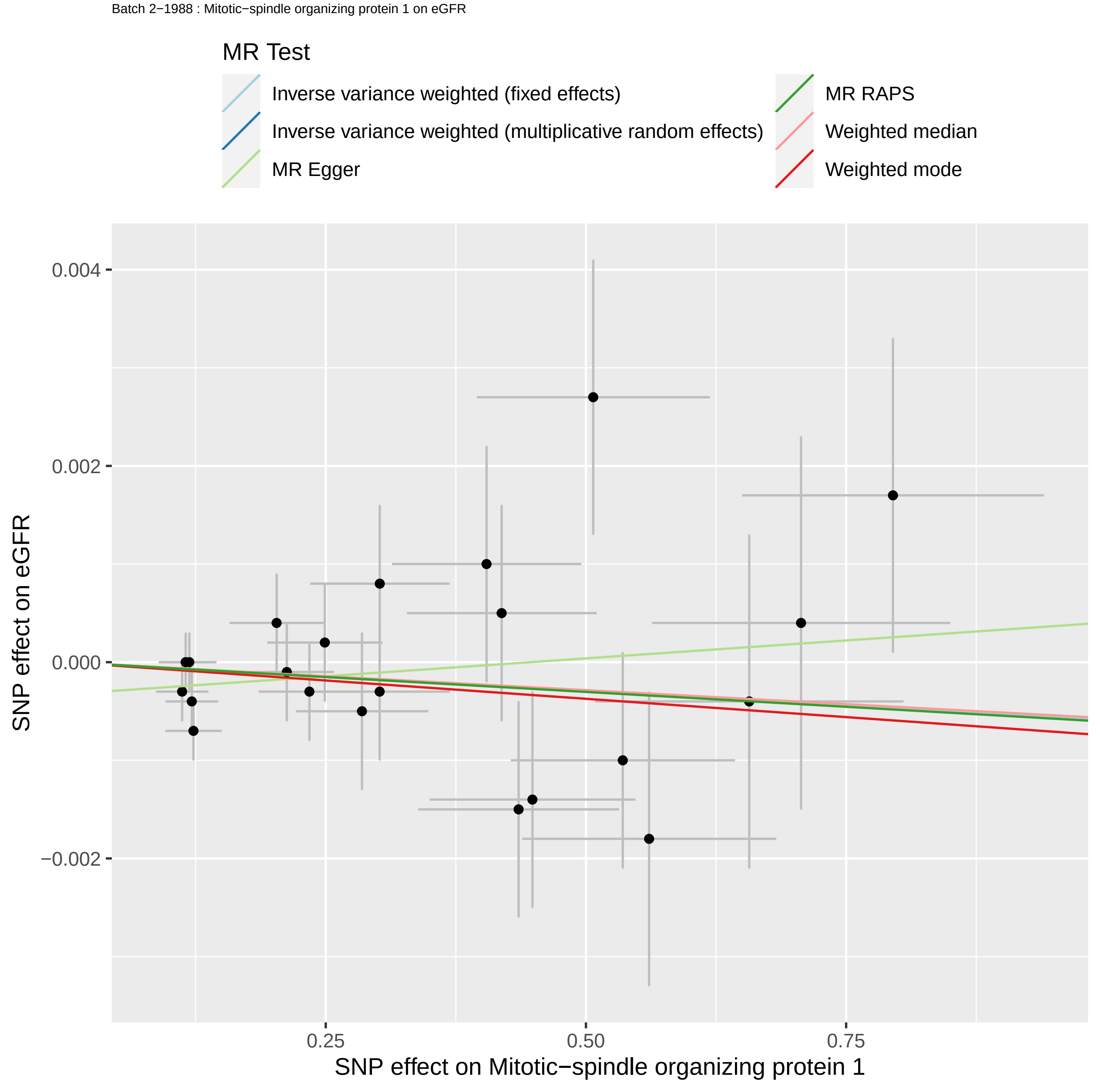
Batch 2−1988 : Mitotic−spindle organizing protein 1 on eGFR
MR Test
Inverse variance weighted (fixed effects)
Inverse variance weighted (multiplicative random effects)
MR Egger
MR RAPS
Weighted median
Weighted mode
0.004
0.002
0.000
−0.002
0.25
0.50
0.75
SNP effect on eGFR
SNP effect on Mitotic−spindle organizing protein 1

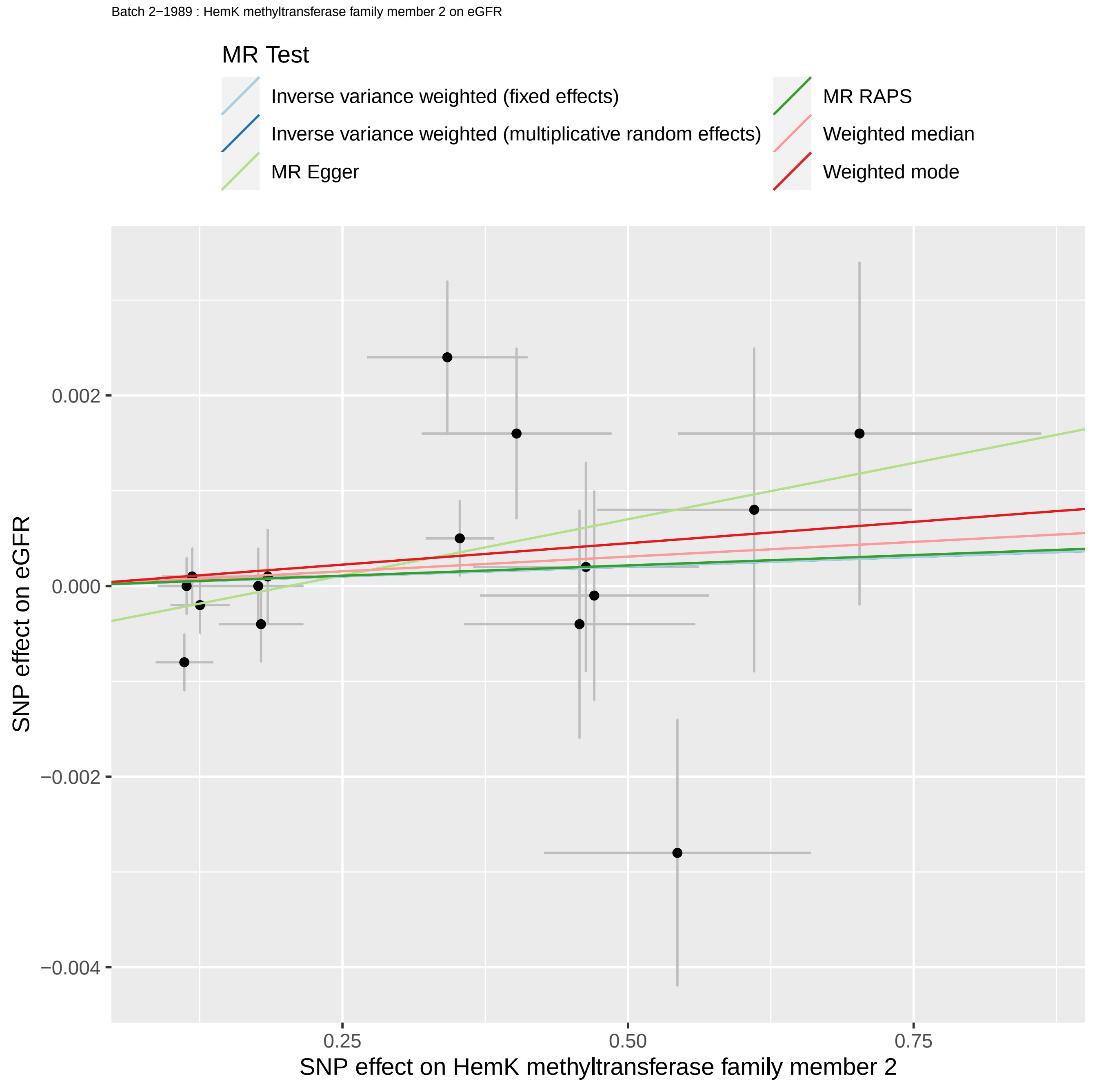
Batch 2−1989 : HemK methyltransferase family member 2 on eGFR
MR Test
Inverse variance weighted (fixed effects)
Inverse variance weighted (multiplicative random effects)
MR Egger
MR RAPS
Weighted median
Weighted mode
0.002
0.000
−0.002
−0.004
SNP effect on eGFR
0.25
0.50
0.75
SNP effect on HemK methyltransferase family member 2

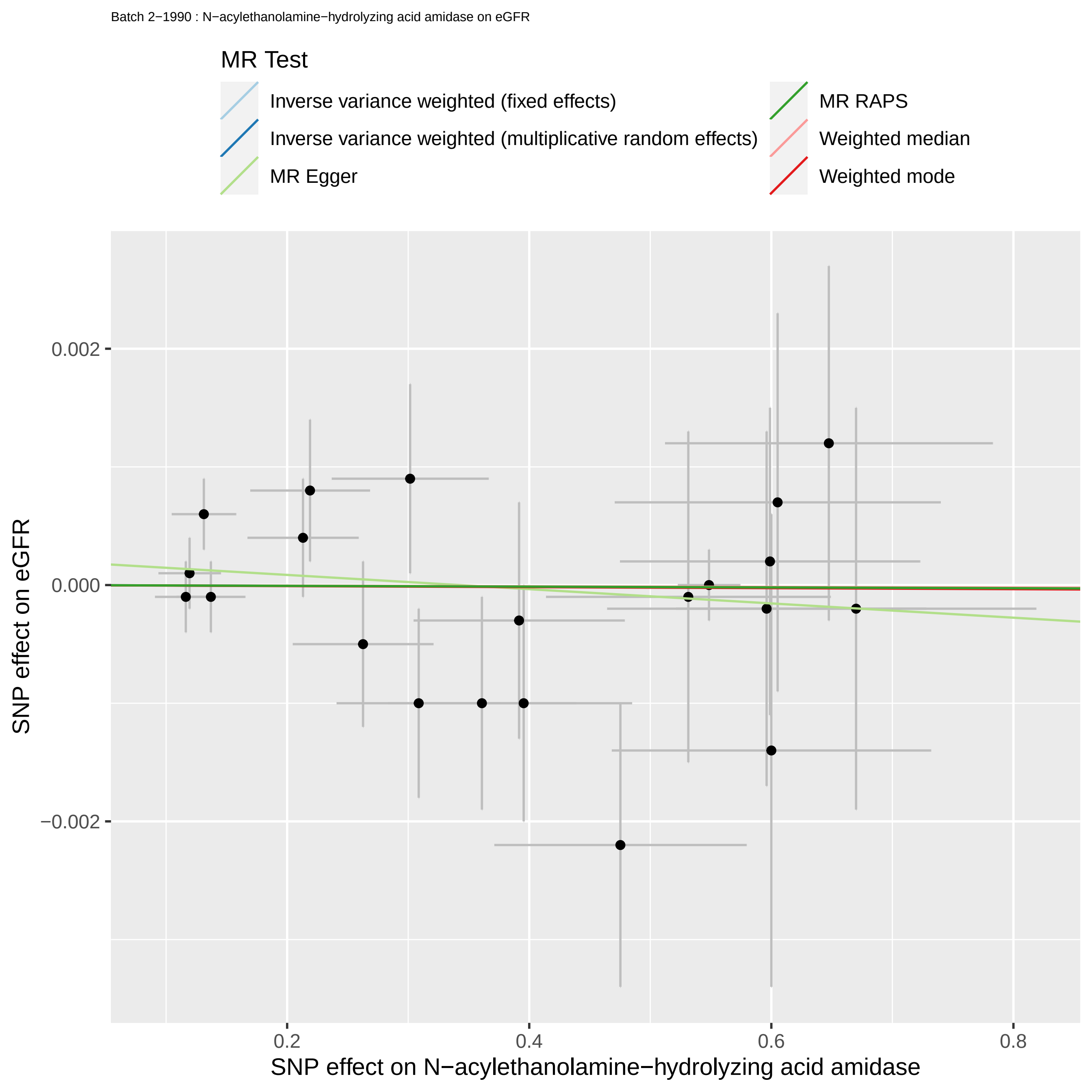
Batch 2−1990 : N−acylethanolamine−hydrolyzing acid amidase on eGFR
MR Test
Inverse variance weighted (fixed effects)
Inverse variance weighted (multiplicative random effects)
MR Egger
MR RAPS
Weighted median
Weighted mode
0.002
0.000
−0.002
0.2
0.4
0.6
0.8
SNP effect on eGFR
SNP effect on N−acylethanolamine−hydrolyzing acid amidase

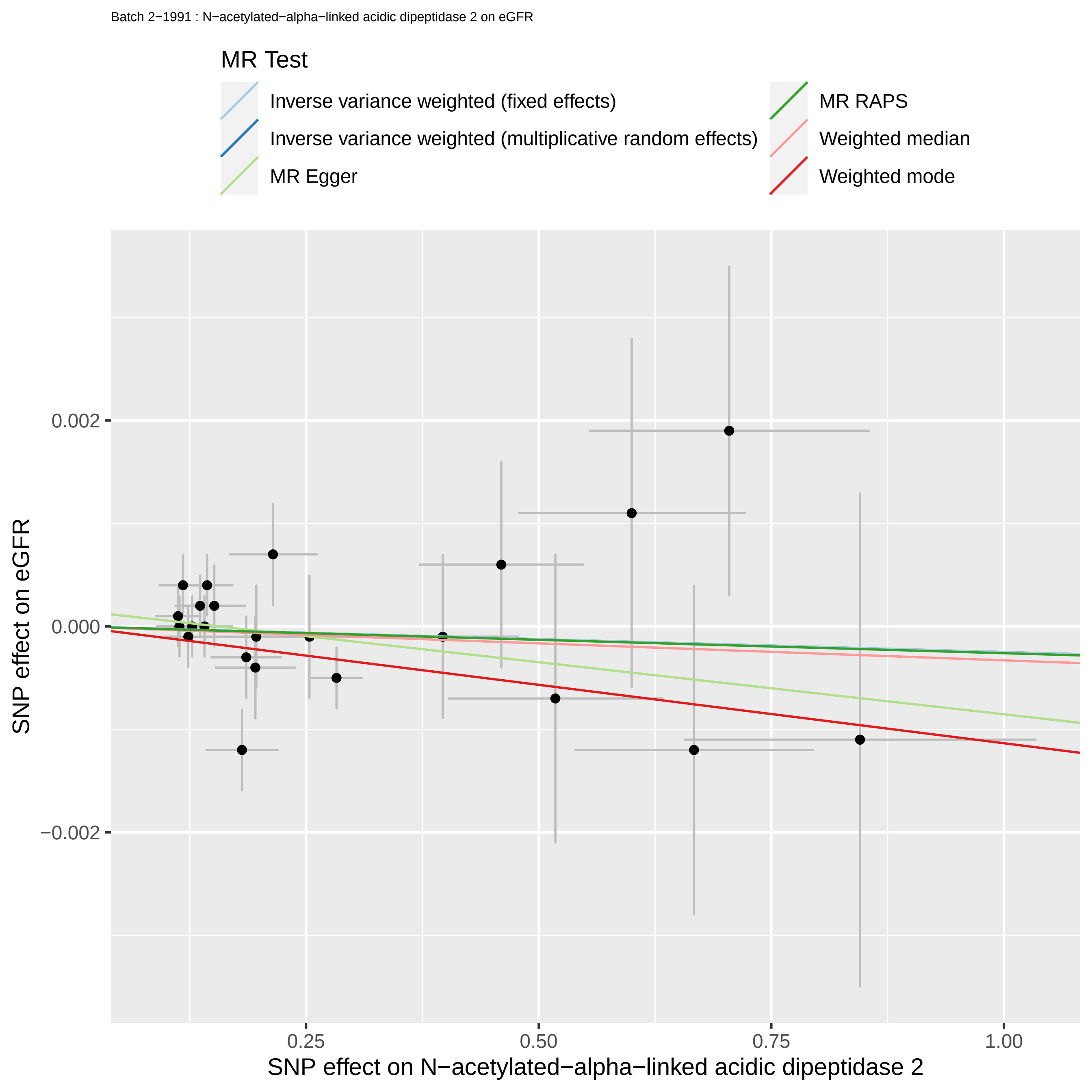

Batch 2−1991 : N−acetylated−alpha−linked acidic dipeptidase 2 on eGFR
MR Test
Inverse variance weighted (fixed effects)
Inverse variance weighted (multiplicative random effects)
MR Egger
MR RAPS
Weighted median
Weighted mode
0.002
0.000
−0.002
SNP effect on eGFR
0.25
0.50
0.75
1.00
SNP effect on N−acetylated−alpha−linked acidic dipeptidase 2

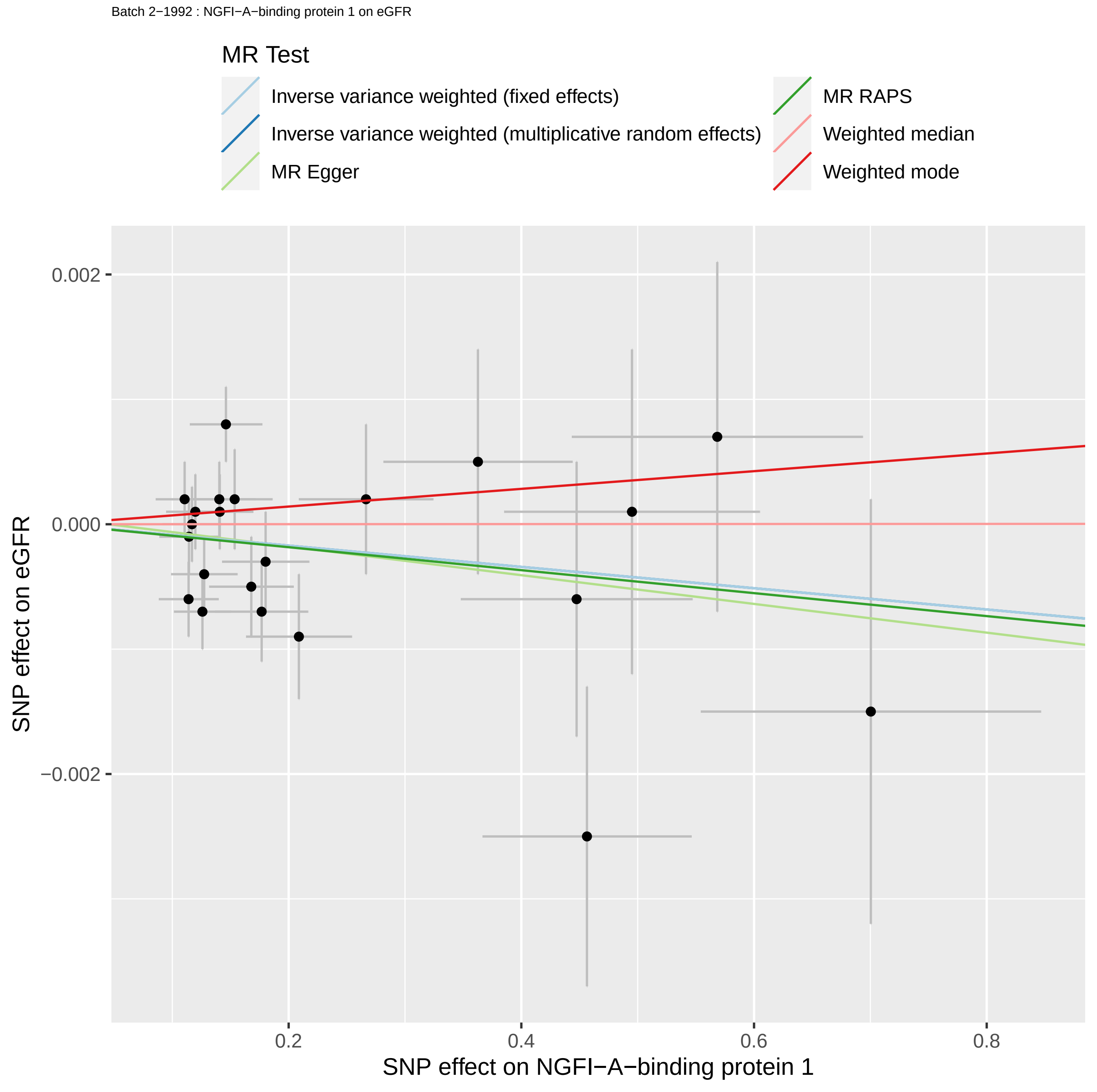

Batch 2−1992 : NGFI−A−binding protein 1 on eGFR
MR Test
Inverse variance weighted (fixed effects)
Inverse variance weighted (multiplicative random effects)
MR Egger
MR RAPS
Weighted median
Weighted mode
0.002
0.000
−0.002
SNP effect on eGFR
0.2
0.4
0.6
0.8
SNP effect on NGFI−A−binding protein 1

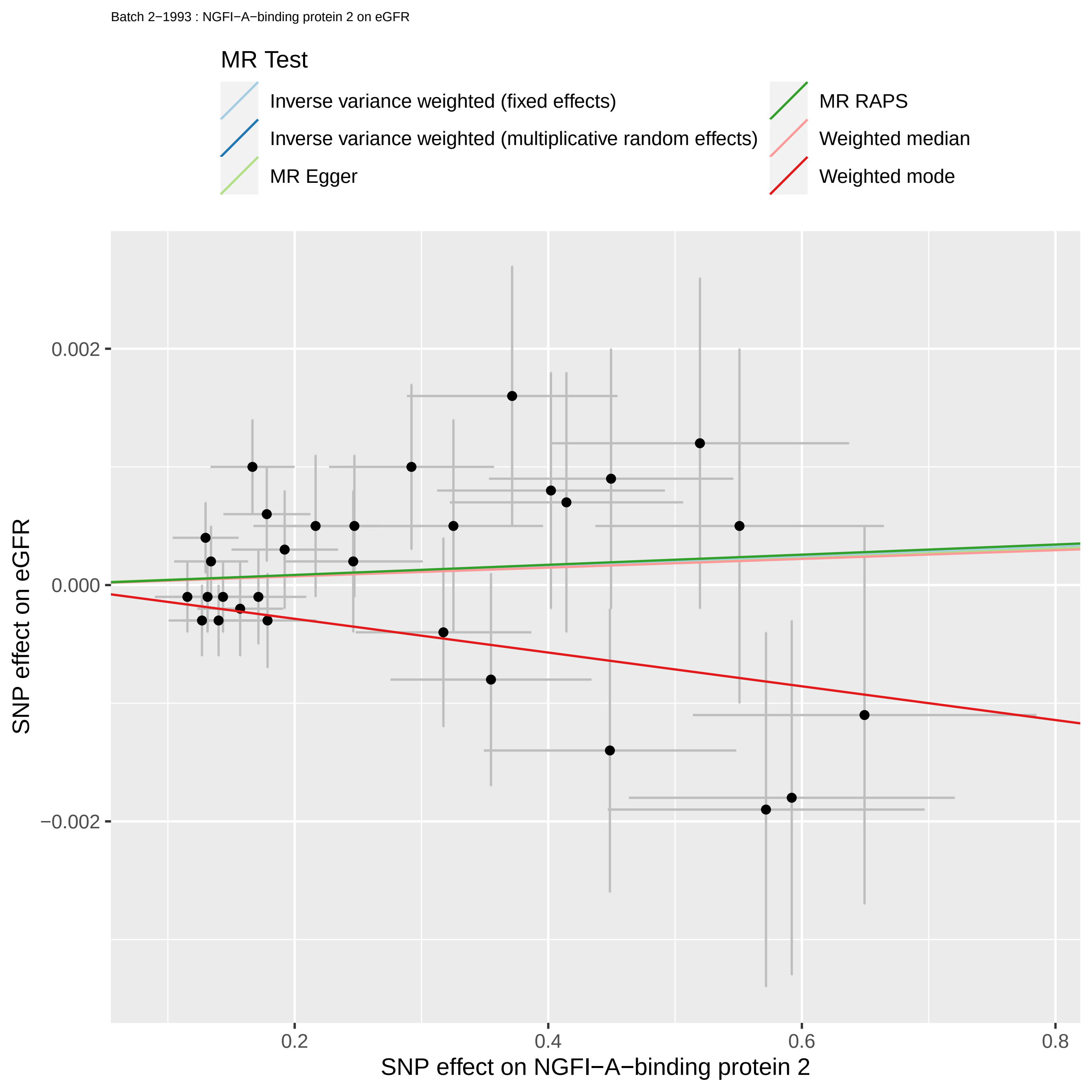
Batch 2−1993 : NGFI−A−binding protein 2 on eGFR
MR Test
Inverse variance weighted (fixed effects)
Inverse variance weighted (multiplicative random effects)
MR Egger
MR RAPS
Weighted median
Weighted mode
0.002
0.000
−0.002
0.2
0.4
0.6
0.8
SNP effect on eGFR
SNP effect on NGFI−A−binding protein 2

Batch 2−1994 : NAD kinase on eGFR

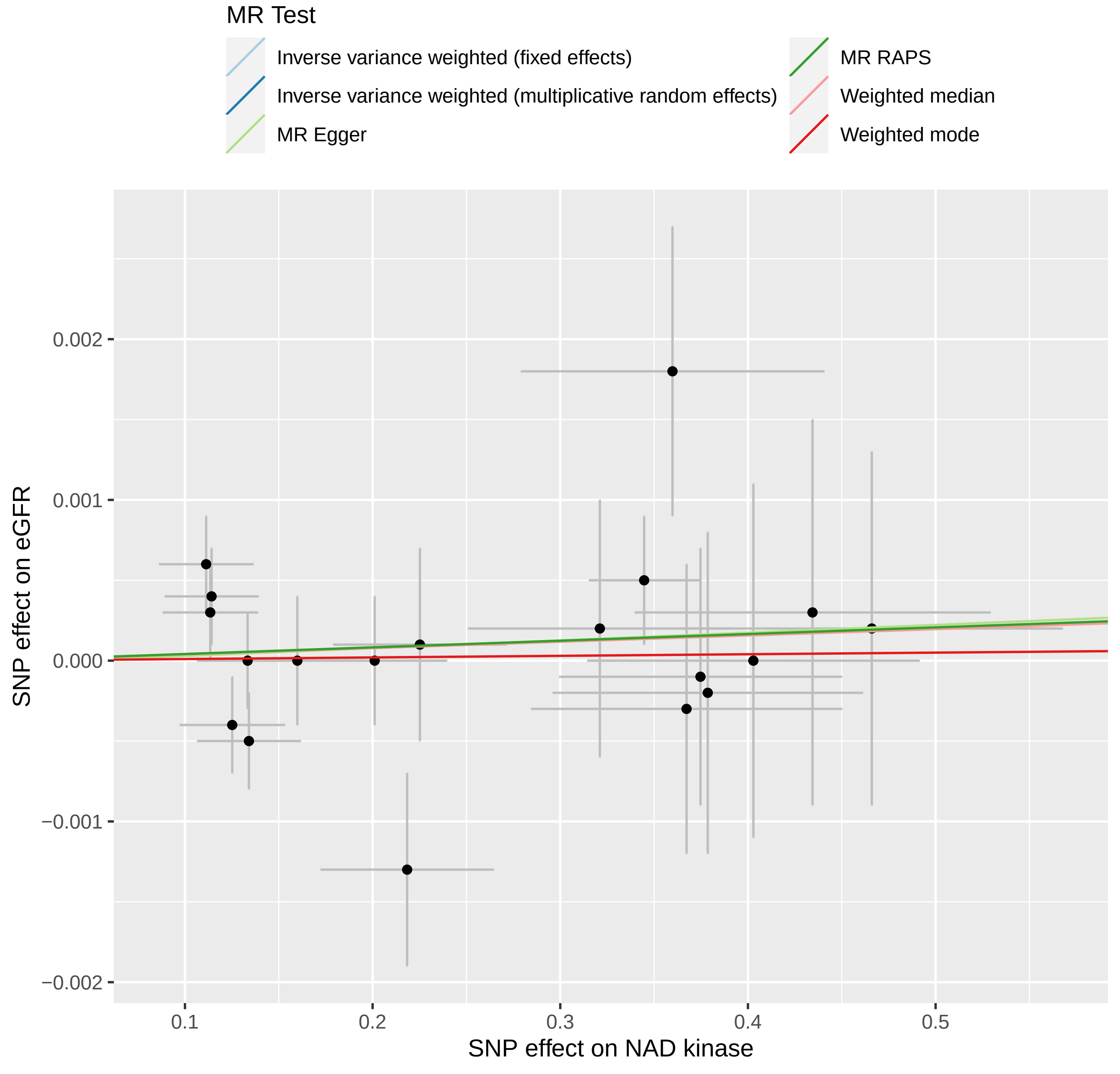

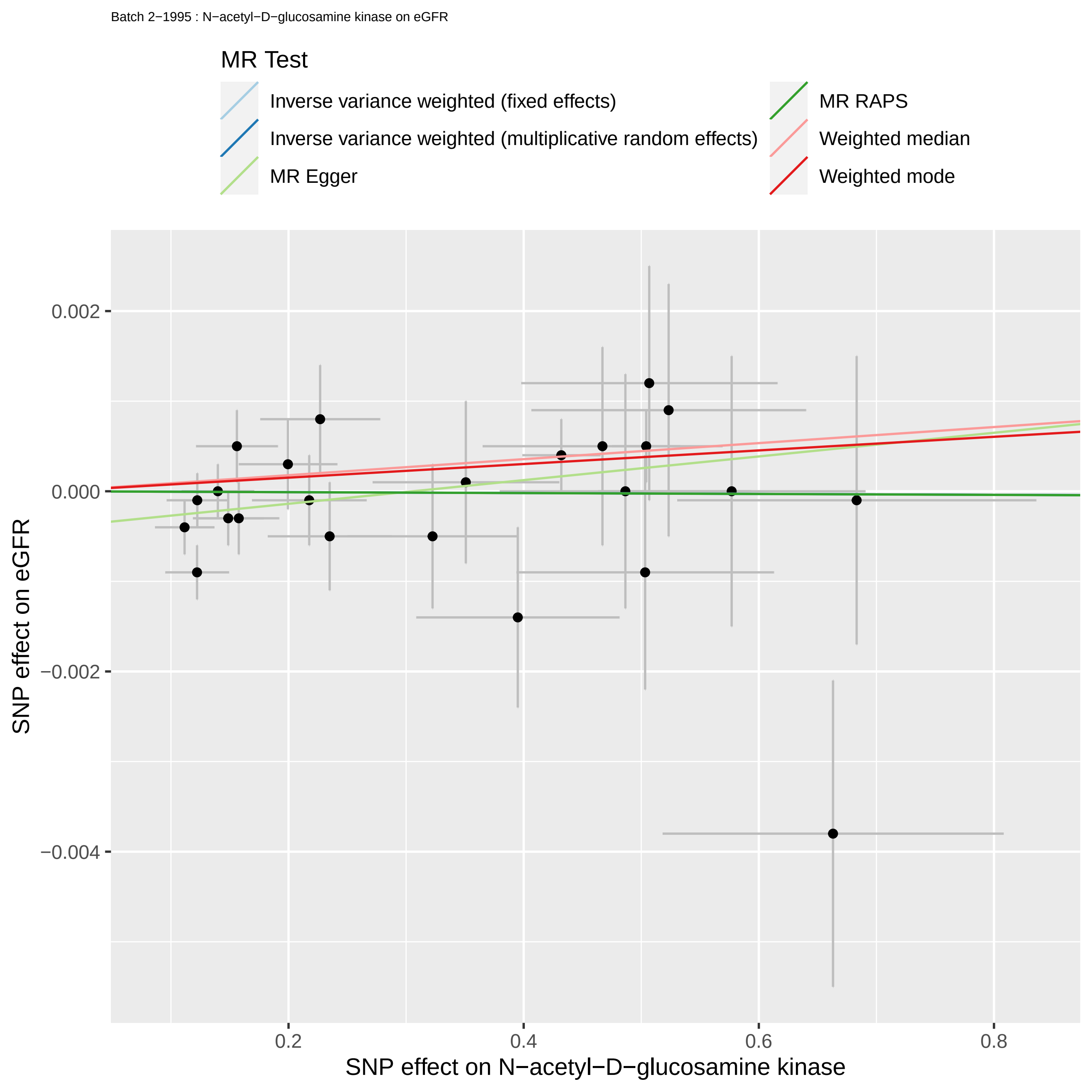

Batch 2−1995 : N−acetyl−D−glucosamine kinase on eGFR
MR Test
Inverse variance weighted (fixed effects)
Inverse variance weighted (multiplicative random effects)
MR Egger
MR RAPS
Weighted median
Weighted mode
0.002
0.000
−0.002
−0.004
0.2
0.4
0.6
0.8
SNP effect on eGFR
SNP effect on N−acetyl−D−glucosamine kinase

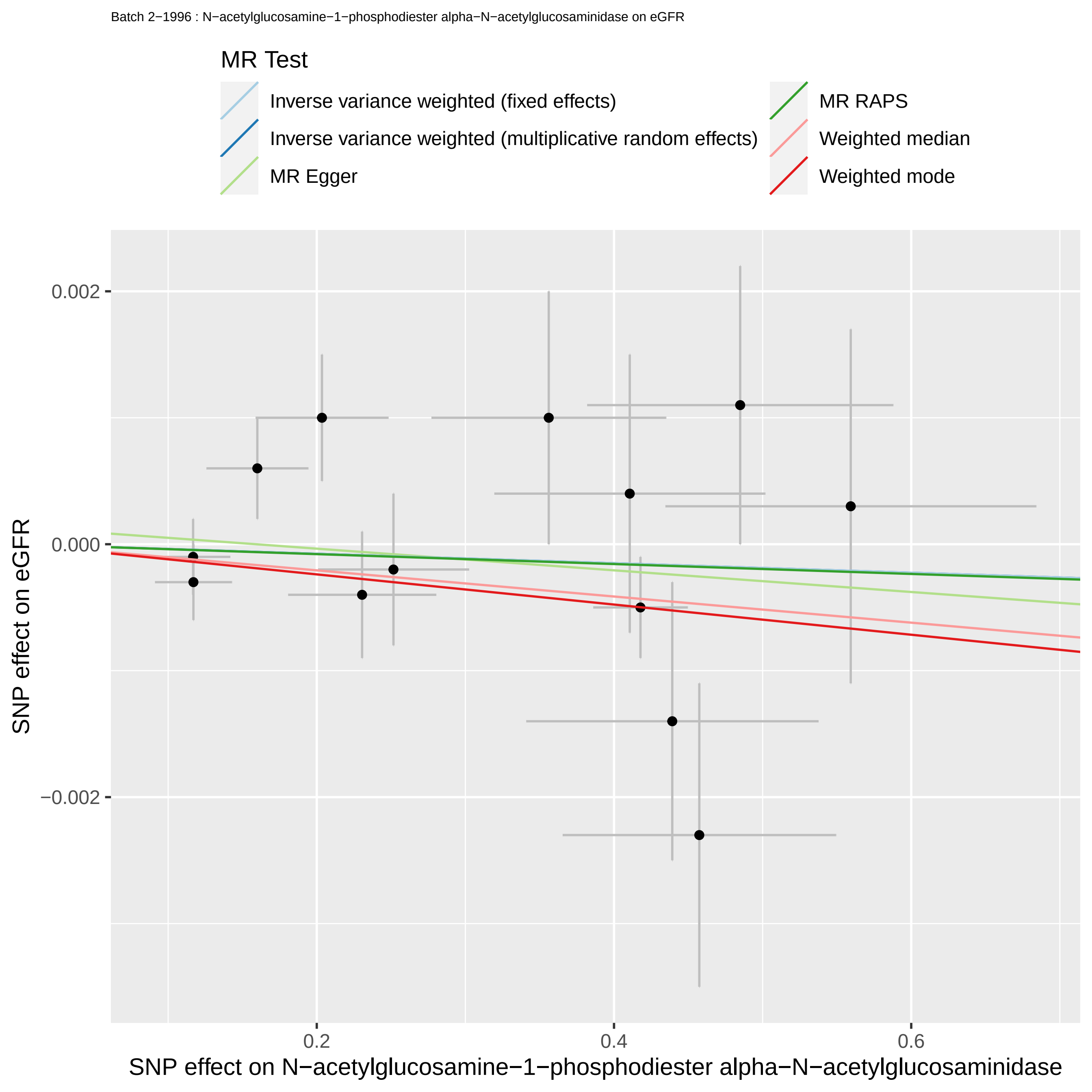

Batch 2−1996 : N−acetylglucosamine−1−phosphodiester alpha−N−acetylglucosaminidase on eGFR
MR Test
Inverse variance weighted (fixed effects)
Inverse variance weighted (multiplicative random effects)
MR Egger
MR RAPS
Weighted median
Weighted mode
0.002
0.000
−0.002
0.2
0.4
0.6
SNP effect on eGFR
SNP effect on N−acetylglucosamine−1−phosphodiester alpha−N−acetylglucosaminidase

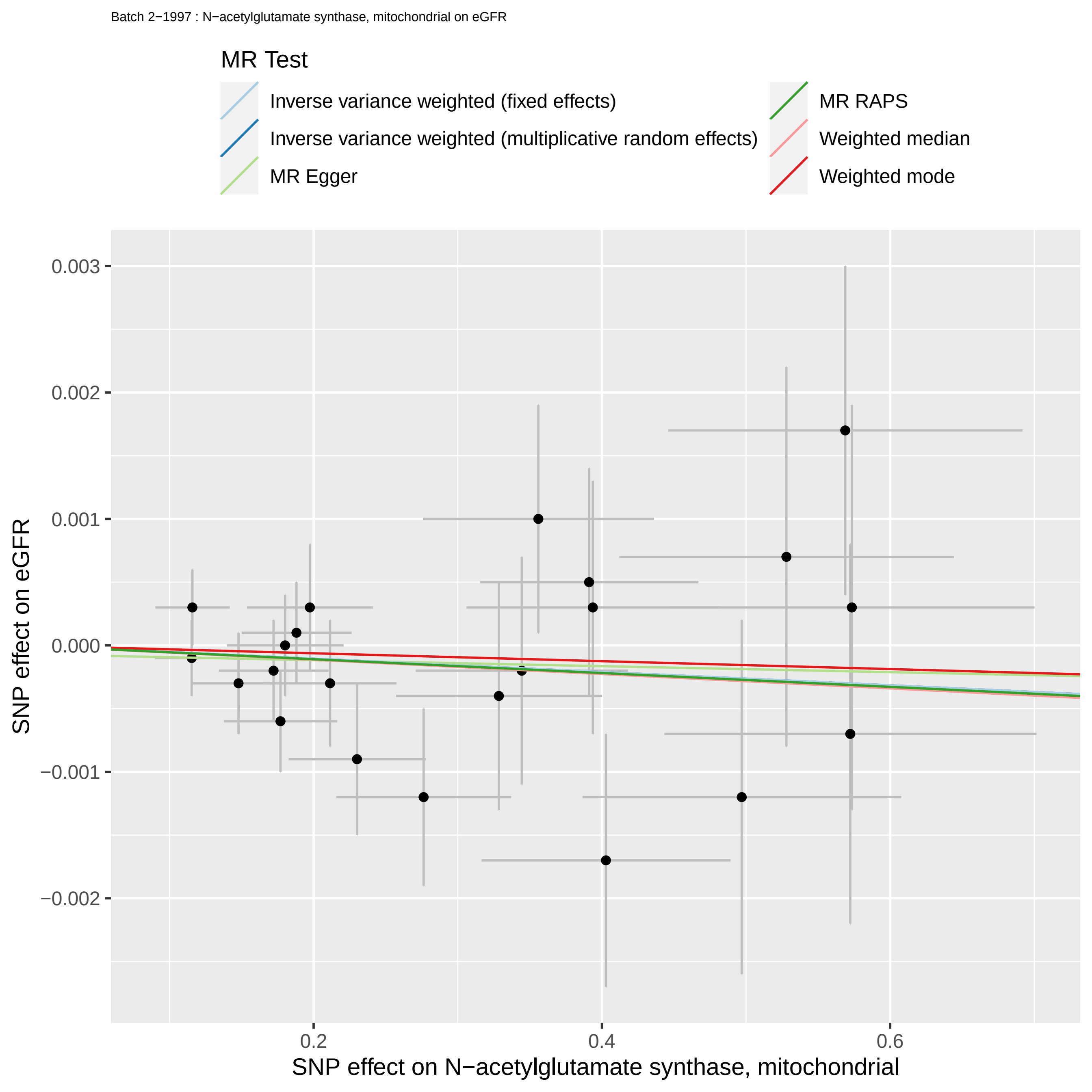
Batch 2−1997 : N−acetylglutamate synthase, mitochondrial on eGFR
MR Test
Inverse variance weighted (fixed effects)
Inverse variance weighted (multiplicative random effects)
MR Egger
MR RAPS
Weighted median
Weighted mode
0.003
0.002
0.001
0.000
−0.001
−0.002
0.2
0.4
0.6
SNP effect on eGFR
SNP effect on N−acetylglutamate synthase, mitochondrial

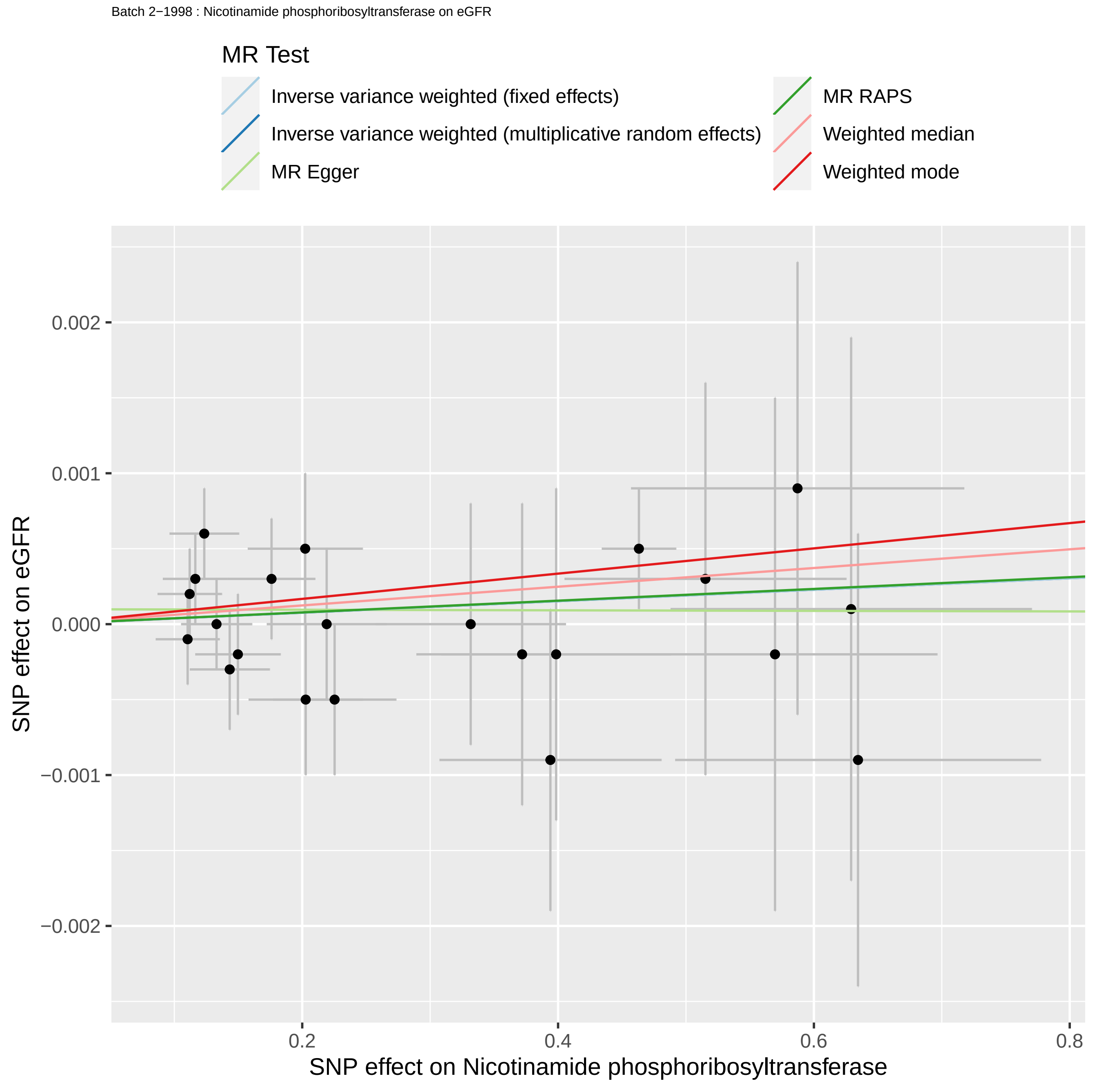

Batch 2−1998 : Nicotinamide phosphoribosyltransferase on eGFR
MR Test
Inverse variance weighted (fixed effects)
Inverse variance weighted (multiplicative random effects)
MR Egger
MR RAPS
Weighted median
Weighted mode
0.002
0.001
0.000
−0.001
−0.002
SNP effect on eGFR
0.2
0.4
0.6
0.8
SNP effect on Nicotinamide phosphoribosyltransferase

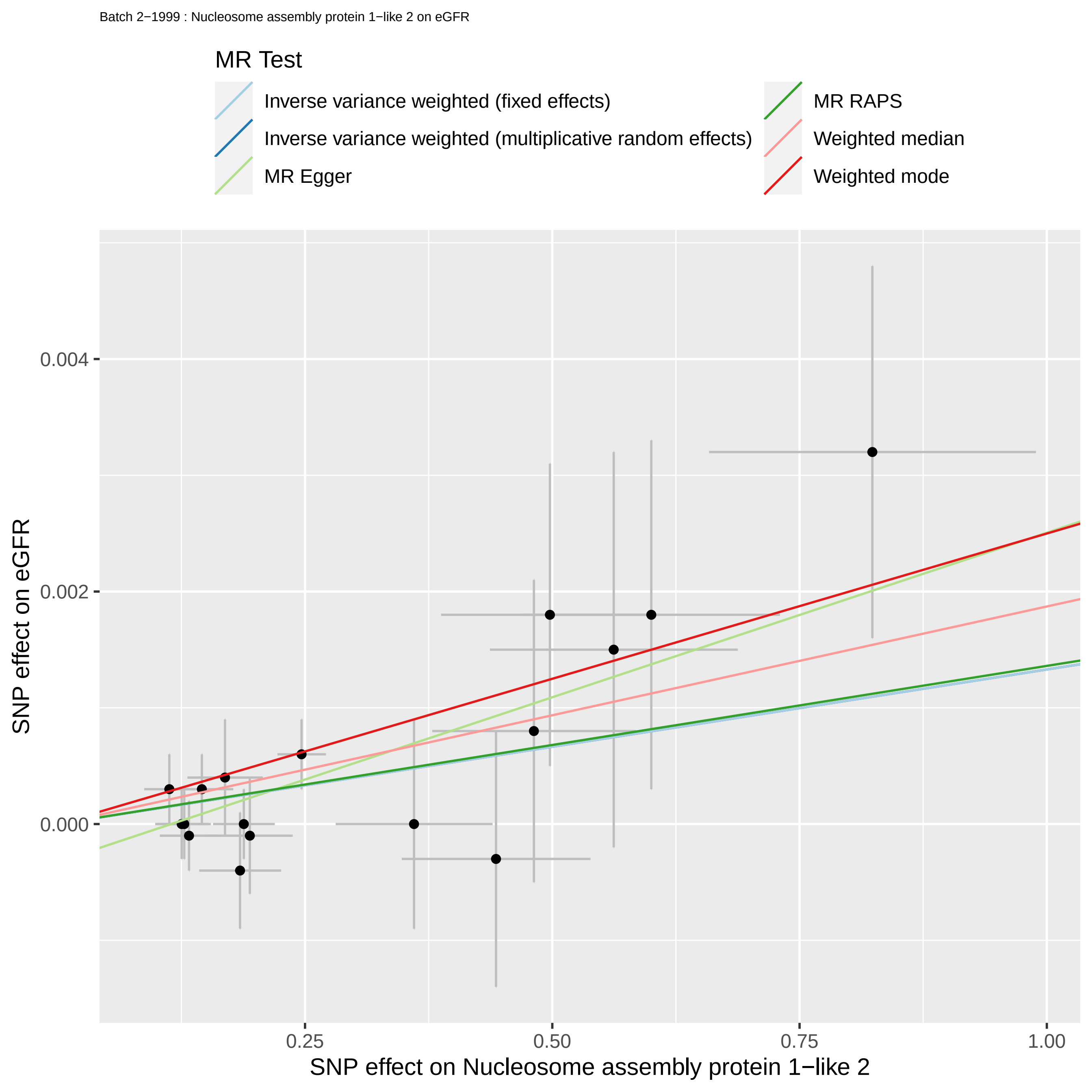

Batch 2−1999 : Nucleosome assembly protein 1−like 2 on eGFR
MR Test
Inverse variance weighted (fixed effects)
Inverse variance weighted (multiplicative random effects)
MR Egger
MR RAPS
Weighted median
Weighted mode
0.004
0.002
0.000
0.25
0.50
0.75
1.00
SNP effect on eGFR
SNP effect on Nucleosome assembly protein 1−like 2

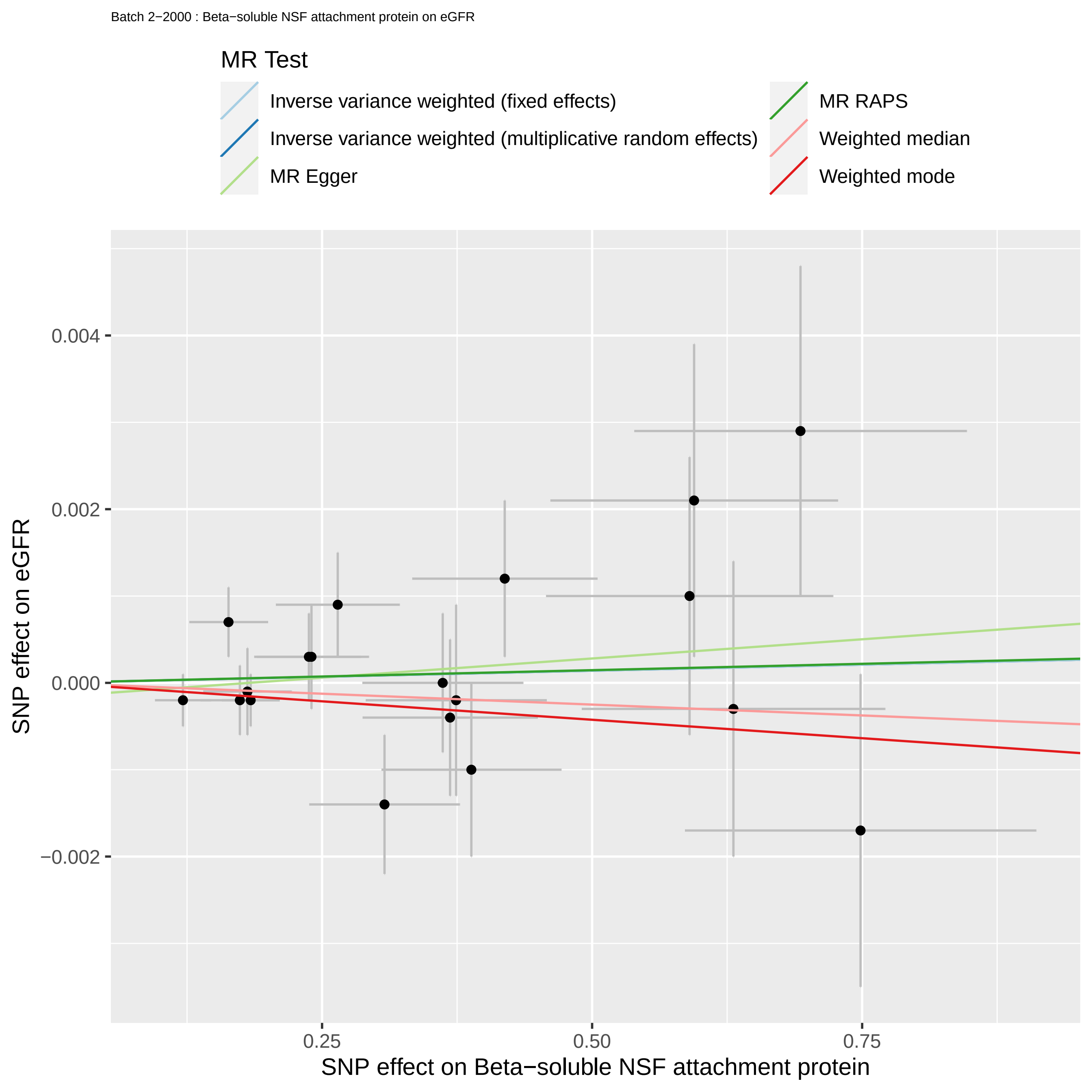

Batch 2−2000 : Beta−soluble NSF attachment protein on eGFR
MR Test
Inverse variance weighted (fixed effects)
Inverse variance weighted (multiplicative random effects)
MR Egger
MR RAPS
Weighted median
Weighted mode
0.004
0.002
0.000
−0.002
SNP effect on eGFR
0.25
0.50
0.75
SNP effect on Beta−soluble NSF attachment protein

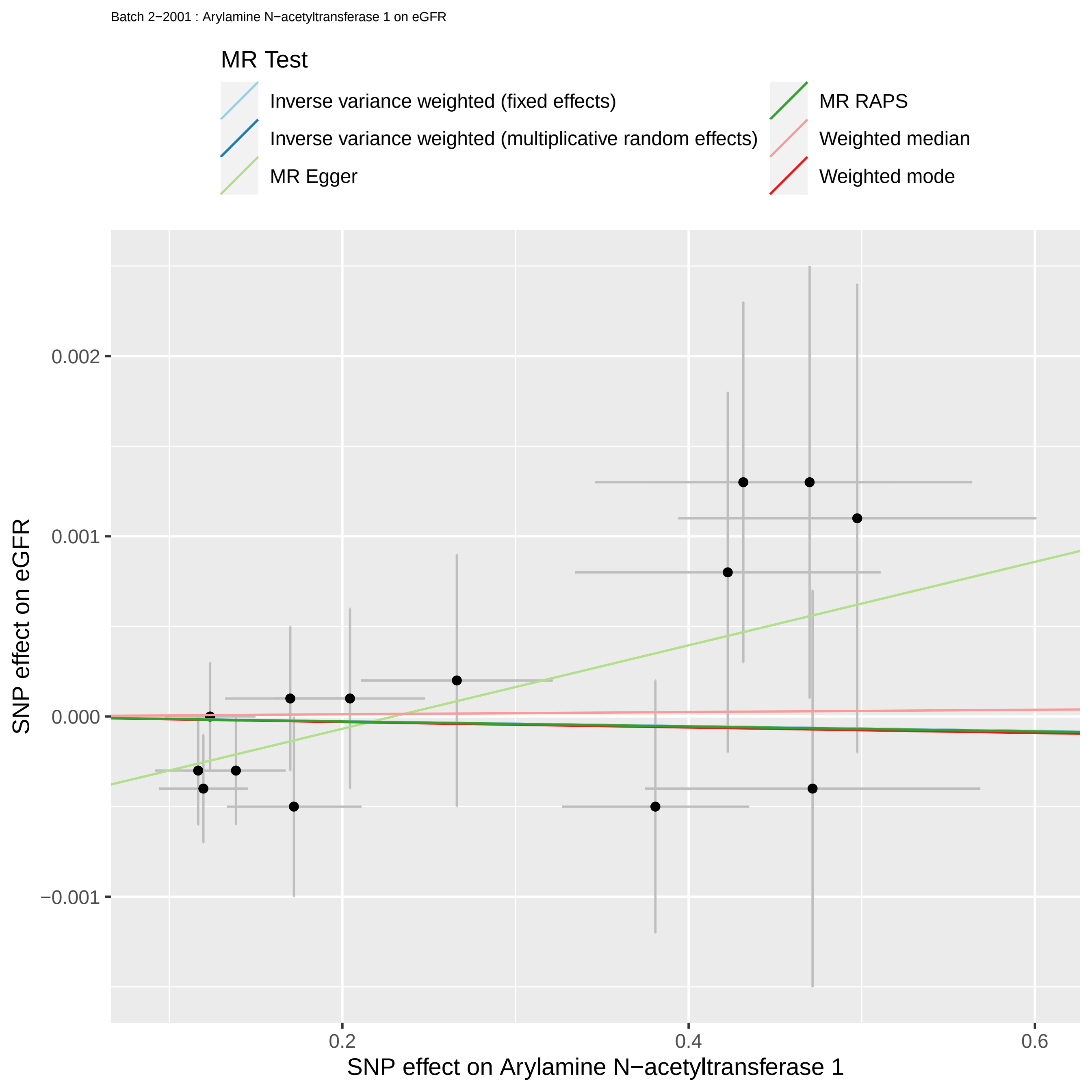

Batch 2−2001 : Arylamine N−acetyltransferase 1 on eGFR
MR Test
Inverse variance weighted (fixed effects)
Inverse variance weighted (multiplicative random effects)
MR Egger
MR RAPS
Weighted median
Weighted mode
0.002
0.001
0.000
−0.001
0.2
0.4
0.6
SNP effect on eGFR
SNP effect on Arylamine N−acetyltransferase 1

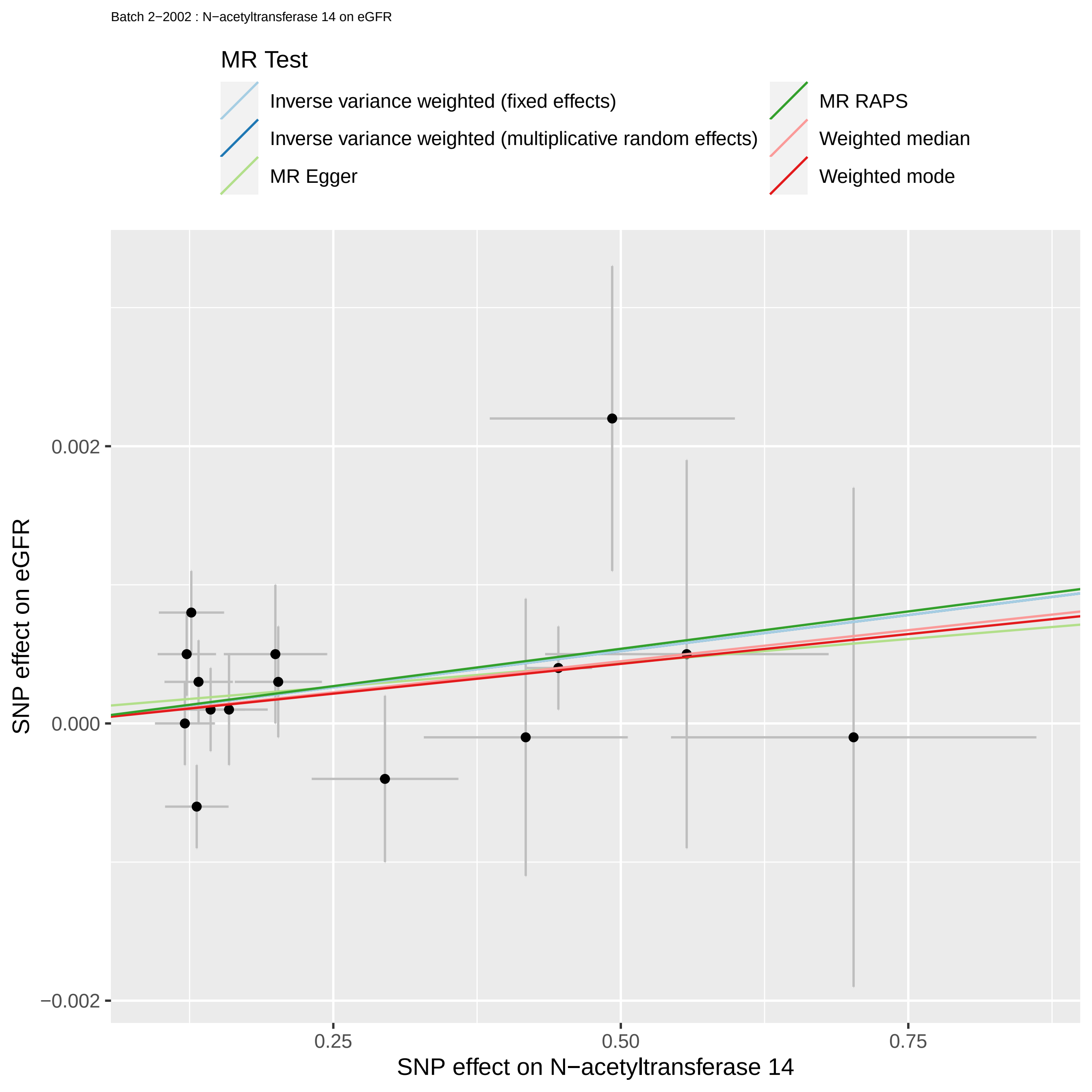
Batch 2−2002 : N−acetyltransferase 14 on eGFR
MR Test
Inverse variance weighted (fixed effects)
Inverse variance weighted (multiplicative random effects)
MR Egger
MR RAPS
Weighted median
Weighted mode
0.002
0.000
−0.002
0.25
0.50
0.75
SNP effect on eGFR
SNP effect on N−acetyltransferase 14

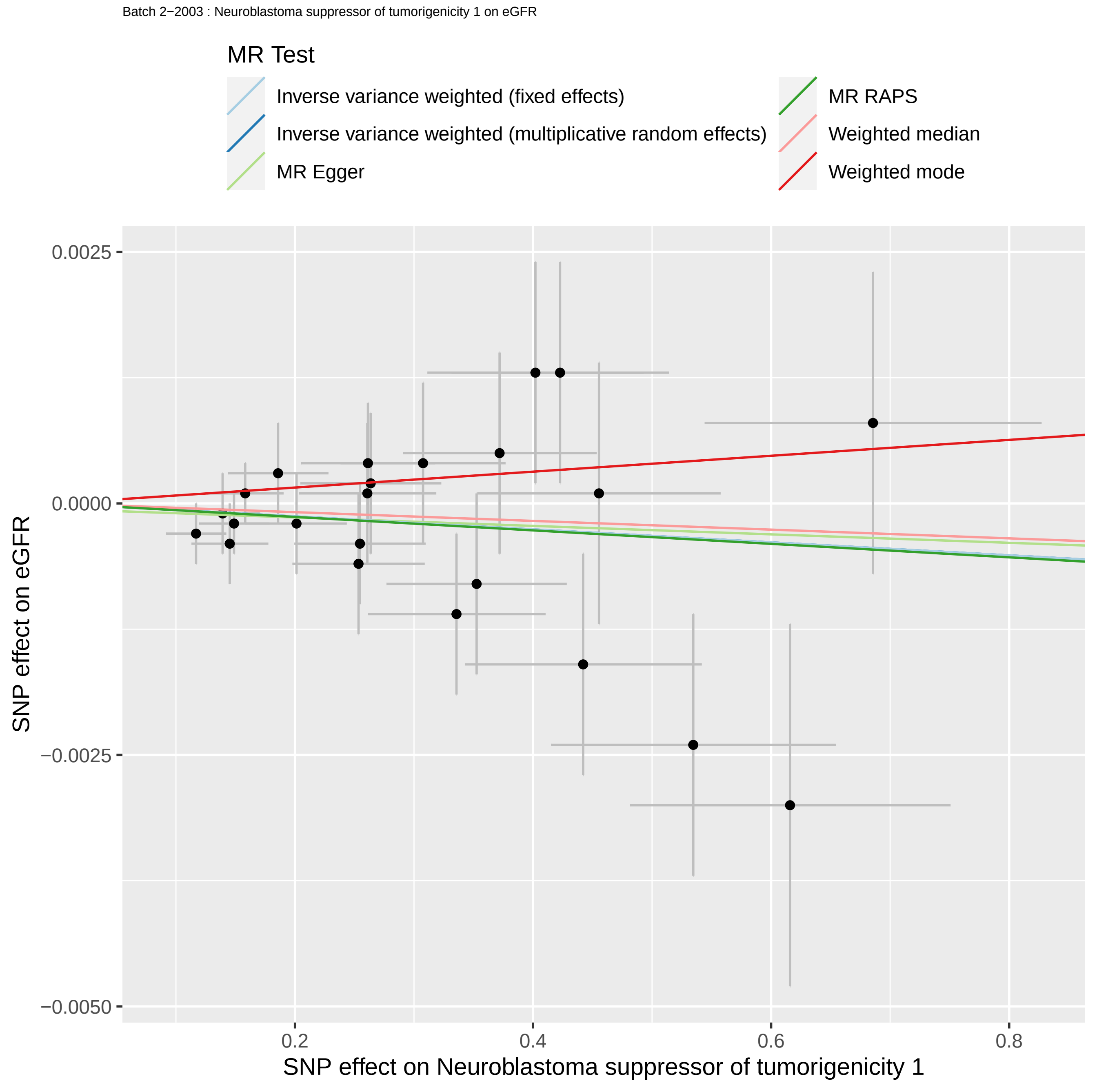
Batch 2−2003 : Neuroblastoma suppressor of tumorigenicity 1 on eGFR
MR Test
Inverse variance weighted (fixed effects)
Inverse variance weighted (multiplicative random effects)
MR Egger
MR RAPS
Weighted median
Weighted mode
0.0025
0.0000
−0.0025
−0.0050
0.2
0.4
0.6
0.8
SNP effect on eGFR
SNP effect on Neuroblastoma suppressor of tumorigenicity 1

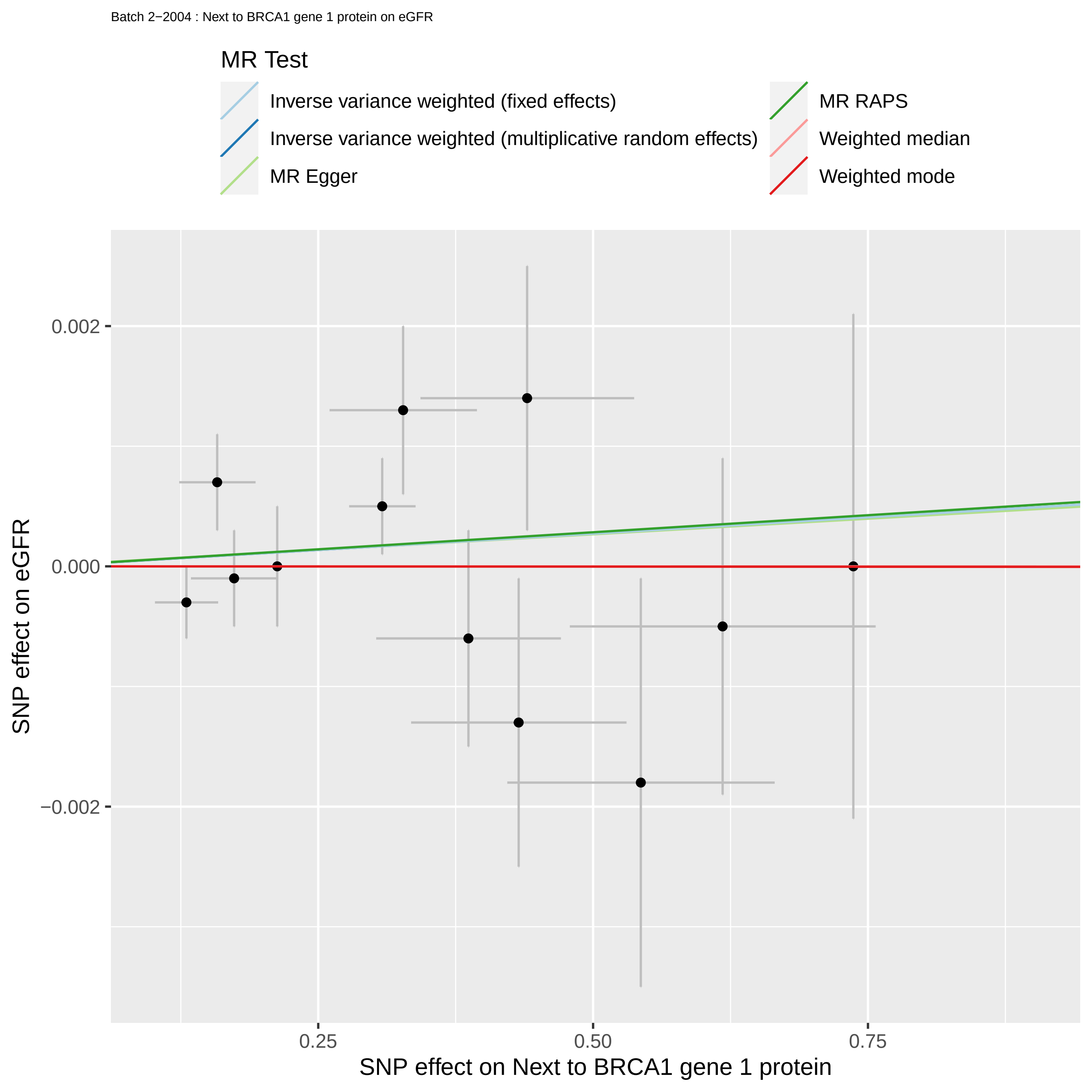

Batch 2−2004 : Next to BRCA1 gene 1 protein on eGFR
MR Test
Inverse variance weighted (fixed effects)
Inverse variance weighted (multiplicative random effects)
MR Egger
MR RAPS
Weighted median
Weighted mode
0.002
0.000
−0.002
0.25
0.50
0.75
SNP effect on eGFR
SNP effect on Next to BRCA1 gene 1 protein

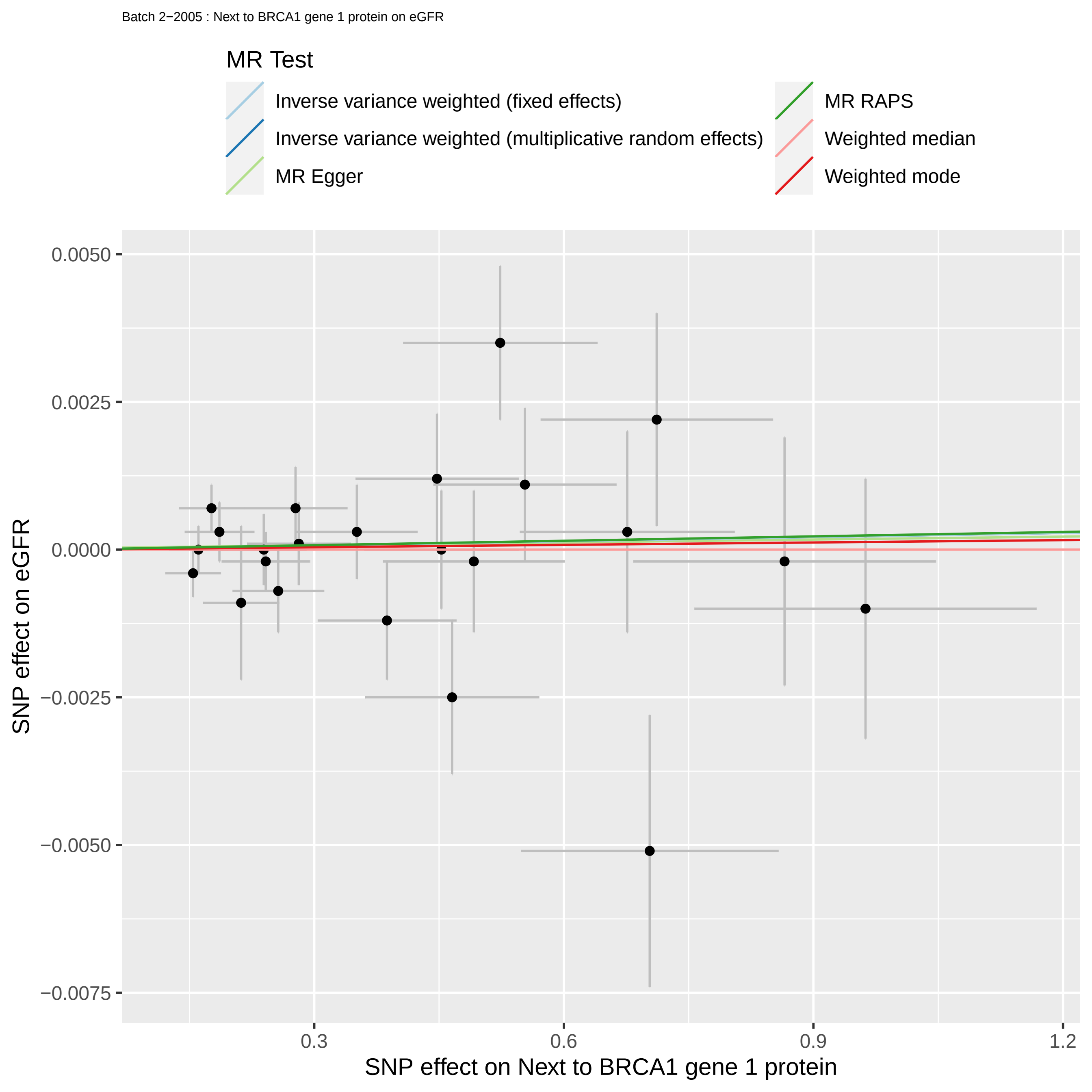

Batch 2−2005 : Next to BRCA1 gene 1 protein on eGFR
MR Test
Inverse variance weighted (fixed effects)
Inverse variance weighted (multiplicative random effects)
MR Egger
MR RAPS
Weighted median
Weighted mode
0.0050
0.0025
0.0000
−0.0025
−0.0050
−0.0075
0.3
0.6
0.9
1.2
SNP effect on eGFR
SNP effect on Next to BRCA1 gene 1 protein

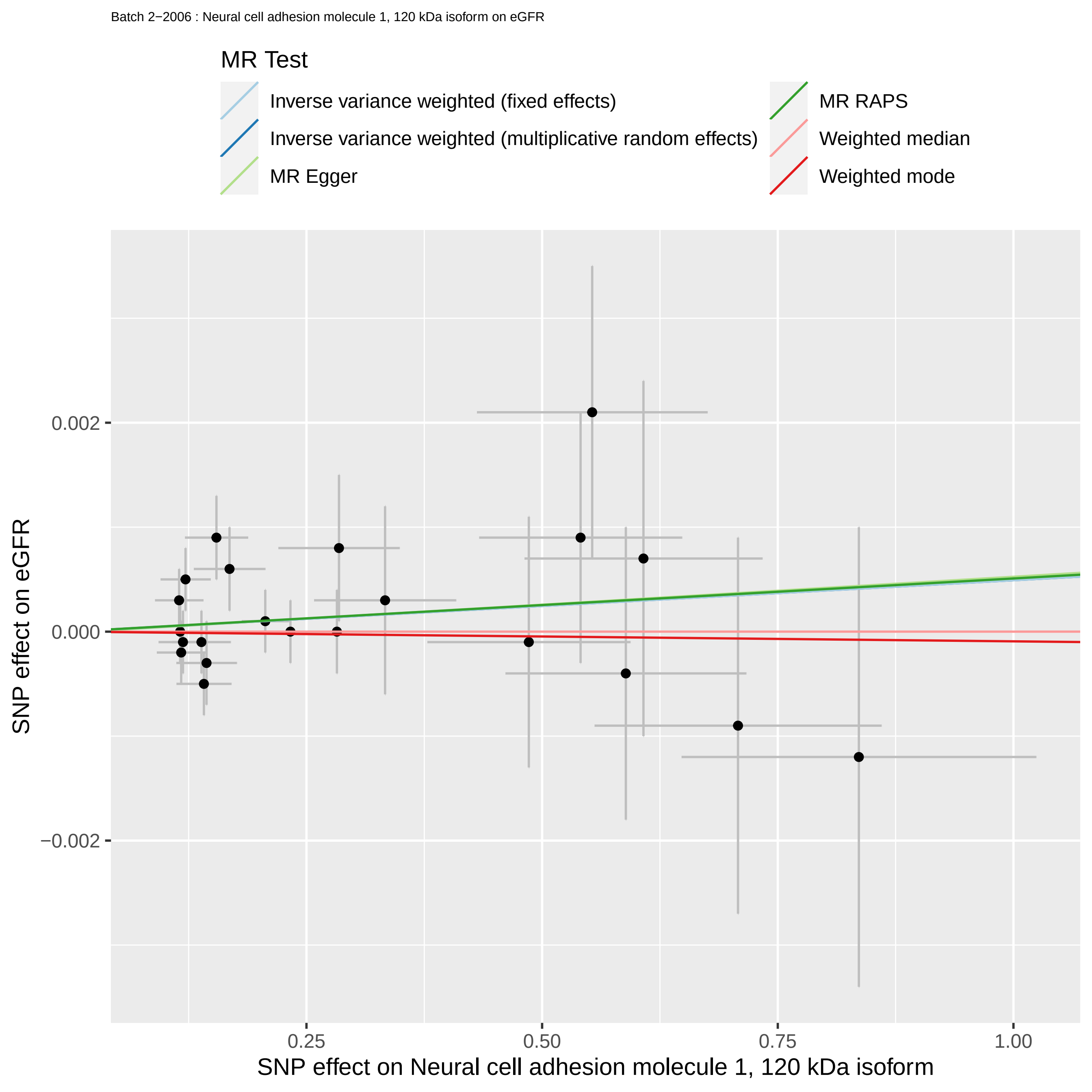

Batch 2−2006 : Neural cell adhesion molecule 1, 120 kDa isoform on eGFR
MR Test
Inverse variance weighted (fixed effects)
Inverse variance weighted (multiplicative random effects)
MR Egger
MR RAPS
Weighted median
Weighted mode
0.002
0.000
−0.002
0.25
0.50
0.75
1.00
SNP effect on eGFR
SNP effect on Neural cell adhesion molecule 1, 120 kDa isoform

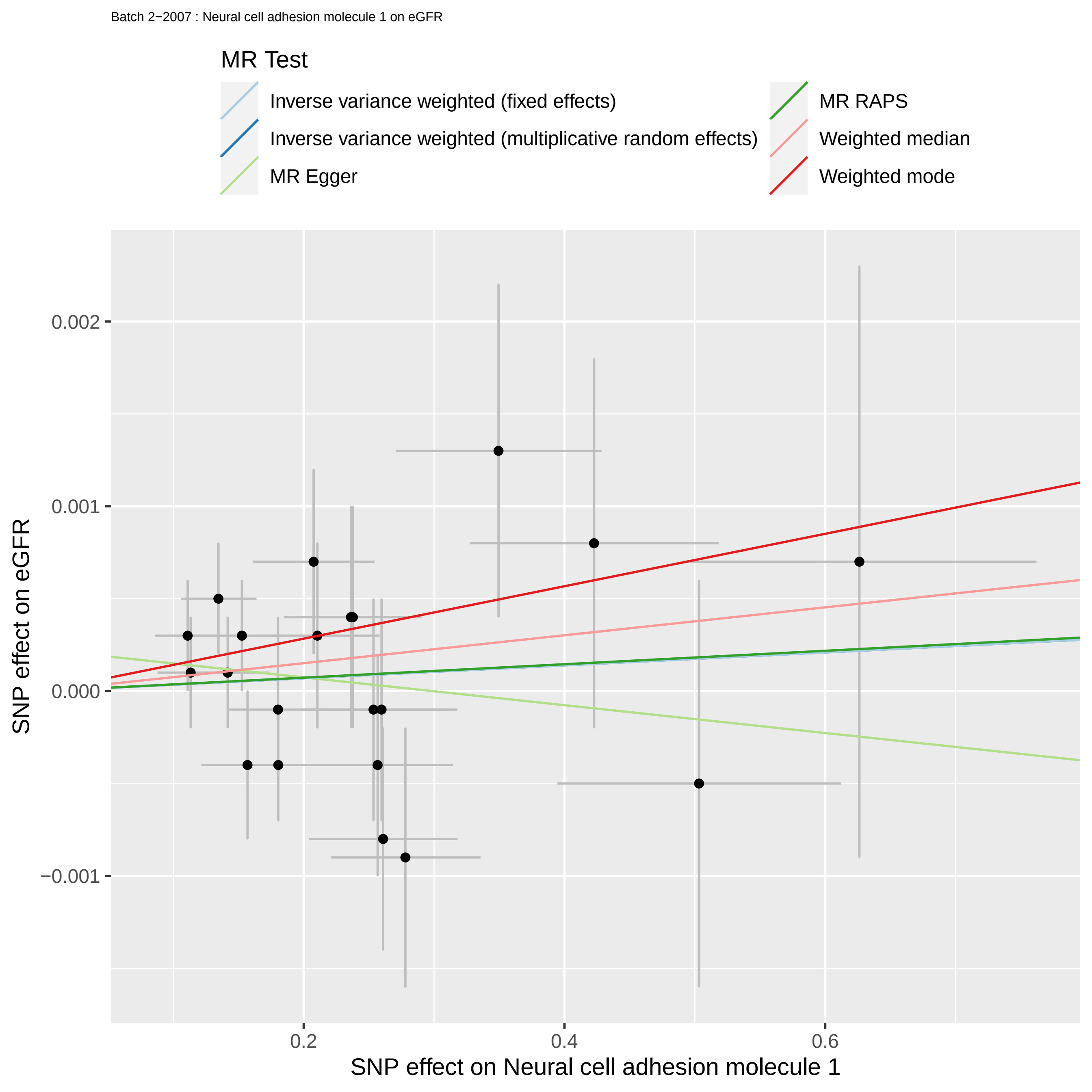

Batch 2−2007 : Neural cell adhesion molecule 1 on eGFR
MR Test
Inverse variance weighted (fixed effects)
Inverse variance weighted (multiplicative random effects)
MR Egger
MR RAPS
Weighted median
Weighted mode
0.002
0.001
0.000
−0.001
0.2
0.4
0.6
SNP effect on eGFR
SNP effect on Neural cell adhesion molecule 1

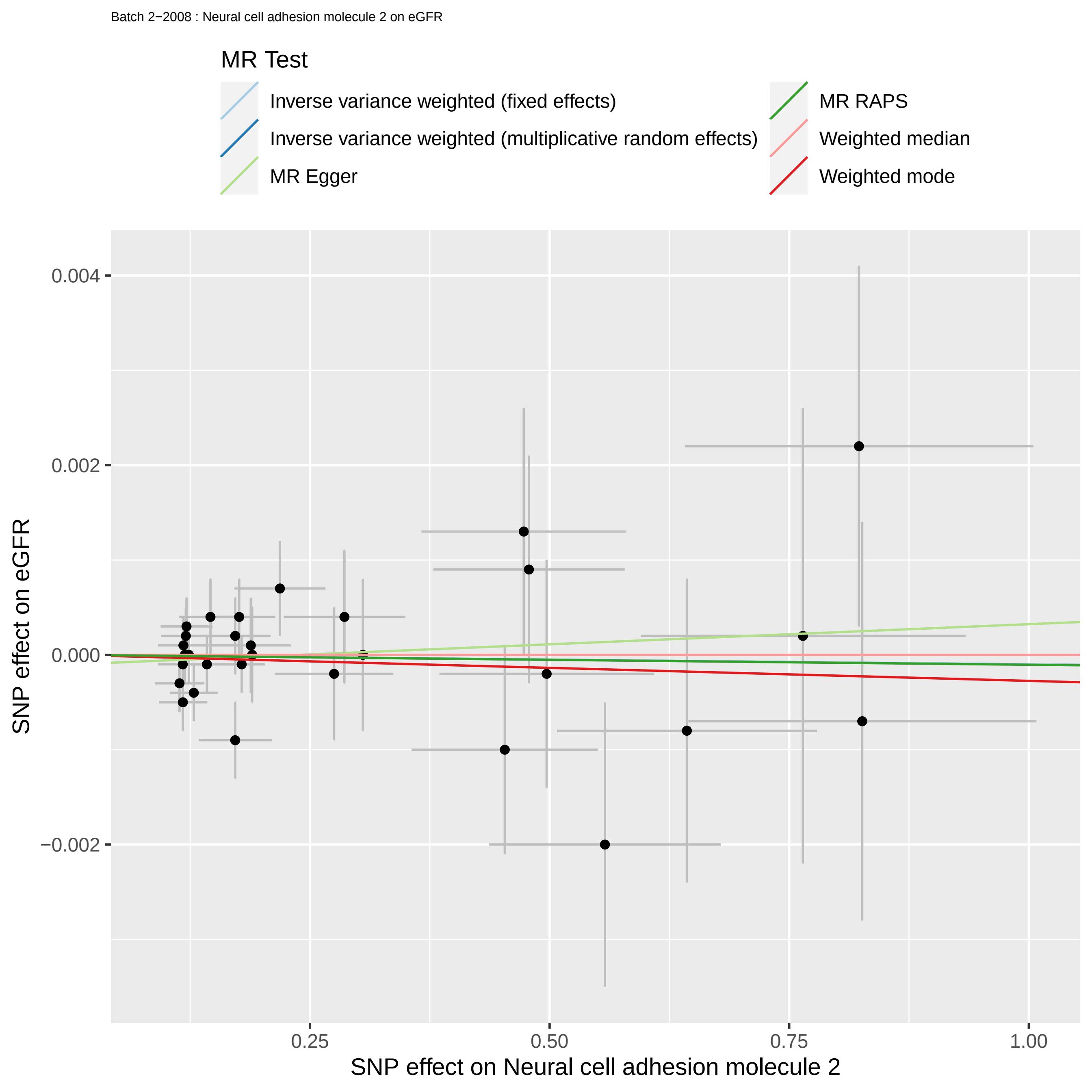
Batch 2−2008 : Neural cell adhesion molecule 2 on eGFR
MR Test
Inverse variance weighted (fixed effects)
Inverse variance weighted (multiplicative random effects)
MR Egger
MR RAPS
Weighted median
Weighted mode
0.004
0.002
0.000
−0.002
0.25
0.50
0.75
1.00
SNP effect on eGFR
SNP effect on Neural cell adhesion molecule 2

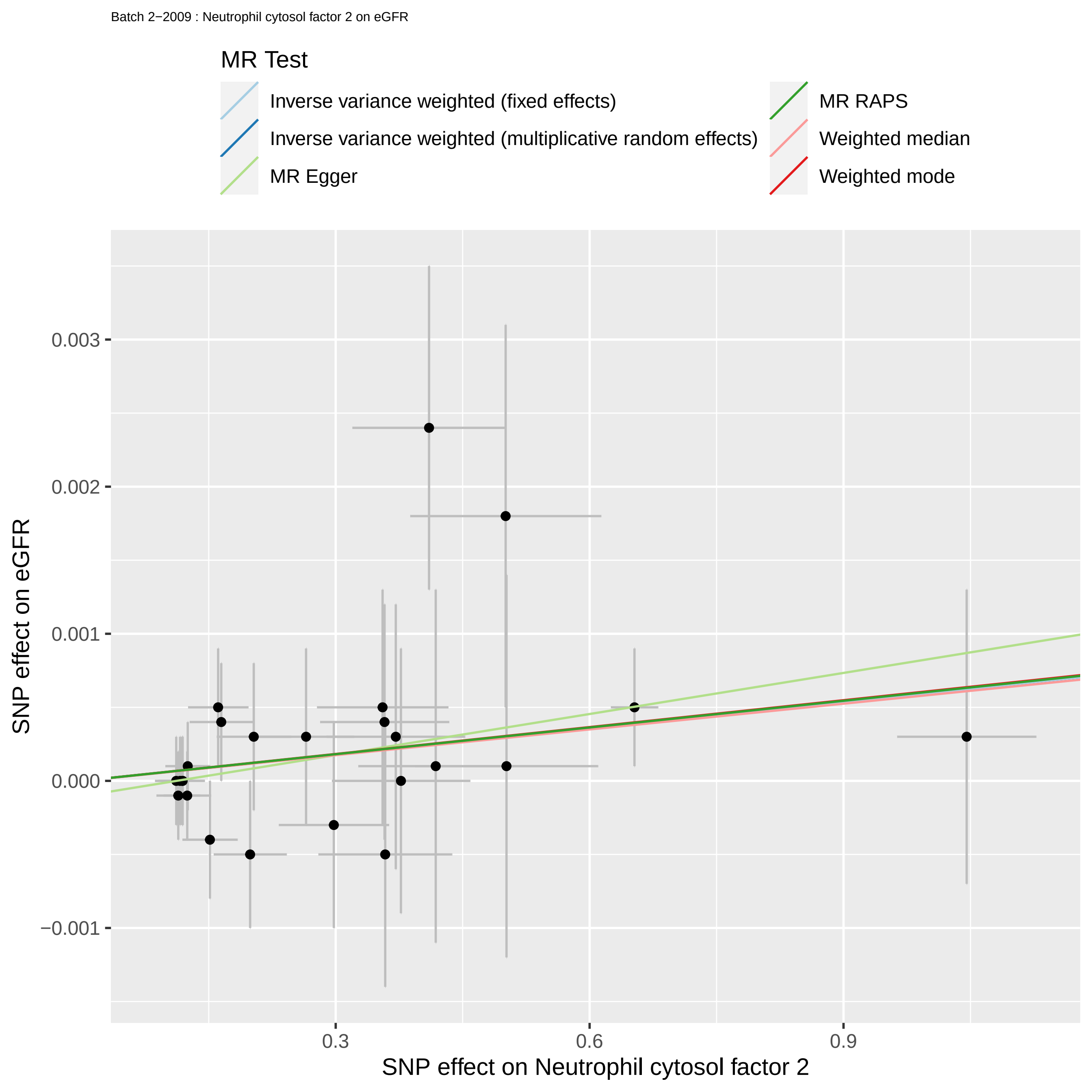
Batch 2−2009 : Neutrophil cytosol factor 2 on eGFR
MR Test
Inverse variance weighted (fixed effects)
Inverse variance weighted (multiplicative random effects)
MR Egger
MR RAPS
Weighted median
Weighted mode
0.003
0.002
0.001
0.000
−0.001
0.3
0.6
0.9
SNP effect on eGFR
SNP effect on Neutrophil cytosol factor 2

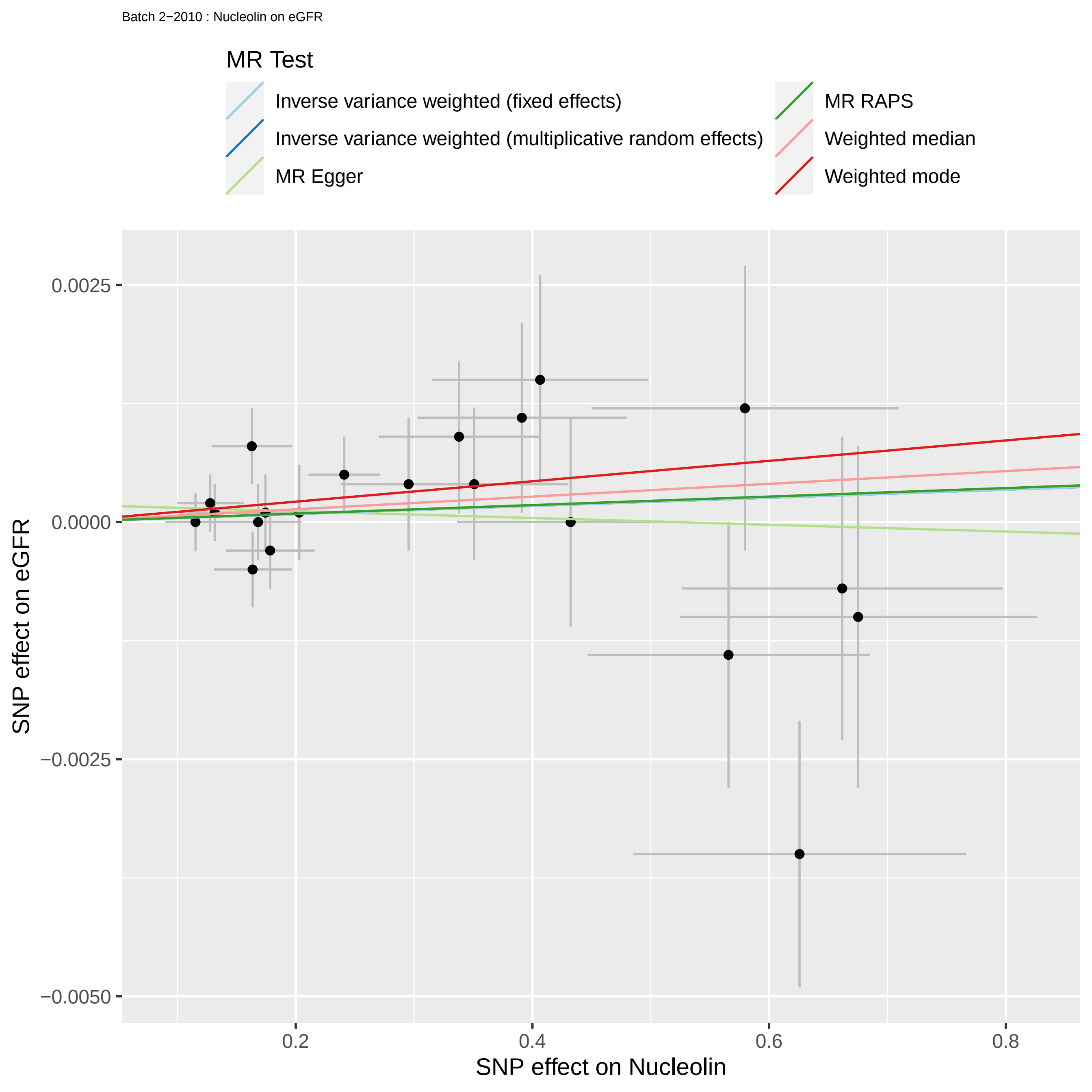

Batch 2−2010 : Nucleolin on eGFR
MR Test
Inverse variance weighted (fixed effects)
Inverse variance weighted (multiplicative random effects)
MR Egger
MR RAPS
Weighted median
Weighted mode
0.0025
0.0000
−0.0025
−0.0050
SNP effect on eGFR
0.2
0.4
0.6
0.8
SNP effect on Nucleolin

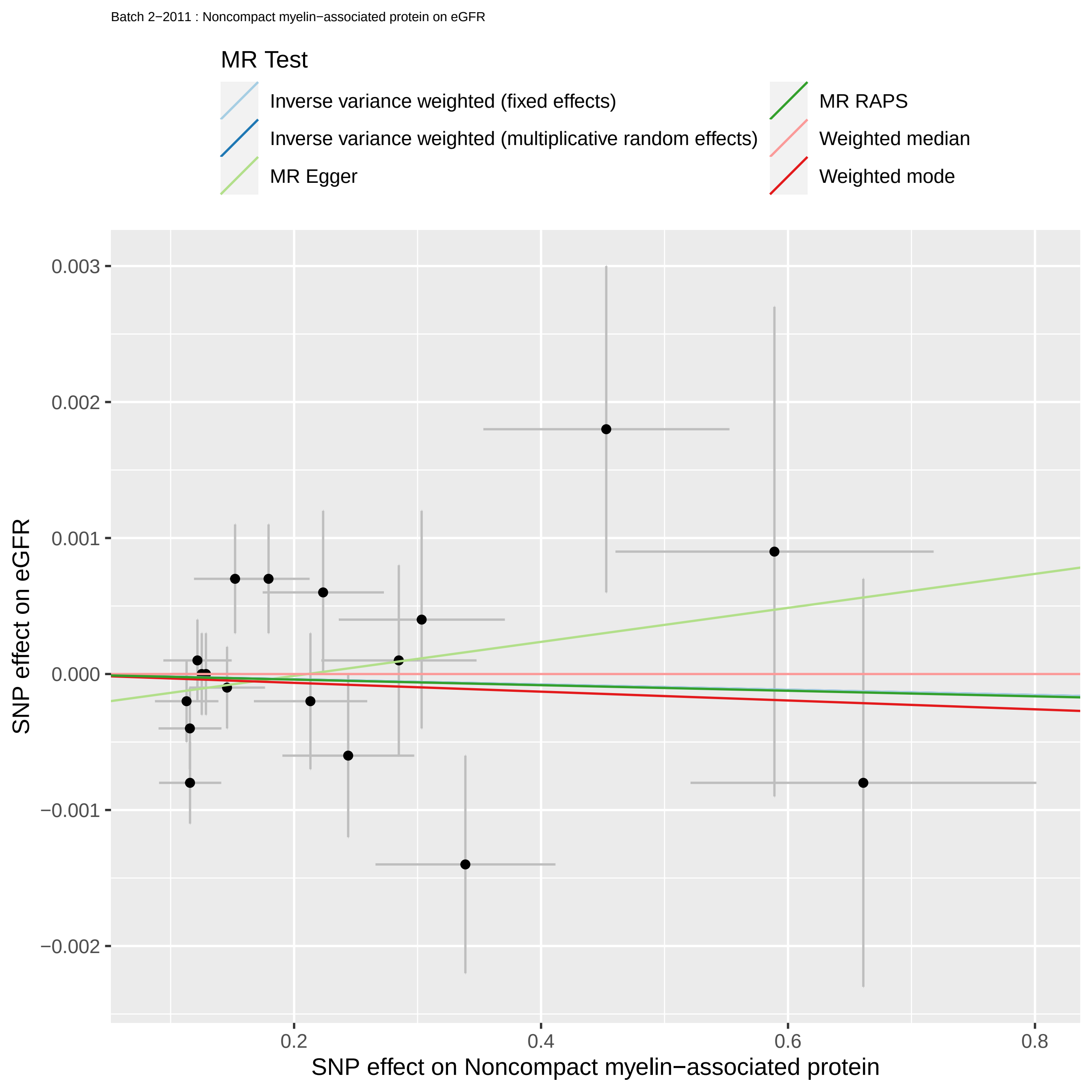

Batch 2−2011 : Noncompact myelin−associated protein on eGFR
MR Test
Inverse variance weighted (fixed effects)
Inverse variance weighted (multiplicative random effects)
MR Egger
MR RAPS
Weighted median
Weighted mode
SNP effect on eGFR
0.003
0.002
0.001
0.000
−0.001
−0.002
0.2
0.4
0.6
0.8
SNP effect on Noncompact myelin−associated protein

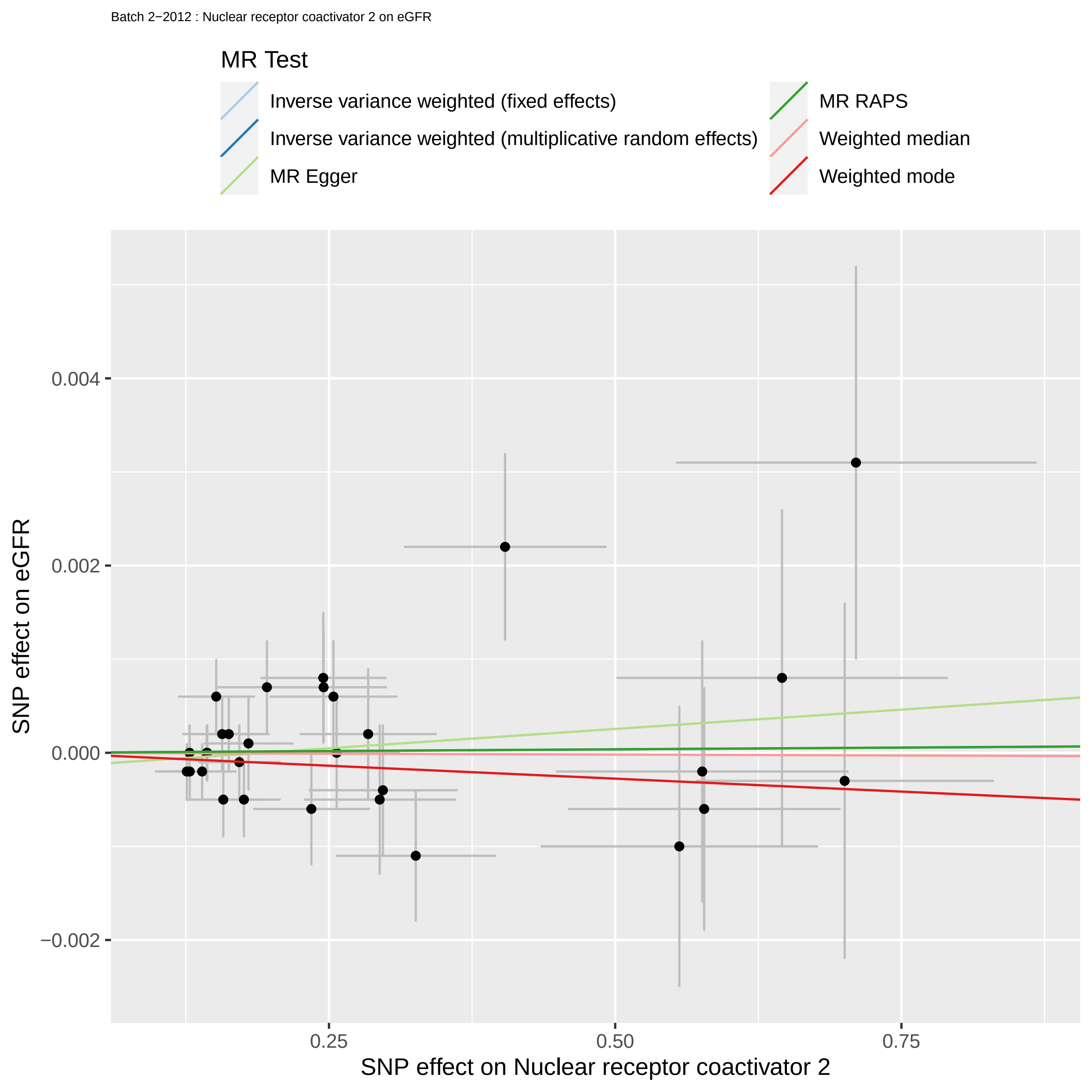

Batch 2−2012 : Nuclear receptor coactivator 2 on eGFR
MR Test
Inverse variance weighted (fixed effects)
Inverse variance weighted (multiplicative random effects)
MR Egger
MR RAPS
Weighted median
Weighted mode
SNP effect on eGFR
0.004
0.002
0.000
−0.002
0.25
0.50
0.75
SNP effect on Nuclear receptor coactivator 2

Batch 2−2013 : Natural cytotoxicity triggering receptor 1 on eGFR

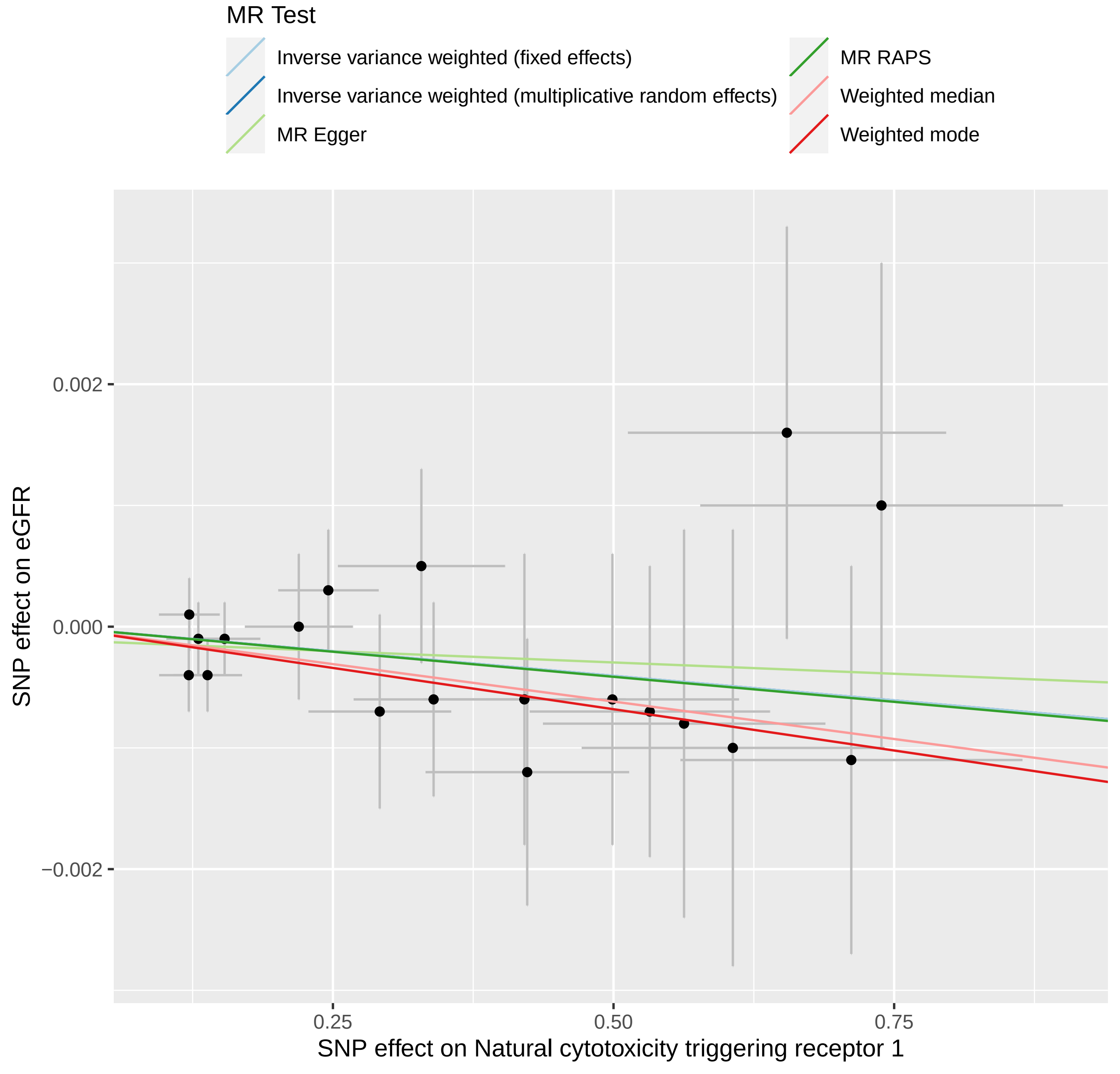

Batch 2−2014 : Natural cytotoxicity triggering receptor 1 on eGFR

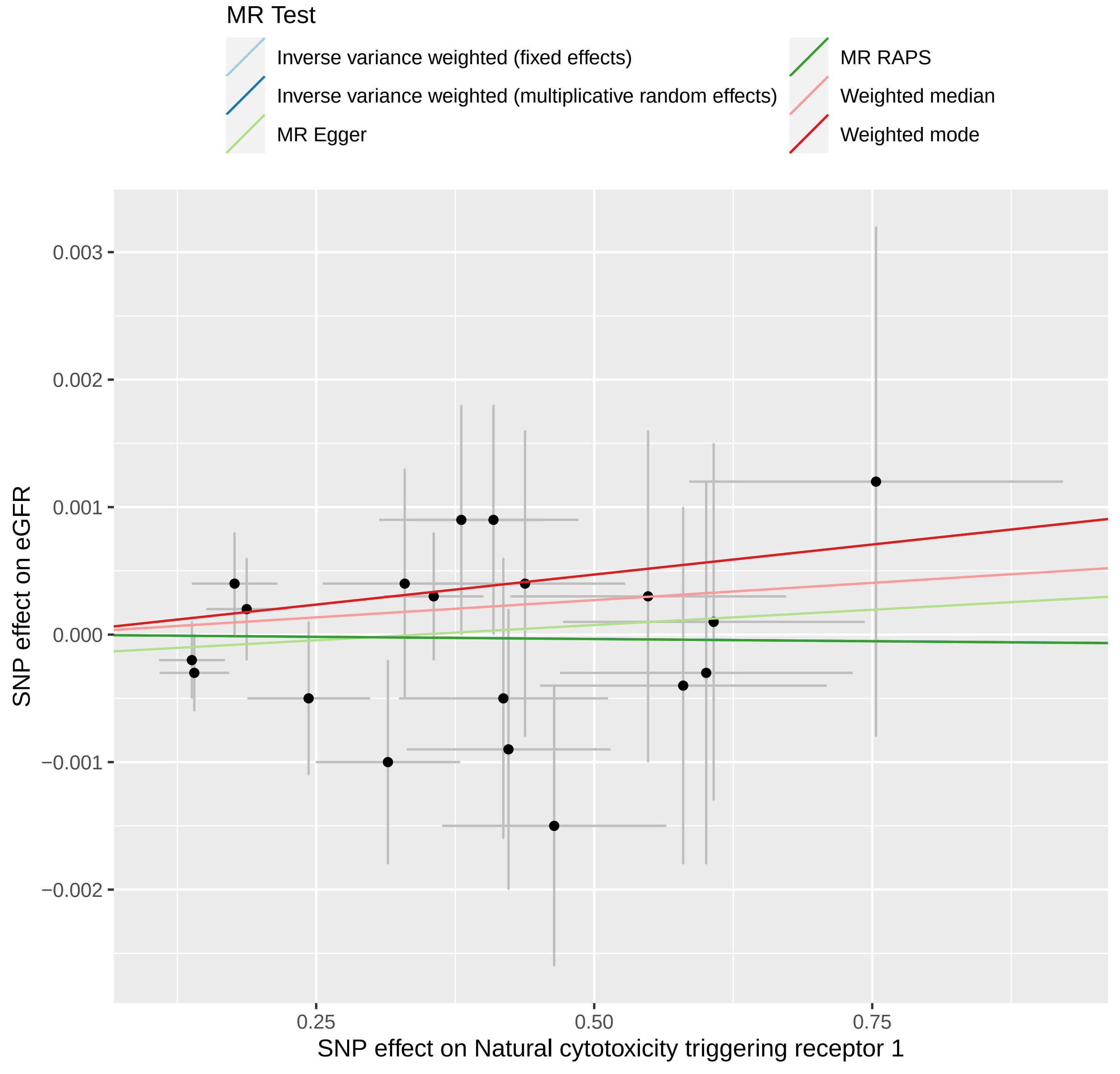

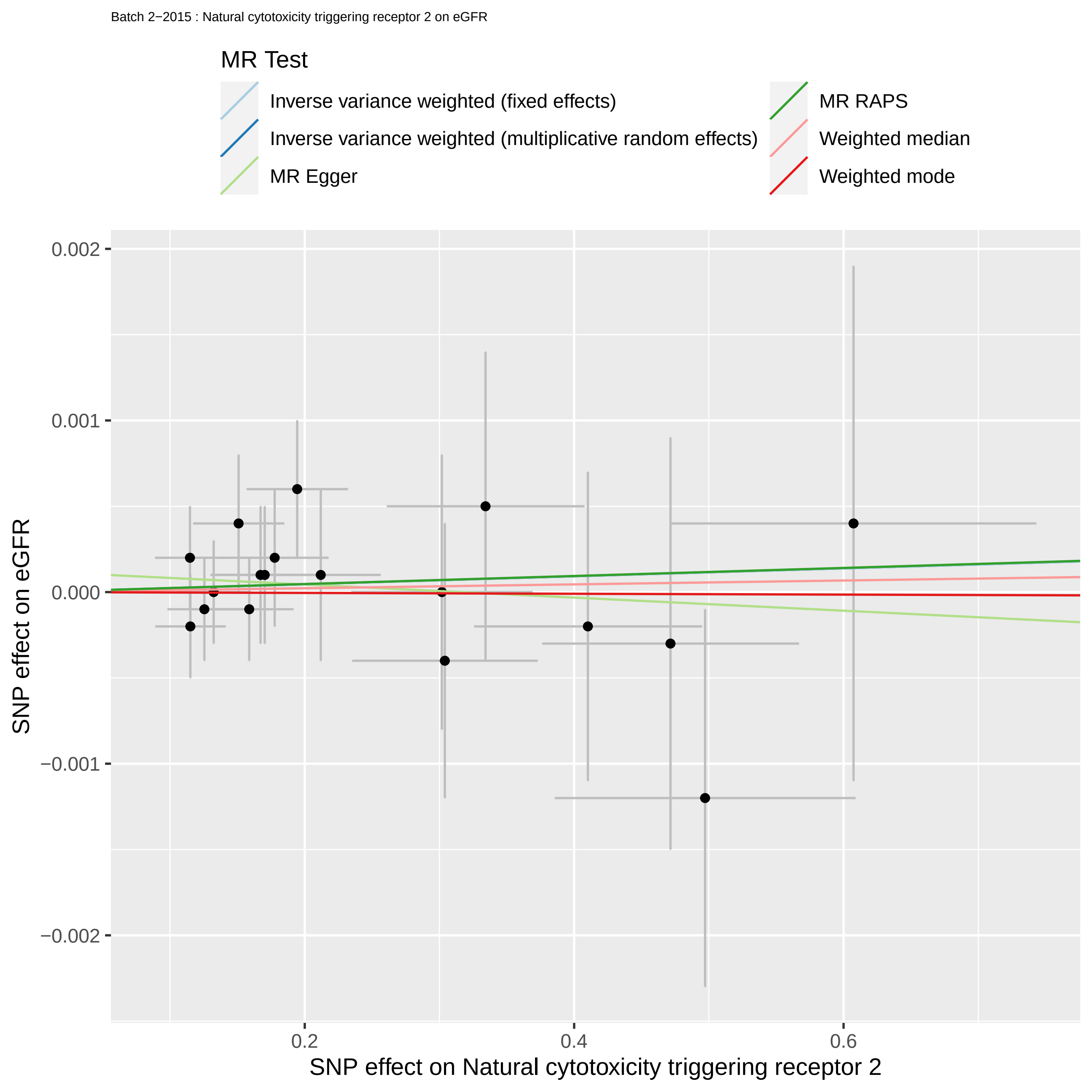
Batch 2−2015 : Natural cytotoxicity triggering receptor 2 on eGFR
MR Test
Inverse variance weighted (fixed effects)
Inverse variance weighted (multiplicative random effects)
MR Egger
MR RAPS
Weighted median
Weighted mode
0.002
0.001
0.000
−0.001
−0.002
SNP effect on eGFR
0.2
0.4
0.6
SNP effect on Natural cytotoxicity triggering receptor 2

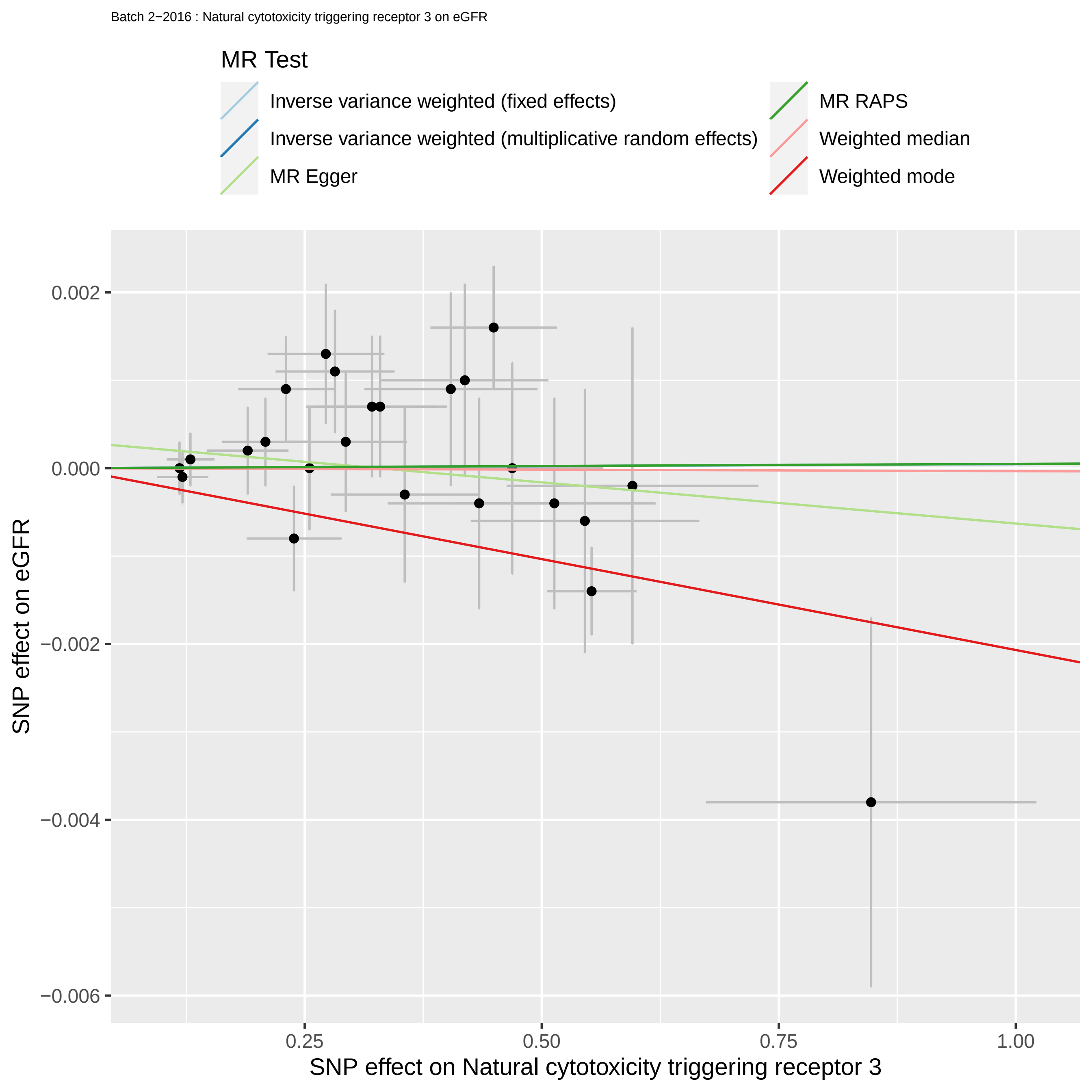
Batch 2−2016 : Natural cytotoxicity triggering receptor 3 on eGFR
MR Test
Inverse variance weighted (fixed effects)
Inverse variance weighted (multiplicative random effects)
MR Egger
MR RAPS
Weighted median
Weighted mode
0.002
0.000
−0.002
−0.004
−0.006
SNP effect on eGFR
0.25
0.50
0.75
1.00
SNP effect on Natural cytotoxicity triggering receptor 3

Batch 2−2017 : Natural cytotoxicity triggering receptor 3 ligand 1 on eGFR

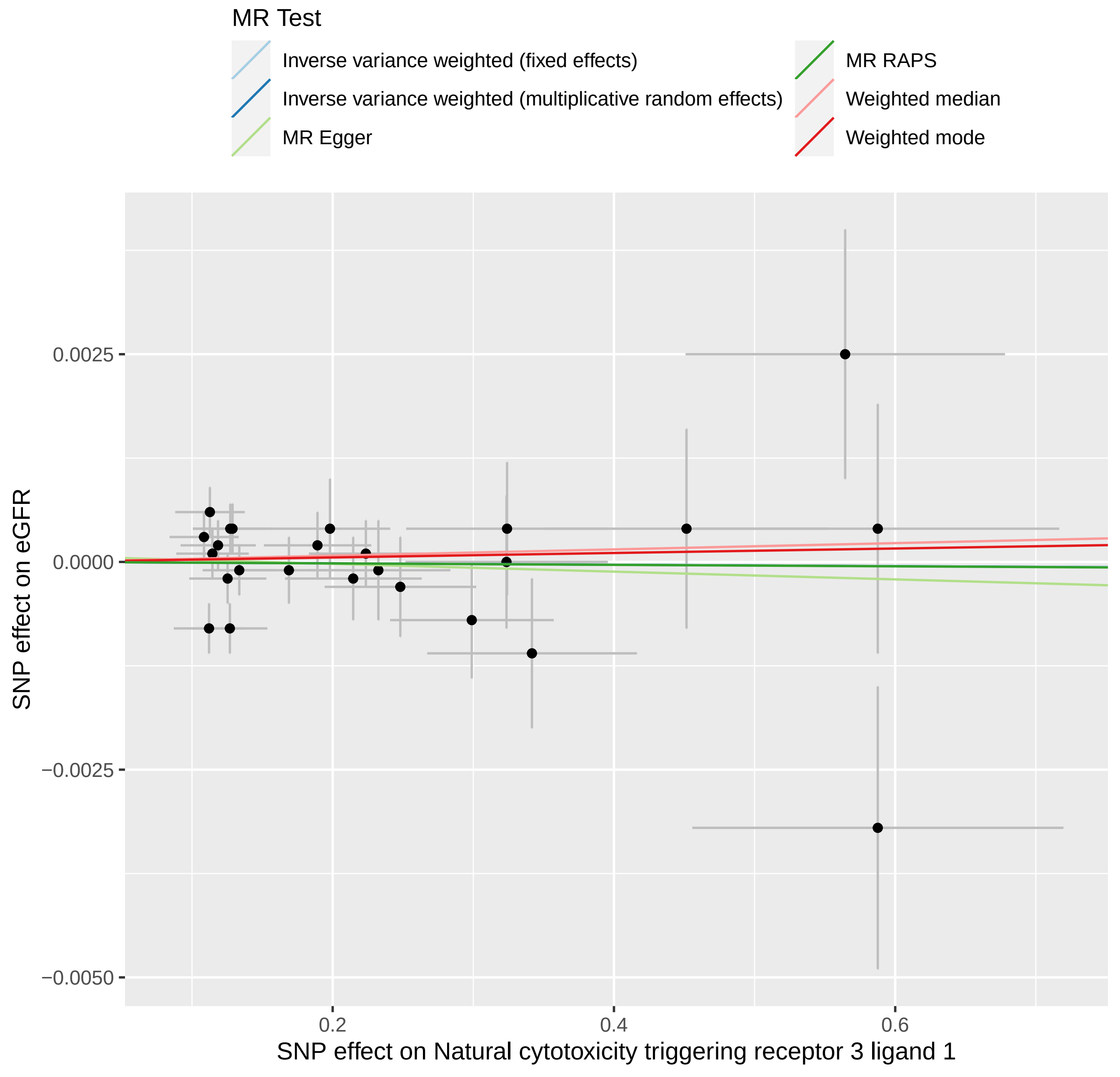

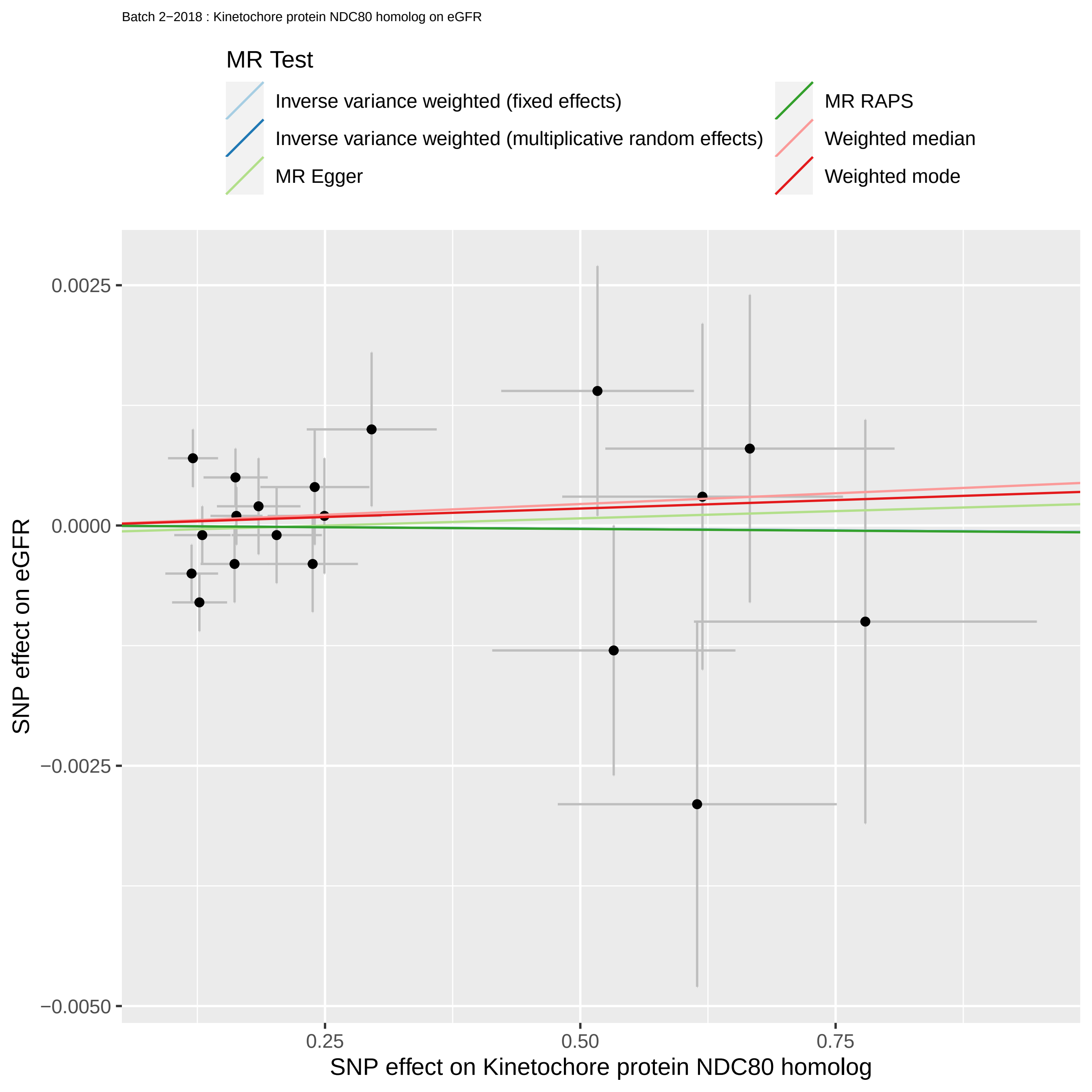

Batch 2−2018 : Kinetochore protein NDC80 homolog on eGFR
MR Test
Inverse variance weighted (fixed effects)
Inverse variance weighted (multiplicative random effects)
MR Egger
MR RAPS
Weighted median
Weighted mode
0.0025
0.0000
−0.0025
−0.0050
0.25
0.50
0.75
SNP effect on eGFR
SNP effect on Kinetochore protein NDC80 homolog

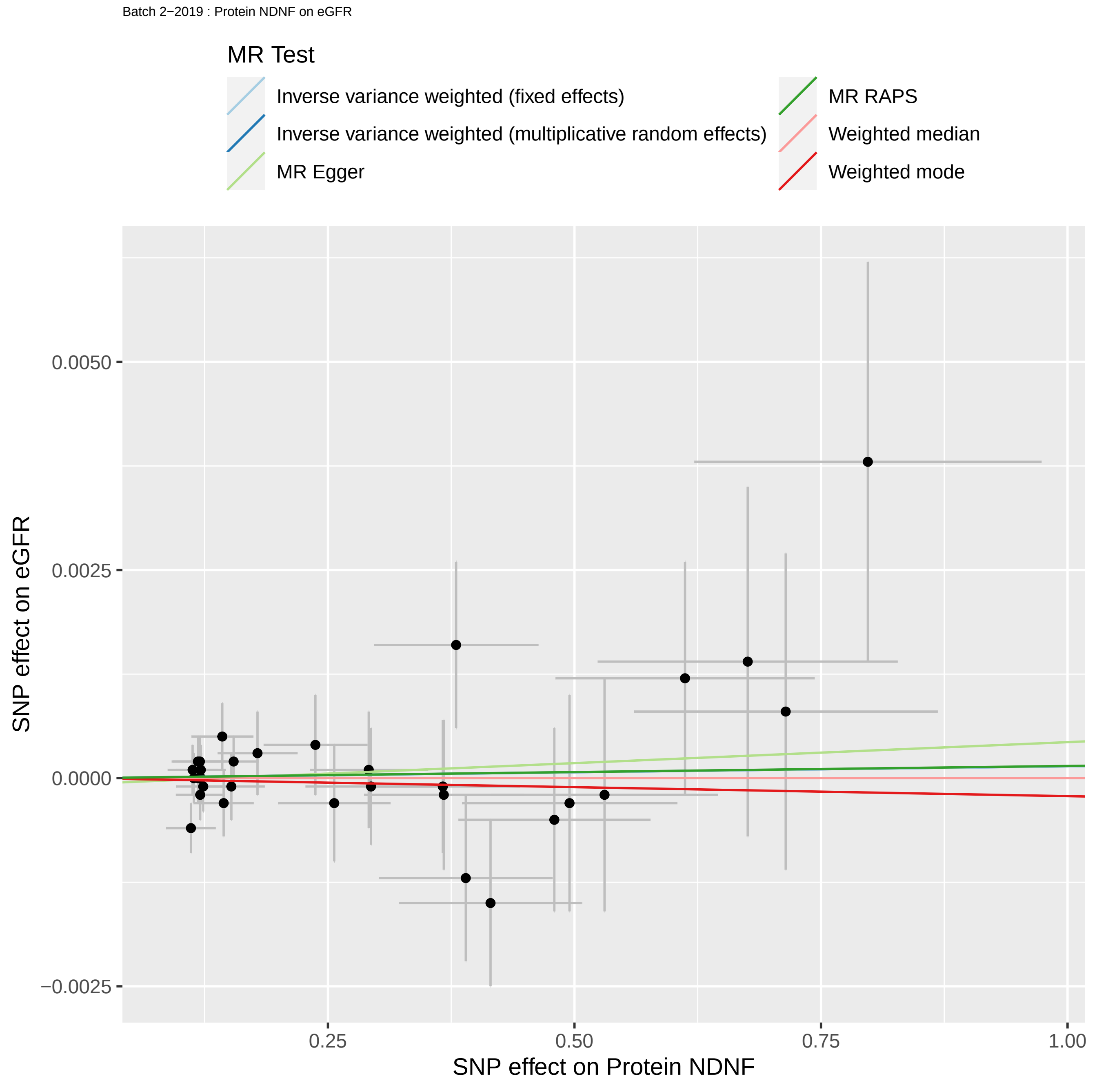

Batch 2−2019 : Protein NDNF on eGFR
MR Test
Inverse variance weighted (fixed effects)
Inverse variance weighted (multiplicative random effects)
MR Egger
MR RAPS
Weighted median
Weighted mode
0.0050
0.0025
0.0000
−0.0025
0.25
0.50
0.75
1.00
SNP effect on eGFR
SNP effect on Protein NDNF

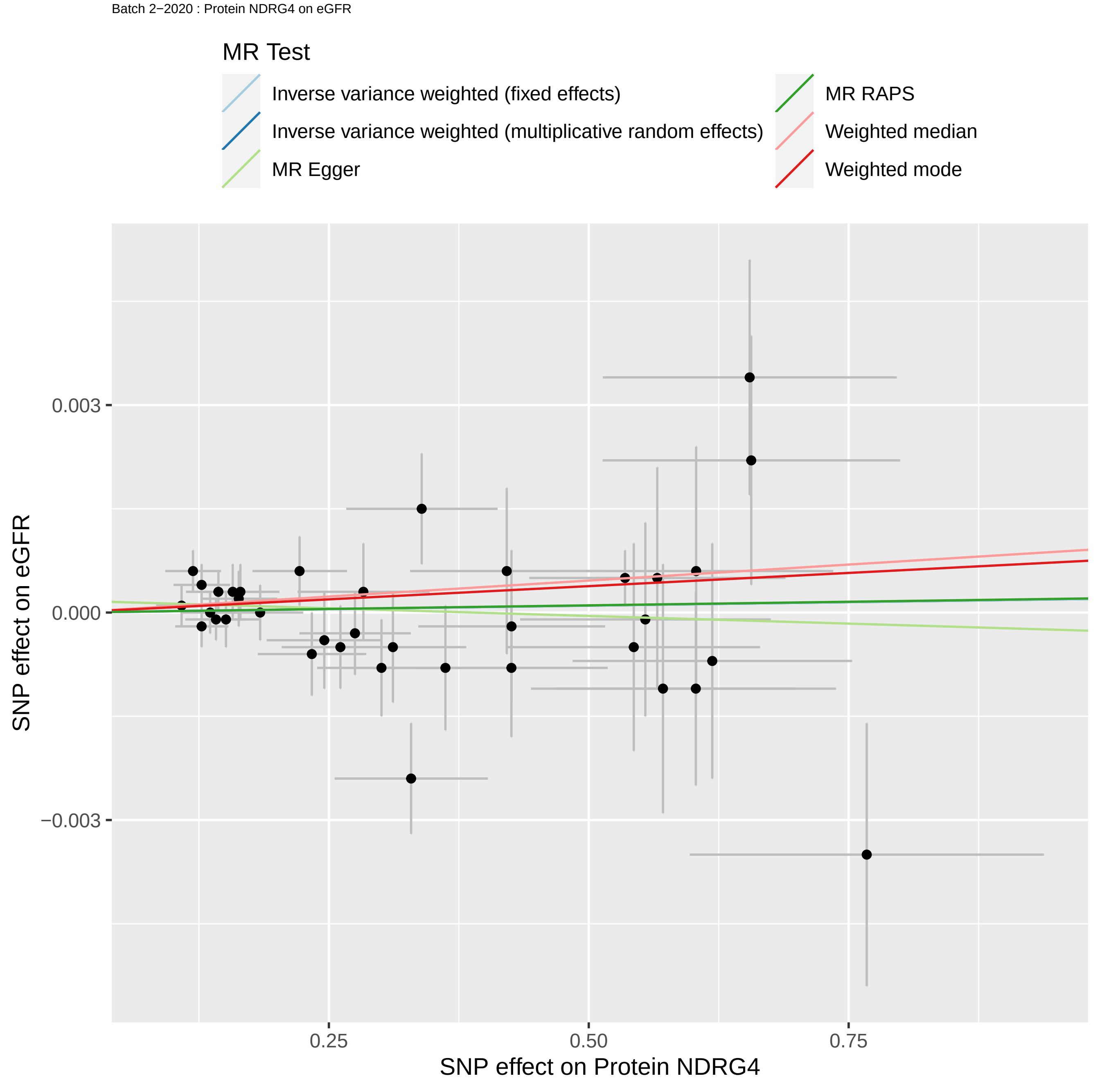

Batch 2−2020 : Protein NDRG4 on eGFR
MR Test
Inverse variance weighted (fixed effects)
Inverse variance weighted (multiplicative random effects)
MR Egger
MR RAPS
Weighted median
Weighted mode
0.003
0.000
−0.003
0.25
0.50
0.75
SNP effect on eGFR
SNP effect on Protein NDRG4

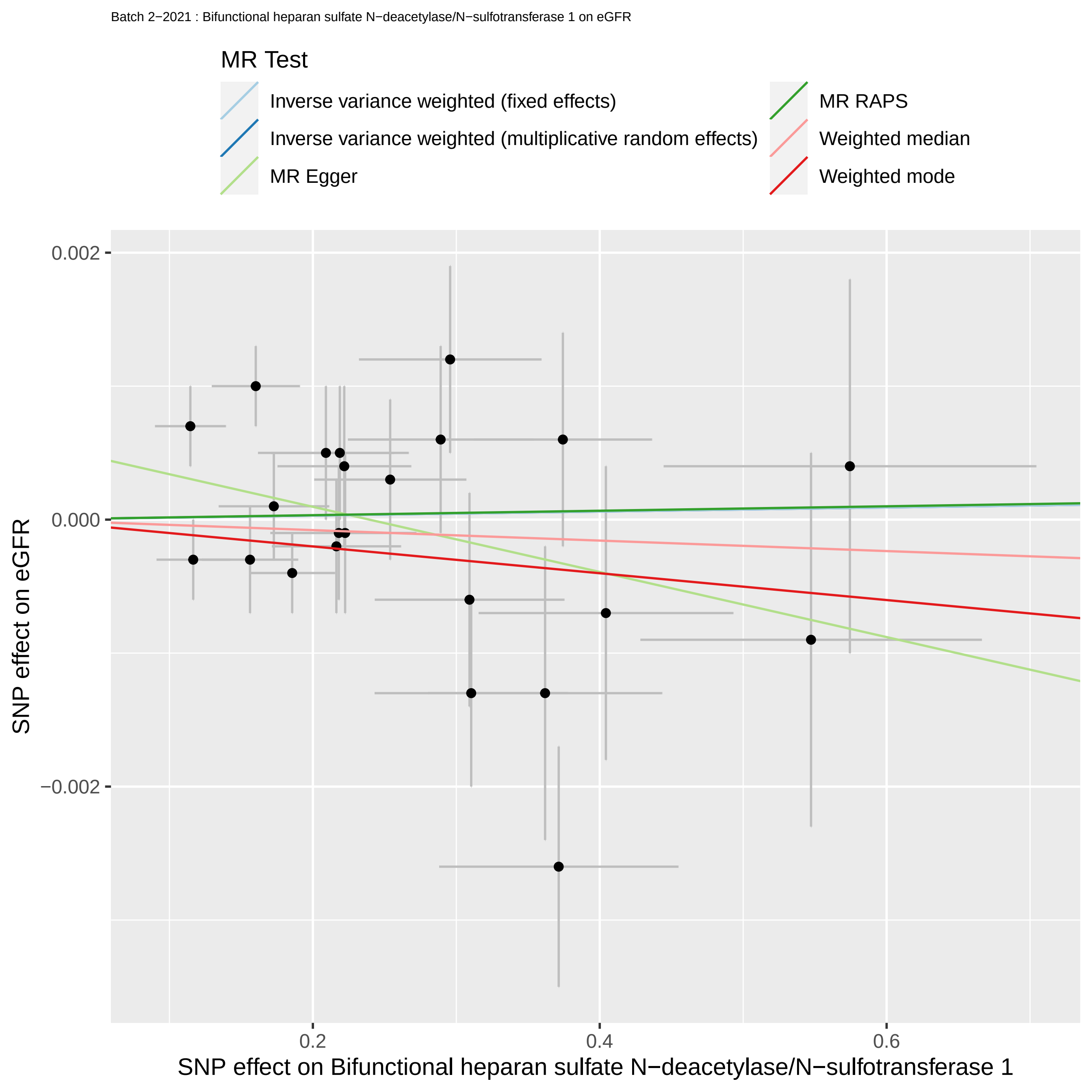

Batch 2−2021 : Bifunctional heparan sulfate N−deacetylase/N−sulfotransferase 1 on eGFR
MR Test
Inverse variance weighted (fixed effects)
Inverse variance weighted (multiplicative random effects)
MR Egger
MR RAPS
Weighted median
Weighted mode
0.002
0.000
−0.002
0.2
0.4
0.6
SNP effect on eGFR
SNP effect on Bifunctional heparan sulfate N−deacetylase/N−sulfotransferase 1

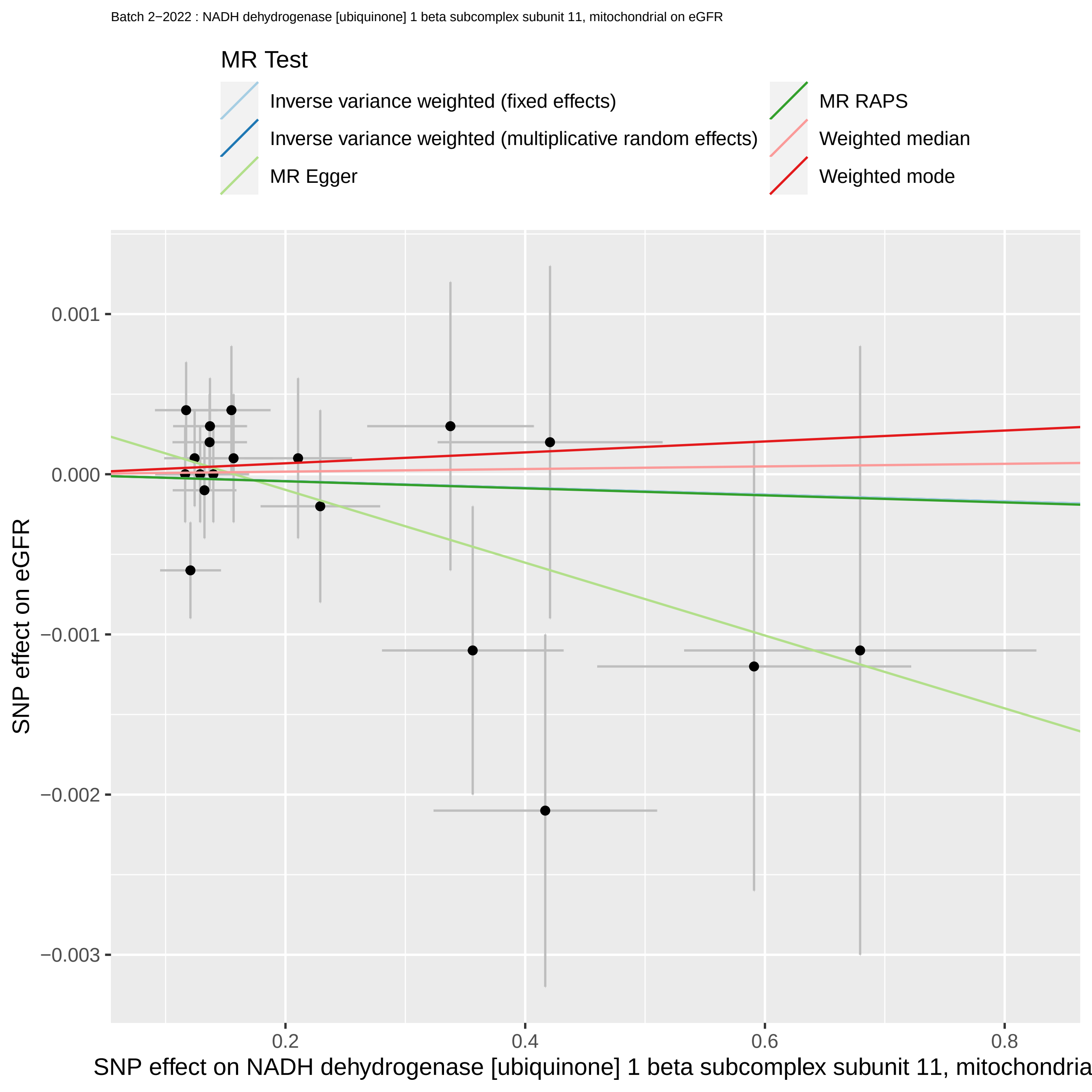

Batch 2−2022 : NADH dehydrogenase [ubiquinone] 1 beta subcomplex subunit 11, mitochondrial on eGFR
MR Test
Inverse variance weighted (fixed effects)
Inverse variance weighted (multiplicative random effects)
MR Egger
MR RAPS
Weighted median
Weighted mode
0.001
0.000
−0.001
−0.002
−0.003
SNP effect on eGFR
0.2
0.4
0.6
0.8
SNP effect on NADH dehydrogenase [ubiquinone] 1 beta subcomplex subunit 11, mitochondria

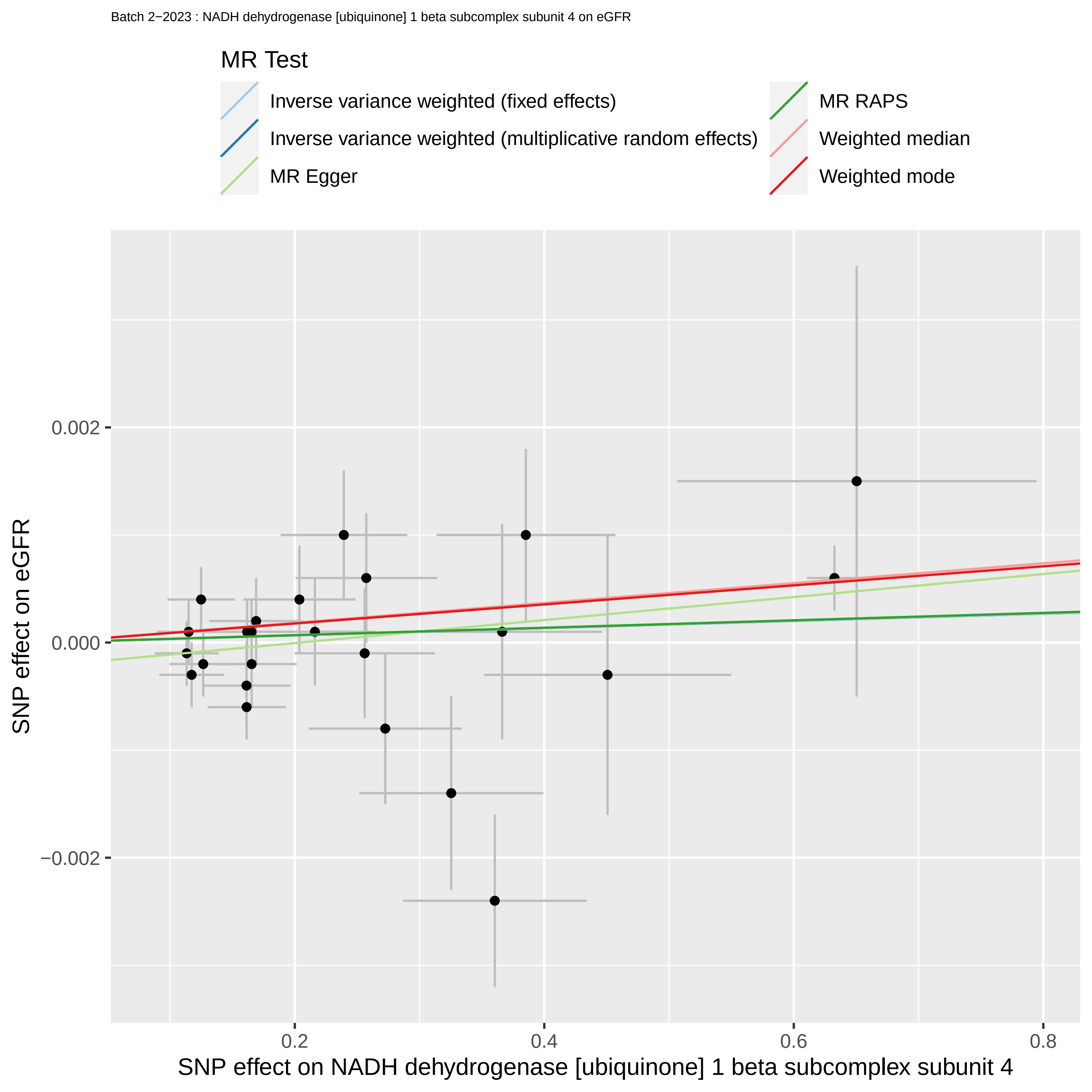

Batch 2−2023 : NADH dehydrogenase [ubiquinone] 1 beta subcomplex subunit 4 on eGFR
MR Test
Inverse variance weighted (fixed effects)
Inverse variance weighted (multiplicative random effects)
MR Egger
MR RAPS
Weighted median
Weighted mode
0.002
0.000
−0.002
0.2
0.4
0.6
0.8
SNP effect on eGFR
SNP effect on NADH dehydrogenase [ubiquinone] 1 beta subcomplex subunit 4

Batch 2−2024 : NADH dehydrogenase [ubiquinone] 1 beta subcomplex subunit 8, mitochondrial on eGFR

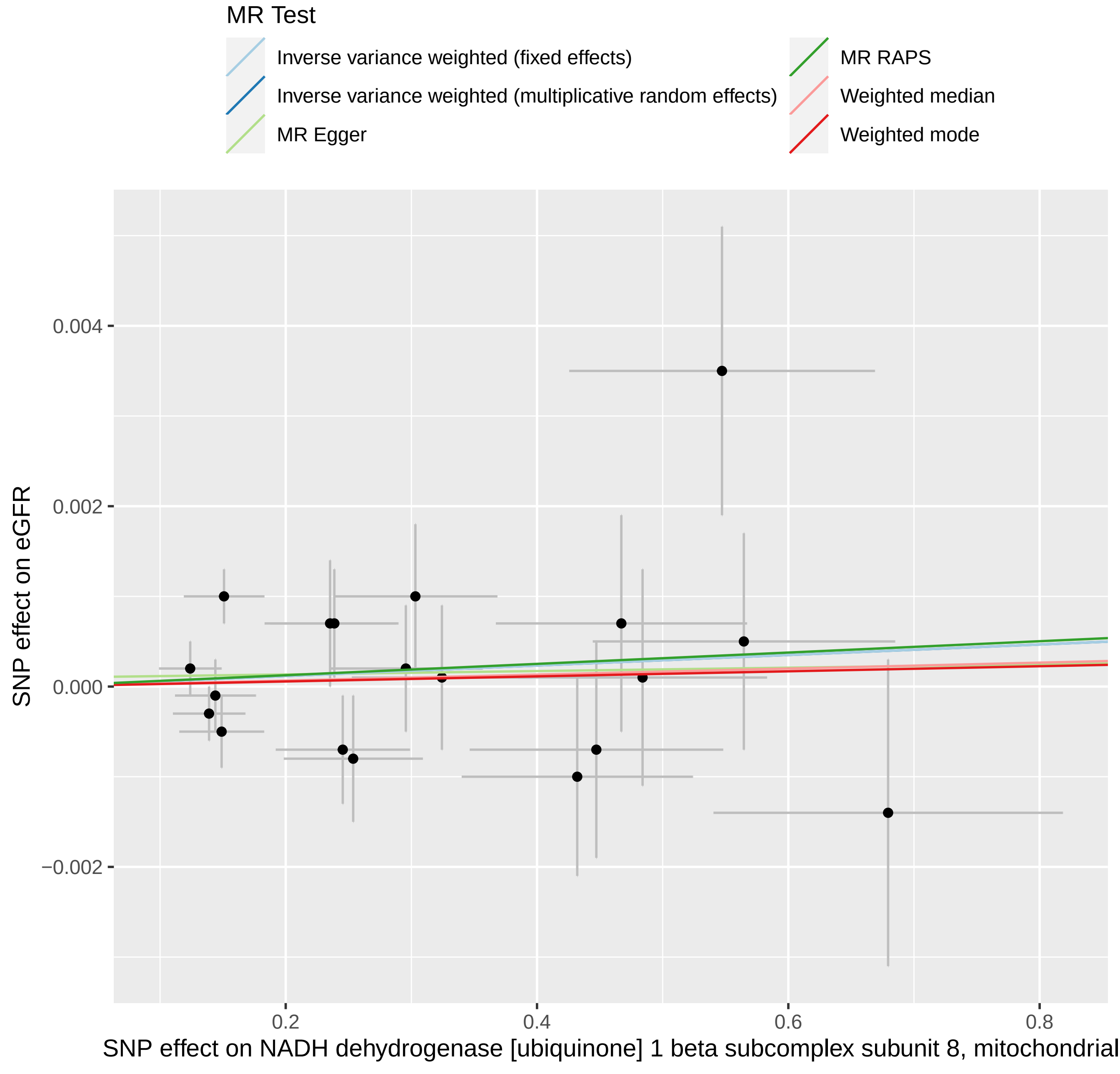

Batch 2−2025 : NADH dehydrogenase [ubiquinone] iron−sulfur protein 4, mitochondrial on eGFR

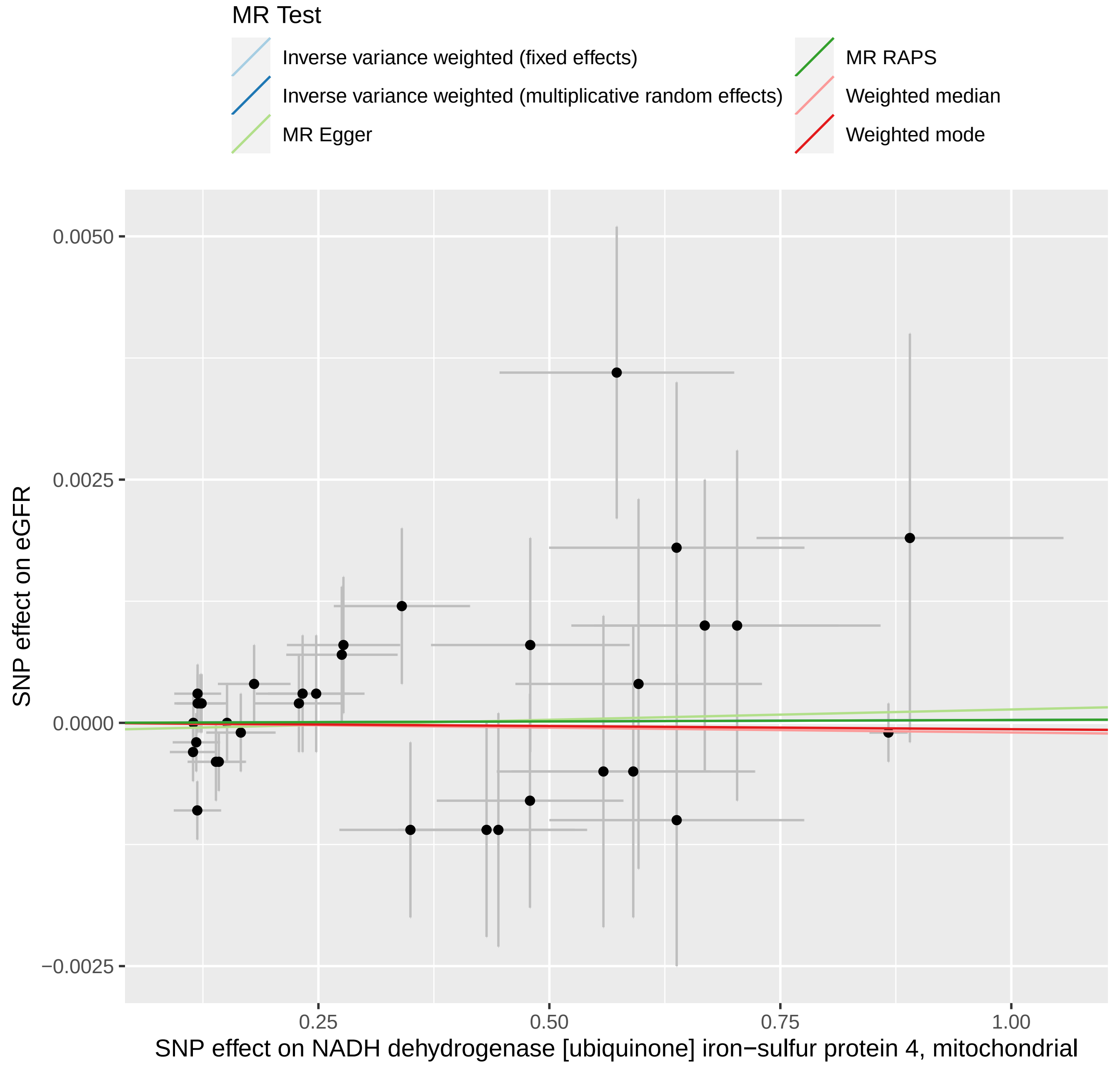

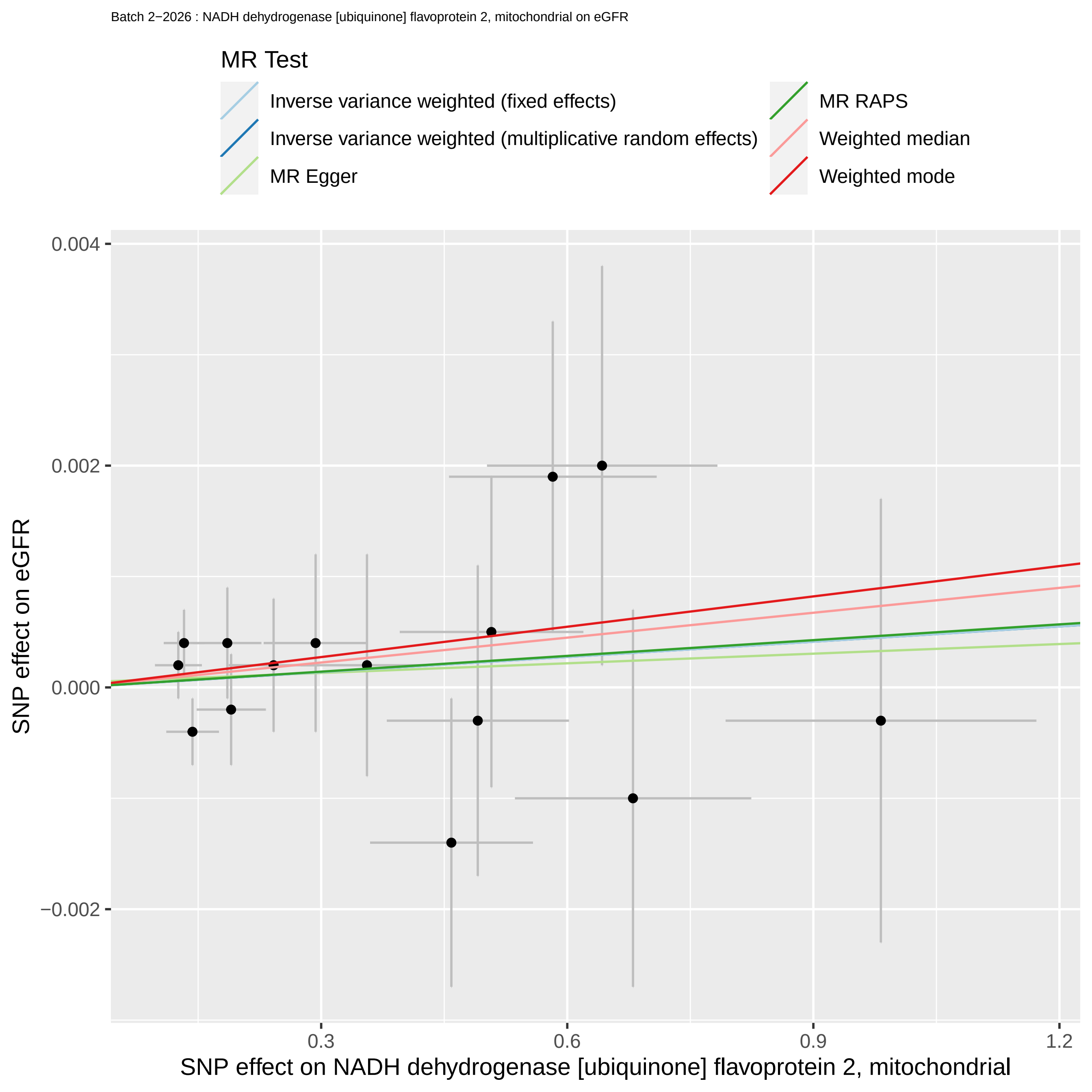

Batch 2−2026 : NADH dehydrogenase [ubiquinone] flavoprotein 2, mitochondrial on eGFR
MR Test
Inverse variance weighted (fixed effects)
Inverse variance weighted (multiplicative random effects)
MR Egger
MR RAPS
Weighted median
Weighted mode
0.004
0.002
0.000
−0.002
SNP effect on eGFR
0.3
0.6
0.9
1.2
SNP effect on NADH dehydrogenase [ubiquinone] flavoprotein 2, mitochondrial

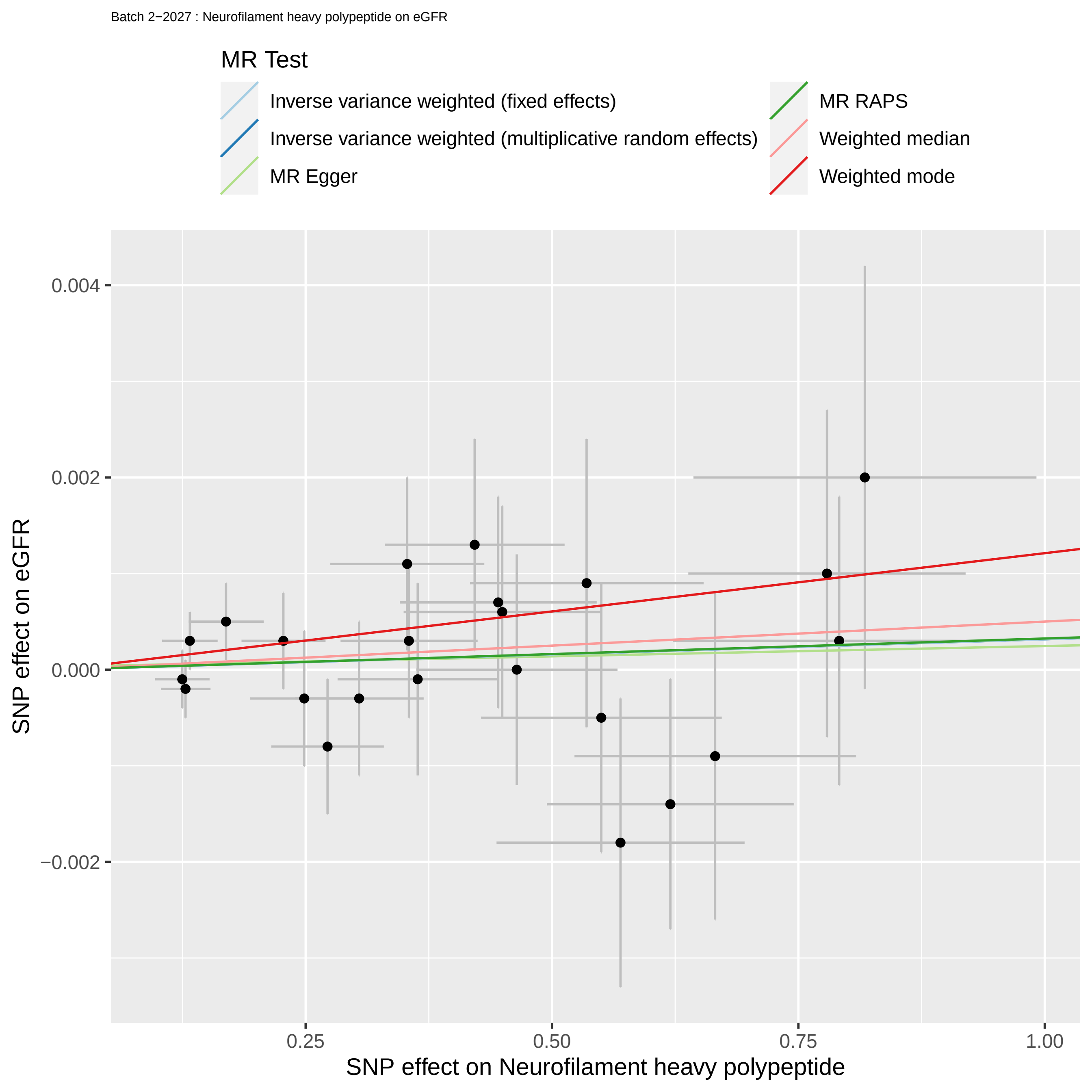

Batch 2−2027 : Neurofilament heavy polypeptide on eGFR
MR Test
Inverse variance weighted (fixed effects)
Inverse variance weighted (multiplicative random effects)
MR Egger
MR RAPS
Weighted median
Weighted mode
0.004
0.002
0.000
−0.002
0.25
0.50
0.75
1.00
SNP effect on eGFR
SNP effect on Neurofilament heavy polypeptide

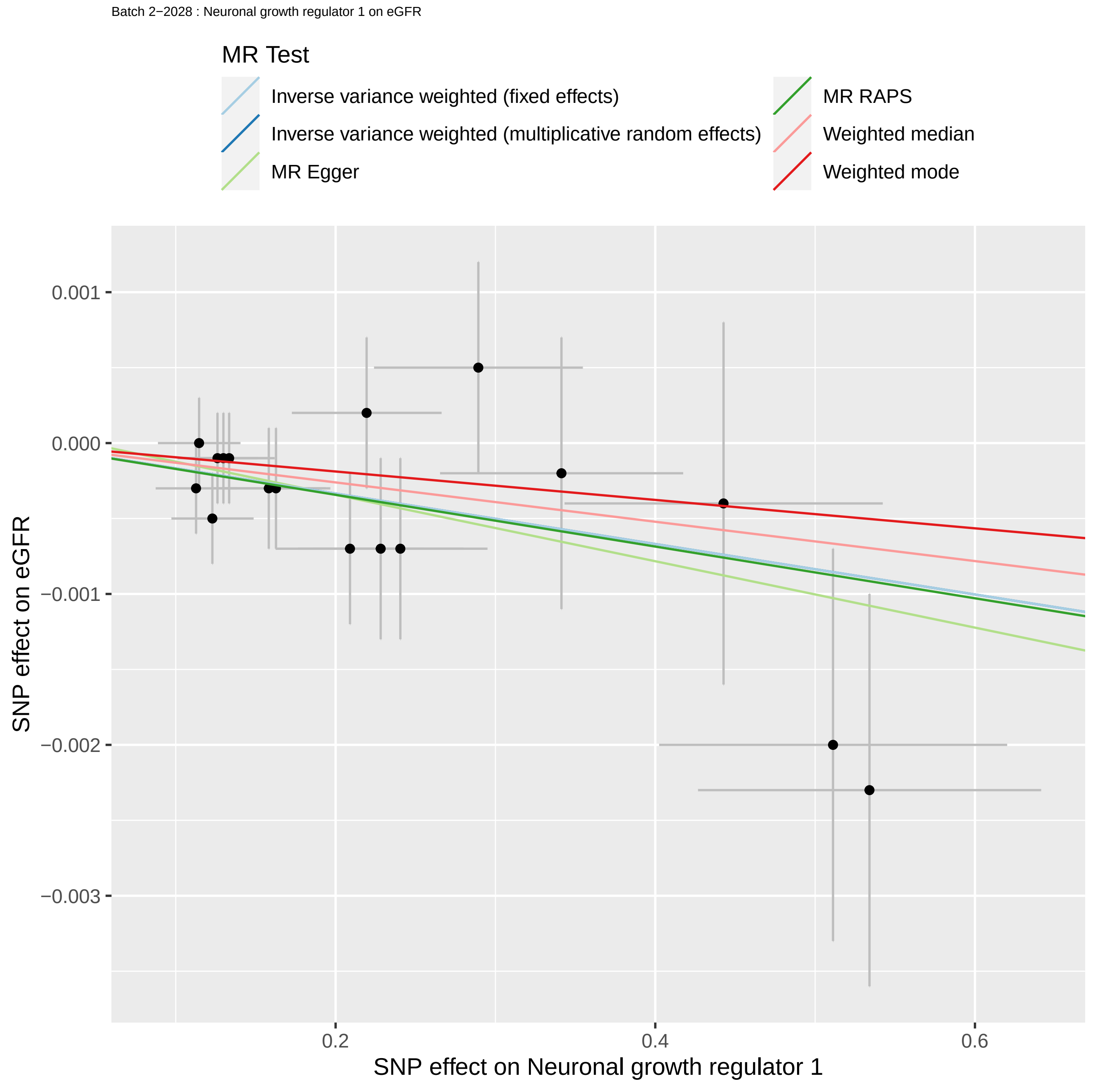
Batch 2−2028 : Neuronal growth regulator 1 on eGFR
MR Test
Inverse variance weighted (fixed effects)
Inverse variance weighted (multiplicative random effects)
MR Egger
MR RAPS
Weighted median
Weighted mode
0.001
0.000
−0.001
−0.002
−0.003
0.2
0.4
0.6
SNP effect on eGFR
SNP effect on Neuronal growth regulator 1

Batch 2−2029 : Neuronal growth regulator 1 on eGFR

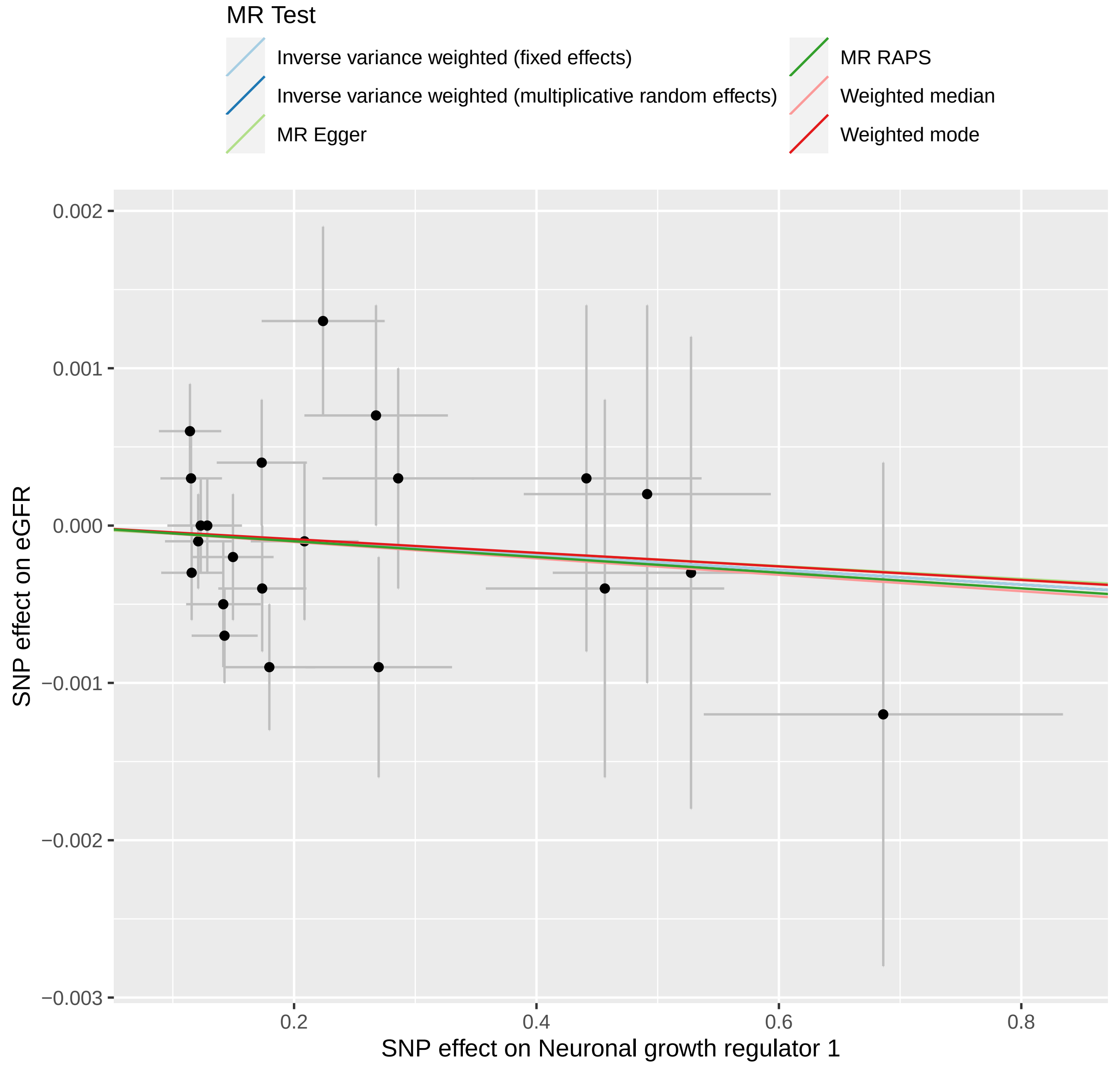

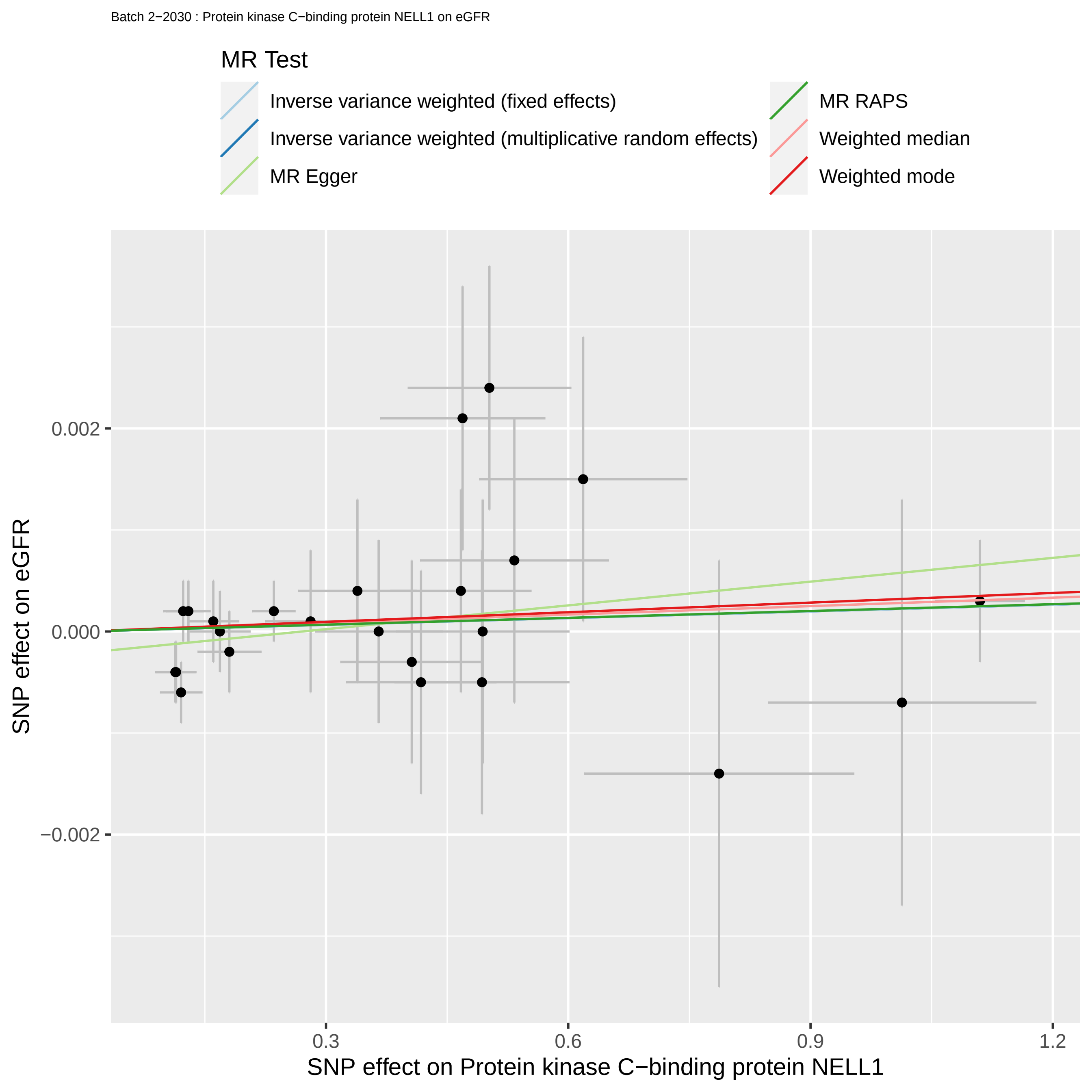
Batch 2−2030 : Protein kinase C−binding protein NELL1 on eGFR
MR Test
Inverse variance weighted (fixed effects)
Inverse variance weighted (multiplicative random effects)
MR Egger
MR RAPS
Weighted median
Weighted mode
SNP effect on eGFR
0.002
0.000
−0.002
0.3
0.6
0.9
1.2
SNP effect on Protein kinase C−binding protein NELL1

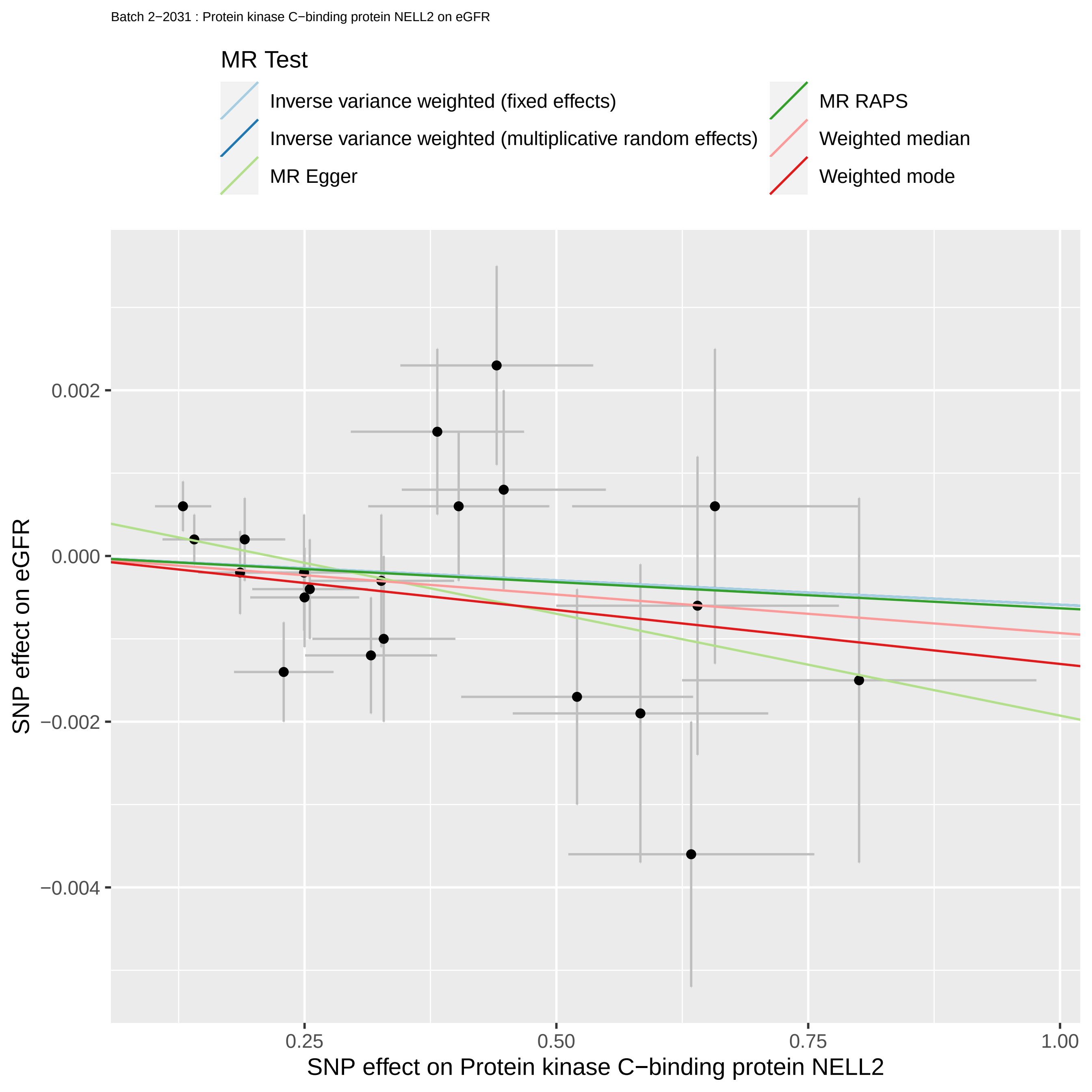
Batch 2−2031 : Protein kinase C−binding protein NELL2 on eGFR
MR Test
Inverse variance weighted (fixed effects)
Inverse variance weighted (multiplicative random effects)
MR Egger
MR RAPS
Weighted median
Weighted mode
SNP effect on eGFR
0.002
0.000
−0.002
−0.004
0.25
0.50
0.75
1.00
SNP effect on Protein kinase C−binding protein NELL2

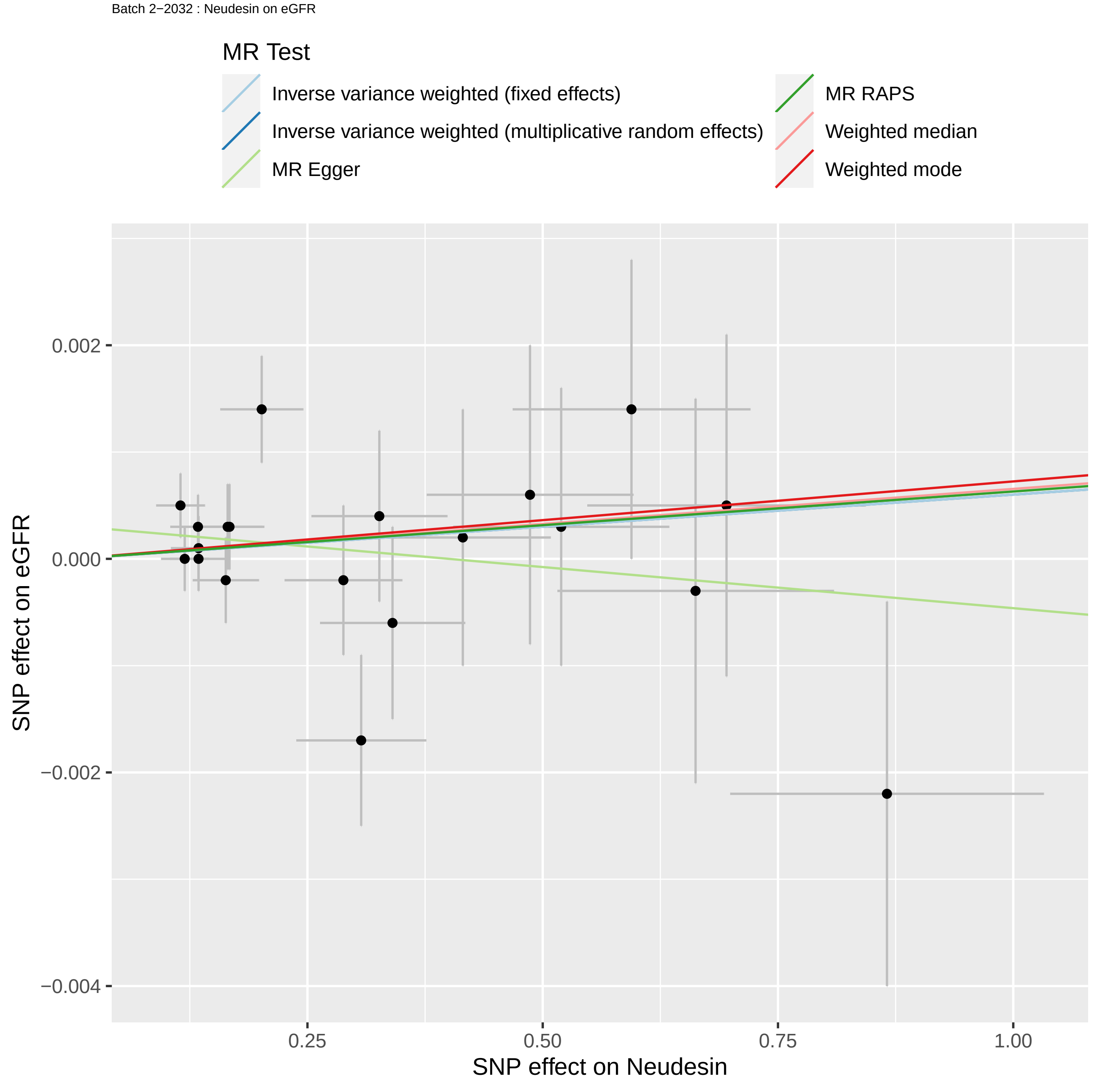

Batch 2−2032 : Neudesin on eGFR
MR Test
Inverse variance weighted (fixed effects)
Inverse variance weighted (multiplicative random effects)
MR Egger
MR RAPS
Weighted median
Weighted mode
0.002
0.000
−0.002
−0.004
SNP effect on eGFR
0.25
0.50
0.75
1.00
SNP effect on Neudesin

Batch 2−2033 : Neogenin on eGFR

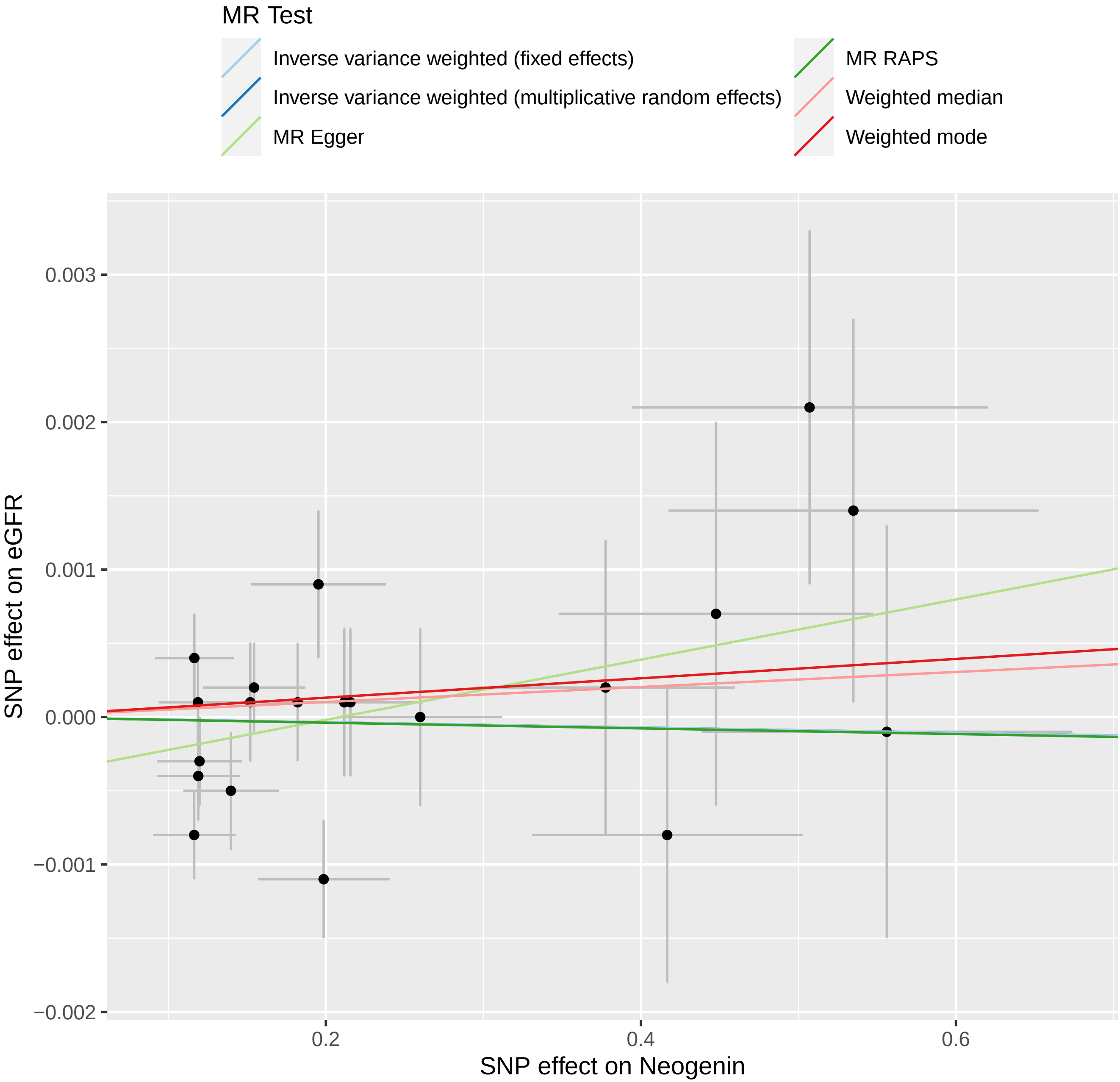

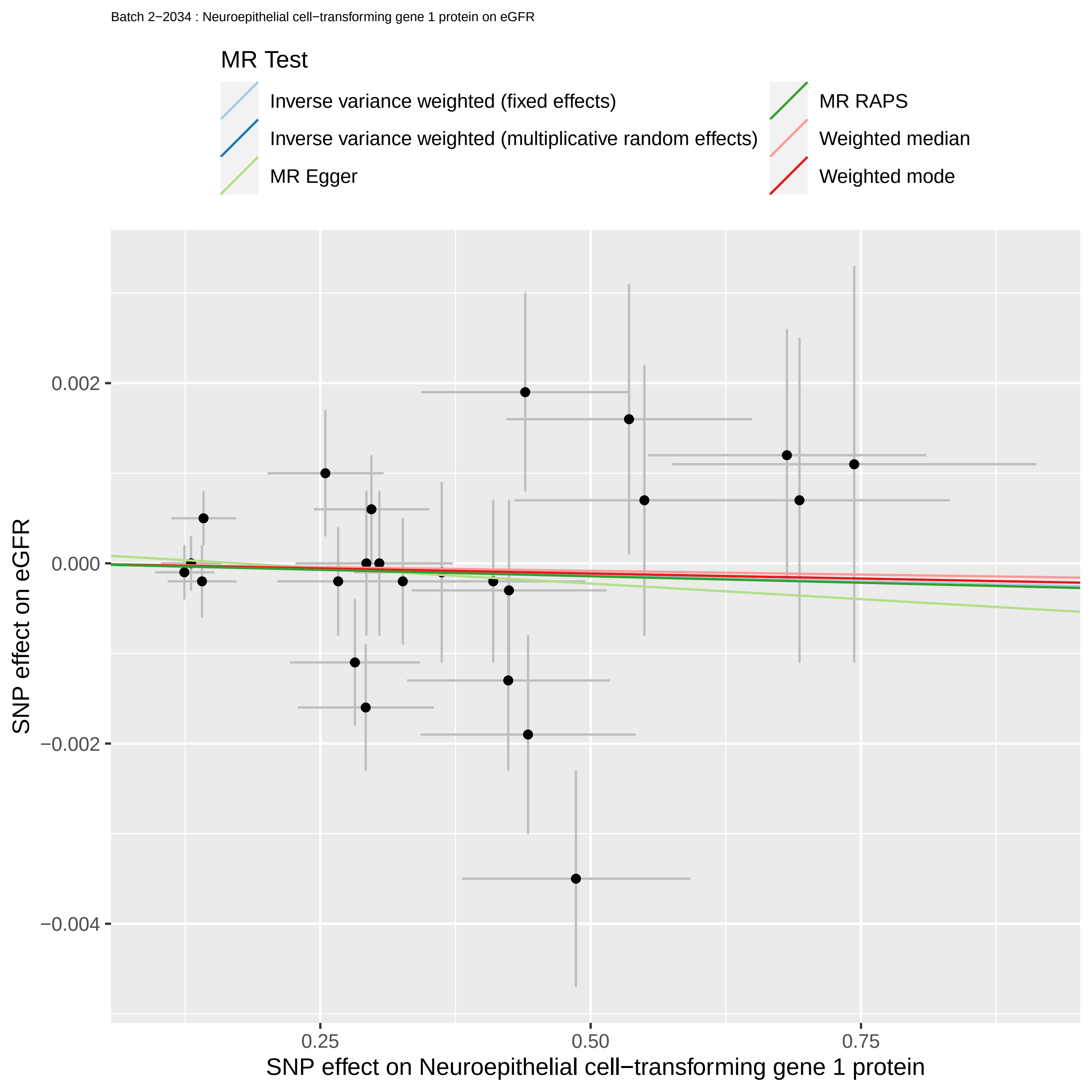
Batch 2−2034 : Neuroepithelial cell−transforming gene 1 protein on eGFR
MR Test
Inverse variance weighted (fixed effects)
Inverse variance weighted (multiplicative random effects)
MR Egger
MR RAPS
Weighted median
Weighted mode
0.002
0.000
−0.002
−0.004
0.25
0.50
0.75
SNP effect on eGFR
SNP effect on Neuroepithelial cell−transforming gene 1 protein

Batch 2−2035 : Neuropilin and tolloid−like protein 1 on eGFR

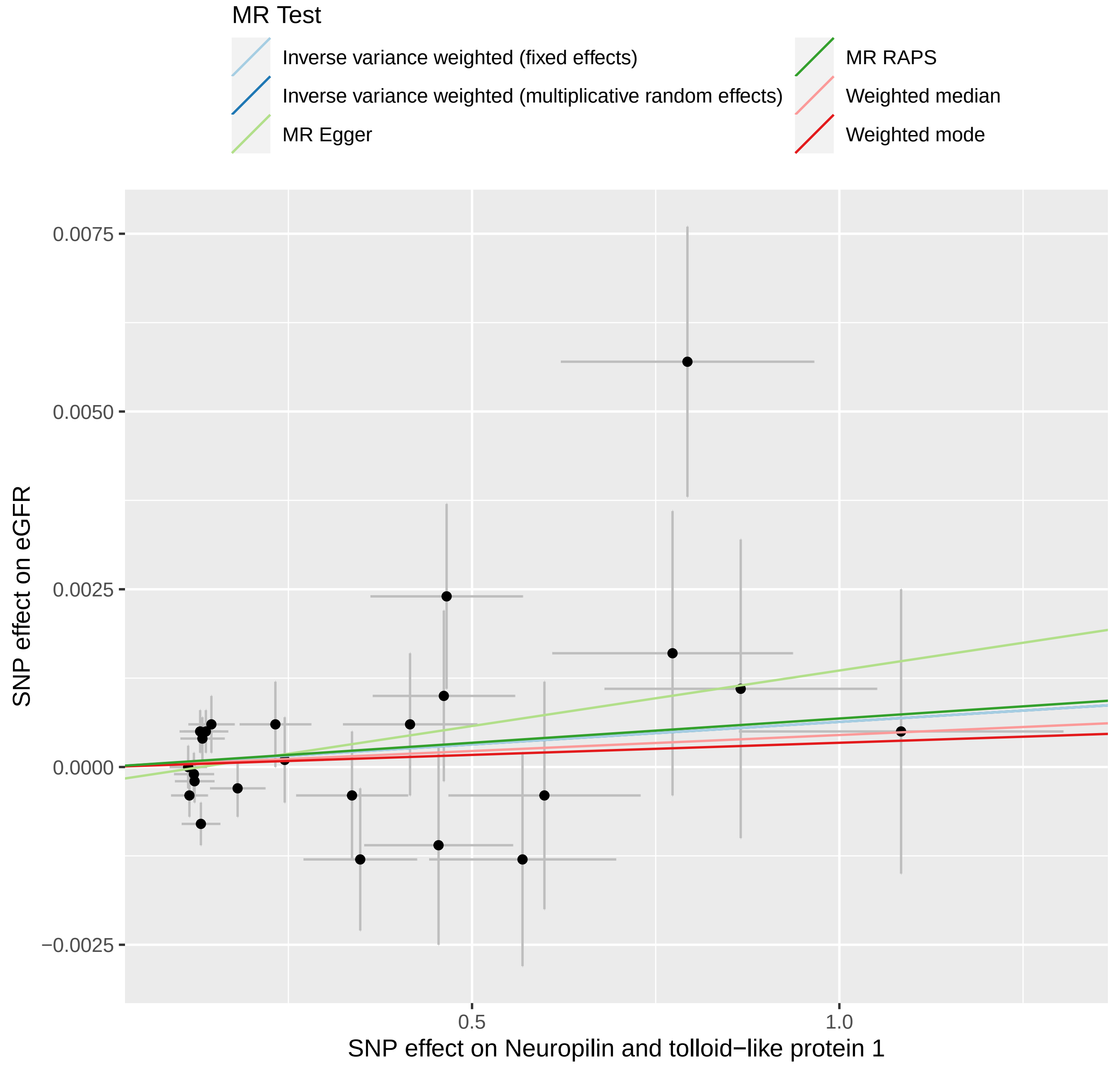

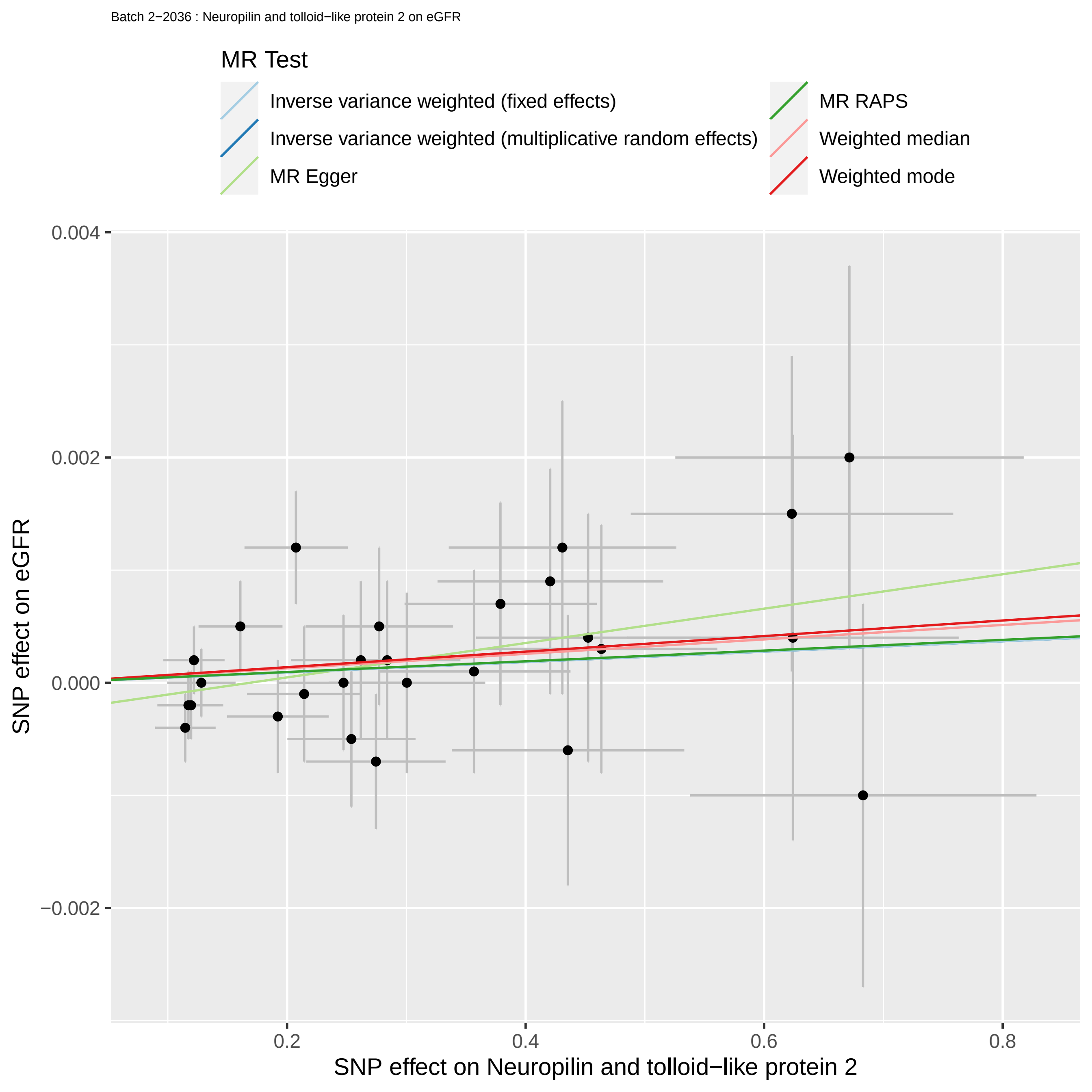
Batch 2−2036 : Neuropilin and tolloid−like protein 2 on eGFR
MR Test
Inverse variance weighted (fixed effects)
Inverse variance weighted (multiplicative random effects)
MR Egger
MR RAPS
Weighted median
Weighted mode
0.004
0.002
0.000
−0.002
0.2
0.4
0.6
0.8
SNP effect on eGFR
SNP effect on Neuropilin and tolloid−like protein 2

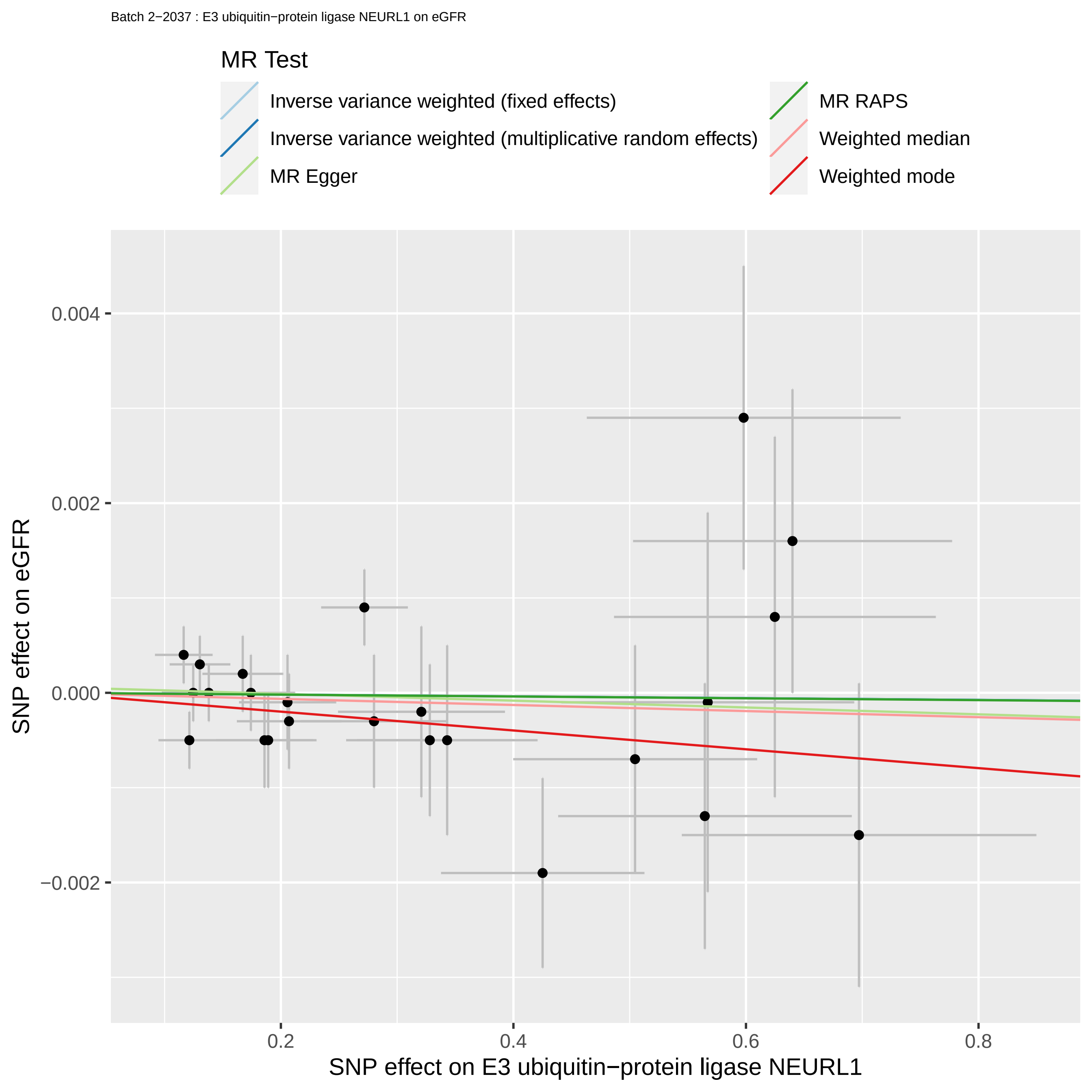
Batch 2−2037 : E3 ubiquitin−protein ligase NEURL1 on eGFR
MR Test
Inverse variance weighted (fixed effects)
Inverse variance weighted (multiplicative random effects)
MR Egger
MR RAPS
Weighted median
Weighted mode
0.004
0.002
0.000
−0.002
0.2
0.4
0.6
0.8
SNP effect on eGFR
SNP effect on E3 ubiquitin−protein ligase NEURL1

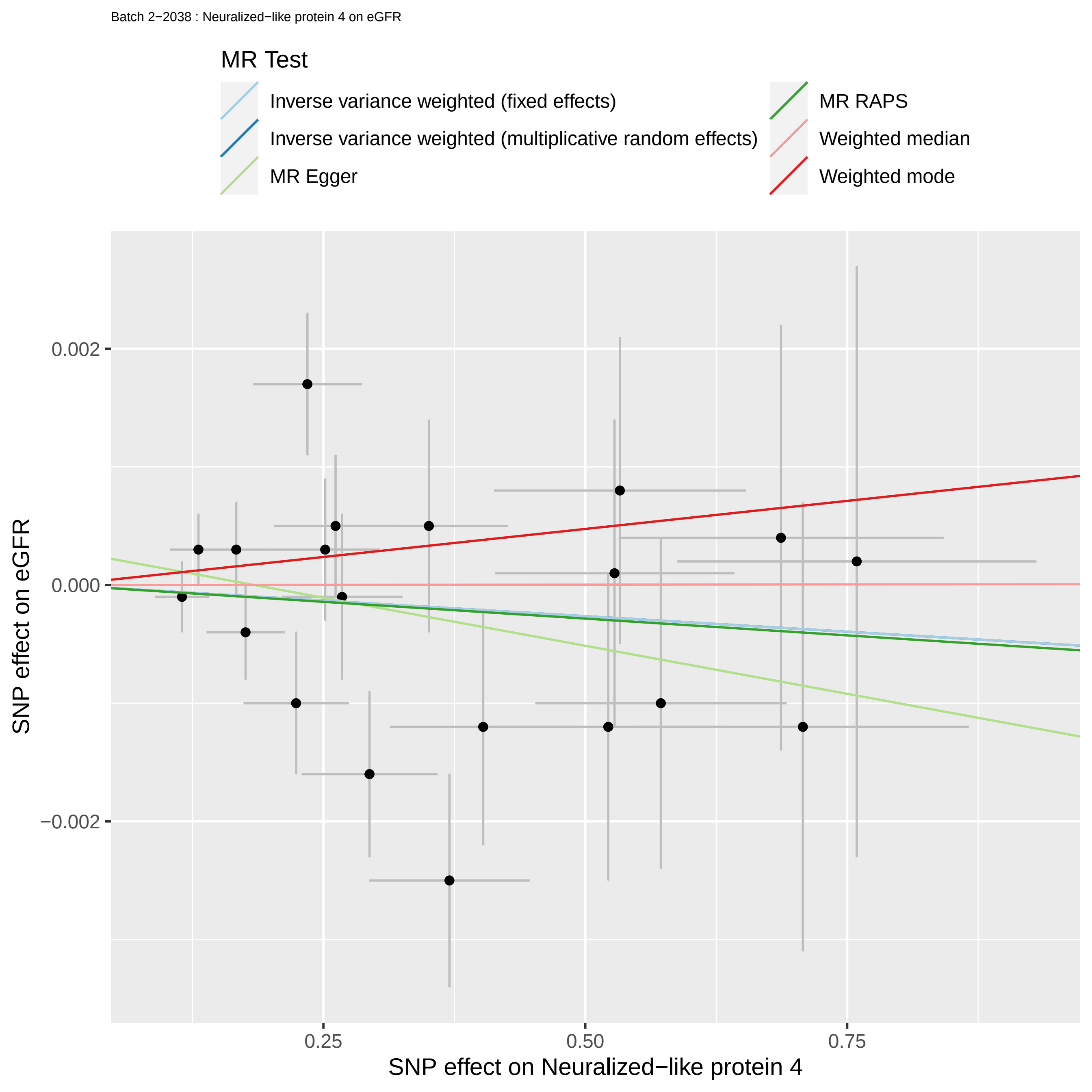

Batch 2−2038 : Neuralized−like protein 4 on eGFR
MR Test
Inverse variance weighted (fixed effects)
Inverse variance weighted (multiplicative random effects)
MR Egger
MR RAPS
Weighted median
Weighted mode
0.002
0.000
−0.002
0.25
0.50
0.75
SNP effect on eGFR
SNP effect on Neuralized−like protein 4

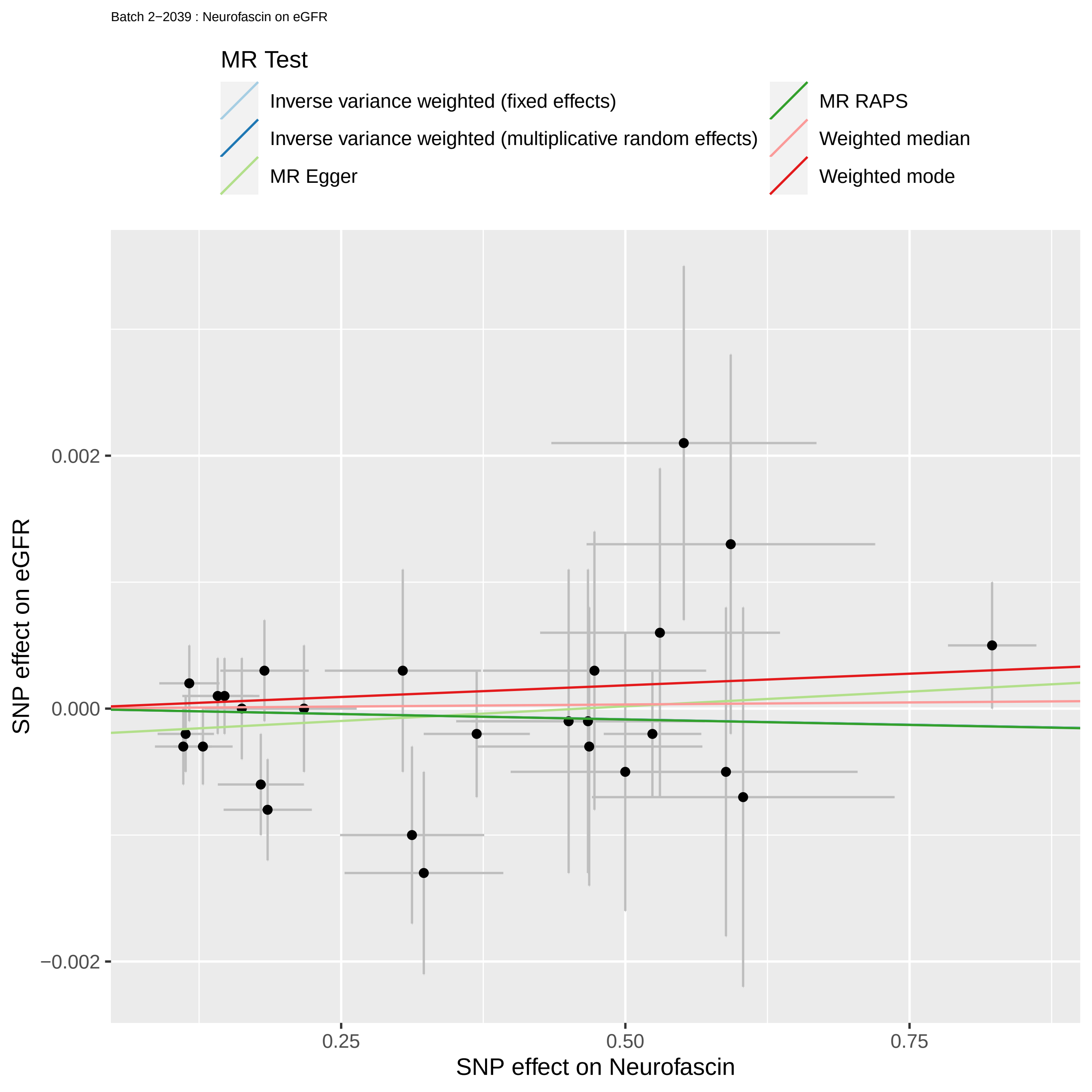

Batch 2−2039 : Neurofascin on eGFR
MR Test
Inverse variance weighted (fixed effects)
Inverse variance weighted (multiplicative random effects)
MR Egger
MR RAPS
Weighted median
Weighted mode
0.002
0.000
−0.002
0.25
0.50
0.75
SNP effect on eGFR
SNP effect on Neurofascin

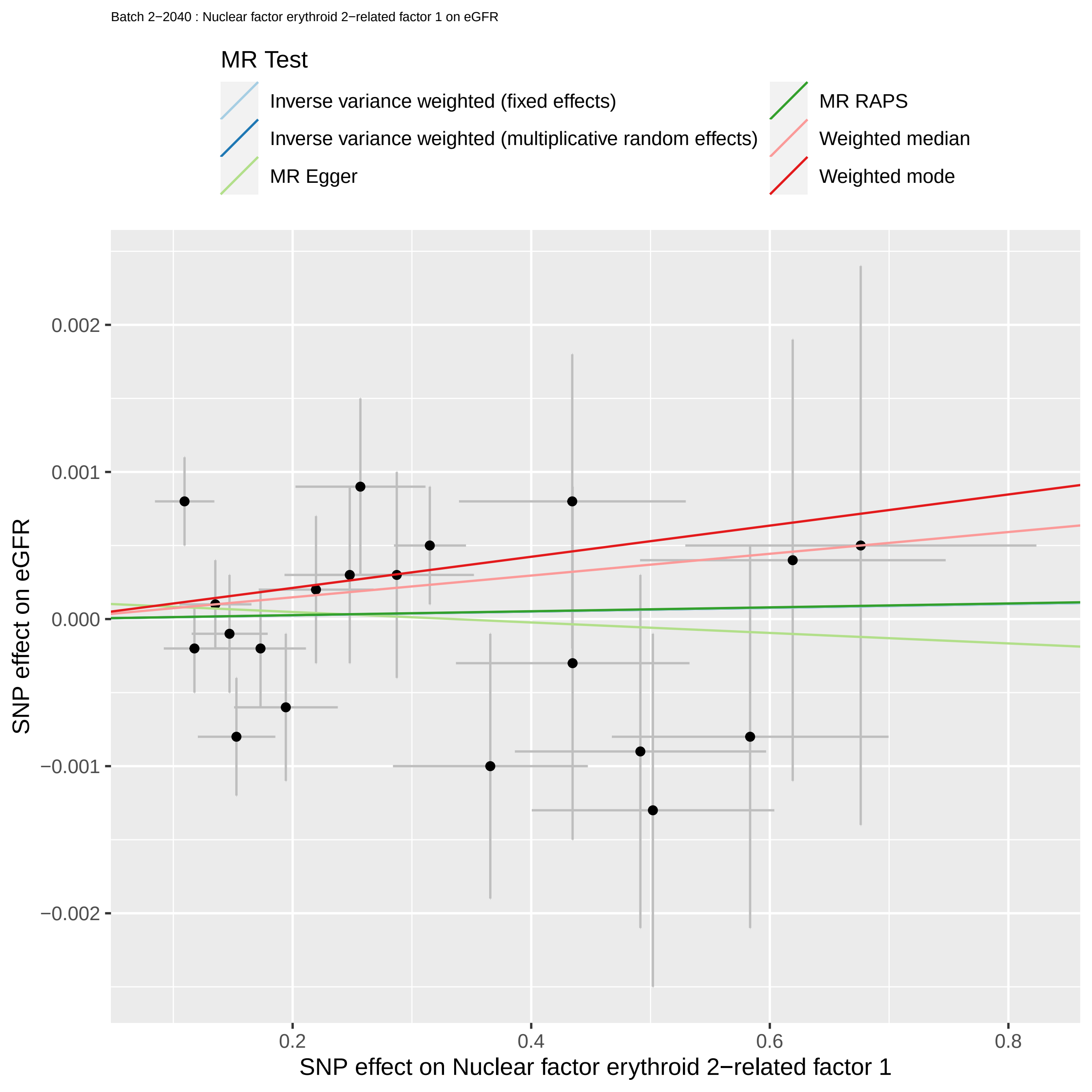

Batch 2−2040 : Nuclear factor erythroid 2−related factor 1 on eGFR
MR Test
Inverse variance weighted (fixed effects)
Inverse variance weighted (multiplicative random effects)
MR Egger
MR RAPS
Weighted median
Weighted mode
0.002
0.001
0.000
−0.001
−0.002
SNP effect on eGFR
0.2
0.4
0.6
0.8
SNP effect on Nuclear factor erythroid 2−related factor 1

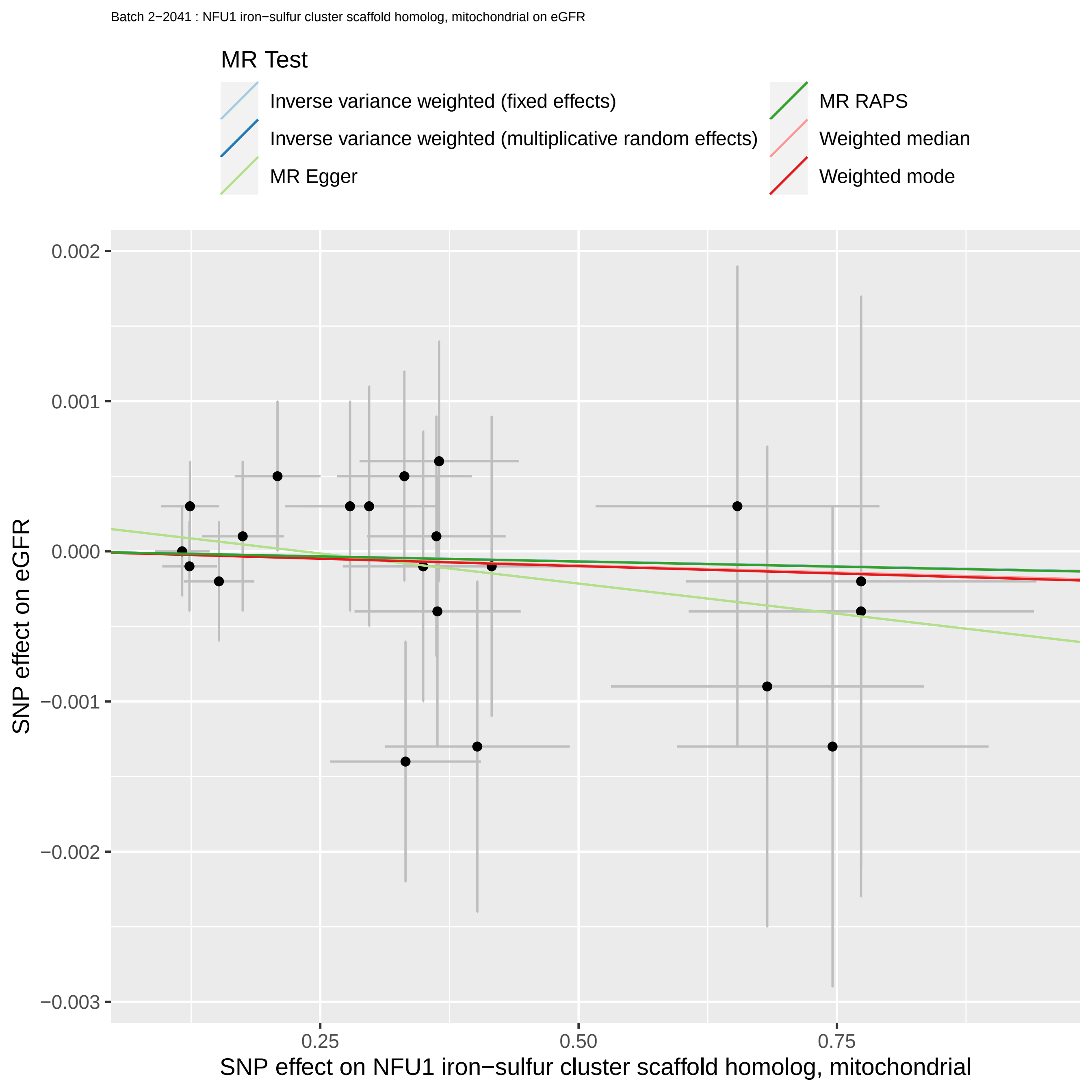
Batch 2−2041 : NFU1 iron−sulfur cluster scaffold homolog, mitochondrial on eGFR
MR Test
Inverse variance weighted (fixed effects)
Inverse variance weighted (multiplicative random effects)
MR Egger
MR RAPS
Weighted median
Weighted mode
0.002
0.001
0.000
−0.001
−0.002
−0.003
SNP effect on eGFR
0.25
0.50
0.75
SNP effect on NFU1 iron−sulfur cluster scaffold homolog, mitochondrial

Batch 2−2042 : beta−nerve growth factor on eGFR

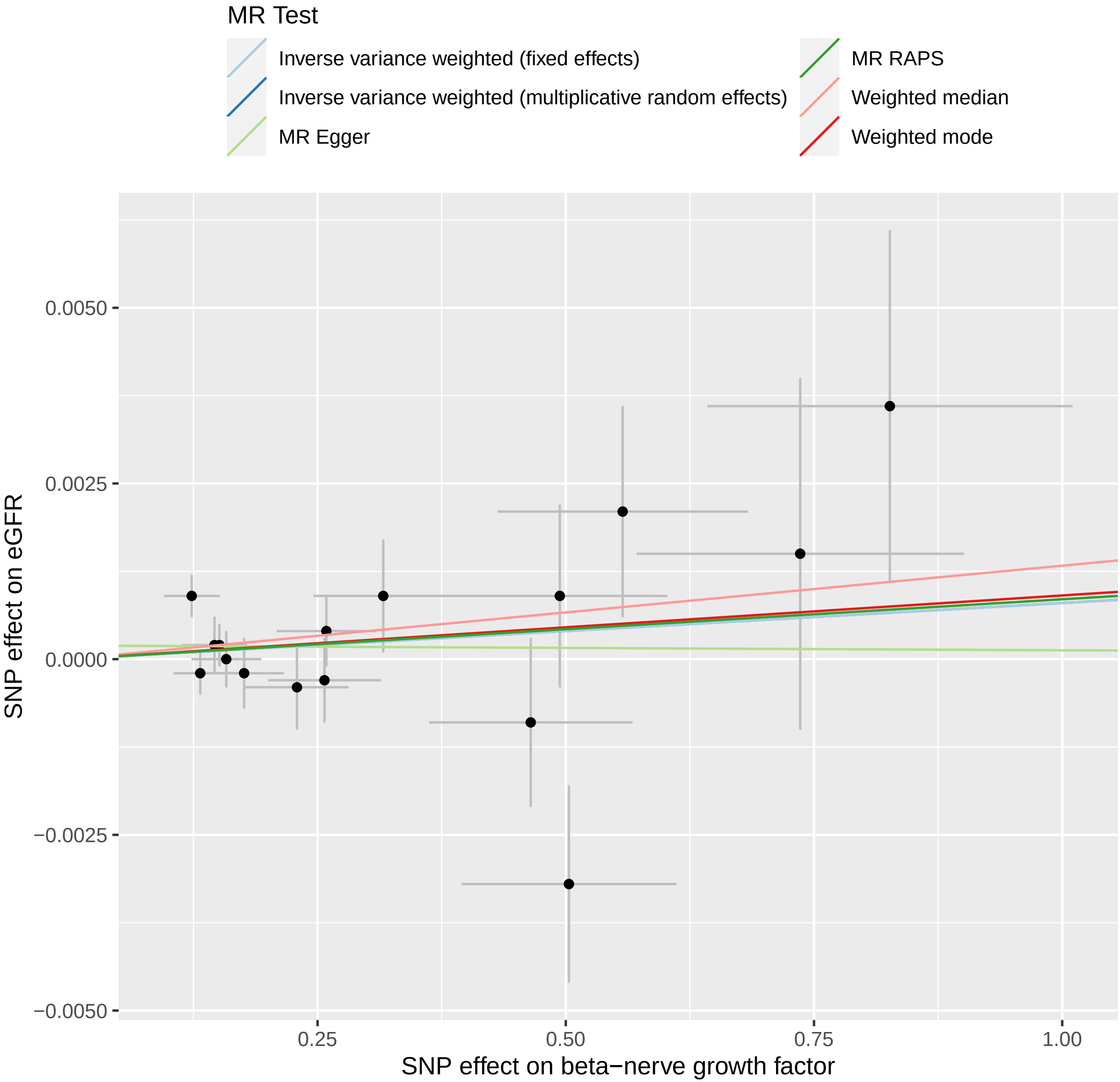

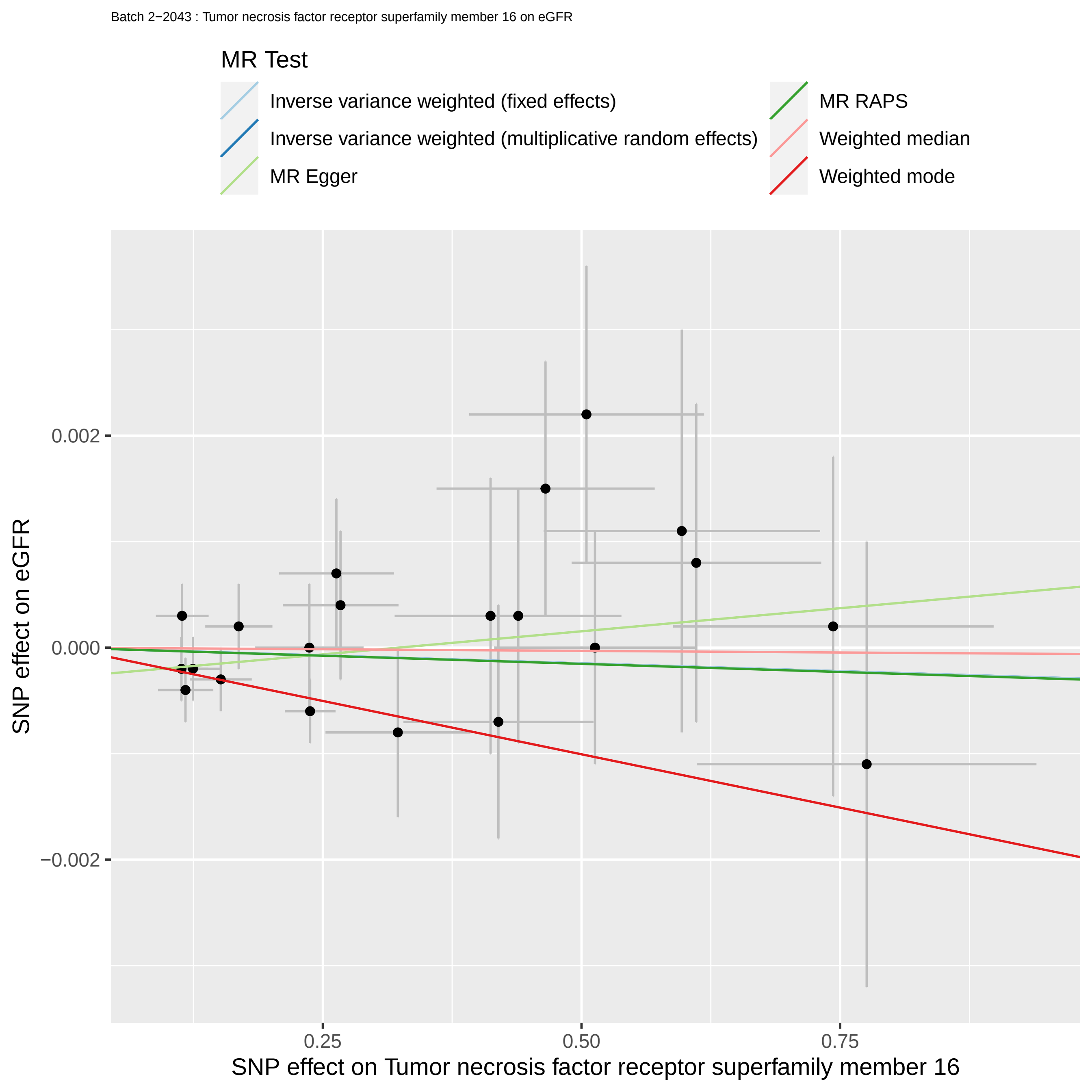
Batch 2−2043 : Tumor necrosis factor receptor superfamily member 16 on eGFR
MR Test
Inverse variance weighted (fixed effects)
Inverse variance weighted (multiplicative random effects)
MR Egger
MR RAPS
Weighted median
Weighted mode
0.002
0.000
−0.002
0.25
0.50
0.75
SNP effect on eGFR
SNP effect on Tumor necrosis factor receptor superfamily member 16

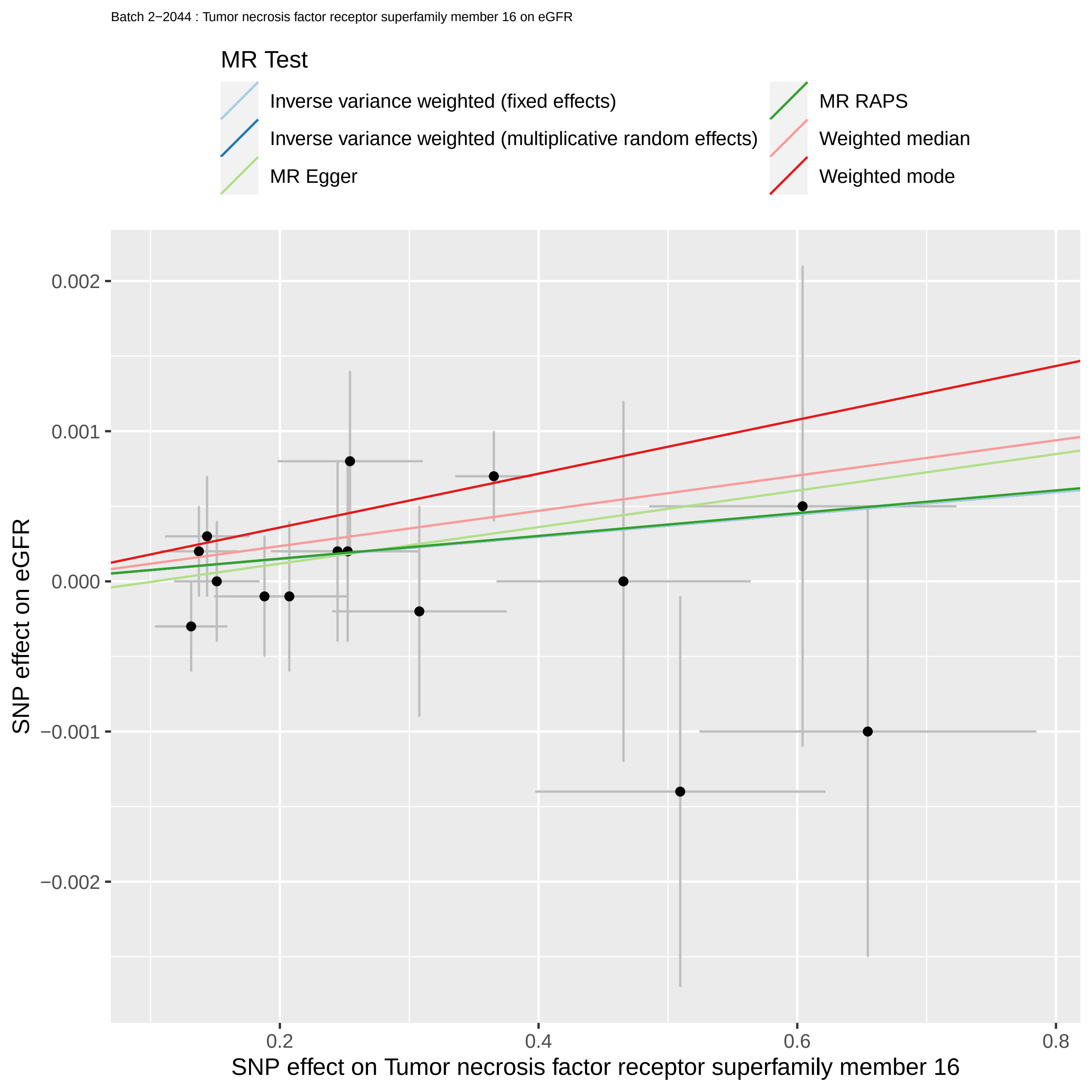

Batch 2−2044 : Tumor necrosis factor receptor superfamily member 16 on eGFR
MR Test
Inverse variance weighted (fixed effects)
Inverse variance weighted (multiplicative random effects)
MR Egger
MR RAPS
Weighted median
Weighted mode
0.002
0.001
0.000
−0.001
−0.002
0.2
0.4
0.6
0.8
SNP effect on eGFR
SNP effect on Tumor necrosis factor receptor superfamily member 16

Batch 2−2045 : Neugrin on eGFR

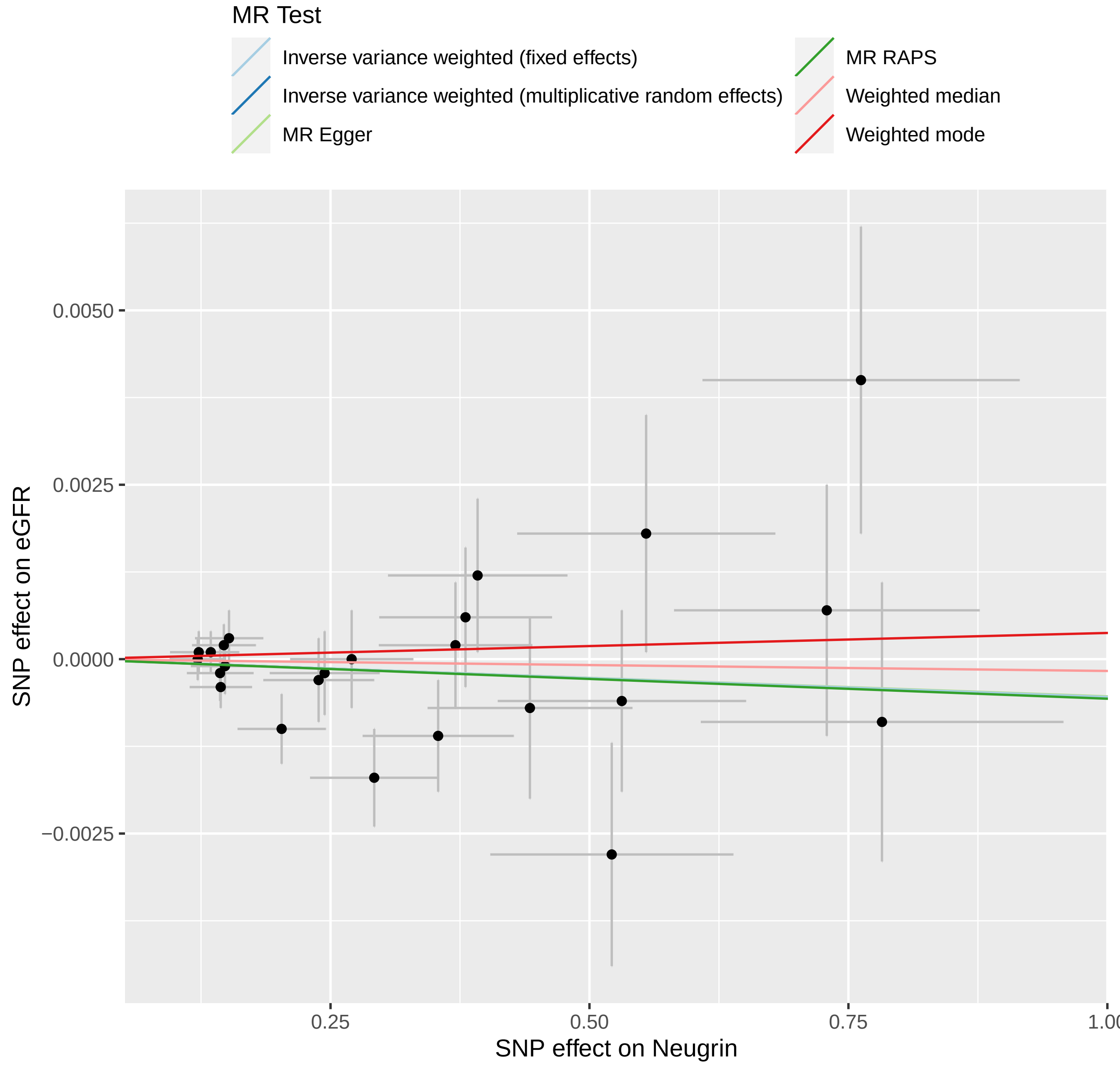

Batch 2−2046 : Non−homologous end−joining factor 1 on eGFR

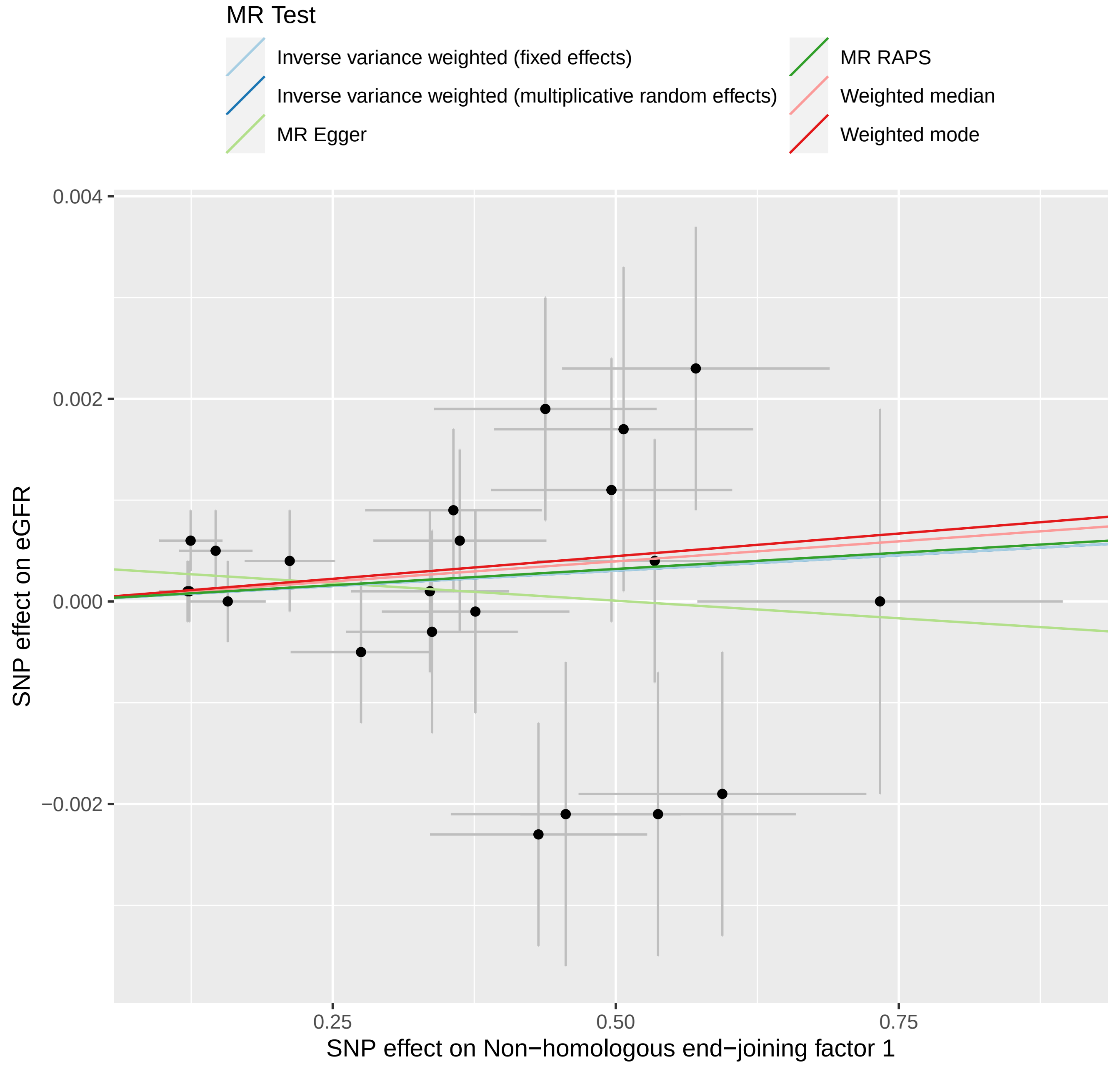

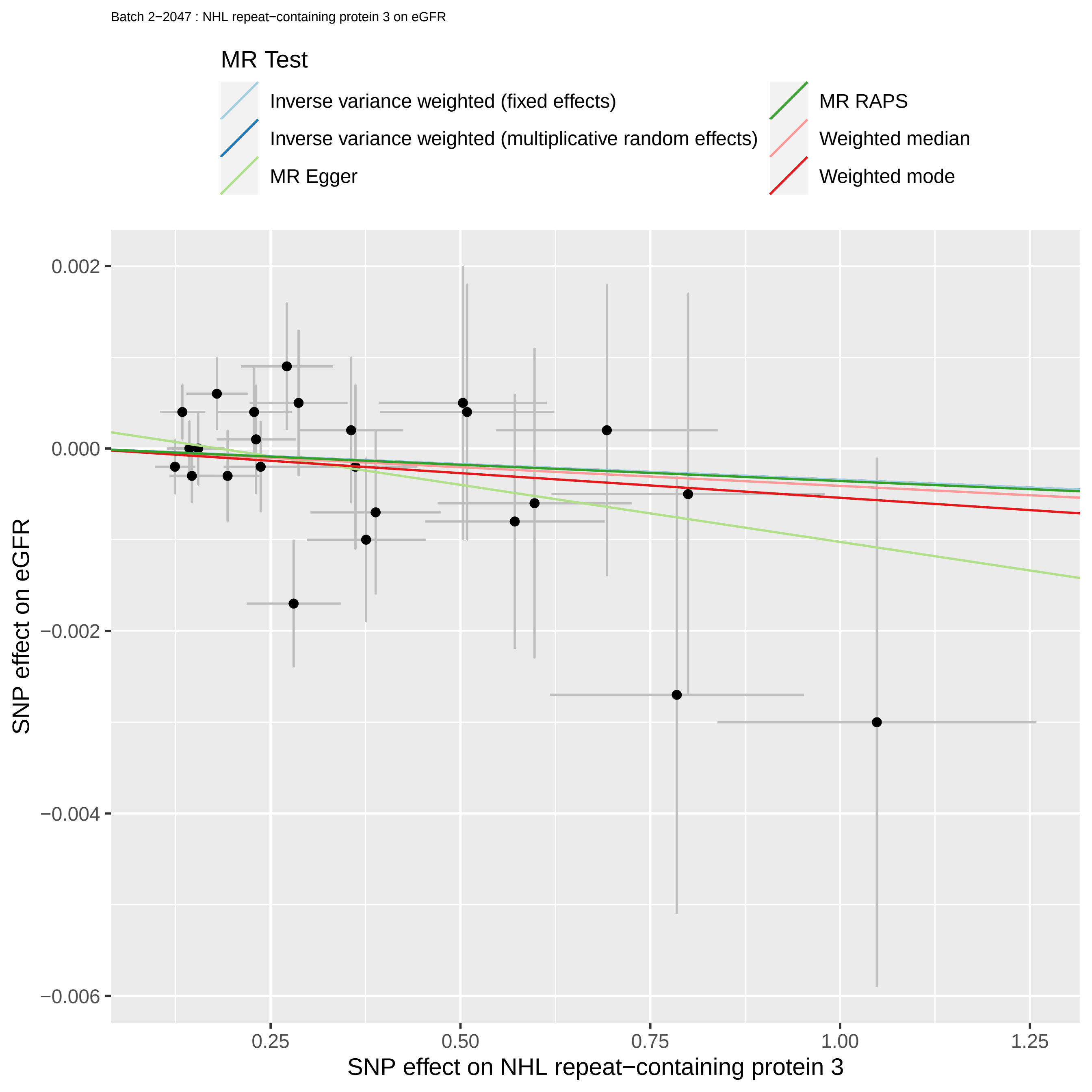
Batch 2−2047 : NHL repeat−containing protein 3 on eGFR
MR Test
Inverse variance weighted (fixed effects)
Inverse variance weighted (multiplicative random effects)
MR Egger
MR RAPS
Weighted median
Weighted mode
0.002
0.000
−0.002
−0.004
−0.006
0.25
0.50
0.75
1.00
1.25
SNP effect on eGFR
SNP effect on NHL repeat−containing protein 3

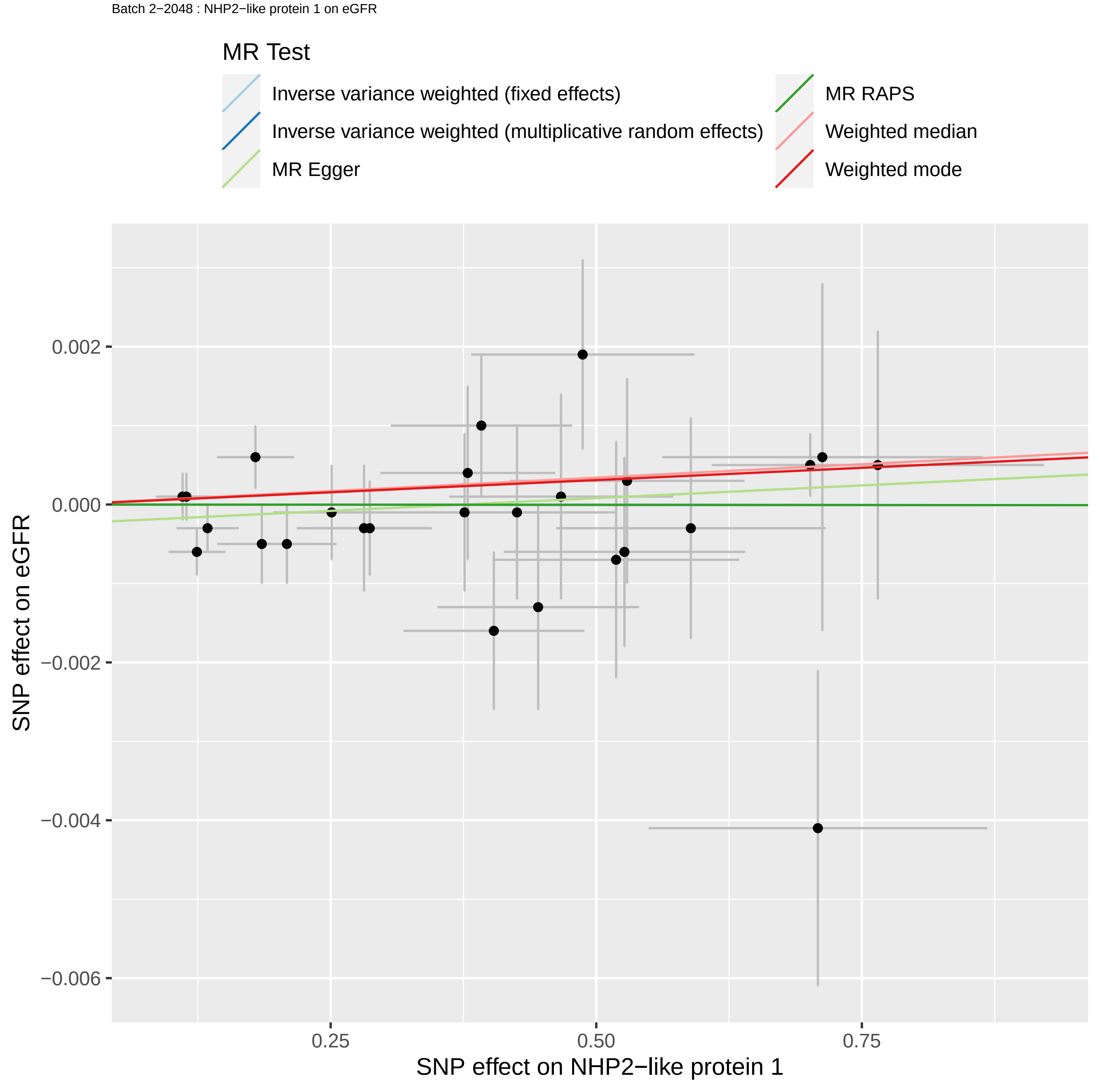

Batch 2−2048 : NHP2−like protein 1 on eGFR
MR Test
Inverse variance weighted (fixed effects)
Inverse variance weighted (multiplicative random effects)
MR Egger
MR RAPS
Weighted median
Weighted mode
0.002
0.000
−0.002
−0.004
−0.006
0.25
0.50
0.75
SNP effect on eGFR
SNP effect on NHP2−like protein 1

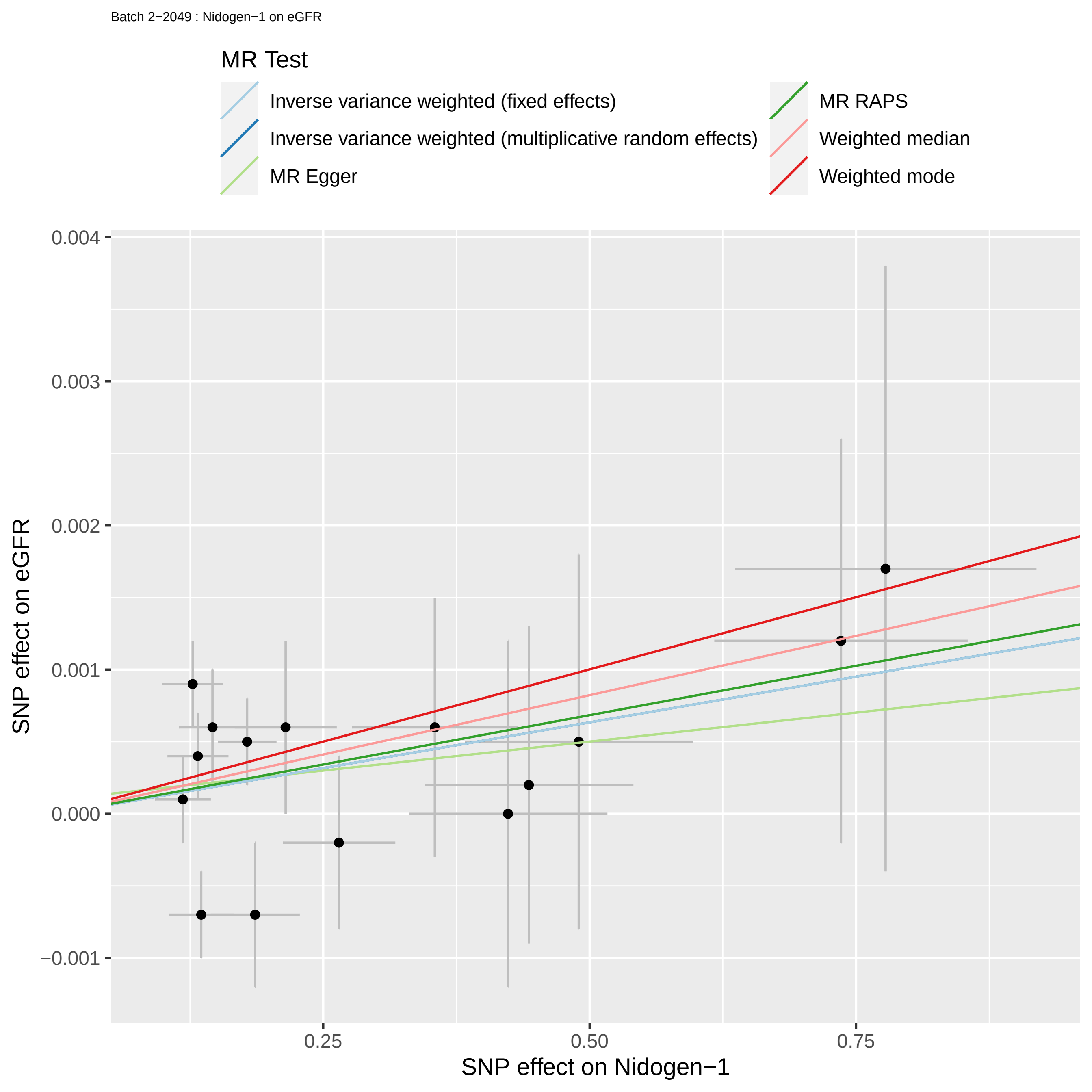

Batch 2−2049 : Nidogen−1 on eGFR
MR Test
Inverse variance weighted (fixed effects)
Inverse variance weighted (multiplicative random effects)
MR Egger
MR RAPS
Weighted median
Weighted mode
0.004
0.003
0.002
0.001
0.000
−0.001
0.25
0.50
0.75
SNP effect on eGFR
SNP effect on Nidogen−1

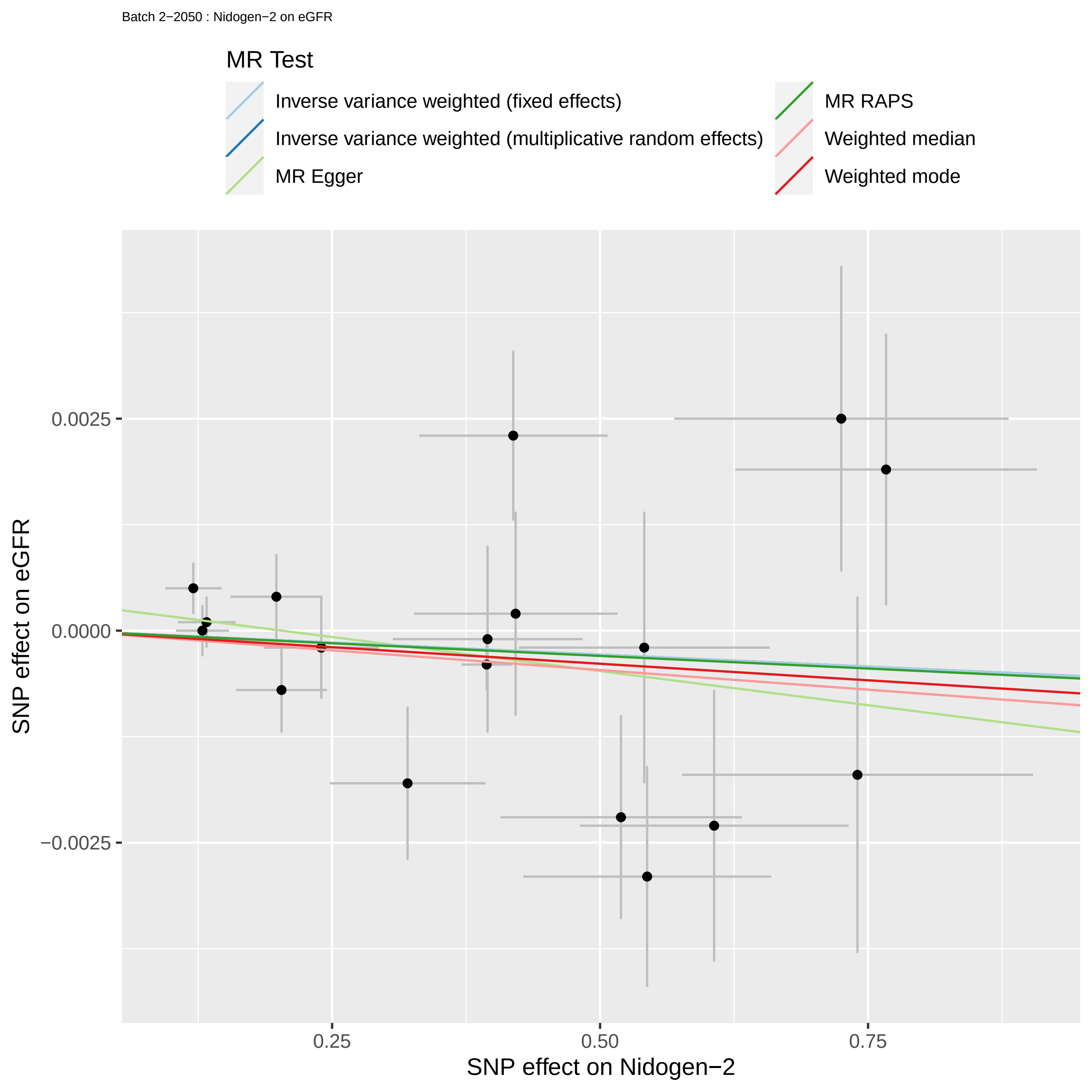

Batch 2−2050 : Nidogen−2 on eGFR
MR Test
Inverse variance weighted (fixed effects)
Inverse variance weighted (multiplicative random effects)
MR Egger
MR RAPS
Weighted median
Weighted mode
0.0025
0.0000
−0.0025
0.25
0.50
0.75
SNP effect on eGFR
SNP effect on Nidogen−2

Batch 2−2051 : Magnesium transporter NIPA4 on eGFR

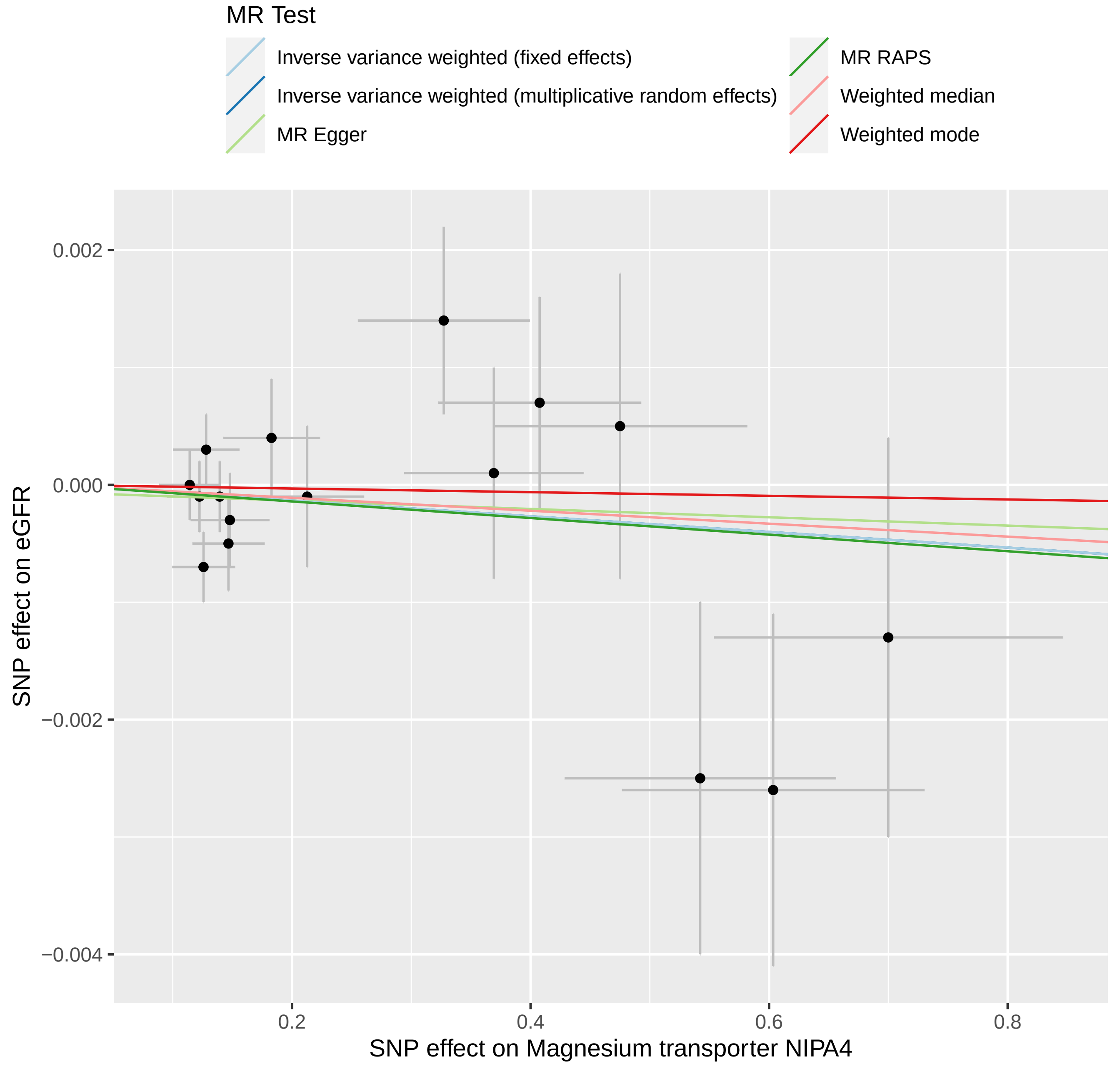

Batch 2−2052 : Nischarin on eGFR

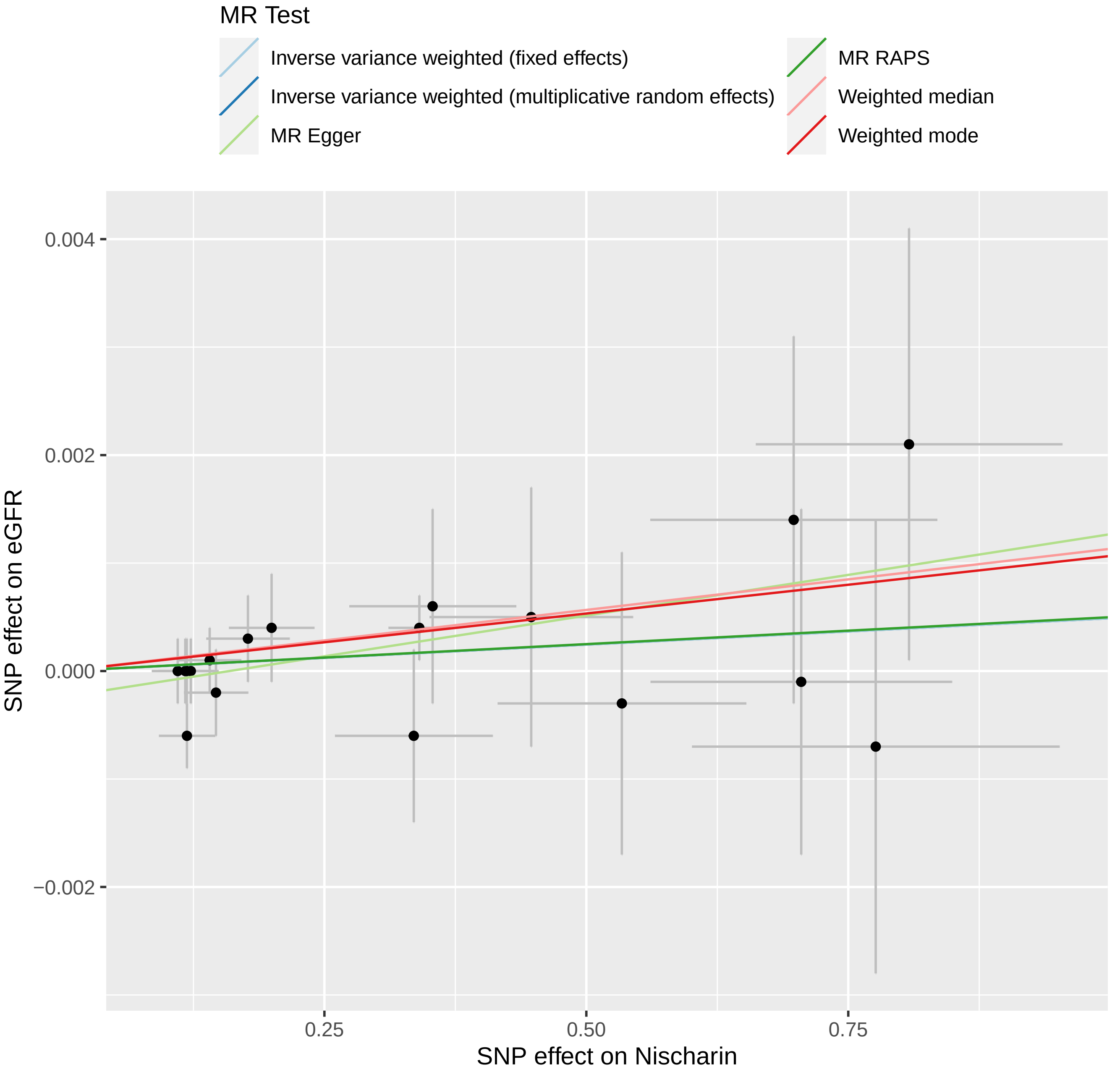

Batch 2−2053 : Neuroligin−1 on eGFR

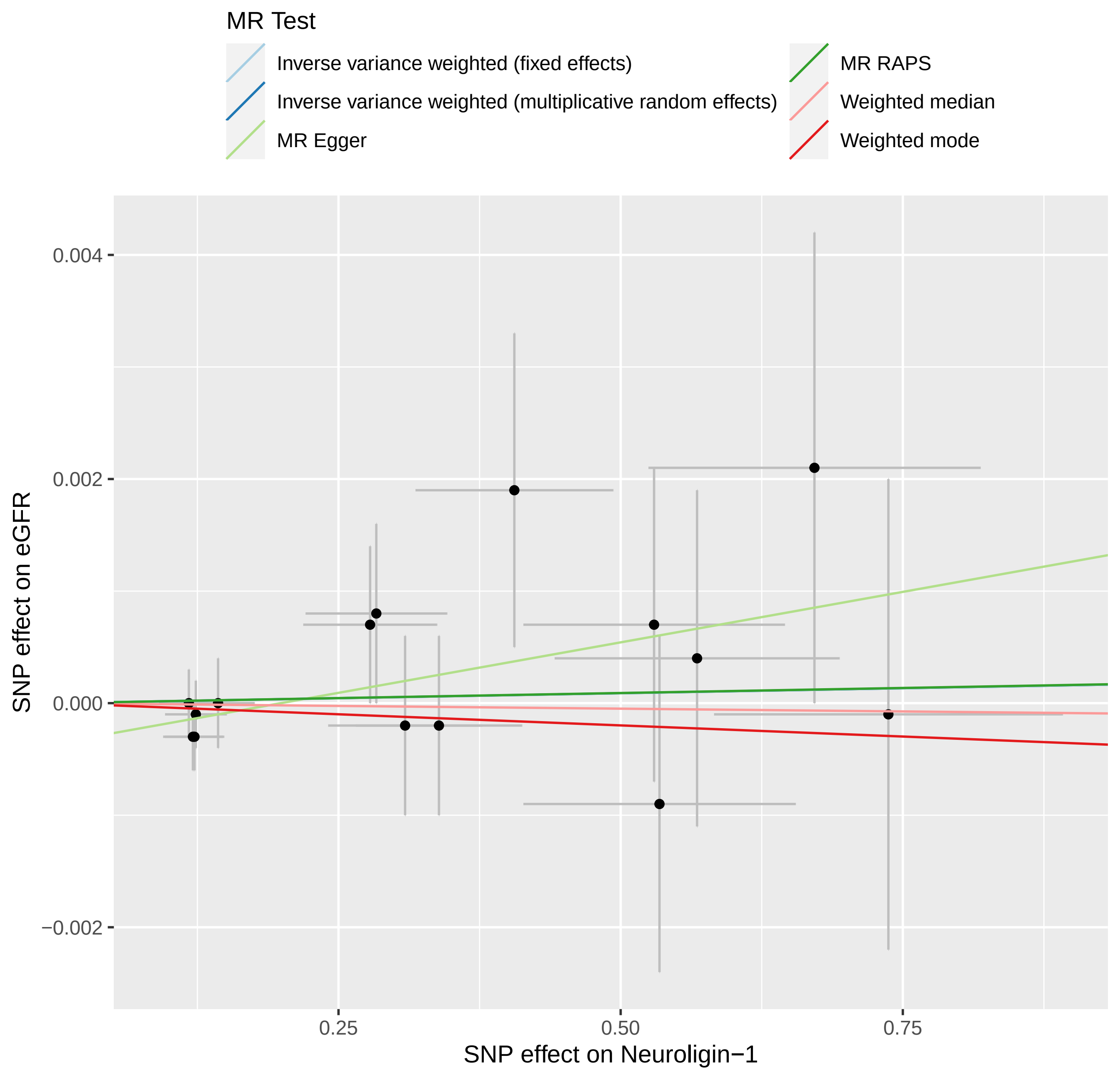

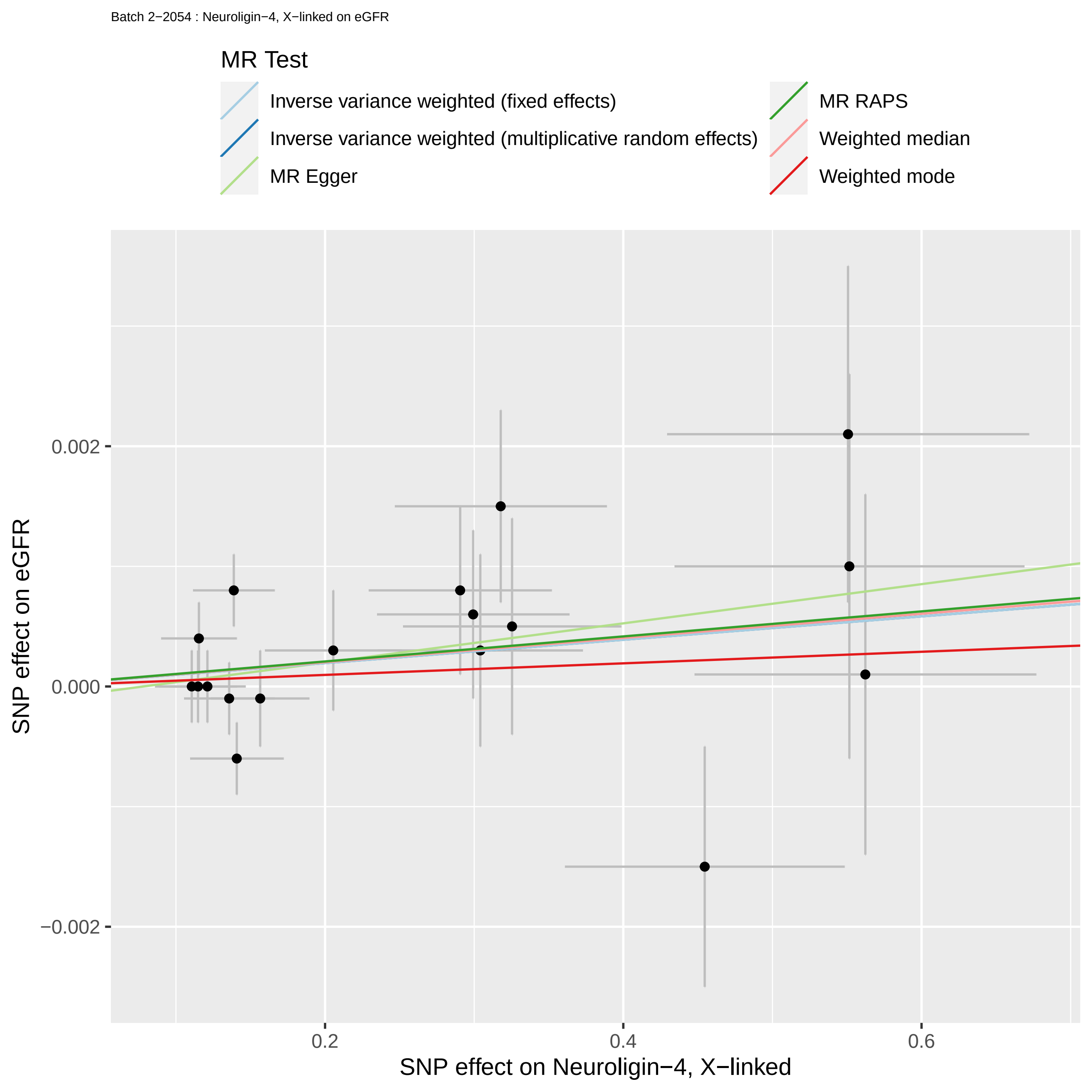

Batch 2−2054 : Neuroligin−4, X−linked on eGFR
MR Test
Inverse variance weighted (fixed effects)
Inverse variance weighted (multiplicative random effects)
MR Egger
MR RAPS
Weighted median
Weighted mode
0.002
0.000
−0.002
0.2
0.4
0.6
SNP effect on eGFR
SNP effect on Neuroligin−4, X−linked

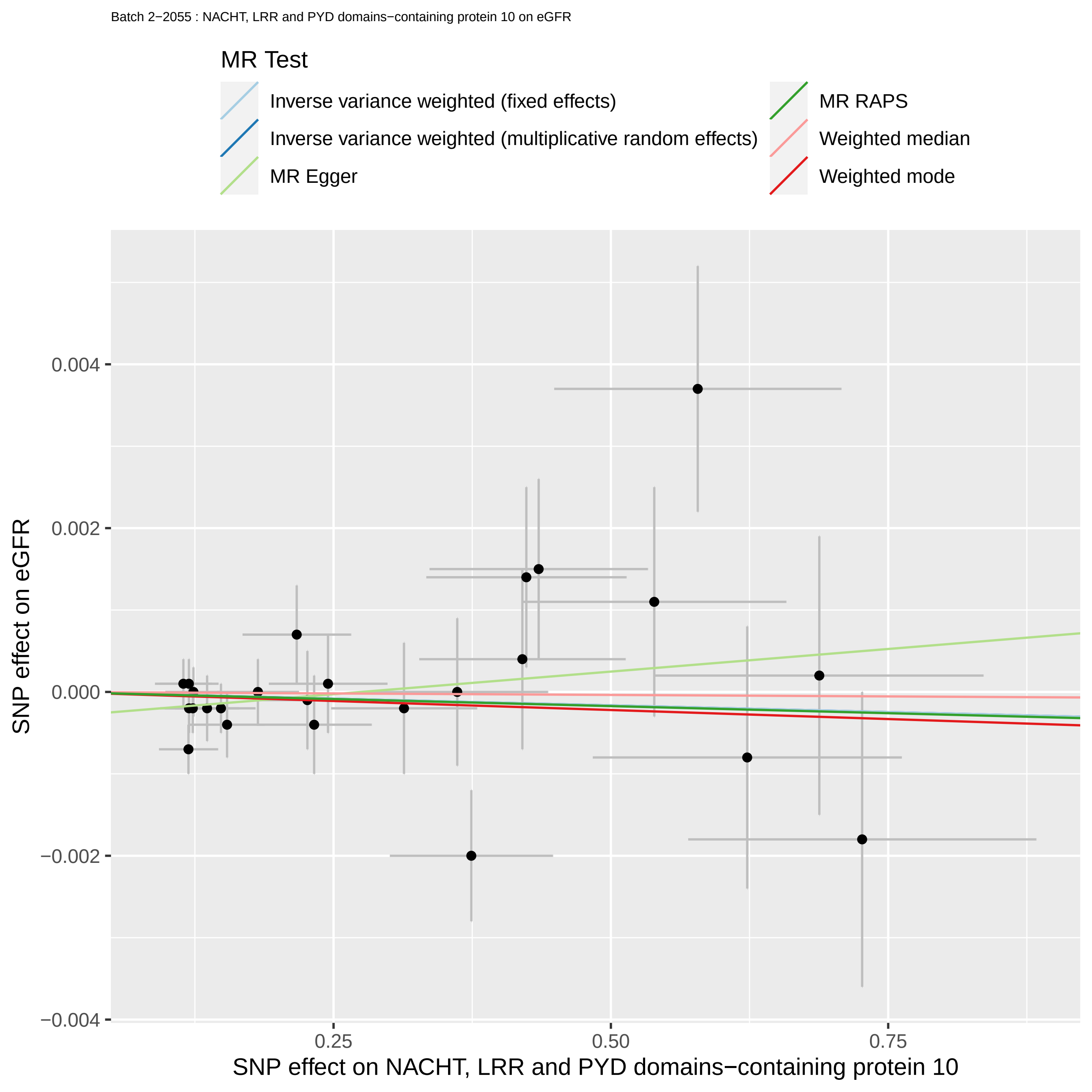
Batch 2−2055 : NACHT, LRR and PYD domains−containing protein 10 on eGFR
MR Test
Inverse variance weighted (fixed effects)
Inverse variance weighted (multiplicative random effects)
MR Egger
MR RAPS
Weighted median
Weighted mode
0.004
0.002
0.000
−0.002
−0.004
0.25
0.50
0.75
SNP effect on eGFR
SNP effect on NACHT, LRR and PYD domains−containing protein 10

Batch 2−2056 : NACHT, LRR and PYD domains−containing protein 1 on eGFR

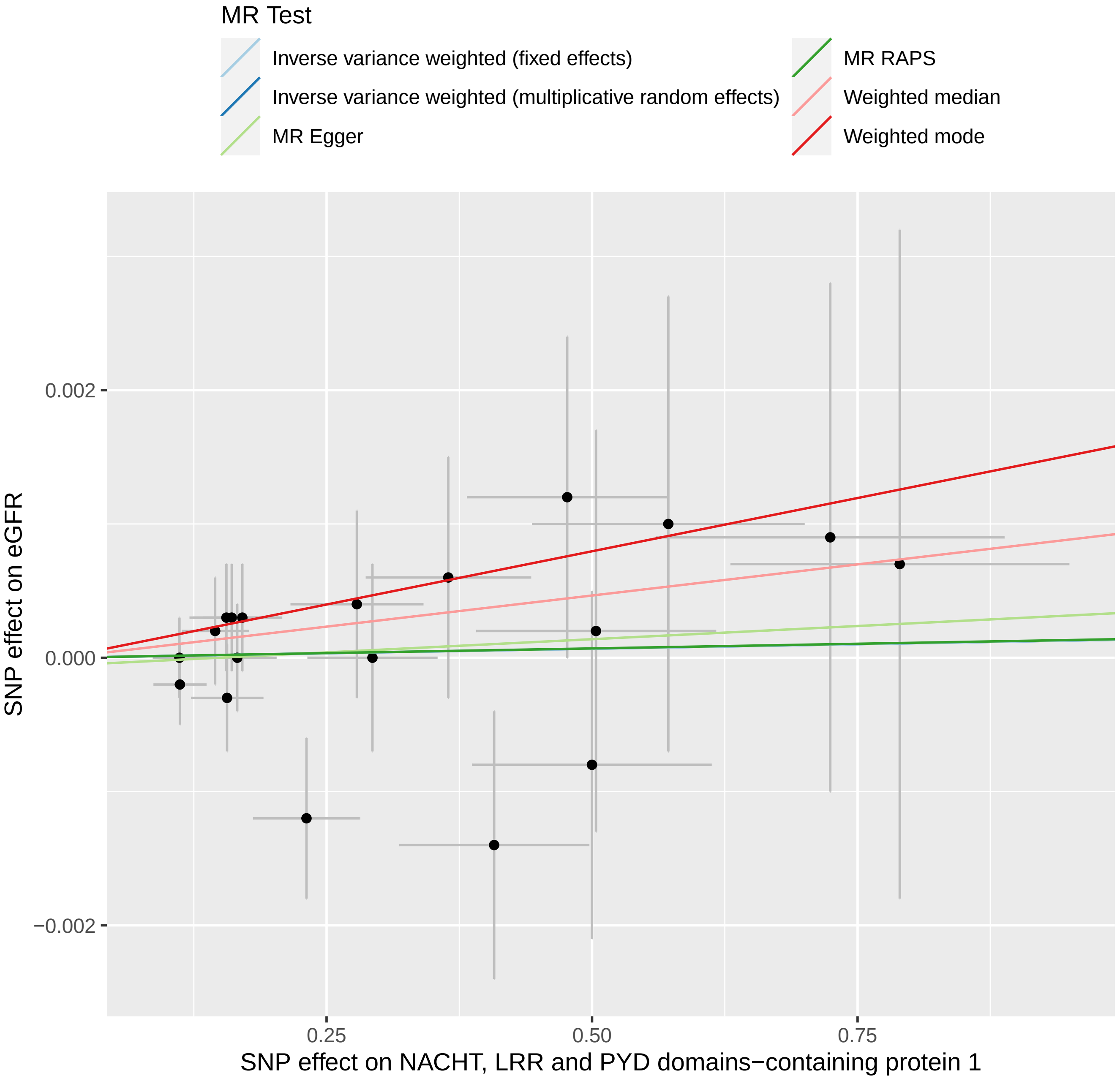

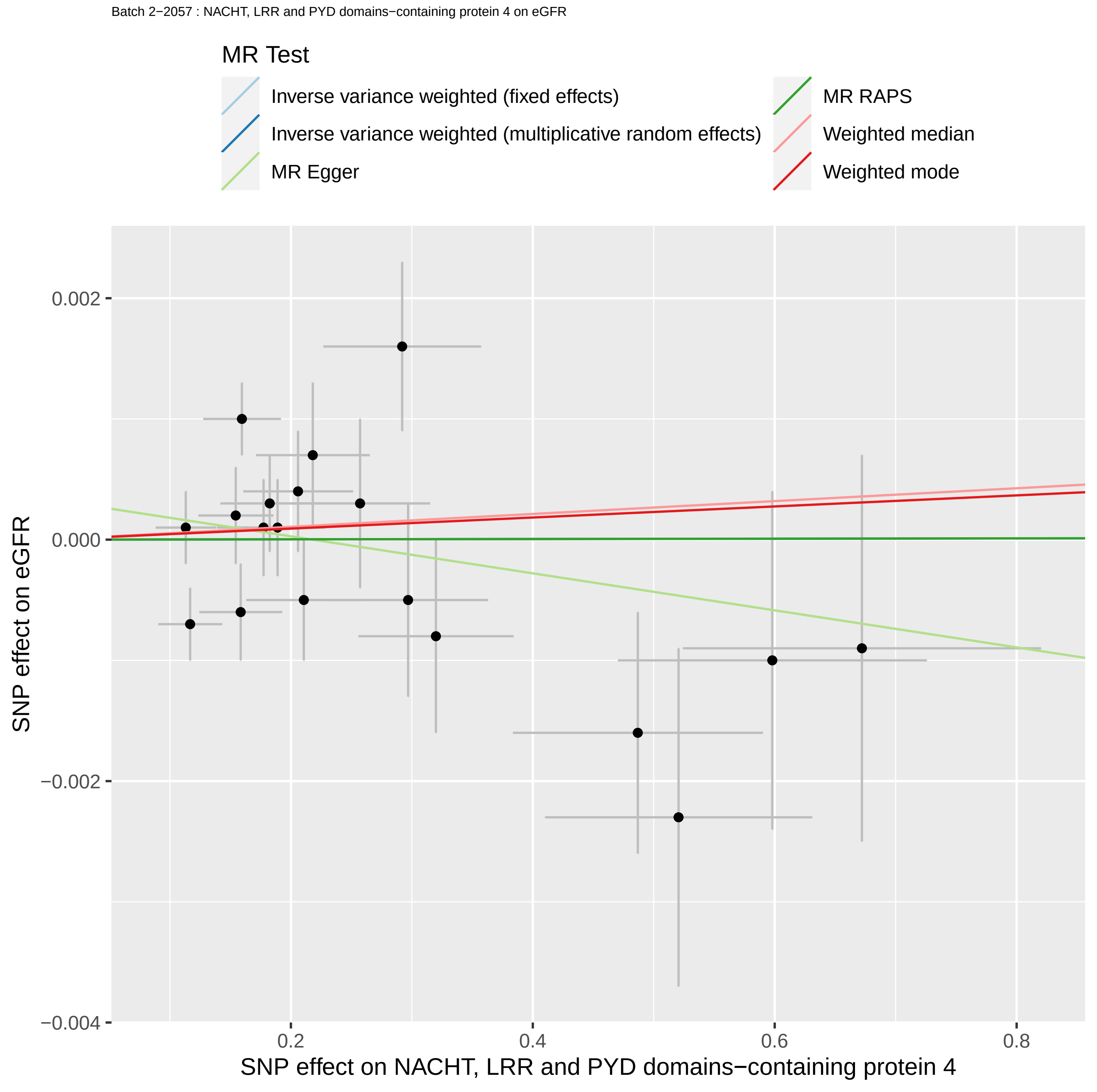
Batch 2−2057 : NACHT, LRR and PYD domains−containing protein 4 on eGFR
MR Test
Inverse variance weighted (fixed effects)
Inverse variance weighted (multiplicative random effects)
MR Egger
MR RAPS
Weighted median
Weighted mode
0.002
0.000
−0.002
−0.004
0.2
0.4
0.6
0.8
SNP effect on eGFR
SNP effect on NACHT, LRR and PYD domains−containing protein 4

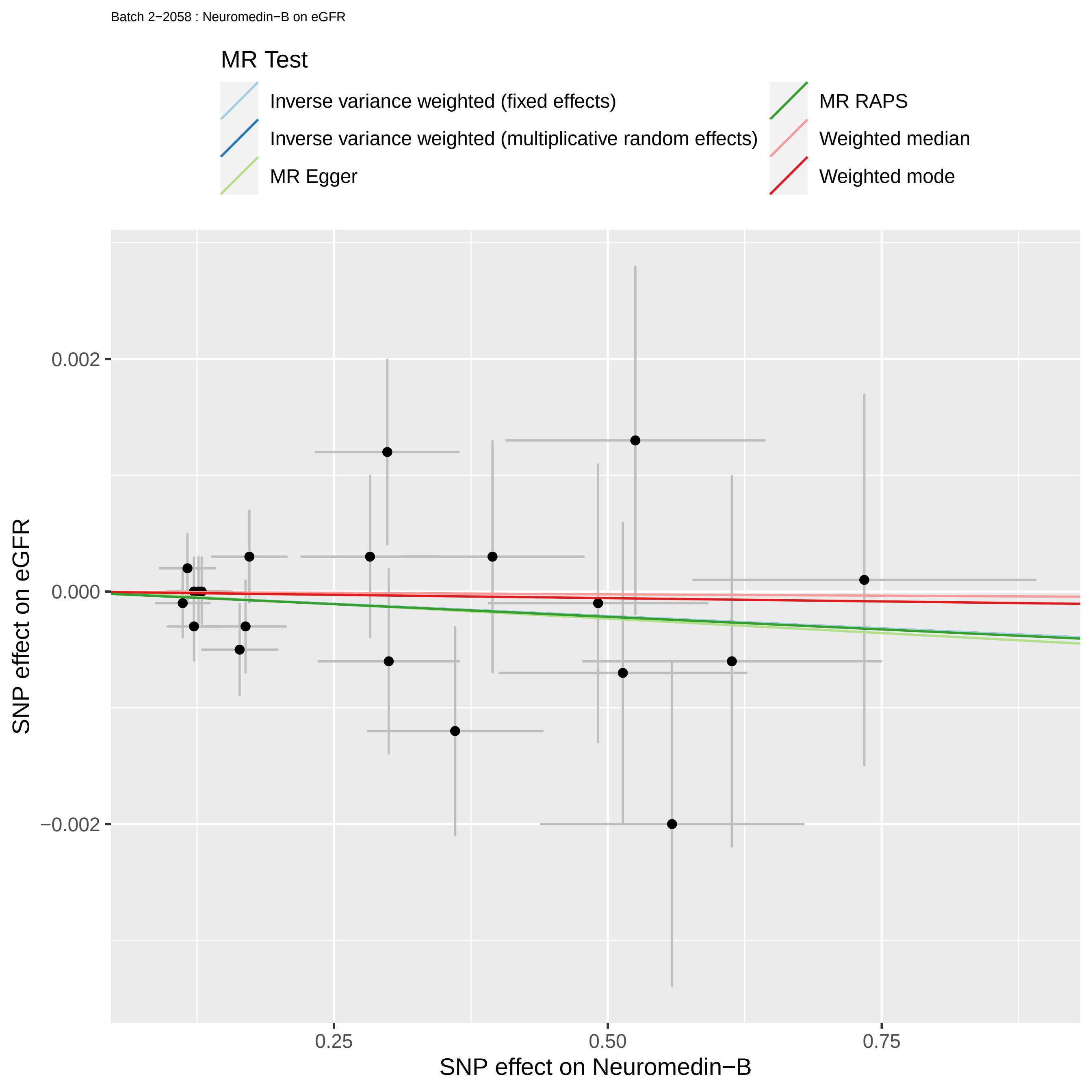
Batch 2−2058 : Neuromedin−B on eGFR
MR Test
Inverse variance weighted (fixed effects)
Inverse variance weighted (multiplicative random effects)
MR Egger
MR RAPS
Weighted median
Weighted mode
0.002
0.000
−0.002
SNP effect on eGFR
0.25
0.50
0.75
SNP effect on Neuromedin−B

Batch 2−2059 : Nucleoside diphosphate kinase A on eGFR

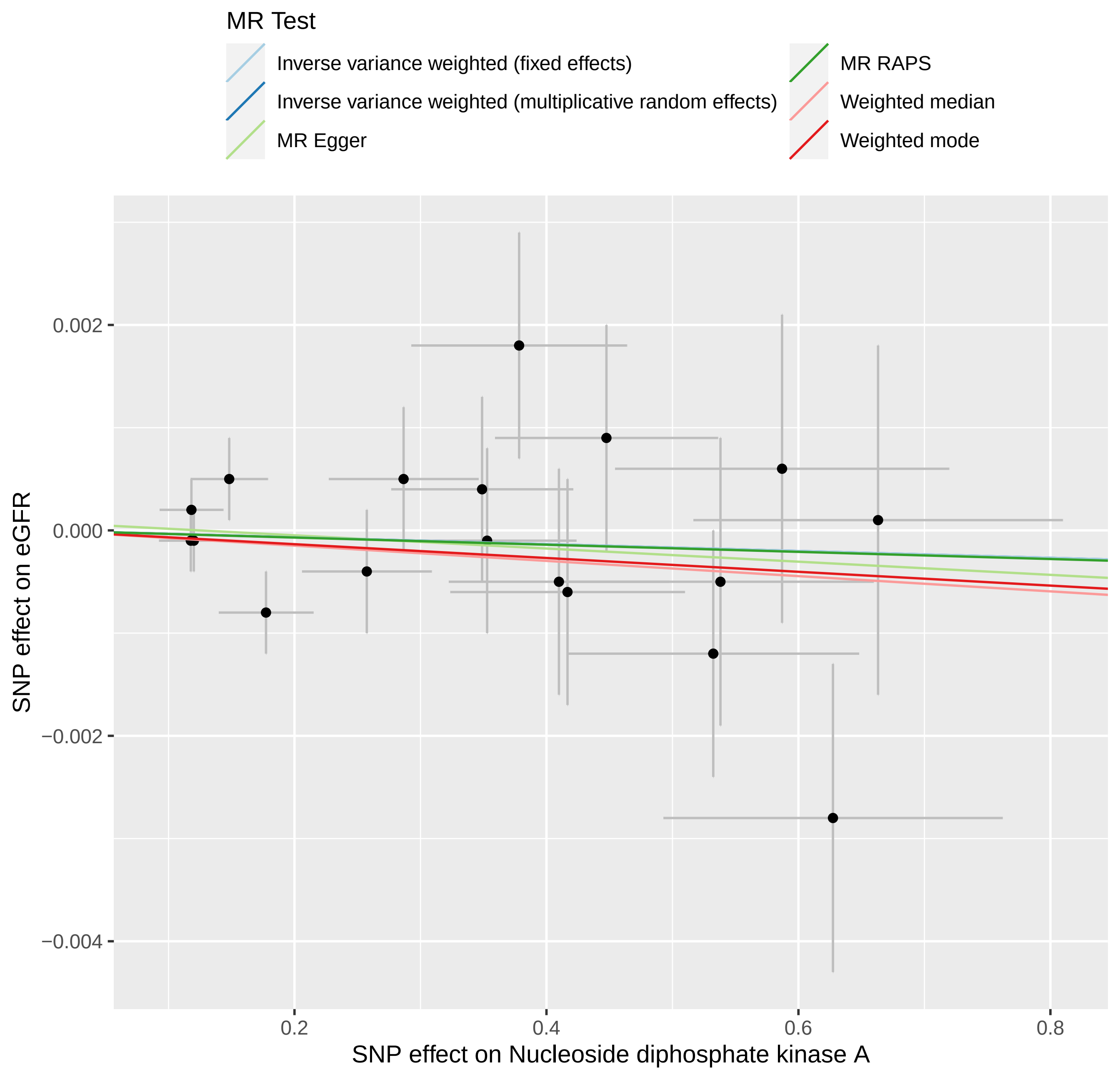

Batch 2−2060 : Nucleoside diphosphate kinase B on eGFR

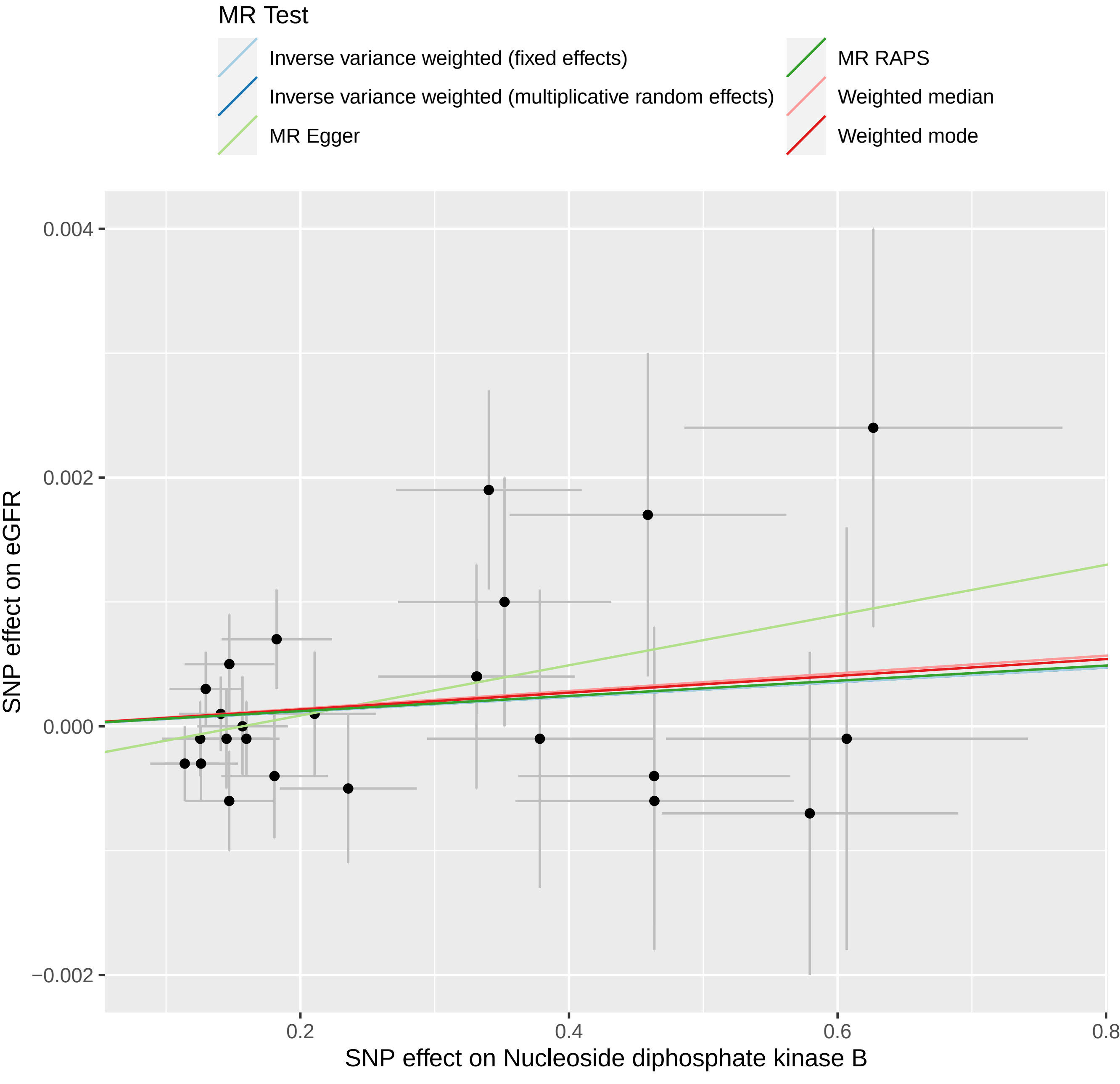

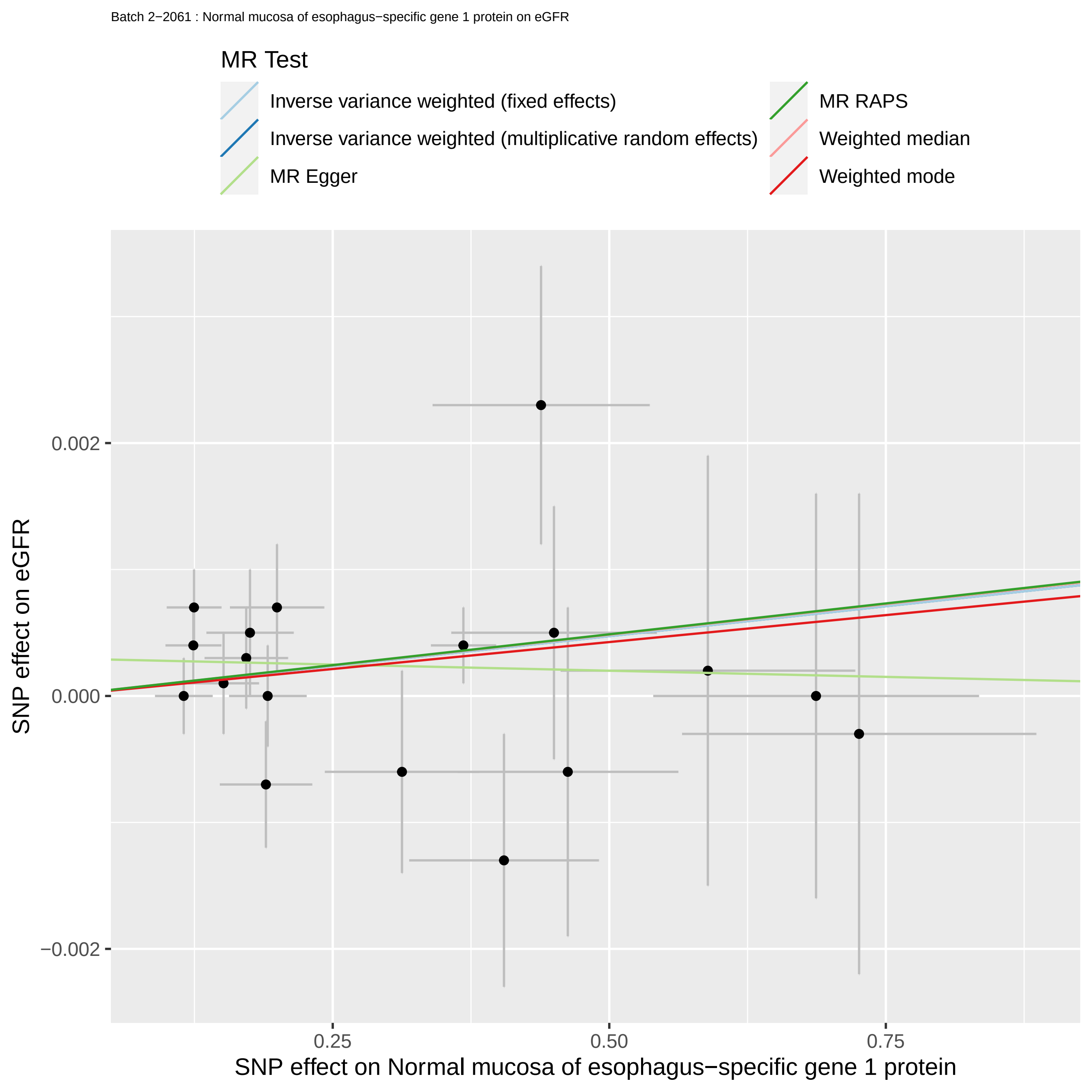

Batch 2−2061 : Normal mucosa of esophagus−specific gene 1 protein on eGFR
MR Test
Inverse variance weighted (fixed effects)
Inverse variance weighted (multiplicative random effects)
MR Egger
MR RAPS
Weighted median
Weighted mode
0.002
0.000
−0.002
0.25
0.50
0.75
SNP effect on eGFR
SNP effect on Normal mucosa of esophagus−specific gene 1 protein

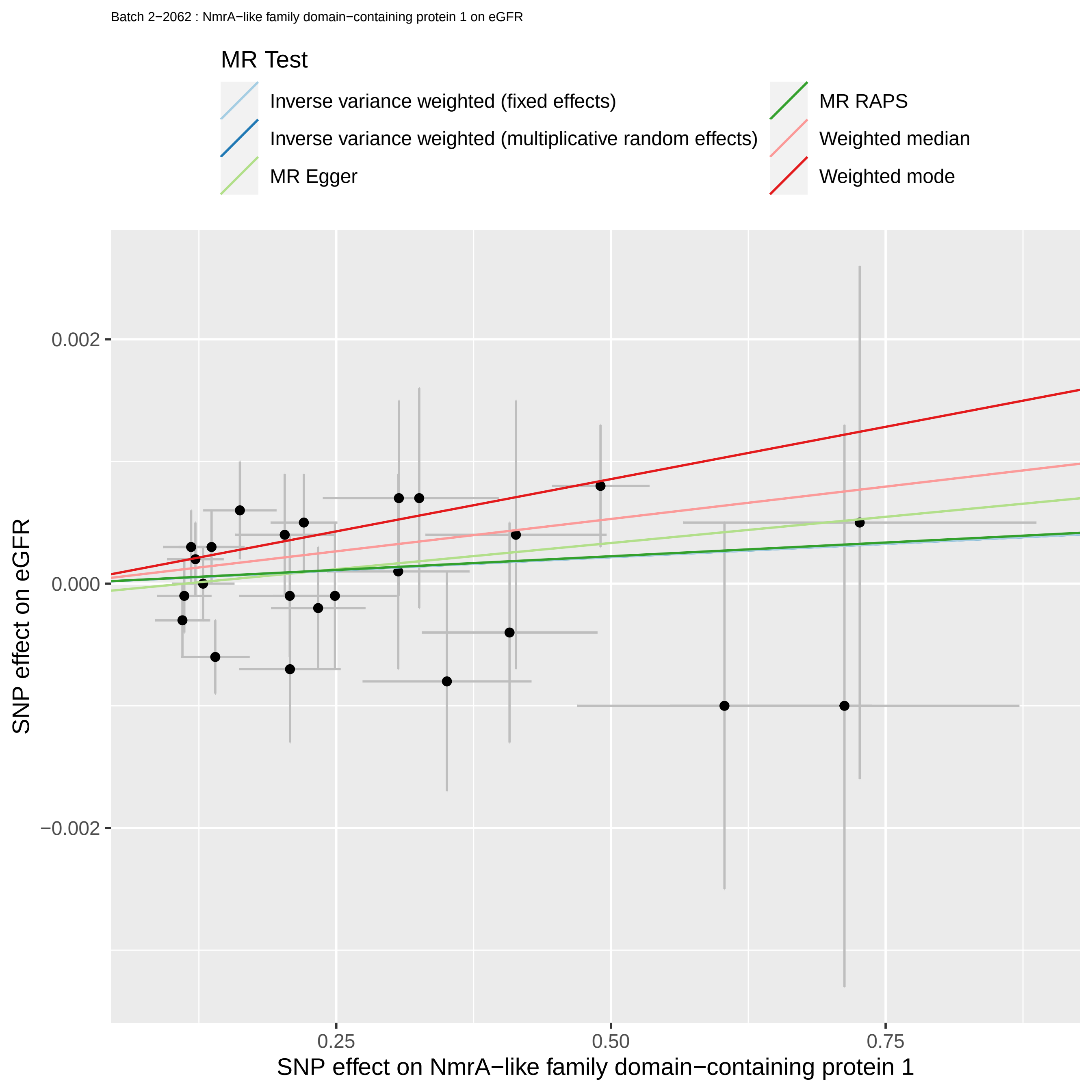

Batch 2−2062 : NmrA−like family domain−containing protein 1 on eGFR
MR Test
Inverse variance weighted (fixed effects)
Inverse variance weighted (multiplicative random effects)
MR Egger
MR RAPS
Weighted median
Weighted mode
0.002
0.000
−0.002
SNP effect on eGFR
0.25
0.50
0.75
SNP effect on NmrA−like family domain−containing protein 1

Batch 2−2063 : Glycylpeptide N−tetradecanoyltransferase 1 on eGFR

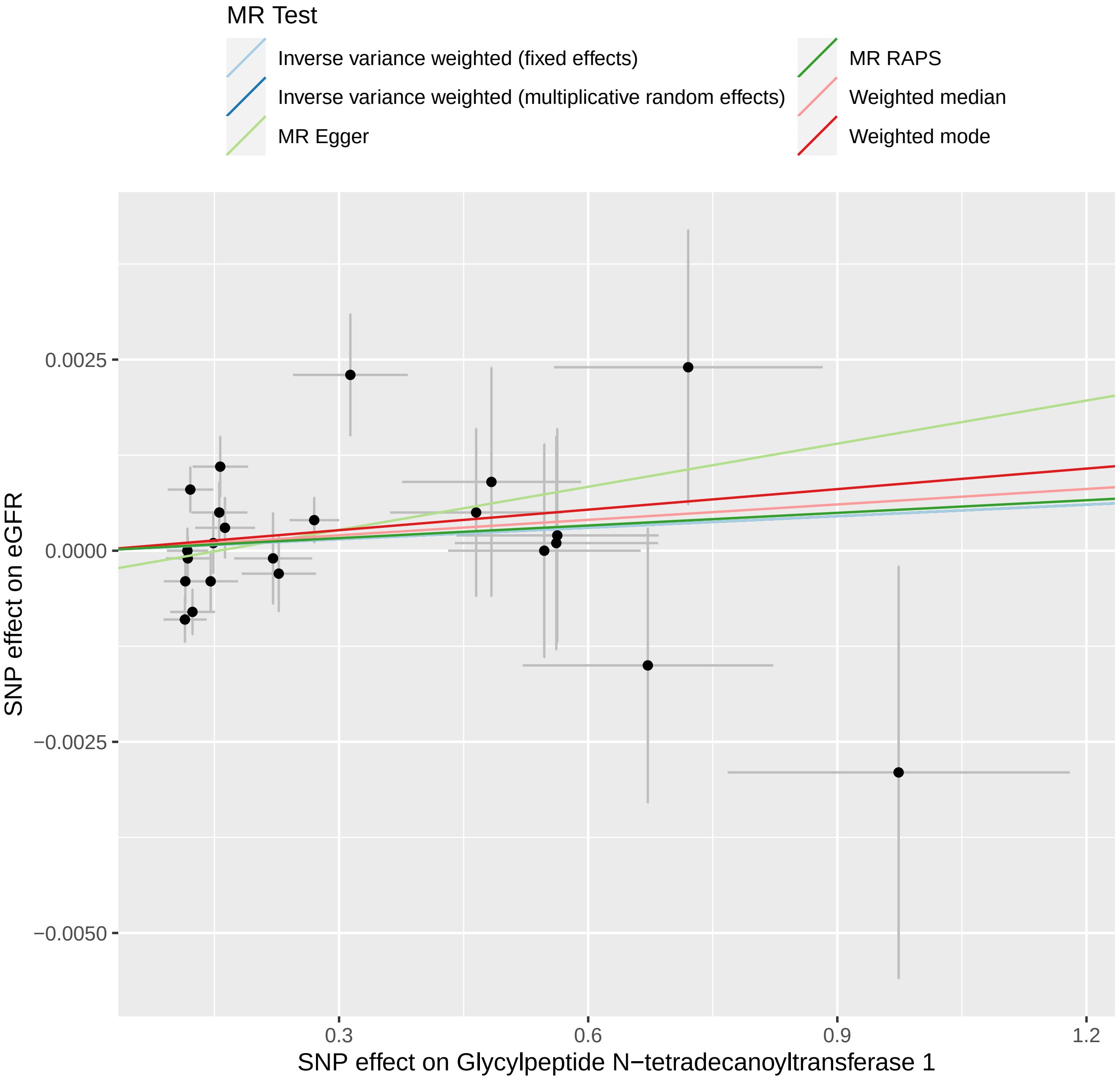

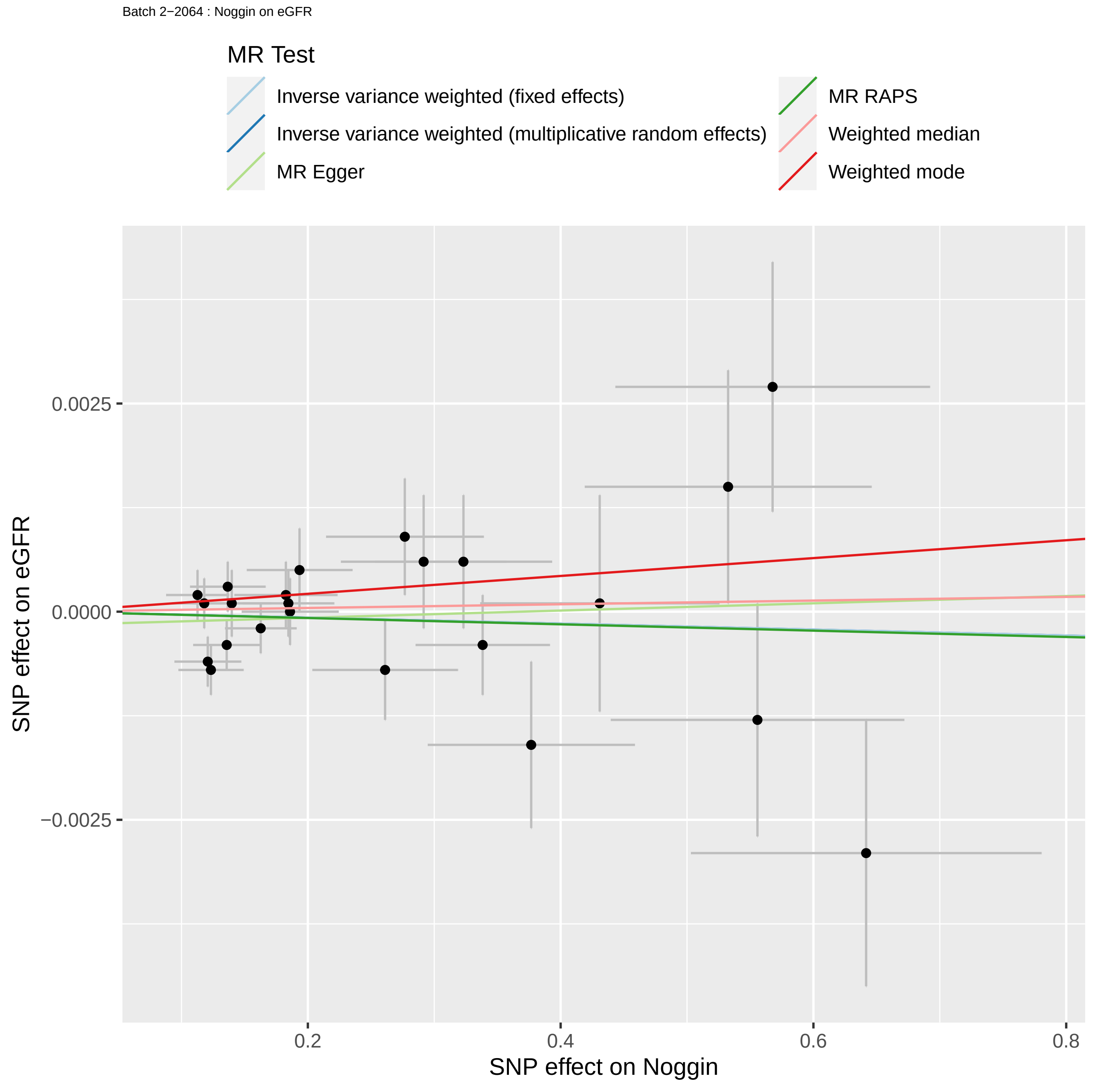
Batch 2−2064 : Noggin on eGFR
MR Test
Inverse variance weighted (fixed effects)
Inverse variance weighted (multiplicative random effects)
MR Egger
MR RAPS
Weighted median
Weighted mode
0.0025
0.0000
−0.0025
0.2
0.4
0.6
0.8
SNP effect on Noggin
SNP effect on eGFR

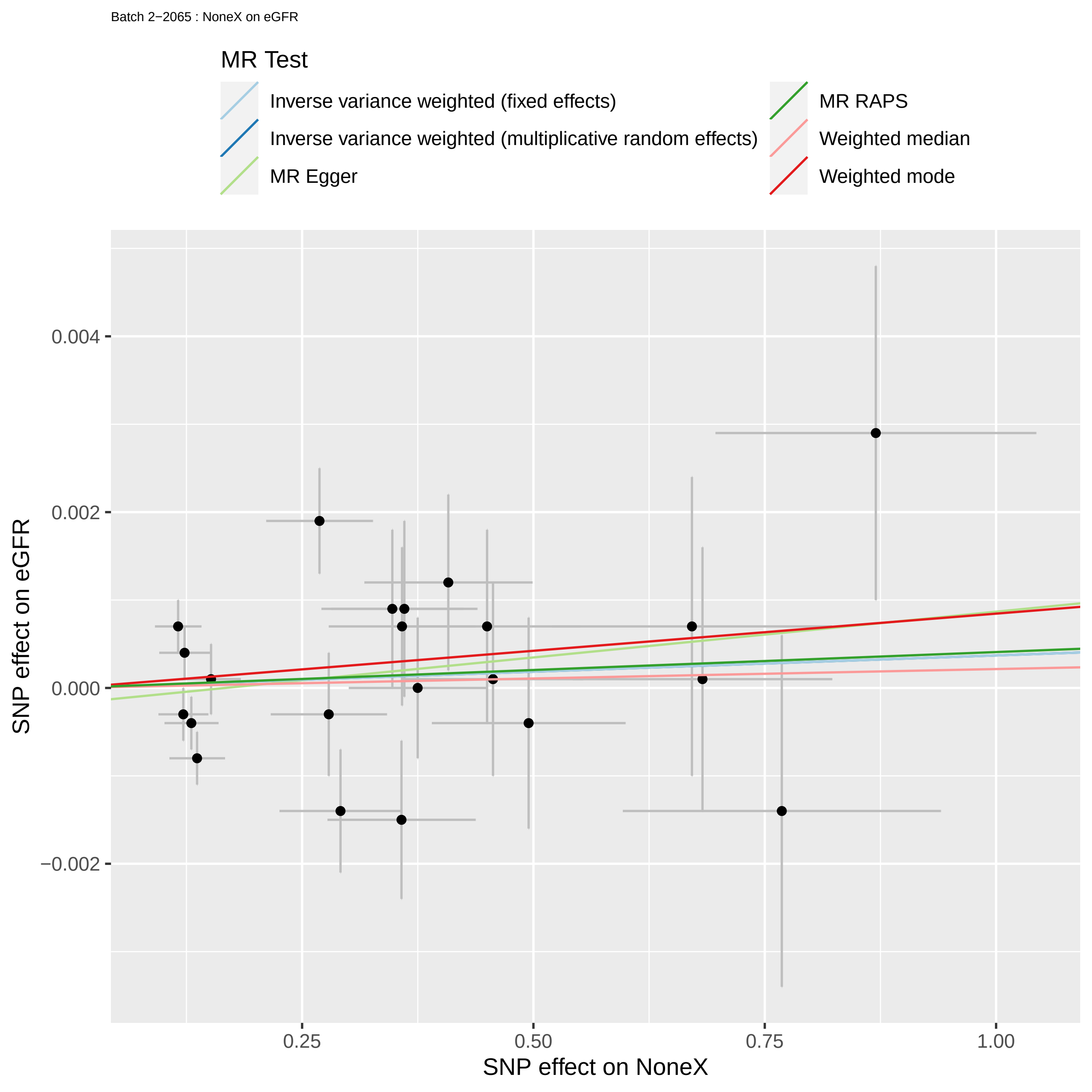

Batch 2−2065 : NoneX on eGFR
MR Test
Inverse variance weighted (fixed effects)
Inverse variance weighted (multiplicative random effects)
MR Egger
MR RAPS
Weighted median
Weighted mode
0.004
0.002
0.000
−0.002
0.25
0.50
0.75
1.00
SNP effect on eGFR
SNP effect on NoneX

Batch 2−2066 : NoneX on eGFR

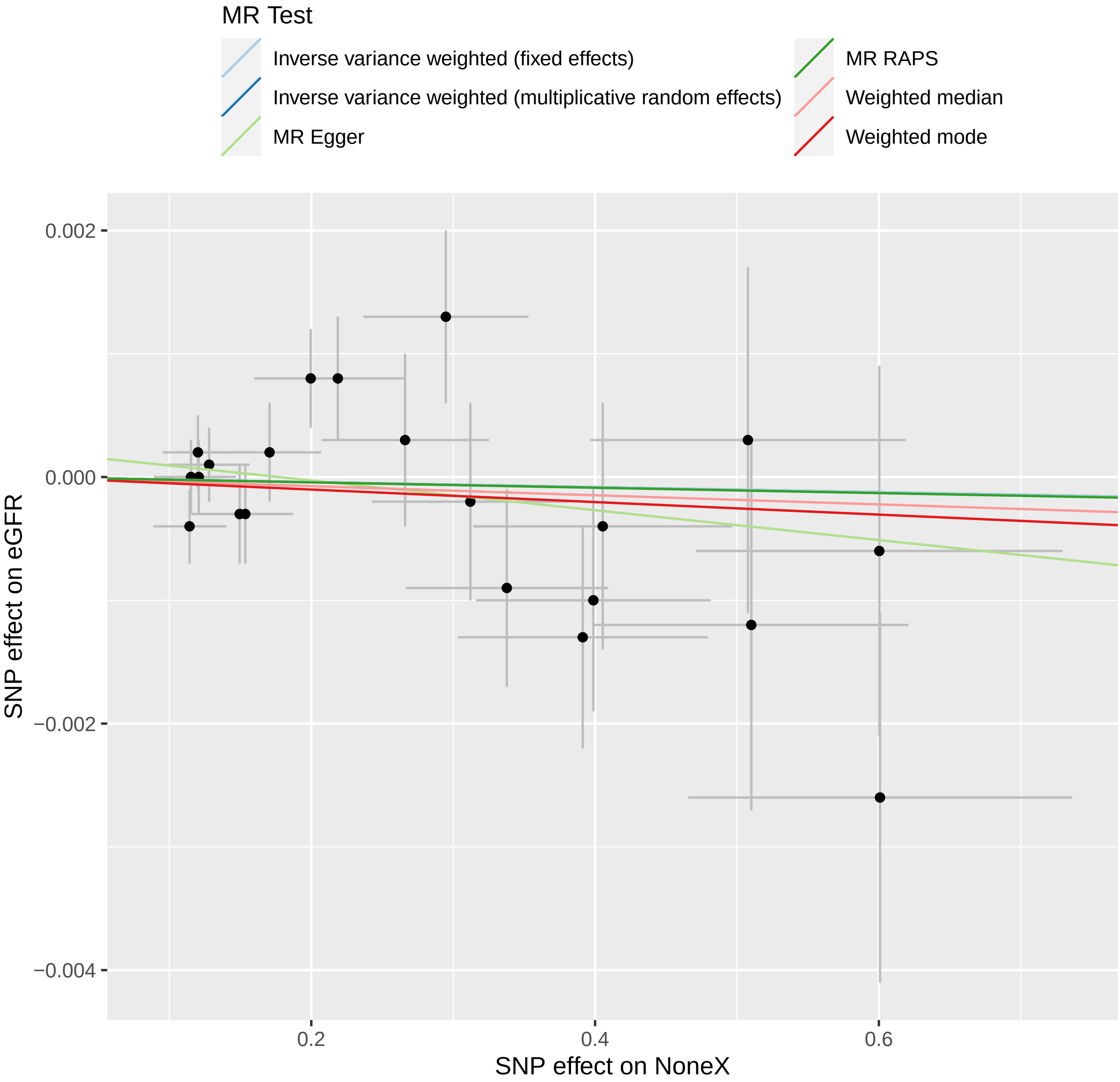

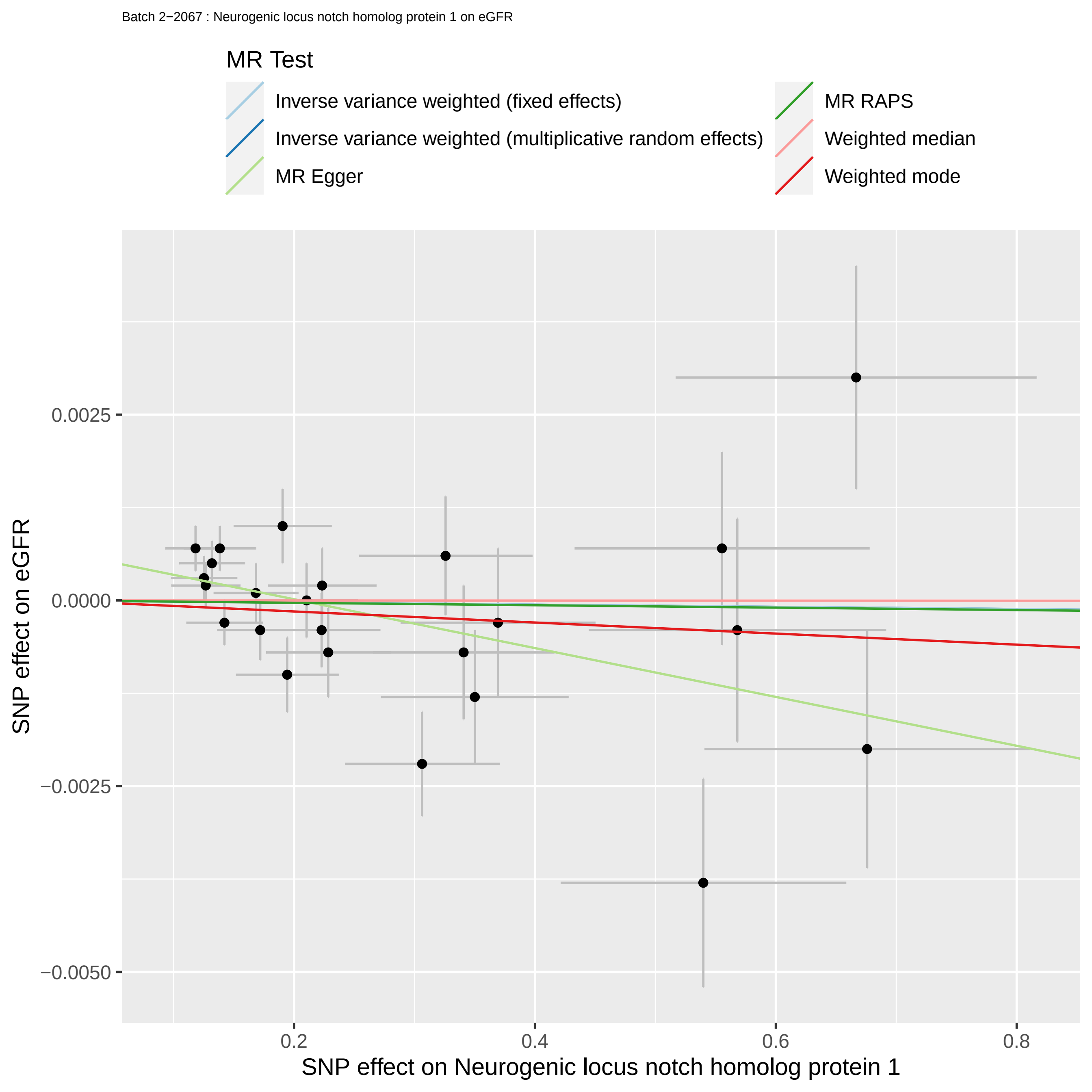

Batch 2−2067 : Neurogenic locus notch homolog protein 1 on eGFR
MR Test
Inverse variance weighted (fixed effects)
Inverse variance weighted (multiplicative random effects)
MR Egger
MR RAPS
Weighted median
Weighted mode
0.0025
0.0000
−0.0025
−0.0050
0.2
0.4
0.6
0.8
SNP effect on eGFR
SNP effect on Neurogenic locus notch homolog protein 1

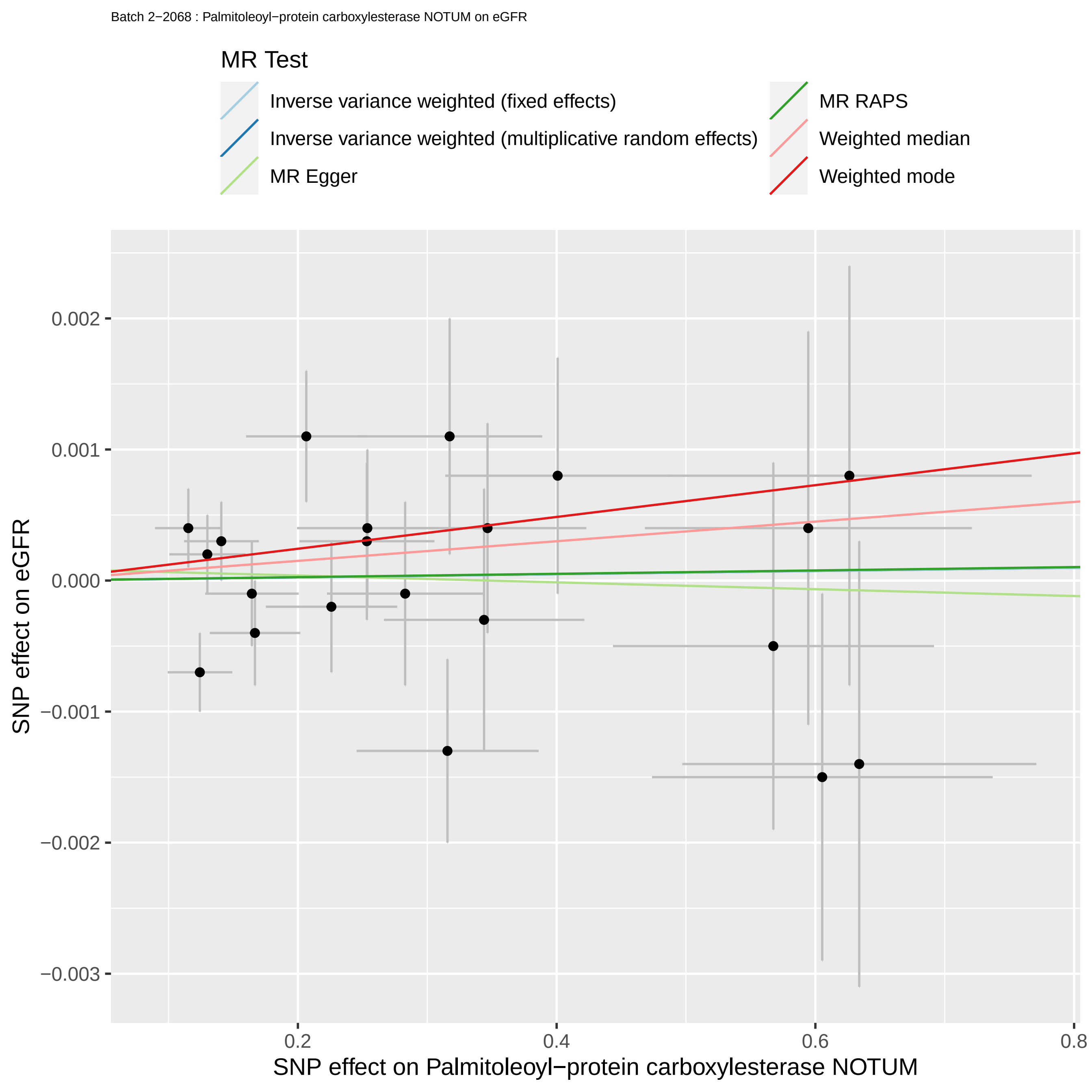

Batch 2−2068 : Palmitoleoyl−protein carboxylesterase NOTUM on eGFR
MR Test
Inverse variance weighted (fixed effects)
Inverse variance weighted (multiplicative random effects)
MR Egger
MR RAPS
Weighted median
Weighted mode
0.002
0.001
0.000
−0.001
−0.002
−0.003
SNP effect on eGFR
0.2
0.4
0.6
0.8
SNP effect on Palmitoleoyl−protein carboxylesterase NOTUM

Batch 2−2069 : Protein NOV homolog on eGFR

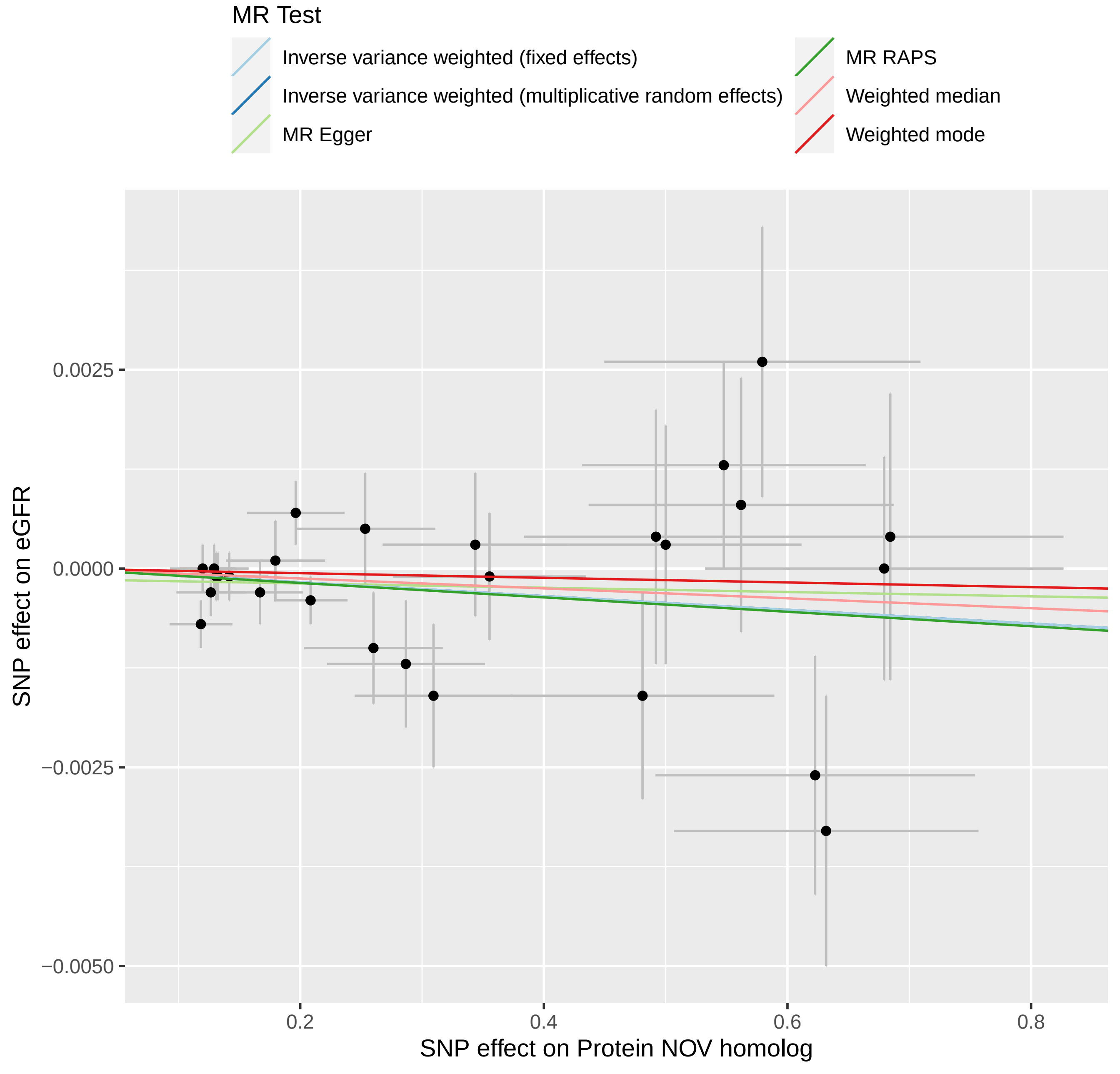

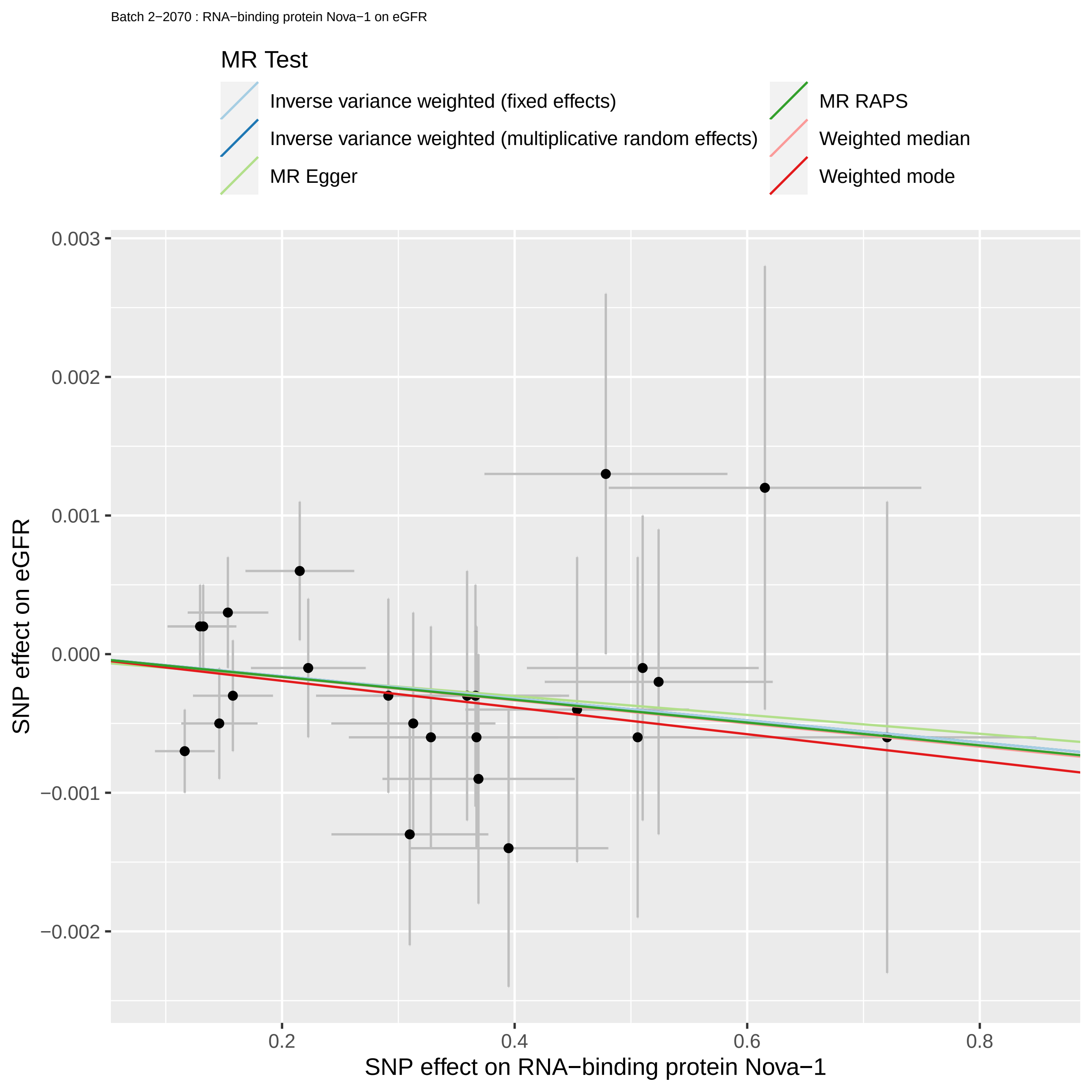

Batch 2−2070 : RNA−binding protein Nova−1 on eGFR
MR Test
Inverse variance weighted (fixed effects)
Inverse variance weighted (multiplicative random effects)
MR Egger
MR RAPS
Weighted median
Weighted mode
0.003
0.002
0.001
0.000
−0.001
−0.002
SNP effect on eGFR
0.2
0.4
0.6
0.8
SNP effect on RNA−binding protein Nova−1

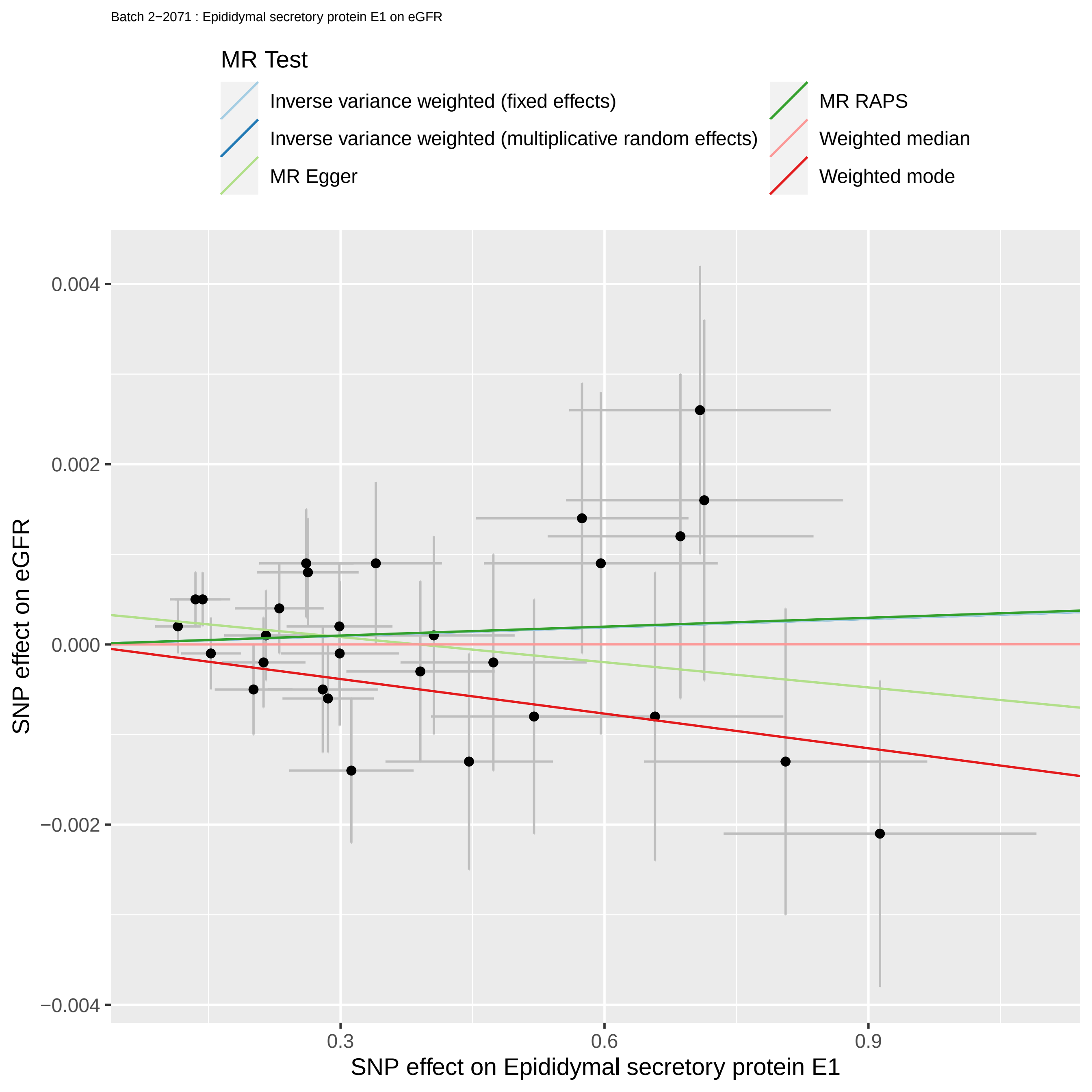

Batch 2−2071 : Epididymal secretory protein E1 on eGFR
MR Test
Inverse variance weighted (fixed effects)
Inverse variance weighted (multiplicative random effects)
MR Egger
MR RAPS
Weighted median
Weighted mode
0.004
0.002
0.000
−0.002
−0.004
SNP effect on eGFR
0.3
0.6
0.9
SNP effect on Epididymal secretory protein E1

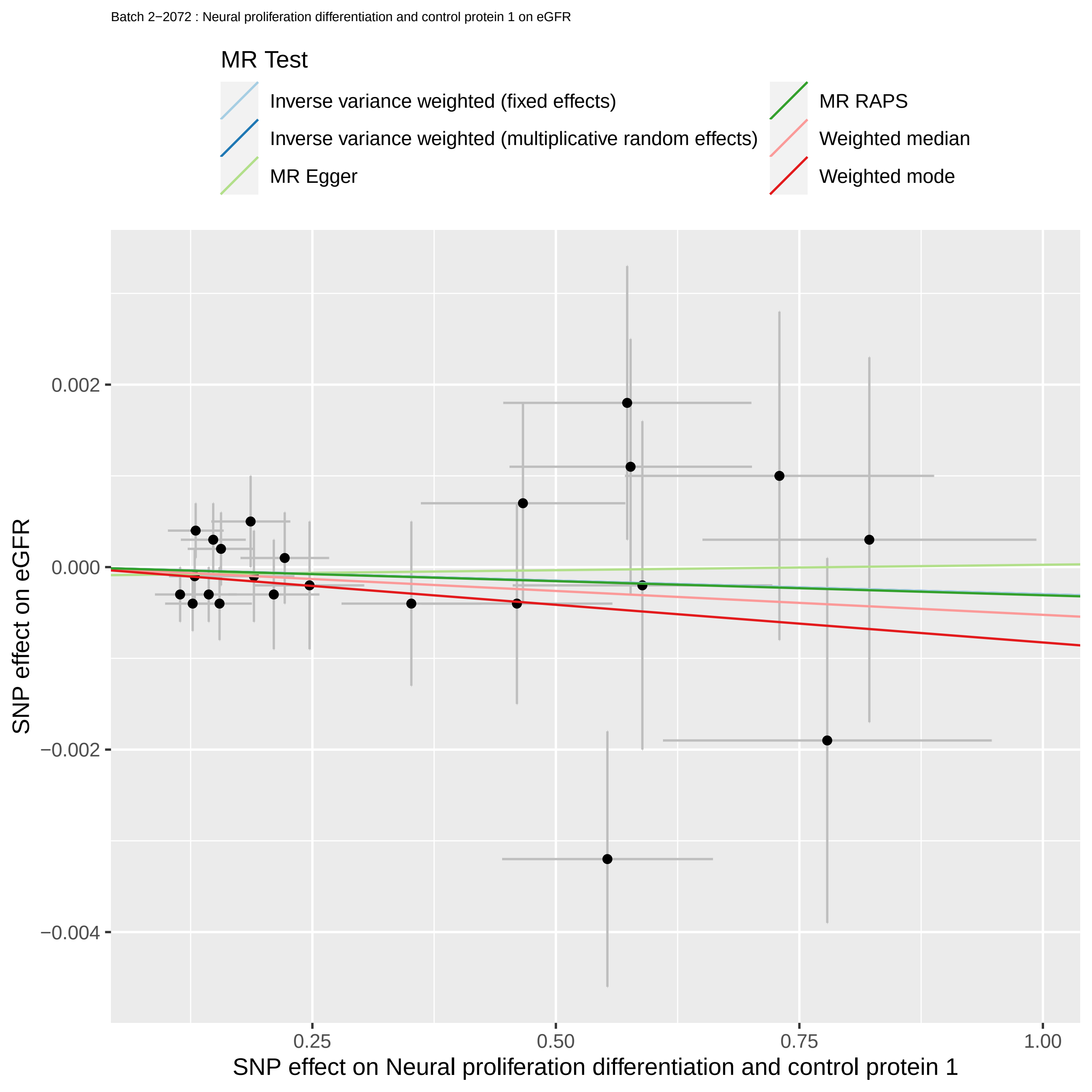

Batch 2−2072 : Neural proliferation differentiation and control protein 1 on eGFR
MR Test
Inverse variance weighted (fixed effects)
Inverse variance weighted (multiplicative random effects)
MR Egger
MR RAPS
Weighted median
Weighted mode
0.002
0.000
−0.002
−0.004
0.25
0.50
0.75
1.00
SNP effect on eGFR
SNP effect on Neural proliferation differentiation and control protein 1

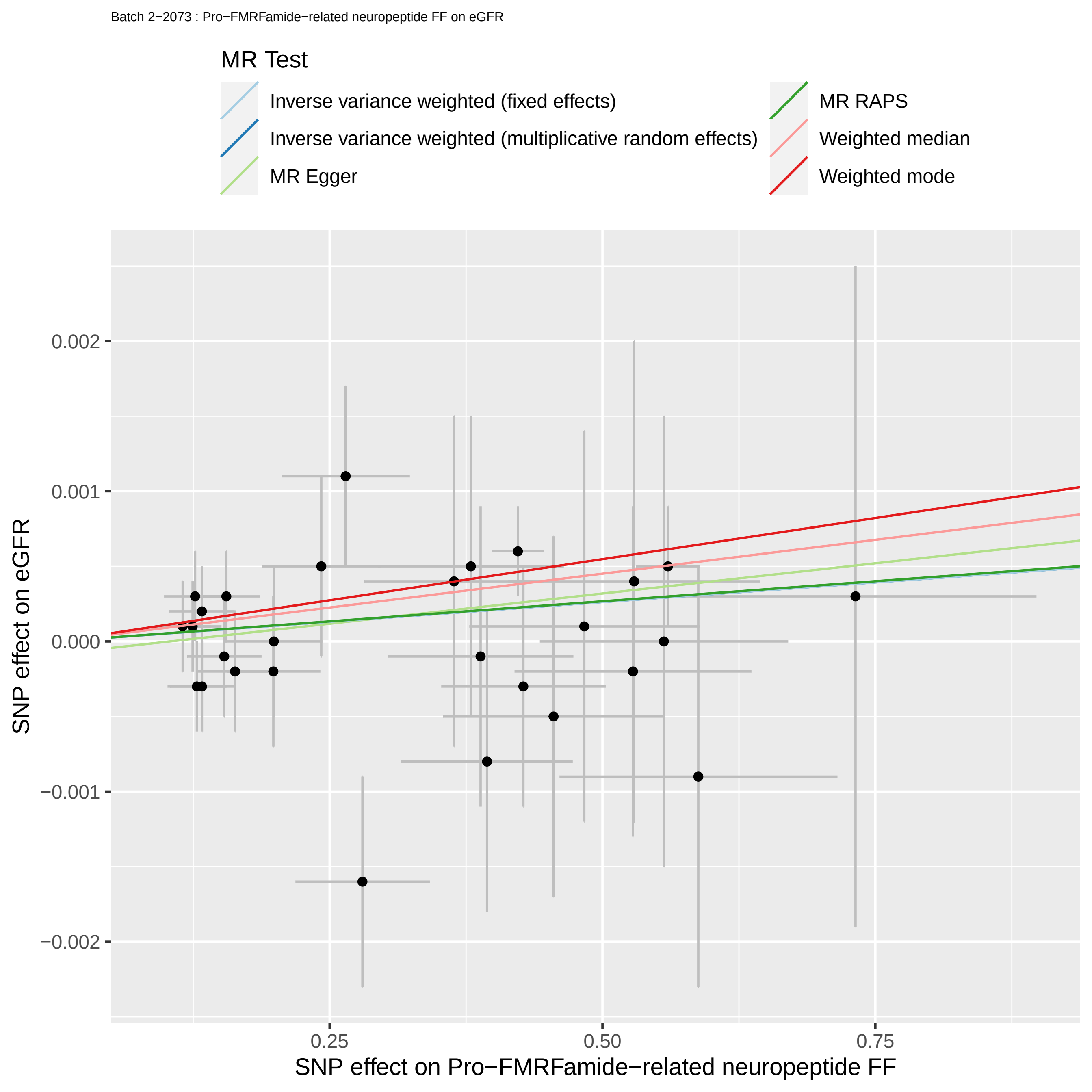

Batch 2−2073 : Pro−FMRFamide−related neuropeptide FF on eGFR
MR Test
Inverse variance weighted (fixed effects)
Inverse variance weighted (multiplicative random effects)
MR Egger
MR RAPS
Weighted median
Weighted mode
0.002
0.001
0.000
−0.001
−0.002
0.25
0.50
0.75
SNP effect on eGFR
SNP effect on Pro−FMRFamide−related neuropeptide FF

Batch 2−2074 : Nuclear pore complex−interacting protein family member B3 on eGFR

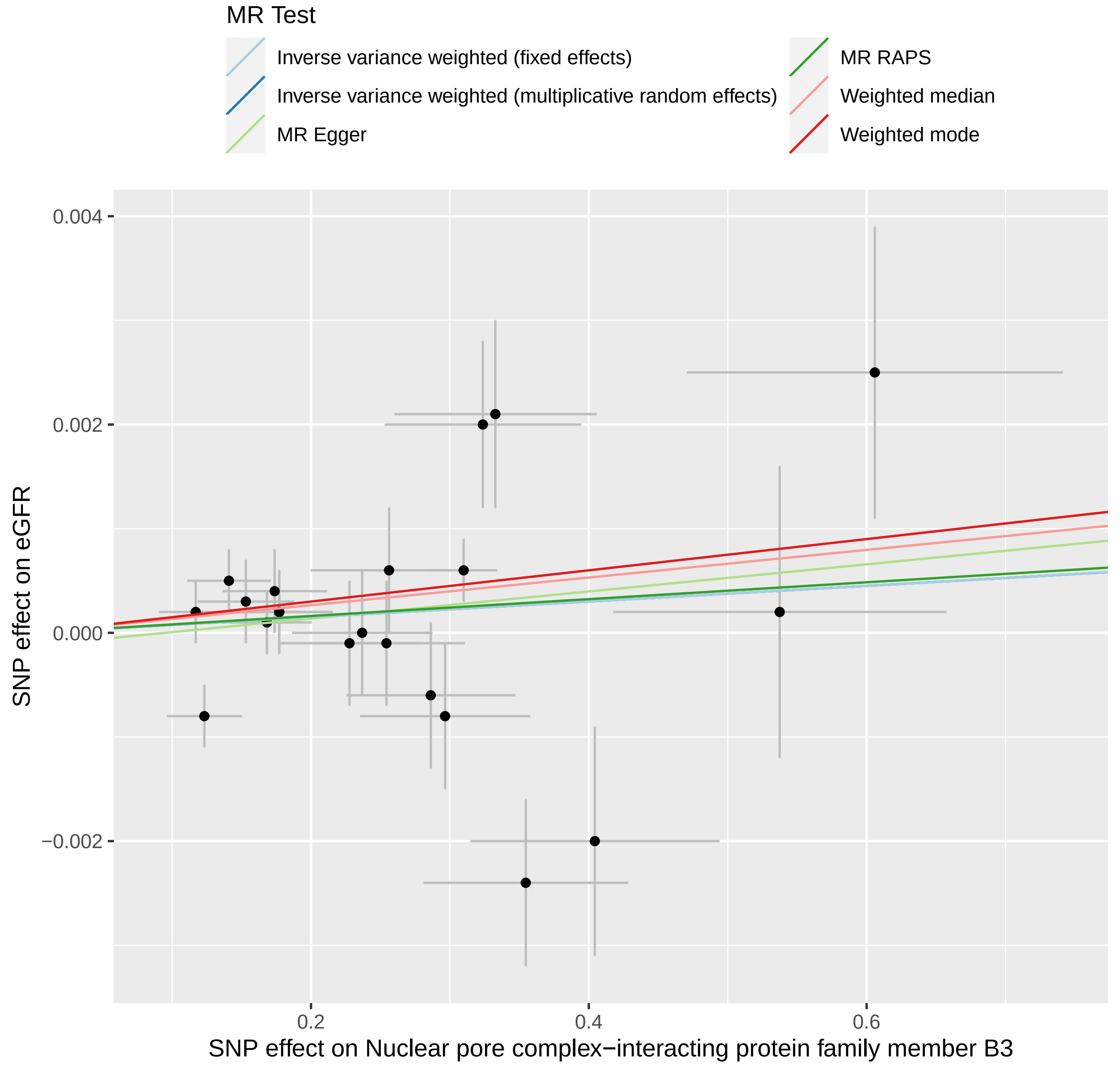

Batch 2−2075 : Nuclear protein localization protein 4 homolog on eGFR

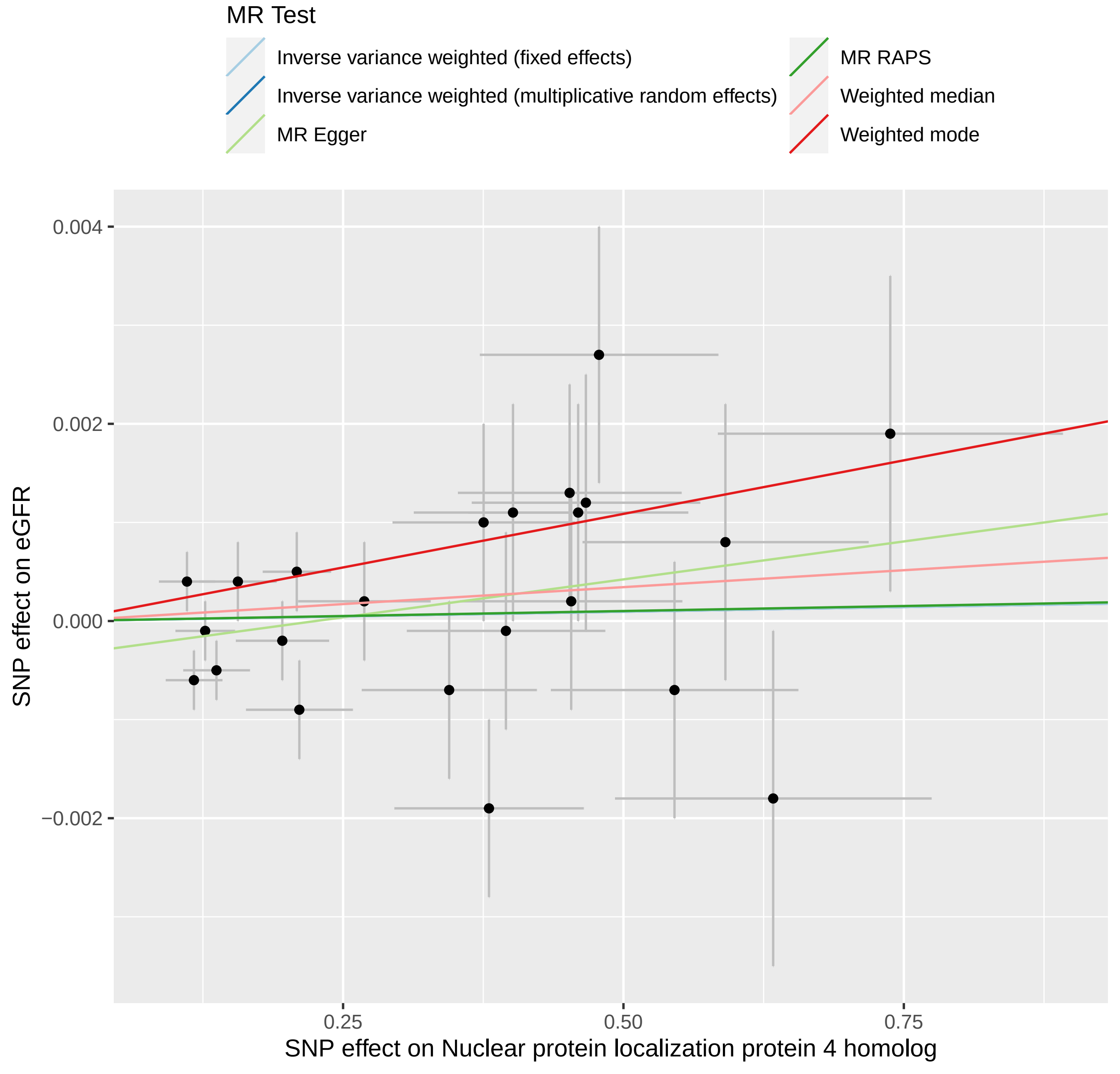

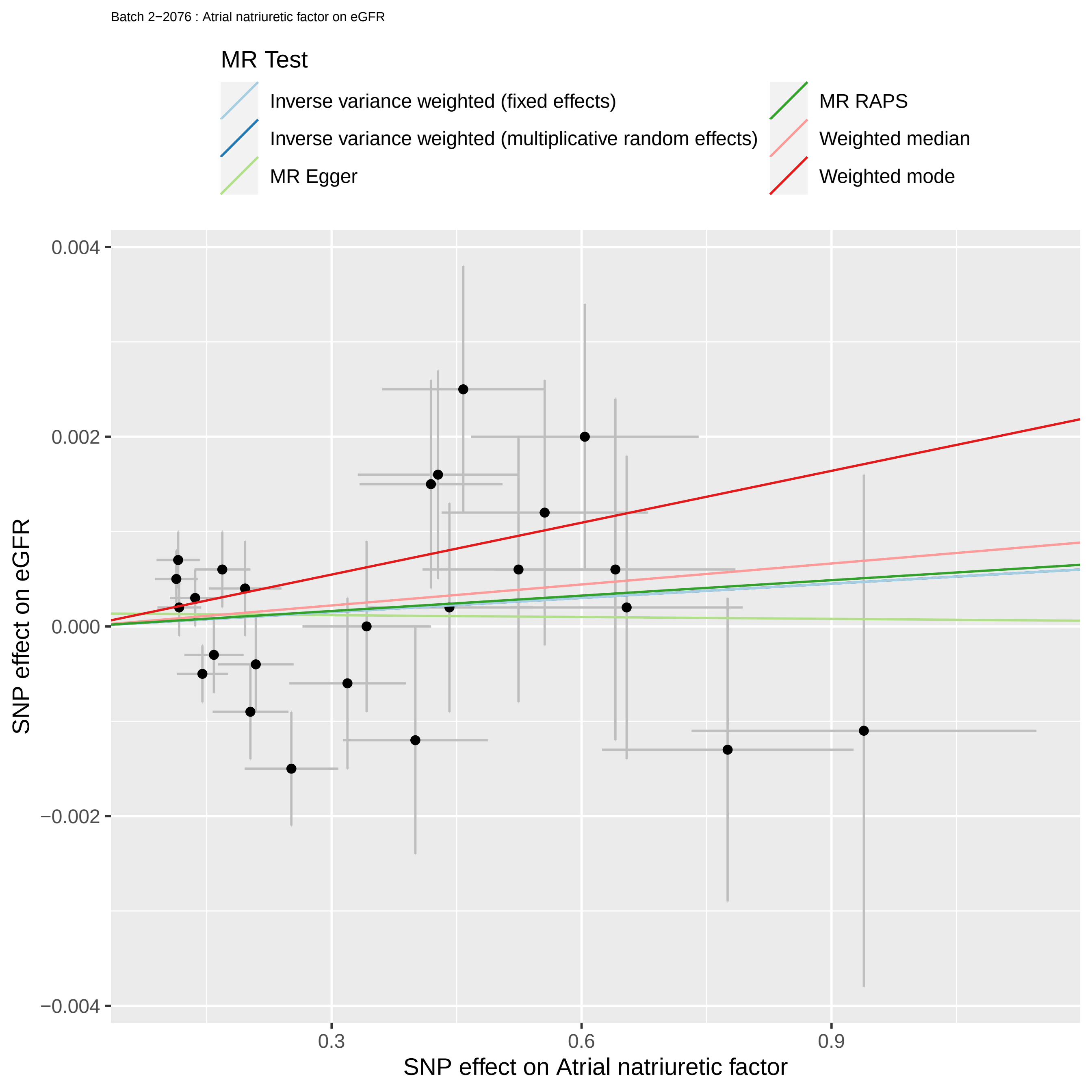

Batch 2−2076 : Atrial natriuretic factor on eGFR
MR Test
Inverse variance weighted (fixed effects)
Inverse variance weighted (multiplicative random effects)
MR Egger
MR RAPS
Weighted median
Weighted mode
0.004
0.002
0.000
−0.002
−0.004
0.3
0.6
0.9
SNP effect on eGFR
SNP effect on Atrial natriuretic factor

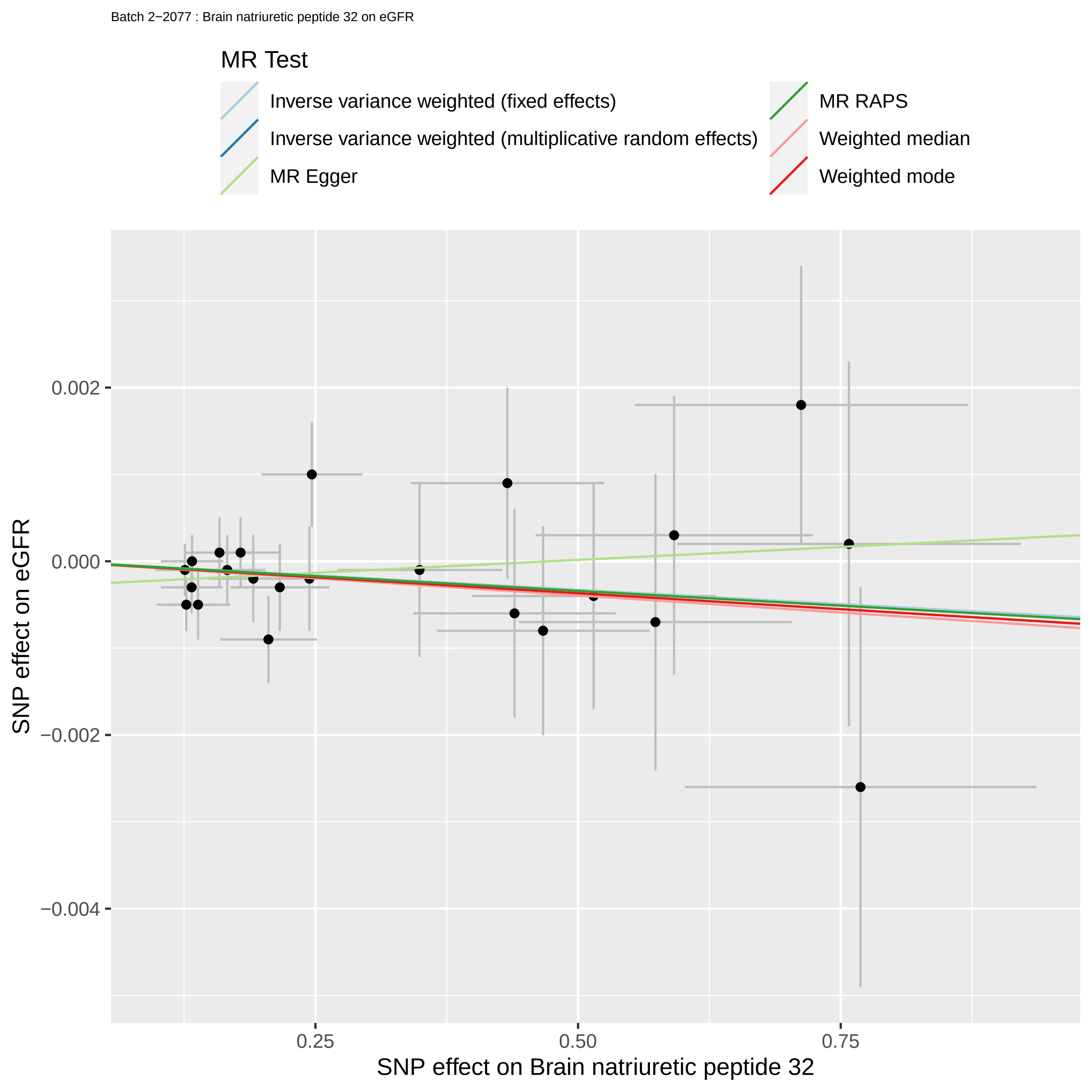

Batch 2−2077 : Brain natriuretic peptide 32 on eGFR
MR Test
Inverse variance weighted (fixed effects)
Inverse variance weighted (multiplicative random effects)
MR Egger
MR RAPS
Weighted median
Weighted mode
0.002
0.000
−0.002
−0.004
0.25
0.50
0.75
SNP effect on eGFR
SNP effect on Brain natriuretic peptide 32

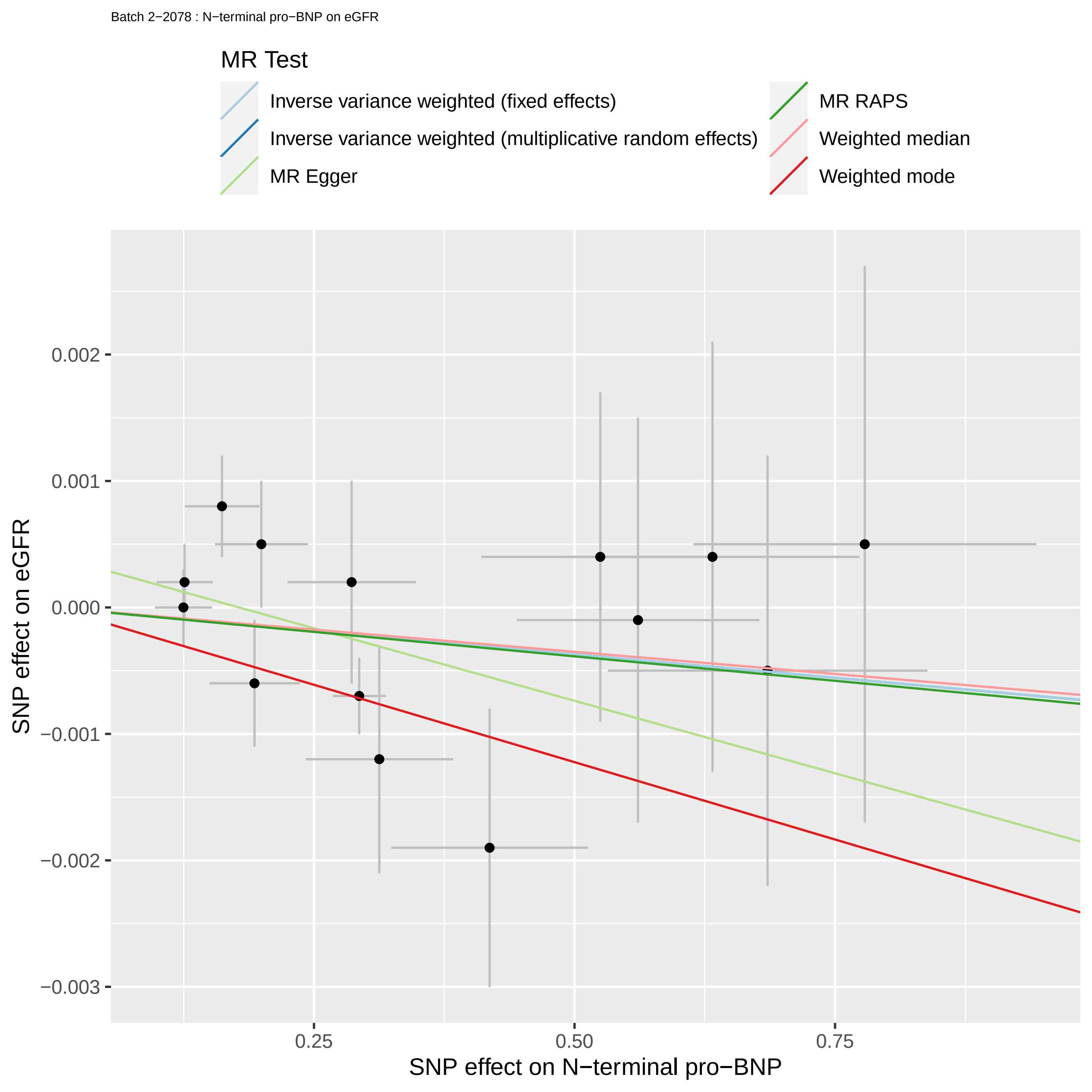
Batch 2−2078 : N−terminal pro−BNP on eGFR
MR Test
Inverse variance weighted (fixed effects)
Inverse variance weighted (multiplicative random effects)
MR Egger
MR RAPS
Weighted median
Weighted mode
SNP effect on eGFR
0.002
0.001
0.000
−0.001
−0.002
−0.003
0.25
0.50
0.75
SNP effect on N−terminal pro−BNP

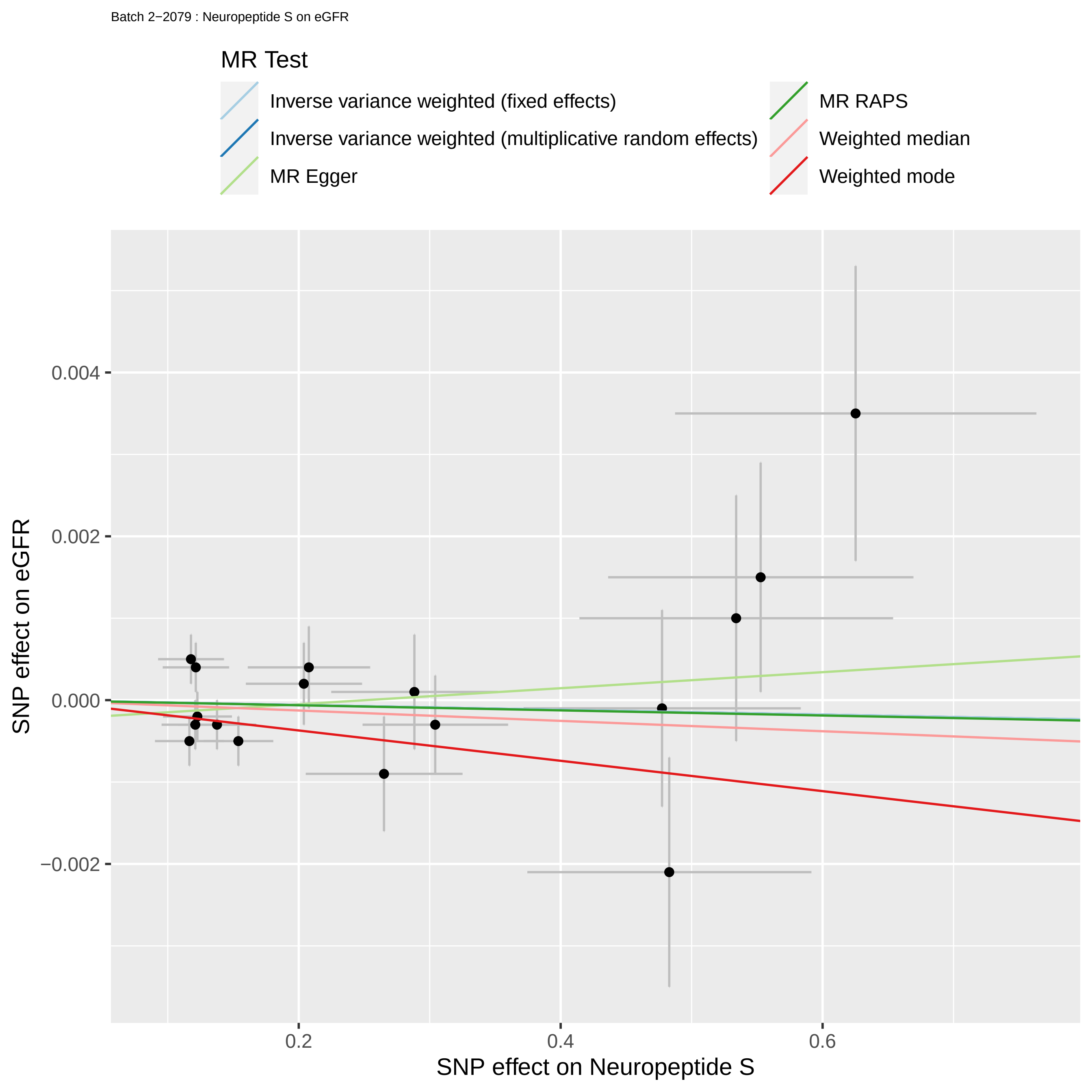
Batch 2−2079 : Neuropeptide S on eGFR
MR Test
Inverse variance weighted (fixed effects)
Inverse variance weighted (multiplicative random effects)
MR Egger
MR RAPS
Weighted median
Weighted mode
0.004
0.002
0.000
−0.002
0.2
0.4
0.6
SNP effect on eGFR
SNP effect on Neuropeptide S

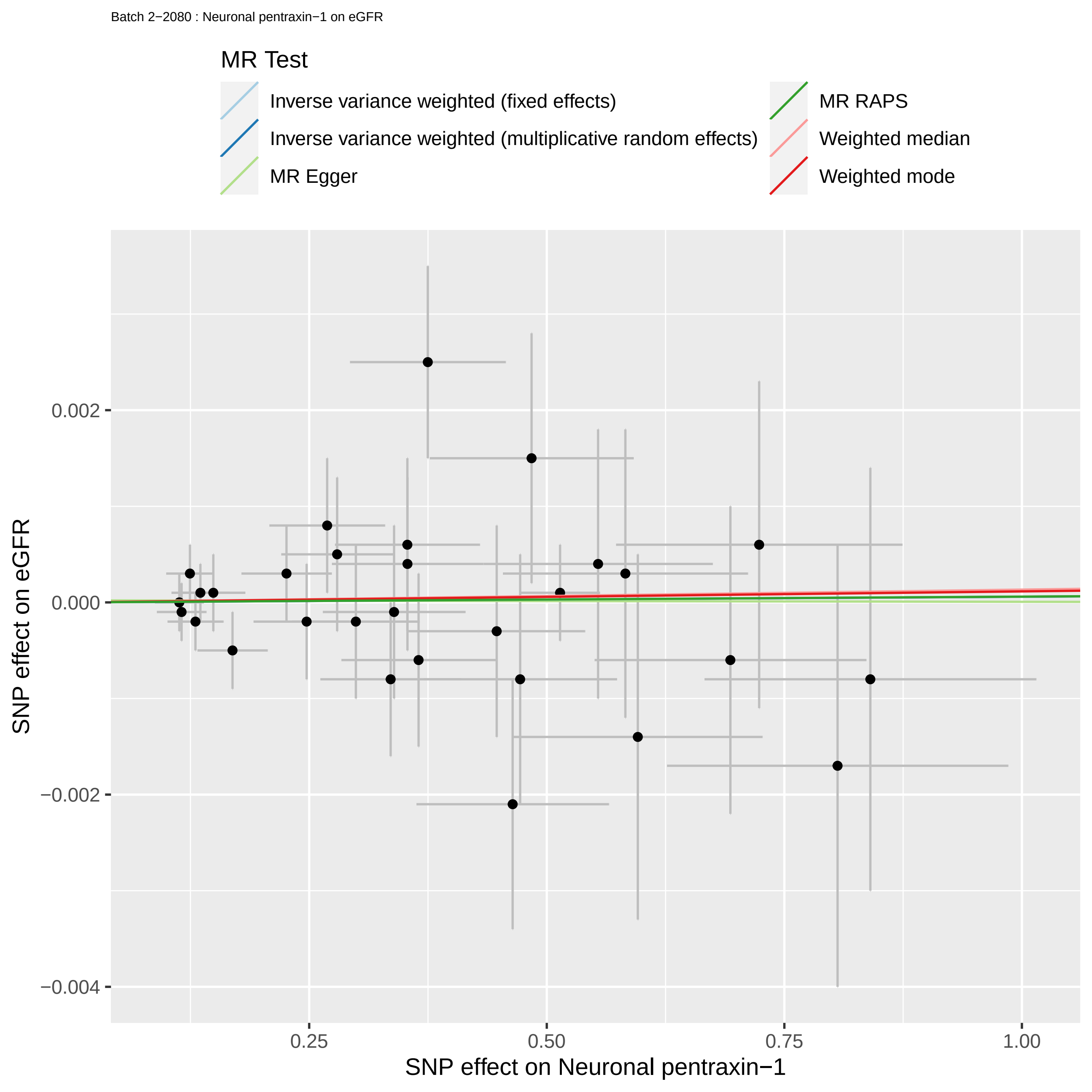

Batch 2−2080 : Neuronal pentraxin−1 on eGFR
MR Test
Inverse variance weighted (fixed effects)
Inverse variance weighted (multiplicative random effects)
MR Egger
MR RAPS
Weighted median
Weighted mode
0.002
0.000
−0.002
−0.004
0.25
0.50
0.75
1.00
SNP effect on eGFR
SNP effect on Neuronal pentraxin−1

Batch 2−2081 : Neuronal pentraxin−2 on eGFR

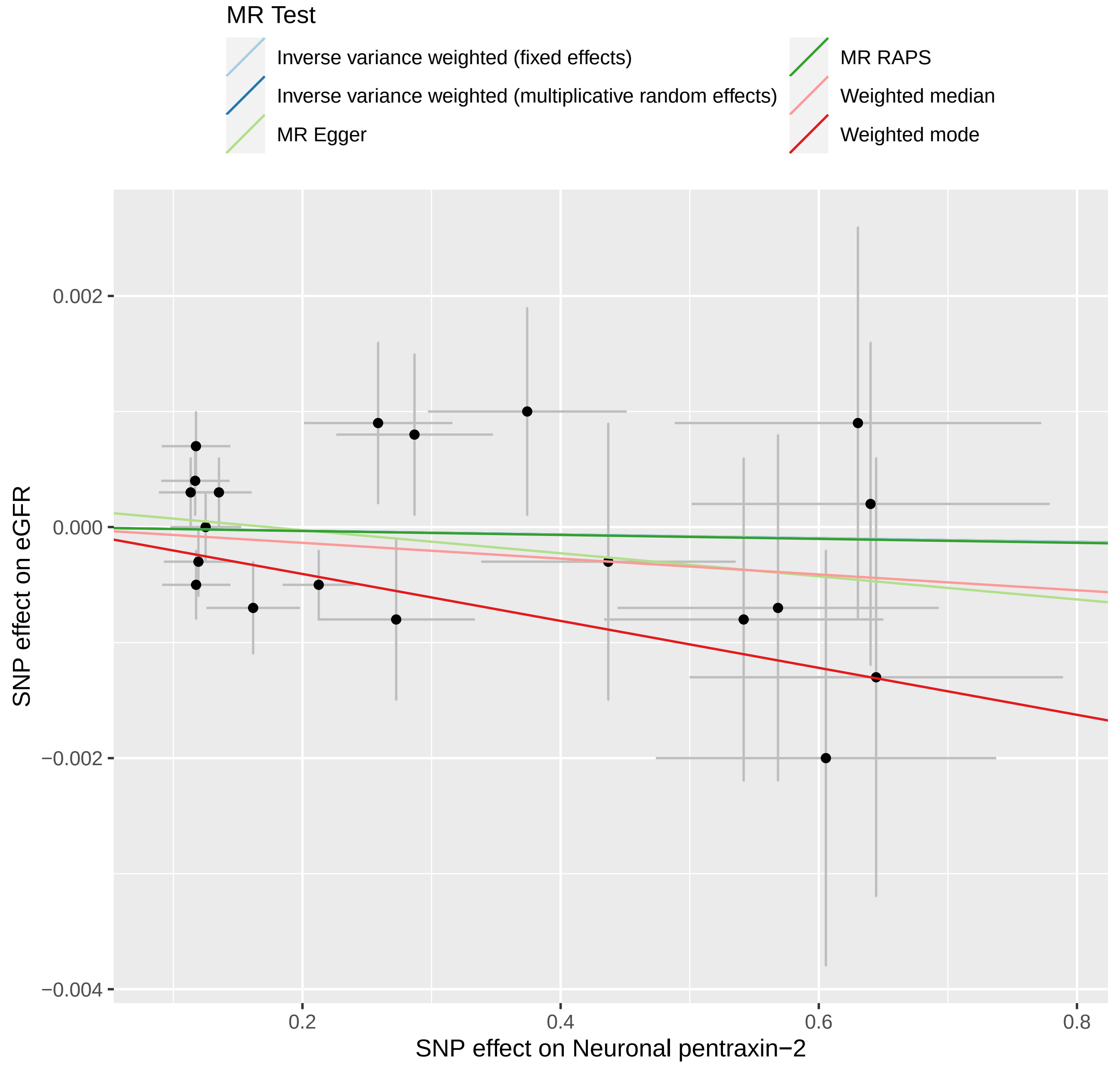

Batch 2−2082 : Neuropeptide W on eGFR

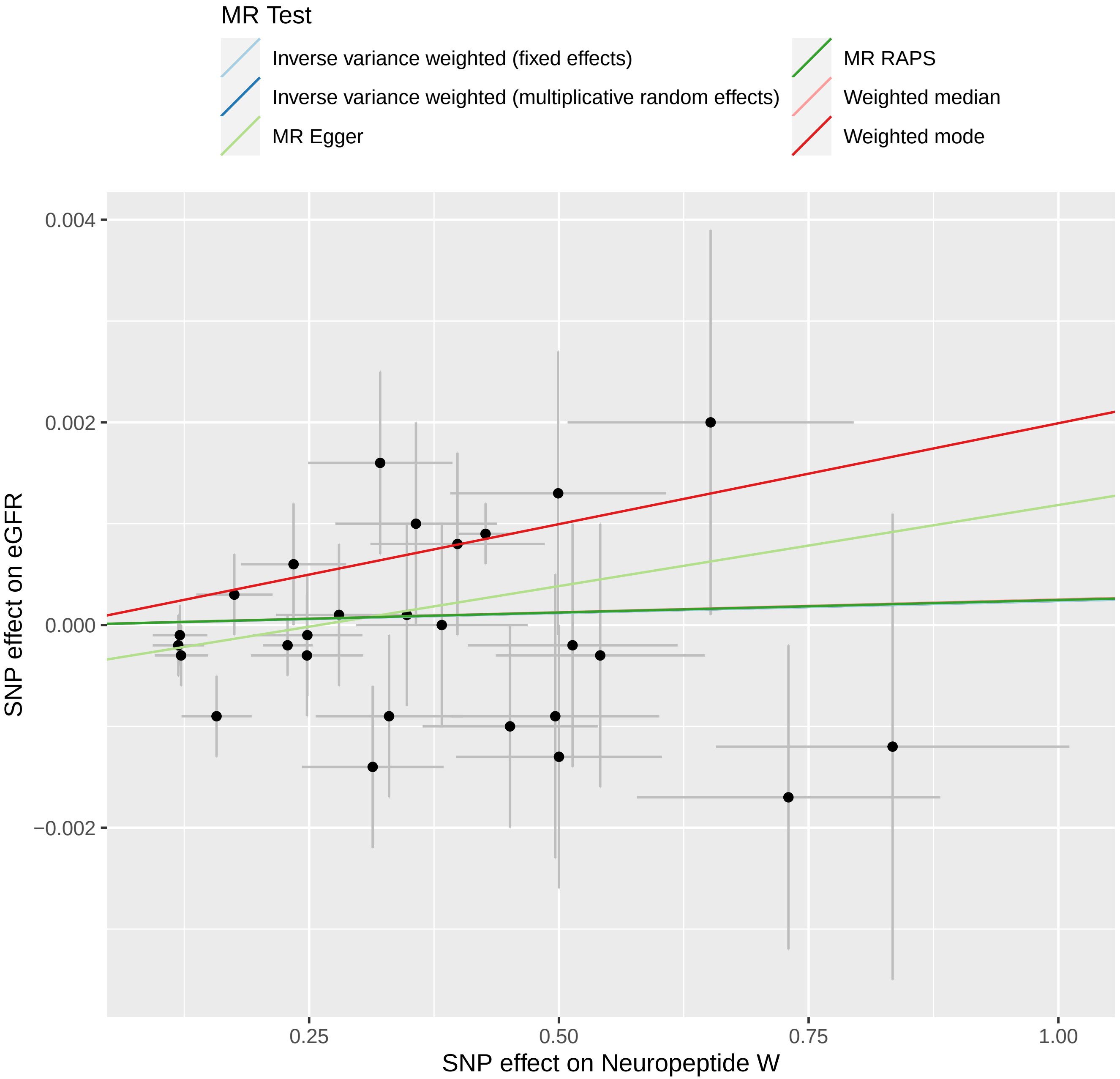

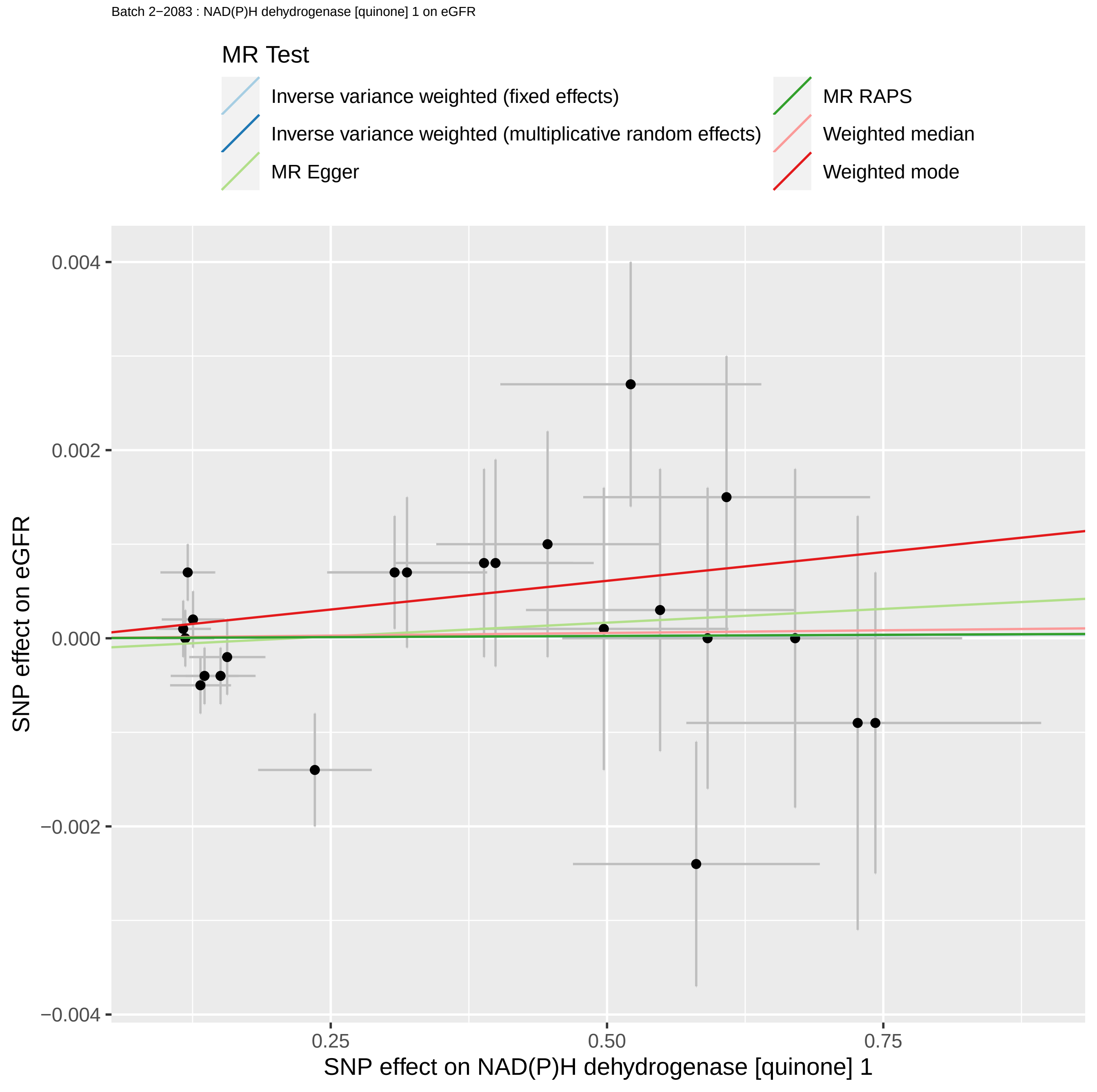

Batch 2−2083 : NAD(P)H dehydrogenase [quinone] 1 on eGFR
MR Test
Inverse variance weighted (fixed effects)
Inverse variance weighted (multiplicative random effects)
MR Egger
MR RAPS
Weighted median
Weighted mode
0.004
0.002
0.000
−0.002
−0.004
0.25
0.50
0.75
SNP effect on eGFR
SNP effect on NAD(P)H dehydrogenase [quinone] 1

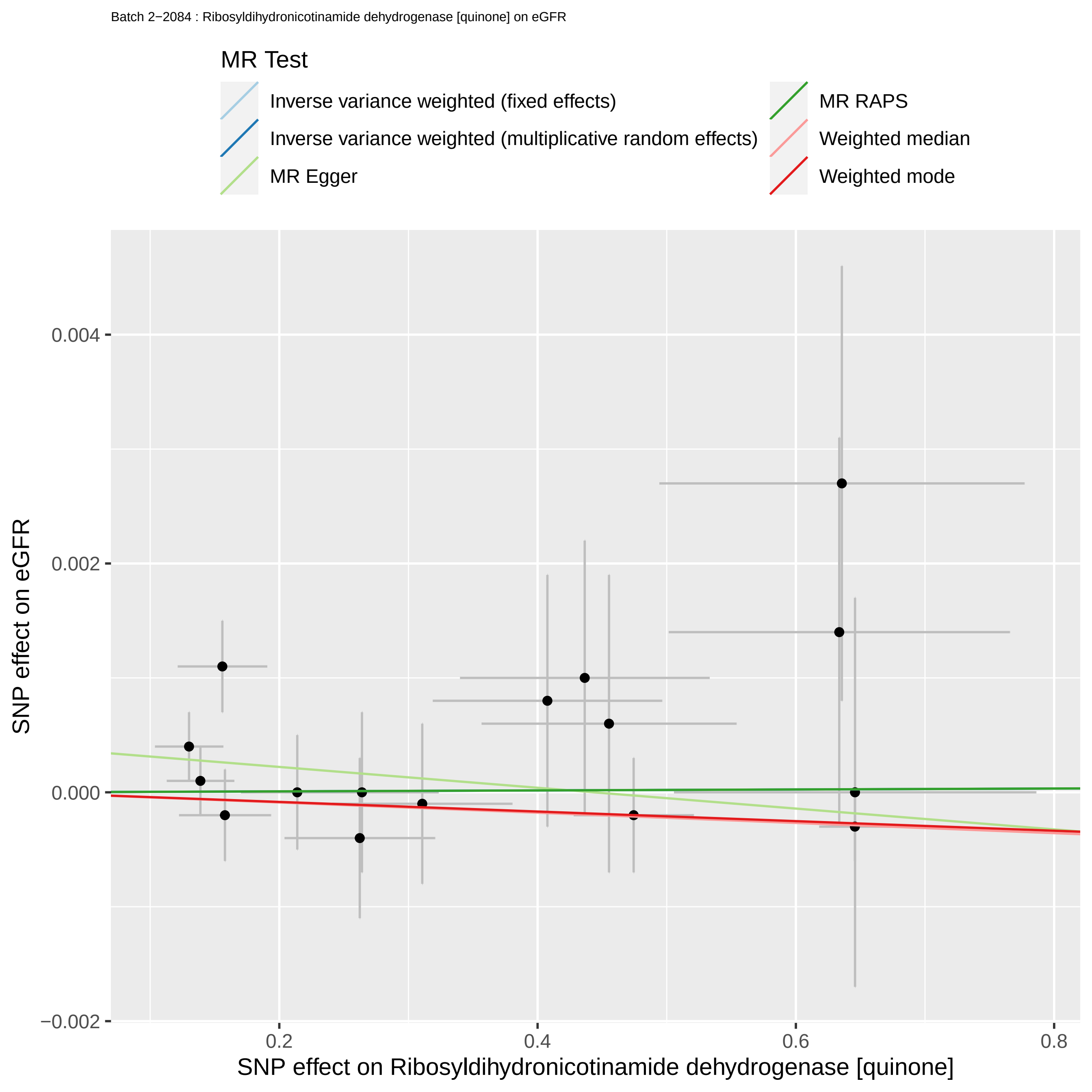
Batch 2−2084 : Ribosyldihydronicotinamide dehydrogenase [quinone] on eGFR
MR Test
Inverse variance weighted (fixed effects)
Inverse variance weighted (multiplicative random effects)
MR Egger
MR RAPS
Weighted median
Weighted mode
0.004
0.002
0.000
−0.002
0.2
0.4
0.6
0.8
SNP effect on eGFR
SNP effect on Ribosyldihydronicotinamide dehydrogenase [quinone]

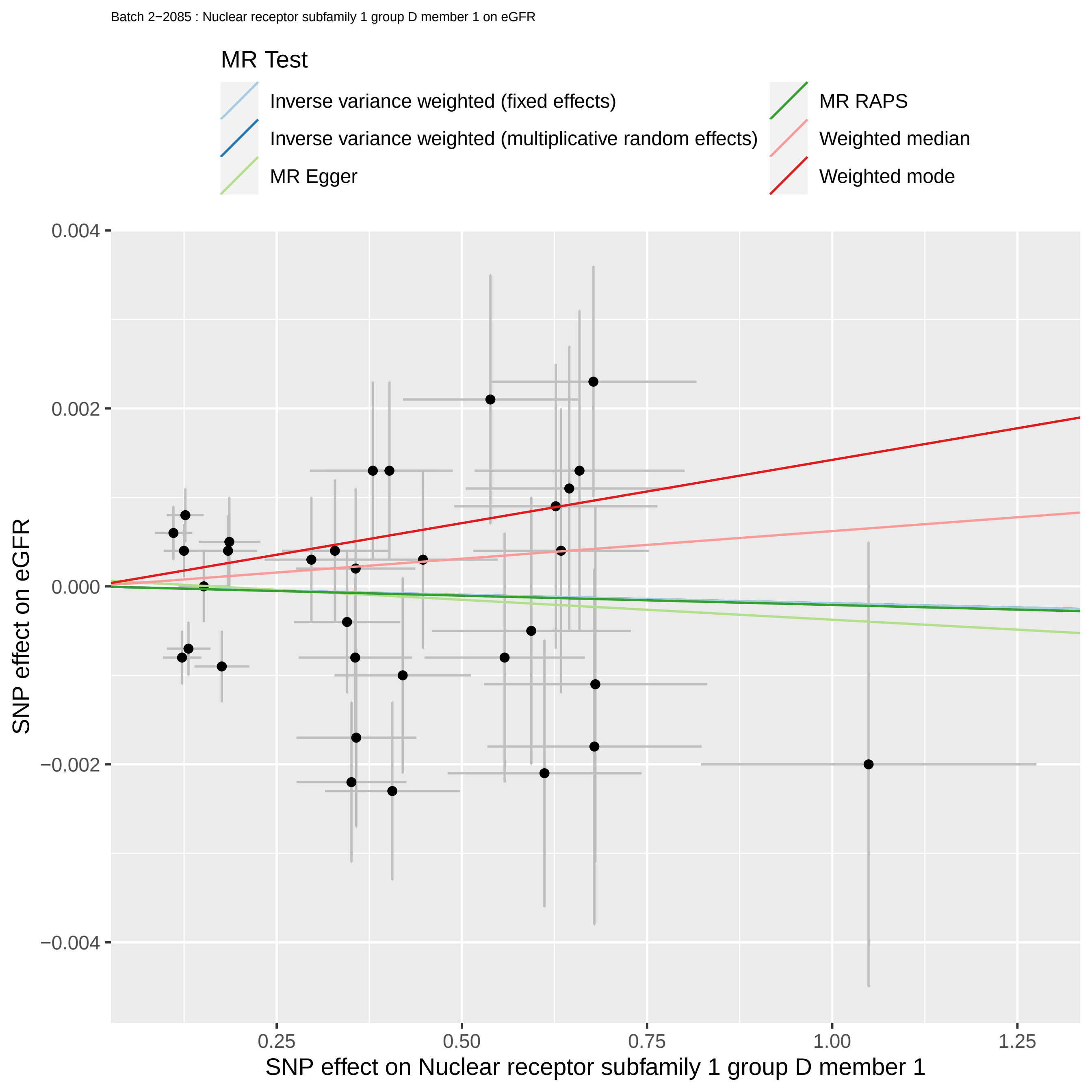

Batch 2−2085 : Nuclear receptor subfamily 1 group D member 1 on eGFR
MR Test
Inverse variance weighted (fixed effects)
Inverse variance weighted (multiplicative random effects)
MR Egger
MR RAPS
Weighted median
Weighted mode
0.004
0.002
0.000
−0.002
−0.004
SNP effect on eGFR
0.25
0.50
0.75
1.00
1.25
SNP effect on Nuclear receptor subfamily 1 group D member 1

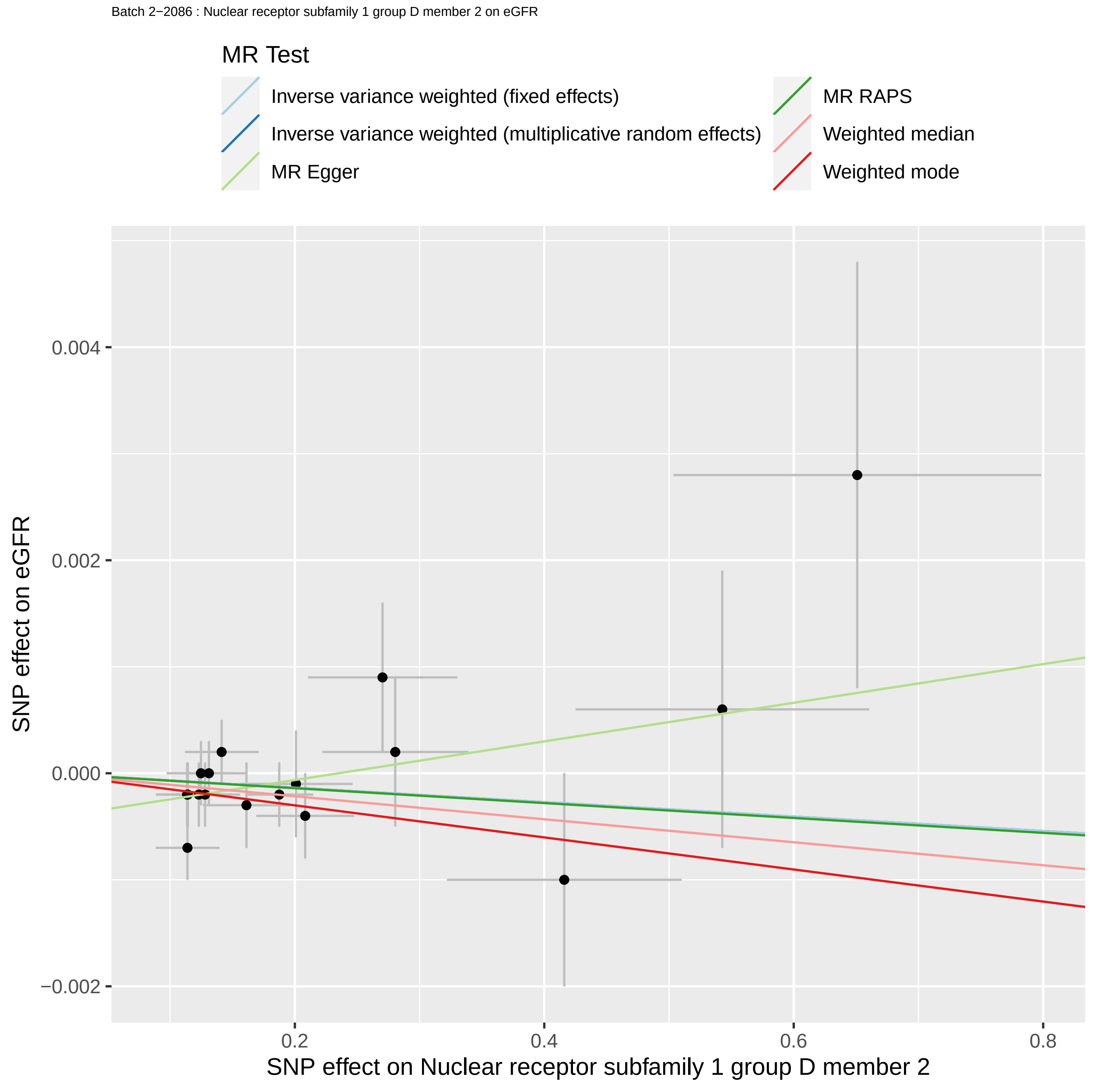

Batch 2−2086 : Nuclear receptor subfamily 1 group D member 2 on eGFR
MR Test
Inverse variance weighted (fixed effects)
Inverse variance weighted (multiplicative random effects)
MR Egger
MR RAPS
Weighted median
Weighted mode
0.004
0.002
0.000
−0.002
0.2
0.4
0.6
0.8
SNP effect on eGFR
SNP effect on Nuclear receptor subfamily 1 group D member 2

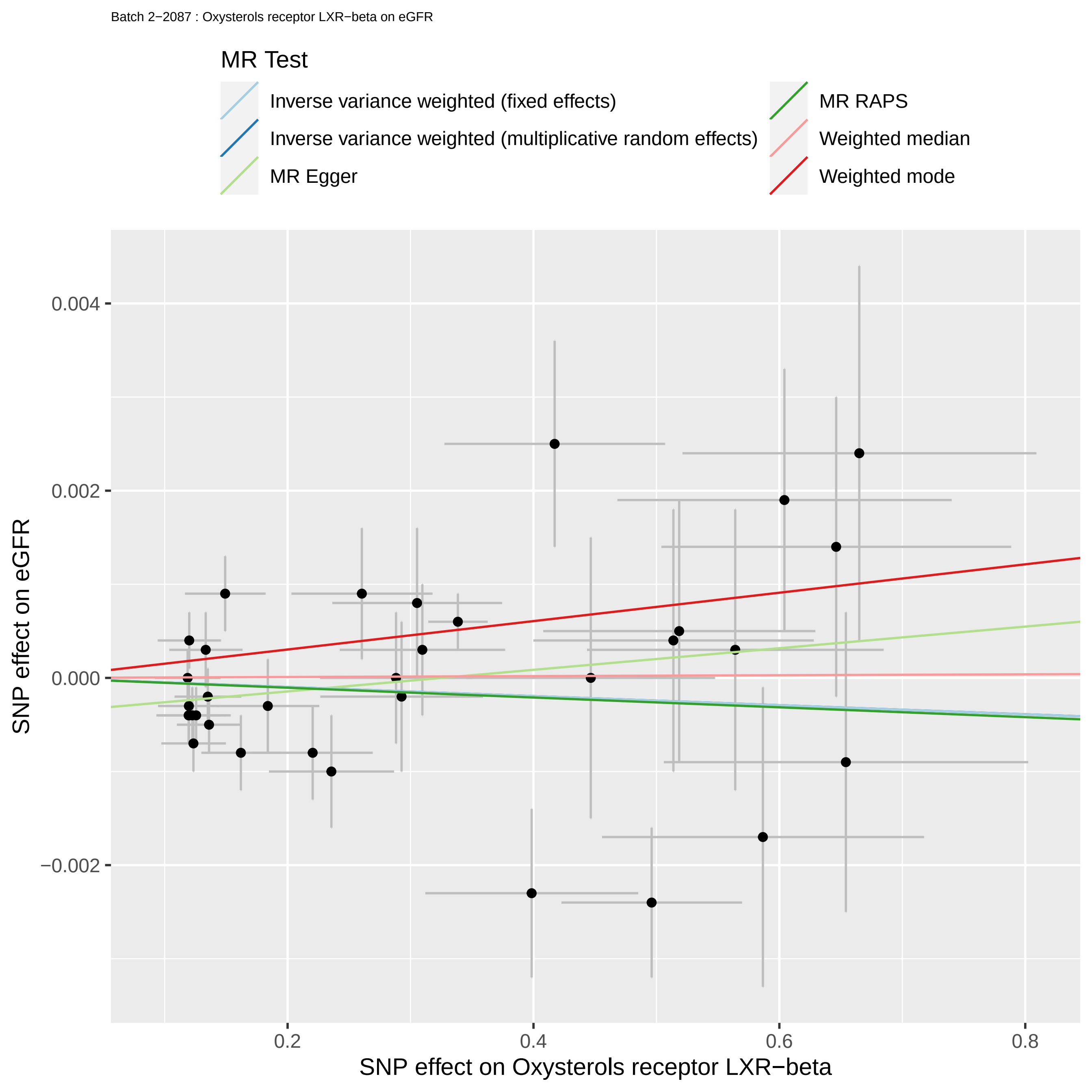

Batch 2−2087 : Oxysterols receptor LXR−beta on eGFR
MR Test
Inverse variance weighted (fixed effects)
Inverse variance weighted (multiplicative random effects)
MR Egger
MR RAPS
Weighted median
Weighted mode
0.004
0.002
0.000
−0.002
SNP effect on eGFR
0.2
0.4
0.6
0.8
SNP effect on Oxysterols receptor LXR−beta

Batch 2−2088 : Glucocorticoid receptor on eGFR

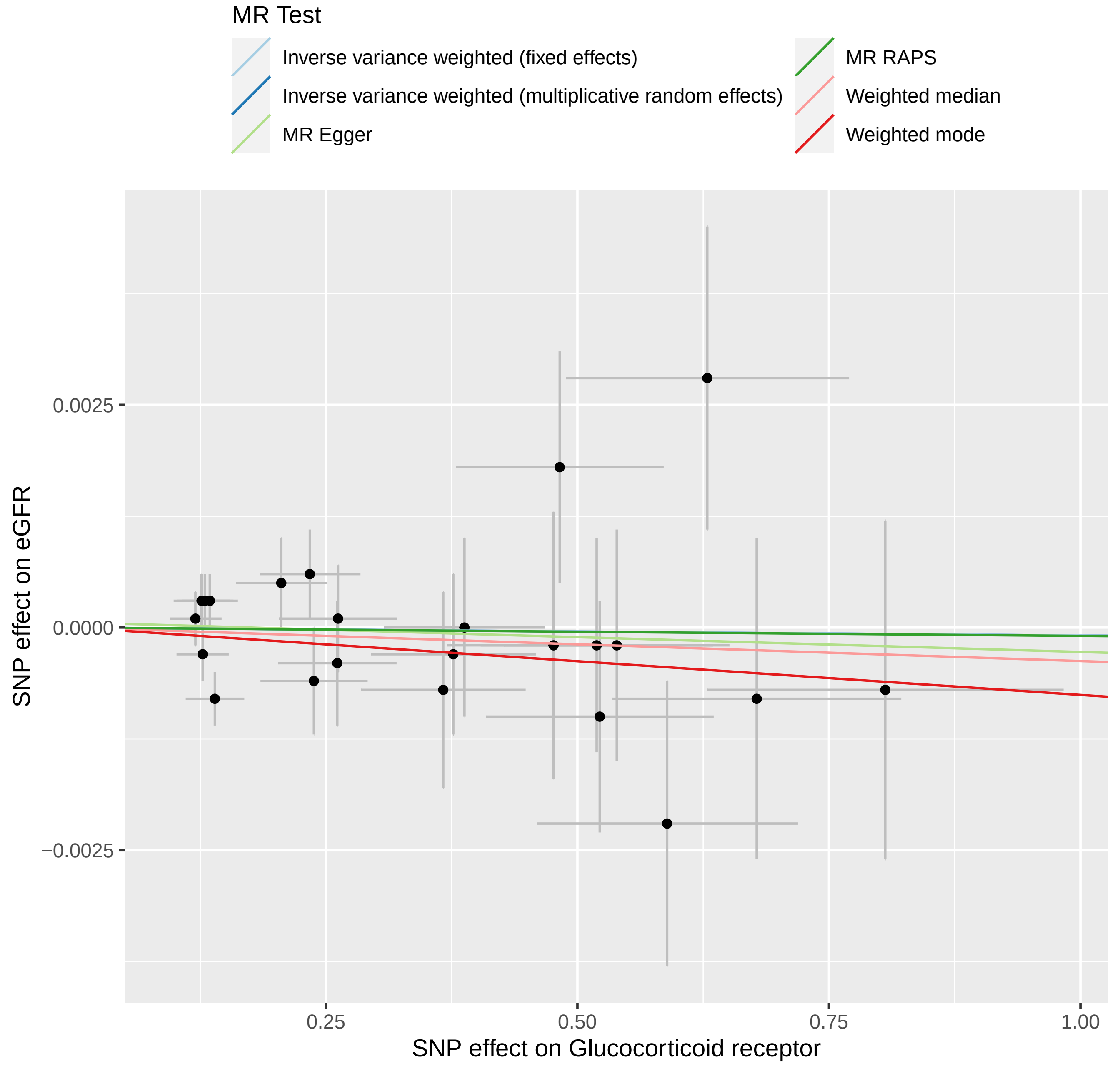

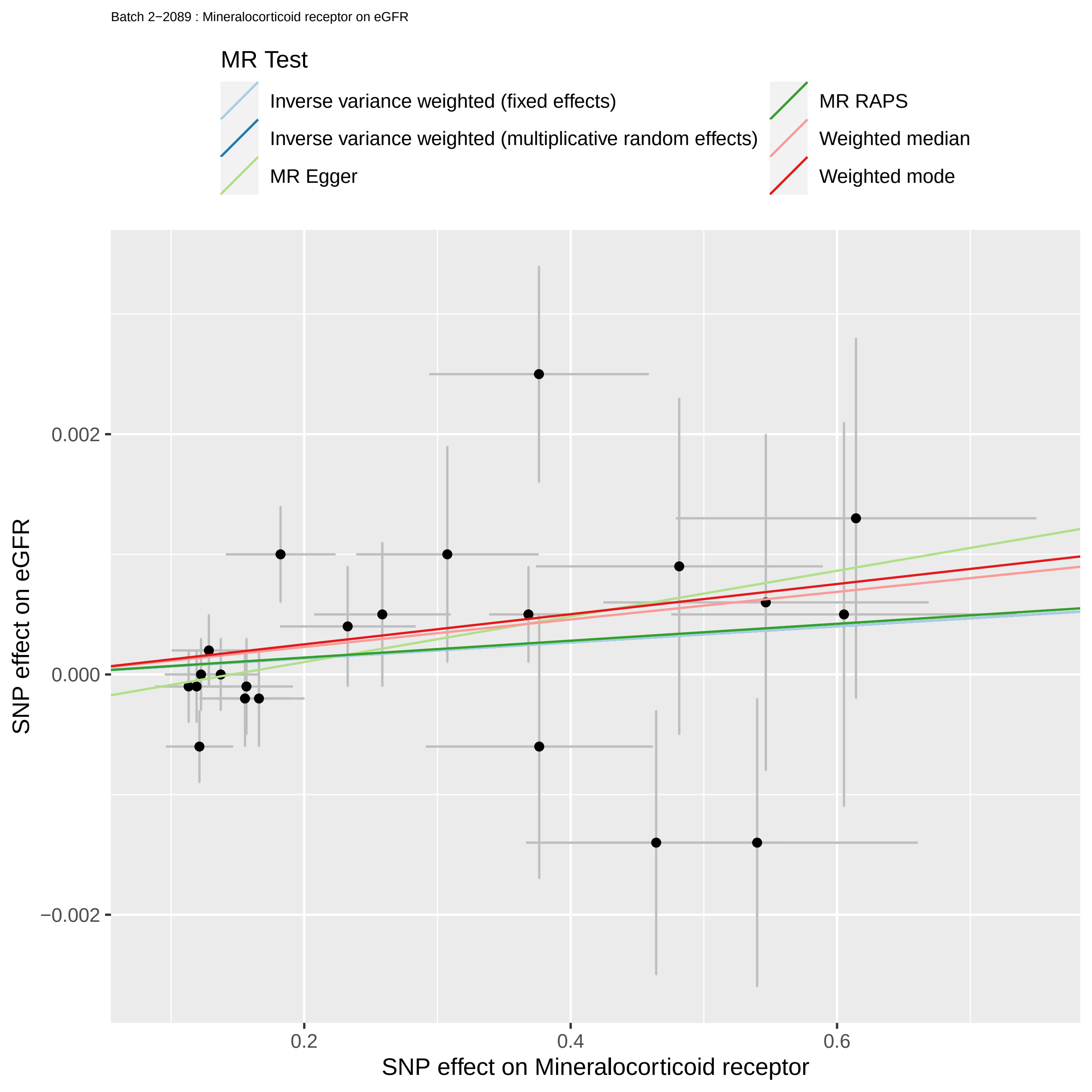
Batch 2−2089 : Mineralocorticoid receptor on eGFR
MR Test
Inverse variance weighted (fixed effects)
Inverse variance weighted (multiplicative random effects)
MR Egger
MR RAPS
Weighted median
Weighted mode
0.002
0.000
−0.002
0.2
0.4
0.6
SNP effect on eGFR
SNP effect on Mineralocorticoid receptor

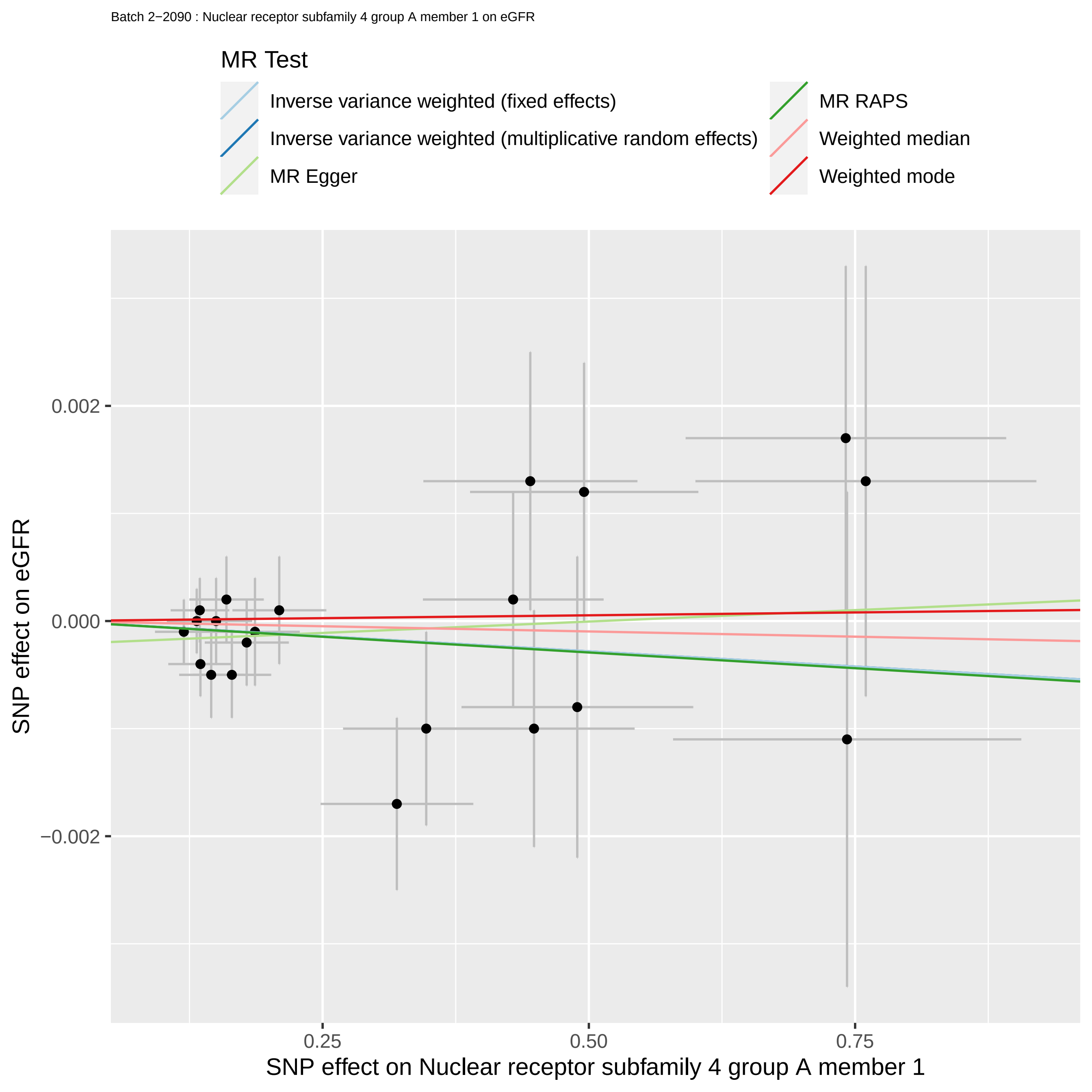
Batch 2−2090 : Nuclear receptor subfamily 4 group A member 1 on eGFR
MR Test
Inverse variance weighted (fixed effects)
Inverse variance weighted (multiplicative random effects)
MR Egger
MR RAPS
Weighted median
Weighted mode
0.002
0.000
−0.002
0.25
0.50
0.75
SNP effect on eGFR
SNP effect on Nuclear receptor subfamily 4 group A member 1

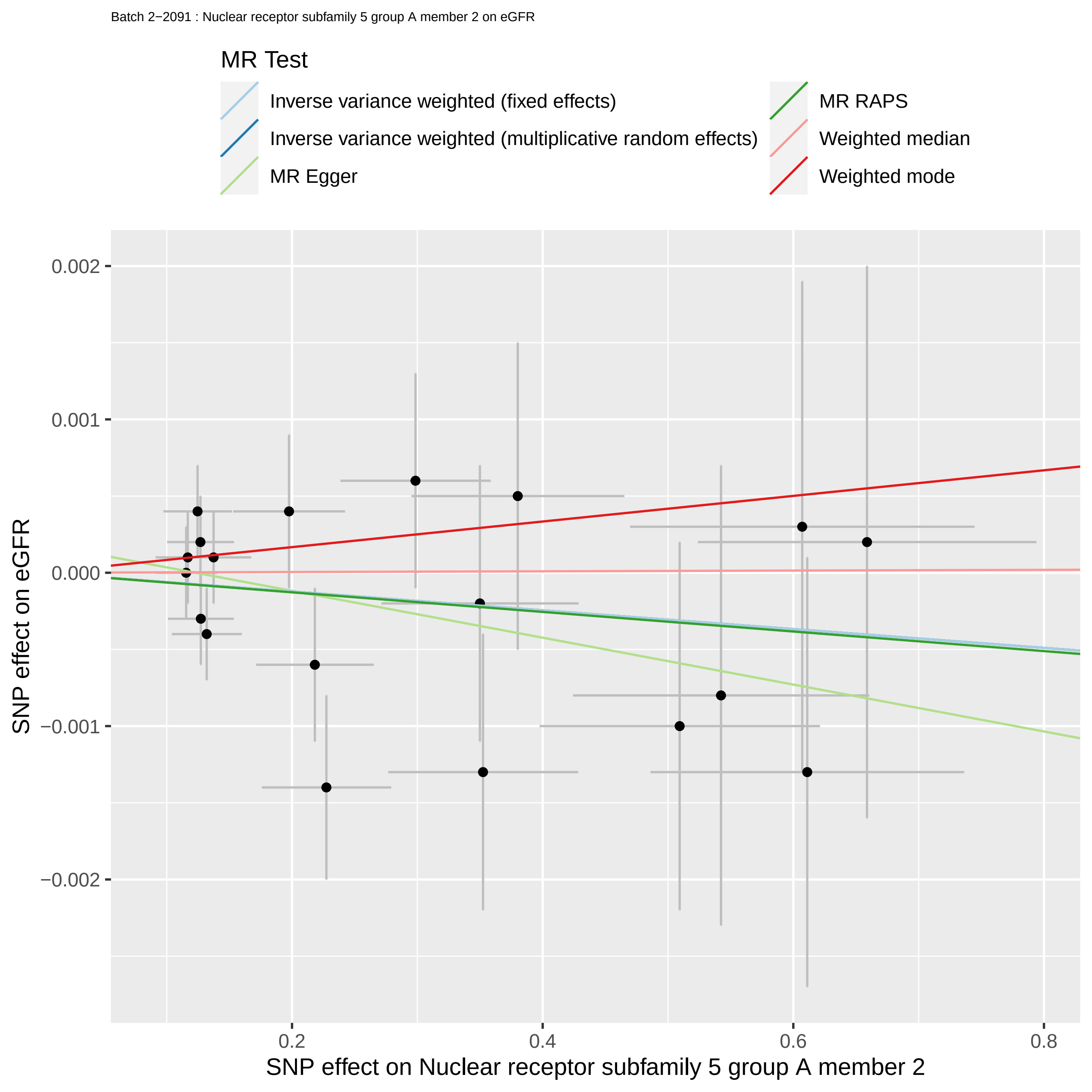
Batch 2−2091 : Nuclear receptor subfamily 5 group A member 2 on eGFR
MR Test
Inverse variance weighted (fixed effects)
Inverse variance weighted (multiplicative random effects)
MR Egger
MR RAPS
Weighted median
Weighted mode
0.002
0.001
0.000
−0.001
−0.002
0.2
0.4
0.6
0.8
SNP effect on eGFR
SNP effect on Nuclear receptor subfamily 5 group A member 2

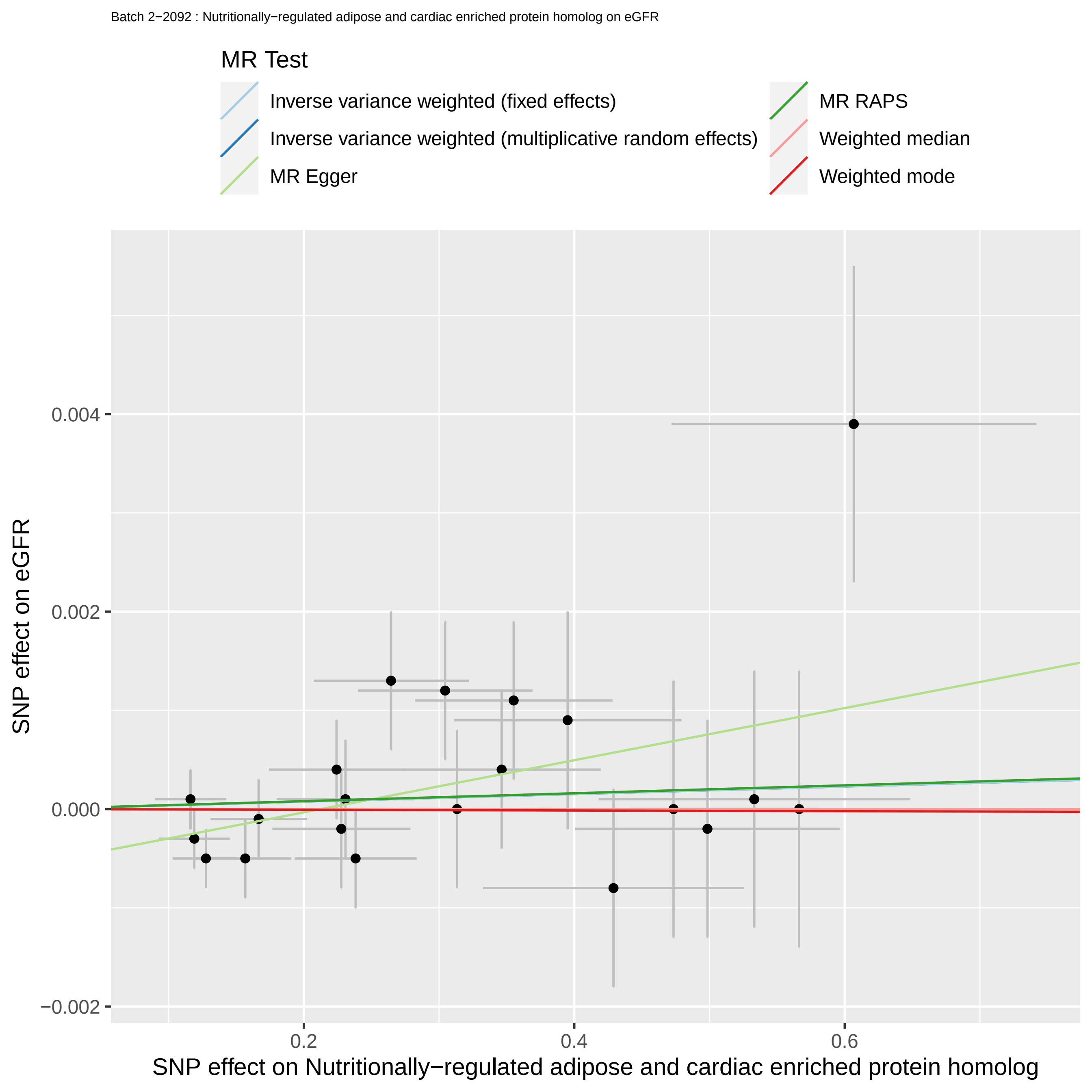
Batch 2−2092 : Nutritionally−regulated adipose and cardiac enriched protein homolog on eGFR
MR Test
Inverse variance weighted (fixed effects)
Inverse variance weighted (multiplicative random effects)
MR Egger
MR RAPS
Weighted median
Weighted mode
SNP effect on eGFR
0.004
0.002
0.000
−0.002
0.2
0.4
0.6
SNP effect on Nutritionally−regulated adipose and cardiac enriched protein homolog

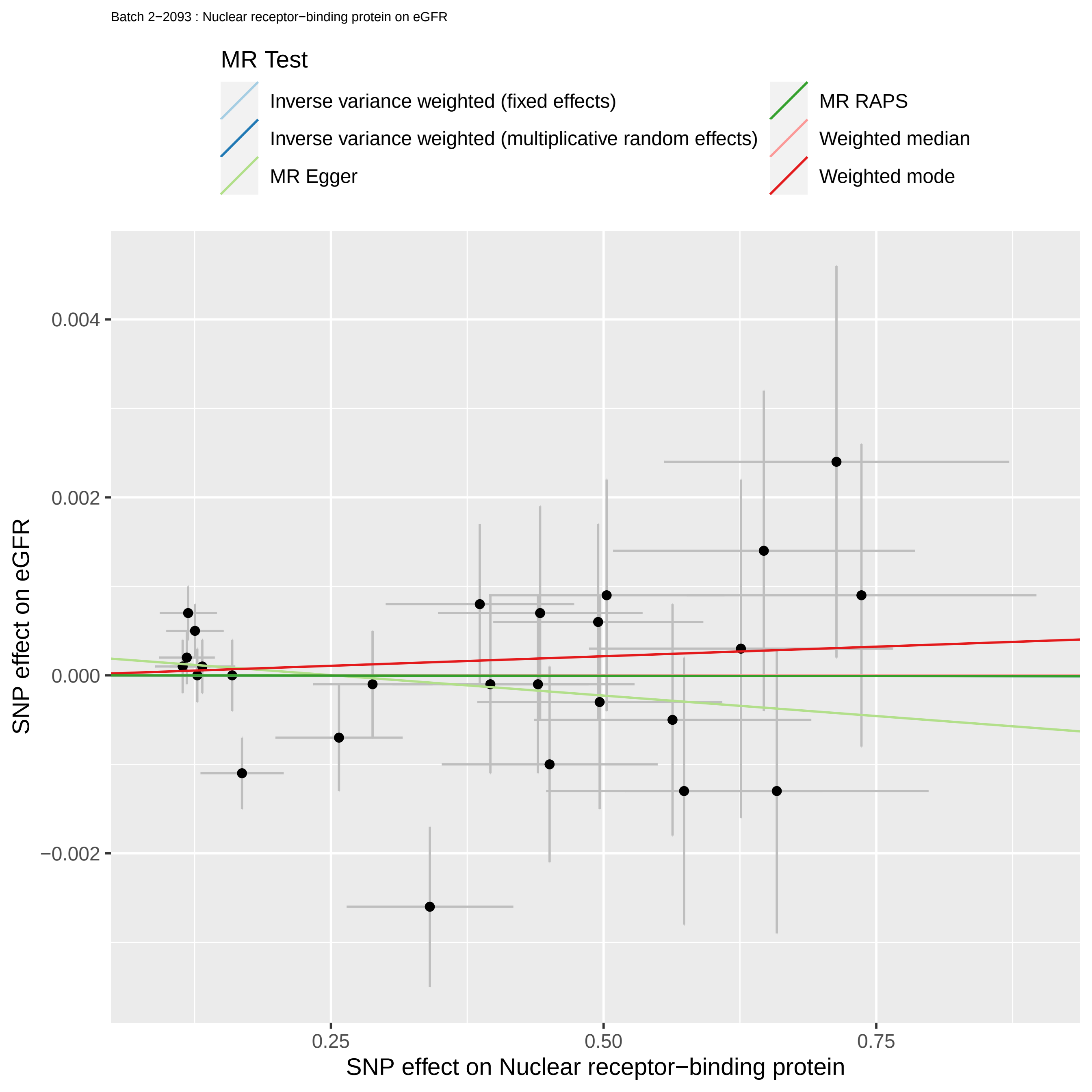

Batch 2−2093 : Nuclear receptor−binding protein on eGFR
MR Test
Inverse variance weighted (fixed effects)
Inverse variance weighted (multiplicative random effects)
MR Egger
MR RAPS
Weighted median
Weighted mode
0.004
0.002
0.000
−0.002
0.25
0.50
0.75
SNP effect on eGFR
SNP effect on Nuclear receptor−binding protein

Batch 2−2094 : Neuronal cell adhesion molecule on eGFR

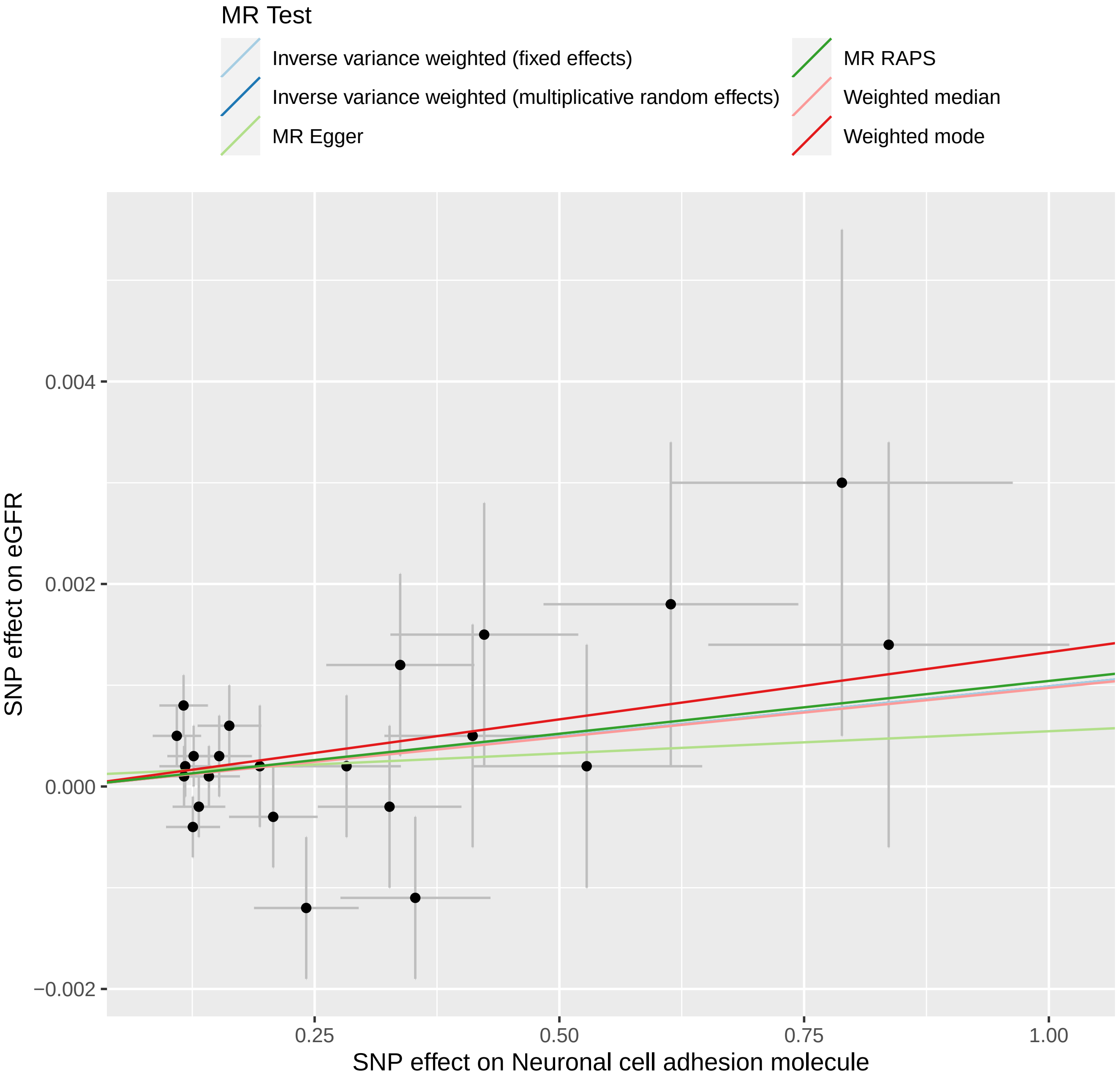

Batch 2−2095 : Neuregulin−1 on eGFR

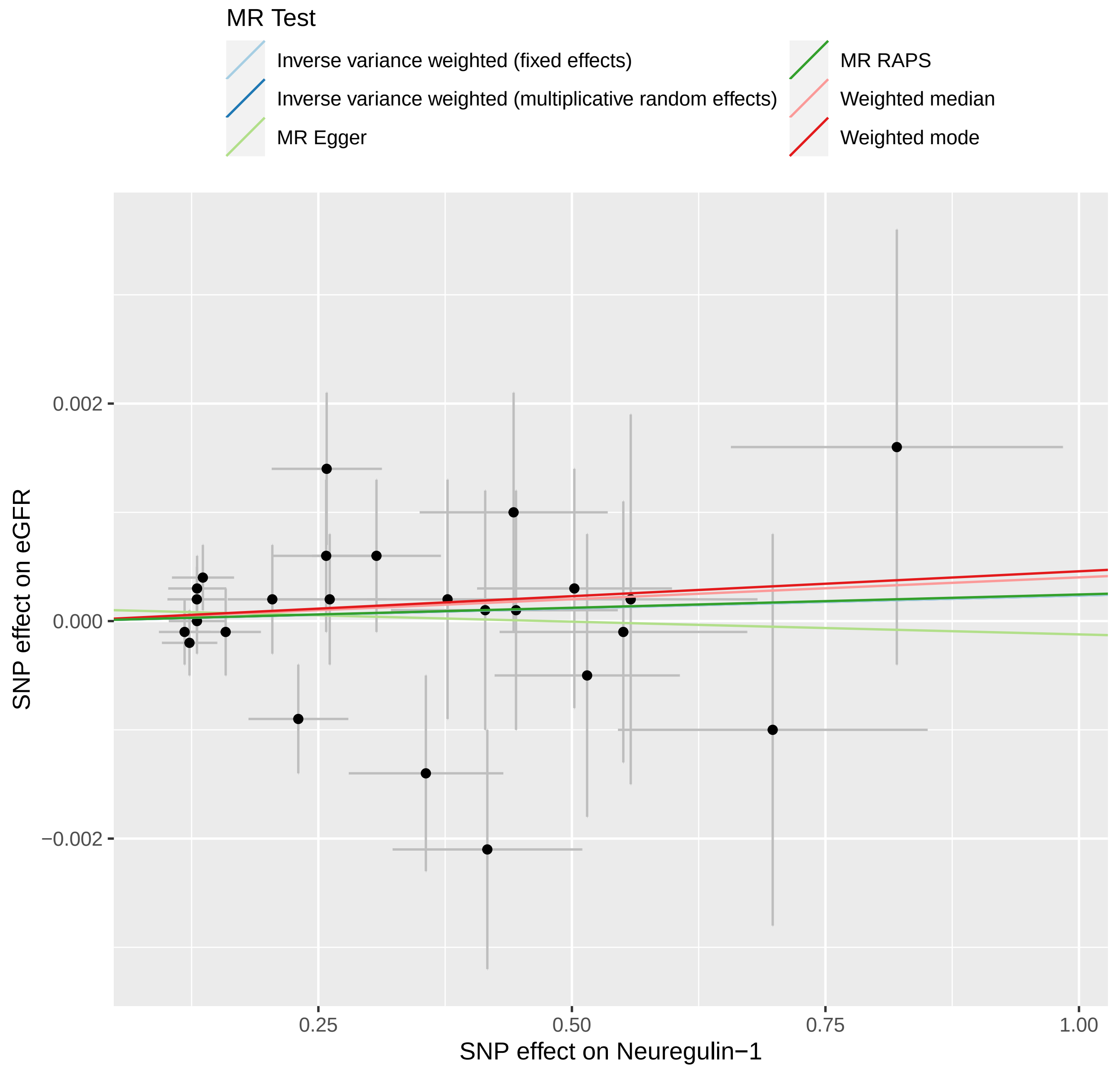

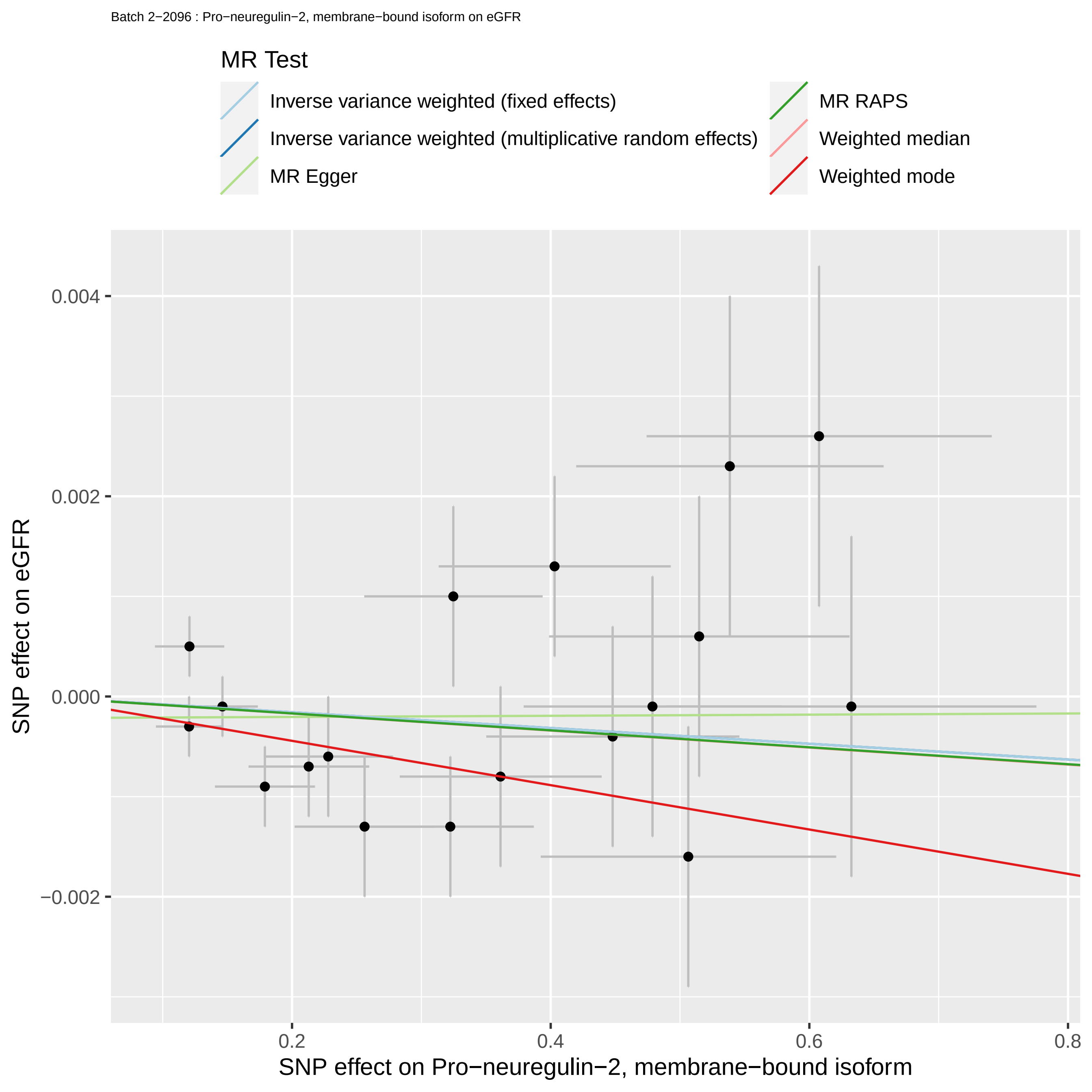

Batch 2−2096 : Pro−neuregulin−2, membrane−bound isoform on eGFR
MR Test
Inverse variance weighted (fixed effects)
Inverse variance weighted (multiplicative random effects)
MR Egger
MR RAPS
Weighted median
Weighted mode
0.004
0.002
0.000
−0.002
0.2
0.4
0.6
0.8
SNP effect on eGFR
SNP effect on Pro−neuregulin−2, membrane−bound isoform

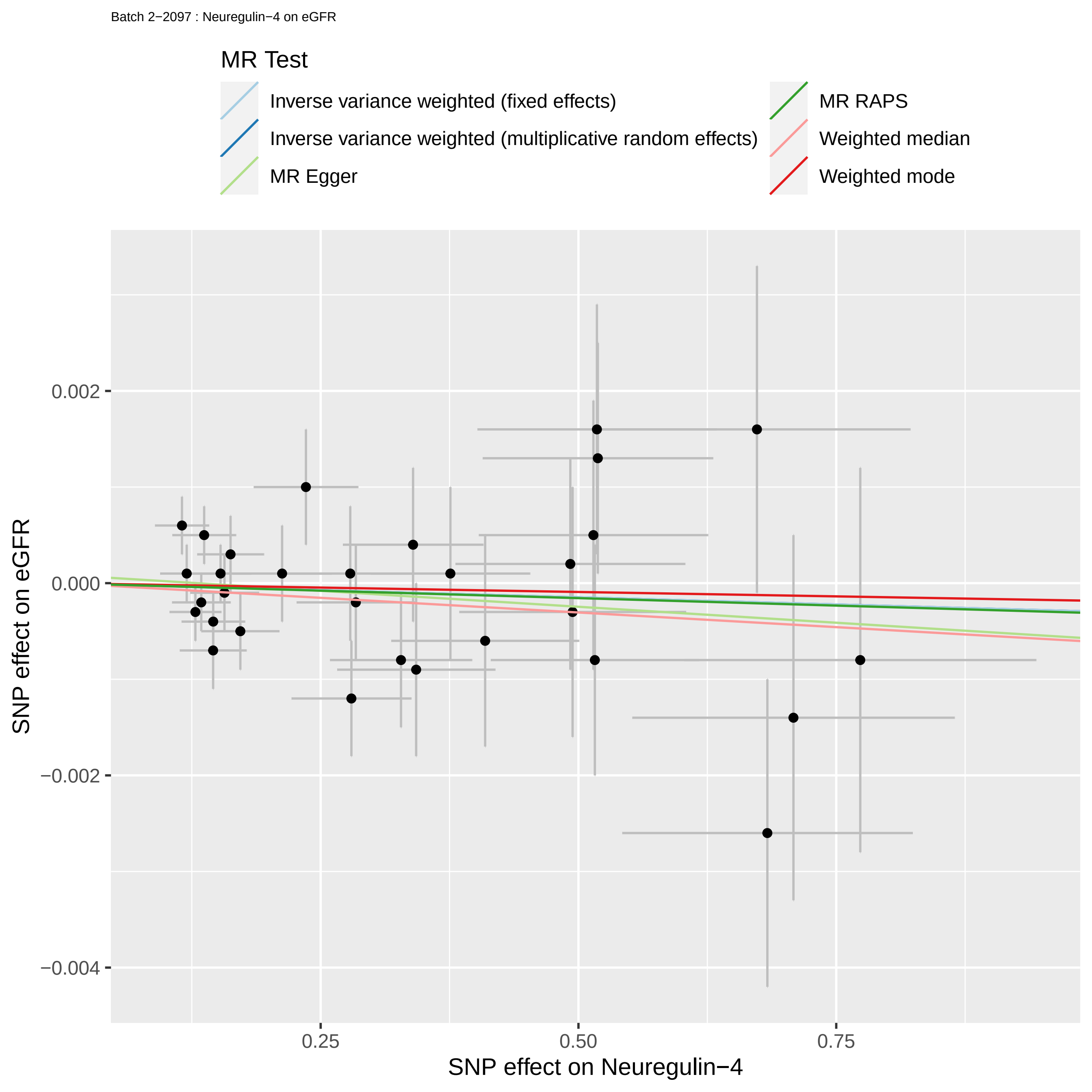

Batch 2−2097 : Neuregulin−4 on eGFR
MR Test
Inverse variance weighted (fixed effects)
Inverse variance weighted (multiplicative random effects)
MR Egger
MR RAPS
Weighted median
Weighted mode
0.002
0.000
−0.002
−0.004
0.25
0.50
0.75
SNP effect on eGFR
SNP effect on Neuregulin−4

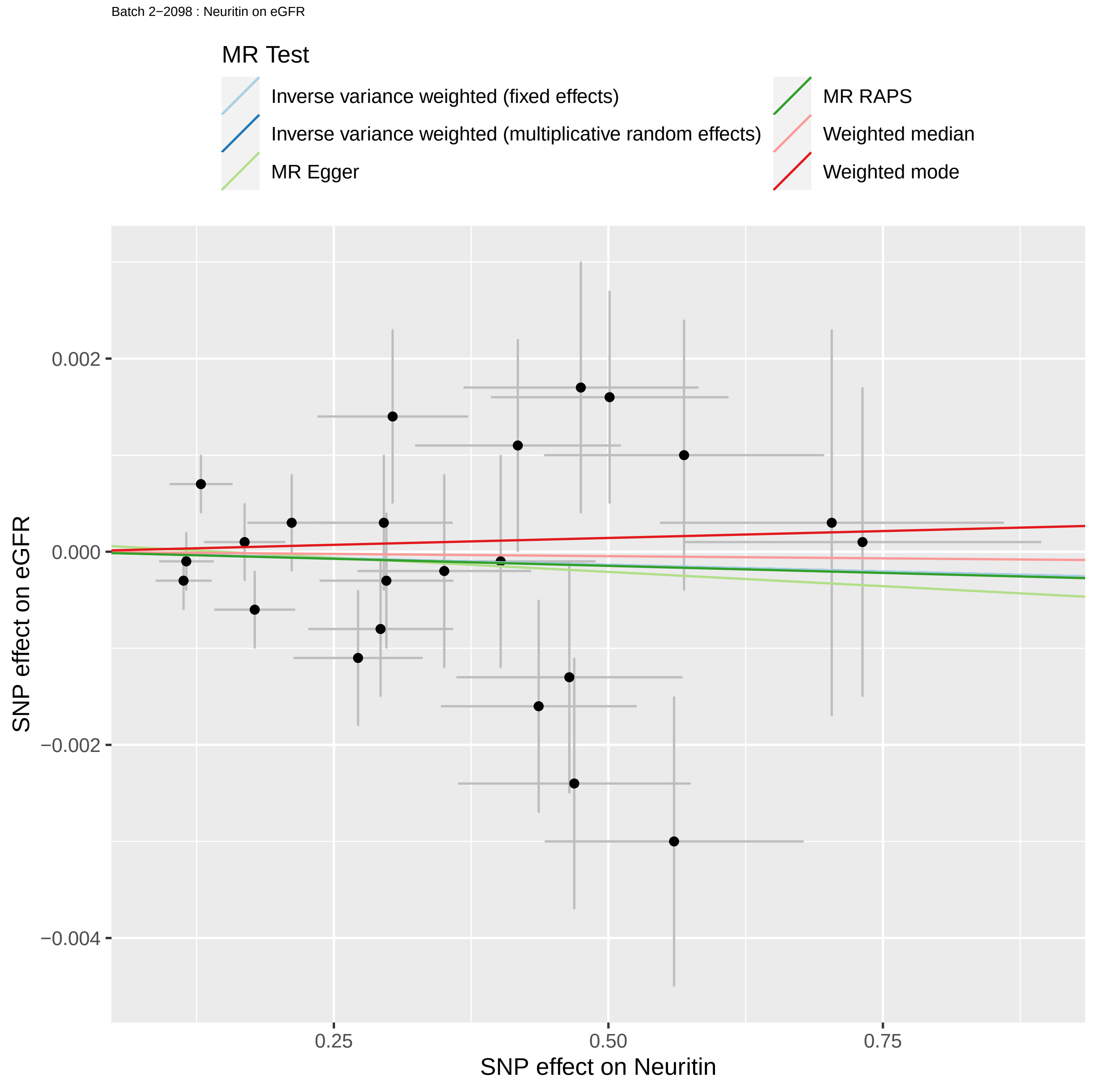

Batch 2−2098 : Neuritin on eGFR
MR Test
Inverse variance weighted (fixed effects)
Inverse variance weighted (multiplicative random effects)
MR Egger
MR RAPS
Weighted median
Weighted mode
0.002
0.000
−0.002
−0.004
0.25
0.50
0.75
SNP effect on eGFR
SNP effect on Neuritin

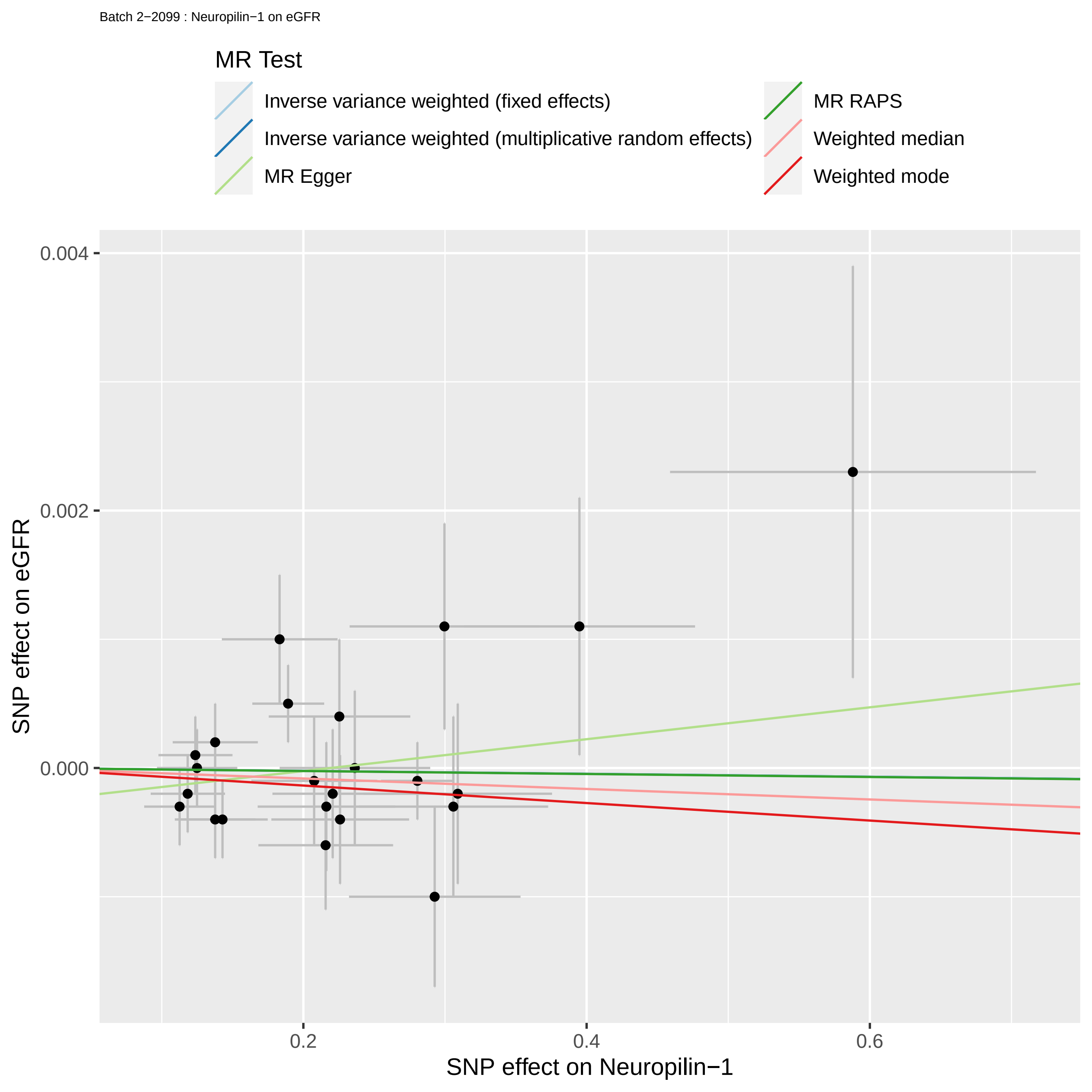

Batch 2−2099 : Neuropilin−1 on eGFR
MR Test
Inverse variance weighted (fixed effects)
Inverse variance weighted (multiplicative random effects)
MR Egger
MR RAPS
Weighted median
Weighted mode
0.004
0.002
0.000
0.2
0.4
0.6
SNP effect on eGFR
SNP effect on Neuropilin−1

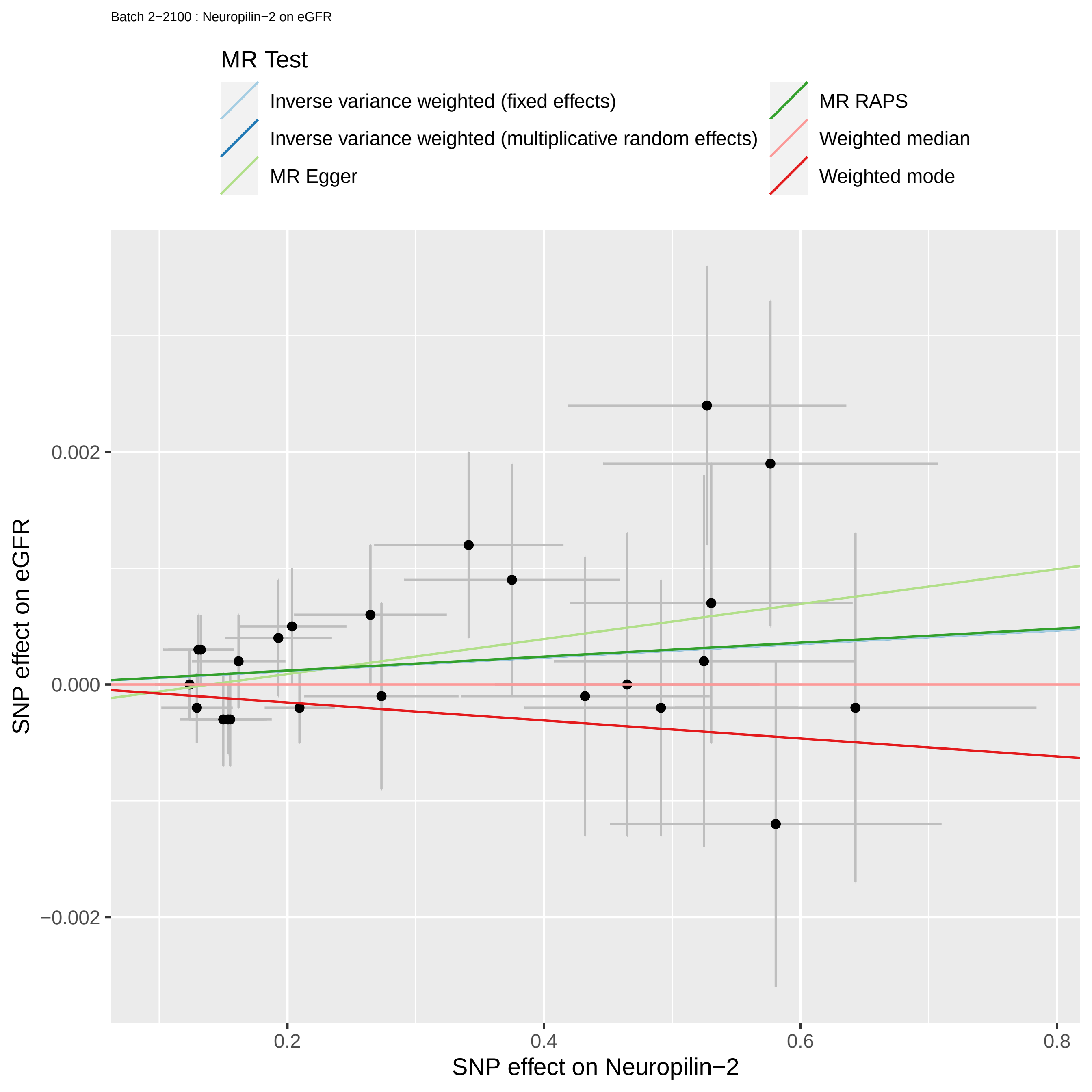

Batch 2−2100 : Neuropilin−2 on eGFR
MR Test
Inverse variance weighted (fixed effects)
Inverse variance weighted (multiplicative random effects)
MR Egger
MR RAPS
Weighted median
Weighted mode
0.002
0.000
−0.002
0.2
0.4
0.6
0.8
SNP effect on eGFR
SNP effect on Neuropilin−2

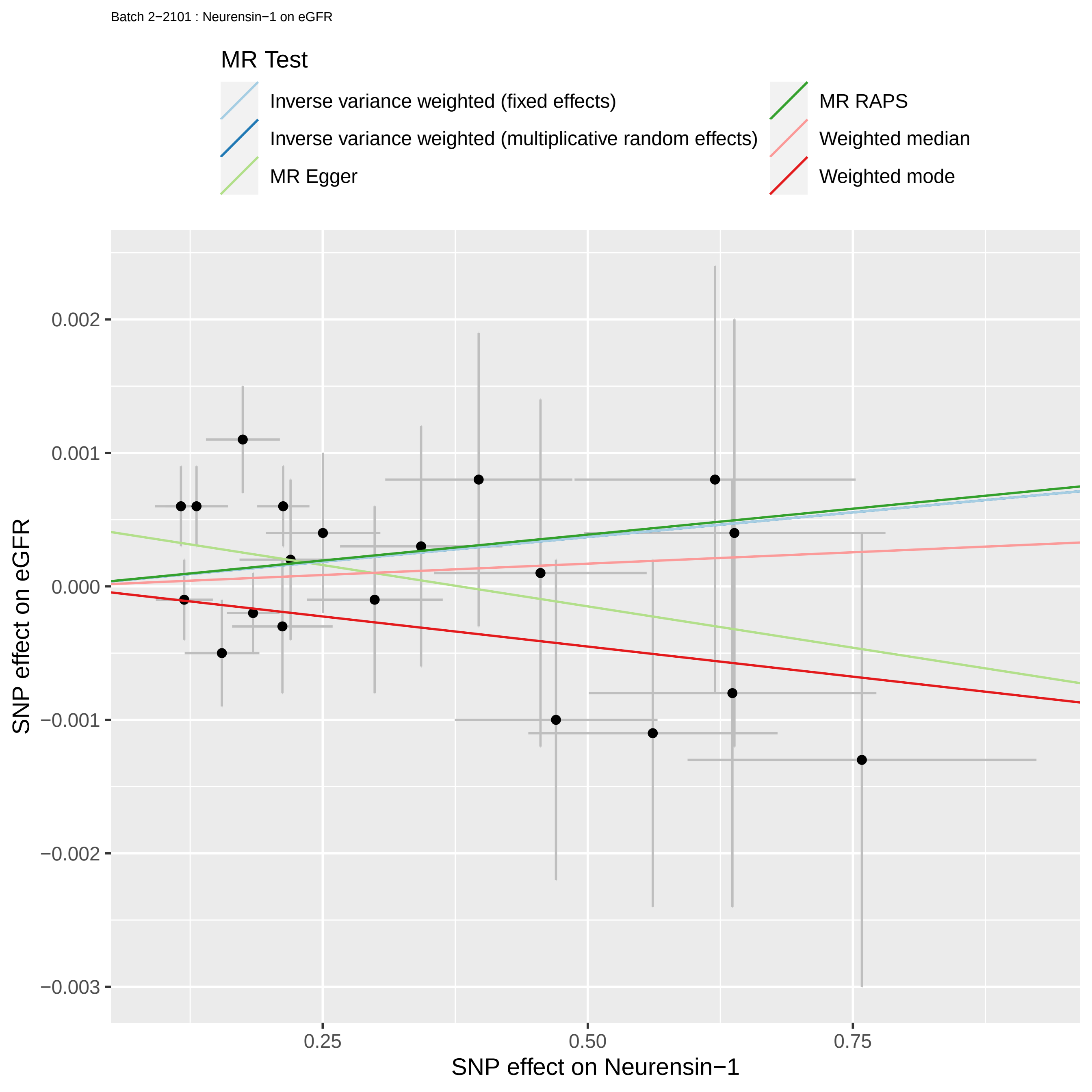

Batch 2−2101 : Neurensin−1 on eGFR
MR Test
Inverse variance weighted (fixed effects)
Inverse variance weighted (multiplicative random effects)
MR Egger
MR RAPS
Weighted median
Weighted mode
SNP effect on eGFR
0.002
0.001
0.000
−0.001
−0.002
−0.003
0.25
0.50
0.75
SNP effect on Neurensin−1

Batch 2−2102 : Neurexin−1−beta on eGFR

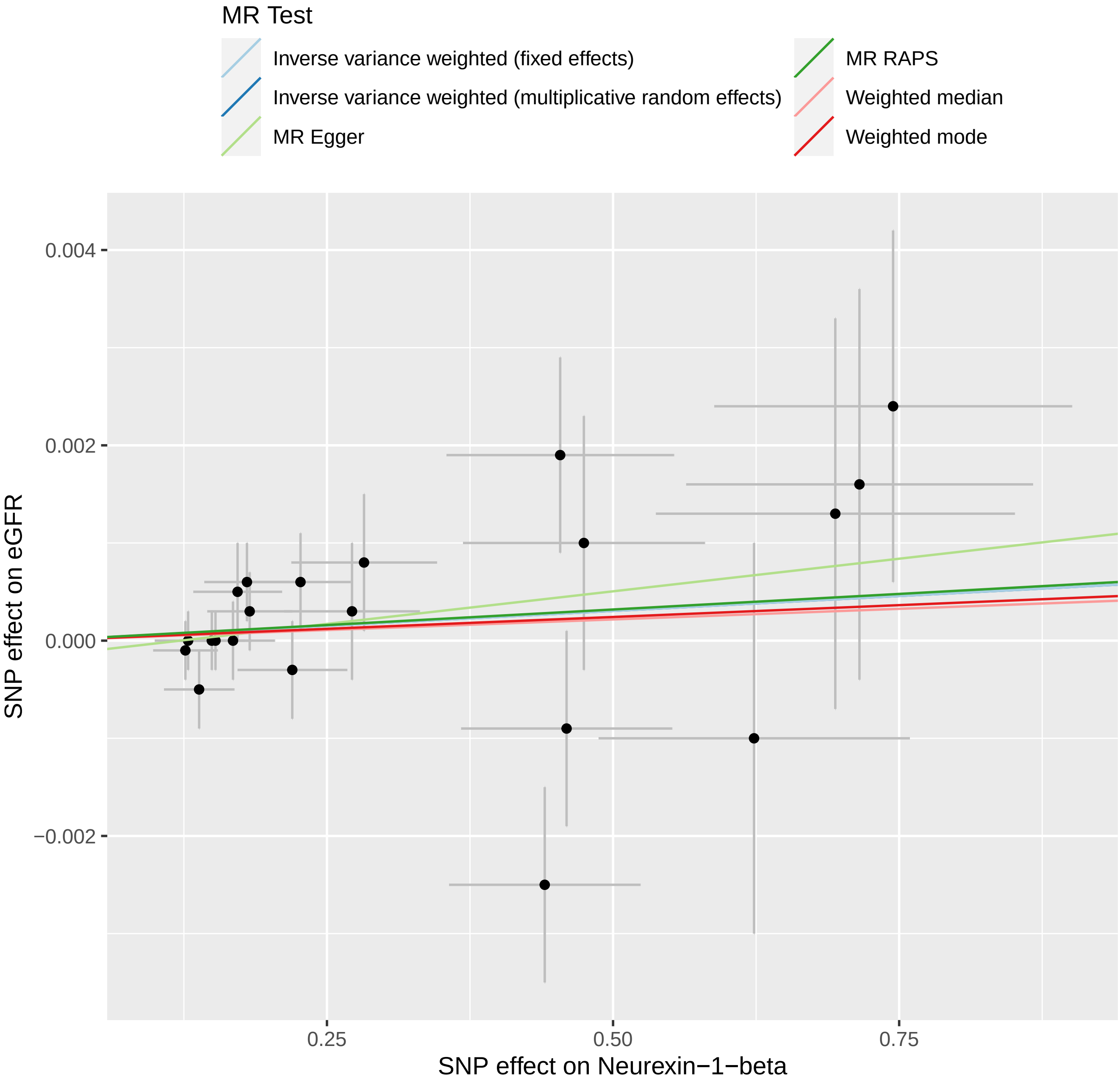

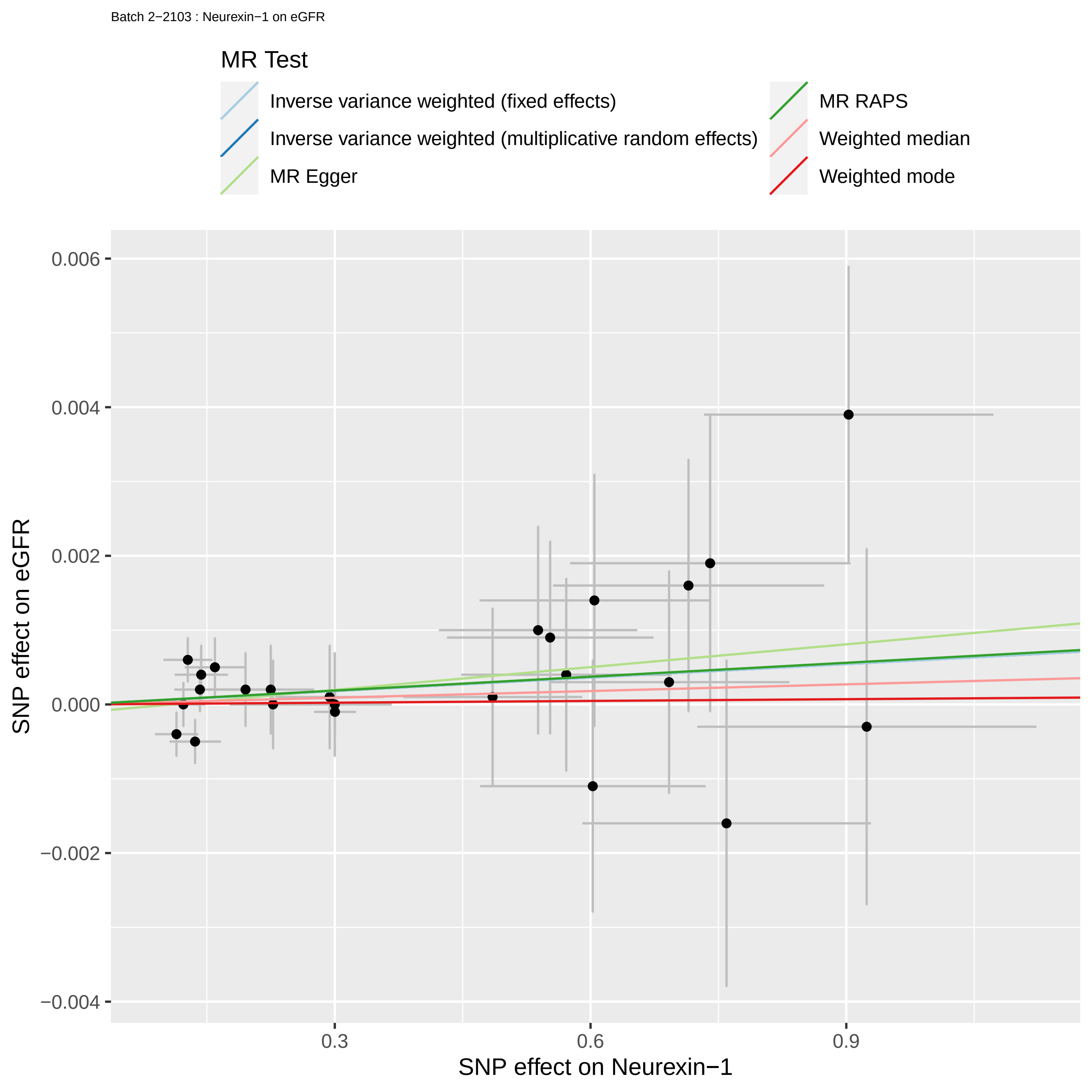
Batch 2−2103 : Neurexin−1 on eGFR
MR Test
Inverse variance weighted (fixed effects)
Inverse variance weighted (multiplicative random effects)
MR Egger
MR RAPS
Weighted median
Weighted mode
0.006
0.004
0.002
0.000
−0.002
−0.004
SNP effect on eGFR
0.3
0.6
0.9
SNP effect on Neurexin−1

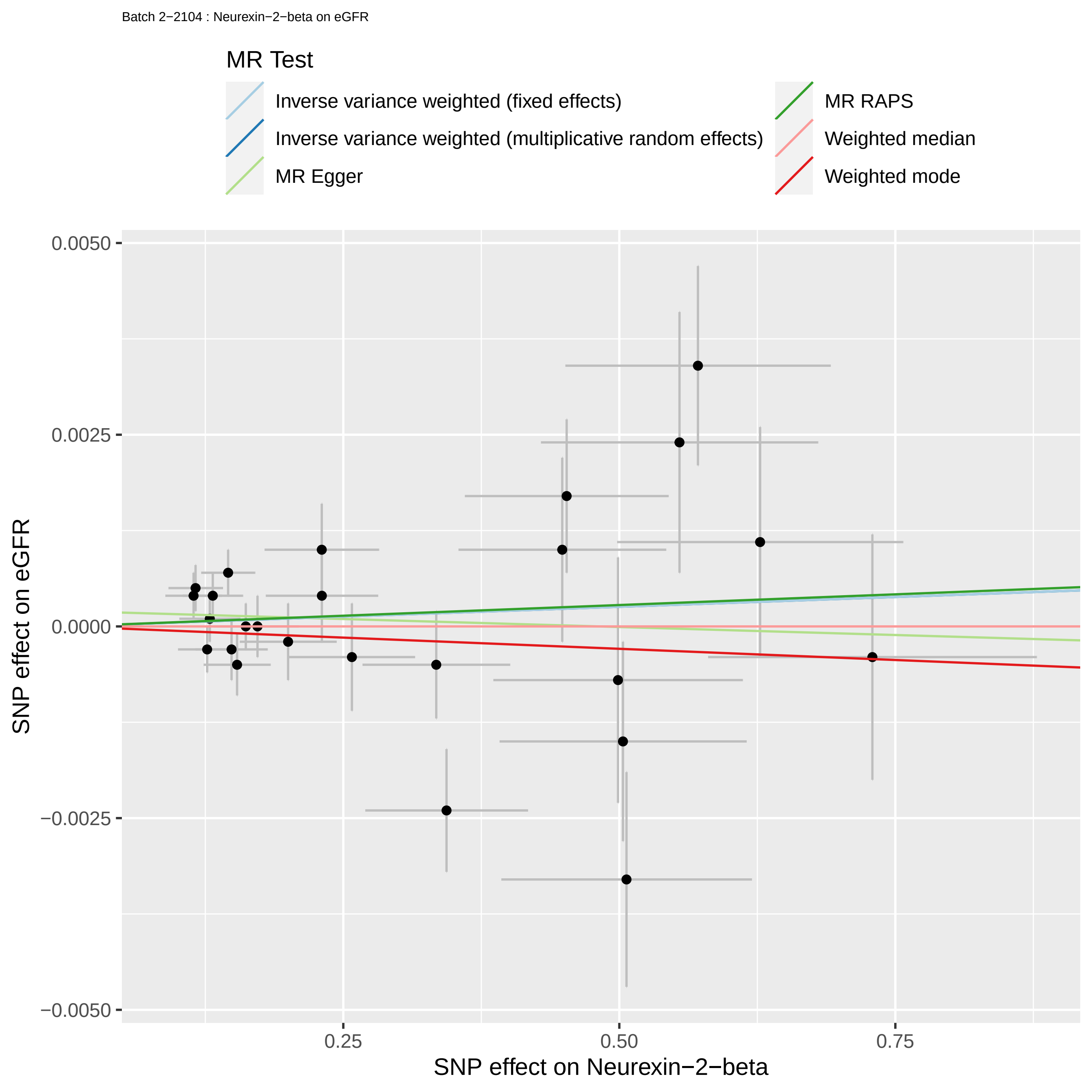

Batch 2−2104 : Neurexin−2−beta on eGFR
MR Test
Inverse variance weighted (fixed effects)
Inverse variance weighted (multiplicative random effects)
MR Egger
MR RAPS
Weighted median
Weighted mode
0.0050
0.0025
0.0000
−0.0025
−0.0050
SNP effect on eGFR
0.25
0.50
0.75
SNP effect on Neurexin−2−beta

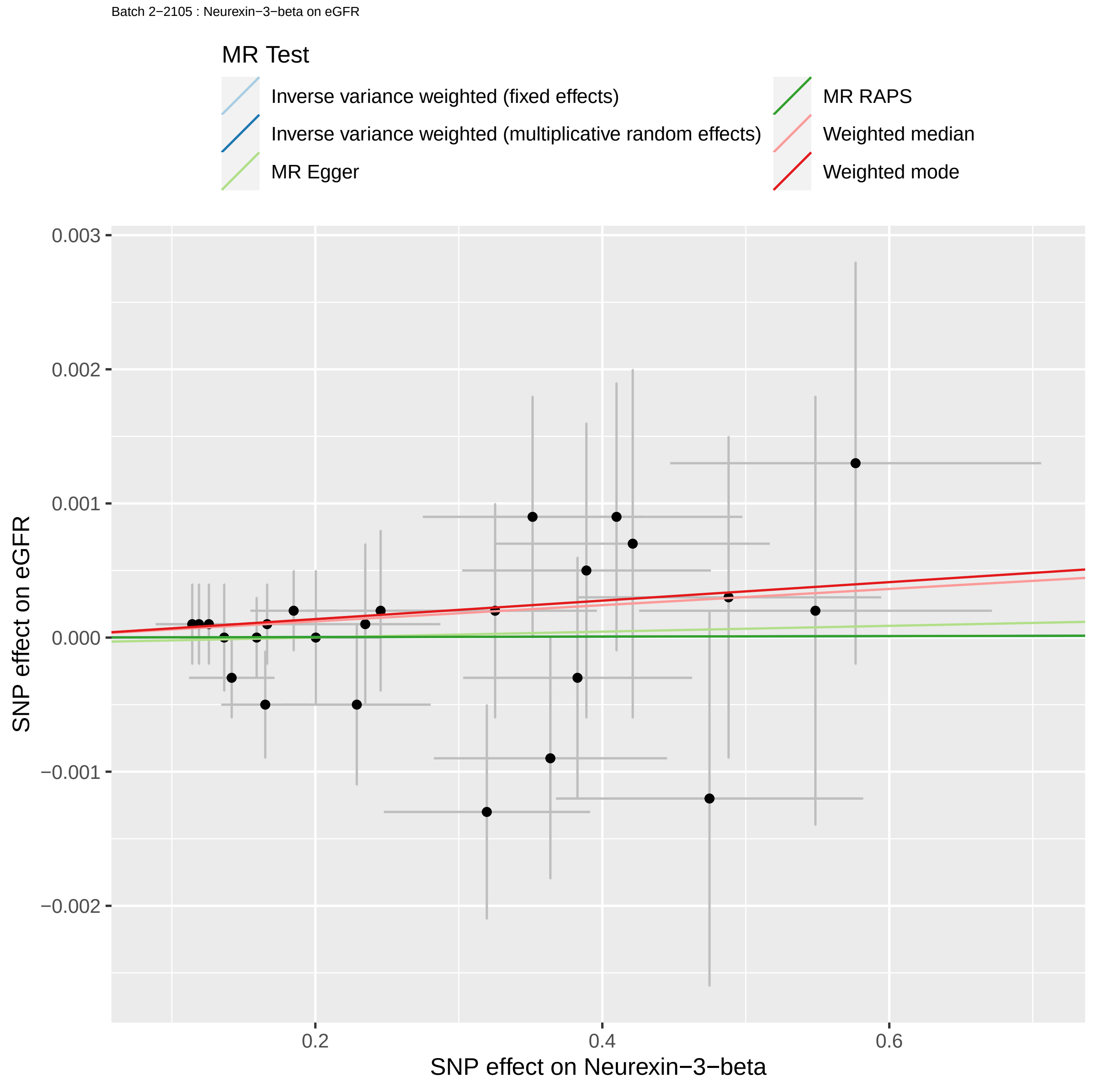

Batch 2−2105 : Neurexin−3−beta on eGFR
MR Test
Inverse variance weighted (fixed effects)
Inverse variance weighted (multiplicative random effects)
MR Egger
MR RAPS
Weighted median
Weighted mode
0.003
0.002
0.001
0.000
−0.001
−0.002
SNP effect on eGFR
0.2
0.4
0.6
SNP effect on Neurexin−3−beta

Batch 2−2106 : Neurexin−3−beta on eGFR

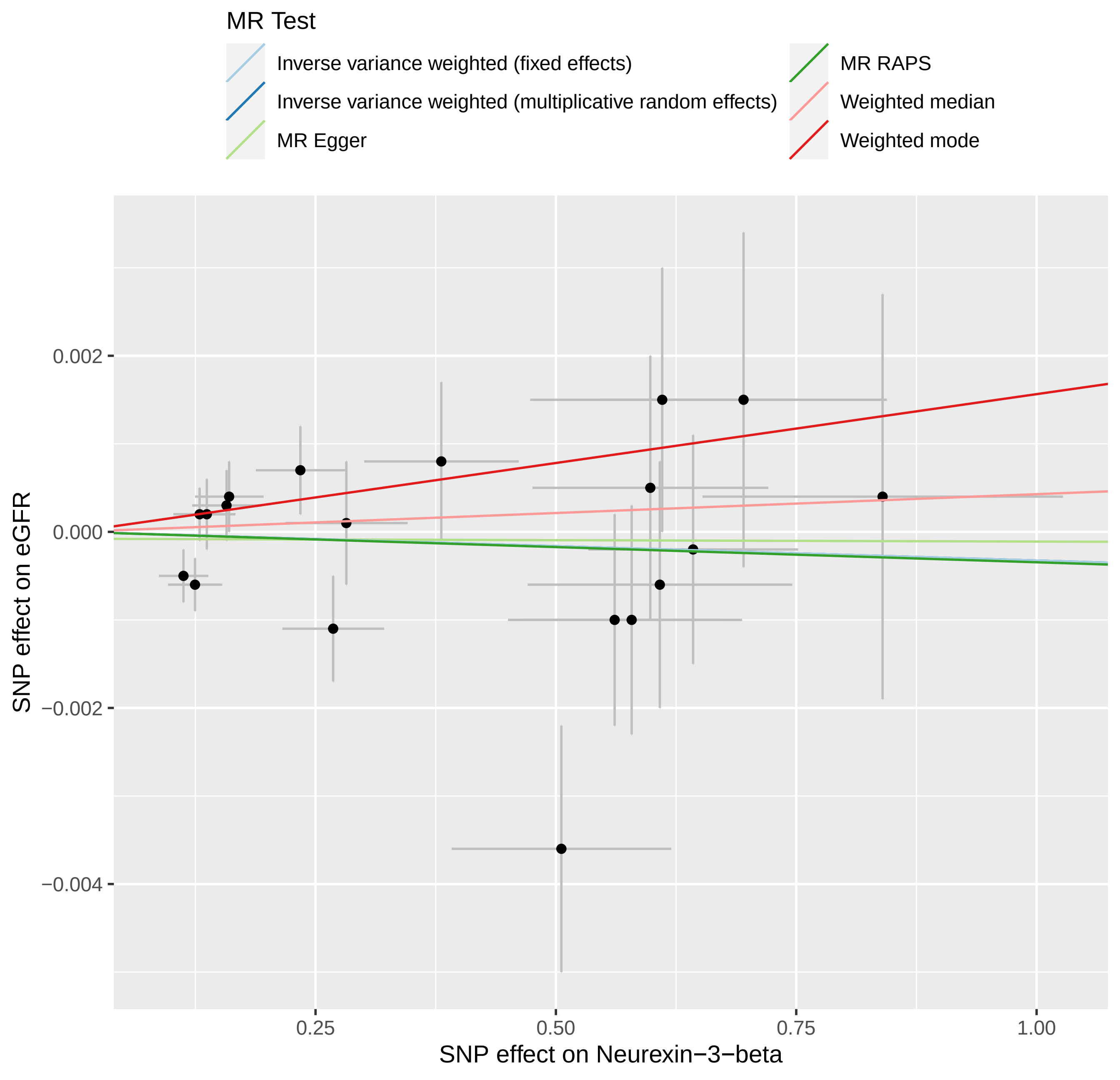

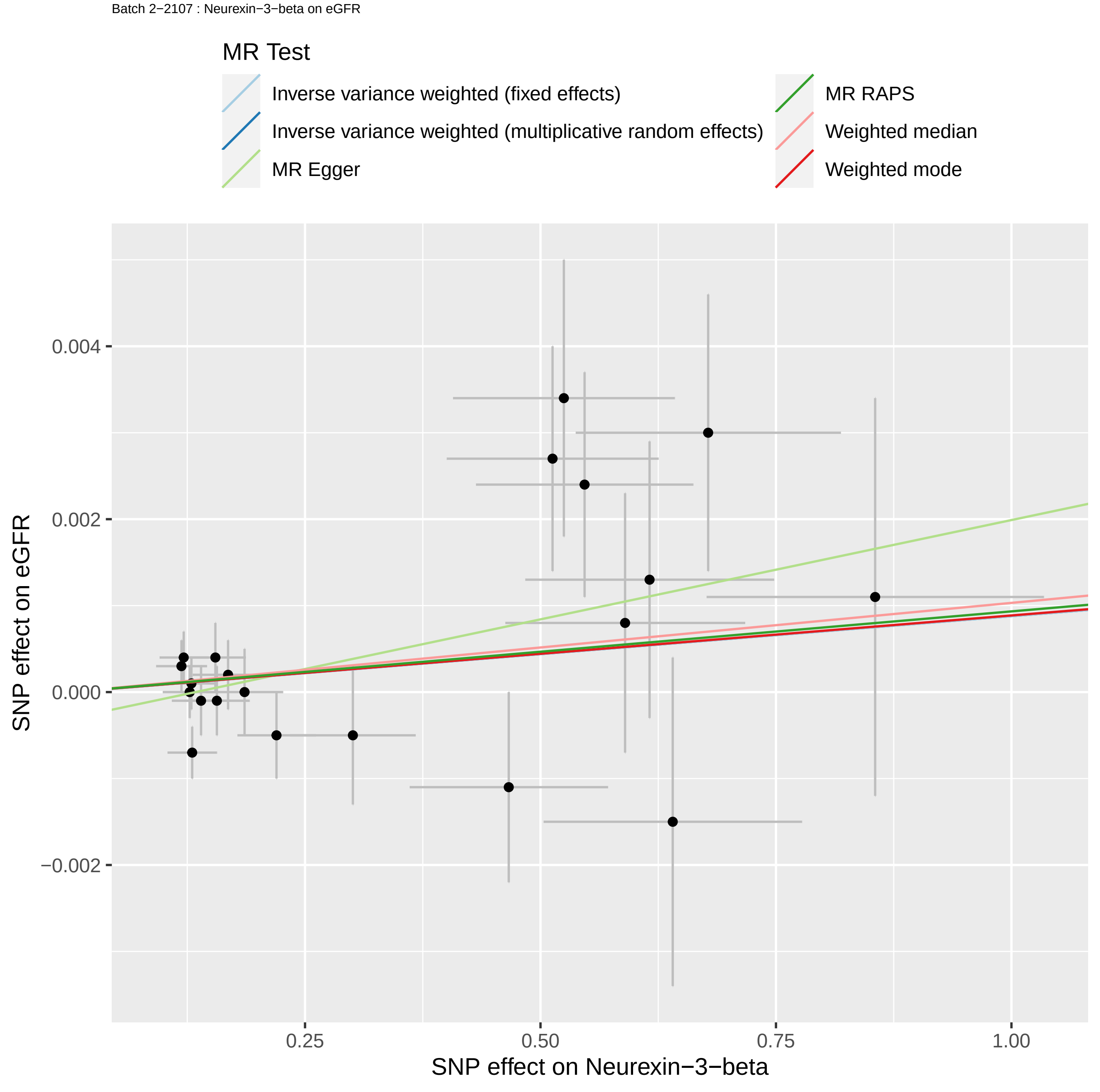

Batch 2−2107 : Neurexin−3−beta on eGFR
MR Test
Inverse variance weighted (fixed effects)
Inverse variance weighted (multiplicative random effects)
MR Egger
MR RAPS
Weighted median
Weighted mode
0.004
0.002
0.000
−0.002
SNP effect on eGFR
0.25
0.50
0.75
1.00
SNP effect on Neurexin−3−beta

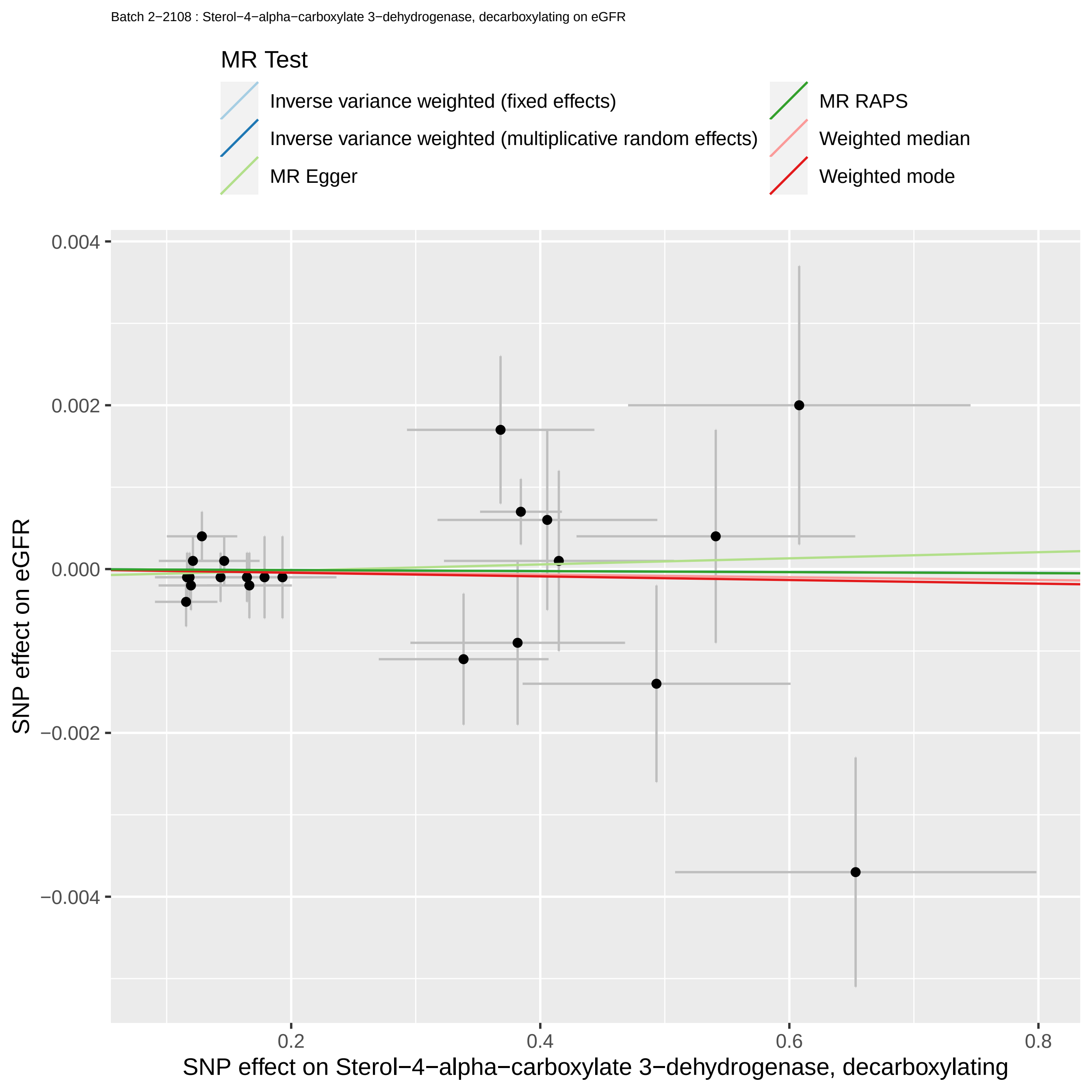

Batch 2−2108 : Sterol−4−alpha−carboxylate 3−dehydrogenase, decarboxylating on eGFR
MR Test
Inverse variance weighted (fixed effects)
Inverse variance weighted (multiplicative random effects)
MR Egger
MR RAPS
Weighted median
Weighted mode
0.004
0.002
0.000
−0.002
−0.004
SNP effect on eGFR
0.2
0.4
0.6
0.8
SNP effect on Sterol−4−alpha−carboxylate 3−dehydrogenase, decarboxylating

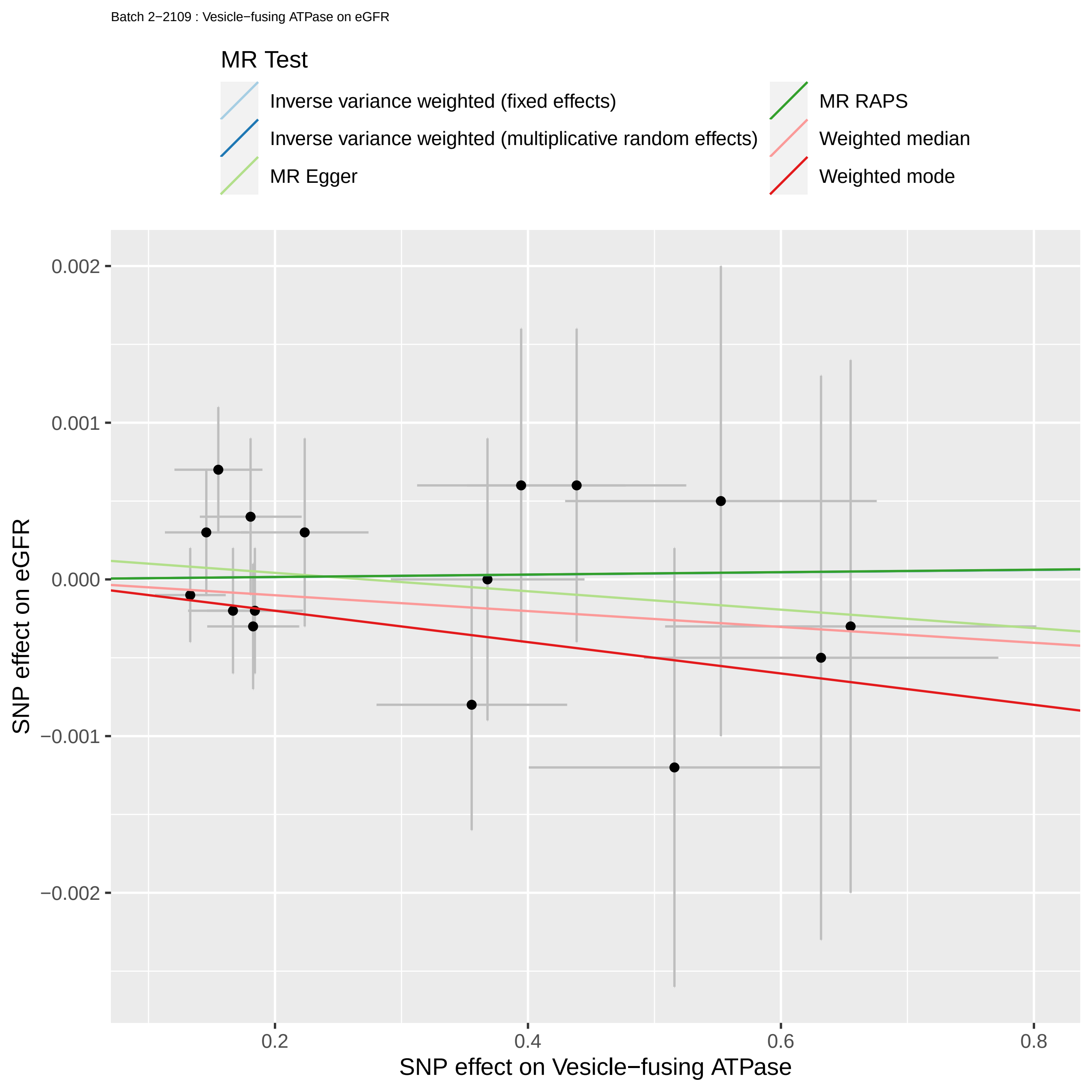

Batch 2−2109 : Vesicle−fusing ATPase on eGFR
MR Test
Inverse variance weighted (fixed effects)
Inverse variance weighted (multiplicative random effects)
MR Egger
MR RAPS
Weighted median
Weighted mode
0.002
0.001
0.000
−0.001
−0.002
SNP effect on eGFR
0.2
0.4
0.6
0.8
SNP effect on Vesicle−fusing ATPase

Batch 2−2110 : Neuron−specific protein family member 1 on eGFR

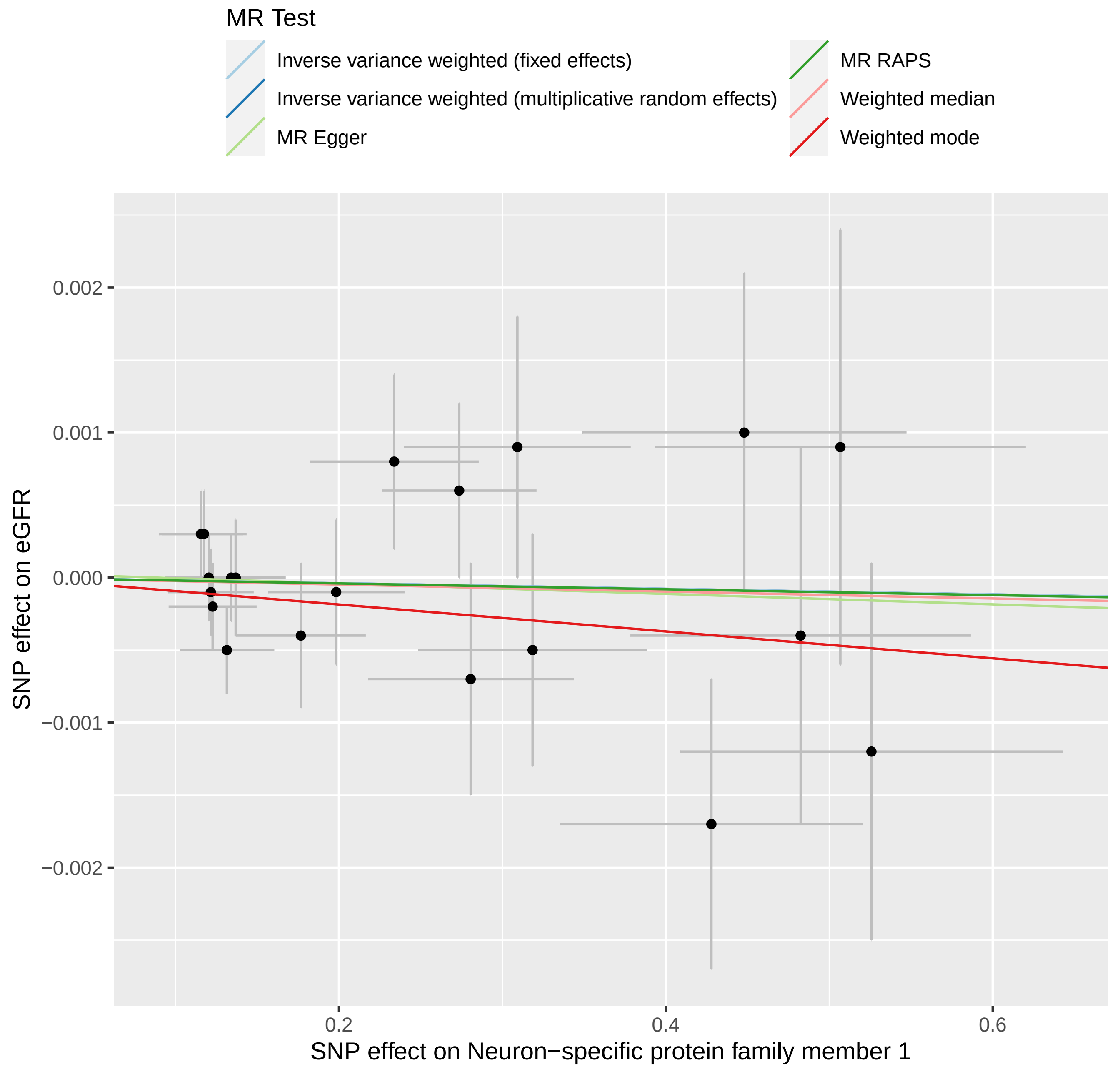

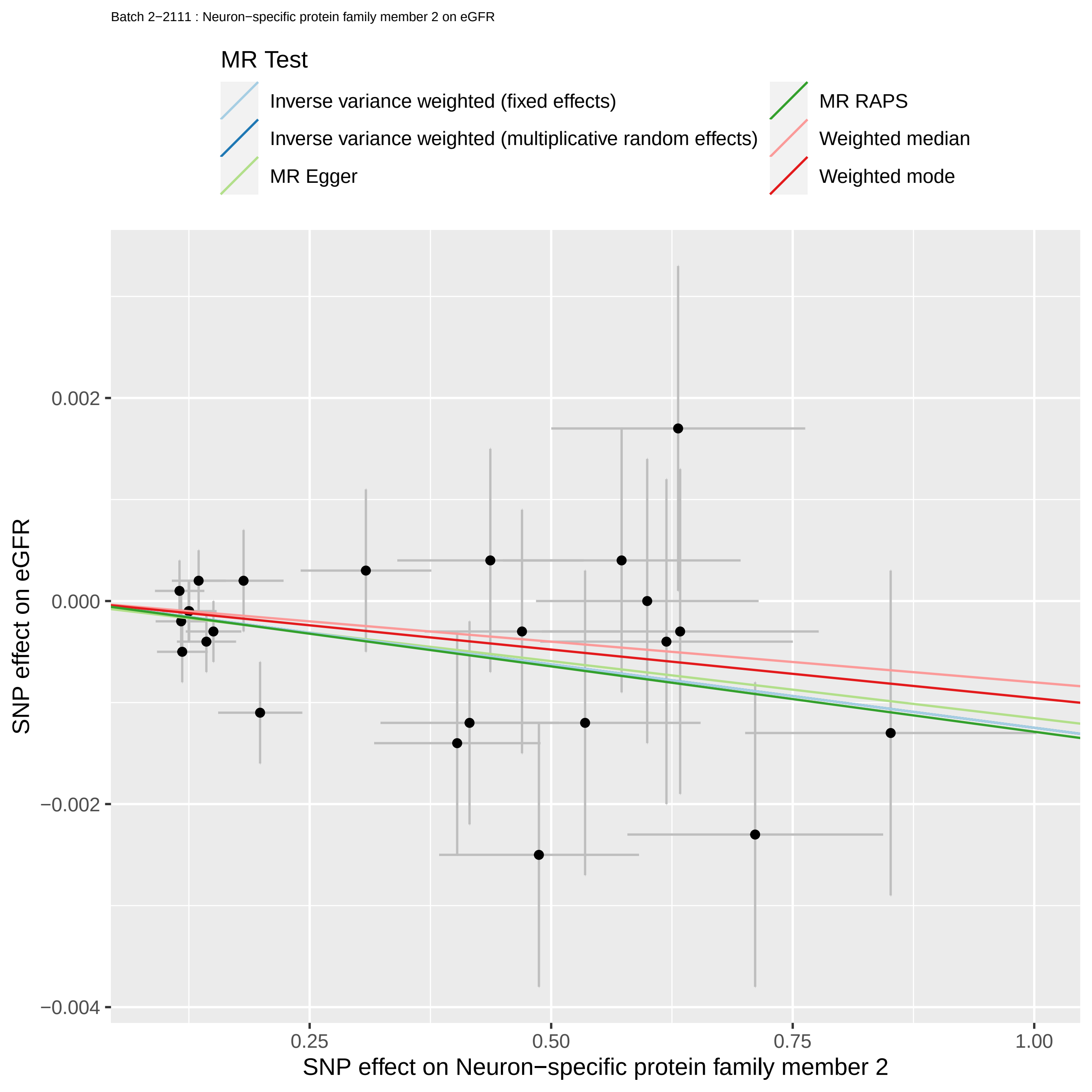

Batch 2−2111 : Neuron−specific protein family member 2 on eGFR
MR Test
Inverse variance weighted (fixed effects)
Inverse variance weighted (multiplicative random effects)
MR Egger
MR RAPS
Weighted median
Weighted mode
0.002
0.000
−0.002
−0.004
SNP effect on eGFR
0.25
0.50
0.75
1.00
SNP effect on Neuron−specific protein family member 2

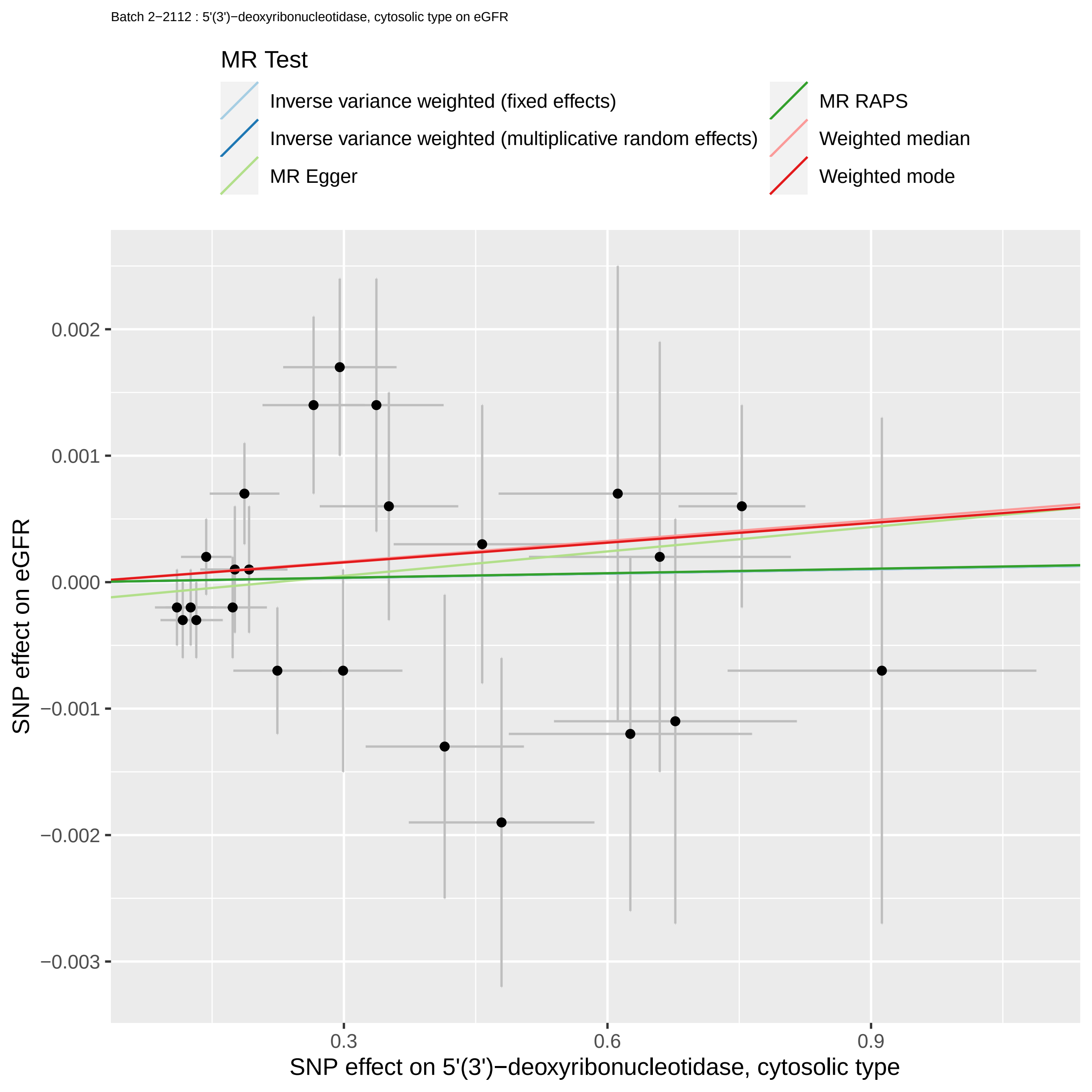
Batch 2−2112 : 5'(3')−deoxyribonucleotidase, cytosolic type on eGFR
MR Test
Inverse variance weighted (fixed effects)
Inverse variance weighted (multiplicative random effects)
MR Egger
MR RAPS
Weighted median
Weighted mode
SNP effect on eGFR
0.002
0.001
0.000
−0.001
−0.002
−0.003
0.3
0.6
0.9
SNP effect on 5'(3')−deoxyribonucleotidase, cytosolic type

Batch 2−2113 : 5'−Nucleotidase on eGFR

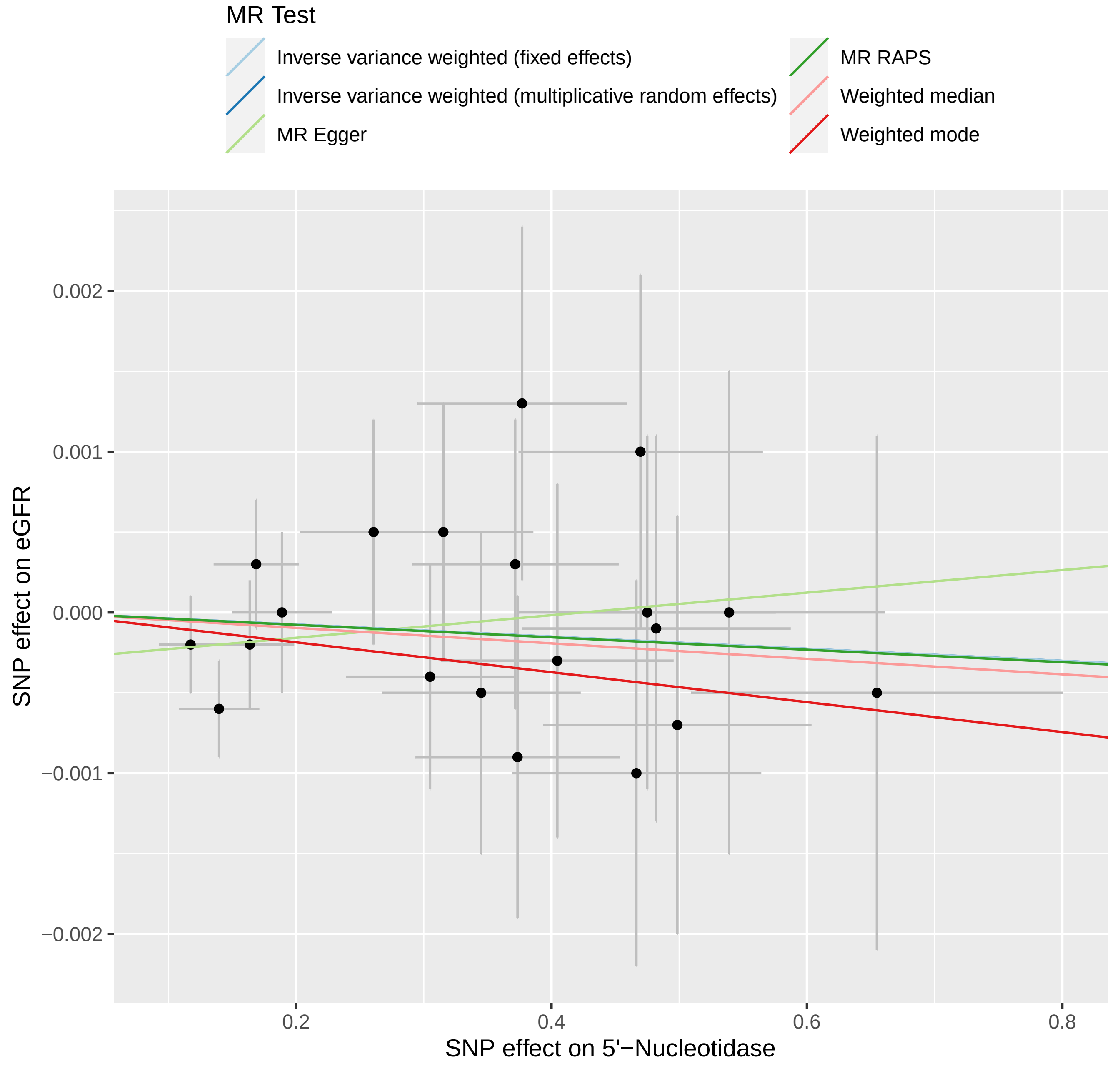

Batch 2−2114 : Neurotrophin−3 on eGFR

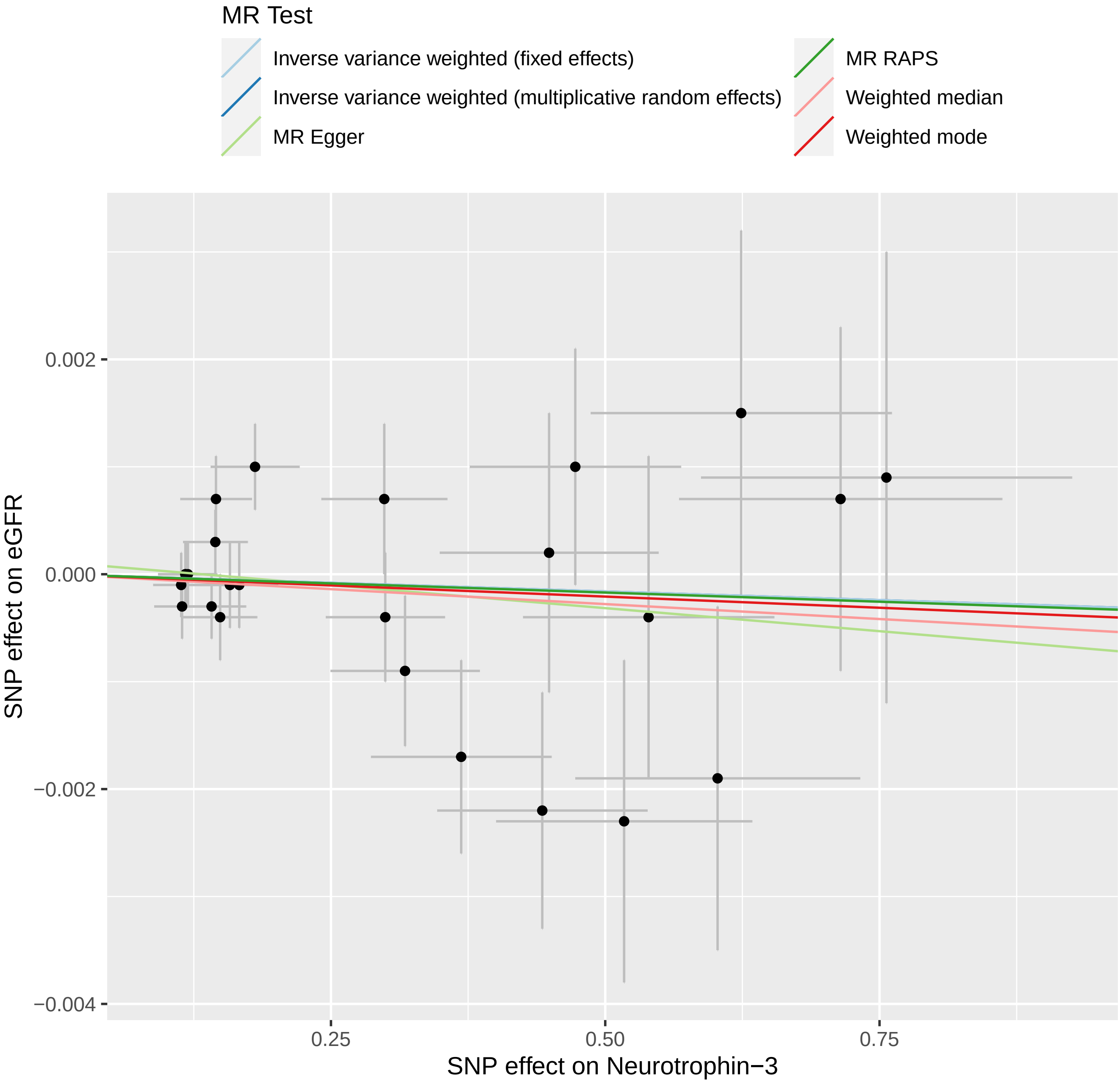

Batch 2−2115 : Neurotrophin−4 on eGFR

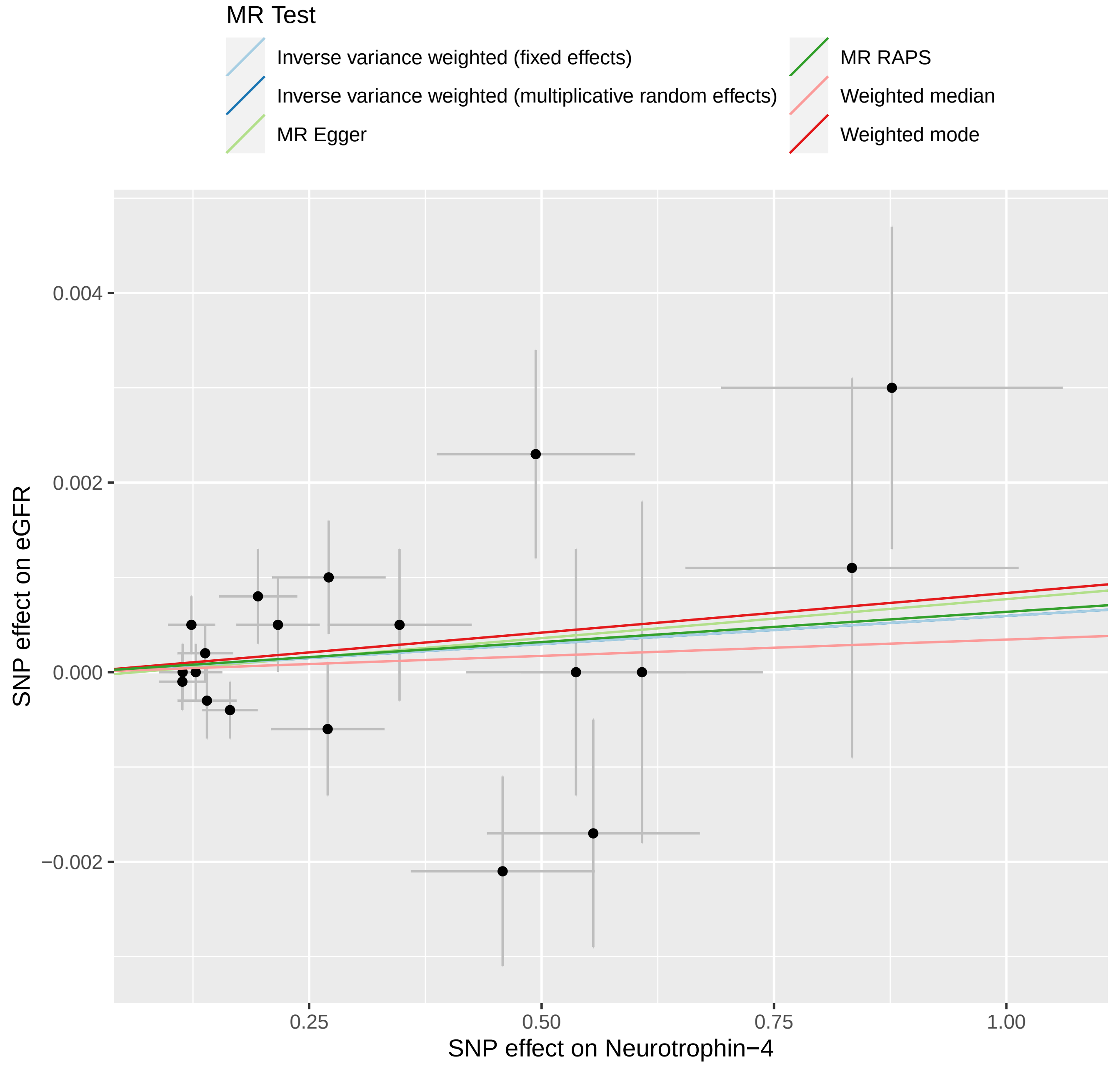

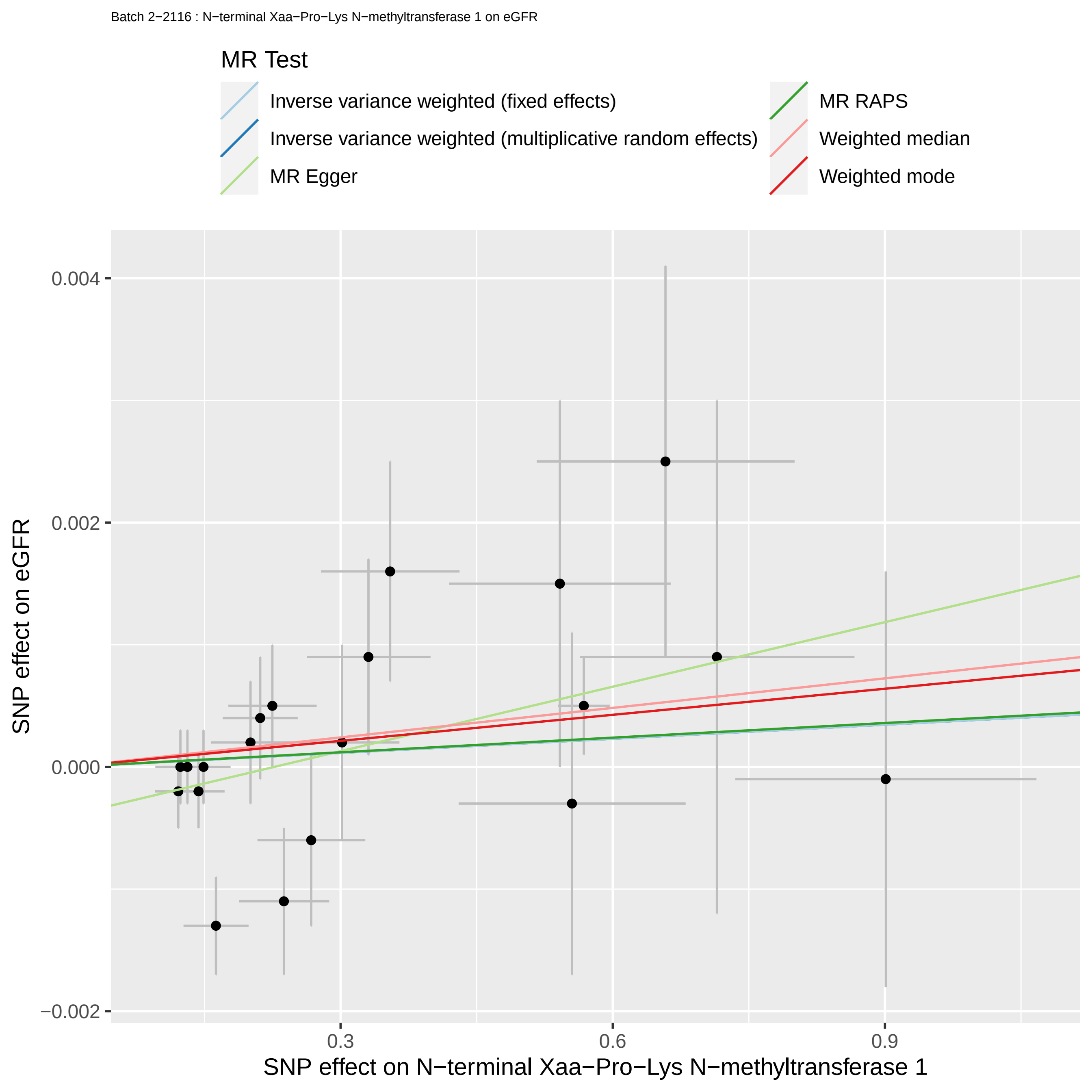

Batch 2−2116 : N−terminal Xaa−Pro−Lys N−methyltransferase 1 on eGFR
MR Test
Inverse variance weighted (fixed effects)
Inverse variance weighted (multiplicative random effects)
MR Egger
MR RAPS
Weighted median
Weighted mode
0.004
0.002
0.000
−0.002
0.3
0.6
0.9
SNP effect on eGFR
SNP effect on N−terminal Xaa−Pro−Lys N−methyltransferase 1

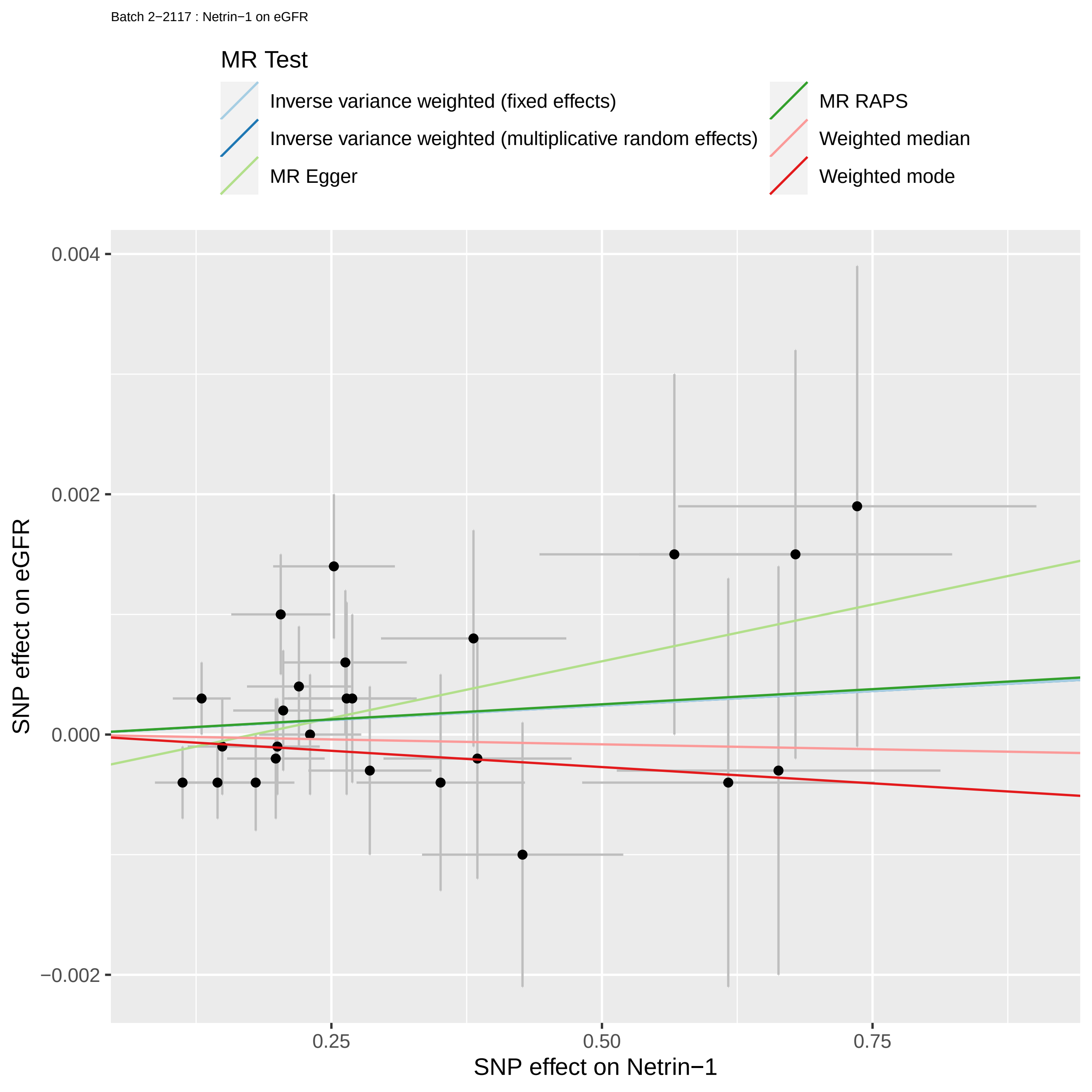

Batch 2−2117 : Netrin−1 on eGFR
MR Test
Inverse variance weighted (fixed effects)
Inverse variance weighted (multiplicative random effects)
MR Egger
MR RAPS
Weighted median
Weighted mode
0.004
0.002
0.000
−0.002
0.25
0.50
0.75
SNP effect on eGFR
SNP effect on Netrin−1

Batch 2−2118 : Netrin−1 on eGFR

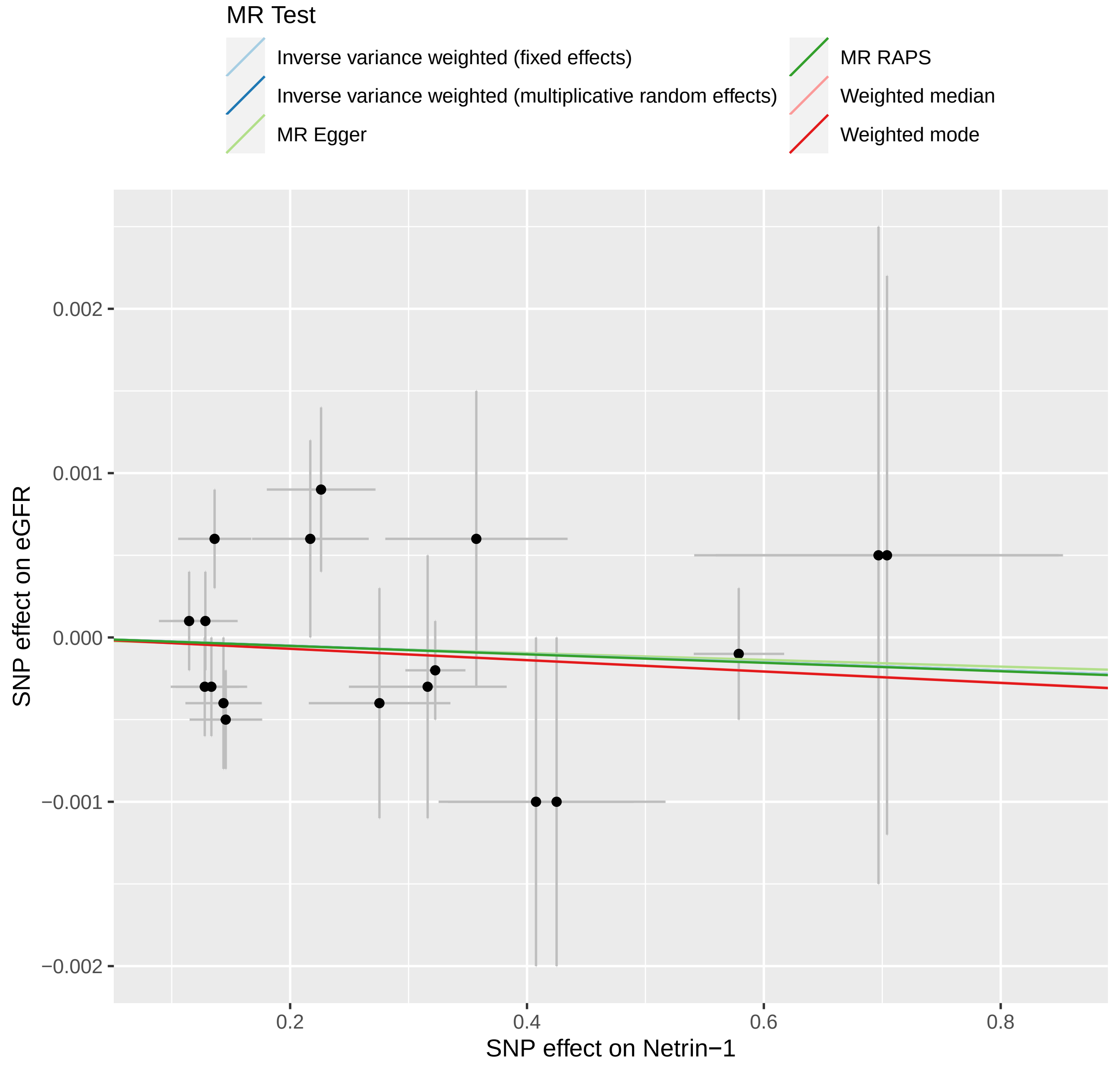

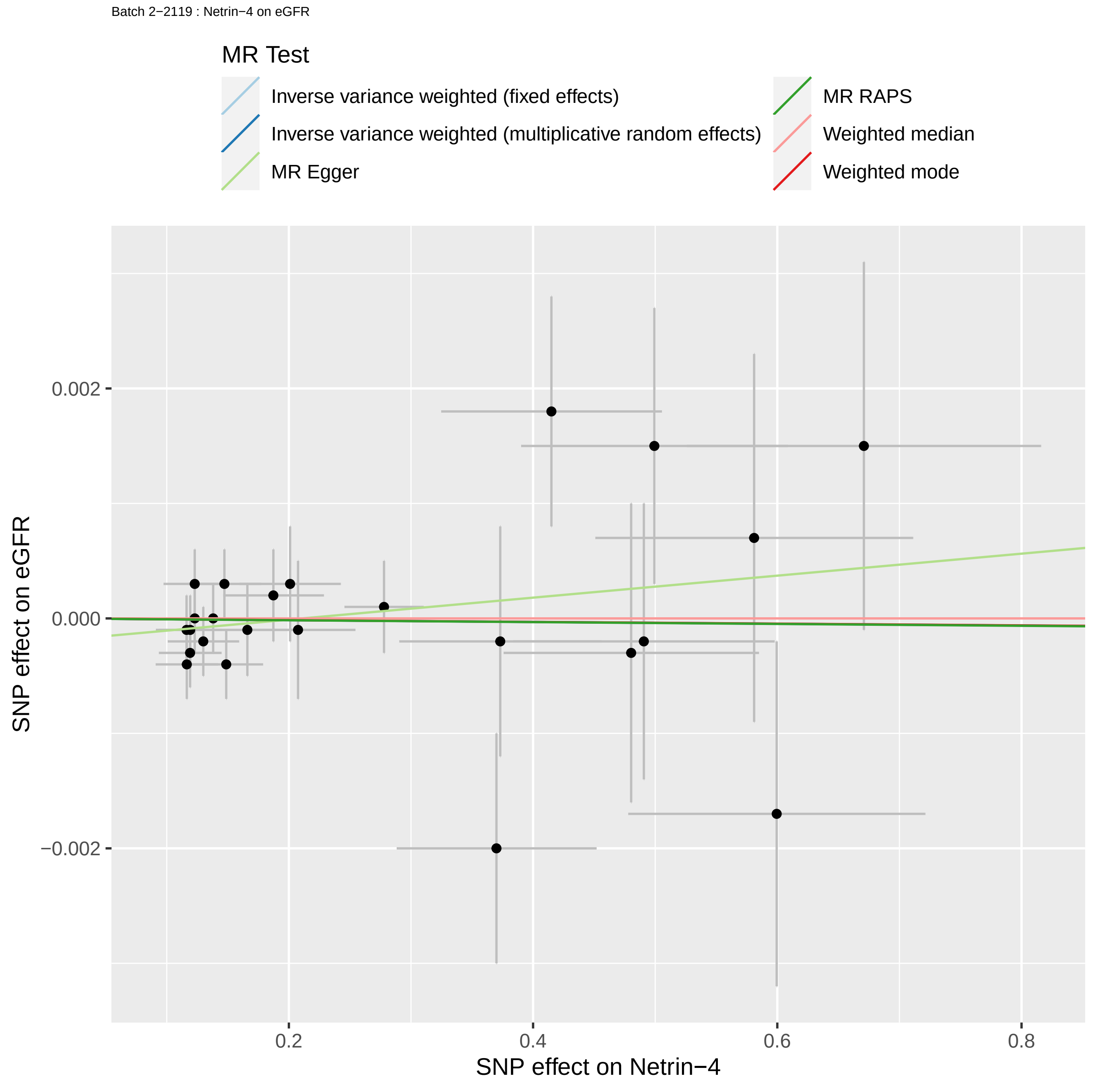
Batch 2−2119 : Netrin−4 on eGFR
MR Test
Inverse variance weighted (fixed effects)
Inverse variance weighted (multiplicative random effects)
MR Egger
MR RAPS
Weighted median
Weighted mode
0.002
0.000
−0.002
0.2
0.4
0.6
0.8
SNP effect on eGFR
SNP effect on Netrin−4

Batch 2−2120 : Netrin−G1 on eGFR

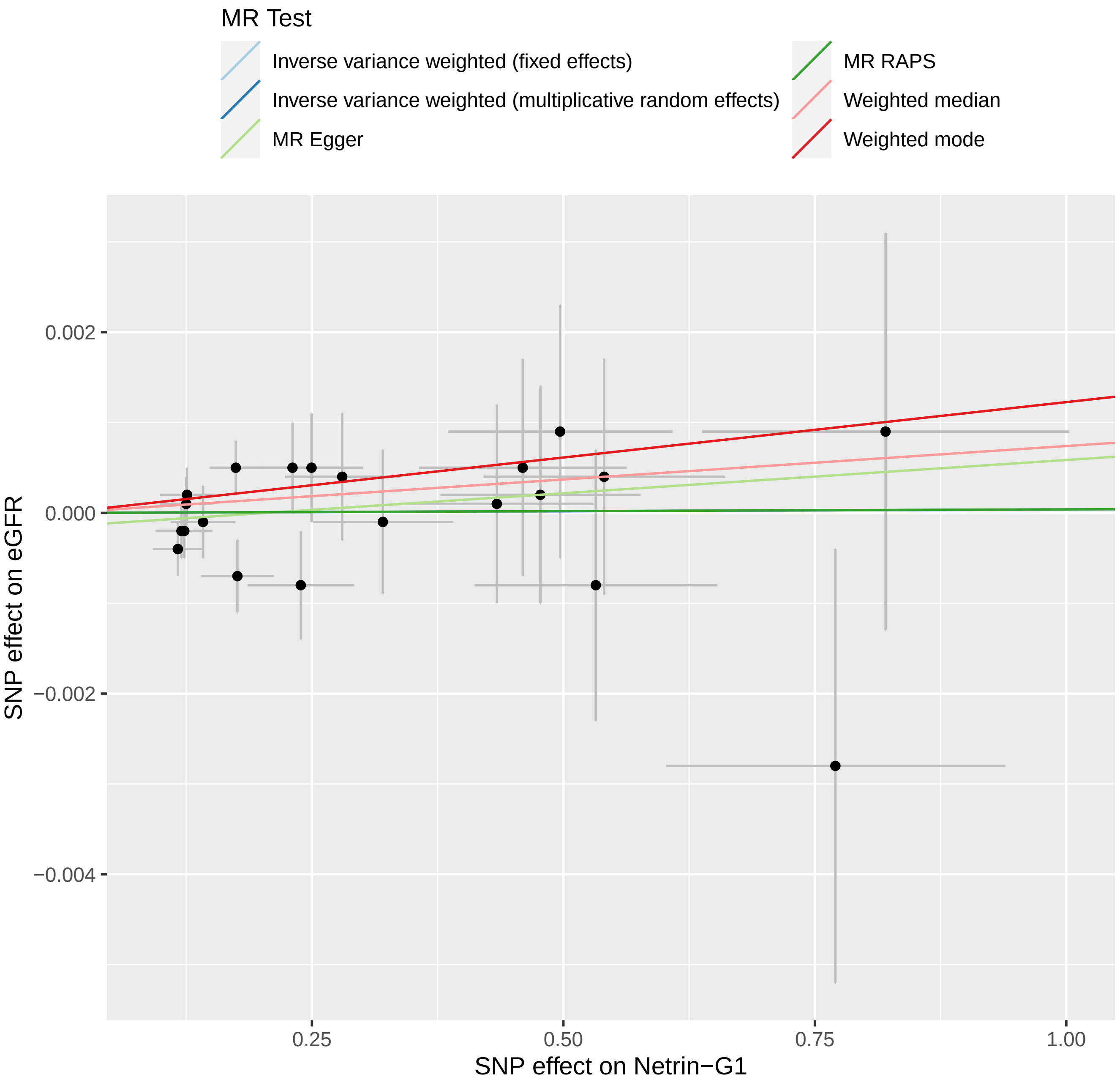

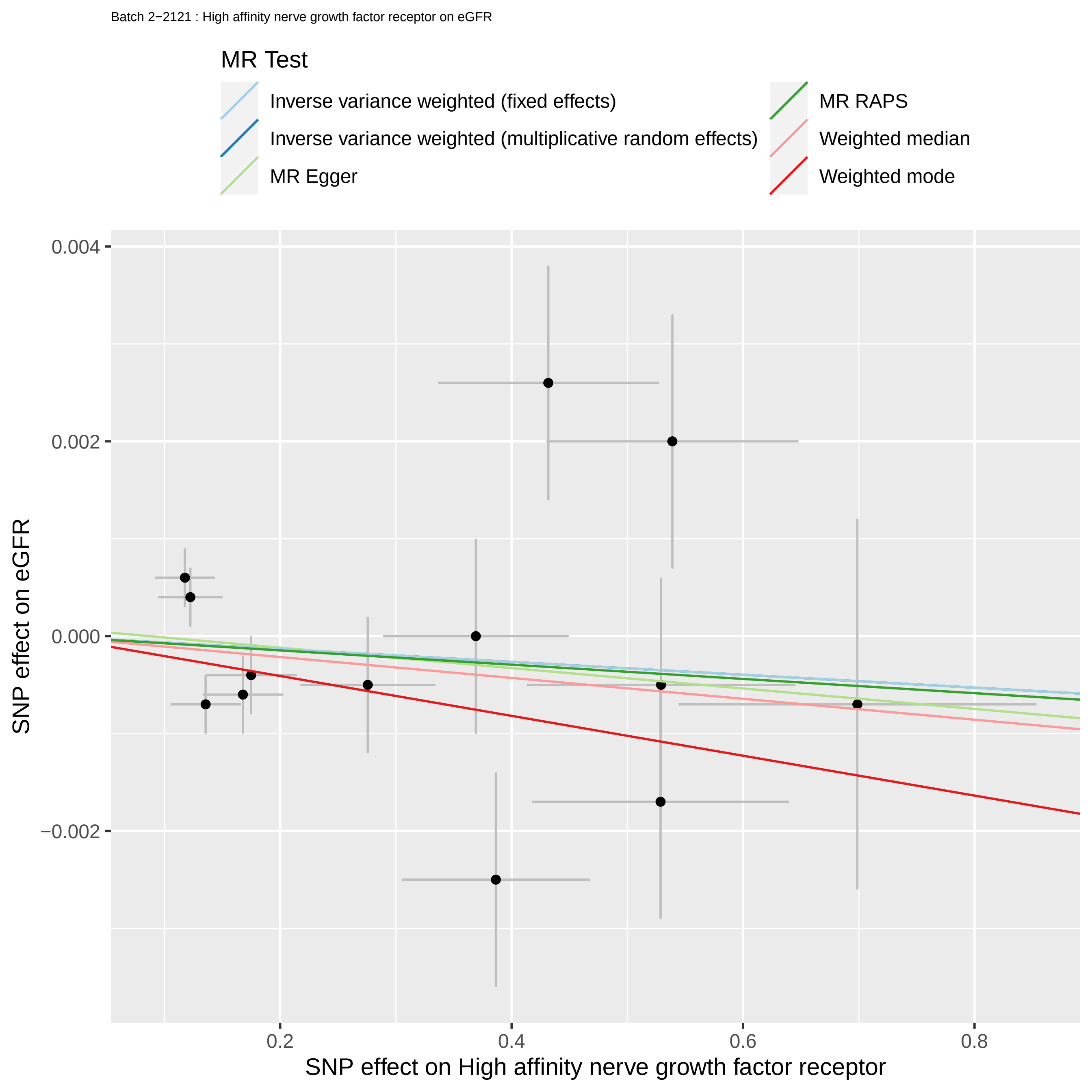
Batch 2−2121 : High affinity nerve growth factor receptor on eGFR
MR Test
Inverse variance weighted (fixed effects)
Inverse variance weighted (multiplicative random effects)
MR Egger
MR RAPS
Weighted median
Weighted mode
0.004
0.002
0.000
−0.002
0.2
0.4
0.6
0.8
SNP effect on eGFR
SNP effect on High affinity nerve growth factor receptor

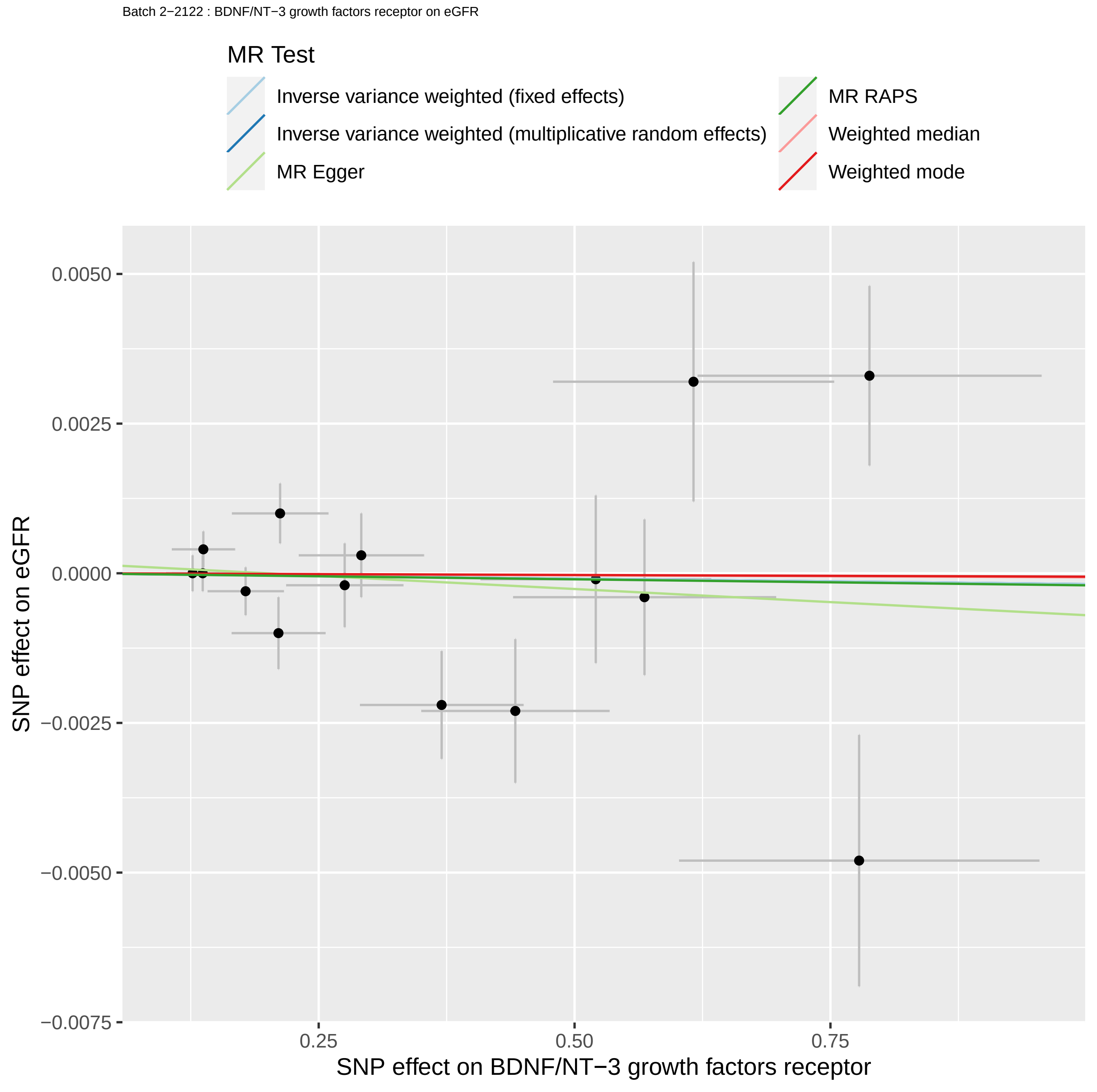
Batch 2−2122 : BDNF/NT−3 growth factors receptor on eGFR
MR Test
Inverse variance weighted (fixed effects)
Inverse variance weighted (multiplicative random effects)
MR Egger
MR RAPS
Weighted median
Weighted mode
0.0050
0.0025
0.0000
−0.0025
−0.0050
−0.0075
0.25
0.50
0.75
SNP effect on eGFR
SNP effect on BDNF/NT−3 growth factors receptor

Batch 2−2123 : NT−3 growth factor receptor on eGFR

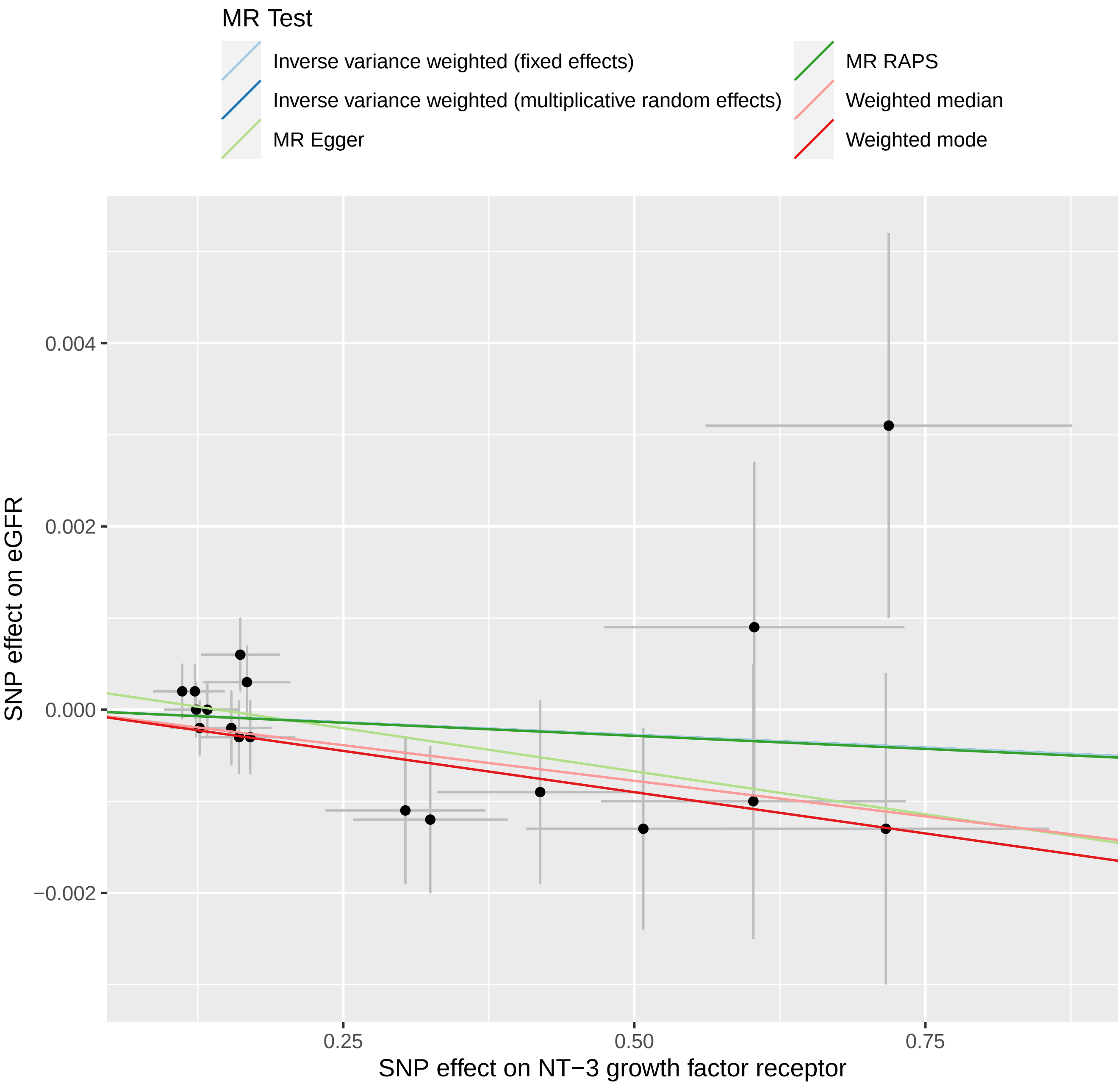

Batch 2−2124 : Neurotensin/neuromedin N on eGFR

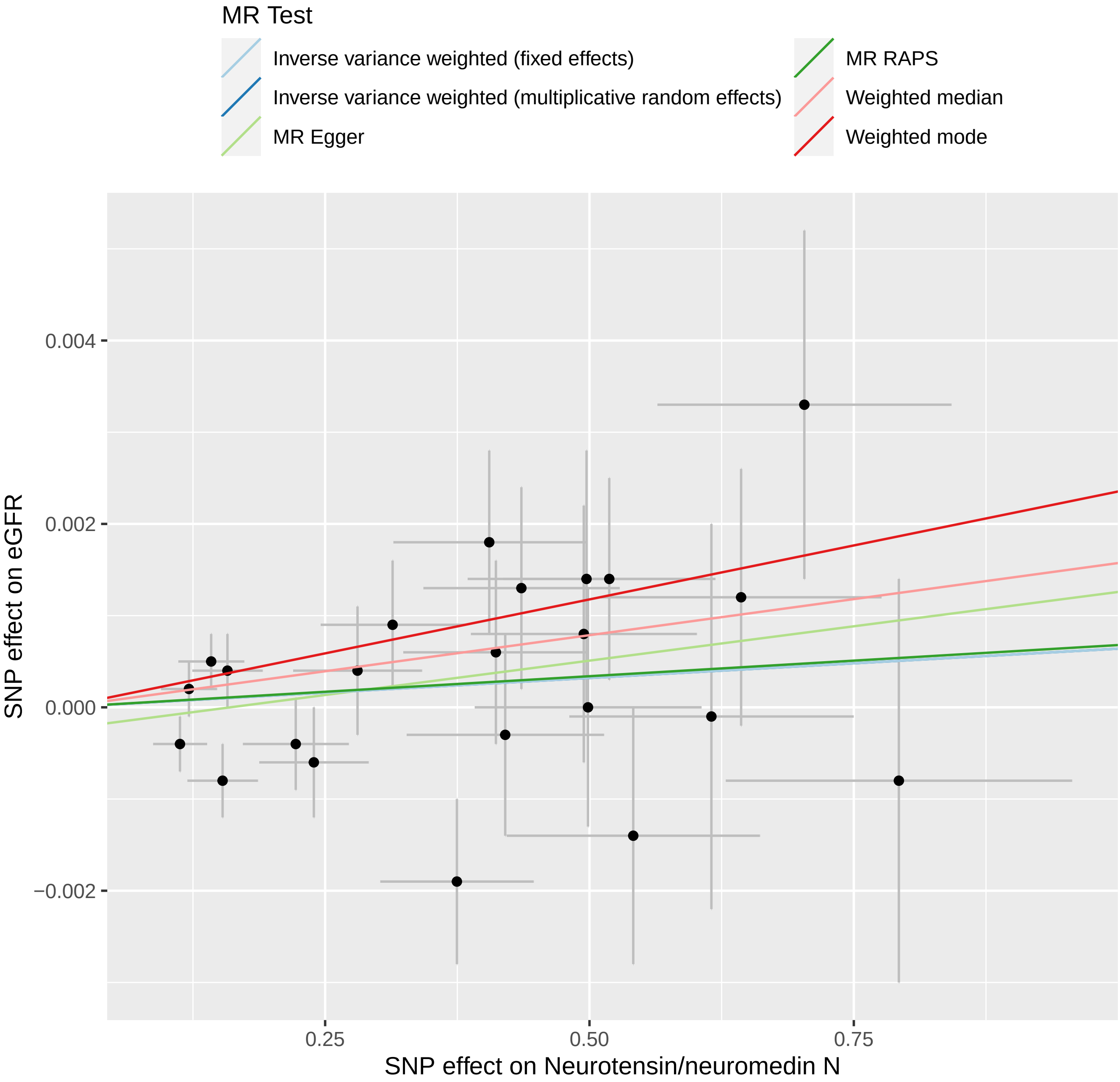

Batch 2−2125 : Nuclear migration protein nudC on eGFR

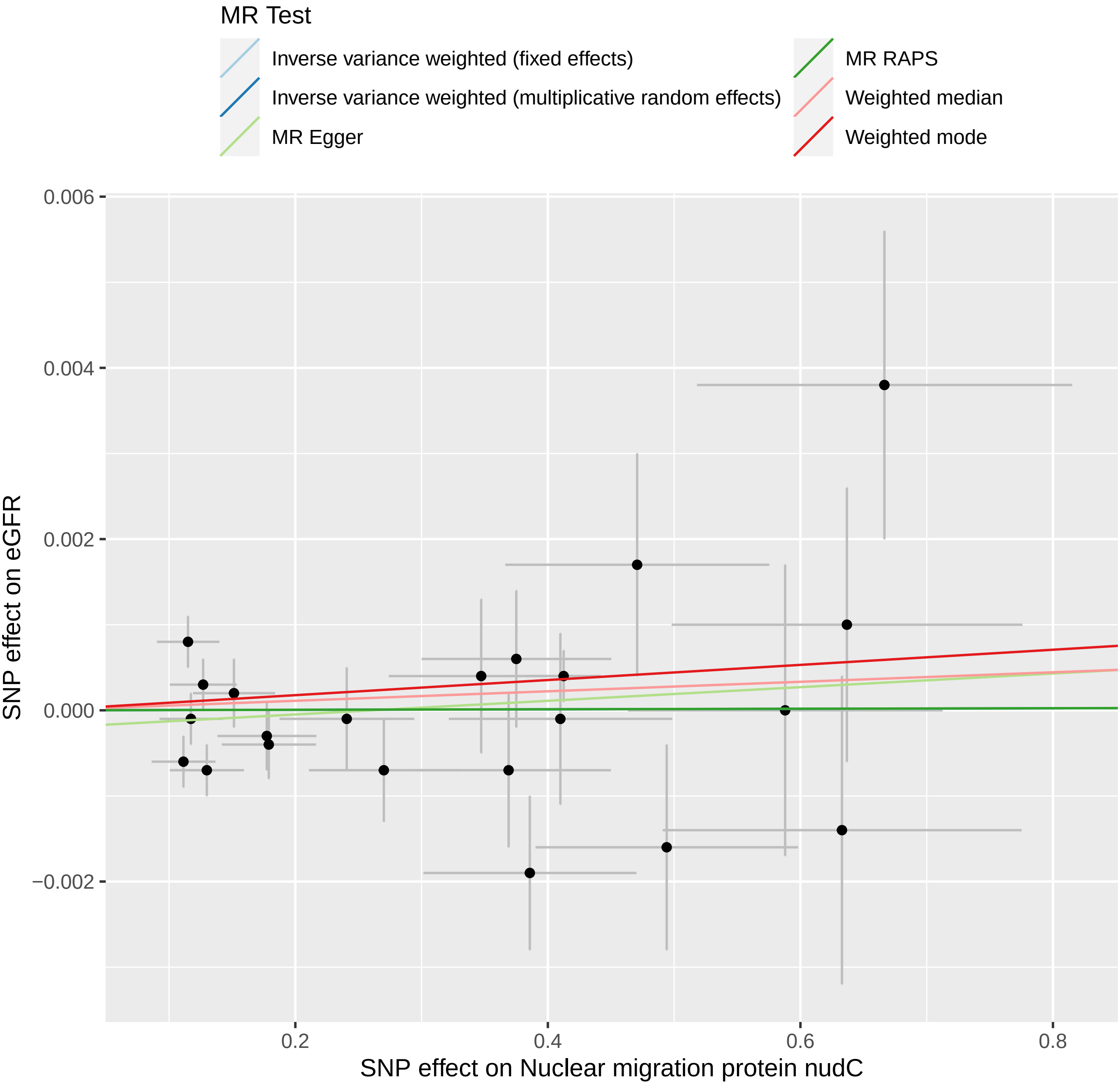

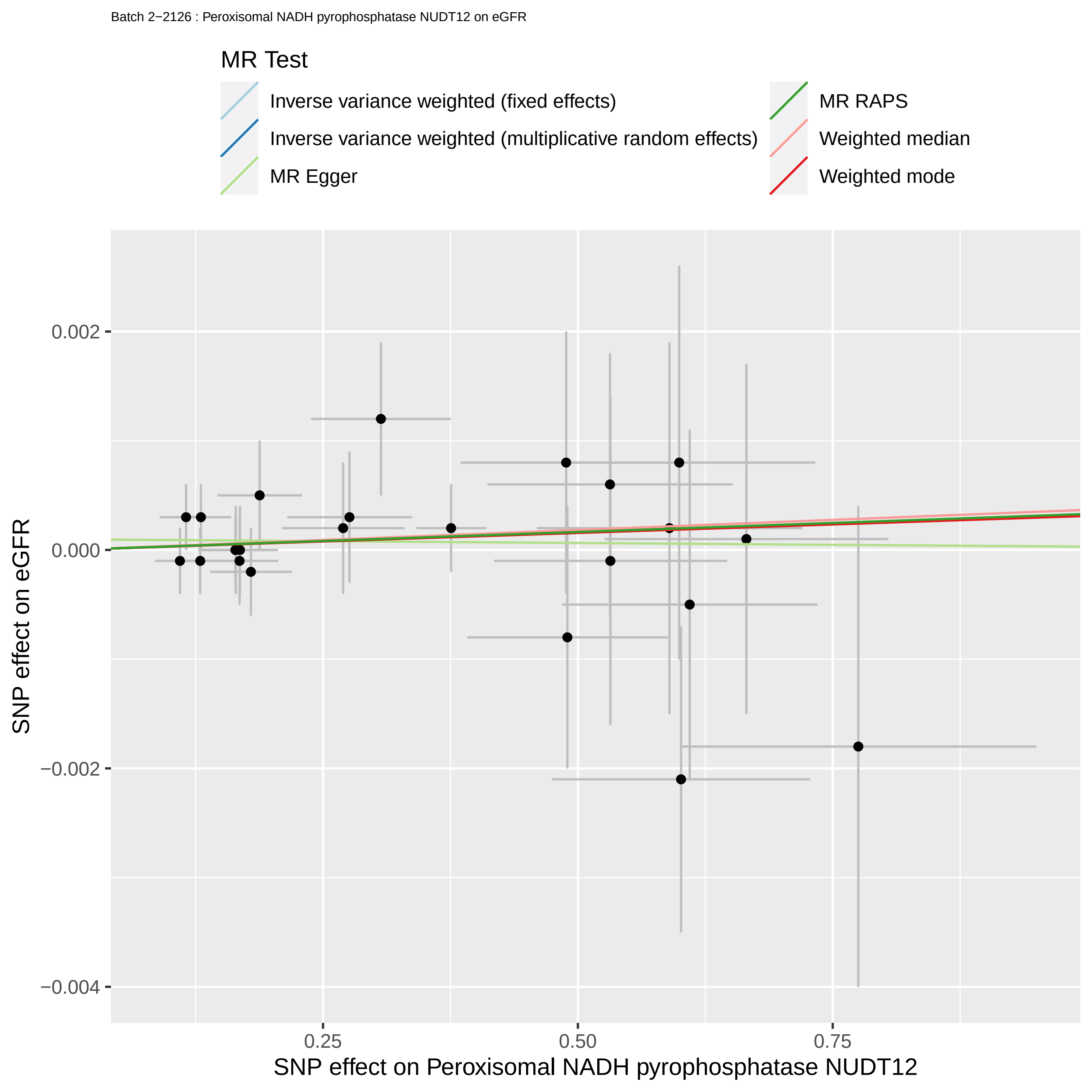

Batch 2−2126 : Peroxisomal NADH pyrophosphatase NUDT12 on eGFR
MR Test
Inverse variance weighted (fixed effects)
Inverse variance weighted (multiplicative random effects)
MR Egger
MR RAPS
Weighted median
Weighted mode
0.002
0.000
−0.002
−0.004
0.25
0.50
0.75
SNP effect on eGFR
SNP effect on Peroxisomal NADH pyrophosphatase NUDT12

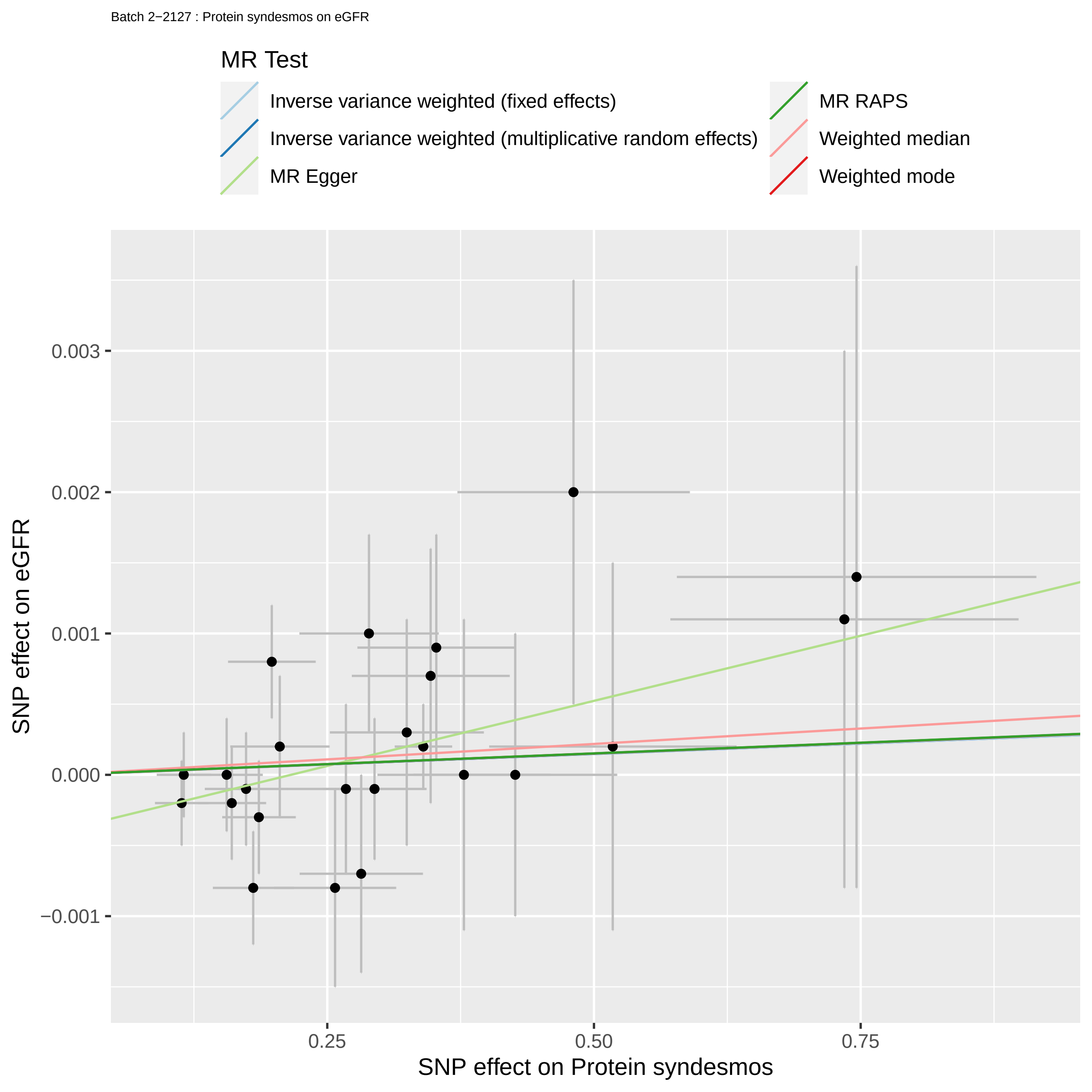

Batch 2−2127 : Protein syndesmos on eGFR
MR Test
Inverse variance weighted (fixed effects)
Inverse variance weighted (multiplicative random effects)
MR Egger
MR RAPS
Weighted median
Weighted mode
0.003
0.002
0.001
0.000
−0.001
SNP effect on eGFR
0.25
0.50
0.75
SNP effect on Protein syndesmos

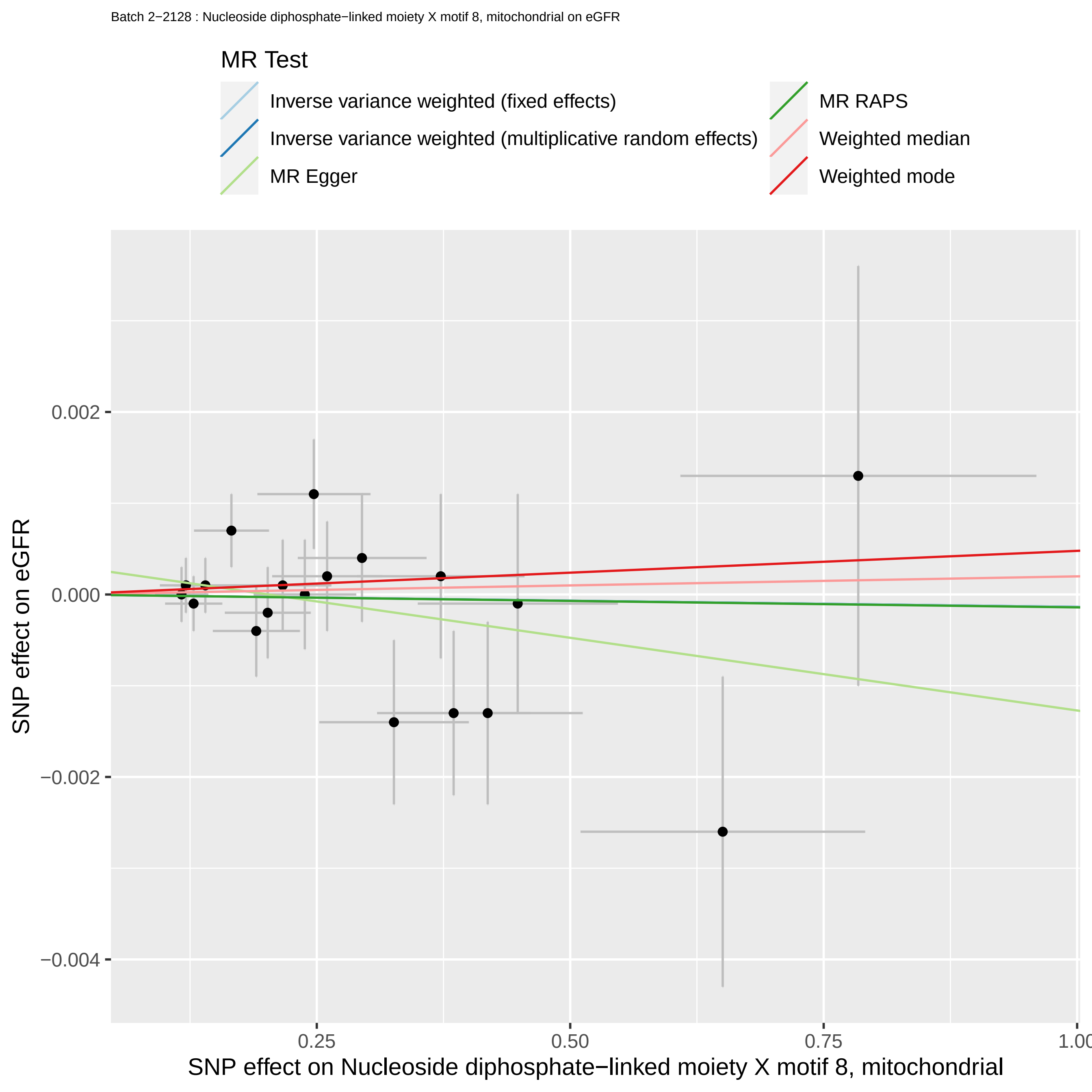

Batch 2−2128 : Nucleoside diphosphate−linked moiety X motif 8, mitochondrial on eGFR
MR Test
Inverse variance weighted (fixed effects)
Inverse variance weighted (multiplicative random effects)
MR Egger
MR RAPS
Weighted median
Weighted mode
0.002
0.000
−0.002
−0.004
0.25
0.50
0.75
1.00
SNP effect on eGFR
SNP effect on Nucleoside diphosphate−linked moiety X motif 8, mitochondrial

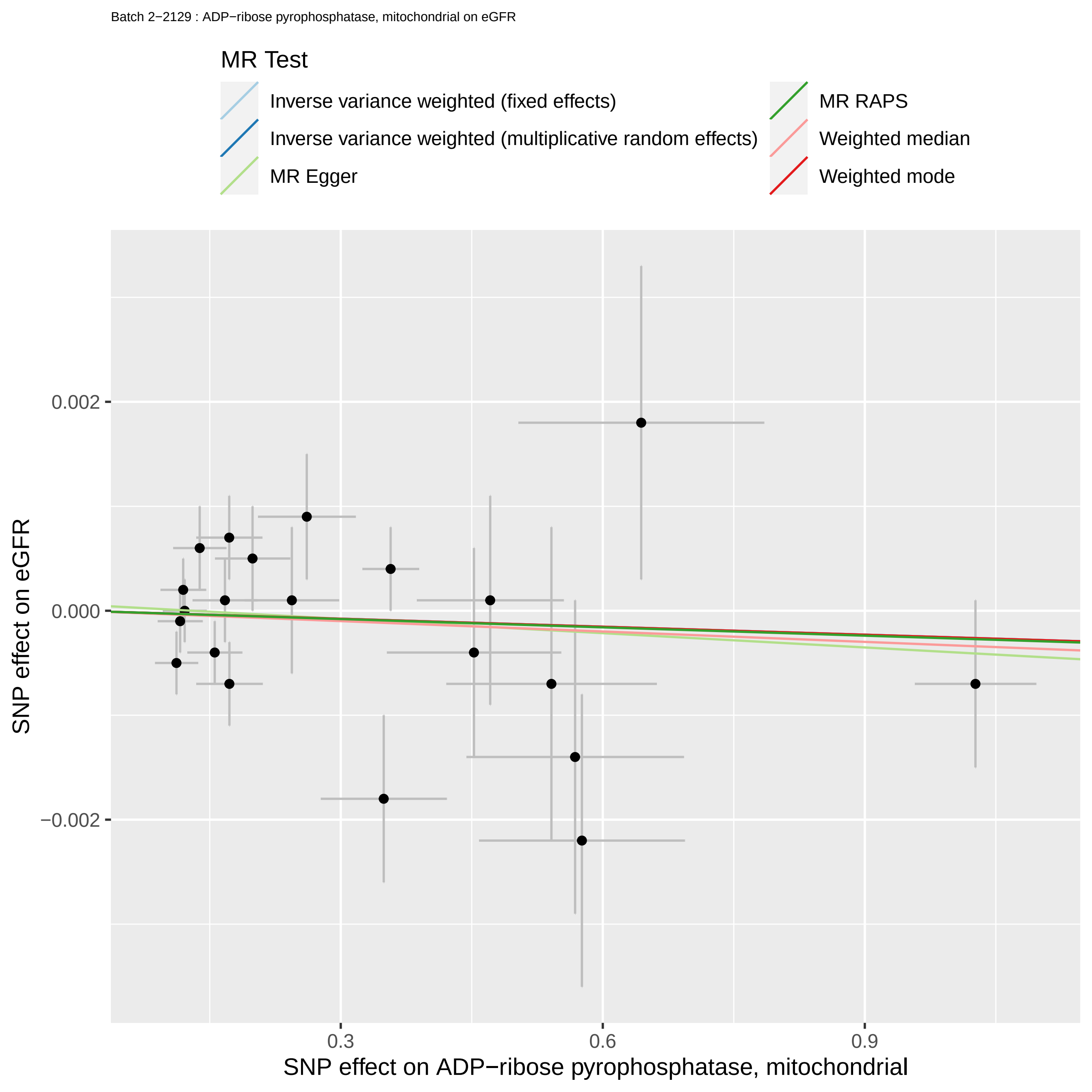

Batch 2−2129 : ADP−ribose pyrophosphatase, mitochondrial on eGFR
MR Test
Inverse variance weighted (fixed effects)
Inverse variance weighted (multiplicative random effects)
MR Egger
MR RAPS
Weighted median
Weighted mode
0.002
0.000
−0.002
0.3
0.6
0.9
SNP effect on eGFR
SNP effect on ADP−ribose pyrophosphatase, mitochondrial

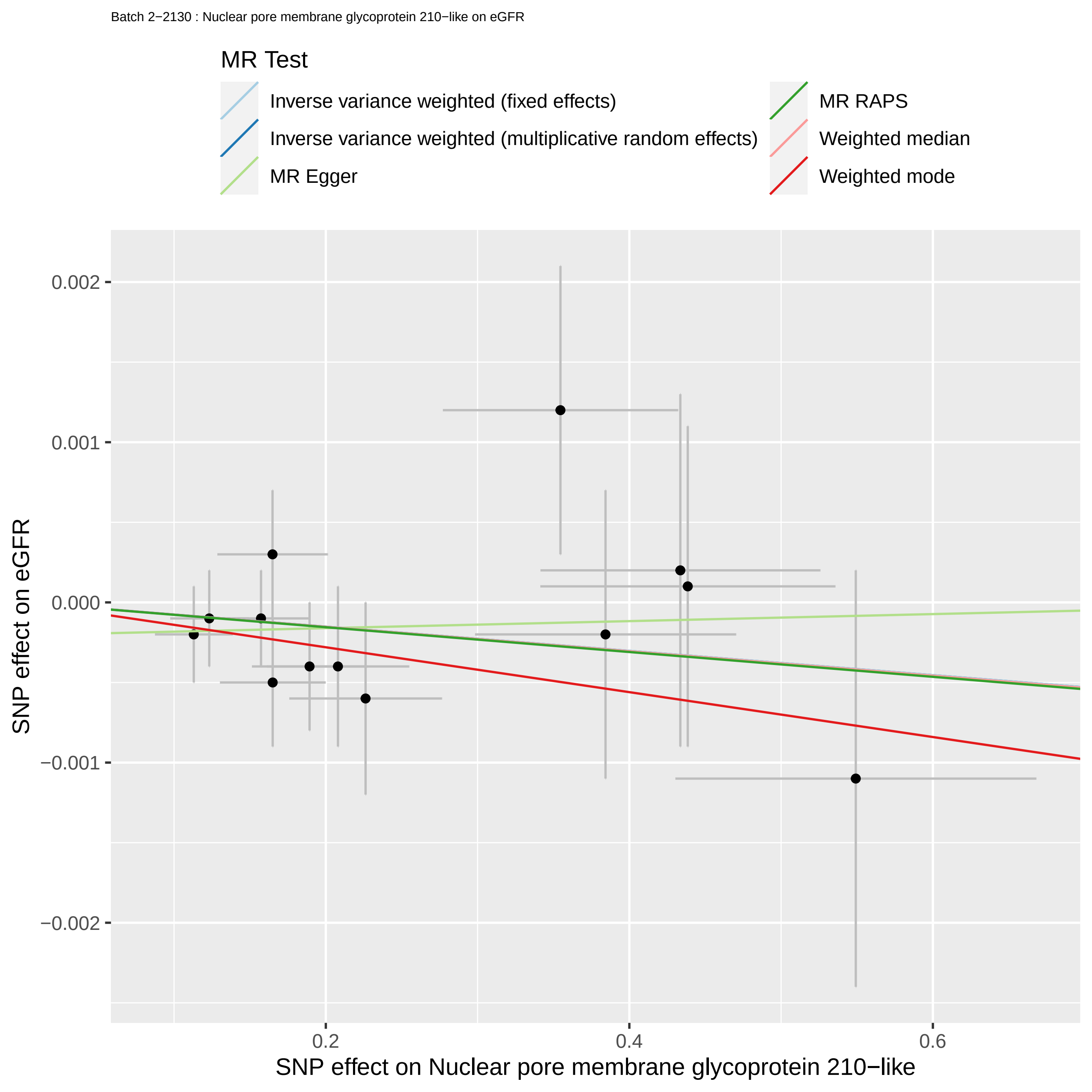

Batch 2−2130 : Nuclear pore membrane glycoprotein 210−like on eGFR
MR Test
Inverse variance weighted (fixed effects)
Inverse variance weighted (multiplicative random effects)
MR Egger
MR RAPS
Weighted median
Weighted mode
0.002
0.001
0.000
−0.001
−0.002
0.2
0.4
0.6
SNP effect on eGFR
SNP effect on Nuclear pore membrane glycoprotein 210−like

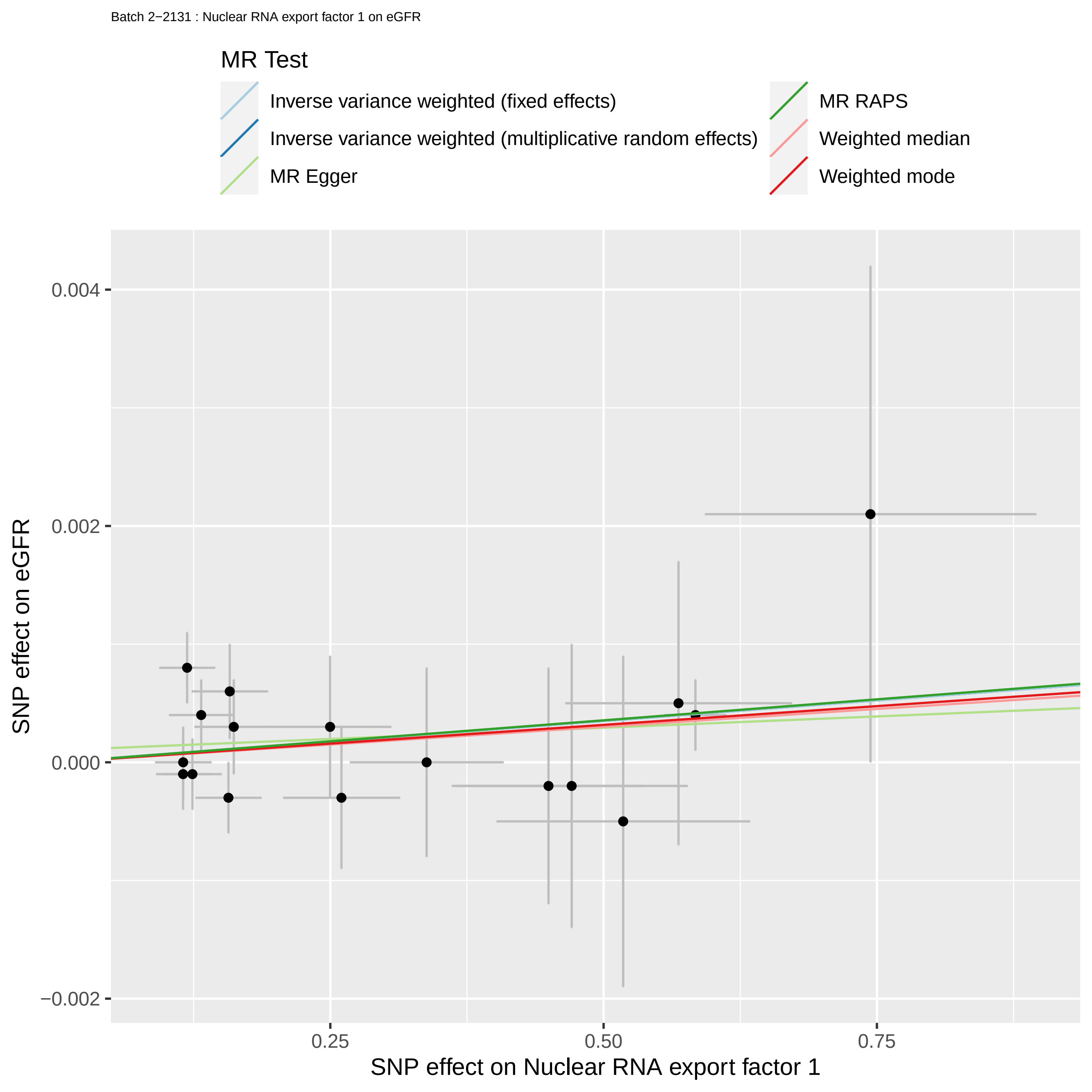
Batch 2−2131 : Nuclear RNA export factor 1 on eGFR
MR Test
Inverse variance weighted (fixed effects)
Inverse variance weighted (multiplicative random effects)
MR Egger
MR RAPS
Weighted median
Weighted mode
0.004
0.002
0.000
−0.002
SNP effect on eGFR
0.25
0.50
0.75
SNP effect on Nuclear RNA export factor 1

Batch 2−2132 : Neurexophilin−1 on eGFR

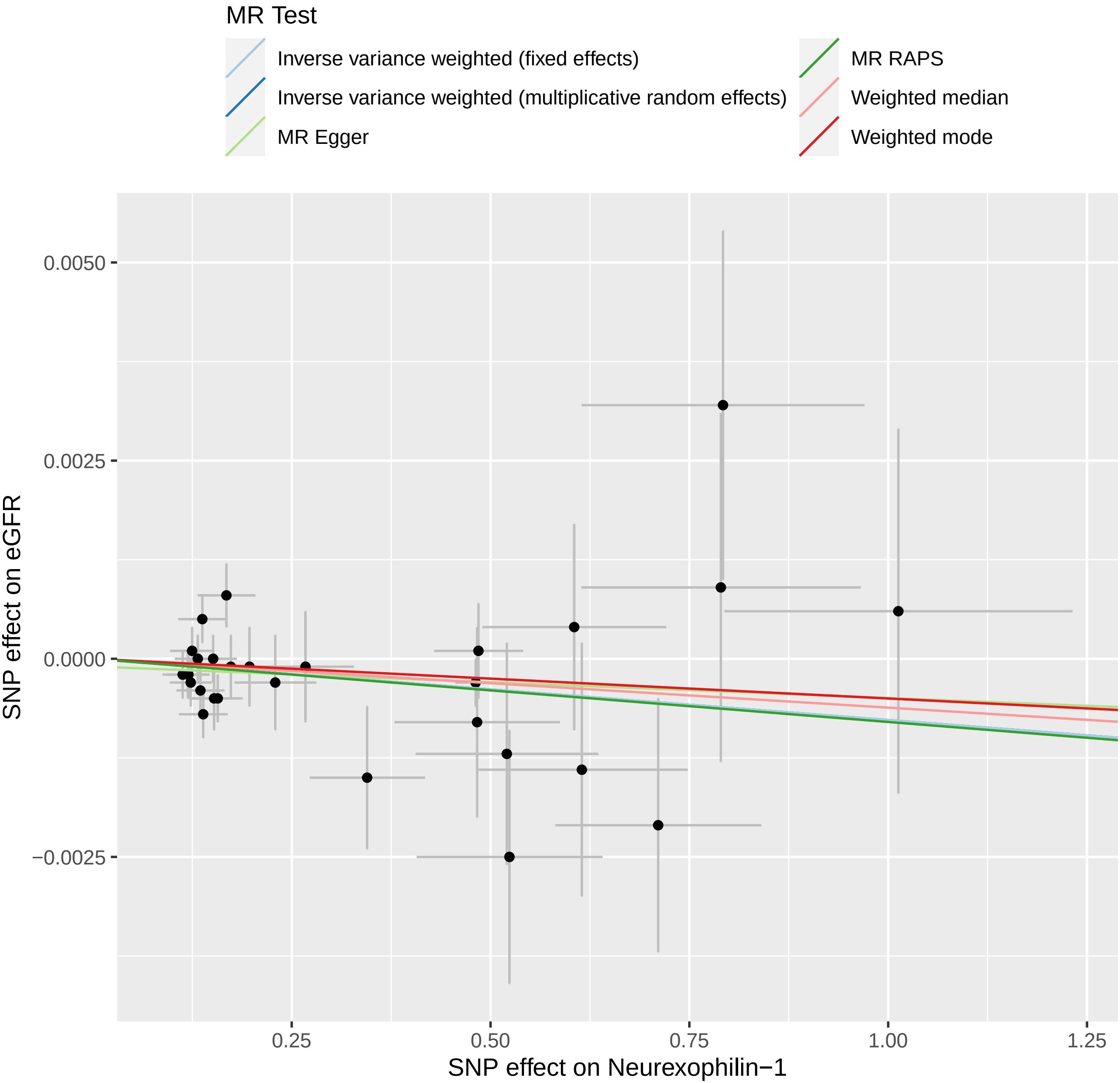

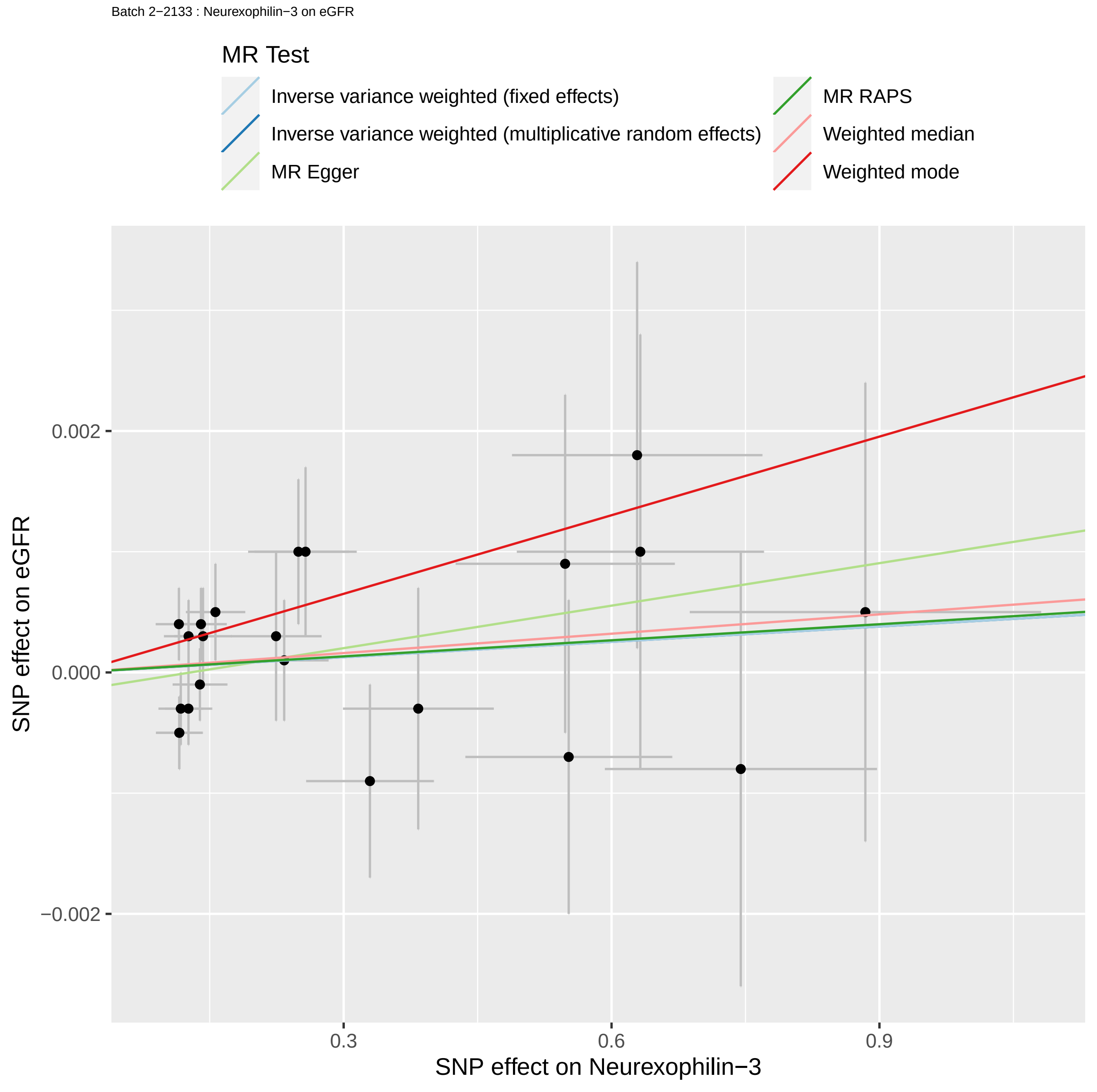

Batch 2−2133 : Neurexophilin−3 on eGFR
MR Test
Inverse variance weighted (fixed effects)
Inverse variance weighted (multiplicative random effects)
MR Egger
MR RAPS
Weighted median
Weighted mode
0.002
0.000
−0.002
0.3
0.6
0.9
SNP effect on eGFR
SNP effect on Neurexophilin−3

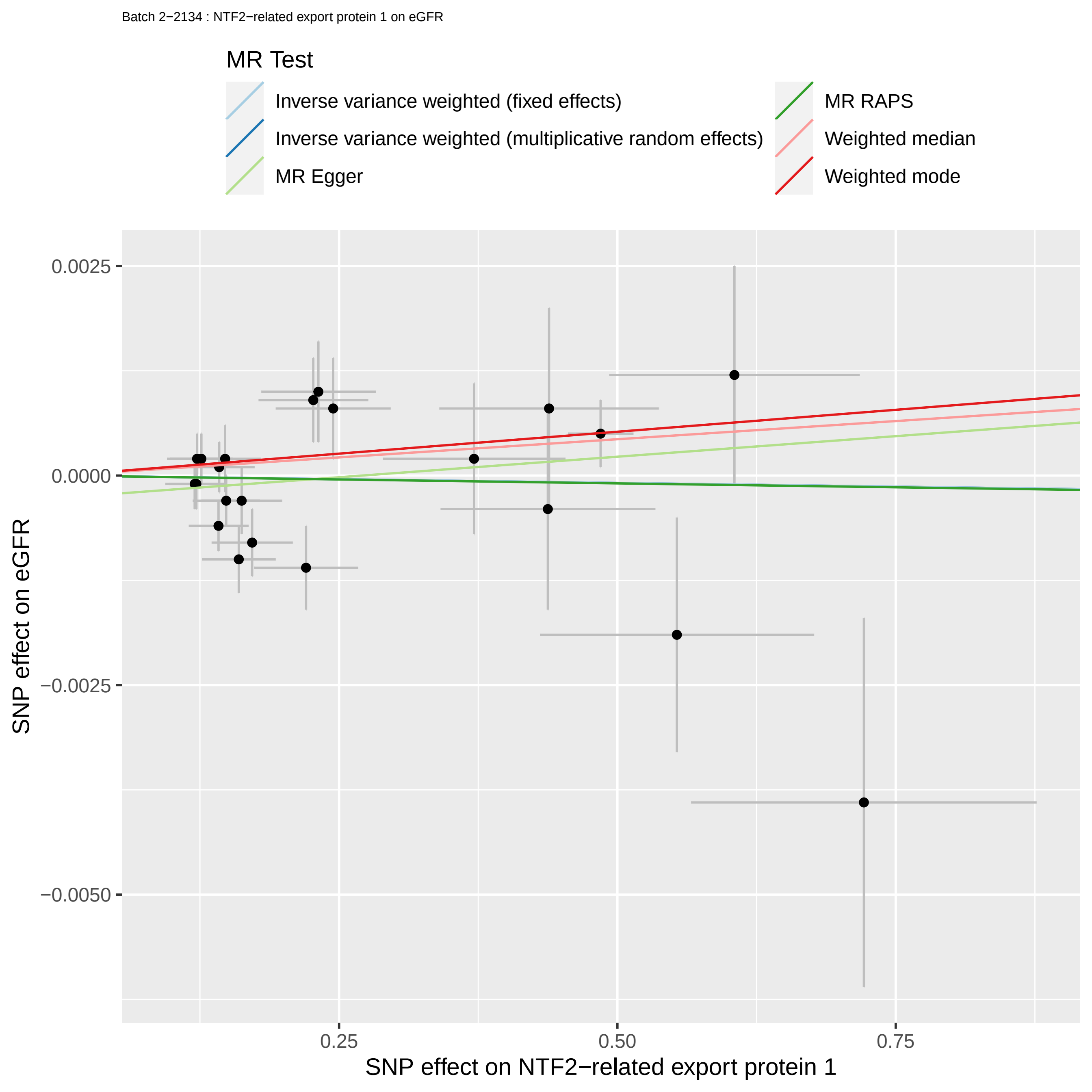

Batch 2−2134 : NTF2−related export protein 1 on eGFR
MR Test
Inverse variance weighted (fixed effects)
Inverse variance weighted (multiplicative random effects)
MR Egger
MR RAPS
Weighted median
Weighted mode
0.0025
0.0000
−0.0025
−0.0050
0.25
0.50
0.75
SNP effect on eGFR
SNP effect on NTF2−related export protein 1

Batch 2−2135 : Out at first protein homolog on eGFR

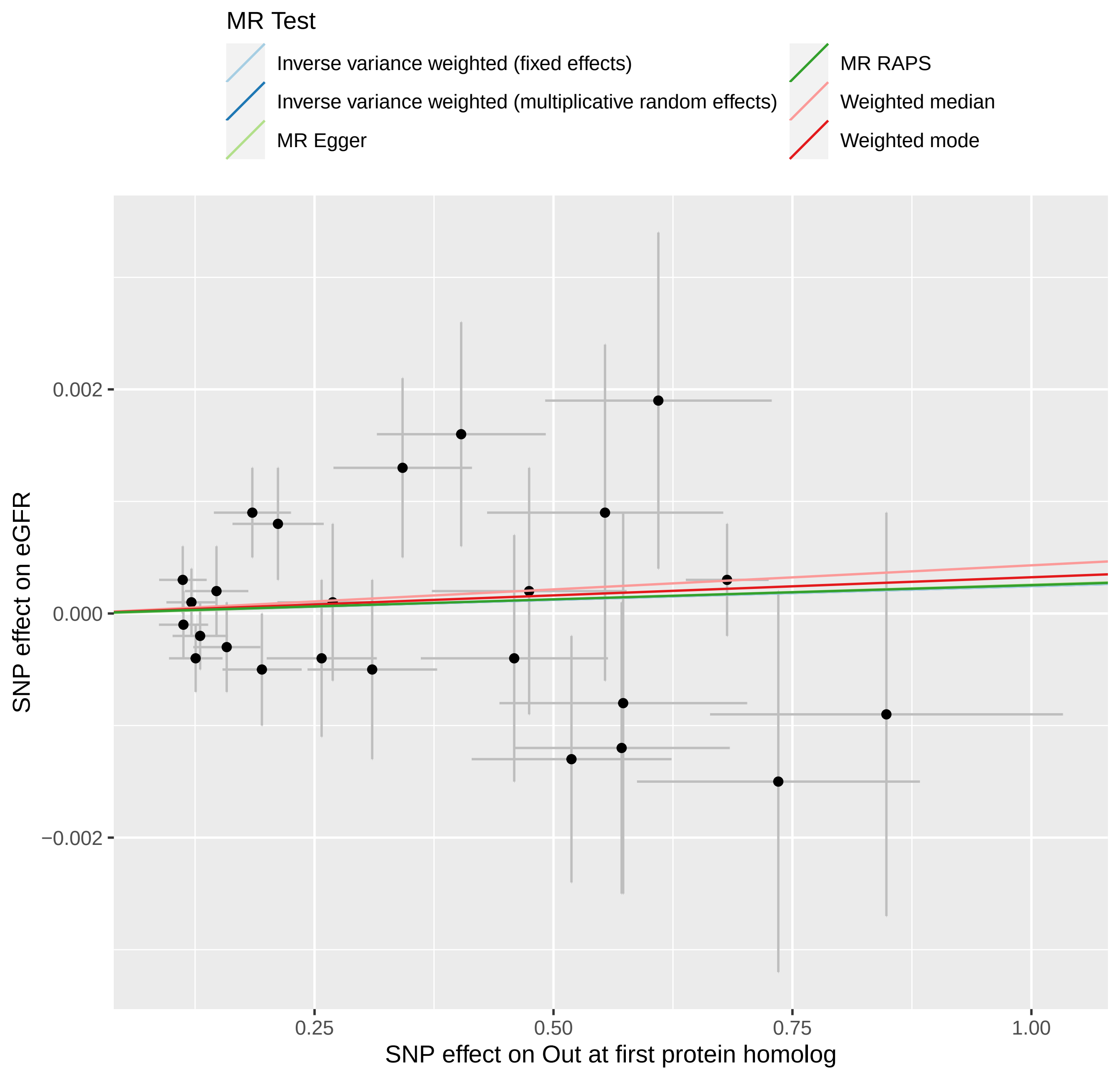

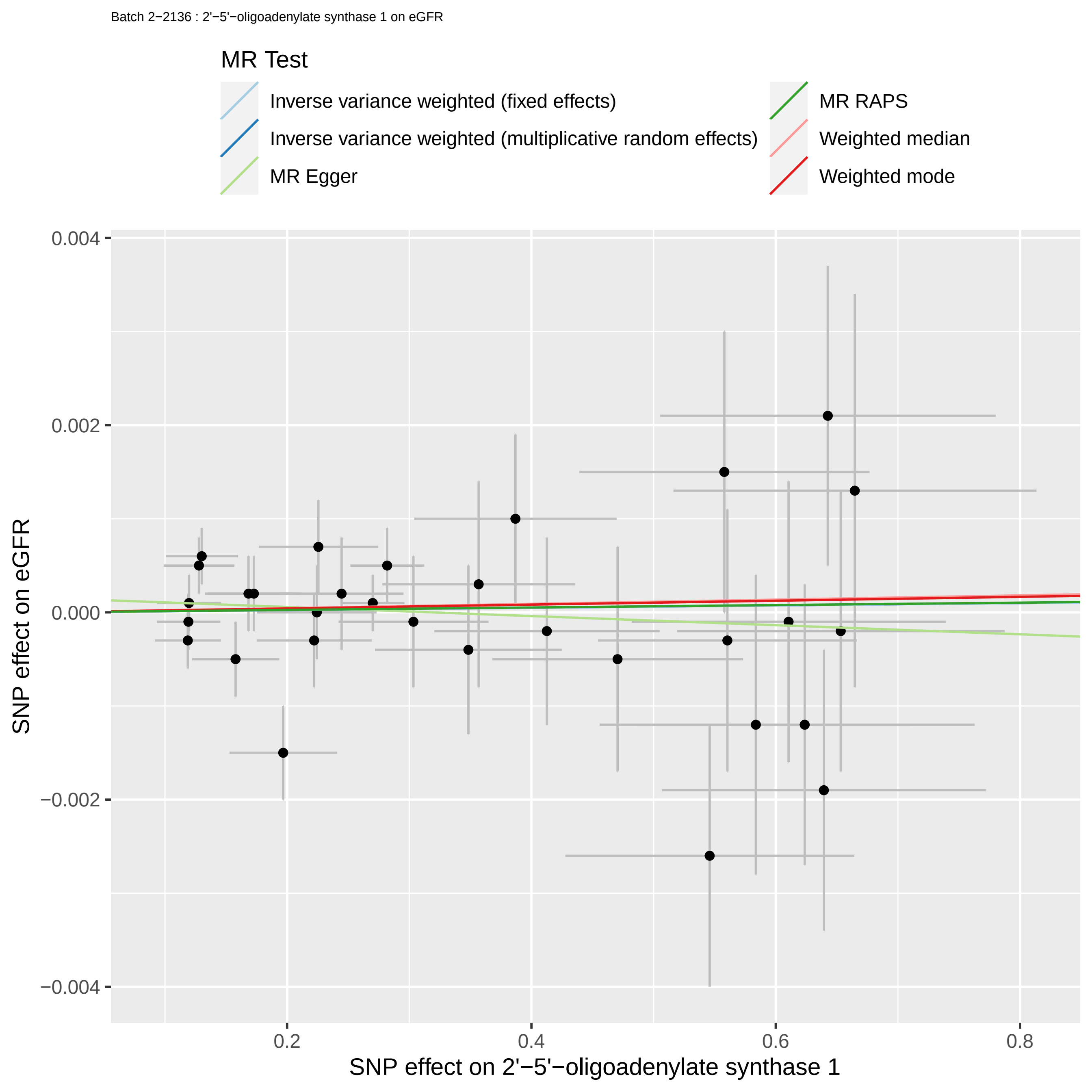

Batch 2−2136 : 2'−5'−oligoadenylate synthase 1 on eGFR
MR Test
Inverse variance weighted (fixed effects)
Inverse variance weighted (multiplicative random effects)
MR Egger
MR RAPS
Weighted median
Weighted mode
0.004
0.002
0.000
−0.002
−0.004
SNP effect on eGFR
0.2
0.4
0.6
0.8
SNP effect on 2'−5'−oligoadenylate synthase 1

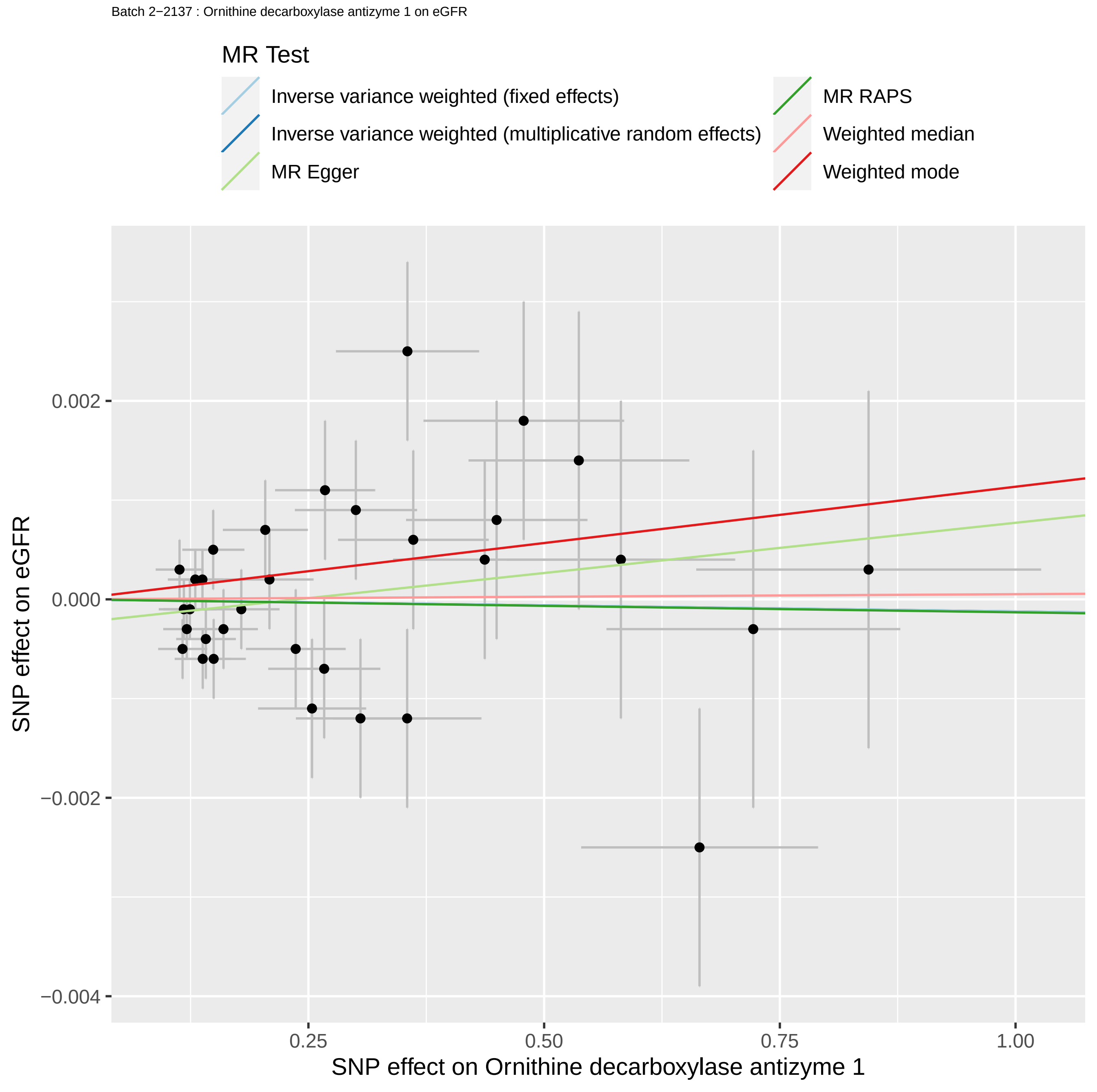
Batch 2−2137 : Ornithine decarboxylase antizyme 1 on eGFR
MR Test
Inverse variance weighted (fixed effects)
Inverse variance weighted (multiplicative random effects)
MR Egger
MR RAPS
Weighted median
Weighted mode
0.002
0.000
−0.002
−0.004
0.25
0.50
0.75
1.00
SNP effect on eGFR
SNP effect on Ornithine decarboxylase antizyme 1

Batch 2−2138 : Odorant−binding protein 2a on eGFR

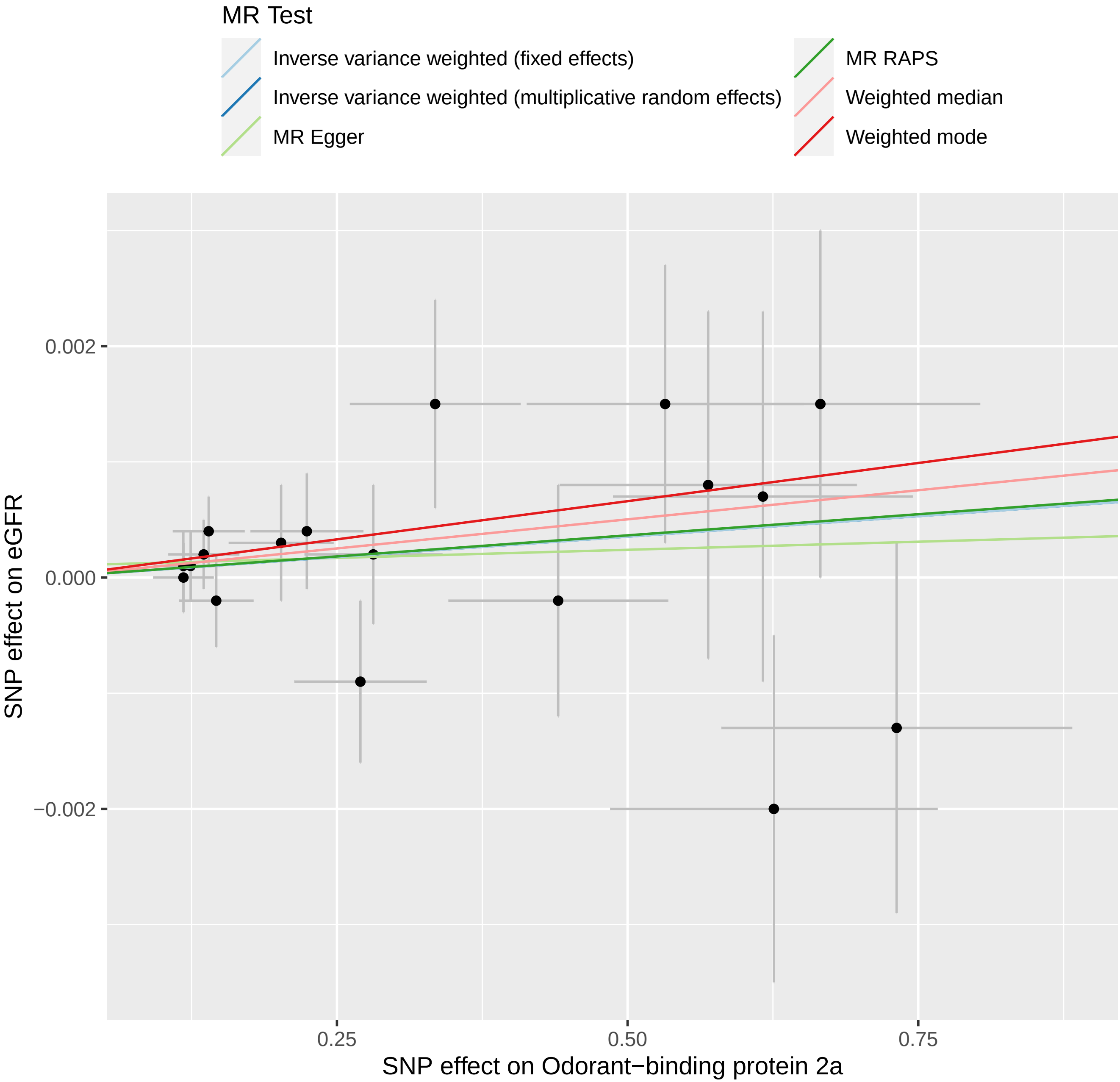

Batch 2−2139 : Odorant−binding protein 2b on eGFR

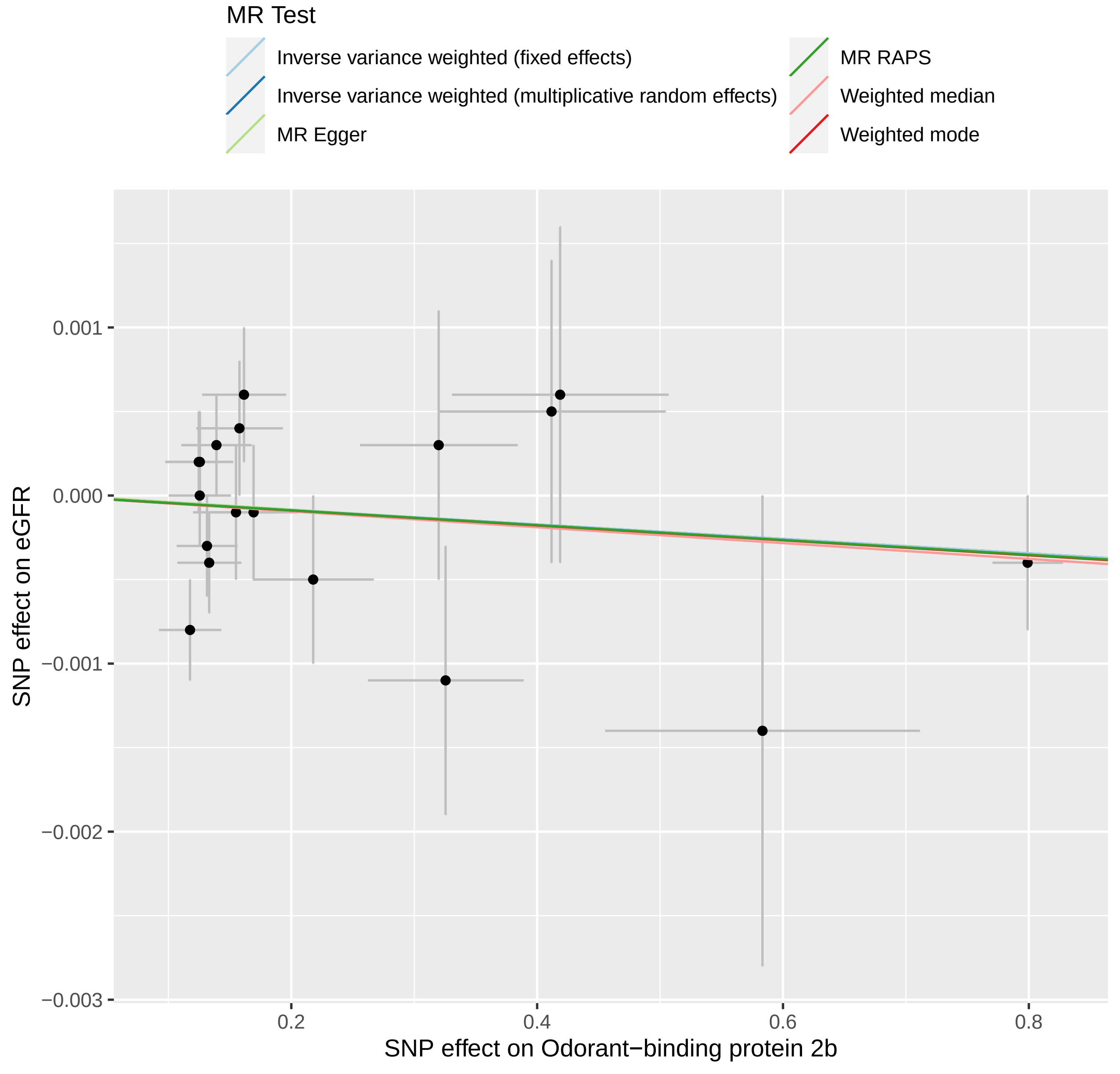

Batch 2−2140 : OCIA domain−containing protein 1 on eGFR

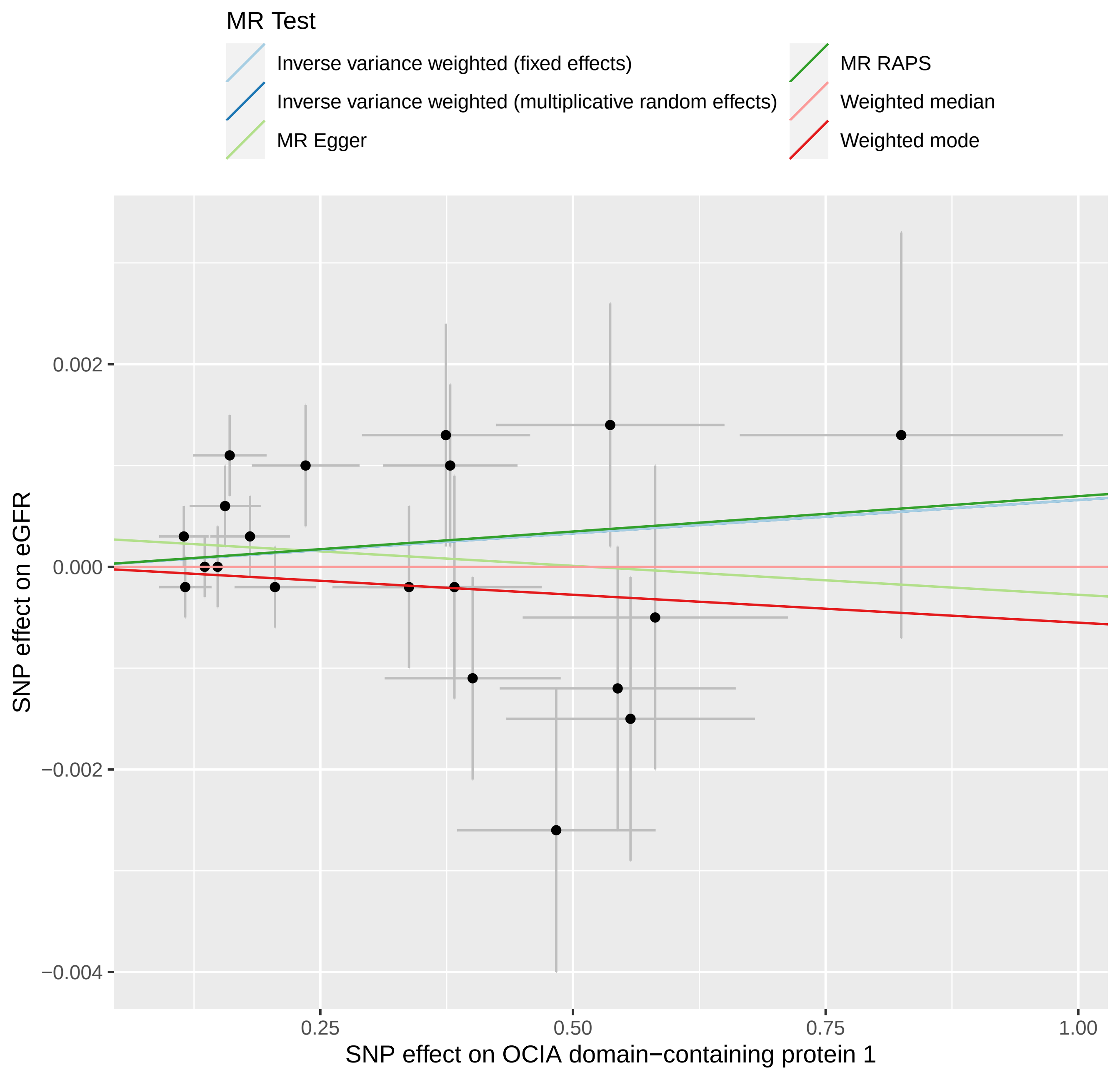

Batch 2−2141 : Inositol polyphosphate 5−phosphatase OCRL−1 on eGFR

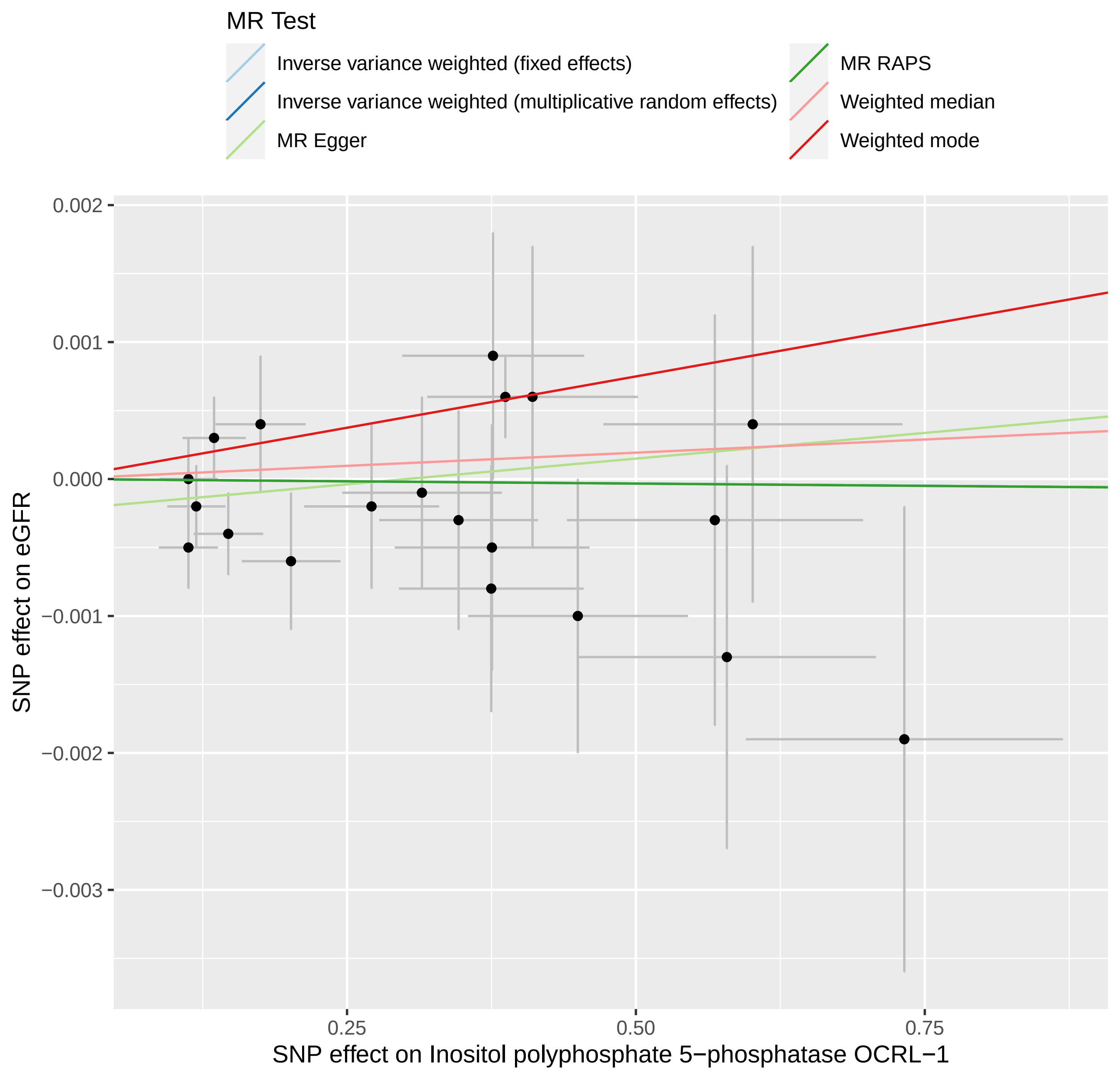

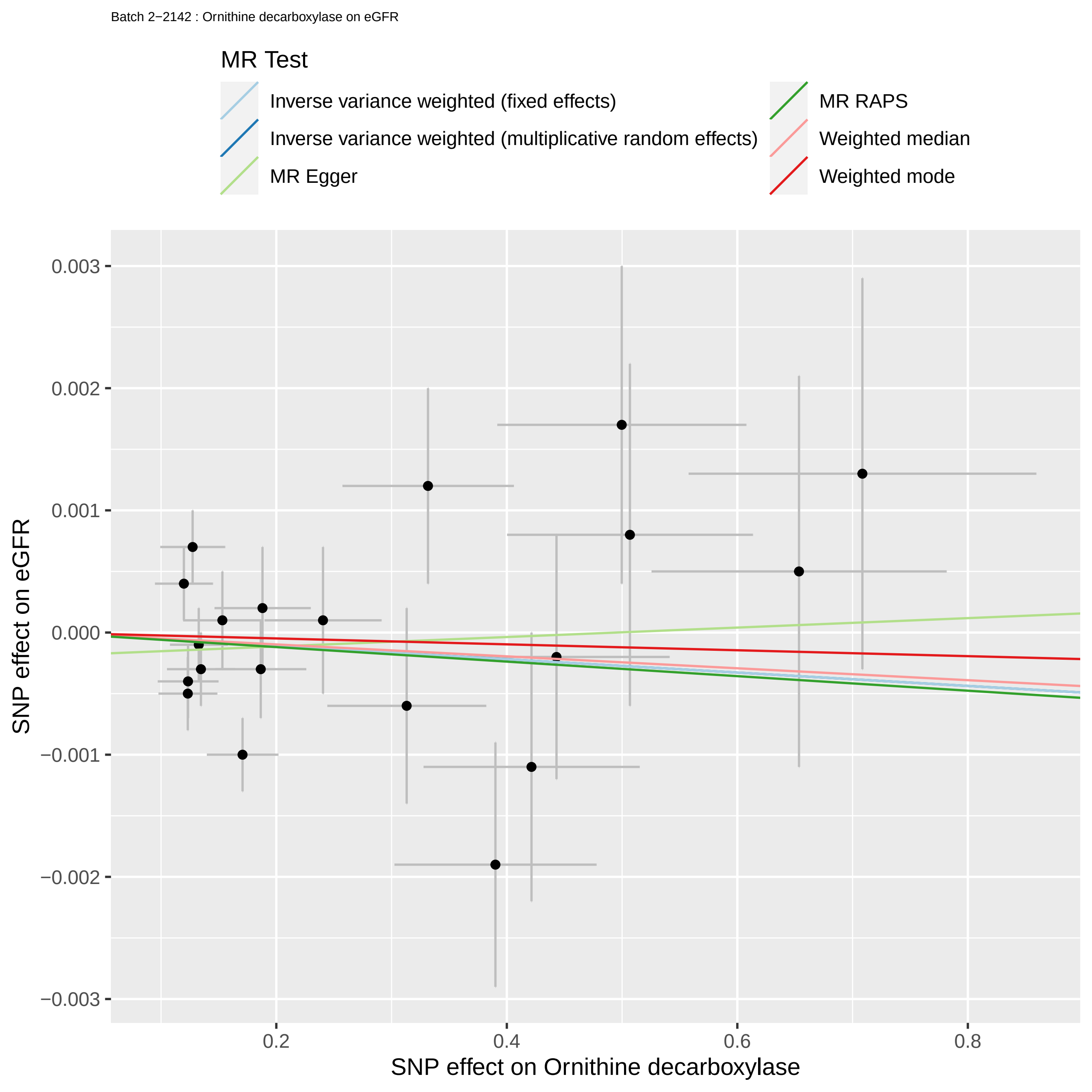
Batch 2−2142 : Ornithine decarboxylase on eGFR
MR Test
Inverse variance weighted (fixed effects)
Inverse variance weighted (multiplicative random effects)
MR Egger
MR RAPS
Weighted median
Weighted mode
0.003
0.002
0.001
0.000
−0.001
−0.002
−0.003
SNP effect on eGFR
0.2
0.4
0.6
0.8
SNP effect on Ornithine decarboxylase

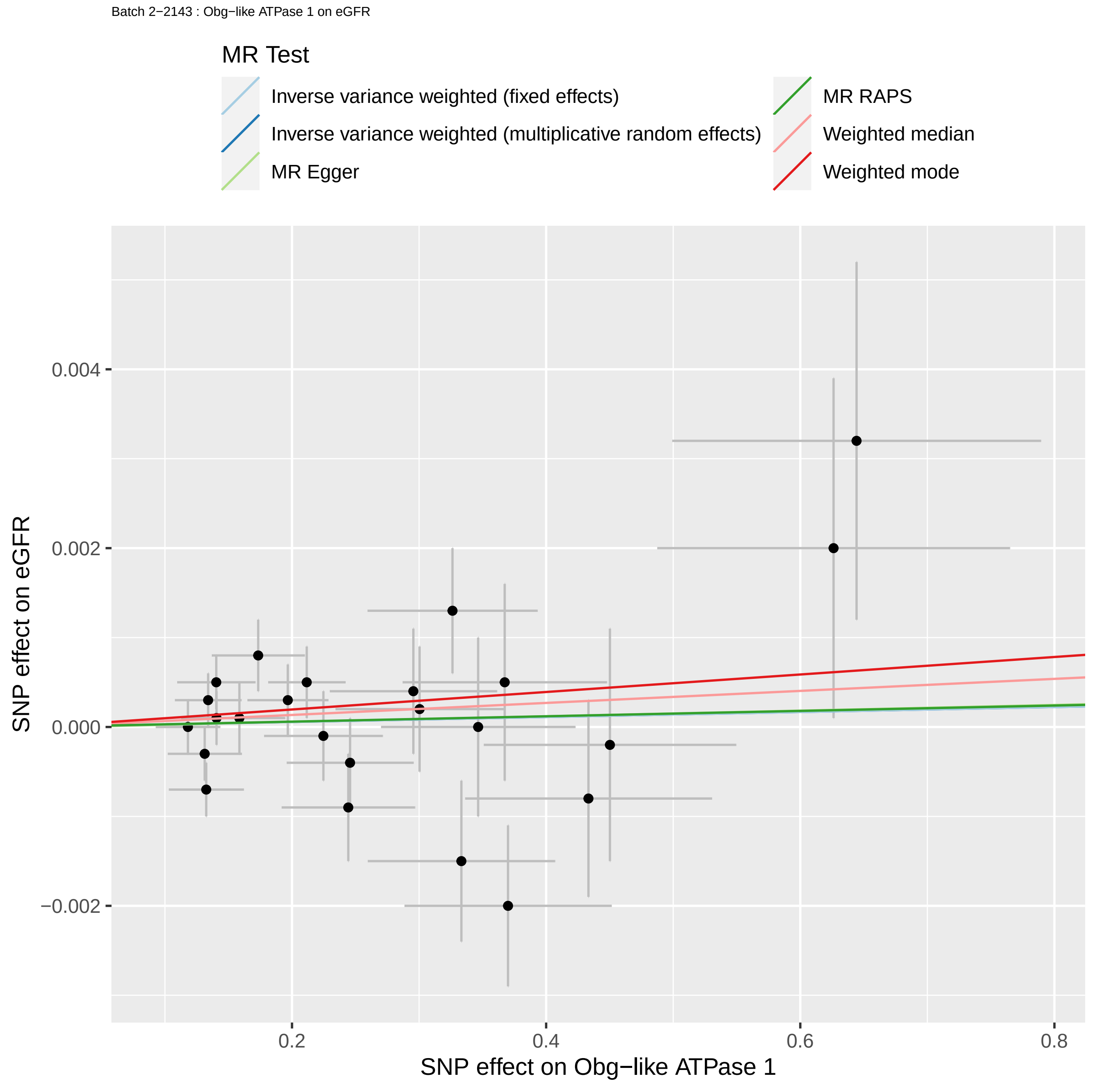

Batch 2−2143 : Obg−like ATPase 1 on eGFR
MR Test
Inverse variance weighted (fixed effects)
Inverse variance weighted (multiplicative random effects)
MR Egger
MR RAPS
Weighted median
Weighted mode
0.004
0.002
0.000
−0.002
0.2
0.4
0.6
0.8
SNP effect on eGFR
SNP effect on Obg−like ATPase 1

Batch 2−2144 : Olfactomedin−like protein 3 on eGFR

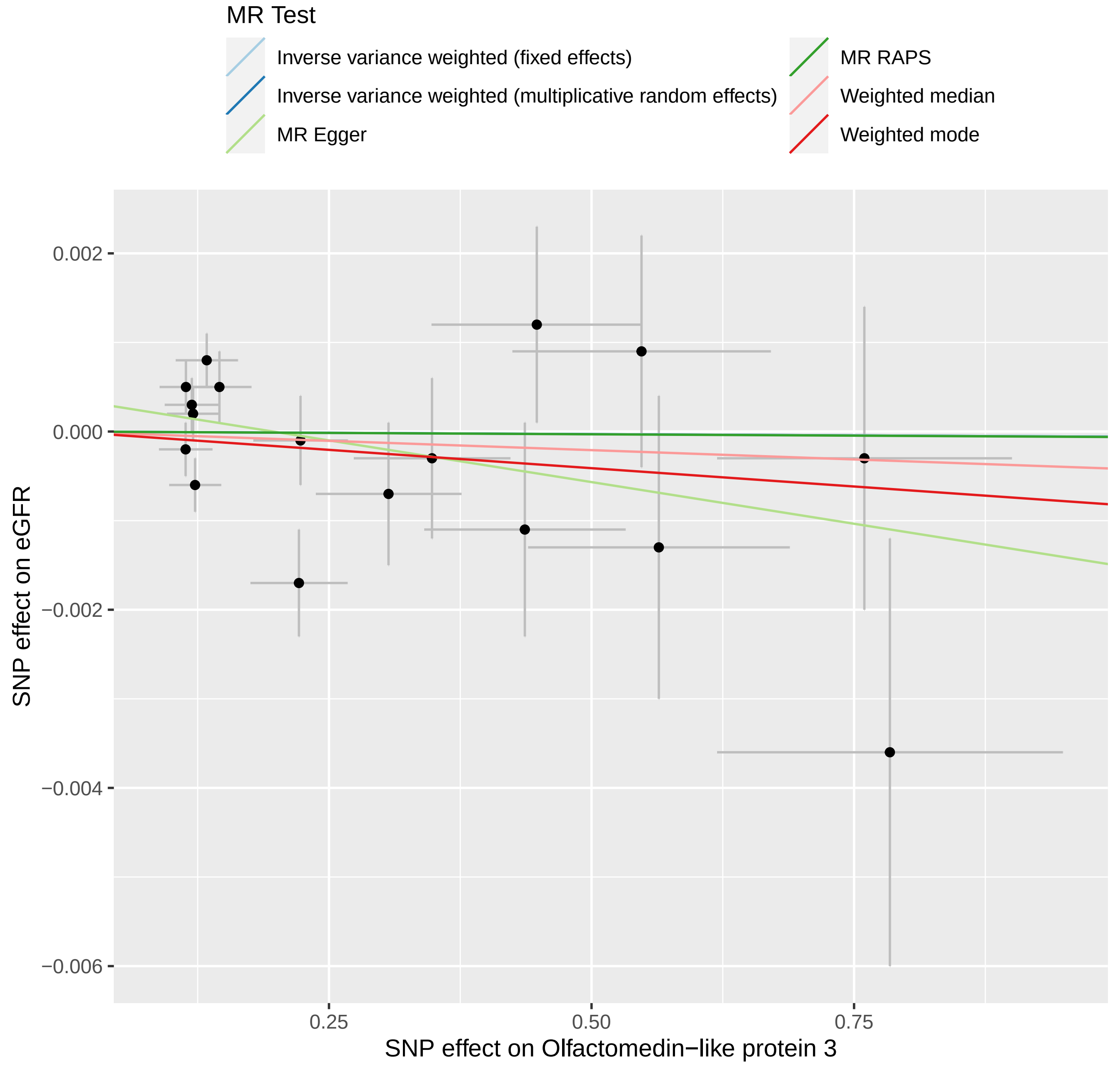

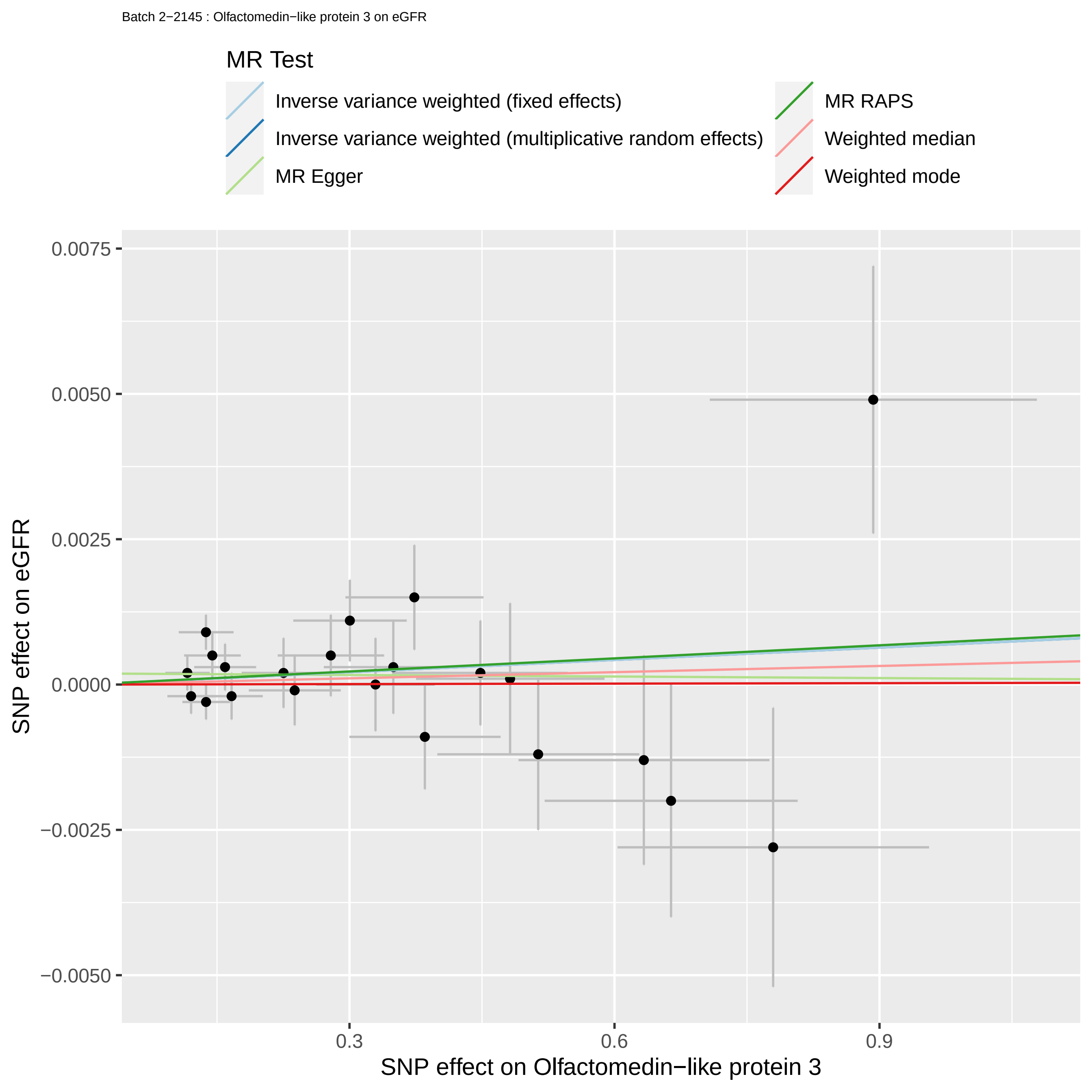
Batch 2−2145 : Olfactomedin−like protein 3 on eGFR
MR Test
Inverse variance weighted (fixed effects)
Inverse variance weighted (multiplicative random effects)
MR Egger
MR RAPS
Weighted median
Weighted mode
0.0075
0.0050
0.0025
0.0000
−0.0025
−0.0050
SNP effect on eGFR
0.3
0.6
0.9
SNP effect on Olfactomedin−like protein 3

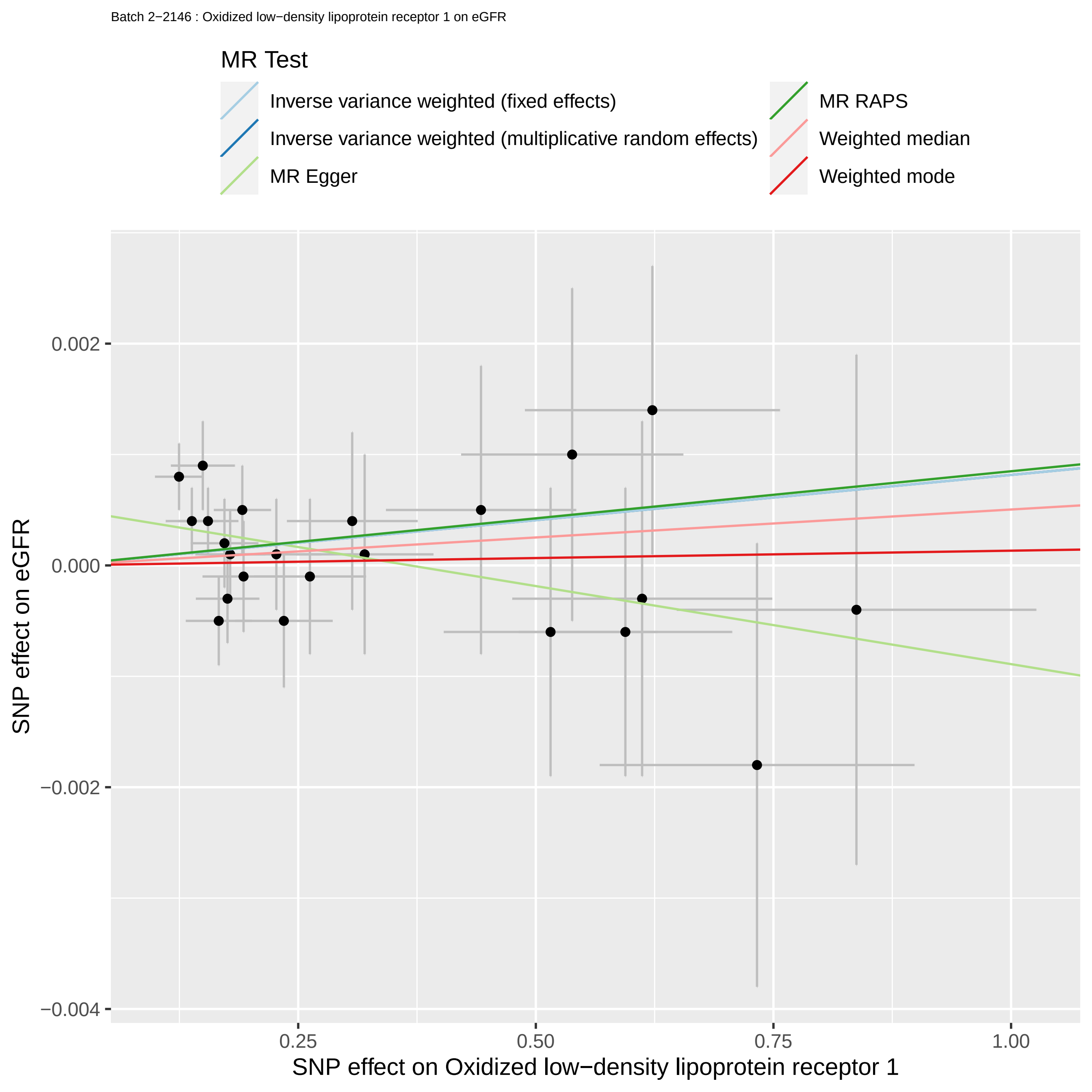
Batch 2−2146 : Oxidized low−density lipoprotein receptor 1 on eGFR
MR Test
Inverse variance weighted (fixed effects)
Inverse variance weighted (multiplicative random effects)
MR Egger
MR RAPS
Weighted median
Weighted mode
0.002
0.000
−0.002
−0.004
0.25
0.50
0.75
1.00
SNP effect on eGFR
SNP effect on Oxidized low−density lipoprotein receptor 1

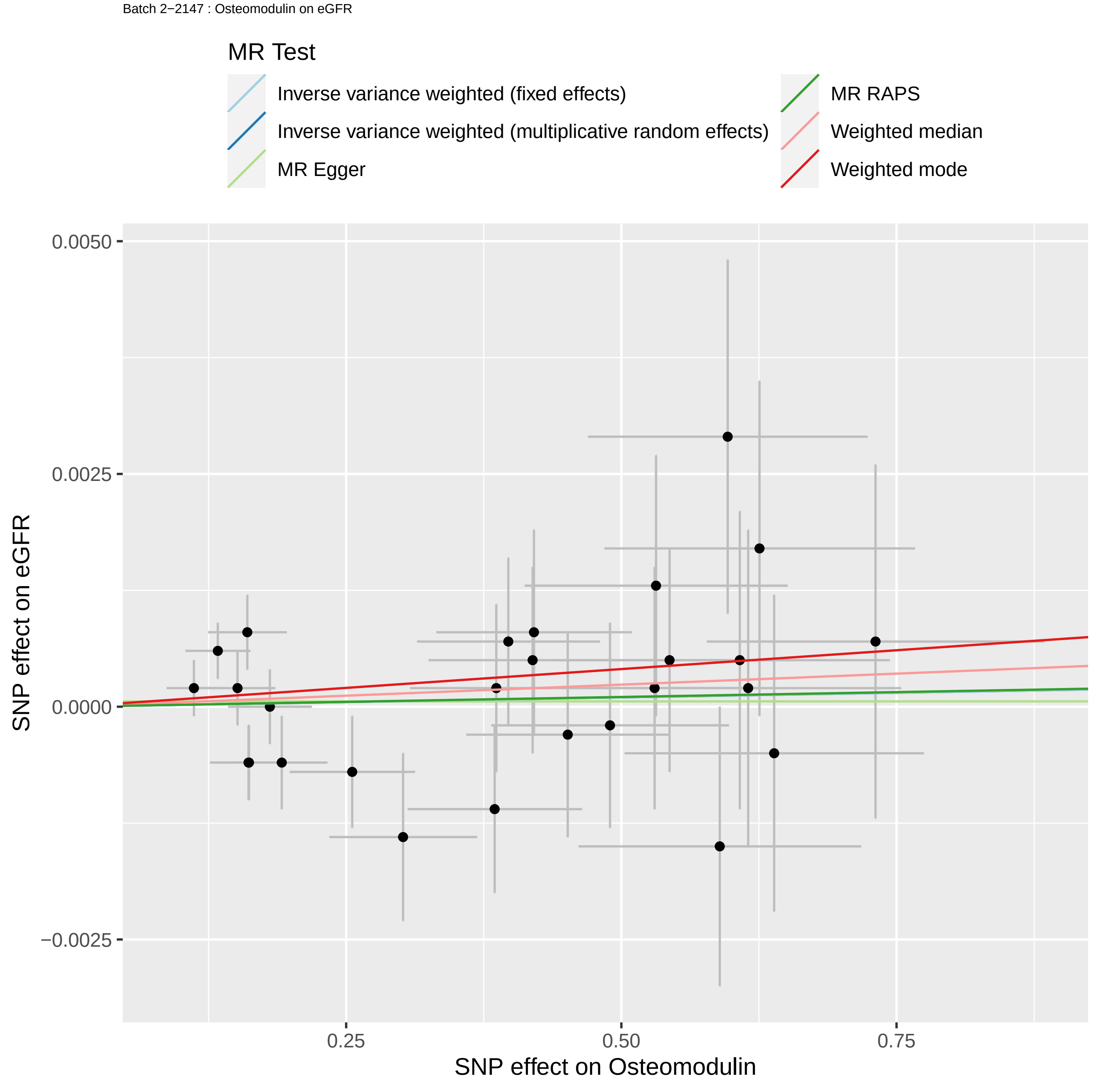
Batch 2−2147 : Osteomodulin on eGFR
MR Test
Inverse variance weighted (fixed effects)
Inverse variance weighted (multiplicative random effects)
MR Egger
MR RAPS
Weighted median
Weighted mode
0.0050
0.0025
0.0000
−0.0025
0.25
0.50
0.75
SNP effect on eGFR
SNP effect on Osteomodulin

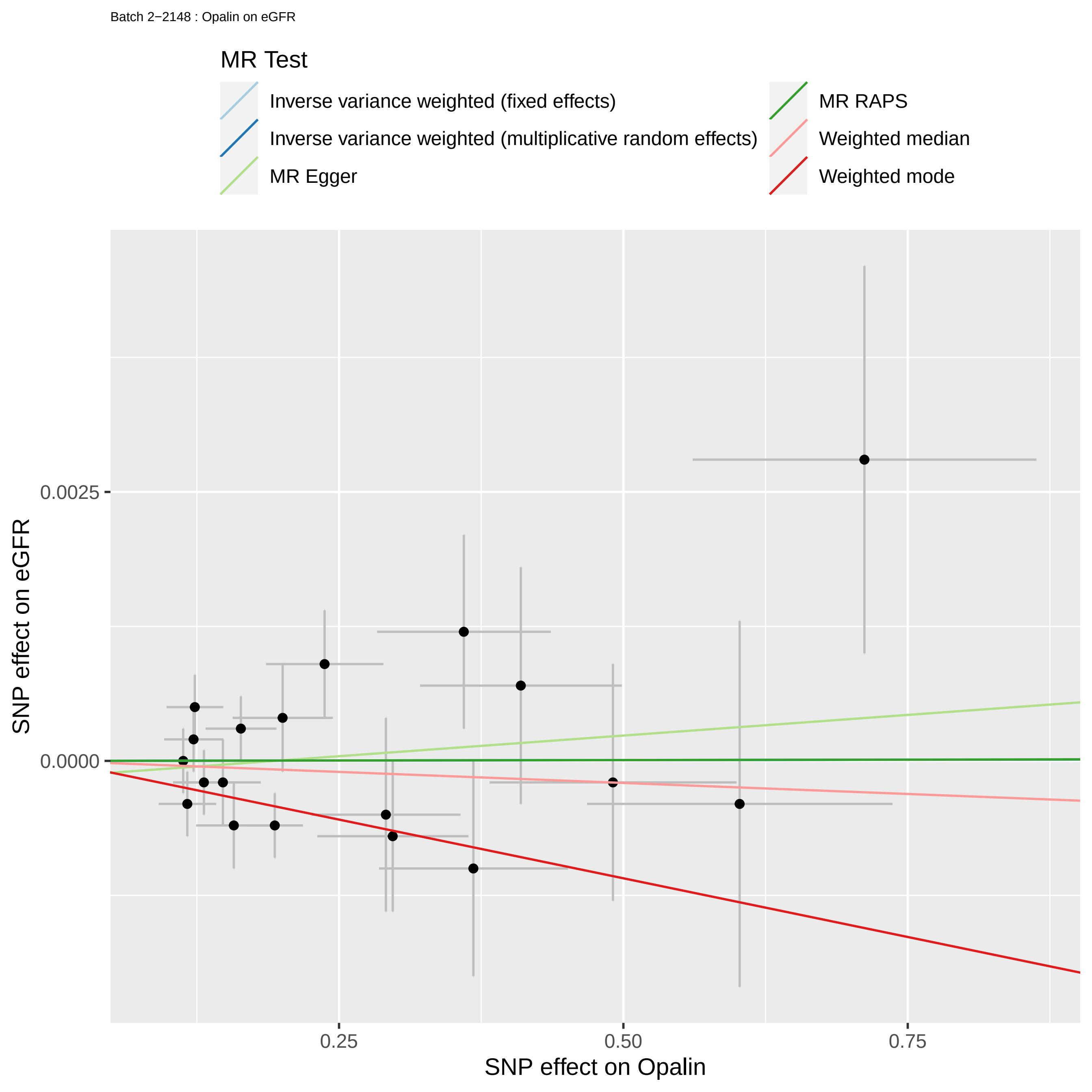

Batch 2−2148 : Opalin on eGFR
MR Test
Inverse variance weighted (fixed effects)
Inverse variance weighted (multiplicative random effects)
MR Egger
MR RAPS
Weighted median
Weighted mode
0.0025
0.0000
0.25
0.50
0.75
SNP effect on eGFR
SNP effect on Opalin

Batch 2−2149 : Opioid−binding protein/cell adhesion molecule on eGFR

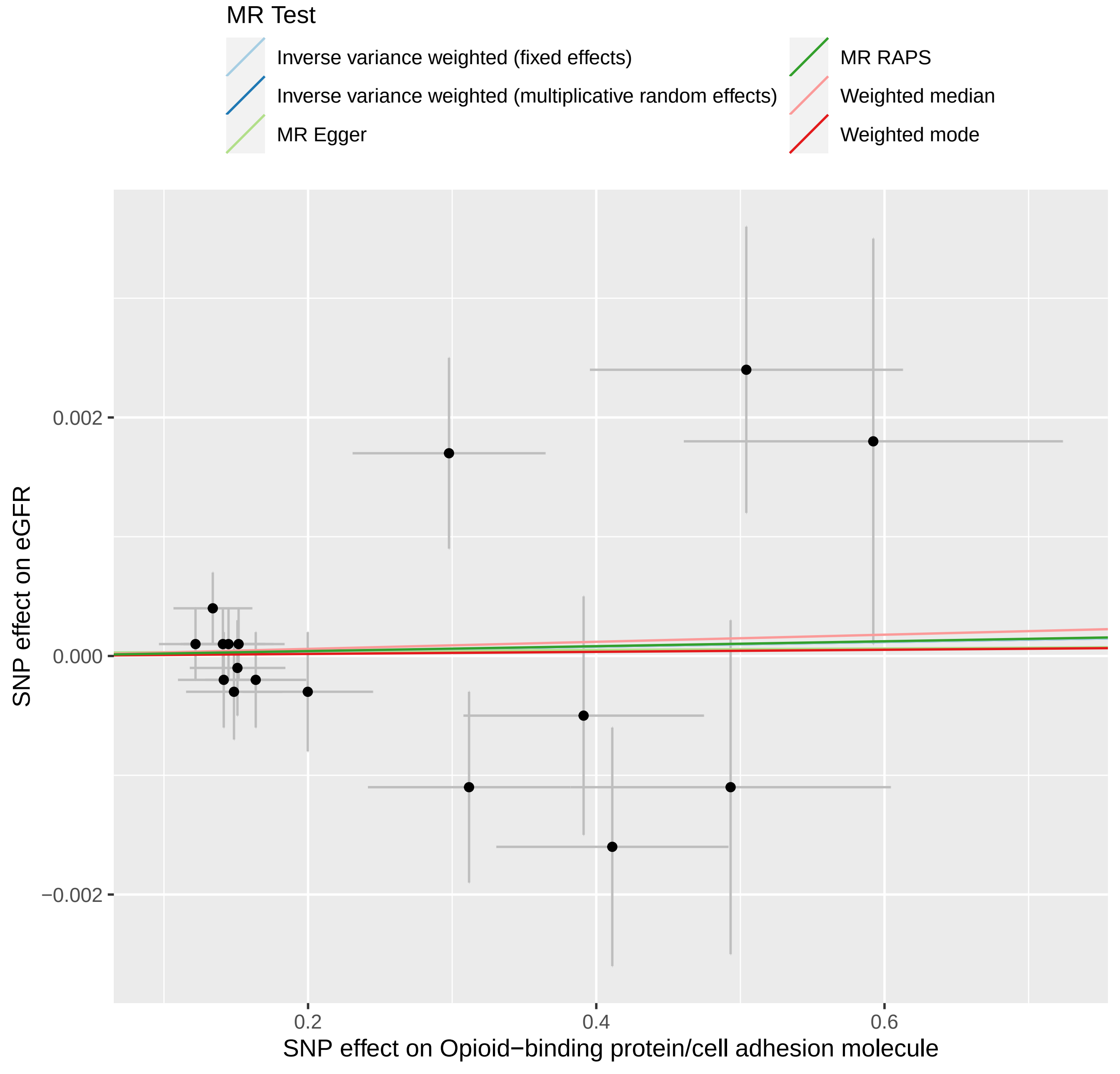

Batch 2−2150 : Oligophrenin−1 on eGFR

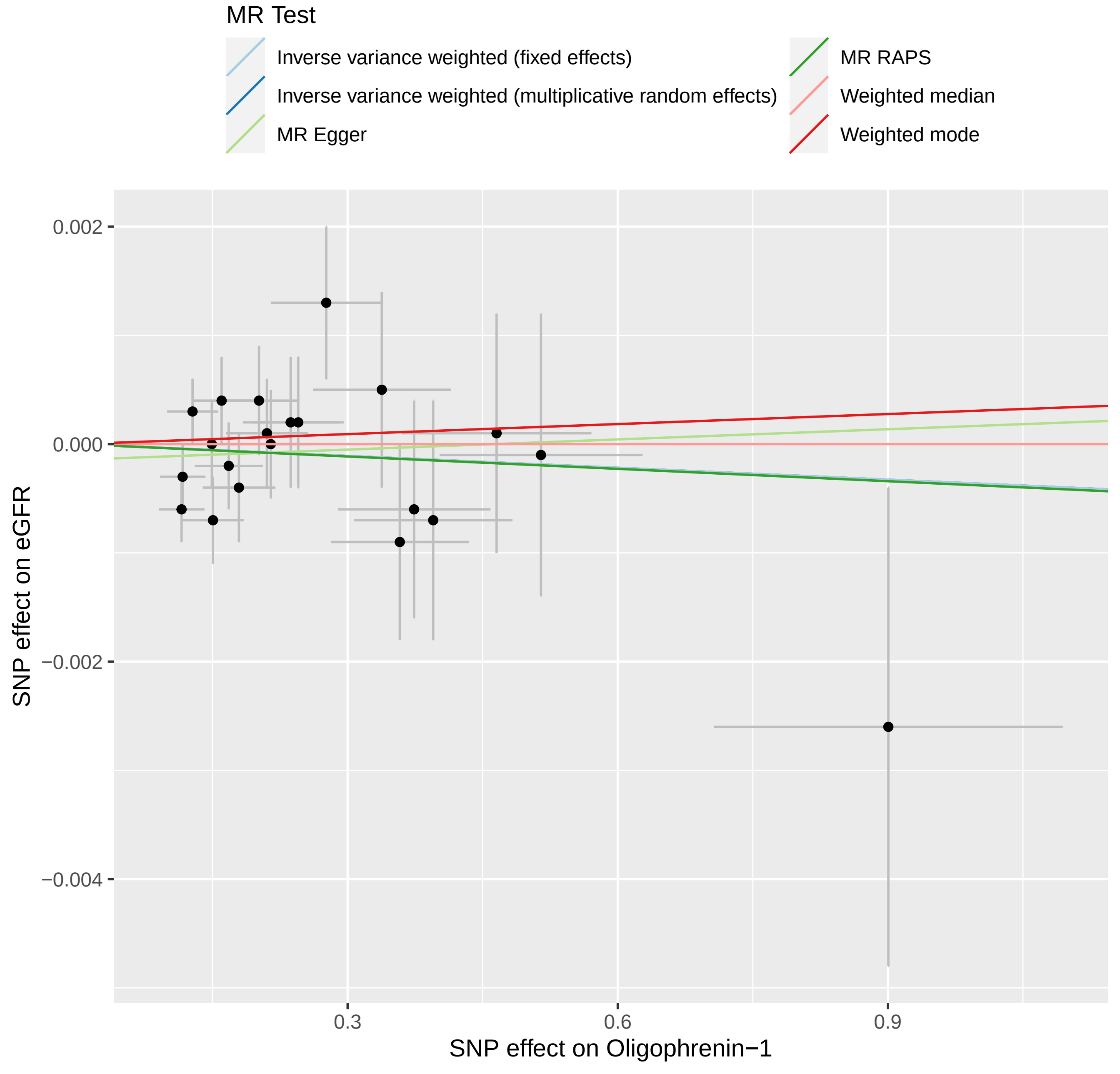

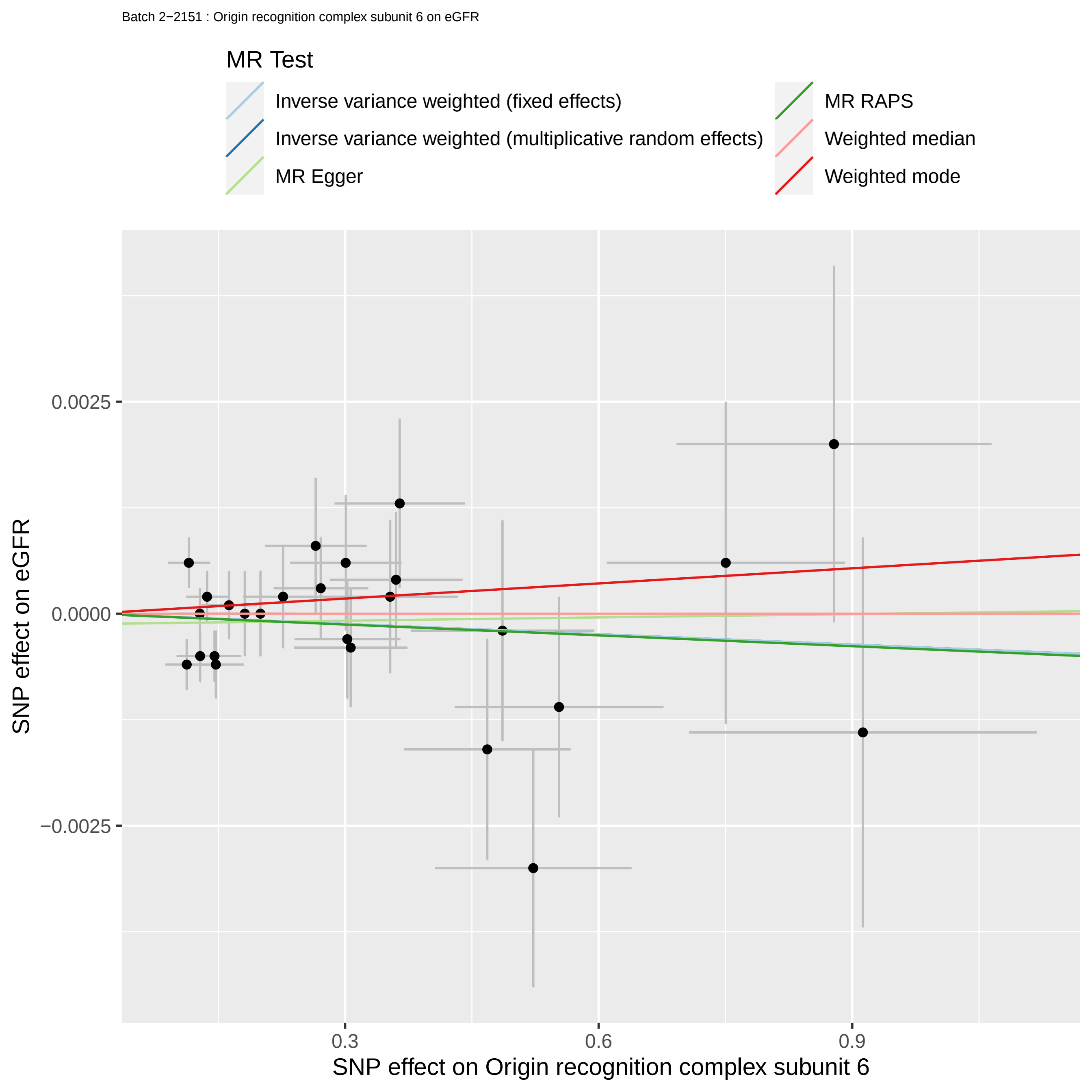

Batch 2−2151 : Origin recognition complex subunit 6 on eGFR
MR Test
Inverse variance weighted (fixed effects)
Inverse variance weighted (multiplicative random effects)
MR Egger
MR RAPS
Weighted median
Weighted mode
0.0025
0.0000
−0.0025
0.3
0.6
0.9
SNP effect on eGFR
SNP effect on Origin recognition complex subunit 6

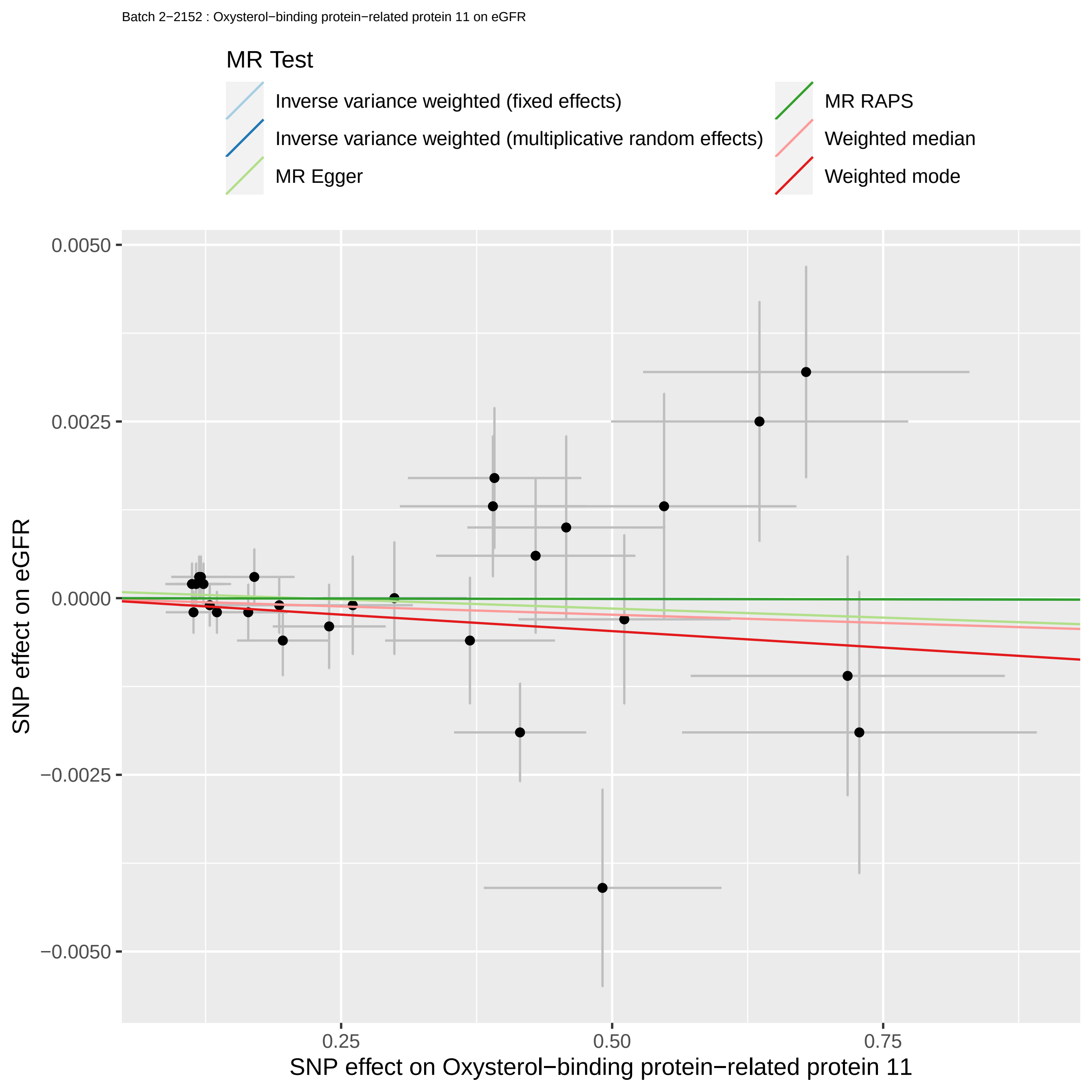

Batch 2−2152 : Oxysterol−binding protein−related protein 11 on eGFR
MR Test
Inverse variance weighted (fixed effects)
Inverse variance weighted (multiplicative random effects)
MR Egger
MR RAPS
Weighted median
Weighted mode
0.0050
0.0025
0.0000
−0.0025
−0.0050
SNP effect on eGFR
0.25
0.50
0.75
SNP effect on Oxysterol−binding protein−related protein 11

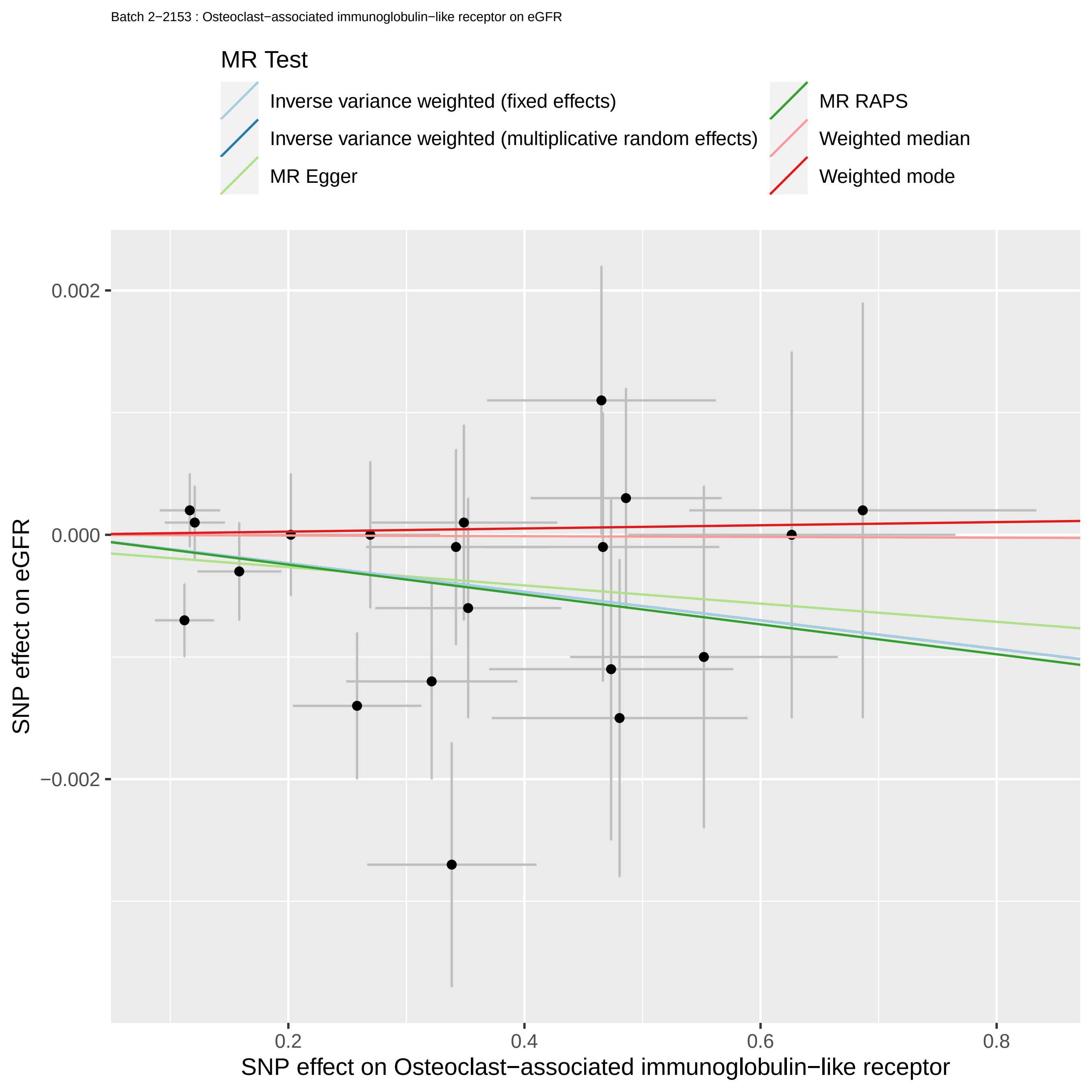
Batch 2−2153 : Osteoclast−associated immunoglobulin−like receptor on eGFR
MR Test
Inverse variance weighted (fixed effects)
Inverse variance weighted (multiplicative random effects)
MR Egger
MR RAPS
Weighted median
Weighted mode
0.002
0.000
−0.002
0.2
0.4
0.6
0.8
SNP effect on eGFR
SNP effect on Osteoclast−associated immunoglobulin−like receptor

Batch 2−2154 : Oncostatin−M on eGFR

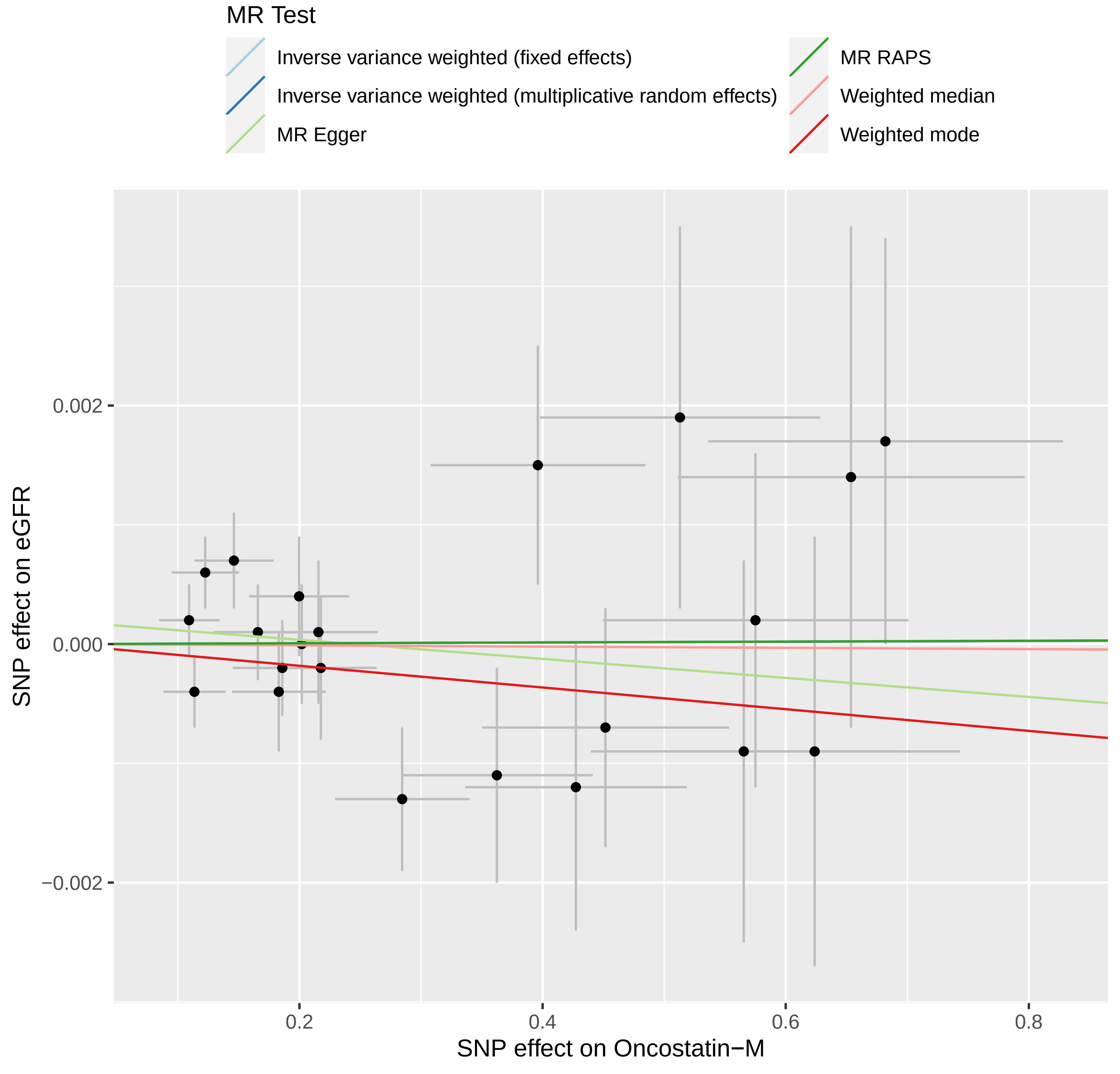

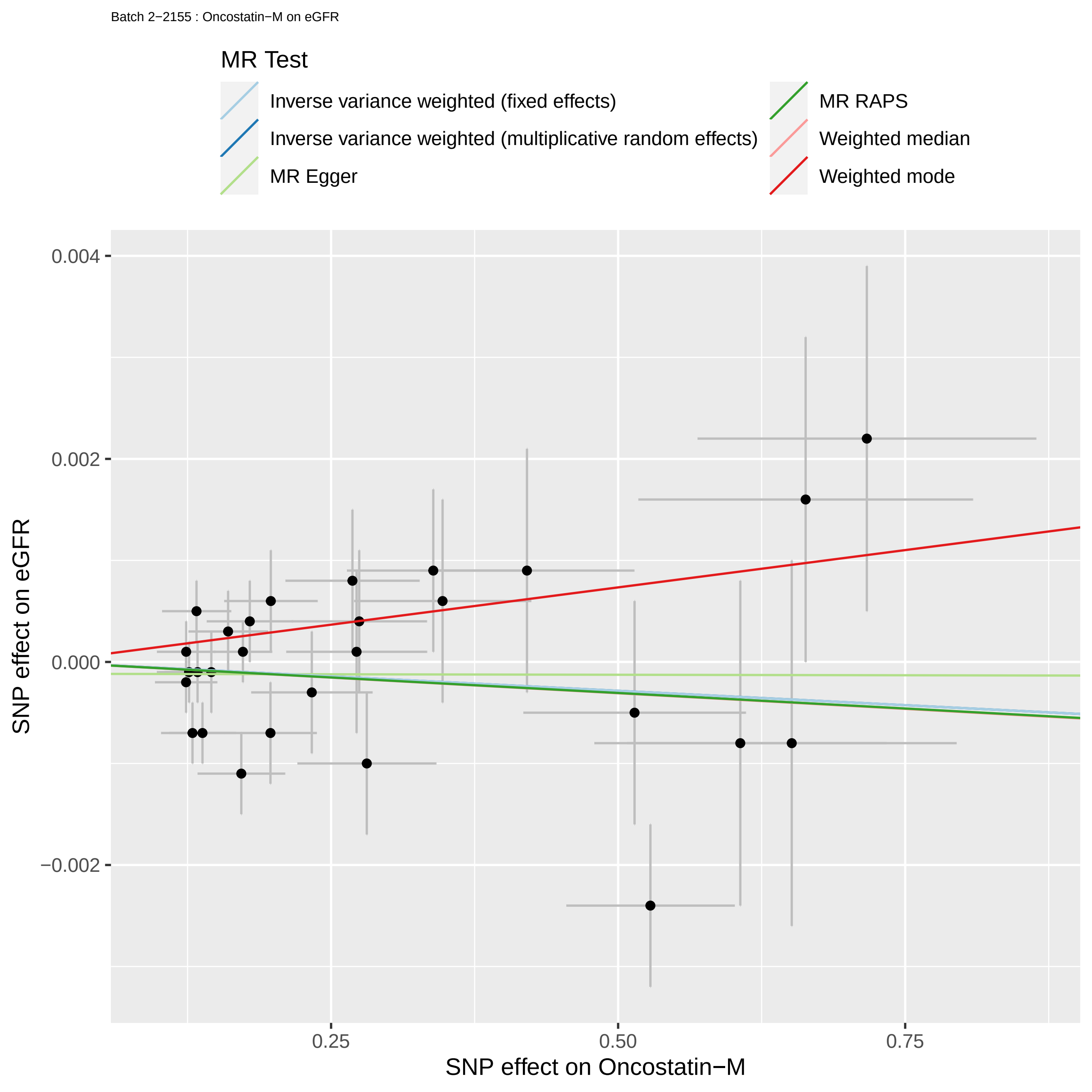

Batch 2−2155 : Oncostatin−M on eGFR
MR Test
Inverse variance weighted (fixed effects)
Inverse variance weighted (multiplicative random effects)
MR Egger
MR RAPS
Weighted median
Weighted mode
0.004
0.002
0.000
−0.002
SNP effect on eGFR
0.25
0.50
0.75
SNP effect on Oncostatin−M

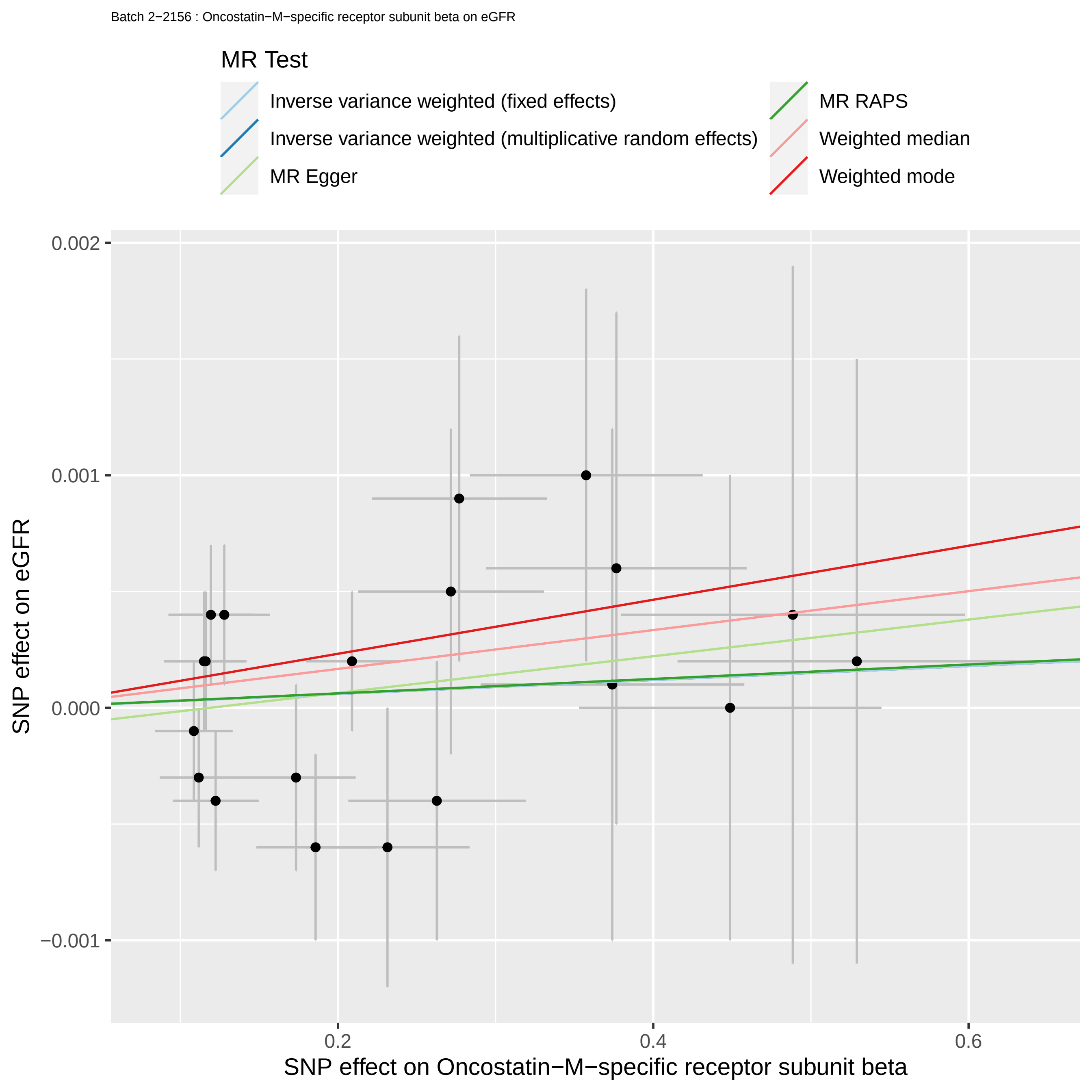

Batch 2−2156 : Oncostatin−M−specific receptor subunit beta on eGFR
MR Test
Inverse variance weighted (fixed effects)
Inverse variance weighted (multiplicative random effects)
MR Egger
MR RAPS
Weighted median
Weighted mode
0.002
0.001
0.000
−0.001
0.2
0.4
0.6
SNP effect on eGFR
SNP effect on Oncostatin−M−specific receptor subunit beta

Batch 2−2157 : Osteocrin on eGFR

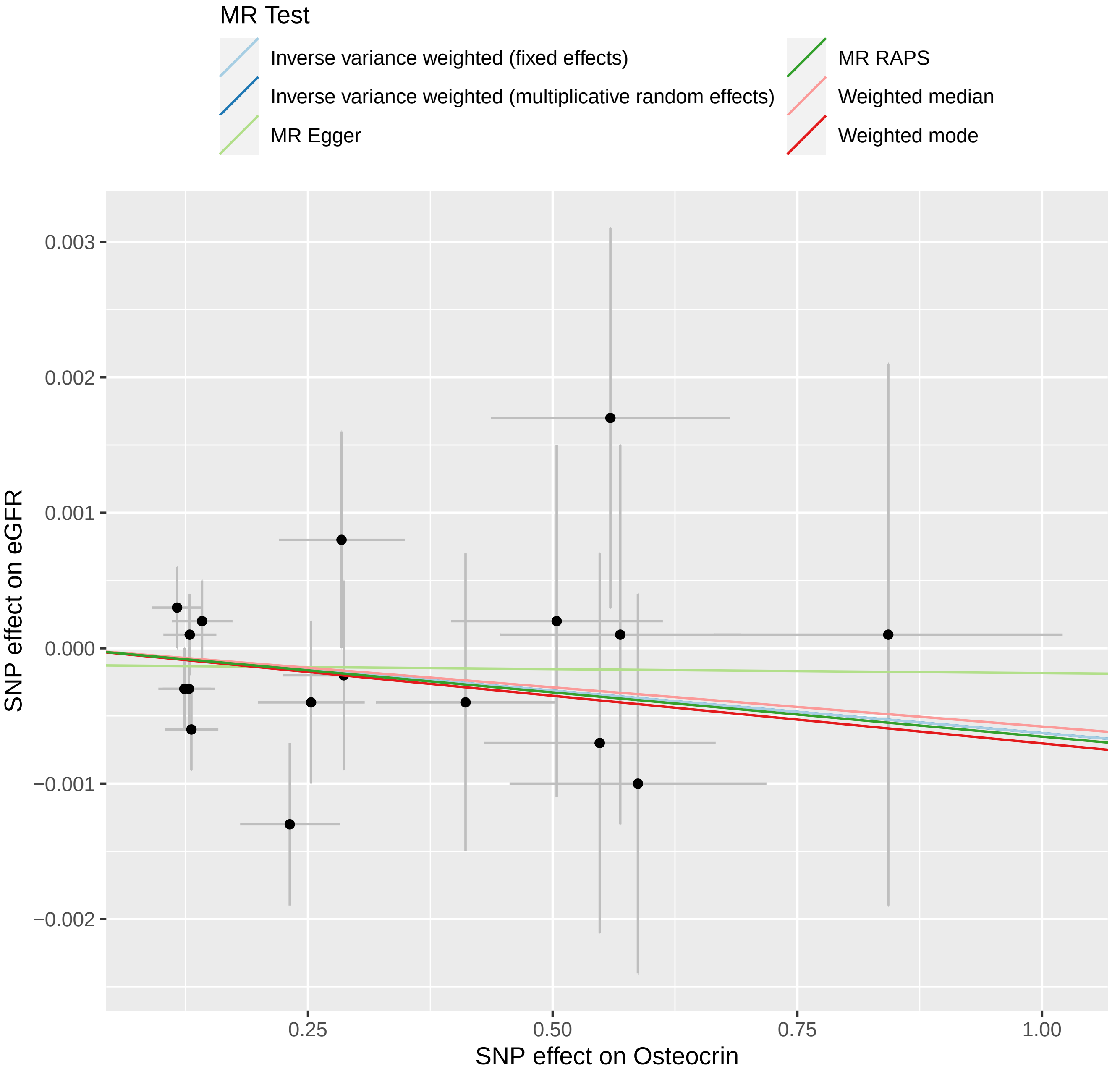

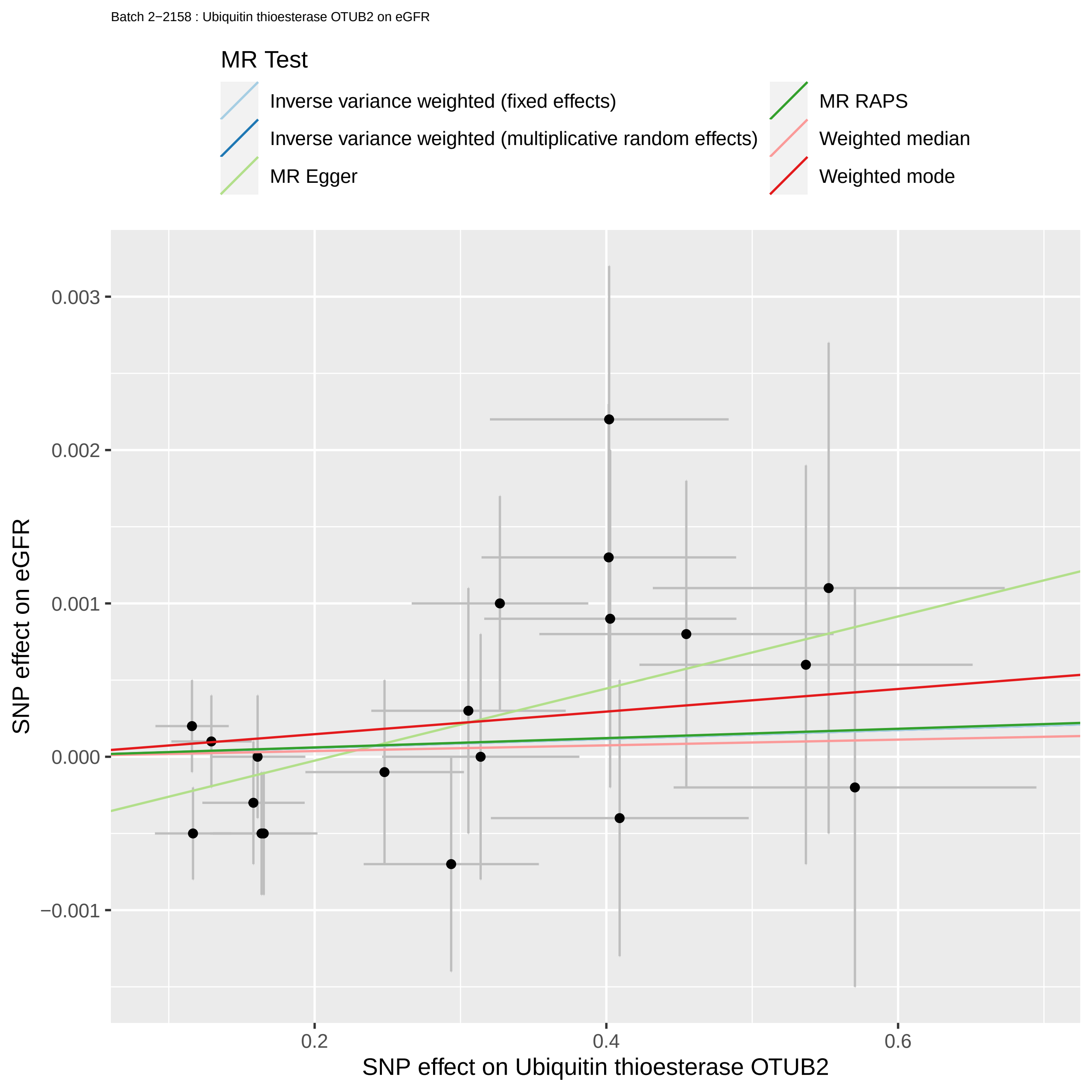

Batch 2−2158 : Ubiquitin thioesterase OTUB2 on eGFR
MR Test
Inverse variance weighted (fixed effects)
Inverse variance weighted (multiplicative random effects)
MR Egger
MR RAPS
Weighted median
Weighted mode
0.003
0.002
0.001
0.000
−0.001
SNP effect on eGFR
0.2
0.4
0.6
SNP effect on Ubiquitin thioesterase OTUB2

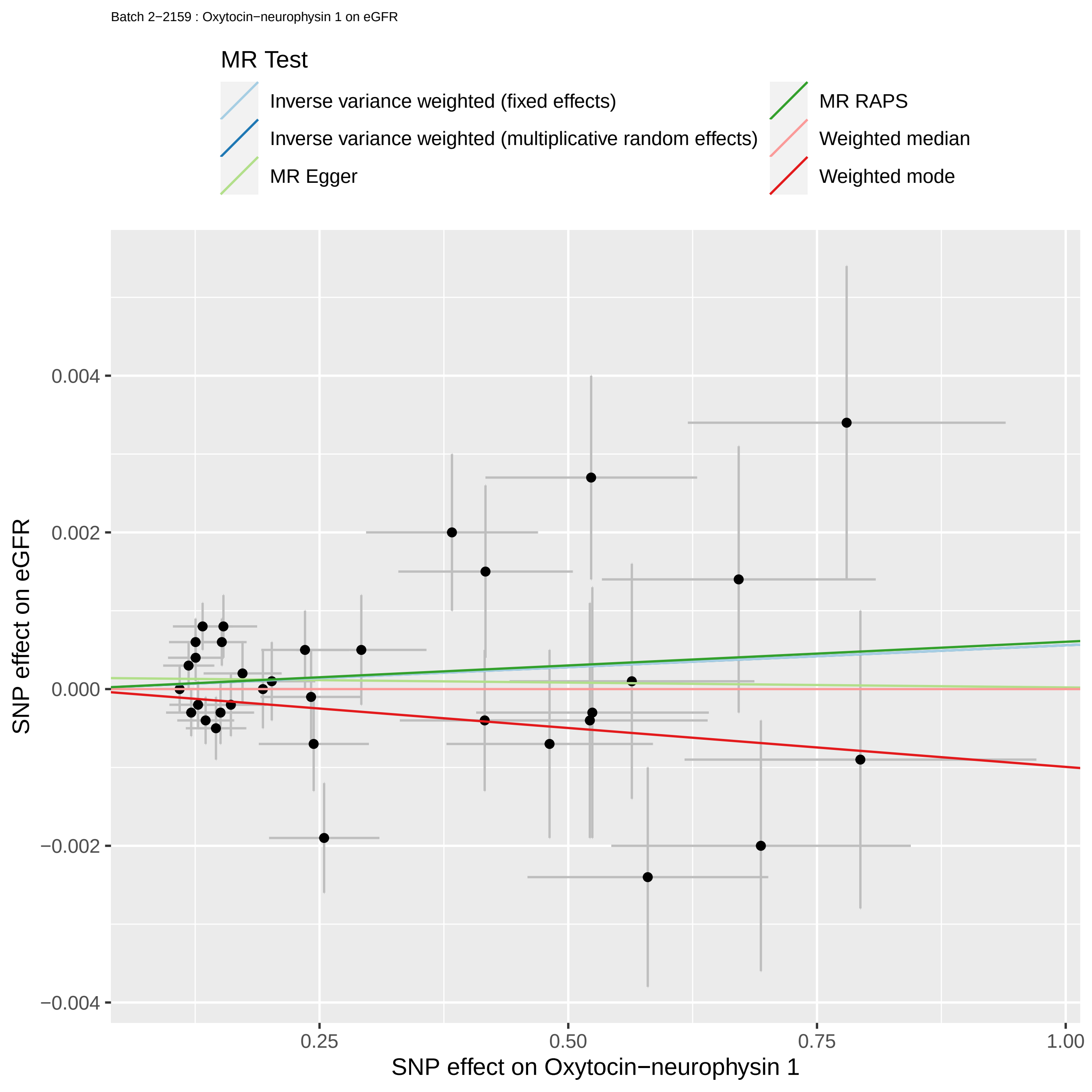
Batch 2−2159 : Oxytocin−neurophysin 1 on eGFR
MR Test
Inverse variance weighted (fixed effects)
Inverse variance weighted (multiplicative random effects)
MR Egger
MR RAPS
Weighted median
Weighted mode
0.004
0.002
0.000
−0.002
−0.004
SNP effect on eGFR
0.25
0.50
0.75
1.00
SNP effect on Oxytocin−neurophysin 1

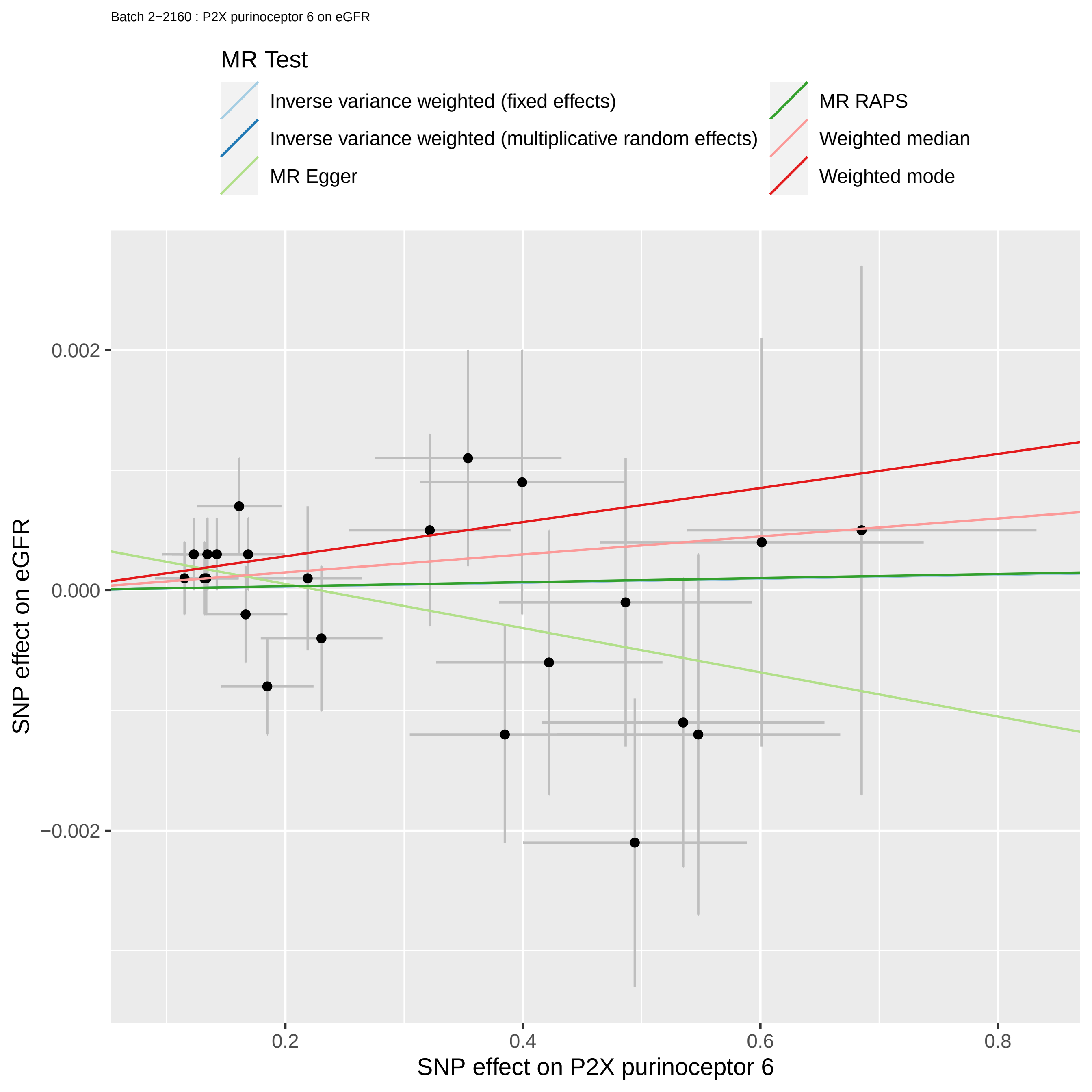

Batch 2−2160 : P2X purinoceptor 6 on eGFR
MR Test
Inverse variance weighted (fixed effects)
Inverse variance weighted (multiplicative random effects)
MR Egger
MR RAPS
Weighted median
Weighted mode
0.002
0.000
−0.002
0.2
0.4
0.6
0.8
SNP effect on eGFR
SNP effect on P2X purinoceptor 6

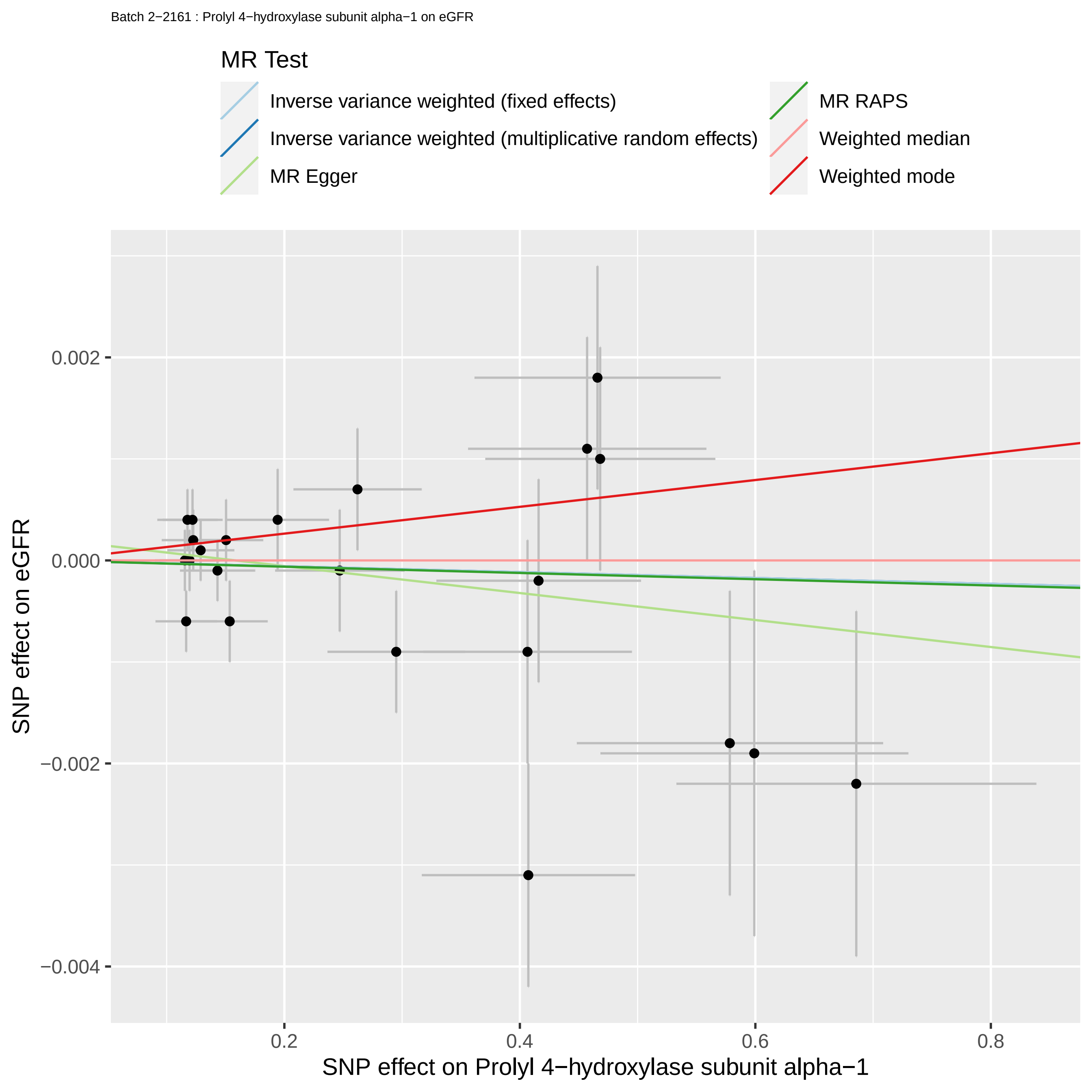

Batch 2−2161 : Prolyl 4−hydroxylase subunit alpha−1 on eGFR
MR Test
Inverse variance weighted (fixed effects)
Inverse variance weighted (multiplicative random effects)
MR Egger
MR RAPS
Weighted median
Weighted mode
0.002
0.000
−0.002
−0.004
SNP effect on eGFR
0.2
0.4
0.6
0.8
SNP effect on Prolyl 4−hydroxylase subunit alpha−1

Batch 2−2162 : Prolyl 4−hydroxylase subunit alpha−2 on eGFR

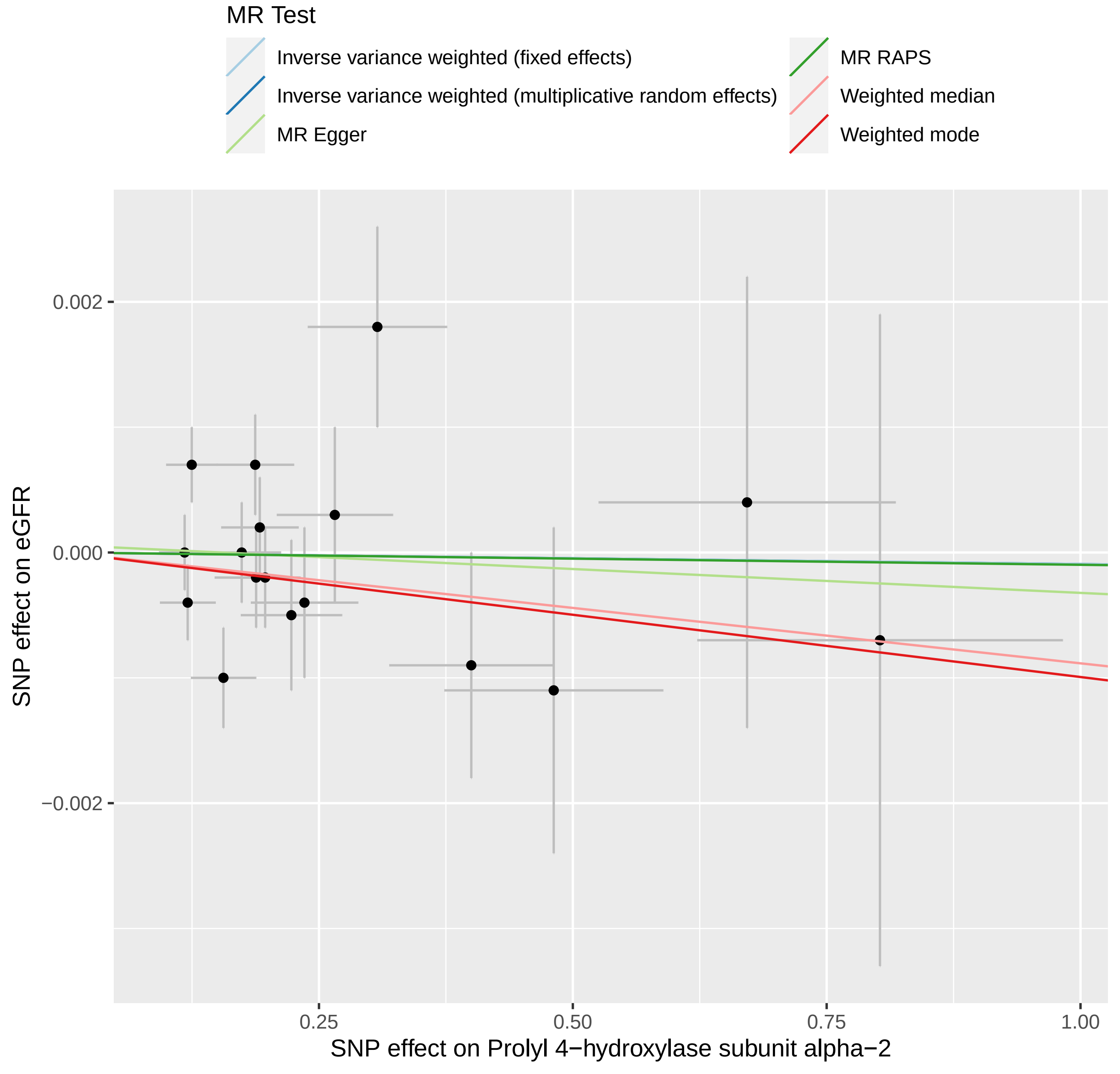

Batch 2−2163 : Polyadenylate−binding protein 3 on eGFR

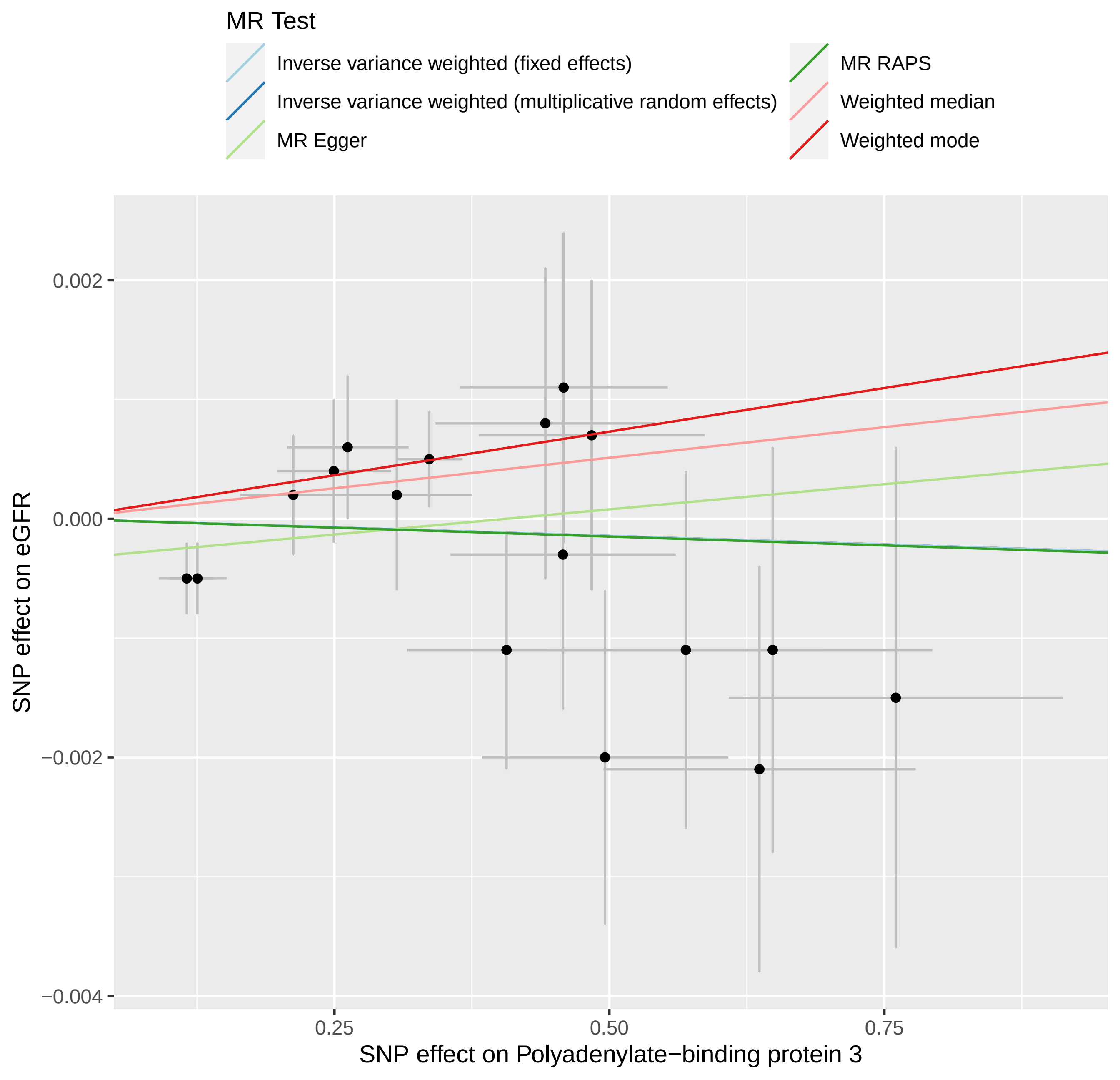

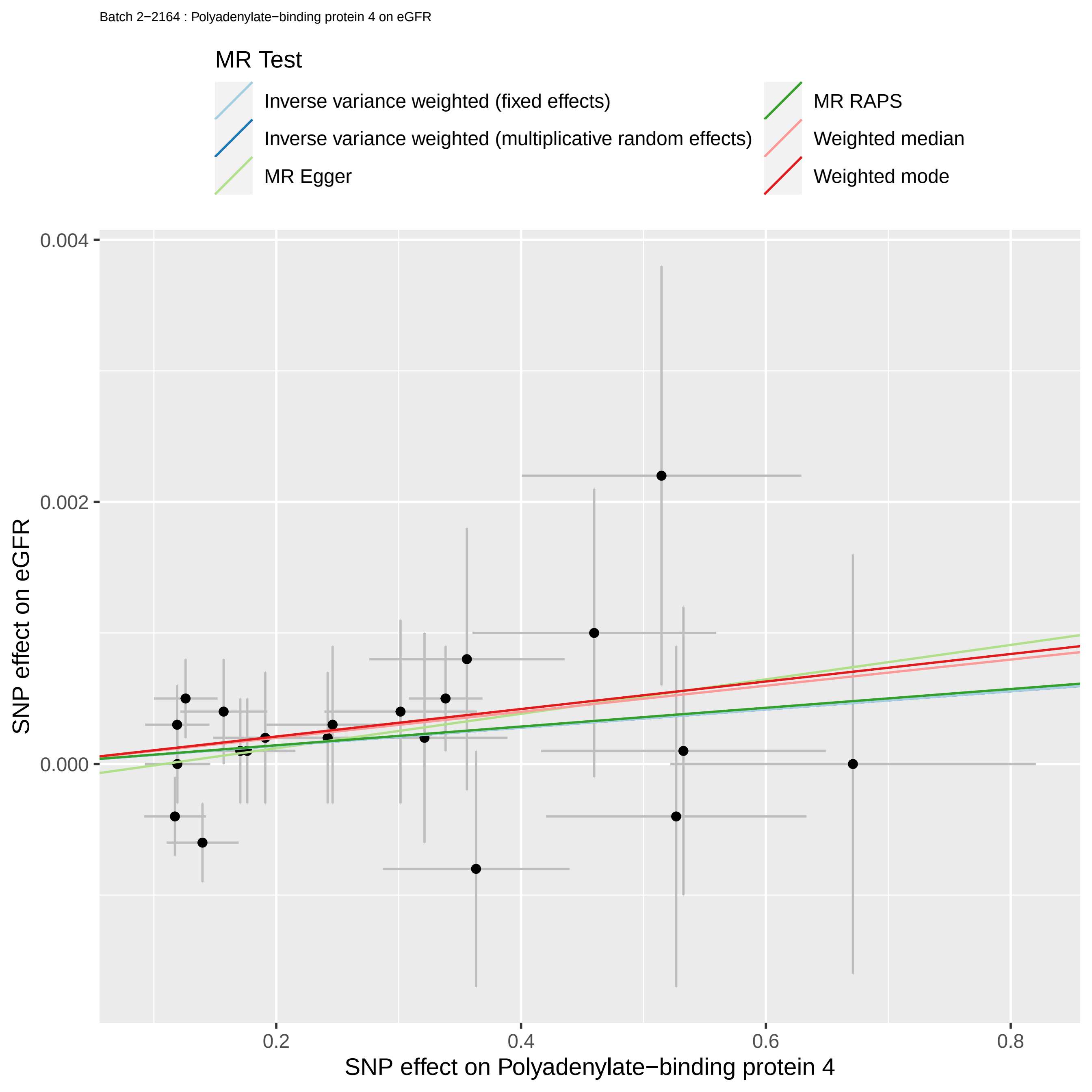

Batch 2−2164 : Polyadenylate−binding protein 4 on eGFR
MR Test
Inverse variance weighted (fixed effects)
Inverse variance weighted (multiplicative random effects)
MR Egger
MR RAPS
Weighted median
Weighted mode
0.004
0.002
0.000
0.2
0.4
0.6
0.8
SNP effect on eGFR
SNP effect on Polyadenylate−binding protein 4

Batch 2−2165 : Marginal zone B− and B1−cell−specific protein on eGFR

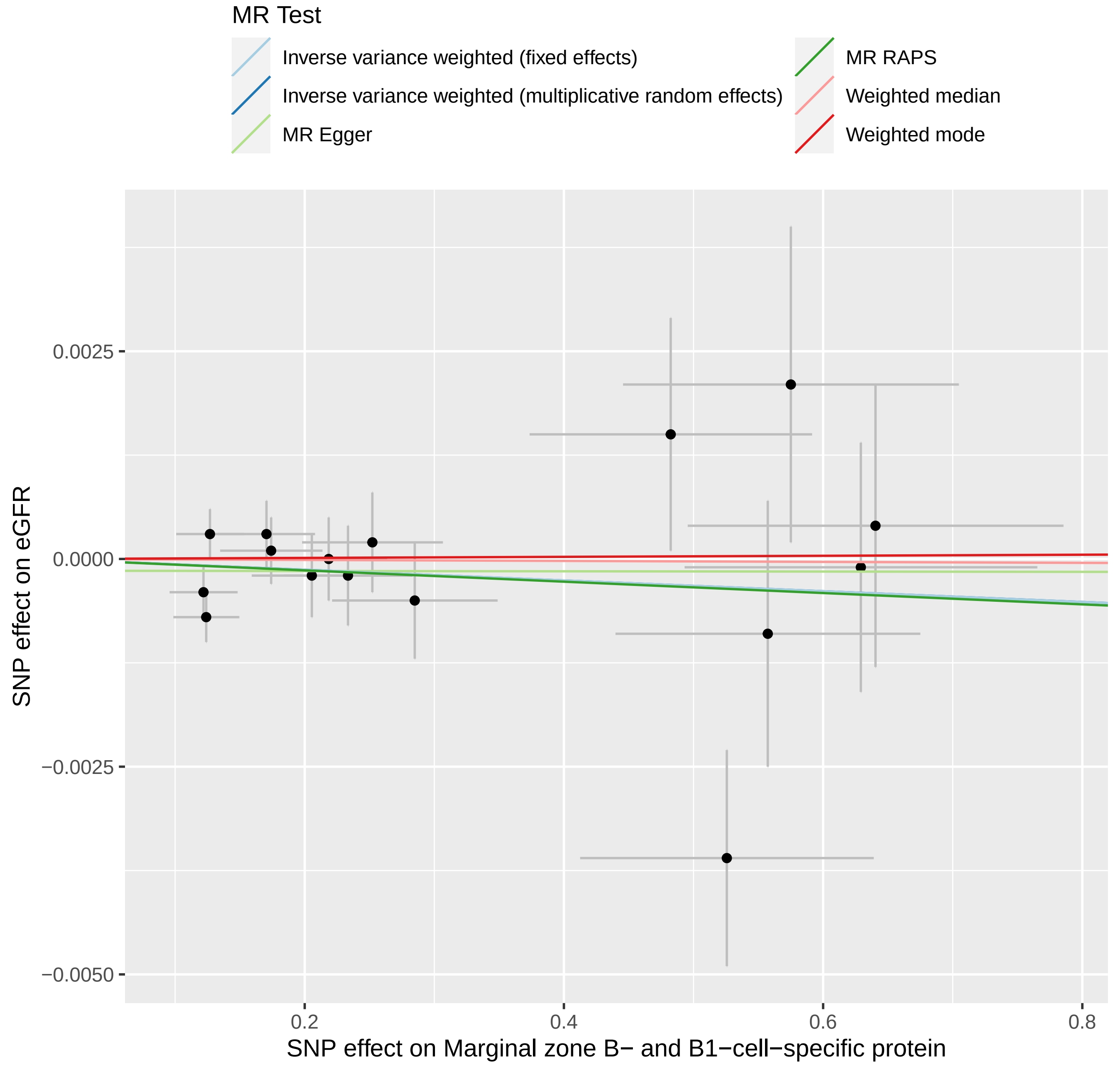

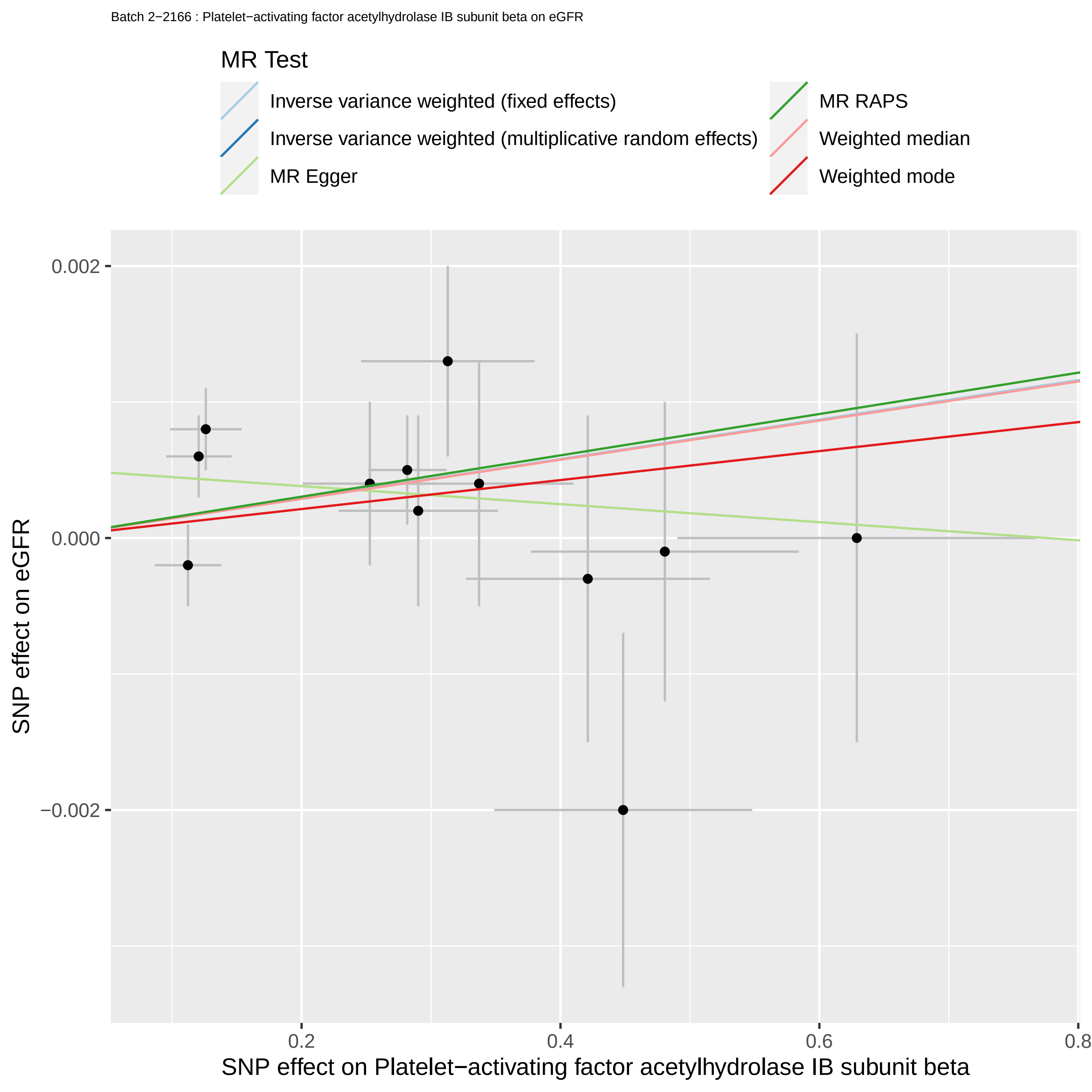
Batch 2−2166 : Platelet−activating factor acetylhydrolase IB subunit beta on eGFR
MR Test
Inverse variance weighted (fixed effects)
Inverse variance weighted (multiplicative random effects)
MR Egger
MR RAPS
Weighted median
Weighted mode
0.002
0.000
−0.002
0.2
0.4
0.6
0.8
SNP effect on eGFR
SNP effect on Platelet−activating factor acetylhydrolase IB subunit beta

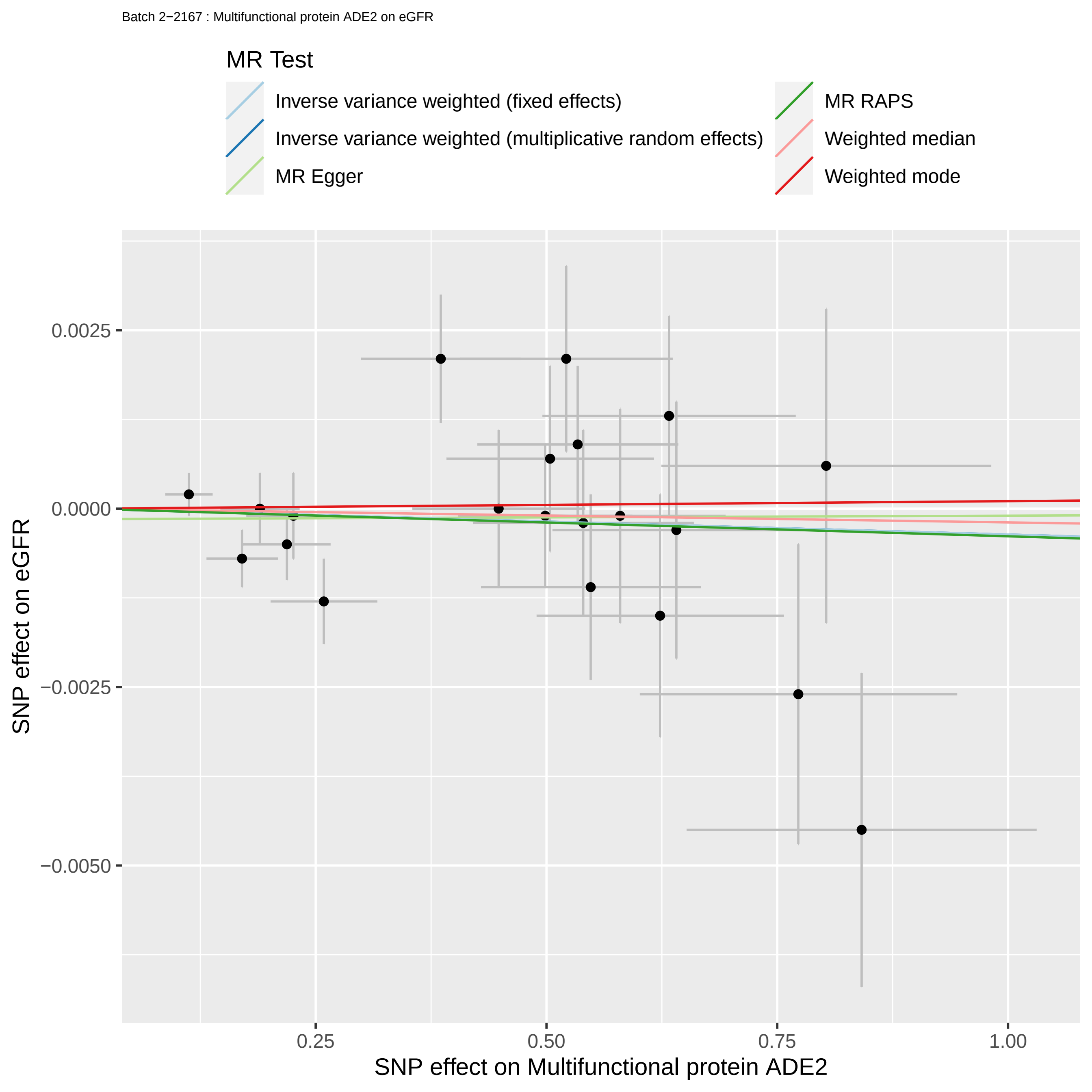

Batch 2−2167 : Multifunctional protein ADE2 on eGFR
MR Test
Inverse variance weighted (fixed effects)
Inverse variance weighted (multiplicative random effects)
MR Egger
MR RAPS
Weighted median
Weighted mode
0.0025
0.0000
−0.0025
−0.0050
0.25
0.50
0.75
1.00
SNP effect on eGFR
SNP effect on Multifunctional protein ADE2

Batch 2−2168 : Polyadenylate−binding protein−interacting protein 1 on eGFR

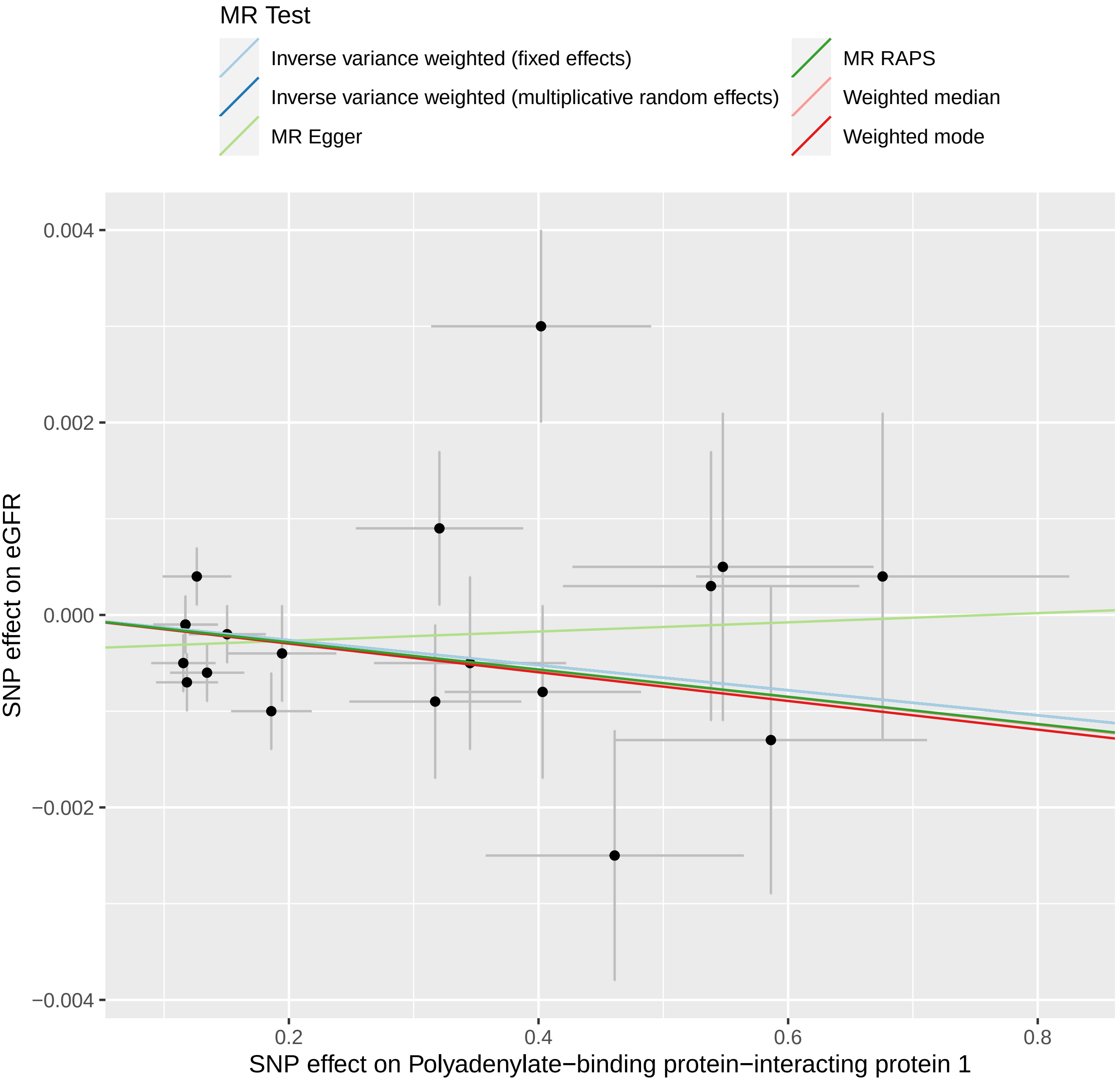

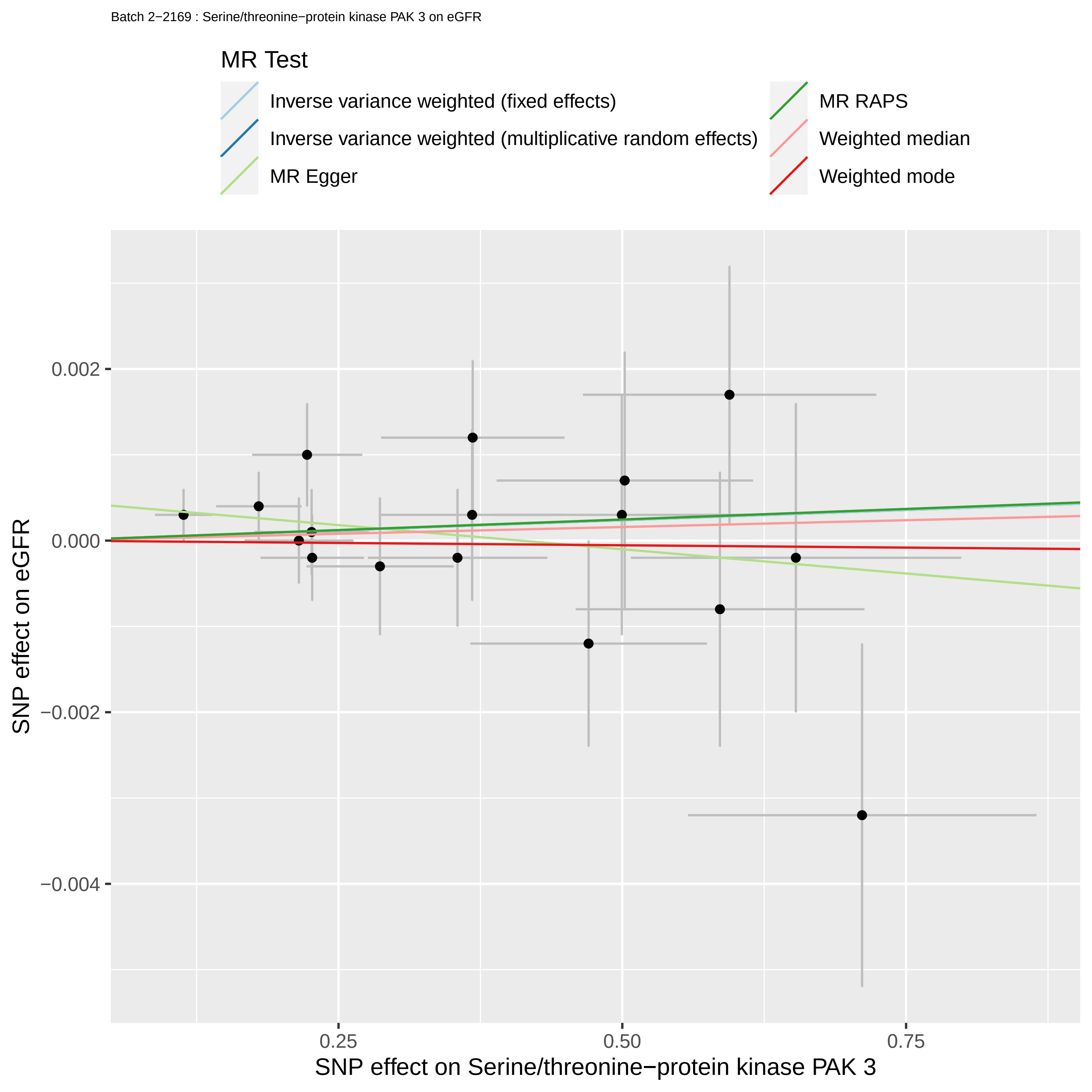

Batch 2−2169 : Serine/threonine−protein kinase PAK 3 on eGFR
MR Test
Inverse variance weighted (fixed effects)
Inverse variance weighted (multiplicative random effects)
MR Egger
MR RAPS
Weighted median
Weighted mode
0.002
0.000
−0.002
−0.004
SNP effect on eGFR
0.25
0.50
0.75
SNP effect on Serine/threonine−protein kinase PAK 3

Batch 2−2170 : Serine/threonine−protein kinase PAK 4 on eGFR

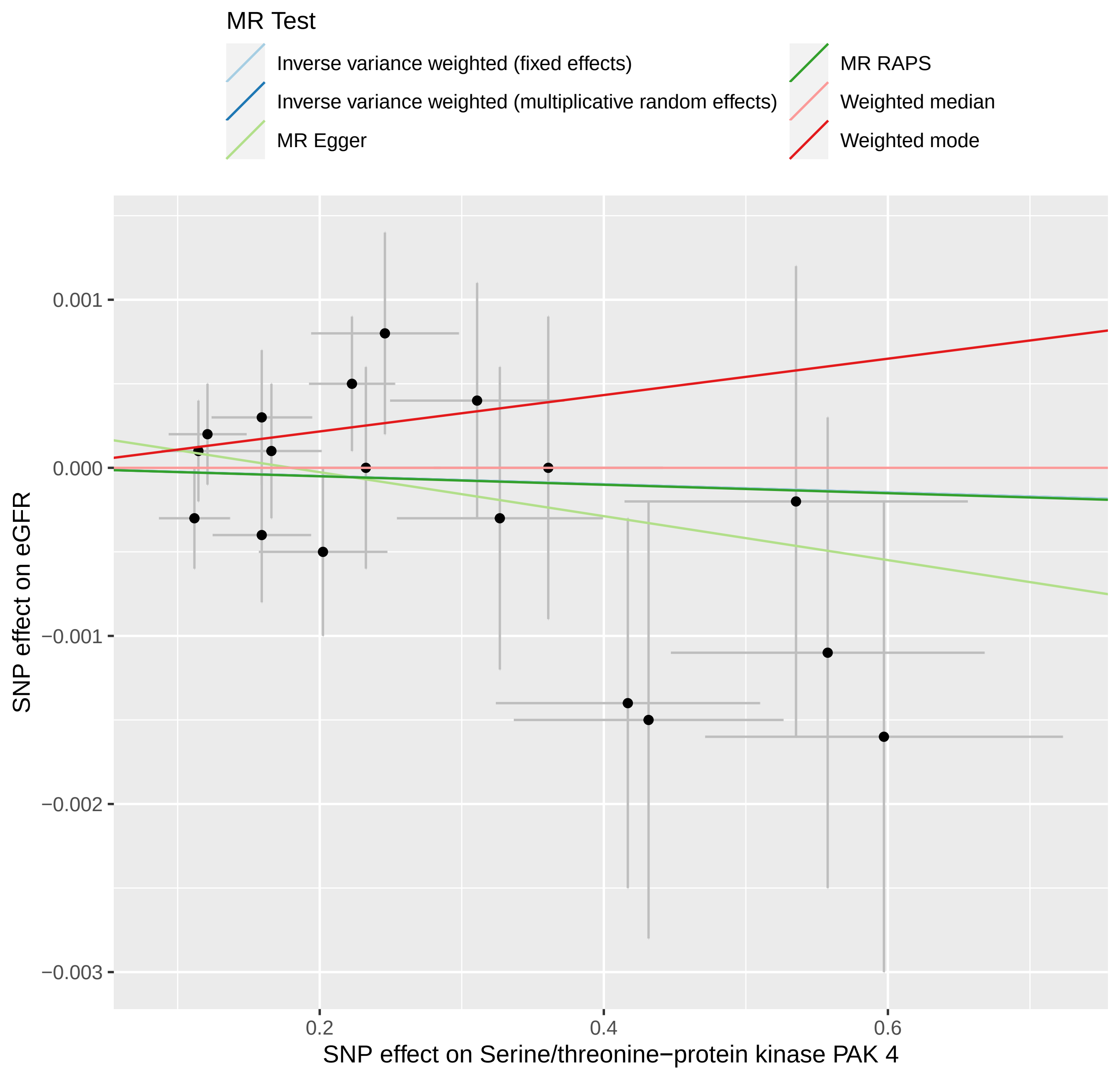

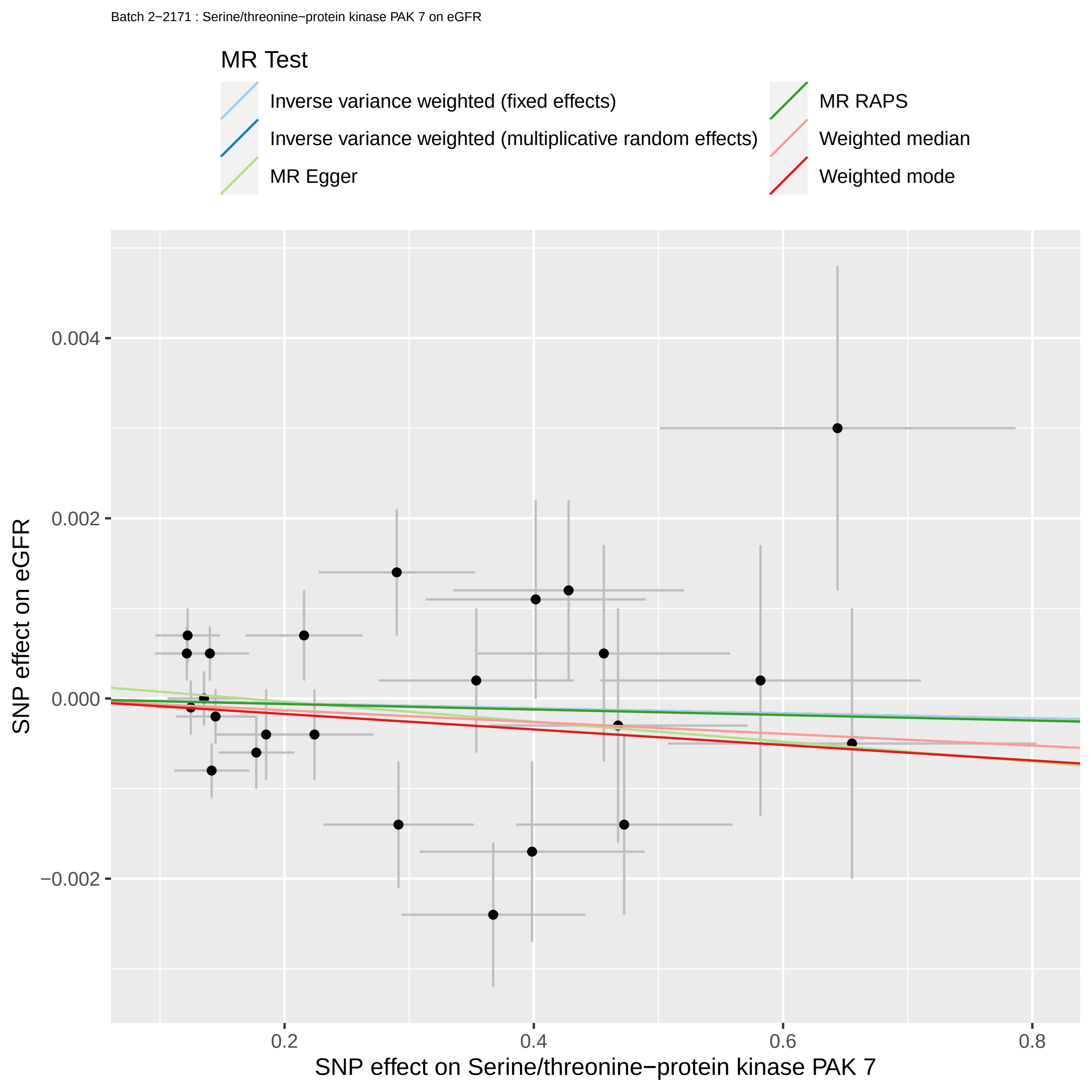

Batch 2−2171 : Serine/threonine−protein kinase PAK 7 on eGFR
MR Test
Inverse variance weighted (fixed effects)
Inverse variance weighted (multiplicative random effects)
MR Egger
MR RAPS
Weighted median
Weighted mode
0.004
0.002
0.000
−0.002
SNP effect on eGFR
0.2
0.4
0.6
0.8
SNP effect on Serine/threonine−protein kinase PAK 7

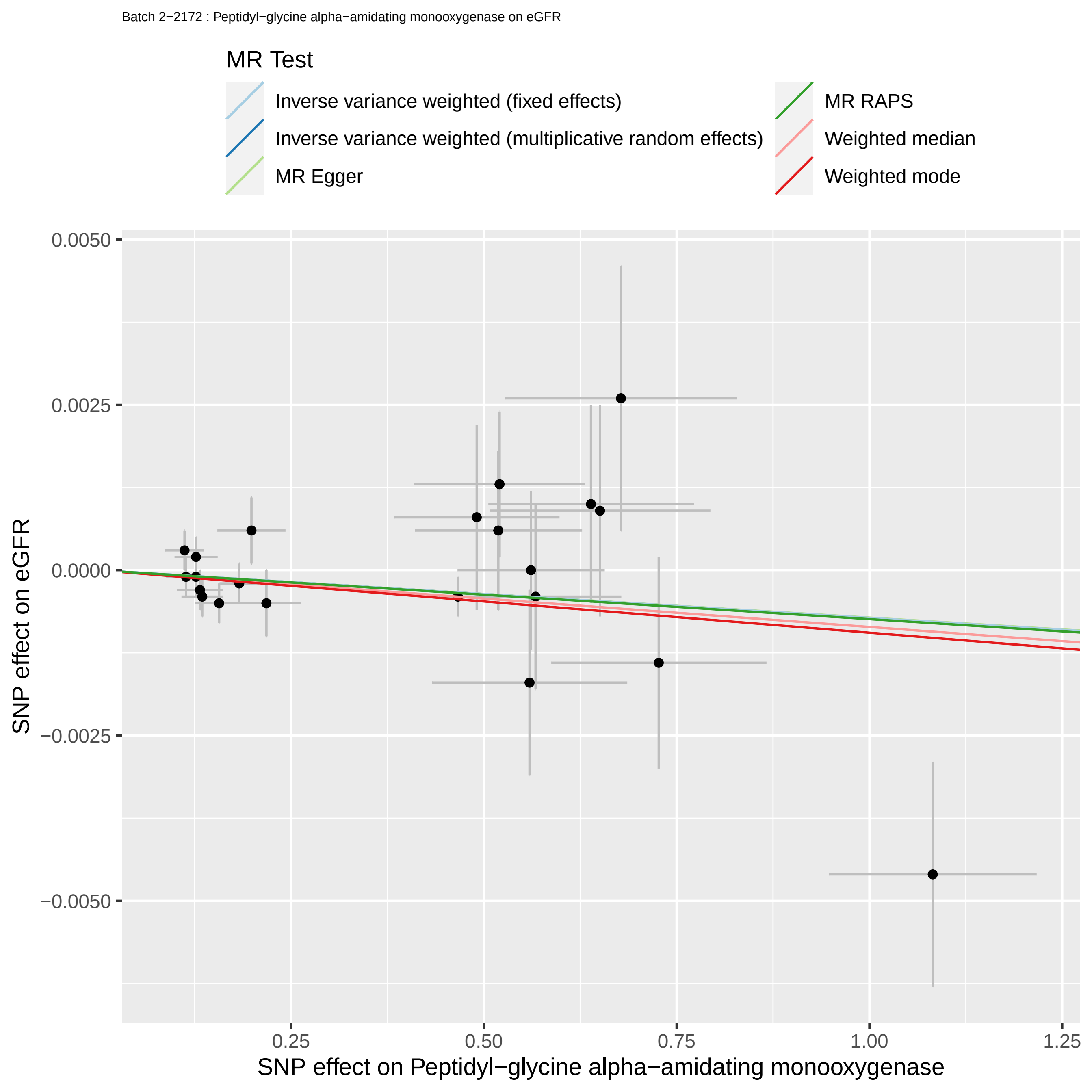
Batch 2−2172 : Peptidyl−glycine alpha−amidating monooxygenase on eGFR
MR Test
Inverse variance weighted (fixed effects)
Inverse variance weighted (multiplicative random effects)
MR Egger
MR RAPS
Weighted median
Weighted mode
0.0050
0.0025
0.0000
−0.0025
−0.0050
SNP effect on eGFR
0.25
0.50
0.75
1.00
1.25
SNP effect on Peptidyl−glycine alpha−amidating monooxygenase

Batch 2−2173 : Pantothenate kinase 3 on eGFR

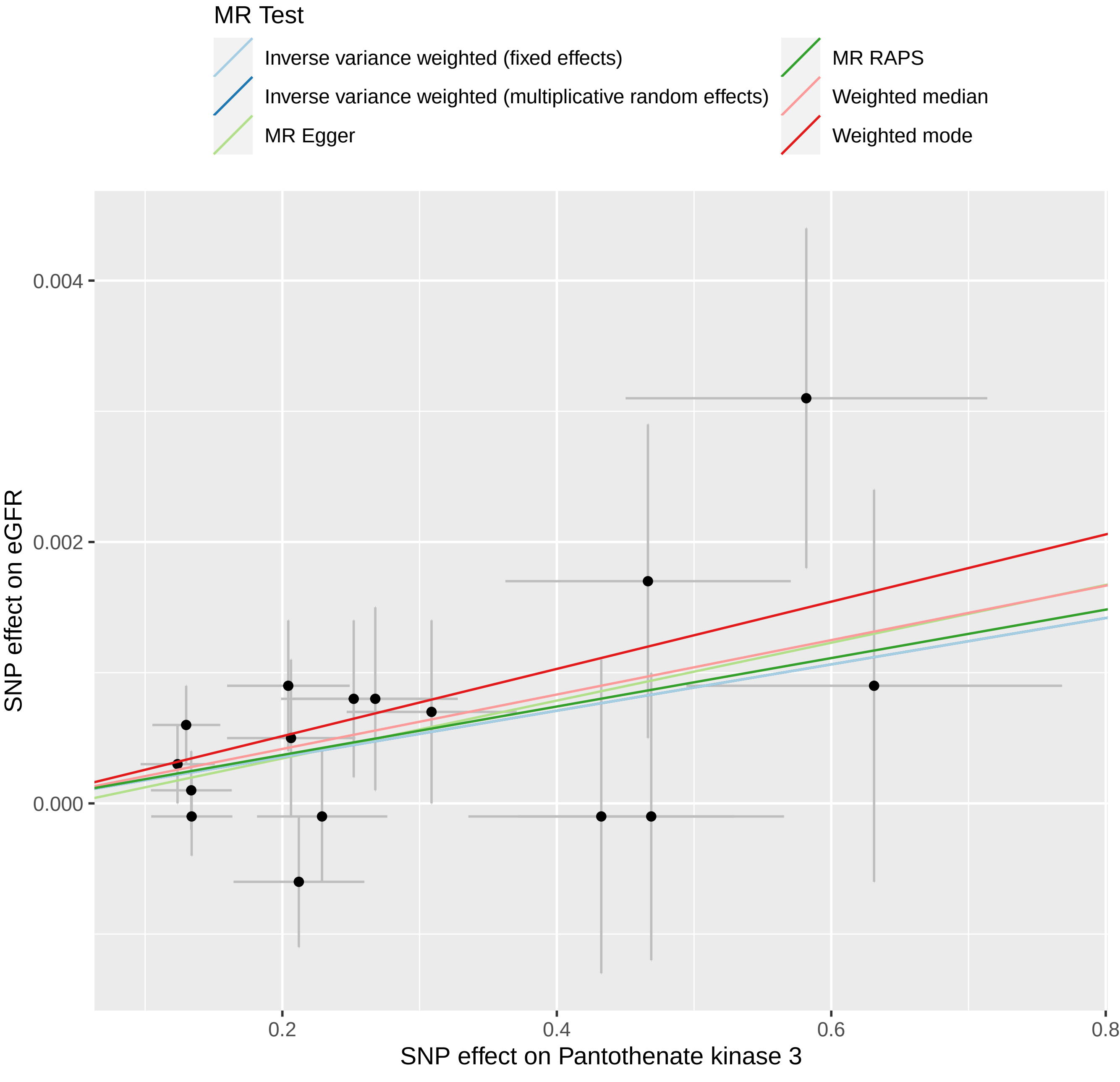

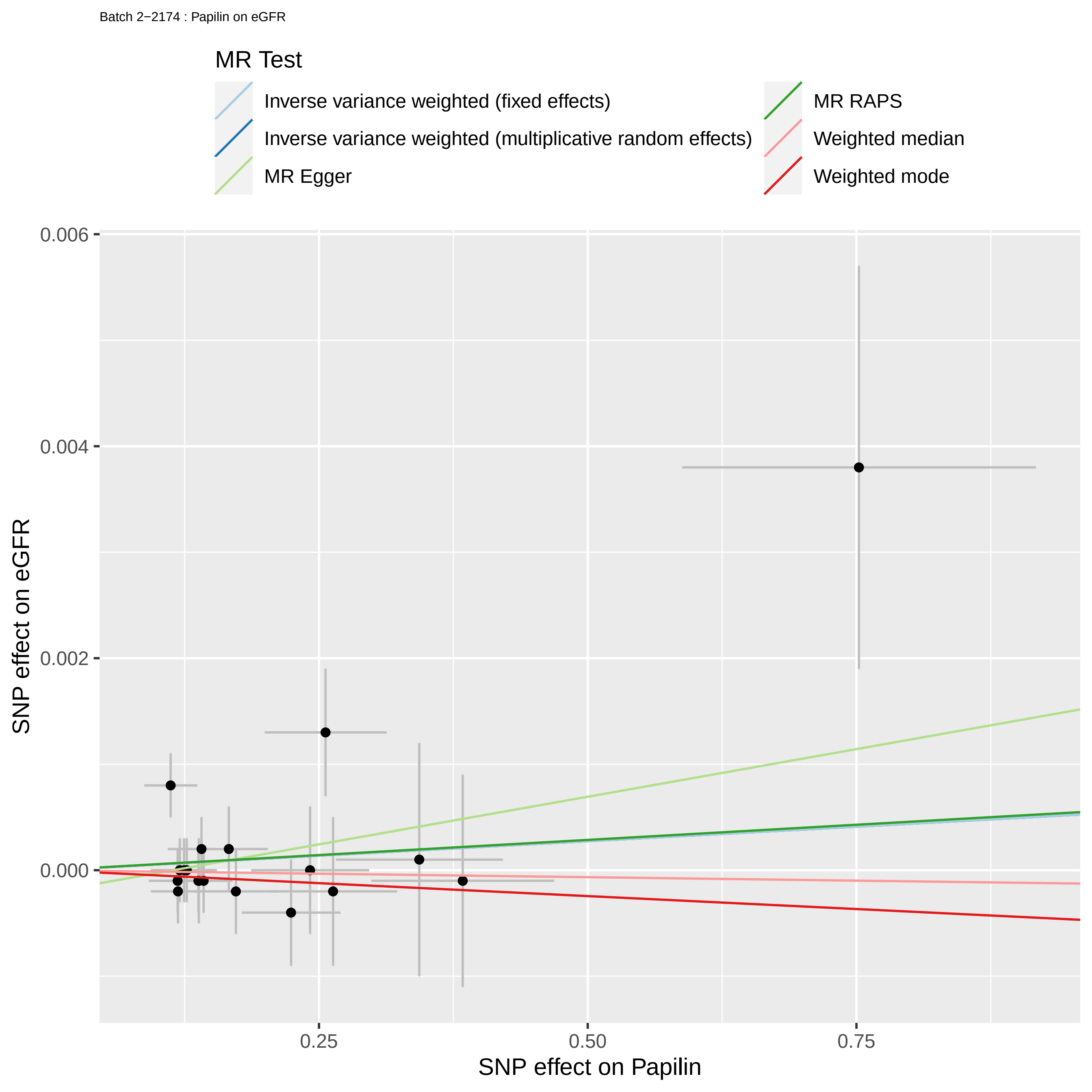

Batch 2−2174 : Papilin on eGFR
MR Test
Inverse variance weighted (fixed effects)
Inverse variance weighted (multiplicative random effects)
MR Egger
MR RAPS
Weighted median
Weighted mode
0.006
0.004
0.002
0.000
0.25
0.50
0.75
SNP effect on eGFR
SNP effect on Papilin

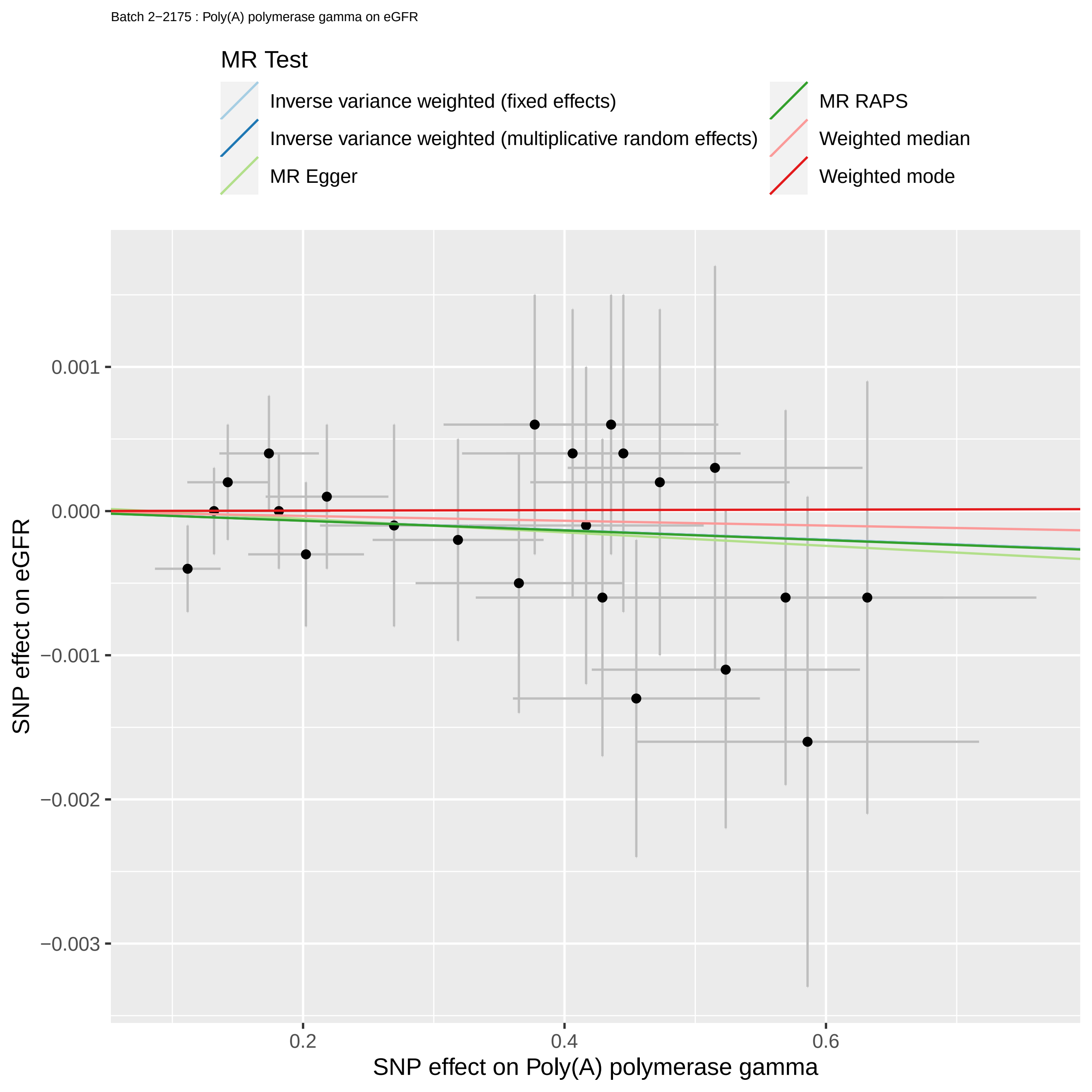

Batch 2−2175 : Poly(A) polymerase gamma on eGFR
MR Test
Inverse variance weighted (fixed effects)
Inverse variance weighted (multiplicative random effects)
MR Egger
MR RAPS
Weighted median
Weighted mode
0.001
0.000
−0.001
−0.002
−0.003
SNP effect on eGFR
0.2
0.4
0.6
SNP effect on Poly(A) polymerase gamma

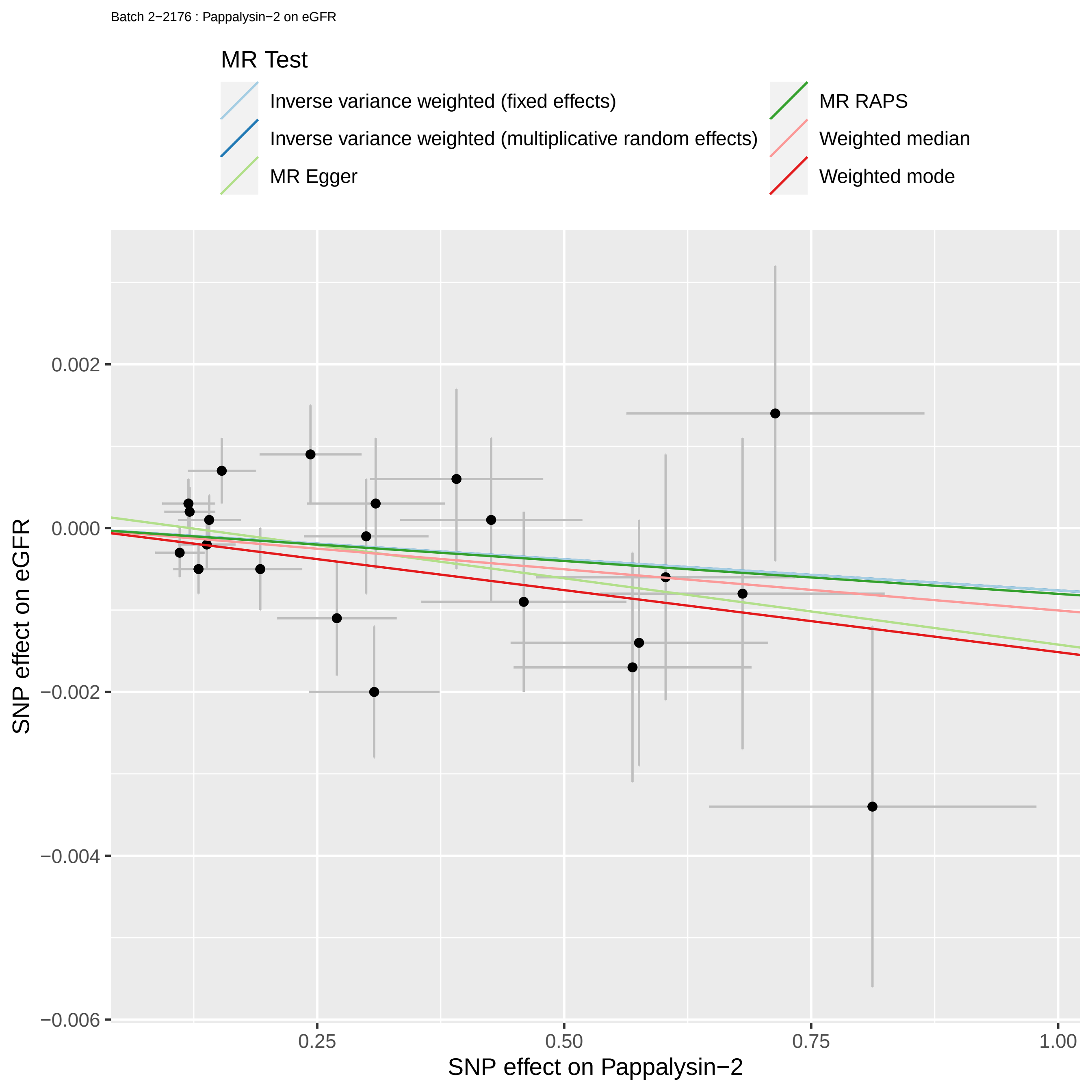
Batch 2−2176 : Pappalysin−2 on eGFR
MR Test
Inverse variance weighted (fixed effects)
Inverse variance weighted (multiplicative random effects)
MR Egger
MR RAPS
Weighted median
Weighted mode
0.002
0.000
−0.002
−0.004
−0.006
0.25
0.50
0.75
1.00
SNP effect on eGFR
SNP effect on Pappalysin−2

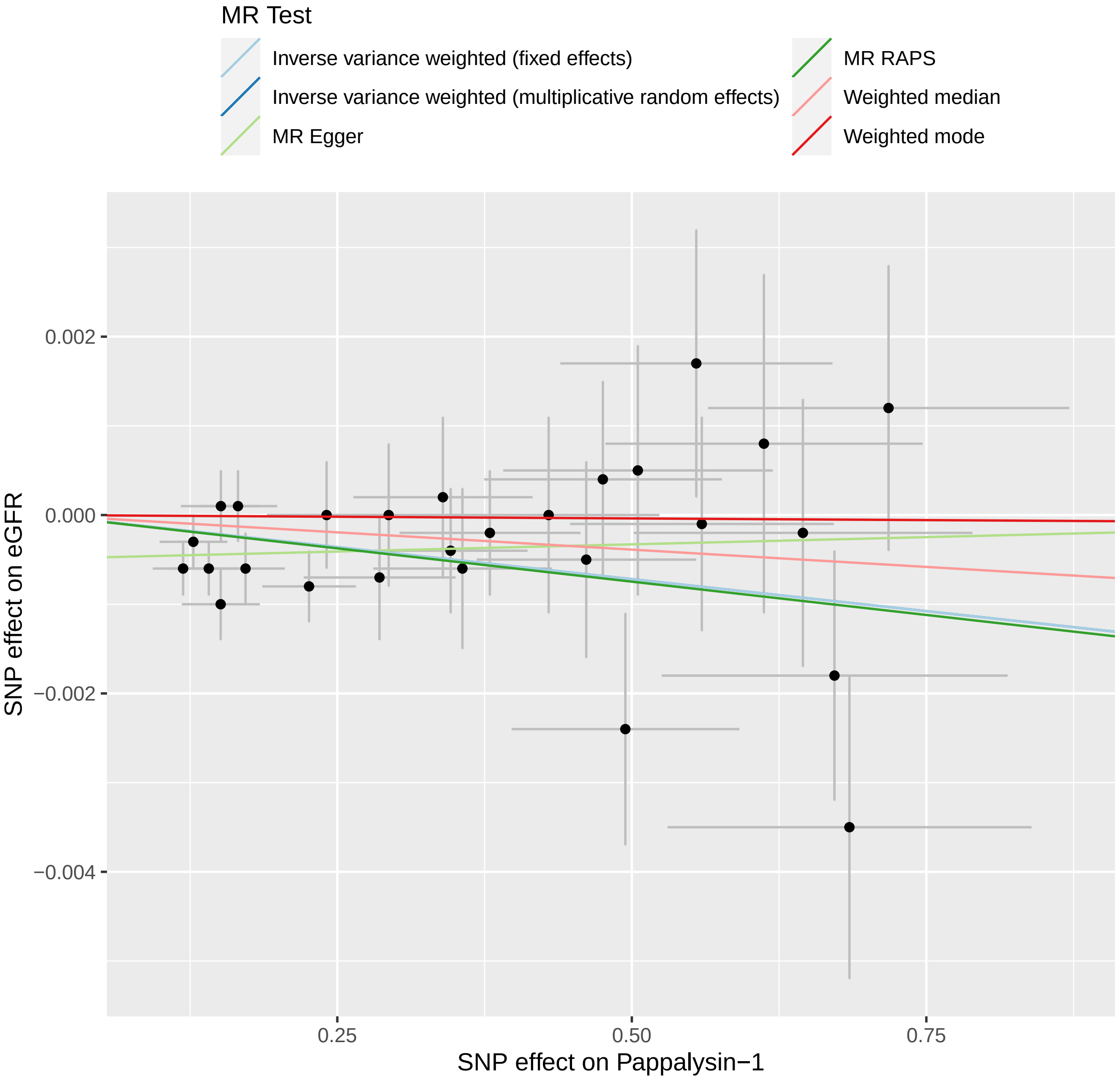
MR Test
Inverse variance weighted (fixed effects)
Inverse variance weighted (multiplicative random effects)
MR Egger
MR RAPS
Weighted median
Weighted mode
0.002
0.000
−0.002
−0.004
SNP effect on eGFR
0.25
0.50
0.75
SNP effect on Pappalysin−1

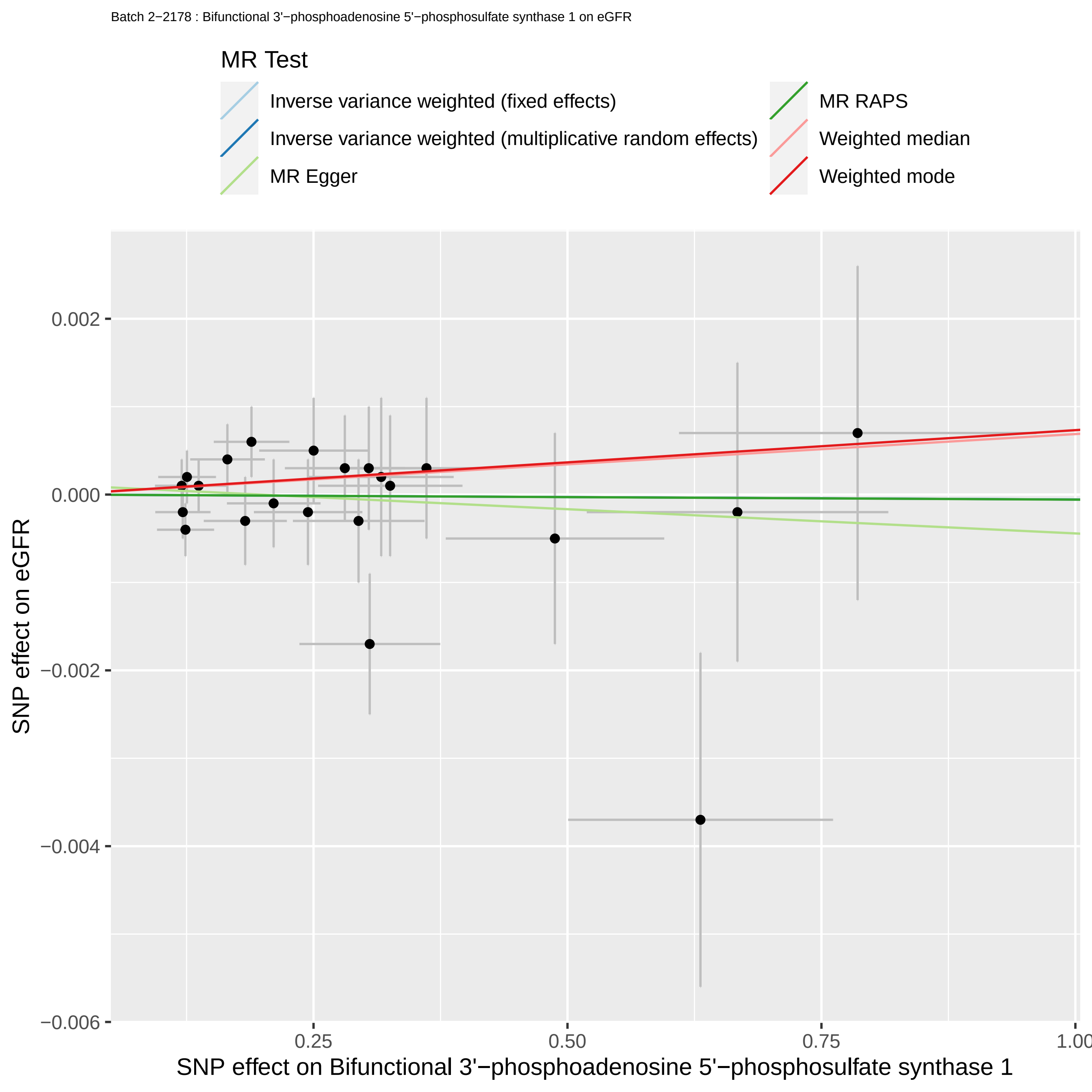

Batch 2−2178 : Bifunctional 3'−phosphoadenosine 5'−phosphosulfate synthase 1 on eGFR
MR Test
Inverse variance weighted (fixed effects)
Inverse variance weighted (multiplicative random effects)
MR Egger
MR RAPS
Weighted median
Weighted mode
0.002
0.000
−0.002
−0.004
−0.006
0.25
0.50
0.75
1.00
SNP effect on eGFR
SNP effect on Bifunctional 3'−phosphoadenosine 5'−phosphosulfate synthase 1

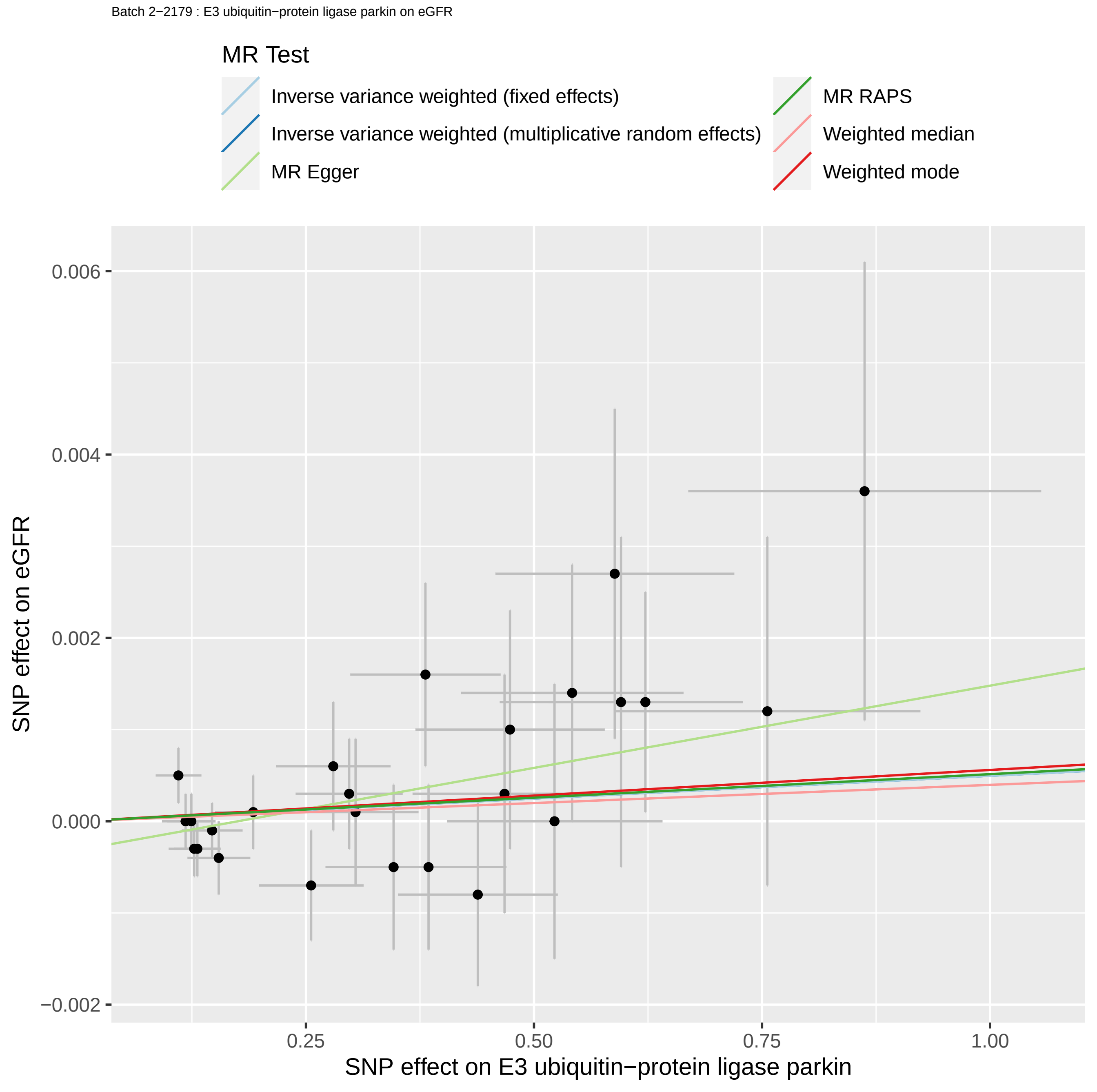

Batch 2−2179 : E3 ubiquitin−protein ligase parkin on eGFR
MR Test
Inverse variance weighted (fixed effects)
Inverse variance weighted (multiplicative random effects)
MR Egger
MR RAPS
Weighted median
Weighted mode
0.006
0.004
0.002
0.000
−0.002
0.25
0.50
0.75
1.00
SNP effect on eGFR
SNP effect on E3 ubiquitin−protein ligase parkin

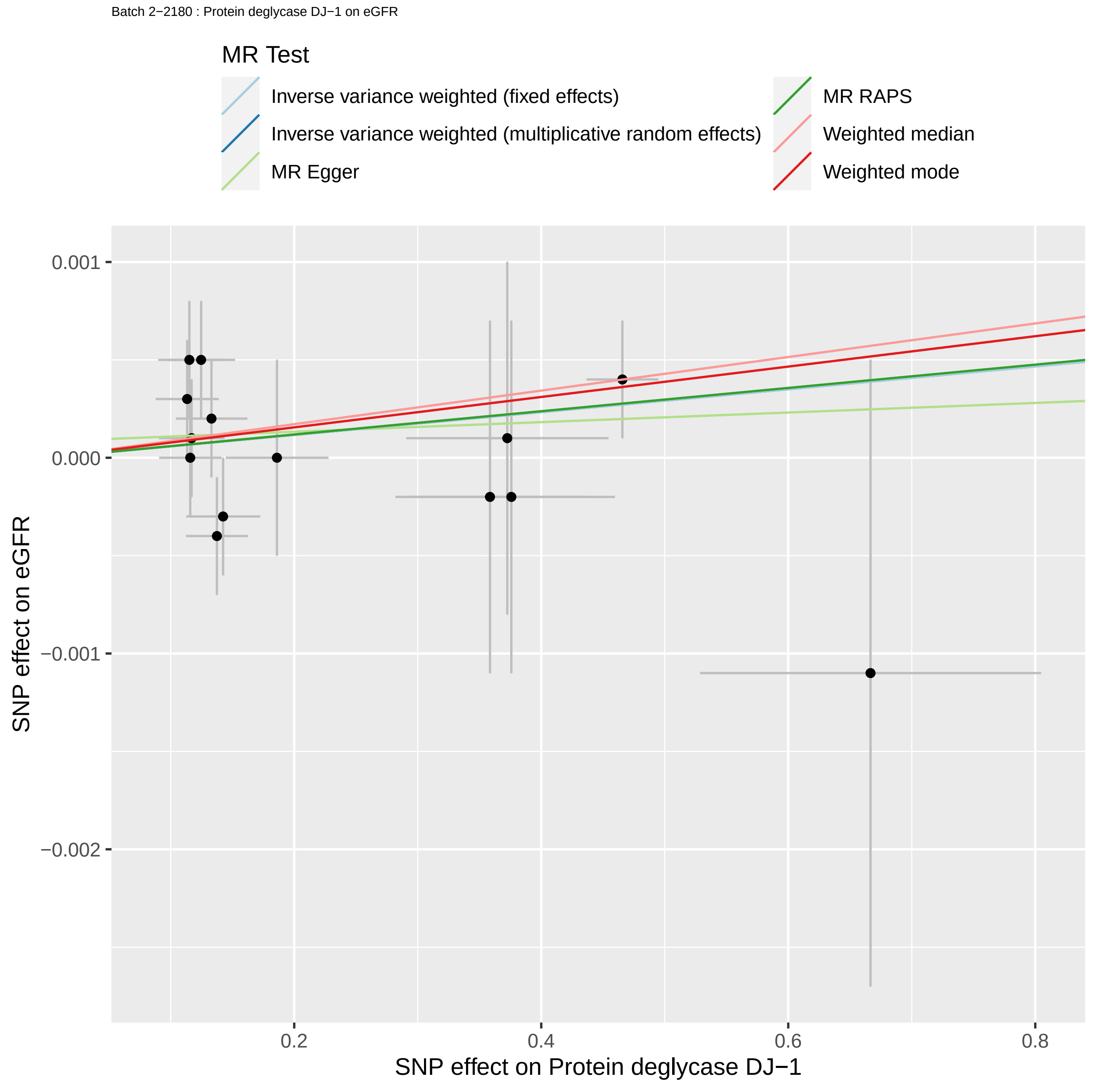
Batch 2−2180 : Protein deglycase DJ−1 on eGFR
MR Test
Inverse variance weighted (fixed effects)
Inverse variance weighted (multiplicative random effects)
MR Egger
MR RAPS
Weighted median
Weighted mode
0.001
0.000
−0.001
−0.002
SNP effect on eGFR
0.2
0.4
0.6
0.8
SNP effect on Protein deglycase DJ−1

Batch 2−2181 : Protein deglycase DJ−1 on eGFR

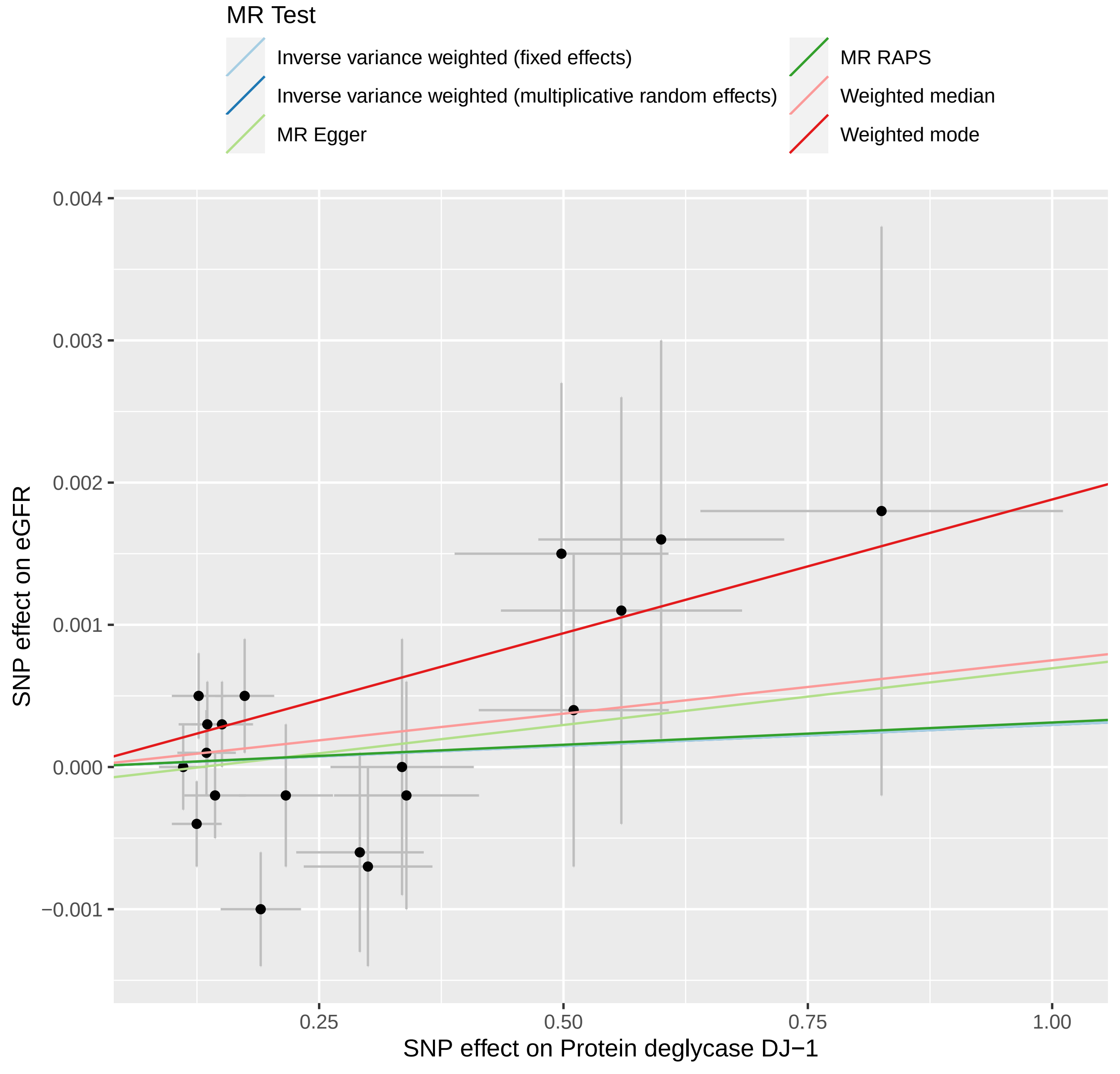

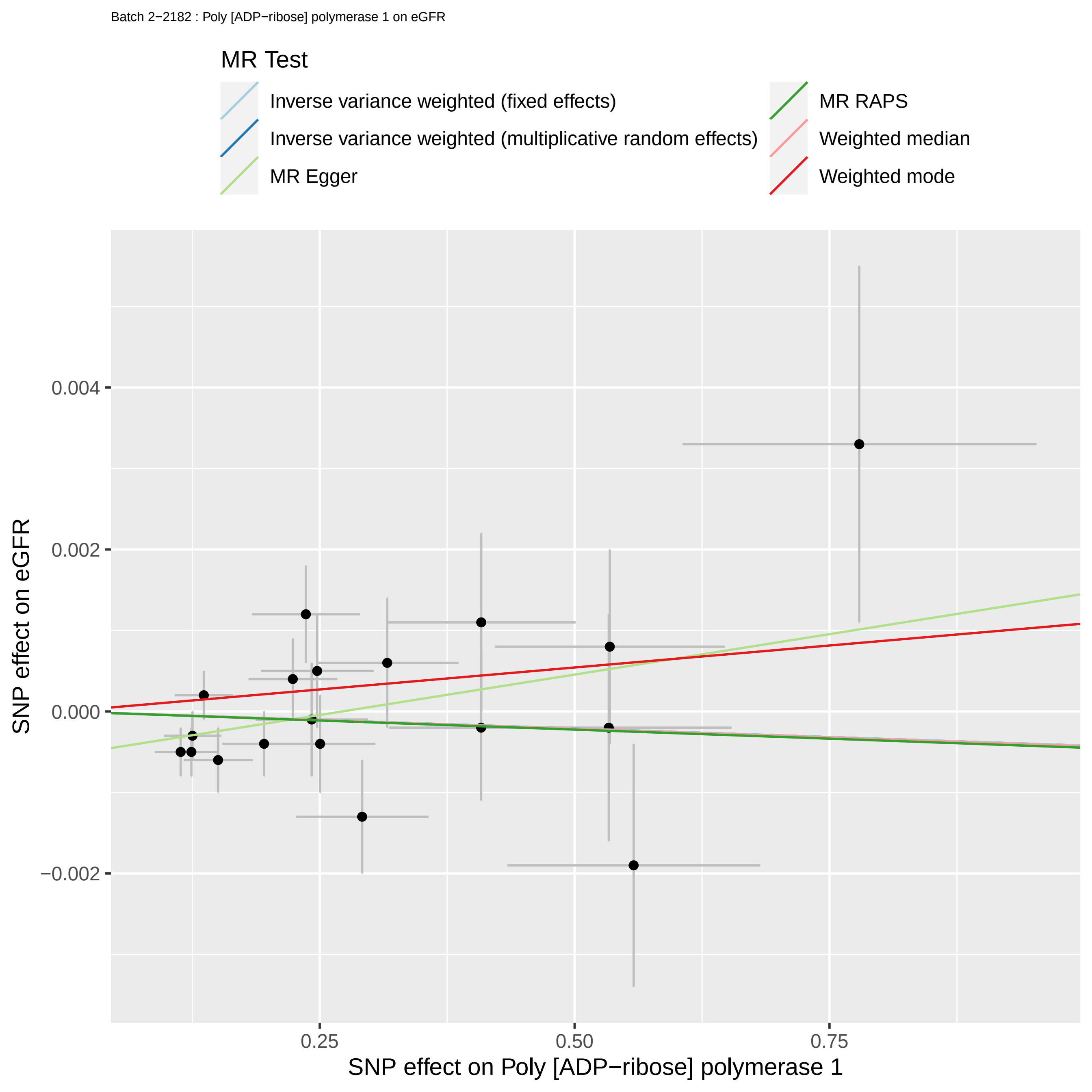
Batch 2−2182 : Poly [ADP−ribose] polymerase 1 on eGFR
MR Test
Inverse variance weighted (fixed effects)
Inverse variance weighted (multiplicative random effects)
MR Egger
MR RAPS
Weighted median
Weighted mode
0.004
0.002
0.000
−0.002
0.25
0.50
0.75
SNP effect on eGFR
SNP effect on Poly [ADP−ribose] polymerase 1

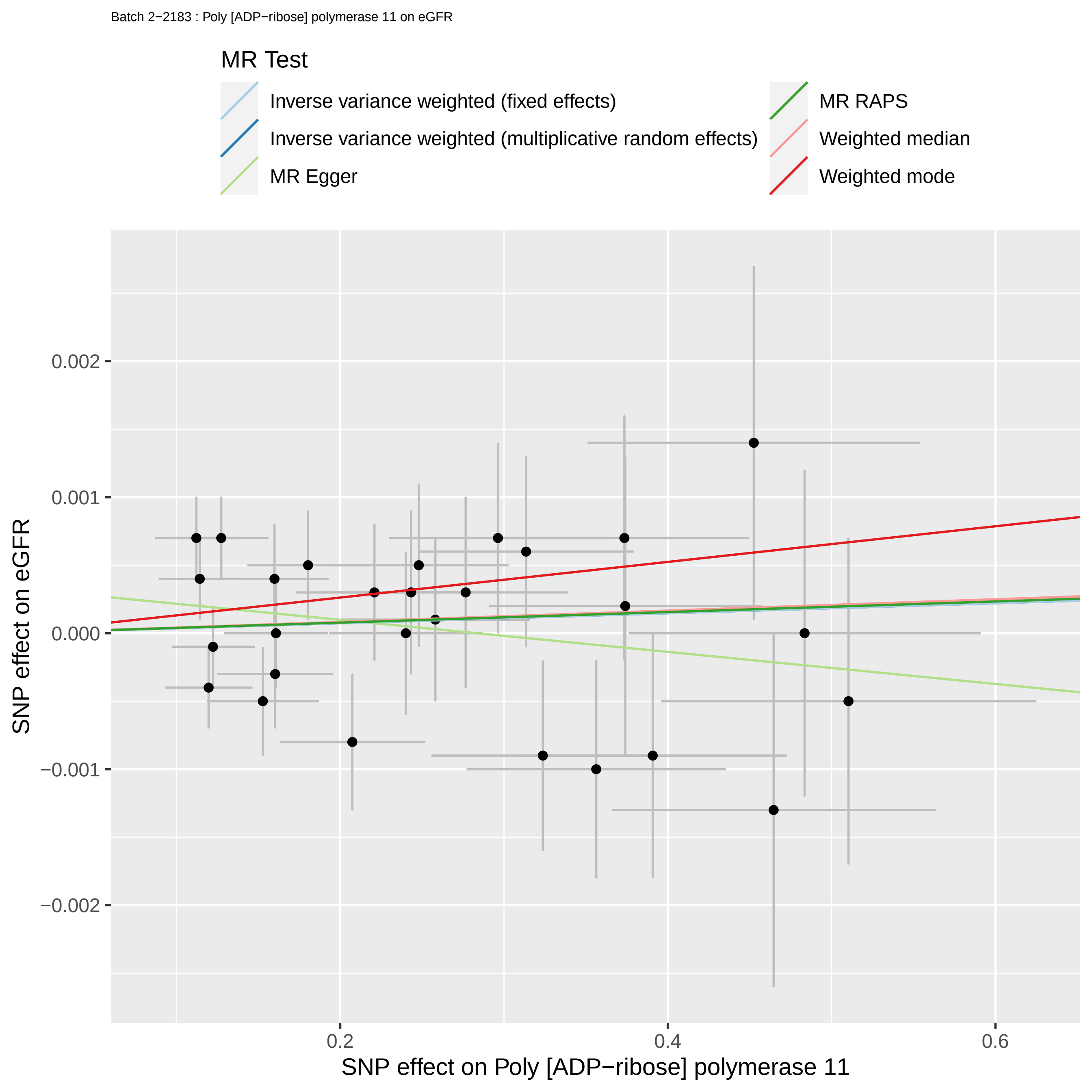

Batch 2−2183 : Poly [ADP−ribose] polymerase 11 on eGFR
MR Test
Inverse variance weighted (fixed effects)
Inverse variance weighted (multiplicative random effects)
MR Egger
MR RAPS
Weighted median
Weighted mode
0.002
0.001
0.000
−0.001
−0.002
0.2
0.4
0.6
SNP effect on eGFR
SNP effect on Poly [ADP−ribose] polymerase 11

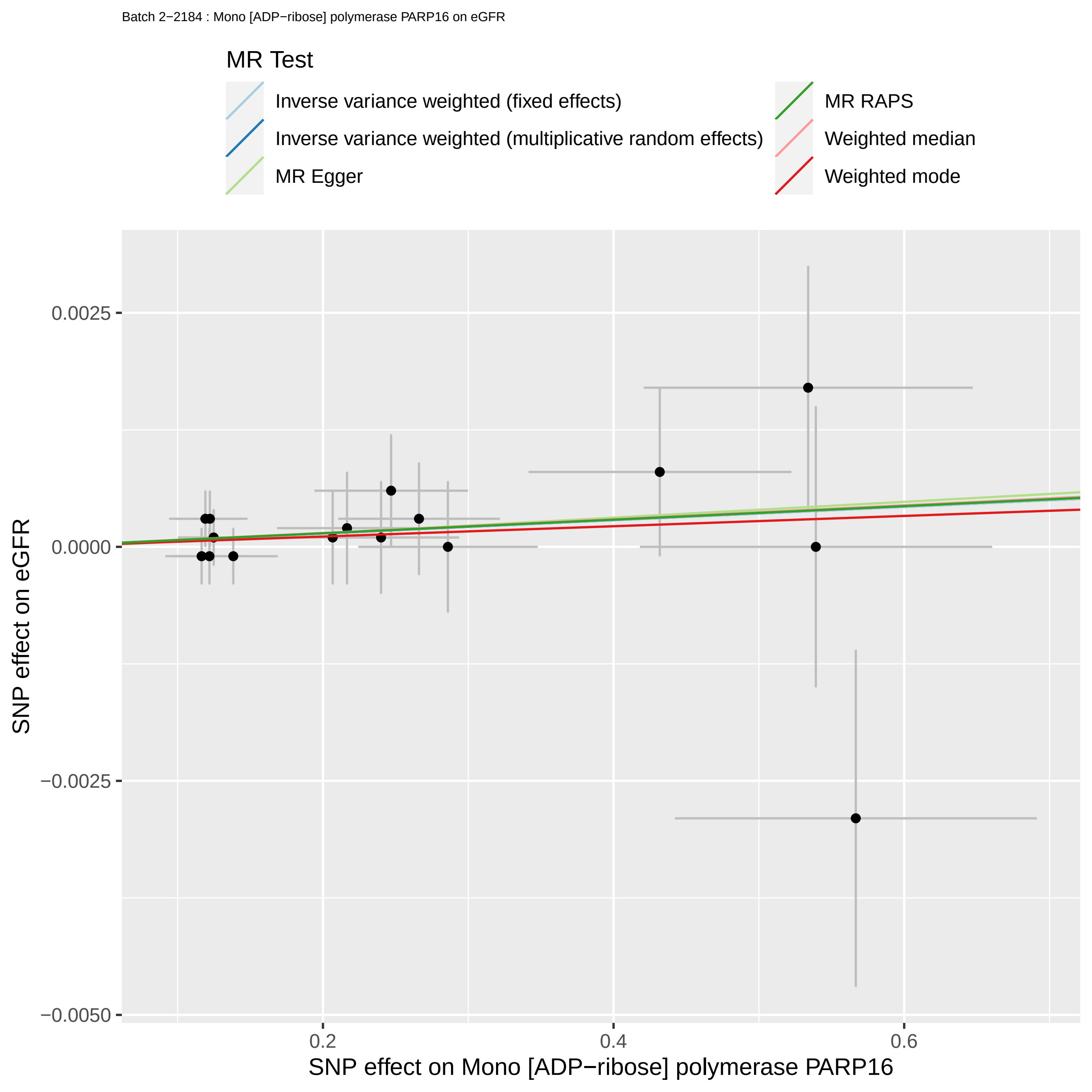

Batch 2−2184 : Mono [ADP−ribose] polymerase PARP16 on eGFR
MR Test
Inverse variance weighted (fixed effects)
Inverse variance weighted (multiplicative random effects)
MR Egger
MR RAPS
Weighted median
Weighted mode
0.0025
0.0000
−0.0025
−0.0050
0.2
0.4
0.6
SNP effect on eGFR
SNP effect on Mono [ADP−ribose] polymerase PARP16

Batch 2−2185 : Alpha−parvin on eGFR

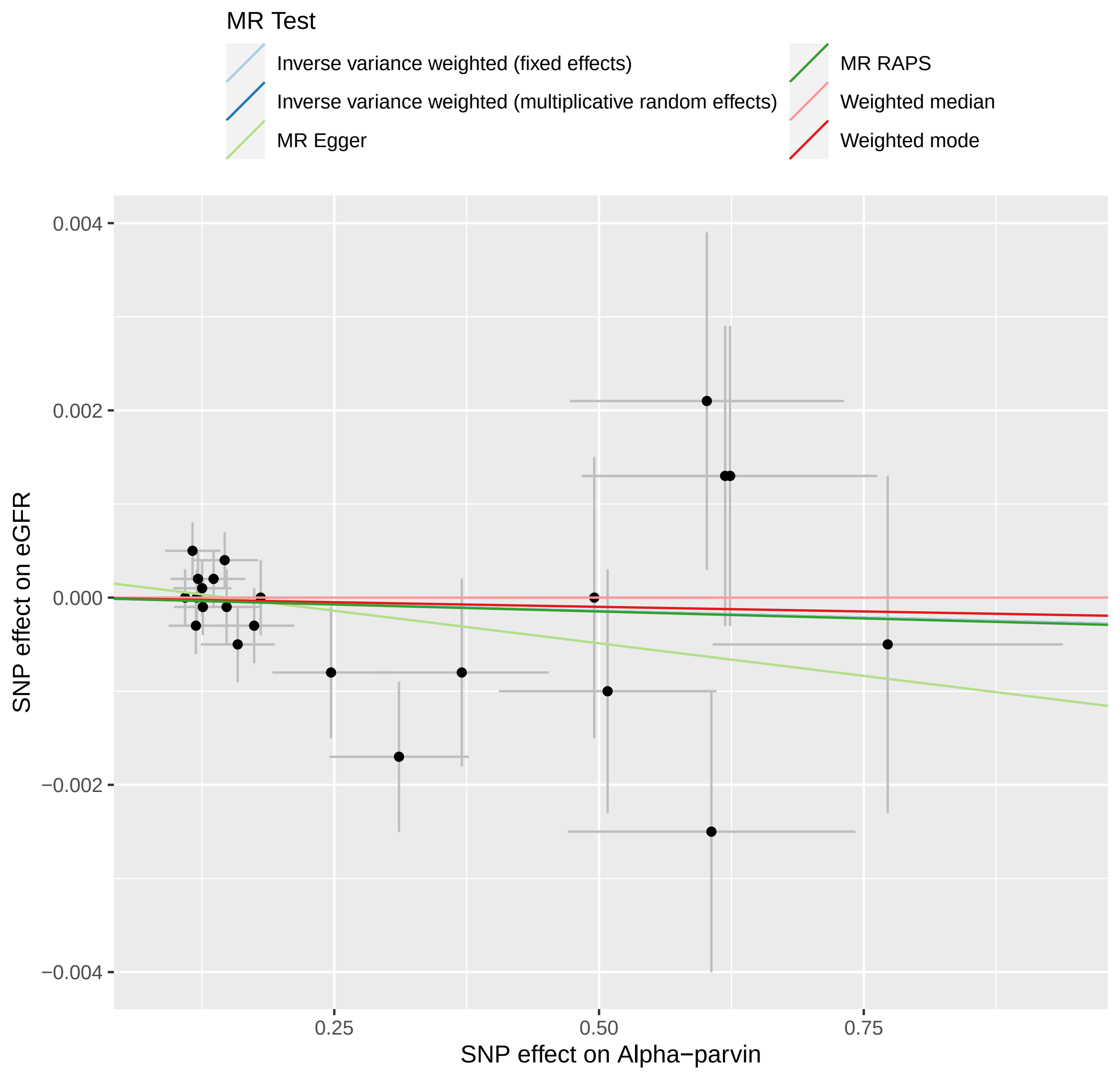

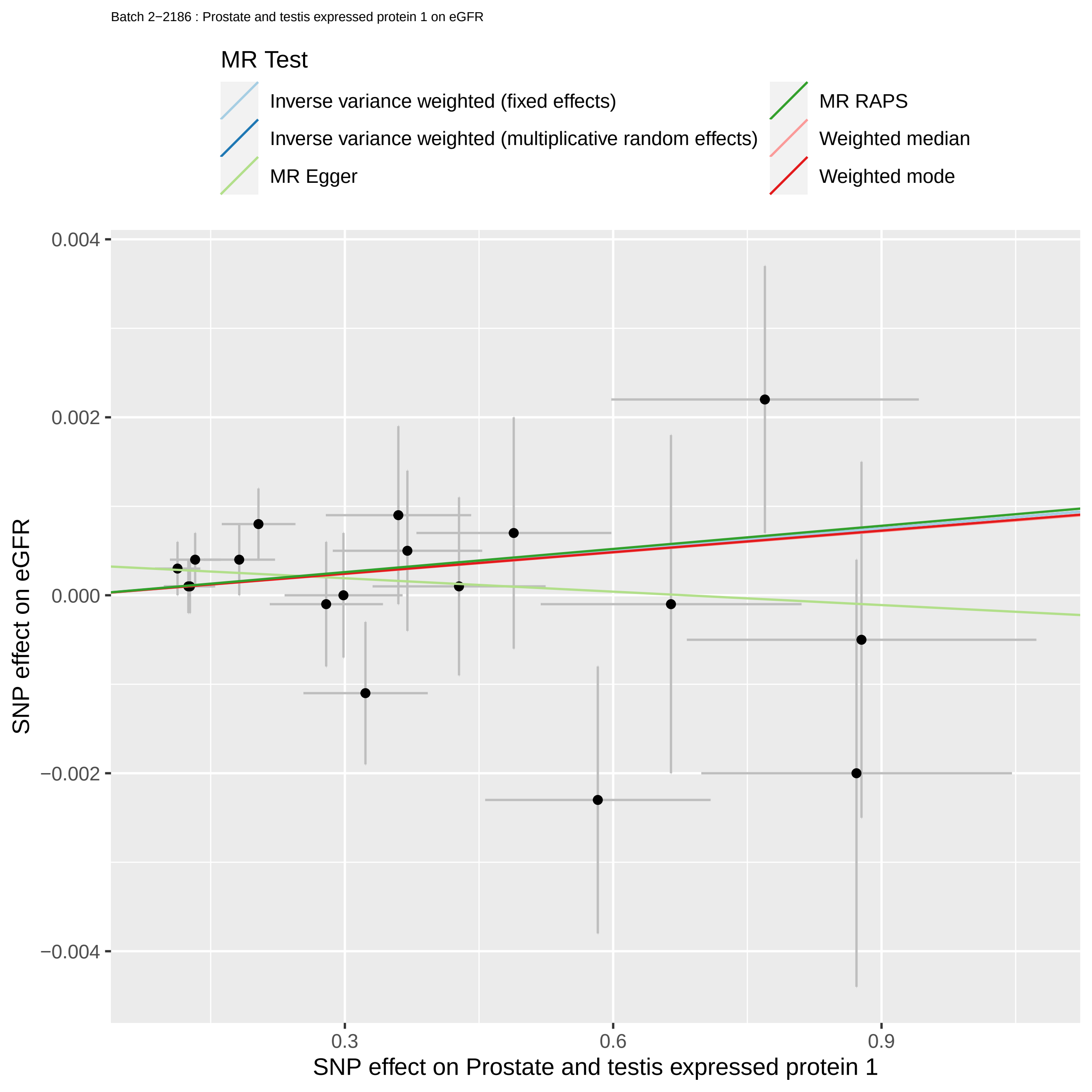

Batch 2−2186 : Prostate and testis expressed protein 1 on eGFR
MR Test
Inverse variance weighted (fixed effects)
Inverse variance weighted (multiplicative random effects)
MR Egger
MR RAPS
Weighted median
Weighted mode
0.004
0.002
0.000
−0.002
−0.004
0.3
0.6
0.9
SNP effect on eGFR
SNP effect on Prostate and testis expressed protein 1

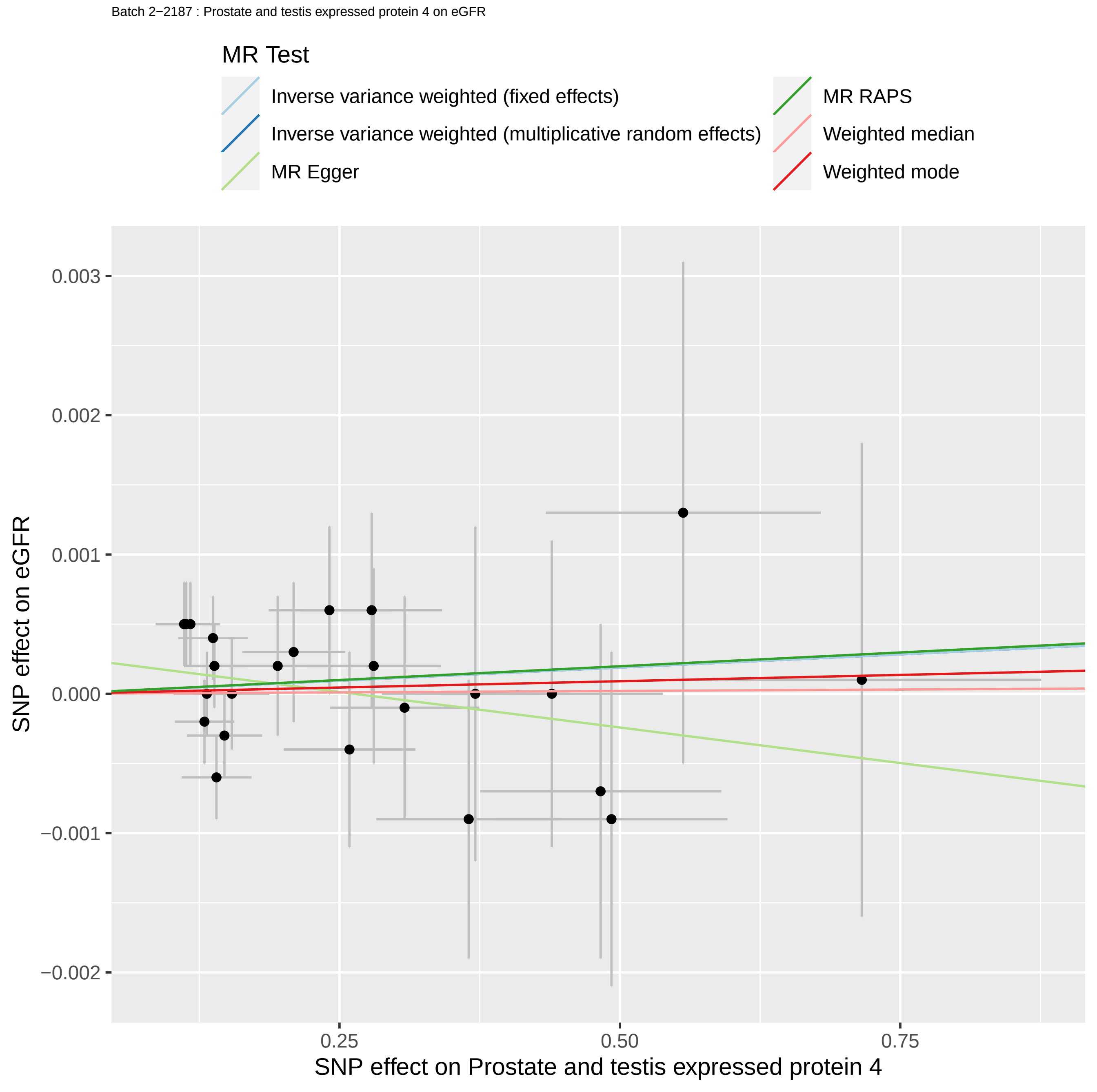

Batch 2−2187 : Prostate and testis expressed protein 4 on eGFR
MR Test
Inverse variance weighted (fixed effects)
Inverse variance weighted (multiplicative random effects)
MR Egger
MR RAPS
Weighted median
Weighted mode
0.003
0.002
0.001
0.000
−0.001
−0.002
SNP effect on eGFR
0.25
0.50
0.75
SNP effect on Prostate and testis expressed protein 4

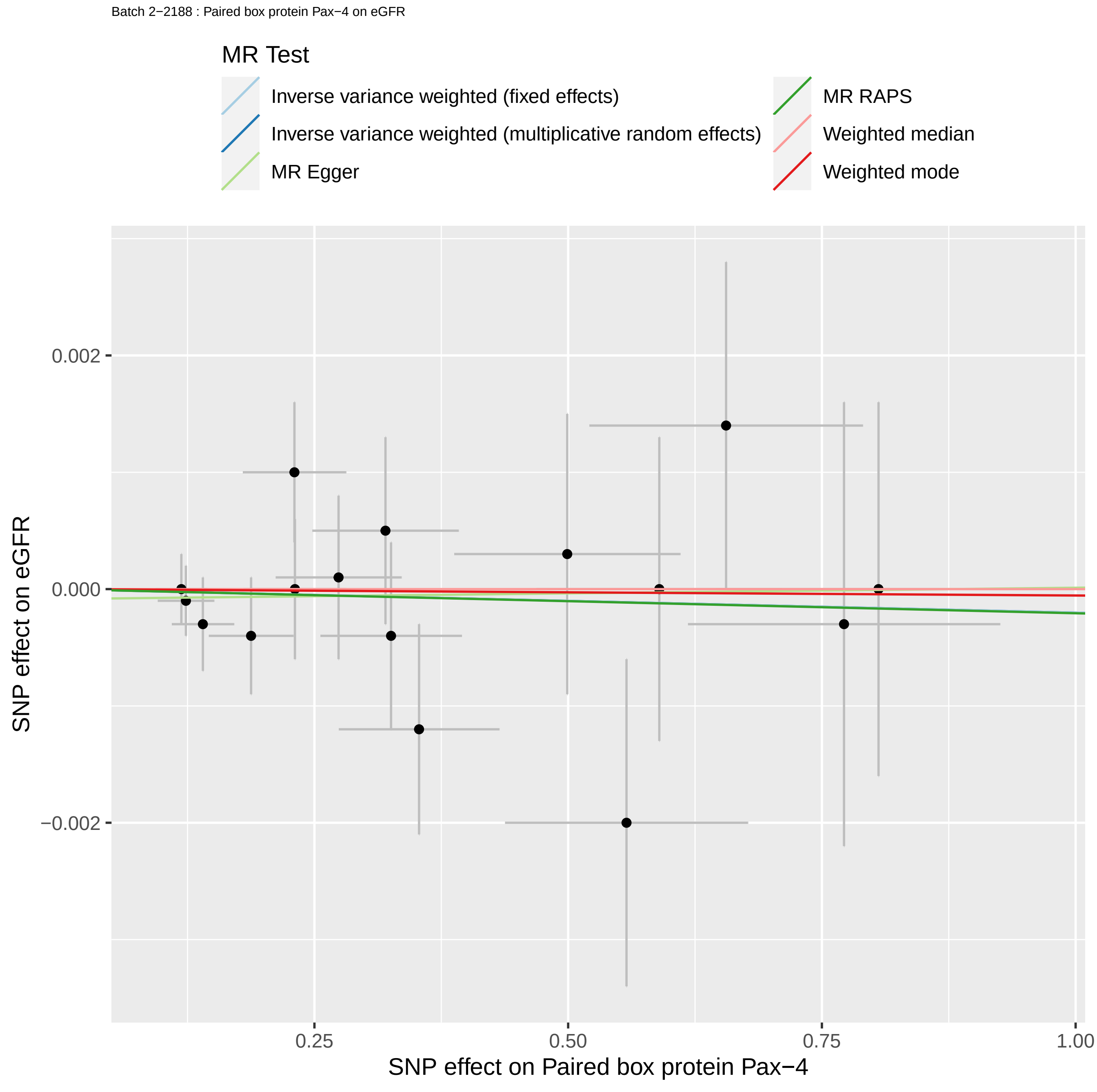
Batch 2−2188 : Paired box protein Pax−4 on eGFR
MR Test
Inverse variance weighted (fixed effects)
Inverse variance weighted (multiplicative random effects)
MR Egger
MR RAPS
Weighted median
Weighted mode
0.002
0.000
−0.002
0.25
0.50
0.75
1.00
SNP effect on eGFR
SNP effect on Paired box protein Pax−4

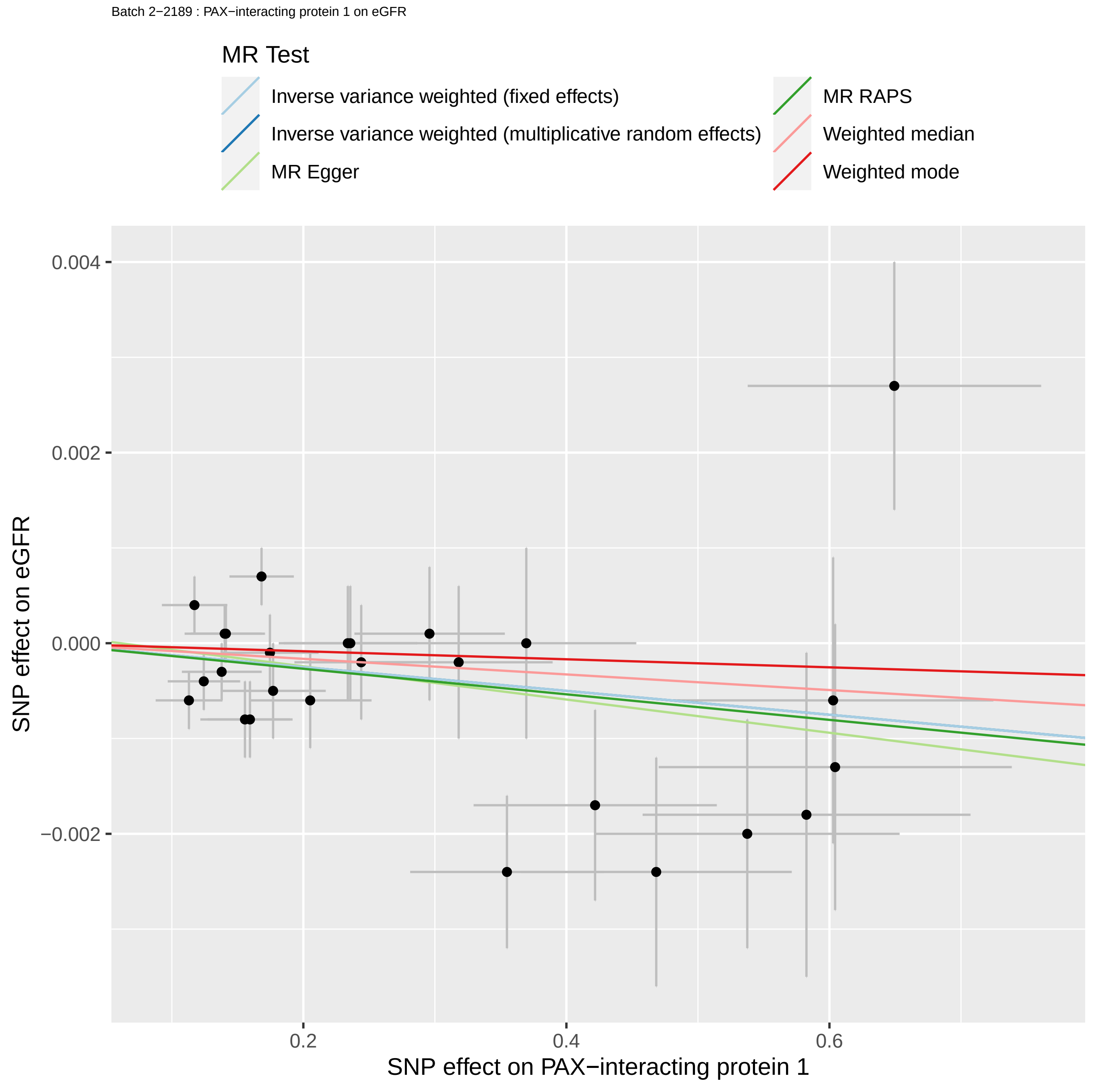
Batch 2−2189 : PAX−interacting protein 1 on eGFR
MR Test
Inverse variance weighted (fixed effects)
Inverse variance weighted (multiplicative random effects)
MR Egger
MR RAPS
Weighted median
Weighted mode
0.004
0.002
0.000
−0.002
0.2
0.4
0.6
SNP effect on eGFR
SNP effect on PAX−interacting protein 1

Batch 2−2190 : Pyruvate carboxylase, mitochondrial on eGFR

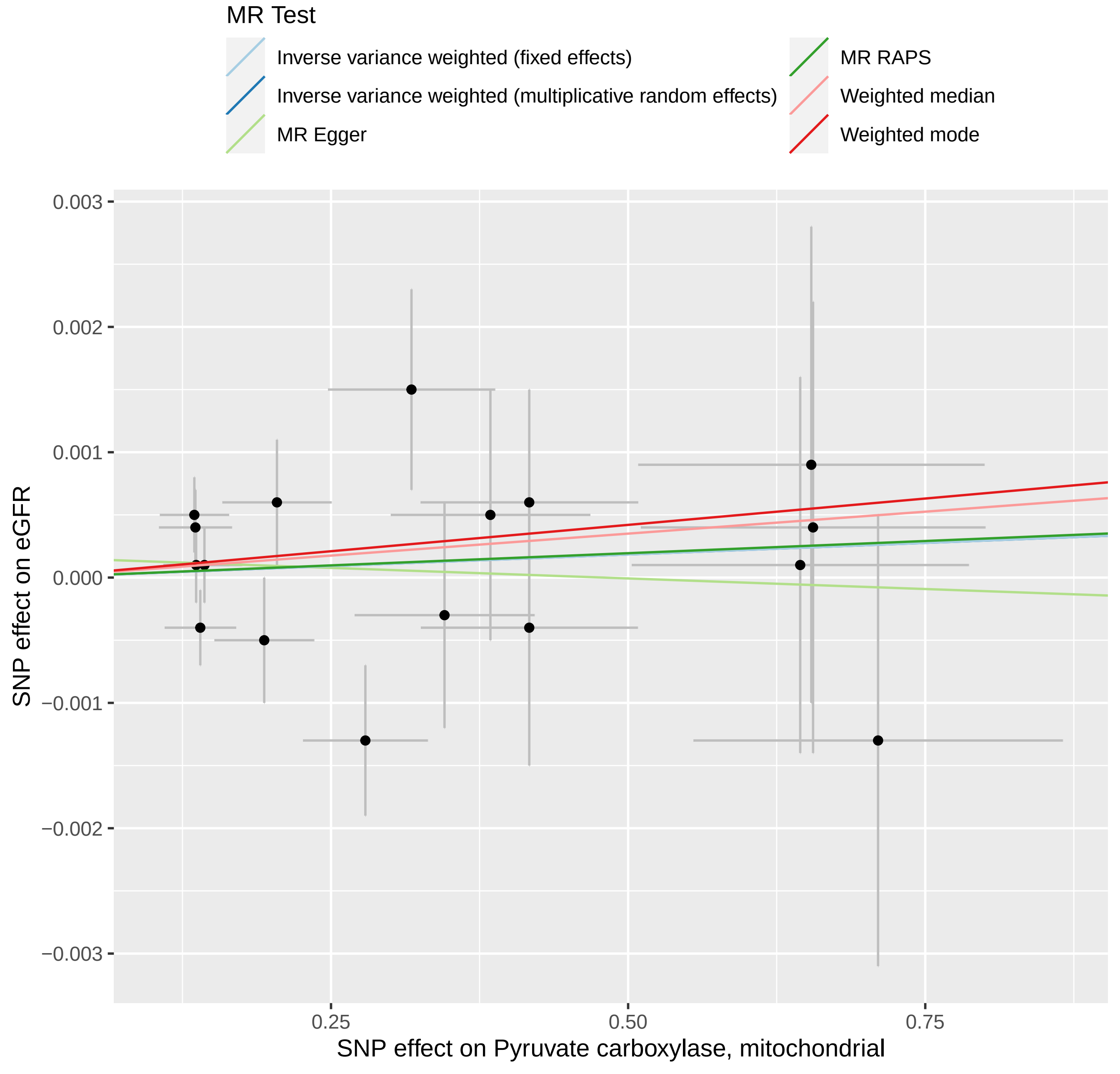

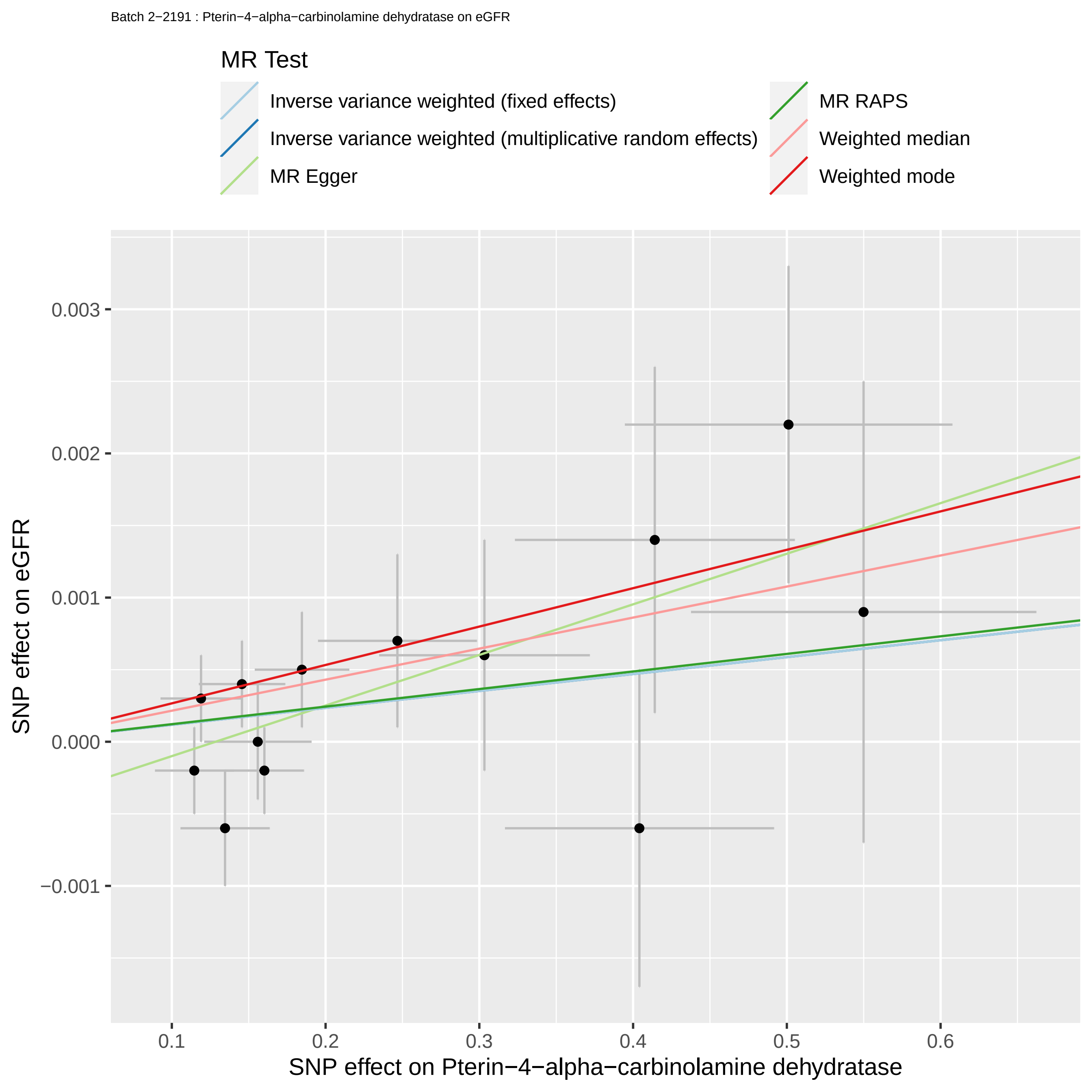

Batch 2−2191 : Pterin−4−alpha−carbinolamine dehydratase on eGFR
MR Test
Inverse variance weighted (fixed effects)
Inverse variance weighted (multiplicative random effects)
MR Egger
MR RAPS
Weighted median
Weighted mode
0.003
0.002
0.001
0.000
−0.001
0.1
0.2
0.3
0.4
0.5
0.6
SNP effect on eGFR
SNP effect on Pterin−4−alpha−carbinolamine dehydratase

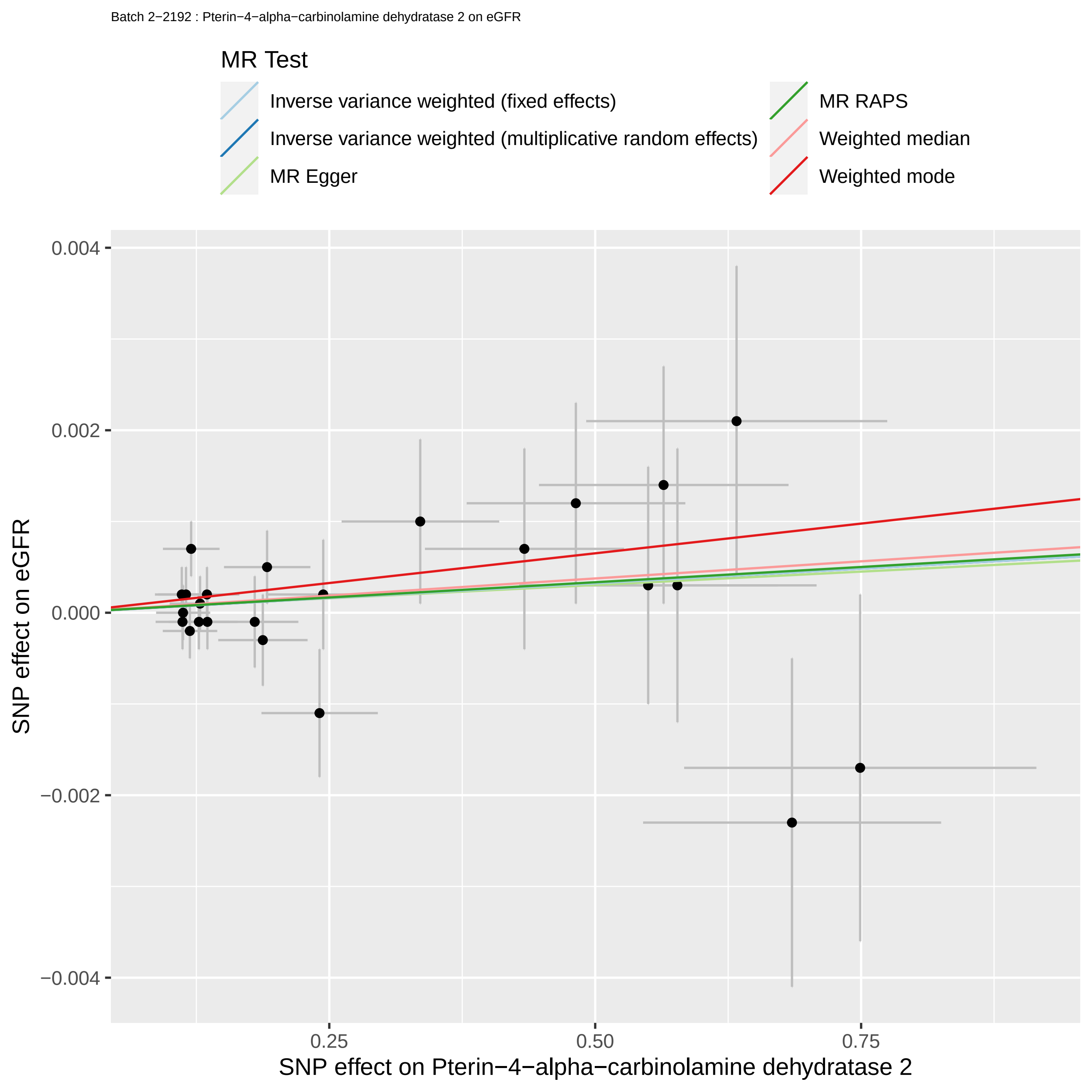
Batch 2−2192 : Pterin−4−alpha−carbinolamine dehydratase 2 on eGFR
MR Test
Inverse variance weighted (fixed effects)
Inverse variance weighted (multiplicative random effects)
MR Egger
MR RAPS
Weighted median
Weighted mode
0.004
0.002
0.000
−0.002
−0.004
0.25
0.50
0.75
SNP effect on eGFR
SNP effect on Pterin−4−alpha−carbinolamine dehydratase 2

Batch 2−2193 : Poly(rC)−binding protein 1 on eGFR

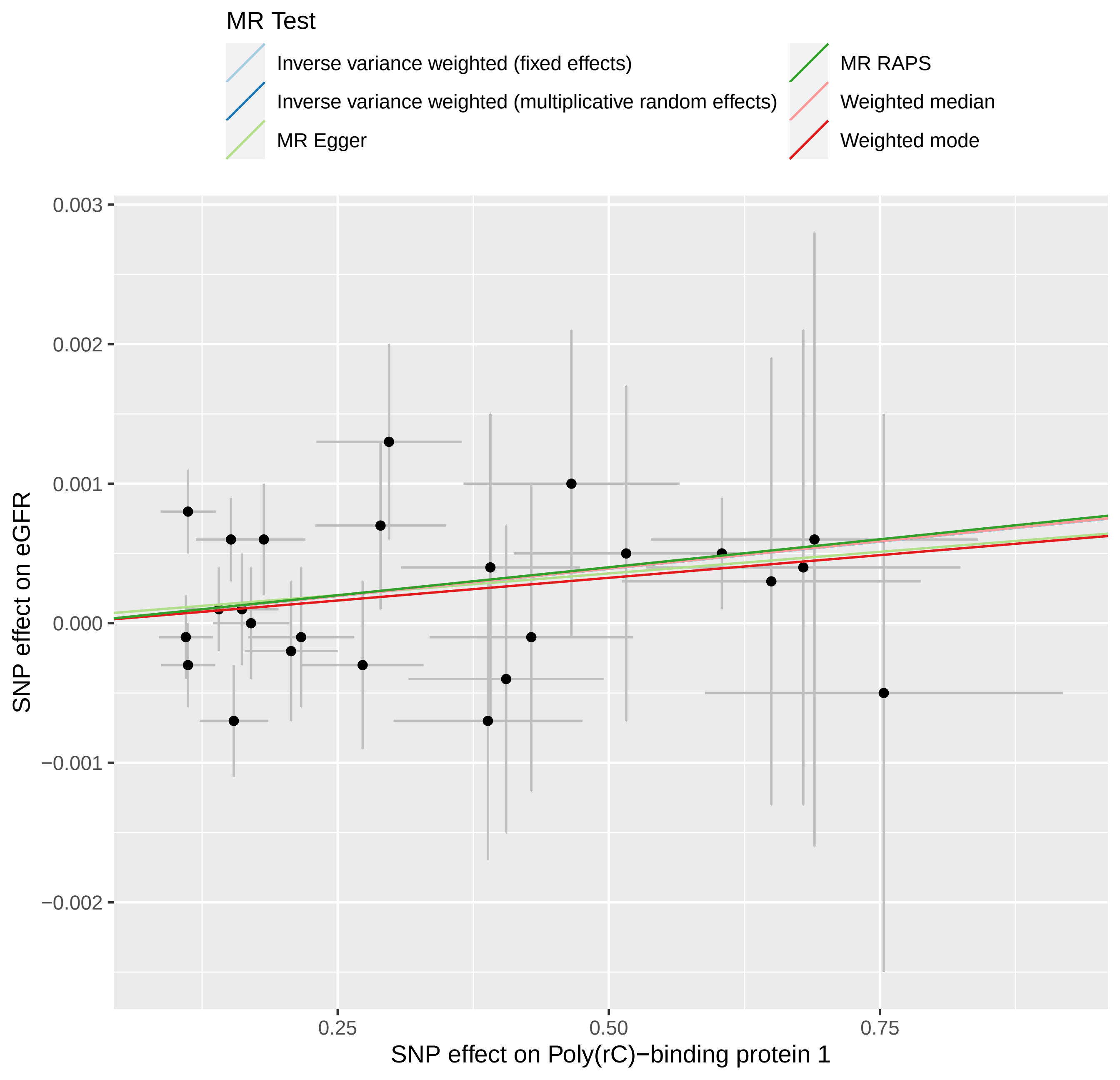

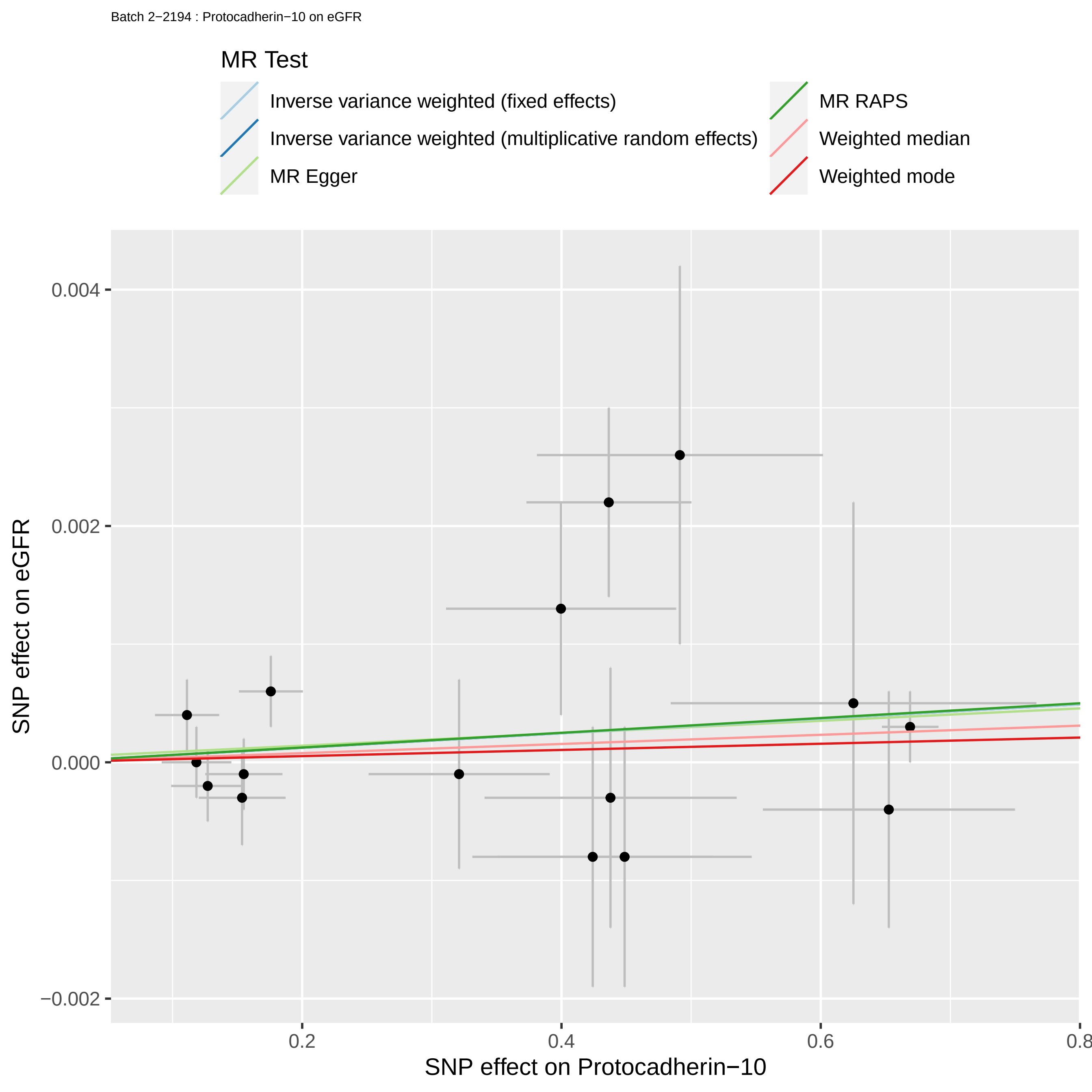
Batch 2−2194 : Protocadherin−10 on eGFR
MR Test
Inverse variance weighted (fixed effects)
Inverse variance weighted (multiplicative random effects)
MR Egger
MR RAPS
Weighted median
Weighted mode
0.004
0.002
0.000
−0.002
SNP effect on eGFR
0.2
0.4
0.6
0.8
SNP effect on Protocadherin−10

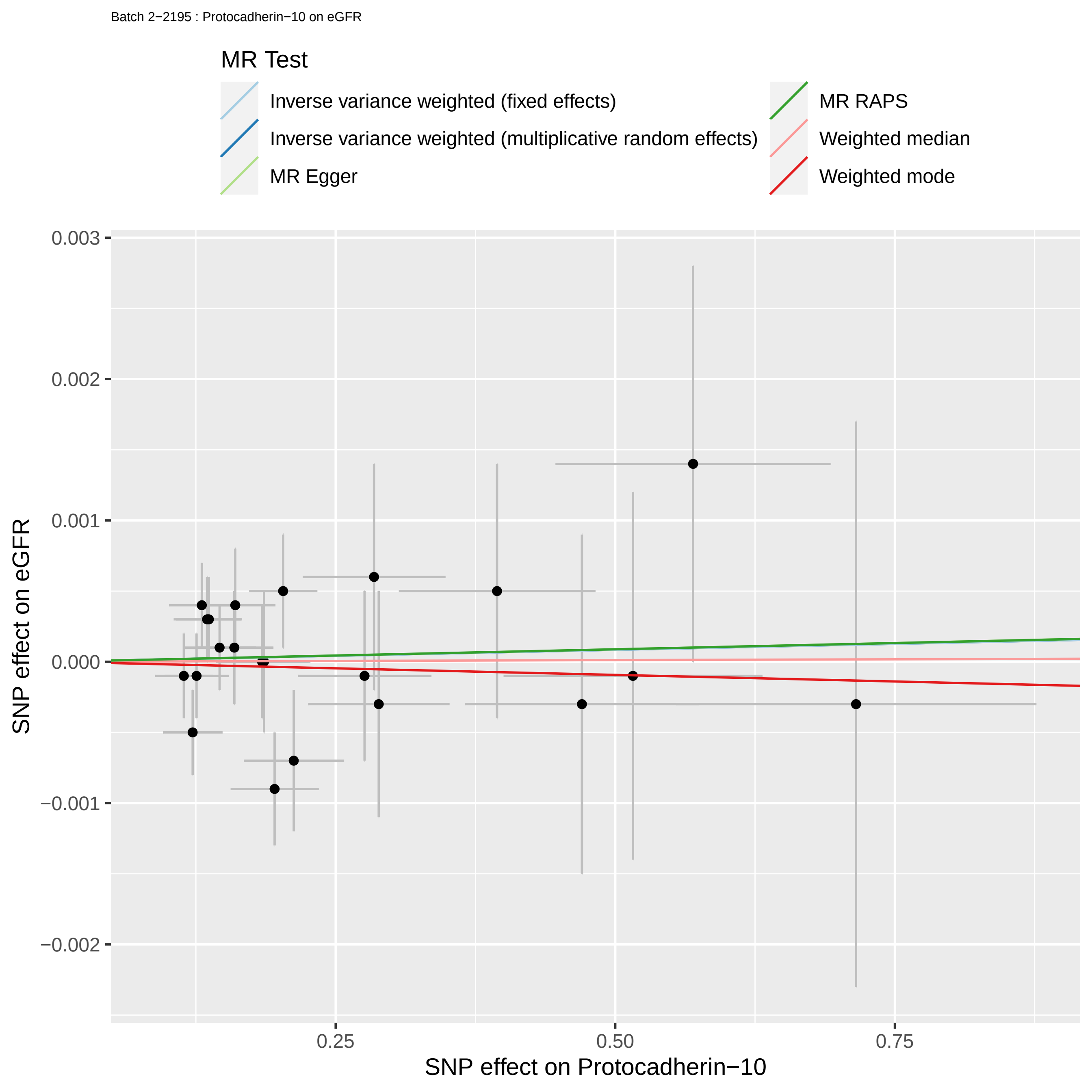

Batch 2−2195 : Protocadherin−10 on eGFR
MR Test
Inverse variance weighted (fixed effects)
Inverse variance weighted (multiplicative random effects)
MR Egger
MR RAPS
Weighted median
Weighted mode
0.003
0.002
0.001
0.000
−0.001
−0.002
SNP effect on eGFR
0.25
0.50
0.75
SNP effect on Protocadherin−10

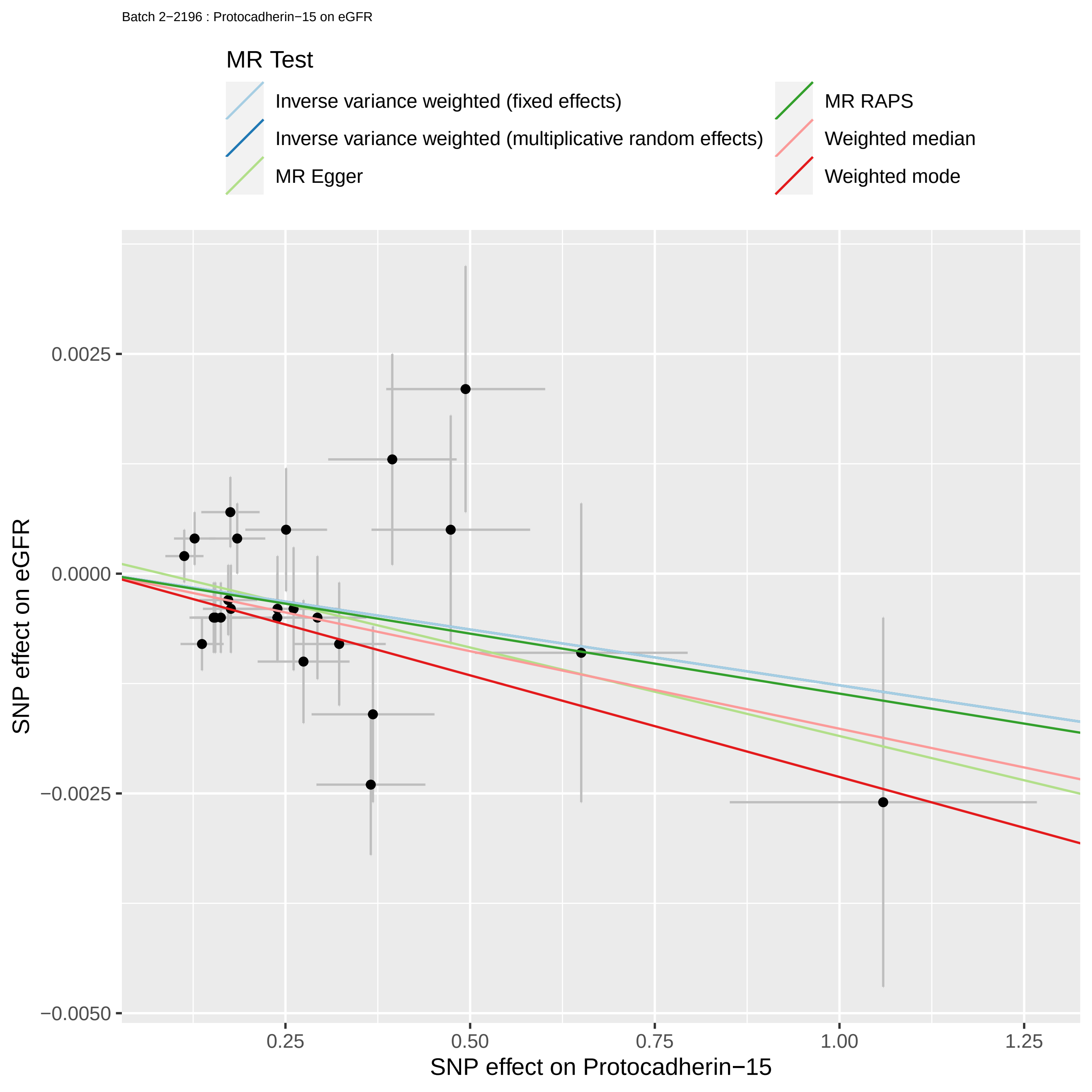
Batch 2−2196 : Protocadherin−15 on eGFR
MR Test
Inverse variance weighted (fixed effects)
Inverse variance weighted (multiplicative random effects)
MR Egger
MR RAPS
Weighted median
Weighted mode
0.0025
0.0000
−0.0025
−0.0050
0.25
0.50
0.75
1.00
1.25
SNP effect on eGFR
SNP effect on Protocadherin−15

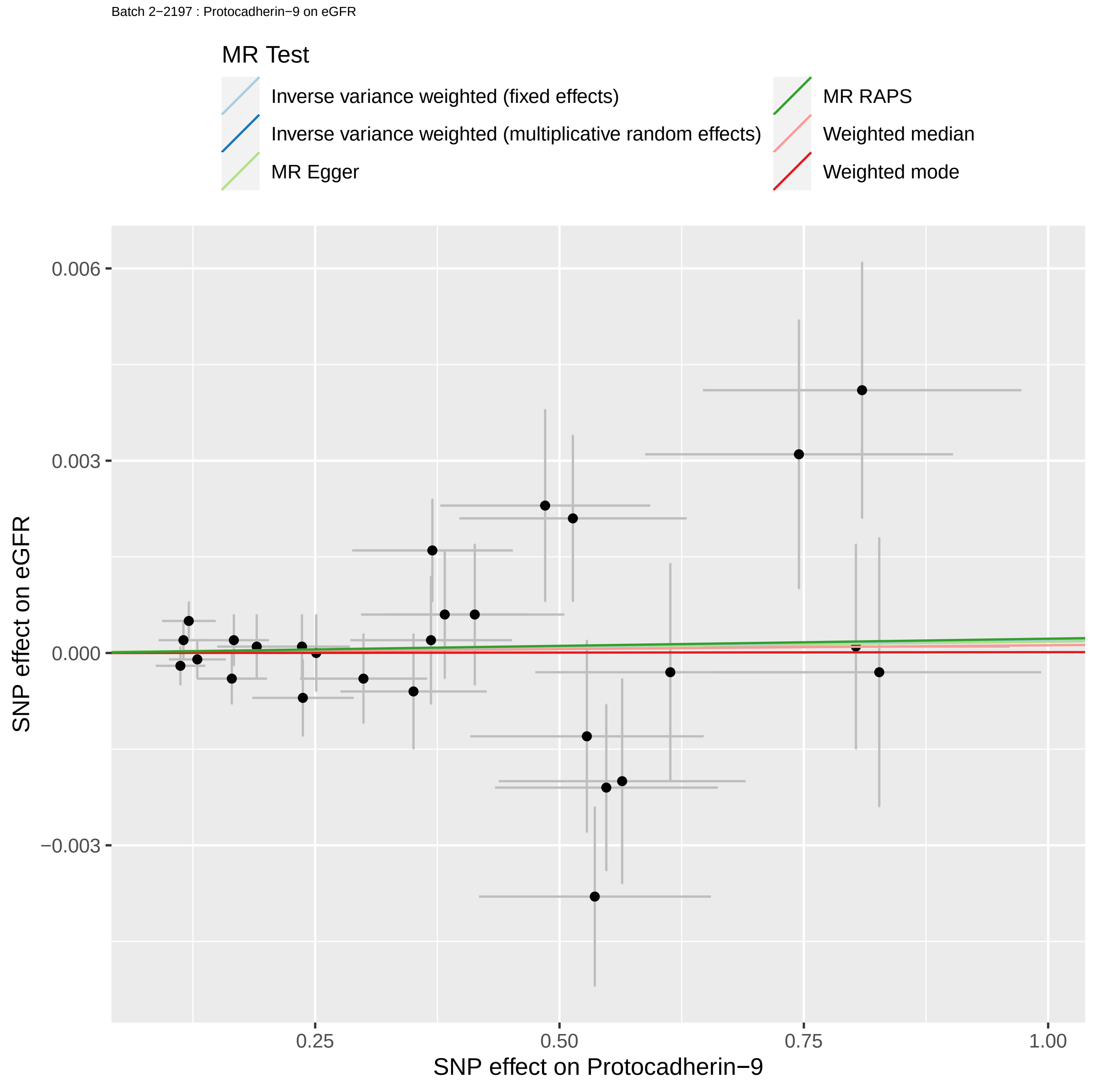
Batch 2−2197 : Protocadherin−9 on eGFR
MR Test
Inverse variance weighted (fixed effects)
Inverse variance weighted (multiplicative random effects)
MR Egger
MR RAPS
Weighted median
Weighted mode
0.006
0.003
0.000
−0.003
0.25
0.50
0.75
1.00
SNP effect on eGFR
SNP effect on Protocadherin−9

Batch 2−2198 : Protocadherin alpha−4 on eGFR

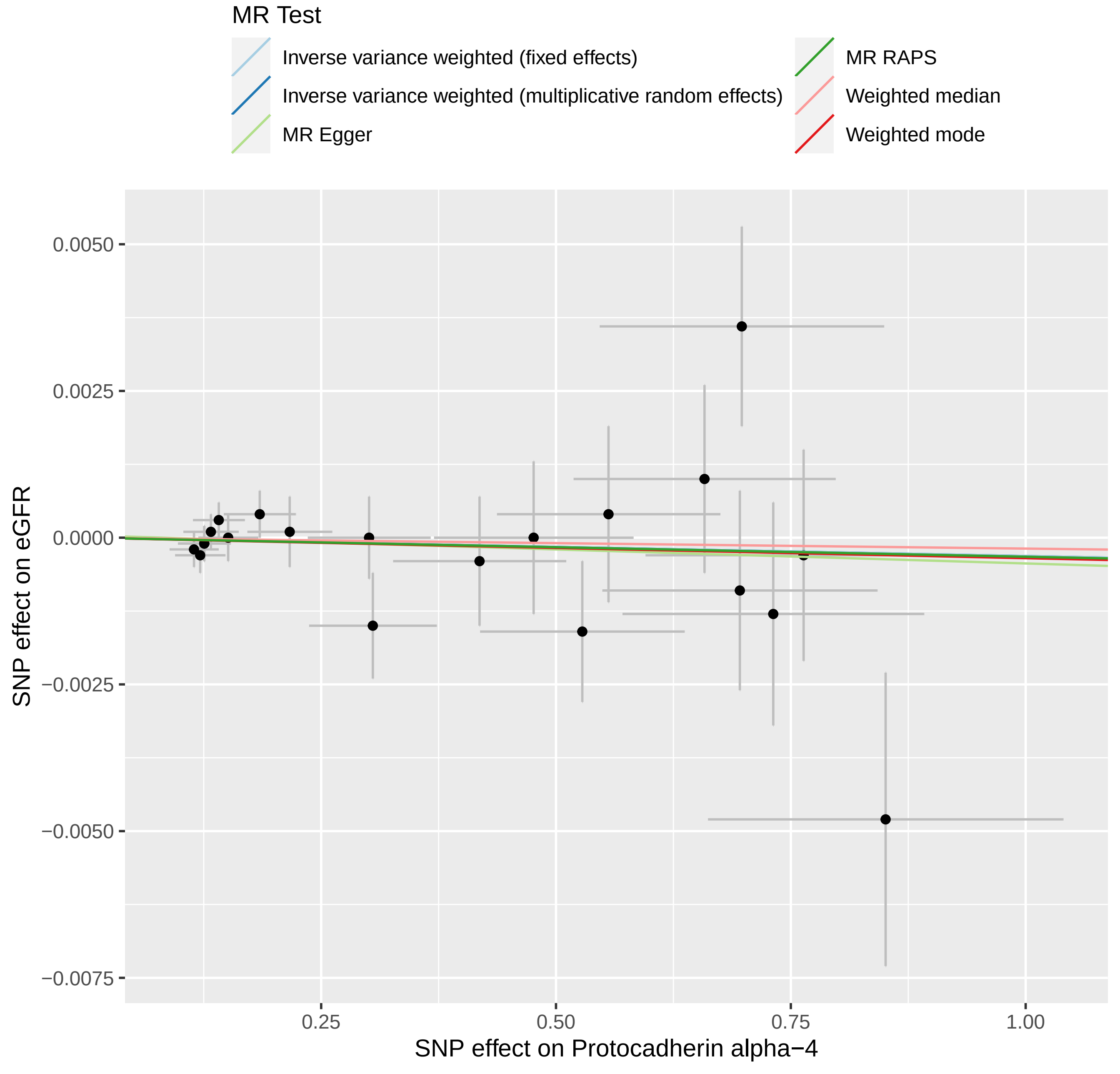

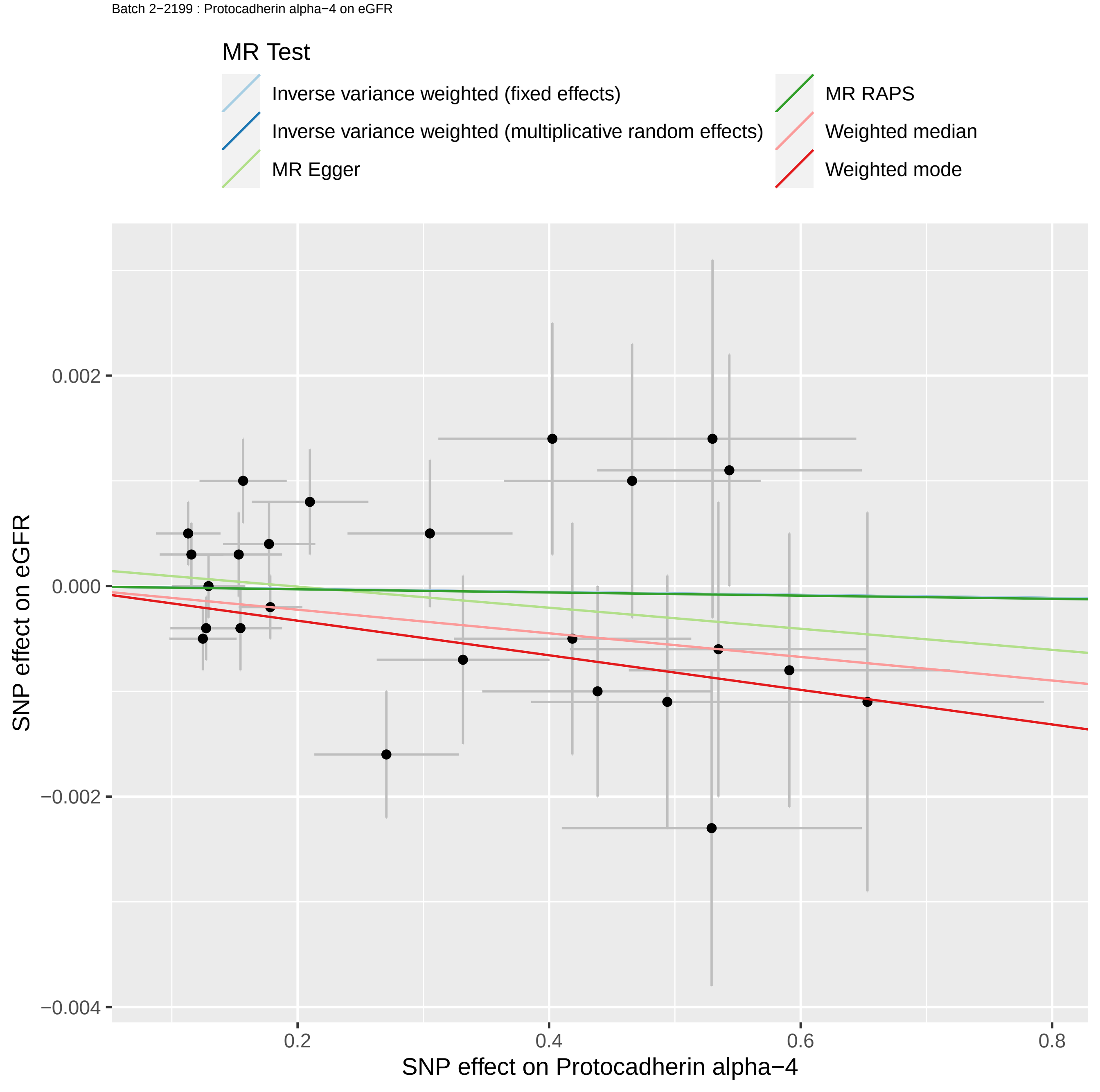

Batch 2−2199 : Protocadherin alpha−4 on eGFR
MR Test
Inverse variance weighted (fixed effects)
Inverse variance weighted (multiplicative random effects)
MR Egger
MR RAPS
Weighted median
Weighted mode
0.002
0.000
−0.002
−0.004
0.2
0.4
0.6
0.8
SNP effect on eGFR
SNP effect on Protocadherin alpha−4

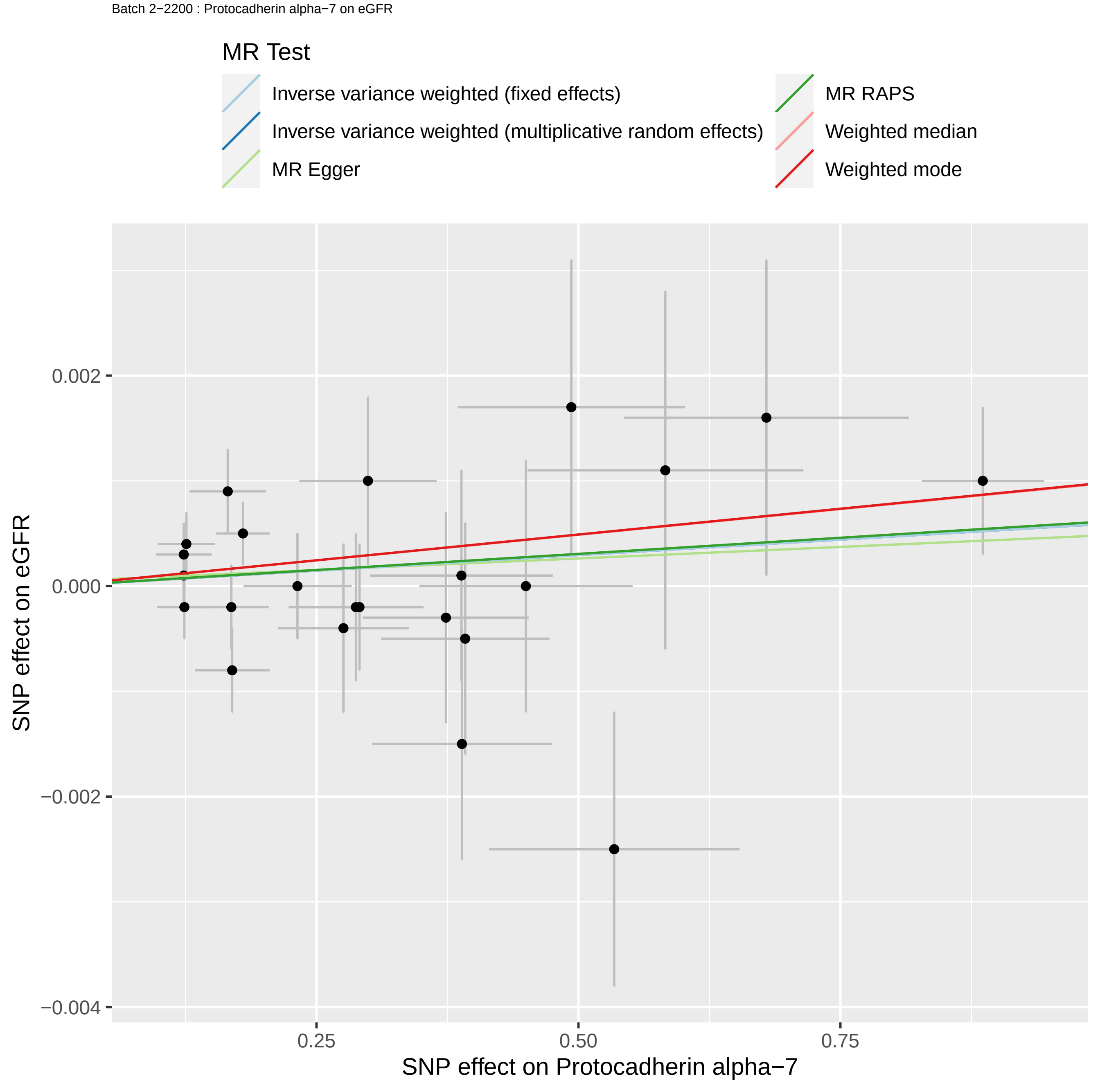

Batch 2−2200 : Protocadherin alpha−7 on eGFR
MR Test
Inverse variance weighted (fixed effects)
Inverse variance weighted (multiplicative random effects)
MR Egger
MR RAPS
Weighted median
Weighted mode
0.002
0.000
−0.002
−0.004
SNP effect on eGFR
0.25
0.50
0.75
SNP effect on Protocadherin alpha−7

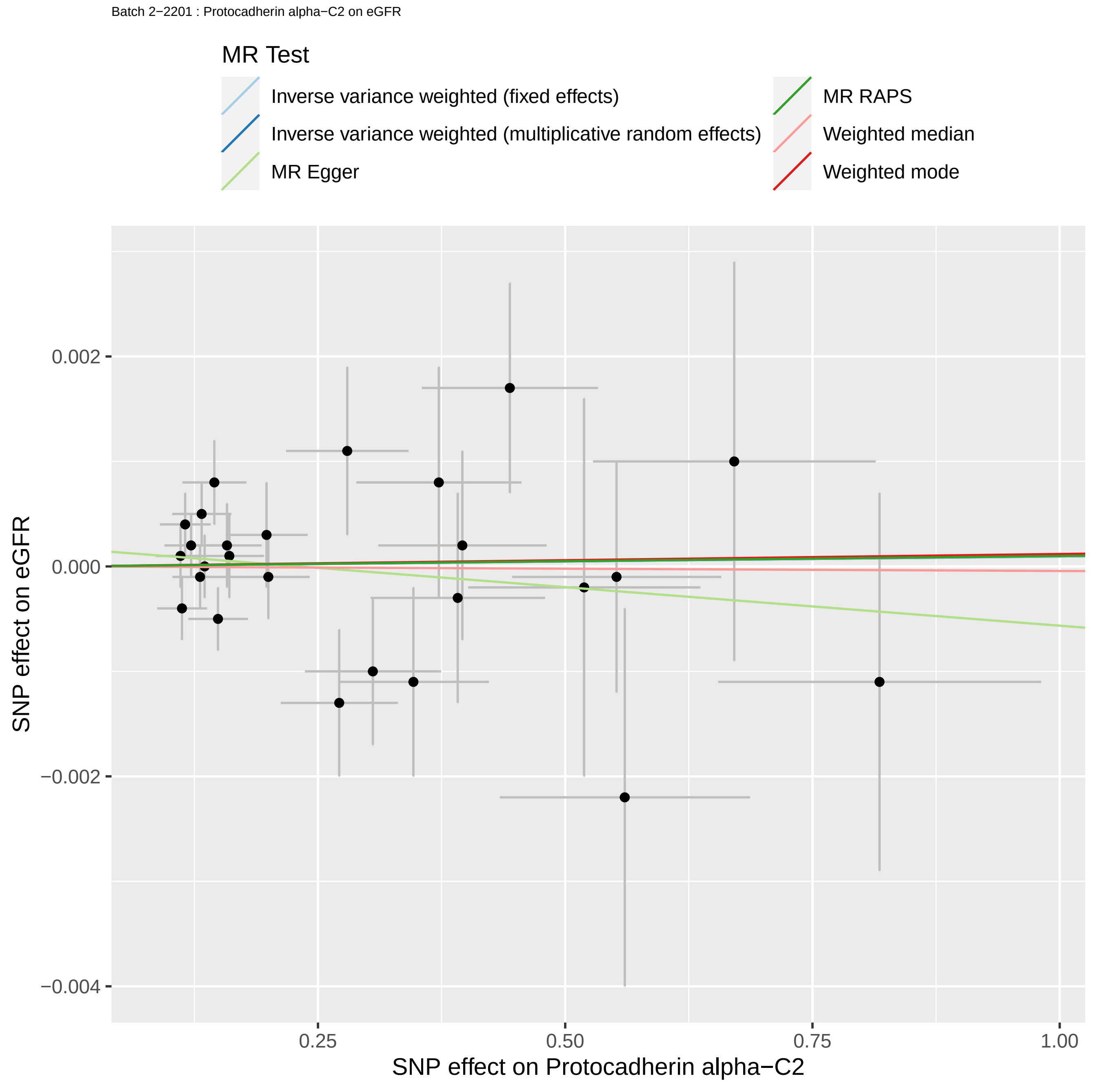

Batch 2−2201 : Protocadherin alpha−C2 on eGFR
MR Test
Inverse variance weighted (fixed effects)
Inverse variance weighted (multiplicative random effects)
MR Egger
MR RAPS
Weighted median
Weighted mode
0.002
0.000
−0.002
−0.004
SNP effect on eGFR
0.25
0.50
0.75
1.00
SNP effect on Protocadherin alpha−C2

Batch 2−2202 : Protocadherin beta−10 on eGFR

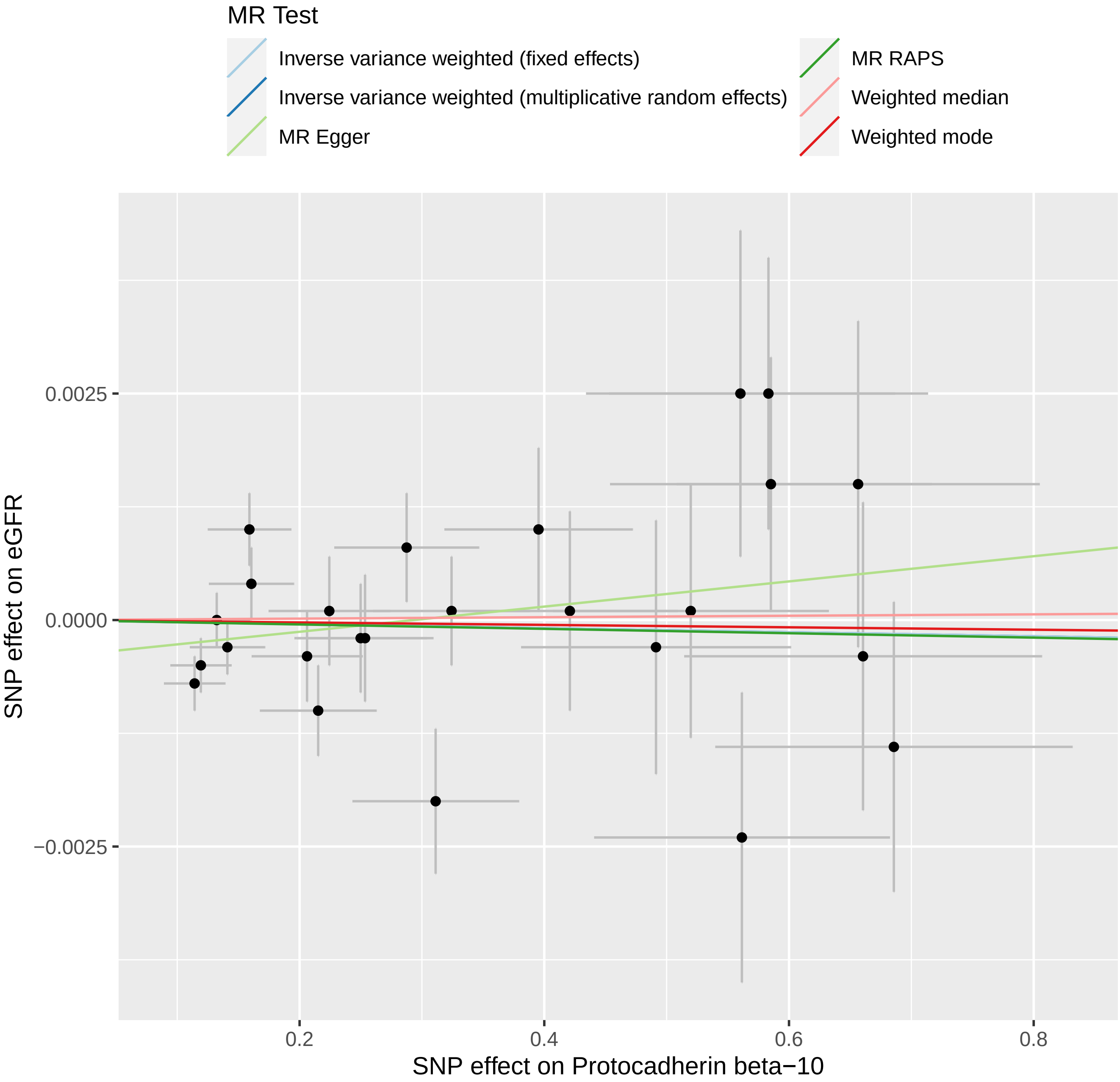

Batch 2−2203 : Protocadherin beta−1 on eGFR

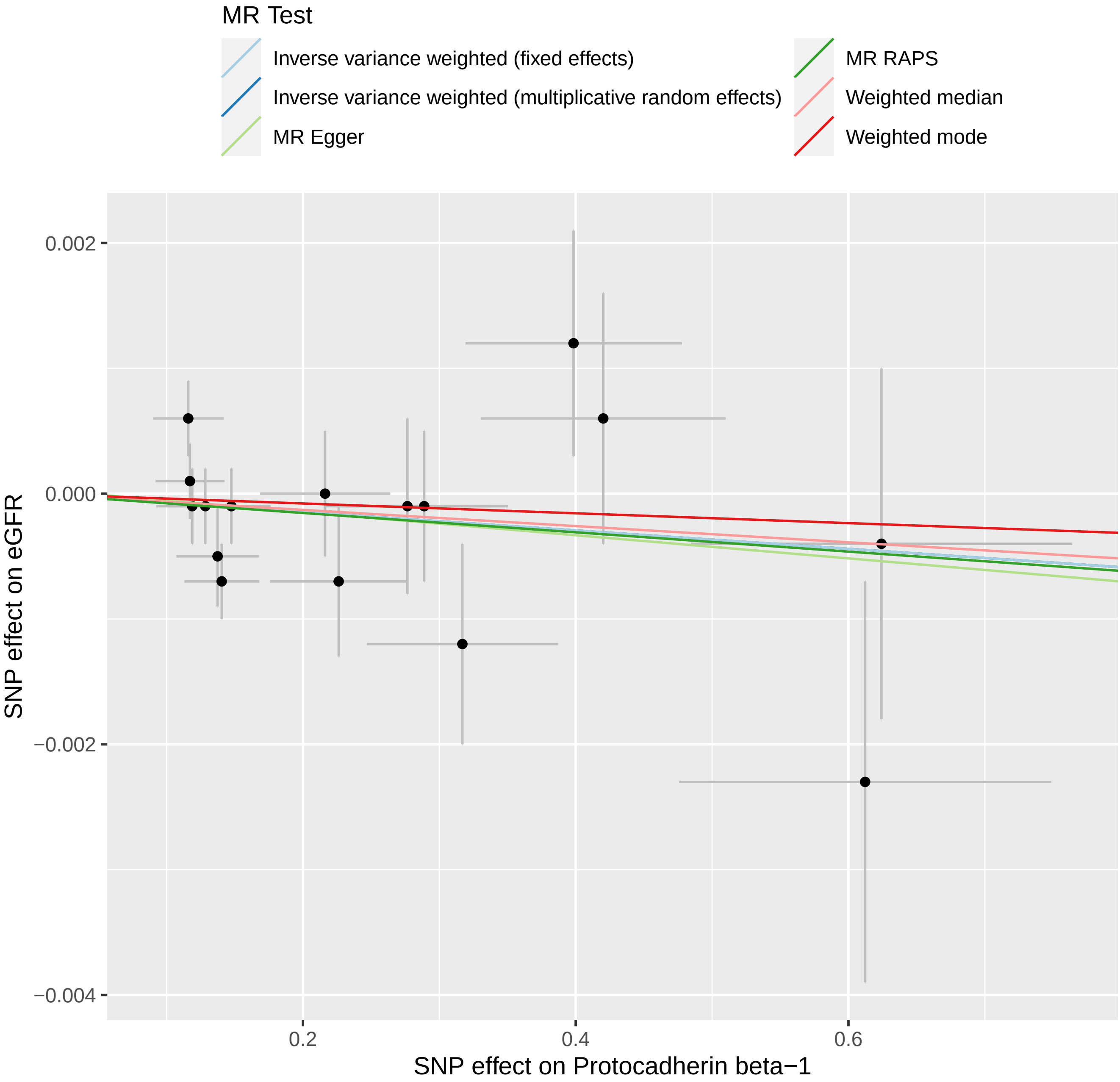

Batch 2−2204 : Protocadherin beta−2 on eGFR

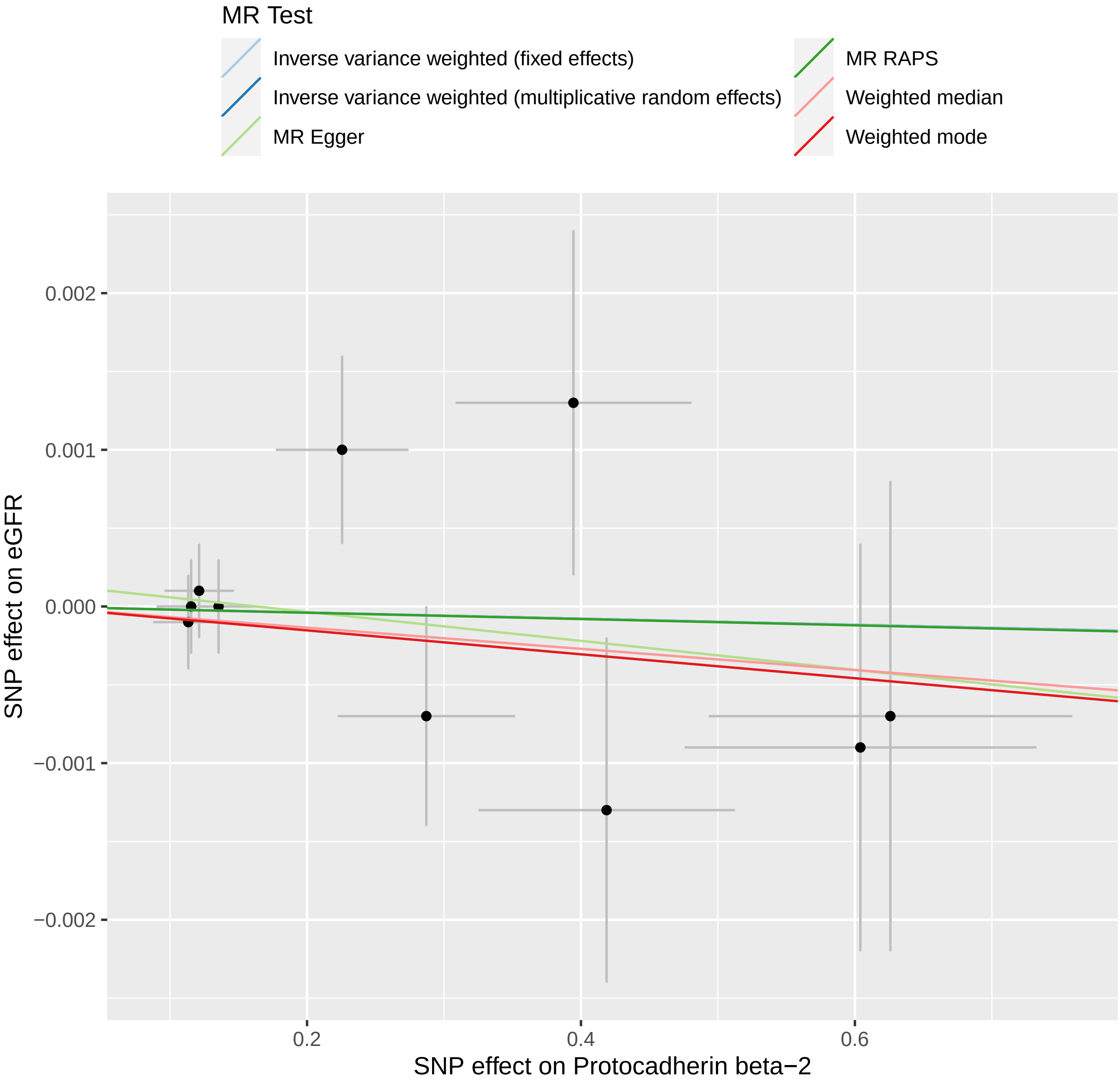

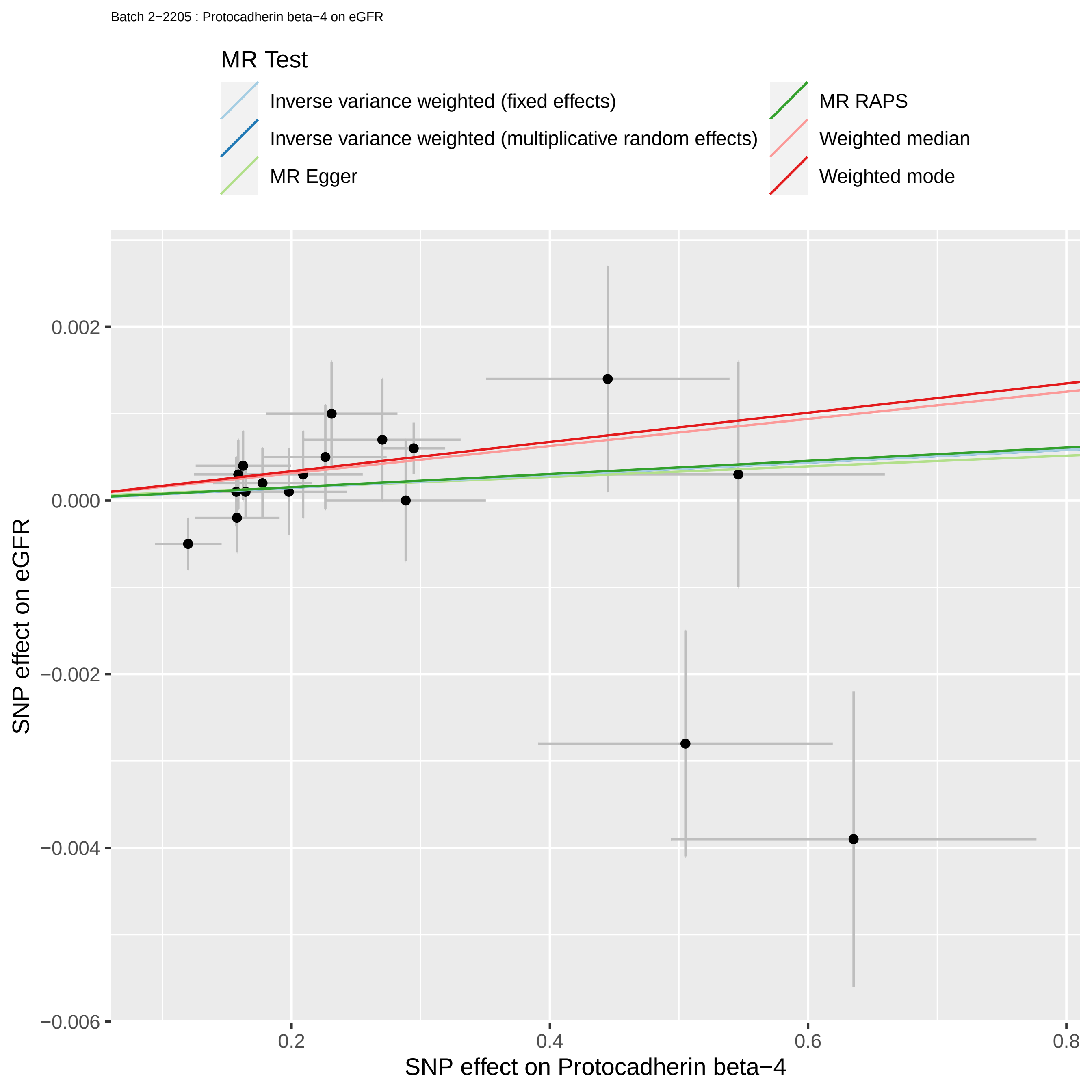
Batch 2−2205 : Protocadherin beta−4 on eGFR
MR Test
Inverse variance weighted (fixed effects)
Inverse variance weighted (multiplicative random effects)
MR Egger
MR RAPS
Weighted median
Weighted mode
0.002
0.000
−0.002
−0.004
−0.006
0.2
0.4
0.6
0.8
SNP effect on eGFR
SNP effect on Protocadherin beta−4

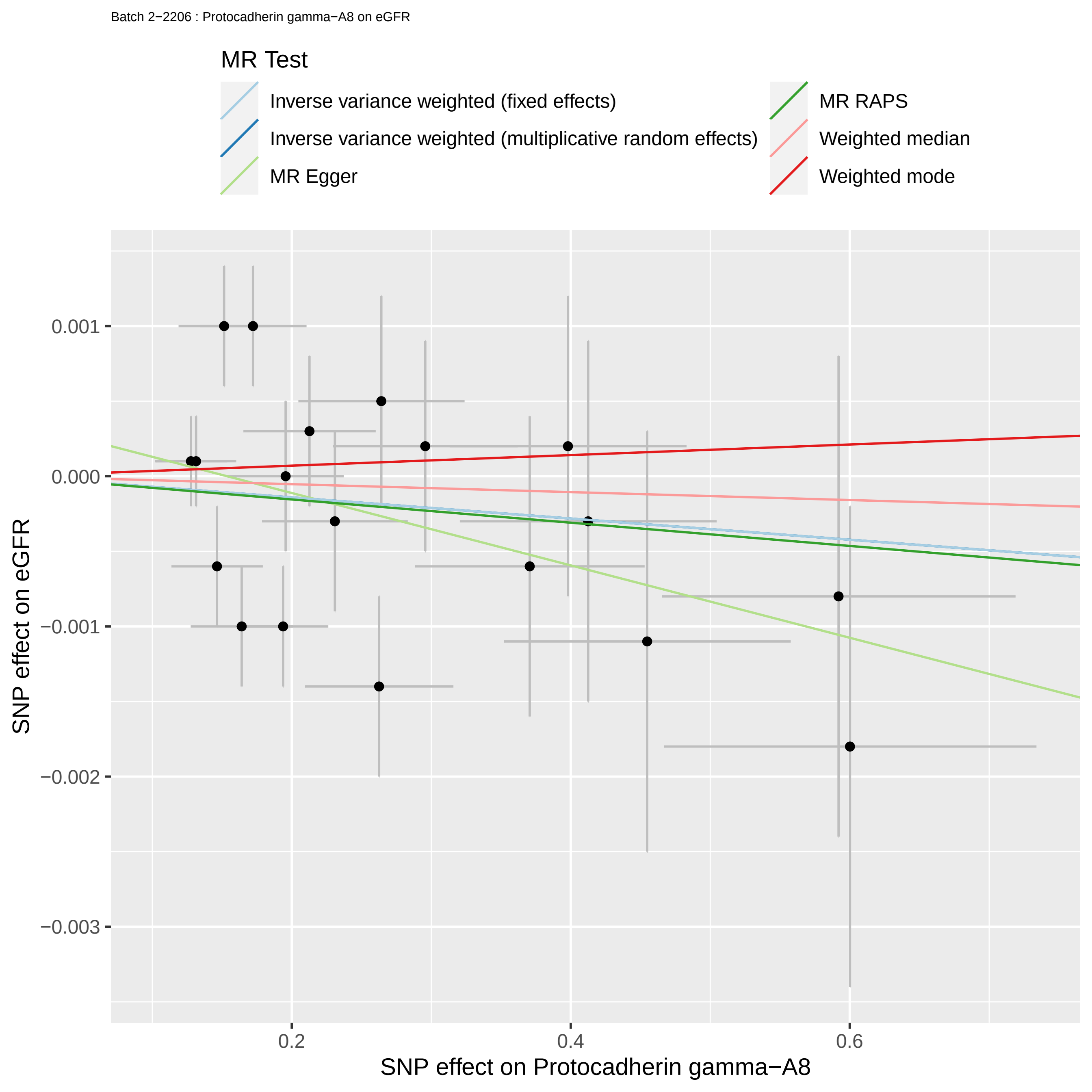

Batch 2−2206 : Protocadherin gamma−A8 on eGFR
MR Test
Inverse variance weighted (fixed effects)
Inverse variance weighted (multiplicative random effects)
MR Egger
MR RAPS
Weighted median
Weighted mode
0.001
0.000
−0.001
−0.002
−0.003
SNP effect on eGFR
0.2
0.4
0.6
SNP effect on Protocadherin gamma−A8

Batch 2−2207 : Protocadherin gamma−C5 on eGFR

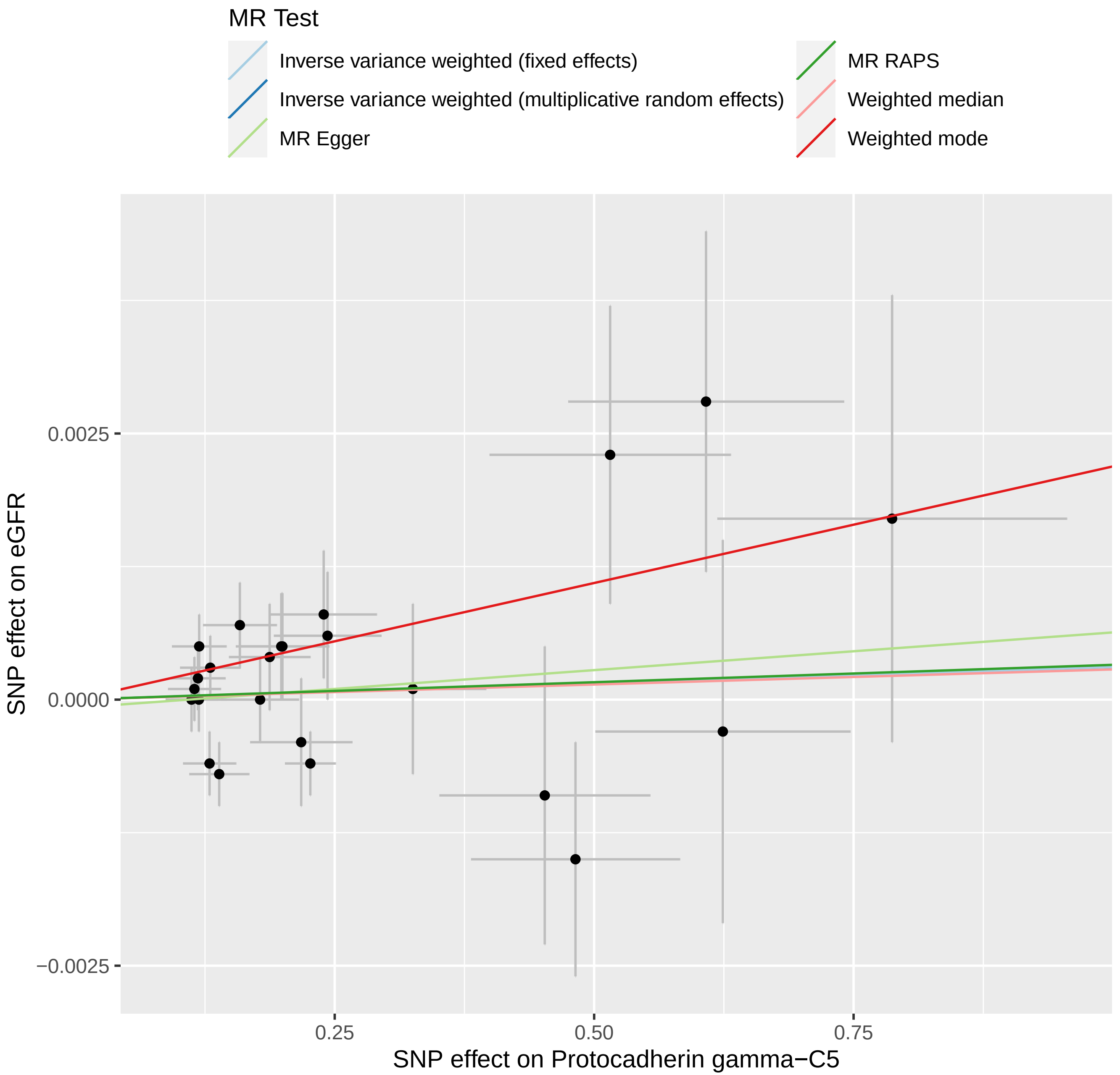

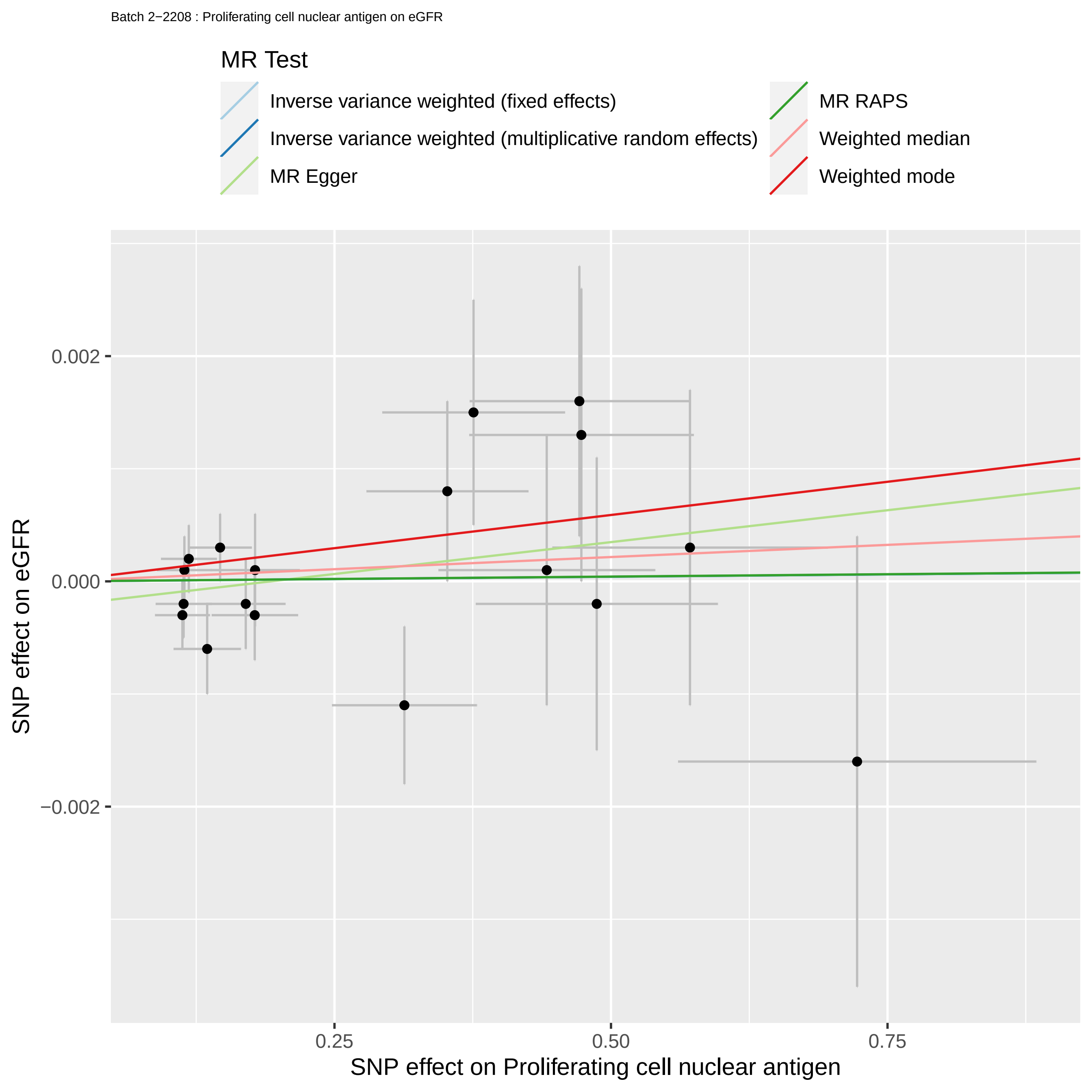
Batch 2−2208 : Proliferating cell nuclear antigen on eGFR
MR Test
Inverse variance weighted (fixed effects)
Inverse variance weighted (multiplicative random effects)
MR Egger
MR RAPS
Weighted median
Weighted mode
0.002
0.000
−0.002
SNP effect on eGFR
0.25
0.50
0.75
SNP effect on Proliferating cell nuclear antigen

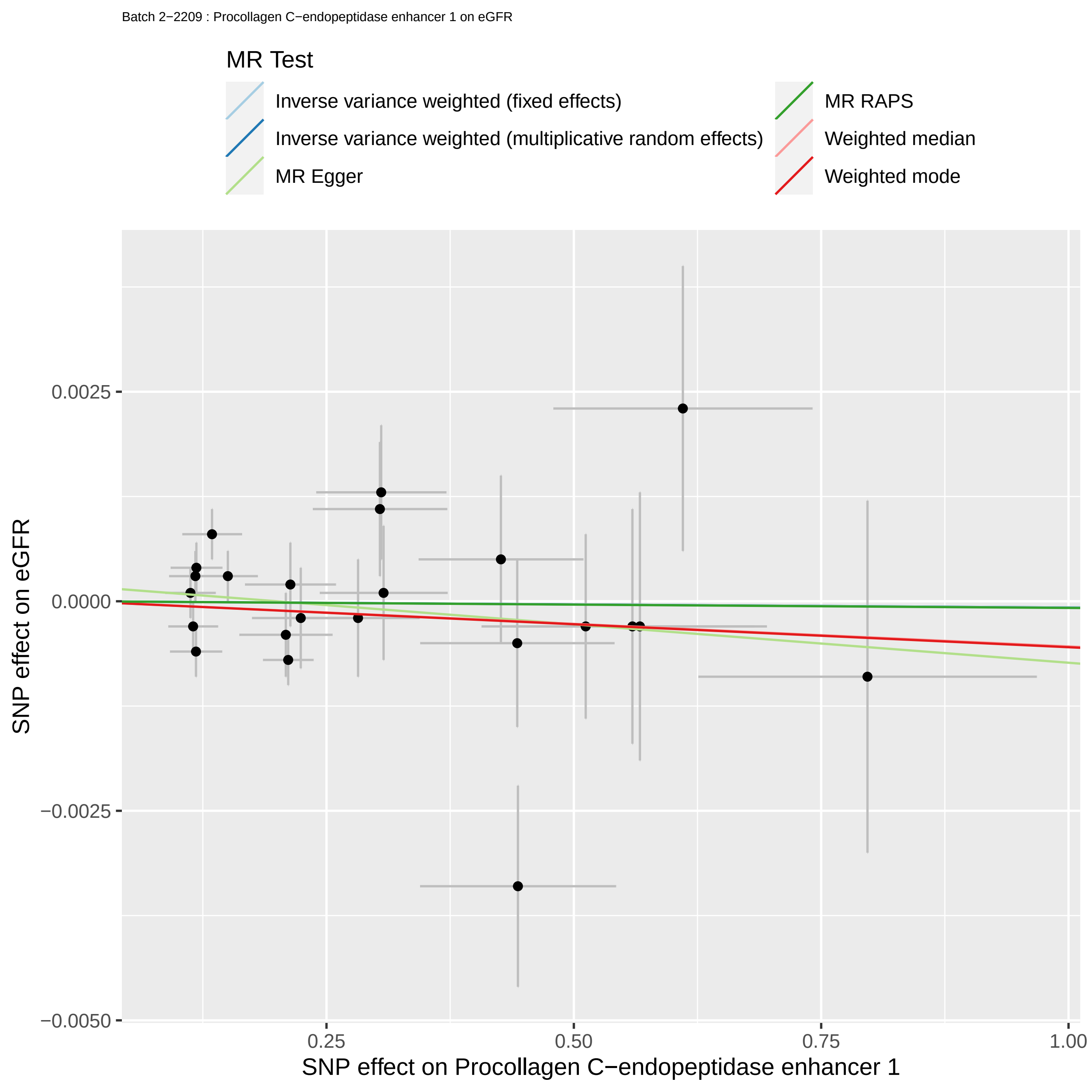

Batch 2−2209 : Procollagen C−endopeptidase enhancer 1 on eGFR
MR Test
Inverse variance weighted (fixed effects)
Inverse variance weighted (multiplicative random effects)
MR Egger
MR RAPS
Weighted median
Weighted mode
0.0025
0.0000
−0.0025
−0.0050
0.25
0.50
0.75
1.00
SNP effect on eGFR
SNP effect on Procollagen C−endopeptidase enhancer 1

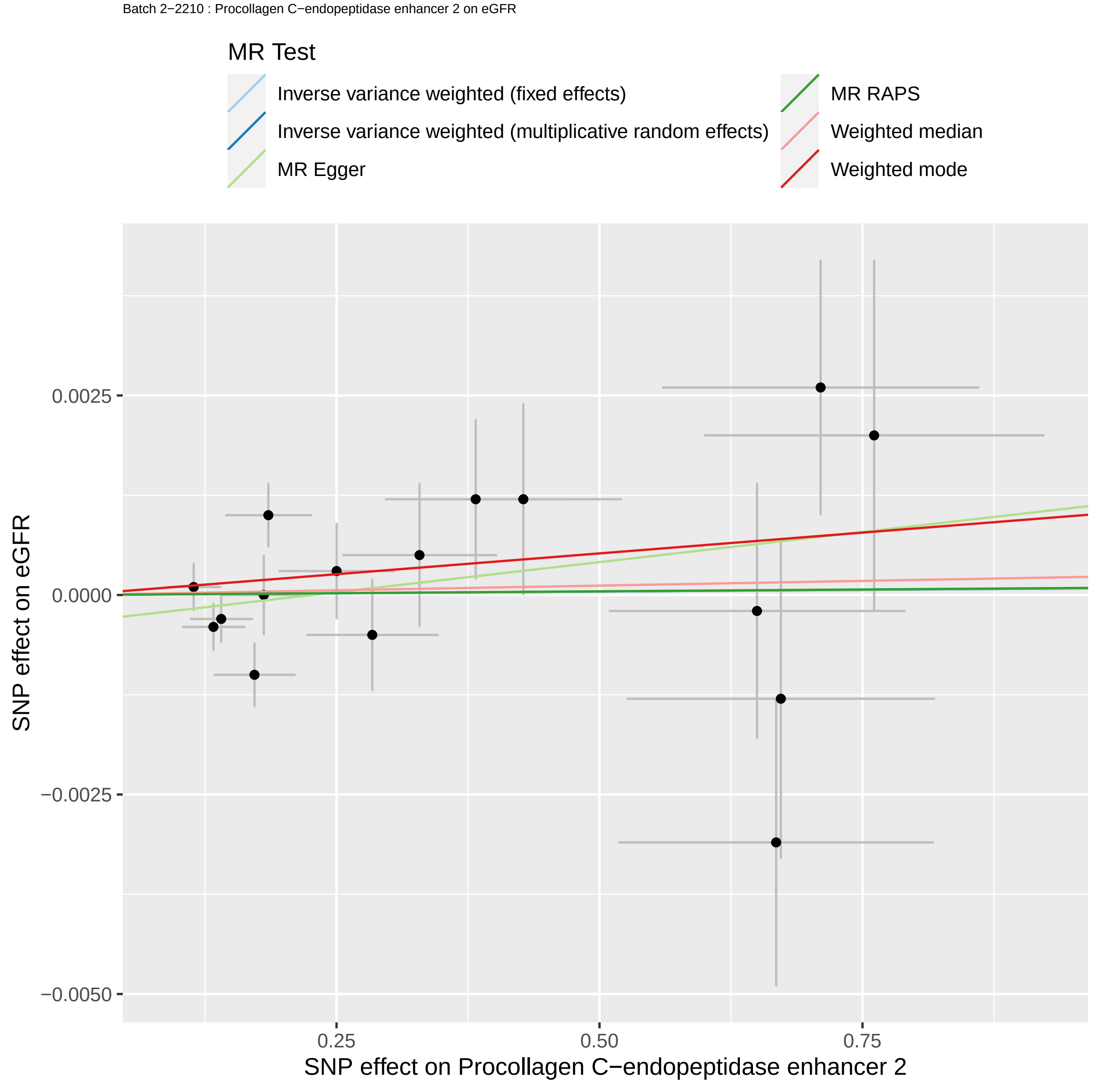

Batch 2−2210 : Procollagen C−endopeptidase enhancer 2 on eGFR
MR Test
Inverse variance weighted (fixed effects)
Inverse variance weighted (multiplicative random effects)
MR Egger
MR RAPS
Weighted median
Weighted mode
0.0025
0.0000
−0.0025
−0.0050
0.25
0.50
0.75
SNP effect on eGFR
SNP effect on Procollagen C−endopeptidase enhancer 2

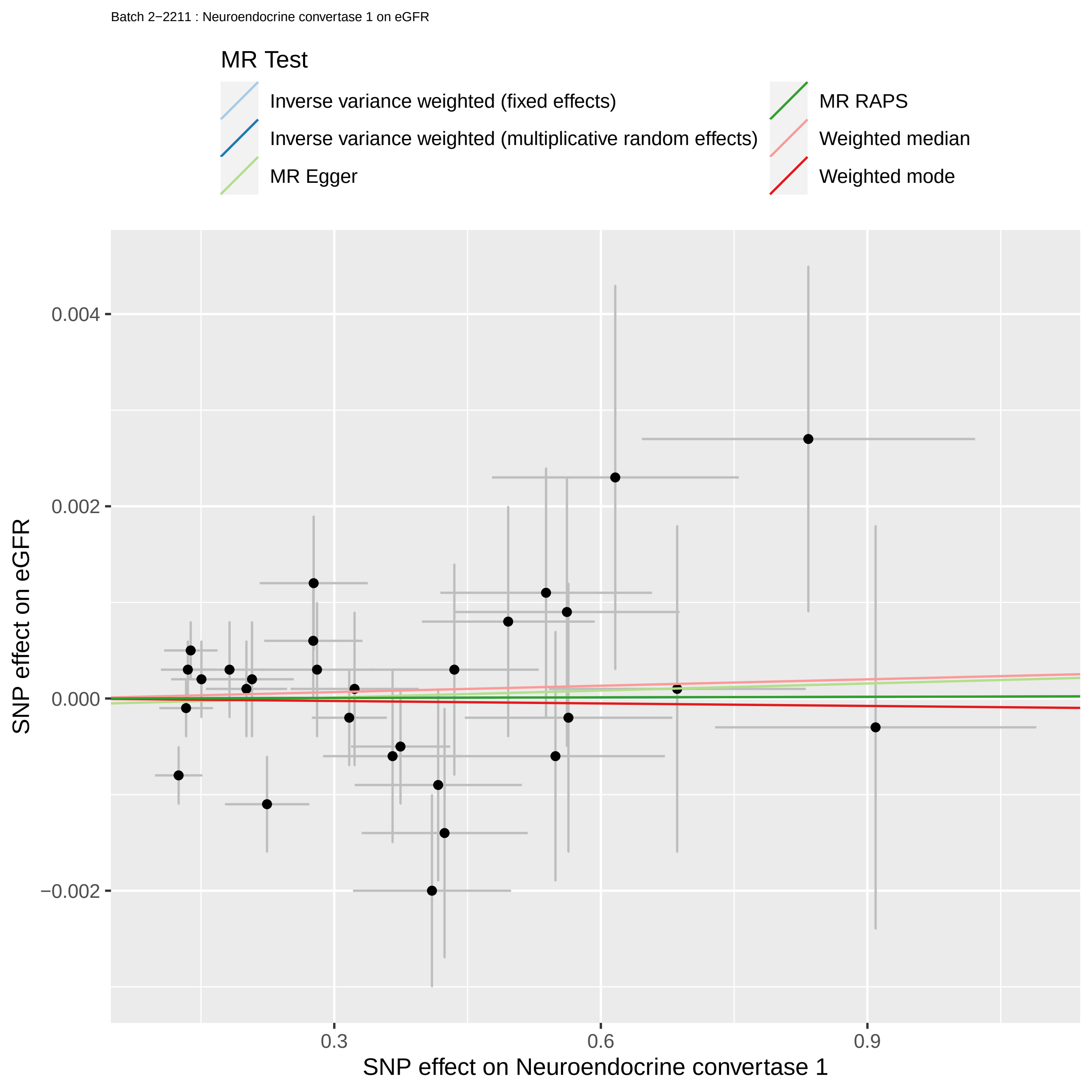

Batch 2−2211 : Neuroendocrine convertase 1 on eGFR
MR Test
Inverse variance weighted (fixed effects)
Inverse variance weighted (multiplicative random effects)
MR Egger
MR RAPS
Weighted median
Weighted mode
0.004
0.002
0.000
−0.002
0.3
0.6
0.9
SNP effect on eGFR
SNP effect on Neuroendocrine convertase 1

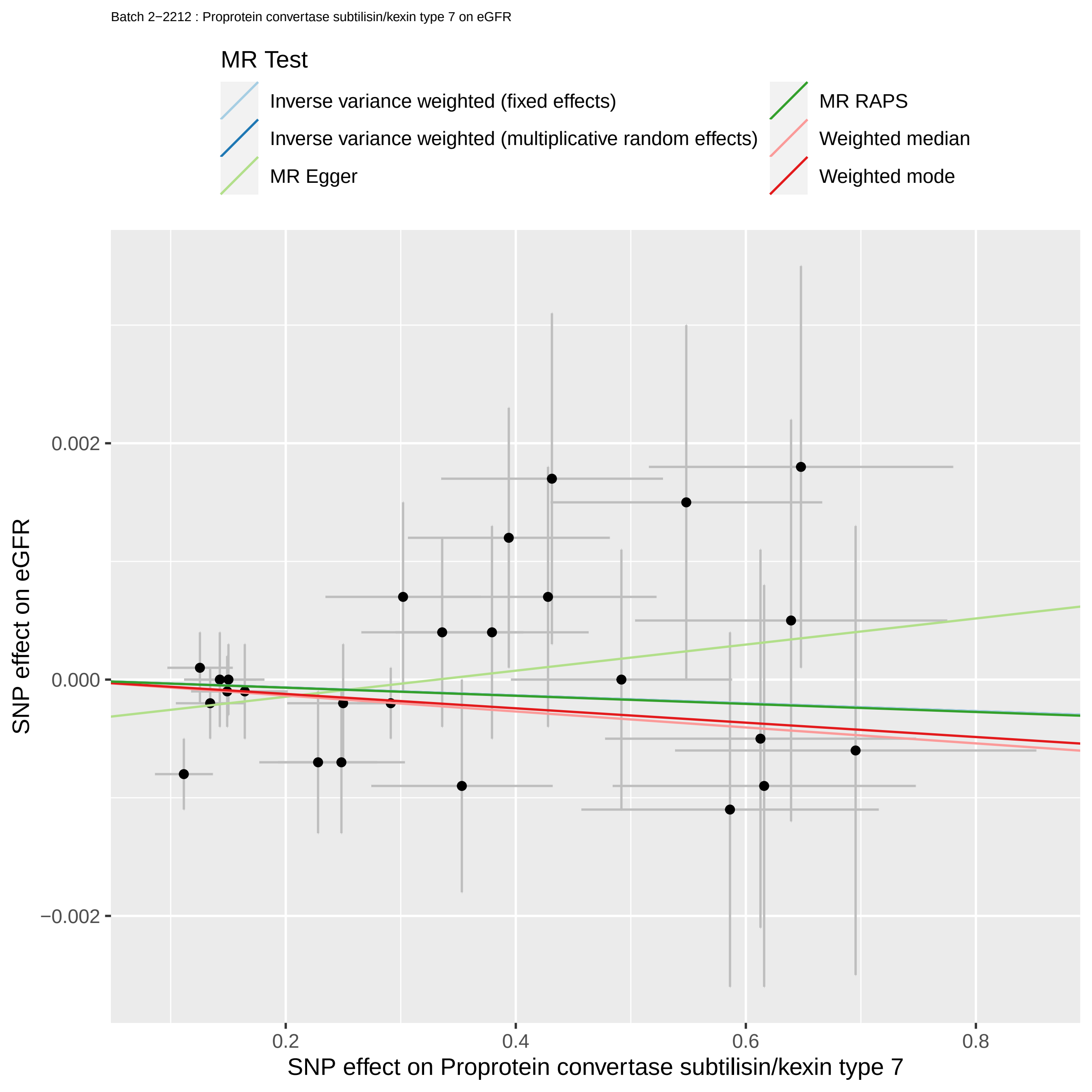
Batch 2−2212 : Proprotein convertase subtilisin/kexin type 7 on eGFR
MR Test
Inverse variance weighted (fixed effects)
Inverse variance weighted (multiplicative random effects)
MR Egger
MR RAPS
Weighted median
Weighted mode
SNP effect on eGFR
0.002
0.000
−0.002
0.2
0.4
0.6
0.8
SNP effect on Proprotein convertase subtilisin/kexin type 7

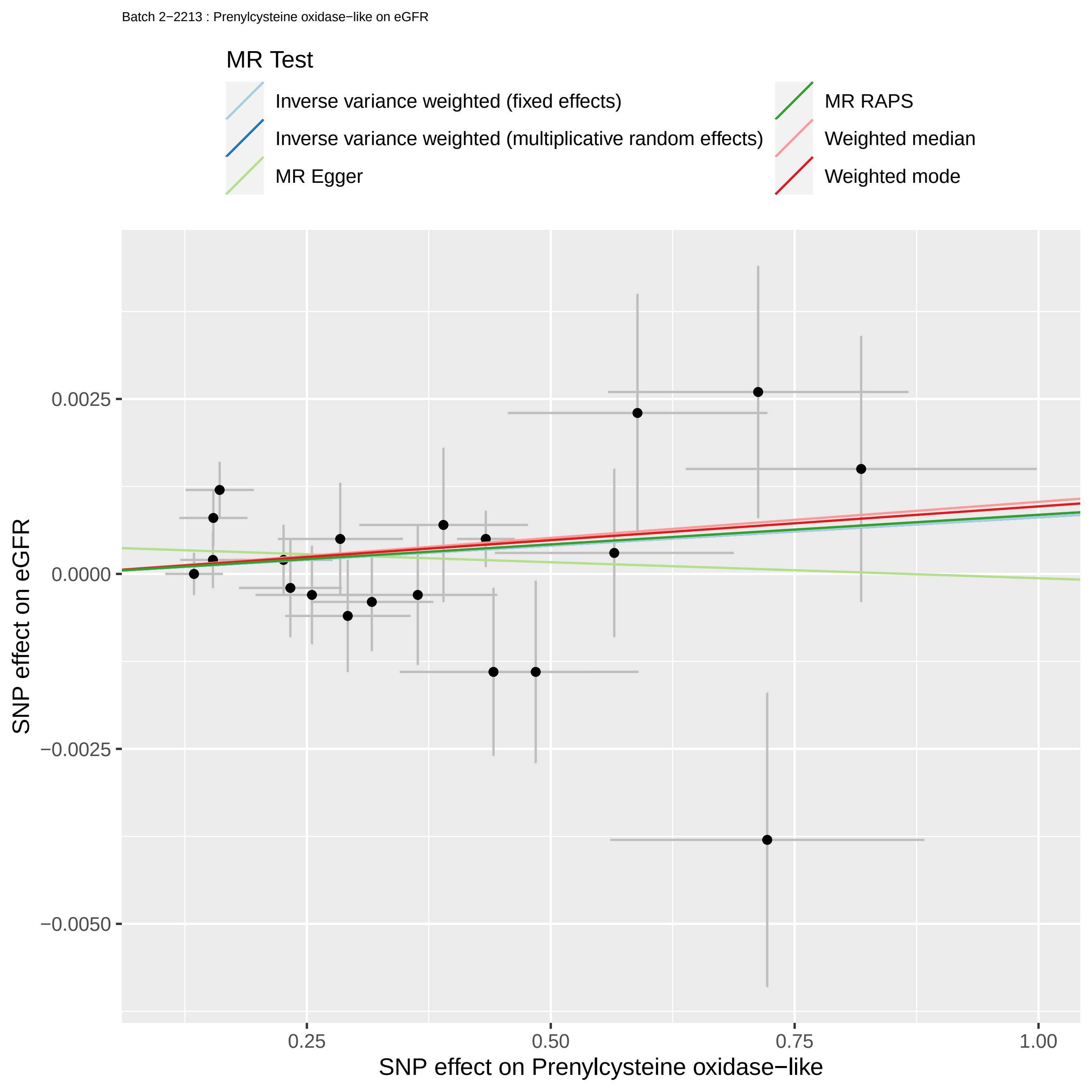

Batch 2−2213 : Prenylcysteine oxidase−like on eGFR
MR Test
Inverse variance weighted (fixed effects)
Inverse variance weighted (multiplicative random effects)
MR Egger
MR RAPS
Weighted median
Weighted mode
0.0025
0.0000
−0.0025
−0.0050
SNP effect on eGFR
0.25
0.50
0.75
1.00
SNP effect on Prenylcysteine oxidase−like

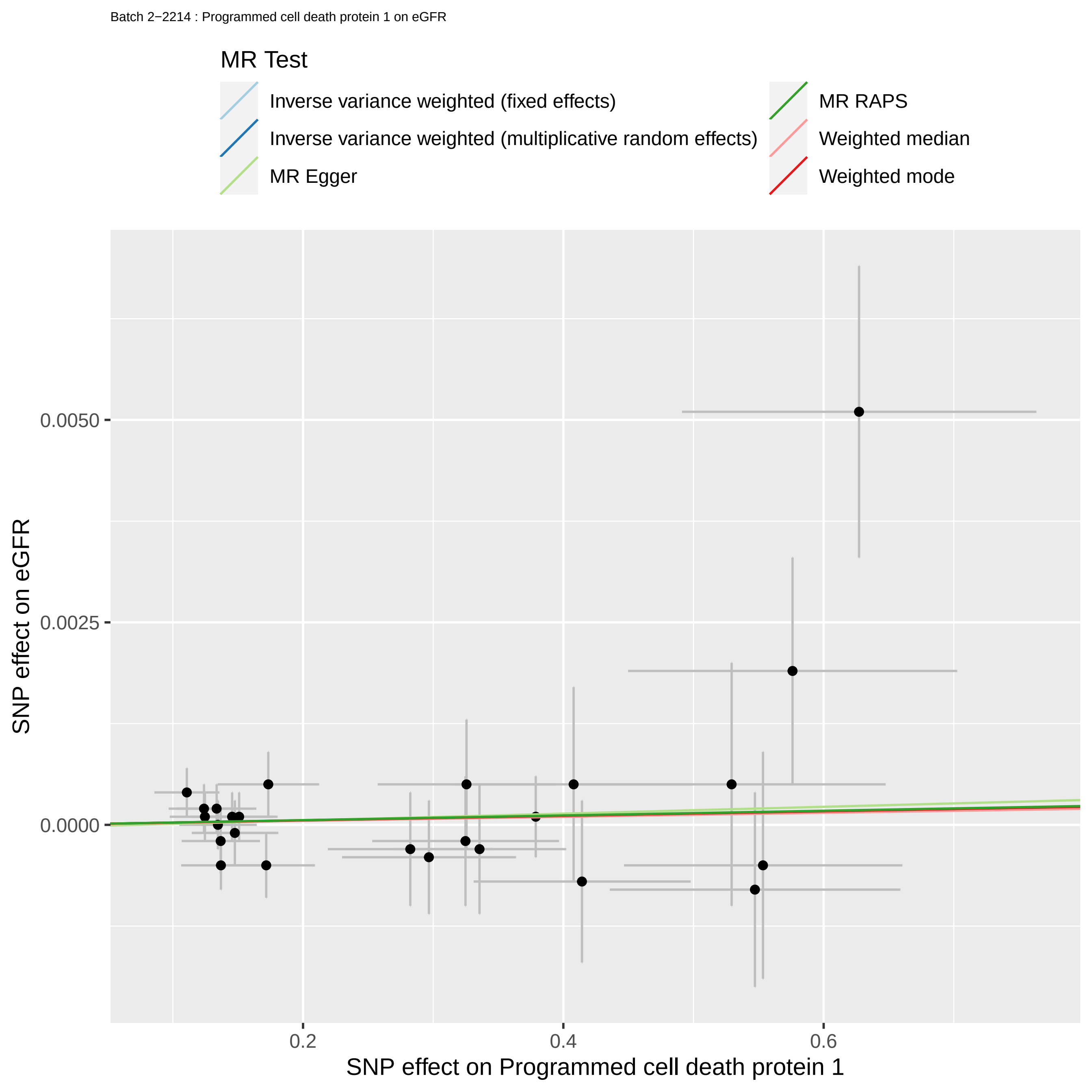
Batch 2−2214 : Programmed cell death protein 1 on eGFR
MR Test
Inverse variance weighted (fixed effects)
Inverse variance weighted (multiplicative random effects)
MR Egger
MR RAPS
Weighted median
Weighted mode
0.0050
0.0025
0.0000
0.2
0.4
0.6
SNP effect on eGFR
SNP effect on Programmed cell death protein 1

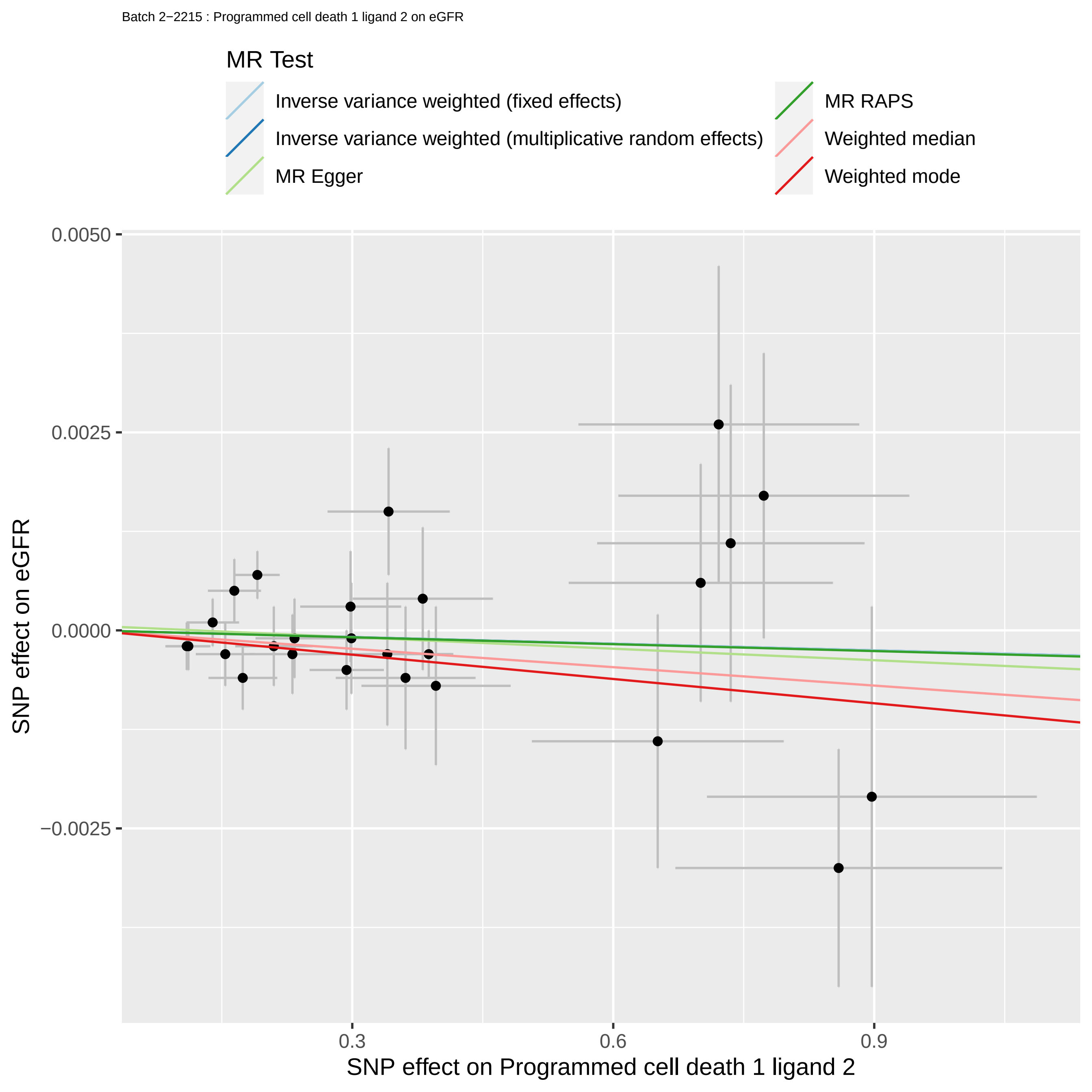
Batch 2−2215 : Programmed cell death 1 ligand 2 on eGFR
MR Test
Inverse variance weighted (fixed effects)
Inverse variance weighted (multiplicative random effects)
MR Egger
MR RAPS
Weighted median
Weighted mode
0.0050
0.0025
0.0000
−0.0025
SNP effect on eGFR
0.3
0.6
0.9
SNP effect on Programmed cell death 1 ligand 2

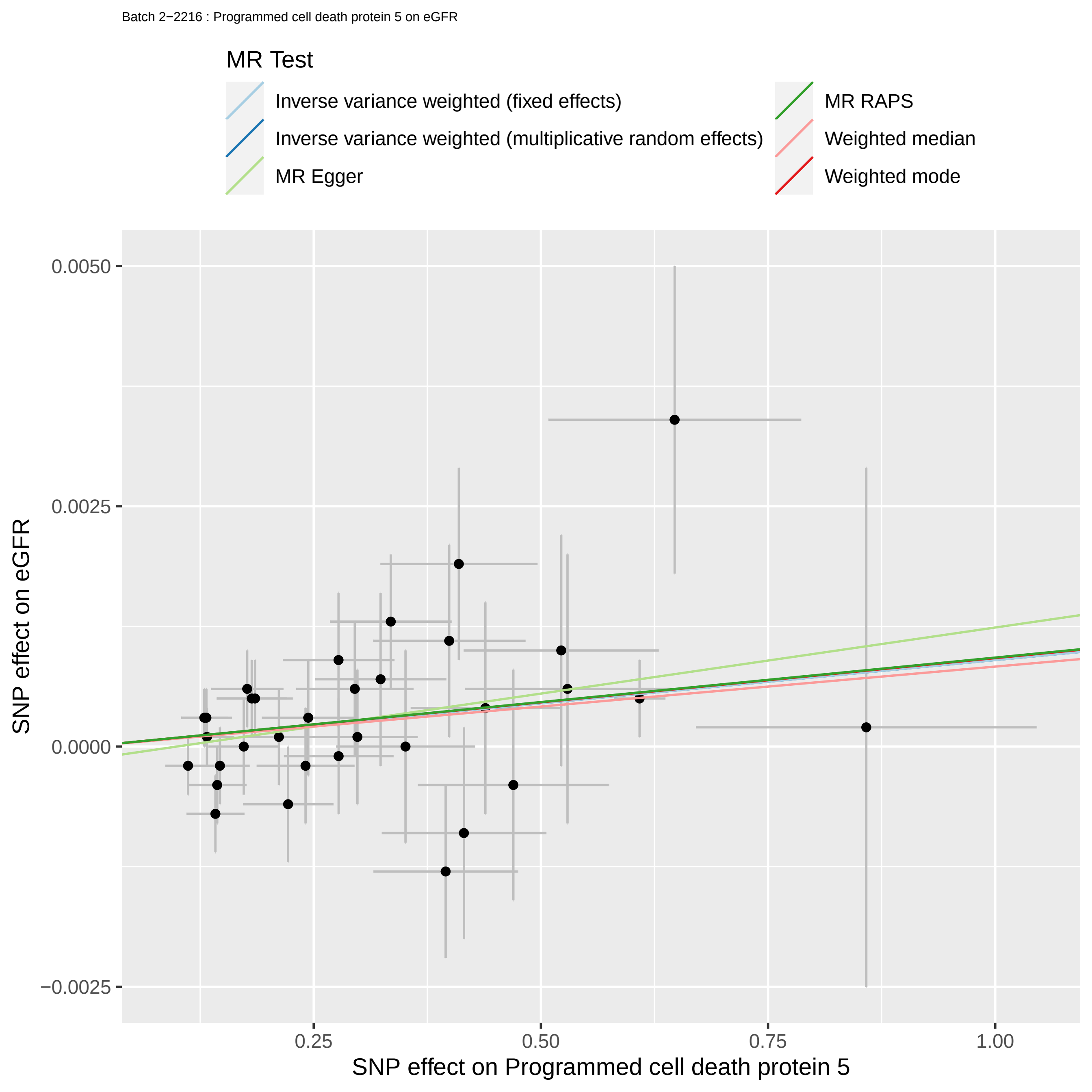

Batch 2−2216 : Programmed cell death protein 5 on eGFR
MR Test
Inverse variance weighted (fixed effects)
Inverse variance weighted (multiplicative random effects)
MR Egger
MR RAPS
Weighted median
Weighted mode
0.0050
0.0025
0.0000
−0.0025
0.25
0.50
0.75
1.00
SNP effect on eGFR
SNP effect on Programmed cell death protein 5

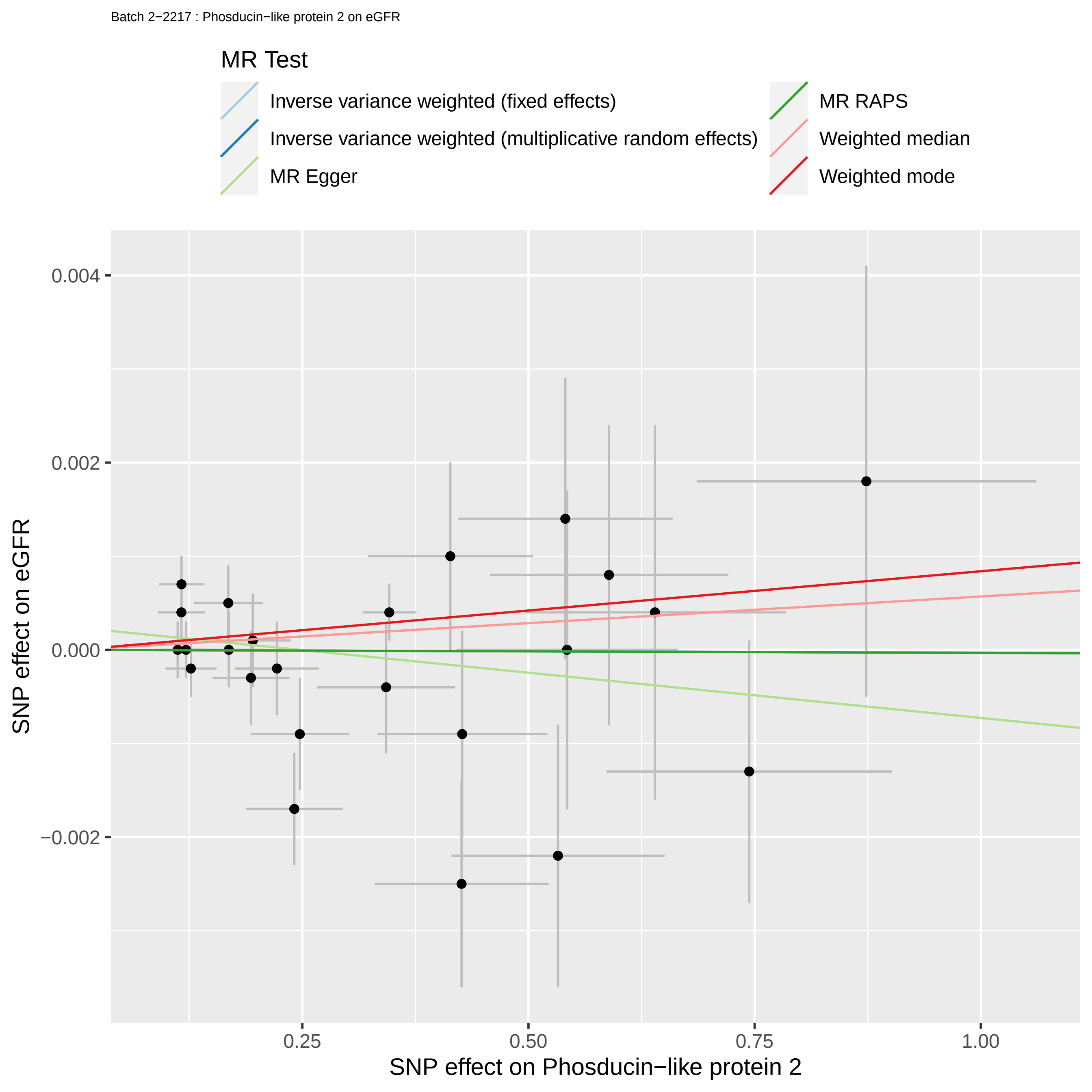

Batch 2−2217 : Phosducin−like protein 2 on eGFR
MR Test
Inverse variance weighted (fixed effects)
Inverse variance weighted (multiplicative random effects)
MR Egger
MR RAPS
Weighted median
Weighted mode
SNP effect on eGFR
0.004
0.002
0.000
−0.002
0.25
0.50
0.75
1.00
SNP effect on Phosducin−like protein 2

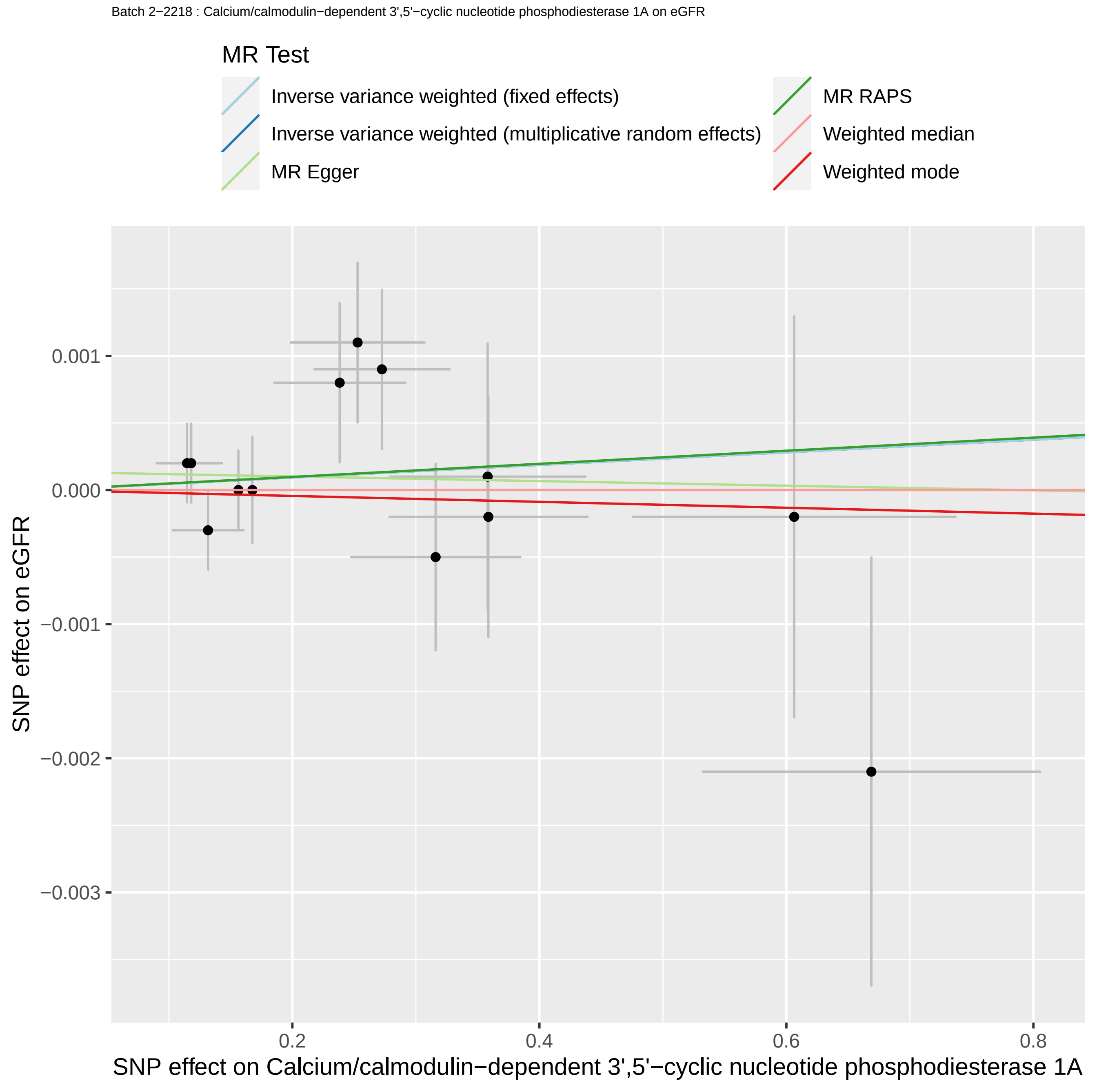

Batch 2−2218 : Calcium/calmodulin−dependent 3',5'−cyclic nucleotide phosphodiesterase 1A on eGFR
MR Test
Inverse variance weighted (fixed effects)
Inverse variance weighted (multiplicative random effects)
MR Egger
MR RAPS
Weighted median
Weighted mode
0.001
0.000
−0.001
−0.002
−0.003
0.2
0.4
0.6
0.8
SNP effect on eGFR
SNP effect on Calcium/calmodulin−dependent 3',5'−cyclic nucleotide phosphodiesterase 1A

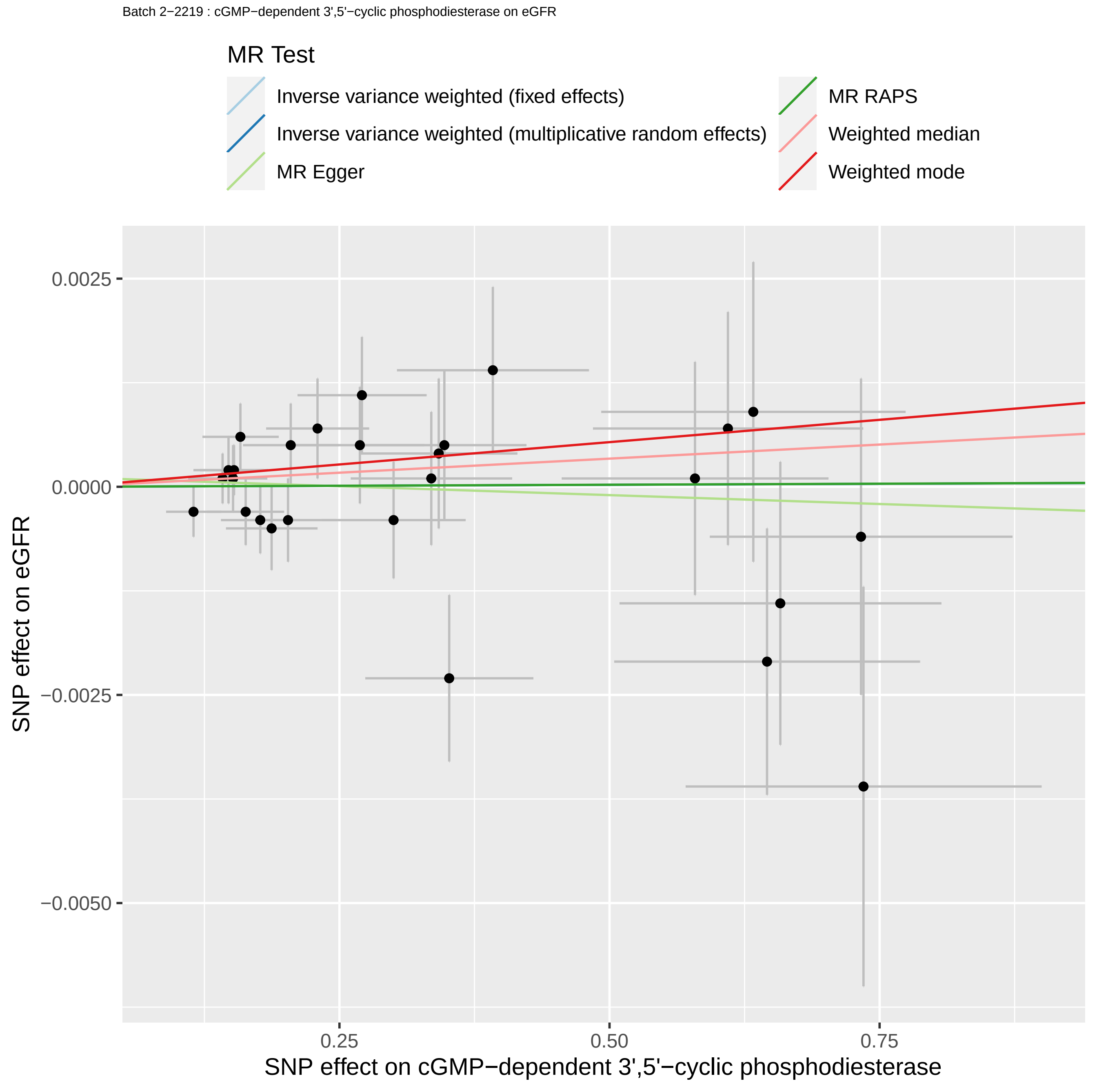
Batch 2−2219 : cGMP−dependent 3',5'−cyclic phosphodiesterase on eGFR
MR Test
Inverse variance weighted (fixed effects)
Inverse variance weighted (multiplicative random effects)
MR Egger
MR RAPS
Weighted median
Weighted mode
0.0025
0.0000
−0.0025
−0.0050
0.25
0.50
0.75
SNP effect on eGFR
SNP effect on cGMP−dependent 3',5'−cyclic phosphodiesterase

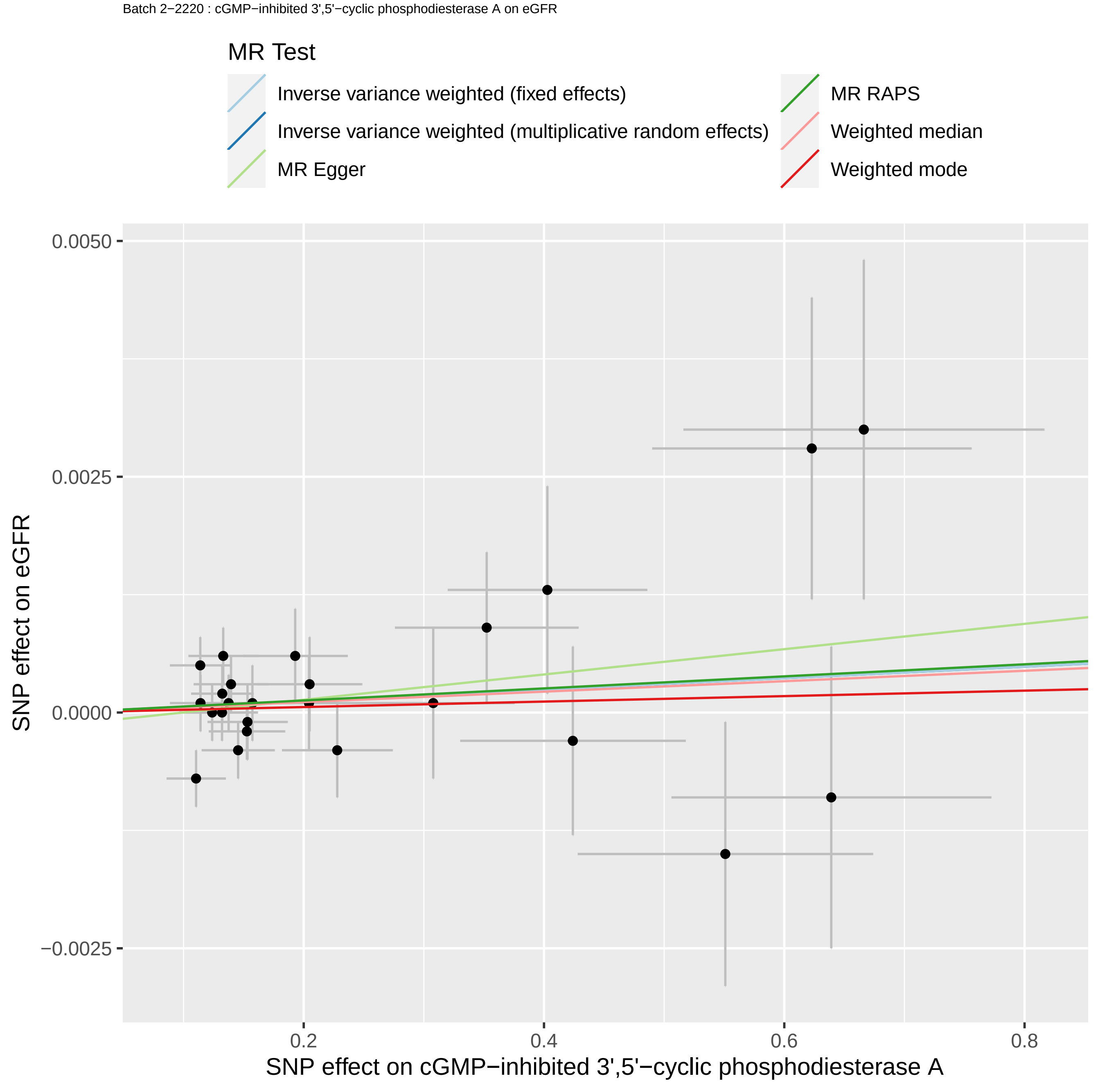

Batch 2−2220 : cGMP−inhibited 3',5'−cyclic phosphodiesterase A on eGFR
MR Test
Inverse variance weighted (fixed effects)
Inverse variance weighted (multiplicative random effects)
MR Egger
MR RAPS
Weighted median
Weighted mode
0.0050
0.0025
0.0000
−0.0025
0.2
0.4
0.6
0.8
SNP effect on eGFR
SNP effect on cGMP−inhibited 3',5'−cyclic phosphodiesterase A

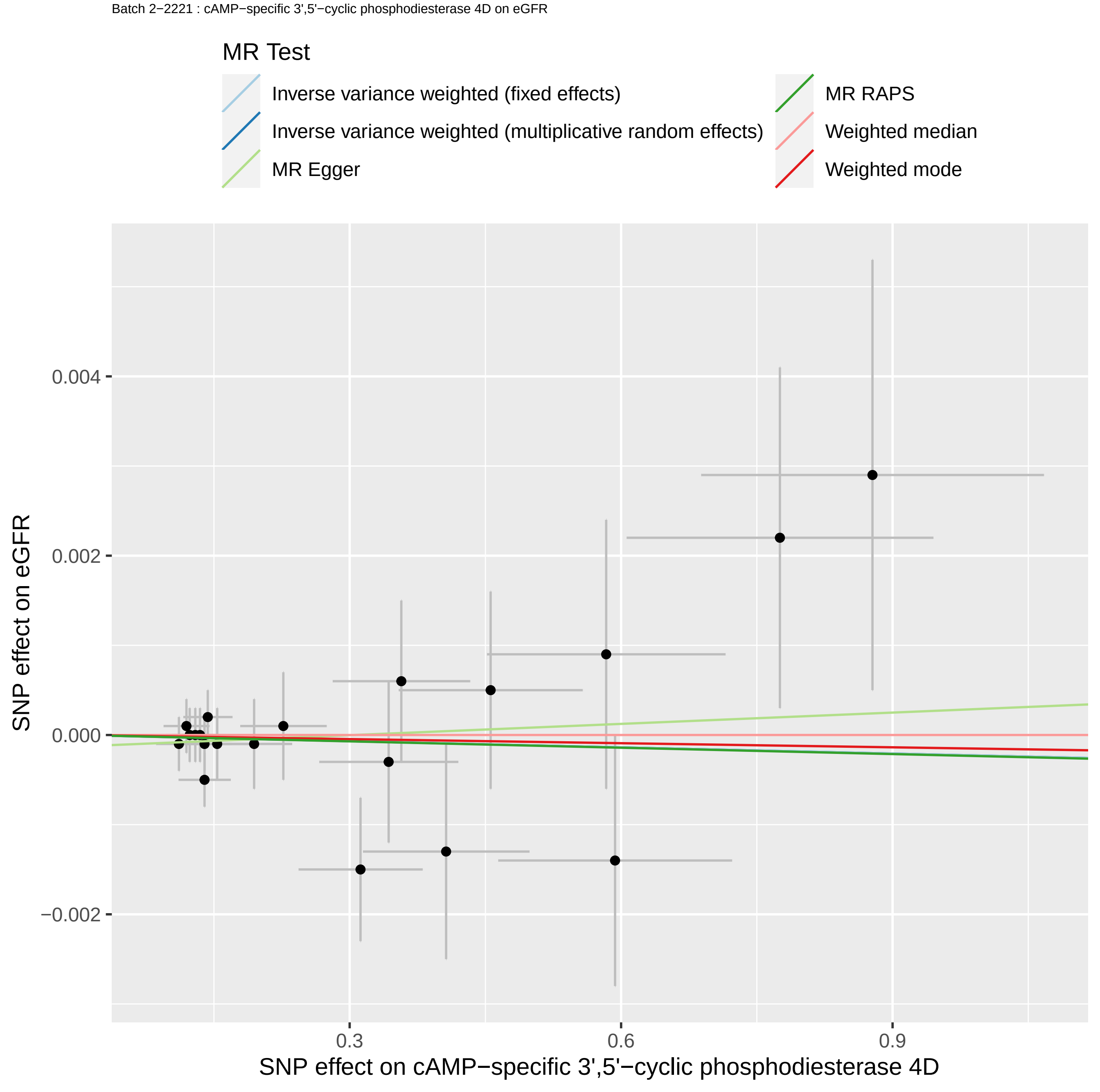

Batch 2−2221 : cAMP−specific 3',5'−cyclic phosphodiesterase 4D on eGFR
MR Test
Inverse variance weighted (fixed effects)
Inverse variance weighted (multiplicative random effects)
MR Egger
MR RAPS
Weighted median
Weighted mode
0.004
0.002
0.000
−0.002
0.3
0.6
0.9
SNP effect on eGFR
SNP effect on cAMP−specific 3',5'−cyclic phosphodiesterase 4D

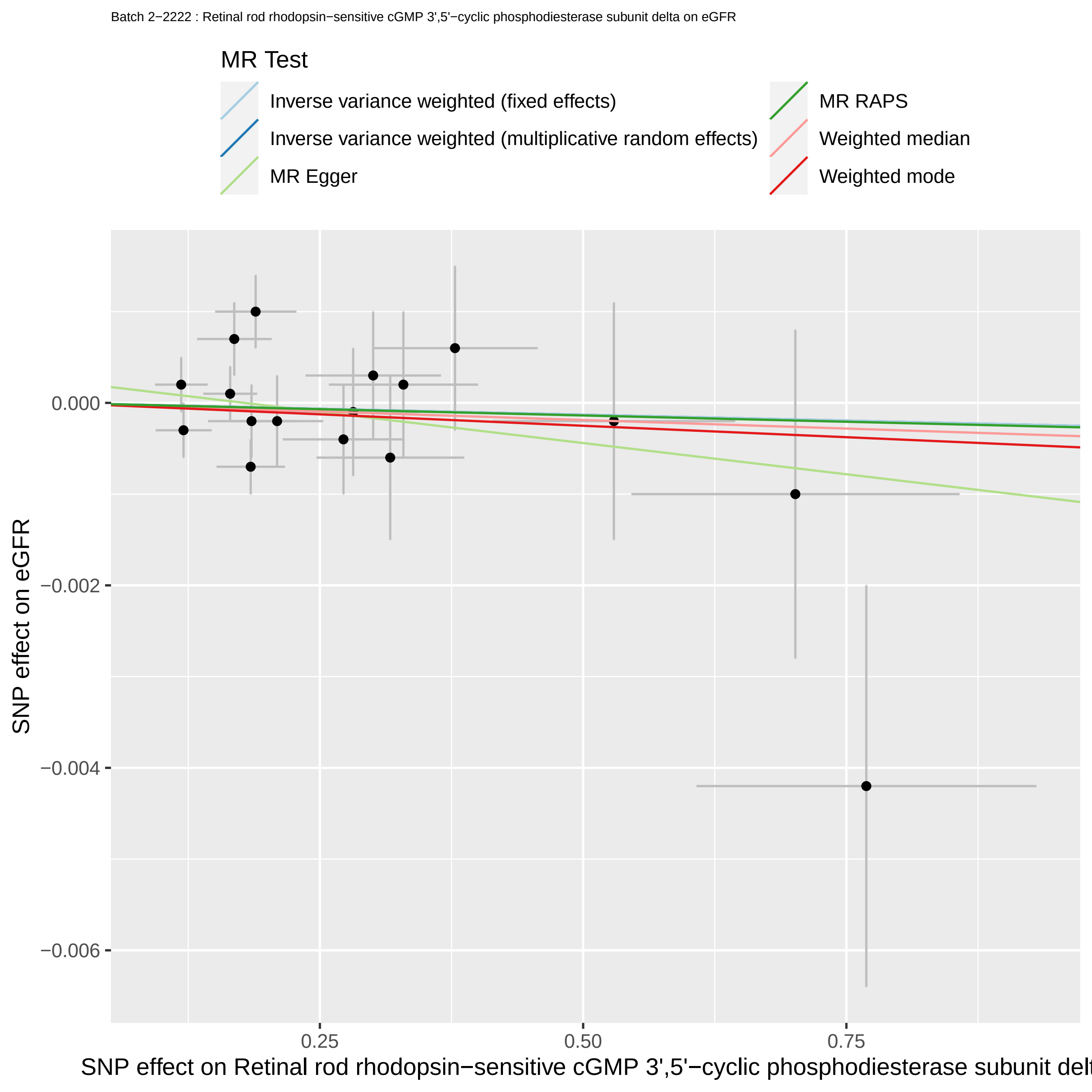
Batch 2−2222 : Retinal rod rhodopsin−sensitive cGMP 3',5'−cyclic phosphodiesterase subunit delta on eGFR
MR Test
Inverse variance weighted (fixed effects)
Inverse variance weighted (multiplicative random effects)
MR Egger
MR RAPS
Weighted median
Weighted mode
0.000
−0.002
−0.004
−0.006
SNP effect on eGFR
0.25
0.50
0.75
SNP effect on Retinal rod rhodopsin−sensitive cGMP 3',5'−cyclic phosphodiesterase subunit delt

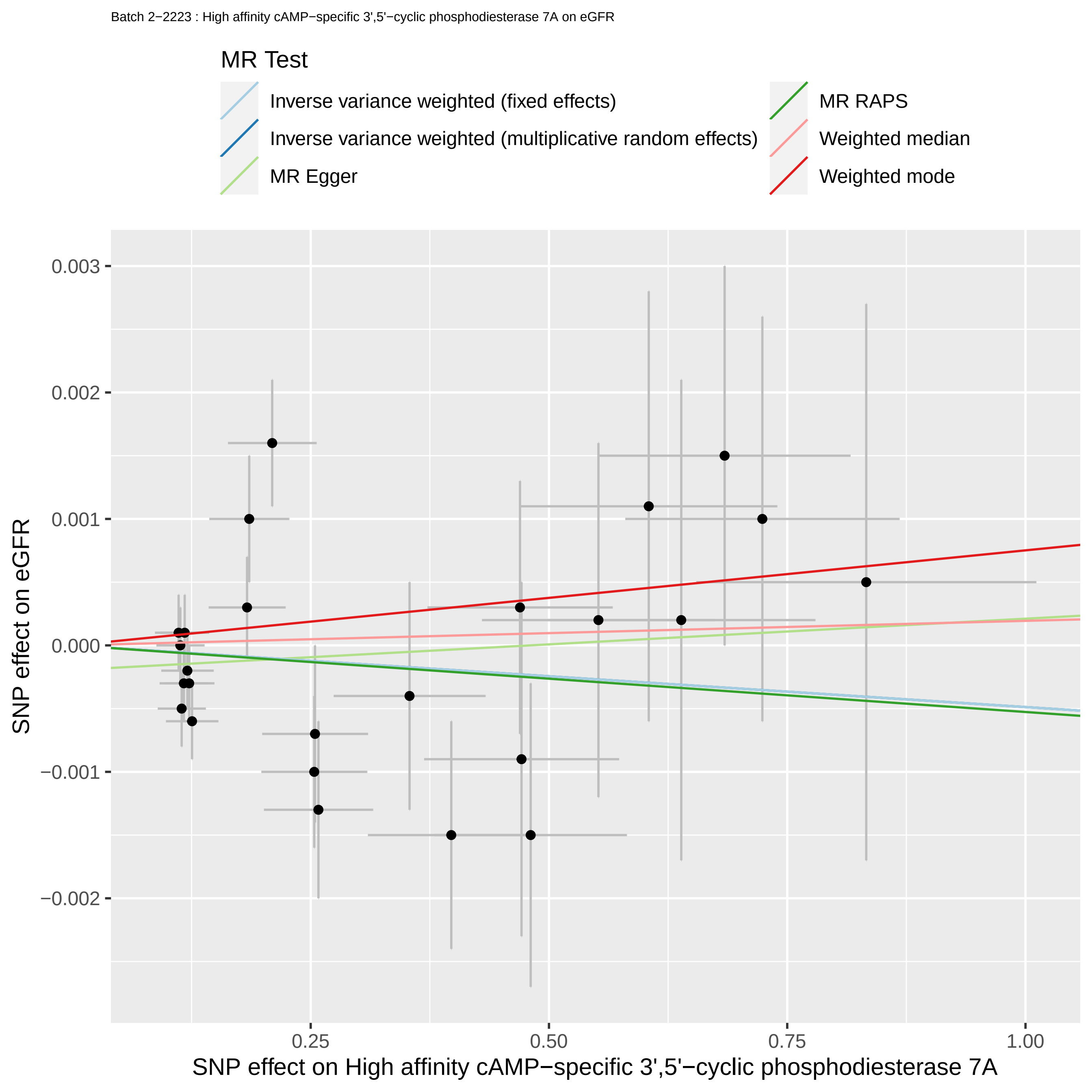

Batch 2−2223 : High affinity cAMP−specific 3',5'−cyclic phosphodiesterase 7A on eGFR
MR Test
Inverse variance weighted (fixed effects)
Inverse variance weighted (multiplicative random effects)
MR Egger
MR RAPS
Weighted median
Weighted mode
SNP effect on eGFR
0.003
0.002
0.001
0.000
−0.001
−0.002
0.25
0.50
0.75
1.00
SNP effect on High affinity cAMP−specific 3',5'−cyclic phosphodiesterase 7A

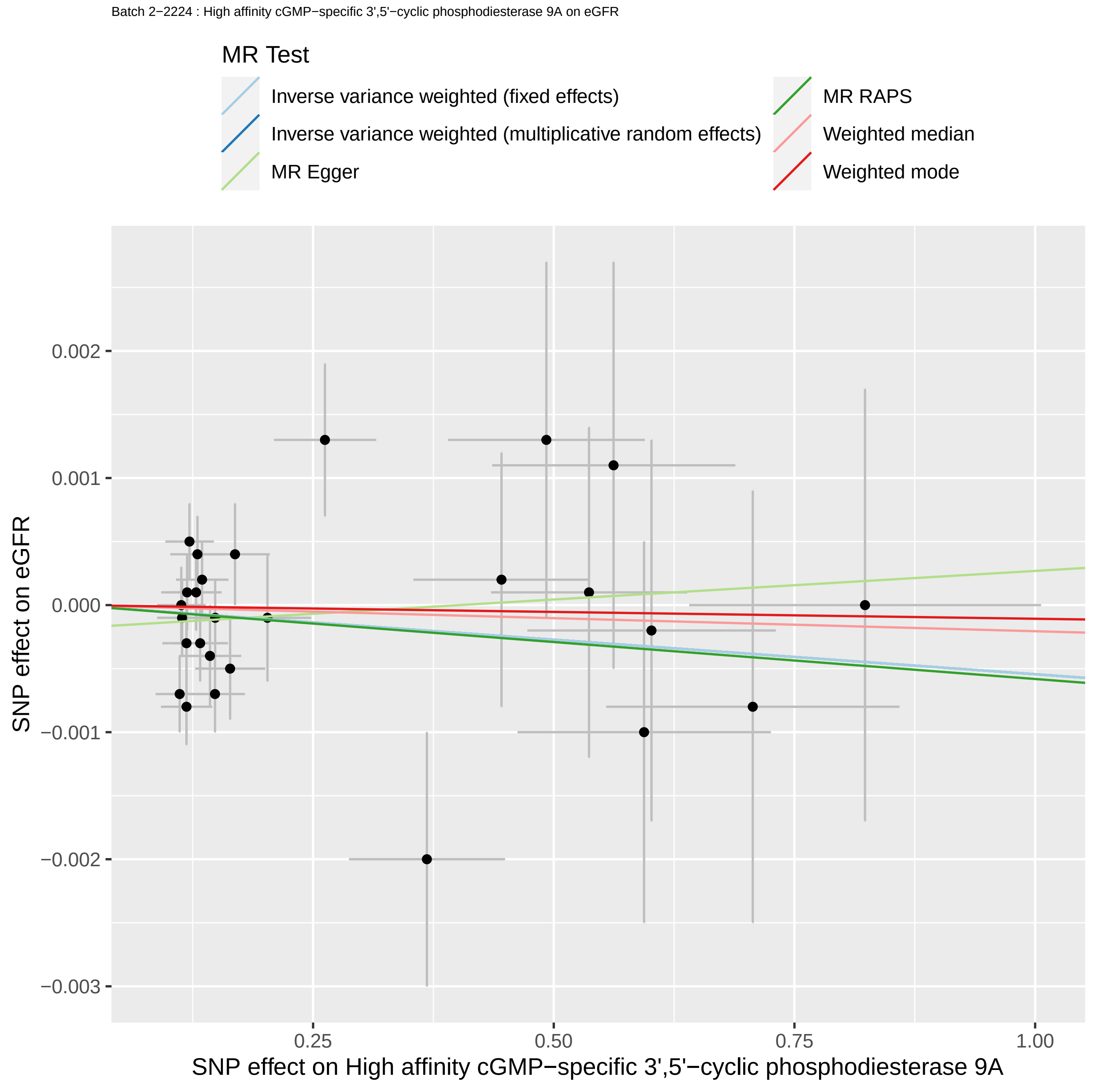

Batch 2−2224 : High affinity cGMP−specific 3',5'−cyclic phosphodiesterase 9A on eGFR
MR Test
Inverse variance weighted (fixed effects)
Inverse variance weighted (multiplicative random effects)
MR Egger
MR RAPS
Weighted median
Weighted mode
0.002
0.001
0.000
−0.001
−0.002
−0.003
0.25
0.50
0.75
1.00
SNP effect on eGFR
SNP effect on High affinity cGMP−specific 3',5'−cyclic phosphodiesterase 9A

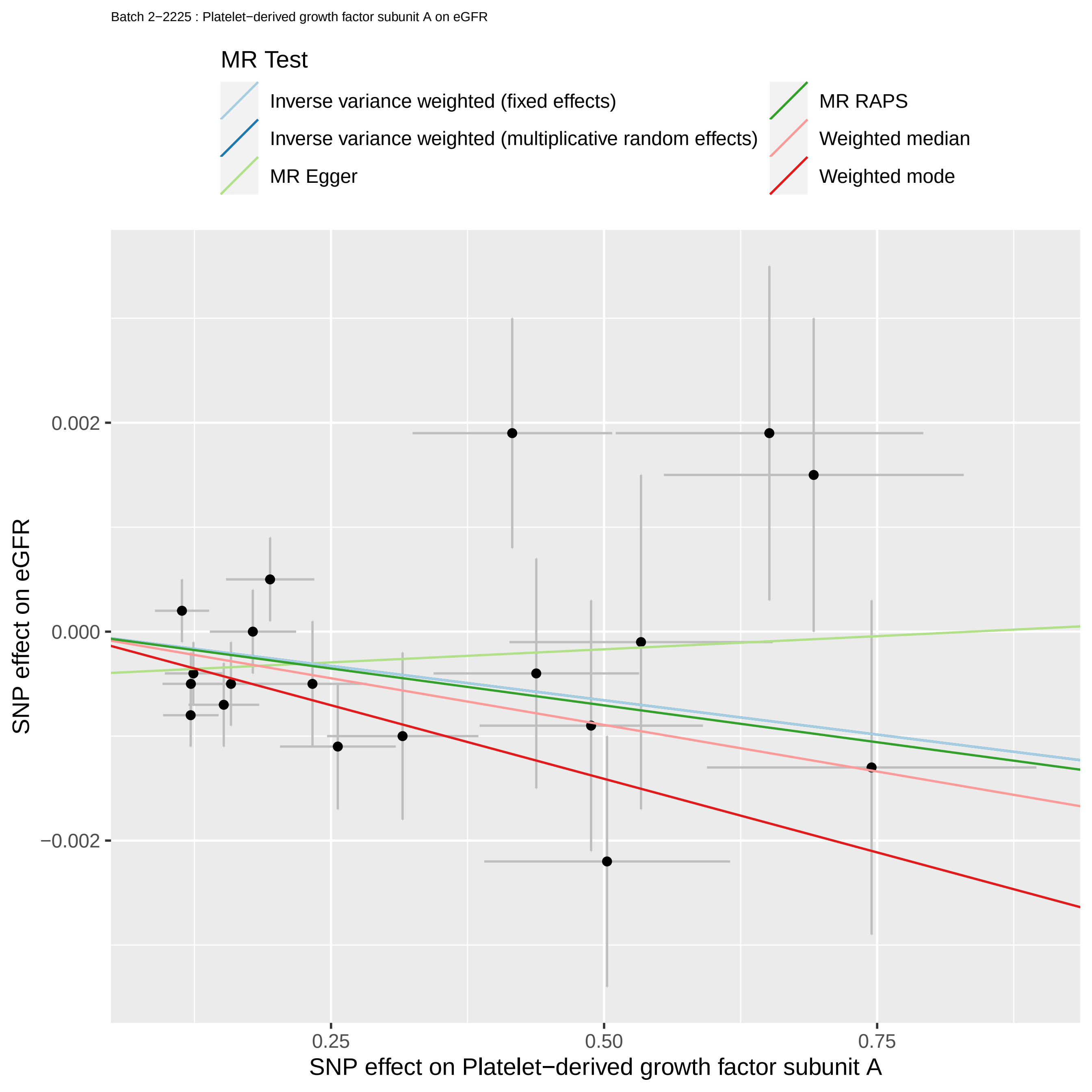

Batch 2−2225 : Platelet−derived growth factor subunit A on eGFR
MR Test
Inverse variance weighted (fixed effects)
Inverse variance weighted (multiplicative random effects)
MR Egger
MR RAPS
Weighted median
Weighted mode
0.002
0.000
−0.002
0.25
0.50
0.75
SNP effect on eGFR
SNP effect on Platelet−derived growth factor subunit A

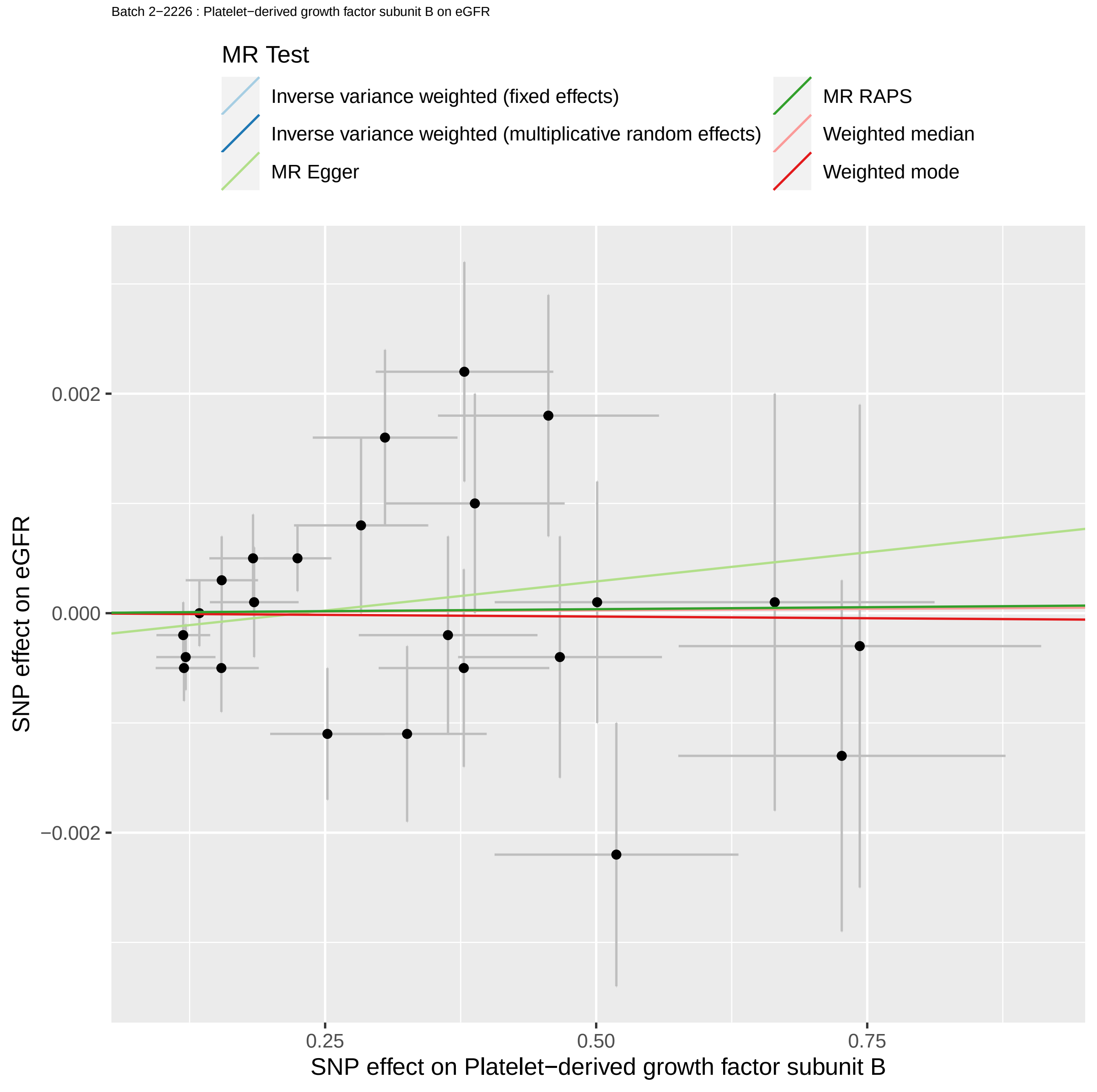
Batch 2−2226 : Platelet−derived growth factor subunit B on eGFR
MR Test
Inverse variance weighted (fixed effects)
Inverse variance weighted (multiplicative random effects)
MR Egger
MR RAPS
Weighted median
Weighted mode
0.002
0.000
−0.002
0.25
0.50
0.75
SNP effect on eGFR
SNP effect on Platelet−derived growth factor subunit B

Batch 2−2227 : Platelet−derived growth factor C on eGFR

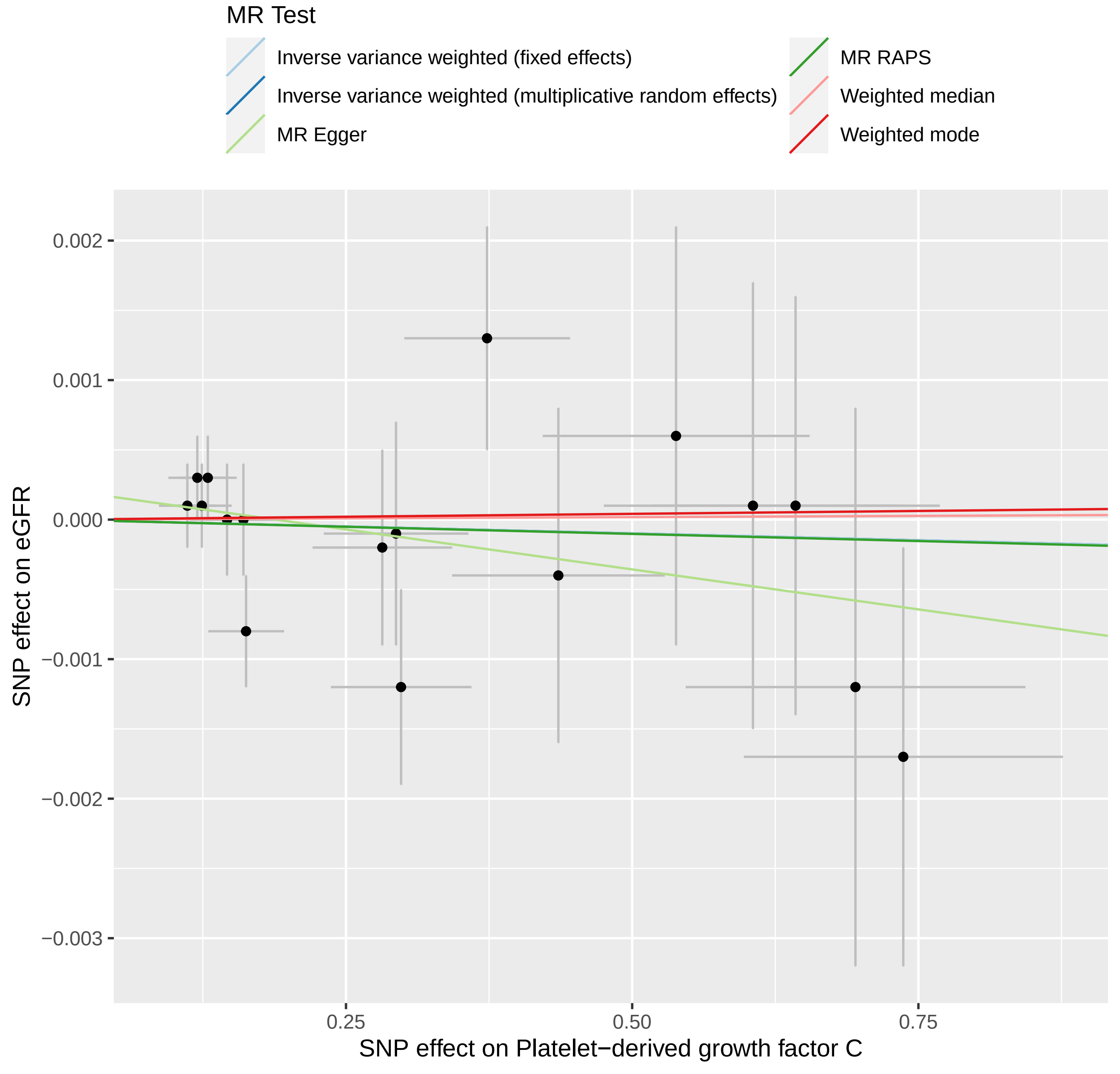

Batch 2−2228 : Platelet−derived growth factor D on eGFR

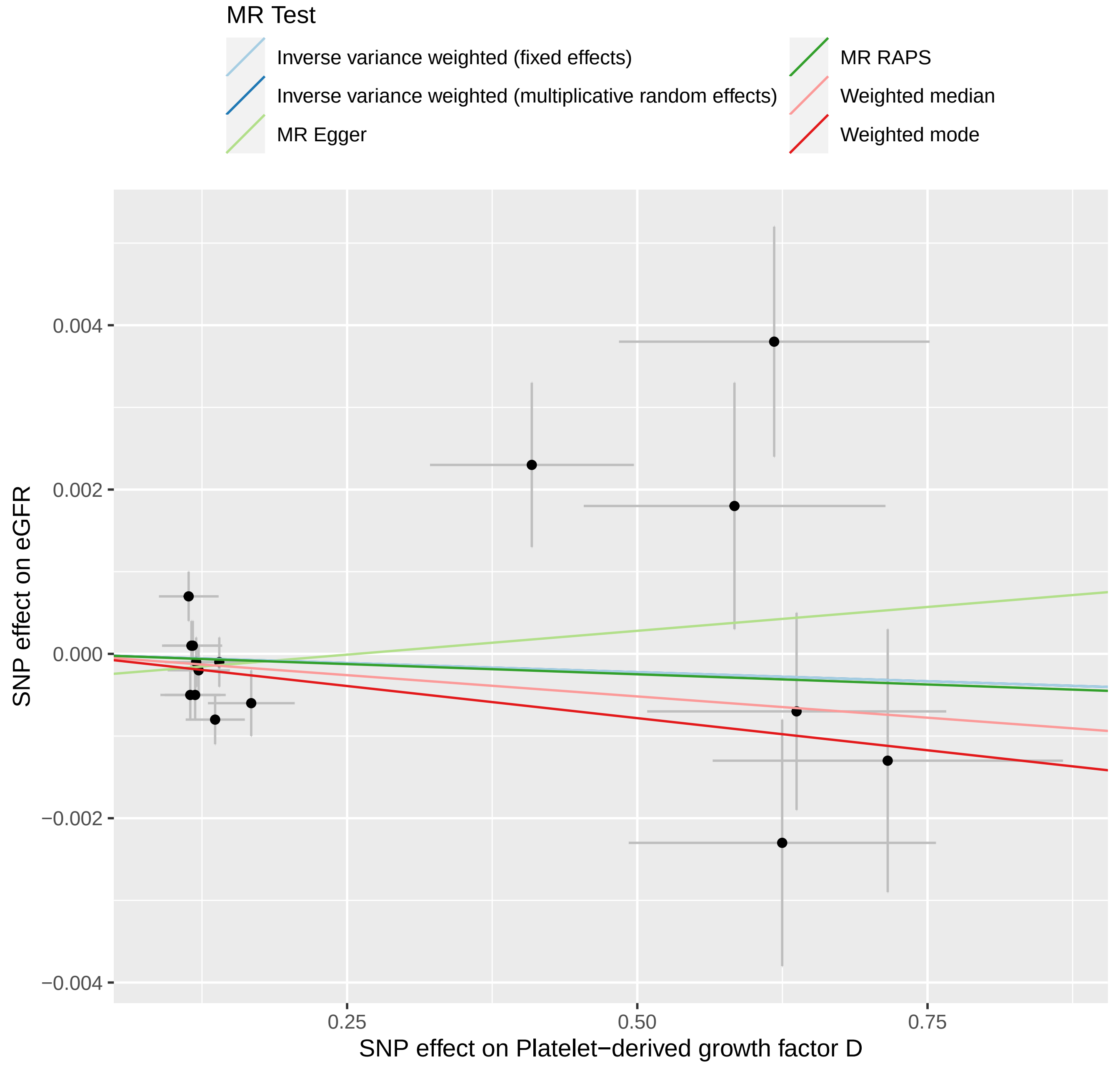

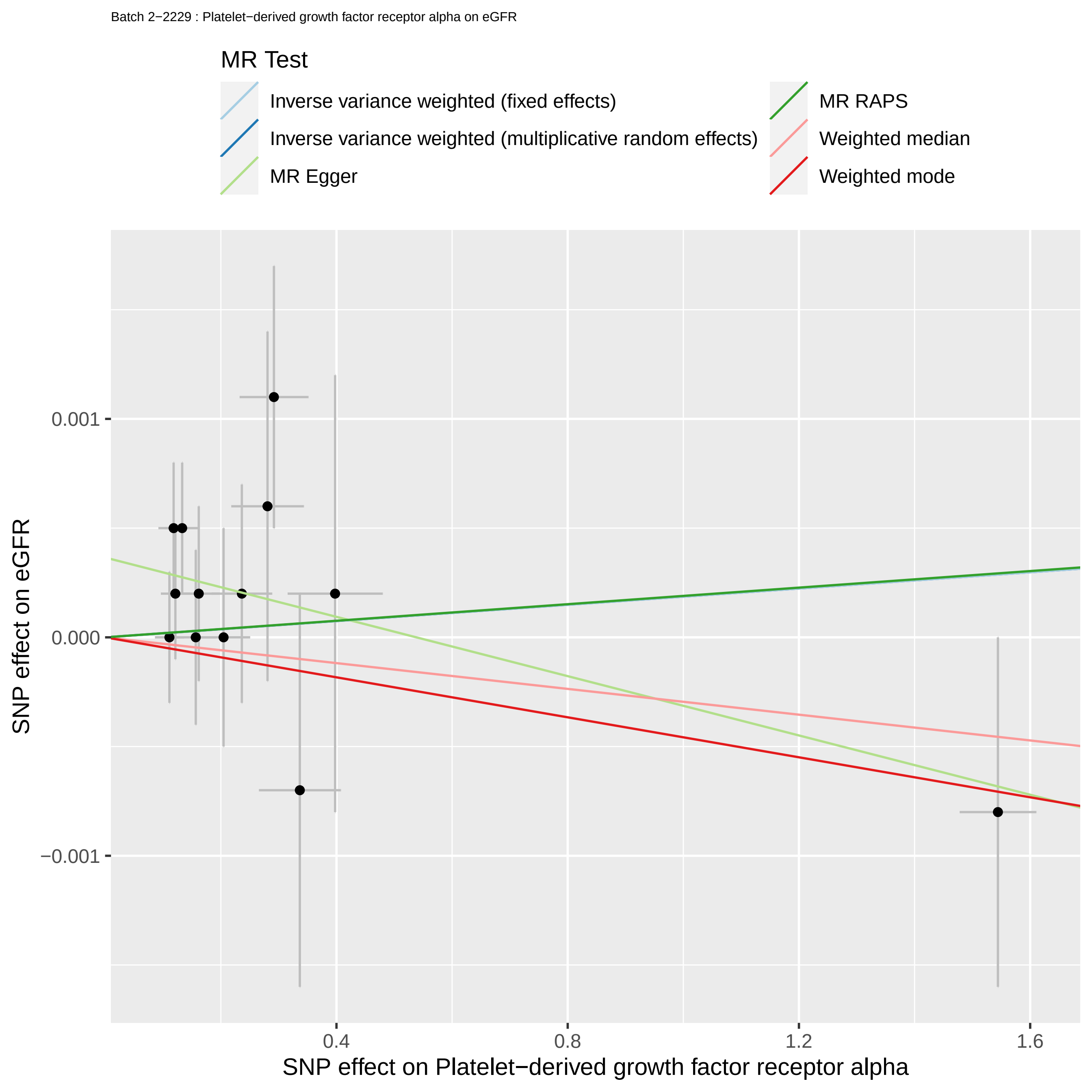

Batch 2−2229 : Platelet−derived growth factor receptor alpha on eGFR
MR Test
Inverse variance weighted (fixed effects)
Inverse variance weighted (multiplicative random effects)
MR Egger
MR RAPS
Weighted median
Weighted mode
0.001
0.000
−0.001
SNP effect on eGFR
0.4
0.8
1.2
1.6
SNP effect on Platelet−derived growth factor receptor alpha

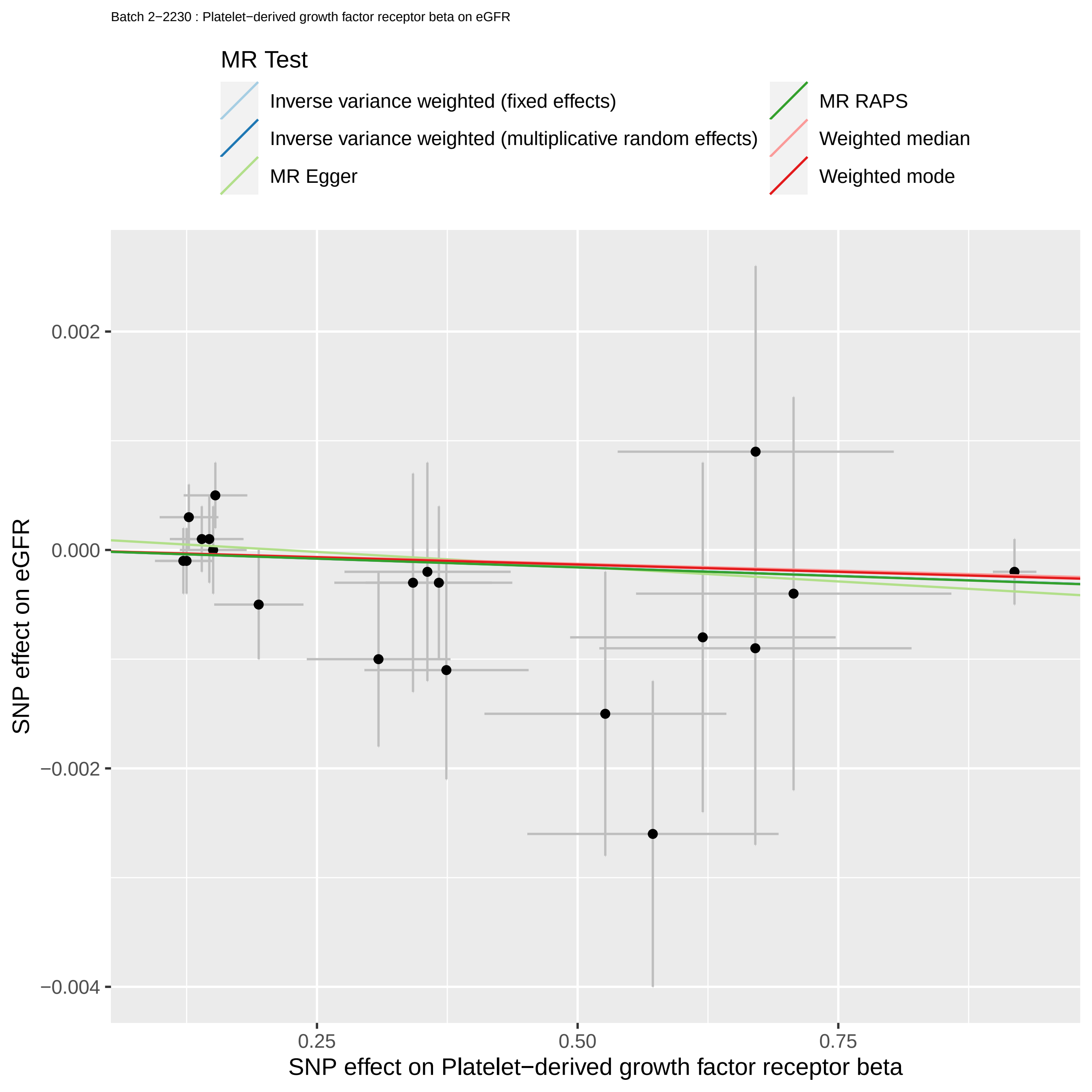

Batch 2−2230 : Platelet−derived growth factor receptor beta on eGFR
MR Test
Inverse variance weighted (fixed effects)
Inverse variance weighted (multiplicative random effects)
MR Egger
MR RAPS
Weighted median
Weighted mode
0.002
0.000
−0.002
−0.004
0.25
0.50
0.75
SNP effect on Platelet−derived growth factor receptor beta
SNP effect on eGFR

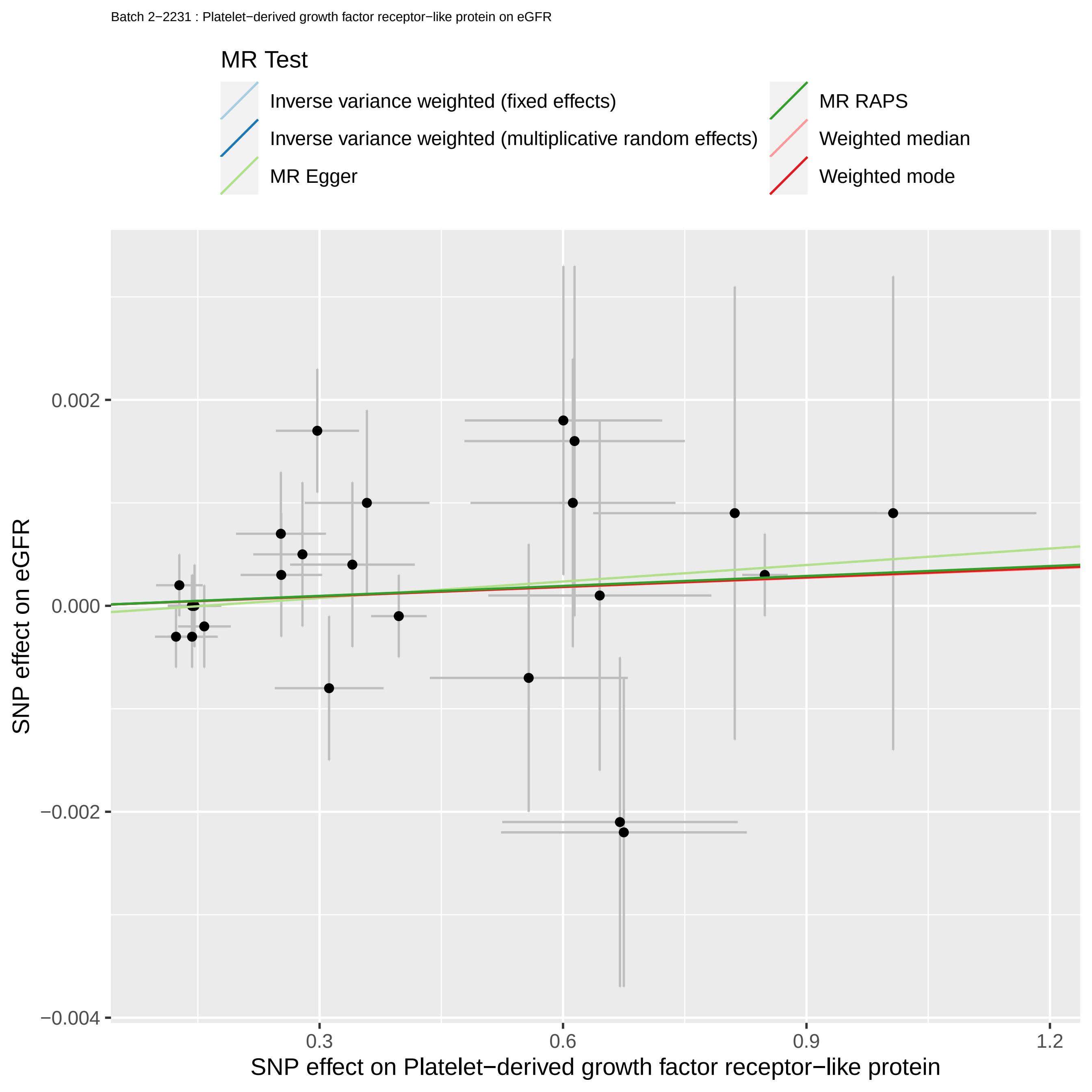
Batch 2−2231 : Platelet−derived growth factor receptor−like protein on eGFR
MR Test
Inverse variance weighted (fixed effects)
Inverse variance weighted (multiplicative random effects)
MR Egger
MR RAPS
Weighted median
Weighted mode
0.002
0.000
−0.002
−0.004
0.3
0.6
0.9
1.2
SNP effect on eGFR
SNP effect on Platelet−derived growth factor receptor−like protein

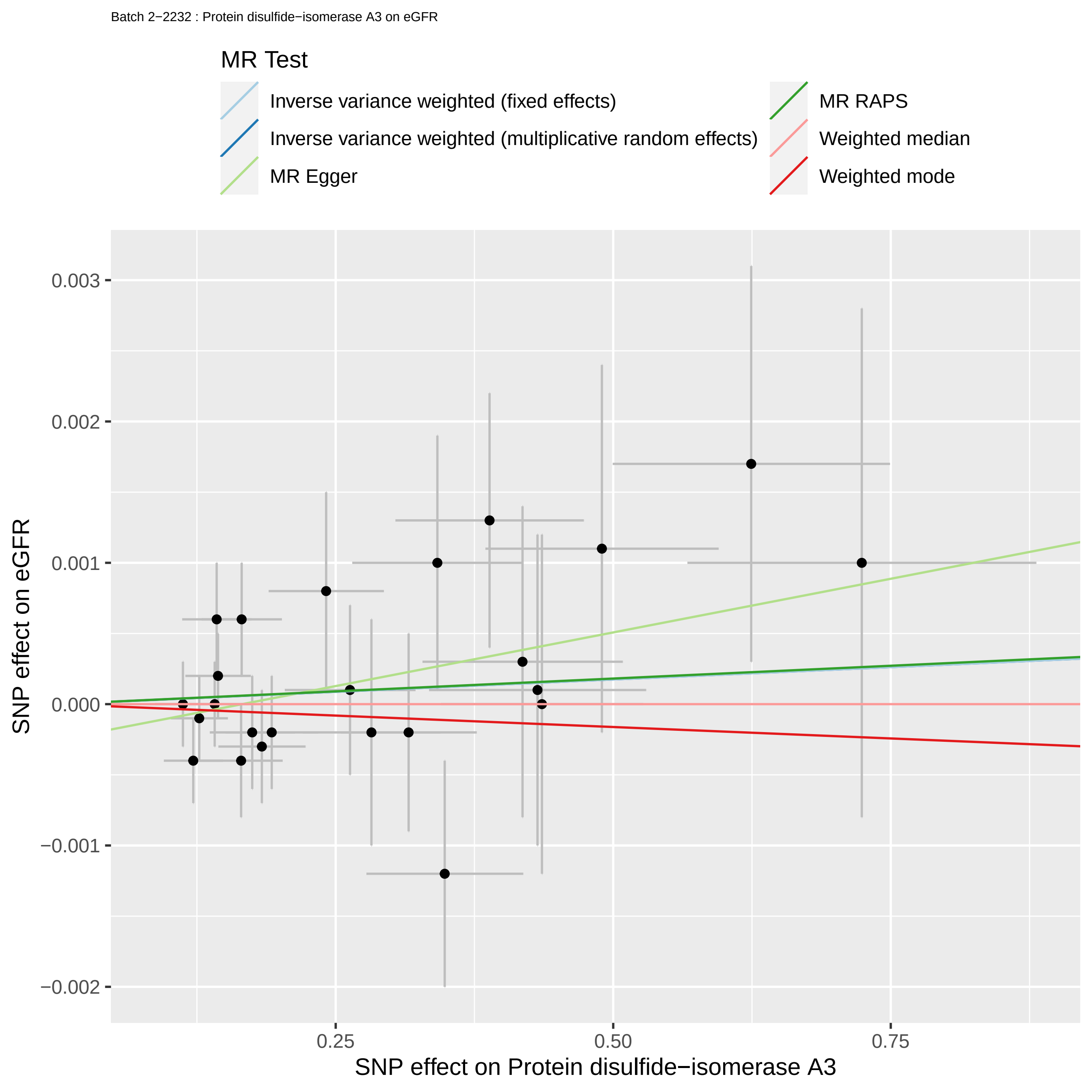
Batch 2−2232 : Protein disulfide−isomerase A3 on eGFR
MR Test
Inverse variance weighted (fixed effects)
Inverse variance weighted (multiplicative random effects)
MR Egger
MR RAPS
Weighted median
Weighted mode
SNP effect on eGFR
0.003
0.002
0.001
0.000
−0.001
−0.002
0.25
0.50
0.75
SNP effect on Protein disulfide−isomerase A3

Batch 2−2233 : Protein disulfide−isomerase A5 on eGFR

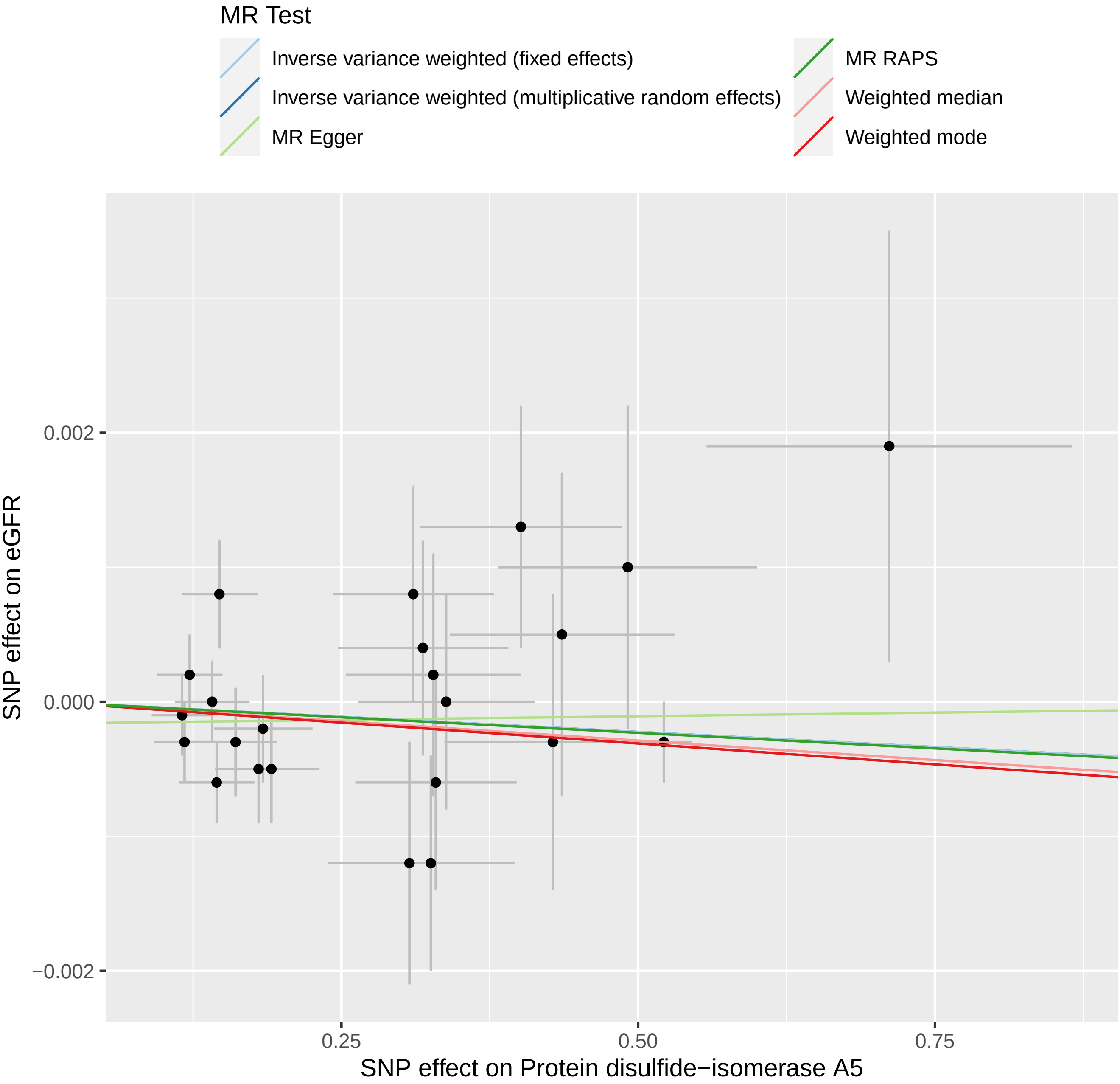

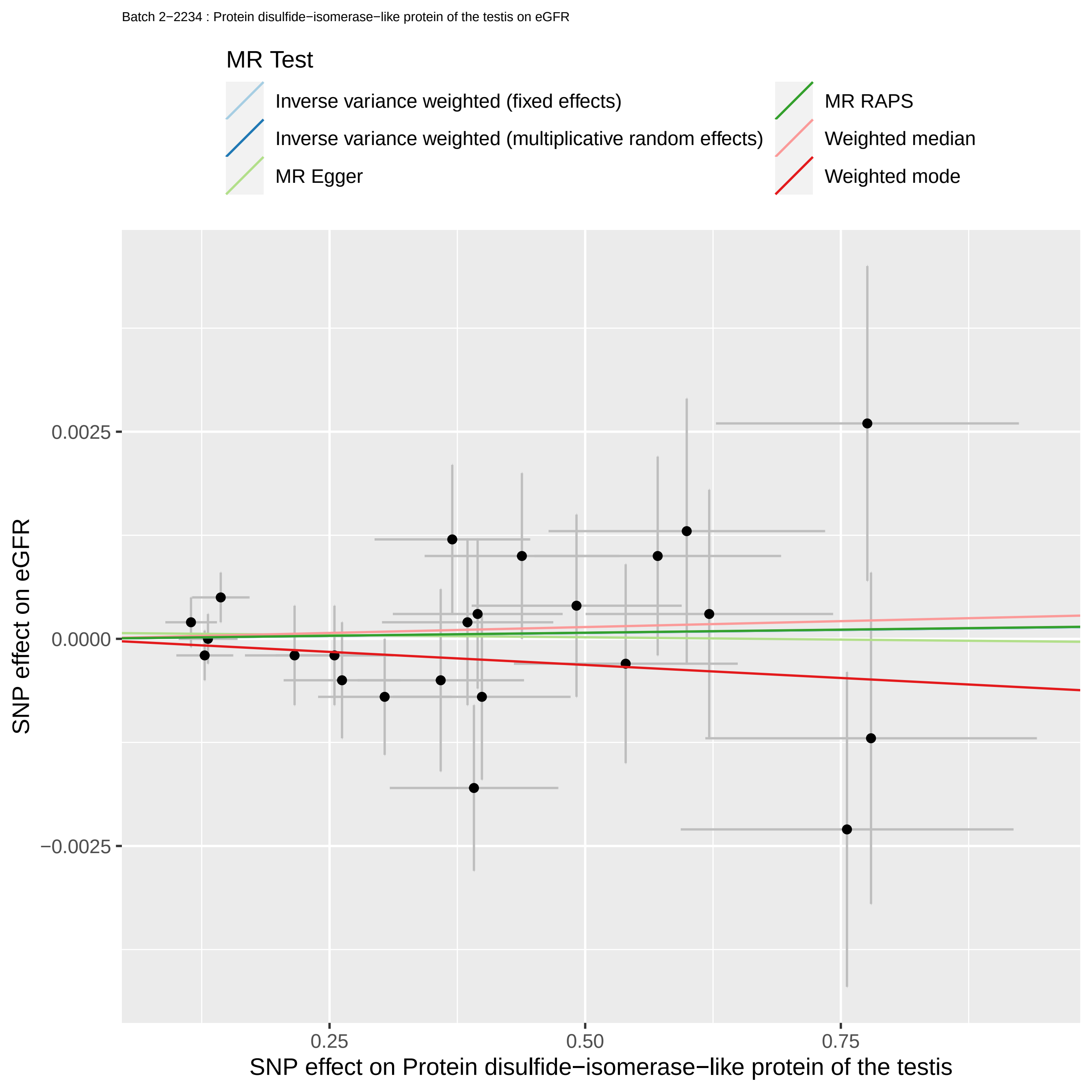
Batch 2−2234 : Protein disulfide−isomerase−like protein of the testis on eGFR
MR Test
Inverse variance weighted (fixed effects)
Inverse variance weighted (multiplicative random effects)
MR Egger
MR RAPS
Weighted median
Weighted mode
0.0025
0.0000
−0.0025
0.25
0.50
0.75
SNP effect on eGFR
SNP effect on Protein disulfide−isomerase−like protein of the testis

Batch 2−2235 : [Pyruvate dehydrogenase (acetyl−transferring)] kinase isozyme 1, mitochondrial on eGFR

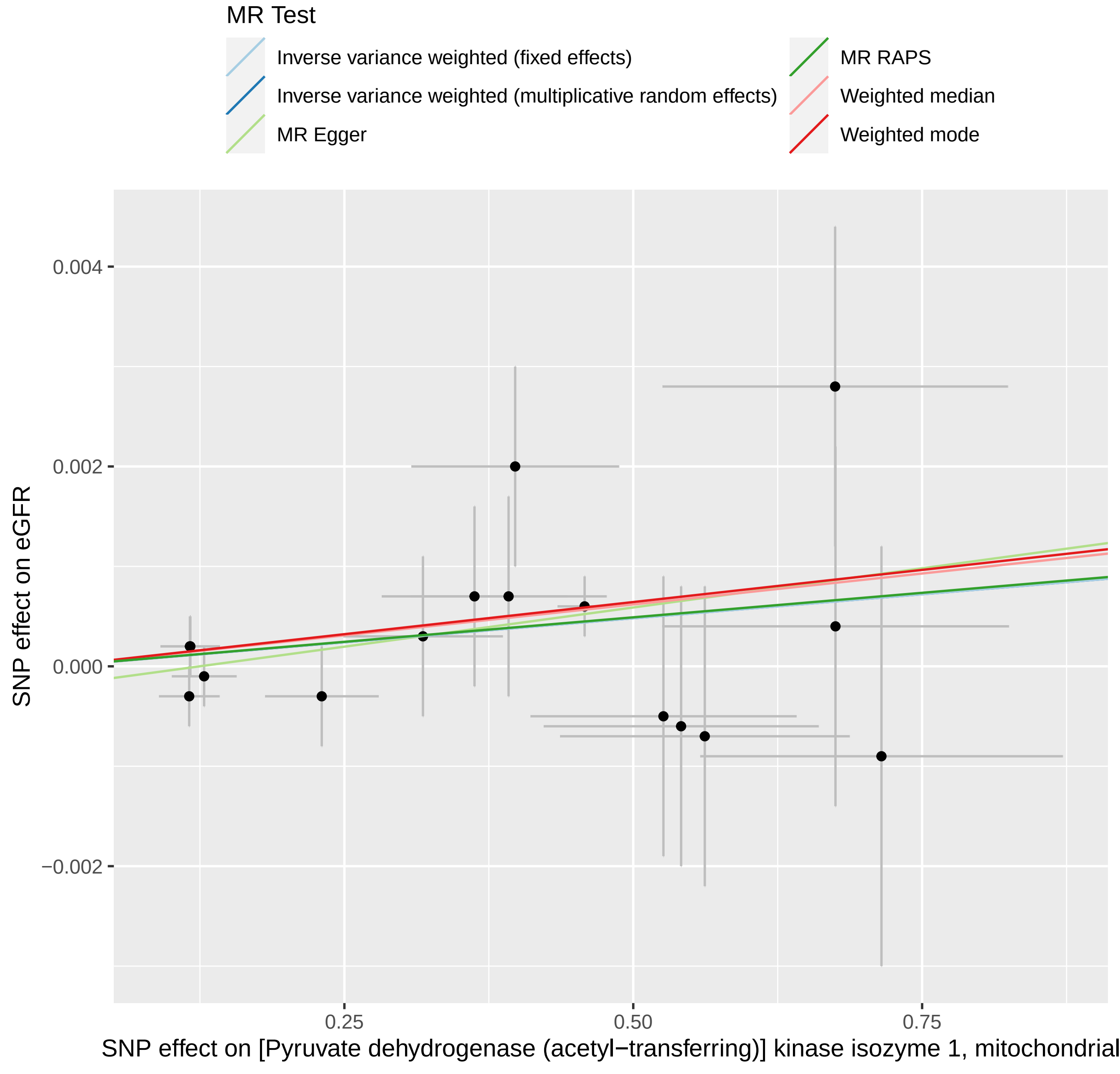

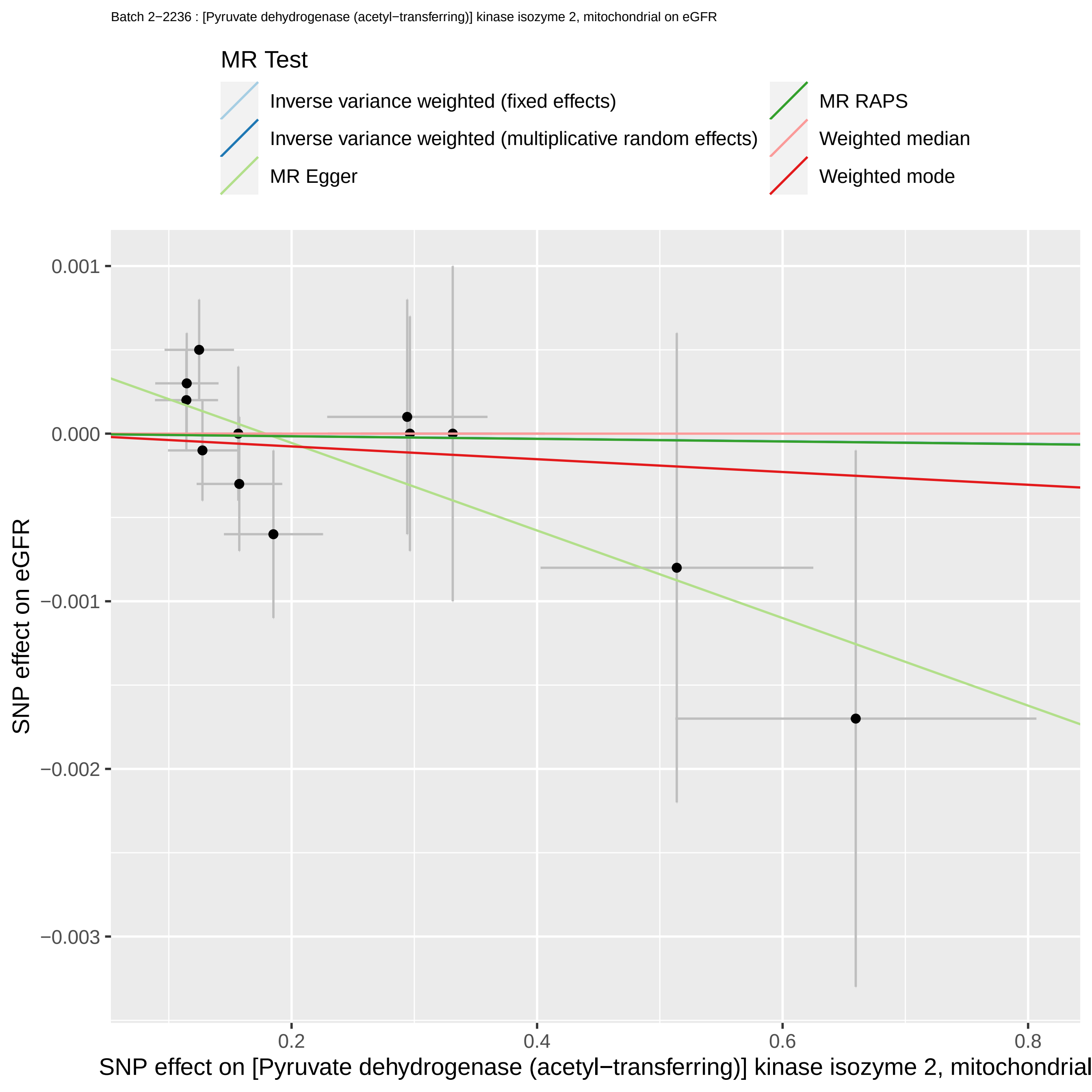

Batch 2−2236 : [Pyruvate dehydrogenase (acetyl−transferring)] kinase isozyme 2, mitochondrial on eGFR
MR Test
Inverse variance weighted (fixed effects)
Inverse variance weighted (multiplicative random effects)
MR Egger
MR RAPS
Weighted median
Weighted mode
0.001
0.000
−0.001
−0.002
−0.003
SNP effect on eGFR
0.2
0.4
0.6
0.8
SNP effect on [Pyruvate dehydrogenase (acetyl−transferring)] kinase isozyme 2, mitochondrial

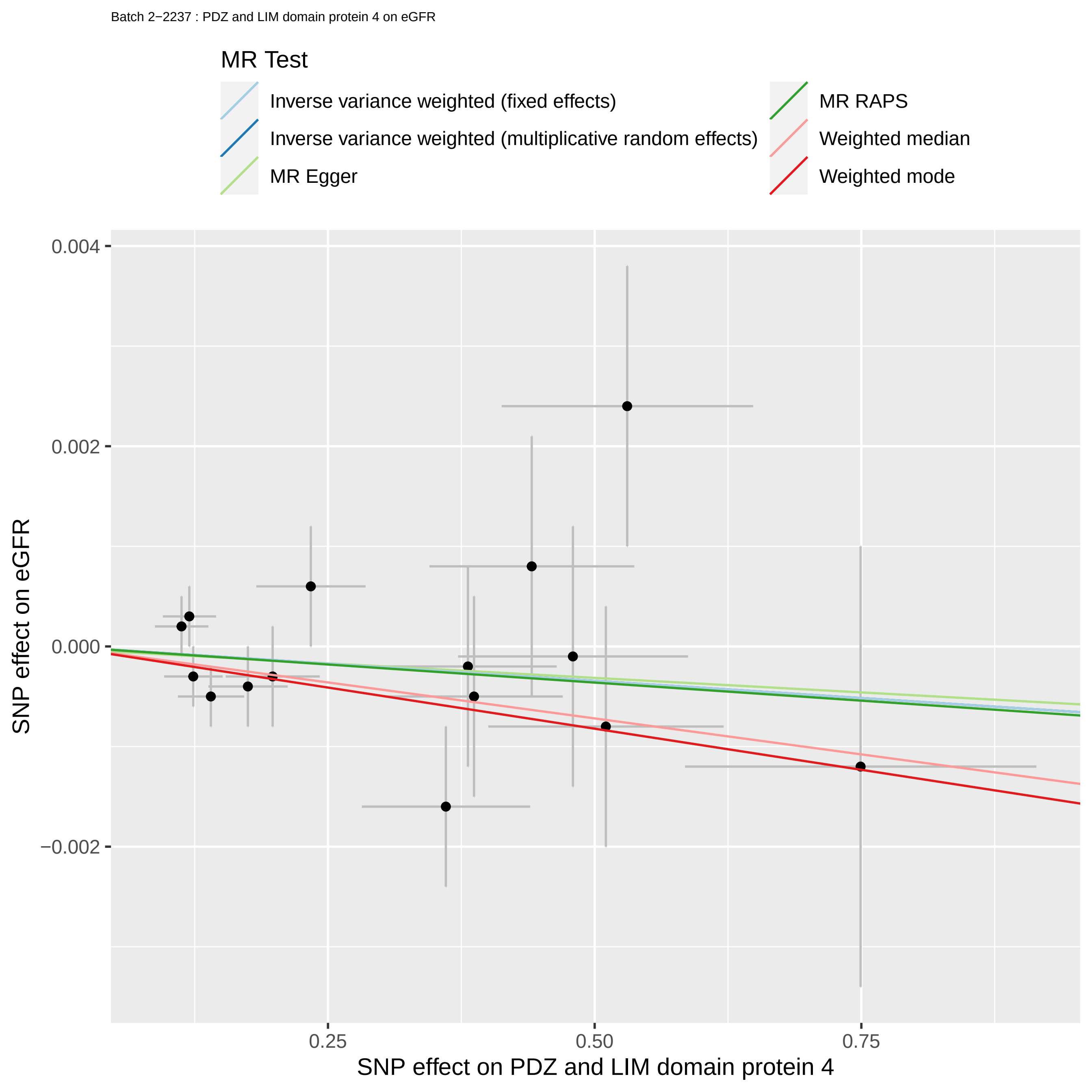

Batch 2−2237 : PDZ and LIM domain protein 4 on eGFR
MR Test
Inverse variance weighted (fixed effects)
Inverse variance weighted (multiplicative random effects)
MR Egger
MR RAPS
Weighted median
Weighted mode
0.004
0.002
0.000
−0.002
SNP effect on eGFR
0.25
0.50
0.75
SNP effect on PDZ and LIM domain protein 4

Batch 2−2238 : p53 and DNA damage−regulated protein 1 on eGFR

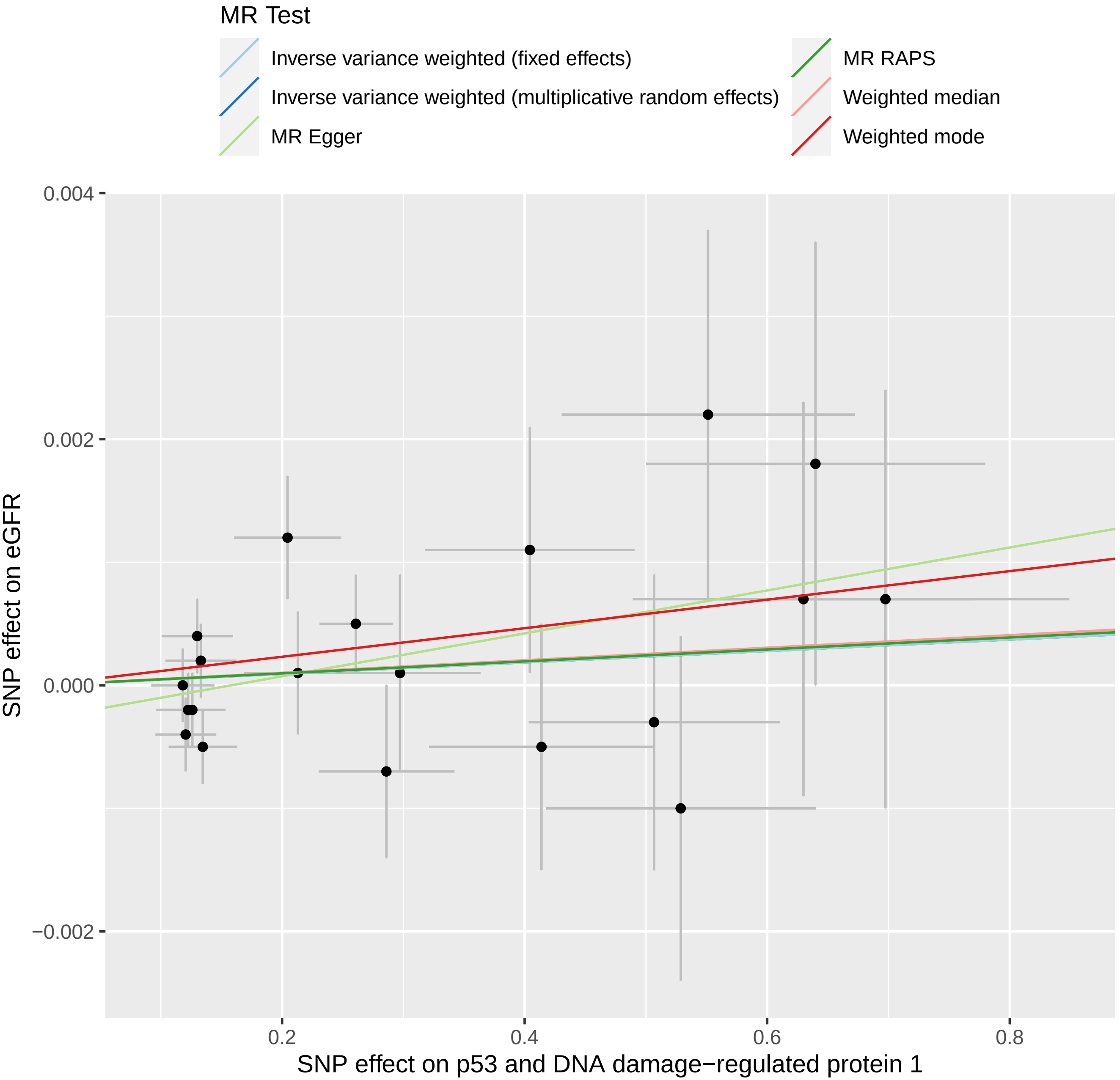

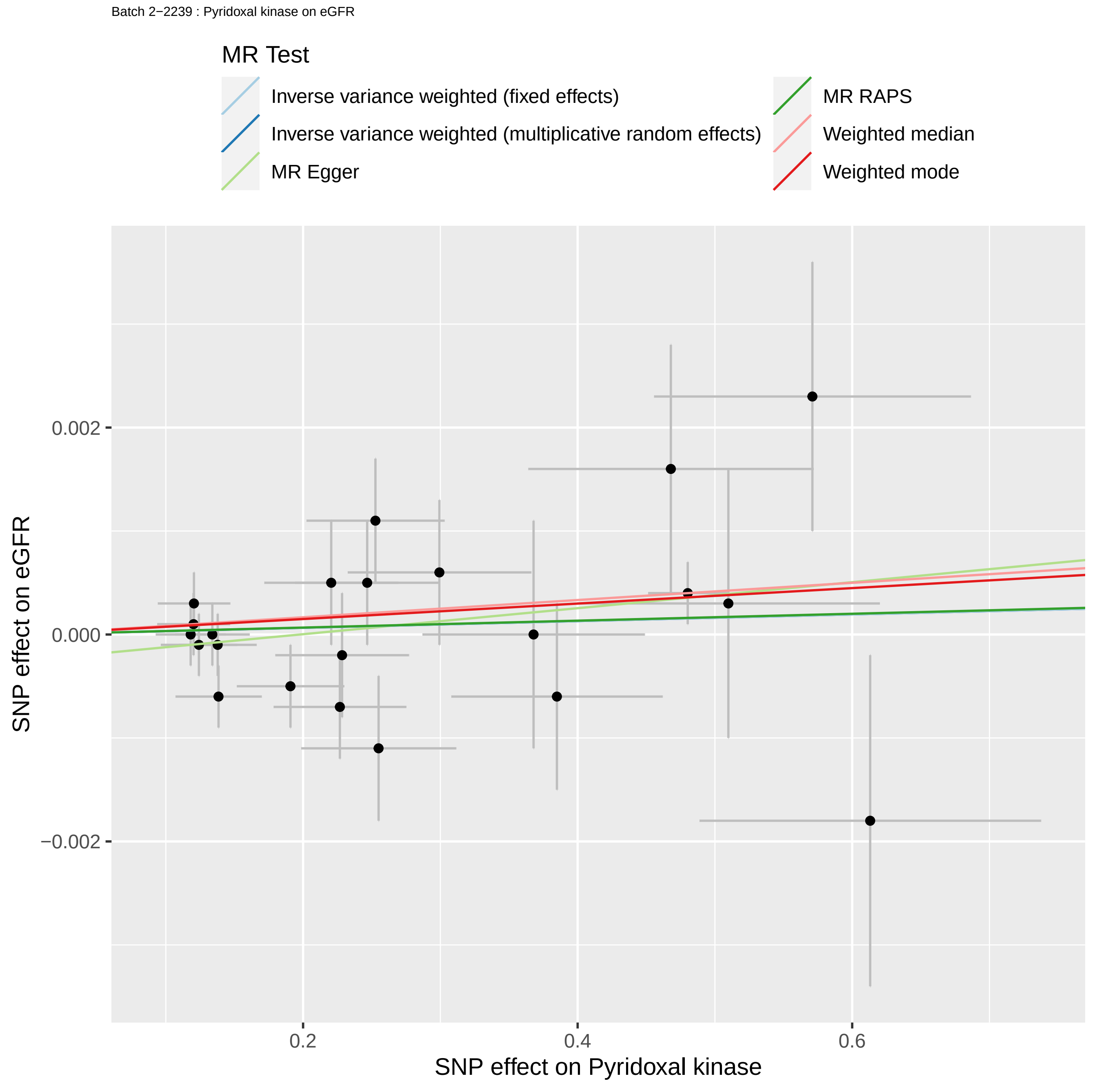

Batch 2−2239 : Pyridoxal kinase on eGFR
MR Test
Inverse variance weighted (fixed effects)
Inverse variance weighted (multiplicative random effects)
MR Egger
MR RAPS
Weighted median
Weighted mode
0.002
0.000
−0.002
0.2
0.4
0.6
SNP effect on eGFR
SNP effect on Pyridoxal kinase

Batch 2−2240 : Pyridoxal phosphate phosphatase on eGFR

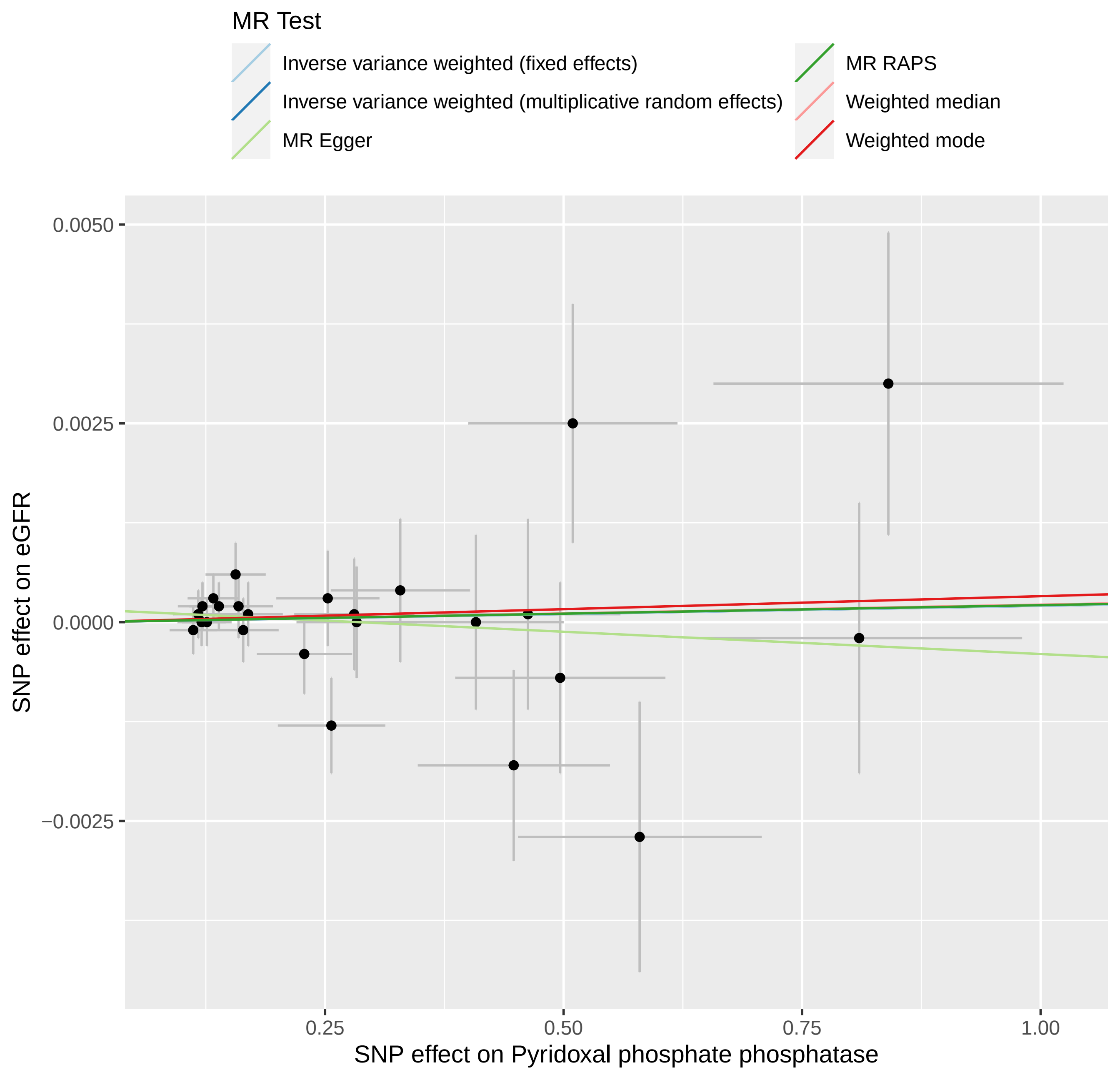

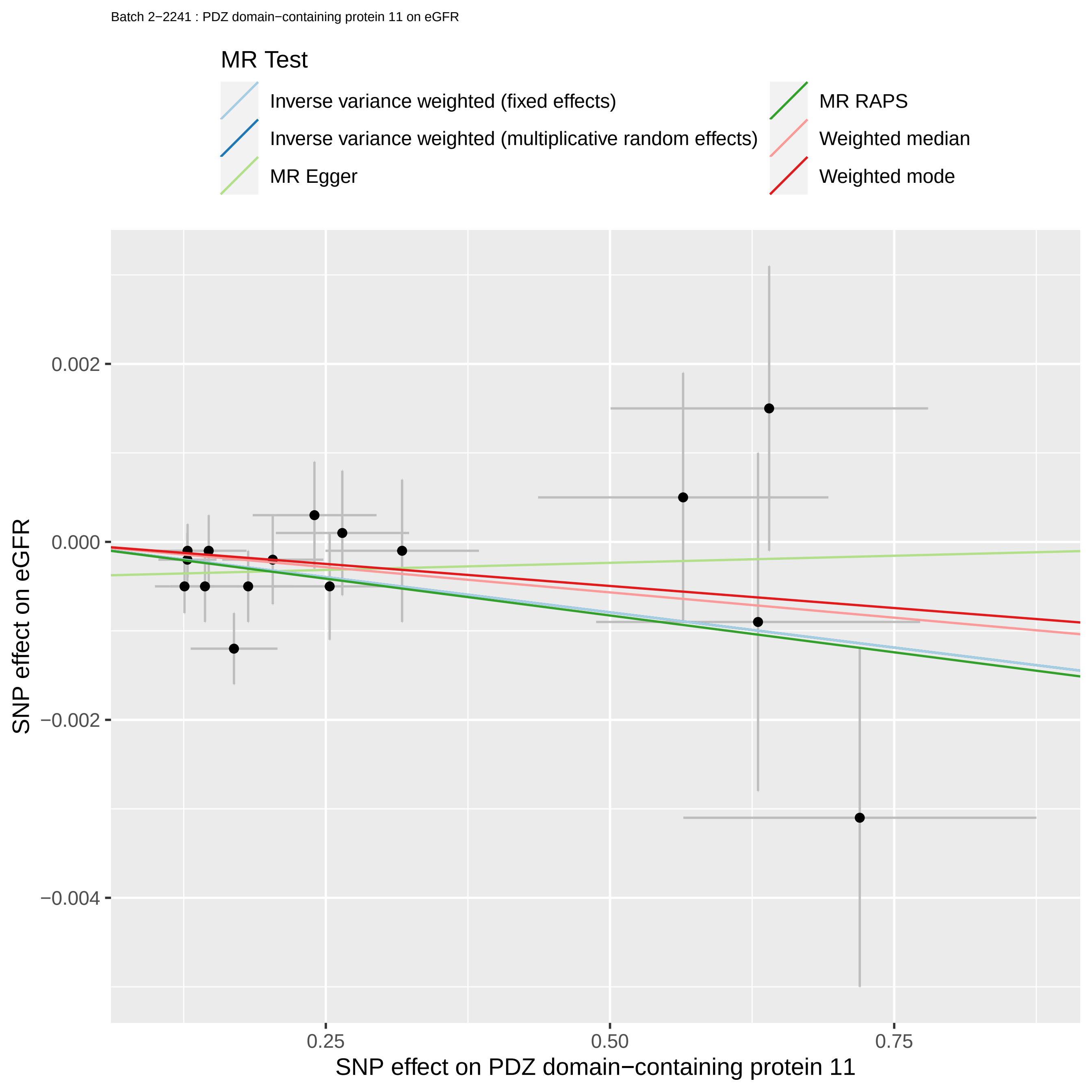

Batch 2−2241 : PDZ domain−containing protein 11 on eGFR
MR Test
Inverse variance weighted (fixed effects)
Inverse variance weighted (multiplicative random effects)
MR Egger
MR RAPS
Weighted median
Weighted mode
0.002
0.000
−0.002
−0.004
0.25
0.50
0.75
SNP effect on eGFR
SNP effect on PDZ domain−containing protein 11

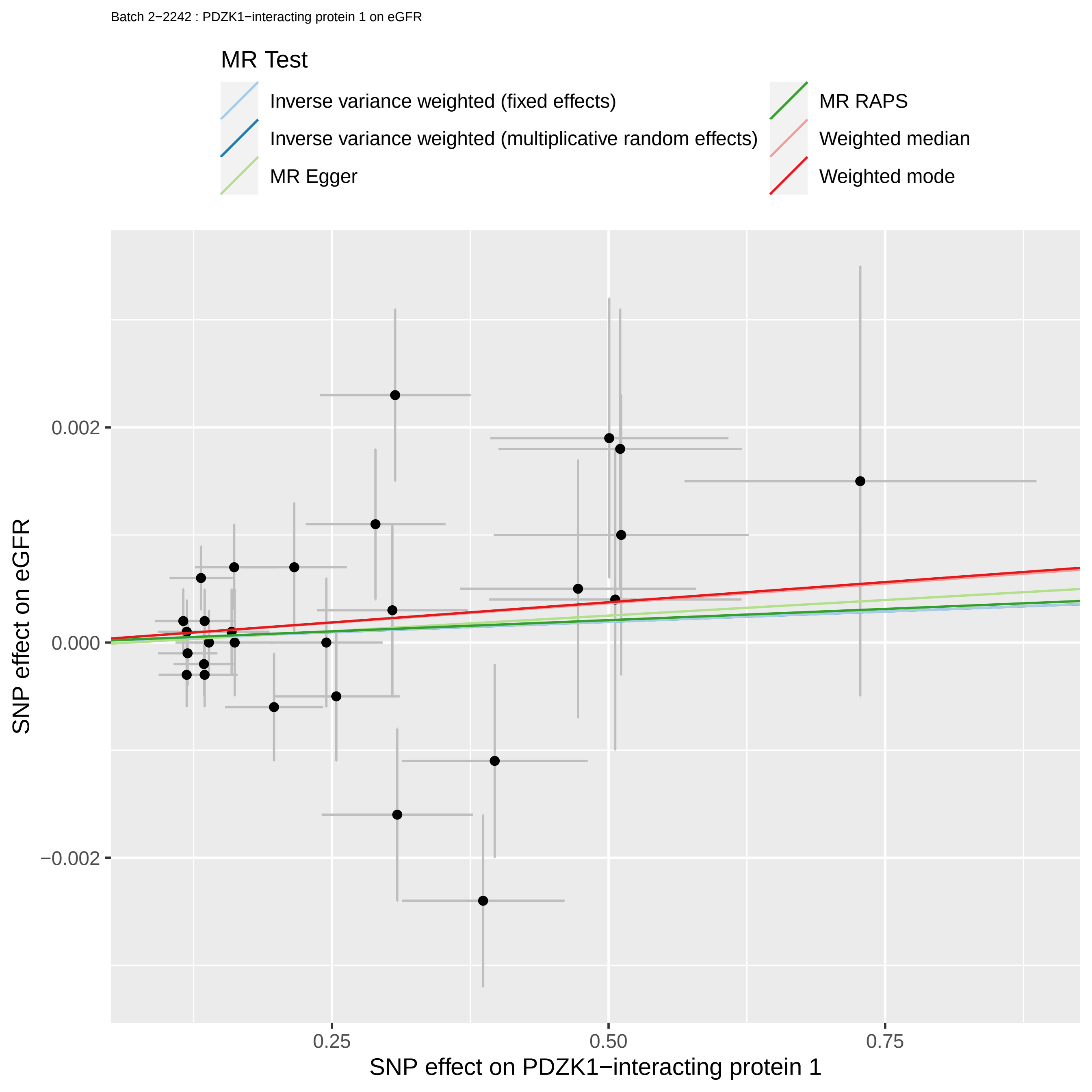

Batch 2−2242 : PDZK1−interacting protein 1 on eGFR
MR Test
Inverse variance weighted (fixed effects)
Inverse variance weighted (multiplicative random effects)
MR Egger
MR RAPS
Weighted median
Weighted mode
0.002
0.000
−0.002
0.25
0.50
0.75
SNP effect on eGFR
SNP effect on PDZK1−interacting protein 1

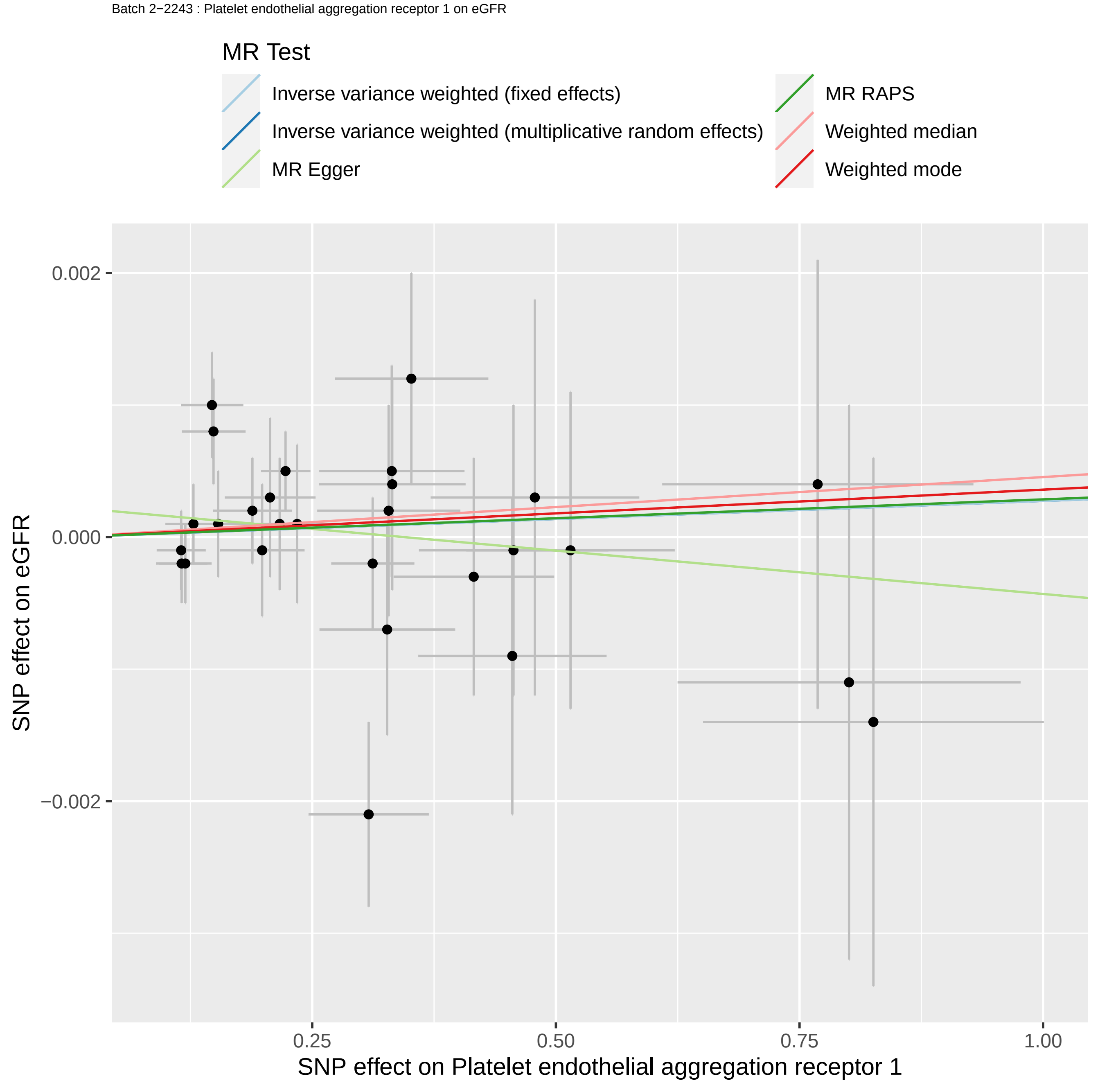
Batch 2−2243 : Platelet endothelial aggregation receptor 1 on eGFR
MR Test
Inverse variance weighted (fixed effects)
Inverse variance weighted (multiplicative random effects)
MR Egger
MR RAPS
Weighted median
Weighted mode
0.002
0.000
−0.002
0.25
0.50
0.75
1.00
SNP effect on eGFR
SNP effect on Platelet endothelial aggregation receptor 1

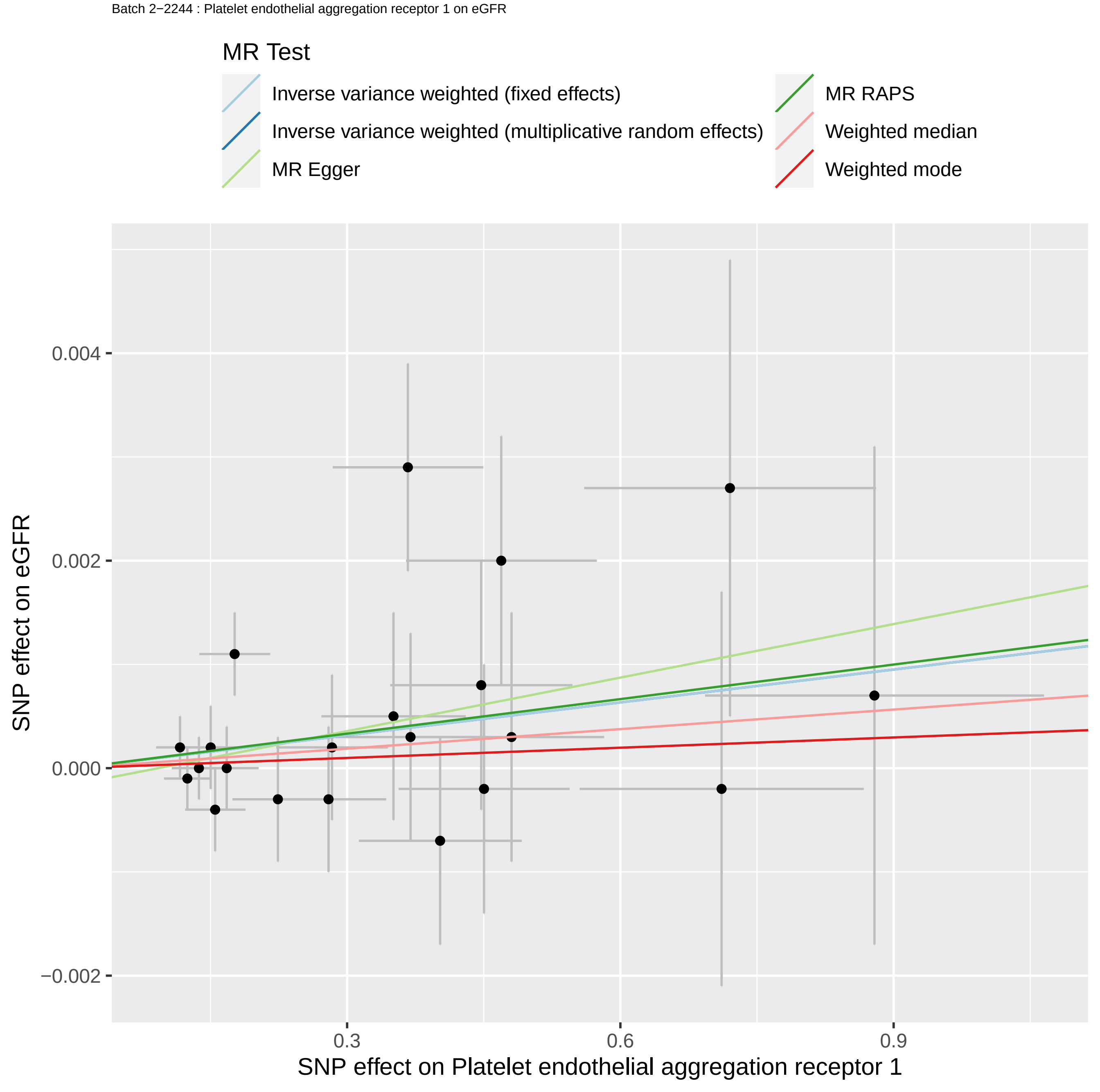

Batch 2−2244 : Platelet endothelial aggregation receptor 1 on eGFR
MR Test
Inverse variance weighted (fixed effects)
Inverse variance weighted (multiplicative random effects)
MR Egger
MR RAPS
Weighted median
Weighted mode
SNP effect on eGFR
0.004
0.002
0.000
−0.002
0.3
0.6
0.9
SNP effect on Platelet endothelial aggregation receptor 1

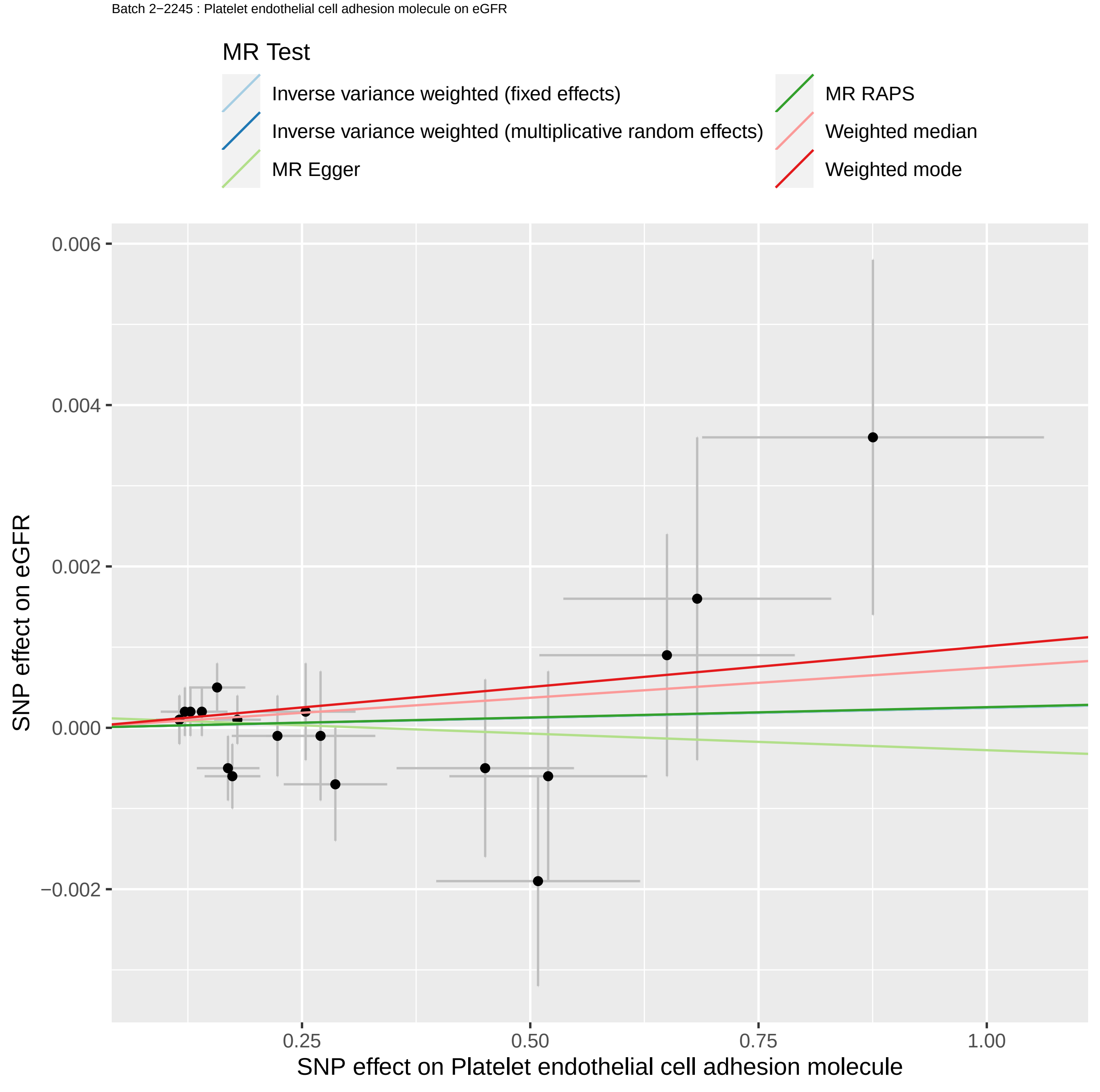

Batch 2−2245 : Platelet endothelial cell adhesion molecule on eGFR
MR Test
Inverse variance weighted (fixed effects)
Inverse variance weighted (multiplicative random effects)
MR Egger
MR RAPS
Weighted median
Weighted mode
SNP effect on eGFR
0.006
0.004
0.002
0.000
−0.002
0.25
0.50
0.75
1.00
SNP effect on Platelet endothelial cell adhesion molecule

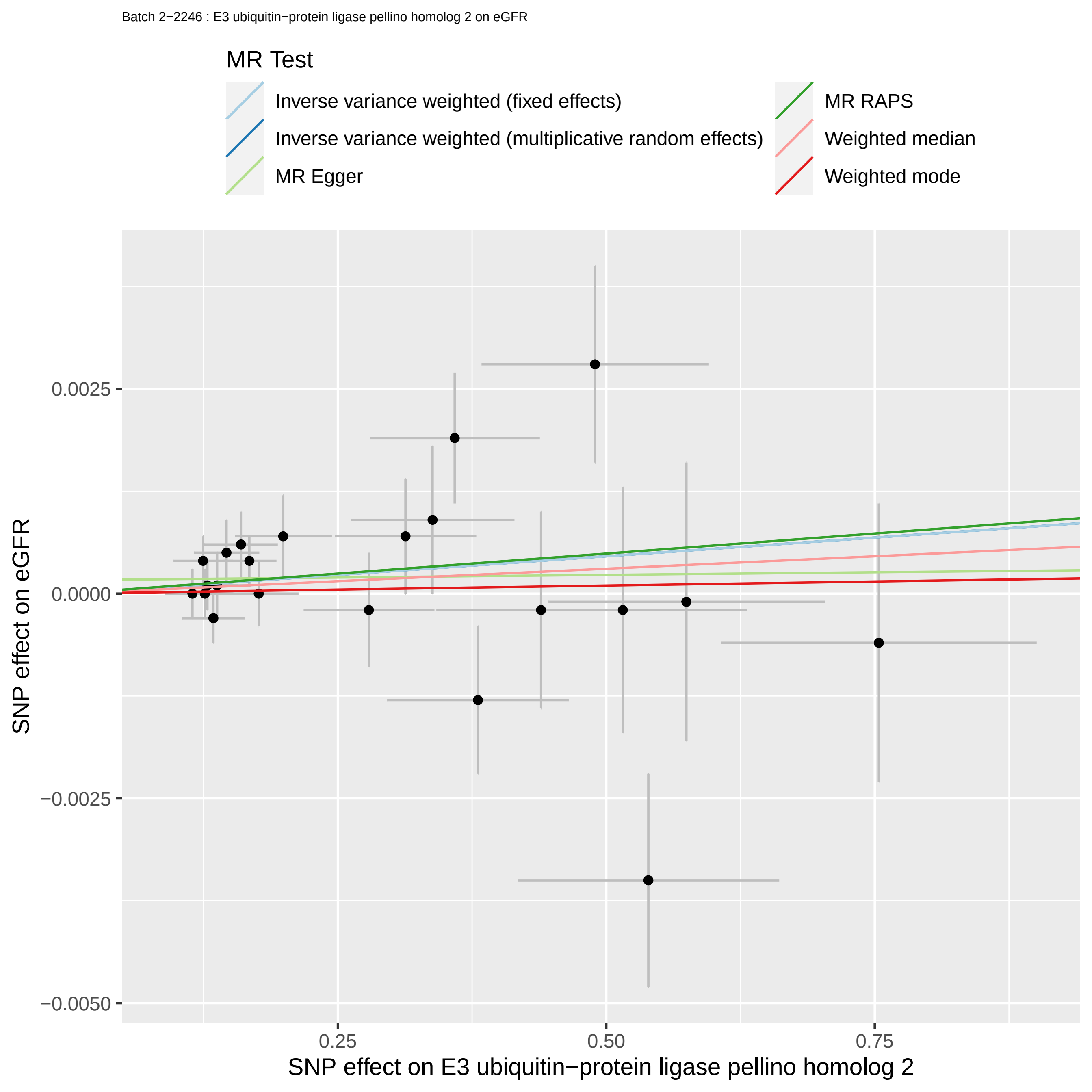

Batch 2−2246 : E3 ubiquitin−protein ligase pellino homolog 2 on eGFR
MR Test
Inverse variance weighted (fixed effects)
Inverse variance weighted (multiplicative random effects)
MR Egger
MR RAPS
Weighted median
Weighted mode
0.0025
0.0000
−0.0025
−0.0050
0.25
0.50
0.75
SNP effect on eGFR
SNP effect on E3 ubiquitin−protein ligase pellino homolog 2

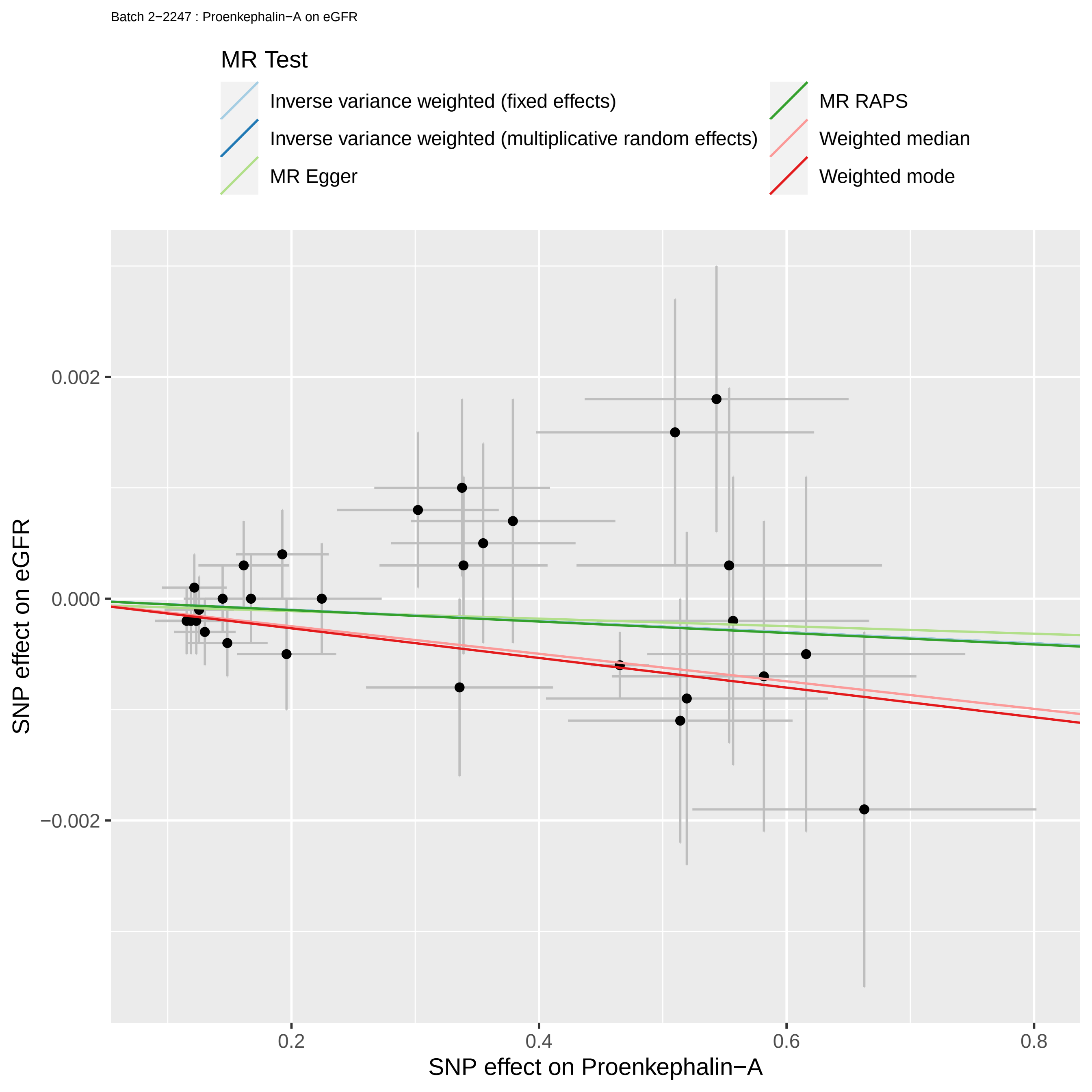

Batch 2−2247 : Proenkephalin−A on eGFR
MR Test
Inverse variance weighted (fixed effects)
Inverse variance weighted (multiplicative random effects)
MR Egger
MR RAPS
Weighted median
Weighted mode
0.002
0.000
−0.002
0.2
0.4
0.6
0.8
SNP effect on eGFR
SNP effect on Proenkephalin−A

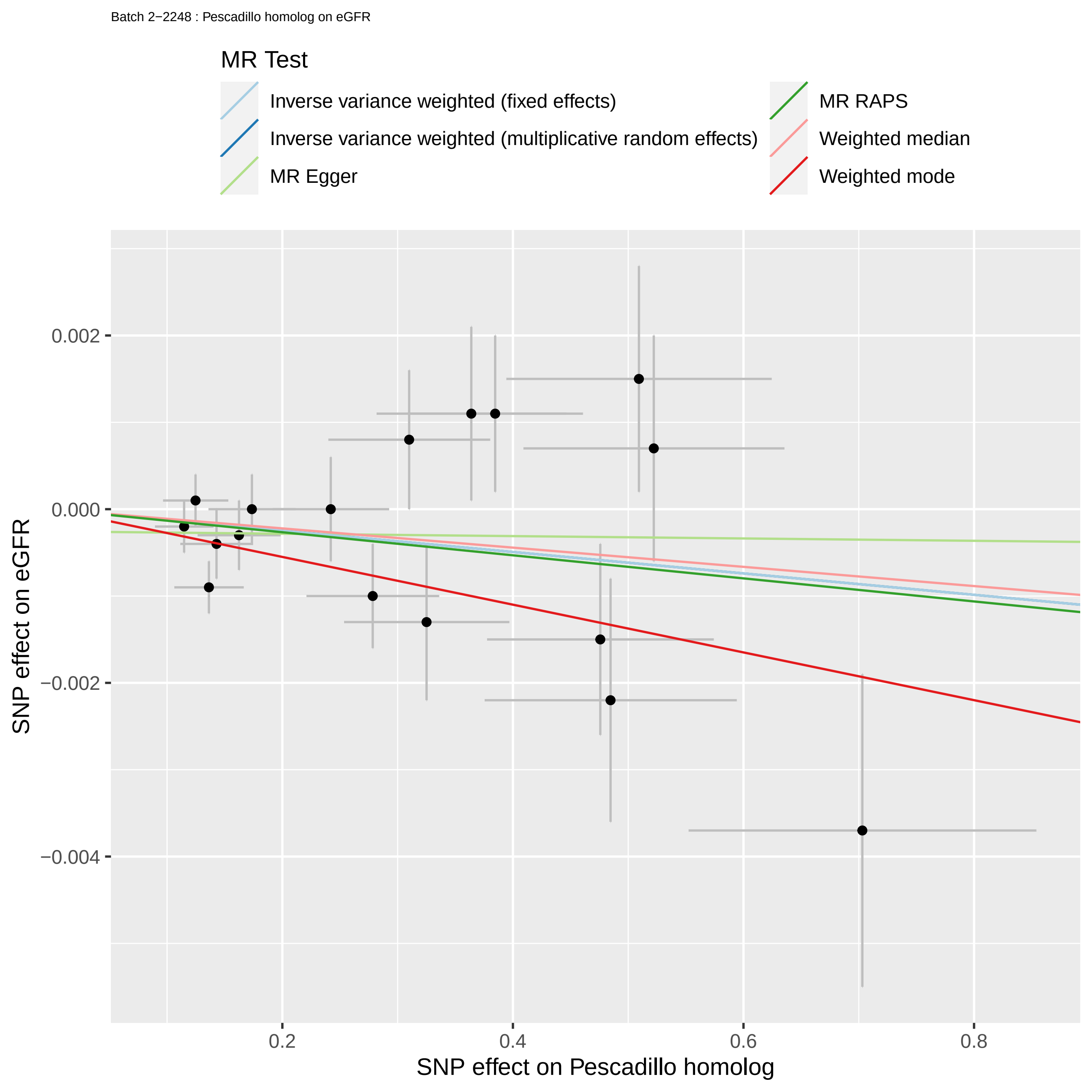
Batch 2−2248 : Pescadillo homolog on eGFR
MR Test
Inverse variance weighted (fixed effects)
Inverse variance weighted (multiplicative random effects)
MR Egger
MR RAPS
Weighted median
Weighted mode
0.002
0.000
−0.002
−0.004
0.2
0.4
0.6
0.8
SNP effect on eGFR
SNP effect on Pescadillo homolog

Batch 2−2249 : Peroxisomal membrane protein PEX14 on eGFR

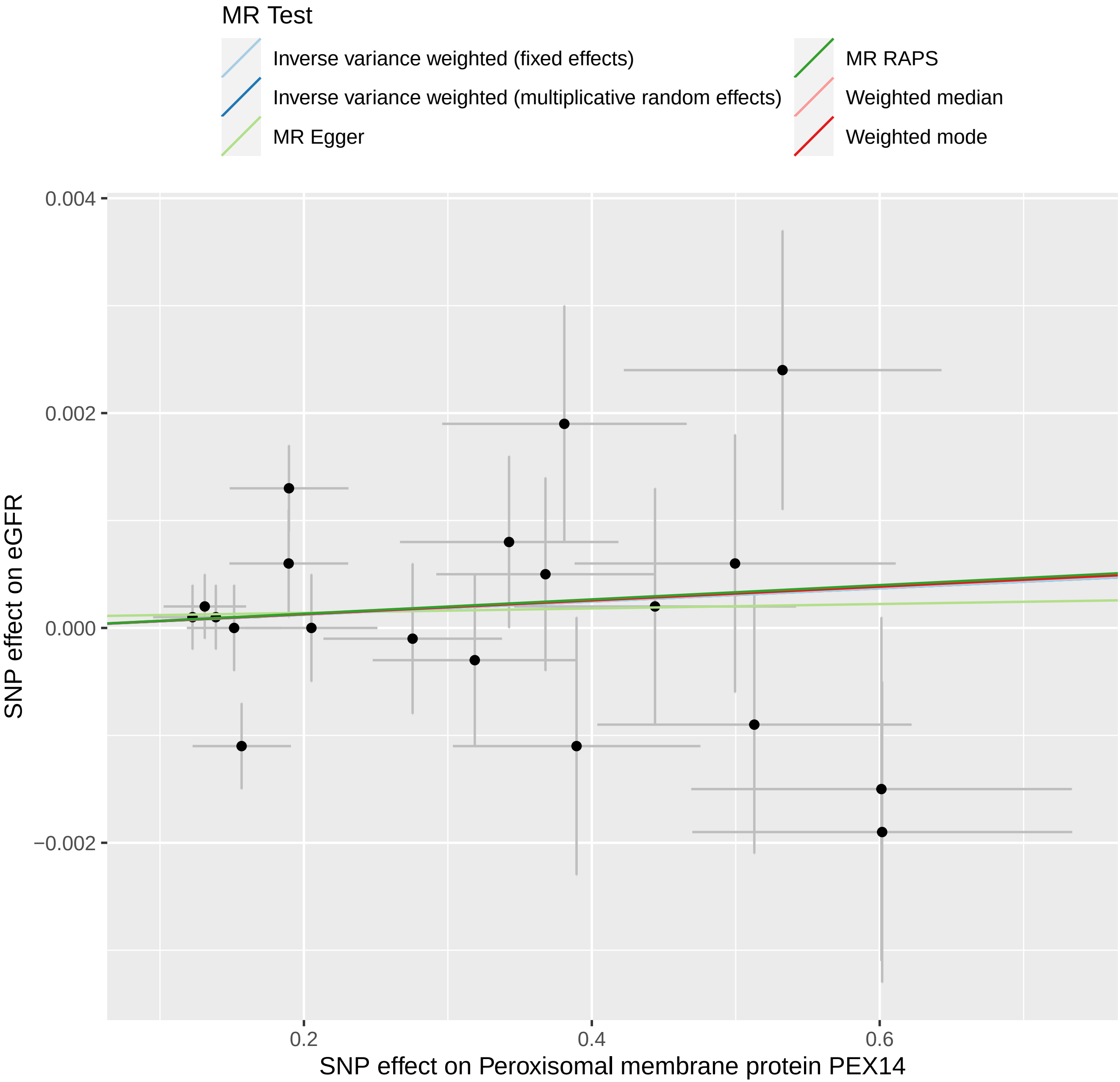

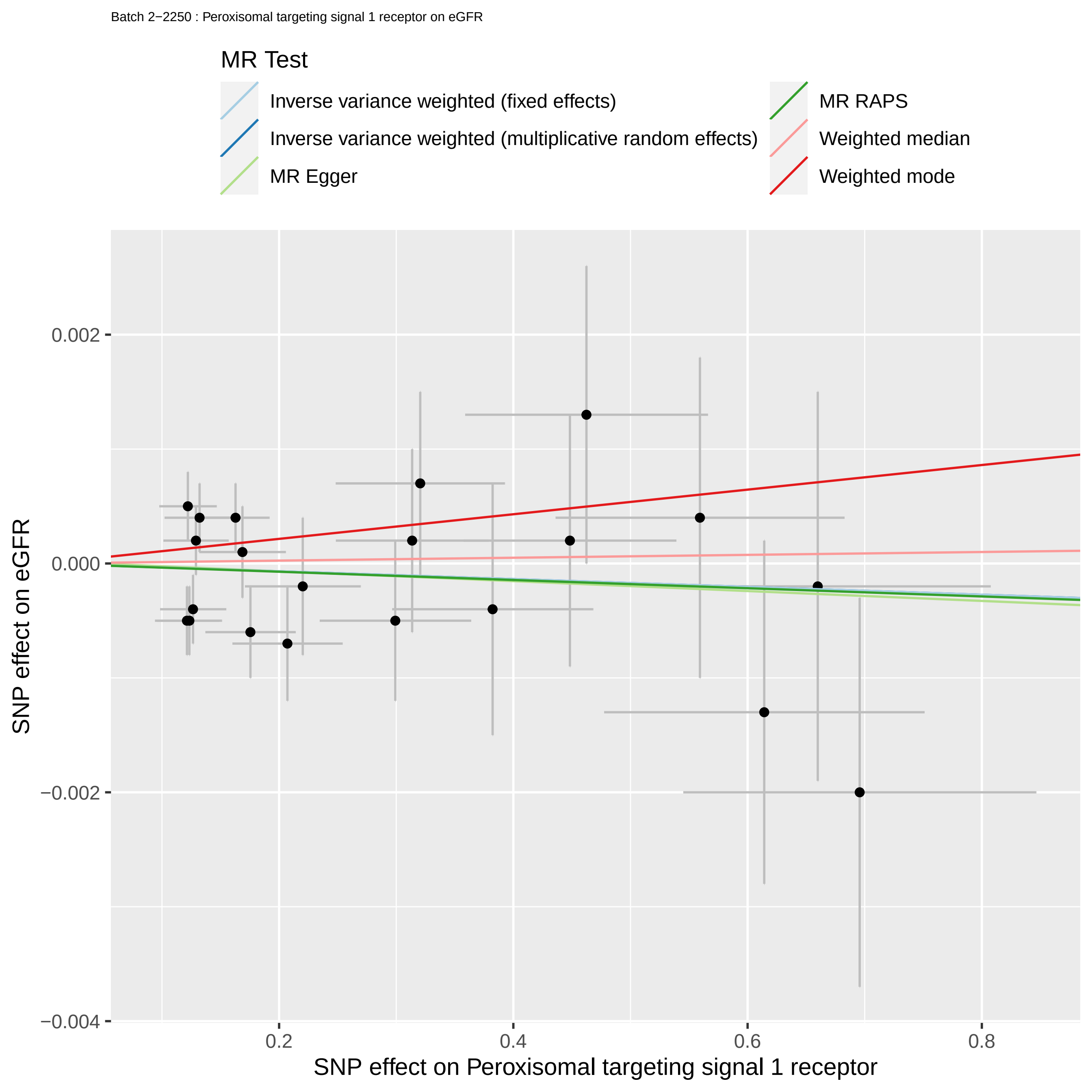
Batch 2−2250 : Peroxisomal targeting signal 1 receptor on eGFR
MR Test
Inverse variance weighted (fixed effects)
Inverse variance weighted (multiplicative random effects)
MR Egger
MR RAPS
Weighted median
Weighted mode
0.002
0.000
−0.002
−0.004
0.2
0.4
0.6
0.8
SNP effect on eGFR
SNP effect on Peroxisomal targeting signal 1 receptor

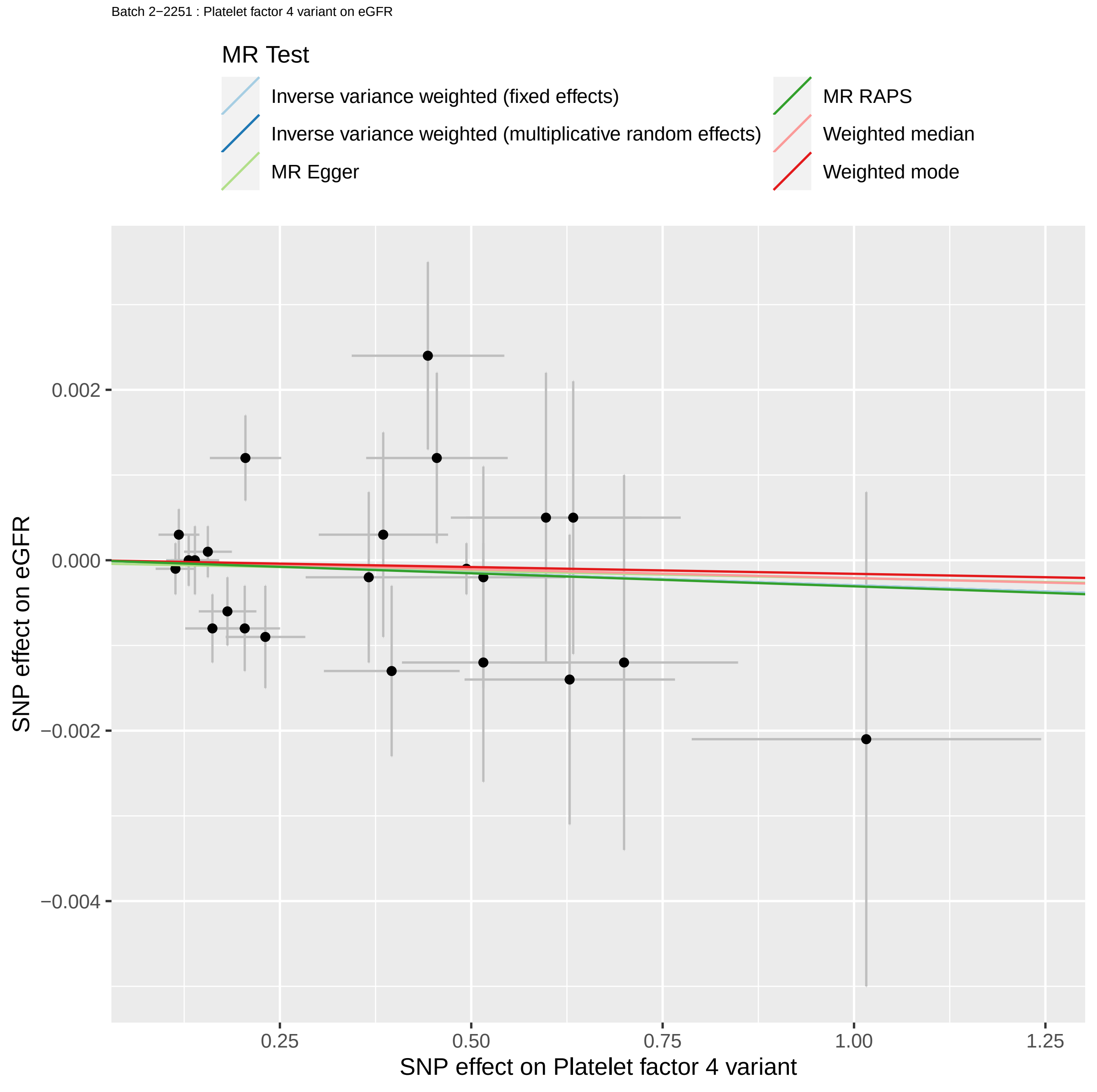

Batch 2−2251 : Platelet factor 4 variant on eGFR
MR Test
Inverse variance weighted (fixed effects)
Inverse variance weighted (multiplicative random effects)
MR Egger
MR RAPS
Weighted median
Weighted mode
0.002
0.000
−0.002
−0.004
SNP effect on eGFR
0.25
0.50
0.75
1.00
1.25
SNP effect on Platelet factor 4 variant

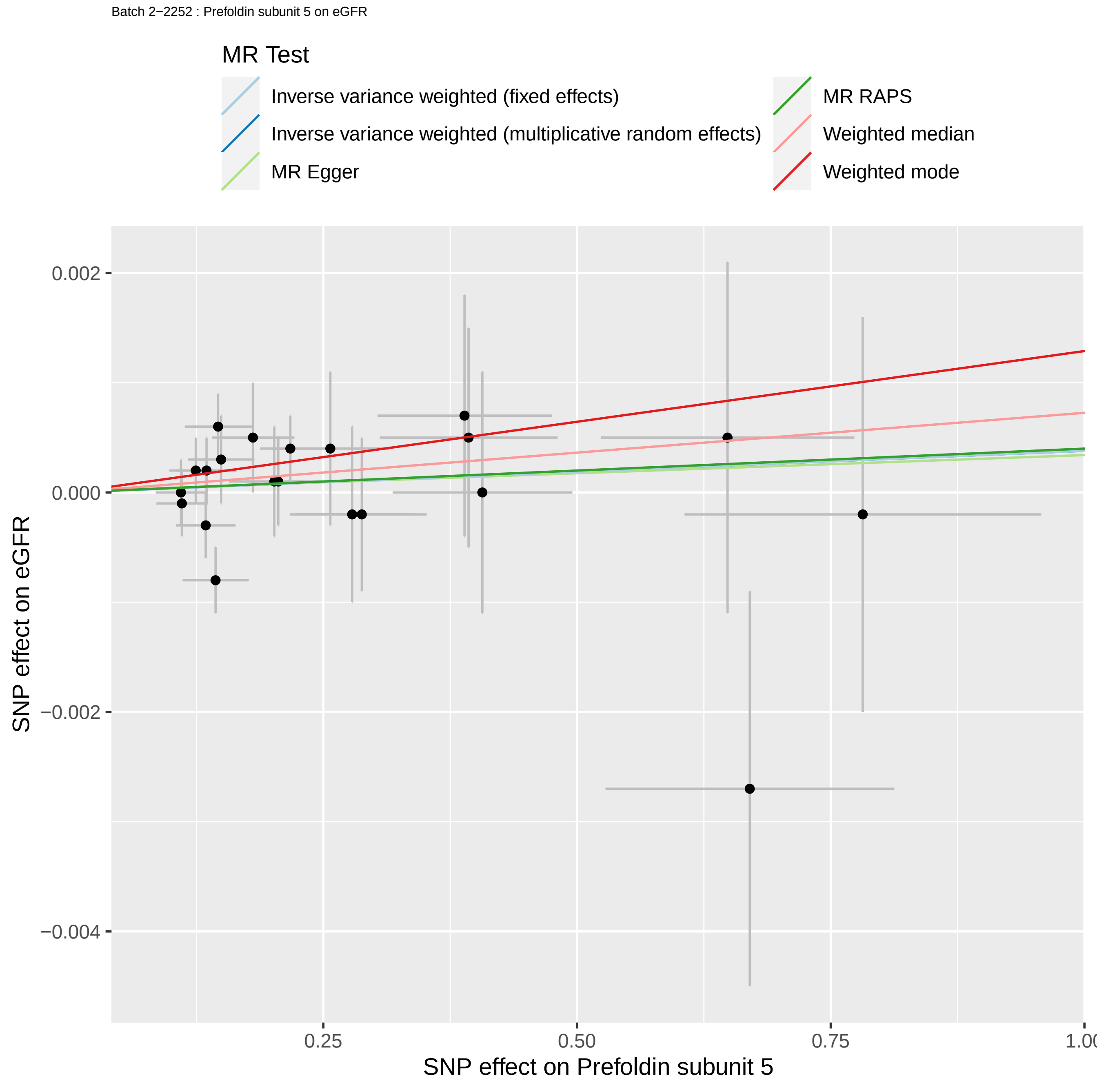

Batch 2−2252 : Prefoldin subunit 5 on eGFR
MR Test
Inverse variance weighted (fixed effects)
Inverse variance weighted (multiplicative random effects)
MR Egger
MR RAPS
Weighted median
Weighted mode
0.002
0.000
−0.002
−0.004
0.25
0.50
0.75
1.00
SNP effect on eGFR
SNP effect on Prefoldin subunit 5

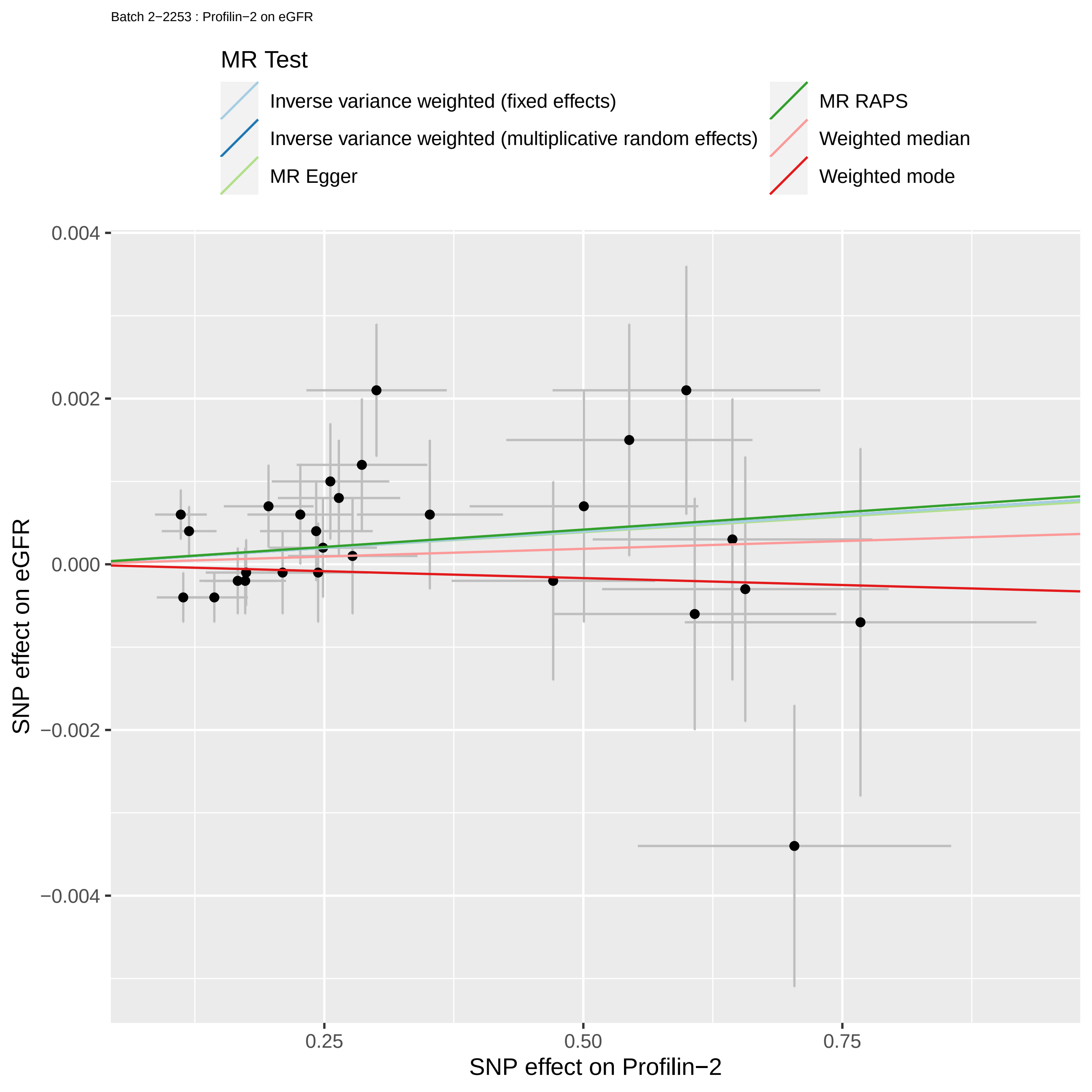
Batch 2−2253 : Profilin−2 on eGFR
MR Test
Inverse variance weighted (fixed effects)
Inverse variance weighted (multiplicative random effects)
MR Egger
MR RAPS
Weighted median
Weighted mode
0.004
0.002
0.000
−0.002
−0.004
SNP effect on eGFR
0.25
0.50
0.75
SNP effect on Profilin−2

Batch 2−2254 : Placenta growth factor on eGFR

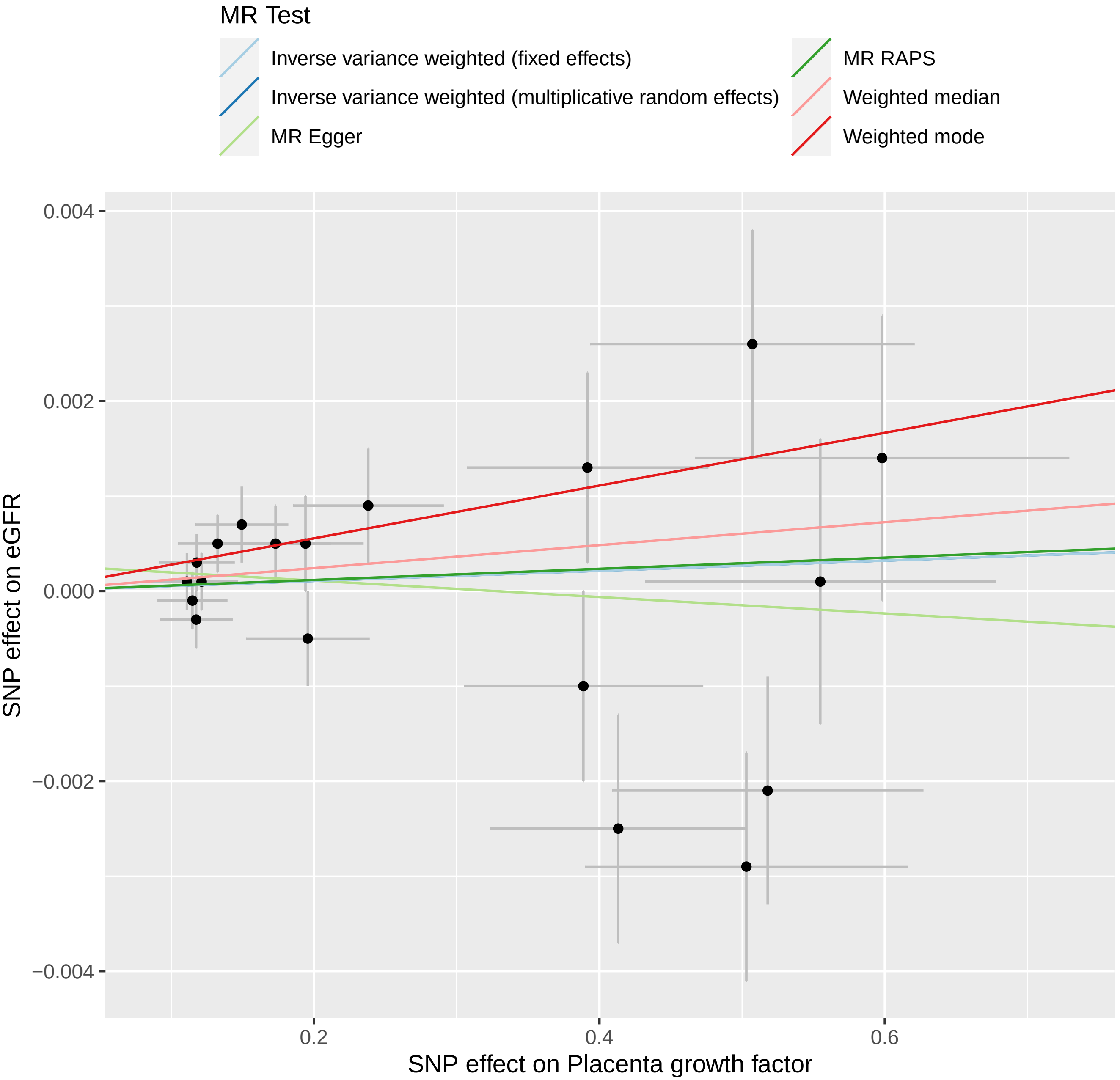

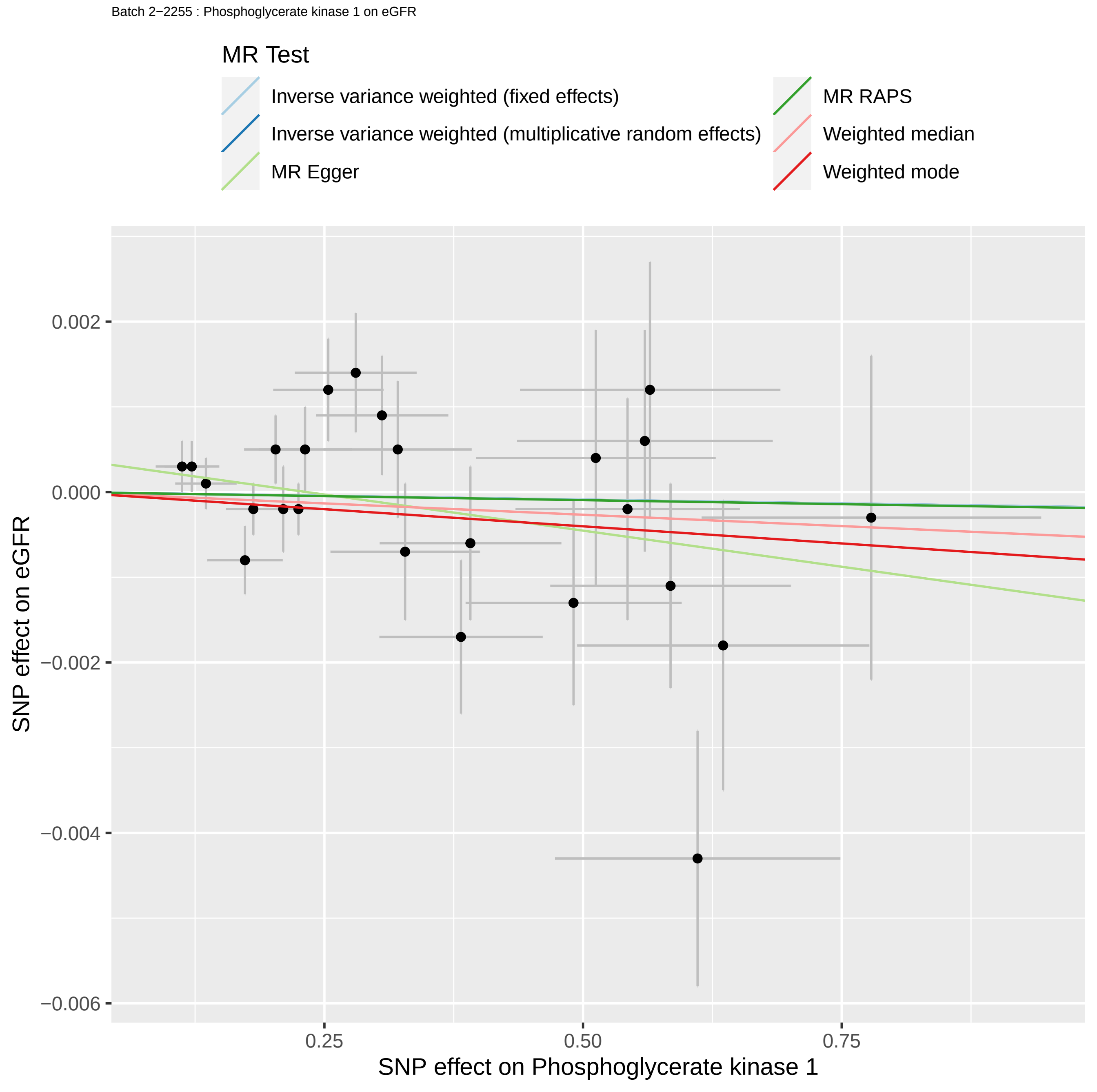

Batch 2−2255 : Phosphoglycerate kinase 1 on eGFR
MR Test
Inverse variance weighted (fixed effects)
Inverse variance weighted (multiplicative random effects)
MR Egger
MR RAPS
Weighted median
Weighted mode
0.002
0.000
−0.002
−0.004
−0.006
0.25
0.50
0.75
SNP effect on eGFR
SNP effect on Phosphoglycerate kinase 1

Batch 2−2256 : Phosphoglycerate kinase 2 on eGFR

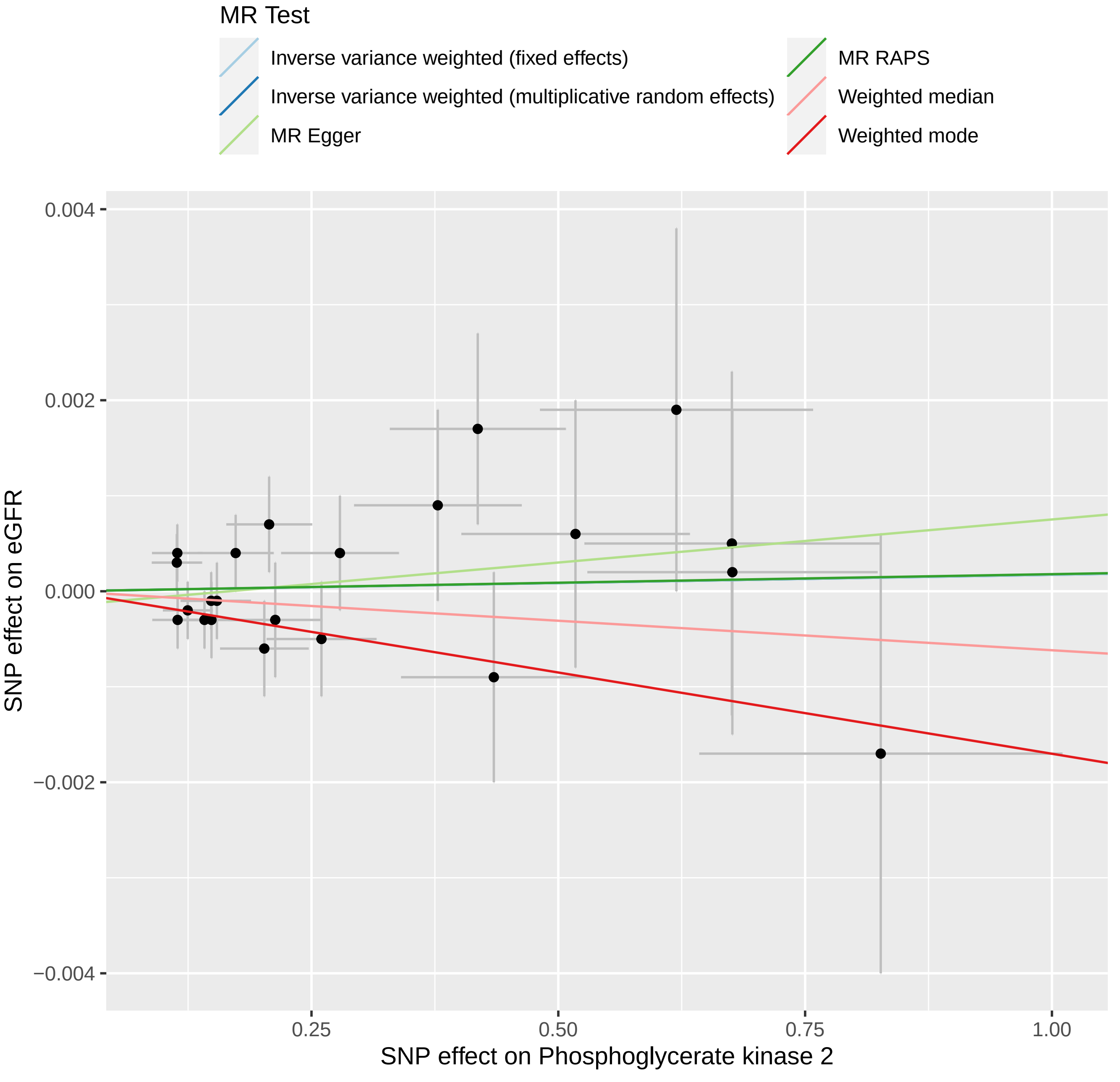

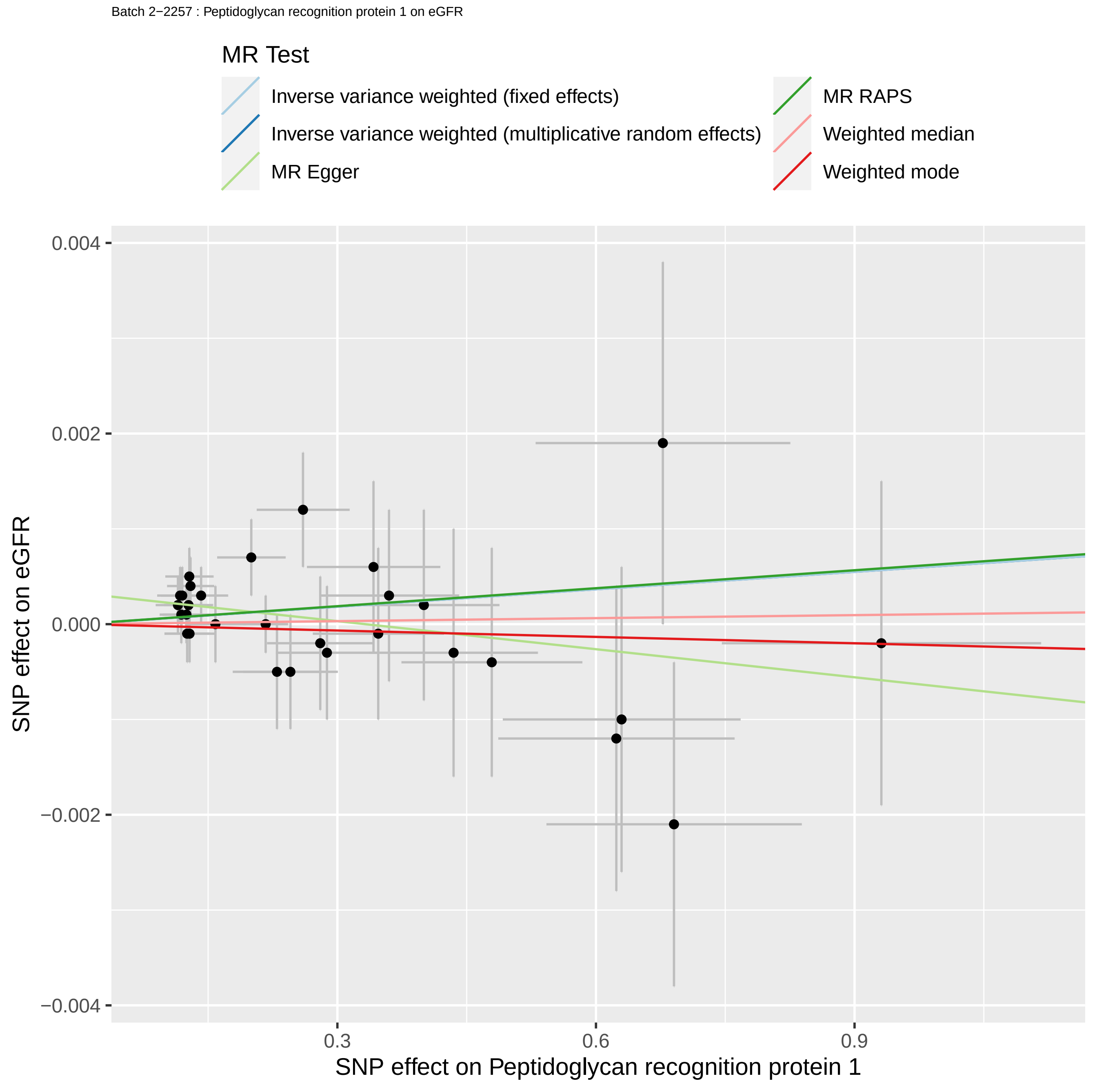
Batch 2−2257 : Peptidoglycan recognition protein 1 on eGFR
MR Test
Inverse variance weighted (fixed effects)
Inverse variance weighted (multiplicative random effects)
MR Egger
MR RAPS
Weighted median
Weighted mode
0.004
0.002
0.000
−0.002
−0.004
0.3
0.6
0.9
SNP effect on eGFR
SNP effect on Peptidoglycan recognition protein 1

Batch 2−2258 : Phosphoglucomutase−1 on eGFR

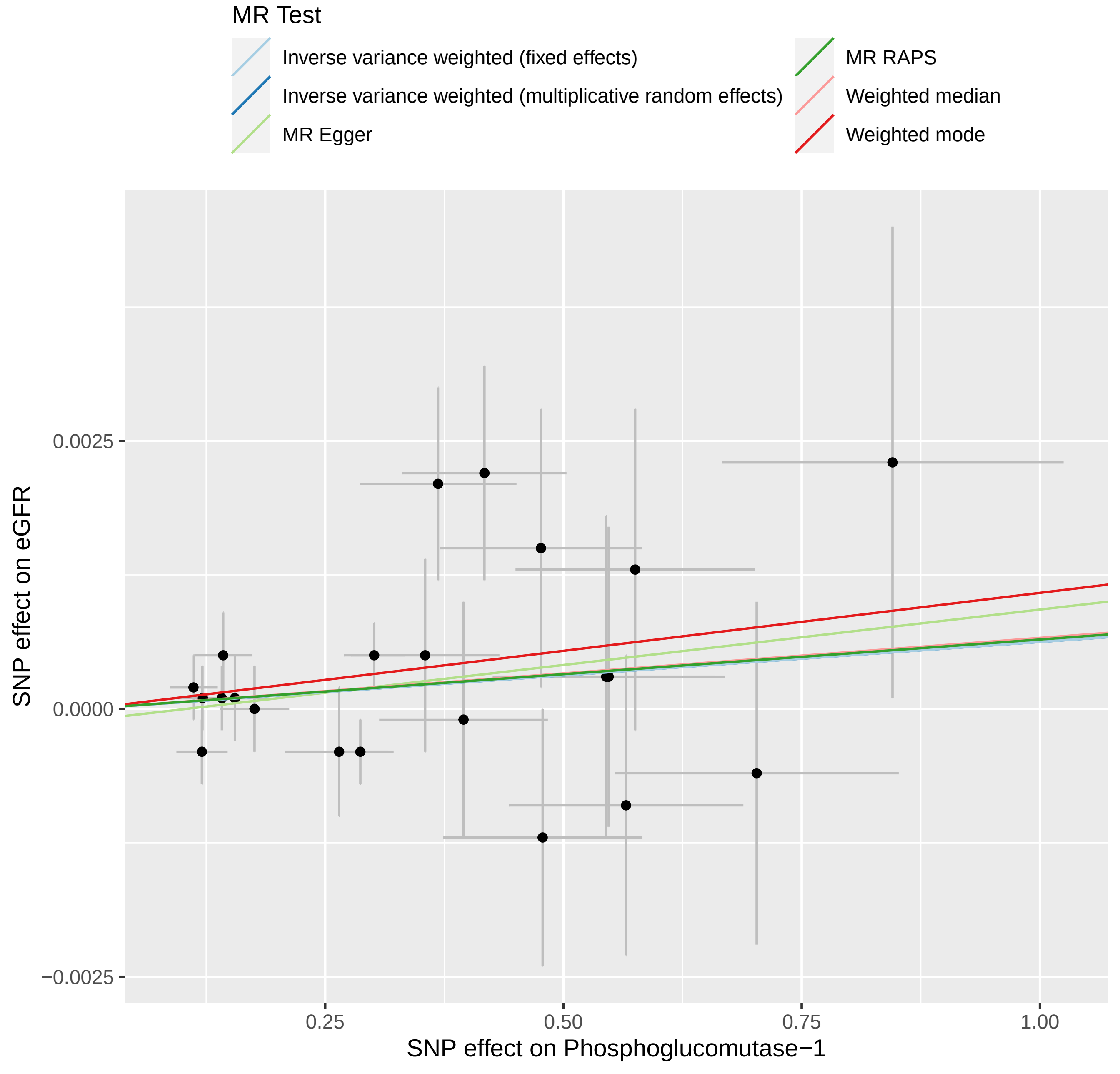

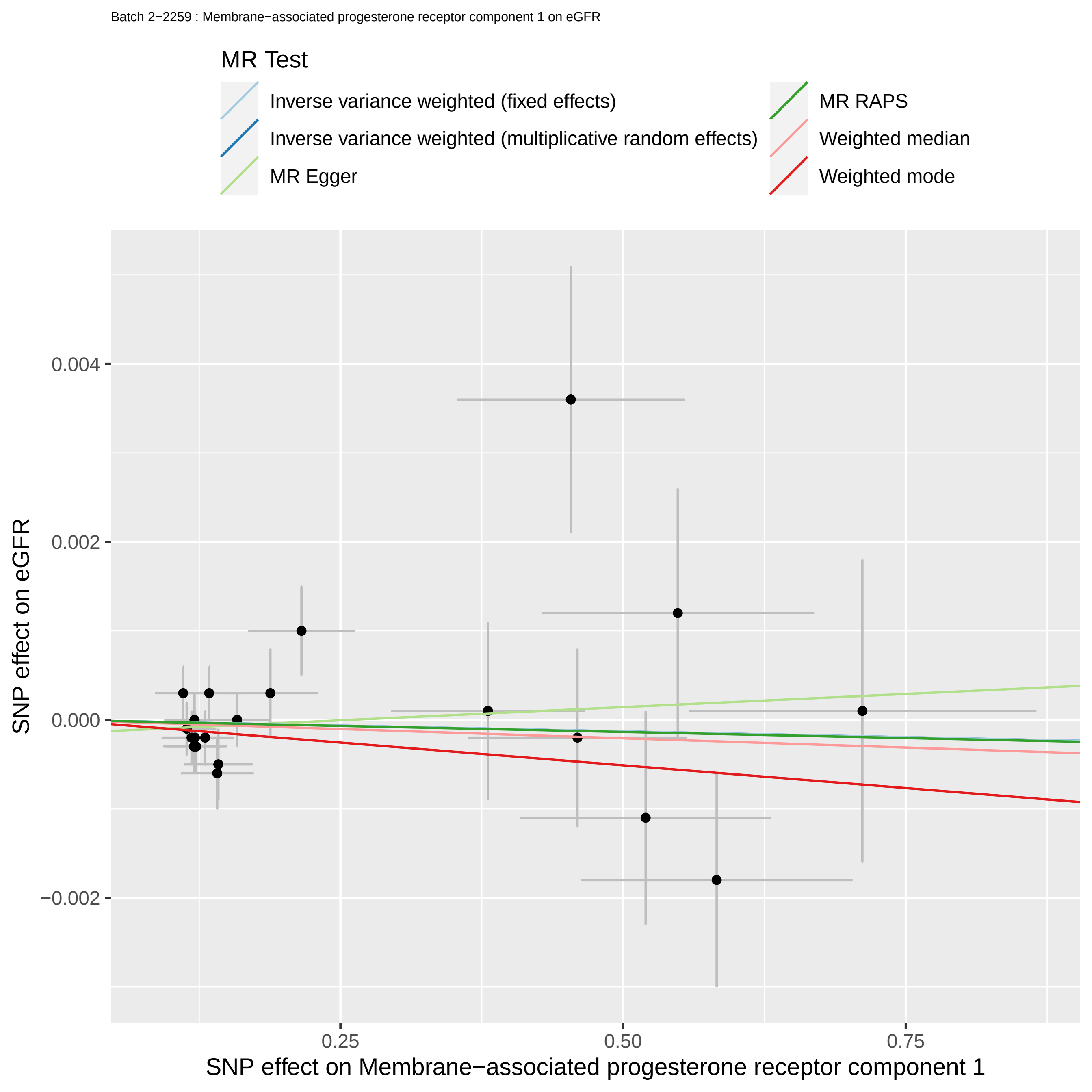
Batch 2−2259 : Membrane−associated progesterone receptor component 1 on eGFR
MR Test
Inverse variance weighted (fixed effects)
Inverse variance weighted (multiplicative random effects)
MR Egger
MR RAPS
Weighted median
Weighted mode
0.004
0.002
0.000
−0.002
SNP effect on eGFR
0.25
0.50
0.75
SNP effect on Membrane−associated progesterone receptor component 1

Batch 2−2260 : Membrane−associated progesterone receptor component 2 on eGFR

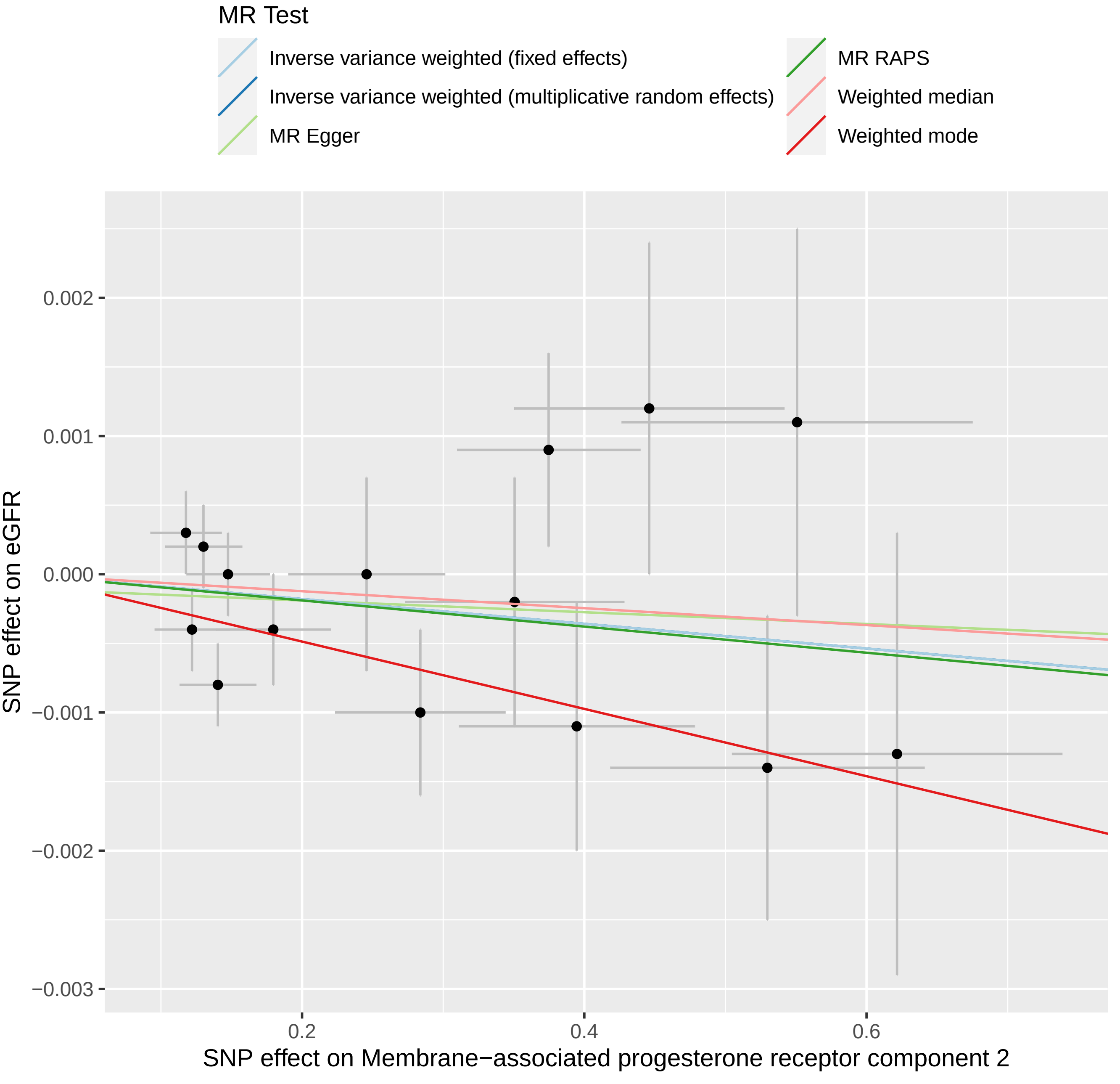

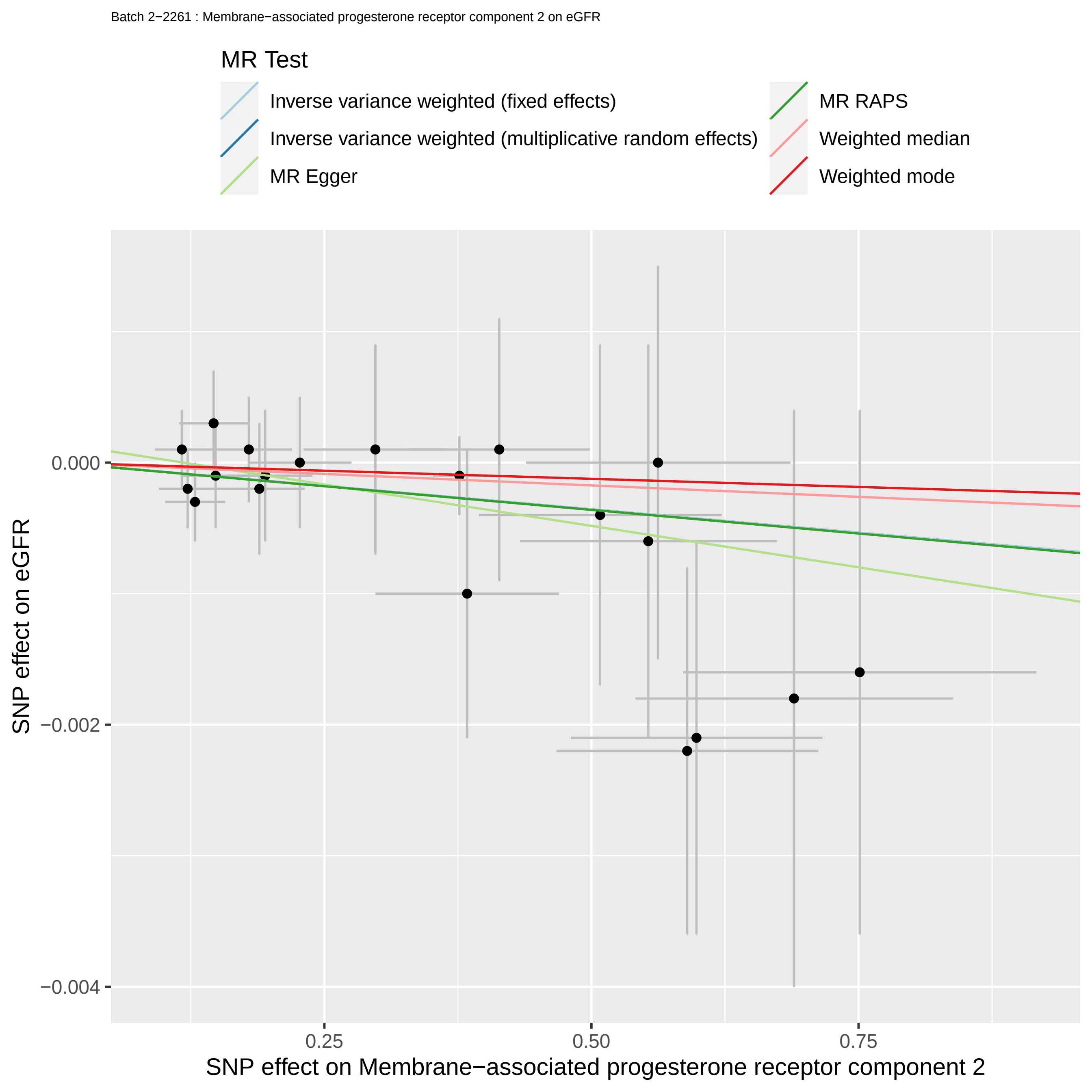
Batch 2−2261 : Membrane−associated progesterone receptor component 2 on eGFR
MR Test
Inverse variance weighted (fixed effects)
Inverse variance weighted (multiplicative random effects)
MR Egger
MR RAPS
Weighted median
Weighted mode
0.000
−0.002
−0.004
0.25
0.50
0.75
SNP effect on eGFR
SNP effect on Membrane−associated progesterone receptor component 2

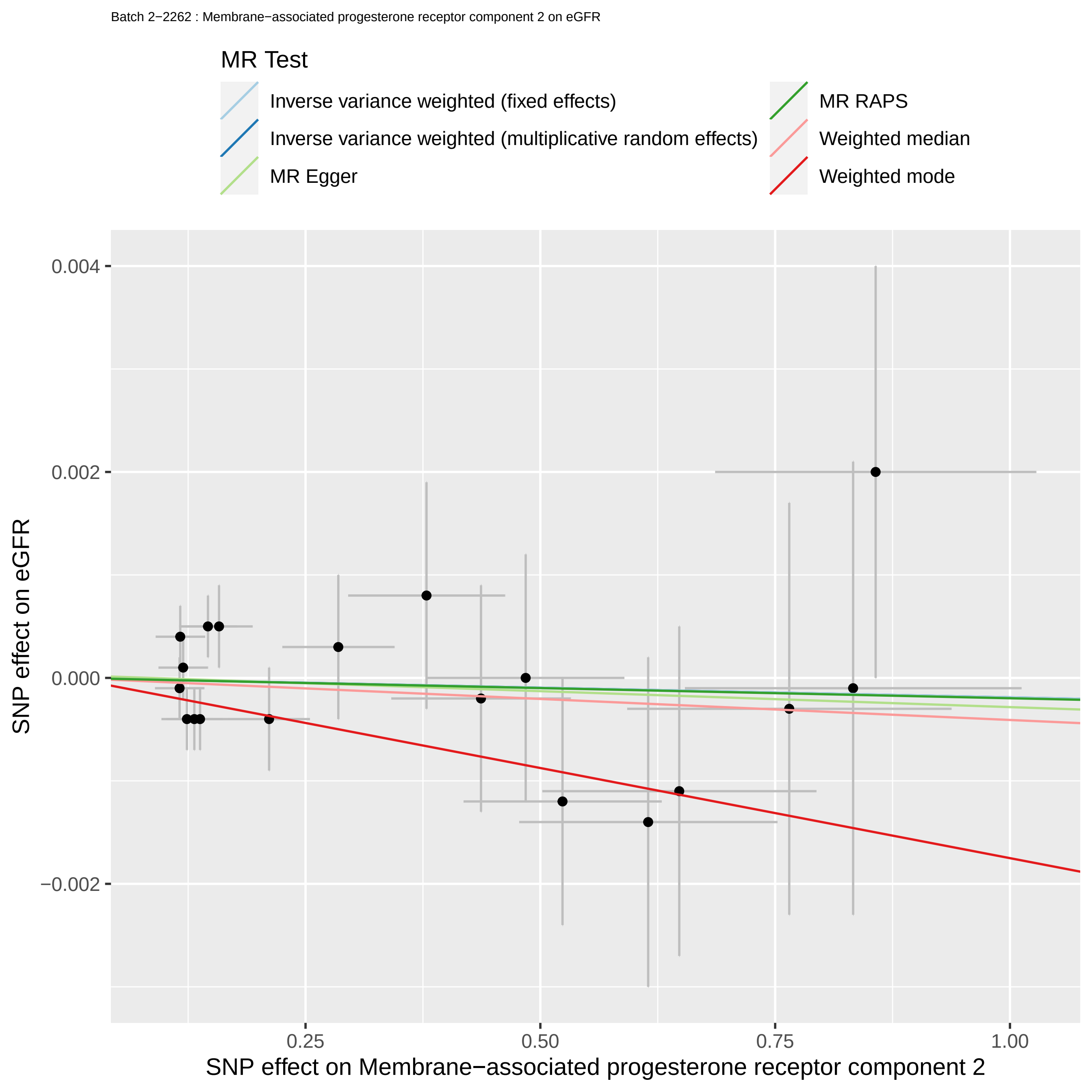

Batch 2−2262 : Membrane−associated progesterone receptor component 2 on eGFR
MR Test
Inverse variance weighted (fixed effects)
Inverse variance weighted (multiplicative random effects)
MR Egger
MR RAPS
Weighted median
Weighted mode
0.004
0.002
0.000
−0.002
SNP effect on eGFR
0.25
0.50
0.75
1.00
SNP effect on Membrane−associated progesterone receptor component 2

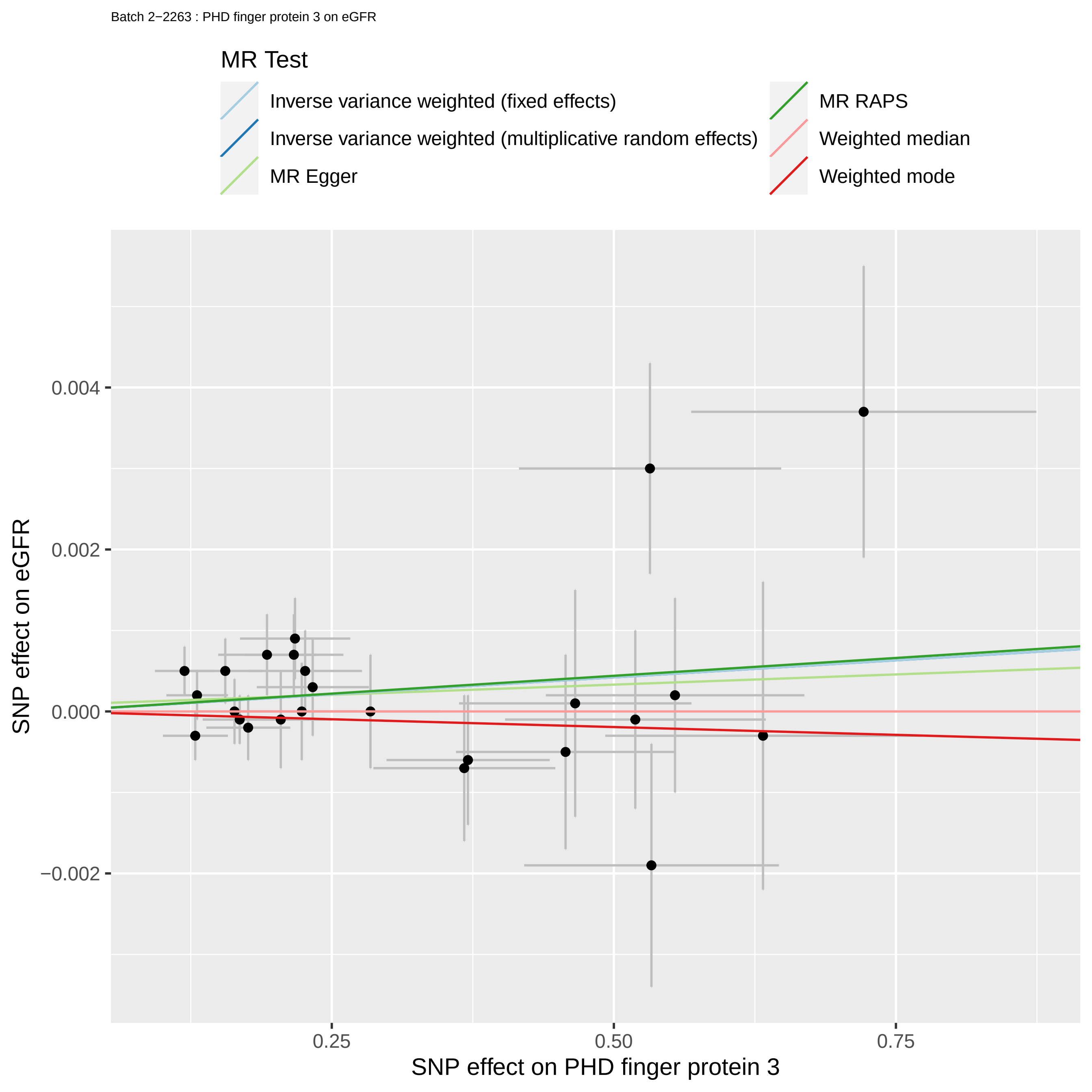
Batch 2−2263 : PHD finger protein 3 on eGFR
MR Test
Inverse variance weighted (fixed effects)
Inverse variance weighted (multiplicative random effects)
MR Egger
MR RAPS
Weighted median
Weighted mode
0.004
0.002
0.000
−0.002
0.25
0.50
0.75
SNP effect on eGFR
SNP effect on PHD finger protein 3

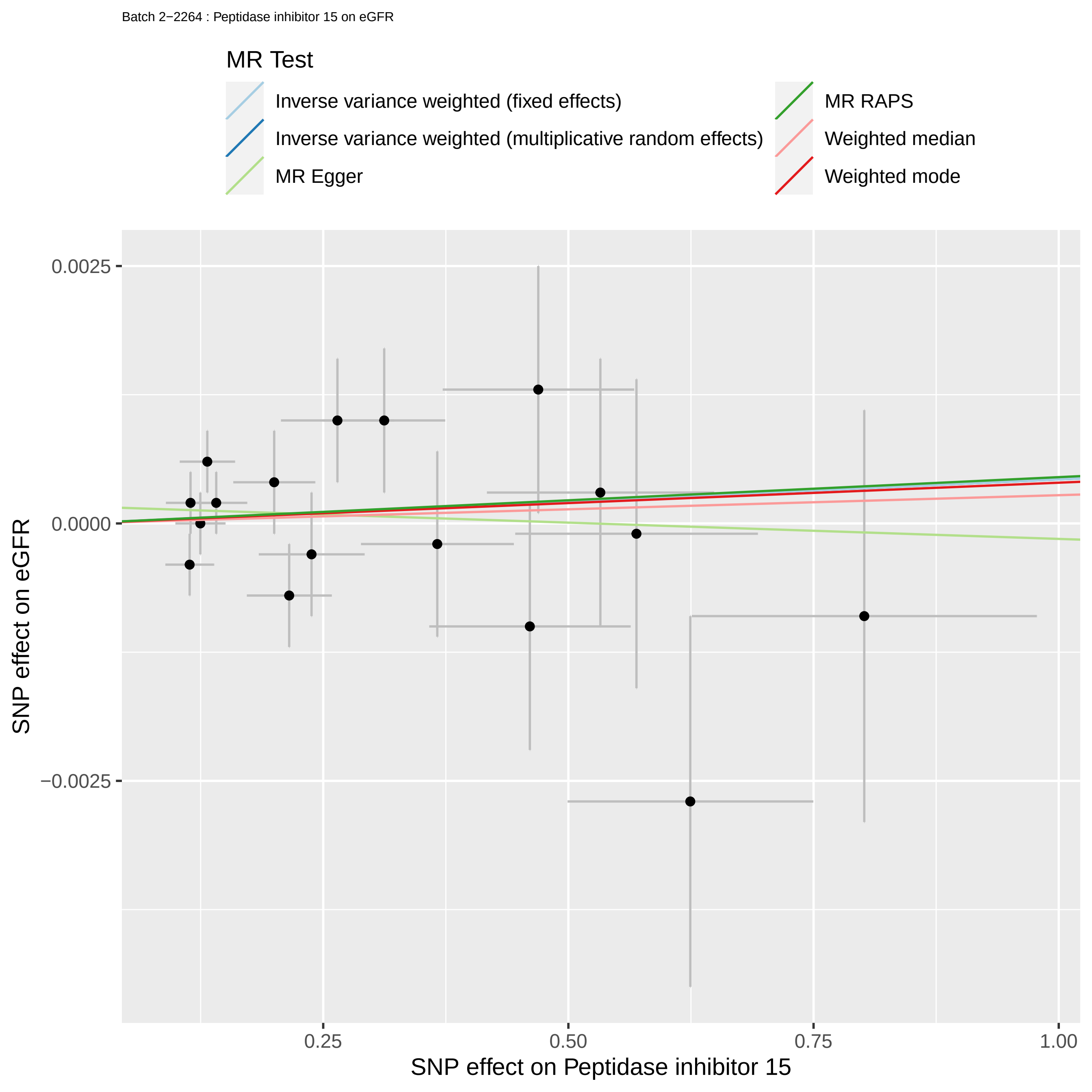

Batch 2−2264 : Peptidase inhibitor 15 on eGFR
MR Test
Inverse variance weighted (fixed effects)
Inverse variance weighted (multiplicative random effects)
MR Egger
MR RAPS
Weighted median
Weighted mode
0.0025
0.0000
−0.0025
SNP effect on eGFR
0.25
0.50
0.75
1.00
SNP effect on Peptidase inhibitor 15

Batch 2−2265 : Elafin on eGFR

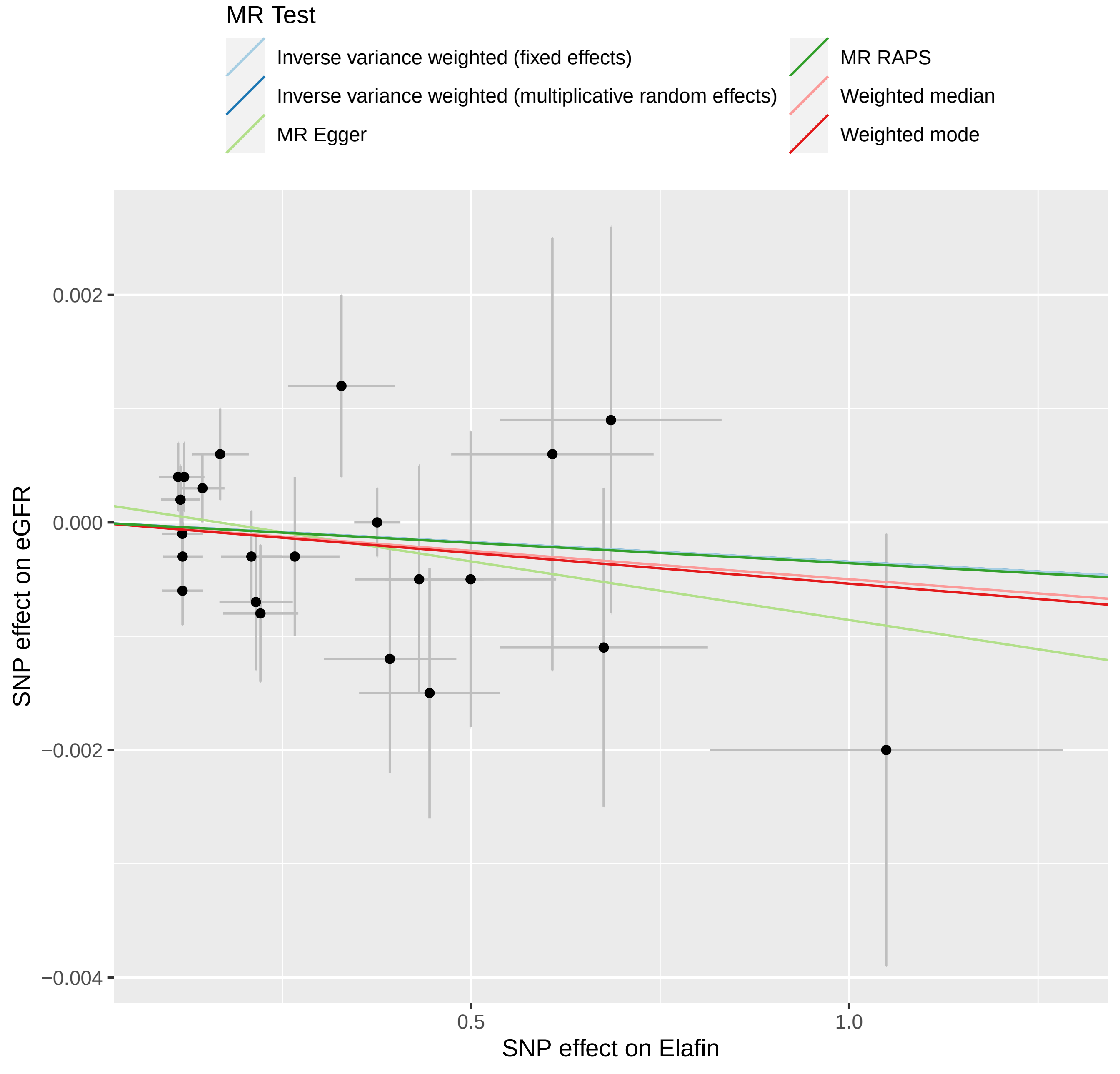

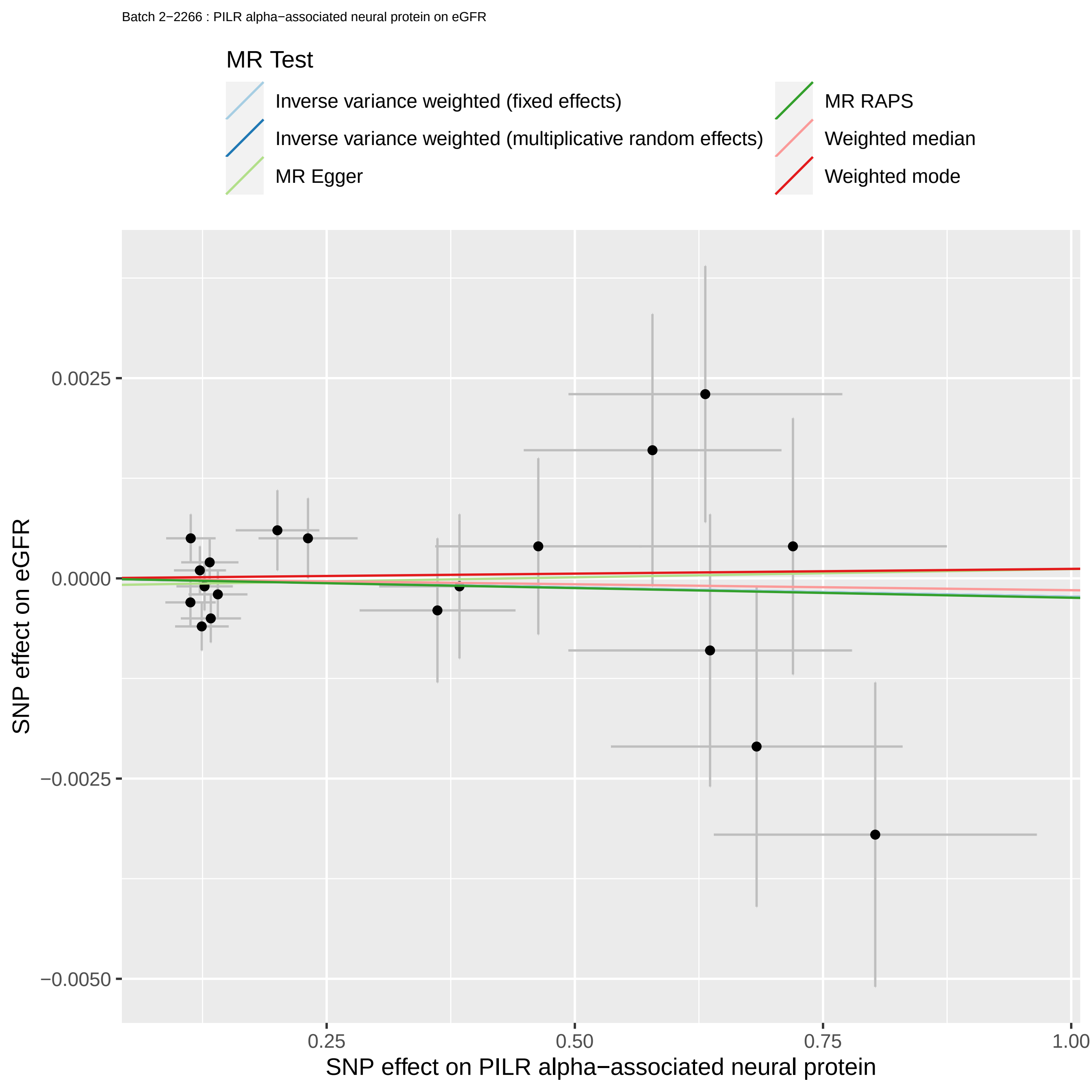
Batch 2−2266 : PILR alpha−associated neural protein on eGFR
MR Test
Inverse variance weighted (fixed effects)
Inverse variance weighted (multiplicative random effects)
MR Egger
MR RAPS
Weighted median
Weighted mode
0.0025
0.0000
−0.0025
−0.0050
SNP effect on eGFR
0.25
0.50
0.75
1.00
SNP effect on PILR alpha−associated neural protein

Batch 2−2267 : PILR alpha−associated neural protein on eGFR

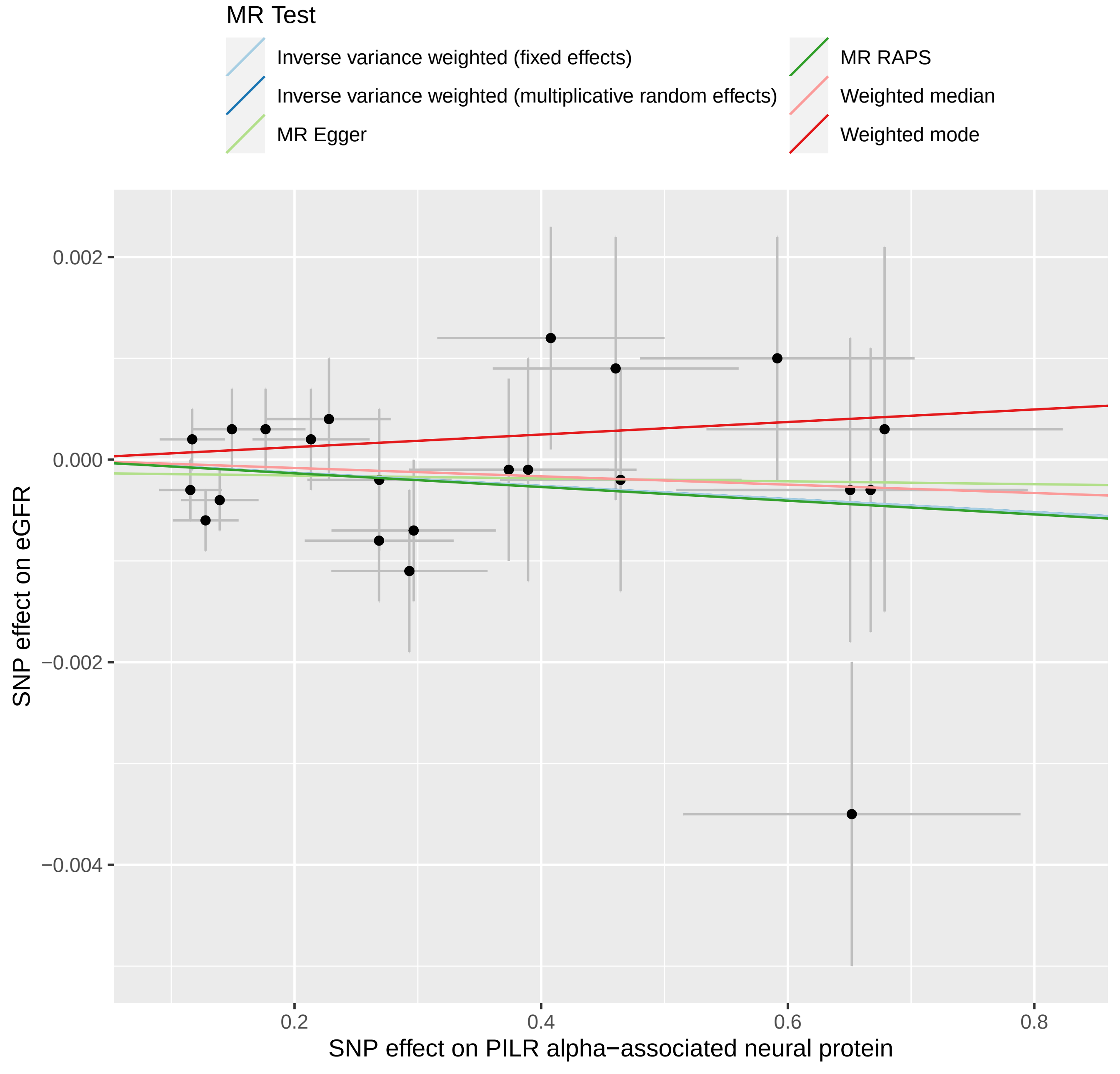

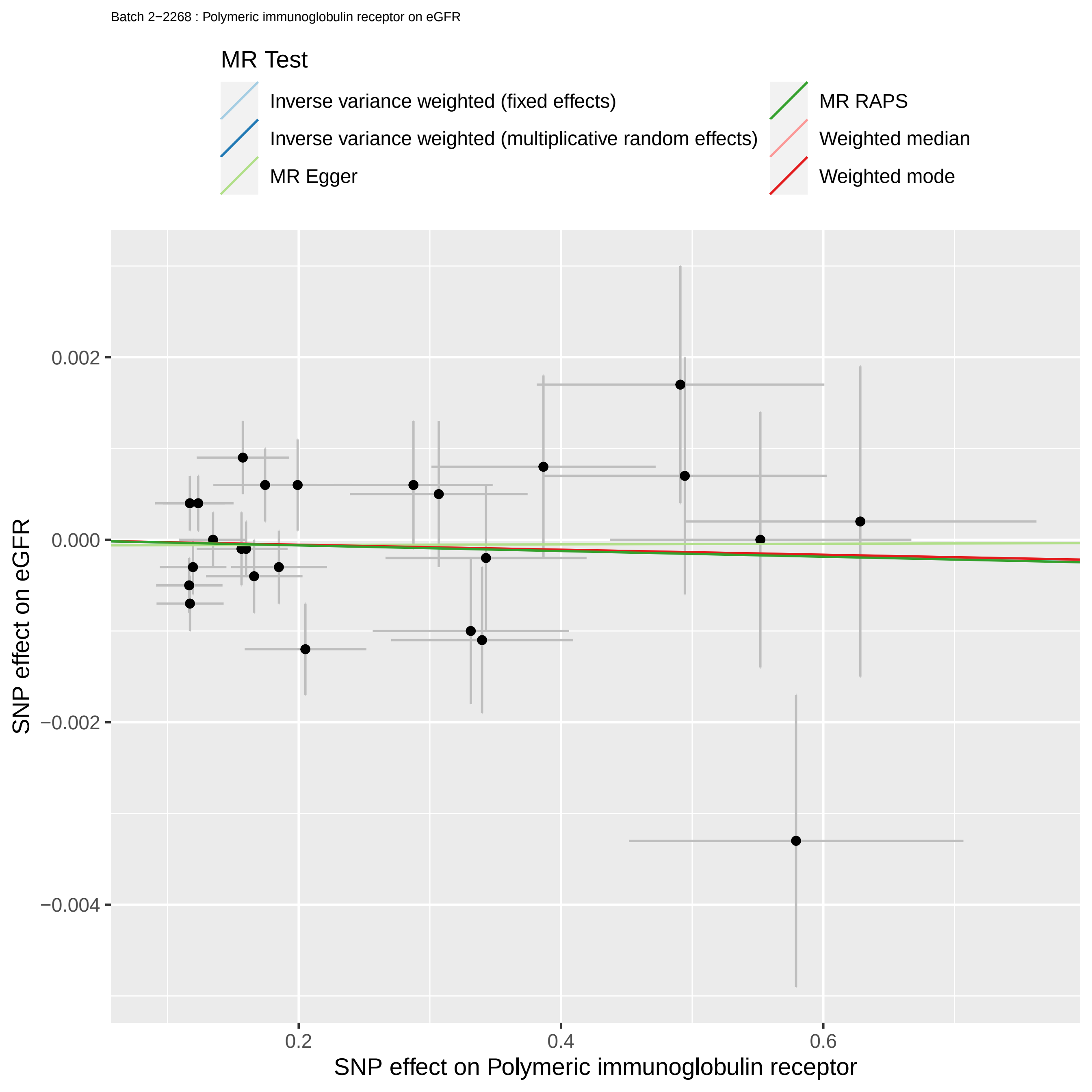

Batch 2−2268 : Polymeric immunoglobulin receptor on eGFR
MR Test
Inverse variance weighted (fixed effects)
Inverse variance weighted (multiplicative random effects)
MR Egger
MR RAPS
Weighted median
Weighted mode
0.002
0.000
−0.002
−0.004
SNP effect on eGFR
0.2
0.4
0.6
SNP effect on Polymeric immunoglobulin receptor

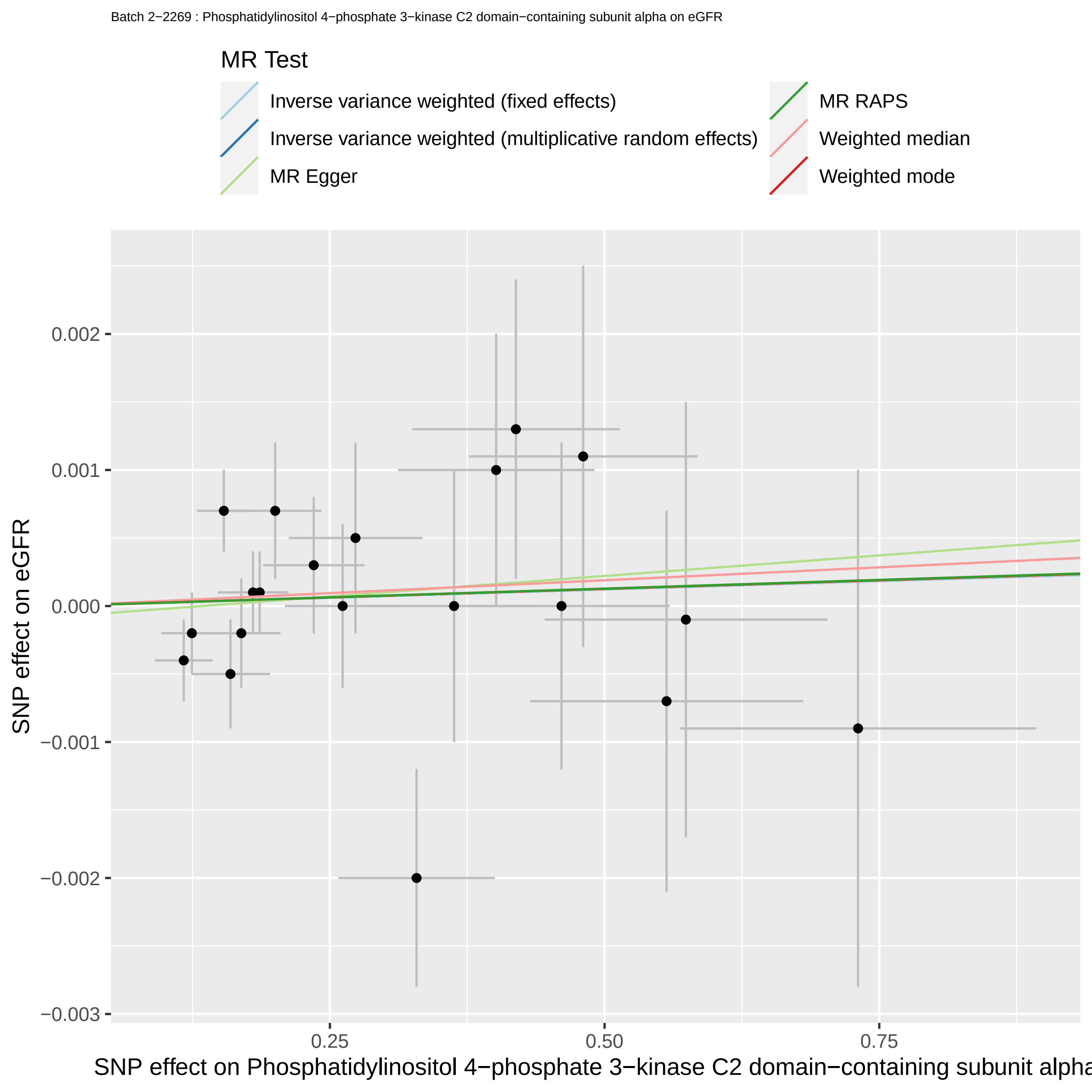

Batch 2−2269 : Phosphatidylinositol 4−phosphate 3−kinase C2 domain−containing subunit alpha on eGFR
MR Test
Inverse variance weighted (fixed effects)
Inverse variance weighted (multiplicative random effects)
MR Egger
MR RAPS
Weighted median
Weighted mode
0.002
0.001
0.000
−0.001
−0.002
−0.003
SNP effect on eGFR
0.25
0.50
0.75
SNP effect on Phosphatidylinositol 4−phosphate 3−kinase C2 domain−containing subunit alpha

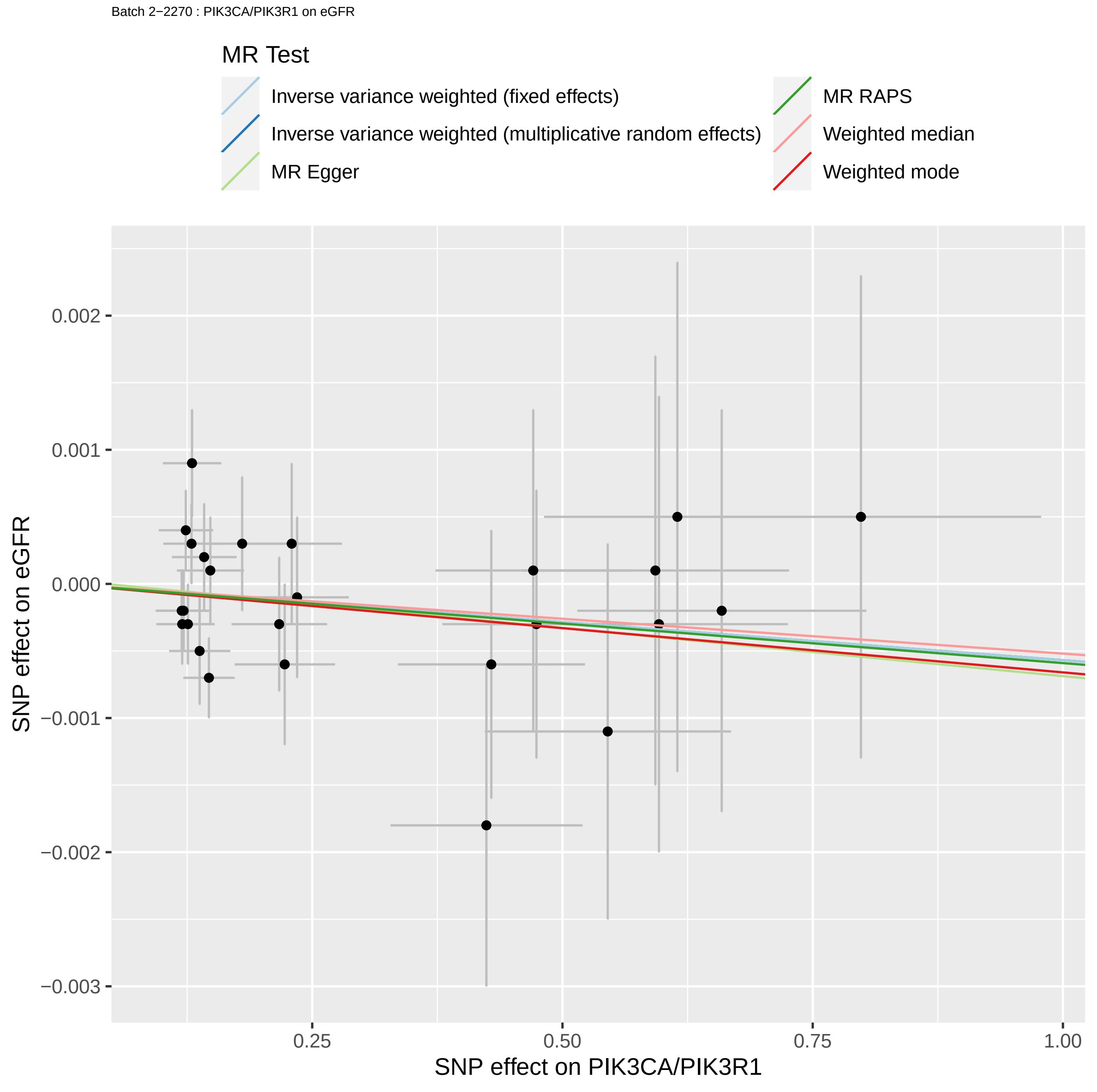

Batch 2−2270 : PIK3CA/PIK3R1 on eGFR
MR Test
Inverse variance weighted (fixed effects)
Inverse variance weighted (multiplicative random effects)
MR Egger
MR RAPS
Weighted median
Weighted mode
SNP effect on eGFR
0.002
0.001
0.000
−0.001
−0.002
−0.003
0.25
0.50
0.75
1.00
SNP effect on PIK3CA/PIK3R1

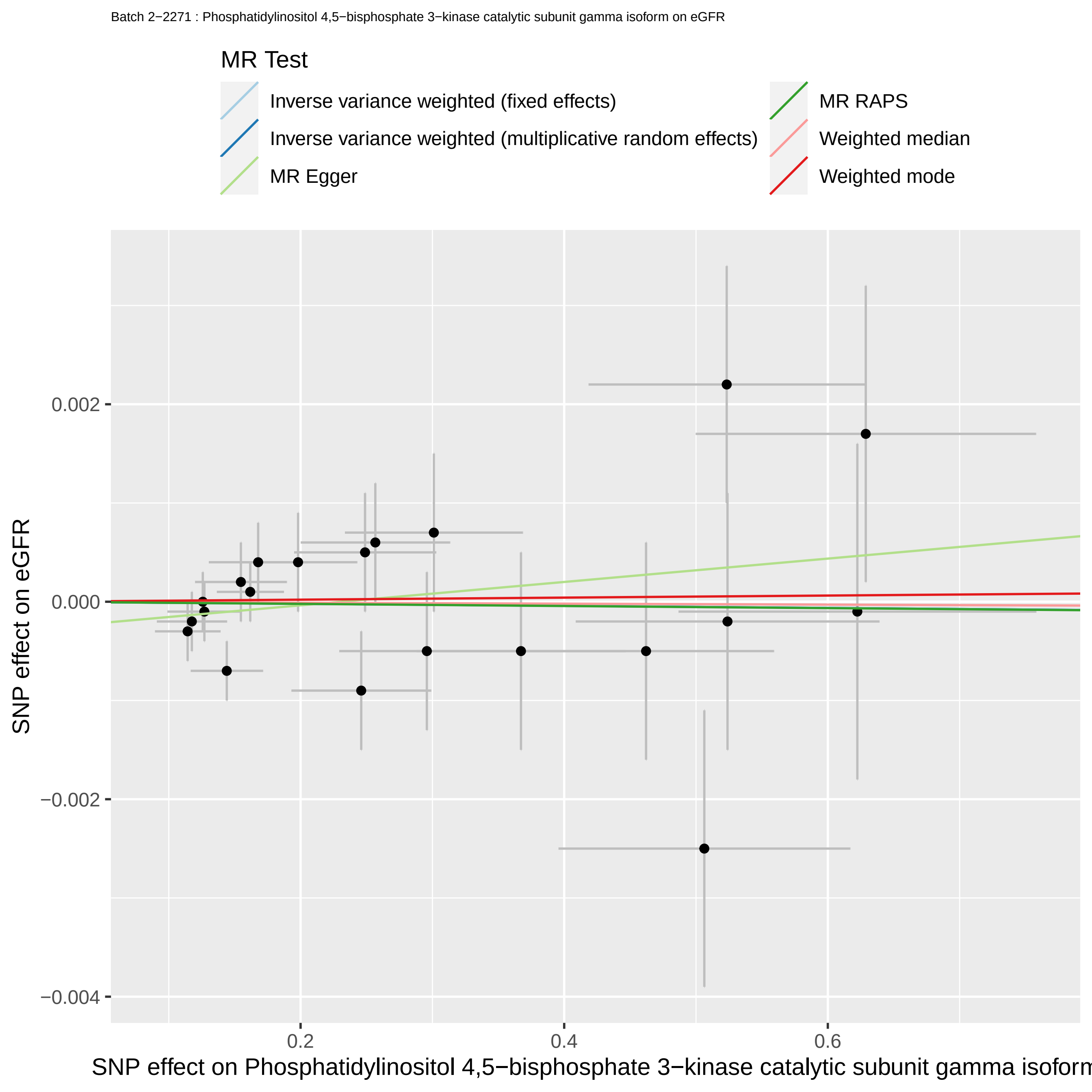

Batch 2−2271 : Phosphatidylinositol 4,5−bisphosphate 3−kinase catalytic subunit gamma isoform on eGFR
MR Test
Inverse variance weighted (fixed effects)
Inverse variance weighted (multiplicative random effects)
MR Egger
MR RAPS
Weighted median
Weighted mode
0.002
0.000
−0.002
−0.004
0.2
0.4
0.6
SNP effect on eGFR
SNP effect on Phosphatidylinositol 4,5−bisphosphate 3−kinase catalytic subunit gamma isoform

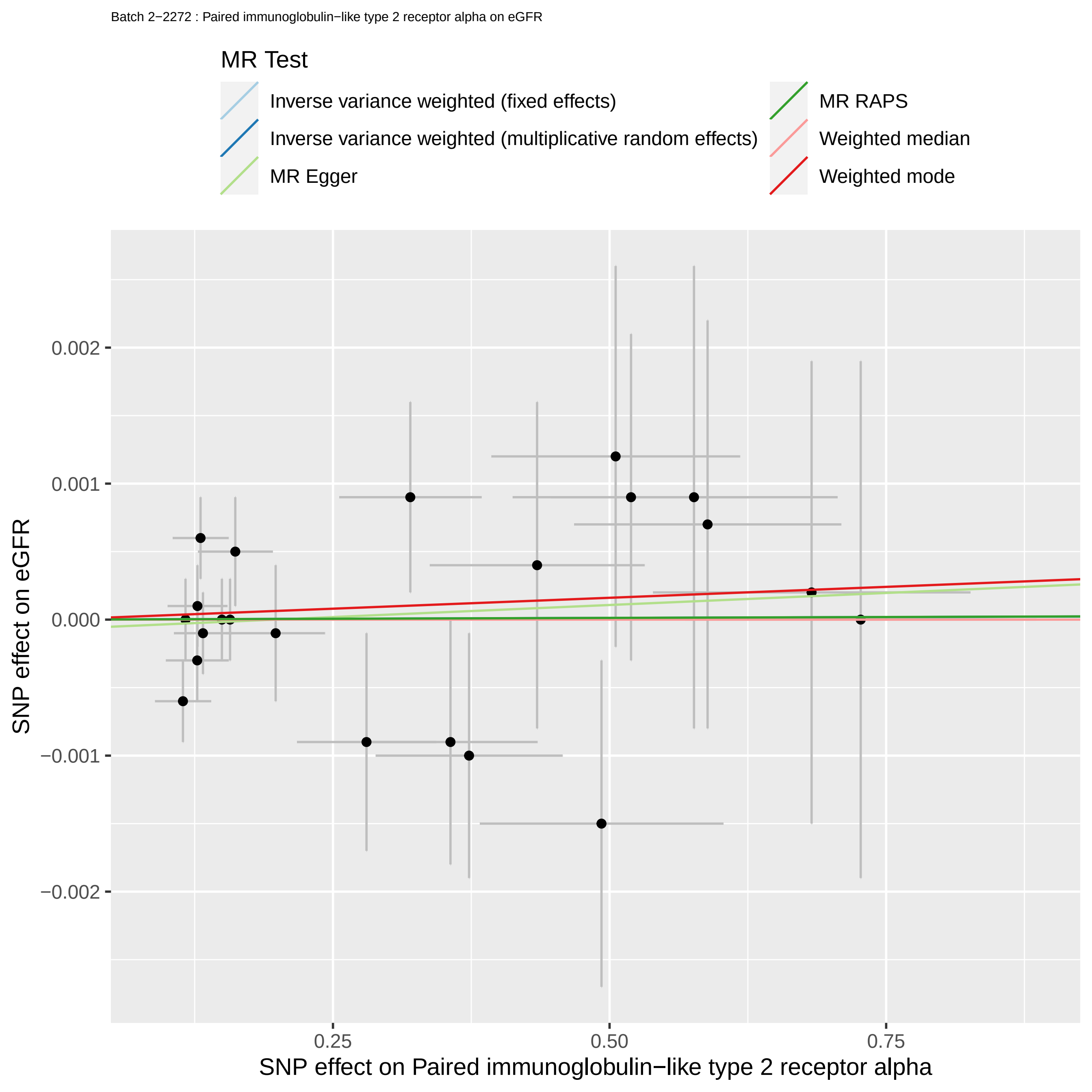

Batch 2−2272 : Paired immunoglobulin−like type 2 receptor alpha on eGFR
MR Test
Inverse variance weighted (fixed effects)
Inverse variance weighted (multiplicative random effects)
MR Egger
MR RAPS
Weighted median
Weighted mode
0.002
0.001
0.000
−0.001
−0.002
0.25
0.50
0.75
SNP effect on eGFR
SNP effect on Paired immunoglobulin−like type 2 receptor alpha

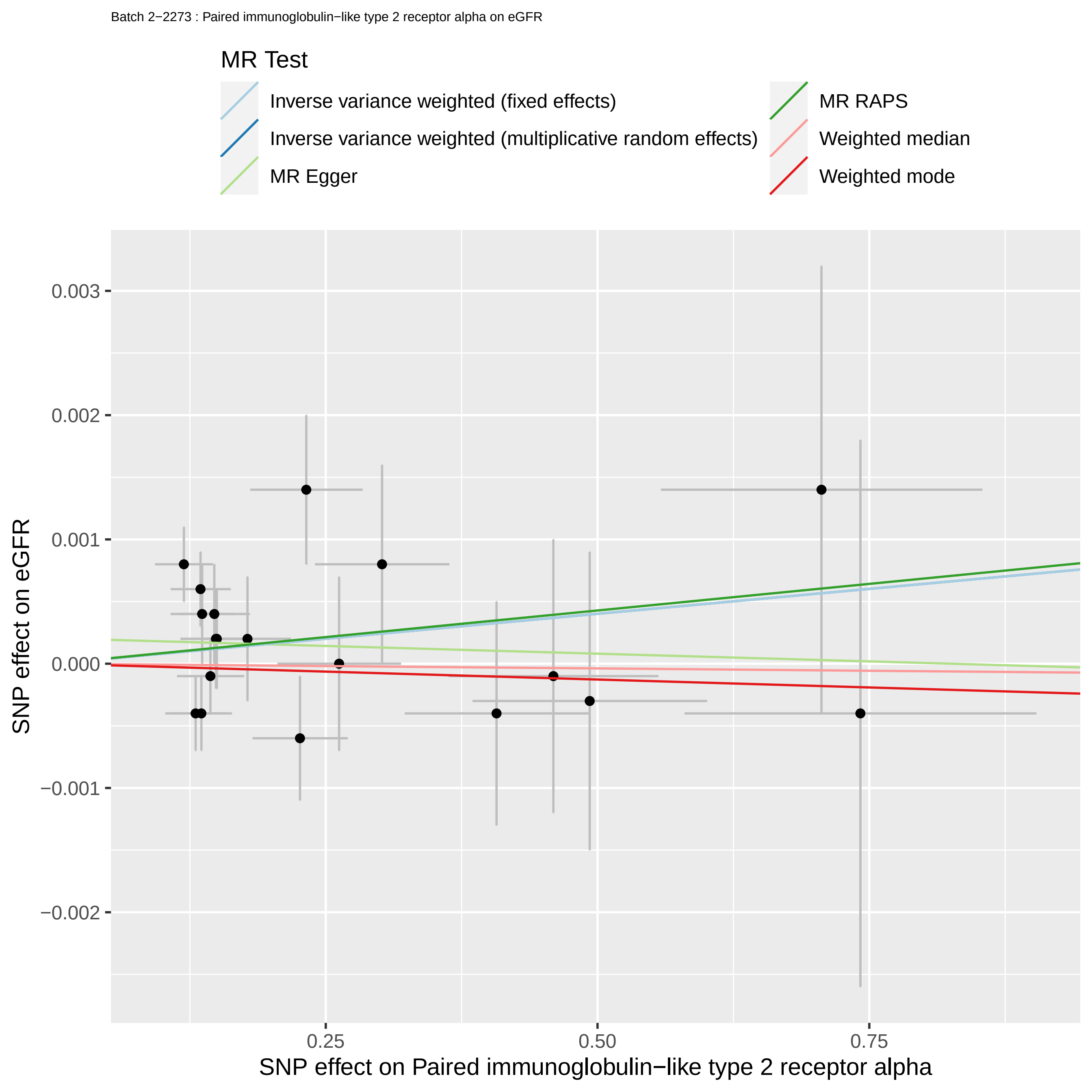

Batch 2−2273 : Paired immunoglobulin−like type 2 receptor alpha on eGFR
MR Test
Inverse variance weighted (fixed effects)
Inverse variance weighted (multiplicative random effects)
MR Egger
MR RAPS
Weighted median
Weighted mode
0.003
0.002
0.001
0.000
−0.001
−0.002
SNP effect on eGFR
0.25
0.50
0.75
SNP effect on Paired immunoglobulin−like type 2 receptor alpha

Batch 2−2274 : Serine/threonine−protein kinase pim−1 on eGFR

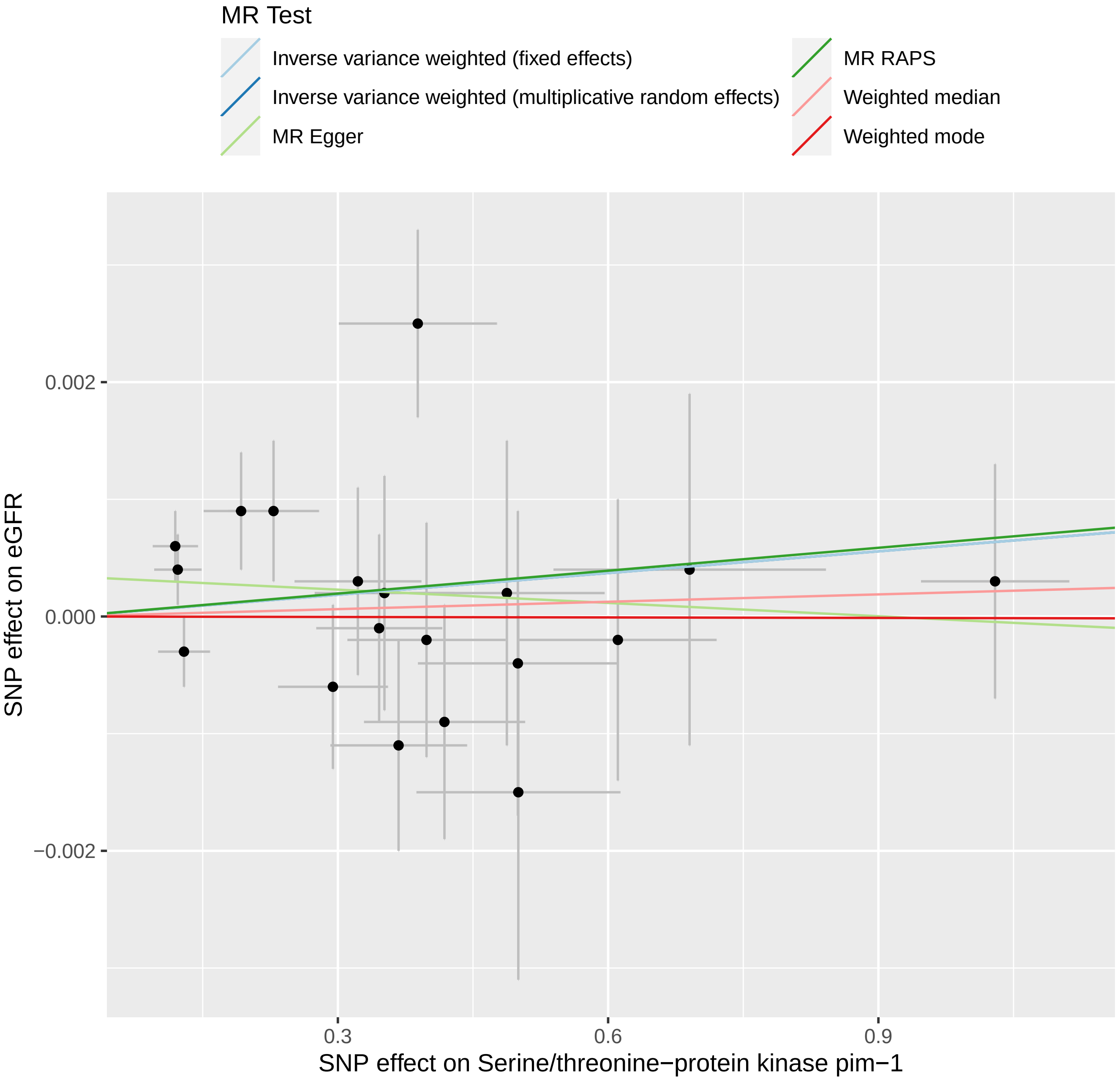

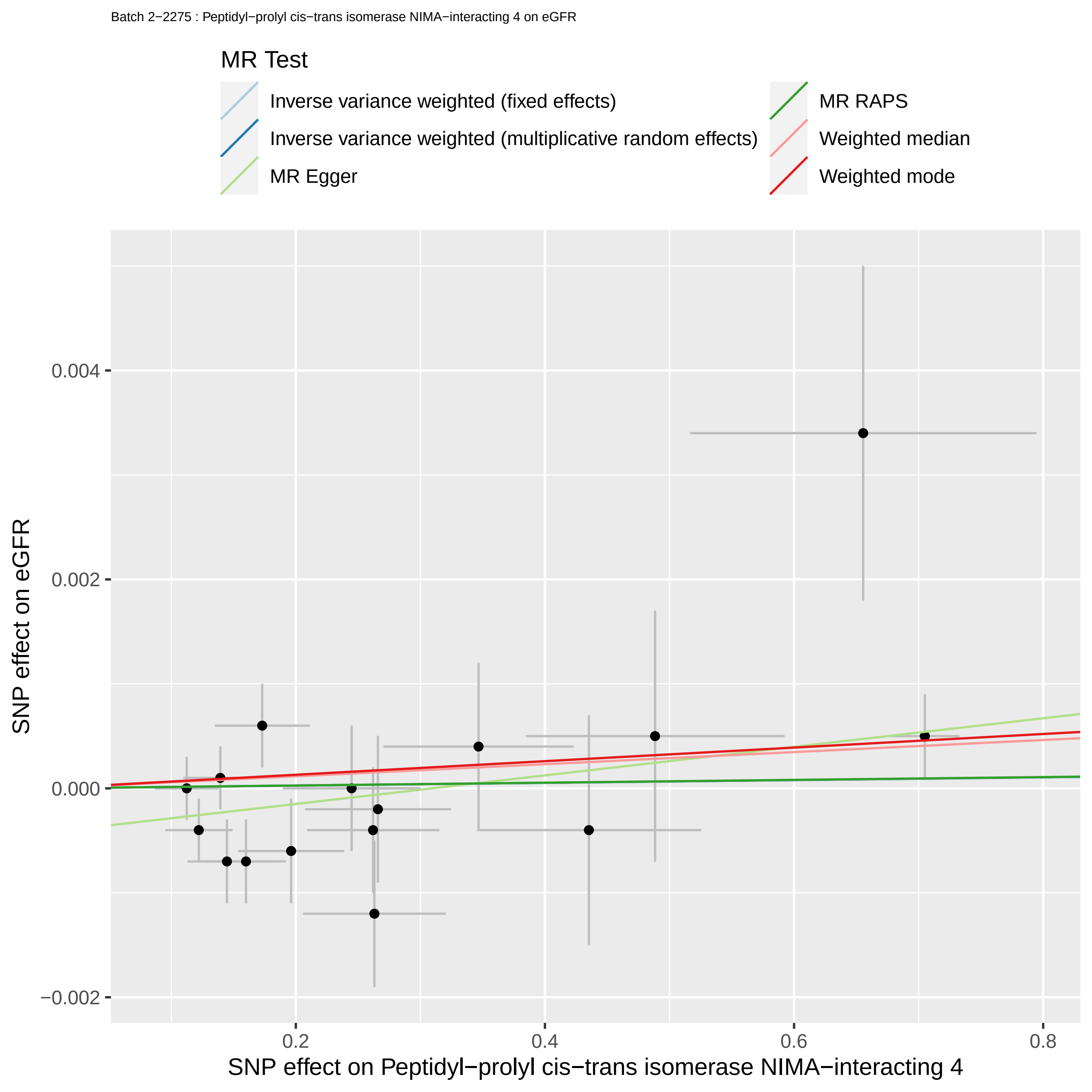

Batch 2−2275 : Peptidyl−prolyl cis−trans isomerase NIMA−interacting 4 on eGFR
MR Test
Inverse variance weighted (fixed effects)
Inverse variance weighted (multiplicative random effects)
MR Egger
MR RAPS
Weighted median
Weighted mode
SNP effect on eGFR
0.004
0.002
0.000
−0.002
0.2
0.4
0.6
0.8
SNP effect on Peptidyl−prolyl cis−trans isomerase NIMA−interacting 4

Batch 2−2276 : Phosphatidylinositol 5−phosphate 4−kinase type−2 alpha on eGFR

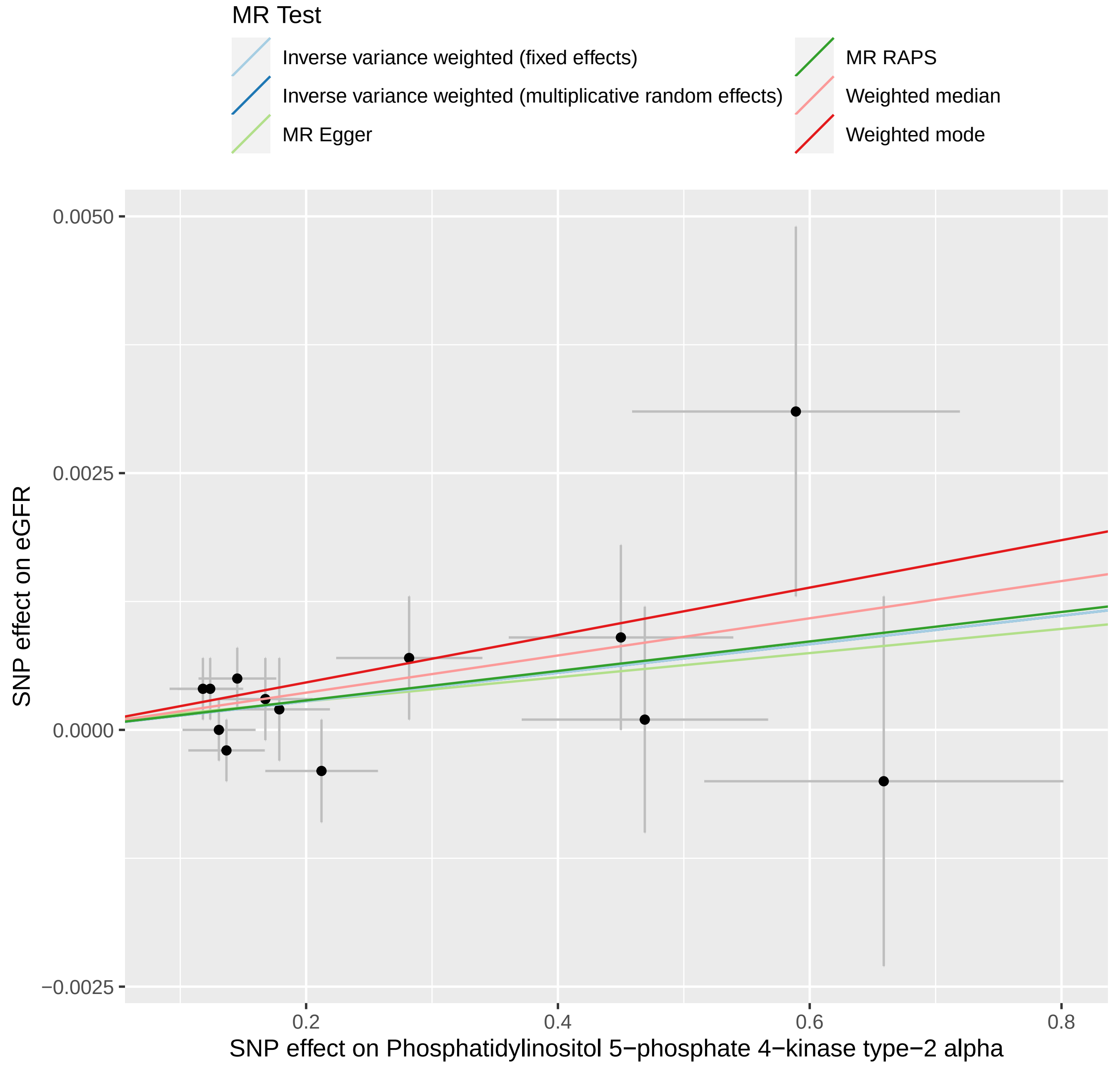

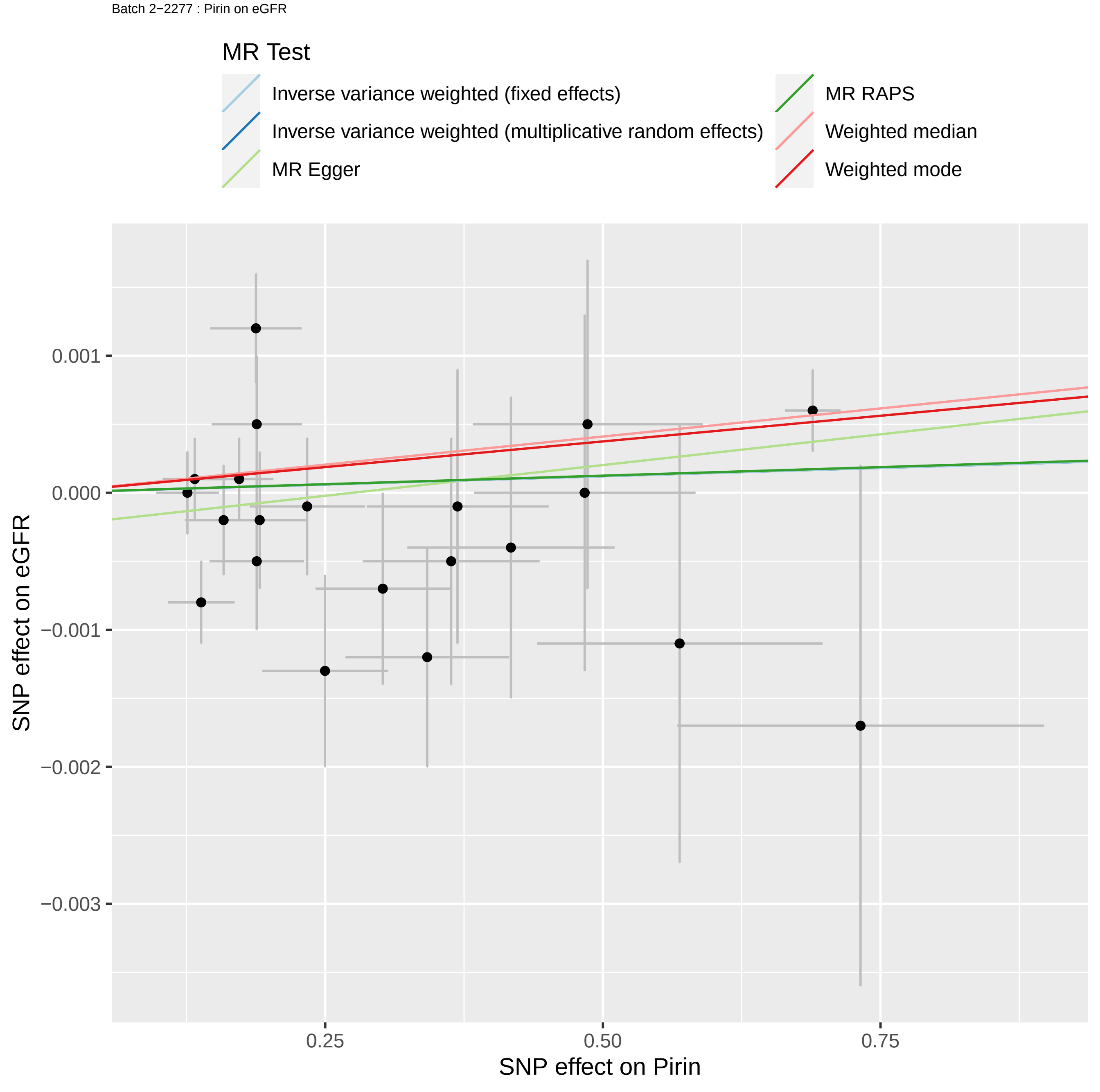
Batch 2−2277 : Pirin on eGFR
MR Test
Inverse variance weighted (fixed effects)
Inverse variance weighted (multiplicative random effects)
MR Egger
MR RAPS
Weighted median
Weighted mode
0.001
0.000
−0.001
−0.002
−0.003
SNP effect on eGFR
0.25
0.50
0.75
SNP effect on Pirin

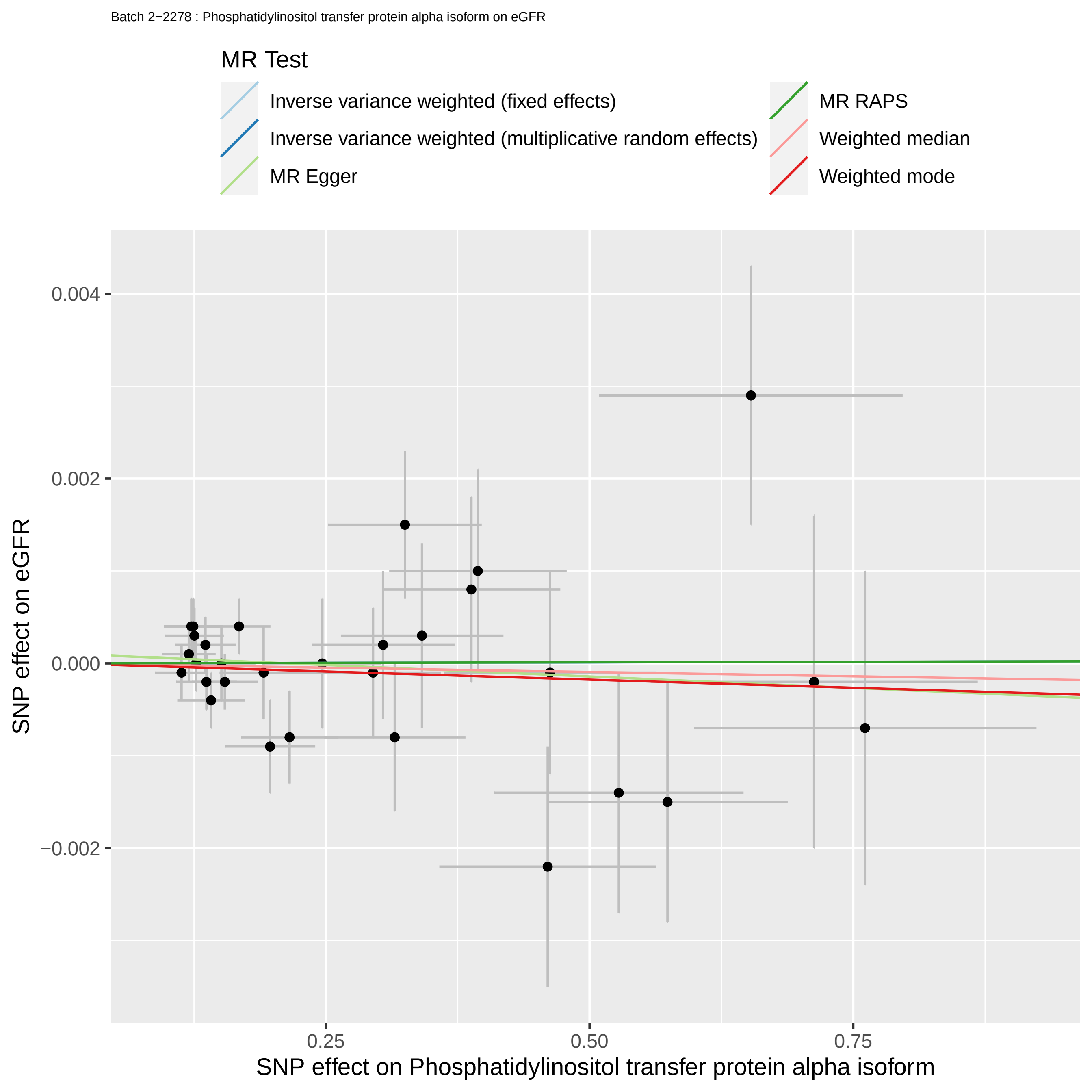
Batch 2−2278 : Phosphatidylinositol transfer protein alpha isoform on eGFR
MR Test
Inverse variance weighted (fixed effects)
Inverse variance weighted (multiplicative random effects)
MR Egger
MR RAPS
Weighted median
Weighted mode
0.004
0.002
0.000
−0.002
0.25
0.50
0.75
SNP effect on eGFR
SNP effect on Phosphatidylinositol transfer protein alpha isoform

Batch 2−2279 : Phosphatidylinositol transfer protein beta isoform on eGFR

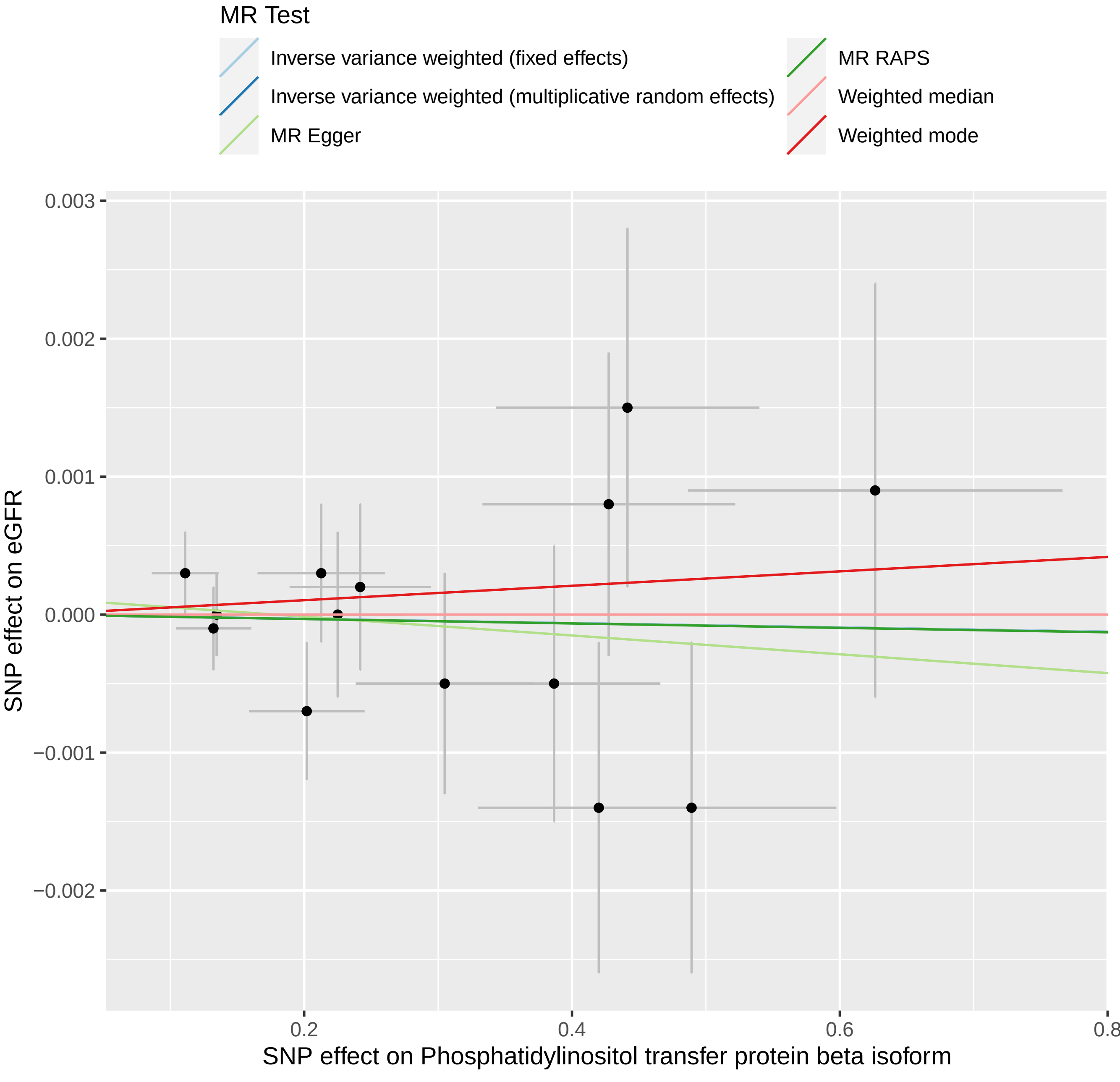

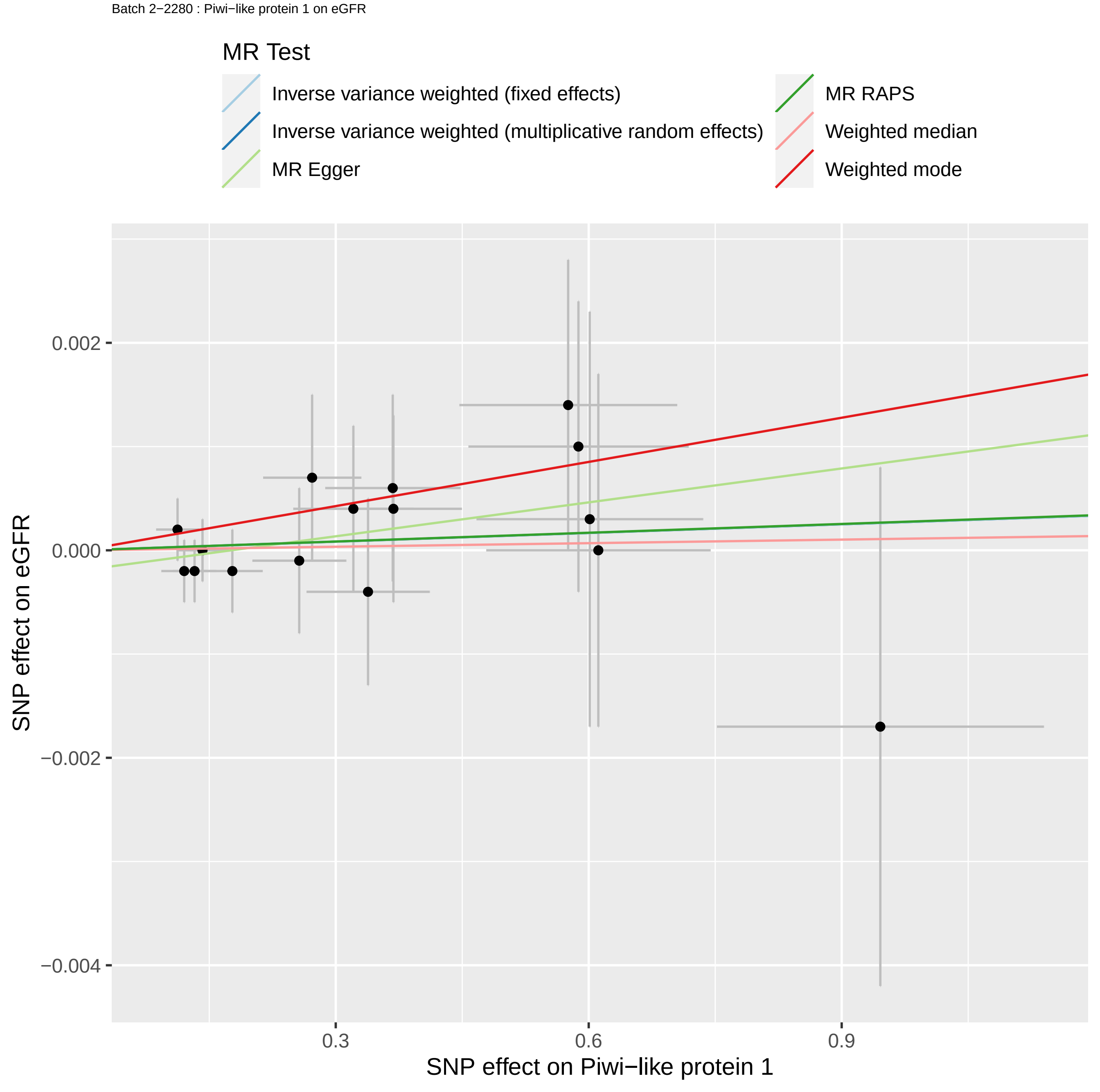

Batch 2−2280 : Piwi−like protein 1 on eGFR
MR Test
Inverse variance weighted (fixed effects)
Inverse variance weighted (multiplicative random effects)
MR Egger
MR RAPS
Weighted median
Weighted mode
0.002
0.000
−0.002
−0.004
0.3
0.6
0.9
SNP effect on eGFR
SNP effect on Piwi−like protein 1

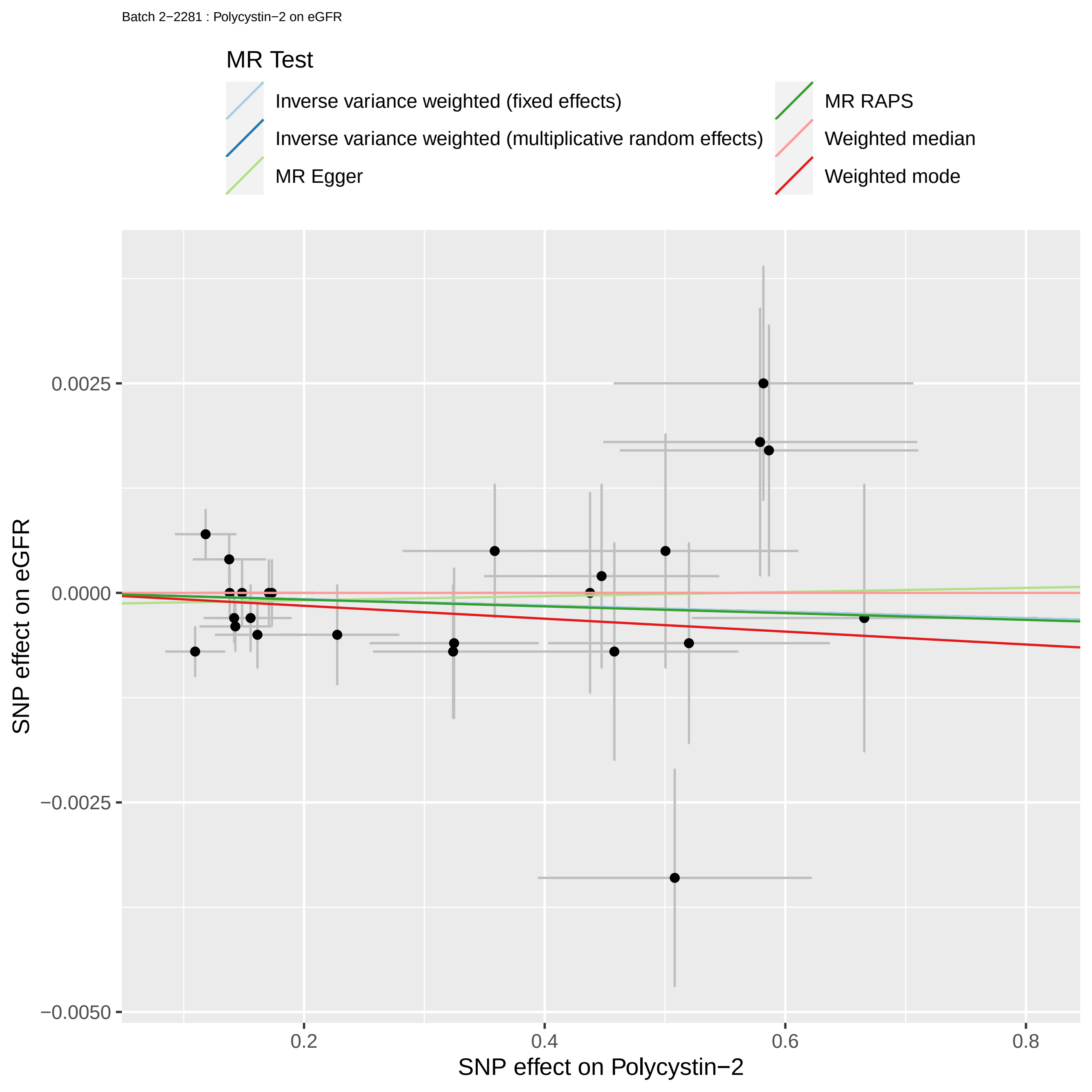
Batch 2−2281 : Polycystin−2 on eGFR
MR Test
Inverse variance weighted (fixed effects)
Inverse variance weighted (multiplicative random effects)
MR Egger
MR RAPS
Weighted median
Weighted mode
0.0025
0.0000
−0.0025
−0.0050
0.2
0.4
0.6
0.8
SNP effect on eGFR
SNP effect on Polycystin−2

Batch 2−2282 : Pyruvate kinase PKLR on eGFR

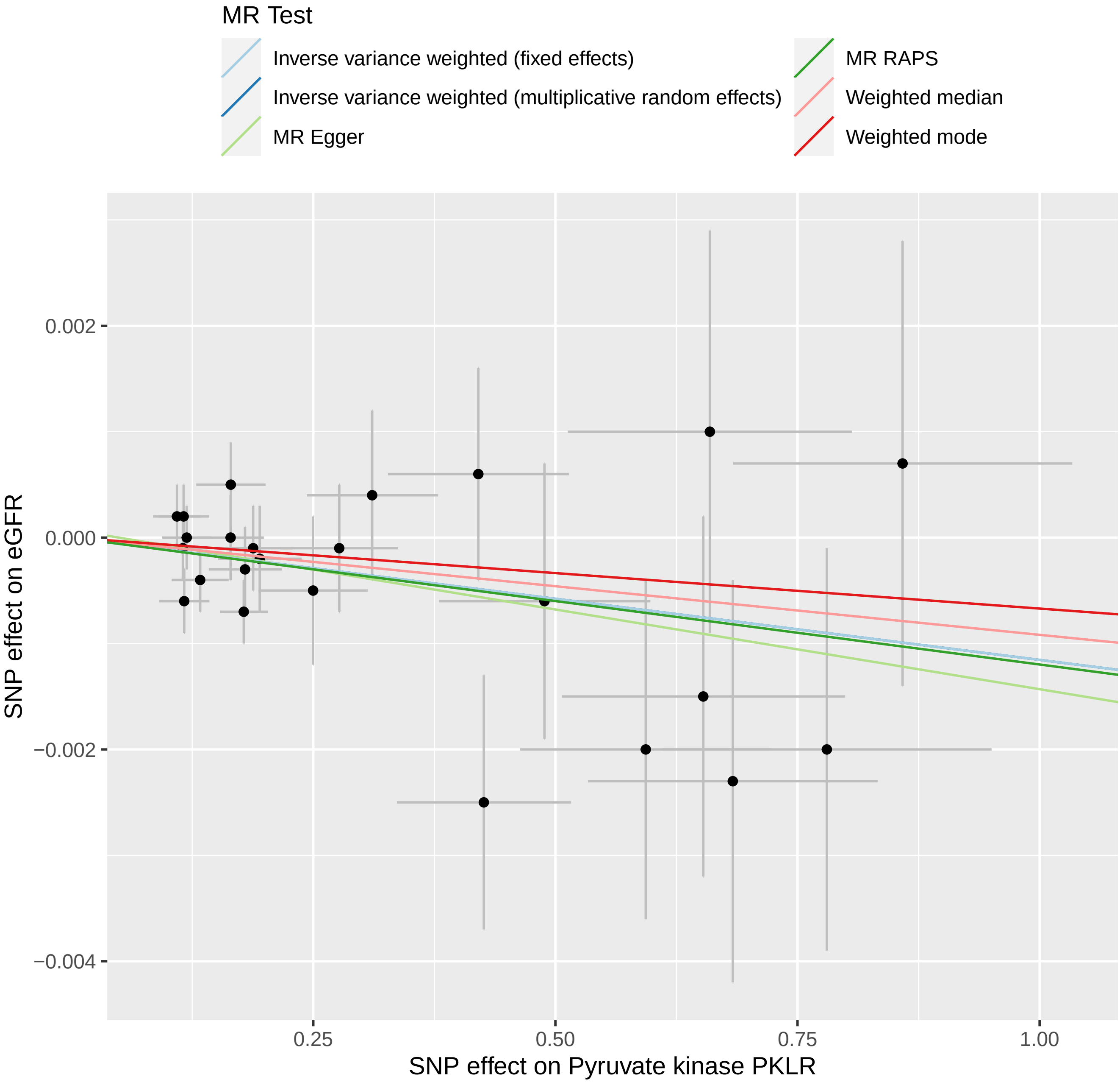

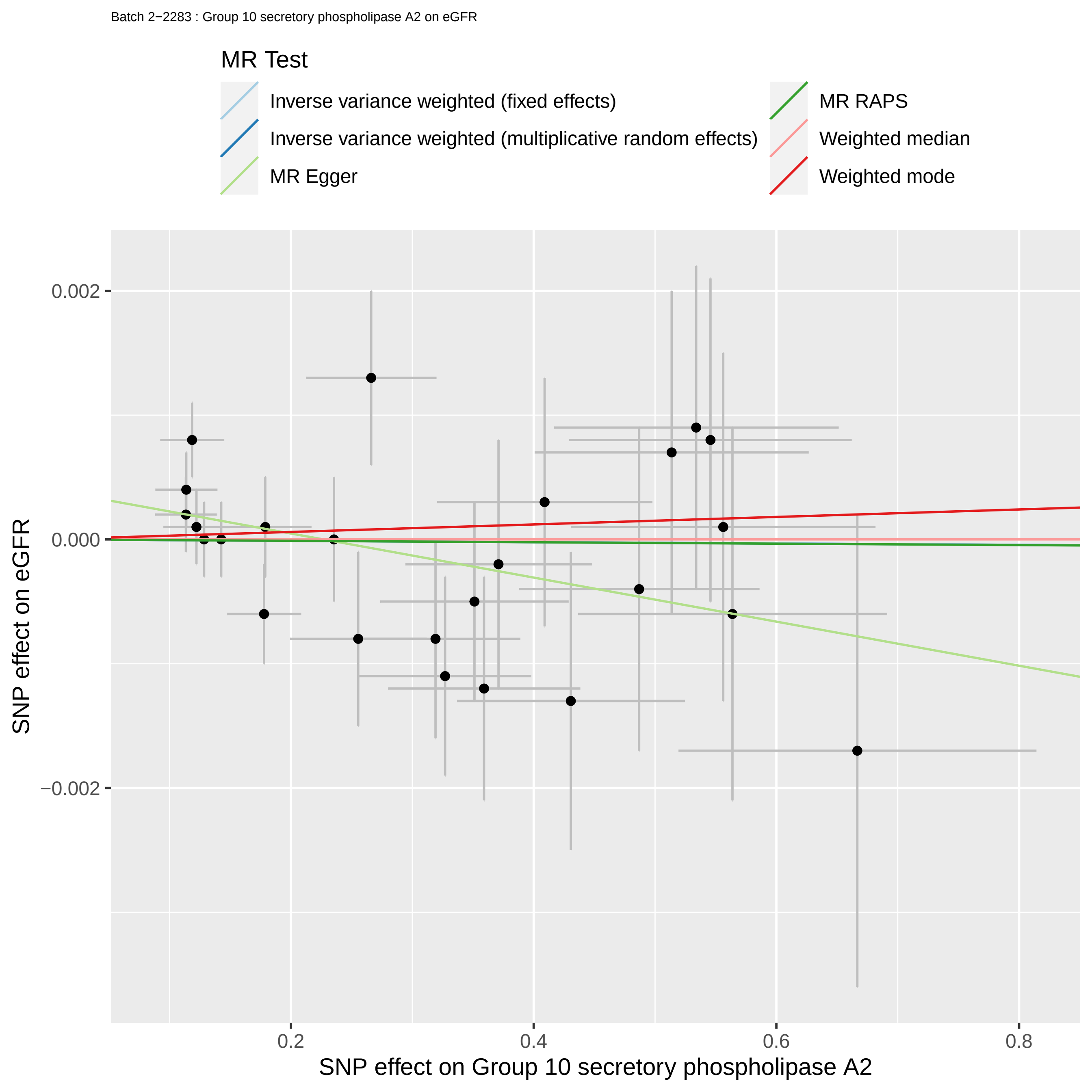

Batch 2−2283 : Group 10 secretory phospholipase A2 on eGFR
MR Test
Inverse variance weighted (fixed effects)
Inverse variance weighted (multiplicative random effects)
MR Egger
MR RAPS
Weighted median
Weighted mode
0.002
0.000
−0.002
0.2
0.4
0.6
0.8
SNP effect on eGFR
SNP effect on Group 10 secretory phospholipase A2

Batch 2−2284 : Group XIIB secretory phospholipase A2−like protein on eGFR

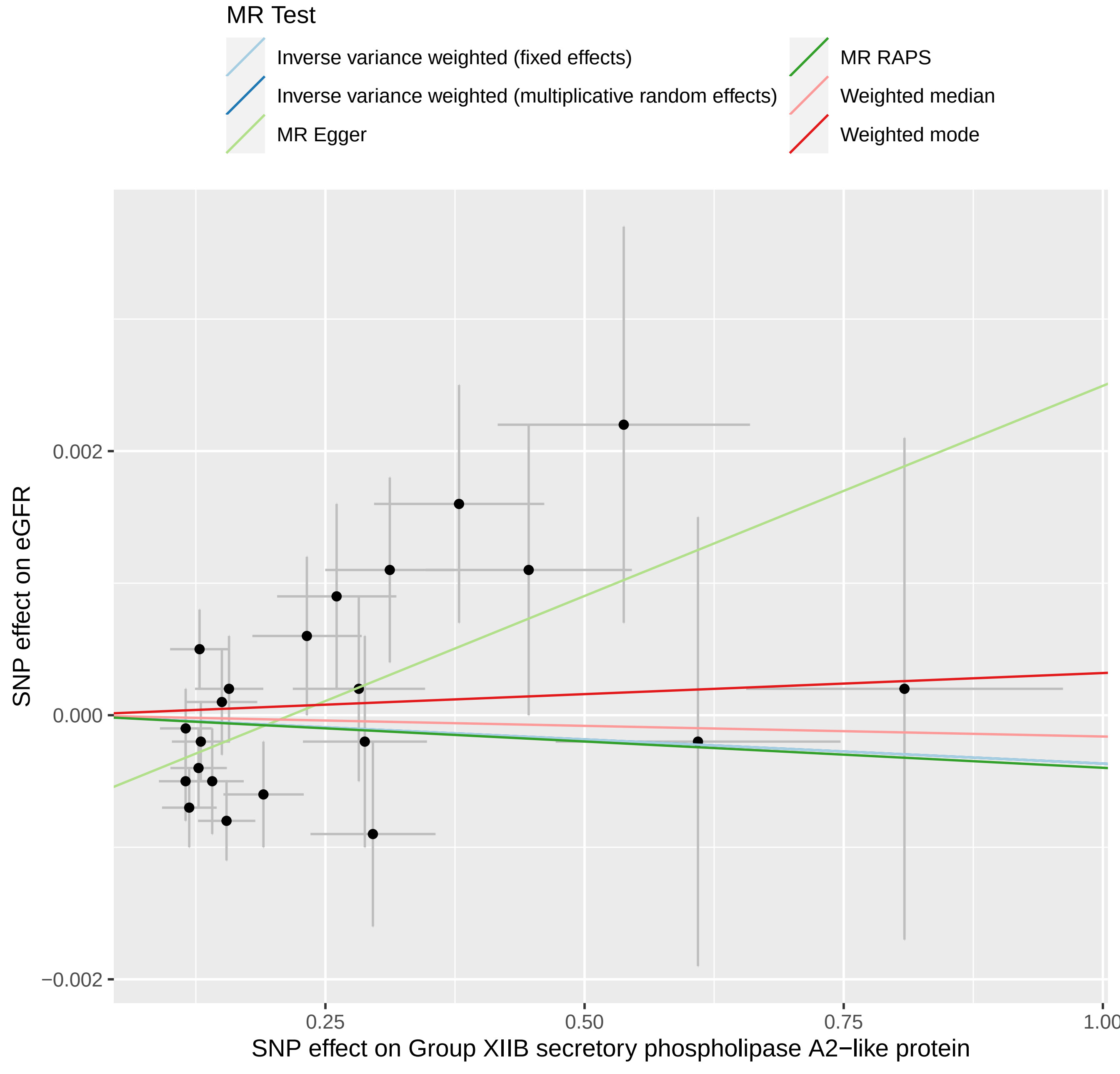

Batch 2−2285 : HRAS−like suppressor 3 on eGFR

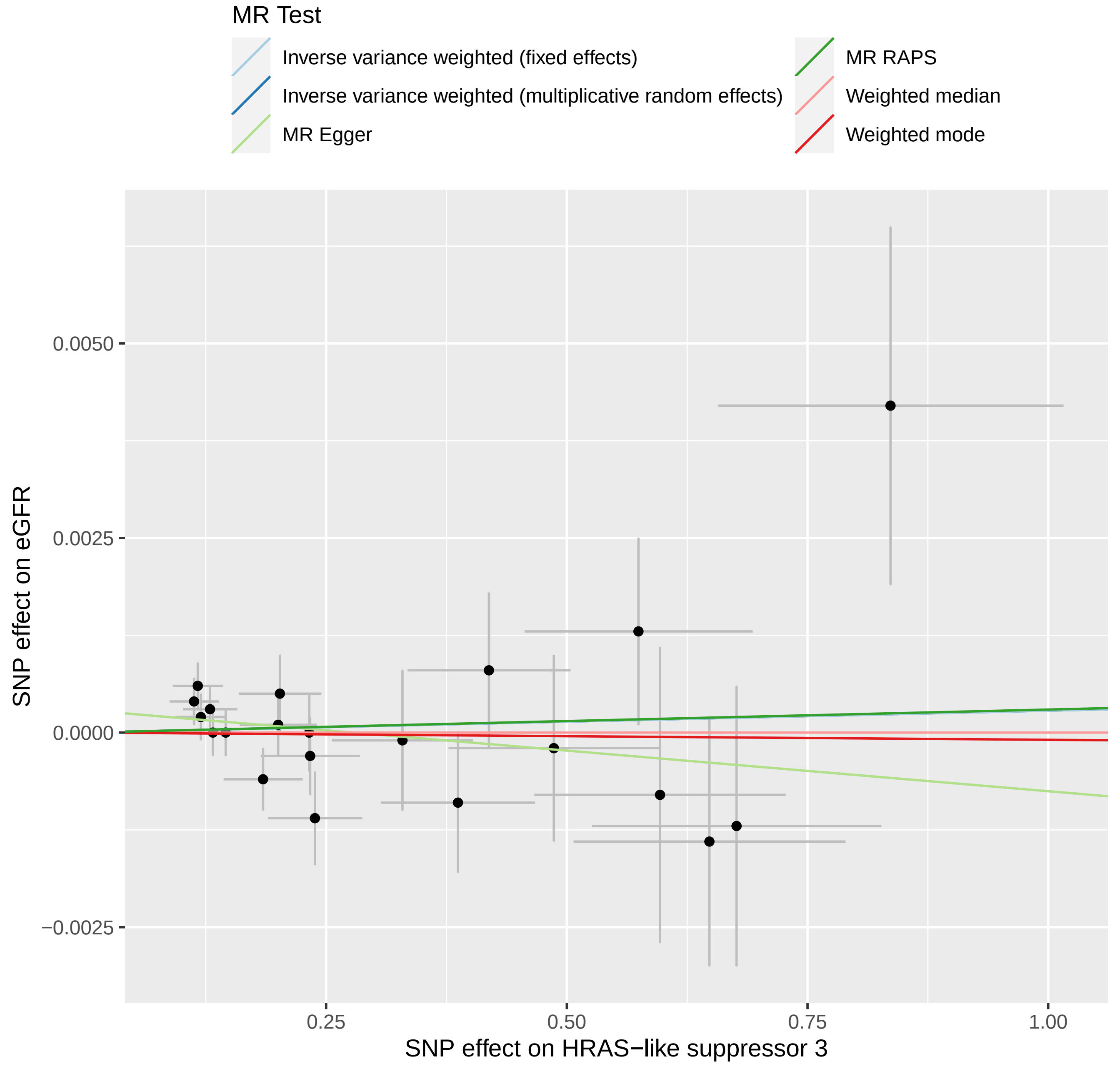

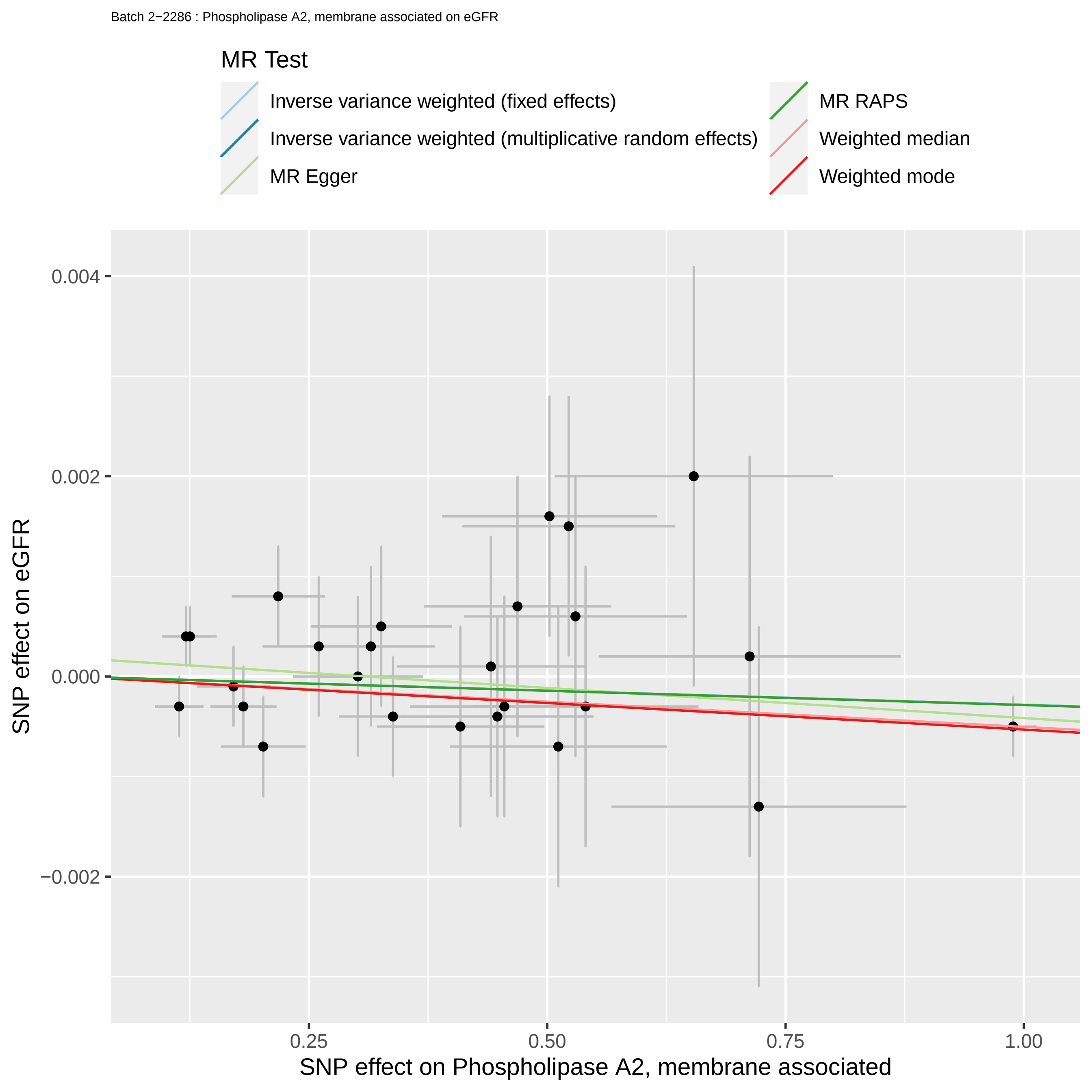
Batch 2−2286 : Phospholipase A2, membrane associated on eGFR
MR Test
Inverse variance weighted (fixed effects)
Inverse variance weighted (multiplicative random effects)
MR Egger
MR RAPS
Weighted median
Weighted mode
0.004
0.002
0.000
−0.002
0.25
0.50
0.75
1.00
SNP effect on eGFR
SNP effect on Phospholipase A2, membrane associated

Batch 2−2287 : Putative inactive group IIC secretory phospholipase A2 on eGFR

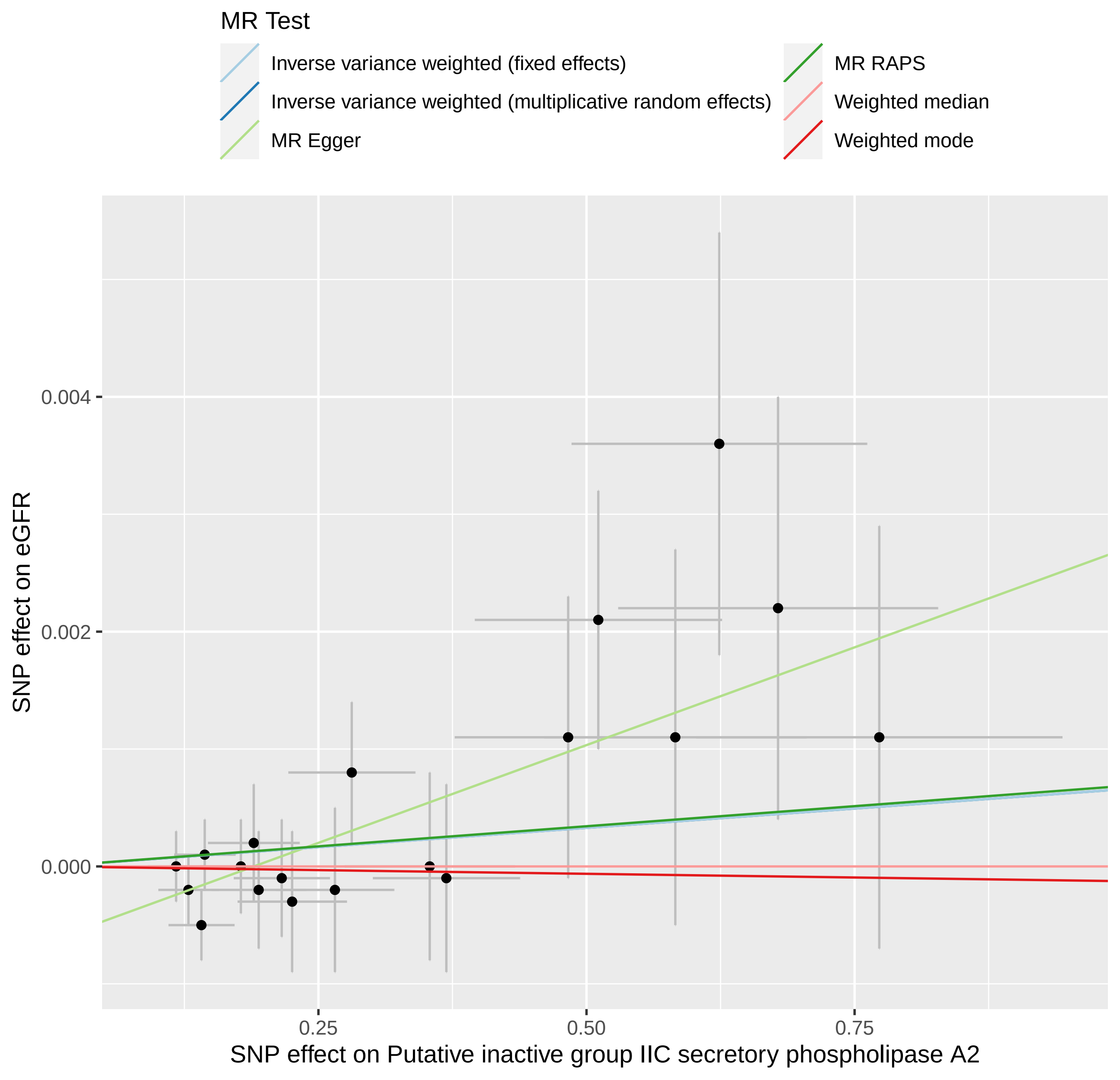

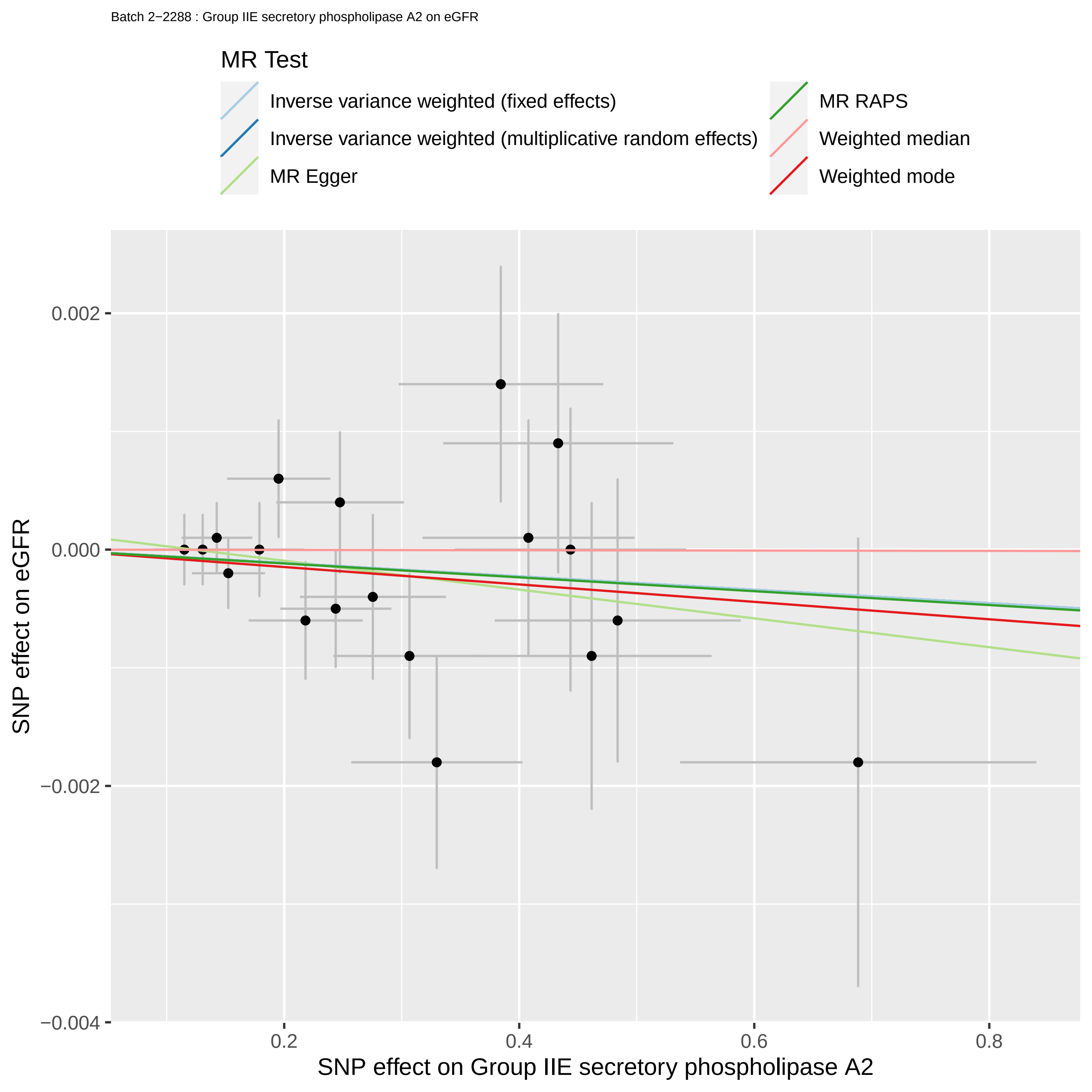

Batch 2−2288 : Group IIE secretory phospholipase A2 on eGFR
MR Test
Inverse variance weighted (fixed effects)
Inverse variance weighted (multiplicative random effects)
MR Egger
MR RAPS
Weighted median
Weighted mode
0.002
0.000
−0.002
−0.004
0.2
0.4
0.6
0.8
SNP effect on eGFR
SNP effect on Group IIE secretory phospholipase A2

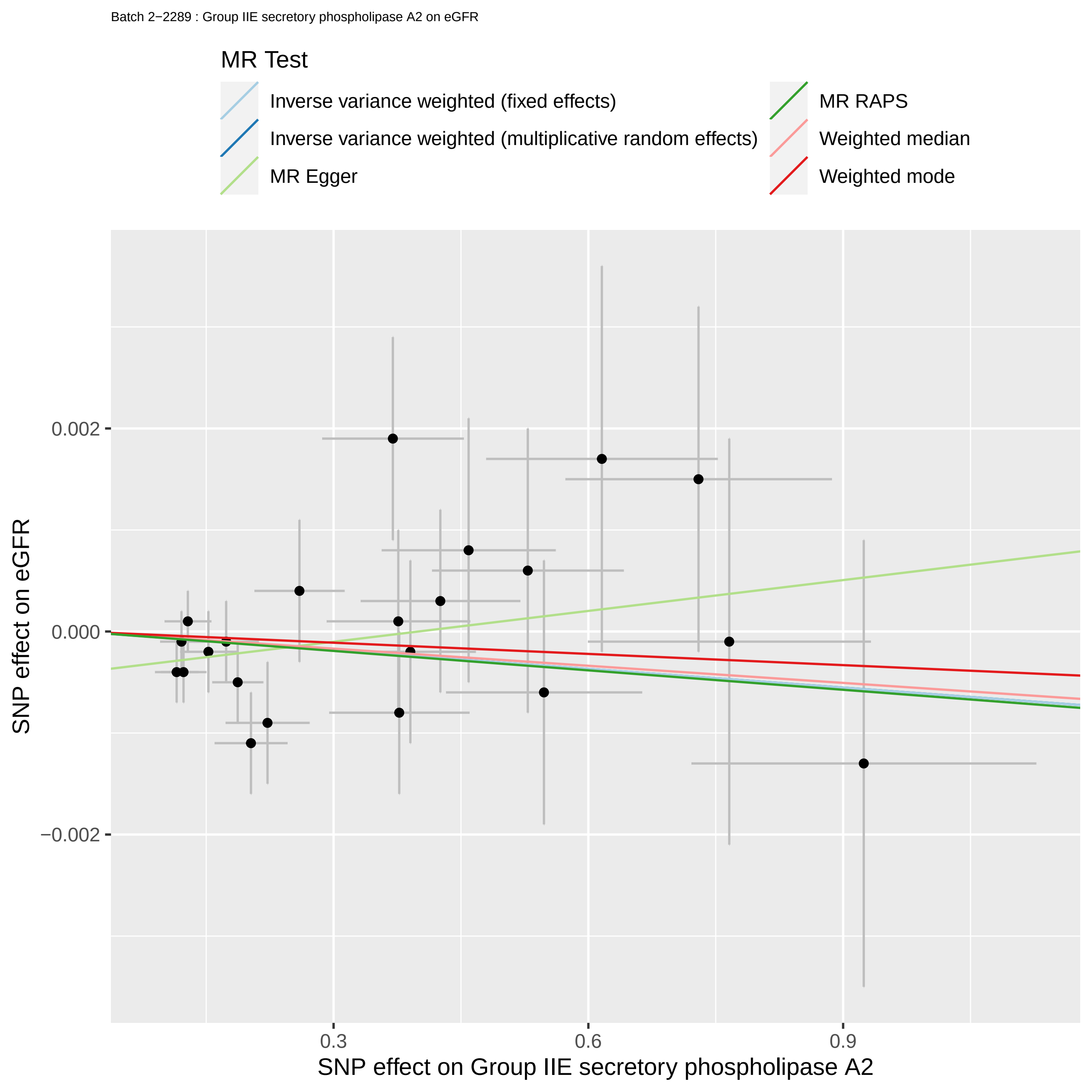

Batch 2−2289 : Group IIE secretory phospholipase A2 on eGFR
MR Test
Inverse variance weighted (fixed effects)
Inverse variance weighted (multiplicative random effects)
MR Egger
MR RAPS
Weighted median
Weighted mode
0.002
0.000
−0.002
0.3
0.6
0.9
SNP effect on eGFR
SNP effect on Group IIE secretory phospholipase A2

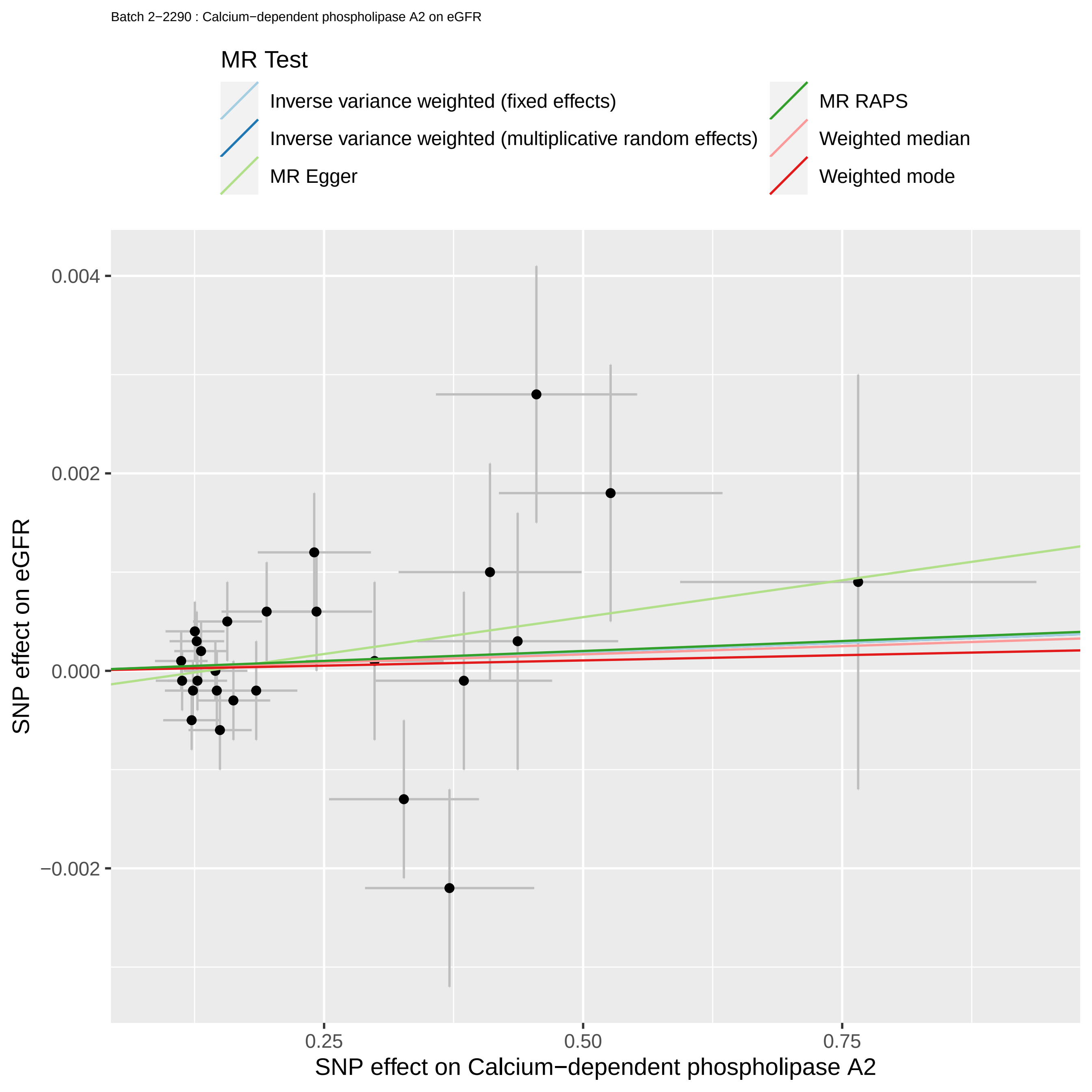

Batch 2−2290 : Calcium−dependent phospholipase A2 on eGFR
MR Test
Inverse variance weighted (fixed effects)
Inverse variance weighted (multiplicative random effects)
MR Egger
MR RAPS
Weighted median
Weighted mode
0.004
0.002
0.000
−0.002
0.25
0.50
0.75
SNP effect on eGFR
SNP effect on Calcium−dependent phospholipase A2

Batch 2−2291 : Secretory phospholipase A2 receptor on eGFR

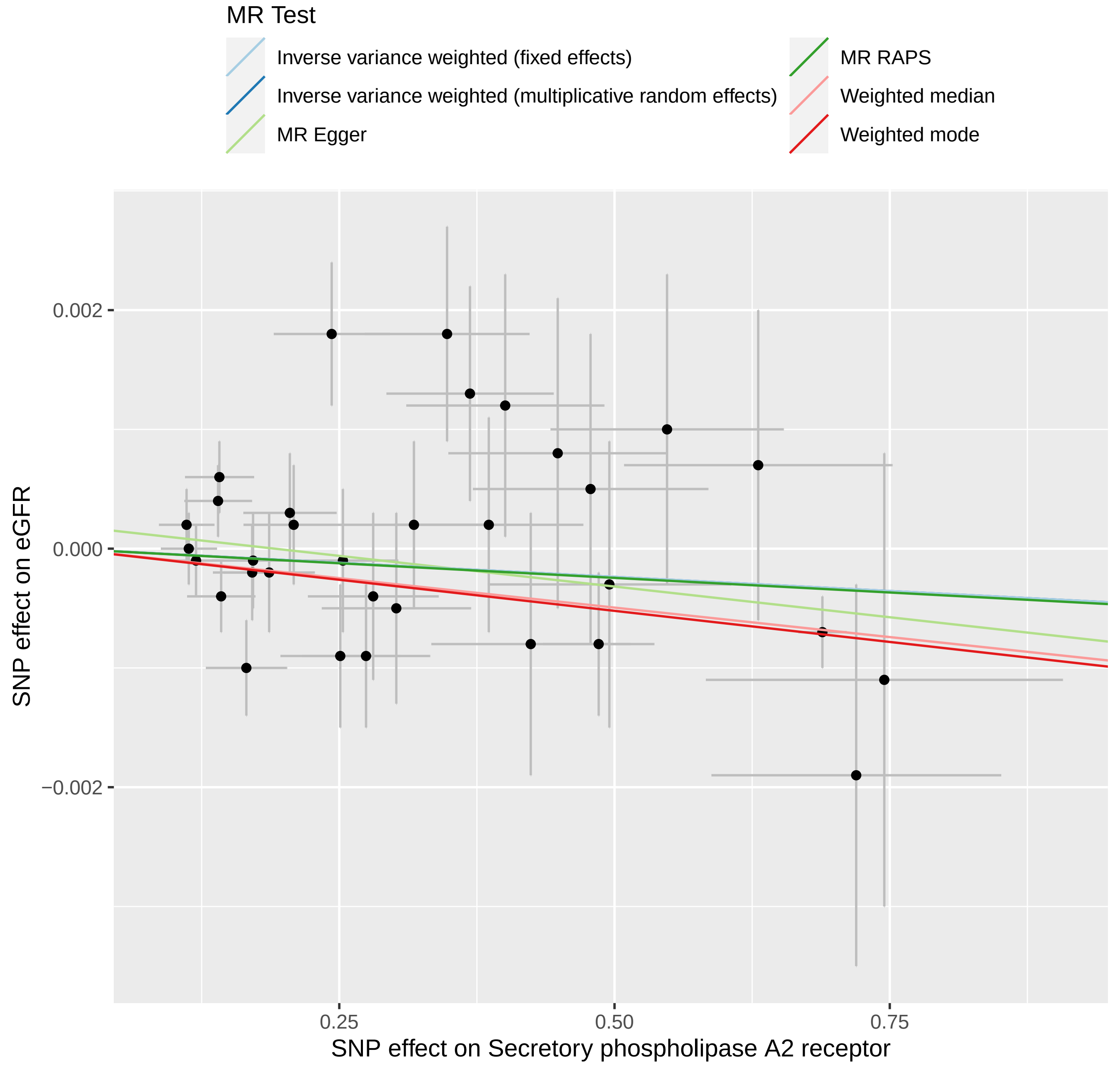

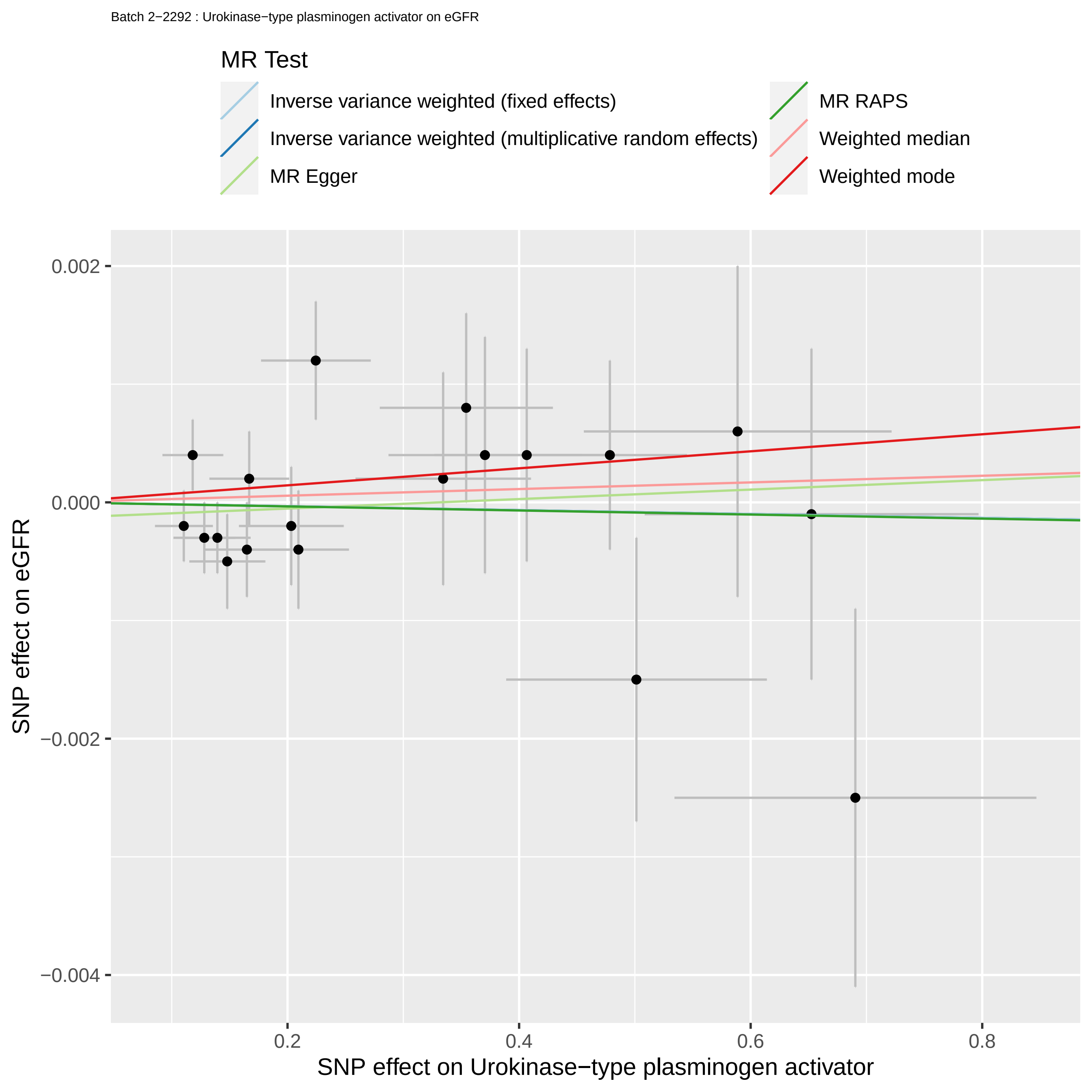
Batch 2−2292 : Urokinase−type plasminogen activator on eGFR
MR Test
Inverse variance weighted (fixed effects)
Inverse variance weighted (multiplicative random effects)
MR Egger
MR RAPS
Weighted median
Weighted mode
0.002
0.000
−0.002
−0.004
SNP effect on eGFR
0.2
0.4
0.6
0.8
SNP effect on Urokinase−type plasminogen activator

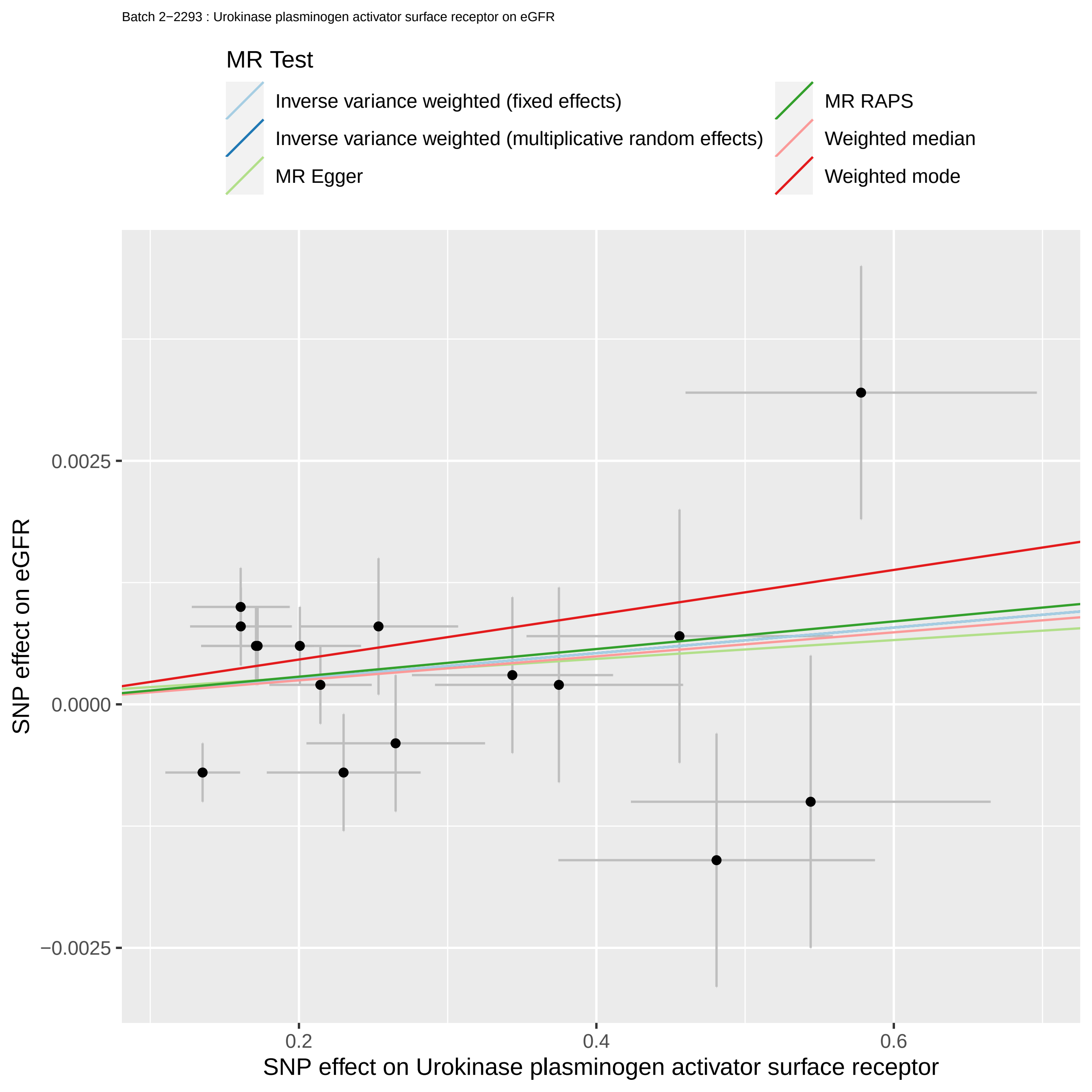

Batch 2−2293 : Urokinase plasminogen activator surface receptor on eGFR
MR Test
Inverse variance weighted (fixed effects)
Inverse variance weighted (multiplicative random effects)
MR Egger
MR RAPS
Weighted median
Weighted mode
0.0025
0.0000
−0.0025
0.2
0.4
0.6
SNP effect on eGFR
SNP effect on Urokinase plasminogen activator surface receptor

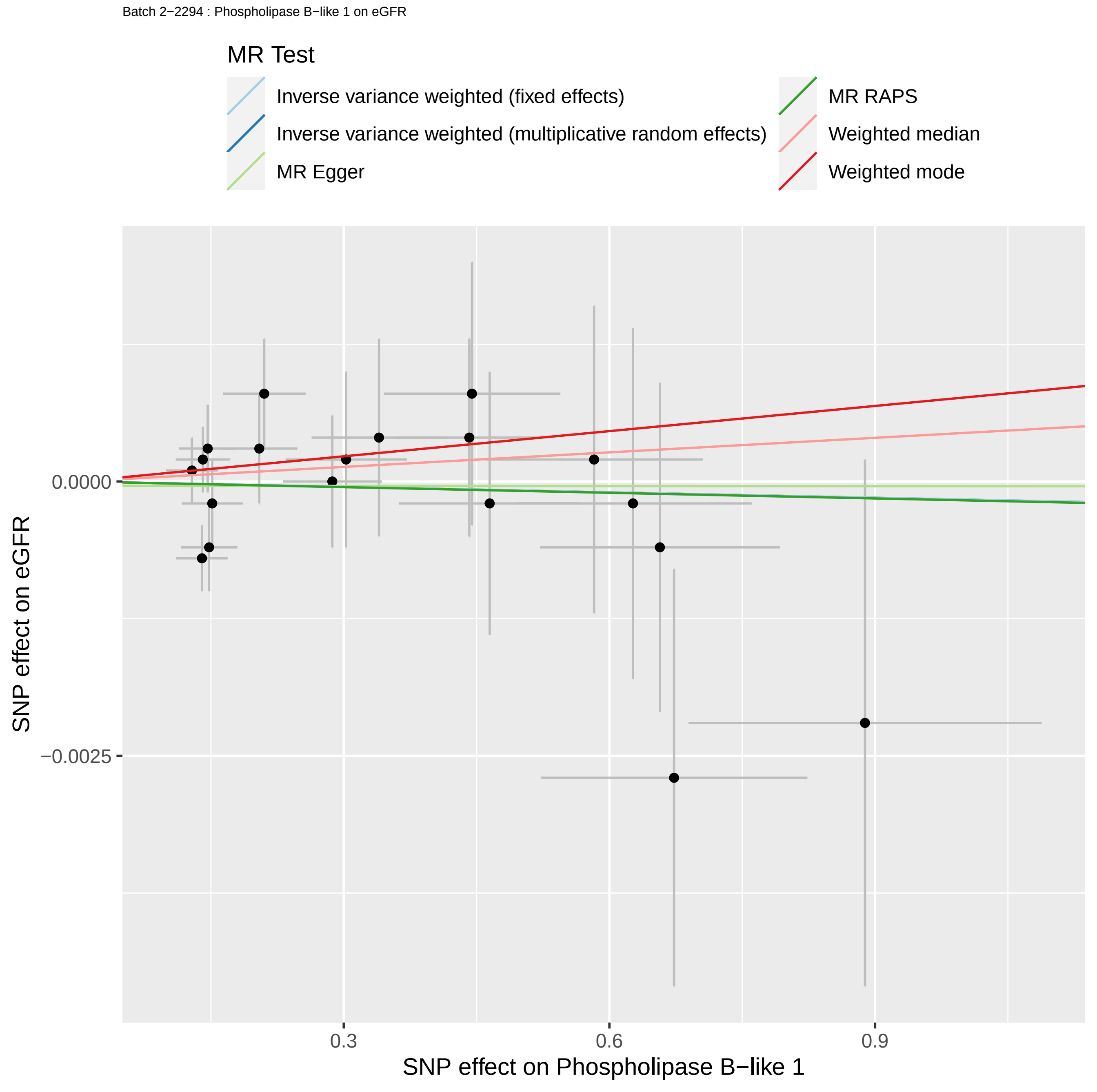

Batch 2−2294 : Phospholipase B−like 1 on eGFR
MR Test
Inverse variance weighted (fixed effects)
Inverse variance weighted (multiplicative random effects)
MR Egger
MR RAPS
Weighted median
Weighted mode
0.0000
−0.0025
0.3
0.6
0.9
SNP effect on eGFR
SNP effect on Phospholipase B−like 1

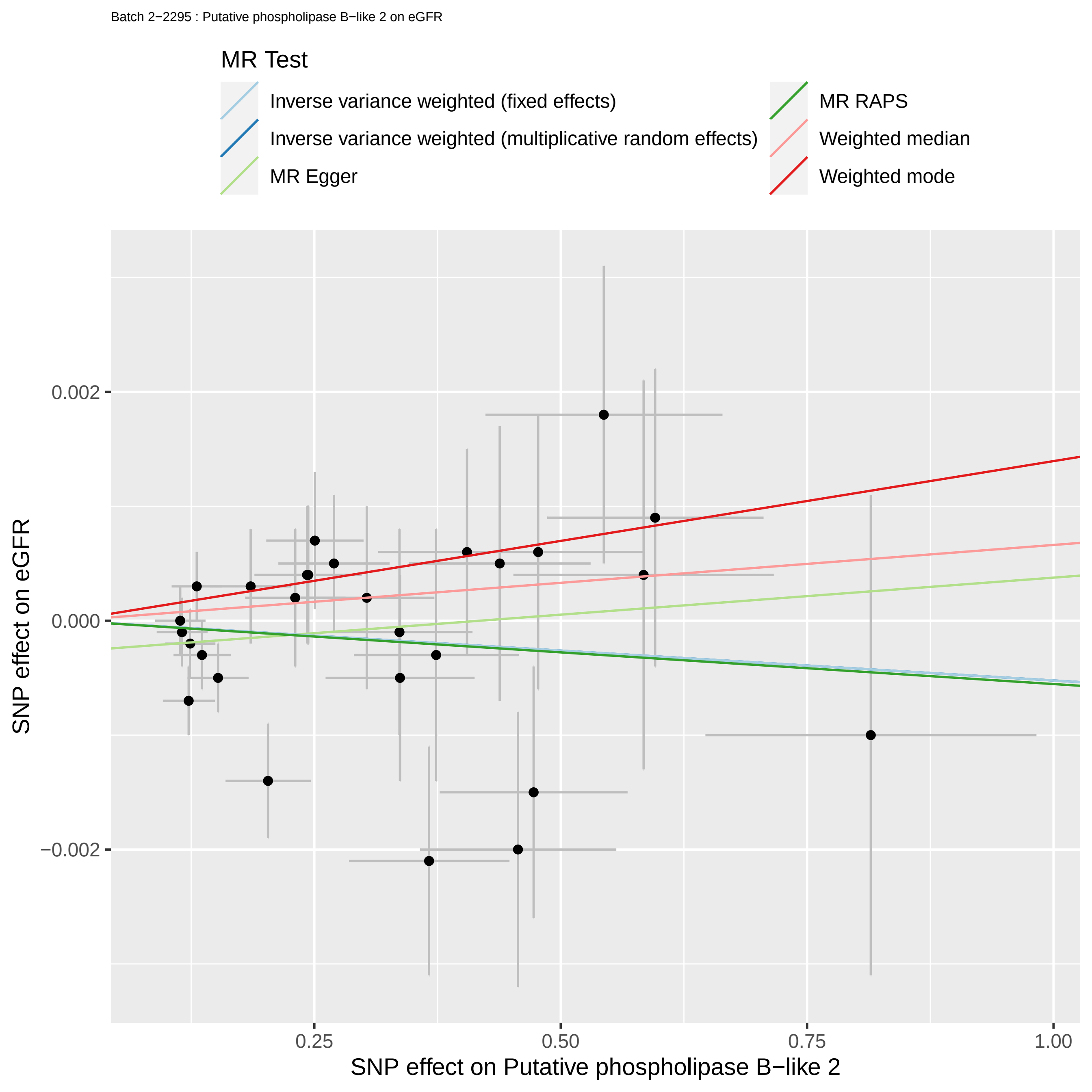

Batch 2−2295 : Putative phospholipase B−like 2 on eGFR
MR Test
Inverse variance weighted (fixed effects)
Inverse variance weighted (multiplicative random effects)
MR Egger
MR RAPS
Weighted median
Weighted mode
0.002
0.000
−0.002
SNP effect on eGFR
0.25
0.50
0.75
1.00
SNP effect on Putative phospholipase B−like 2

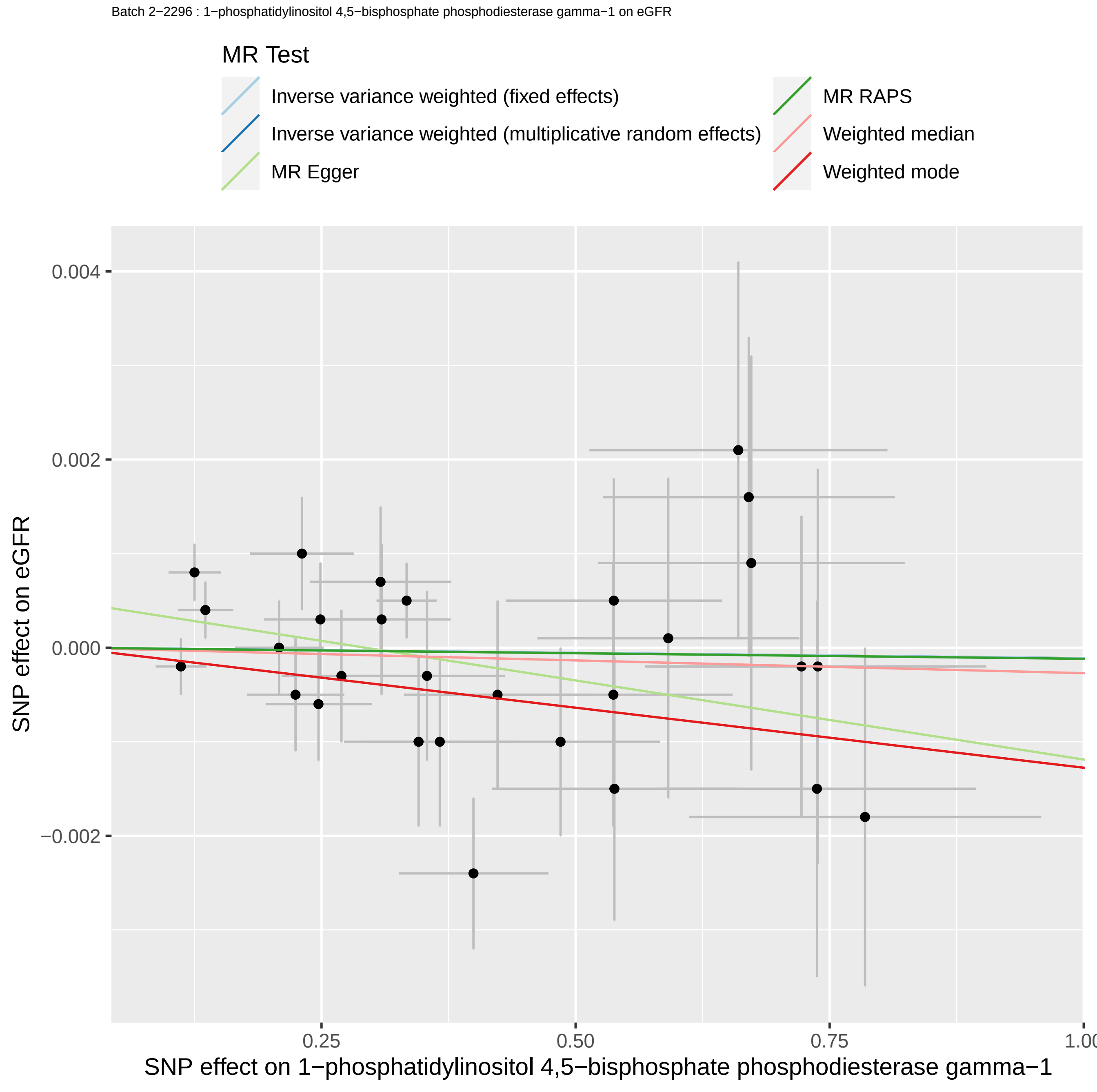

Batch 2−2296 : 1−phosphatidylinositol 4,5−bisphosphate phosphodiesterase gamma−1 on eGFR
MR Test
Inverse variance weighted (fixed effects)
Inverse variance weighted (multiplicative random effects)
MR Egger
MR RAPS
Weighted median
Weighted mode
0.004
0.002
0.000
−0.002
SNP effect on eGFR
0.25
0.50
0.75
1.00
SNP effect on 1−phosphatidylinositol 4,5−bisphosphate phosphodiesterase gamma−1

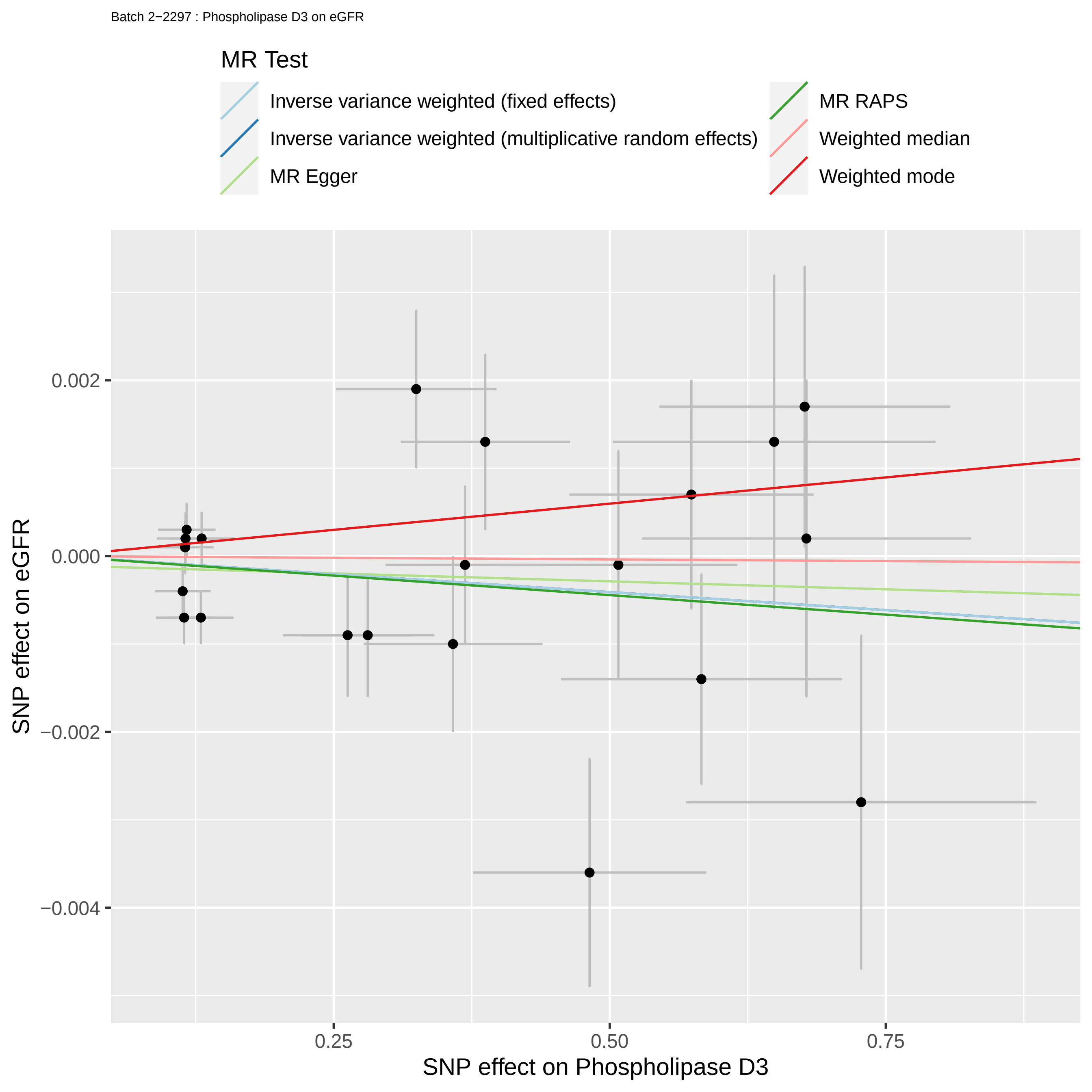

Batch 2−2297 : Phosphopilase D3 on eGFR
MR Test
Inverse variance weighted (fixed effects)
Inverse variance weighted (multiplicative random effects)
MR Egger
MR RAPS
Weighted median
Weighted mode
0.002
0.000
−0.002
−0.004
0.25
0.50
0.75
SNP effect on eGFR
SNP effect on Phospholipase D3

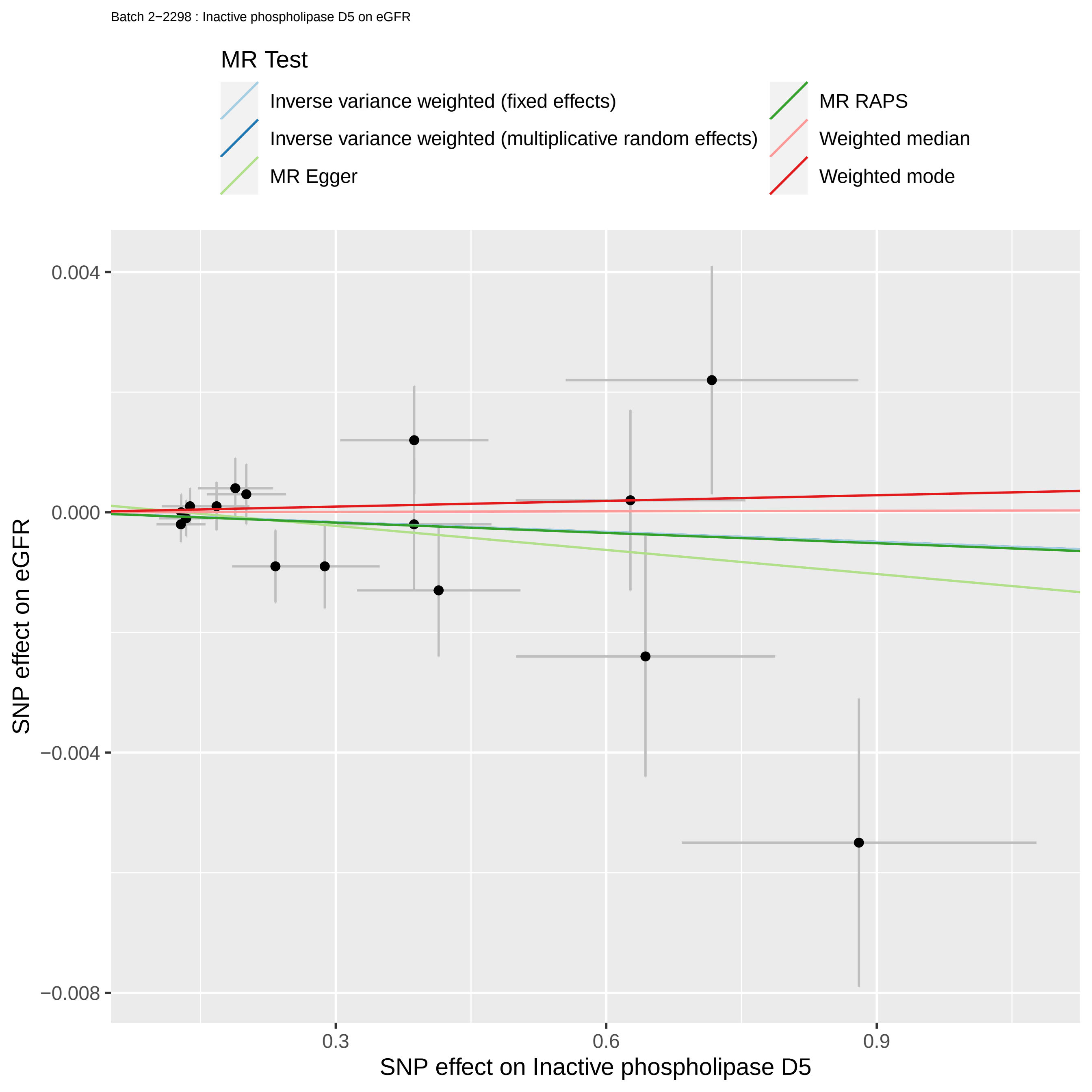

Batch 2−2298 : Inactive phospholipase D5 on eGFR
MR Test
Inverse variance weighted (fixed effects)
Inverse variance weighted (multiplicative random effects)
MR Egger
MR RAPS
Weighted median
Weighted mode
0.004
0.000
−0.004
−0.008
0.3
0.6
0.9
SNP effect on eGFR
SNP effect on Inactive phospholipase D5

Batch 2−2299 : Pleckstrin homology domain−containing family A member 1 on eGFR

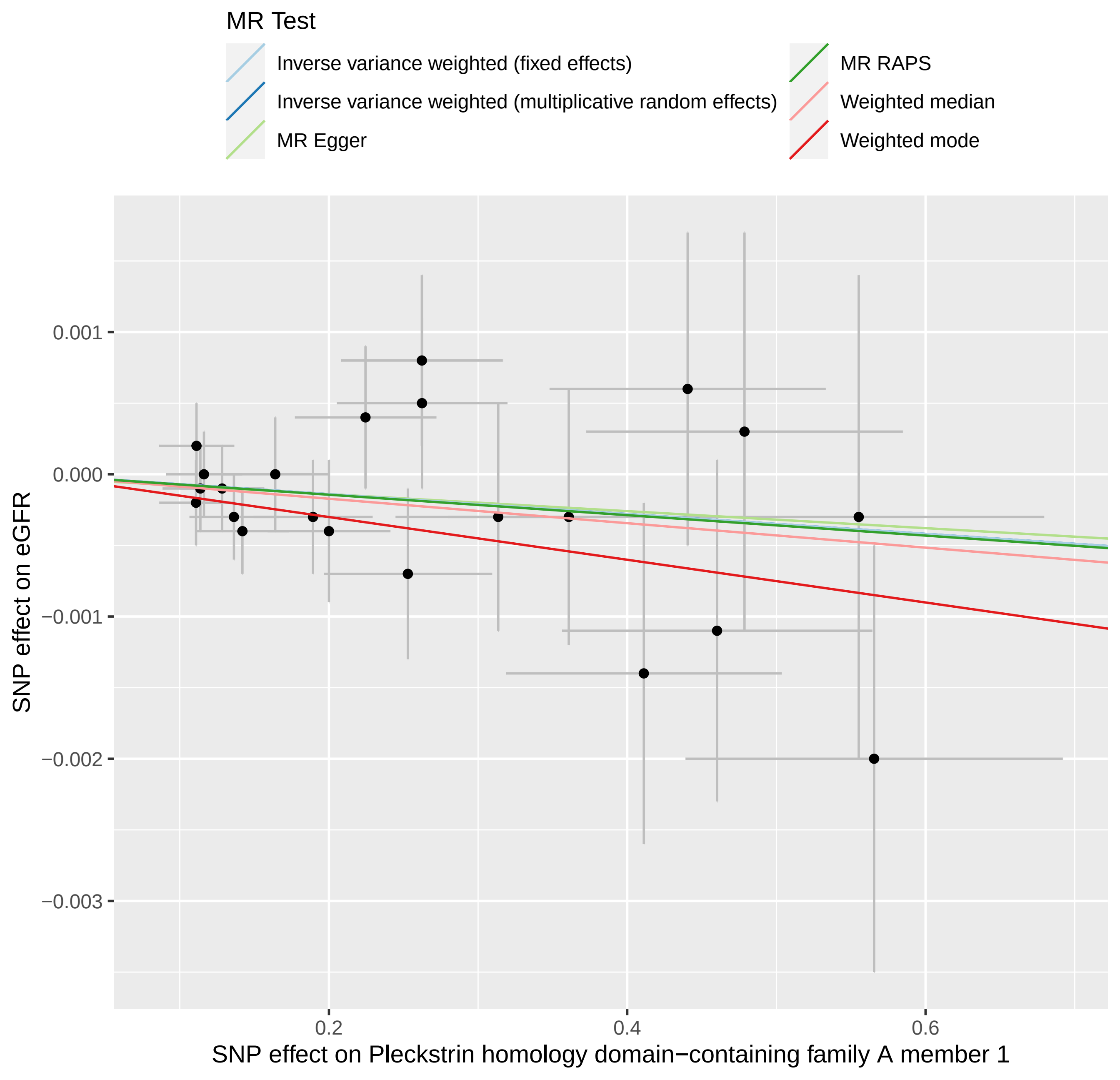

Batch 2−2300 : Pleckstrin homology domain−containing family A member 7 on eGFR

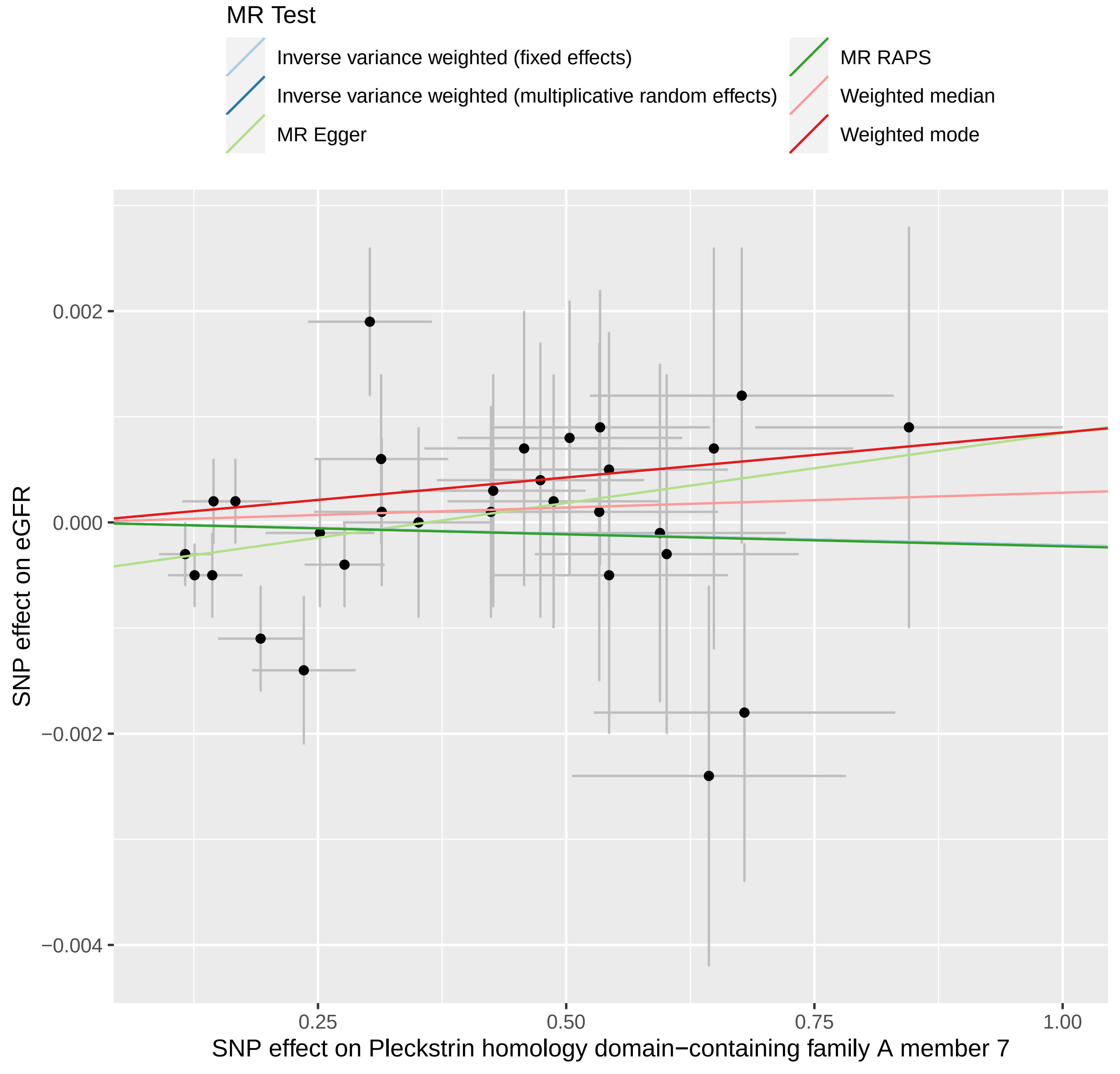

Batch 2−2301 : Angiostatin on eGFR

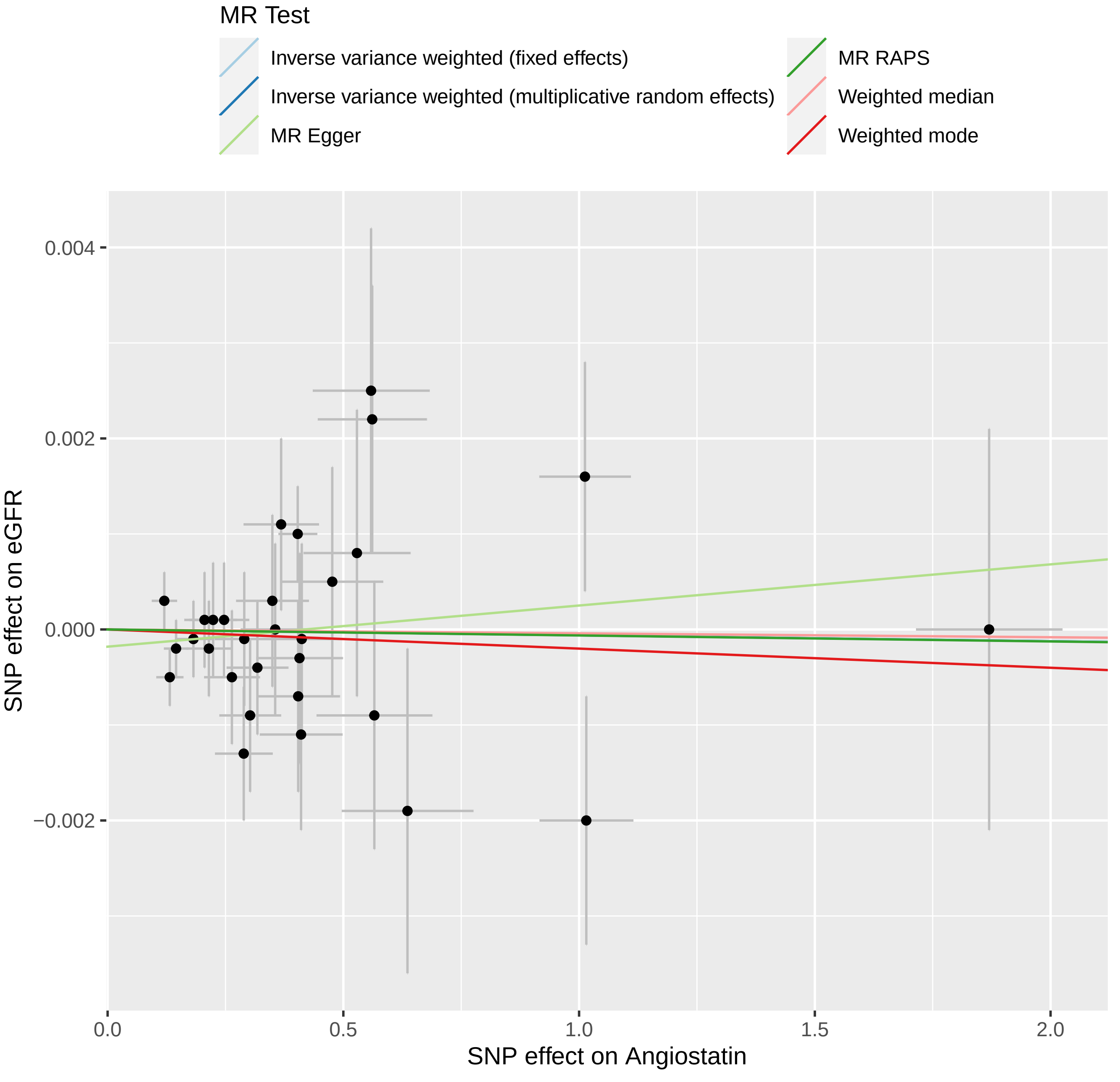

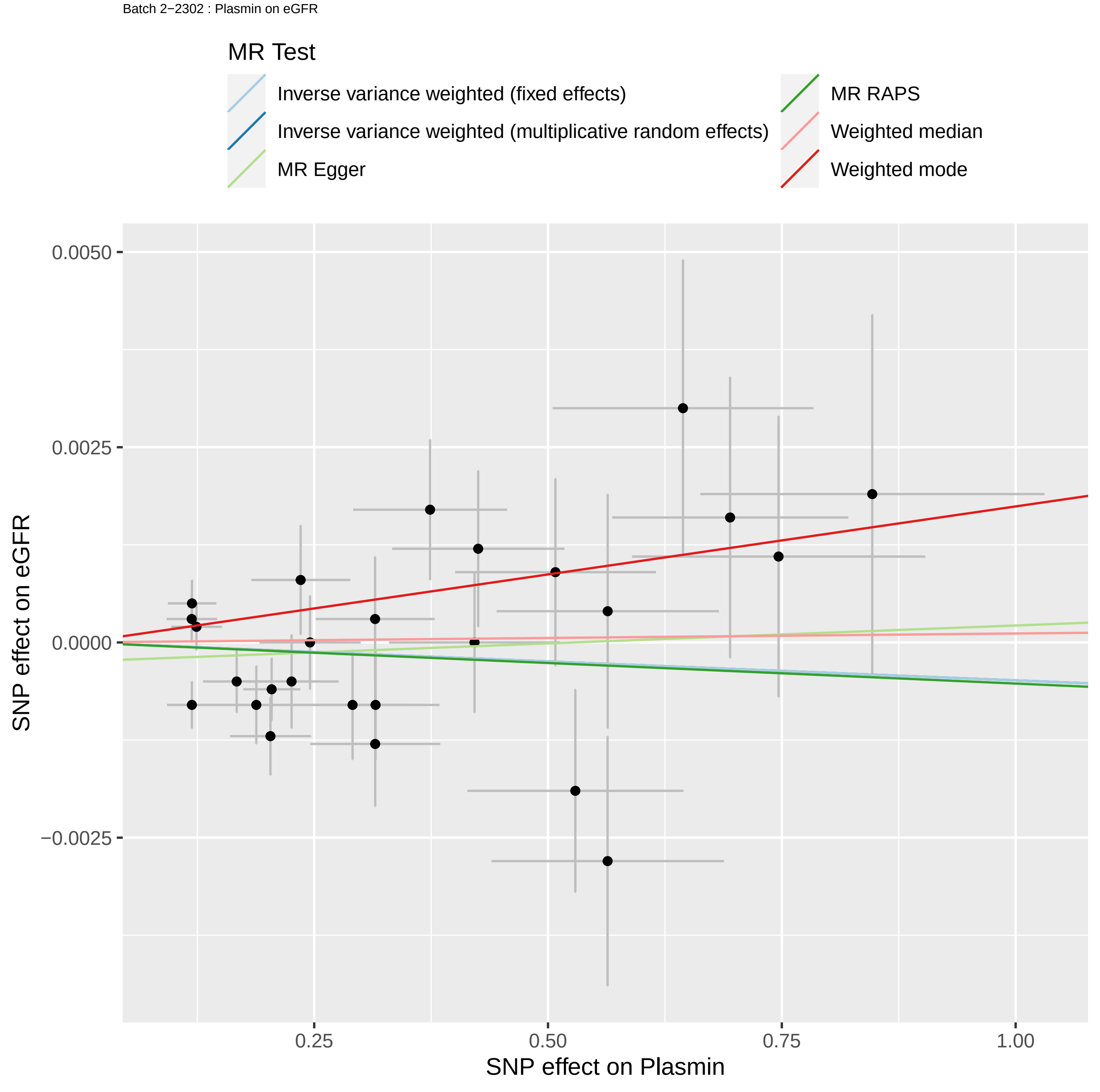

Batch 2−2302 : Plasmin on eGFR
MR Test
Inverse variance weighted (fixed effects)
Inverse variance weighted (multiplicative random effects)
MR Egger
MR RAPS
Weighted median
Weighted mode
0.0050
0.0025
0.0000
−0.0025
0.25
0.50
0.75
1.00
SNP effect on eGFR
SNP effect on Plasmin

Batch 2−2303 : Serine/threonine−protein kinase PLK1 on eGFR

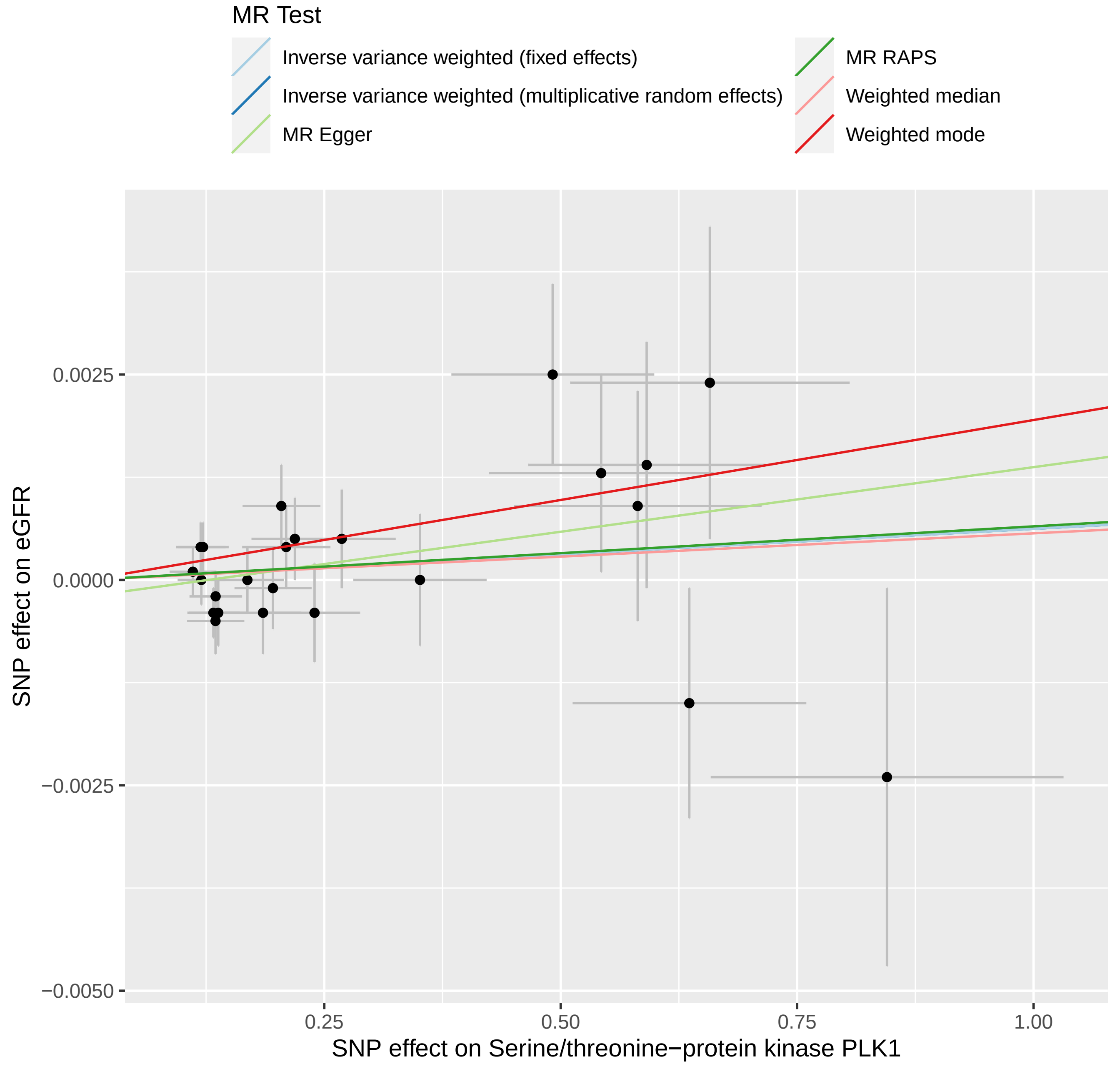

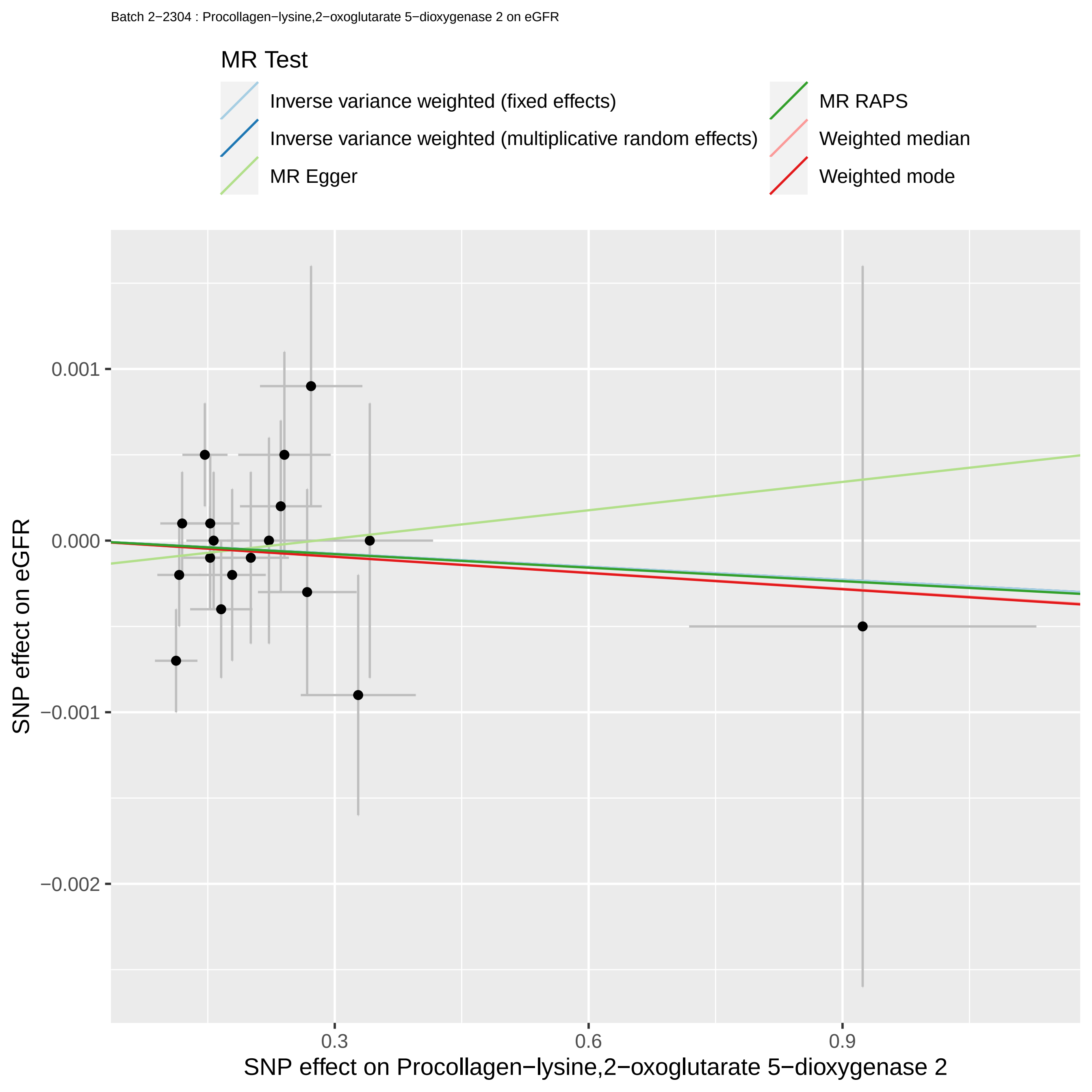

Batch 2−2304 : Procollagen−lysine,2−oxoglutarate 5−dioxygenase 2 on eGFR
MR Test
Inverse variance weighted (fixed effects)
Inverse variance weighted (multiplicative random effects)
MR Egger
MR RAPS
Weighted median
Weighted mode
0.001
0.000
−0.001
−0.002
SNP effect on eGFR
0.3
0.6
0.9
SNP effect on Procollagen−lysine,2−oxoglutarate 5−dioxygenase 2

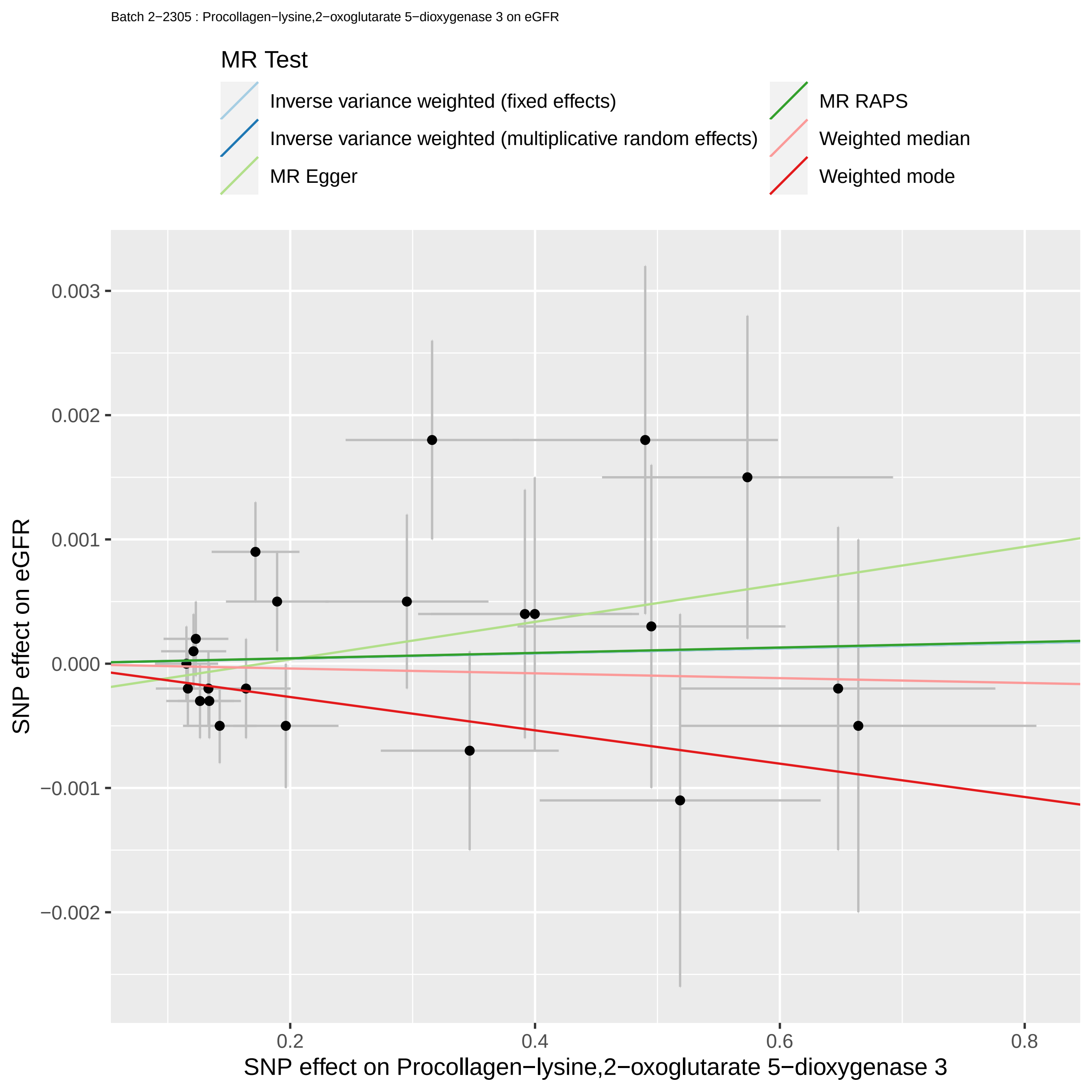
Batch 2−2305 : Procollagen−lysine,2−oxoglutarate 5−dioxygenase 3 on eGFR
MR Test
Inverse variance weighted (fixed effects)
Inverse variance weighted (multiplicative random effects)
MR Egger
MR RAPS
Weighted median
Weighted mode
0.003
0.002
0.001
0.000
−0.001
−0.002
0.2
0.4
0.6
0.8
SNP effect on eGFR
SNP effect on Procollagen−lysine,2−oxoglutarate 5−dioxygenase 3

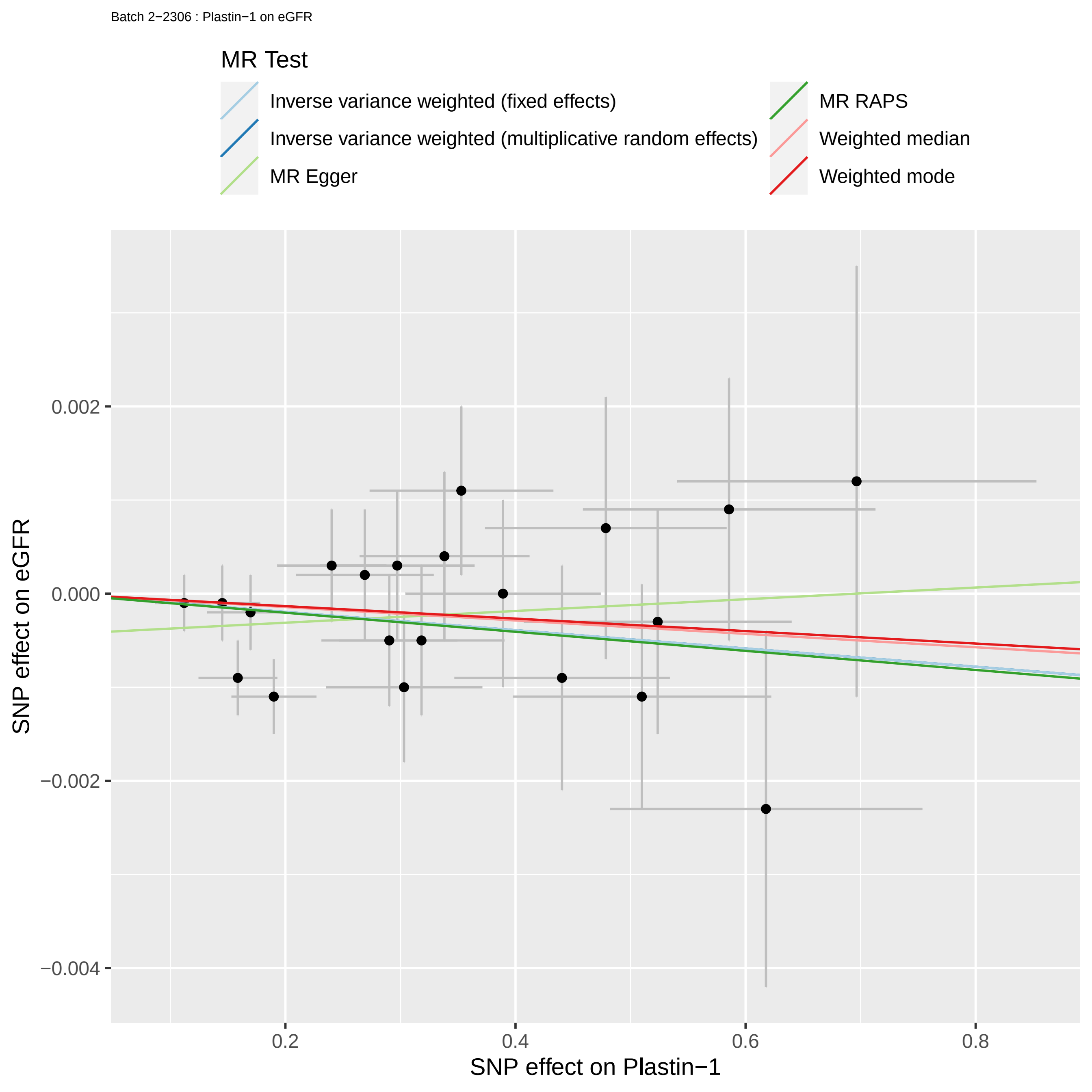

Batch 2−2306 : Plastin−1 on eGFR
MR Test
Inverse variance weighted (fixed effects)
Inverse variance weighted (multiplicative random effects)
MR Egger
MR RAPS
Weighted median
Weighted mode
0.002
0.000
−0.002
−0.004
SNP effect on eGFR
0.2
0.4
0.6
0.8
SNP effect on Plastin−1

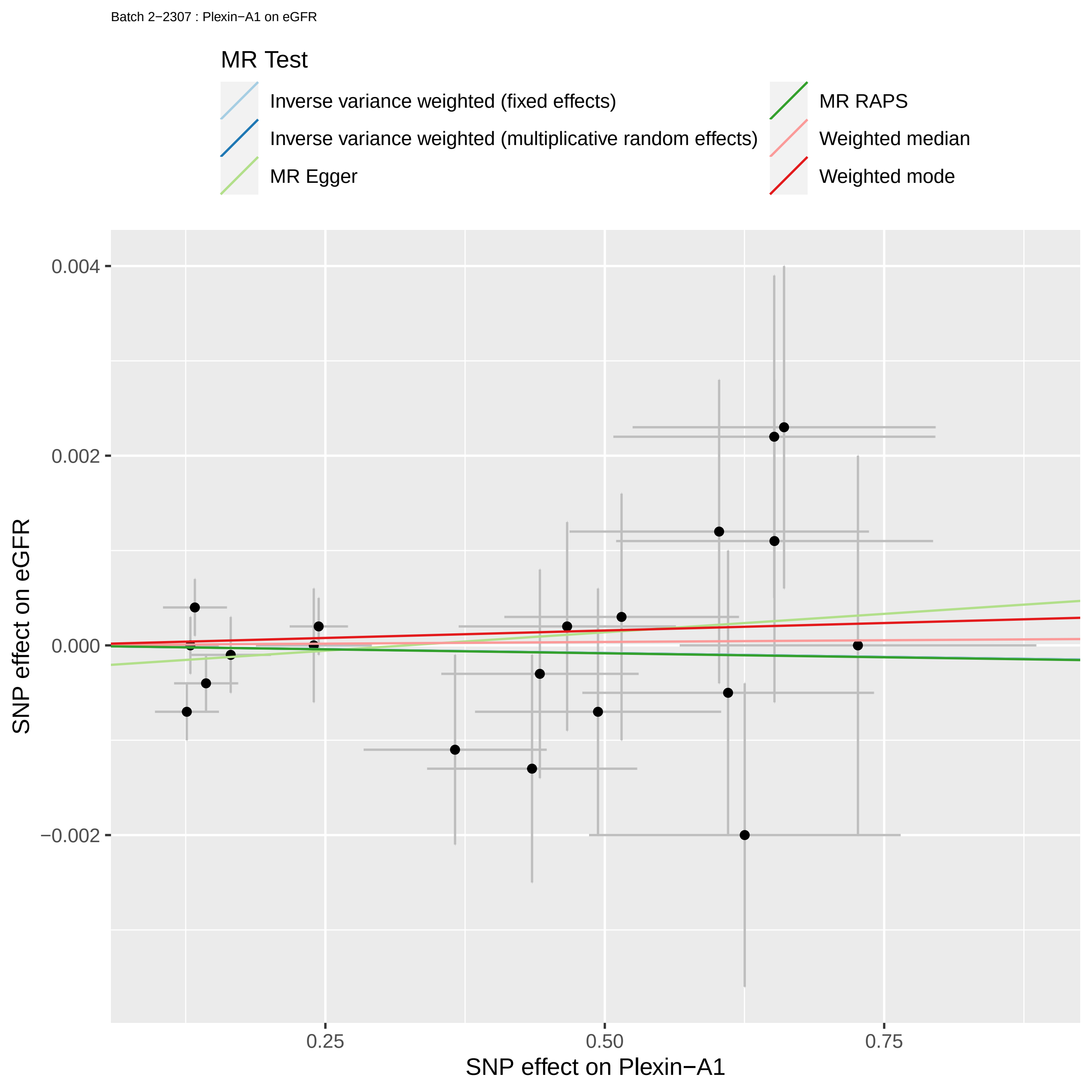

Batch 2−2307 : Plexin−A1 on eGFR
MR Test
Inverse variance weighted (fixed effects)
Inverse variance weighted (multiplicative random effects)
MR Egger
MR RAPS
Weighted median
Weighted mode
0.004
0.002
0.000
−0.002
0.25
0.50
0.75
SNP effect on eGFR
SNP effect on Plexin−A1

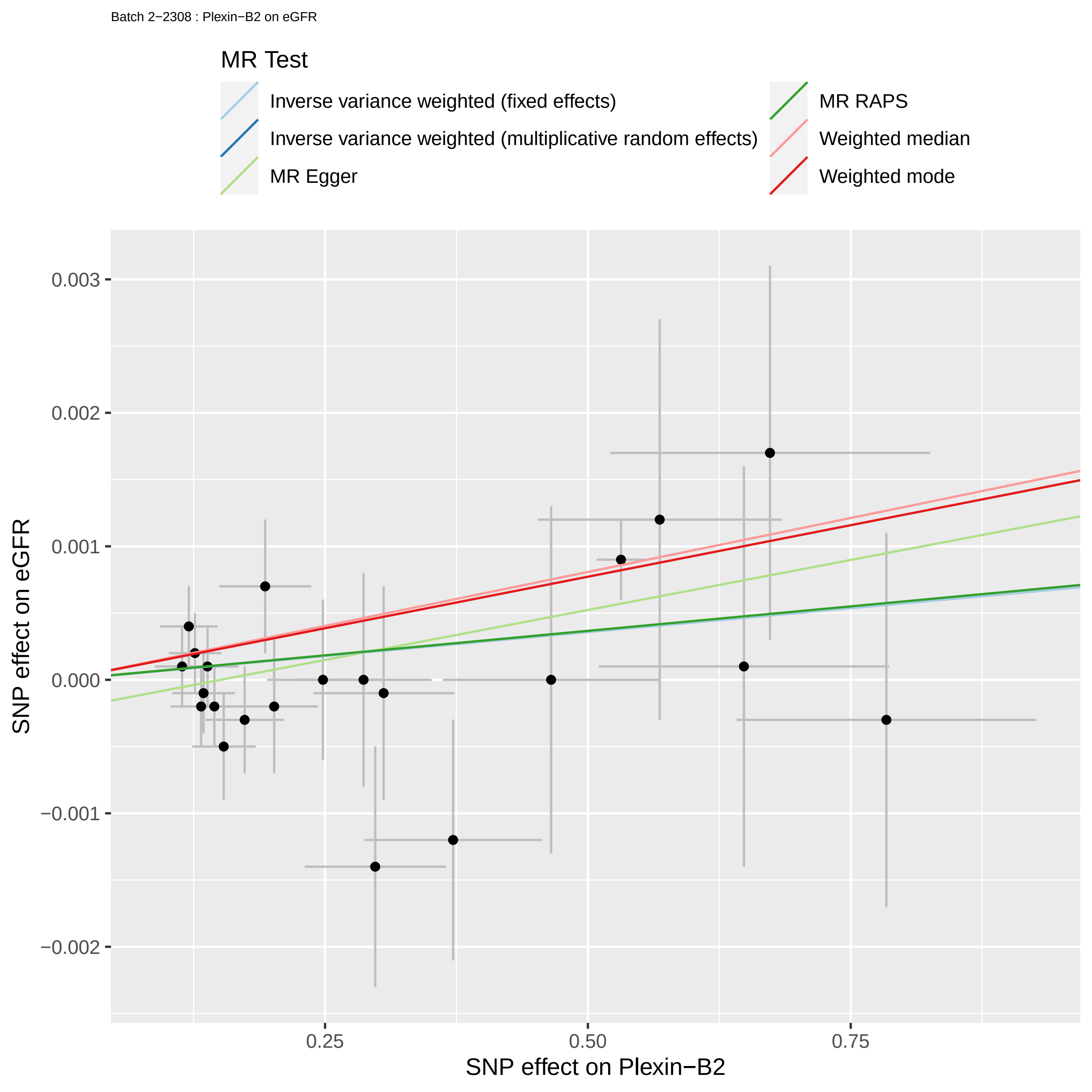
Batch 2−2308 : Plexin−B2 on eGFR
MR Test
Inverse variance weighted (fixed effects)
Inverse variance weighted (multiplicative random effects)
MR Egger
MR RAPS
Weighted median
Weighted mode
0.003
0.002
0.001
0.000
−0.001
−0.002
SNP effect on eGFR
0.25
0.50
0.75
SNP effect on Plexin−B2

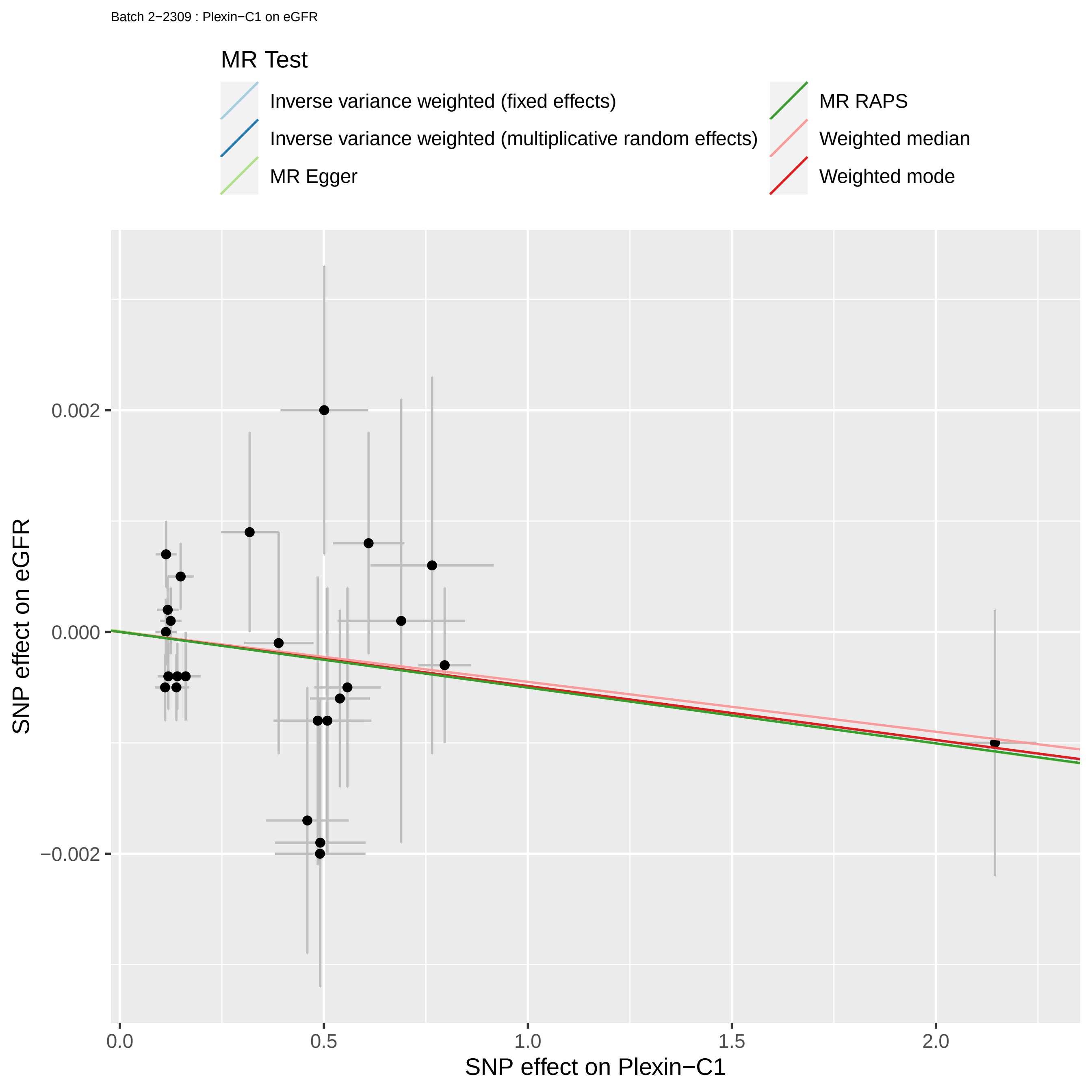

Batch 2−2309 : Plexin−C1 on eGFR
MR Test
Inverse variance weighted (fixed effects)
Inverse variance weighted (multiplicative random effects)
MR Egger
MR RAPS
Weighted median
Weighted mode
0.002
0.000
−0.002
0.0
0.5
1.0
1.5
2.0
SNP effect on eGFR
SNP effect on Plexin−C1

Batch 2−2310 : Melanocyte protein PMEL on eGFR

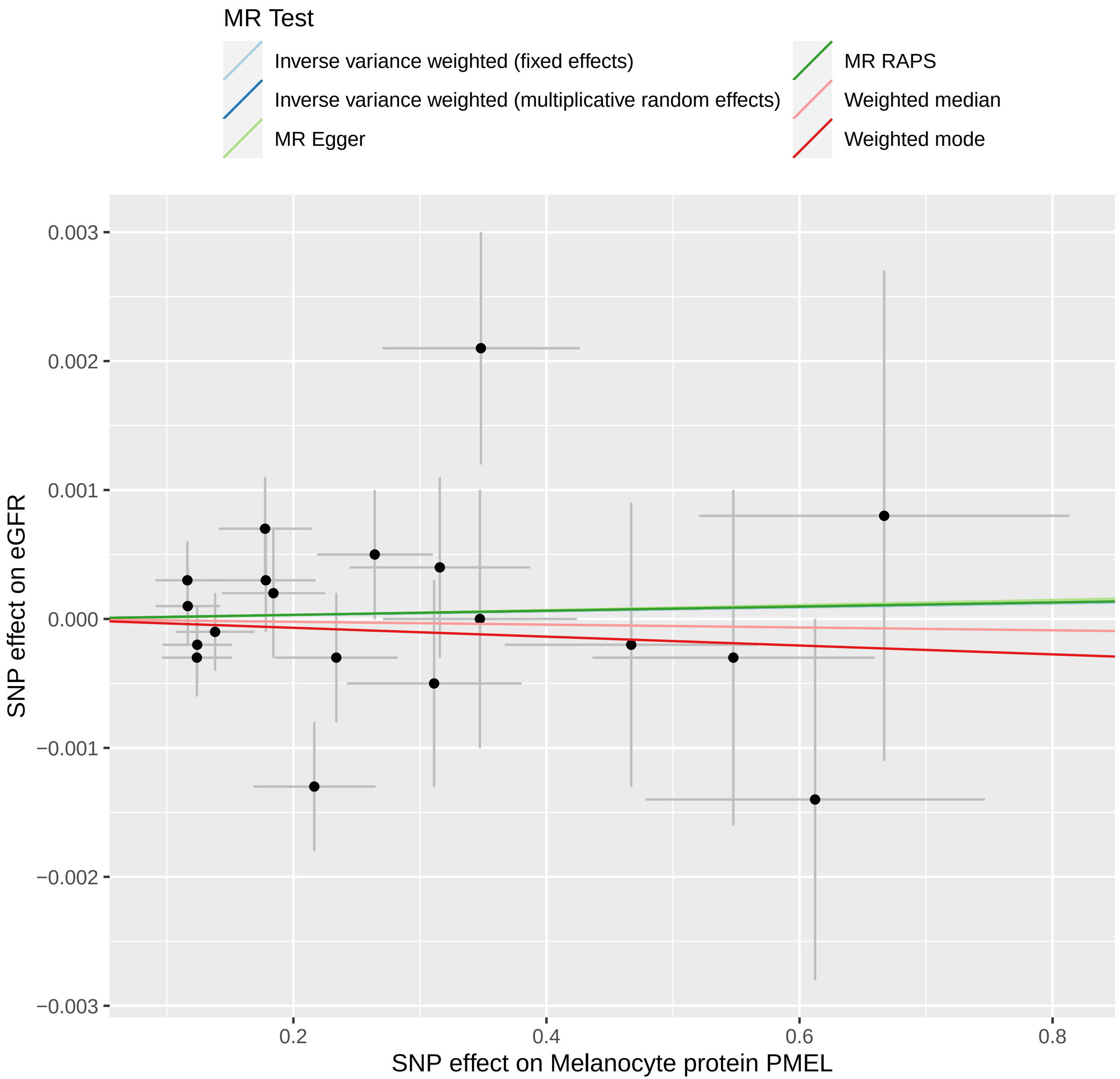

Batch 2−2311 : Protein TMEPAI on eGFR

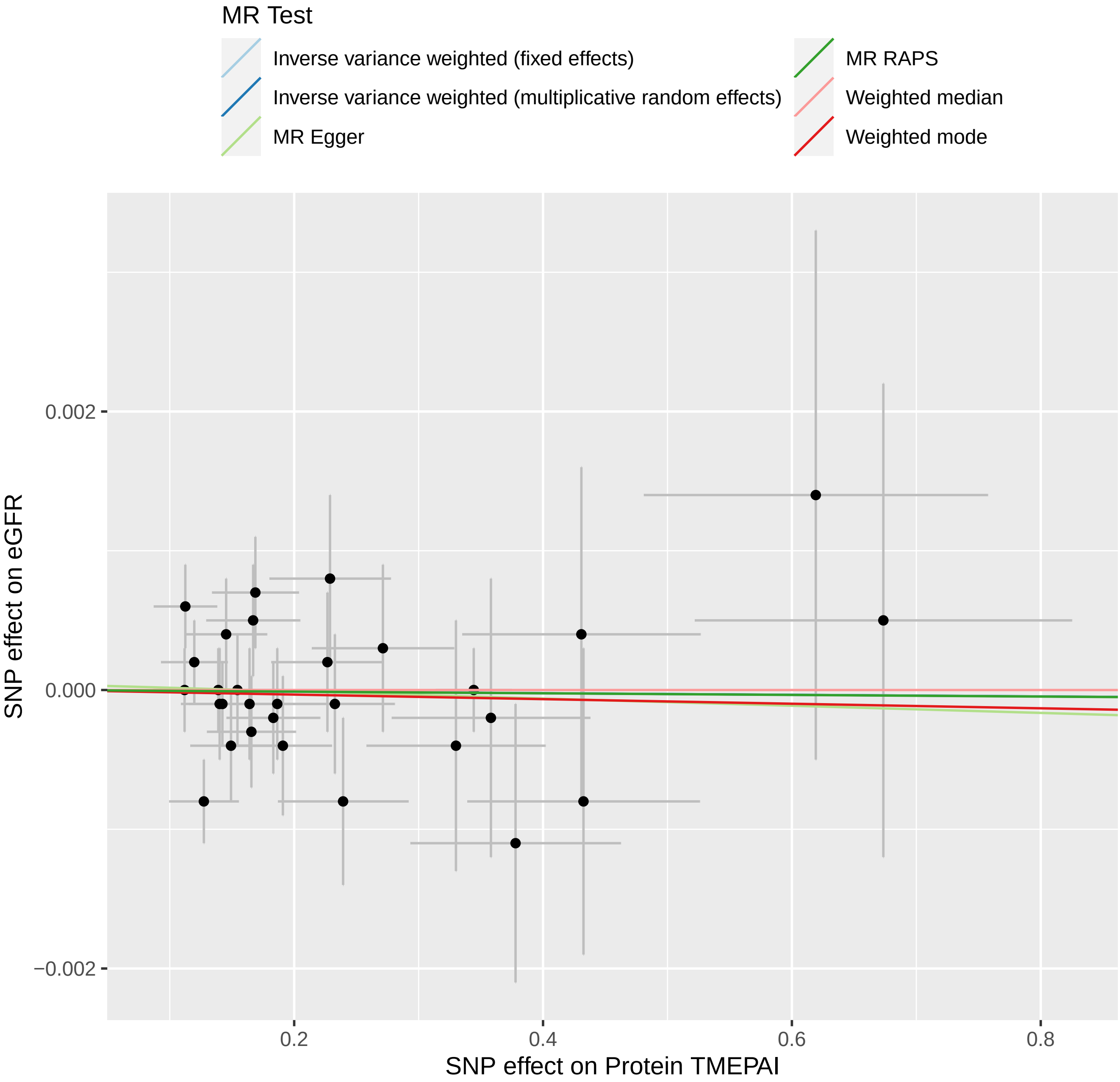

Batch 2−2312 : Mismatch repair endonuclease PMS2 on eGFR

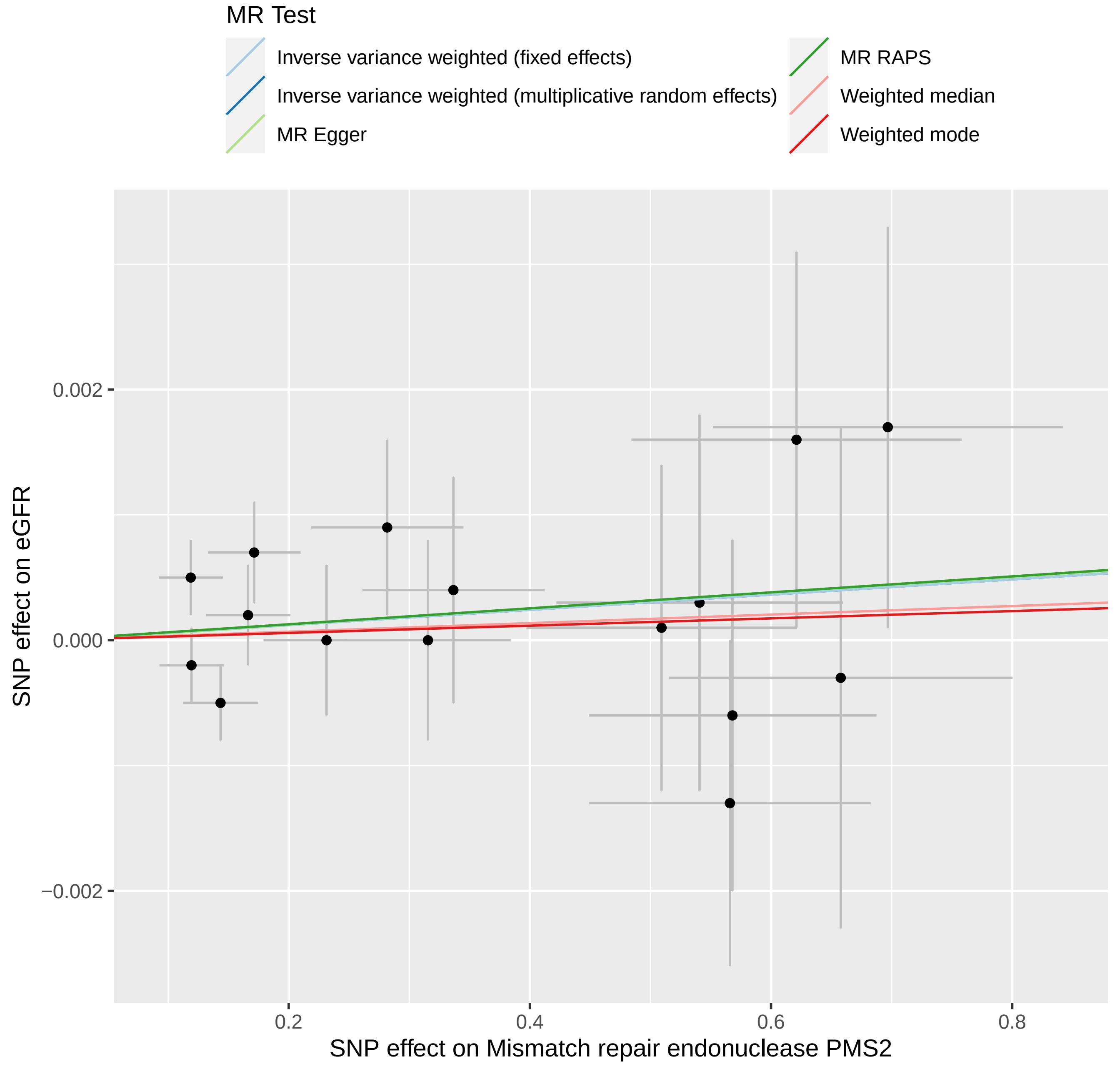

Batch 2−2313 : Phosphomevalonate kinase on eGFR

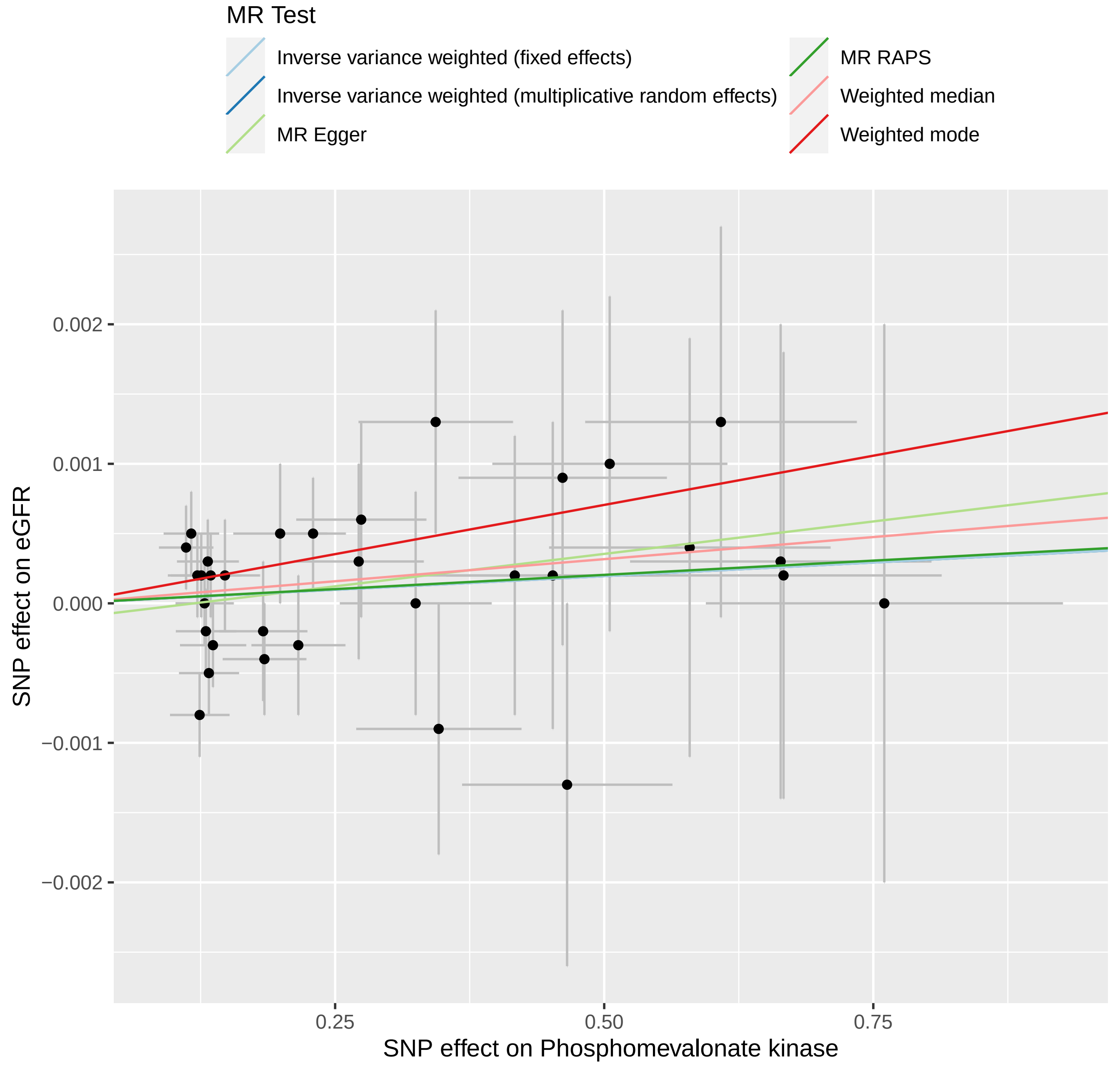

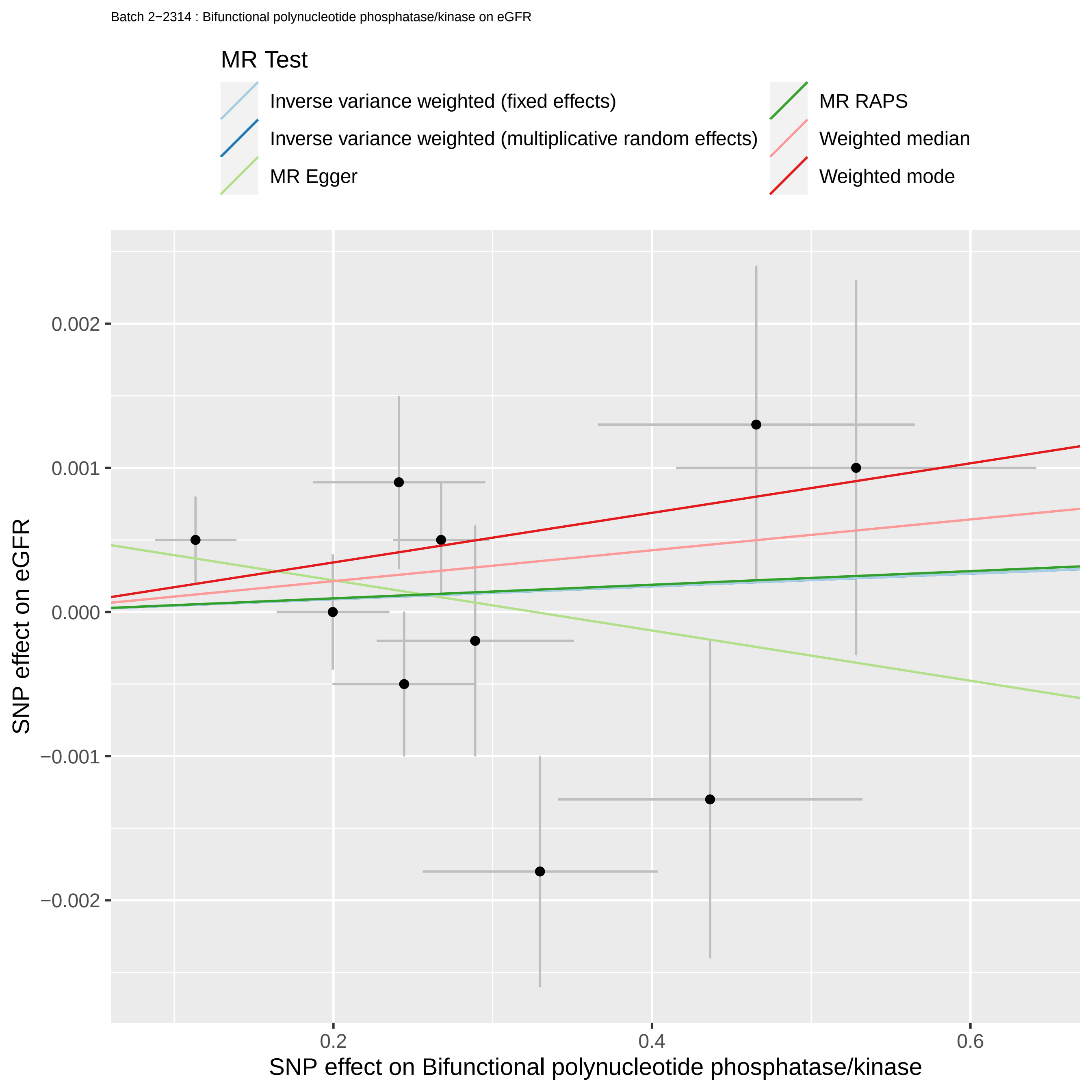
Batch 2−2314 : Bifunctional polynucleotide phosphatase/kinase on eGFR
MR Test
Inverse variance weighted (fixed effects)
Inverse variance weighted (multiplicative random effects)
MR Egger
MR RAPS
Weighted median
Weighted mode
0.002
0.001
0.000
−0.001
−0.002
0.2
0.4
0.6
SNP effect on eGFR
SNP effect on Bifunctional polynucleotide phosphatase/kinase

Batch 2−2315 : Inactive pancreatic lipase−related protein 1 on eGFR

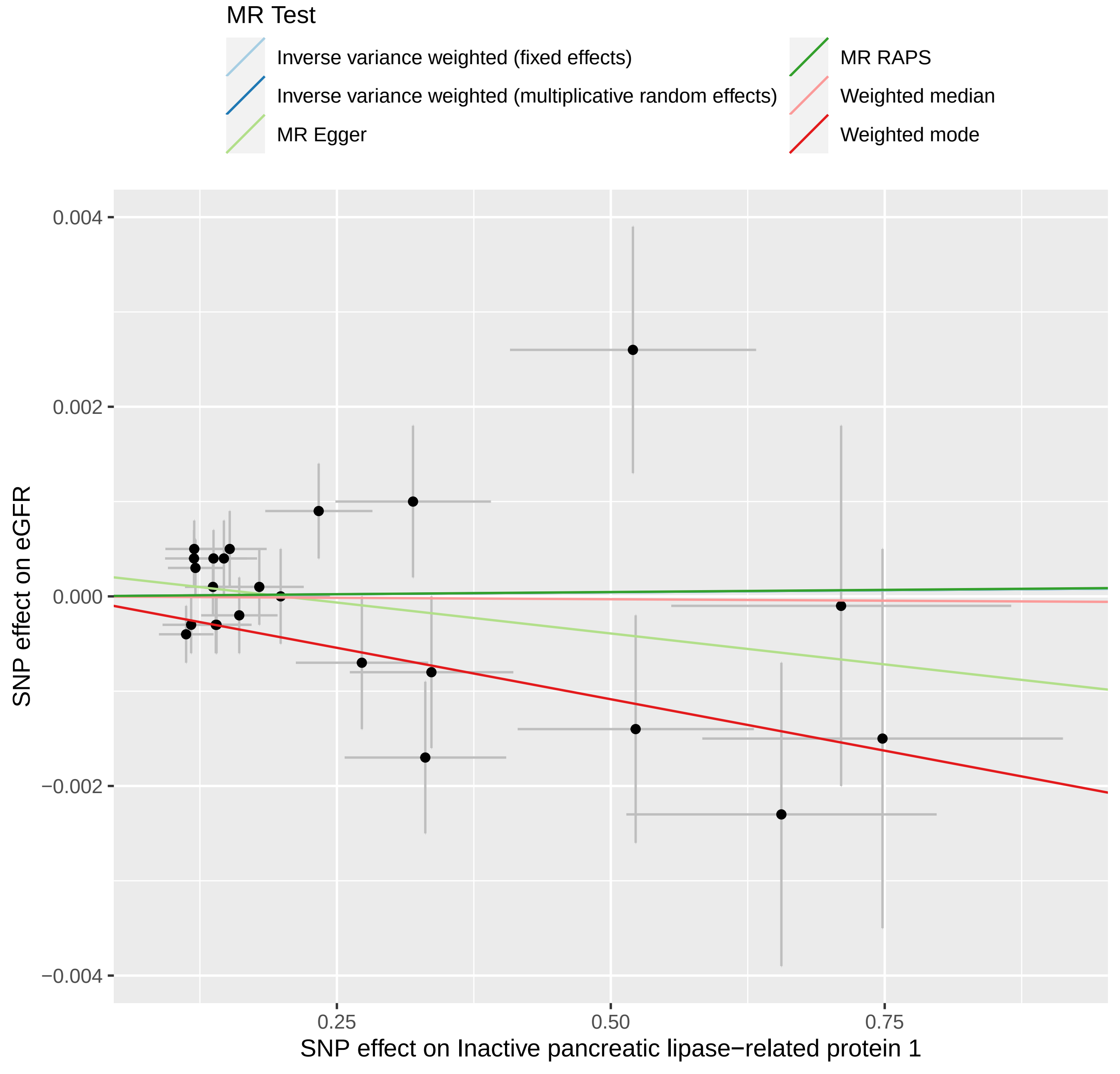

Batch 2−2316 : Purine nucleoside phosphorylase on eGFR

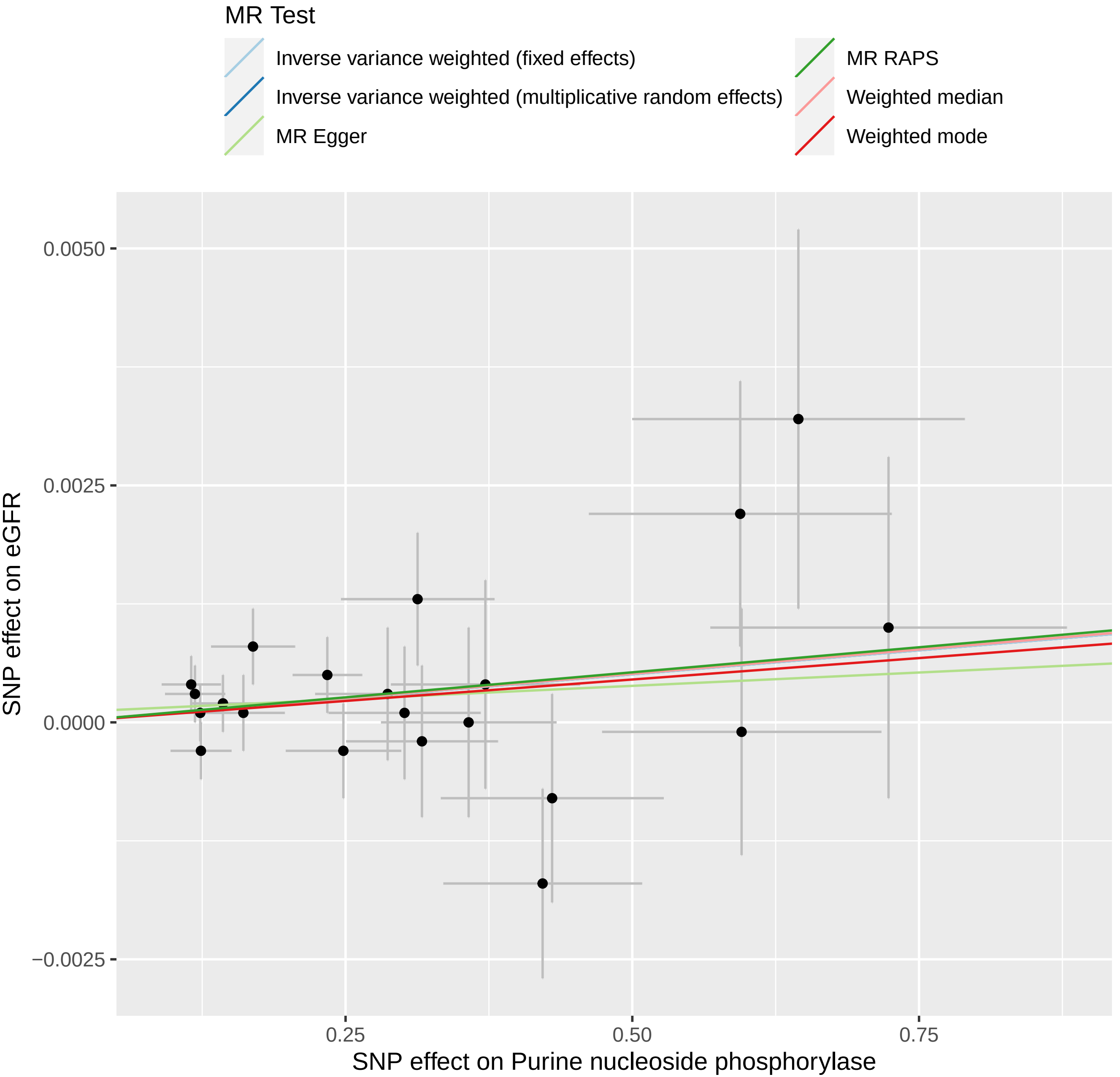

Batch 2−2317 : Podocalyxin on eGFR

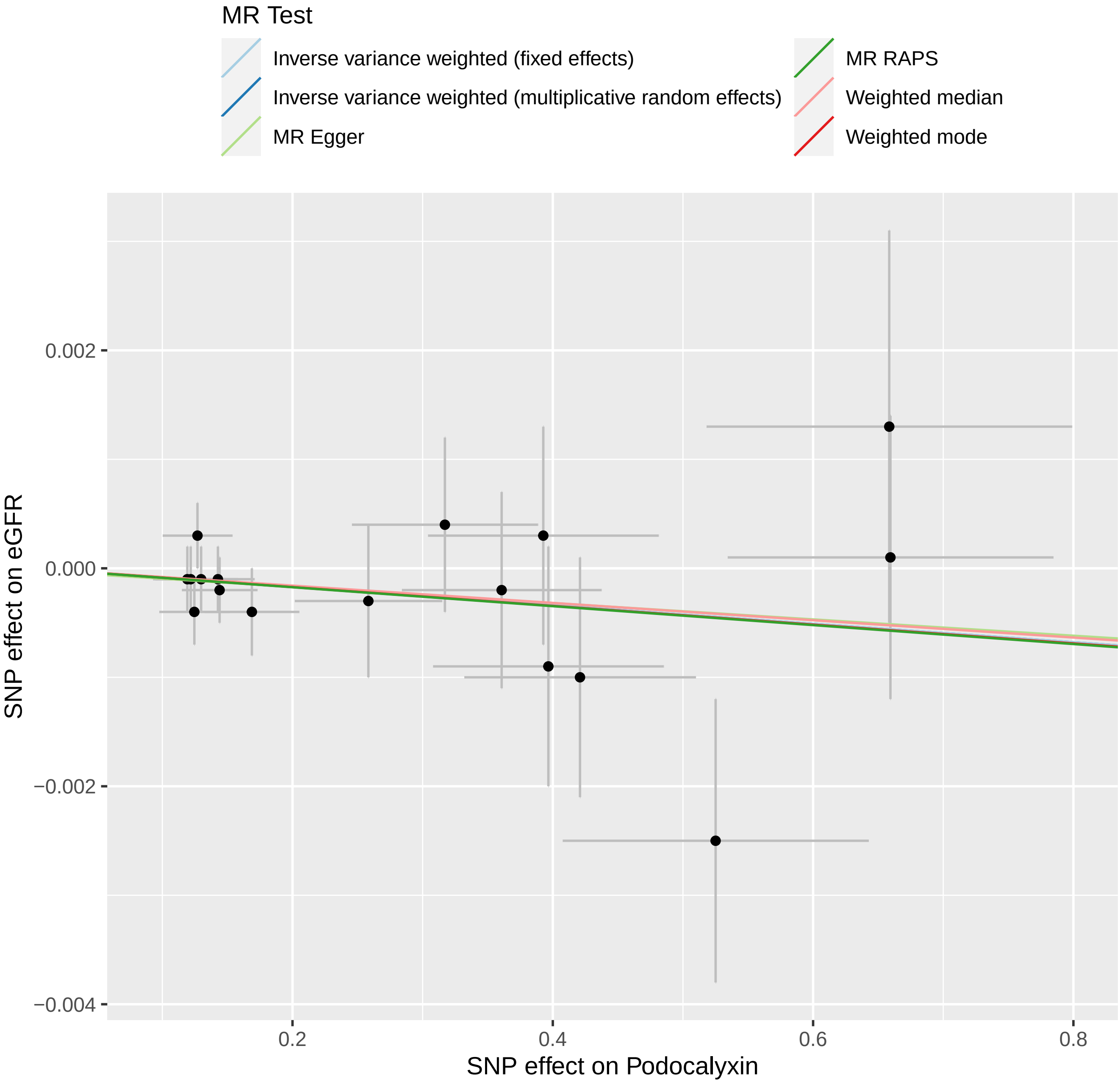

Batch 2−2318 : GDP−fucose protein O−fucosyltransferase 1 on eGFR

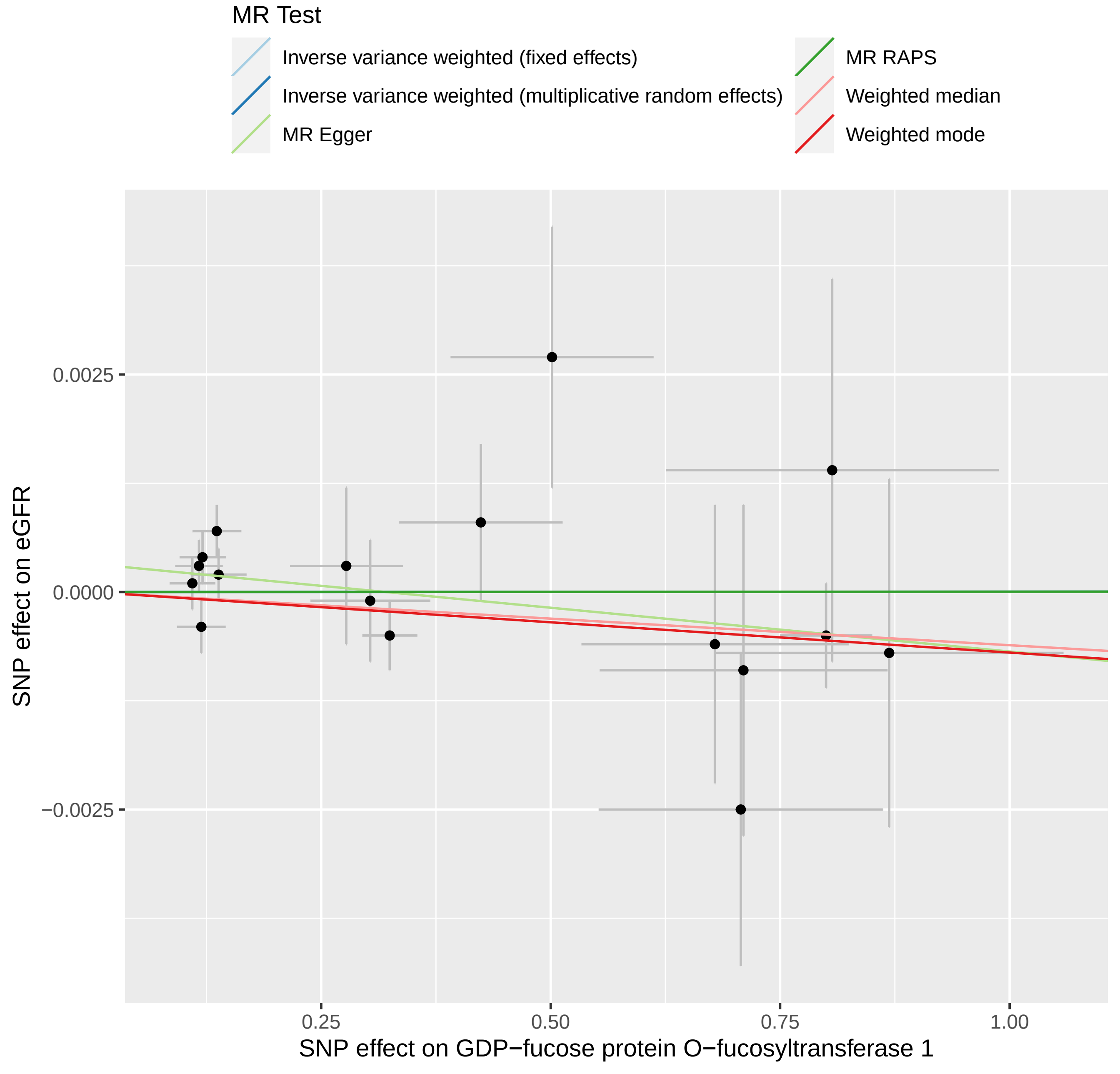

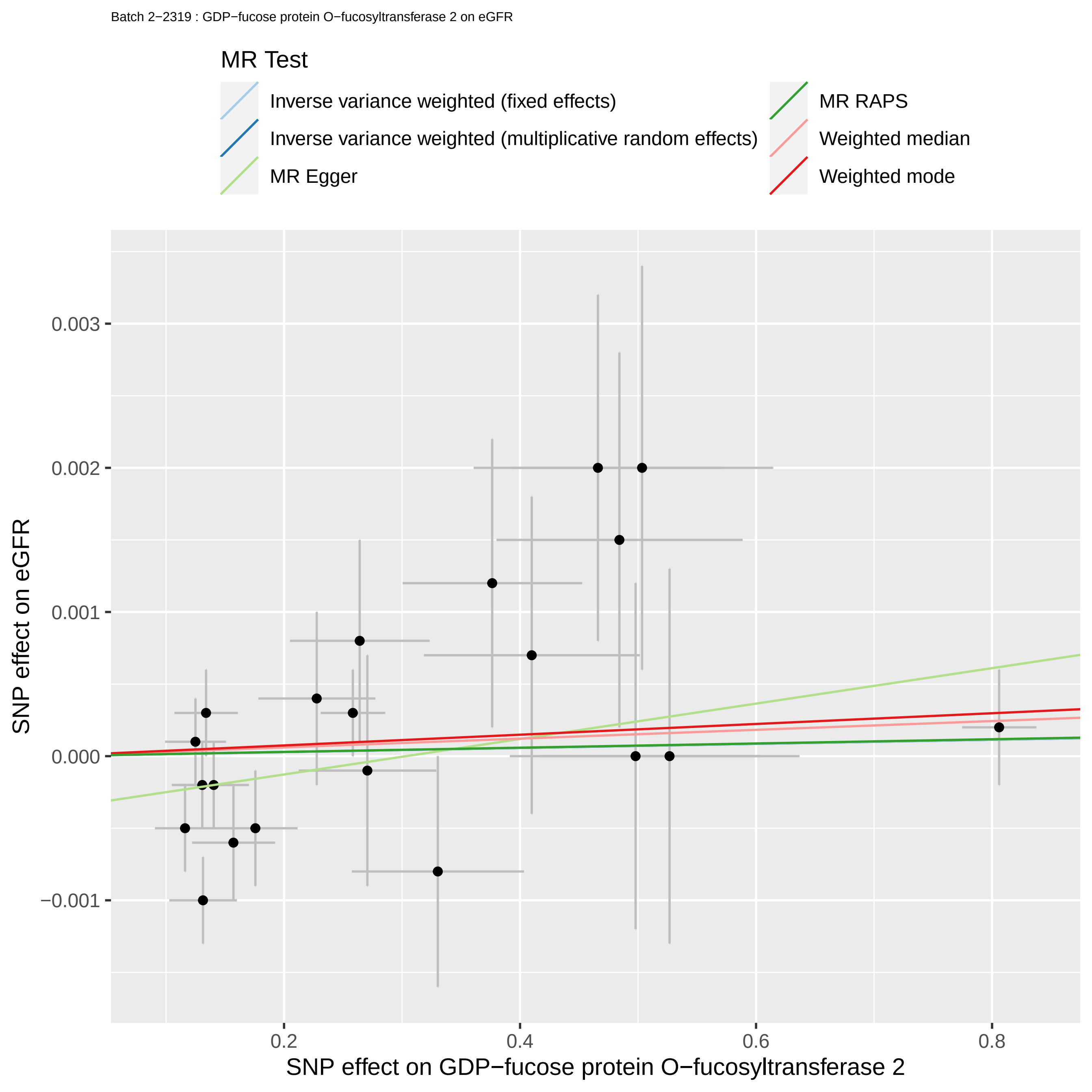

Batch 2−2319 : GDP−fucose protein O−fucosyltransferase 2 on eGFR
MR Test
Inverse variance weighted (fixed effects)
Inverse variance weighted (multiplicative random effects)
MR Egger
MR RAPS
Weighted median
Weighted mode
0.003
0.002
0.001
0.000
−0.001
0.2
0.4
0.6
0.8
SNP effect on eGFR
SNP effect on GDP−fucose protein O−fucosyltransferase 2

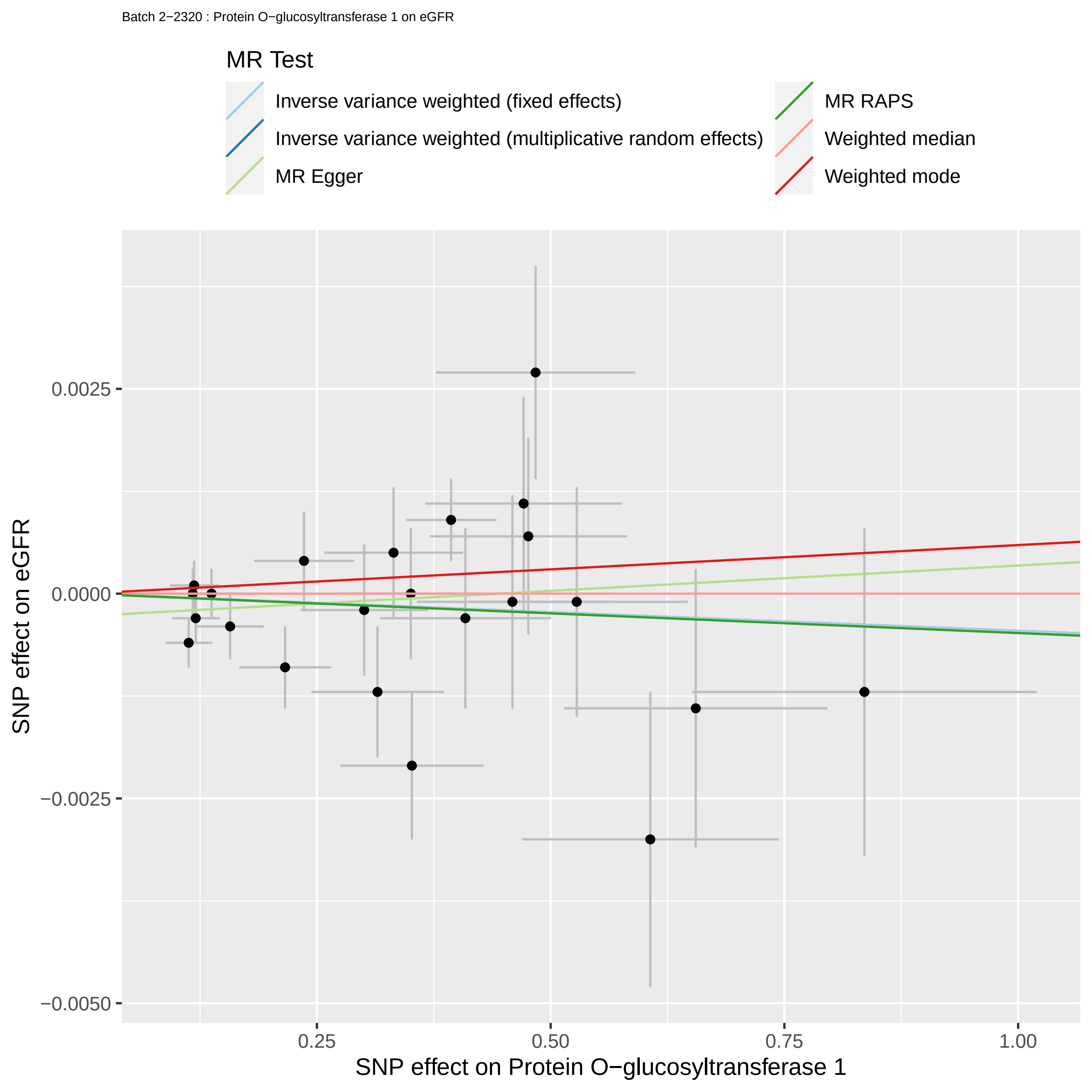

Batch 2−2320 : Protein O−glucosyltransferase 1 on eGFR
MR Test
Inverse variance weighted (fixed effects)
Inverse variance weighted (multiplicative random effects)
MR Egger
MR RAPS
Weighted median
Weighted mode
0.0025
0.0000
−0.0025
−0.0050
0.25
0.50
0.75
1.00
SNP effect on eGFR
SNP effect on Protein O−glucosyltransferase 1

Batch 2−2321 : DNA polymerase eta on eGFR

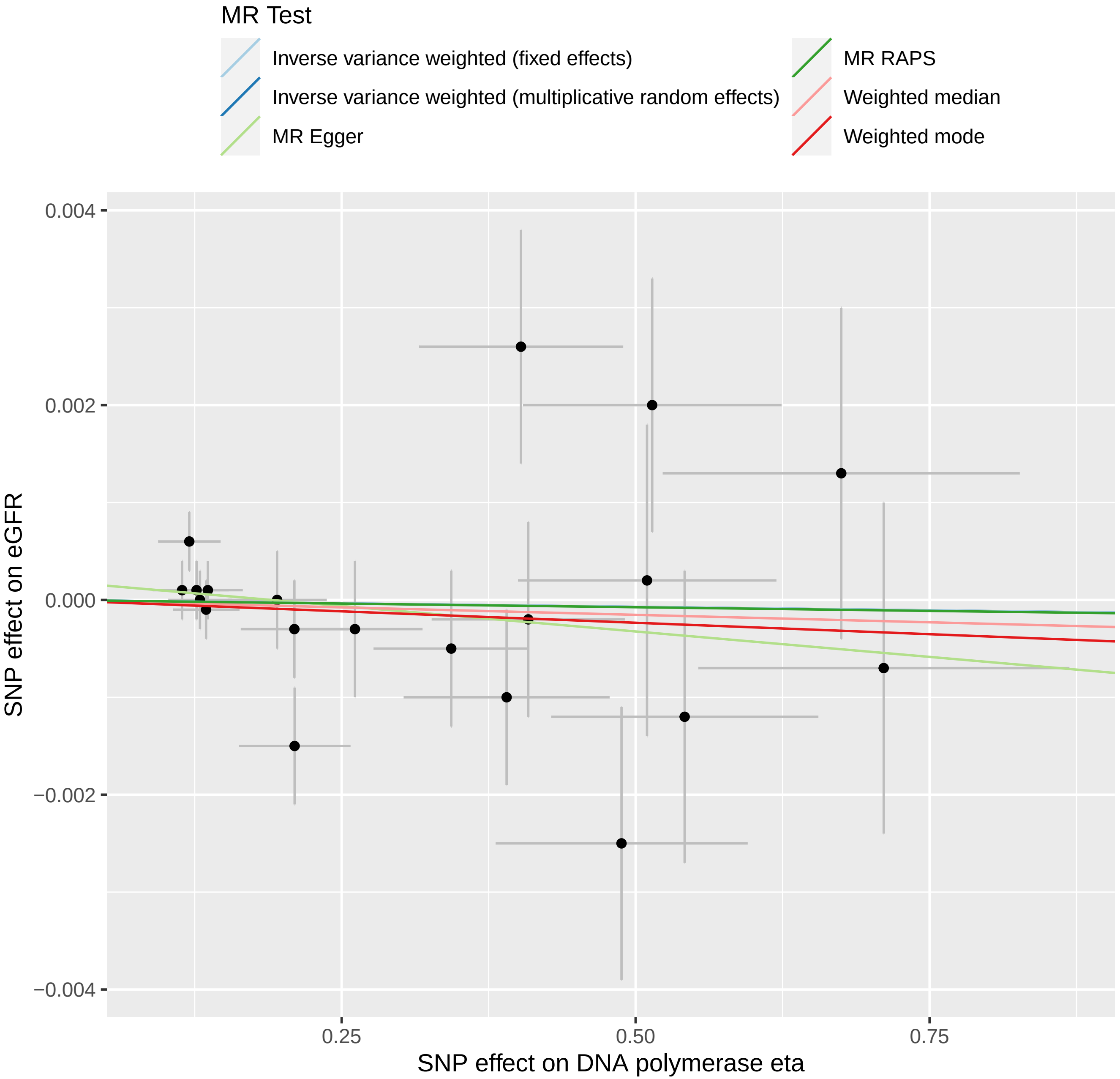

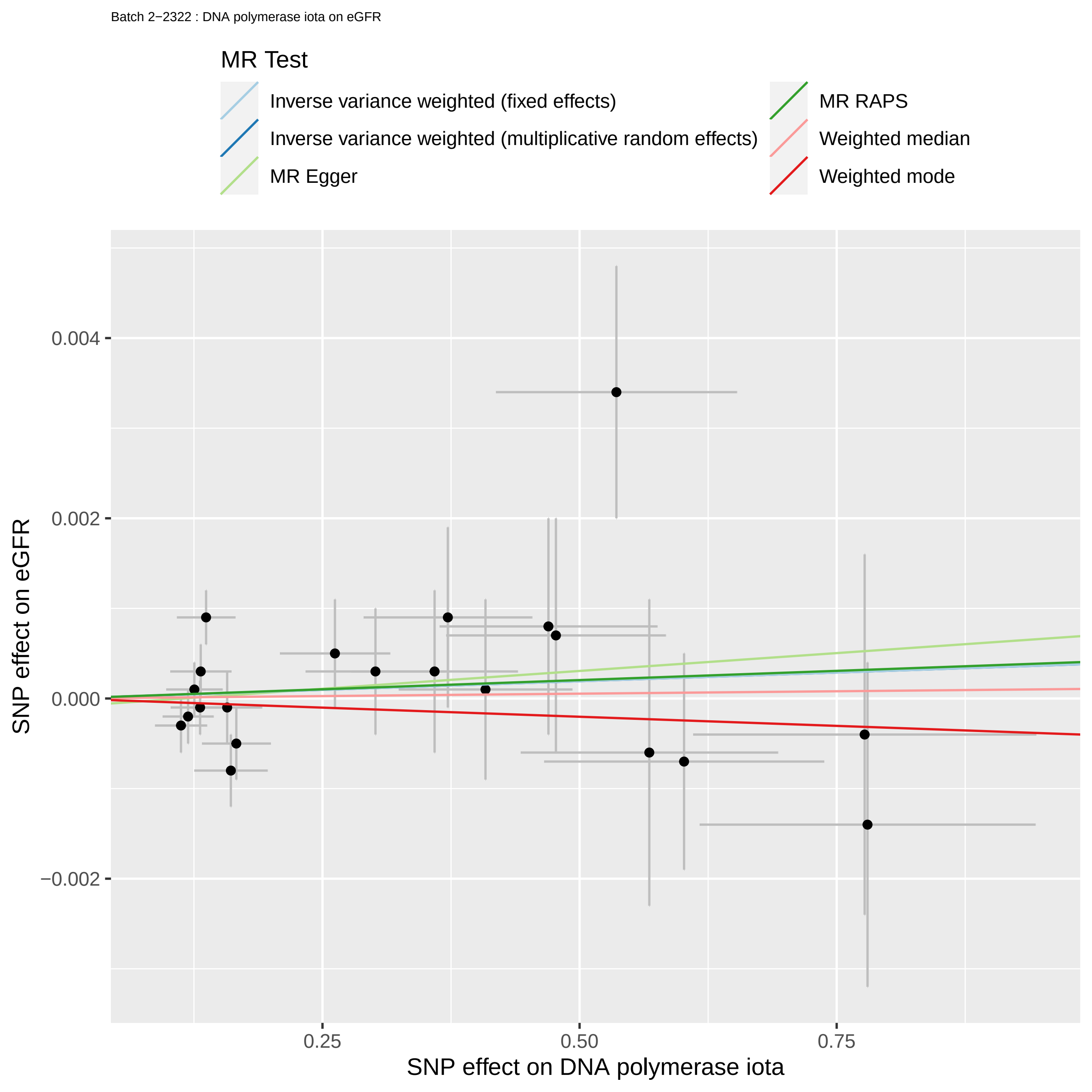

Batch 2−2322 : DNA polymerase iota on eGFR
MR Test
Inverse variance weighted (fixed effects)
Inverse variance weighted (multiplicative random effects)
MR Egger
MR RAPS
Weighted median
Weighted mode
0.004
0.002
0.000
−0.002
0.25
0.50
0.75
SNP effect on eGFR
SNP effect on DNA polymerase iota

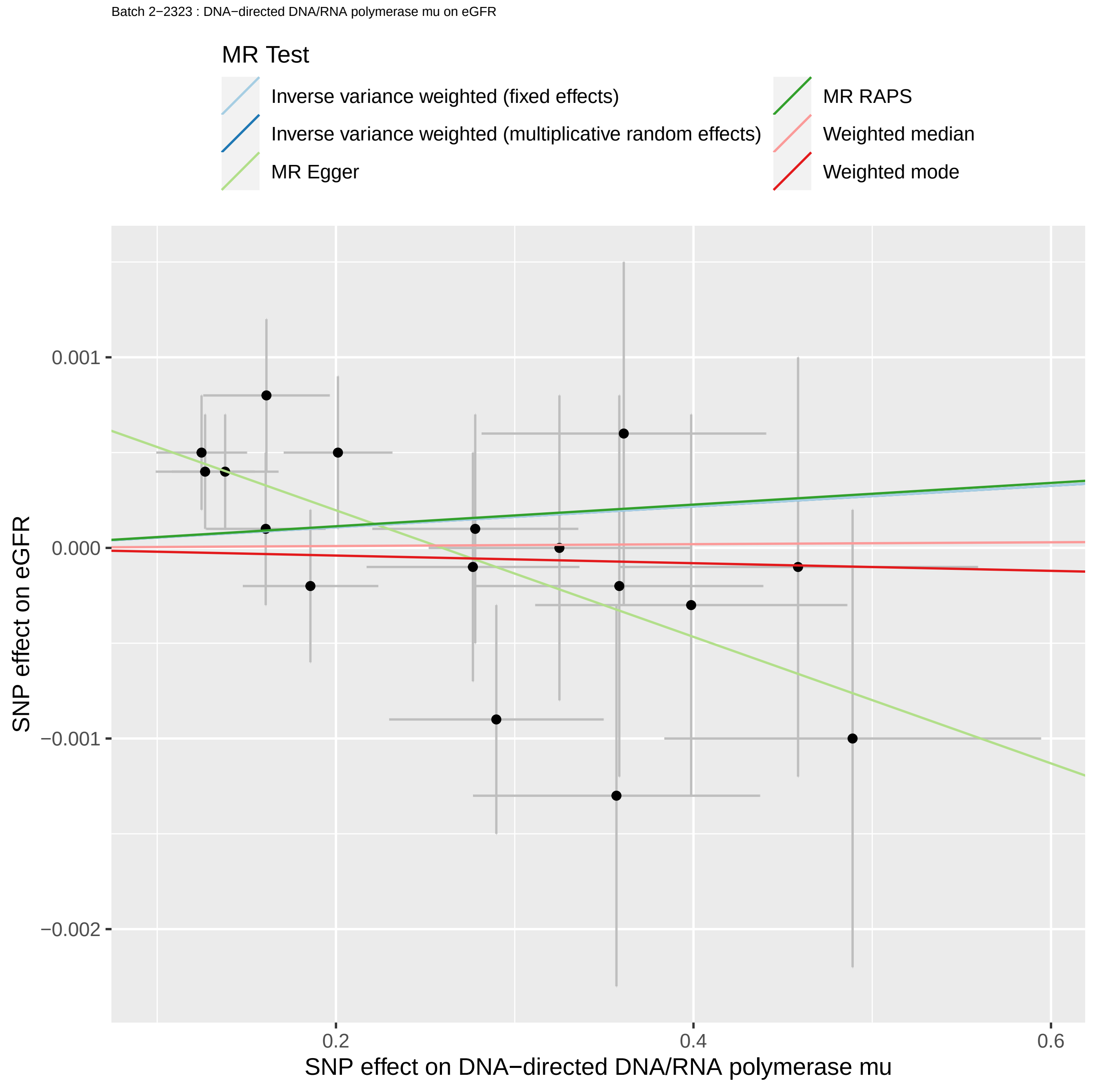
Batch 2−2323 : DNA−directed DNA/RNA polymerase mu on eGFR
MR Test
Inverse variance weighted (fixed effects)
Inverse variance weighted (multiplicative random effects)
MR Egger
MR RAPS
Weighted median
Weighted mode
0.001
0.000
−0.001
−0.002
SNP effect on eGFR
0.2
0.4
0.6
SNP effect on DNA−directed DNA/RNA polymerase mu

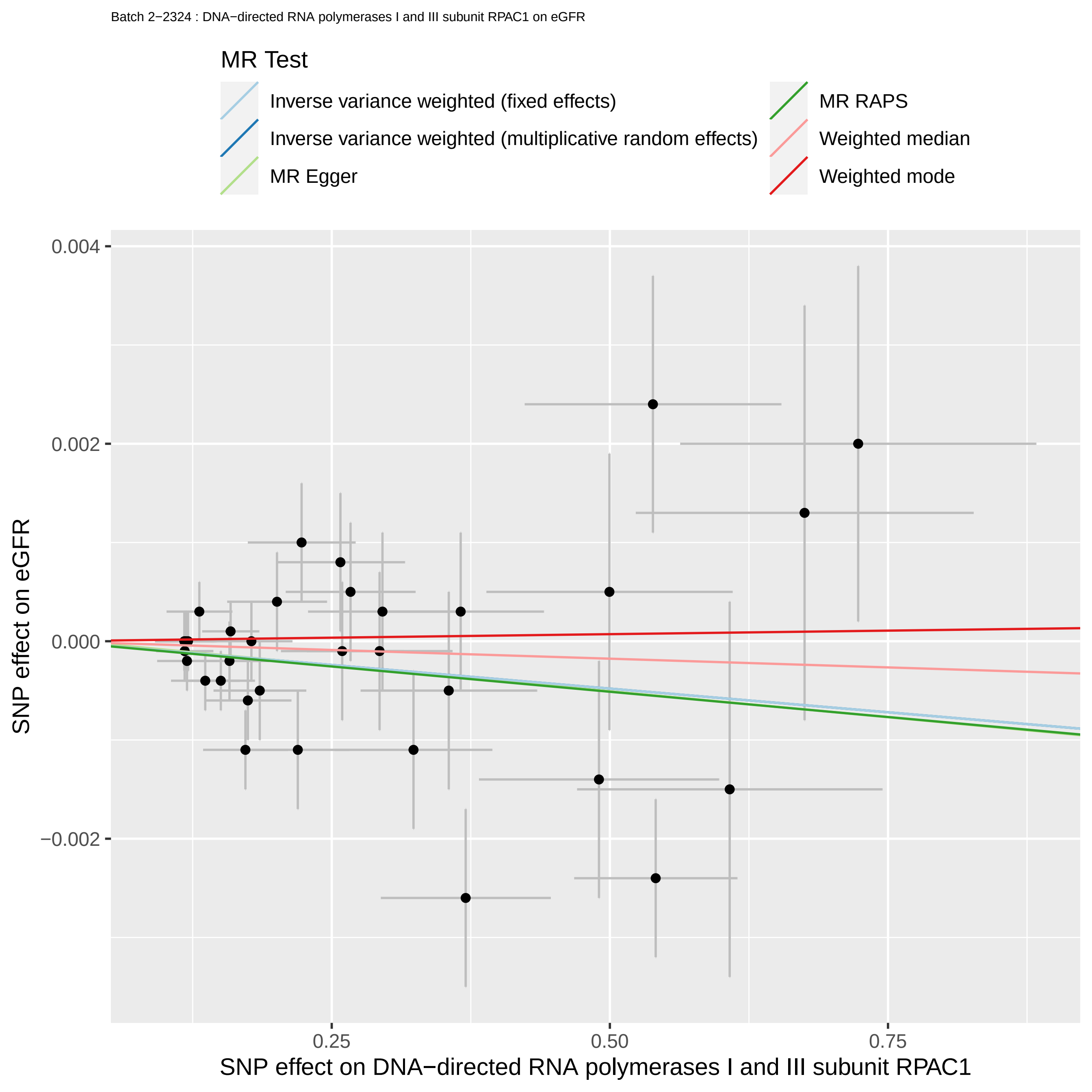

Batch 2−2324 : DNA−directed RNA polymerases I and III subunit RPAC1 on eGFR
MR Test
Inverse variance weighted (fixed effects)
Inverse variance weighted (multiplicative random effects)
MR Egger
MR RAPS
Weighted median
Weighted mode
0.004
0.002
0.000
−0.002
SNP effect on eGFR
0.25
0.50
0.75
SNP effect on DNA−directed RNA polymerases I and III subunit RPAC1

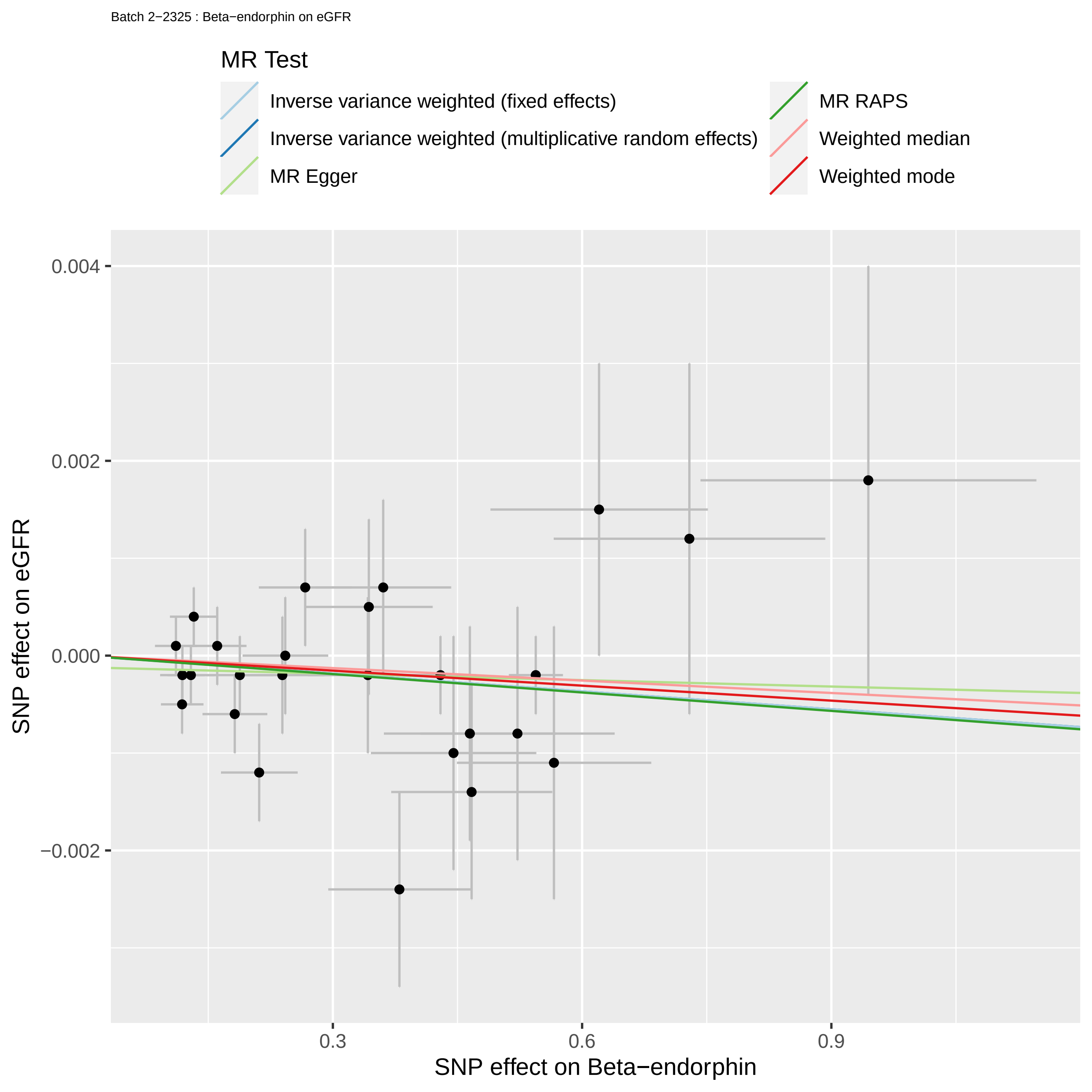

Batch 2−2325 : Beta−endorphin on eGFR
MR Test
Inverse variance weighted (fixed effects)
Inverse variance weighted (multiplicative random effects)
MR Egger
MR RAPS
Weighted median
Weighted mode
0.004
0.002
0.000
−0.002
0.3
0.6
0.9
SNP effect on eGFR
SNP effect on Beta−endorphin

Batch 2−2326 : Corticotropin on eGFR

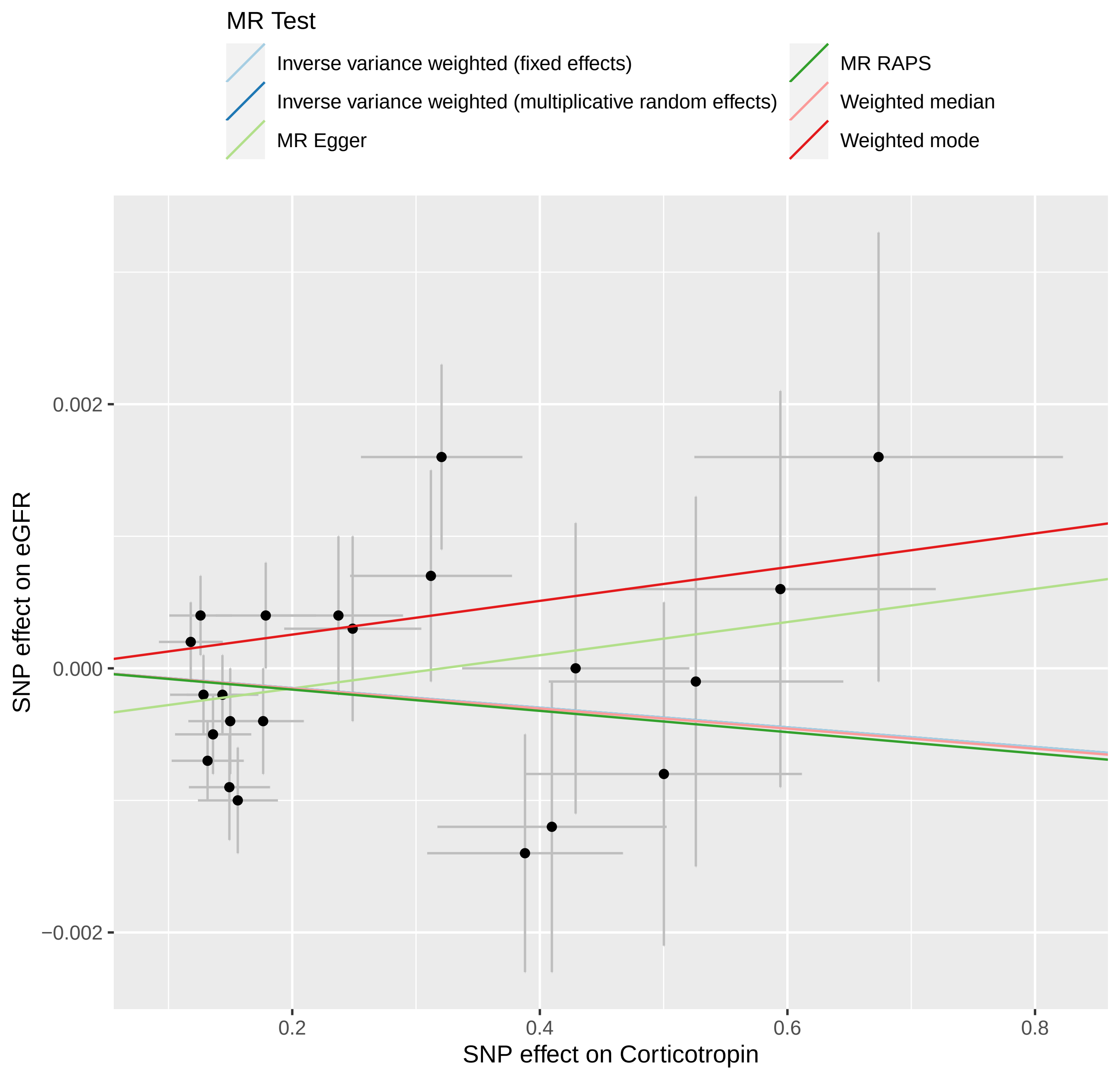

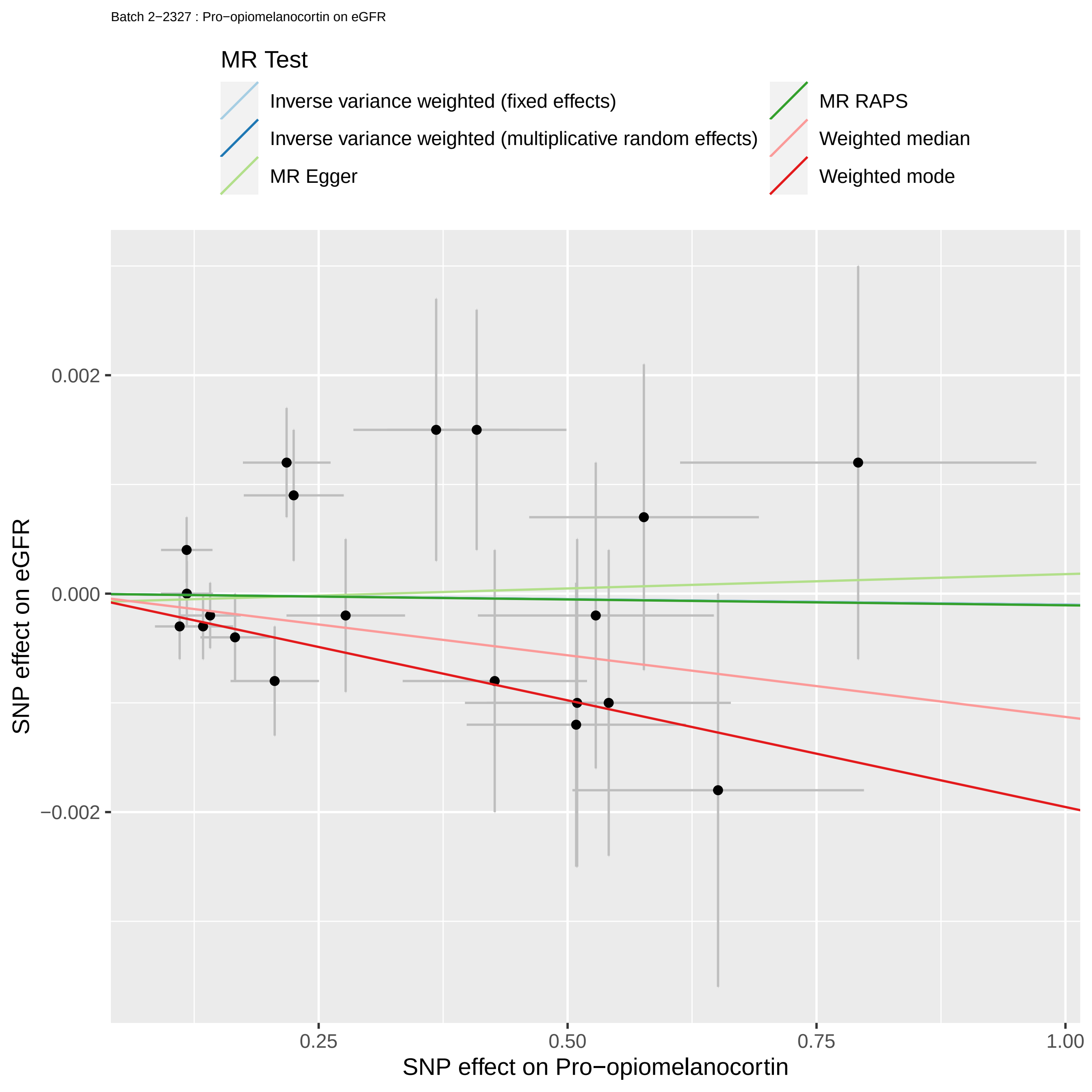

Batch 2−2327 : Pro−opiomelanocortin on eGFR
MR Test
Inverse variance weighted (fixed effects)
Inverse variance weighted (multiplicative random effects)
MR Egger
MR RAPS
Weighted median
Weighted mode
0.002
0.000
−0.002
0.25
0.50
0.75
1.00
SNP effect on eGFR
SNP effect on Pro−opiomelanocortin

Batch 2−2328 : Protein O−linked−mannose beta−1,2−N−acetylglucosaminyltransferase 1 on eGFR

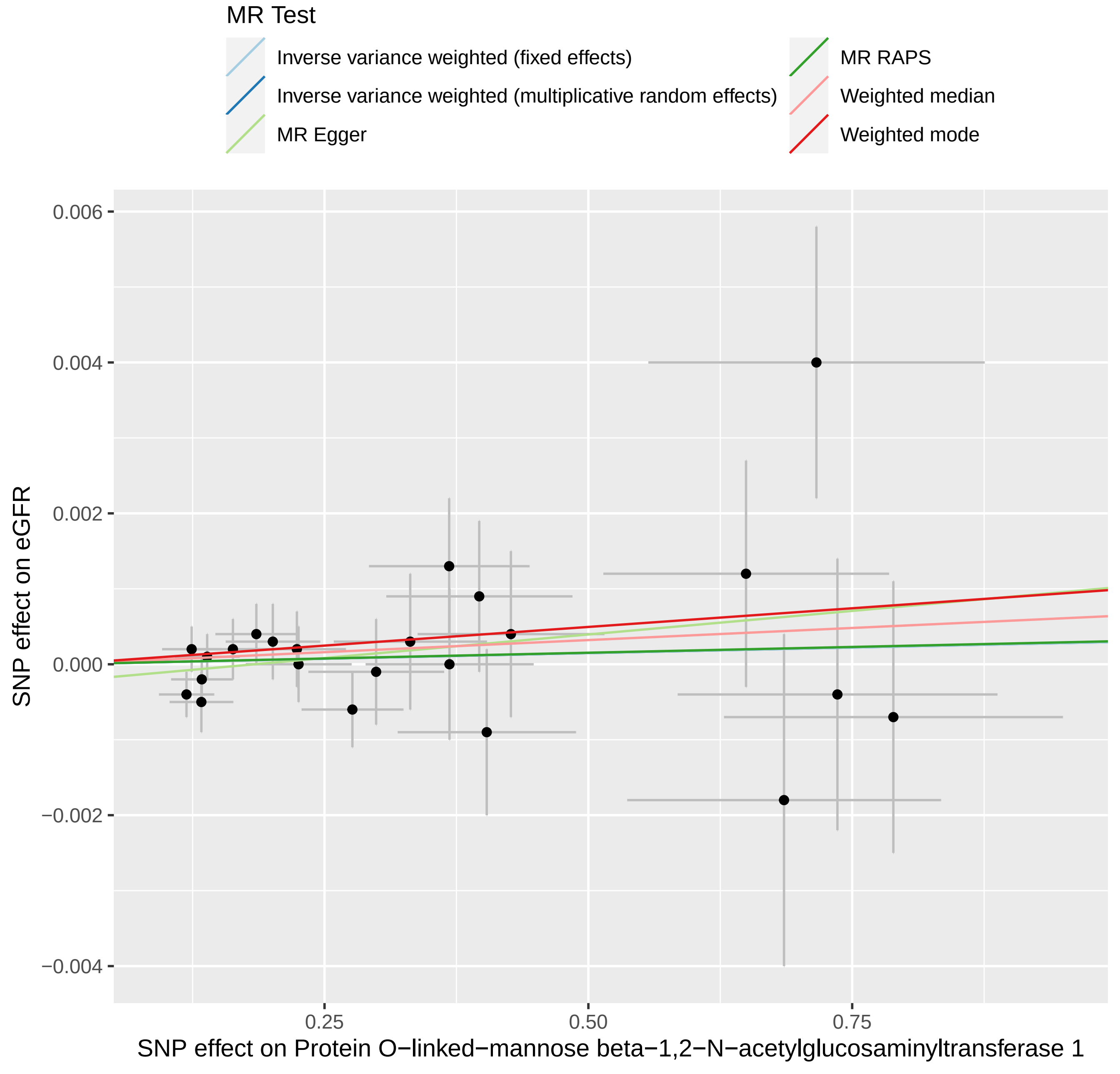

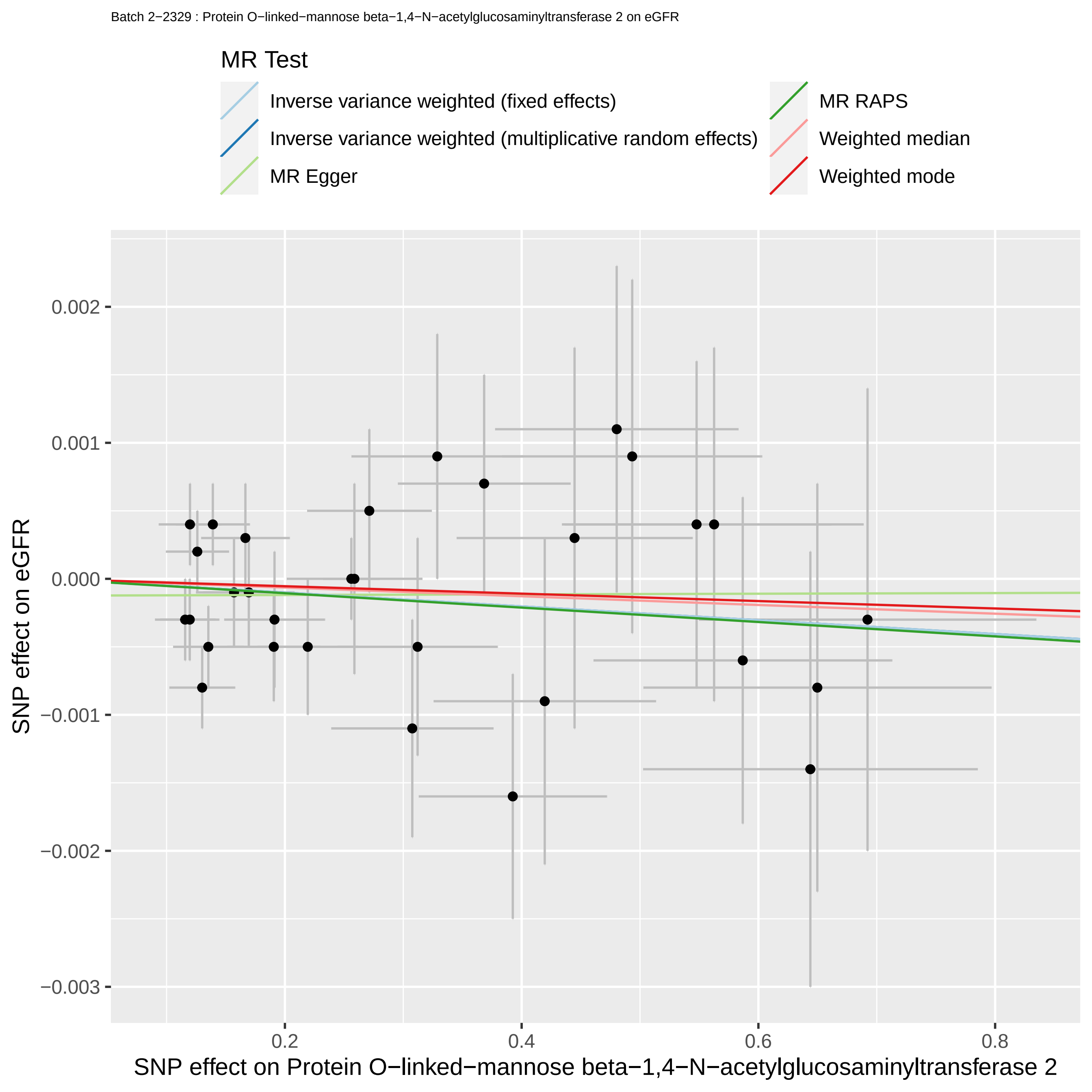
Batch 2−2329 : Protein O−linked−mannose beta−1,4−N−acetylglucosaminyltransferase 2 on eGFR
MR Test
Inverse variance weighted (fixed effects)
Inverse variance weighted (multiplicative random effects)
MR Egger
MR RAPS
Weighted median
Weighted mode
0.002
0.001
0.000
−0.001
−0.002
−0.003
0.2
0.4
0.6
0.8
SNP effect on eGFR
SNP effect on Protein O−linked−mannose beta−1,4−N−acetylglucosaminyltransferase 2

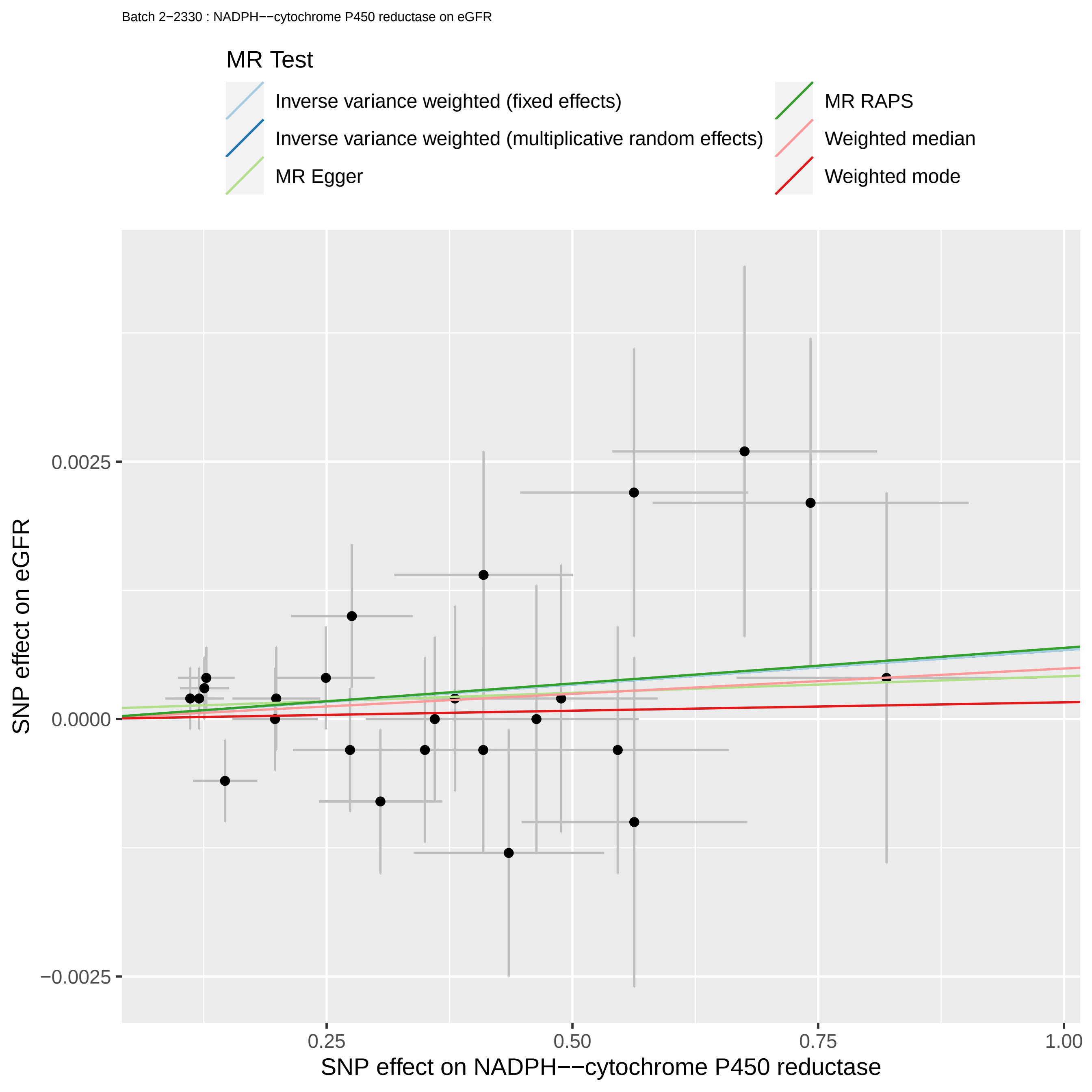

Batch 2−2330 : NADPH−−cytochrome P450 reductase on eGFR
MR Test
Inverse variance weighted (fixed effects)
Inverse variance weighted (multiplicative random effects)
MR Egger
MR RAPS
Weighted median
Weighted mode
0.0025
0.0000
−0.0025
0.25
0.50
0.75
1.00
SNP effect on eGFR
SNP effect on NADPH−−cytochrome P450 reductase

Batch 2−2331 : Periostin on eGFR

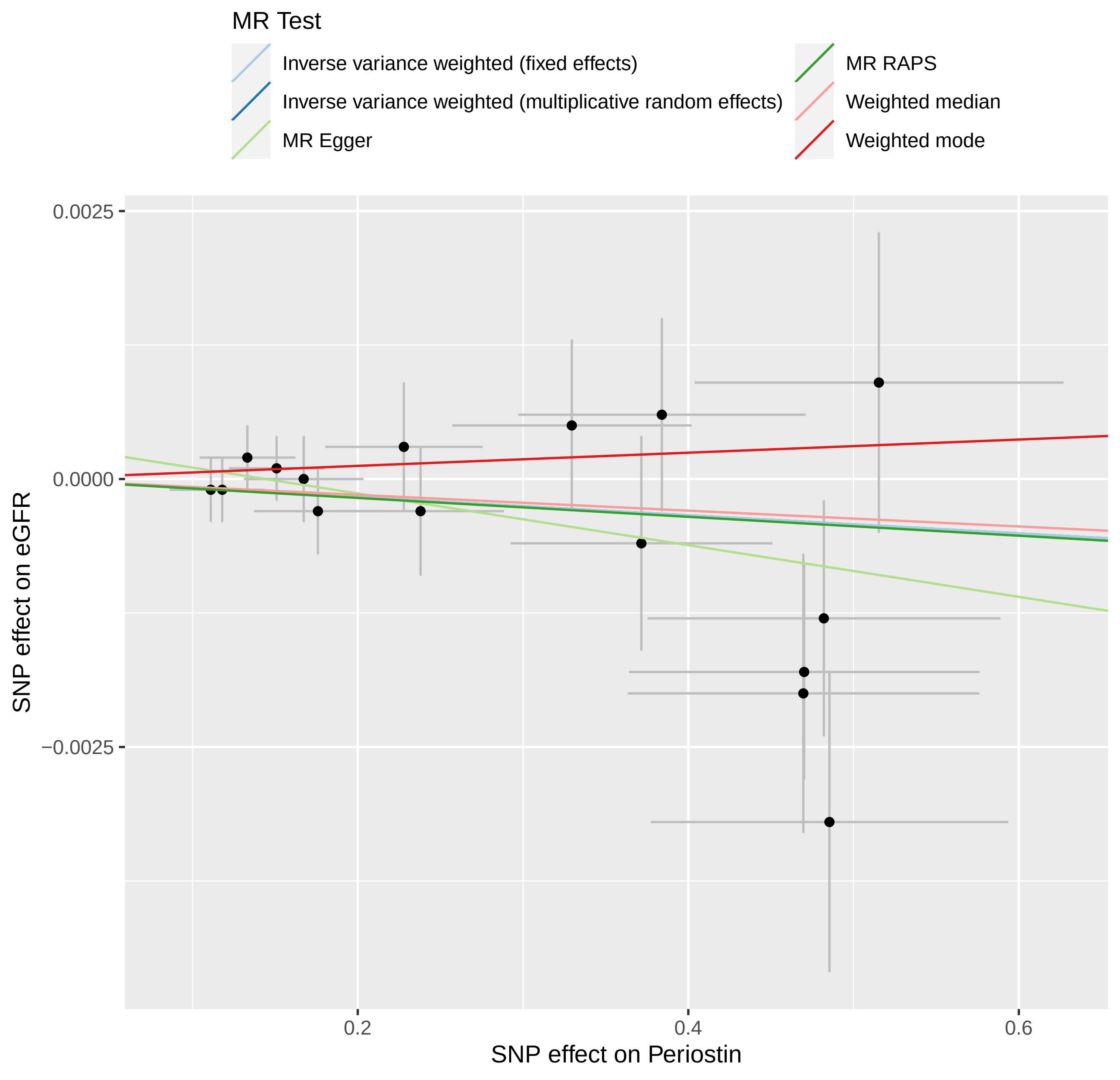

Batch 2−2332 : Periostin on eGFR

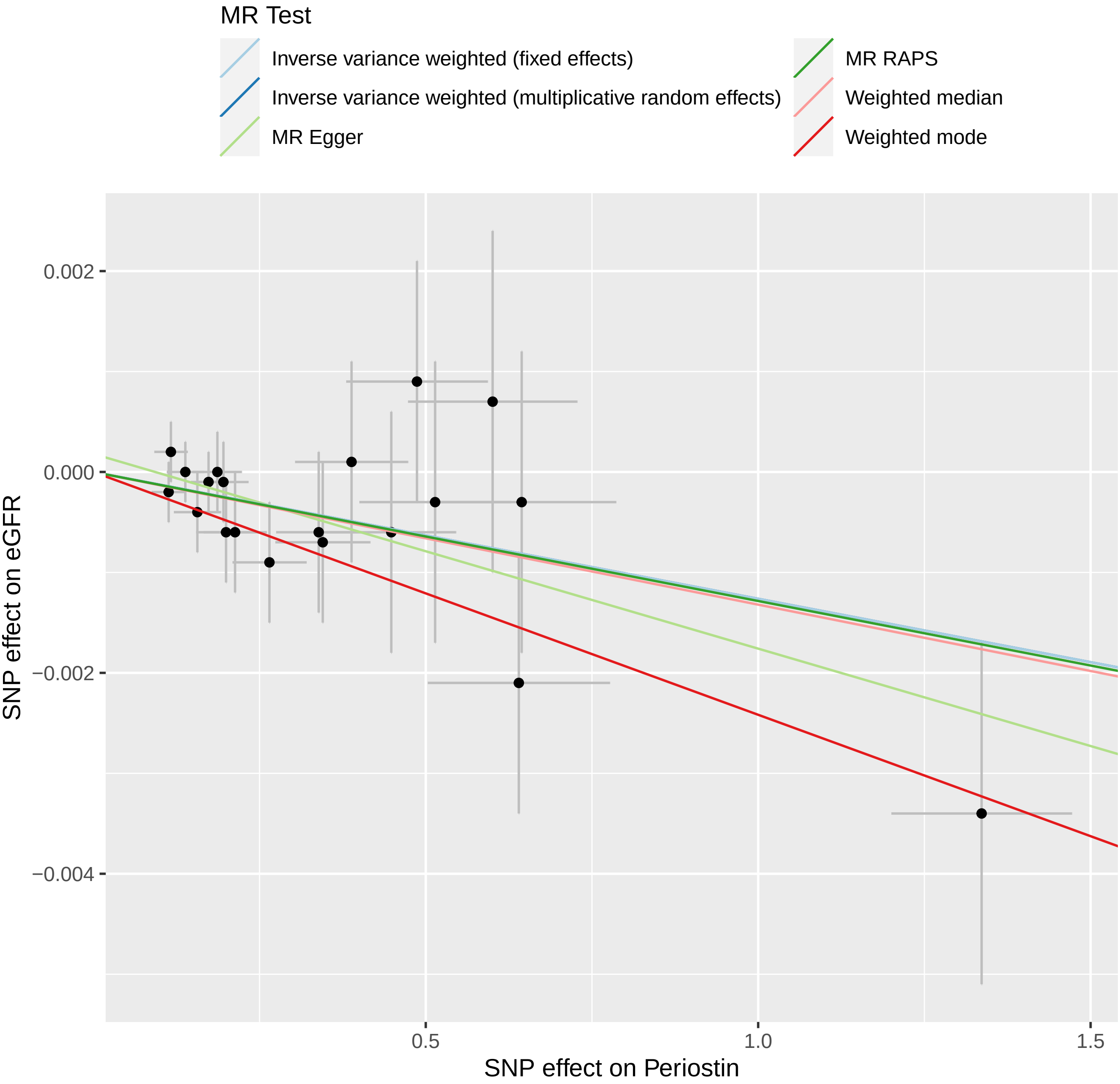

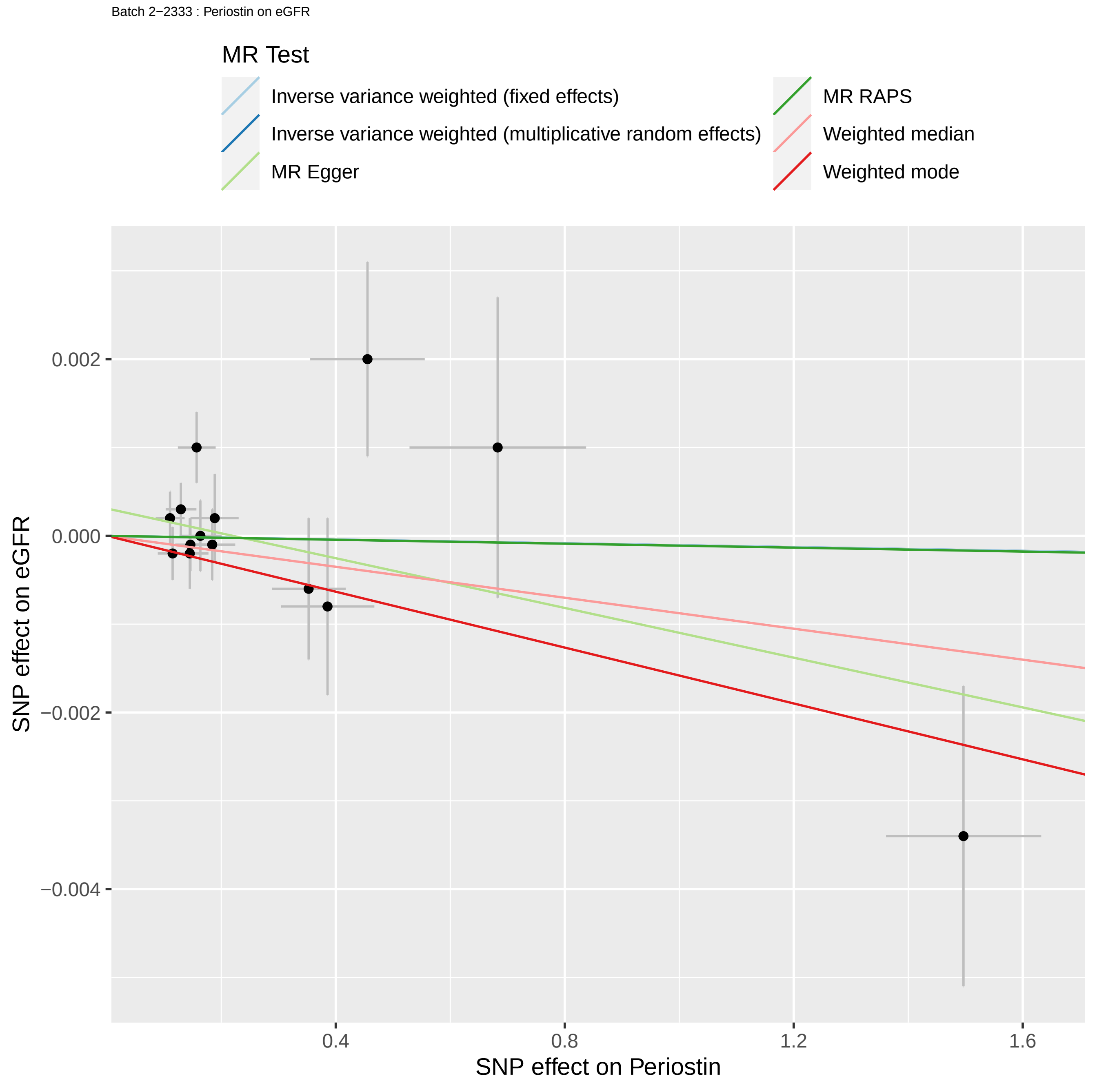

Batch 2−2333 : Periostin on eGFR
MR Test
Inverse variance weighted (fixed effects)
Inverse variance weighted (multiplicative random effects)
MR Egger
MR RAPS
Weighted median
Weighted mode
0.002
0.000
−0.002
−0.004
0.4
0.8
1.2
1.6
SNP effect on eGFR
SNP effect on Periostin

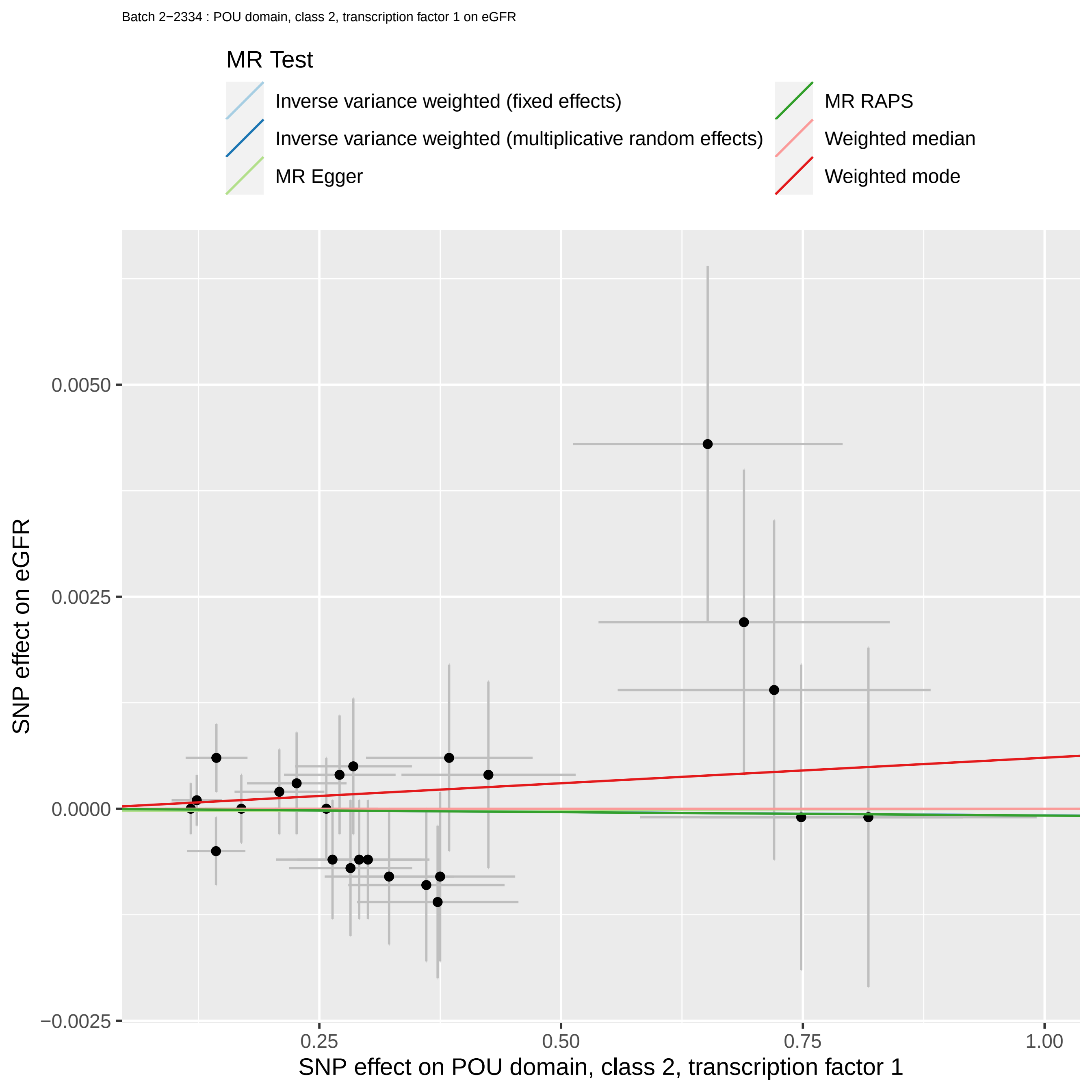
Batch 2−2334 : POU domain, class 2, transcription factor 1 on eGFR
MR Test
Inverse variance weighted (fixed effects)
Inverse variance weighted (multiplicative random effects)
MR Egger
MR RAPS
Weighted median
Weighted mode
0.0050
0.0025
0.0000
−0.0025
0.25
0.50
0.75
1.00
SNP effect on eGFR
SNP effect on POU domain, class 2, transcription factor 1

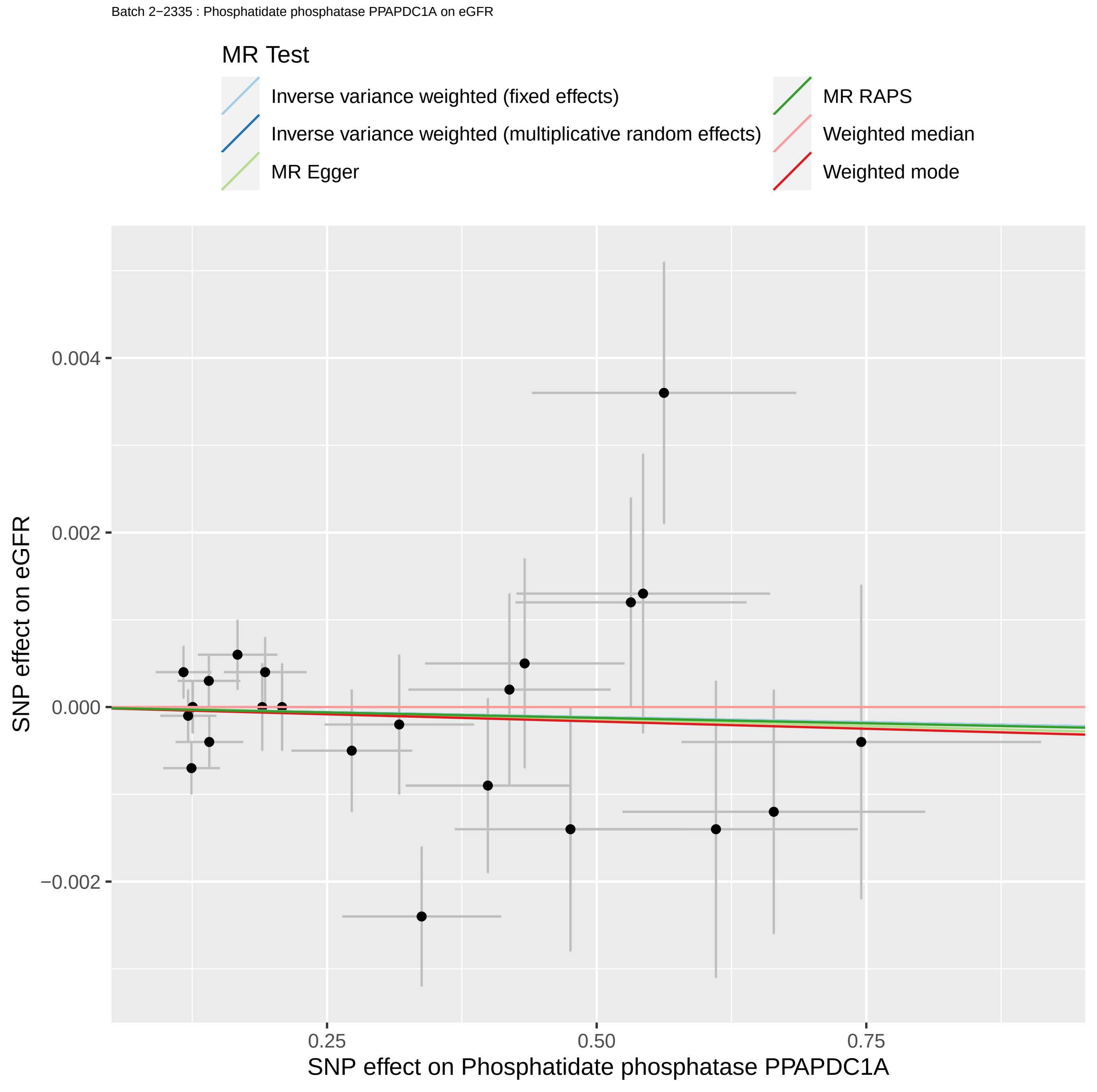
Batch 2−2335 : Phosphatidate phosphatase PPAPDC1A on eGFR
MR Test
Inverse variance weighted (fixed effects)
Inverse variance weighted (multiplicative random effects)
MR Egger
MR RAPS
Weighted median
Weighted mode
0.004
0.002
0.000
−0.002
0.25
0.50
0.75
SNP effect on eGFR
SNP effect on Phosphatidate phosphatase PPAPDC1A

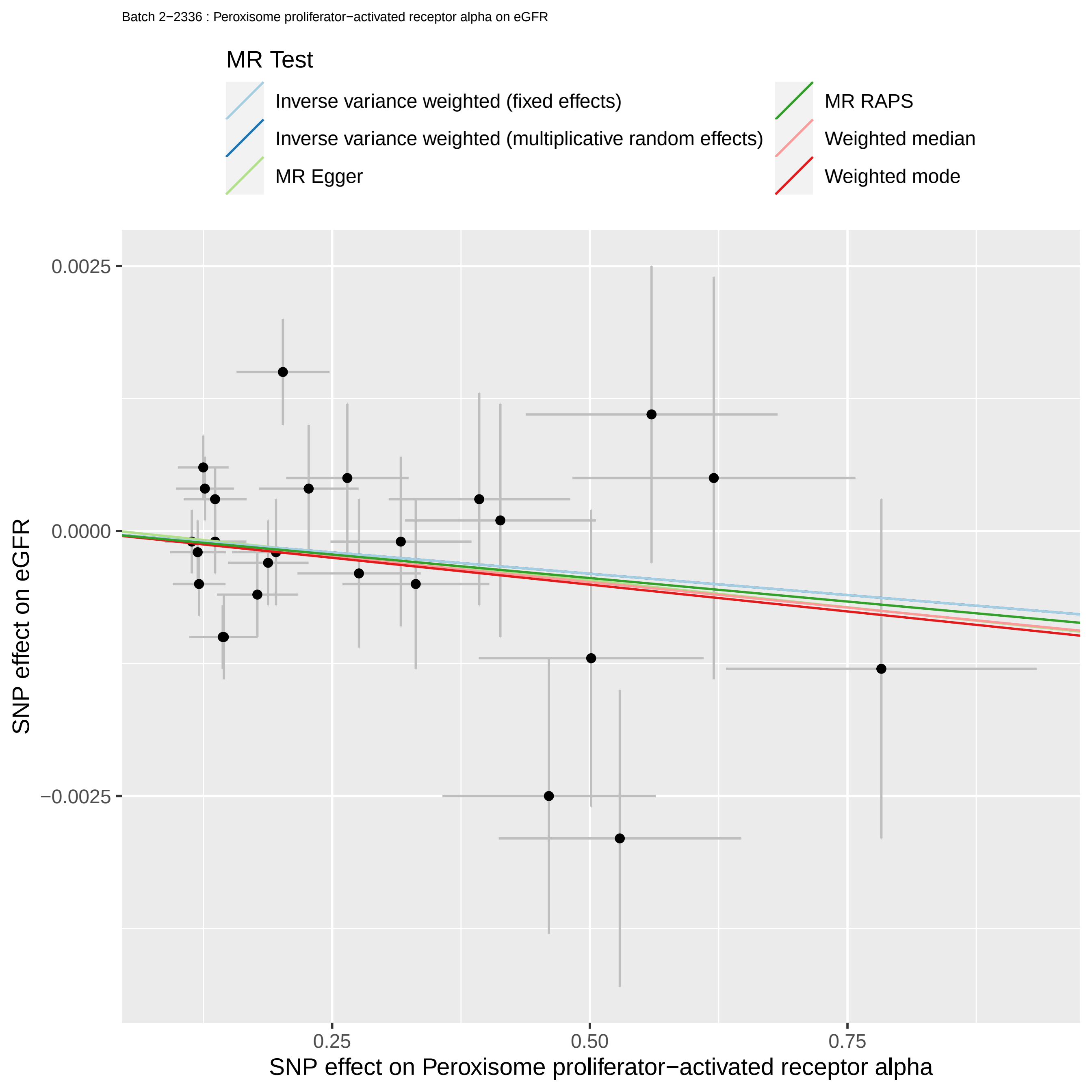
Batch 2−2336 : Peroxisome proliferator−activated receptor alpha on eGFR
MR Test
Inverse variance weighted (fixed effects)
Inverse variance weighted (multiplicative random effects)
MR Egger
MR RAPS
Weighted median
Weighted mode
0.0025
0.0000
−0.0025
0.25
0.50
0.75
SNP effect on eGFR
SNP effect on Peroxisome proliferator−activated receptor alpha

Batch 2−2337 : Neutrophil−activating peptide 2 on eGFR

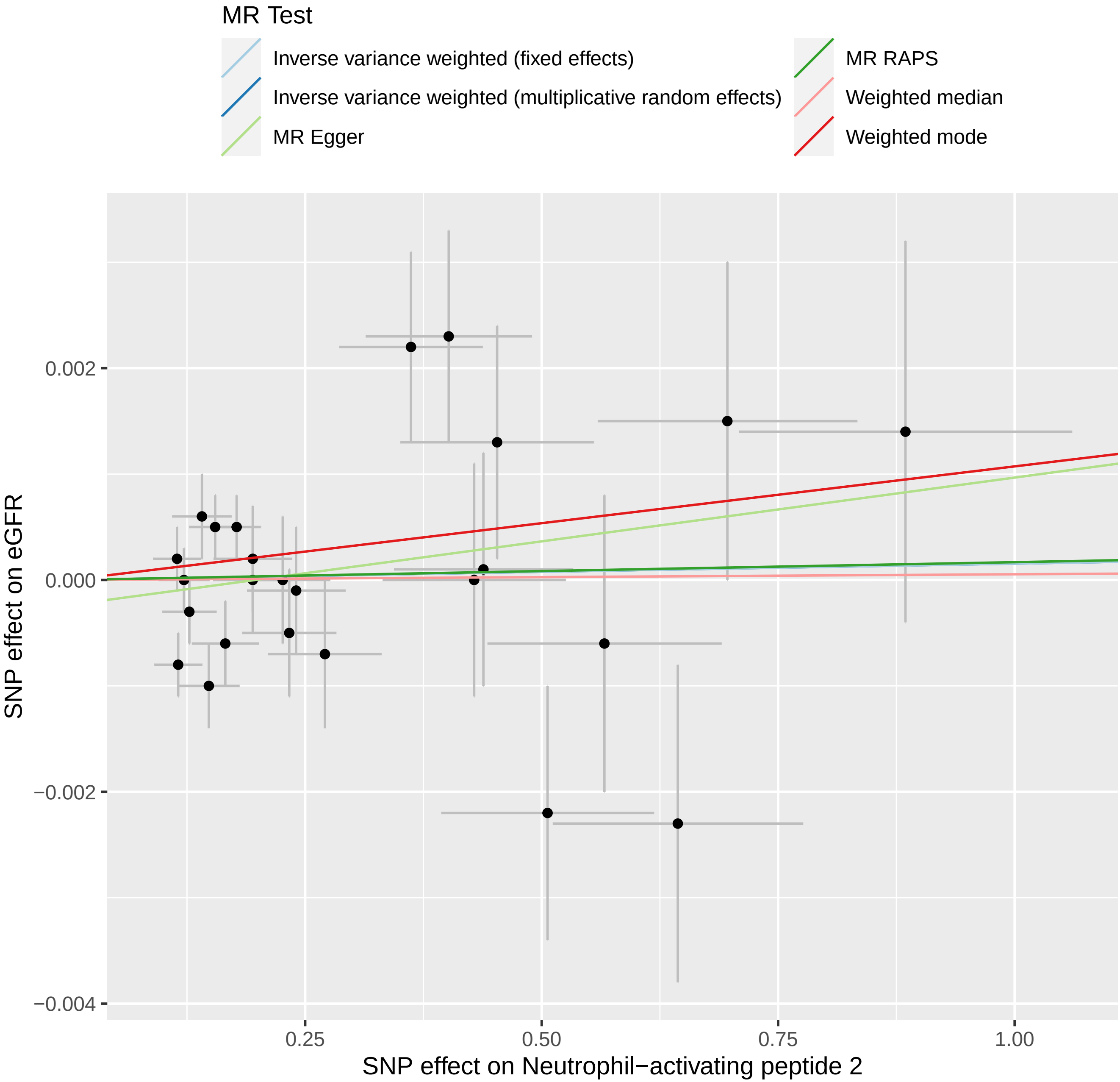

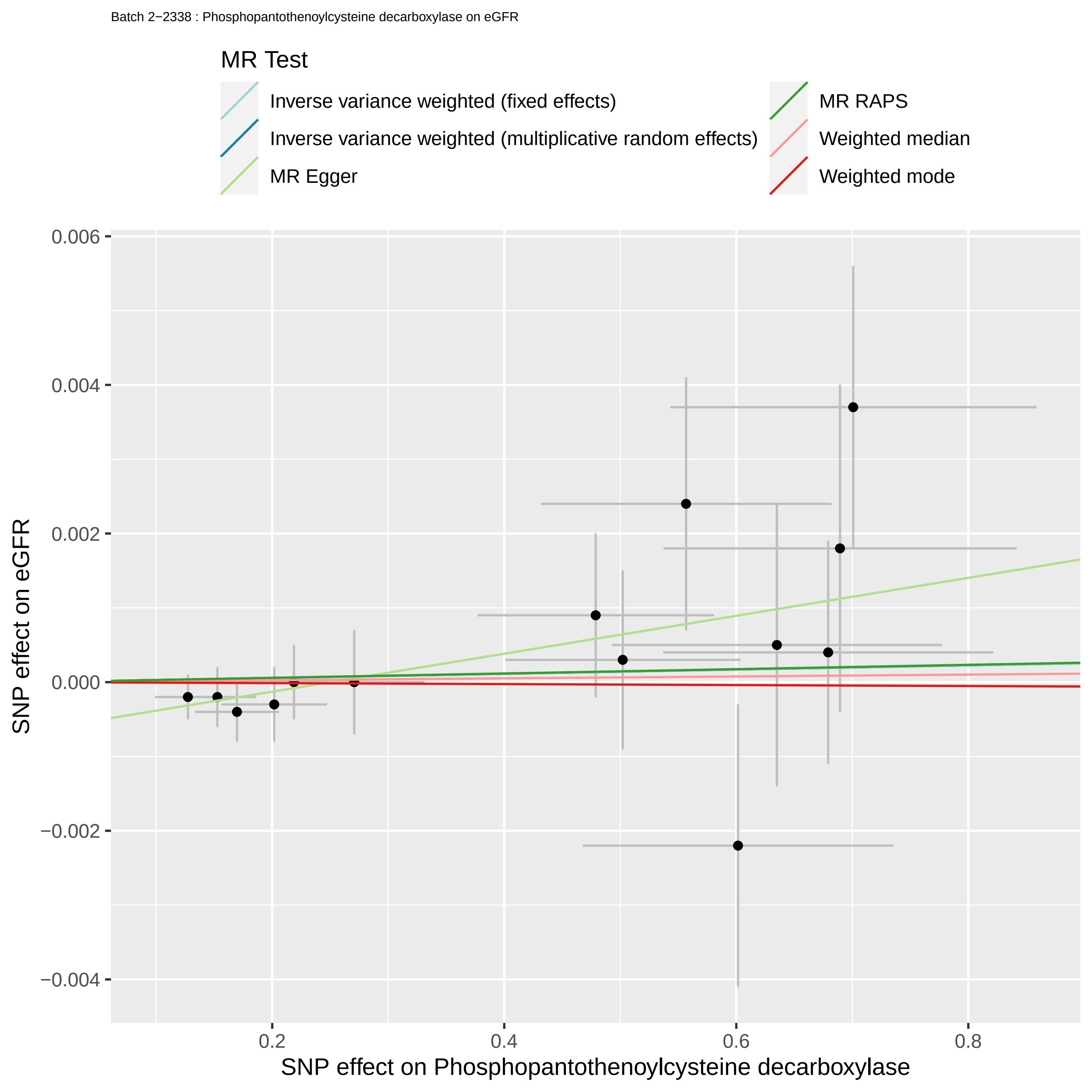

Batch 2−2338 : Phosphopantothenoylcysteine decarboxylase on eGFR
MR Test
Inverse variance weighted (fixed effects)
Inverse variance weighted (multiplicative random effects)
MR Egger
MR RAPS
Weighted median
Weighted mode
SNP effect on eGFR
0.006
0.004
0.002
0.000
−0.002
−0.004
0.2
0.4
0.6
0.8
SNP effect on Phosphopantothenoylcysteine decarboxylase

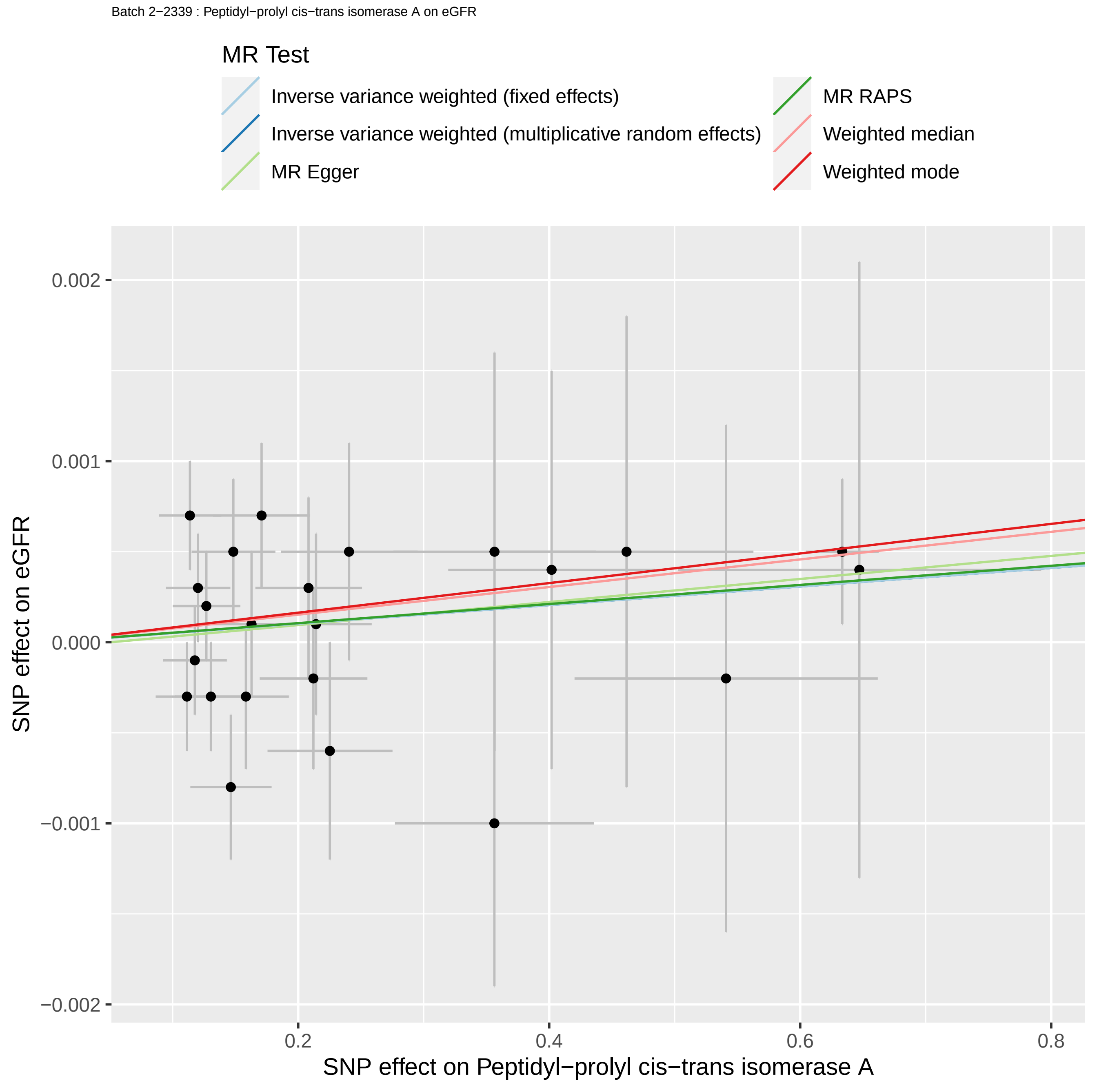
Batch 2−2339 : Peptidyl−prolyl cis−trans isomerase A on eGFR
MR Test
Inverse variance weighted (fixed effects)
Inverse variance weighted (multiplicative random effects)
MR Egger
MR RAPS
Weighted median
Weighted mode
SNP effect on eGFR
0.002
0.001
0.000
−0.001
−0.002
0.2
0.4
0.6
0.8
SNP effect on Peptidyl−prolyl cis−trans isomerase A

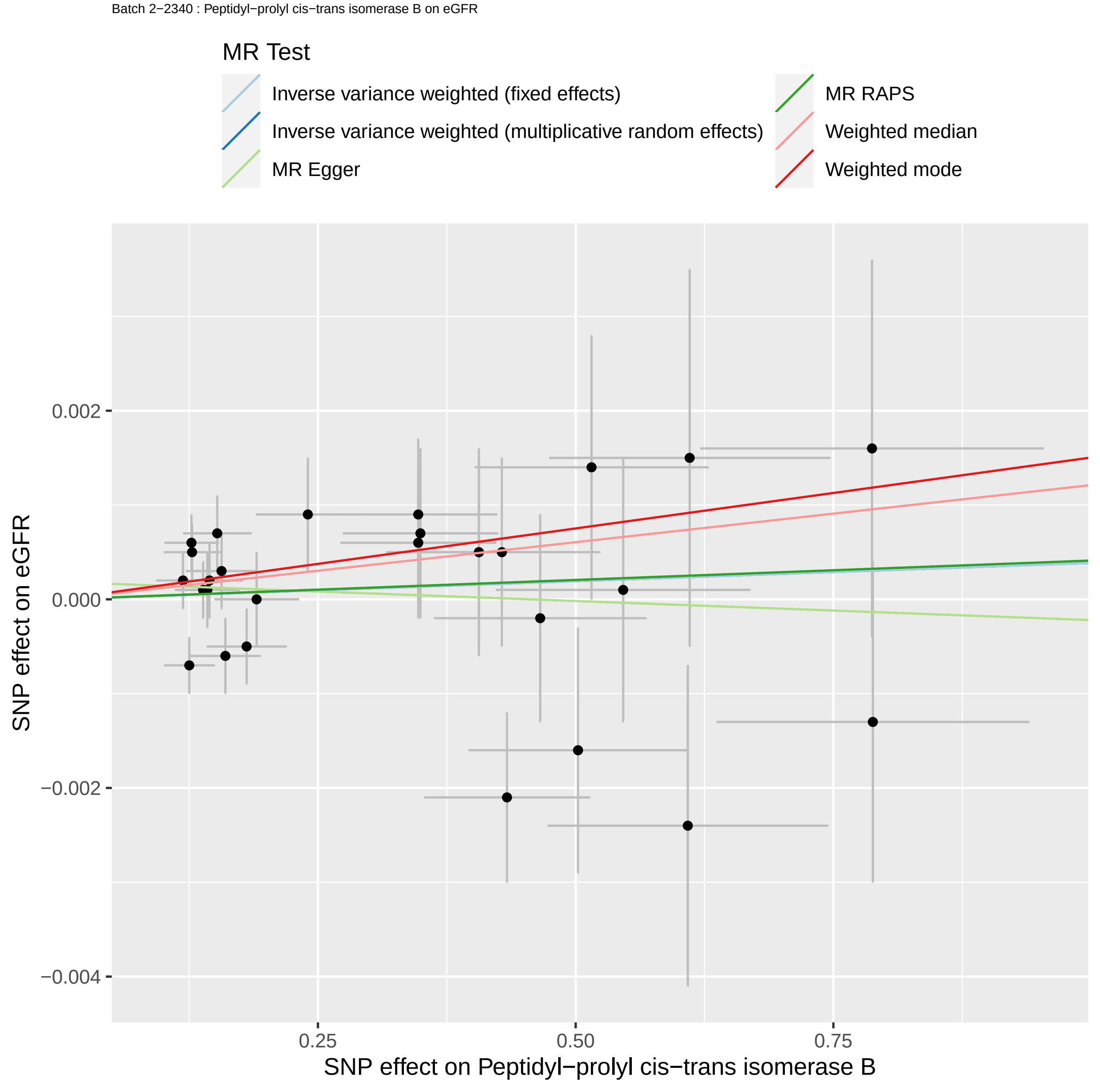

Batch 2−2340 : Peptidyl−prolyl cis−trans isomerase B on eGFR
MR Test
Inverse variance weighted (fixed effects)
Inverse variance weighted (multiplicative random effects)
MR Egger
MR RAPS
Weighted median
Weighted mode
0.002
0.000
−0.002
−0.004
0.25
0.50
0.75
SNP effect on eGFR
SNP effect on Peptidyl−prolyl cis−trans isomerase B

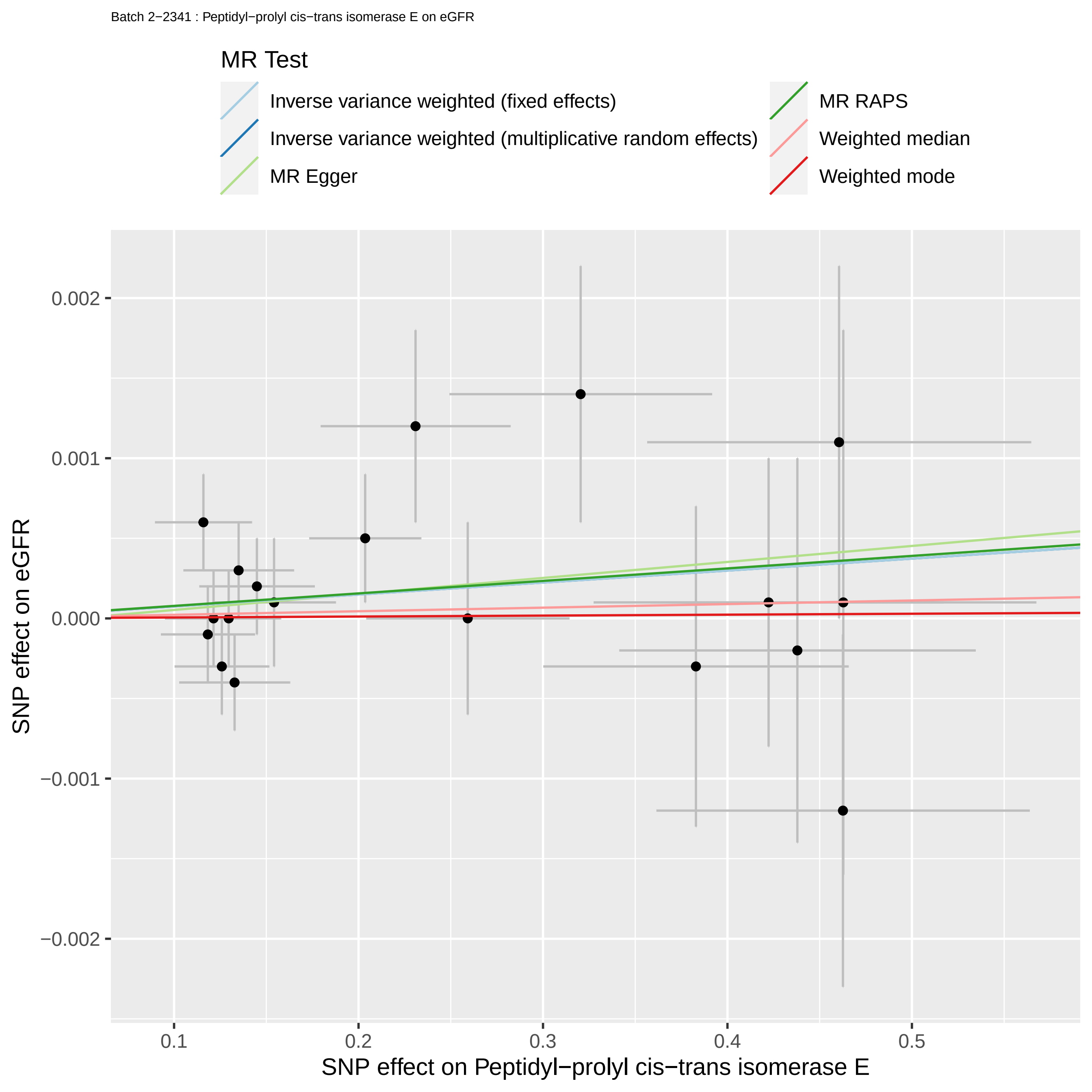

Batch 2−2341 : Peptidyl−prolyl cis−trans isomerase E on eGFR
MR Test
Inverse variance weighted (fixed effects)
Inverse variance weighted (multiplicative random effects)
MR Egger
MR RAPS
Weighted median
Weighted mode
0.002
0.001
0.000
−0.001
−0.002
SNP effect on eGFR
0.1
0.2
0.3
0.4
0.5
SNP effect on Peptidyl−prolyl cis−trans isomerase E

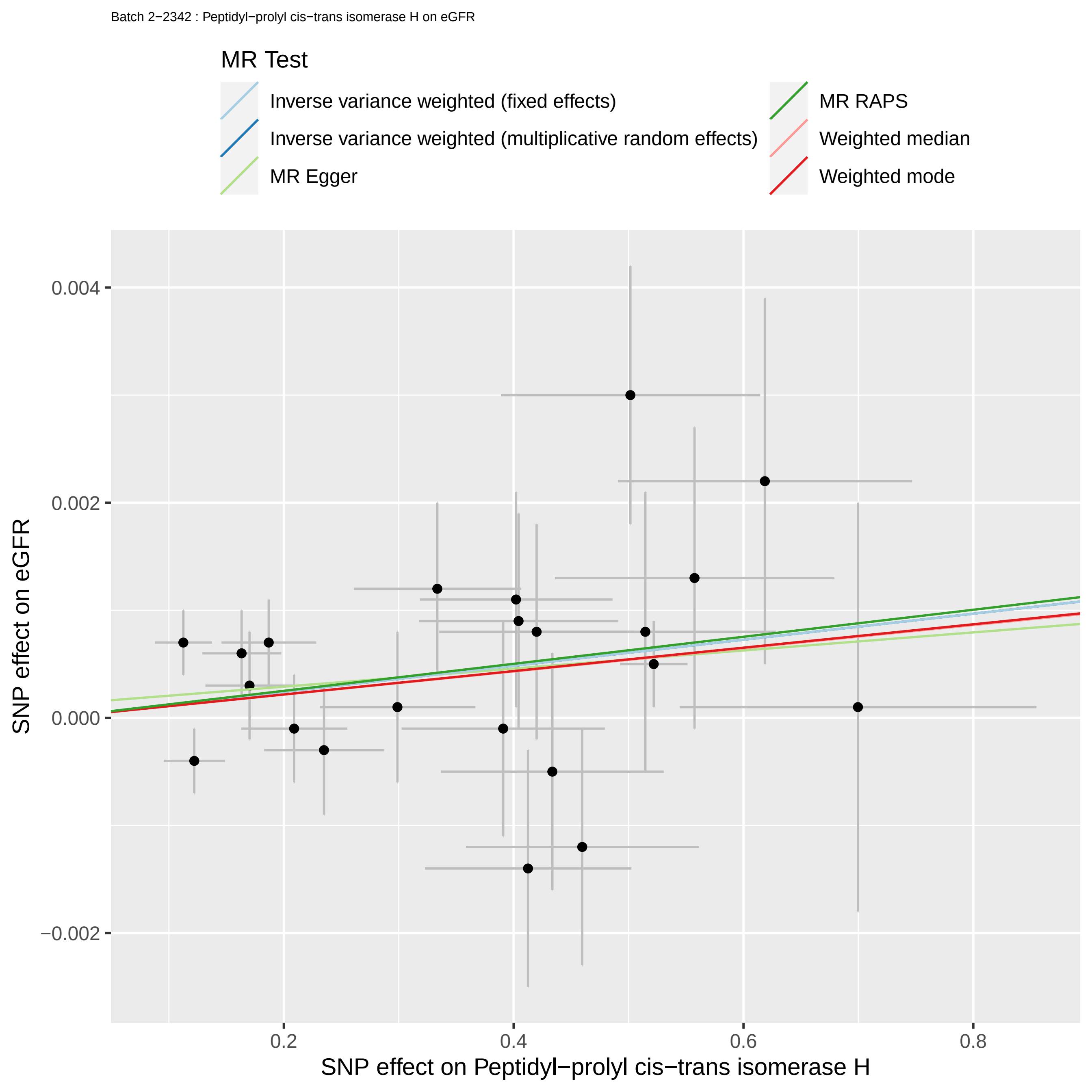
Batch 2−2342 : Peptidyl−prolyl cis−trans isomerase H on eGFR
MR Test
Inverse variance weighted (fixed effects)
Inverse variance weighted (multiplicative random effects)
MR Egger
MR RAPS
Weighted median
Weighted mode
0.004
0.002
0.000
−0.002
SNP effect on eGFR
0.2
0.4
0.6
0.8
SNP effect on Peptidyl−prolyl cis−trans isomerase H

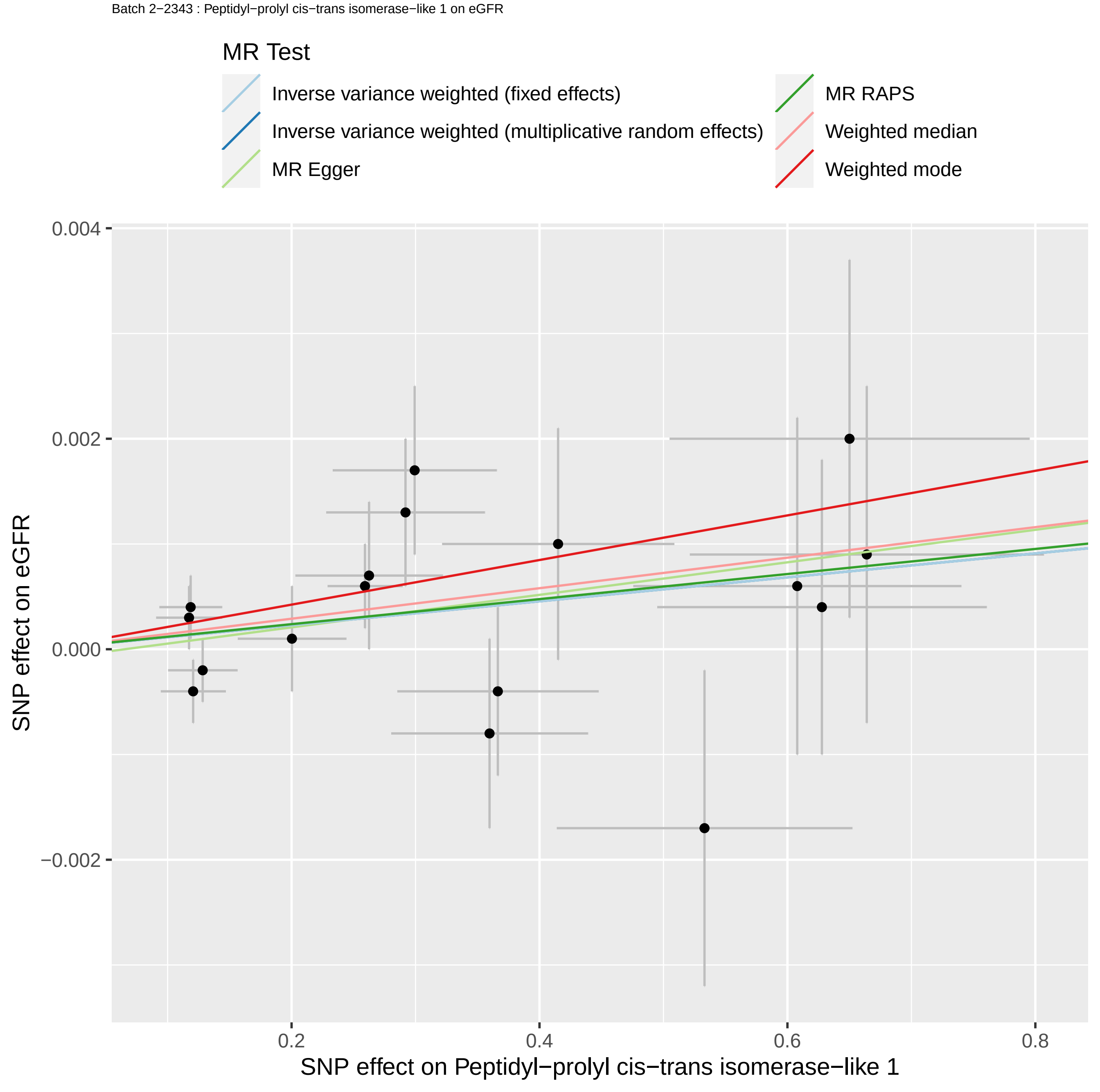
Batch 2−2343 : Peptidyl−prolyl cis−trans isomerase−like 1 on eGFR
MR Test
Inverse variance weighted (fixed effects)
Inverse variance weighted (multiplicative random effects)
MR Egger
MR RAPS
Weighted median
Weighted mode
0.004
0.002
0.000
−0.002
0.2
0.4
0.6
0.8
SNP effect on eGFR
SNP effect on Peptidyl−prolyl cis−trans isomerase−like 1

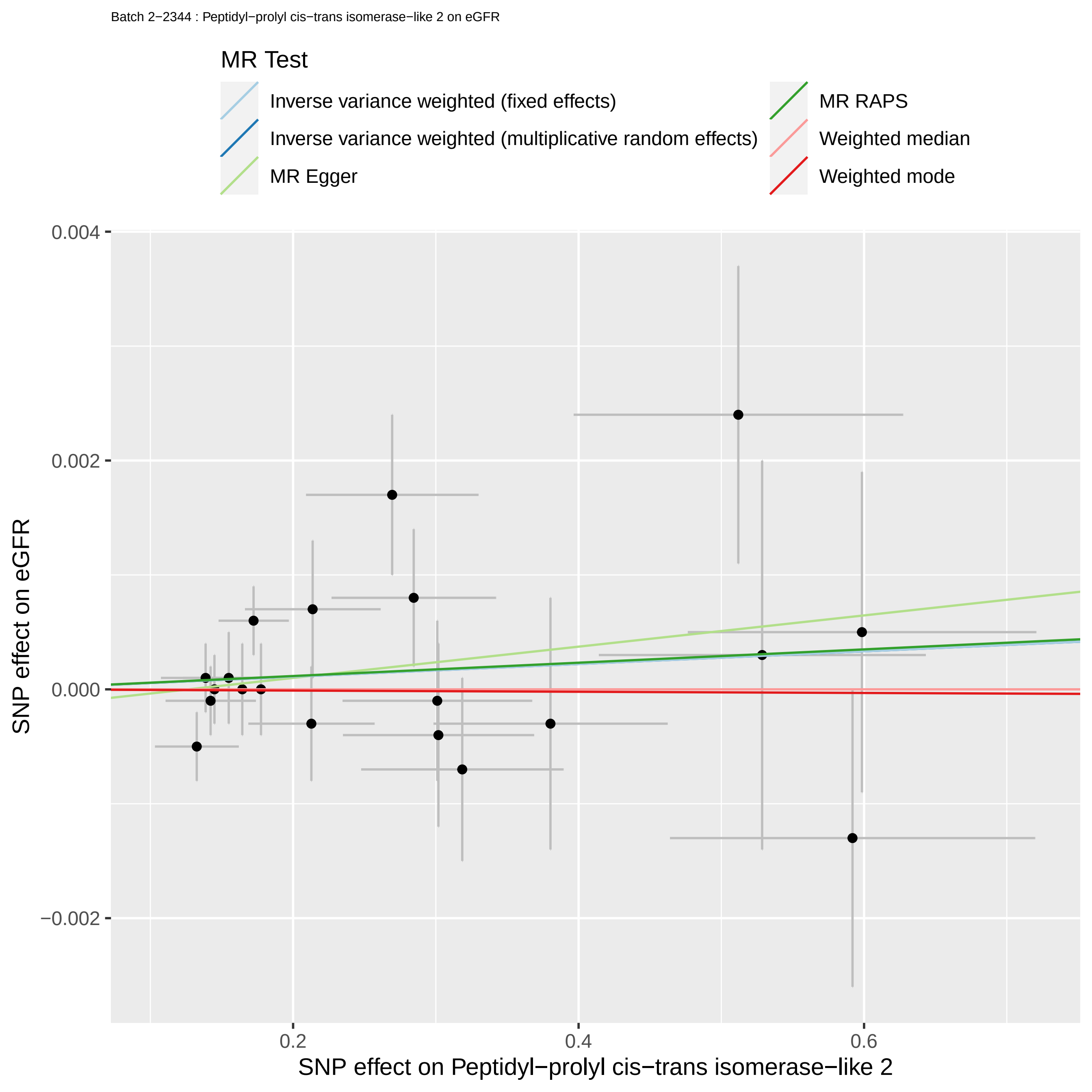
Batch 2−2344 : Peptidyl−prolyl cis−trans isomerase−like 2 on eGFR
MR Test
Inverse variance weighted (fixed effects)
Inverse variance weighted (multiplicative random effects)
MR Egger
MR RAPS
Weighted median
Weighted mode
0.004
0.002
0.000
−0.002
0.2
0.4
0.6
SNP effect on eGFR
SNP effect on Peptidyl−prolyl cis−trans isomerase−like 2

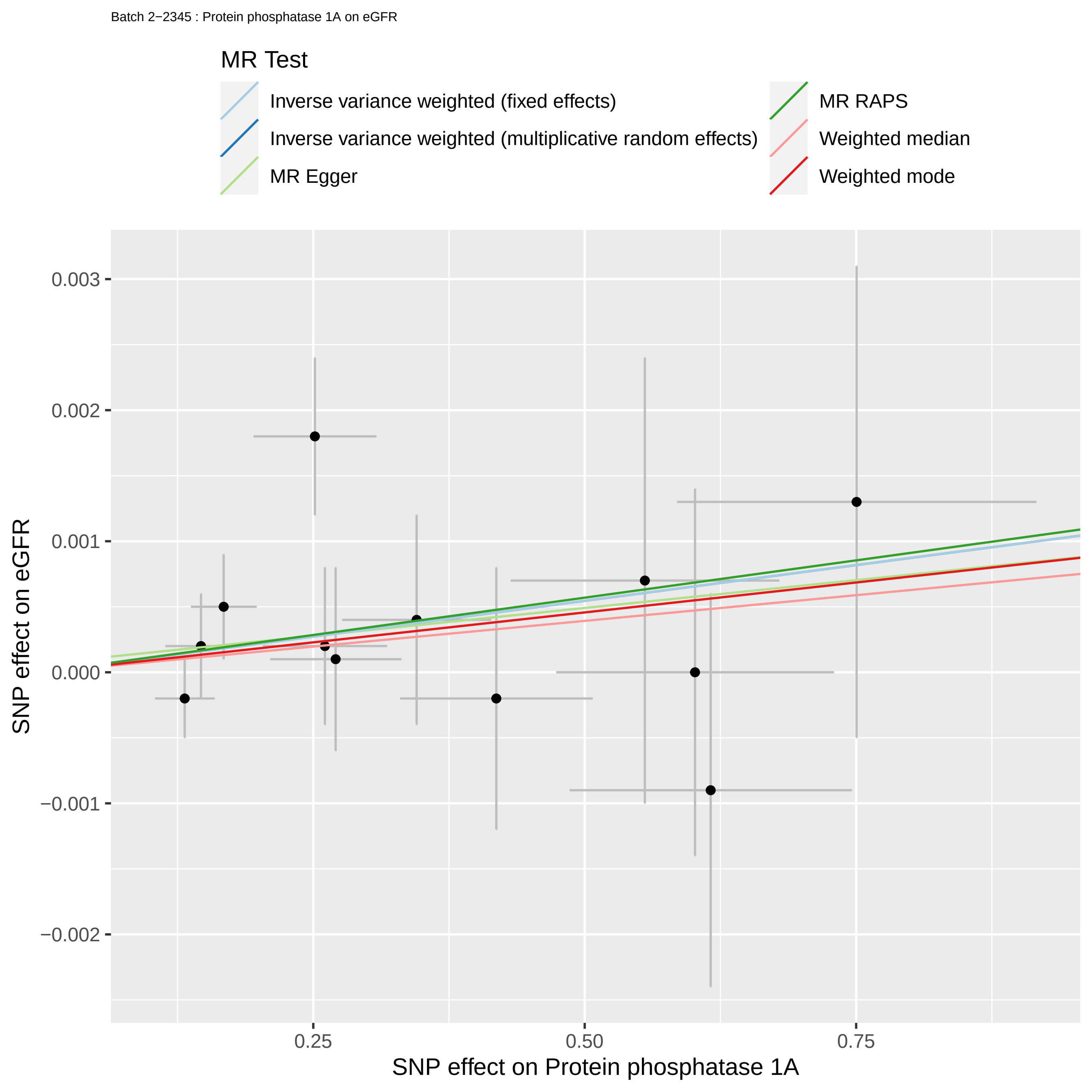

Batch 2−2345 : Protein phosphatase 1A on eGFR
MR Test
Inverse variance weighted (fixed effects)
Inverse variance weighted (multiplicative random effects)
MR Egger
MR RAPS
Weighted median
Weighted mode
0.003
0.002
0.001
0.000
−0.001
−0.002
SNP effect on eGFR
0.25
0.50
0.75
SNP effect on Protein phosphatase 1A

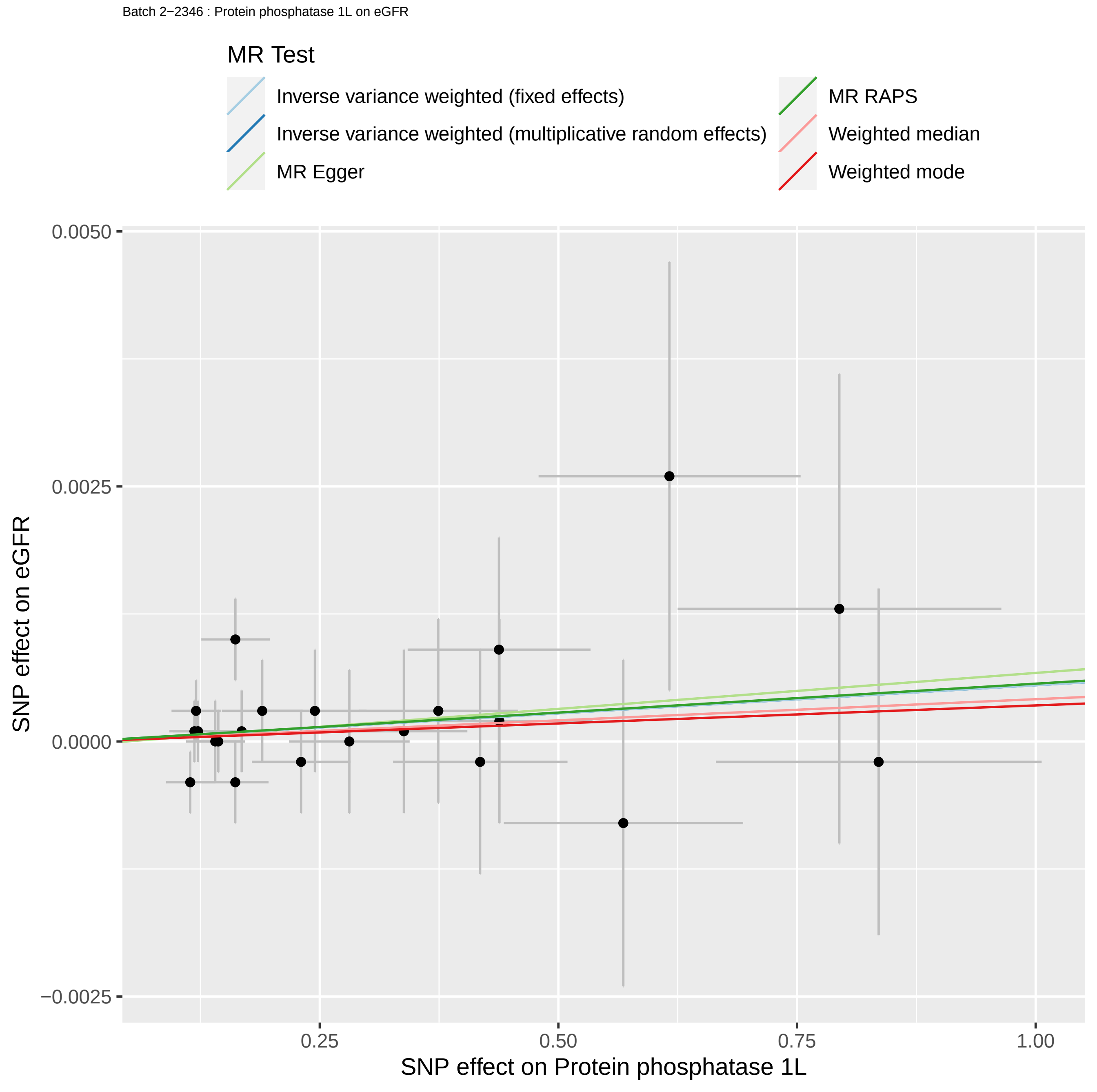
Batch 2−2346 : Protein phosphatase 1L on eGFR
MR Test
Inverse variance weighted (fixed effects)
Inverse variance weighted (multiplicative random effects)
MR Egger
MR RAPS
Weighted median
Weighted mode
0.0050
0.0025
0.0000
−0.0025
0.25
0.50
0.75
1.00
SNP effect on eGFR
SNP effect on Protein phosphatase 1L

Batch 2−2347 : Protein phosphatase 1 regulatory subunit 3B on eGFR

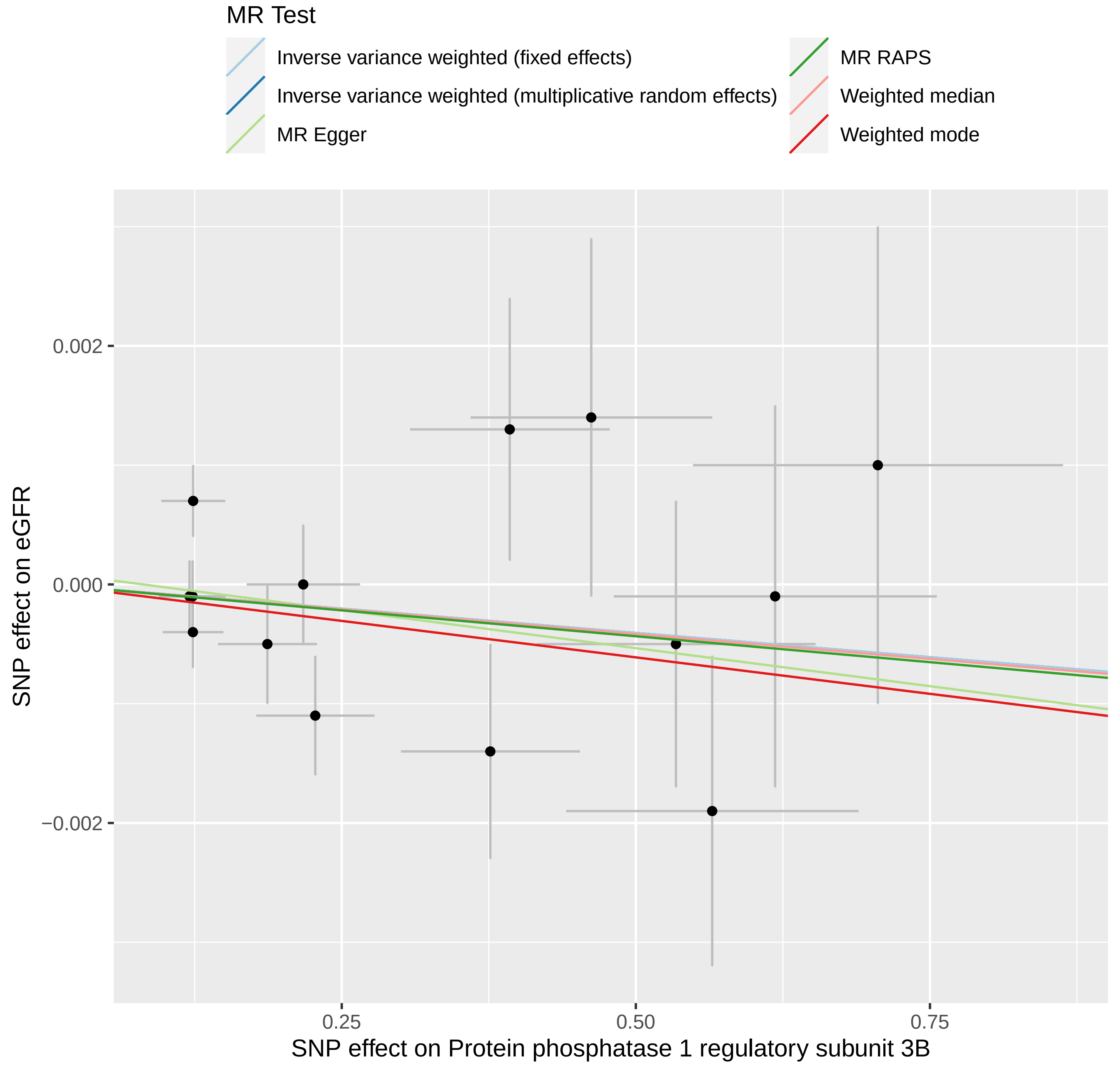

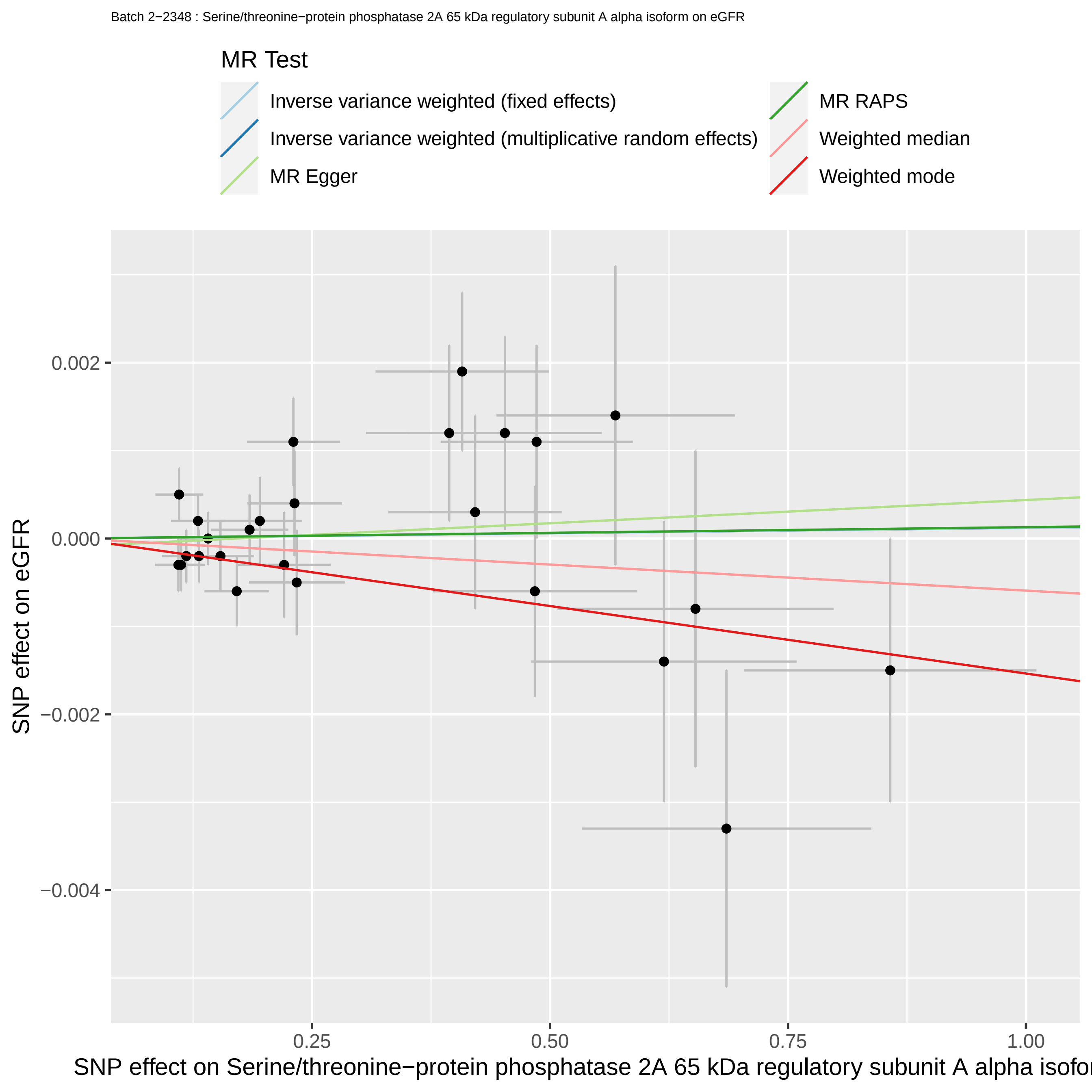

Batch 2−2348 : Serine/threonine−protein phosphatase 2A 65 kDa regulatory subunit A alpha isoform on eGFR
MR Test
Inverse variance weighted (fixed effects)
Inverse variance weighted (multiplicative random effects)
MR Egger
MR RAPS
Weighted median
Weighted mode
0.002
0.000
−0.002
−0.004
0.25
0.50
0.75
1.00
SNP effect on eGFR
SNP effect on Serine/threonine−protein phosphatase 2A 65 kDa regulatory subunit A alpha isofor

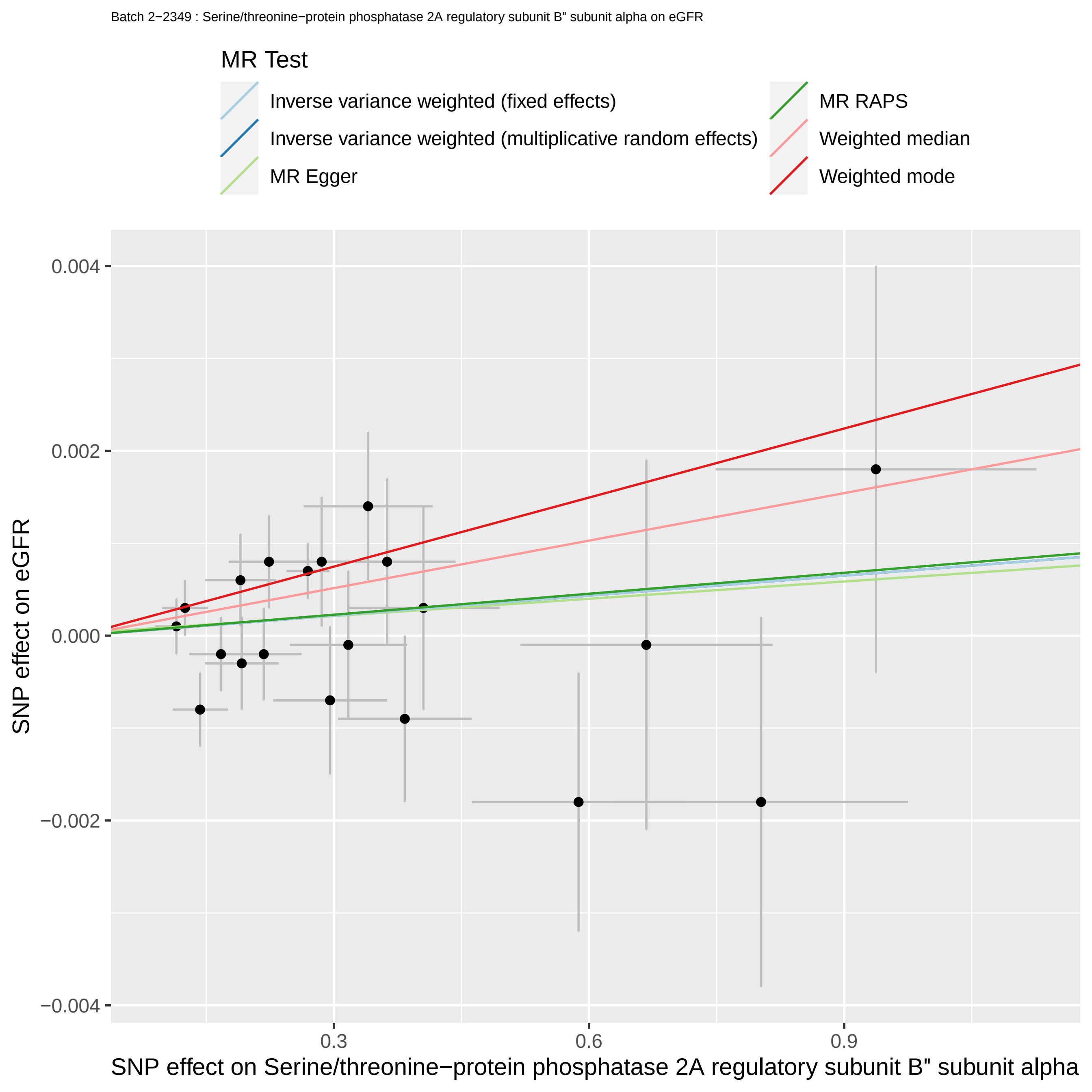
Batch 2−2349 : Serine/threonine−protein phosphatase 2A regulatory subunit B" subunit alpha on eGFR
MR Test
Inverse variance weighted (fixed effects)
Inverse variance weighted (multiplicative random effects)
MR Egger
MR RAPS
Weighted median
Weighted mode
SNP effect on eGFR
0.004
0.002
0.000
−0.002
−0.004
0.3
0.6
0.9
SNP effect on Serine/threonine−protein phosphatase 2A regulatory subunit B" subunit alpha

Batch 2−2350 : Calcineurin subunit B type 1 on eGFR

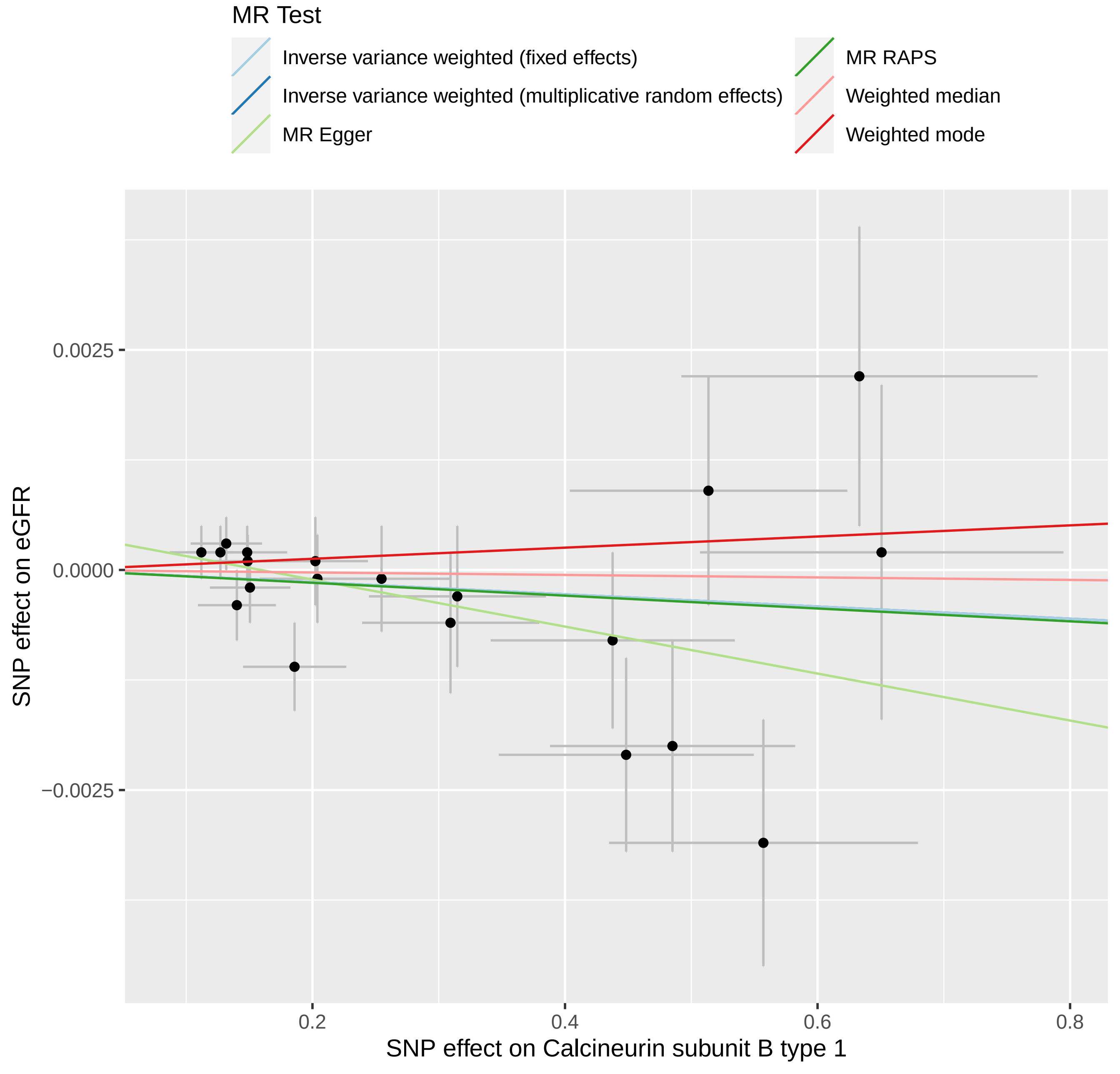

Batch 2−2351 : Palmitoyl−protein thioesterase 1 on eGFR

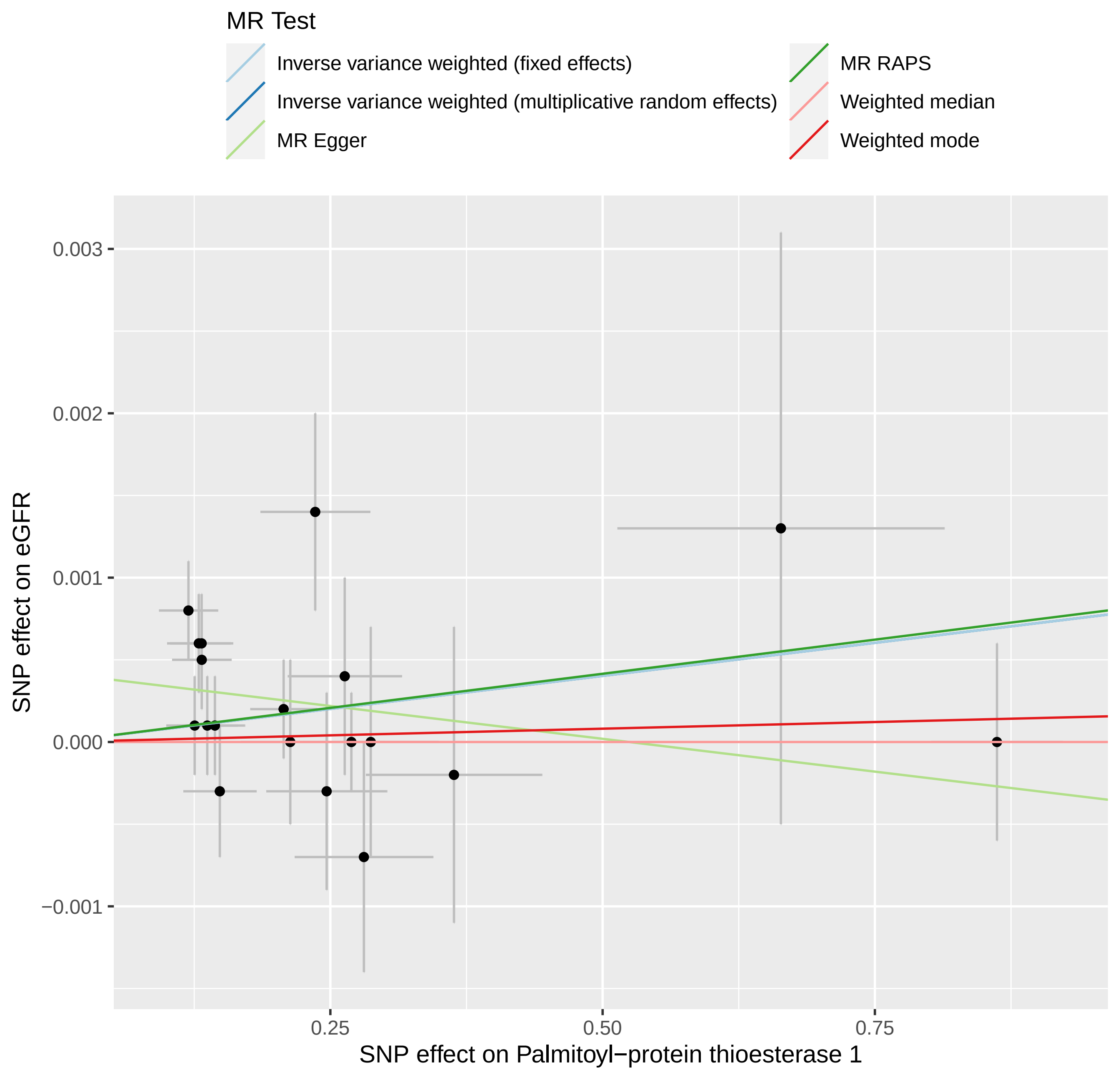

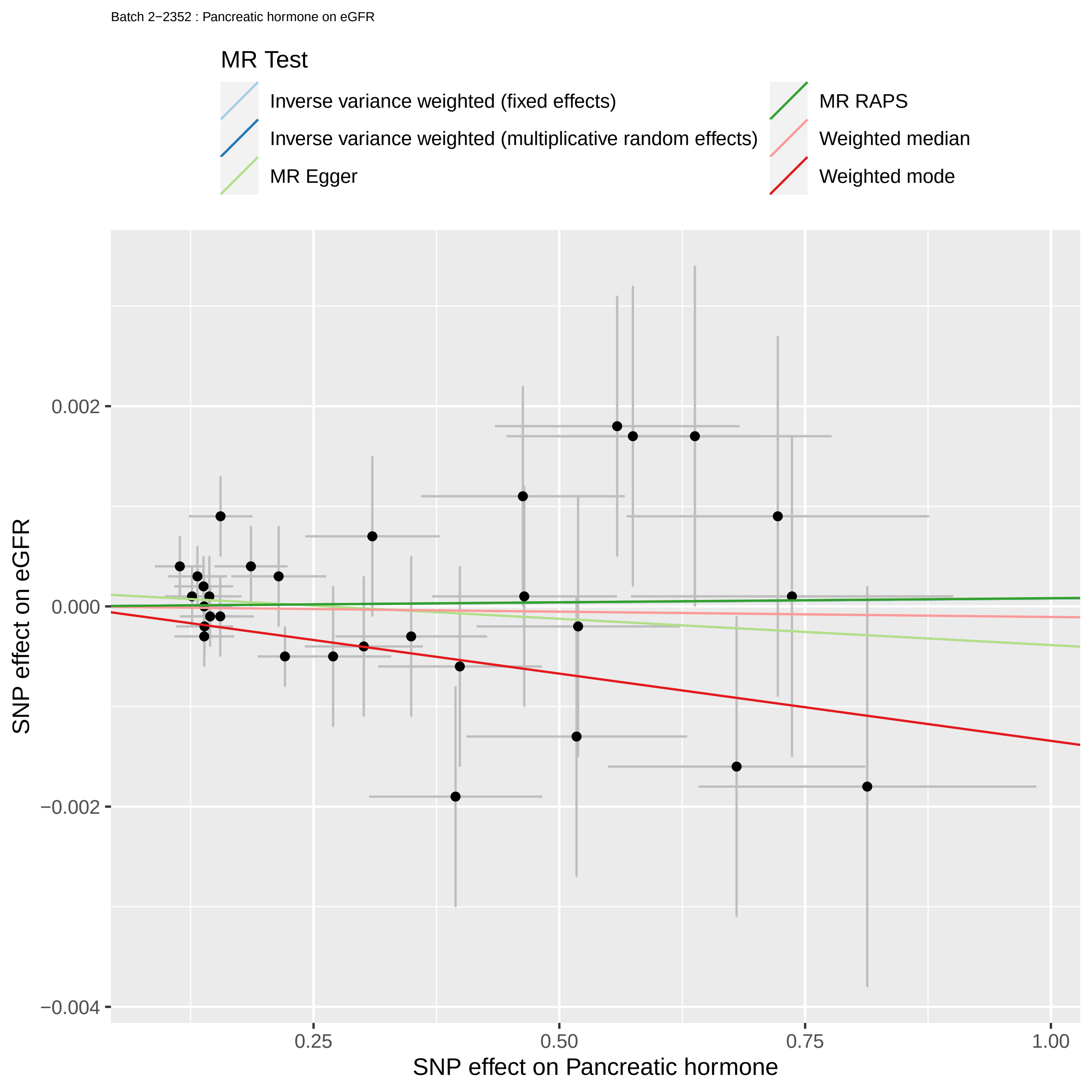
Batch 2−2352 : Pancreatic hormone on eGFR
MR Test
Inverse variance weighted (fixed effects)
Inverse variance weighted (multiplicative random effects)
MR Egger
MR RAPS
Weighted median
Weighted mode
0.002
0.000
−0.002
−0.004
0.25
0.50
0.75
1.00
SNP effect on eGFR
SNP effect on Pancreatic hormone

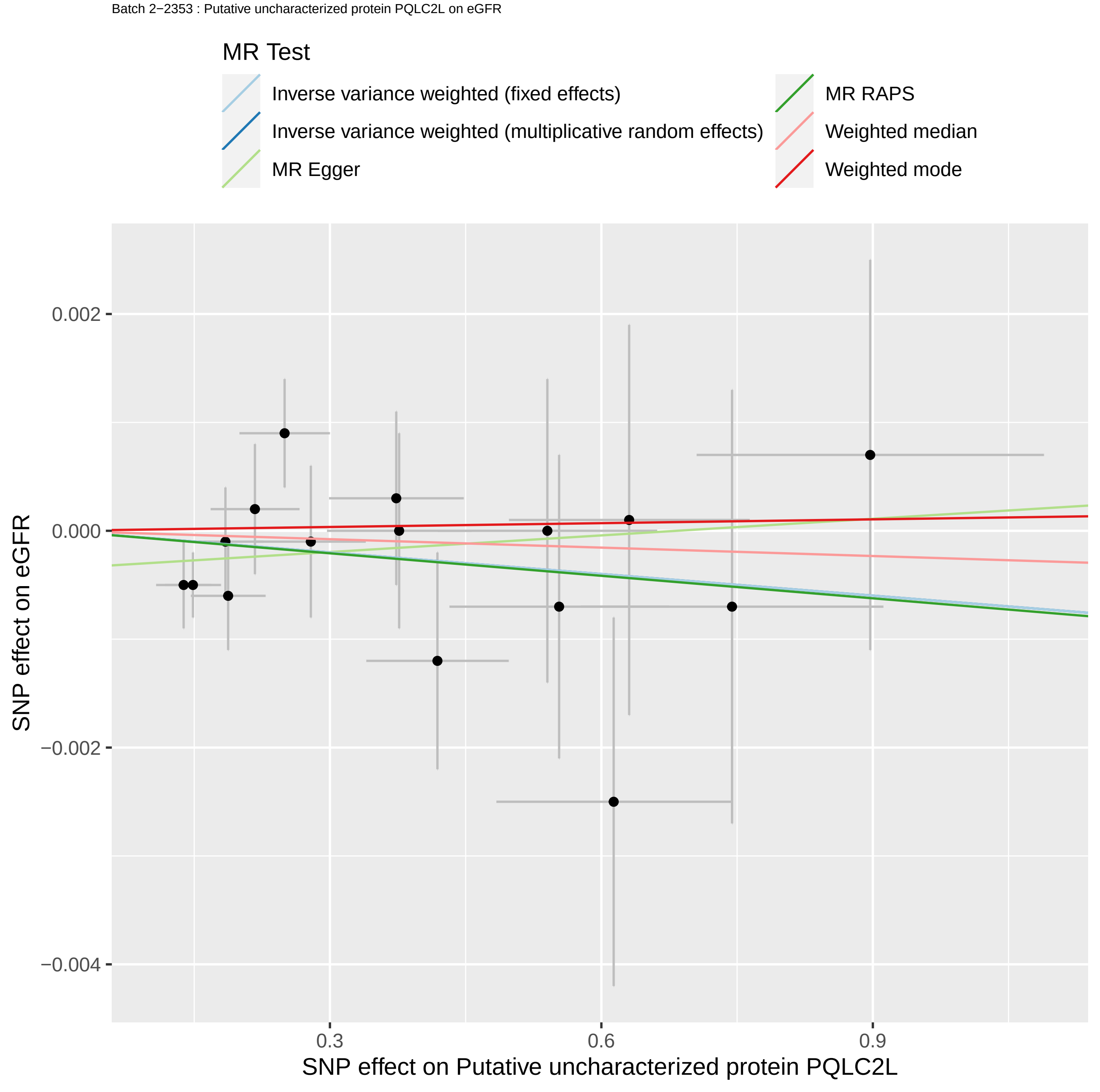

Batch 2−2353 : Putative uncharacterized protein PQLC2L on eGFR
MR Test
Inverse variance weighted (fixed effects)
Inverse variance weighted (multiplicative random effects)
MR Egger
MR RAPS
Weighted median
Weighted mode
0.002
0.000
−0.002
−0.004
0.3
0.6
0.9
SNP effect on eGFR
SNP effect on Putative uncharacterized protein PQLC2L

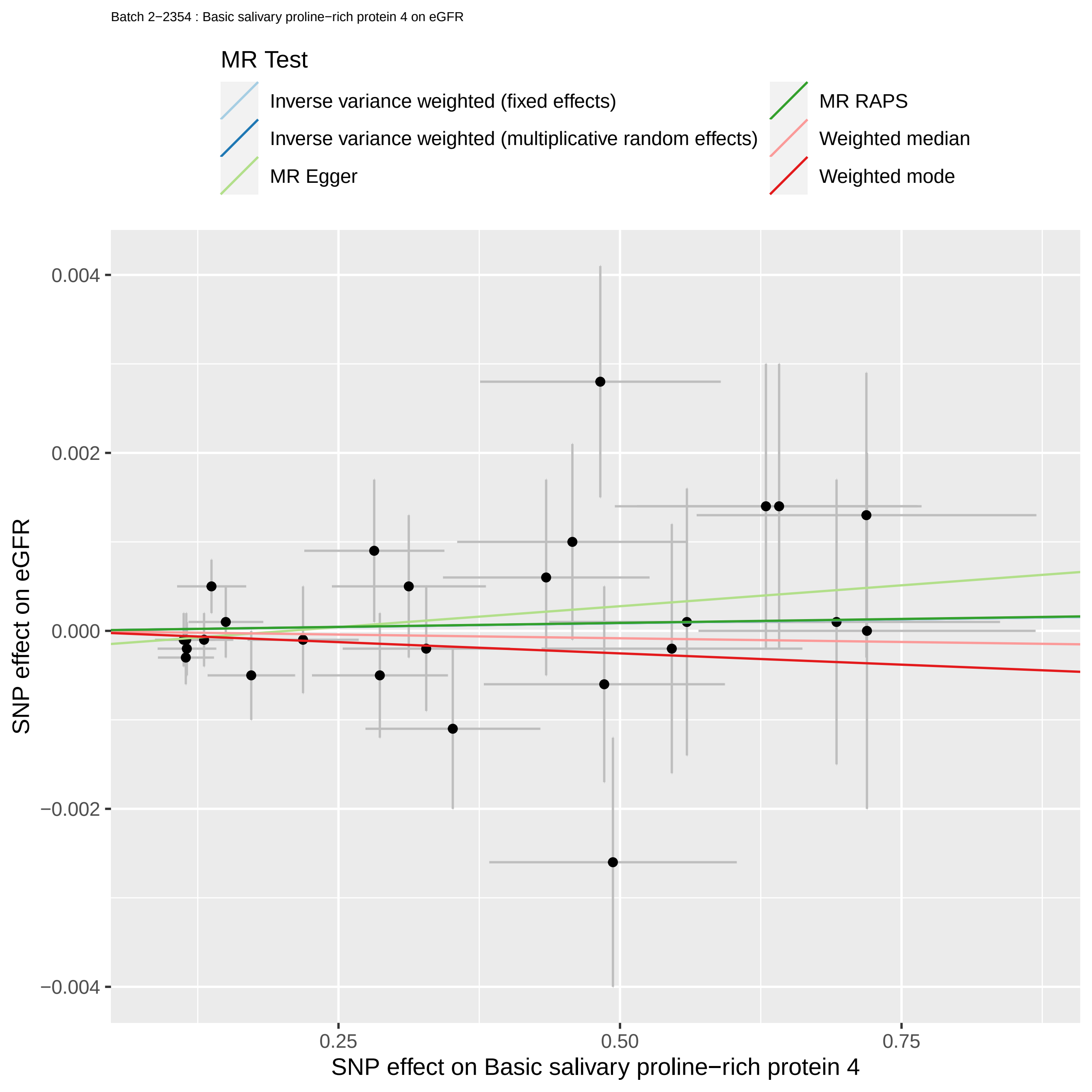
Batch 2−2354 : Basic salivary proline−rich protein 4 on eGFR
MR Test
Inverse variance weighted (fixed effects)
Inverse variance weighted (multiplicative random effects)
MR Egger
MR RAPS
Weighted median
Weighted mode
0.004
0.002
0.000
−0.002
−0.004
0.25
0.50
0.75
SNP effect on eGFR
SNP effect on Basic salivary proline−rich protein 4

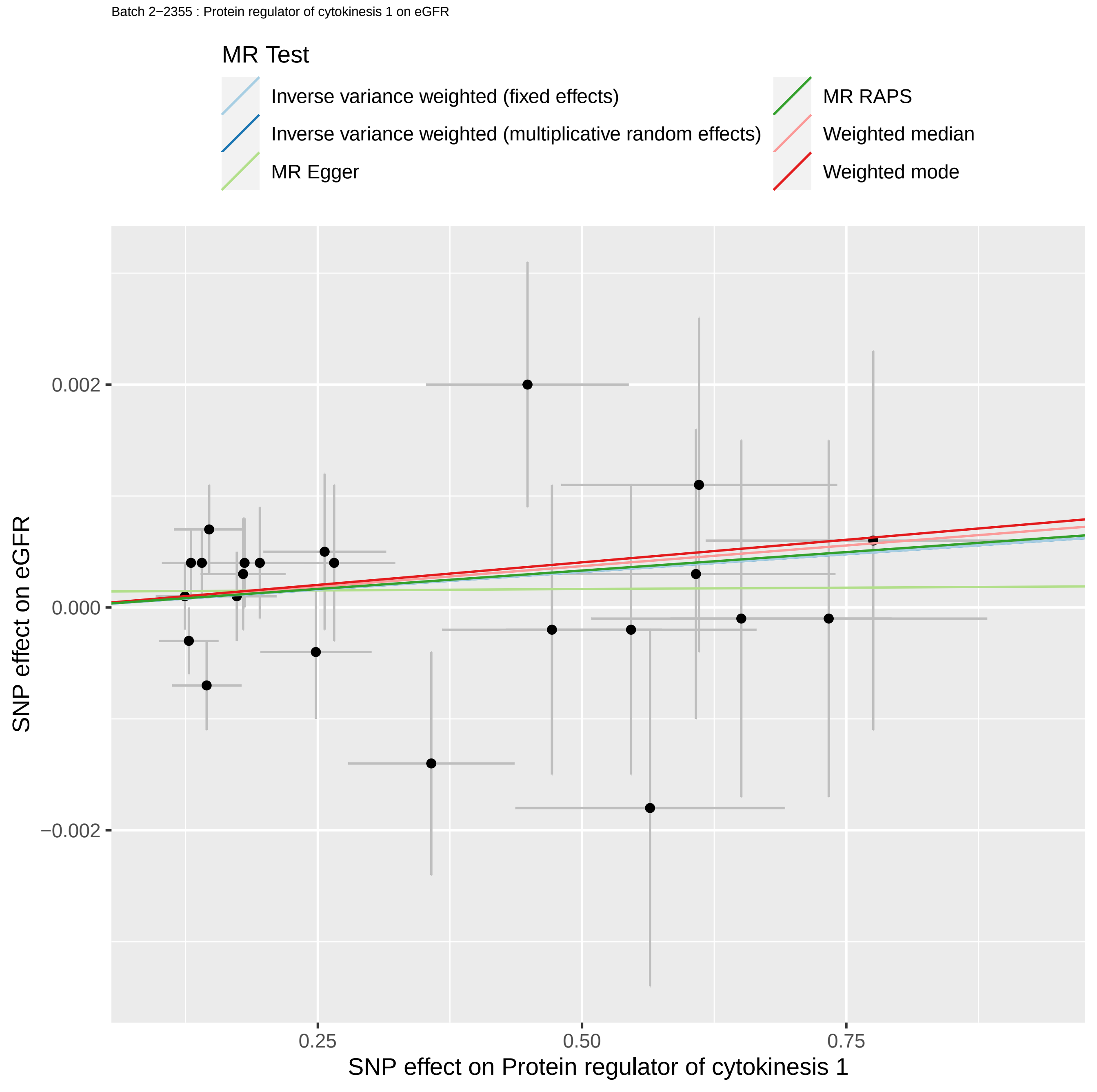

Batch 2−2355 : Protein regulator of cytokinesis 1 on eGFR
MR Test
Inverse variance weighted (fixed effects)
Inverse variance weighted (multiplicative random effects)
MR Egger
MR RAPS
Weighted median
Weighted mode
0.002
0.000
−0.002
0.25
0.50
0.75
SNP effect on eGFR
SNP effect on Protein regulator of cytokinesis 1

Batch 2−2356 : Lysosomal Pro−X carboxypeptidase on eGFR

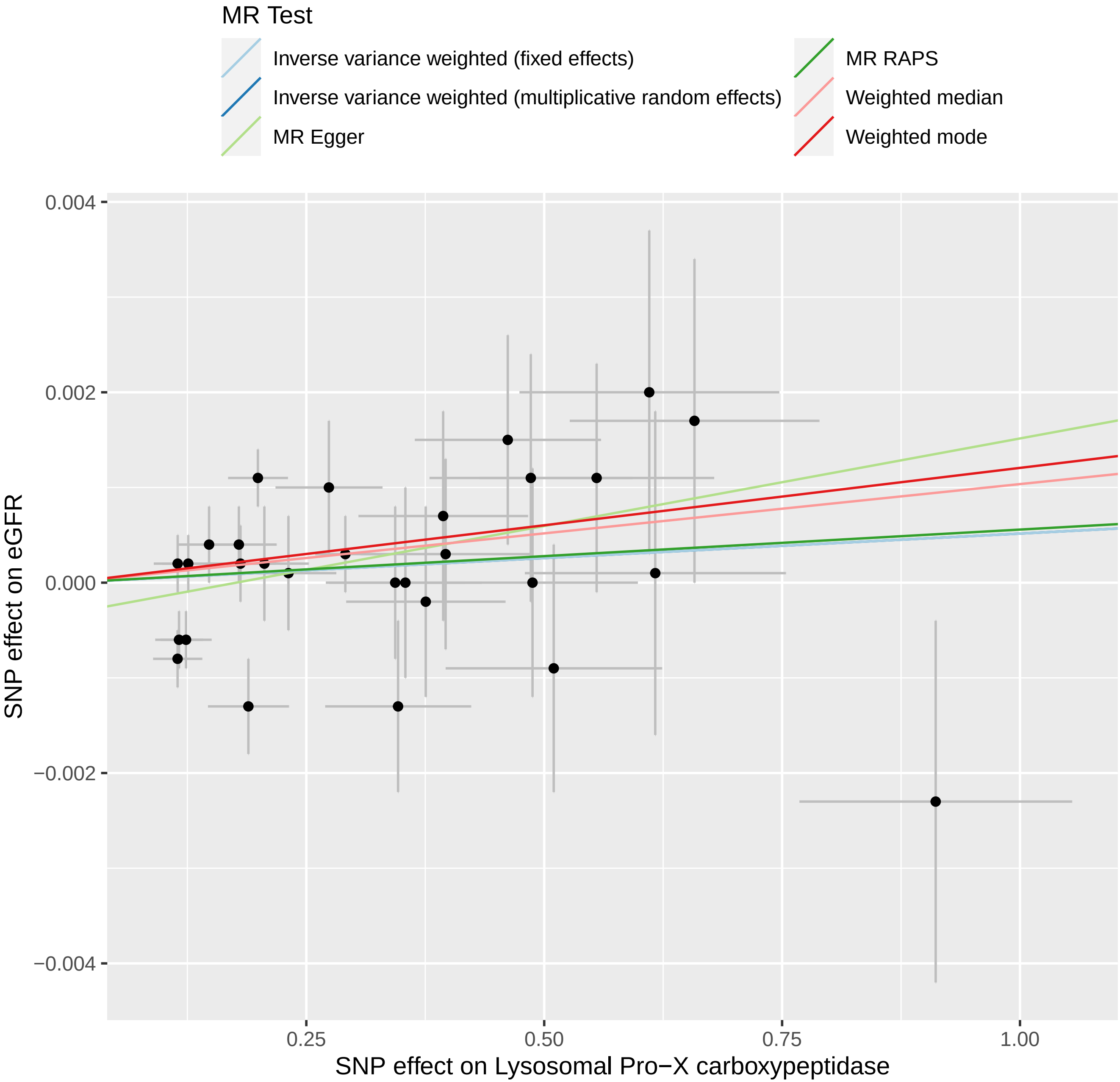

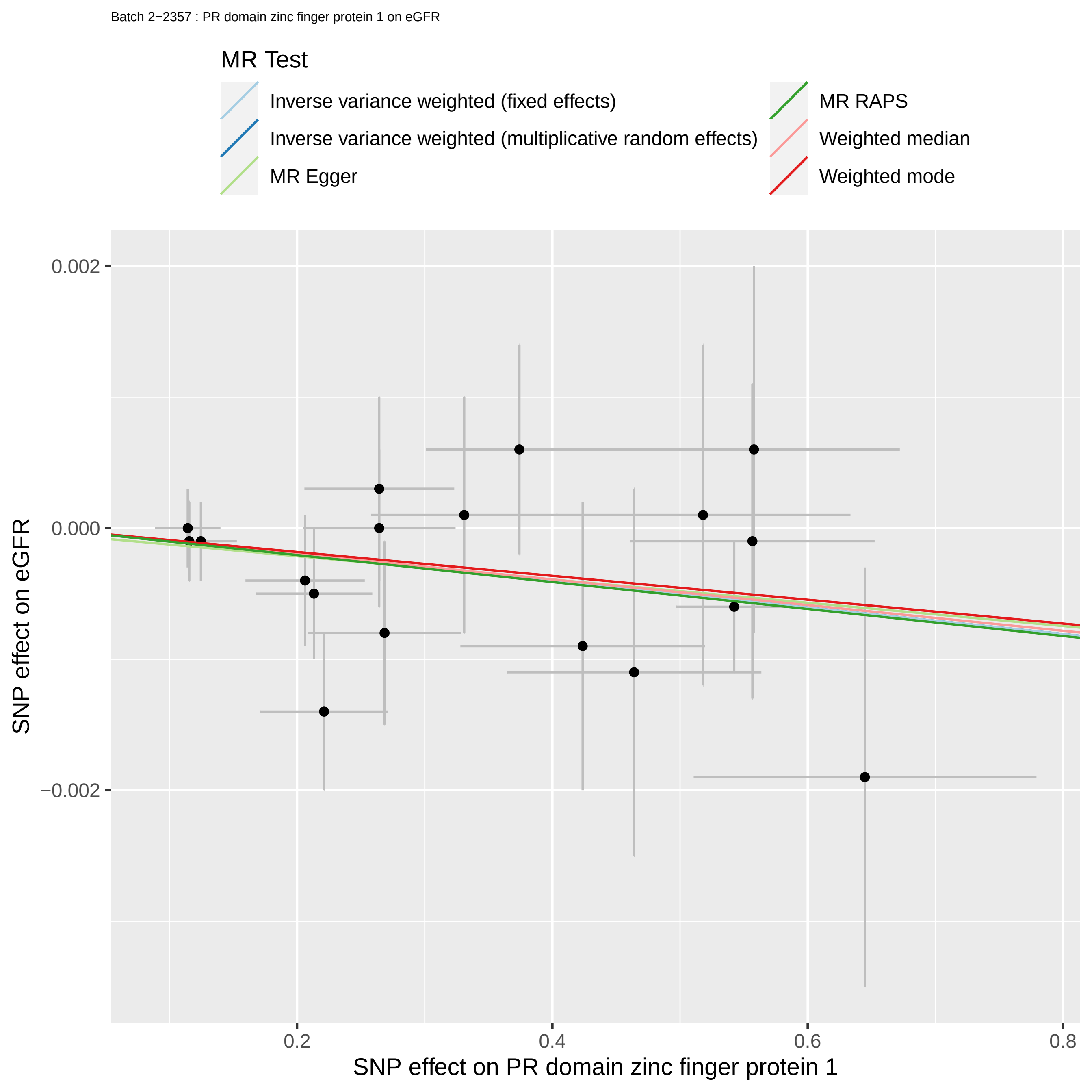

Batch 2−2357 : PR domain zinc finger protein 1 on eGFR
MR Test
Inverse variance weighted (fixed effects)
Inverse variance weighted (multiplicative random effects)
MR Egger
MR RAPS
Weighted median
Weighted mode
0.002
0.000
−0.002
SNP effect on eGFR
0.2
0.4
0.6
0.8
SNP effect on PR domain zinc finger protein 1

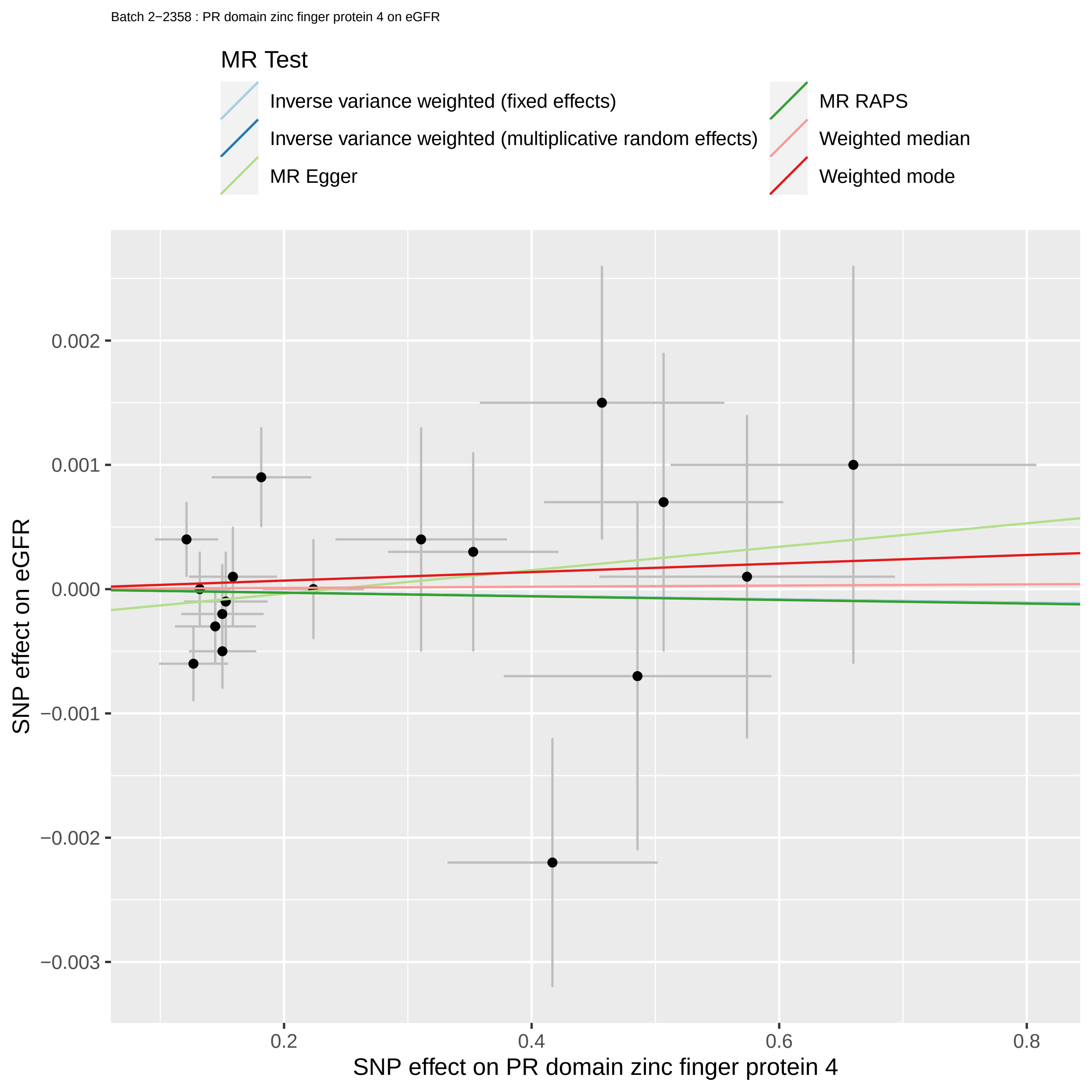

Batch 2−2358 : PR domain zinc finger protein 4 on eGFR
MR Test
Inverse variance weighted (fixed effects)
Inverse variance weighted (multiplicative random effects)
MR Egger
MR RAPS
Weighted median
Weighted mode
0.002
0.001
0.000
−0.001
−0.002
−0.003
SNP effect on eGFR
0.2
0.4
0.6
0.8
SNP effect on PR domain zinc finger protein 4

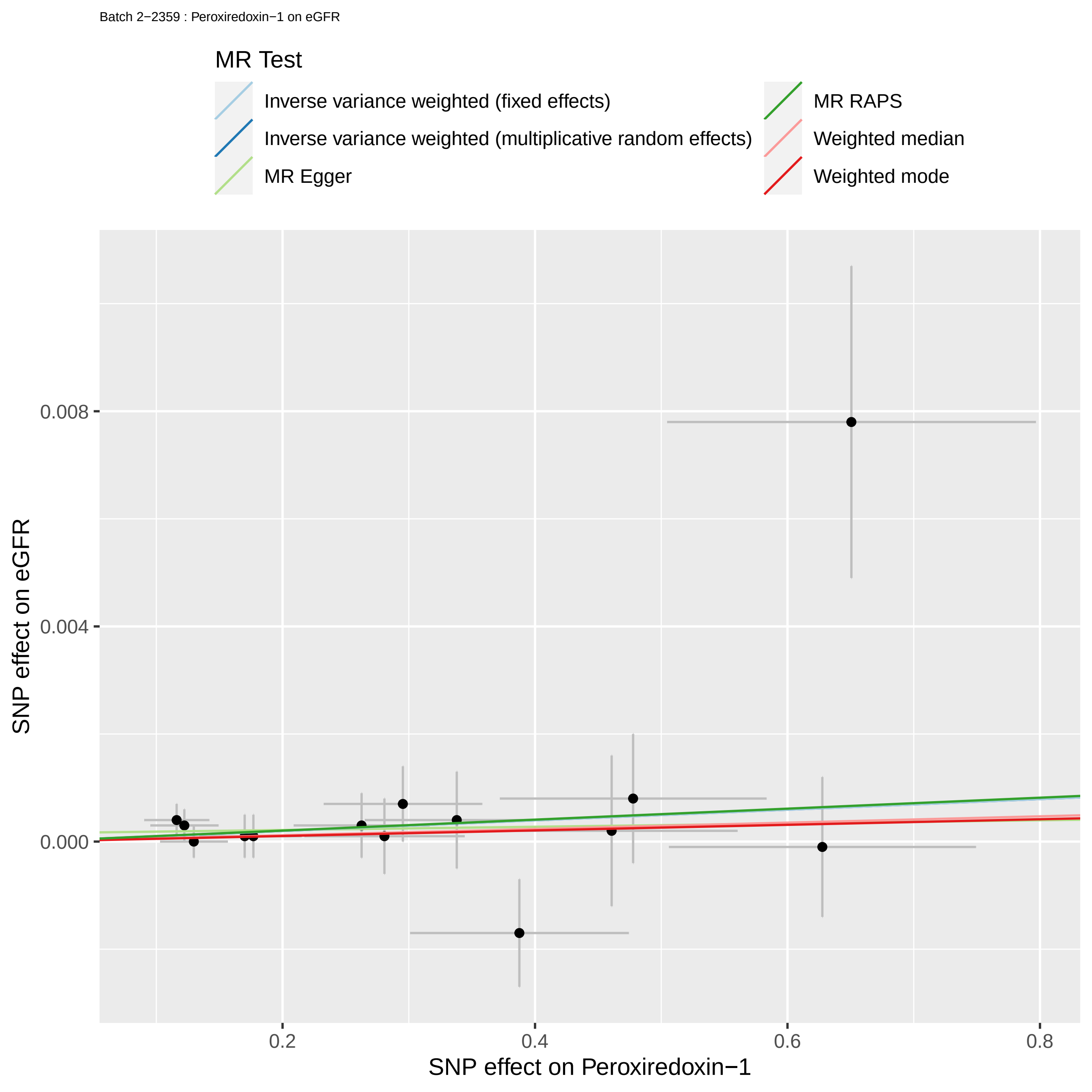
Batch 2−2359 : Peroxiredoxin−1 on eGFR
MR Test
Inverse variance weighted (fixed effects)
Inverse variance weighted (multiplicative random effects)
MR Egger
MR RAPS
Weighted median
Weighted mode
0.008
0.004
0.000
0.2
0.4
0.6
0.8
SNP effect on eGFR
SNP effect on Peroxiredoxin−1

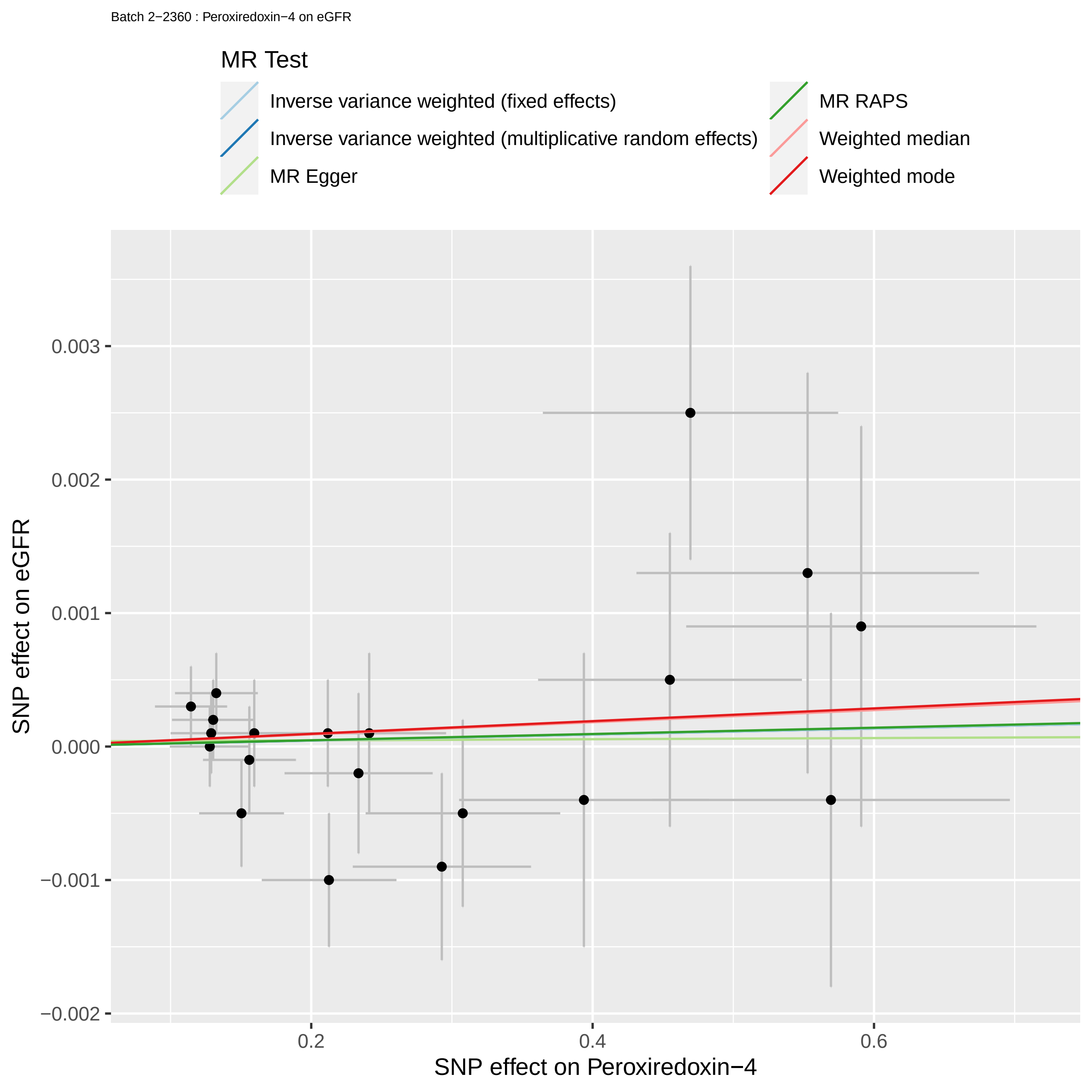
Batch 2−2360 : Peroxiredoxin−4 on eGFR
MR Test
Inverse variance weighted (fixed effects)
Inverse variance weighted (multiplicative random effects)
MR Egger
MR RAPS
Weighted median
Weighted mode
0.003
0.002
0.001
0.000
−0.001
−0.002
0.2
0.4
0.6
SNP effect on eGFR
SNP effect on Peroxiredoxin−4

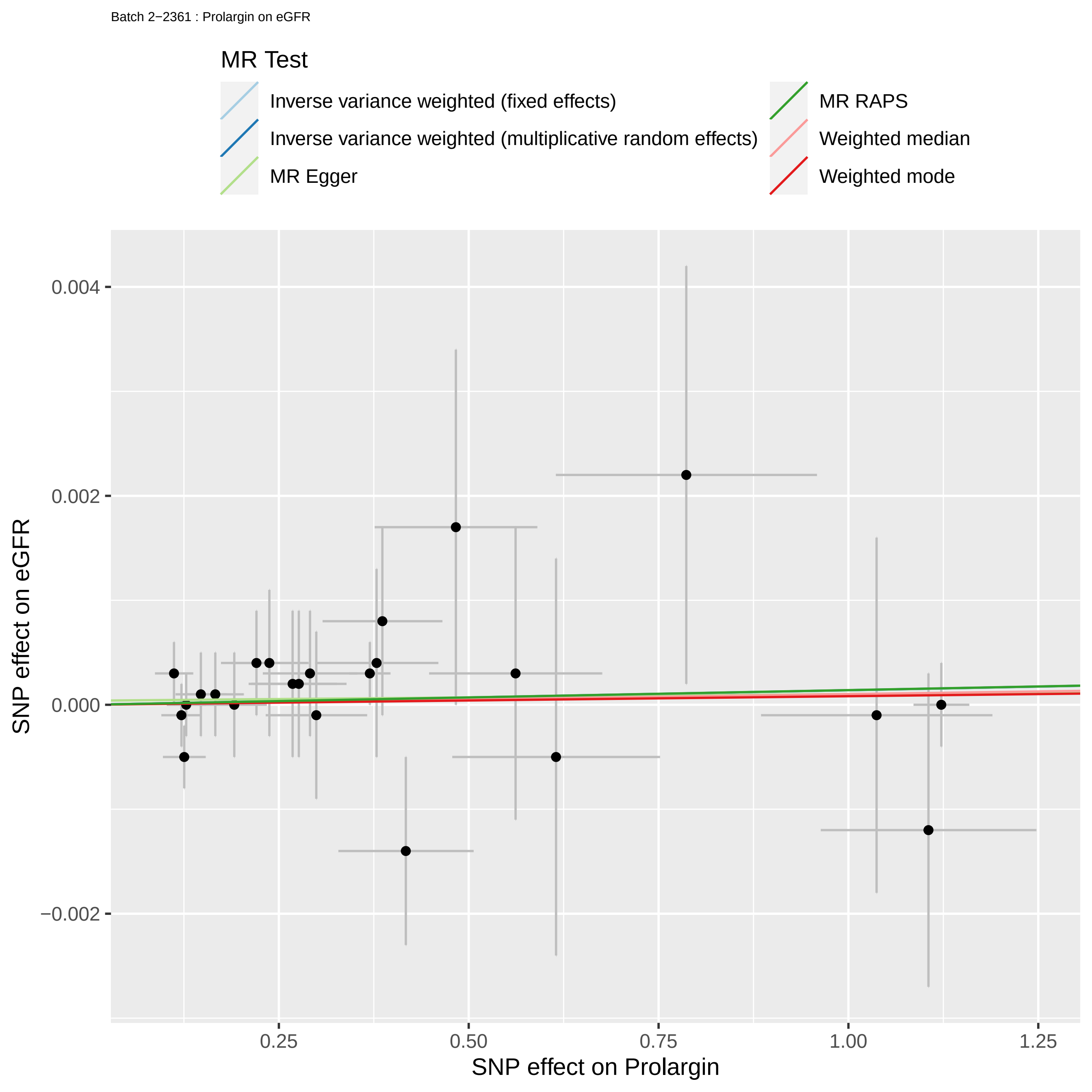

Batch 2−2361 : Prolargin on eGFR
MR Test
Inverse variance weighted (fixed effects)
Inverse variance weighted (multiplicative random effects)
MR Egger
MR RAPS
Weighted median
Weighted mode
0.004
0.002
0.000
−0.002
SNP effect on eGFR
0.25
0.50
0.75
1.00
1.25
SNP effect on Prolargin

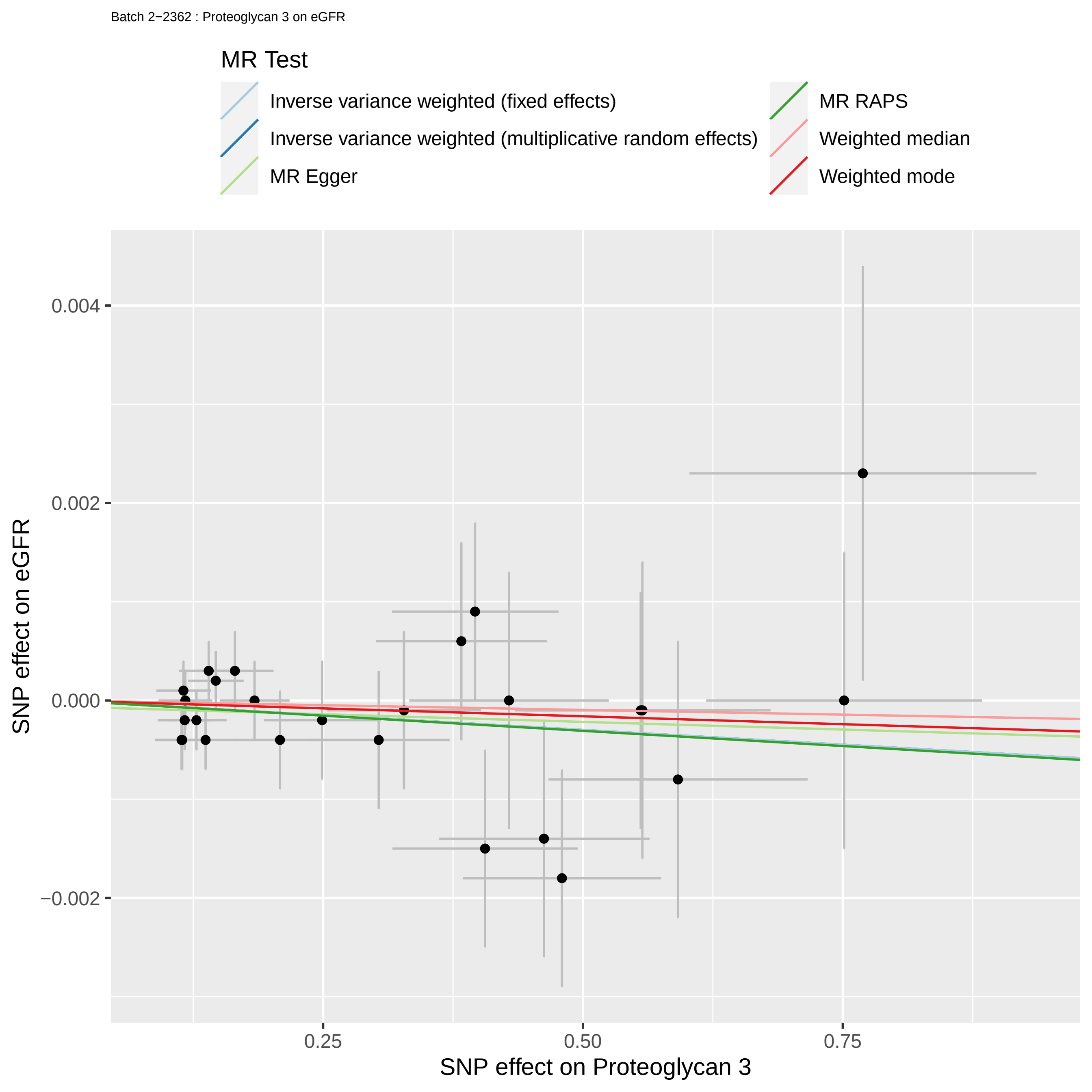
Batch 2−2362 : Proteoglycan 3 on eGFR
MR Test
Inverse variance weighted (fixed effects)
Inverse variance weighted (multiplicative random effects)
MR Egger
MR RAPS
Weighted median
Weighted mode
0.004
0.002
0.000
−0.002
0.25
0.50
0.75
SNP effect on eGFR
SNP effect on Proteoglycan 3

Batch 2−2363 : Salivary acidic proline−rich phosphoprotein 1/2 on eGFR

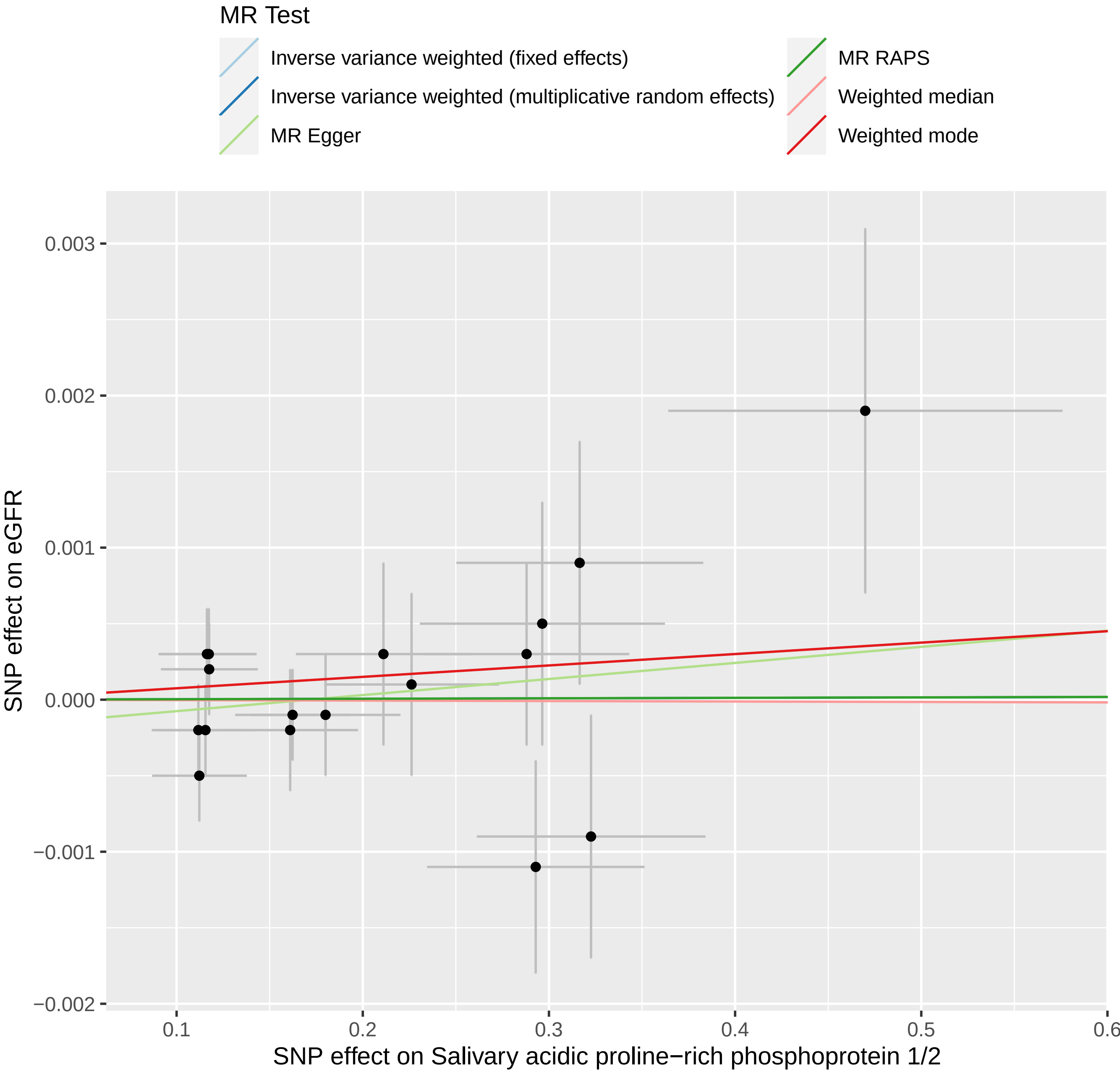

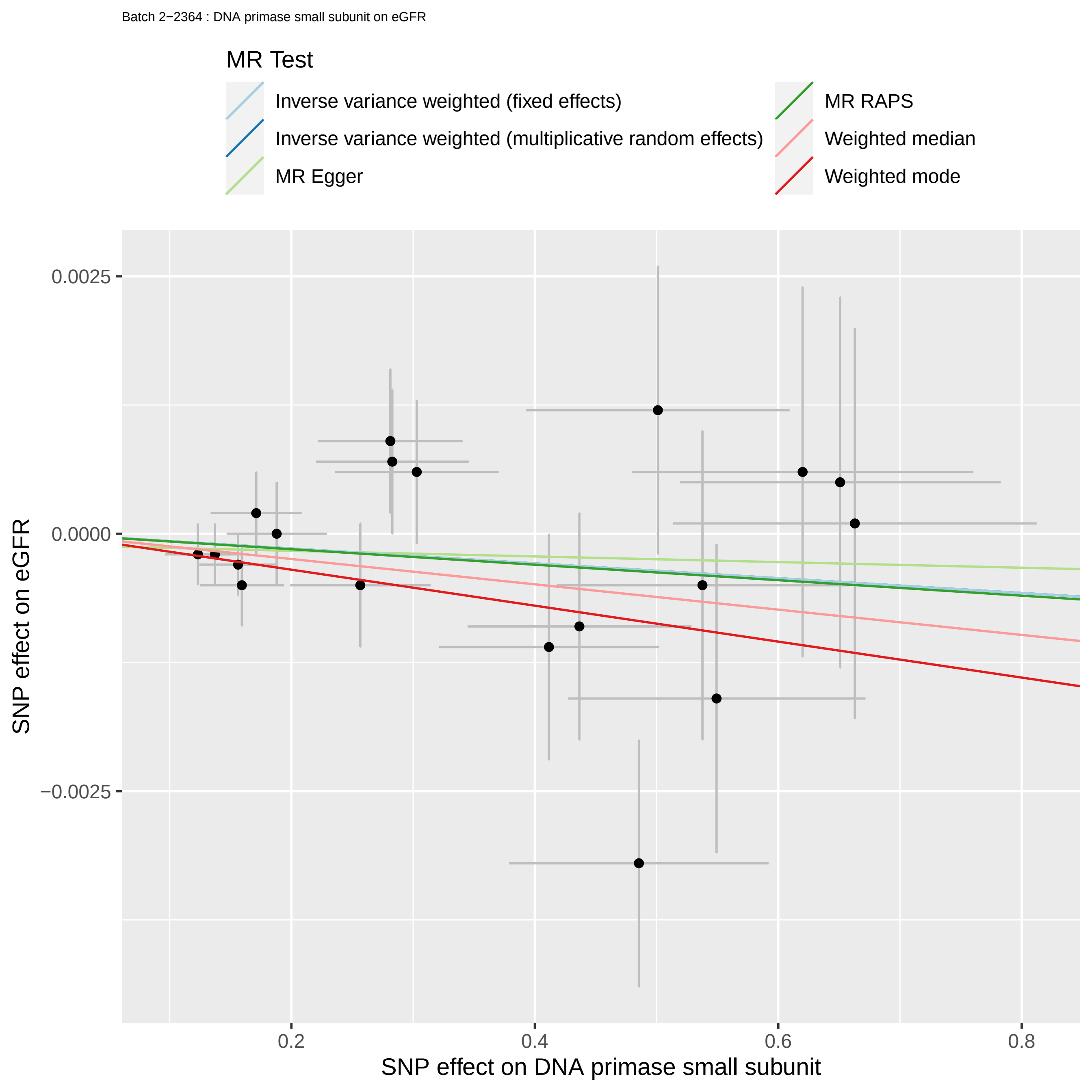

Batch 2−2364 : DNA primase small subunit on eGFR
MR Test
Inverse variance weighted (fixed effects)
Inverse variance weighted (multiplicative random effects)
MR Egger
MR RAPS
Weighted median
Weighted mode
0.0025
0.0000
−0.0025
0.2
0.4
0.6
0.8
SNP effect on eGFR
SNP effect on DNA primase small subunit

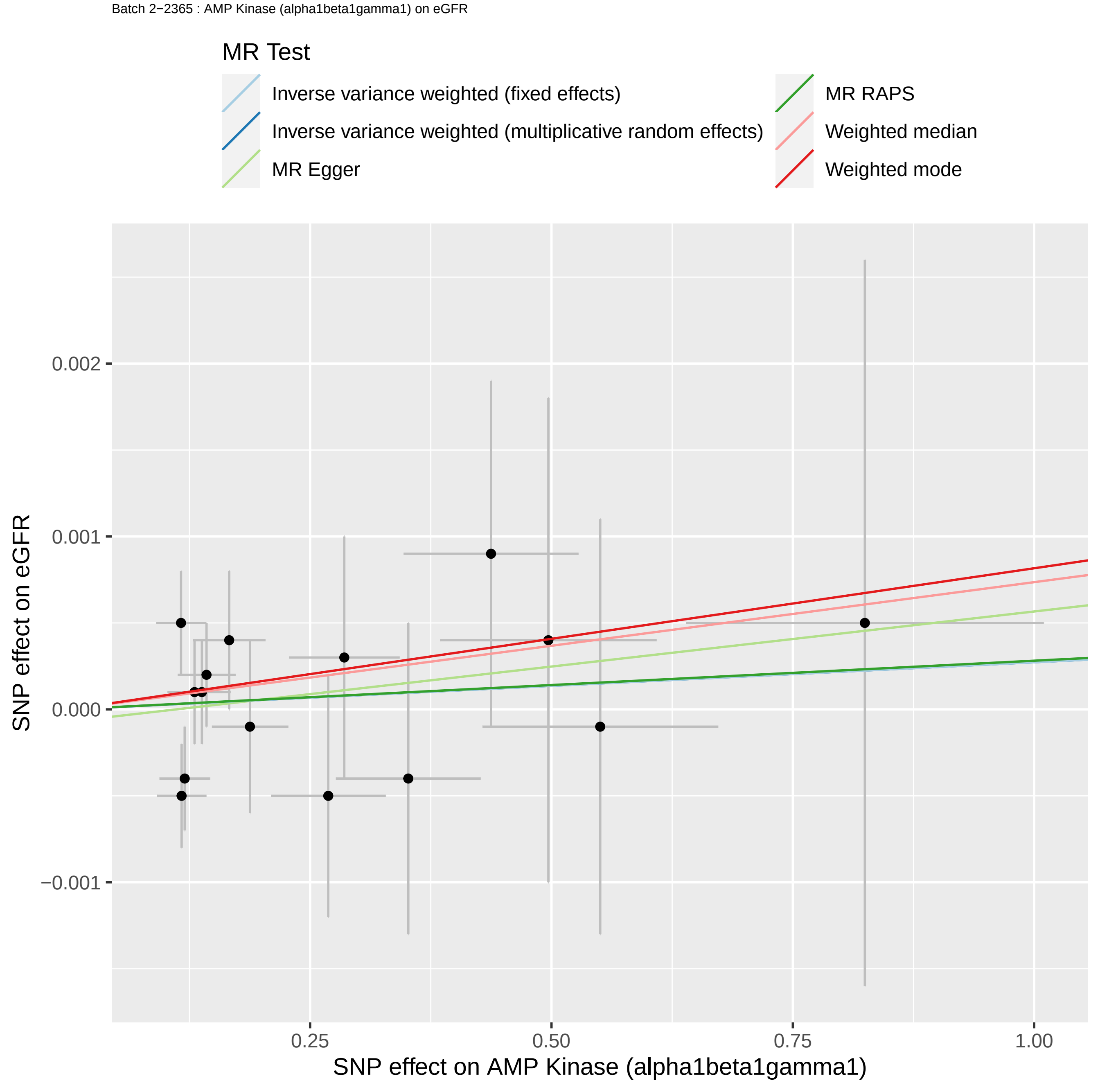

Batch 2−2365 : AMP Kinase (alpha1beta1gamma1) on eGFR
MR Test
Inverse variance weighted (fixed effects)
Inverse variance weighted (multiplicative random effects)
MR Egger
MR RAPS
Weighted median
Weighted mode
SNP effect on eGFR
0.002
0.001
0.000
−0.001
0.25
0.50
0.75
1.00
SNP effect on AMP Kinase (alpha1beta1gamma1)

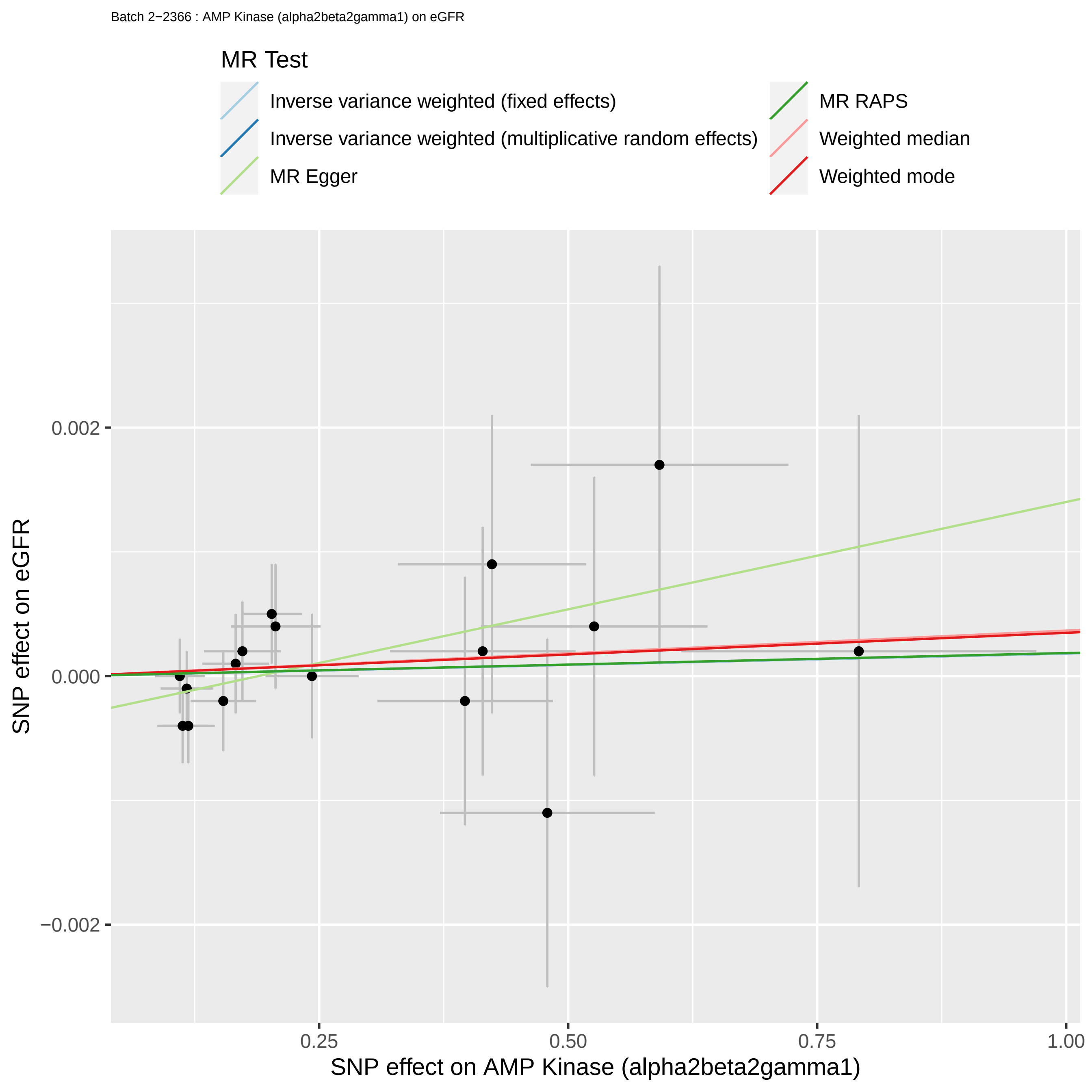

Batch 2−2366 : AMP Kinase (alpha2beta2gamma1) on eGFR
MR Test
Inverse variance weighted (fixed effects)
Inverse variance weighted (multiplicative random effects)
MR Egger
MR RAPS
Weighted median
Weighted mode
SNP effect on eGFR
0.002
0.000
−0.002
0.25
0.50
0.75
1.00
SNP effect on AMP Kinase (alpha2beta2gamma1)

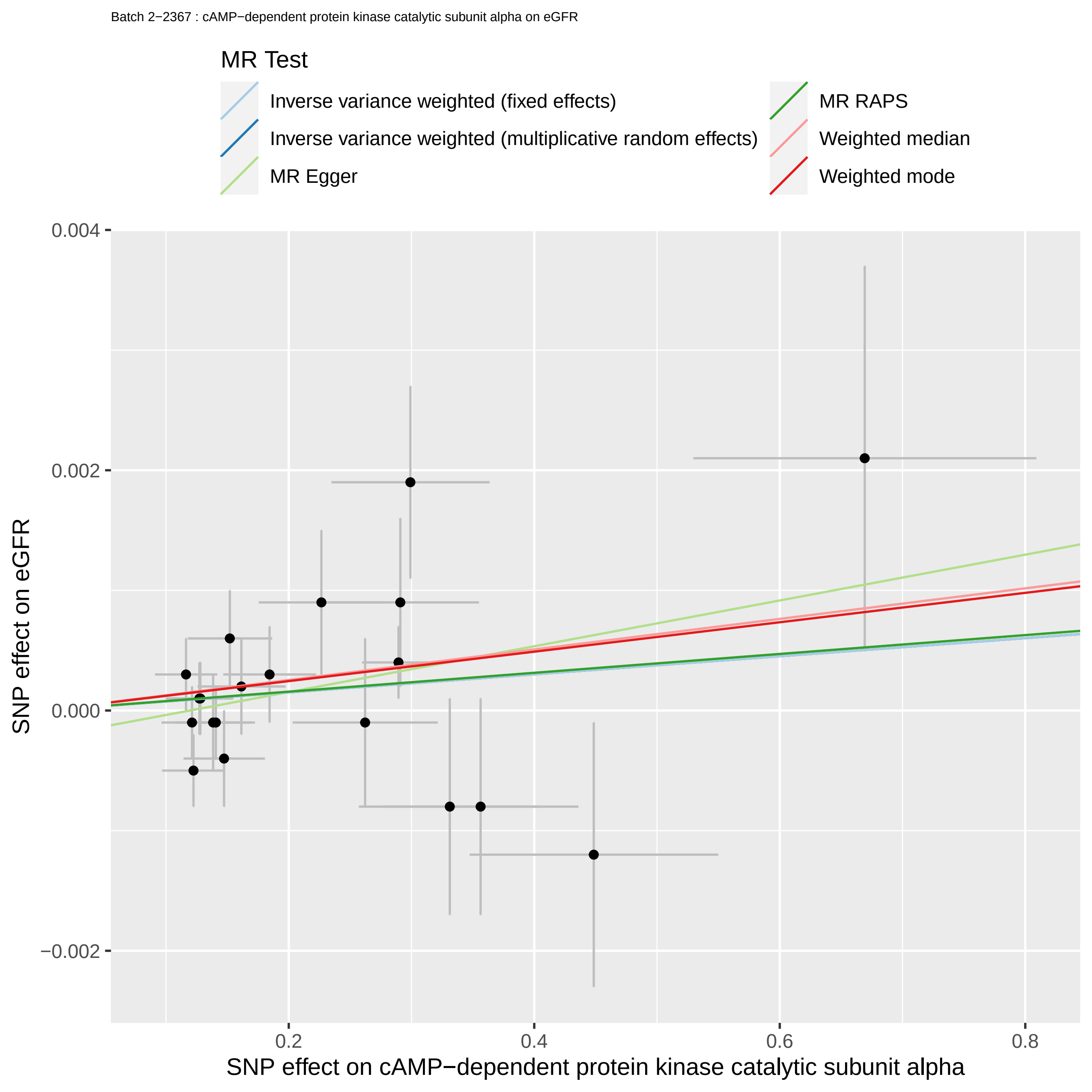

Batch 2−2367 : cAMP−dependent protein kinase catalytic subunit alpha on eGFR
MR Test
Inverse variance weighted (fixed effects)
Inverse variance weighted (multiplicative random effects)
MR Egger
MR RAPS
Weighted median
Weighted mode
0.004
0.002
0.000
−0.002
0.2
0.4
0.6
0.8
SNP effect on eGFR
SNP effect on cAMP−dependent protein kinase catalytic subunit alpha

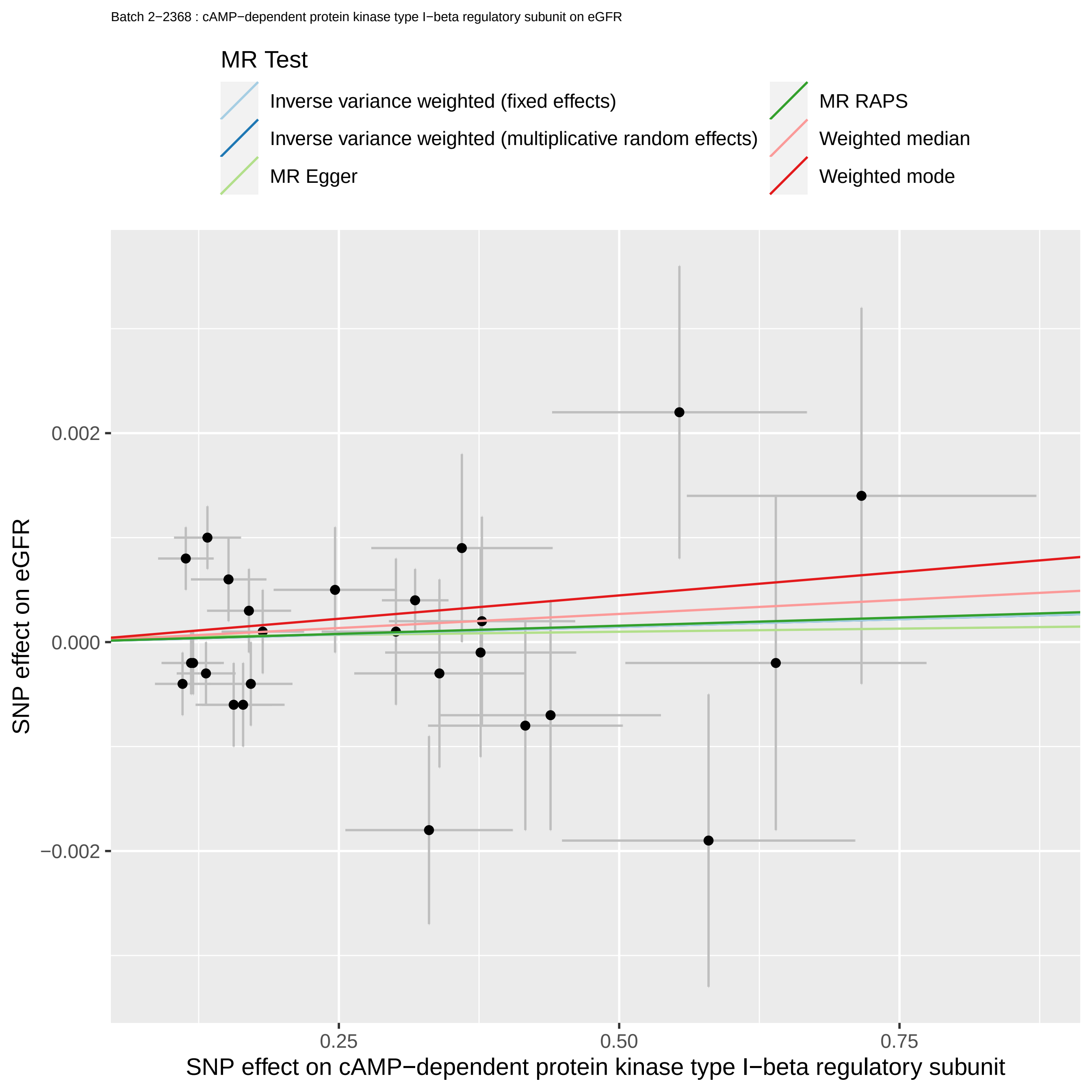

Batch 2−2368 : cAMP−dependent protein kinase type I−beta regulatory subunit on eGFR
MR Test
Inverse variance weighted (fixed effects)
Inverse variance weighted (multiplicative random effects)
MR Egger
MR RAPS
Weighted median
Weighted mode
0.002
0.000
−0.002
0.25
0.50
0.75
SNP effect on eGFR
SNP effect on cAMP−dependent protein kinase type I−beta regulatory subunit

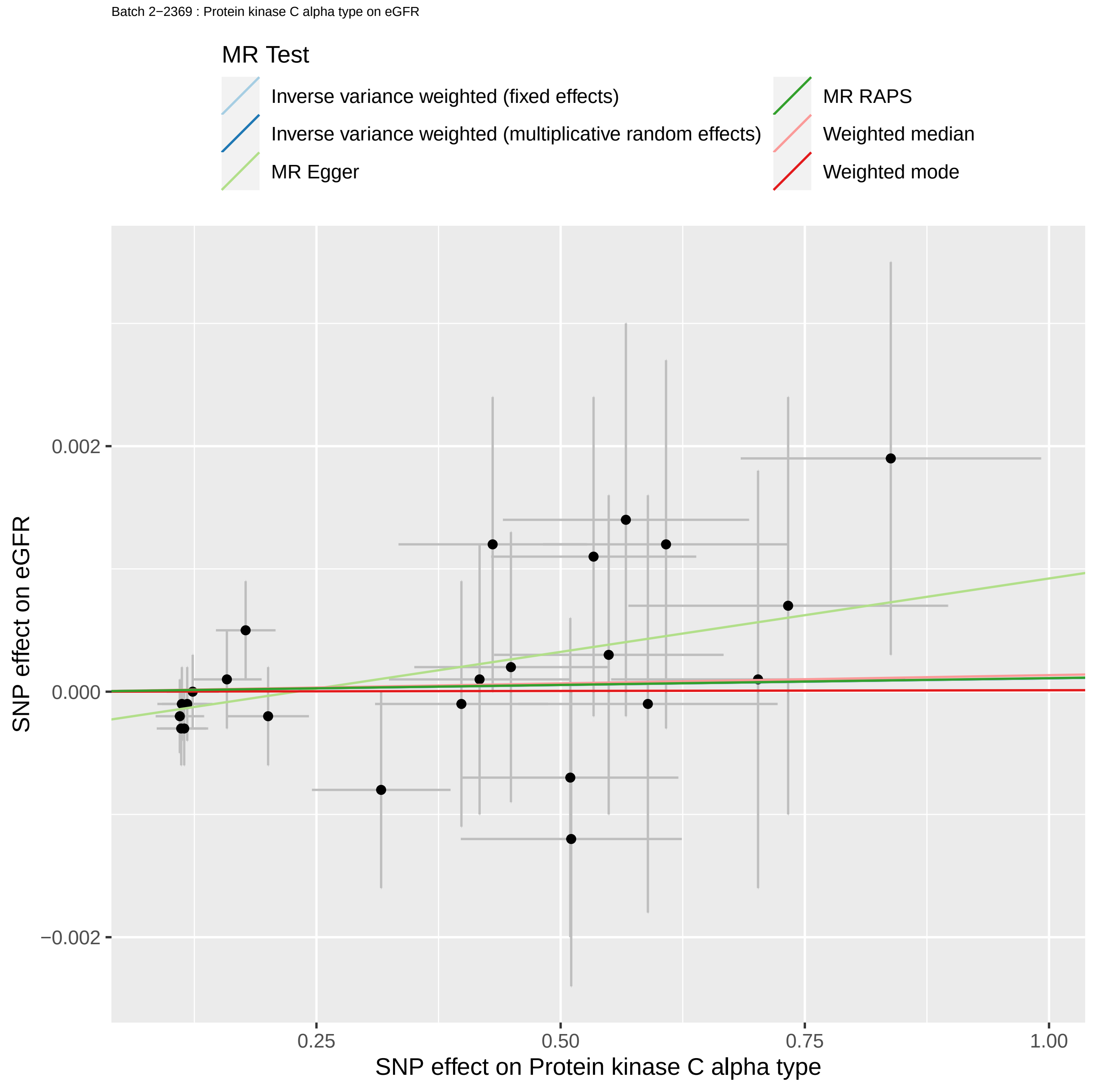
Batch 2−2369 : Protein kinase C alpha type on eGFR
MR Test
Inverse variance weighted (fixed effects)
Inverse variance weighted (multiplicative random effects)
MR Egger
MR RAPS
Weighted median
Weighted mode
0.002
0.000
−0.002
0.25
0.50
0.75
1.00
SNP effect on eGFR
SNP effect on Protein kinase C alpha type

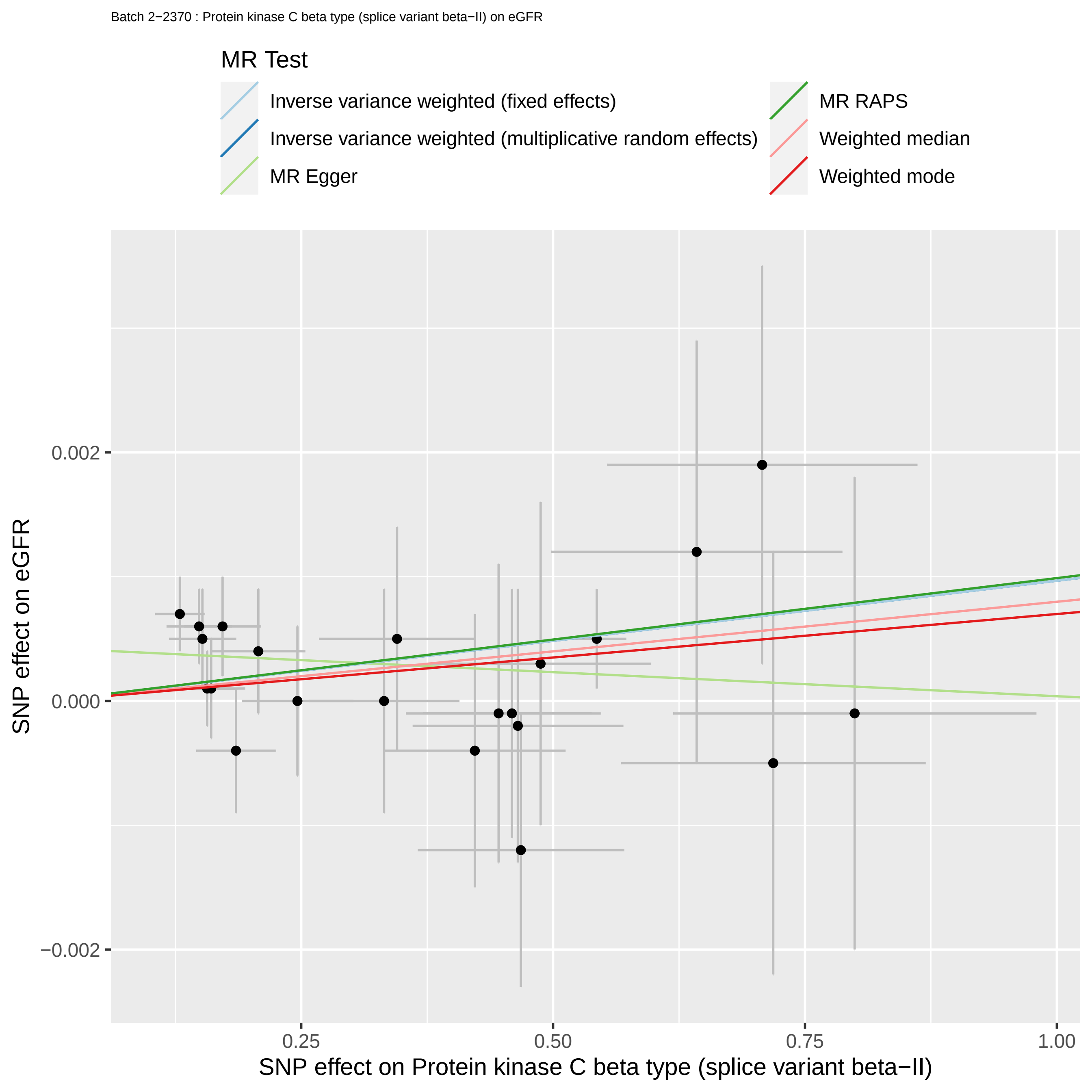

Batch 2−2370 : Protein kinase C beta type (splice variant beta−II) on eGFR
MR Test
Inverse variance weighted (fixed effects)
Inverse variance weighted (multiplicative random effects)
MR Egger
MR RAPS
Weighted median
Weighted mode
SNP effect on eGFR
0.002
0.000
−0.002
0.25
0.50
0.75
1.00
SNP effect on Protein kinase C beta type (splice variant beta−II)

Batch 2−2371 : Protein kinase C iota type on eGFR

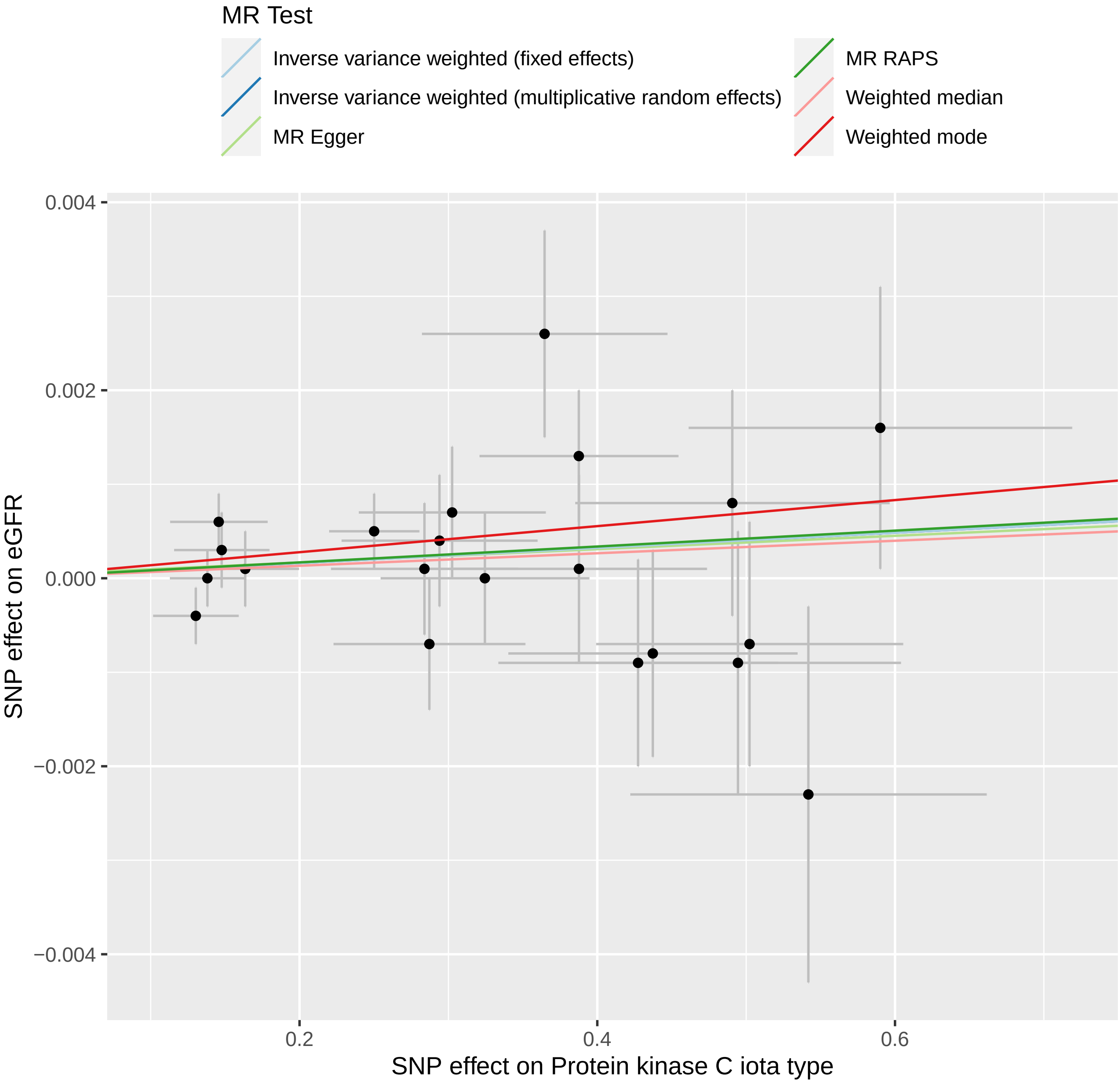

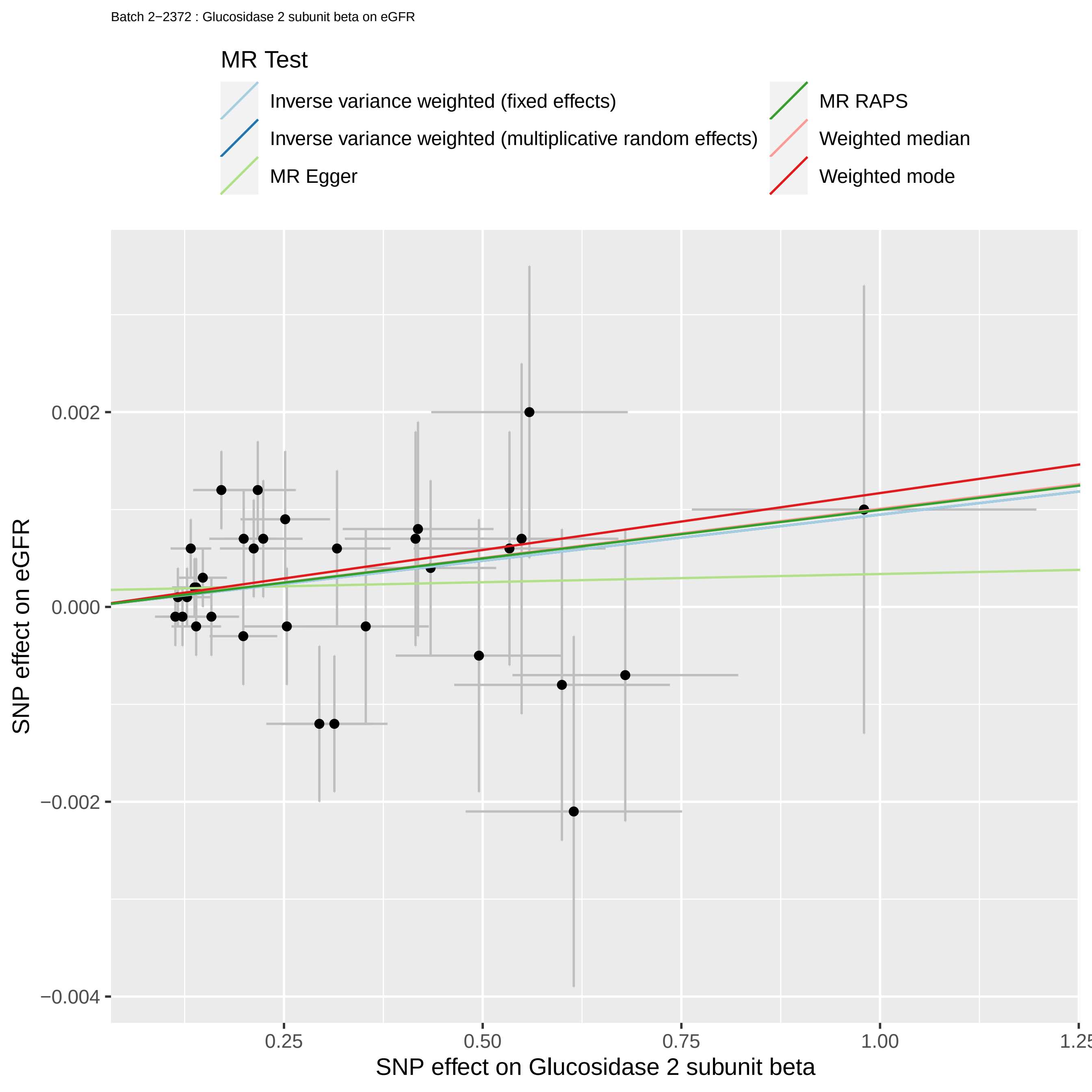

Batch 2−2372 : Glucosidase 2 subunit beta on eGFR
MR Test
Inverse variance weighted (fixed effects)
Inverse variance weighted (multiplicative random effects)
MR Egger
MR RAPS
Weighted median
Weighted mode
0.002
0.000
−0.002
−0.004
SNP effect on eGFR
0.25
0.50
0.75
1.00
1.25
SNP effect on Glucosidase 2 subunit beta

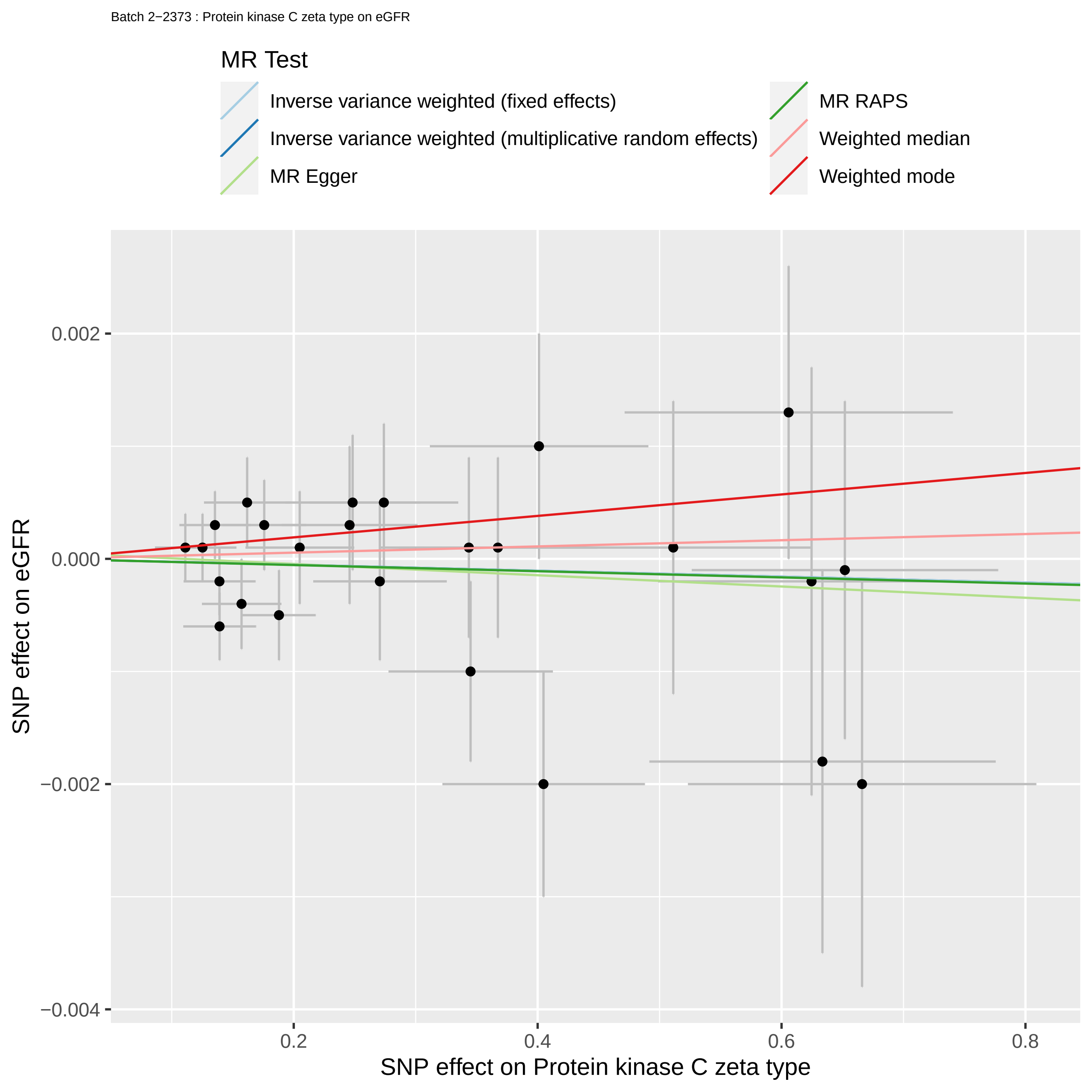

Batch 2−2373 : Protein kinase C zeta type on eGFR
MR Test
Inverse variance weighted (fixed effects)
Inverse variance weighted (multiplicative random effects)
MR Egger
MR RAPS
Weighted median
Weighted mode
0.002
0.000
−0.002
−0.004
SNP effect on eGFR
0.2
0.4
0.6
0.8
SNP effect on Protein kinase C zeta type

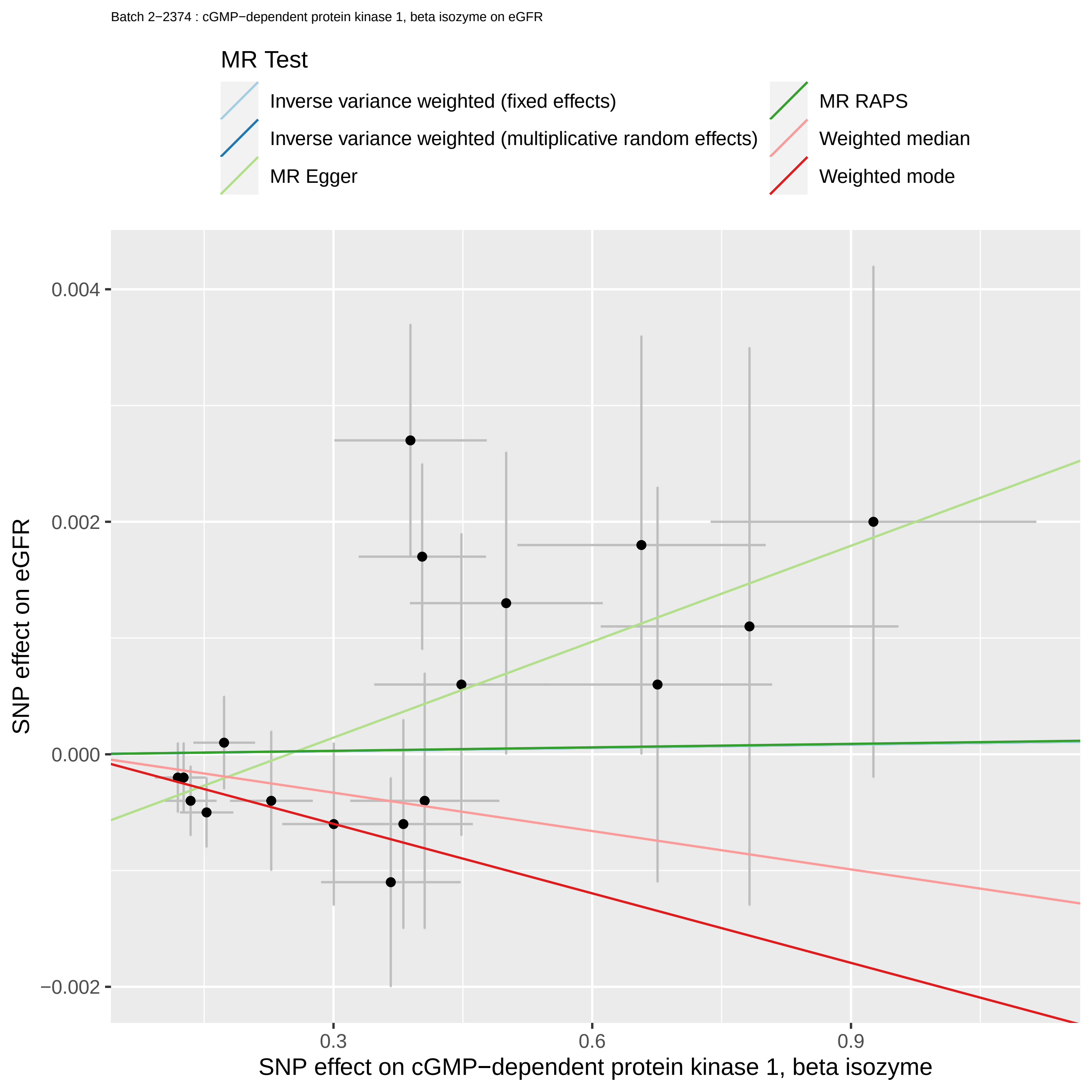
Batch 2−2374 : cGMP−dependent protein kinase 1, beta isozyme on eGFR
MR Test
Inverse variance weighted (fixed effects)
Inverse variance weighted (multiplicative random effects)
MR Egger
MR RAPS
Weighted median
Weighted mode
0.004
0.002
0.000
−0.002
0.3
0.6
0.9
SNP effect on eGFR
SNP effect on cGMP−dependent protein kinase 1, beta isozyme

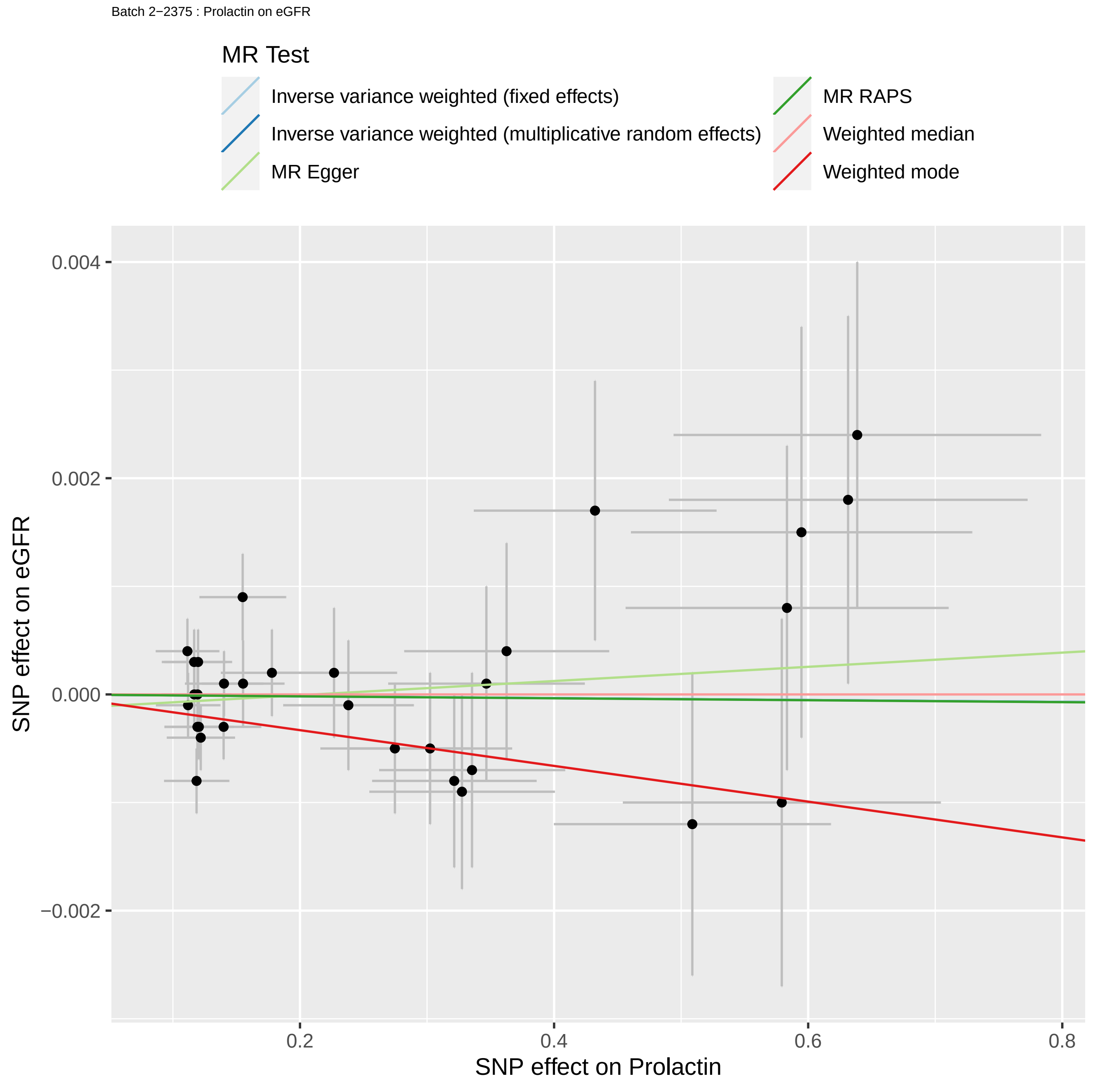

Batch 2−2375 : Prolactin on eGFR
MR Test
Inverse variance weighted (fixed effects)
Inverse variance weighted (multiplicative random effects)
MR Egger
MR RAPS
Weighted median
Weighted mode
0.004
0.002
0.000
−0.002
SNP effect on eGFR
0.2
0.4
0.6
0.8
SNP effect on Prolactin

Batch 2−2376 : Prolactin−releasing peptide on eGFR

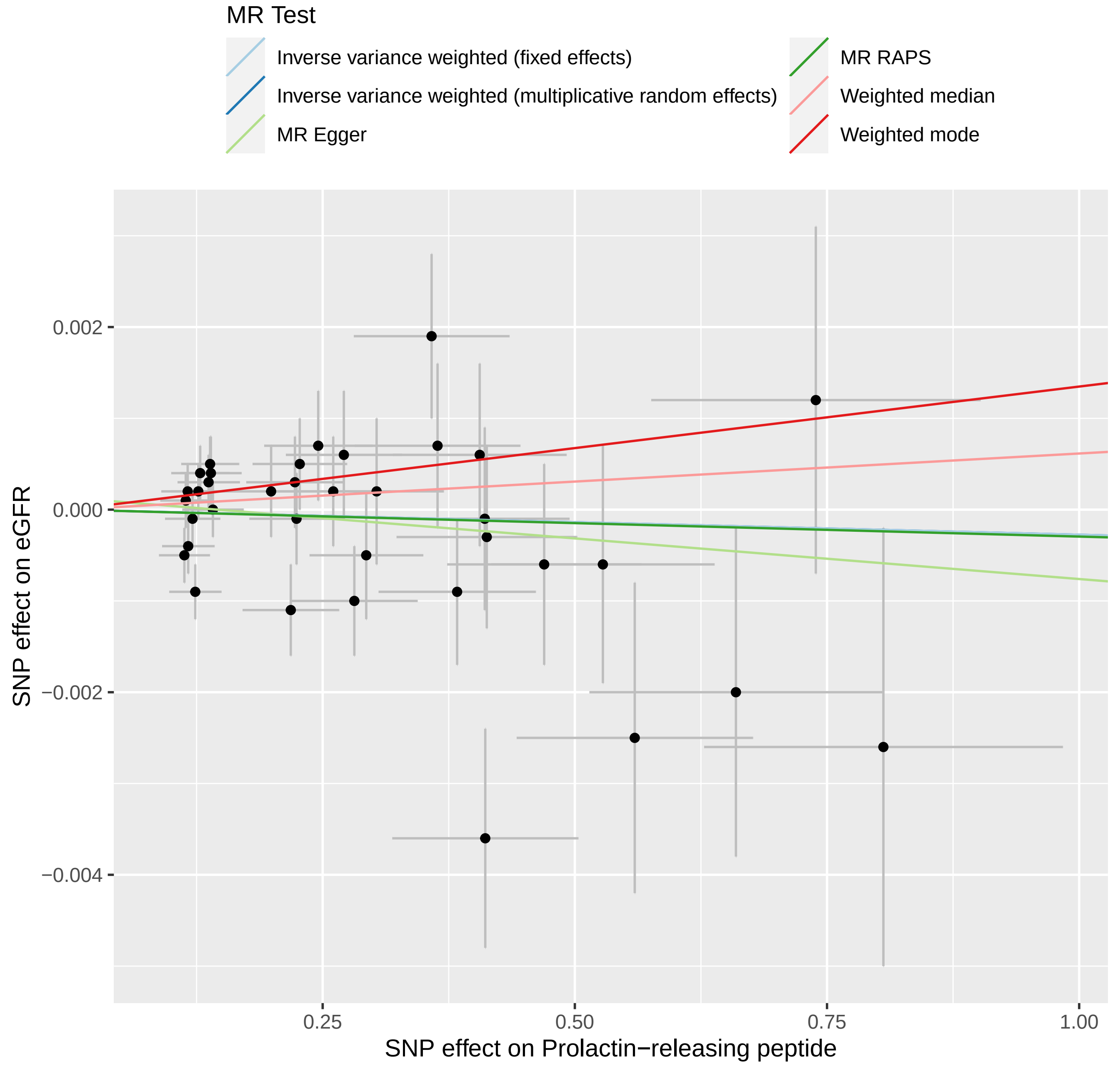

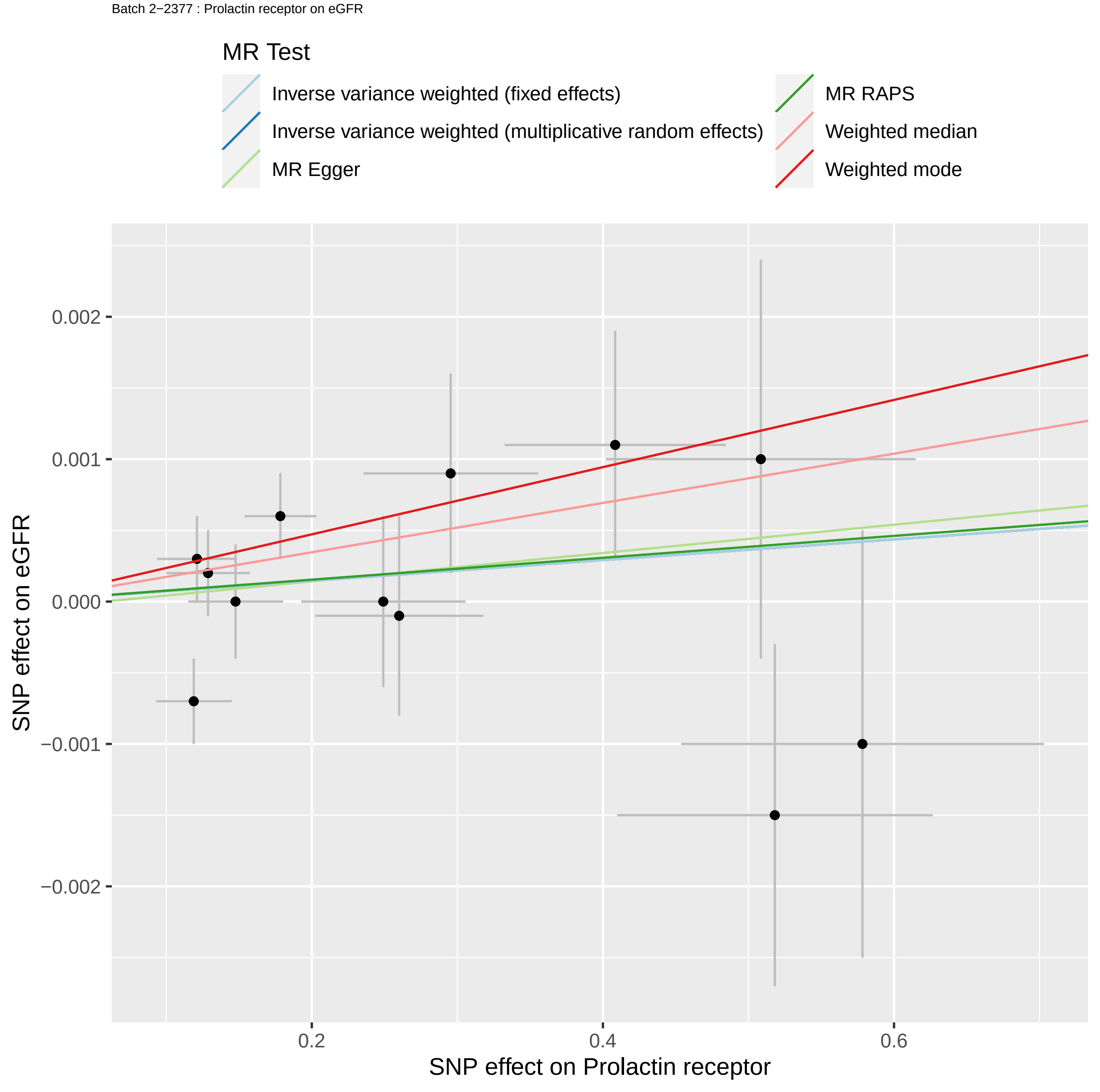
Batch 2−2377 : Prolactin receptor on eGFR
MR Test
Inverse variance weighted (fixed effects)
Inverse variance weighted (multiplicative random effects)
MR Egger
MR RAPS
Weighted median
Weighted mode
0.002
0.001
0.000
−0.001
−0.002
SNP effect on eGFR
0.2
0.4
0.6
SNP effect on Prolactin receptor

Batch 2−2378 : Protein arginine N−methyltransferase 3 on eGFR

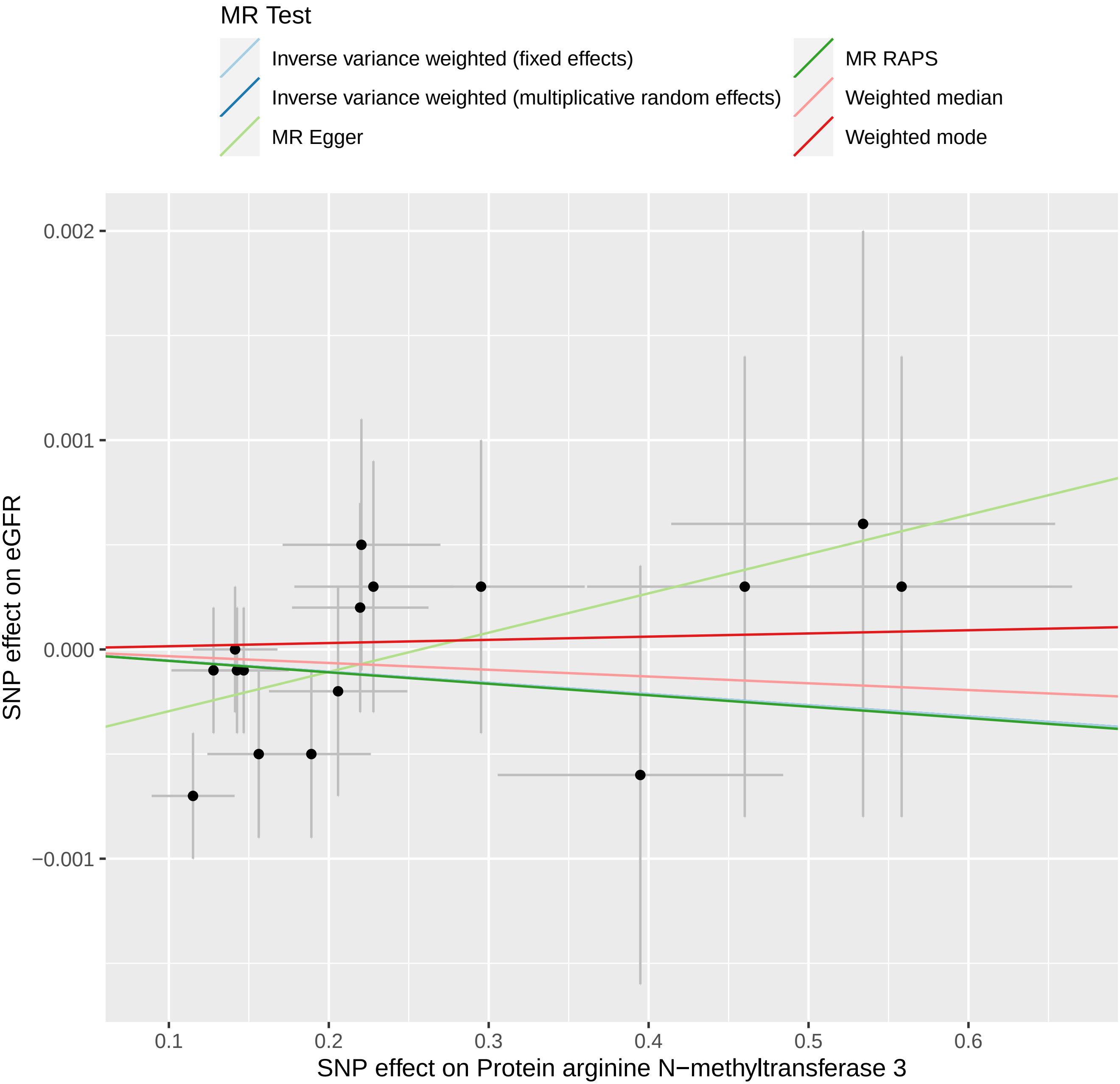

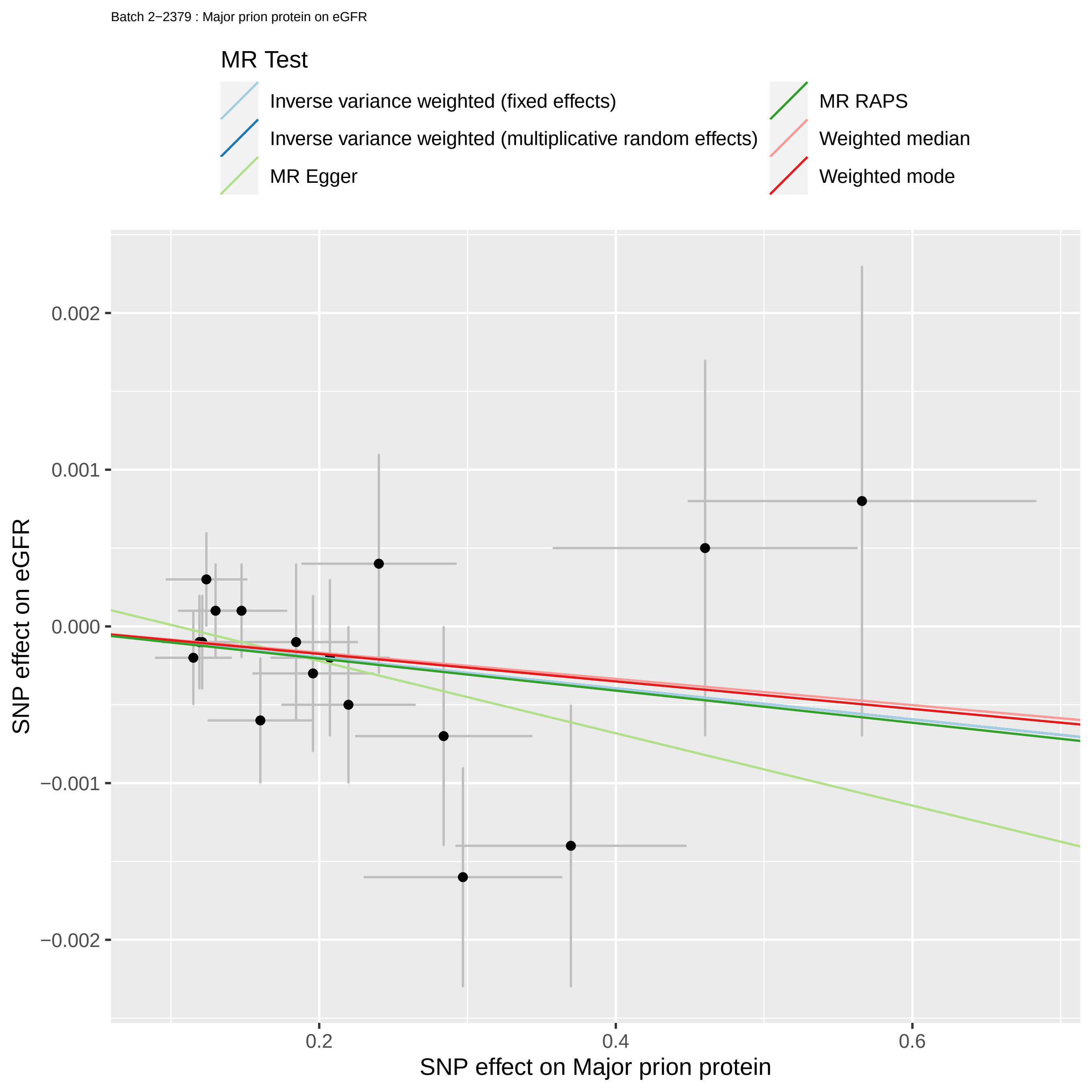

Batch 2−2379 : Major prion protein on eGFR
MR Test
Inverse variance weighted (fixed effects)
Inverse variance weighted (multiplicative random effects)
MR Egger
MR RAPS
Weighted median
Weighted mode
0.002
0.001
0.000
−0.001
−0.002
0.2
0.4
0.6
SNP effect on eGFR
SNP effect on Major prion protein

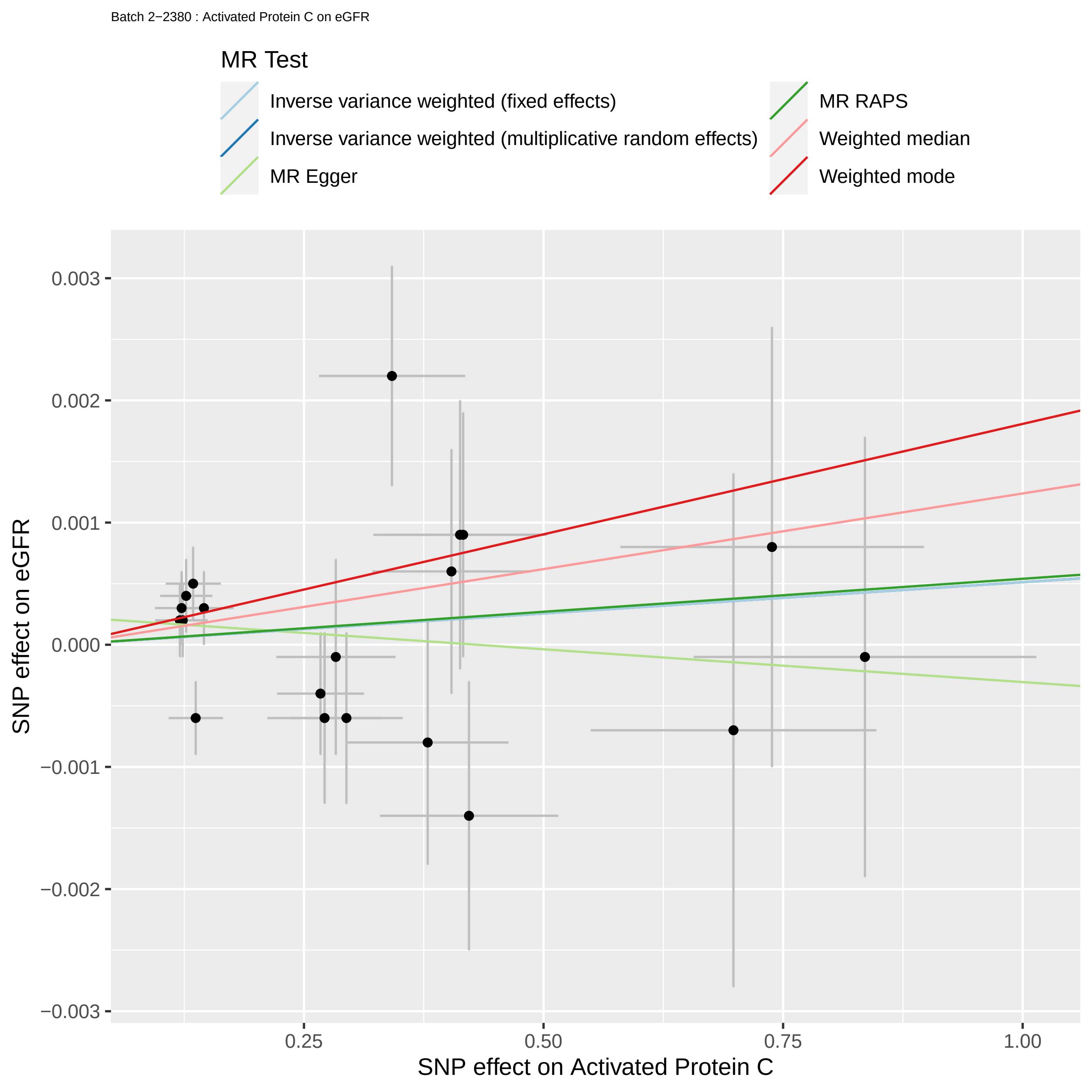
Batch 2−2380 : Activated Protein C on eGFR
MR Test
Inverse variance weighted (fixed effects)
Inverse variance weighted (multiplicative random effects)
MR Egger
MR RAPS
Weighted median
Weighted mode
SNP effect on eGFR
0.003
0.002
0.001
0.000
−0.001
−0.002
−0.003
0.25
0.50
0.75
1.00
SNP effect on Activated Protein C

Batch 2−2381 : Activated Protein C on eGFR

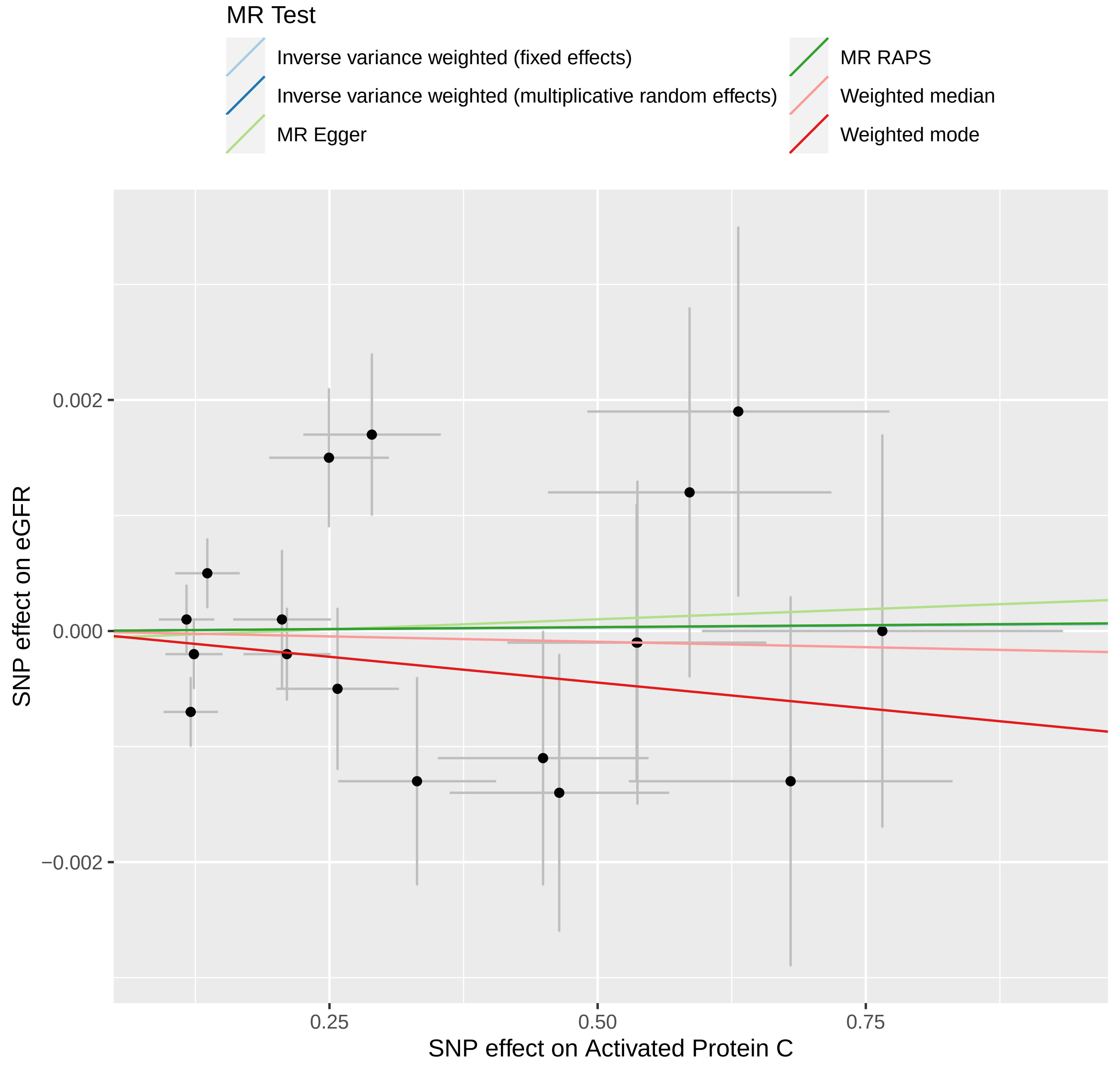

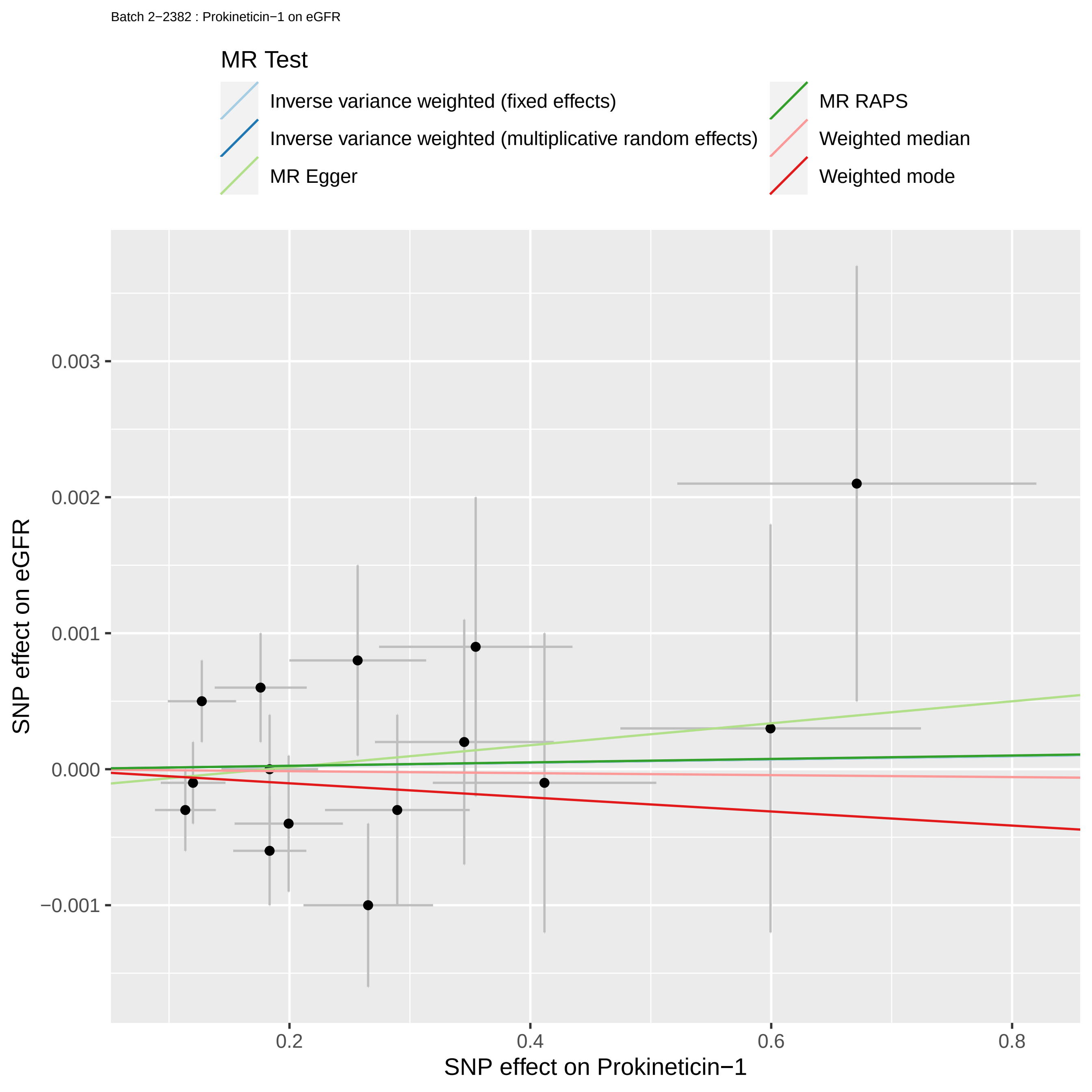

Batch 2−2382 : Prokineticin−1 on eGFR
MR Test
Inverse variance weighted (fixed effects)
Inverse variance weighted (multiplicative random effects)
MR Egger
MR RAPS
Weighted median
Weighted mode
0.003
0.002
0.001
0.000
−0.001
0.2
0.4
0.6
0.8
SNP effect on eGFR
SNP effect on Prokineticin−1

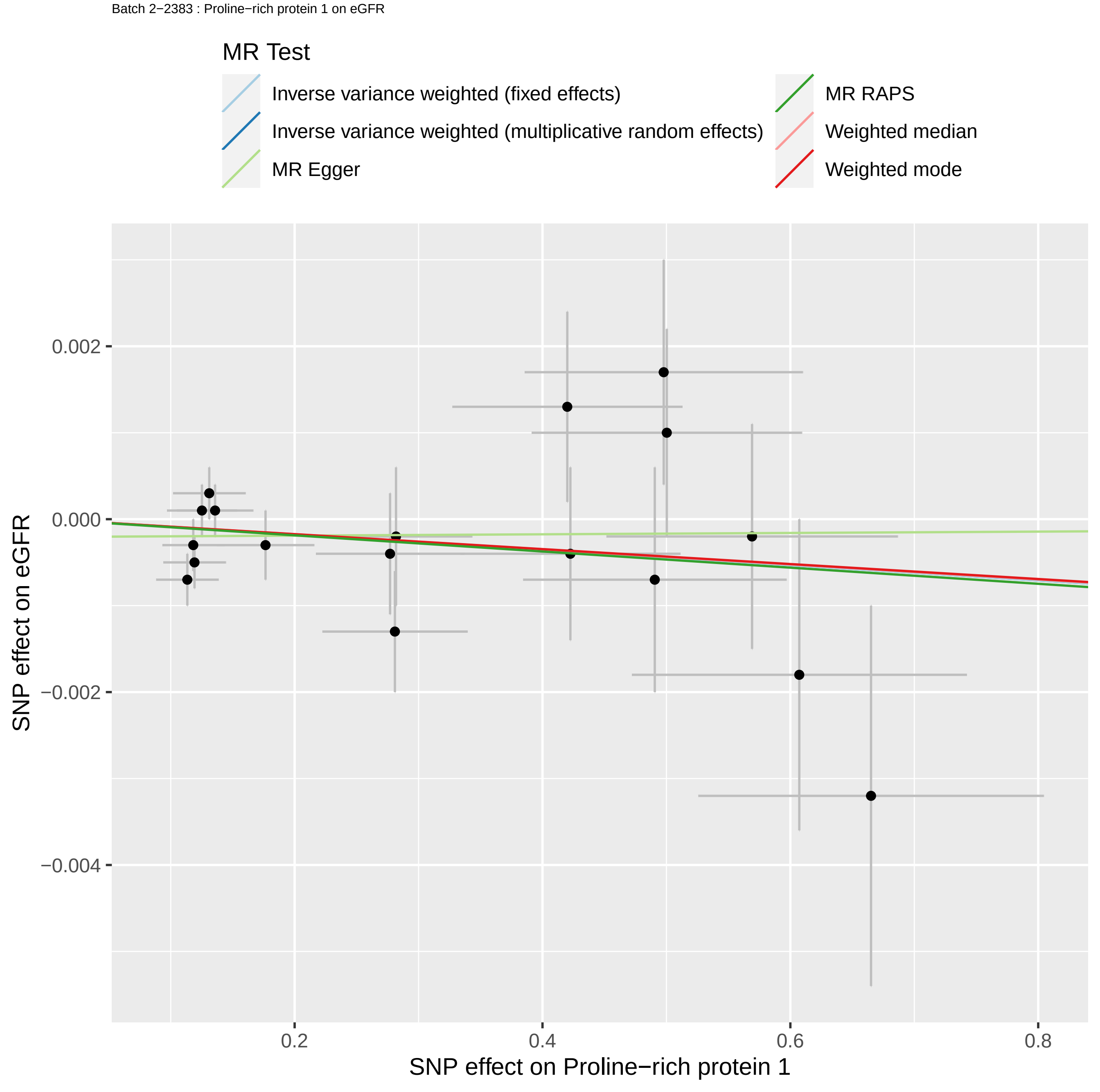

Batch 2−2383 : Proline−rich protein 1 on eGFR
MR Test
Inverse variance weighted (fixed effects)
Inverse variance weighted (multiplicative random effects)
MR Egger
MR RAPS
Weighted median
Weighted mode
0.002
0.000
−0.002
−0.004
SNP effect on eGFR
0.2
0.4
0.6
0.8
SNP effect on Proline−rich protein 1

Batch 2−2384 : Pre−mRNA−processing factor 6 on eGFR

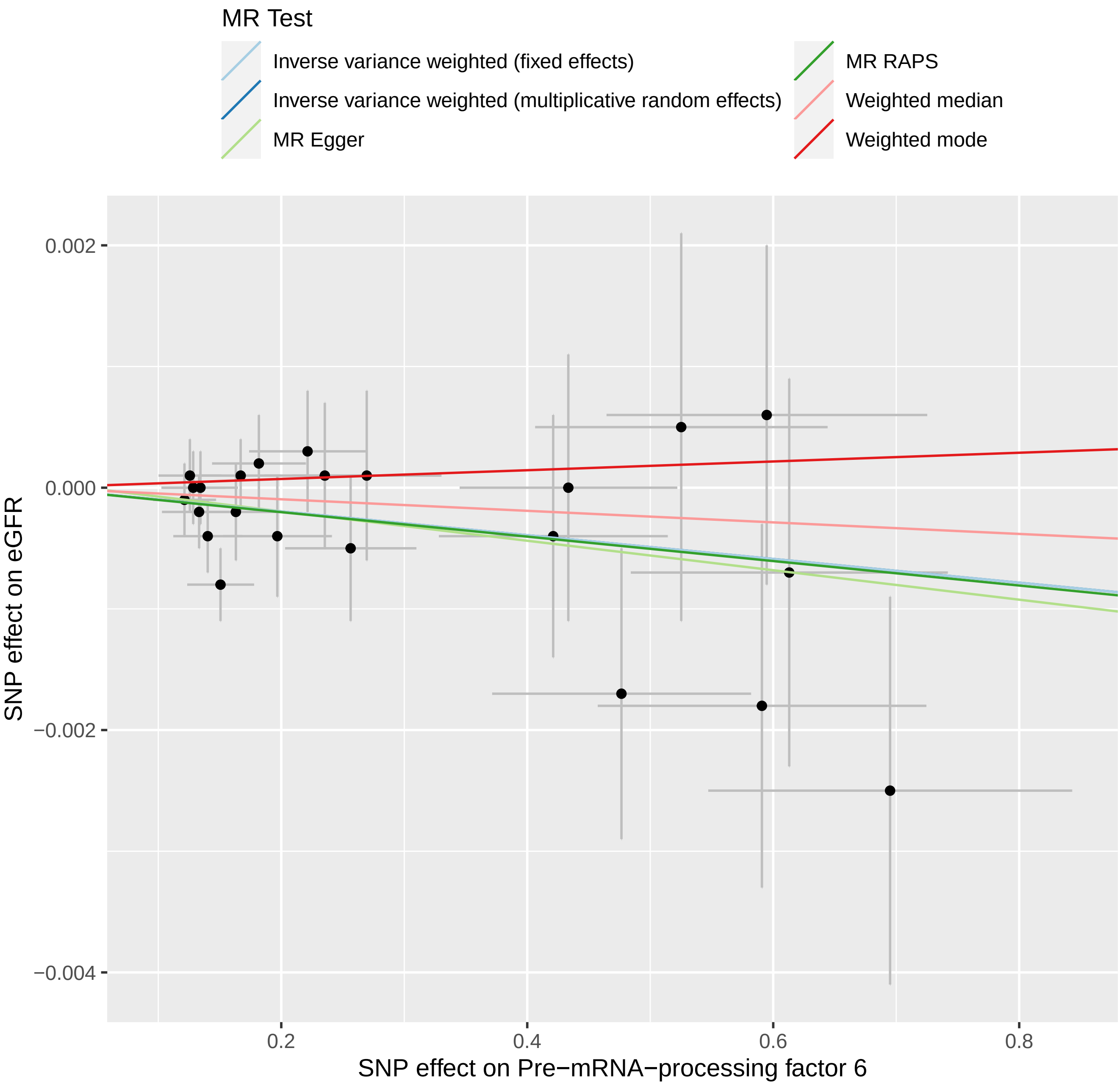

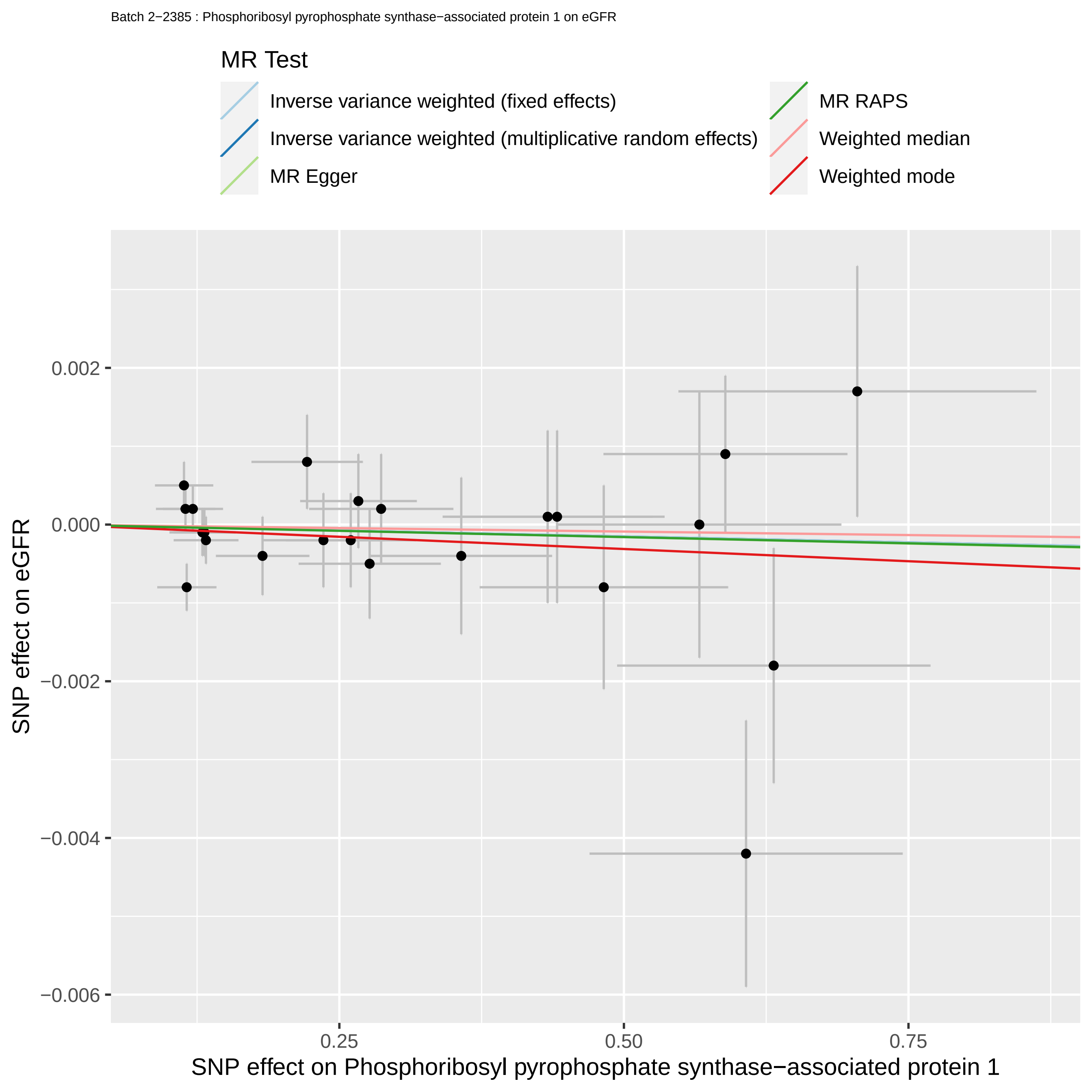
Batch 2−2385 : Phosphoribosyl pyrophosphate synthase−associated protein 1 on eGFR
MR Test
Inverse variance weighted (fixed effects)
Inverse variance weighted (multiplicative random effects)
MR Egger
MR RAPS
Weighted median
Weighted mode
0.002
0.000
−0.002
−0.004
−0.006
0.25
0.50
0.75
SNP effect on eGFR
SNP effect on Phosphoribosyl pyrophosphate synthase−associated protein 1

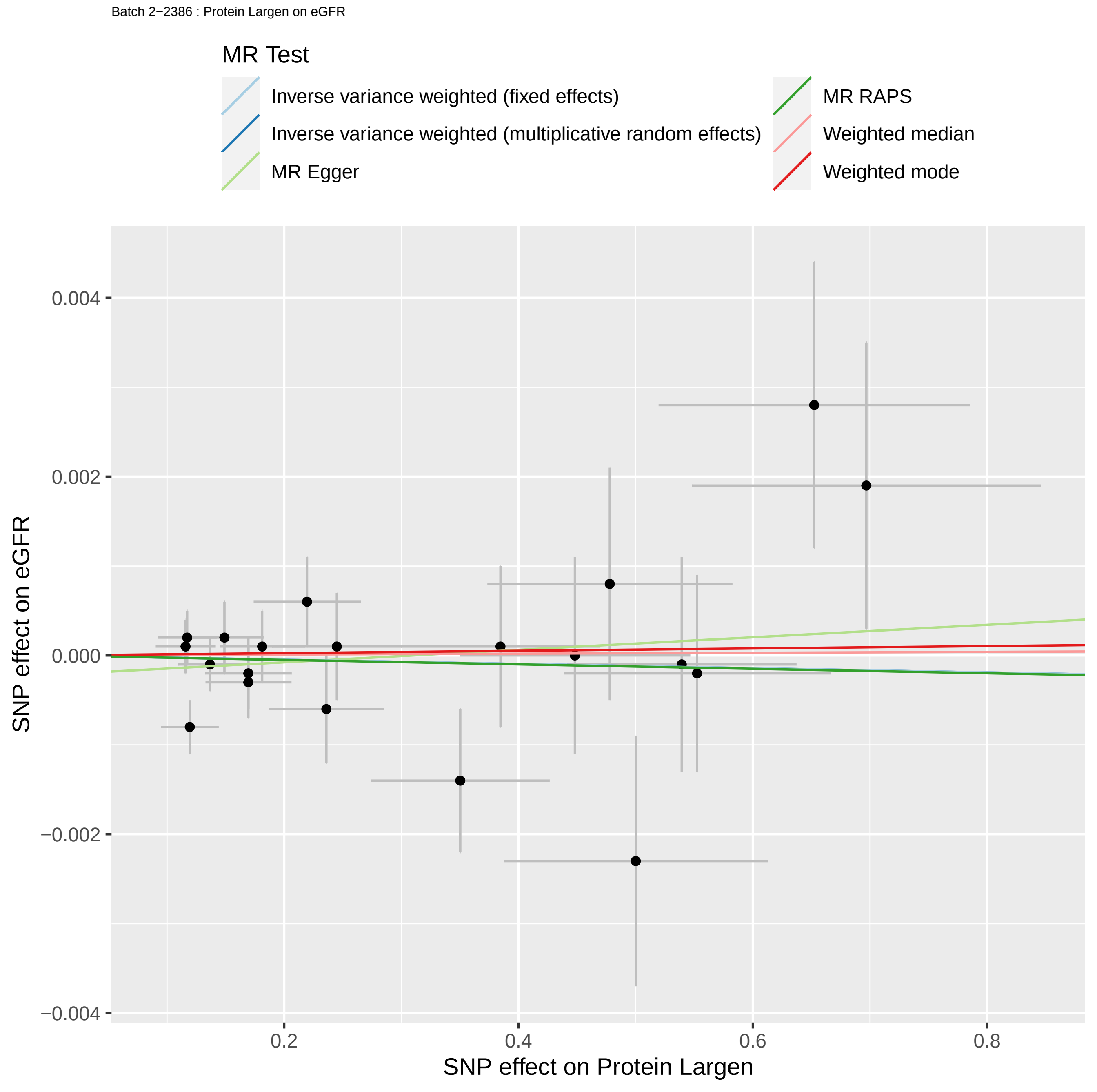

Batch 2−2386 : Protein Largen on eGFR
MR Test
Inverse variance weighted (fixed effects)
Inverse variance weighted (multiplicative random effects)
MR Egger
MR RAPS
Weighted median
Weighted mode
0.004
0.002
0.000
−0.002
−0.004
0.2
0.4
0.6
0.8
SNP effect on eGFR
SNP effect on Protein Largen

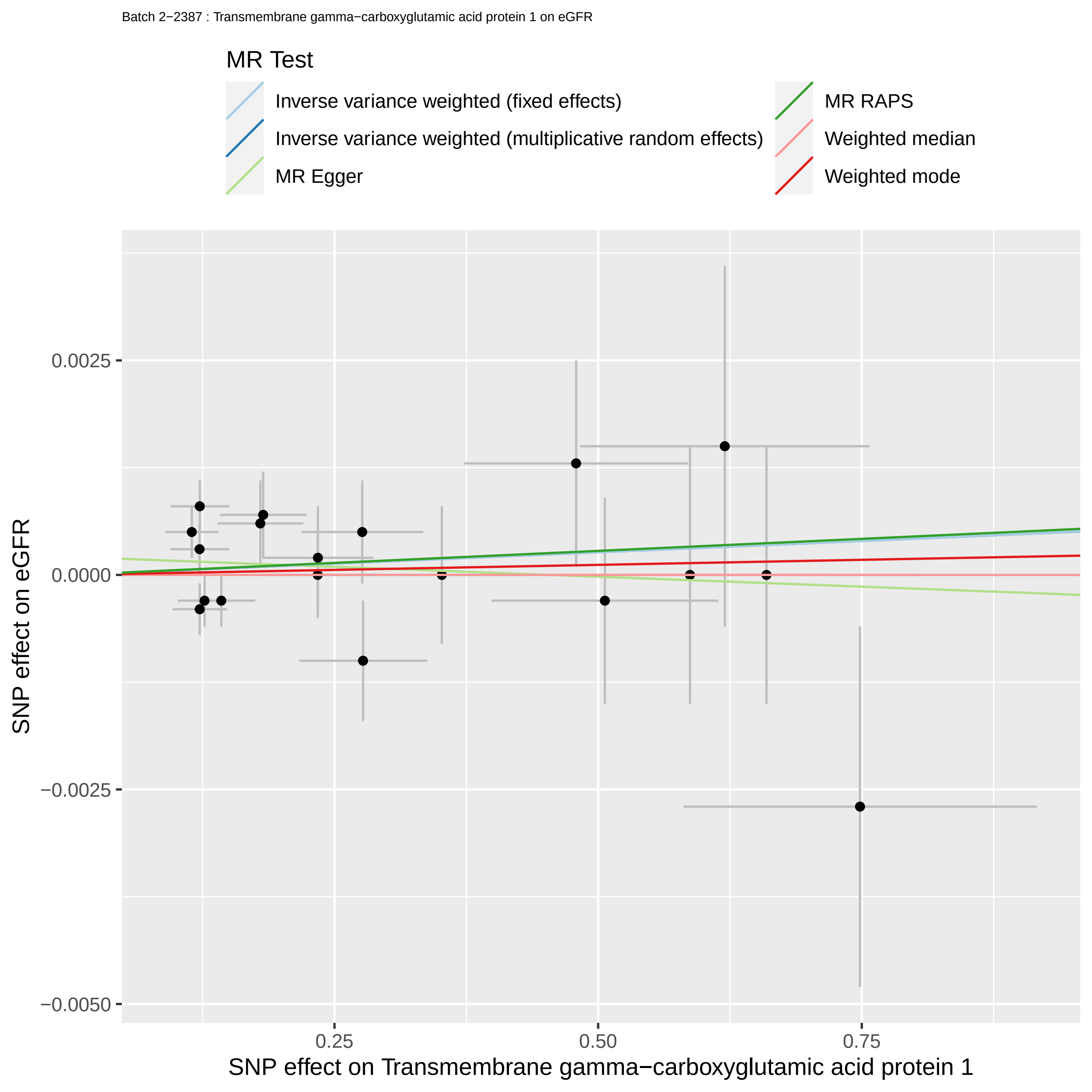

Batch 2−2387 : Transmembrane gamma−carboxyglutamic acid protein 1 on eGFR
MR Test
Inverse variance weighted (fixed effects)
Inverse variance weighted (multiplicative random effects)
MR Egger
MR RAPS
Weighted median
Weighted mode
0.0025
0.0000
−0.0025
−0.0050
0.25
0.50
0.75
SNP effect on eGFR
SNP effect on Transmembrane gamma−carboxyglutamic acid protein 1

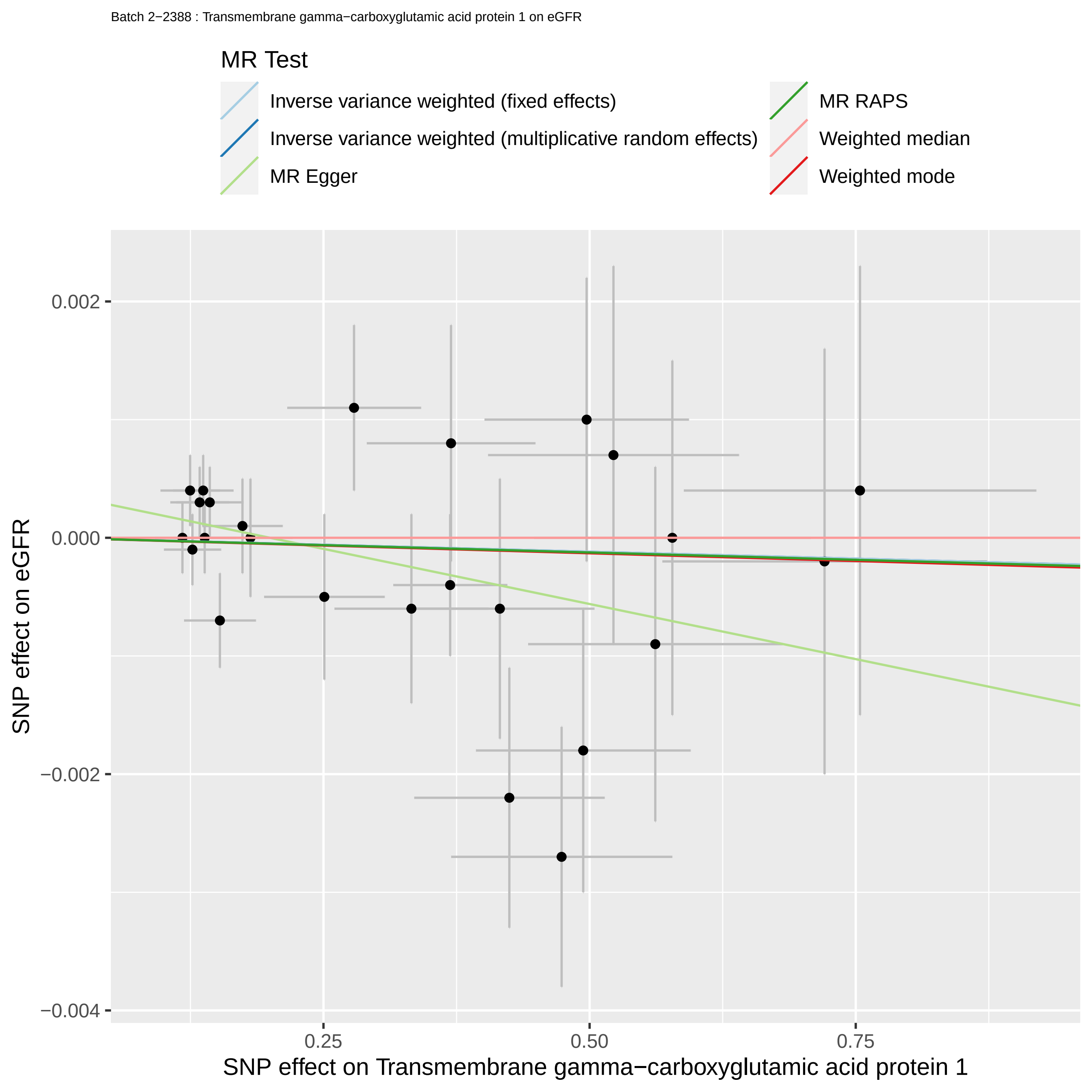

Batch 2−2388 : Transmembrane gamma−carboxyglutamic acid protein 1 on eGFR
MR Test
Inverse variance weighted (fixed effects)
Inverse variance weighted (multiplicative random effects)
MR Egger
MR RAPS
Weighted median
Weighted mode
0.002
0.000
−0.002
−0.004
0.25
0.50
0.75
SNP effect on eGFR
SNP effect on Transmembrane gamma−carboxyglutamic acid protein 1

Batch 2−2389 : Transmembrane gamma−carboxyglutamic acid protein 4 on eGFR

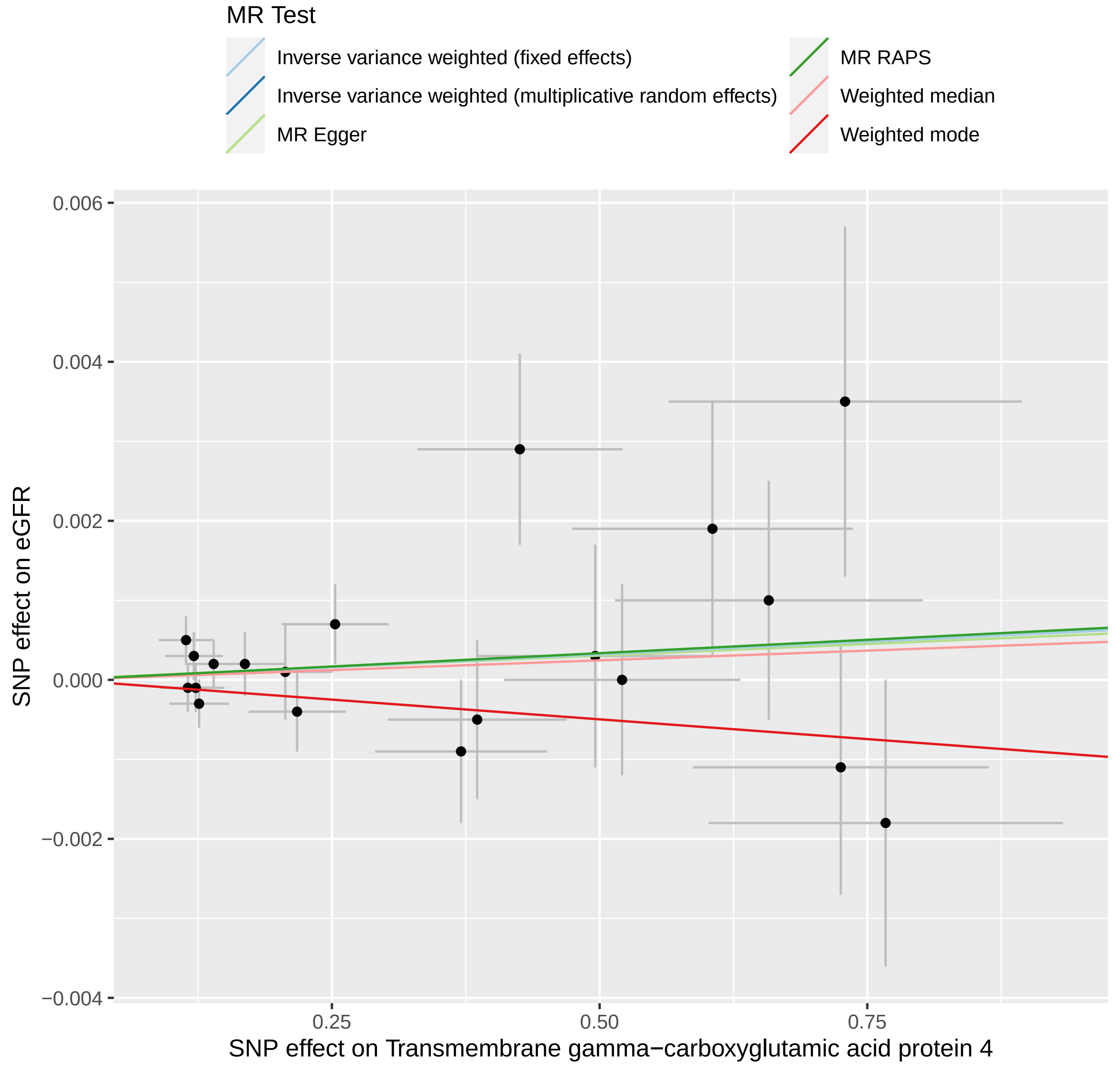

Batch 2−2390 : Trypsin−1 on eGFR

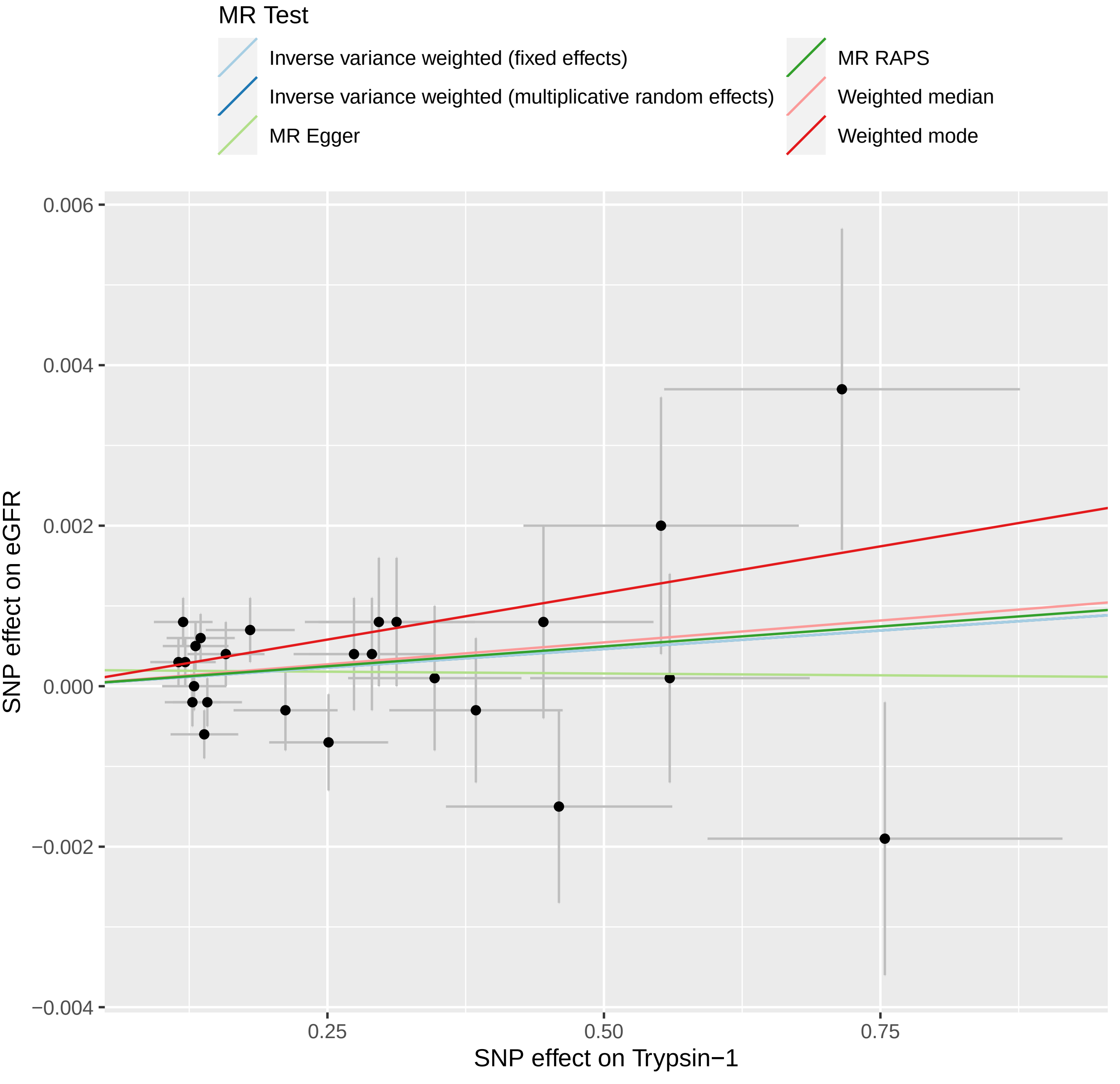

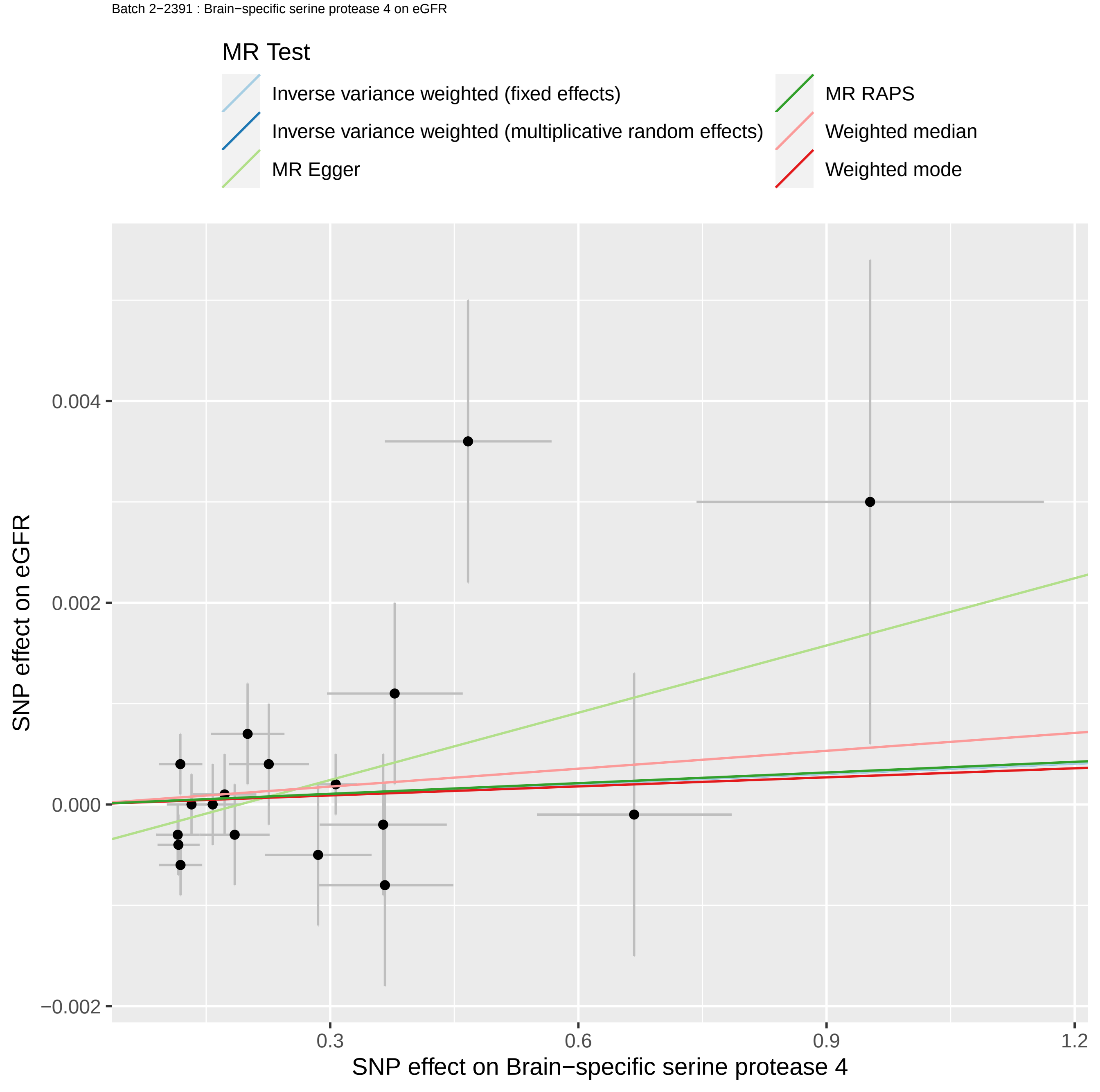

Batch 2−2391 : Brain−specific serine protease 4 on eGFR
MR Test
Inverse variance weighted (fixed effects)
Inverse variance weighted (multiplicative random effects)
MR Egger
MR RAPS
Weighted median
Weighted mode
0.004
0.002
0.000
−0.002
SNP effect on eGFR
0.3
0.6
0.9
1.2
SNP effect on Brain−specific serine protease 4

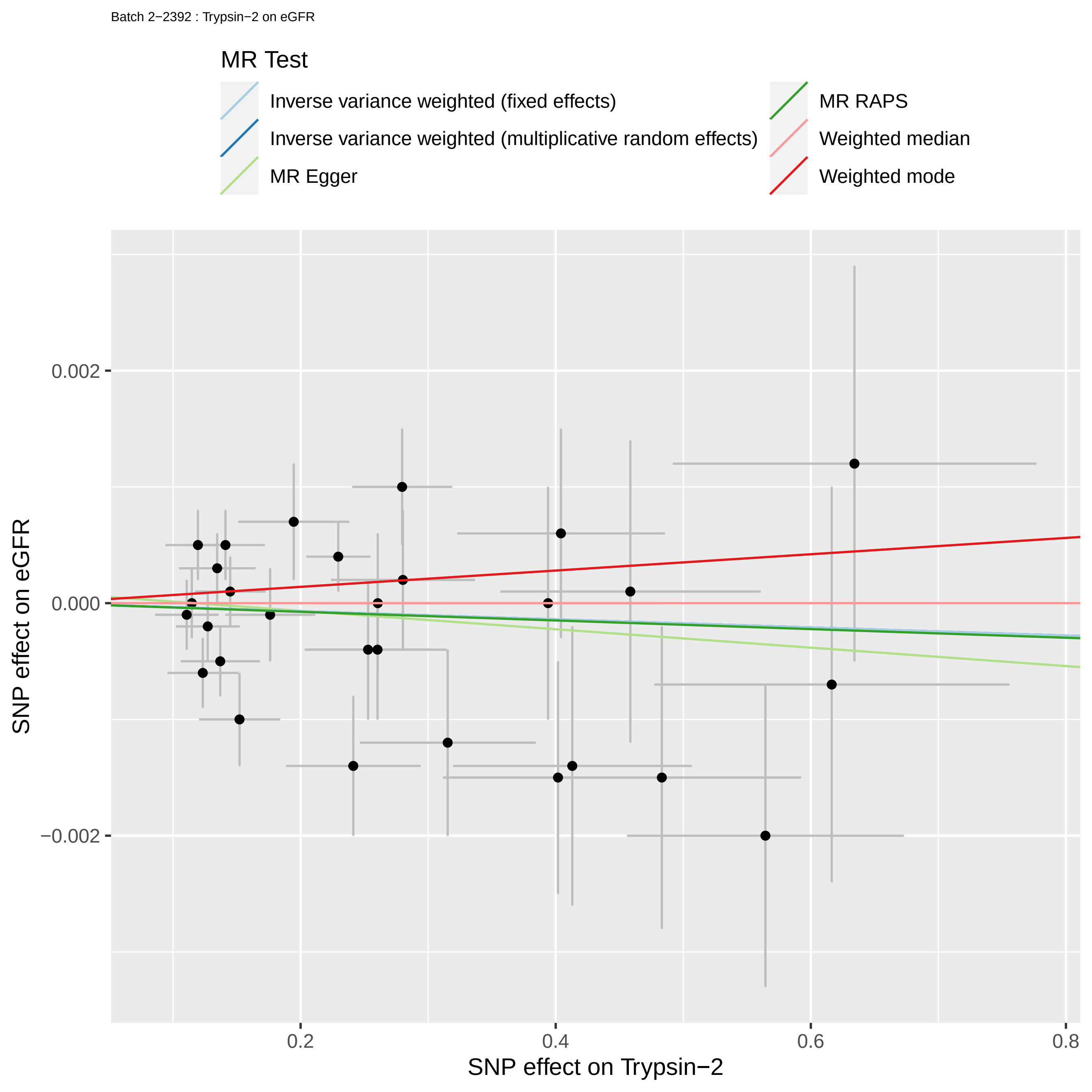

Batch 2−2392 : Trypsin−2 on eGFR
MR Test
Inverse variance weighted (fixed effects)
Inverse variance weighted (multiplicative random effects)
MR Egger
MR RAPS
Weighted median
Weighted mode
0.002
0.000
−0.002
SNP effect on eGFR
0.2
0.4
0.6
0.8
SNP effect on Trypsin−2

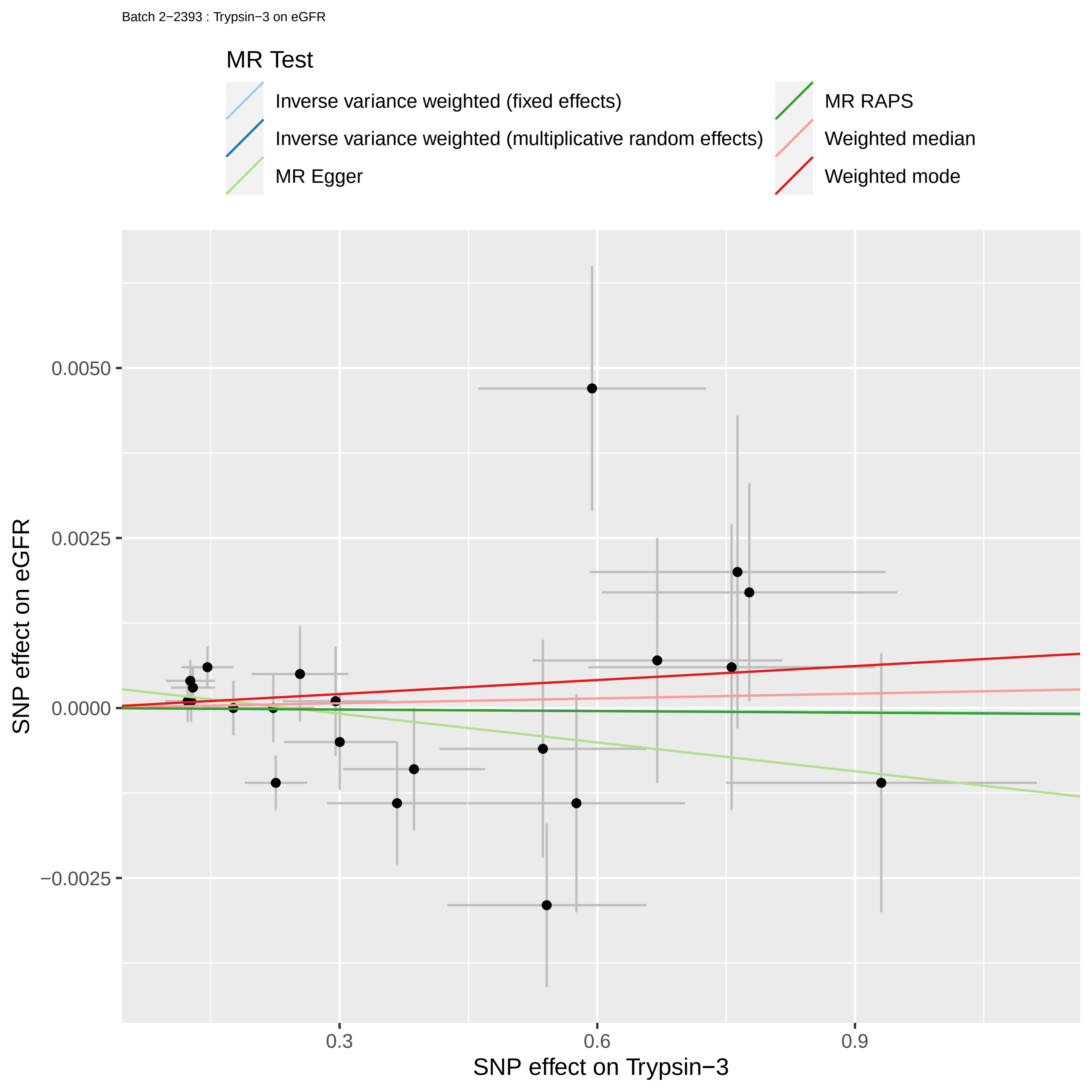
Batch 2−2393 : Trypsin−3 on eGFR
MR Test
Inverse variance weighted (fixed effects)
Inverse variance weighted (multiplicative random effects)
MR Egger
MR RAPS
Weighted median
Weighted mode
0.0050
0.0025
0.0000
−0.0025
0.3
0.6
0.9
SNP effect on Trypsin−3
SNP effect on eGFR

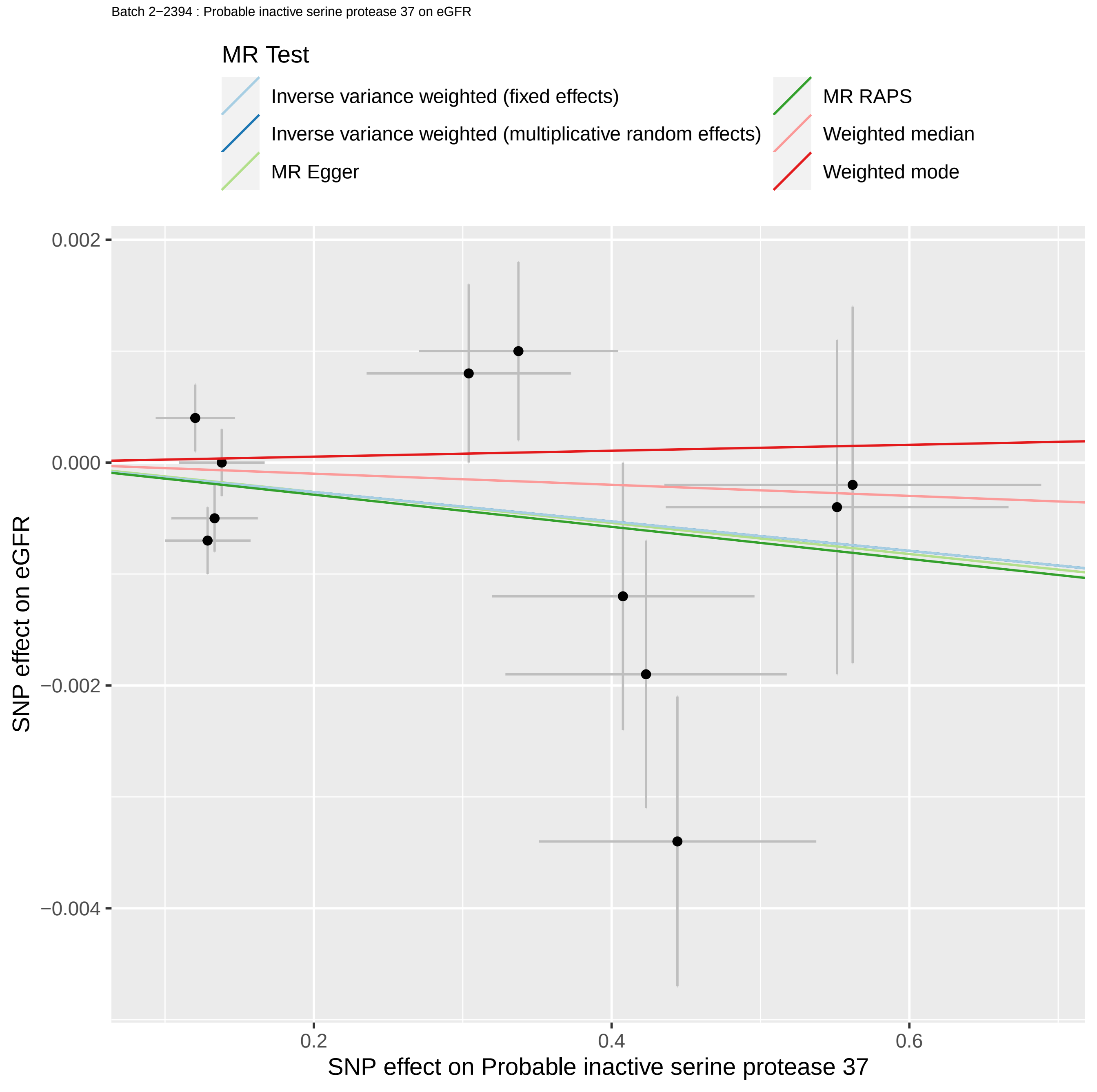
Batch 2−2394 : Probable inactive serine protease 37 on eGFR
MR Test
Inverse variance weighted (fixed effects)
Inverse variance weighted (multiplicative random effects)
MR Egger
MR RAPS
Weighted median
Weighted mode
0.002
0.000
−0.002
−0.004
SNP effect on eGFR
0.2
0.4
0.6
SNP effect on Probable inactive serine protease 37

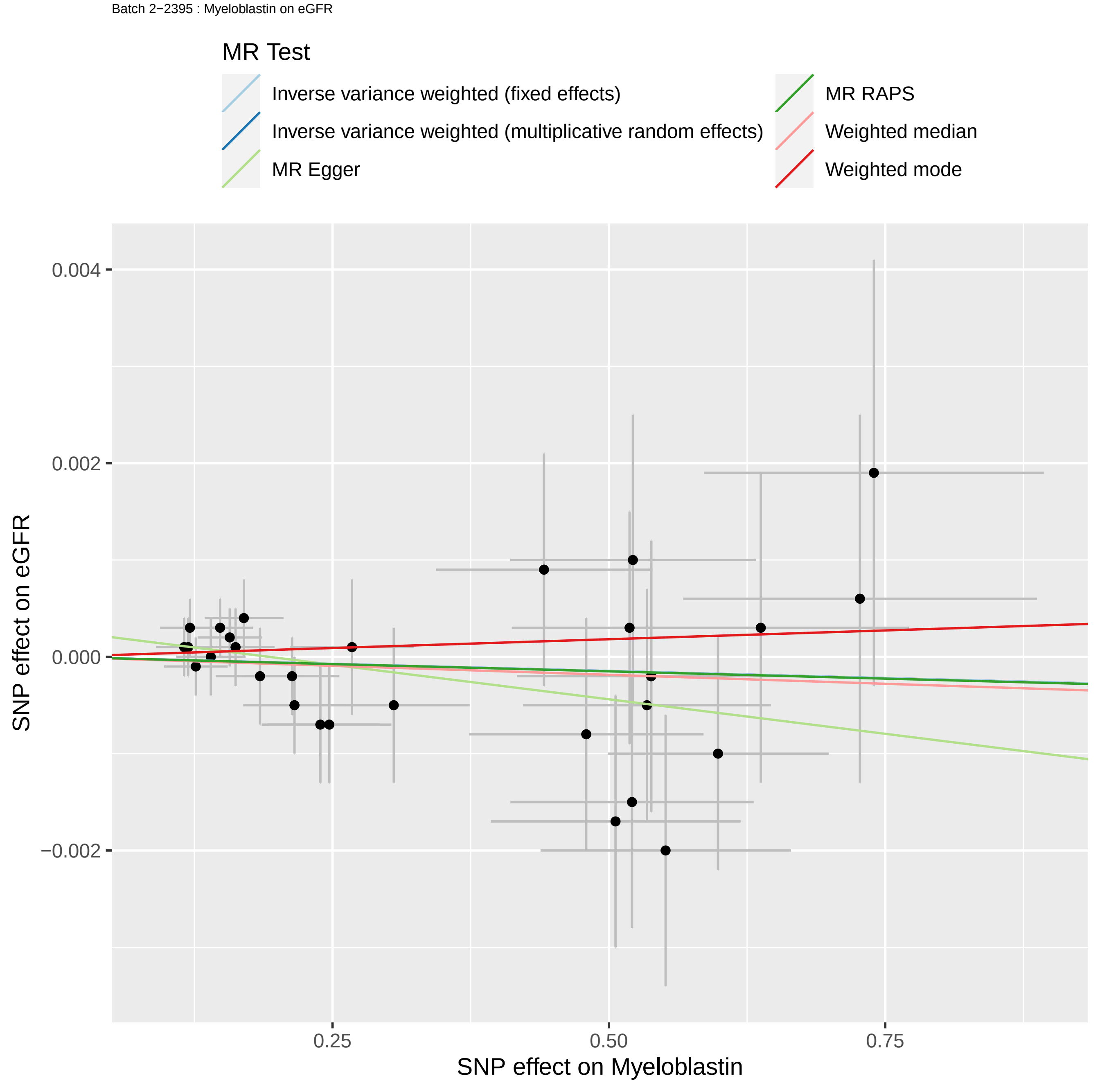
Batch 2−2395 : Myeloblastin on eGFR
MR Test
Inverse variance weighted (fixed effects)
Inverse variance weighted (multiplicative random effects)
MR Egger
MR RAPS
Weighted median
Weighted mode
0.004
0.002
0.000
−0.002
0.25
0.50
0.75
SNP effect on eGFR
SNP effect on Myeloblastin

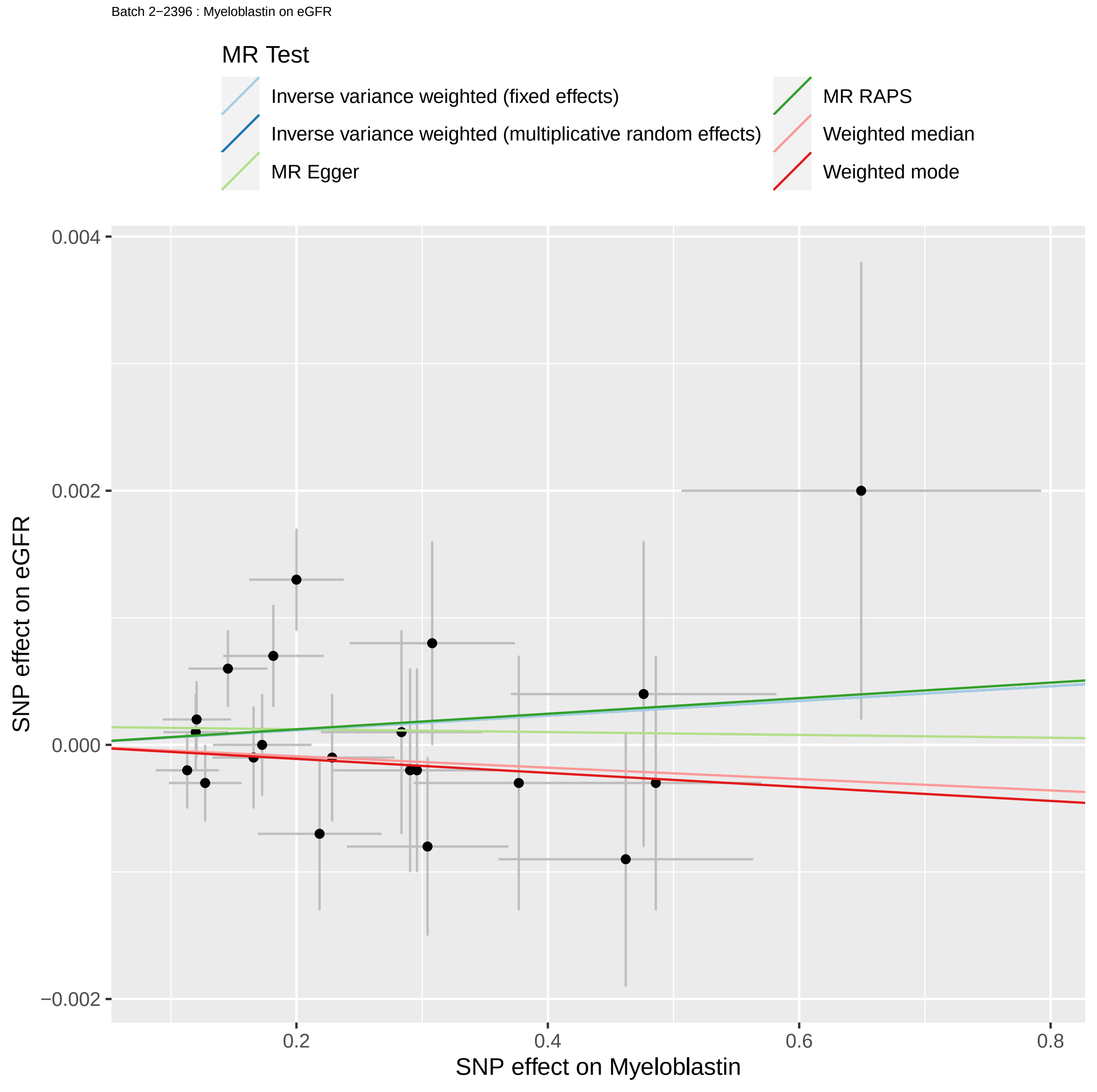
Batch 2−2396 : Myeloblastin on eGFR
MR Test
Inverse variance weighted (fixed effects)
Inverse variance weighted (multiplicative random effects)
MR Egger
MR RAPS
Weighted median
Weighted mode
0.004
0.002
0.000
−0.002
SNP effect on eGFR
0.2
0.4
0.6
0.8
SNP effect on Myeloblastin

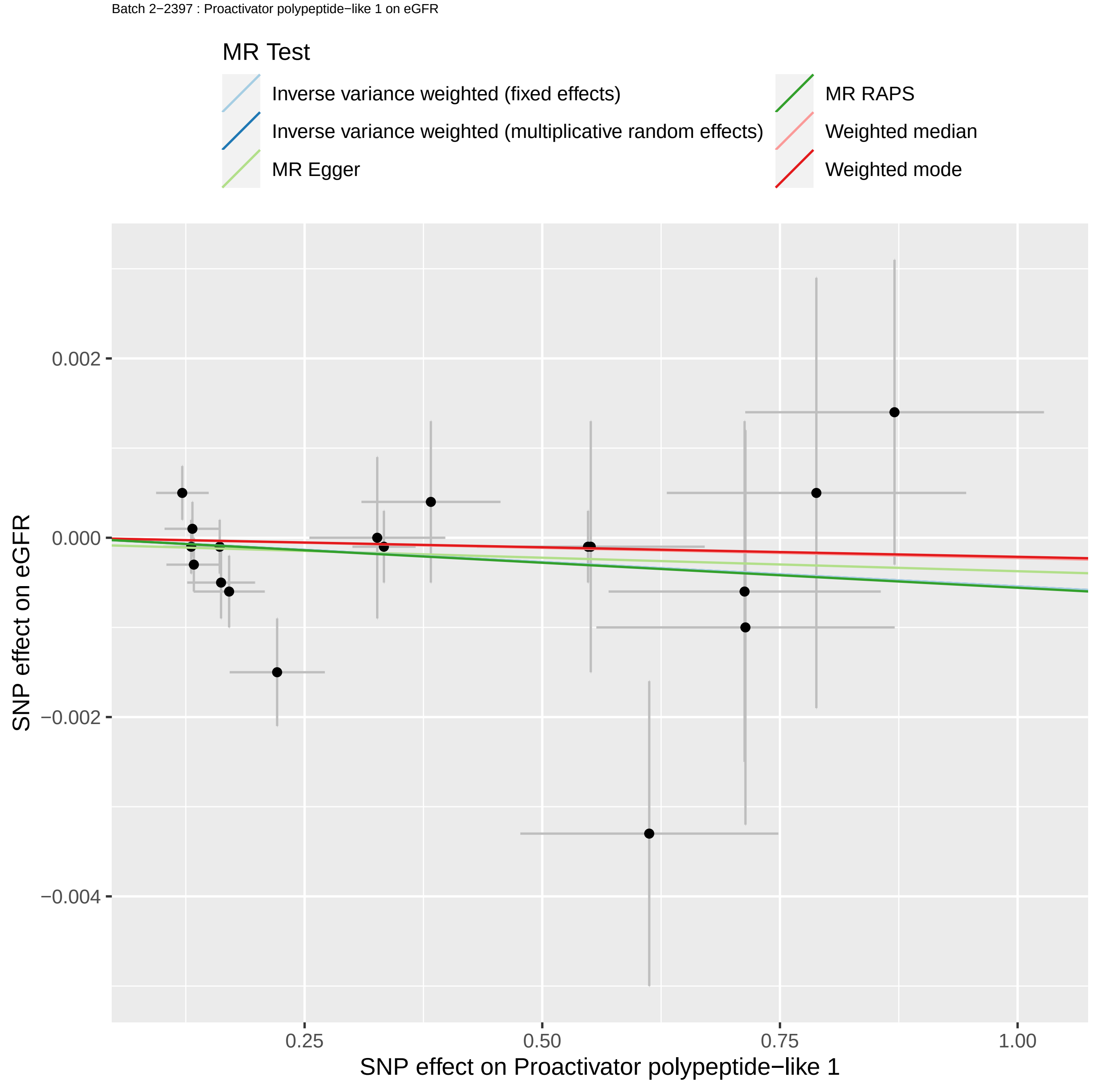

Batch 2−2397 : Proactivator polypeptide−like 1 on eGFR
MR Test
Inverse variance weighted (fixed effects)
Inverse variance weighted (multiplicative random effects)
MR Egger
MR RAPS
Weighted median
Weighted mode
0.002
0.000
−0.002
−0.004
SNP effect on eGFR
0.25
0.50
0.75
1.00
SNP effect on Proactivator polypeptide−like 1

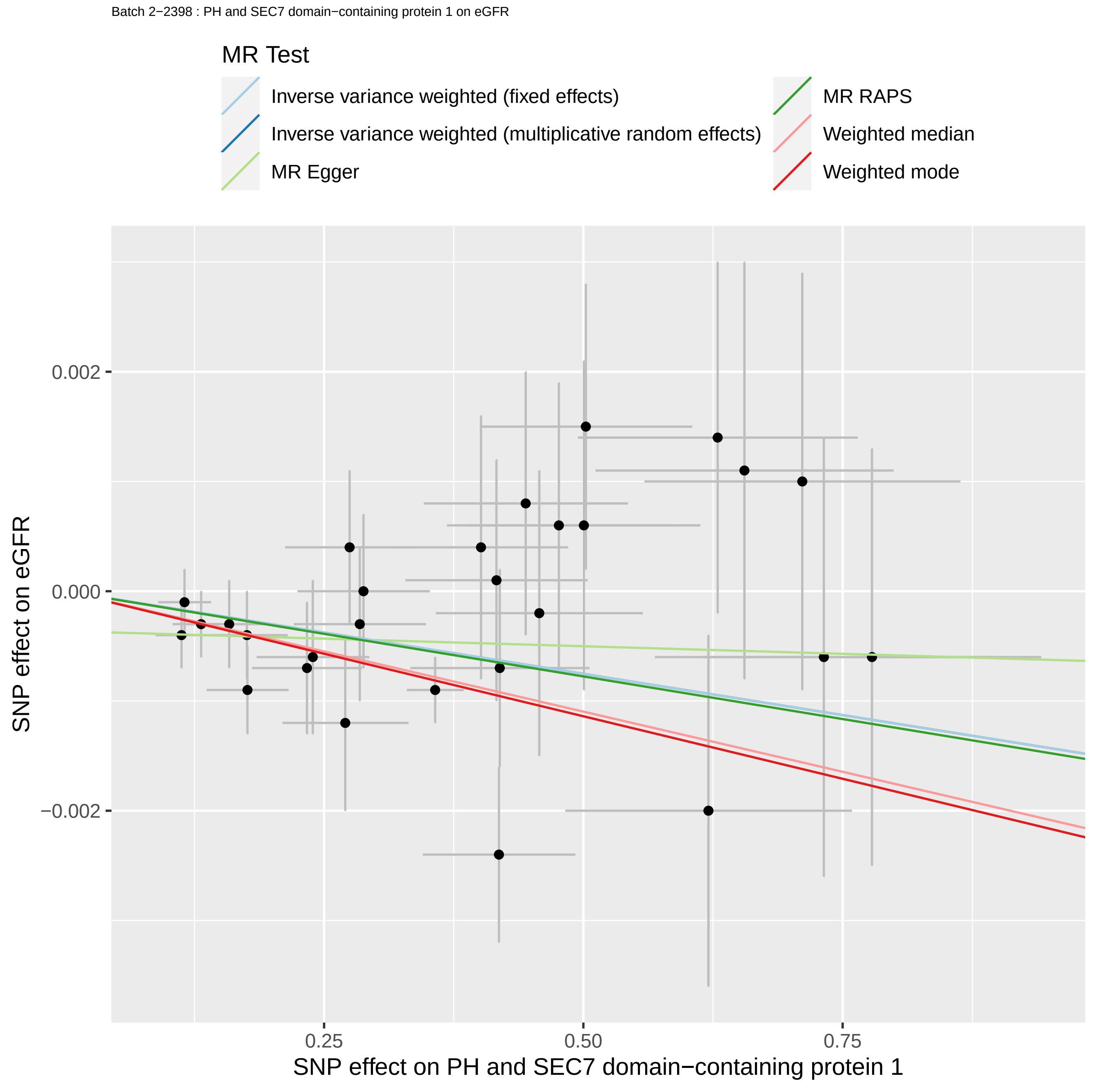

Batch 2−2398 : PH and SEC7 domain−containing protein 1 on eGFR
MR Test
Inverse variance weighted (fixed effects)
Inverse variance weighted (multiplicative random effects)
MR Egger
MR RAPS
Weighted median
Weighted mode
0.002
0.000
−0.002
0.25
0.50
0.75
SNP effect on eGFR
SNP effect on PH and SEC7 domain−containing protein 1

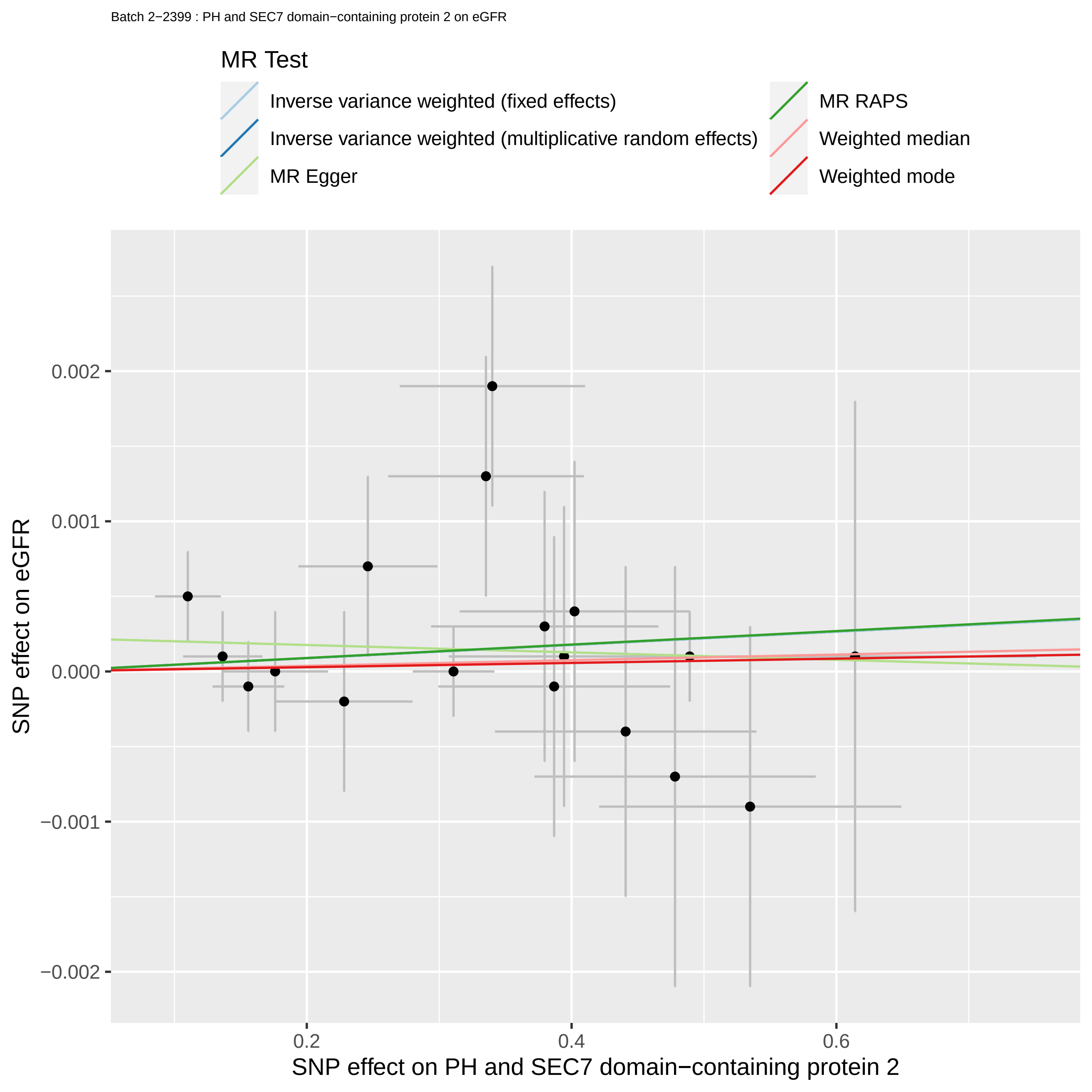
Batch 2−2399 : PH and SEC7 domain−containing protein 2 on eGFR
MR Test
Inverse variance weighted (fixed effects)
Inverse variance weighted (multiplicative random effects)
MR Egger
MR RAPS
Weighted median
Weighted mode
0.002
0.001
0.000
−0.001
−0.002
SNP effect on eGFR
0.2
0.4
0.6
SNP effect on PH and SEC7 domain−containing protein 2

Batch 2−2400 : Pregnancy−specific beta−1−glycoprotein 11 on eGFR

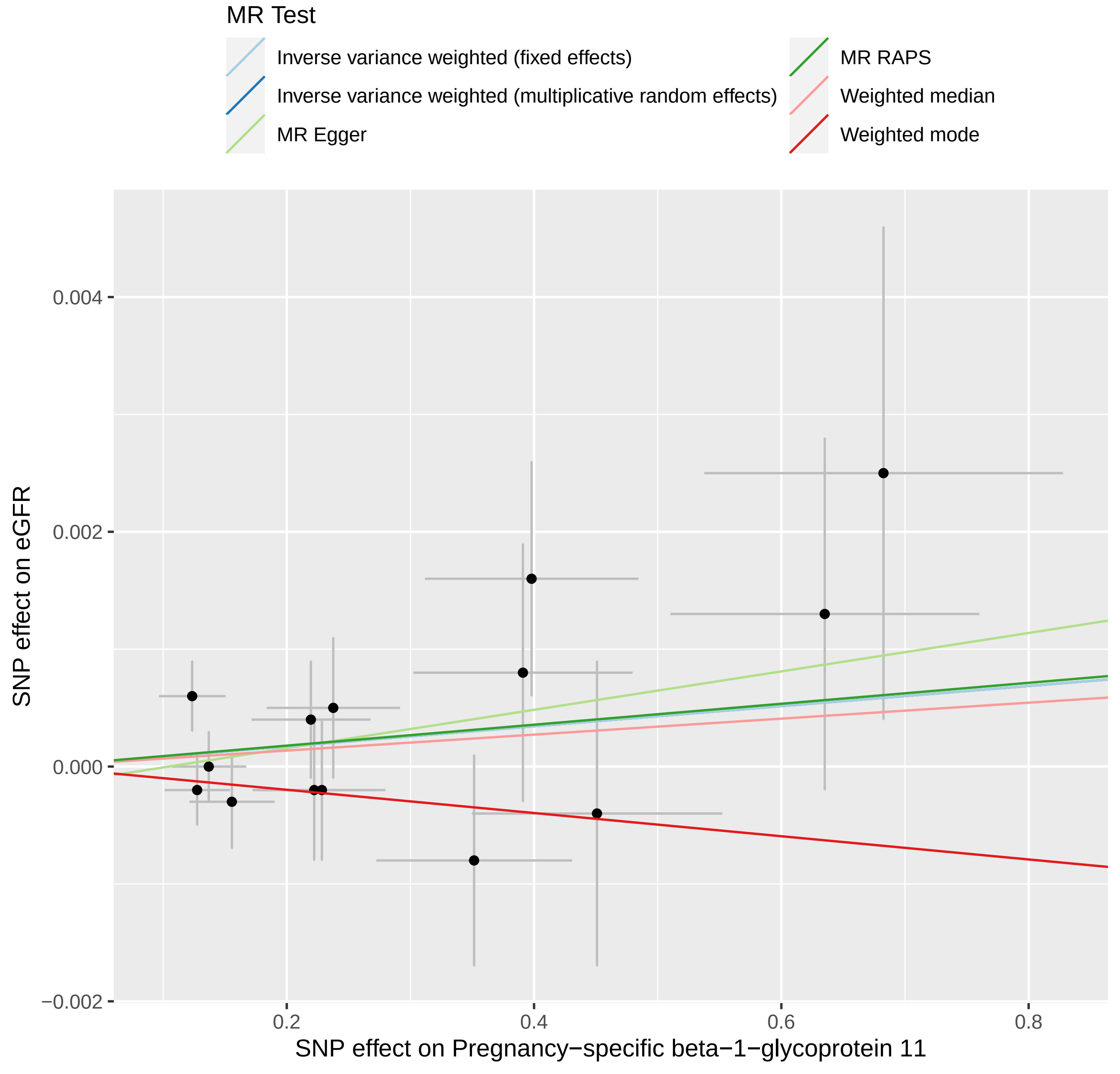

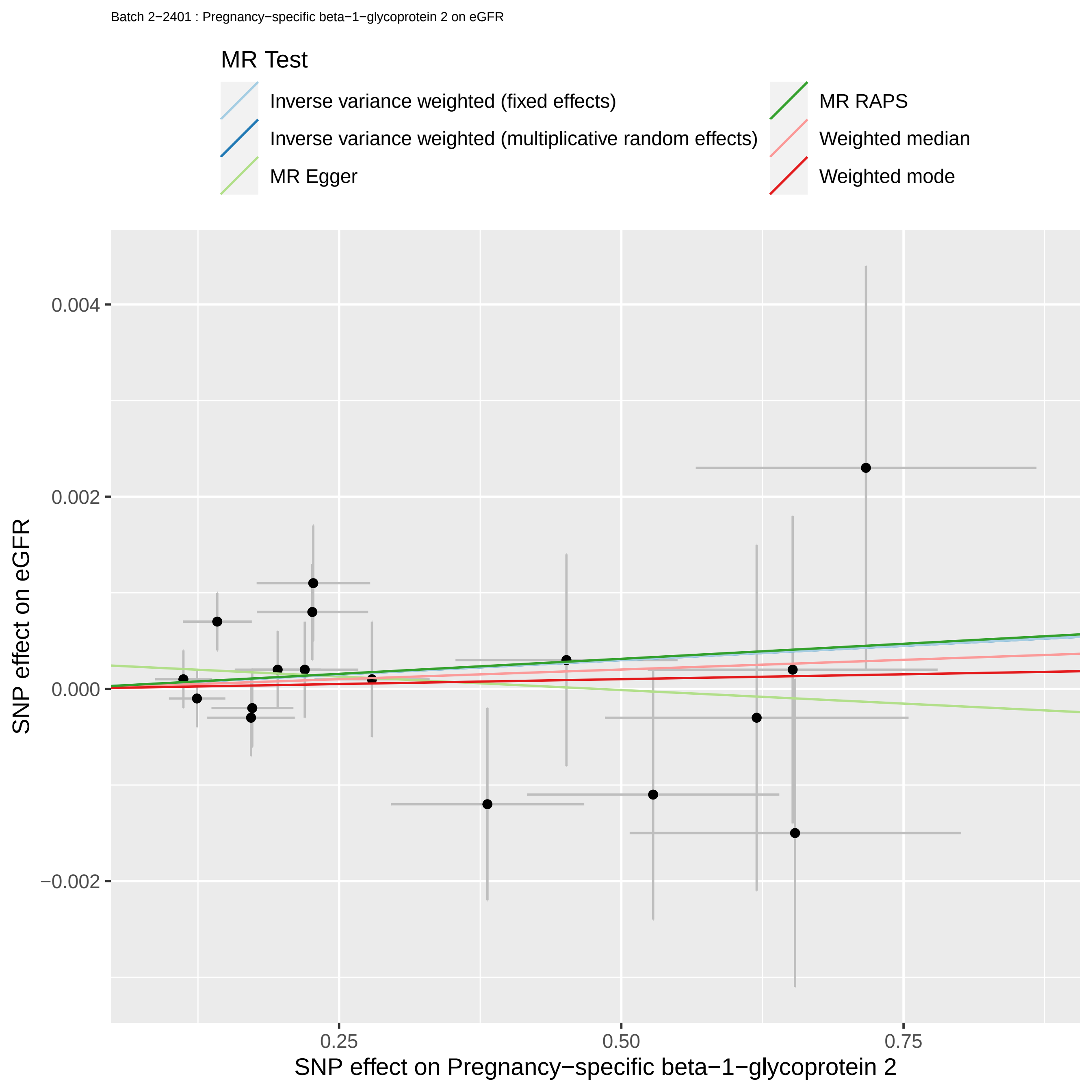
Batch 2−2401 : Pregnancy−specific beta−1−glycoprotein 2 on eGFR
MR Test
Inverse variance weighted (fixed effects)
Inverse variance weighted (multiplicative random effects)
MR Egger
MR RAPS
Weighted median
Weighted mode
0.004
0.002
0.000
−0.002
0.25
0.50
0.75
SNP effect on eGFR
SNP effect on Pregnancy−specific beta−1−glycoprotein 2

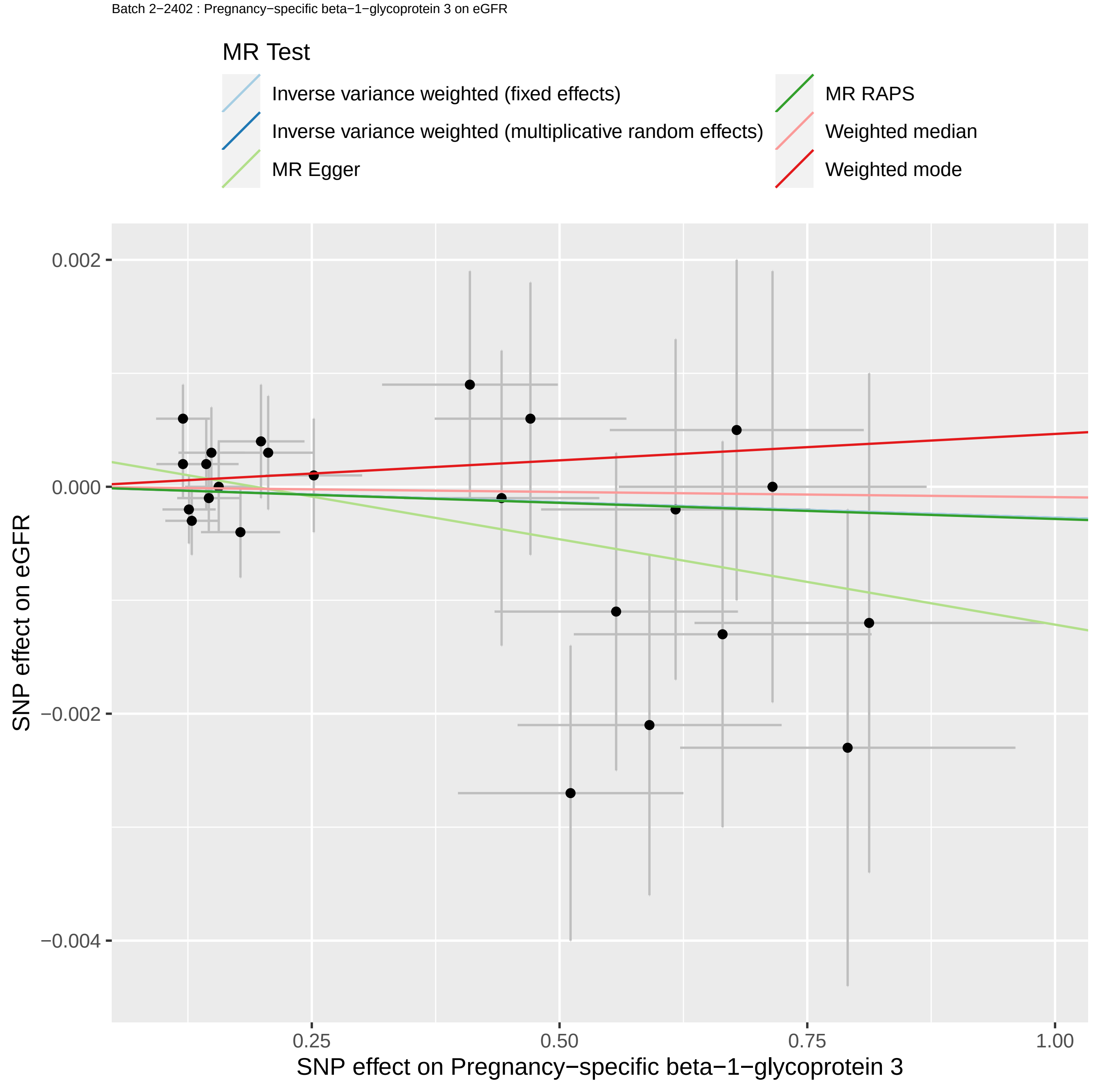
Batch 2−2402 : Pregnancy−specific beta−1−glycoprotein 3 on eGFR
MR Test
Inverse variance weighted (fixed effects)
Inverse variance weighted (multiplicative random effects)
MR Egger
MR RAPS
Weighted median
Weighted mode
0.002
0.000
−0.002
−0.004
0.25
0.50
0.75
1.00
SNP effect on eGFR
SNP effect on Pregnancy−specific beta−1−glycoprotein 3

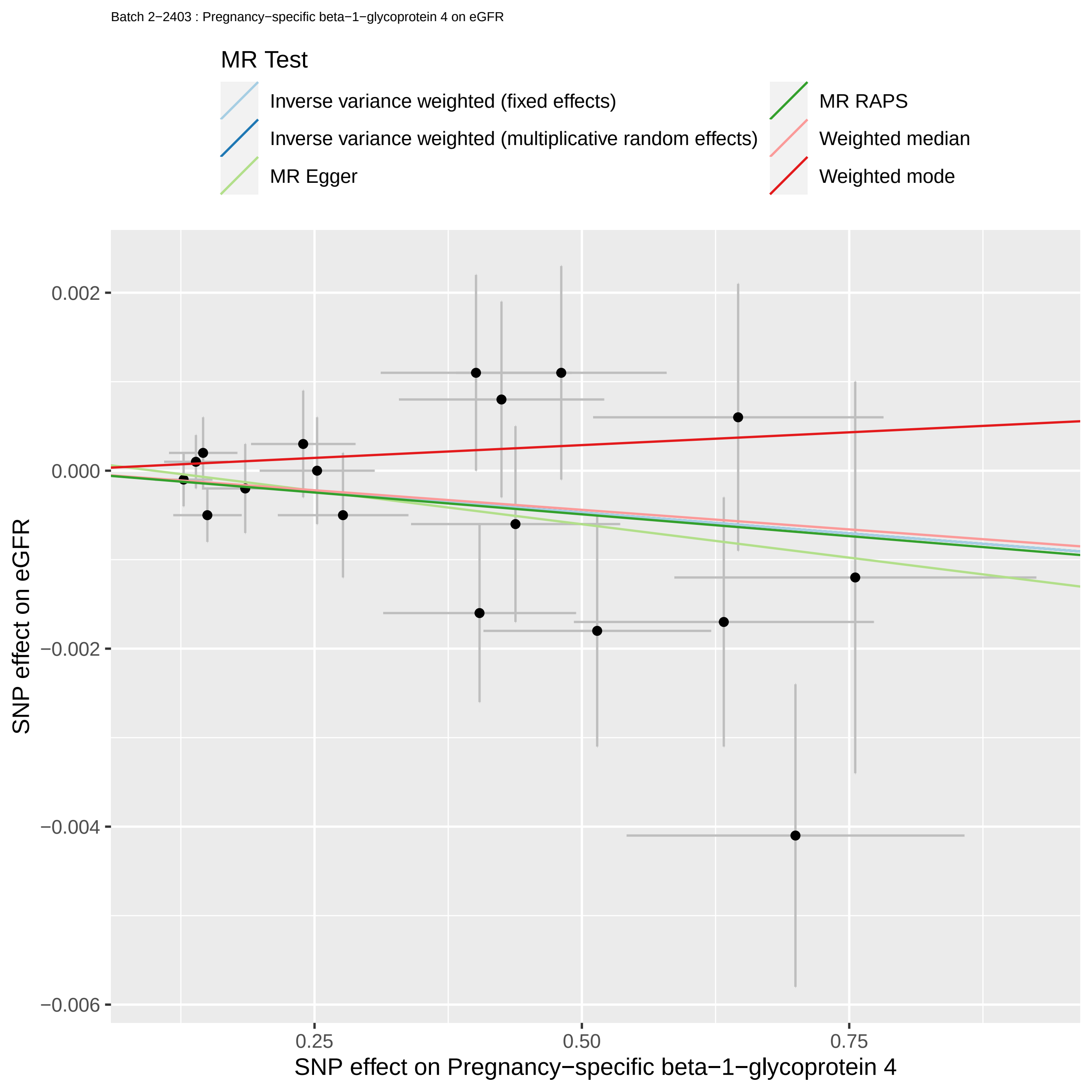
Batch 2−2403 : Pregnancy−specific beta−1−glycoprotein 4 on eGFR
MR Test
Inverse variance weighted (fixed effects)
Inverse variance weighted (multiplicative random effects)
MR Egger
MR RAPS
Weighted median
Weighted mode
0.002
0.000
−0.002
−0.004
−0.006
SNP effect on eGFR
0.25
0.50
0.75
SNP effect on Pregnancy−specific beta−1−glycoprotein 4

Batch 2−2404 : Pregnancy−specific beta−1−glycoprotein 5 on eGFR

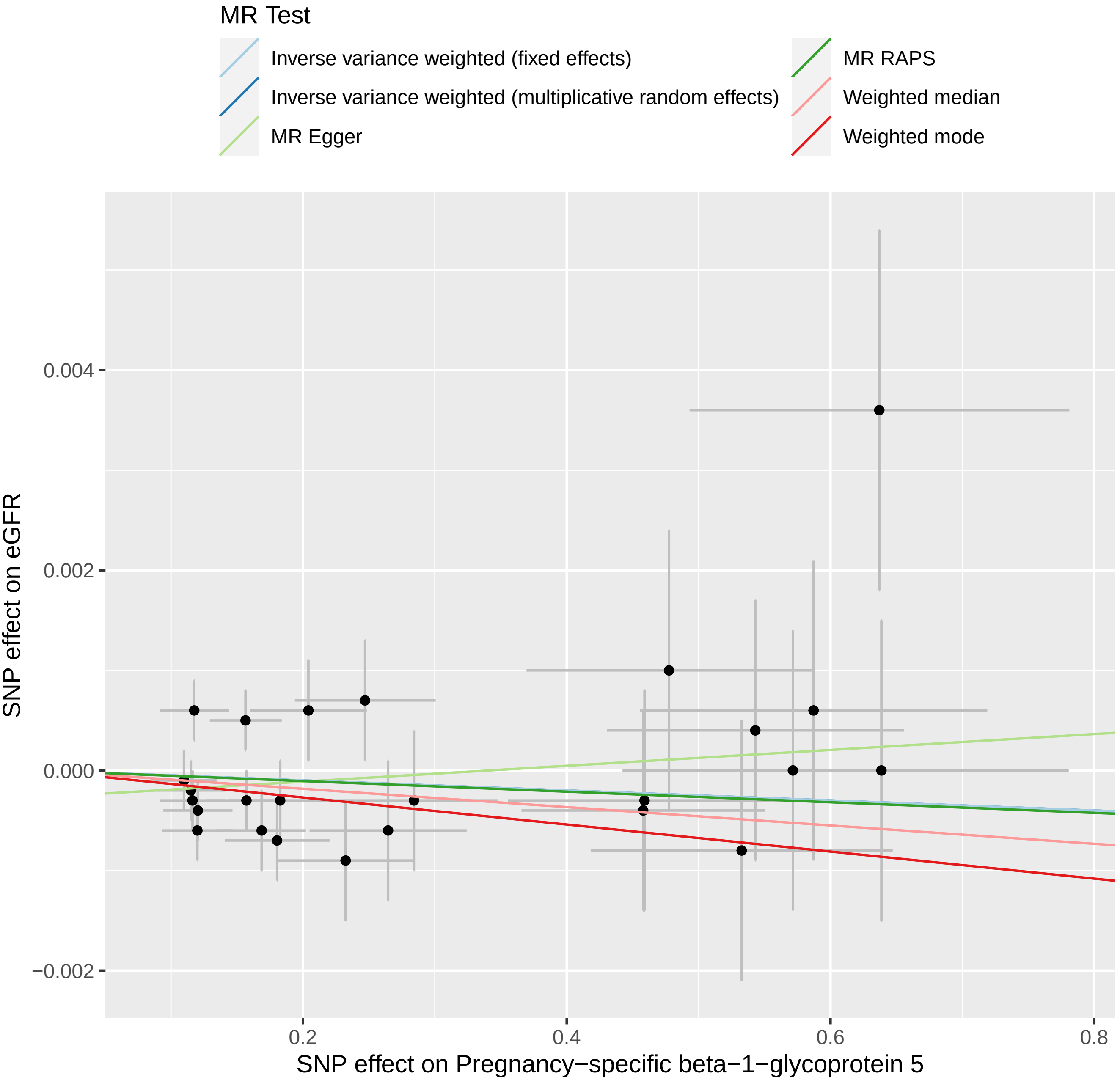

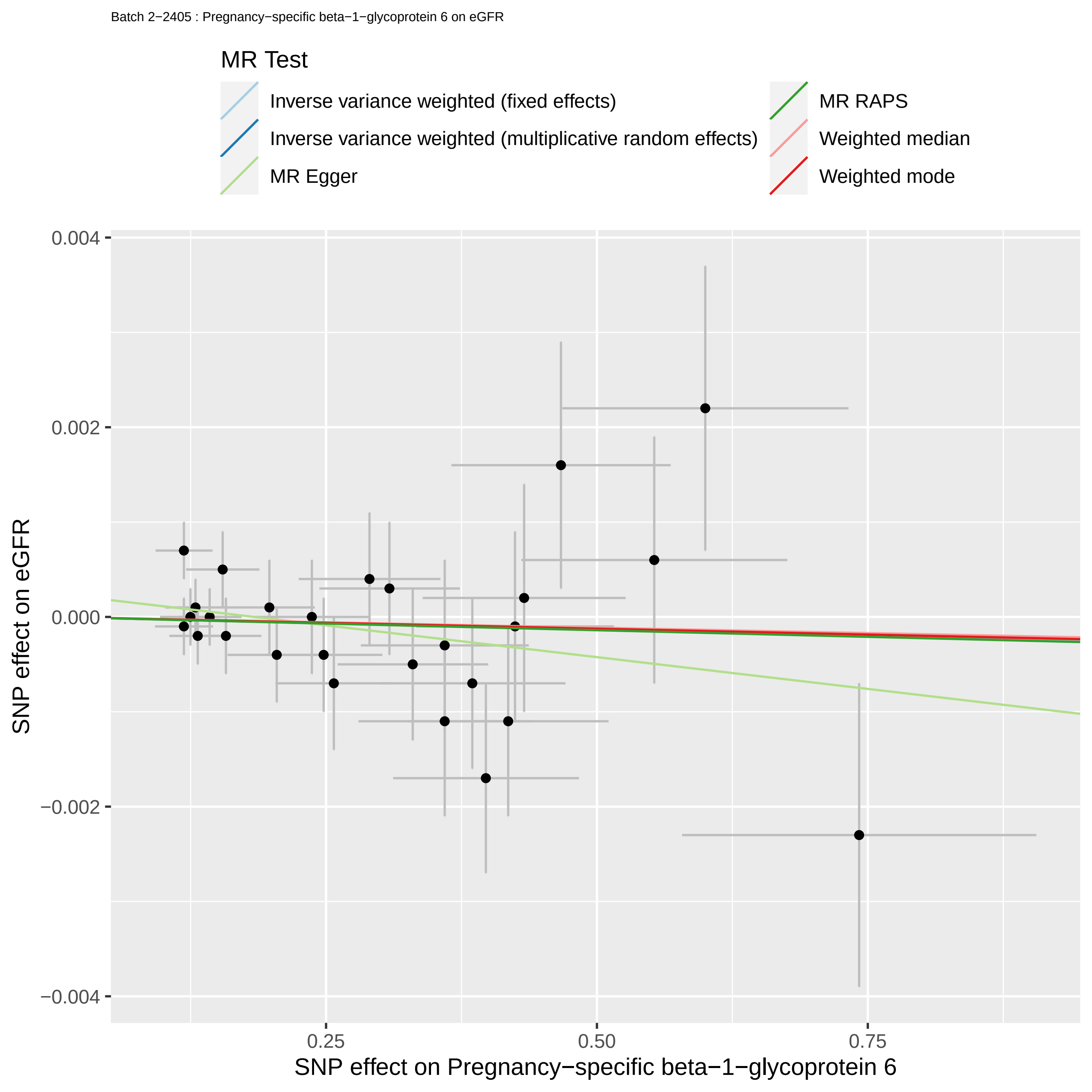

Batch 2−2405 : Pregnancy−specific beta−1−glycoprotein 6 on eGFR
MR Test
Inverse variance weighted (fixed effects)
Inverse variance weighted (multiplicative random effects)
MR Egger
MR RAPS
Weighted median
Weighted mode
0.004
0.002
0.000
−0.002
−0.004
0.25
0.50
0.75
SNP effect on eGFR
SNP effect on Pregnancy−specific beta−1−glycoprotein 6

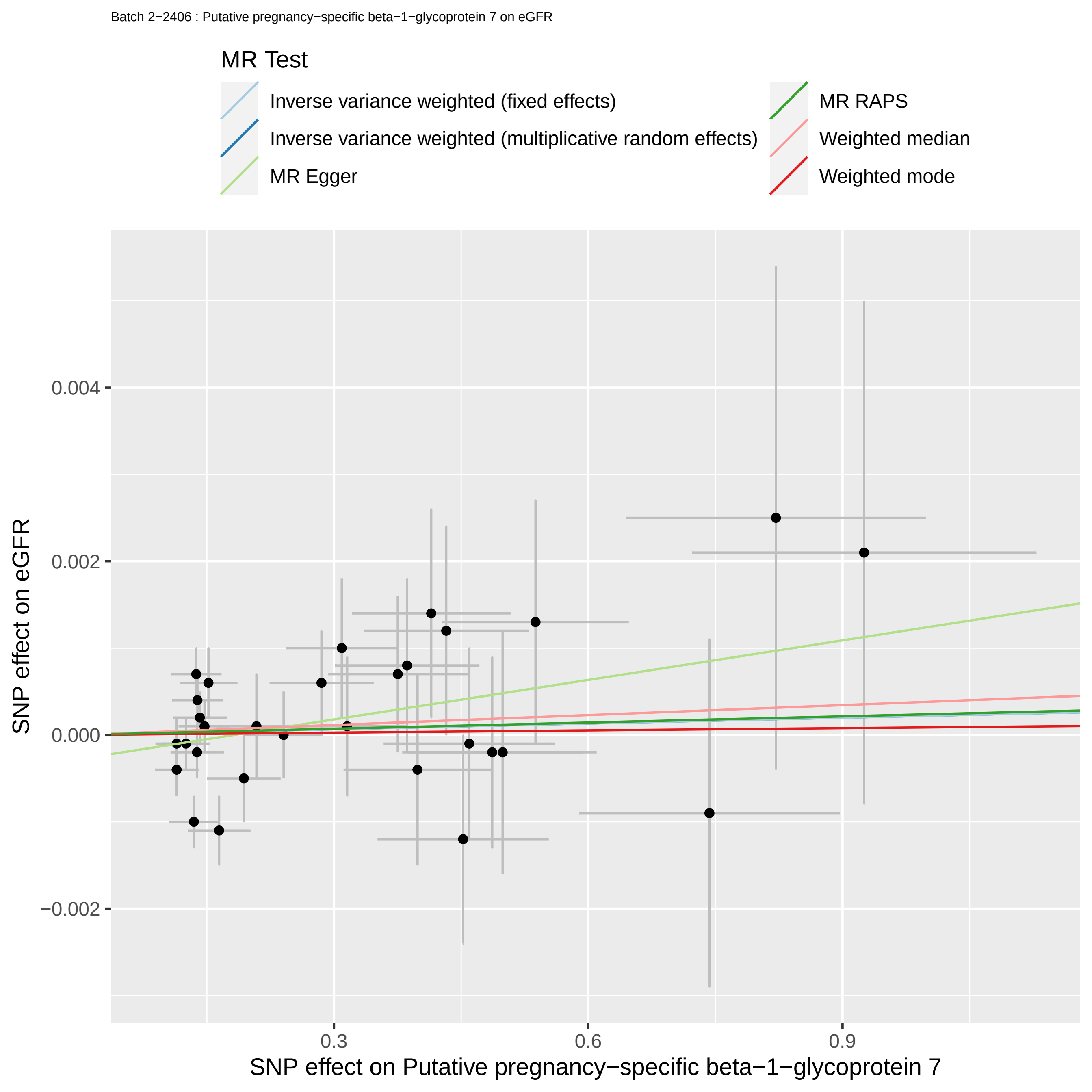

Batch 2−2406 : Putative pregnancy−specific beta−1−glycoprotein 7 on eGFR
MR Test
Inverse variance weighted (fixed effects)
Inverse variance weighted (multiplicative random effects)
MR Egger
MR RAPS
Weighted median
Weighted mode
0.004
0.002
0.000
−0.002
0.3
0.6
0.9
SNP effect on eGFR
SNP effect on Putative pregnancy−specific beta−1−glycoprotein 7

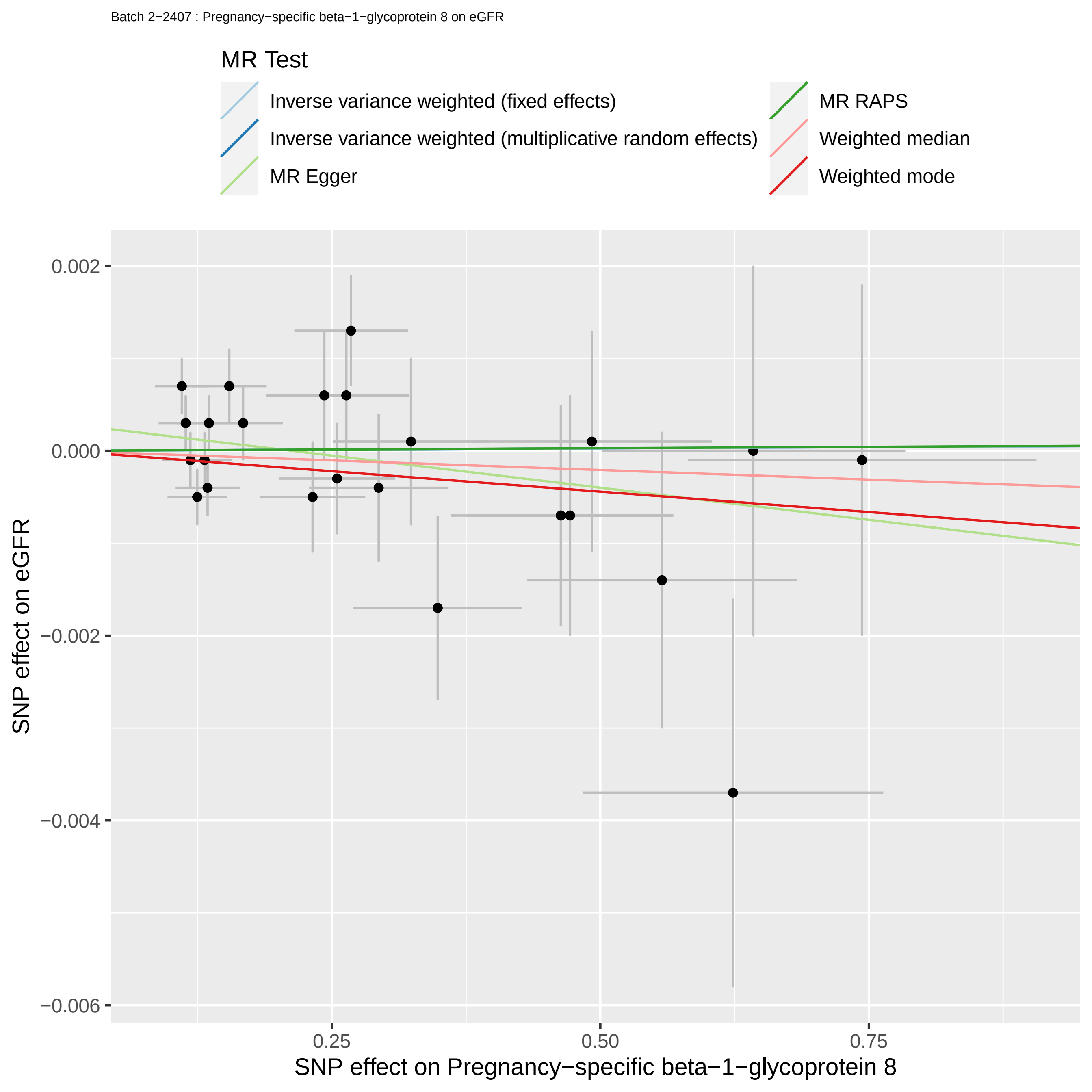

Batch 2−2407 : Pregnancy−specific beta−1−glycoprotein 8 on eGFR
MR Test
Inverse variance weighted (fixed effects)
Inverse variance weighted (multiplicative random effects)
MR Egger
MR RAPS
Weighted median
Weighted mode
0.002
0.000
−0.002
−0.004
−0.006
0.25
0.50
0.75
SNP effect on eGFR
SNP effect on Pregnancy−specific beta−1−glycoprotein 8

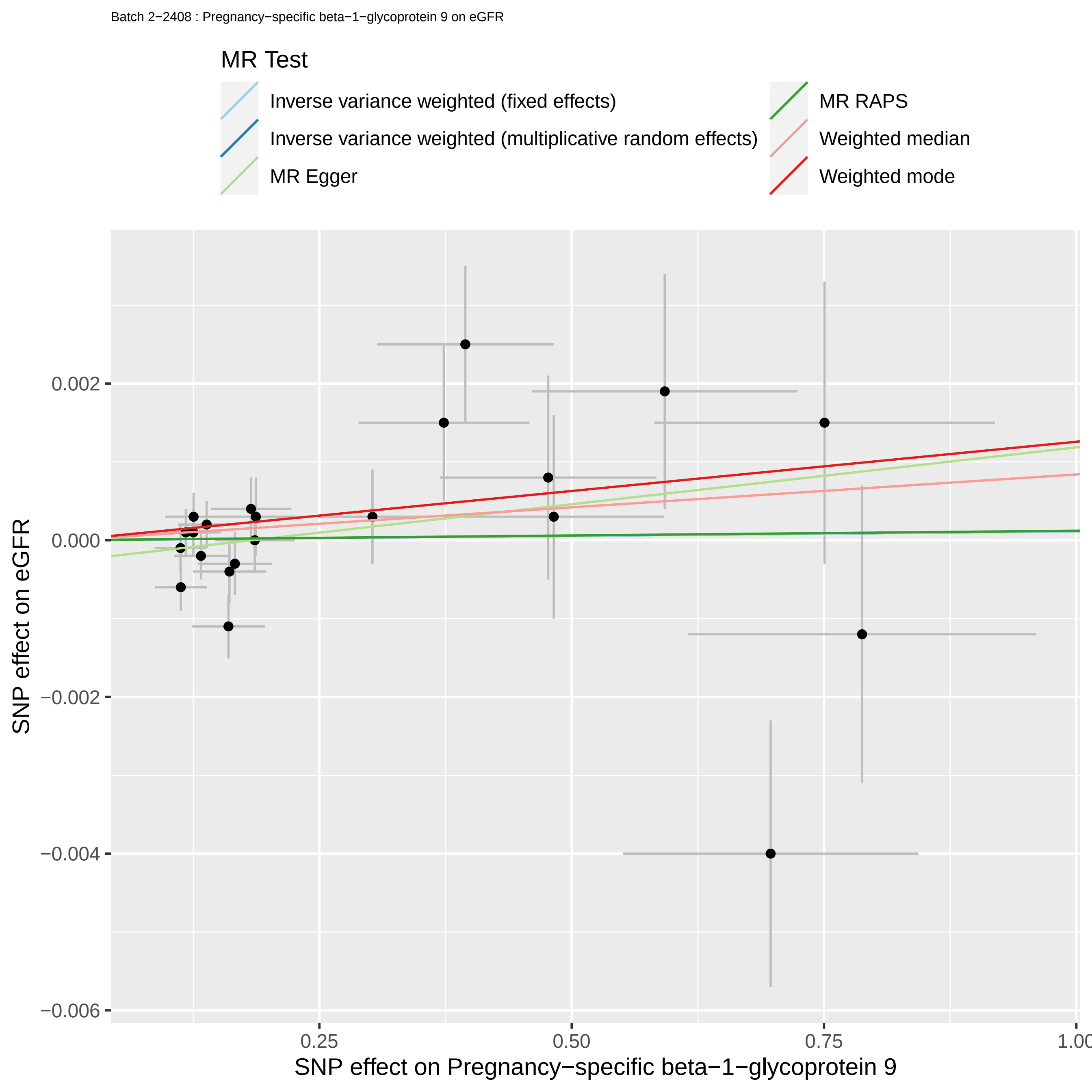
Batch 2−2408 : Pregnancy−specific beta−1−glycoprotein 9 on eGFR
MR Test
Inverse variance weighted (fixed effects)
Inverse variance weighted (multiplicative random effects)
MR Egger
MR RAPS
Weighted median
Weighted mode
0.002
0.000
−0.002
−0.004
−0.006
0.25
0.50
0.75
1.00
SNP effect on eGFR
SNP effect on Pregnancy−specific beta−1−glycoprotein 9

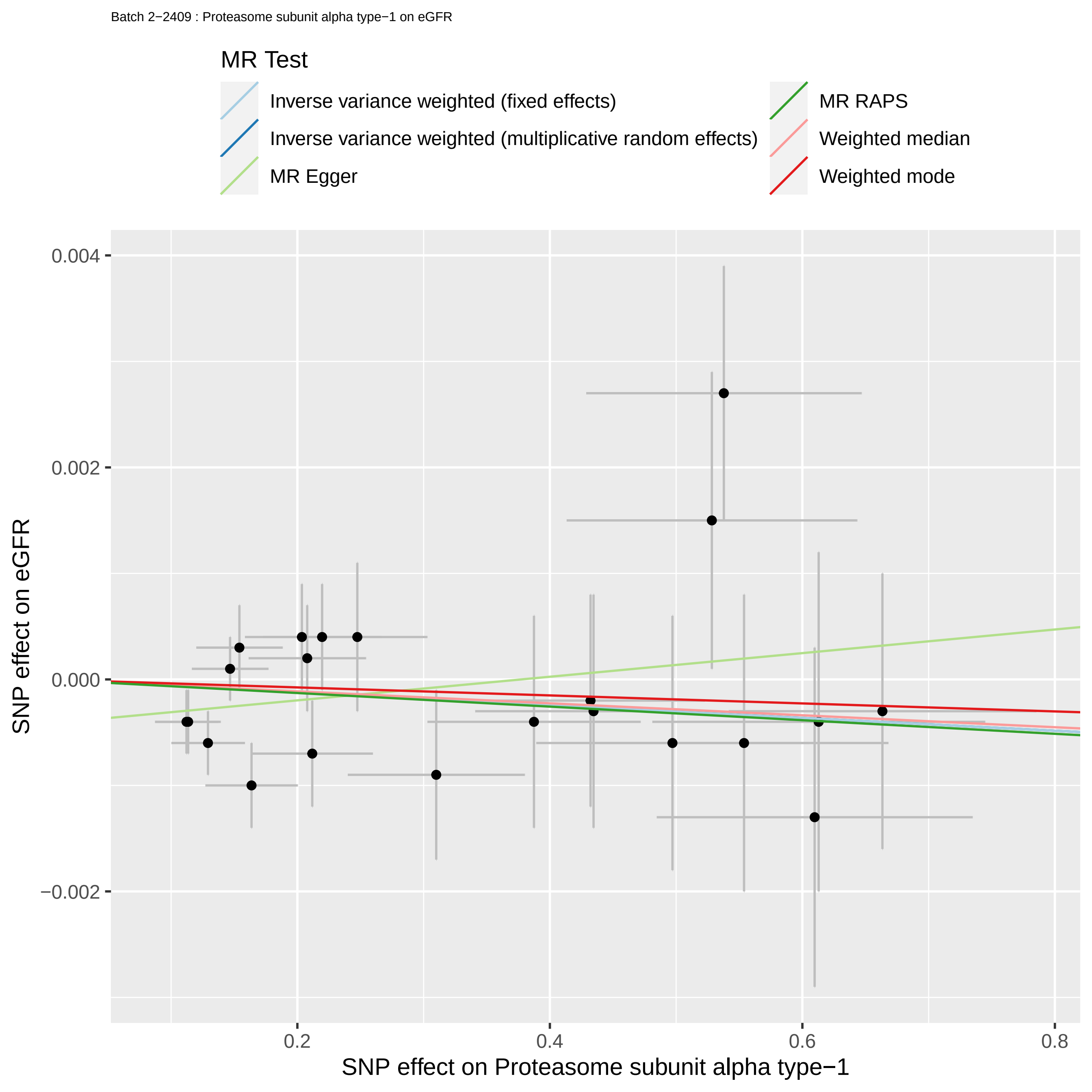

Batch 2−2409 : Proteasome subunit alpha type−1 on eGFR
MR Test
Inverse variance weighted (fixed effects)
Inverse variance weighted (multiplicative random effects)
MR Egger
MR RAPS
Weighted median
Weighted mode
0.004
0.002
0.000
−0.002
SNP effect on eGFR
0.2
0.4
0.6
0.8
SNP effect on Proteasome subunit alpha type−1

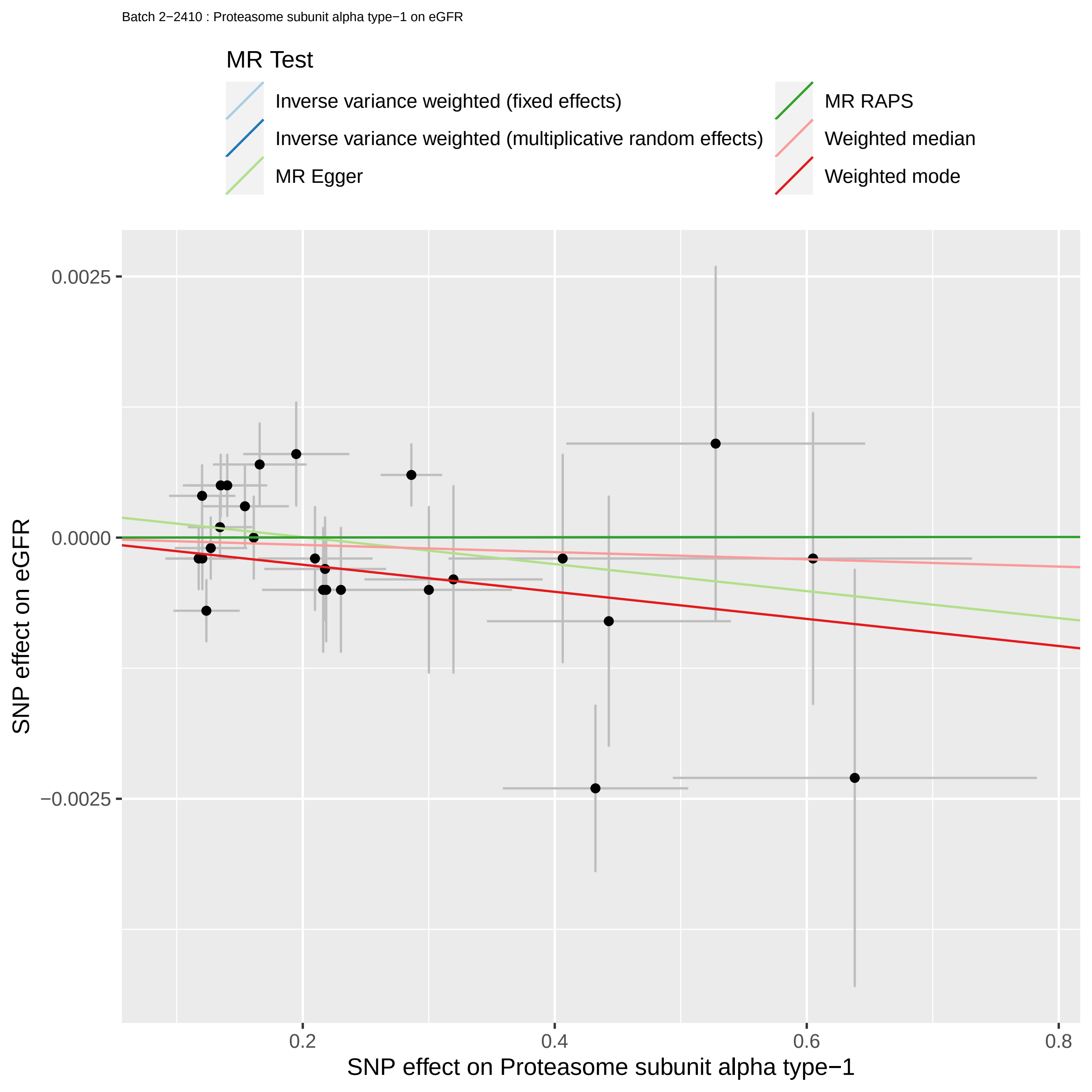

Batch 2−2410 : Proteasome subunit alpha type−1 on eGFR
MR Test
Inverse variance weighted (fixed effects)
Inverse variance weighted (multiplicative random effects)
MR Egger
MR RAPS
Weighted median
Weighted mode
0.0025
0.0000
−0.0025
SNP effect on eGFR
0.2
0.4
0.6
0.8
SNP effect on Proteasome subunit alpha type−1

Batch 2−2411 : Proteasome subunit alpha type−2 on eGFR

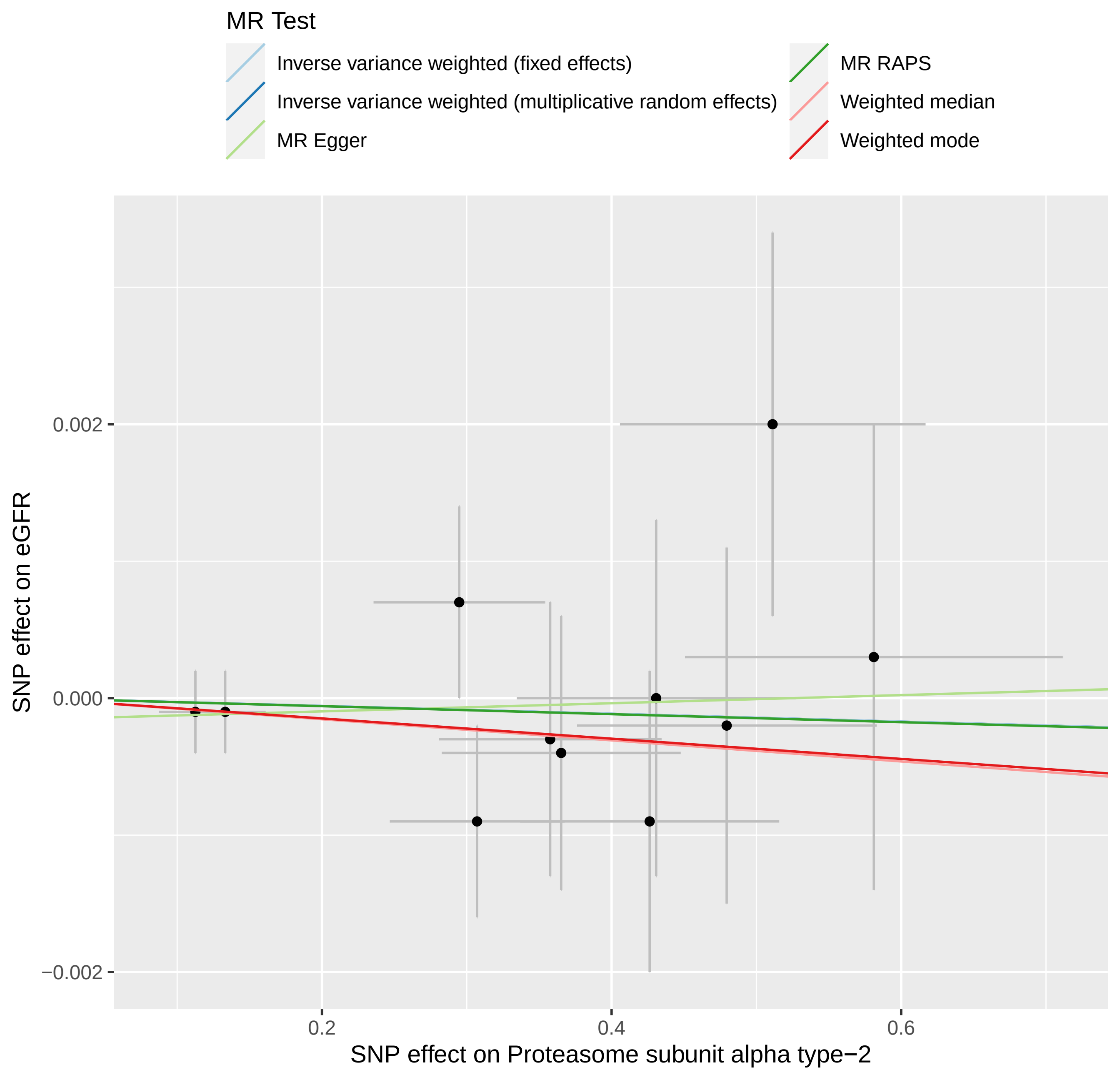

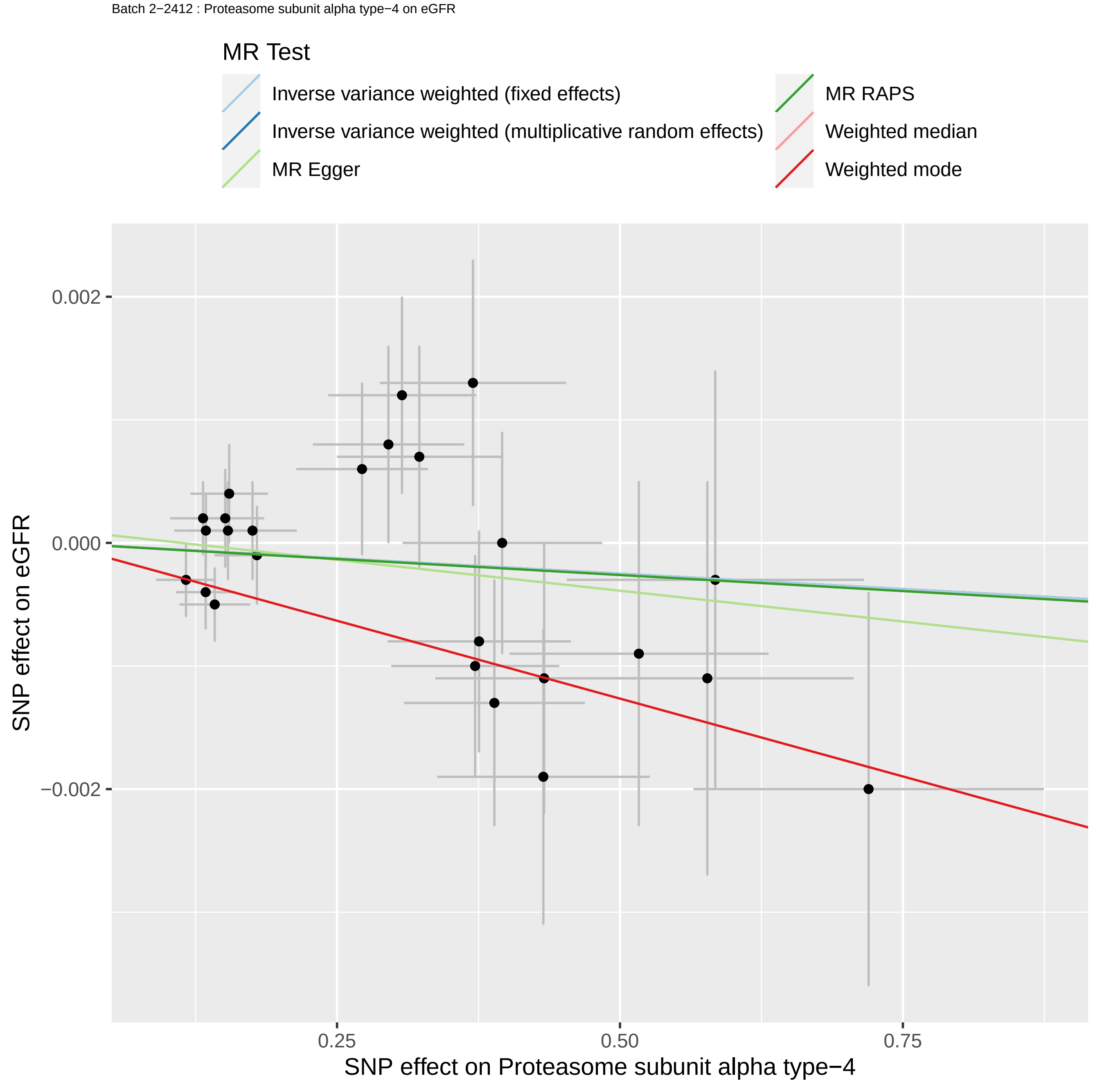

Batch 2−2412 : Proteasome subunit alpha type−4 on eGFR
MR Test
Inverse variance weighted (fixed effects)
Inverse variance weighted (multiplicative random effects)
MR Egger
MR RAPS
Weighted median
Weighted mode
0.002
0.000
−0.002
0.25
0.50
0.75
SNP effect on eGFR
SNP effect on Proteasome subunit alpha type−4

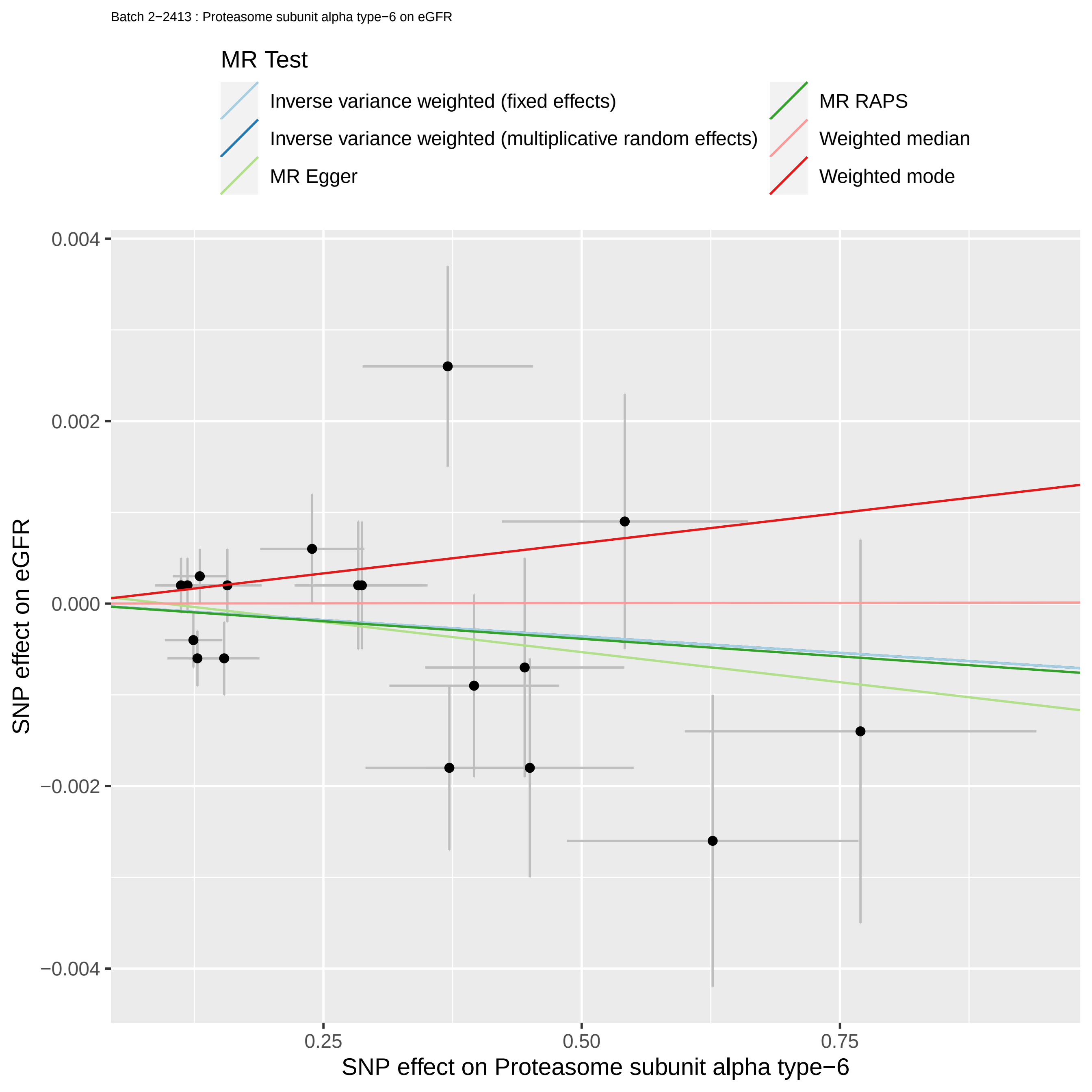

Batch 2−2413 : Proteasome subunit alpha type−6 on eGFR
MR Test
Inverse variance weighted (fixed effects)
Inverse variance weighted (multiplicative random effects)
MR Egger
MR RAPS
Weighted median
Weighted mode
0.004
0.002
0.000
−0.002
−0.004
SNP effect on eGFR
0.25
0.50
0.75
SNP effect on Proteasome subunit alpha type−6

Batch 2−2414 : Proteasome subunit beta type−5 on eGFR

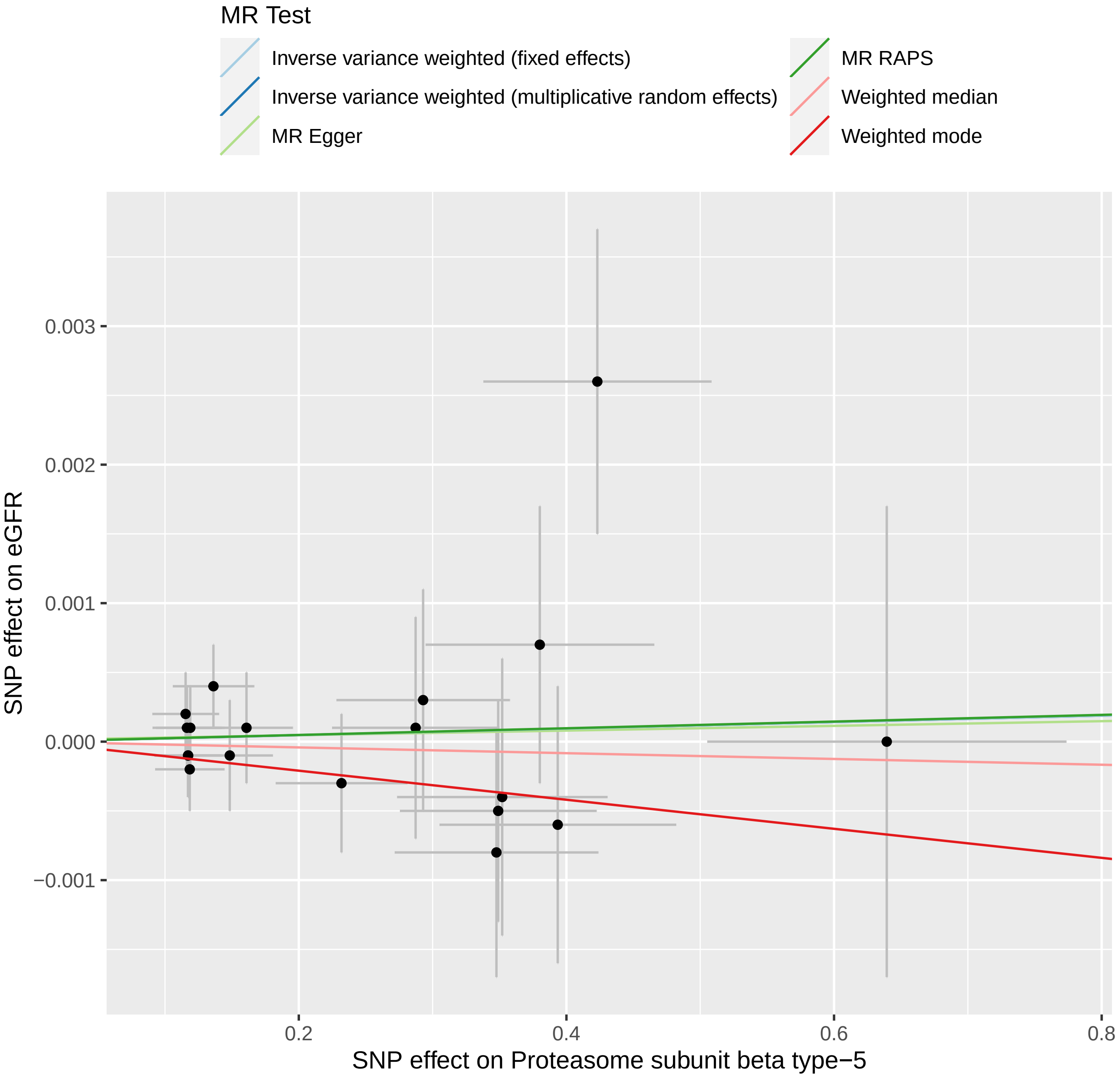

Batch 2−2415 : 26S proteasome non−ATPase regulatory subunit 11 on eGFR

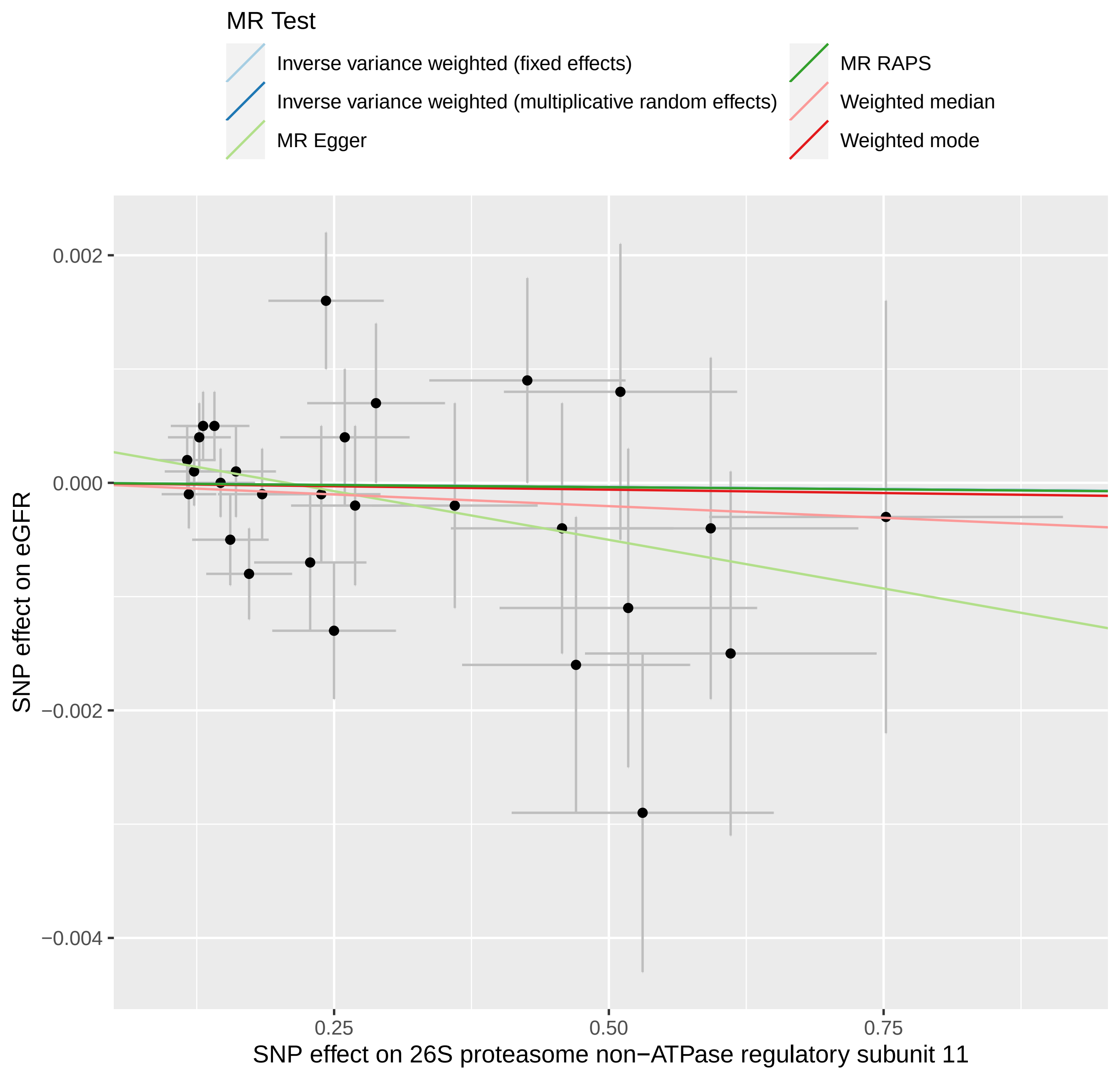

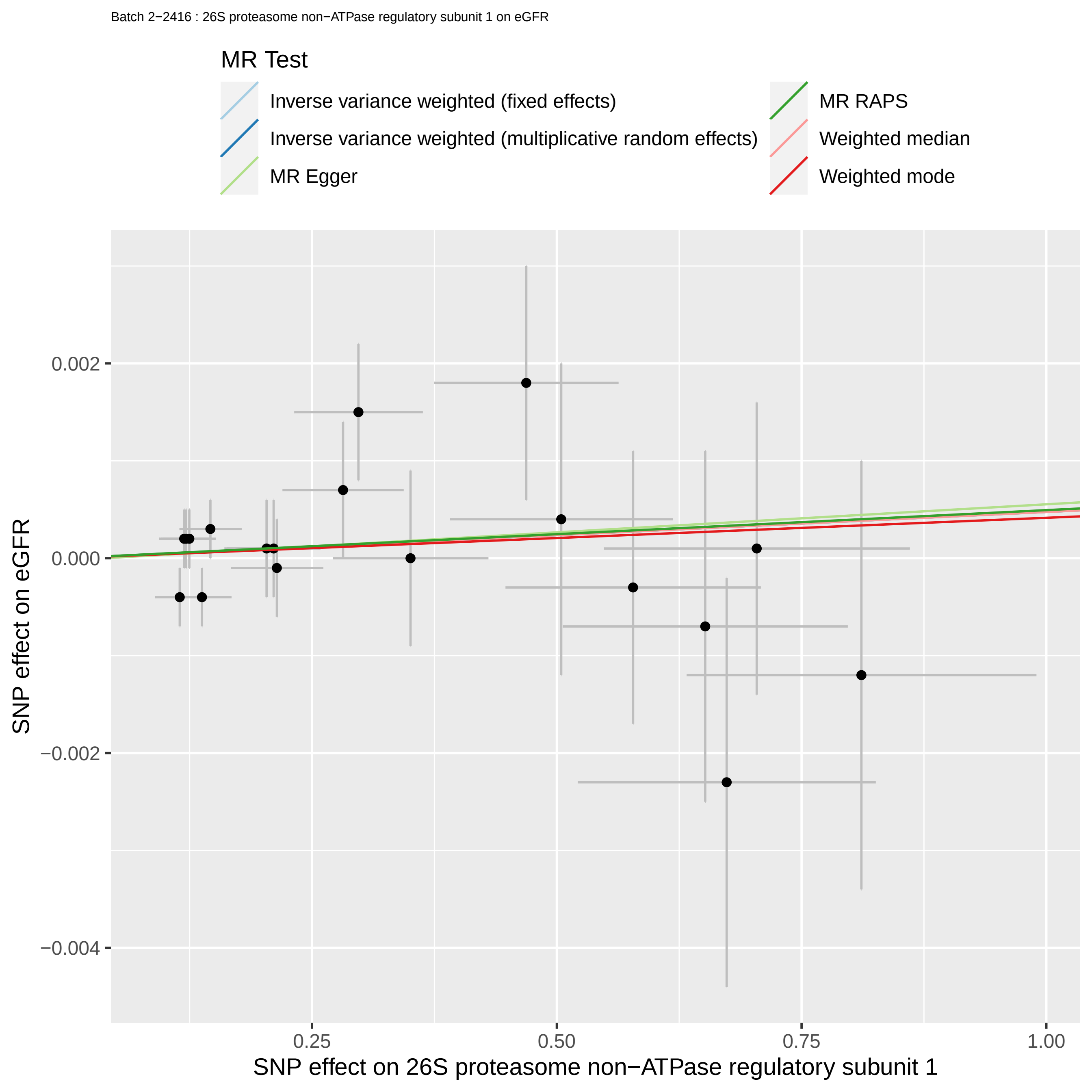

Batch 2−2416 : 26S proteasome non−ATPase regulatory subunit 1 on eGFR
MR Test
Inverse variance weighted (fixed effects)
Inverse variance weighted (multiplicative random effects)
MR Egger
MR RAPS
Weighted median
Weighted mode
0.002
0.000
−0.002
−0.004
SNP effect on eGFR
0.25
0.50
0.75
1.00
SNP effect on 26S proteasome non−ATPase regulatory subunit 1

Batch 2−2417 : 26S proteasome non−ATPase regulatory subunit 4 on eGFR

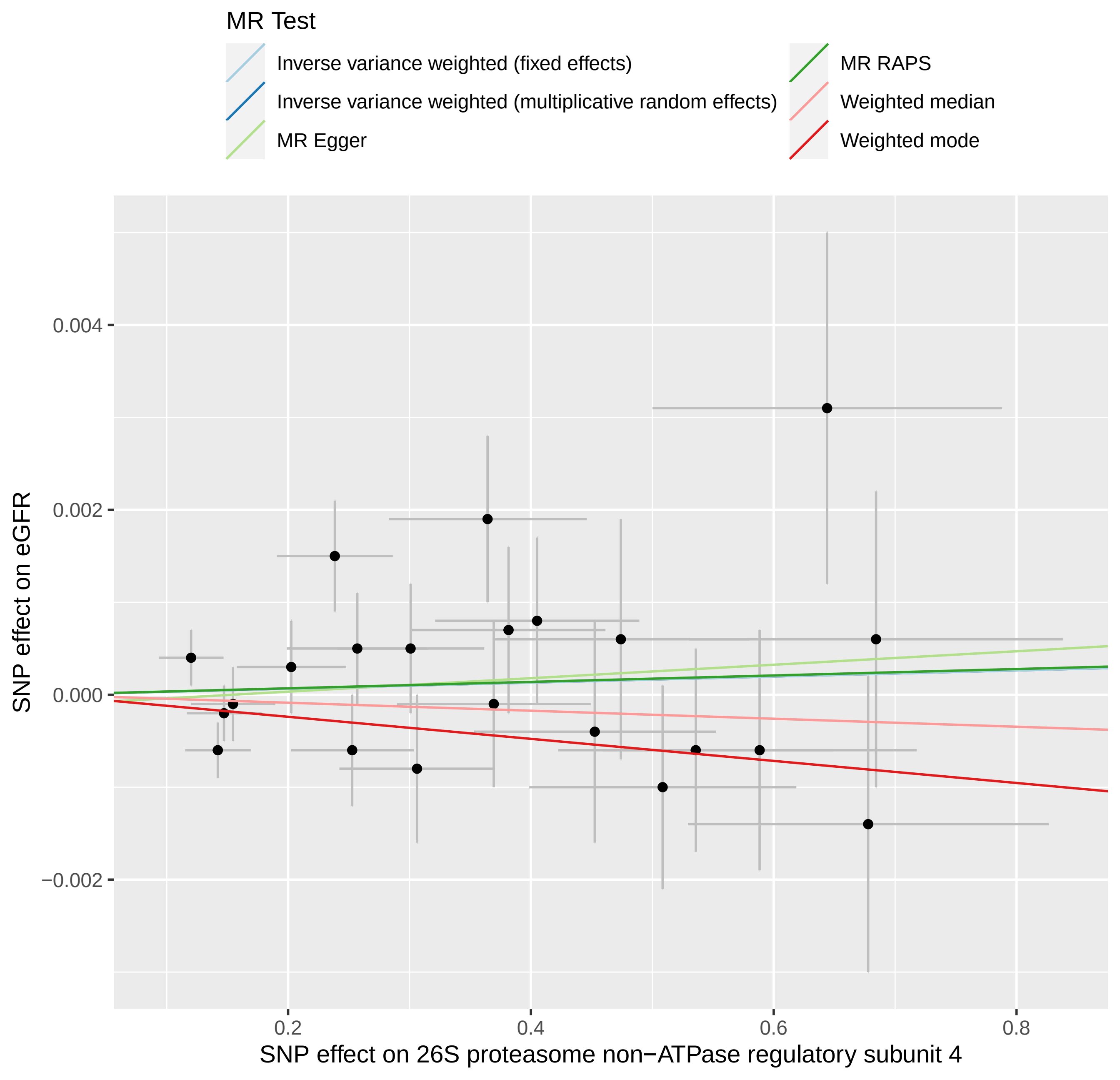

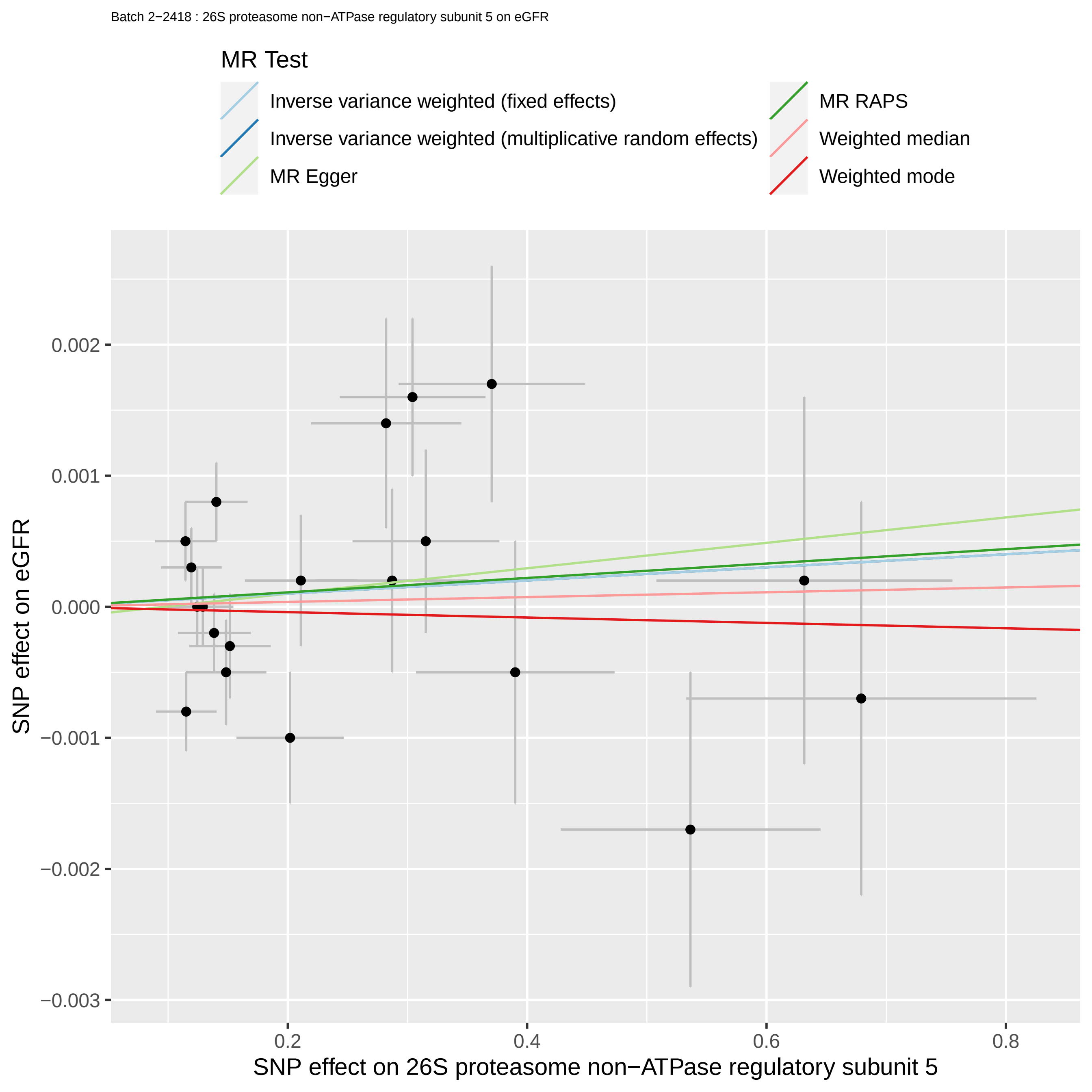

Batch 2−2418 : 26S proteasome non−ATPase regulatory subunit 5 on eGFR
MR Test
Inverse variance weighted (fixed effects)
Inverse variance weighted (multiplicative random effects)
MR Egger
MR RAPS
Weighted median
Weighted mode
0.002
0.001
0.000
−0.001
−0.002
−0.003
SNP effect on eGFR
0.2
0.4
0.6
0.8
SNP effect on 26S proteasome non−ATPase regulatory subunit 5

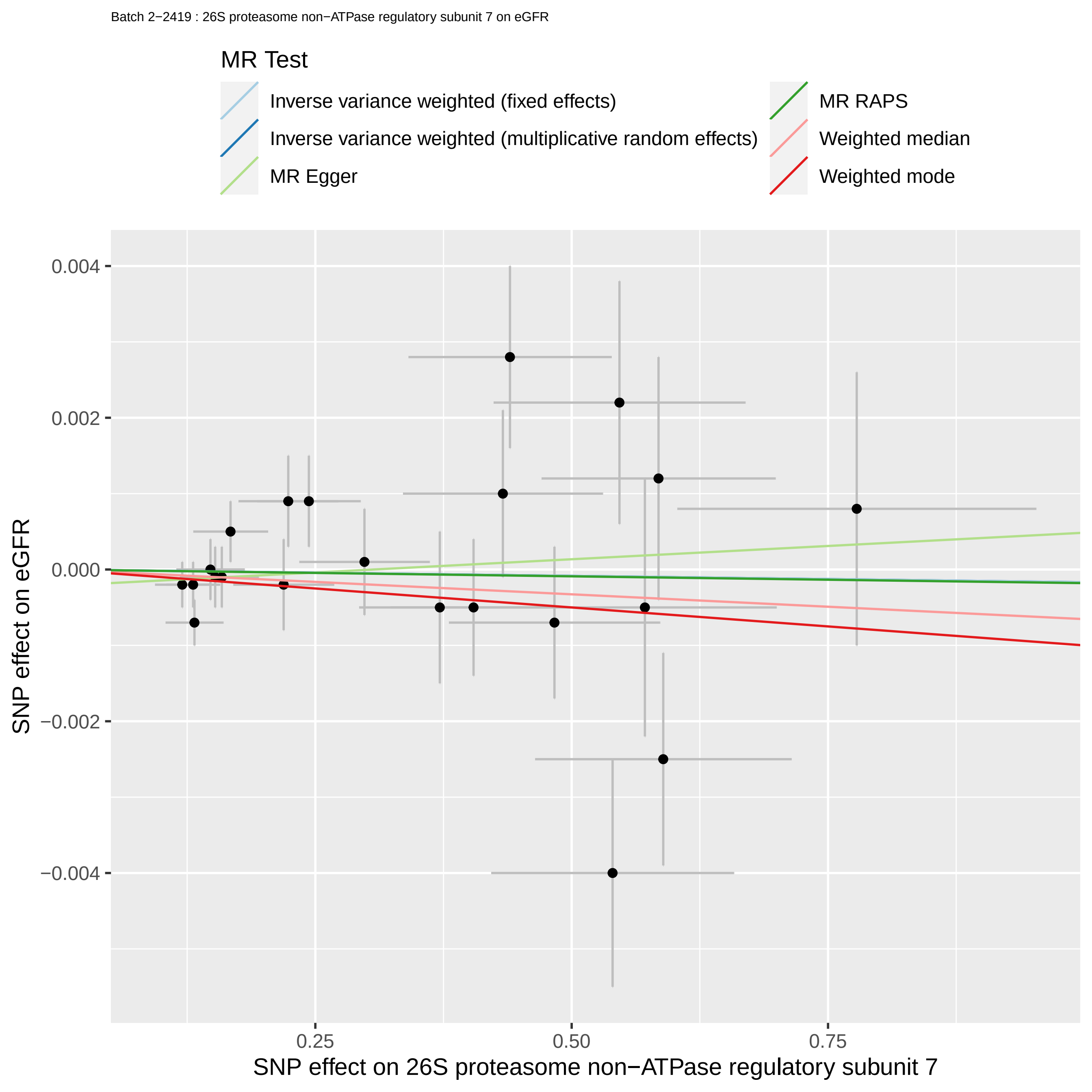
Batch 2−2419 : 26S proteasome non−ATPase regulatory subunit 7 on eGFR
MR Test
Inverse variance weighted (fixed effects)
Inverse variance weighted (multiplicative random effects)
MR Egger
MR RAPS
Weighted median
Weighted mode
0.004
0.002
0.000
−0.002
−0.004
0.25
0.50
0.75
SNP effect on eGFR
SNP effect on 26S proteasome non−ATPase regulatory subunit 7

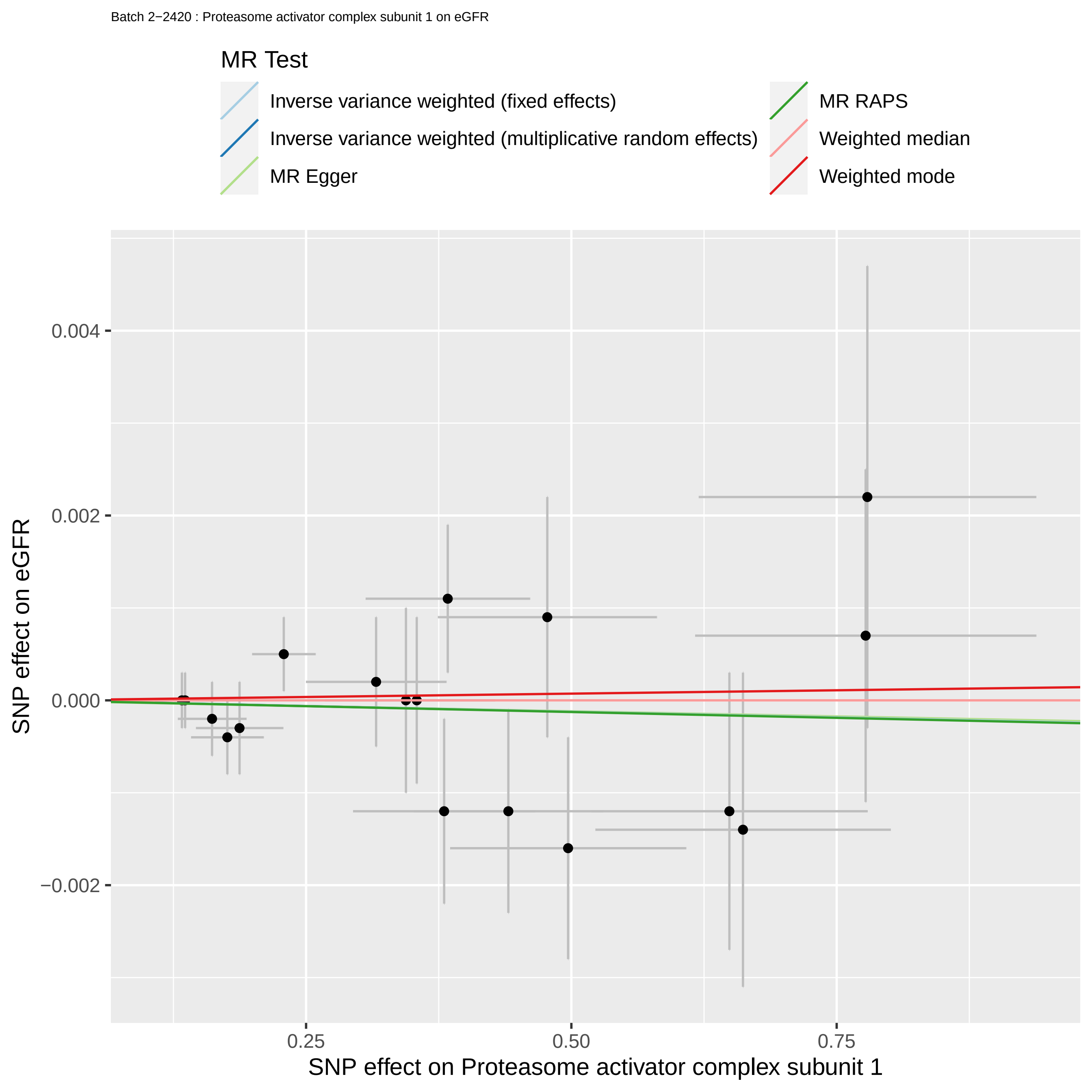

Batch 2−2420 : Proteasome activator complex subunit 1 on eGFR
MR Test
Inverse variance weighted (fixed effects)
Inverse variance weighted (multiplicative random effects)
MR Egger
MR RAPS
Weighted median
Weighted mode
0.004
0.002
0.000
−0.002
0.25
0.50
0.75
SNP effect on eGFR
SNP effect on Proteasome activator complex subunit 1

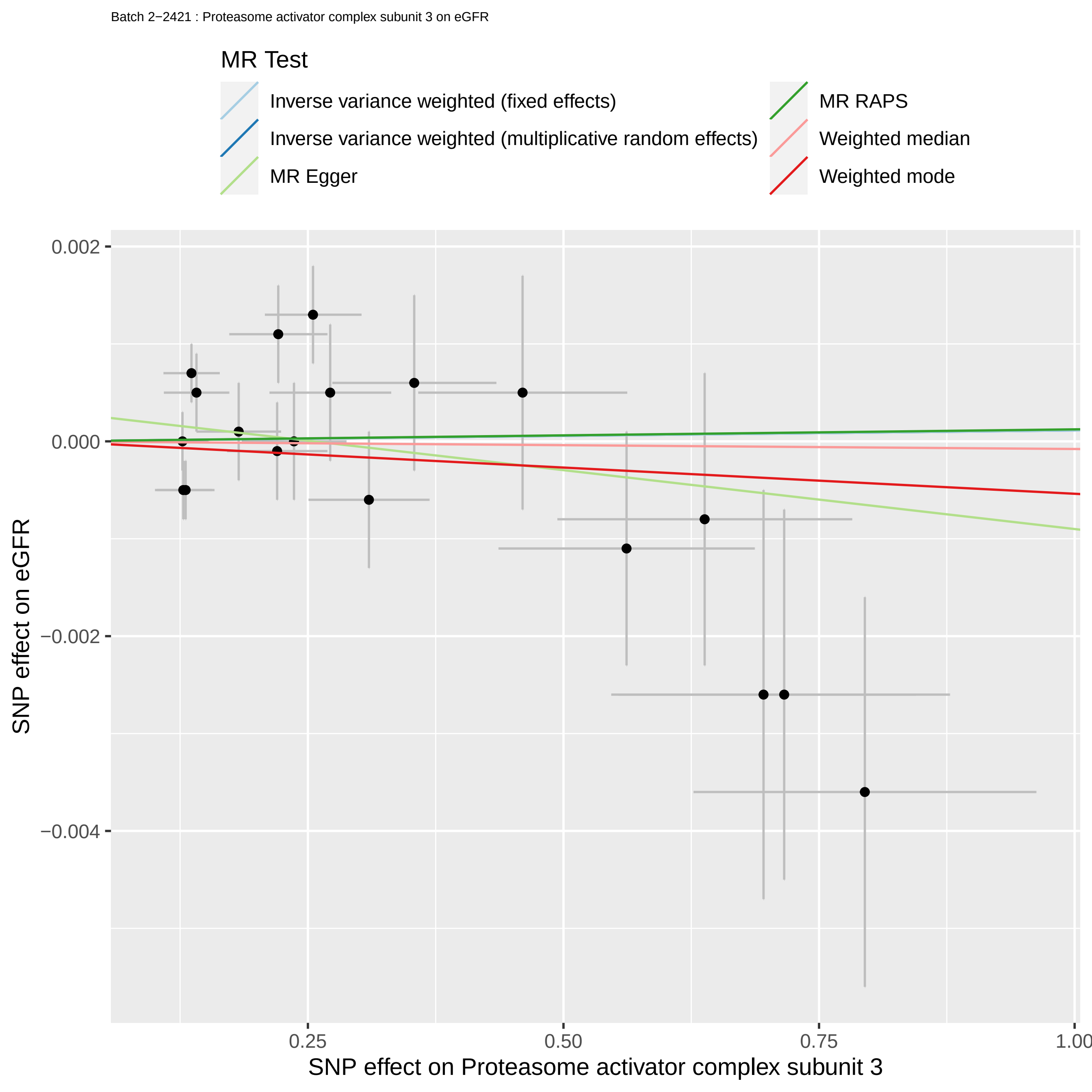
Batch 2−2421 : Proteasome activator complex subunit 3 on eGFR
MR Test
Inverse variance weighted (fixed effects)
Inverse variance weighted (multiplicative random effects)
MR Egger
MR RAPS
Weighted median
Weighted mode
0.002
0.000
−0.002
−0.004
SNP effect on eGFR
0.25
0.50
0.75
1.00
SNP effect on Proteasome activator complex subunit 3

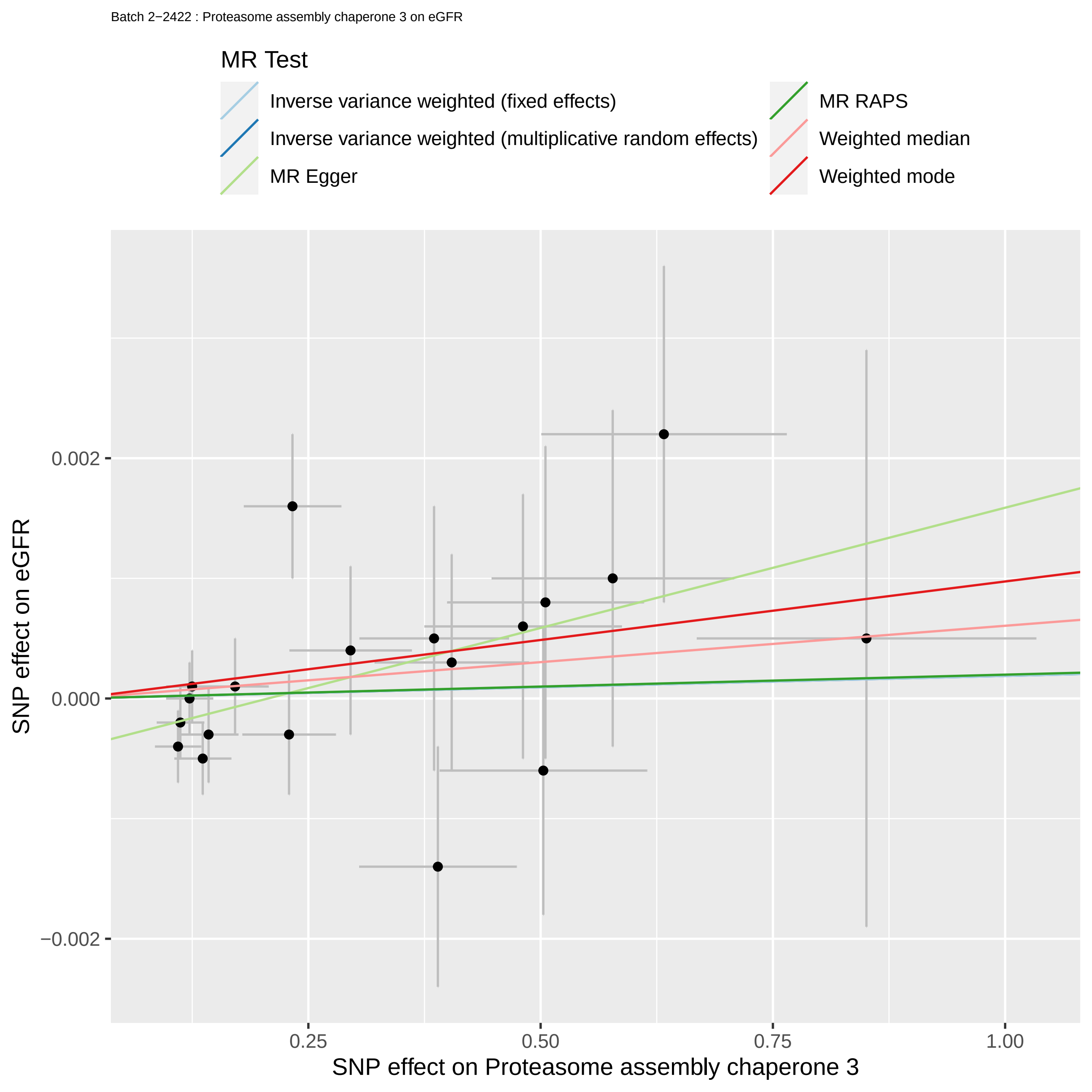

Batch 2−2422 : Proteasome assembly chaperone 3 on eGFR
MR Test
Inverse variance weighted (fixed effects)
Inverse variance weighted (multiplicative random effects)
MR Egger
MR RAPS
Weighted median
Weighted mode
SNP effect on eGFR
0.002
0.000
−0.002
0.25
0.50
0.75
1.00
SNP effect on Proteasome assembly chaperone 3

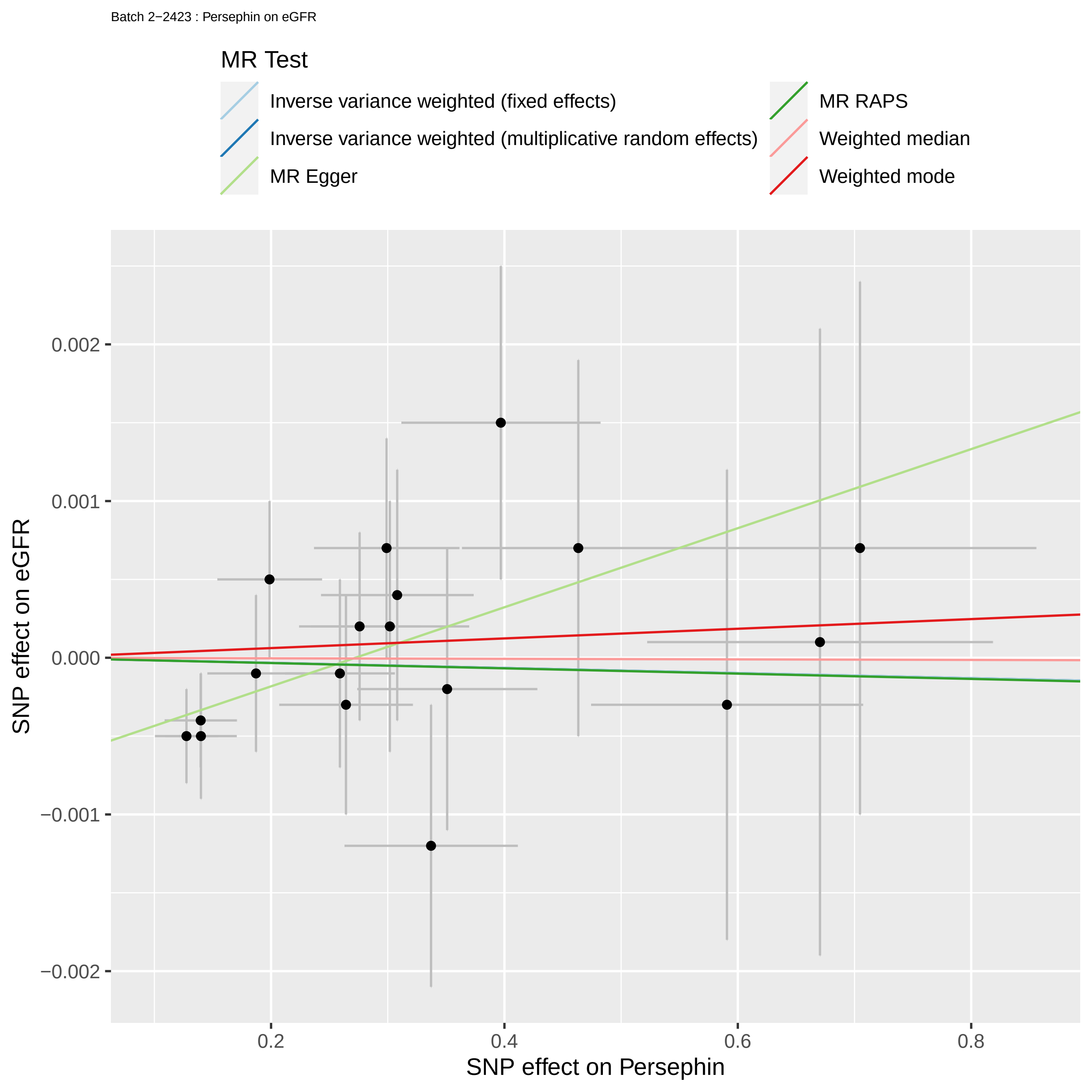
Batch 2−2423 : Persephin on eGFR
MR Test
Inverse variance weighted (fixed effects)
Inverse variance weighted (multiplicative random effects)
MR Egger
MR RAPS
Weighted median
Weighted mode
0.002
0.001
0.000
−0.001
−0.002
0.2
0.4
0.6
0.8
SNP effect on eGFR
SNP effect on Persephin

Batch 2−2424 : Patched domain−containing protein 3 on eGFR

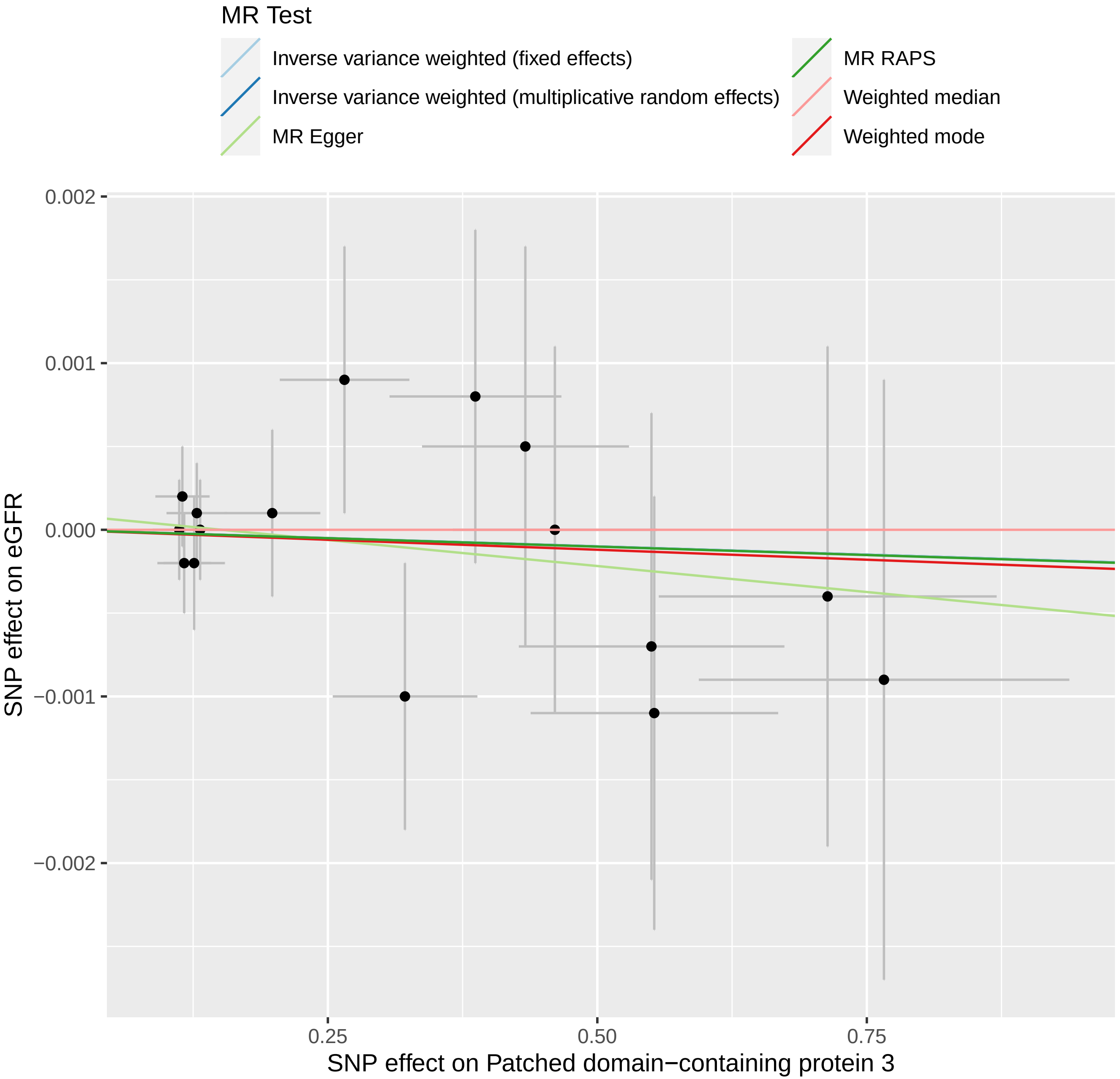

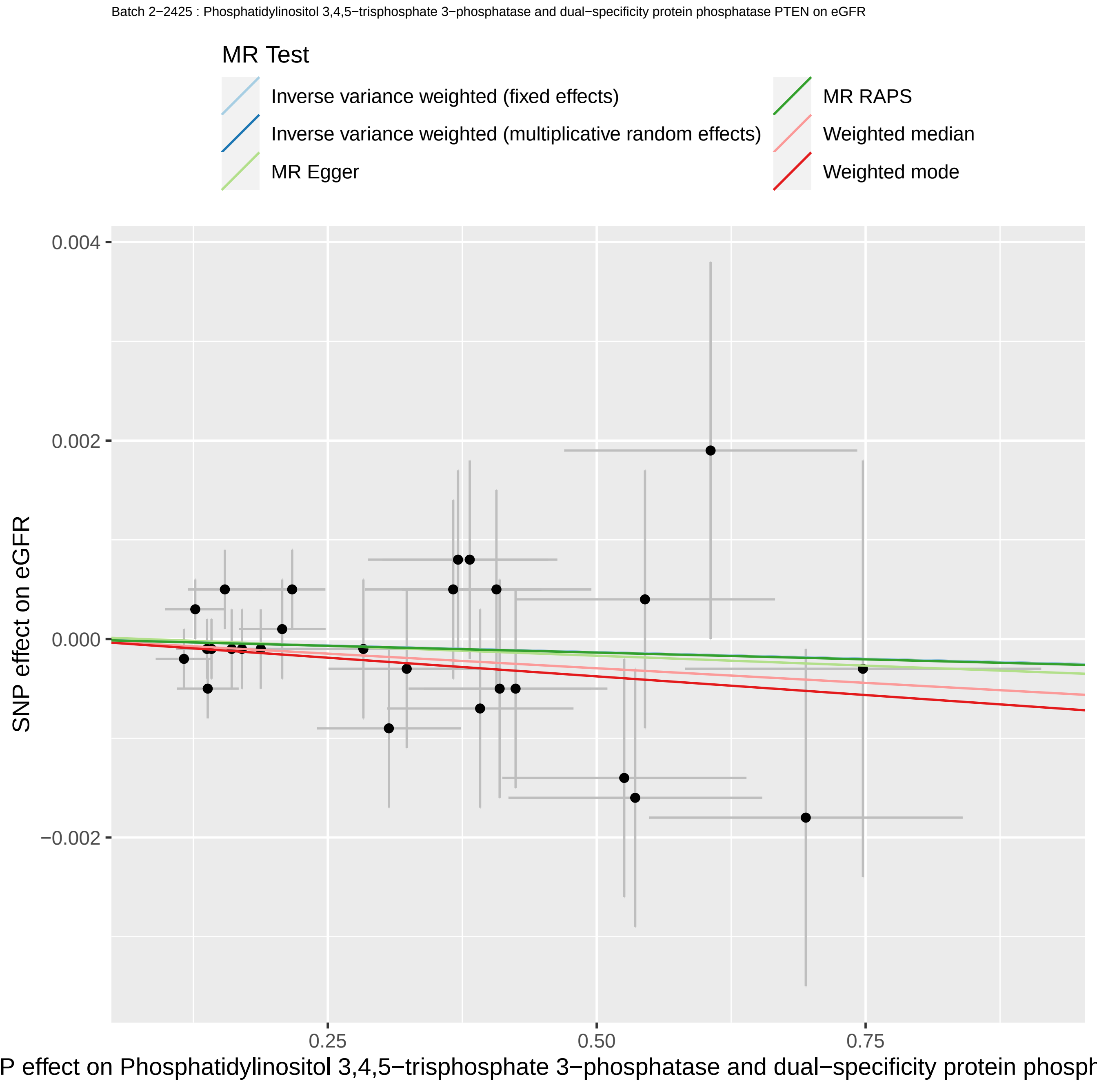

Batch 2−2425 : Phosphatidylinositol 3,4,5−trisphosphate 3−phosphatase and dual−specificity protein phosphatase PTEN on eGFR
MR Test
Inverse variance weighted (fixed effects)
Inverse variance weighted (multiplicative random effects)
MR Egger
MR RAPS
Weighted median
Weighted mode
0.004
0.002
0.000
−0.002
SNP effect on eGFR
0.25
0.50
0.75
P effect on Phosphatidylinositol 3,4,5−trisphosphate 3−phosphatase and dual−specificity protein phosph

Batch 2−2426 : Prostaglandin−H2 D−isomerase on eGFR

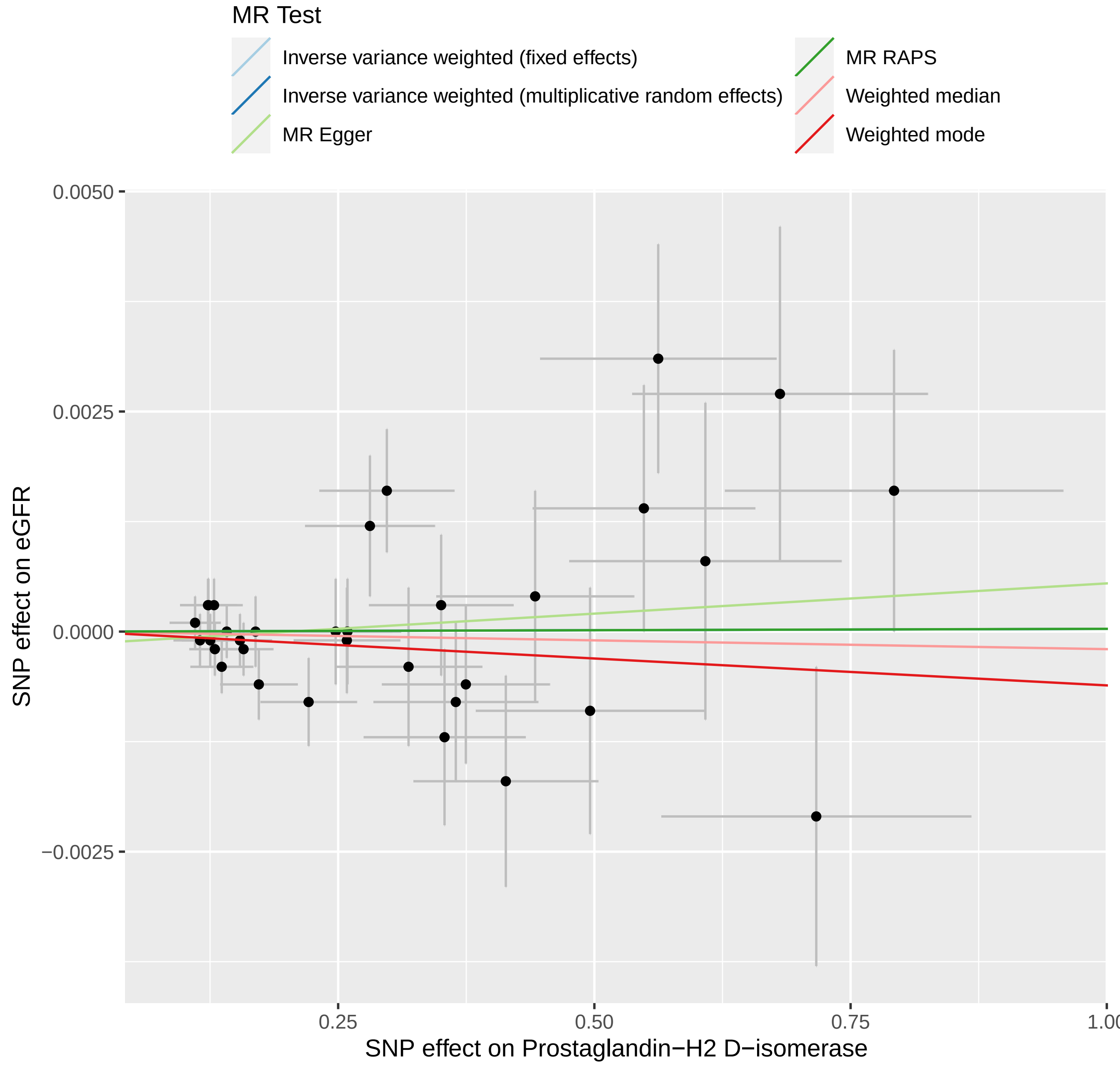

Batch 2−2427 : Prostaglandin F2 receptor negative regulator on eGFR

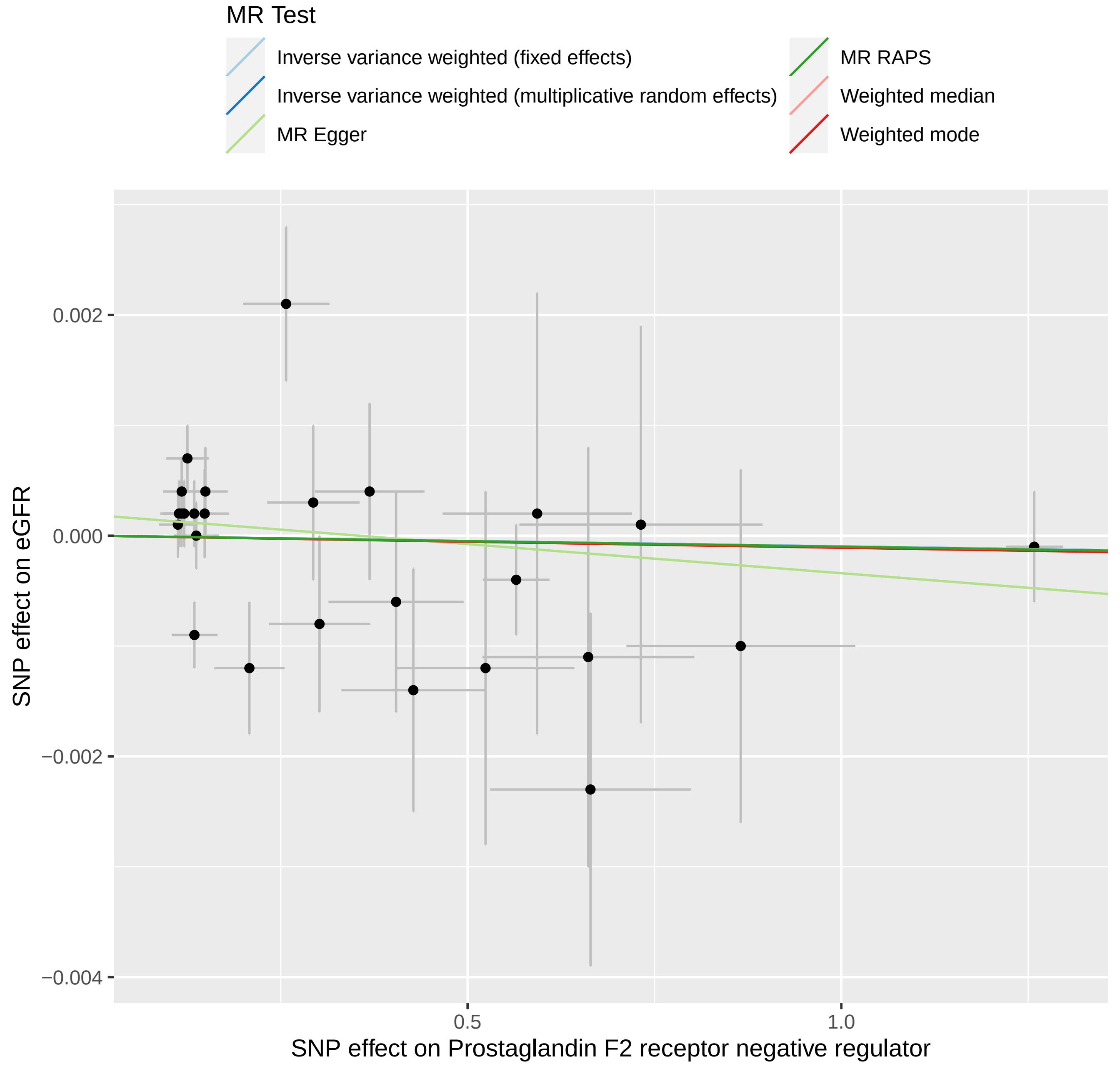

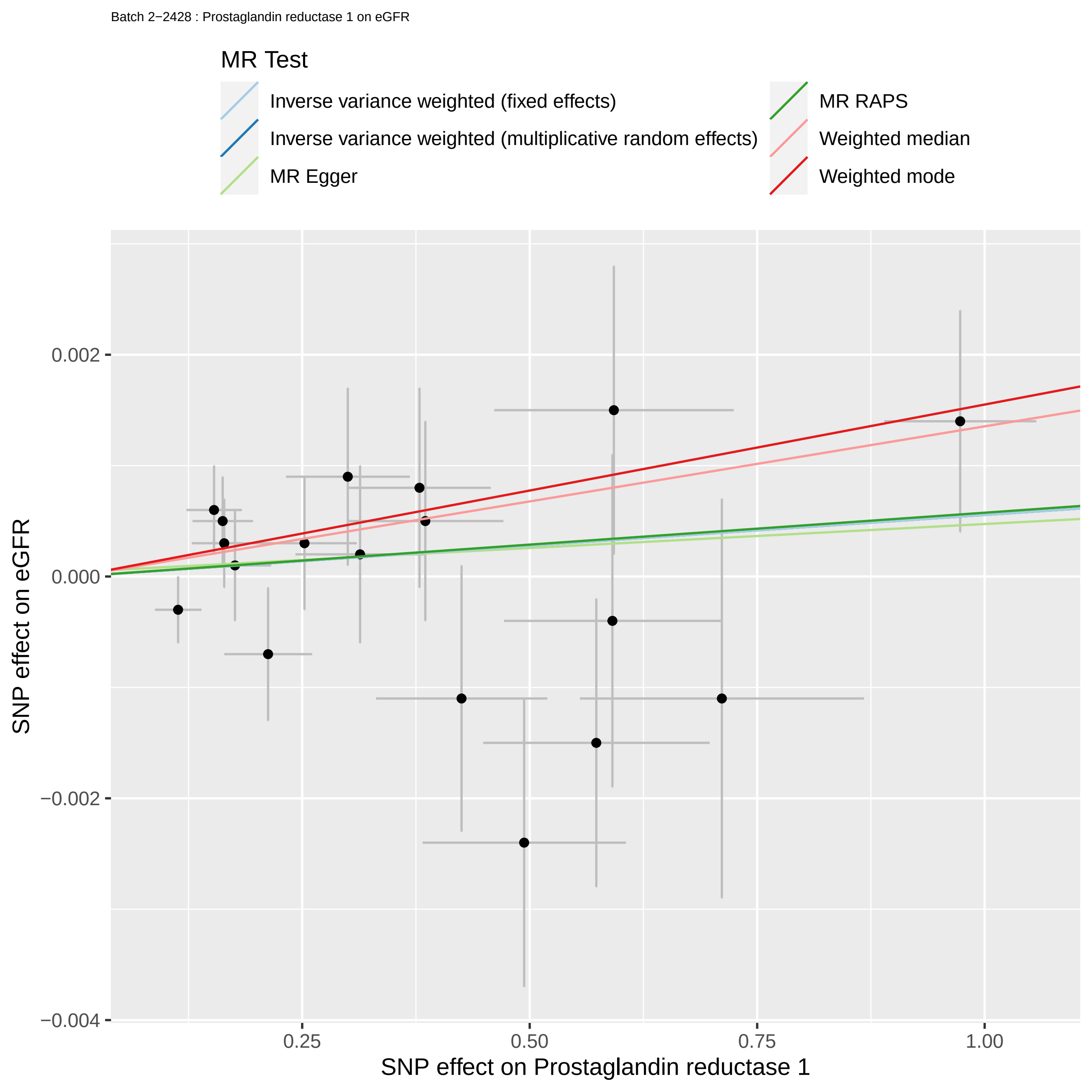

Batch 2−2428 : Prostaglandin reductase 1 on eGFR
MR Test
Inverse variance weighted (fixed effects)
Inverse variance weighted (multiplicative random effects)
MR Egger
MR RAPS
Weighted median
Weighted mode
0.002
0.000
−0.002
−0.004
SNP effect on eGFR
0.25
0.50
0.75
1.00
SNP effect on Prostaglandin reductase 1

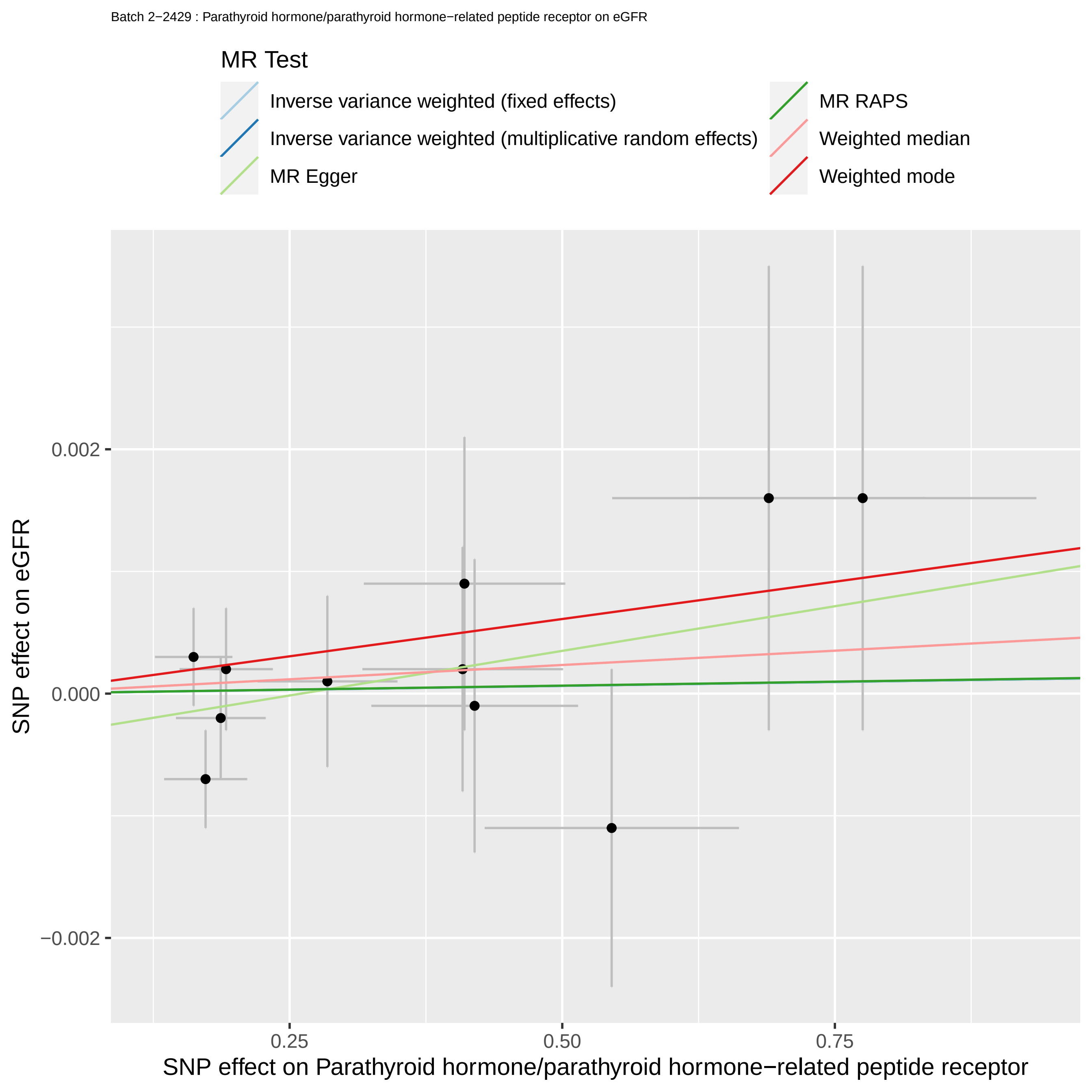

Batch 2−2429 : Parathyroid hormone/parathyroid hormone−related peptide receptor on eGFR
MR Test
Inverse variance weighted (fixed effects)
Inverse variance weighted (multiplicative random effects)
MR Egger
MR RAPS
Weighted median
Weighted mode
0.002
0.000
−0.002
0.25
0.50
0.75
SNP effect on eGFR
SNP effect on Parathyroid hormone/parathyroid hormone−related peptide receptor

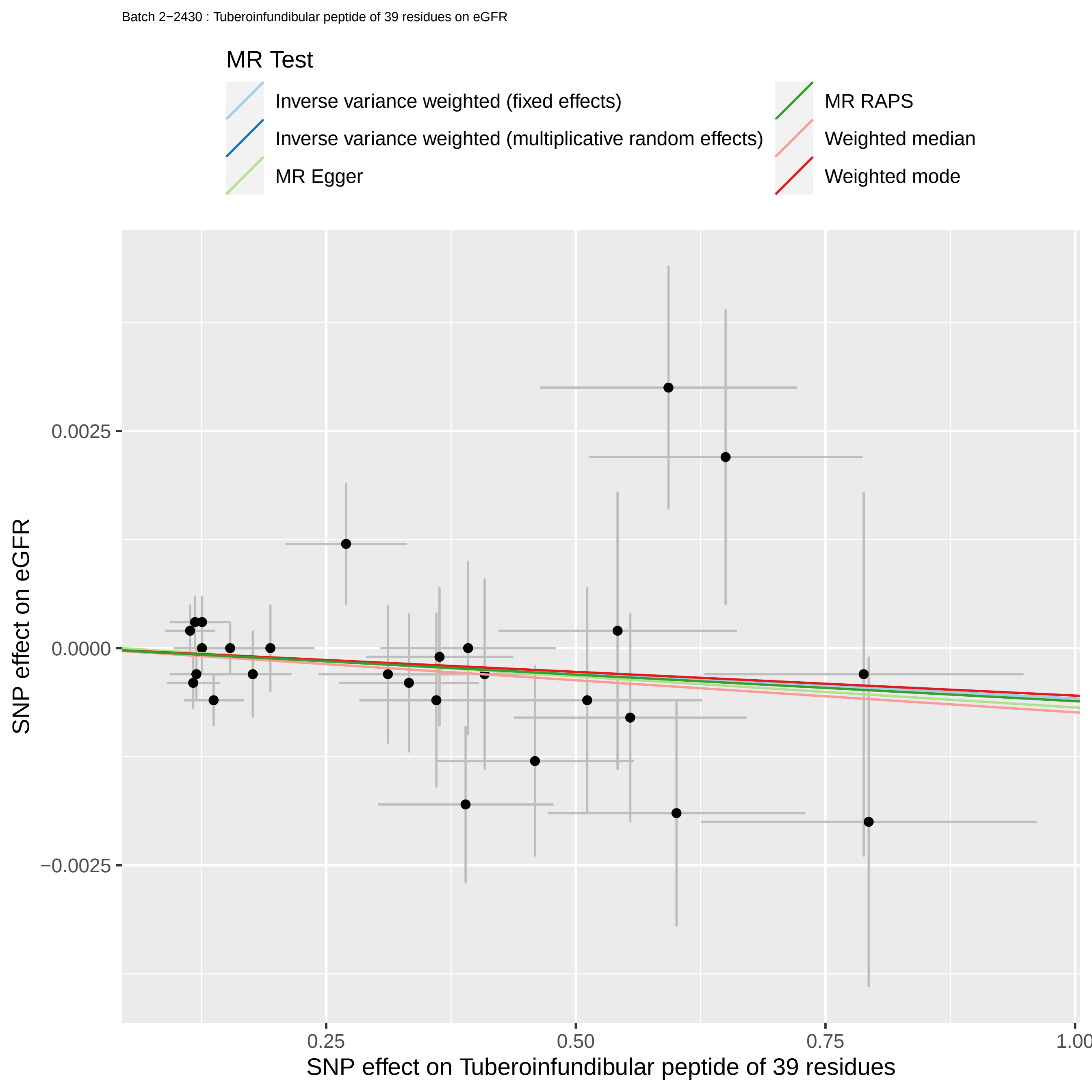

Batch 2−2430 : Tuberoinfundibular peptide of 39 residues on eGFR
MR Test
Inverse variance weighted (fixed effects)
Inverse variance weighted (multiplicative random effects)
MR Egger
MR RAPS
Weighted median
Weighted mode
0.0025
0.0000
−0.0025
SNP effect on eGFR
0.25
0.50
0.75
1.00
SNP effect on Tuberoinfundibular peptide of 39 residues

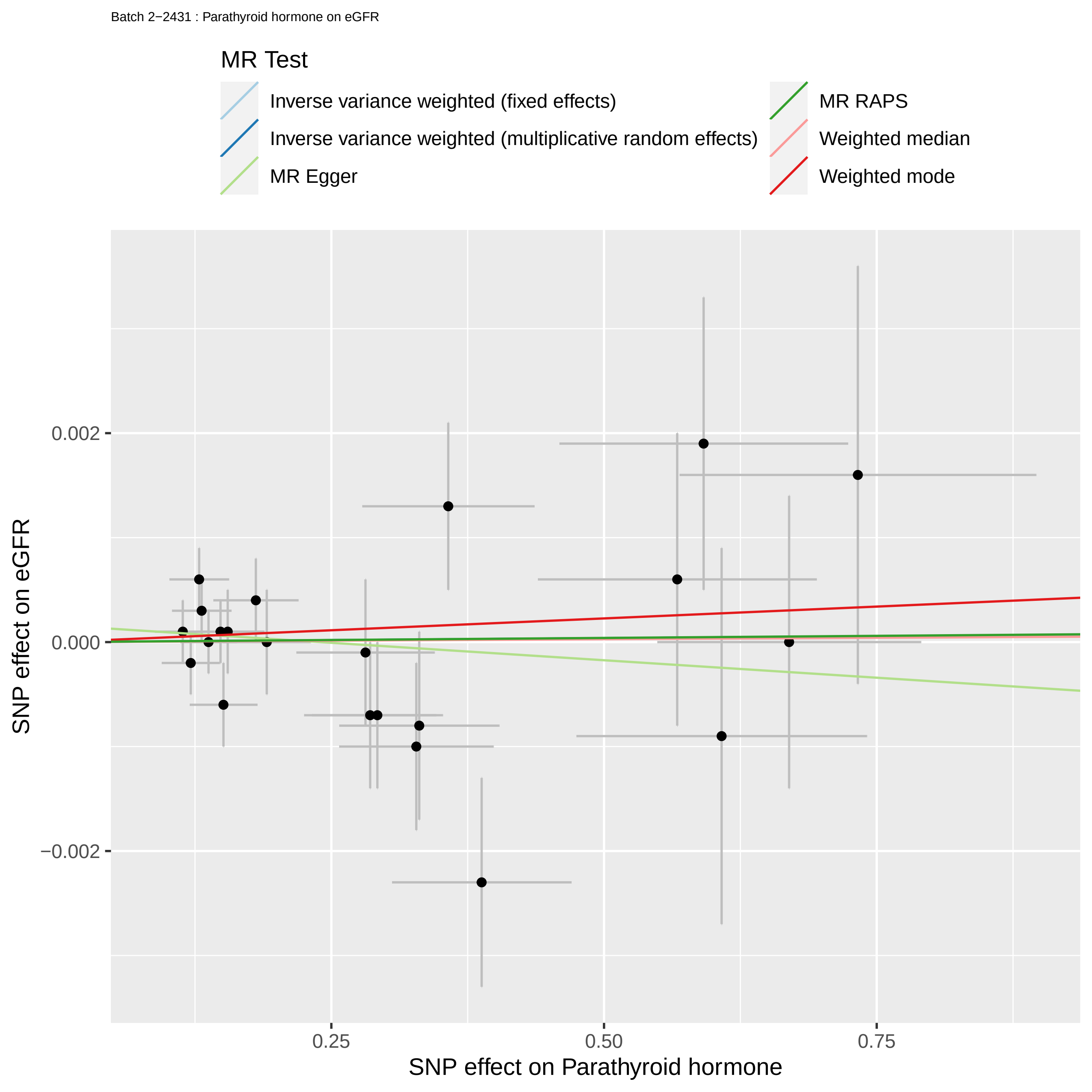

Batch 2−2431 : Parathyroid hormone on eGFR
MR Test
Inverse variance weighted (fixed effects)
Inverse variance weighted (multiplicative random effects)
MR Egger
MR RAPS
Weighted median
Weighted mode
0.002
0.000
−0.002
0.25
0.50
0.75
SNP effect on eGFR
SNP effect on Parathyroid hormone

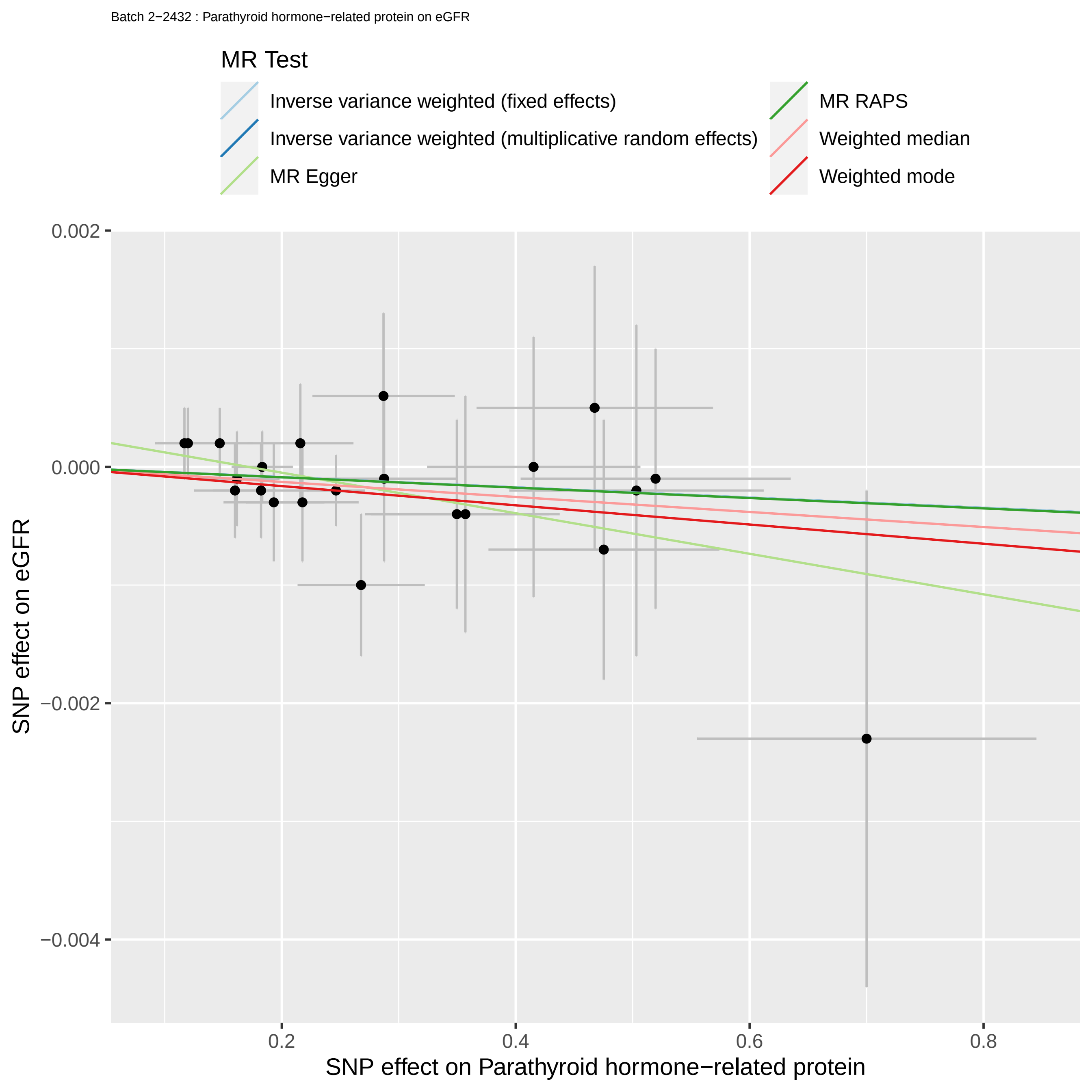

Batch 2−2432 : Parathyroid hormone−related protein on eGFR
MR Test
Inverse variance weighted (fixed effects)
Inverse variance weighted (multiplicative random effects)
MR Egger
MR RAPS
Weighted median
Weighted mode
0.002
0.000
−0.002
−0.004
0.2
0.4
0.6
0.8
SNP effect on eGFR
SNP effect on Parathyroid hormone−related protein

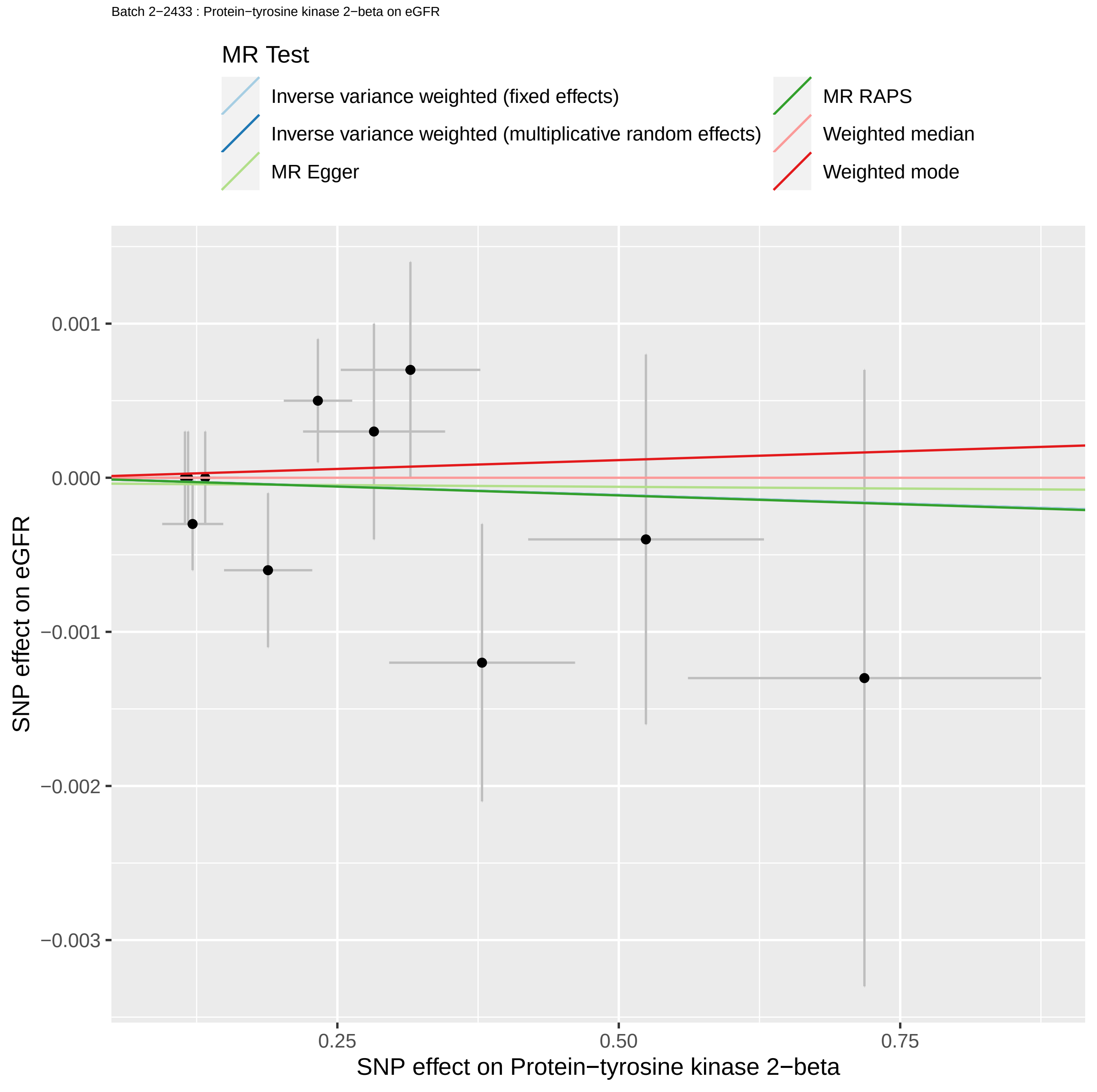
Batch 2−2433 : Protein−tyrosine kinase 2−beta on eGFR
MR Test
Inverse variance weighted (fixed effects)
Inverse variance weighted (multiplicative random effects)
MR Egger
MR RAPS
Weighted median
Weighted mode
0.001
0.000
−0.001
−0.002
−0.003
0.25
0.50
0.75
SNP effect on eGFR
SNP effect on Protein−tyrosine kinase 2−beta

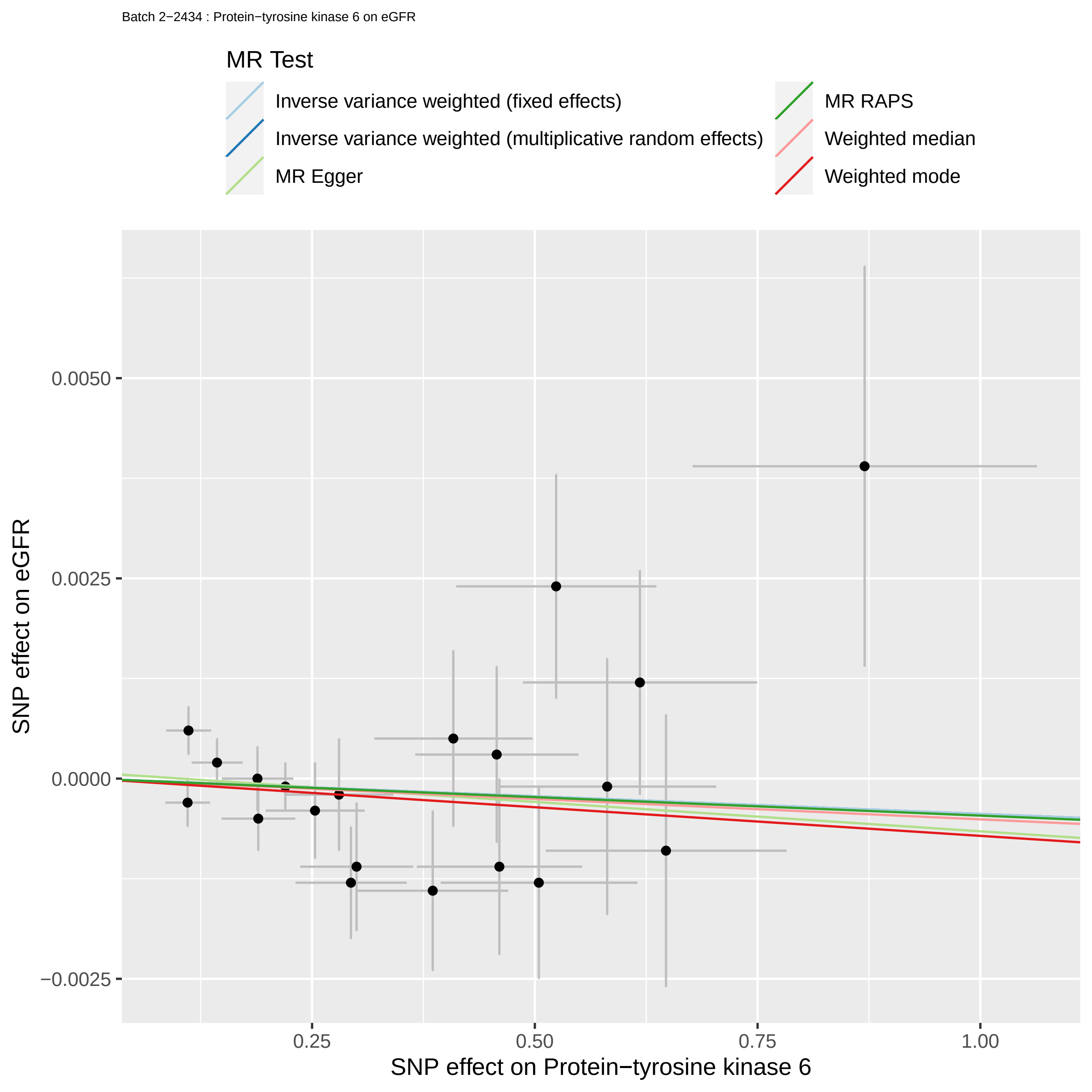
Batch 2−2434 : Protein−tyrosine kinase 6 on eGFR
MR Test
Inverse variance weighted (fixed effects)
Inverse variance weighted (multiplicative random effects)
MR Egger
MR RAPS
Weighted median
Weighted mode
SNP effect on eGFR
0.0050
0.0025
0.0000
−0.0025
0.25
0.50
0.75
1.00
SNP effect on Protein−tyrosine kinase 6

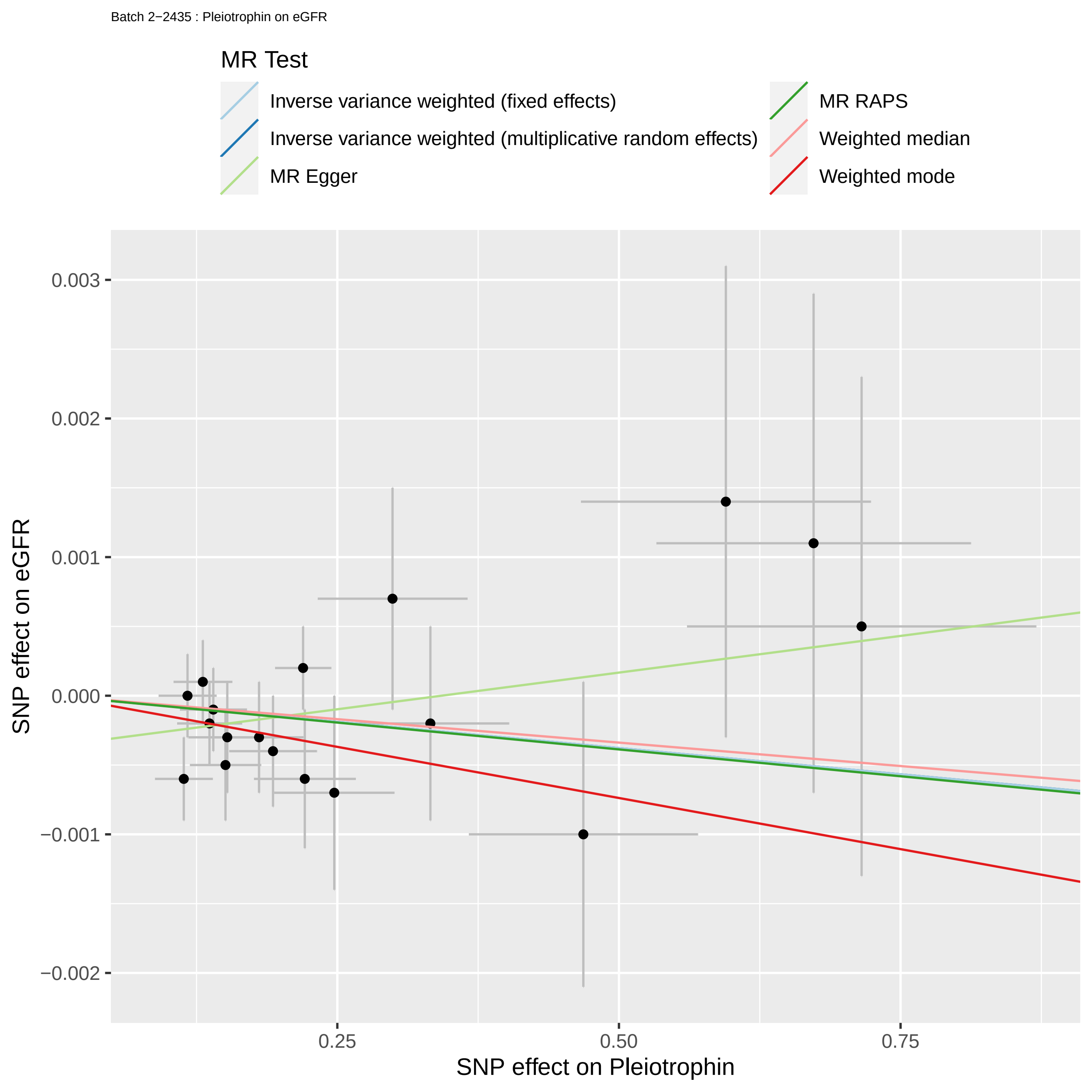

Batch 2−2435 : Pleiotrophin on eGFR
MR Test
Inverse variance weighted (fixed effects)
Inverse variance weighted (multiplicative random effects)
MR Egger
MR RAPS
Weighted median
Weighted mode
0.003
0.002
0.001
0.000
−0.001
−0.002
0.25
0.50
0.75
SNP effect on eGFR
SNP effect on Pleiotrophin

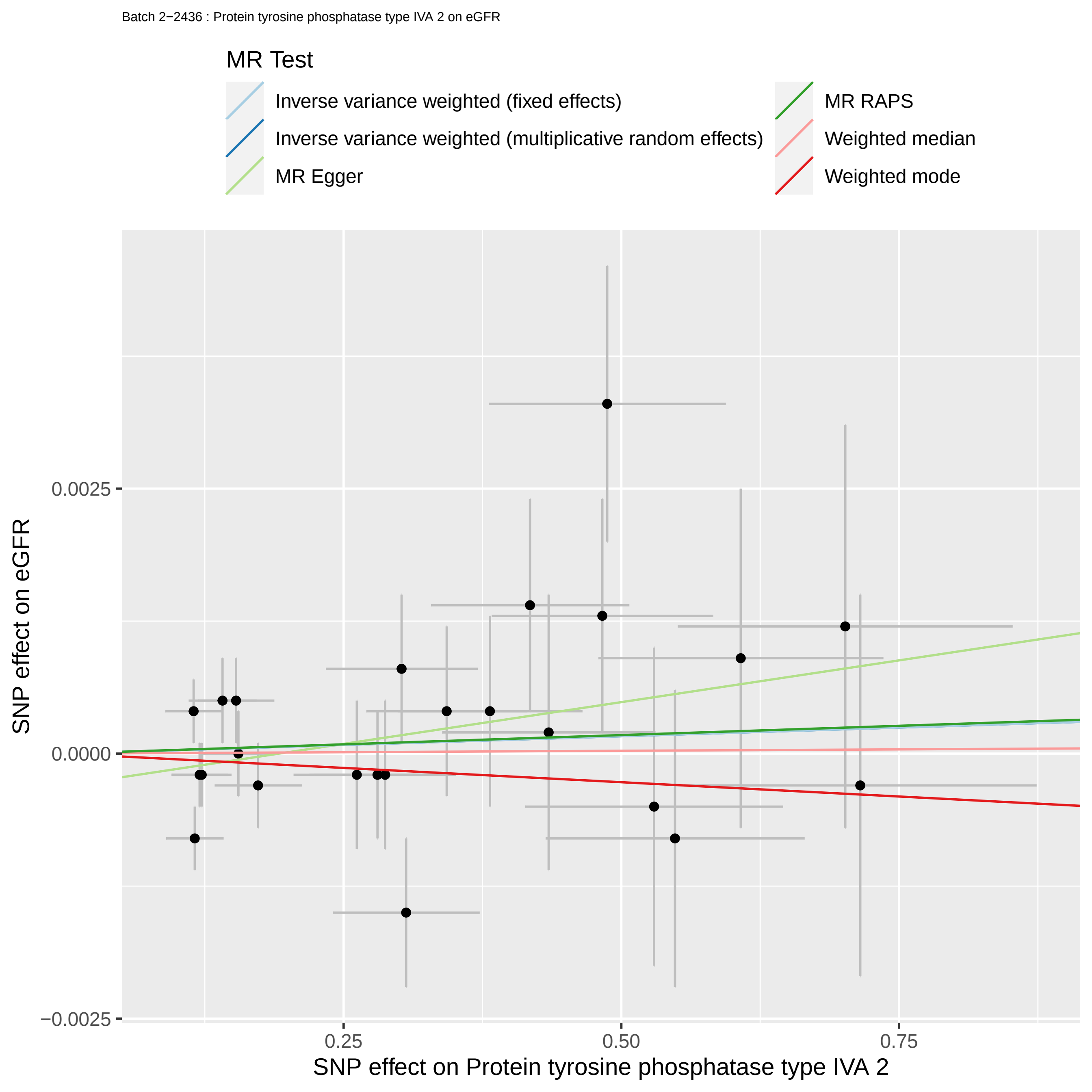
Batch 2−2436 : Protein tyrosine phosphatase type IVA 2 on eGFR
MR Test
Inverse variance weighted (fixed effects)
Inverse variance weighted (multiplicative random effects)
MR Egger
MR RAPS
Weighted median
Weighted mode
0.0025
0.0000
−0.0025
0.25
0.50
0.75
SNP effect on eGFR
SNP effect on Protein tyrosine phosphatase type IVA 2

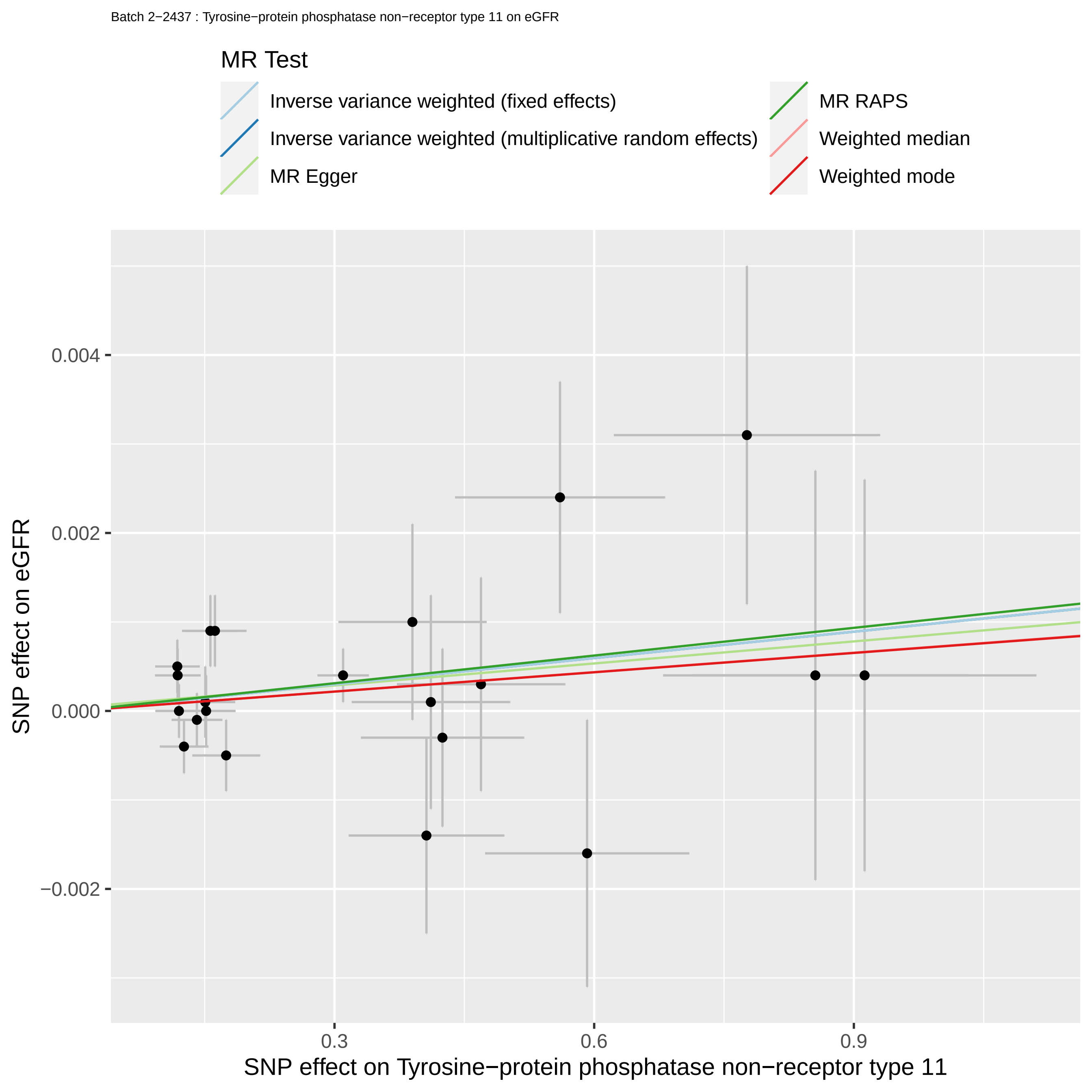

Batch 2−2437 : Tyrosine−protein phosphatase non−receptor type 11 on eGFR
MR Test
Inverse variance weighted (fixed effects)
Inverse variance weighted (multiplicative random effects)
MR Egger
MR RAPS
Weighted median
Weighted mode
0.004
0.002
0.000
−0.002
0.3
0.6
0.9
SNP effect on eGFR
SNP effect on Tyrosine−protein phosphatase non−receptor type 11

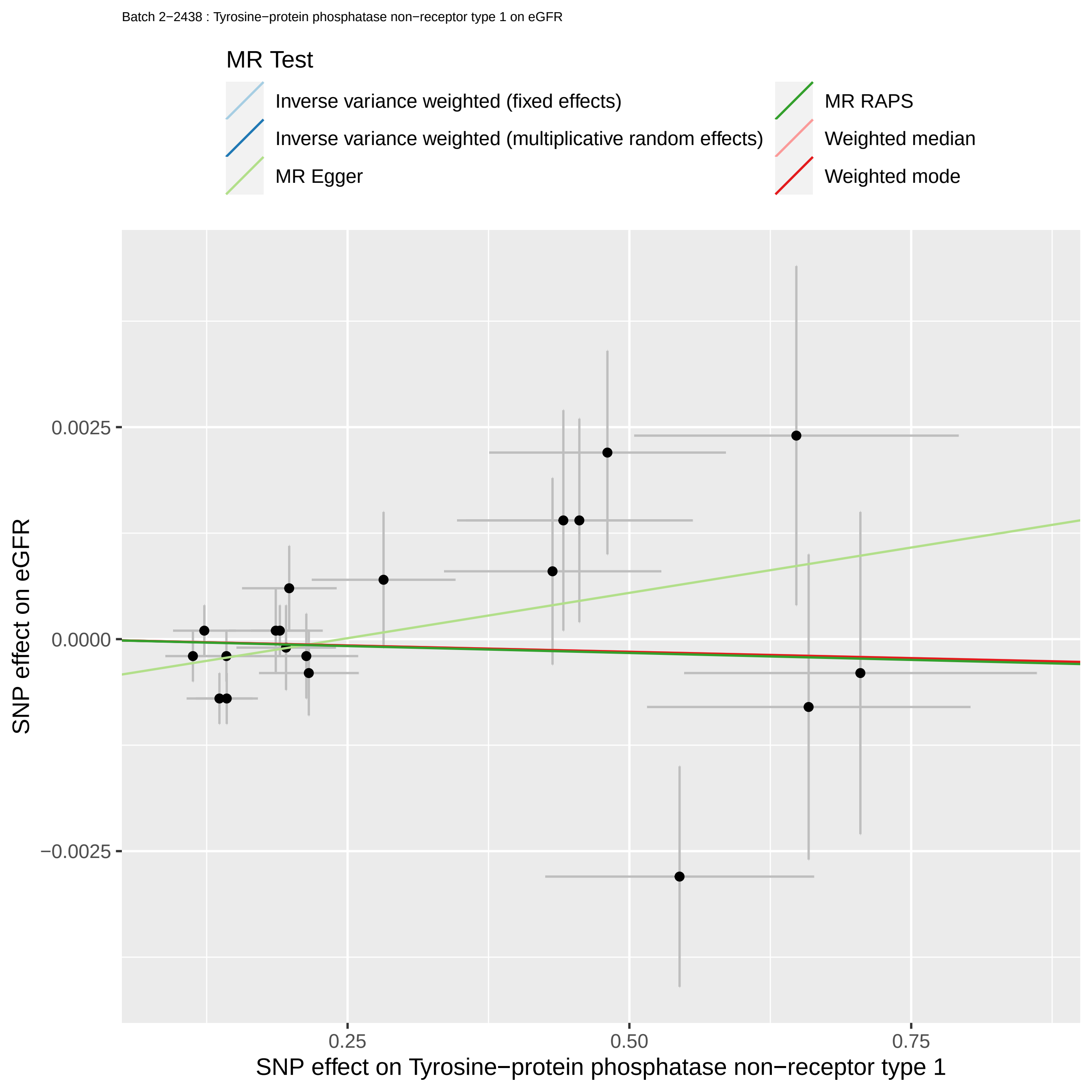
Batch 2−2438 : Tyrosine−protein phosphatase non−receptor type 1 on eGFR
MR Test
Inverse variance weighted (fixed effects)
Inverse variance weighted (multiplicative random effects)
MR Egger
MR RAPS
Weighted median
Weighted mode
0.0025
0.0000
−0.0025
0.25
0.50
0.75
SNP effect on eGFR
SNP effect on Tyrosine−protein phosphatase non−receptor type 1

Batch 2−2439 : Tyrosine−protein phosphatase non−receptor type 2 on eGFR

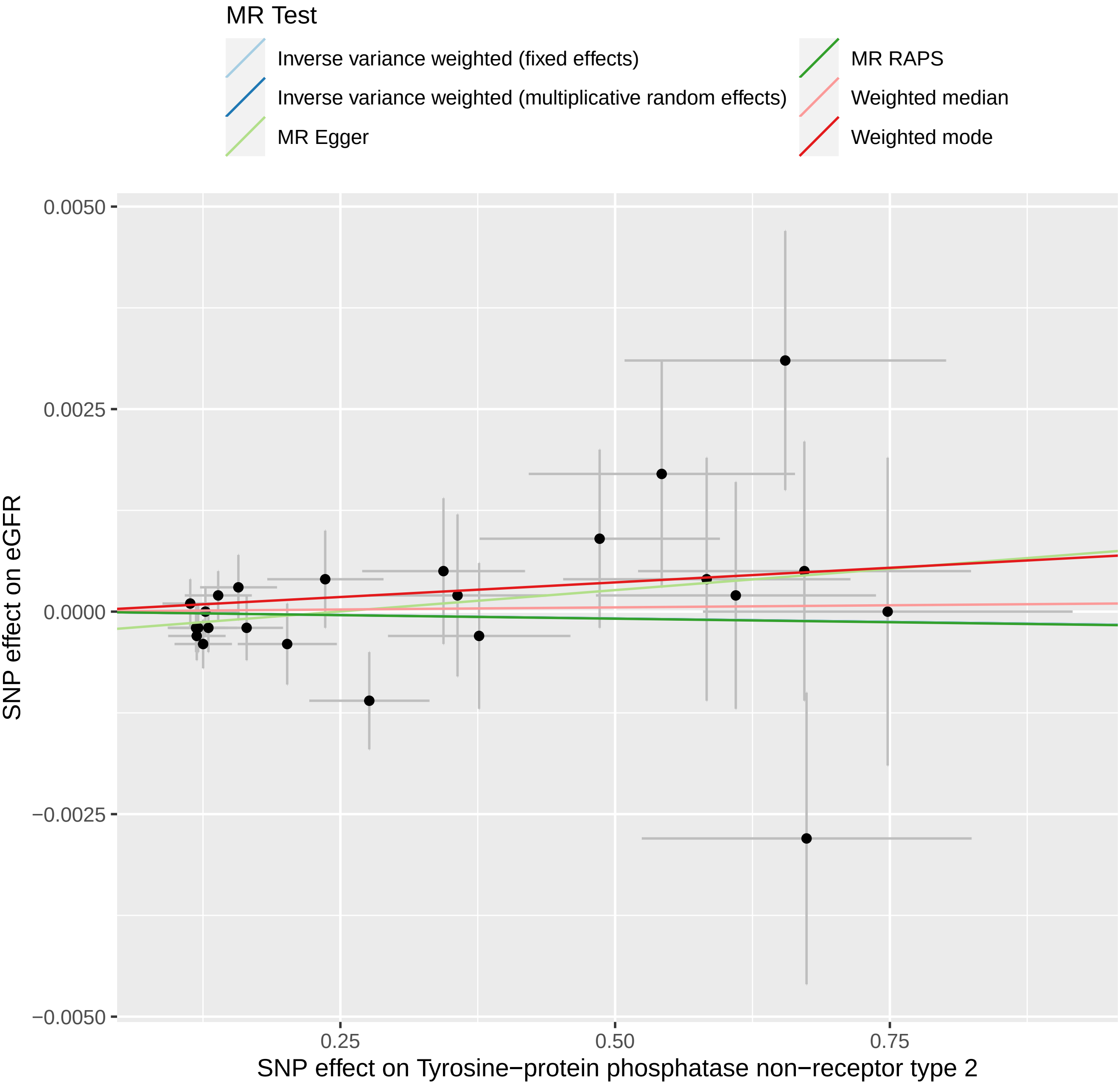

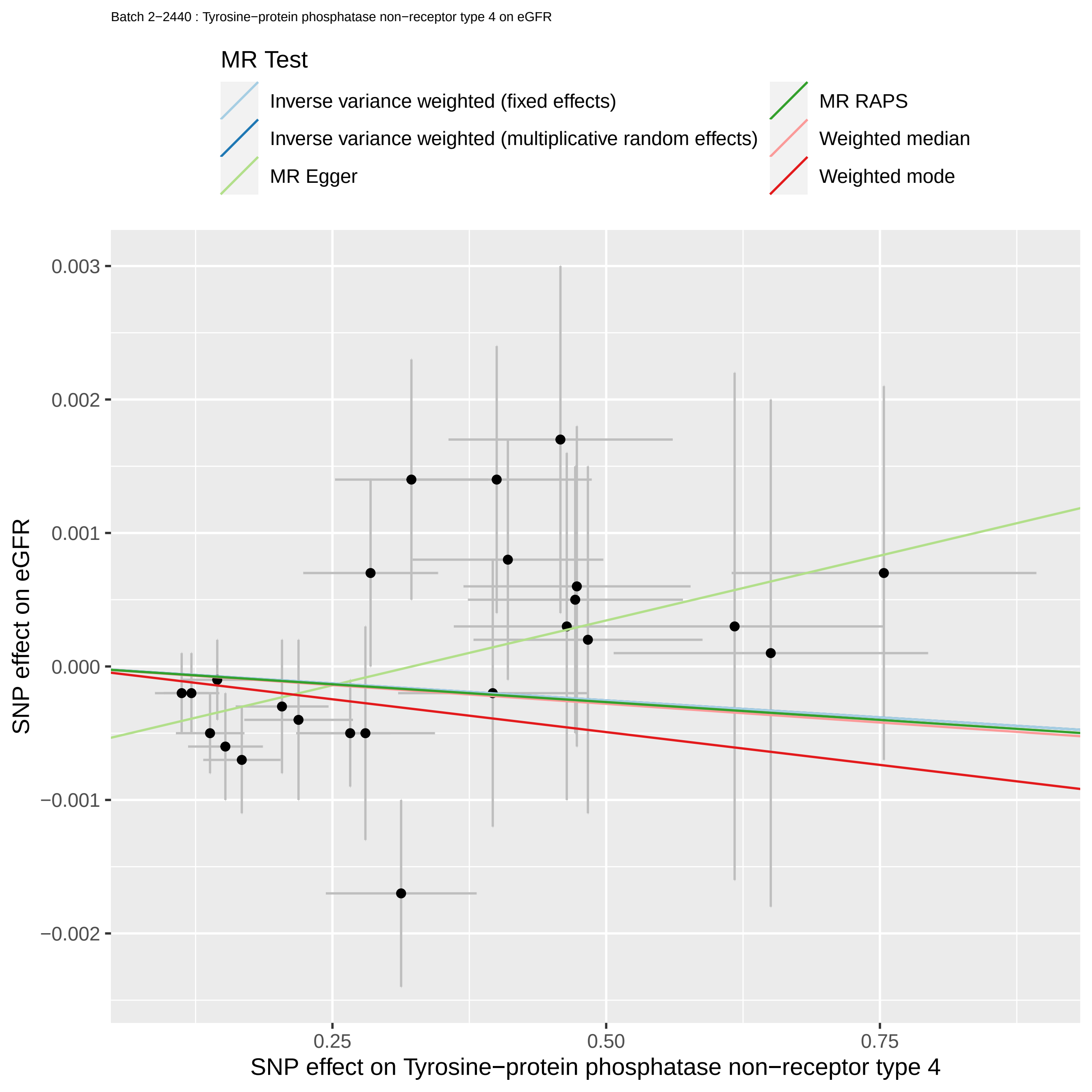

Batch 2−2440 : Tyrosine−protein phosphatase non−receptor type 4 on eGFR
MR Test
Inverse variance weighted (fixed effects)
Inverse variance weighted (multiplicative random effects)
MR Egger
MR RAPS
Weighted median
Weighted mode
SNP effect on eGFR
0.003
0.002
0.001
0.000
−0.001
−0.002
0.25
0.50
0.75
SNP effect on Tyrosine−protein phosphatase non−receptor type 4

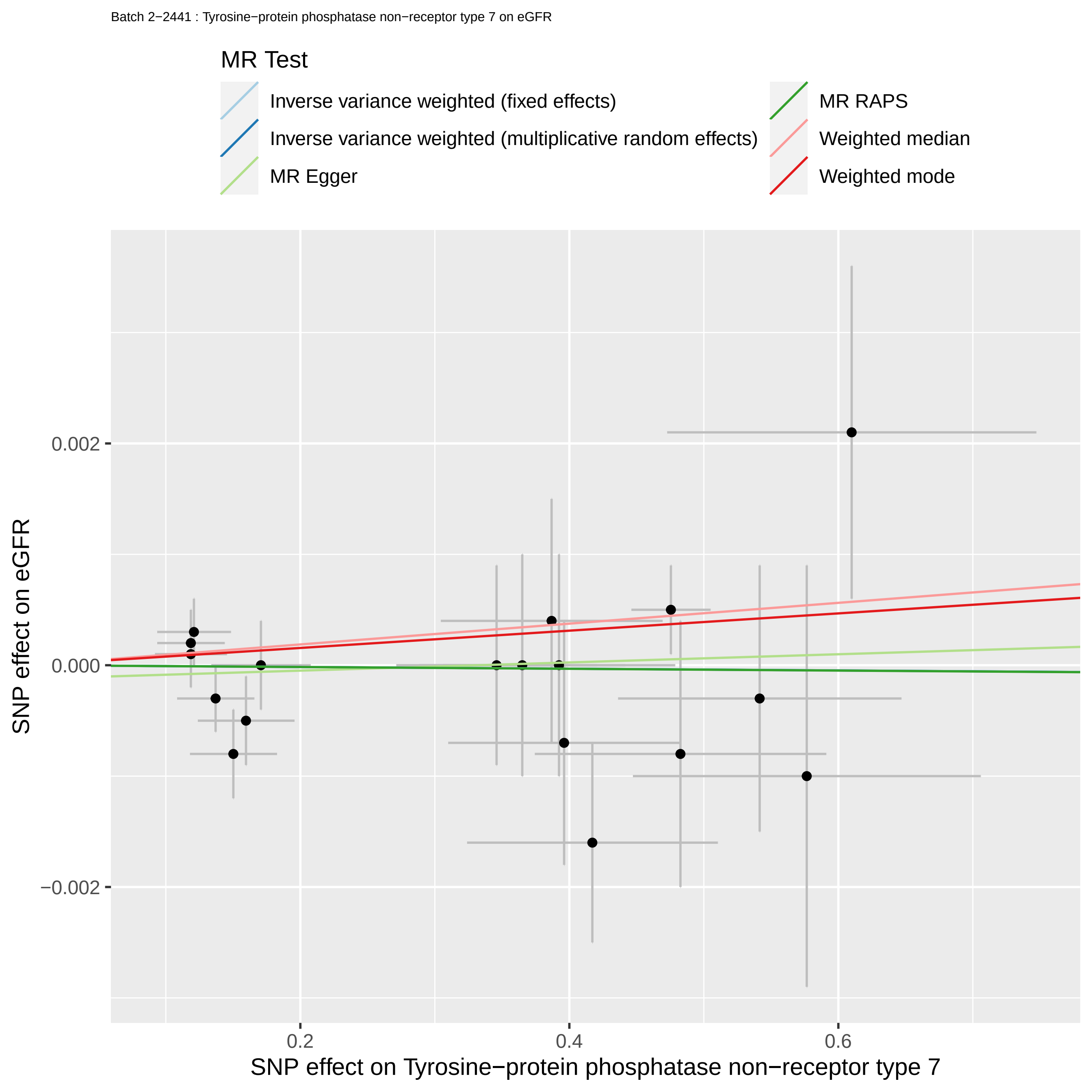

Batch 2−2441 : Tyrosine−protein phosphatase non−receptor type 7 on eGFR
MR Test
Inverse variance weighted (fixed effects)
Inverse variance weighted (multiplicative random effects)
MR Egger
MR RAPS
Weighted median
Weighted mode
0.002
0.000
−0.002
0.2
0.4
0.6
SNP effect on eGFR
SNP effect on Tyrosine−protein phosphatase non−receptor type 7

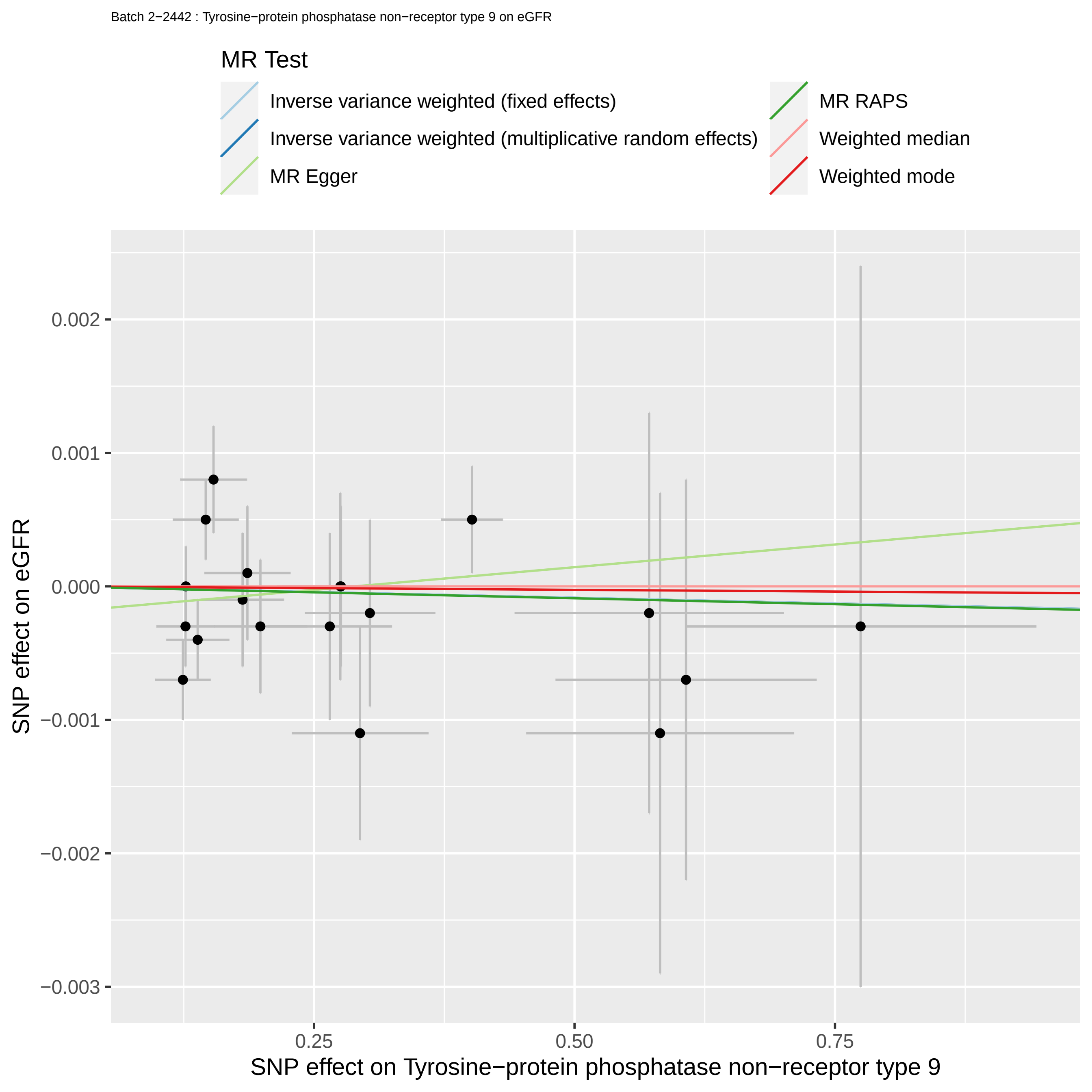
Batch 2−2442 : Tyrosine−protein phosphatase non−receptor type 9 on eGFR
MR Test
Inverse variance weighted (fixed effects)
Inverse variance weighted (multiplicative random effects)
MR Egger
MR RAPS
Weighted median
Weighted mode
0.002
0.001
0.000
−0.001
−0.002
−0.003
0.25
0.50
0.75
SNP effect on eGFR
SNP effect on Tyrosine−protein phosphatase non−receptor type 9

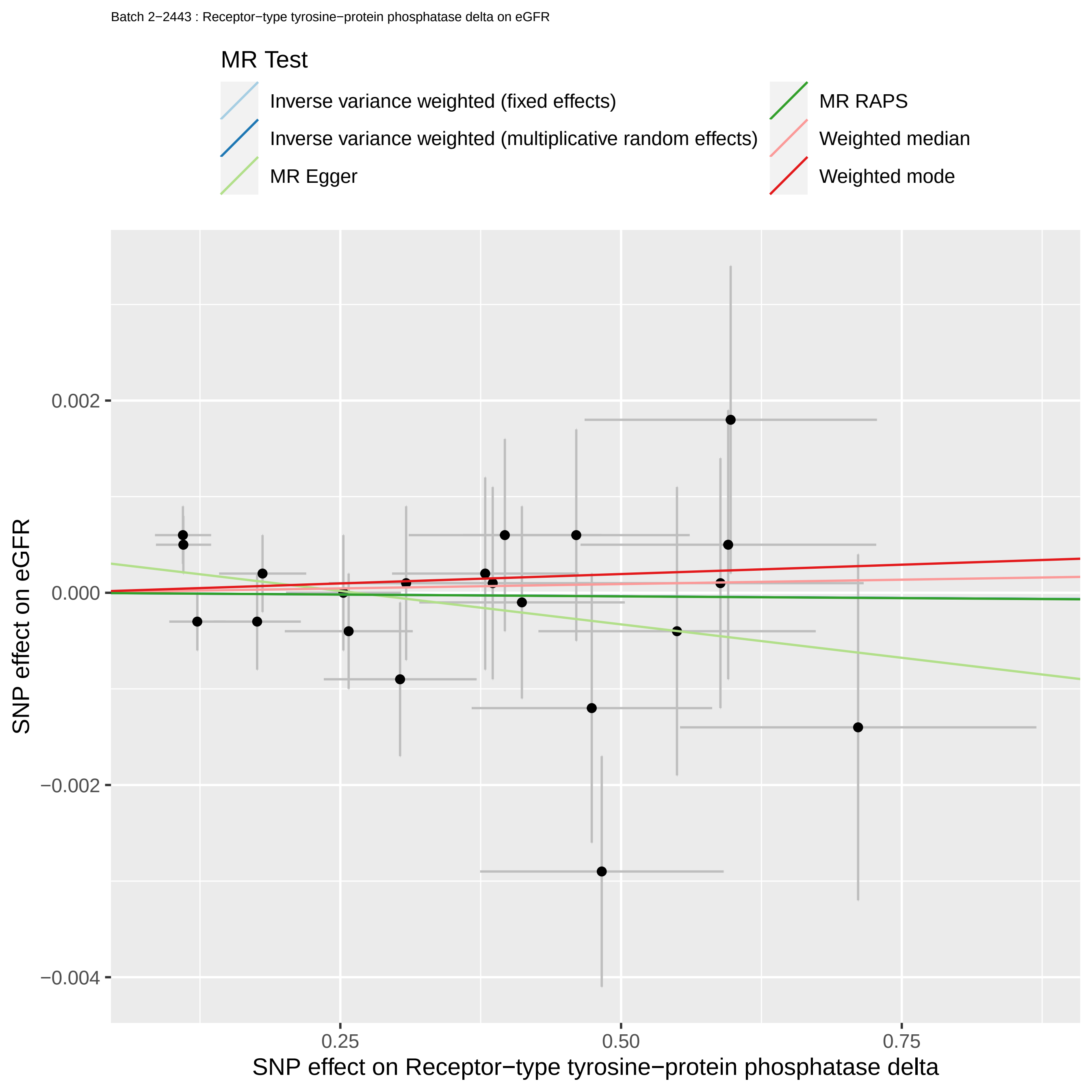
Batch 2−2443 : Receptor−type tyrosine−protein phosphatase delta on eGFR
MR Test
Inverse variance weighted (fixed effects)
Inverse variance weighted (multiplicative random effects)
MR Egger
MR RAPS
Weighted median
Weighted mode
0.002
0.000
−0.002
−0.004
0.25
0.50
0.75
SNP effect on eGFR
SNP effect on Receptor−type tyrosine−protein phosphatase delta

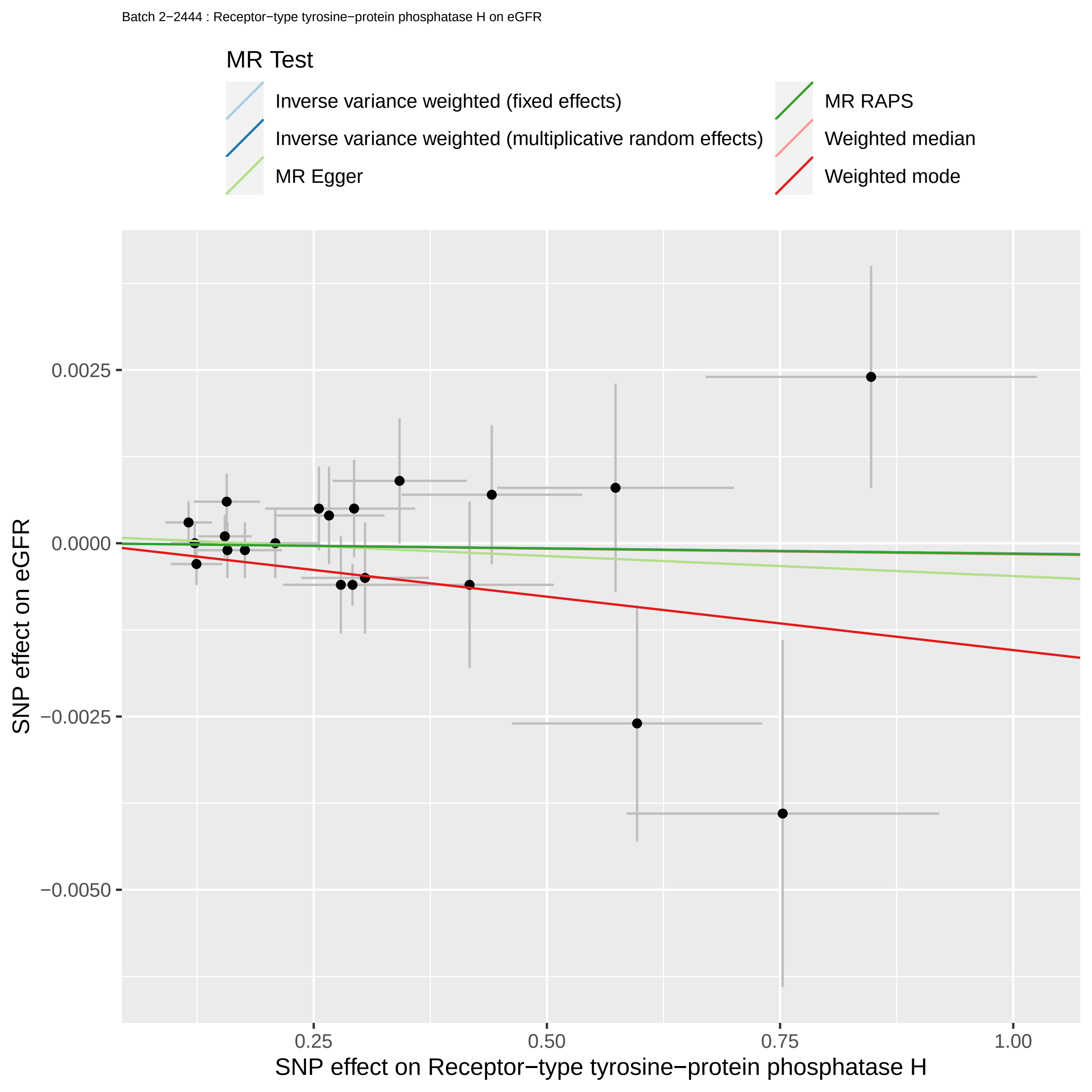

Batch 2−2444 : Receptor−type tyrosine−protein phosphatase H on eGFR
MR Test
Inverse variance weighted (fixed effects)
Inverse variance weighted (multiplicative random effects)
MR Egger
MR RAPS
Weighted median
Weighted mode
0.0025
0.0000
−0.0025
−0.0050
0.25
0.50
0.75
1.00
SNP effect on eGFR
SNP effect on Receptor−type tyrosine−protein phosphatase H

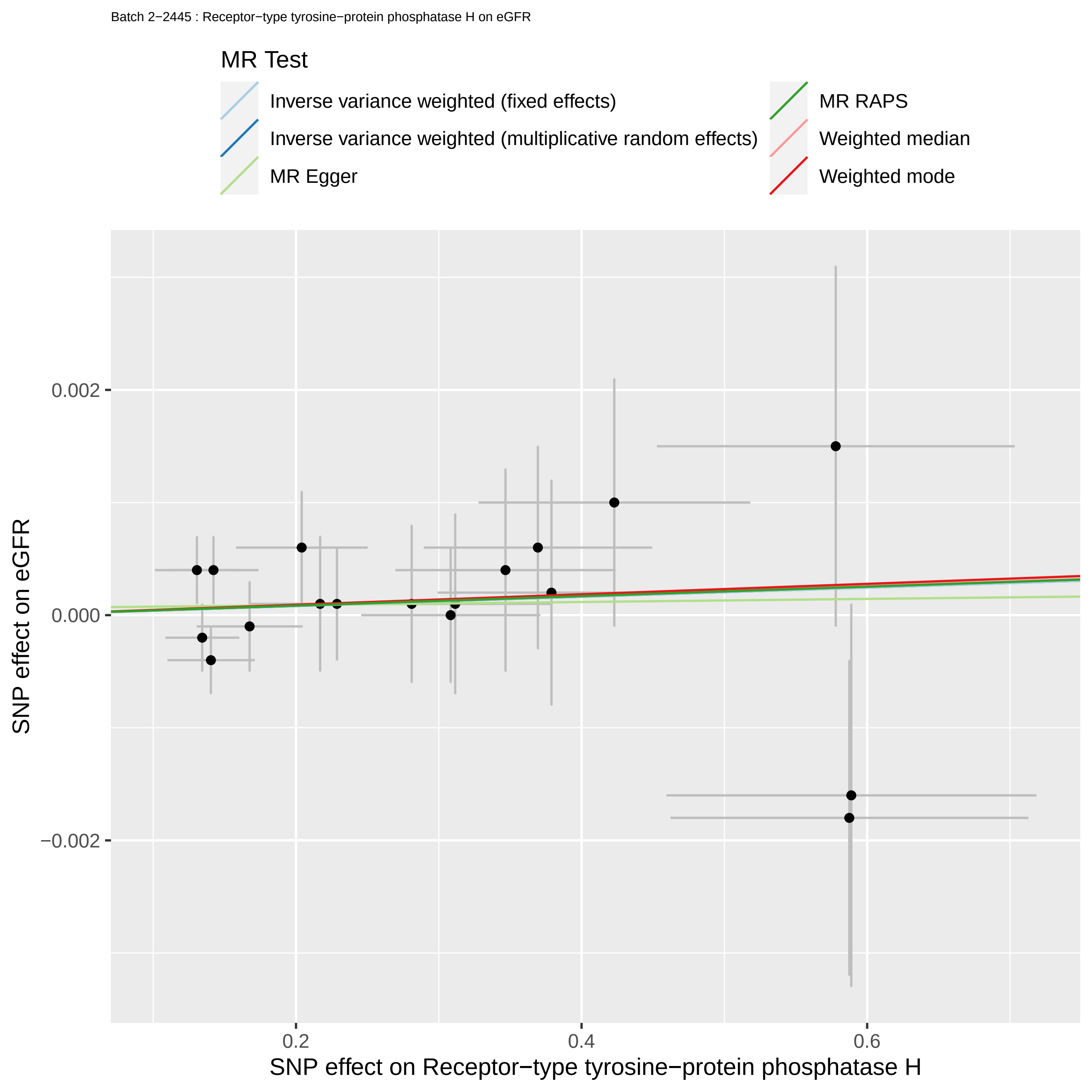

Batch 2−2445 : Receptor−type tyrosine−protein phosphatase H on eGFR
MR Test
Inverse variance weighted (fixed effects)
Inverse variance weighted (multiplicative random effects)
MR Egger
MR RAPS
Weighted median
Weighted mode
0.002
0.000
−0.002
0.2
0.4
0.6
SNP effect on eGFR
SNP effect on Receptor−type tyrosine−protein phosphatase H

Batch 2−2446 : Receptor−type tyrosine−protein phosphatase eta on eGFR

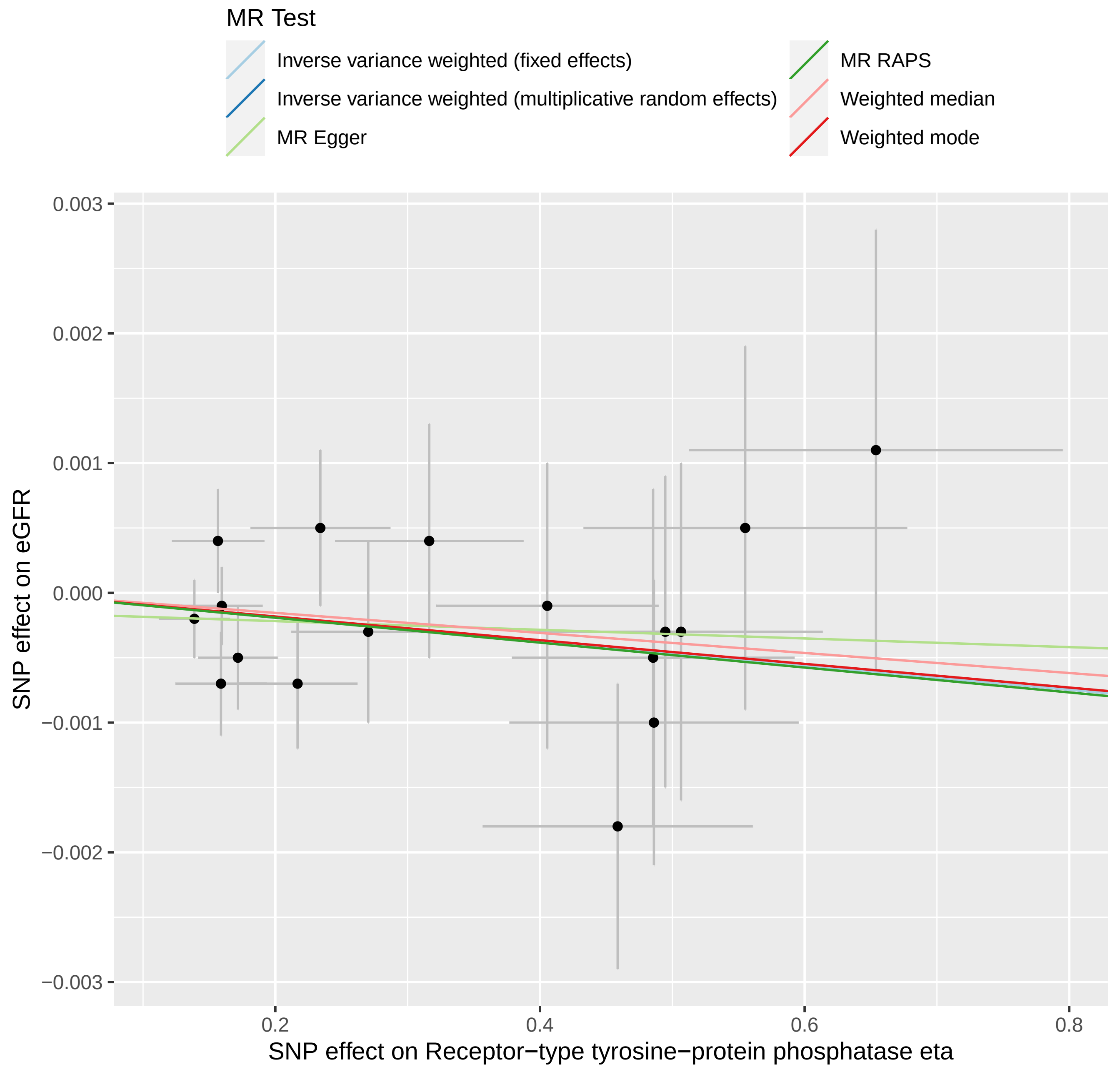

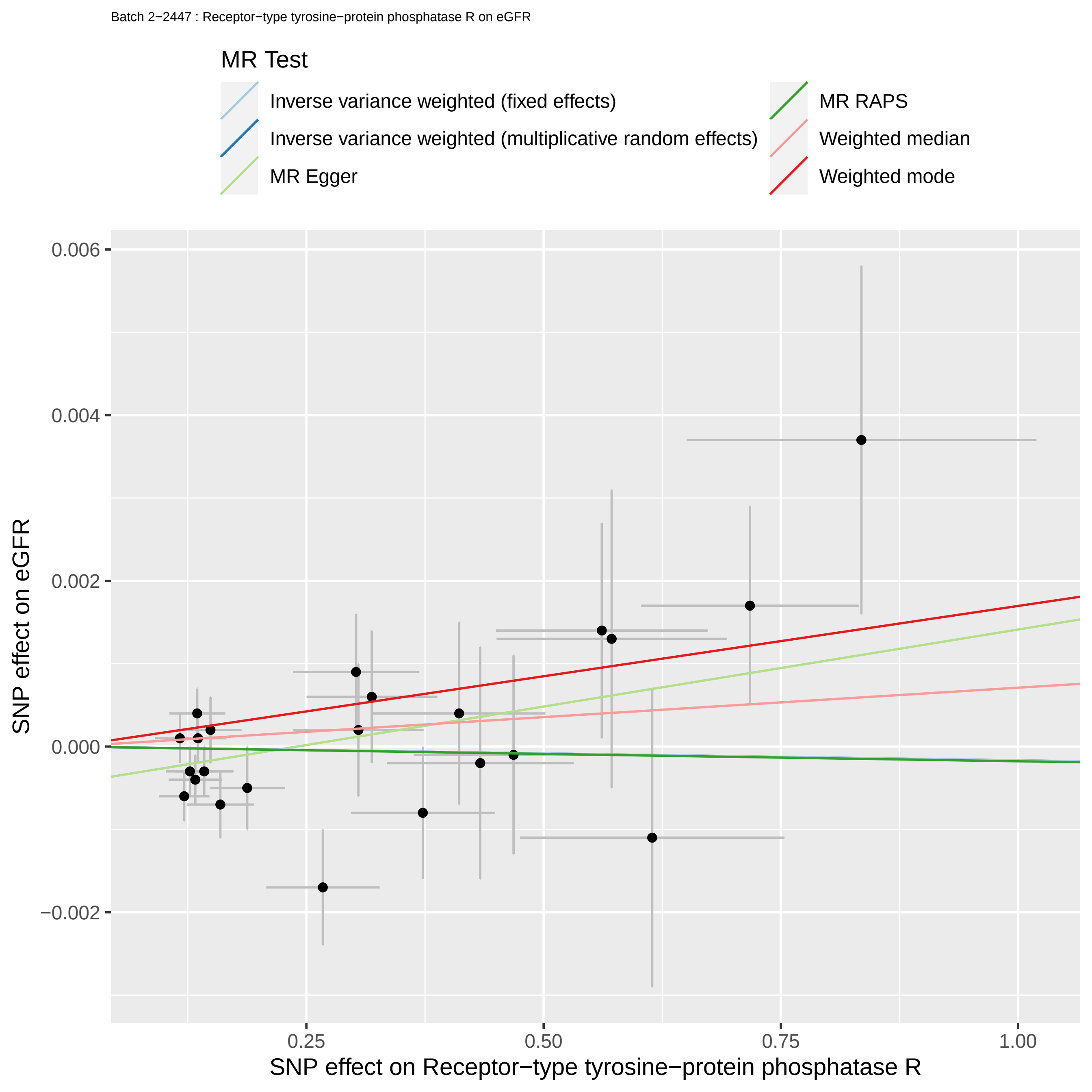
Batch 2−2447 : Receptor−type tyrosine−protein phosphatase R on eGFR
MR Test
Inverse variance weighted (fixed effects)
Inverse variance weighted (multiplicative random effects)
MR Egger
MR RAPS
Weighted median
Weighted mode
SNP effect on eGFR
0.006
0.004
0.002
0.000
−0.002
0.25
0.50
0.75
1.00
SNP effect on Receptor−type tyrosine−protein phosphatase R

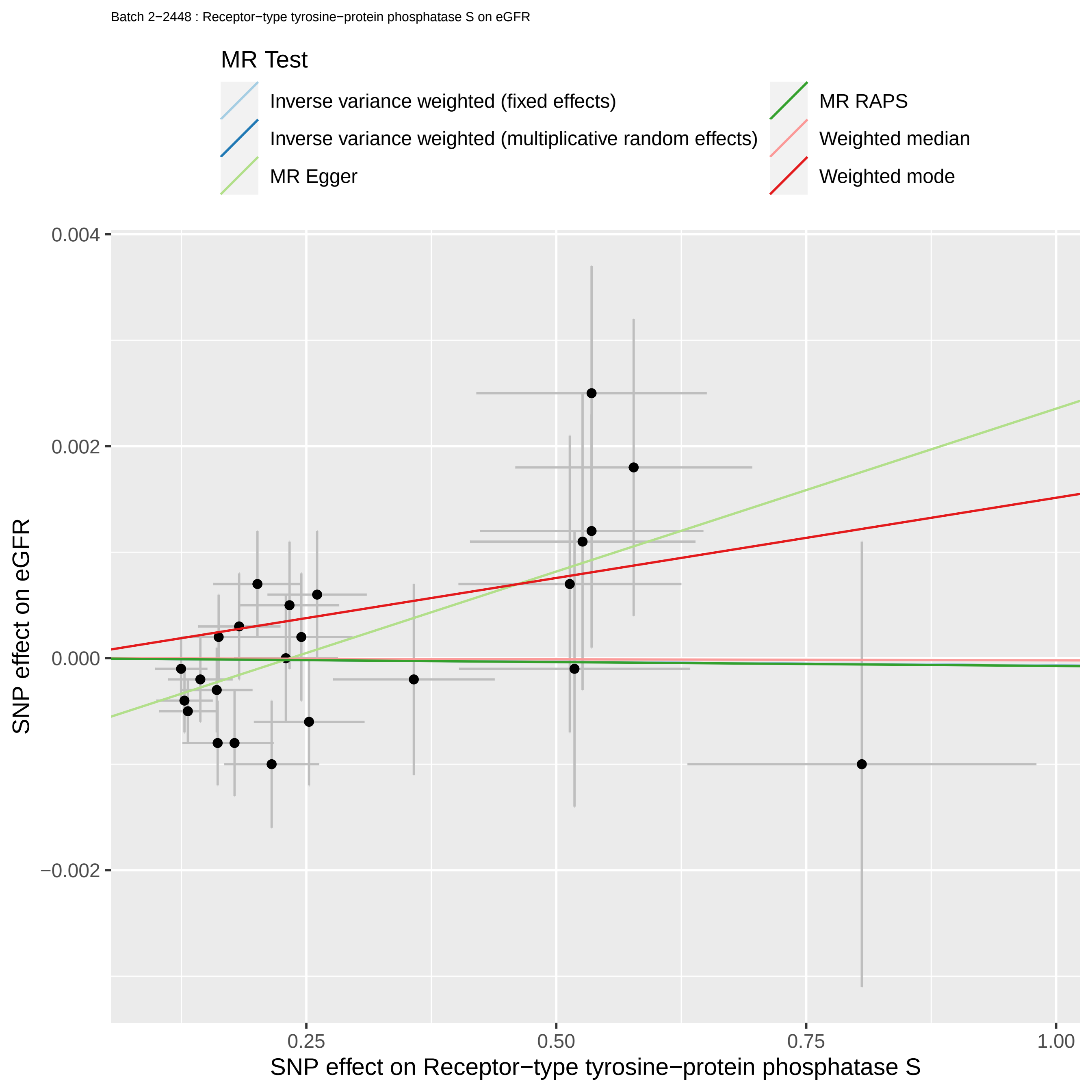

Batch 2−2448 : Receptor−type tyrosine−protein phosphatase S on eGFR
MR Test
Inverse variance weighted (fixed effects)
Inverse variance weighted (multiplicative random effects)
MR Egger
MR RAPS
Weighted median
Weighted mode
0.004
0.002
0.000
−0.002
0.25
0.50
0.75
1.00
SNP effect on eGFR
SNP effect on Receptor−type tyrosine−protein phosphatase S

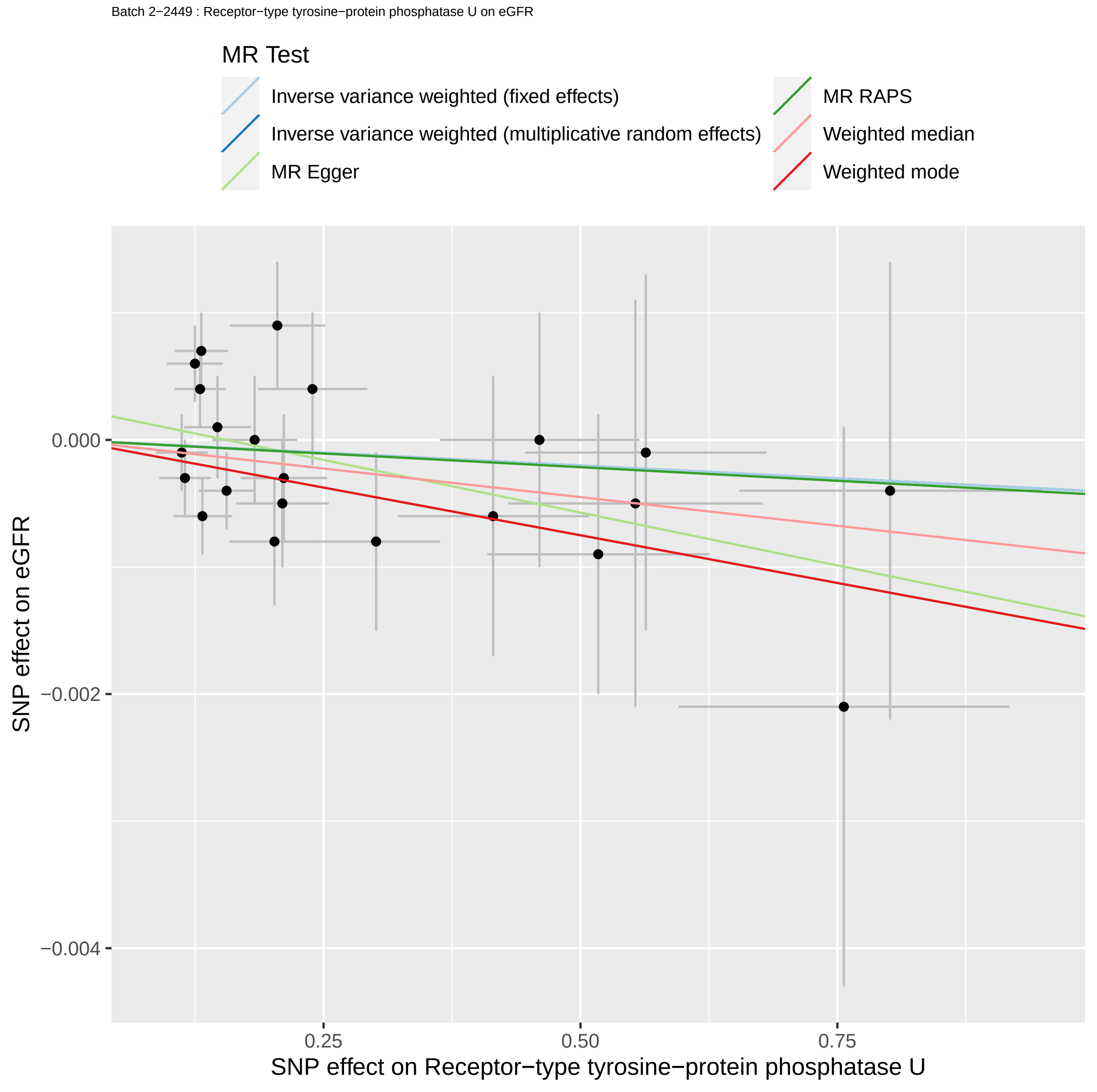

Batch 2−2449 : Receptor−type tyrosine−protein phosphatase U on eGFR
MR Test
Inverse variance weighted (fixed effects)
Inverse variance weighted (multiplicative random effects)
MR Egger
MR RAPS
Weighted median
Weighted mode
SNP effect on eGFR
0.000
−0.002
−0.004
0.25
0.50
0.75
SNP effect on Receptor−type tyrosine−protein phosphatase U

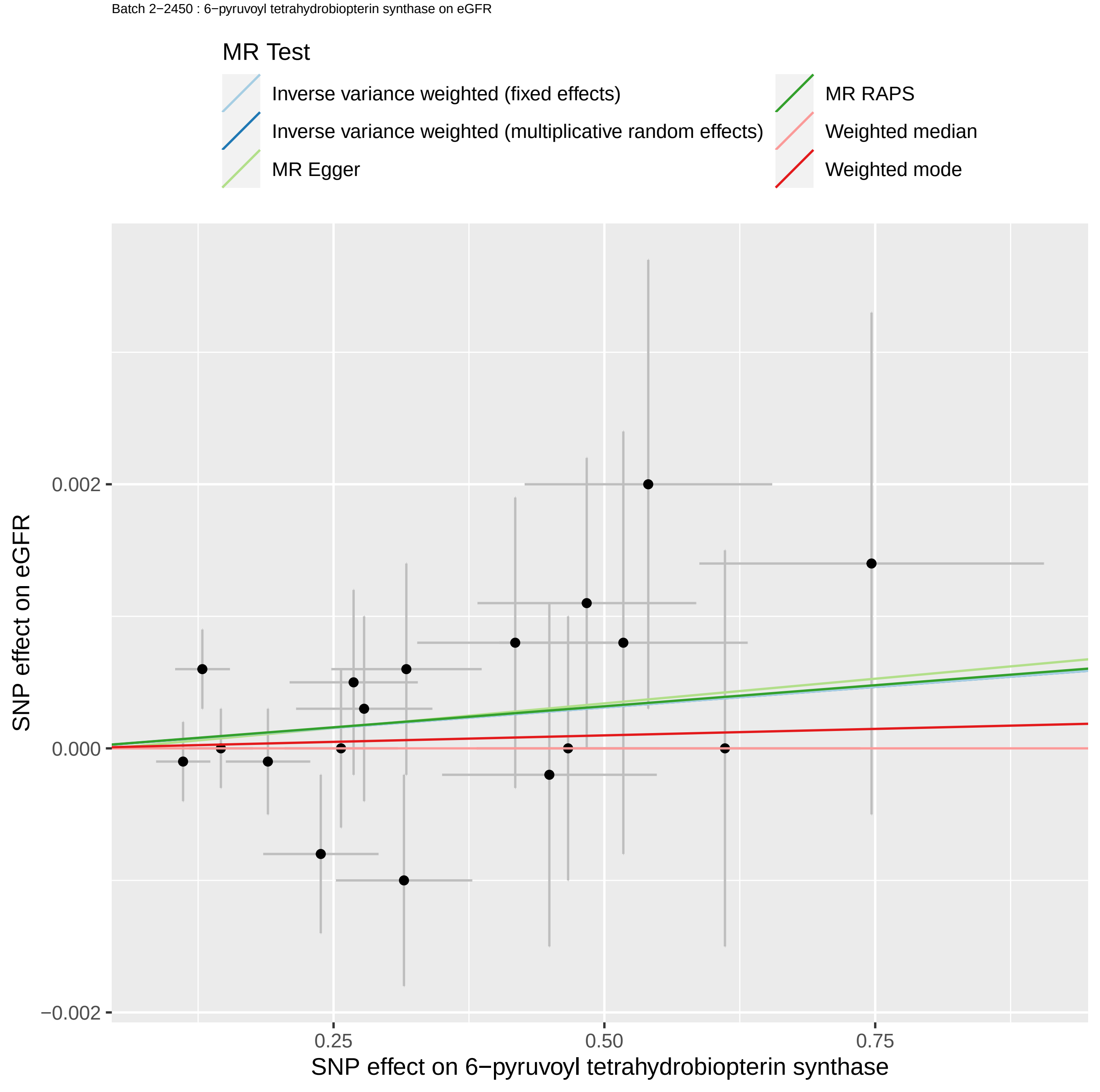

Batch 2−2450 : 6−pyruvoyl tetrahydrobiopterin synthase on eGFR
MR Test
Inverse variance weighted (fixed effects)
Inverse variance weighted (multiplicative random effects)
MR Egger
MR RAPS
Weighted median
Weighted mode
0.002
0.000
−0.002
0.25
0.50
0.75
SNP effect on eGFR
SNP effect on 6−pyruvoyl tetrahydrobiopterin synthase

Batch 2−2451 : Pentraxin−related protein PTX3 on eGFR

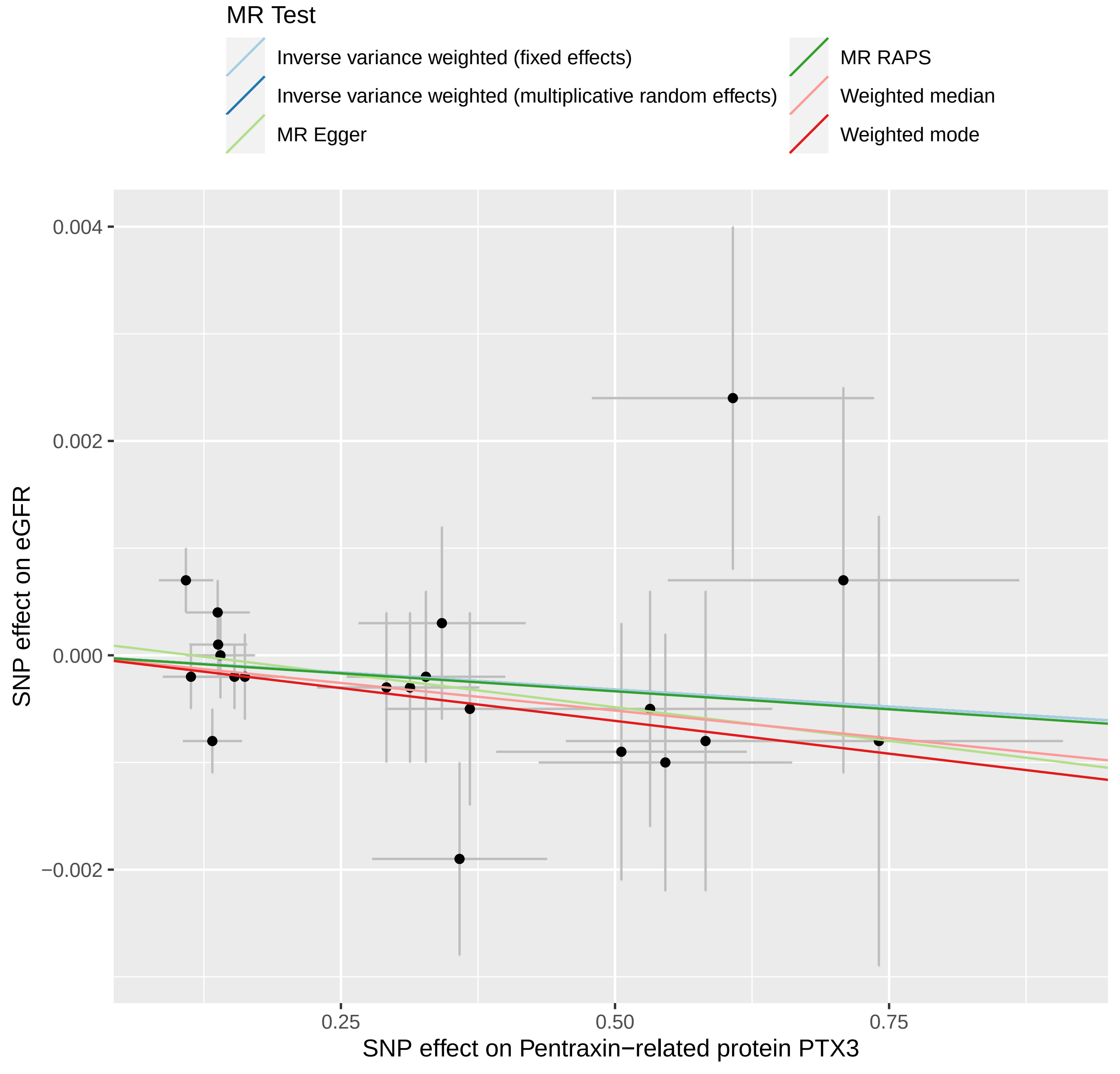

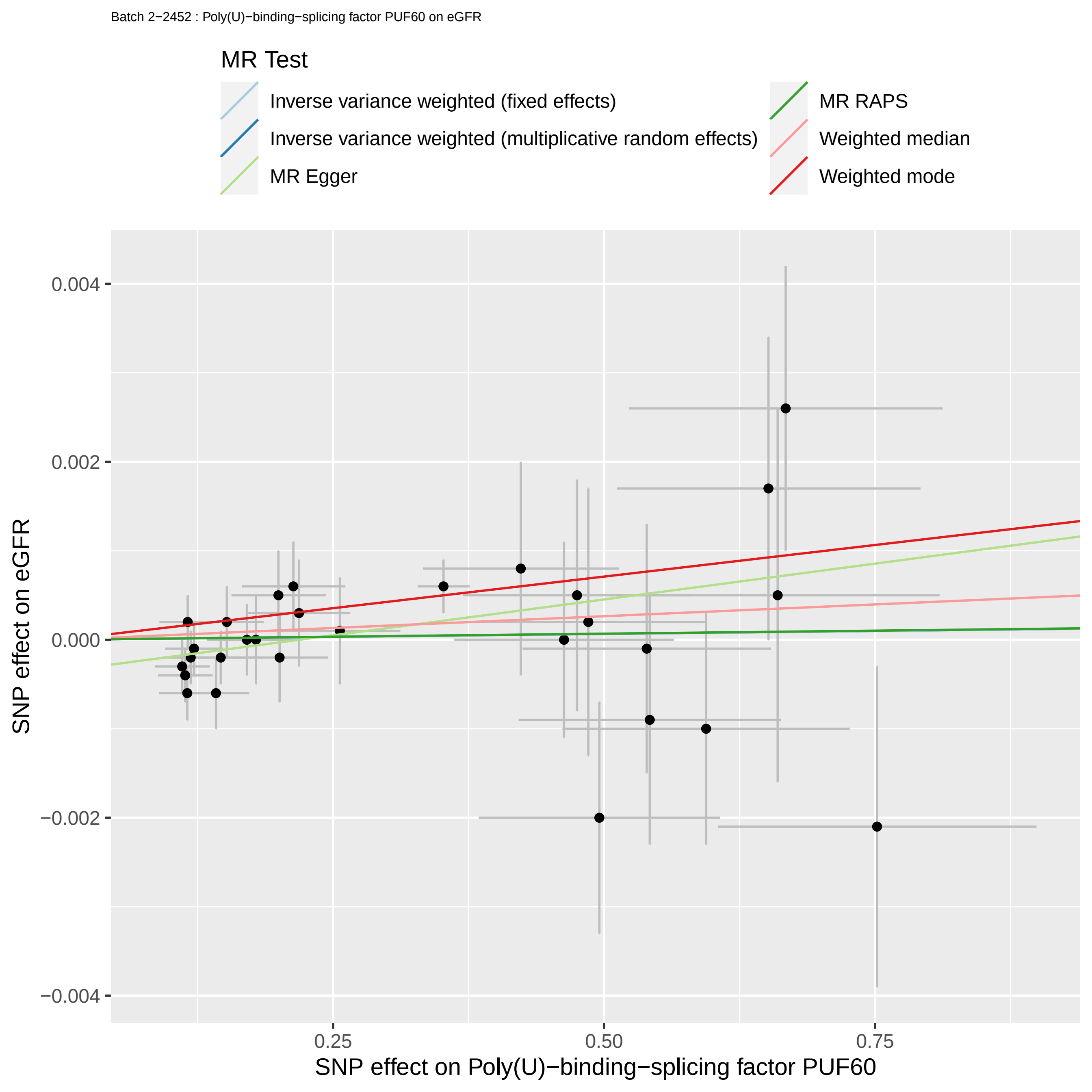

Batch 2−2452 : Poly(U)−binding−splicing factor PUF60 on eGFR
MR Test
Inverse variance weighted (fixed effects)
Inverse variance weighted (multiplicative random effects)
MR Egger
MR RAPS
Weighted median
Weighted mode
0.004
0.002
0.000
−0.002
−0.004
SNP effect on eGFR
0.25
0.50
0.75
SNP effect on Poly(U)−binding−splicing factor PUF60

Batch 2−2453 : Transcriptional activator protein Pur−alpha on eGFR

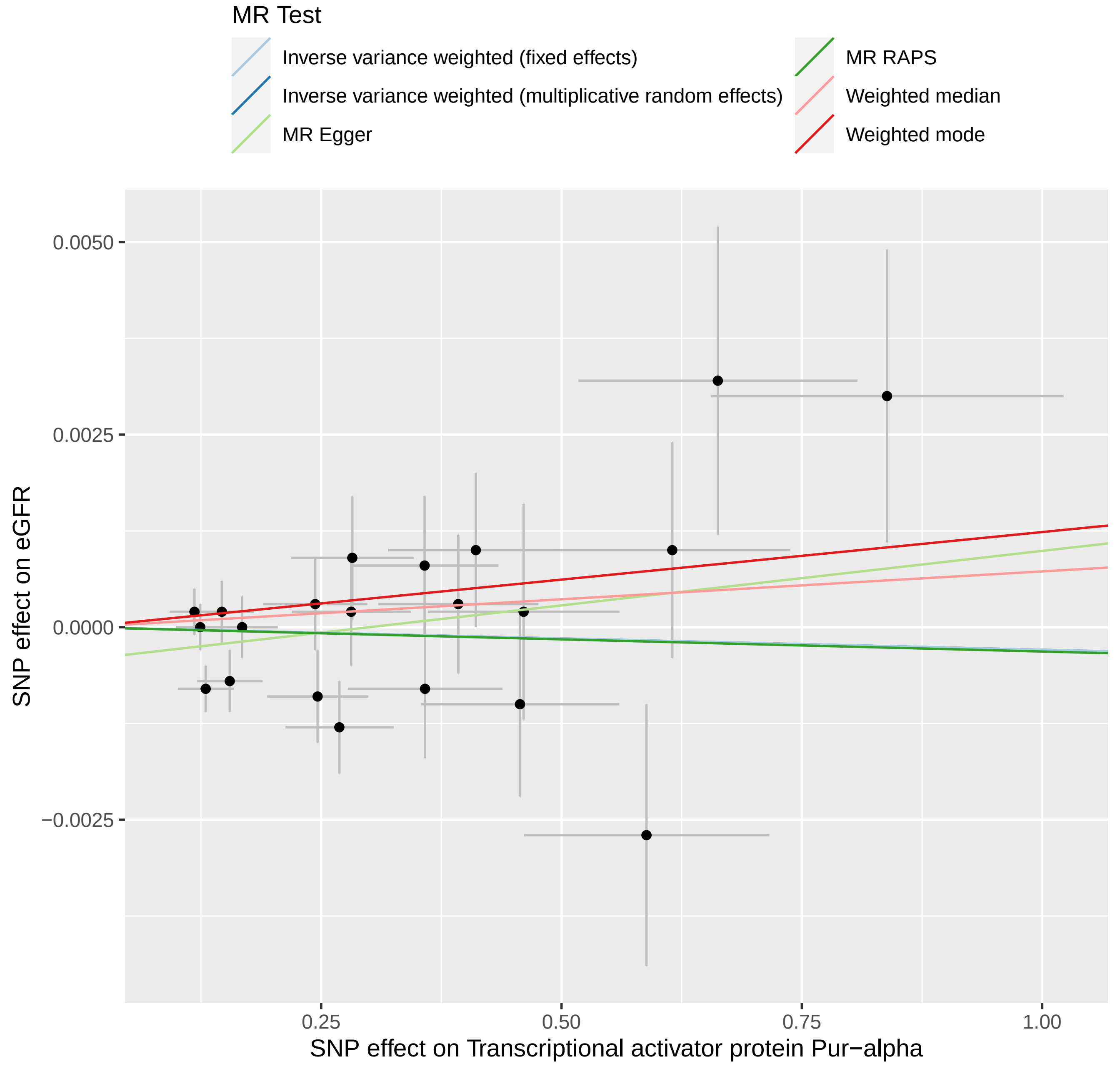

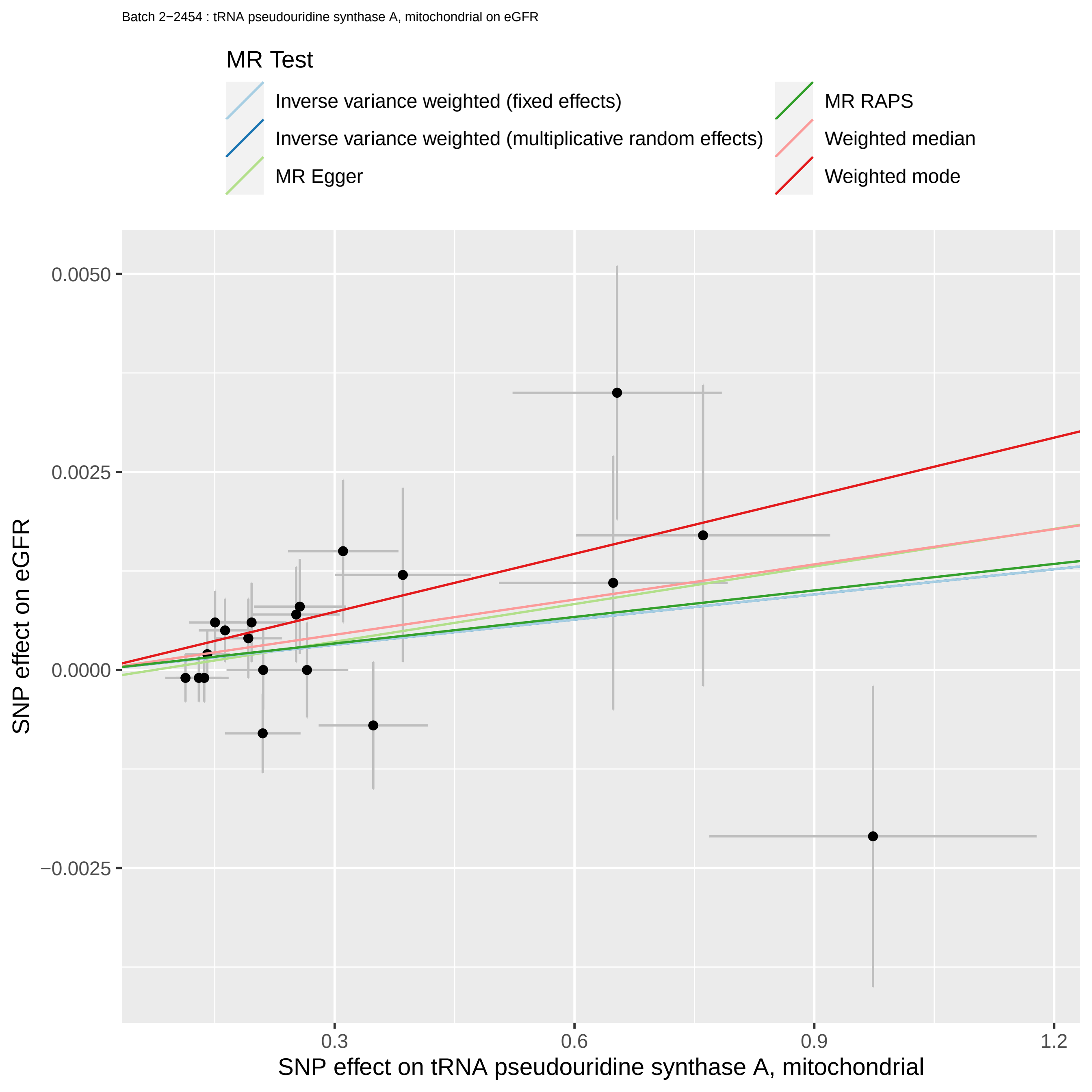

Batch 2−2454 : tRNA pseudouridine synthase A, mitochondrial on eGFR
MR Test
Inverse variance weighted (fixed effects)
Inverse variance weighted (multiplicative random effects)
MR Egger
MR RAPS
Weighted median
Weighted mode
0.0050
0.0025
0.0000
−0.0025
SNP effect on eGFR
0.3
0.6
0.9
1.2
SNP effect on tRNA pseudouridine synthase A, mitochondrial

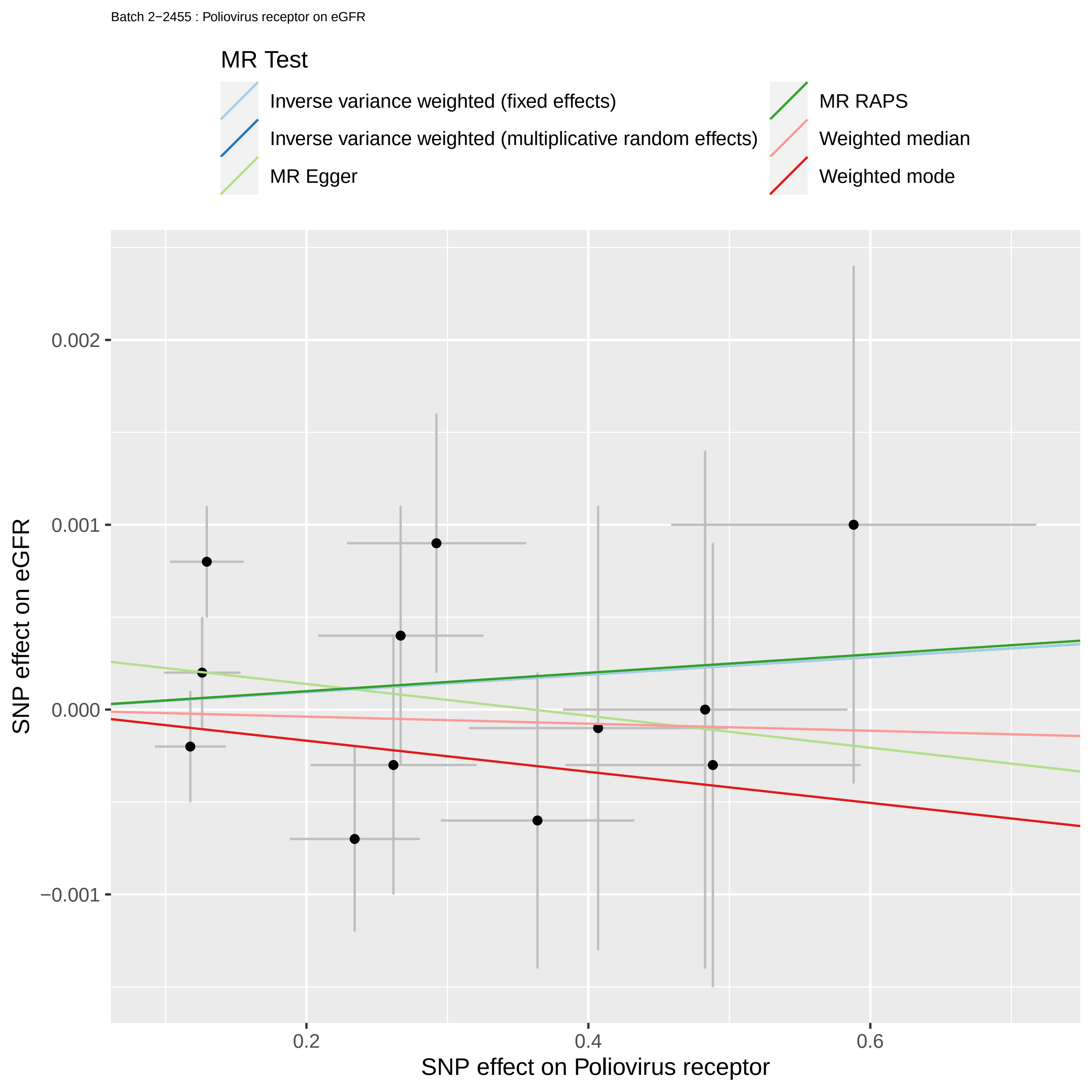
Batch 2−2455 : Poliovirus receptor on eGFR
MR Test
Inverse variance weighted (fixed effects)
Inverse variance weighted (multiplicative random effects)
MR Egger
MR RAPS
Weighted median
Weighted mode
0.002
0.001
0.000
−0.001
SNP effect on eGFR
0.2
0.4
0.6
SNP effect on Poliovirus receptor

Batch 2−2456 : Transmembrane protein PVRIG on eGFR

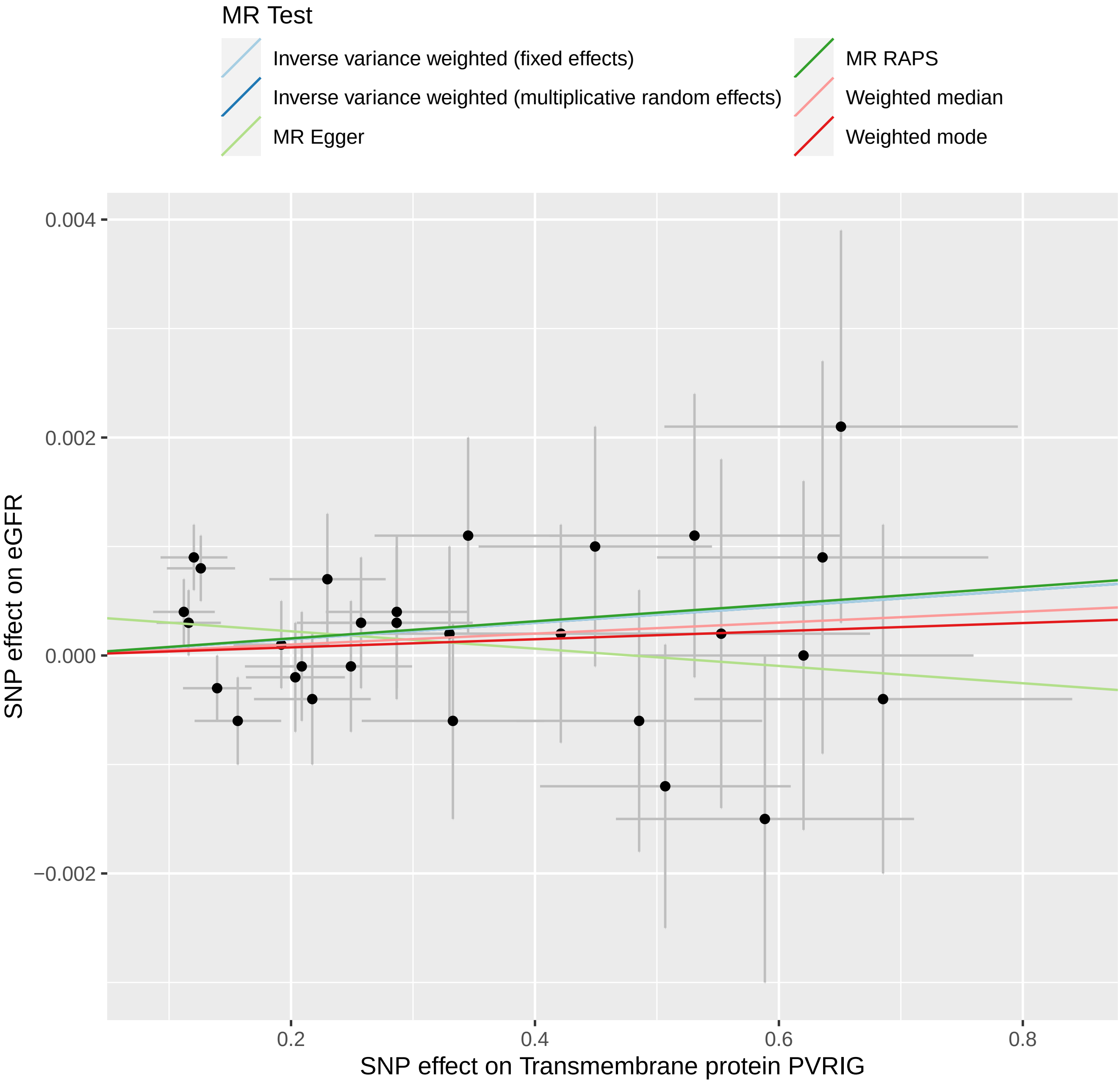

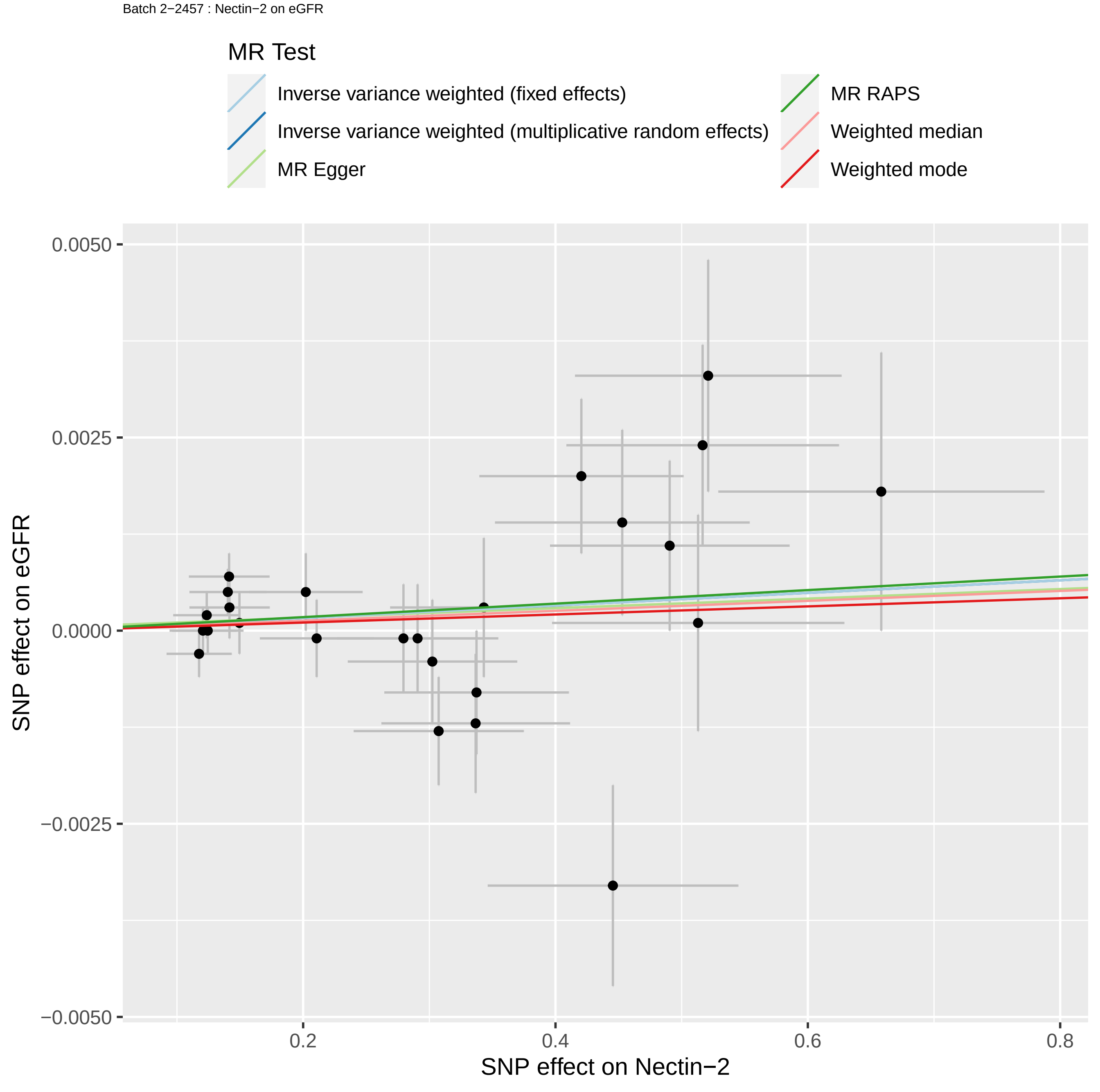
Batch 2−2457 : Nectin−2 on eGFR
MR Test
Inverse variance weighted (fixed effects)
Inverse variance weighted (multiplicative random effects)
MR Egger
MR RAPS
Weighted median
Weighted mode
0.0050
0.0025
0.0000
−0.0025
−0.0050
SNP effect on eGFR
0.2
0.4
0.6
0.8
SNP effect on Nectin−2

Batch 2−2458 : Nectin−3 on eGFR

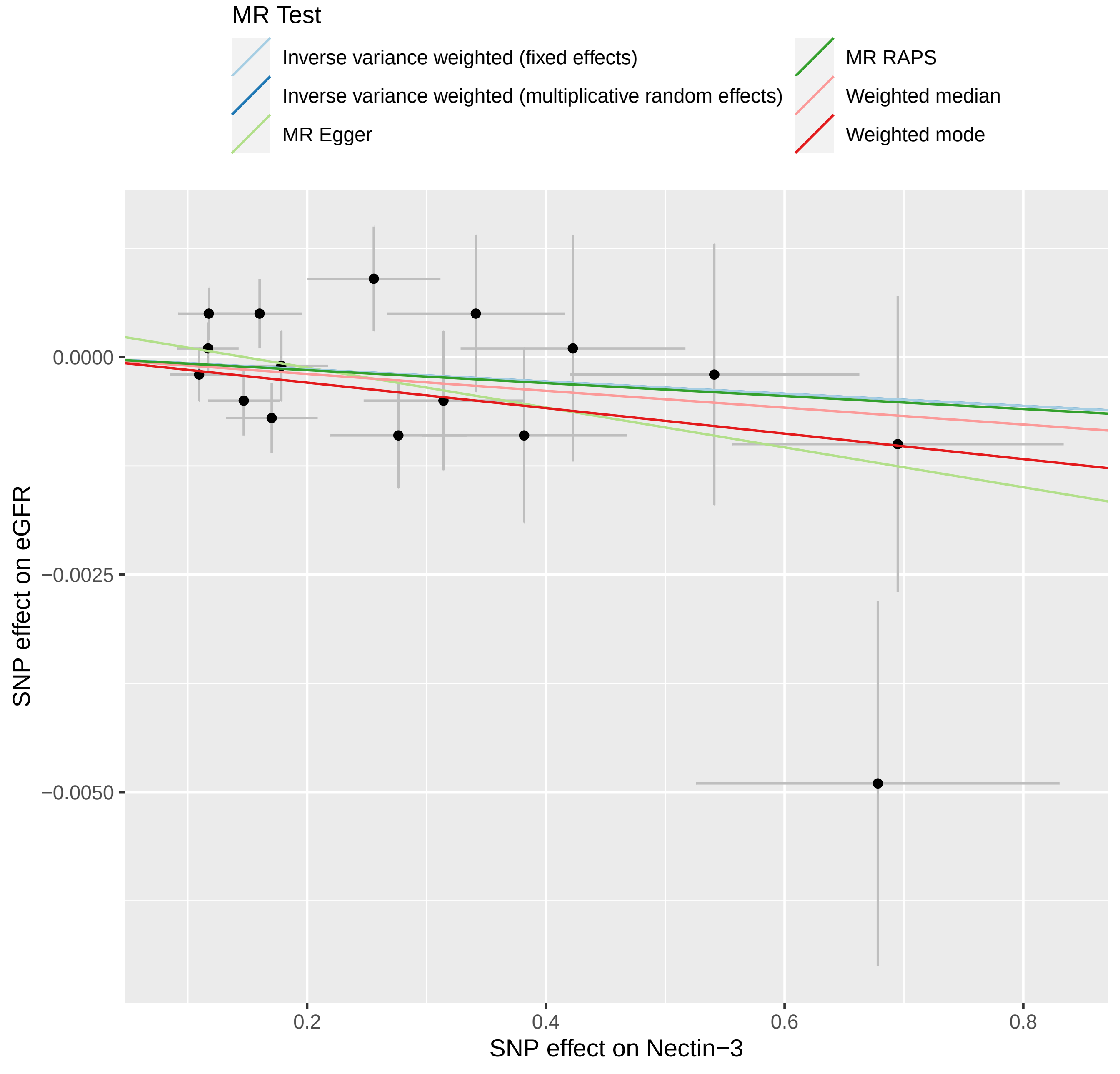

Batch 2−2459 : Nectin−4 on eGFR

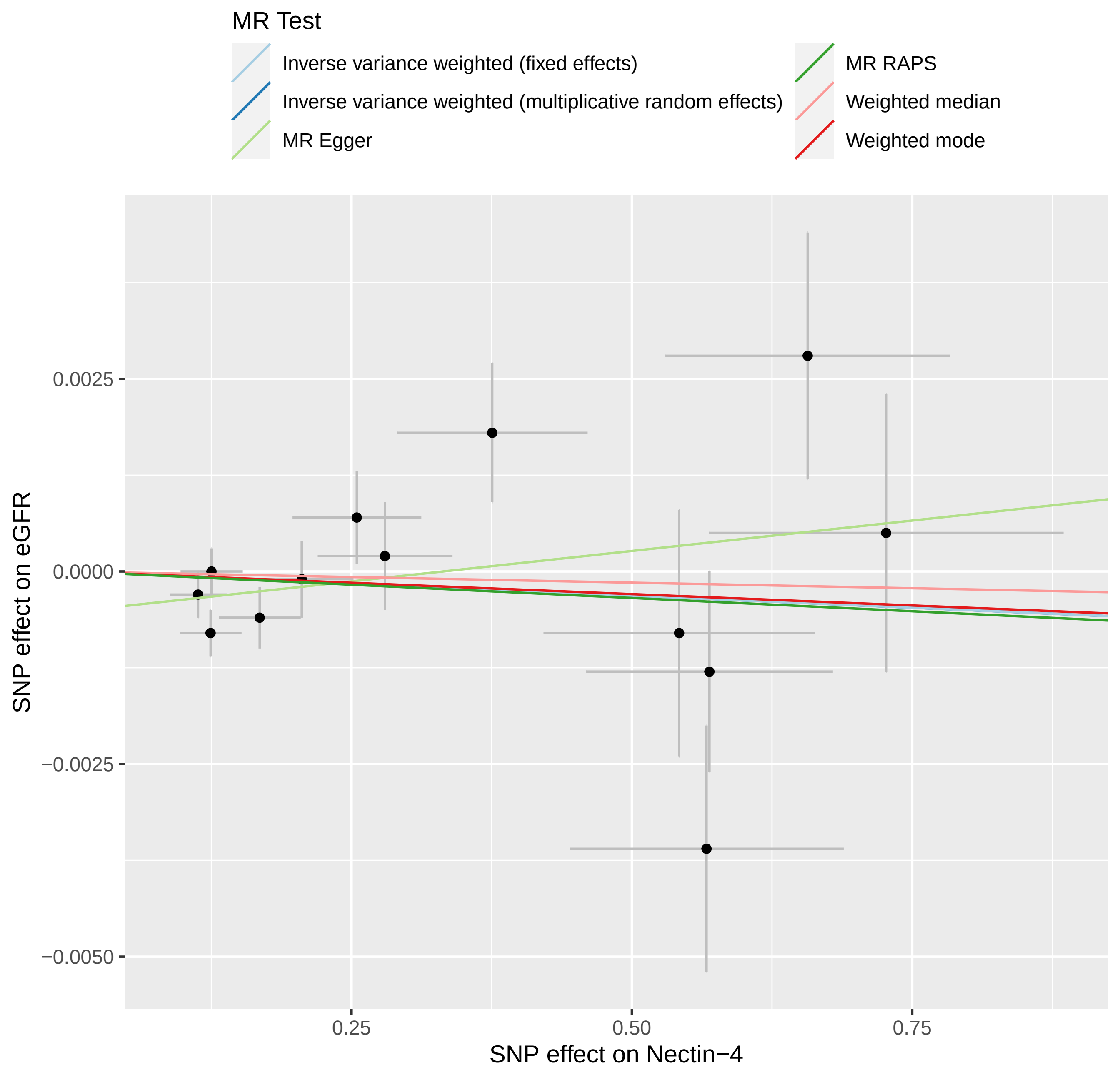

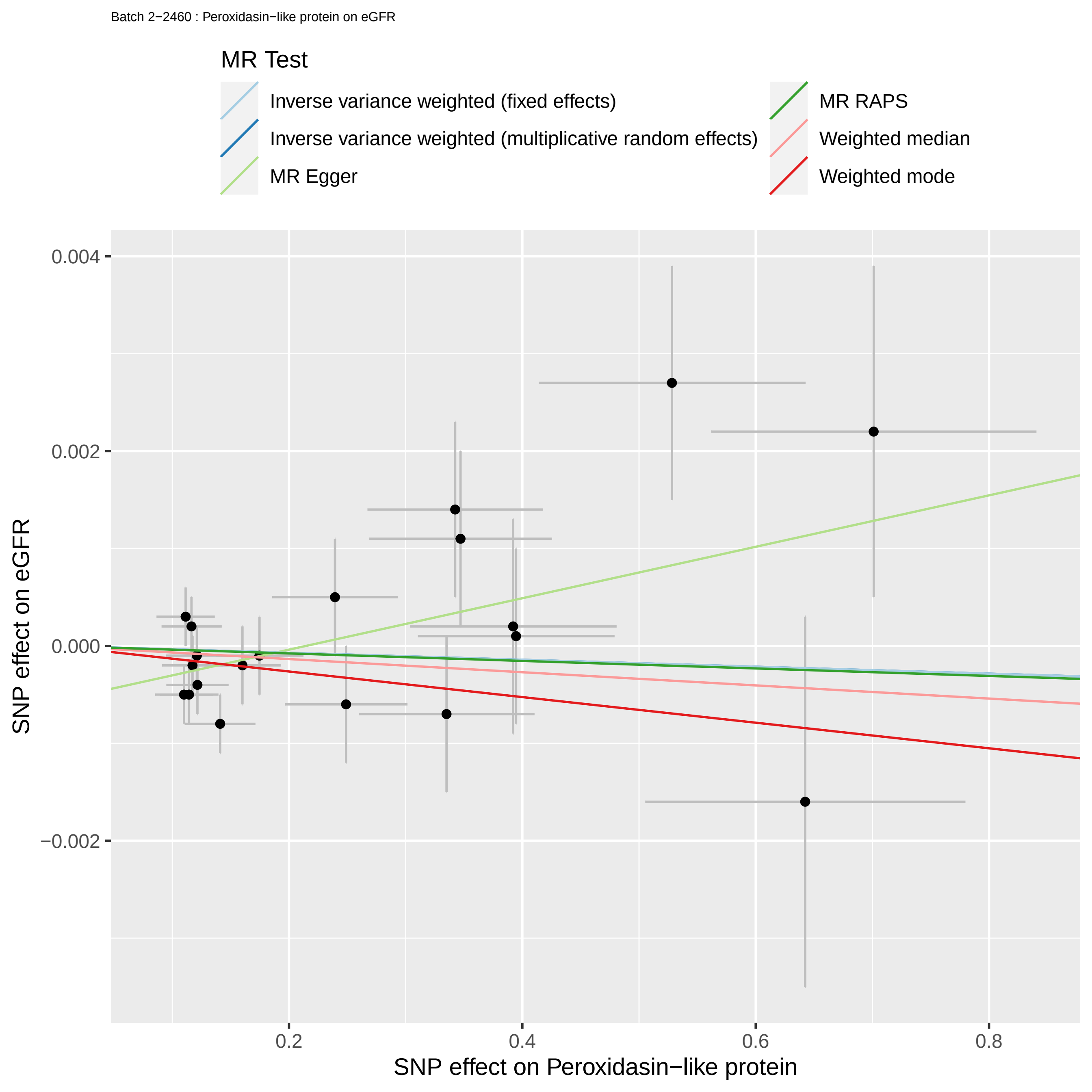
Batch 2−2460 : Peroxidasin−like protein on eGFR
MR Test
Inverse variance weighted (fixed effects)
Inverse variance weighted (multiplicative random effects)
MR Egger
MR RAPS
Weighted median
Weighted mode
0.004
0.002
0.000
−0.002
SNP effect on eGFR
0.2
0.4
0.6
0.8
SNP effect on Peroxidasin−like protein

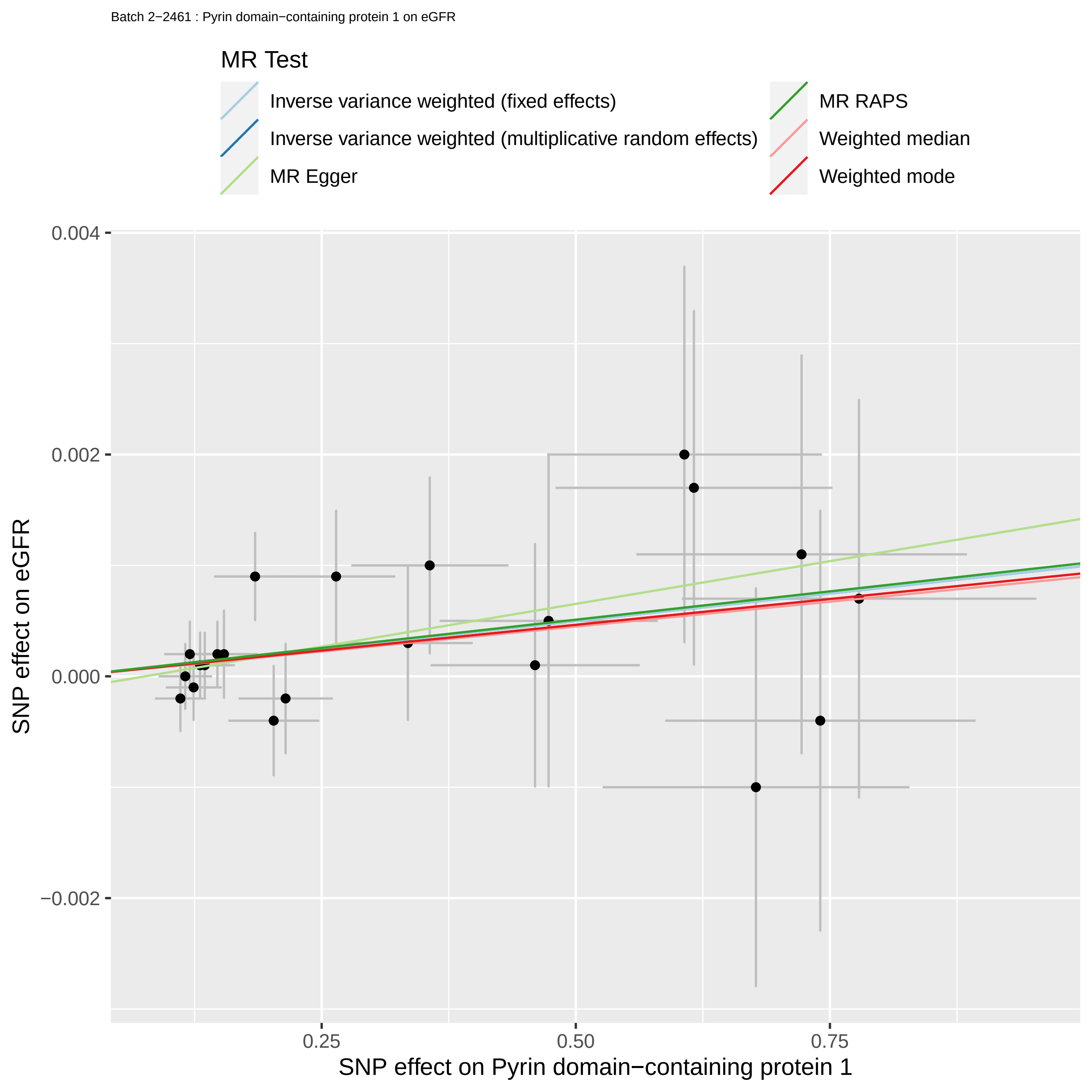

Batch 2−2461 : Pyrin domain−containing protein 1 on eGFR
MR Test
Inverse variance weighted (fixed effects)
Inverse variance weighted (multiplicative random effects)
MR Egger
MR RAPS
Weighted median
Weighted mode
0.004
0.002
0.000
−0.002
SNP effect on eGFR
0.25
0.50
0.75
SNP effect on Pyrin domain−containing protein 1

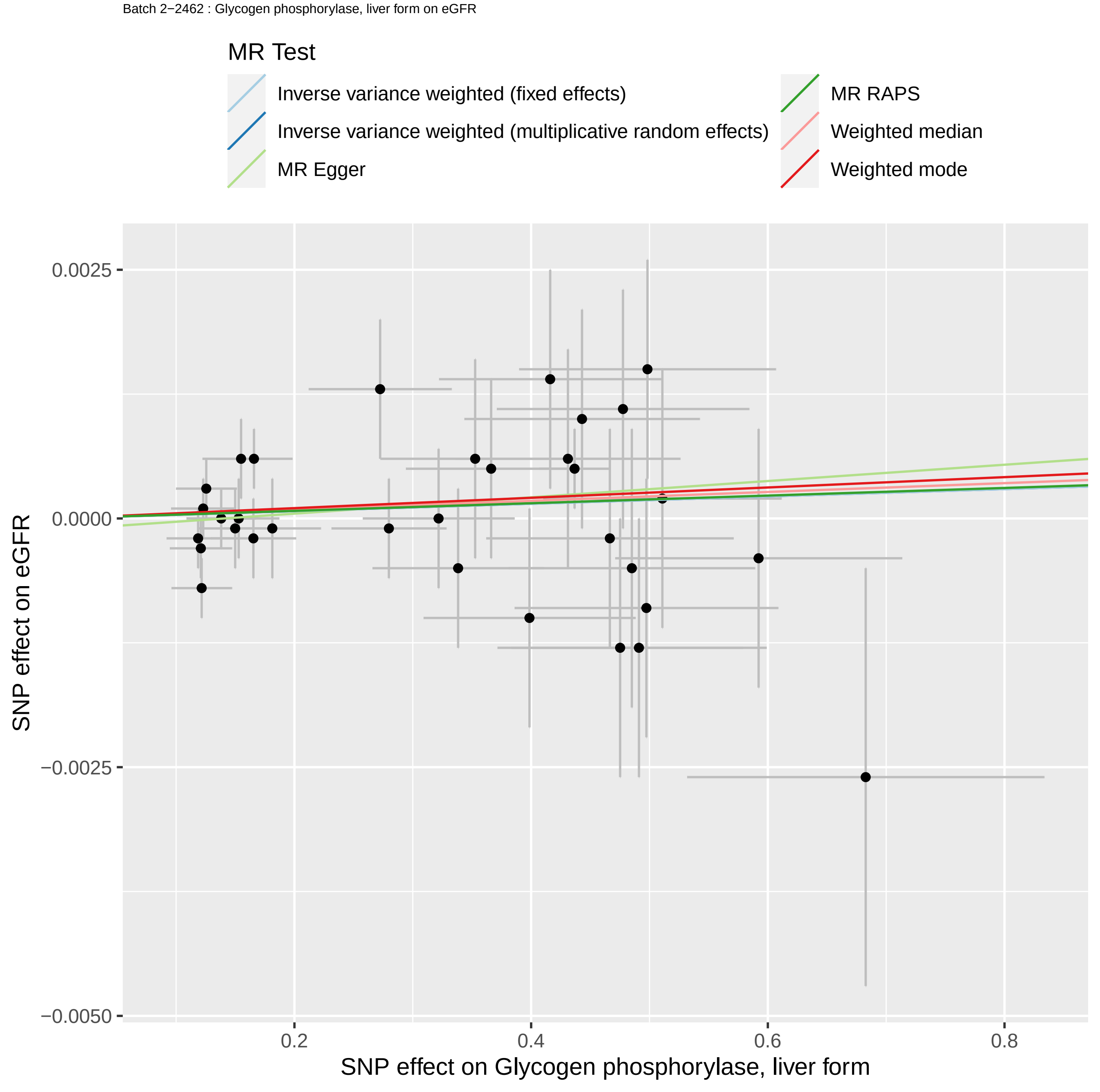

Batch 2−2462 : Glycogen phosphorylase, liver form on eGFR
MR Test
Inverse variance weighted (fixed effects)
Inverse variance weighted (multiplicative random effects)
MR Egger
MR RAPS
Weighted median
Weighted mode
0.0025
0.0000
−0.0025
−0.0050
0.2
0.4
0.6
0.8
SNP effect on eGFR
SNP effect on Glycogen phosphorylase, liver form

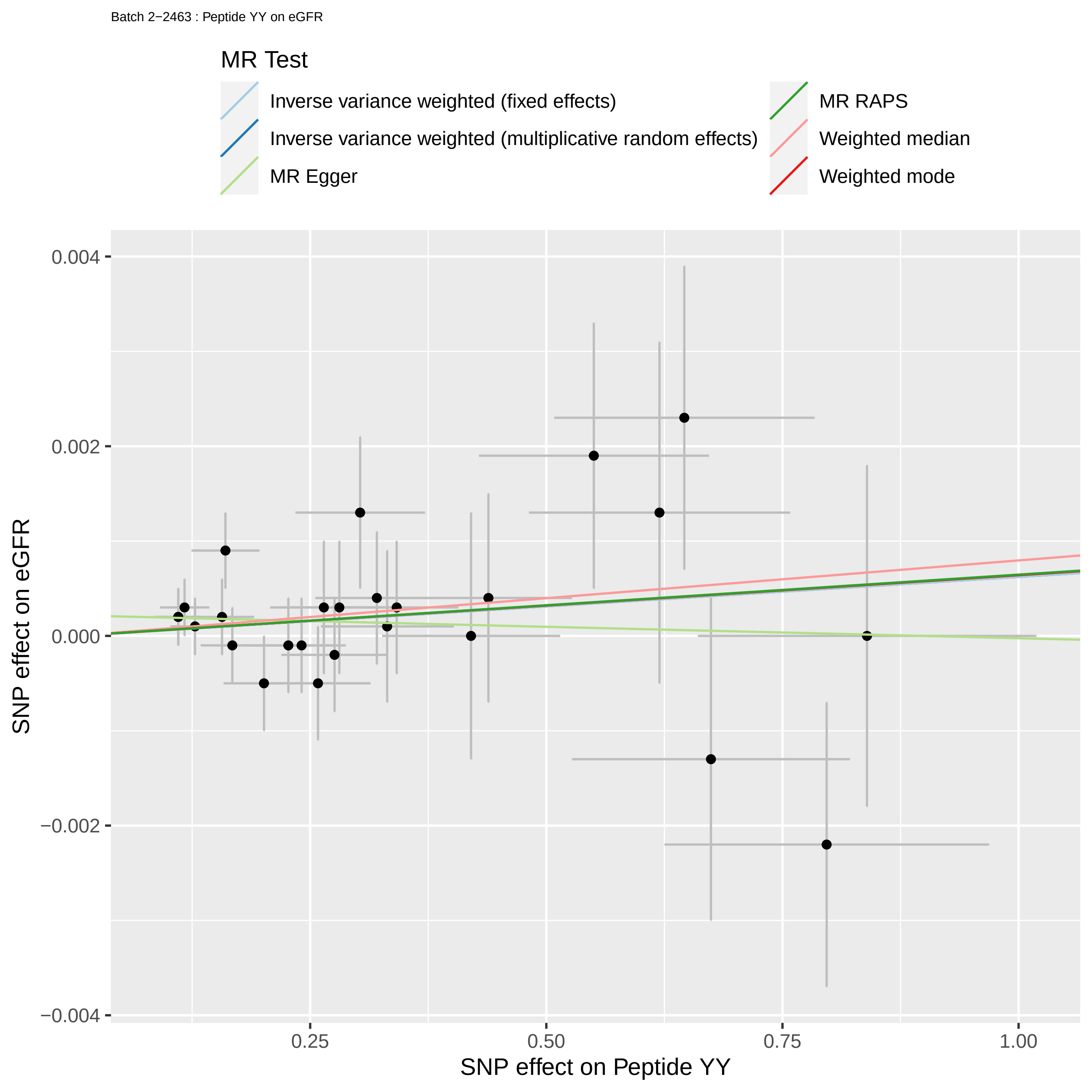

Batch 2−2463 : Peptide YY on eGFR
MR Test
Inverse variance weighted (fixed effects)
Inverse variance weighted (multiplicative random effects)
MR Egger
MR RAPS
Weighted median
Weighted mode
0.004
0.002
0.000
−0.002
−0.004
0.25
0.50
0.75
1.00
SNP effect on eGFR
SNP effect on Peptide YY

Batch 2−2464 : Dihydropteridine reductase on eGFR

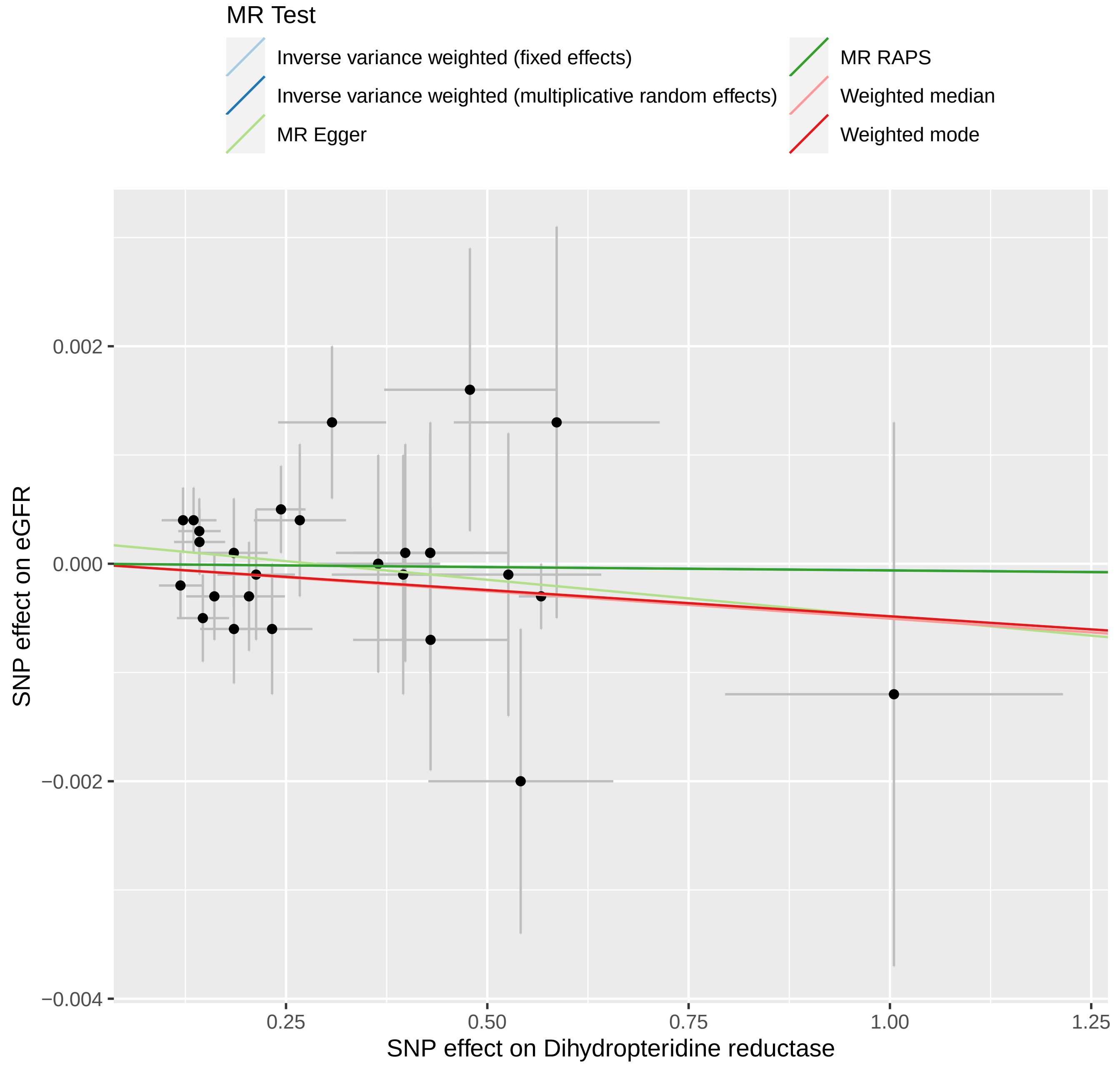

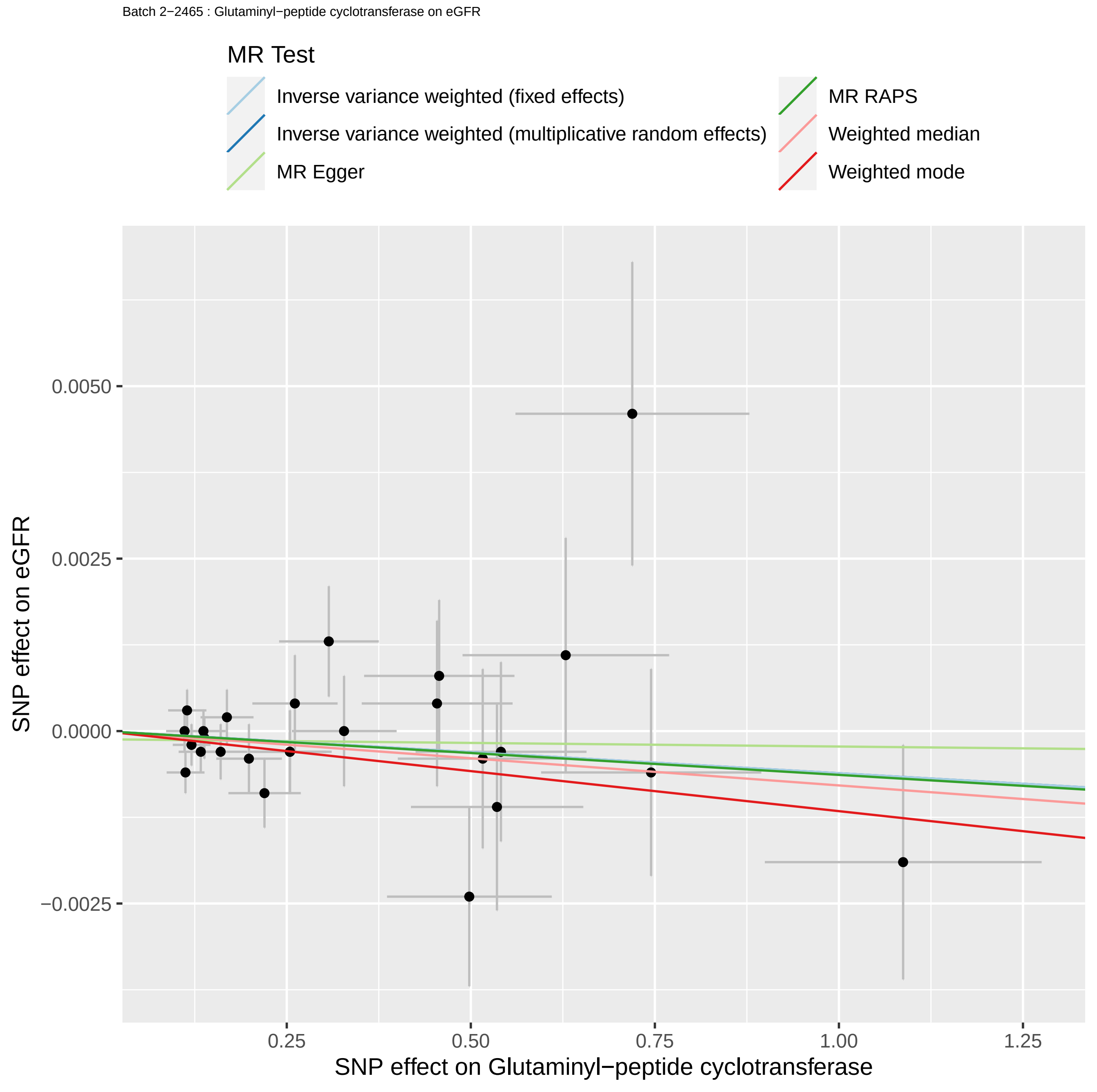
Batch 2−2465 : Glutaminyl−peptide cyclotransferase on eGFR
MR Test
Inverse variance weighted (fixed effects)
Inverse variance weighted (multiplicative random effects)
MR Egger
MR RAPS
Weighted median
Weighted mode
0.0050
0.0025
0.0000
−0.0025
SNP effect on eGFR
0.25
0.50
0.75
1.00
1.25
SNP effect on Glutaminyl−peptide cyclotransferase

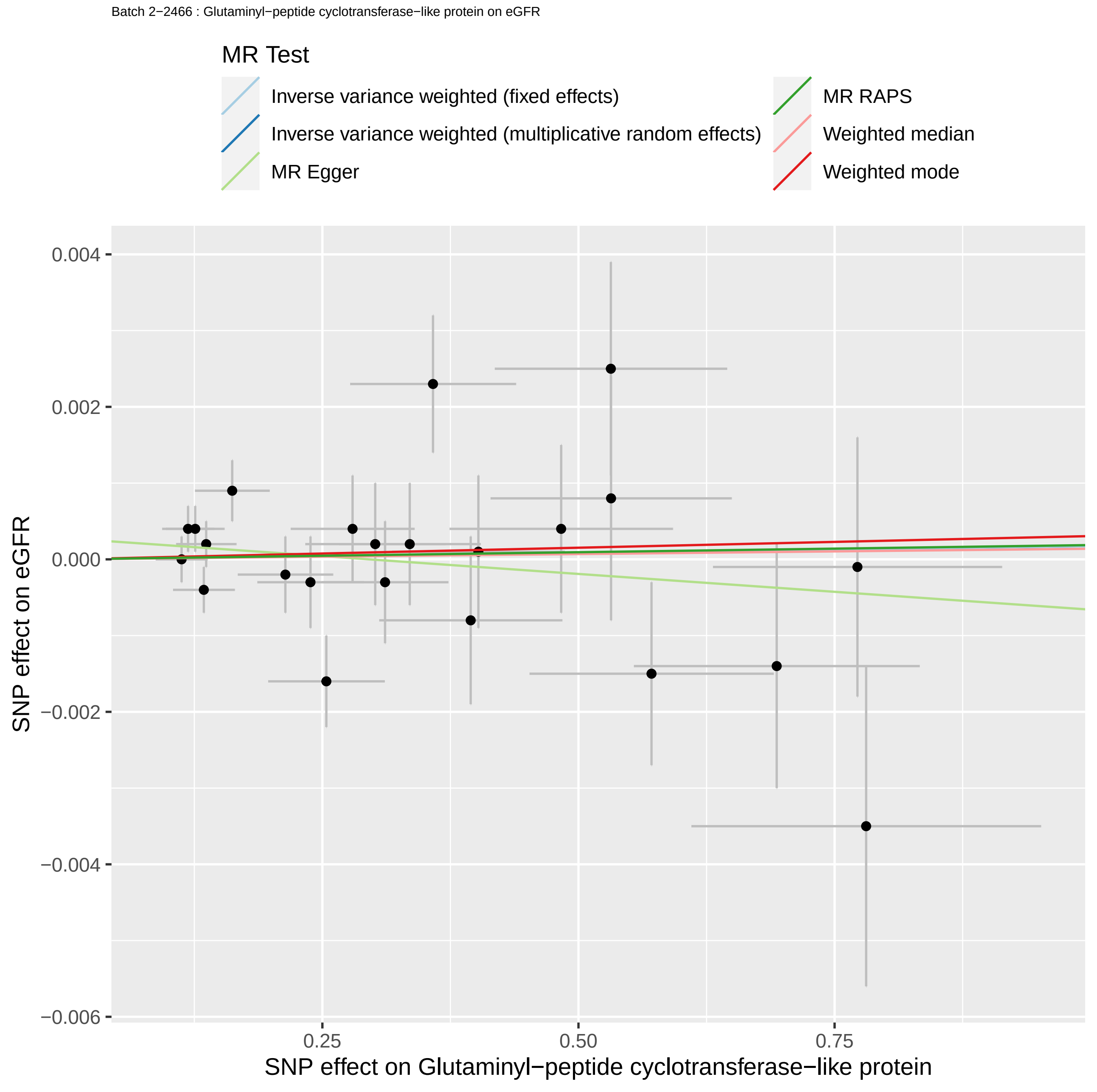

Batch 2−2466 : Glutaminyl−peptide cyclotransferase−like protein on eGFR
MR Test
Inverse variance weighted (fixed effects)
Inverse variance weighted (multiplicative random effects)
MR Egger
MR RAPS
Weighted median
Weighted mode
0.004
0.002
0.000
−0.002
−0.004
−0.006
0.25
0.50
0.75
SNP effect on eGFR
SNP effect on Glutaminyl−peptide cyclotransferase−like protein

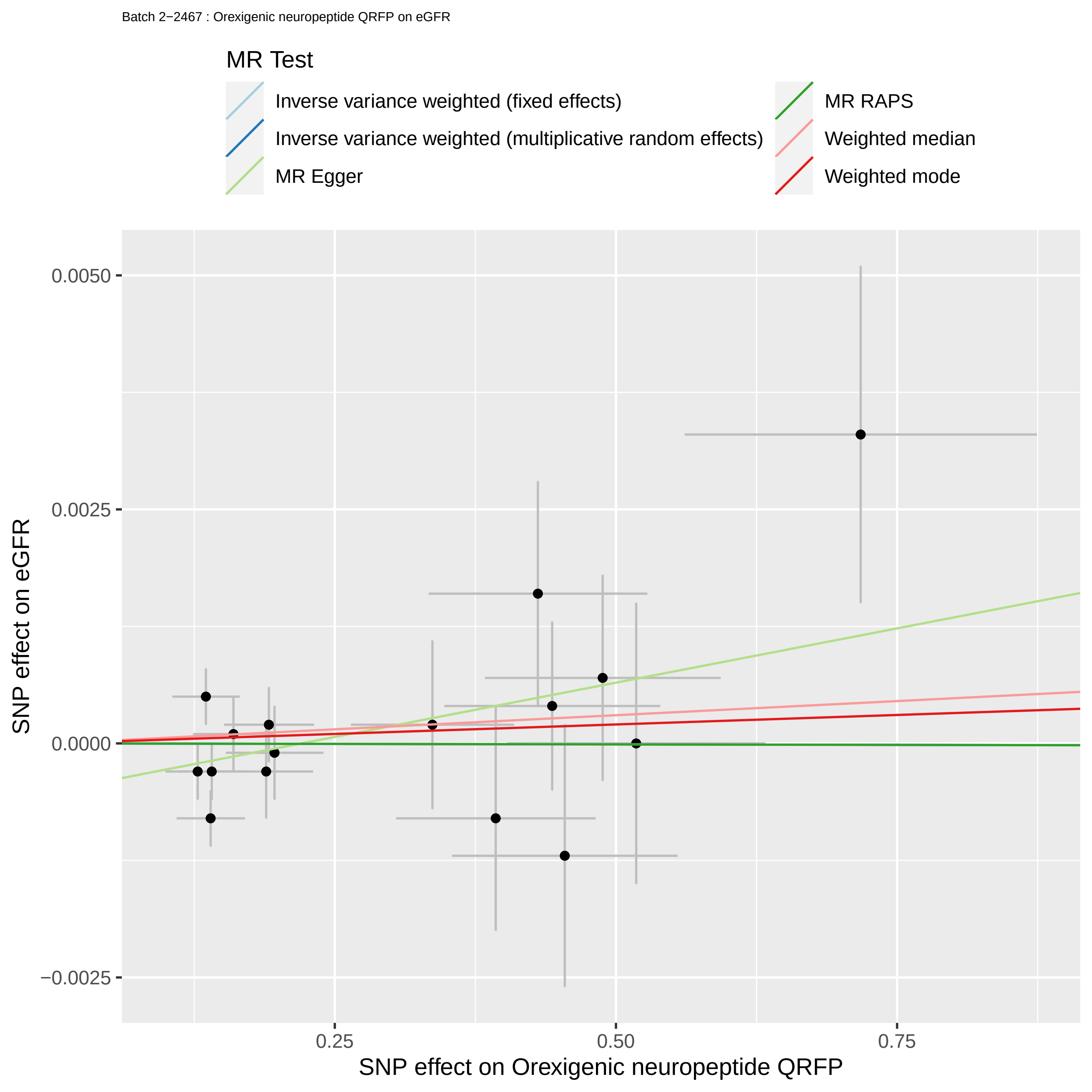

Batch 2−2467 : Orexigenic neuropeptide QRFP on eGFR
MR Test
Inverse variance weighted (fixed effects)
Inverse variance weighted (multiplicative random effects)
MR Egger
MR RAPS
Weighted median
Weighted mode
0.0050
0.0025
0.0000
−0.0025
0.25
0.50
0.75
SNP effect on eGFR
SNP effect on Orexigenic neuropeptide QRFP

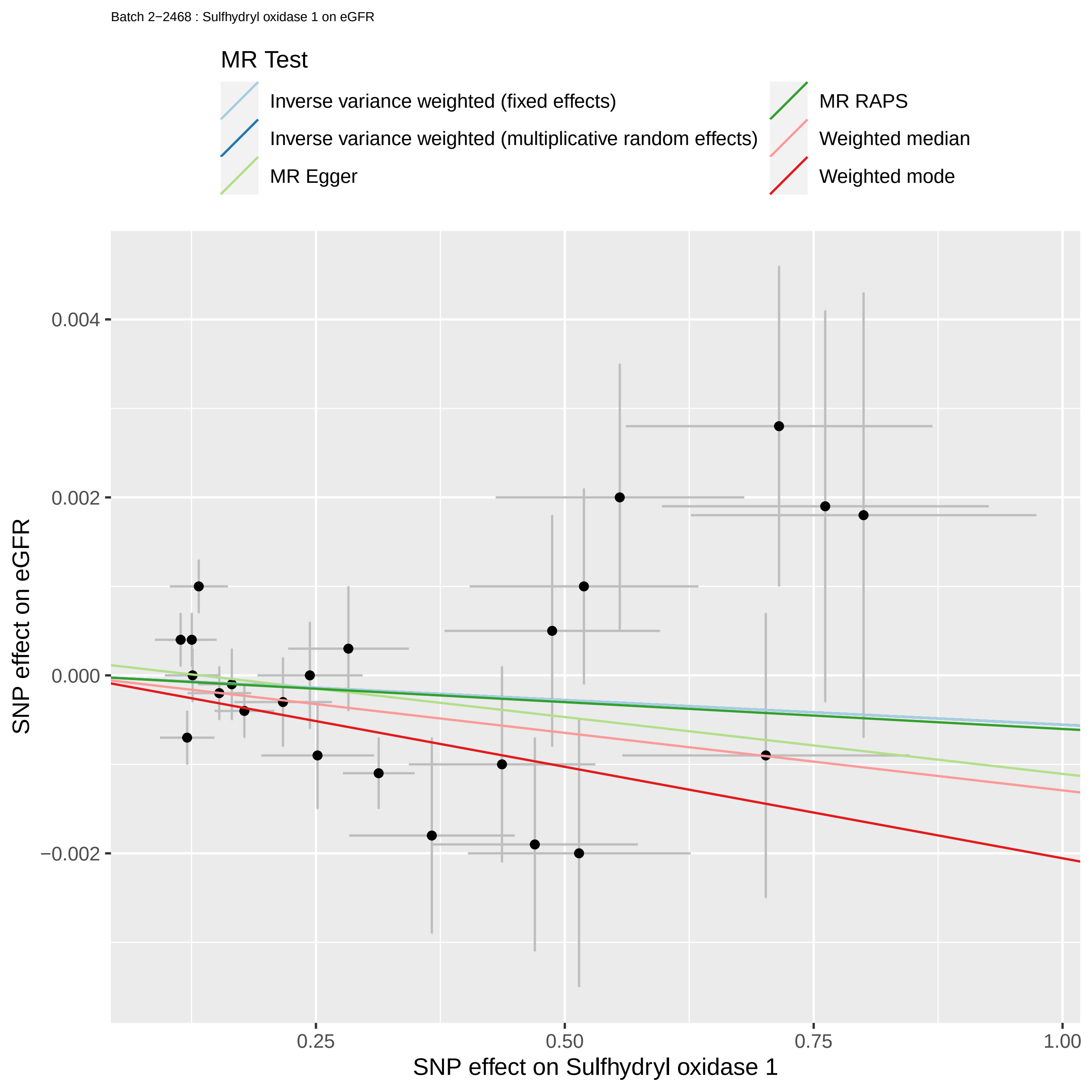

Batch 2−2468 : Sulfhydryl oxidase 1 on eGFR
MR Test
Inverse variance weighted (fixed effects)
Inverse variance weighted (multiplicative random effects)
MR Egger
MR RAPS
Weighted median
Weighted mode
0.004
0.002
0.000
−0.002
SNP effect on eGFR
0.25
0.50
0.75
1.00
SNP effect on Sulfhydryl oxidase 1

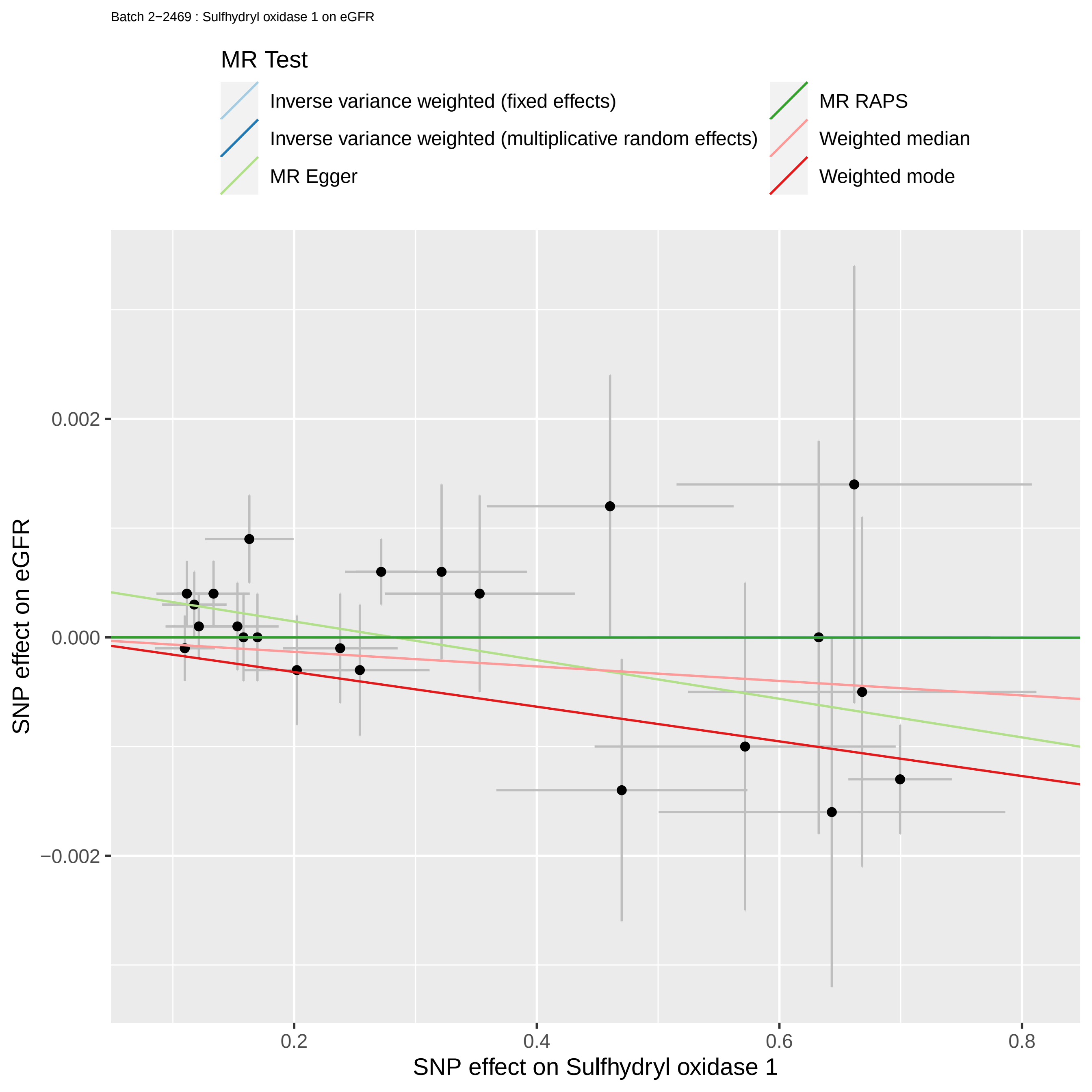

Batch 2−2469 : Sulfhydryl oxidase 1 on eGFR
MR Test
Inverse variance weighted (fixed effects)
Inverse variance weighted (multiplicative random effects)
MR Egger
MR RAPS
Weighted median
Weighted mode
0.002
0.000
−0.002
0.2
0.4
0.6
0.8
SNP effect on eGFR
SNP effect on Sulfhydryl oxidase 1

Batch 2−2470 : Sulfhydryl oxidase 2 on eGFR

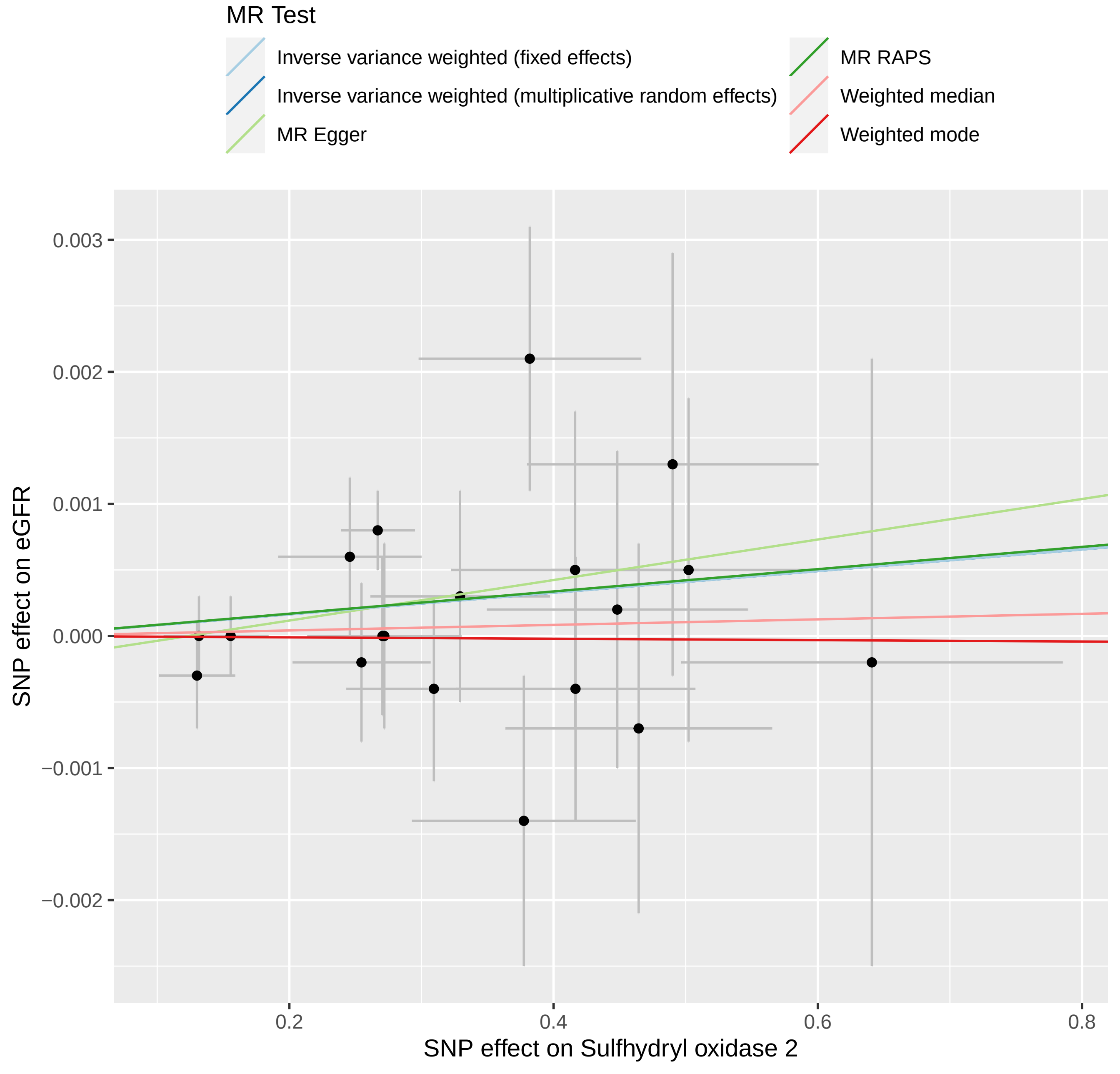

Batch 2−2471 : Ras−related protein Rab−14 on eGFR

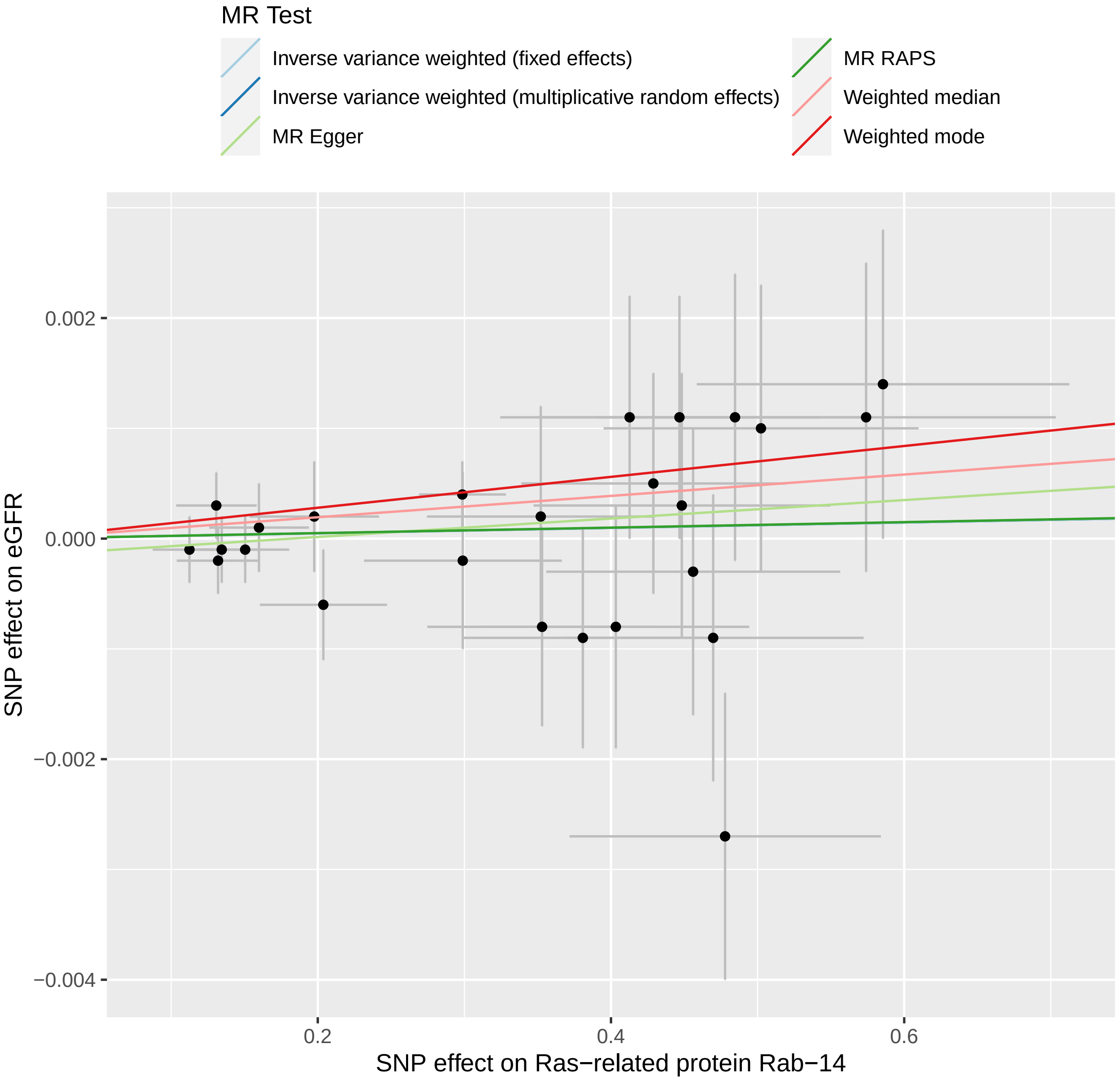

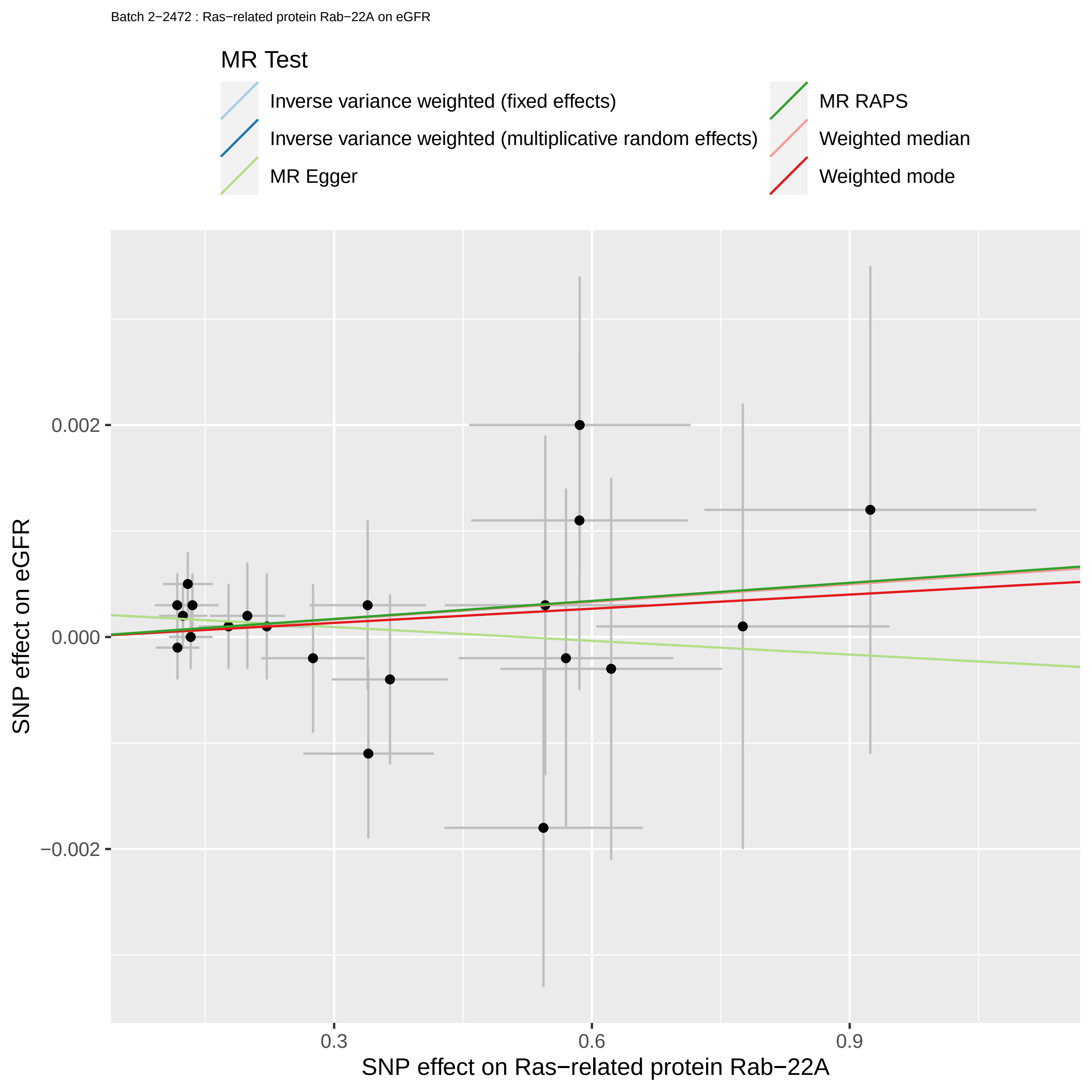
Batch 2−2472 : Ras−related protein Rab−22A on eGFR
MR Test
Inverse variance weighted (fixed effects)
Inverse variance weighted (multiplicative random effects)
MR Egger
MR RAPS
Weighted median
Weighted mode
0.002
0.000
−0.002
0.3
0.6
0.9
SNP effect on eGFR
SNP effect on Ras−related protein Rab−22A

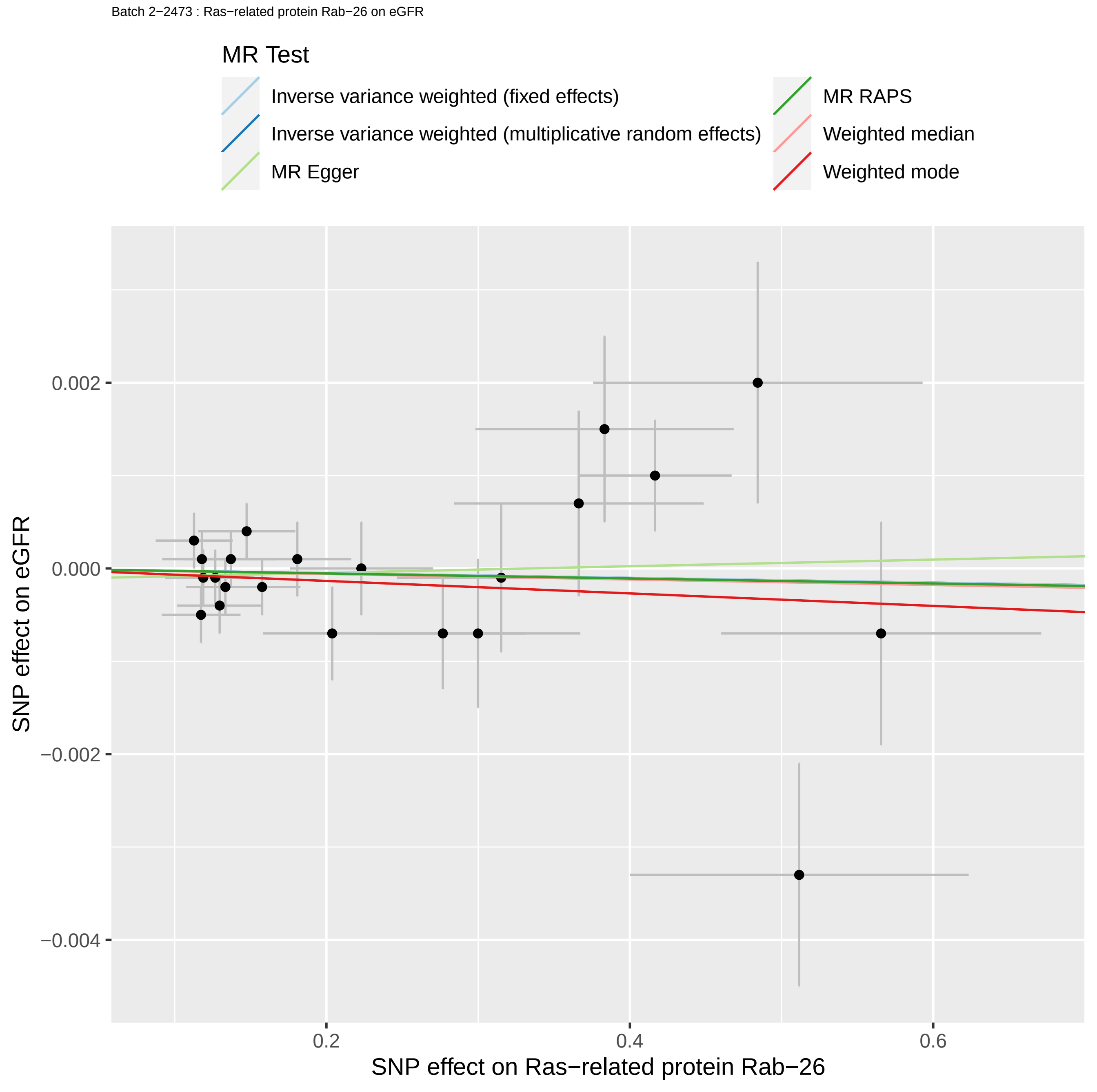

Batch 2−2473 : Ras−related protein Rab−26 on eGFR
MR Test
Inverse variance weighted (fixed effects)
Inverse variance weighted (multiplicative random effects)
MR Egger
MR RAPS
Weighted median
Weighted mode
0.002
0.000
−0.002
−0.004
0.2
0.4
0.6
SNP effect on eGFR
SNP effect on Ras−related protein Rab−26

Batch 2−2474 : Ras−related protein Rab−27A on eGFR

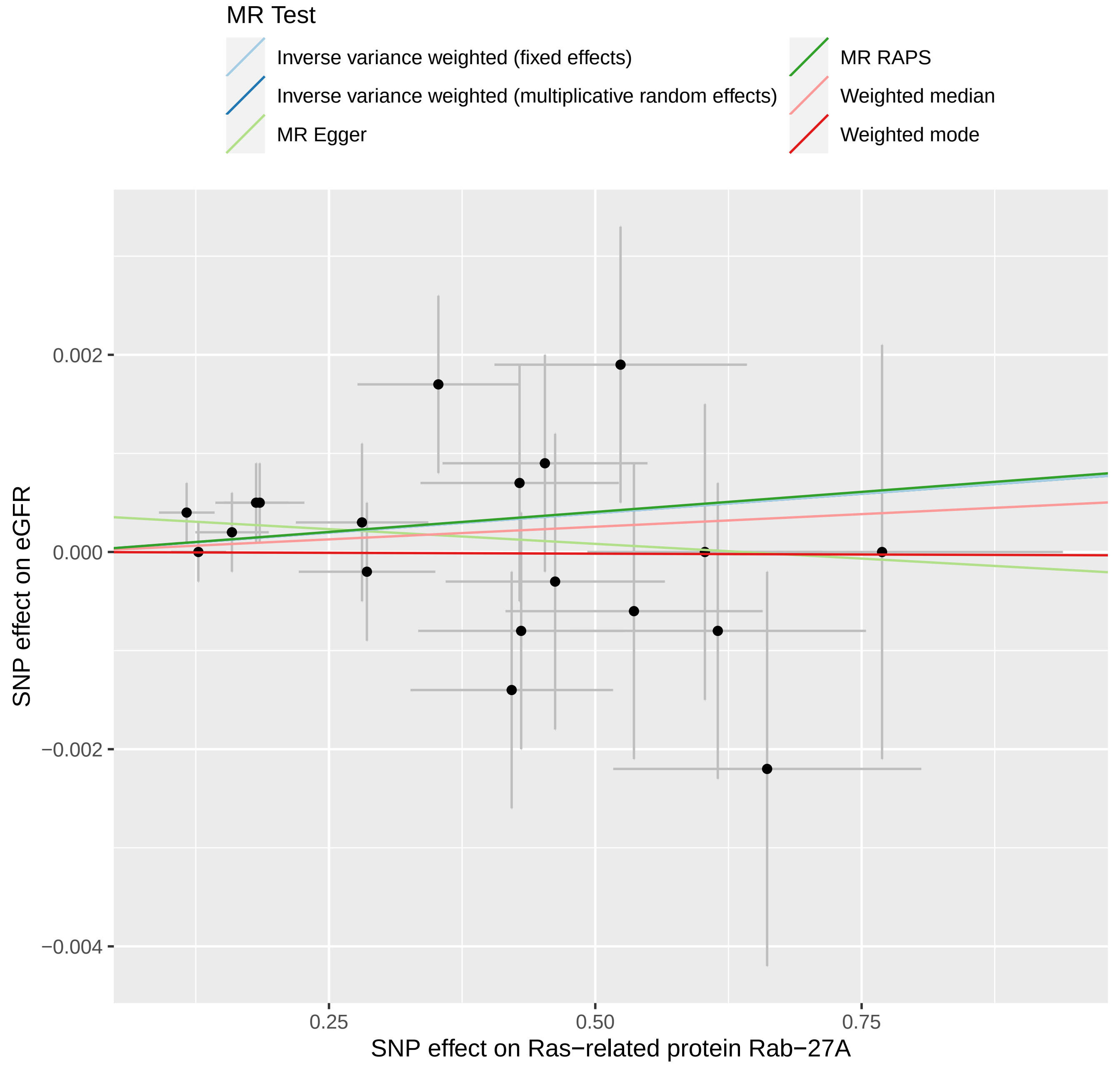

Batch 2−2475 : Ras−related protein Rab−27B on eGFR

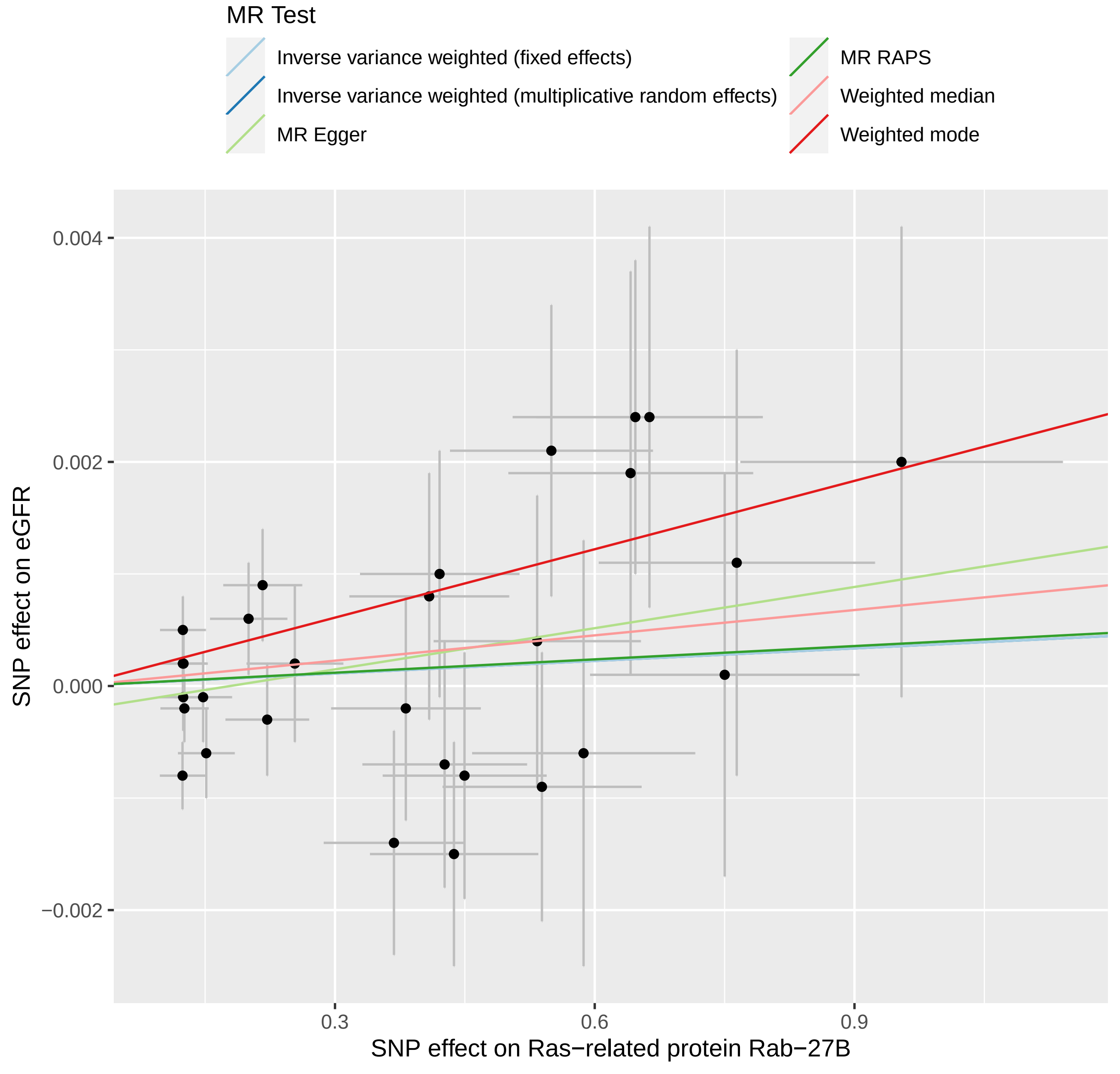

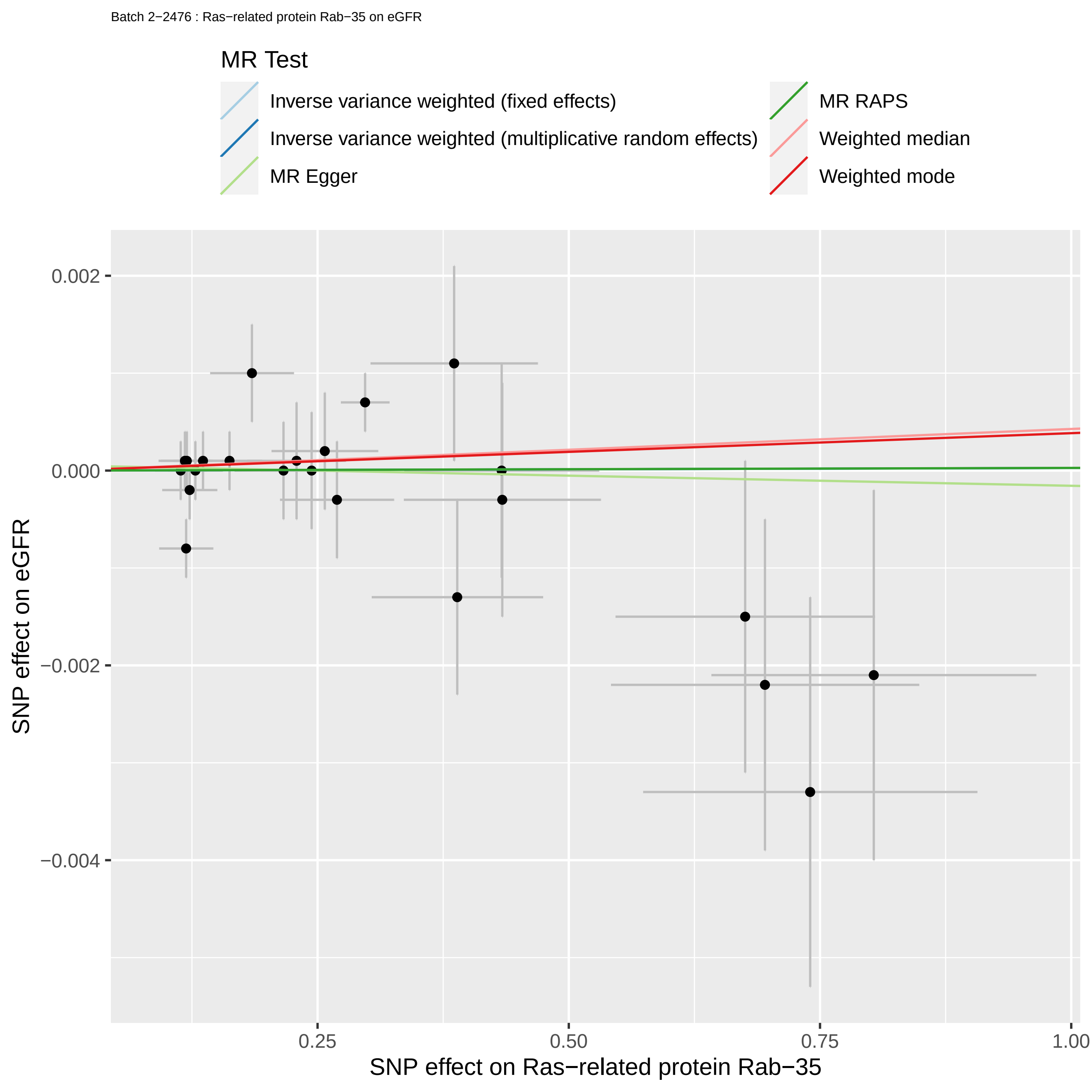
Batch 2−2476 : Ras−related protein Rab−35 on eGFR
MR Test
Inverse variance weighted (fixed effects)
Inverse variance weighted (multiplicative random effects)
MR Egger
MR RAPS
Weighted median
Weighted mode
0.002
0.000
−0.002
−0.004
0.25
0.50
0.75
1.00
SNP effect on eGFR
SNP effect on Ras−related protein Rab−35

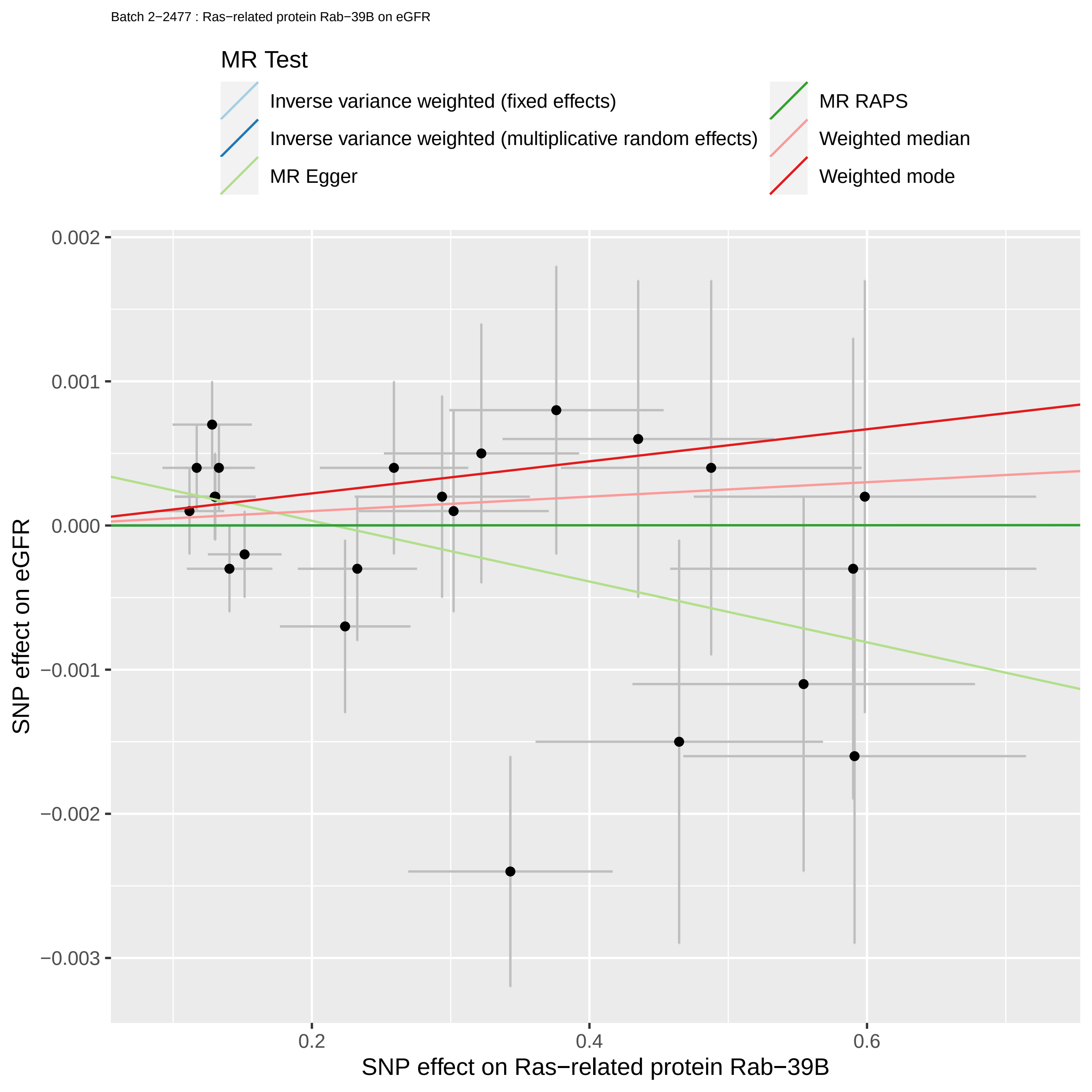

Batch 2−2477 : Ras−related protein Rab−39B on eGFR
MR Test
Inverse variance weighted (fixed effects)
Inverse variance weighted (multiplicative random effects)
MR Egger
MR RAPS
Weighted median
Weighted mode
0.002
0.001
0.000
−0.001
−0.002
−0.003
SNP effect on eGFR
0.2
0.4
0.6
SNP effect on Ras−related protein Rab−39B

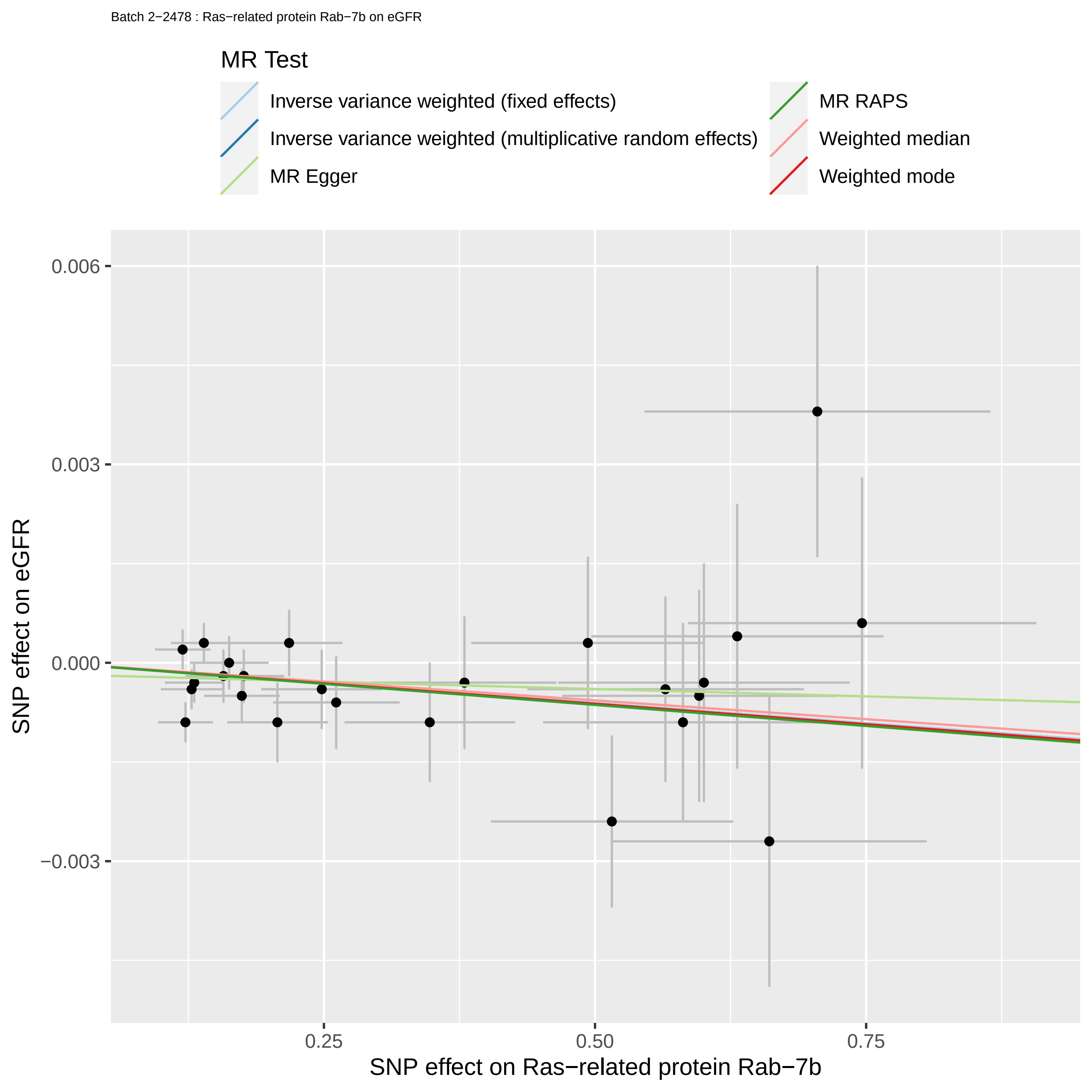

Batch 2−2478 : Ras−related protein Rab−7b on eGFR
MR Test
Inverse variance weighted (fixed effects)
Inverse variance weighted (multiplicative random effects)
MR Egger
MR RAPS
Weighted median
Weighted mode
0.006
0.003
0.000
−0.003
0.25
0.50
0.75
SNP effect on eGFR
SNP effect on Ras−related protein Rab−7b

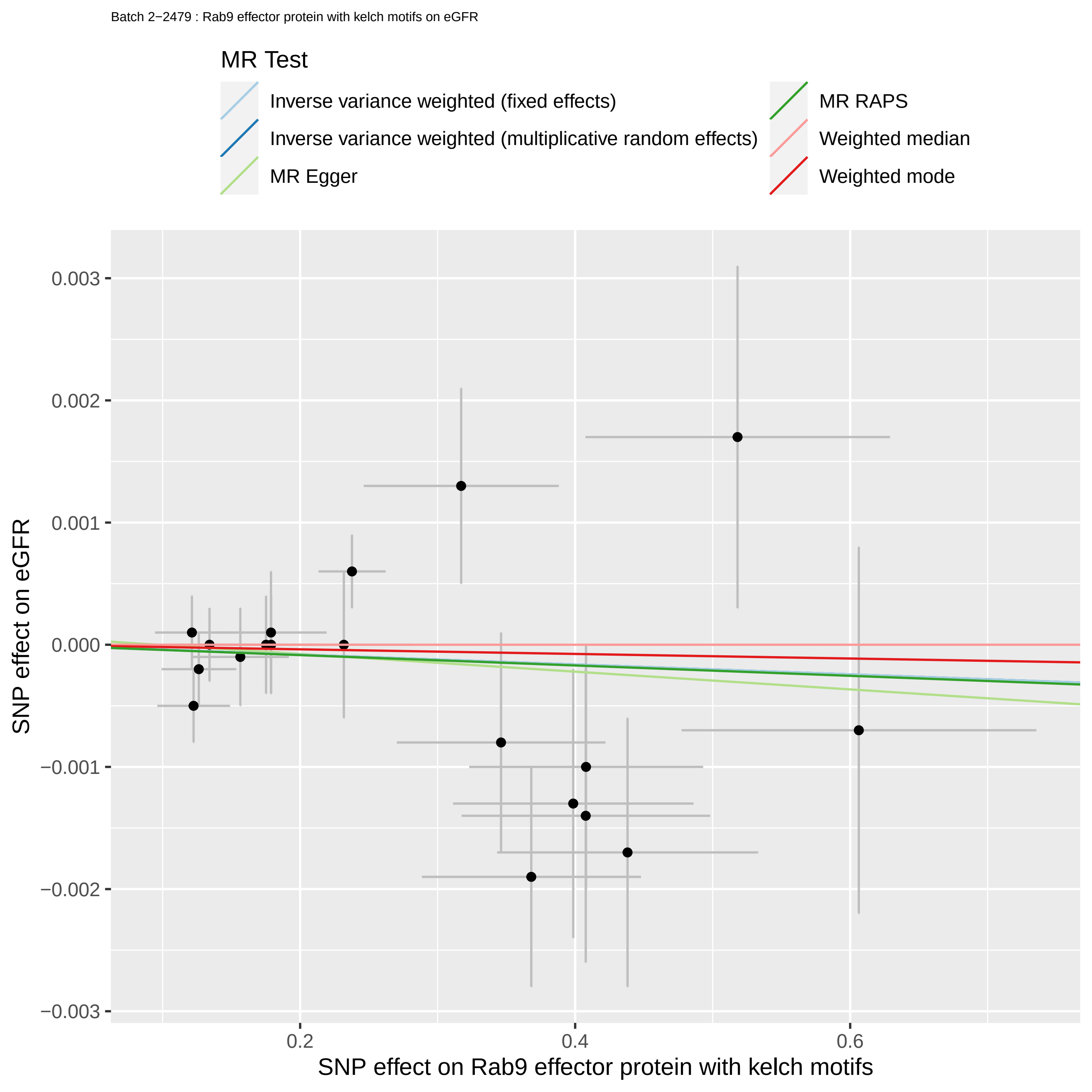

Batch 2−2479 : Rab9 effector protein with kelch motifs on eGFR
MR Test
Inverse variance weighted (fixed effects)
Inverse variance weighted (multiplicative random effects)
MR Egger
MR RAPS
Weighted median
Weighted mode
0.003
0.002
0.001
0.000
−0.001
−0.002
−0.003
0.2
0.4
0.6
SNP effect on eGFR
SNP effect on Rab9 effector protein with kelch motifs

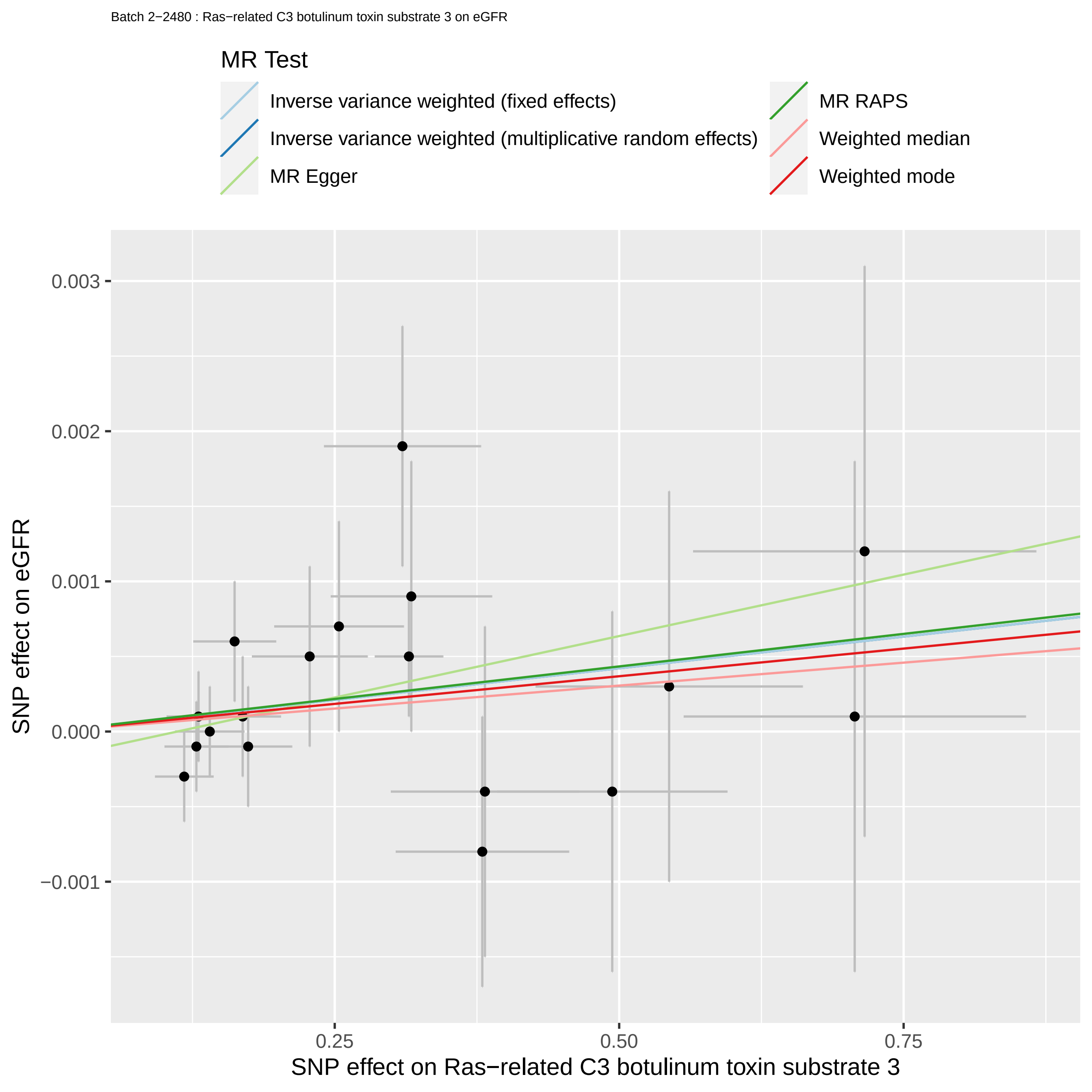

Batch 2−2480 : Ras−related C3 botulinum toxin substrate 3 on eGFR
MR Test
Inverse variance weighted (fixed effects)
Inverse variance weighted (multiplicative random effects)
MR Egger
MR RAPS
Weighted median
Weighted mode
0.003
0.002
0.001
0.000
−0.001
SNP effect on eGFR
0.25
0.50
0.75
SNP effect on Ras−related C3 botulinum toxin substrate 3

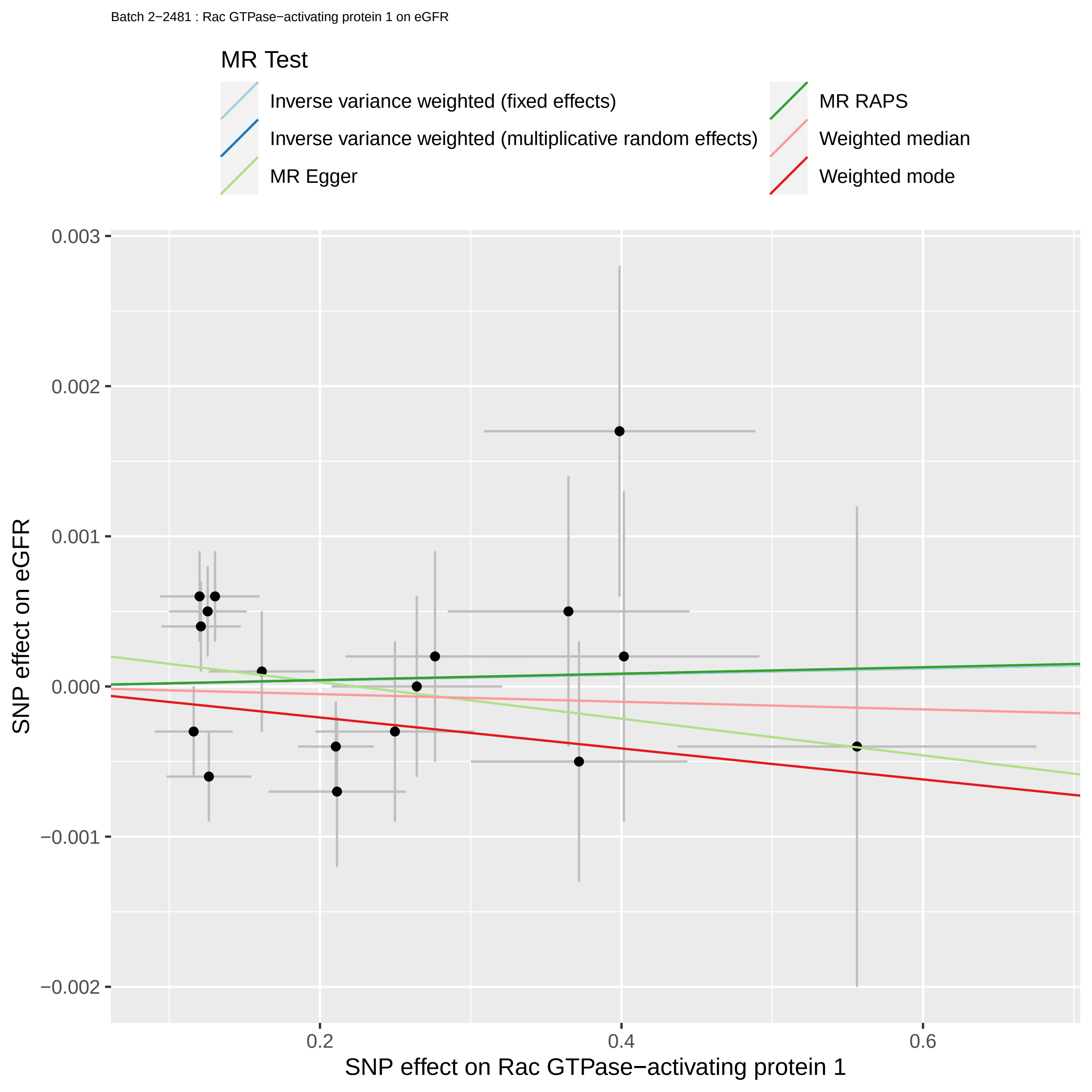

Batch 2−2481 : Rac GTPase−activating protein 1 on eGFR
MR Test
Inverse variance weighted (fixed effects)
Inverse variance weighted (multiplicative random effects)
MR Egger
MR RAPS
Weighted median
Weighted mode
0.003
0.002
0.001
0.000
−0.001
−0.002
0.2
0.4
0.6
SNP effect on eGFR
SNP effect on Rac GTPase−activating protein 1

Batch 2−2482 : Cell cycle checkpoint protein RAD1 on eGFR

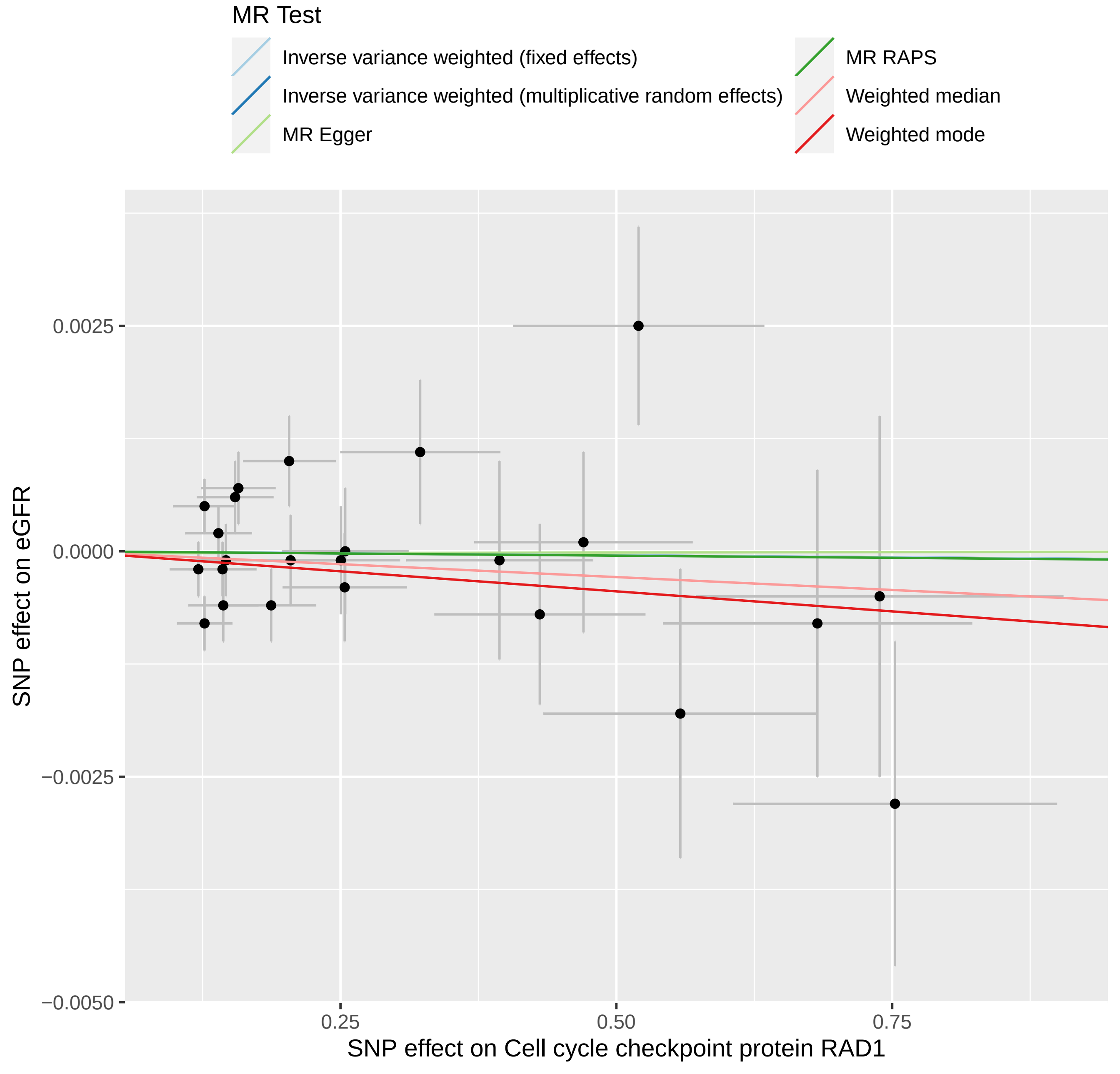

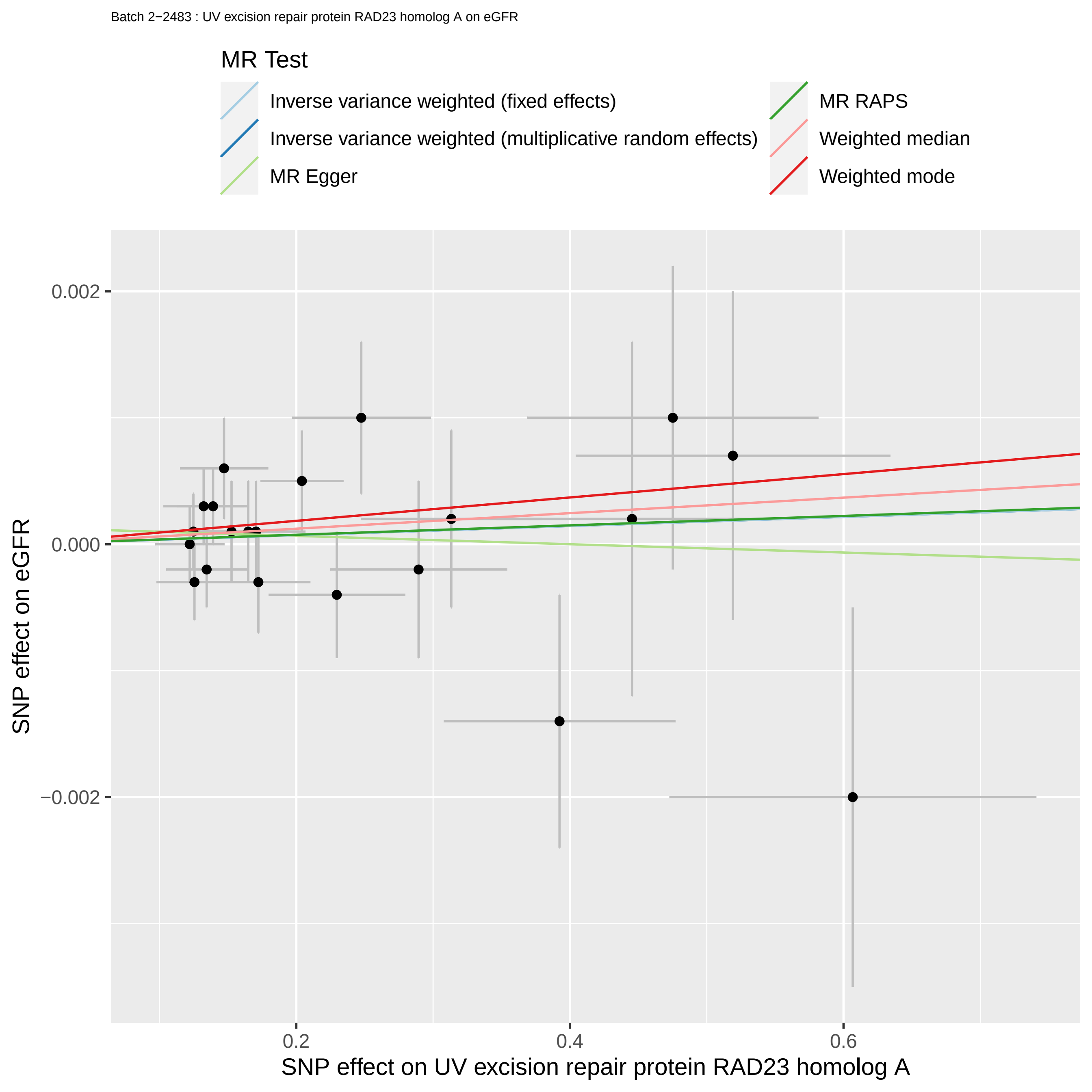

Batch 2−2483 : UV excision repair protein RAD23 homolog A on eGFR
MR Test
Inverse variance weighted (fixed effects)
Inverse variance weighted (multiplicative random effects)
MR Egger
MR RAPS
Weighted median
Weighted mode
0.002
0.000
−0.002
0.2
0.4
0.6
SNP effect on eGFR
SNP effect on UV excision repair protein RAD23 homolog A

Batch 2−2484 : UV excision repair protein RAD23 homolog B on eGFR

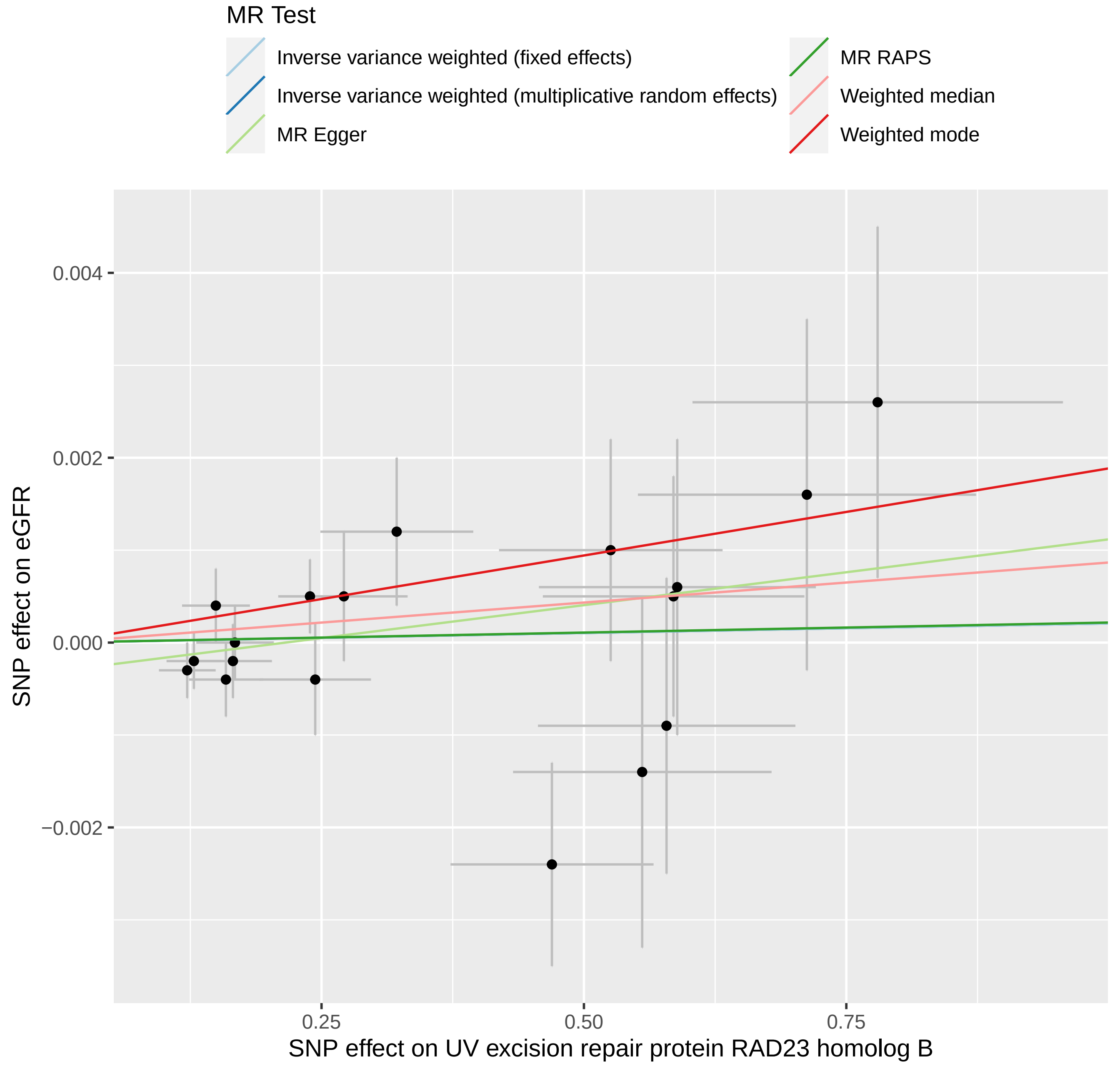

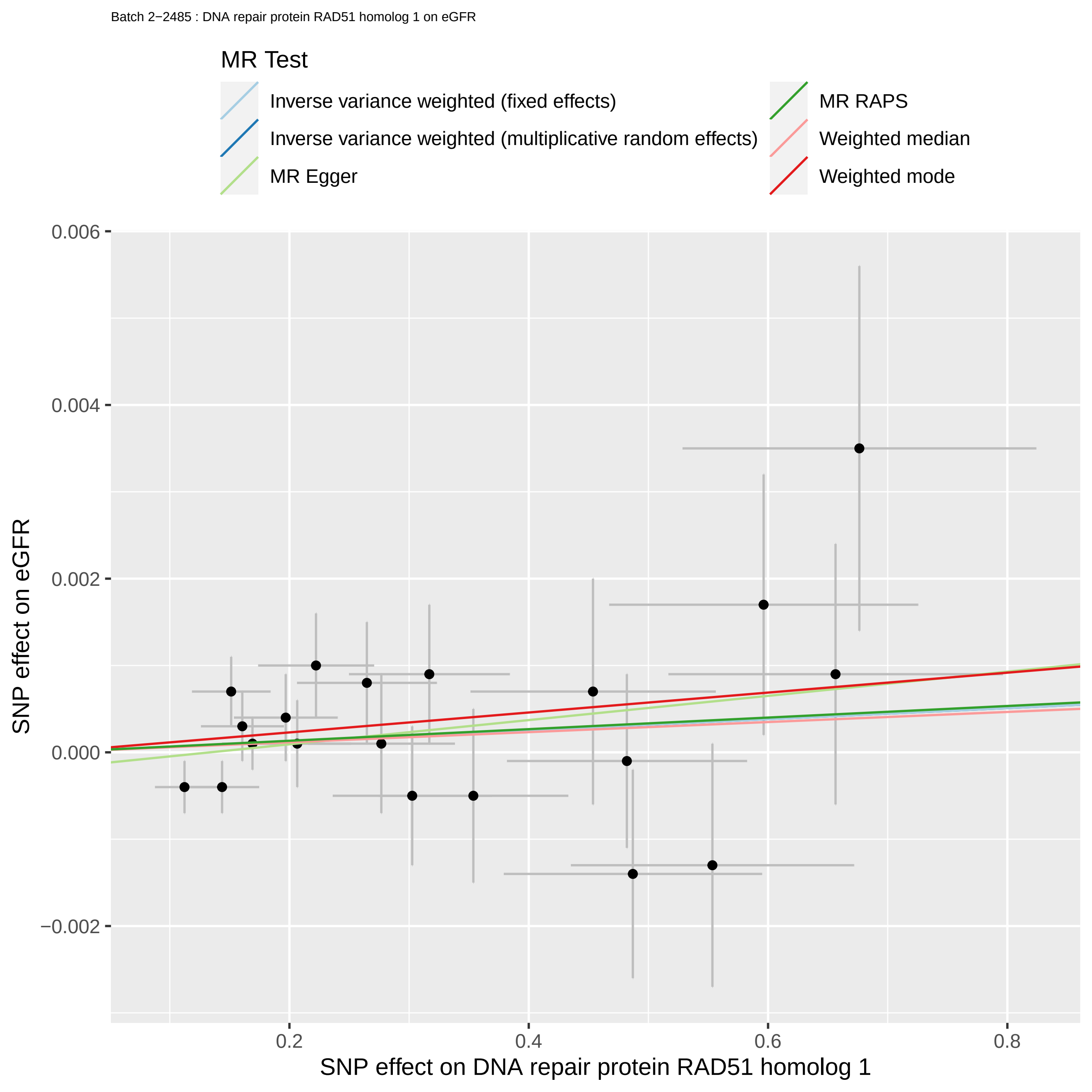

Batch 2−2485 : DNA repair protein RAD51 homolog 1 on eGFR
MR Test
Inverse variance weighted (fixed effects)
Inverse variance weighted (multiplicative random effects)
MR Egger
MR RAPS
Weighted median
Weighted mode
0.006
0.004
0.002
0.000
−0.002
0.2
0.4
0.6
0.8
SNP effect on eGFR
SNP effect on DNA repair protein RAD51 homolog 1

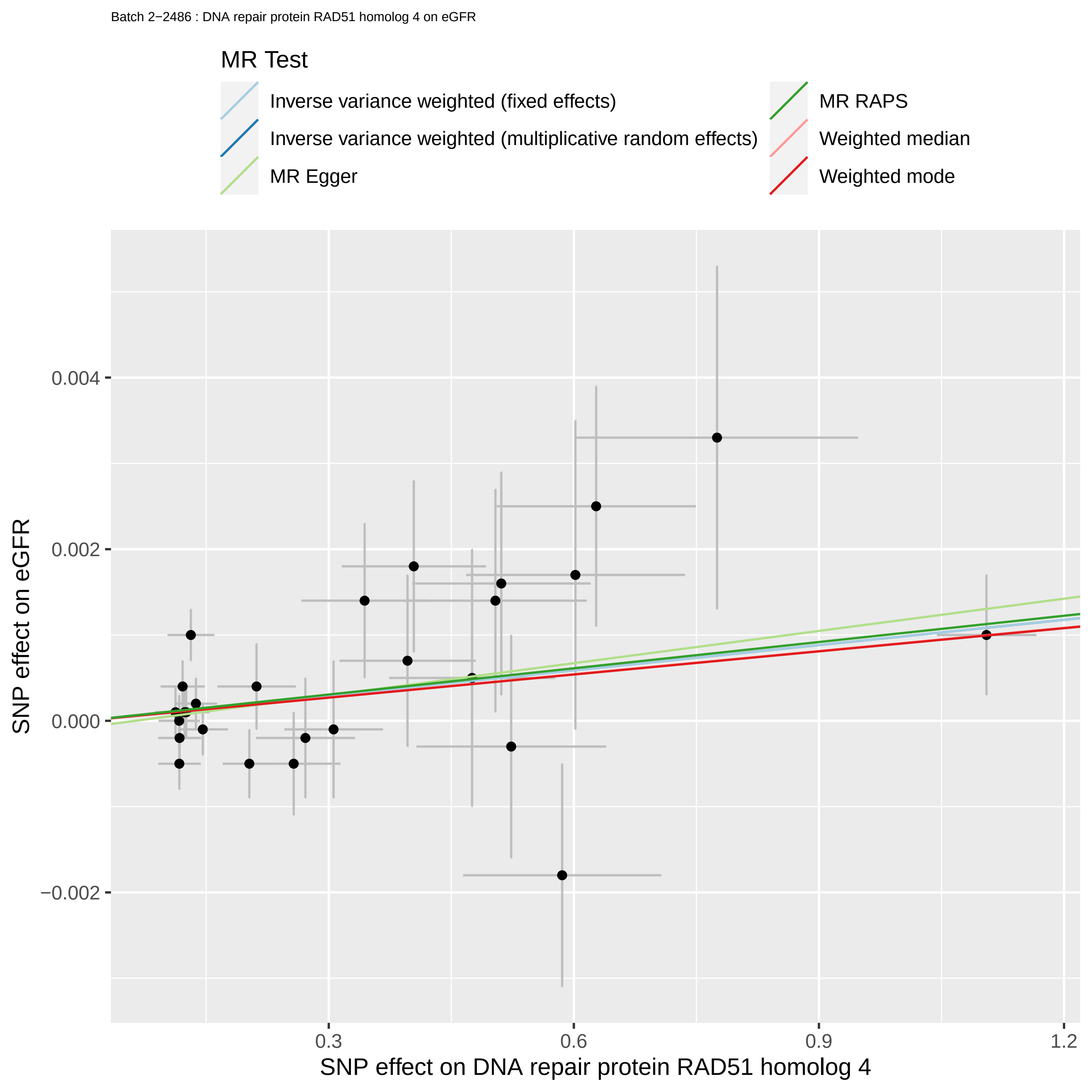
Batch 2−2486 : DNA repair protein RAD51 homolog 4 on eGFR
MR Test
Inverse variance weighted (fixed effects)
Inverse variance weighted (multiplicative random effects)
MR Egger
MR RAPS
Weighted median
Weighted mode
SNP effect on eGFR
0.004
0.002
0.000
−0.002
0.3
0.6
0.9
1.2
SNP effect on DNA repair protein RAD51 homolog 4

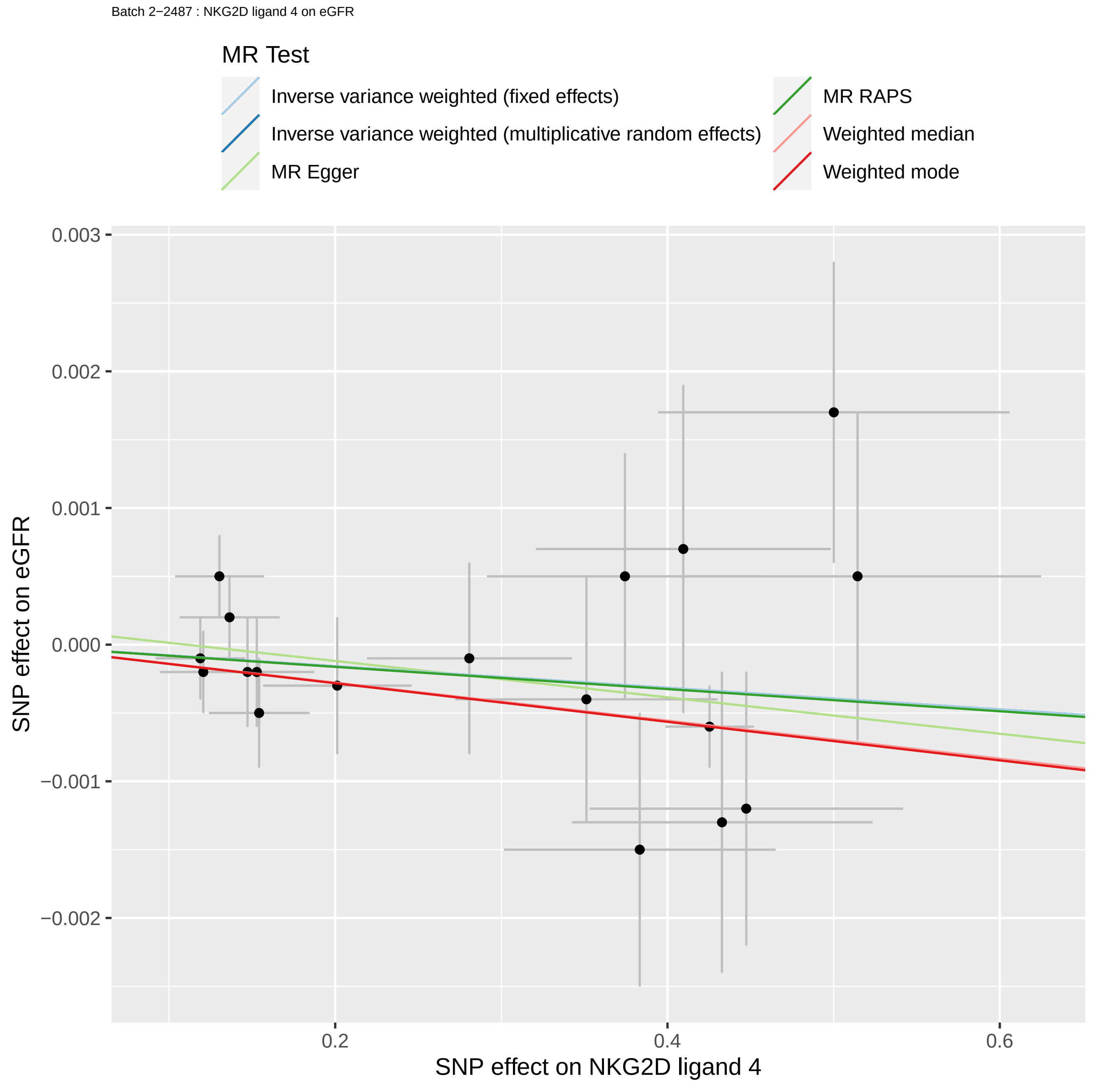

Batch 2−2487 : NKG2D ligand 4 on eGFR
MR Test
Inverse variance weighted (fixed effects)
Inverse variance weighted (multiplicative random effects)
MR Egger
MR RAPS
Weighted median
Weighted mode
0.003
0.002
0.001
0.000
−0.001
−0.002
SNP effect on eGFR
0.2
0.4
0.6
SNP effect on NKG2D ligand 4

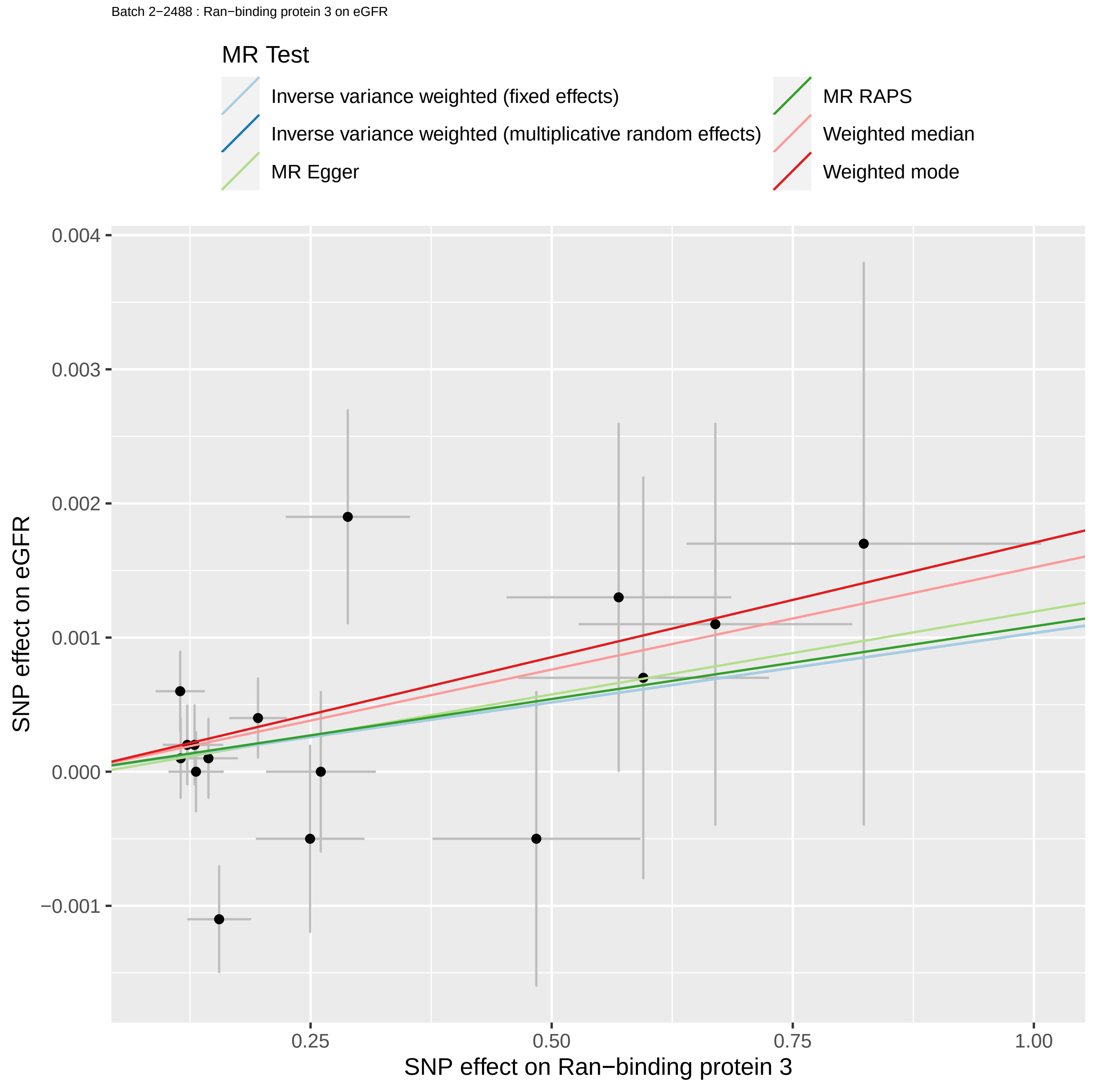
Batch 2−2488 : Ran−binding protein 3 on eGFR
MR Test
Inverse variance weighted (fixed effects)
Inverse variance weighted (multiplicative random effects)
MR Egger
MR RAPS
Weighted median
Weighted mode
0.004
0.003
0.002
0.001
0.000
−0.001
0.25
0.50
0.75
1.00
SNP effect on Ran−binding protein 3
SNP effect on eGFR

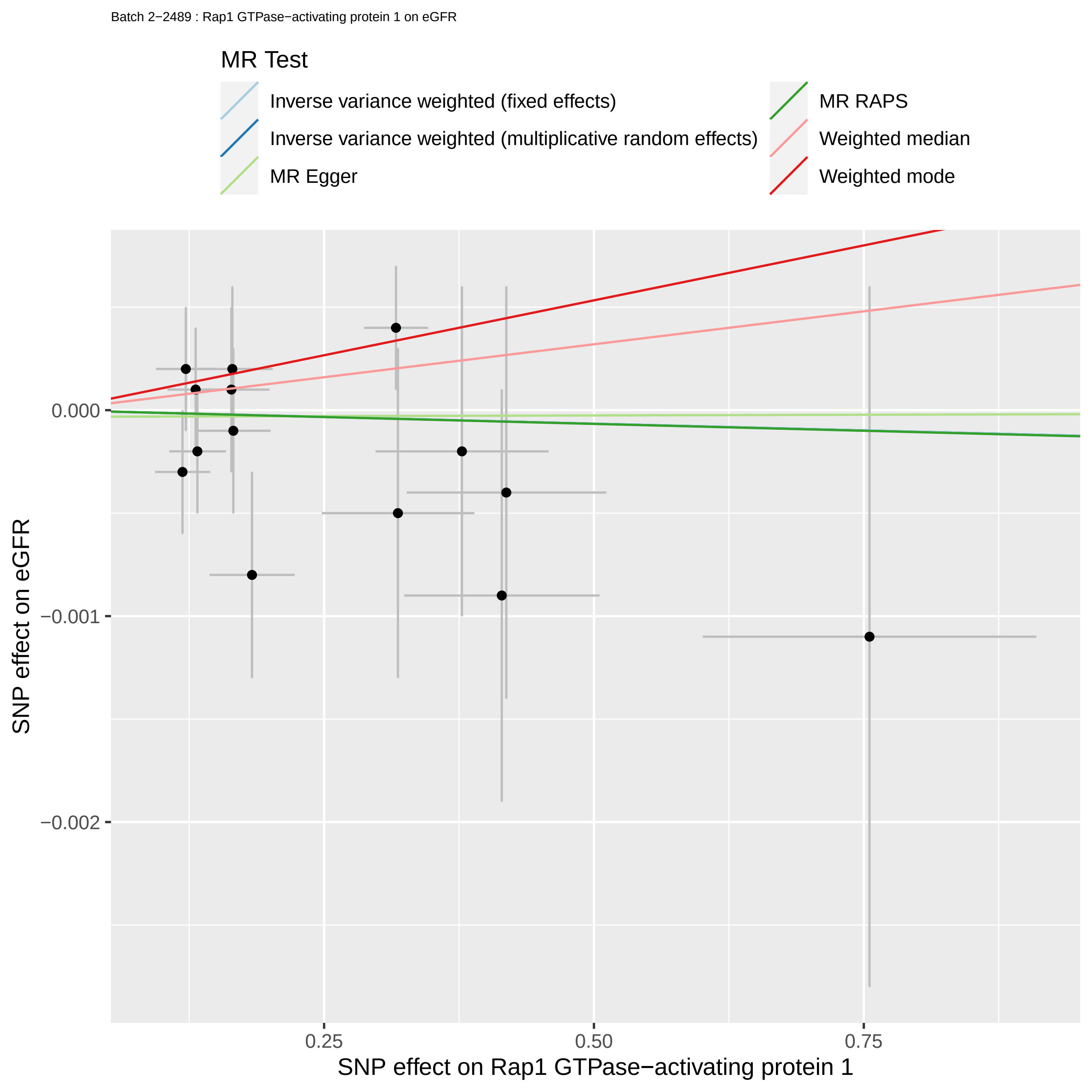
Batch 2−2489 : Rap1 GTPase−activating protein 1 on eGFR
MR Test
Inverse variance weighted (fixed effects)
Inverse variance weighted (multiplicative random effects)
MR Egger
MR RAPS
Weighted median
Weighted mode
0.000
−0.001
−0.002
0.25
0.50
0.75
SNP effect on eGFR
SNP effect on Rap1 GTPase−activating protein 1

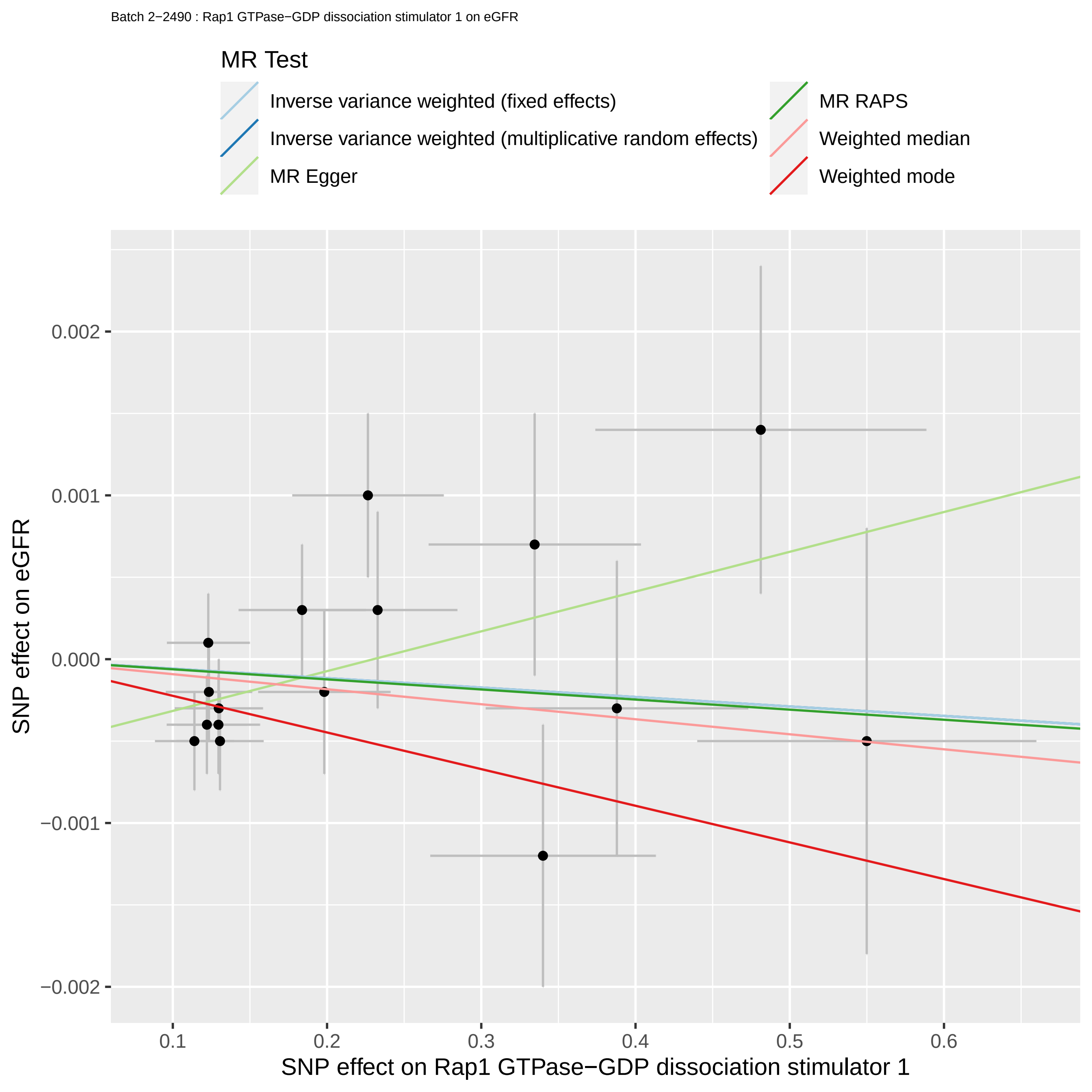

Batch 2−2490 : Rap1 GTPase−GDP dissociation stimulator 1 on eGFR
MR Test
Inverse variance weighted (fixed effects)
Inverse variance weighted (multiplicative random effects)
MR Egger
MR RAPS
Weighted median
Weighted mode
0.002
0.001
0.000
−0.001
−0.002
0.1
0.2
0.3
0.4
0.5
0.6
SNP effect on eGFR
SNP effect on Rap1 GTPase−GDP dissociation stimulator 1

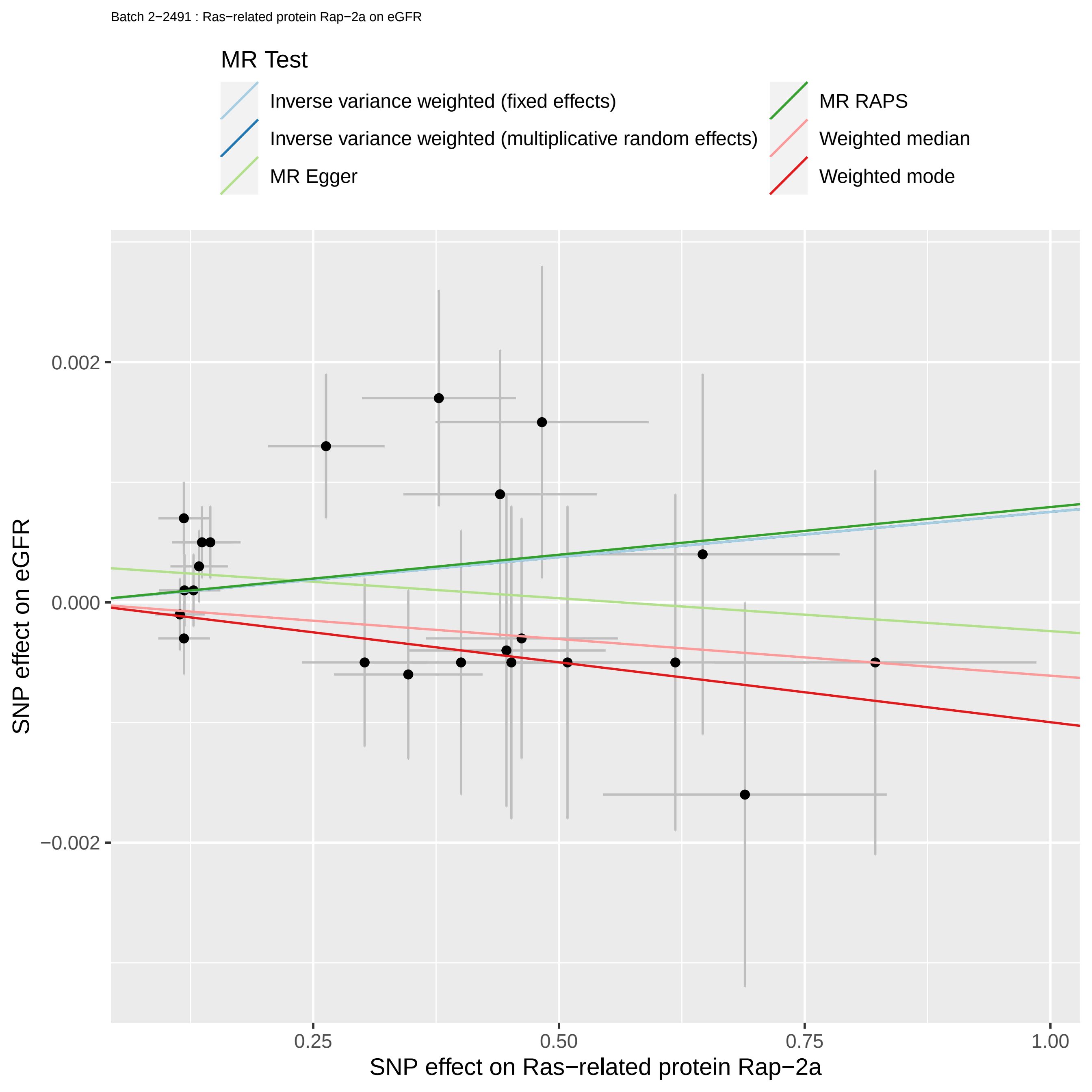
Batch 2−2491 : Ras−related protein Rap−2a on eGFR
MR Test
Inverse variance weighted (fixed effects)
Inverse variance weighted (multiplicative random effects)
MR Egger
MR RAPS
Weighted median
Weighted mode
0.002
0.000
−0.002
0.25
0.50
0.75
1.00
SNP effect on eGFR
SNP effect on Ras−related protein Rap−2a

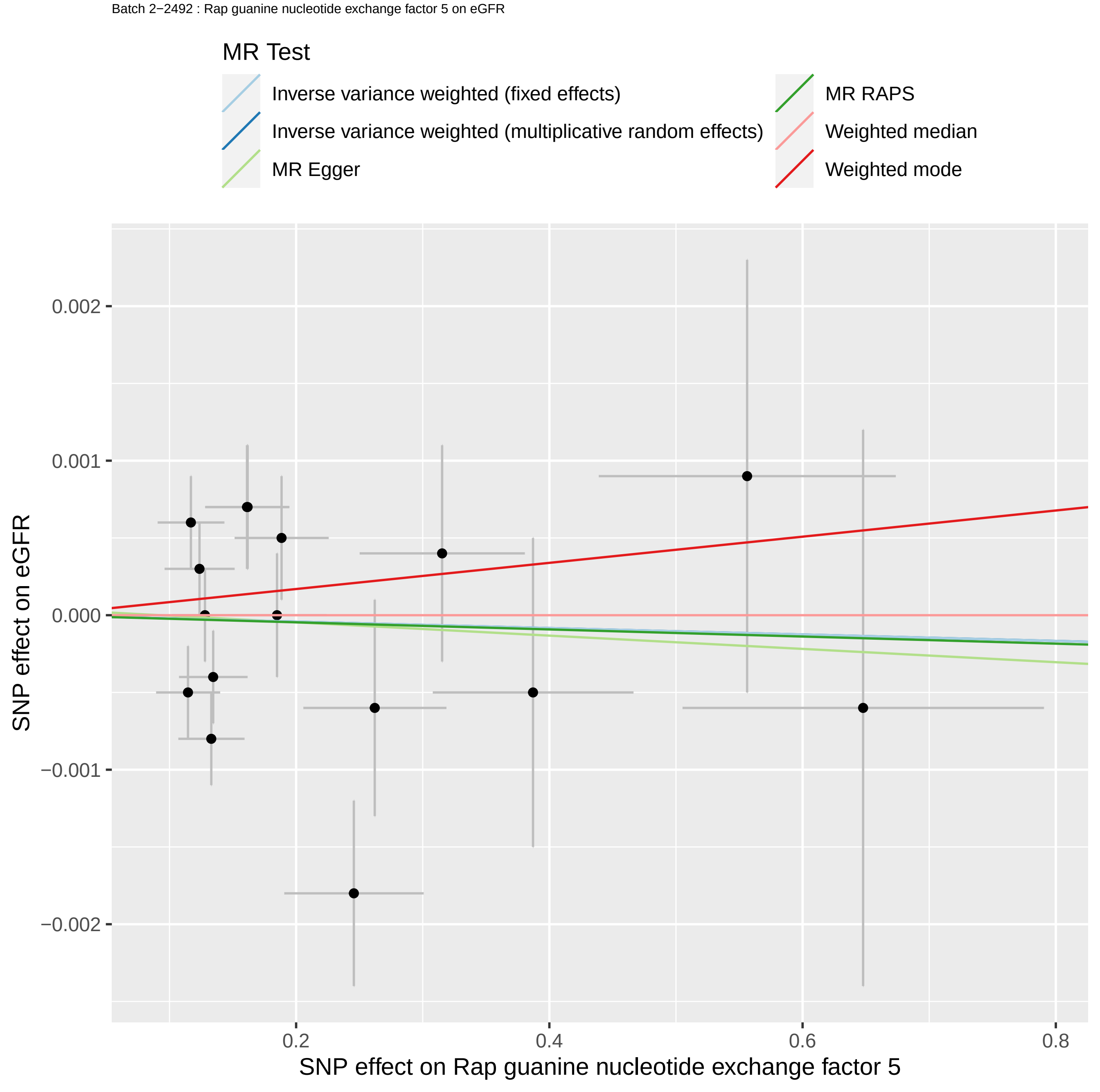
Batch 2−2492 : Rap guanine nucleotide exchange factor 5 on eGFR
MR Test
Inverse variance weighted (fixed effects)
Inverse variance weighted (multiplicative random effects)
MR Egger
MR RAPS
Weighted median
Weighted mode
0.002
0.001
0.000
−0.001
−0.002
SNP effect on eGFR
0.2
0.4
0.6
0.8
SNP effect on Rap guanine nucleotide exchange factor 5

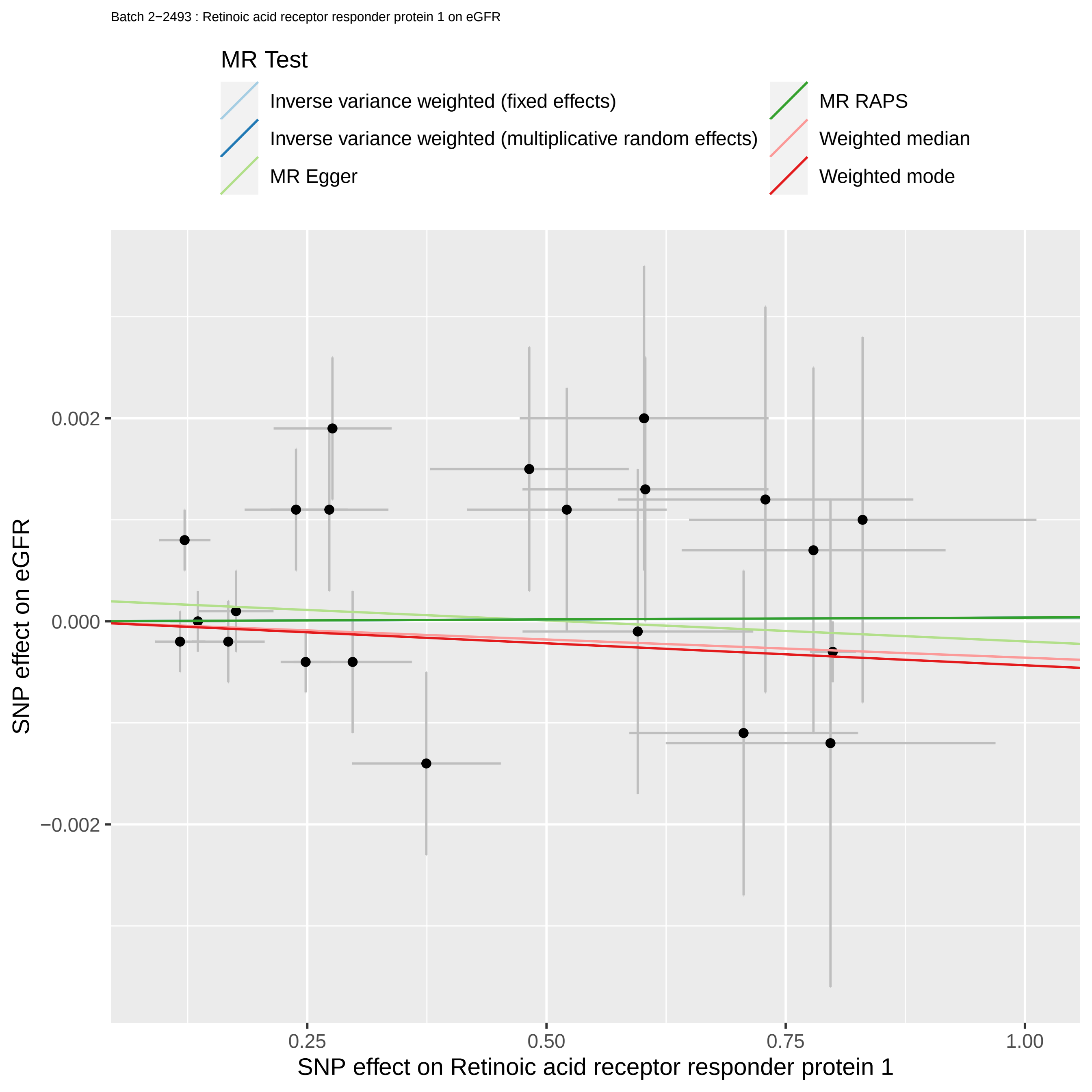

Batch 2−2493 : Retinoic acid receptor responder protein 1 on eGFR
MR Test
Inverse variance weighted (fixed effects)
Inverse variance weighted (multiplicative random effects)
MR Egger
MR RAPS
Weighted median
Weighted mode
0.002
0.000
−0.002
0.25
0.50
0.75
1.00
SNP effect on eGFR
SNP effect on Retinoic acid receptor responder protein 1

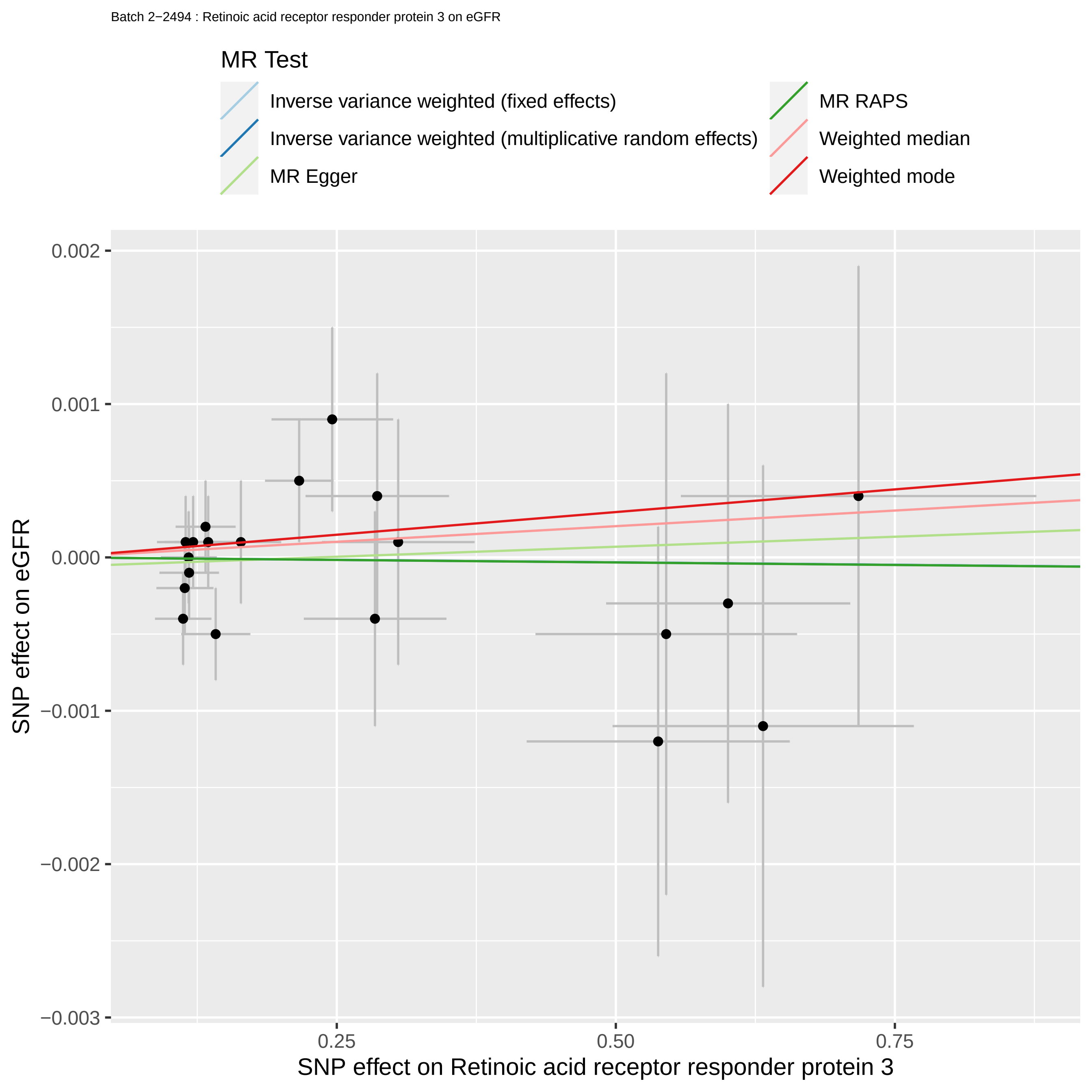

Batch 2−2494 : Retinoic acid receptor responder protein 3 on eGFR
MR Test
Inverse variance weighted (fixed effects)
Inverse variance weighted (multiplicative random effects)
MR Egger
MR RAPS
Weighted median
Weighted mode
0.002
0.001
0.000
−0.001
−0.002
−0.003
0.25
0.50
0.75
SNP effect on eGFR
SNP effect on Retinoic acid receptor responder protein 3

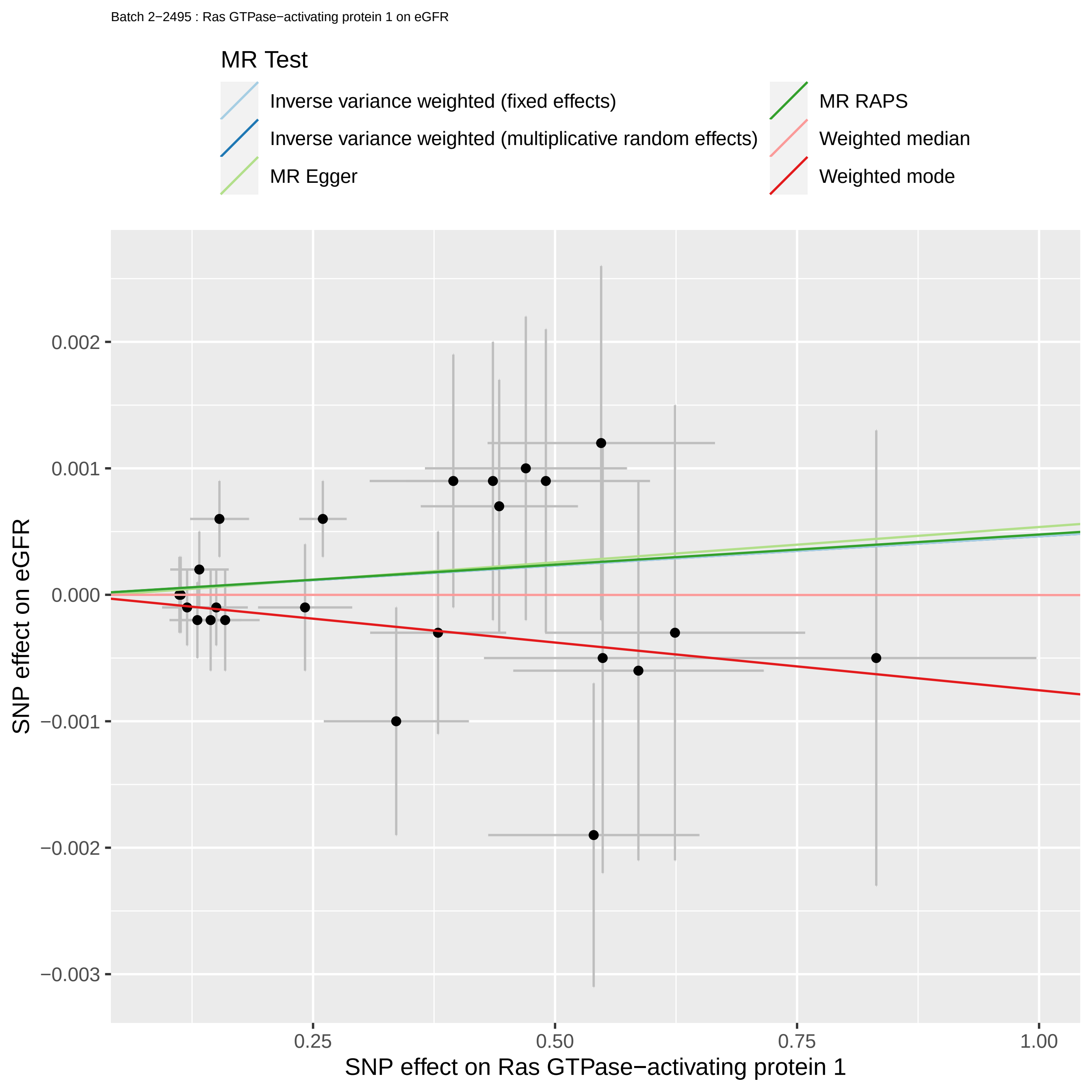

Batch 2−2495 : Ras GTPase−activating protein 1 on eGFR
MR Test
Inverse variance weighted (fixed effects)
Inverse variance weighted (multiplicative random effects)
MR Egger
MR RAPS
Weighted median
Weighted mode
0.002
0.001
0.000
−0.001
−0.002
−0.003
SNP effect on eGFR
0.25
0.50
0.75
1.00
SNP effect on Ras GTPase−activating protein 1

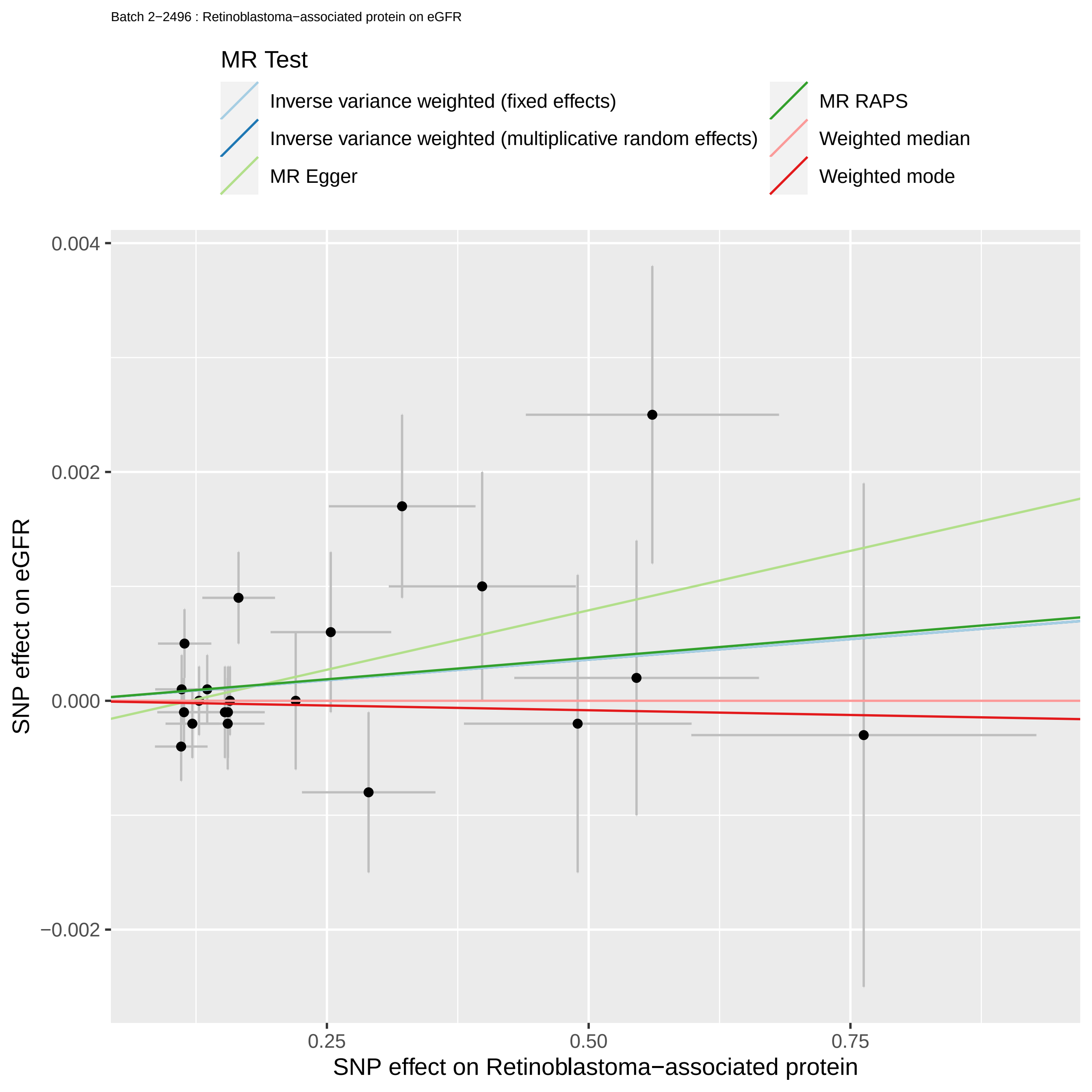
Batch 2−2496 : Retinoblastoma−associated protein on eGFR
MR Test
Inverse variance weighted (fixed effects)
Inverse variance weighted (multiplicative random effects)
MR Egger
MR RAPS
Weighted median
Weighted mode
0.004
0.002
0.000
−0.002
0.25
0.50
0.75
SNP effect on eGFR
SNP effect on Retinoblastoma−associated protein

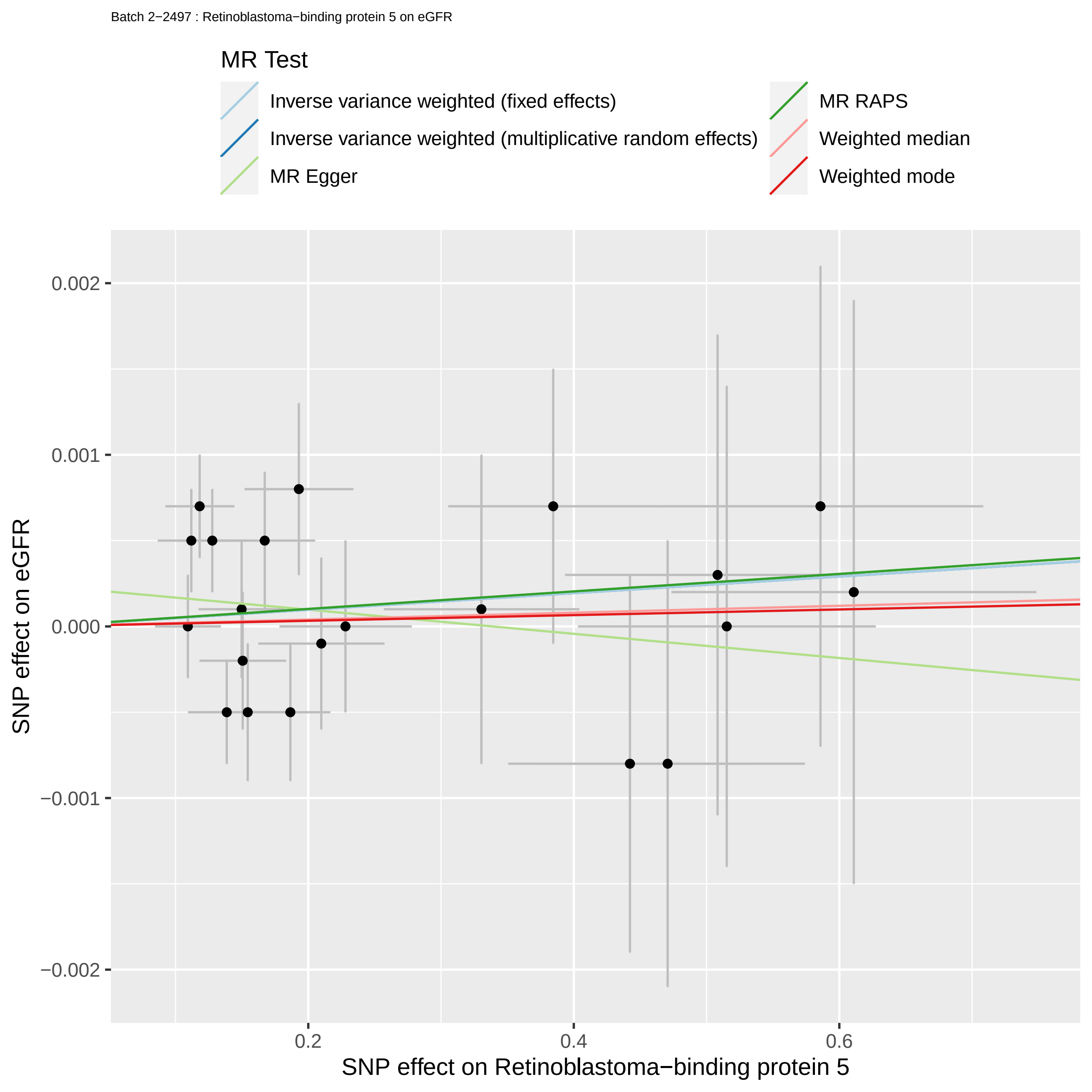

Batch 2−2497 : Retinoblastoma−binding protein 5 on eGFR
MR Test
Inverse variance weighted (fixed effects)
Inverse variance weighted (multiplicative random effects)
MR Egger
MR RAPS
Weighted median
Weighted mode
0.002
0.001
0.000
−0.001
−0.002
0.2
0.4
0.6
SNP effect on eGFR
SNP effect on Retinoblastoma−binding protein 5

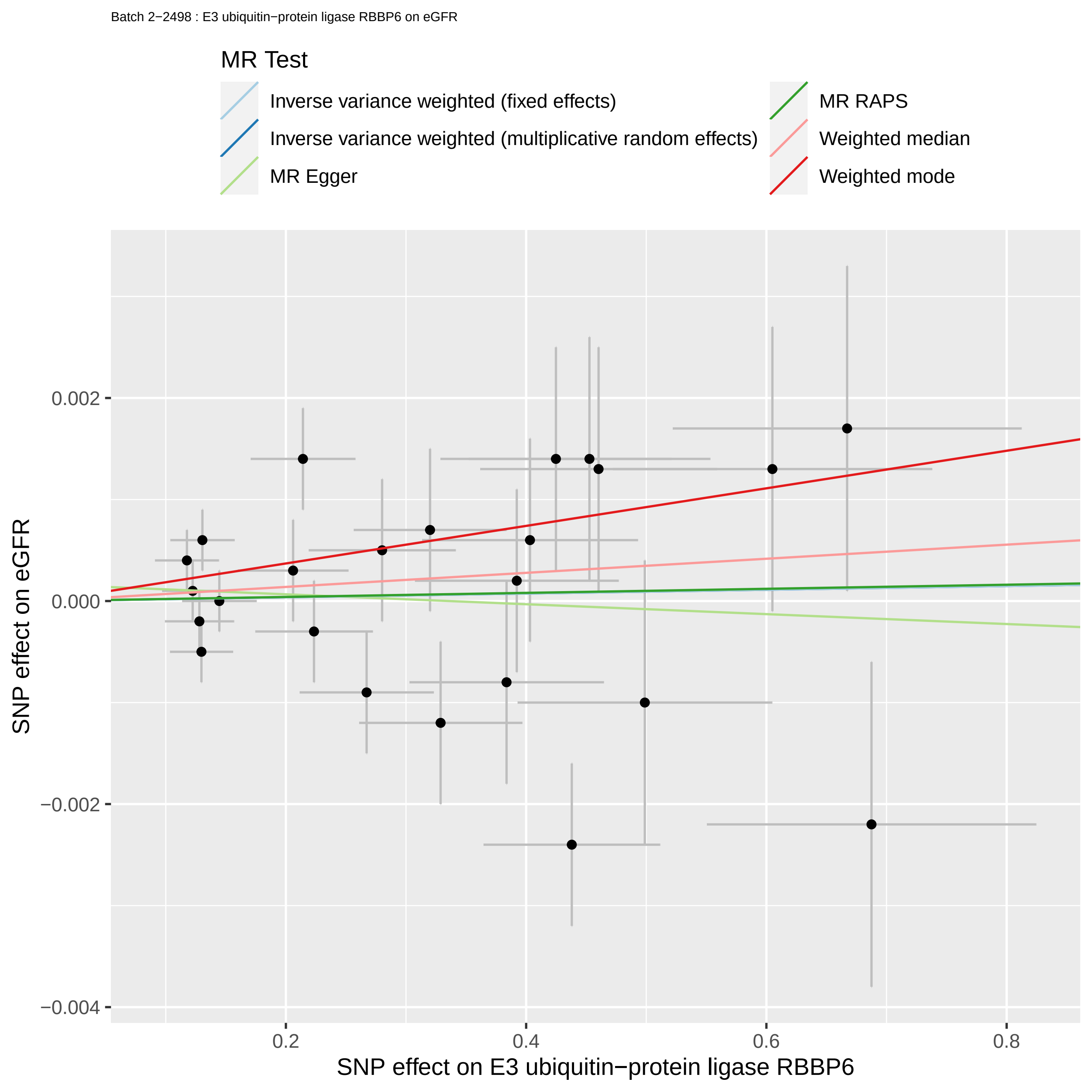

Batch 2−2498 : E3 ubiquitin−protein ligase RBBP6 on eGFR
MR Test
Inverse variance weighted (fixed effects)
Inverse variance weighted (multiplicative random effects)
MR Egger
MR RAPS
Weighted median
Weighted mode
0.002
0.000
−0.002
−0.004
0.2
0.4
0.6
0.8
SNP effect on eGFR
SNP effect on E3 ubiquitin−protein ligase RBBP6

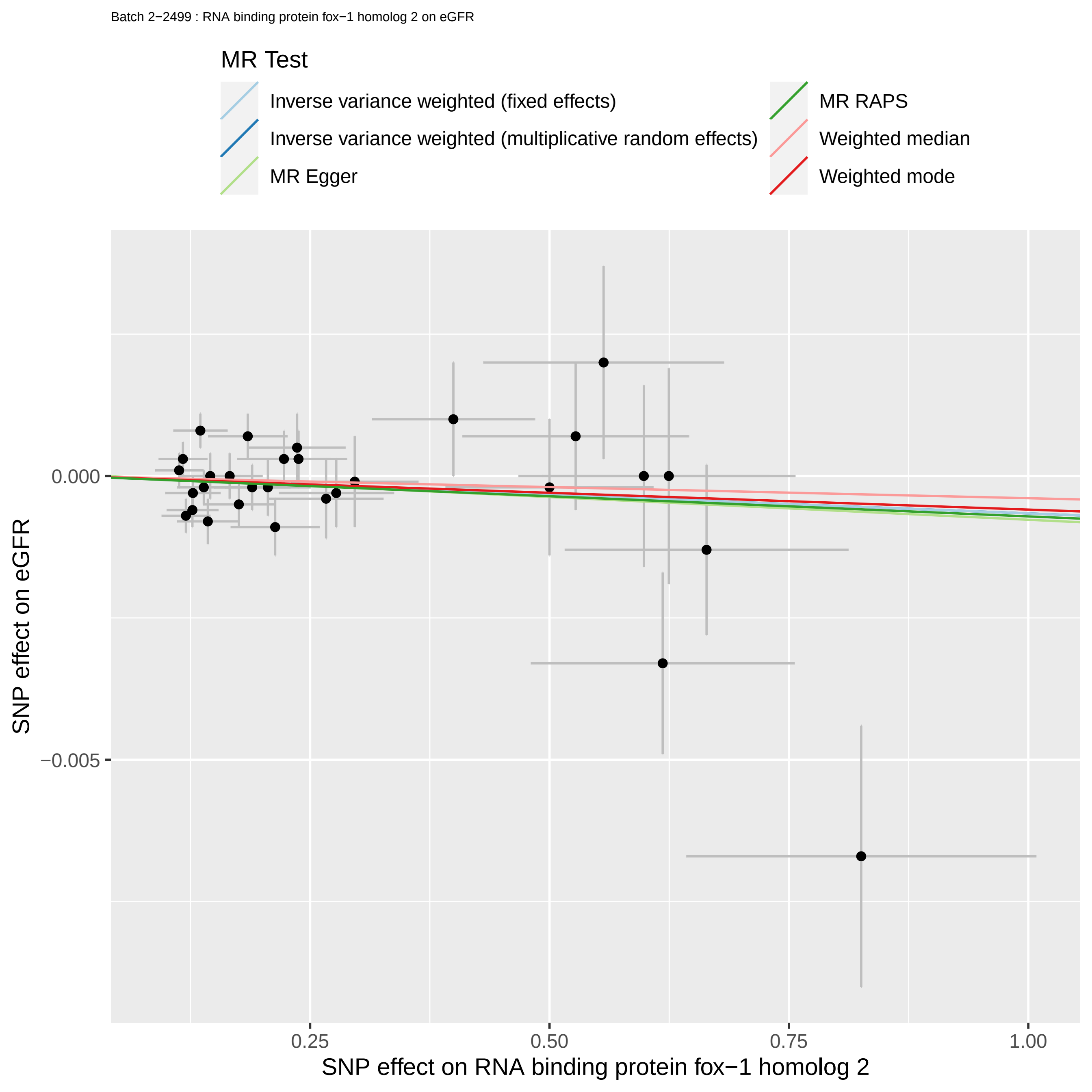

Batch 2−2499 : RNA binding protein fox−1 homolog 2 on eGFR
MR Test
Inverse variance weighted (fixed effects)
Inverse variance weighted (multiplicative random effects)
MR Egger
MR RAPS
Weighted median
Weighted mode
0.000
−0.005
0.25
0.50
0.75
1.00
SNP effect on RNA binding protein fox−1 homolog 2
SNP effect on eGFR

Batch 2−2500 : Retinoblastoma−like protein 1 on eGFR

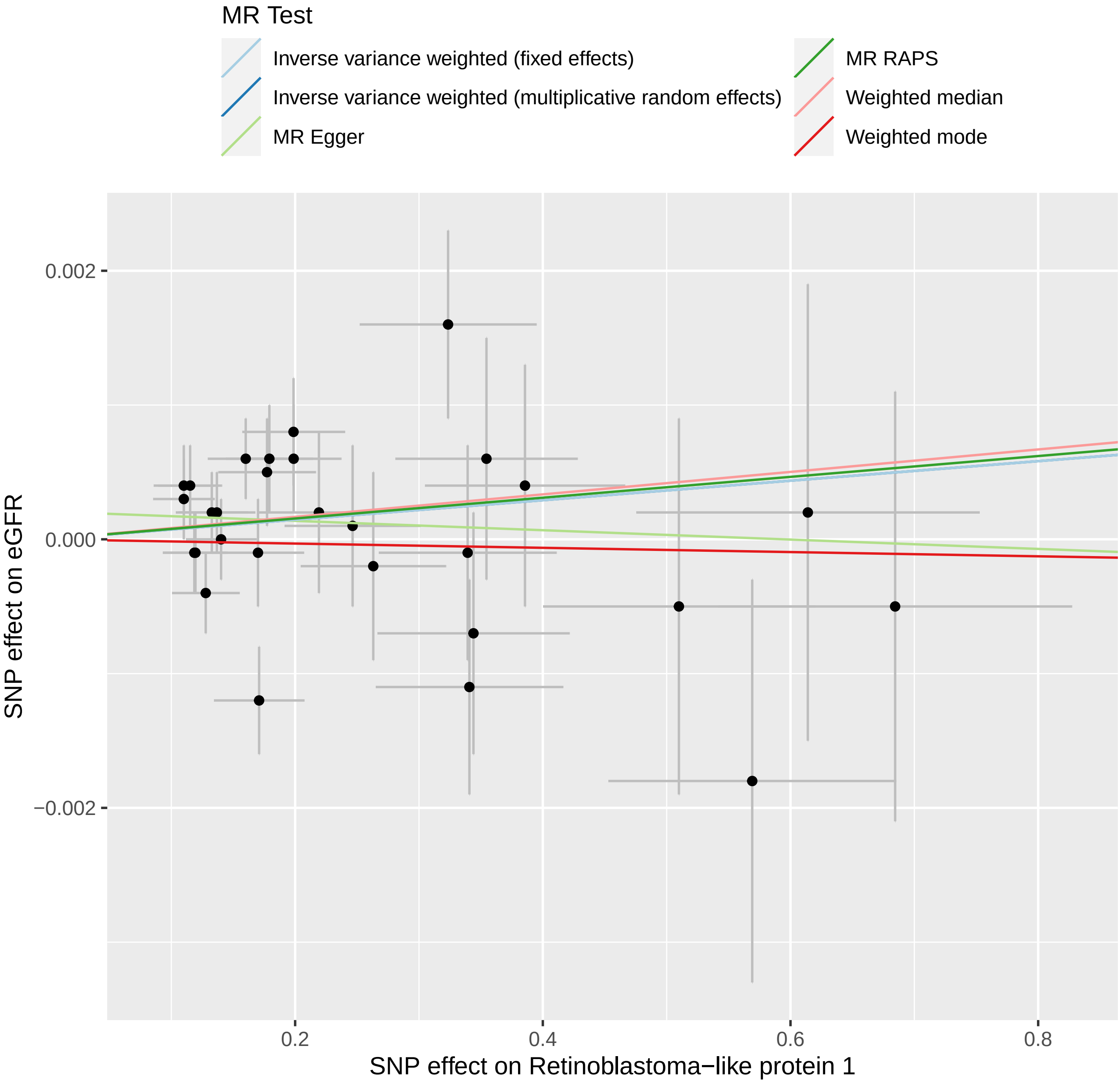

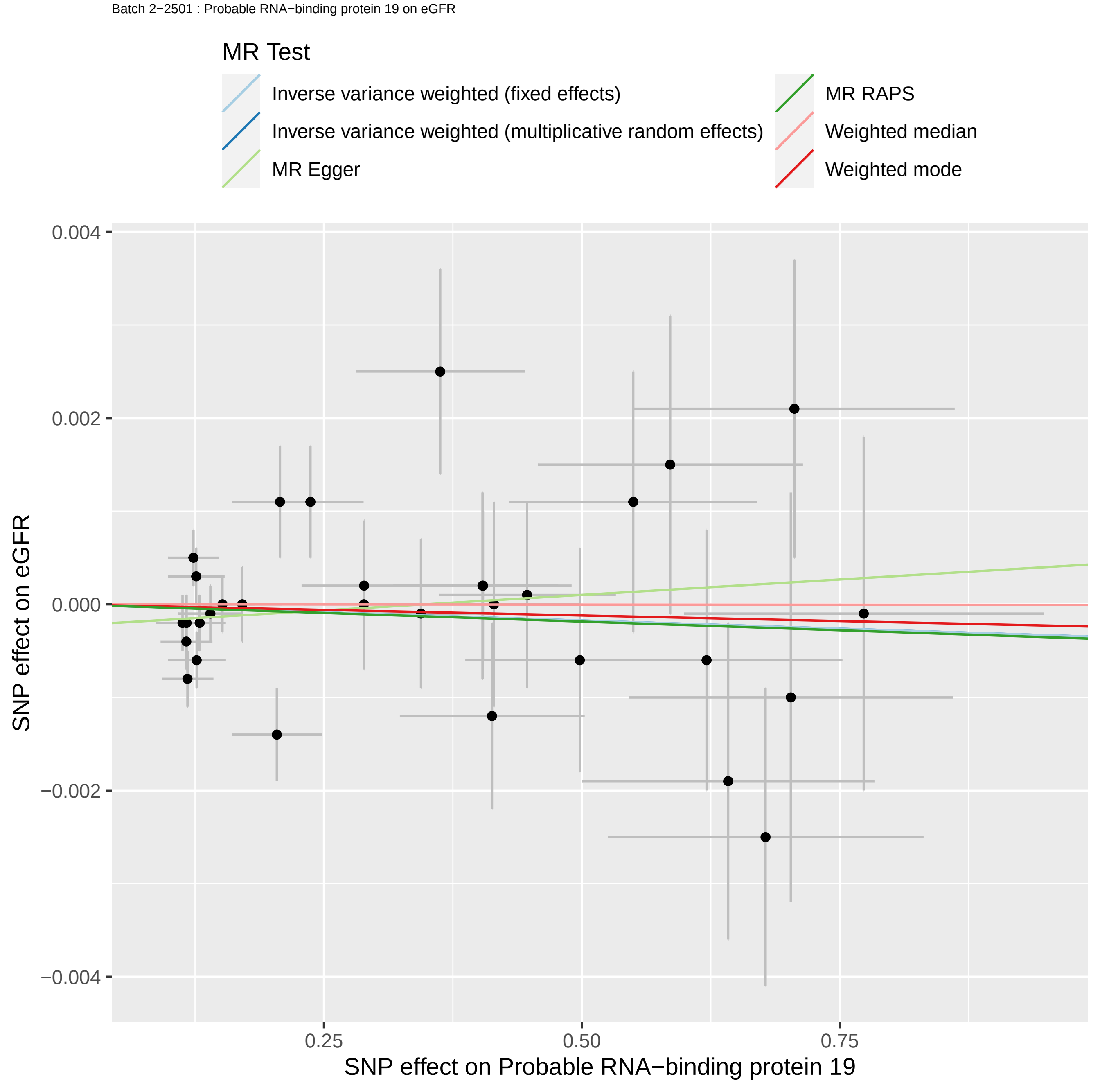

Batch 2−2501 : Probable RNA−binding protein 19 on eGFR
MR Test
Inverse variance weighted (fixed effects)
Inverse variance weighted (multiplicative random effects)
MR Egger
MR RAPS
Weighted median
Weighted mode
0.004
0.002
0.000
−0.002
−0.004
0.25
0.50
0.75
SNP effect on eGFR
SNP effect on Probable RNA−binding protein 19

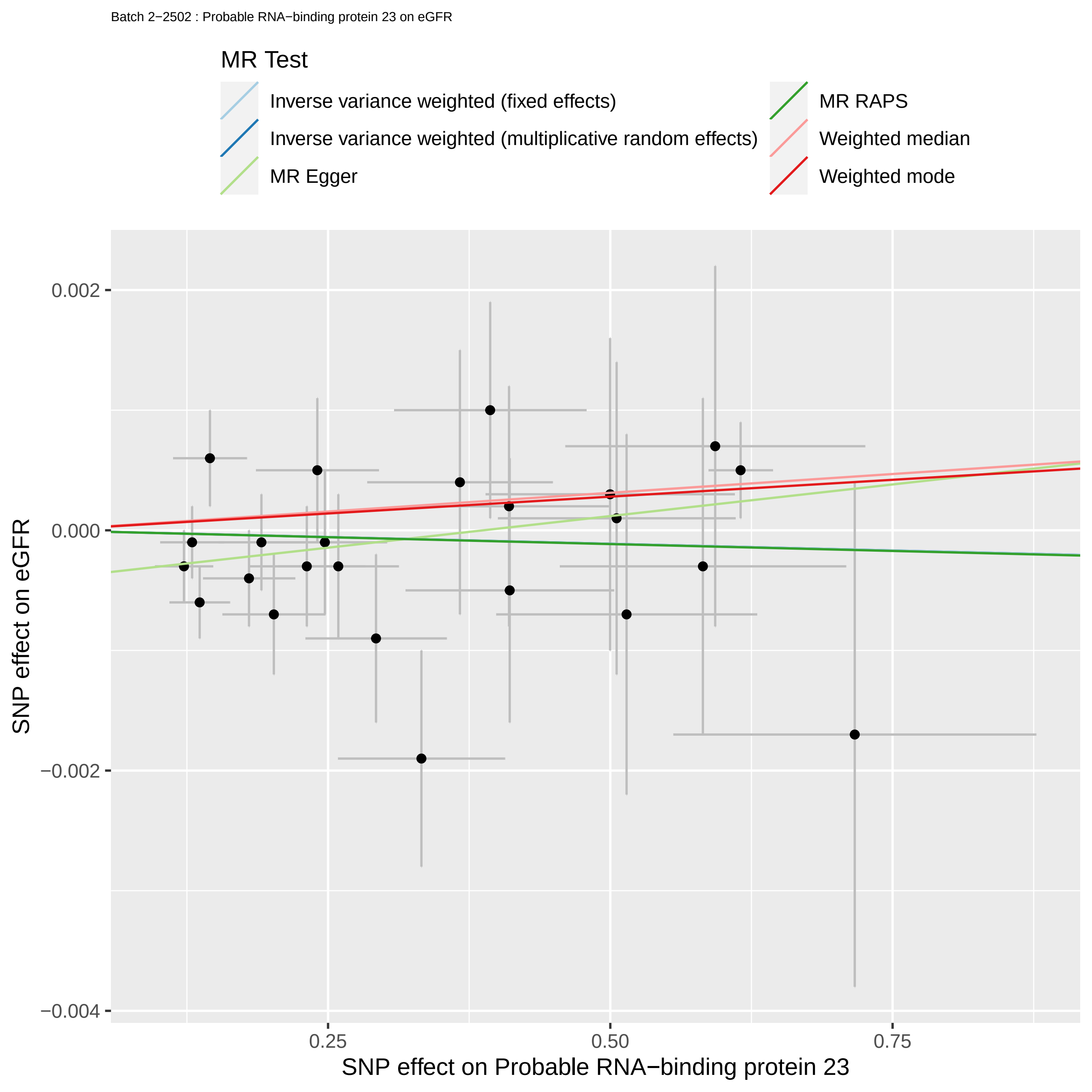

Batch 2−2502 : Probable RNA−binding protein 23 on eGFR
MR Test
Inverse variance weighted (fixed effects)
Inverse variance weighted (multiplicative random effects)
MR Egger
MR RAPS
Weighted median
Weighted mode
0.002
0.000
−0.002
−0.004
0.25
0.50
0.75
SNP effect on eGFR
SNP effect on Probable RNA−binding protein 23

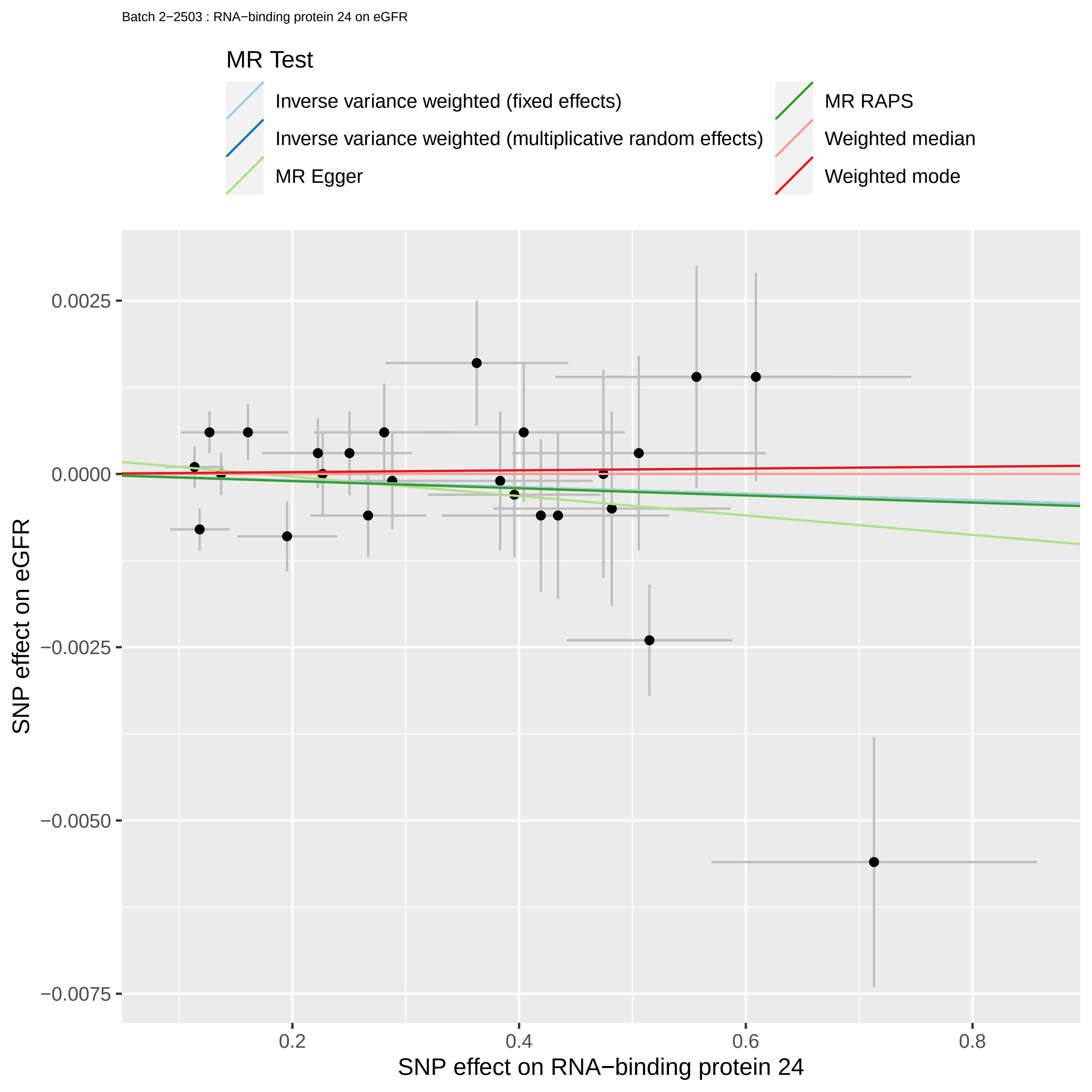

Batch 2−2503 : RNA−binding protein 24 on eGFR
MR Test
Inverse variance weighted (fixed effects)
Inverse variance weighted (multiplicative random effects)
MR Egger
MR RAPS
Weighted median
Weighted mode
0.0025
0.0000
−0.0025
−0.0050
−0.0075
0.2
0.4
0.6
0.8
SNP effect on eGFR
SNP effect on RNA−binding protein 24

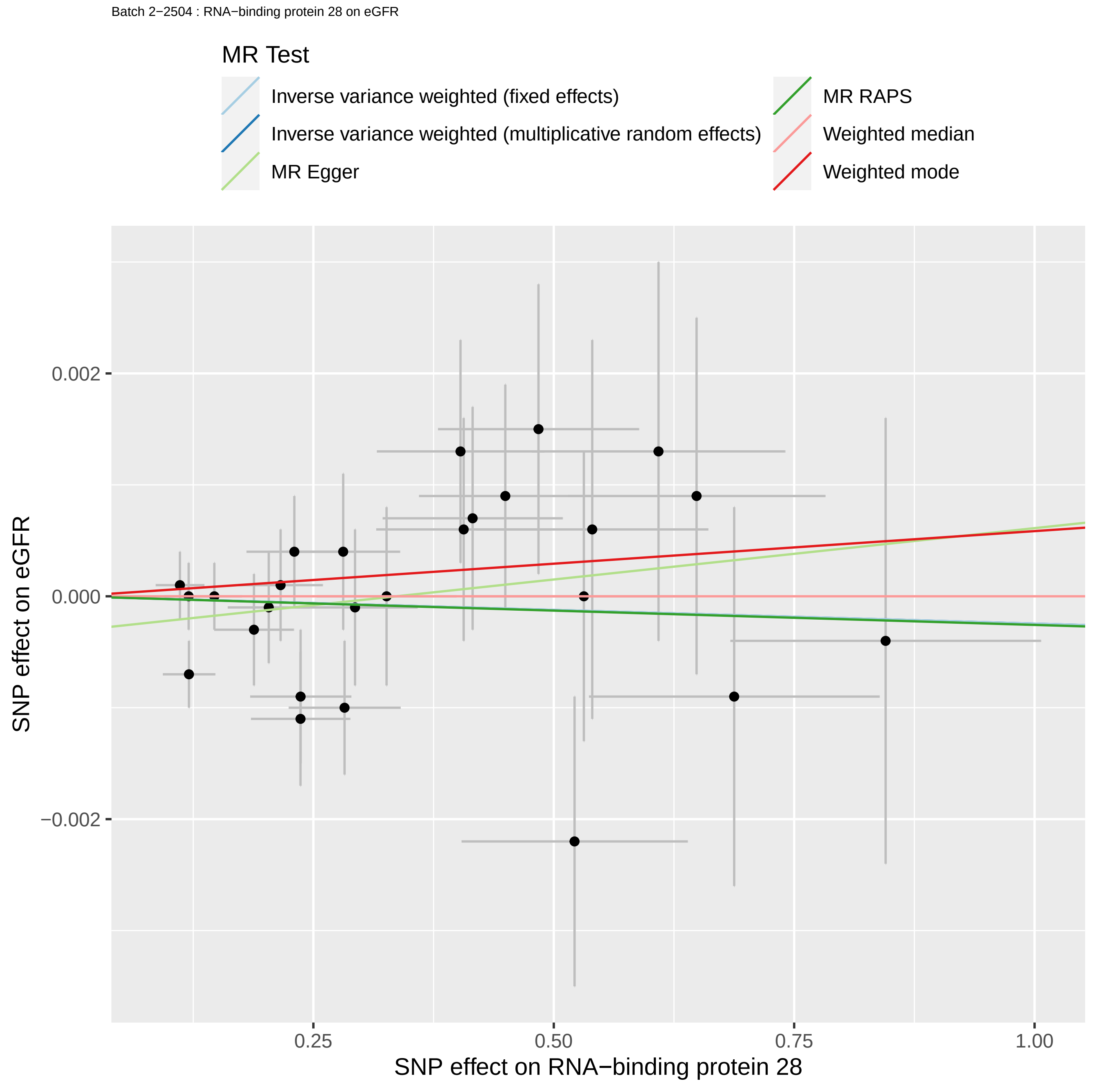
Batch 2−2504 : RNA−binding protein 28 on eGFR
MR Test
Inverse variance weighted (fixed effects)
Inverse variance weighted (multiplicative random effects)
MR Egger
MR RAPS
Weighted median
Weighted mode
0.002
0.000
−0.002
0.25
0.50
0.75
1.00
SNP effect on eGFR
SNP effect on RNA−binding protein 28

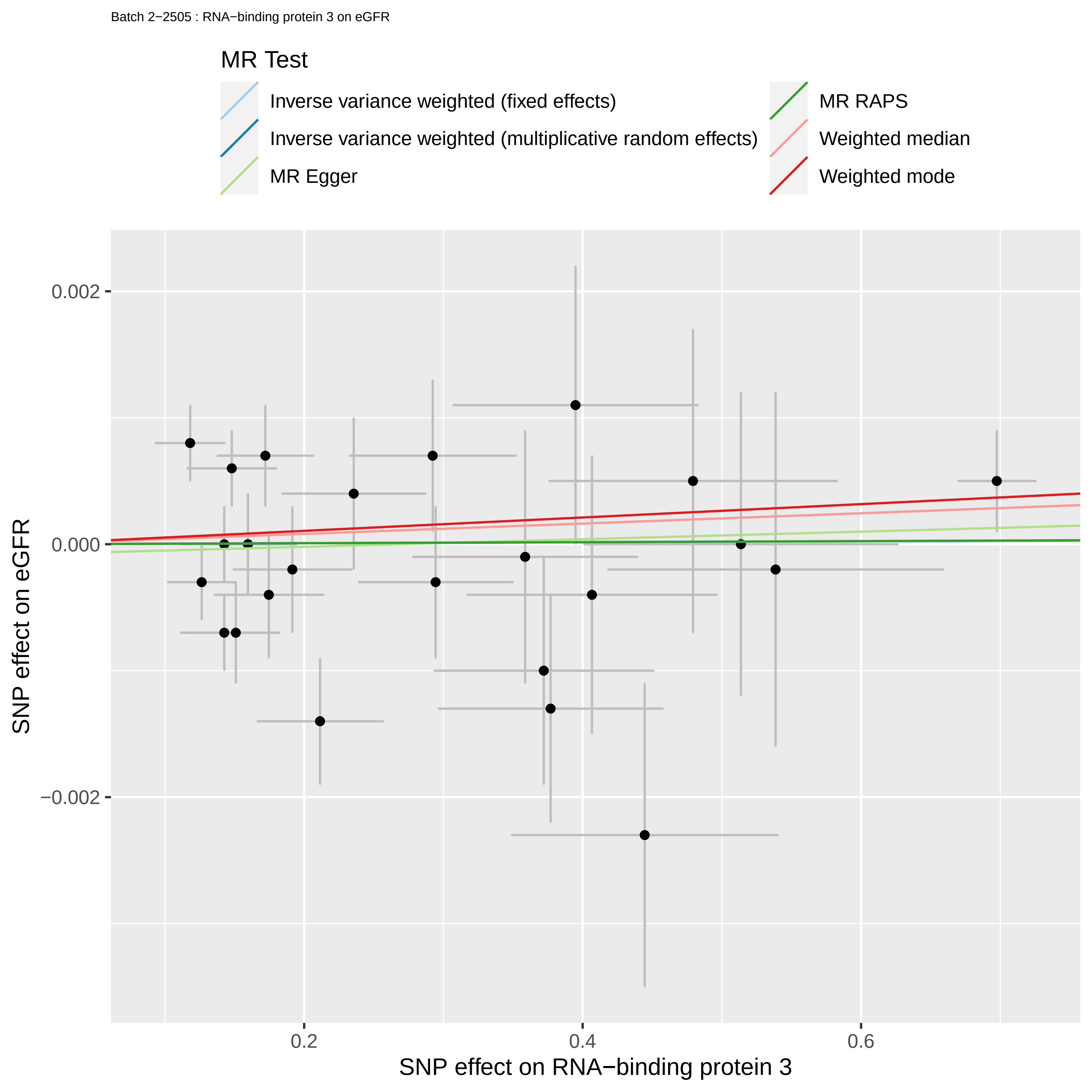

Batch 2−2505 : RNA−binding protein 3 on eGFR
MR Test
Inverse variance weighted (fixed effects)
Inverse variance weighted (multiplicative random effects)
MR Egger
MR RAPS
Weighted median
Weighted mode
0.002
0.000
−0.002
0.2
0.4
0.6
SNP effect on eGFR
SNP effect on RNA−binding protein 3

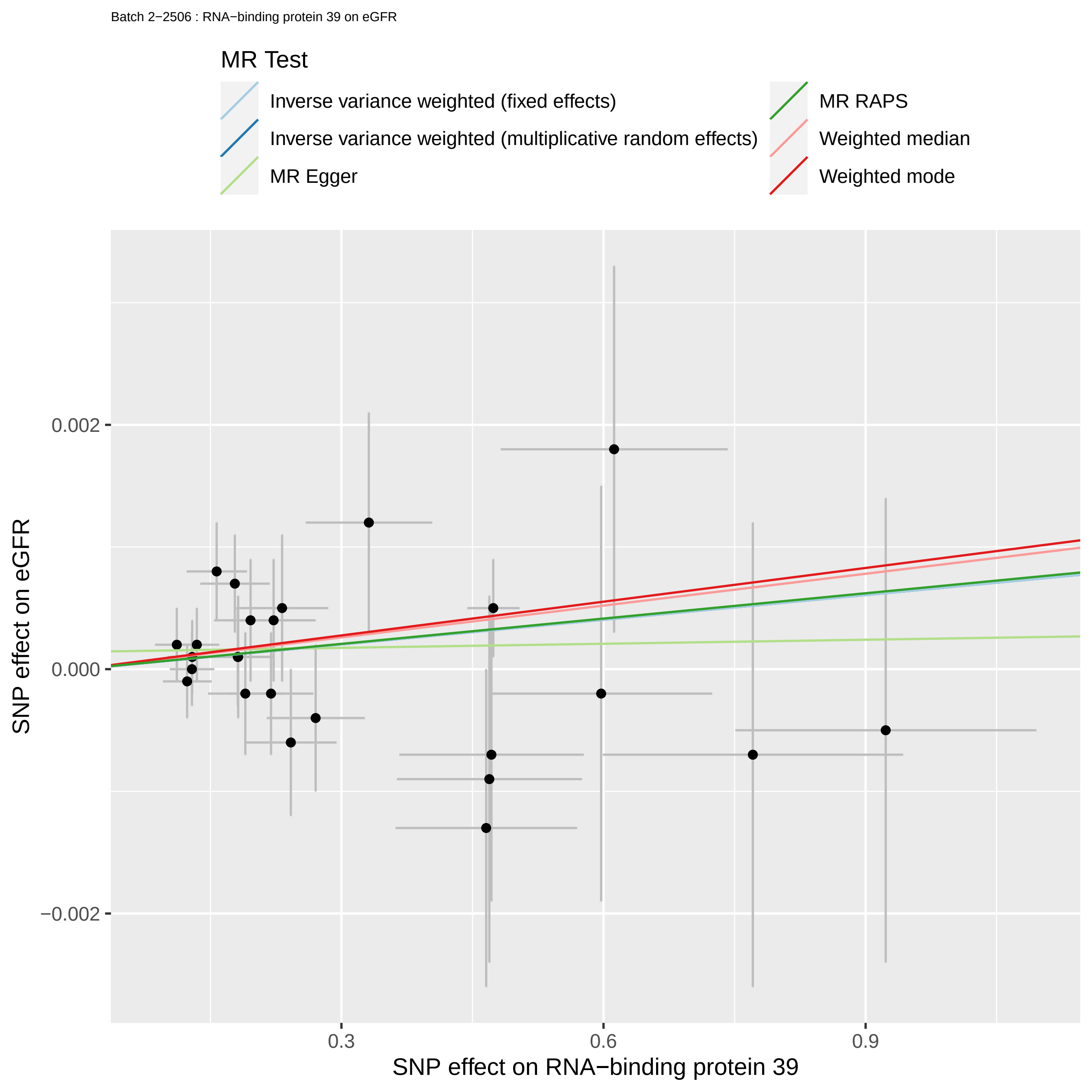

Batch 2−2506 : RNA−binding protein 39 on eGFR
MR Test
Inverse variance weighted (fixed effects)
Inverse variance weighted (multiplicative random effects)
MR Egger
MR RAPS
Weighted median
Weighted mode
0.002
0.000
−0.002
0.3
0.6
0.9
SNP effect on eGFR
SNP effect on RNA−binding protein 39

Batch 2−2507 : Retinol−binding protein 4 on eGFR

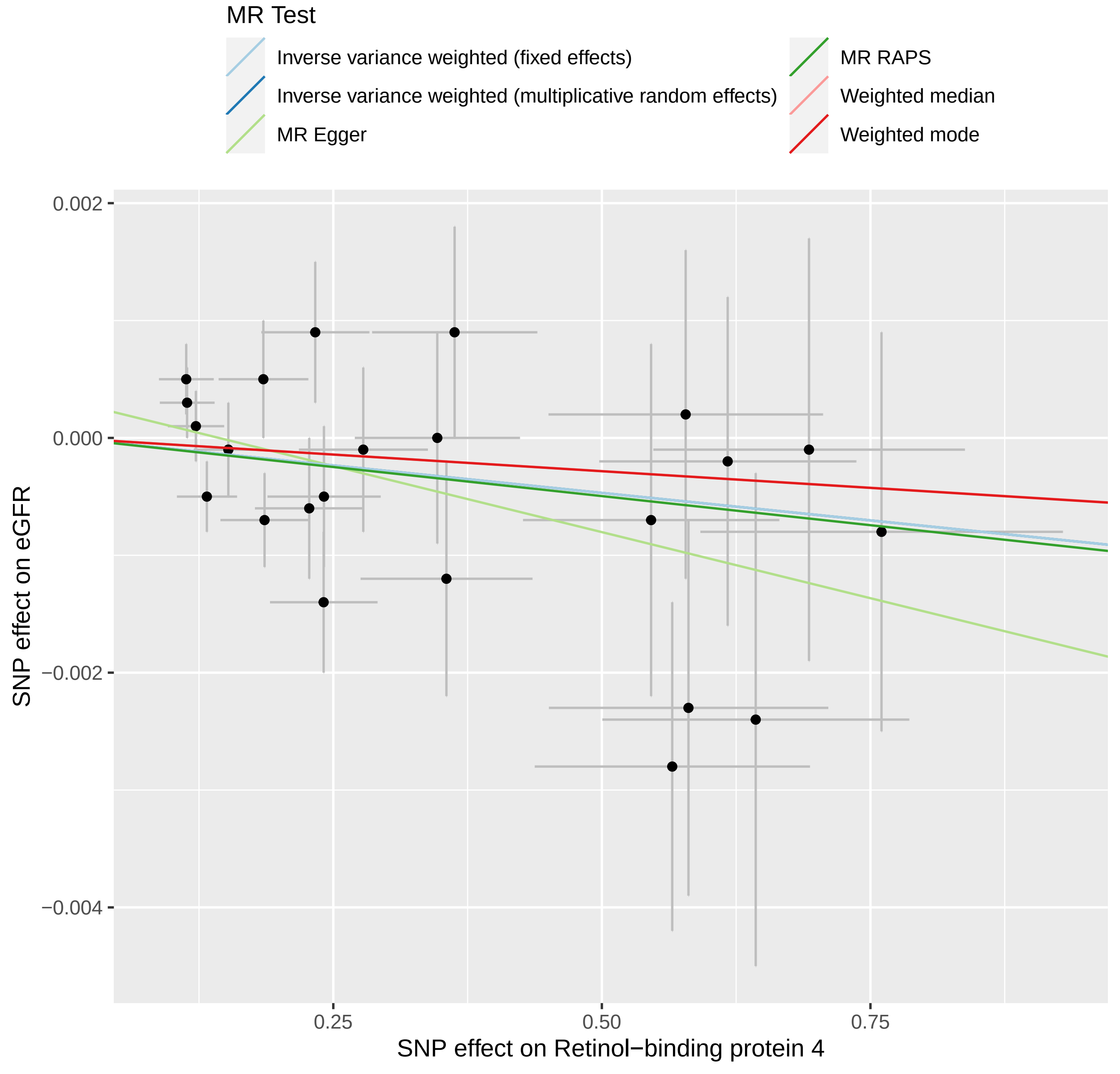

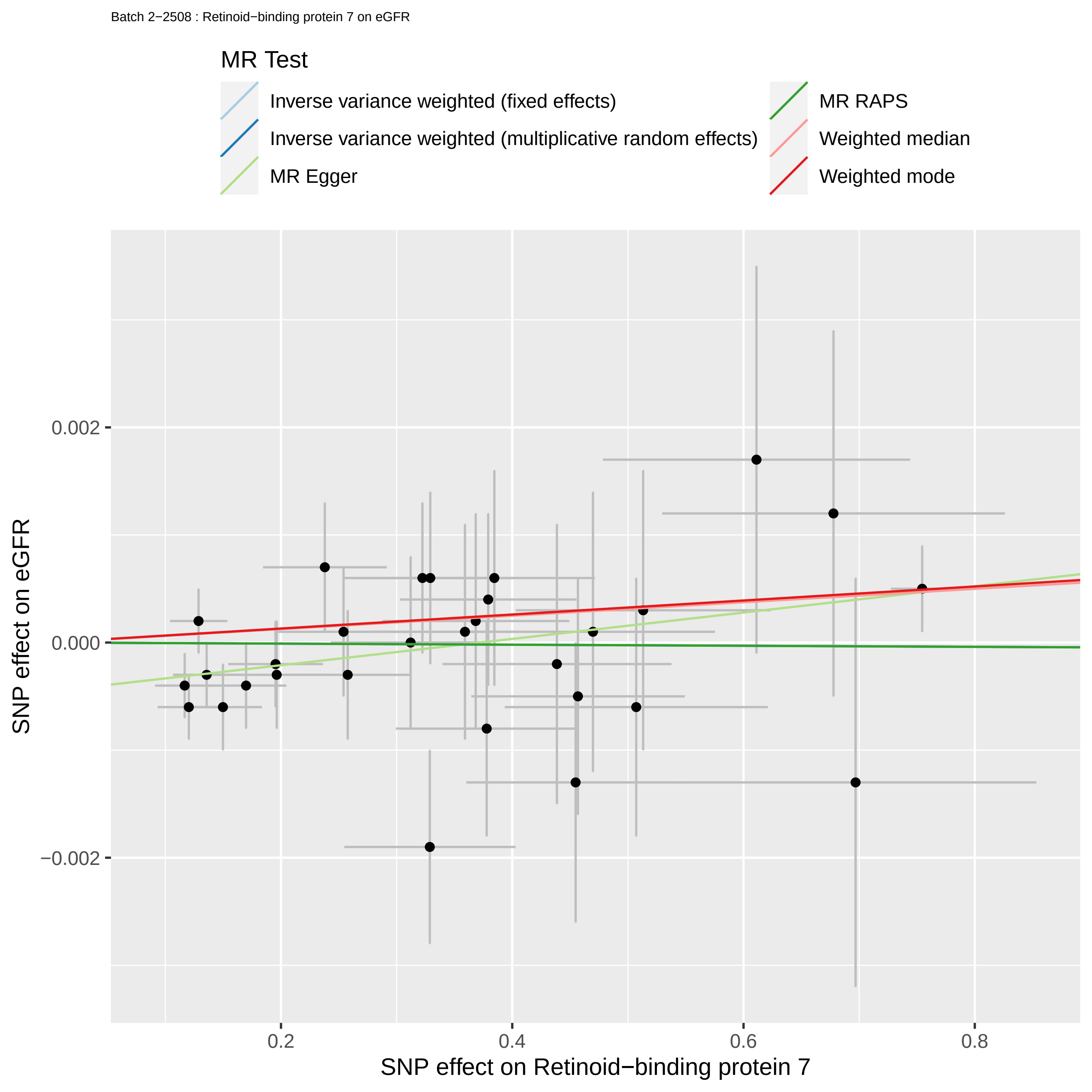

Batch 2−2508 : Retinoid−binding protein 7 on eGFR
MR Test
Inverse variance weighted (fixed effects)
Inverse variance weighted (multiplicative random effects)
MR Egger
MR RAPS
Weighted median
Weighted mode
0.002
0.000
−0.002
0.2
0.4
0.6
0.8
SNP effect on eGFR
SNP effect on Retinoid−binding protein 7

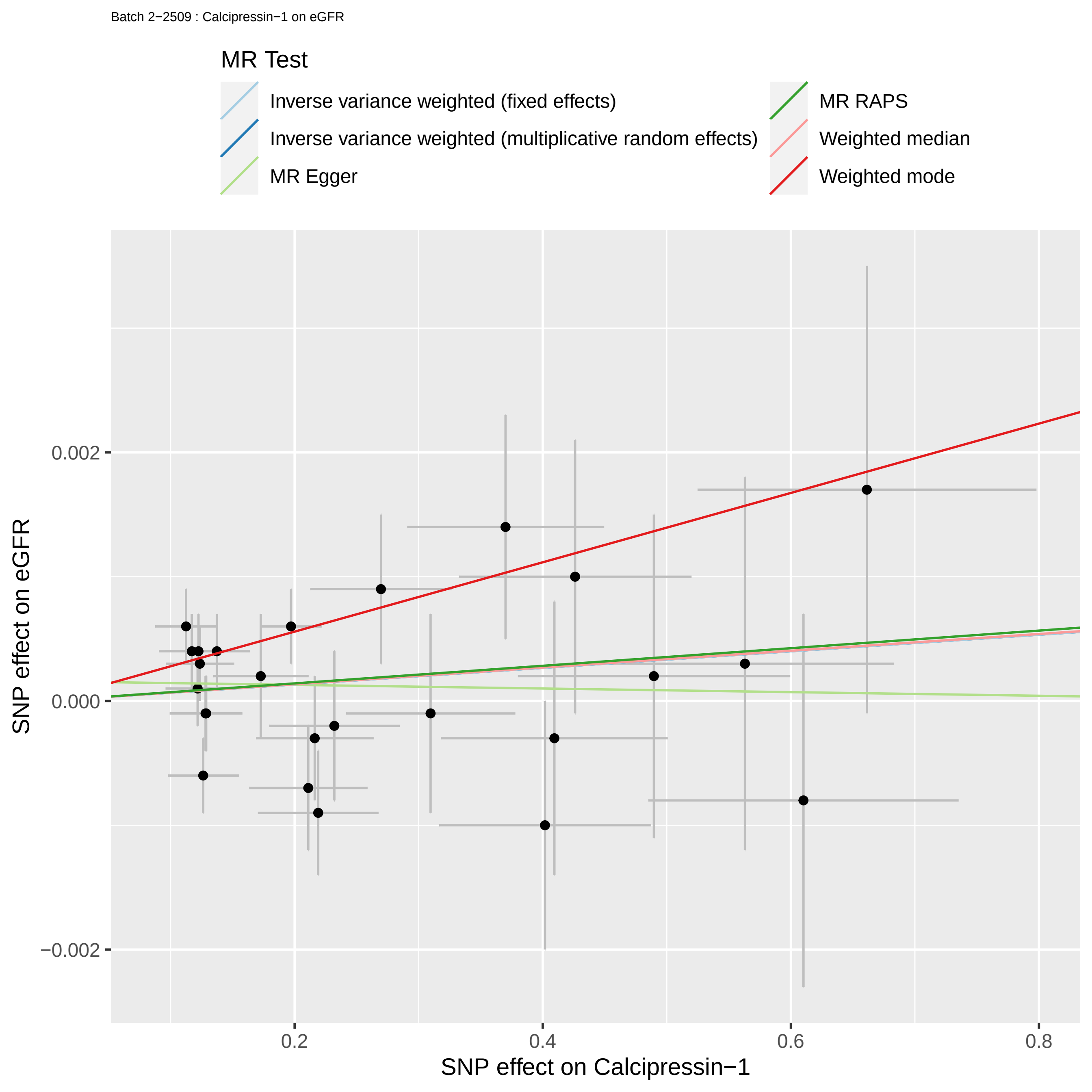

Batch 2−2509 : Calcipressin−1 on eGFR
MR Test
Inverse variance weighted (fixed effects)
Inverse variance weighted (multiplicative random effects)
MR Egger
MR RAPS
Weighted median
Weighted mode
SNP effect on eGFR
0.002
0.000
−0.002
0.2
0.4
0.6
0.8
SNP effect on Calcipressin−1

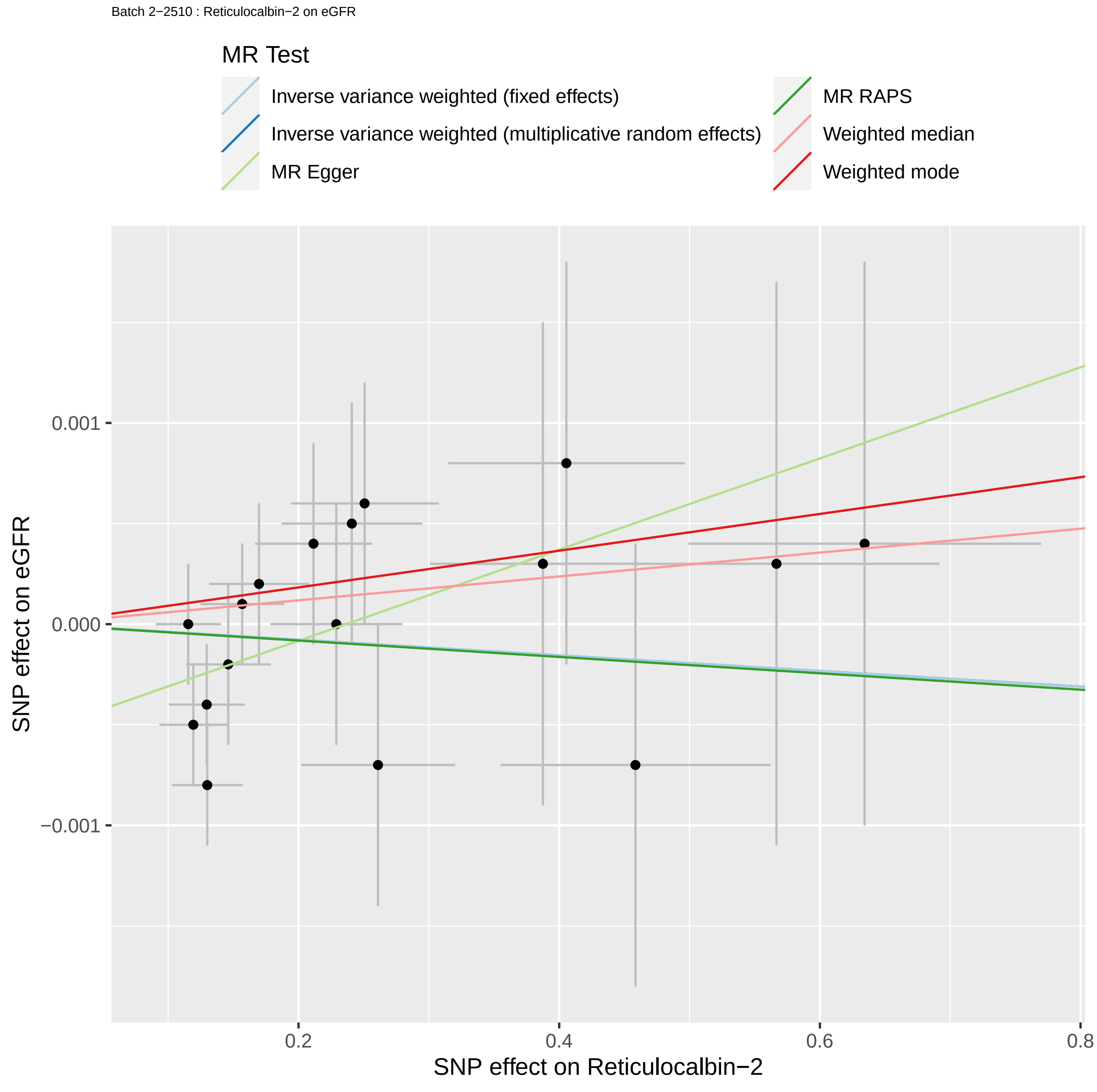
Batch 2−2510 : Reticulocalbin−2 on eGFR
MR Test
Inverse variance weighted (fixed effects)
Inverse variance weighted (multiplicative random effects)
MR Egger
MR RAPS
Weighted median
Weighted mode
SNP effect on eGFR
0.001
0.000
−0.001
SNP effect on Reticulocalbin−2
0.2
0.4
0.6
0.8

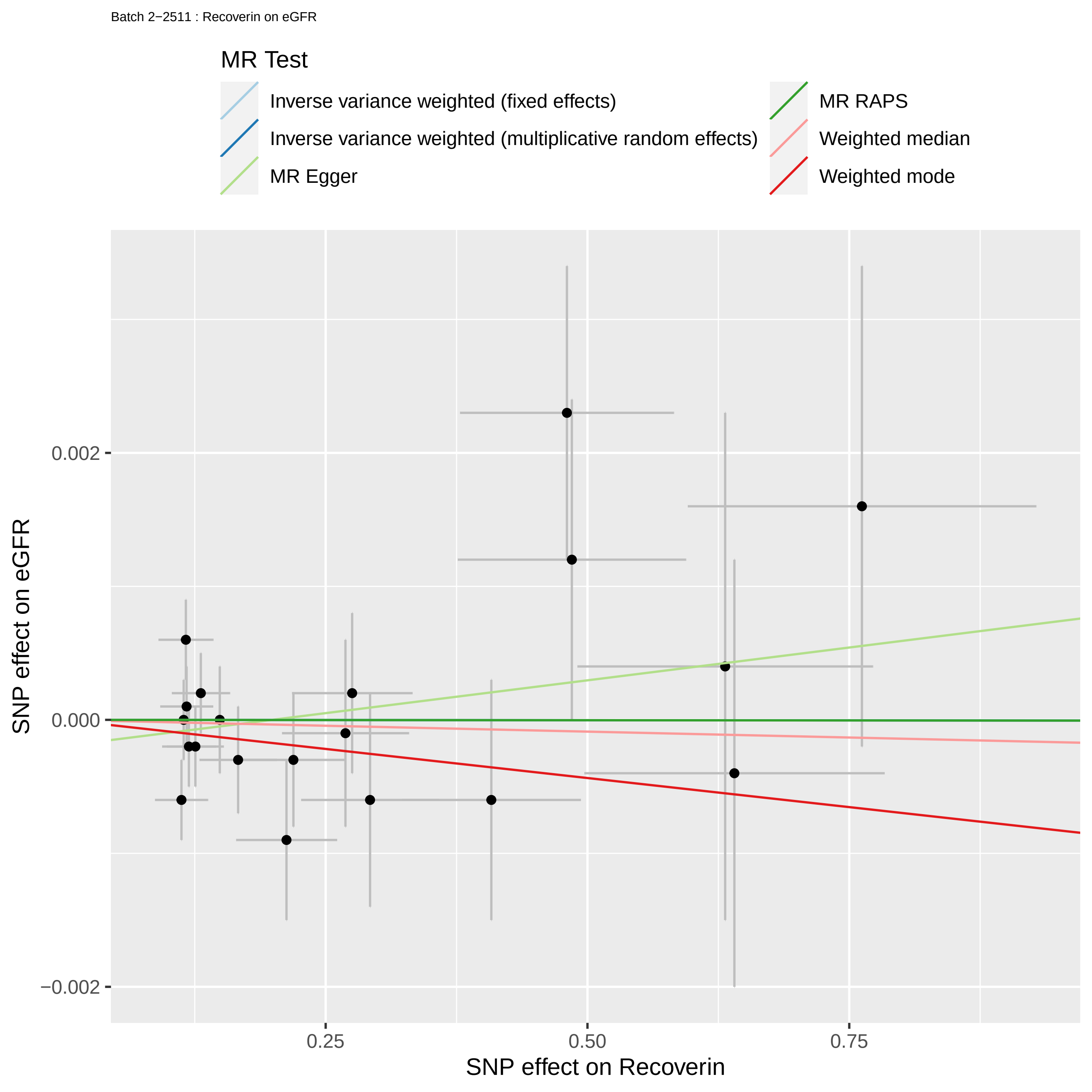

Batch 2−2511 : Recoverin on eGFR
MR Test
Inverse variance weighted (fixed effects)
Inverse variance weighted (multiplicative random effects)
MR Egger
MR RAPS
Weighted median
Weighted mode
0.002
0.000
−0.002
0.25
0.50
0.75
SNP effect on eGFR
SNP effect on Recoverin

Batch 2−2512 : Retinol dehydrogenase 16 on eGFR

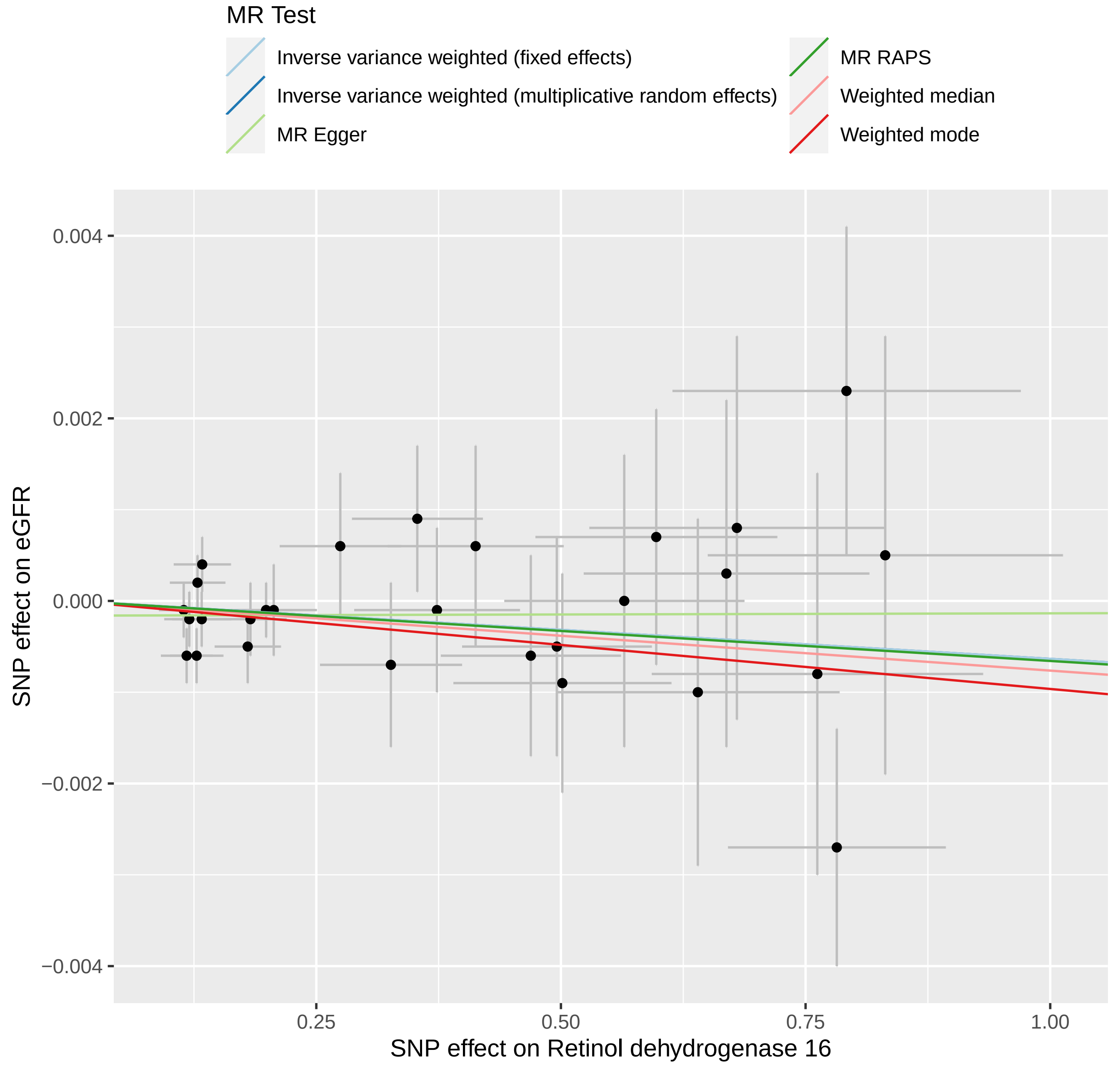

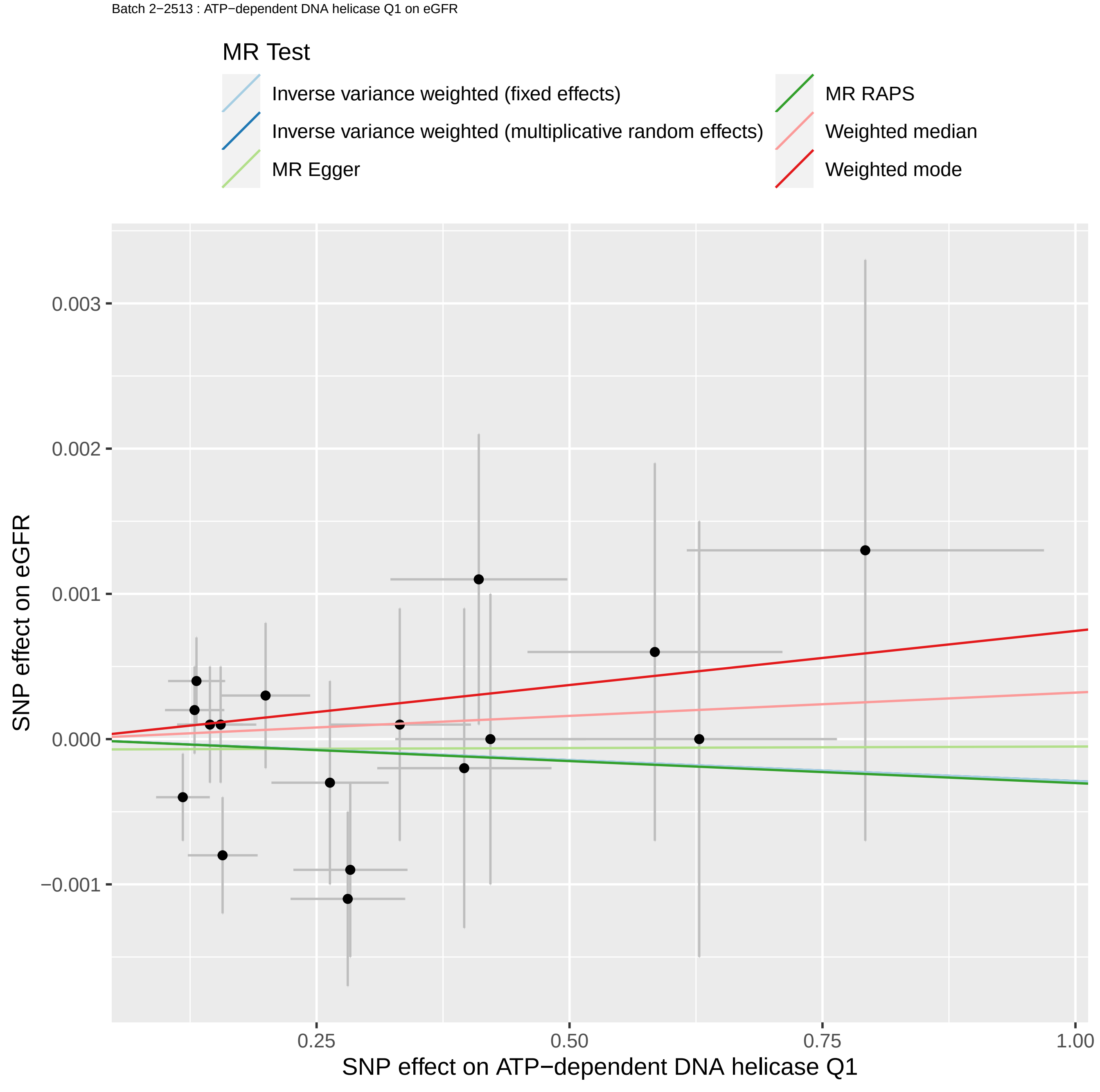

Batch 2−2513 : ATP−dependent DNA helicase Q1 on eGFR
MR Test
Inverse variance weighted (fixed effects)
Inverse variance weighted (multiplicative random effects)
MR Egger
MR RAPS
Weighted median
Weighted mode
0.003
0.002
0.001
0.000
−0.001
0.25
0.50
0.75
1.00
SNP effect on eGFR
SNP effect on ATP−dependent DNA helicase Q1

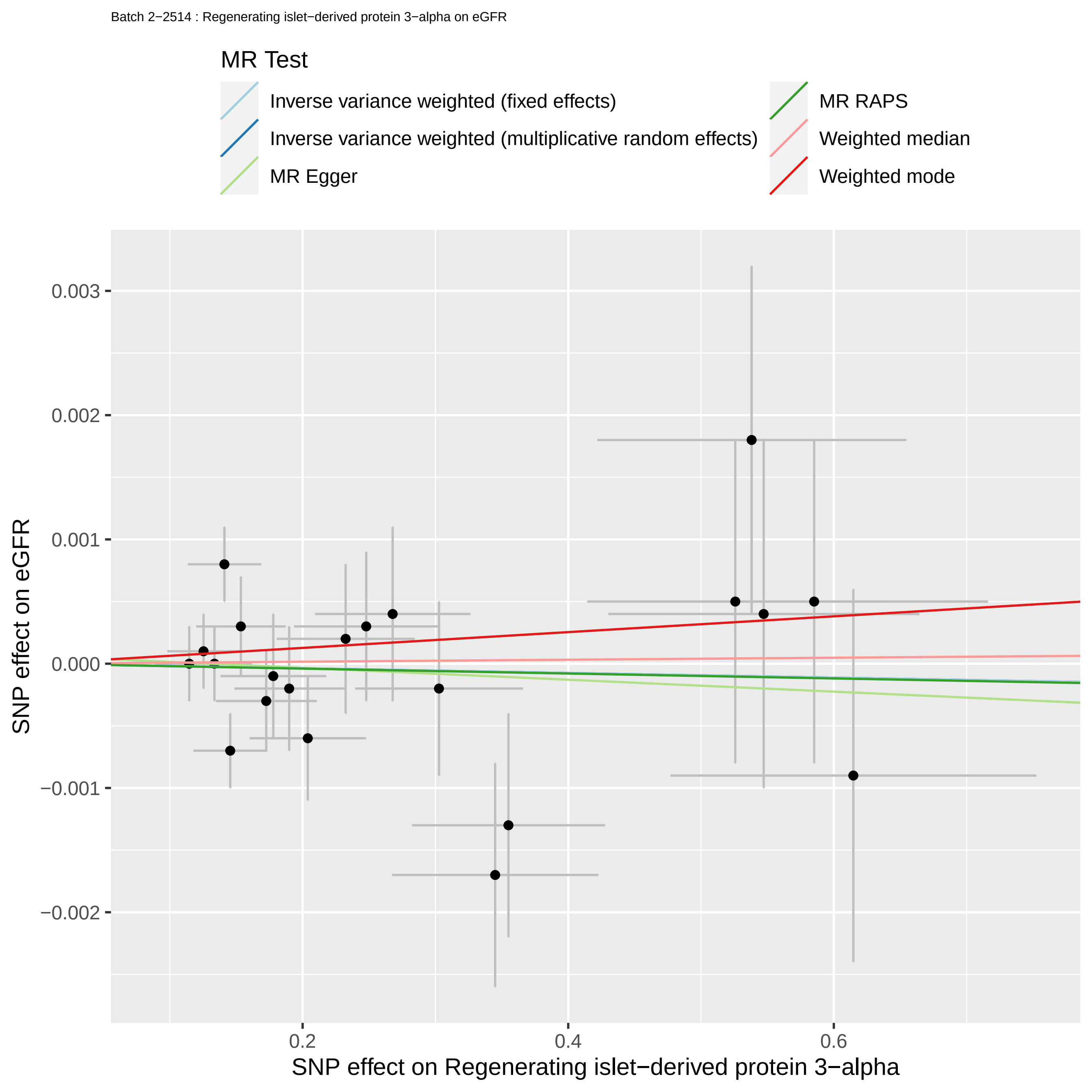

Batch 2−2514 : Regenerating islet−derived protein 3−alpha on eGFR
MR Test
Inverse variance weighted (fixed effects)
Inverse variance weighted (multiplicative random effects)
MR Egger
MR RAPS
Weighted median
Weighted mode
0.003
0.002
0.001
0.000
−0.001
−0.002
SNP effect on eGFR
0.2
0.4
0.6
SNP effect on Regenerating islet−derived protein 3−alpha

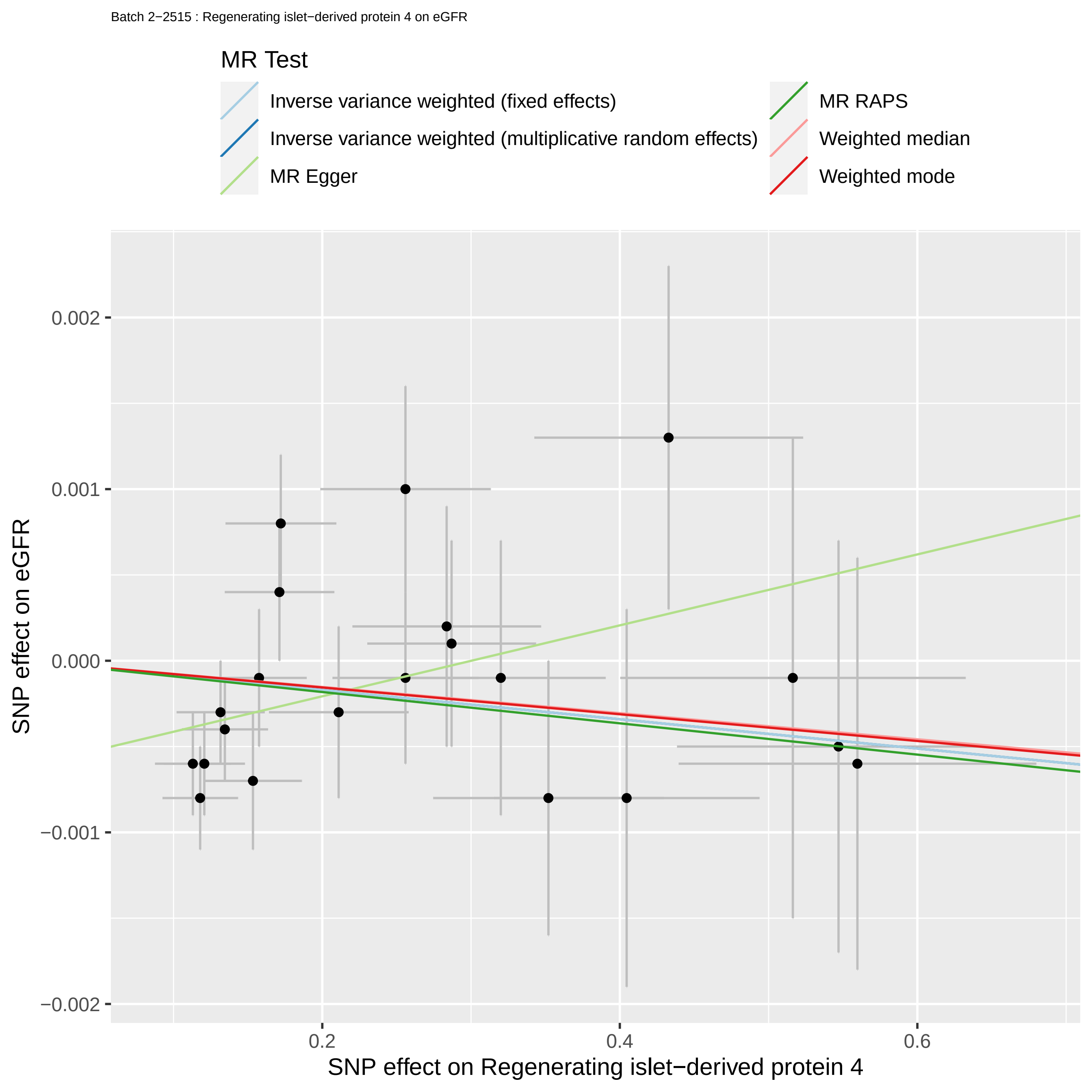

Batch 2−2515 : Regenerating islet−derived protein 4 on eGFR
MR Test
Inverse variance weighted (fixed effects)
Inverse variance weighted (multiplicative random effects)
MR Egger
MR RAPS
Weighted median
Weighted mode
0.002
0.001
0.000
−0.001
−0.002
SNP effect on eGFR
0.2
0.4
0.6
SNP effect on Regenerating islet−derived protein 4

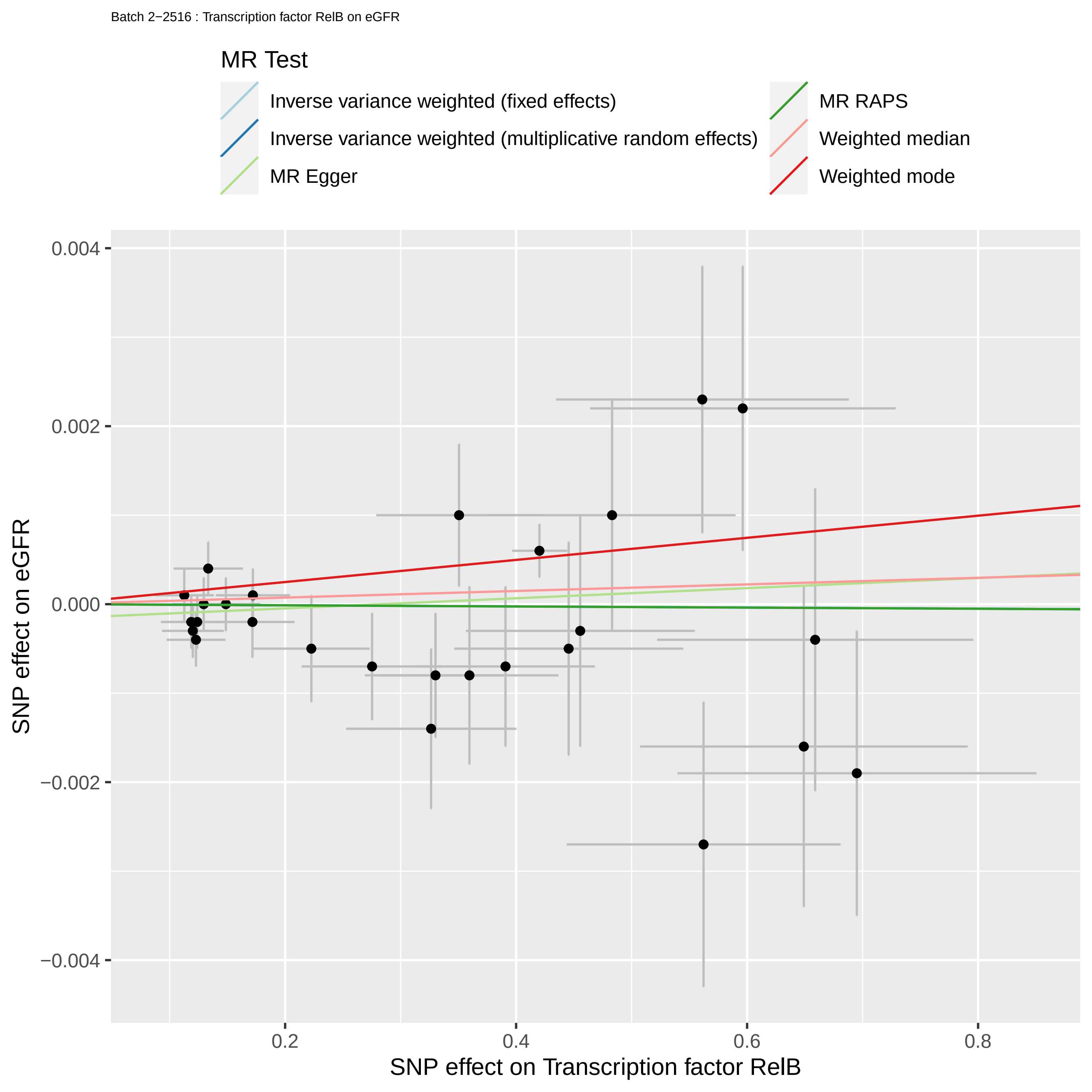

Batch 2−2516 : Transcription factor RelB on eGFR
MR Test
Inverse variance weighted (fixed effects)
Inverse variance weighted (multiplicative random effects)
MR Egger
MR RAPS
Weighted median
Weighted mode
0.004
0.002
0.000
−0.002
−0.004
0.2
0.4
0.6
0.8
SNP effect on eGFR
SNP effect on Transcription factor RelB

Batch 2−2517 : RELT−like protein 1 on eGFR

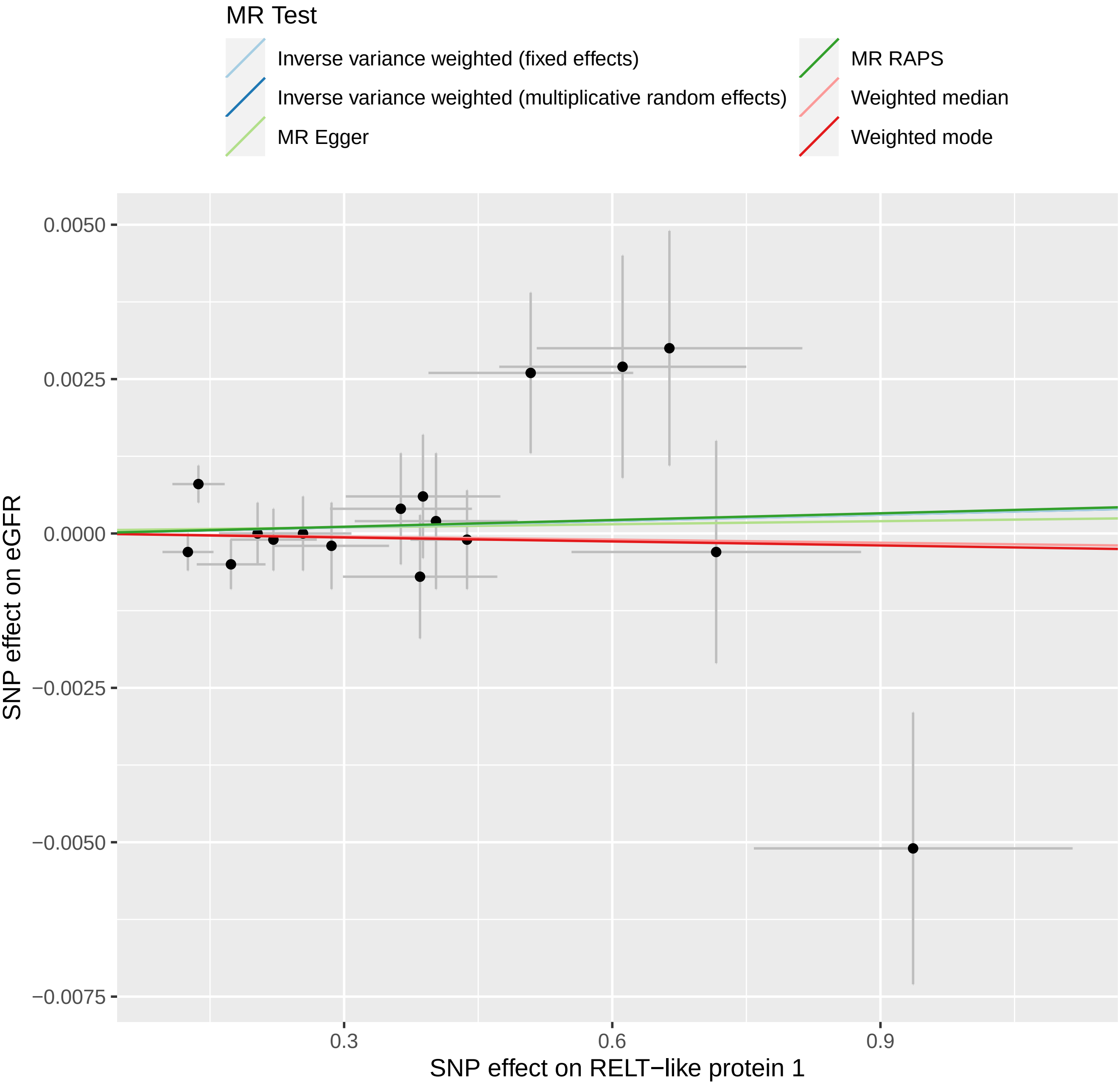

Batch 2−2518 : RELT−like protein 2 on eGFR

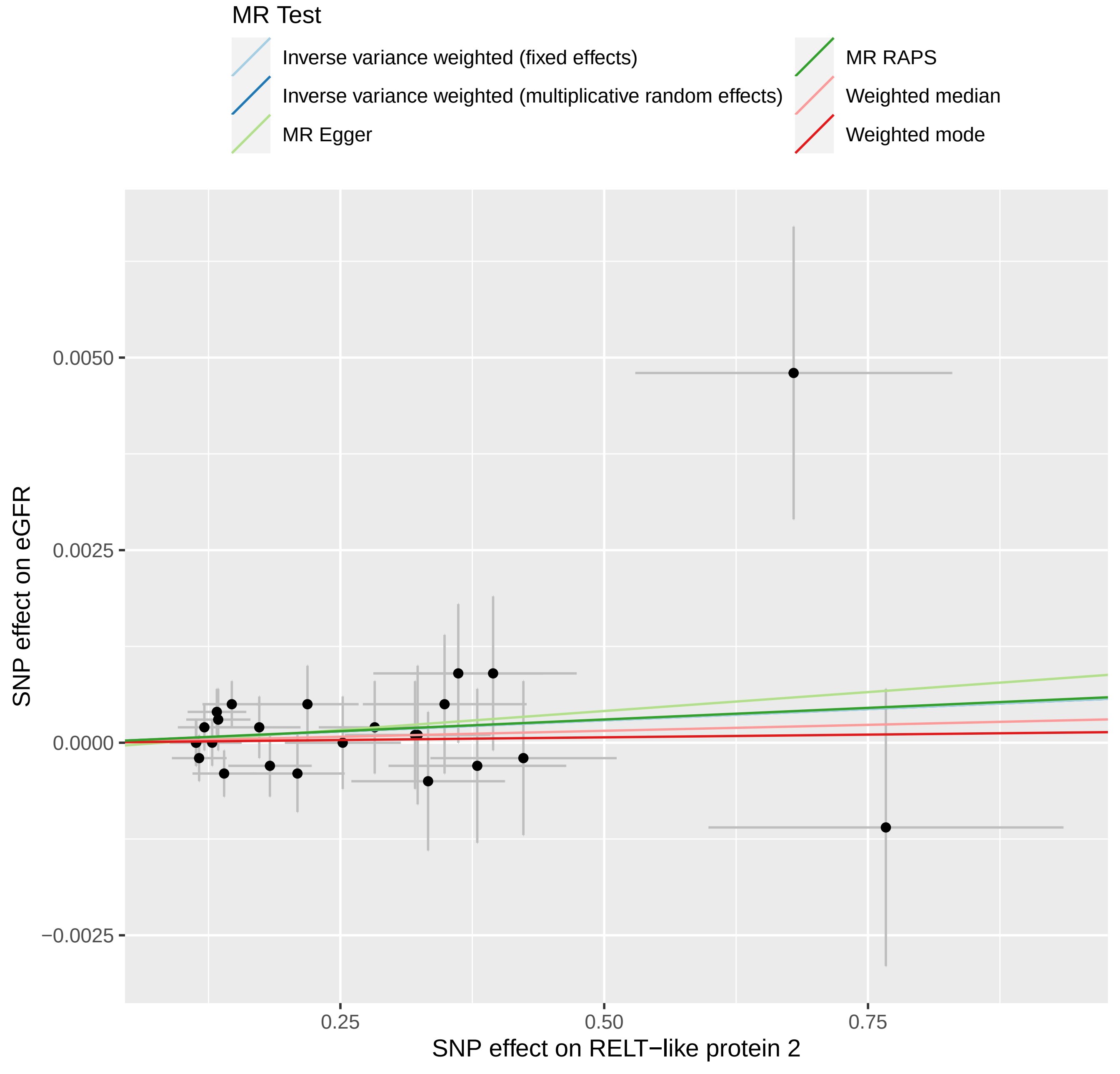

Batch 2−2519 : Tumor necrosis factor receptor superfamily member 19L on eGFR

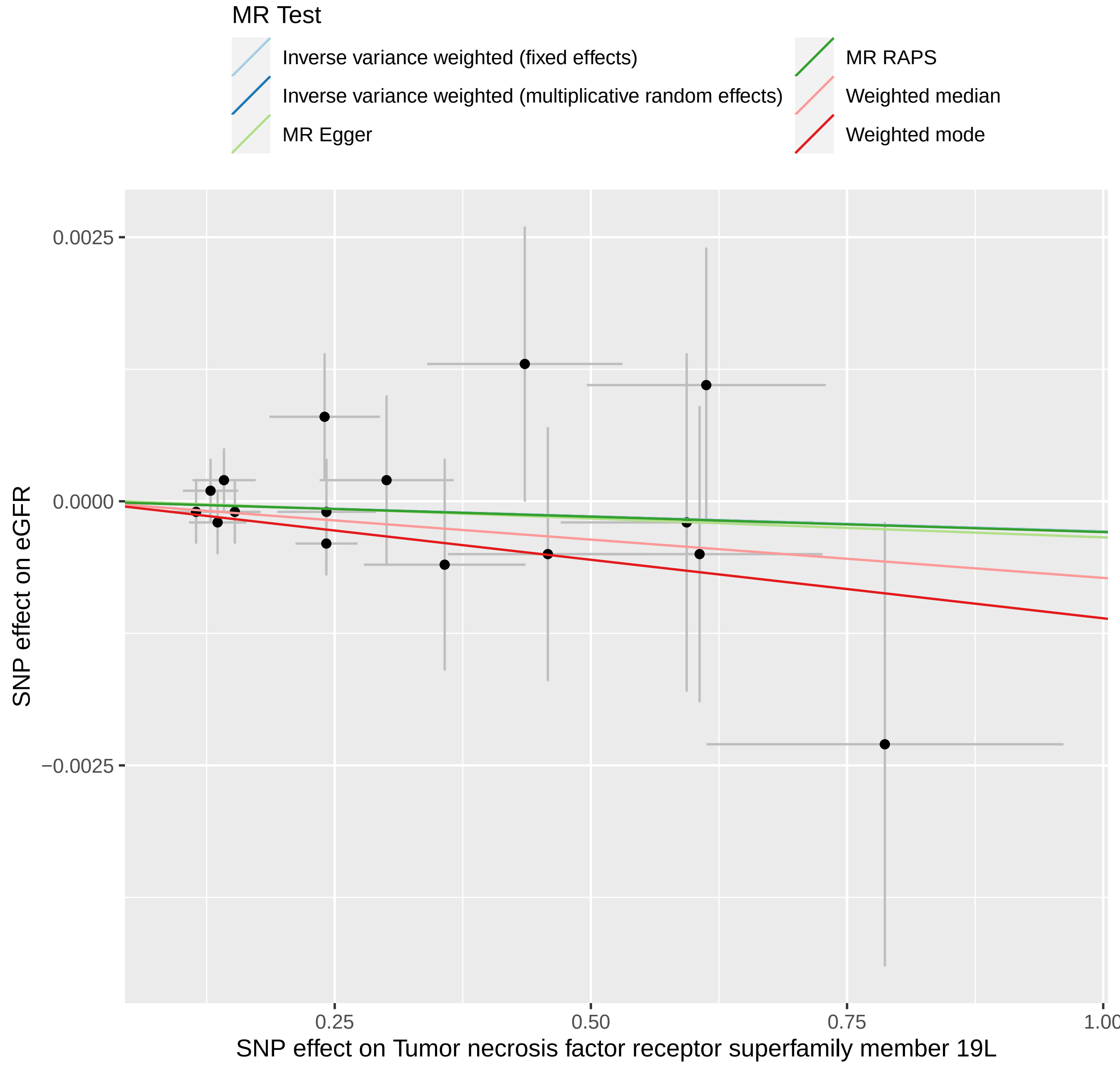

Batch 2−2520 : Tumor necrosis factor receptor superfamily member 19L on eGFR

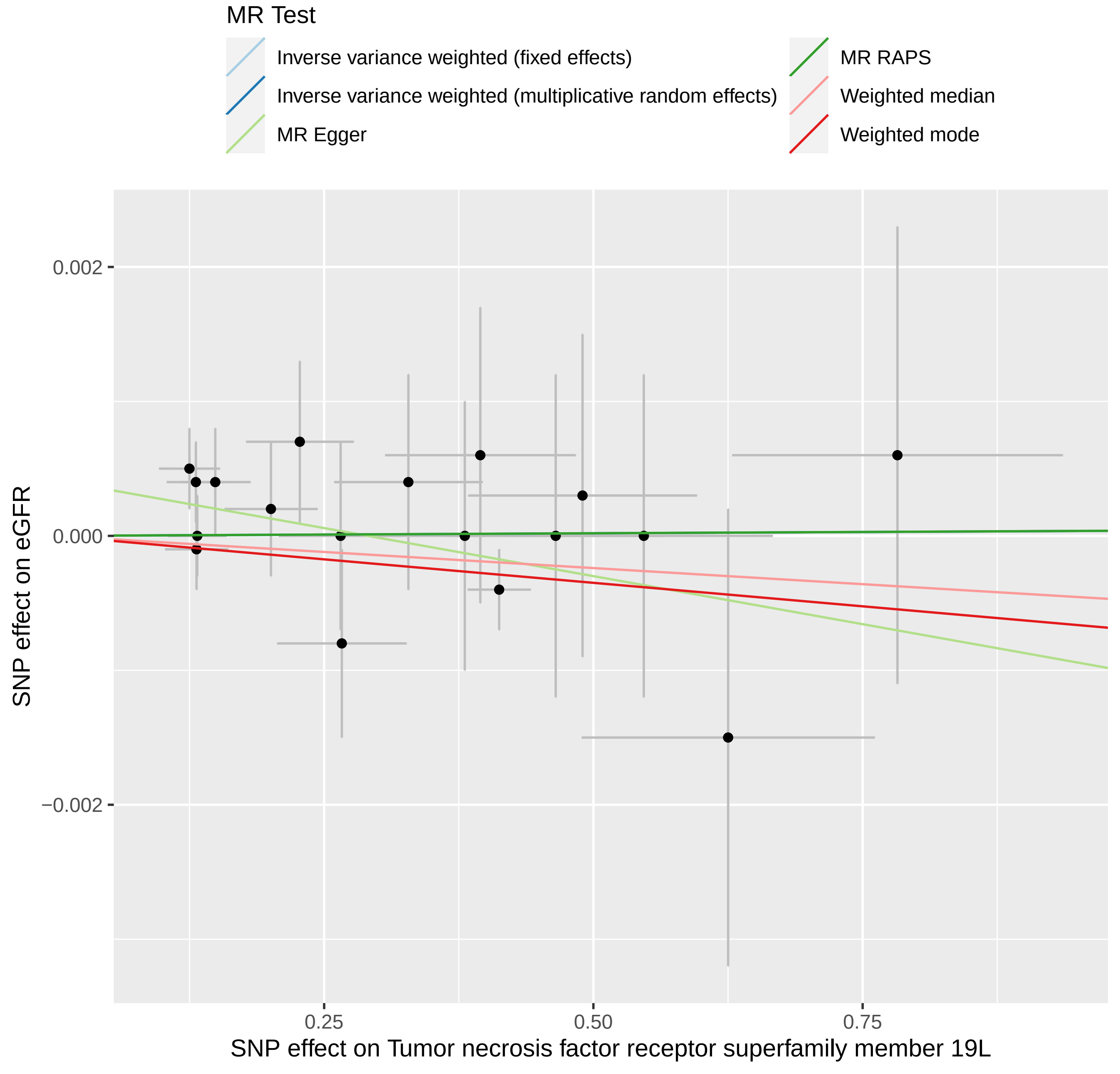

Batch 2−2521 : Renin on eGFR

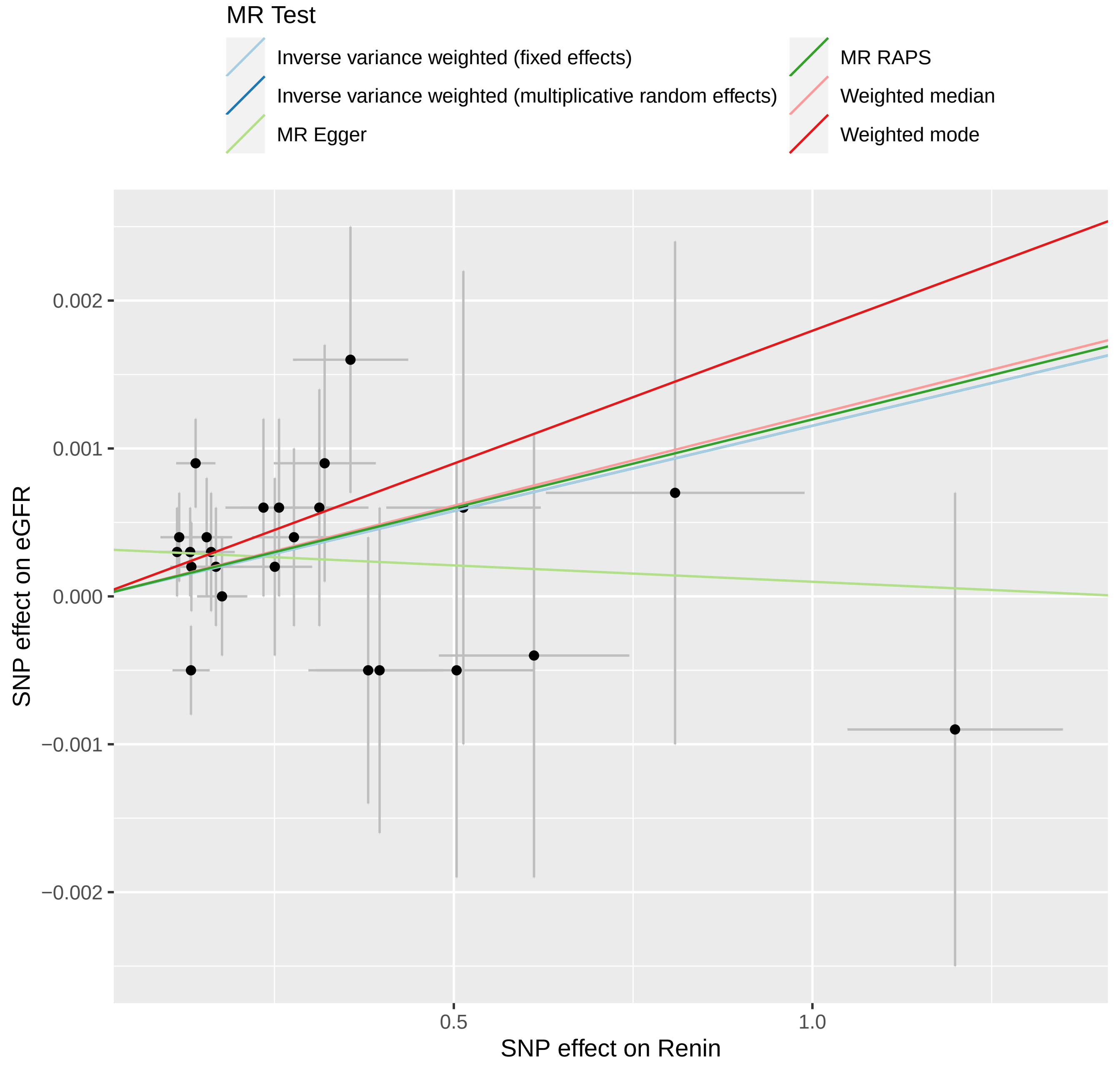

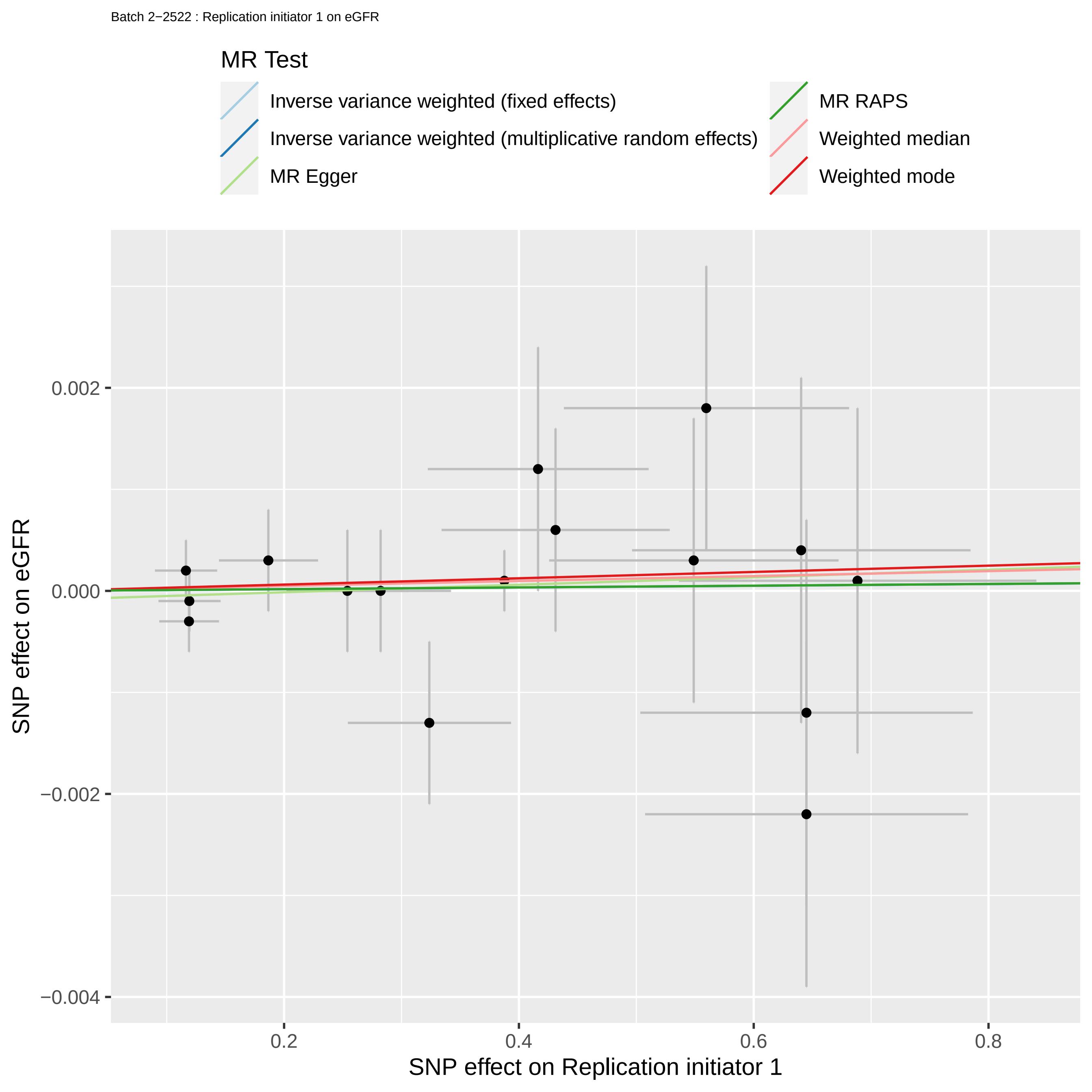

Batch 2−2522 : Replication initiator 1 on eGFR
MR Test
Inverse variance weighted (fixed effects)
Inverse variance weighted (multiplicative random effects)
MR Egger
MR RAPS
Weighted median
Weighted mode
0.002
0.000
−0.002
−0.004
0.2
0.4
0.6
0.8
SNP effect on eGFR
SNP effect on Replication initiator 1

Batch 2−2523 : Proto−oncogene tyrosine−protein kinase receptor Ret on eGFR

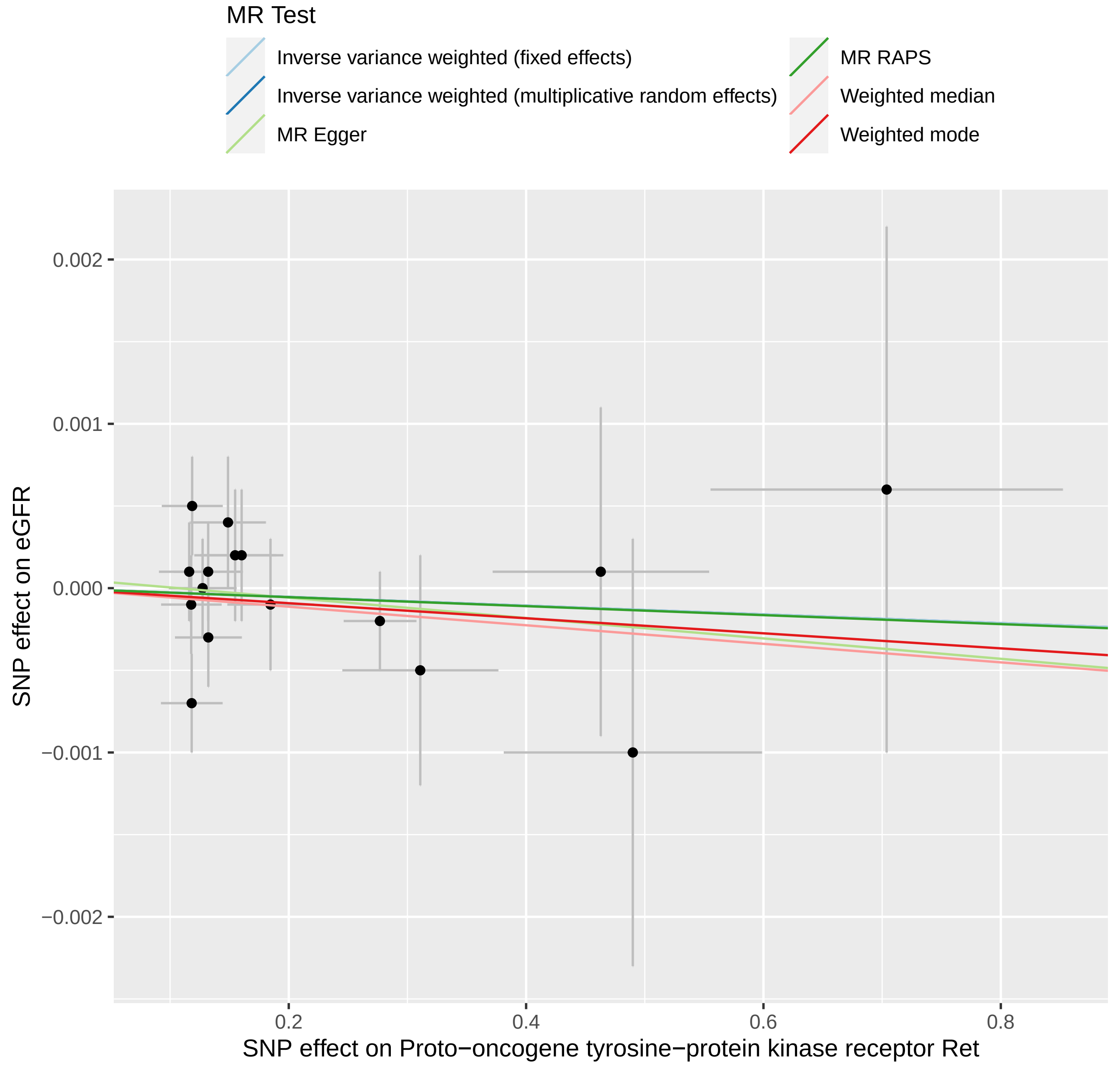

Batch 2−2524 : Resistin on eGFR

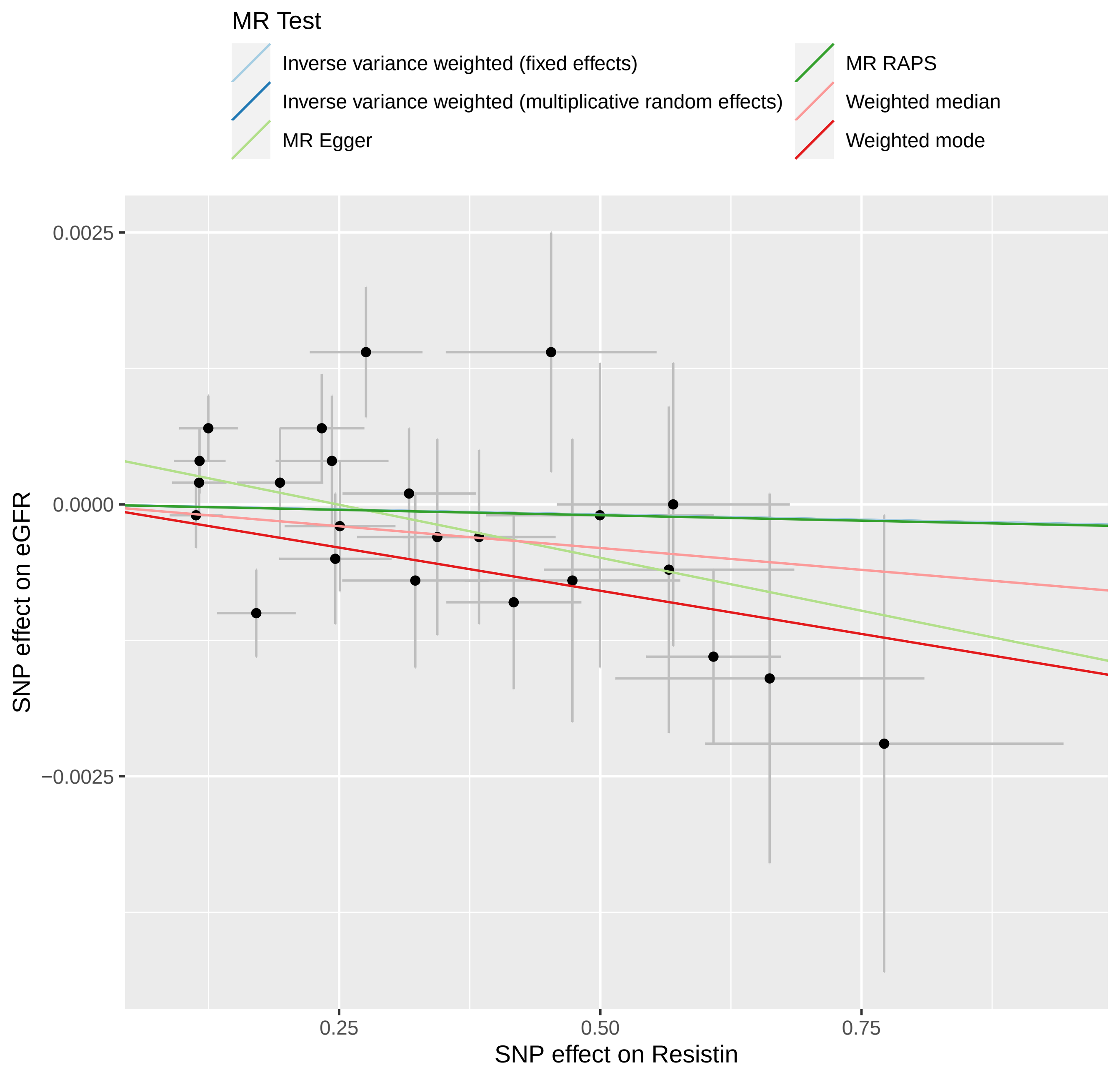

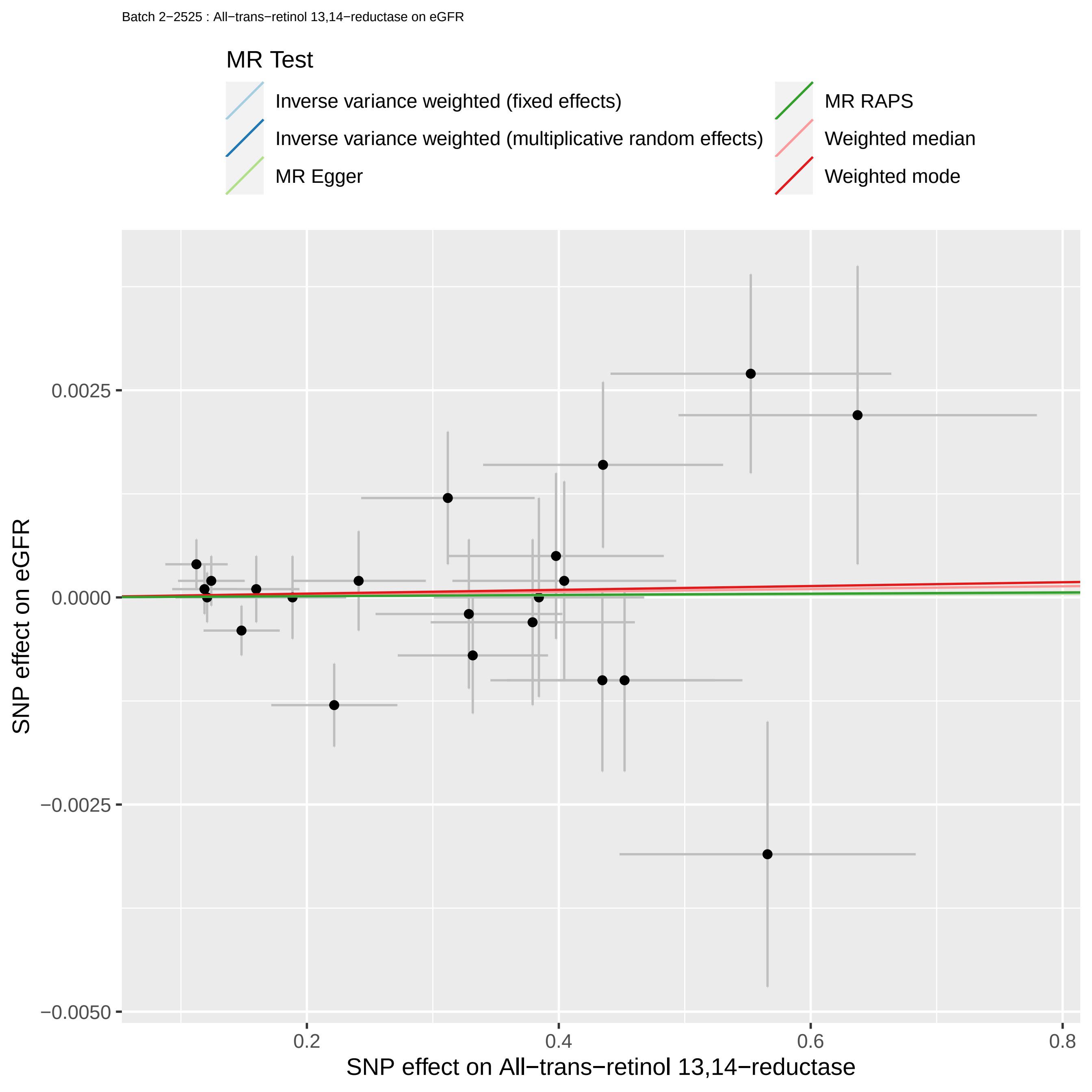
Batch 2−2525 : All−trans−retinol 13,14−reductase on eGFR
MR Test
Inverse variance weighted (fixed effects)
Inverse variance weighted (multiplicative random effects)
MR Egger
MR RAPS
Weighted median
Weighted mode
SNP effect on eGFR
0.0025
0.0000
−0.0025
−0.0050
0.2
0.4
0.6
0.8
SNP effect on All−trans−retinol 13,14−reductase

Batch 2−2526 : Oligoribonuclease, mitochondrial on eGFR

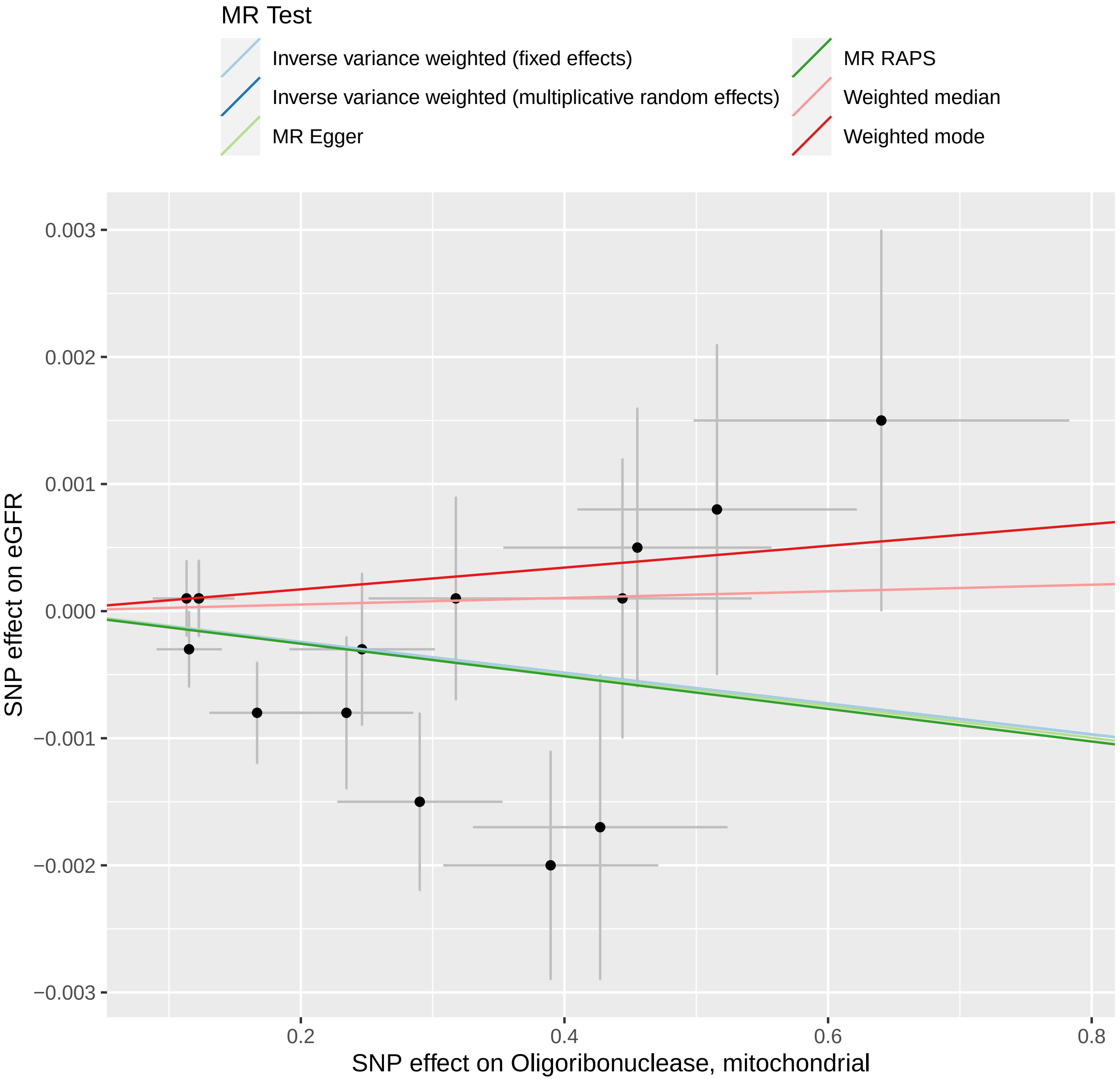

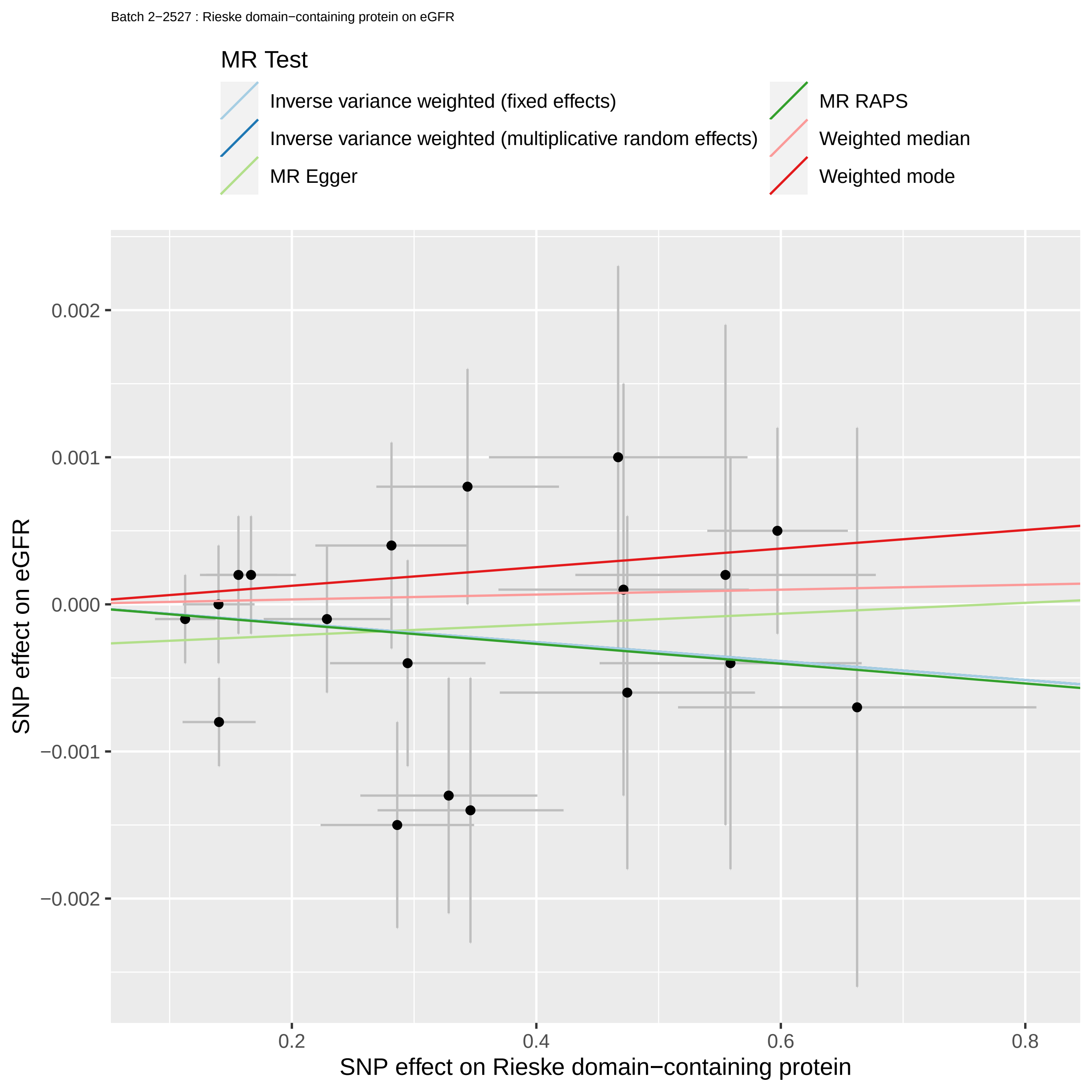

Batch 2−2527 : Rieske domain−containing protein on eGFR
MR Test
Inverse variance weighted (fixed effects)
Inverse variance weighted (multiplicative random effects)
MR Egger
MR RAPS
Weighted median
Weighted mode
0.002
0.001
0.000
−0.001
−0.002
0.2
0.4
0.6
0.8
SNP effect on eGFR
SNP effect on Rieske domain−containing protein

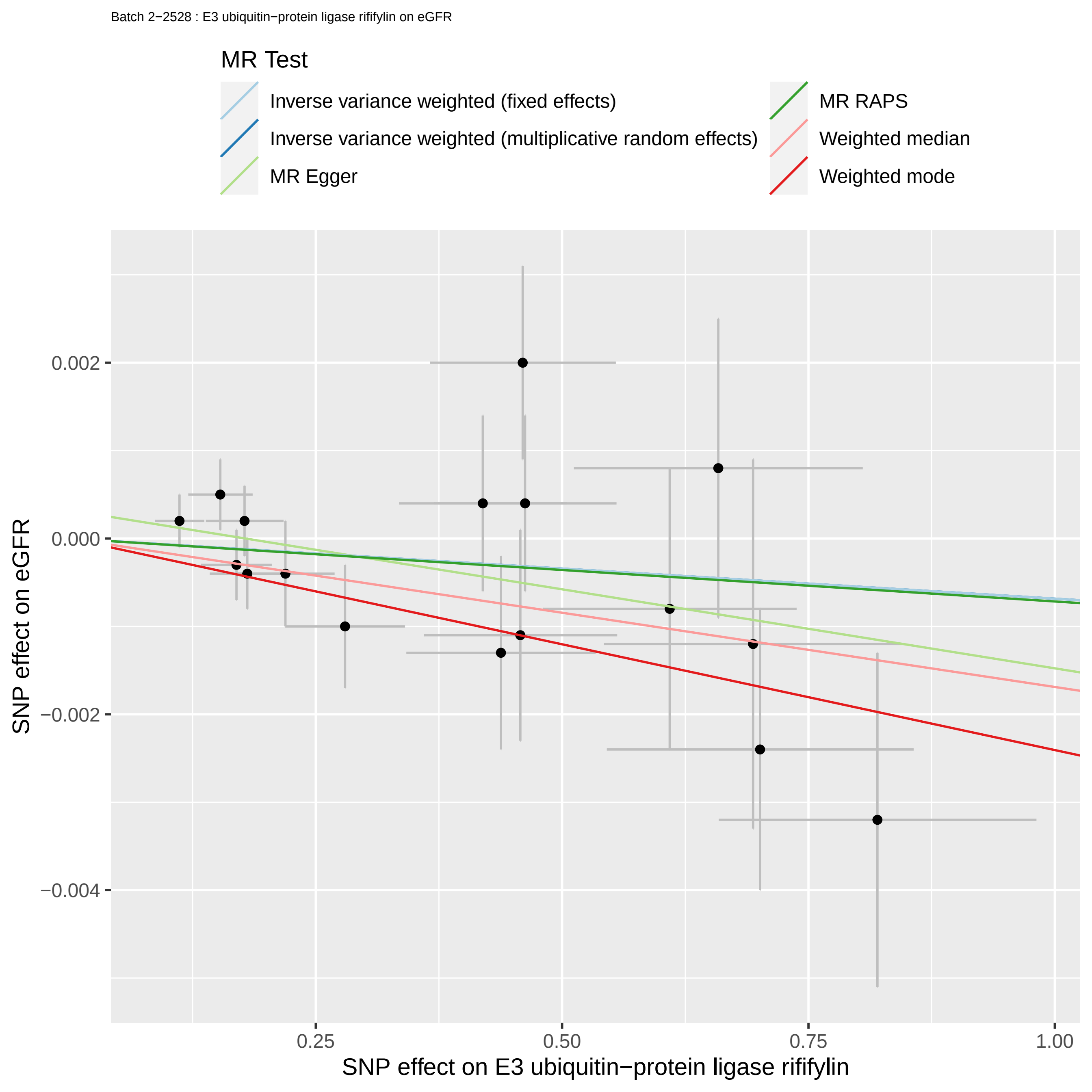

Batch 2−2528 : E3 ubiquitin−protein ligase rififylin on eGFR
MR Test
Inverse variance weighted (fixed effects)
Inverse variance weighted (multiplicative random effects)
MR Egger
MR RAPS
Weighted median
Weighted mode
0.002
0.000
−0.002
−0.004
SNP effect on eGFR
0.25
0.50
0.75
1.00
SNP effect on E3 ubiquitin−protein ligase rififylin

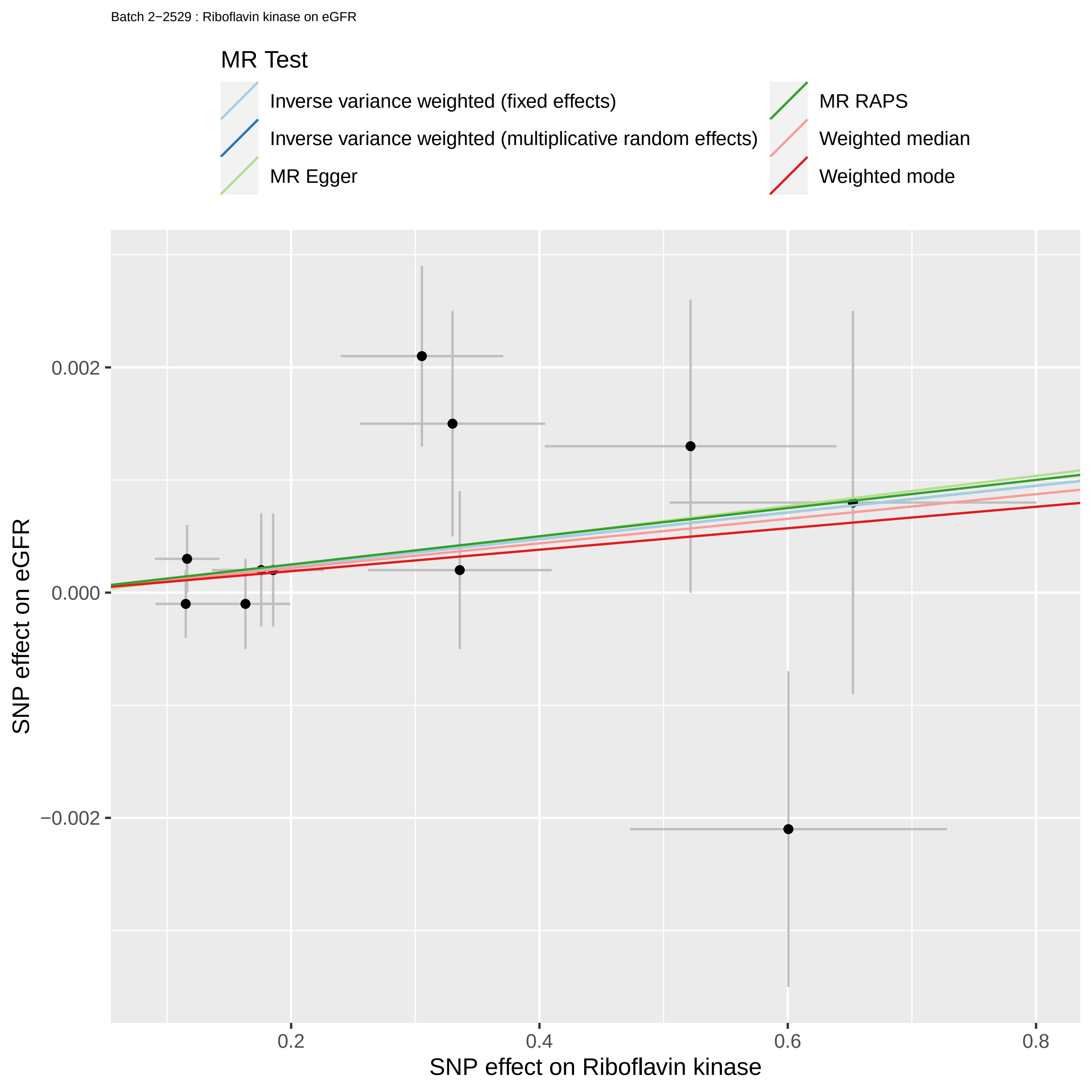
Batch 2−2529 : Riboflavin kinase on eGFR
MR Test
Inverse variance weighted (fixed effects)
Inverse variance weighted (multiplicative random effects)
MR Egger
MR RAPS
Weighted median
Weighted mode
SNP effect on eGFR
0.002
0.000
−0.002
0.2
0.4
0.6
0.8
SNP effect on Riboflavin kinase

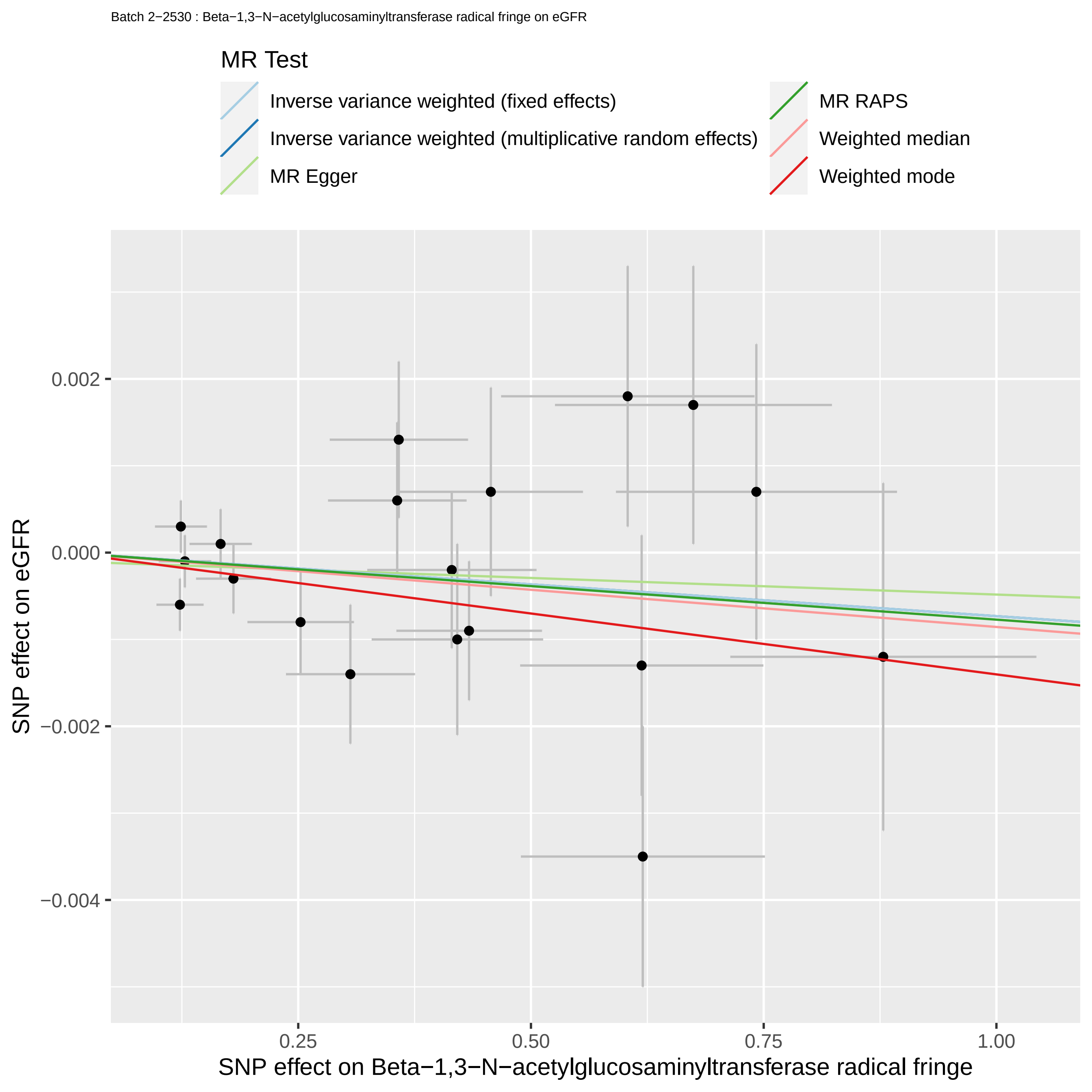

Batch 2−2530 : Beta−1,3−N−acetylglucosaminyltransferase radical fringe on eGFR
MR Test
Inverse variance weighted (fixed effects)
Inverse variance weighted (multiplicative random effects)
MR Egger
MR RAPS
Weighted median
Weighted mode
0.002
0.000
−0.002
−0.004
0.25
0.50
0.75
1.00
SNP effect on eGFR
SNP effect on Beta−1,3−N−acetylglucosaminyltransferase radical fringe

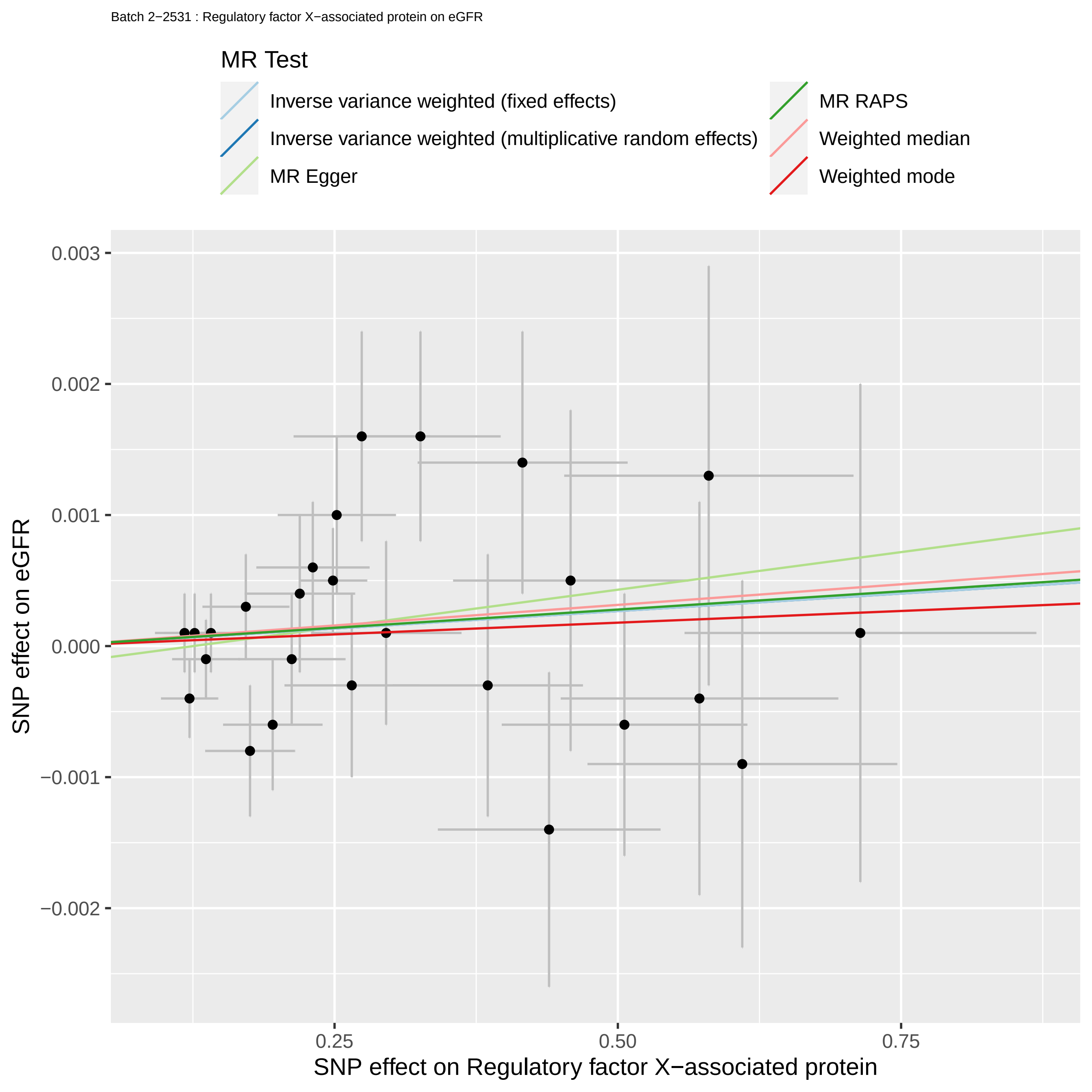

Batch 2−2531 : Regulatory factor X−associated protein on eGFR
MR Test
Inverse variance weighted (fixed effects)
Inverse variance weighted (multiplicative random effects)
MR Egger
MR RAPS
Weighted median
Weighted mode
0.003
0.002
0.001
0.000
−0.001
−0.002
SNP effect on eGFR
0.25
0.50
0.75
SNP effect on Regulatory factor X−associated protein

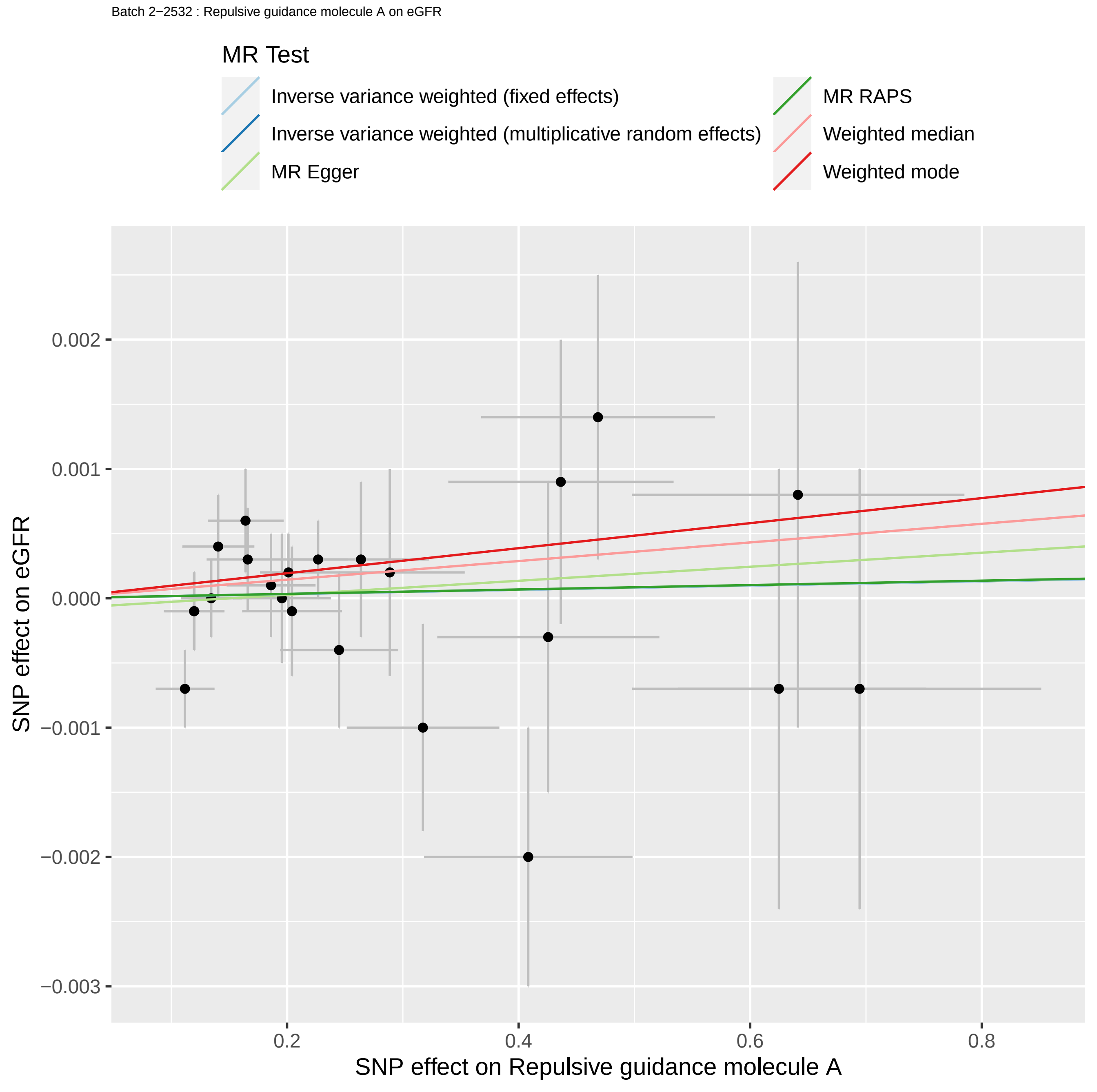

Batch 2−2532 : Repulsive guidance molecule A on eGFR
MR Test
Inverse variance weighted (fixed effects)
Inverse variance weighted (multiplicative random effects)
MR Egger
MR RAPS
Weighted median
Weighted mode
0.002
0.001
0.000
−0.001
−0.002
−0.003
SNP effect on eGFR
0.2
0.4
0.6
0.8
SNP effect on Repulsive guidance molecule A

Batch 2−2533 : RGM domain family member B on eGFR

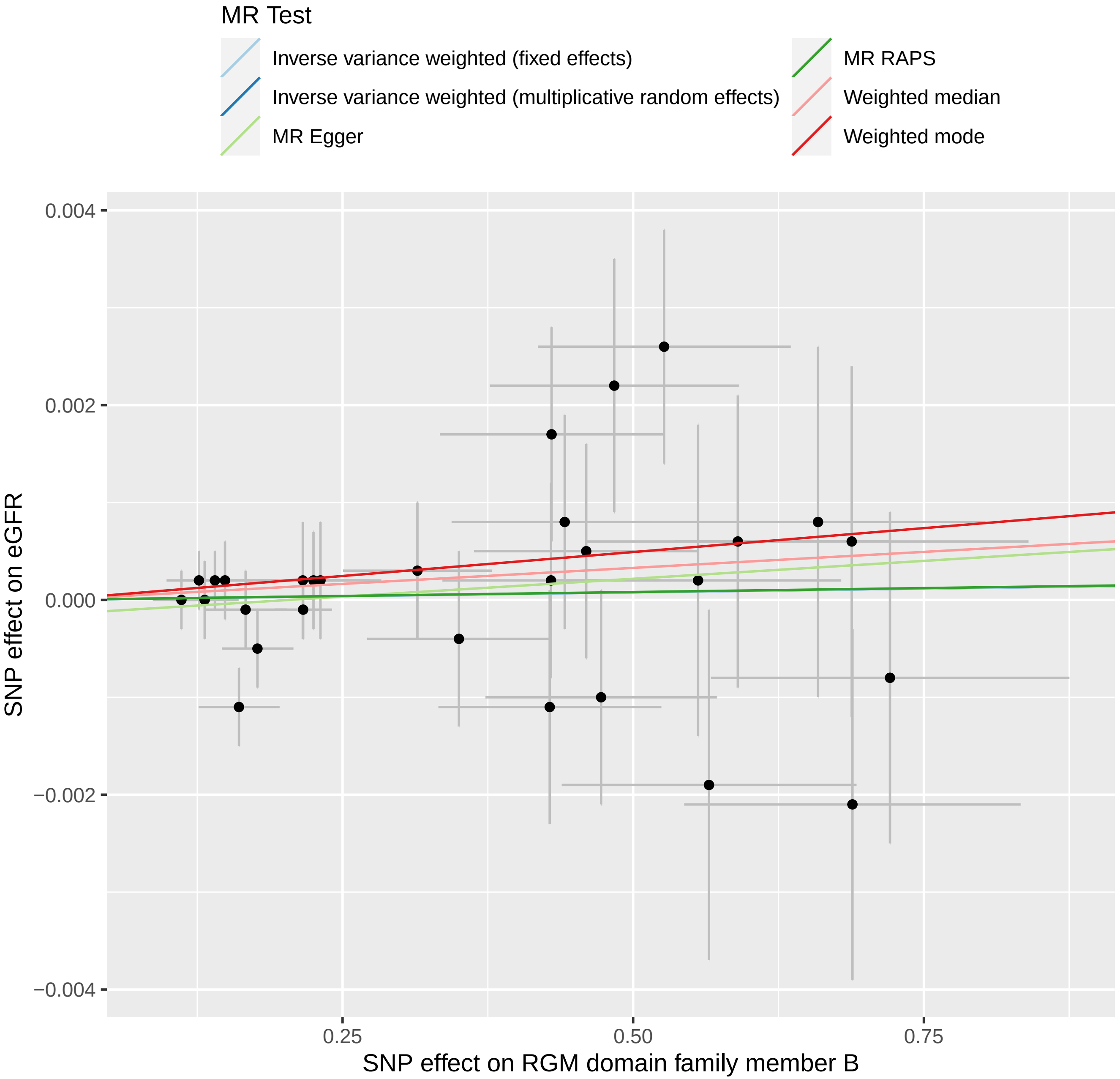

Batch 2−2534 : Regulator of G−protein signaling 10 on eGFR

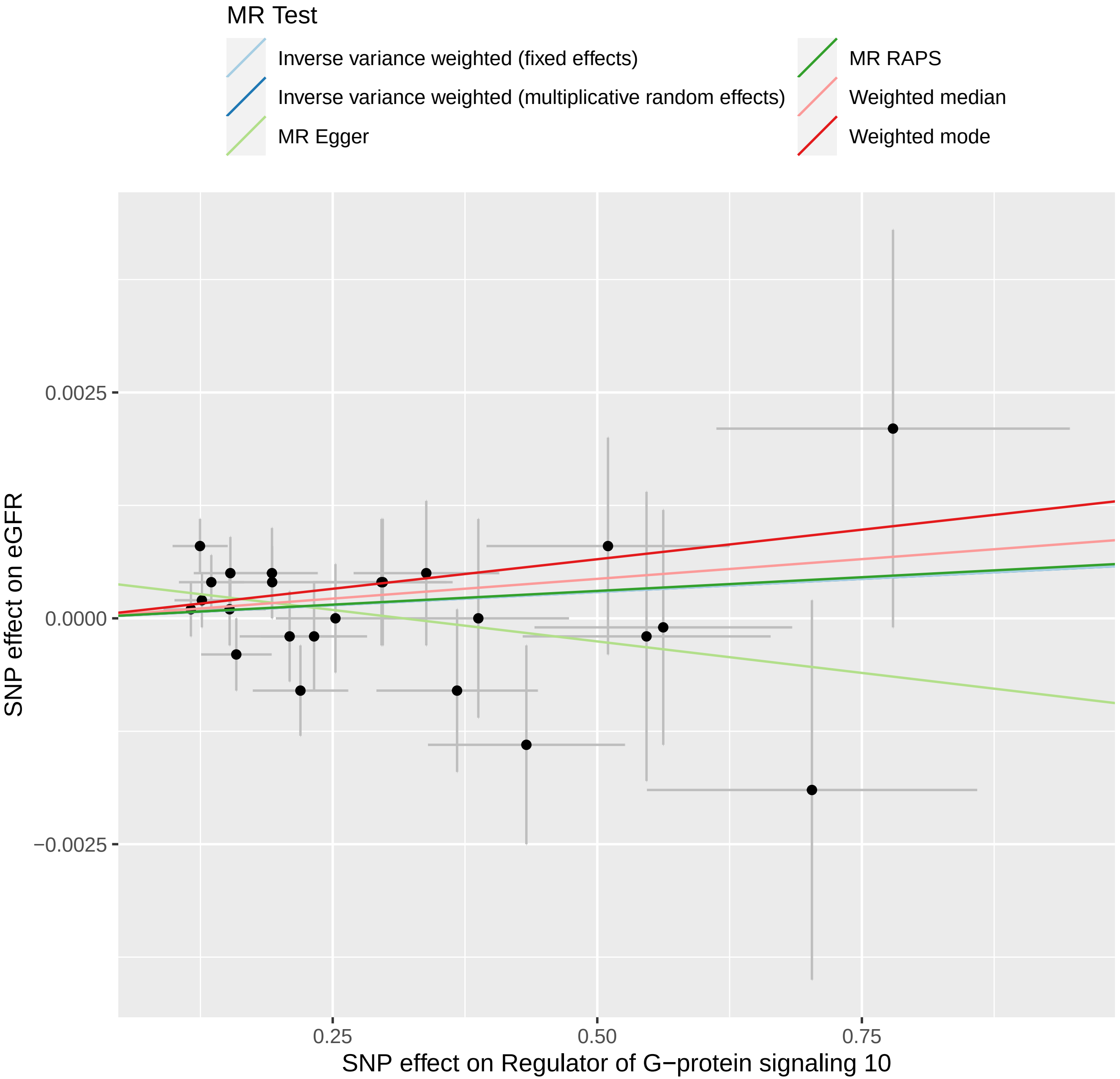

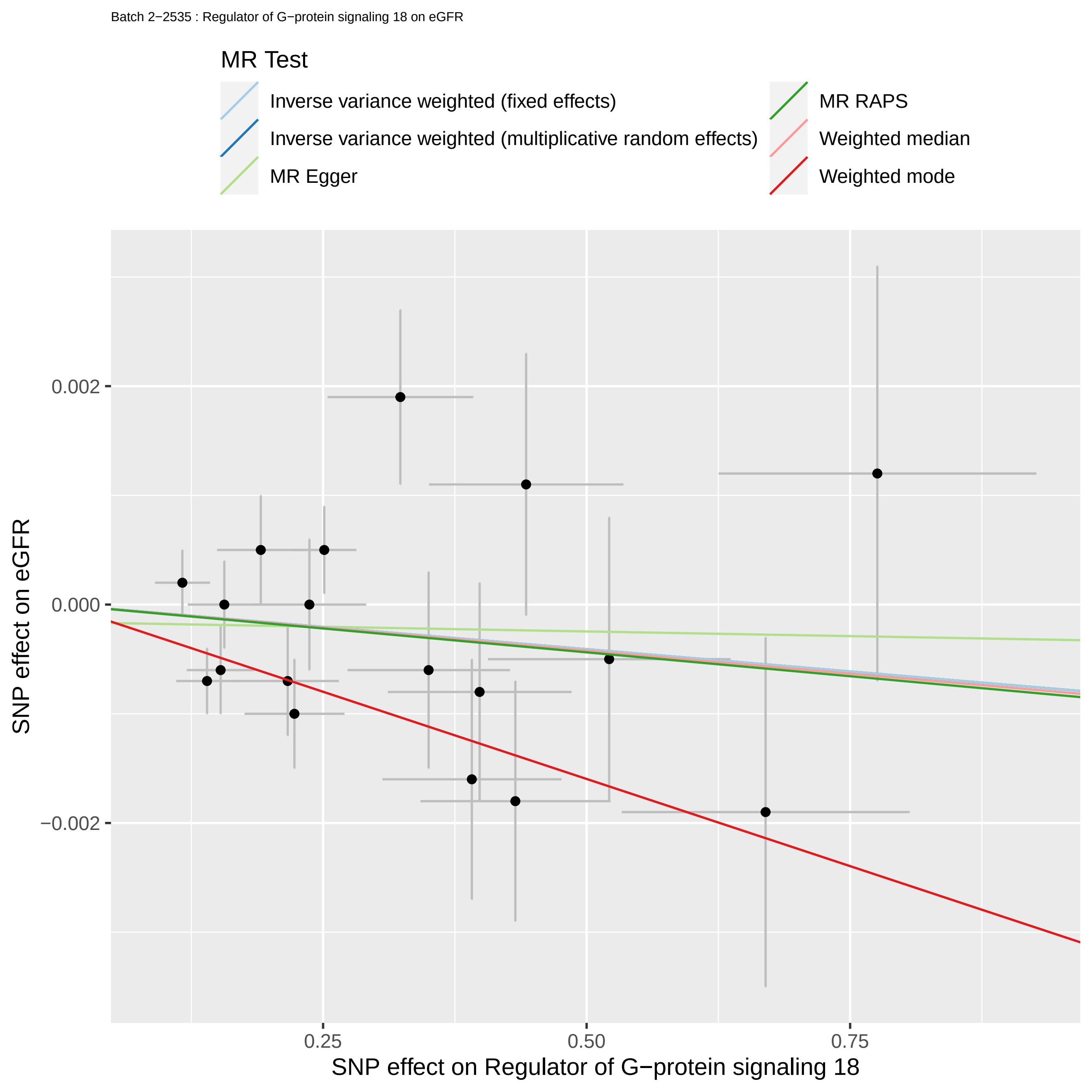

Batch 2−2535 : Regulator of G−protein signaling 18 on eGFR
MR Test
Inverse variance weighted (fixed effects)
Inverse variance weighted (multiplicative random effects)
MR Egger
MR RAPS
Weighted median
Weighted mode
0.002
0.000
−0.002
0.25
0.50
0.75
SNP effect on eGFR
SNP effect on Regulator of G−protein signaling 18

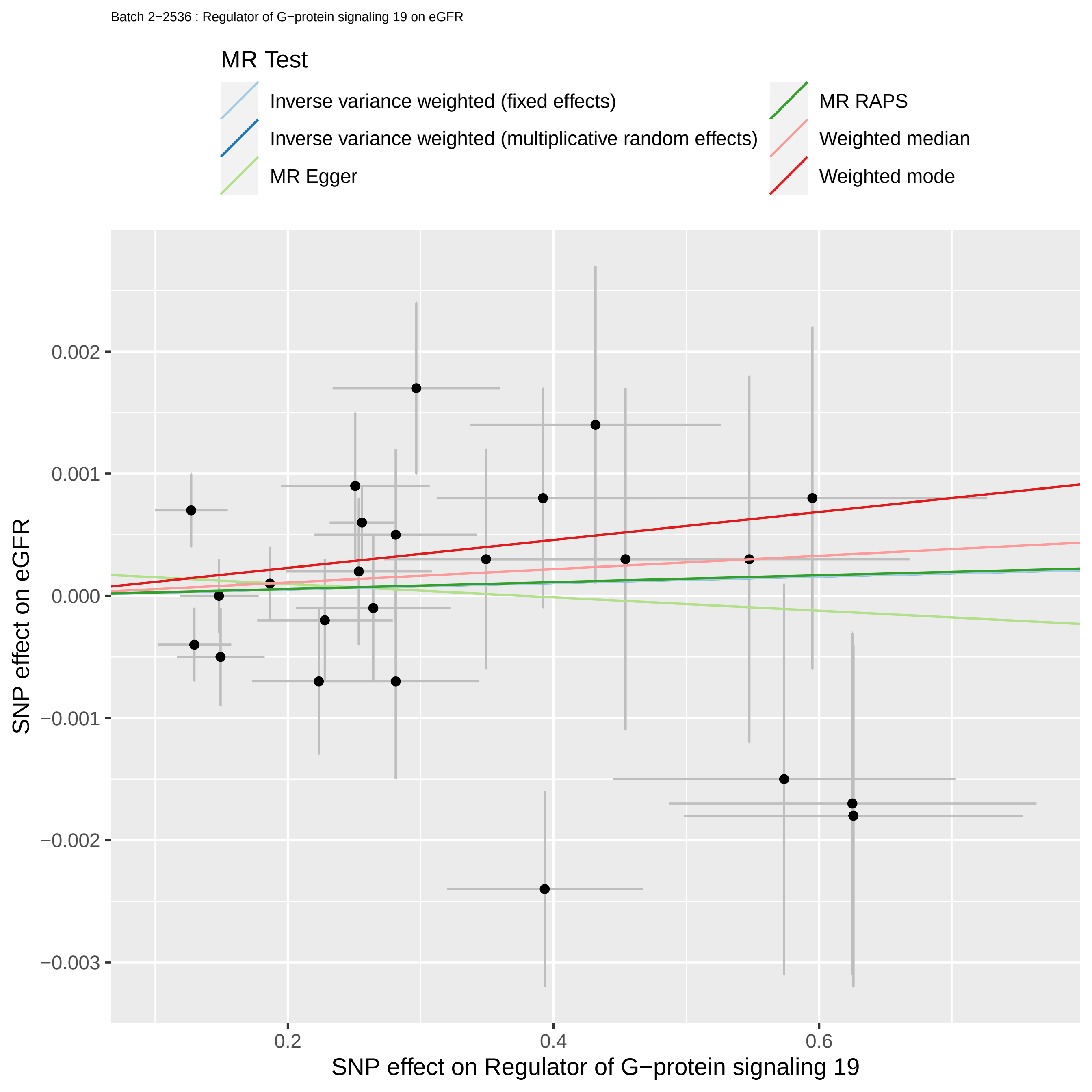
Batch 2−2536 : Regulator of G−protein signaling 19 on eGFR
MR Test
Inverse variance weighted (fixed effects)
Inverse variance weighted (multiplicative random effects)
MR Egger
MR RAPS
Weighted median
Weighted mode
0.002
0.001
0.000
−0.001
−0.002
−0.003
SNP effect on eGFR
0.2
0.4
0.6
SNP effect on Regulator of G−protein signaling 19

Batch 2−2537 : Regulator of G−protein signaling 3 on eGFR

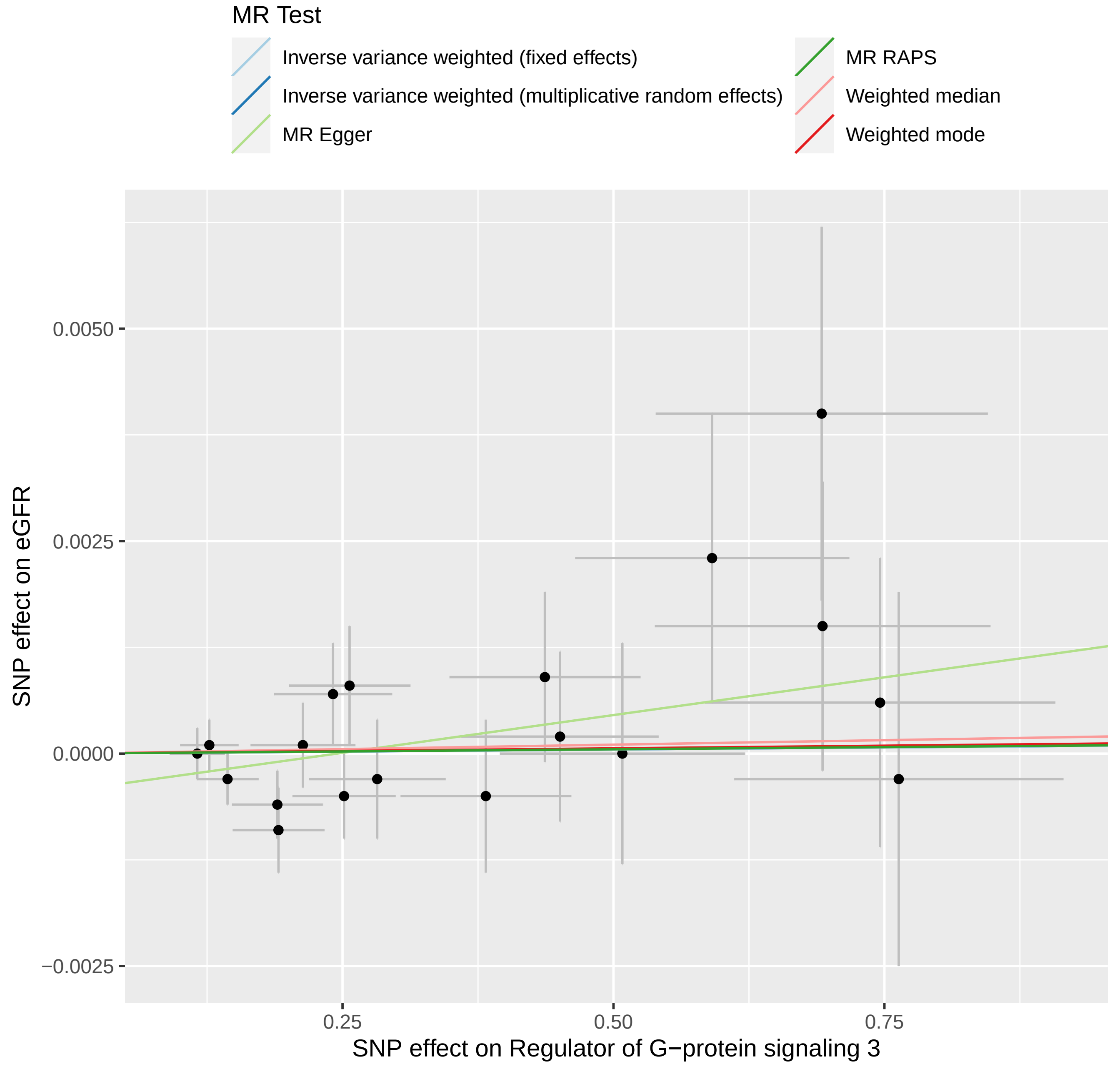

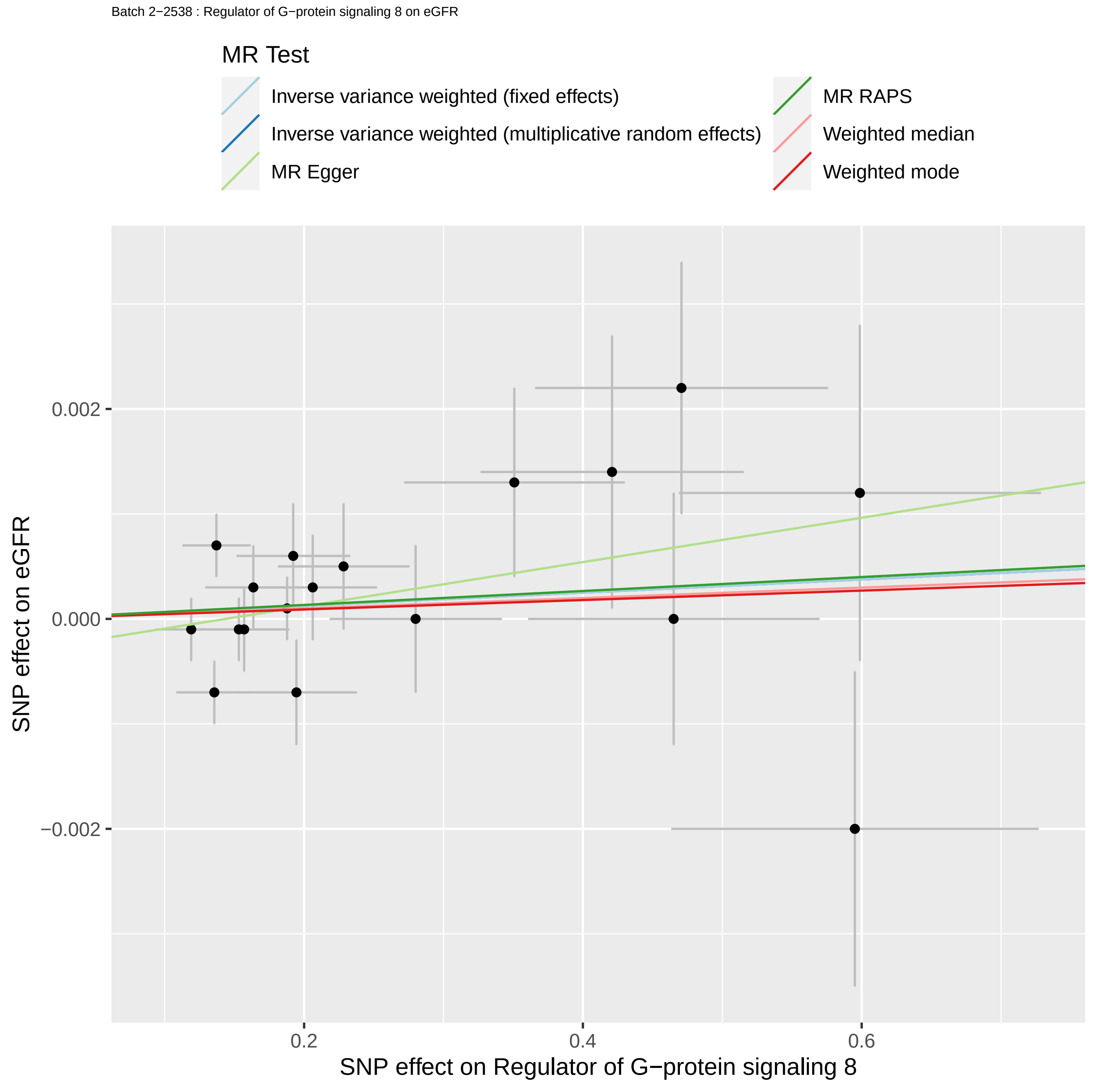
Batch 2−2538 : Regulator of G−protein signaling 8 on eGFR
MR Test
Inverse variance weighted (fixed effects)
Inverse variance weighted (multiplicative random effects)
MR Egger
MR RAPS
Weighted median
Weighted mode
0.002
0.000
−0.002
0.2
0.4
0.6
SNP effect on eGFR
SNP effect on Regulator of G−protein signaling 8

Batch 2−2539 : Transforming protein RhoA on eGFR

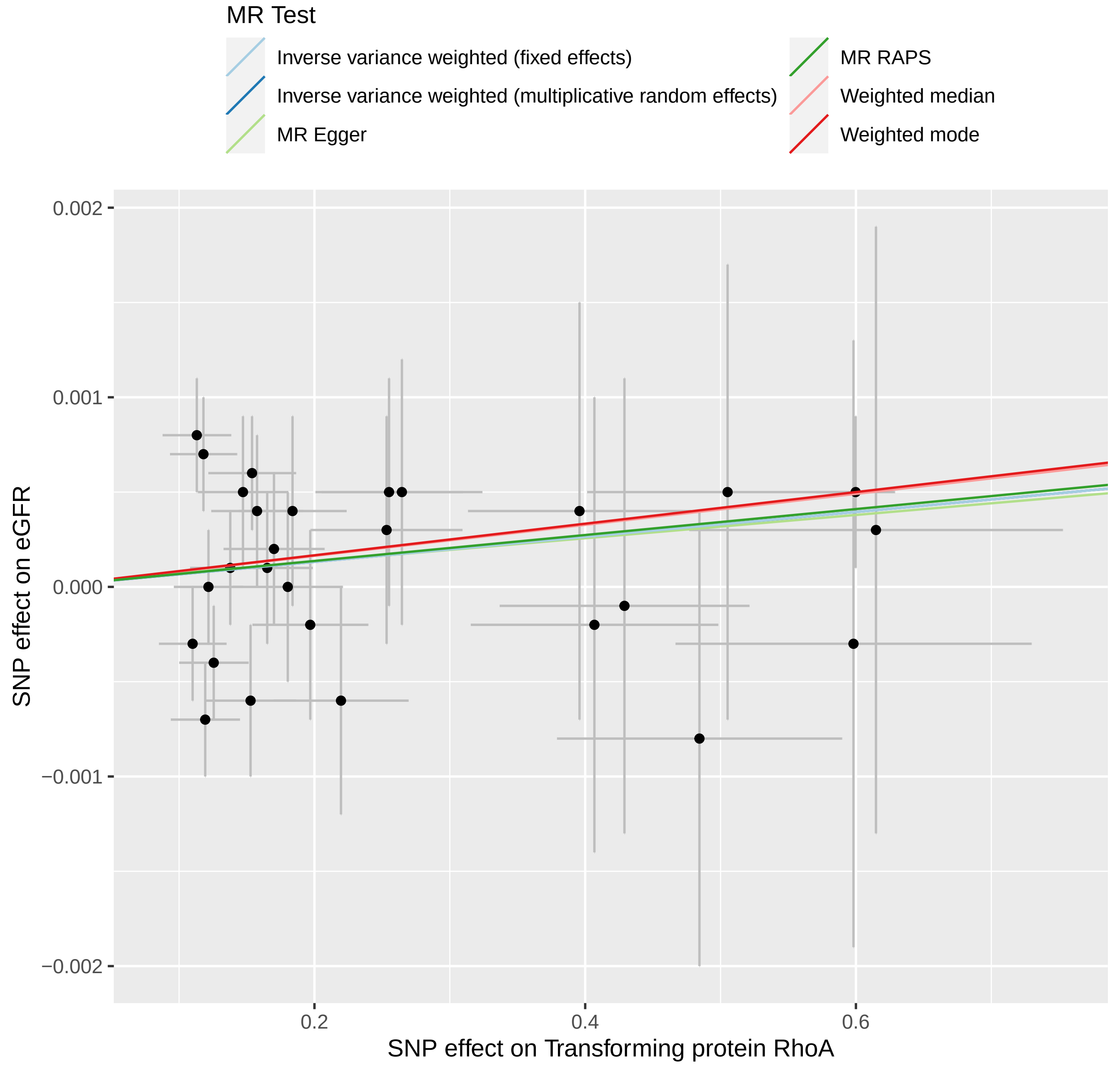

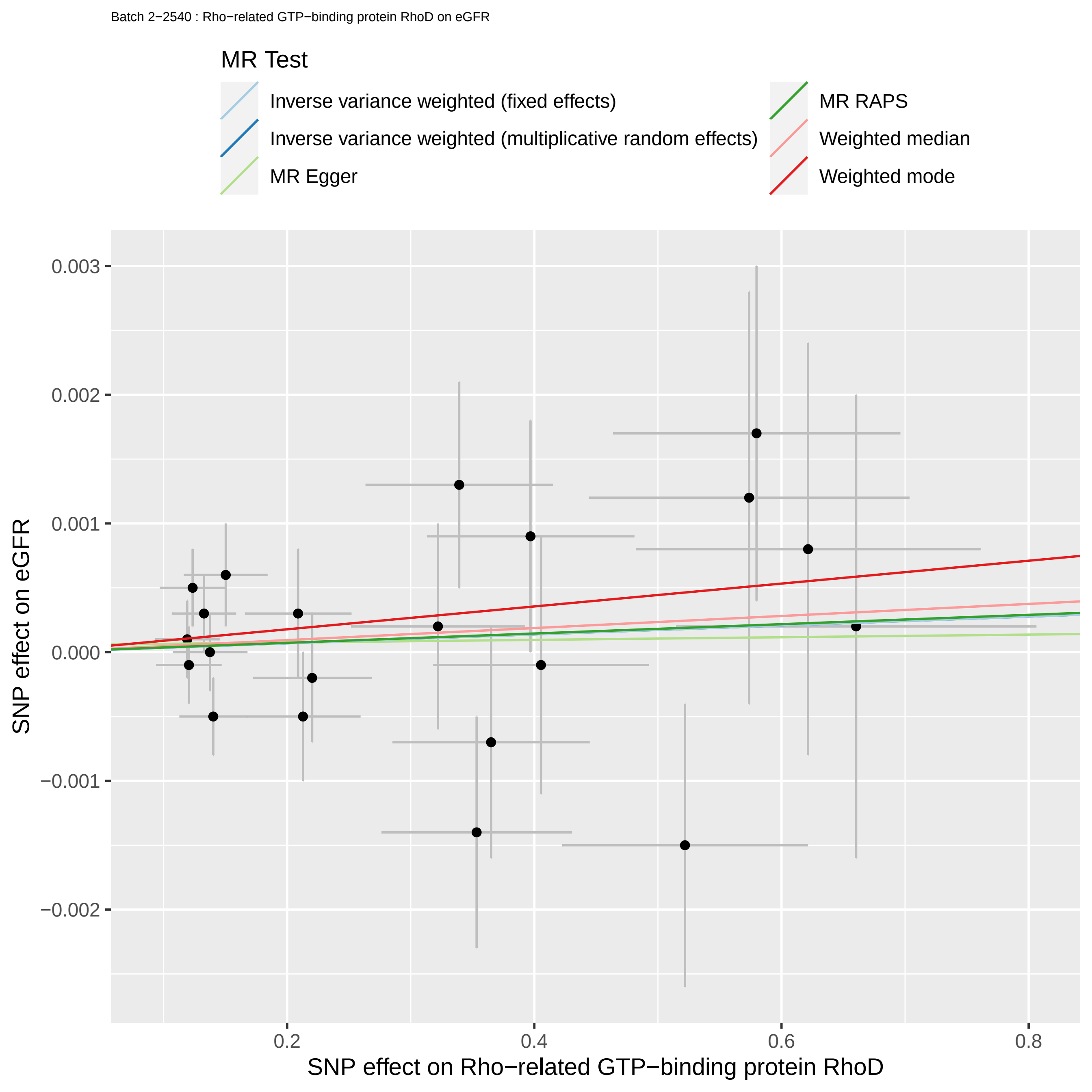
Batch 2−2540 : Rho−related GTP−binding protein RhoD on eGFR
MR Test
Inverse variance weighted (fixed effects)
Inverse variance weighted (multiplicative random effects)
MR Egger
MR RAPS
Weighted median
Weighted mode
0.003
0.002
0.001
0.000
−0.001
−0.002
SNP effect on eGFR
0.2
0.4
0.6
0.8
SNP effect on Rho−related GTP−binding protein RhoD

Batch 2−2541 : Rho−related GTP−binding protein RhoG on eGFR

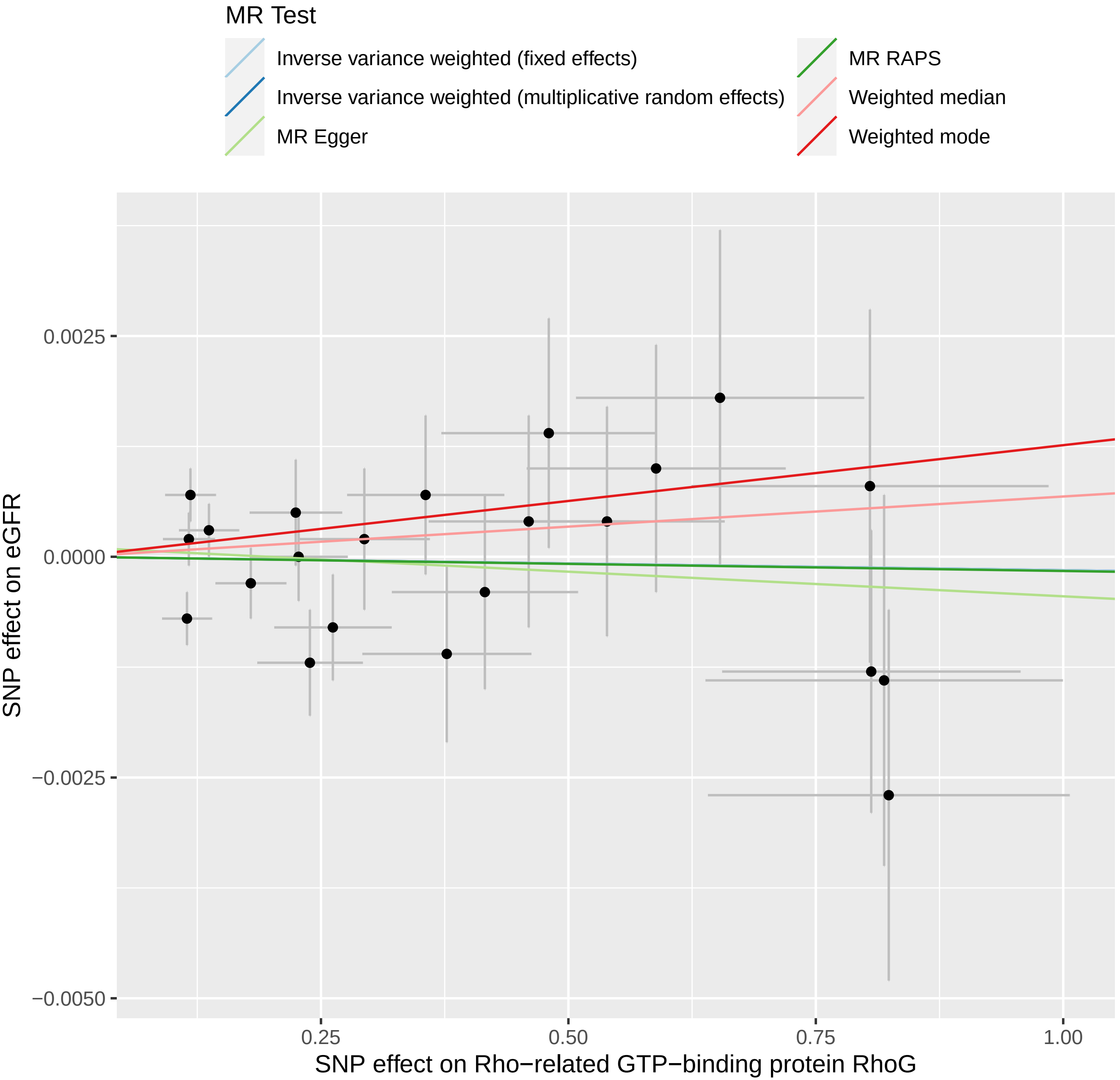

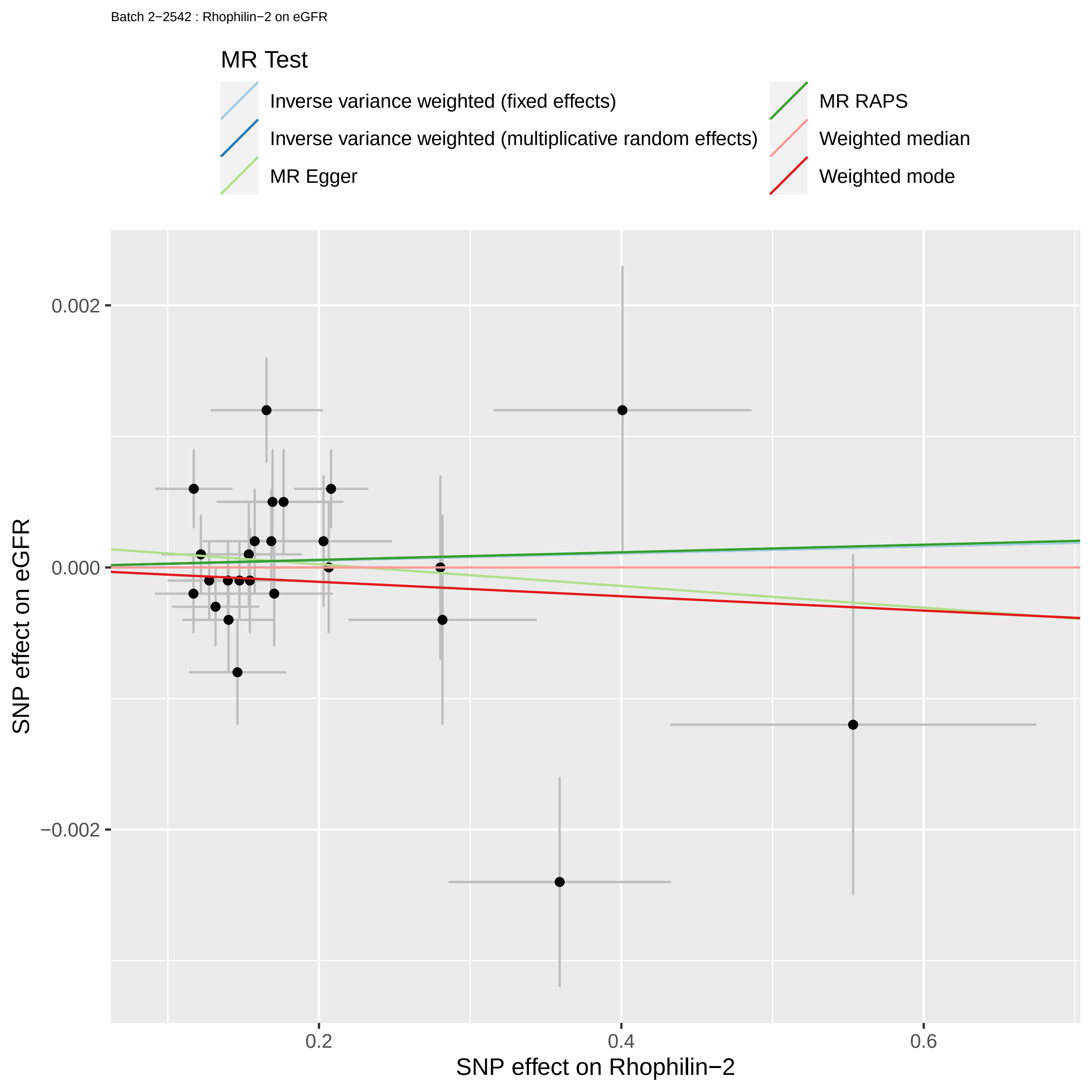

Batch 2−2542 : Rhophilin−2 on eGFR
MR Test
Inverse variance weighted (fixed effects)
Inverse variance weighted (multiplicative random effects)
MR Egger
MR RAPS
Weighted median
Weighted mode
0.002
0.000
−0.002
0.2
0.4
0.6
SNP effect on eGFR
SNP effect on Rhophilin−2

Batch 2−2543 : Protein RIC−3 on eGFR

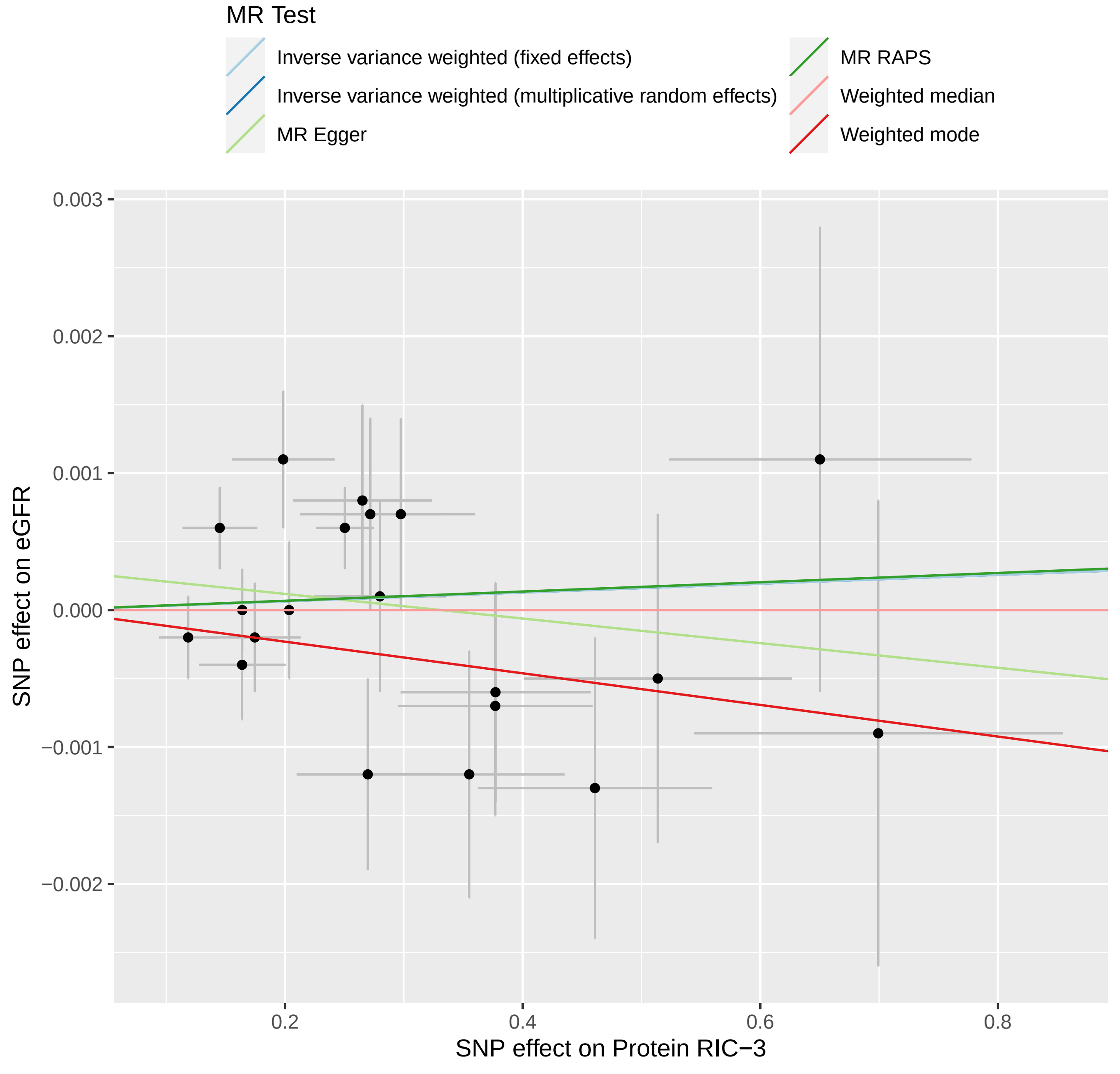

Batch 2−2544 : Protein RIC−3 on eGFR

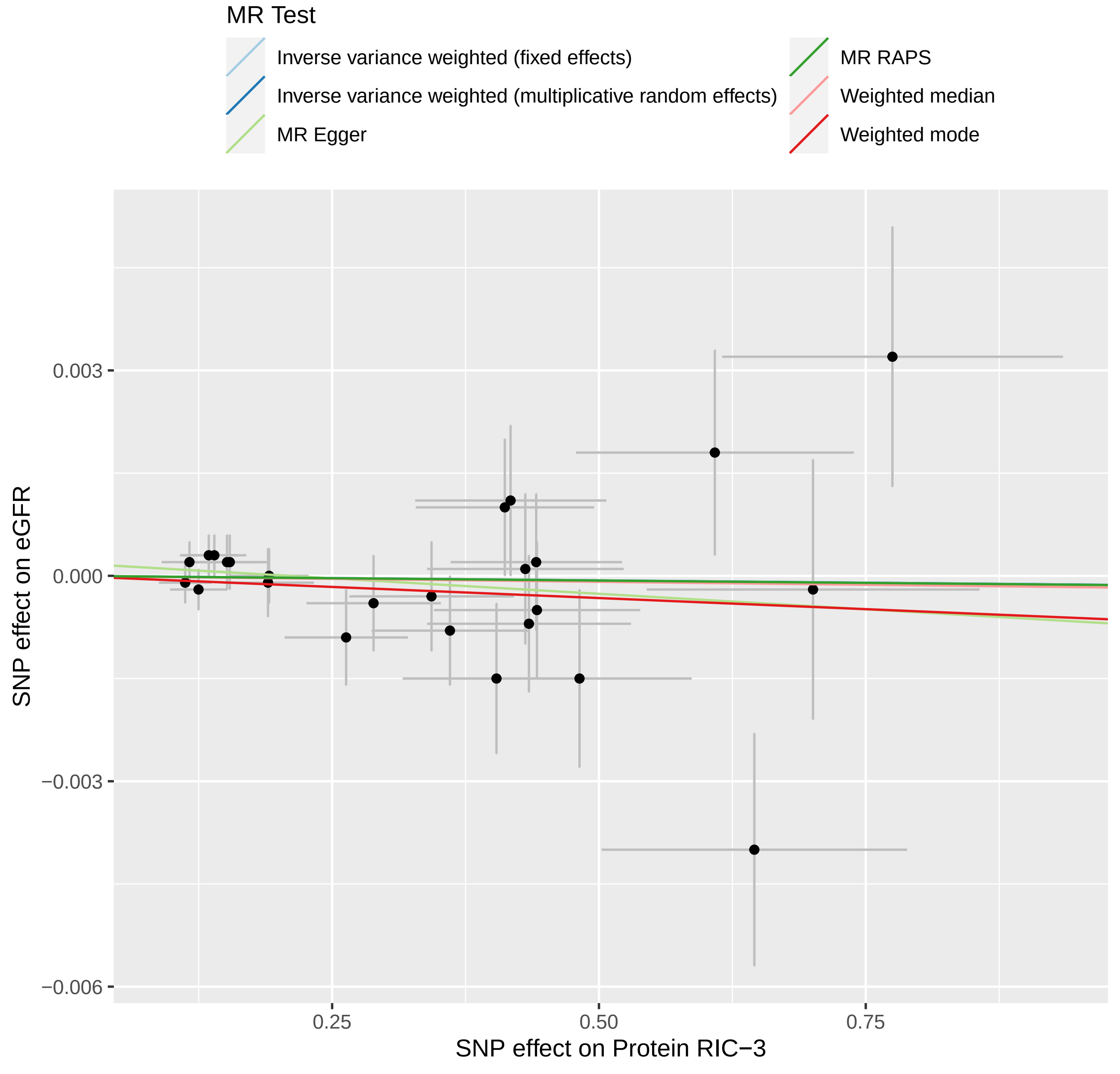

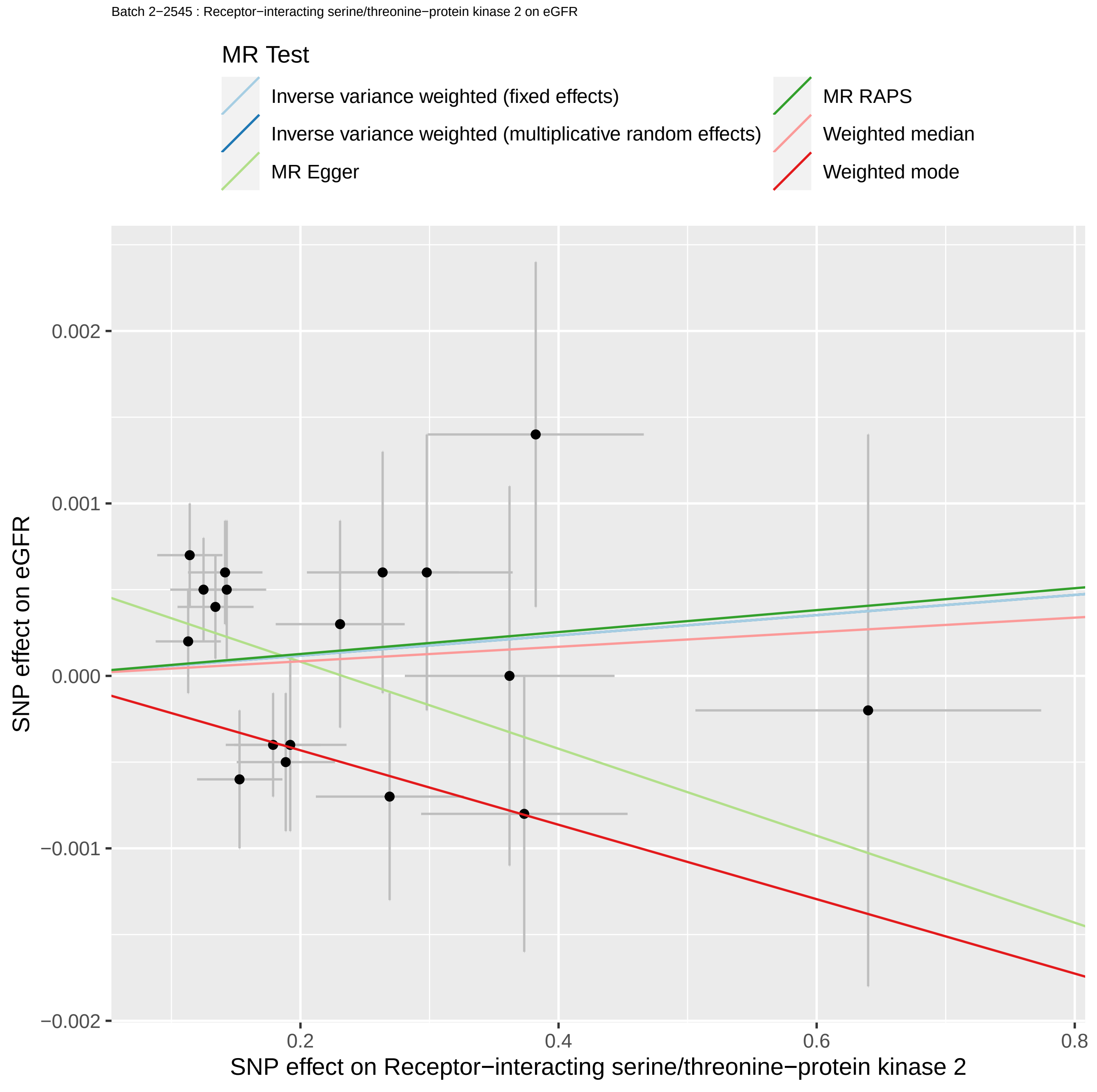

Batch 2−2545 : Receptor−interacting serine/threonine−protein kinase 2 on eGFR
MR Test
Inverse variance weighted (fixed effects)
Inverse variance weighted (multiplicative random effects)
MR Egger
MR RAPS
Weighted median
Weighted mode
0.002
0.001
0.000
−0.001
−0.002
0.2
0.4
0.6
0.8
SNP effect on eGFR
SNP effect on Receptor−interacting serine/threonine−protein kinase 2

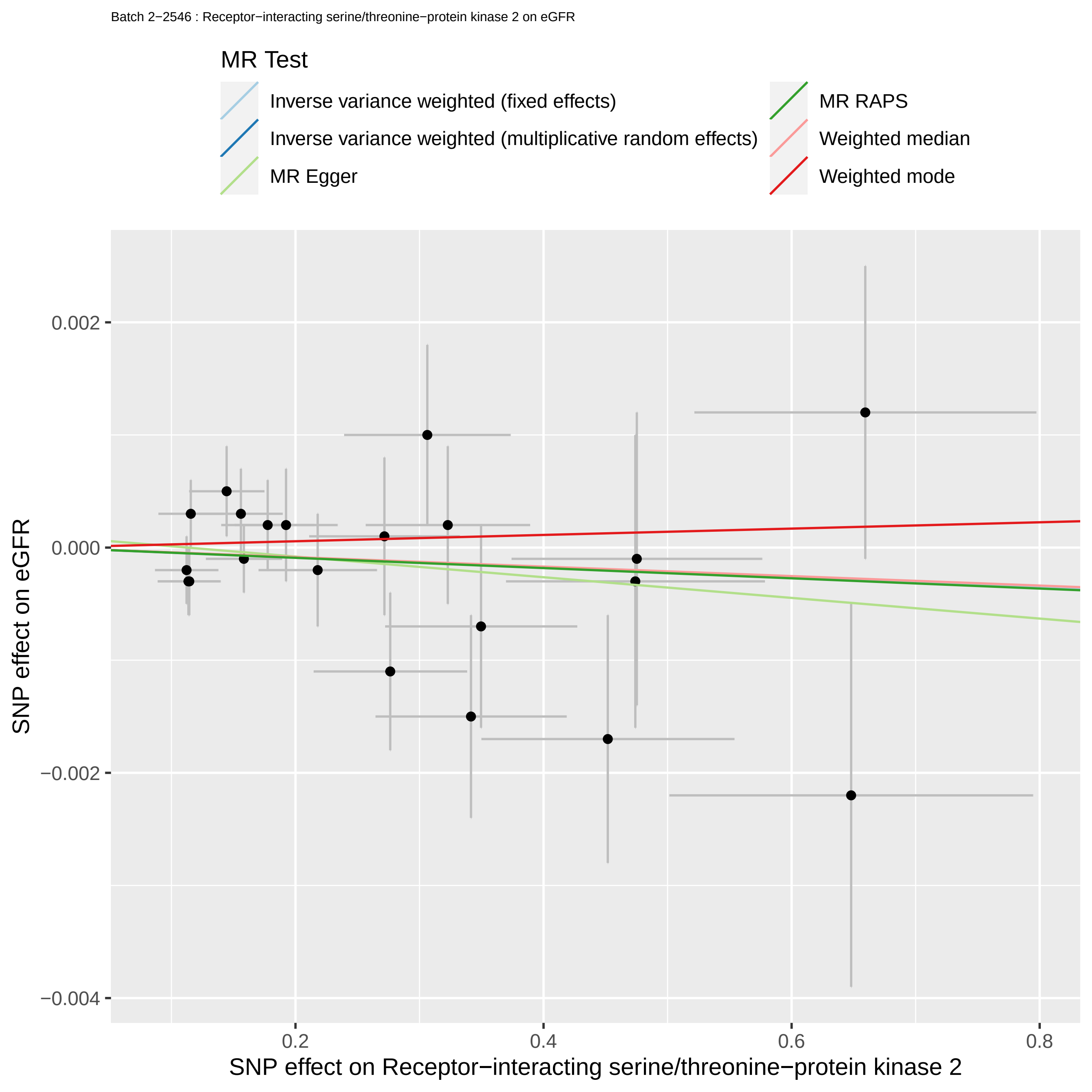

Batch 2−2546 : Receptor−interacting serine/threonine−protein kinase 2 on eGFR
MR Test
Inverse variance weighted (fixed effects)
Inverse variance weighted (multiplicative random effects)
MR Egger
MR RAPS
Weighted median
Weighted mode
0.002
0.000
−0.002
−0.004
0.2
0.4
0.6
0.8
SNP effect on eGFR
SNP effect on Receptor−interacting serine/threonine−protein kinase 2

Batch 2−2547 : Protein ripply1 on eGFR

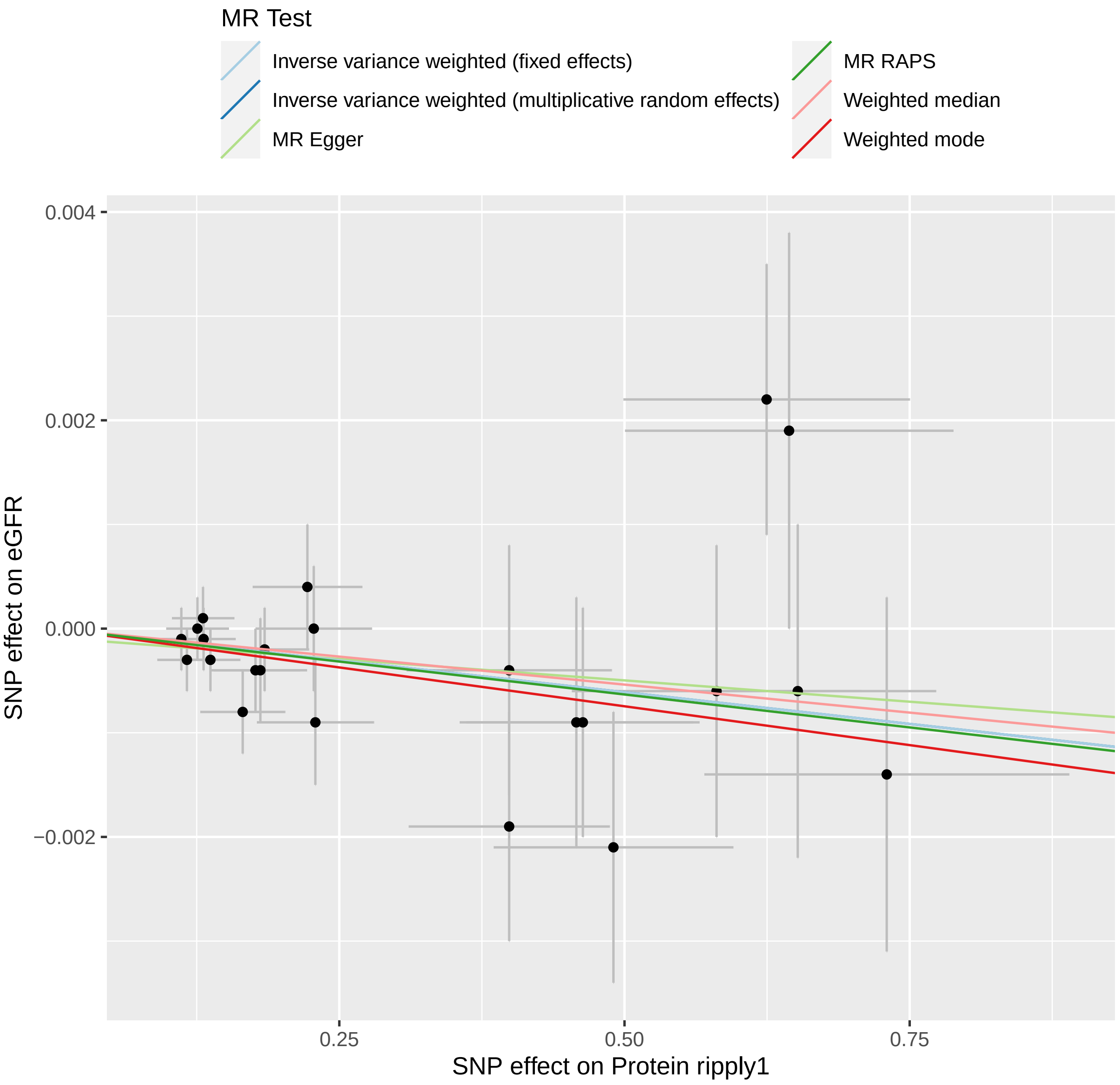

Batch 2−2548 : Retinaldehyde−binding protein 1 on eGFR

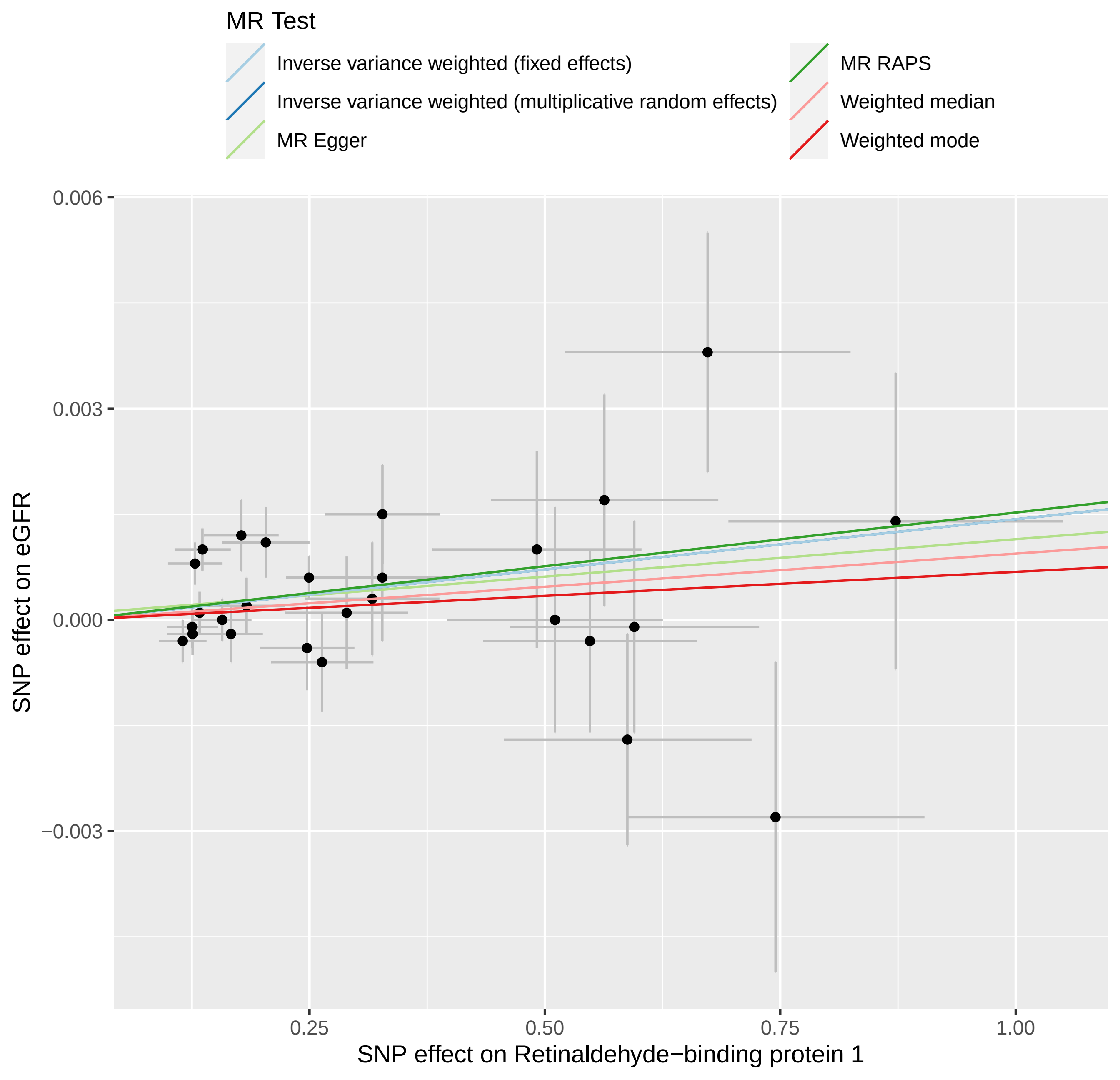

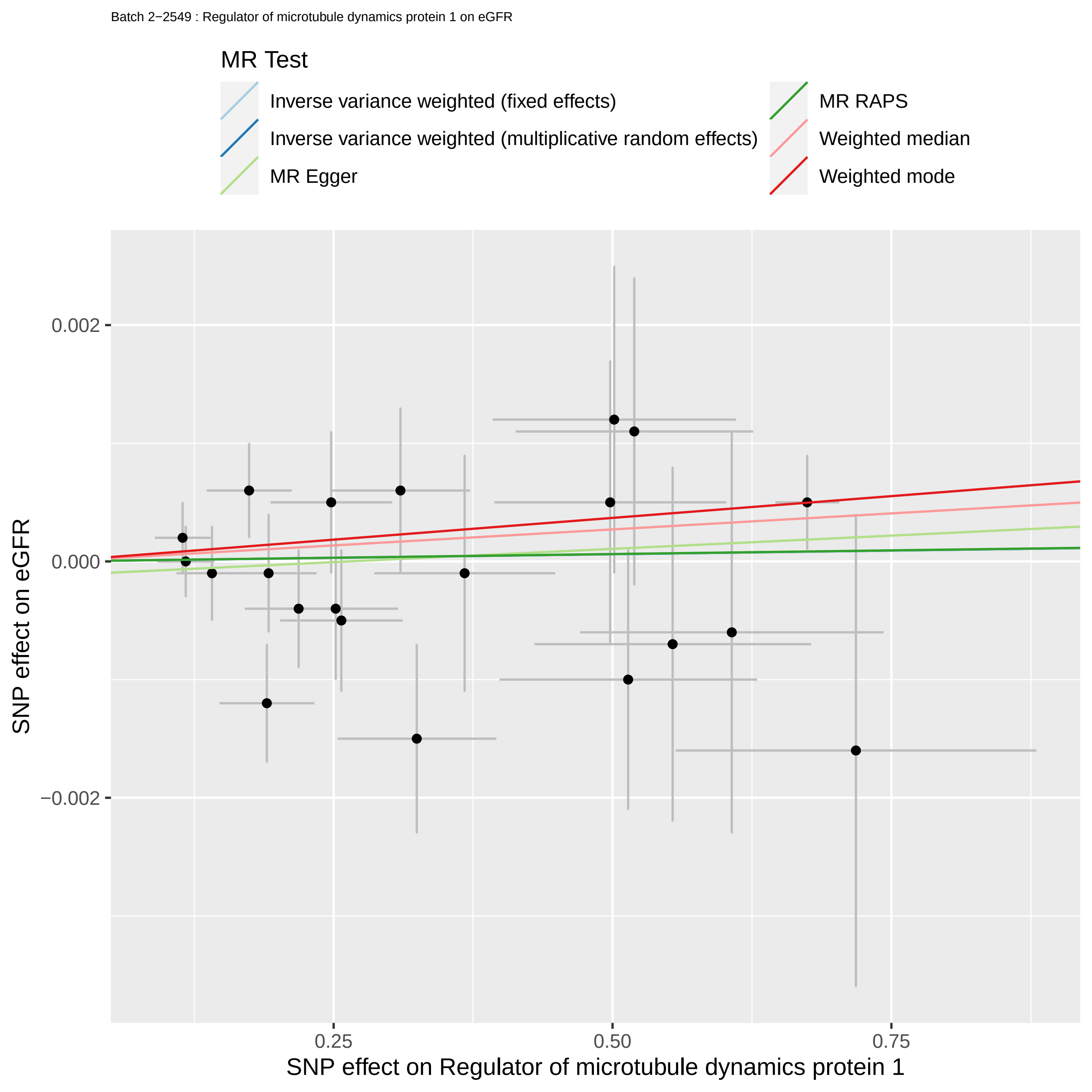
Batch 2−2549 : Regulator of microtubule dynamics protein 1 on eGFR
MR Test
Inverse variance weighted (fixed effects)
Inverse variance weighted (multiplicative random effects)
MR Egger
MR RAPS
Weighted median
Weighted mode
0.002
0.000
−0.002
0.25
0.50
0.75
SNP effect on eGFR
SNP effect on Regulator of microtubule dynamics protein 1

Batch 2−2550 : Regulator of microtubule dynamics protein 3 on eGFR

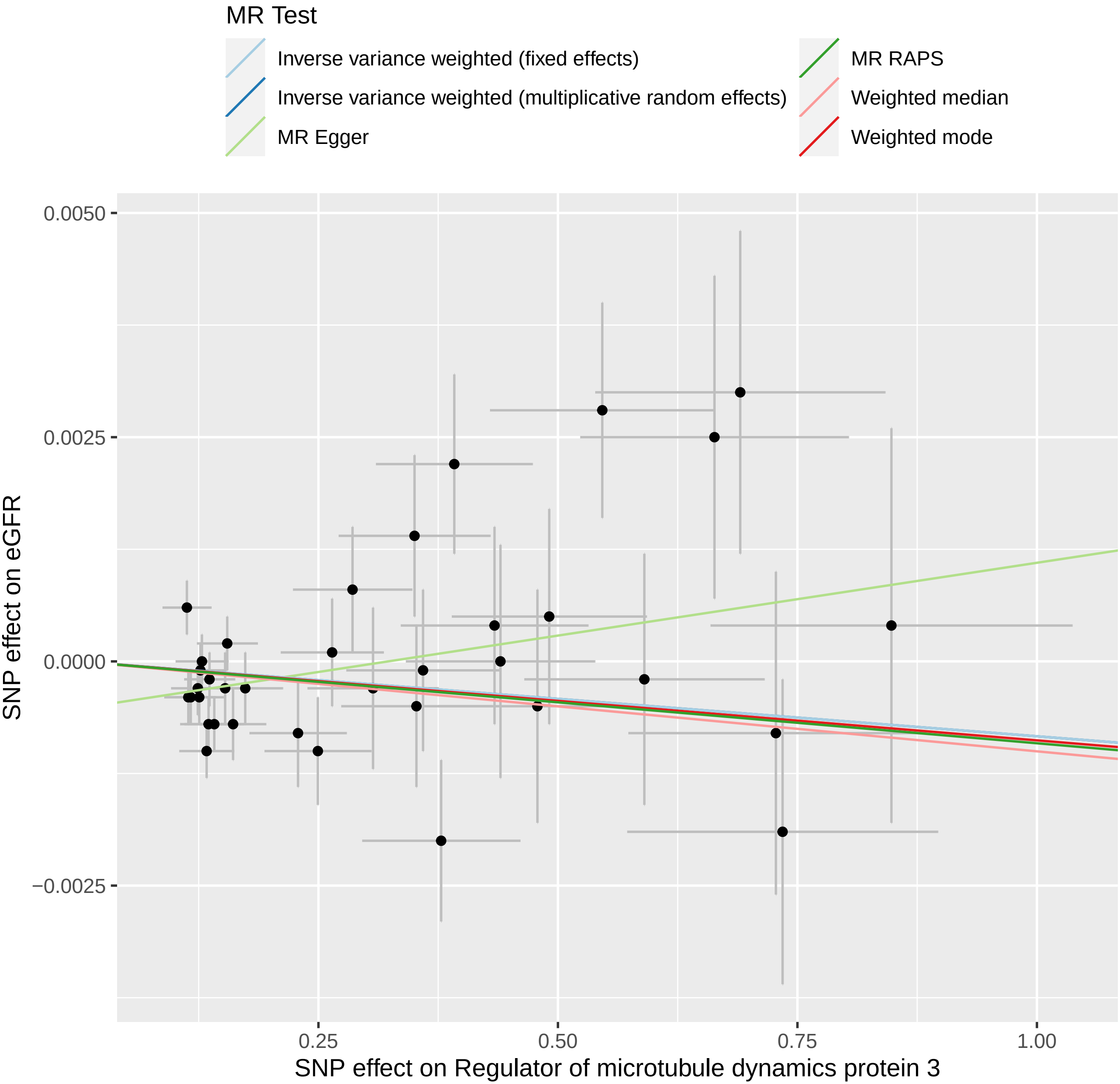

Batch 2−2551 : RecQ−mediated genome instability protein 1 on eGFR

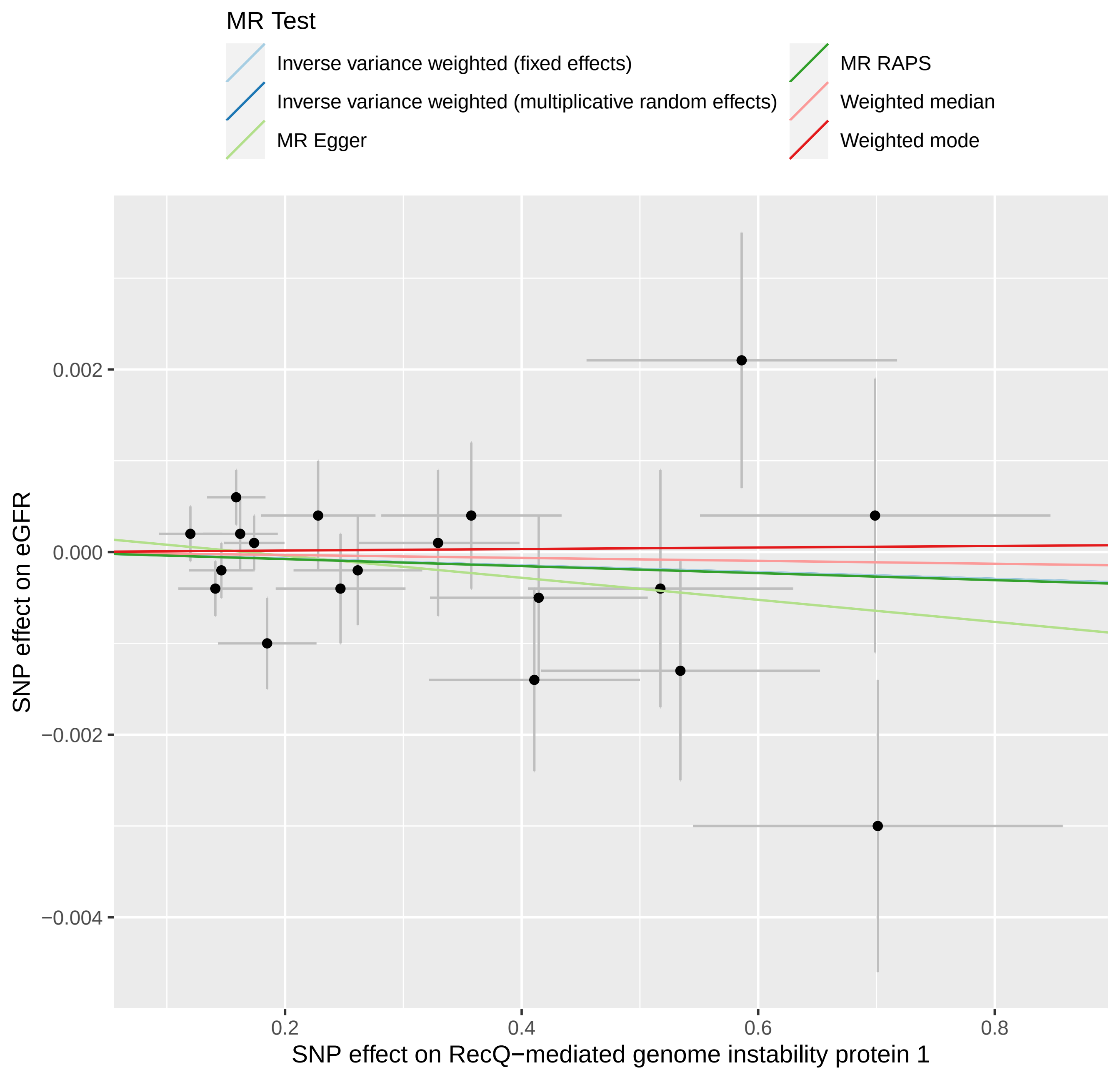

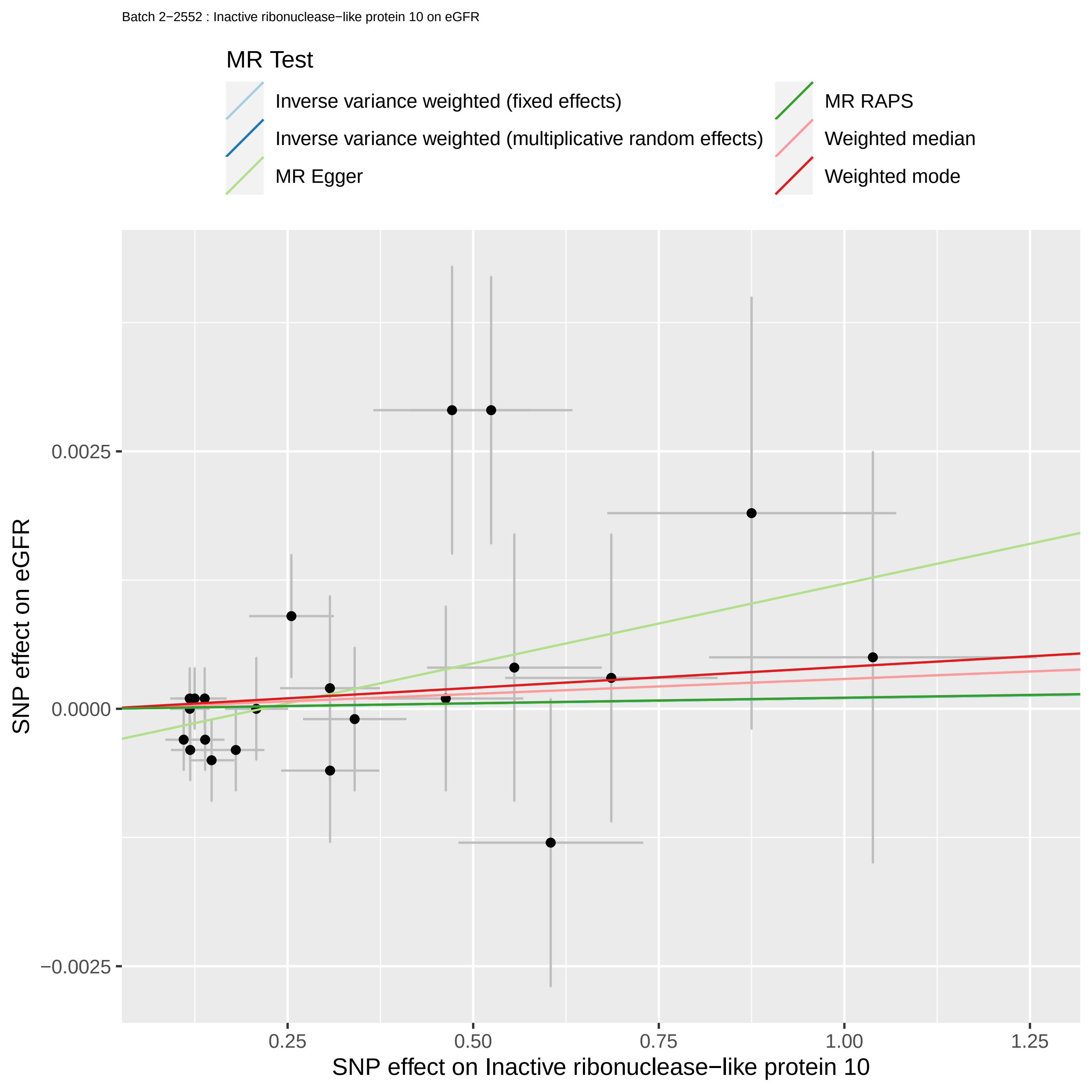
Batch 2−2552 : Inactive ribonuclease−like protein 10 on eGFR
MR Test
Inverse variance weighted (fixed effects)
Inverse variance weighted (multiplicative random effects)
MR Egger
MR RAPS
Weighted median
Weighted mode
0.0025
0.0000
−0.0025
0.25
0.50
0.75
1.00
1.25
SNP effect on eGFR
SNP effect on Inactive ribonuclease−like protein 10

Batch 2−2553 : Probable inactive ribonuclease−like protein 13 on eGFR

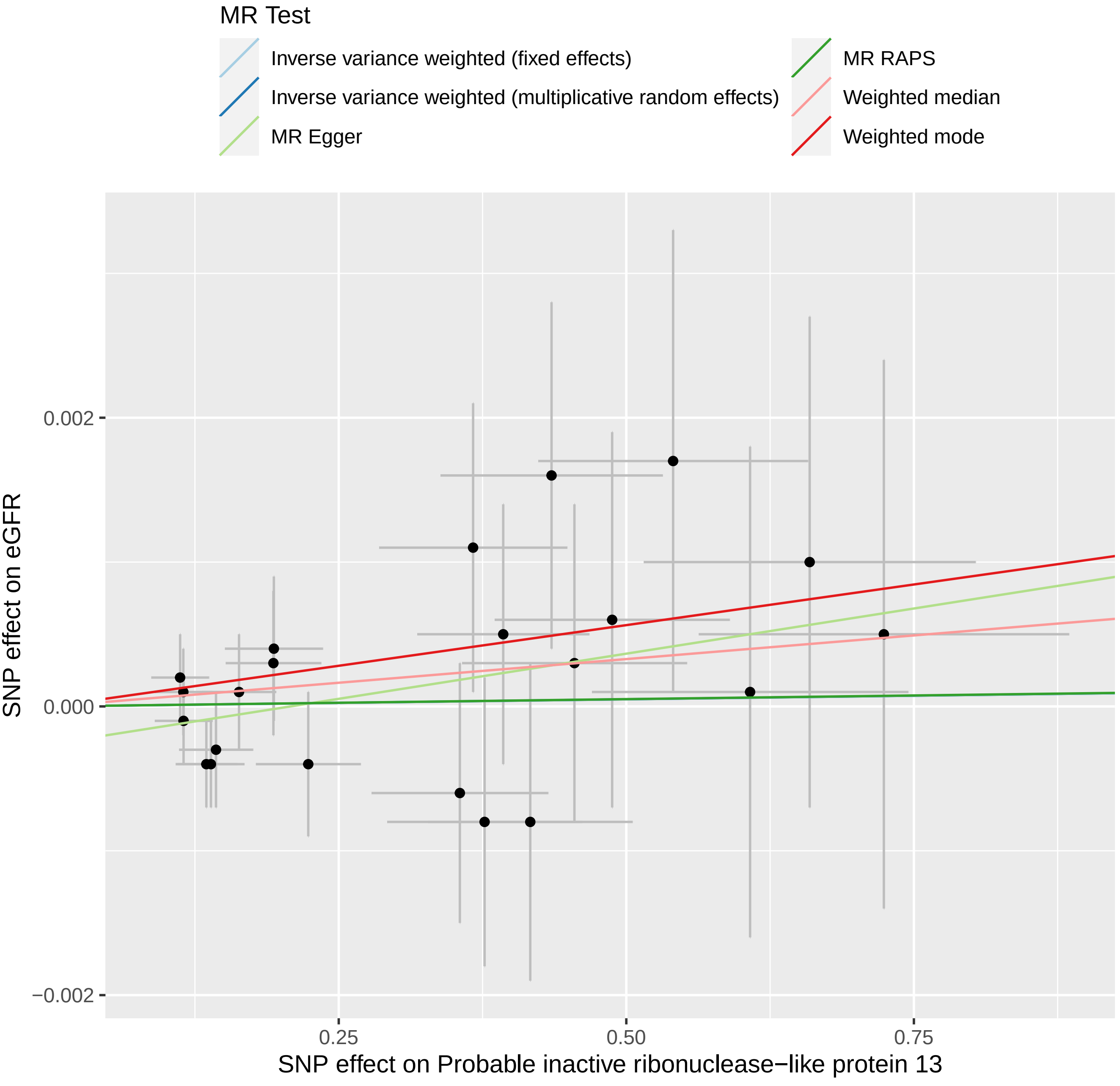

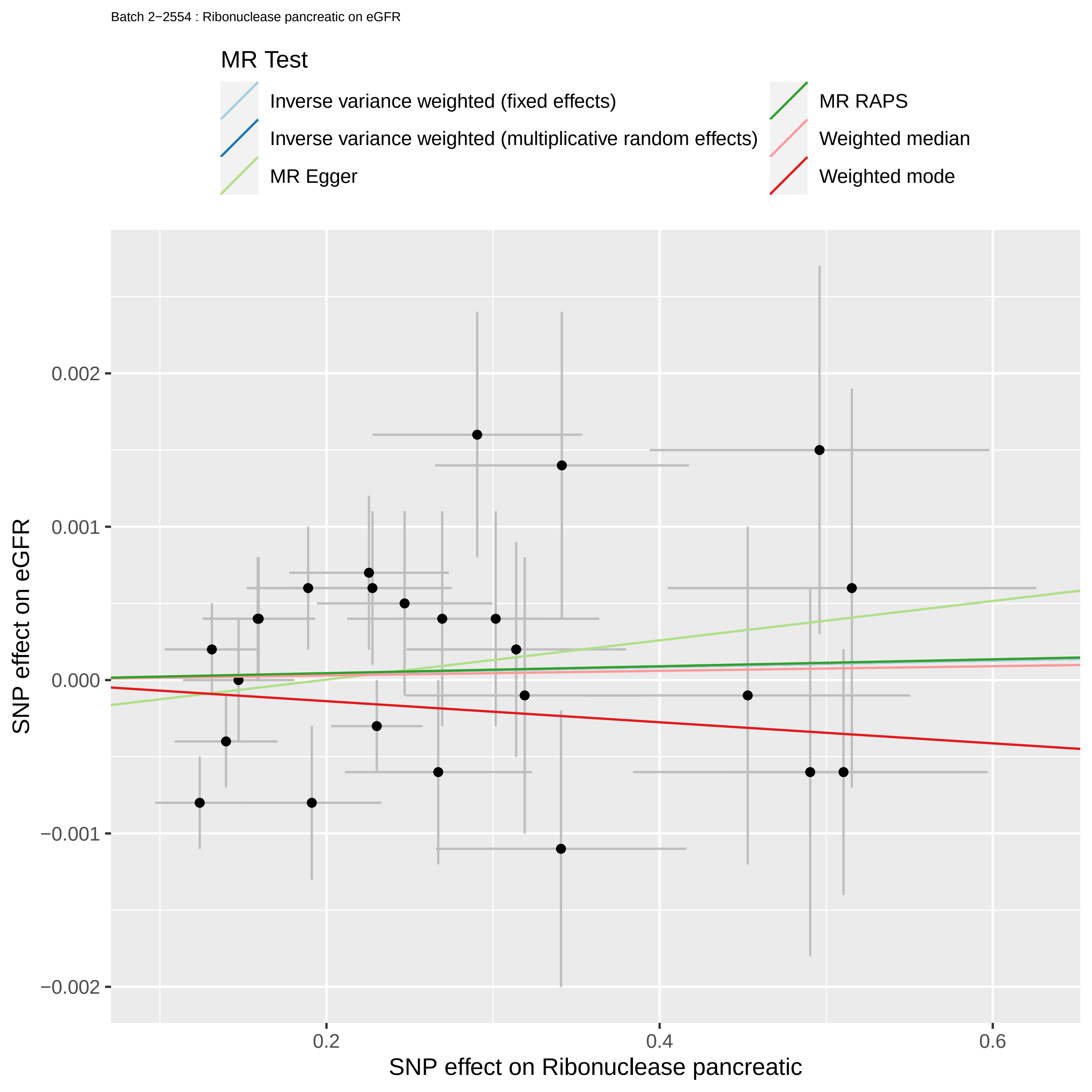
Batch 2−2554 : Ribonuclease pancreatic on eGFR
MR Test
Inverse variance weighted (fixed effects)
Inverse variance weighted (multiplicative random effects)
MR Egger
MR RAPS
Weighted median
Weighted mode
0.002
0.001
0.000
−0.001
−0.002
0.2
0.4
0.6
SNP effect on eGFR
SNP effect on Ribonuclease pancreatic

Batch 2−2555 : Non−secretory ribonuclease on eGFR

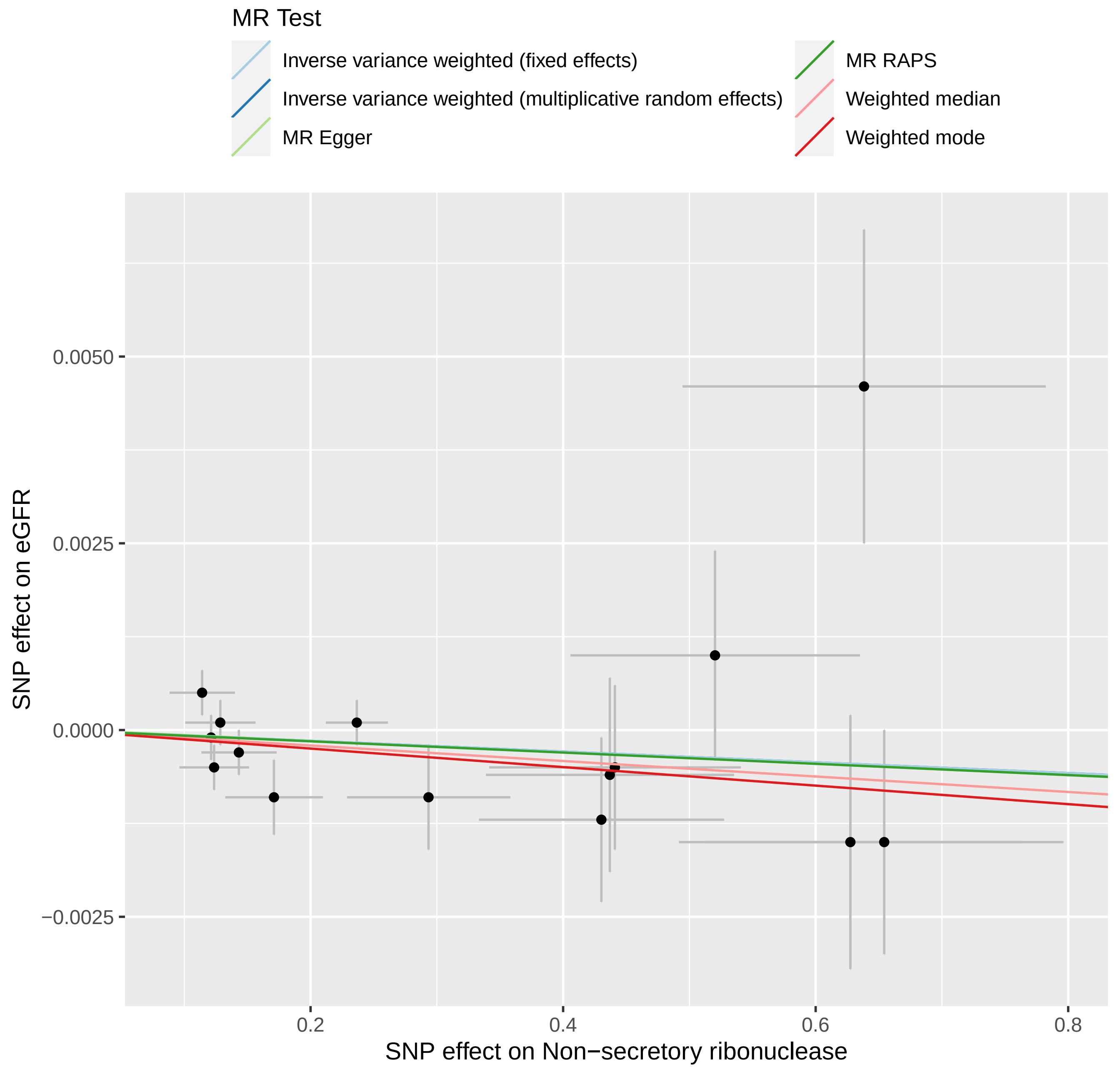

Batch 2−2556 : Ribonuclease 4 on eGFR

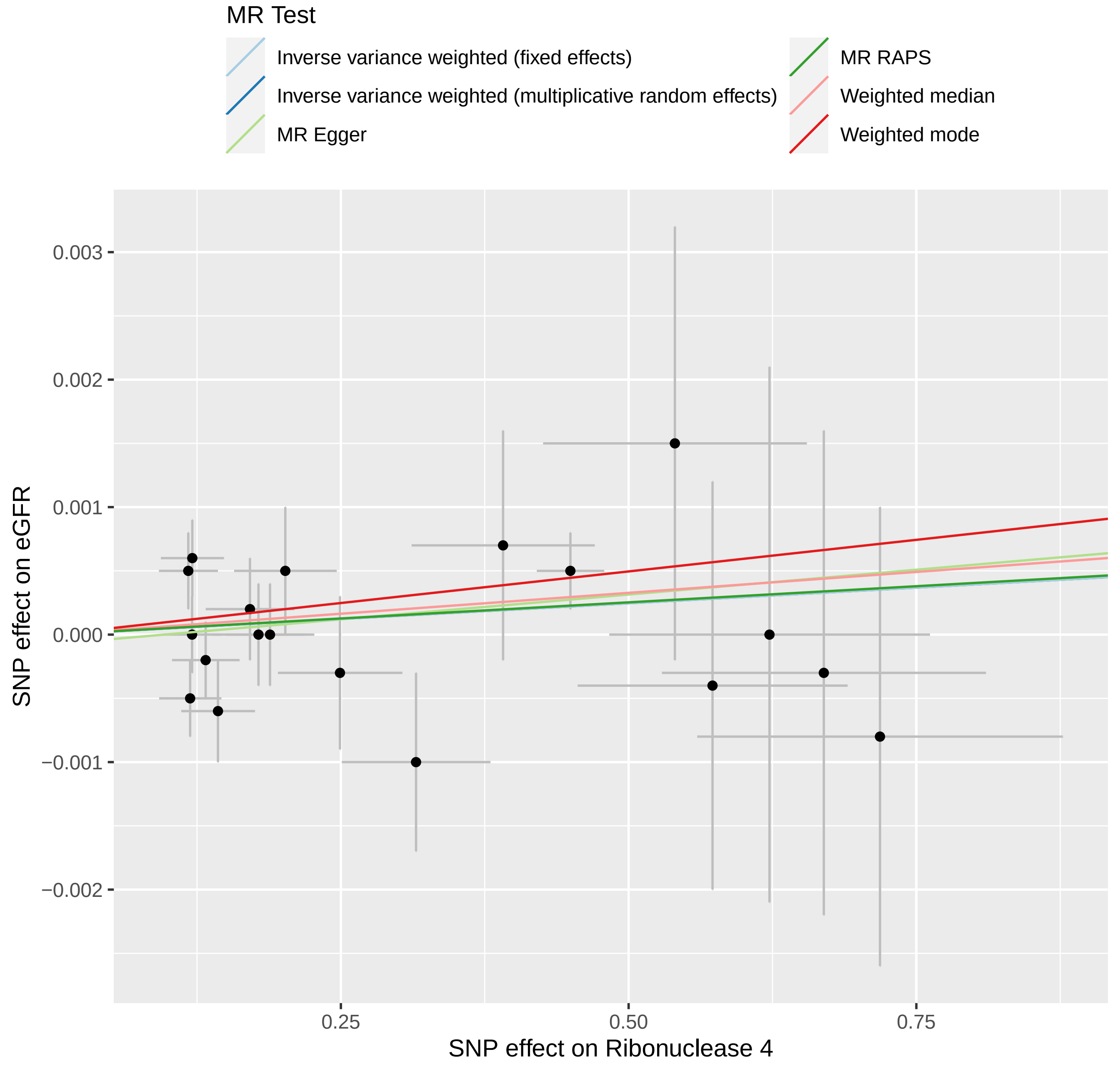

Batch 2−2557 : Ribonuclease K6 on eGFR

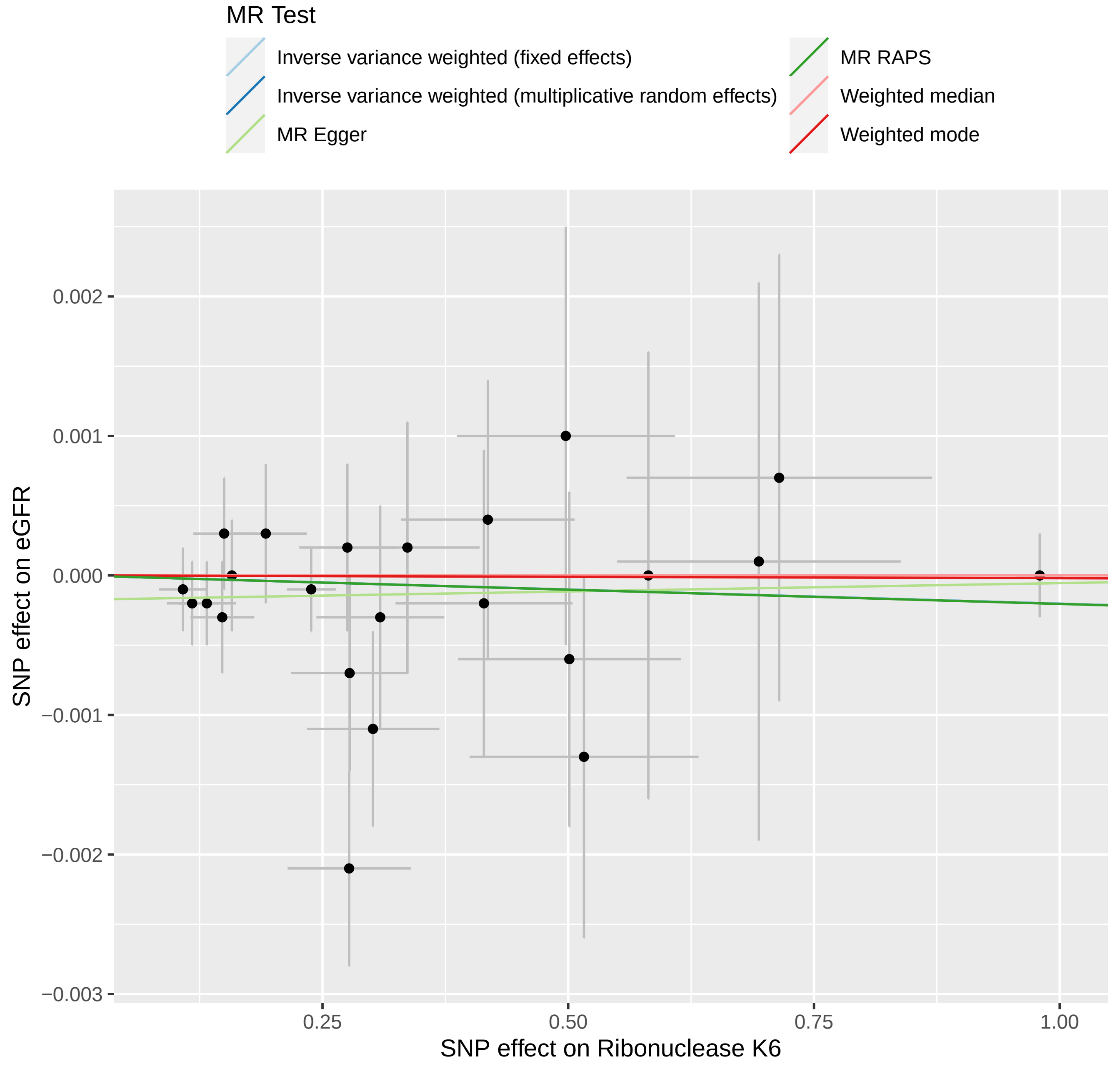

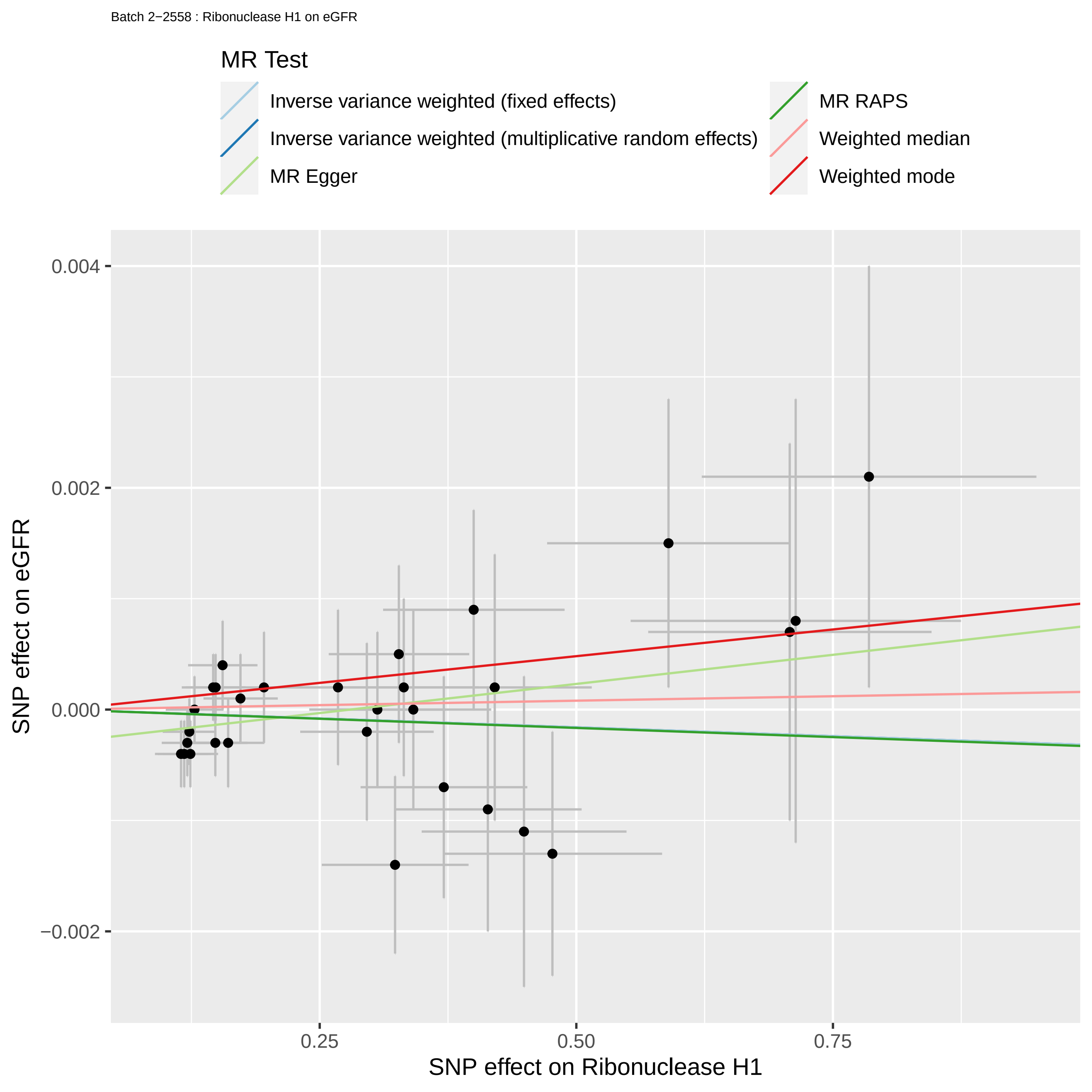

Batch 2−2558 : Ribonuclease H1 on eGFR
MR Test
Inverse variance weighted (fixed effects)
Inverse variance weighted (multiplicative random effects)
MR Egger
MR RAPS
Weighted median
Weighted mode
0.004
0.002
0.000
−0.002
0.25
0.50
0.75
SNP effect on eGFR
SNP effect on Ribonuclease H1

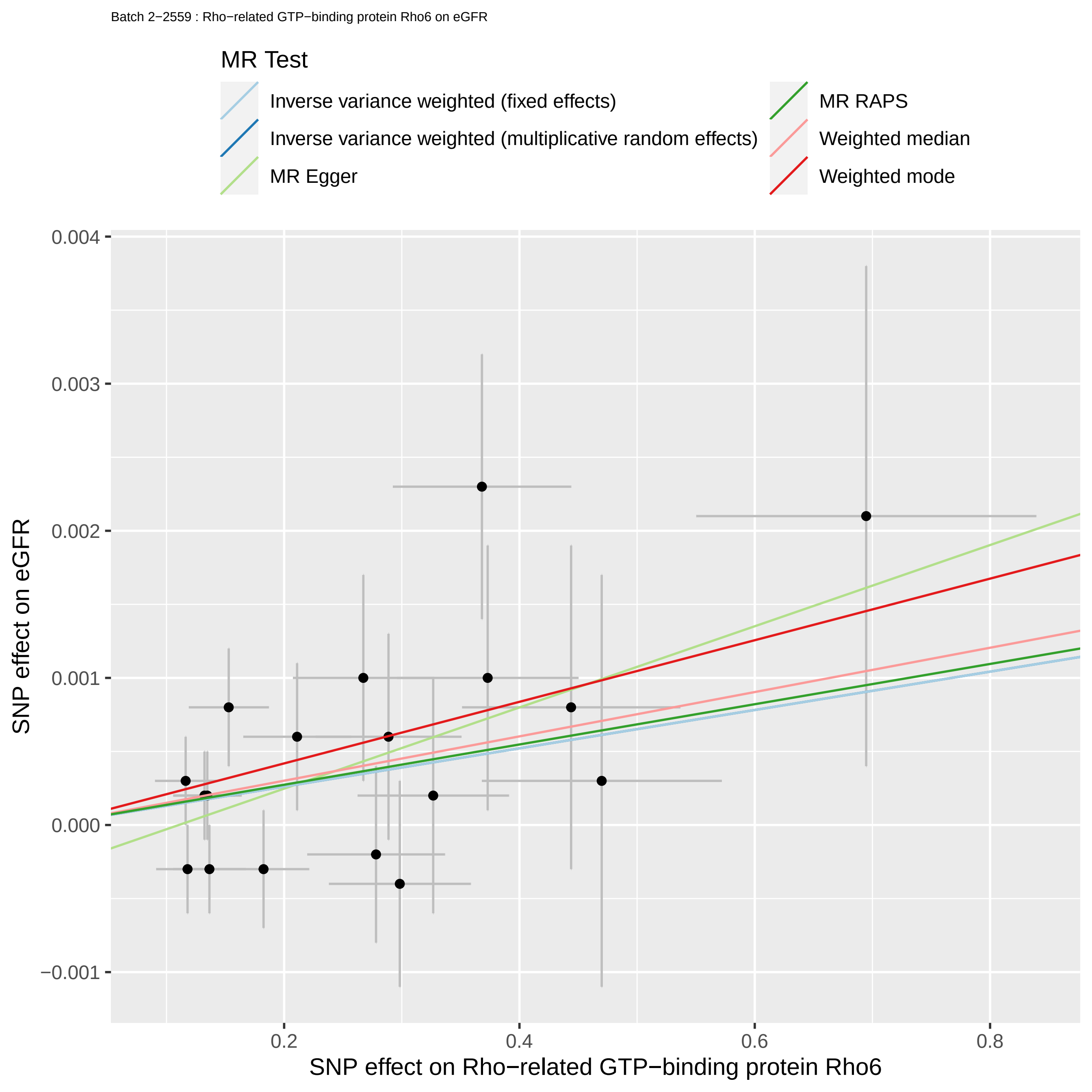

Batch 2−2559 : Rho−related GTP−binding protein Rho6 on eGFR
MR Test
Inverse variance weighted (fixed effects)
Inverse variance weighted (multiplicative random effects)
MR Egger
MR RAPS
Weighted median
Weighted mode
0.004
0.003
0.002
0.001
0.000
−0.001
SNP effect on eGFR
0.2
0.4
0.6
0.8
SNP effect on Rho−related GTP−binding protein Rho6

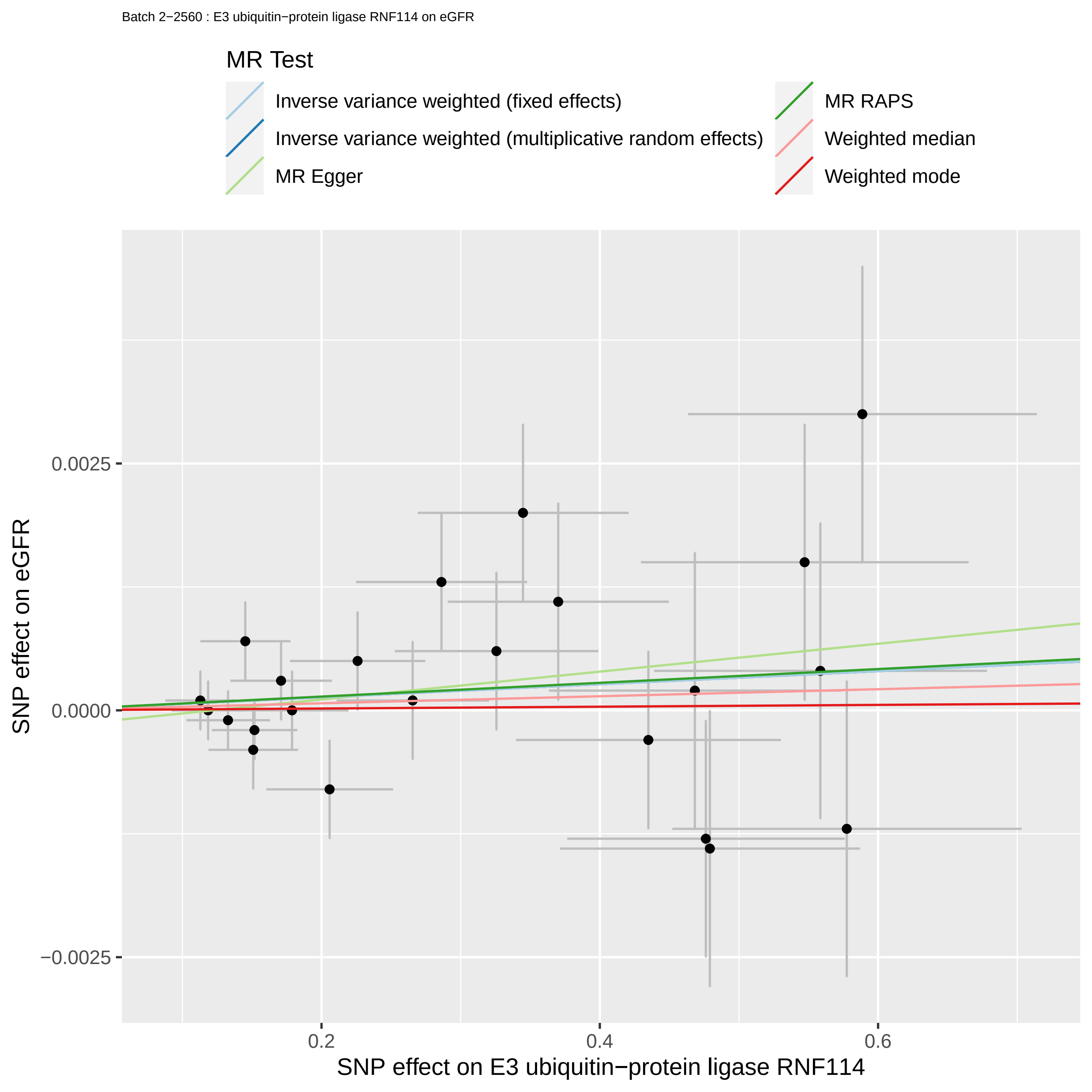

Batch 2−2560 : E3 ubiquitin−protein ligase RNF114 on eGFR
MR Test
Inverse variance weighted (fixed effects)
Inverse variance weighted (multiplicative random effects)
MR Egger
MR RAPS
Weighted median
Weighted mode
0.0025
0.0000
−0.0025
0.2
0.4
0.6
SNP effect on eGFR
SNP effect on E3 ubiquitin−protein ligase RNF114

Batch 2−2561 : RING finger protein 122 on eGFR

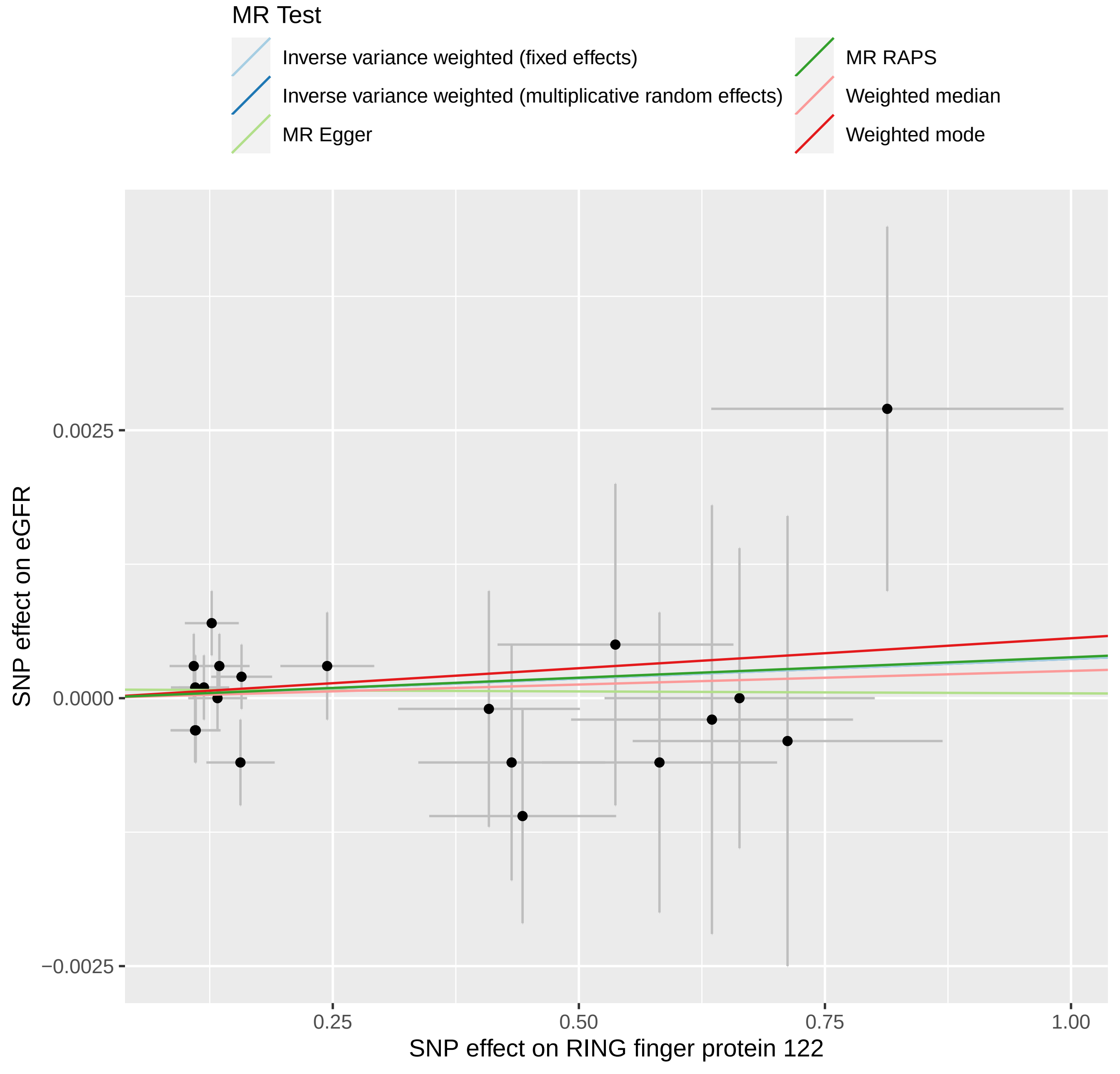

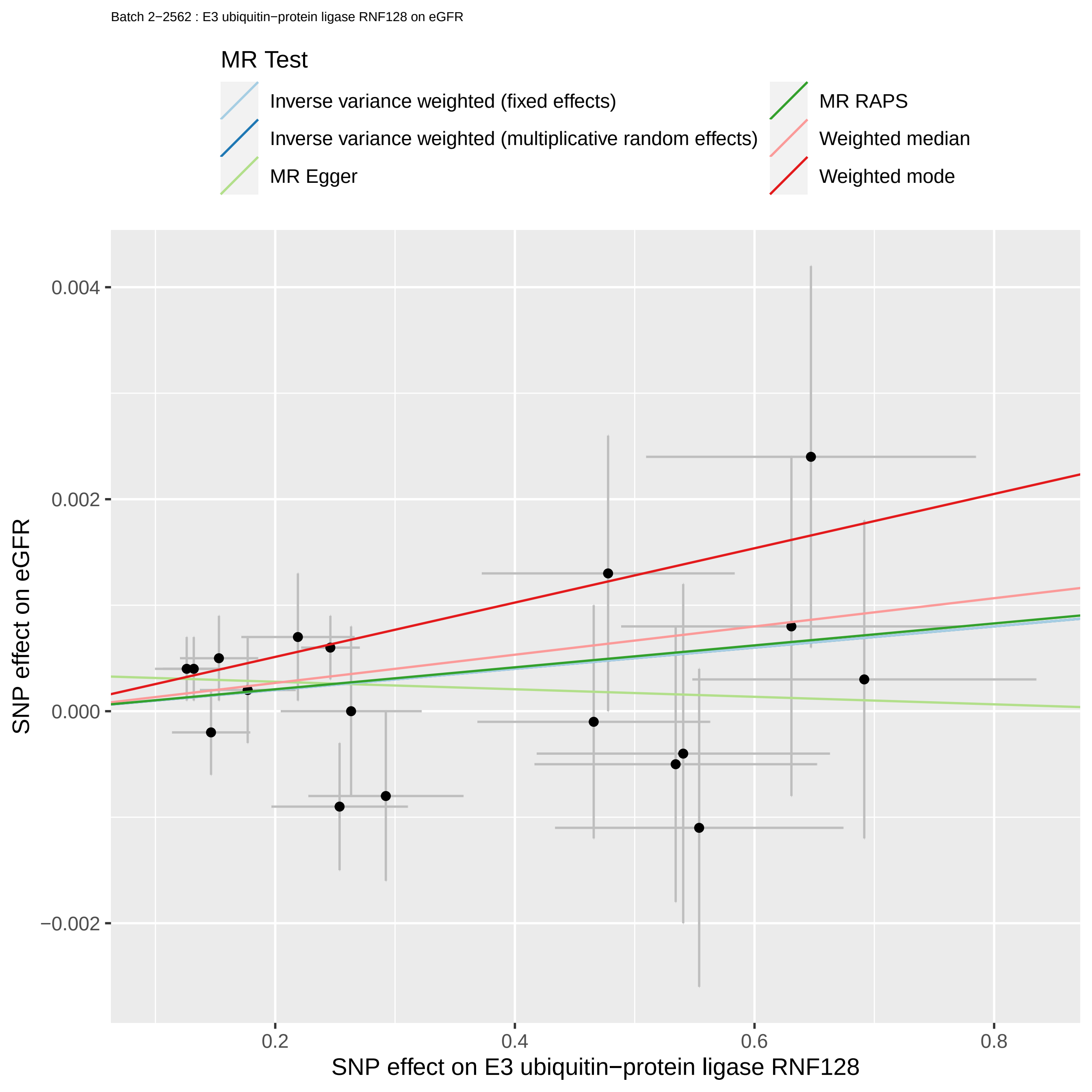

Batch 2−2562 : E3 ubiquitin−protein ligase RNF128 on eGFR
MR Test
Inverse variance weighted (fixed effects)
Inverse variance weighted (multiplicative random effects)
MR Egger
MR RAPS
Weighted median
Weighted mode
0.004
0.002
0.000
−0.002
0.2
0.4
0.6
0.8
SNP effect on eGFR
SNP effect on E3 ubiquitin−protein ligase RNF128

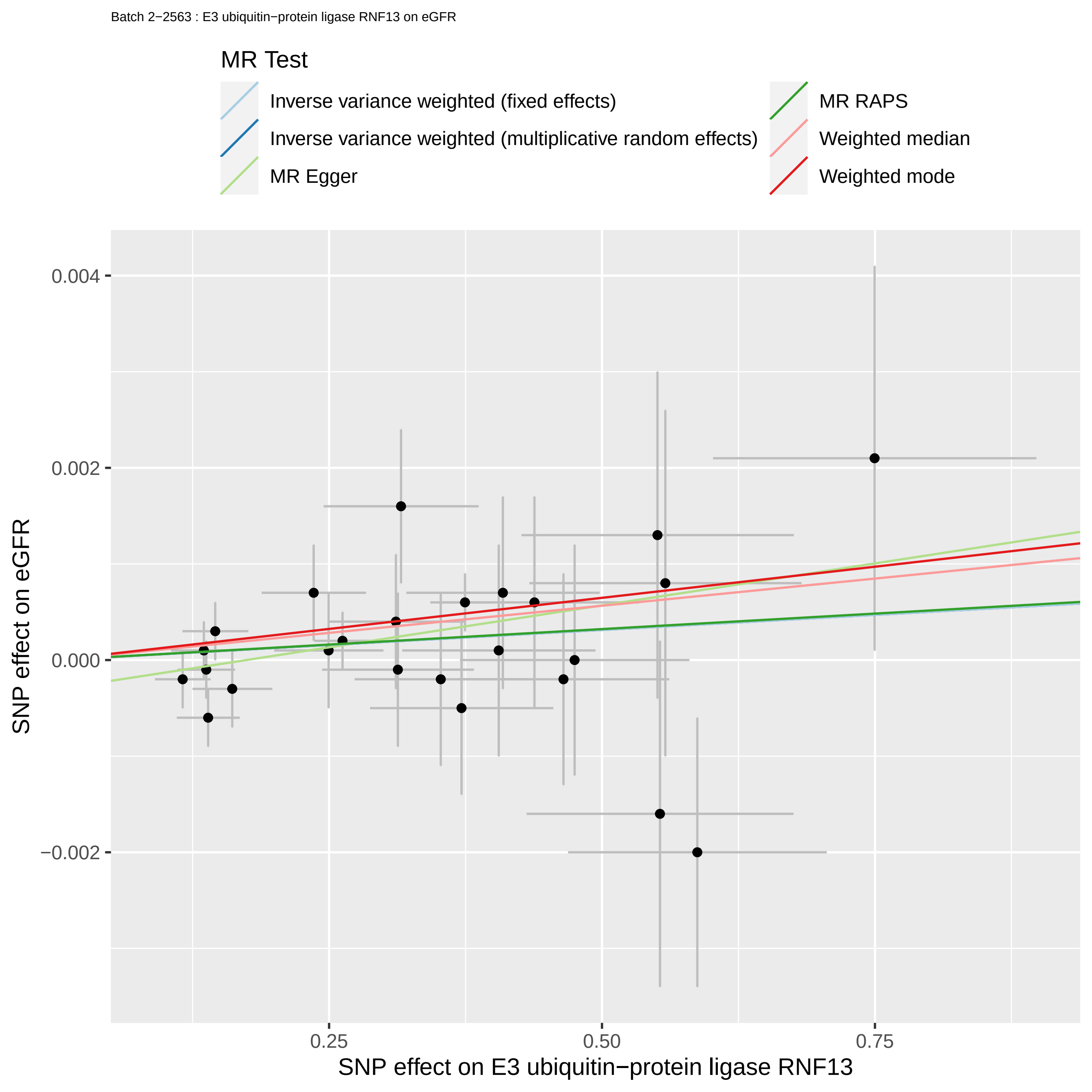

Batch 2−2563 : E3 ubiquitin−protein ligase RNF13 on eGFR
MR Test
Inverse variance weighted (fixed effects)
Inverse variance weighted (multiplicative random effects)
MR Egger
MR RAPS
Weighted median
Weighted mode
0.004
0.002
0.000
−0.002
0.25
0.50
0.75
SNP effect on eGFR
SNP effect on E3 ubiquitin−protein ligase RNF13

Batch 2−2564 : RING finger protein 148 on eGFR

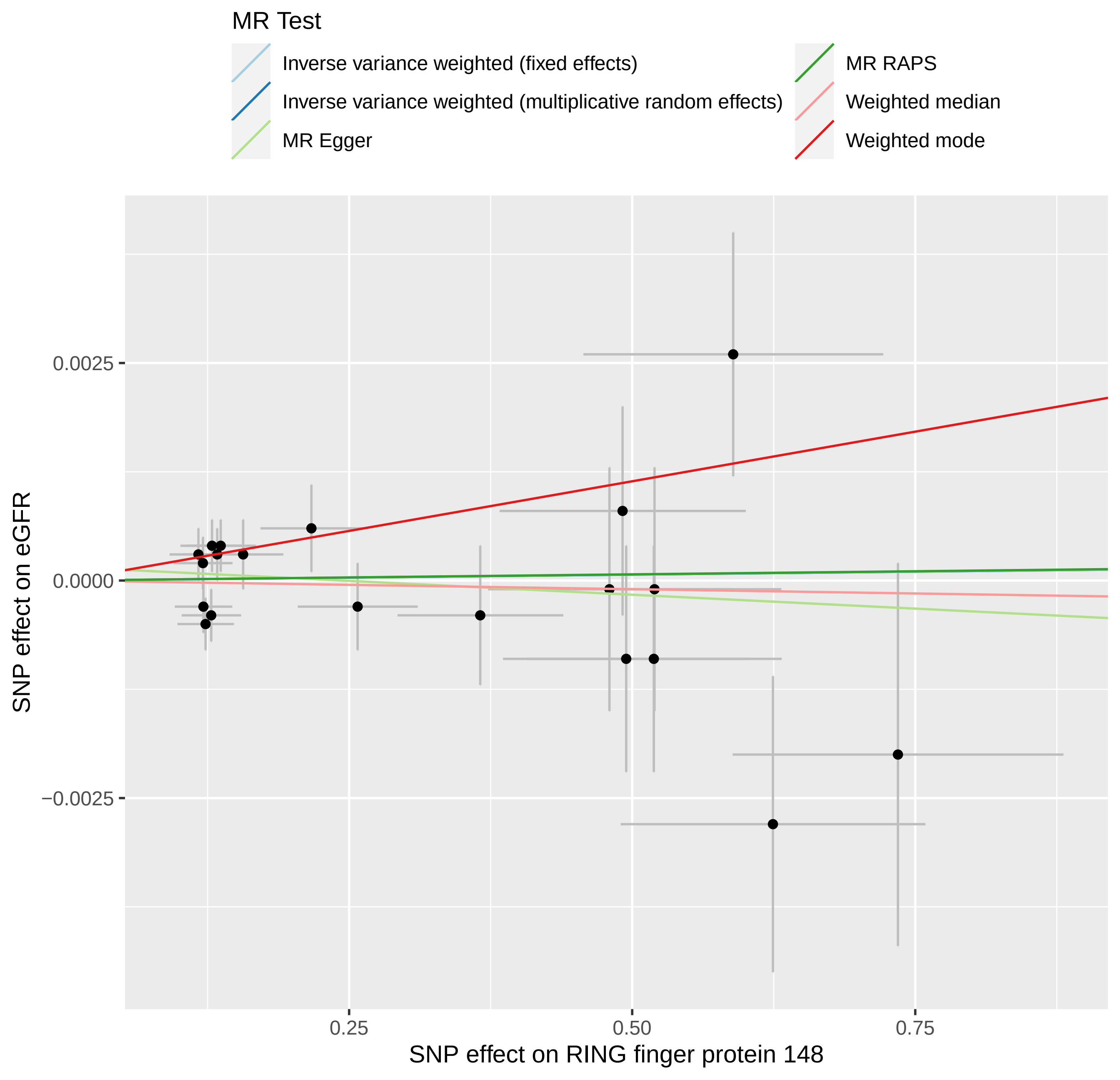

Batch 2−2565 : RING finger protein 148 on eGFR

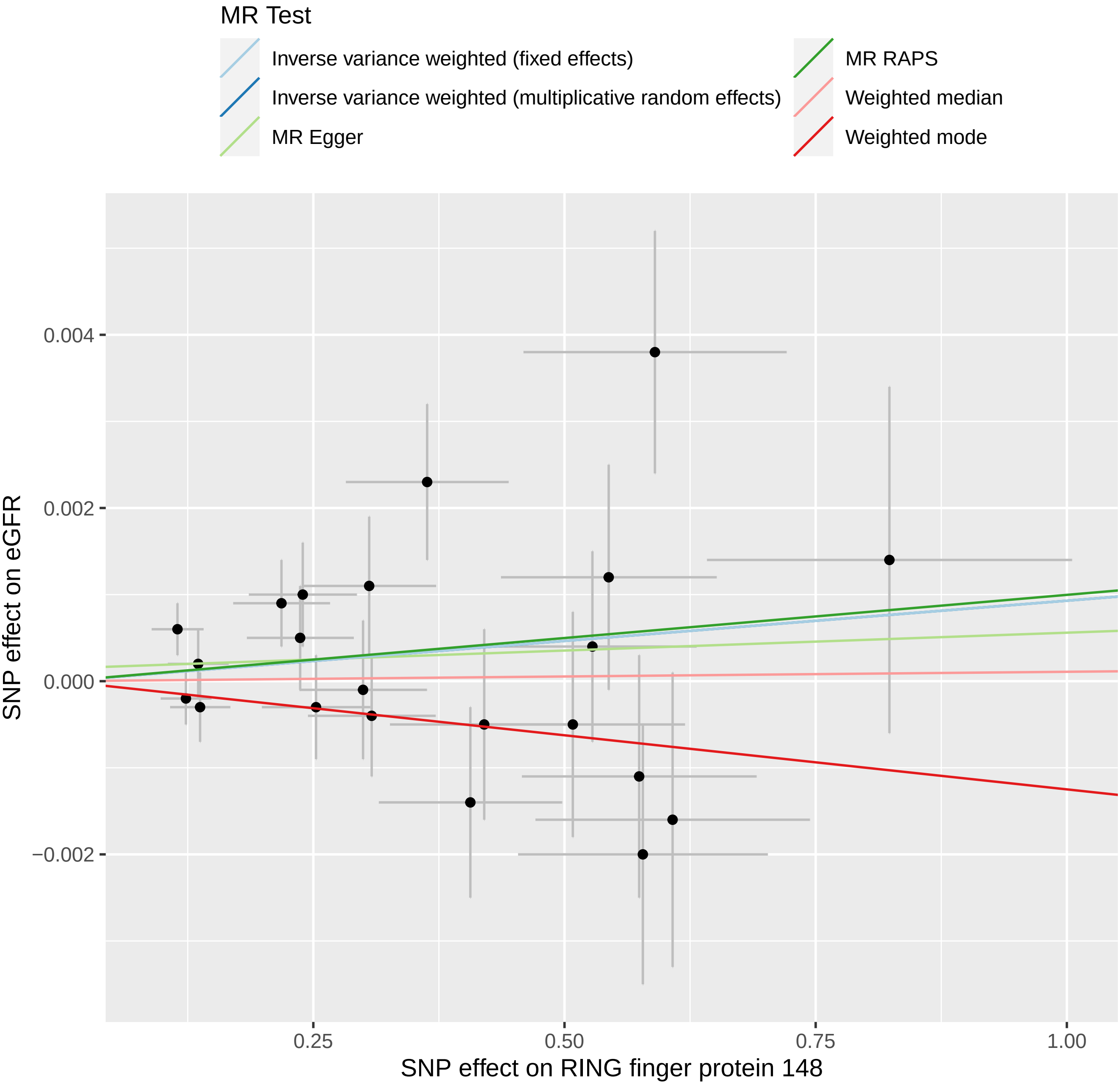

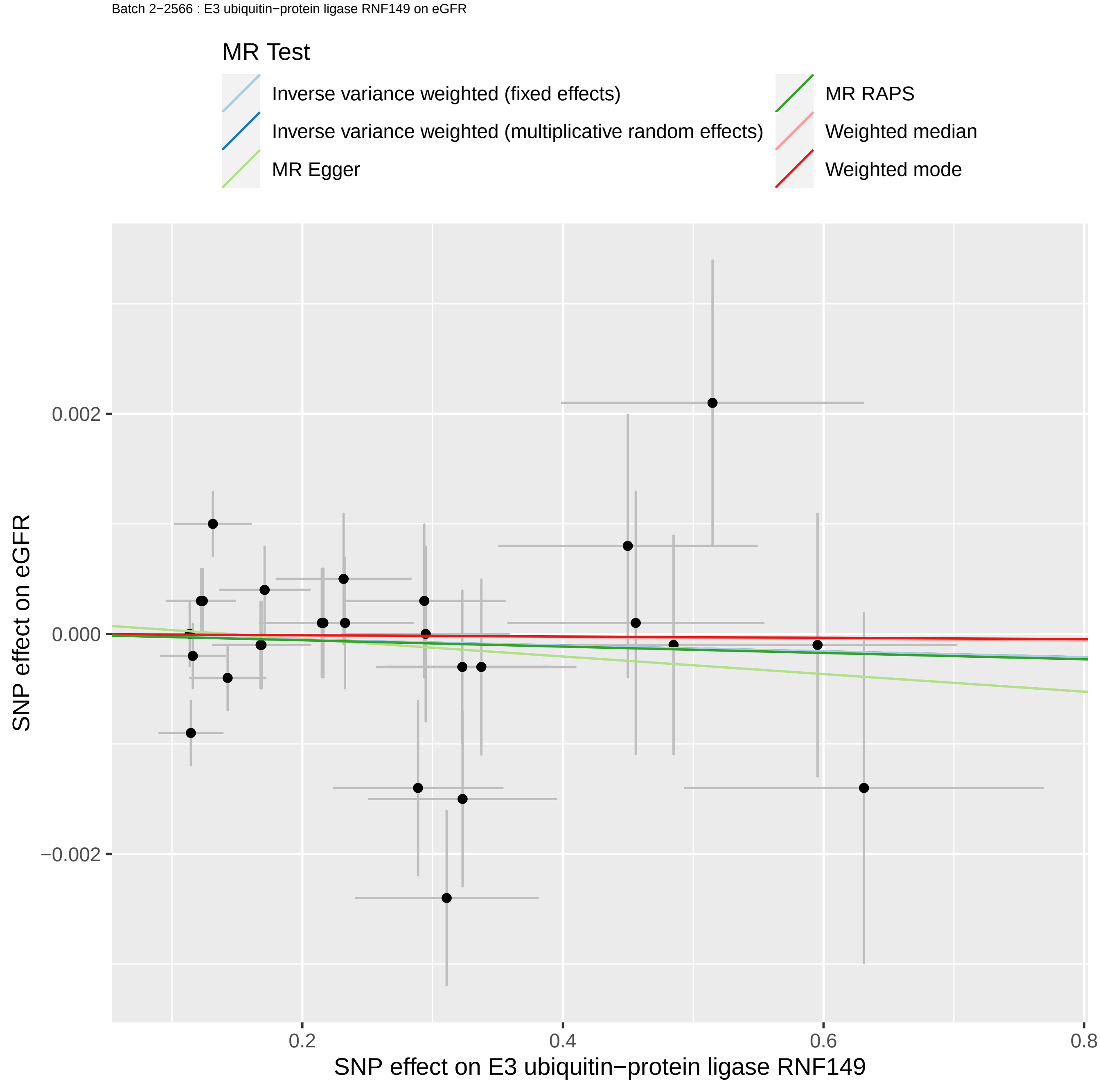
Batch 2−2566 : E3 ubiquitin−protein ligase RNF149 on eGFR
MR Test
Inverse variance weighted (fixed effects)
Inverse variance weighted (multiplicative random effects)
MR Egger
MR RAPS
Weighted median
Weighted mode
0.002
0.000
−0.002
0.2
0.4
0.6
0.8
SNP effect on eGFR
SNP effect on E3 ubiquitin−protein ligase RNF149

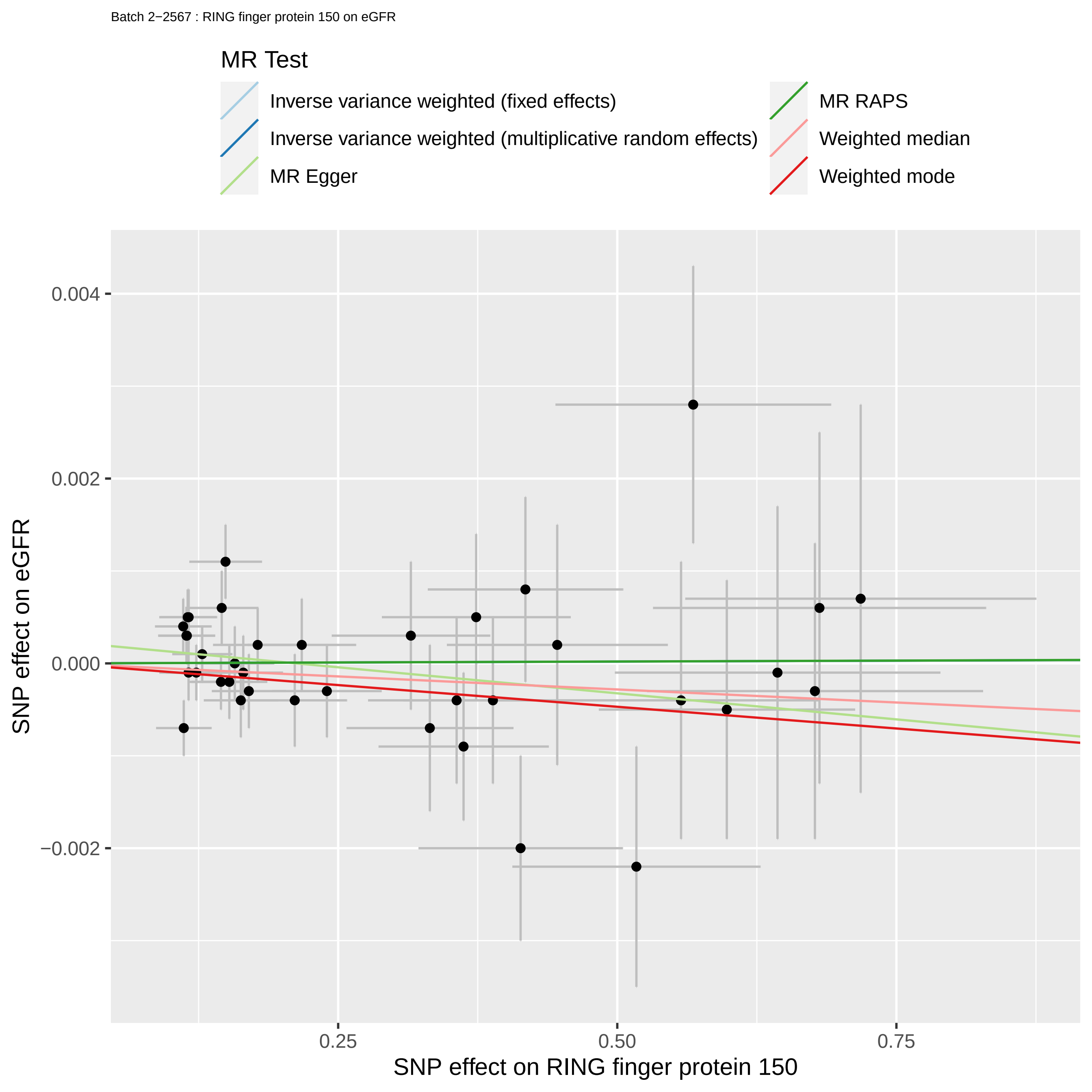
Batch 2−2567 : RING finger protein 150 on eGFR
MR Test
Inverse variance weighted (fixed effects)
Inverse variance weighted (multiplicative random effects)
MR Egger
MR RAPS
Weighted median
Weighted mode
0.004
0.002
0.000
−0.002
0.25
0.50
0.75
SNP effect on eGFR
SNP effect on RING finger protein 150

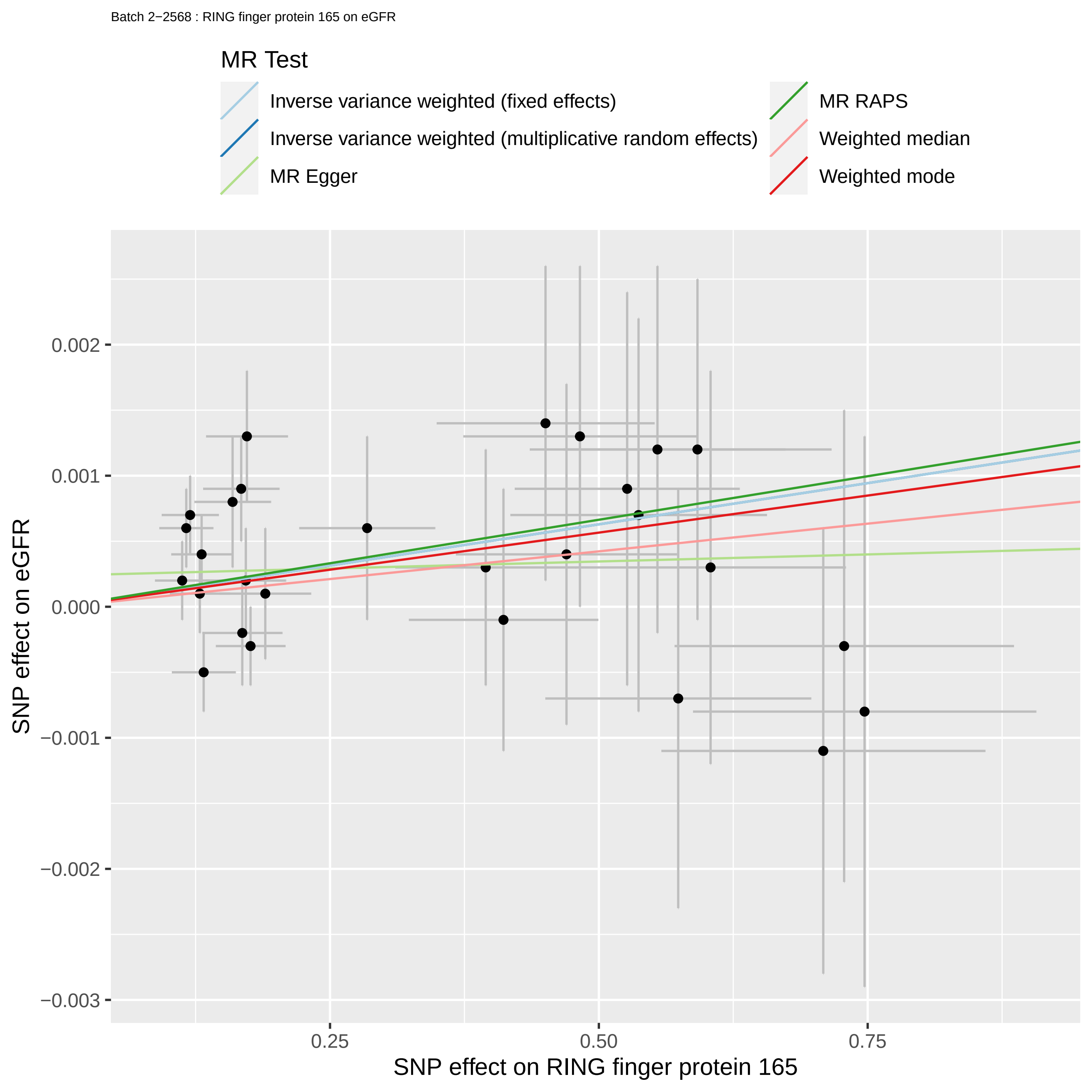

Batch 2−2568 : RING finger protein 165 on eGFR
MR Test
Inverse variance weighted (fixed effects)
Inverse variance weighted (multiplicative random effects)
MR Egger
MR RAPS
Weighted median
Weighted mode
0.002
0.001
0.000
−0.001
−0.002
−0.003
SNP effect on eGFR
0.25
0.50
0.75
SNP effect on RING finger protein 165

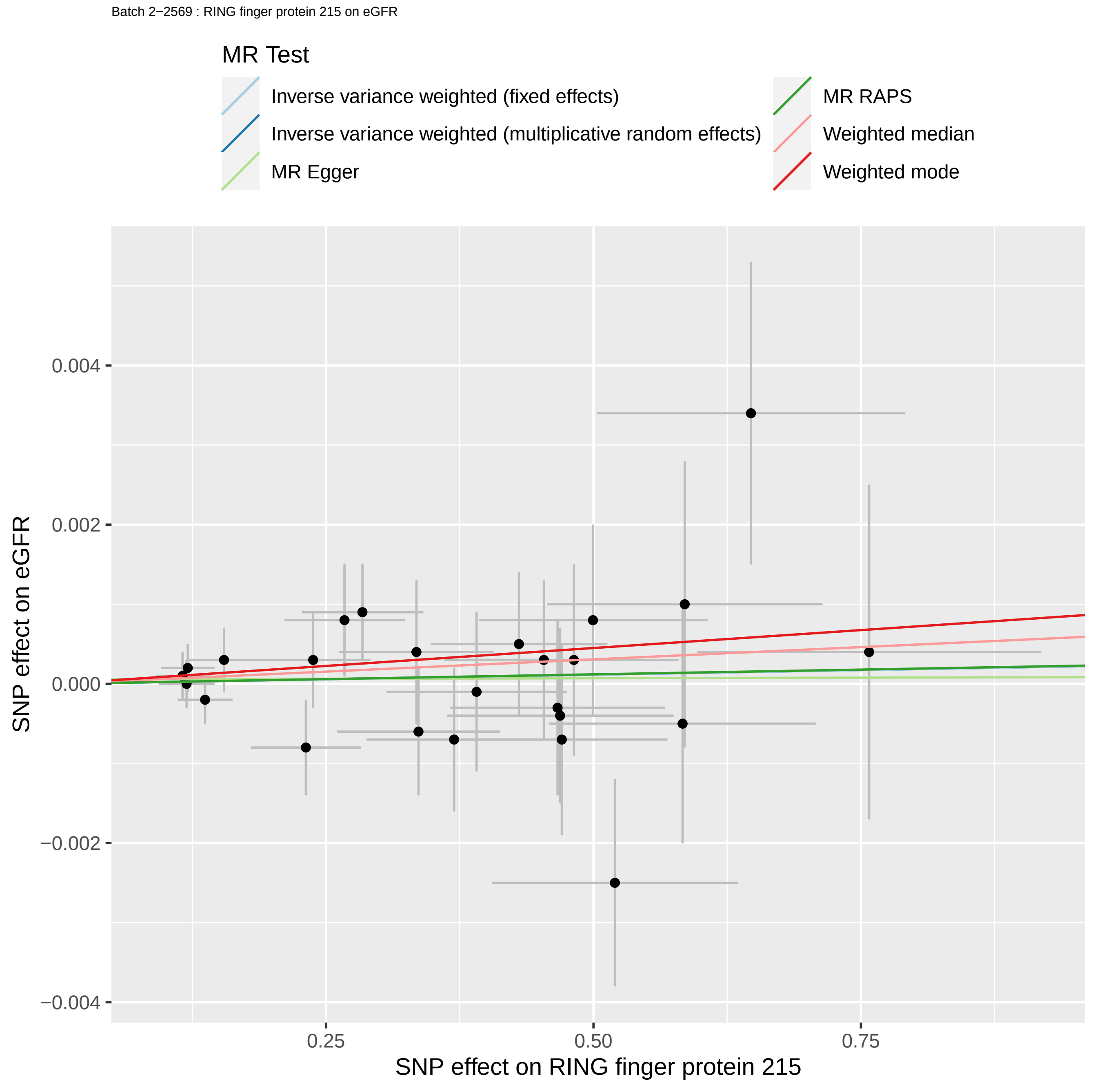

Batch 2−2569 : RING finger protein 215 on eGFR
MR Test
Inverse variance weighted (fixed effects)
Inverse variance weighted (multiplicative random effects)
MR Egger
MR RAPS
Weighted median
Weighted mode
0.004
0.002
0.000
−0.002
−0.004
SNP effect on eGFR
0.25
0.50
0.75
SNP effect on RING finger protein 215

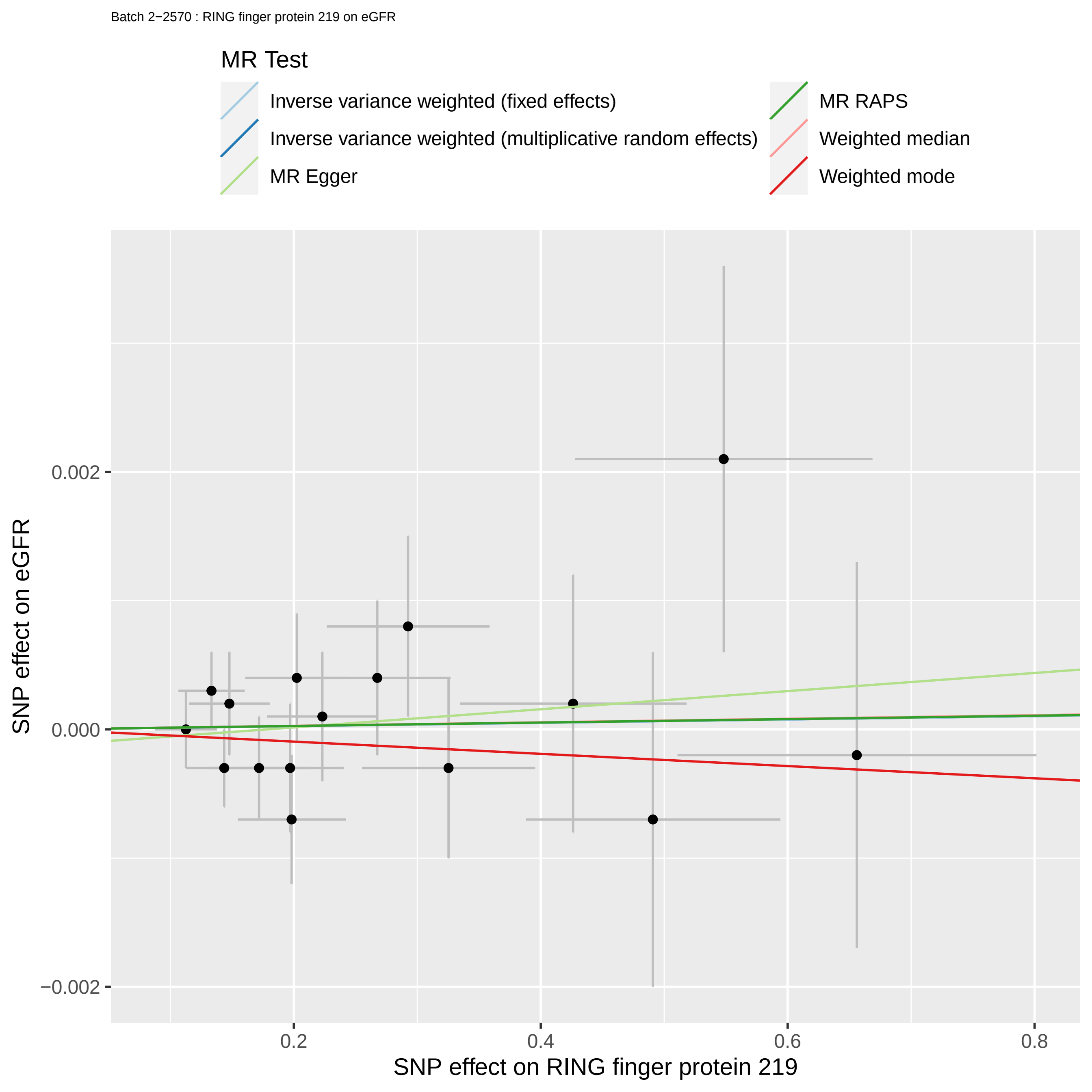

Batch 2−2570 : RING finger protein 219 on eGFR
MR Test
Inverse variance weighted (fixed effects)
Inverse variance weighted (multiplicative random effects)
MR Egger
MR RAPS
Weighted median
Weighted mode
0.002
0.000
−0.002
0.2
0.4
0.6
0.8
SNP effect on eGFR
SNP effect on RING finger protein 219

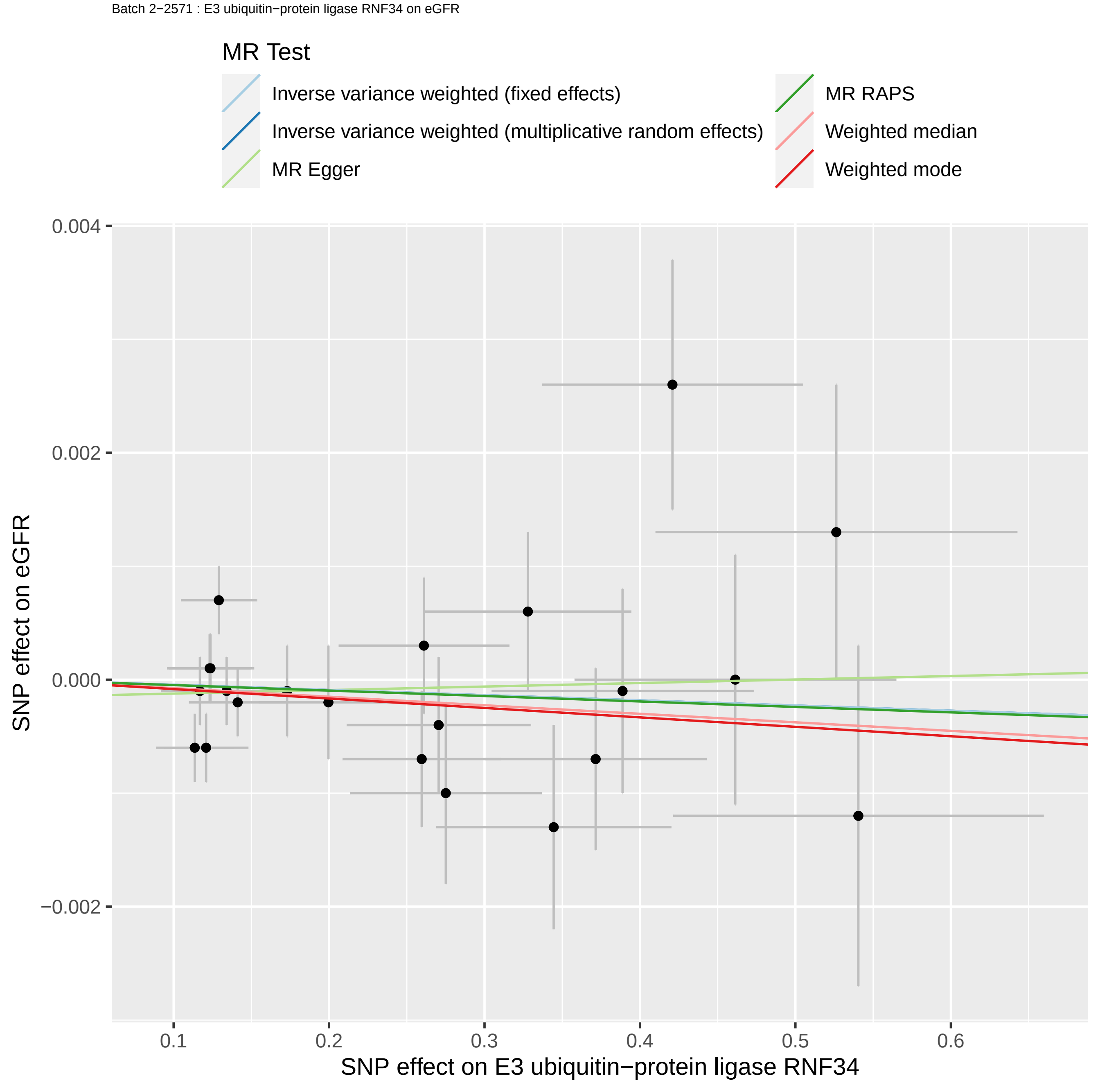
Batch 2−2571 : E3 ubiquitin−protein ligase RNF34 on eGFR
MR Test
Inverse variance weighted (fixed effects)
Inverse variance weighted (multiplicative random effects)
MR Egger
MR RAPS
Weighted median
Weighted mode
0.004
0.002
0.000
−0.002
SNP effect on eGFR
0.1
0.2
0.3
0.4
0.5
0.6
SNP effect on E3 ubiquitin−protein ligase RNF34

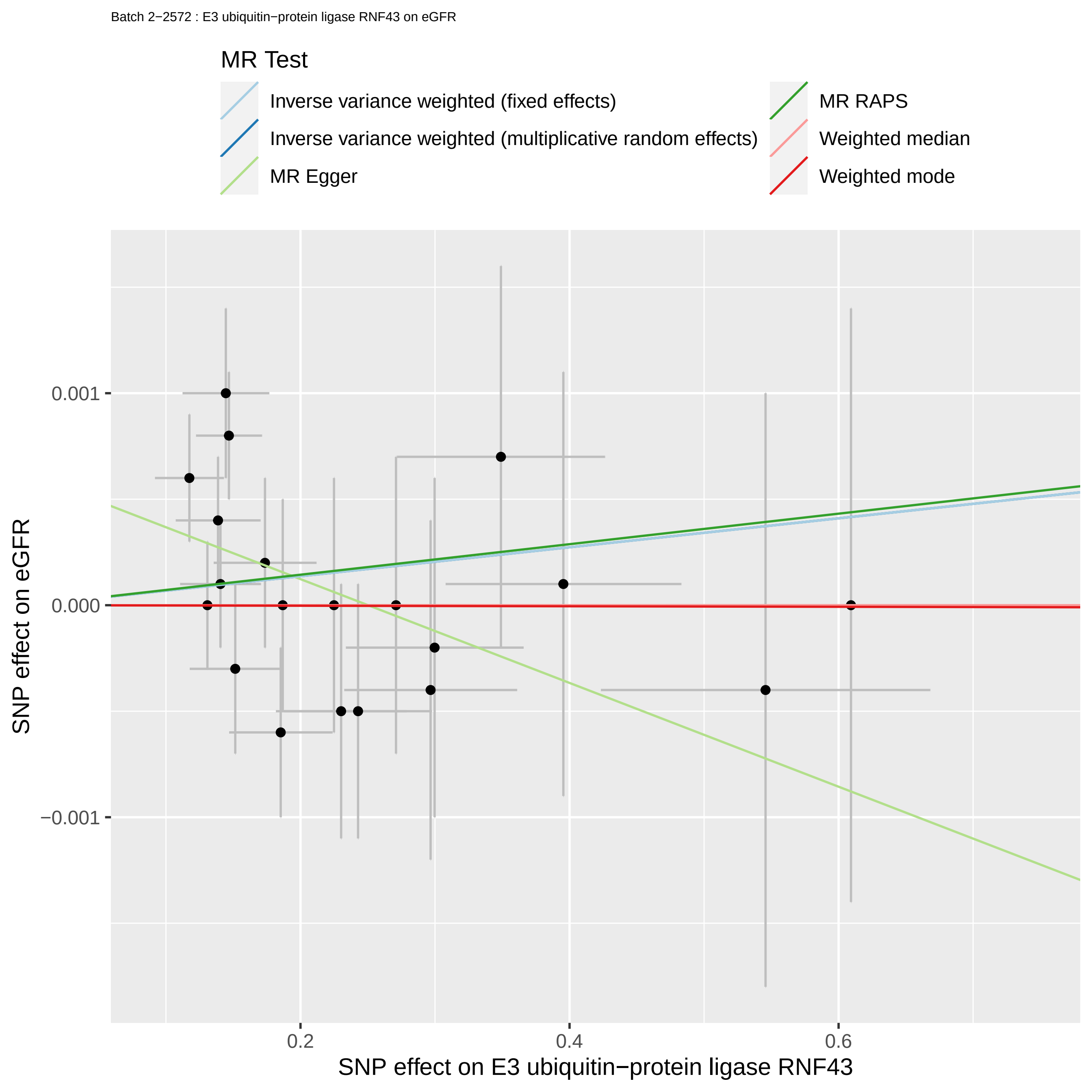

Batch 2−2572 : E3 ubiquitin−protein ligase RNF43 on eGFR
MR Test
Inverse variance weighted (fixed effects)
Inverse variance weighted (multiplicative random effects)
MR Egger
MR RAPS
Weighted median
Weighted mode
0.001
0.000
−0.001
0.2
0.4
0.6
SNP effect on eGFR
SNP effect on E3 ubiquitin−protein ligase RNF43

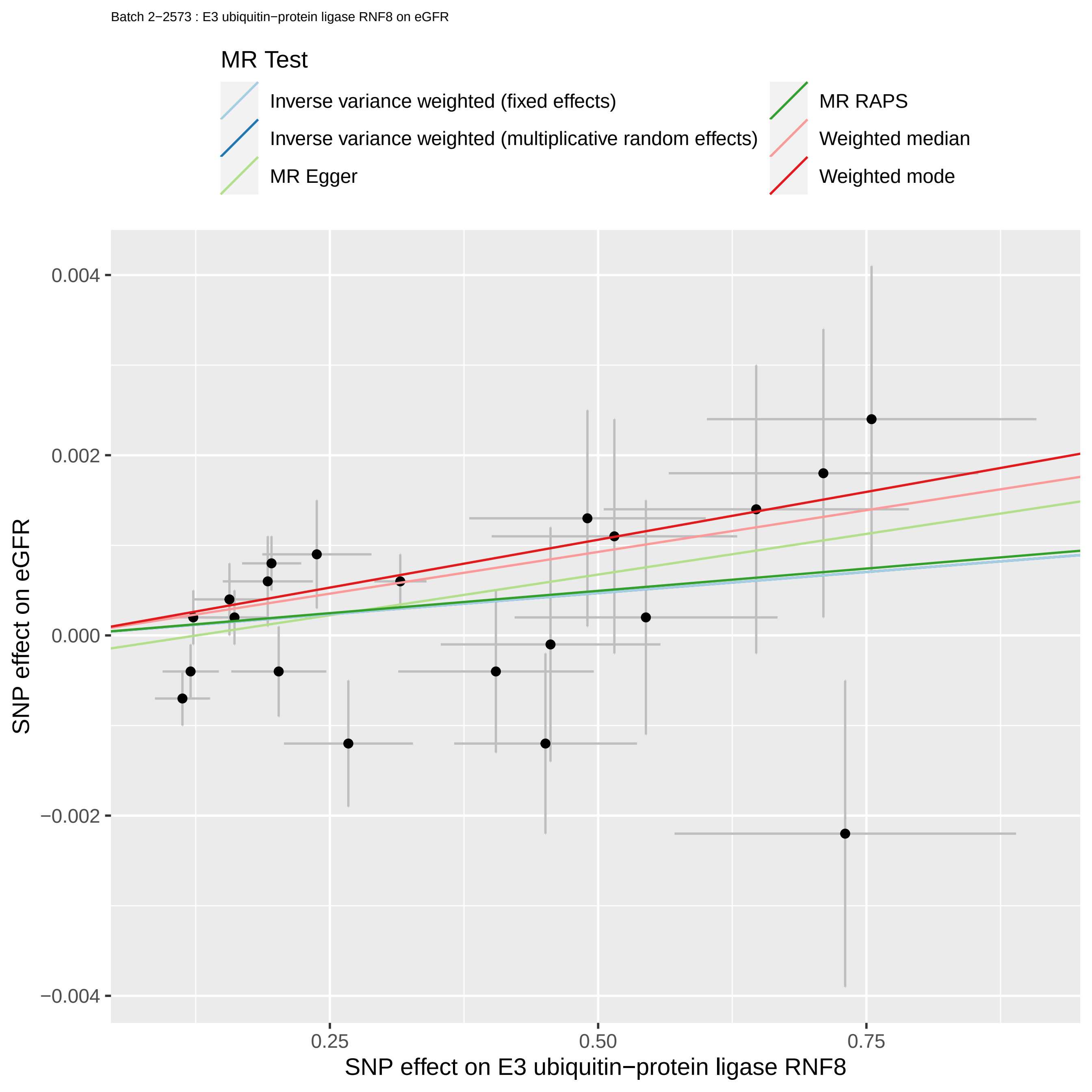

Batch 2−2573 : E3 ubiquitin−protein ligase RNF8 on eGFR
MR Test
Inverse variance weighted (fixed effects)
Inverse variance weighted (multiplicative random effects)
MR Egger
MR RAPS
Weighted median
Weighted mode
0.004
0.002
0.000
−0.002
−0.004
0.25
0.50
0.75
SNP effect on eGFR
SNP effect on E3 ubiquitin−protein ligase RNF8

Batch 2−2574 : mRNA−capping enzyme on eGFR

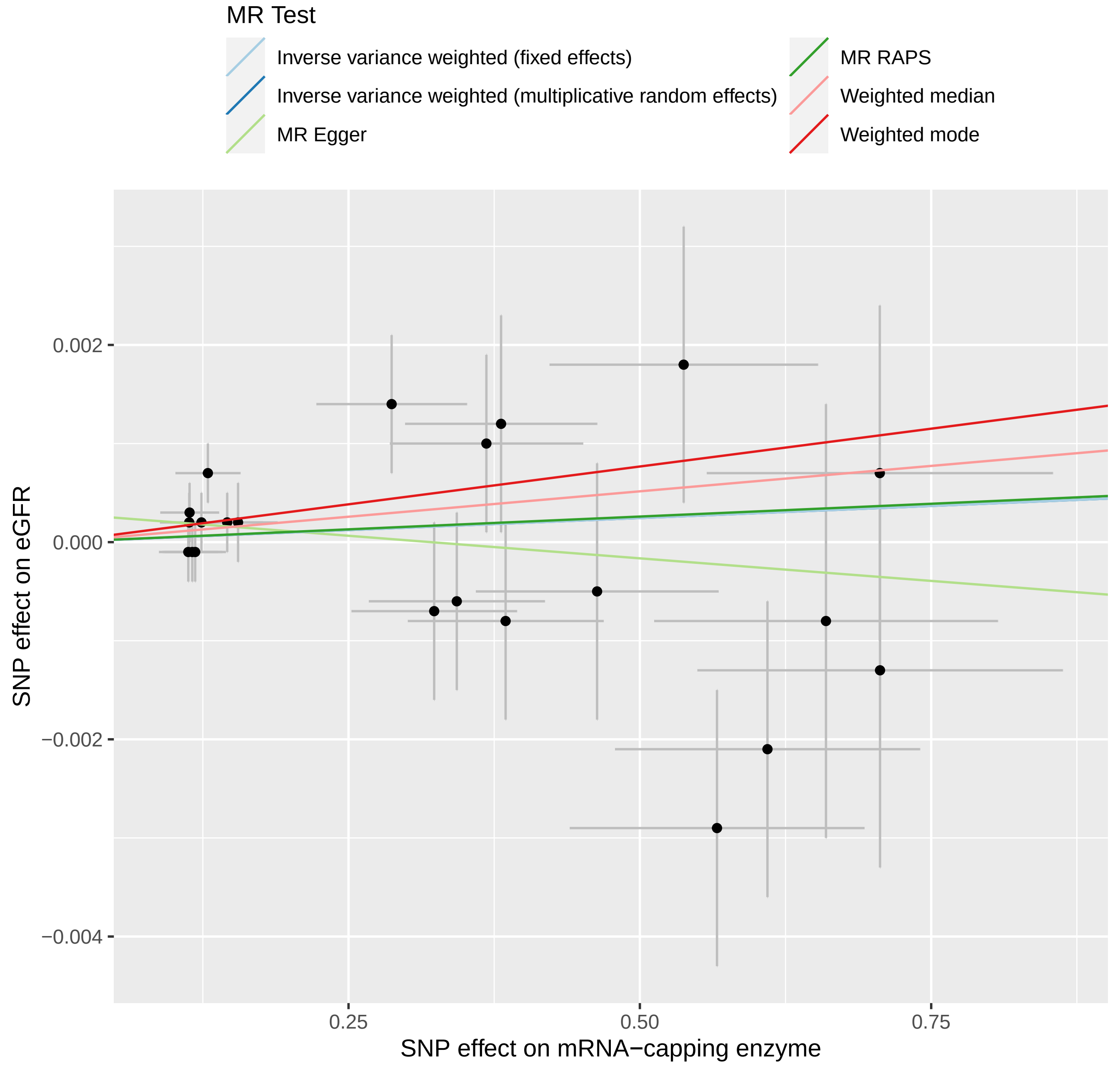

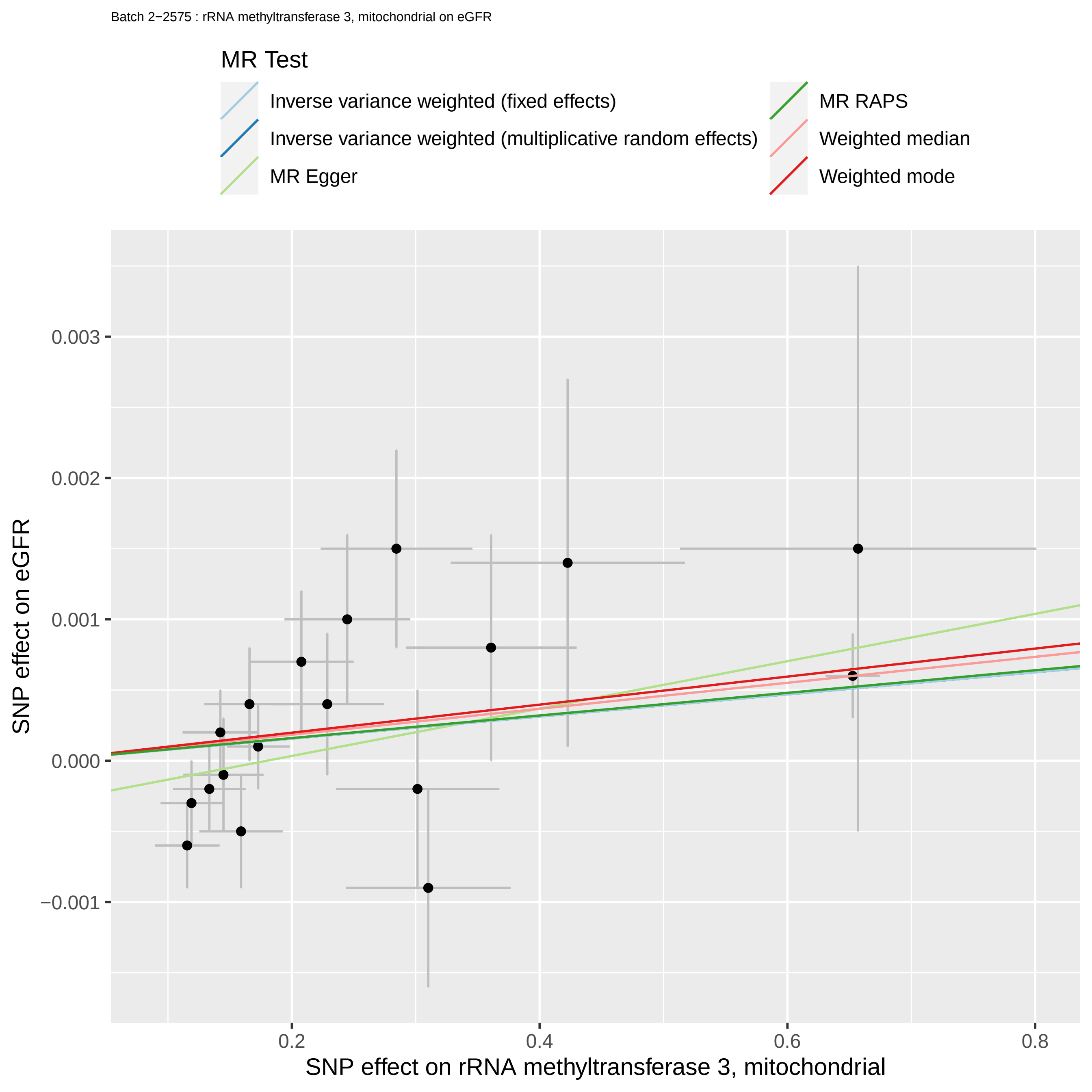

Batch 2−2575 : rRNA methyltransferase 3, mitochondrial on eGFR
MR Test
Inverse variance weighted (fixed effects)
Inverse variance weighted (multiplicative random effects)
MR Egger
MR RAPS
Weighted median
Weighted mode
0.003
0.002
0.001
0.000
−0.001
0.2
0.4
0.6
0.8
SNP effect on eGFR
SNP effect on rRNA methyltransferase 3, mitochondrial

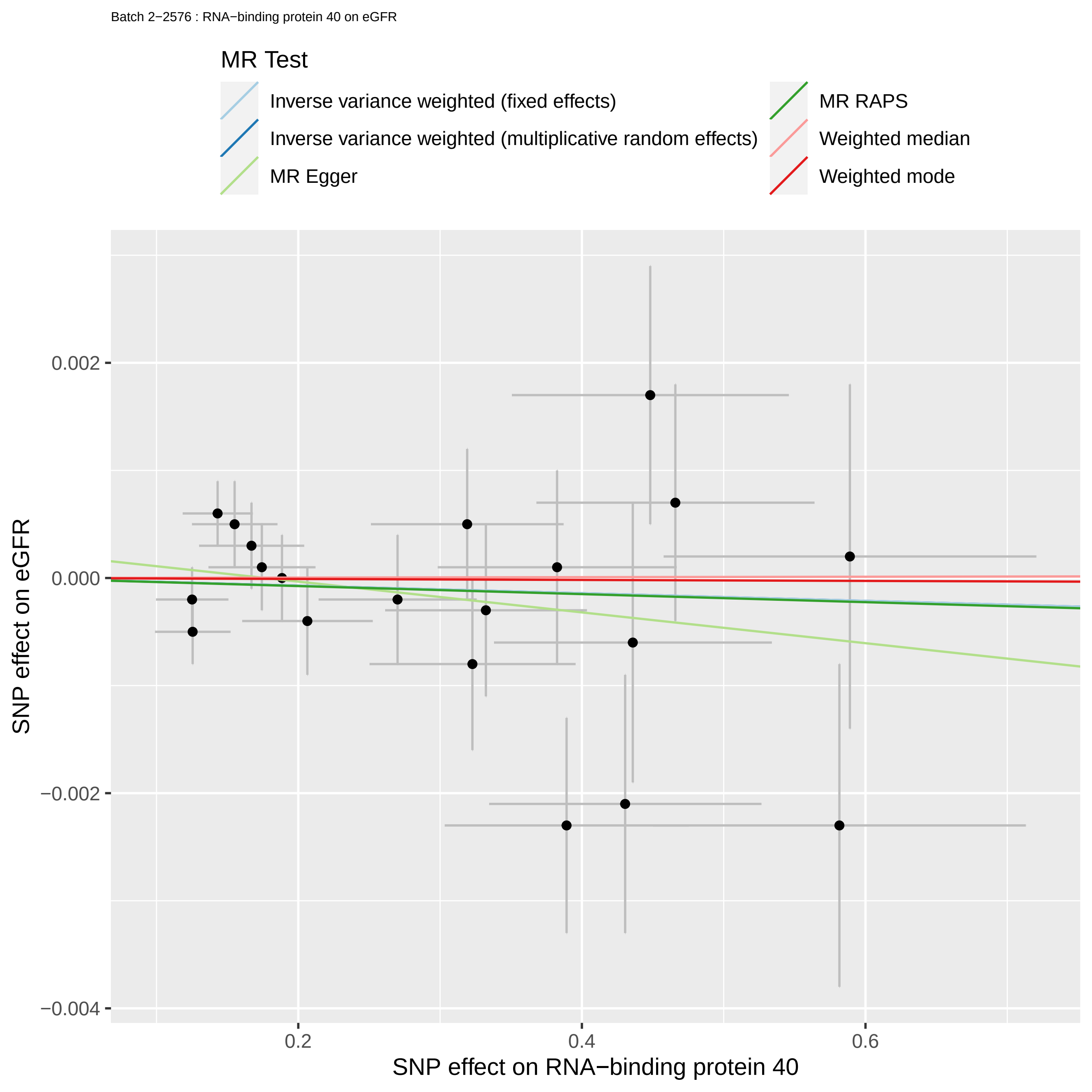
Batch 2−2576 : RNA−binding protein 40 on eGFR
MR Test
Inverse variance weighted (fixed effects)
Inverse variance weighted (multiplicative random effects)
MR Egger
MR RAPS
Weighted median
Weighted mode
0.002
0.000
−0.002
−0.004
SNP effect on eGFR
0.2
0.4
0.6
SNP effect on RNA−binding protein 40

Batch 2−2577 : Roundabout homolog 1 on eGFR

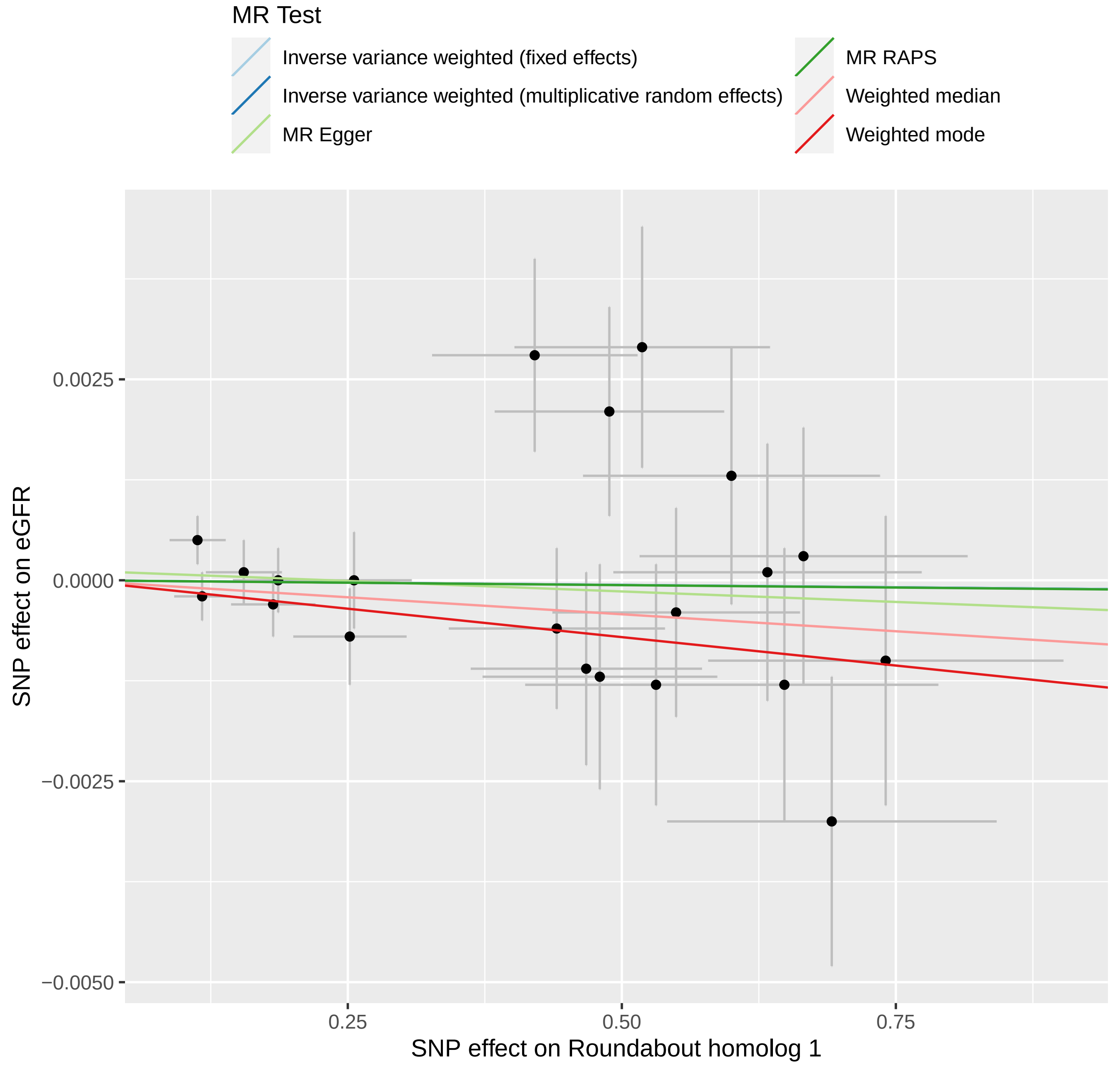

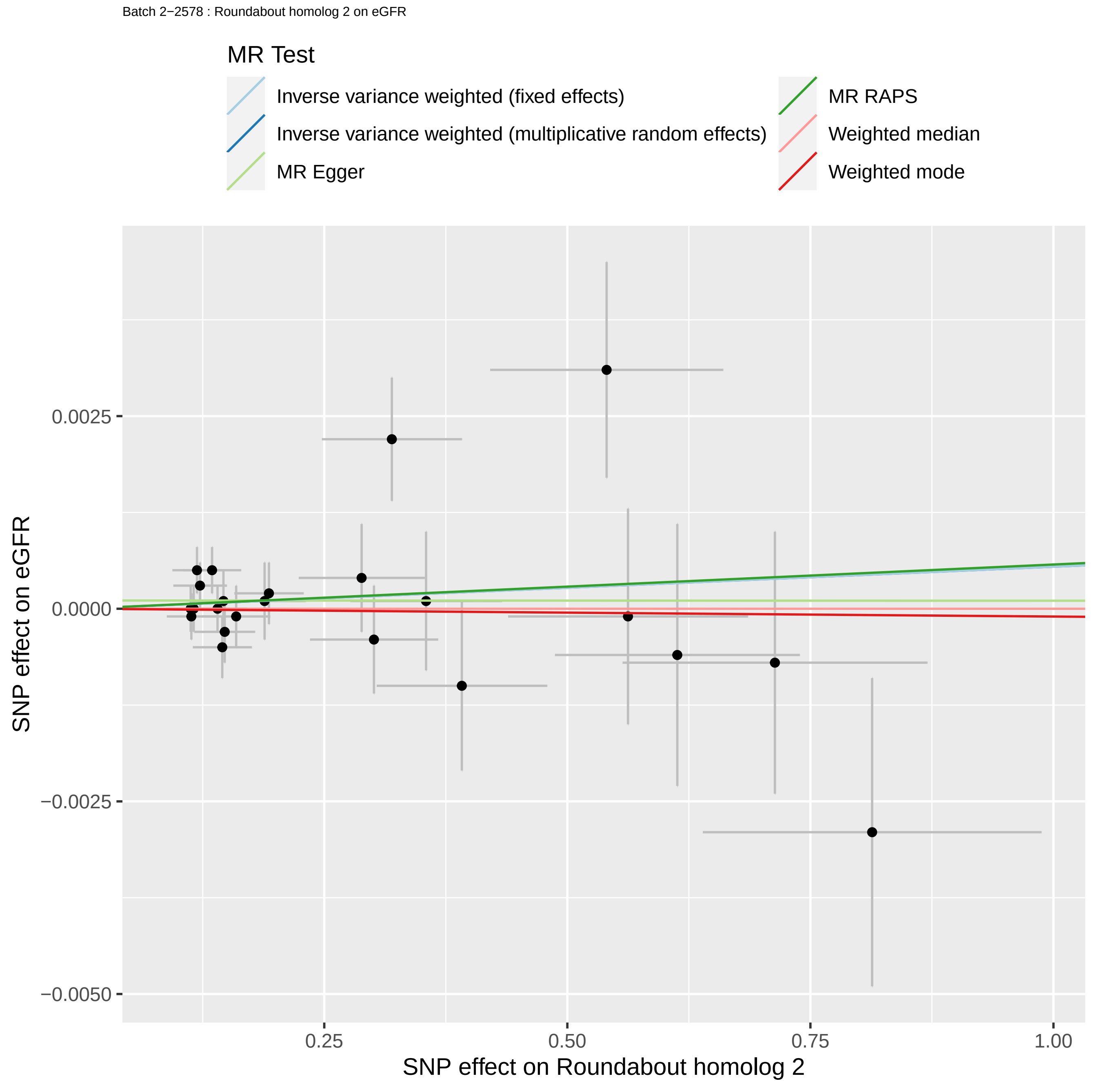

Batch 2−2578 : Roundabout homolog 2 on eGFR
MR Test
Inverse variance weighted (fixed effects)
Inverse variance weighted (multiplicative random effects)
MR Egger
MR RAPS
Weighted median
Weighted mode
0.0025
0.0000
−0.0025
−0.0050
0.25
0.50
0.75
1.00
SNP effect on eGFR
SNP effect on Roundabout homolog 2

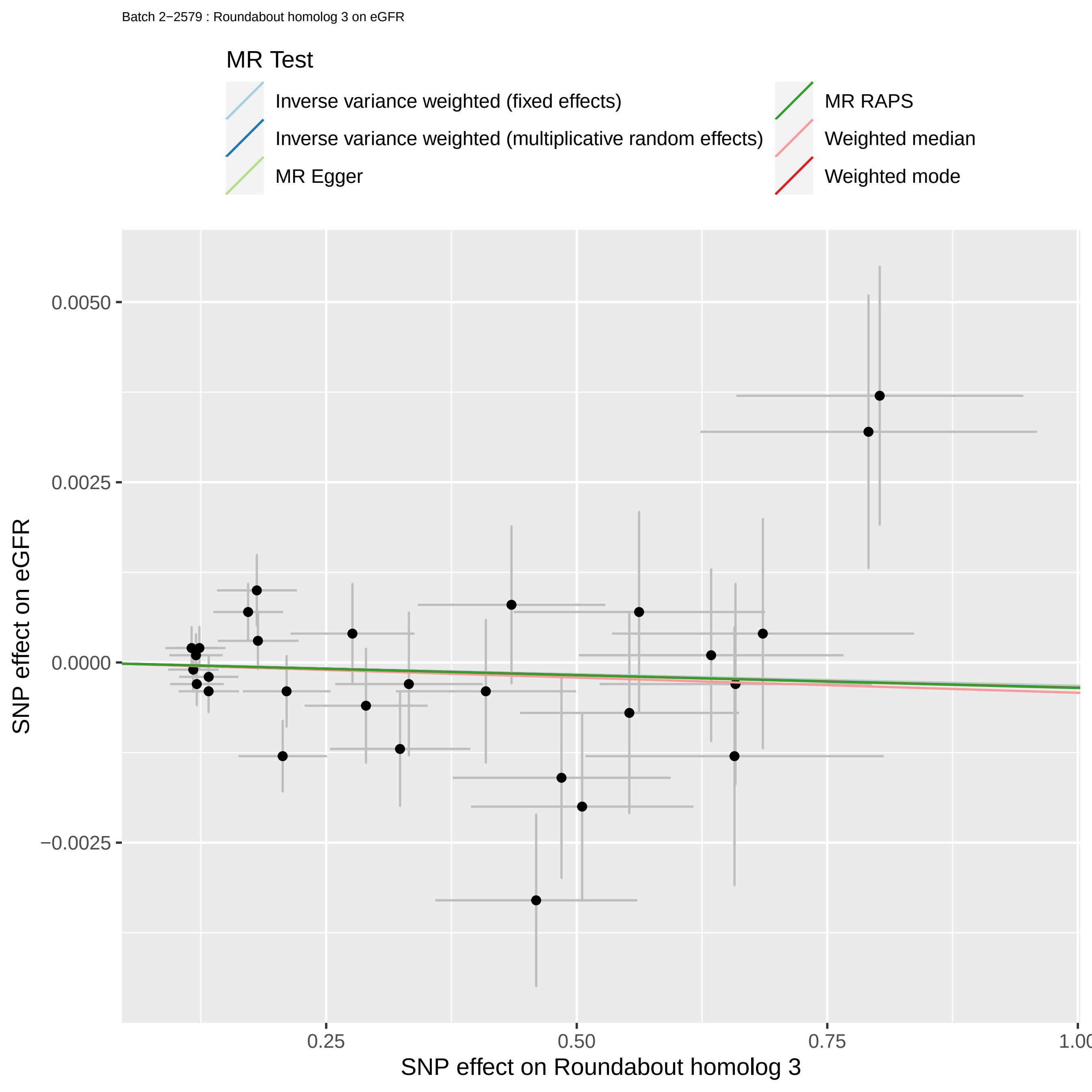

Batch 2−2579 : Roundabout homolog 3 on eGFR
MR Test
Inverse variance weighted (fixed effects)
Inverse variance weighted (multiplicative random effects)
MR Egger
MR RAPS
Weighted median
Weighted mode
0.0050
0.0025
0.0000
−0.0025
SNP effect on eGFR
0.25
0.50
0.75
1.00
SNP effect on Roundabout homolog 3

Batch 2−2580 : Rho−associated protein kinase 2 on eGFR

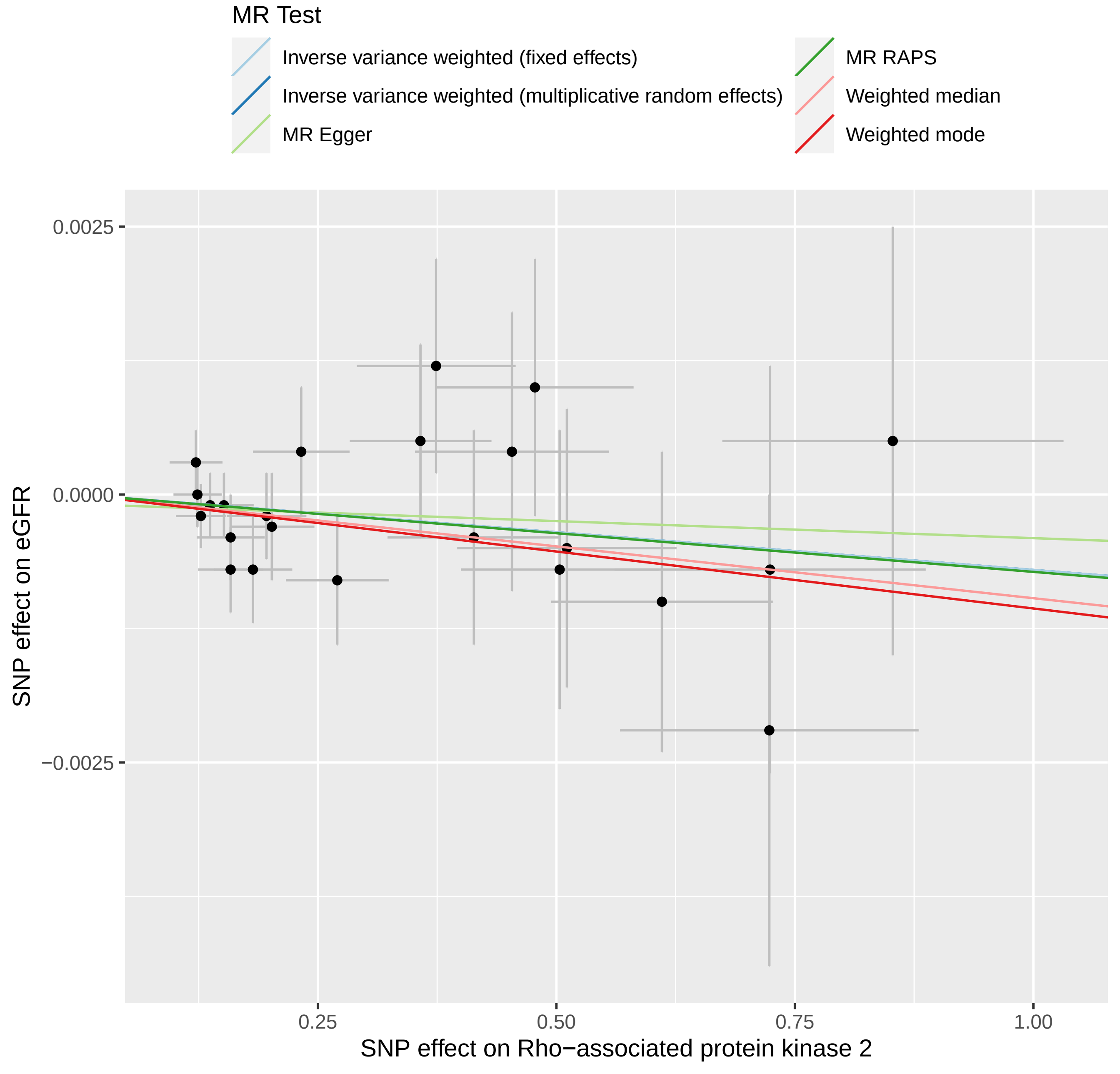

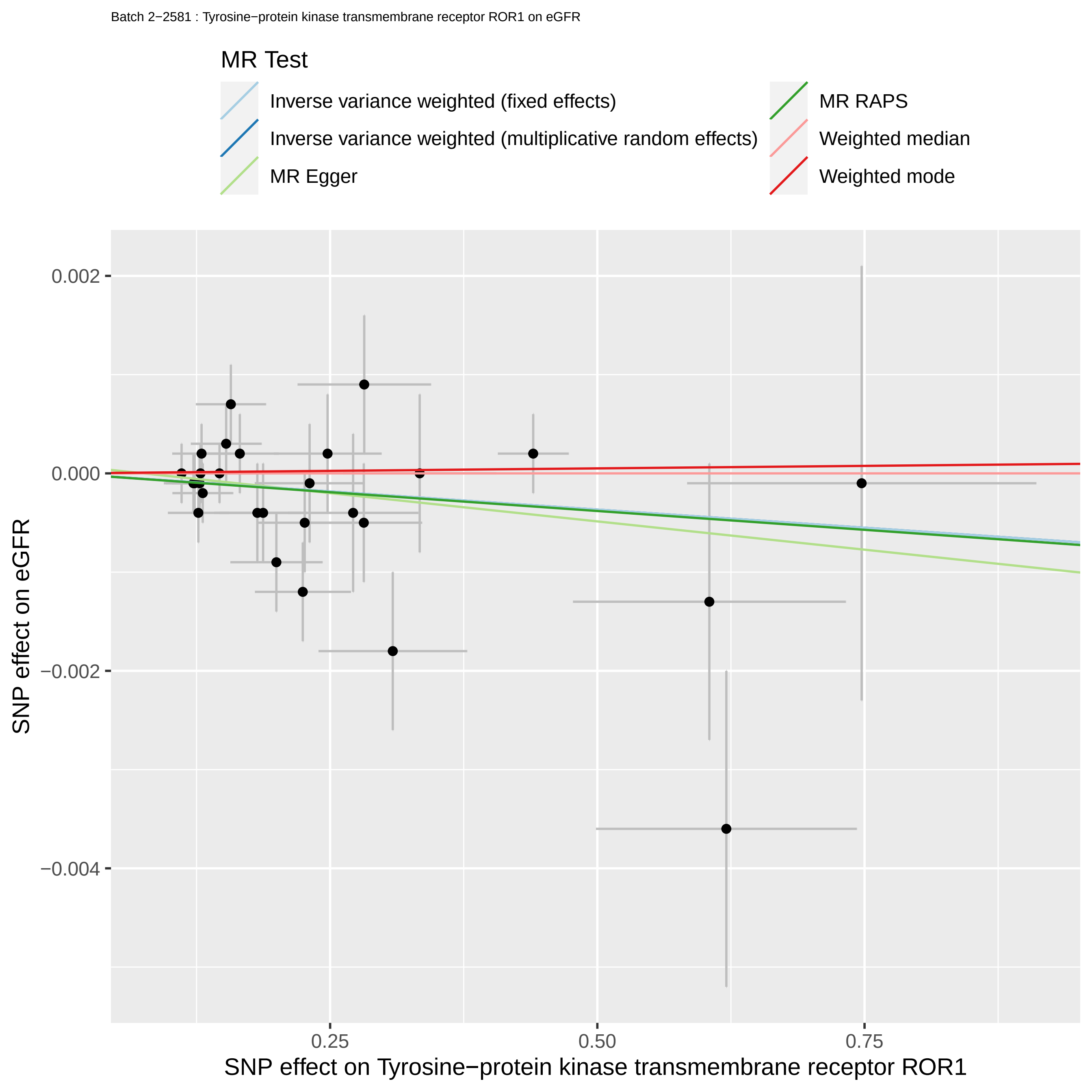

Batch 2−2581 : Tyrosine−protein kinase transmembrane receptor ROR1 on eGFR
MR Test
Inverse variance weighted (fixed effects)
Inverse variance weighted (multiplicative random effects)
MR Egger
MR RAPS
Weighted median
Weighted mode
0.002
0.000
−0.002
−0.004
SNP effect on eGFR
0.25
0.50
0.75
SNP effect on Tyrosine−protein kinase transmembrane receptor ROR1

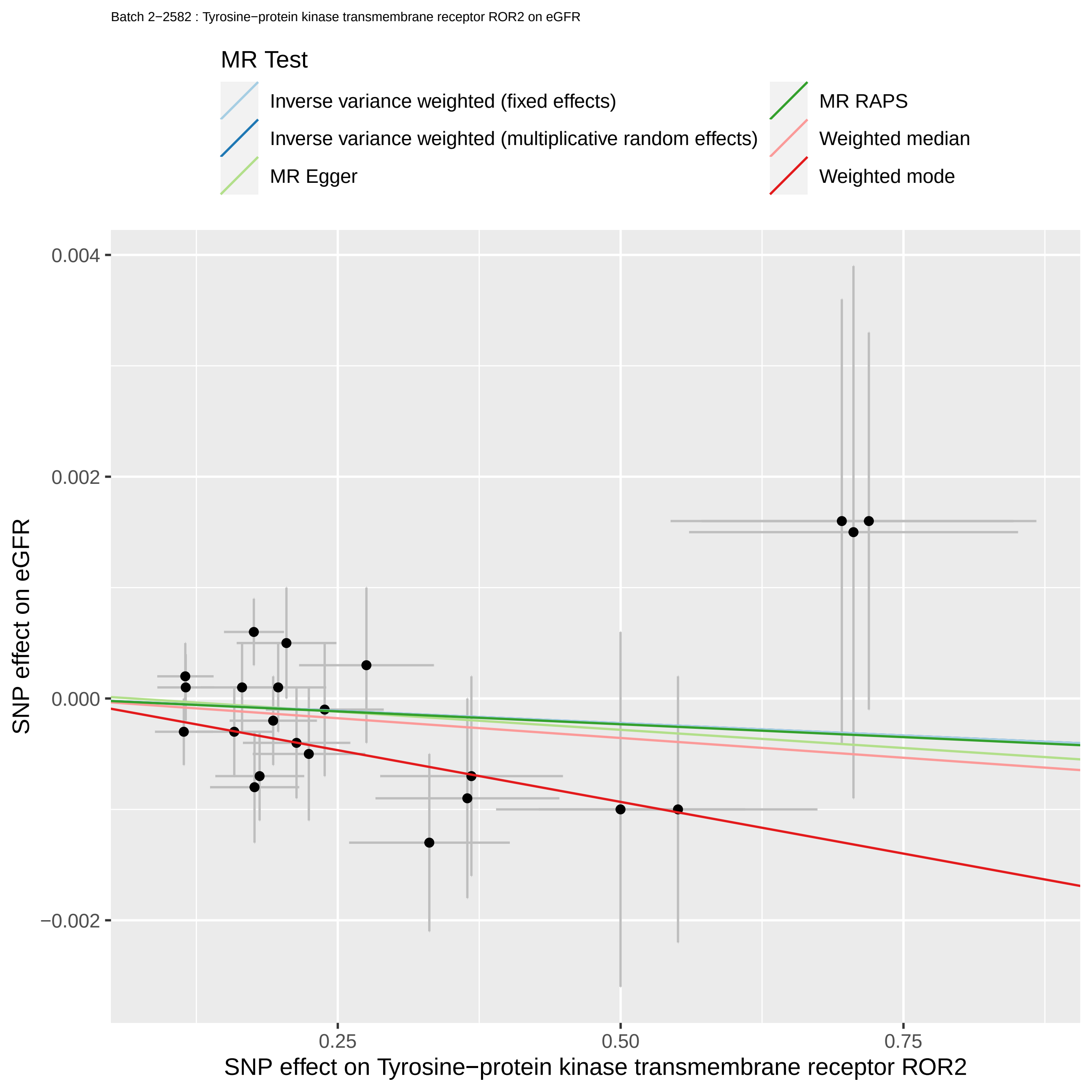
Batch 2−2582 : Tyrosine−protein kinase transmembrane receptor ROR2 on eGFR
MR Test
Inverse variance weighted (fixed effects)
Inverse variance weighted (multiplicative random effects)
MR Egger
MR RAPS
Weighted median
Weighted mode
0.004
0.002
0.000
−0.002
0.25
0.50
0.75
SNP effect on eGFR
SNP effect on Tyrosine−protein kinase transmembrane receptor ROR2

Batch 2−2583 : Nuclear receptor ROR−beta on eGFR

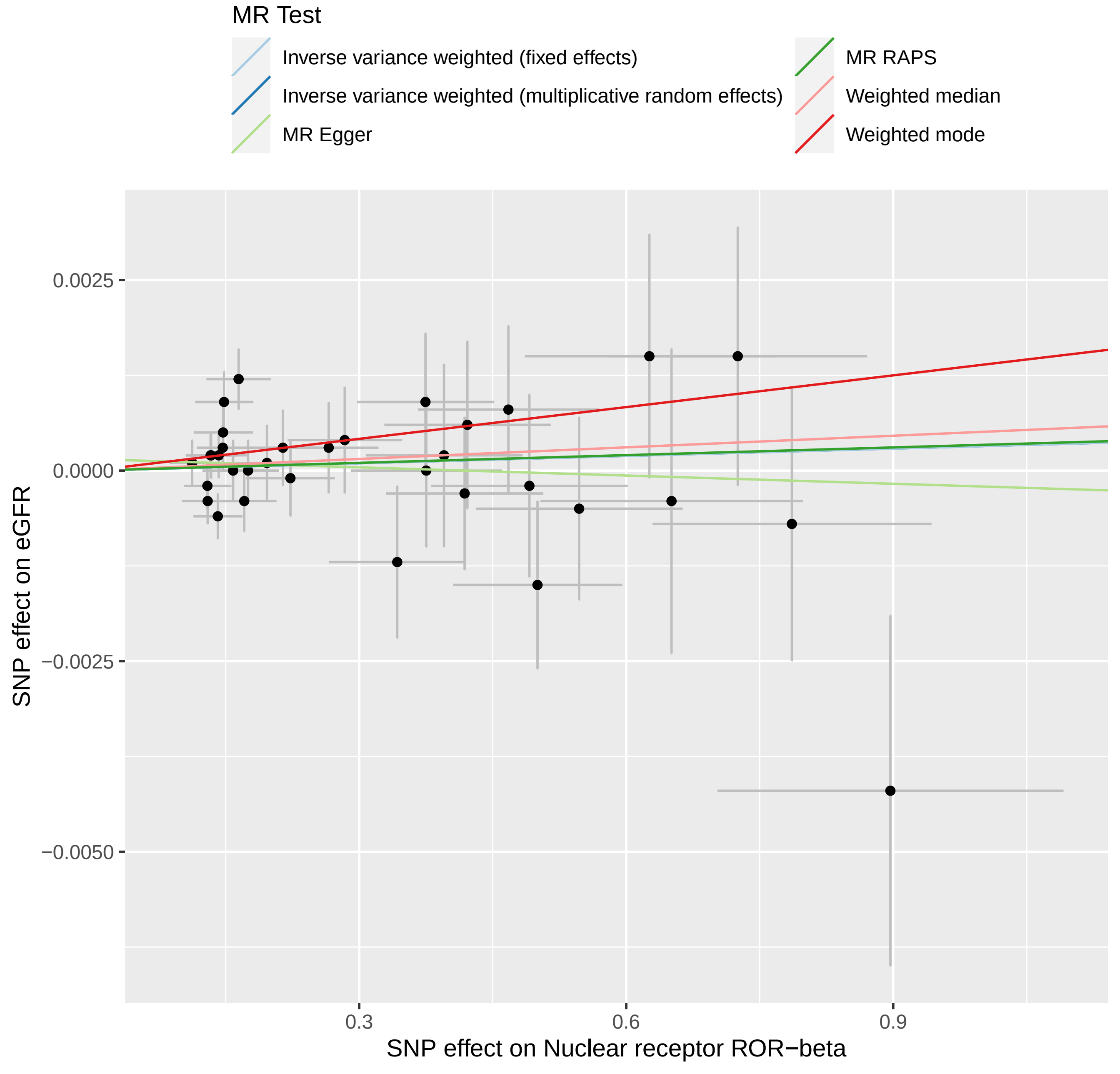

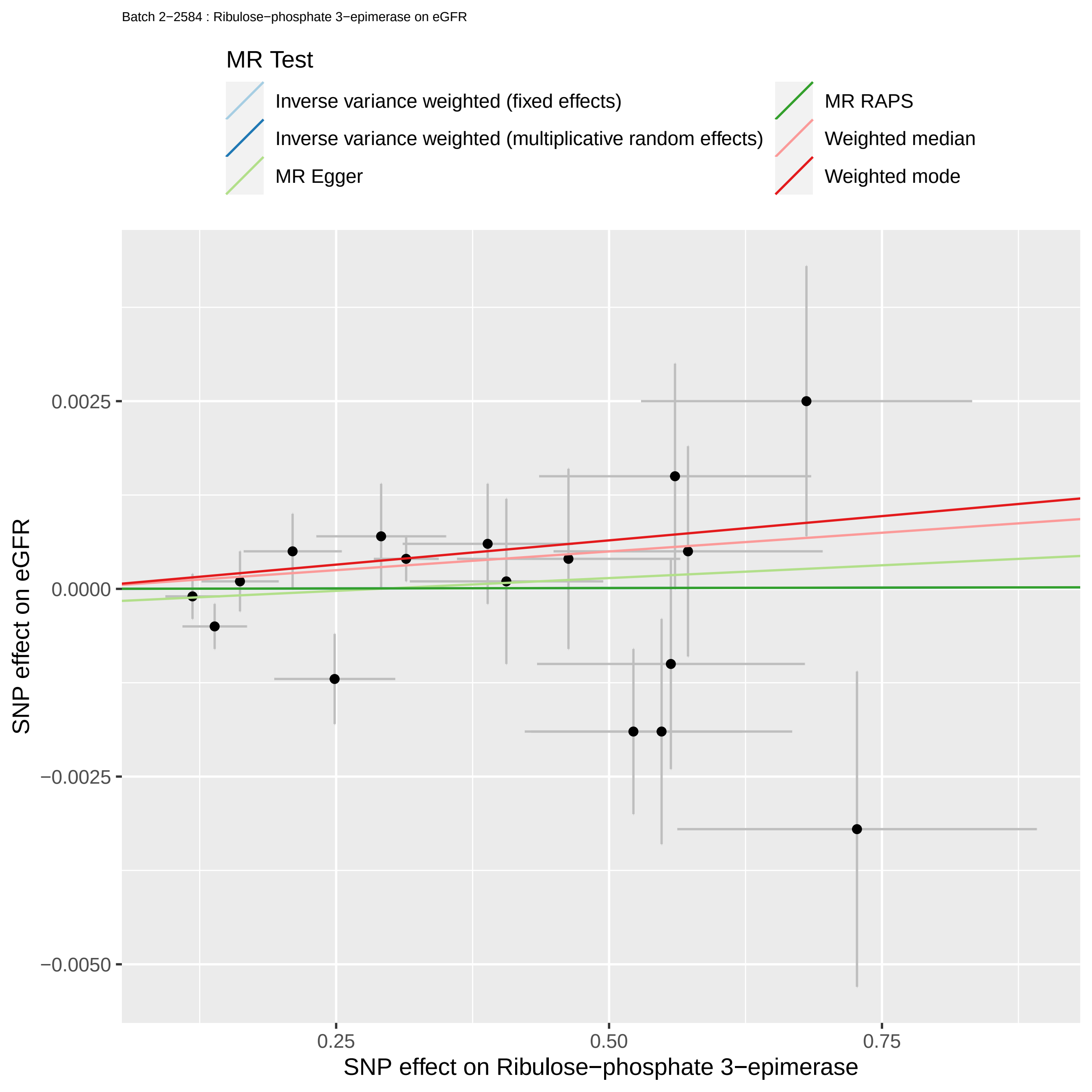
Batch 2−2584 : Ribulose−phosphate 3−epimerase on eGFR
MR Test
Inverse variance weighted (fixed effects)
Inverse variance weighted (multiplicative random effects)
MR Egger
MR RAPS
Weighted median
Weighted mode
0.0025
0.0000
−0.0025
−0.0050
SNP effect on eGFR
0.25
0.50
0.75
SNP effect on Ribulose−phosphate 3−epimerase

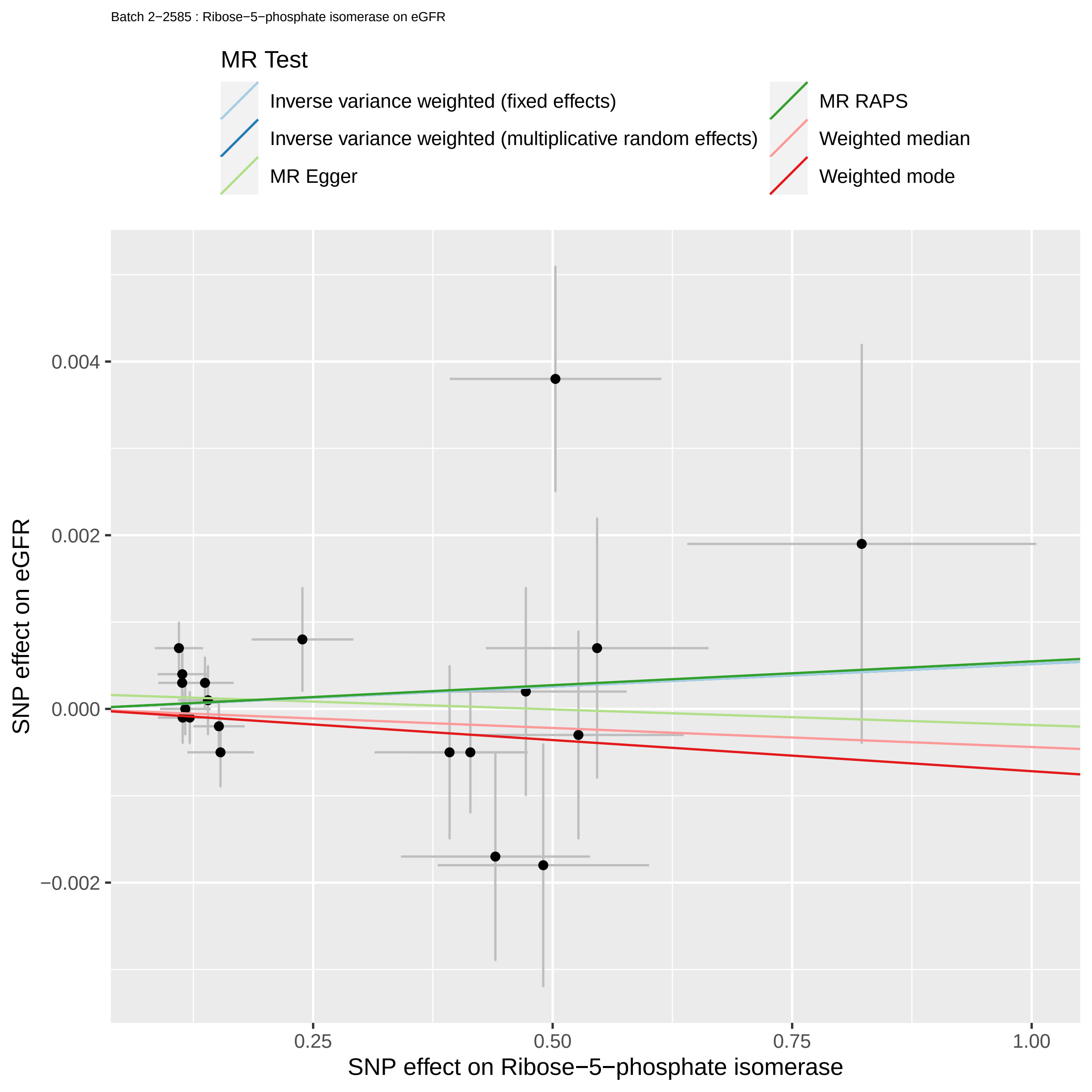
Batch 2−2585 : Ribose−5−phosphate isomerase on eGFR
MR Test
Inverse variance weighted (fixed effects)
Inverse variance weighted (multiplicative random effects)
MR Egger
MR RAPS
Weighted median
Weighted mode
0.004
0.002
0.000
−0.002
0.25
0.50
0.75
1.00
SNP effect on eGFR
SNP effect on Ribose−5−phosphate isomerase

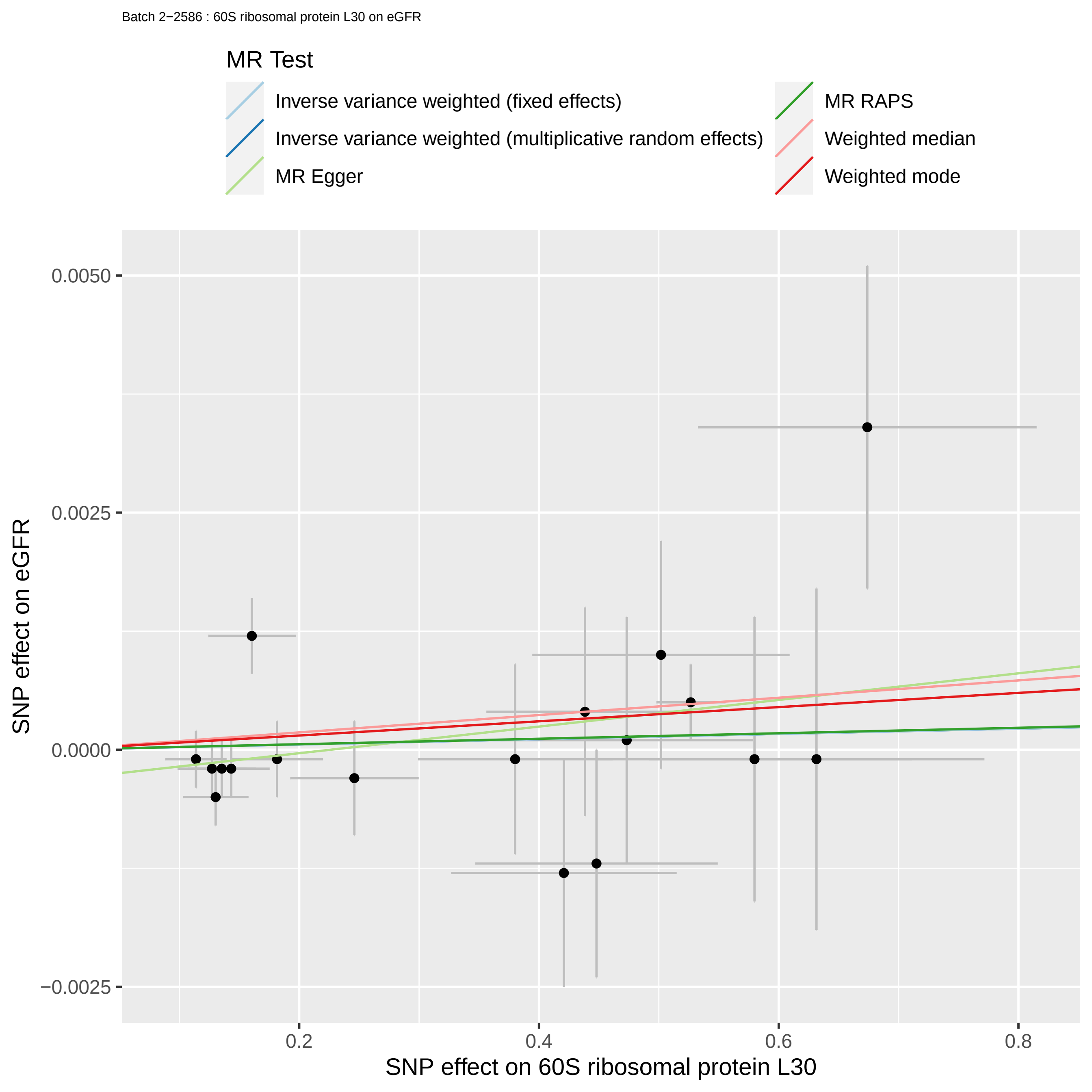
Batch 2−2586 : 60S ribosomal protein L30 on eGFR
MR Test
Inverse variance weighted (fixed effects)
Inverse variance weighted (multiplicative random effects)
MR Egger
MR RAPS
Weighted median
Weighted mode
0.0050
0.0025
0.0000
−0.0025
SNP effect on eGFR
0.2
0.4
0.6
0.8
SNP effect on 60S ribosomal protein L30

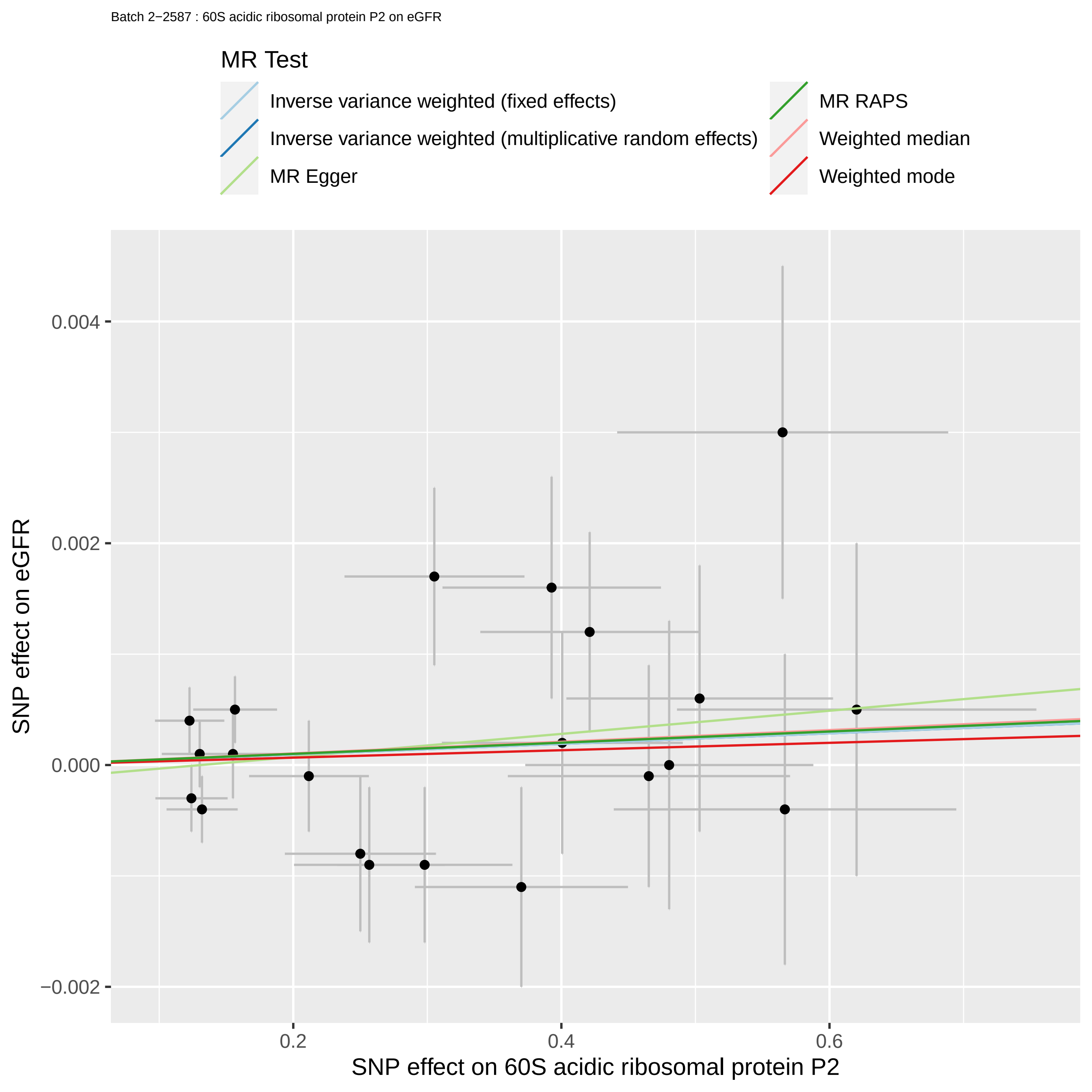

Batch 2−2587 : 60S acidic ribosomal protein P2 on eGFR
MR Test
Inverse variance weighted (fixed effects)
Inverse variance weighted (multiplicative random effects)
MR Egger
MR RAPS
Weighted median
Weighted mode
SNP effect on eGFR
0.004
0.002
0.000
−0.002
0.2
0.4
0.6
SNP effect on 60S acidic ribosomal protein P2

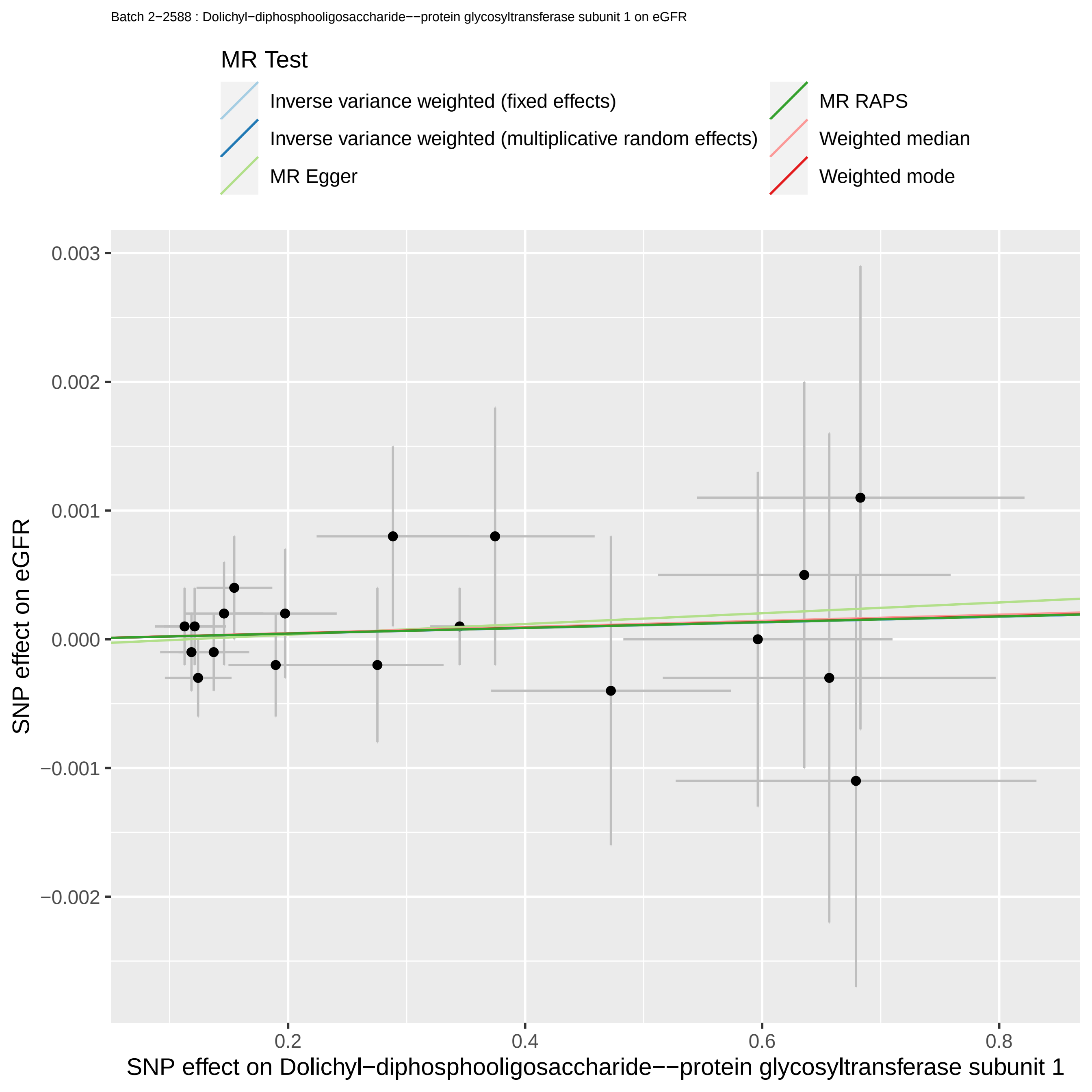

Batch 2−2588 : Dolichyl−diphosphooligosaccharide−−protein glycosyltransferase subunit 1 on eGFR
MR Test
Inverse variance weighted (fixed effects)
Inverse variance weighted (multiplicative random effects)
MR Egger
MR RAPS
Weighted median
Weighted mode
0.003
0.002
0.001
0.000
−0.001
−0.002
SNP effect on eGFR
0.2
0.4
0.6
0.8
SNP effect on Dolichyl−diphosphooligosaccharide−−protein glycosyltransferase subunit 1

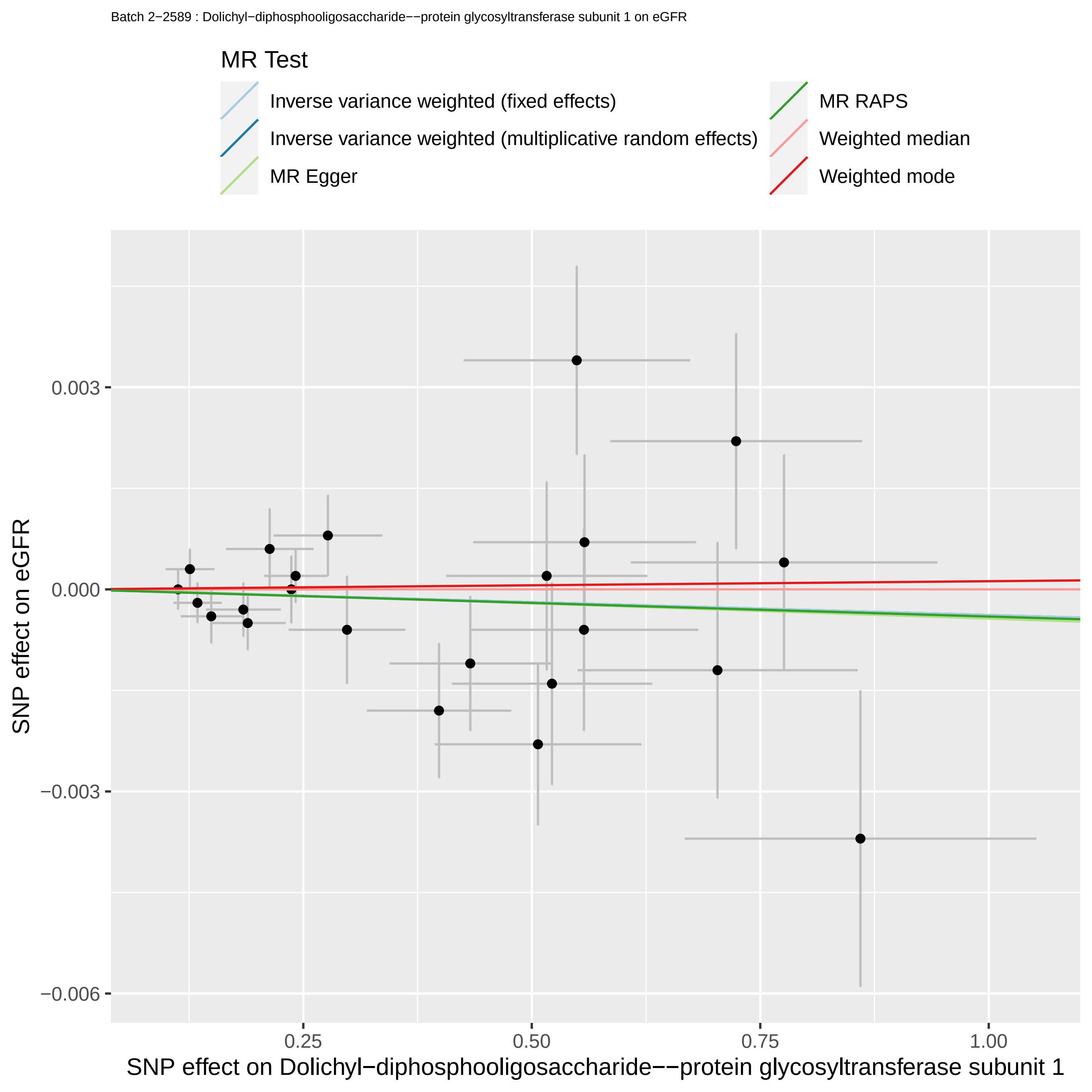

Batch 2−2589 : Dolichyl−diphosphooligosaccharide−−protein glycosyltransferase subunit 1 on eGFR
MR Test
Inverse variance weighted (fixed effects)
Inverse variance weighted (multiplicative random effects)
MR Egger
MR RAPS
Weighted median
Weighted mode
SNP effect on eGFR
0.003
0.000
−0.003
−0.006
0.25
0.50
0.75
1.00
SNP effect on Dolichyl−diphosphooligosaccharide−−protein glycosyltransferase subunit 1

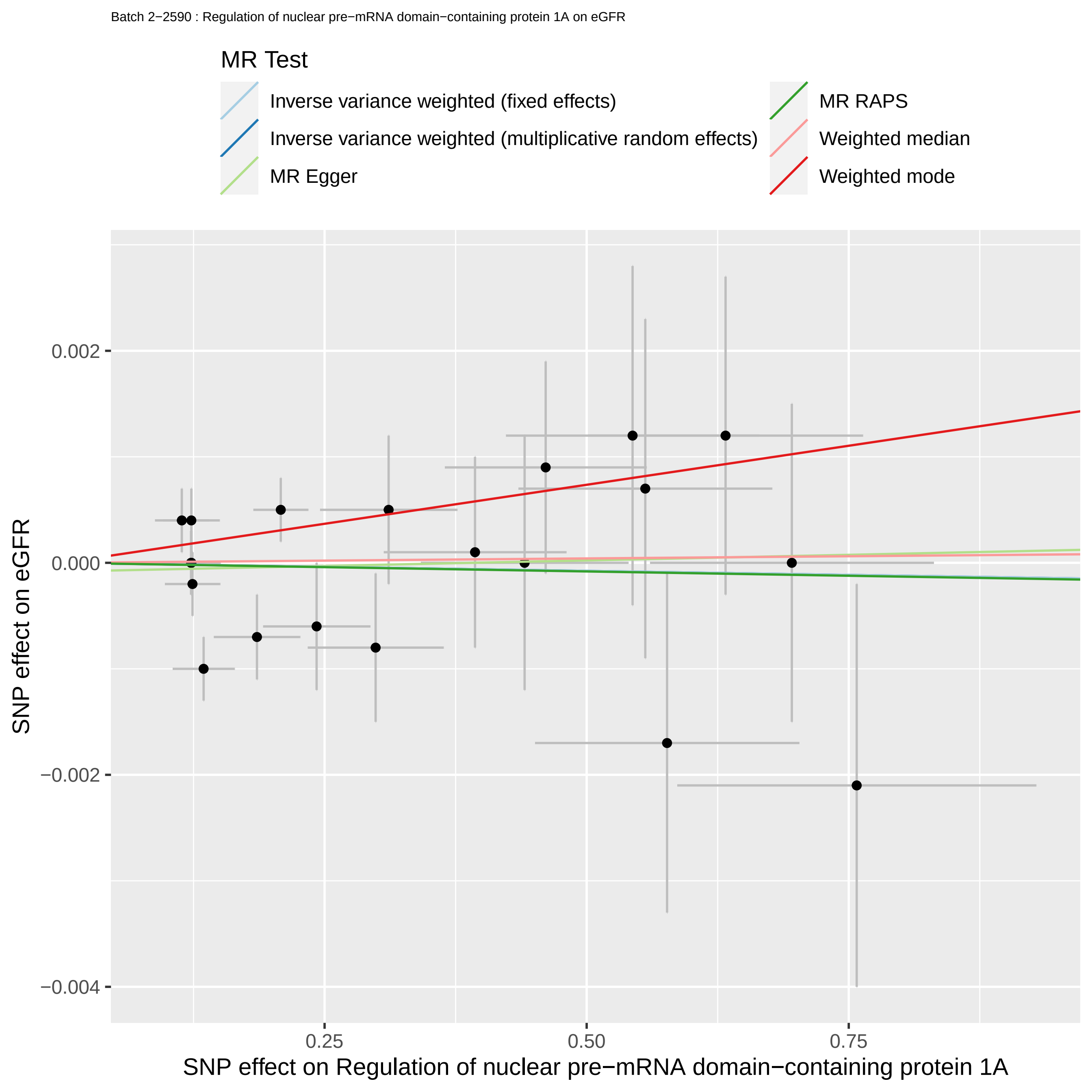

Batch 2−2590 : Regulation of nuclear pre−mRNA domain−containing protein 1A on eGFR
MR Test
Inverse variance weighted (fixed effects)
Inverse variance weighted (multiplicative random effects)
MR Egger
MR RAPS
Weighted median
Weighted mode
0.002
0.000
−0.002
−0.004
0.25
0.50
0.75
SNP effect on eGFR
SNP effect on Regulation of nuclear pre−mRNA domain−containing protein 1A

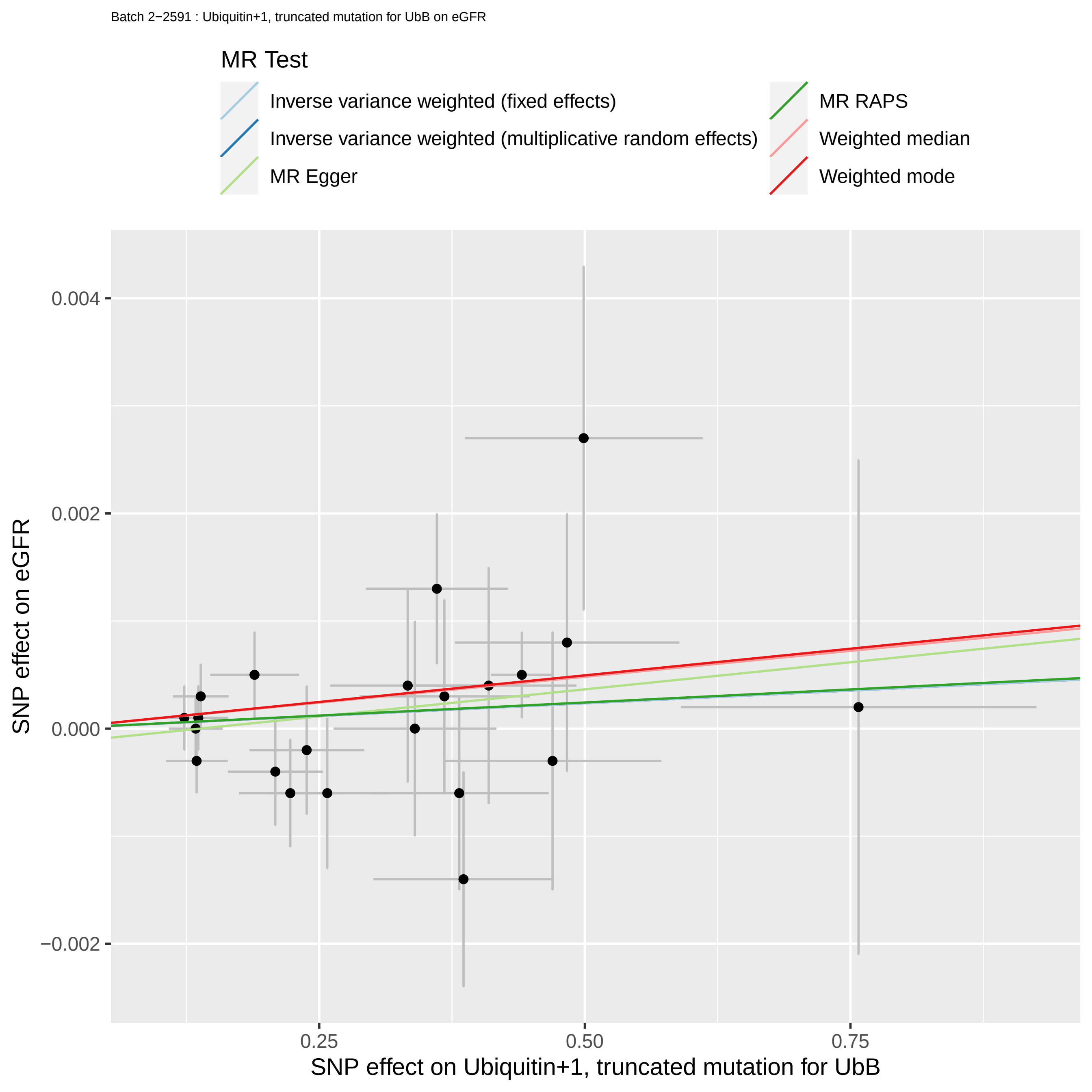
Batch 2−2591 : Ubiquitin+1, truncated mutation for UbB on eGFR
MR Test
Inverse variance weighted (fixed effects)
Inverse variance weighted (multiplicative random effects)
MR Egger
MR RAPS
Weighted median
Weighted mode
0.004
0.002
0.000
−0.002
0.25
0.50
0.75
SNP effect on eGFR
SNP effect on Ubiquitin+1, truncated mutation for UbB

Batch 2−2592 : Ubiquitin on eGFR

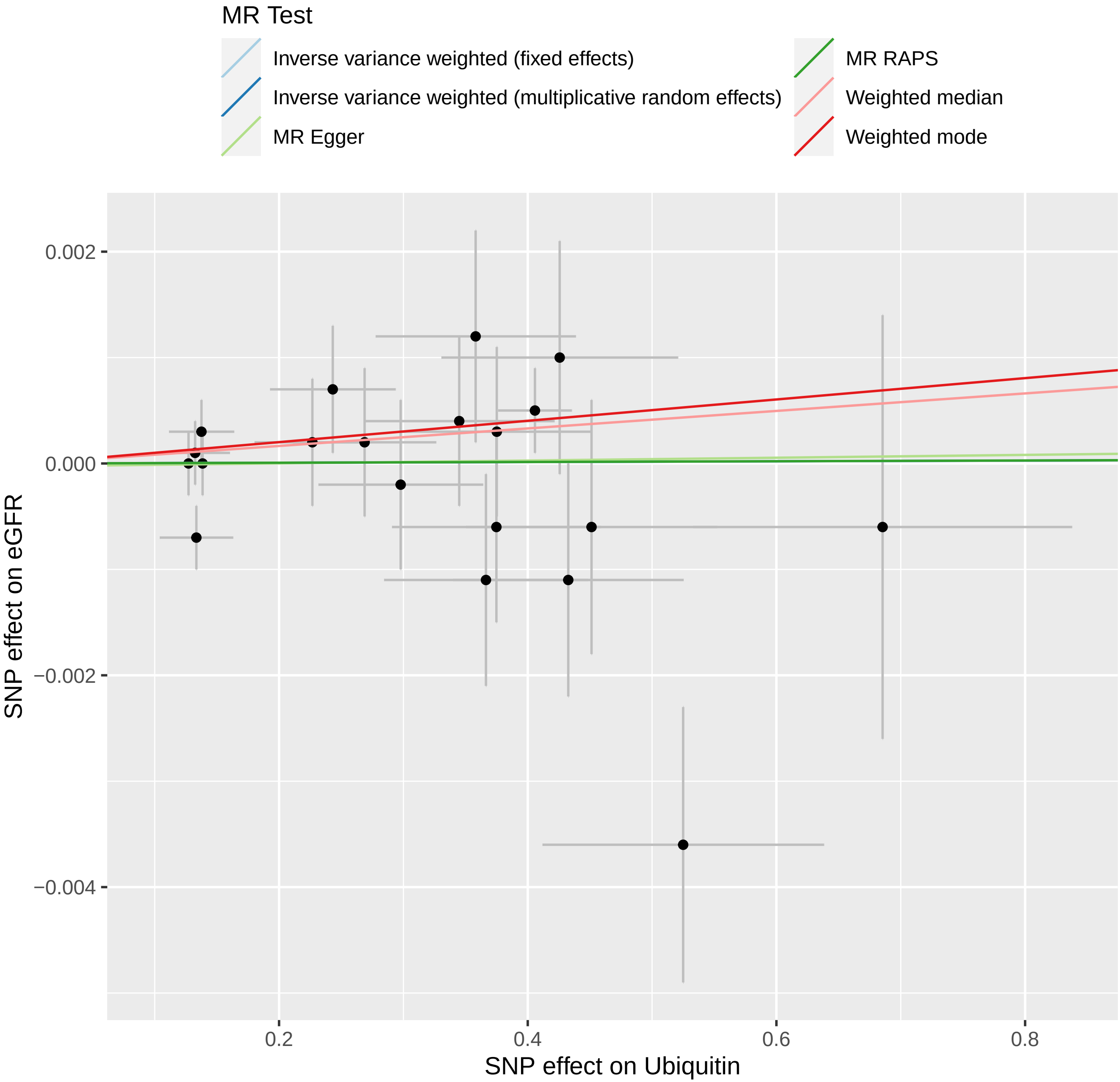

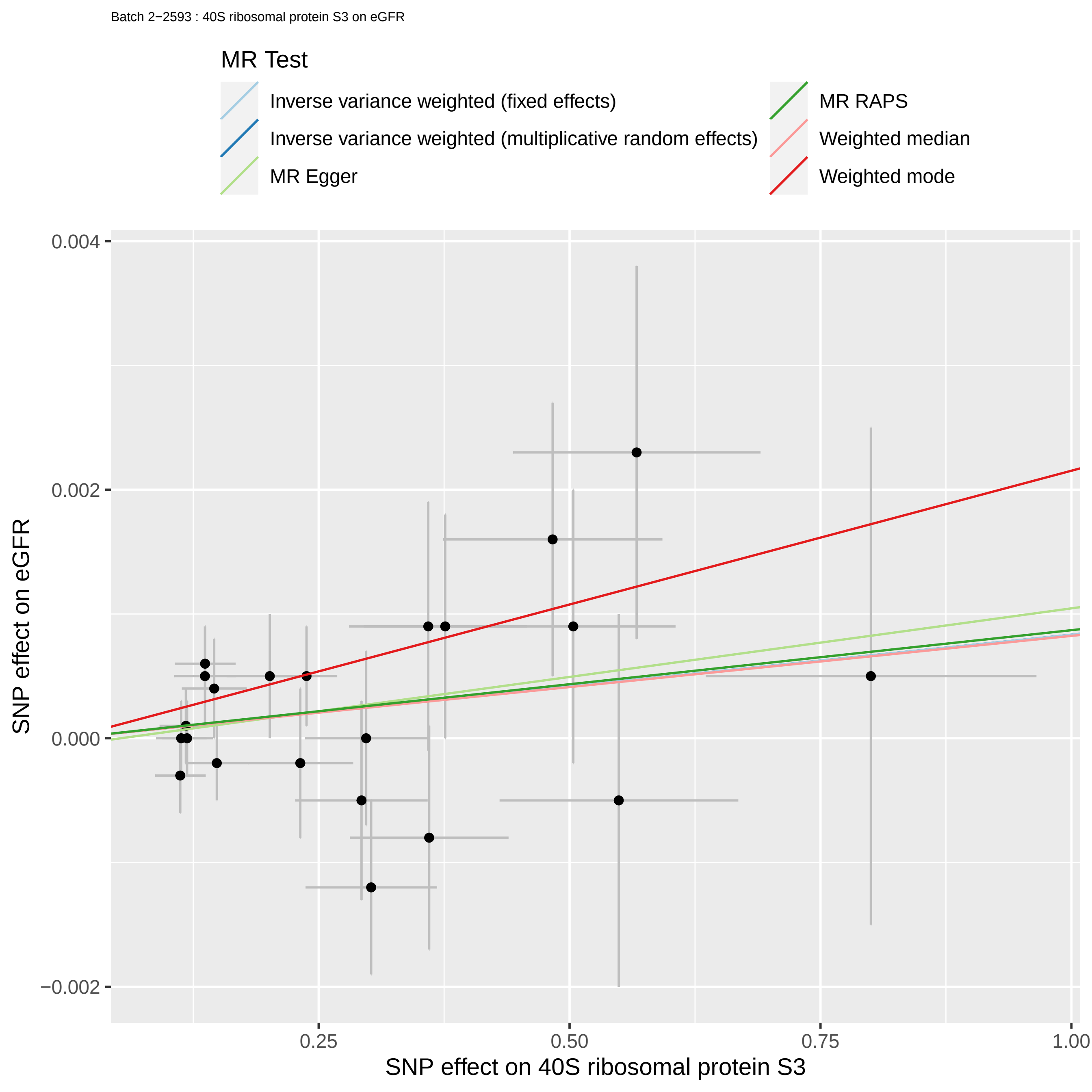
Batch 2−2593 : 40S ribosomal protein S3 on eGFR
MR Test
Inverse variance weighted (fixed effects)
Inverse variance weighted (multiplicative random effects)
MR Egger
MR RAPS
Weighted median
Weighted mode
0.004
0.002
0.000
−0.002
SNP effect on eGFR
0.25
0.50
0.75
1.00
SNP effect on 40S ribosomal protein S3

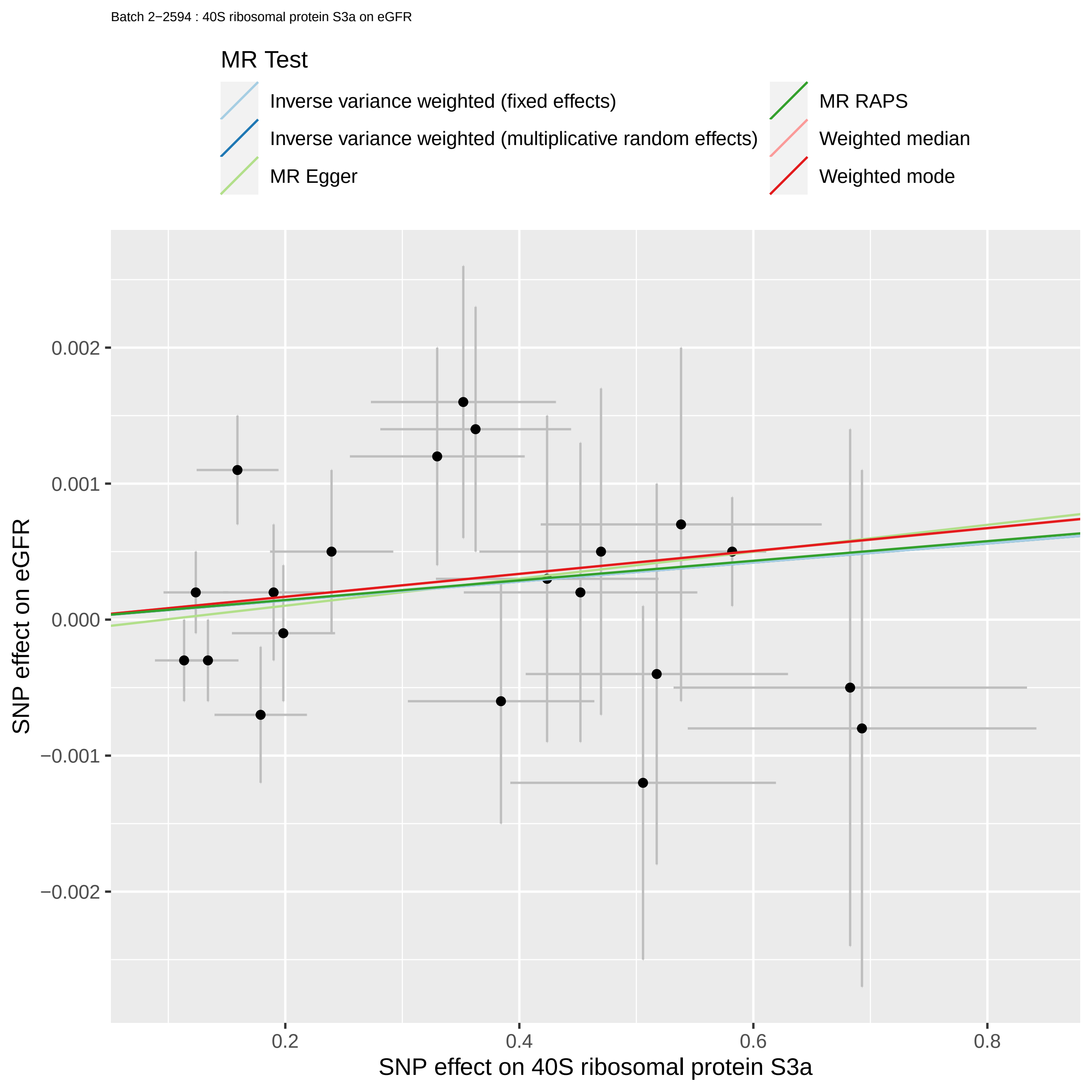
Batch 2−2594 : 40S ribosomal protein S3a on eGFR
MR Test
Inverse variance weighted (fixed effects)
Inverse variance weighted (multiplicative random effects)
MR Egger
MR RAPS
Weighted median
Weighted mode
SNP effect on eGFR
0.002
0.001
0.000
−0.001
−0.002
0.2
0.4
0.6
0.8
SNP effect on 40S ribosomal protein S3a

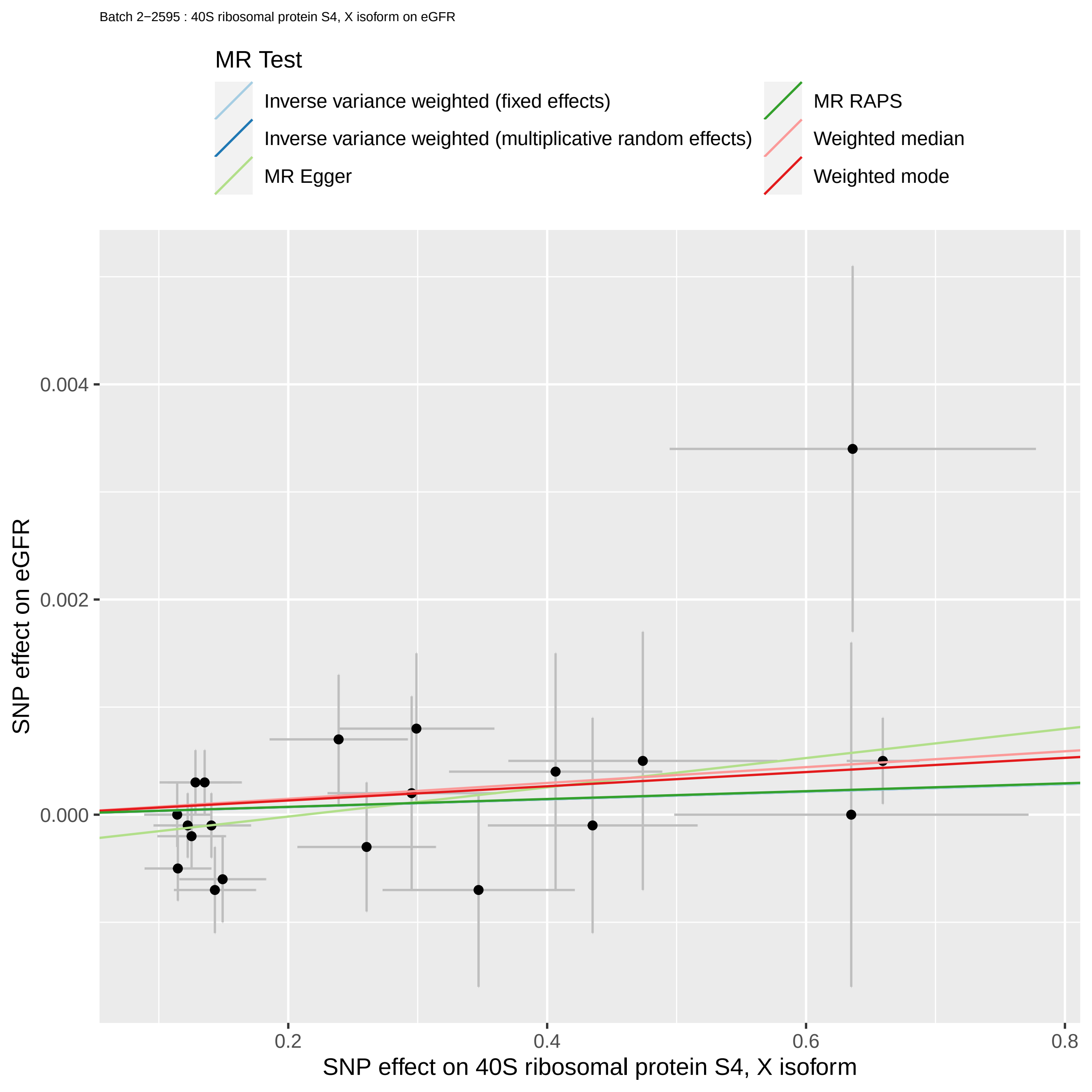
Batch 2−2595 : 40S ribosomal protein S4, X isoform on eGFR
MR Test
Inverse variance weighted (fixed effects)
Inverse variance weighted (multiplicative random effects)
MR Egger
MR RAPS
Weighted median
Weighted mode
0.004
0.002
0.000
0.2
0.4
0.6
0.8
SNP effect on eGFR
SNP effect on 40S ribosomal protein S4, X isoform

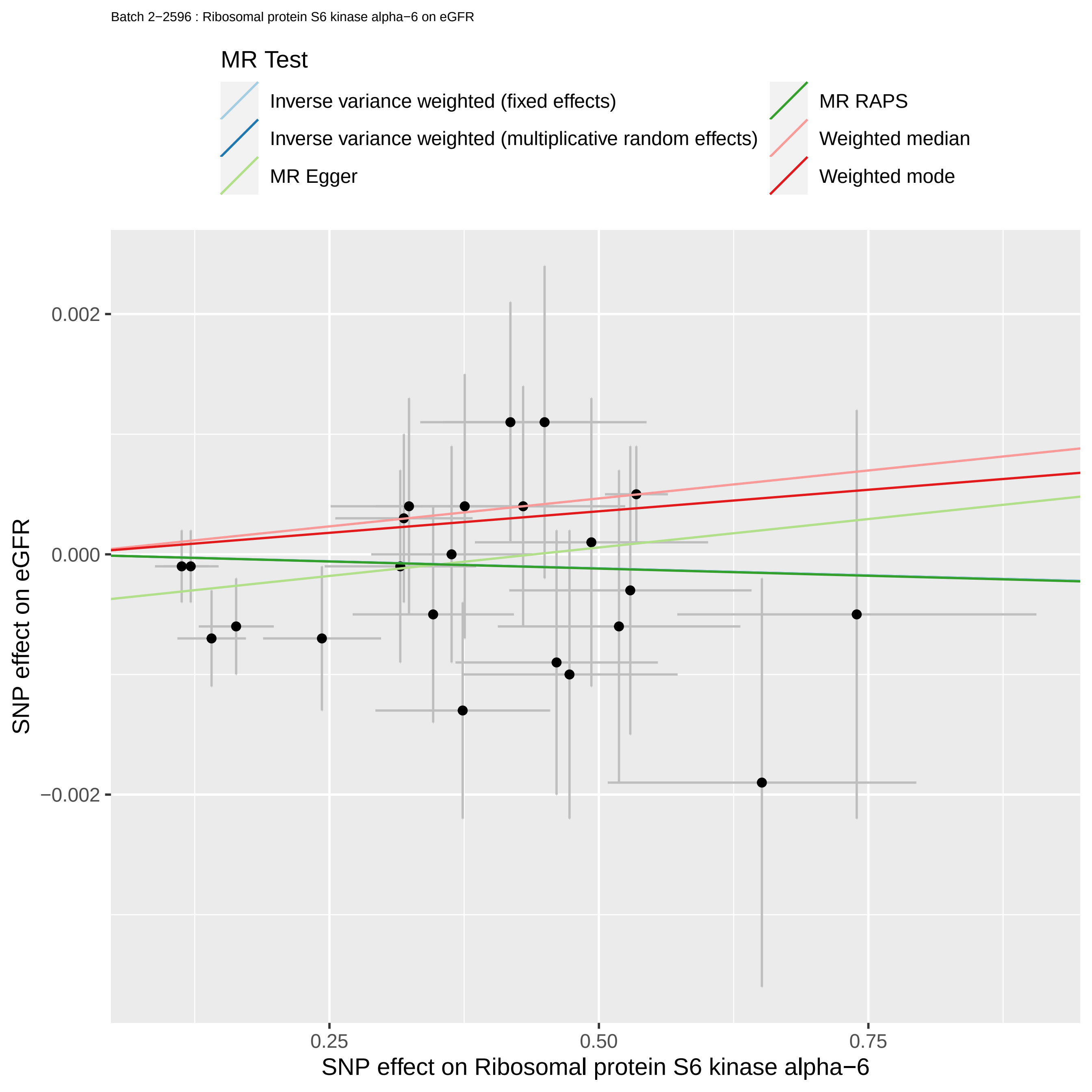

Batch 2−2596 : Ribosomal protein S6 kinase alpha−6 on eGFR
MR Test
Inverse variance weighted (fixed effects)
Inverse variance weighted (multiplicative random effects)
MR Egger
MR RAPS
Weighted median
Weighted mode
0.002
0.000
−0.002
0.25
0.50
0.75
SNP effect on eGFR
SNP effect on Ribosomal protein S6 kinase alpha−6

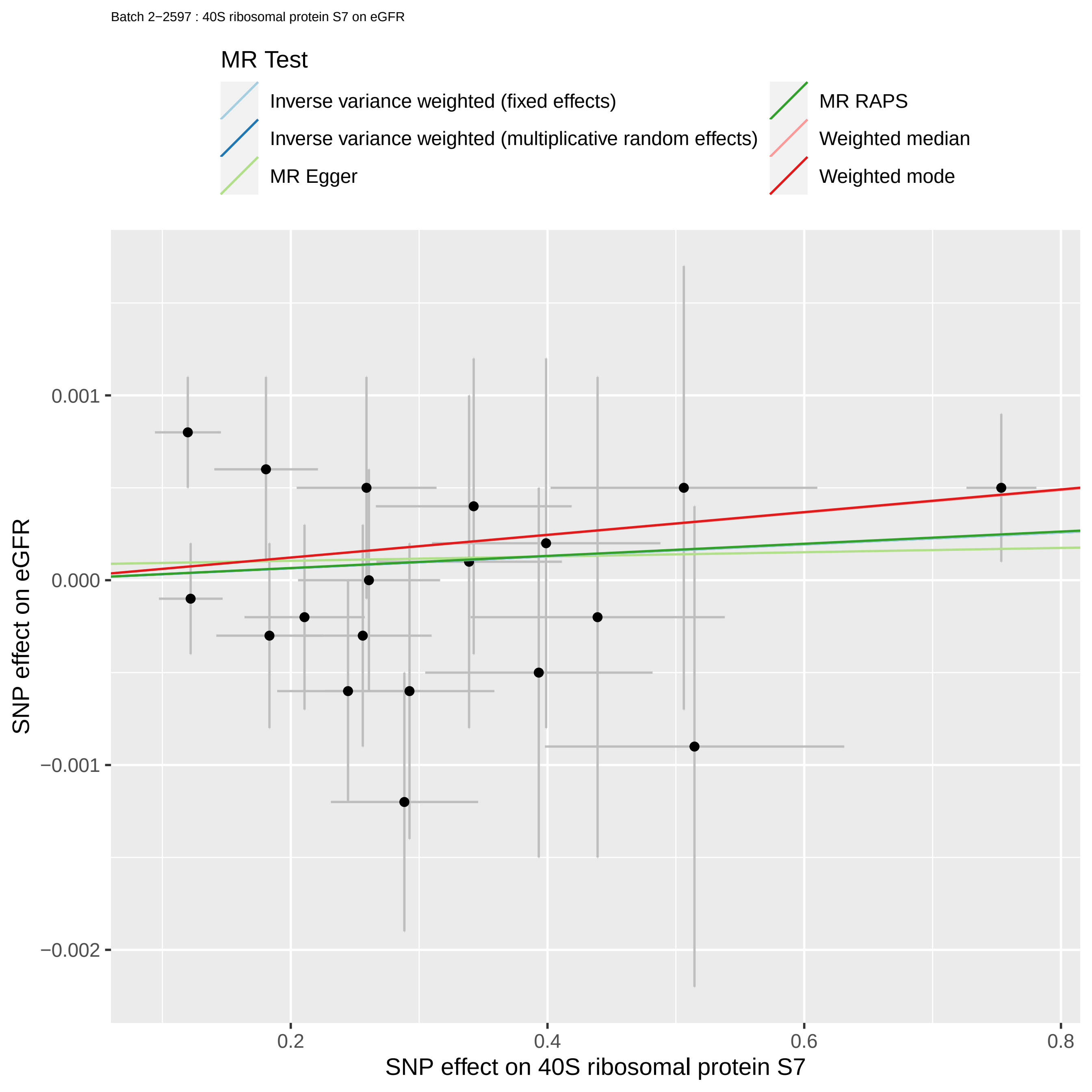

Batch 2−2597 : 40S ribosomal protein S7 on eGFR
MR Test
Inverse variance weighted (fixed effects)
Inverse variance weighted (multiplicative random effects)
MR Egger
MR RAPS
Weighted median
Weighted mode
0.001
0.000
−0.001
−0.002
0.2
0.4
0.6
0.8
SNP effect on 40S ribosomal protein S7
SNP effect on eGFR

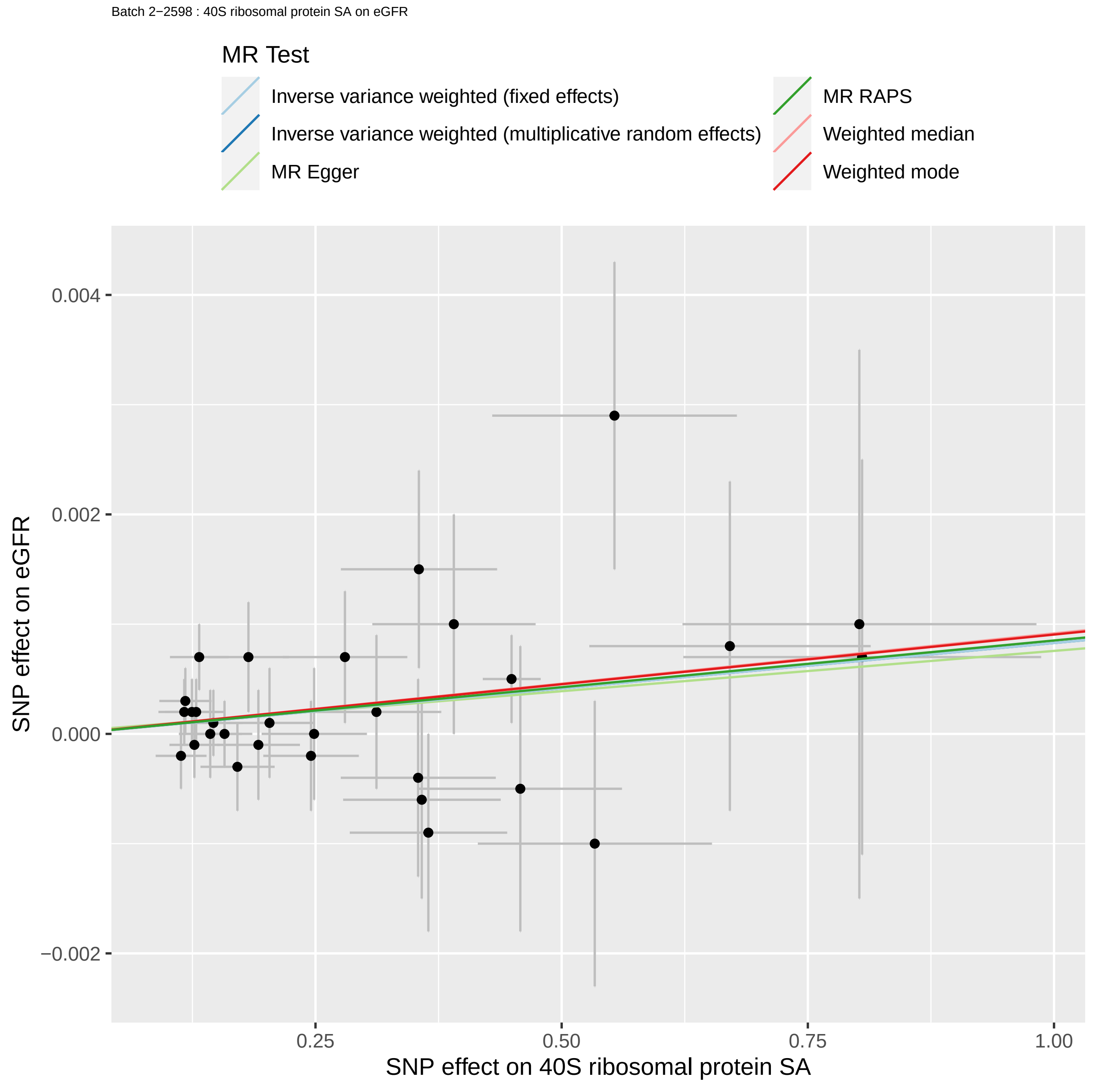

Batch 2−2598 : 40S ribosomal protein SA on eGFR
MR Test
Inverse variance weighted (fixed effects)
Inverse variance weighted (multiplicative random effects)
MR Egger
MR RAPS
Weighted median
Weighted mode
0.004
0.002
0.000
−0.002
SNP effect on eGFR
0.25
0.50
0.75
1.00
SNP effect on 40S ribosomal protein SA

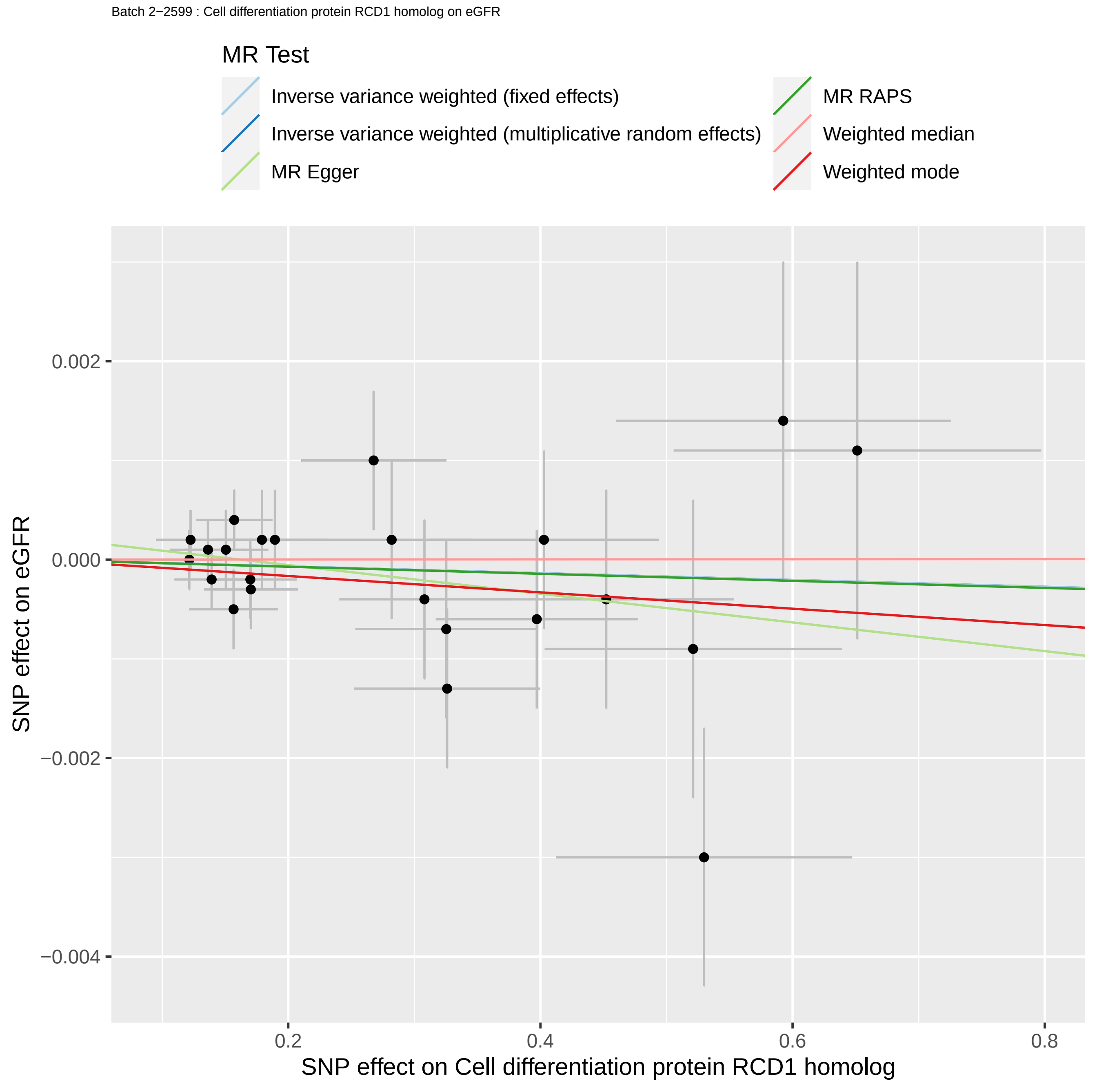
Batch 2−2599 : Cell differentiation protein RCD1 homolog on eGFR
MR Test
Inverse variance weighted (fixed effects)
Inverse variance weighted (multiplicative random effects)
MR Egger
MR RAPS
Weighted median
Weighted mode
0.002
0.000
−0.002
−0.004
SNP effect on eGFR
0.2
0.4
0.6
0.8
SNP effect on Cell differentiation protein RCD1 homolog

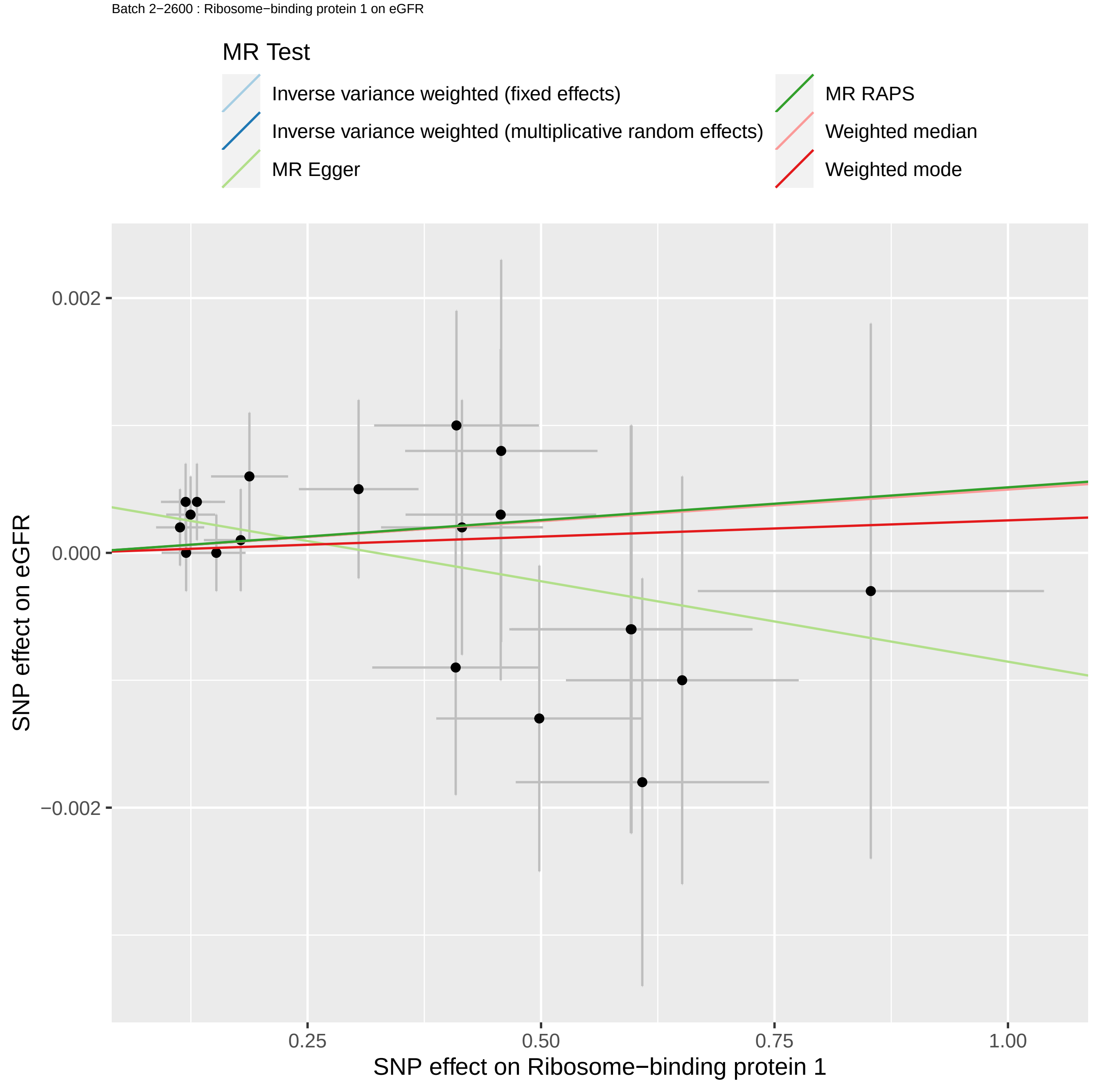

Batch 2−2600 : Ribosome−binding protein 1 on eGFR
MR Test
Inverse variance weighted (fixed effects)
Inverse variance weighted (multiplicative random effects)
MR Egger
MR RAPS
Weighted median
Weighted mode
0.002
0.000
−0.002
0.25
0.50
0.75
1.00
SNP effect on eGFR
SNP effect on Ribosome−binding protein 1

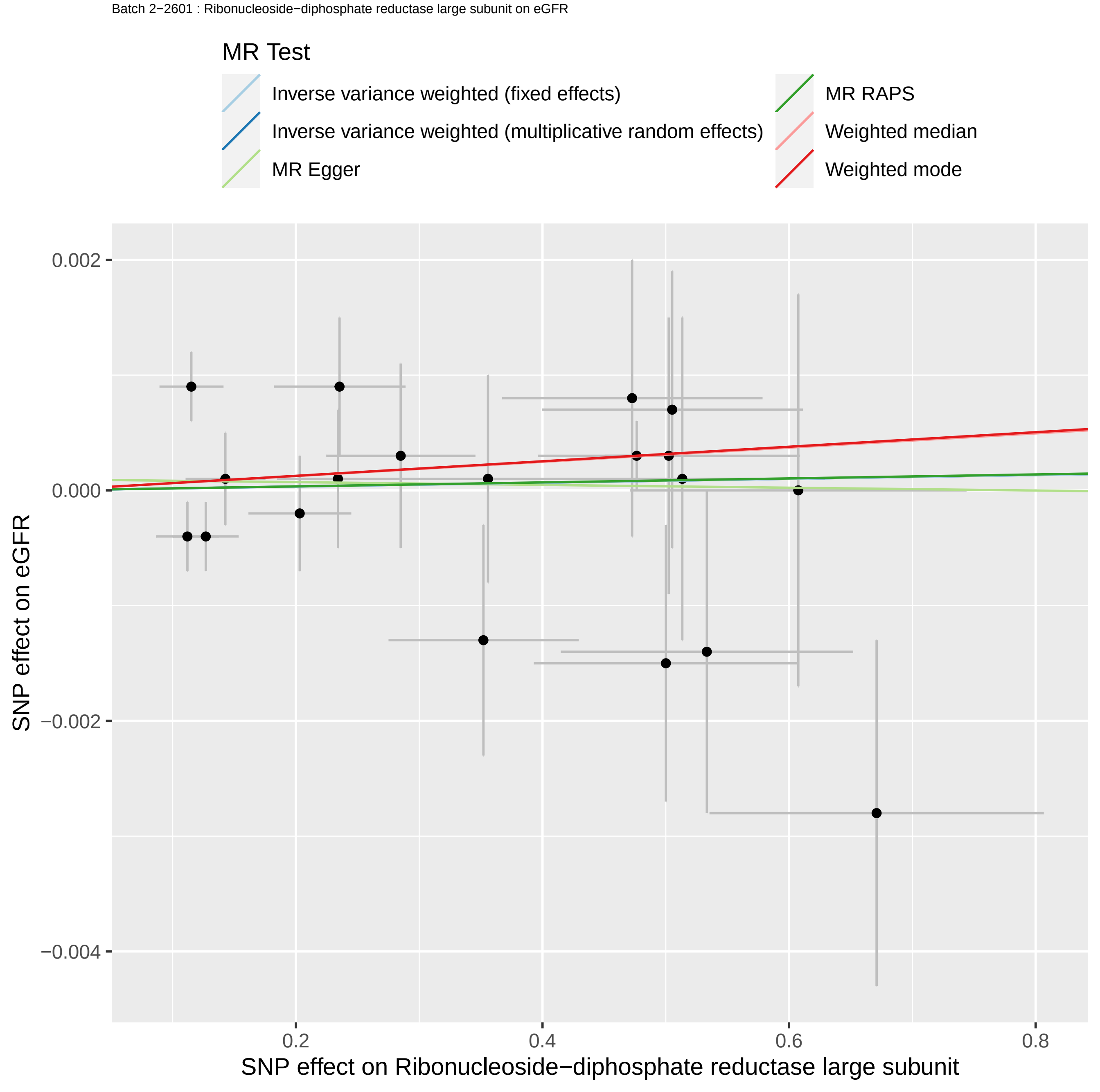
Batch 2−2601 : Ribonucleoside−diphosphate reductase large subunit on eGFR
MR Test
Inverse variance weighted (fixed effects)
Inverse variance weighted (multiplicative random effects)
MR Egger
MR RAPS
Weighted median
Weighted mode
0.002
0.000
−0.002
−0.004
0.2
0.4
0.6
0.8
SNP effect on eGFR
SNP effect on Ribonucleoside−diphosphate reductase large subunit

Batch 2−2602 : Ribonucleoside−diphosphate reductase subunit M2 B on eGFR

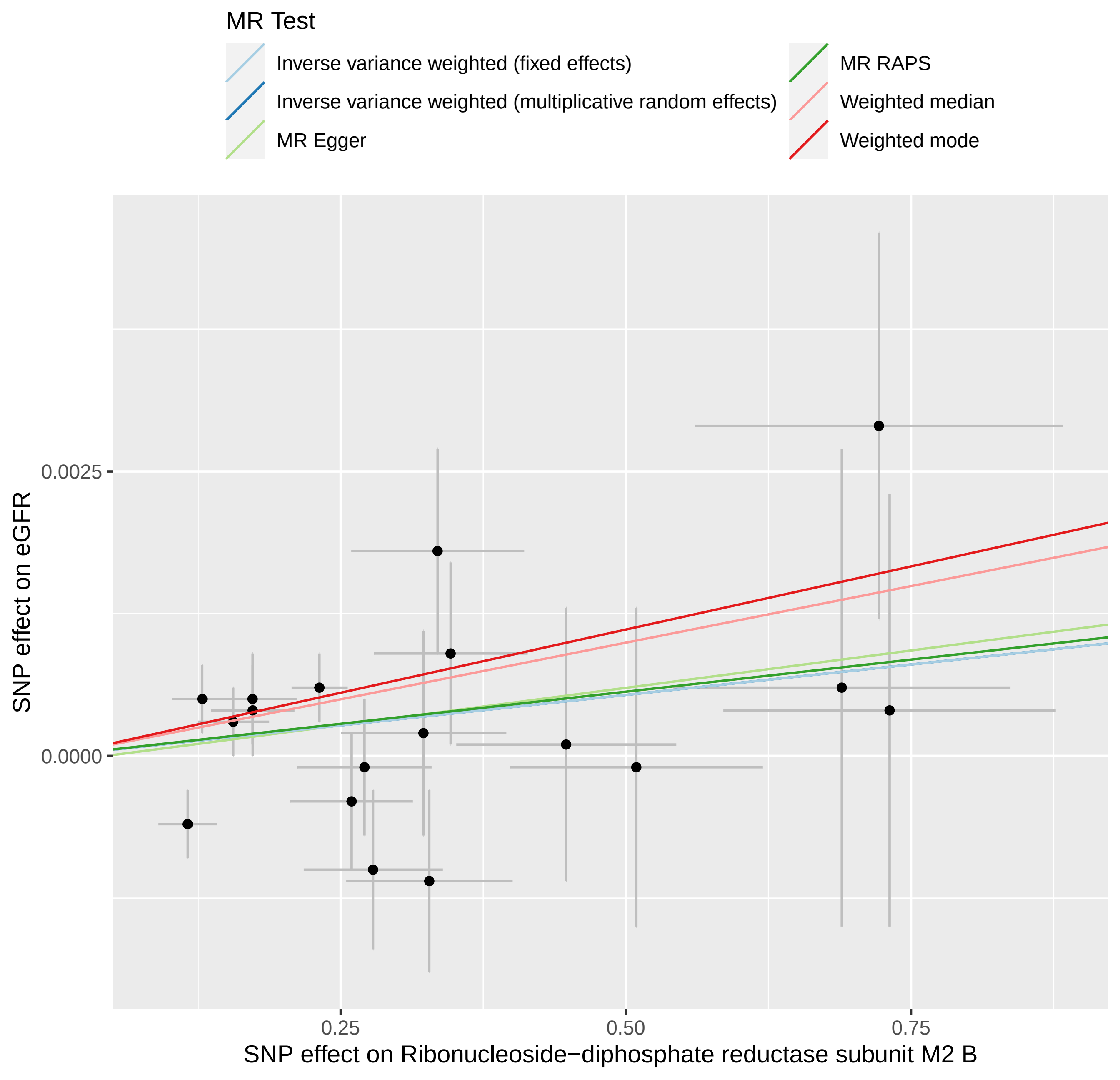

Batch 2−2603 : Ribonucleoside−diphosphate reductase subunit M2 B on eGFR

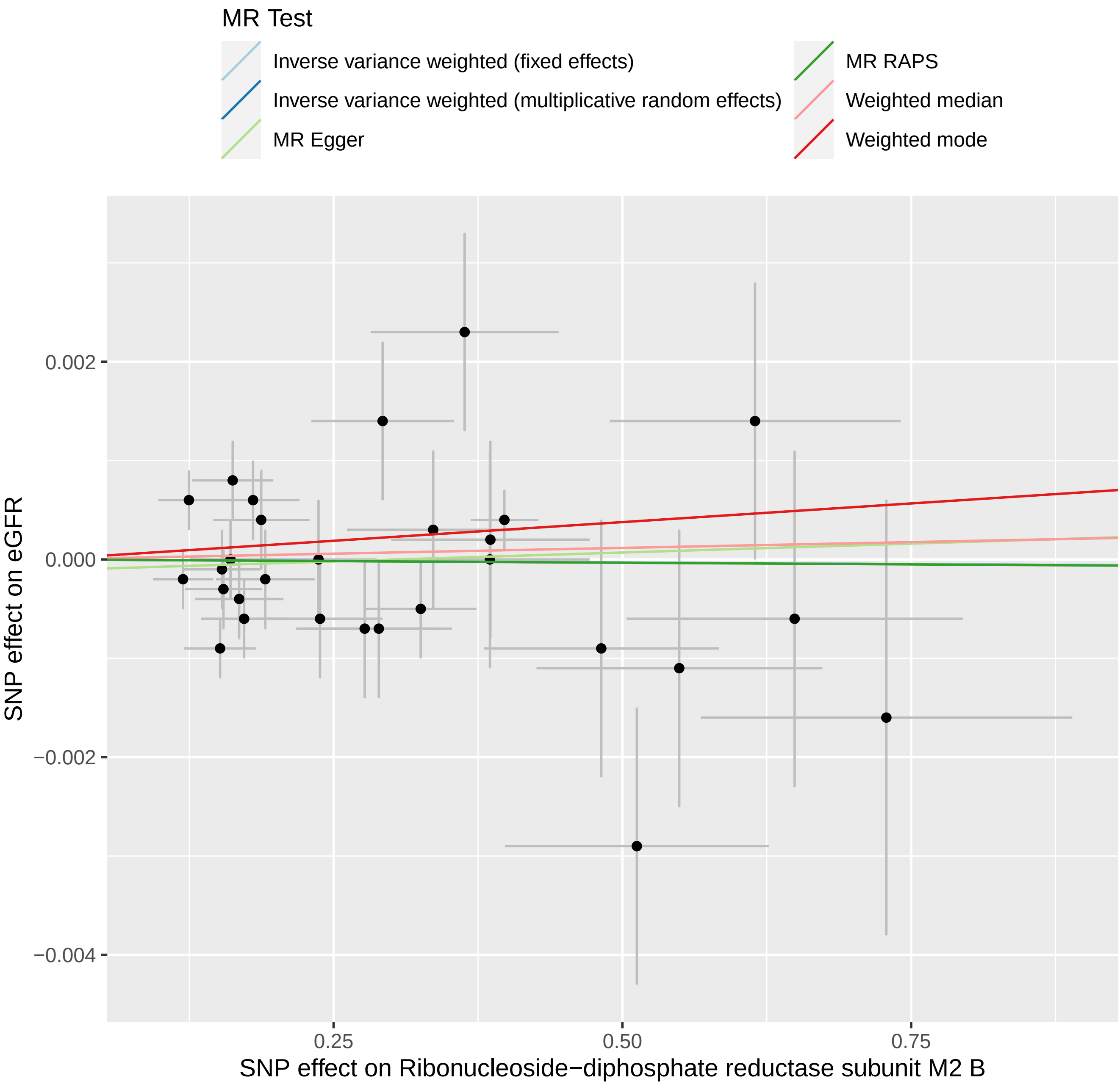

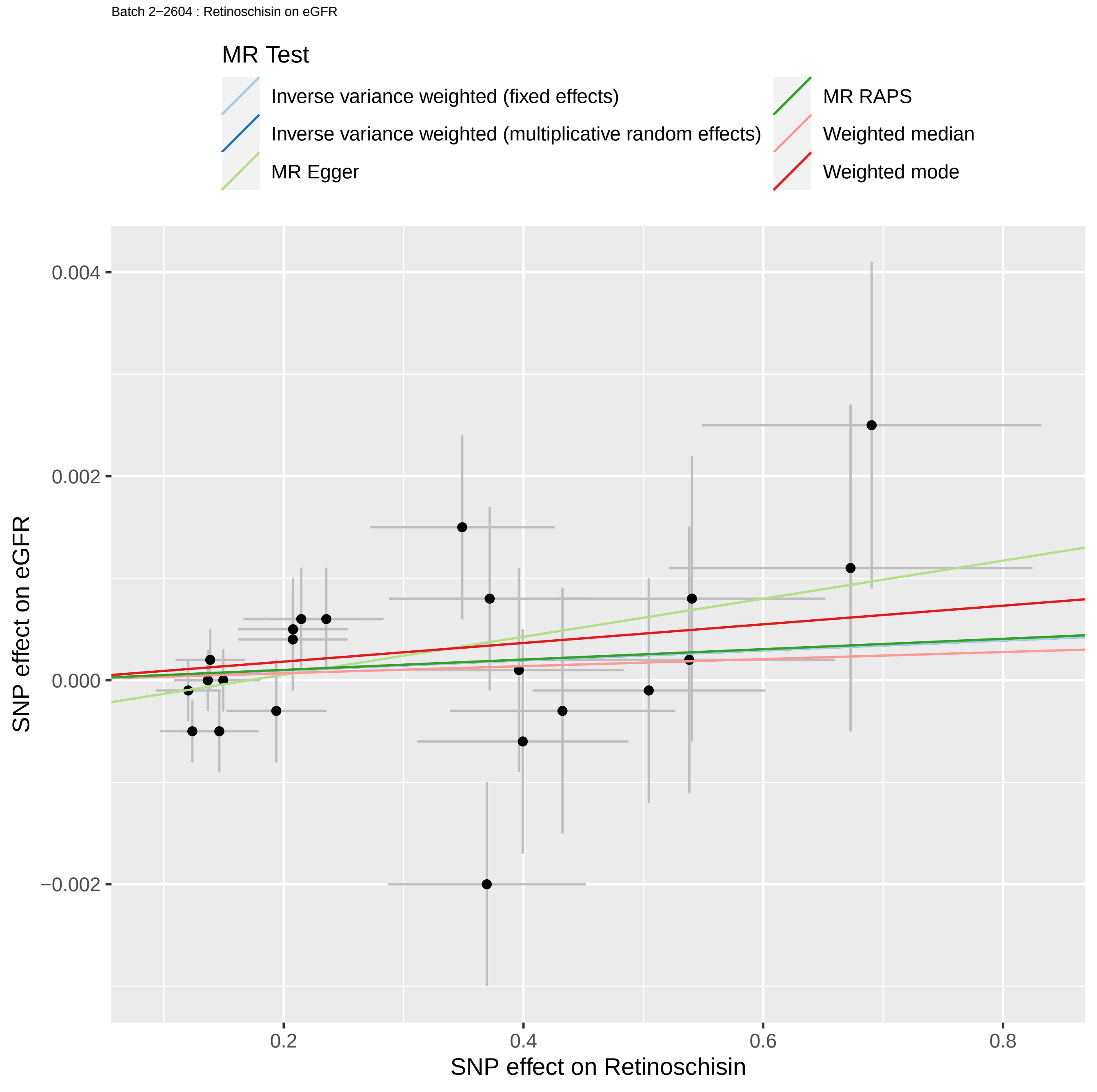

Batch 2−2604 : Retinoschisin on eGFR
MR Test
Inverse variance weighted (fixed effects)
Inverse variance weighted (multiplicative random effects)
MR Egger
MR RAPS
Weighted median
Weighted mode
0.004
0.002
0.000
−0.002
0.2
0.4
0.6
0.8
SNP effect on eGFR
SNP effect on Retinoschisin

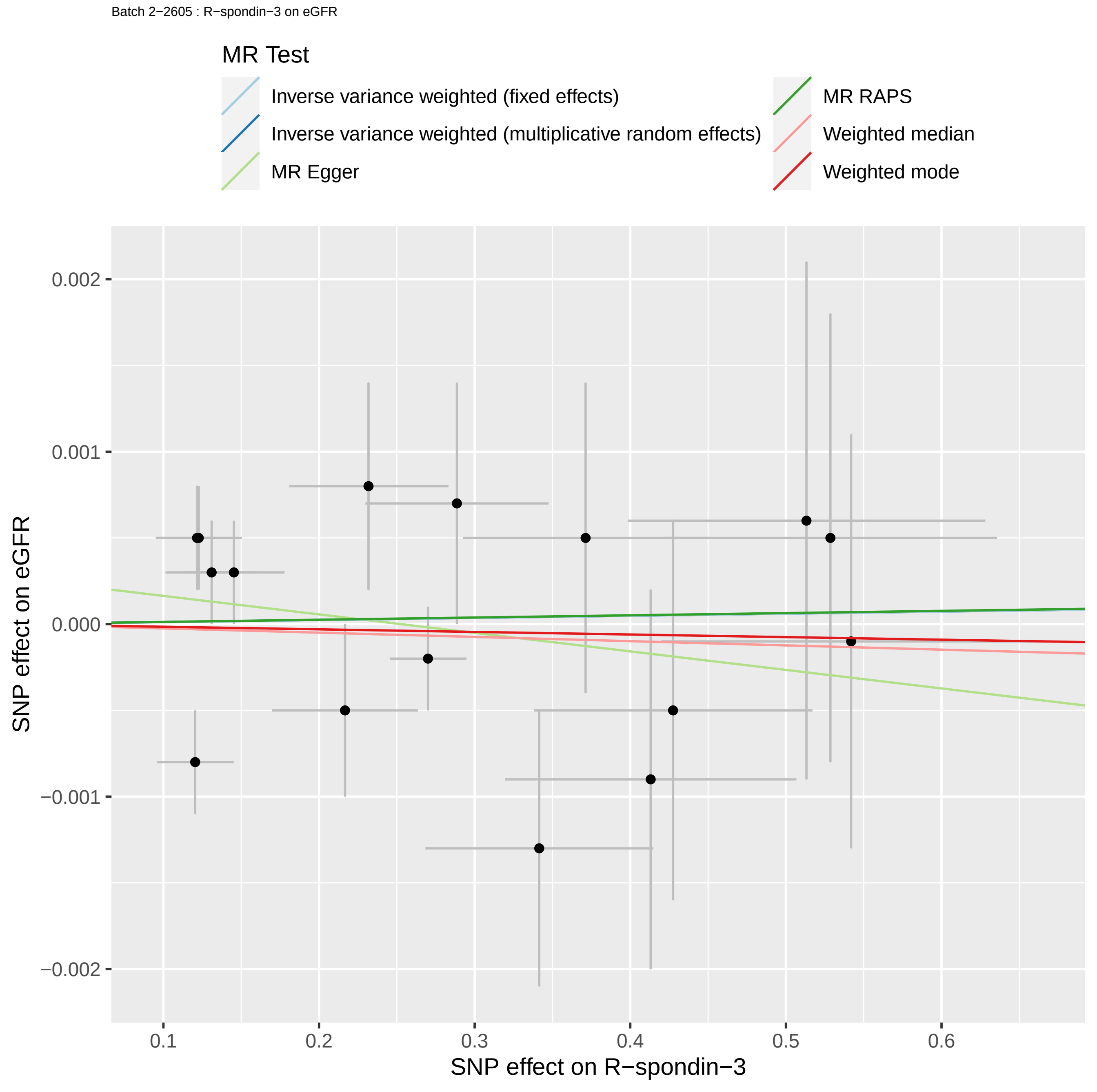

Batch 2−2605 : R−spondin−3 on eGFR
MR Test
Inverse variance weighted (fixed effects)
Inverse variance weighted (multiplicative random effects)
MR Egger
MR RAPS
Weighted median
Weighted mode
0.002
0.001
0.000
−0.001
−0.002
0.1
0.2
0.3
0.4
0.5
0.6
SNP effect on eGFR
SNP effect on R−spondin−3

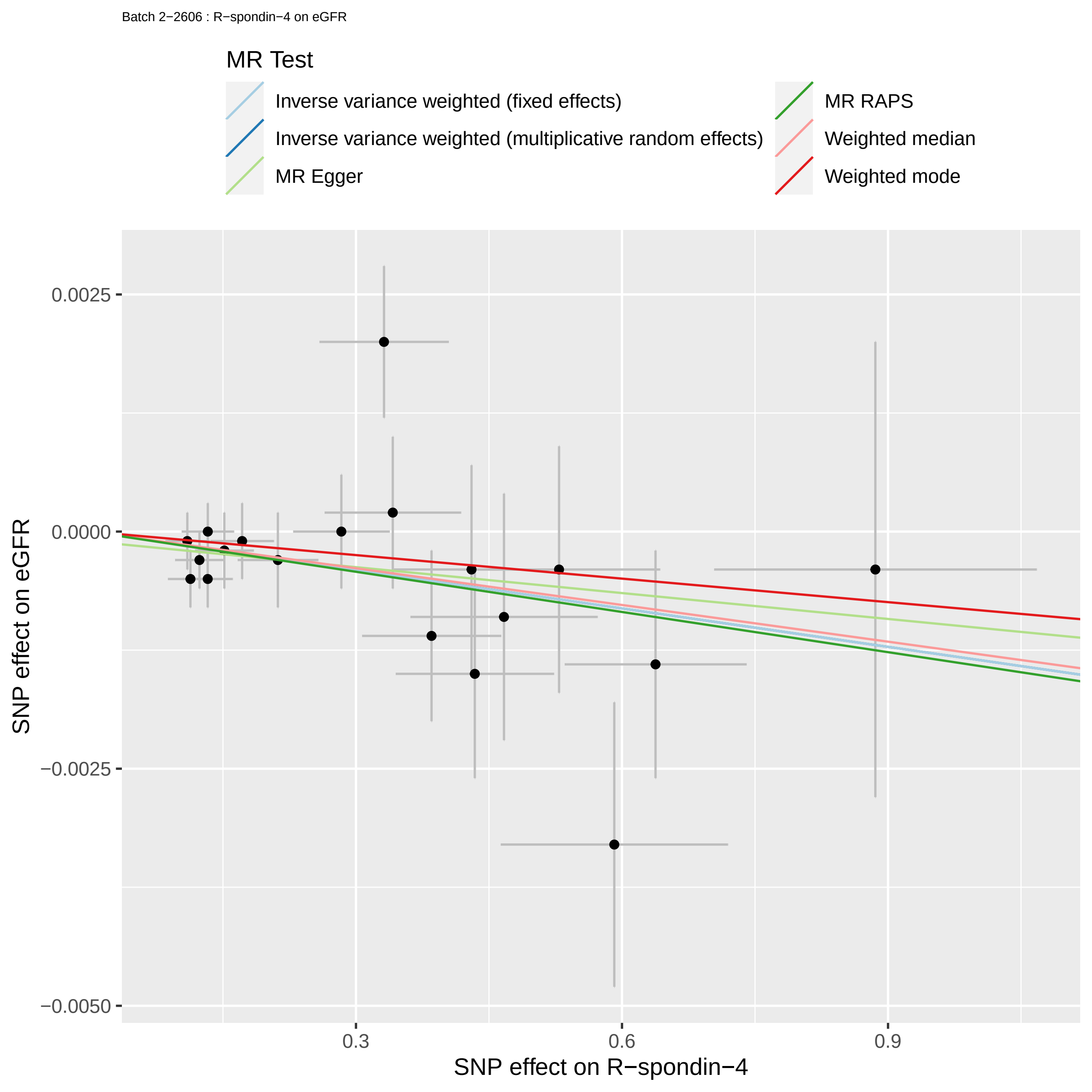

Batch 2−2606 : R−spondin−4 on eGFR
MR Test
Inverse variance weighted (fixed effects)
Inverse variance weighted (multiplicative random effects)
MR Egger
MR RAPS
Weighted median
Weighted mode
0.0025
0.0000
−0.0025
−0.0050
0.3
0.6
0.9
SNP effect on eGFR
SNP effect on R−spondin−4

Batch 2−2607 : Arginine/serine−rich protein 1 on eGFR

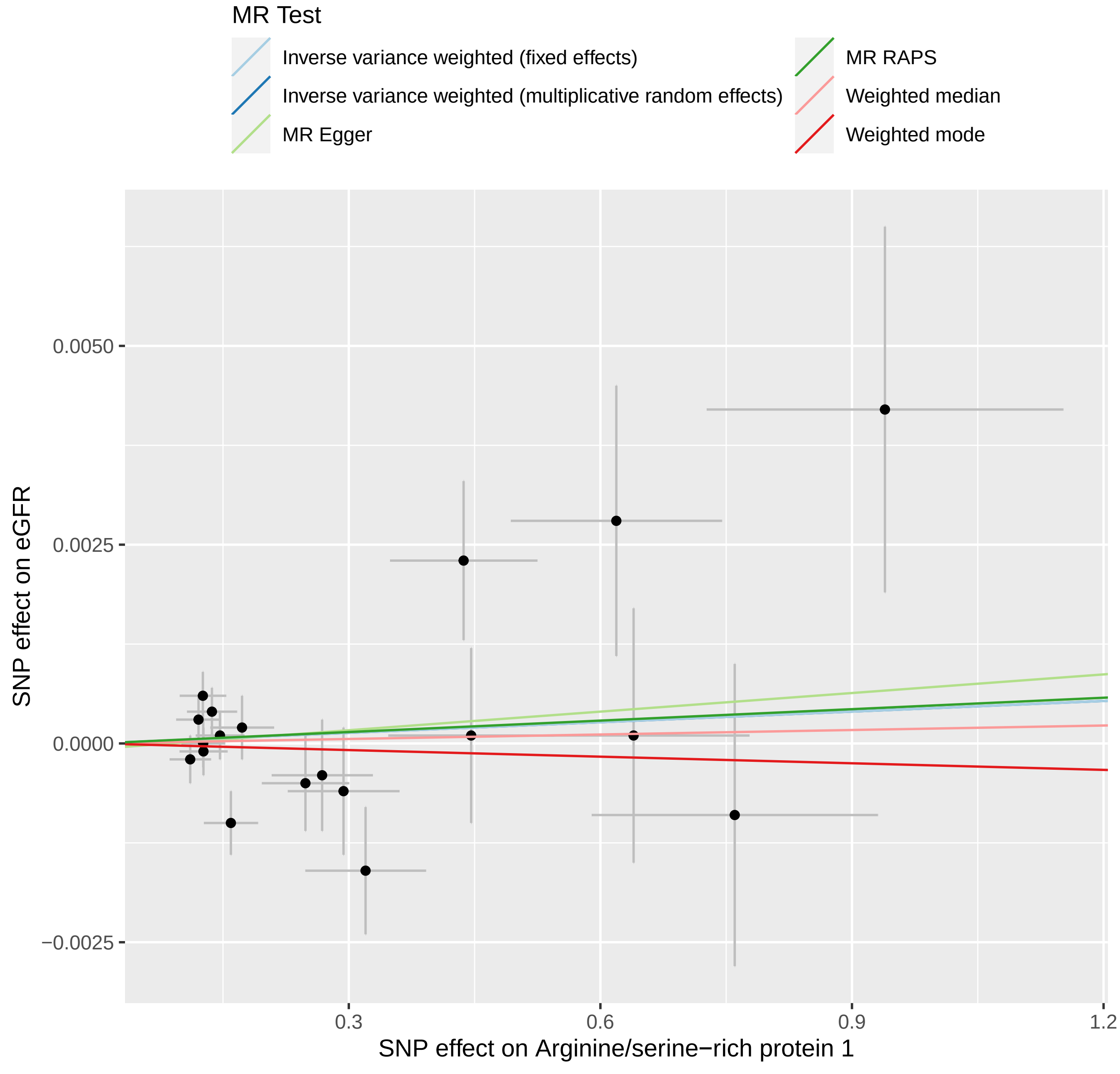

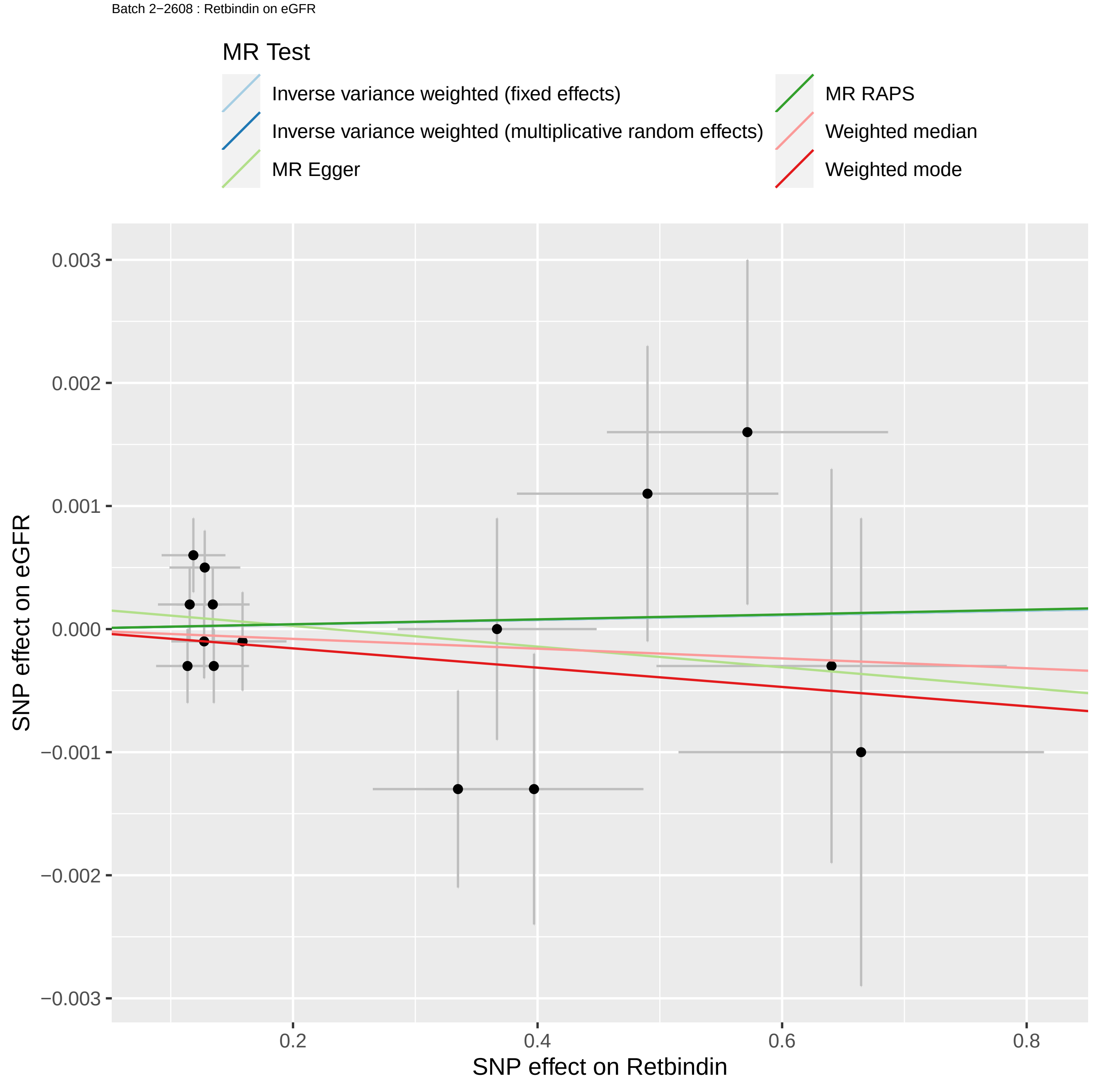
Batch 2−2608 : Retbindin on eGFR
MR Test
Inverse variance weighted (fixed effects)
Inverse variance weighted (multiplicative random effects)
MR Egger
MR RAPS
Weighted median
Weighted mode
0.003
0.002
0.001
0.000
−0.001
−0.002
−0.003
0.2
0.4
0.6
0.8
SNP effect on eGFR
SNP effect on Retbindin

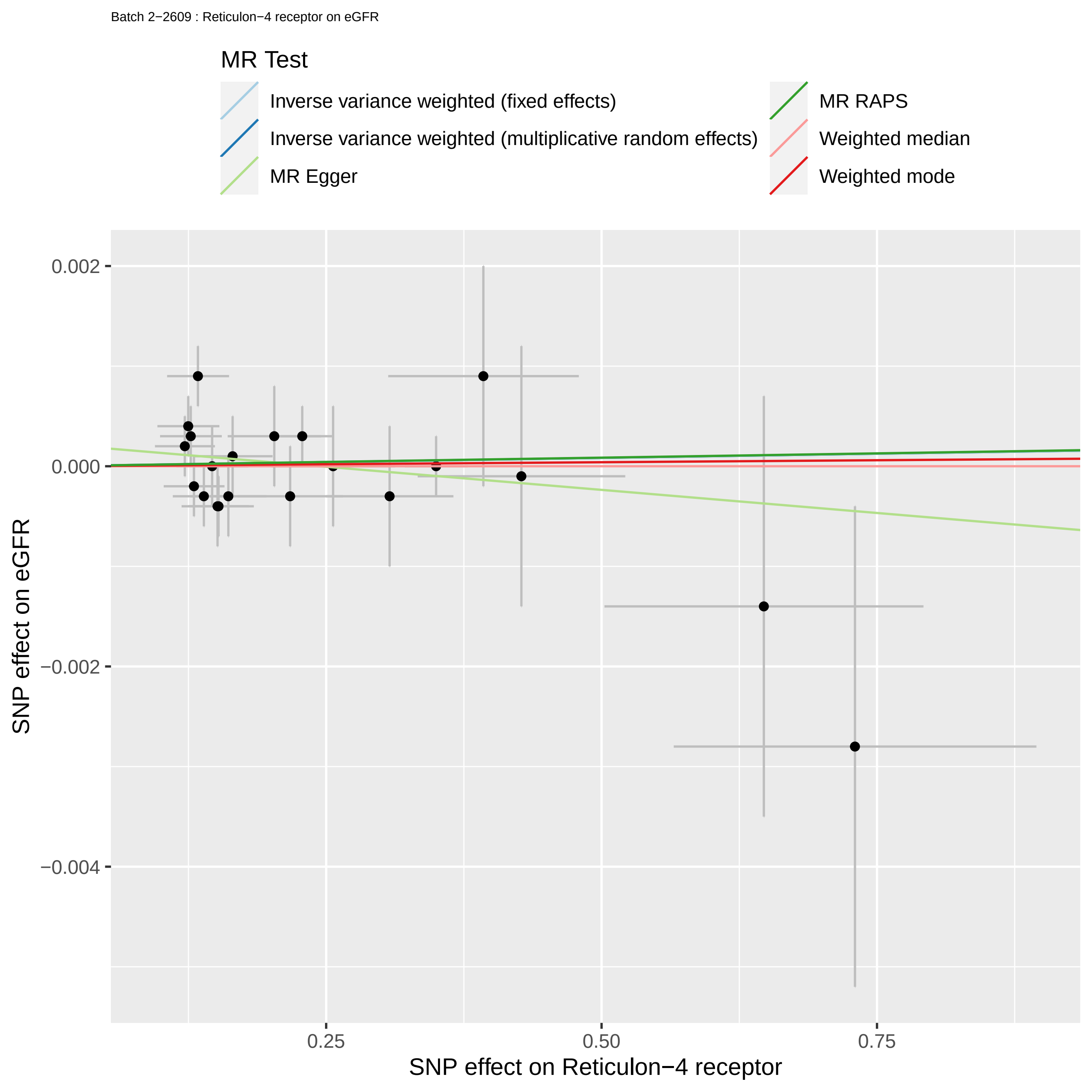
Batch 2−2609 : Reticulon−4 receptor on eGFR
MR Test
Inverse variance weighted (fixed effects)
Inverse variance weighted (multiplicative random effects)
MR Egger
MR RAPS
Weighted median
Weighted mode
0.002
0.000
−0.002
−0.004
0.25
0.50
0.75
SNP effect on eGFR
SNP effect on Reticulon−4 receptor

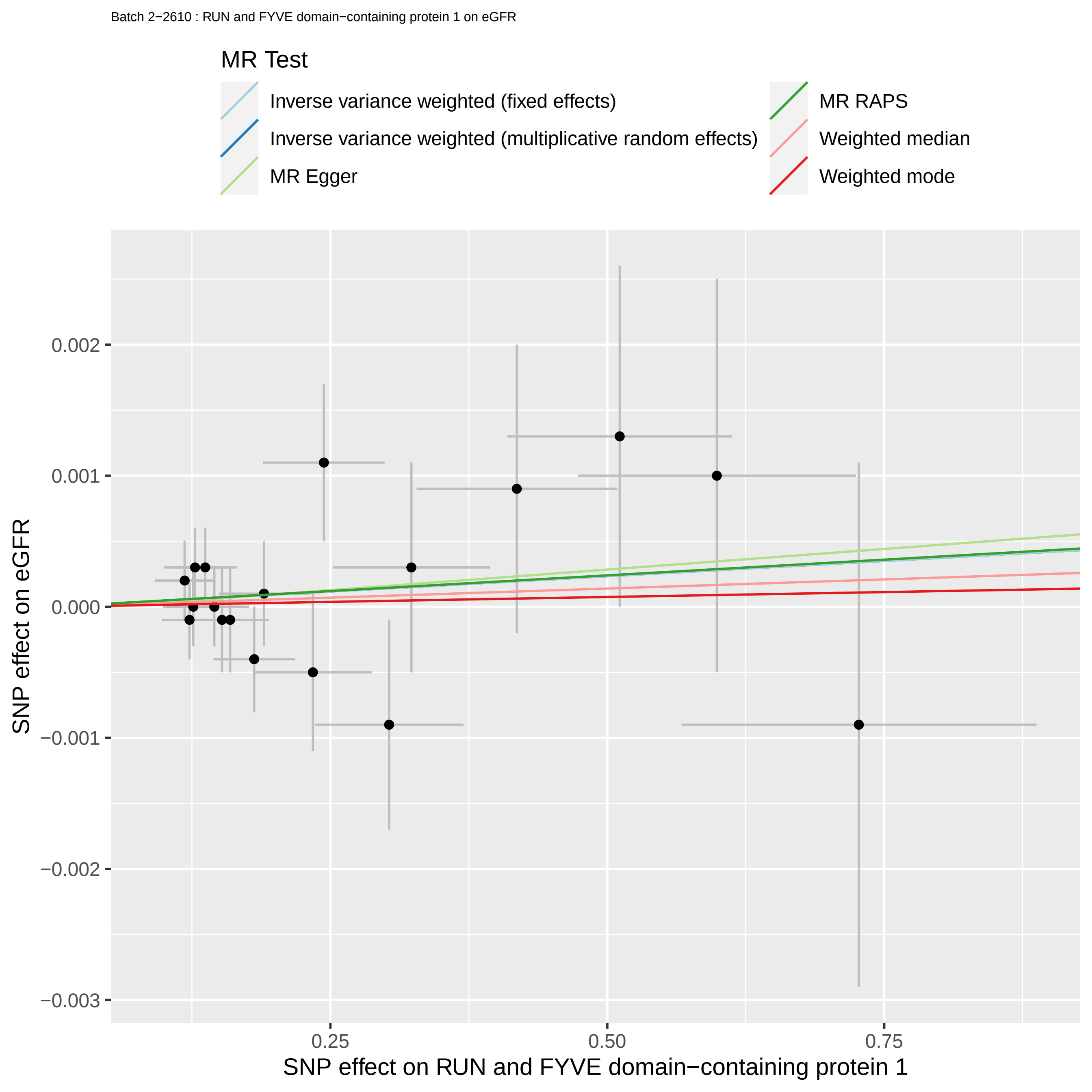
Batch 2−2610 : RUN and FYVE domain−containing protein 1 on eGFR
MR Test
Inverse variance weighted (fixed effects)
Inverse variance weighted (multiplicative random effects)
MR Egger
MR RAPS
Weighted median
Weighted mode
0.002
0.001
0.000
−0.001
−0.002
−0.003
SNP effect on eGFR
0.25
0.50
0.75
SNP effect on RUN and FYVE domain−containing protein 1

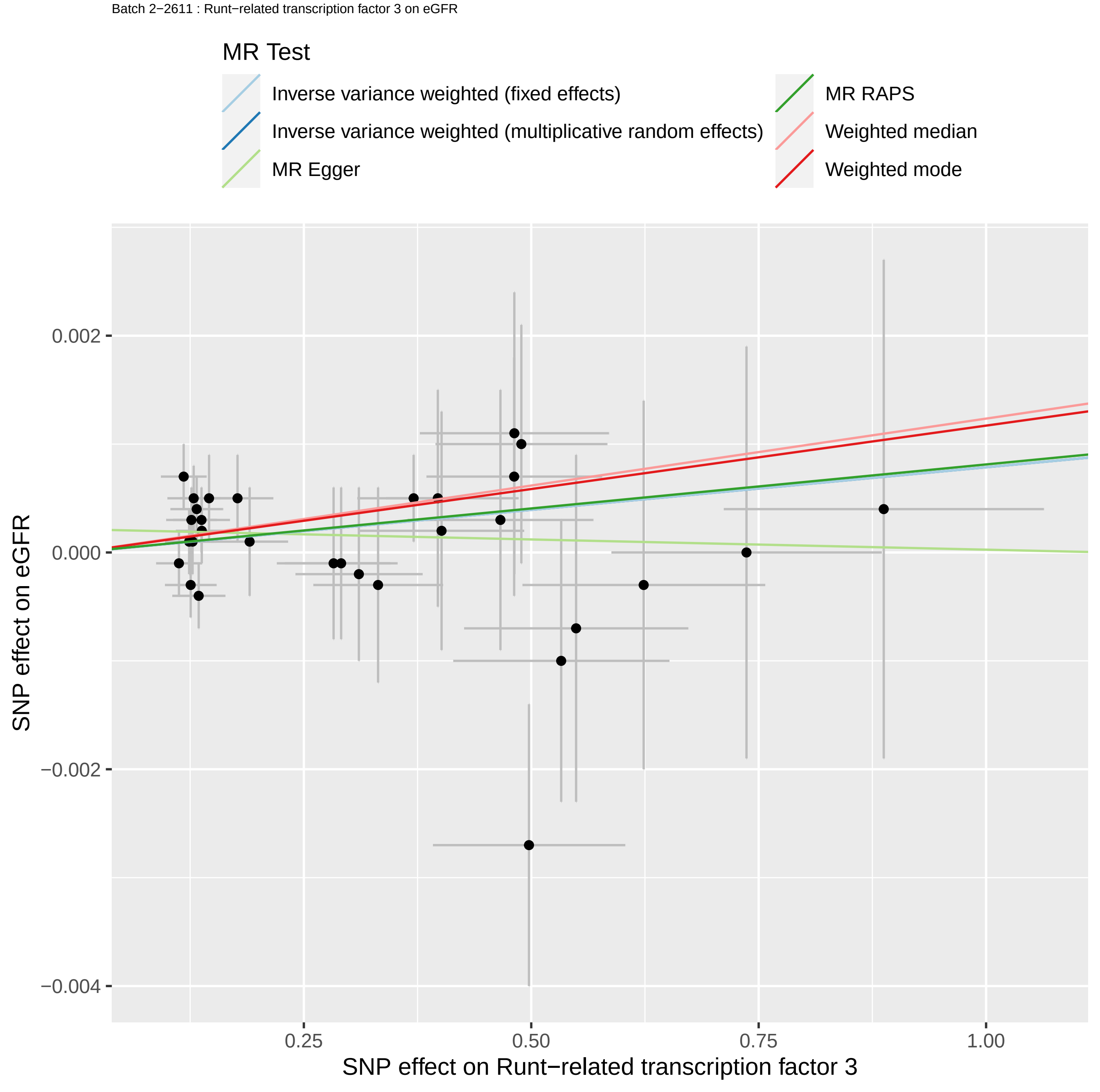

Batch 2−2611 : Runt−related transcription factor 3 on eGFR
MR Test
Inverse variance weighted (fixed effects)
Inverse variance weighted (multiplicative random effects)
MR Egger
MR RAPS
Weighted median
Weighted mode
0.002
0.000
−0.002
−0.004
0.25
0.50
0.75
1.00
SNP effect on eGFR
SNP effect on Runt−related transcription factor 3

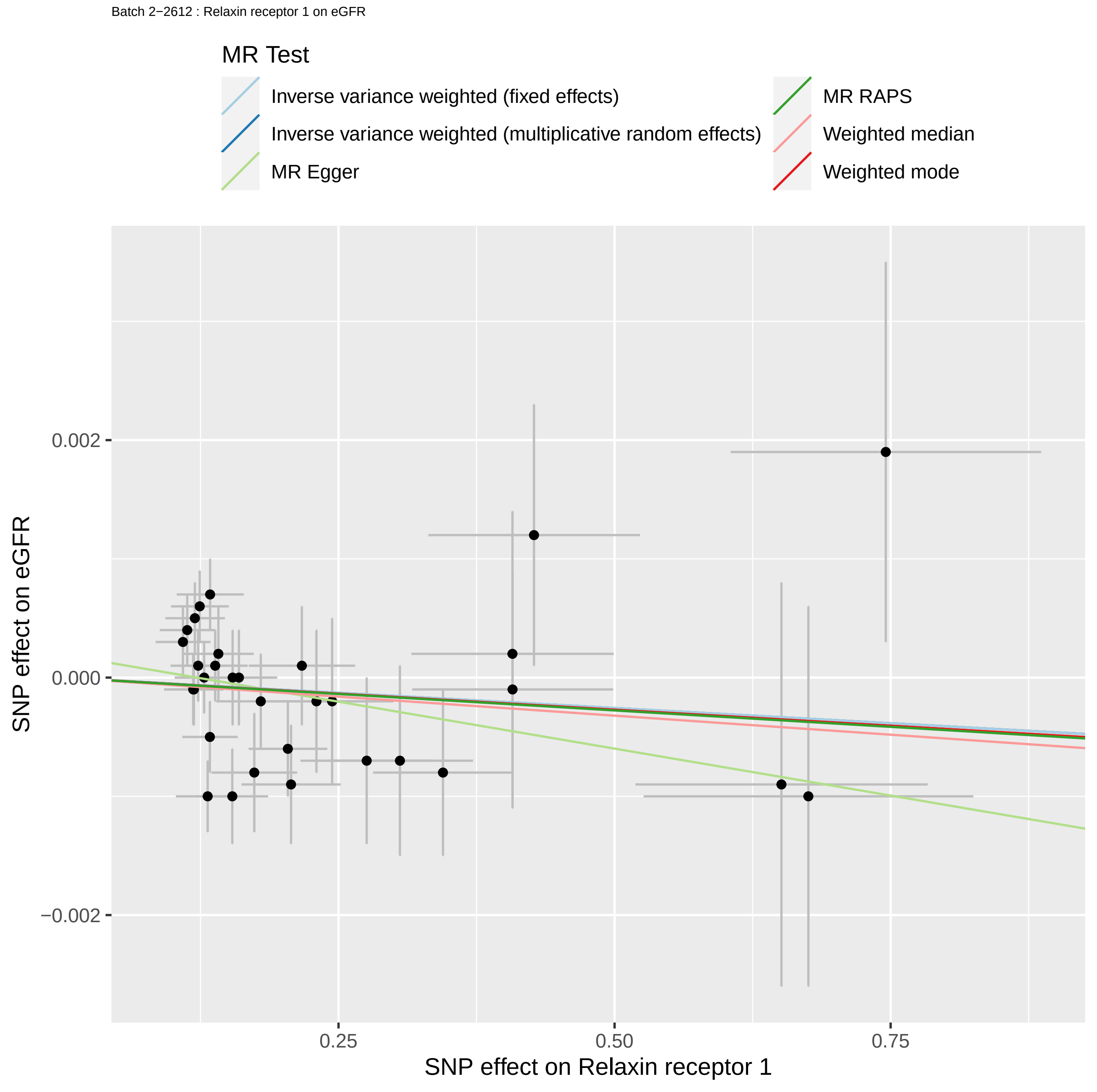
Batch 2−2612 : Relaxin receptor 1 on eGFR
MR Test
Inverse variance weighted (fixed effects)
Inverse variance weighted (multiplicative random effects)
MR Egger
MR RAPS
Weighted median
Weighted mode
0.002
0.000
−0.002
0.25
0.50
0.75
SNP effect on eGFR
SNP effect on Relaxin receptor 1

Batch 2−2613 : Tyrosine−protein kinase RYK on eGFR

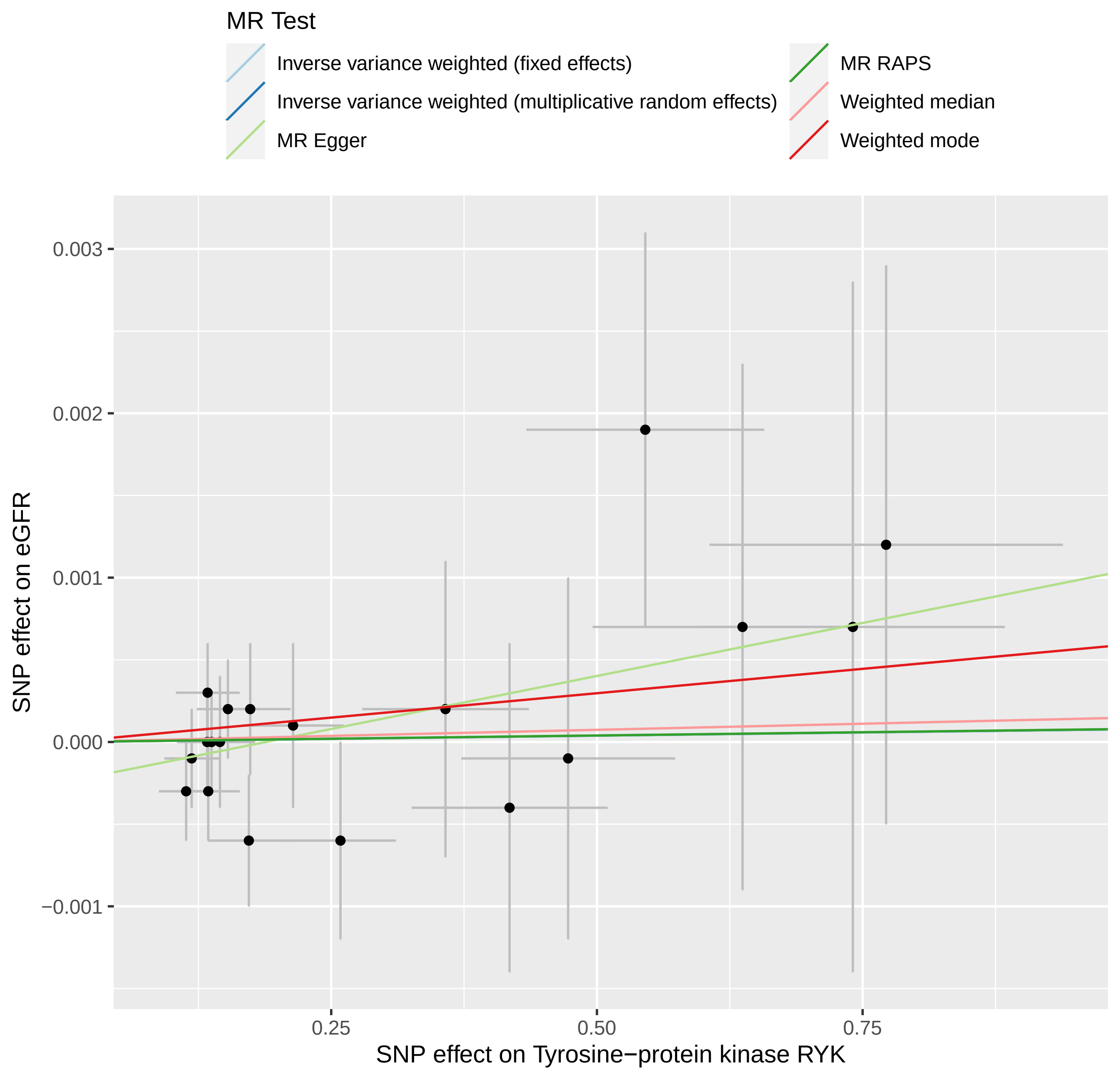

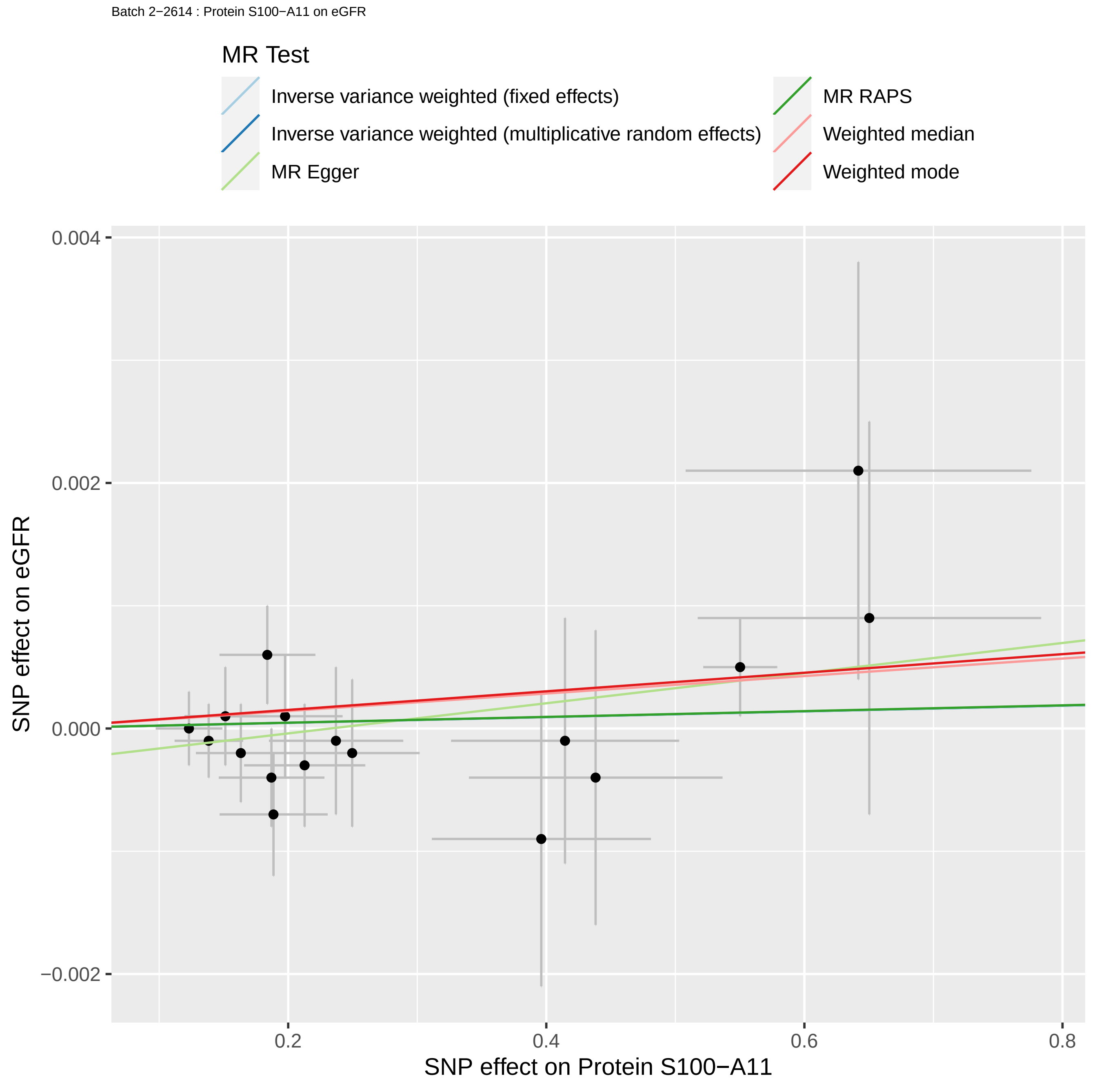
Batch 2−2614 : Protein S100−A11 on eGFR
MR Test
Inverse variance weighted (fixed effects)
Inverse variance weighted (multiplicative random effects)
MR Egger
MR RAPS
Weighted median
Weighted mode
0.004
0.002
0.000
−0.002
SNP effect on eGFR
0.2
0.4
0.6
0.8
SNP effect on Protein S100−A11

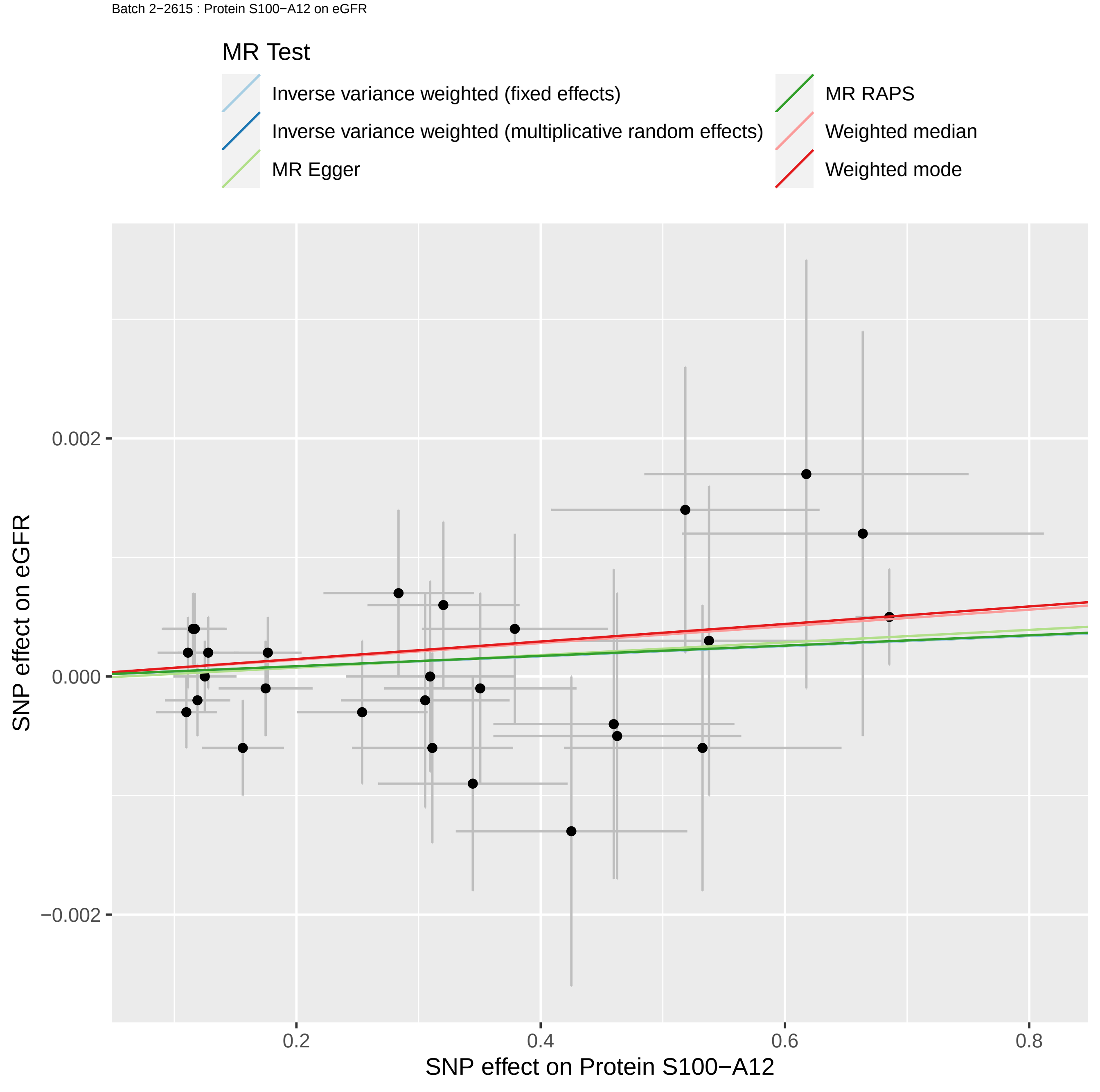
Batch 2−2615 : Protein S100−A12 on eGFR
MR Test
Inverse variance weighted (fixed effects)
Inverse variance weighted (multiplicative random effects)
MR Egger
MR RAPS
Weighted median
Weighted mode
SNP effect on eGFR
0.002
0.000
−0.002
0.2
0.4
0.6
0.8
SNP effect on Protein S100−A12

Batch 2−2616 : Protein S100−A13 on eGFR

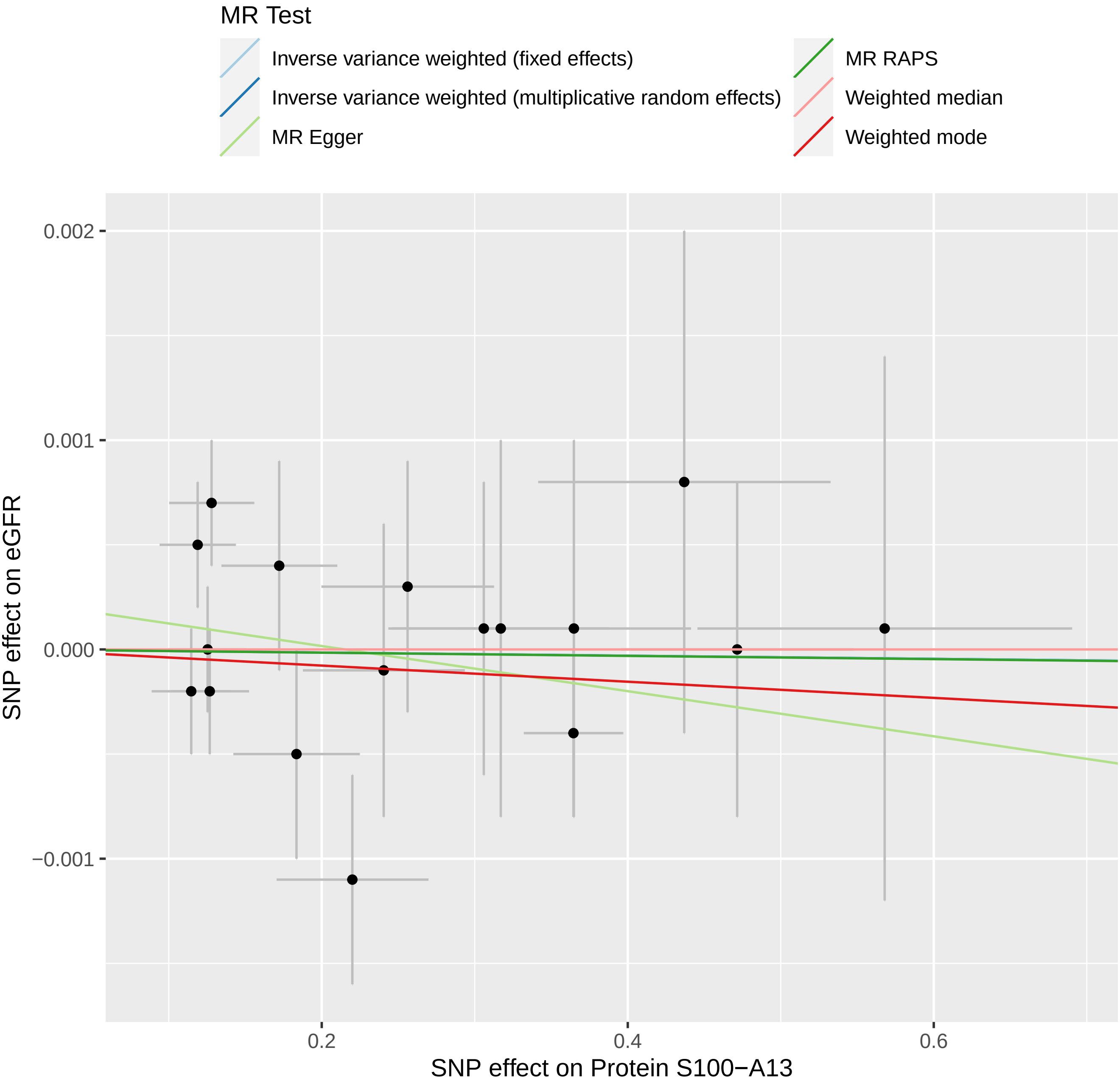

Batch 2−2617 : Protein S100−A2 on eGFR

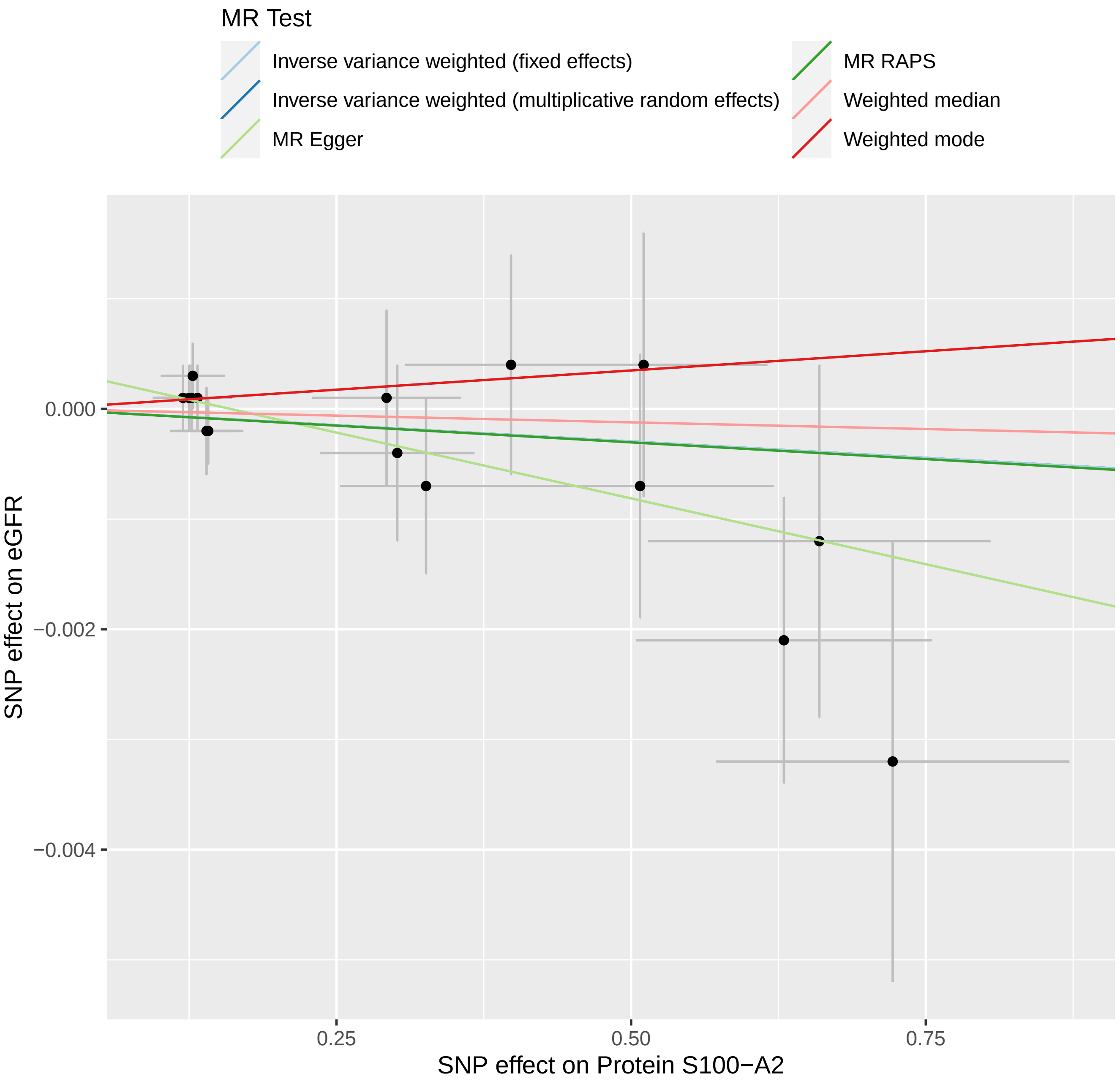

Batch 2−2618 : Protein S100−A4 on eGFR

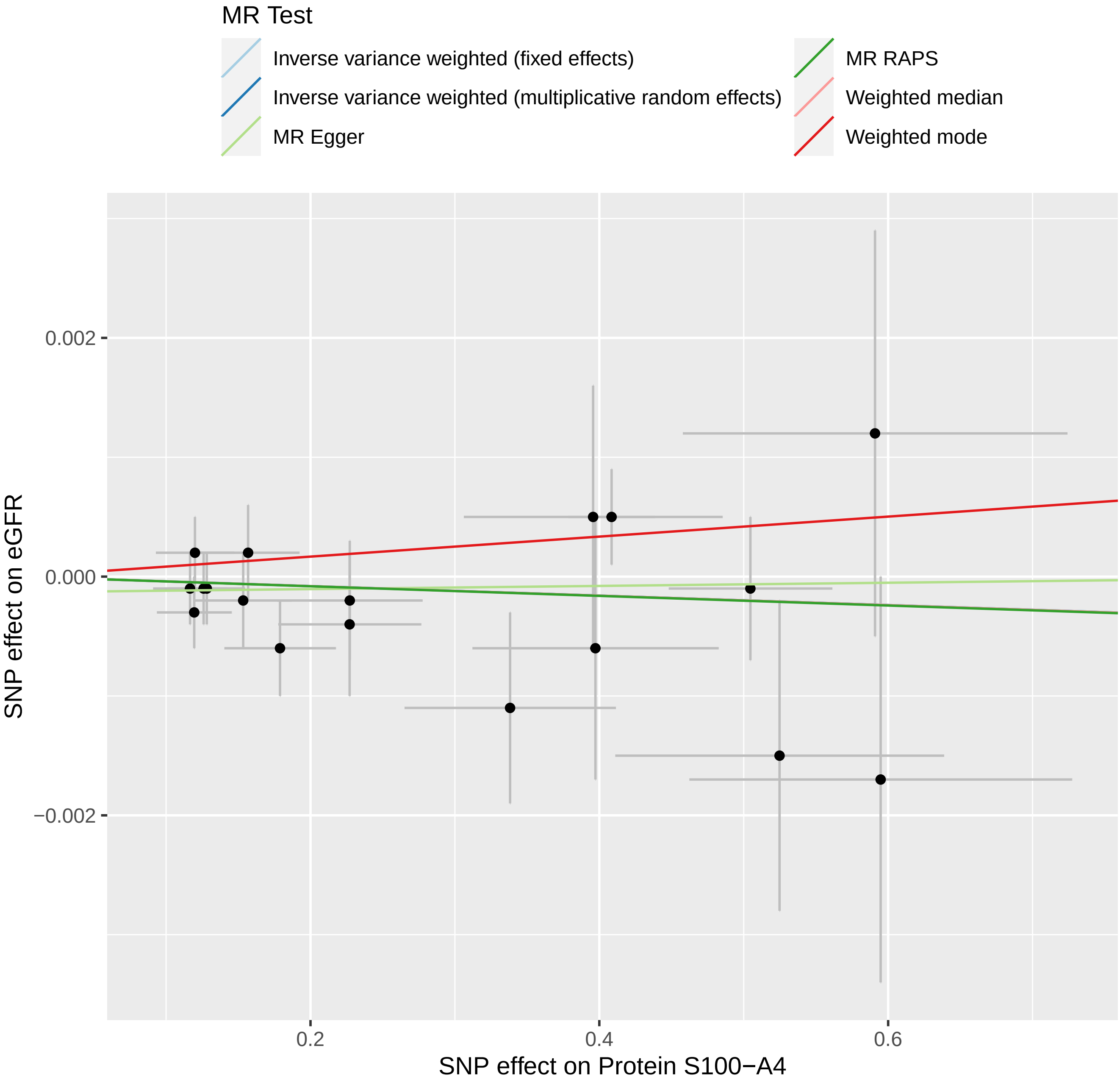

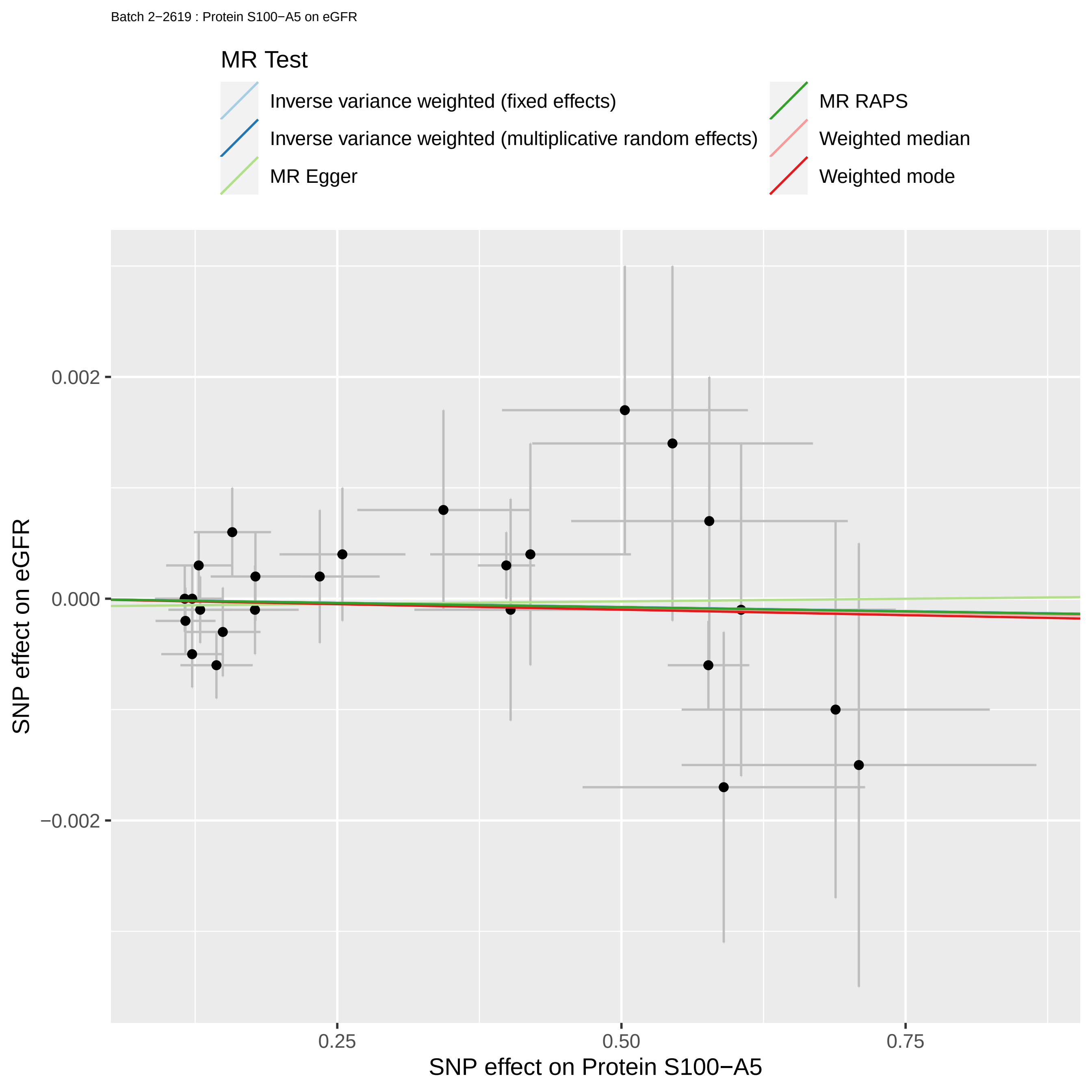
Batch 2−2619 : Protein S100−A5 on eGFR
MR Test
Inverse variance weighted (fixed effects)
Inverse variance weighted (multiplicative random effects)
MR Egger
MR RAPS
Weighted median
Weighted mode
0.002
0.000
−0.002
0.25
0.50
0.75
SNP effect on eGFR
SNP effect on Protein S100−A5

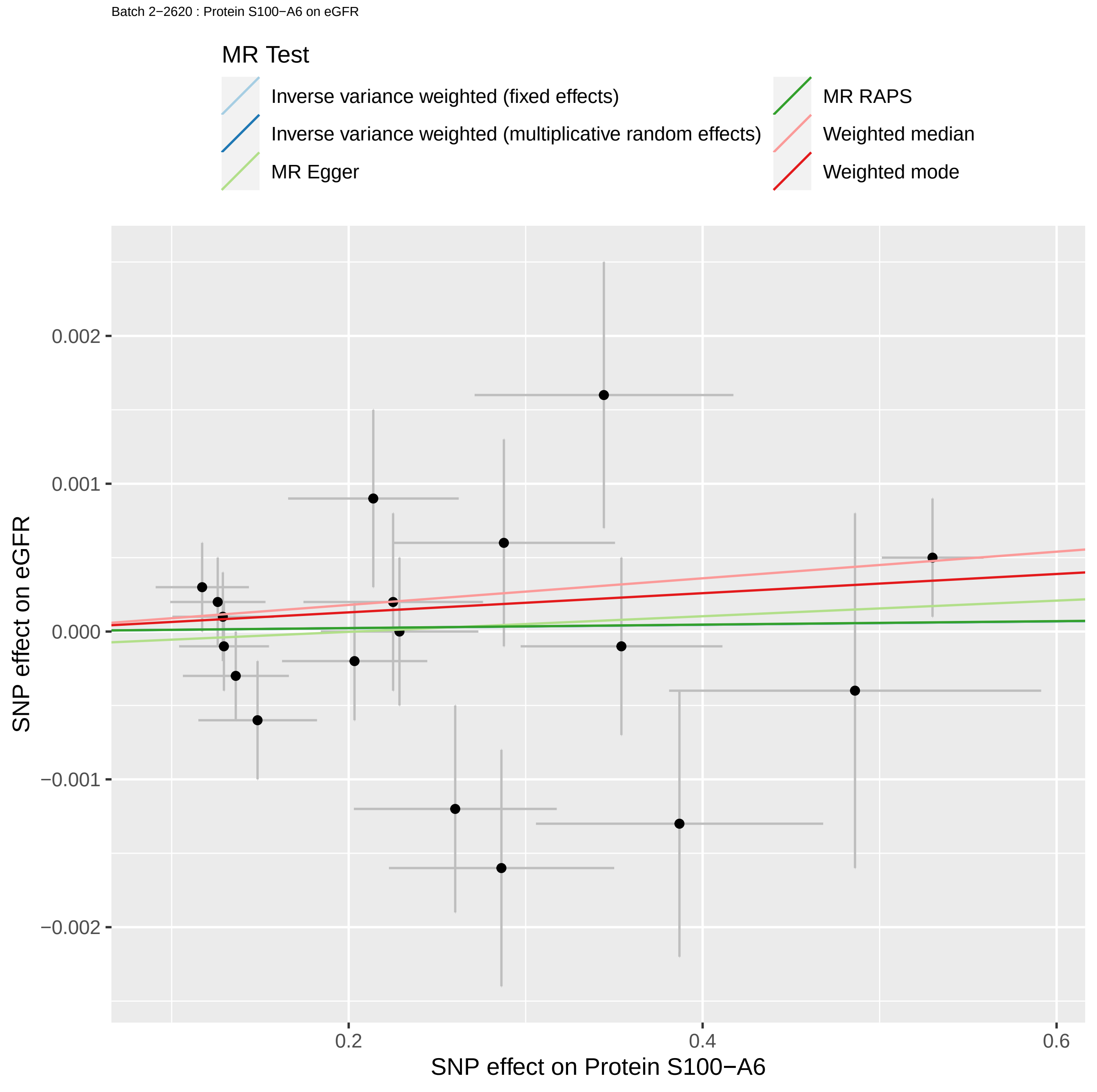

Batch 2−2620 : Protein S100−A6 on eGFR
MR Test
Inverse variance weighted (fixed effects)
Inverse variance weighted (multiplicative random effects)
MR Egger
MR RAPS
Weighted median
Weighted mode
0.002
0.001
0.000
−0.001
−0.002
SNP effect on eGFR
0.2
0.4
0.6
SNP effect on Protein S100−A6

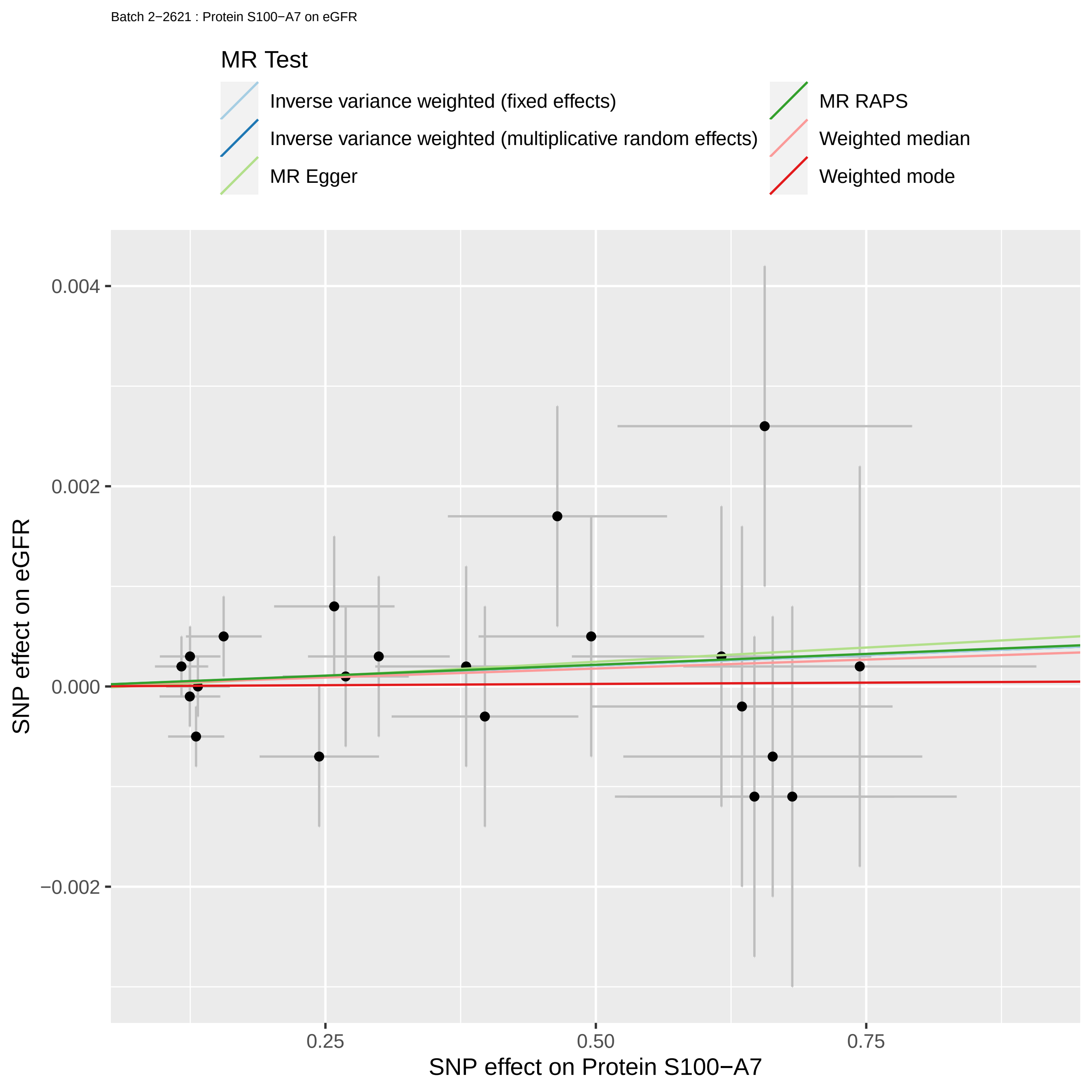

Batch 2−2621 : Protein S100−A7 on eGFR
MR Test
Inverse variance weighted (fixed effects)
Inverse variance weighted (multiplicative random effects)
MR Egger
MR RAPS
Weighted median
Weighted mode
0.004
0.002
0.000
−0.002
0.25
0.50
0.75
SNP effect on eGFR
SNP effect on Protein S100−A7

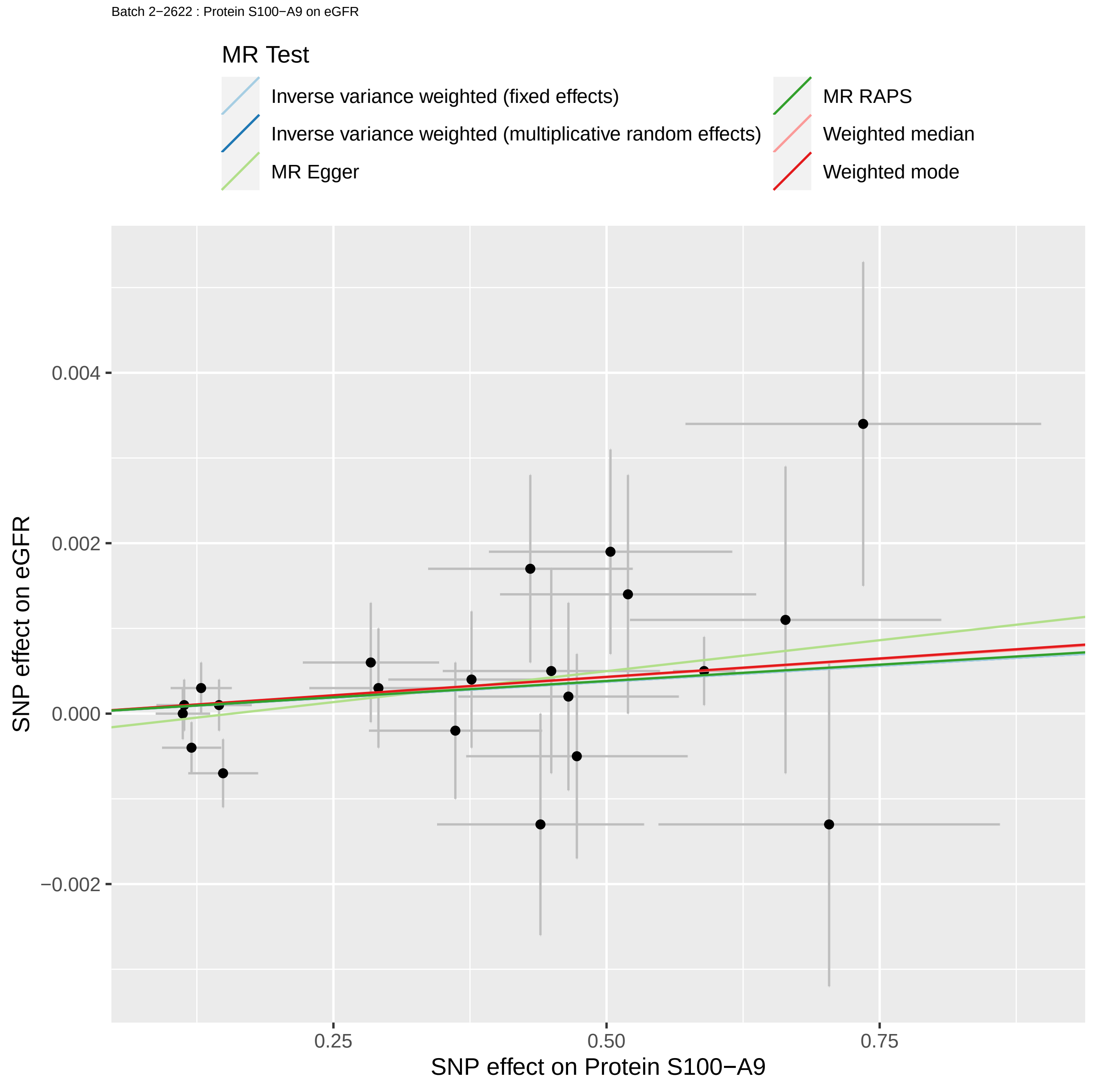
Batch 2−2622 : Protein S100−A9 on eGFR
MR Test
Inverse variance weighted (fixed effects)
Inverse variance weighted (multiplicative random effects)
MR Egger
MR RAPS
Weighted median
Weighted mode
0.004
0.002
0.000
−0.002
0.25
0.50
0.75
SNP effect on eGFR
SNP effect on Protein S100−A9

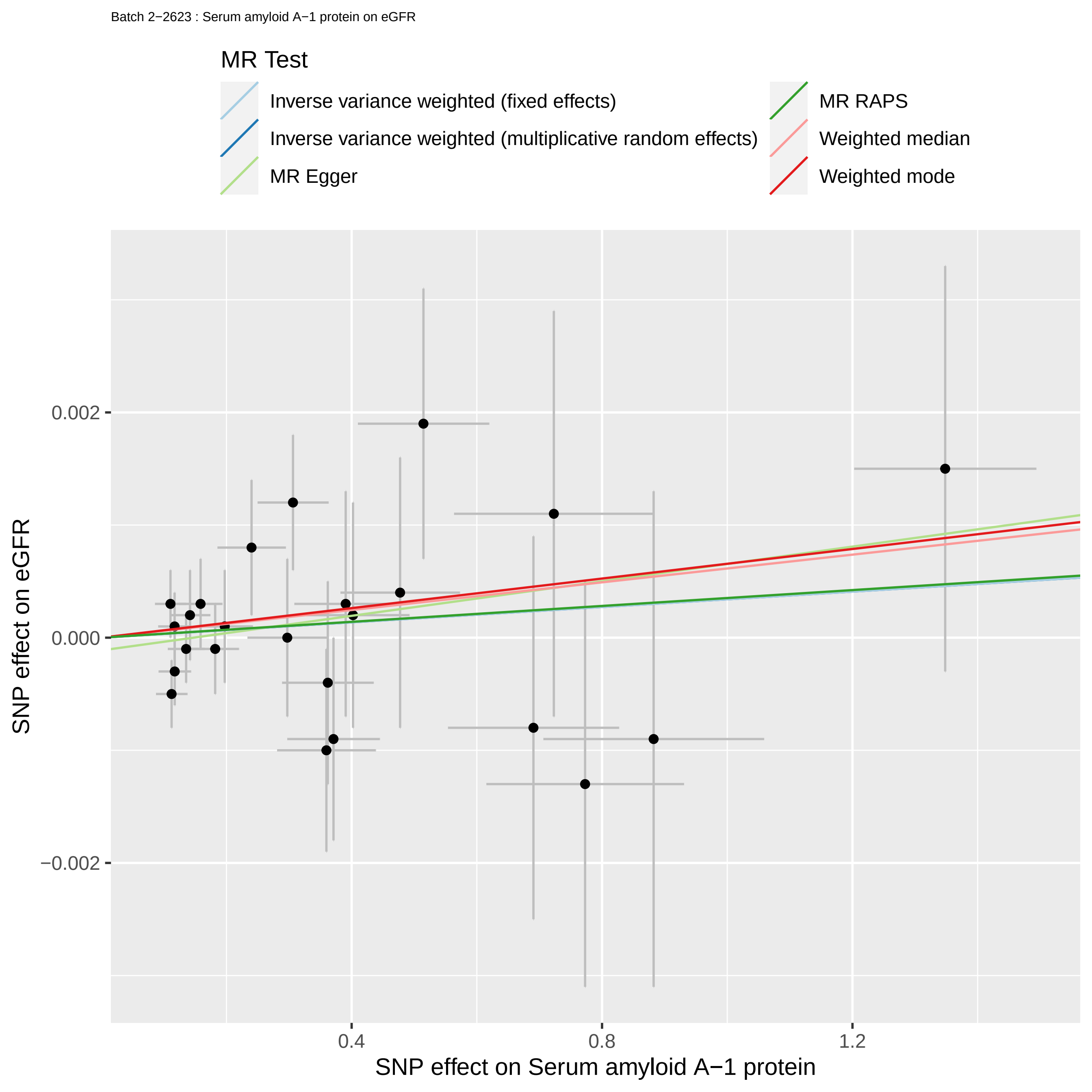

Batch 2−2623 : Serum amyloid A−1 protein on eGFR
MR Test
Inverse variance weighted (fixed effects)
Inverse variance weighted (multiplicative random effects)
MR Egger
MR RAPS
Weighted median
Weighted mode
0.002
0.000
−0.002
0.4
0.8
1.2
SNP effect on eGFR
SNP effect on Serum amyloid A−1 protein

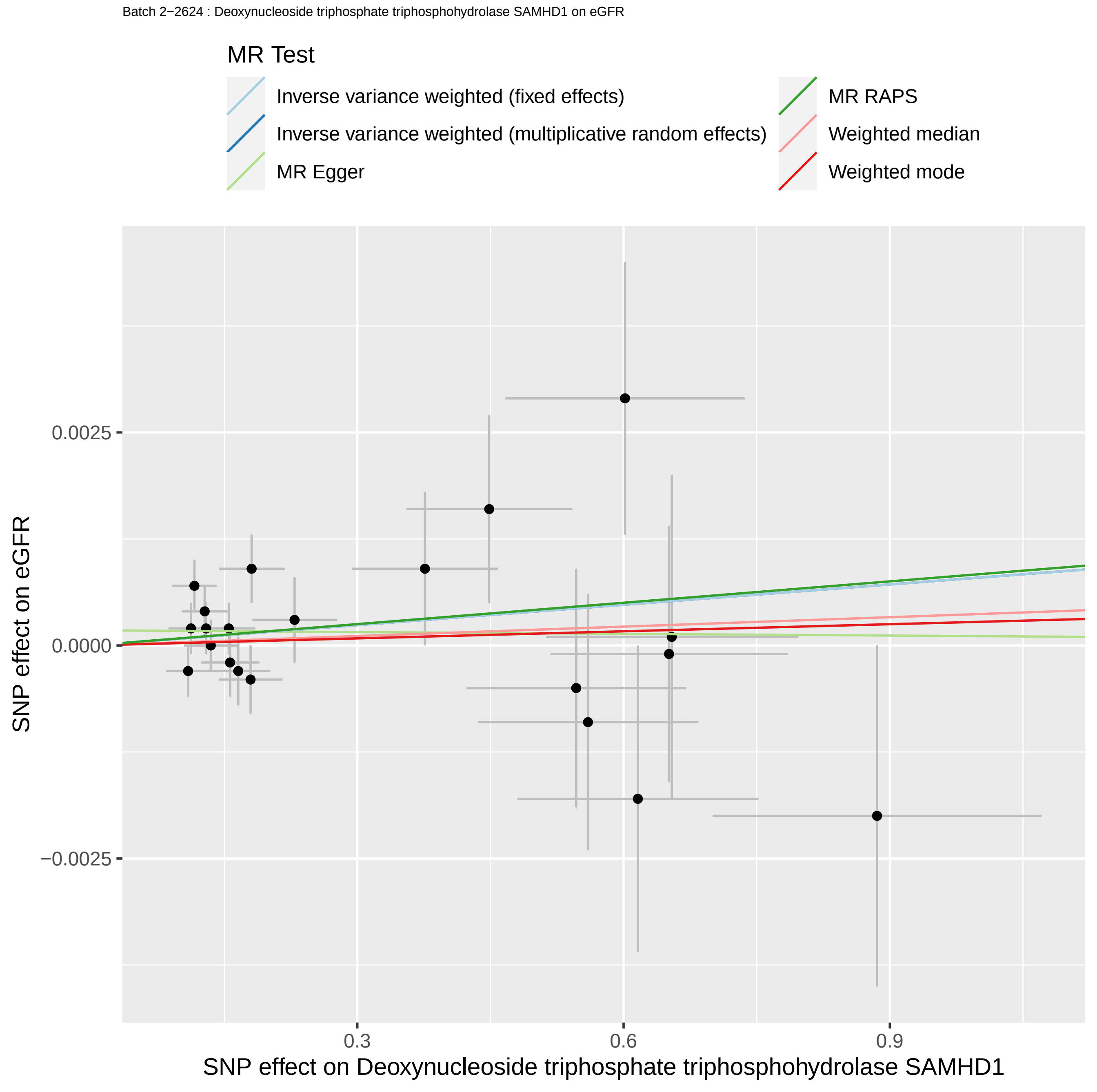
Batch 2−2624 : Deoxynucleoside triphosphate triphosphohydrolase SAMHD1 on eGFR
MR Test
Inverse variance weighted (fixed effects)
Inverse variance weighted (multiplicative random effects)
MR Egger
MR RAPS
Weighted median
Weighted mode
0.0025
0.0000
−0.0025
0.3
0.6
0.9
SNP effect on eGFR
SNP effect on Deoxynucleoside triphosphate triphosphohydrolase SAMHD1

Batch 2−2625 : Histone deacetylase complex subunit SAP18 on eGFR

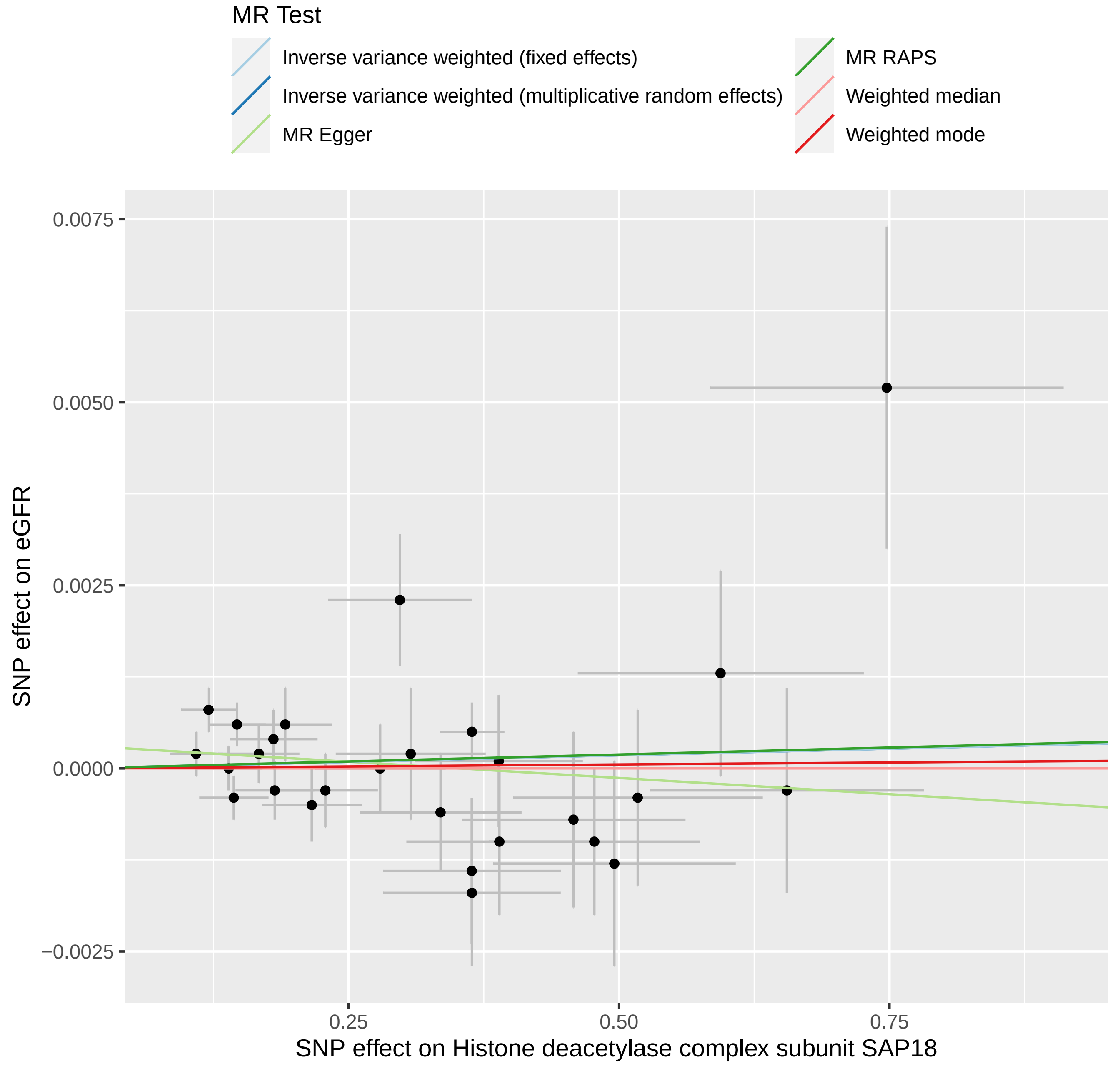

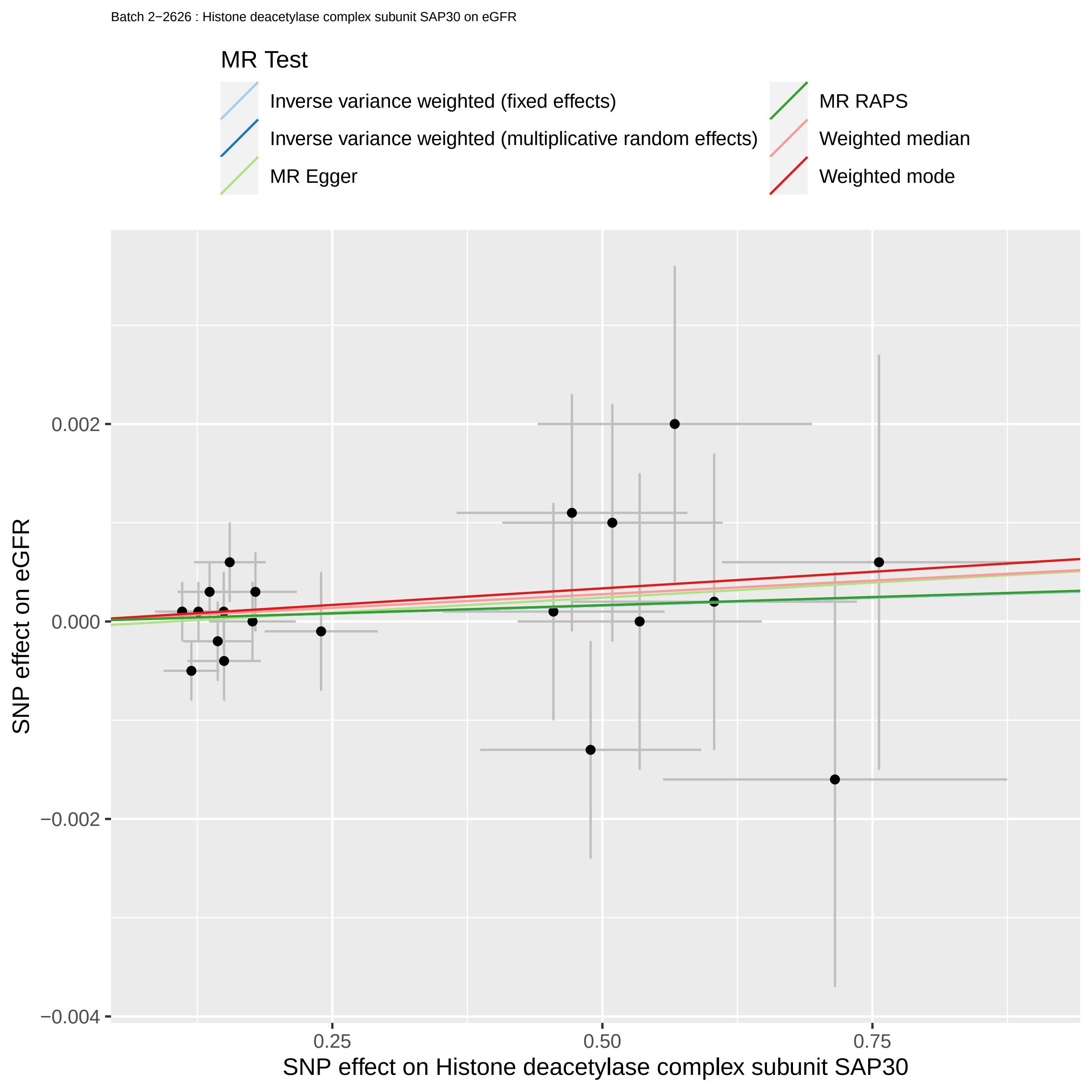

Batch 2−2626 : Histone deacetylase complex subunit SAP30 on eGFR
MR Test
Inverse variance weighted (fixed effects)
Inverse variance weighted (multiplicative random effects)
MR Egger
MR RAPS
Weighted median
Weighted mode
0.002
0.000
−0.002
−0.004
0.25
0.50
0.75
SNP effect on eGFR
SNP effect on Histone deacetylase complex subunit SAP30

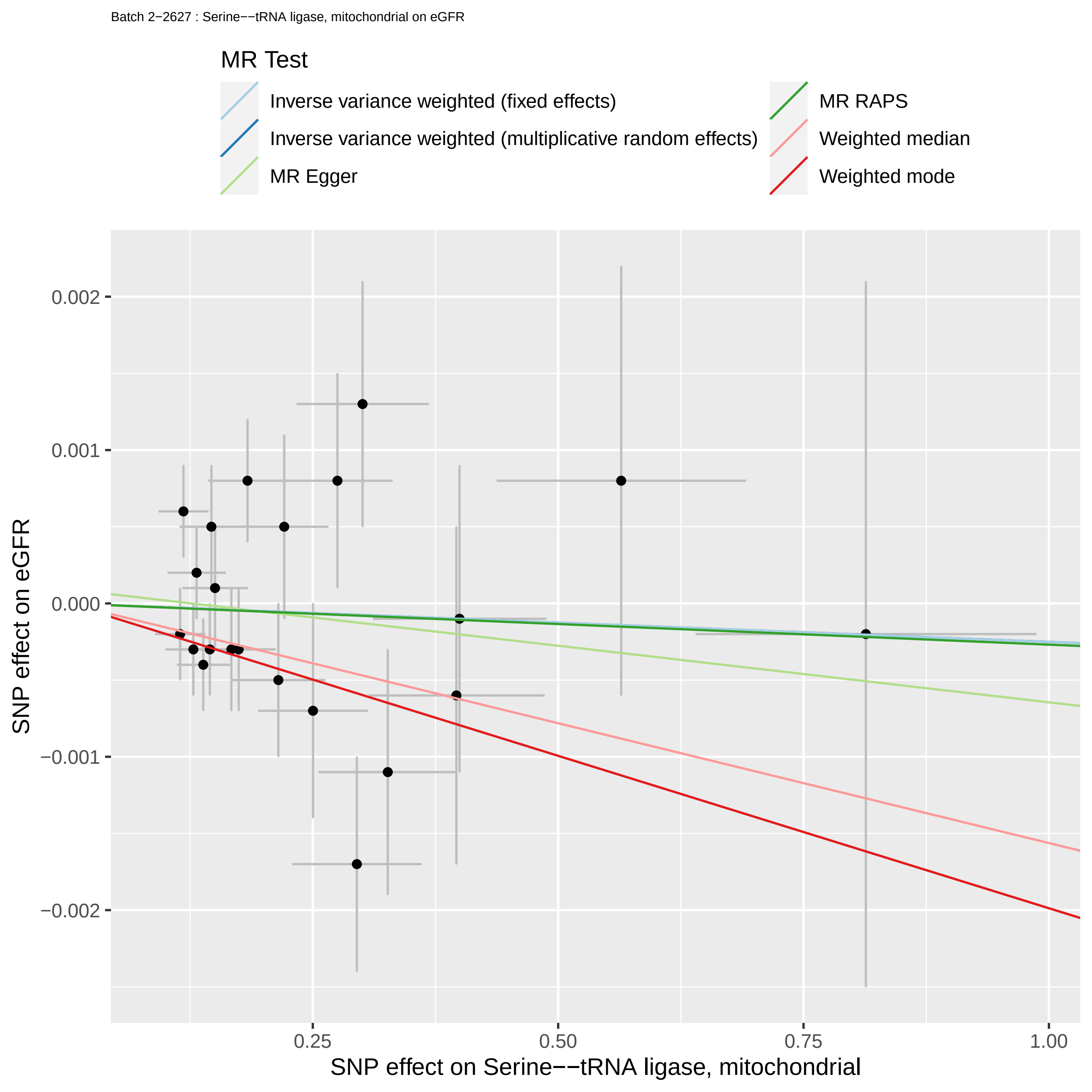

Batch 2−2627 : Serine−−tRNA ligase, mitochondrial on eGFR
MR Test
Inverse variance weighted (fixed effects)
Inverse variance weighted (multiplicative random effects)
MR Egger
MR RAPS
Weighted median
Weighted mode
0.002
0.001
0.000
−0.001
−0.002
SNP effect on eGFR
0.25
0.50
0.75
1.00
SNP effect on Serine−−tRNA ligase, mitochondrial

Batch 2−2628 : Diamine acetyltransferase 1 on eGFR

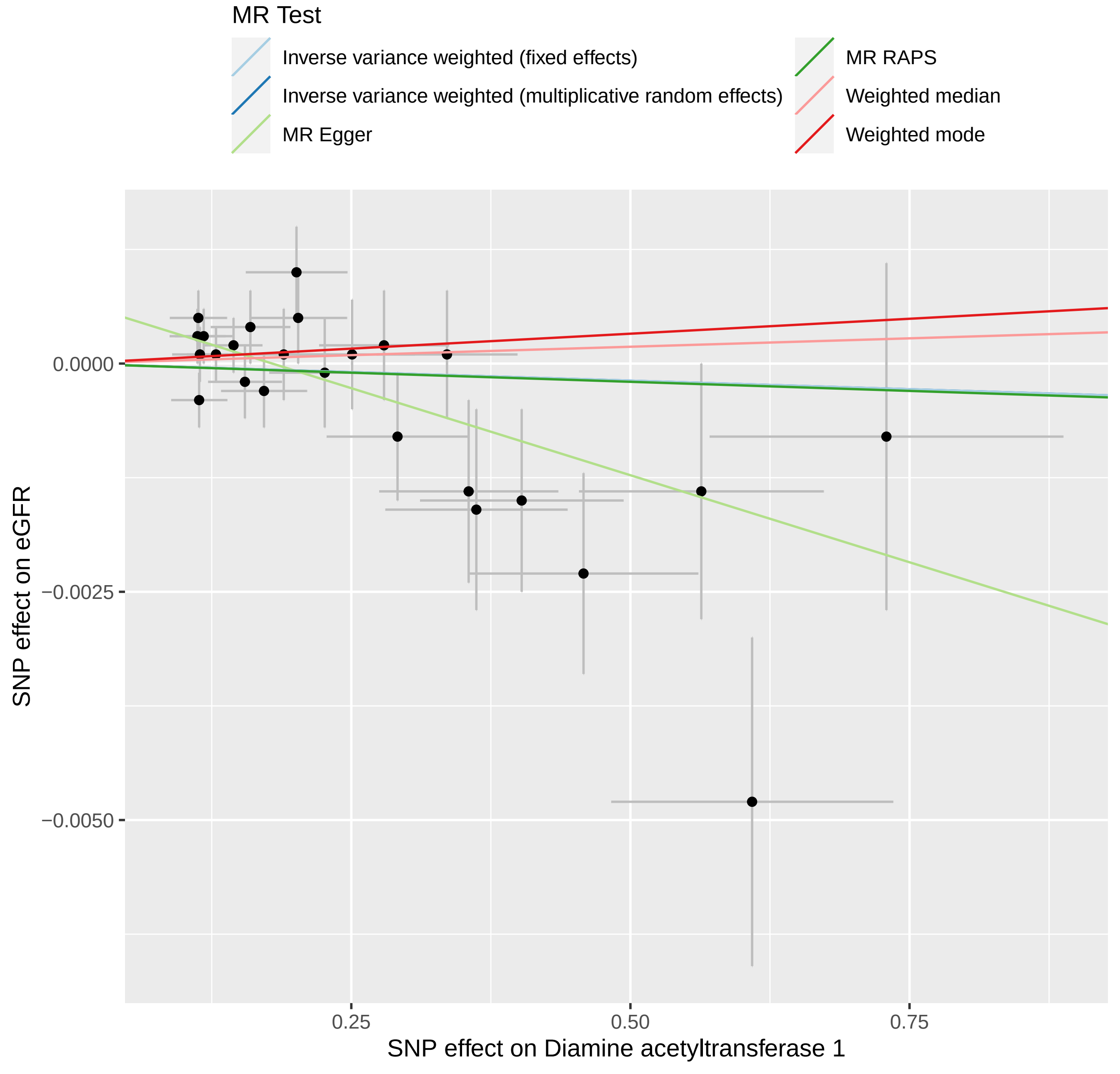

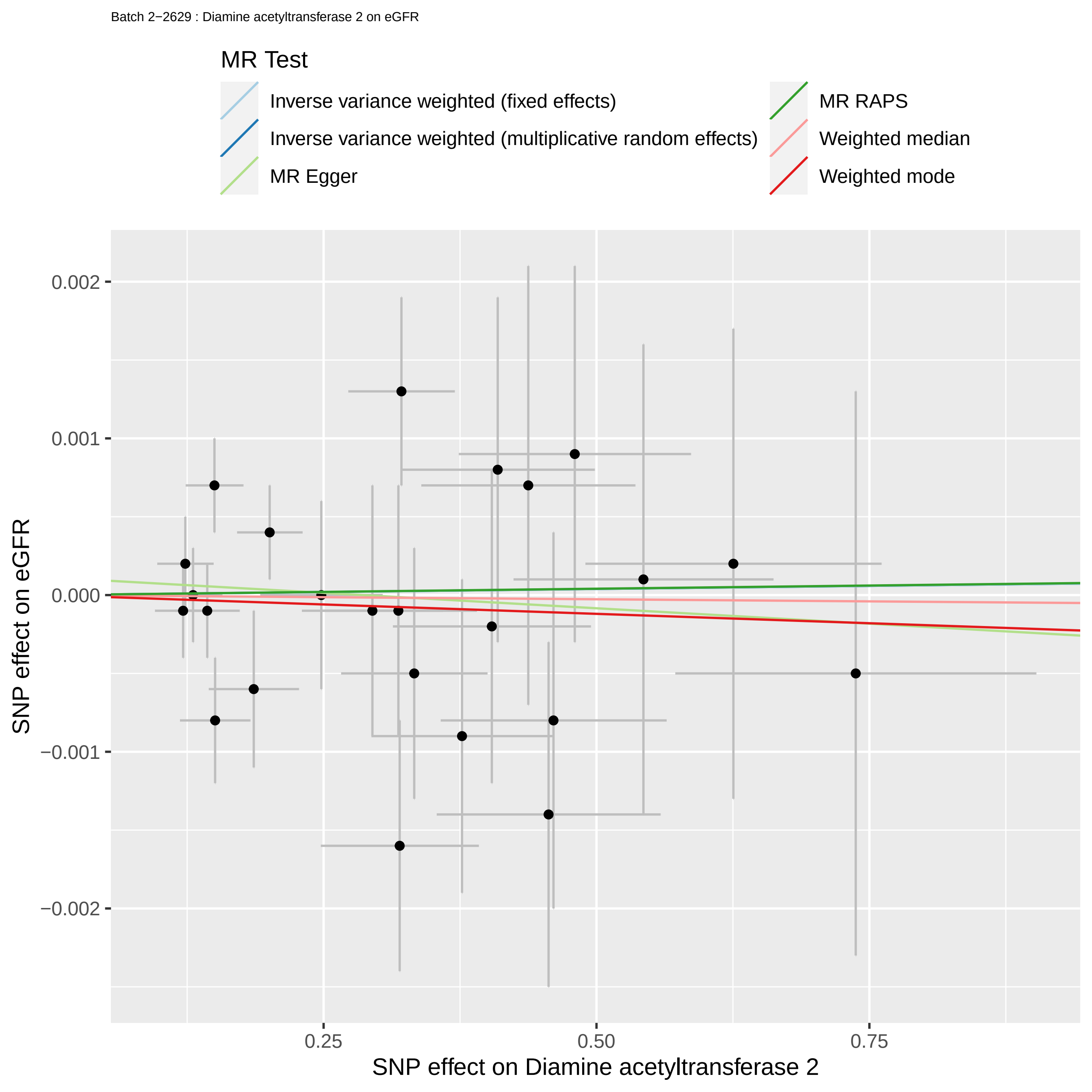
Batch 2−2629 : Diamine acetyltransferase 2 on eGFR
MR Test
Inverse variance weighted (fixed effects)
Inverse variance weighted (multiplicative random effects)
MR Egger
MR RAPS
Weighted median
Weighted mode
0.002
0.001
0.000
−0.001
−0.002
SNP effect on eGFR
0.25
0.50
0.75
SNP effect on Diamine acetyltransferase 2

Batch 2−2630 : DNA−binding protein SATB1 on eGFR

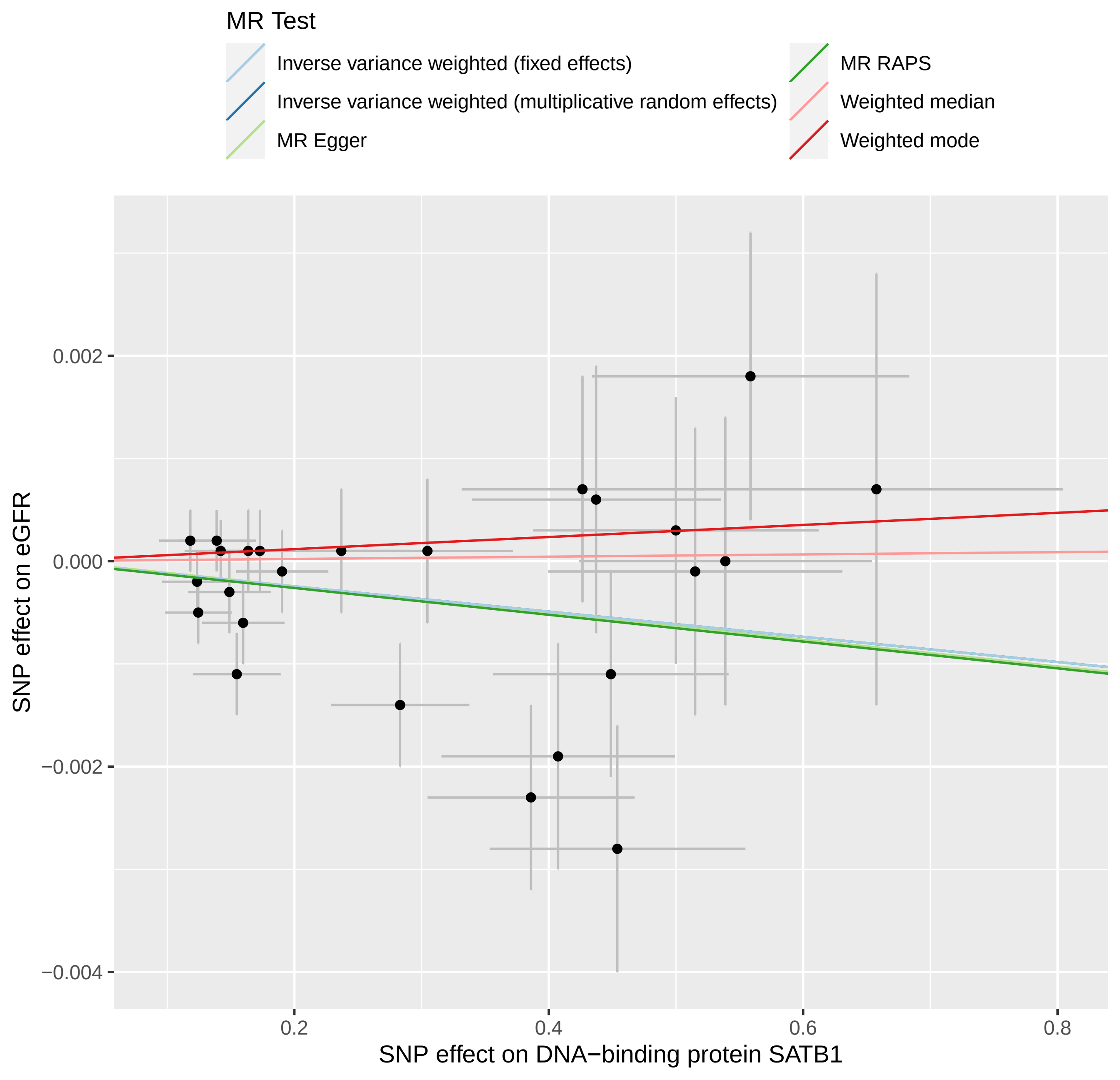

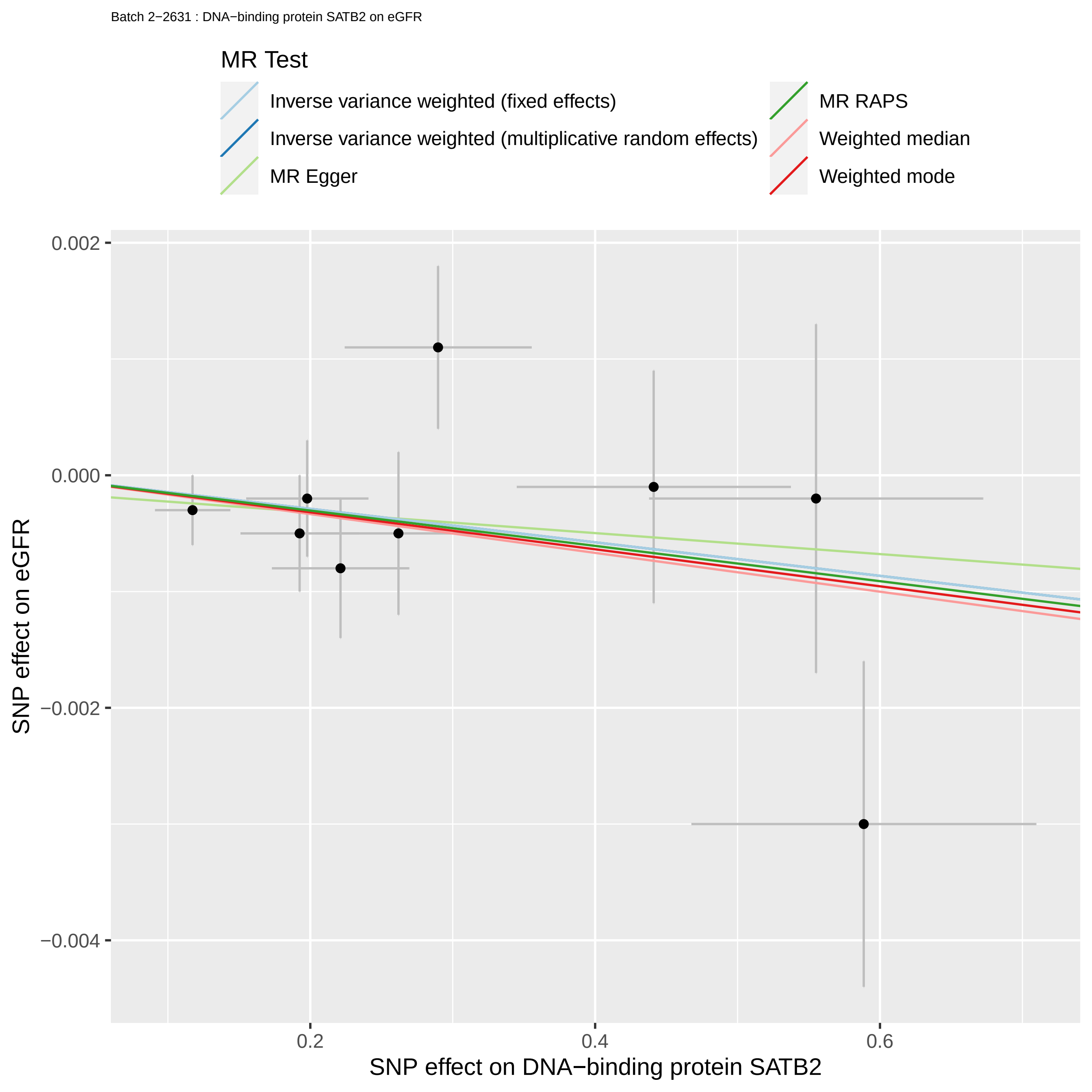
Batch 2−2631 : DNA−binding protein SATB2 on eGFR
MR Test
Inverse variance weighted (fixed effects)
Inverse variance weighted (multiplicative random effects)
MR Egger
MR RAPS
Weighted median
Weighted mode
0.002
0.000
−0.002
−0.004
0.2
0.4
0.6
SNP effect on eGFR
SNP effect on DNA−binding protein SATB2

Batch 2−2632 : SAYSvFN domain−containing protein 1 on eGFR

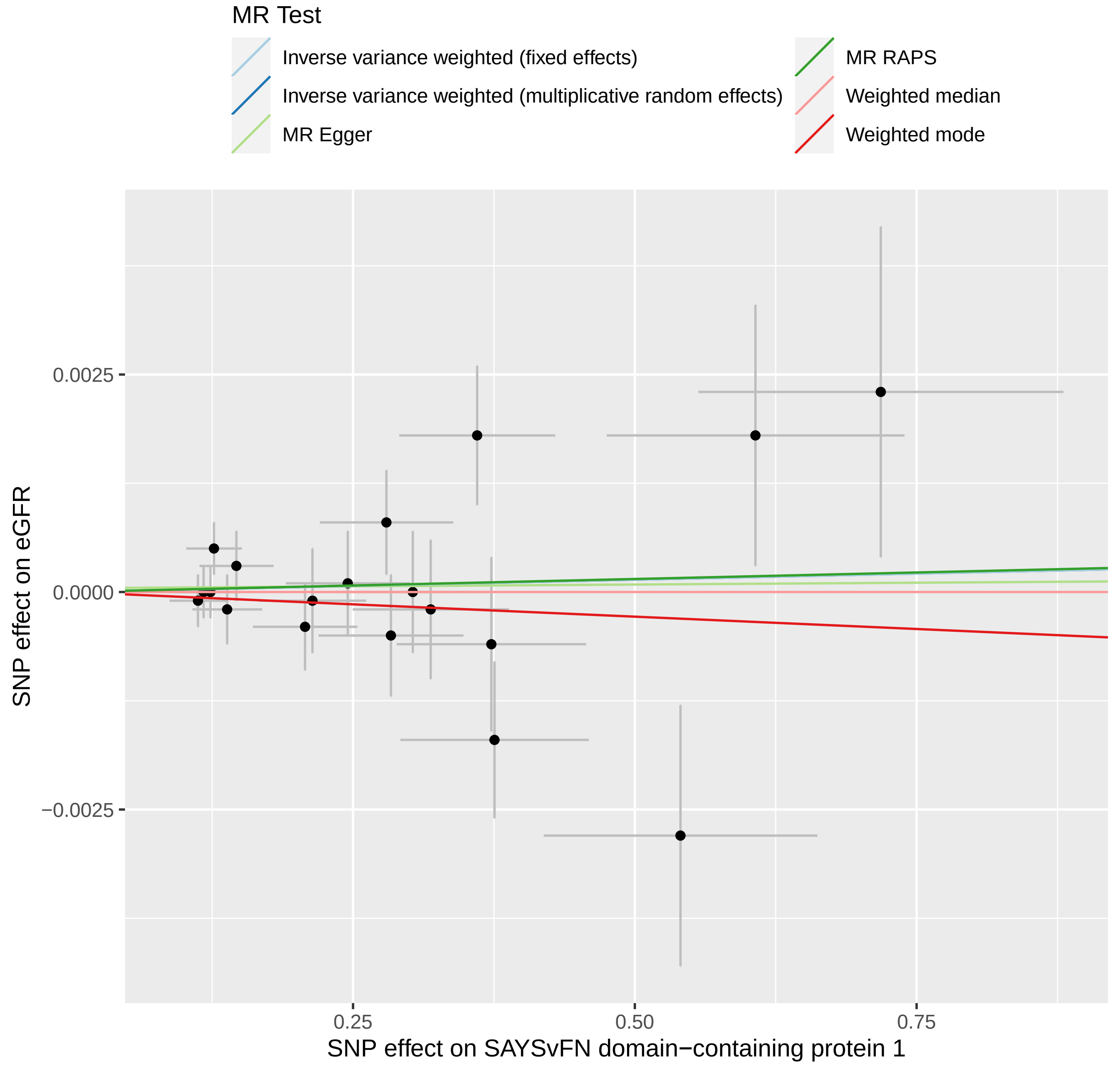

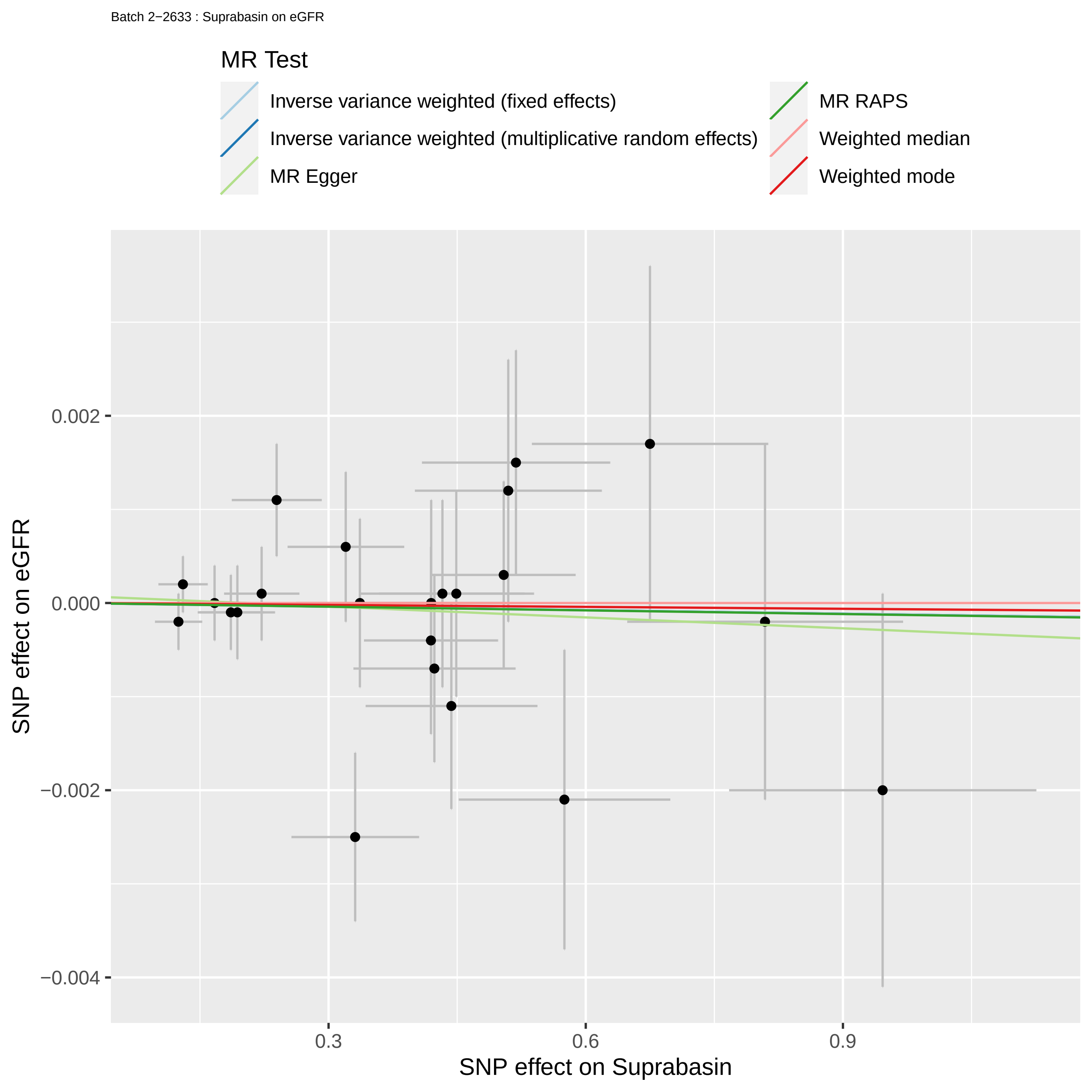
Batch 2−2633 : Suprabasin on eGFR
MR Test
Inverse variance weighted (fixed effects)
Inverse variance weighted (multiplicative random effects)
MR Egger
MR RAPS
Weighted median
Weighted mode
0.002
0.000
−0.002
−0.004
SNP effect on eGFR
0.3
0.6
0.9
SNP effect on Suprabasin

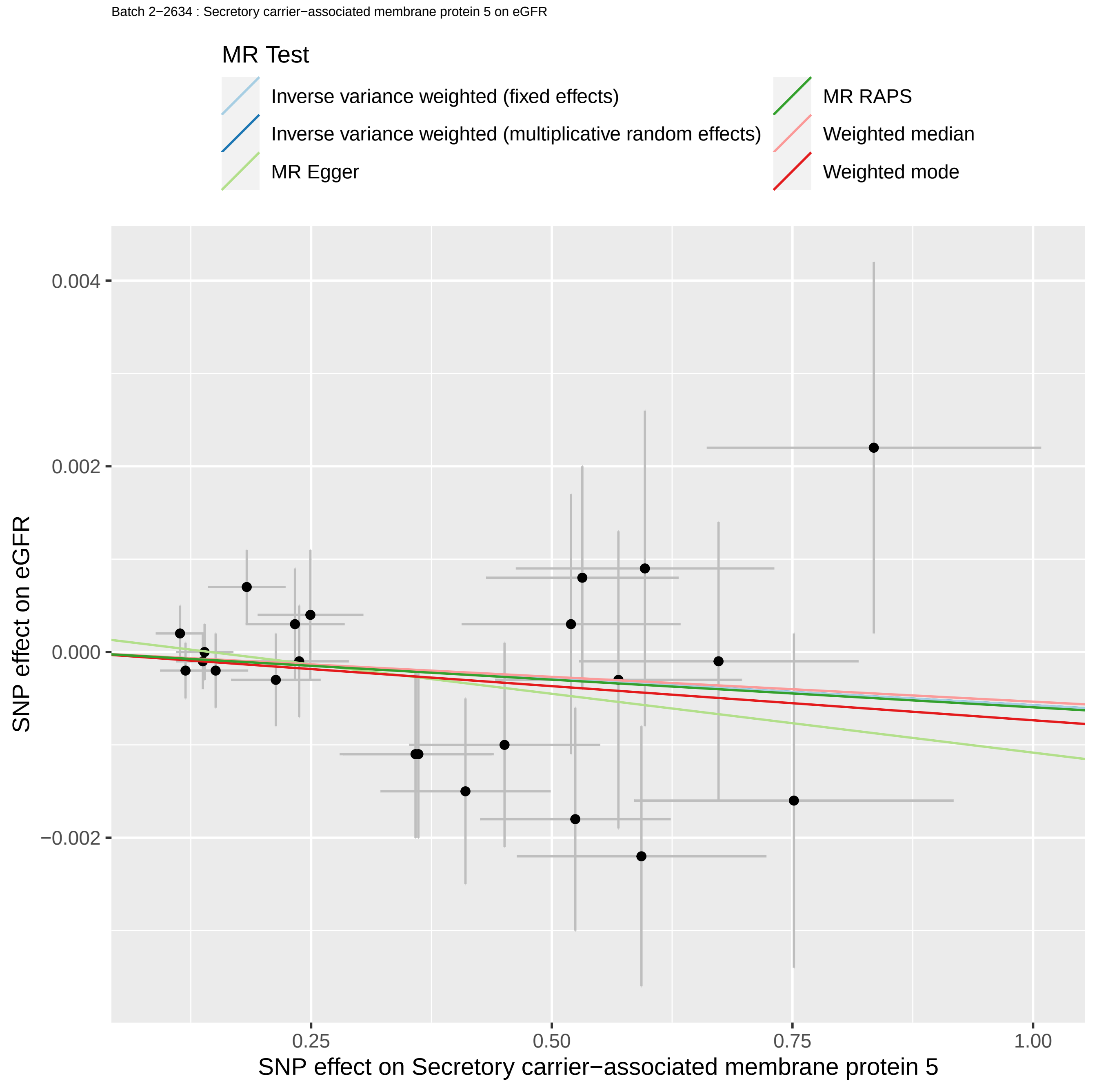

Batch 2−2634 : Secretory carrier−associated membrane protein 5 on eGFR
MR Test
Inverse variance weighted (fixed effects)
Inverse variance weighted (multiplicative random effects)
MR Egger
MR RAPS
Weighted median
Weighted mode
0.004
0.002
0.000
−0.002
0.25
0.50
0.75
1.00
SNP effect on eGFR
SNP effect on Secretory carrier−associated membrane protein 5

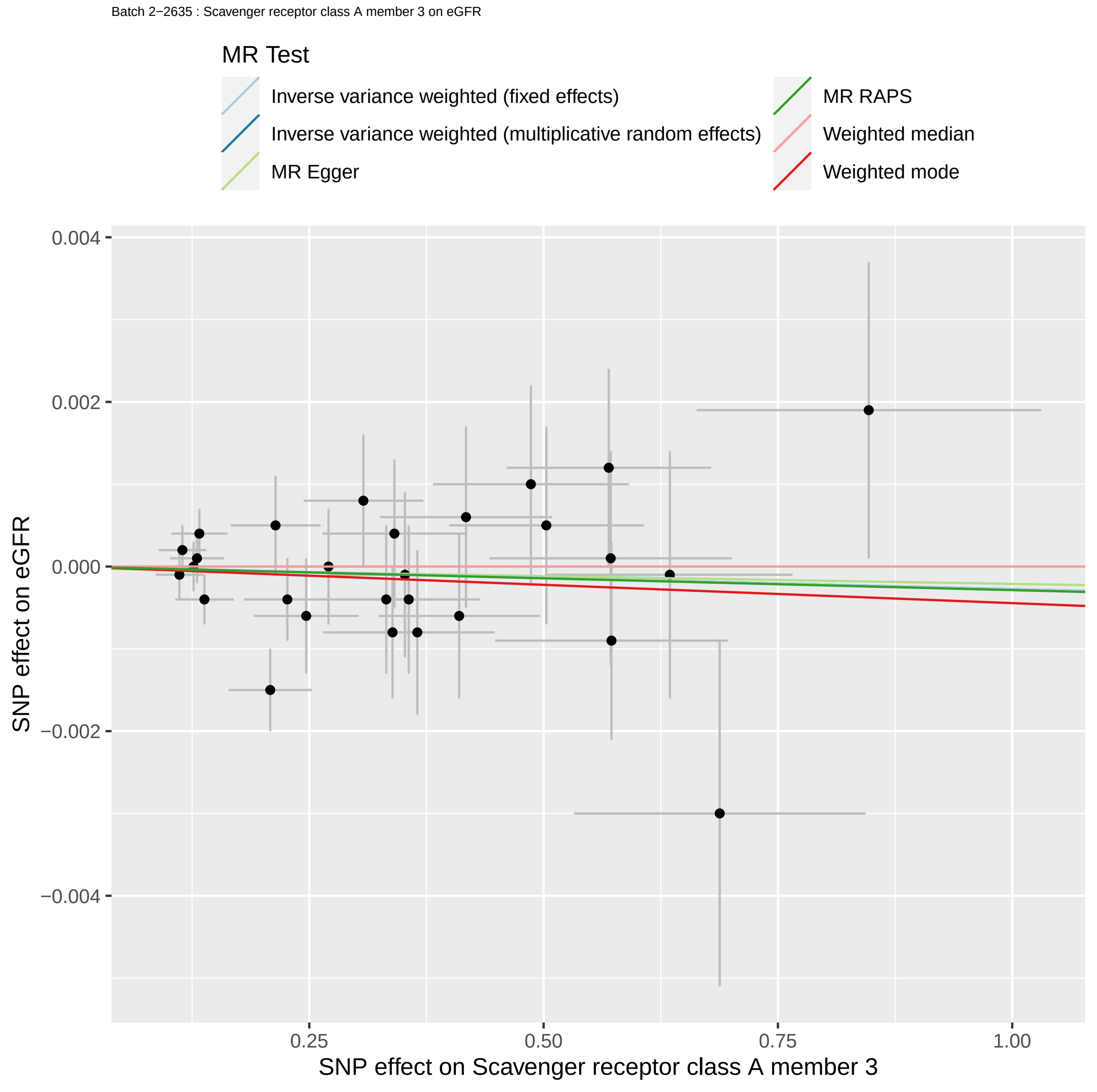

Batch 2−2635 : Scavenger receptor class A member 3 on eGFR
MR Test
Inverse variance weighted (fixed effects)
Inverse variance weighted (multiplicative random effects)
MR Egger
MR RAPS
Weighted median
Weighted mode
0.004
0.002
0.000
−0.002
−0.004
0.25
0.50
0.75
1.00
SNP effect on eGFR
SNP effect on Scavenger receptor class A member 3

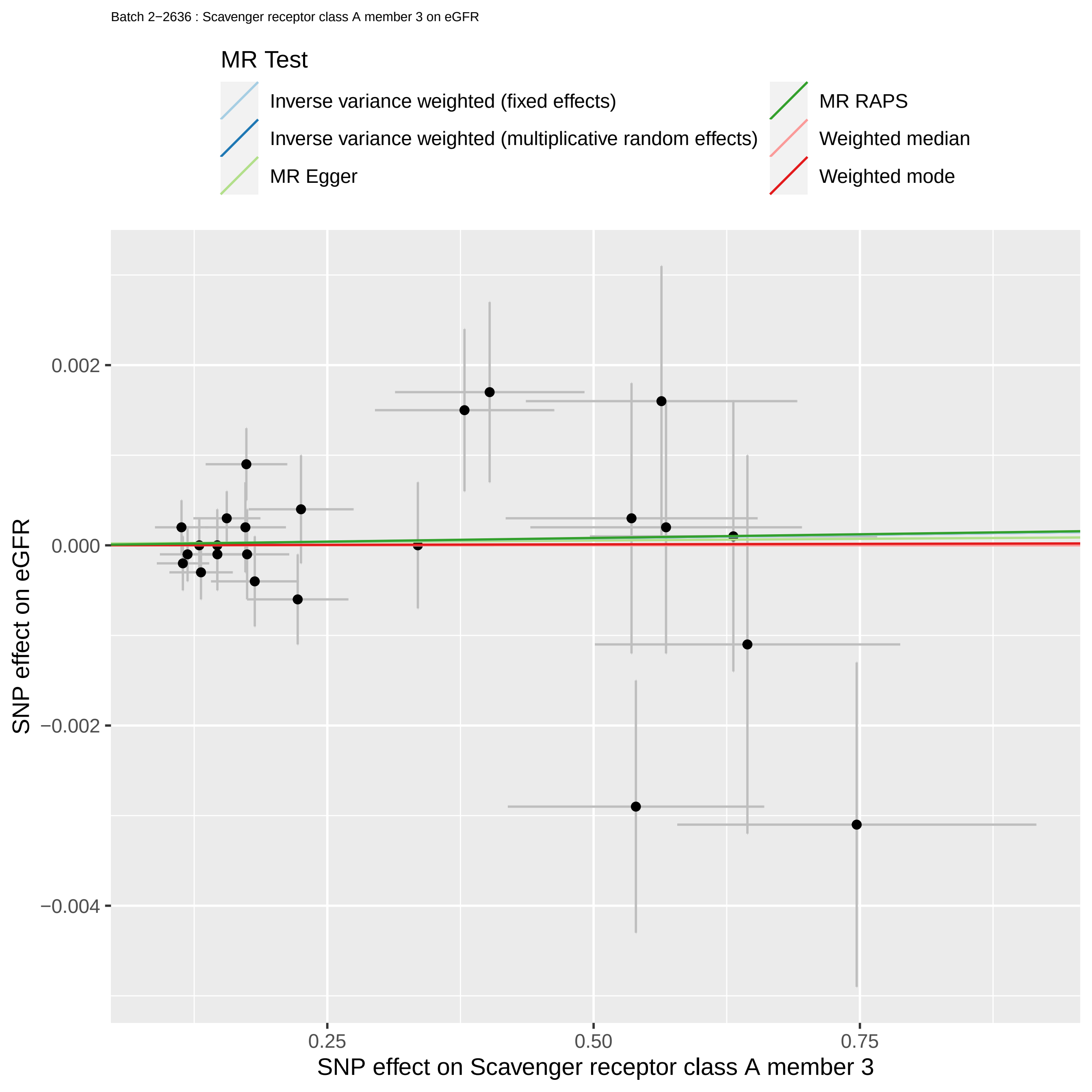

Batch 2−2636 : Scavenger receptor class A member 3 on eGFR
MR Test
Inverse variance weighted (fixed effects)
Inverse variance weighted (multiplicative random effects)
MR Egger
MR RAPS
Weighted median
Weighted mode
0.002
0.000
−0.002
−0.004
SNP effect on eGFR
0.25
0.50
0.75
SNP effect on Scavenger receptor class A member 3

Batch 2−2637 : Scavenger receptor class A member 3 on eGFR

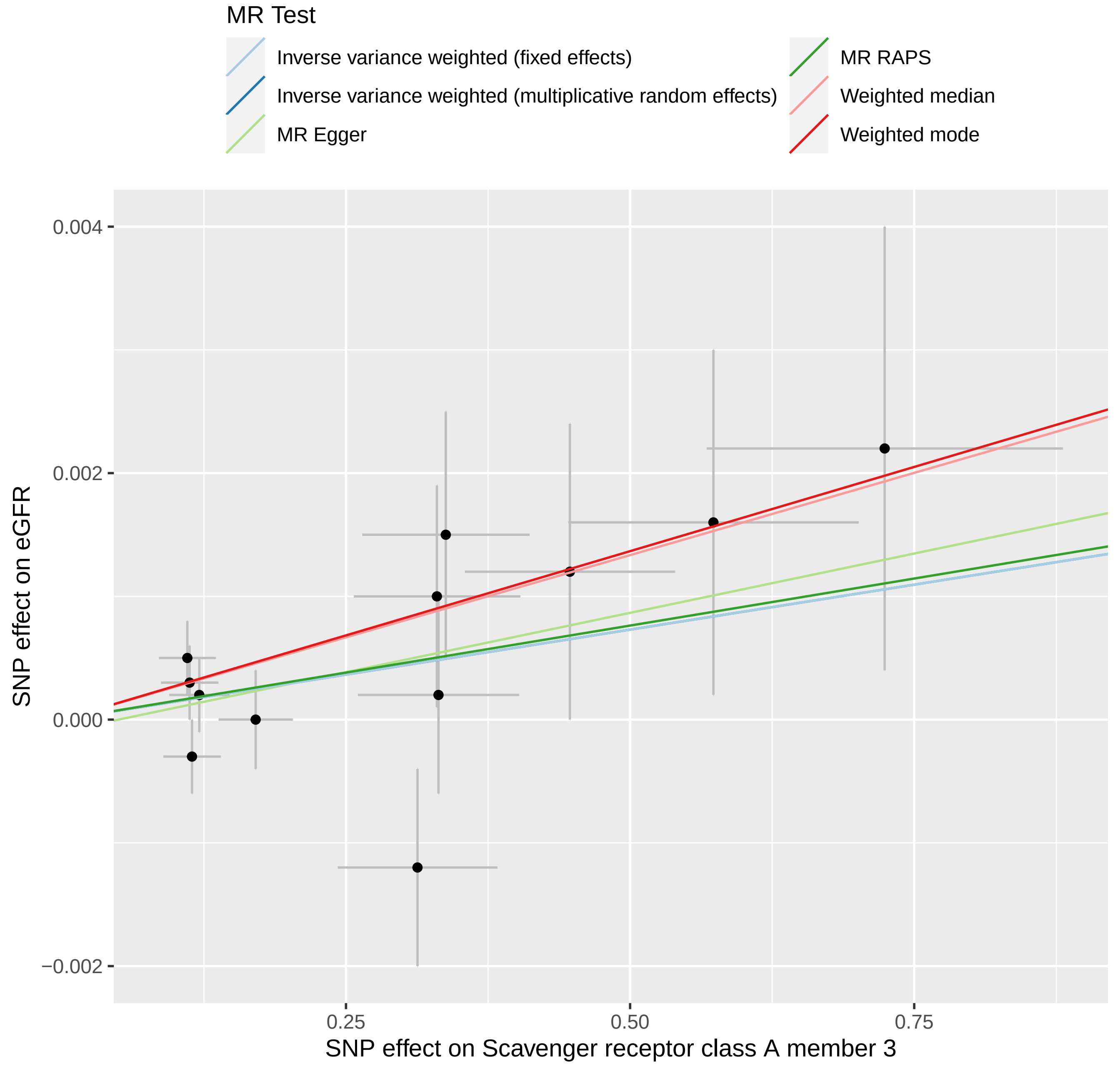

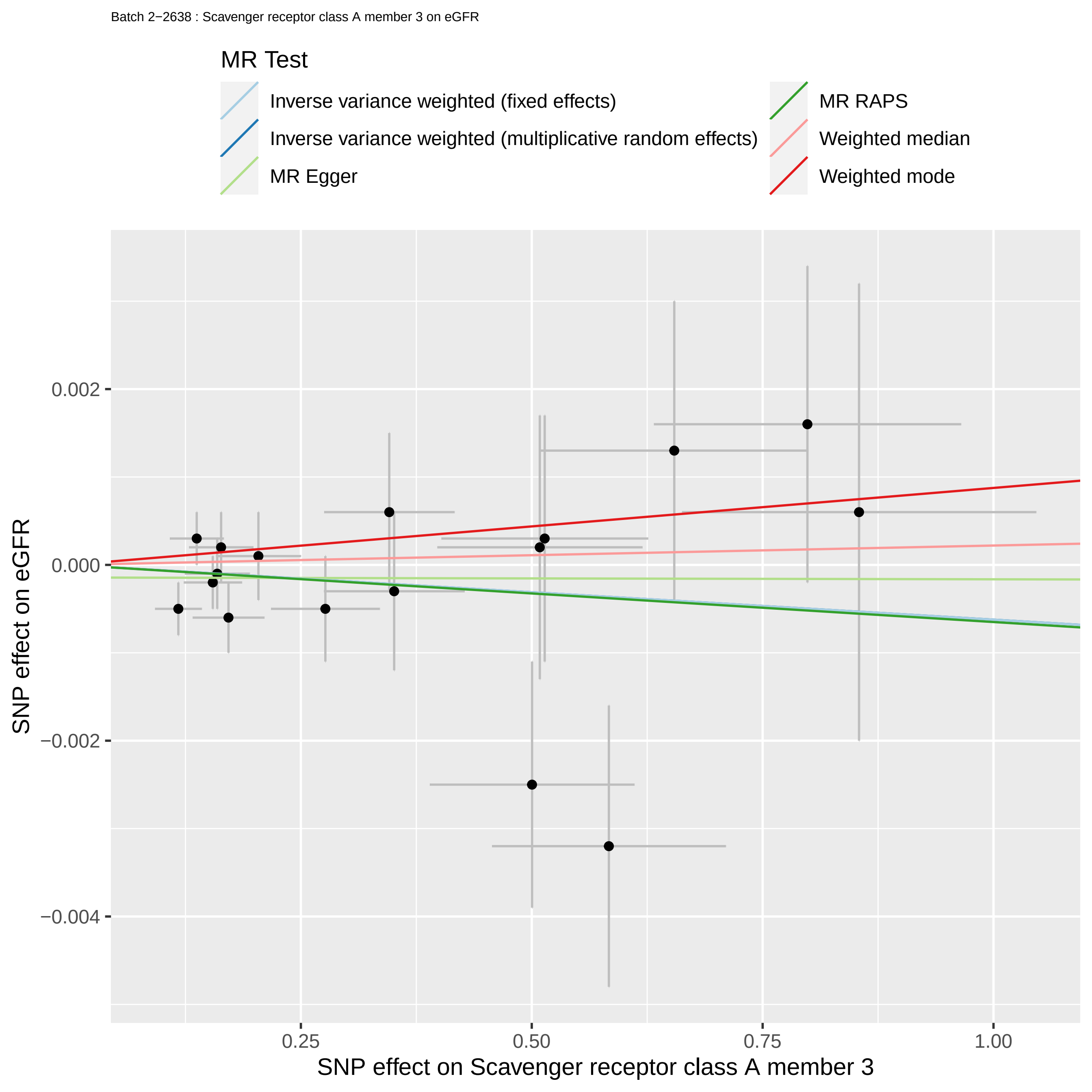

Batch 2−2638 : Scavenger receptor class A member 3 on eGFR
MR Test
Inverse variance weighted (fixed effects)
Inverse variance weighted (multiplicative random effects)
MR Egger
MR RAPS
Weighted median
Weighted mode
0.002
0.000
−0.002
−0.004
0.25
0.50
0.75
1.00
SNP effect on eGFR
SNP effect on Scavenger receptor class A member 3

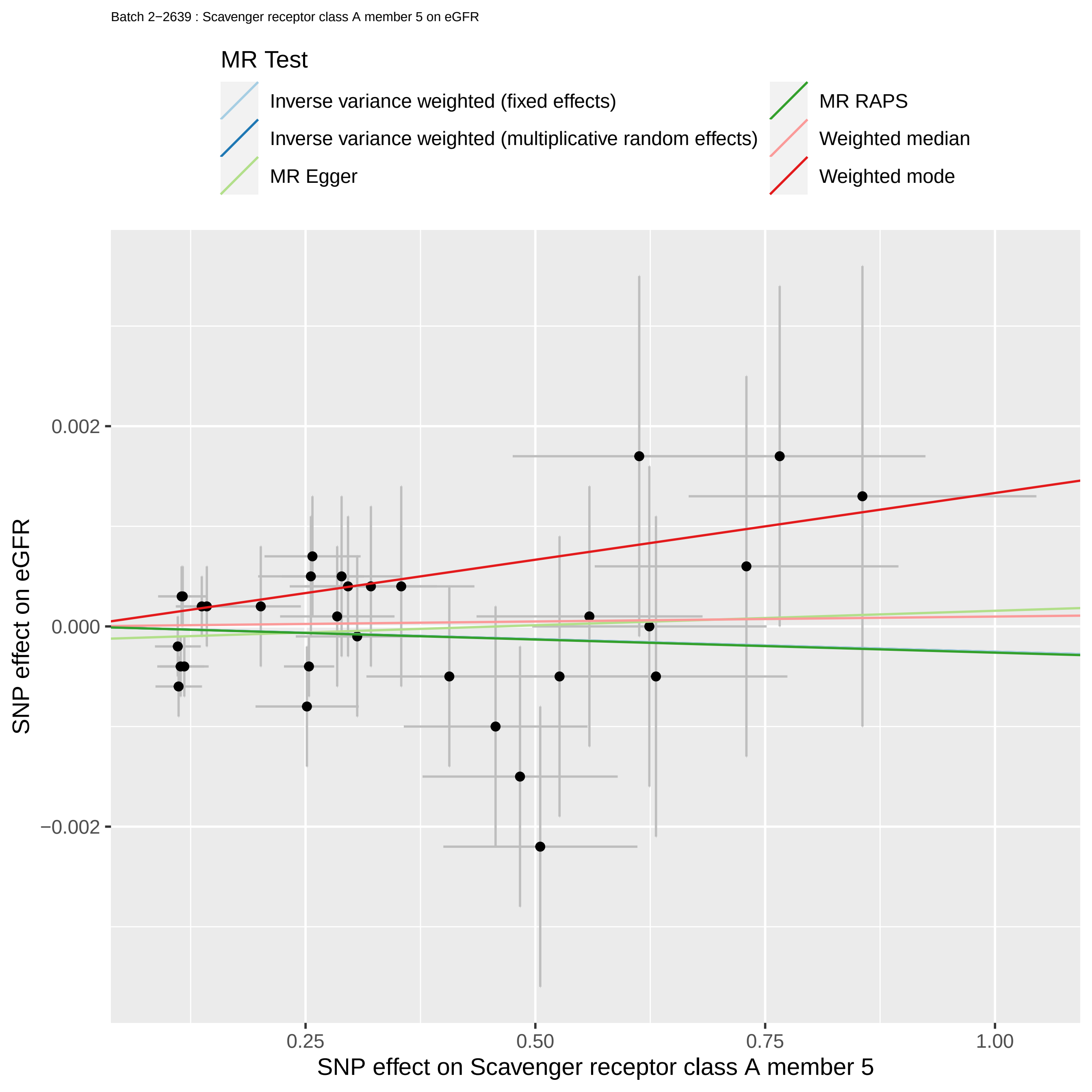

Batch 2−2639 : Scavenger receptor class A member 5 on eGFR
MR Test
Inverse variance weighted (fixed effects)
Inverse variance weighted (multiplicative random effects)
MR Egger
MR RAPS
Weighted median
Weighted mode
0.002
0.000
−0.002
0.25
0.50
0.75
1.00
SNP effect on eGFR
SNP effect on Scavenger receptor class A member 5

Batch 2−2640 : Lysosome membrane protein 2 on eGFR

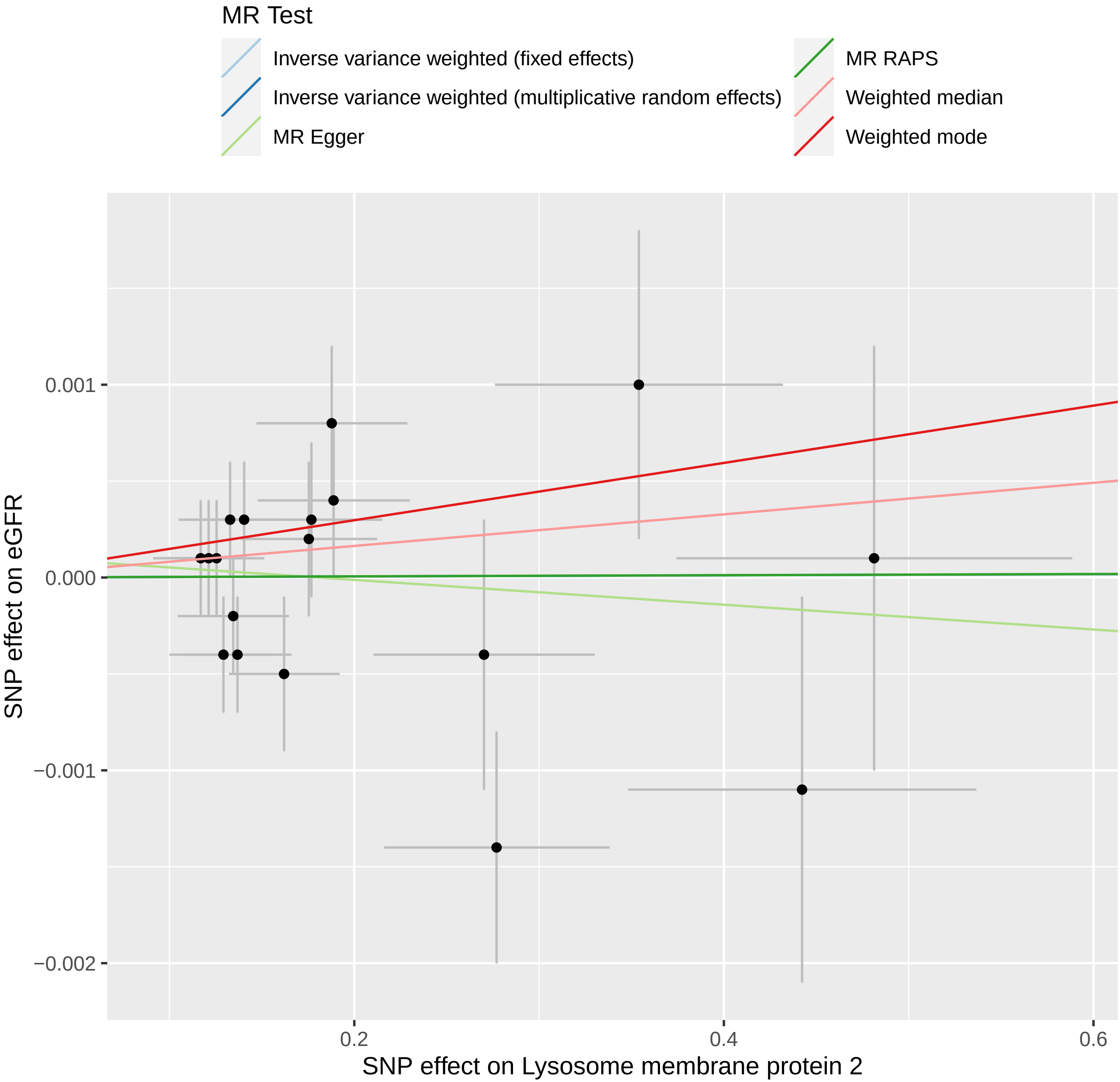

Batch 2−2641 : Scavenger receptor class F member 2 on eGFR

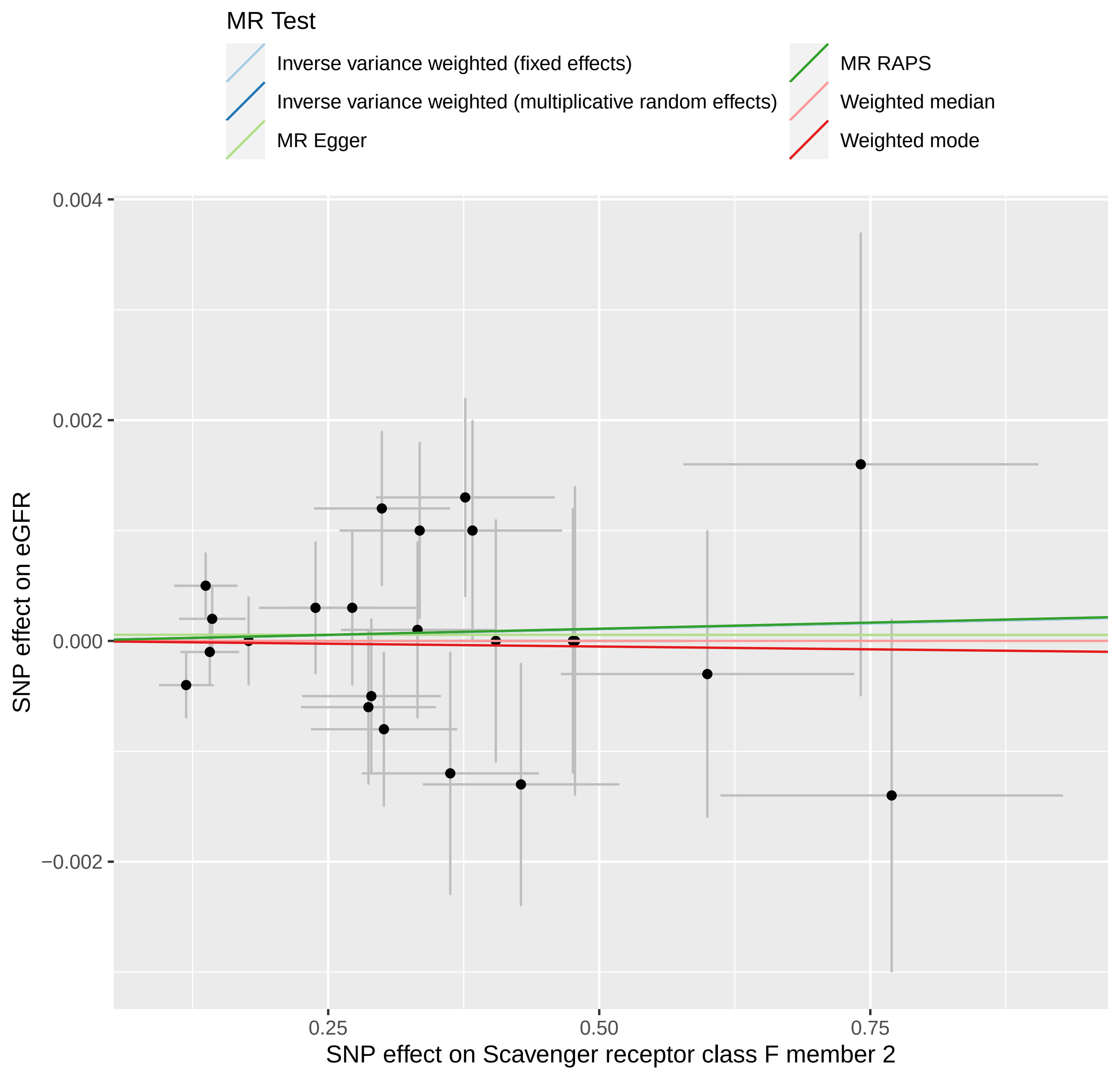

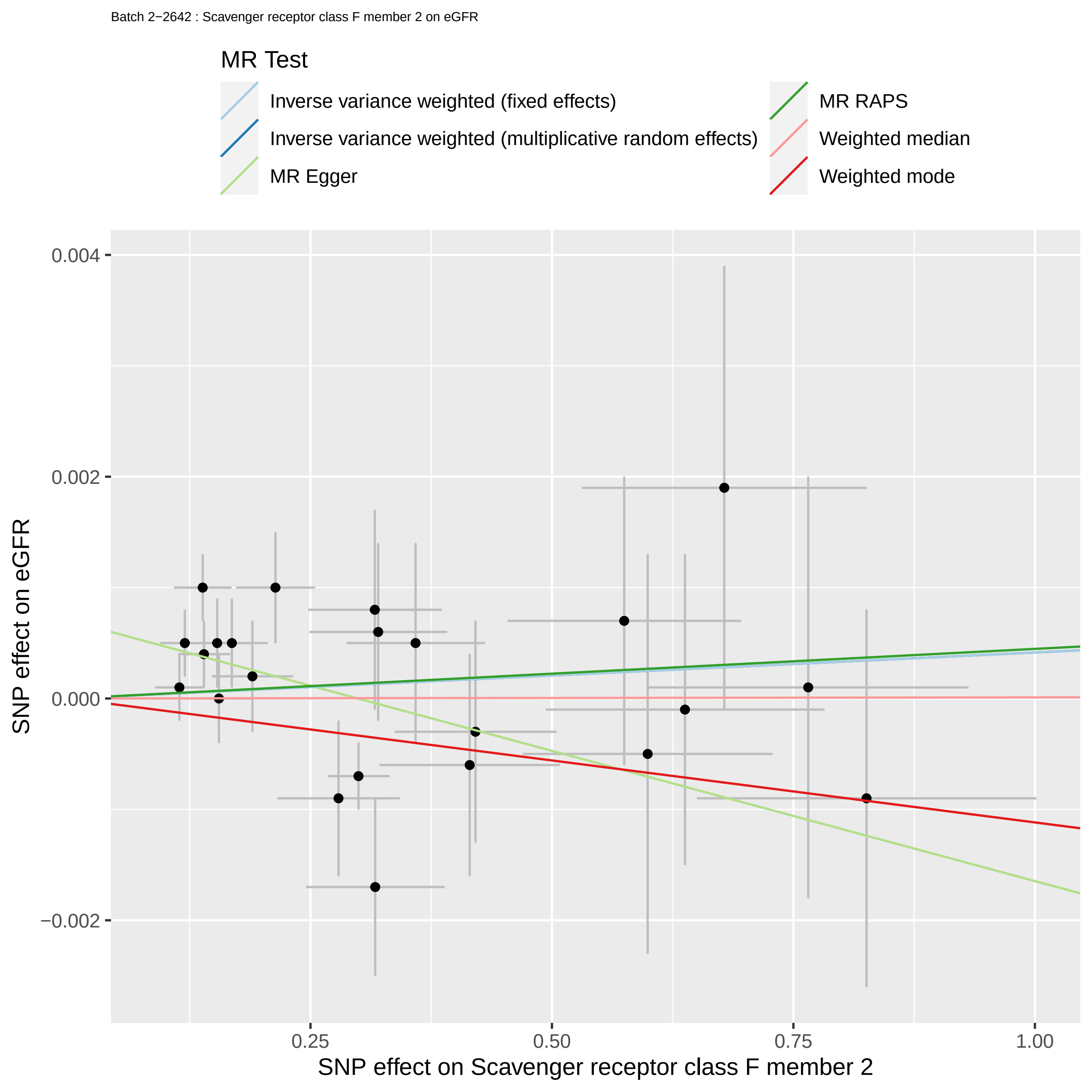

Batch 2−2642 : Scavenger receptor class F member 2 on eGFR
MR Test
Inverse variance weighted (fixed effects)
Inverse variance weighted (multiplicative random effects)
MR Egger
MR RAPS
Weighted median
Weighted mode
0.004
0.002
0.000
−0.002
0.25
0.50
0.75
1.00
SNP effect on eGFR
SNP effect on Scavenger receptor class F member 2

Batch 2−2643 : Scavenger receptor class F member 2 on eGFR

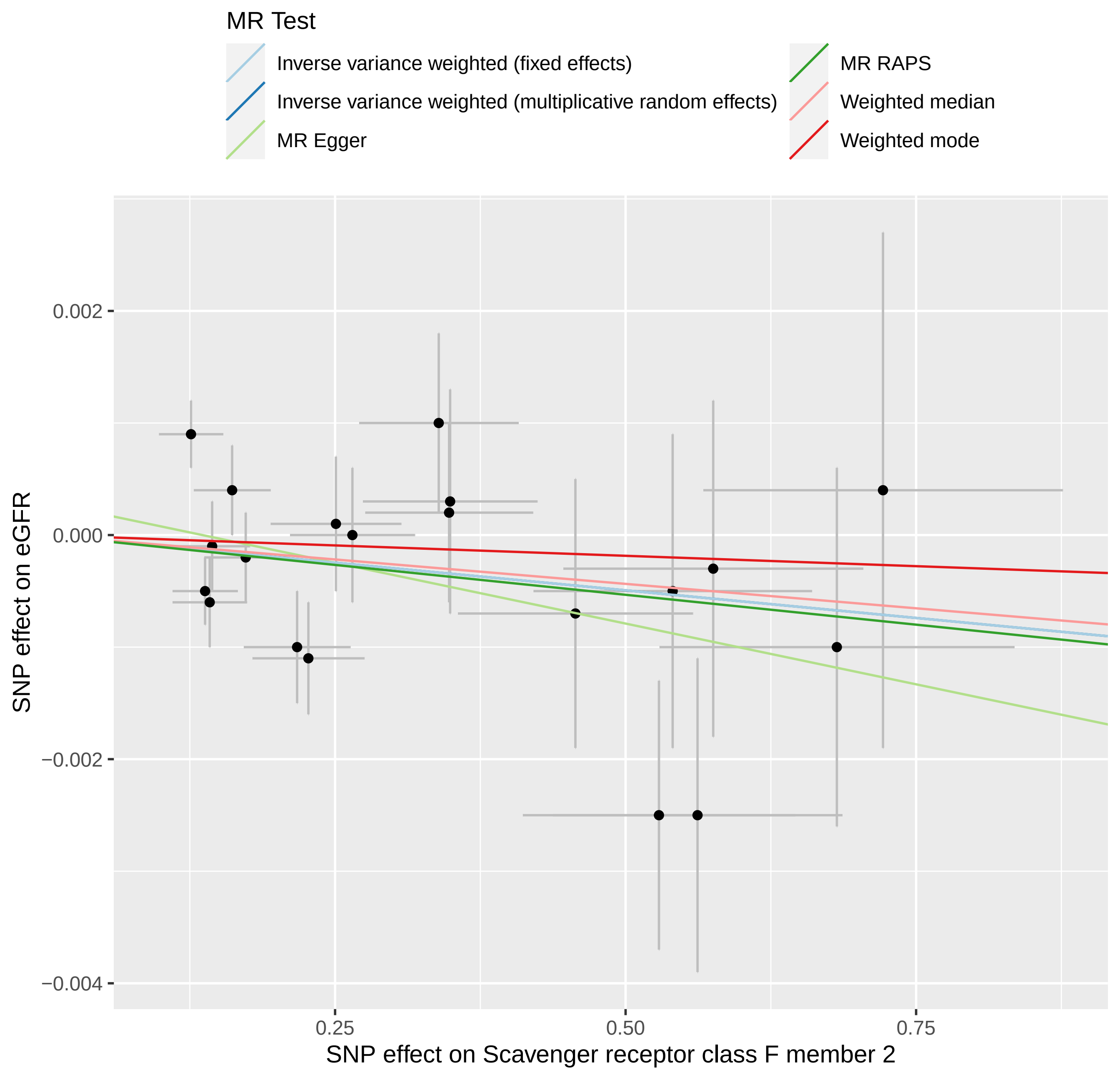

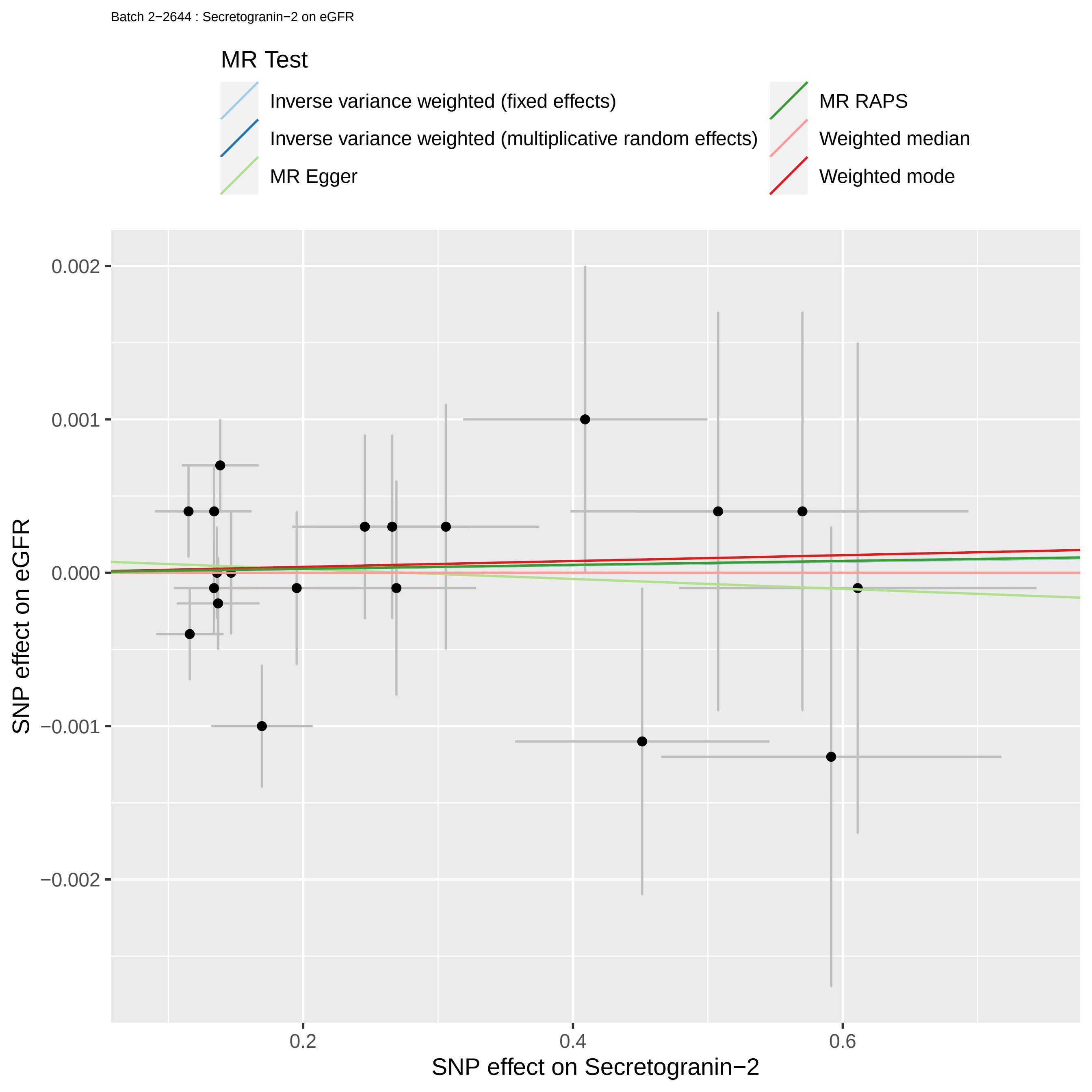

Batch 2−2644 : Secretogranin−2 on eGFR
MR Test
Inverse variance weighted (fixed effects)
Inverse variance weighted (multiplicative random effects)
MR Egger
MR RAPS
Weighted median
Weighted mode
0.002
0.001
0.000
−0.001
−0.002
0.2
0.4
0.6
SNP effect on eGFR
SNP effect on Secretogranin−2

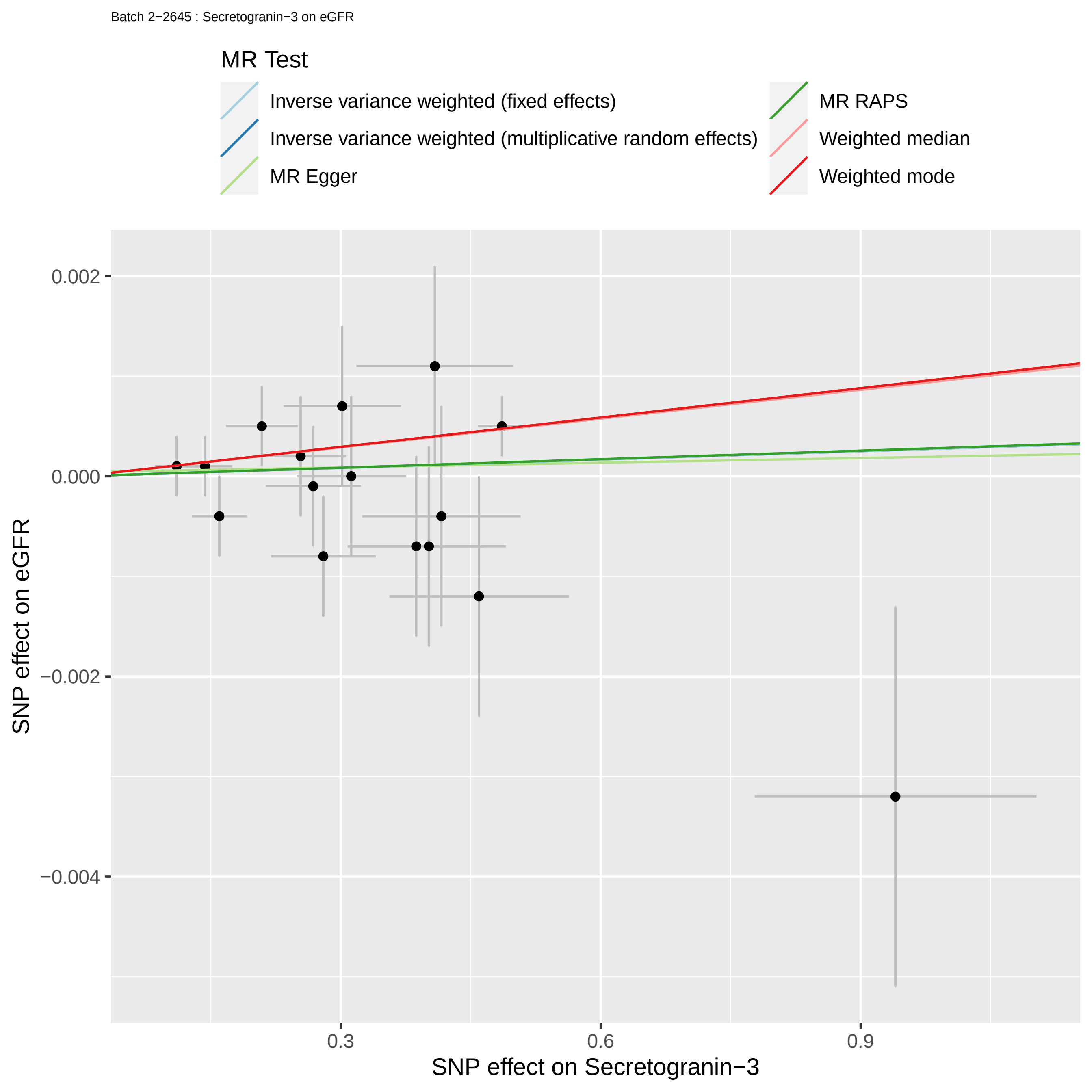

Batch 2−2645 : Secretogranin−3 on eGFR
MR Test
Inverse variance weighted (fixed effects)
Inverse variance weighted (multiplicative random effects)
MR Egger
MR RAPS
Weighted median
Weighted mode
0.002
0.000
−0.002
−0.004
0.3
0.6
0.9
SNP effect on eGFR
SNP effect on Secretogranin−3

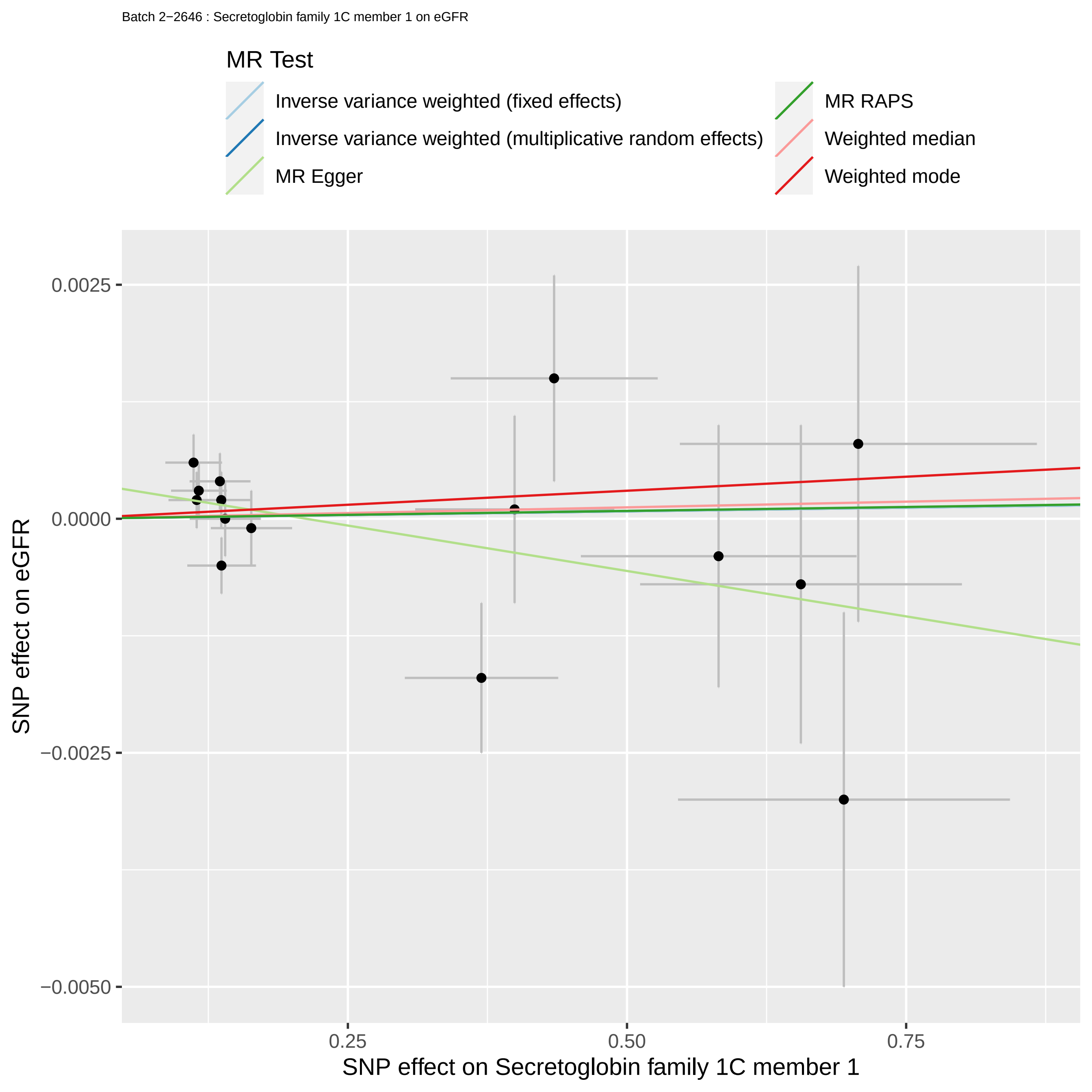

Batch 2−2646 : Secretoglobin family 1C member 1 on eGFR
MR Test
Inverse variance weighted (fixed effects)
Inverse variance weighted (multiplicative random effects)
MR Egger
MR RAPS
Weighted median
Weighted mode
0.0025
0.0000
−0.0025
−0.0050
0.25
0.50
0.75
SNP effect on eGFR
SNP effect on Secretoglobin family 1C member 1

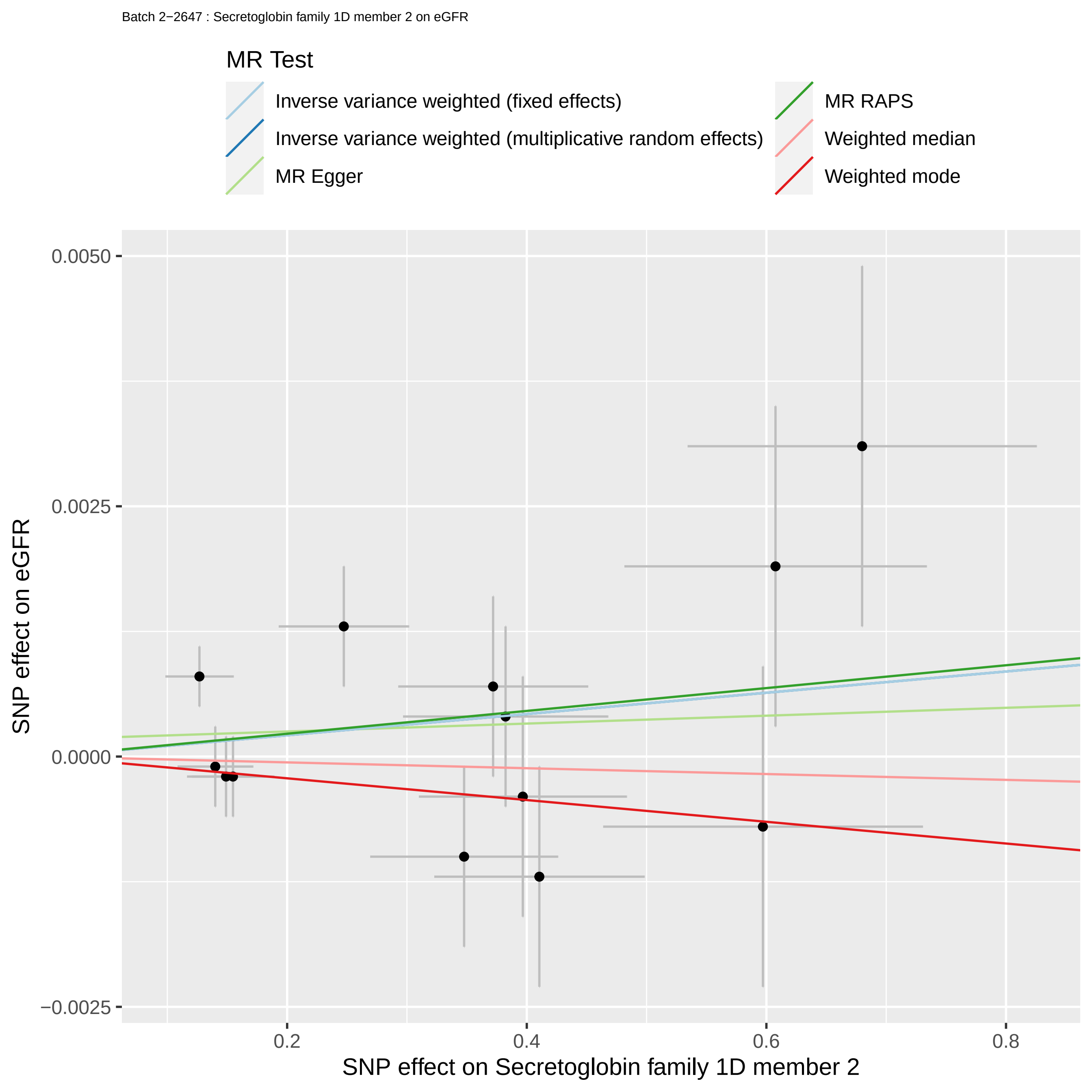
Batch 2−2647 : Secretoglobin family 1D member 2 on eGFR
MR Test
Inverse variance weighted (fixed effects)
Inverse variance weighted (multiplicative random effects)
MR Egger
MR RAPS
Weighted median
Weighted mode
0.0050
0.0025
0.0000
−0.0025
0.2
0.4
0.6
0.8
SNP effect on eGFR
SNP effect on Secretoglobin family 1D member 2

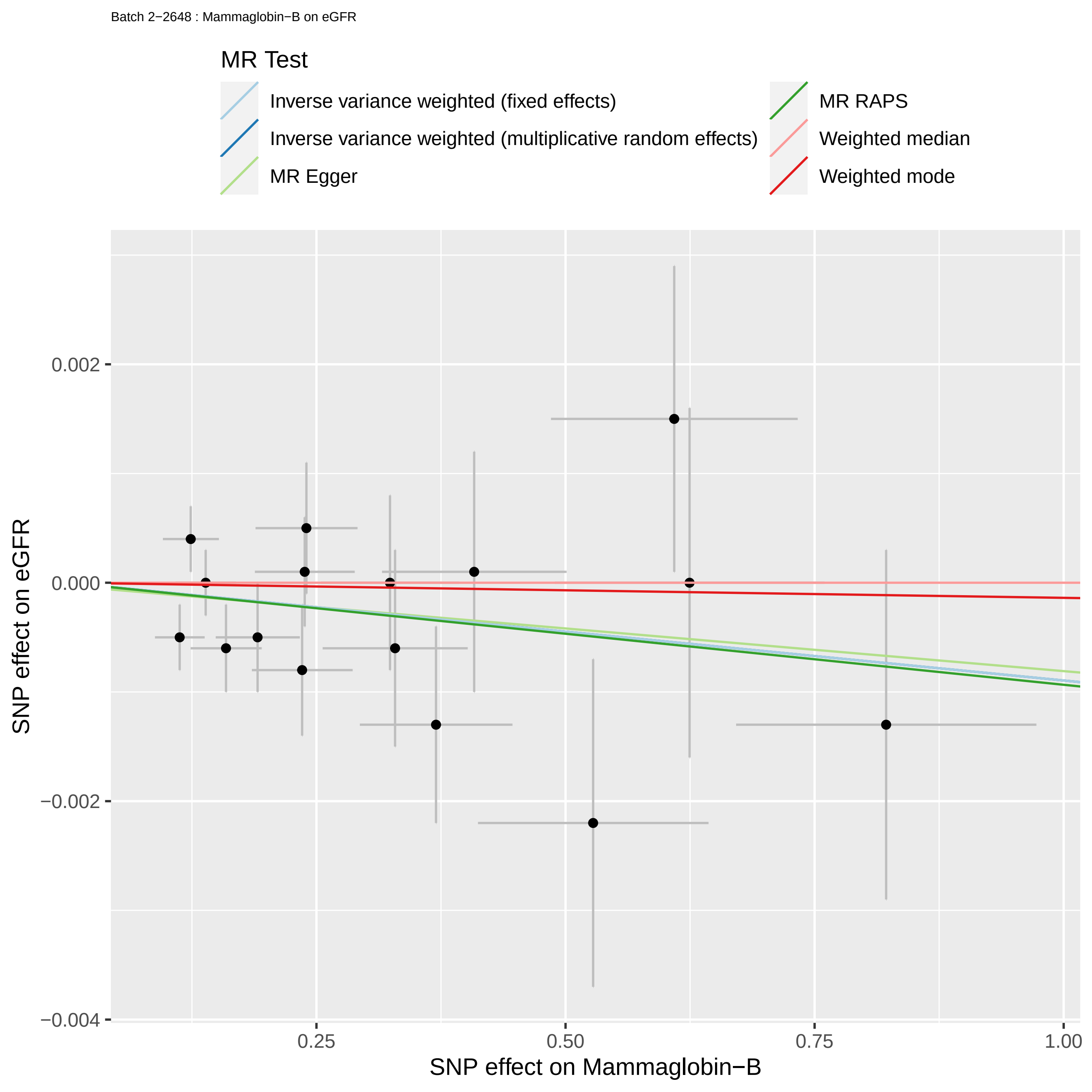
Batch 2−2648 : Mammaglobin−B on eGFR
MR Test
Inverse variance weighted (fixed effects)
Inverse variance weighted (multiplicative random effects)
MR Egger
MR RAPS
Weighted median
Weighted mode
0.002
0.000
−0.002
−0.004
0.25
0.50
0.75
1.00
SNP effect on eGFR
SNP effect on Mammaglobin−B

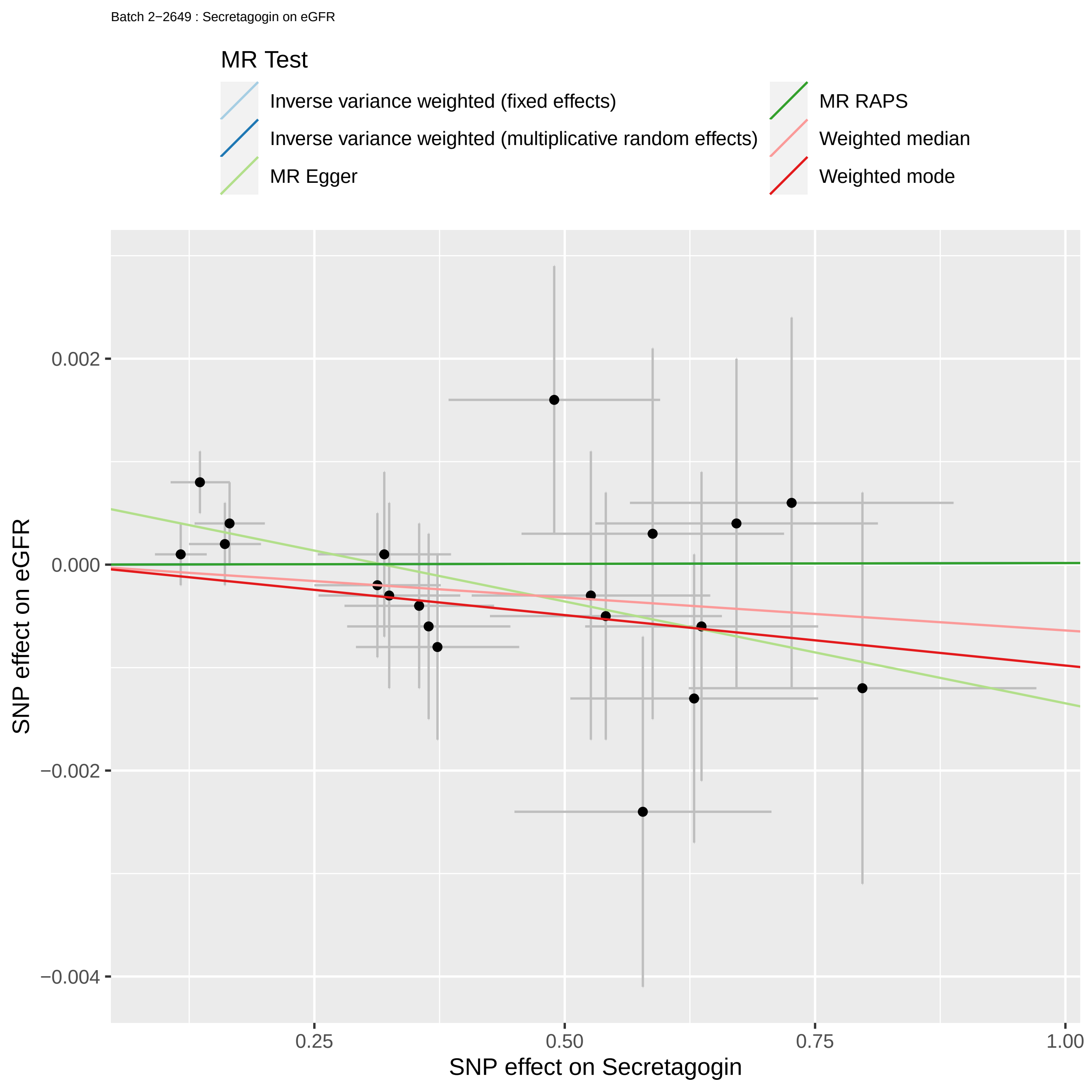

Batch 2−2649 : Secretagogin on eGFR
MR Test
Inverse variance weighted (fixed effects)
Inverse variance weighted (multiplicative random effects)
MR Egger
MR RAPS
Weighted median
Weighted mode
0.002
0.000
−0.002
−0.004
0.25
0.50
0.75
1.00
SNP effect on eGFR
SNP effect on Secretagogin

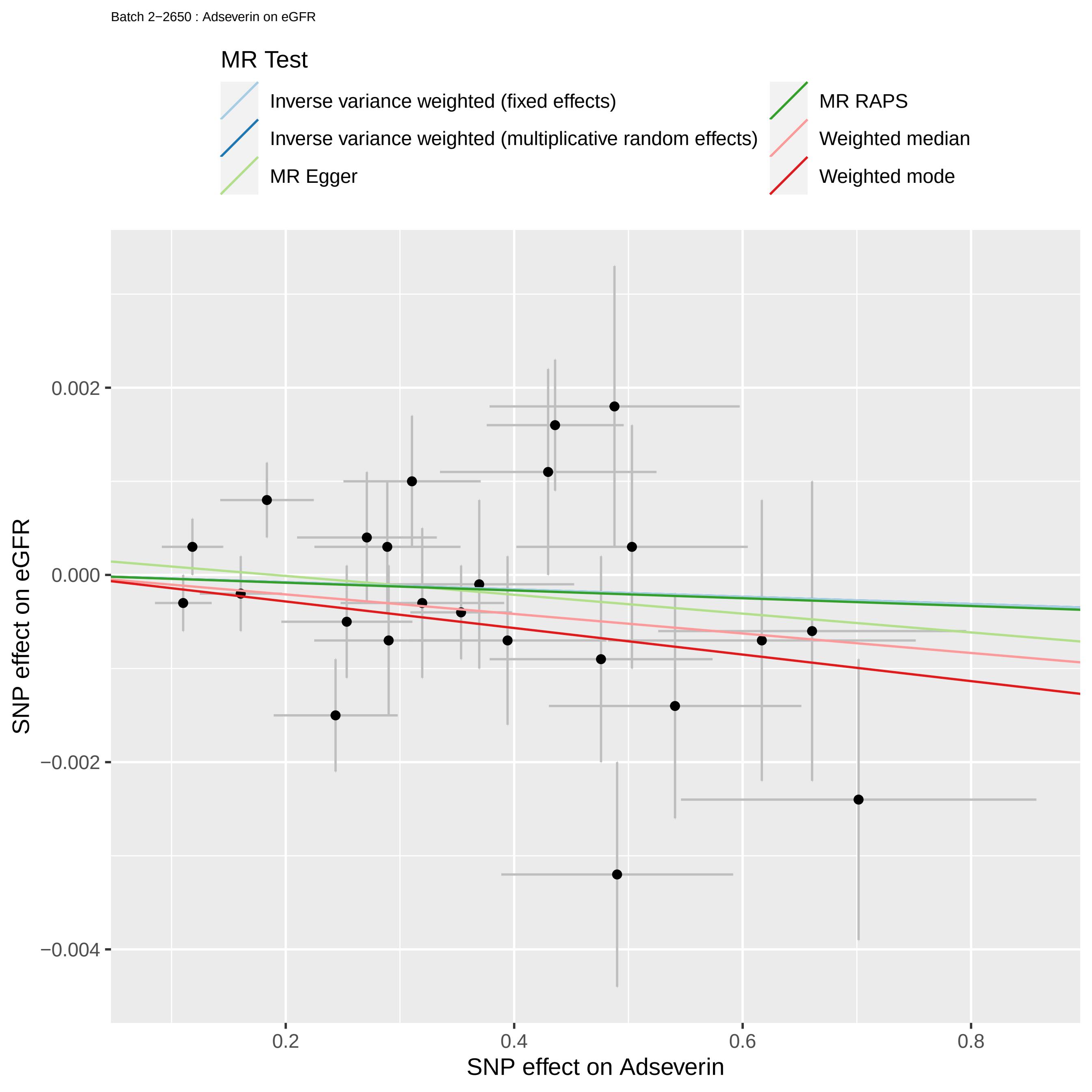

Batch 2−2650 : Adseverin on eGFR
MR Test
Inverse variance weighted (fixed effects)
Inverse variance weighted (multiplicative random effects)
MR Egger
MR RAPS
Weighted median
Weighted mode
0.002
0.000
−0.002
−0.004
0.2
0.4
0.6
0.8
SNP effect on eGFR
SNP effect on Adseverin

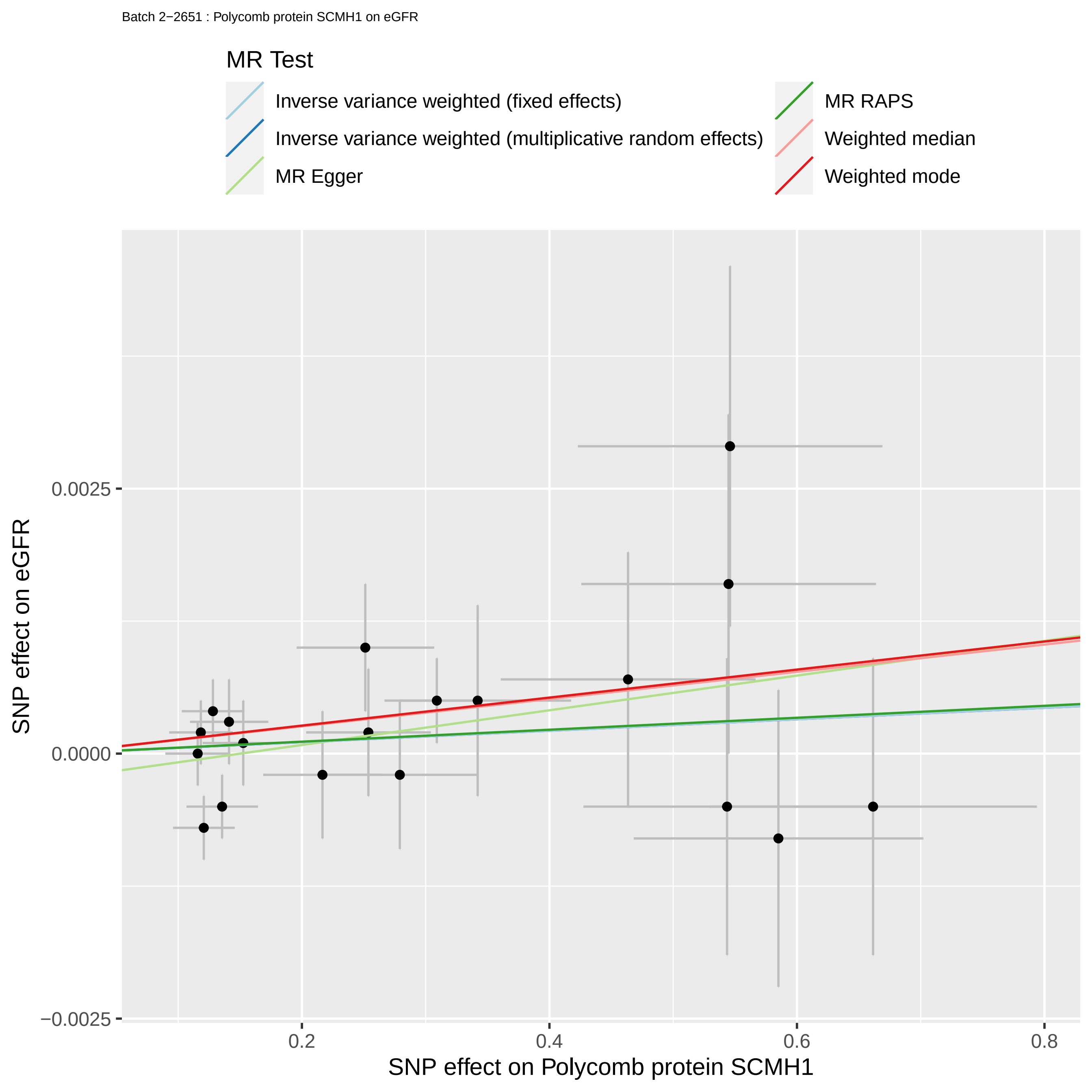

Batch 2−2651 : Polycomb protein SCMH1 on eGFR
MR Test
Inverse variance weighted (fixed effects)
Inverse variance weighted (multiplicative random effects)
MR Egger
MR RAPS
Weighted median
Weighted mode
SNP effect on eGFR
0.0025
0.0000
−0.0025
0.2
0.4
0.6
0.8
SNP effect on Polycomb protein SCMH1

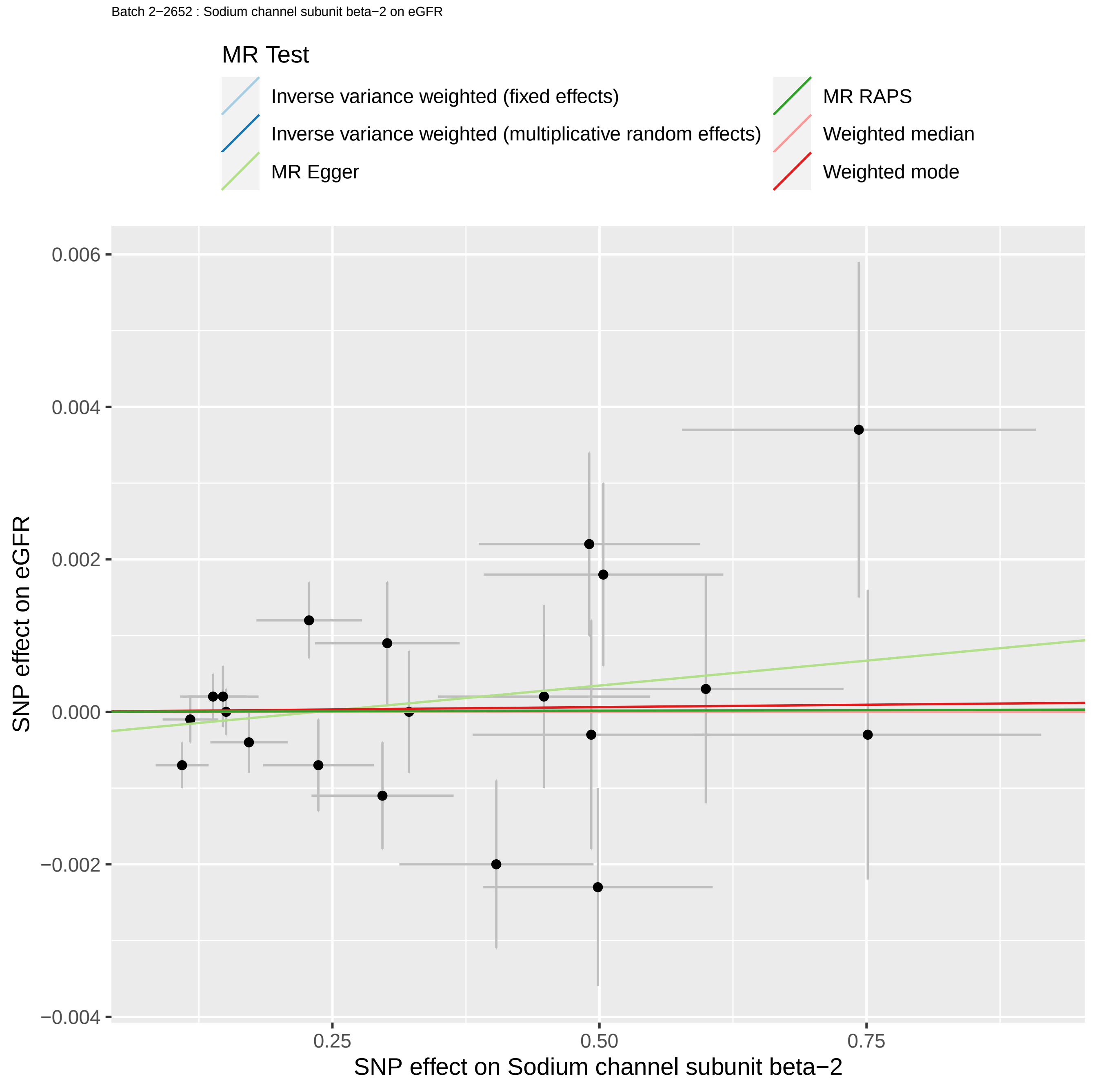

Batch 2−2652 : Sodium channel subunit beta−2 on eGFR
MR Test
Inverse variance weighted (fixed effects)
Inverse variance weighted (multiplicative random effects)
MR Egger
MR RAPS
Weighted median
Weighted mode
0.006
0.004
0.002
0.000
−0.002
−0.004
0.25
0.50
0.75
SNP effect on eGFR
SNP effect on Sodium channel subunit beta−2

Batch 2−2653 : Protein SCO1 homolog, mitochondrial on eGFR

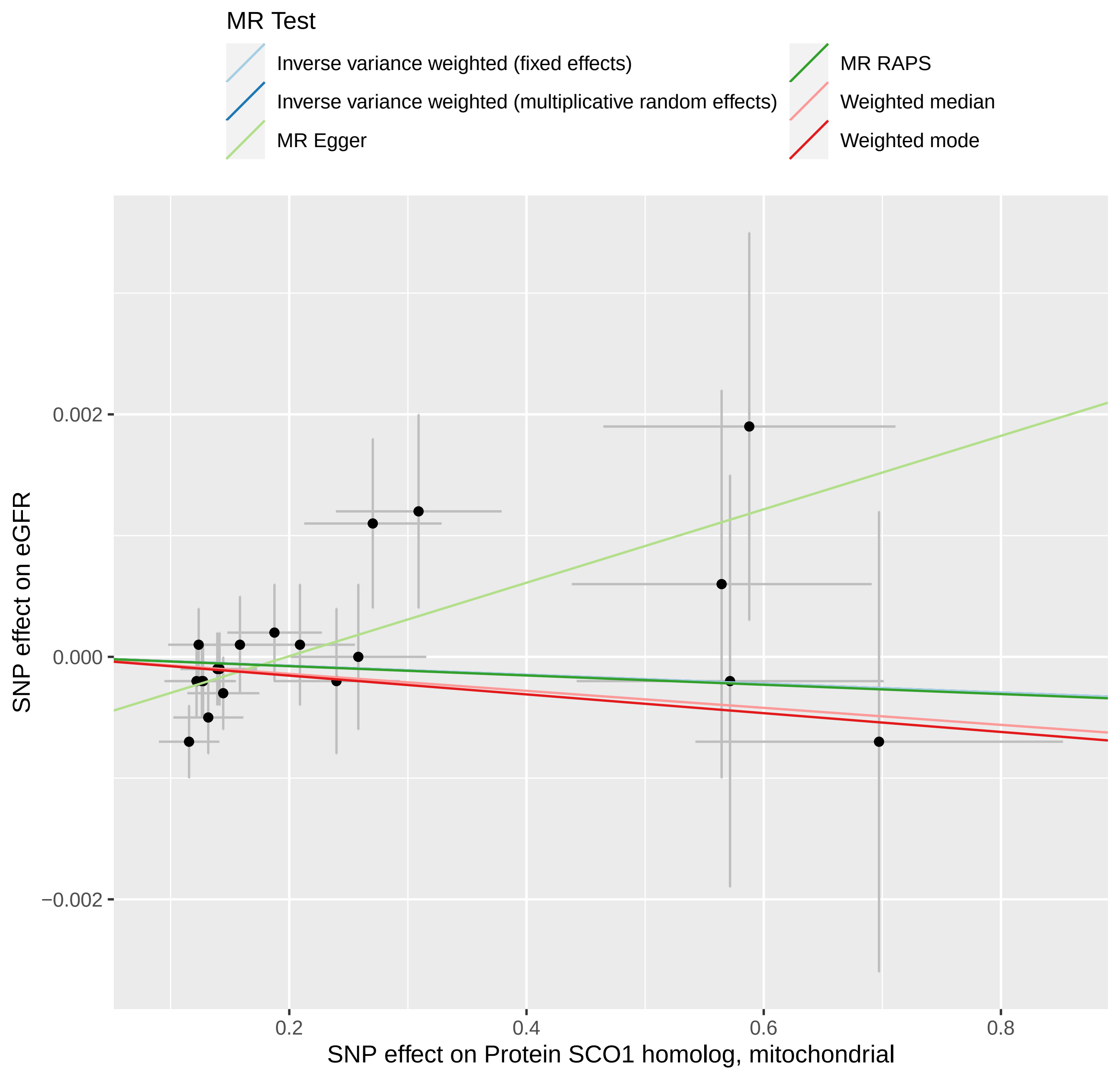

Batch 2−2654 : SCP2 sterol−binding domain−containing protein 1 on eGFR

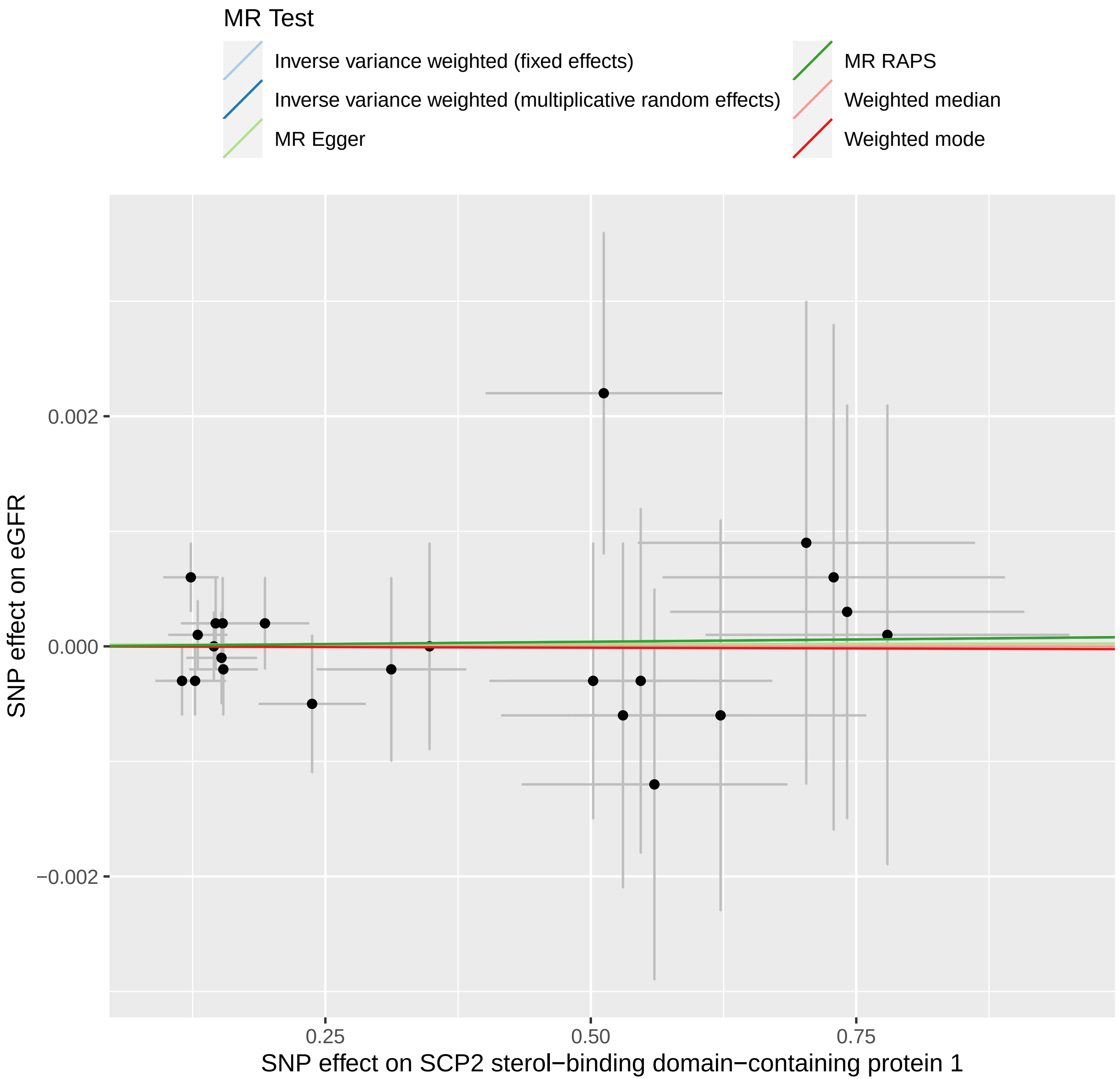

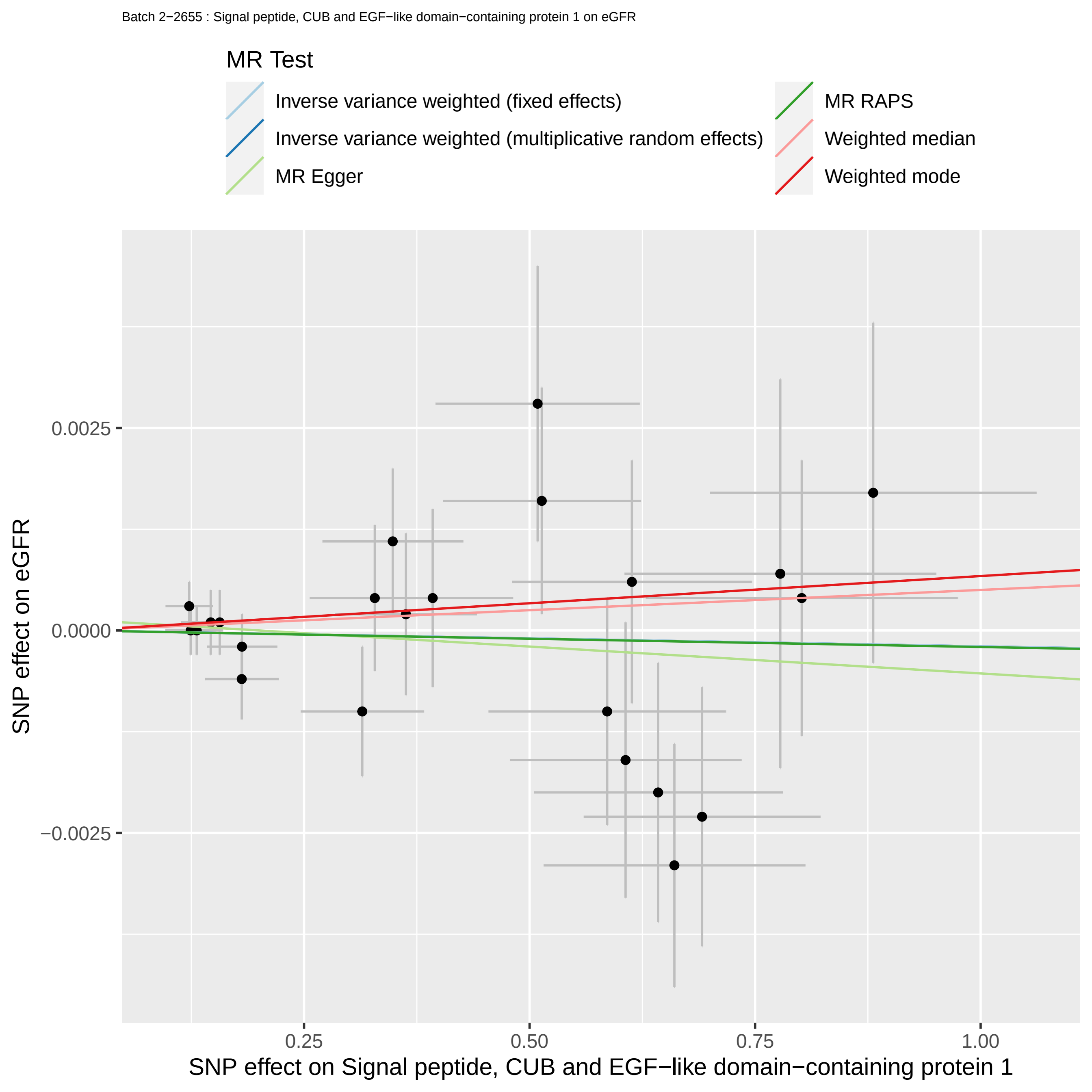

Batch 2−2655 : Signal peptide, CUB and EGF−like domain−containing protein 1 on eGFR
MR Test
Inverse variance weighted (fixed effects)
Inverse variance weighted (multiplicative random effects)
MR Egger
MR RAPS
Weighted median
Weighted mode
0.0025
0.0000
−0.0025
0.25
0.50
0.75
1.00
SNP effect on eGFR
SNP effect on Signal peptide, CUB and EGF−like domain−containing protein 1

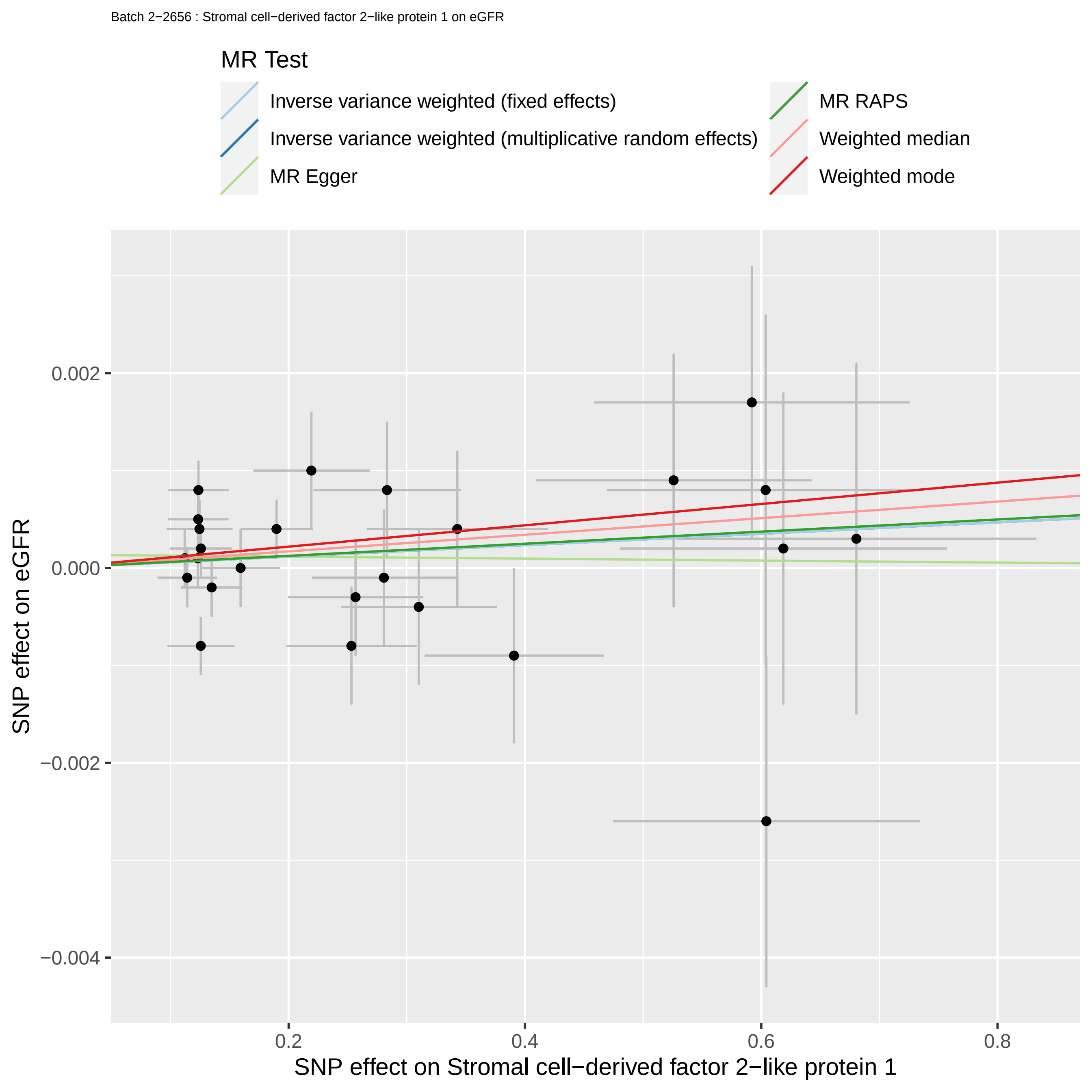
Batch 2−2656 : Stromal cell−derived factor 2−like protein 1 on eGFR
MR Test
Inverse variance weighted (fixed effects)
Inverse variance weighted (multiplicative random effects)
MR Egger
MR RAPS
Weighted median
Weighted mode
0.002
0.000
−0.002
−0.004
0.2
0.4
0.6
0.8
SNP effect on eGFR
SNP effect on Stromal cell−derived factor 2−like protein 1

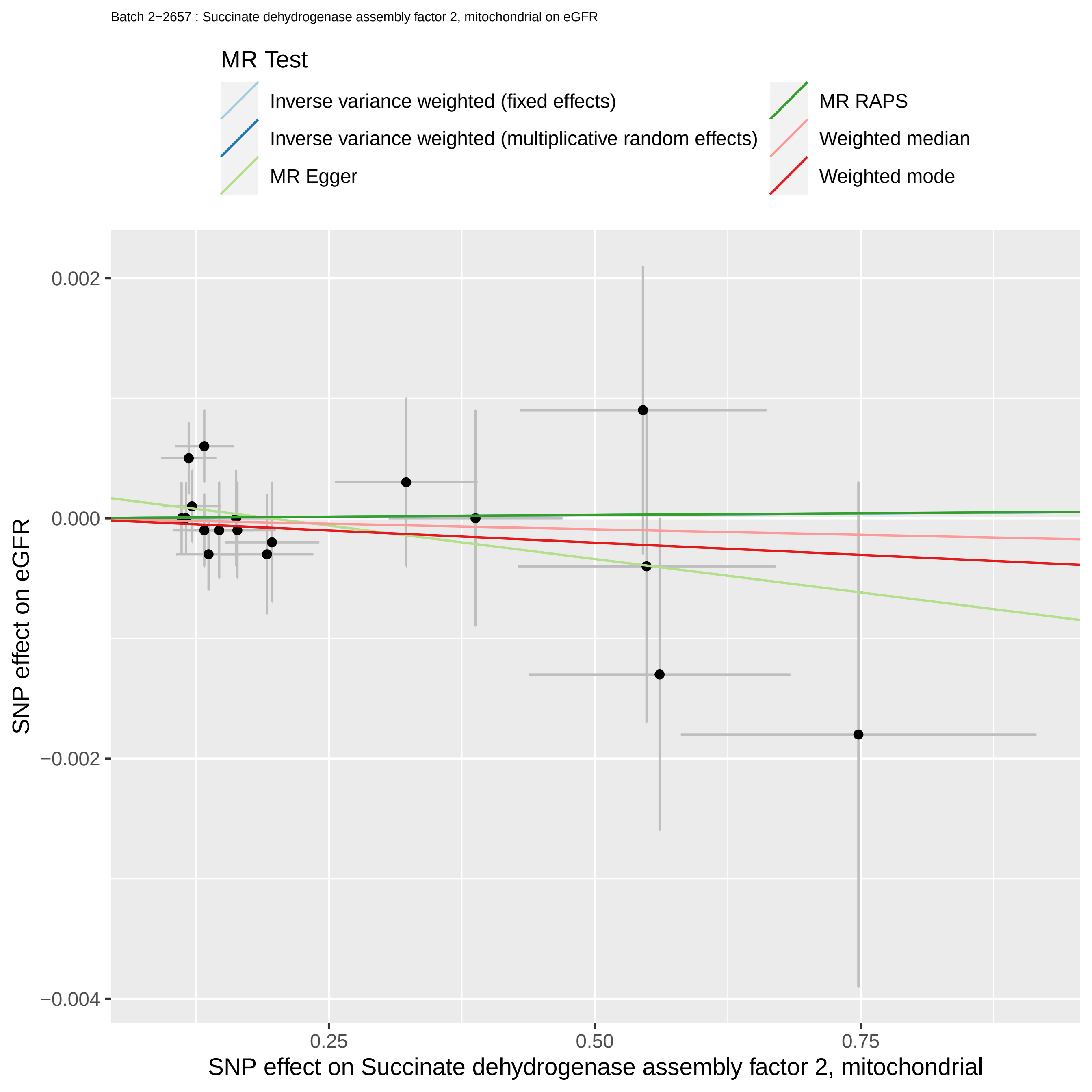

Batch 2−2657 : Succinate dehydrogenase assembly factor 2, mitochondrial on eGFR
MR Test
Inverse variance weighted (fixed effects)
Inverse variance weighted (multiplicative random effects)
MR Egger
MR RAPS
Weighted median
Weighted mode
0.002
0.000
−0.002
−0.004
0.25
0.50
0.75
SNP effect on eGFR
SNP effect on Succinate dehydrogenase assembly factor 2, mitochondrial

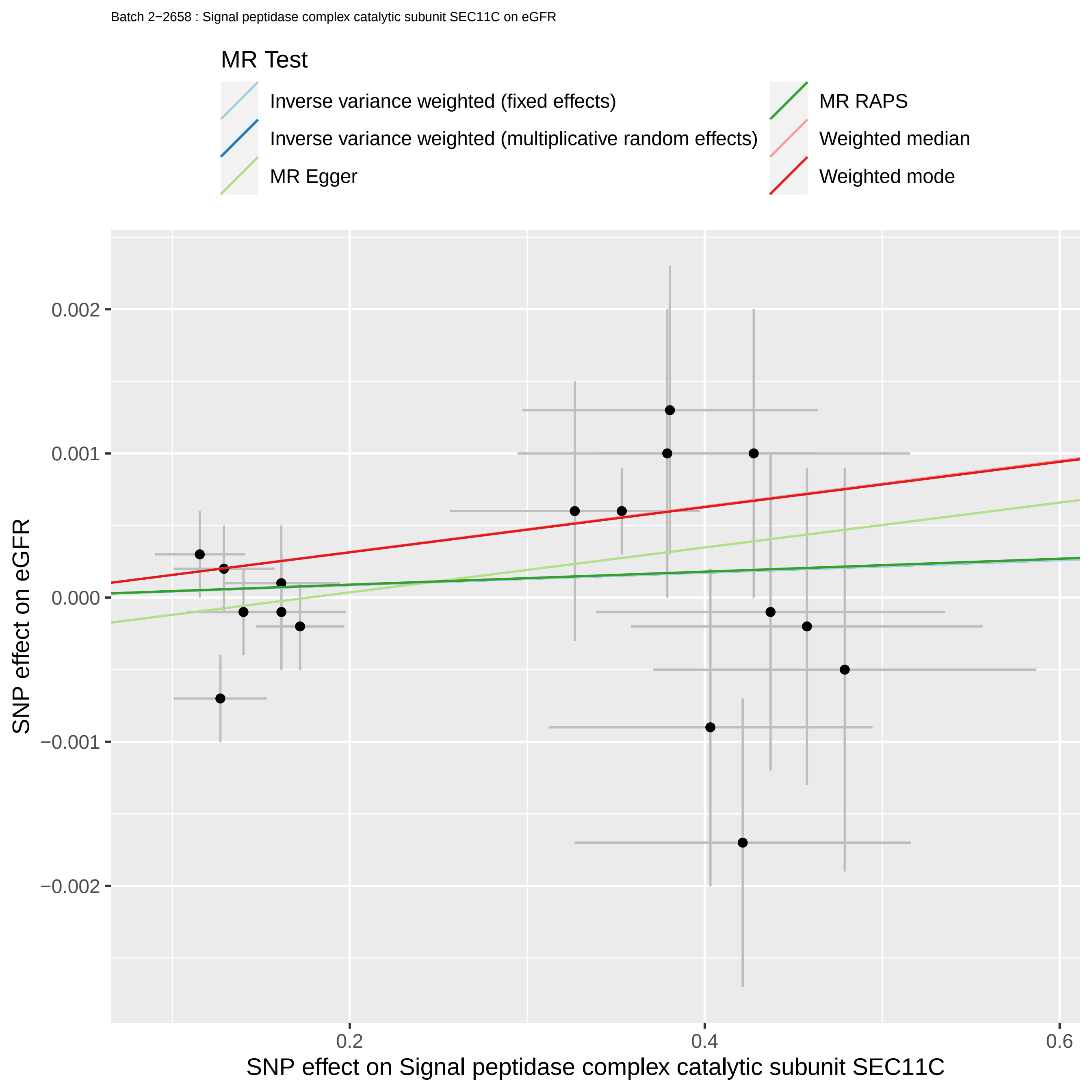
Batch 2−2658 : Signal peptidase complex catalytic subunit SEC11C on eGFR
MR Test
Inverse variance weighted (fixed effects)
Inverse variance weighted (multiplicative random effects)
MR Egger
MR RAPS
Weighted median
Weighted mode
SNP effect on eGFR
0.002
0.001
0.000
−0.001
−0.002
0.2
0.4
0.6
SNP effect on Signal peptidase complex catalytic subunit SEC11C

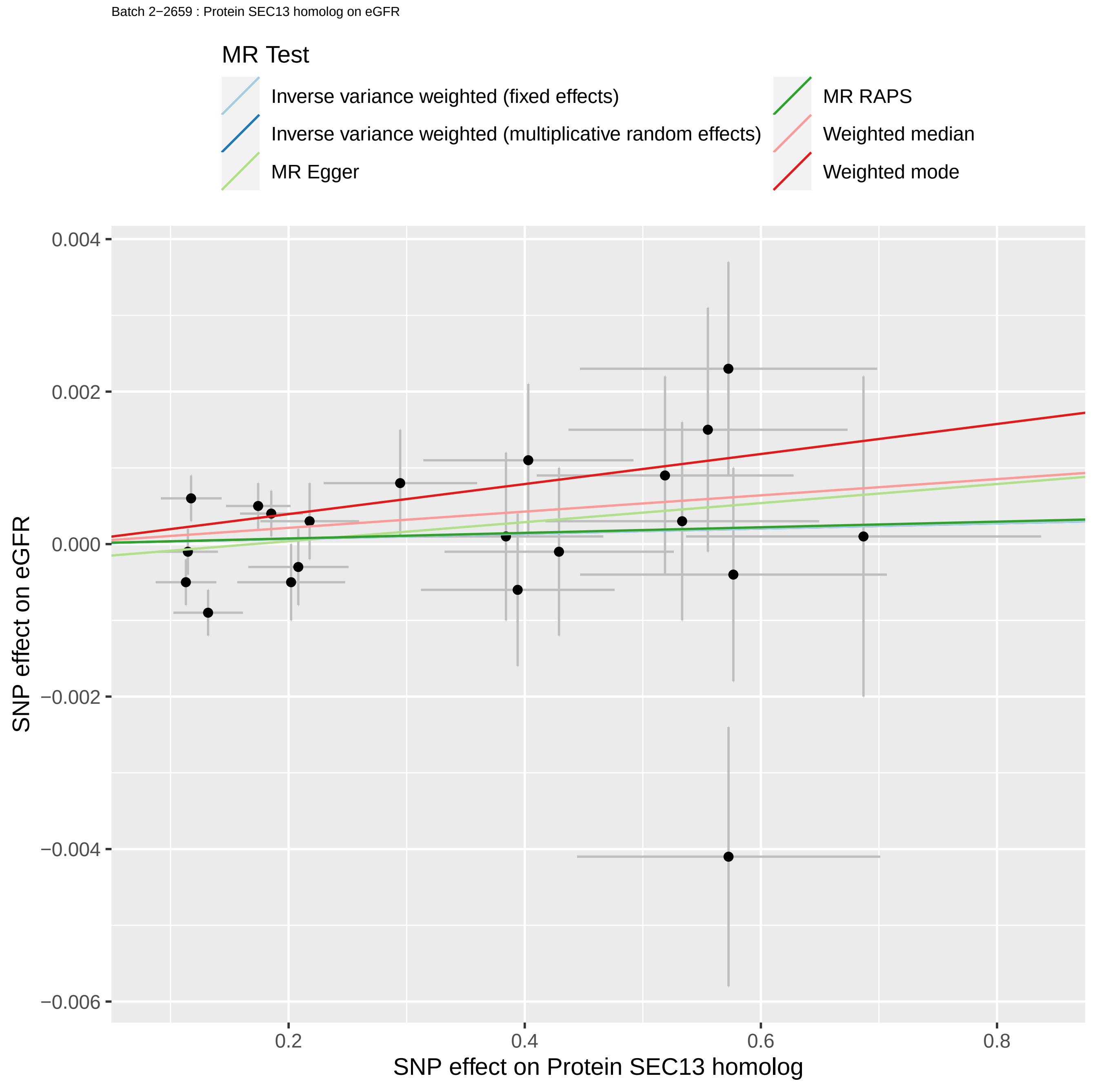
Batch 2−2659 : Protein SEC13 homolog on eGFR
MR Test
Inverse variance weighted (fixed effects)
Inverse variance weighted (multiplicative random effects)
MR Egger
MR RAPS
Weighted median
Weighted mode
0.004
0.002
0.000
−0.002
−0.004
−0.006
0.2
0.4
0.6
0.8
SNP effect on eGFR
SNP effect on Protein SEC13 homolog

Batch 2−2660 : Vesicle−trafficking protein SEC22a on eGFR

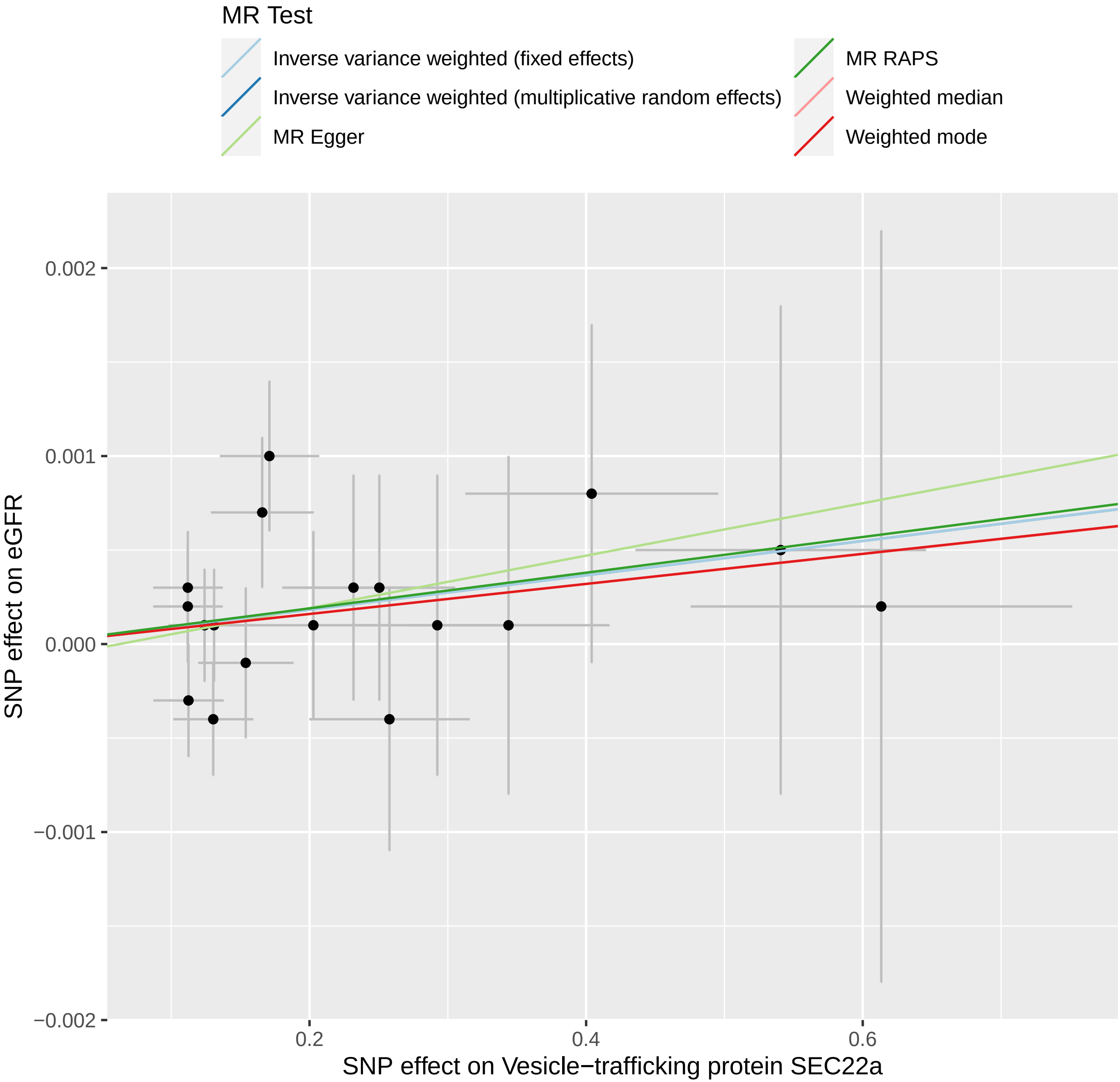

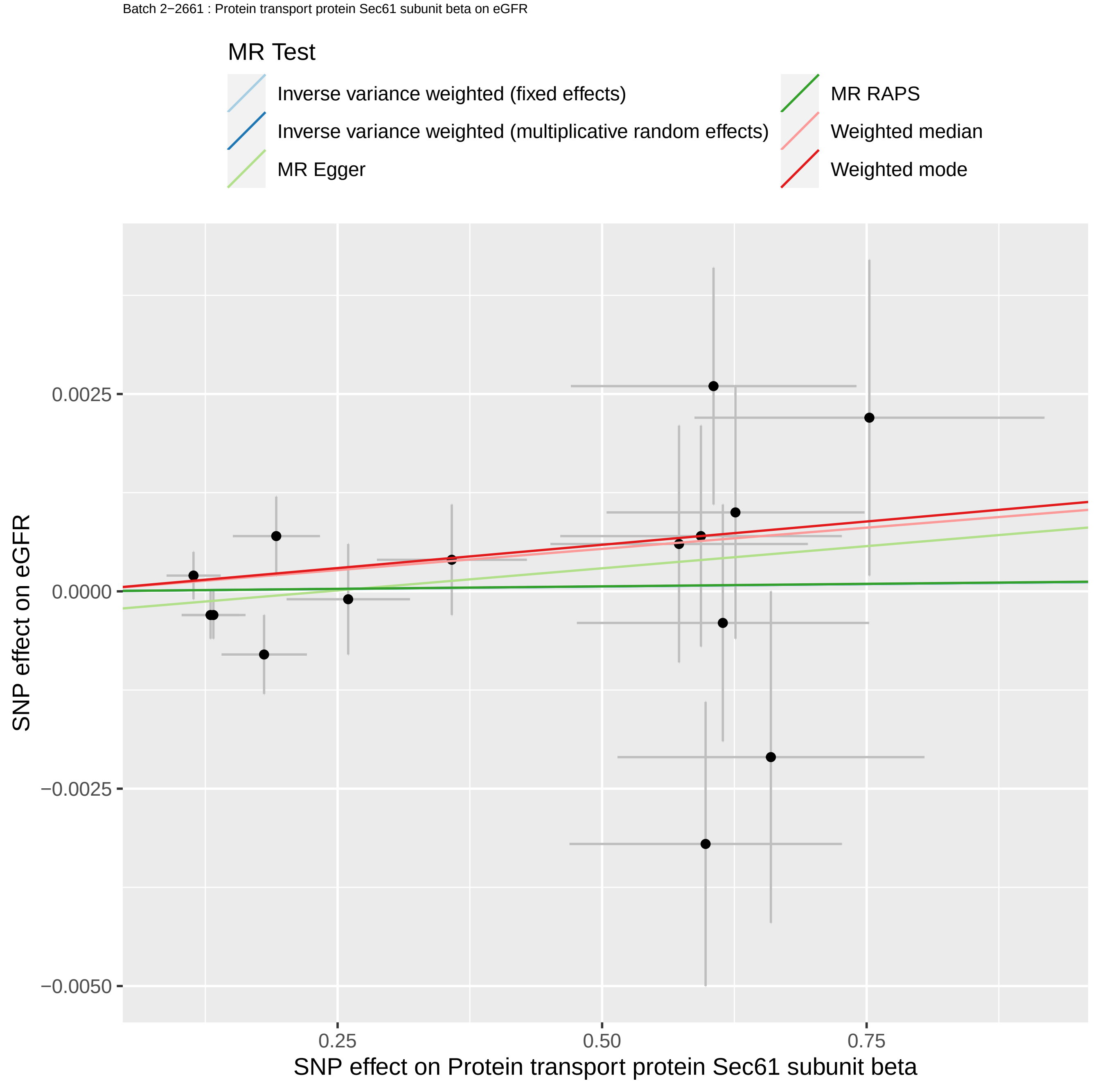

Batch 2−2661 : Protein transport protein Sec61 subunit beta on eGFR
MR Test
Inverse variance weighted (fixed effects)
Inverse variance weighted (multiplicative random effects)
MR Egger
MR RAPS
Weighted median
Weighted mode
0.0025
0.0000
−0.0025
−0.0050
0.25
0.50
0.75
SNP effect on eGFR
SNP effect on Protein transport protein Sec61 subunit beta

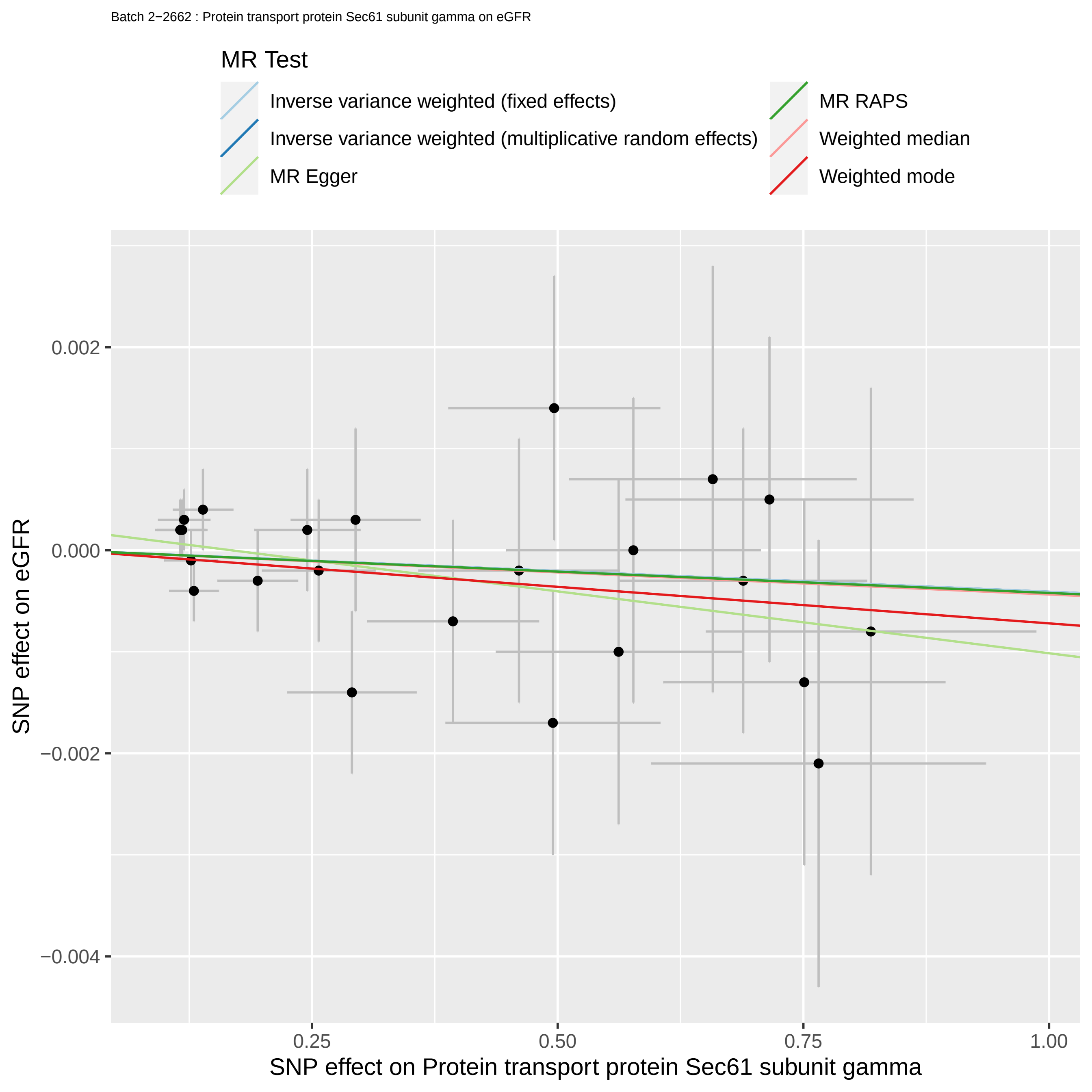

Batch 2−2662 : Protein transport protein Sec61 subunit gamma on eGFR
MR Test
Inverse variance weighted (fixed effects)
Inverse variance weighted (multiplicative random effects)
MR Egger
MR RAPS
Weighted median
Weighted mode
0.002
0.000
−0.002
−0.004
0.25
0.50
0.75
1.00
SNP effect on eGFR
SNP effect on Protein transport protein Sec61 subunit gamma

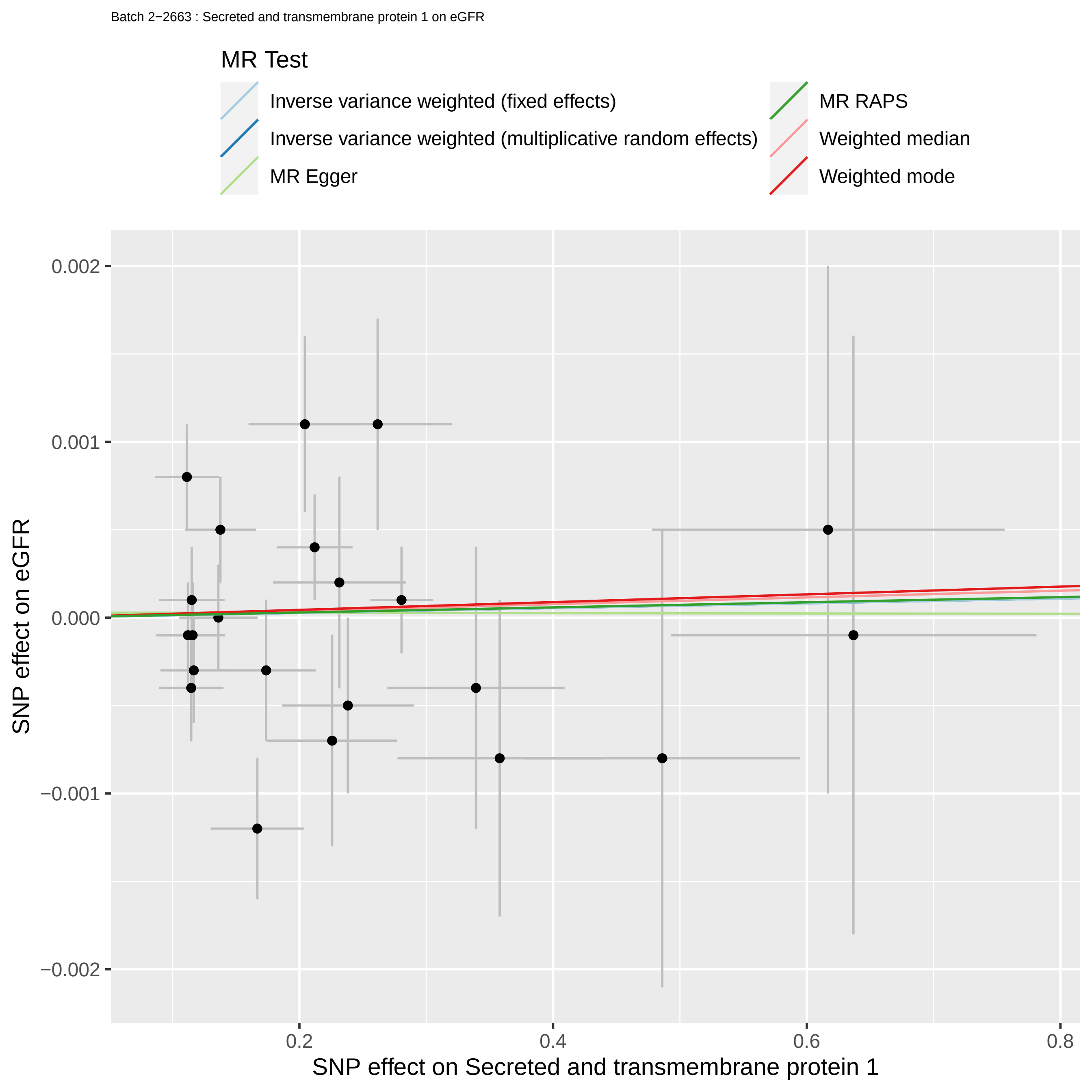
Batch 2−2663 : Secreted and transmembrane protein 1 on eGFR
MR Test
Inverse variance weighted (fixed effects)
Inverse variance weighted (multiplicative random effects)
MR Egger
MR RAPS
Weighted median
Weighted mode
0.002
0.001
0.000
−0.001
−0.002
SNP effect on eGFR
0.2
0.4
0.6
0.8
SNP effect on Secreted and transmembrane protein 1

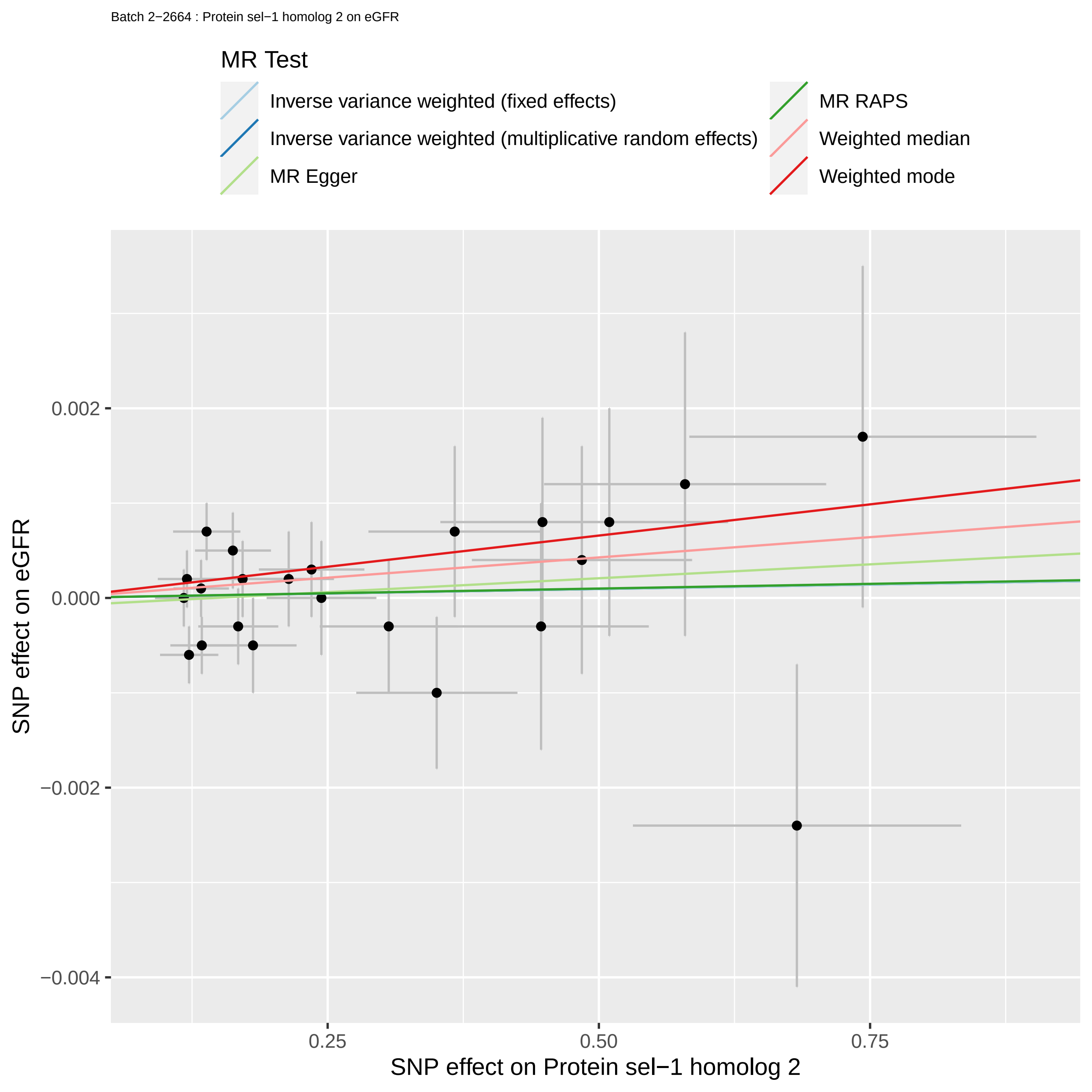
Batch 2−2664 : Protein sel−1 homolog 2 on eGFR
MR Test
Inverse variance weighted (fixed effects)
Inverse variance weighted (multiplicative random effects)
MR Egger
MR RAPS
Weighted median
Weighted mode
0.002
0.000
−0.002
−0.004
0.25
0.50
0.75
SNP effect on eGFR
SNP effect on Protein sel−1 homolog 2

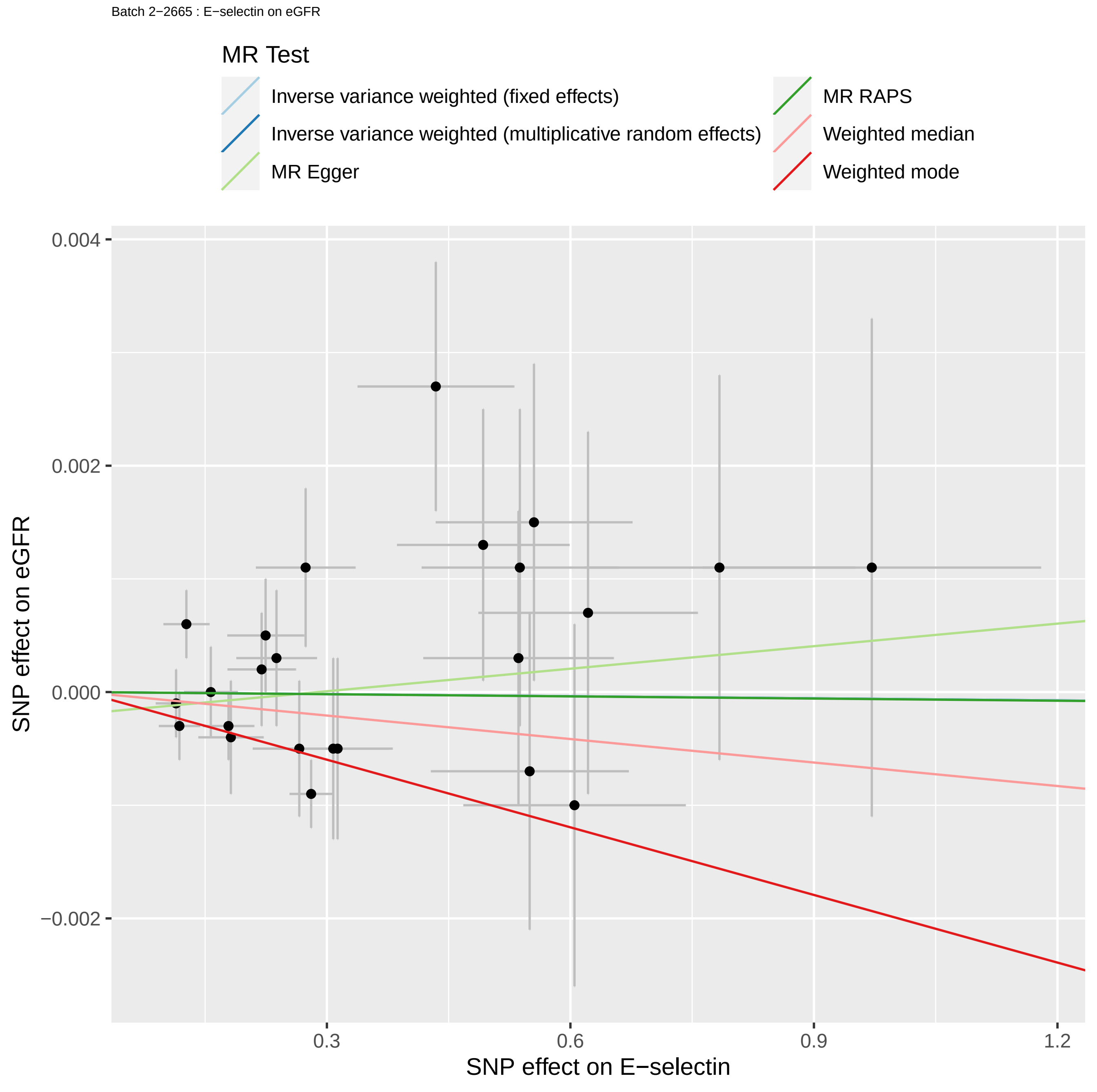
Batch 2−2665 : E−selectin on eGFR
MR Test
Inverse variance weighted (fixed effects)
Inverse variance weighted (multiplicative random effects)
MR Egger
MR RAPS
Weighted median
Weighted mode
0.004
0.002
0.000
−0.002
SNP effect on eGFR
0.3
0.6
0.9
1.2
SNP effect on E−selectin

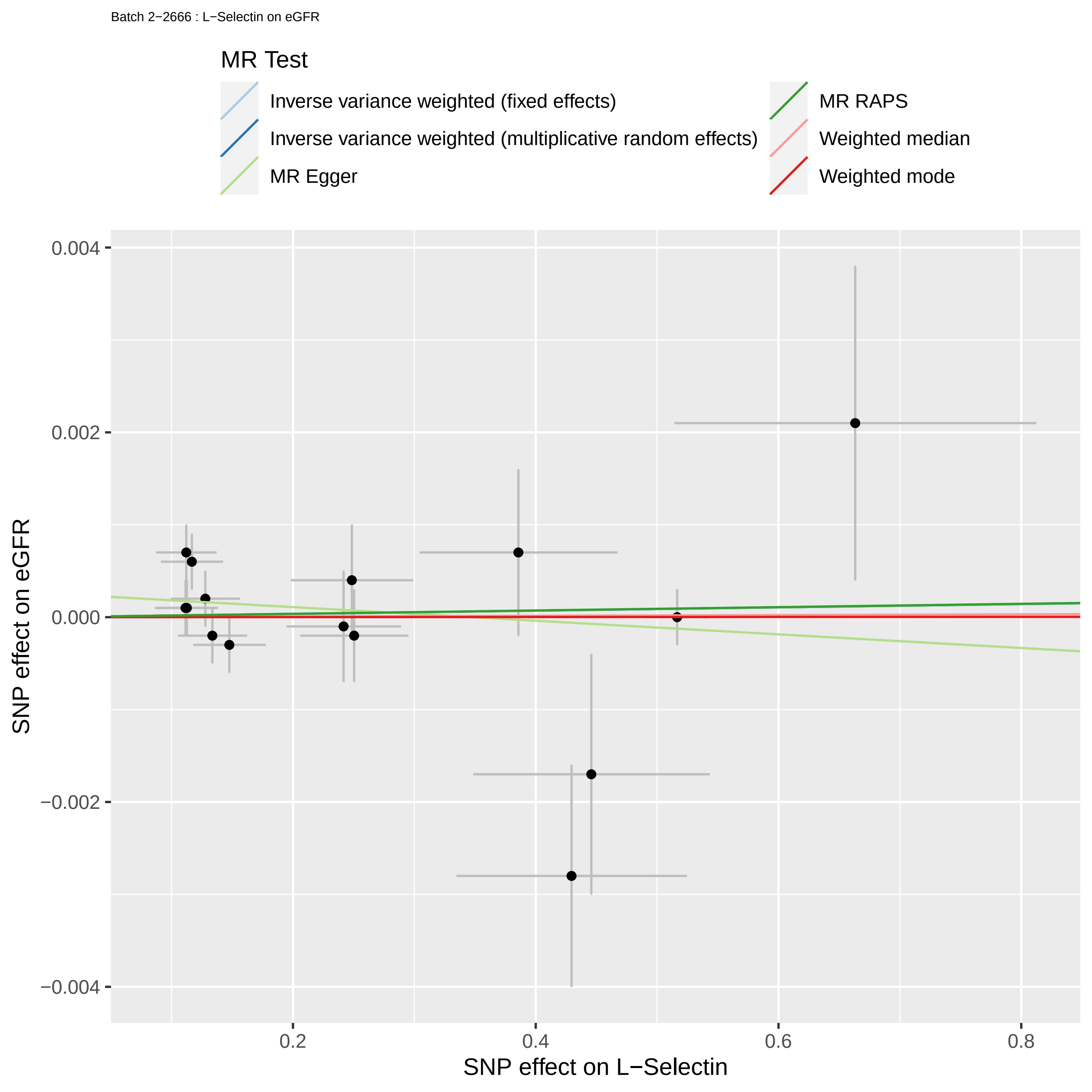
Batch 2−2666 : L−Selectin on eGFR
MR Test
Inverse variance weighted (fixed effects)
Inverse variance weighted (multiplicative random effects)
MR Egger
MR RAPS
Weighted median
Weighted mode
0.004
0.002
0.000
−0.002
−0.004
0.2
0.4
0.6
0.8
SNP effect on eGFR
SNP effect on L−Selectin

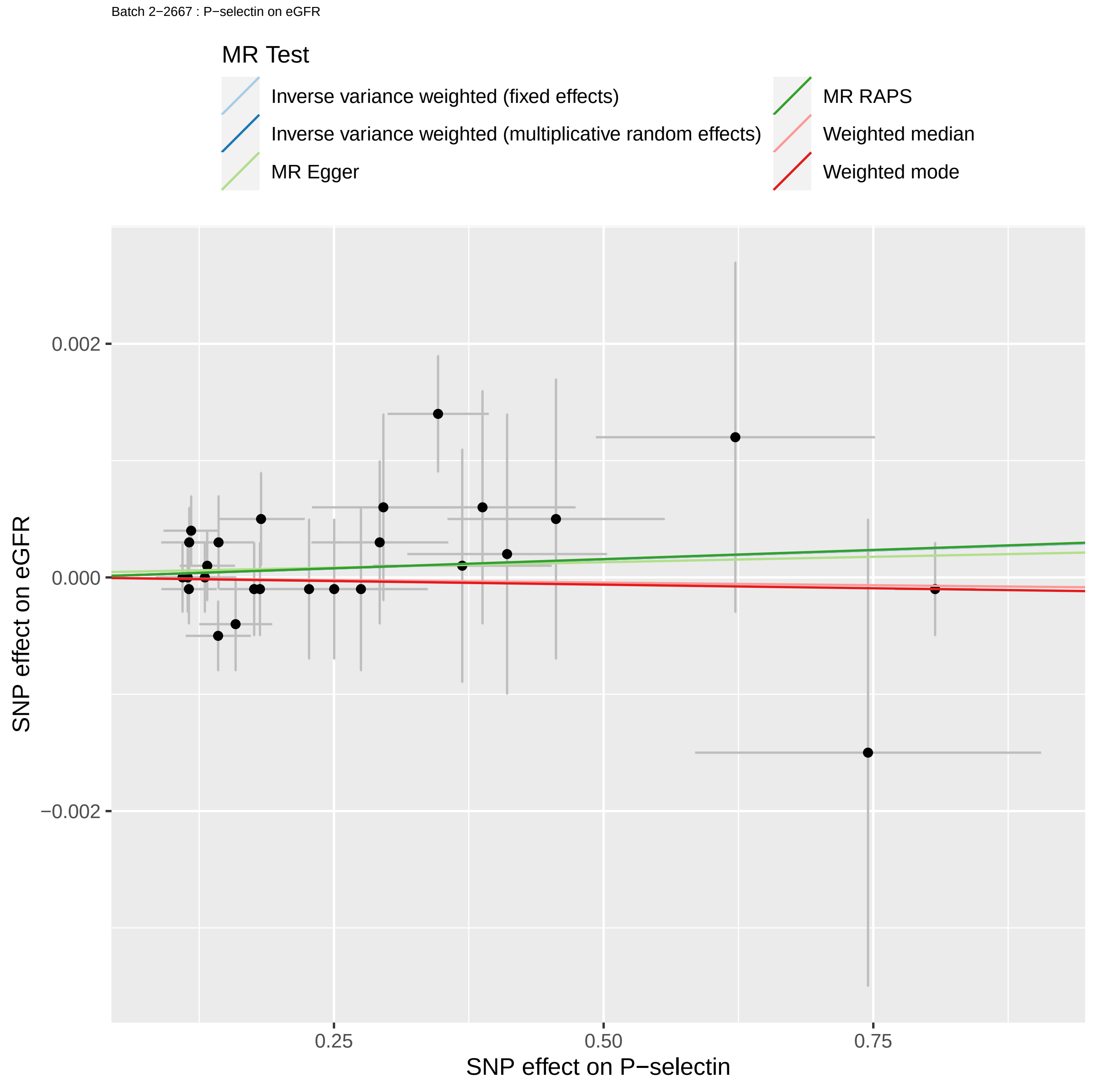

Batch 2−2667 : P−selectin on eGFR
MR Test
Inverse variance weighted (fixed effects)
Inverse variance weighted (multiplicative random effects)
MR Egger
MR RAPS
Weighted median
Weighted mode
0.002
0.000
−0.002
0.25
0.50
0.75
SNP effect on eGFR
SNP effect on P−selectin

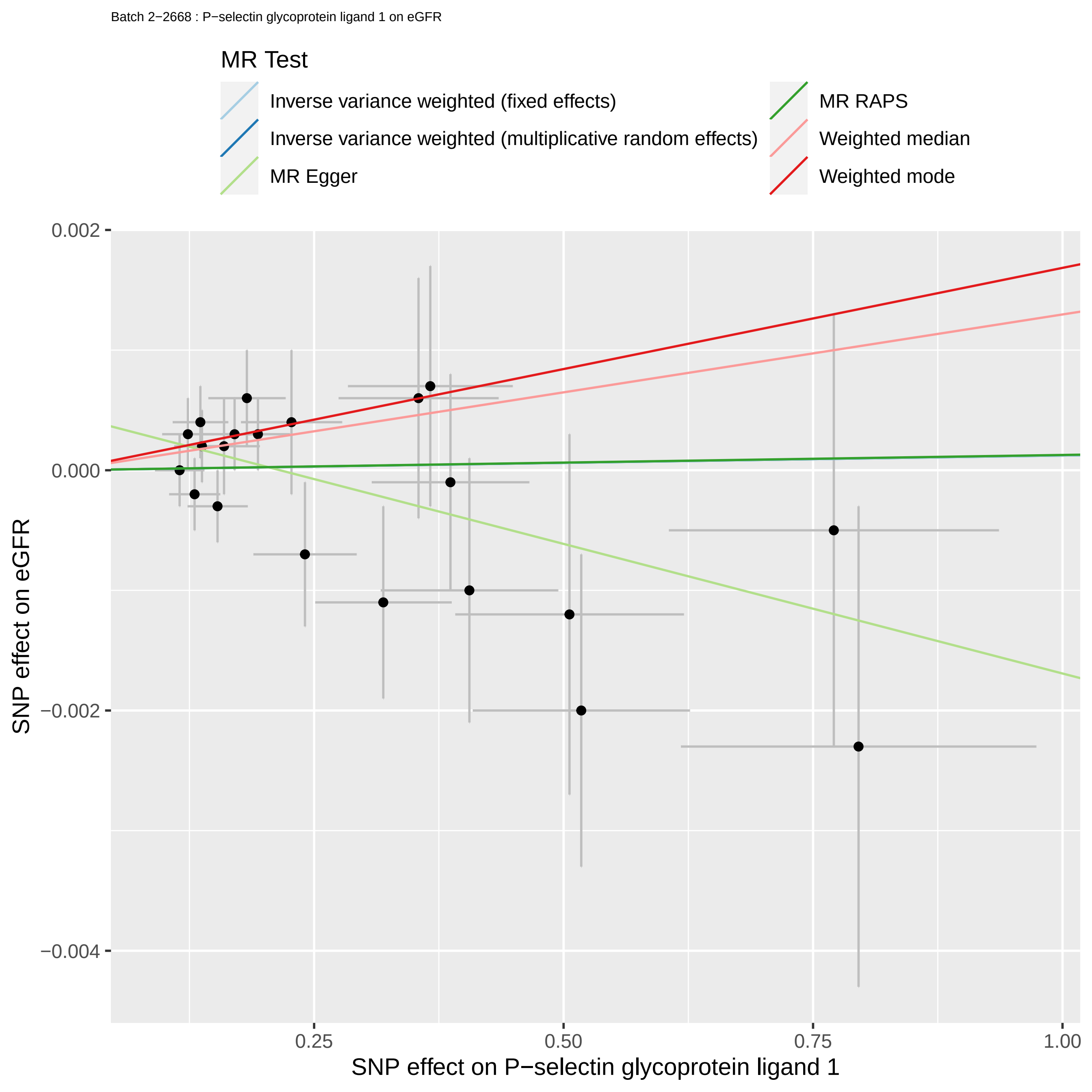

Batch 2−2668 : P−selectin glycoprotein ligand 1 on eGFR
MR Test
Inverse variance weighted (fixed effects)
Inverse variance weighted (multiplicative random effects)
MR Egger
MR RAPS
Weighted median
Weighted mode
0.002
0.000
−0.002
−0.004
SNP effect on eGFR
0.25
0.50
0.75
1.00
SNP effect on P−selectin glycoprotein ligand 1

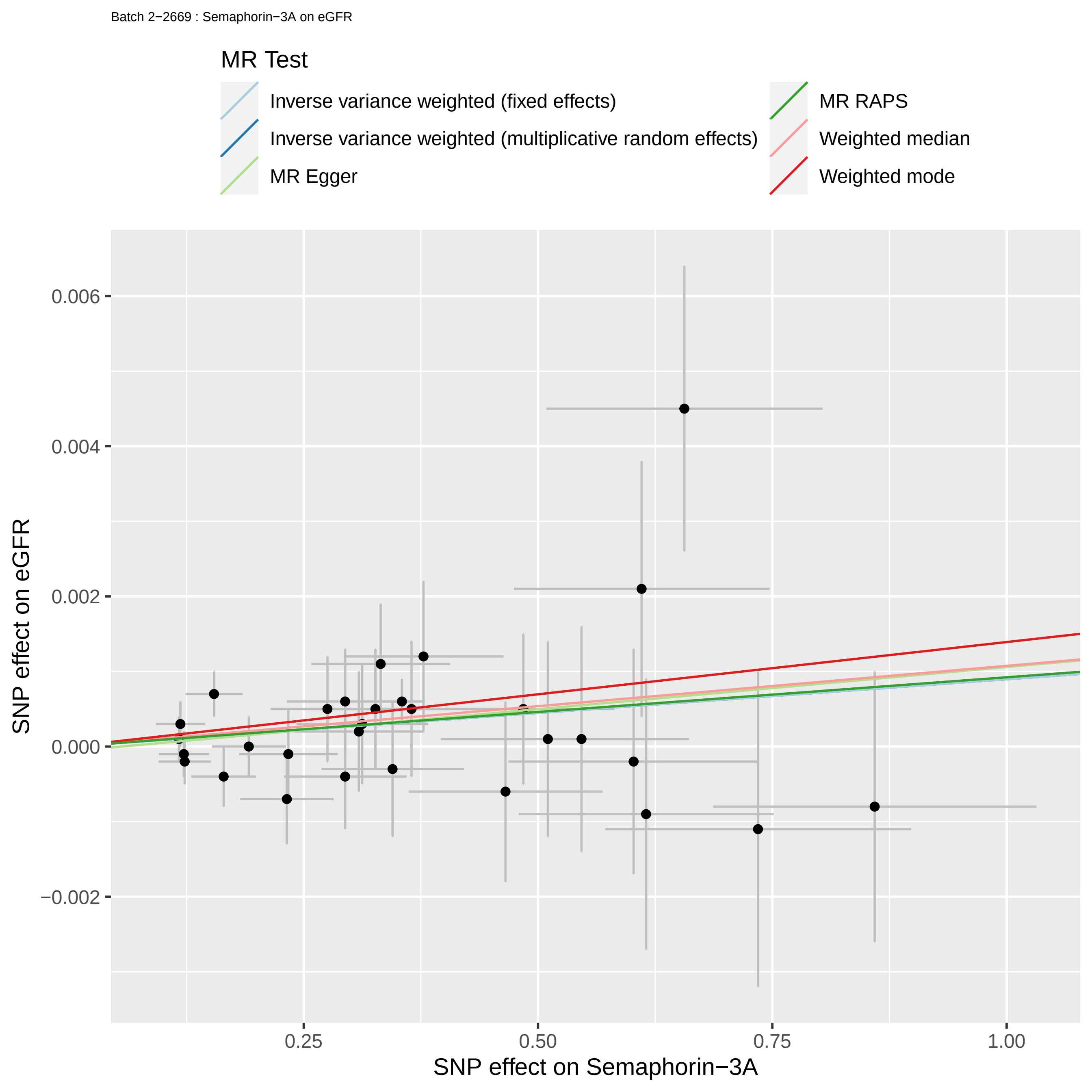

Batch 2−2669 : Semaphorin−3A on eGFR
MR Test
Inverse variance weighted (fixed effects)
Inverse variance weighted (multiplicative random effects)
MR Egger
MR RAPS
Weighted median
Weighted mode
0.006
0.004
0.002
0.000
−0.002
SNP effect on eGFR
0.25
0.50
0.75
1.00
SNP effect on Semaphorin−3A

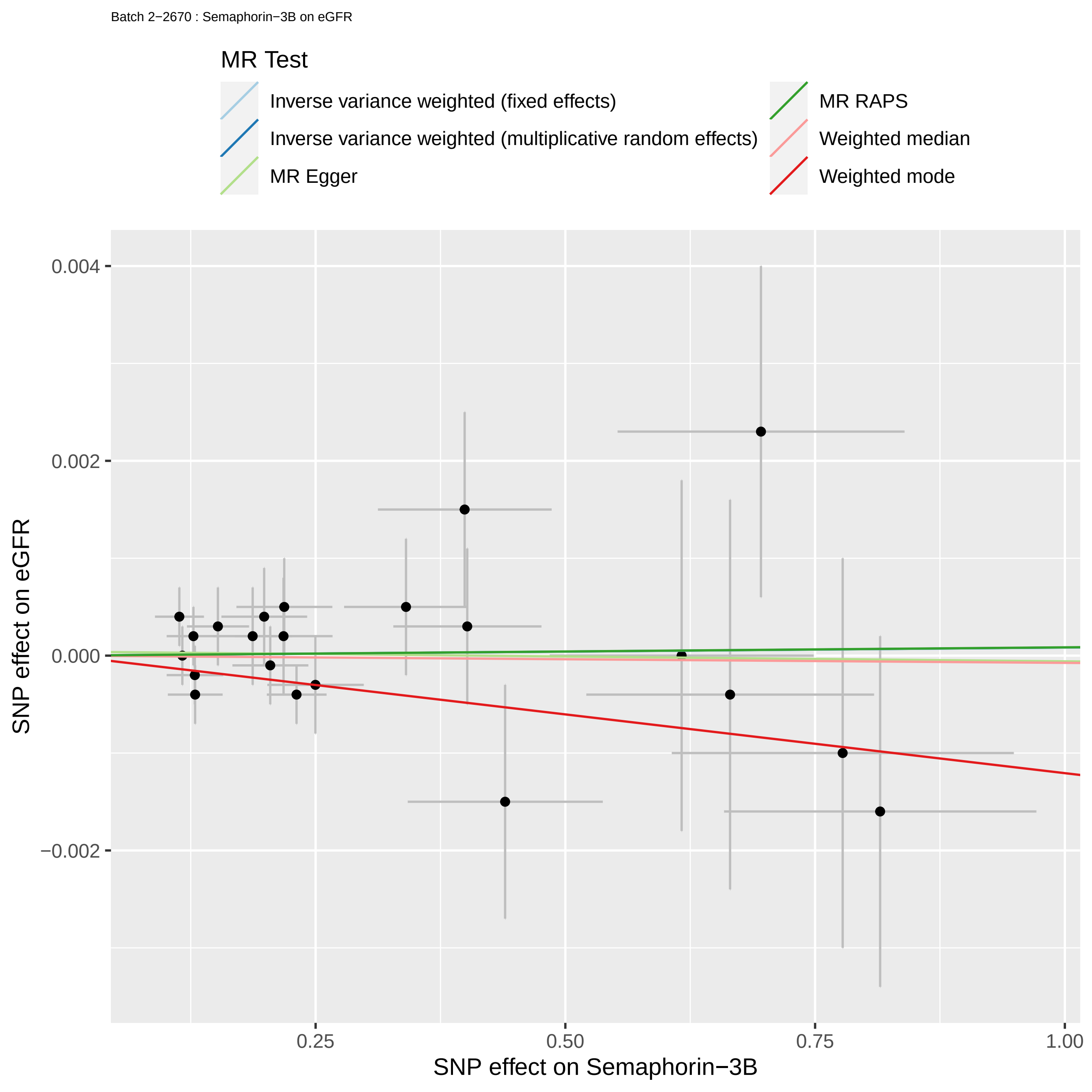

Batch 2−2670 : Semaphorin−3B on eGFR
MR Test
Inverse variance weighted (fixed effects)
Inverse variance weighted (multiplicative random effects)
MR Egger
MR RAPS
Weighted median
Weighted mode
0.004
0.002
0.000
−0.002
0.25
0.50
0.75
1.00
SNP effect on eGFR
SNP effect on Semaphorin−3B

Batch 2−2671 : Semaphorin−3C on eGFR

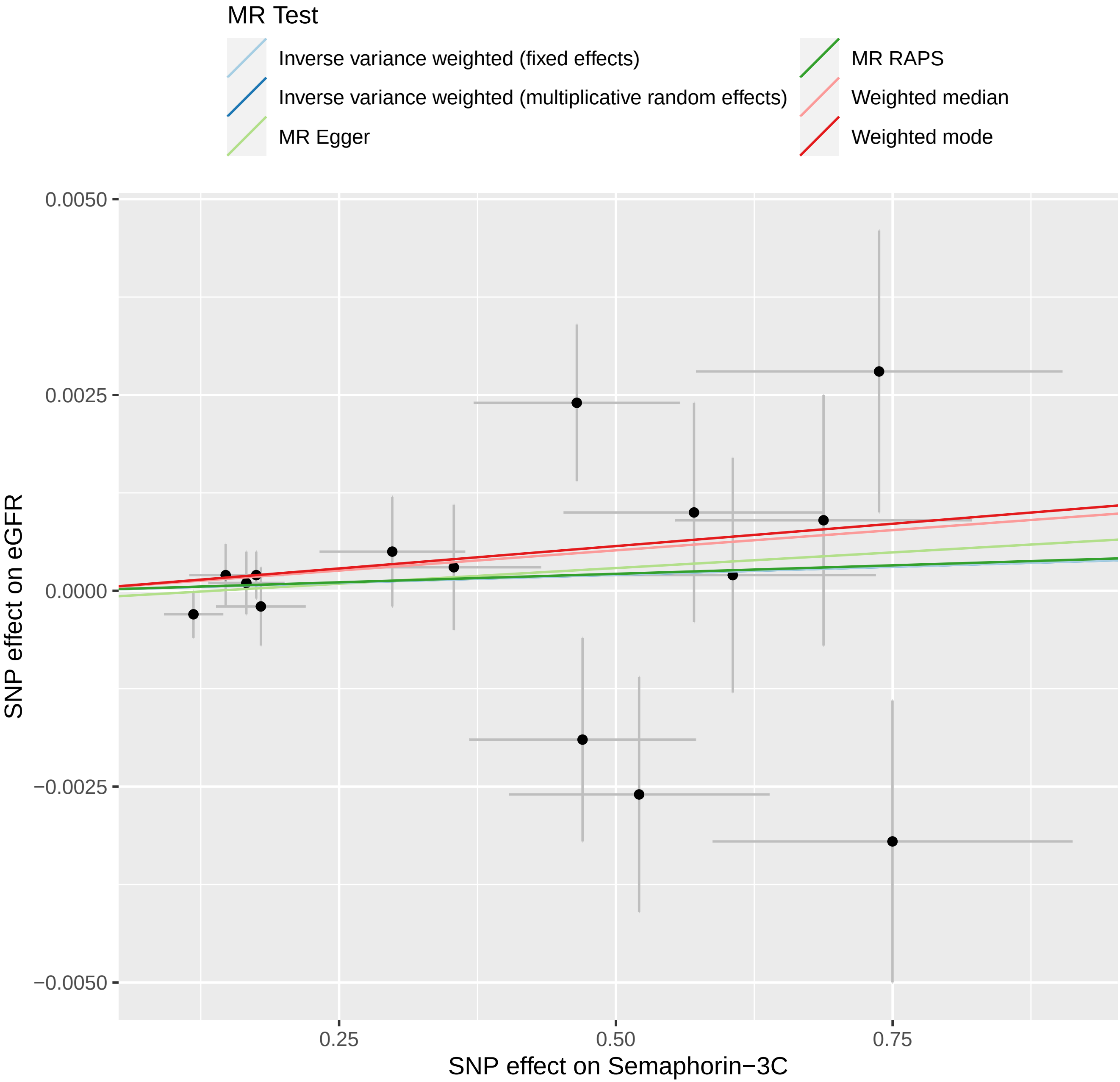

Batch 2−2672 : Semaphorin−3E on eGFR

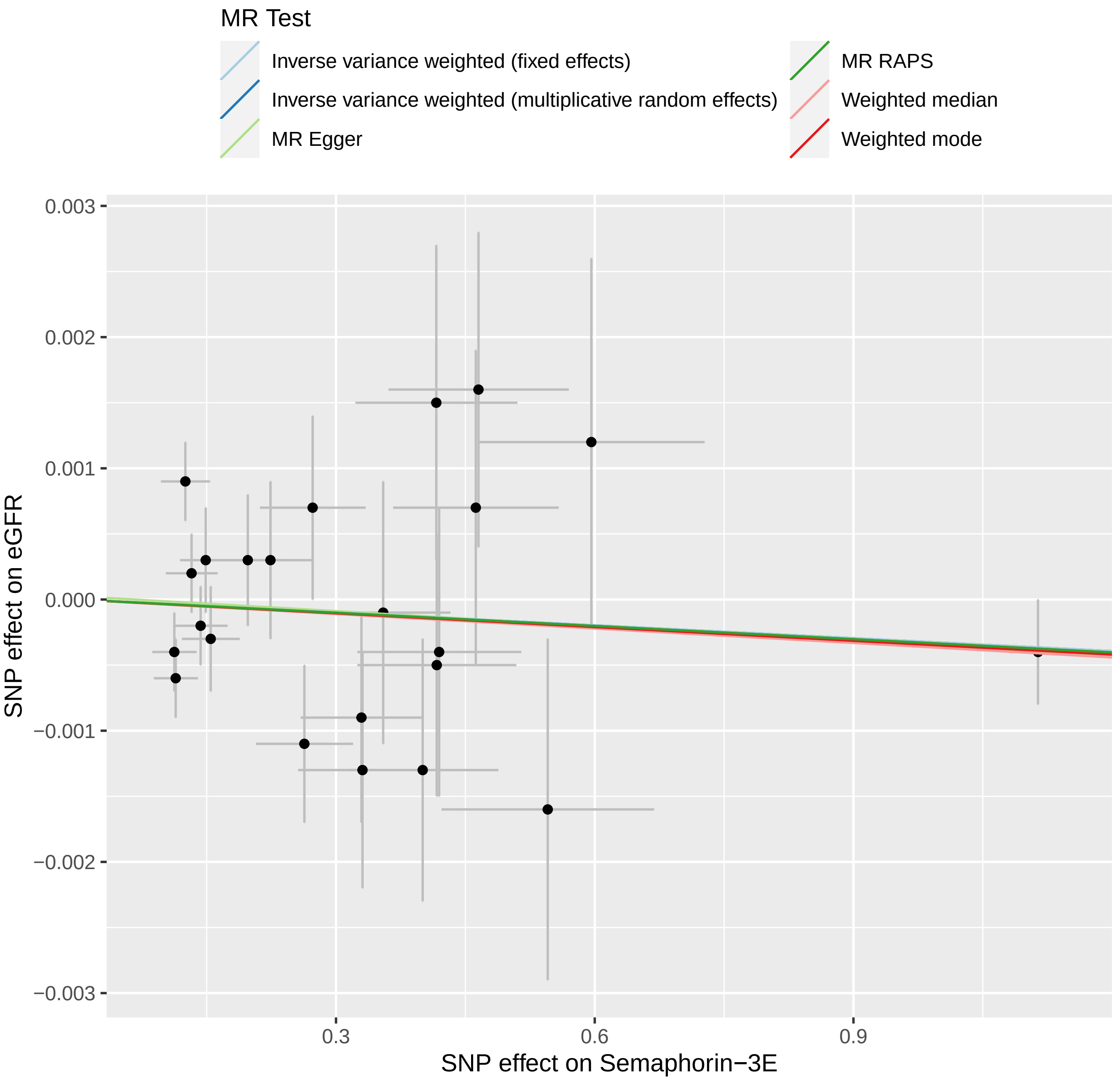

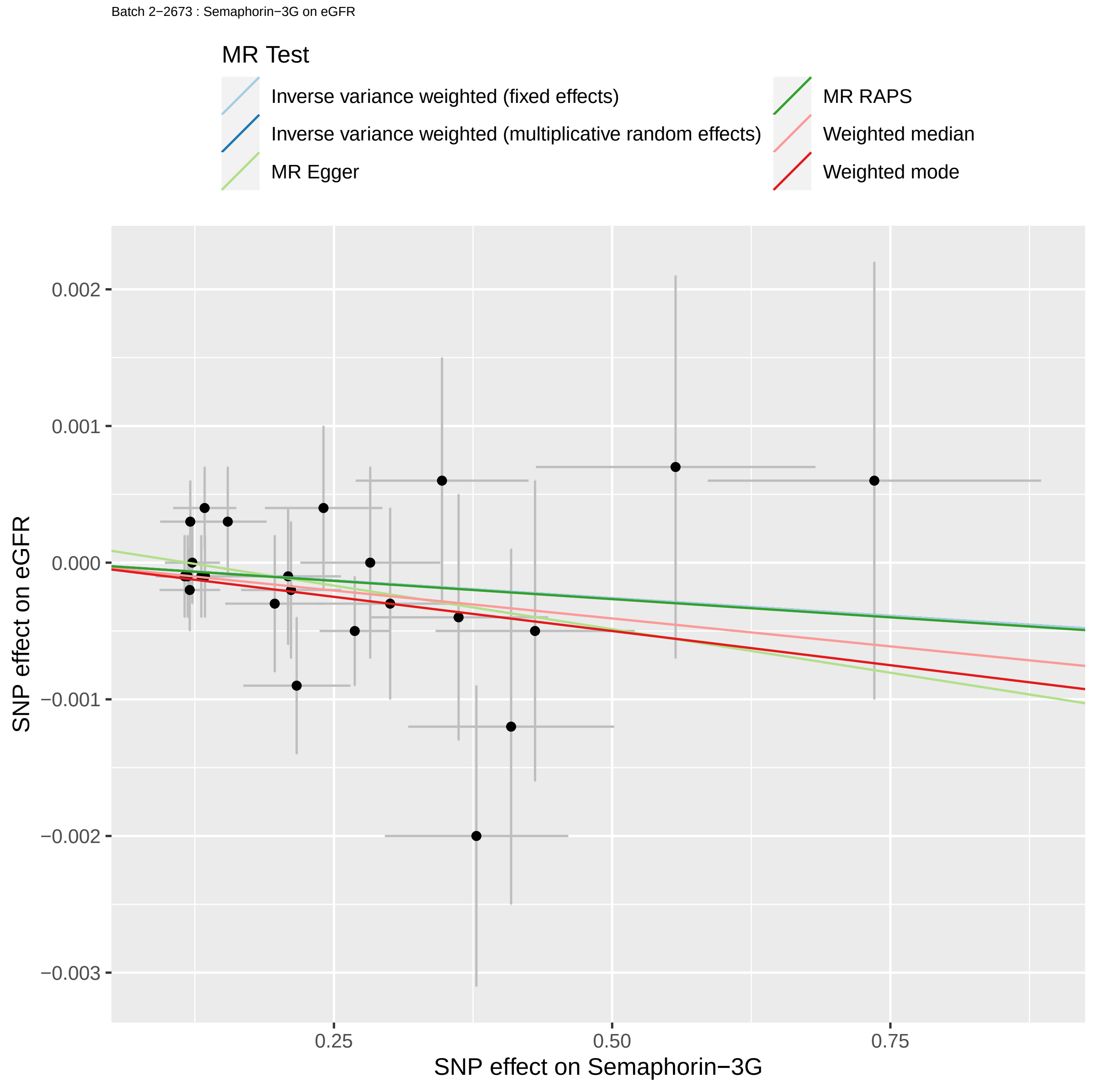

Batch 2−2673 : Semaphorin−3G on eGFR
MR Test
Inverse variance weighted (fixed effects)
Inverse variance weighted (multiplicative random effects)
MR Egger
MR RAPS
Weighted median
Weighted mode
0.002
0.001
0.000
−0.001
−0.002
−0.003
SNP effect on eGFR
0.25
0.50
0.75
SNP effect on Semaphorin−3G

Batch 2−2674 : Semaphorin−4C on eGFR

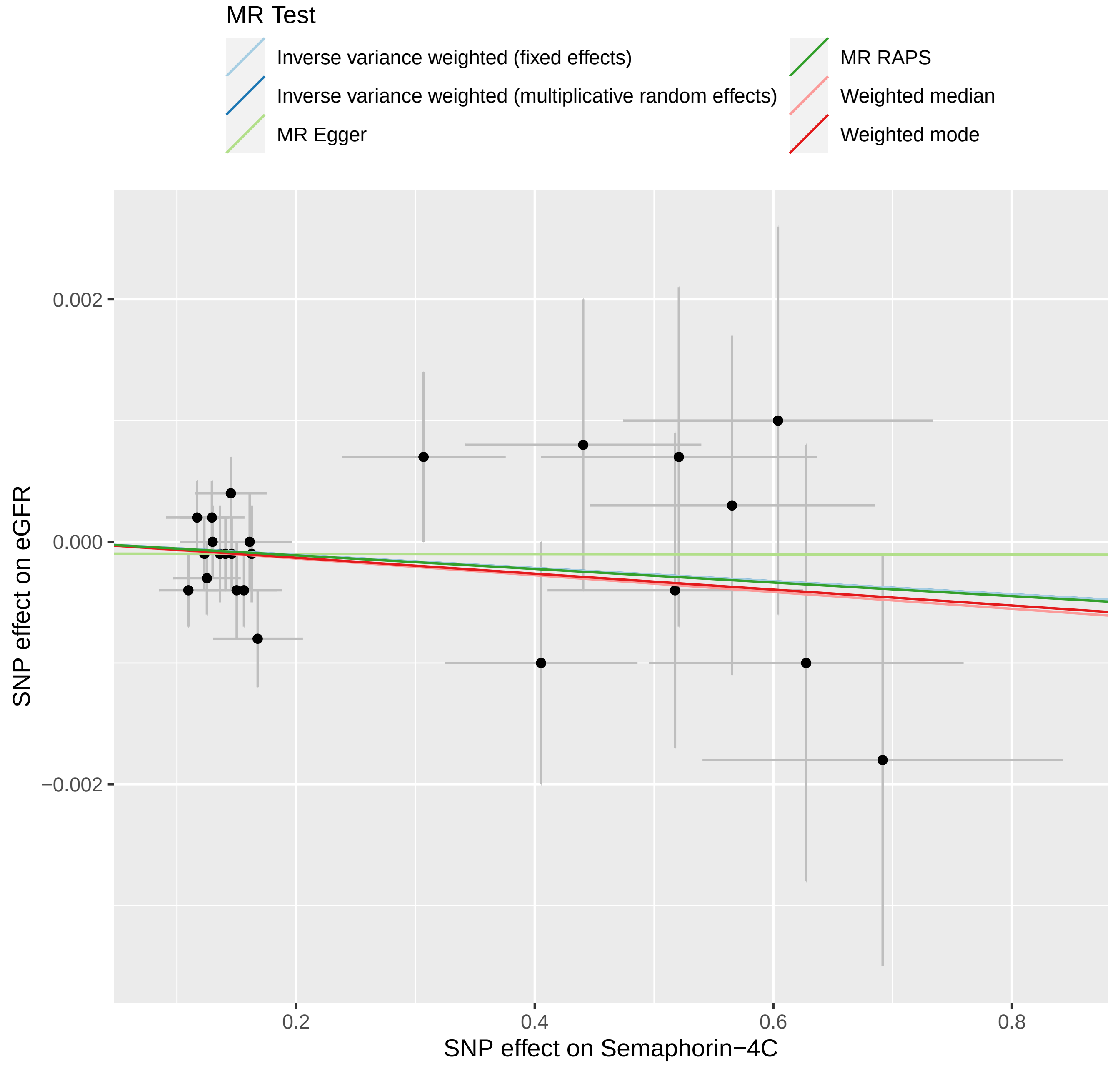

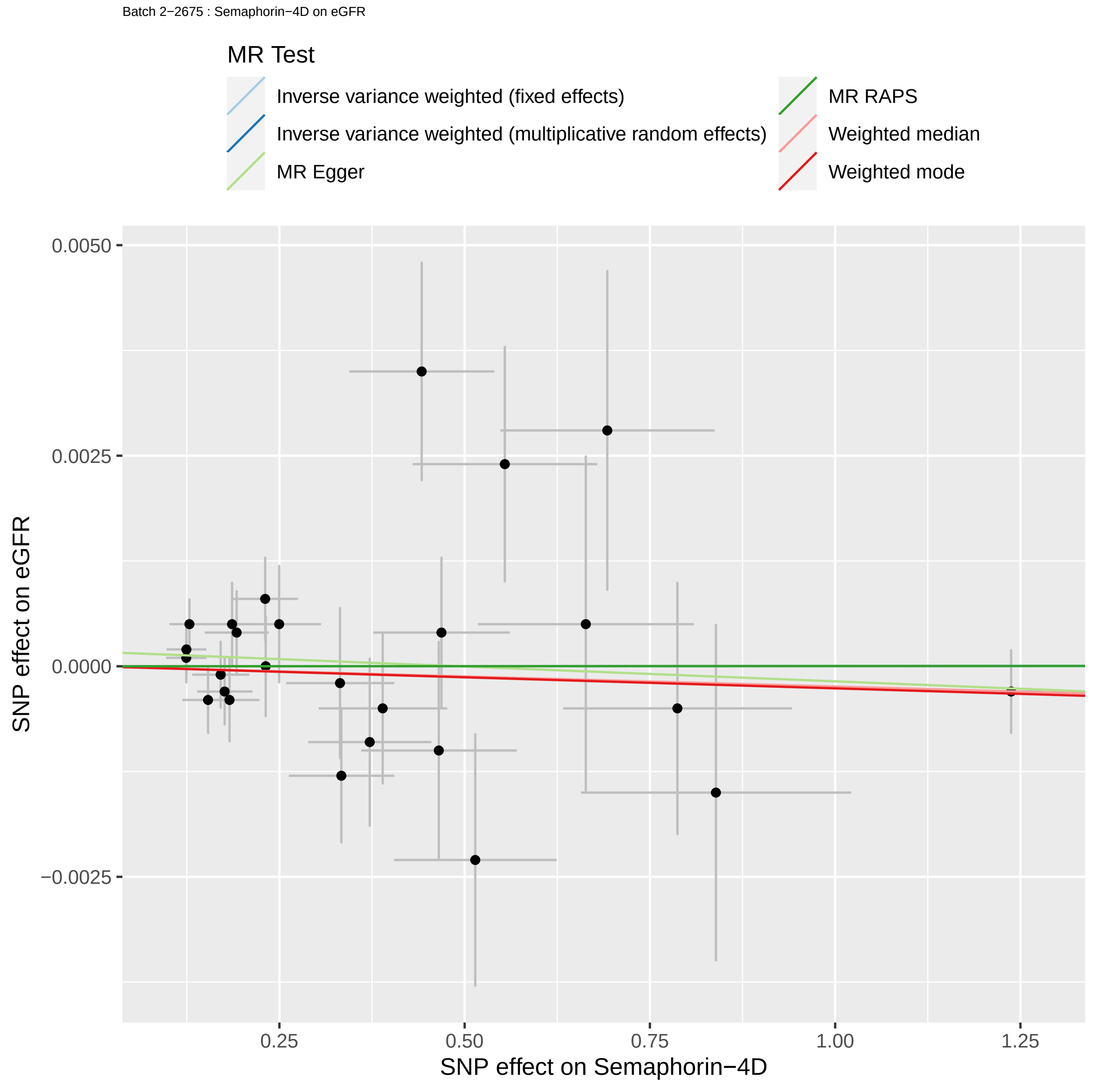

Batch 2−2675 : Semaphorin−4D on eGFR
MR Test
Inverse variance weighted (fixed effects)
Inverse variance weighted (multiplicative random effects)
MR Egger
MR RAPS
Weighted median
Weighted mode
0.0050
0.0025
0.0000
−0.0025
0.25
0.50
0.75
1.00
1.25
SNP effect on eGFR
SNP effect on Semaphorin−4D

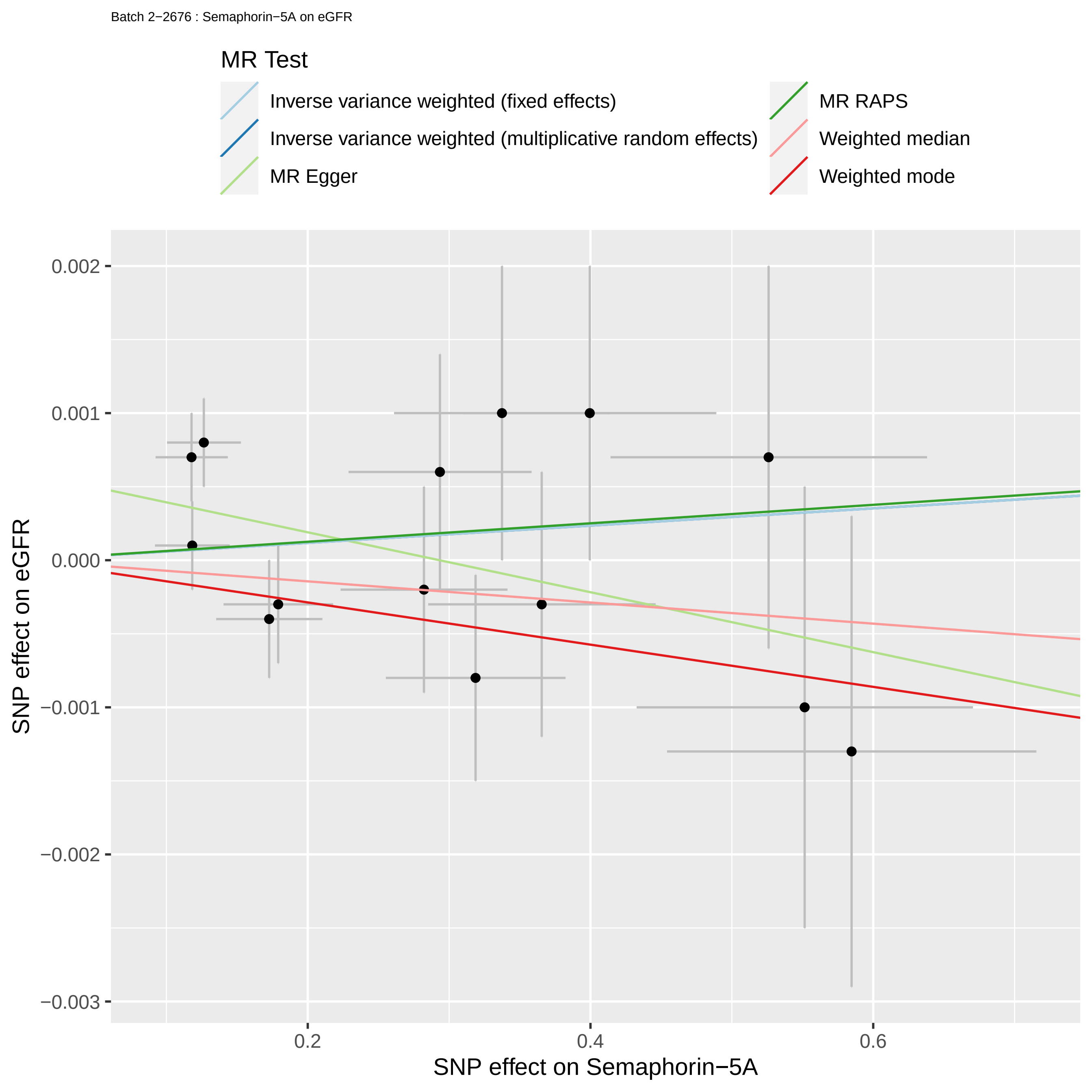

Batch 2−2676 : Semaphorin−5A on eGFR
MR Test
Inverse variance weighted (fixed effects)
Inverse variance weighted (multiplicative random effects)
MR Egger
MR RAPS
Weighted median
Weighted mode
0.002
0.001
0.000
−0.001
−0.002
−0.003
SNP effect on eGFR
0.2
0.4
0.6
SNP effect on Semaphorin−5A

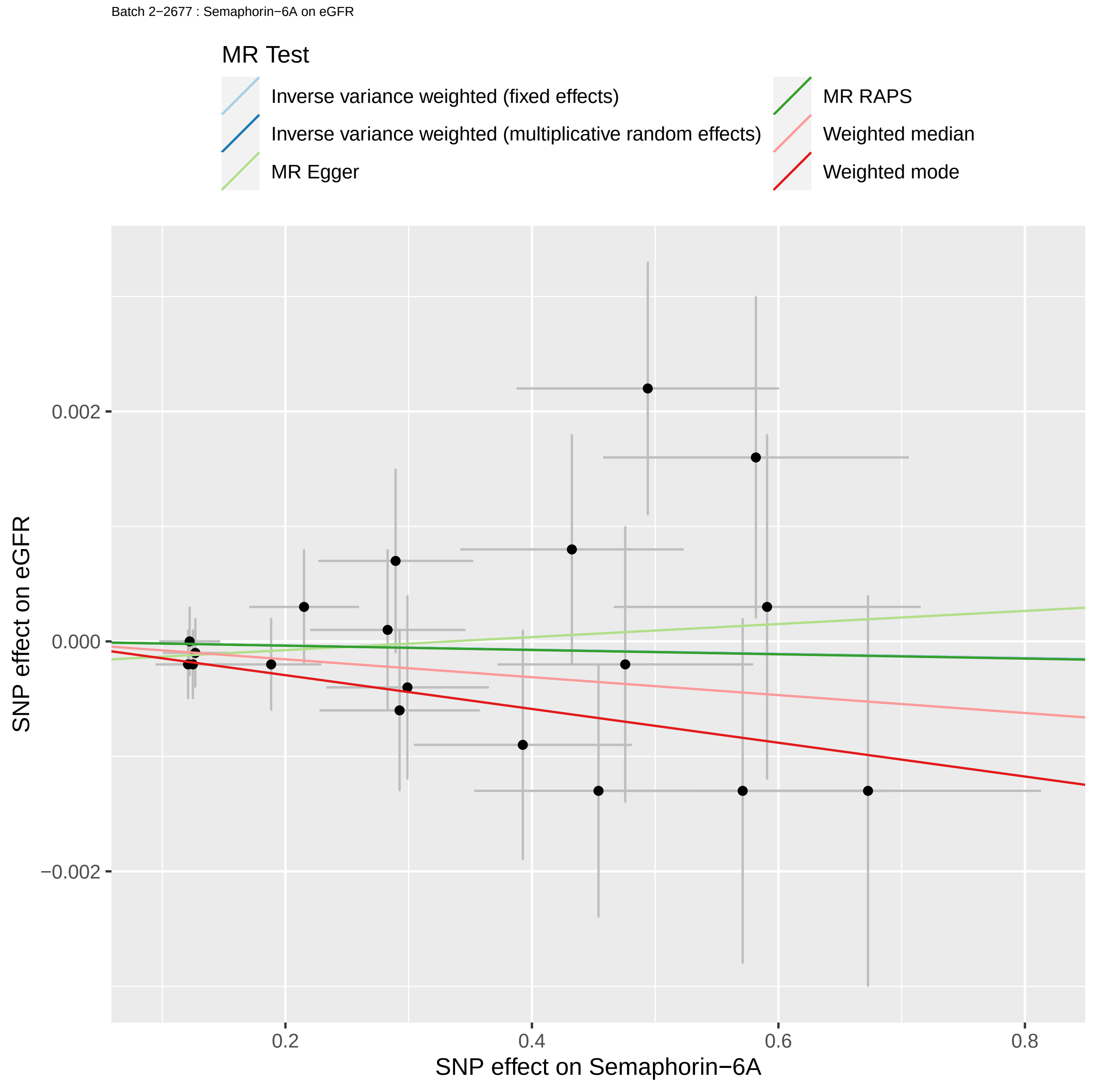

Batch 2−2677 : Semaphorin−6A on eGFR
MR Test
Inverse variance weighted (fixed effects)
Inverse variance weighted (multiplicative random effects)
MR Egger
MR RAPS
Weighted median
Weighted mode
0.002
0.000
−0.002
0.2
0.4
0.6
0.8
SNP effect on eGFR
SNP effect on Semaphorin−6A

Batch 2−2678 : Semaphorin−6A on eGFR

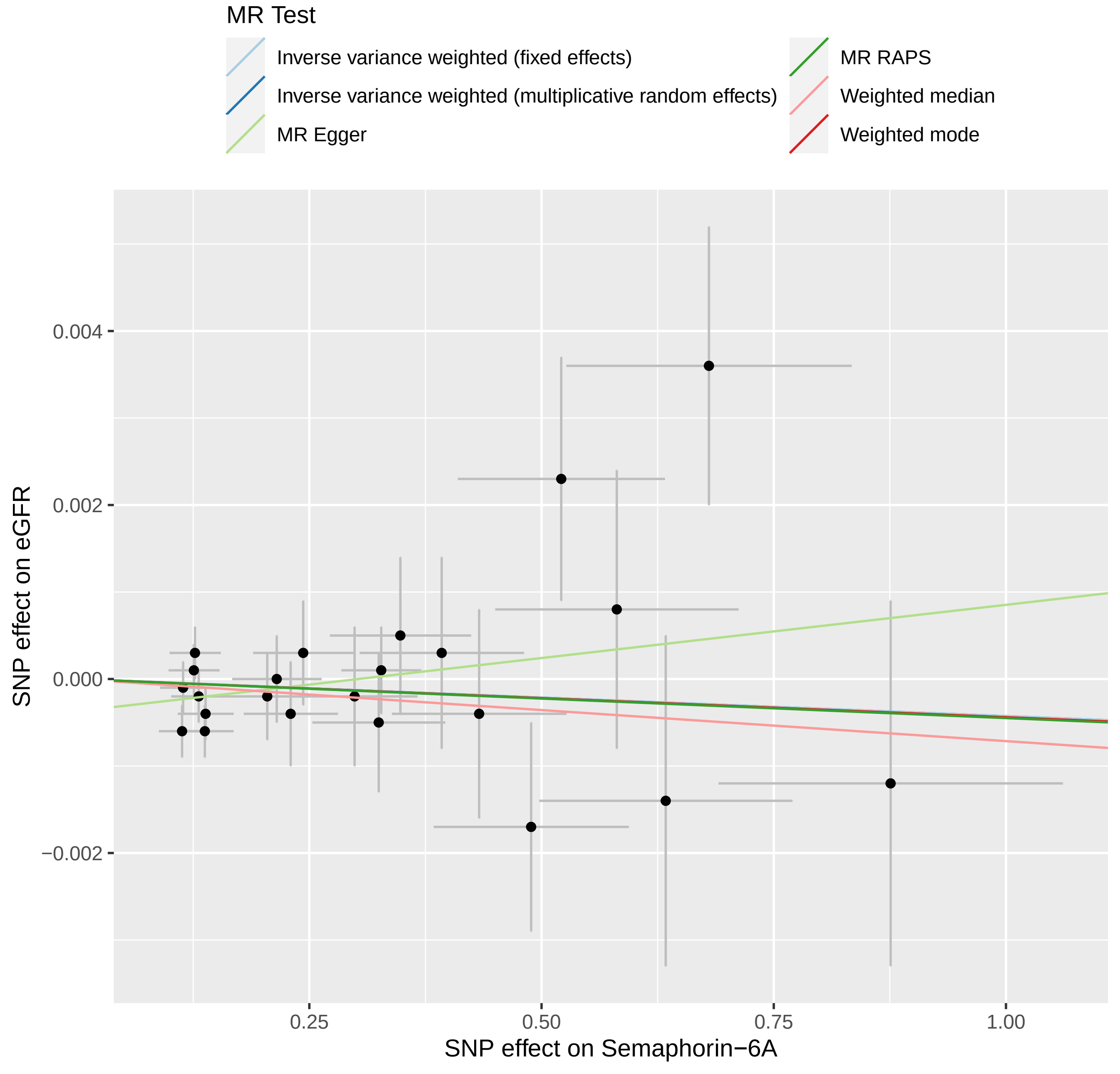

Batch 2−2679 : Semaphorin−6C on eGFR

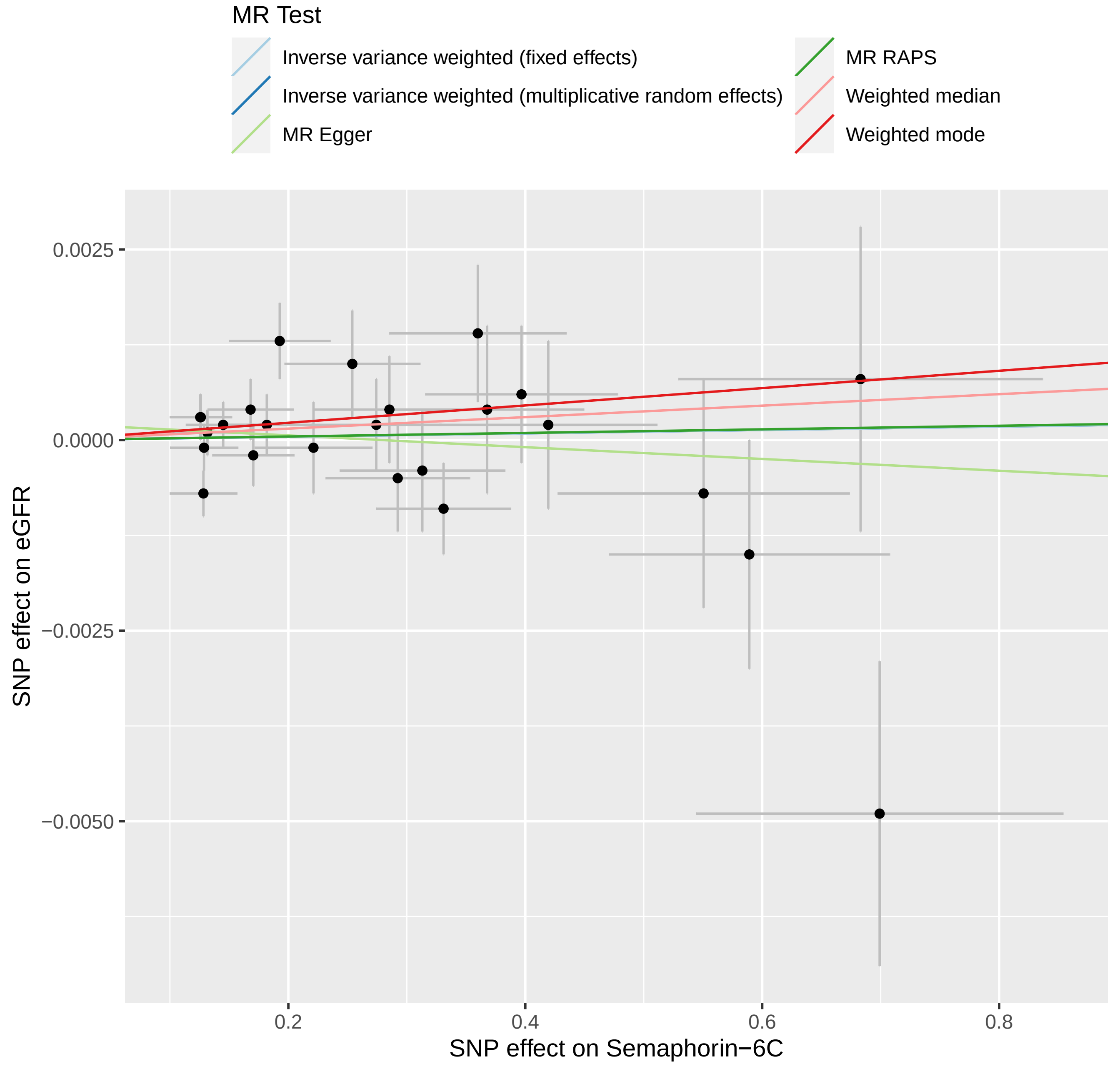

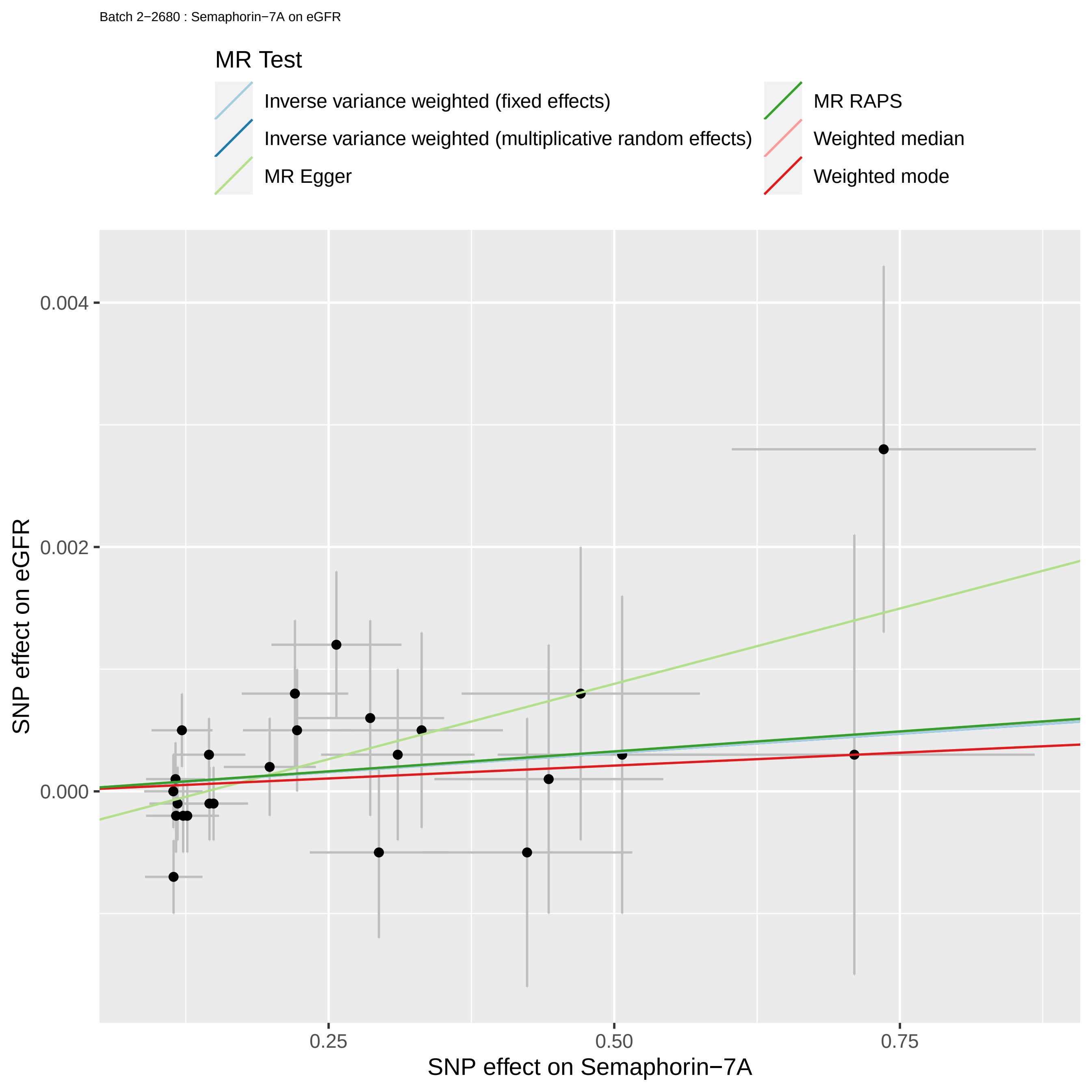

Batch 2−2680 : Semaphorin−7A on eGFR
MR Test
Inverse variance weighted (fixed effects)
Inverse variance weighted (multiplicative random effects)
MR Egger
MR RAPS
Weighted median
Weighted mode
0.004
0.002
0.000
SNP effect on eGFR
0.25
0.50
0.75
SNP effect on Semaphorin−7A

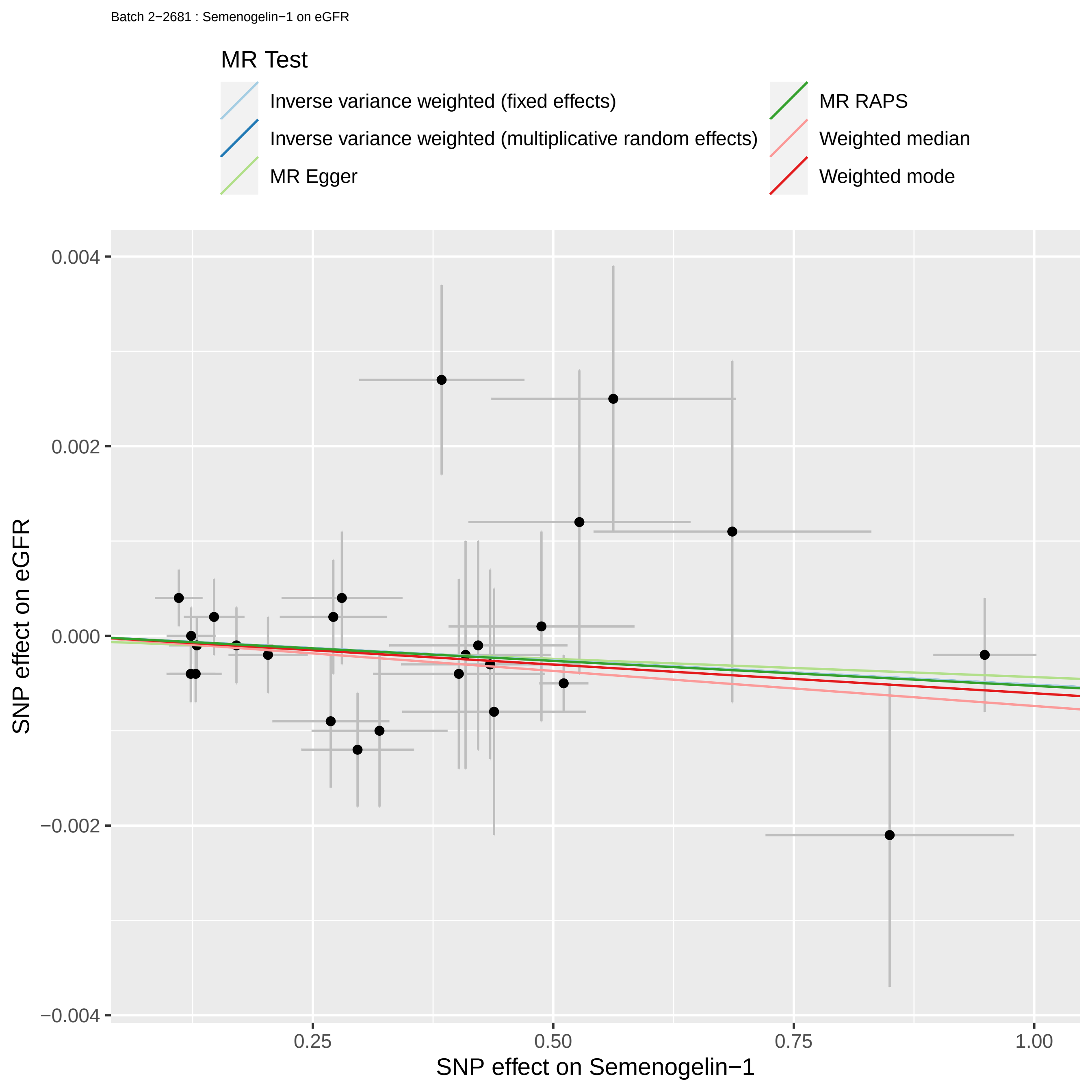

Batch 2−2681 : Semenogelin−1 on eGFR
MR Test
Inverse variance weighted (fixed effects)
Inverse variance weighted (multiplicative random effects)
MR Egger
MR RAPS
Weighted median
Weighted mode
SNP effect on eGFR
0.004
0.002
0.000
−0.002
−0.004
0.25
0.50
0.75
1.00
SNP effect on Semenogelin−1

Batch 2−2682 : Semenogelin−2 on eGFR

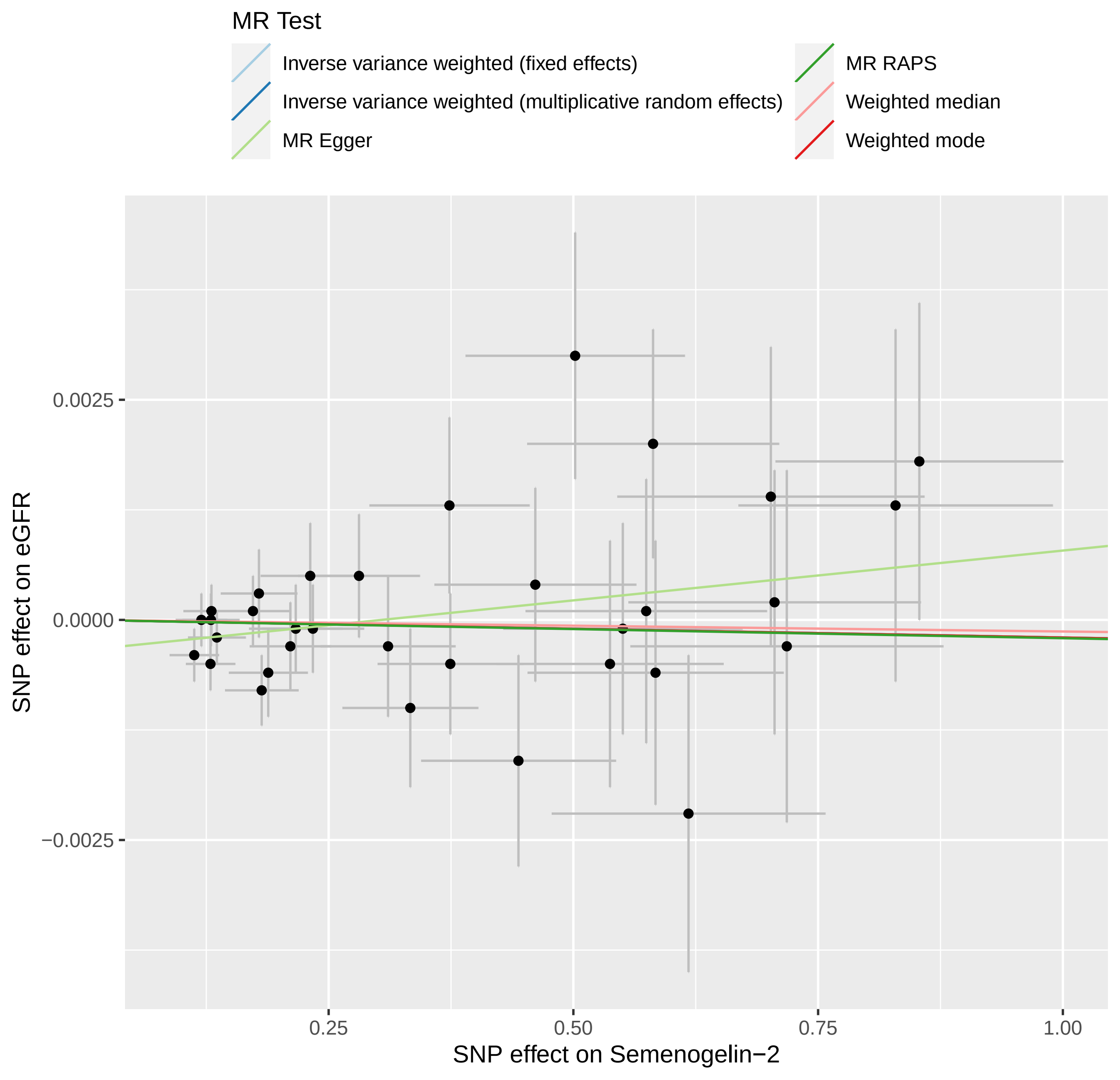

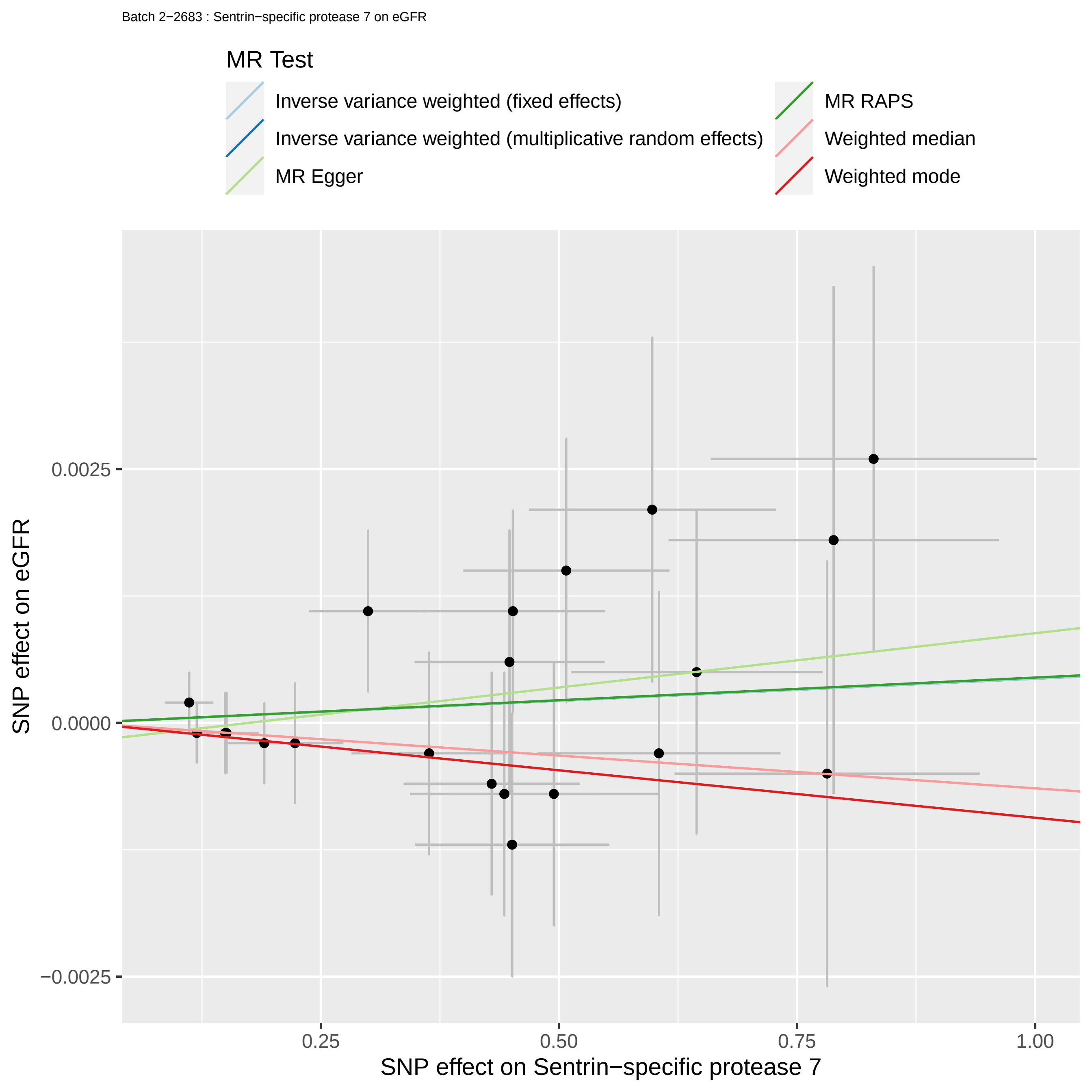
Batch 2−2683 : Sentrin−specific protease 7 on eGFR
MR Test
Inverse variance weighted (fixed effects)
Inverse variance weighted (multiplicative random effects)
MR Egger
MR RAPS
Weighted median
Weighted mode
0.0025
0.0000
−0.0025
0.25
0.50
0.75
1.00
SNP effect on eGFR
SNP effect on Sentrin−specific protease 7

Batch 2−2684 : Selenide, water dikinase 1 on eGFR

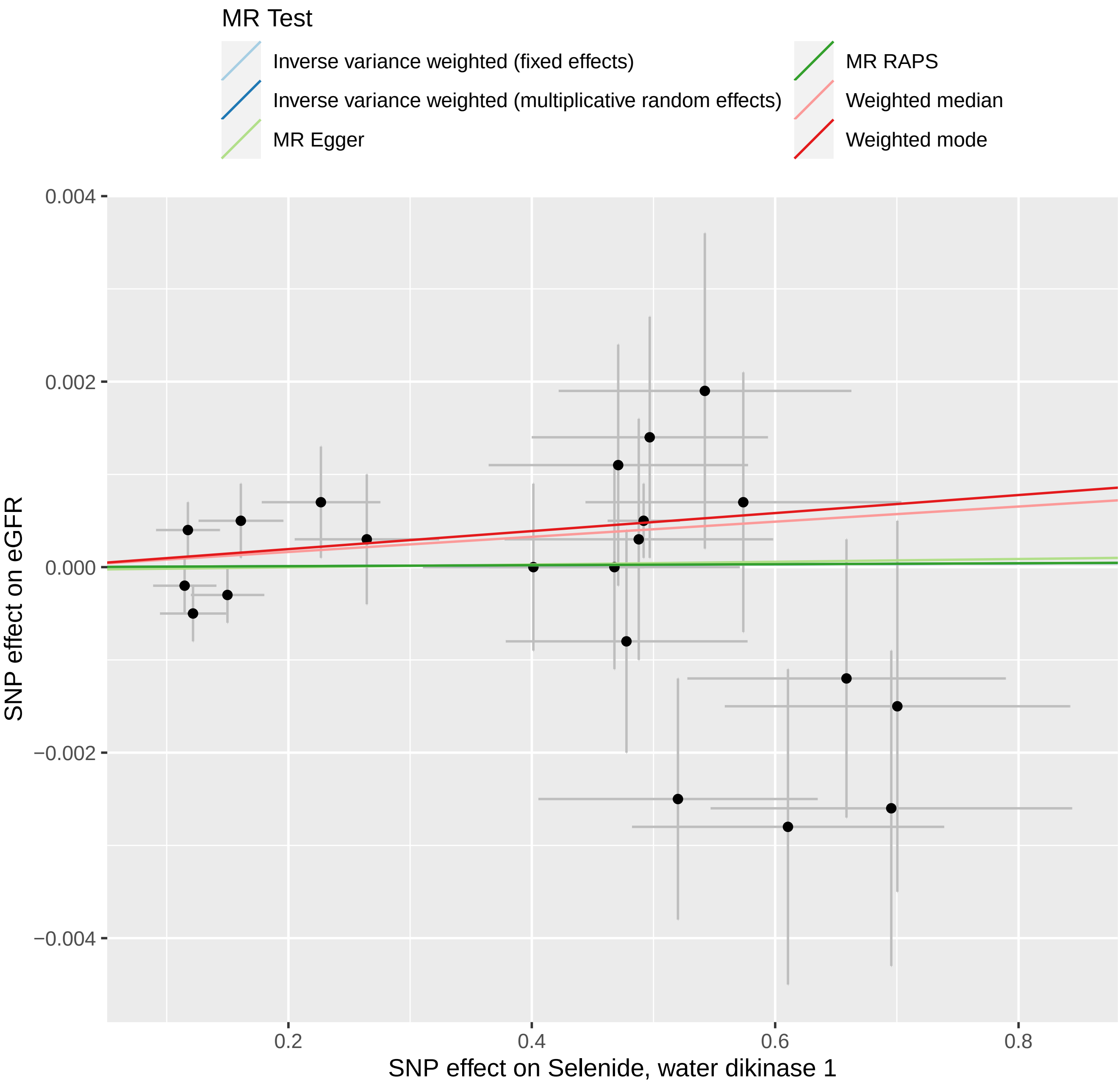

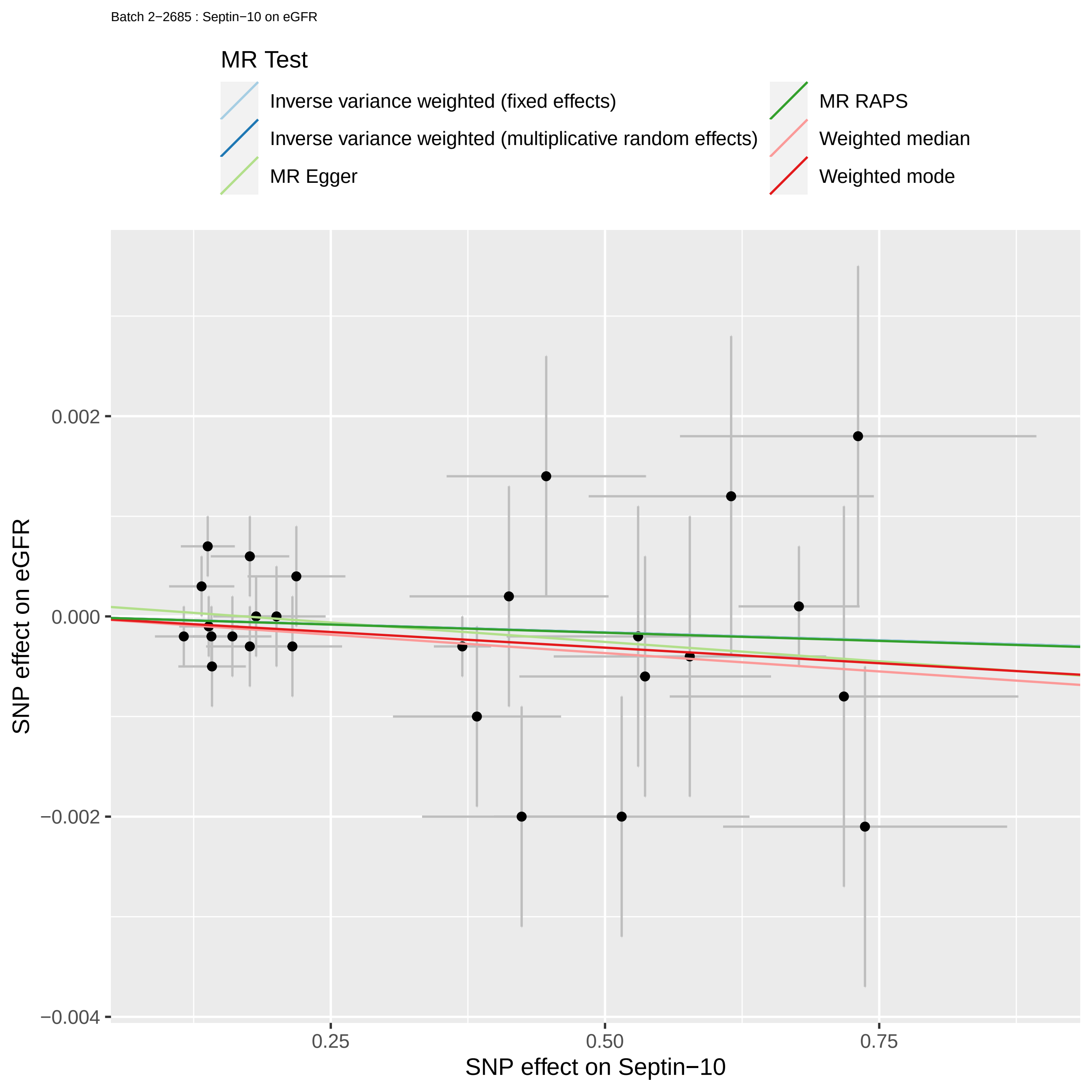

Batch 2−2685 : Septin−10 on eGFR
MR Test
Inverse variance weighted (fixed effects)
Inverse variance weighted (multiplicative random effects)
MR Egger
MR RAPS
Weighted median
Weighted mode
0.002
0.000
−0.002
−0.004
0.25
0.50
0.75
SNP effect on eGFR
SNP effect on Septin−10

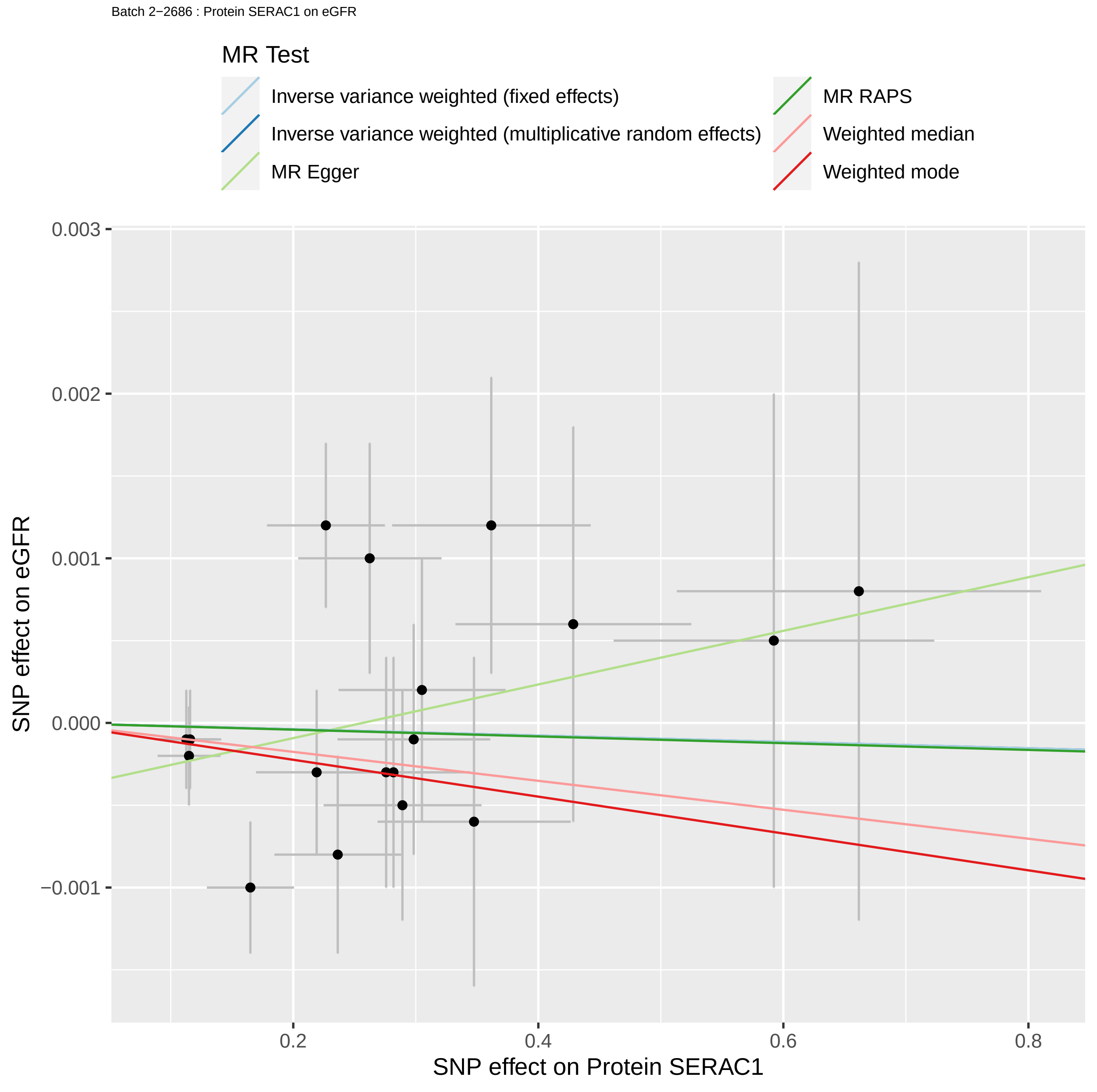
Batch 2−2686 : Protein SERAC1 on eGFR
MR Test
Inverse variance weighted (fixed effects)
Inverse variance weighted (multiplicative random effects)
MR Egger
MR RAPS
Weighted median
Weighted mode
0.003
0.002
0.001
0.000
−0.001
SNP effect on eGFR
0.2
0.4
0.6
0.8
SNP effect on Protein SERAC1

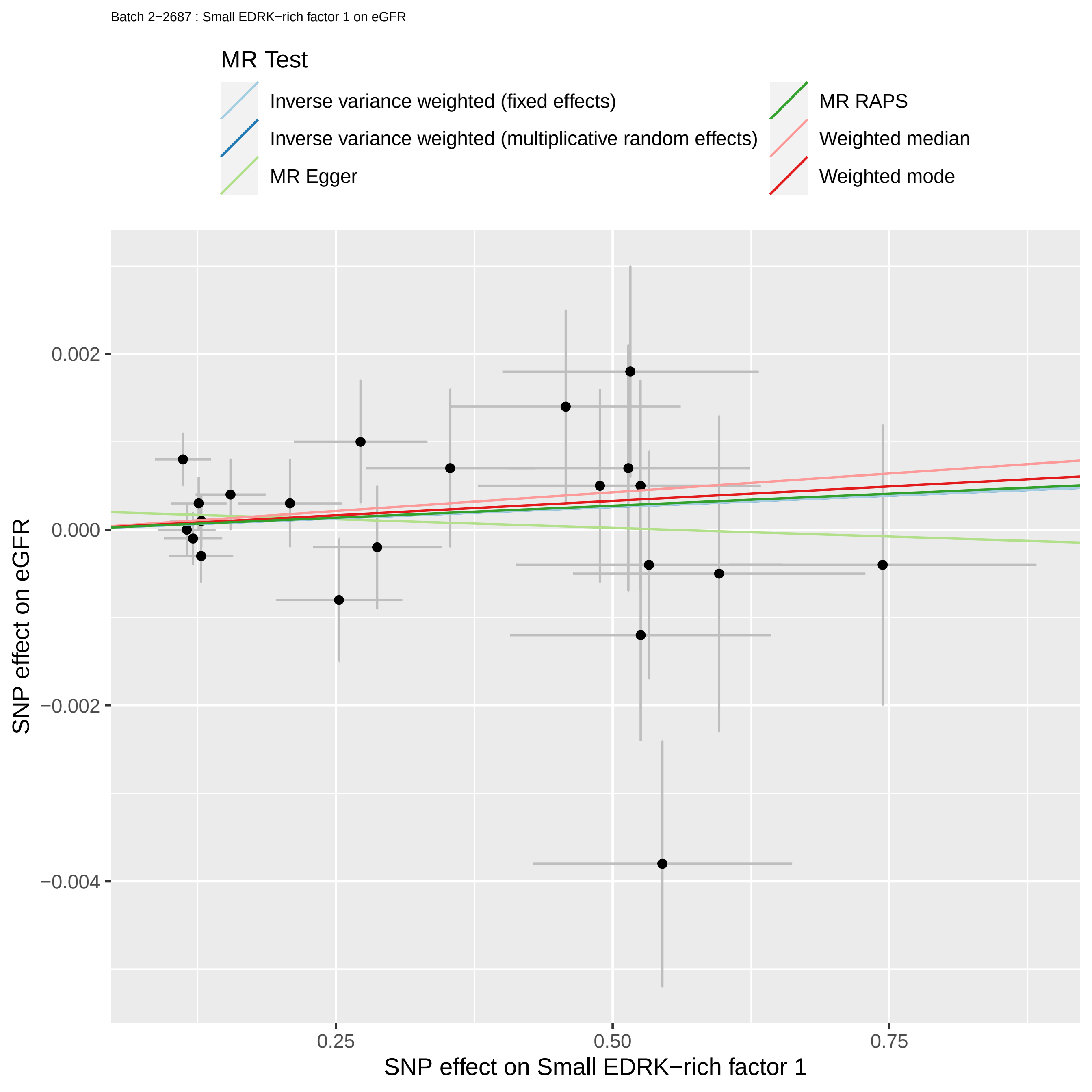
Batch 2−2687 : Small EDRK−rich factor 1 on eGFR
MR Test
Inverse variance weighted (fixed effects)
Inverse variance weighted (multiplicative random effects)
MR Egger
MR RAPS
Weighted median
Weighted mode
0.002
0.000
−0.002
−0.004
0.25
0.50
0.75
SNP effect on eGFR
SNP effect on Small EDRK−rich factor 1

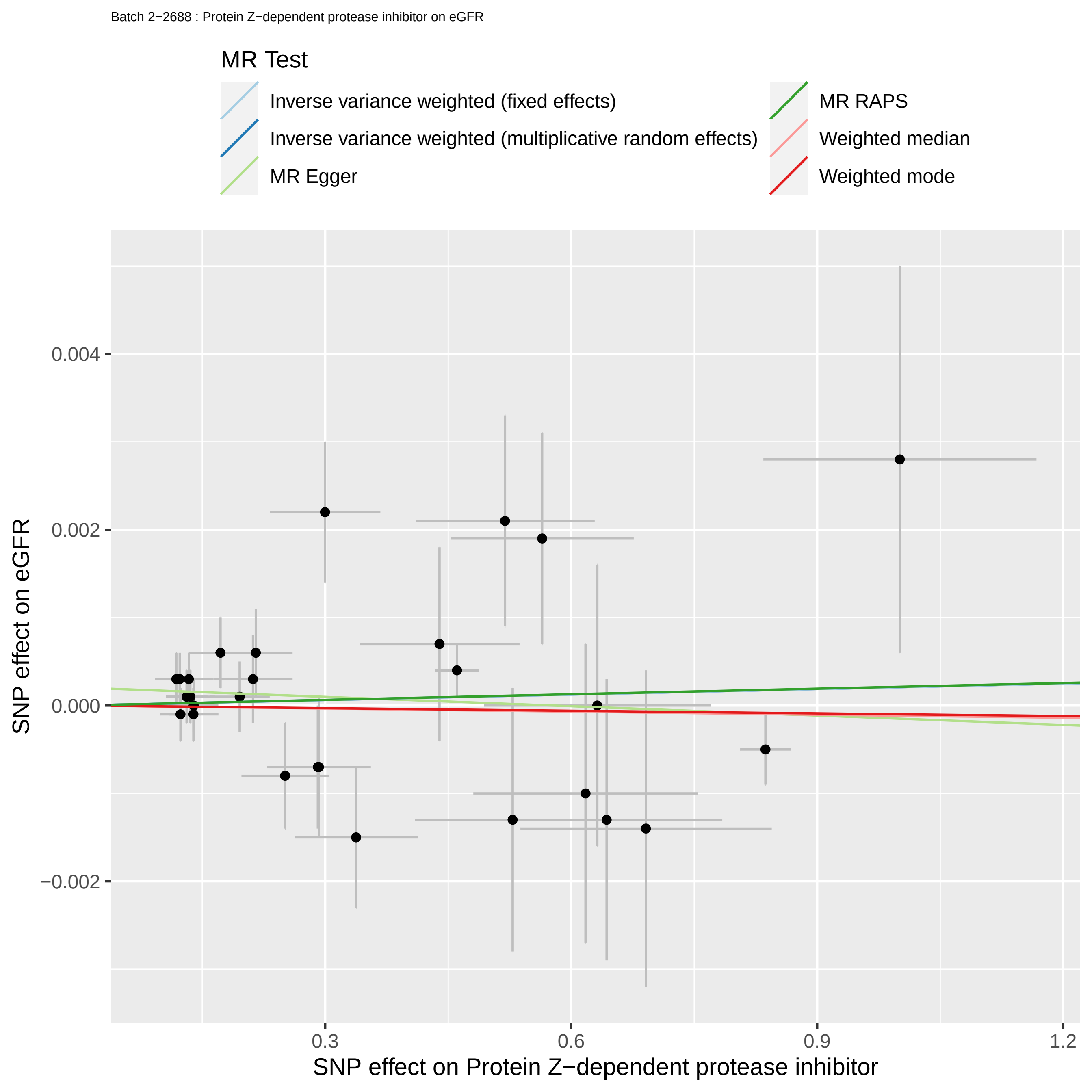
Batch 2−2688 : Protein Z−dependent protease inhibitor on eGFR
MR Test
Inverse variance weighted (fixed effects)
Inverse variance weighted (multiplicative random effects)
MR Egger
MR RAPS
Weighted median
Weighted mode
0.004
0.002
0.000
−0.002
0.3
0.6
0.9
1.2
SNP effect on eGFR
SNP effect on Protein Z−dependent protease inhibitor

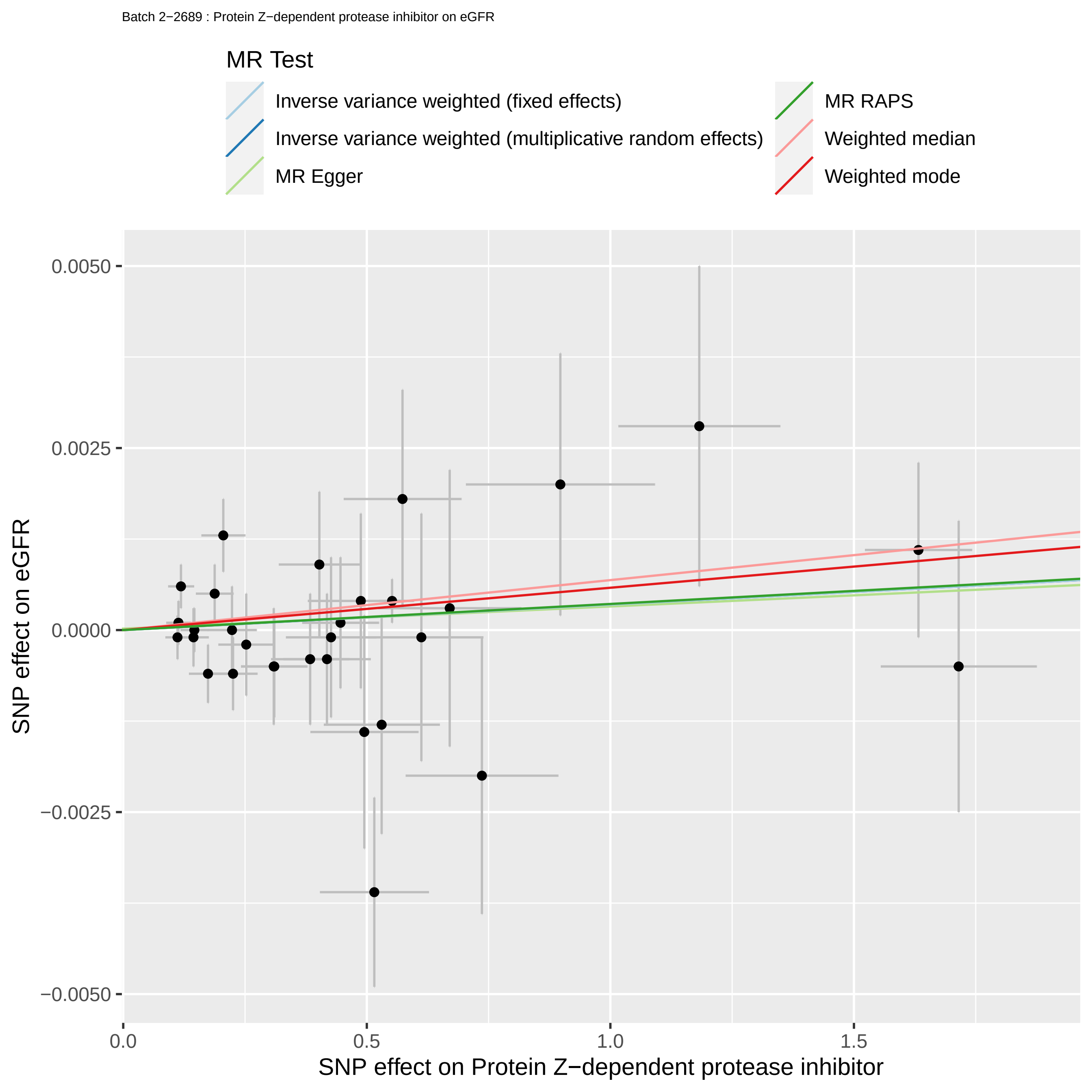
Batch 2−2689 : Protein Z−dependent protease inhibitor on eGFR
MR Test
Inverse variance weighted (fixed effects)
Inverse variance weighted (multiplicative random effects)
MR Egger
MR RAPS
Weighted median
Weighted mode
0.0050
0.0025
0.0000
−0.0025
−0.0050
0.0
0.5
1.0
1.5
SNP effect on eGFR
SNP effect on Protein Z−dependent protease inhibitor

Batch 2−2690 : Serpin A12 on eGFR

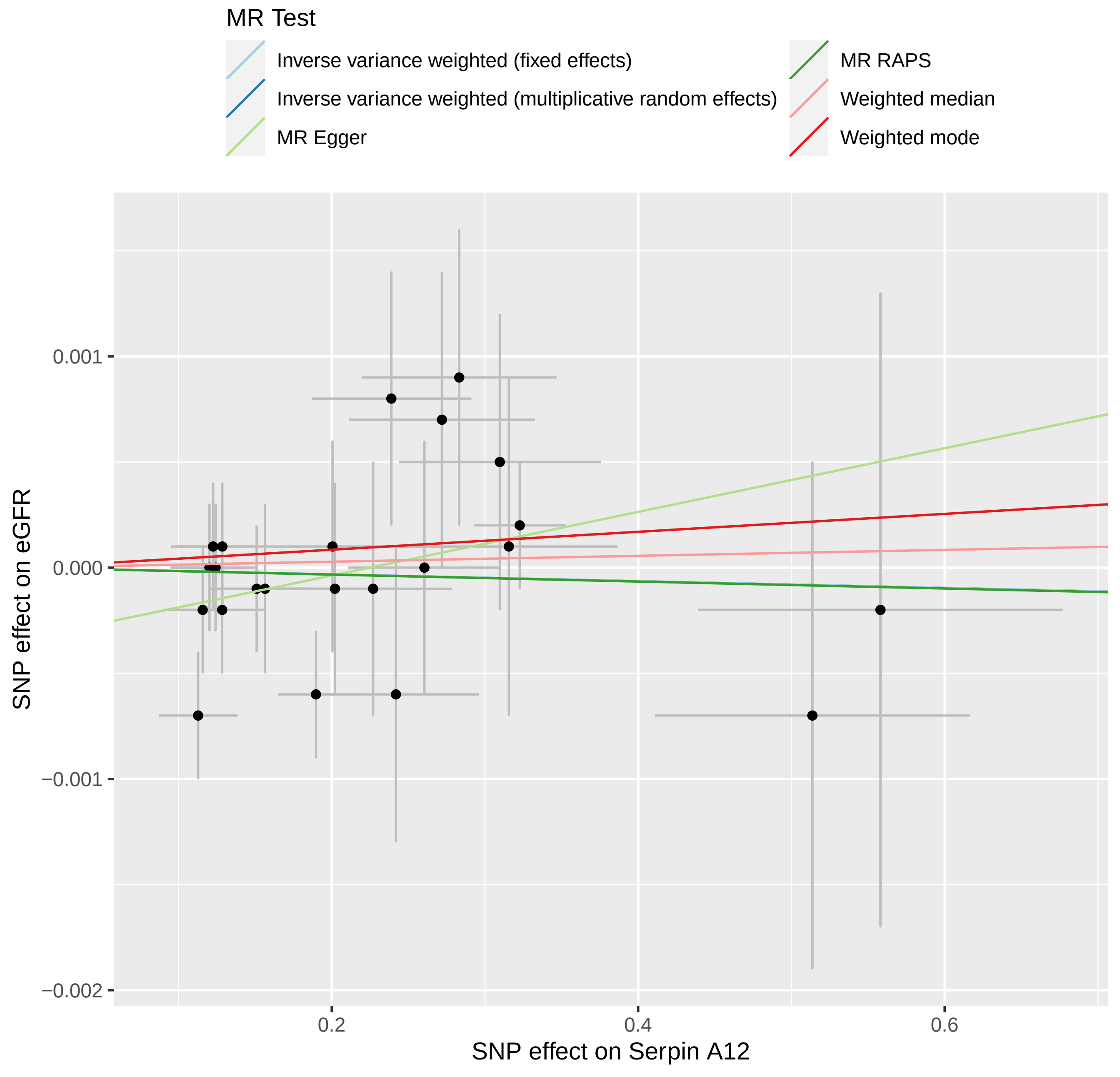

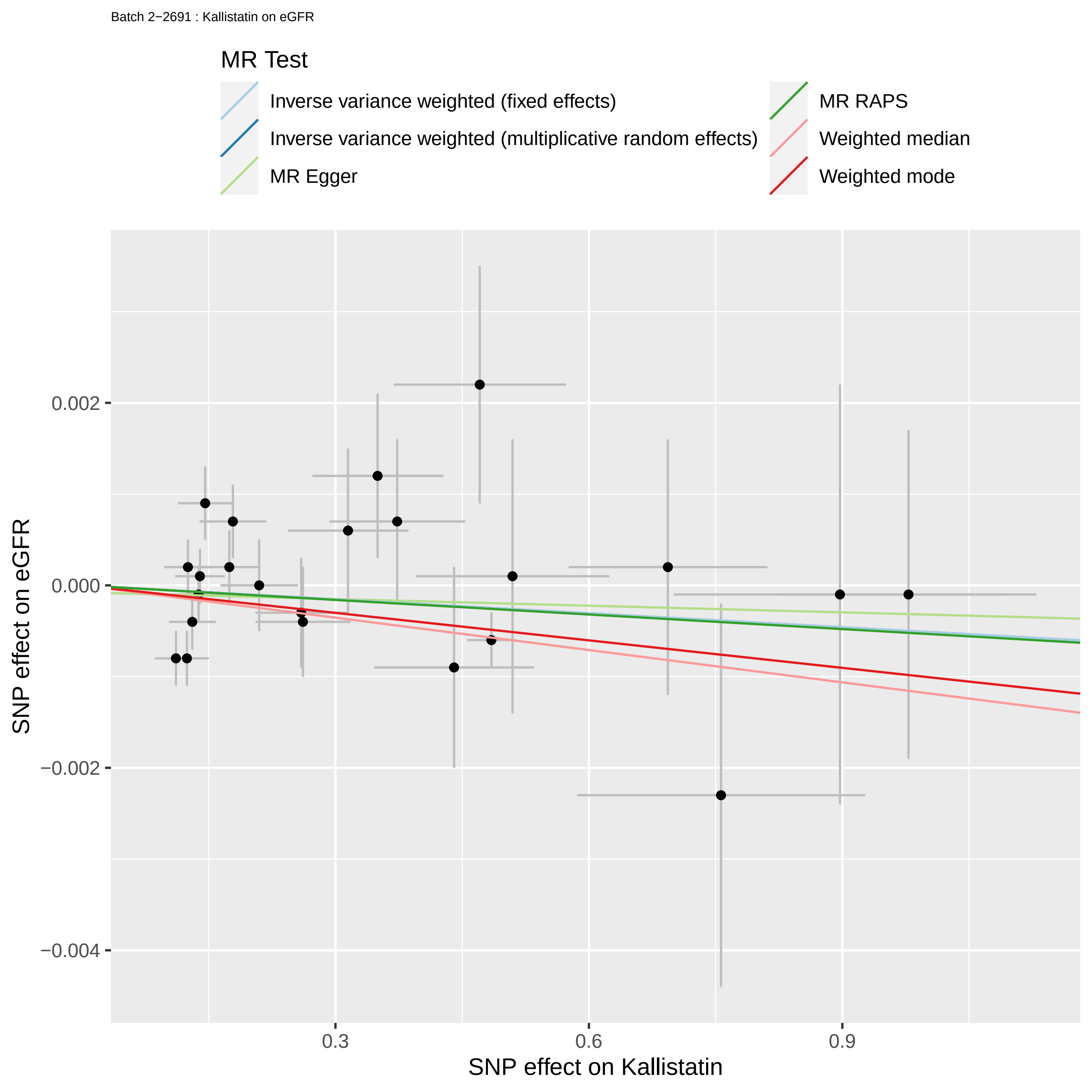

Batch 2−2691 : Kallistatin on eGFR
MR Test
Inverse variance weighted (fixed effects)
Inverse variance weighted (multiplicative random effects)
MR Egger
MR RAPS
Weighted median
Weighted mode
0.002
0.000
−0.002
−0.004
SNP effect on eGFR
0.3
0.6
0.9
SNP effect on Kallistatin

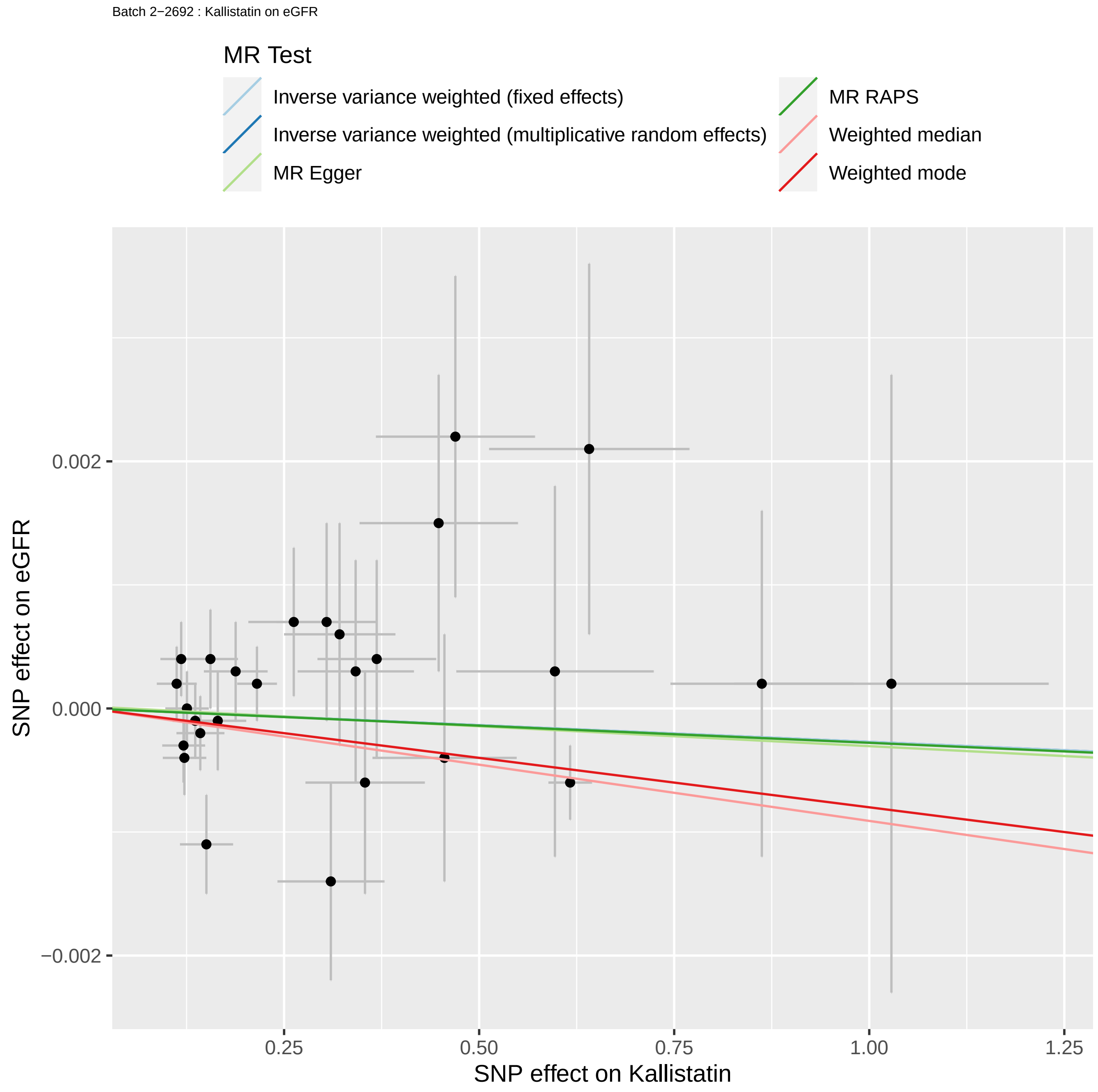

Batch 2−2692 : Kallistatin on eGFR
MR Test
Inverse variance weighted (fixed effects)
Inverse variance weighted (multiplicative random effects)
MR Egger
MR RAPS
Weighted median
Weighted mode
0.002
0.000
−0.002
0.25
0.50
0.75
1.00
1.25
SNP effect on eGFR
SNP effect on Kallistatin

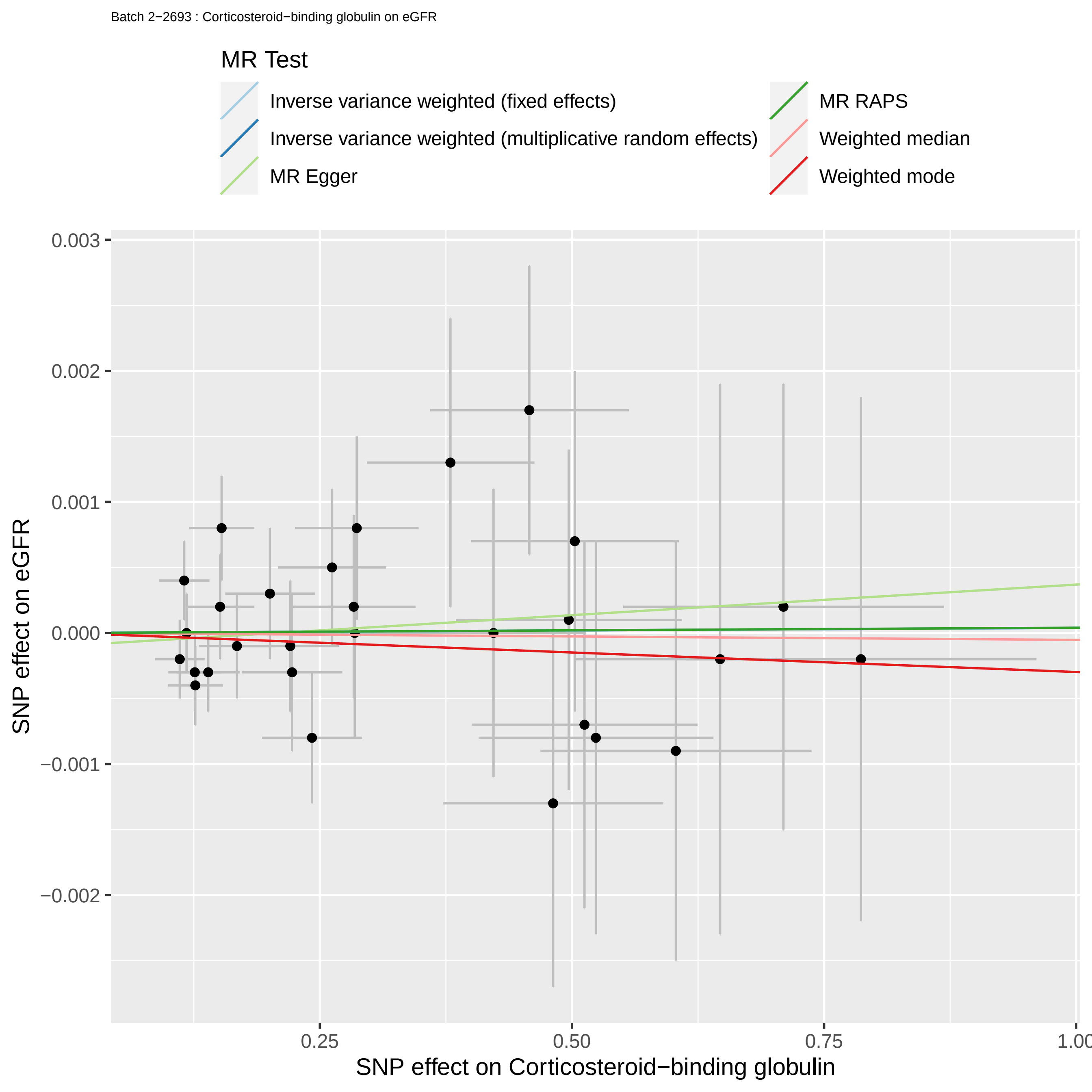
Batch 2−2693 : Corticosteroid−binding globulin on eGFR
MR Test
Inverse variance weighted (fixed effects)
Inverse variance weighted (multiplicative random effects)
MR Egger
MR RAPS
Weighted median
Weighted mode
0.003
0.002
0.001
0.000
−0.001
−0.002
SNP effect on eGFR
0.25
0.50
0.75
1.00
SNP effect on Corticosteroid−binding globulin

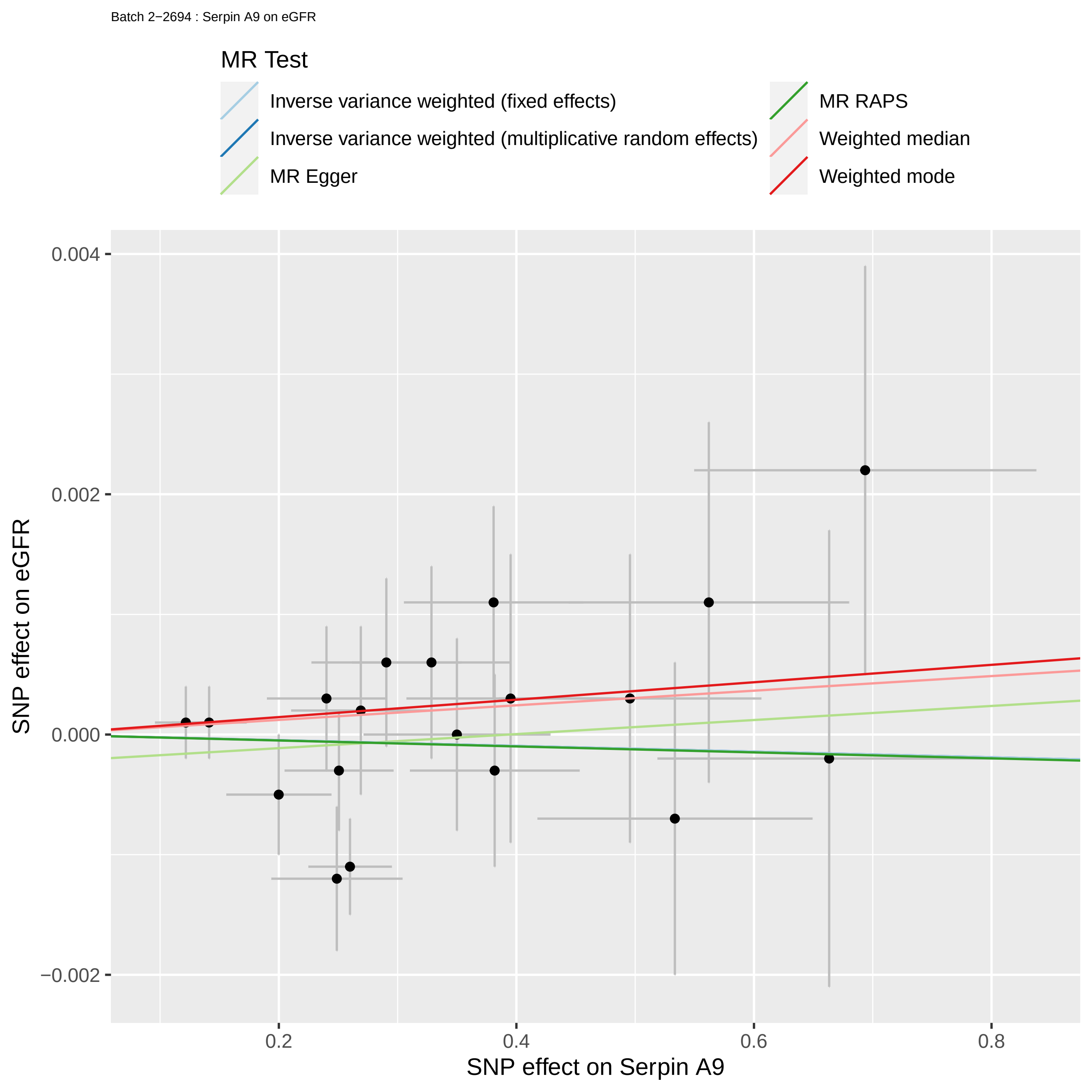
Batch 2−2694 : Serpin A9 on eGFR
MR Test
Inverse variance weighted (fixed effects)
Inverse variance weighted (multiplicative random effects)
MR Egger
MR RAPS
Weighted median
Weighted mode
0.004
0.002
0.000
−0.002
SNP effect on eGFR
0.2
0.4
0.6
0.8
SNP effect on Serpin A9

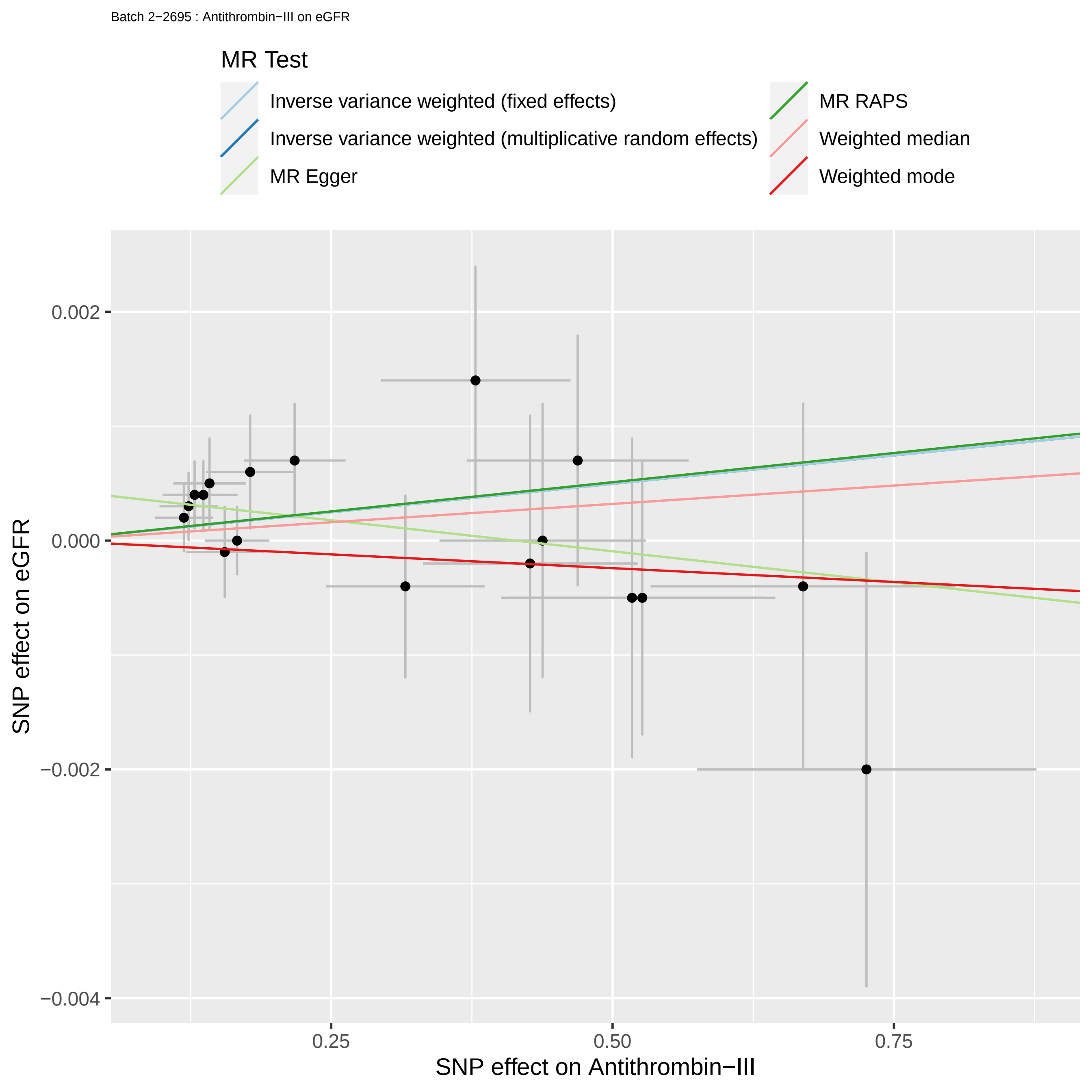

Batch 2−2695 : Antithrombin−III on eGFR
MR Test
Inverse variance weighted (fixed effects)
Inverse variance weighted (multiplicative random effects)
MR Egger
MR RAPS
Weighted median
Weighted mode
0.002
0.000
−0.002
−0.004
0.25
0.50
0.75
SNP effect on eGFR
SNP effect on Antithrombin−III

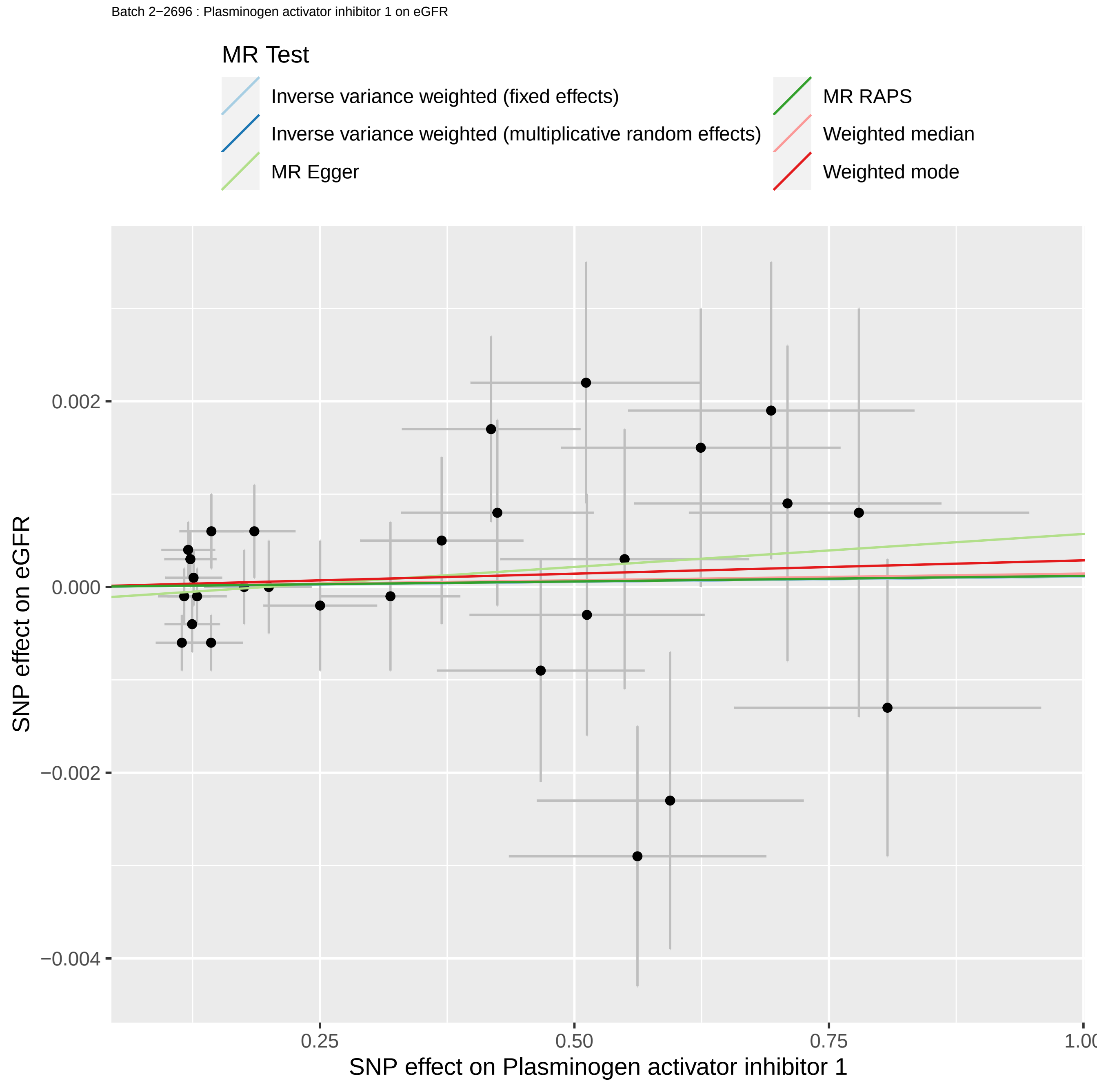

Batch 2−2696 : Plasminogen activator inhibitor 1 on eGFR
MR Test
Inverse variance weighted (fixed effects)
Inverse variance weighted (multiplicative random effects)
MR Egger
MR RAPS
Weighted median
Weighted mode
0.002
0.000
−0.002
−0.004
0.25
0.50
0.75
1.00
SNP effect on eGFR
SNP effect on Plasminogen activator inhibitor 1

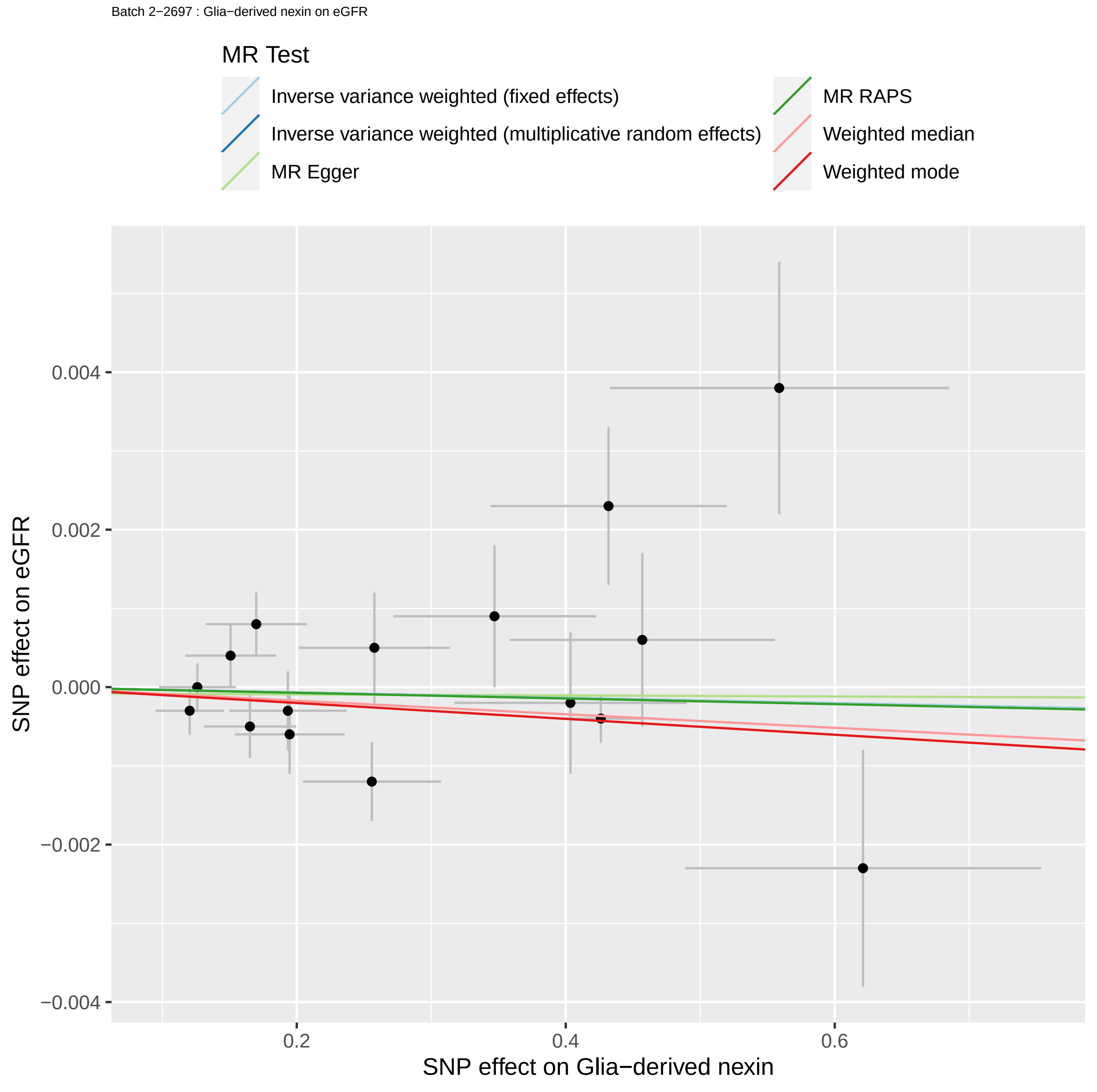

Batch 2−2697 : Glia−derived nexin on eGFR
MR Test
Inverse variance weighted (fixed effects)
Inverse variance weighted (multiplicative random effects)
MR Egger
MR RAPS
Weighted median
Weighted mode
0.004
0.002
0.000
−0.002
−0.004
SNP effect on eGFR
0.2
0.4
0.6
SNP effect on Glia−derived nexin

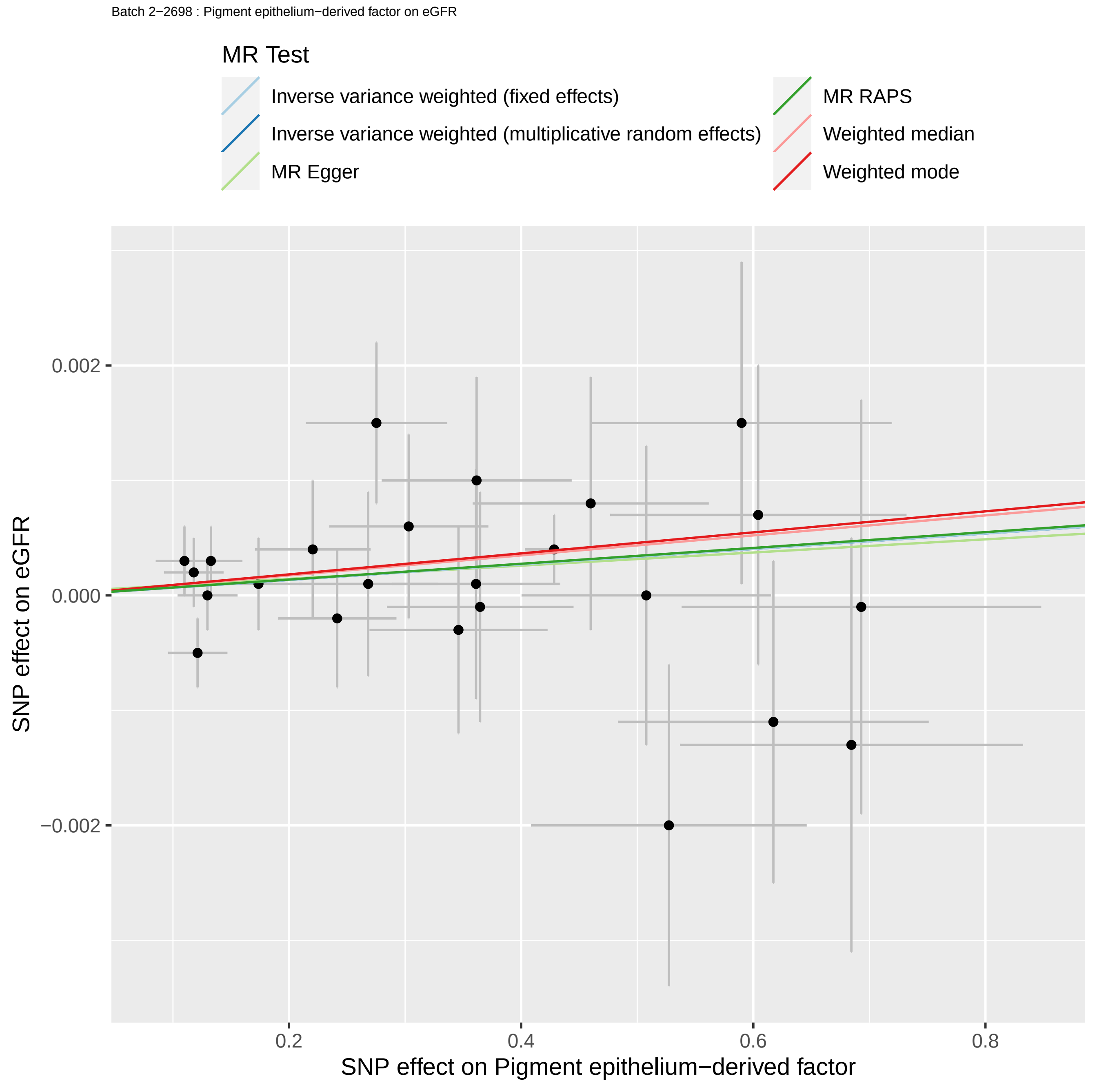

Batch 2−2698 : Pigment epithelium−derived factor on eGFR
MR Test
Inverse variance weighted (fixed effects)
Inverse variance weighted (multiplicative random effects)
MR Egger
MR RAPS
Weighted median
Weighted mode
0.002
0.000
−0.002
SNP effect on eGFR
0.2
0.4
0.6
0.8
SNP effect on Pigment epithelium−derived factor

Batch 2−2699 : Pigment epithelium−derived factor on eGFR

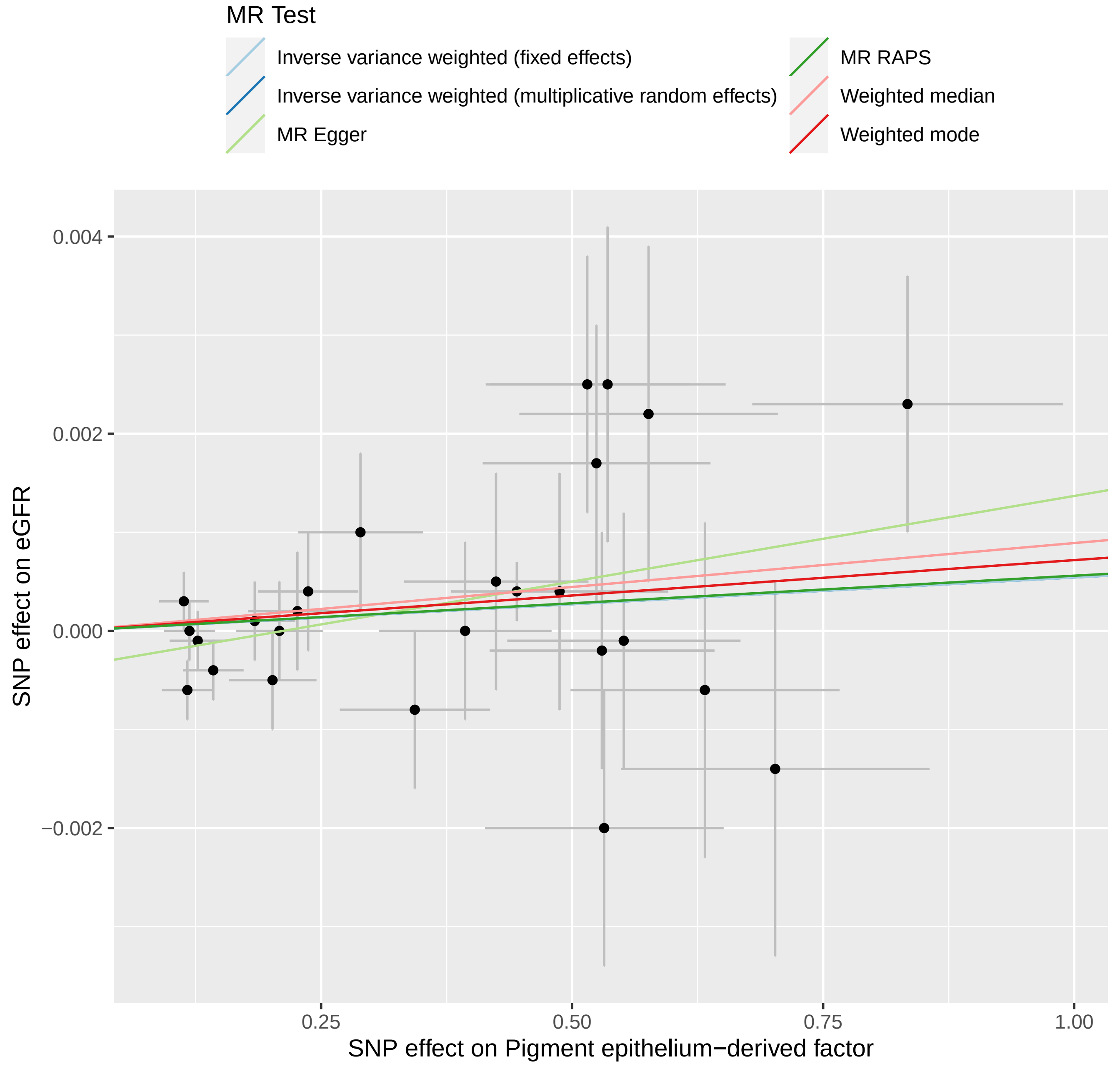

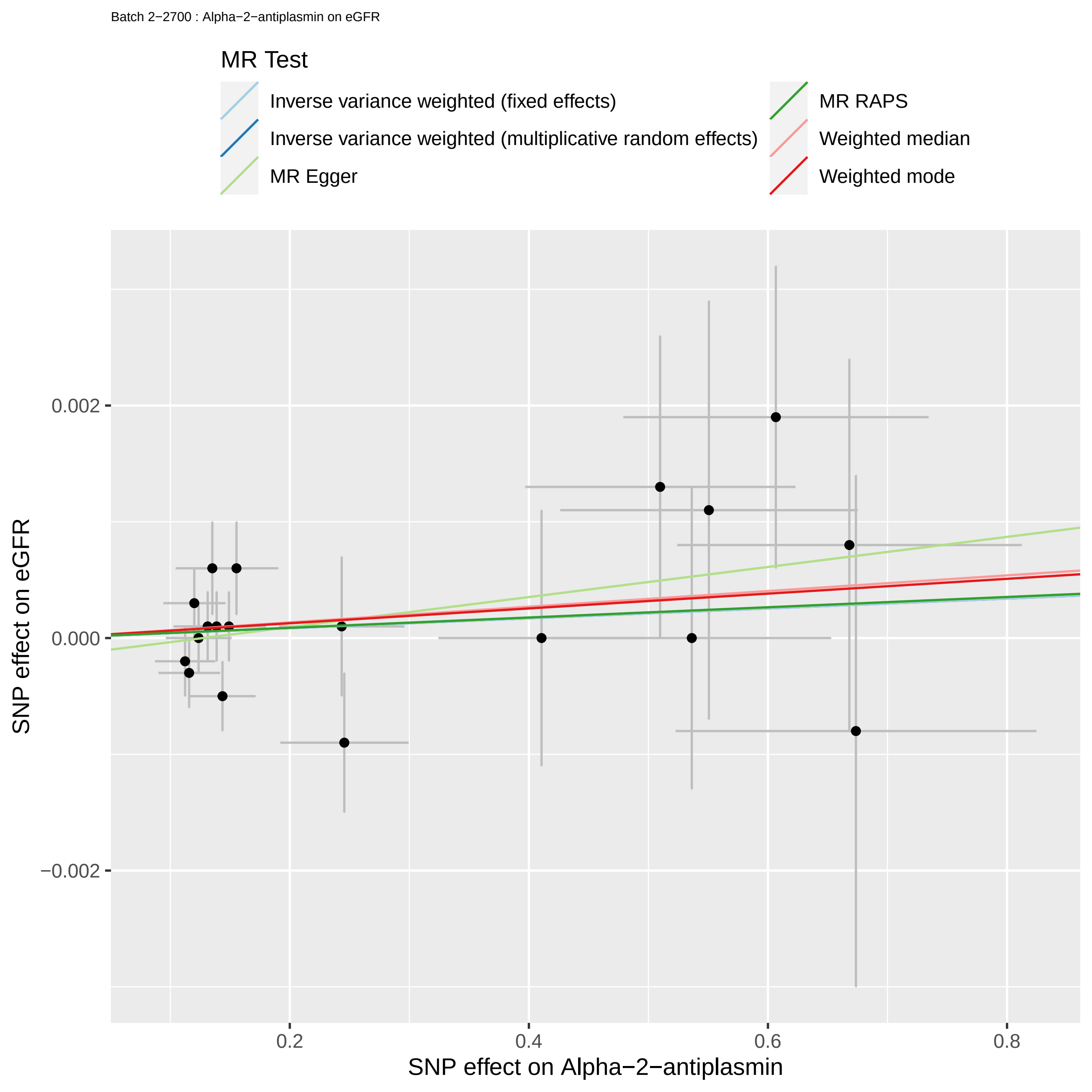

Batch 2−2700 : Alpha−2−antiplasmin on eGFR
MR Test
Inverse variance weighted (fixed effects)
Inverse variance weighted (multiplicative random effects)
MR Egger
MR RAPS
Weighted median
Weighted mode
0.002
0.000
−0.002
0.2
0.4
0.6
0.8
SNP effect on eGFR
SNP effect on Alpha−2−antiplasmin

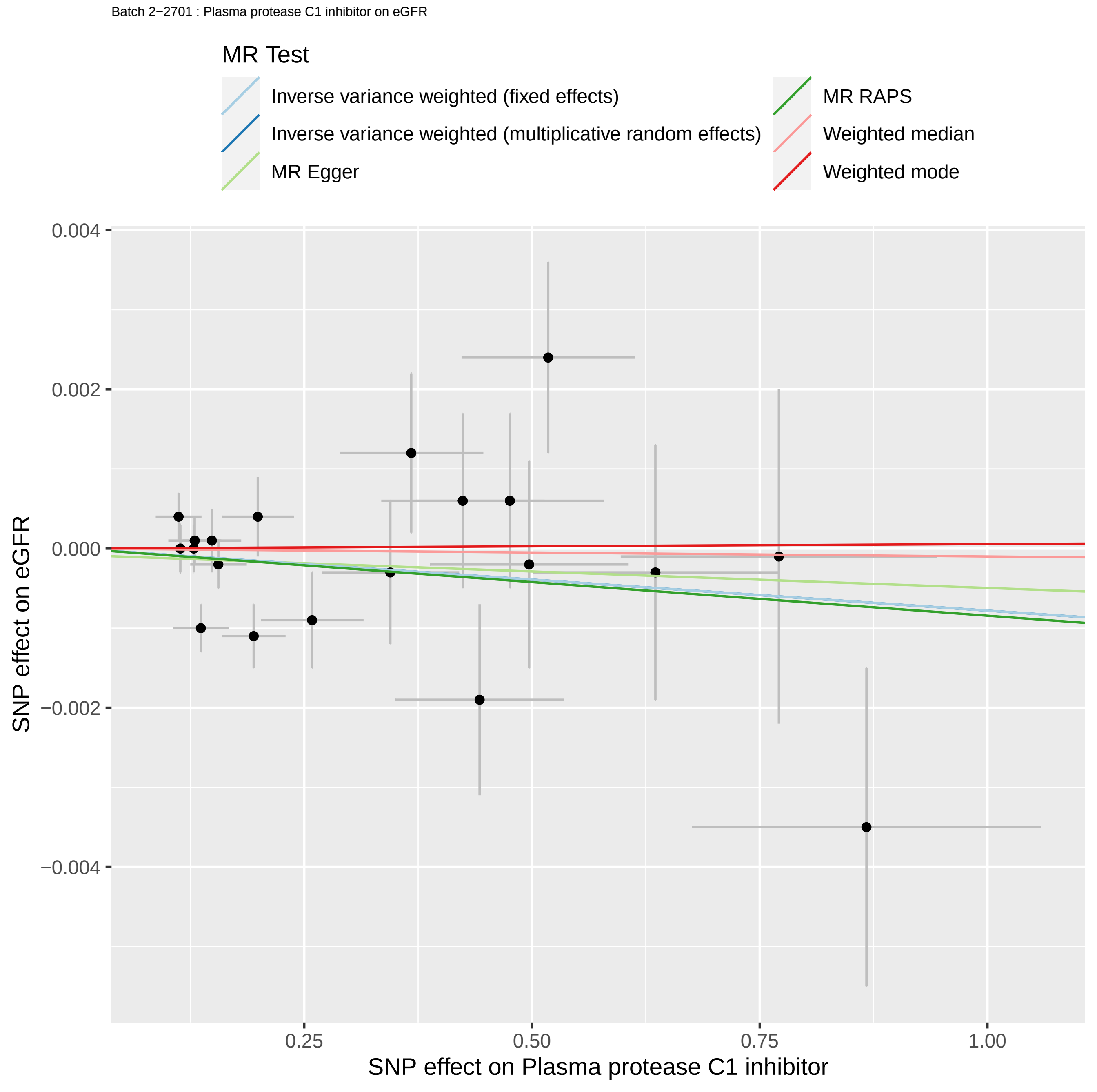

Batch 2−2701 : Plasma protease C1 inhibitor on eGFR
MR Test
Inverse variance weighted (fixed effects)
Inverse variance weighted (multiplicative random effects)
MR Egger
MR RAPS
Weighted median
Weighted mode
0.004
0.002
0.000
−0.002
−0.004
SNP effect on eGFR
0.25
0.50
0.75
1.00
SNP effect on Plasma protease C1 inhibitor

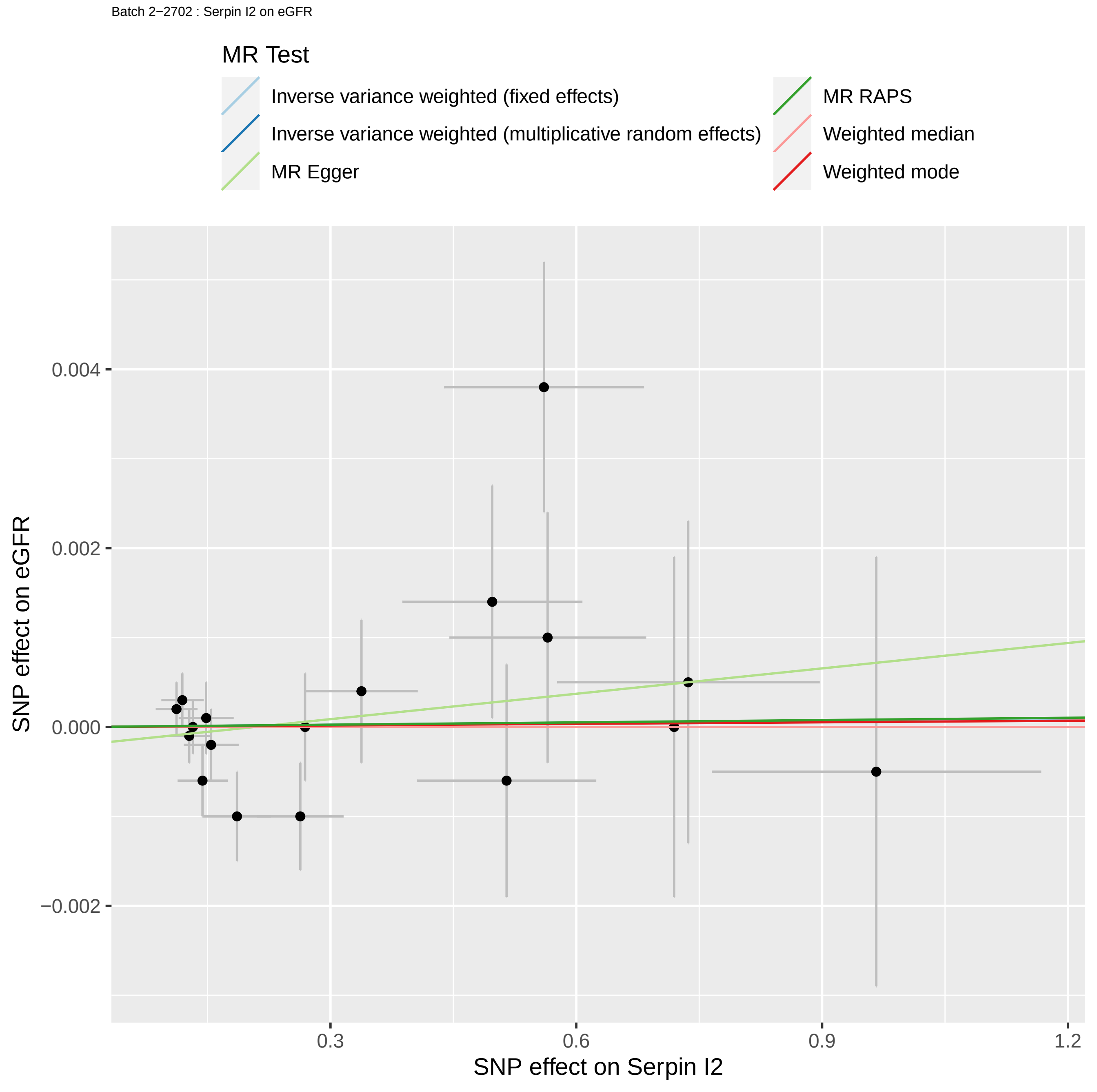

Batch 2−2702 : Serpin I2 on eGFR
MR Test
Inverse variance weighted (fixed effects)
Inverse variance weighted (multiplicative random effects)
MR Egger
MR RAPS
Weighted median
Weighted mode
0.004
0.002
0.000
−0.002
0.3
0.6
0.9
1.2
SNP effect on eGFR
SNP effect on Serpin I2

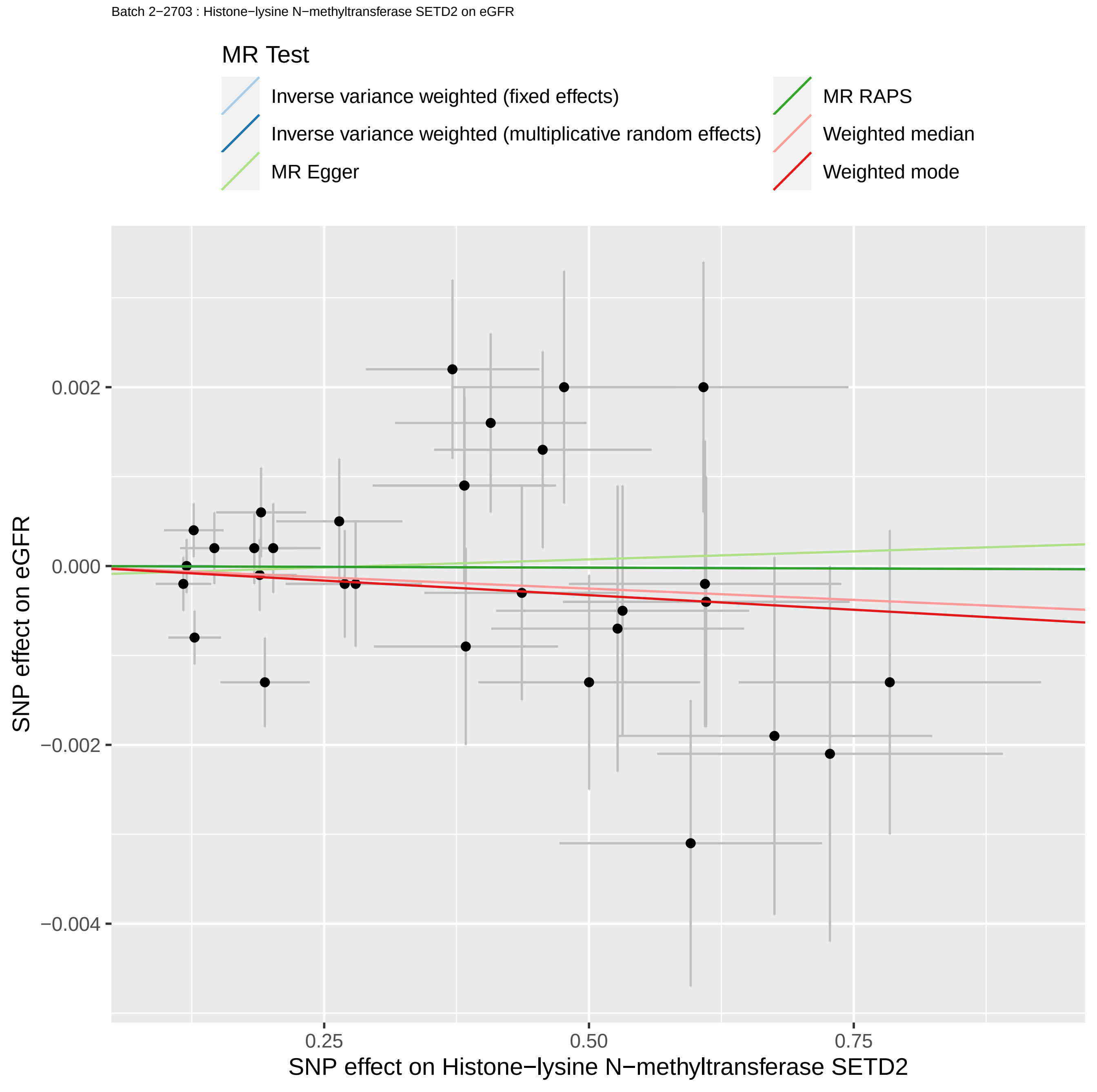

Batch 2−2703 : Histone−lysine N−methyltransferase SETD2 on eGFR
MR Test
Inverse variance weighted (fixed effects)
Inverse variance weighted (multiplicative random effects)
MR Egger
MR RAPS
Weighted median
Weighted mode
0.002
0.000
−0.002
−0.004
0.25
0.50
0.75
SNP effect on eGFR
SNP effect on Histone−lysine N−methyltransferase SETD2

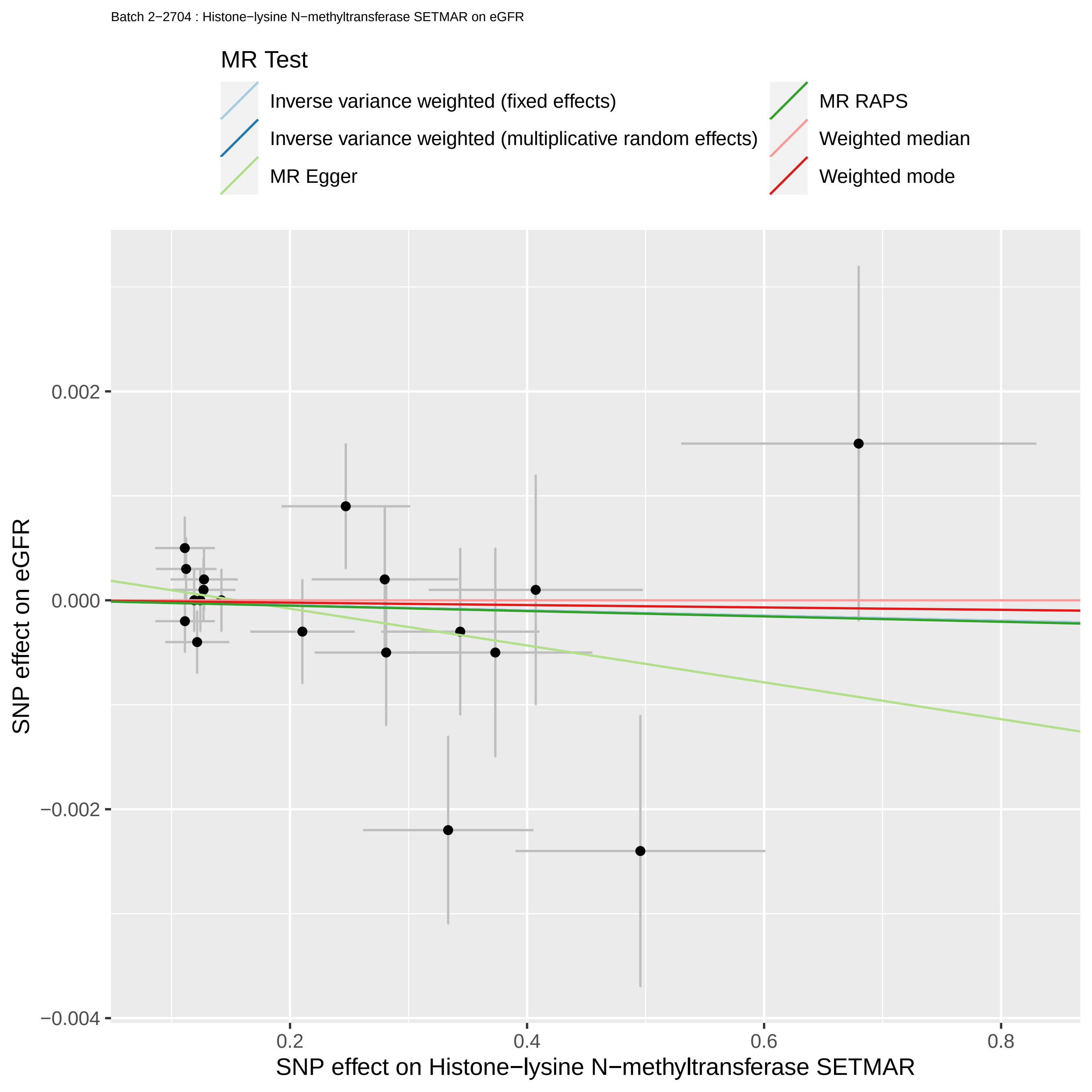

Batch 2−2704 : Histone−lysine N−methyltransferase SETMAR on eGFR
MR Test
Inverse variance weighted (fixed effects)
Inverse variance weighted (multiplicative random effects)
MR Egger
MR RAPS
Weighted median
Weighted mode
0.002
0.000
−0.002
−0.004
0.2
0.4
0.6
0.8
SNP effect on eGFR
SNP effect on Histone−lysine N−methyltransferase SETMAR

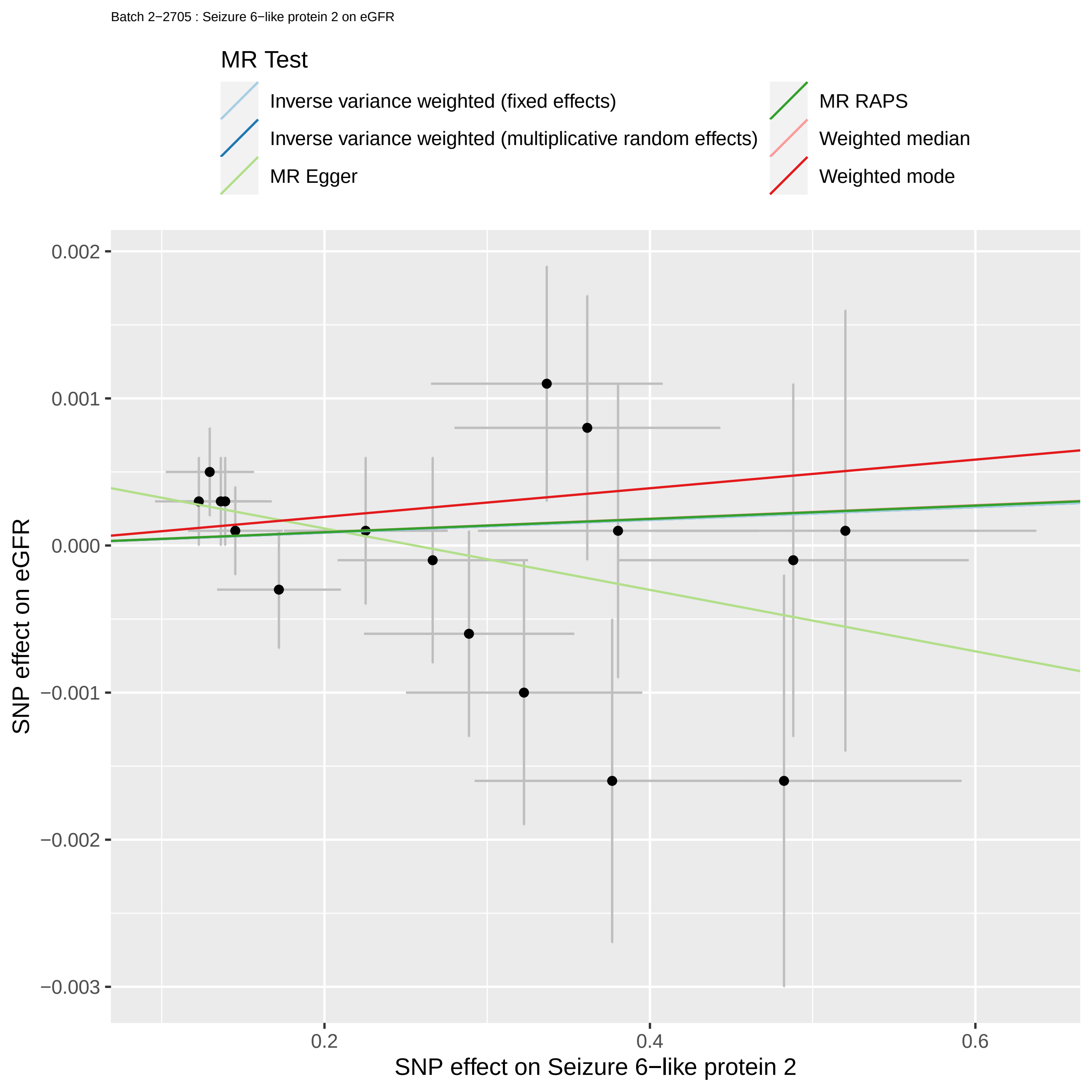

Batch 2−2705 : Seizure 6−like protein 2 on eGFR
MR Test
Inverse variance weighted (fixed effects)
Inverse variance weighted (multiplicative random effects)
MR Egger
MR RAPS
Weighted median
Weighted mode
0.002
0.001
0.000
−0.001
−0.002
−0.003
SNP effect on eGFR
0.2
0.4
0.6
SNP effect on Seizure 6−like protein 2

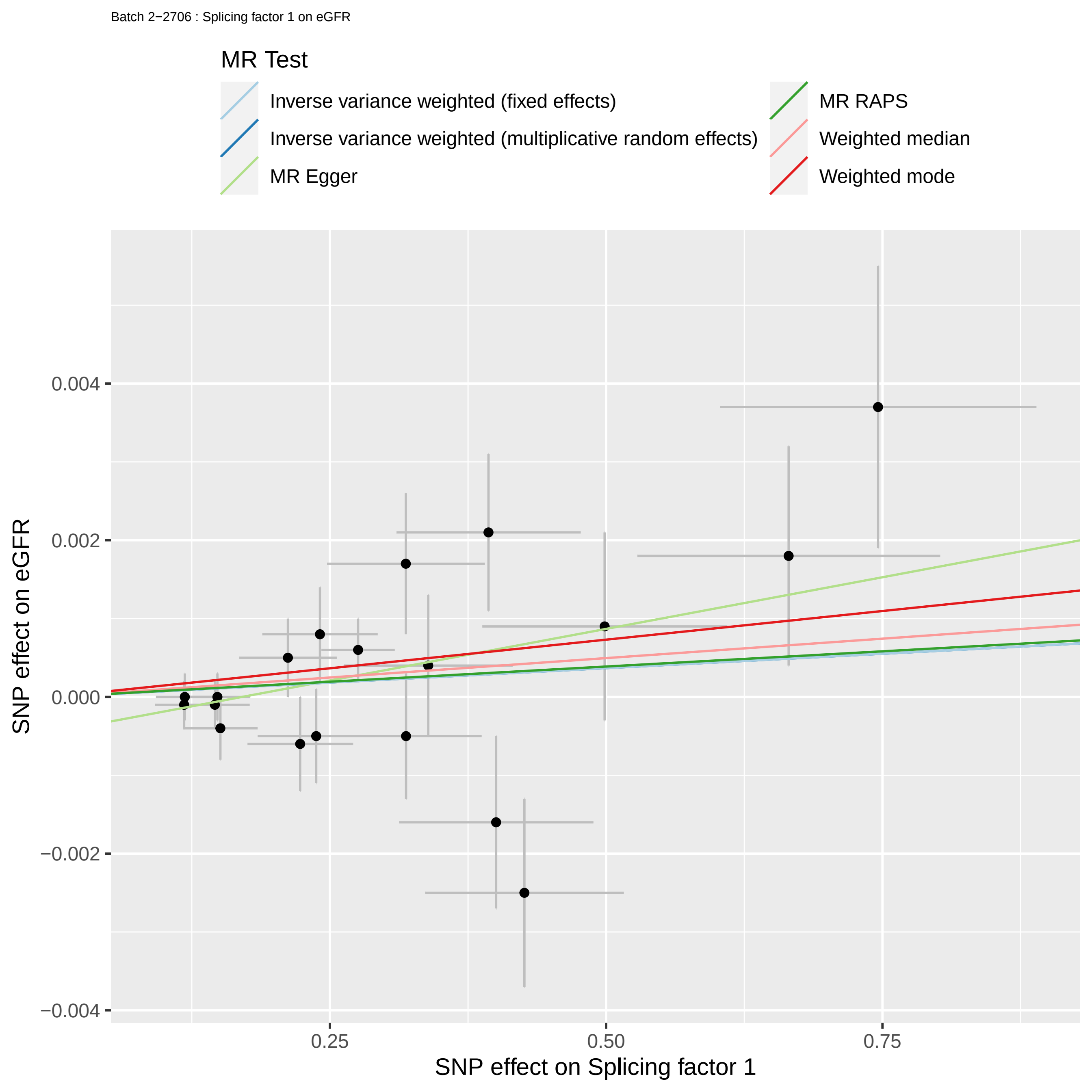
Batch 2−2706 : Splicing factor 1 on eGFR
MR Test
Inverse variance weighted (fixed effects)
Inverse variance weighted (multiplicative random effects)
MR Egger
MR RAPS
Weighted median
Weighted mode
0.004
0.002
0.000
−0.002
−0.004
0.25
0.50
0.75
SNP effect on eGFR
SNP effect on Splicing factor 1

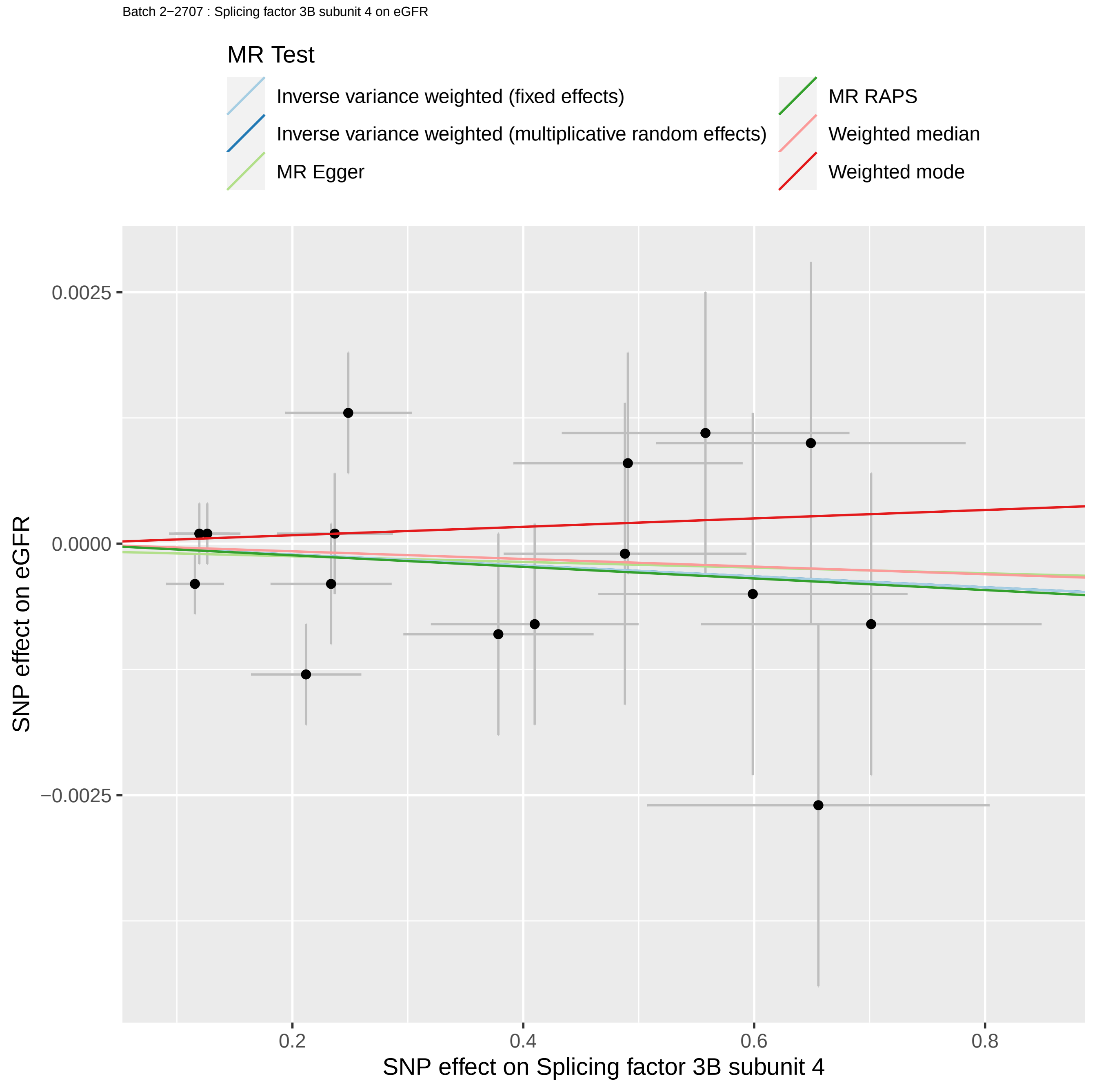

Batch 2−2707 : Splicing factor 3B subunit 4 on eGFR
MR Test
Inverse variance weighted (fixed effects)
Inverse variance weighted (multiplicative random effects)
MR Egger
MR RAPS
Weighted median
Weighted mode
0.0025
0.0000
−0.0025
0.2
0.4
0.6
0.8
SNP effect on eGFR
SNP effect on Splicing factor 3B subunit 4

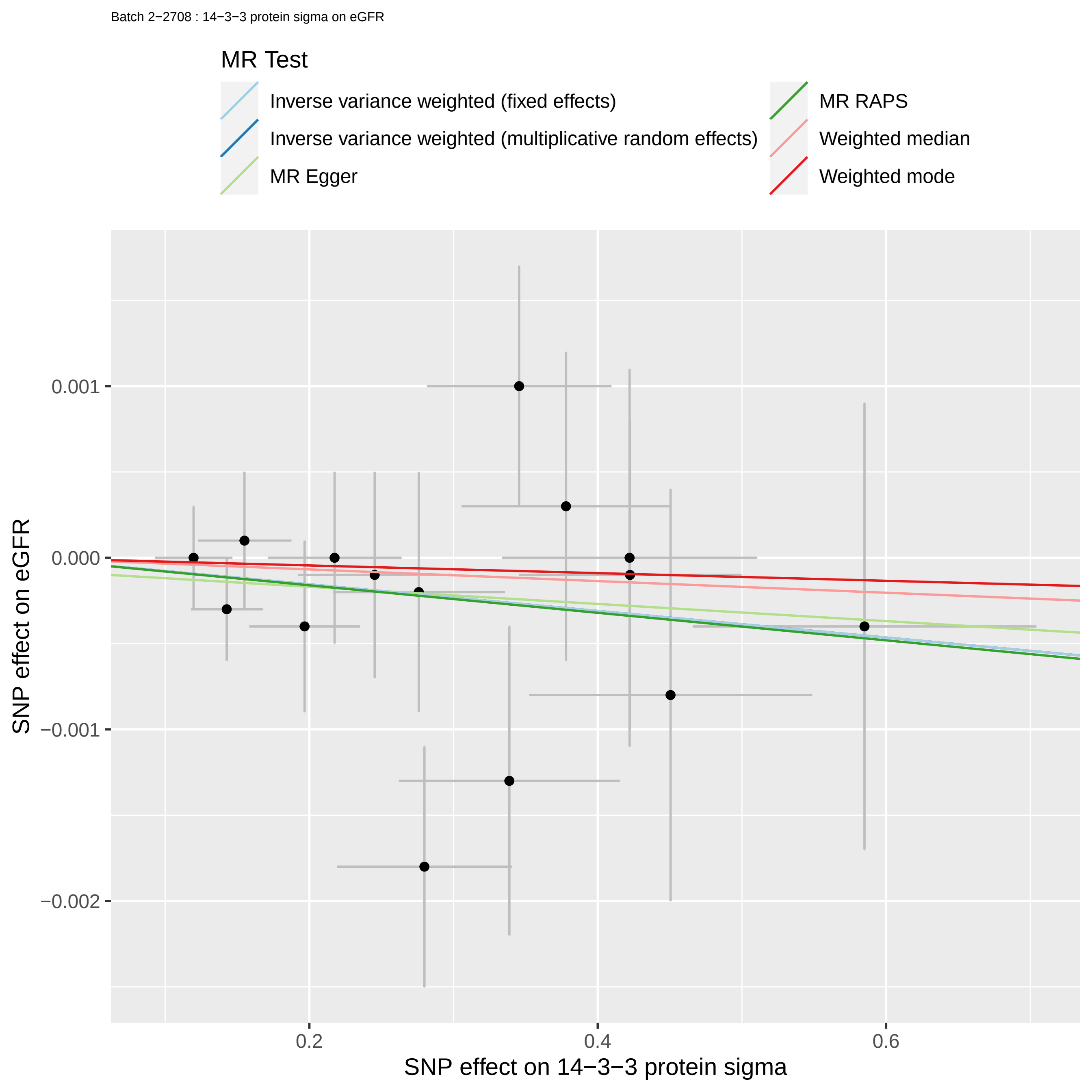

Batch 2−2708 : 14−3−3 protein sigma on eGFR
MR Test
Inverse variance weighted (fixed effects)
Inverse variance weighted (multiplicative random effects)
MR Egger
MR RAPS
Weighted median
Weighted mode
0.001
0.000
−0.001
−0.002
0.2
0.4
0.6
SNP effect on eGFR
SNP effect on 14−3−3 protein sigma

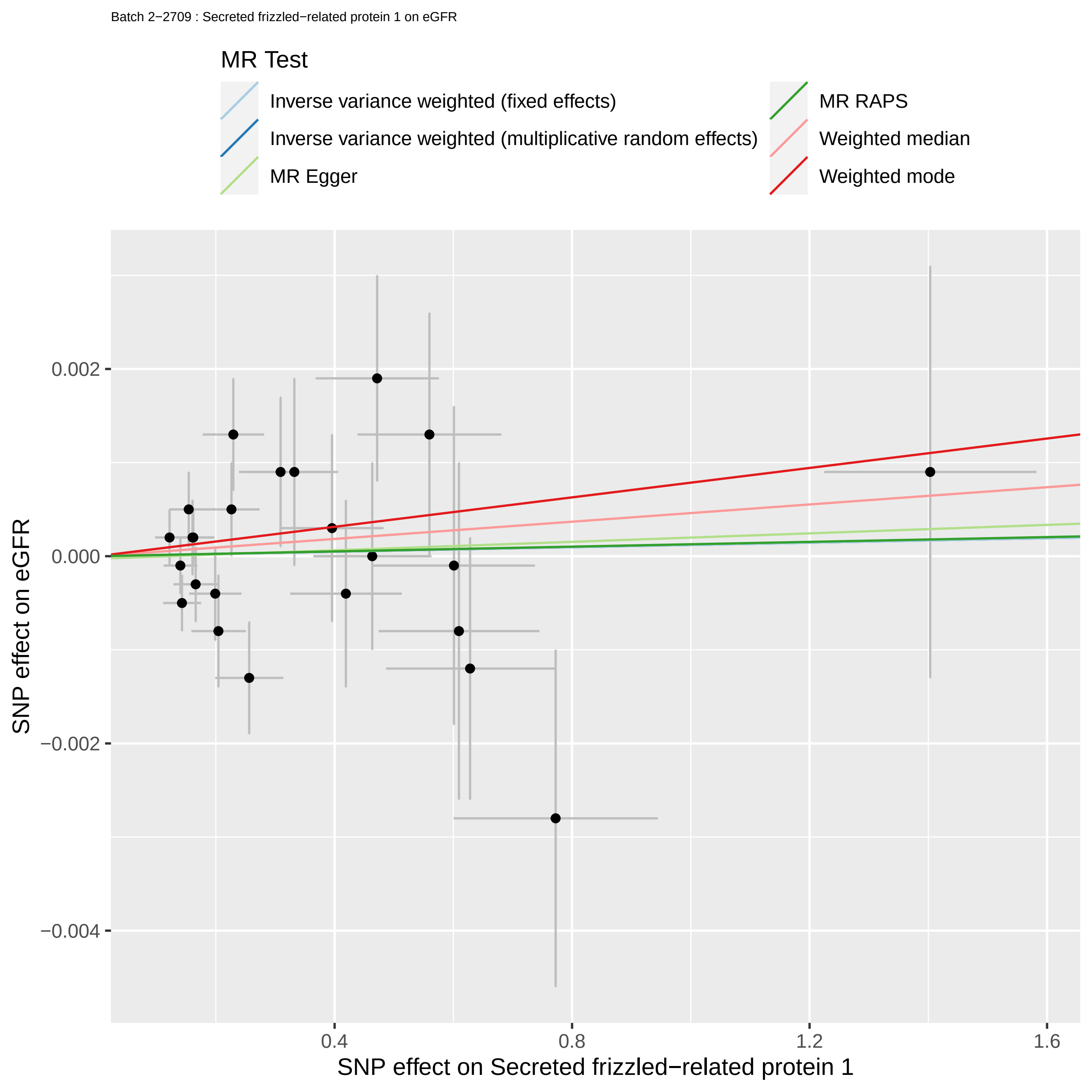

Batch 2−2709 : Secreted frizzled−related protein 1 on eGFR
MR Test
Inverse variance weighted (fixed effects)
Inverse variance weighted (multiplicative random effects)
MR Egger
MR RAPS
Weighted median
Weighted mode
0.002
0.000
−0.002
−0.004
0.4
0.8
1.2
1.6
SNP effect on eGFR
SNP effect on Secreted frizzled−related protein 1

Batch 2−2710 : Secreted frizzled−related protein 2 on eGFR

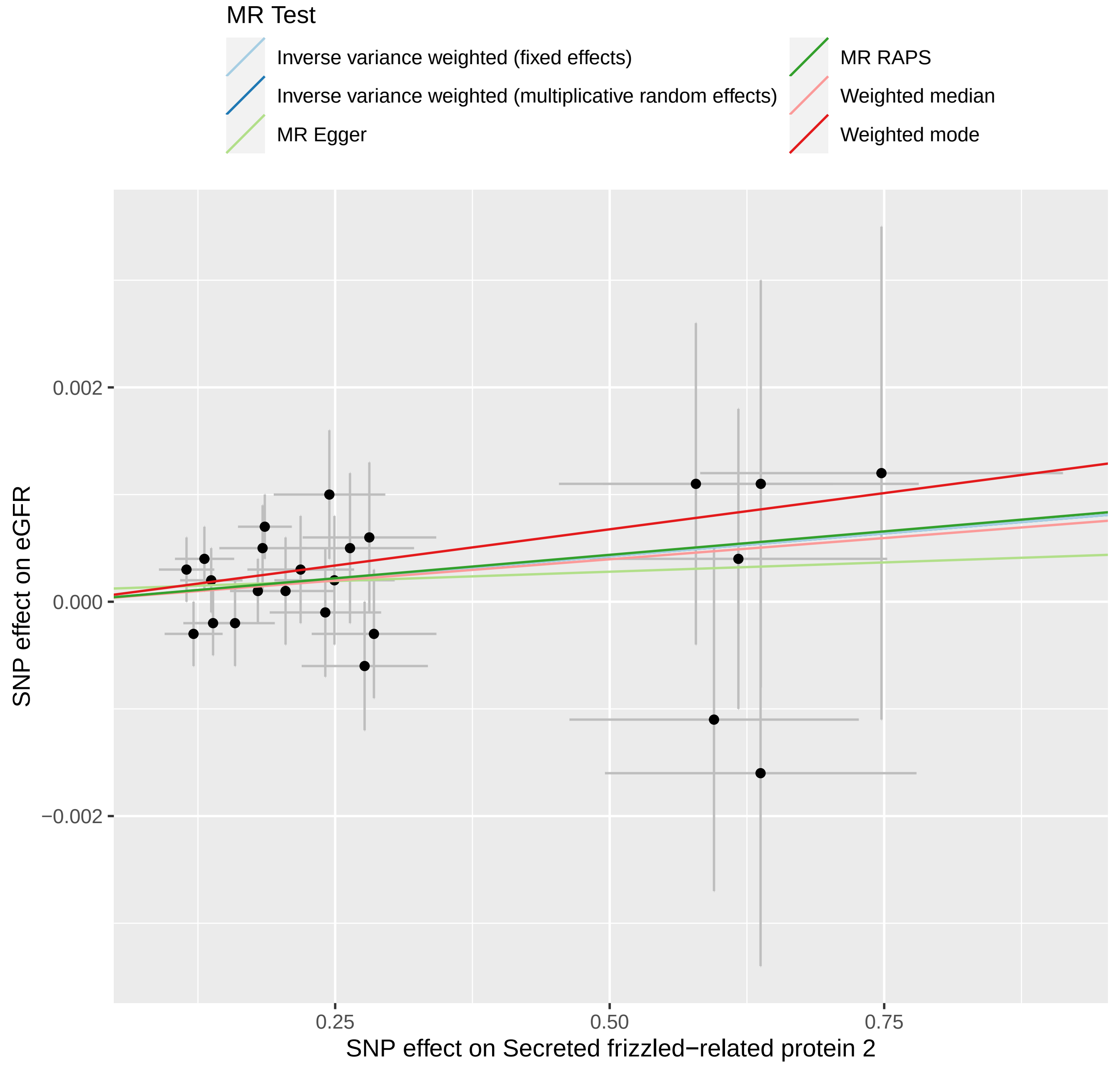

Batch 2−2711 : Pulmonary surfactant−associated protein C on eGFR

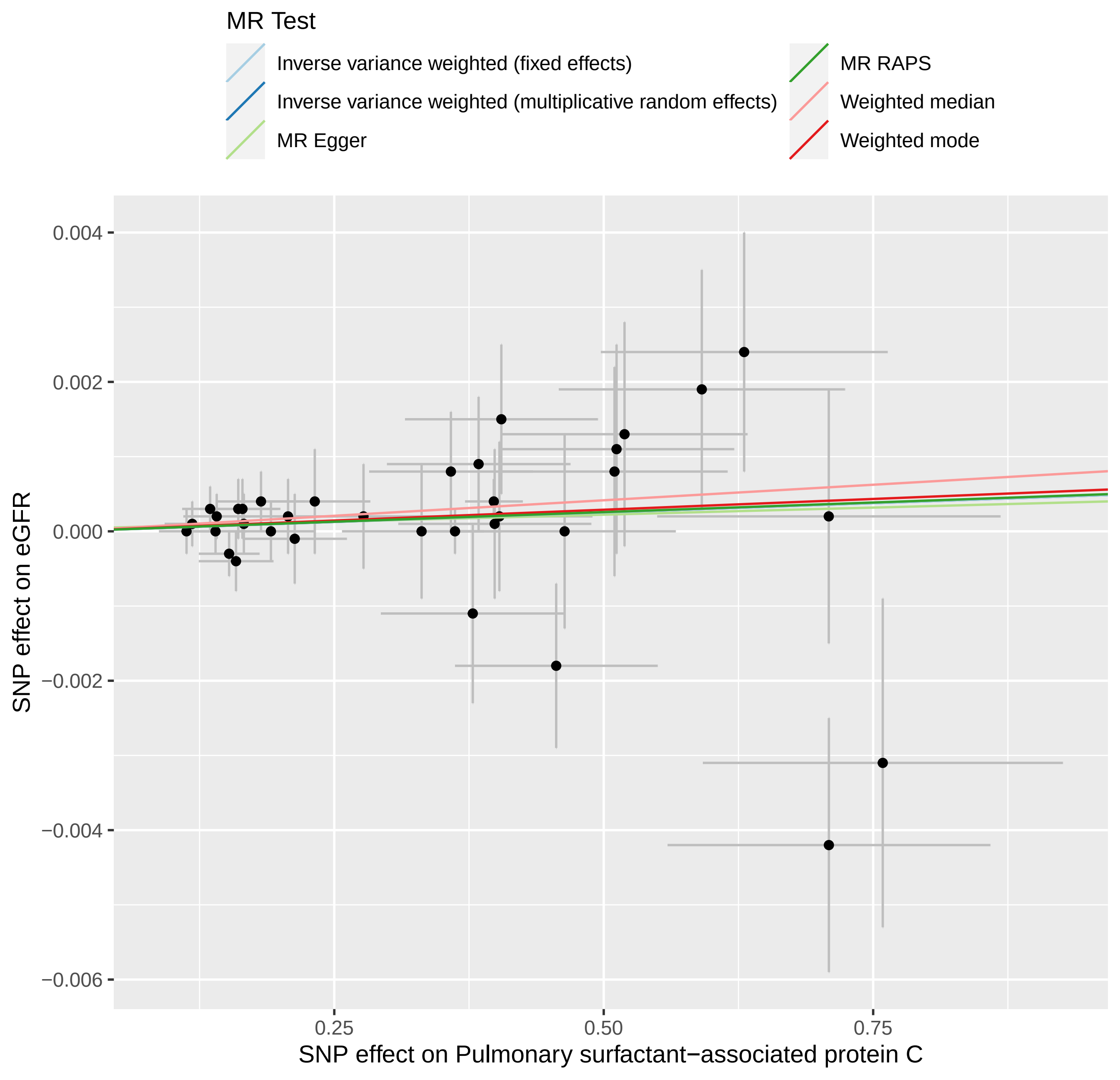

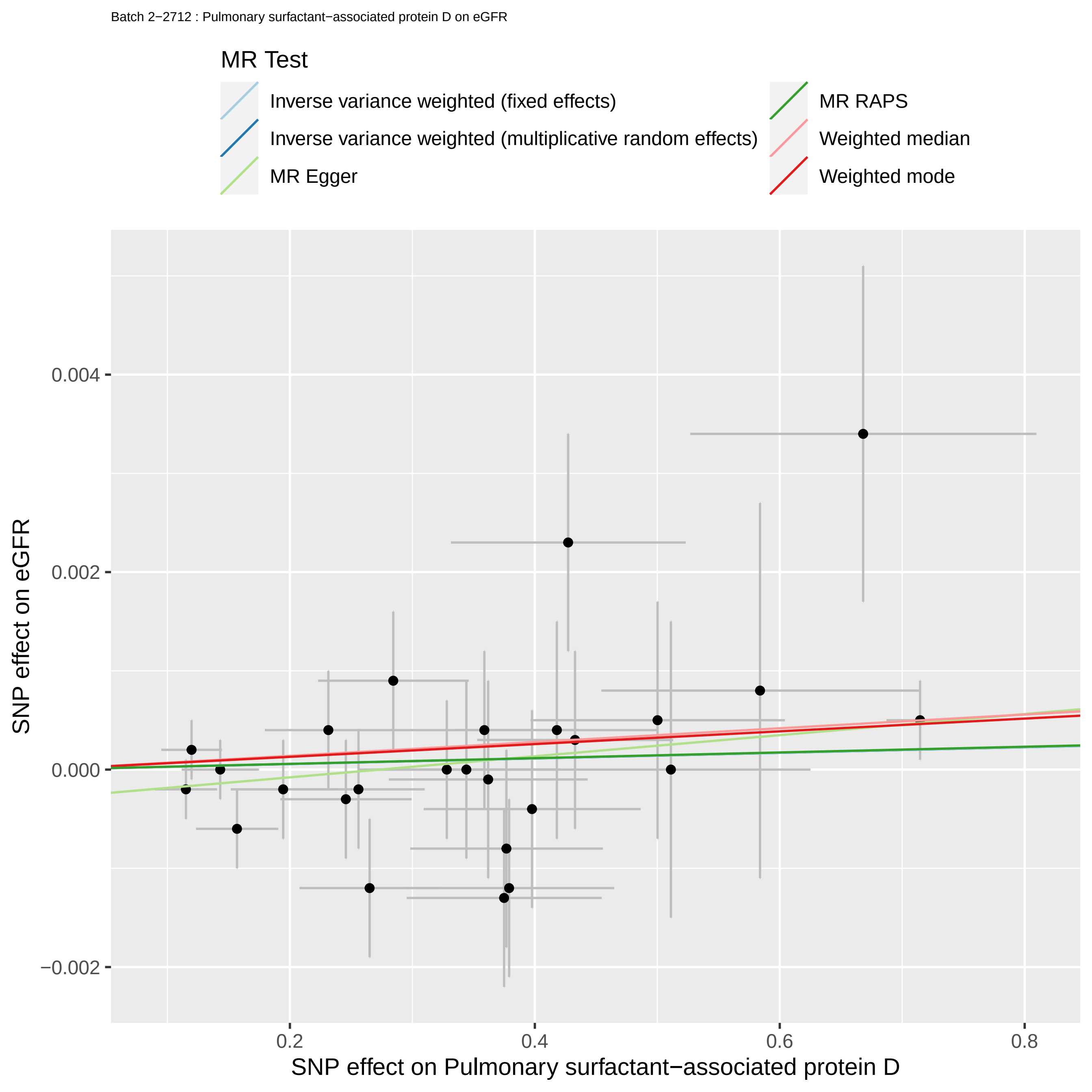
Batch 2−2712 : Pulmonary surfactant−associated protein D on eGFR
MR Test
Inverse variance weighted (fixed effects)
Inverse variance weighted (multiplicative random effects)
MR Egger
MR RAPS
Weighted median
Weighted mode
SNP effect on eGFR
0.004
0.002
0.000
−0.002
0.2
0.4
0.6
0.8
SNP effect on Pulmonary surfactant−associated protein D

Batch 2−2713 : Beta−sarcoglycan on eGFR

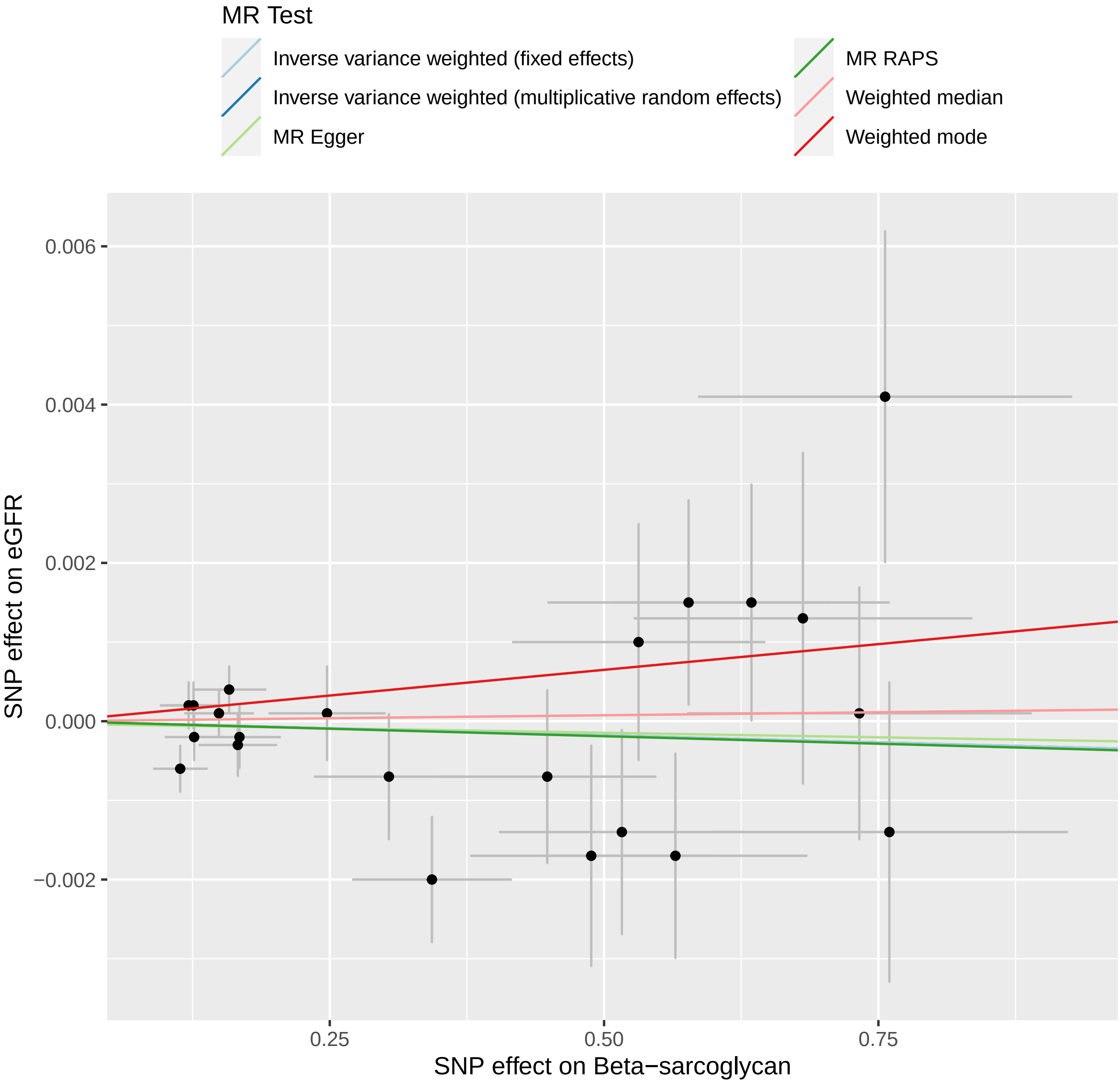

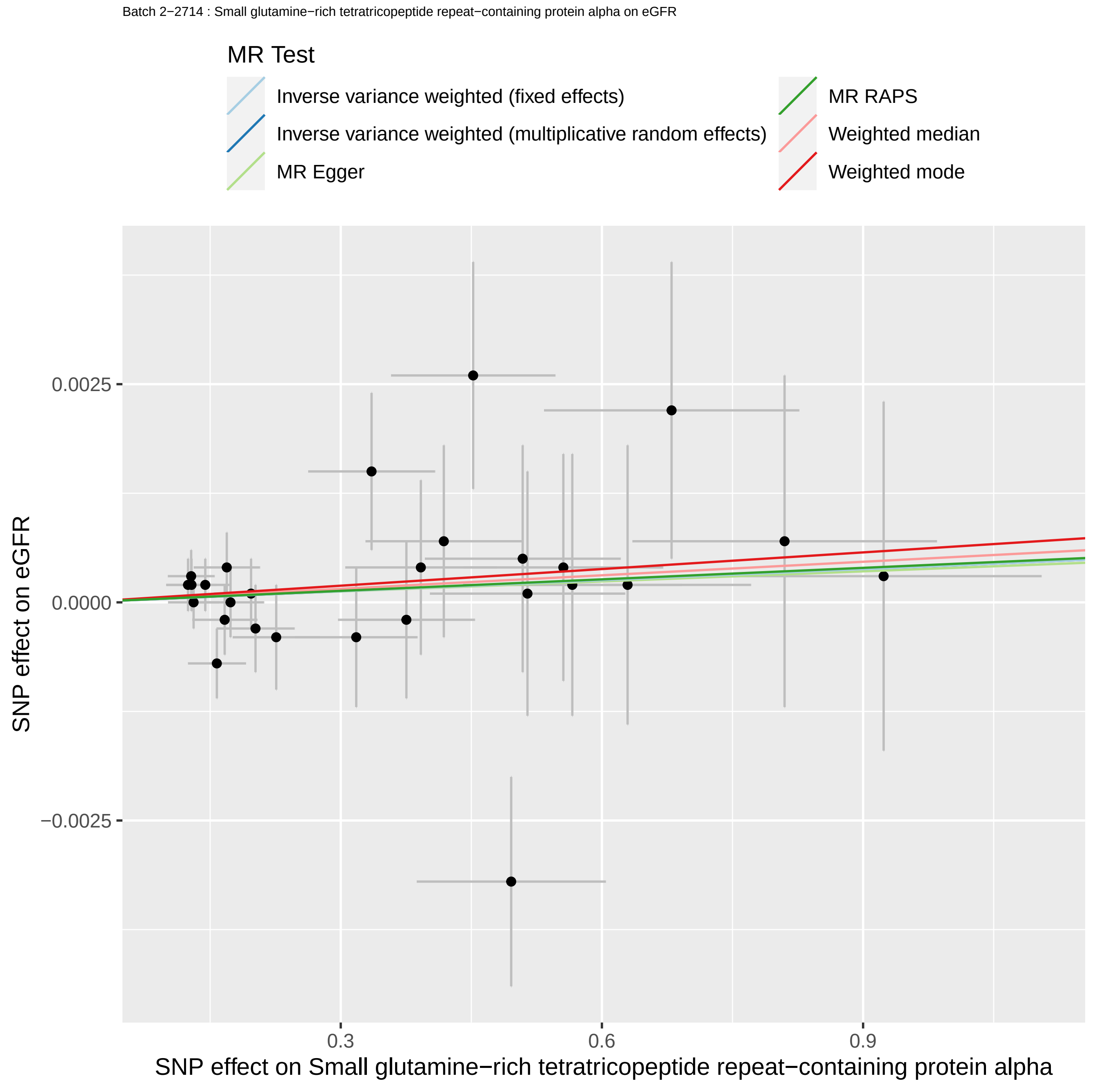
Batch 2−2714 : Small glutamine−rich tetratricopeptide repeat−containing protein alpha on eGFR
MR Test
Inverse variance weighted (fixed effects)
Inverse variance weighted (multiplicative random effects)
MR Egger
MR RAPS
Weighted median
Weighted mode
0.0025
0.0000
−0.0025
0.3
0.6
0.9
SNP effect on eGFR
SNP effect on Small glutamine−rich tetratricopeptide repeat−containing protein alpha

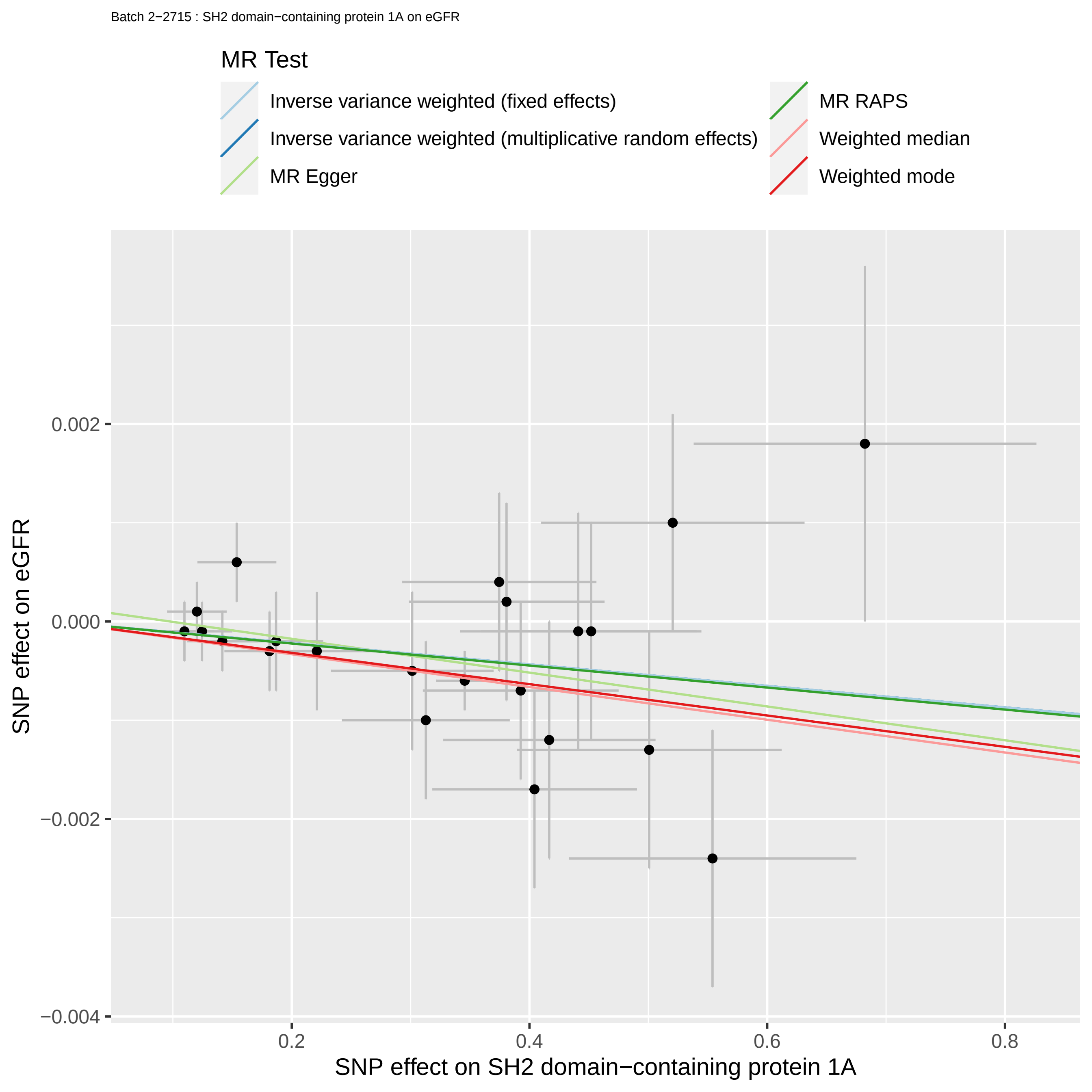
Batch 2−2715 : SH2 domain−containing protein 1A on eGFR
MR Test
Inverse variance weighted (fixed effects)
Inverse variance weighted (multiplicative random effects)
MR Egger
MR RAPS
Weighted median
Weighted mode
0.002
0.000
−0.002
−0.004
SNP effect on eGFR
0.2
0.4
0.6
0.8
SNP effect on SH2 domain−containing protein 1A

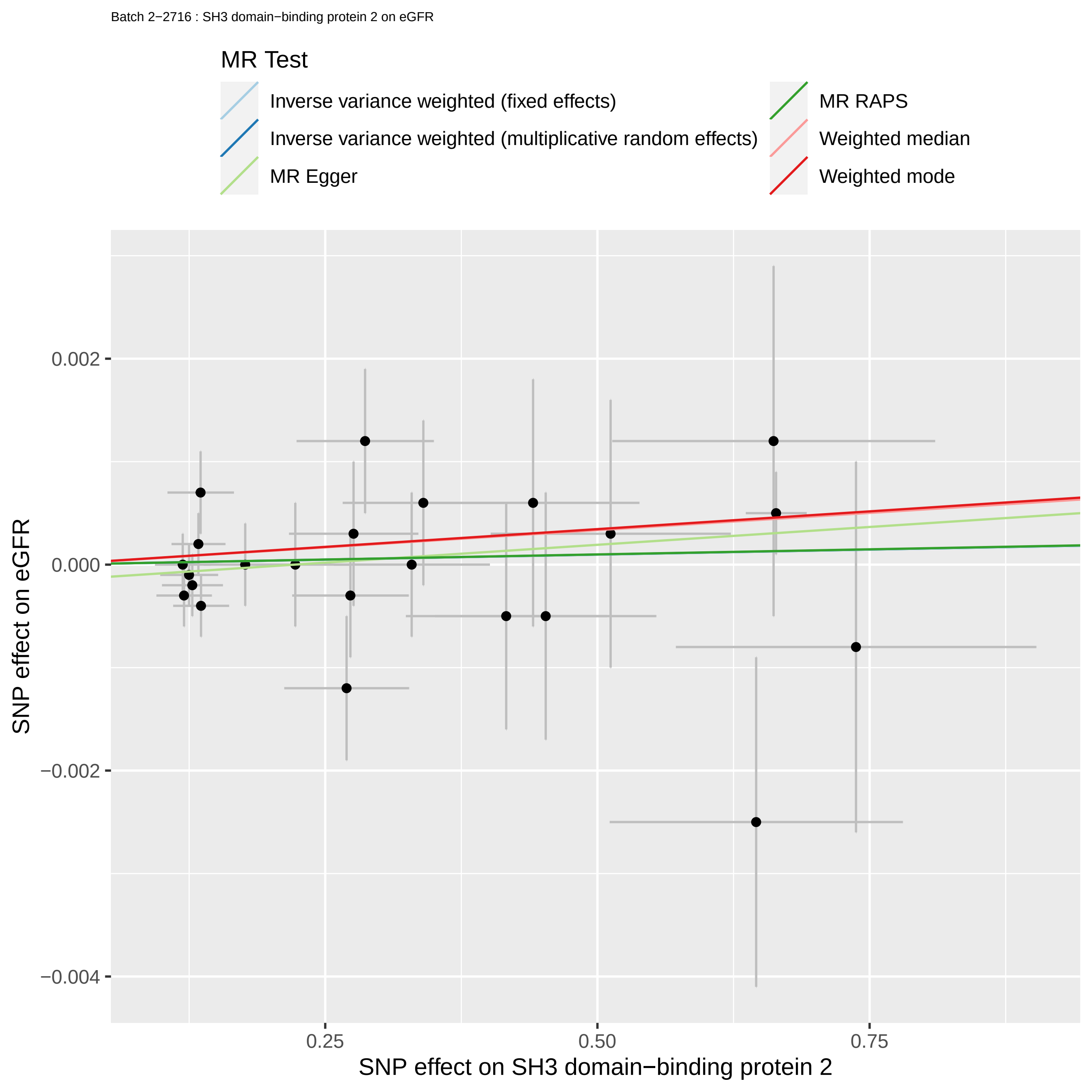

Batch 2−2716 : SH3 domain−binding protein 2 on eGFR
MR Test
Inverse variance weighted (fixed effects)
Inverse variance weighted (multiplicative random effects)
MR Egger
MR RAPS
Weighted median
Weighted mode
0.002
0.000
−0.002
−0.004
SNP effect on eGFR
0.25
0.50
0.75
SNP effect on SH3 domain−binding protein 2

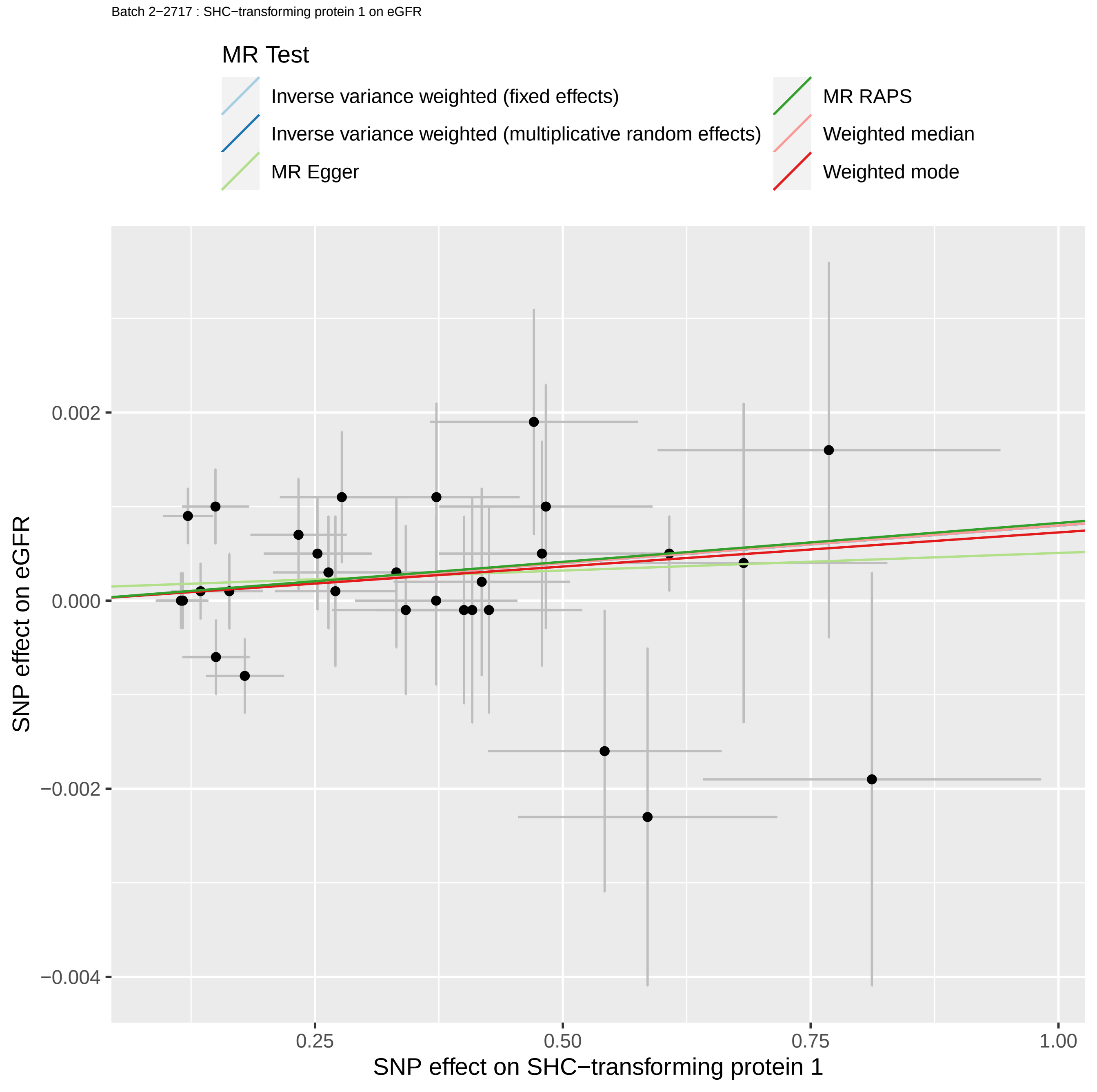
Batch 2−2717 : SHC−transforming protein 1 on eGFR
MR Test
Inverse variance weighted (fixed effects)
Inverse variance weighted (multiplicative random effects)
MR Egger
MR RAPS
Weighted median
Weighted mode
0.002
0.000
−0.002
−0.004
0.25
0.50
0.75
1.00
SNP effect on eGFR
SNP effect on SHC−transforming protein 1

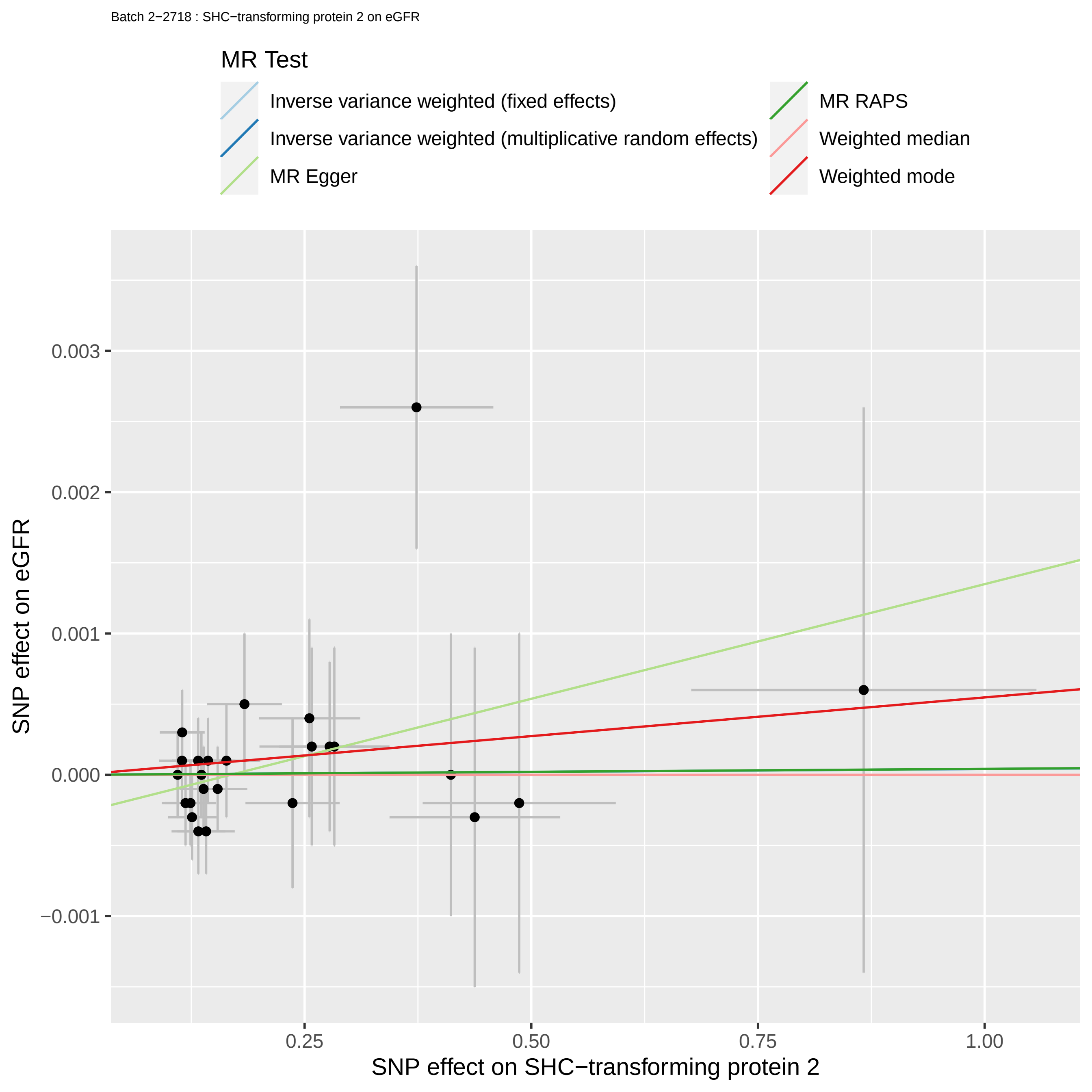

Batch 2−2718 : SHC−transforming protein 2 on eGFR
MR Test
Inverse variance weighted (fixed effects)
Inverse variance weighted (multiplicative random effects)
MR Egger
MR RAPS
Weighted median
Weighted mode
0.003
0.002
0.001
0.000
−0.001
0.25
0.50
0.75
1.00
SNP effect on eGFR
SNP effect on SHC−transforming protein 2

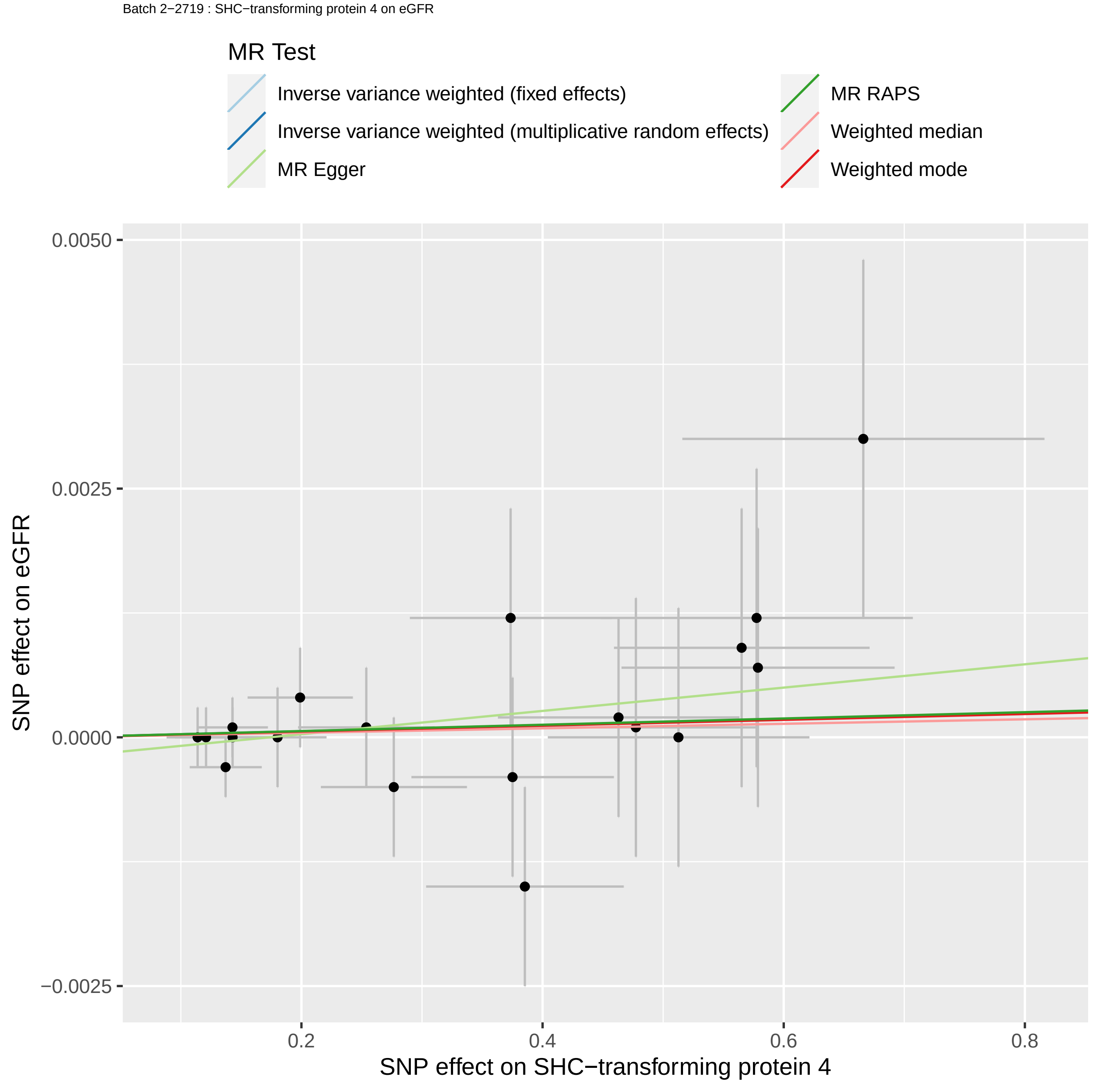
Batch 2−2719 : SHC−transforming protein 4 on eGFR
MR Test
Inverse variance weighted (fixed effects)
Inverse variance weighted (multiplicative random effects)
MR Egger
MR RAPS
Weighted median
Weighted mode
0.0050
0.0025
0.0000
−0.0025
0.2
0.4
0.6
0.8
SNP effect on eGFR
SNP effect on SHC−transforming protein 4

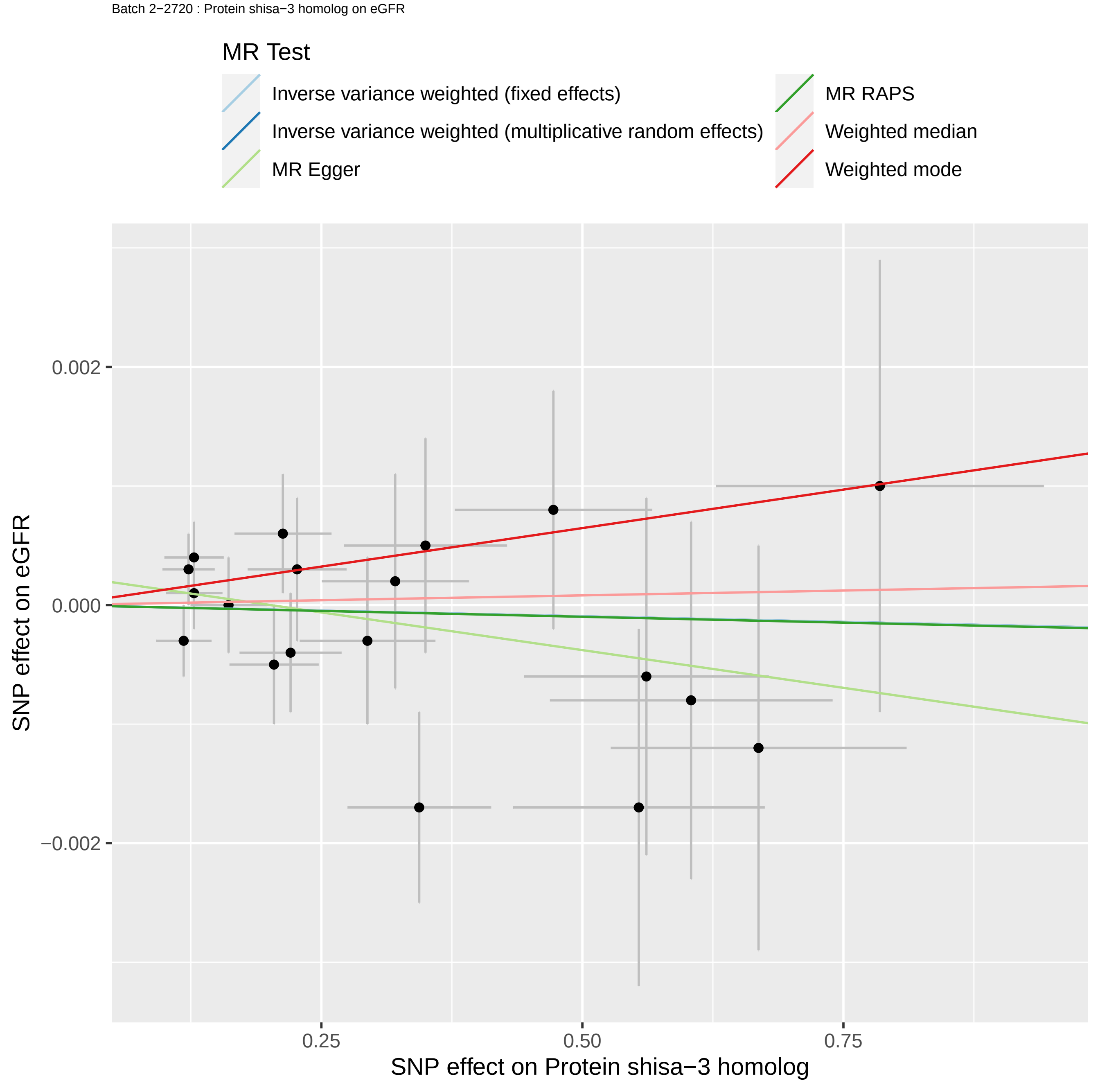
Batch 2−2720 : Protein shisa−3 homolog on eGFR
MR Test
Inverse variance weighted (fixed effects)
Inverse variance weighted (multiplicative random effects)
MR Egger
MR RAPS
Weighted median
Weighted mode
0.002
0.000
−0.002
0.25
0.50
0.75
SNP effect on eGFR
SNP effect on Protein shisa−3 homolog

Batch 2−2721 : Sialate O−acetylesterase on eGFR

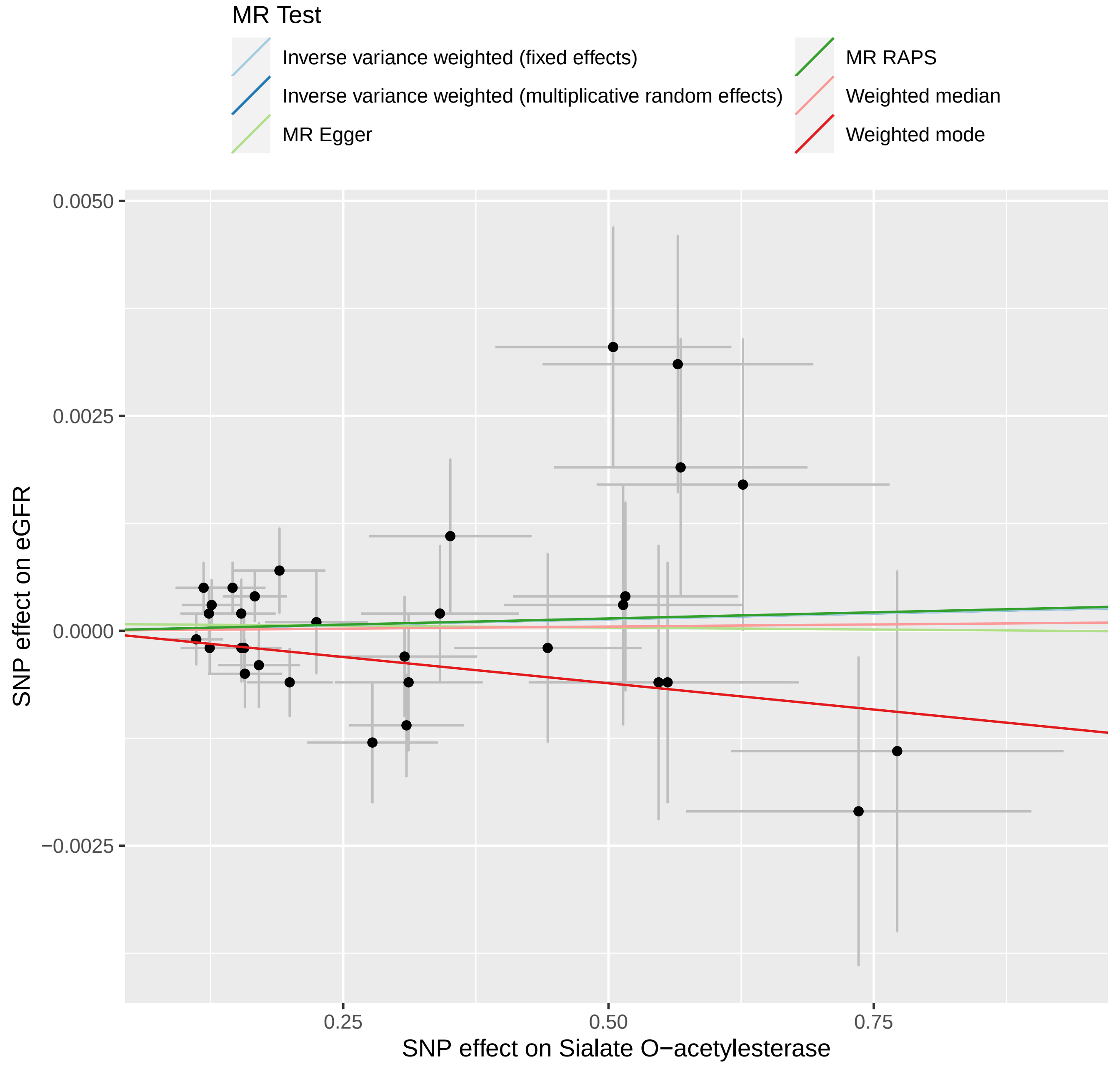

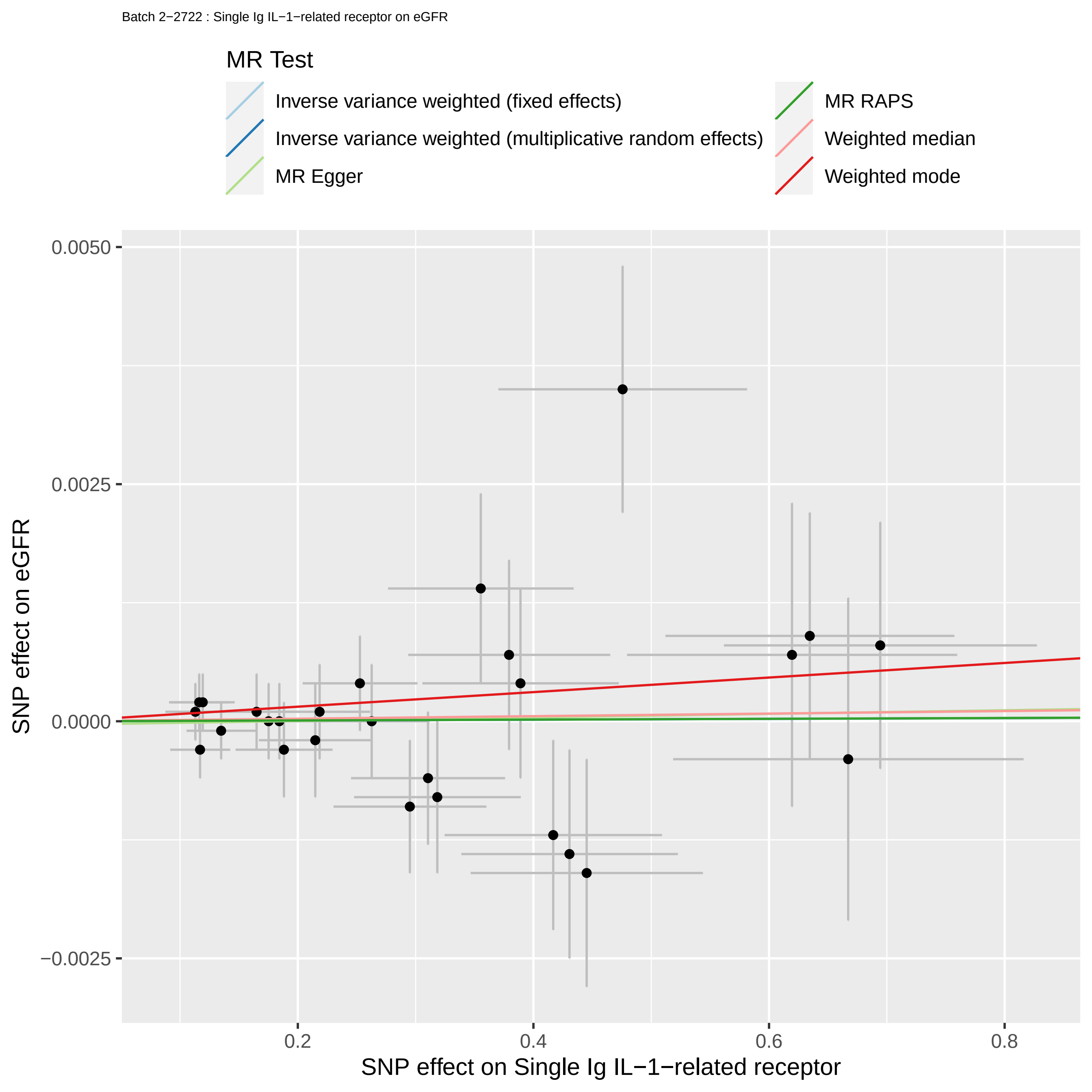

Batch 2−2722 : Single Ig IL−1−related receptor on eGFR
MR Test
Inverse variance weighted (fixed effects)
Inverse variance weighted (multiplicative random effects)
MR Egger
MR RAPS
Weighted median
Weighted mode
0.0050
0.0025
0.0000
−0.0025
0.2
0.4
0.6
0.8
SNP effect on eGFR
SNP effect on Single Ig IL−1−related receptor

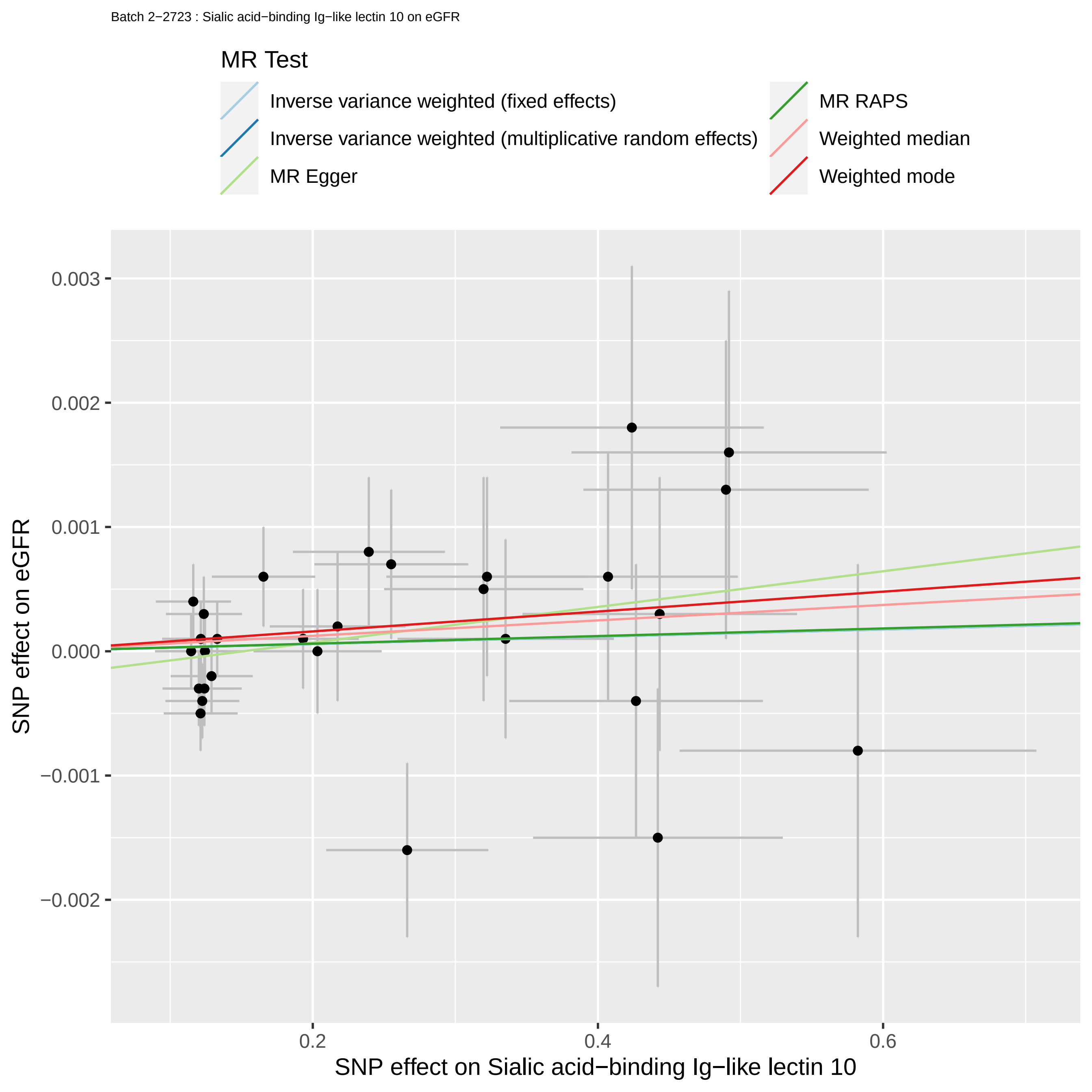

Batch 2−2723 : Sialic acid−binding Ig−like lectin 10 on eGFR
MR Test
Inverse variance weighted (fixed effects)
Inverse variance weighted (multiplicative random effects)
MR Egger
MR RAPS
Weighted median
Weighted mode
0.003
0.002
0.001
0.000
−0.001
−0.002
0.2
0.4
0.6
SNP effect on eGFR
SNP effect on Sialic acid−binding Ig−like lectin 10

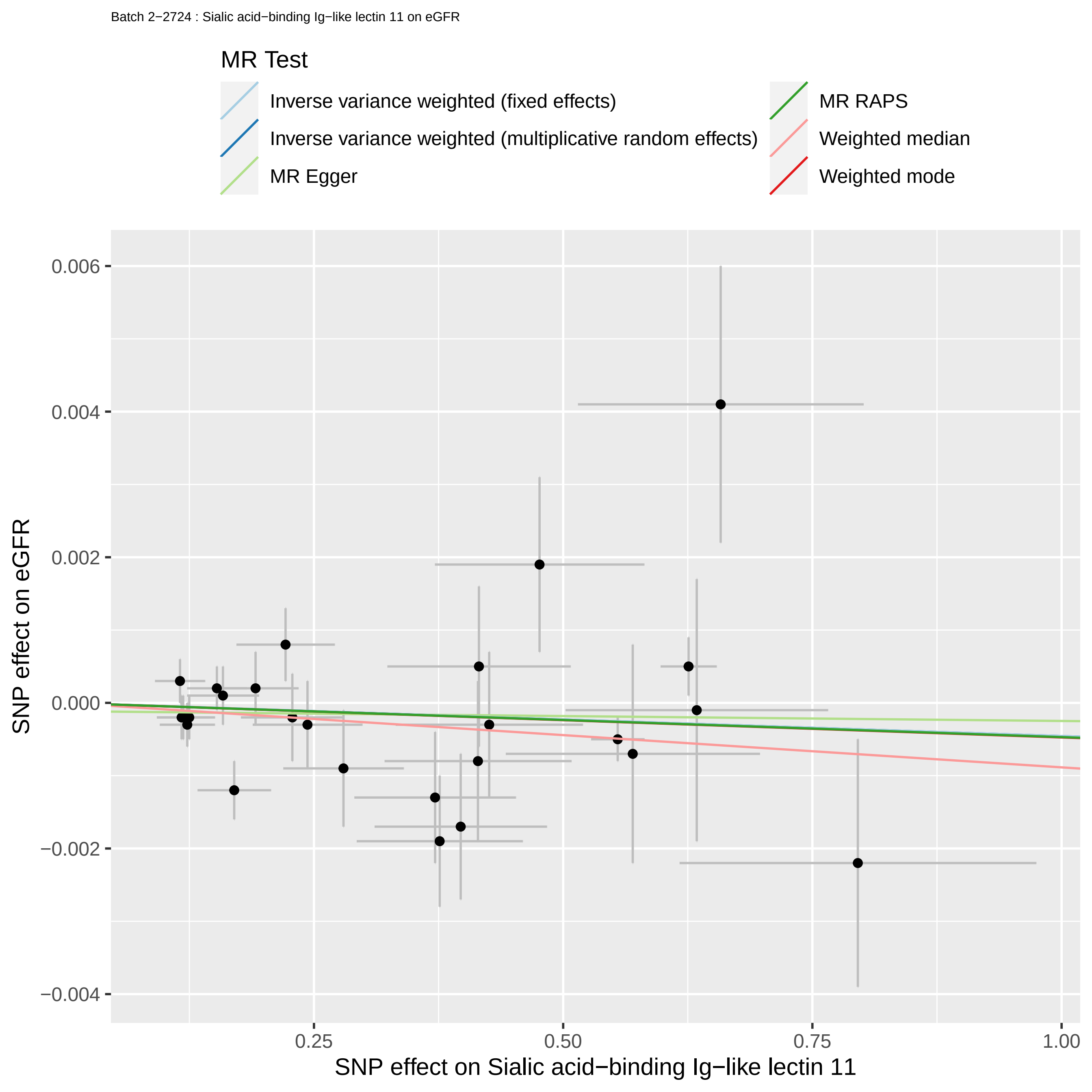

Batch 2−2724 : Sialic acid−binding Ig−like lectin 11 on eGFR
MR Test
Inverse variance weighted (fixed effects)
Inverse variance weighted (multiplicative random effects)
MR Egger
MR RAPS
Weighted median
Weighted mode
0.006
0.004
0.002
0.000
−0.002
−0.004
0.25
0.50
0.75
1.00
SNP effect on eGFR
SNP effect on Sialic acid−binding Ig−like lectin 11

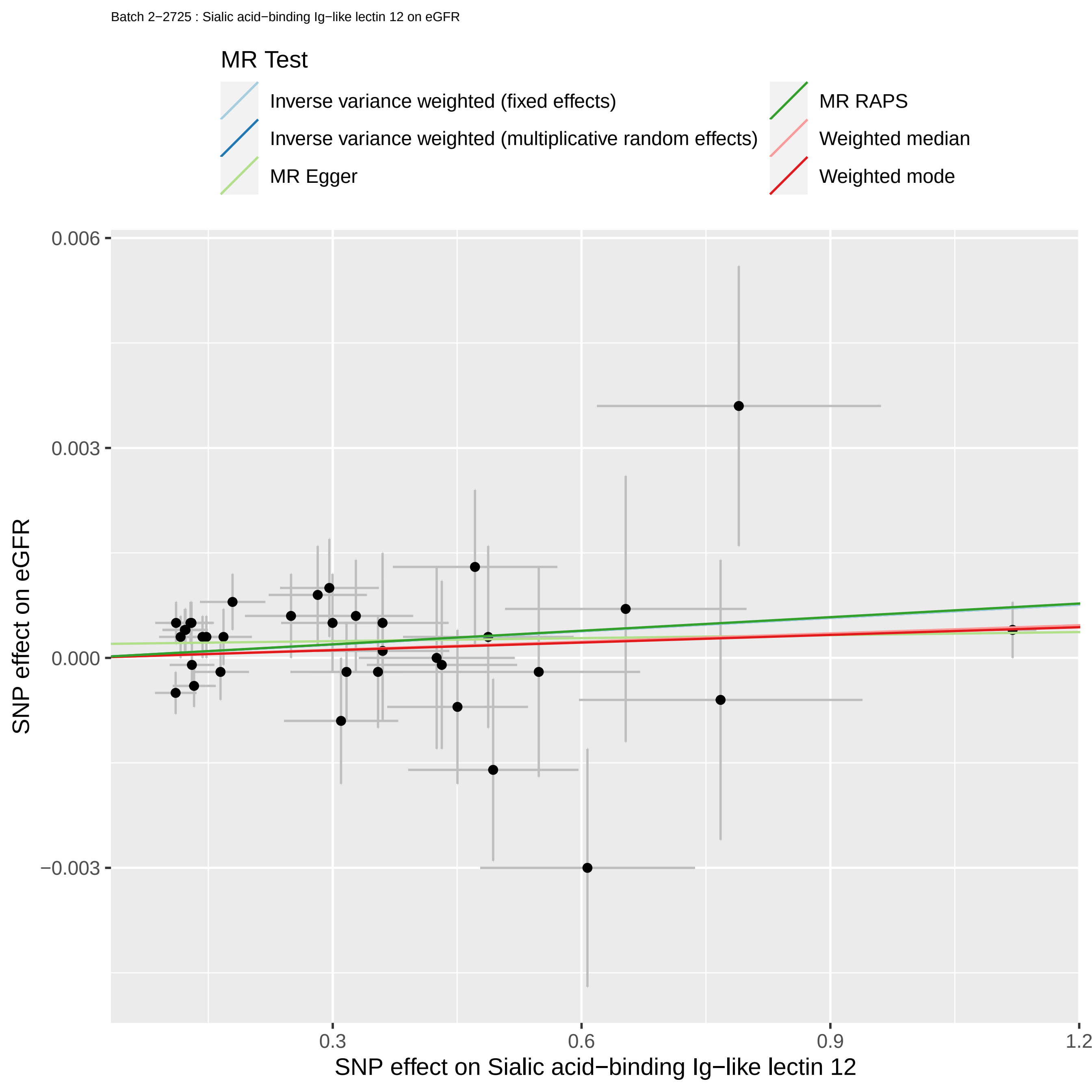
Batch 2−2725 : Sialic acid−binding Ig−like lectin 12 on eGFR
MR Test
Inverse variance weighted (fixed effects)
Inverse variance weighted (multiplicative random effects)
MR Egger
MR RAPS
Weighted median
Weighted mode
0.006
0.003
0.000
−0.003
0.3
0.6
0.9
1.2
SNP effect on eGFR
SNP effect on Sialic acid−binding Ig−like lectin 12

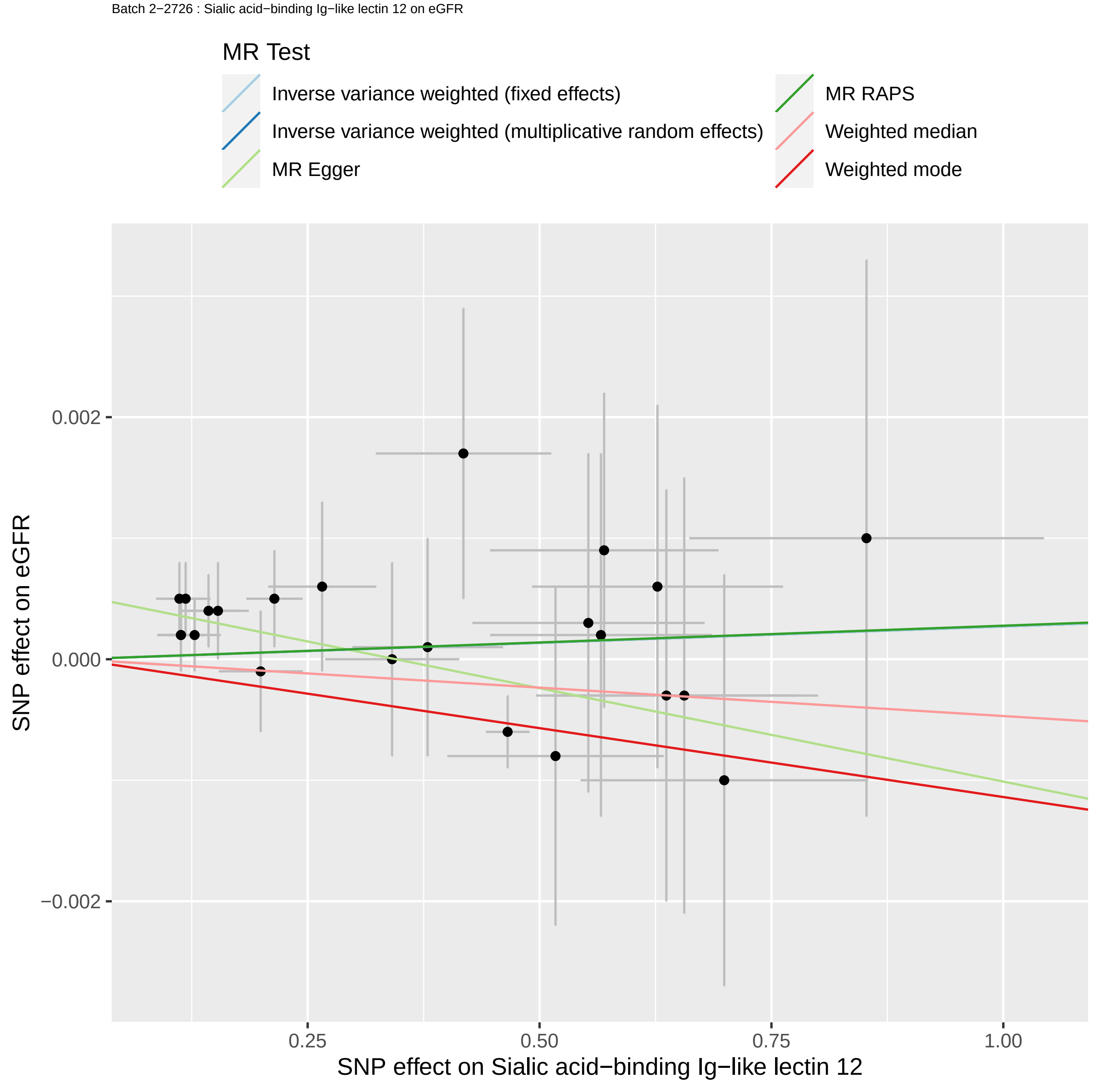

Batch 2−2726 : Sialic acid−binding Ig−like lectin 12 on eGFR
MR Test
Inverse variance weighted (fixed effects)
Inverse variance weighted (multiplicative random effects)
MR Egger
MR RAPS
Weighted median
Weighted mode
0.002
0.000
−0.002
0.25
0.50
0.75
1.00
SNP effect on eGFR
SNP effect on Sialic acid−binding Ig−like lectin 12

Batch 2−2727 : Sialic acid−binding Ig−like lectin 14 on eGFR

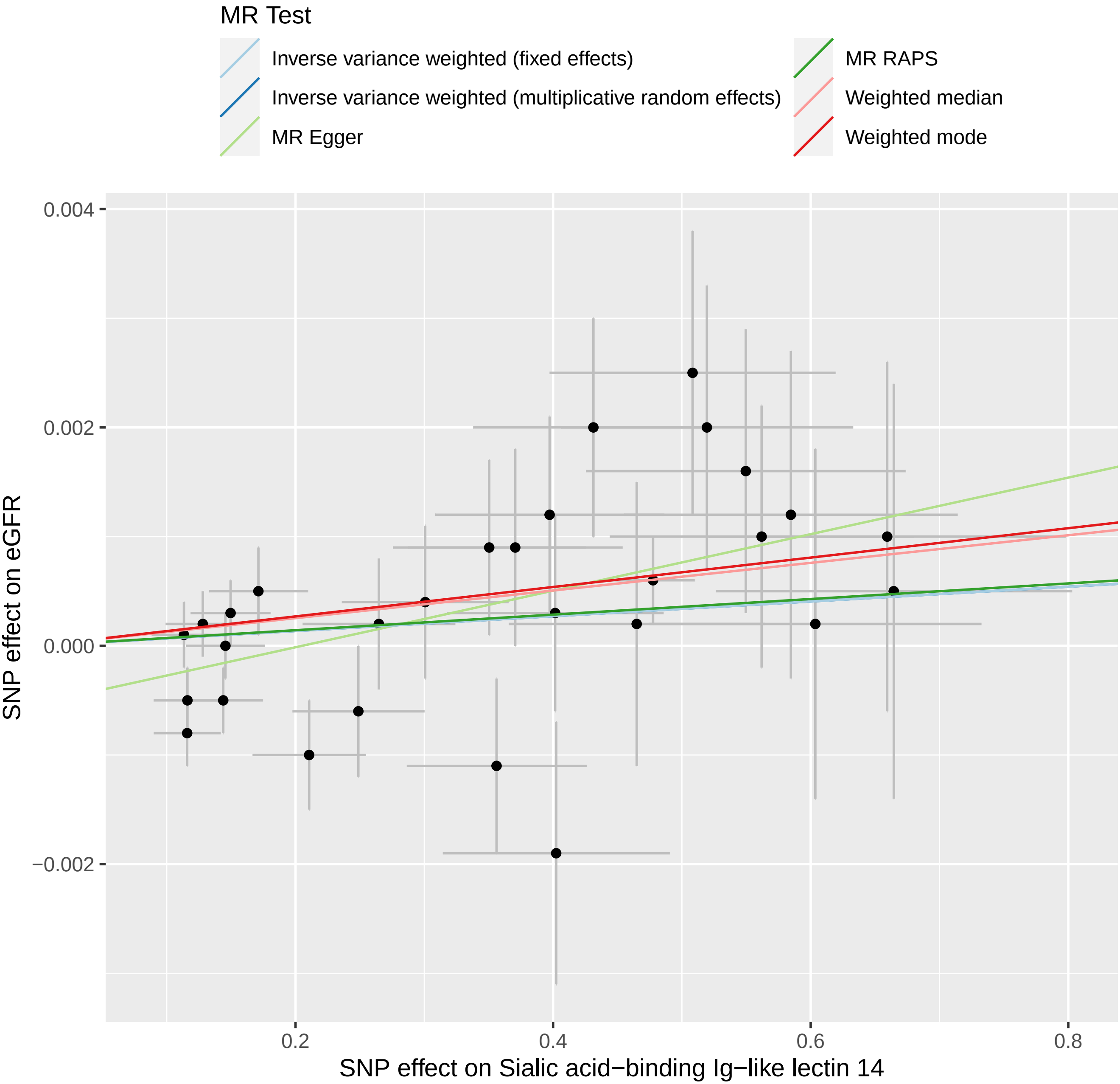

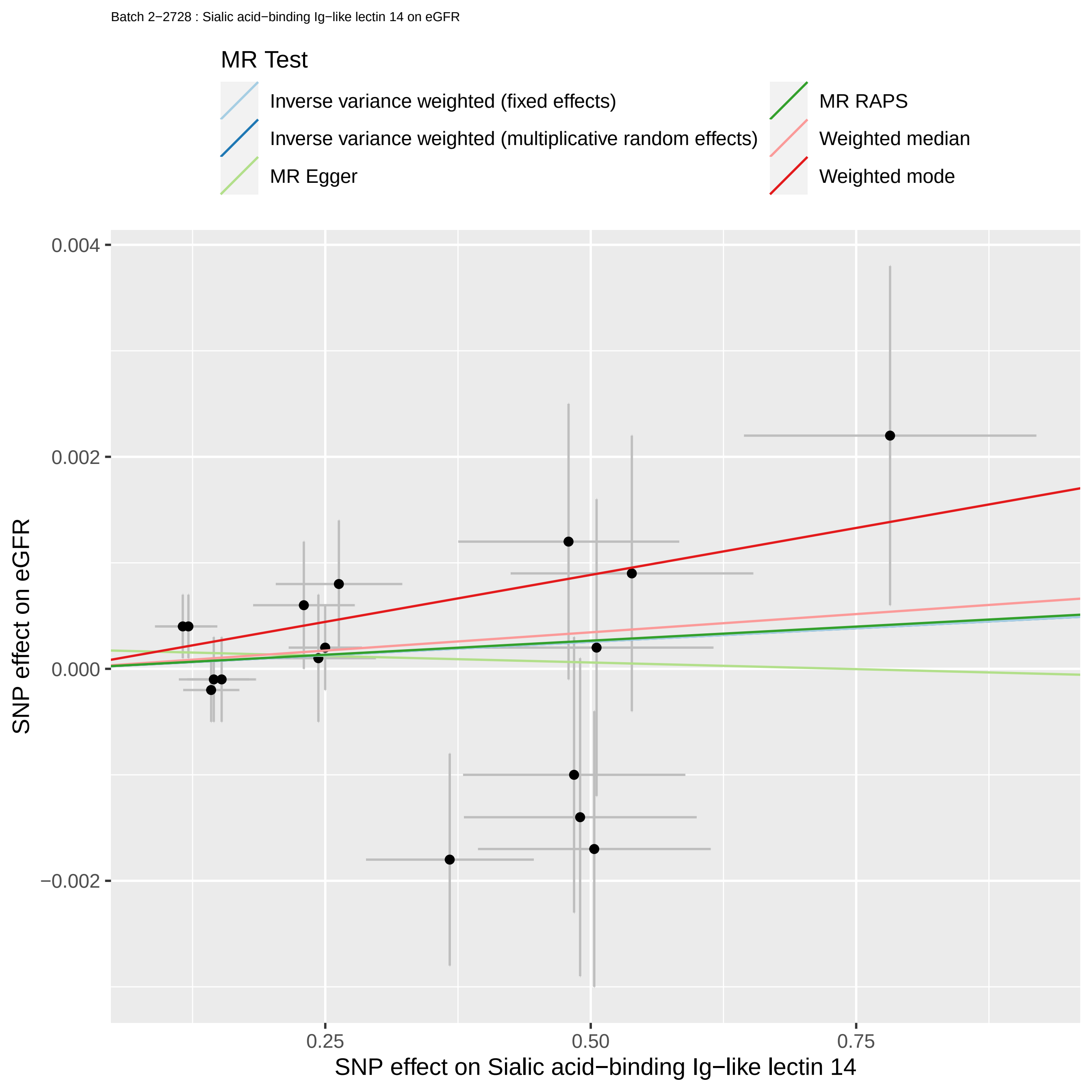

Batch 2−2728 : Sialic acid−binding Ig−like lectin 14 on eGFR
MR Test
Inverse variance weighted (fixed effects)
Inverse variance weighted (multiplicative random effects)
MR Egger
MR RAPS
Weighted median
Weighted mode
0.004
0.002
0.000
−0.002
0.25
0.50
0.75
SNP effect on eGFR
SNP effect on Sialic acid−binding Ig−like lectin 14

Batch 2−2729 : Sialic acid−binding Ig−like lectin 7 on eGFR

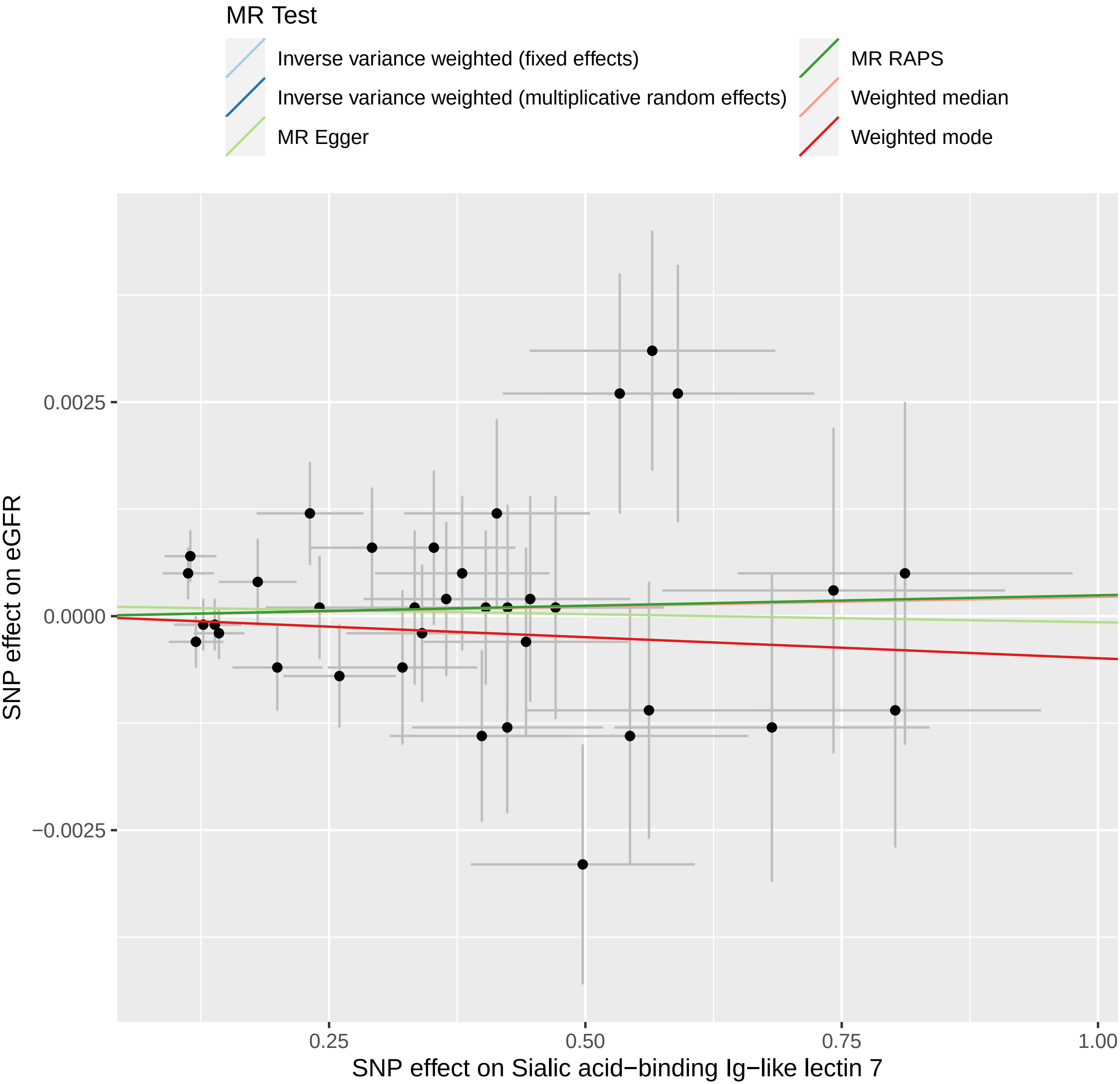

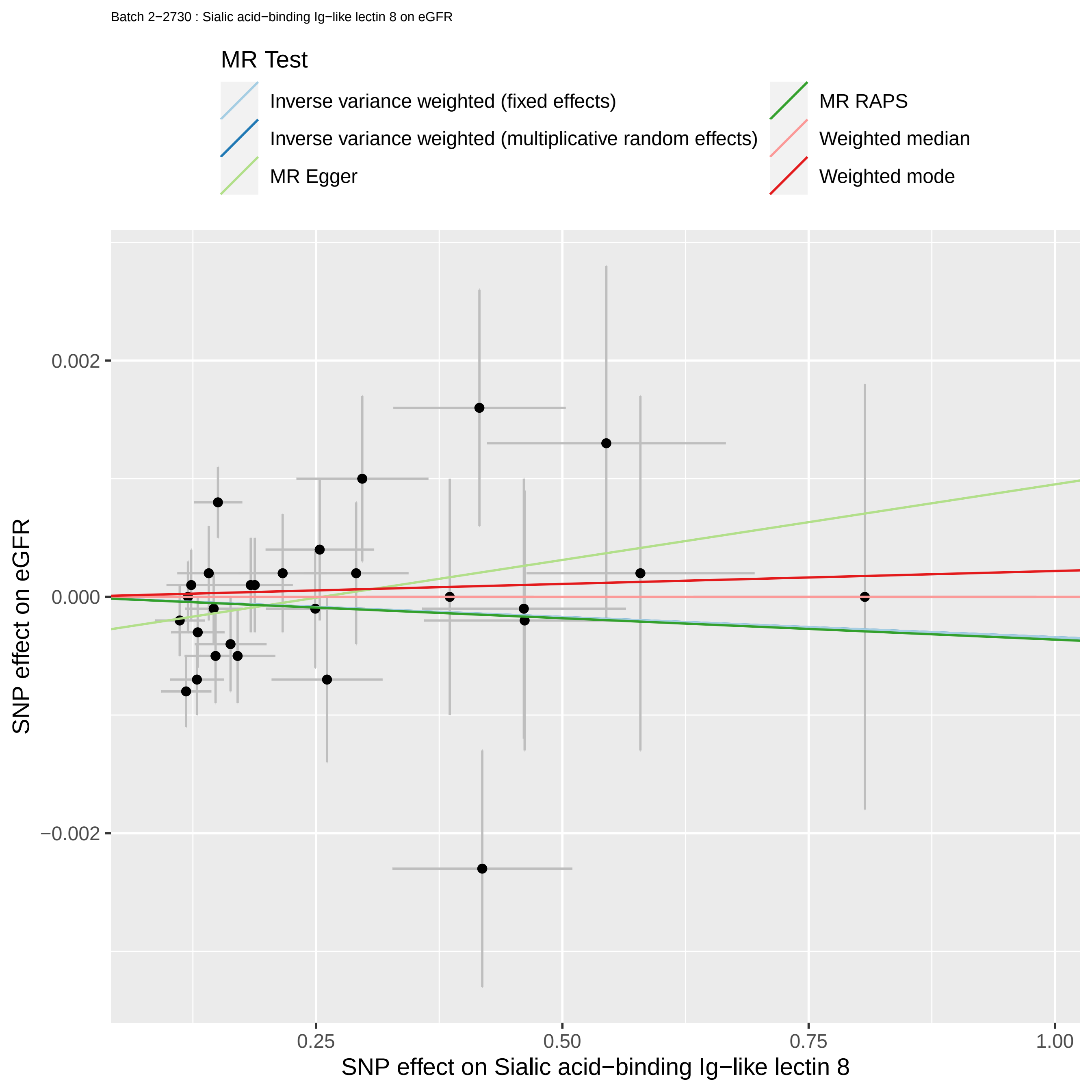

Batch 2−2730 : Sialic acid−binding Ig−like lectin 8 on eGFR
MR Test
Inverse variance weighted (fixed effects)
Inverse variance weighted (multiplicative random effects)
MR Egger
MR RAPS
Weighted median
Weighted mode
0.002
0.000
−0.002
SNP effect on eGFR
0.25
0.50
0.75
1.00
SNP effect on Sialic acid−binding Ig−like lectin 8

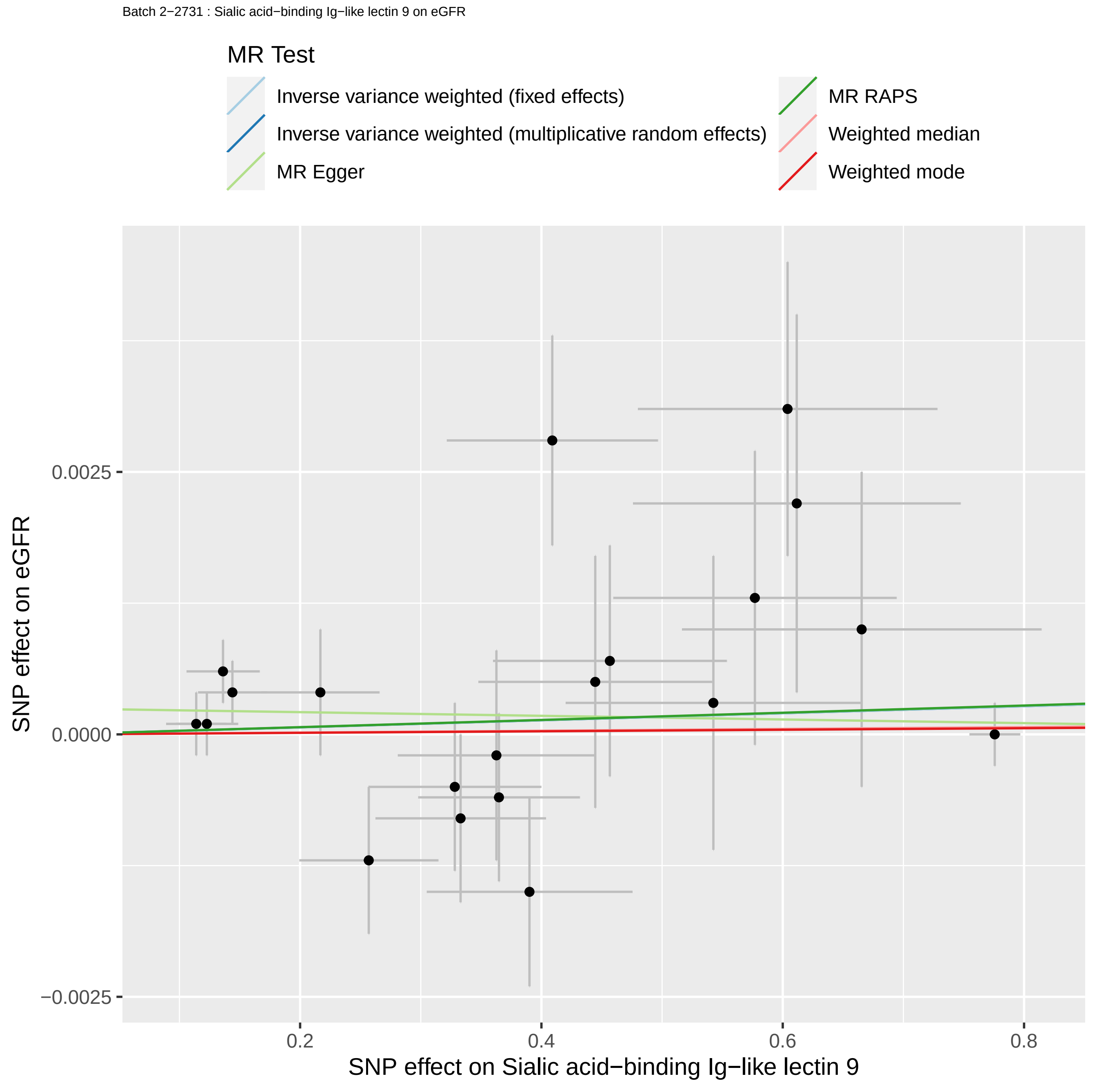
Batch 2−2731 : Sialic acid−binding Ig−like lectin 9 on eGFR
MR Test
Inverse variance weighted (fixed effects)
Inverse variance weighted (multiplicative random effects)
MR Egger
MR RAPS
Weighted median
Weighted mode
0.0025
0.0000
−0.0025
0.2
0.4
0.6
0.8
SNP effect on eGFR
SNP effect on Sialic acid−binding Ig−like lectin 9

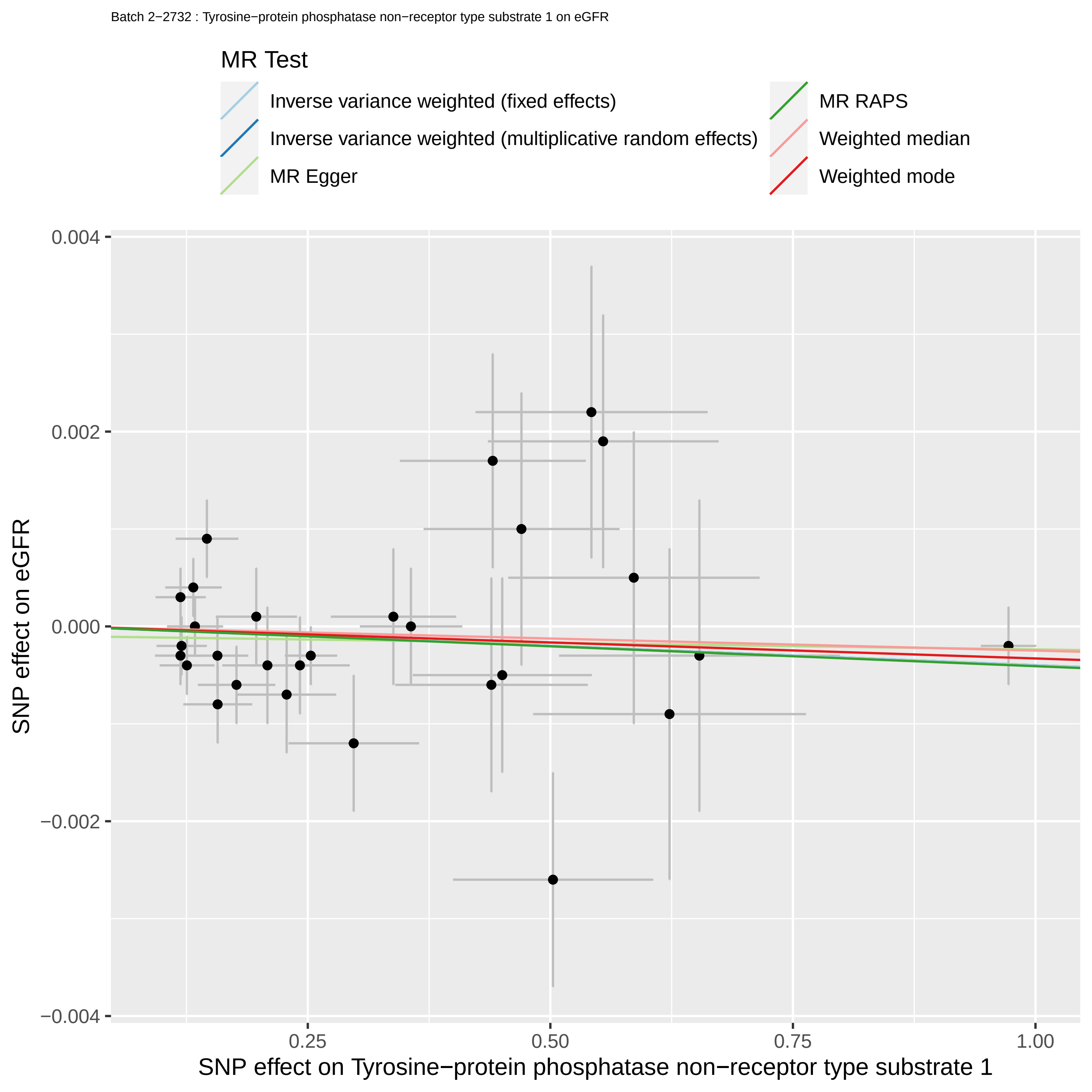
Batch 2−2732 : Tyrosine−protein phosphatase non−receptor type substrate 1 on eGFR
MR Test
Inverse variance weighted (fixed effects)
Inverse variance weighted (multiplicative random effects)
MR Egger
MR RAPS
Weighted median
Weighted mode
0.004
0.002
0.000
−0.002
−0.004
0.25
0.50
0.75
1.00
SNP effect on eGFR
SNP effect on Tyrosine−protein phosphatase non−receptor type substrate 1

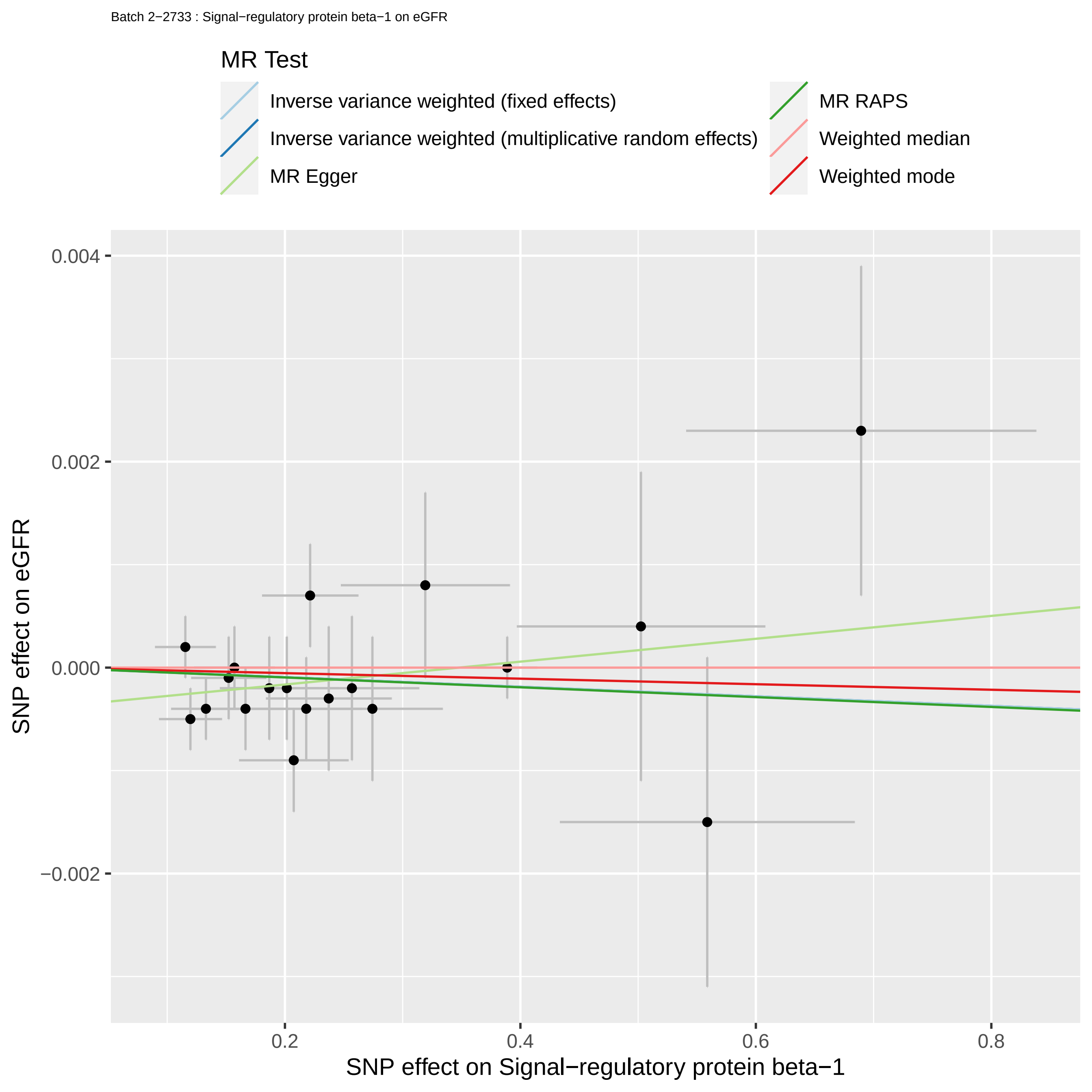
Batch 2−2733 : Signal−regulatory protein beta−1 on eGFR
MR Test
Inverse variance weighted (fixed effects)
Inverse variance weighted (multiplicative random effects)
MR Egger
MR RAPS
Weighted median
Weighted mode
0.004
0.002
0.000
−0.002
SNP effect on eGFR
0.2
0.4
0.6
0.8
SNP effect on Signal−regulatory protein beta−1

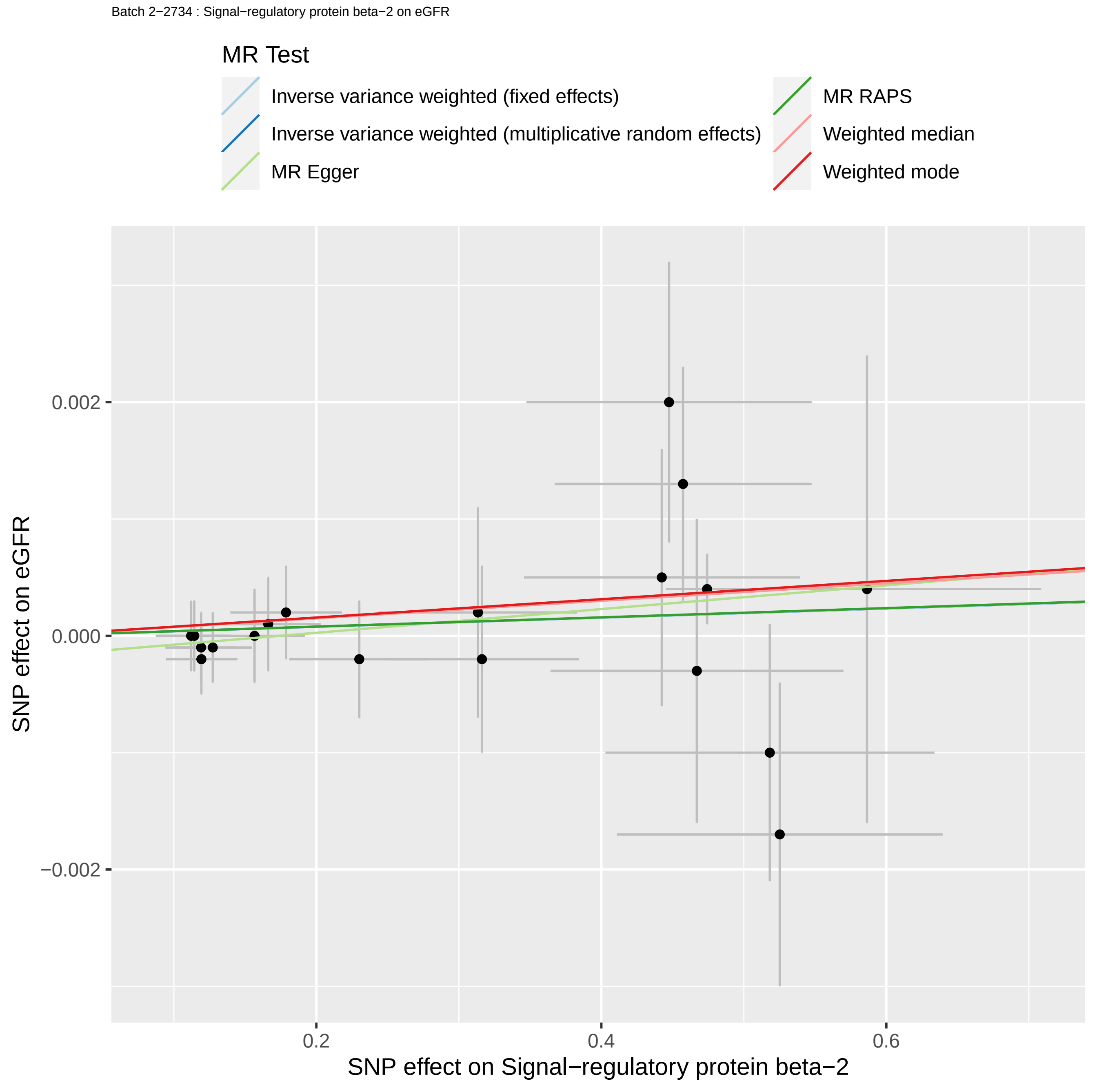
Batch 2−2734 : Signal−regulatory protein beta−2 on eGFR
MR Test
Inverse variance weighted (fixed effects)
Inverse variance weighted (multiplicative random effects)
MR Egger
MR RAPS
Weighted median
Weighted mode
0.002
0.000
−0.002
0.2
0.4
0.6
SNP effect on eGFR
SNP effect on Signal−regulatory protein beta−2

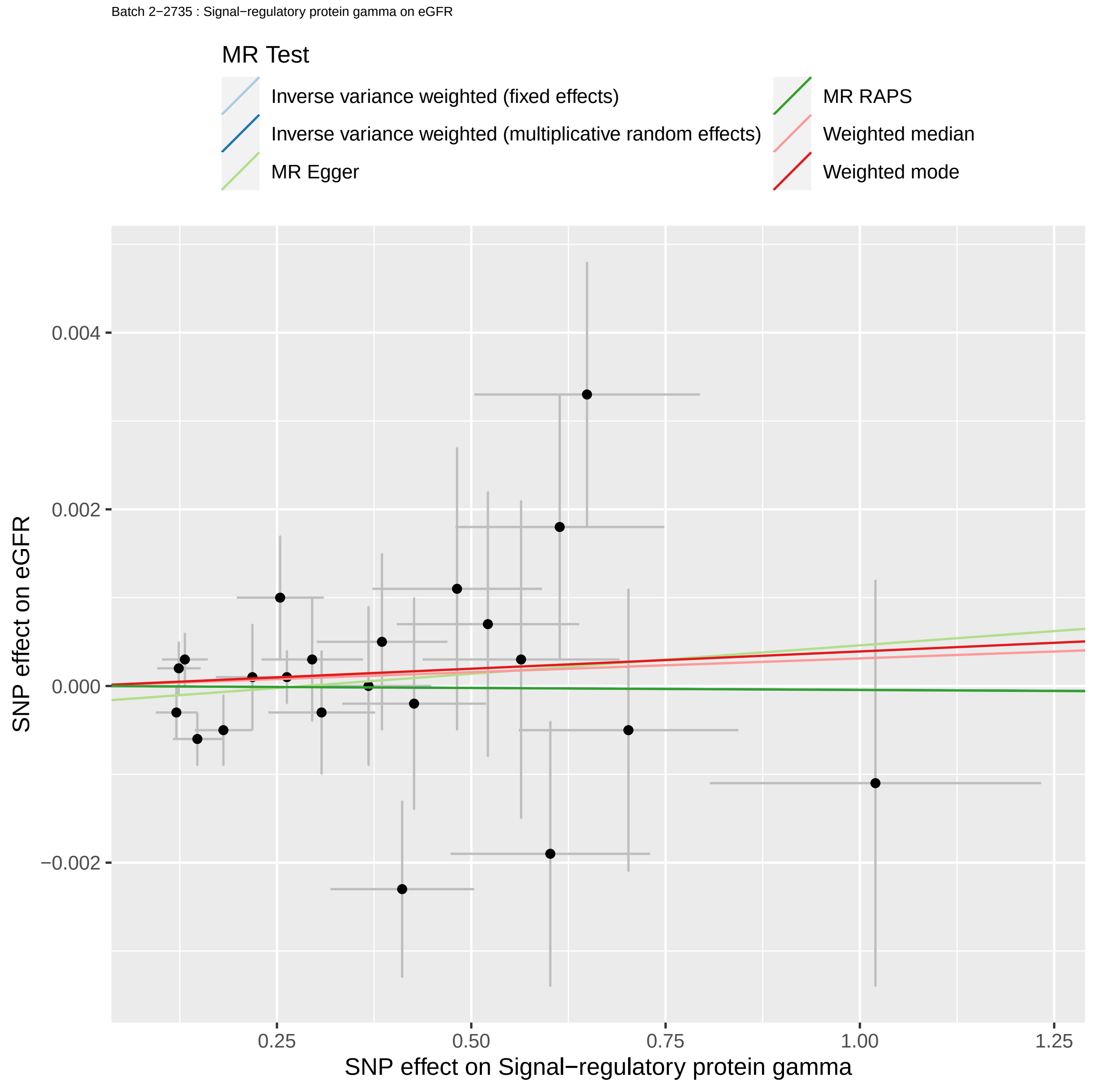

Batch 2−2735 : Signal−regulatory protein gamma on eGFR
MR Test
Inverse variance weighted (fixed effects)
Inverse variance weighted (multiplicative random effects)
MR Egger
MR RAPS
Weighted median
Weighted mode
0.004
0.002
0.000
−0.002
SNP effect on eGFR
0.25
0.50
0.75
1.00
1.25
SNP effect on Signal−regulatory protein gamma

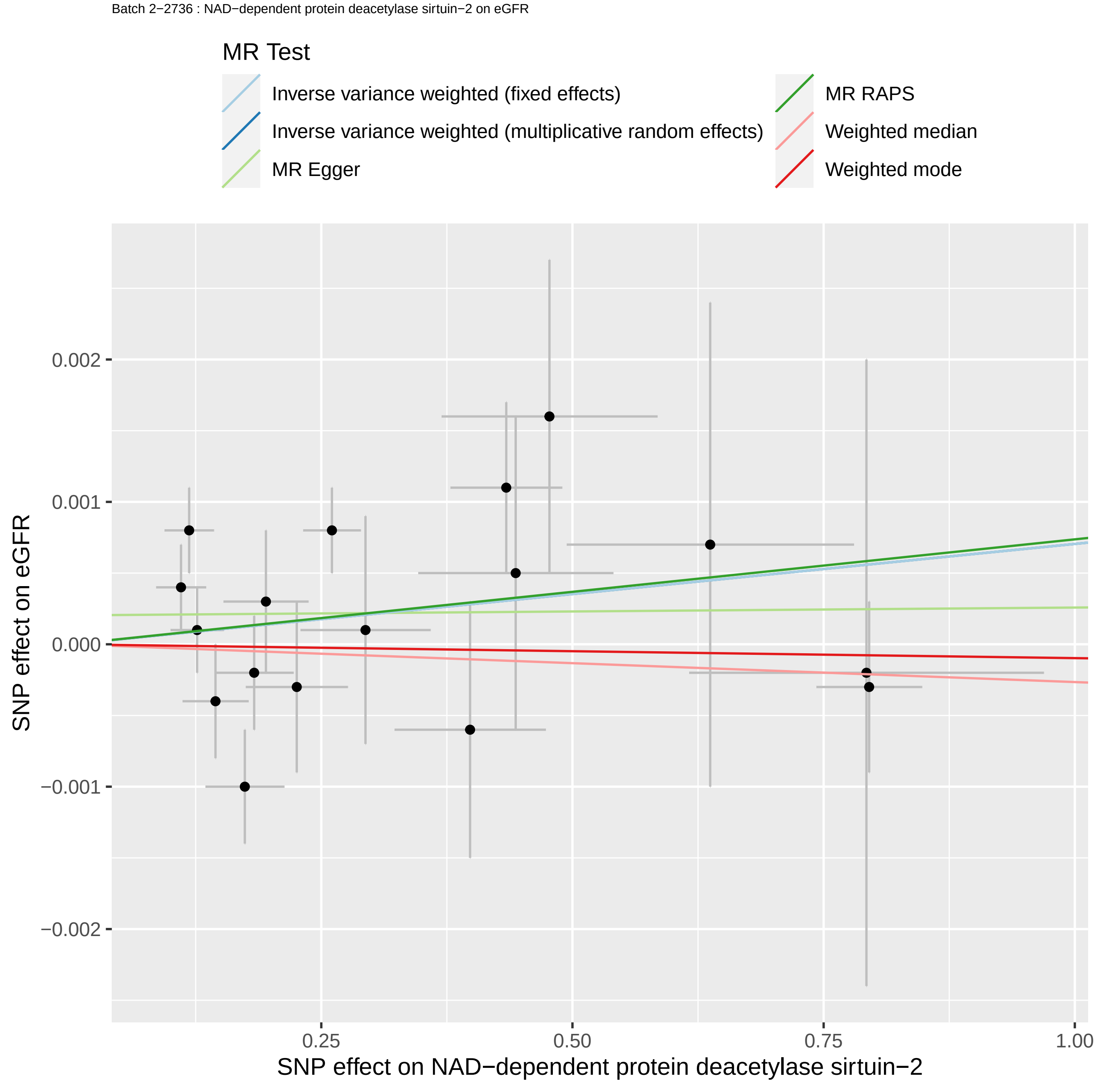

Batch 2−2736 : NAD−dependent protein deacetylase sirtuin−2 on eGFR
MR Test
Inverse variance weighted (fixed effects)
Inverse variance weighted (multiplicative random effects)
MR Egger
MR RAPS
Weighted median
Weighted mode
0.002
0.001
0.000
−0.001
−0.002
0.25
0.50
0.75
1.00
SNP effect on eGFR
SNP effect on NAD−dependent protein deacetylase sirtuin−2

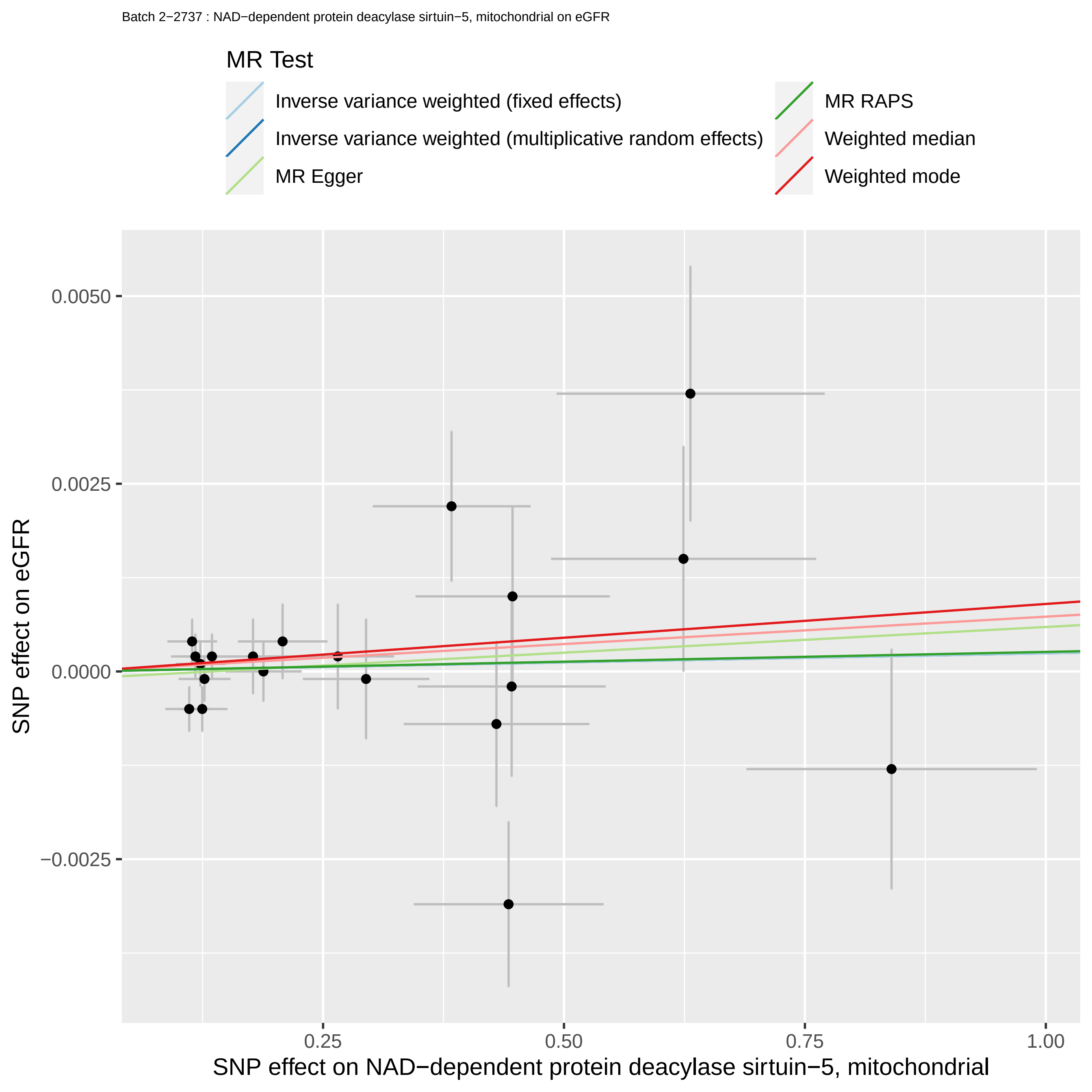

Batch 2−2737 : NAD−dependent protein deacylase sirtuin−5, mitochondrial on eGFR
MR Test
Inverse variance weighted (fixed effects)
Inverse variance weighted (multiplicative random effects)
MR Egger
MR RAPS
Weighted median
Weighted mode
0.0050
0.0025
0.0000
−0.0025
0.25
0.50
0.75
1.00
SNP effect on eGFR
SNP effect on NAD−dependent protein deacylase sirtuin−5, mitochondrial

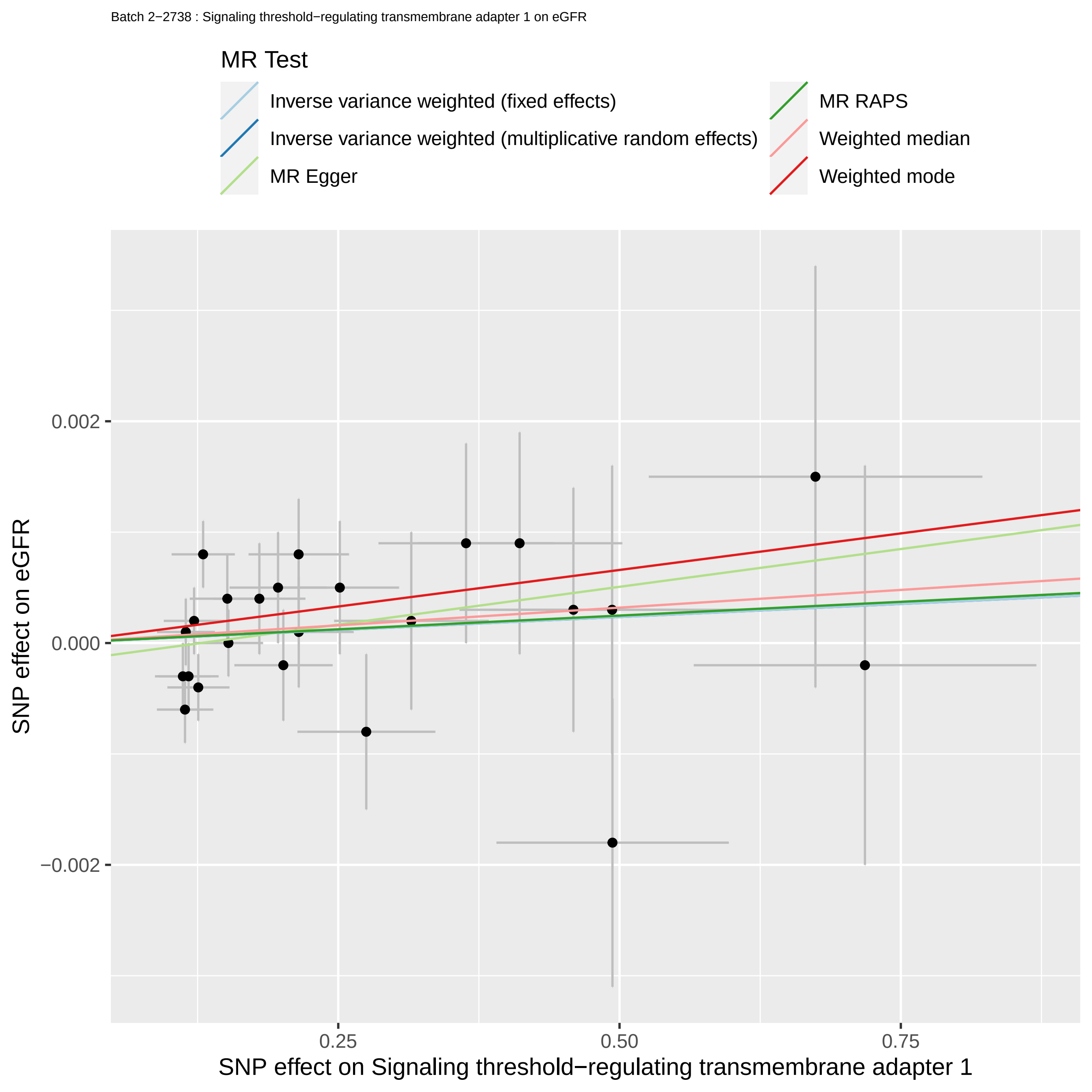
Batch 2−2738 : Signaling threshold−regulating transmembrane adapter 1 on eGFR
MR Test
Inverse variance weighted (fixed effects)
Inverse variance weighted (multiplicative random effects)
MR Egger
MR RAPS
Weighted median
Weighted mode
0.002
0.000
−0.002
SNP effect on eGFR
0.25
0.50
0.75
SNP effect on Signaling threshold−regulating transmembrane adapter 1

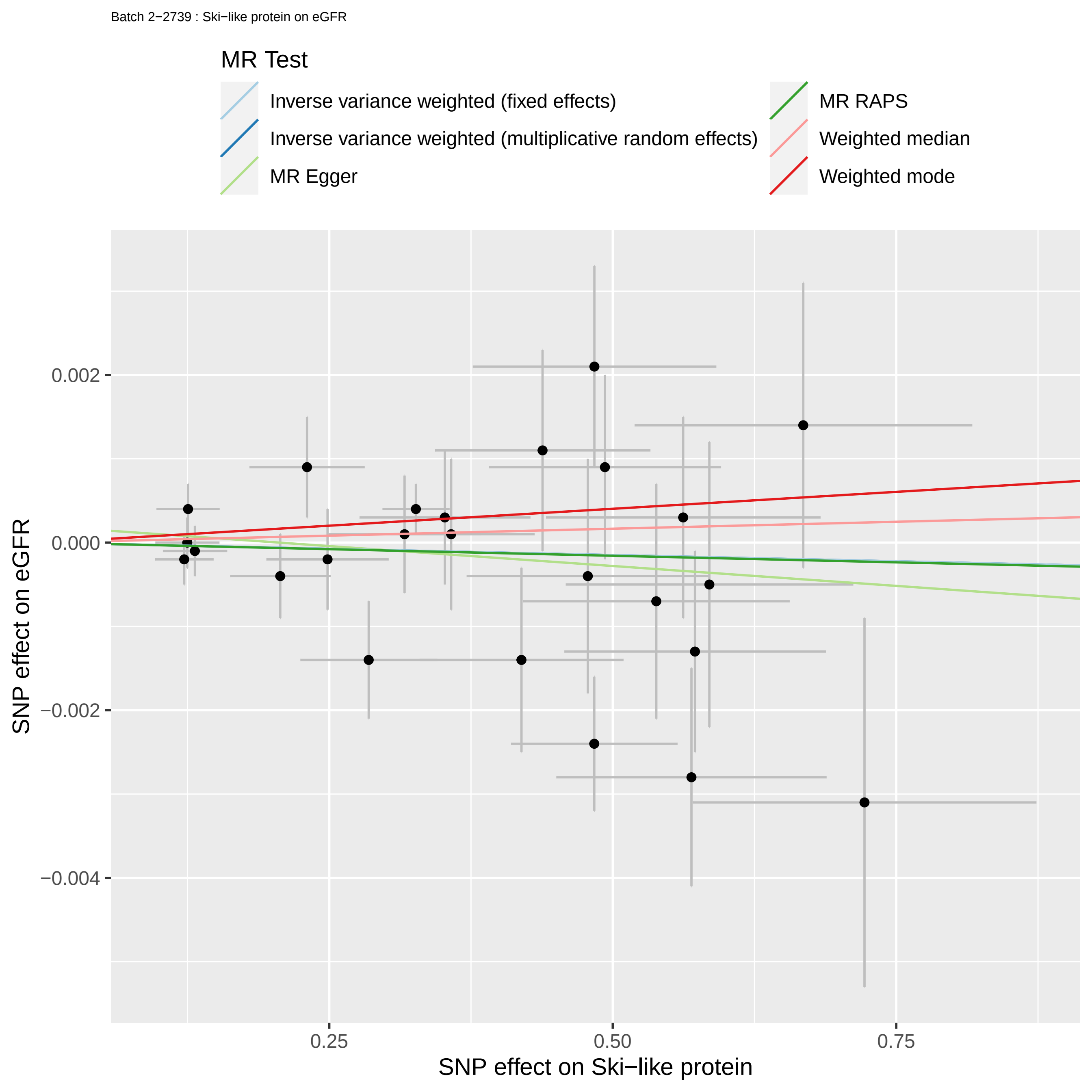

Batch 2−2739 : Ski−like protein on eGFR
MR Test
Inverse variance weighted (fixed effects)
Inverse variance weighted (multiplicative random effects)
MR Egger
MR RAPS
Weighted median
Weighted mode
0.002
0.000
−0.002
−0.004
0.25
0.50
0.75
SNP effect on eGFR
SNP effect on Ski−like protein

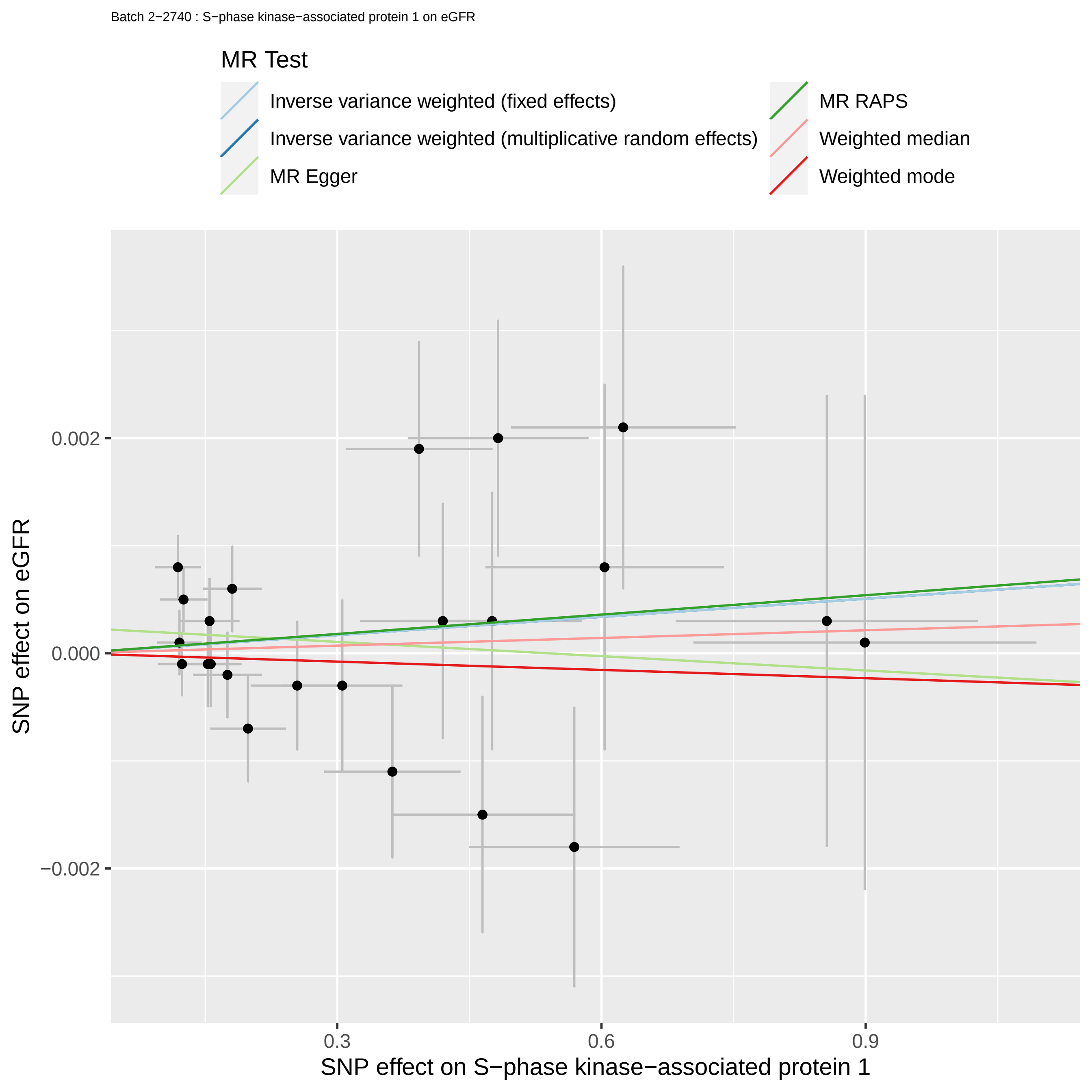

Batch 2−2740 : S−phase kinase−associated protein 1 on eGFR
MR Test
Inverse variance weighted (fixed effects)
Inverse variance weighted (multiplicative random effects)
MR Egger
MR RAPS
Weighted median
Weighted mode
0.002
0.000
−0.002
SNP effect on eGFR
0.3
0.6
0.9
SNP effect on S−phase kinase−associated protein 1

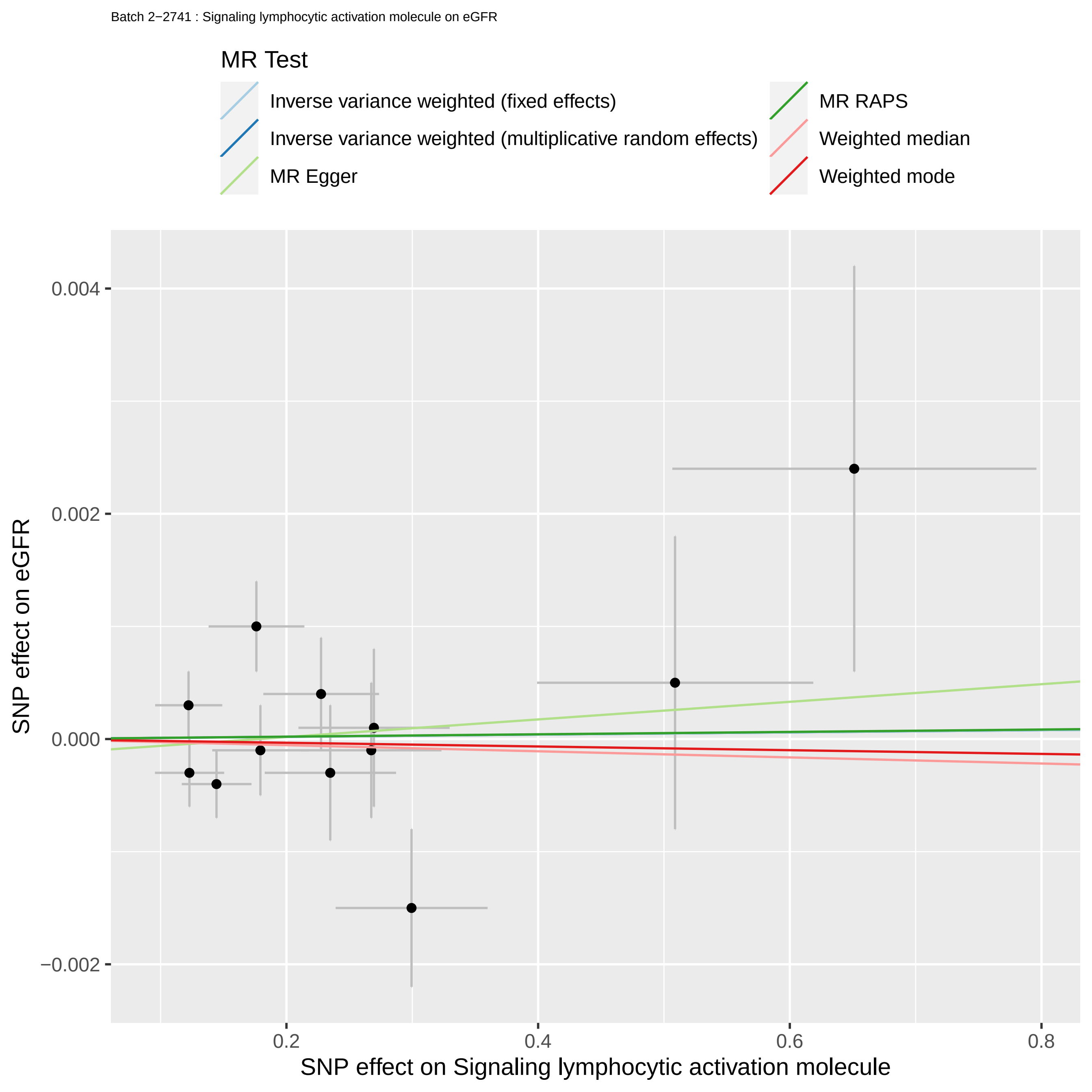
Batch 2−2741 : Signaling lymphocytic activation molecule on eGFR
MR Test
Inverse variance weighted (fixed effects)
Inverse variance weighted (multiplicative random effects)
MR Egger
MR RAPS
Weighted median
Weighted mode
0.004
0.002
0.000
−0.002
0.2
0.4
0.6
0.8
SNP effect on eGFR
SNP effect on Signaling lymphocytic activation molecule

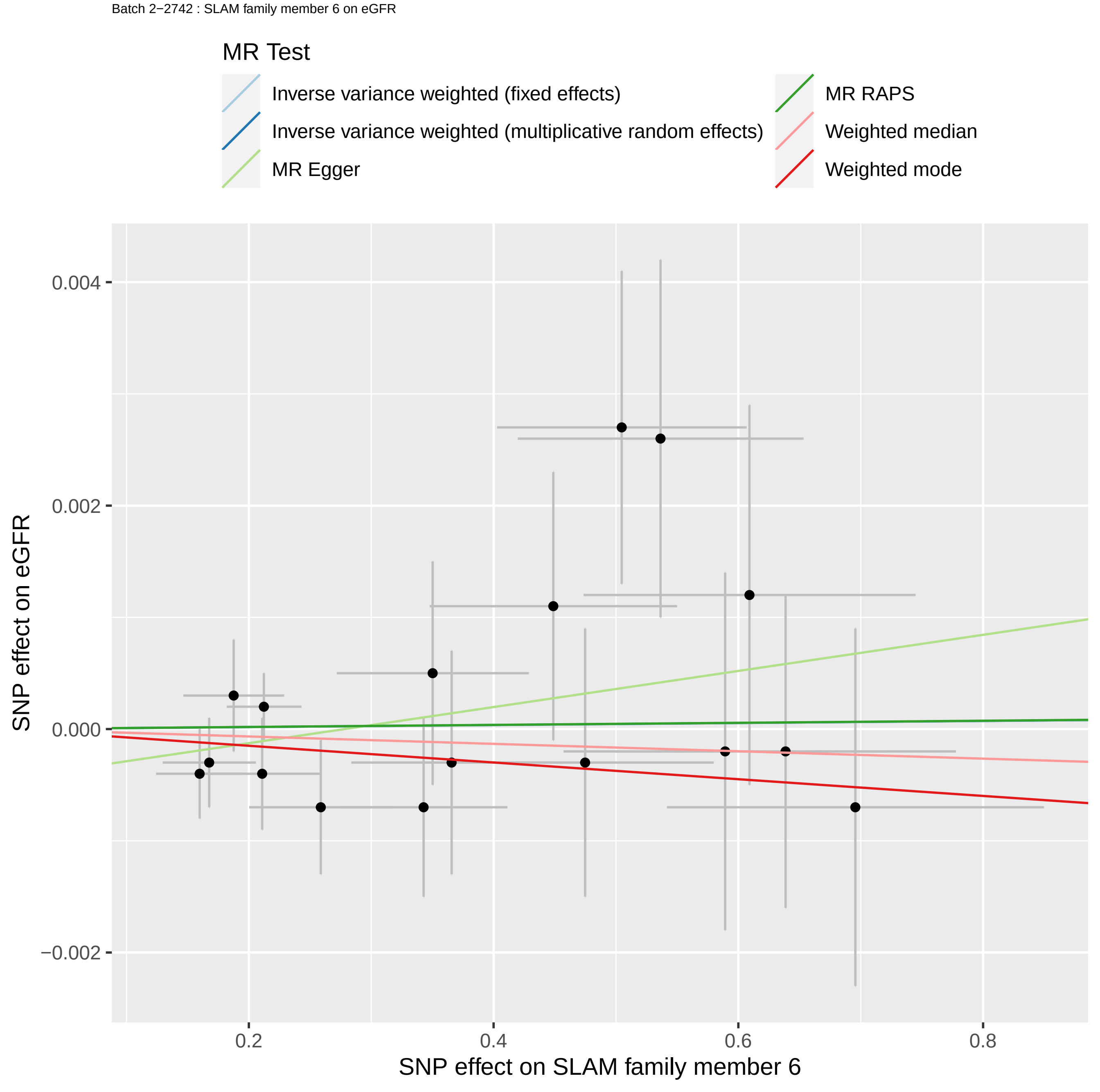
Batch 2−2742 : SLAM family member 6 on eGFR
MR Test
Inverse variance weighted (fixed effects)
Inverse variance weighted (multiplicative random effects)
MR Egger
MR RAPS
Weighted median
Weighted mode
0.004
0.002
0.000
−0.002
0.2
0.4
0.6
0.8
SNP effect on eGFR
SNP effect on SLAM family member 6

Batch 2−2743 : SLAM family member 7 on eGFR

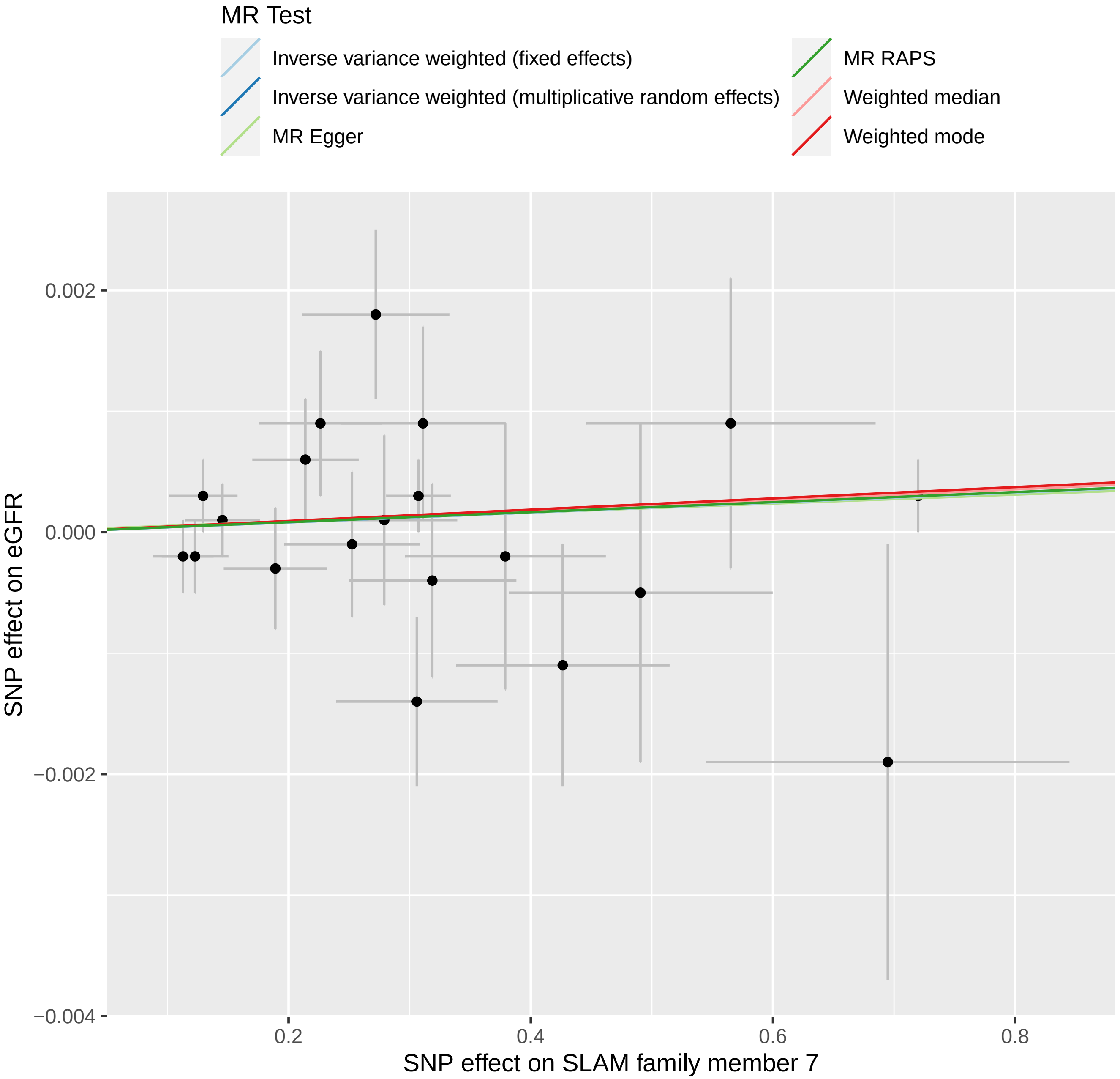

Batch 2−2744 : SLAM family member 7 on eGFR

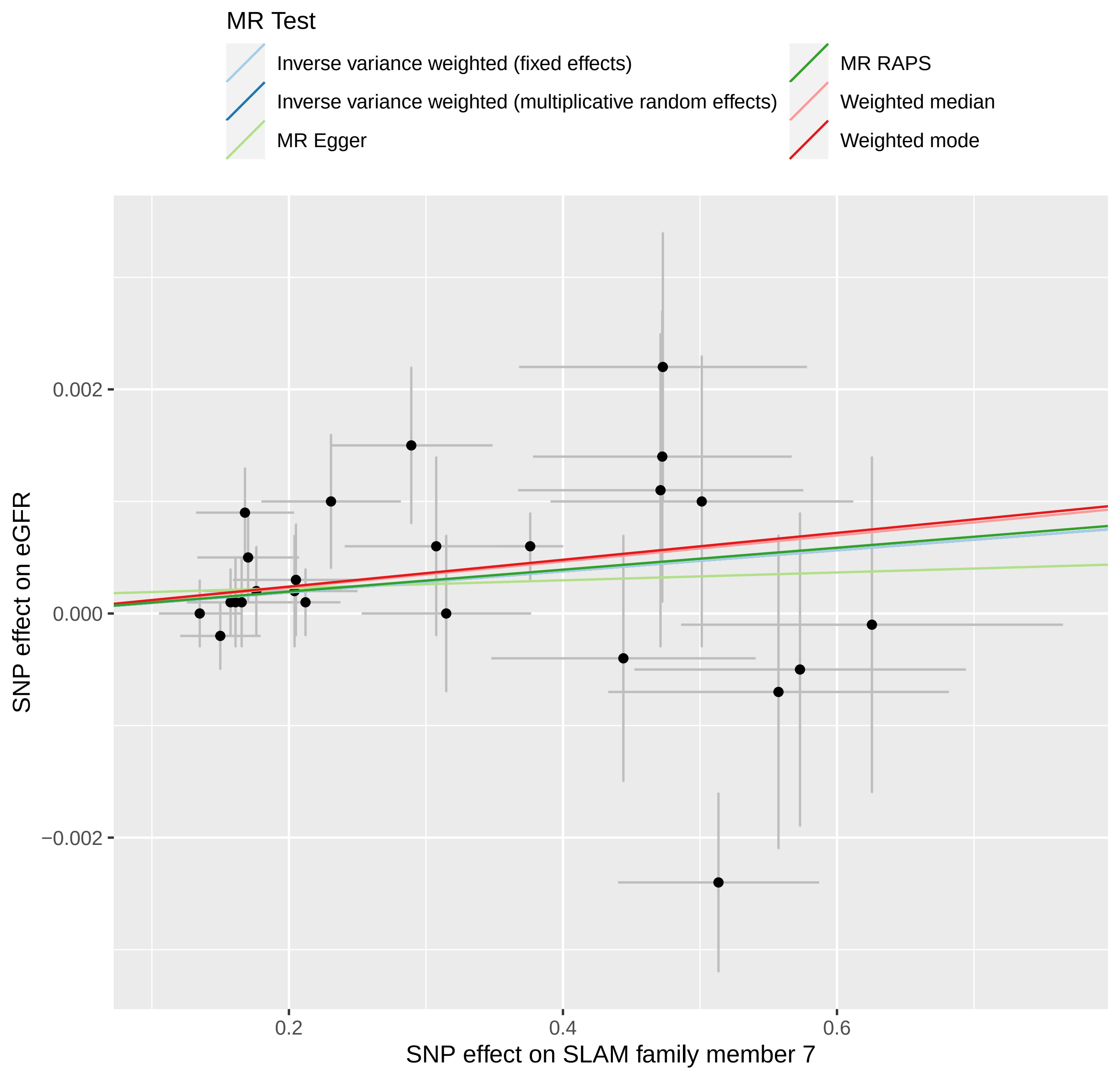

Batch 2−2745 : SLAM family member 8 on eGFR

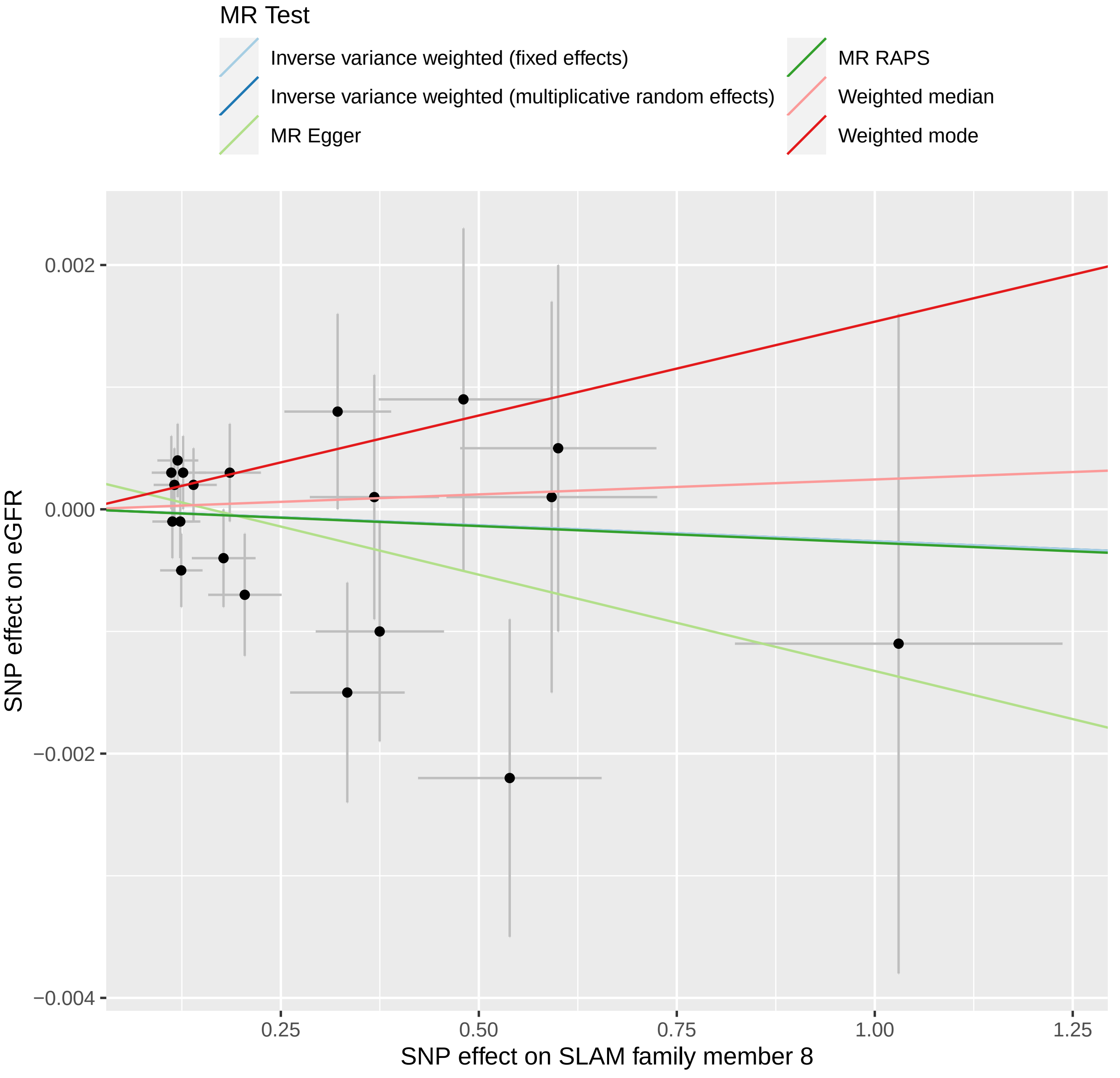

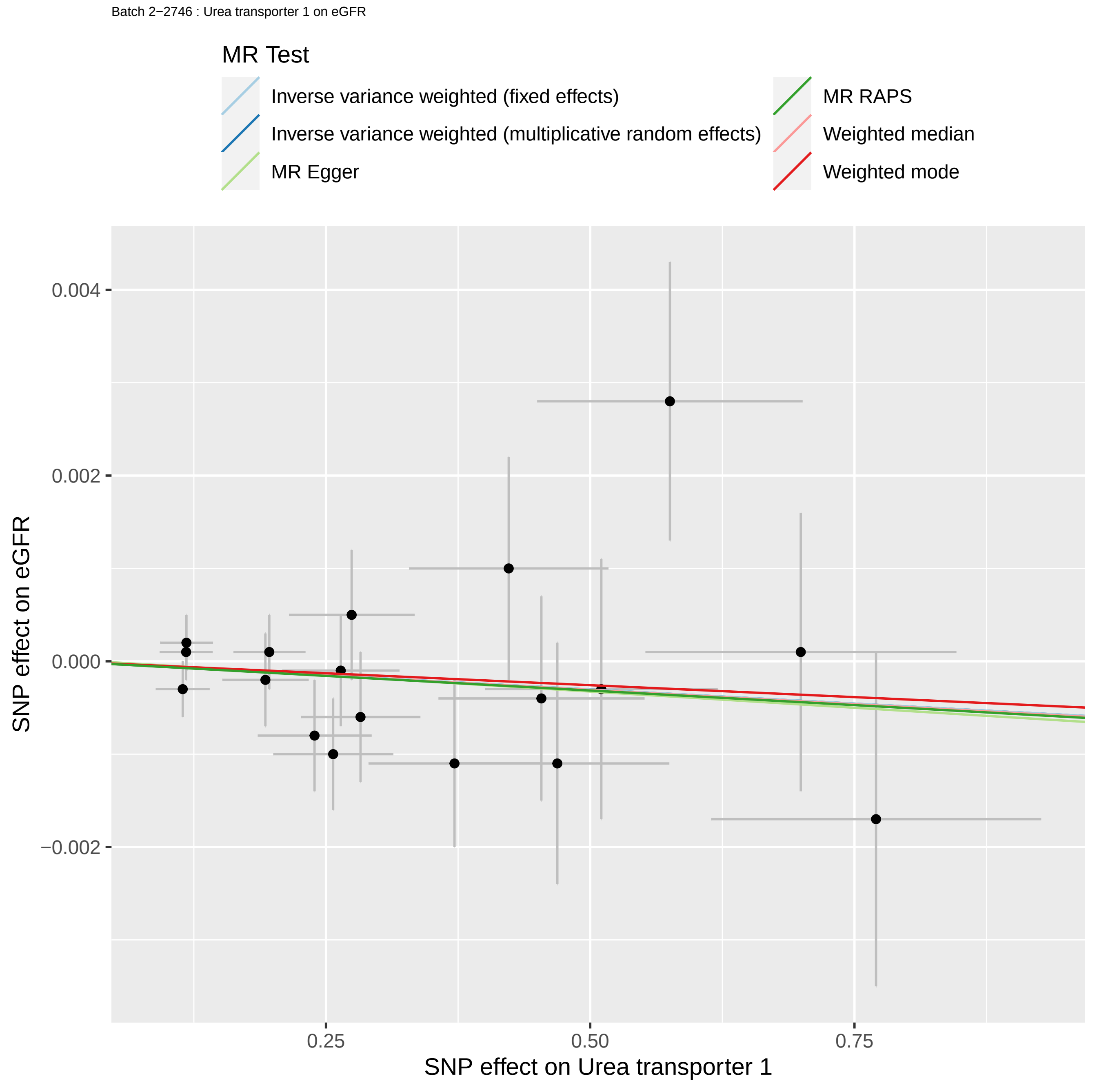
Batch 2−2746 : Urea transporter 1 on eGFR
MR Test
Inverse variance weighted (fixed effects)
Inverse variance weighted (multiplicative random effects)
MR Egger
MR RAPS
Weighted median
Weighted mode
0.004
0.002
0.000
−0.002
0.25
0.50
0.75
SNP effect on eGFR
SNP effect on Urea transporter 1

Batch 2−2747 : Urea transporter 2 on eGFR

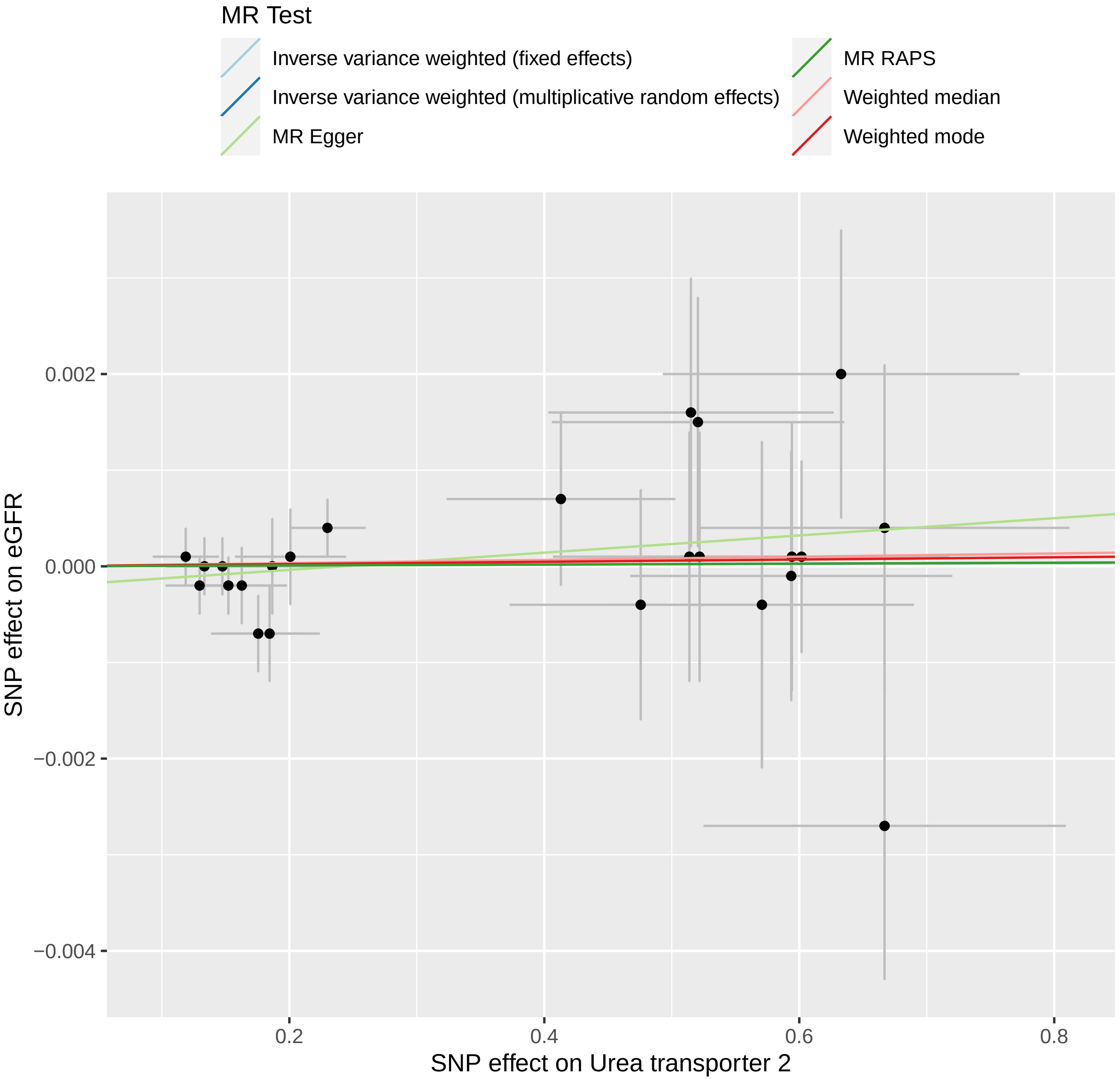

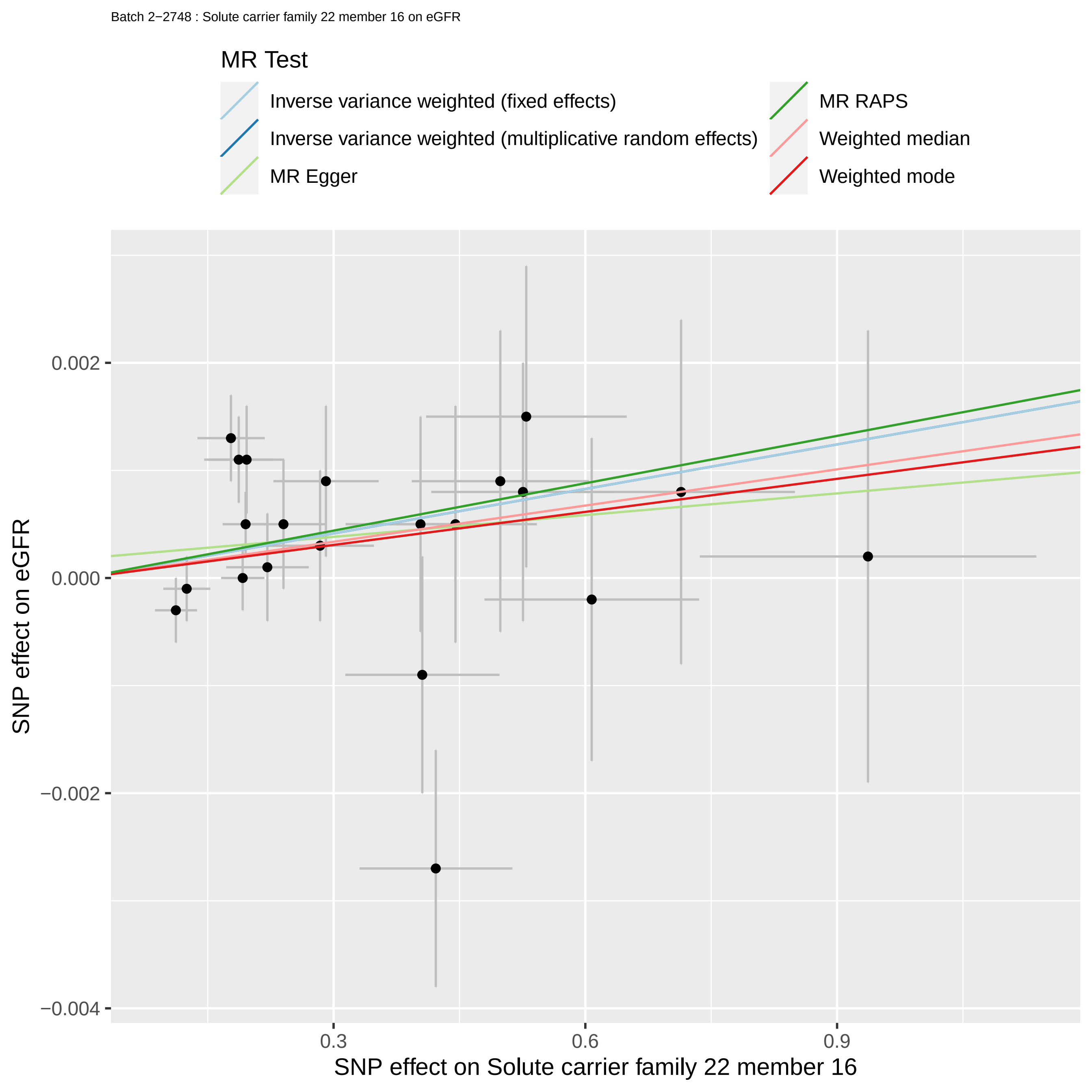

Batch 2−2748 : Solute carrier family 22 member 16 on eGFR
MR Test
Inverse variance weighted (fixed effects)
Inverse variance weighted (multiplicative random effects)
MR Egger
MR RAPS
Weighted median
Weighted mode
0.002
0.000
−0.002
−0.004
SNP effect on eGFR
0.3
0.6
0.9
SNP effect on Solute carrier family 22 member 16

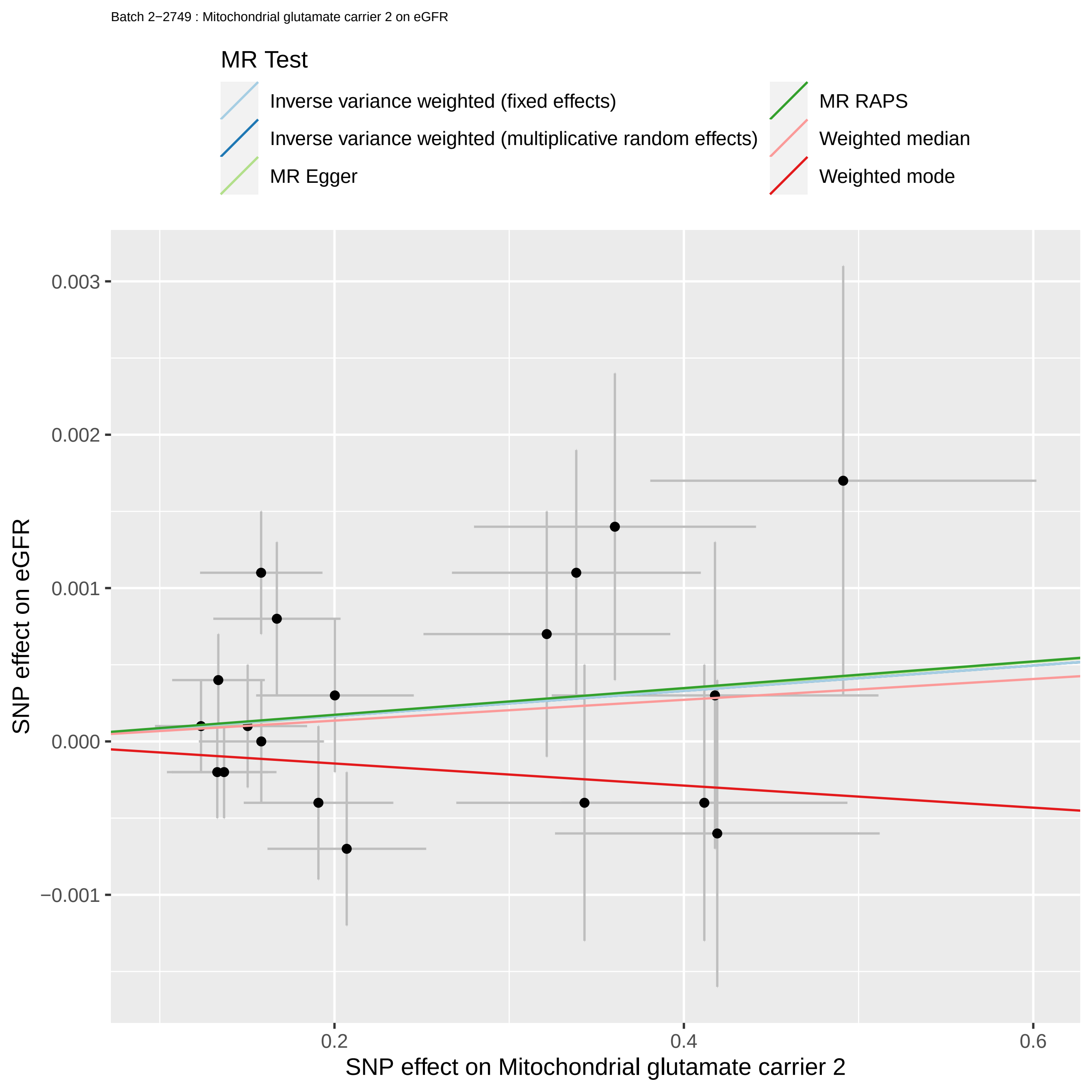
Batch 2−2749 : Mitochondrial glutamate carrier 2 on eGFR
MR Test
Inverse variance weighted (fixed effects)
Inverse variance weighted (multiplicative random effects)
MR Egger
MR RAPS
Weighted median
Weighted mode
0.003
0.002
0.001
0.000
−0.001
0.2
0.4
0.6
SNP effect on eGFR
SNP effect on Mitochondrial glutamate carrier 2

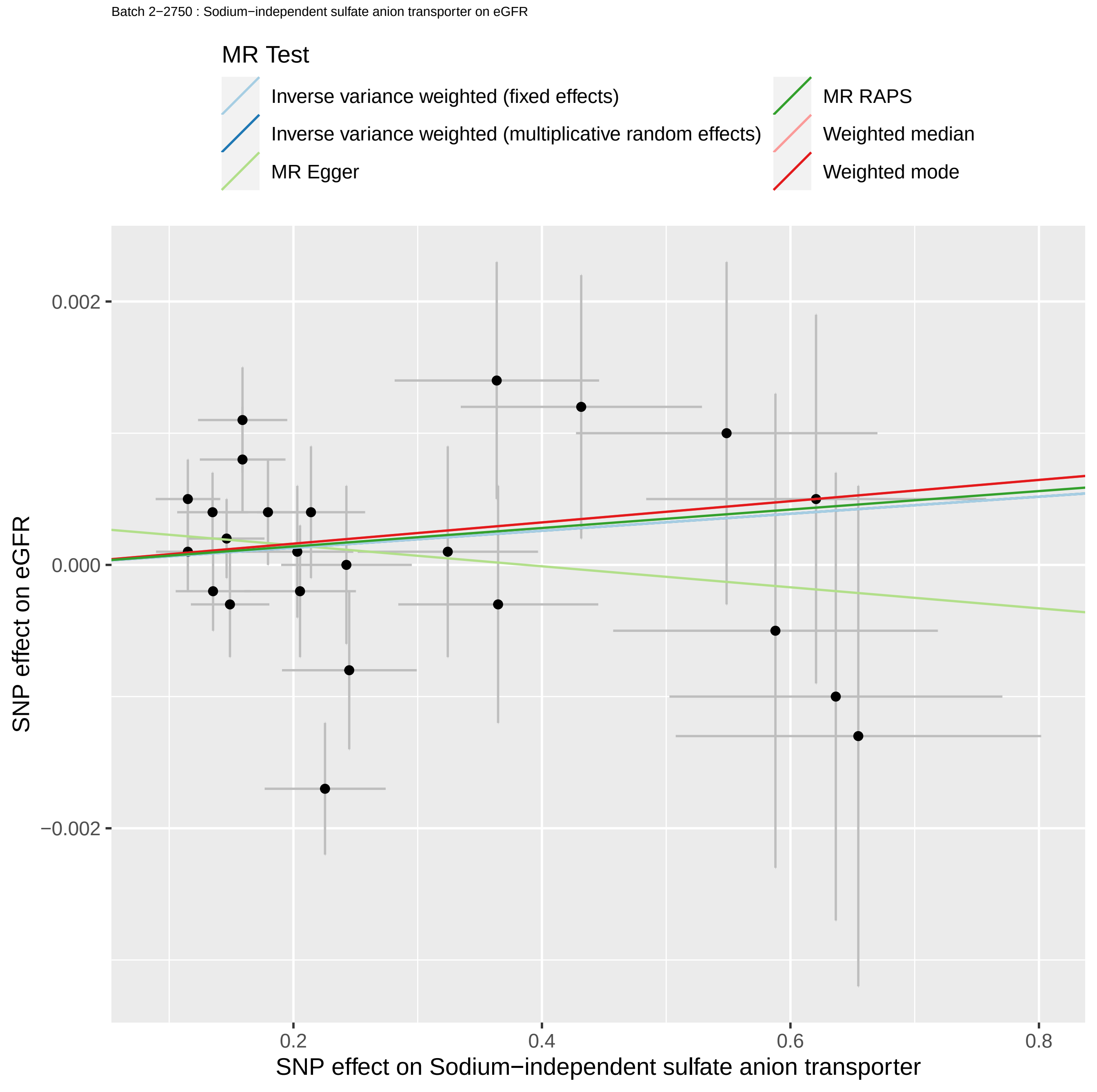

Batch 2−2750 : Sodium−independent sulfate anion transporter on eGFR
MR Test
Inverse variance weighted (fixed effects)
Inverse variance weighted (multiplicative random effects)
MR Egger
MR RAPS
Weighted median
Weighted mode
0.002
0.000
−0.002
0.2
0.4
0.6
0.8
SNP effect on eGFR
SNP effect on Sodium−independent sulfate anion transporter

Batch 2−2751 : Prestin on eGFR

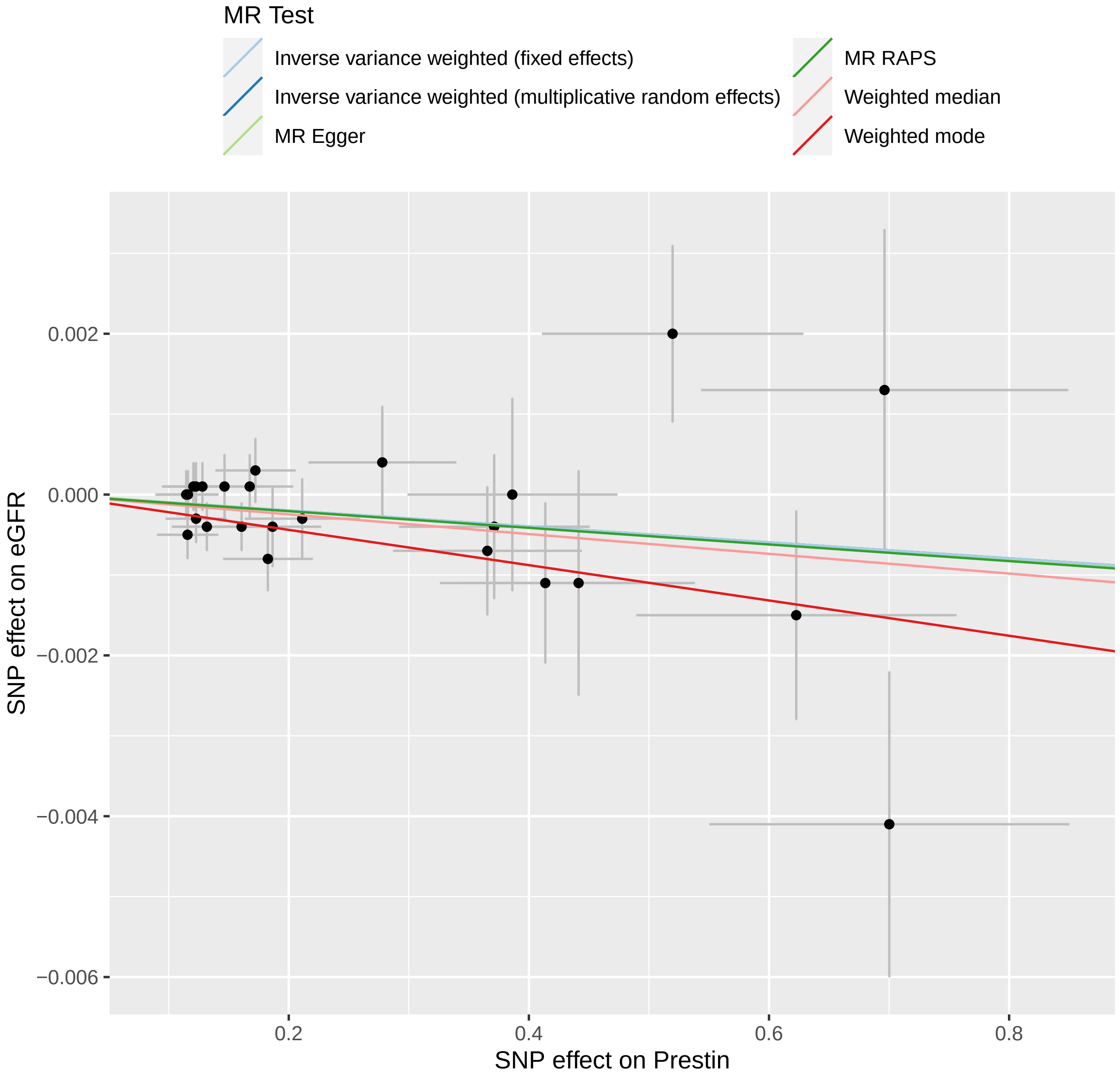

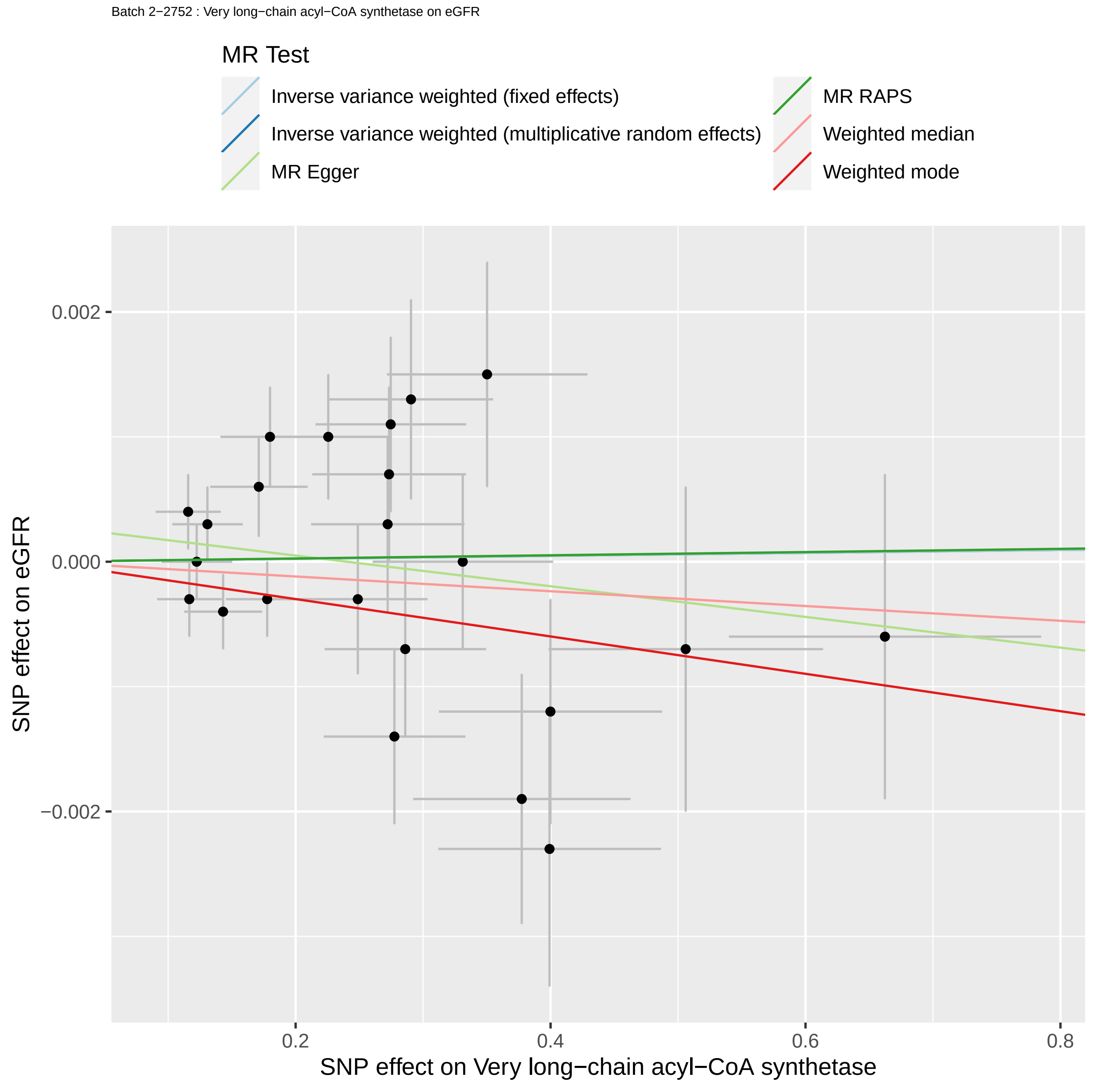
Batch 2−2752 : Very long−chain acyl−CoA synthetase on eGFR
MR Test
Inverse variance weighted (fixed effects)
Inverse variance weighted (multiplicative random effects)
MR Egger
MR RAPS
Weighted median
Weighted mode
0.002
0.000
−0.002
0.2
0.4
0.6
0.8
SNP effect on eGFR
SNP effect on Very long−chain acyl−CoA synthetase

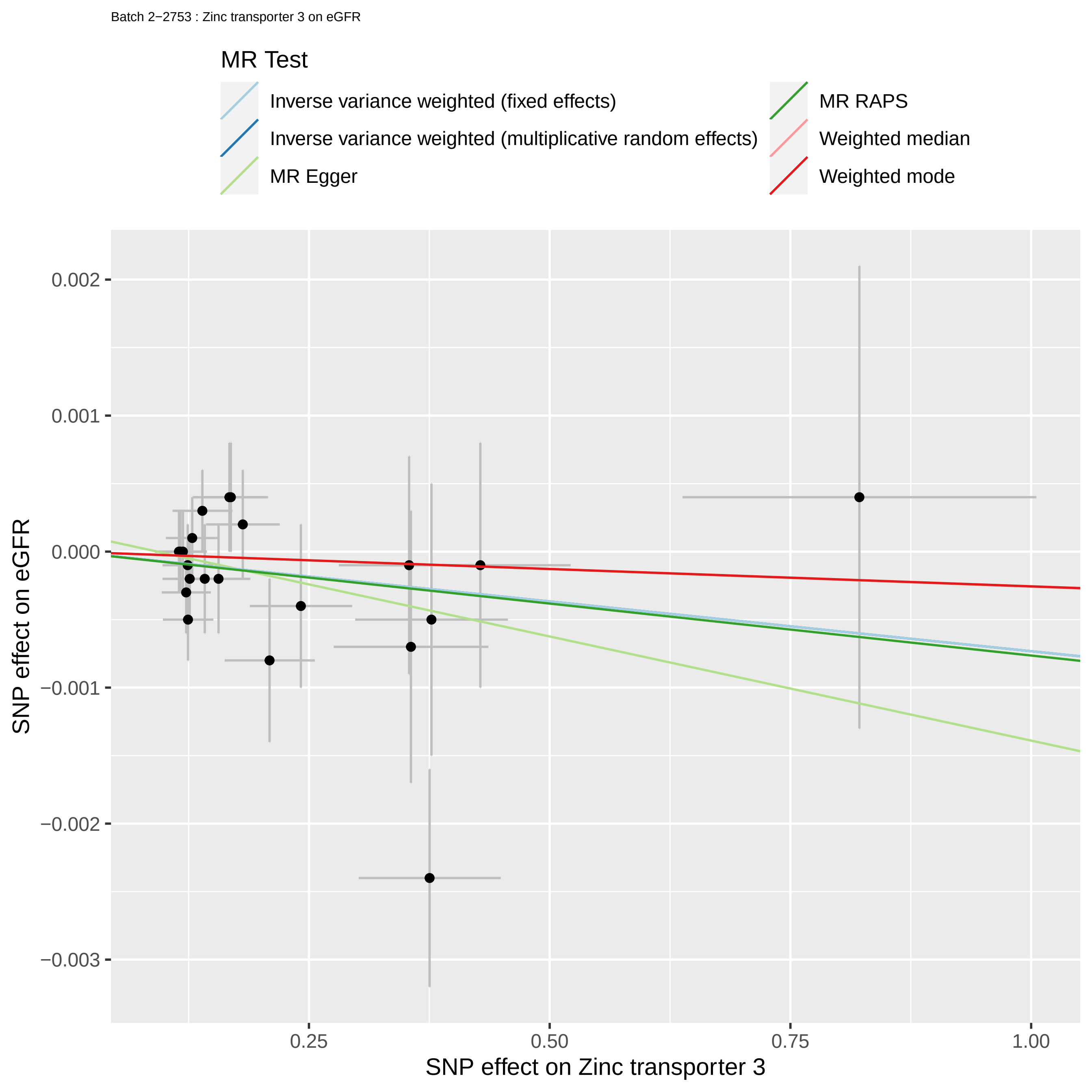

Batch 2−2753 : Zinc transporter 3 on eGFR
MR Test
Inverse variance weighted (fixed effects)
Inverse variance weighted (multiplicative random effects)
MR Egger
MR RAPS
Weighted median
Weighted mode
0.002
0.001
0.000
−0.001
−0.002
−0.003
0.25
0.50
0.75
1.00
SNP effect on eGFR
SNP effect on Zinc transporter 3

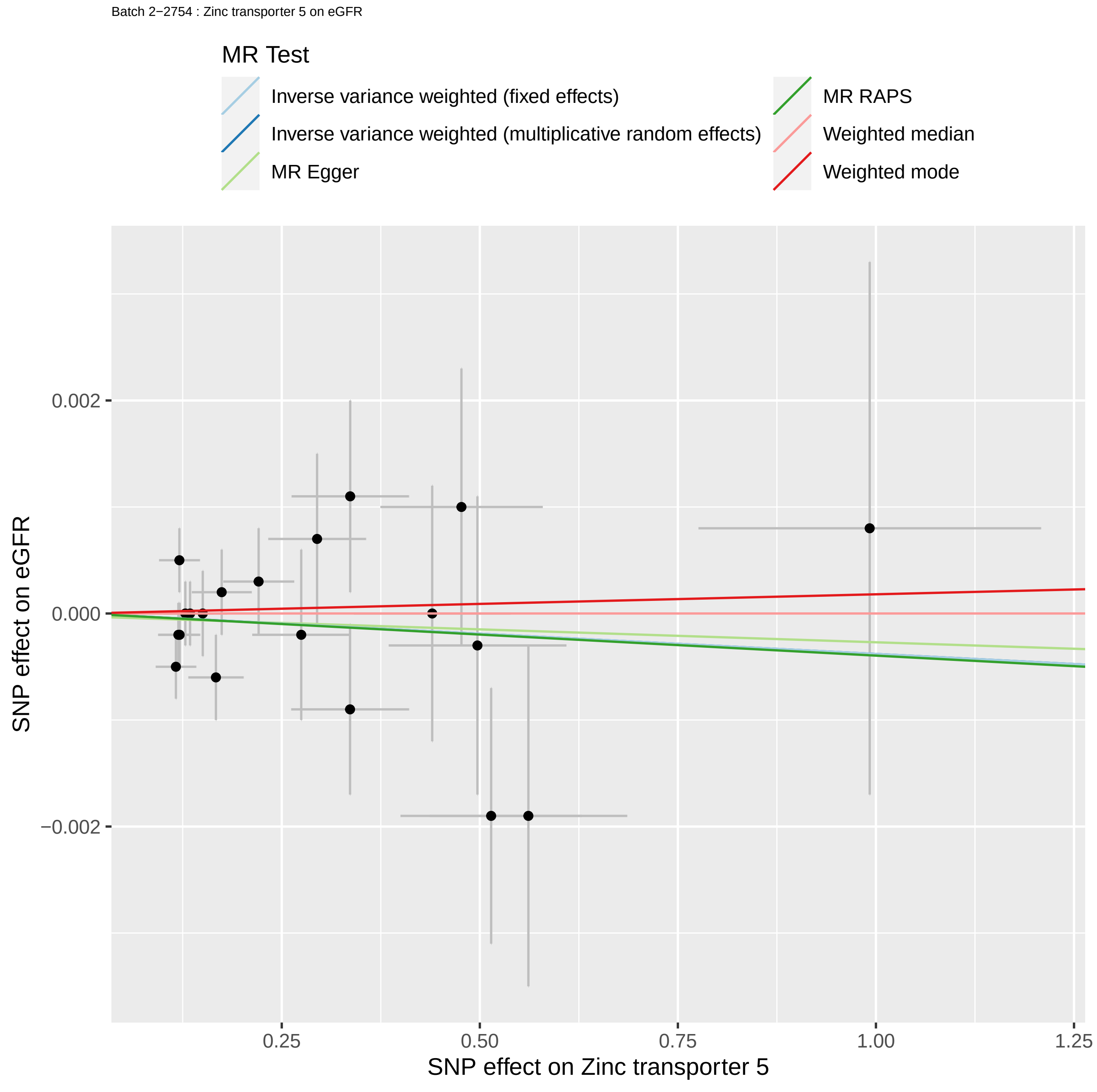

Batch 2−2754 : Zinc transporter 5 on eGFR
MR Test
Inverse variance weighted (fixed effects)
Inverse variance weighted (multiplicative random effects)
MR Egger
MR RAPS
Weighted median
Weighted mode
0.002
0.000
−0.002
SNP effect on eGFR
0.25
0.50
0.75
1.00
1.25
SNP effect on Zinc transporter 5

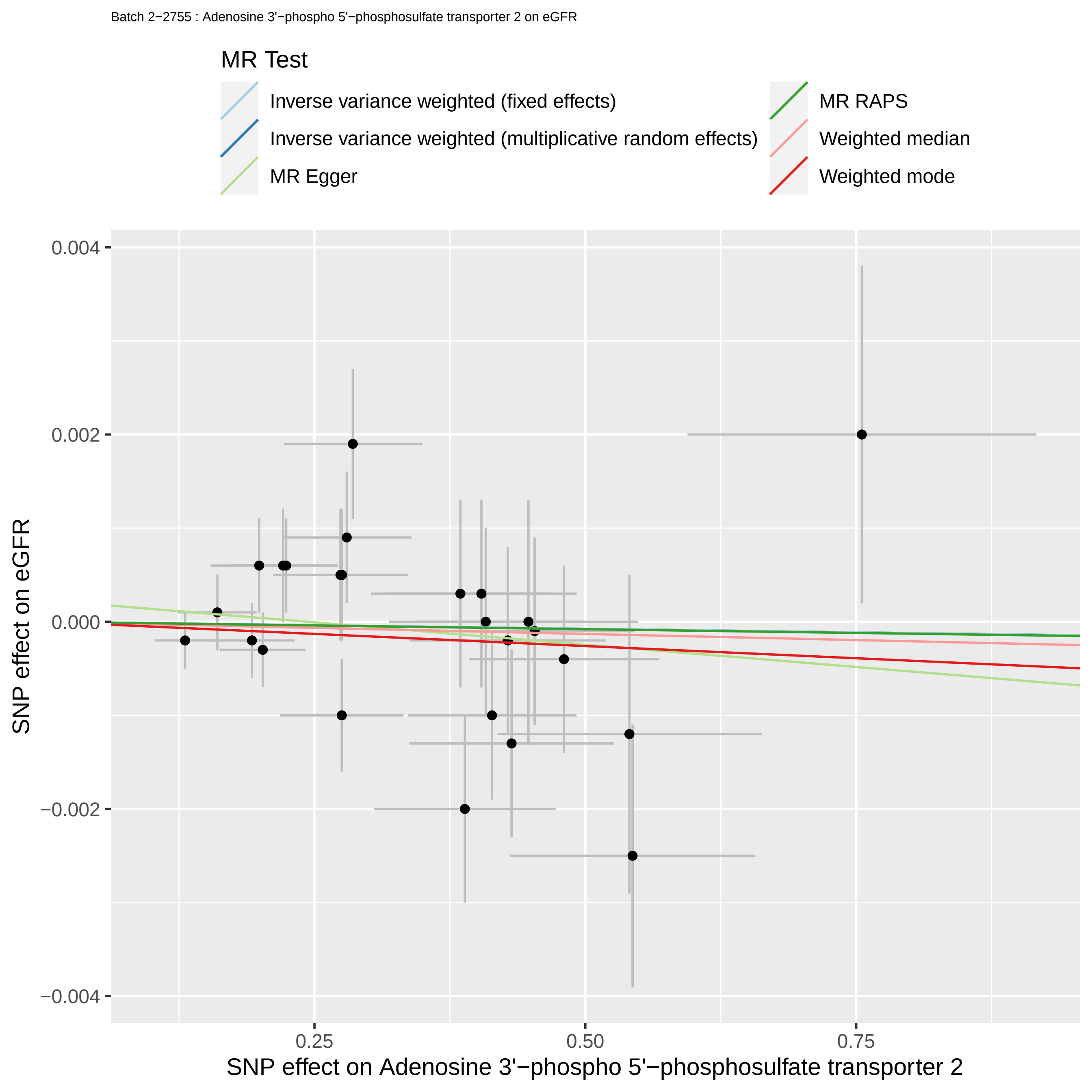

Batch 2−2755 : Adenosine 3'−phospho 5'−phosphosulfate transporter 2 on eGFR
MR Test
Inverse variance weighted (fixed effects)
Inverse variance weighted (multiplicative random effects)
MR Egger
MR RAPS
Weighted median
Weighted mode
0.004
0.002
0.000
−0.002
−0.004
0.25
0.50
0.75
SNP effect on eGFR
SNP effect on Adenosine 3'−phospho 5'−phosphosulfate transporter 2

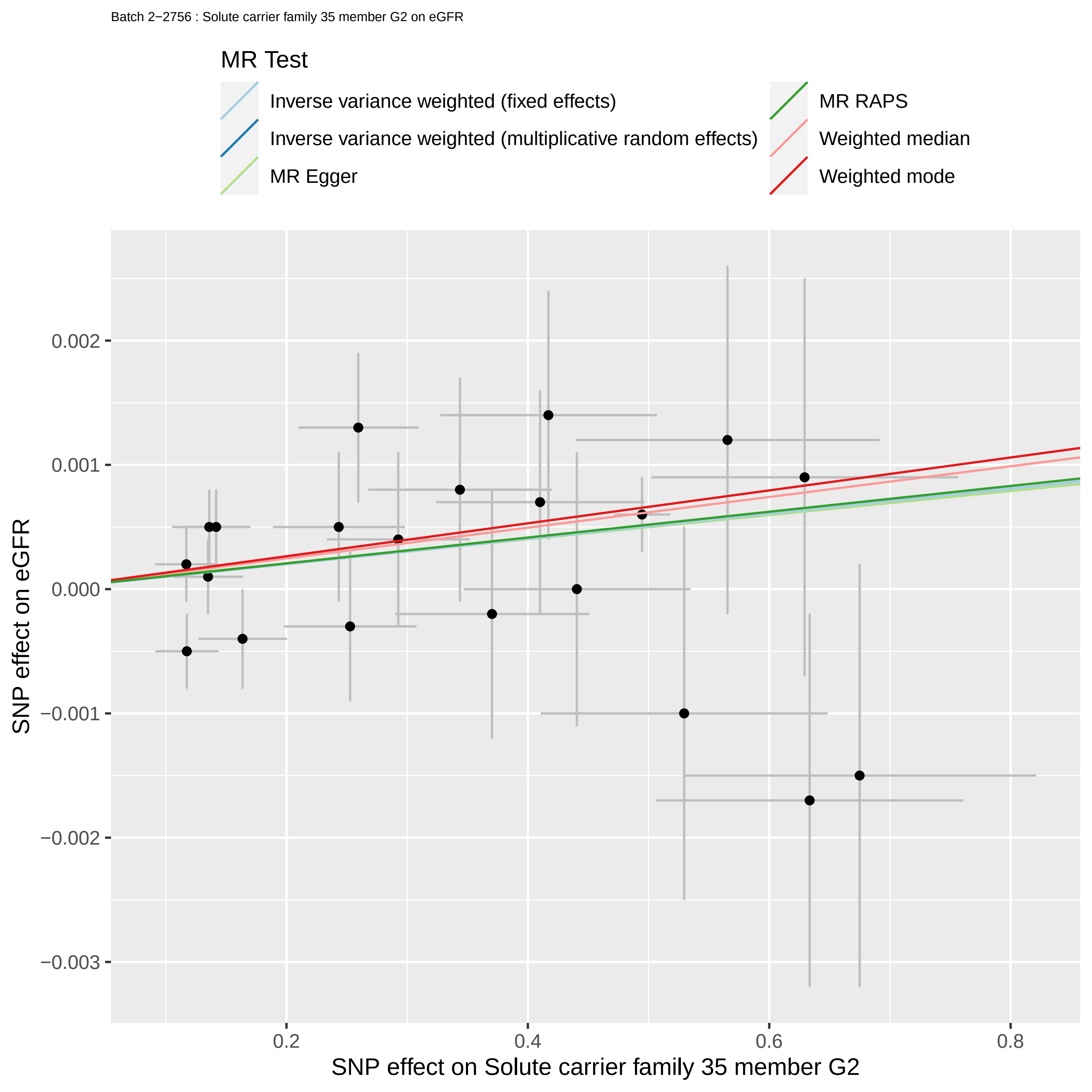

Batch 2−2756 : Solute carrier family 35 member G2 on eGFR
MR Test
Inverse variance weighted (fixed effects)
Inverse variance weighted (multiplicative random effects)
MR Egger
MR RAPS
Weighted median
Weighted mode
SNP effect on eGFR
0.002
0.001
0.000
−0.001
−0.002
−0.003
0.2
0.4
0.6
0.8
SNP effect on Solute carrier family 35 member G2

Batch 2−2757 : Solute carrier family 41 member 2 on eGFR

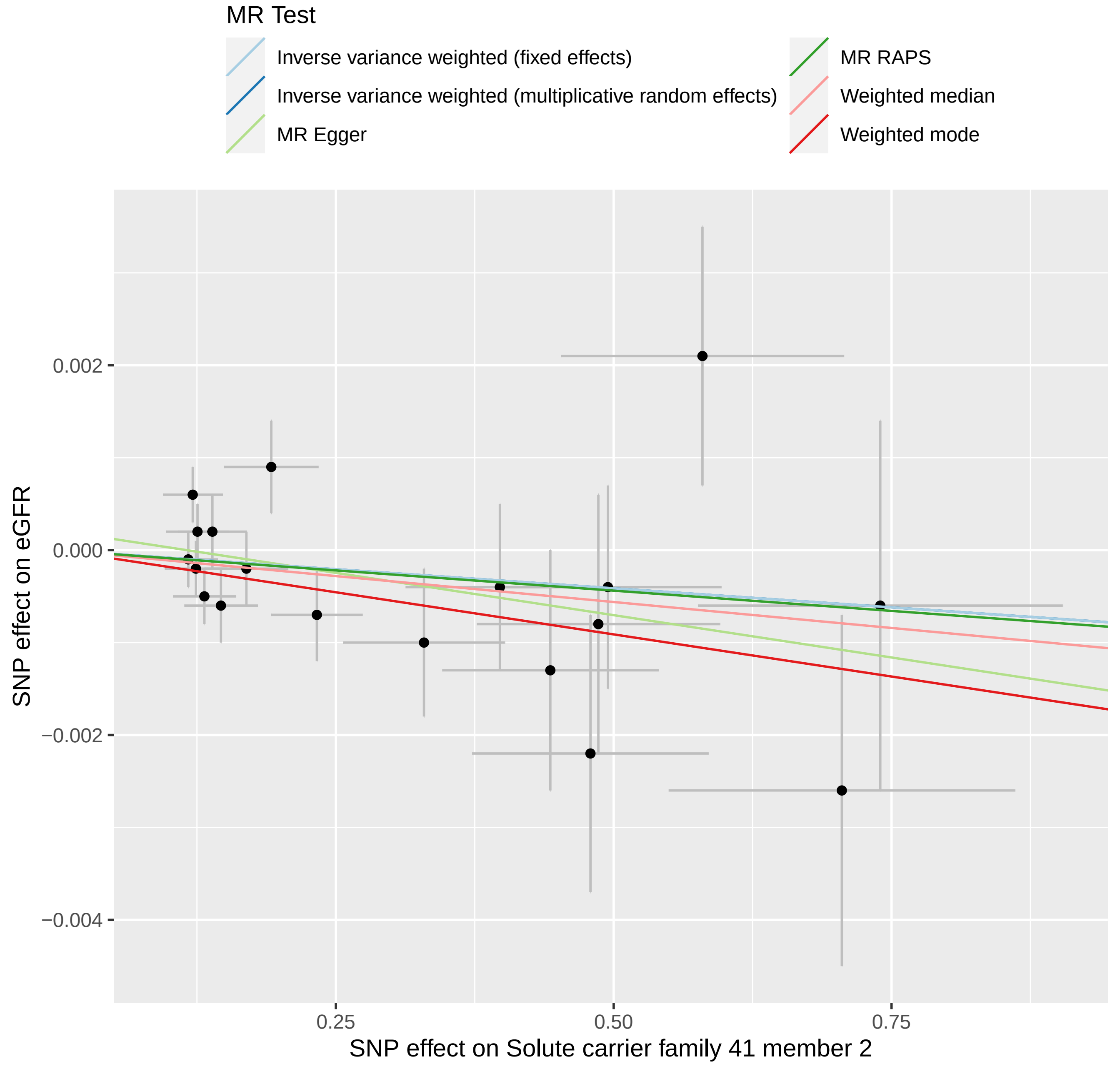

Batch 2−2758 : Electroneutral sodium bicarbonate exchanger 1 on eGFR

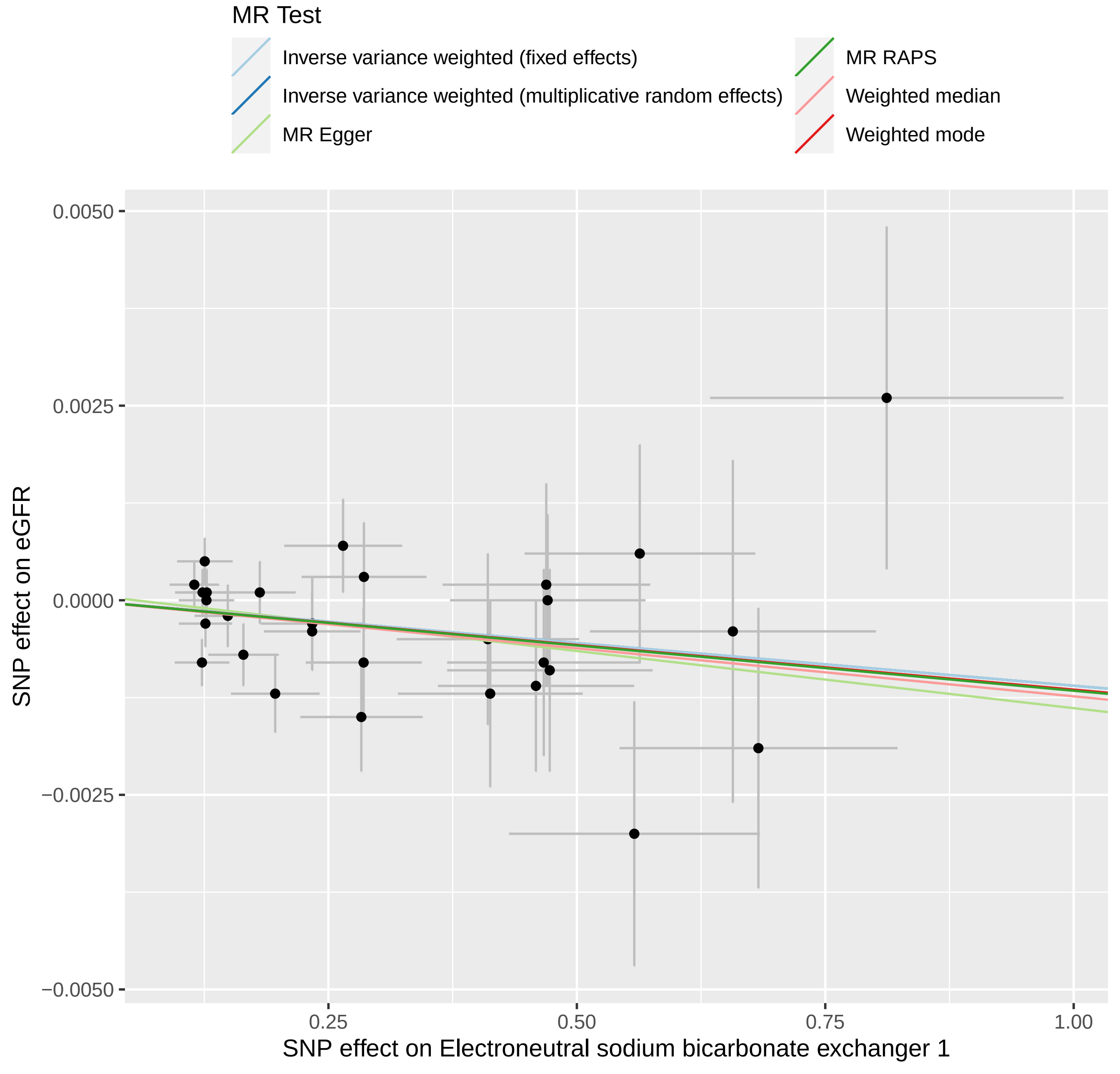

Batch 2−2759 : Sodium/iodide cotransporter on eGFR

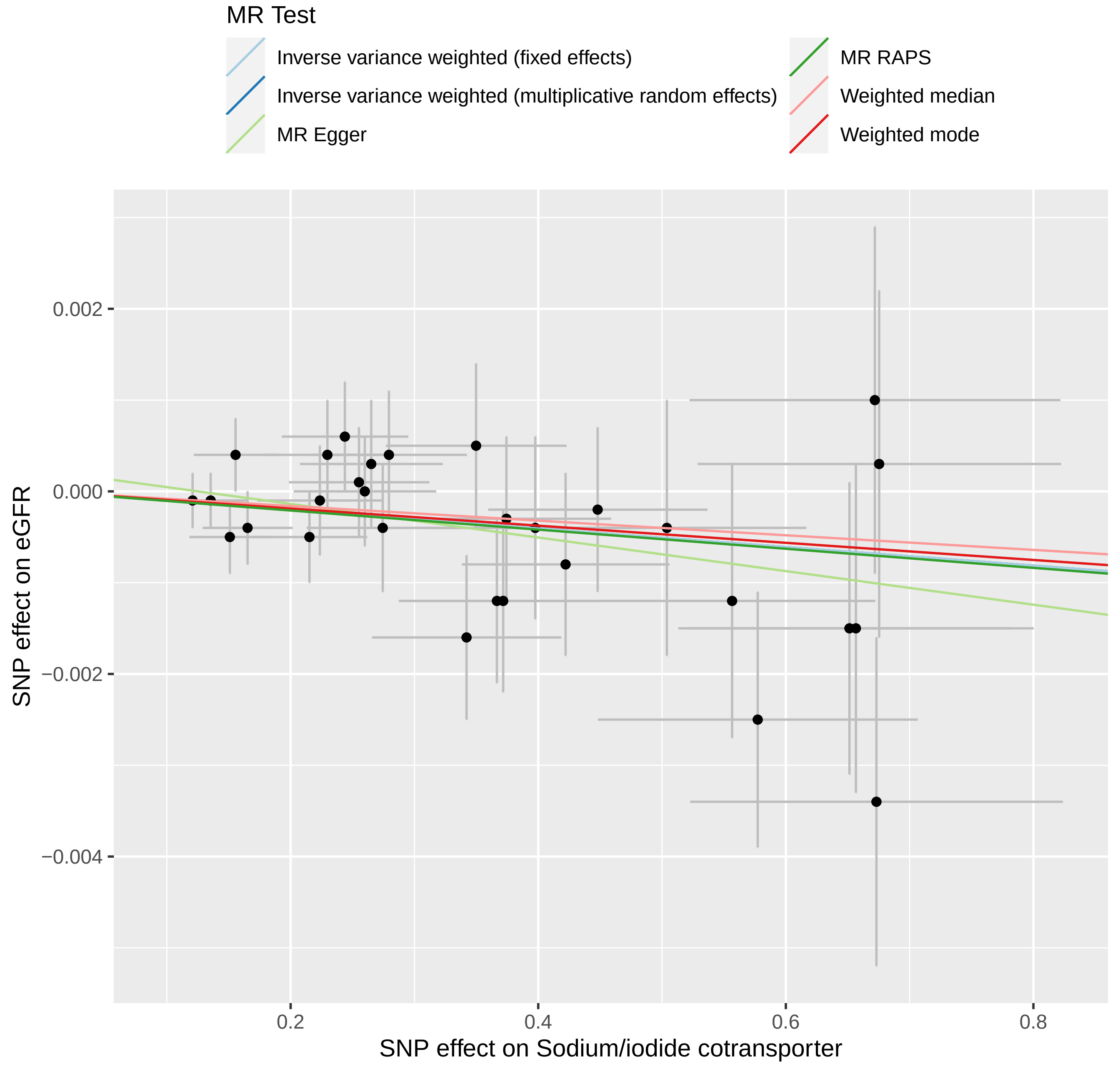

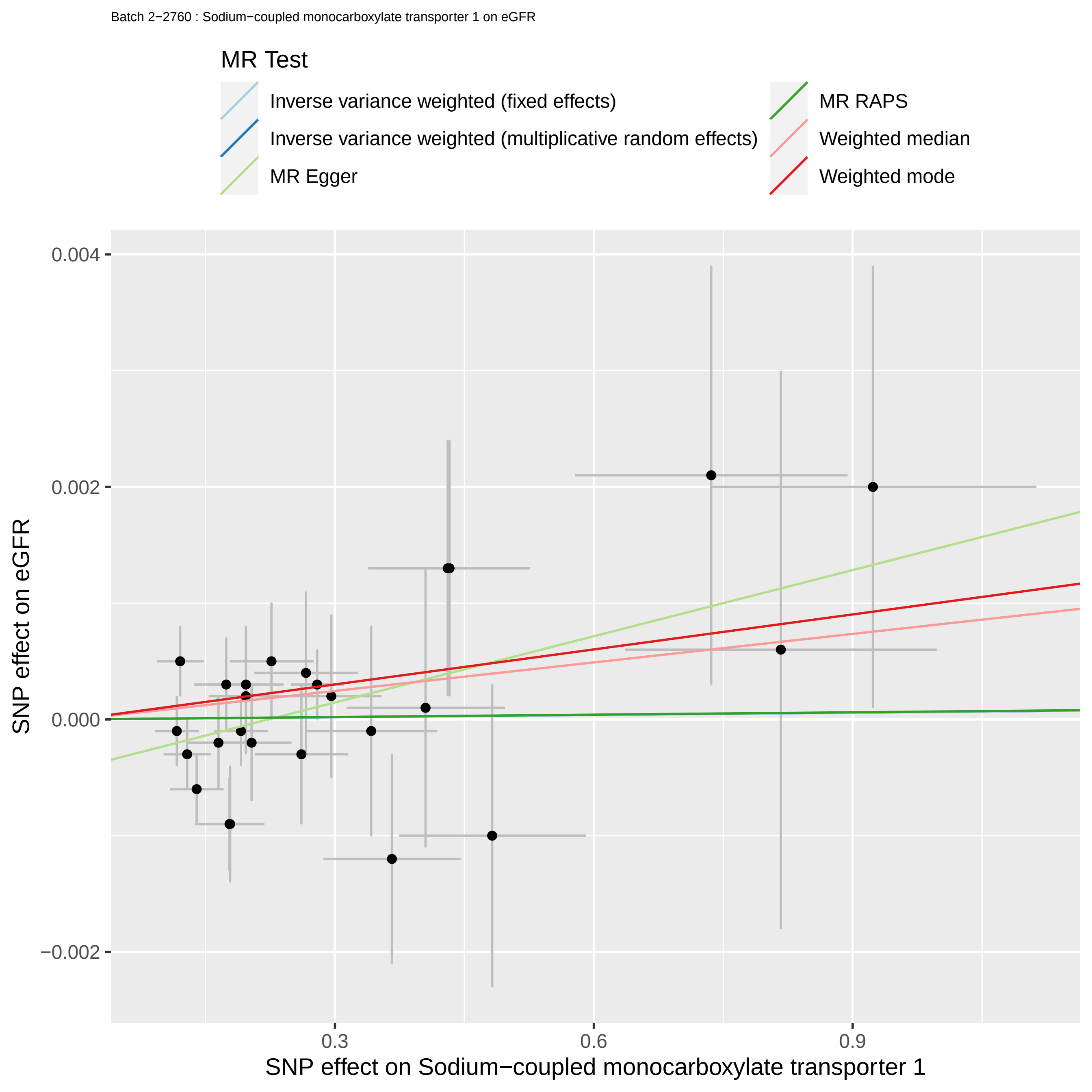

Batch 2−2760 : Sodium−coupled monocarboxylate transporter 1 on eGFR
MR Test
Inverse variance weighted (fixed effects)
Inverse variance weighted (multiplicative random effects)
MR Egger
MR RAPS
Weighted median
Weighted mode
0.004
0.002
0.000
−0.002
SNP effect on eGFR
0.3
0.6
0.9
SNP effect on Sodium−coupled monocarboxylate transporter 1

Batch 2−2761 : Sodium− and chloride−dependent neutral and basic amino acid transporter B(0+) on eGFR

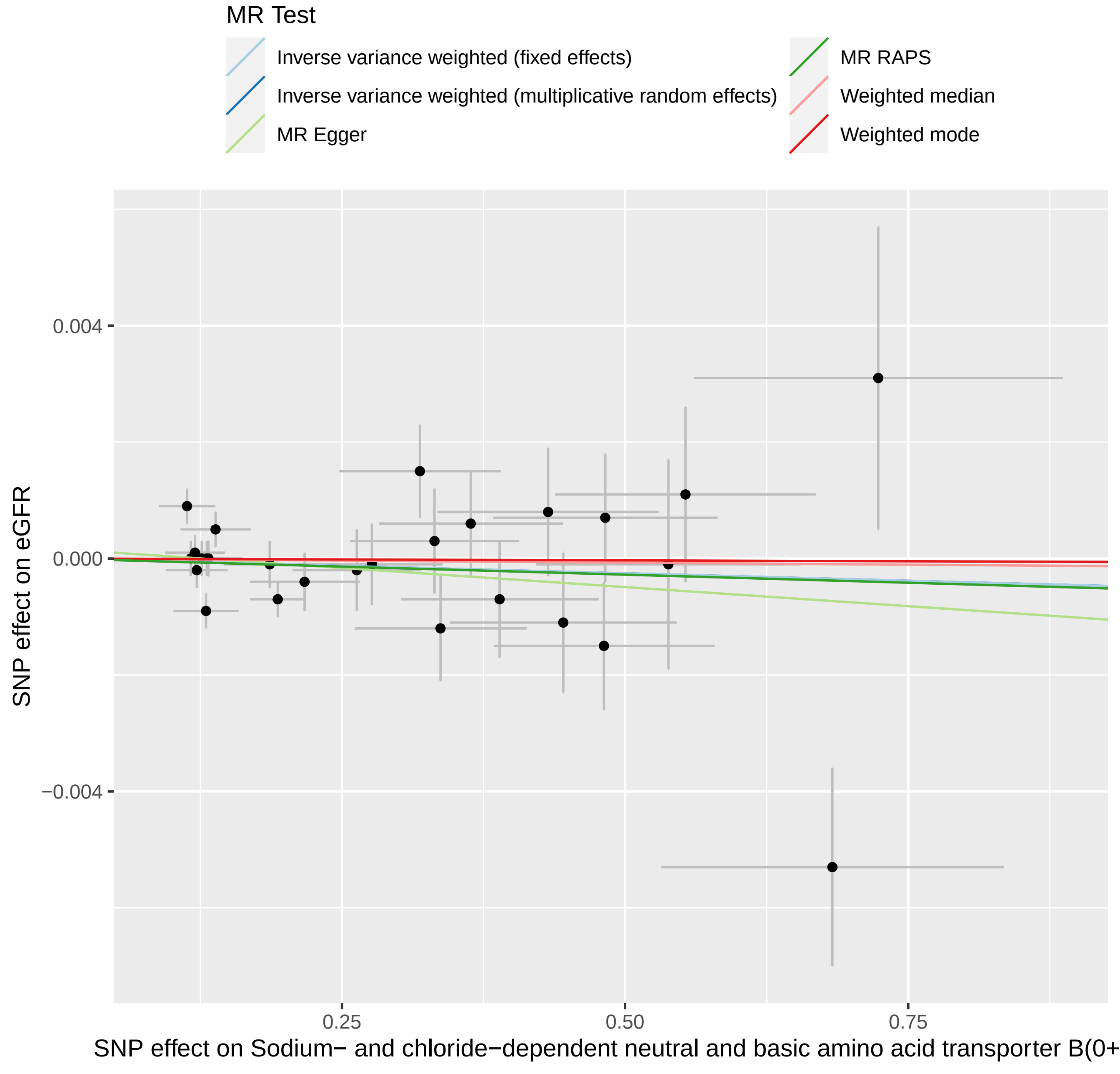

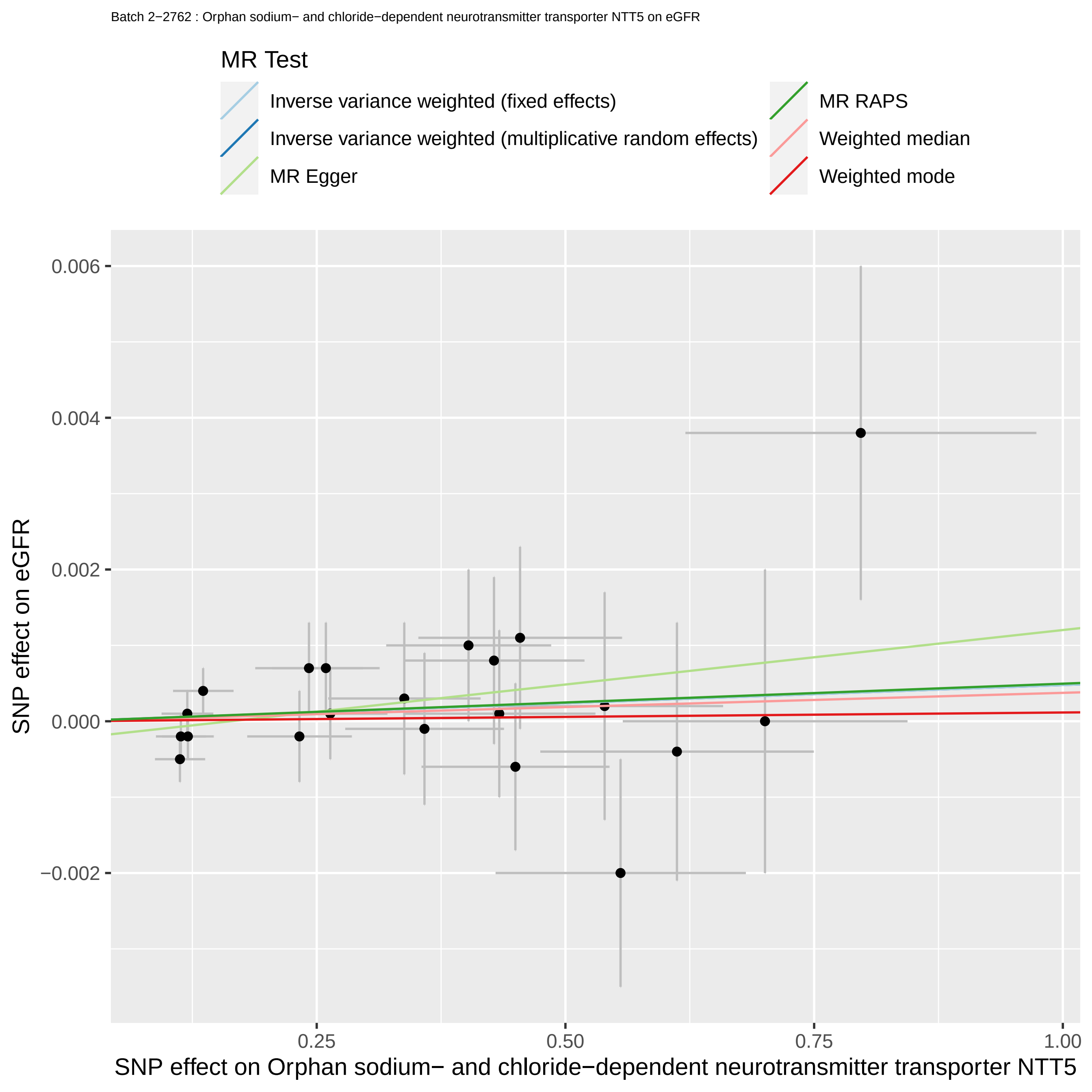

Batch 2−2762 : Orphan sodium− and chloride−dependent neurotransmitter transporter NTT5 on eGFR
MR Test
Inverse variance weighted (fixed effects)
Inverse variance weighted (multiplicative random effects)
MR Egger
MR RAPS
Weighted median
Weighted mode
0.006
0.004
0.002
0.000
−0.002
0.25
0.50
0.75
1.00
SNP effect on eGFR
SNP effect on Orphan sodium− and chloride−dependent neurotransmitter transporter NTT5

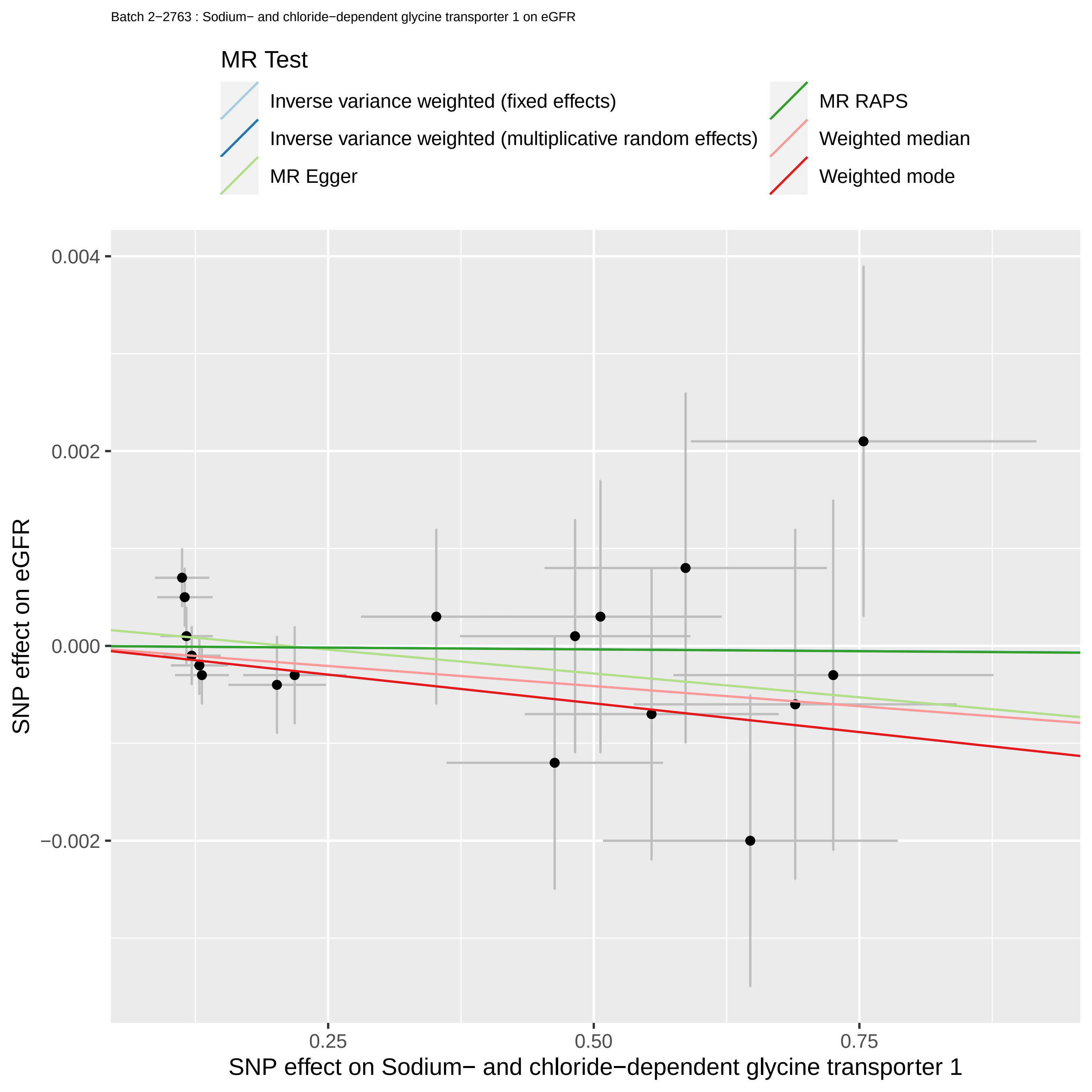
Batch 2−2763 : Sodium− and chloride−dependent glycine transporter 1 on eGFR
MR Test
Inverse variance weighted (fixed effects)
Inverse variance weighted (multiplicative random effects)
MR Egger
MR RAPS
Weighted median
Weighted mode
0.004
0.002
0.000
−0.002
0.25
0.50
0.75
SNP effect on eGFR
SNP effect on Sodium− and chloride−dependent glycine transporter 1

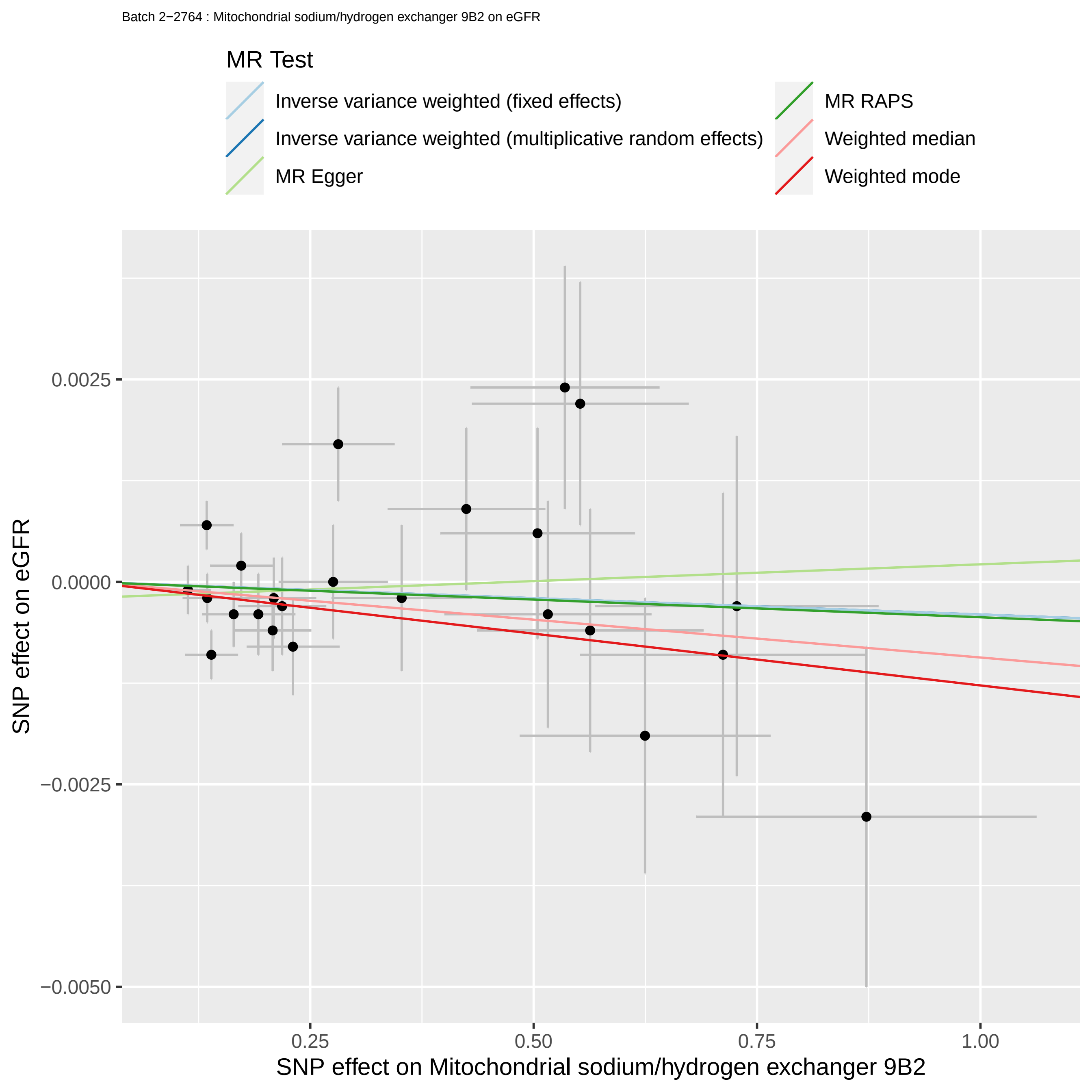

Batch 2−2764 : Mitochondrial sodium/hydrogen exchanger 9B2 on eGFR
MR Test
Inverse variance weighted (fixed effects)
Inverse variance weighted (multiplicative random effects)
MR Egger
MR RAPS
Weighted median
Weighted mode
0.0025
0.0000
−0.0025
−0.0050
SNP effect on eGFR
0.25
0.50
0.75
1.00
SNP effect on Mitochondrial sodium/hydrogen exchanger 9B2

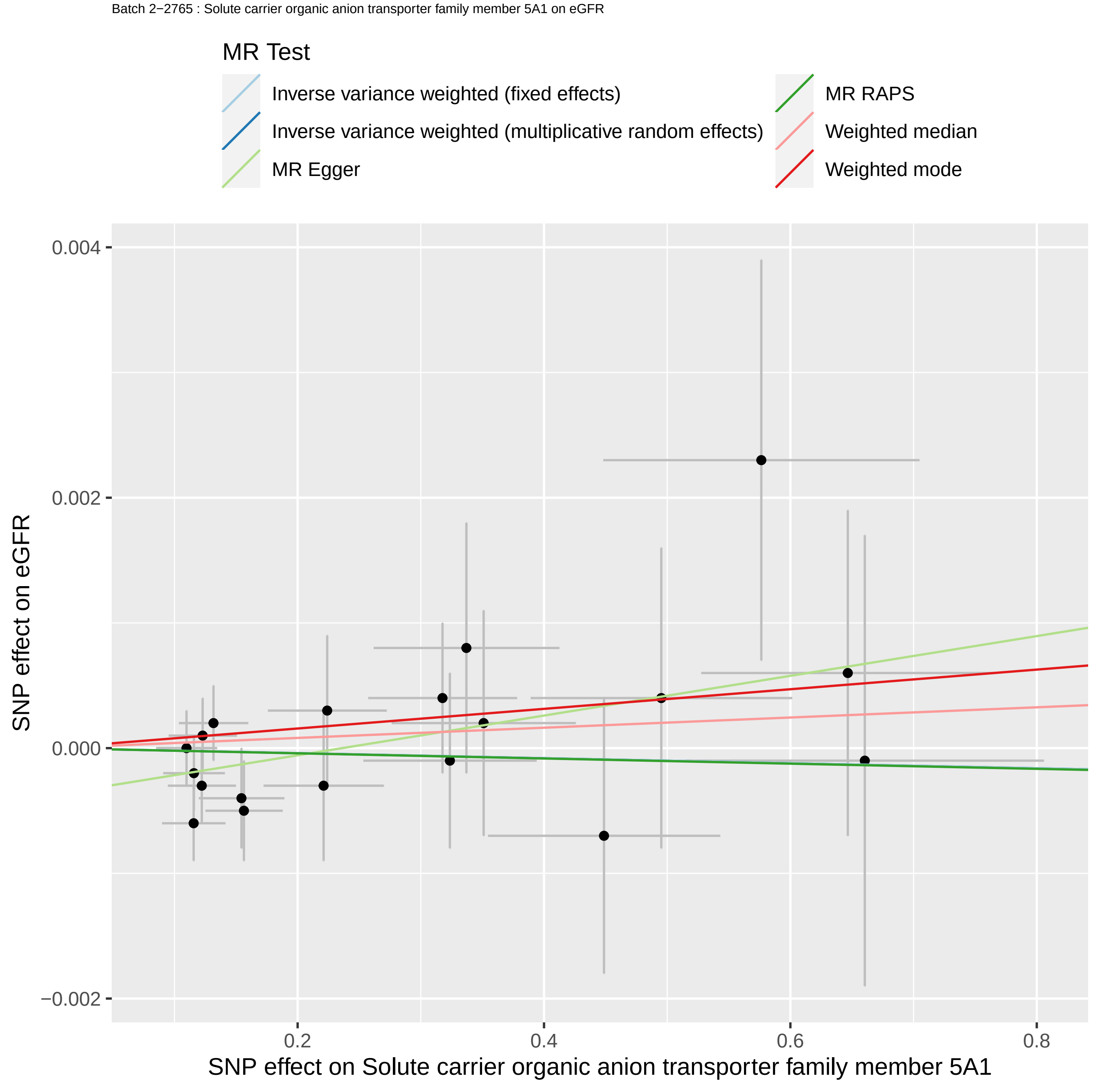
Batch 2−2765 : Solute carrier organic anion transporter family member 5A1 on eGFR
MR Test
Inverse variance weighted (fixed effects)
Inverse variance weighted (multiplicative random effects)
MR Egger
MR RAPS
Weighted median
Weighted mode
0.004
0.002
0.000
−0.002
SNP effect on eGFR
0.2
0.4
0.6
0.8
SNP effect on Solute carrier organic anion transporter family member 5A1

Batch 2−2766 : SLIT and NTRK−like protein 1 on eGFR

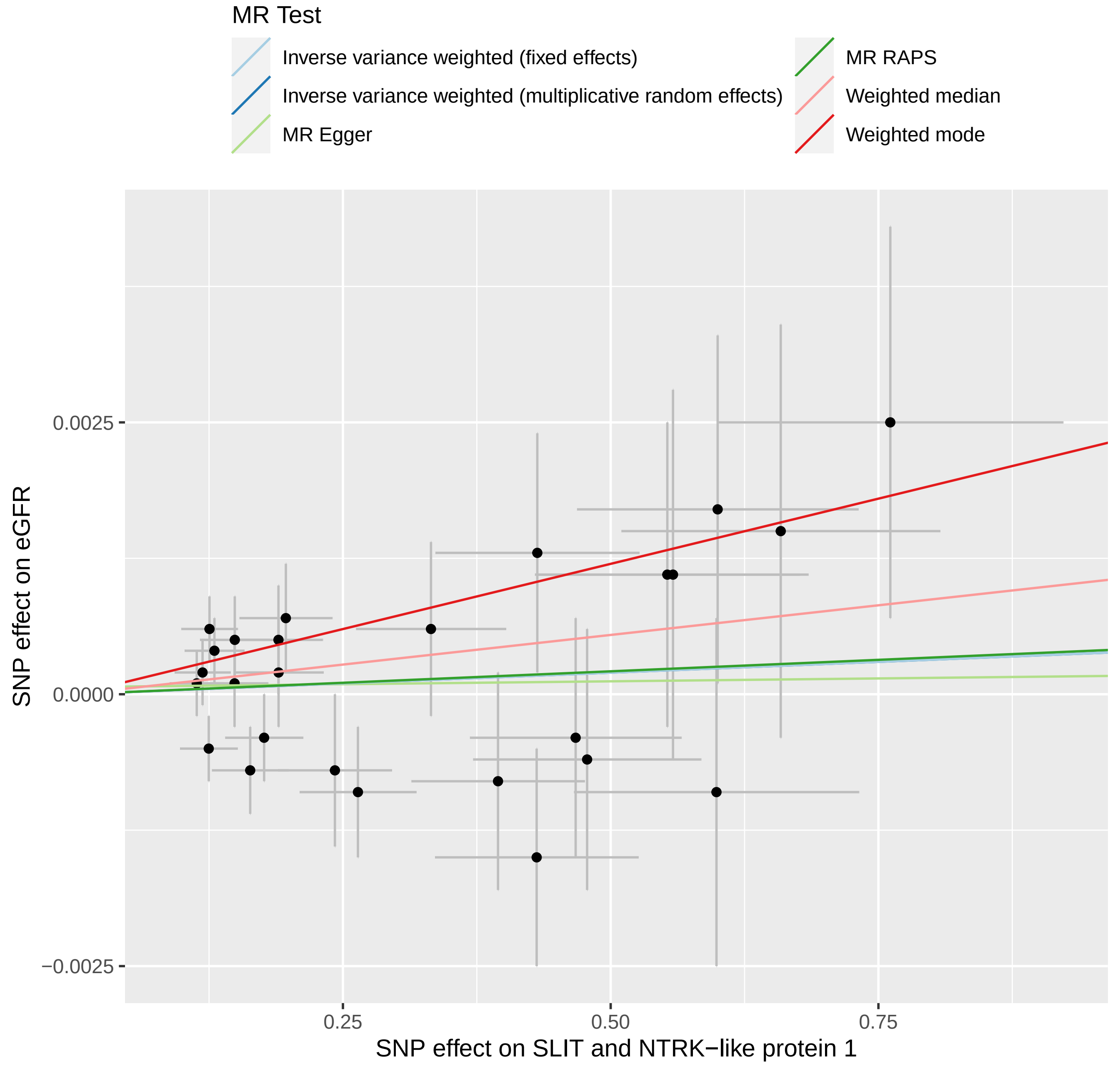

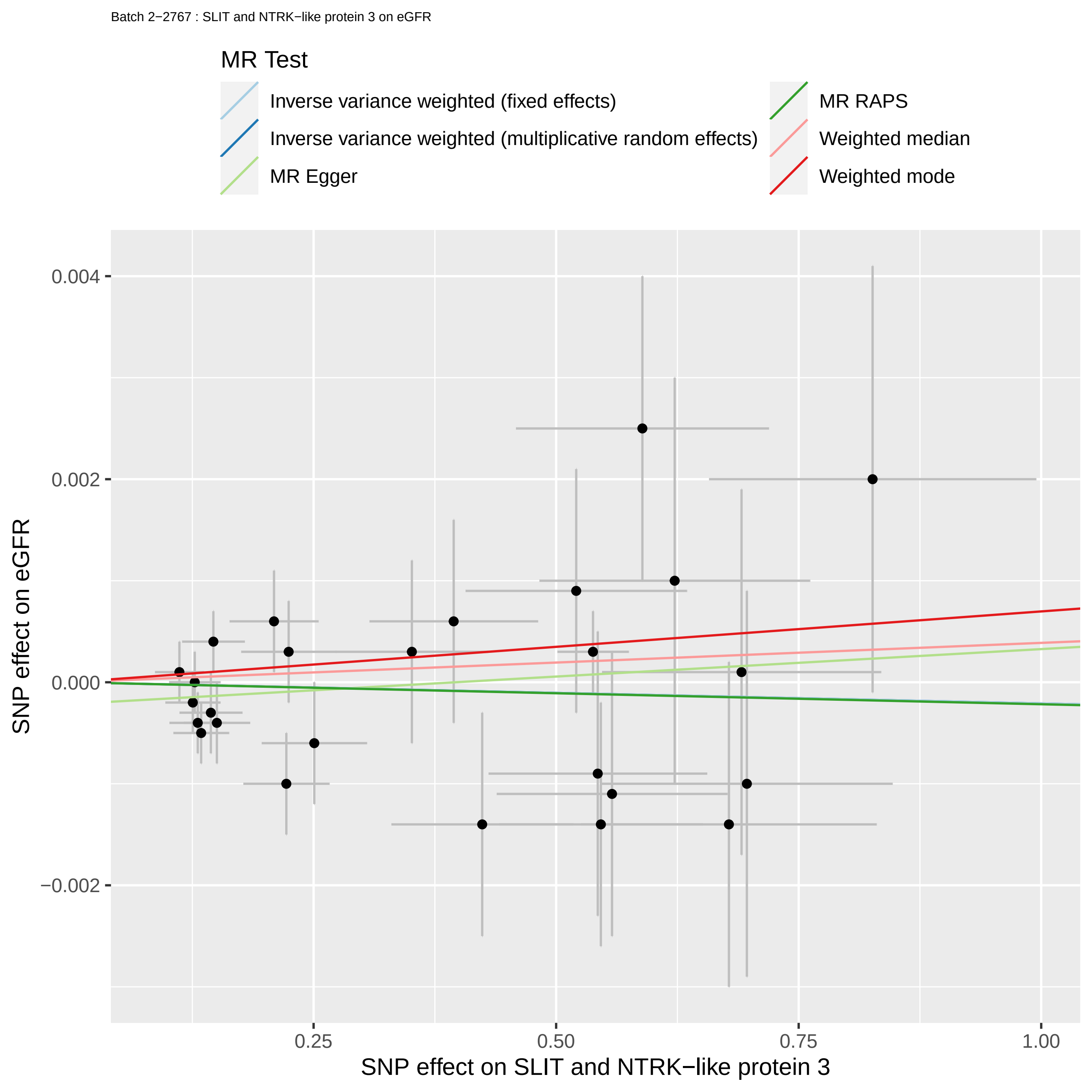

Batch 2−2767 : SLIT and NTRK−like protein 3 on eGFR
MR Test
Inverse variance weighted (fixed effects)
Inverse variance weighted (multiplicative random effects)
MR Egger
MR RAPS
Weighted median
Weighted mode
SNP effect on eGFR
0.004
0.002
0.000
−0.002
0.25
0.50
0.75
1.00
SNP effect on SLIT and NTRK−like protein 3

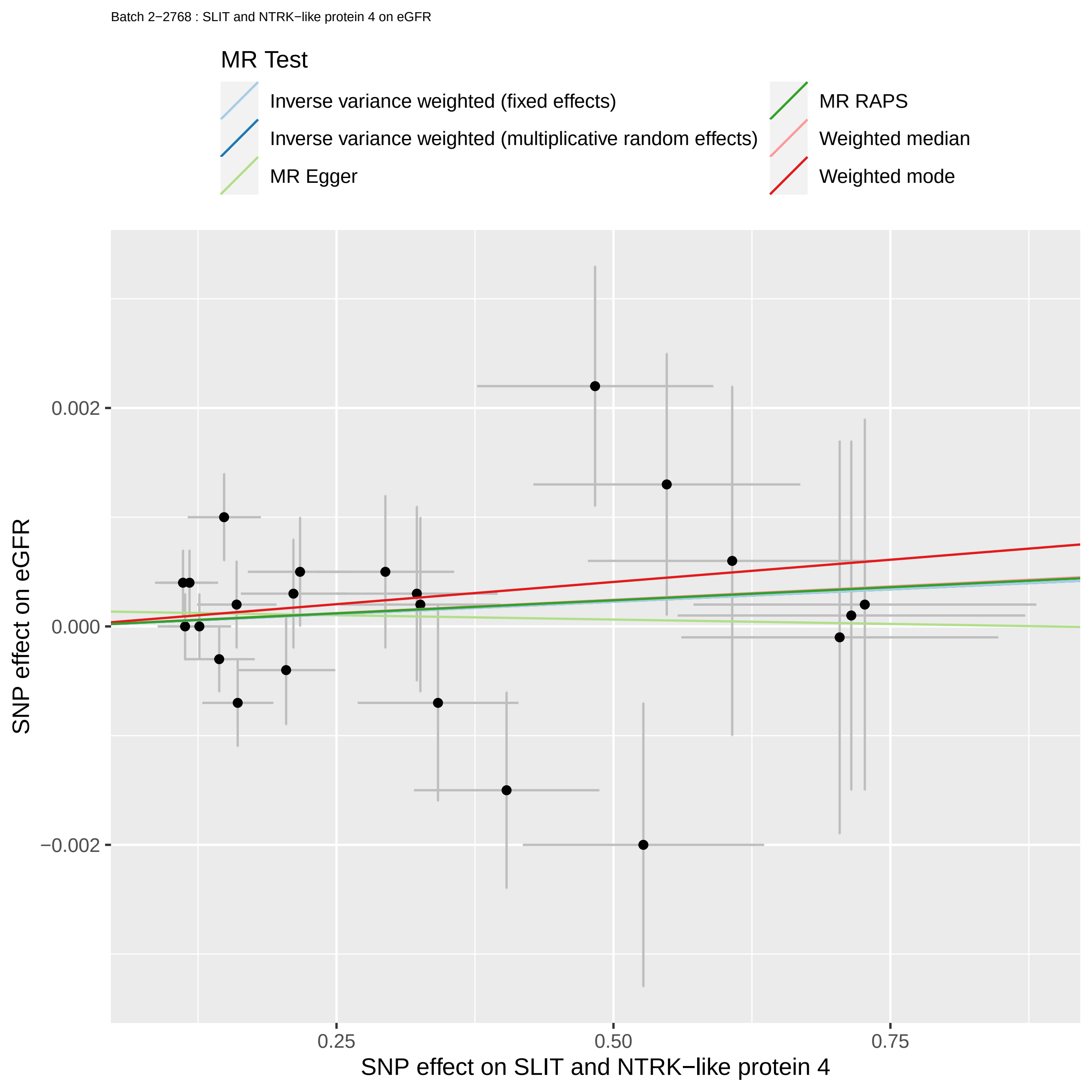
Batch 2−2768 : SLIT and NTRK−like protein 4 on eGFR
MR Test
Inverse variance weighted (fixed effects)
Inverse variance weighted (multiplicative random effects)
MR Egger
MR RAPS
Weighted median
Weighted mode
0.002
0.000
−0.002
0.25
0.50
0.75
SNP effect on SLIT and NTRK−like protein 4
SNP effect on eGFR

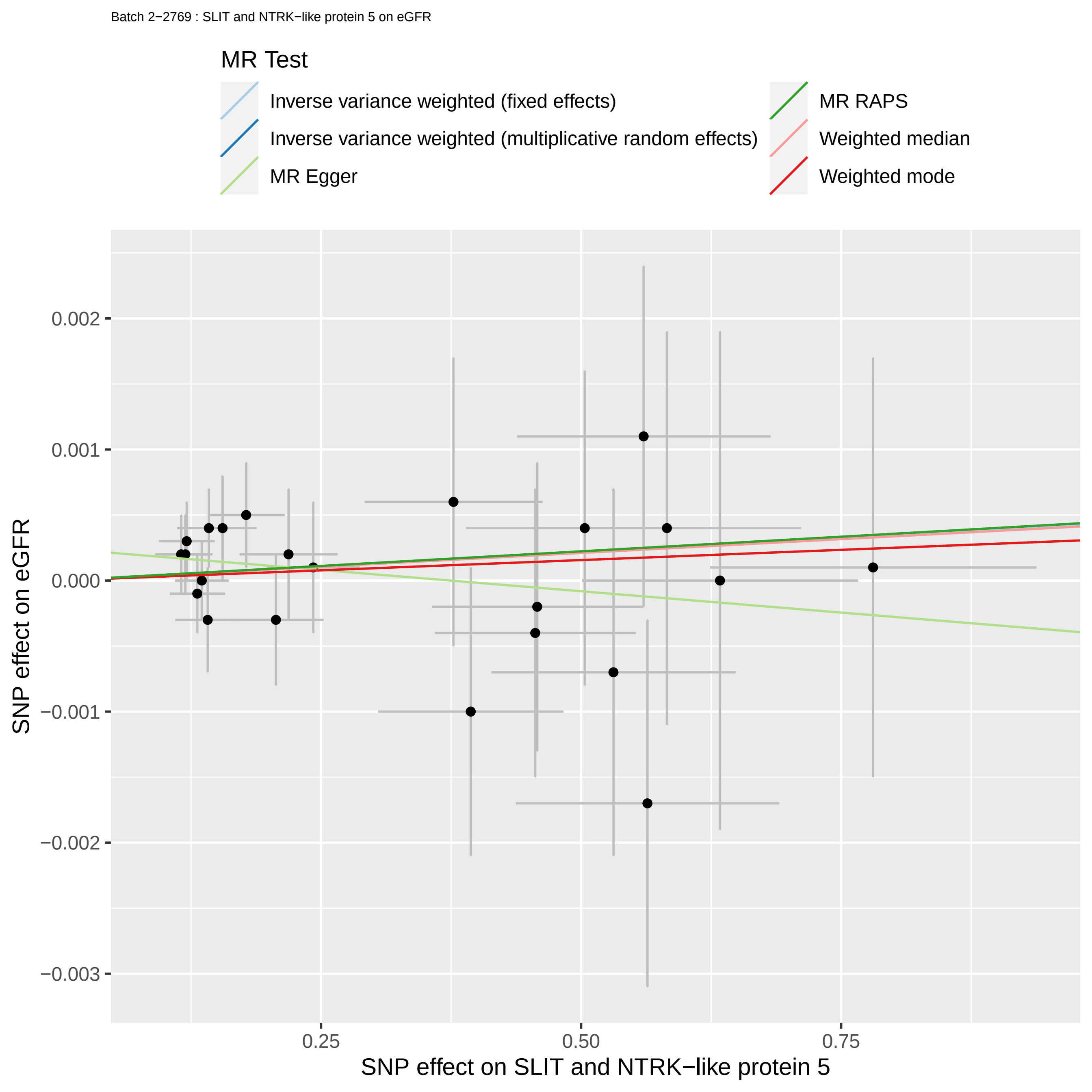
Batch 2−2769 : SLIT and NTRK−like protein 5 on eGFR
MR Test
Inverse variance weighted (fixed effects)
Inverse variance weighted (multiplicative random effects)
MR Egger
MR RAPS
Weighted median
Weighted mode
0.002
0.001
0.000
−0.001
−0.002
−0.003
0.25
0.50
0.75
SNP effect on eGFR
SNP effect on SLIT and NTRK−like protein 5

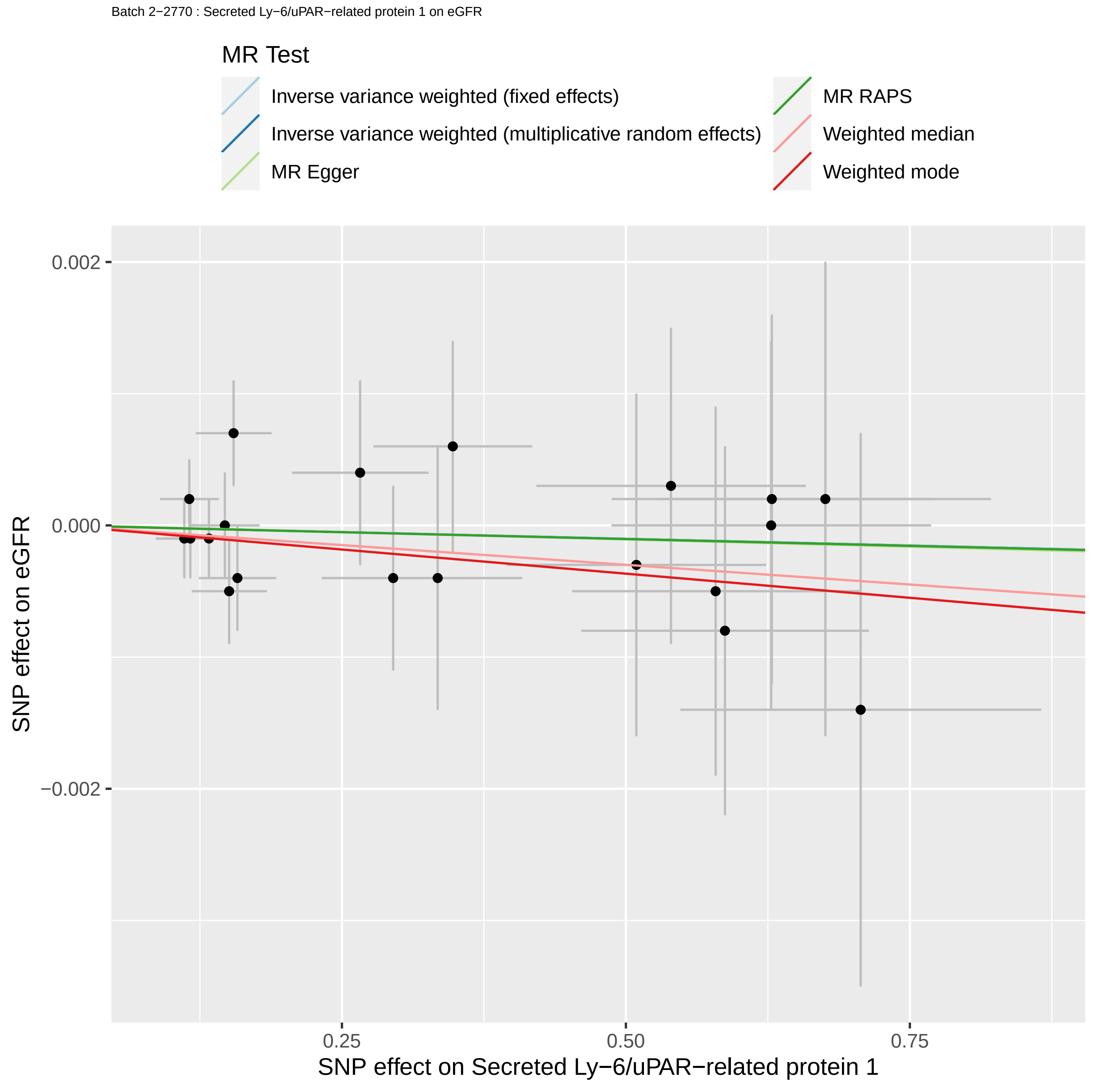
Batch 2−2770 : Secreted Ly−6/uPAR−related protein 1 on eGFR
MR Test
Inverse variance weighted (fixed effects)
Inverse variance weighted (multiplicative random effects)
MR Egger
MR RAPS
Weighted median
Weighted mode
0.002
0.000
−0.002
0.25
0.50
0.75
SNP effect on eGFR
SNP effect on Secreted Ly−6/uPAR−related protein 1

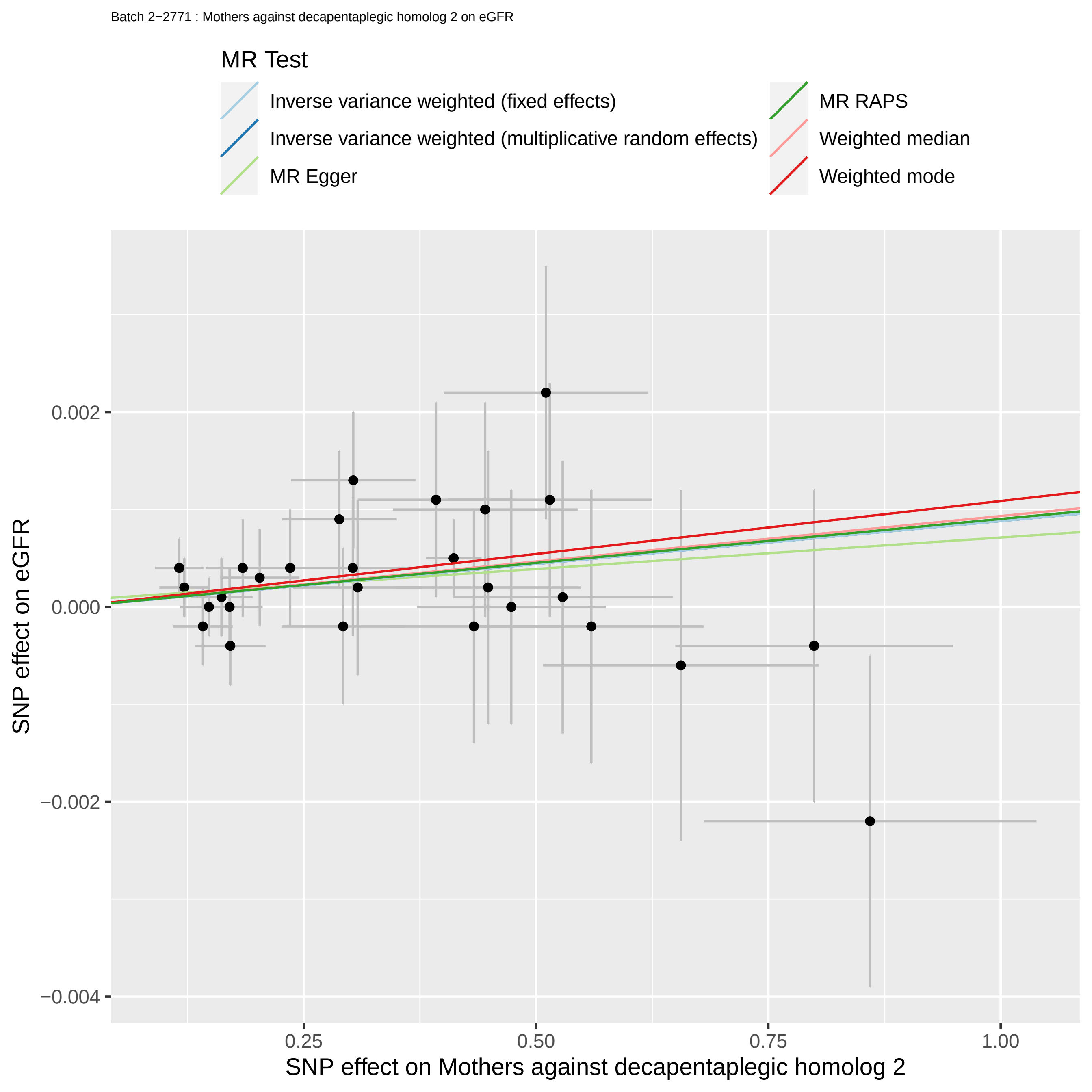

Batch 2−2771 : Mothers against decapentaplegic homolog 2 on eGFR
MR Test
Inverse variance weighted (fixed effects)
Inverse variance weighted (multiplicative random effects)
MR Egger
MR RAPS
Weighted median
Weighted mode
0.002
0.000
−0.002
−0.004
SNP effect on eGFR
0.25
0.50
0.75
1.00
SNP effect on Mothers against decapentaplegic homolog 2

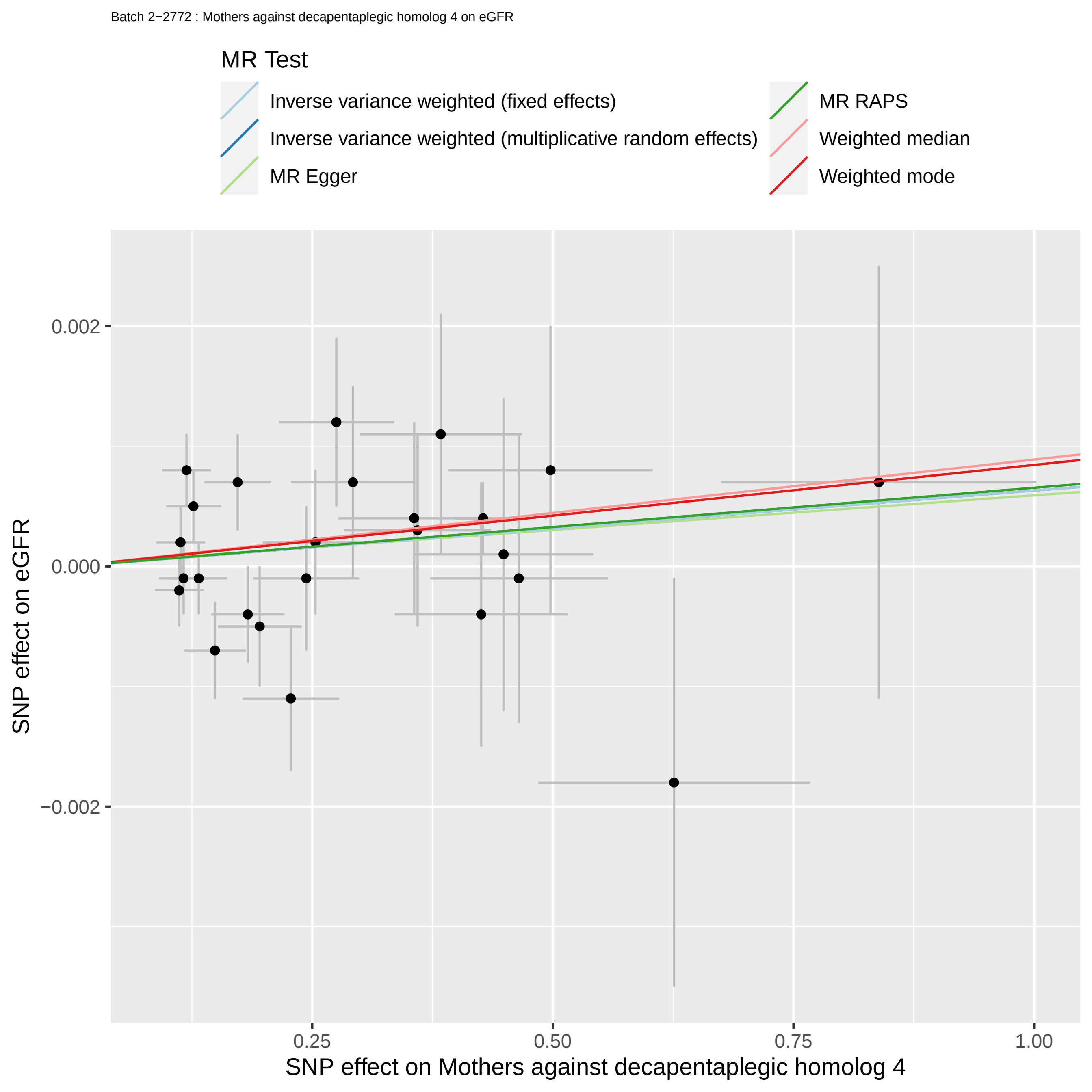

Batch 2−2772 : Mothers against decapentaplegic homolog 4 on eGFR
MR Test
Inverse variance weighted (fixed effects)
Inverse variance weighted (multiplicative random effects)
MR Egger
MR RAPS
Weighted median
Weighted mode
0.002
0.000
−0.002
SNP effect on eGFR
0.25
0.50
0.75
1.00
SNP effect on Mothers against decapentaplegic homolog 4

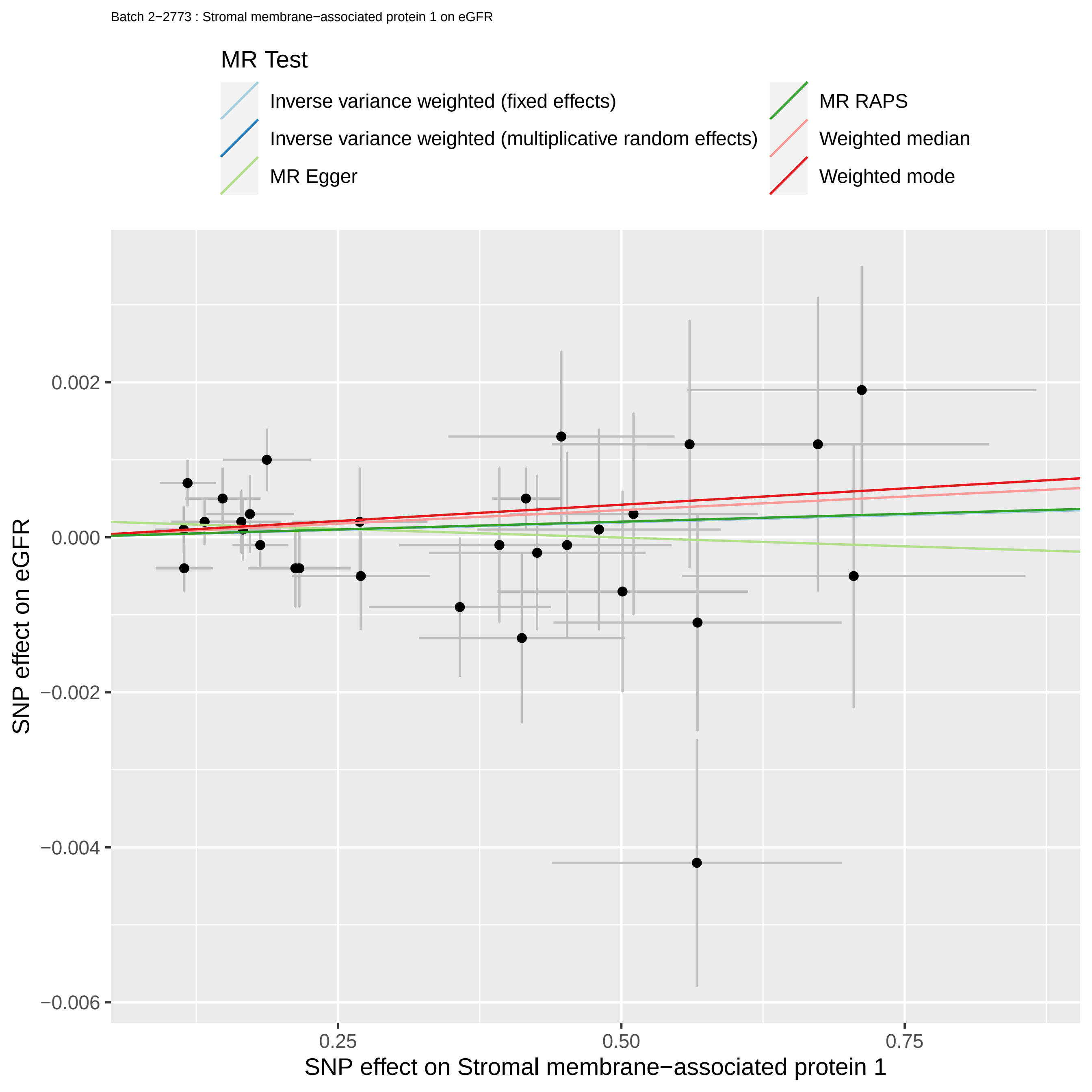

Batch 2−2773 : Stromal membrane−associated protein 1 on eGFR
MR Test
Inverse variance weighted (fixed effects)
Inverse variance weighted (multiplicative random effects)
MR Egger
MR RAPS
Weighted median
Weighted mode
0.002
0.000
−0.002
−0.004
−0.006
SNP effect on eGFR
0.25
0.50
0.75
SNP effect on Stromal membrane−associated protein 1

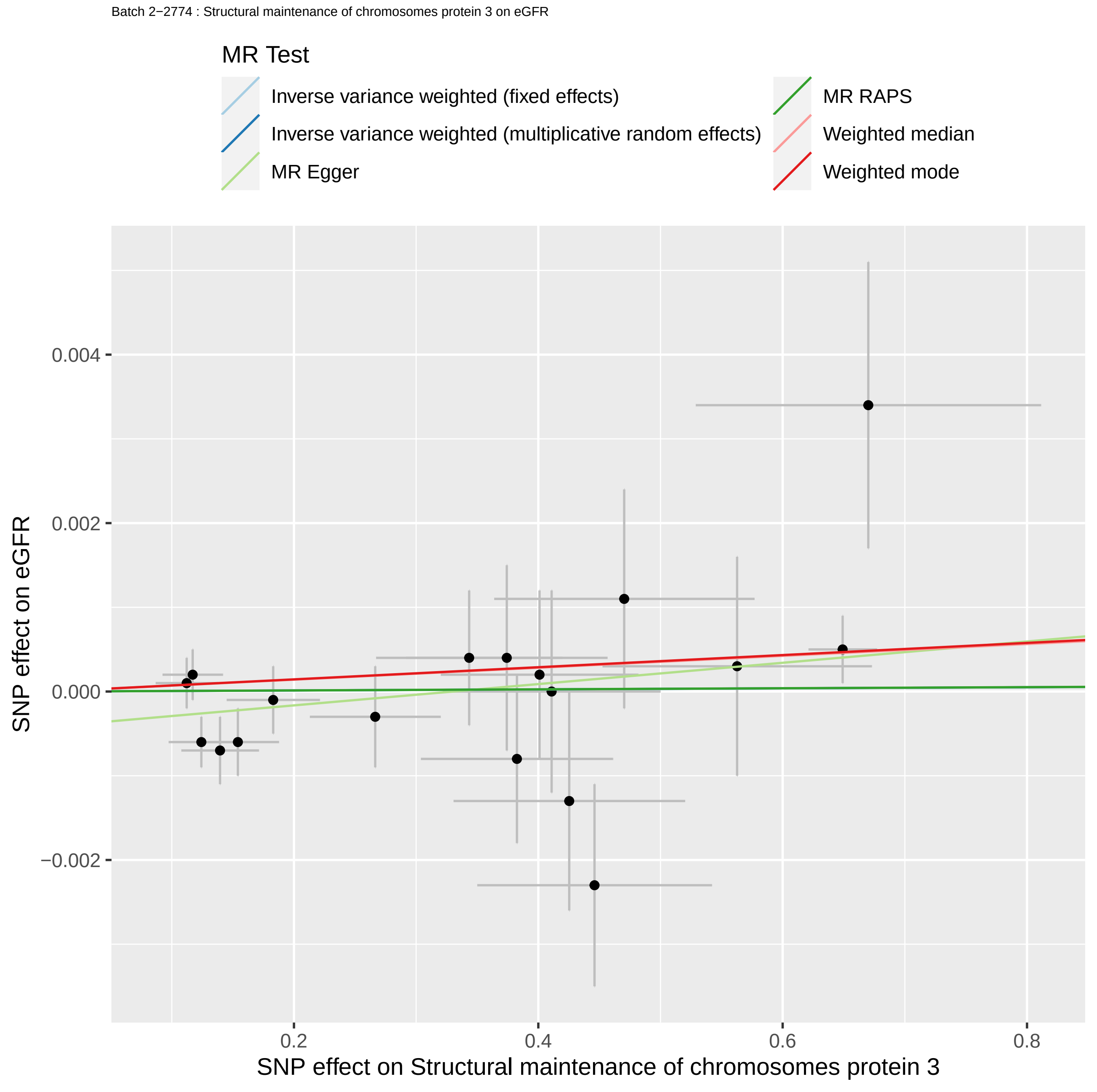

Batch 2−2774 : Structural maintenance of chromosomes protein 3 on eGFR
MR Test
Inverse variance weighted (fixed effects)
Inverse variance weighted (multiplicative random effects)
MR Egger
MR RAPS
Weighted median
Weighted mode
0.004
0.002
0.000
−0.002
SNP effect on eGFR
0.2
0.4
0.6
0.8
SNP effect on Structural maintenance of chromosomes protein 3

Batch 2−2775 : Single−pass membrane and coiled−coil domain−containing protein 2 on eGFR

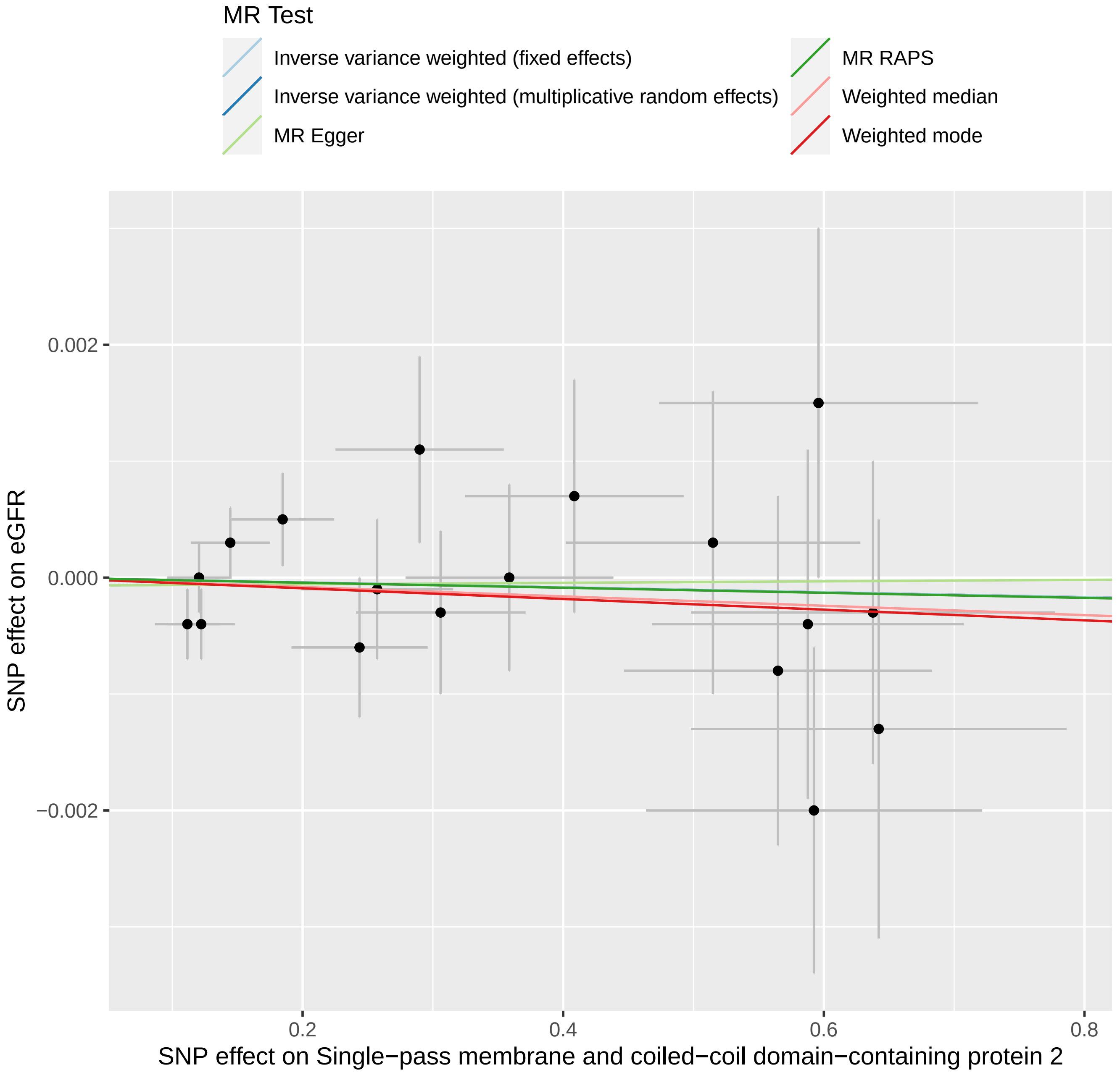

Batch 2−2776 : Essential MCU regulator, mitochondrial on eGFR

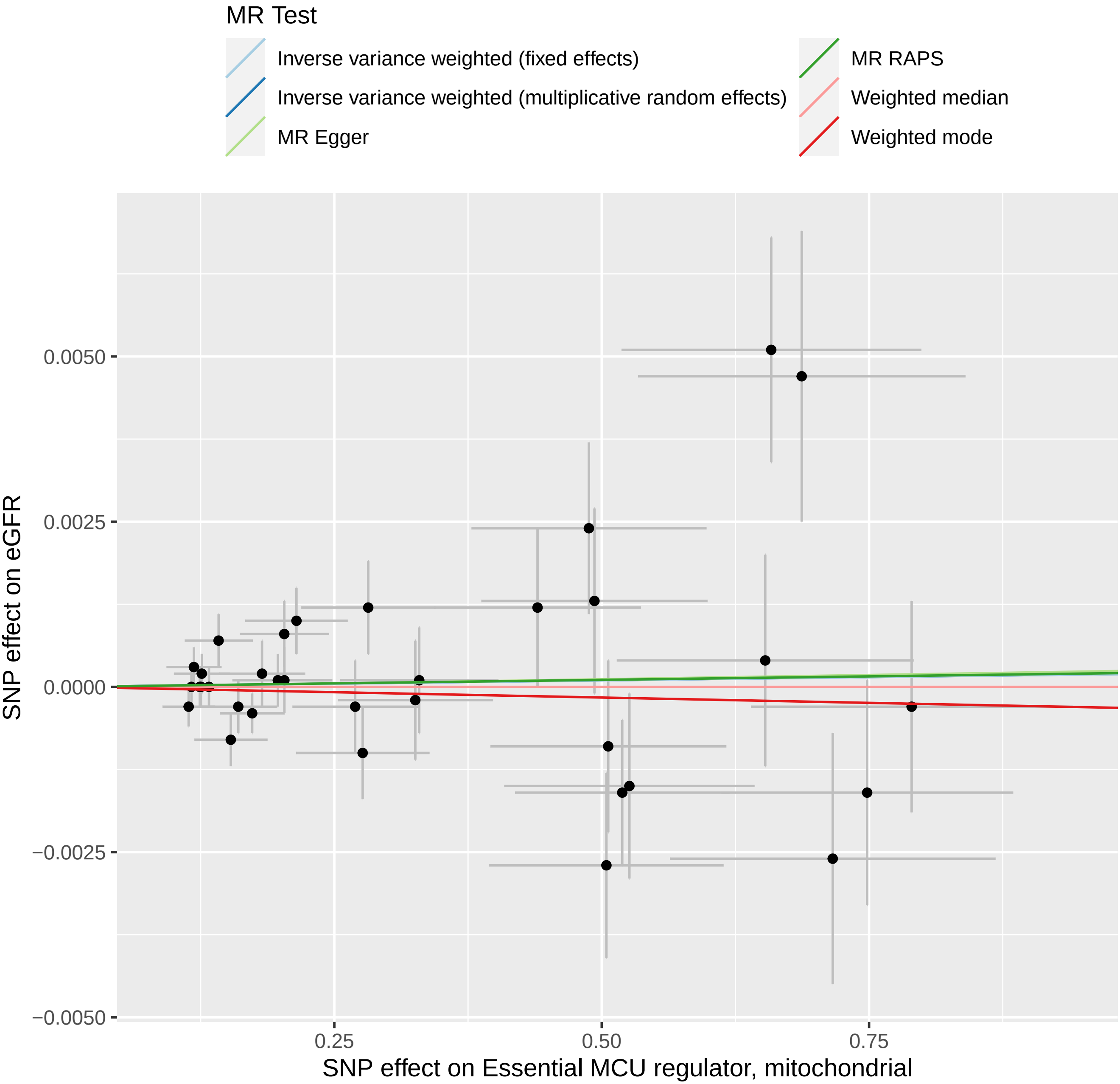

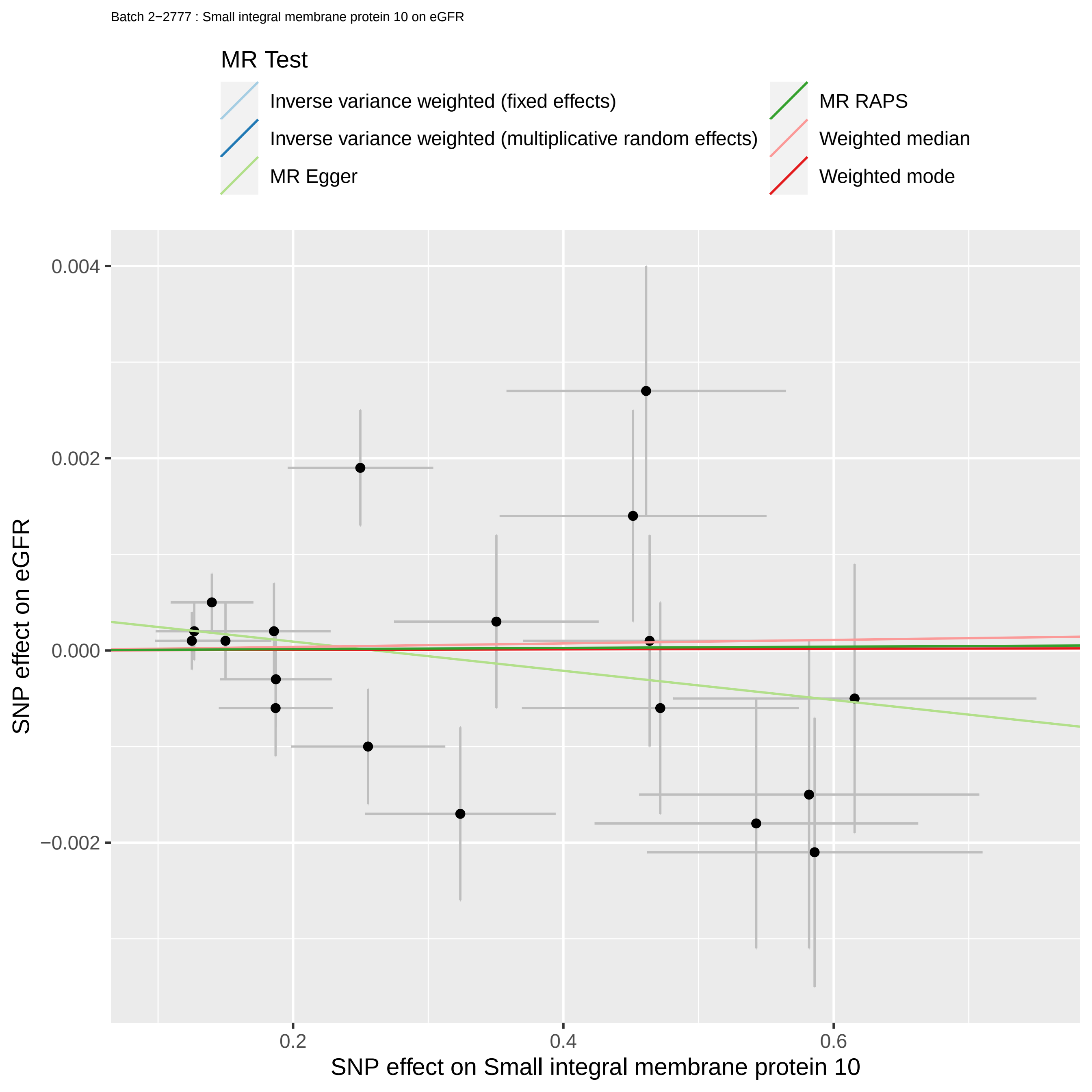

Batch 2−2777 : Small integral membrane protein 10 on eGFR
MR Test
Inverse variance weighted (fixed effects)
Inverse variance weighted (multiplicative random effects)
MR Egger
MR RAPS
Weighted median
Weighted mode
0.004
0.002
0.000
−0.002
SNP effect on eGFR
0.2
0.4
0.6
SNP effect on Small integral membrane protein 10

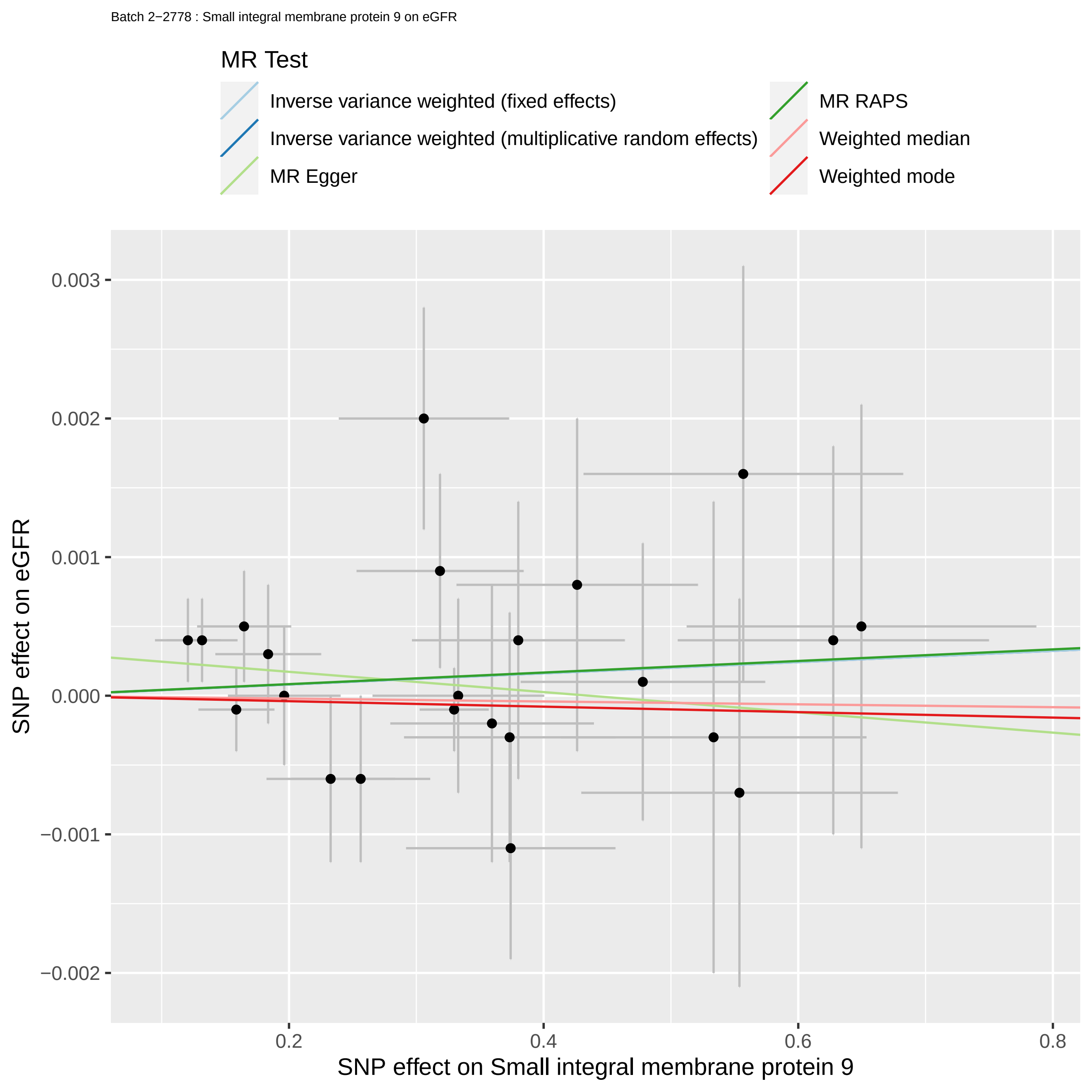
Batch 2−2778 : Small integral membrane protein 9 on eGFR
MR Test
Inverse variance weighted (fixed effects)
Inverse variance weighted (multiplicative random effects)
MR Egger
MR RAPS
Weighted median
Weighted mode
SNP effect on eGFR
0.003
0.002
0.001
0.000
−0.001
−0.002
SNP effect on Small integral membrane protein 9
0.2
0.4
0.6
0.8

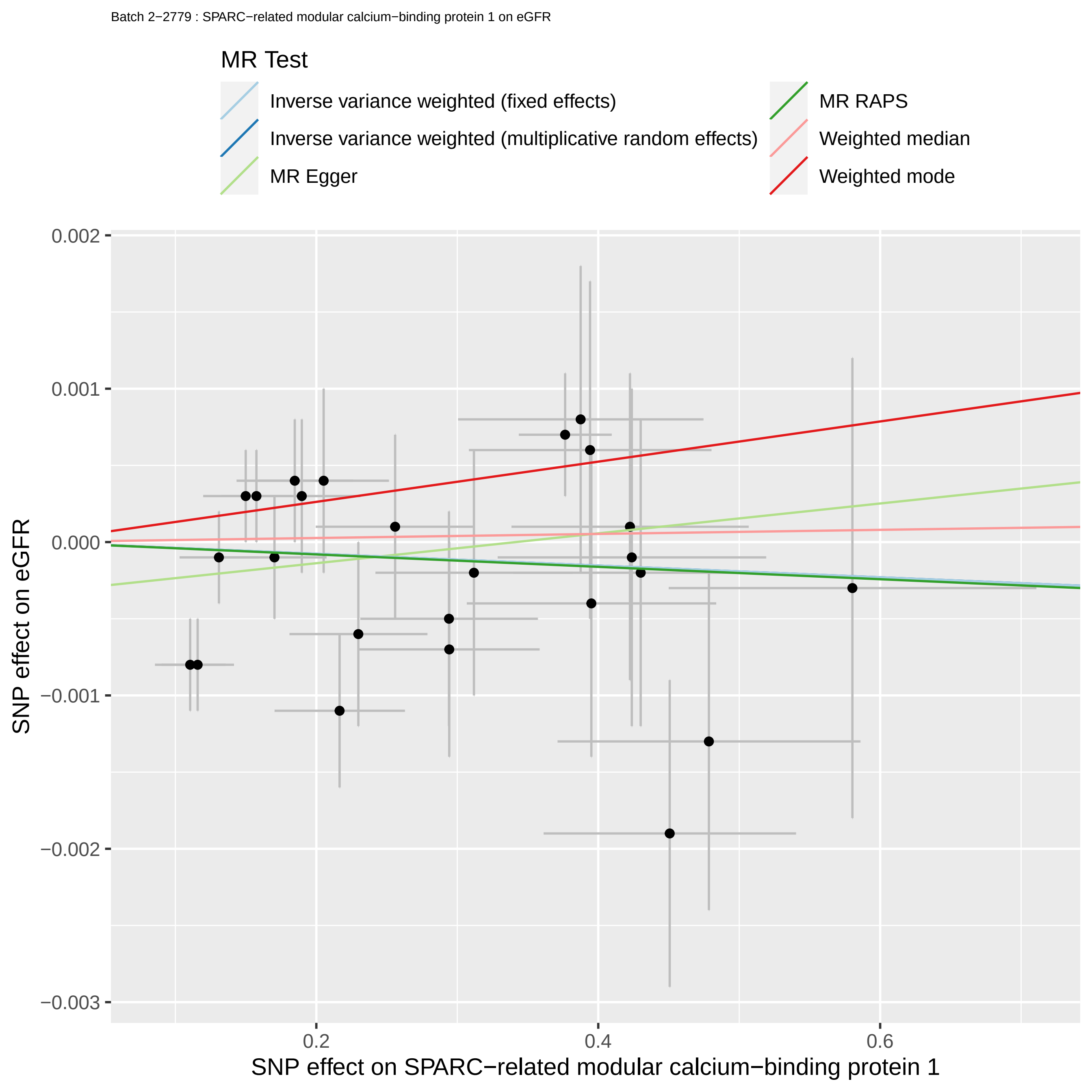

Batch 2−2779 : SPARC−related modular calcium−binding protein 1 on eGFR
MR Test
Inverse variance weighted (fixed effects)
Inverse variance weighted (multiplicative random effects)
MR Egger
MR RAPS
Weighted median
Weighted mode
0.002
0.001
0.000
−0.001
−0.002
−0.003
SNP effect on eGFR
0.2
0.4
0.6
SNP effect on SPARC−related modular calcium−binding protein 1

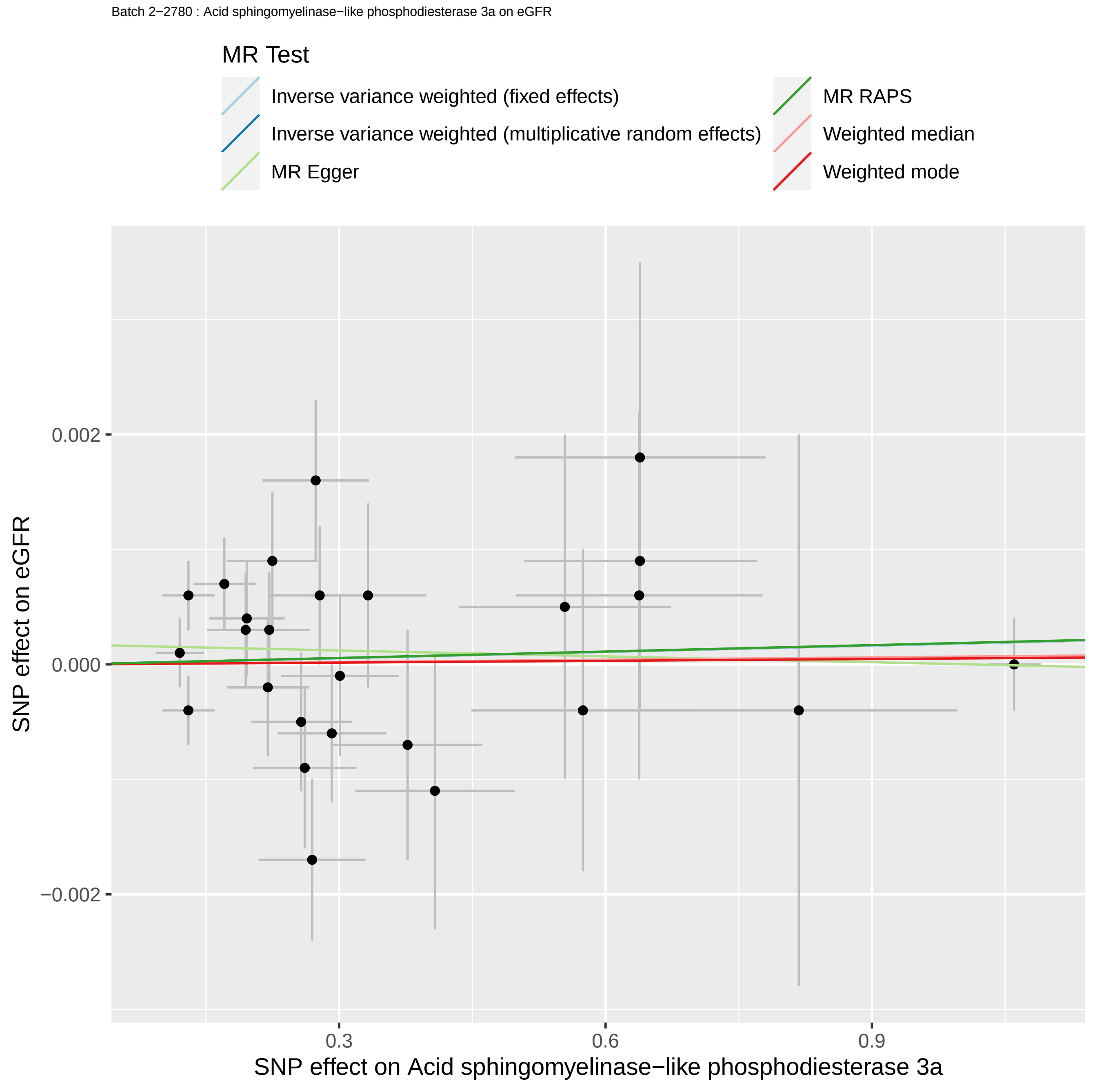

Batch 2−2780 : Acid sphingomyelinase−like phosphodiesterase 3a on eGFR
MR Test
Inverse variance weighted (fixed effects)
Inverse variance weighted (multiplicative random effects)
MR Egger
MR RAPS
Weighted median
Weighted mode
0.002
0.000
−0.002
0.3
0.6
0.9
SNP effect on eGFR
SNP effect on Acid sphingomyelinase−like phosphodiesterase 3a

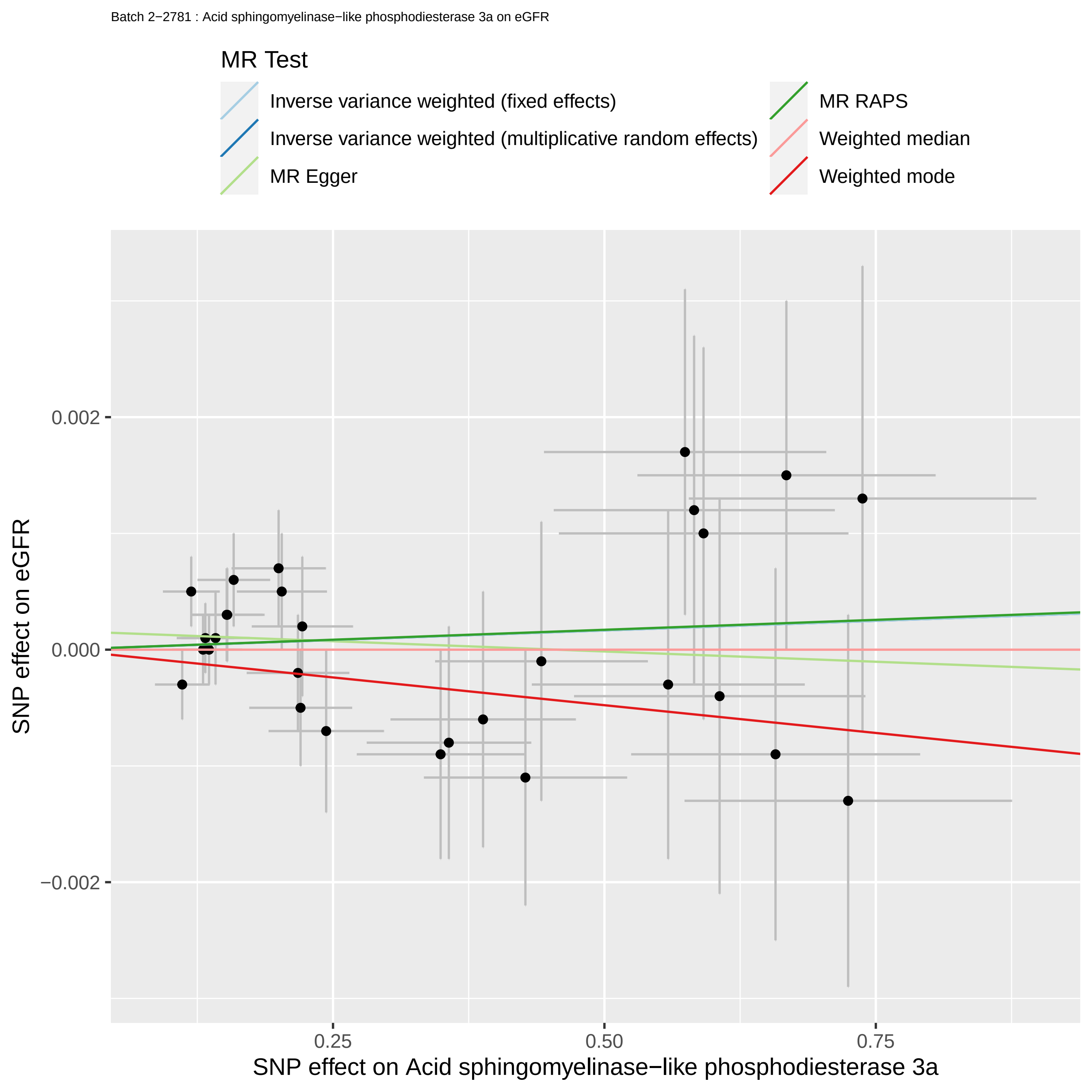

Batch 2−2781 : Acid sphingomyelinase−like phosphodiesterase 3a on eGFR
MR Test
Inverse variance weighted (fixed effects)
Inverse variance weighted (multiplicative random effects)
MR Egger
MR RAPS
Weighted median
Weighted mode
0.002
0.000
−0.002
SNP effect on eGFR
0.25
0.50
0.75
SNP effect on Acid sphingomyelinase−like phosphodiesterase 3a

Batch 2−2782 : Submaxillary gland androgen−regulated protein 3A on eGFR

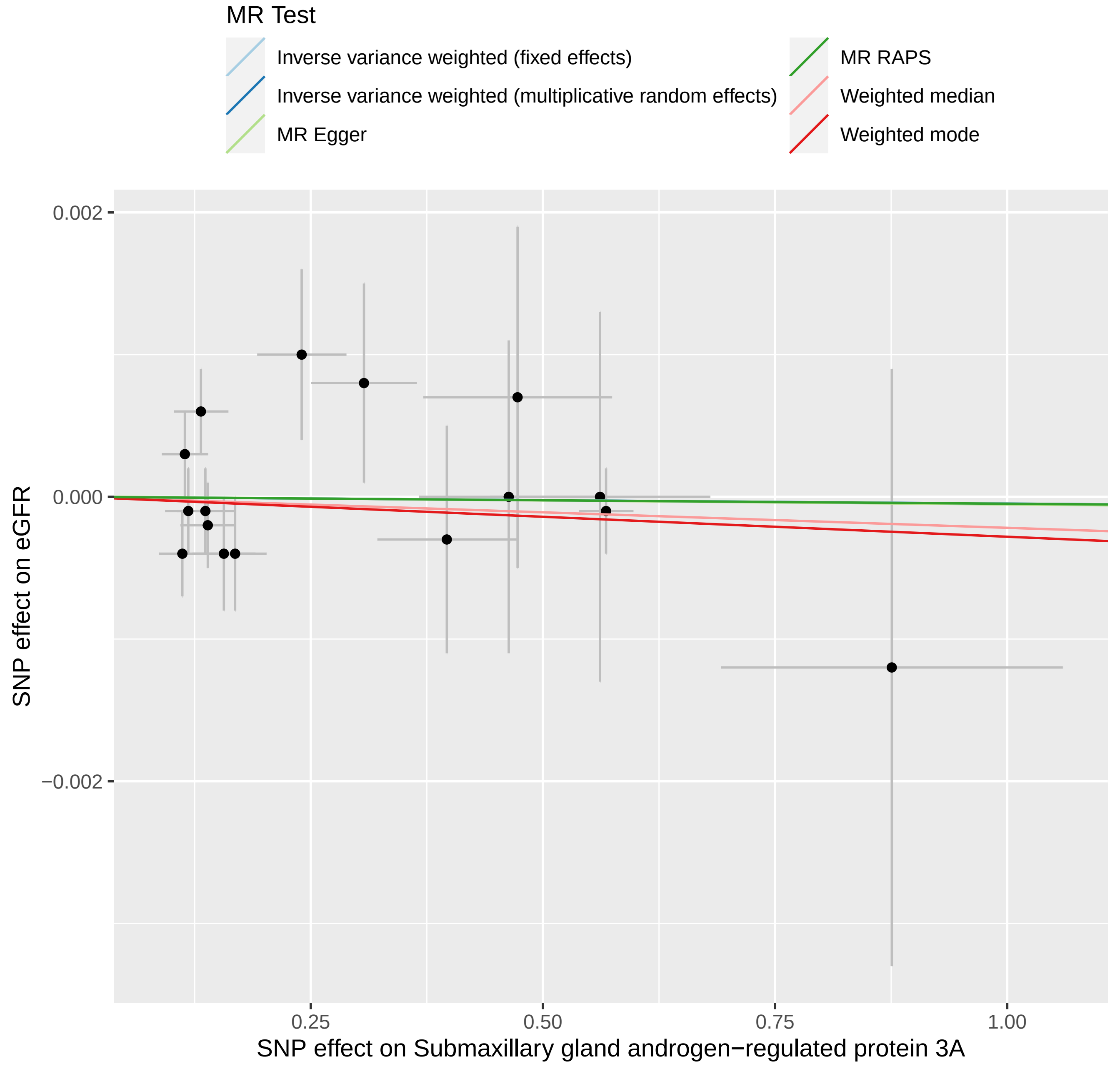

Batch 2−2783 : Submaxillary gland androgen−regulated protein 3B on eGFR

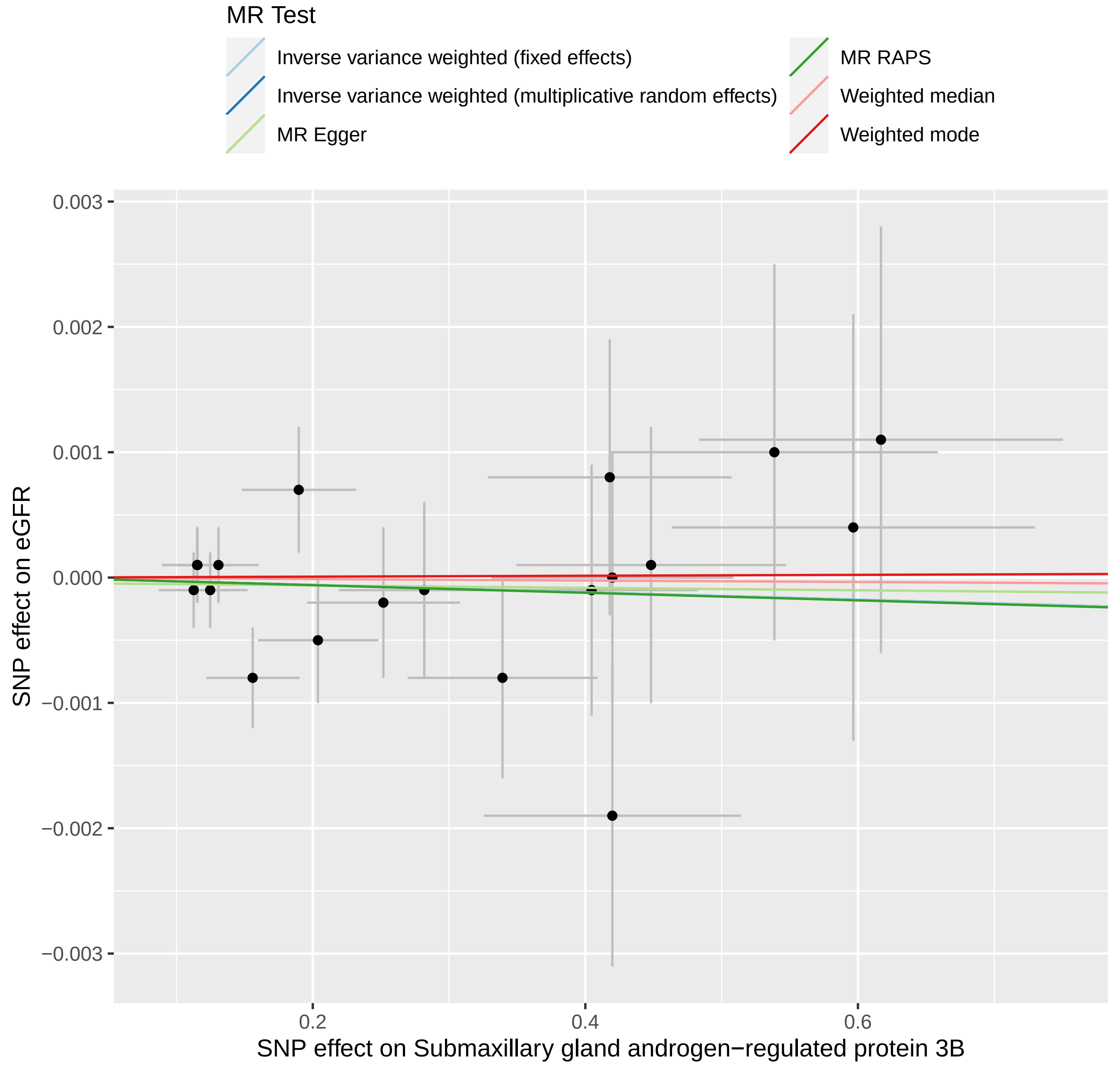

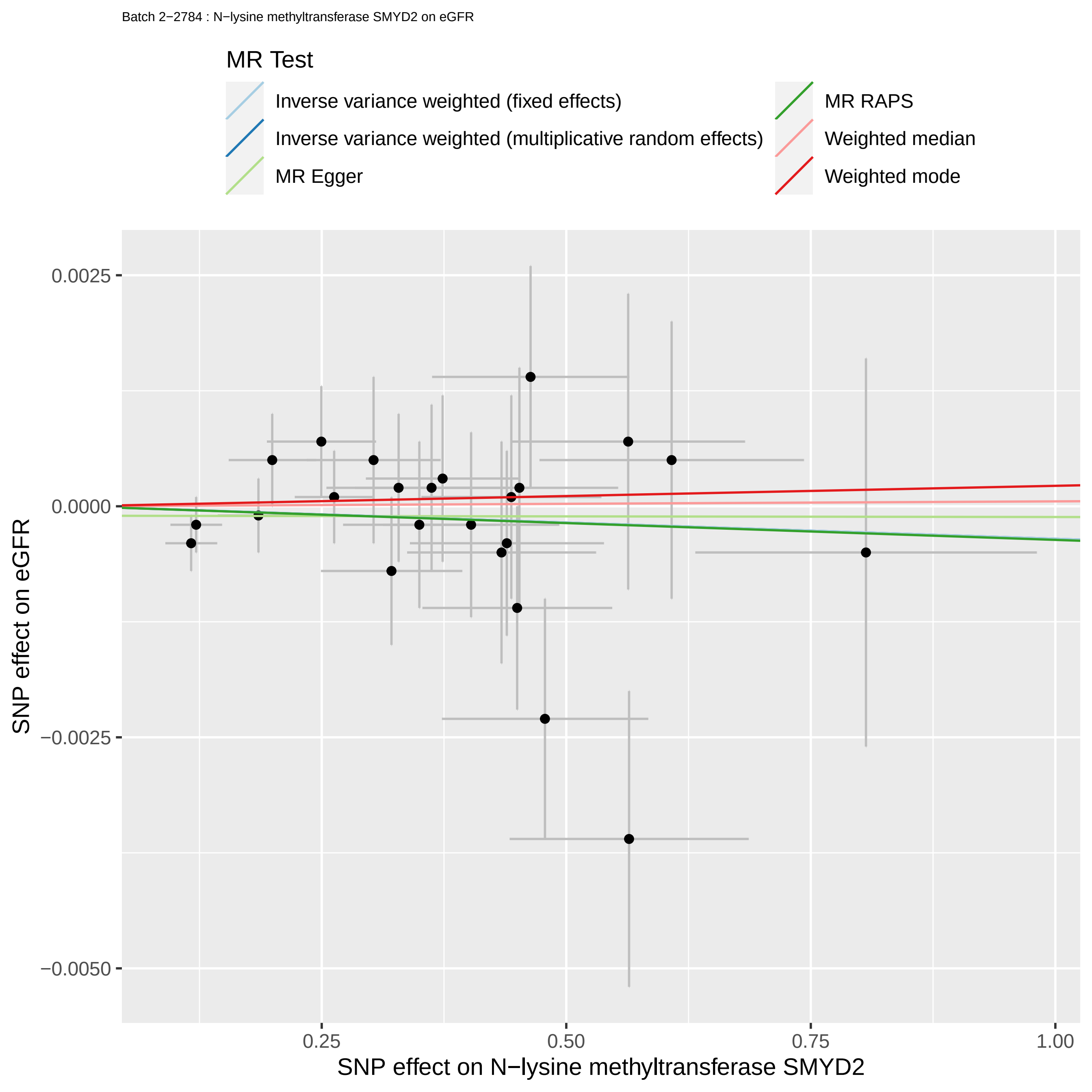

Batch 2−2784 : N−lysine methyltransferase SMYD2 on eGFR
MR Test
Inverse variance weighted (fixed effects)
Inverse variance weighted (multiplicative random effects)
MR Egger
MR RAPS
Weighted median
Weighted mode
0.0025
0.0000
−0.0025
−0.0050
0.25
0.50
0.75
1.00
SNP effect on eGFR
SNP effect on N−lysine methyltransferase SMYD2

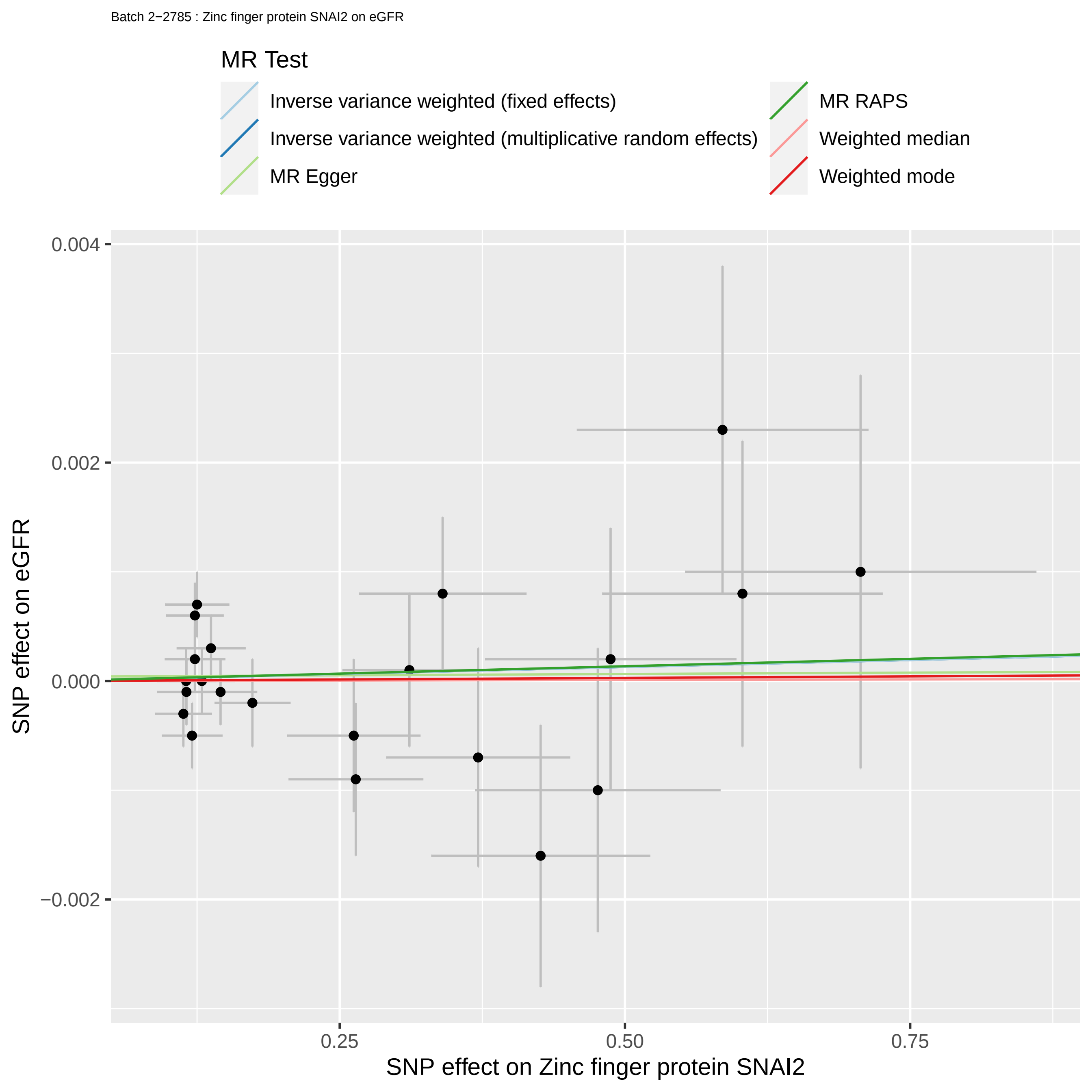
Batch 2−2785 : Zinc finger protein SNAI2 on eGFR
MR Test
Inverse variance weighted (fixed effects)
Inverse variance weighted (multiplicative random effects)
MR Egger
MR RAPS
Weighted median
Weighted mode
0.004
0.002
0.000
−0.002
0.25
0.50
0.75
SNP effect on eGFR
SNP effect on Zinc finger protein SNAI2

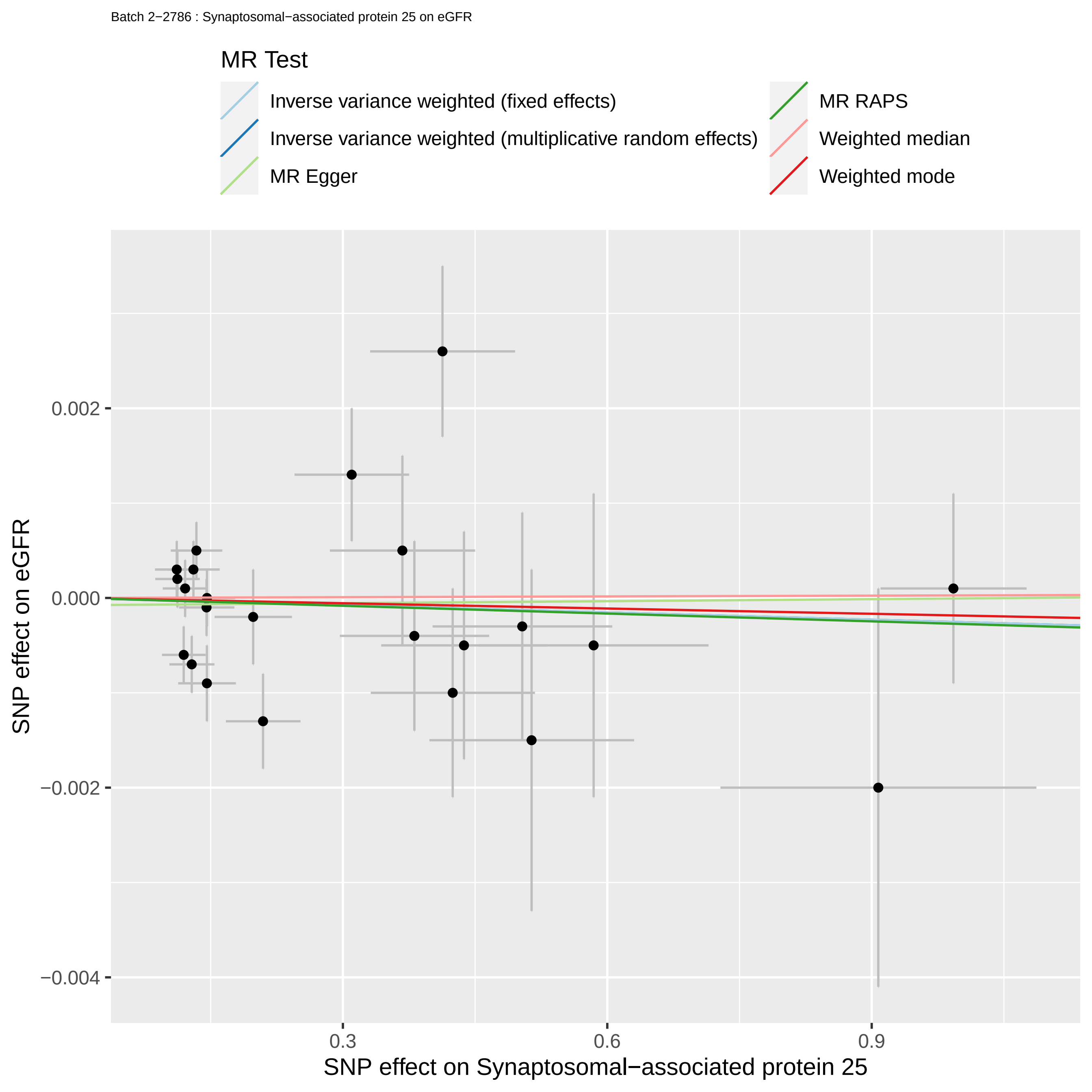

Batch 2−2786 : Synaptosomal−associated protein 25 on eGFR
MR Test
Inverse variance weighted (fixed effects)
Inverse variance weighted (multiplicative random effects)
MR Egger
MR RAPS
Weighted median
Weighted mode
0.002
0.000
−0.002
−0.004
0.3
0.6
0.9
SNP effect on eGFR
SNP effect on Synaptosomal−associated protein 25

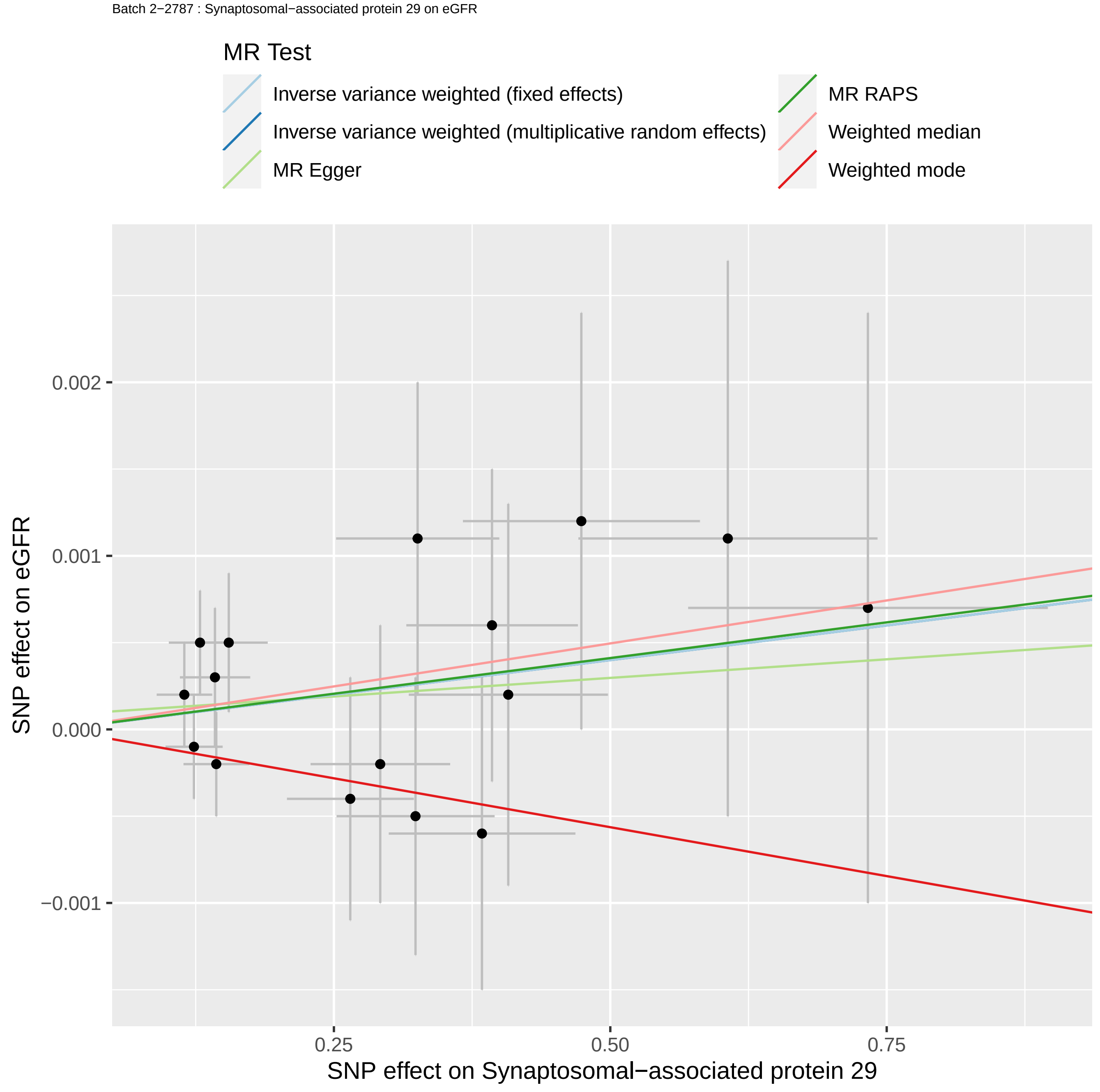

Batch 2−2787 : Synaptosomal−associated protein 29 on eGFR
MR Test
Inverse variance weighted (fixed effects)
Inverse variance weighted (multiplicative random effects)
MR Egger
MR RAPS
Weighted median
Weighted mode
0.002
0.001
0.000
−0.001
0.25
0.50
0.75
SNP effect on eGFR
SNP effect on Synaptosomal−associated protein 29

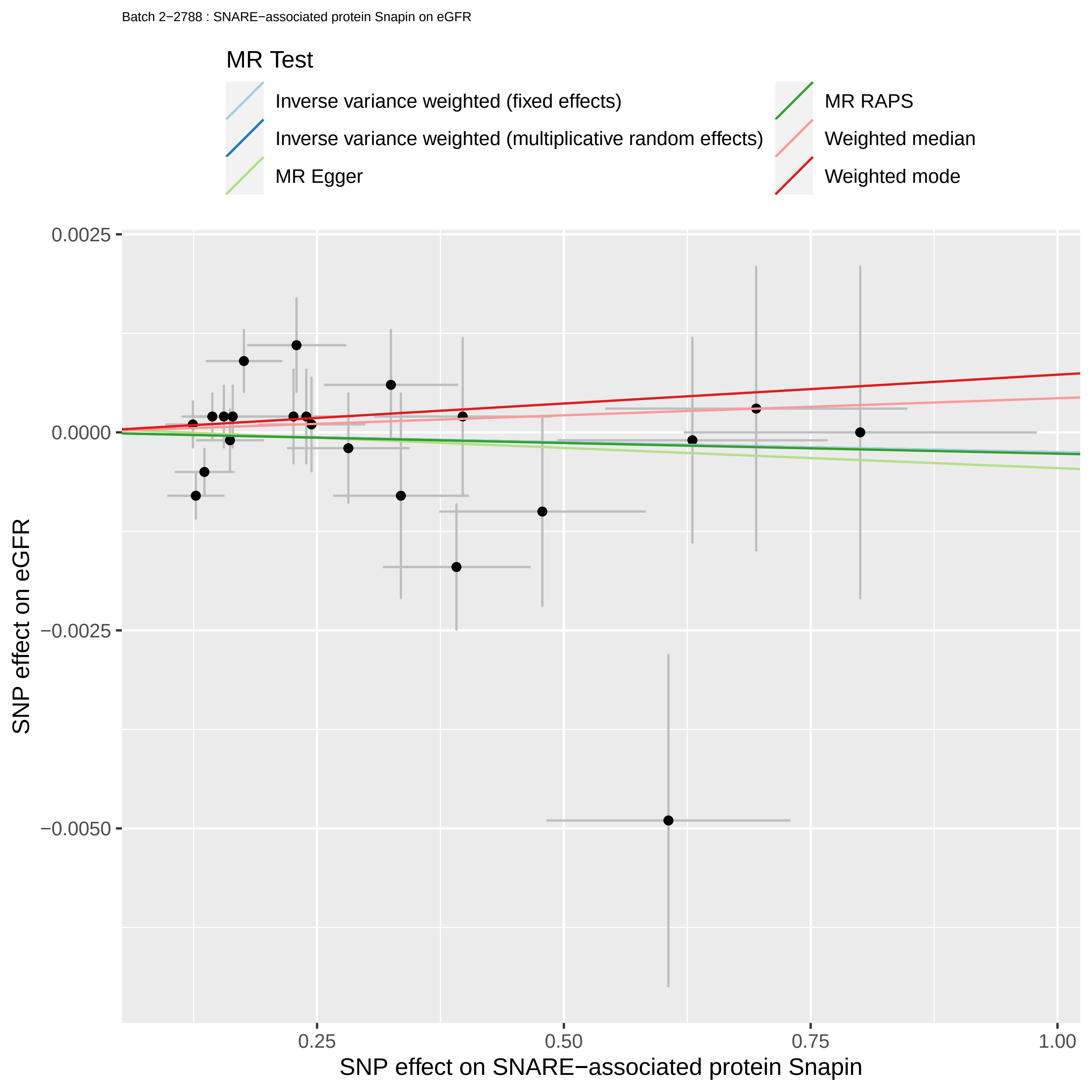
Batch 2−2788 : SNARE−associated protein Snapin on eGFR
MR Test
Inverse variance weighted (fixed effects)
Inverse variance weighted (multiplicative random effects)
MR Egger
MR RAPS
Weighted median
Weighted mode
0.0025
0.0000
−0.0025
−0.0050
SNP effect on eGFR
0.25
0.50
0.75
1.00
SNP effect on SNARE−associated protein Snapin

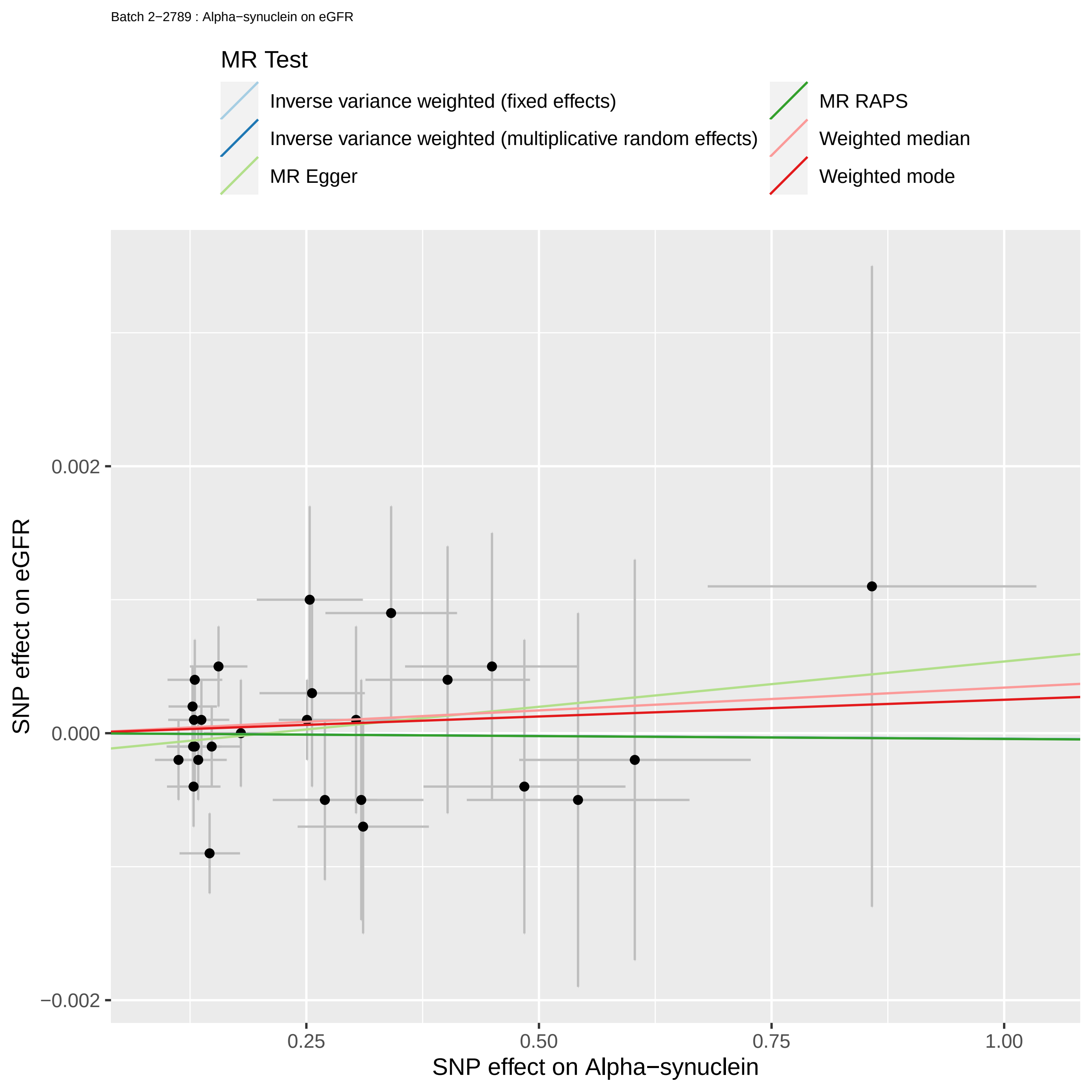
Batch 2−2789 : Alpha−synuclein on eGFR
MR Test
Inverse variance weighted (fixed effects)
Inverse variance weighted (multiplicative random effects)
MR Egger
MR RAPS
Weighted median
Weighted mode
0.002
0.000
−0.002
0.25
0.50
0.75
1.00
SNP effect on eGFR
SNP effect on Alpha−synuclein

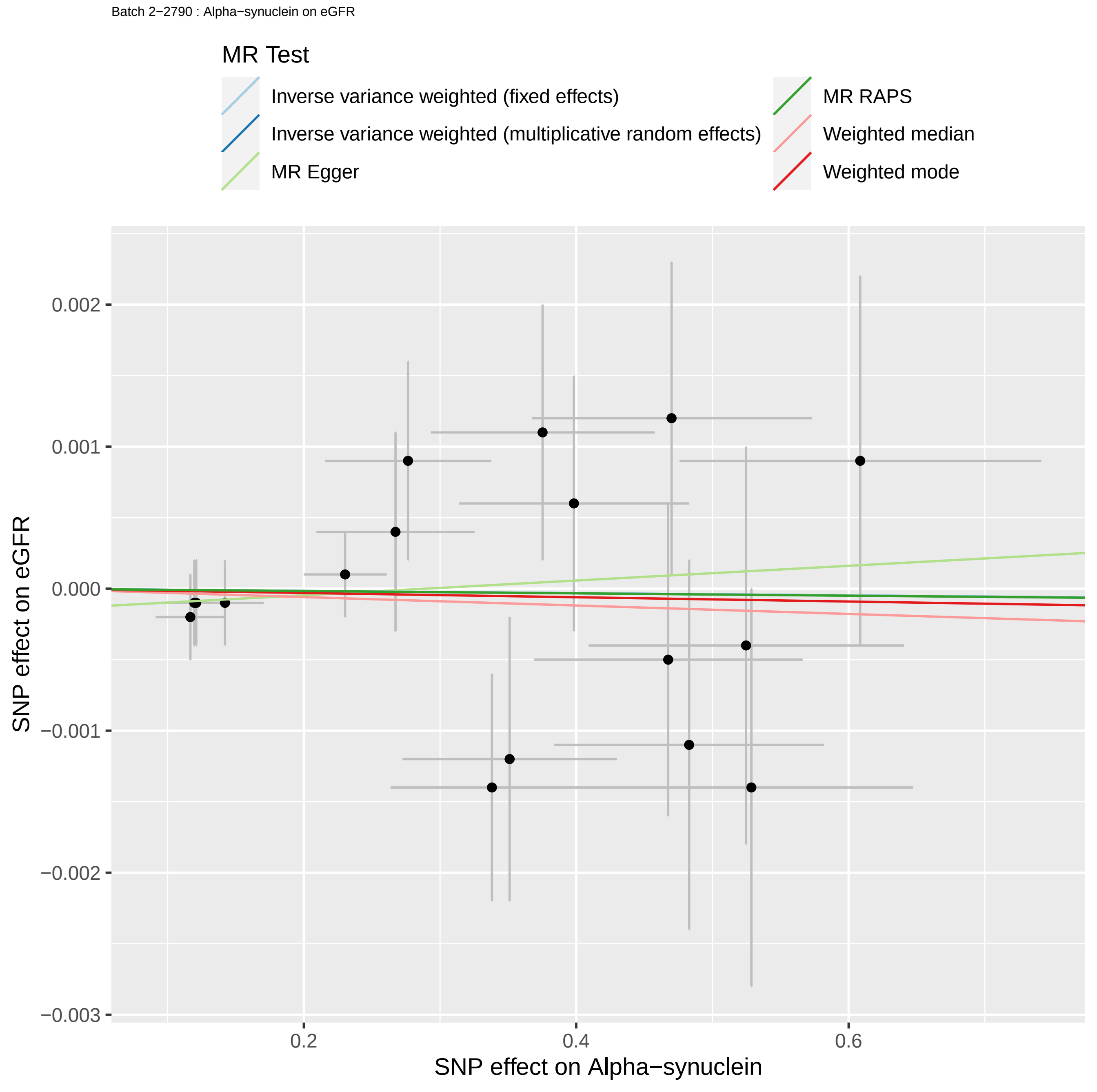

Batch 2−2790 : Alpha−synuclein on eGFR
MR Test
Inverse variance weighted (fixed effects)
Inverse variance weighted (multiplicative random effects)
MR Egger
MR RAPS
Weighted median
Weighted mode
0.002
0.001
0.000
−0.001
−0.002
−0.003
0.2
0.4
0.6
SNP effect on eGFR
SNP effect on Alpha−synuclein

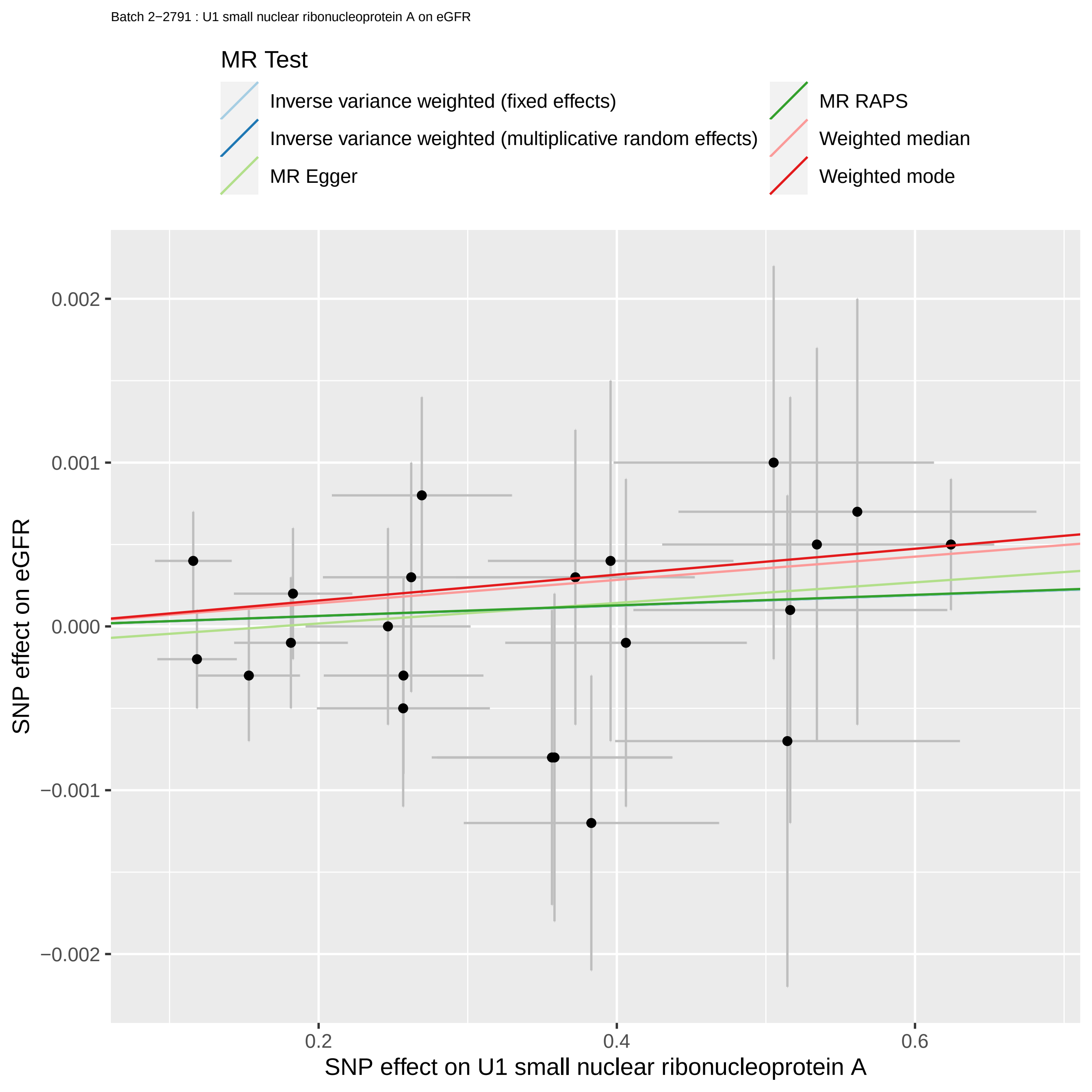
Batch 2−2791 : U1 small nuclear ribonucleoprotein A on eGFR
MR Test
Inverse variance weighted (fixed effects)
Inverse variance weighted (multiplicative random effects)
MR Egger
MR RAPS
Weighted median
Weighted mode
0.002
0.001
0.000
−0.001
−0.002
0.2
0.4
0.6
SNP effect on eGFR
SNP effect on U1 small nuclear ribonucleoprotein A

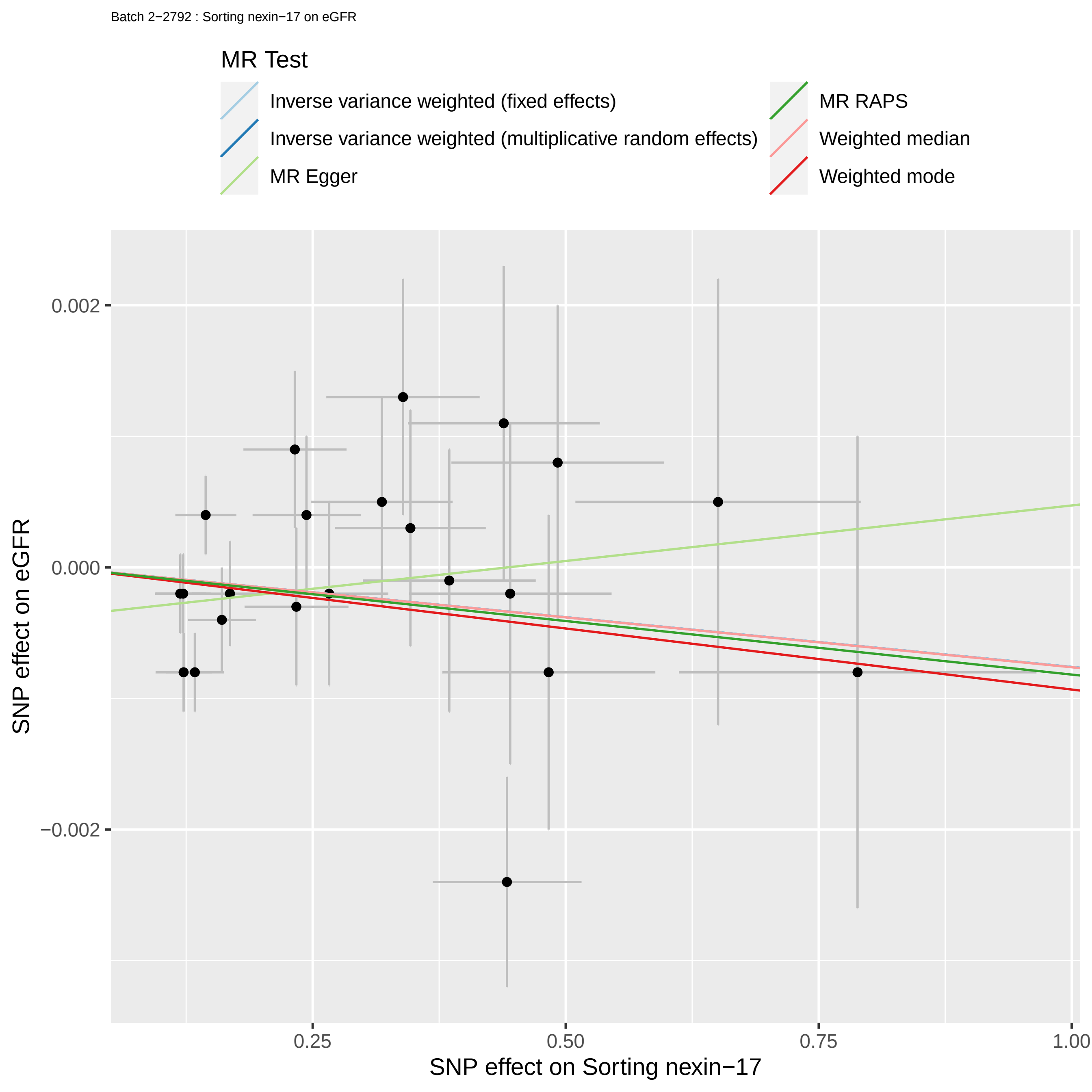
Batch 2−2792 : Sorting nexin−17 on eGFR
MR Test
Inverse variance weighted (fixed effects)
Inverse variance weighted (multiplicative random effects)
MR Egger
MR RAPS
Weighted median
Weighted mode
0.002
0.000
−0.002
0.25
0.50
0.75
1.00
SNP effect on eGFR
SNP effect on Sorting nexin−17

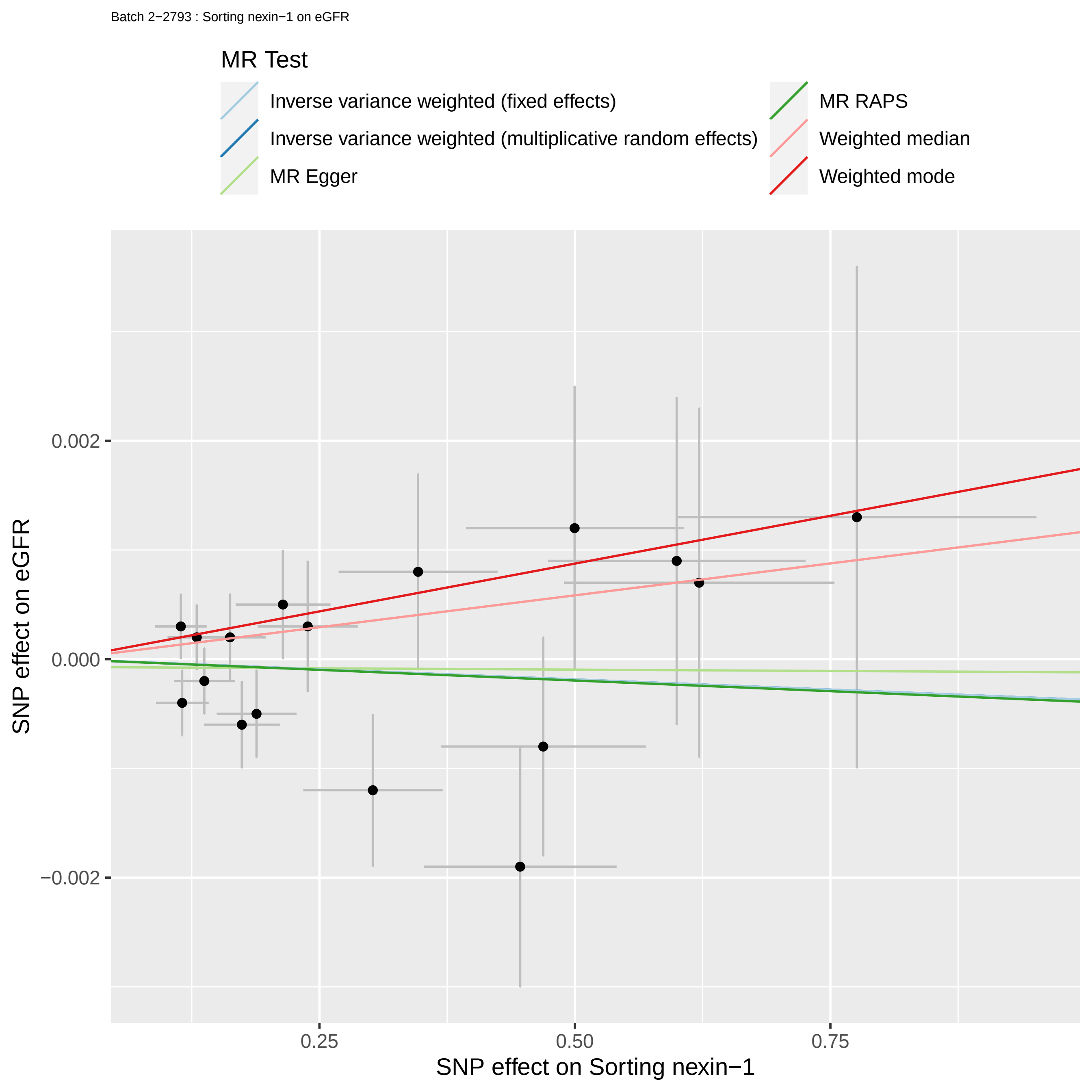

Batch 2−2793 : Sorting nexin−1 on eGFR
MR Test
Inverse variance weighted (fixed effects)
Inverse variance weighted (multiplicative random effects)
MR Egger
MR RAPS
Weighted median
Weighted mode
0.002
0.000
−0.002
0.25
0.50
0.75
SNP effect on eGFR
SNP effect on Sorting nexin−1

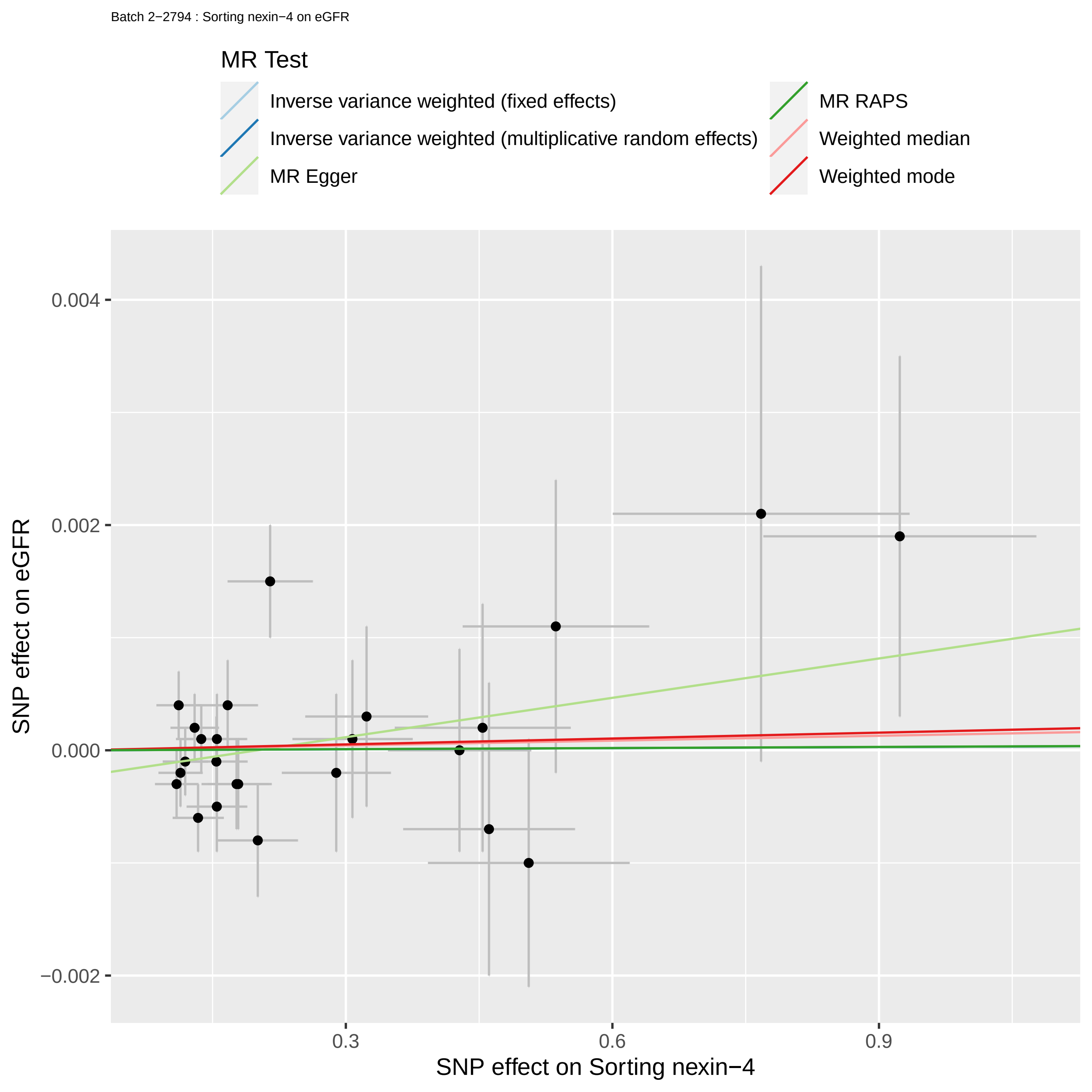

Batch 2−2794 : Sorting nexin−4 on eGFR
MR Test
Inverse variance weighted (fixed effects)
Inverse variance weighted (multiplicative random effects)
MR Egger
MR RAPS
Weighted median
Weighted mode
0.004
0.002
0.000
−0.002
0.3
0.6
0.9
SNP effect on eGFR
SNP effect on Sorting nexin−4

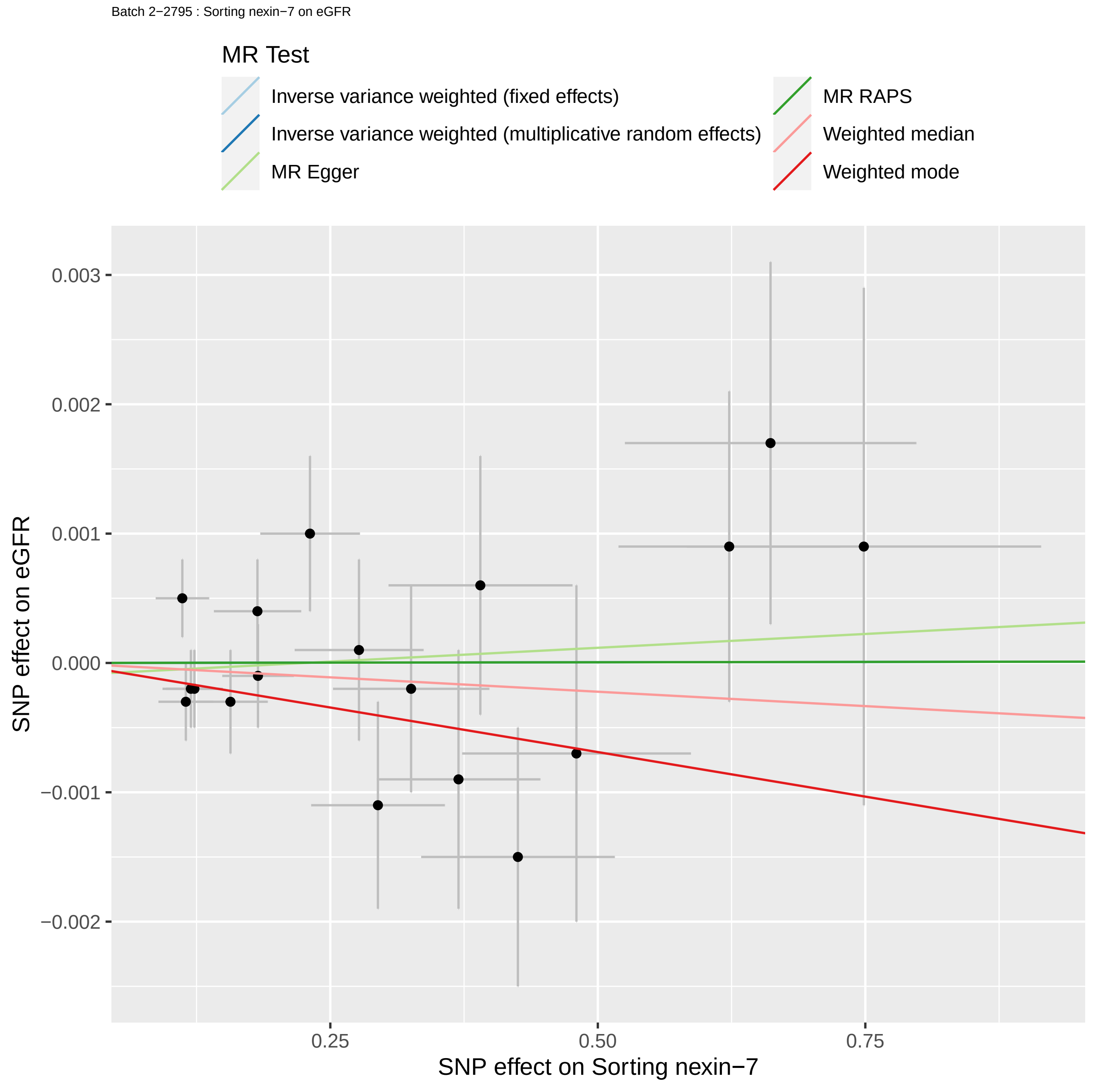

Batch 2−2795 : Sorting nexin−7 on eGFR
MR Test
Inverse variance weighted (fixed effects)
Inverse variance weighted (multiplicative random effects)
MR Egger
MR RAPS
Weighted median
Weighted mode
0.003
0.002
0.001
0.000
−0.001
−0.002
0.25
0.50
0.75
SNP effect on eGFR
SNP effect on Sorting nexin−7

Batch 2−2796 : Sorting nexin−8 on eGFR

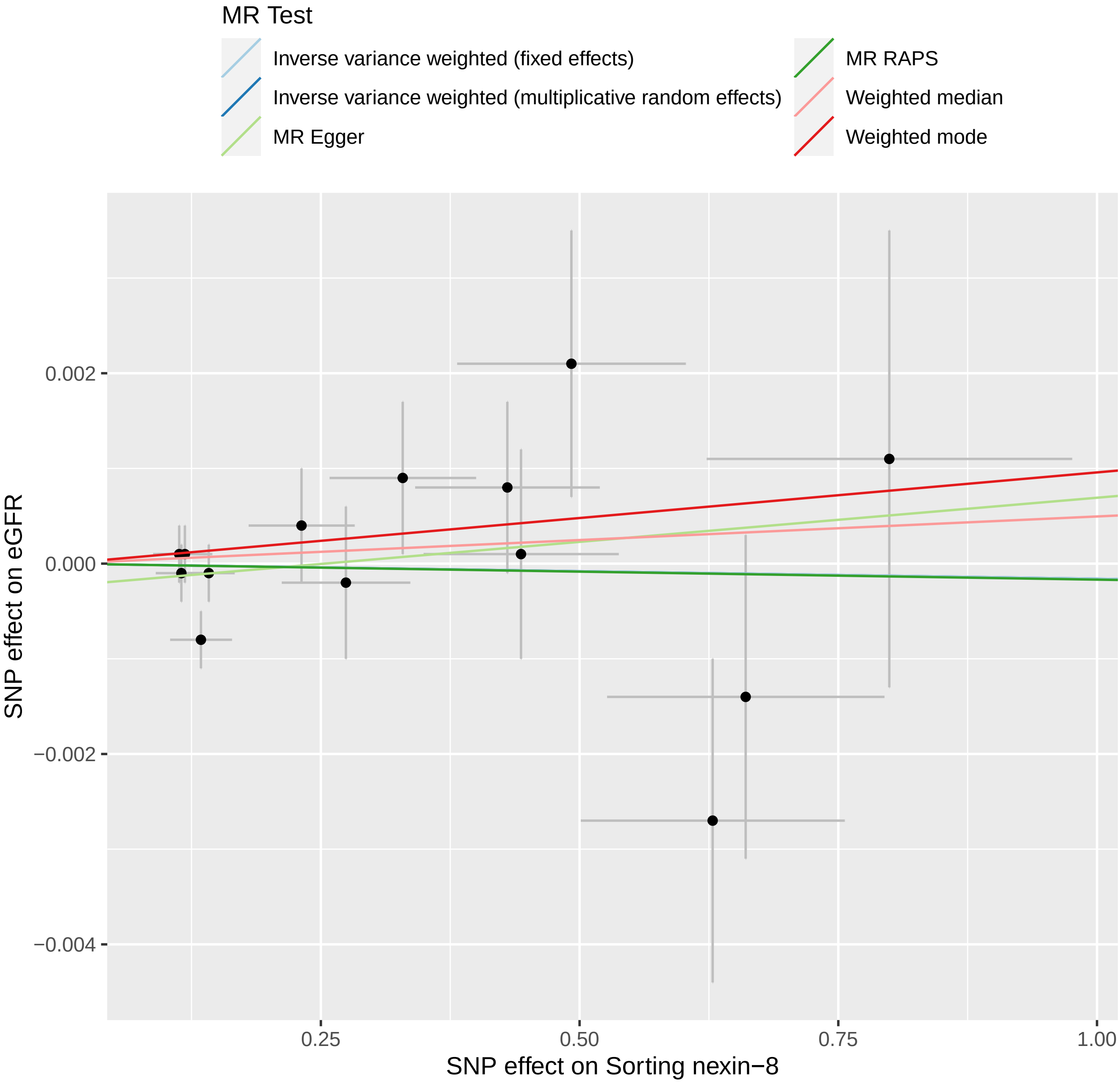

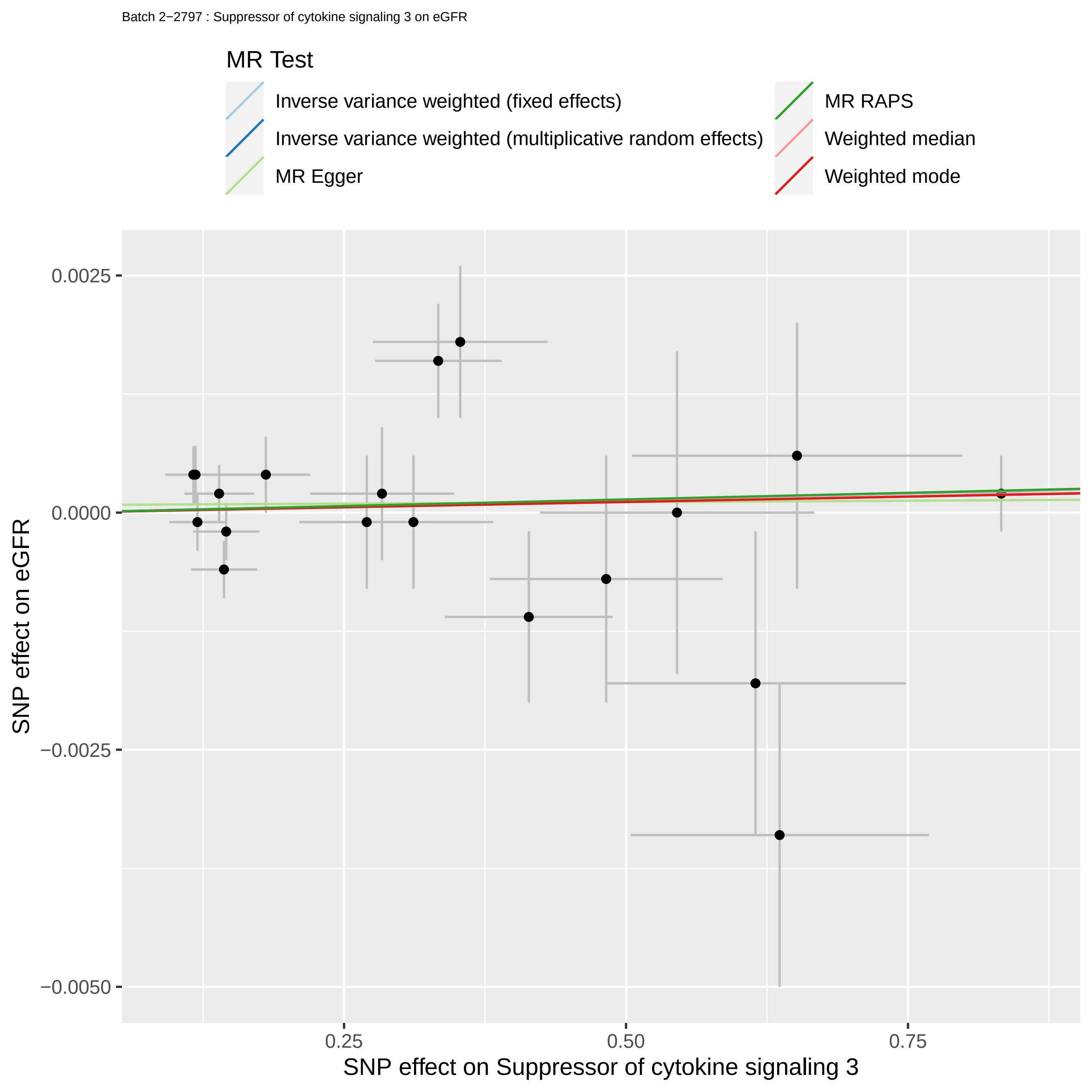

Batch 2−2797 : Suppressor of cytokine signaling 3 on eGFR
MR Test
Inverse variance weighted (fixed effects)
Inverse variance weighted (multiplicative random effects)
MR Egger
MR RAPS
Weighted median
Weighted mode
0.0025
0.0000
−0.0025
−0.0050
0.25
0.50
0.75
SNP effect on eGFR
SNP effect on Suppressor of cytokine signaling 3

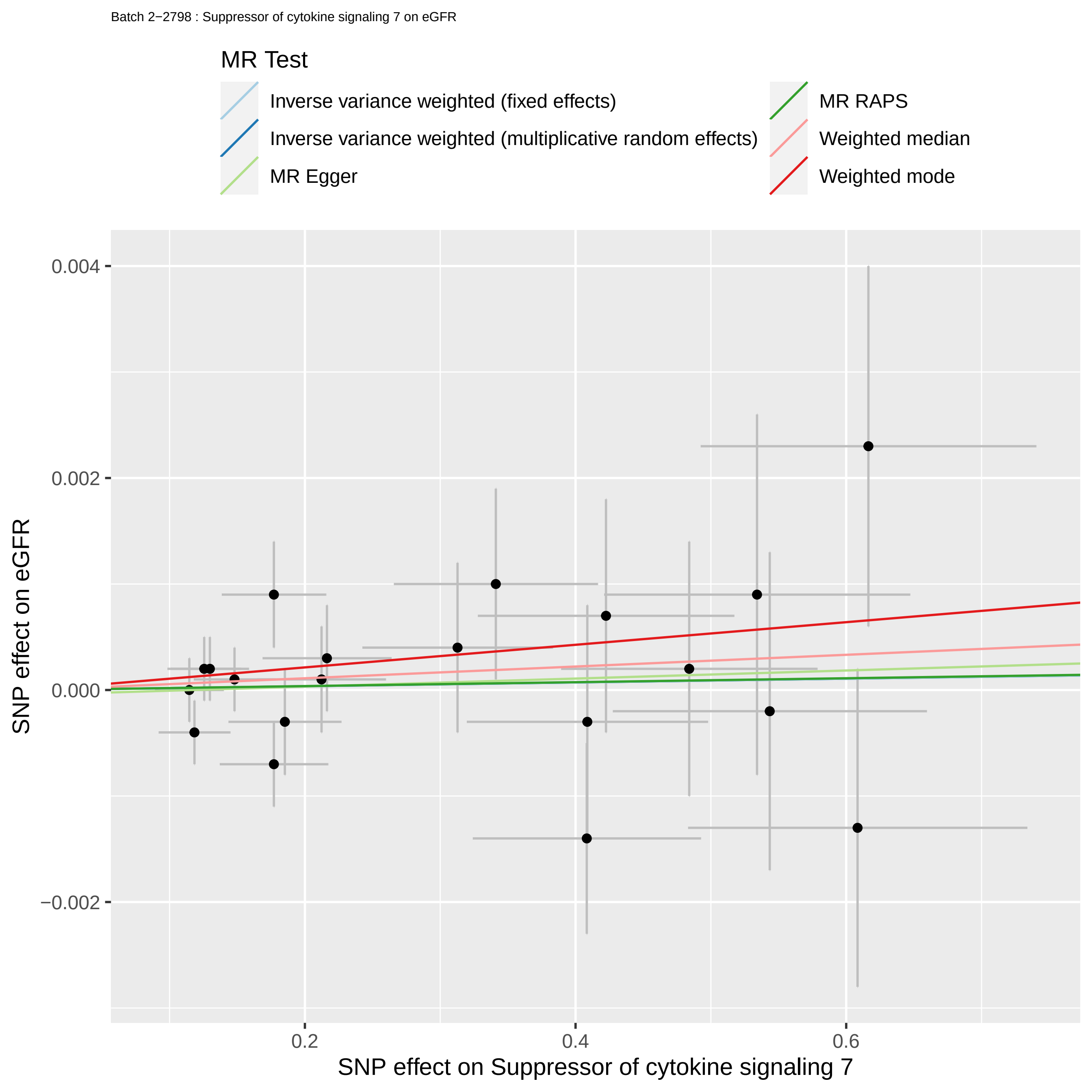
Batch 2−2798 : Suppressor of cytokine signaling 7 on eGFR
MR Test
Inverse variance weighted (fixed effects)
Inverse variance weighted (multiplicative random effects)
MR Egger
MR RAPS
Weighted median
Weighted mode
0.004
0.002
0.000
−0.002
0.2
0.4
0.6
SNP effect on eGFR
SNP effect on Suppressor of cytokine signaling 7

Batch 2−2799 : Superoxide dismutase [Mn], mitochondrial on eGFR

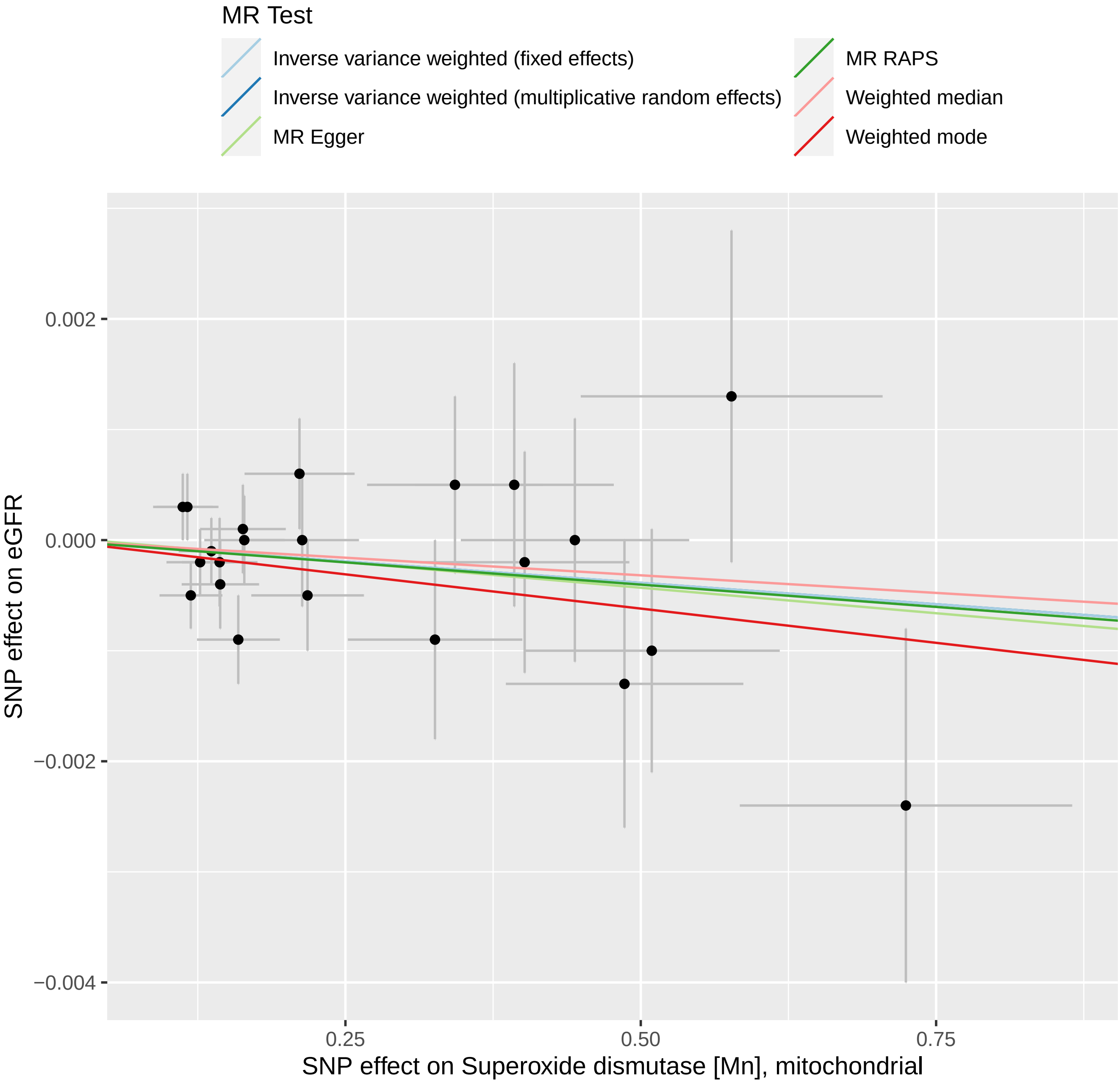

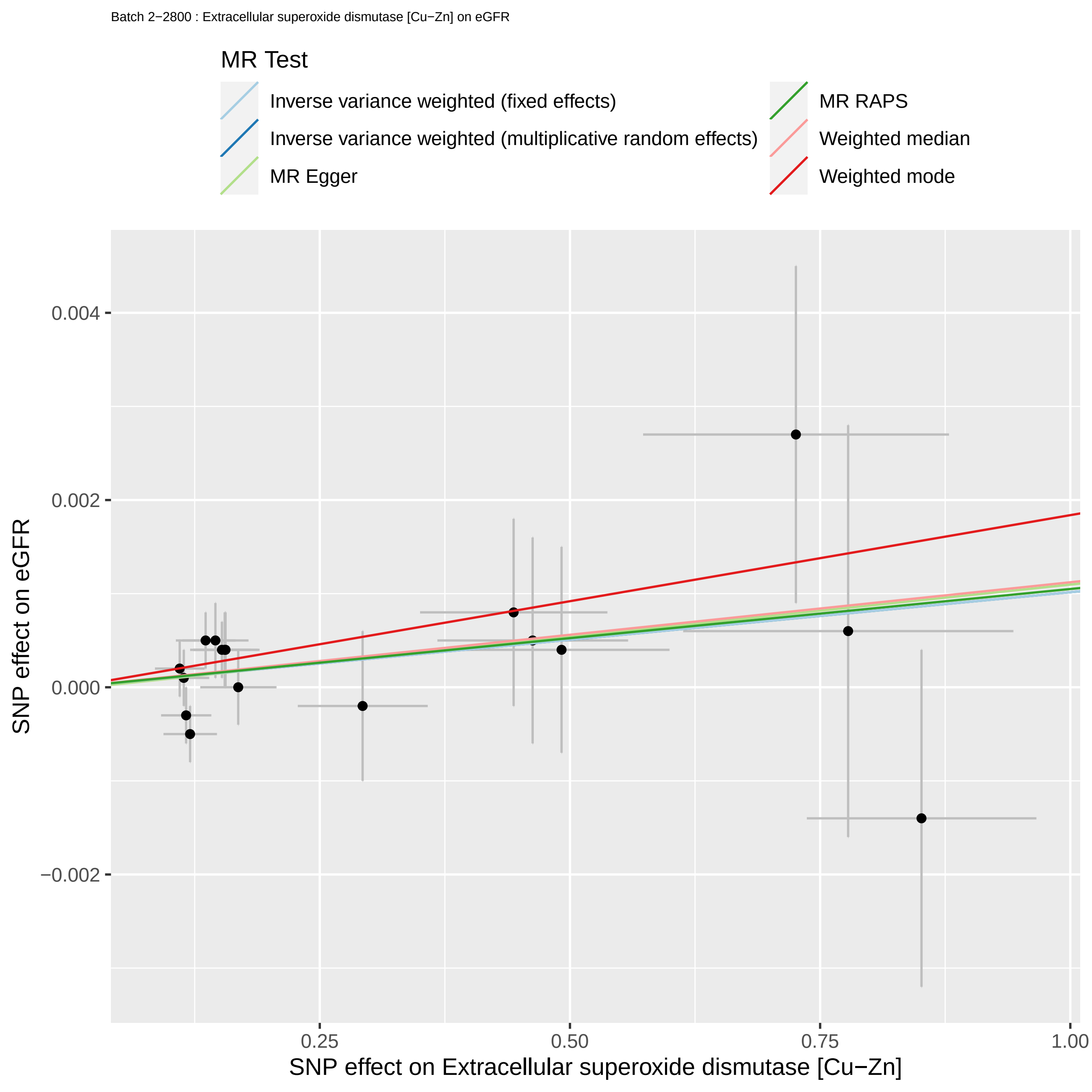
Batch 2−2800 : Extracellular superoxide dismutase [Cu−Zn] on eGFR
MR Test
Inverse variance weighted (fixed effects)
Inverse variance weighted (multiplicative random effects)
MR Egger
MR RAPS
Weighted median
Weighted mode
0.004
0.002
0.000
−0.002
0.25
0.50
0.75
1.00
SNP effect on eGFR
SNP effect on Extracellular superoxide dismutase [Cu−Zn]

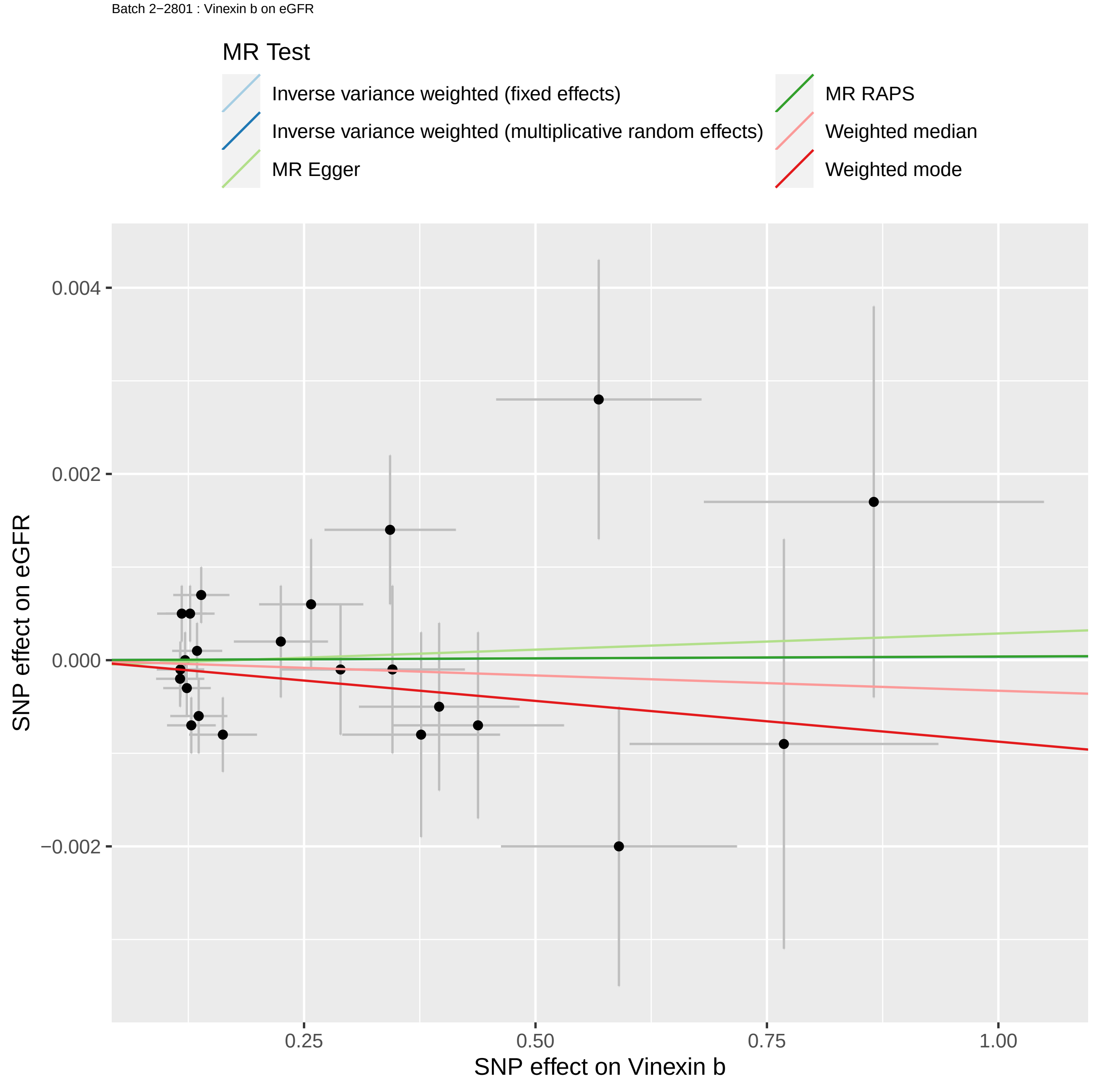

Batch 2−2801 : Vinexin b on eGFR
MR Test
Inverse variance weighted (fixed effects)
Inverse variance weighted (multiplicative random effects)
MR Egger
MR RAPS
Weighted median
Weighted mode
0.004
0.002
0.000
−0.002
0.25
0.50
0.75
1.00
SNP effect on eGFR
SNP effect on Vinexin b

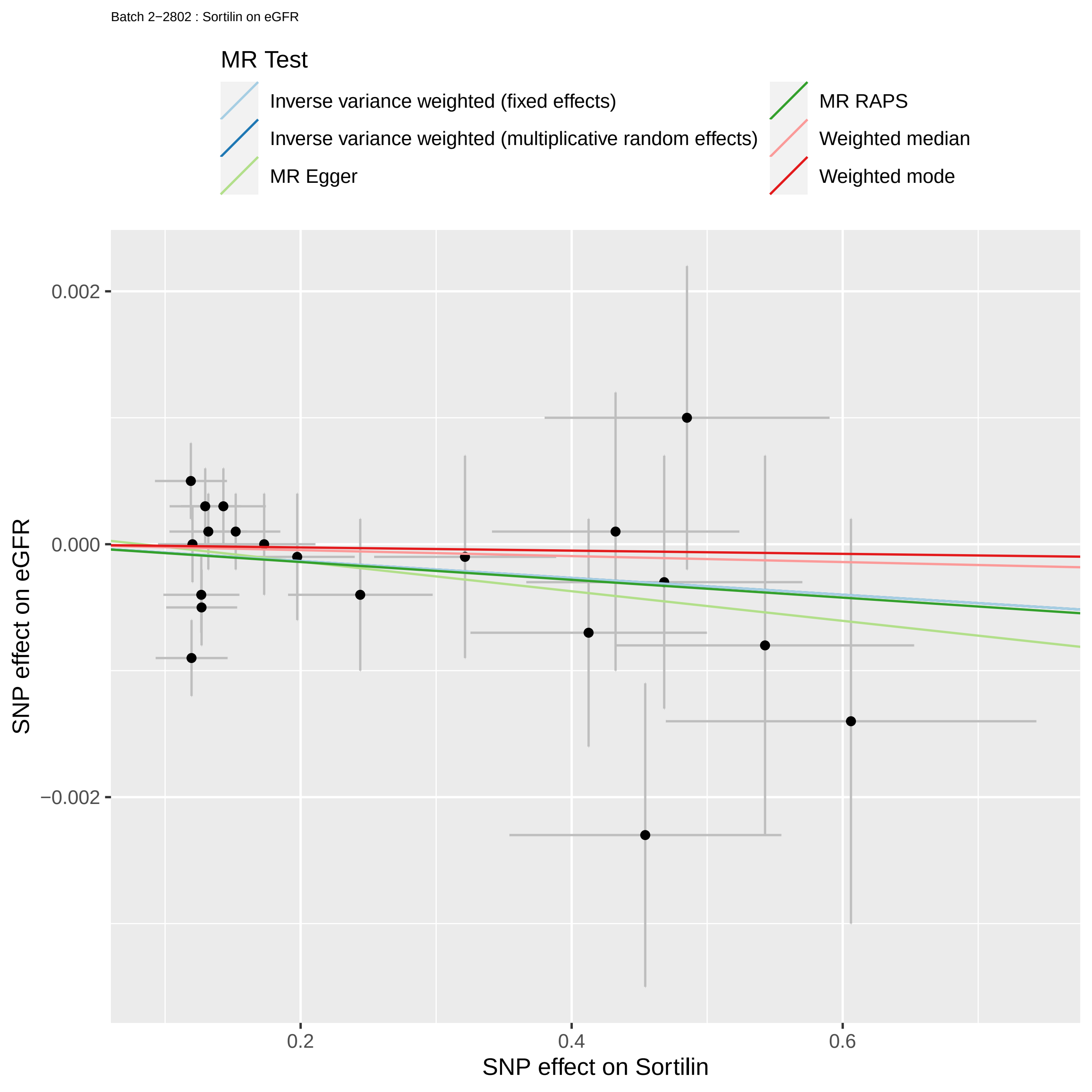

Batch 2−2802 : Sortilin on eGFR
MR Test
Inverse variance weighted (fixed effects)
Inverse variance weighted (multiplicative random effects)
MR Egger
MR RAPS
Weighted median
Weighted mode
0.002
0.000
−0.002
0.2
0.4
0.6
SNP effect on eGFR
SNP effect on Sortilin

Batch 2−2803 : Sclerostin on eGFR

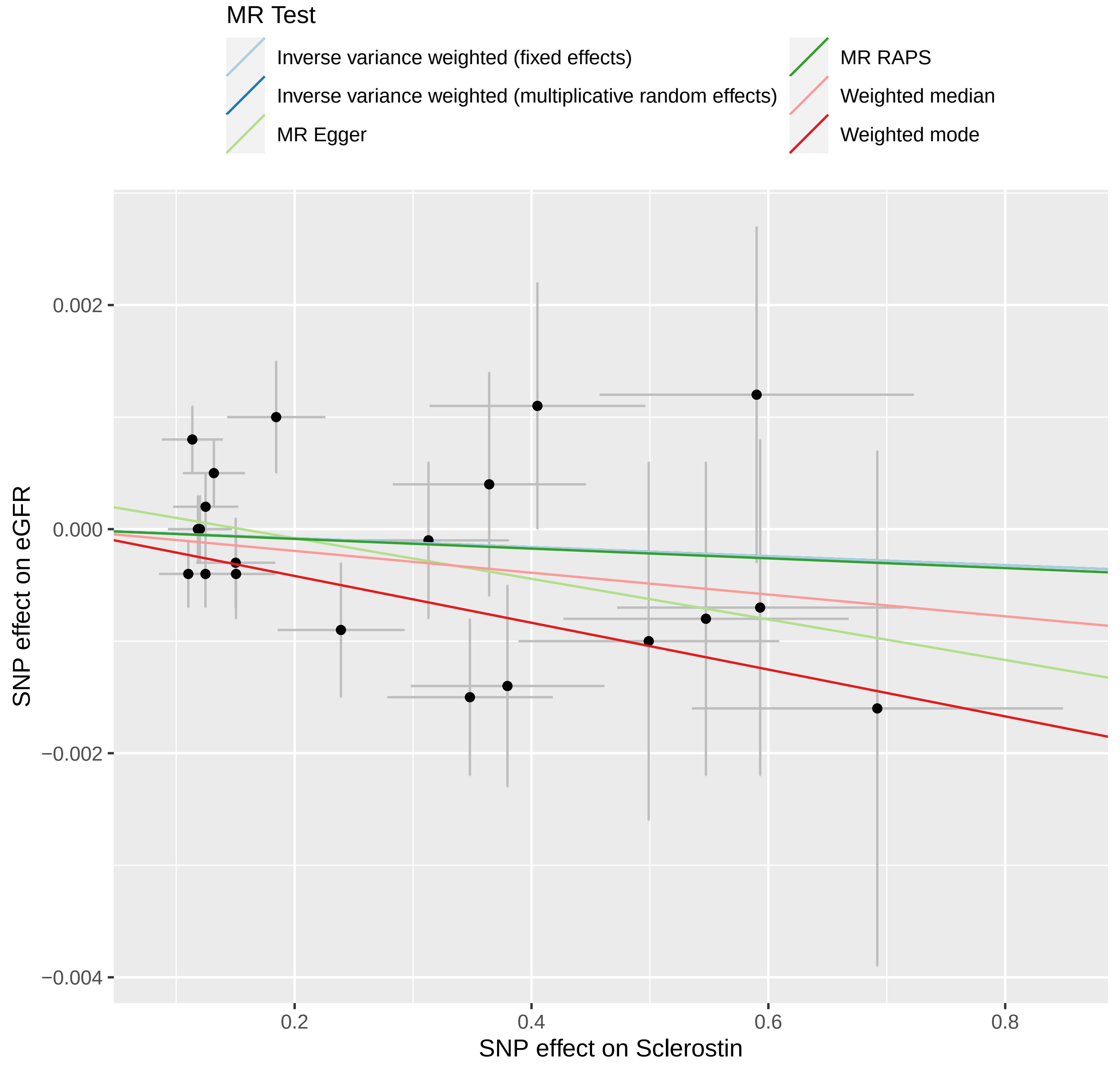

Batch 2−2804 : Sperm acrosome membrane−associated protein 3 on eGFR

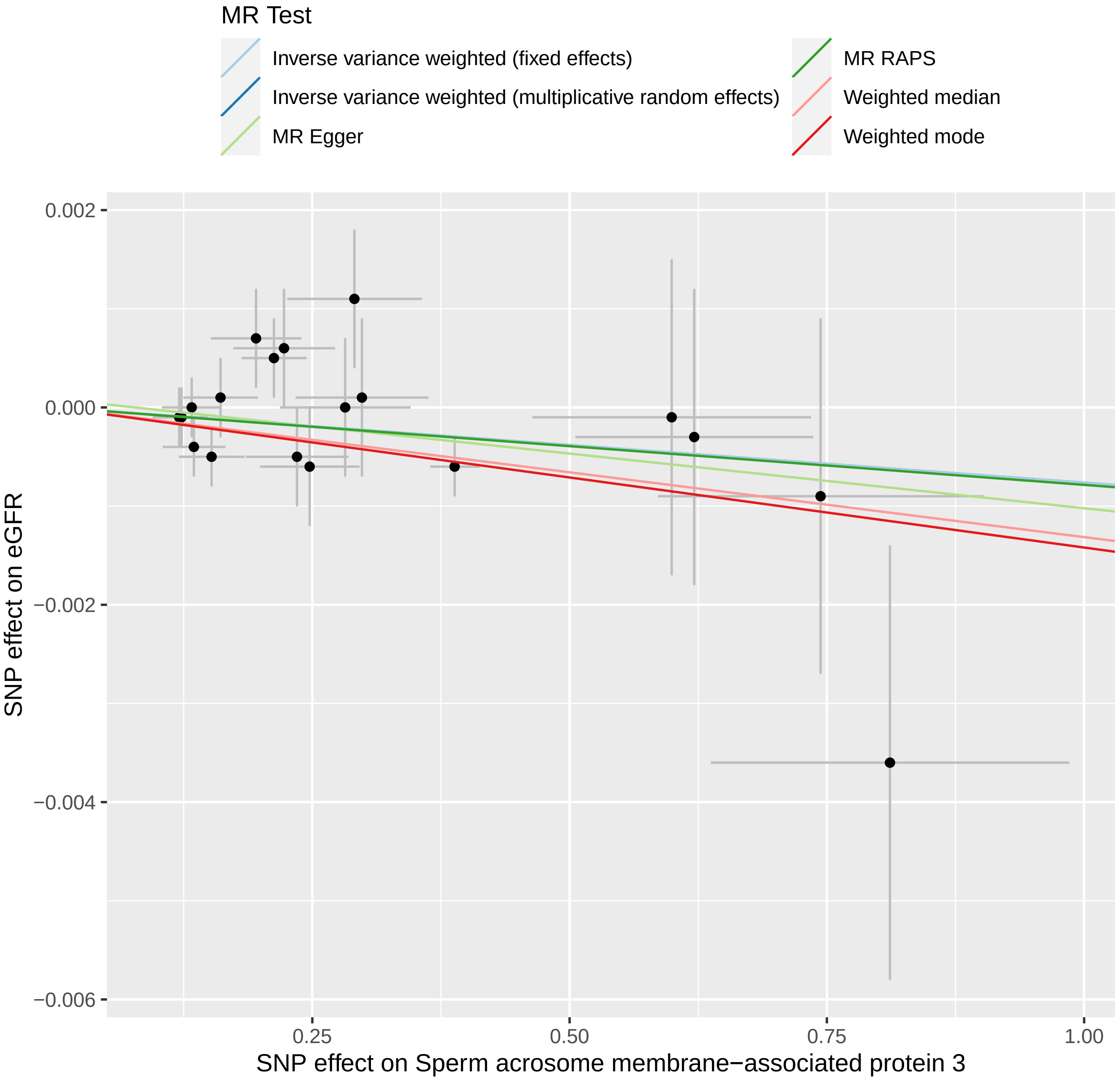

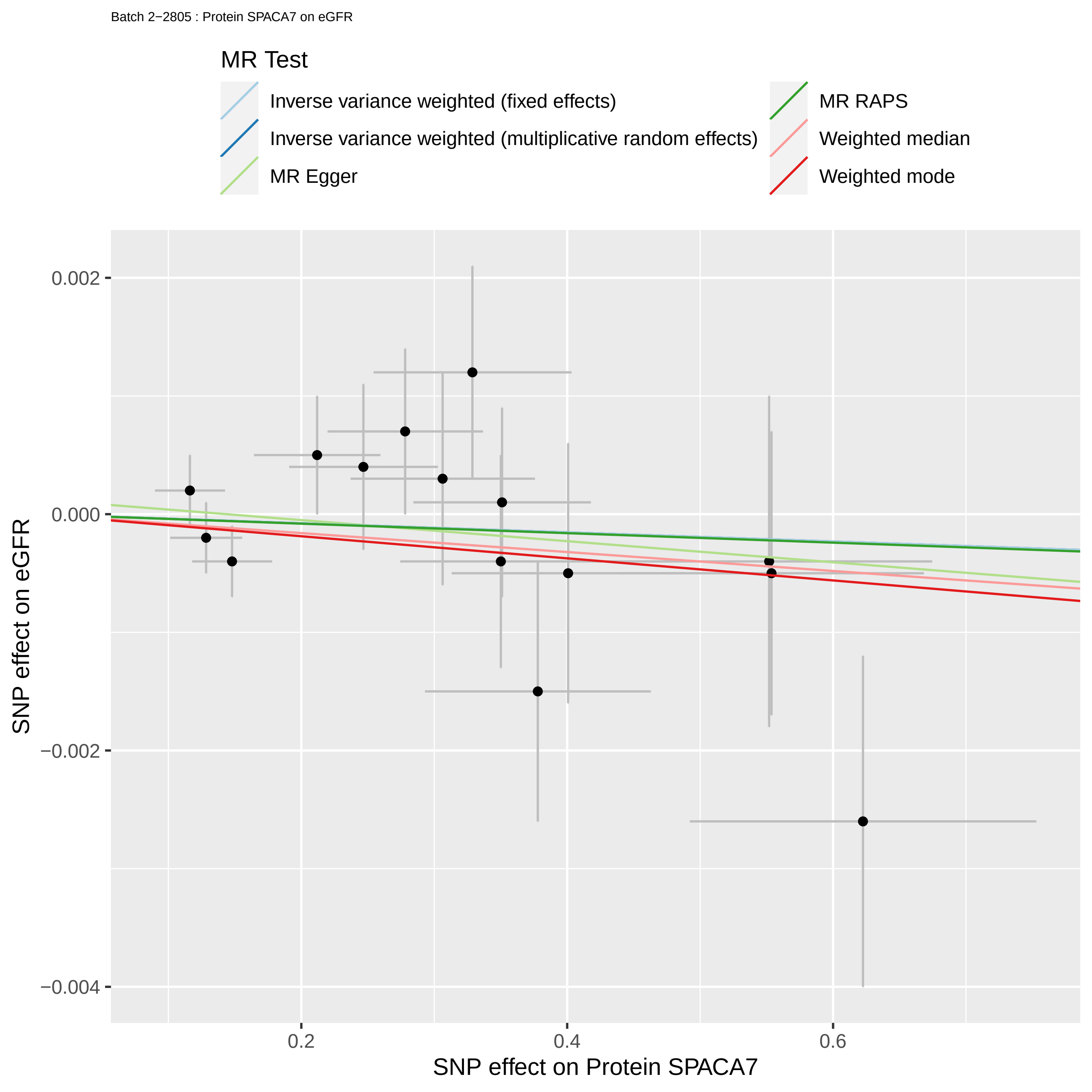

Batch 2−2805 : Protein SPACA7 on eGFR
MR Test
Inverse variance weighted (fixed effects)
Inverse variance weighted (multiplicative random effects)
MR Egger
MR RAPS
Weighted median
Weighted mode
0.002
0.000
−0.002
−0.004
SNP effect on eGFR
0.2
0.4
0.6
SNP effect on Protein SPACA7

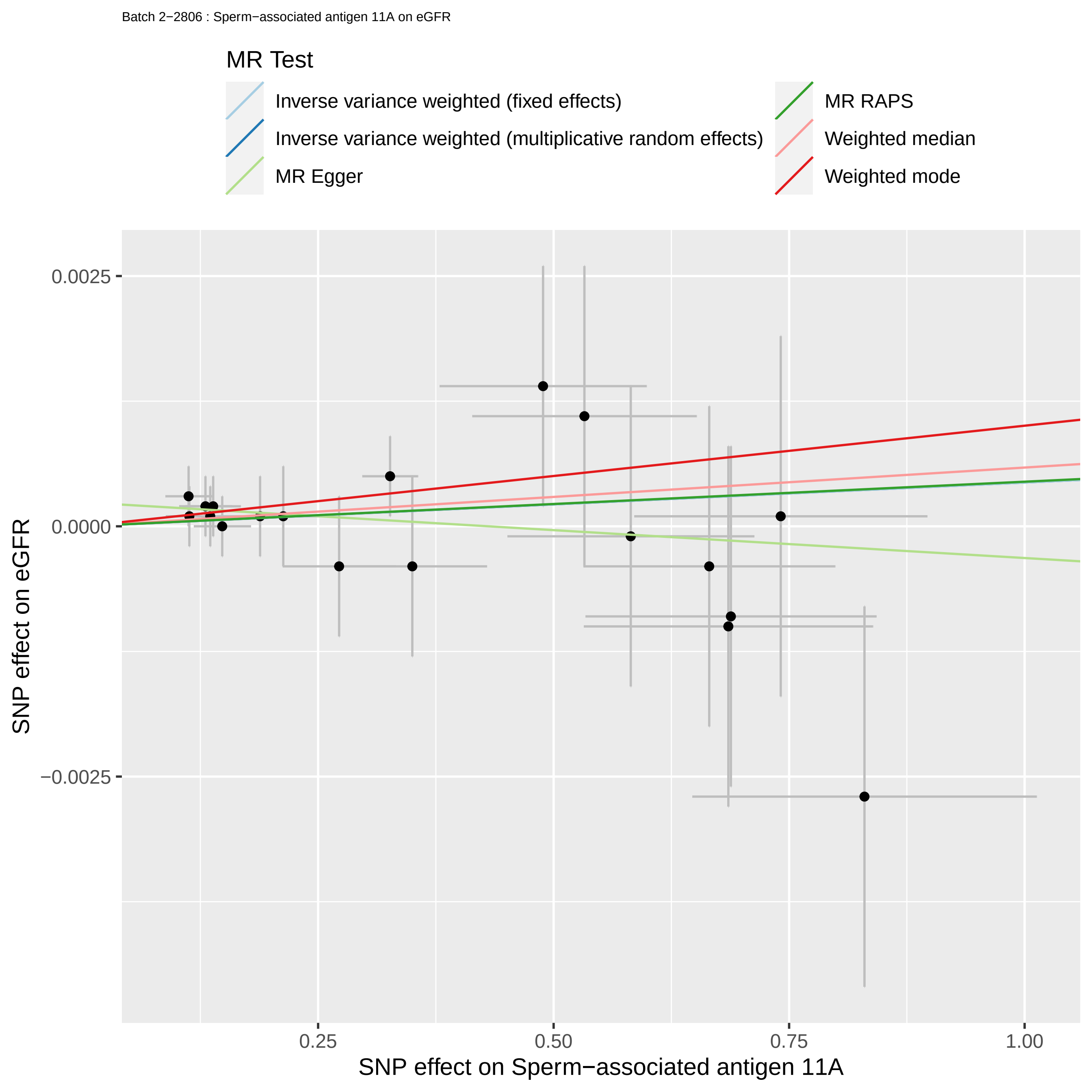

Batch 2−2806 : Sperm−associated antigen 11A on eGFR
MR Test
Inverse variance weighted (fixed effects)
Inverse variance weighted (multiplicative random effects)
MR Egger
MR RAPS
Weighted median
Weighted mode
0.0025
0.0000
−0.0025
SNP effect on eGFR
0.25
0.50
0.75
1.00
SNP effect on Sperm−associated antigen 11A

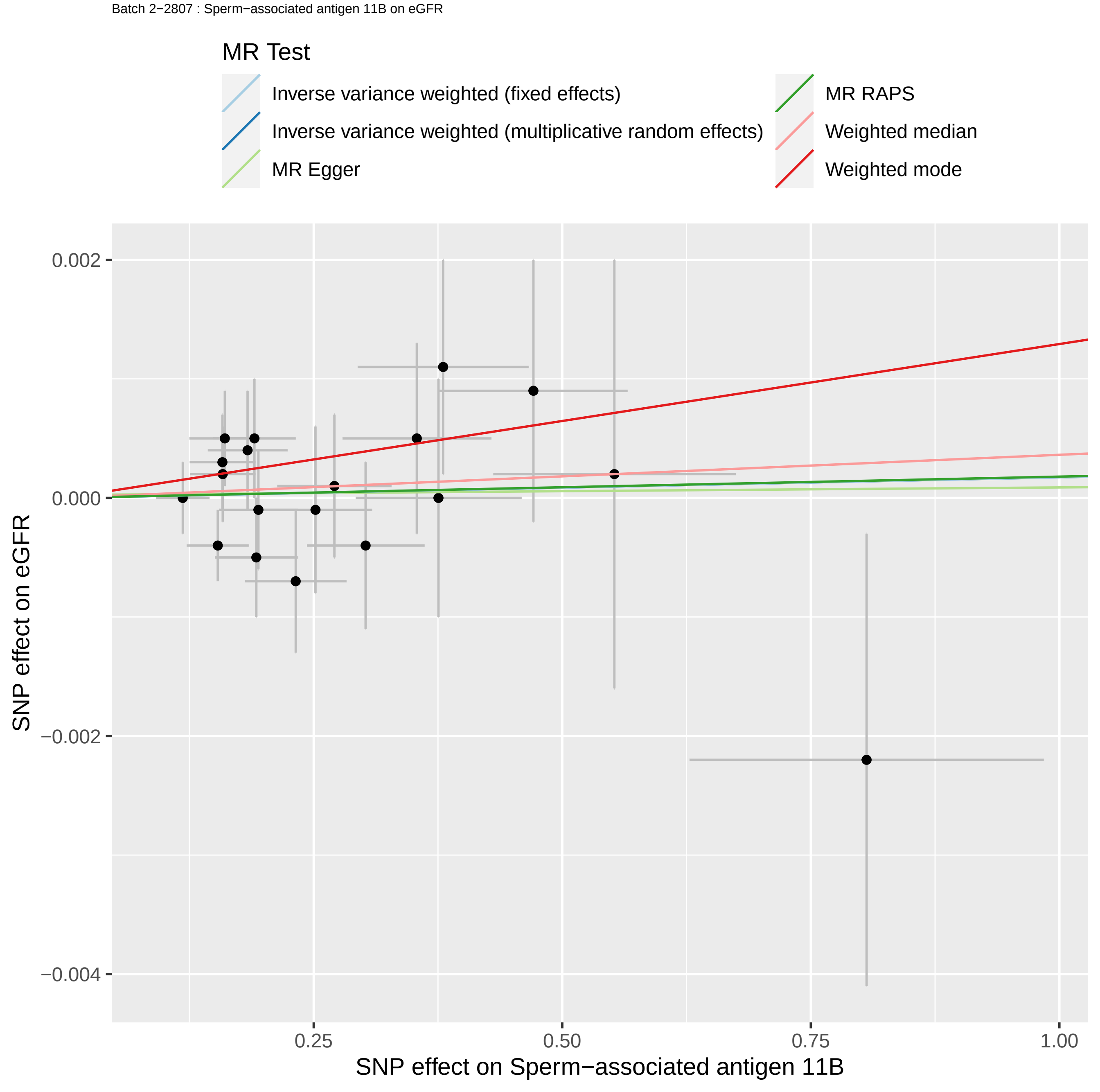

Batch 2−2807 : Sperm−associated antigen 11B on eGFR
MR Test
Inverse variance weighted (fixed effects)
Inverse variance weighted (multiplicative random effects)
MR Egger
MR RAPS
Weighted median
Weighted mode
0.002
0.000
−0.002
−0.004
SNP effect on eGFR
0.25
0.50
0.75
1.00
SNP effect on Sperm−associated antigen 11B

Batch 2−2808 : SPARC on eGFR

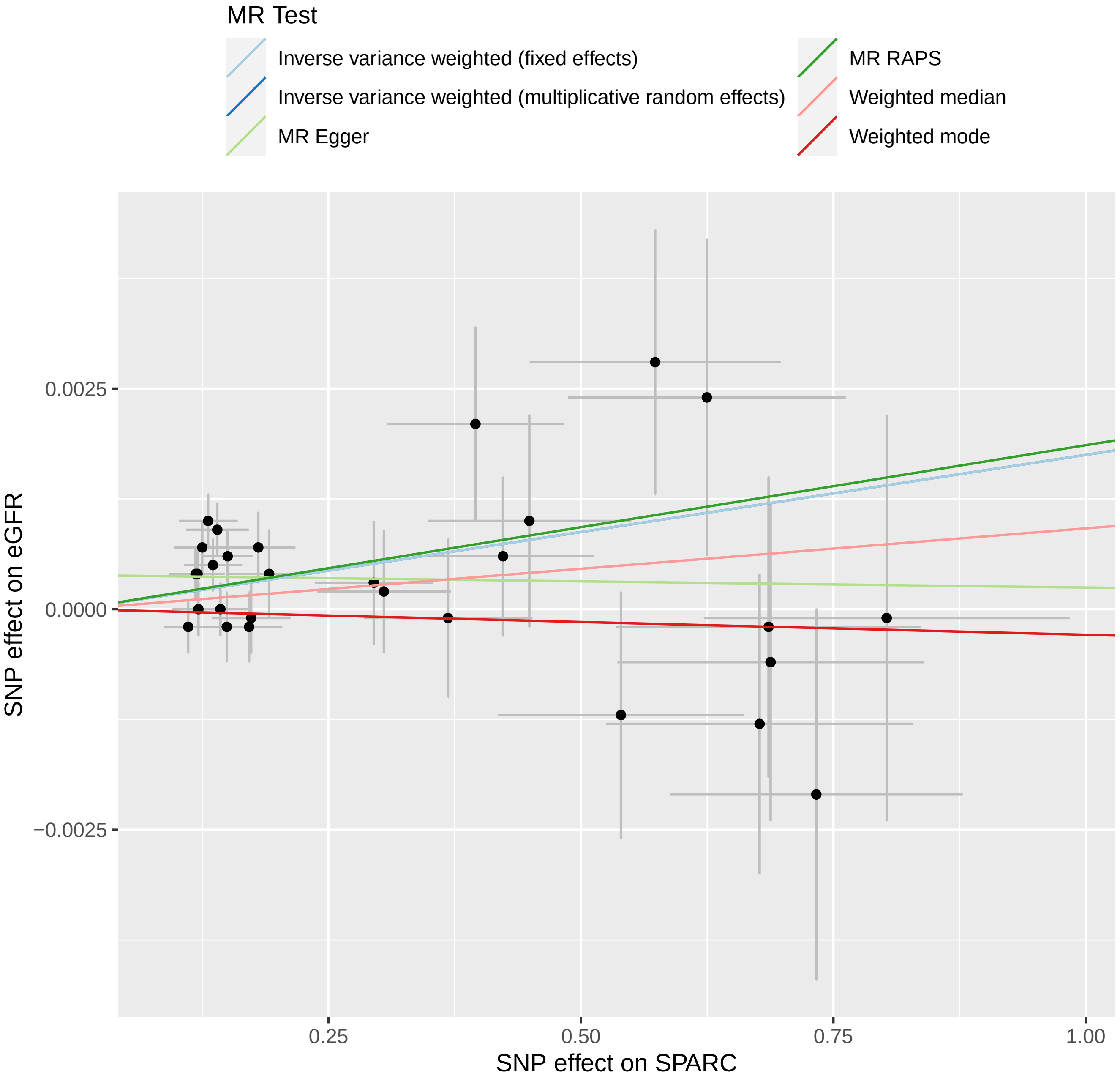

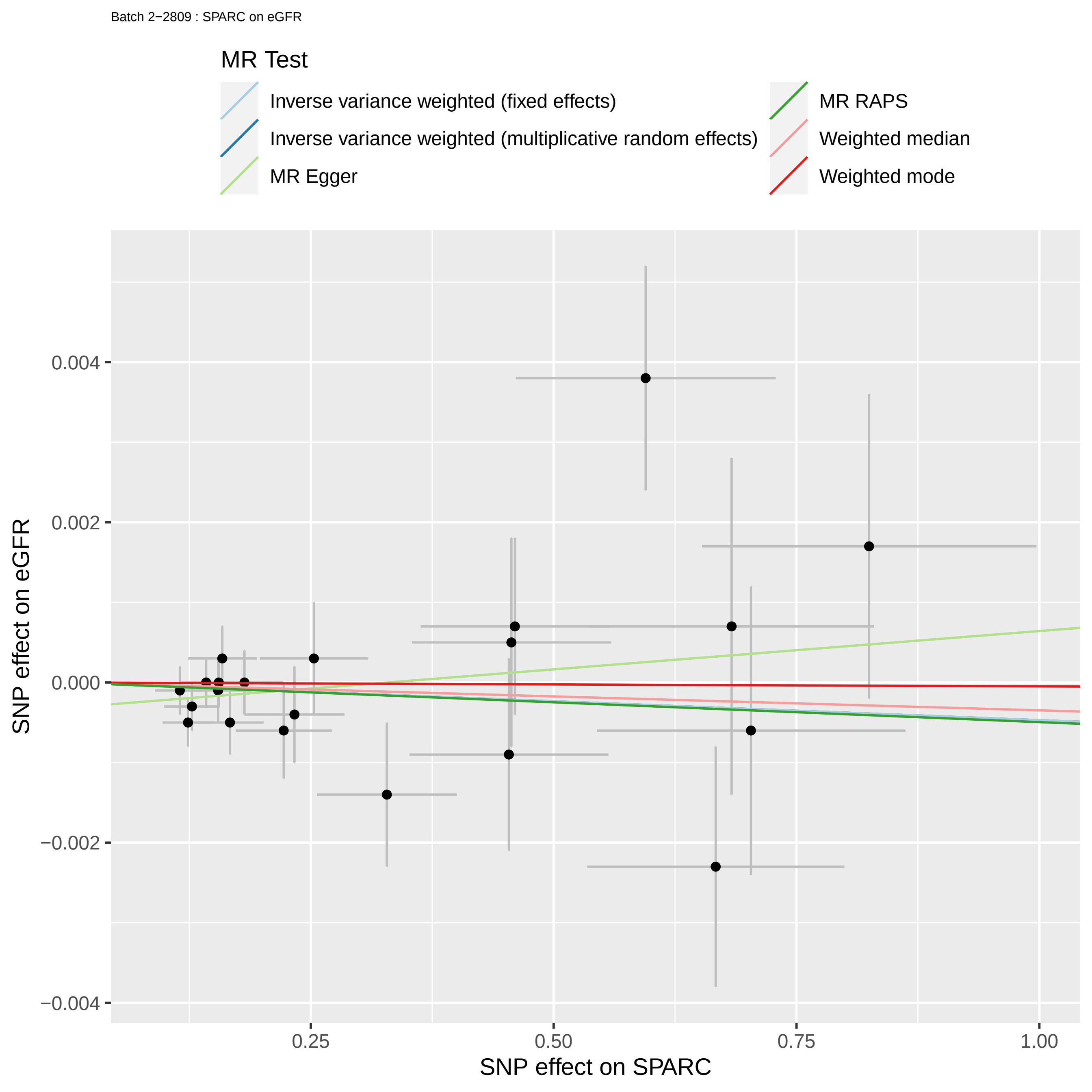
Batch 2−2809 : SPARC on eGFR
MR Test
Inverse variance weighted (fixed effects)
Inverse variance weighted (multiplicative random effects)
MR Egger
MR RAPS
Weighted median
Weighted mode
0.004
0.002
0.000
−0.002
−0.004
0.25
0.50
0.75
1.00
SNP effect on SPARC
SNP effect on eGFR

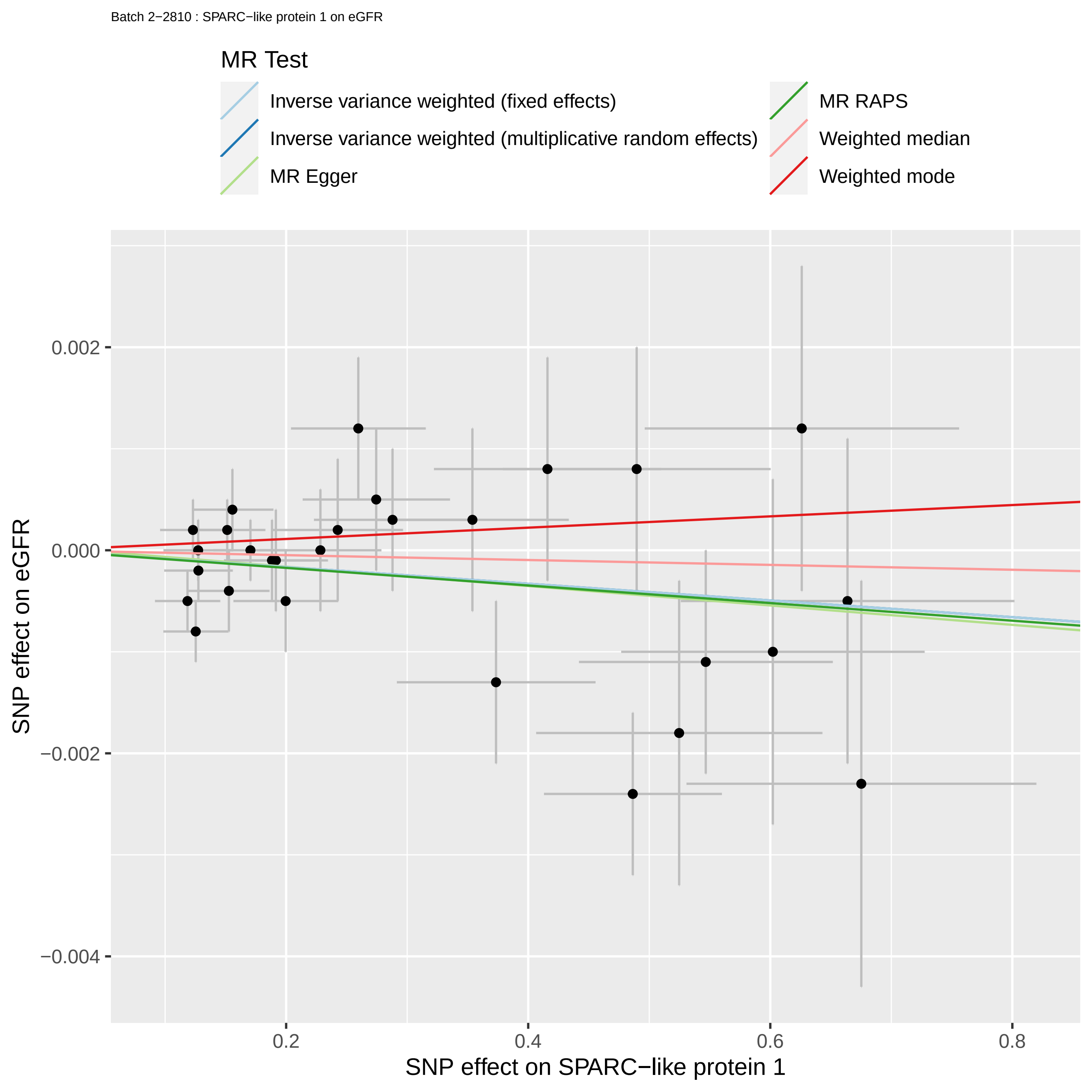
Batch 2−2810 : SPARC−like protein 1 on eGFR
MR Test
Inverse variance weighted (fixed effects)
Inverse variance weighted (multiplicative random effects)
MR Egger
MR RAPS
Weighted median
Weighted mode
SNP effect on eGFR
0.002
0.000
−0.002
−0.004
0.2
0.4
0.6
0.8
SNP effect on SPARC−like protein 1

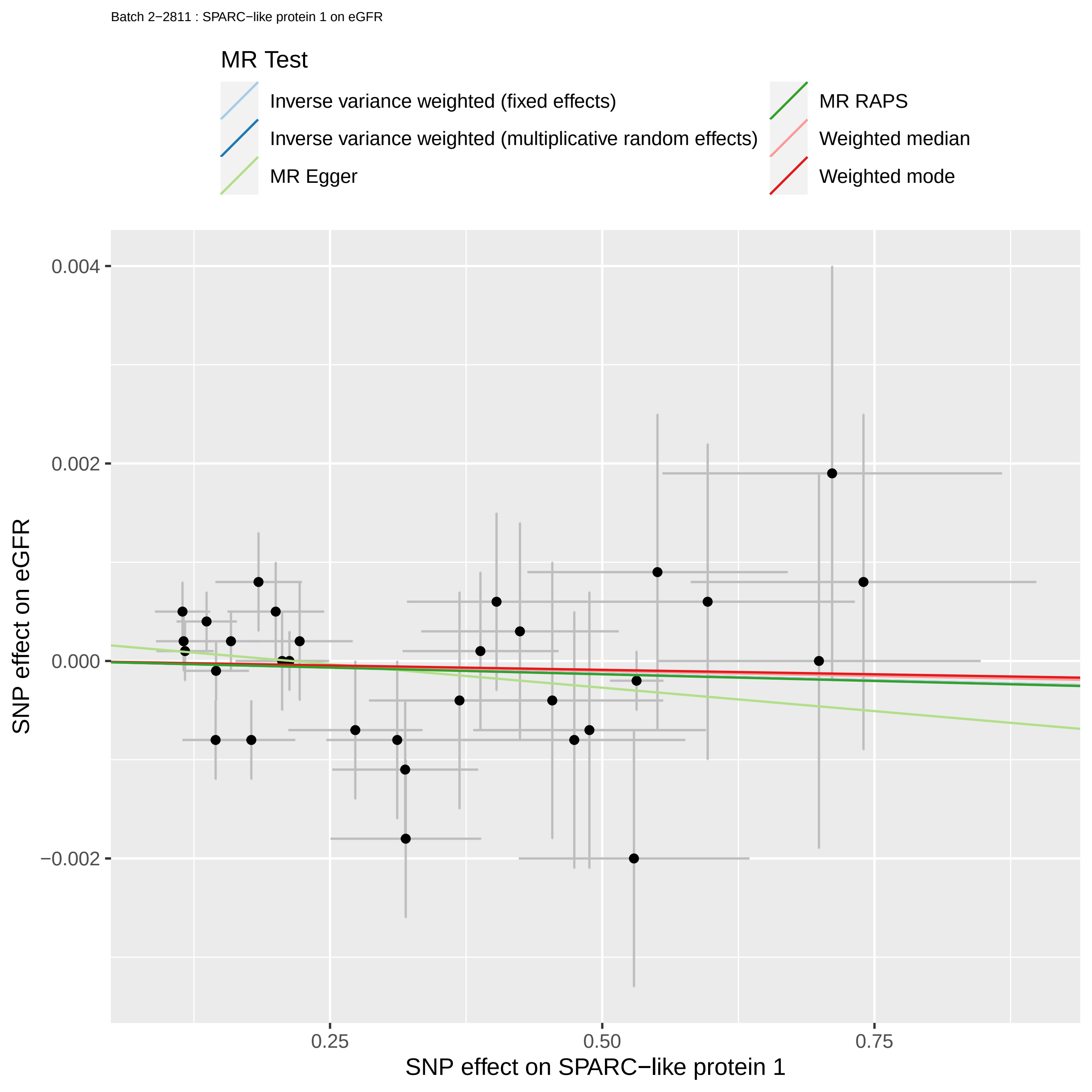

Batch 2−2811 : SPARC−like protein 1 on eGFR
MR Test
Inverse variance weighted (fixed effects)
Inverse variance weighted (multiplicative random effects)
MR Egger
MR RAPS
Weighted median
Weighted mode
0.004
0.002
0.000
−0.002
SNP effect on eGFR
0.25
0.50
0.75
SNP effect on SPARC−like protein 1

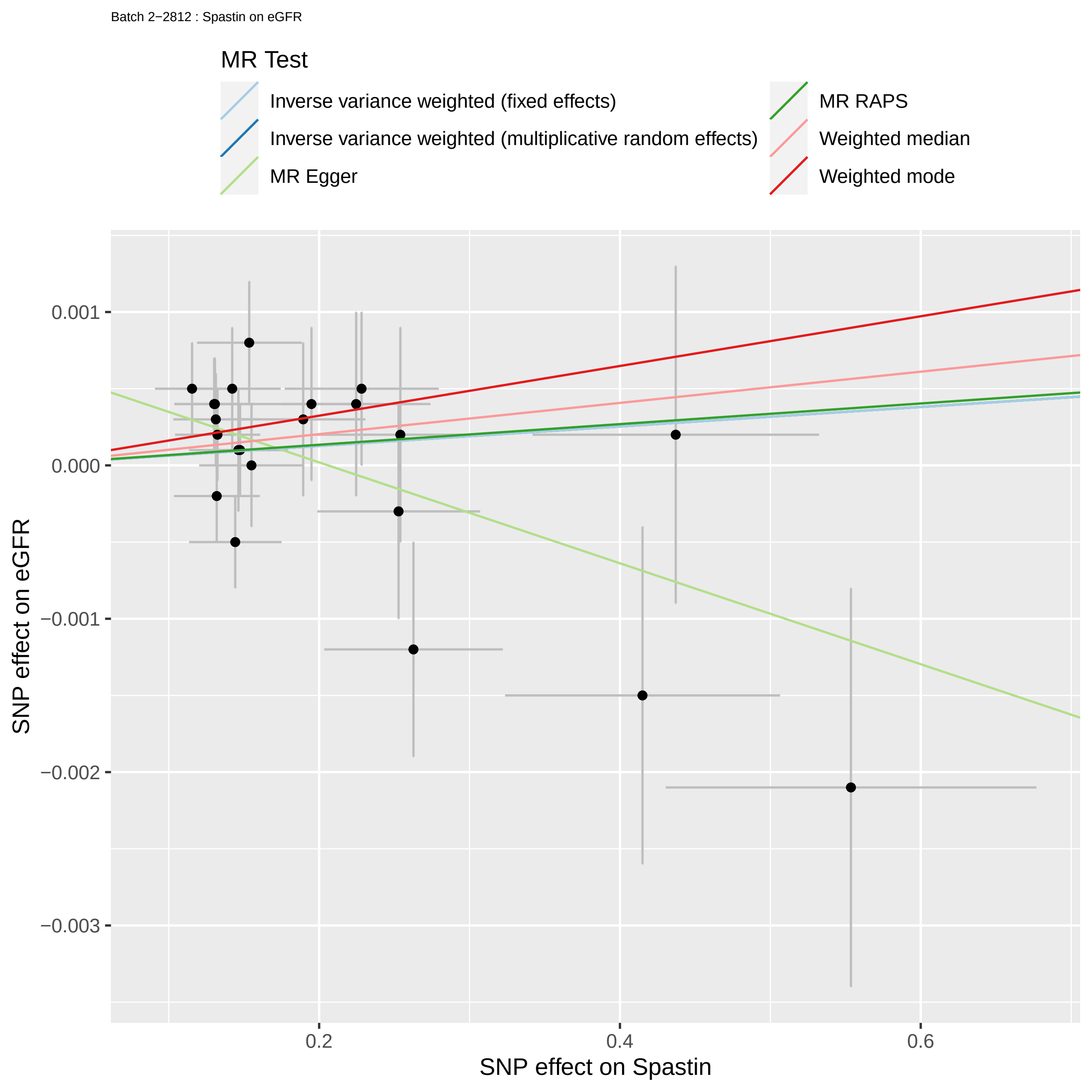

Batch 2−2812 : Spastin on eGFR
MR Test
Inverse variance weighted (fixed effects)
Inverse variance weighted (multiplicative random effects)
MR Egger
MR RAPS
Weighted median
Weighted mode
0.001
0.000
−0.001
−0.002
−0.003
0.2
0.4
0.6
SNP effect on eGFR
SNP effect on Spastin

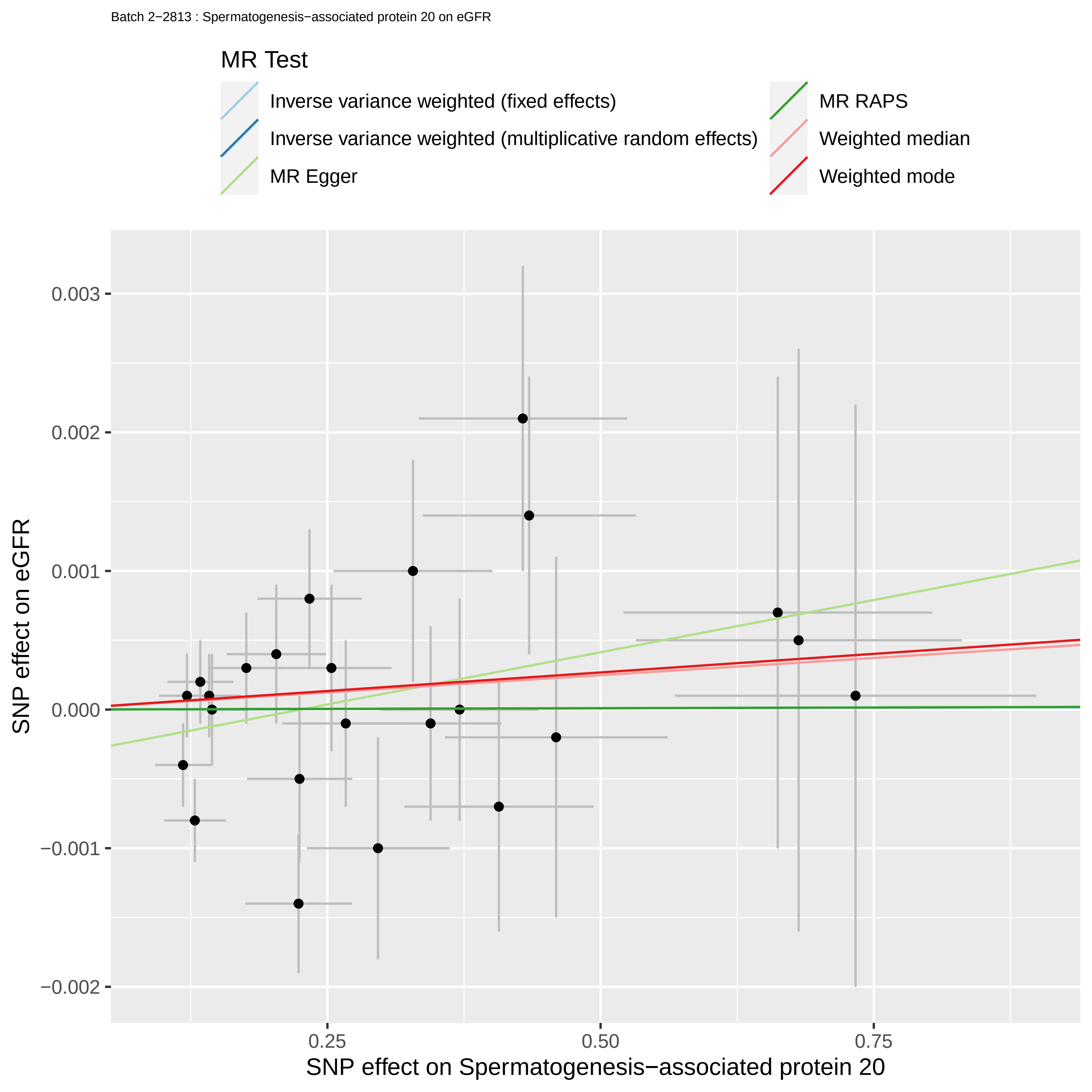
Batch 2−2813 : Spermatogenesis−associated protein 20 on eGFR
MR Test
Inverse variance weighted (fixed effects)
Inverse variance weighted (multiplicative random effects)
MR Egger
MR RAPS
Weighted median
Weighted mode
0.003
0.002
0.001
0.000
−0.001
−0.002
0.25
0.50
0.75
SNP effect on eGFR
SNP effect on Spermatogenesis−associated protein 20

Batch 2−2814 : Spermatogenesis−associated protein 9 on eGFR

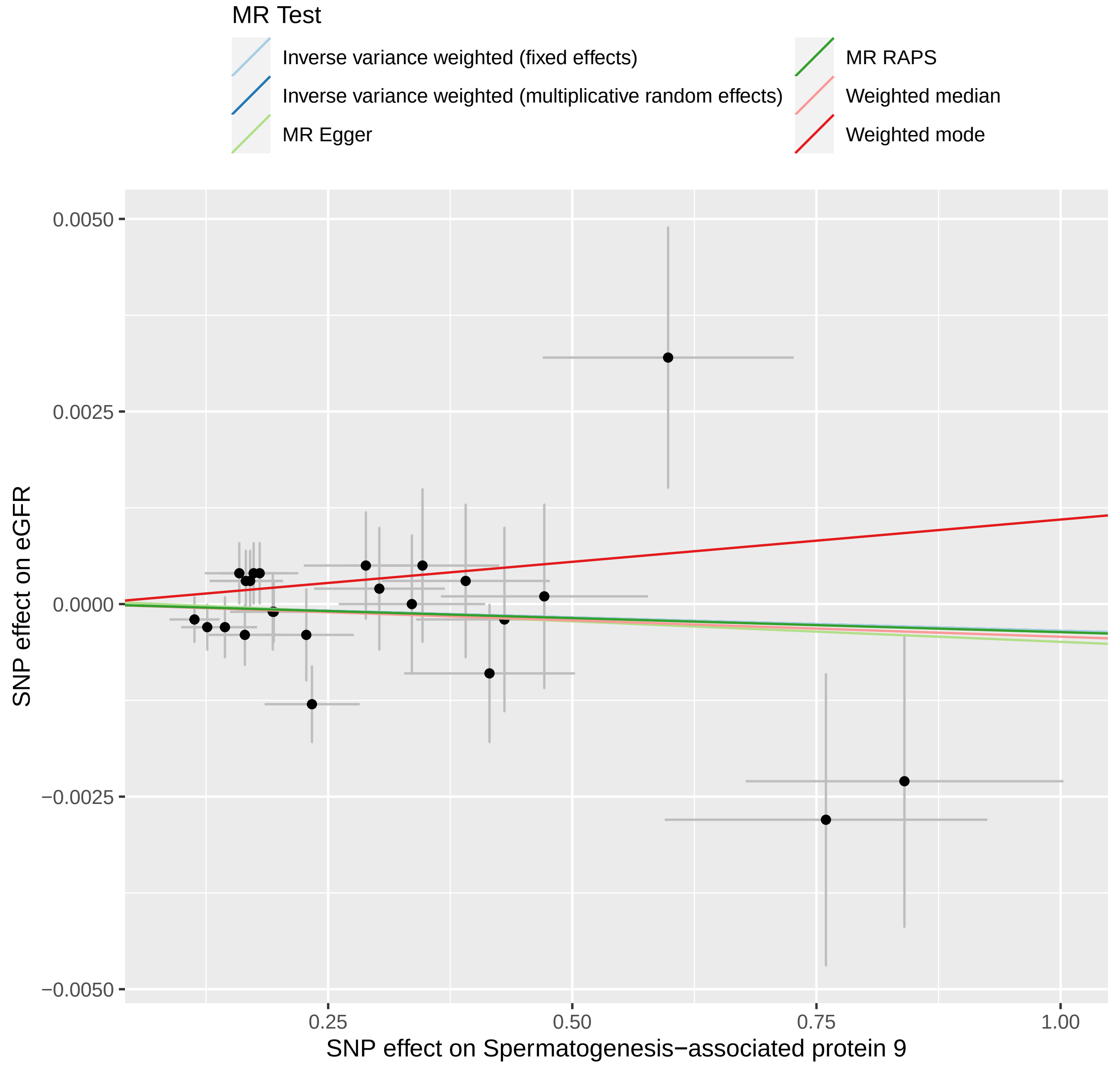

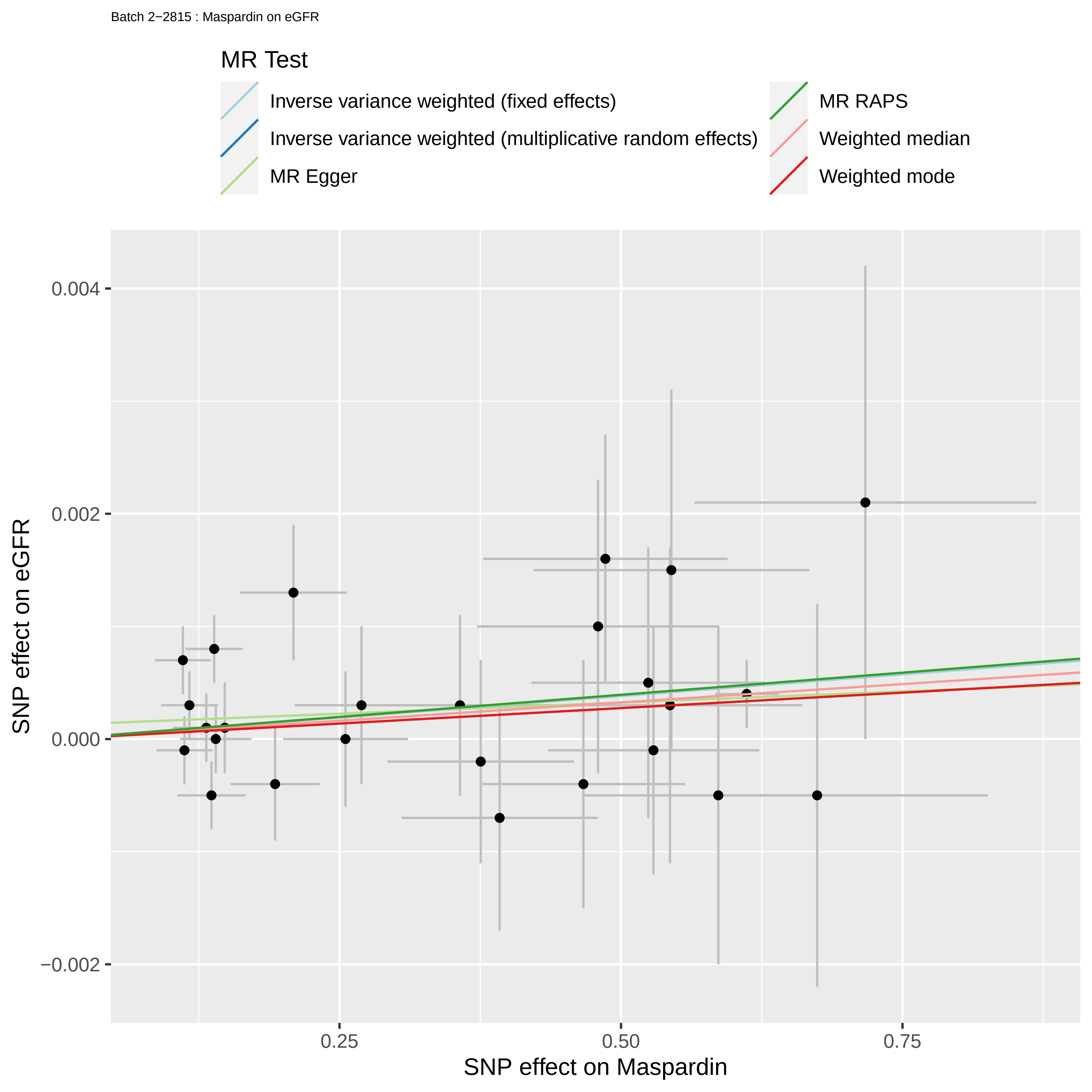

Batch 2−2815 : Maspardin on eGFR
MR Test
Inverse variance weighted (fixed effects)
Inverse variance weighted (multiplicative random effects)
MR Egger
MR RAPS
Weighted median
Weighted mode
0.004
0.002
0.000
−0.002
0.25
0.50
0.75
SNP effect on eGFR
SNP effect on Maspardin

Batch 2−2816 : Sphingosine kinase 2 on eGFR

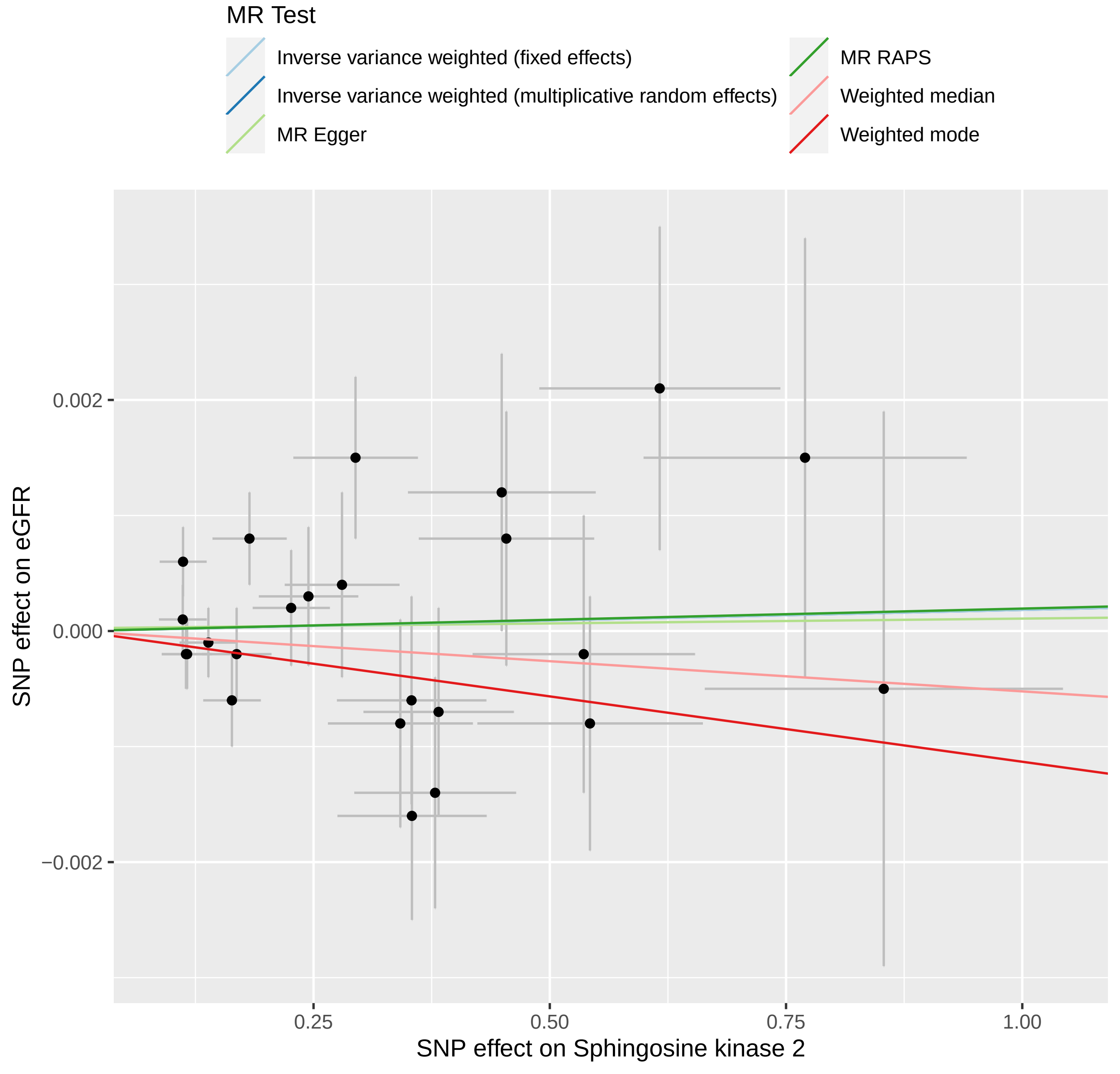

Batch 2−2817 : Serine protease inhibitor Kazal−type 13 on eGFR

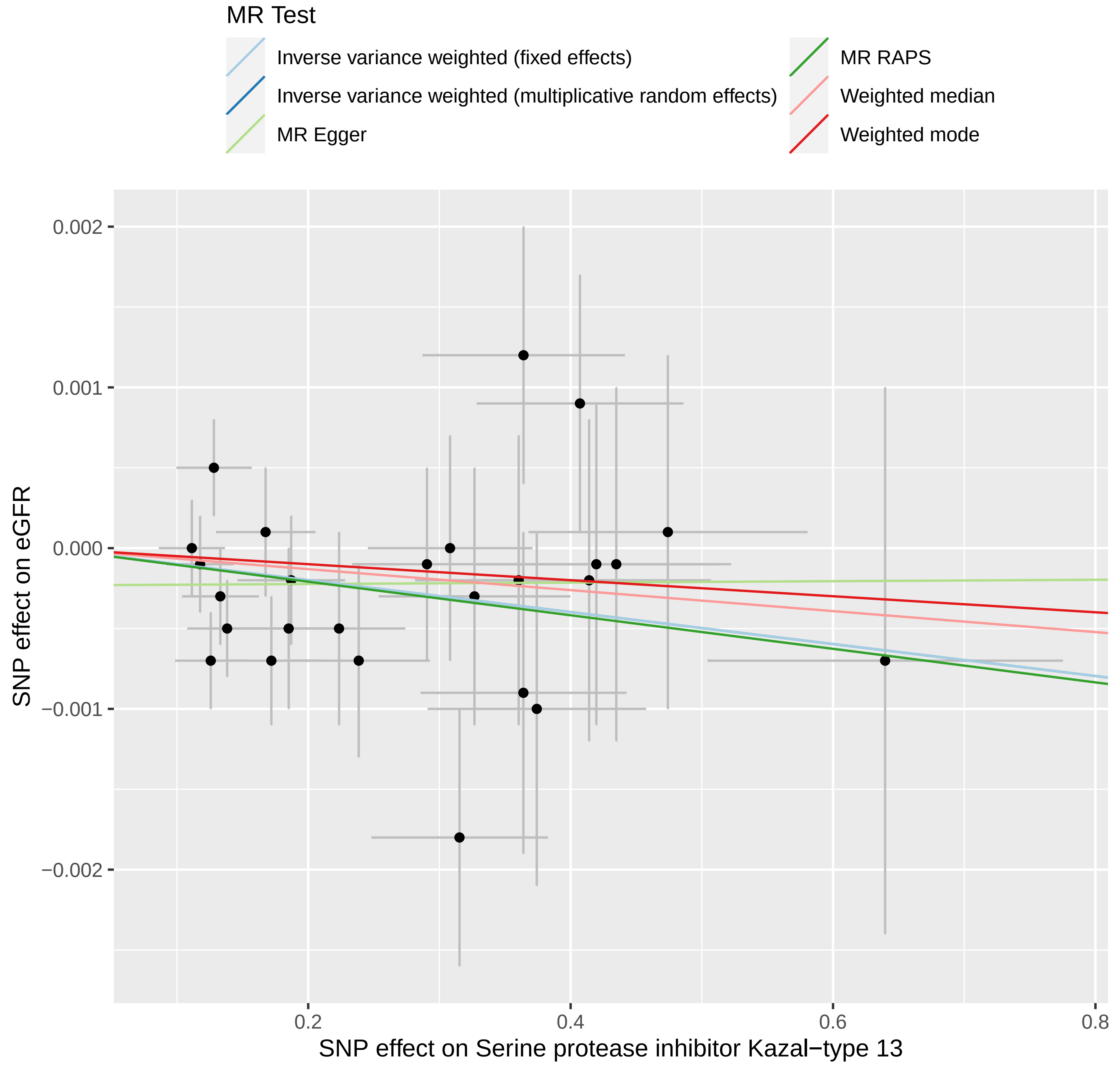

Batch 2−2818 : Serine protease inhibitor Kazal−type 2 on eGFR

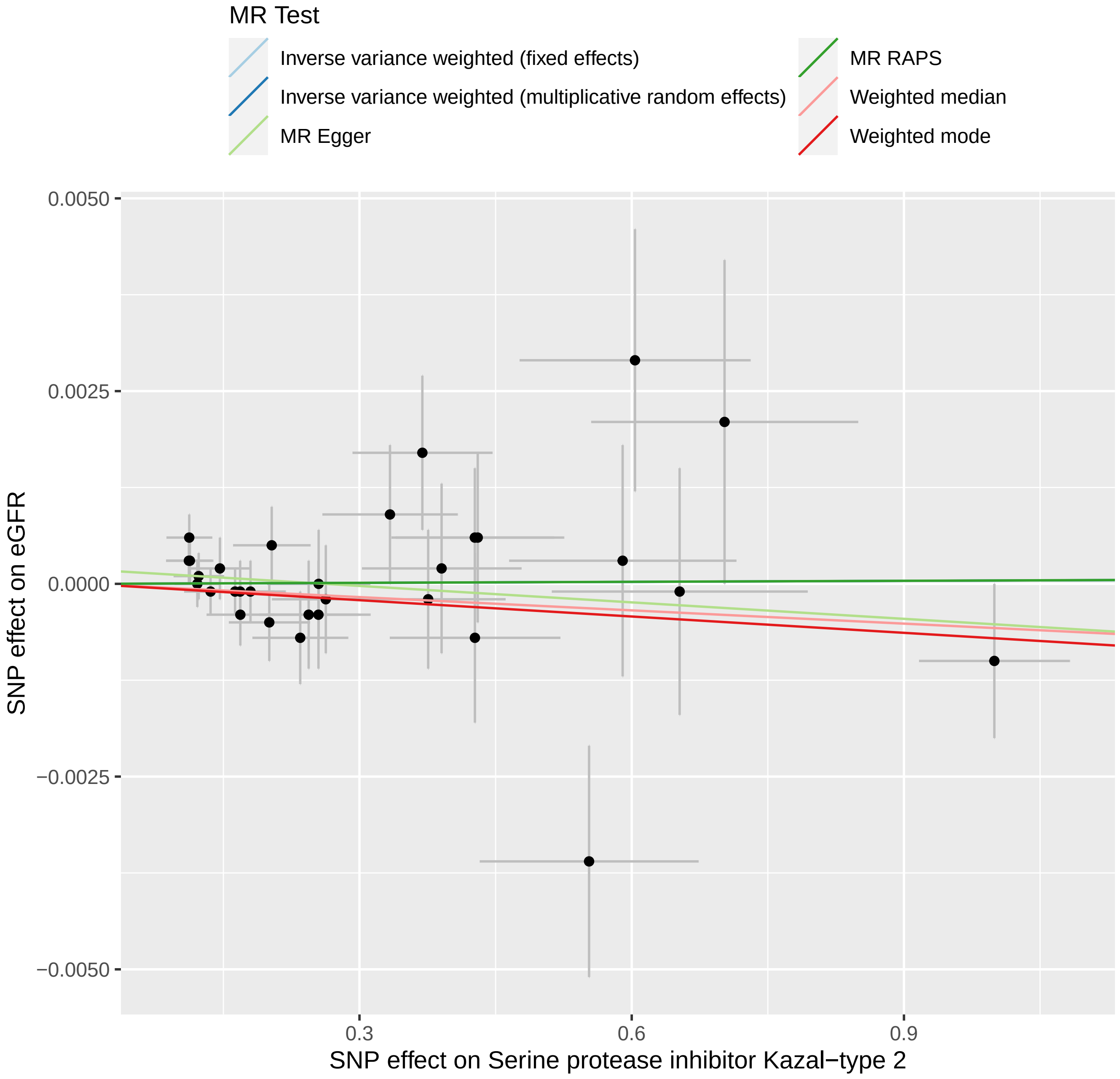

Batch 2−2819 : Serine protease inhibitor Kazal−type 5 on eGFR

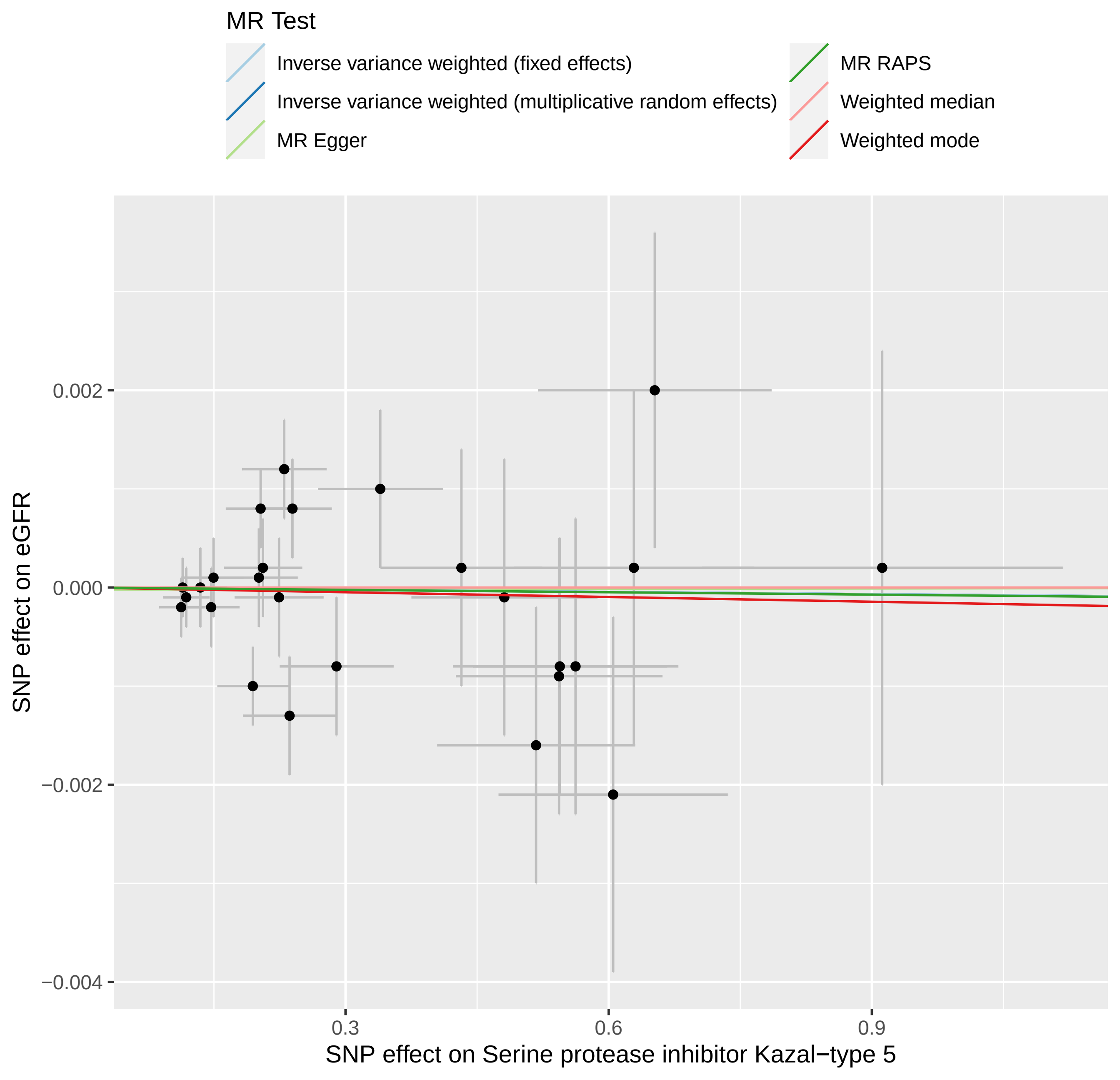

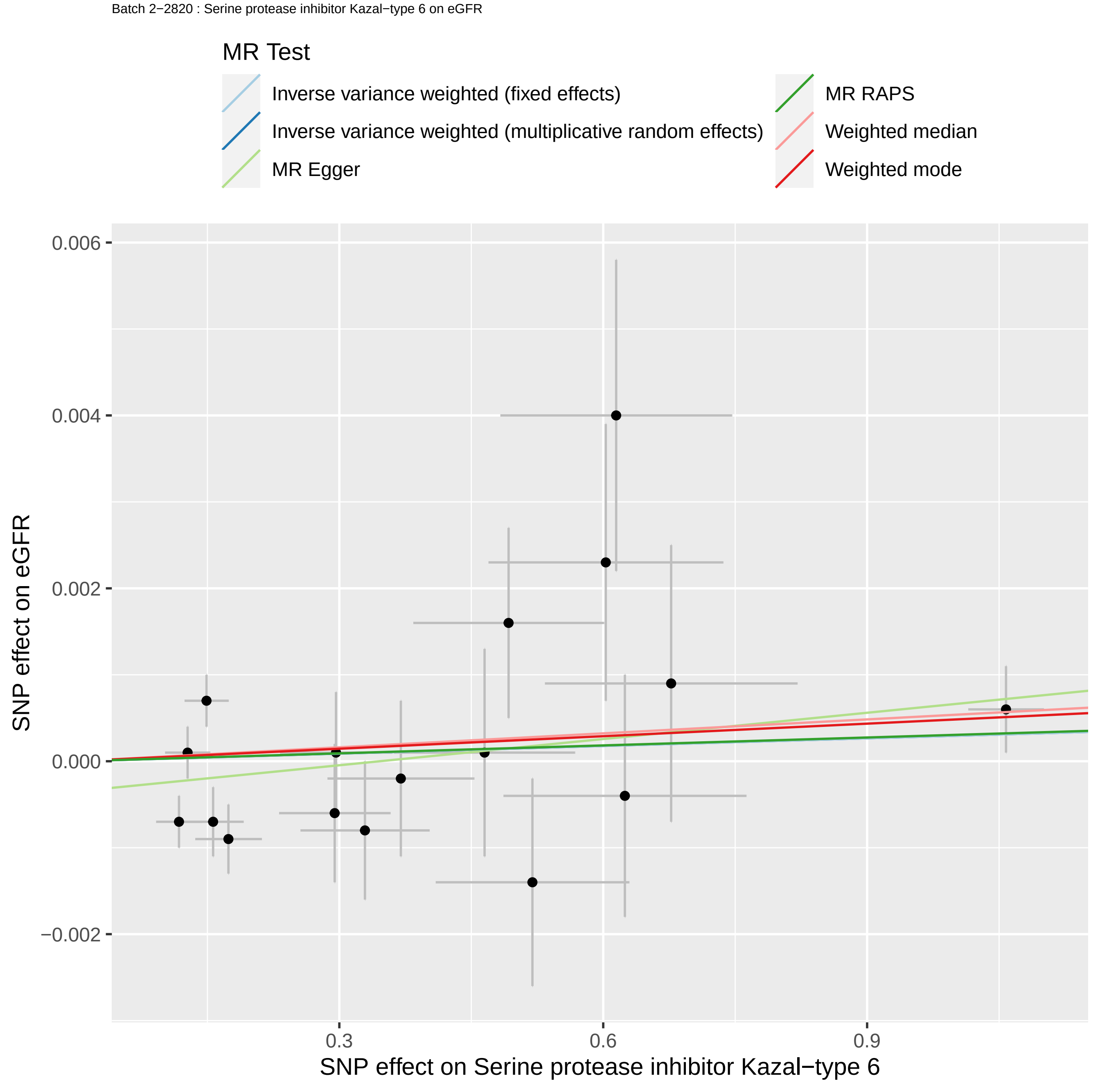

Batch 2−2820 : Serine protease inhibitor Kazal−type 6 on eGFR
MR Test
Inverse variance weighted (fixed effects)
Inverse variance weighted (multiplicative random effects)
MR Egger
MR RAPS
Weighted median
Weighted mode
0.006
0.004
0.002
0.000
−0.002
SNP effect on eGFR
0.3
0.6
0.9
SNP effect on Serine protease inhibitor Kazal−type 6

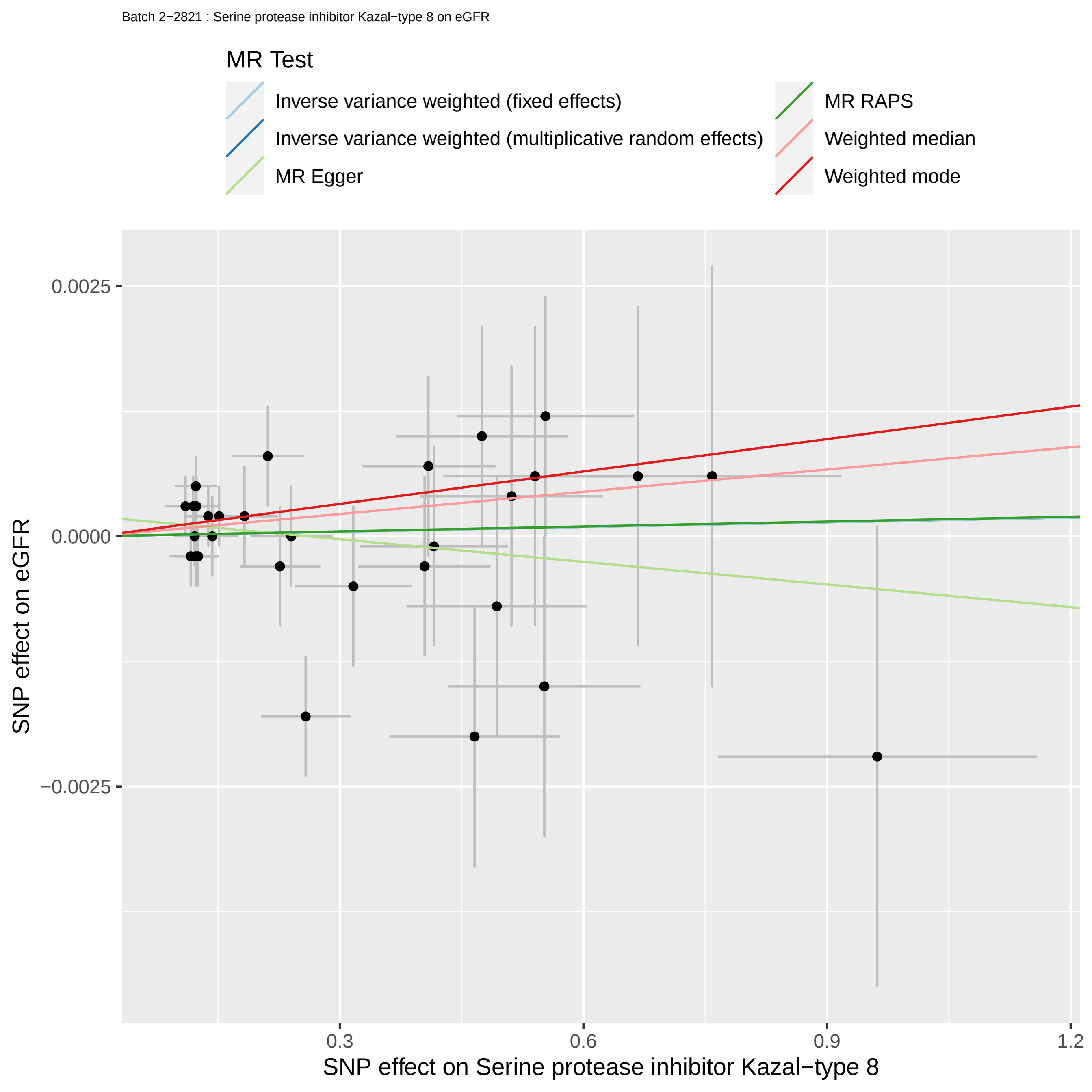
Batch 2−2821 : Serine protease inhibitor Kazal−type 8 on eGFR
MR Test
Inverse variance weighted (fixed effects)
Inverse variance weighted (multiplicative random effects)
MR Egger
MR RAPS
Weighted median
Weighted mode
0.0025
0.0000
−0.0025
SNP effect on eGFR
0.3
0.6
0.9
1.2
SNP effect on Serine protease inhibitor Kazal−type 8

Batch 2−2822 : Serine protease inhibitor Kazal−type 9 on eGFR

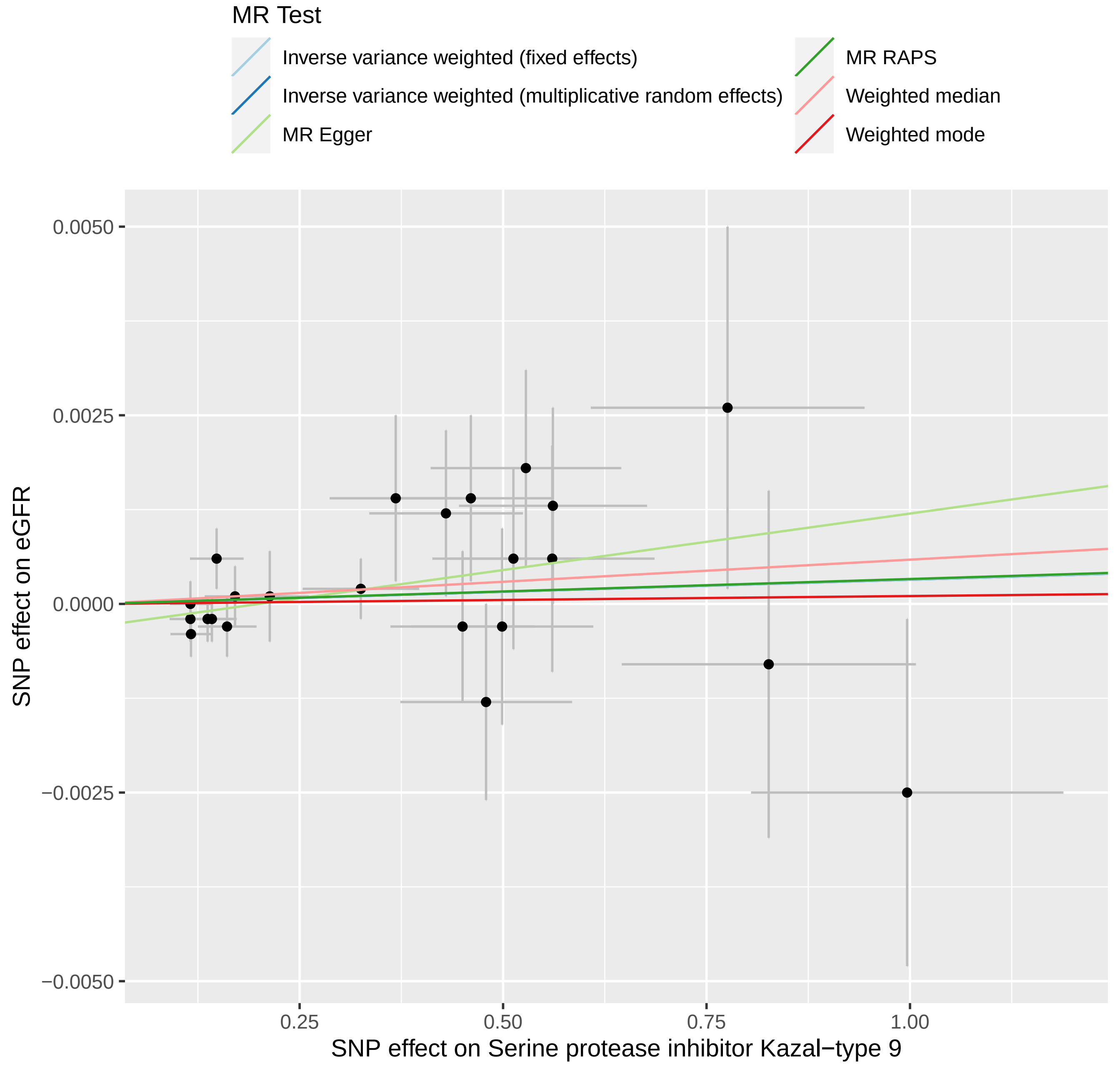

Batch 2−2823 : Kunitz−type protease inhibitor 1 on eGFR

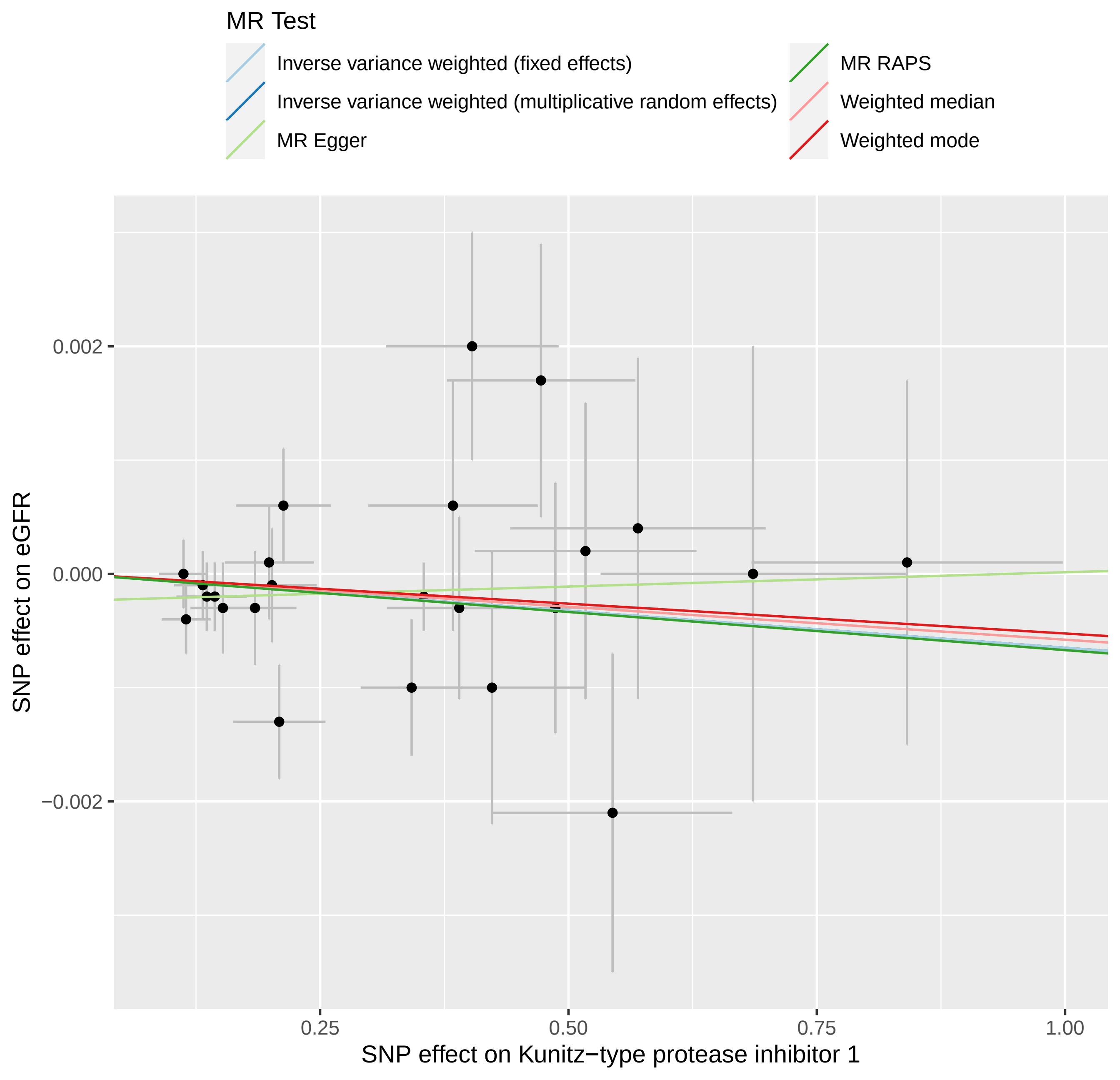

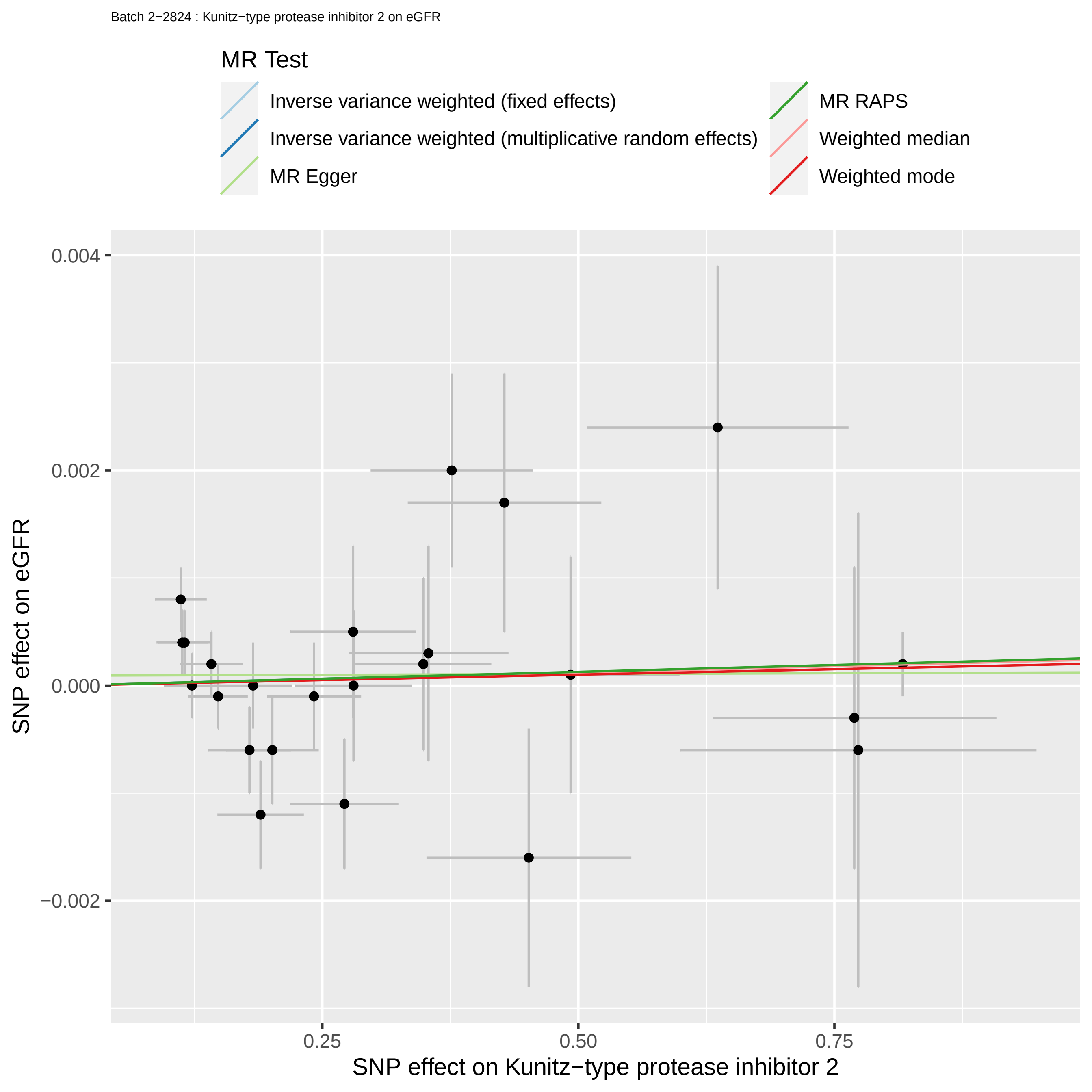
Batch 2−2824 : Kunitz−type protease inhibitor 2 on eGFR
MR Test
Inverse variance weighted (fixed effects)
Inverse variance weighted (multiplicative random effects)
MR Egger
MR RAPS
Weighted median
Weighted mode
0.004
0.002
0.000
−0.002
0.25
0.50
0.75
SNP effect on eGFR
SNP effect on Kunitz−type protease inhibitor 2

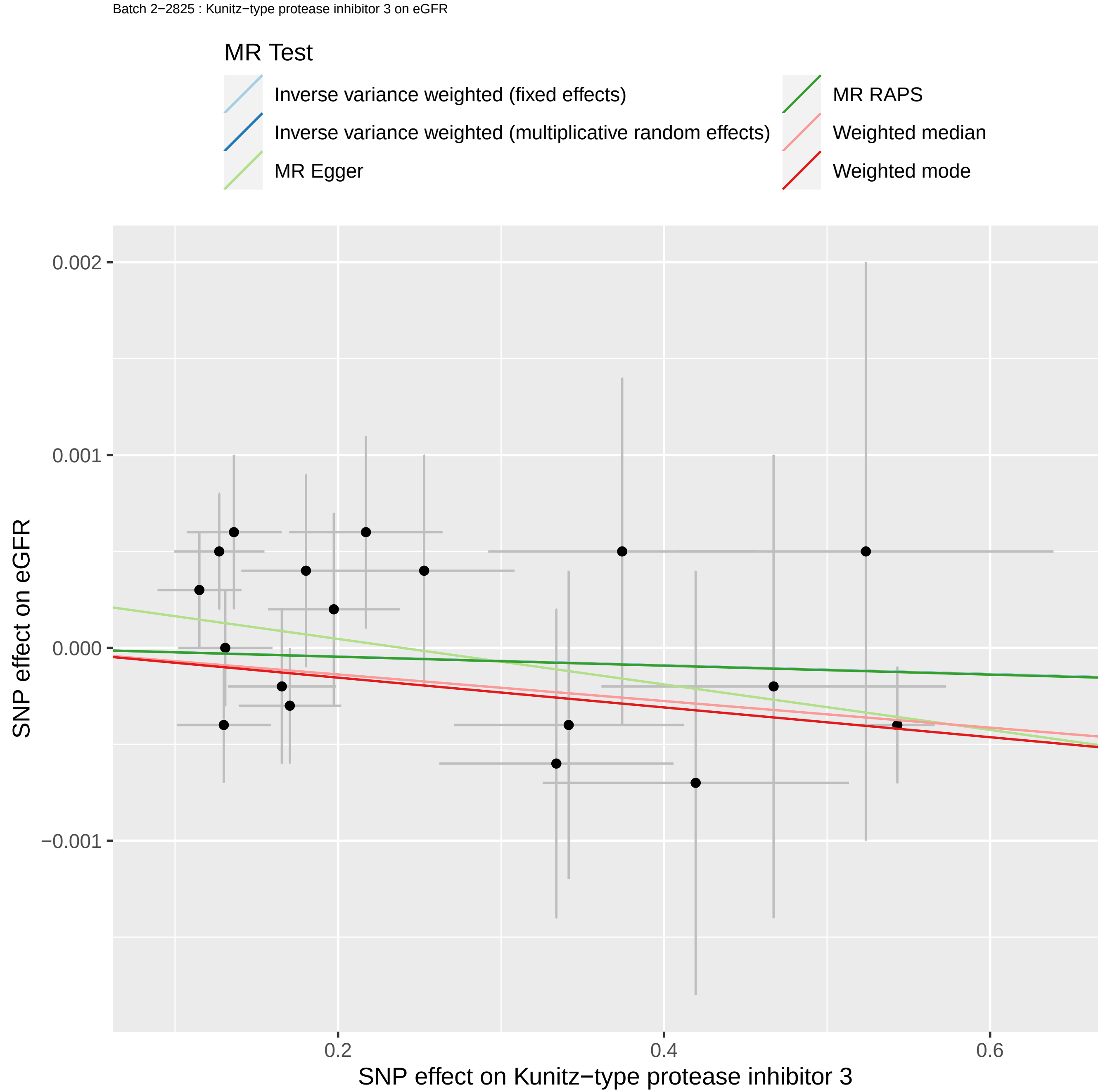

Batch 2−2825 : Kunitz−type protease inhibitor 3 on eGFR
MR Test
Inverse variance weighted (fixed effects)
Inverse variance weighted (multiplicative random effects)
MR Egger
MR RAPS
Weighted median
Weighted mode
0.002
0.001
0.000
−0.001
0.2
0.4
0.6
SNP effect on eGFR
SNP effect on Kunitz−type protease inhibitor 3

Batch 2−2826 : Testican−1 on eGFR

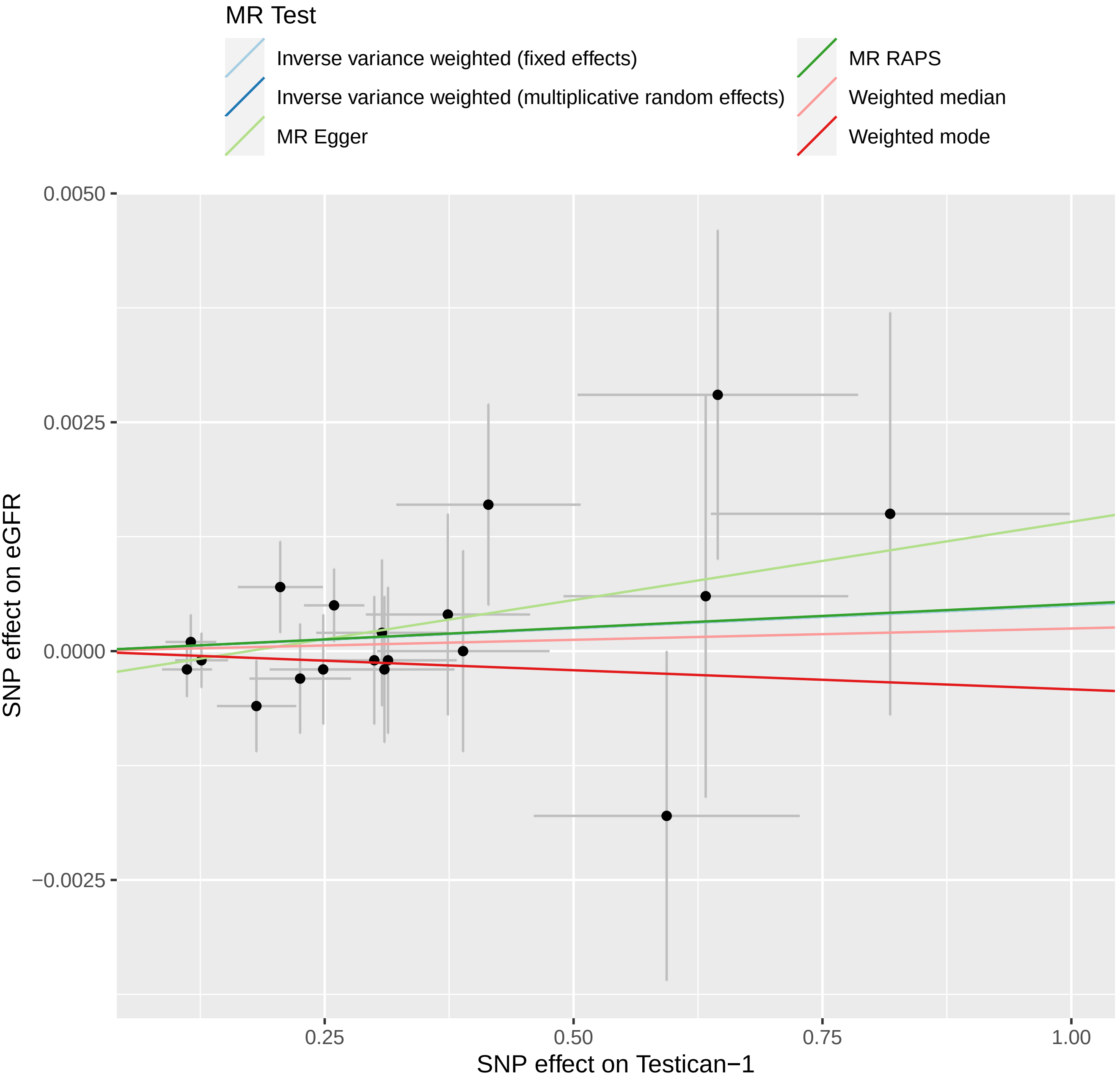

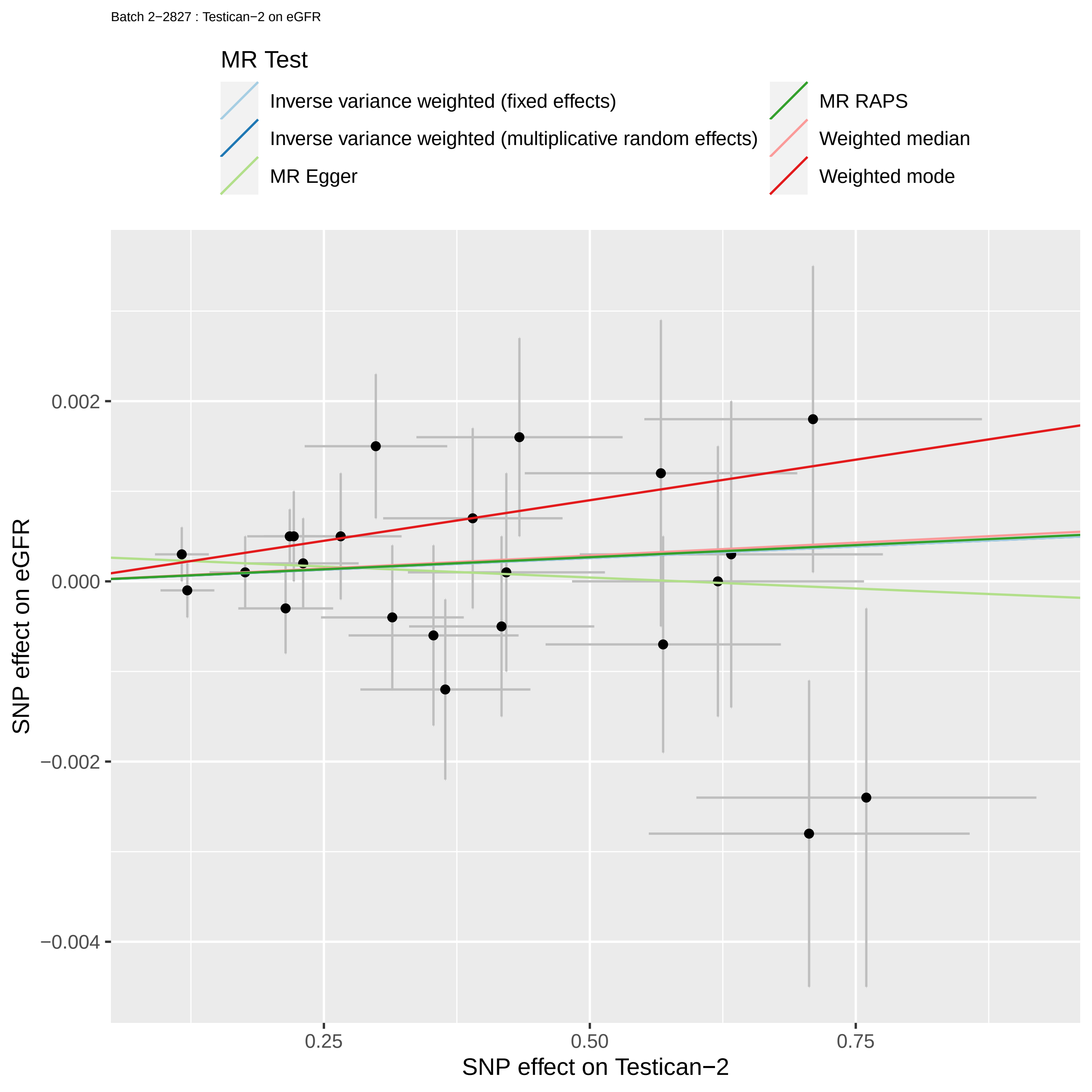

Batch 2−2827 : Testican−2 on eGFR
MR Test
Inverse variance weighted (fixed effects)
Inverse variance weighted (multiplicative random effects)
MR Egger
MR RAPS
Weighted median
Weighted mode
0.002
0.000
−0.002
−0.004
0.25
0.50
0.75
SNP effect on eGFR
SNP effect on Testican−2

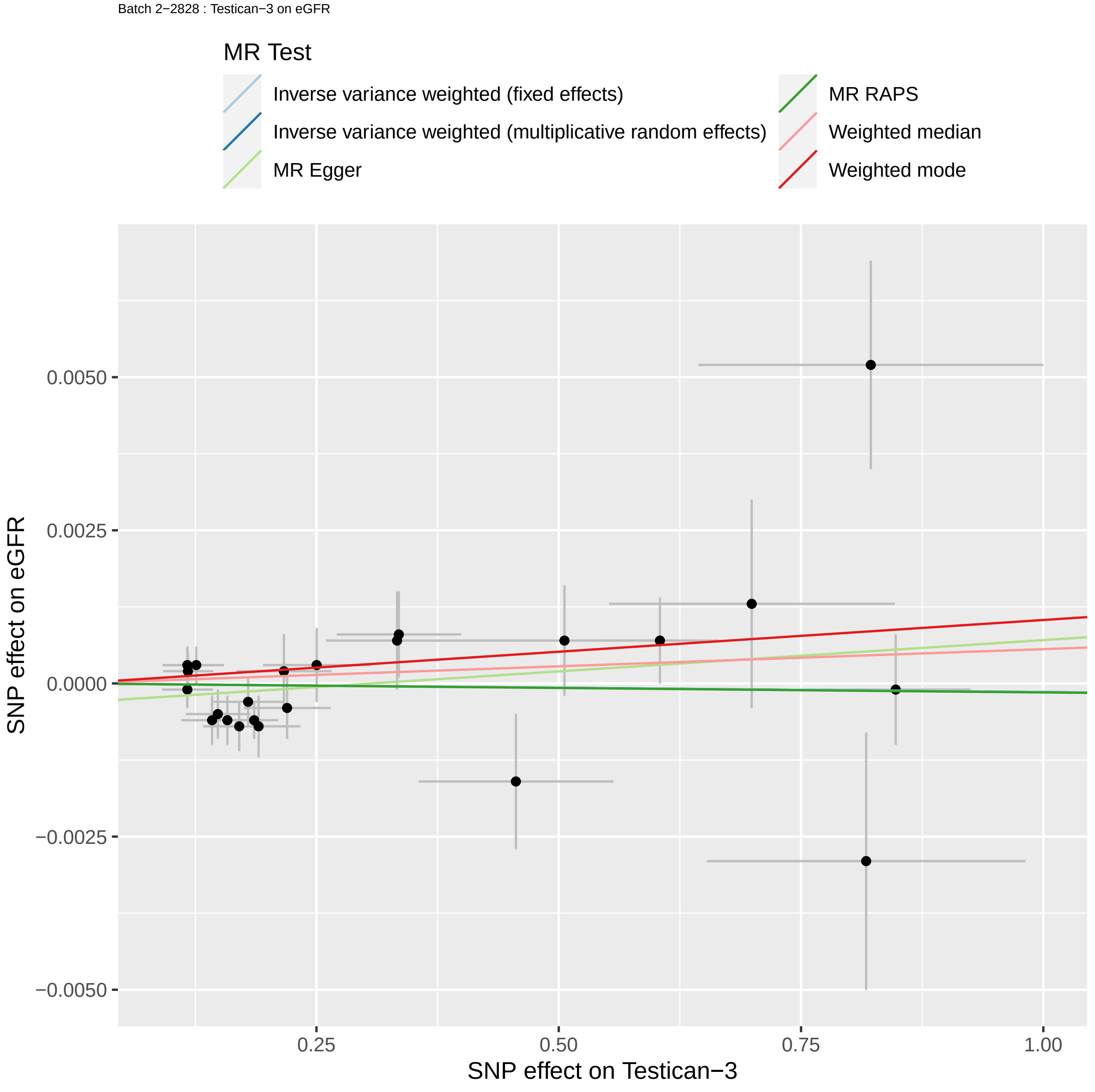

Batch 2−2828 : Testican−3 on eGFR
MR Test
Inverse variance weighted (fixed effects)
Inverse variance weighted (multiplicative random effects)
MR Egger
MR RAPS
Weighted median
Weighted mode
0.0050
0.0025
0.0000
−0.0025
−0.0050
0.25
0.50
0.75
1.00
SNP effect on eGFR
SNP effect on Testican−3

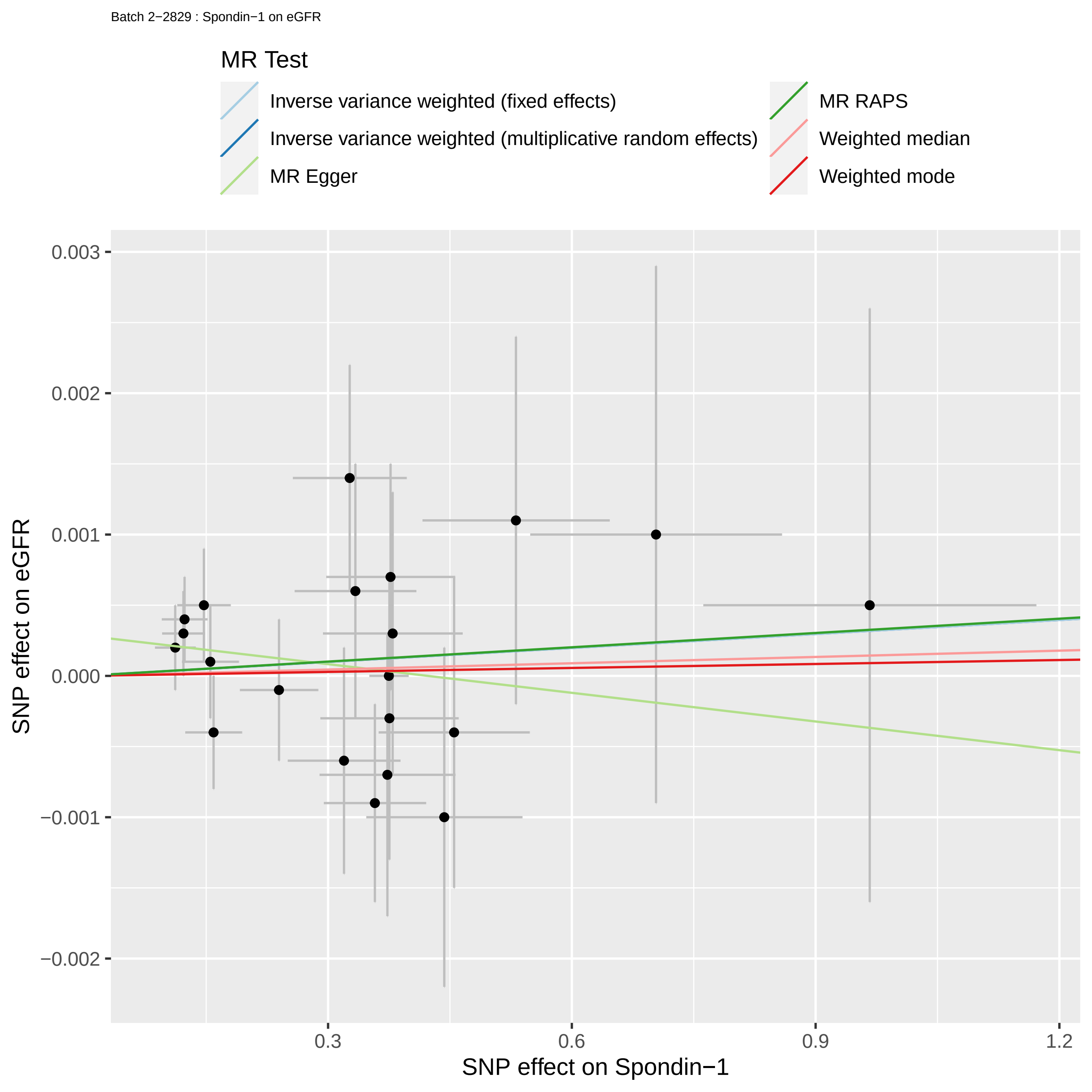

Batch 2−2829 : Spondin−1 on eGFR
MR Test
Inverse variance weighted (fixed effects)
Inverse variance weighted (multiplicative random effects)
MR Egger
MR RAPS
Weighted median
Weighted mode
0.003
0.002
0.001
0.000
−0.001
−0.002
0.3
0.6
0.9
1.2
SNP effect on Spondin−1
SNP effect on eGFR

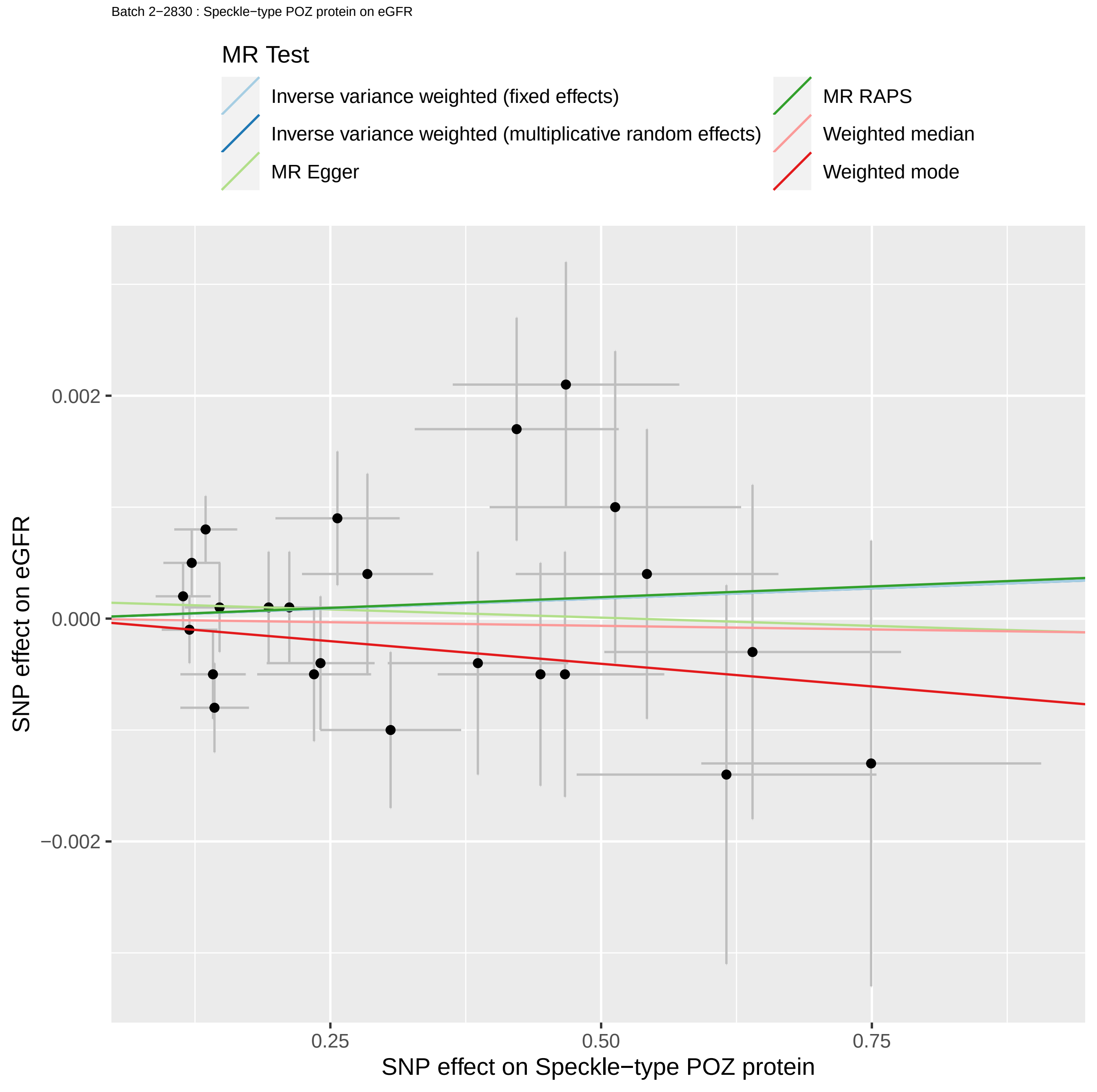

Batch 2−2830 : Speckle−type POZ protein on eGFR
MR Test
Inverse variance weighted (fixed effects)
Inverse variance weighted (multiplicative random effects)
MR Egger
MR RAPS
Weighted median
Weighted mode
0.002
0.000
−0.002
0.25
0.50
0.75
SNP effect on eGFR
SNP effect on Speckle−type POZ protein

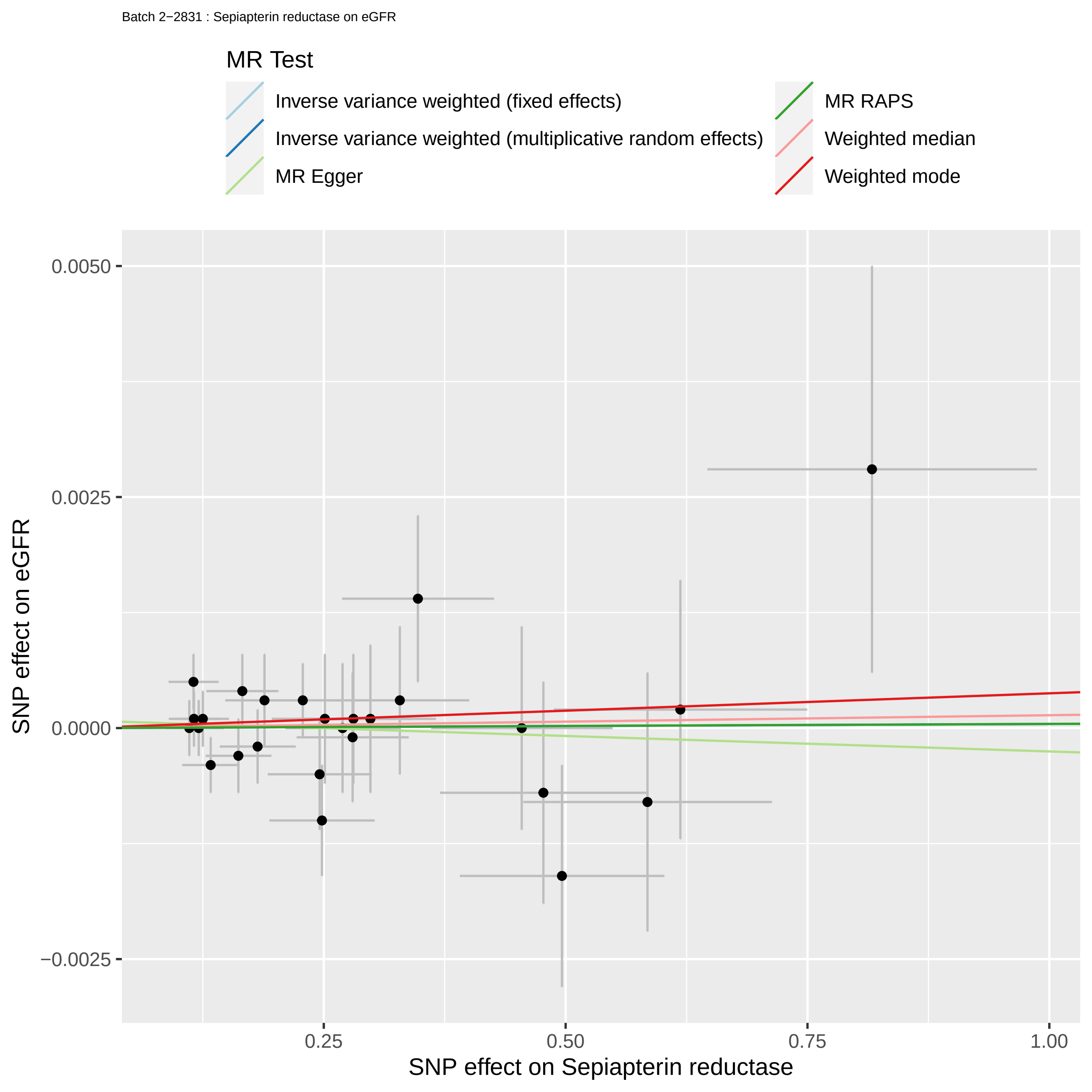

Batch 2−2831 : Sepiapterin reductase on eGFR
MR Test
Inverse variance weighted (fixed effects)
Inverse variance weighted (multiplicative random effects)
MR Egger
MR RAPS
Weighted median
Weighted mode
0.0050
0.0025
0.0000
−0.0025
SNP effect on eGFR
0.25
0.50
0.75
1.00
SNP effect on Sepiapterin reductase

Batch 2−2832 : Sprouty−related, EVH1 domain−containing protein 1 on eGFR

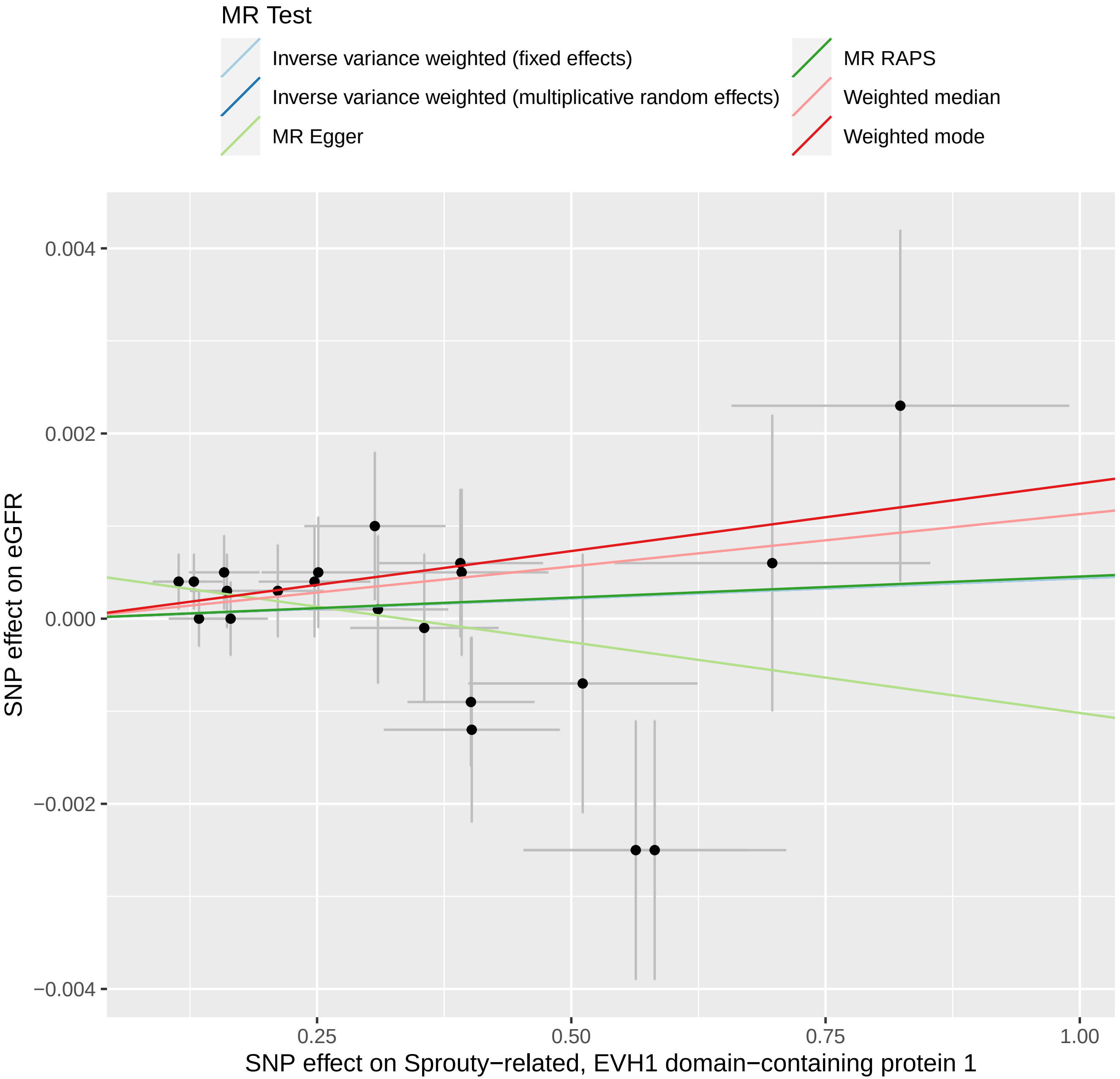

Batch 2−2833 : Protein sprouty homolog 1 on eGFR

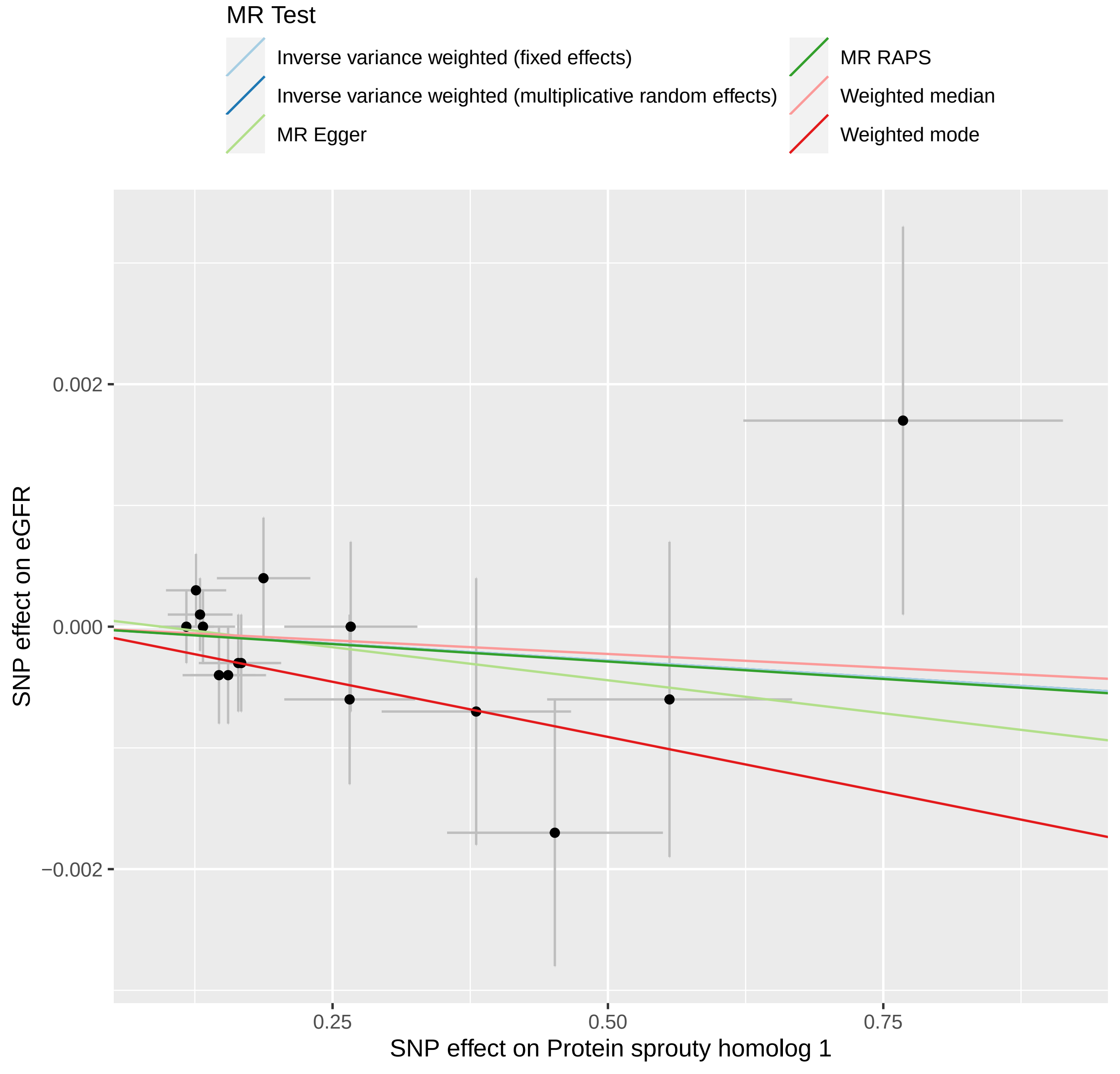

Batch 2−2834 : SPRY domain−containing SOCS box protein 1 on eGFR

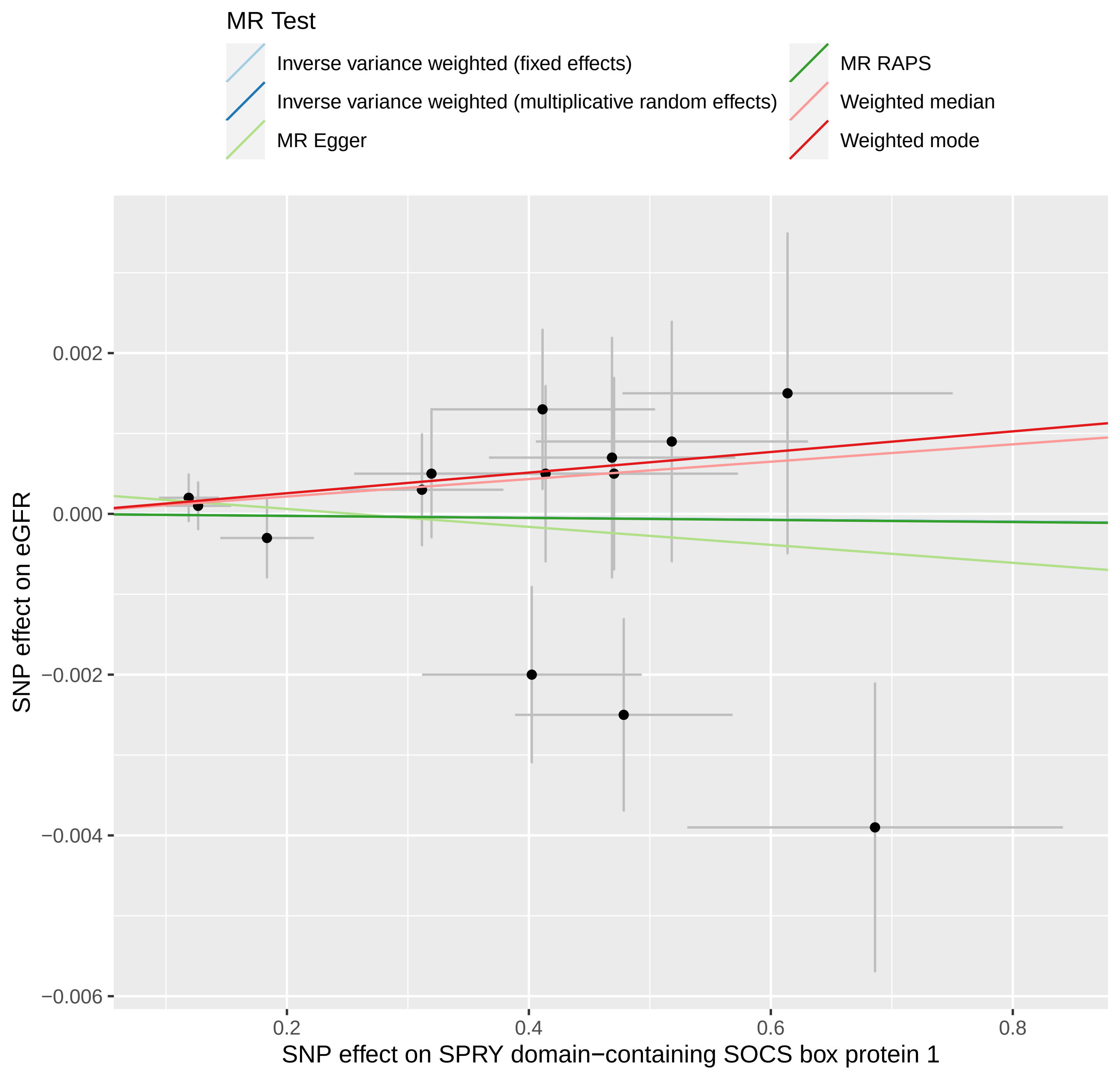

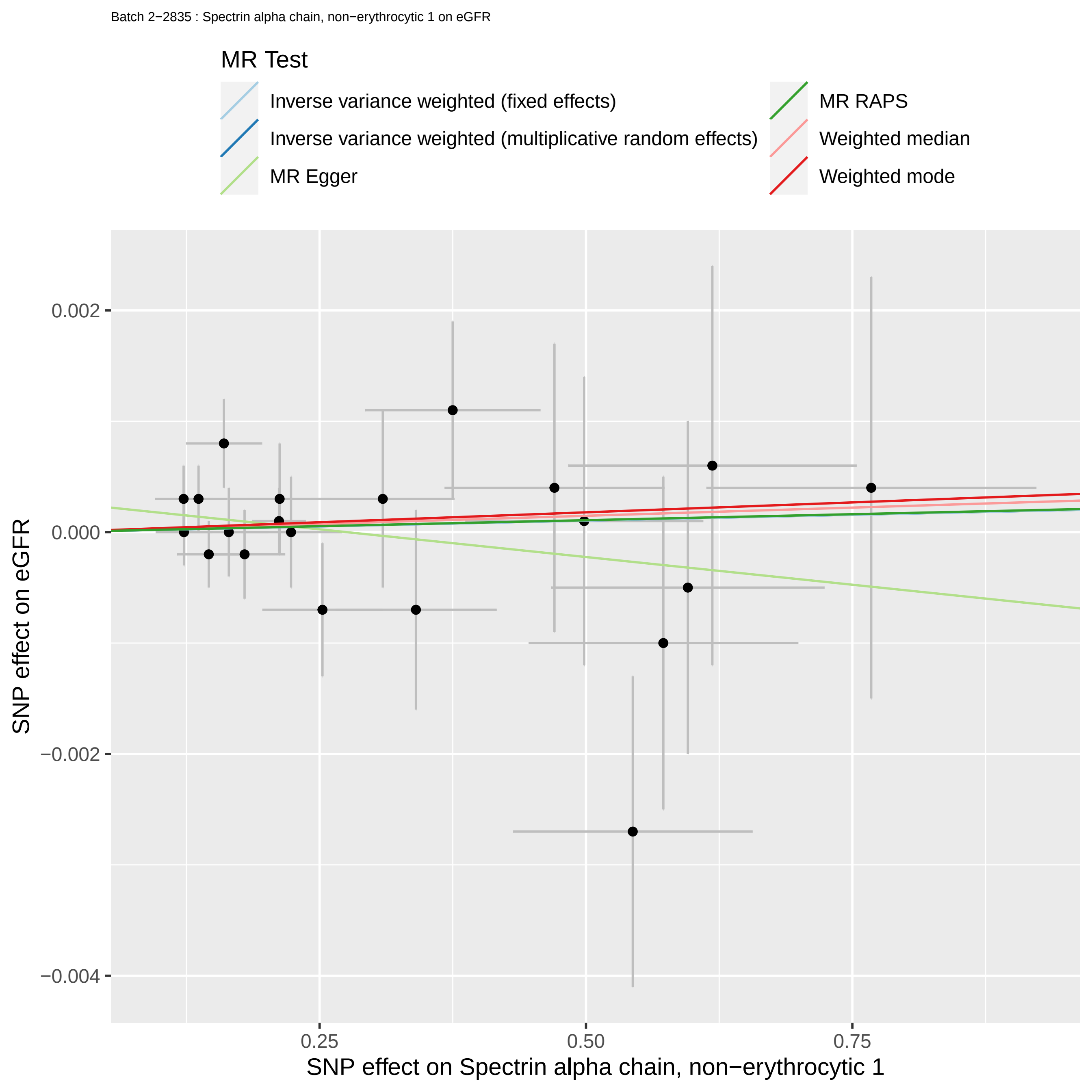

Batch 2−2835 : Spectrin alpha chain, non−erythrocytic 1 on eGFR
MR Test
Inverse variance weighted (fixed effects)
Inverse variance weighted (multiplicative random effects)
MR Egger
MR RAPS
Weighted median
Weighted mode
0.002
0.000
−0.002
−0.004
SNP effect on eGFR
0.25
0.50
0.75
SNP effect on Spectrin alpha chain, non−erythrocytic 1

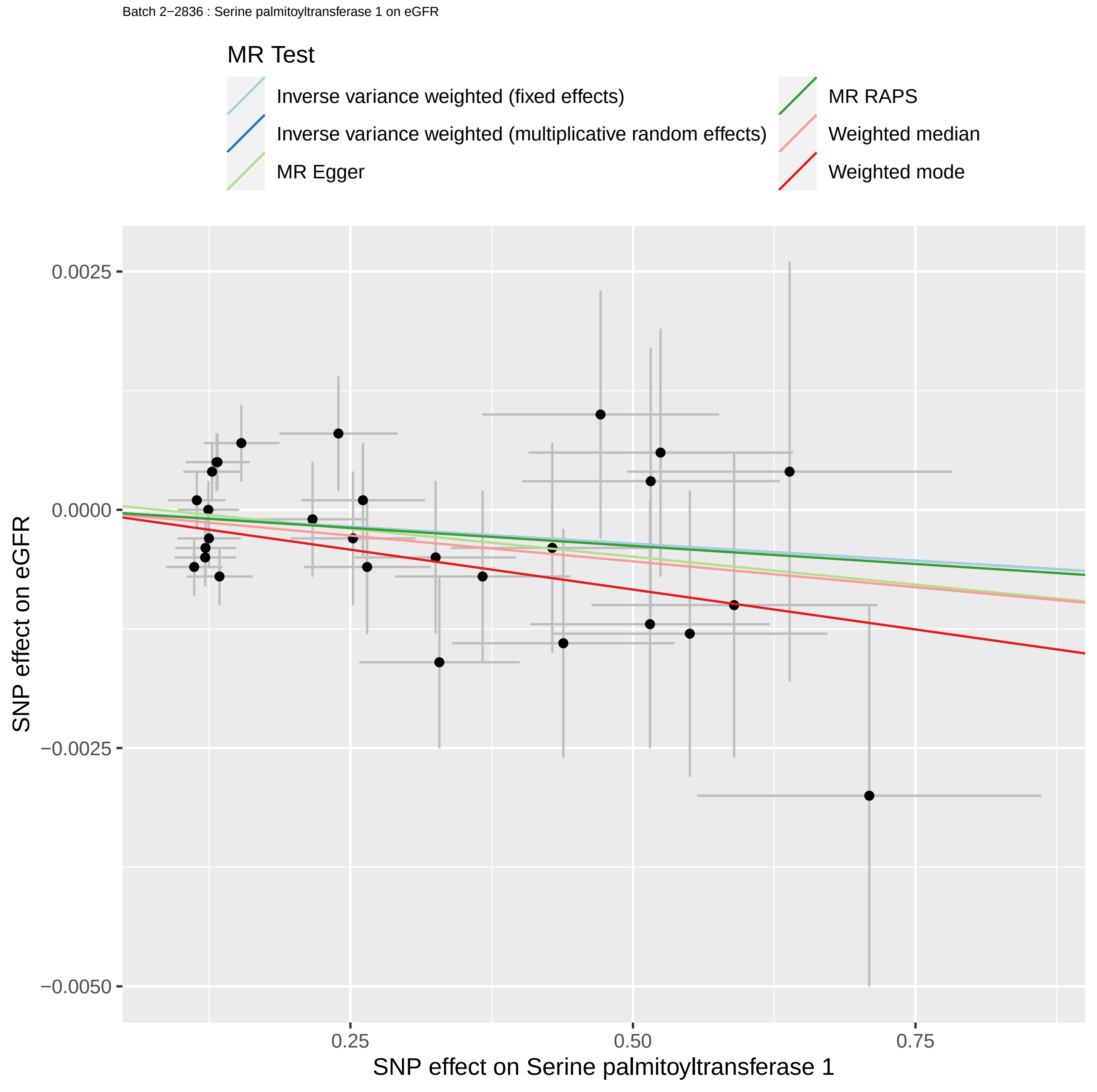

Batch 2−2836 : Serine palmitoyltransferase 1 on eGFR
MR Test
Inverse variance weighted (fixed effects)
Inverse variance weighted (multiplicative random effects)
MR Egger
MR RAPS
Weighted median
Weighted mode
0.0025
0.0000
−0.0025
−0.0050
SNP effect on eGFR
0.25
0.50
0.75
SNP effect on Serine palmitoyltransferase 1

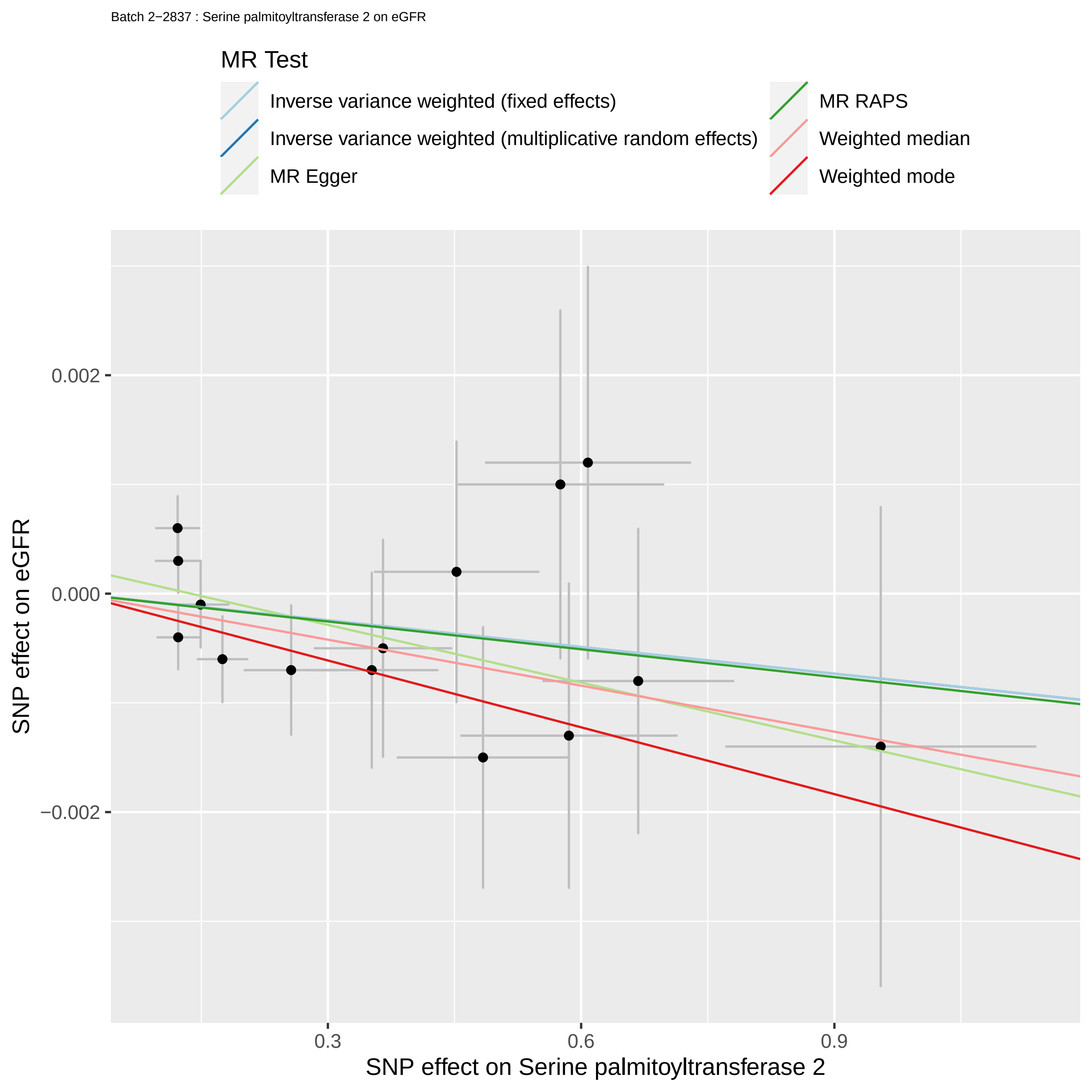

Batch 2−2837 : Serine palmitoyltransferase 2 on eGFR
MR Test
Inverse variance weighted (fixed effects)
Inverse variance weighted (multiplicative random effects)
MR Egger
MR RAPS
Weighted median
Weighted mode
0.002
0.000
−0.002
SNP effect on eGFR
0.3
0.6
0.9
SNP effect on Serine palmitoyltransferase 2

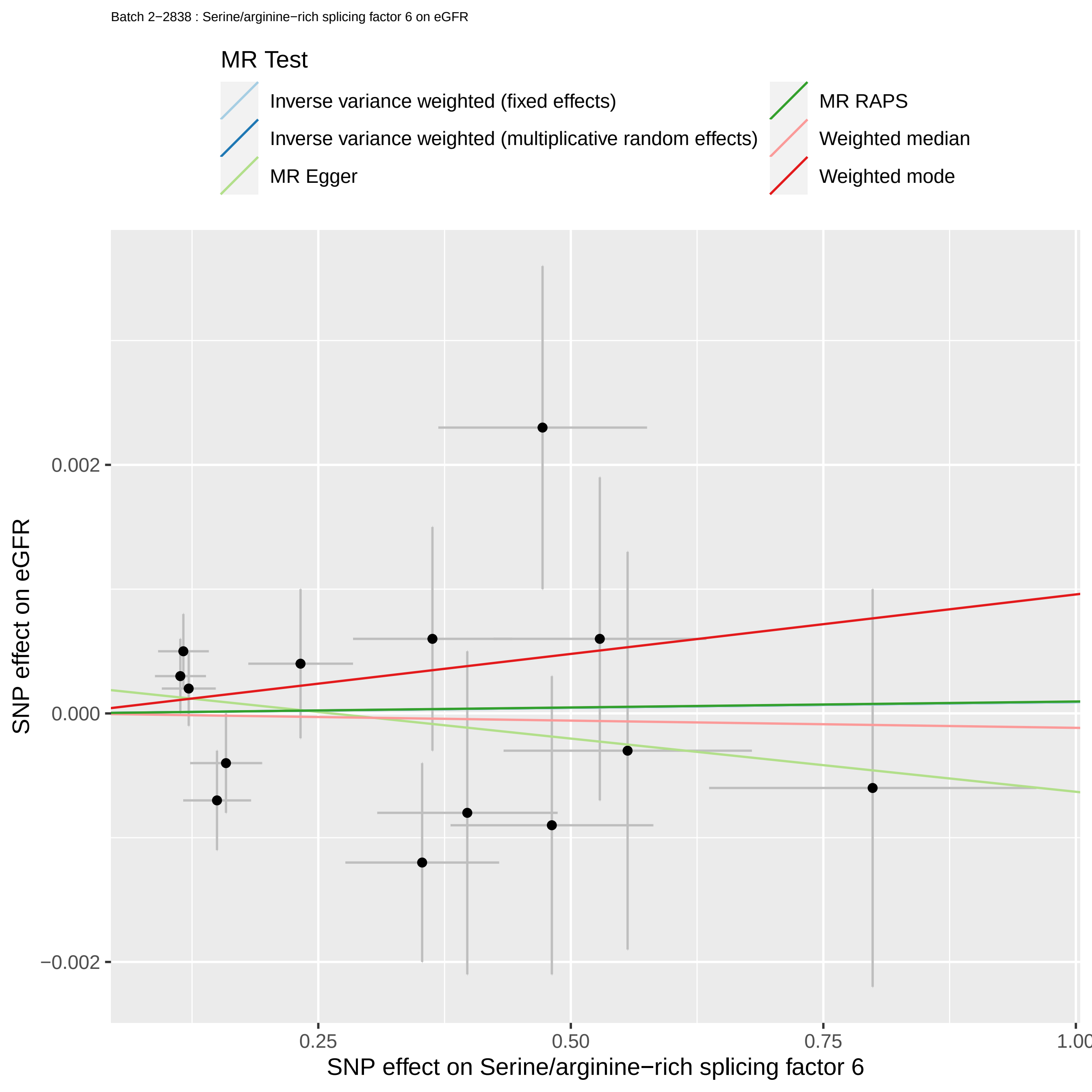
Batch 2−2838 : Serine/arginine−rich splicing factor 6 on eGFR
MR Test
Inverse variance weighted (fixed effects)
Inverse variance weighted (multiplicative random effects)
MR Egger
MR RAPS
Weighted median
Weighted mode
0.002
0.000
−0.002
0.25
0.50
0.75
1.00
SNP effect on eGFR
SNP effect on Serine/arginine−rich splicing factor 6

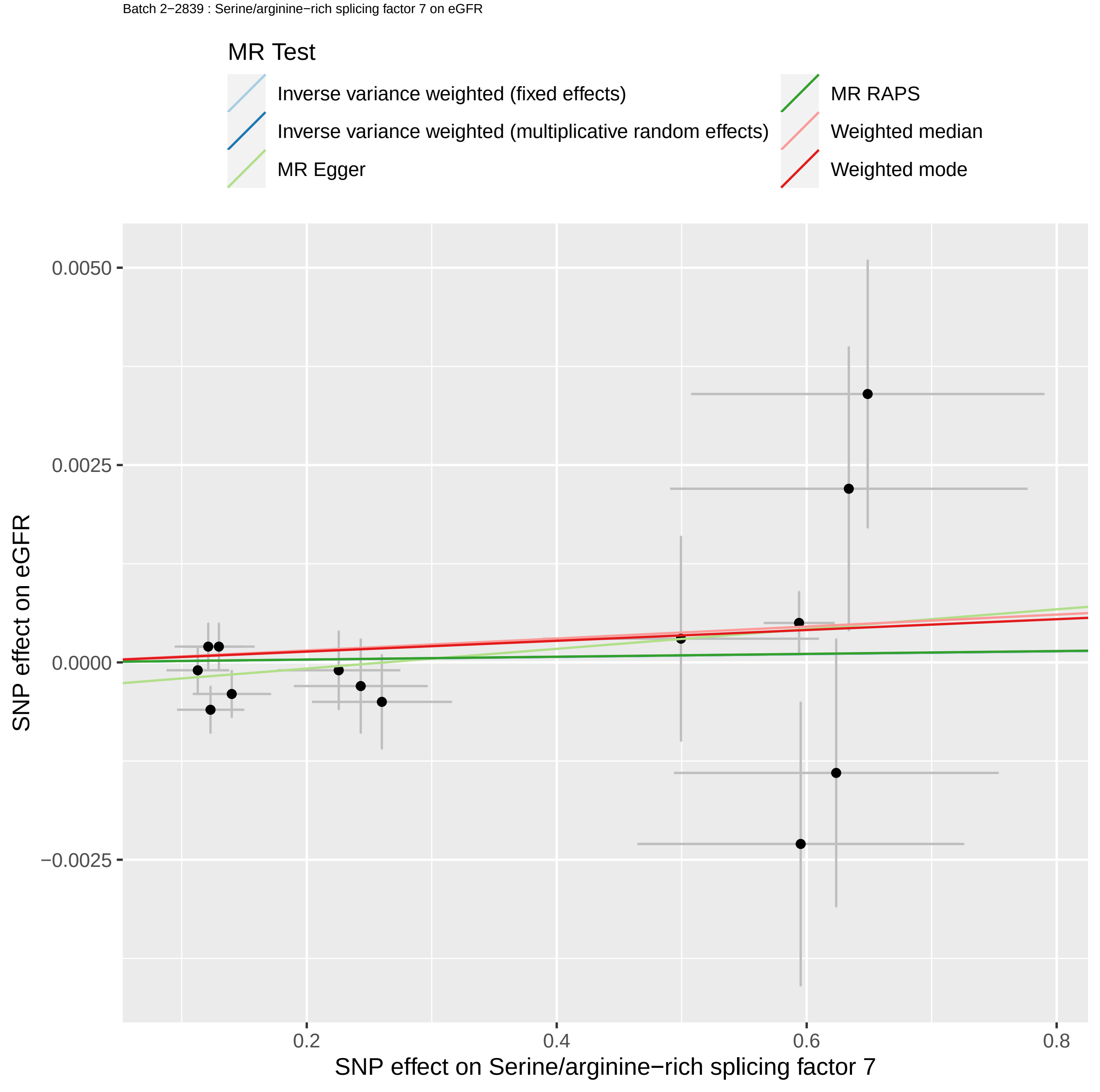

Batch 2−2839 : Serine/arginine−rich splicing factor 7 on eGFR
MR Test
Inverse variance weighted (fixed effects)
Inverse variance weighted (multiplicative random effects)
MR Egger
MR RAPS
Weighted median
Weighted mode
0.0050
0.0025
0.0000
−0.0025
0.2
0.4
0.6
0.8
SNP effect on eGFR
SNP effect on Serine/arginine−rich splicing factor 7

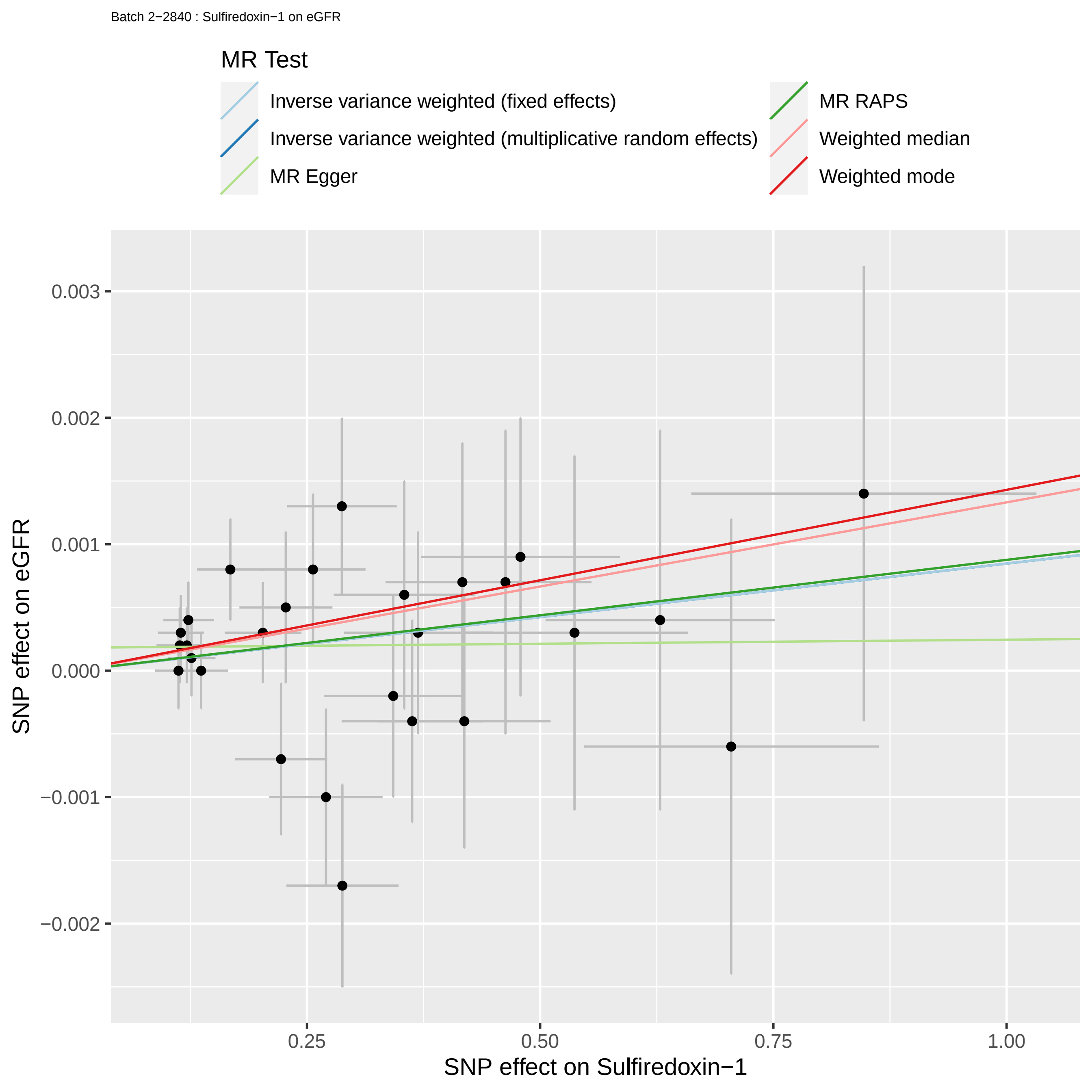

Batch 2−2840 : Sulfiredoxin−1 on eGFR
MR Test
Inverse variance weighted (fixed effects)
Inverse variance weighted (multiplicative random effects)
MR Egger
MR RAPS
Weighted median
Weighted mode
0.003
0.002
0.001
0.000
−0.001
−0.002
SNP effect on eGFR
0.25
0.50
0.75
1.00
SNP effect on Sulfiredoxin−1

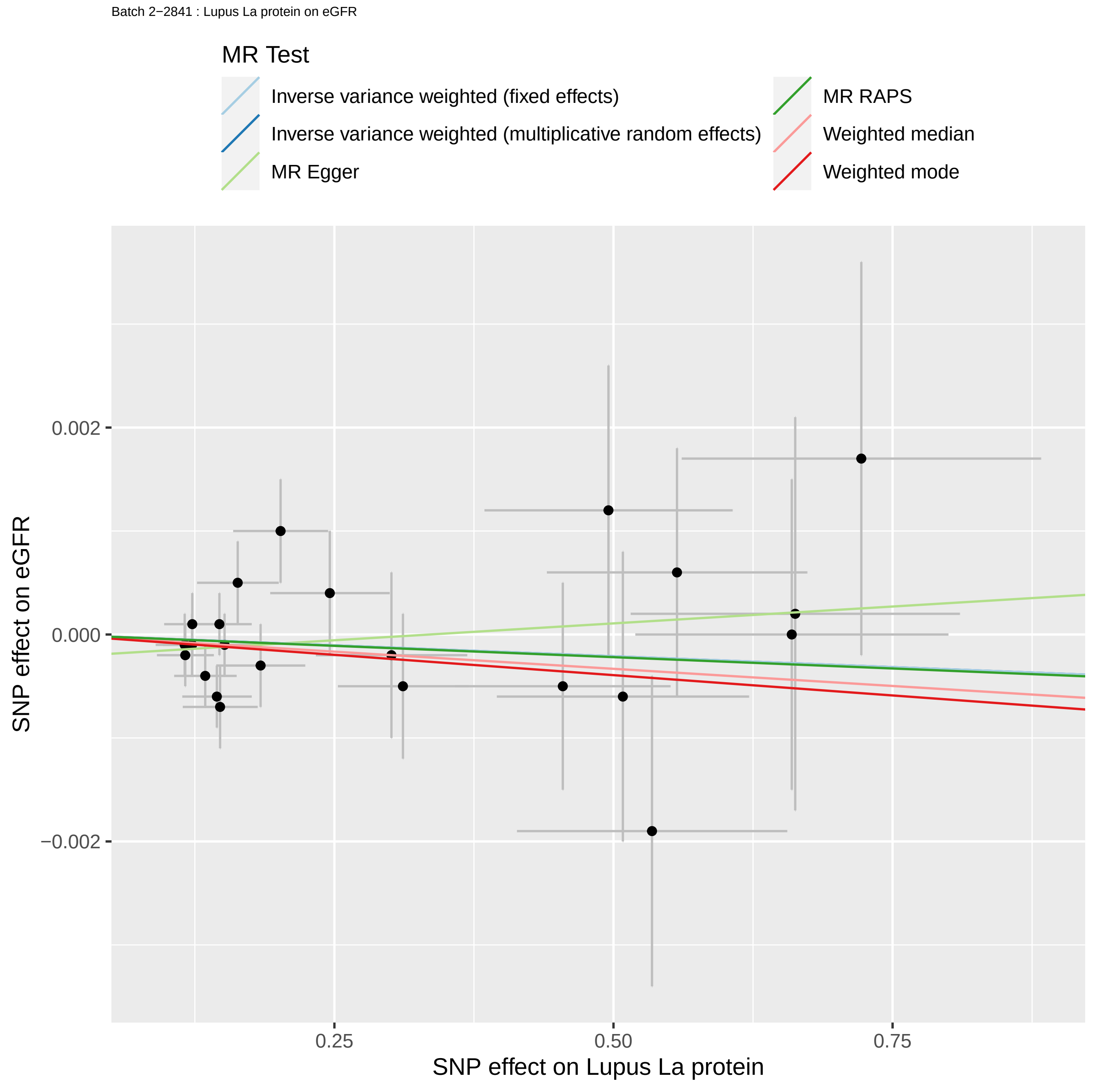

Batch 2−2841 : Lupus La protein on eGFR
MR Test
Inverse variance weighted (fixed effects)
Inverse variance weighted (multiplicative random effects)
MR Egger
MR RAPS
Weighted median
Weighted mode
0.002
0.000
−0.002
0.25
0.50
0.75
SNP effect on eGFR
SNP effect on Lupus La protein

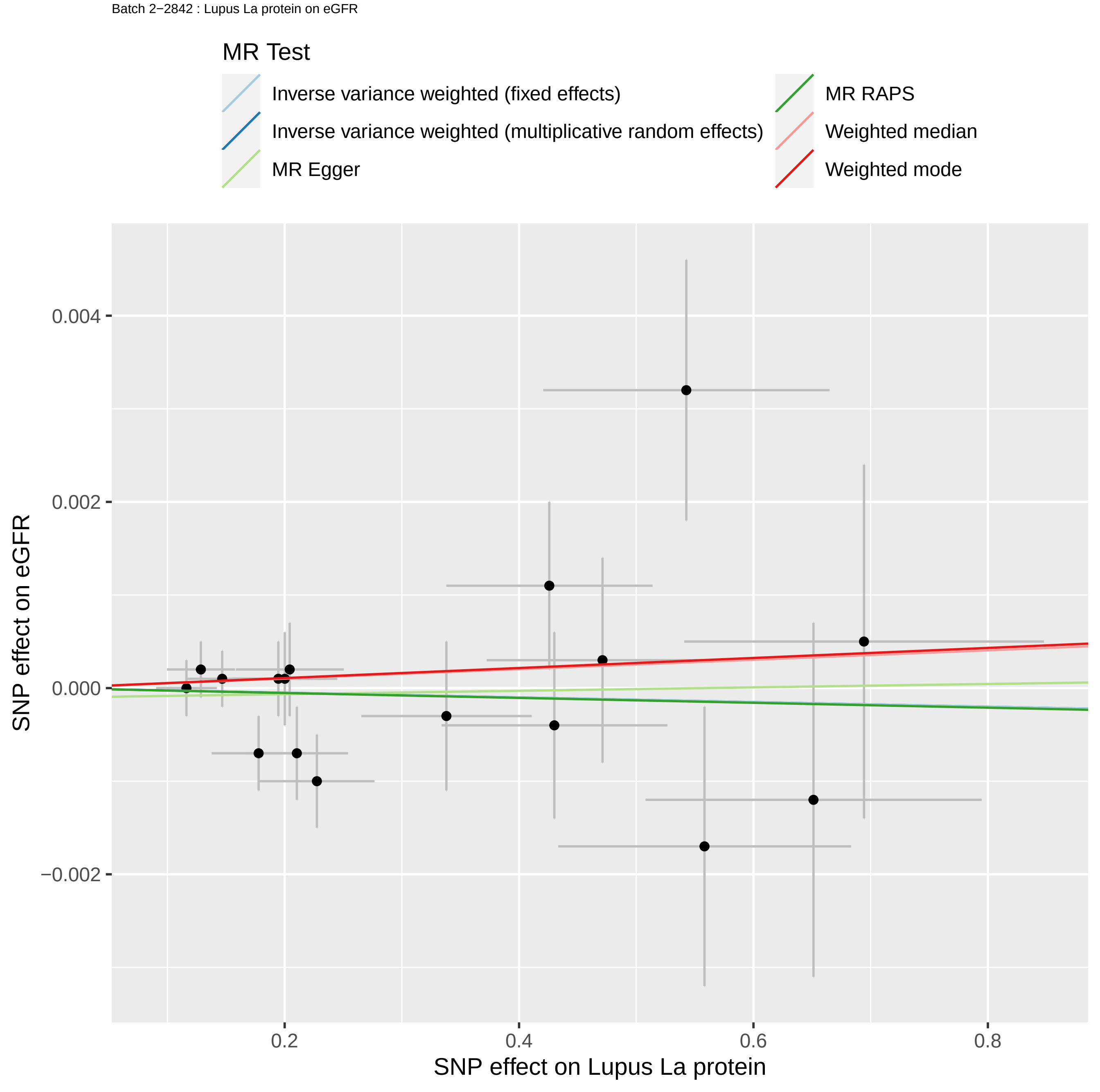

Batch 2−2842 : Lupus La protein on eGFR
MR Test
Inverse variance weighted (fixed effects)
Inverse variance weighted (multiplicative random effects)
MR Egger
MR RAPS
Weighted median
Weighted mode
0.004
0.002
0.000
−0.002
SNP effect on eGFR
0.2
0.4
0.6
0.8
SNP effect on Lupus La protein

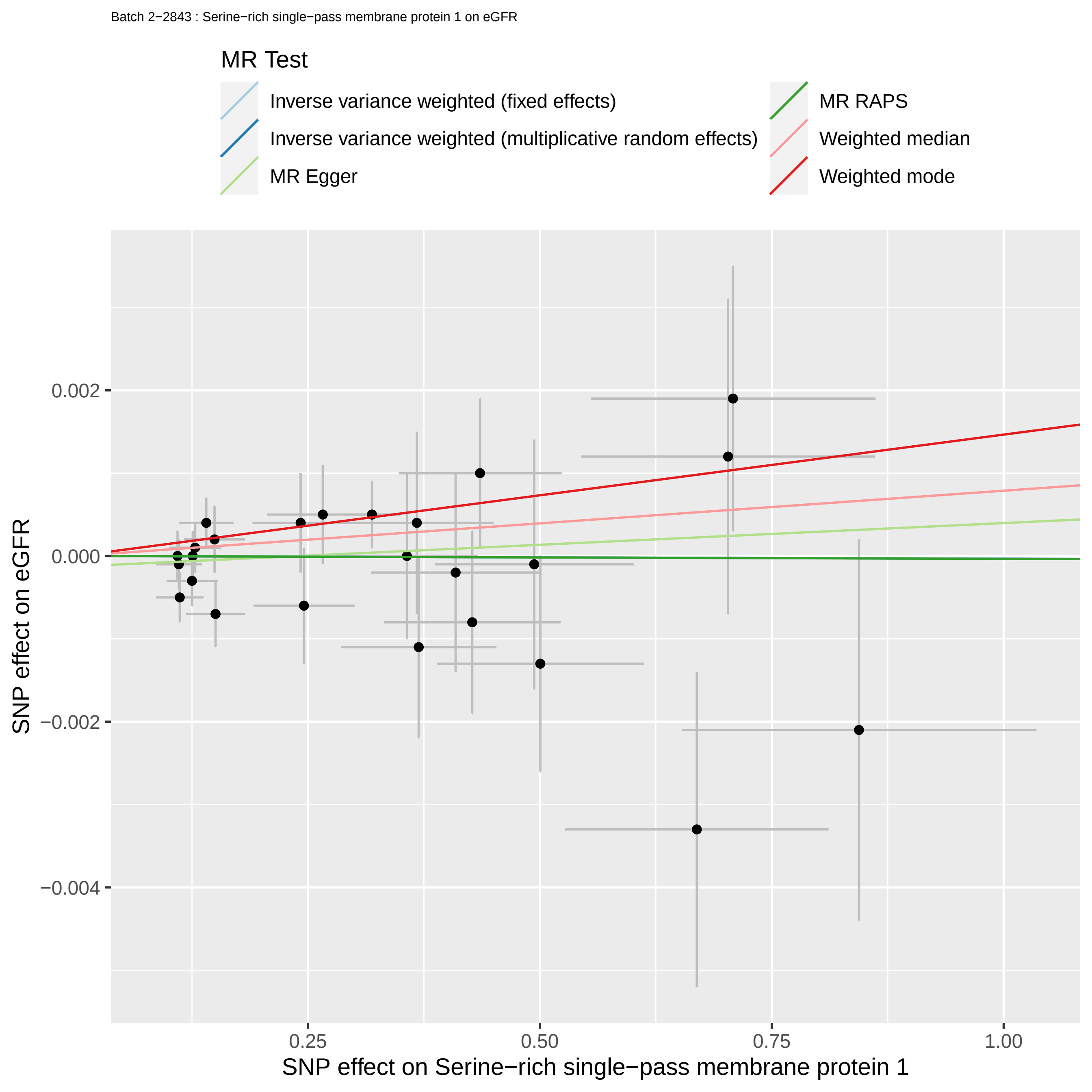

Batch 2−2843 : Serine−rich single−pass membrane protein 1 on eGFR
MR Test
Inverse variance weighted (fixed effects)
Inverse variance weighted (multiplicative random effects)
MR Egger
MR RAPS
Weighted median
Weighted mode
0.002
0.000
−0.002
−0.004
SNP effect on eGFR
0.25
0.50
0.75
1.00
SNP effect on Serine−rich single−pass membrane protein 1

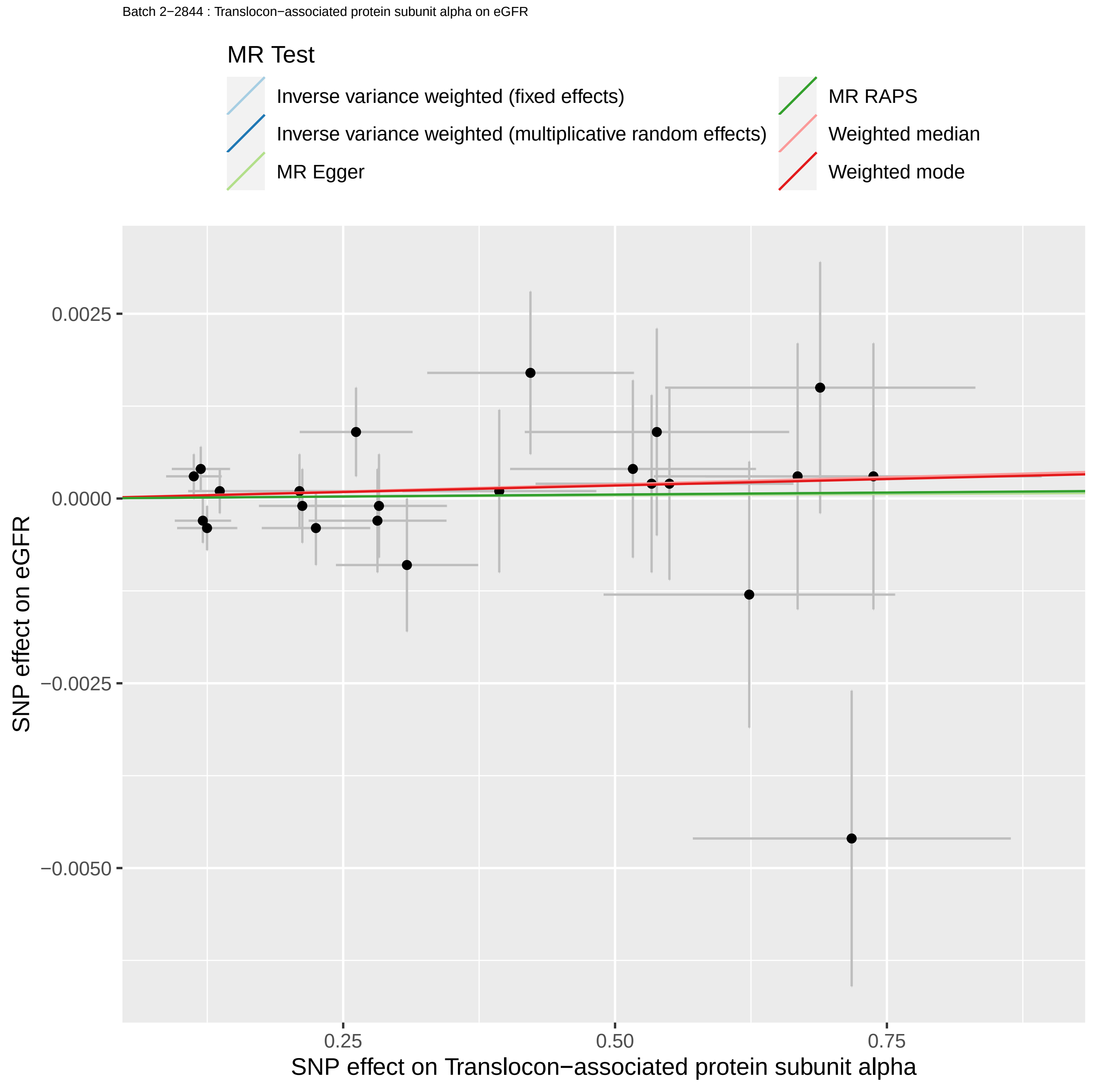

Batch 2−2844 : Translocon−associated protein subunit alpha on eGFR
MR Test
Inverse variance weighted (fixed effects)
Inverse variance weighted (multiplicative random effects)
MR Egger
MR RAPS
Weighted median
Weighted mode
0.0025
0.0000
−0.0025
−0.0050
0.25
0.50
0.75
SNP effect on eGFR
SNP effect on Translocon−associated protein subunit alpha

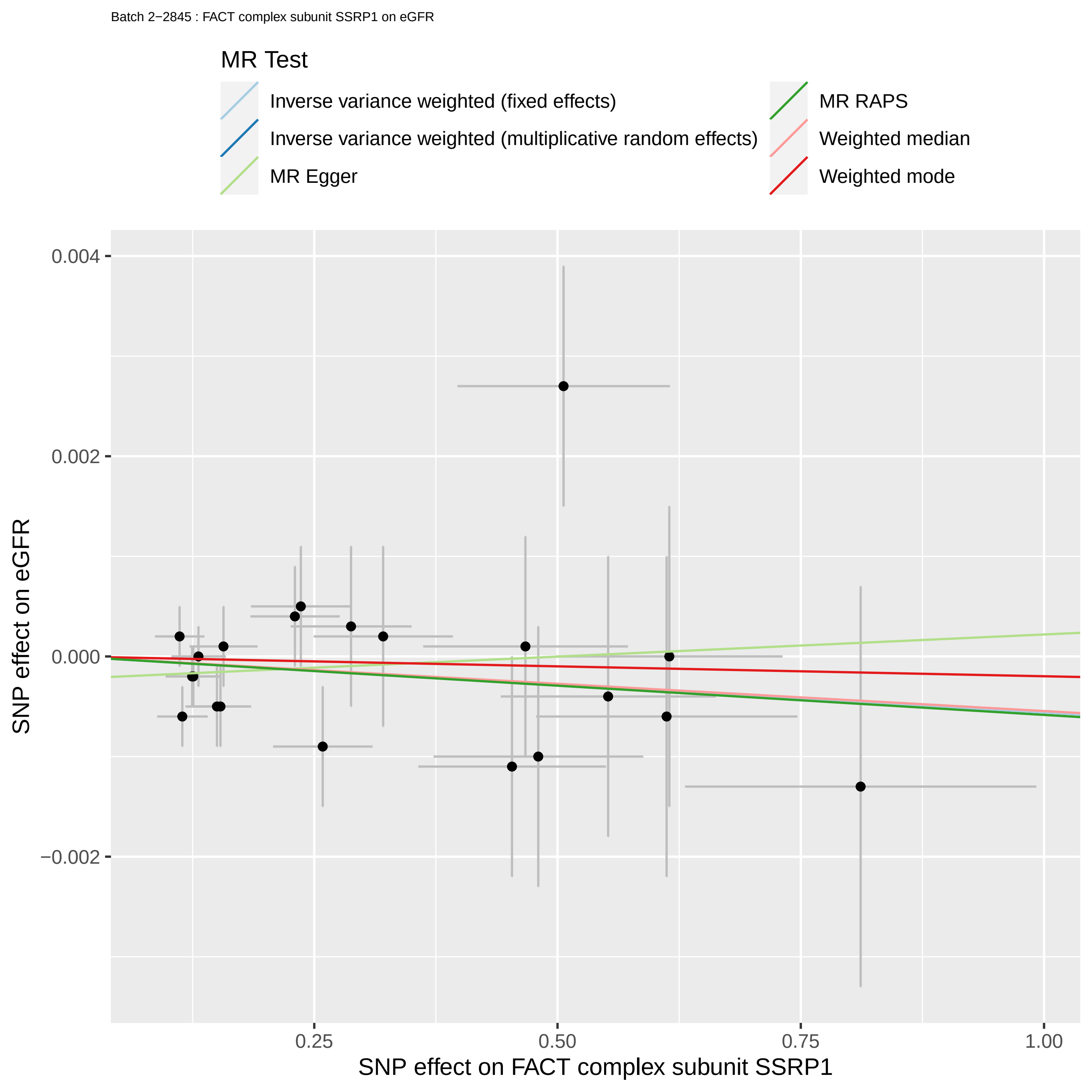
Batch 2−2845 : FACT complex subunit SSRP1 on eGFR
MR Test
Inverse variance weighted (fixed effects)
Inverse variance weighted (multiplicative random effects)
MR Egger
MR RAPS
Weighted median
Weighted mode
0.004
0.002
0.000
−0.002
SNP effect on eGFR
0.25
0.50
0.75
1.00
SNP effect on FACT complex subunit SSRP1

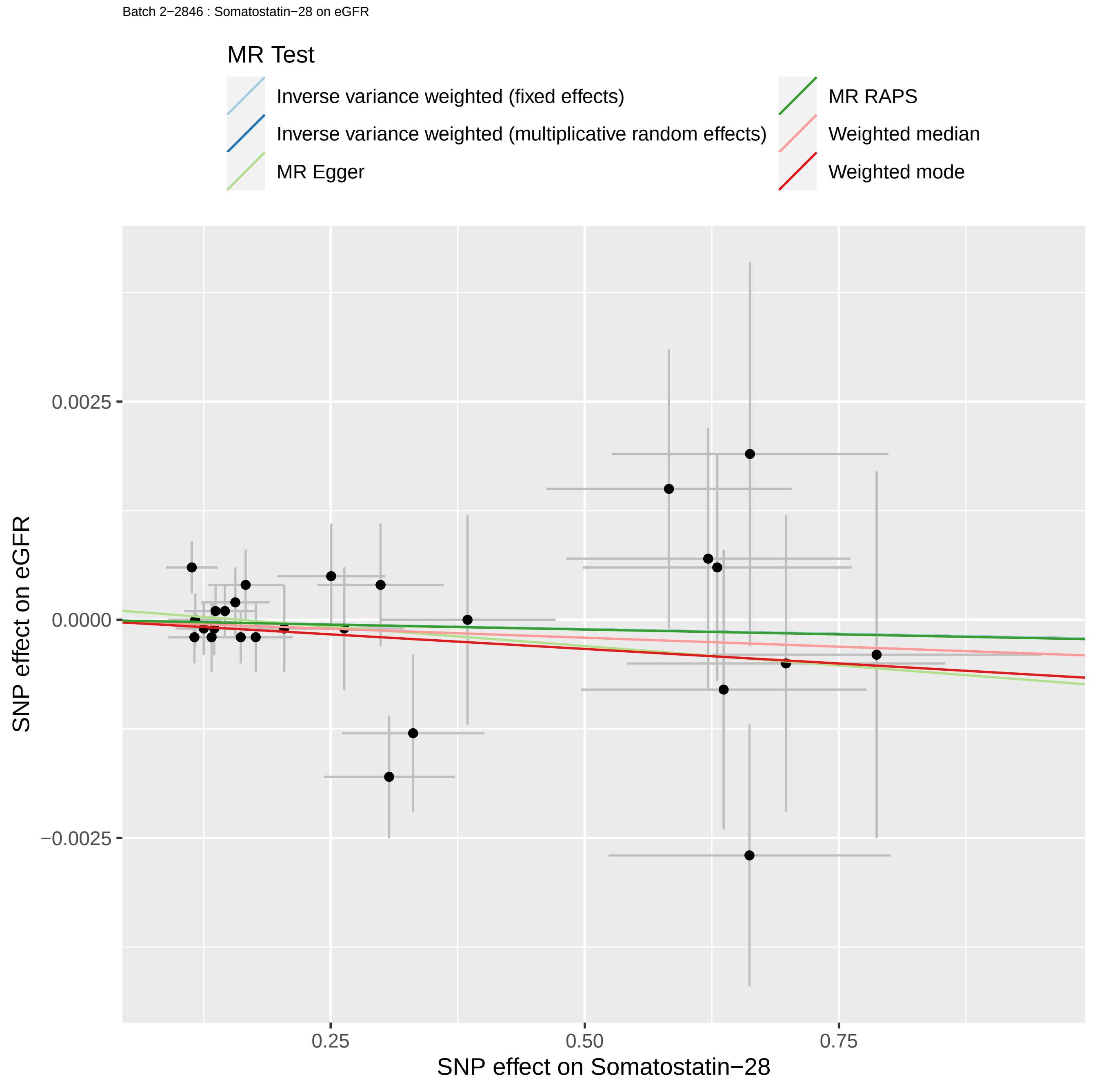

Batch 2−2846 : Somatostatin−28 on eGFR
MR Test
Inverse variance weighted (fixed effects)
Inverse variance weighted (multiplicative random effects)
MR Egger
MR RAPS
Weighted median
Weighted mode
0.0025
0.0000
−0.0025
0.25
0.50
0.75
SNP effect on Somatostatin−28
SNP effect on eGFR

Batch 2−2847 : RNA polymerase II subunit A C−terminal domain phosphatase SSU72 on eGFR

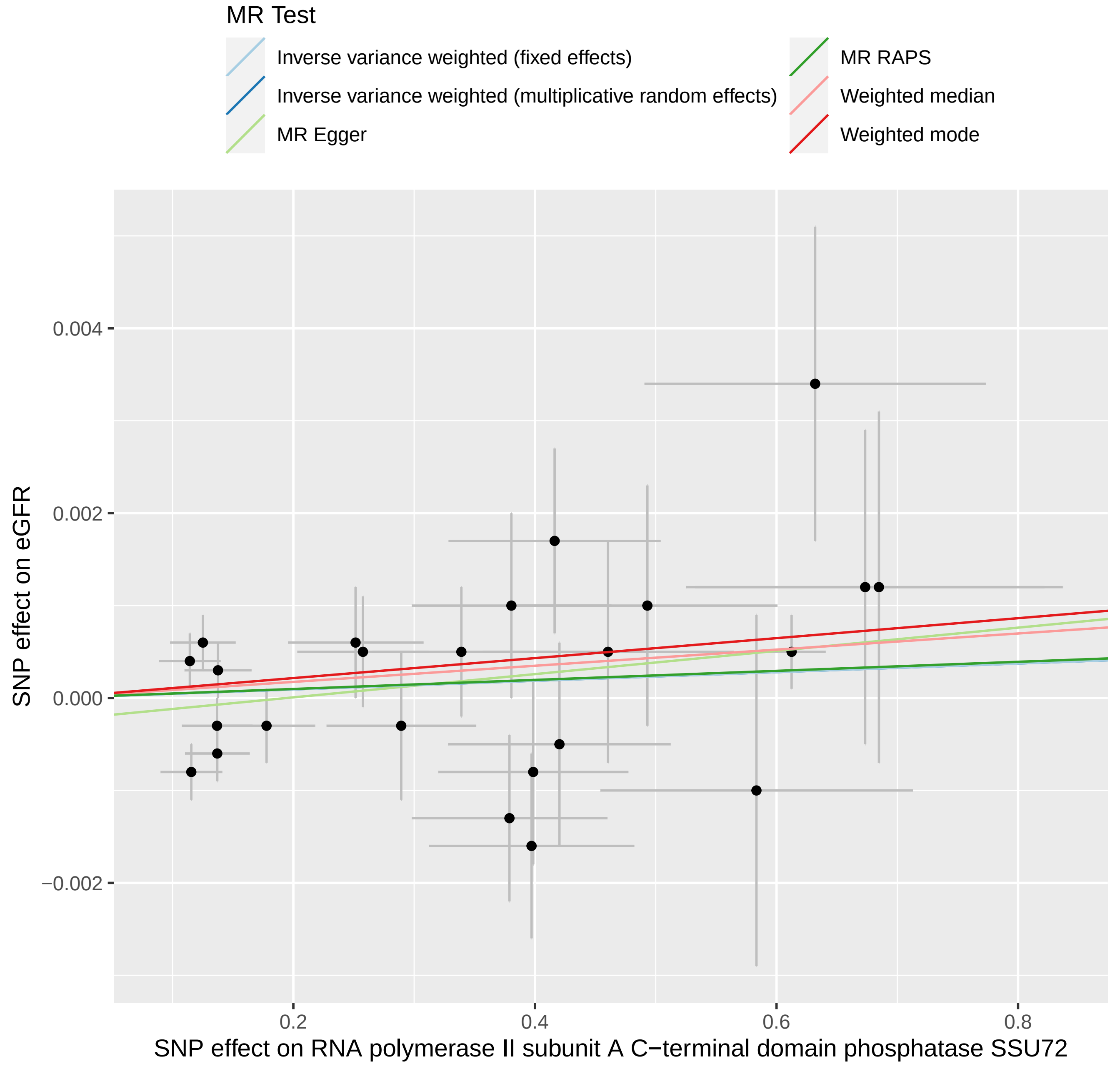

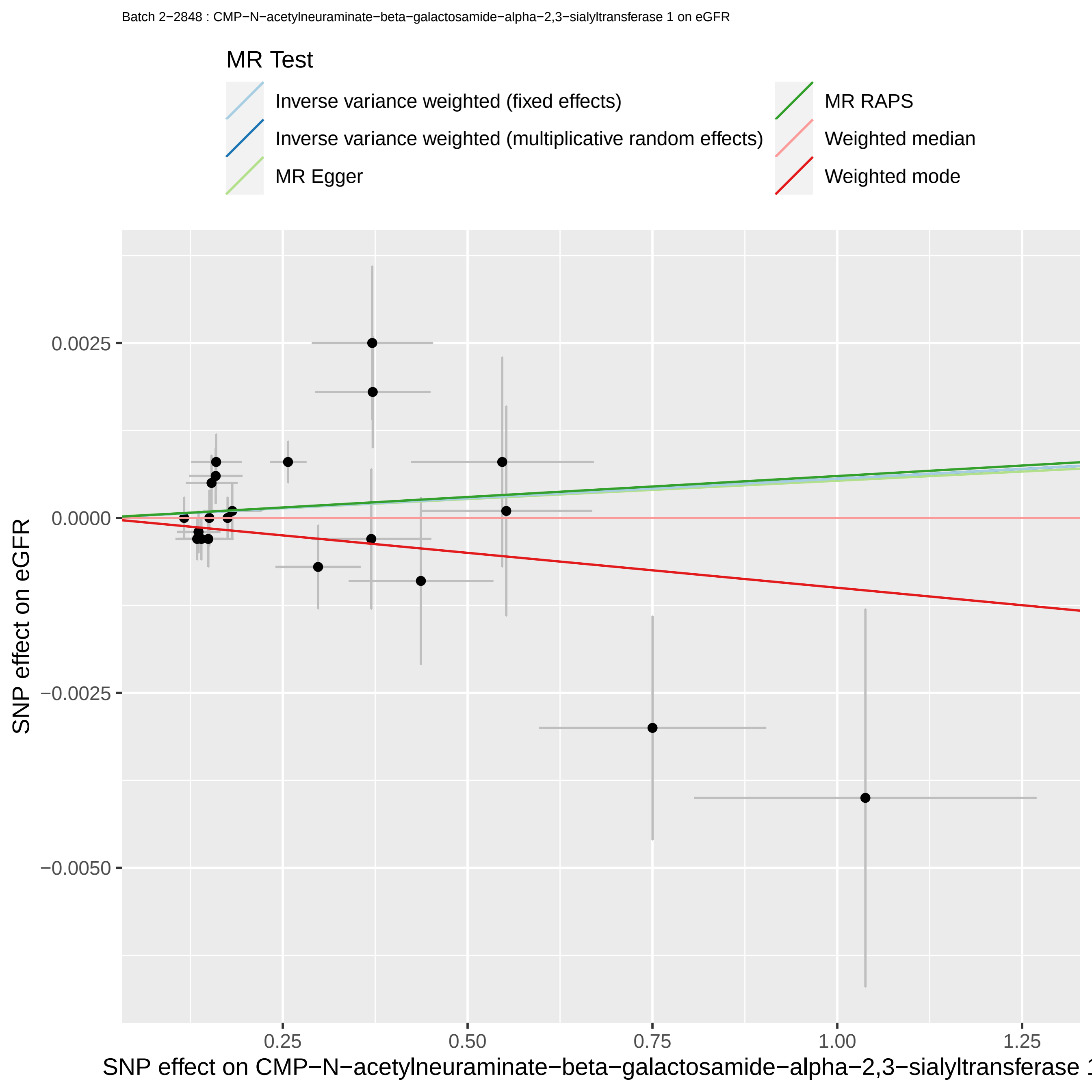
Batch 2−2848 : CMP−N−acetylneuraminate−beta−galactosamide−alpha−2,3−sialyltransferase 1 on eGFR
MR Test
Inverse variance weighted (fixed effects)
Inverse variance weighted (multiplicative random effects)
MR Egger
MR RAPS
Weighted median
Weighted mode
0.0025
0.0000
−0.0025
−0.0050
0.25
0.50
0.75
1.00
1.25
SNP effect on eGFR
SNP effect on CMP−N−acetylneuraminate−beta−galactosamide−alpha−2,3−sialyltransferase 1

Batch 2−2849 : CMP−N−acetylneuraminate−beta−galactosamide−alpha−2,3−sialyltransferase 2 on eGFR

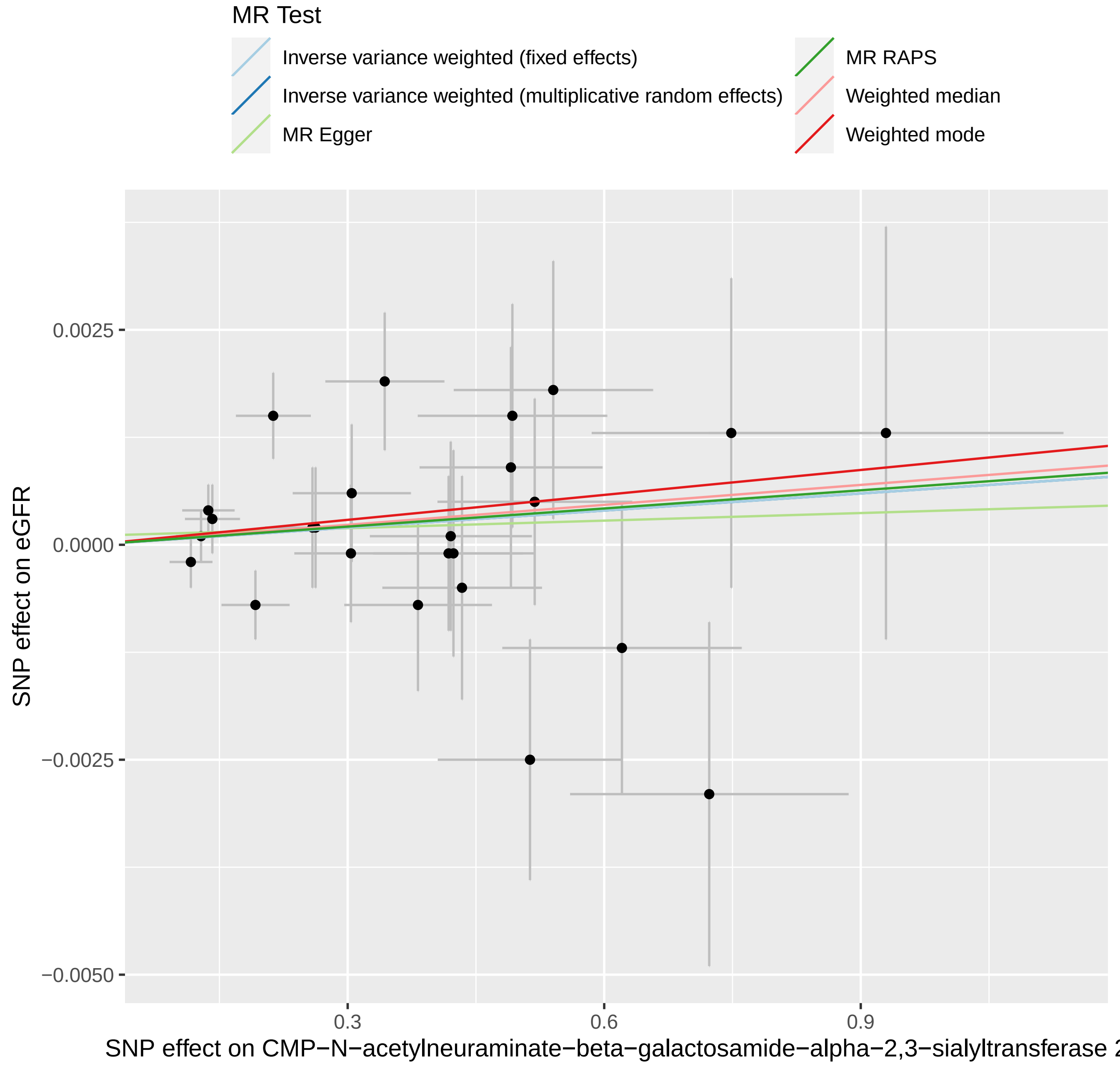

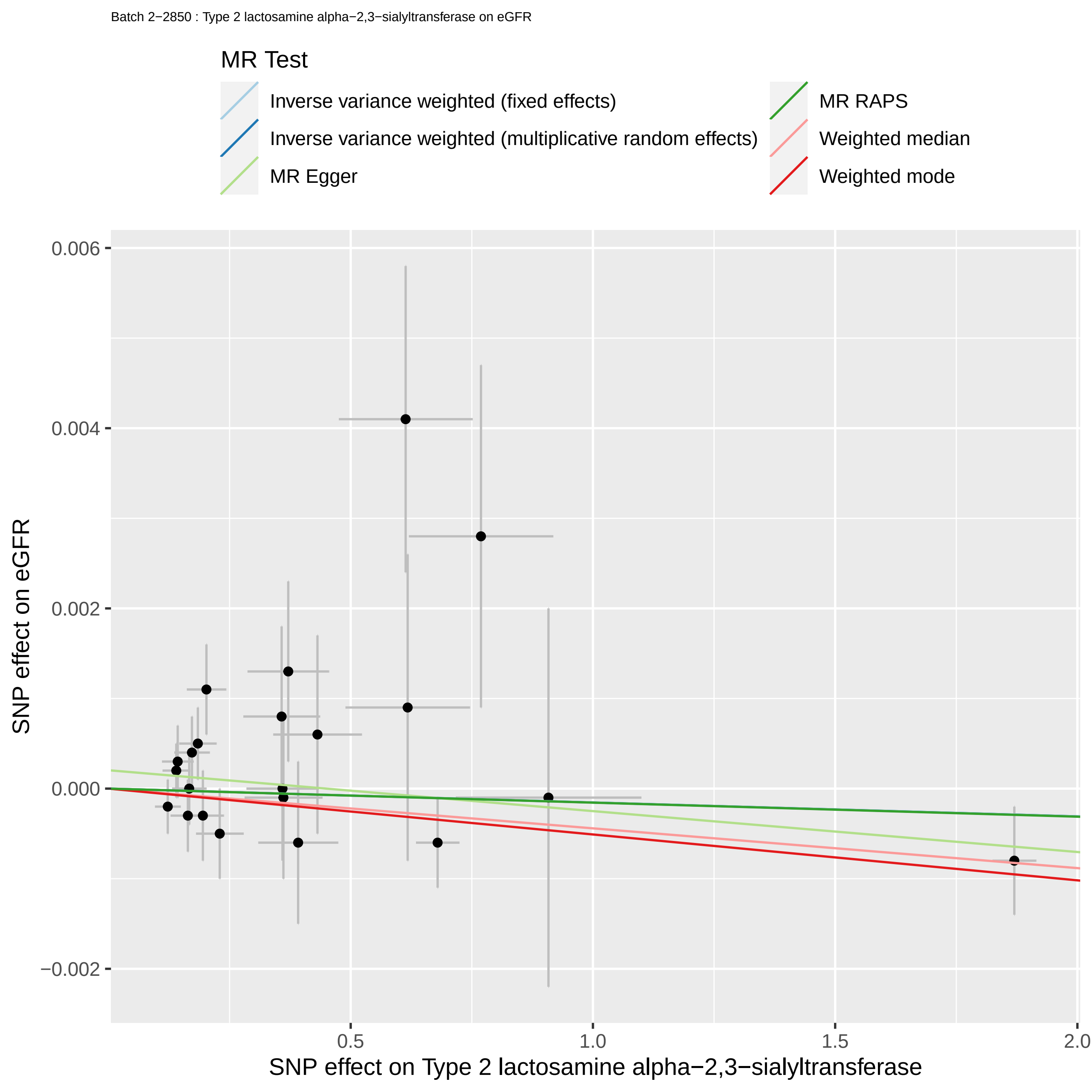
Batch 2−2850 : Type 2 lactosamine alpha−2,3−sialyltransferase on eGFR
MR Test
Inverse variance weighted (fixed effects)
Inverse variance weighted (multiplicative random effects)
MR Egger
MR RAPS
Weighted median
Weighted mode
0.006
0.004
0.002
0.000
−0.002
0.5
1.0
1.5
2.0
SNP effect on eGFR
SNP effect on Type 2 lactosamine alpha−2,3−sialyltransferase

Batch 2−2851 : Beta−galactoside alpha−2,6−sialyltransferase 1 on eGFR

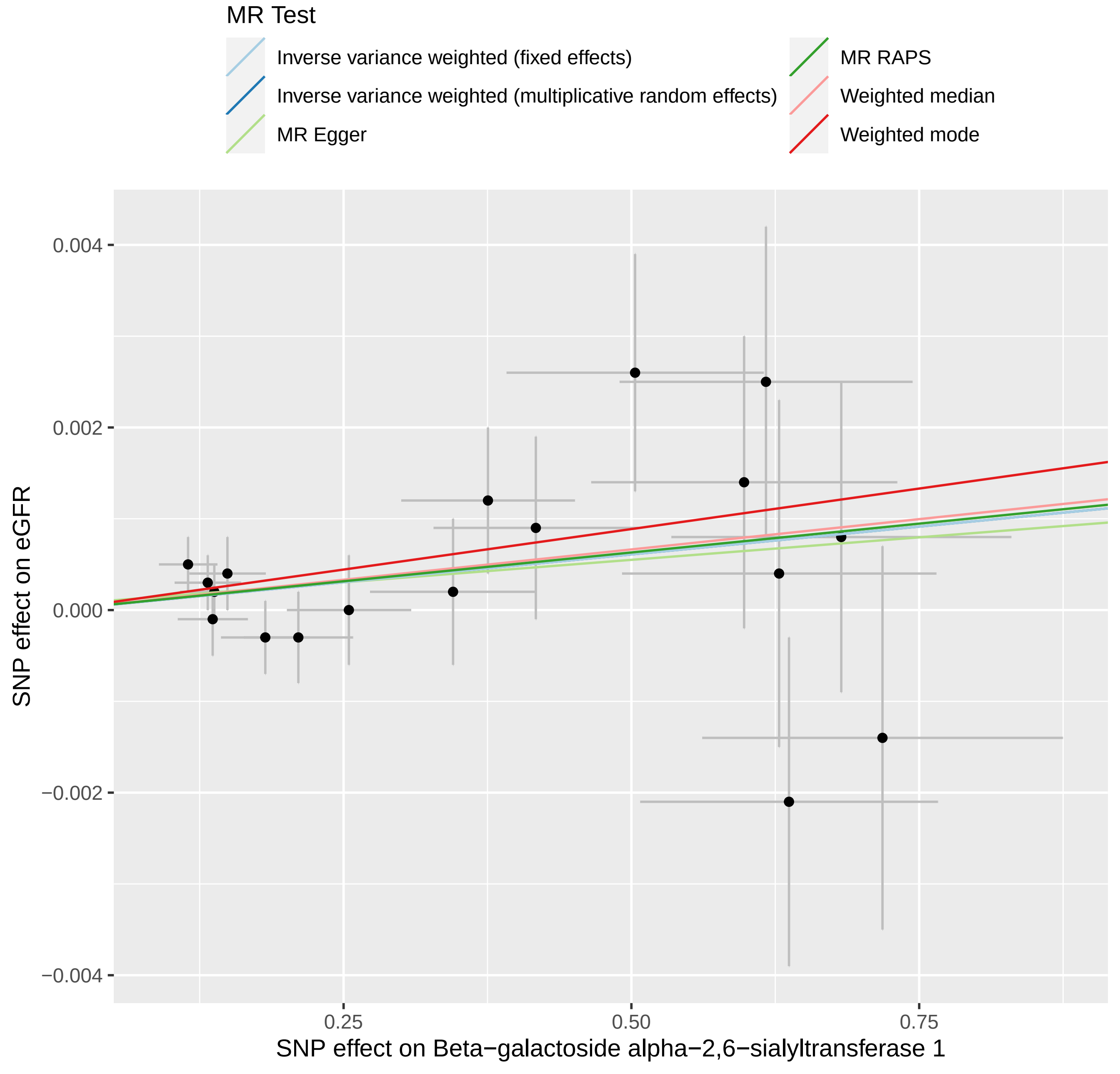

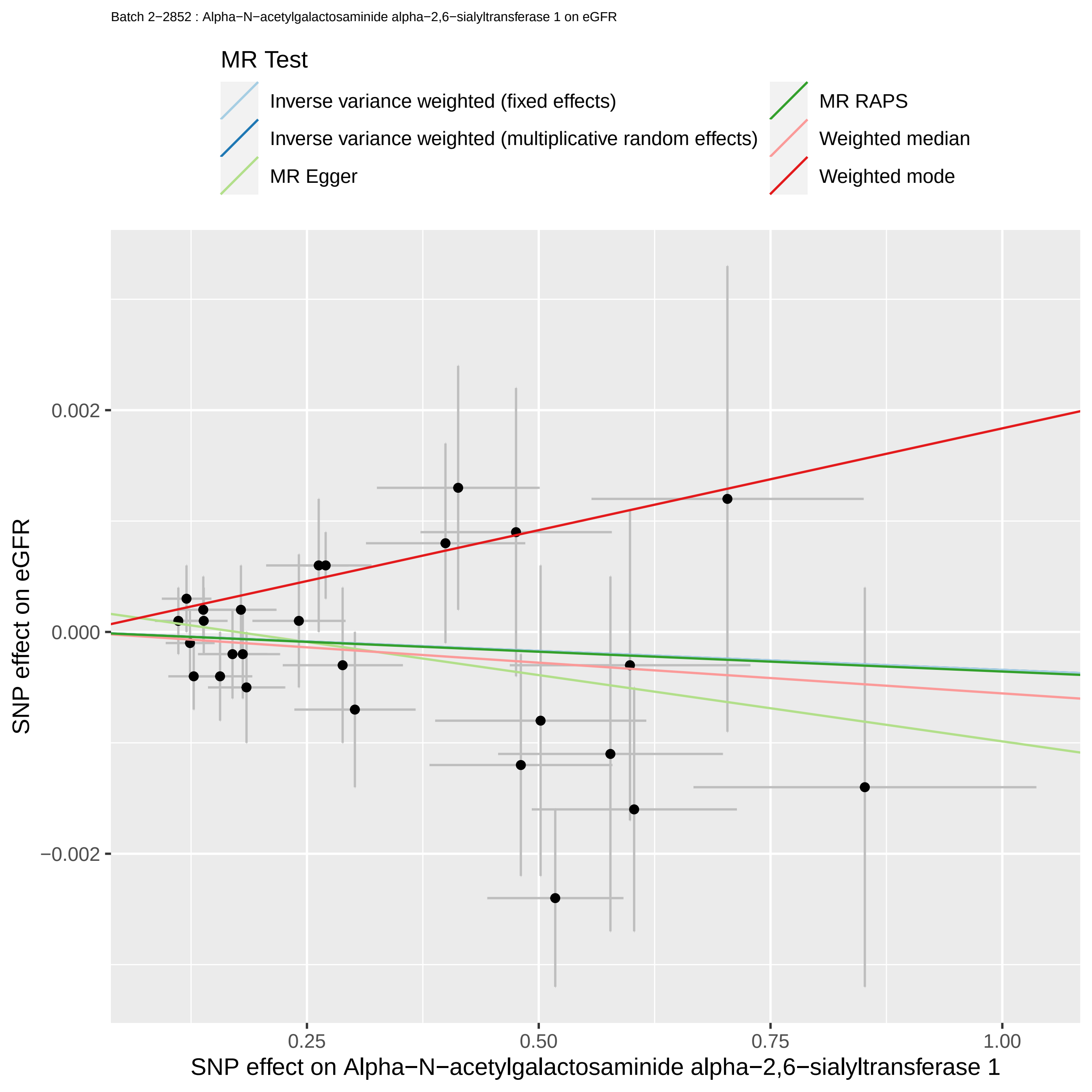

Batch 2−2852 : Alpha−N−acetylgalactosaminide alpha−2,6−sialyltransferase 1 on eGFR
MR Test
Inverse variance weighted (fixed effects)
Inverse variance weighted (multiplicative random effects)
MR Egger
MR RAPS
Weighted median
Weighted mode
0.002
0.000
−0.002
0.25
0.50
0.75
1.00
SNP effect on eGFR
SNP effect on Alpha−N−acetylgalactosaminide alpha−2,6−sialyltransferase 1

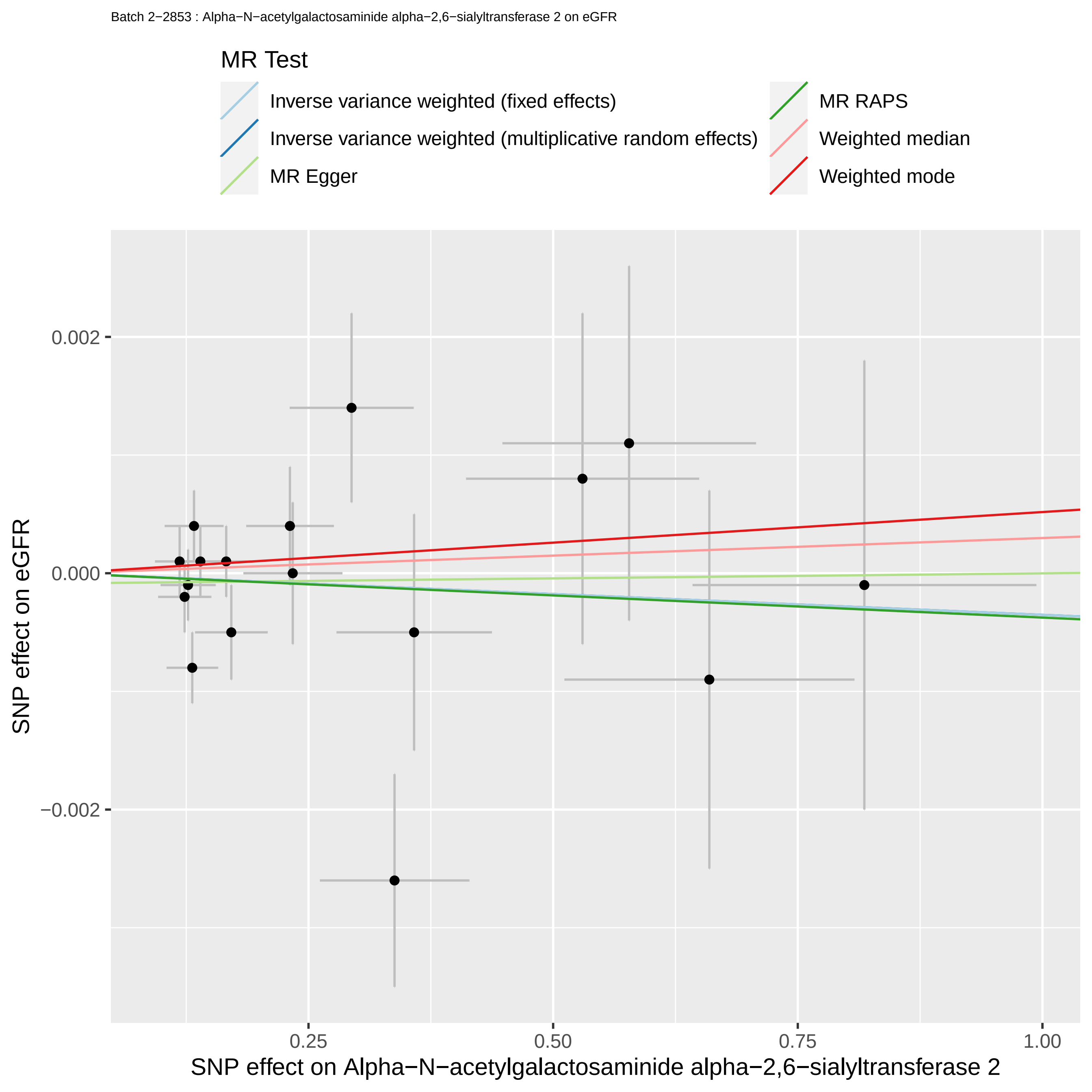

Batch 2−2853 : Alpha−N−acetylgalactosaminide alpha−2,6−sialyltransferase 2 on eGFR
MR Test
Inverse variance weighted (fixed effects)
Inverse variance weighted (multiplicative random effects)
MR Egger
MR RAPS
Weighted median
Weighted mode
0.002
0.000
−0.002
SNP effect on eGFR
0.25
0.50
0.75
1.00
SNP effect on Alpha−N−acetylgalactosaminide alpha−2,6−sialyltransferase 2

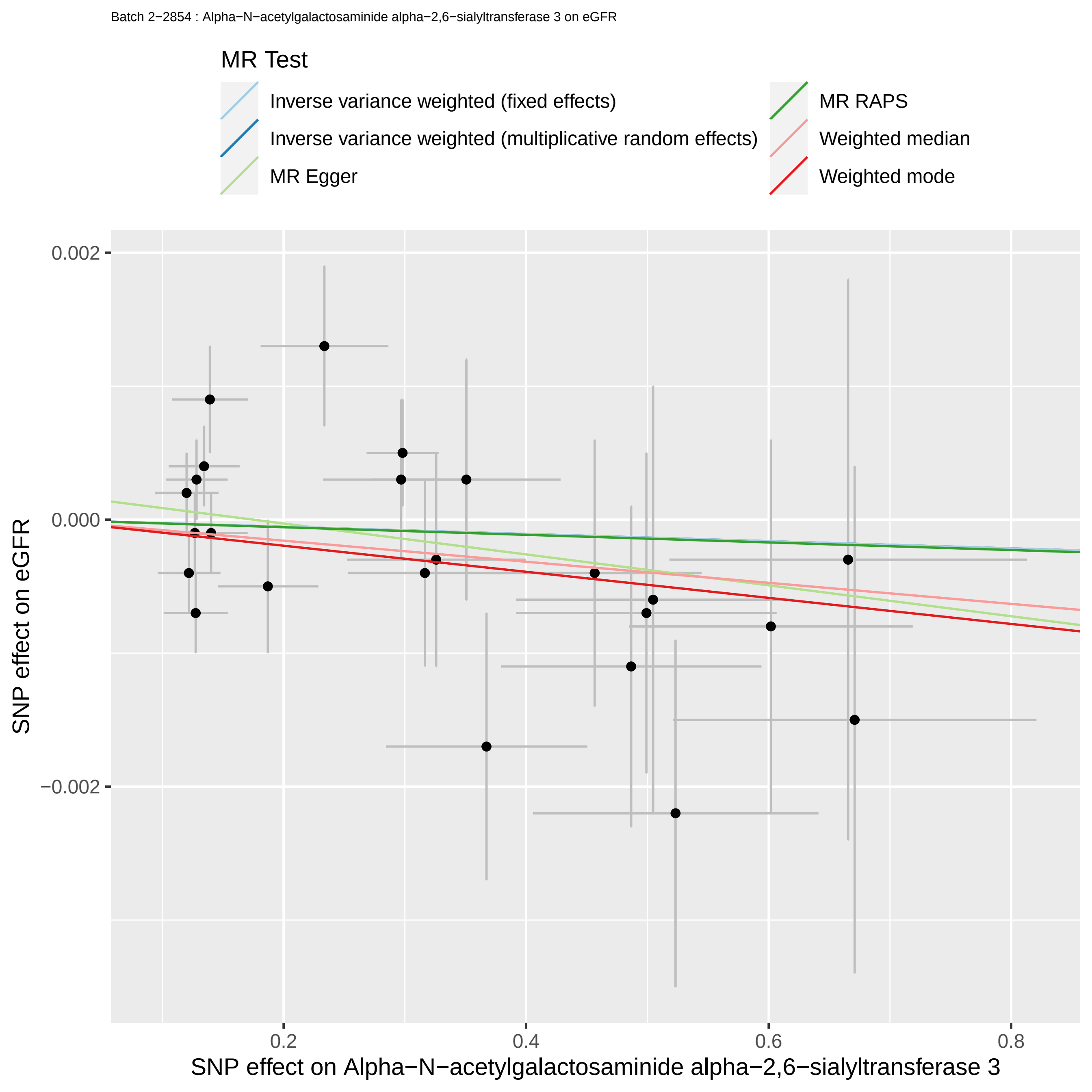

Batch 2−2854 : Alpha−N−acetylgalactosaminide alpha−2,6−sialyltransferase 3 on eGFR
MR Test
Inverse variance weighted (fixed effects)
Inverse variance weighted (multiplicative random effects)
MR Egger
MR RAPS
Weighted median
Weighted mode
0.002
0.000
−0.002
0.2
0.4
0.6
0.8
SNP effect on eGFR
SNP effect on Alpha−N−acetylgalactosaminide alpha−2,6−sialyltransferase 3

Batch 2−2855 : Alpha−N−acetylgalactosaminide alpha−2,6−sialyltransferase 3 on eGFR

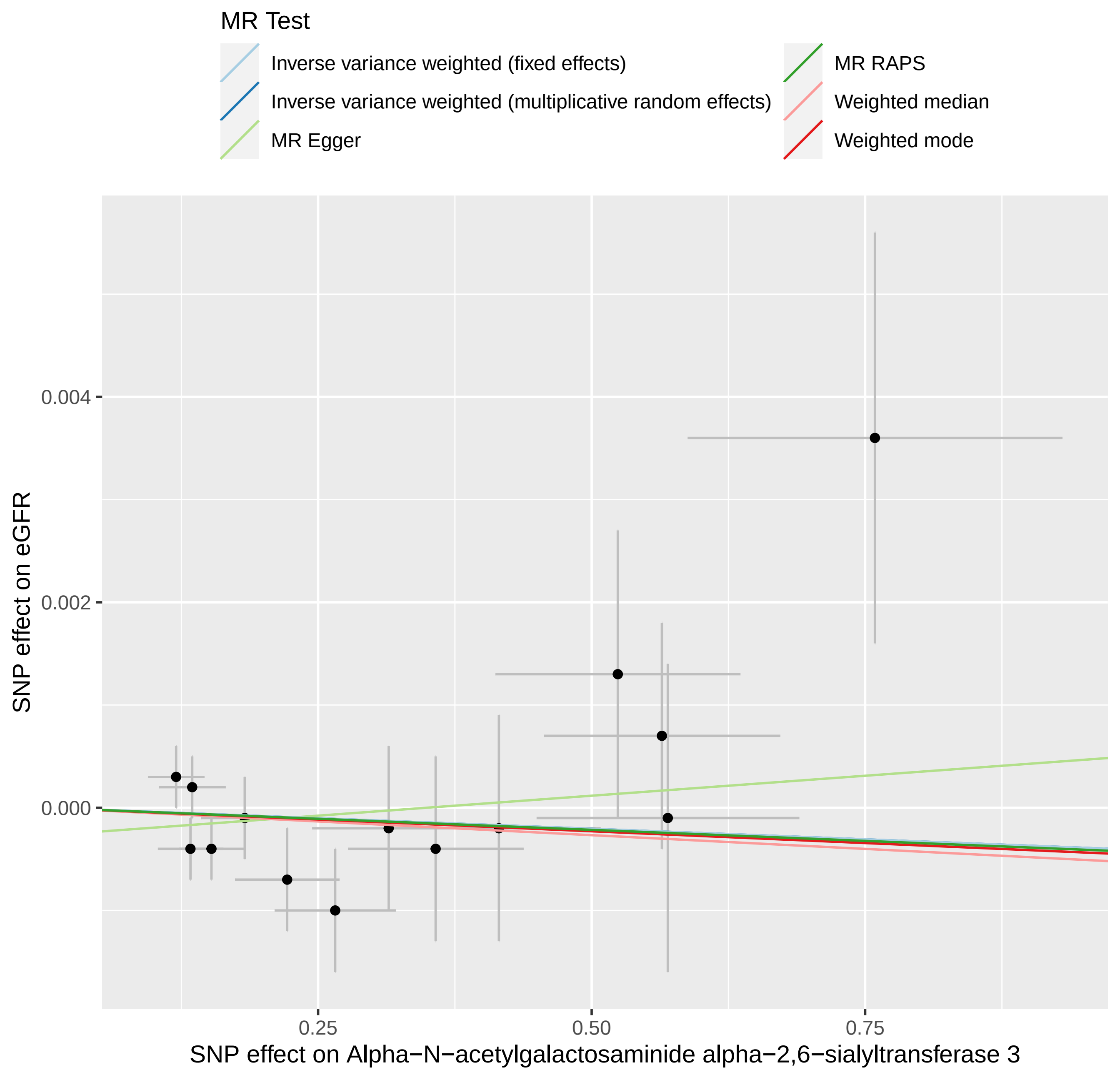

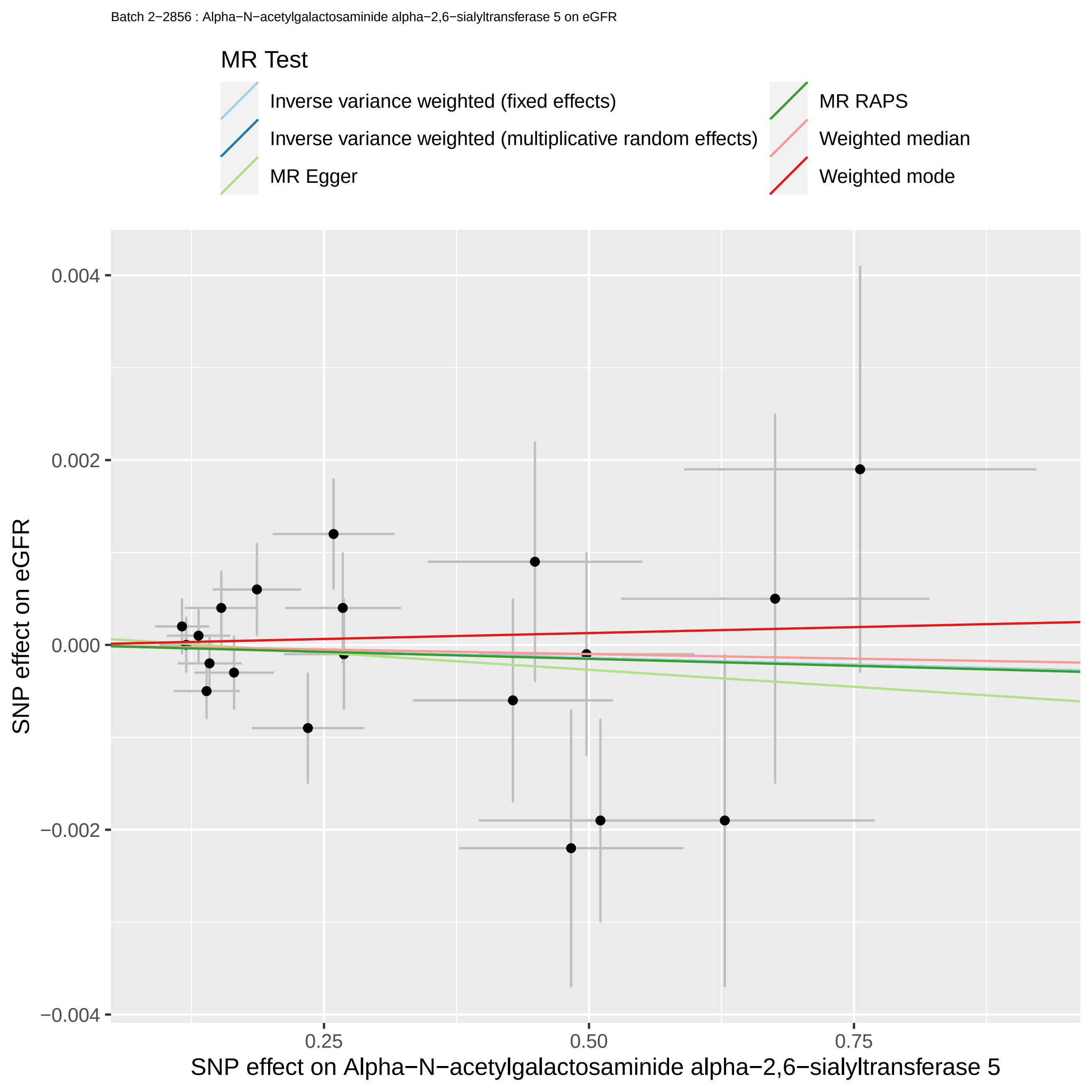

Batch 2−2856 : Alpha−N−acetylgalactosaminide alpha−2,6−sialyltransferase 5 on eGFR
MR Test
Inverse variance weighted (fixed effects)
Inverse variance weighted (multiplicative random effects)
MR Egger
MR RAPS
Weighted median
Weighted mode
0.004
0.002
0.000
−0.002
−0.004
0.25
0.50
0.75
SNP effect on eGFR
SNP effect on Alpha−N−acetylgalactosaminide alpha−2,6−sialyltransferase 5

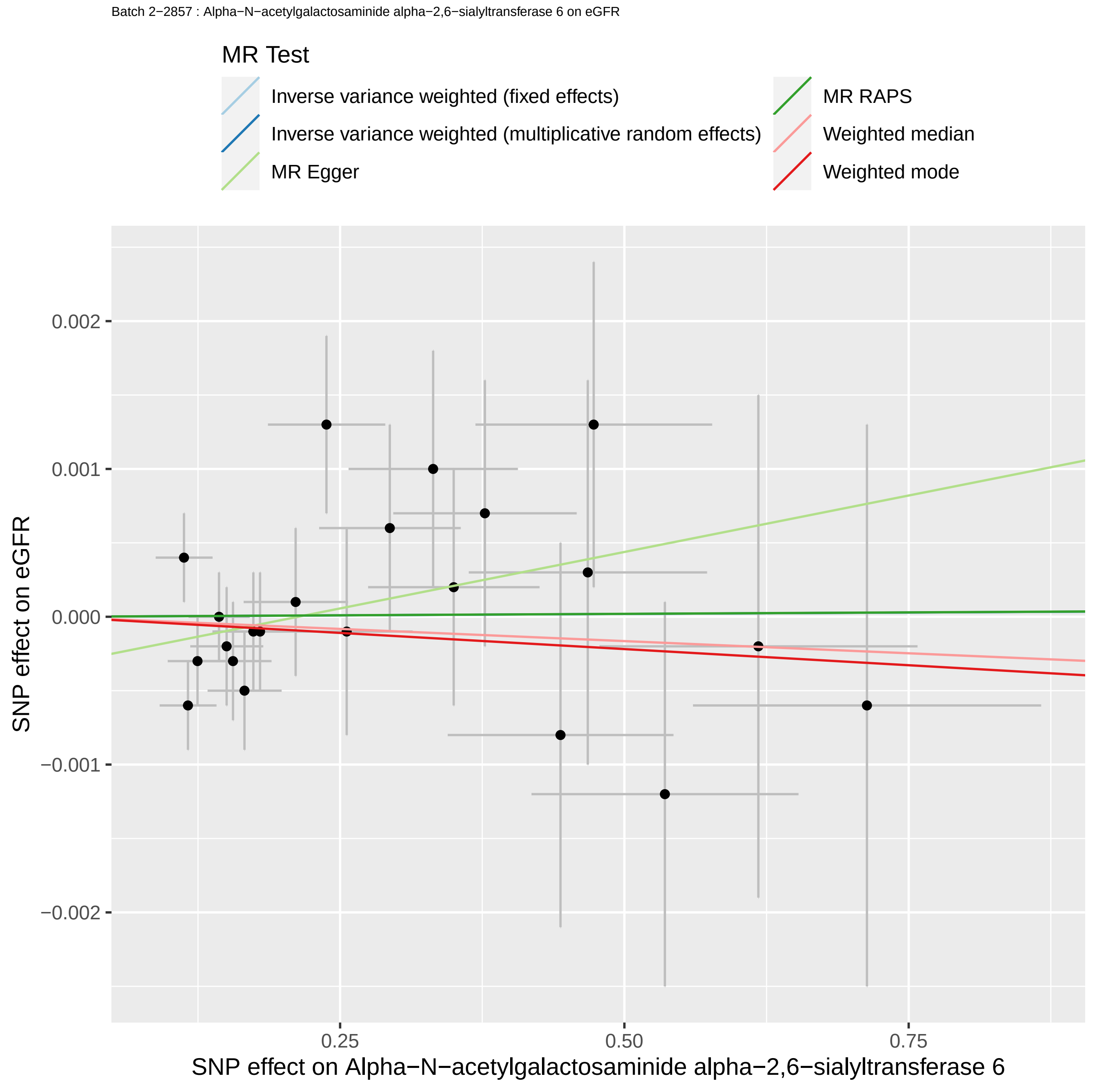
Batch 2−2857 : Alpha−N−acetylgalactosaminide alpha−2,6−sialyltransferase 6 on eGFR
MR Test
Inverse variance weighted (fixed effects)
Inverse variance weighted (multiplicative random effects)
MR Egger
MR RAPS
Weighted median
Weighted mode
0.002
0.001
0.000
−0.001
−0.002
0.25
0.50
0.75
SNP effect on eGFR
SNP effect on Alpha−N−acetylgalactosaminide alpha−2,6−sialyltransferase 6

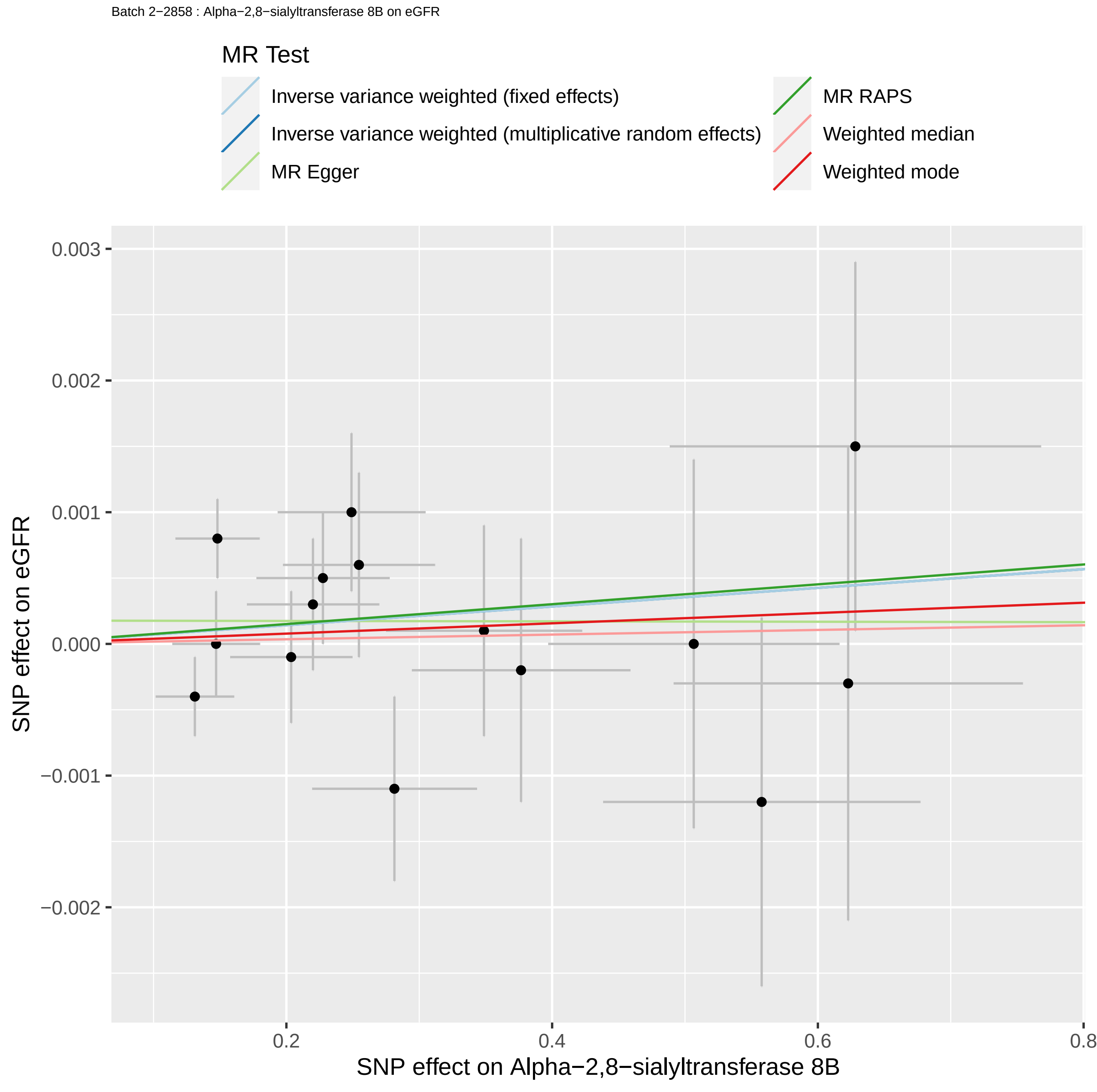
Batch 2−2858 : Alpha−2,8−sialyltransferase 8B on eGFR
MR Test
Inverse variance weighted (fixed effects)
Inverse variance weighted (multiplicative random effects)
MR Egger
MR RAPS
Weighted median
Weighted mode
0.003
0.002
0.001
0.000
−0.001
−0.002
SNP effect on eGFR
0.2
0.4
0.6
0.8
SNP effect on Alpha−2,8−sialyltransferase 8B

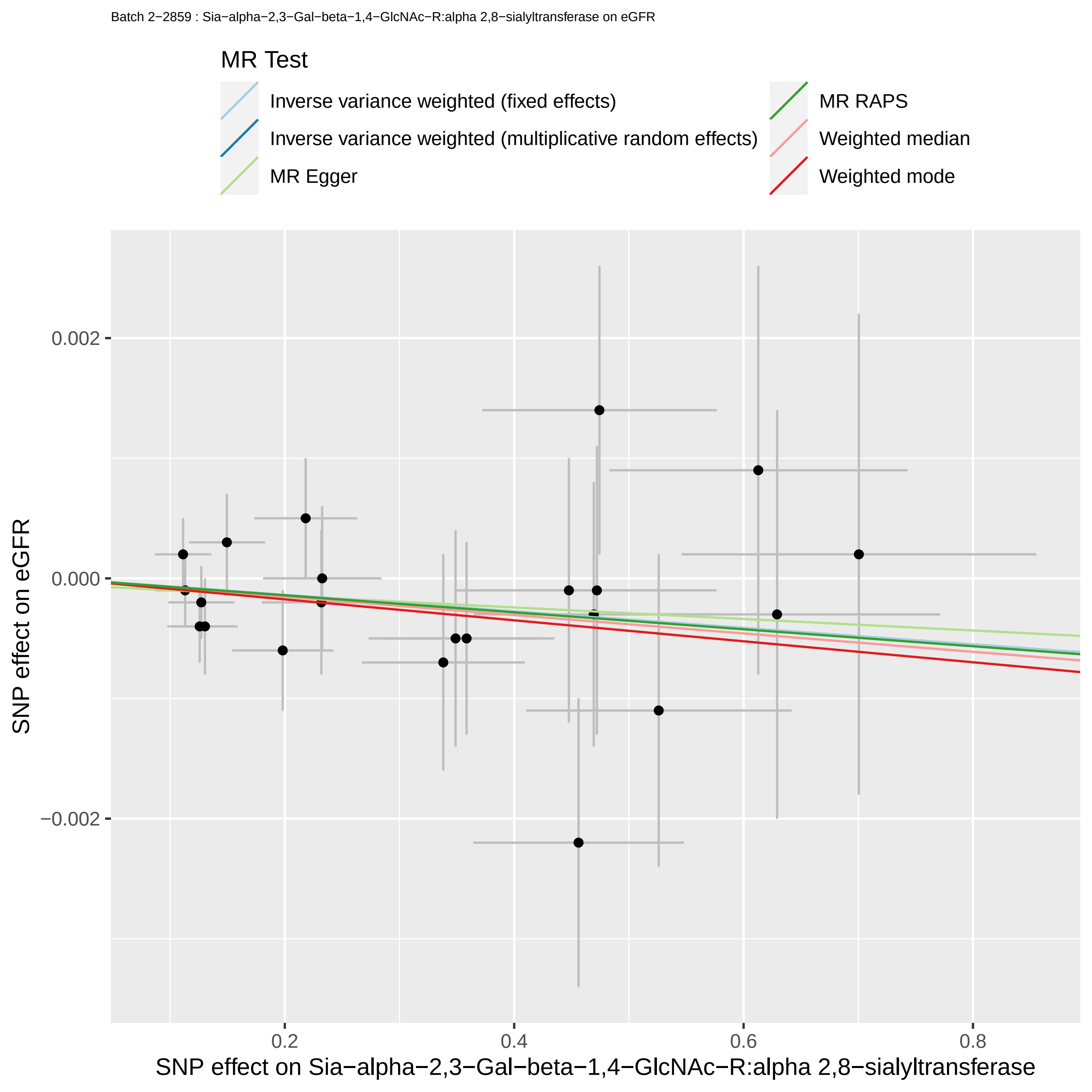
Batch 2−2859 : Sia−alpha−2,3−Gal−beta−1,4−GlcNAc−R:alpha 2,8−sialyltransferase on eGFR
MR Test
Inverse variance weighted (fixed effects)
Inverse variance weighted (multiplicative random effects)
MR Egger
MR RAPS
Weighted median
Weighted mode
0.002
0.000
−0.002
0.2
0.4
0.6
0.8
SNP effect on eGFR
SNP effect on Sia−alpha−2,3−Gal−beta−1,4−GlcNAc−R:alpha 2,8−sialyltransferase

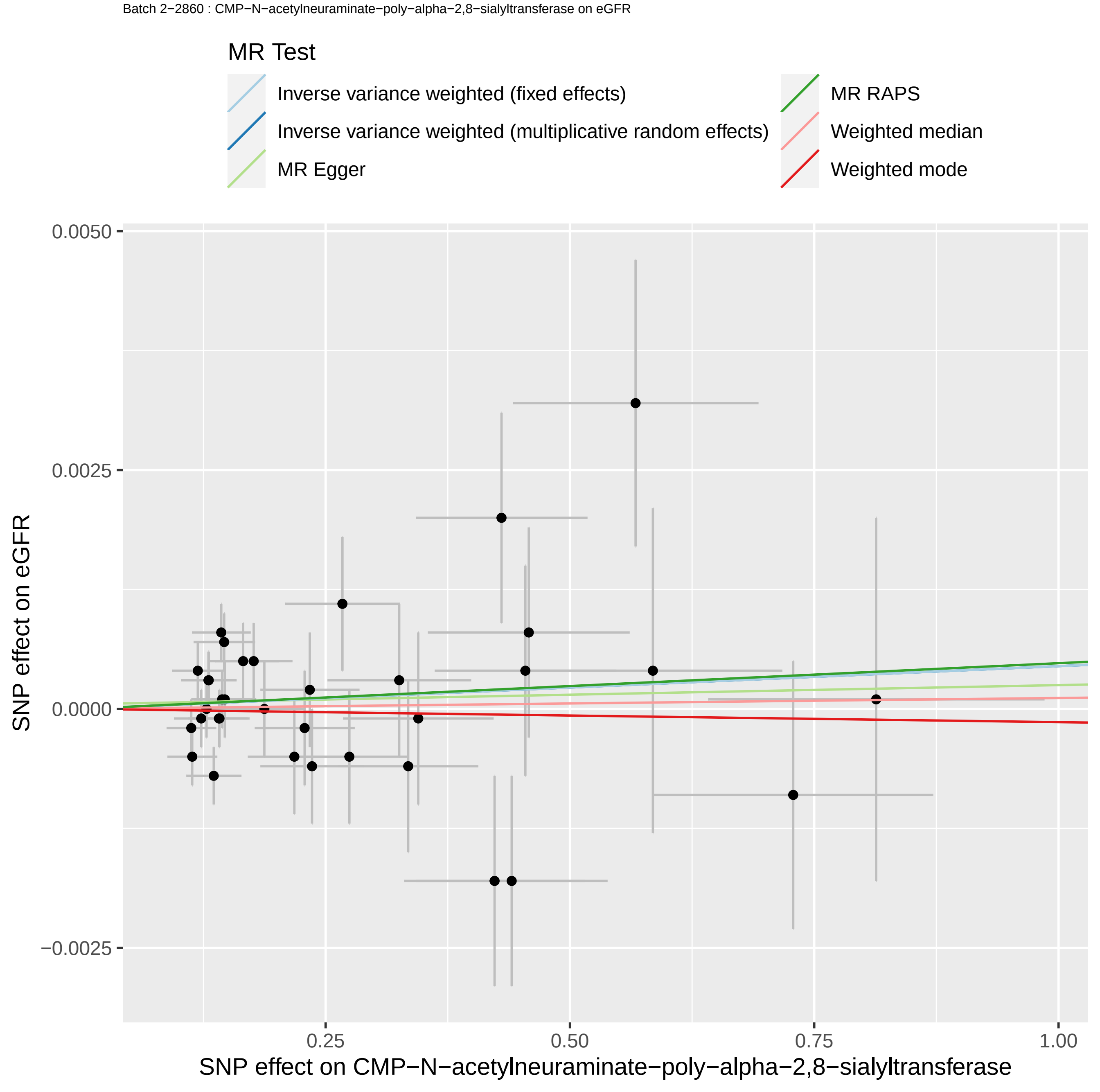
Batch 2−2860 : CMP−N−acetylneuraminate−poly−alpha−2,8−sialyltransferase on eGFR
MR Test
Inverse variance weighted (fixed effects)
Inverse variance weighted (multiplicative random effects)
MR Egger
MR RAPS
Weighted median
Weighted mode
0.0050
0.0025
0.0000
−0.0025
0.25
0.50
0.75
1.00
SNP effect on eGFR
SNP effect on CMP−N−acetylneuraminate−poly−alpha−2,8−sialyltransferase

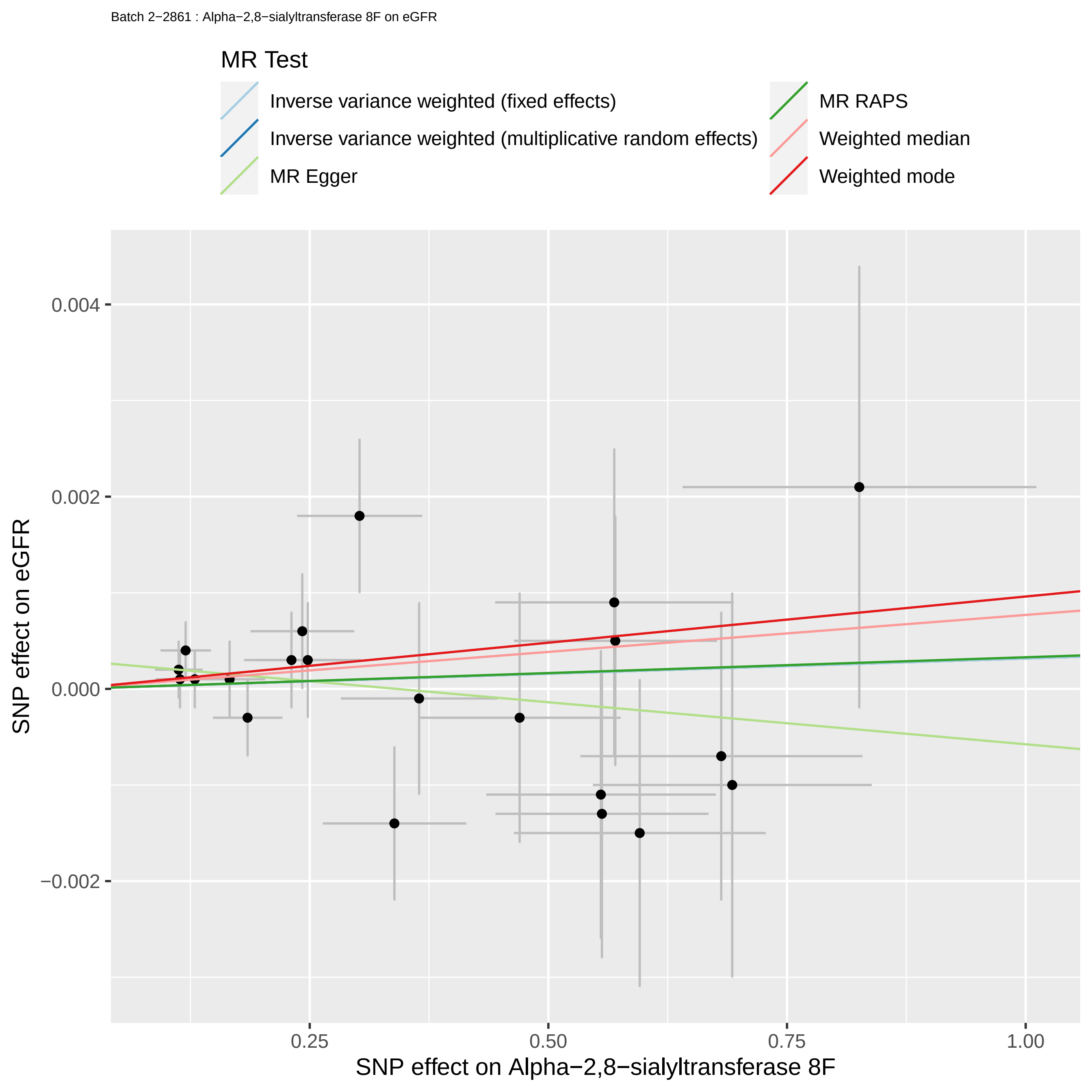
Batch 2−2861 : Alpha−2,8−sialyltransferase 8F on eGFR
MR Test
Inverse variance weighted (fixed effects)
Inverse variance weighted (multiplicative random effects)
MR Egger
MR RAPS
Weighted median
Weighted mode
0.004
0.002
0.000
−0.002
0.25
0.50
0.75
1.00
SNP effect on eGFR
SNP effect on Alpha−2,8−sialyltransferase 8F

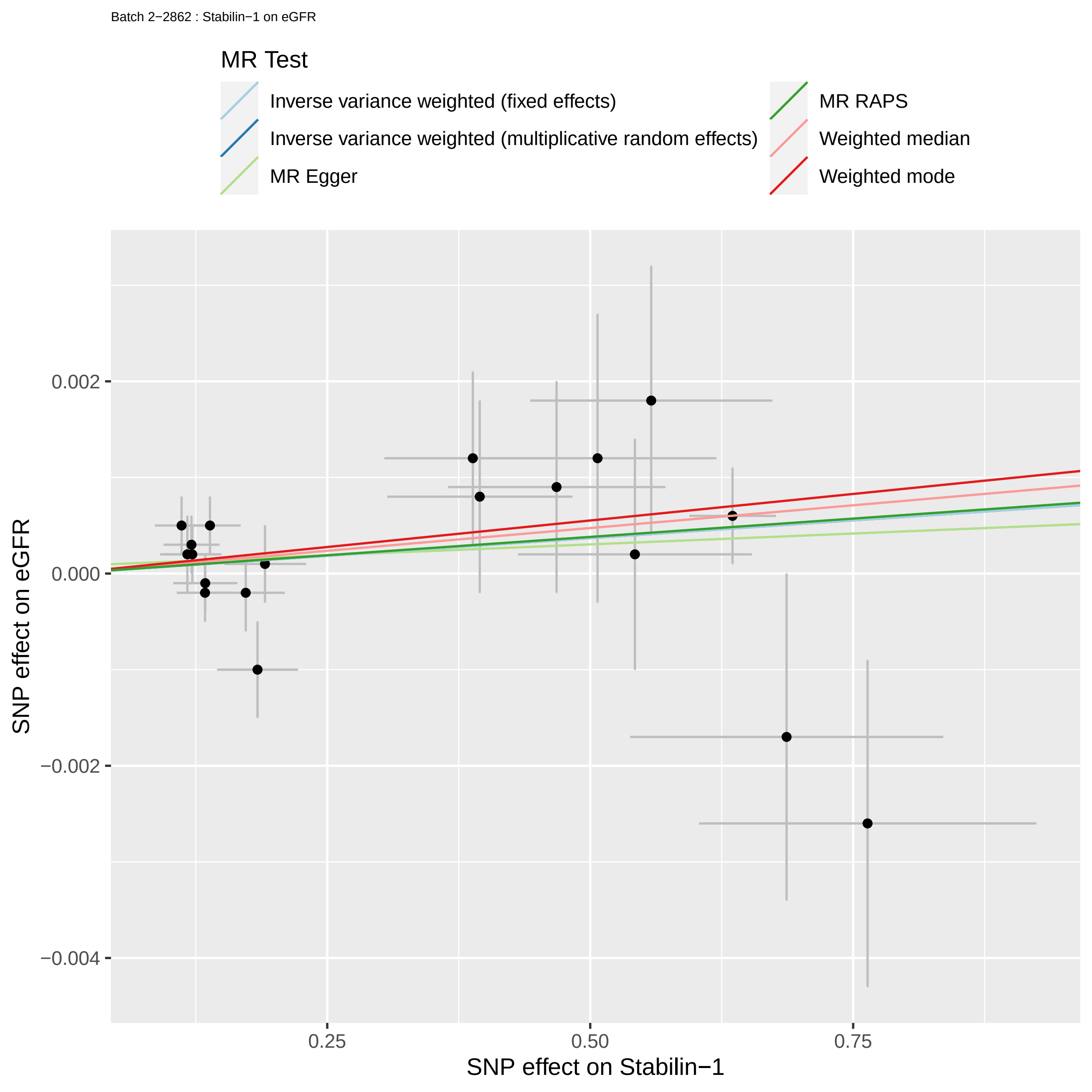
Batch 2−2862 : Stabilin−1 on eGFR
MR Test
Inverse variance weighted (fixed effects)
Inverse variance weighted (multiplicative random effects)
MR Egger
MR RAPS
Weighted median
Weighted mode
0.002
0.000
−0.002
−0.004
0.25
0.50
0.75
SNP effect on eGFR
SNP effect on Stabilin−1

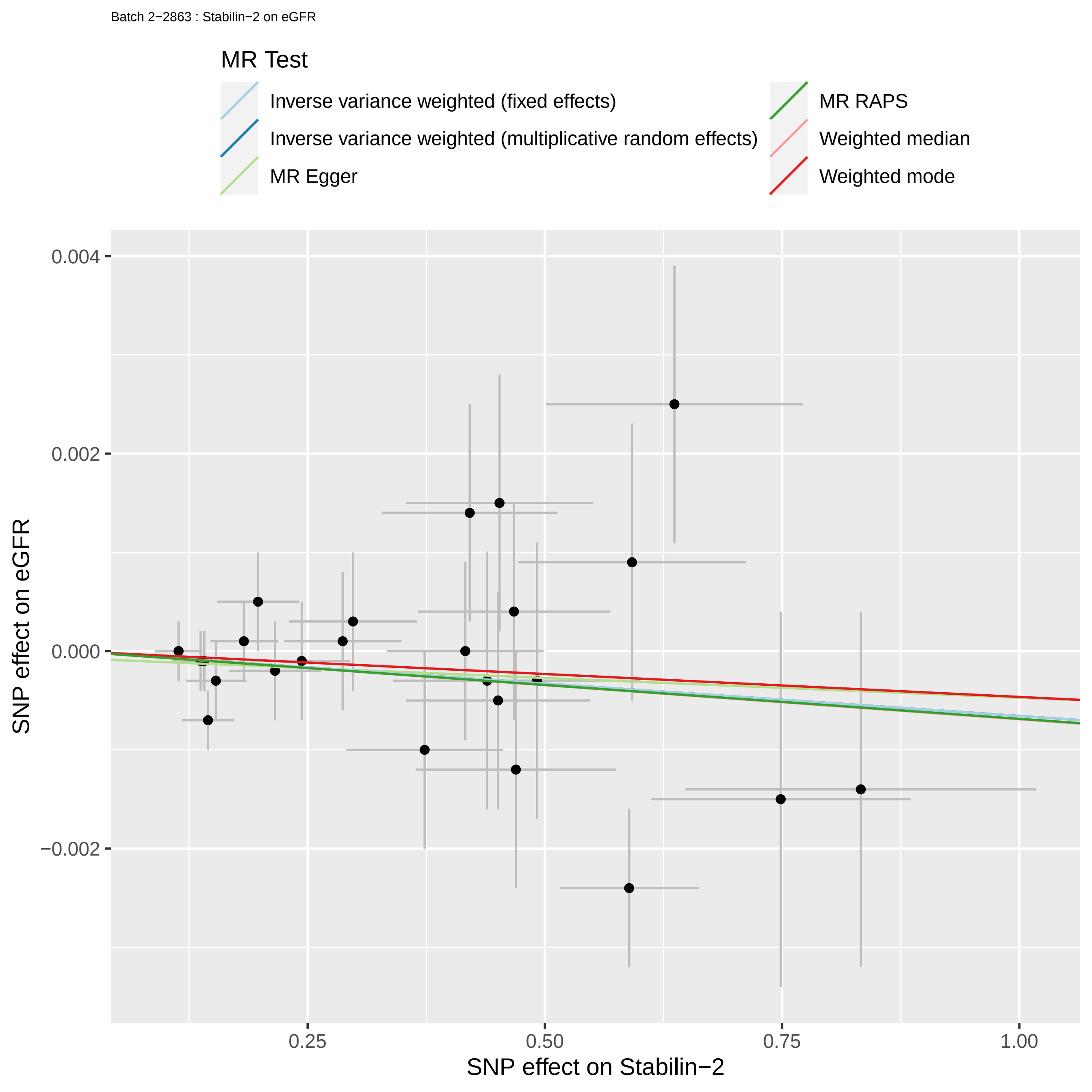

Batch 2−2863 : Stabilin−2 on eGFR
MR Test
Inverse variance weighted (fixed effects)
Inverse variance weighted (multiplicative random effects)
MR Egger
MR RAPS
Weighted median
Weighted mode
0.004
0.002
0.000
−0.002
0.25
0.50
0.75
1.00
SNP effect on eGFR
SNP effect on Stabilin−2

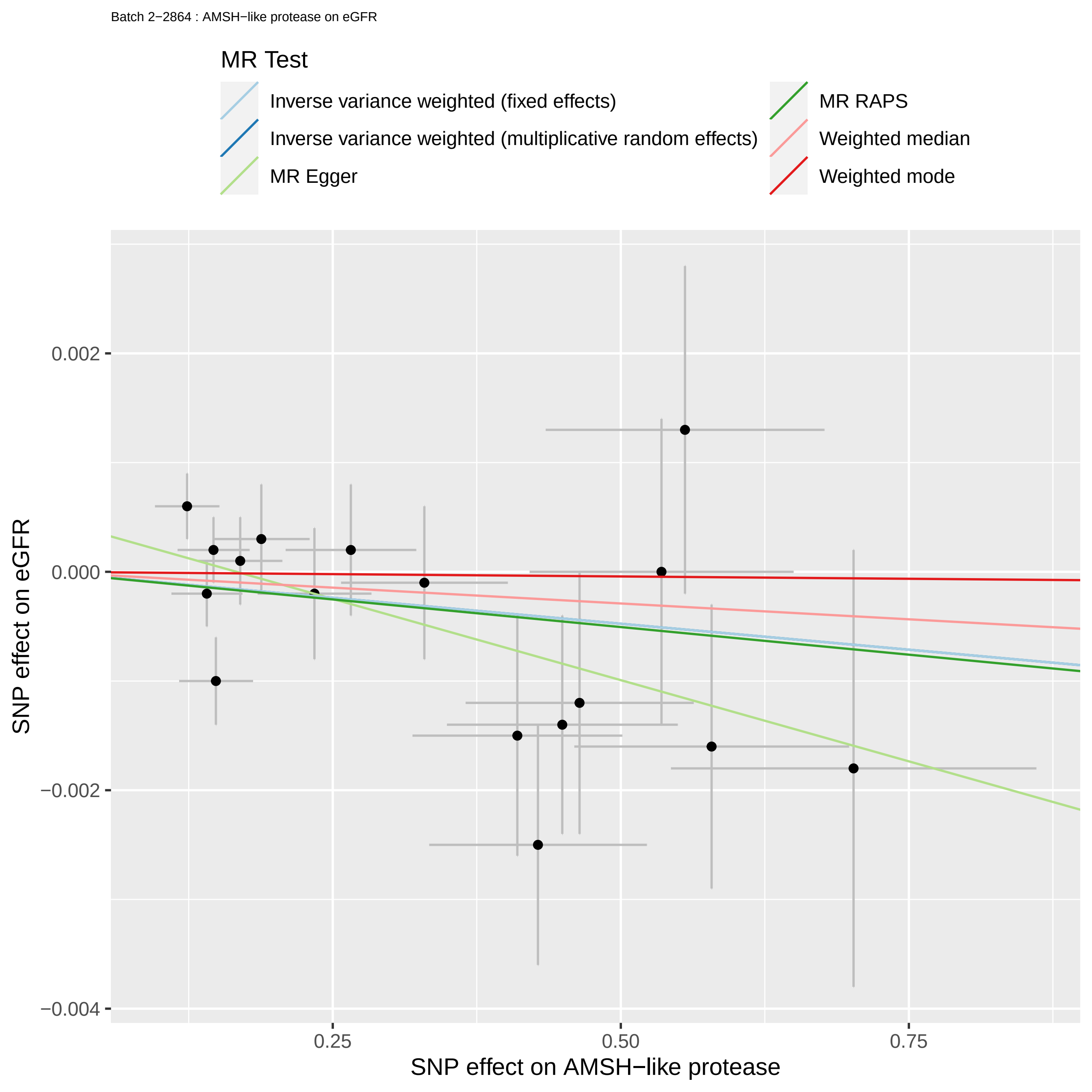

Batch 2−2864 : AMSH−like protease on eGFR
MR Test
Inverse variance weighted (fixed effects)
Inverse variance weighted (multiplicative random effects)
MR Egger
MR RAPS
Weighted median
Weighted mode
0.002
0.000
−0.002
−0.004
0.25
0.50
0.75
SNP effect on eGFR
SNP effect on AMSH−like protease

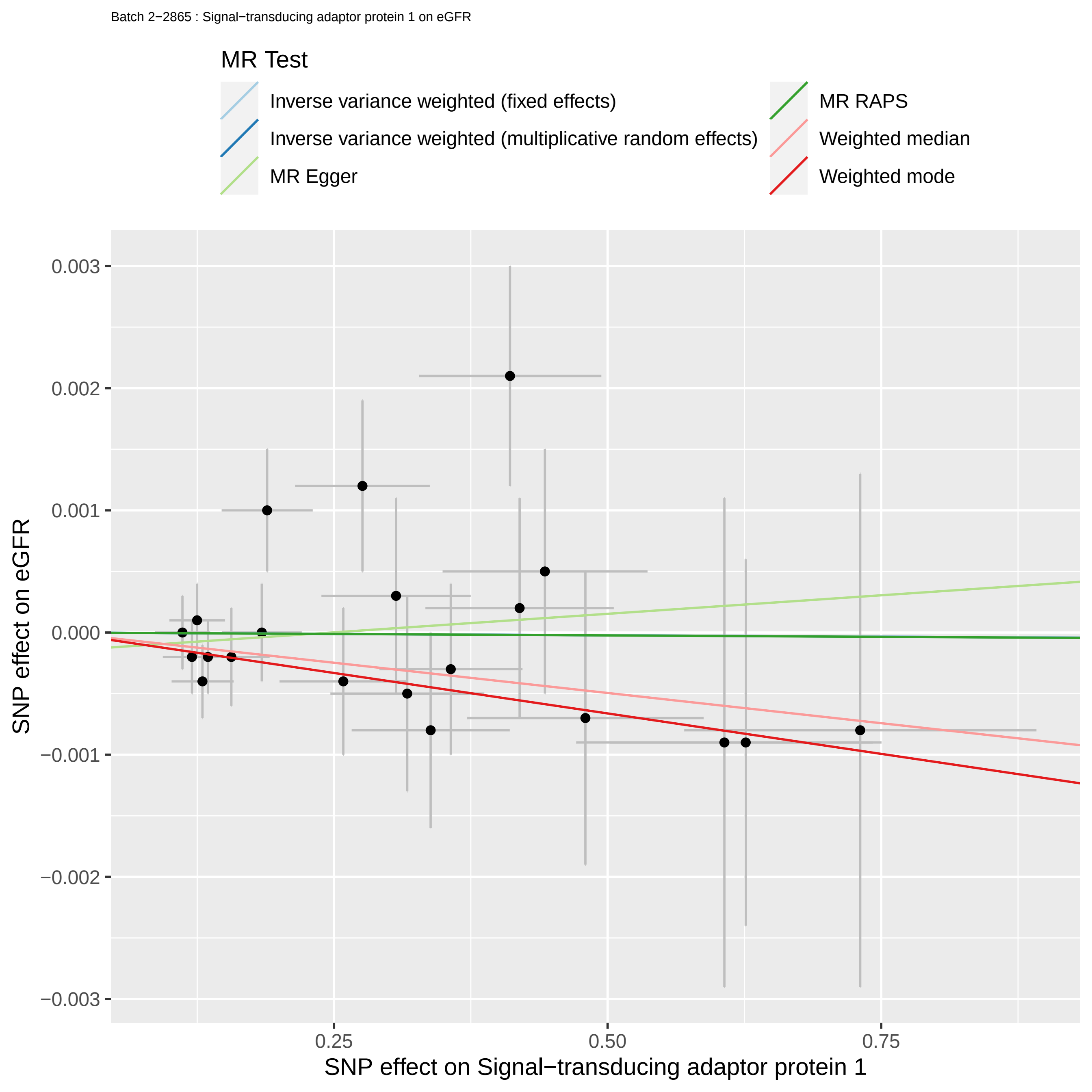

Batch 2−2865 : Signal−transducing adaptor protein 1 on eGFR
MR Test
Inverse variance weighted (fixed effects)
Inverse variance weighted (multiplicative random effects)
MR Egger
MR RAPS
Weighted median
Weighted mode
0.003
0.002
0.001
0.000
−0.001
−0.002
−0.003
SNP effect on eGFR
0.25
0.50
0.75
SNP effect on Signal−transducing adaptor protein 1

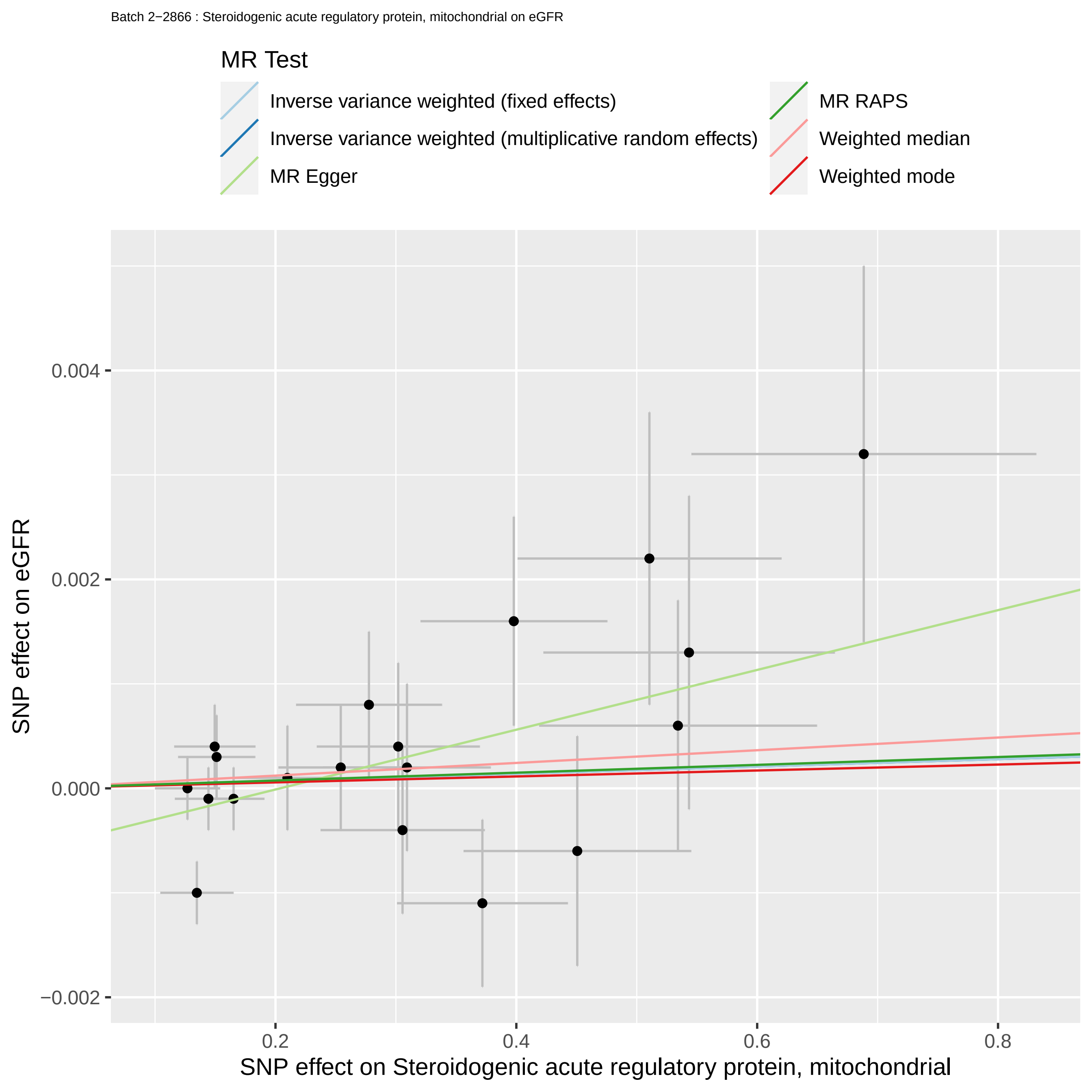
Batch 2−2866 : Steroidogenic acute regulatory protein, mitochondrial on eGFR
MR Test
Inverse variance weighted (fixed effects)
Inverse variance weighted (multiplicative random effects)
MR Egger
MR RAPS
Weighted median
Weighted mode
0.004
0.002
0.000
−0.002
0.2
0.4
0.6
0.8
SNP effect on eGFR
SNP effect on Steroidogenic acute regulatory protein, mitochondrial

Batch 2−2867 : Signal transducer and activator of transcription 1−alpha/beta on eGFR

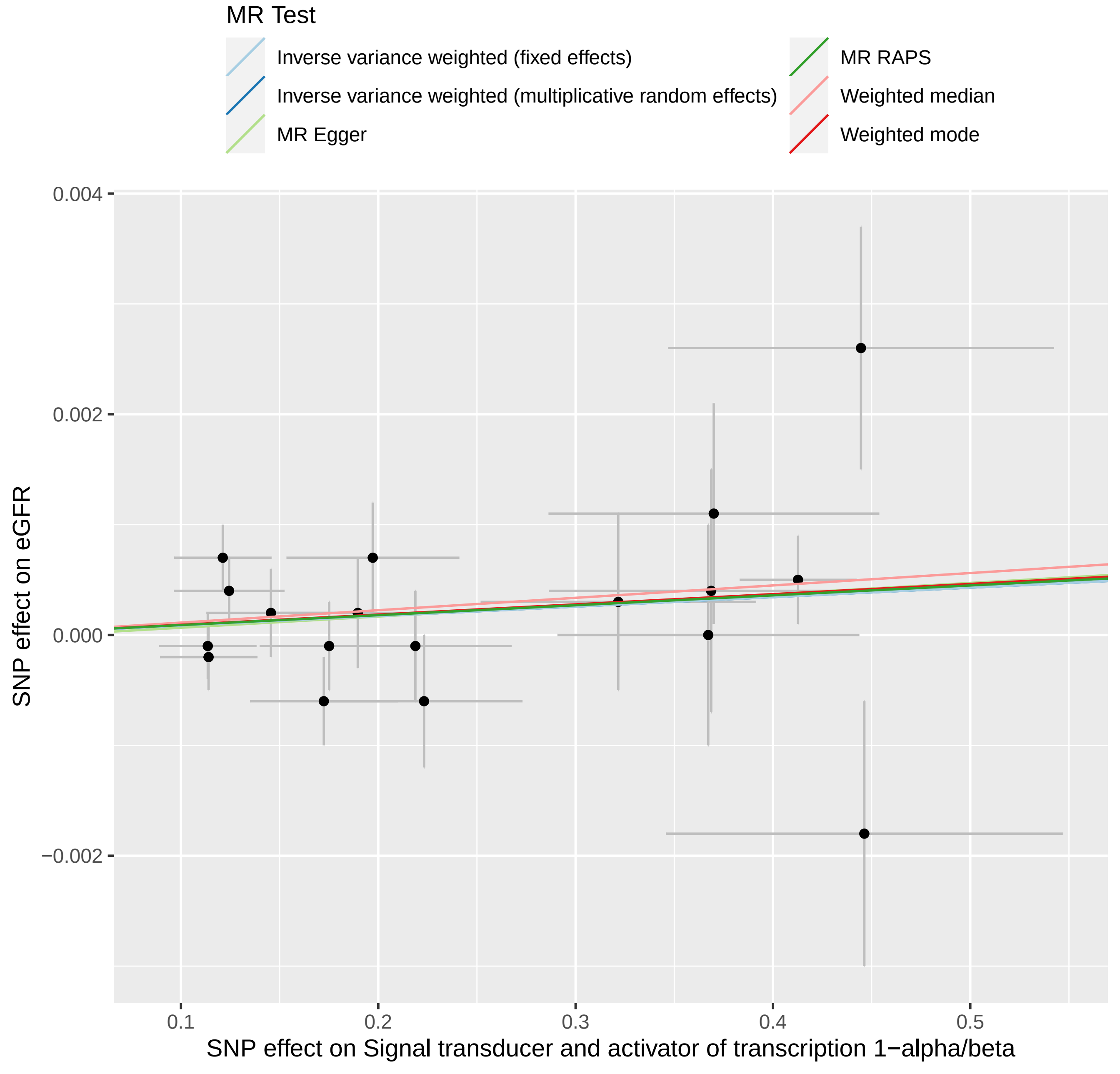

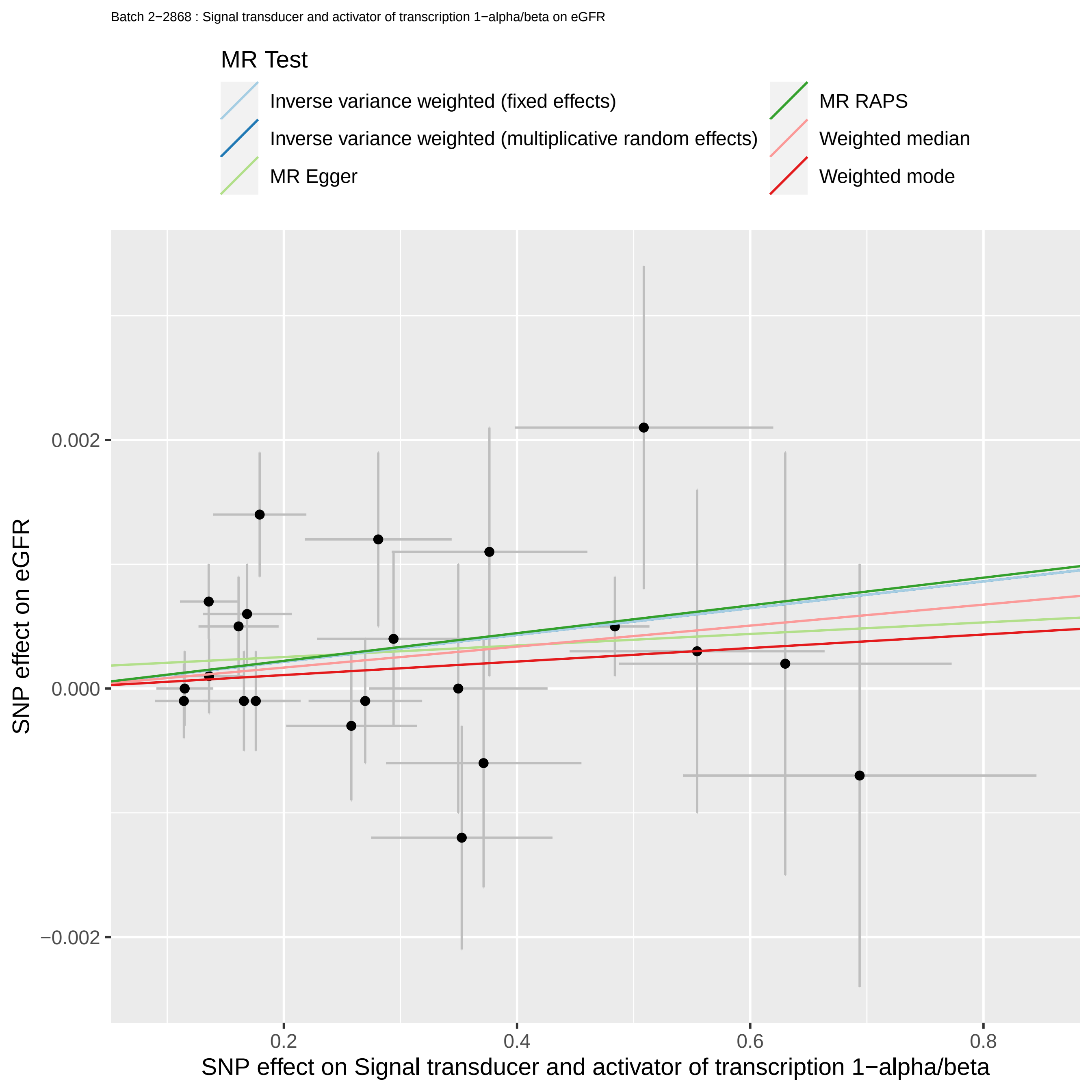
Batch 2−2868 : Signal transducer and activator of transcription 1−alpha/beta on eGFR
MR Test
Inverse variance weighted (fixed effects)
Inverse variance weighted (multiplicative random effects)
MR Egger
MR RAPS
Weighted median
Weighted mode
0.002
0.000
−0.002
0.2
0.4
0.6
0.8
SNP effect on eGFR
SNP effect on Signal transducer and activator of transcription 1−alpha/beta

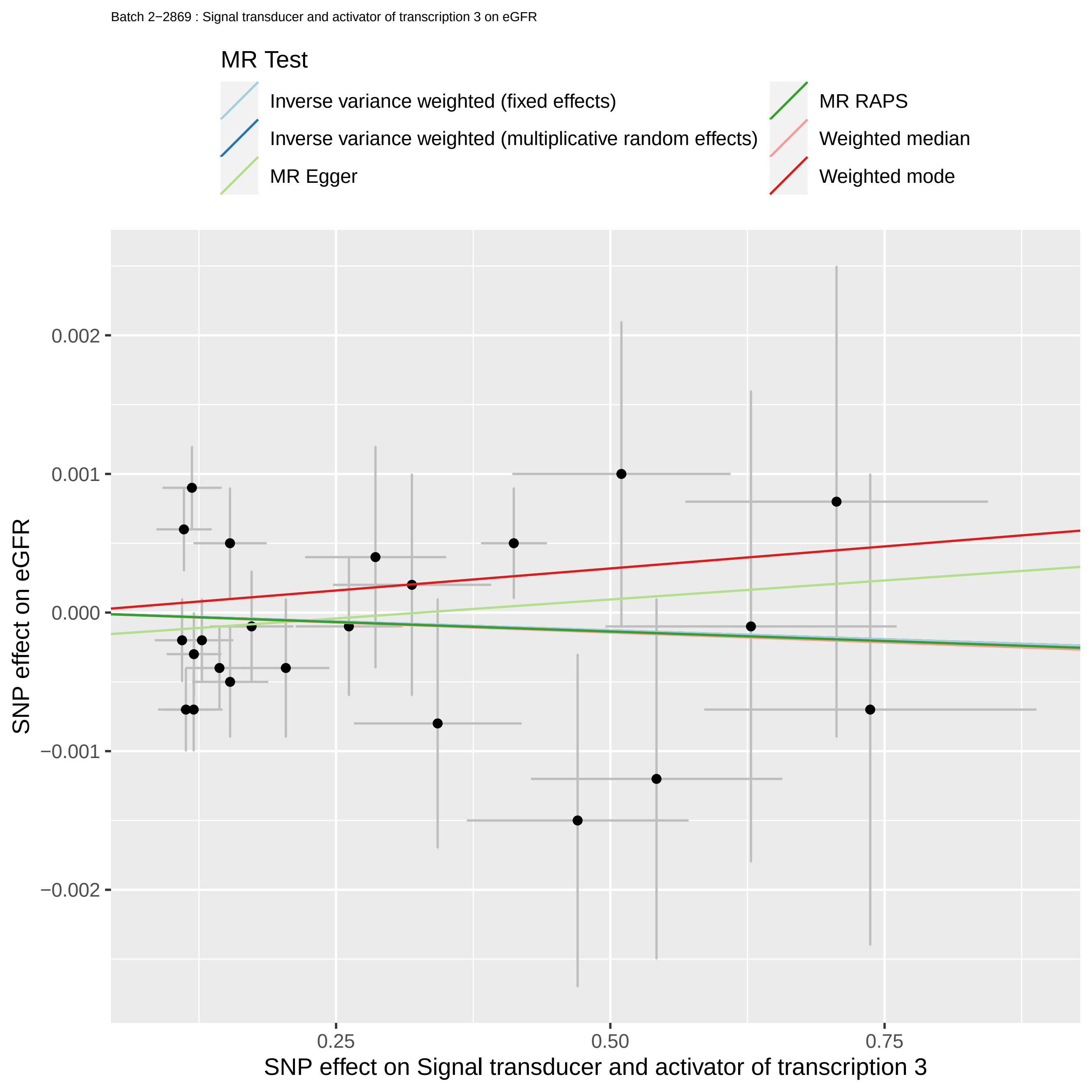
Batch 2−2869 : Signal transducer and activator of transcription 3 on eGFR
MR Test
Inverse variance weighted (fixed effects)
Inverse variance weighted (multiplicative random effects)
MR Egger
MR RAPS
Weighted median
Weighted mode
0.002
0.001
0.000
−0.001
−0.002
0.25
0.50
0.75
SNP effect on eGFR
SNP effect on Signal transducer and activator of transcription 3

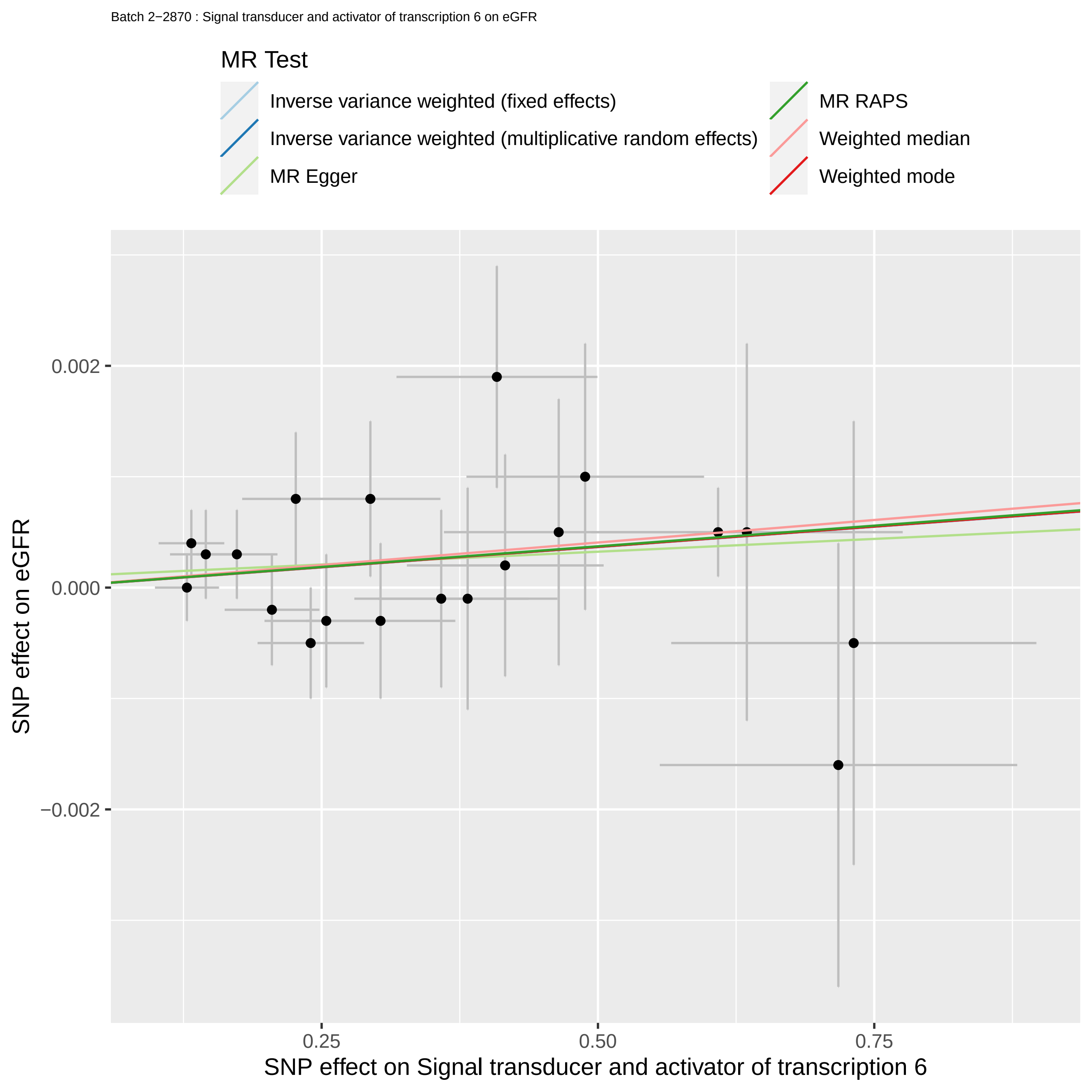

Batch 2−2870 : Signal transducer and activator of transcription 6 on eGFR
MR Test
Inverse variance weighted (fixed effects)
Inverse variance weighted (multiplicative random effects)
MR Egger
MR RAPS
Weighted median
Weighted mode
0.002
0.000
−0.002
0.25
0.50
0.75
SNP effect on eGFR
SNP effect on Signal transducer and activator of transcription 6

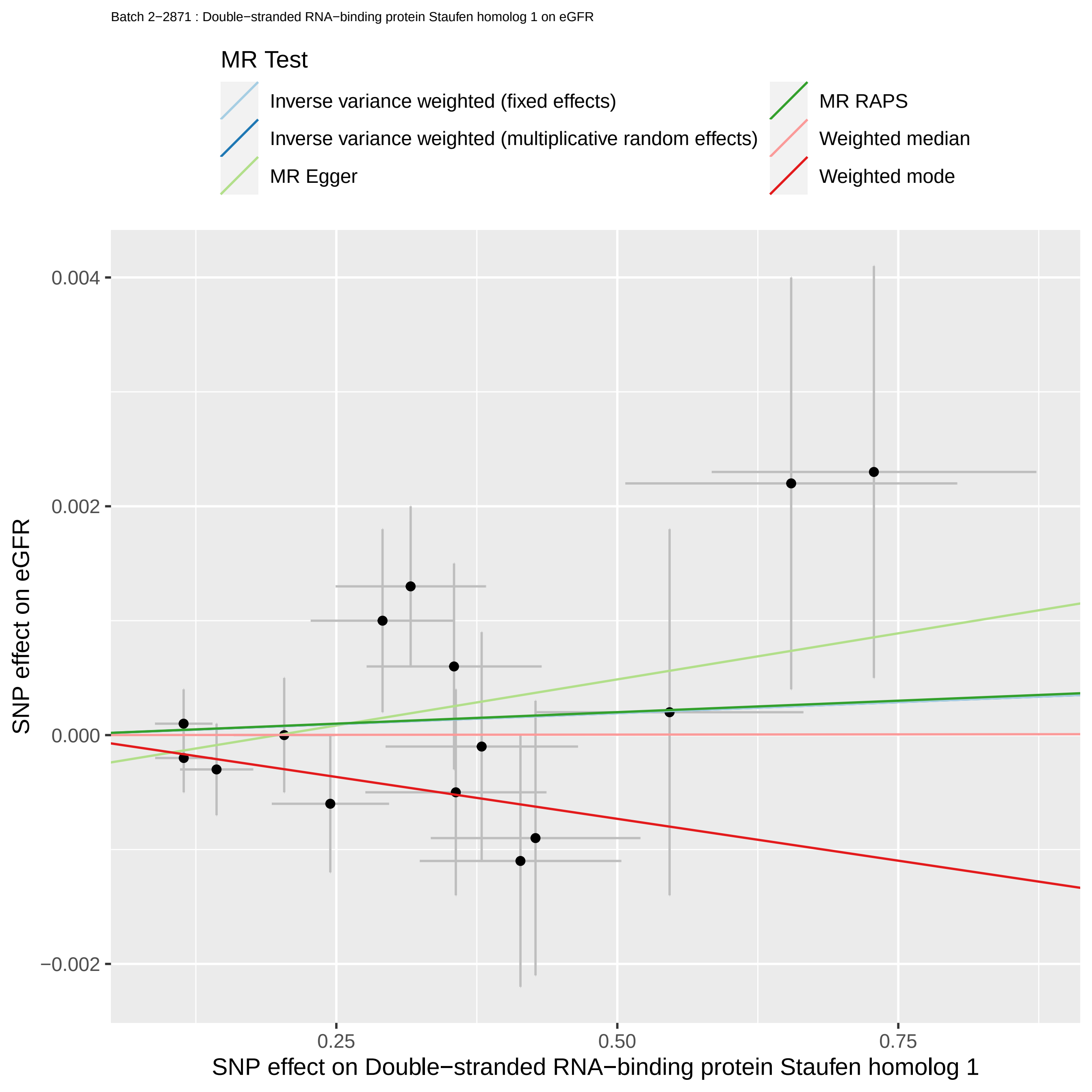
Batch 2−2871 : Double−stranded RNA−binding protein Staufen homolog 1 on eGFR
MR Test
Inverse variance weighted (fixed effects)
Inverse variance weighted (multiplicative random effects)
MR Egger
MR RAPS
Weighted median
Weighted mode
SNP effect on eGFR
0.004
0.002
0.000
−0.002
0.25
0.50
0.75
SNP effect on Double−stranded RNA−binding protein Staufen homolog 1

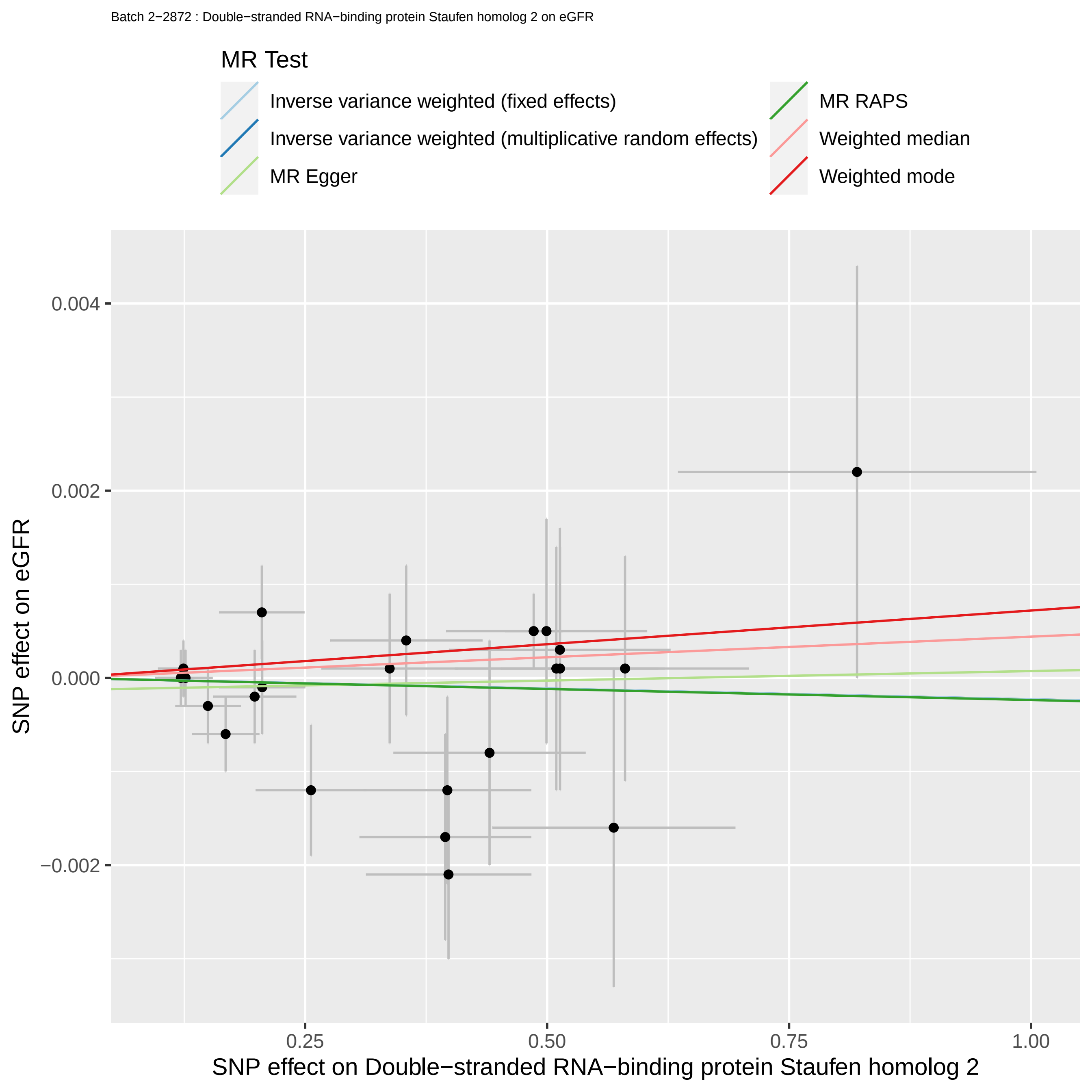

Batch 2−2872 : Double−stranded RNA−binding protein Staufen homolog 2 on eGFR
MR Test
Inverse variance weighted (fixed effects)
Inverse variance weighted (multiplicative random effects)
MR Egger
MR RAPS
Weighted median
Weighted mode
SNP effect on eGFR
0.004
0.002
0.000
−0.002
0.25
0.50
0.75
1.00
SNP effect on Double−stranded RNA−binding protein Staufen homolog 2

Batch 2−2873 : Stanniocalcin−1 on eGFR

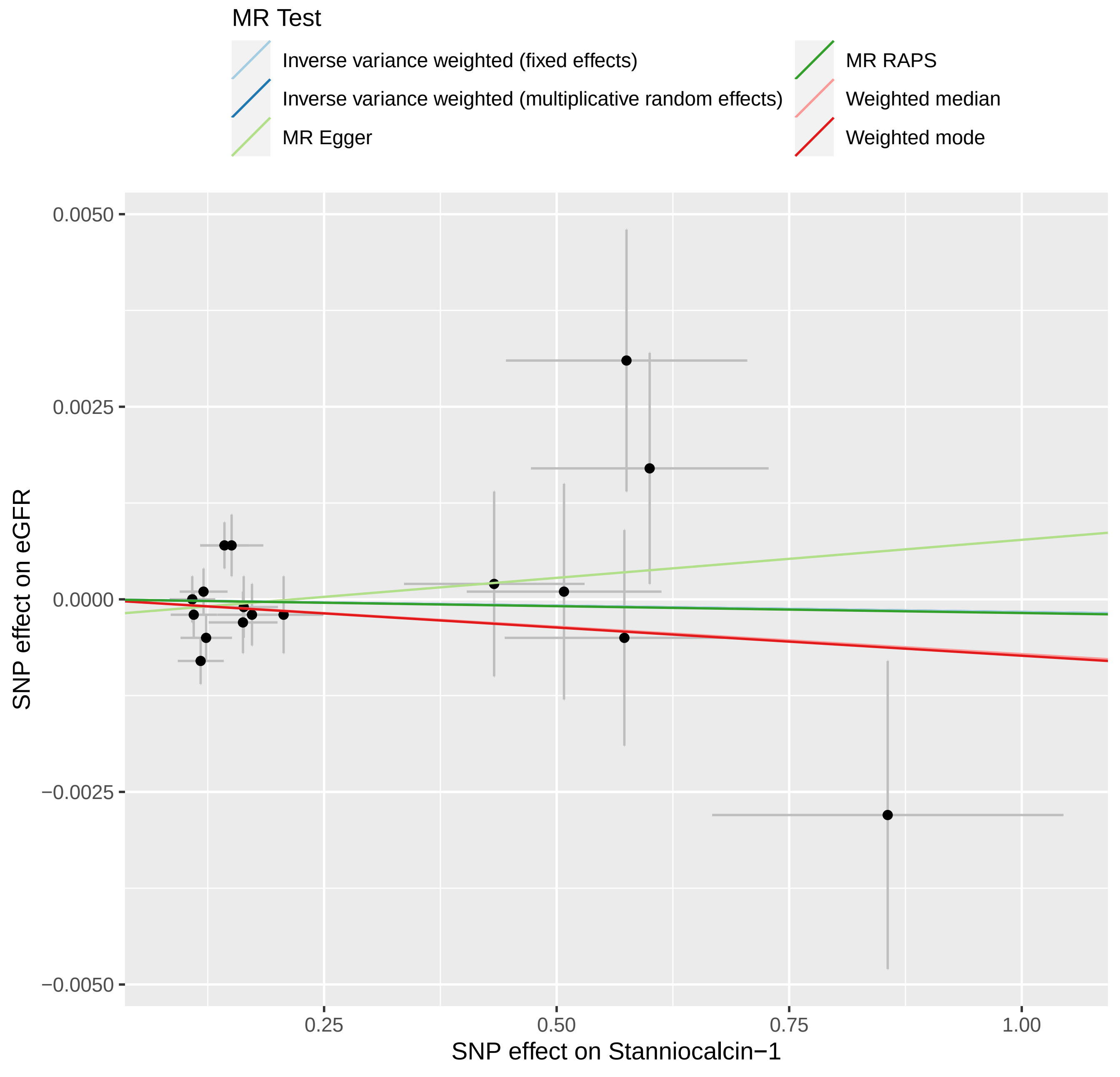

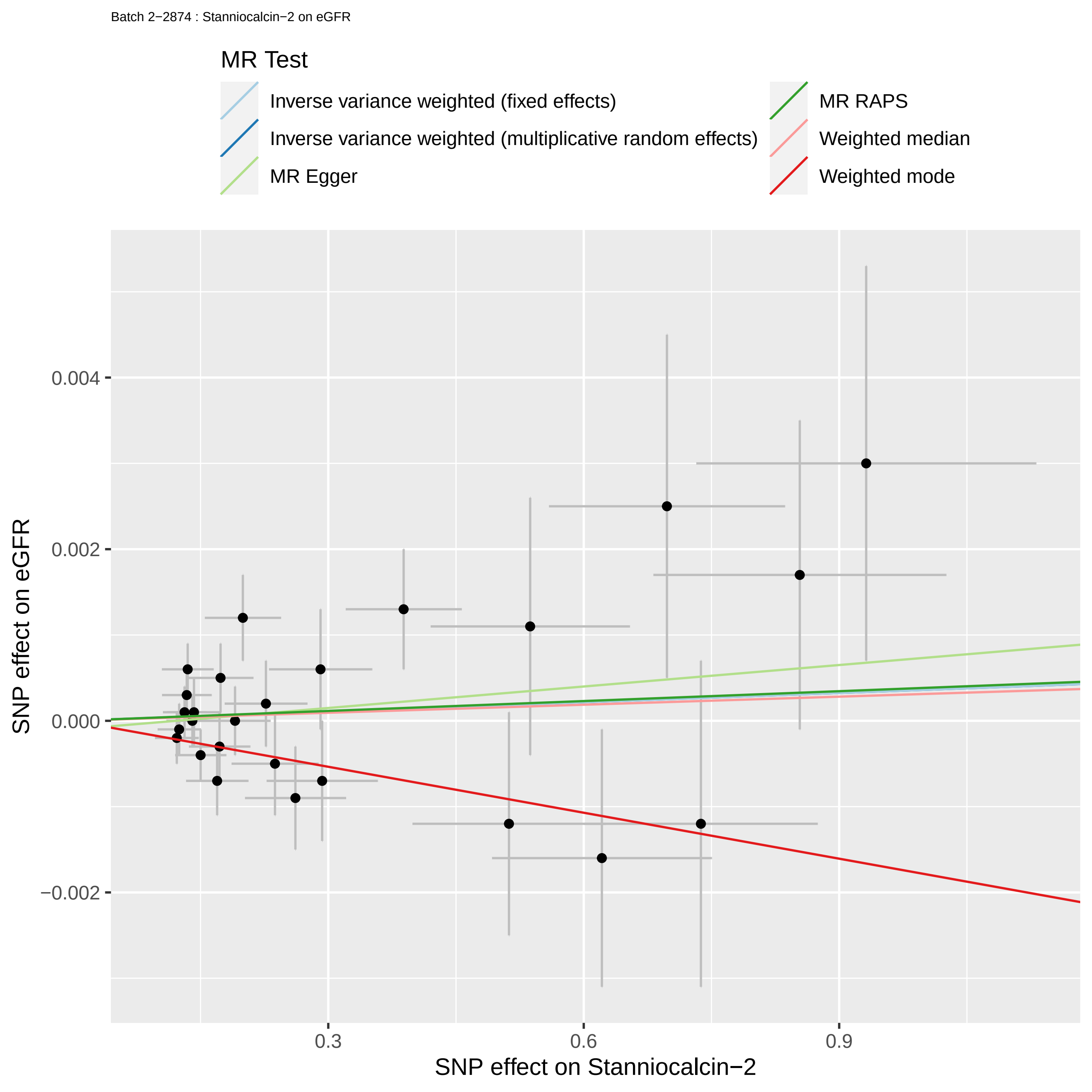
Batch 2−2874 : Stanniocalcin−2 on eGFR
MR Test
Inverse variance weighted (fixed effects)
Inverse variance weighted (multiplicative random effects)
MR Egger
MR RAPS
Weighted median
Weighted mode
0.004
0.002
0.000
−0.002
SNP effect on eGFR
0.3
0.6
0.9
SNP effect on Stanniocalcin−2

Batch 2−2875 : Stromal interaction molecule 1 on eGFR

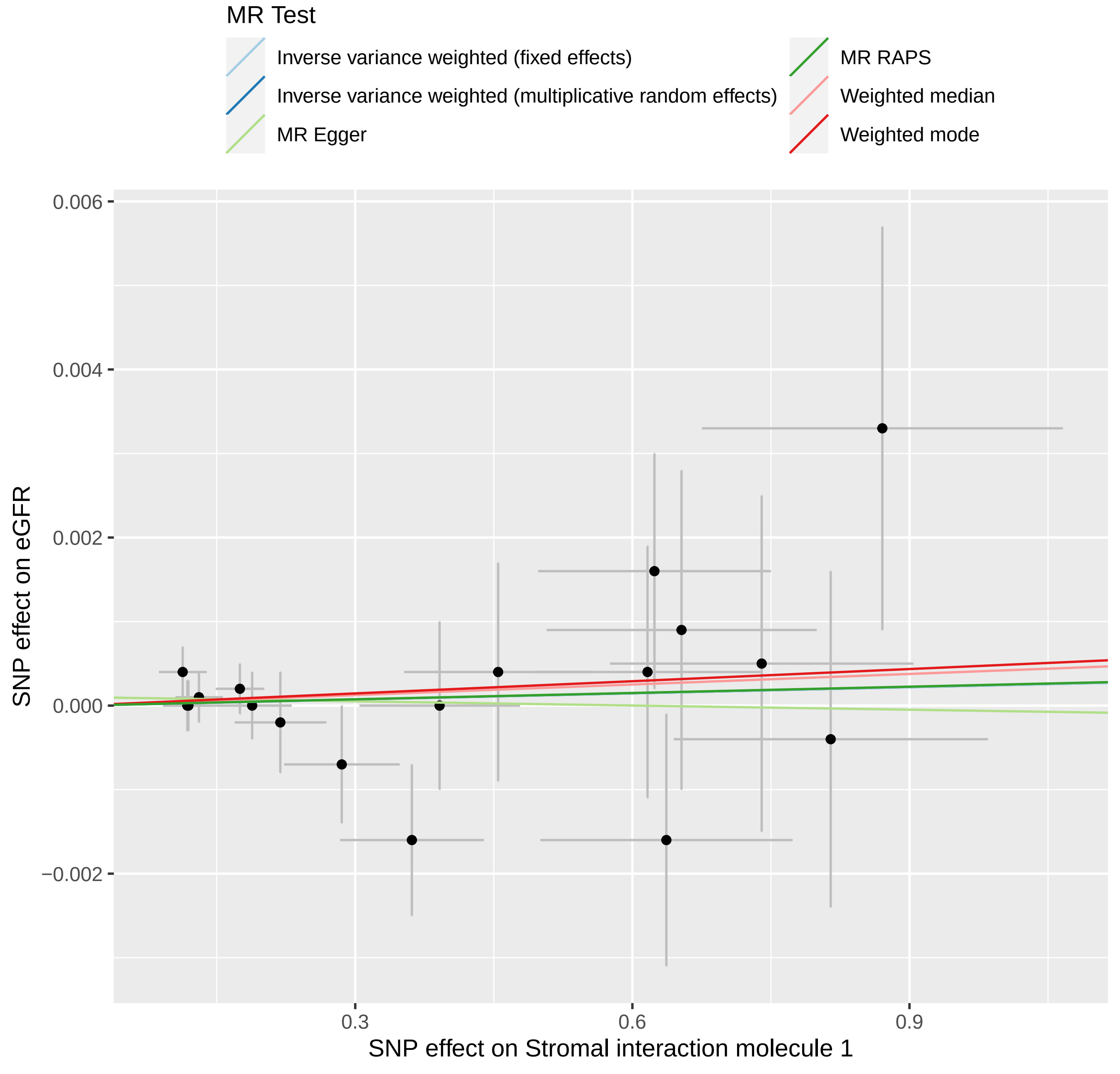

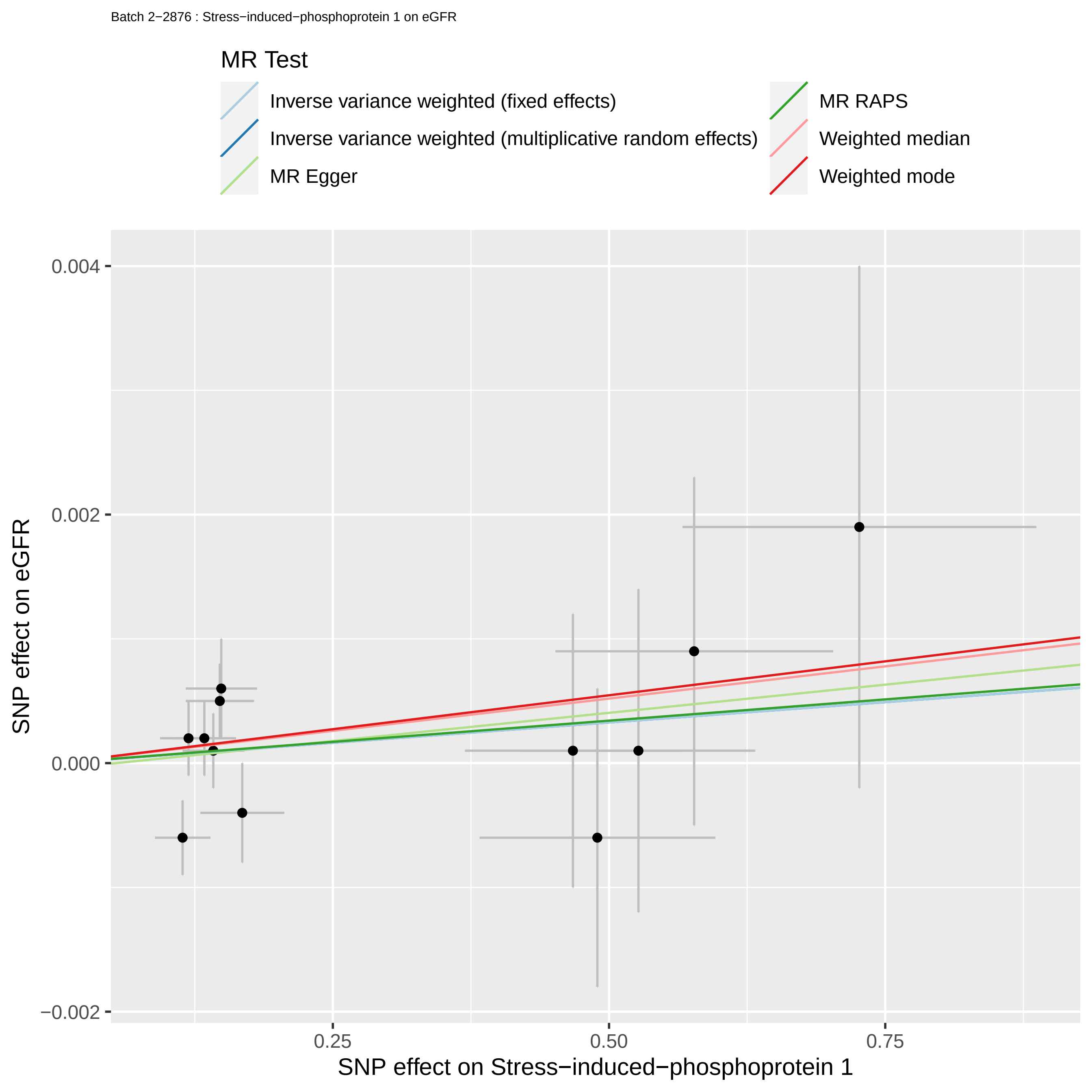

Batch 2−2876 : Stress−induced−phosphoprotein 1 on eGFR
MR Test
Inverse variance weighted (fixed effects)
Inverse variance weighted (multiplicative random effects)
MR Egger
MR RAPS
Weighted median
Weighted mode
0.004
0.002
0.000
−0.002
0.25
0.50
0.75
SNP effect on eGFR
SNP effect on Stress−induced−phosphoprotein 1

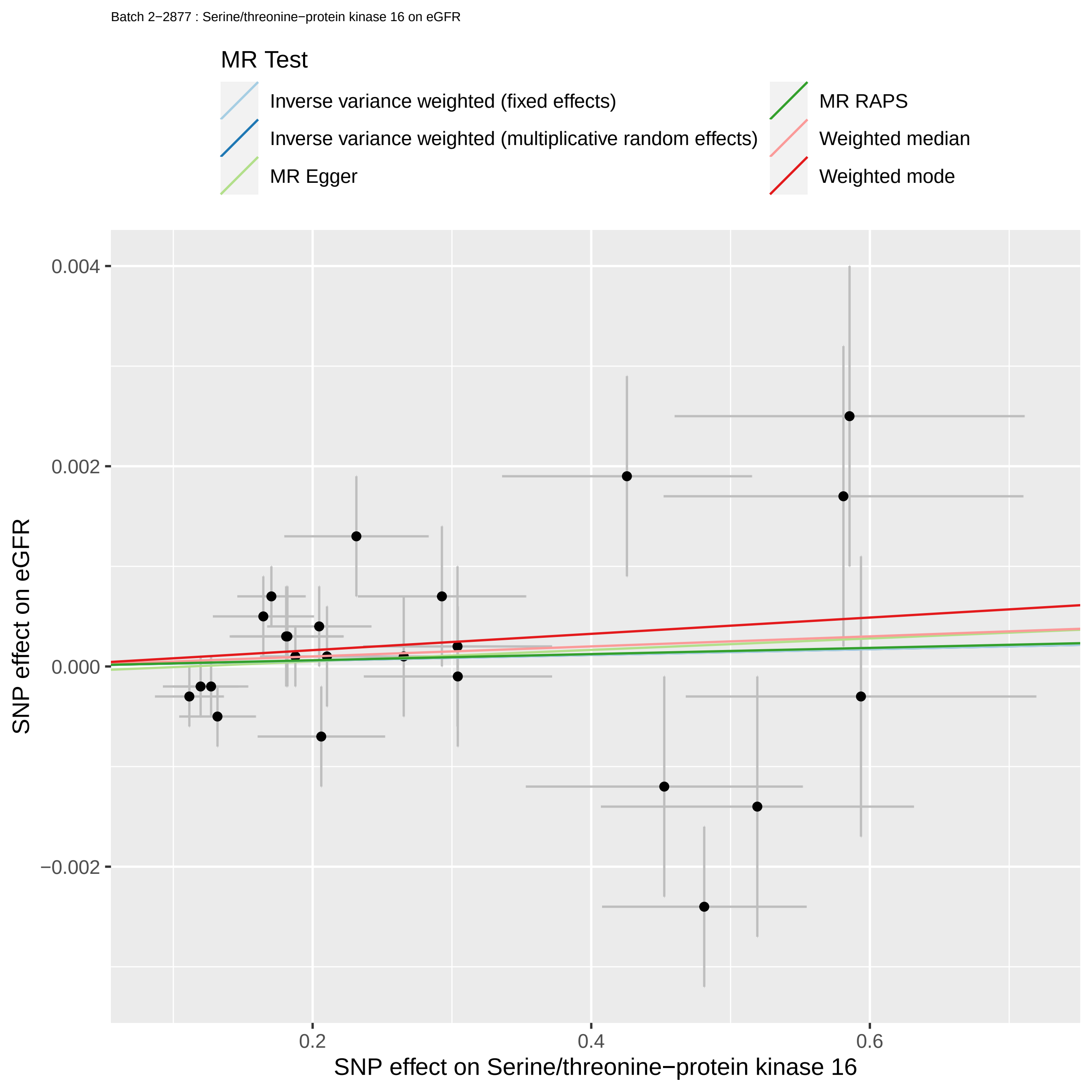

Batch 2−2877 : Serine/threonine−protein kinase 16 on eGFR
MR Test
Inverse variance weighted (fixed effects)
Inverse variance weighted (multiplicative random effects)
MR Egger
MR RAPS
Weighted median
Weighted mode
0.004
0.002
0.000
−0.002
0.2
0.4
0.6
SNP effect on eGFR
SNP effect on Serine/threonine−protein kinase 16

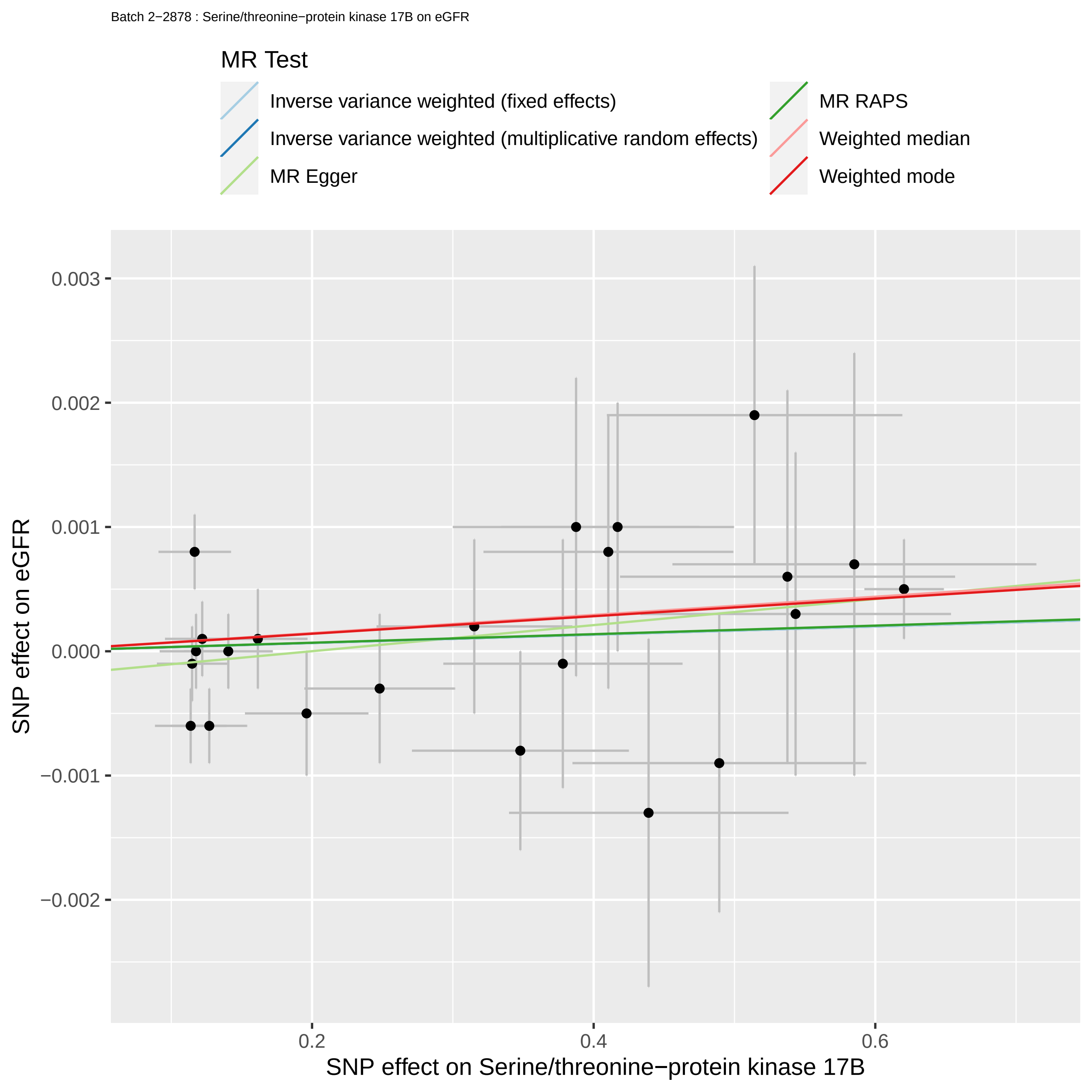

Batch 2−2878 : Serine/threonine−protein kinase 17B on eGFR
MR Test
Inverse variance weighted (fixed effects)
Inverse variance weighted (multiplicative random effects)
MR Egger
MR RAPS
Weighted median
Weighted mode
0.003
0.002
0.001
0.000
−0.001
−0.002
0.2
0.4
0.6
SNP effect on eGFR
SNP effect on Serine/threonine−protein kinase 17B

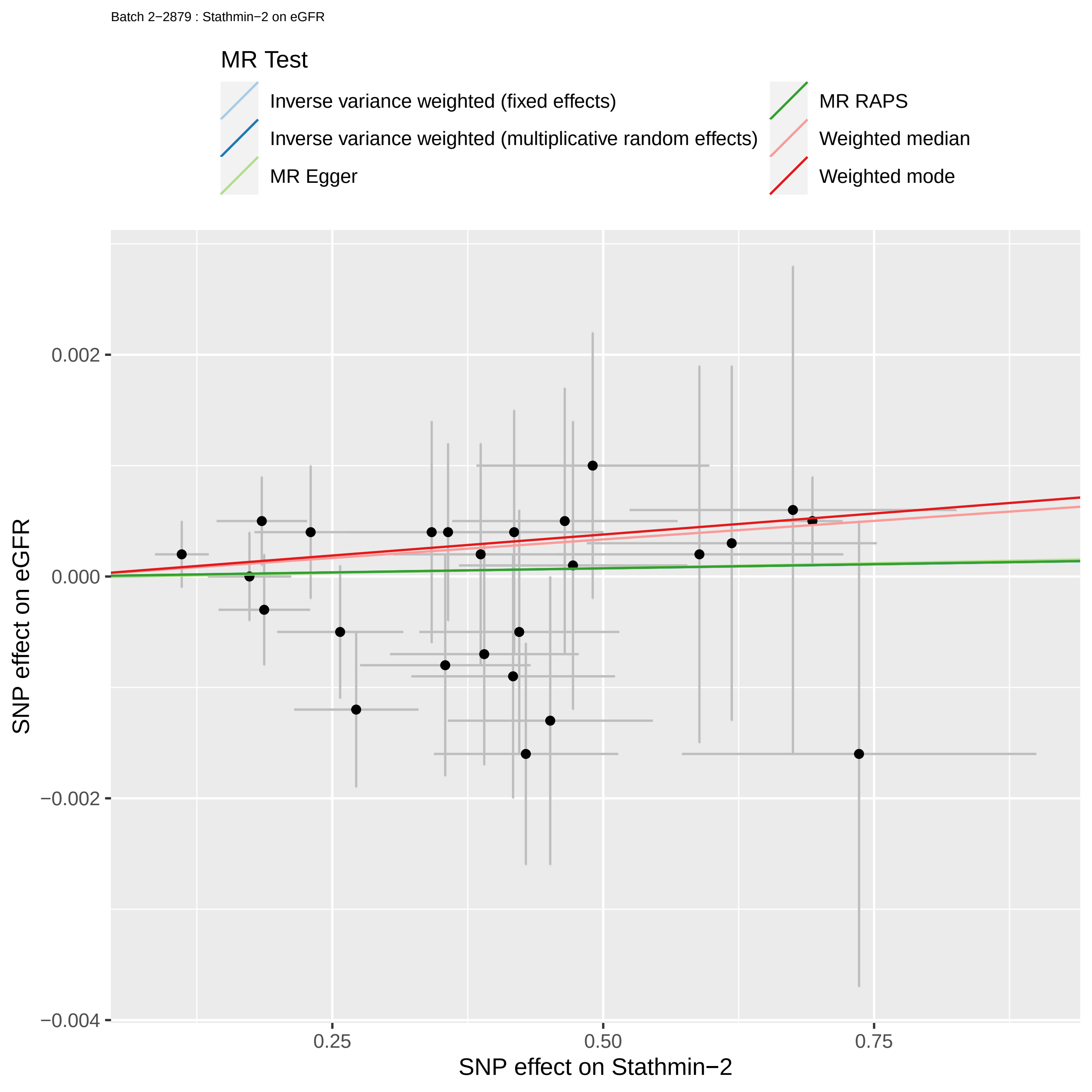

Batch 2−2879 : Stathmin−2 on eGFR
MR Test
Inverse variance weighted (fixed effects)
Inverse variance weighted (multiplicative random effects)
MR Egger
MR RAPS
Weighted median
Weighted mode
0.002
0.000
−0.002
−0.004
SNP effect on eGFR
0.25
0.50
0.75
SNP effect on Stathmin−2

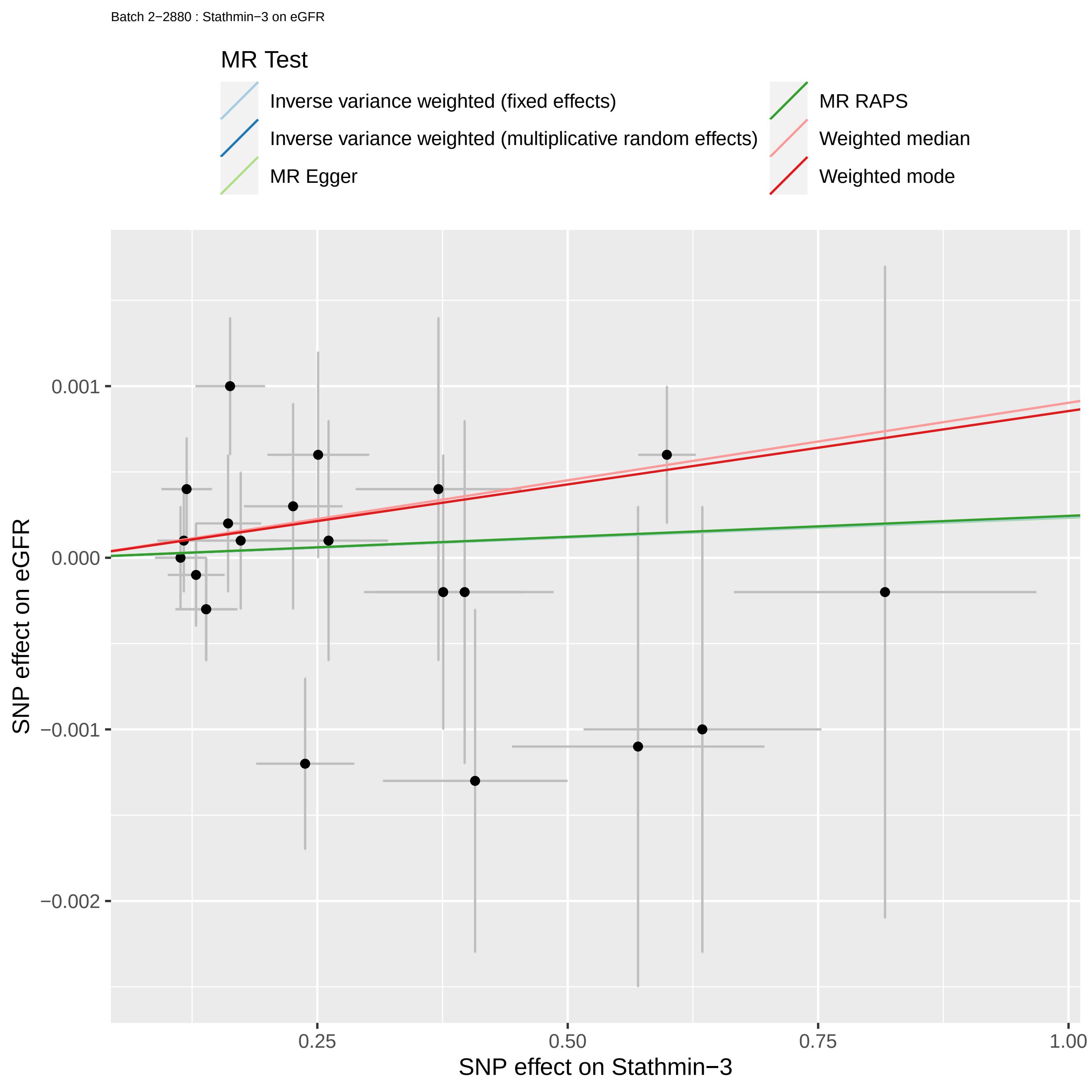
Batch 2−2880 : Stathmin−3 on eGFR
MR Test
Inverse variance weighted (fixed effects)
Inverse variance weighted (multiplicative random effects)
MR Egger
MR RAPS
Weighted median
Weighted mode
0.001
0.000
−0.001
−0.002
0.25
0.50
0.75
1.00
SNP effect on eGFR
SNP effect on Stathmin−3

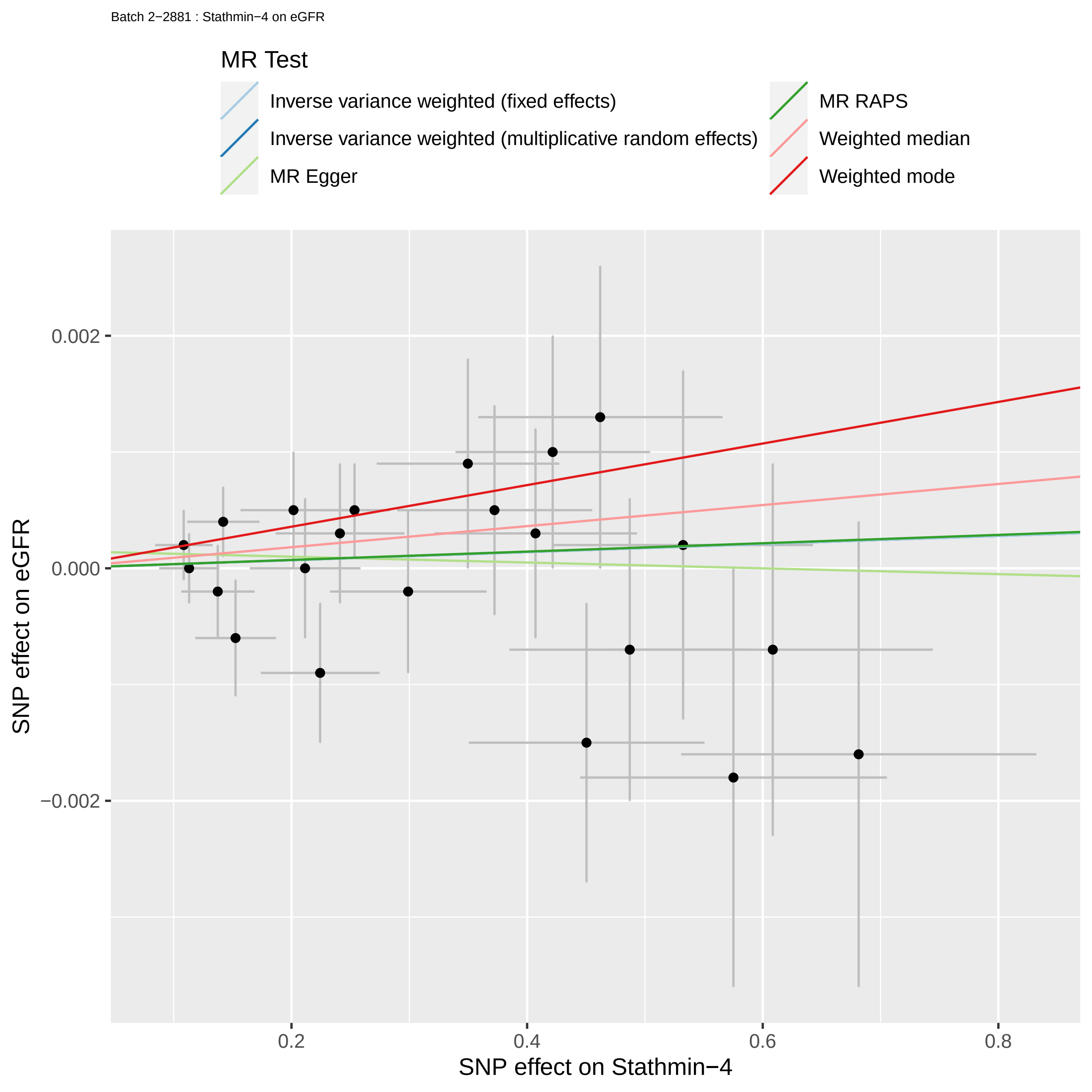
Batch 2−2881 : Stathmin−4 on eGFR
MR Test
Inverse variance weighted (fixed effects)
Inverse variance weighted (multiplicative random effects)
MR Egger
MR RAPS
Weighted median
Weighted mode
0.002
0.000
−0.002
SNP effect on eGFR
0.2
0.4
0.6
0.8
SNP effect on Stathmin−4

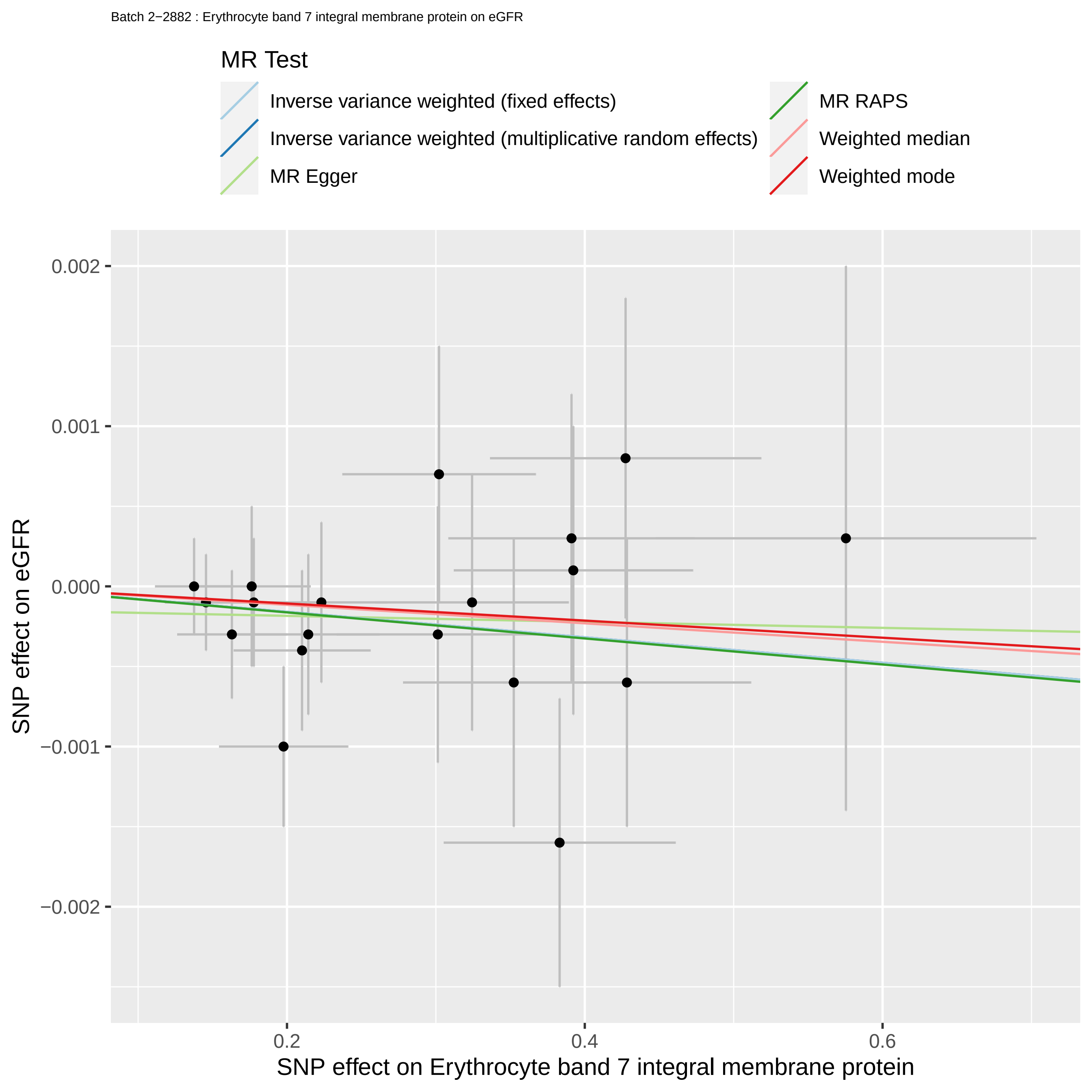

Batch 2−2882 : Erythrocyte band 7 integral membrane protein on eGFR
MR Test
Inverse variance weighted (fixed effects)
Inverse variance weighted (multiplicative random effects)
MR Egger
MR RAPS
Weighted median
Weighted mode
0.002
0.001
0.000
−0.001
−0.002
0.2
0.4
0.6
SNP effect on eGFR
SNP effect on Erythrocyte band 7 integral membrane protein

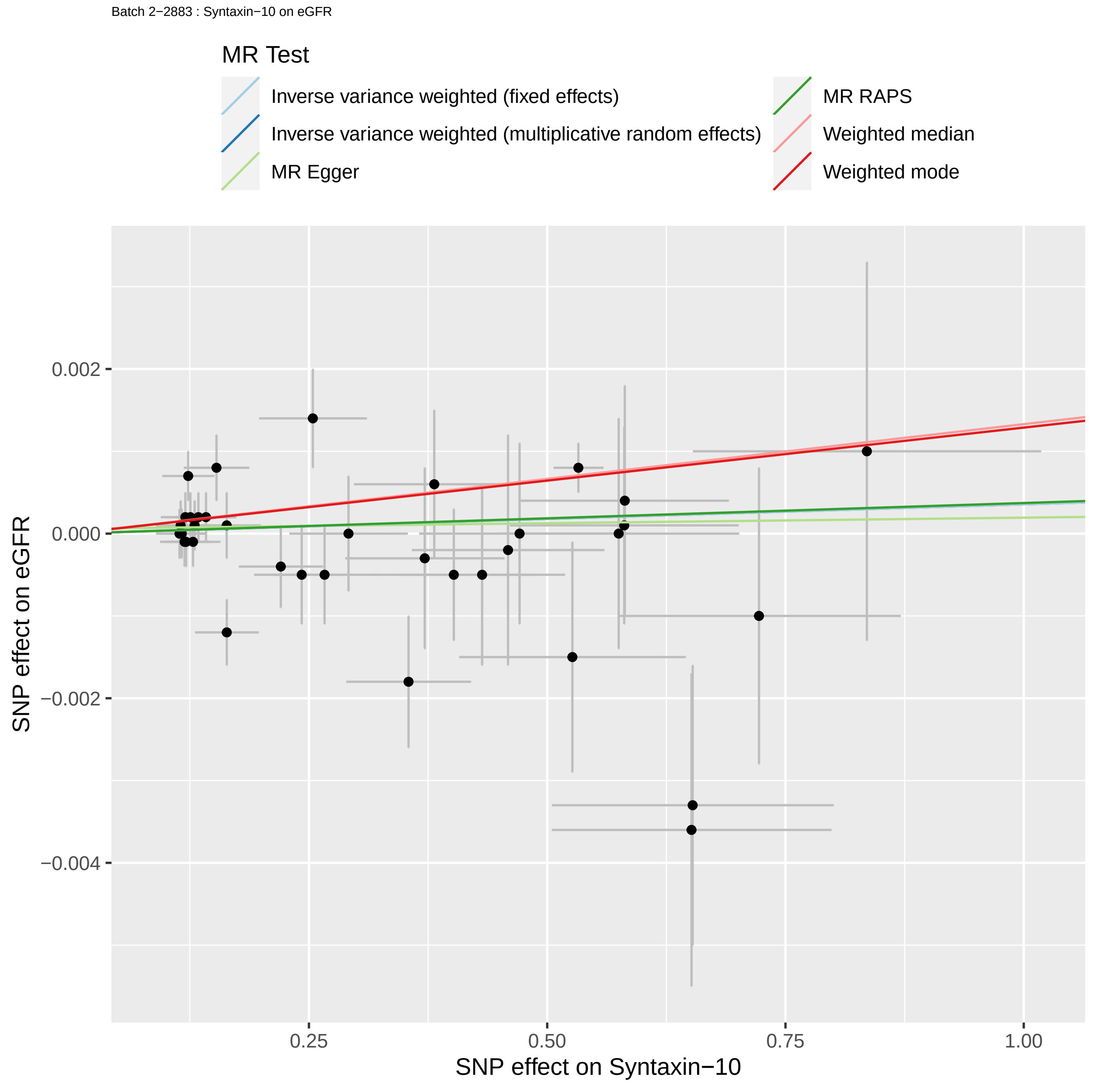

Batch 2−2883 : Syntaxin−10 on eGFR
MR Test
Inverse variance weighted (fixed effects)
Inverse variance weighted (multiplicative random effects)
MR Egger
MR RAPS
Weighted median
Weighted mode
0.002
0.000
−0.002
−0.004
0.25
0.50
0.75
1.00
SNP effect on eGFR
SNP effect on Syntaxin−10

Batch 2−2884 : Syntaxin−18 on eGFR

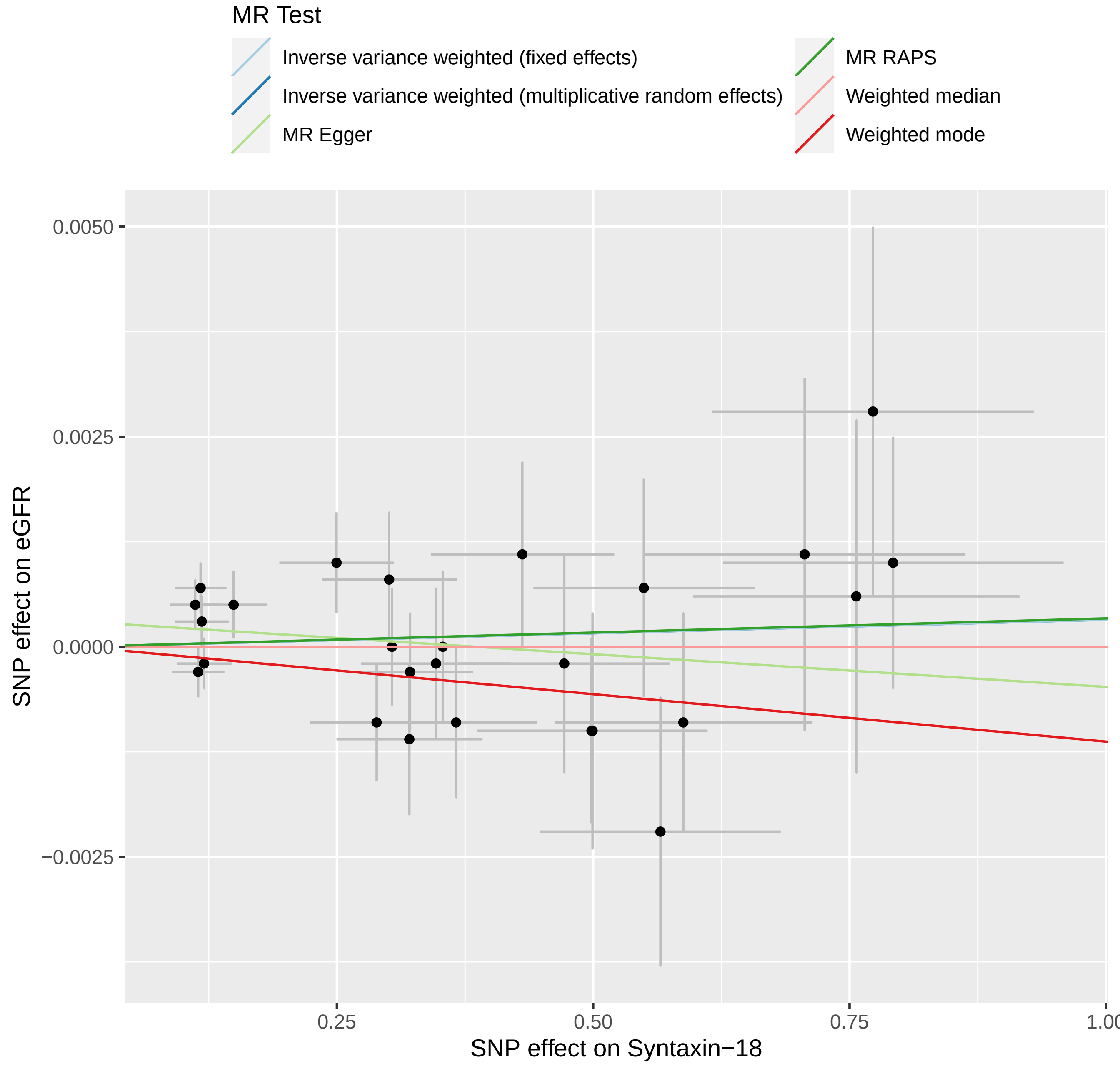

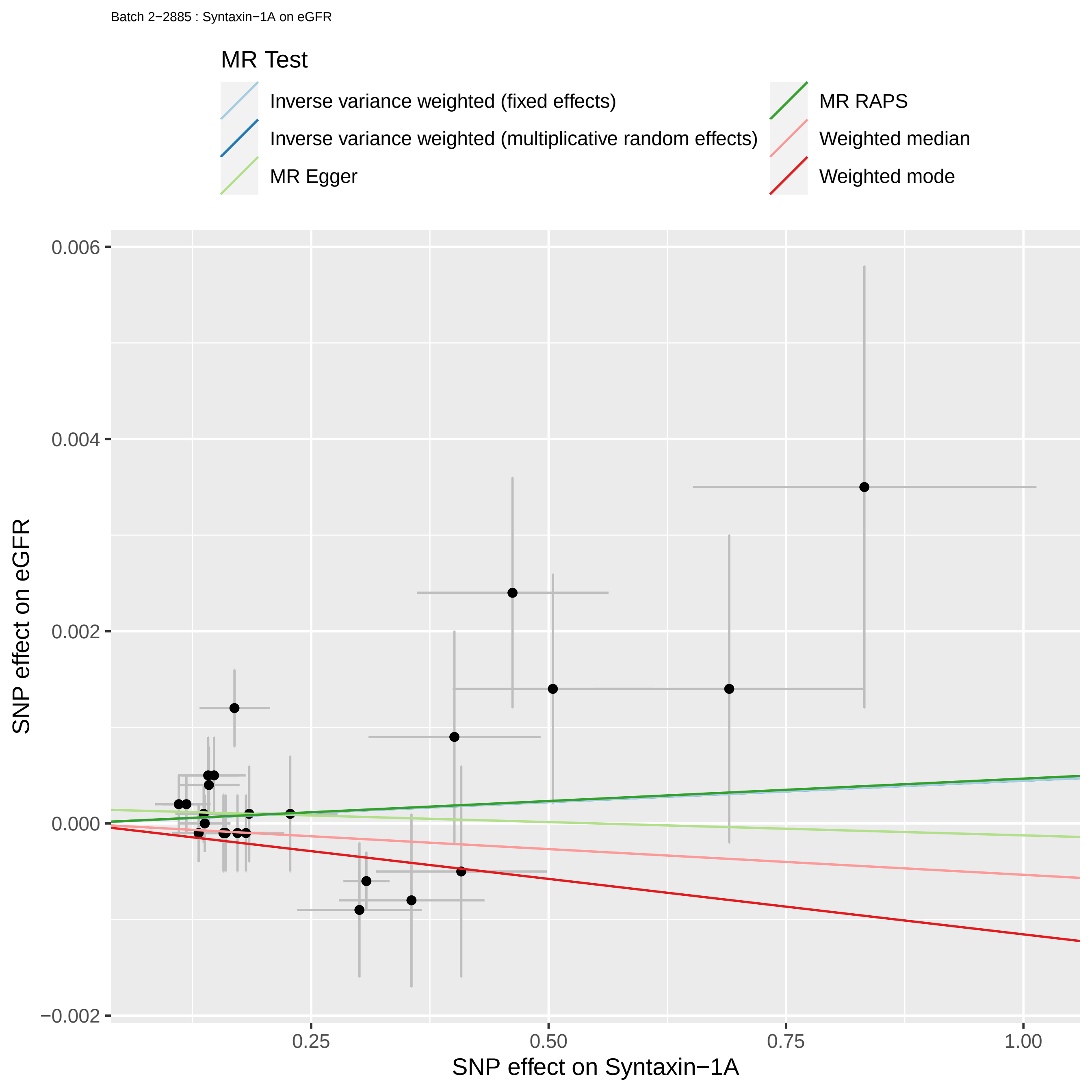
Batch 2−2885 : Syntaxin−1A on eGFR
MR Test
Inverse variance weighted (fixed effects)
Inverse variance weighted (multiplicative random effects)
MR Egger
MR RAPS
Weighted median
Weighted mode
0.006
0.004
0.002
0.000
−0.002
0.25
0.50
0.75
1.00
SNP effect on eGFR
SNP effect on Syntaxin−1A

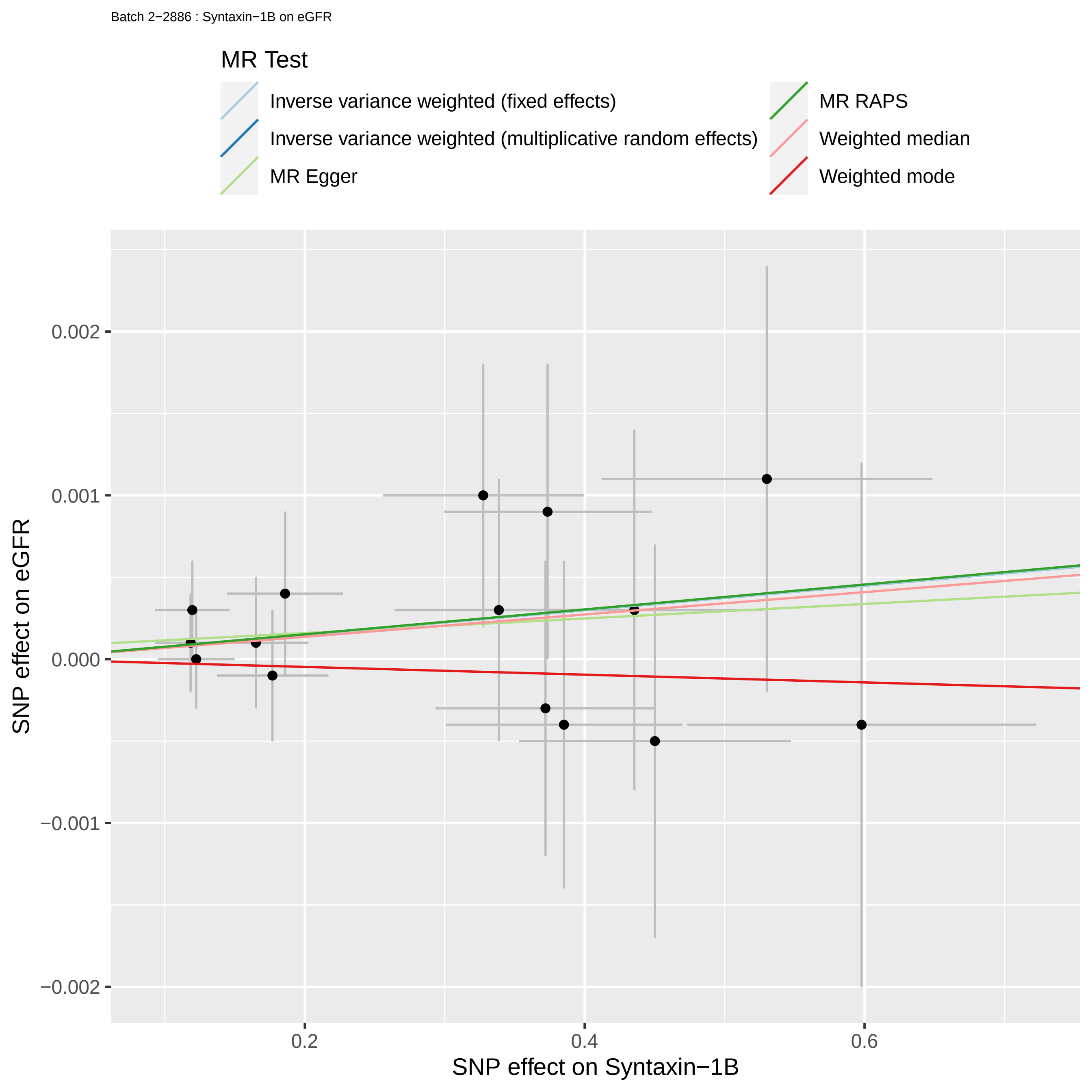
Batch 2−2886 : Syntaxin−1B on eGFR
MR Test
Inverse variance weighted (fixed effects)
Inverse variance weighted (multiplicative random effects)
MR Egger
MR RAPS
Weighted median
Weighted mode
0.002
0.001
0.000
−0.001
−0.002
0.2
0.4
0.6
SNP effect on eGFR
SNP effect on Syntaxin−1B

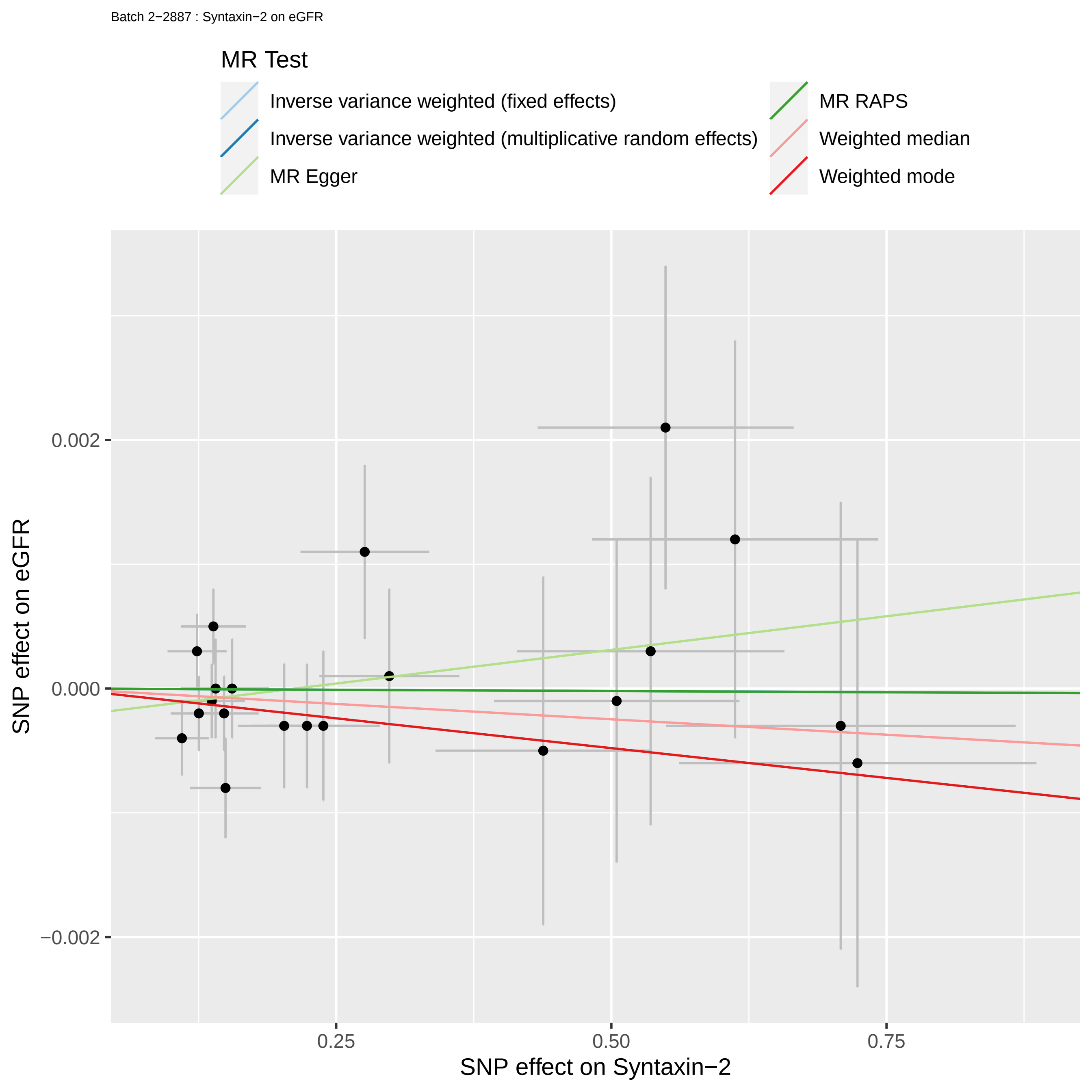
Batch 2−2887 : Syntaxin−2 on eGFR
MR Test
Inverse variance weighted (fixed effects)
Inverse variance weighted (multiplicative random effects)
MR Egger
MR RAPS
Weighted median
Weighted mode
0.002
0.000
−0.002
0.25
0.50
0.75
SNP effect on eGFR
SNP effect on Syntaxin−2

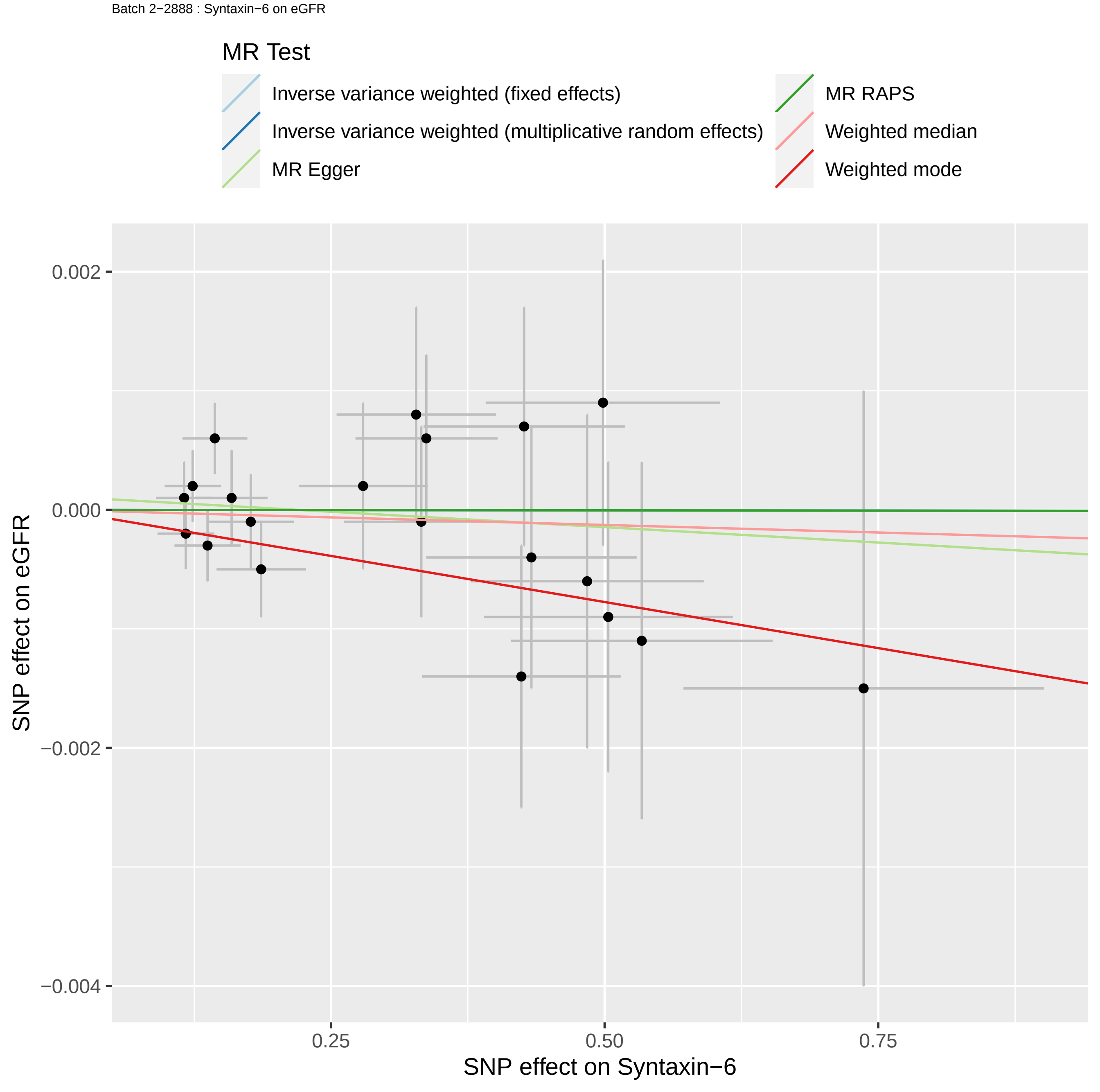
Batch 2−2888 : Syntaxin−6 on eGFR
MR Test
Inverse variance weighted (fixed effects)
Inverse variance weighted (multiplicative random effects)
MR Egger
MR RAPS
Weighted median
Weighted mode
0.002
0.000
−0.002
−0.004
SNP effect on eGFR
0.25
0.50
0.75
SNP effect on Syntaxin−6

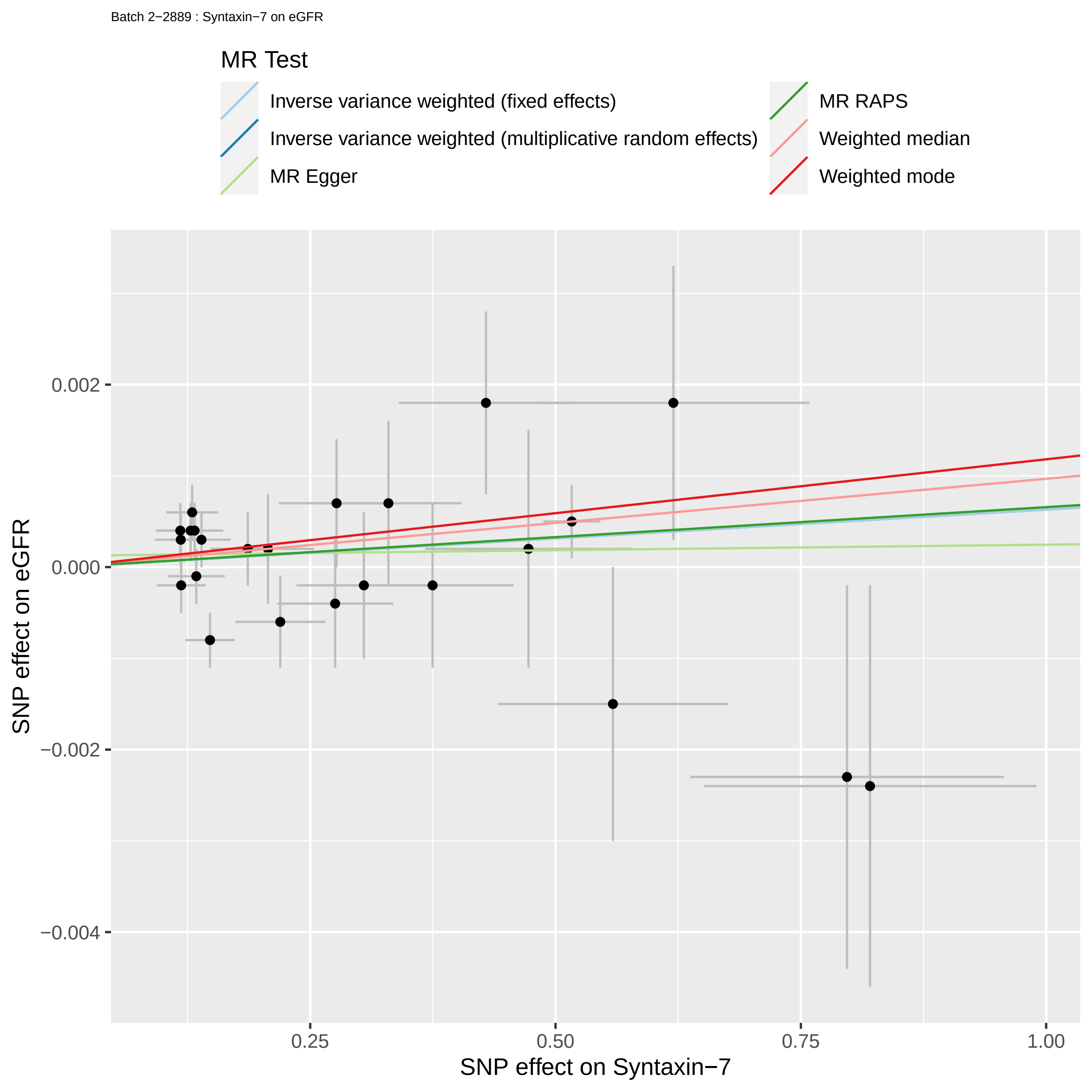

Batch 2−2889 : Syntaxin−7 on eGFR
MR Test
Inverse variance weighted (fixed effects)
Inverse variance weighted (multiplicative random effects)
MR Egger
MR RAPS
Weighted median
Weighted mode
0.002
0.000
−0.002
−0.004
SNP effect on eGFR
0.25
0.50
0.75
1.00
SNP effect on Syntaxin−7

Batch 2−2890 : Syntaxin−8 on eGFR

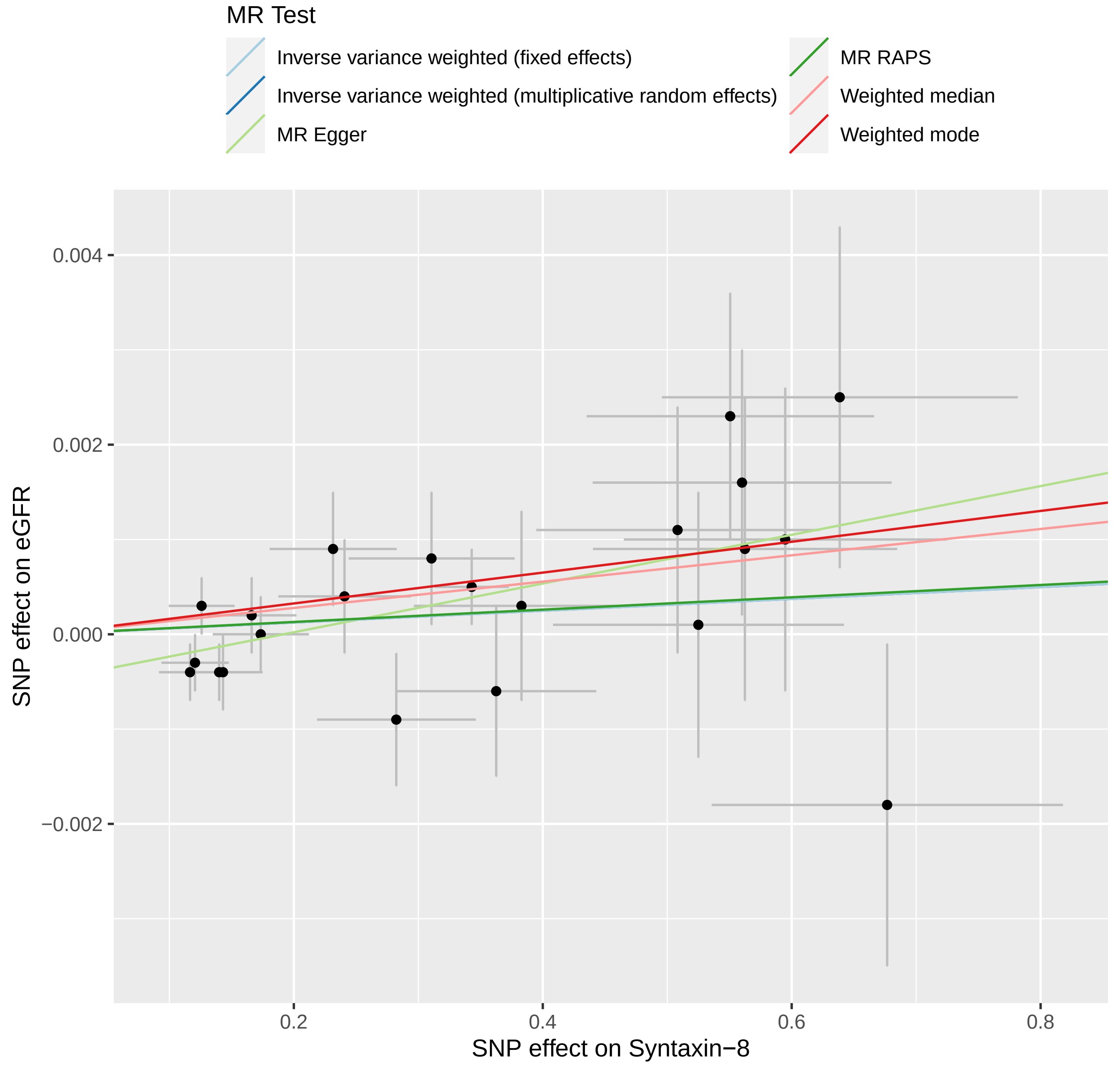

Batch 2−2891 : Extracellular sulfatase Sulf−2 on eGFR

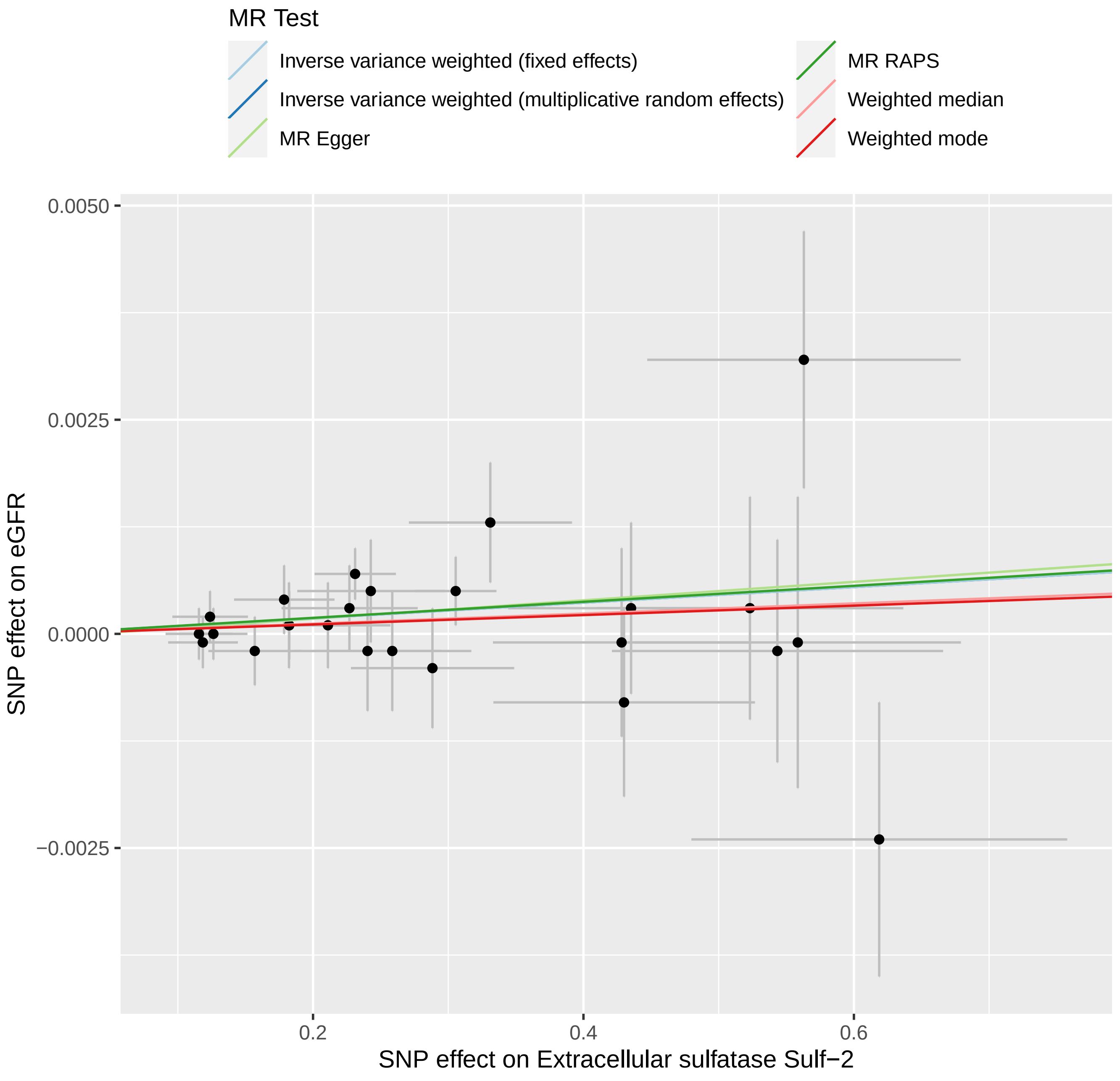

Batch 2−2892 : Estrogen sulfotransferase on eGFR

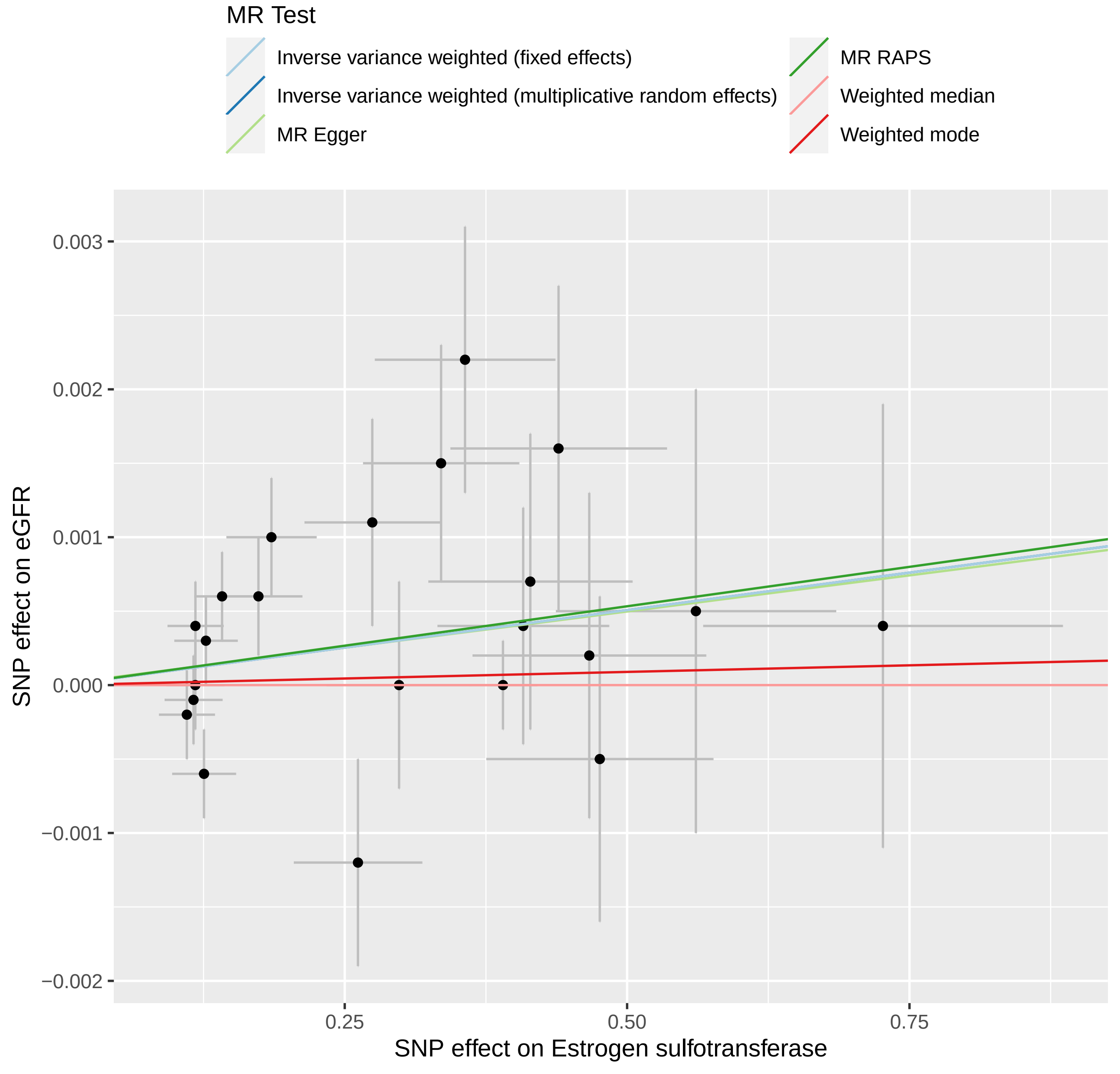

Batch 2−2893 : Bile salt sulfotransferase on eGFR

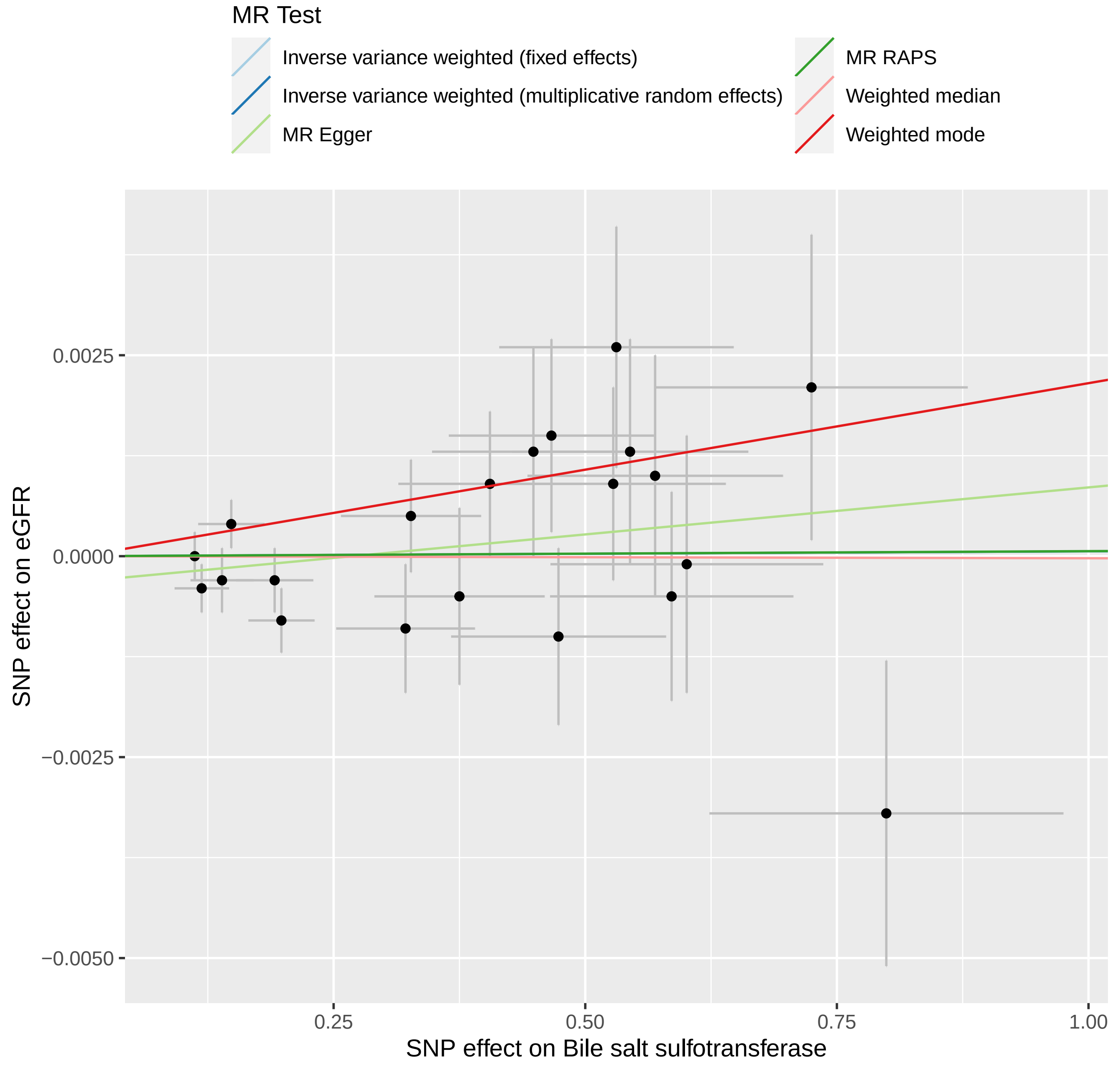

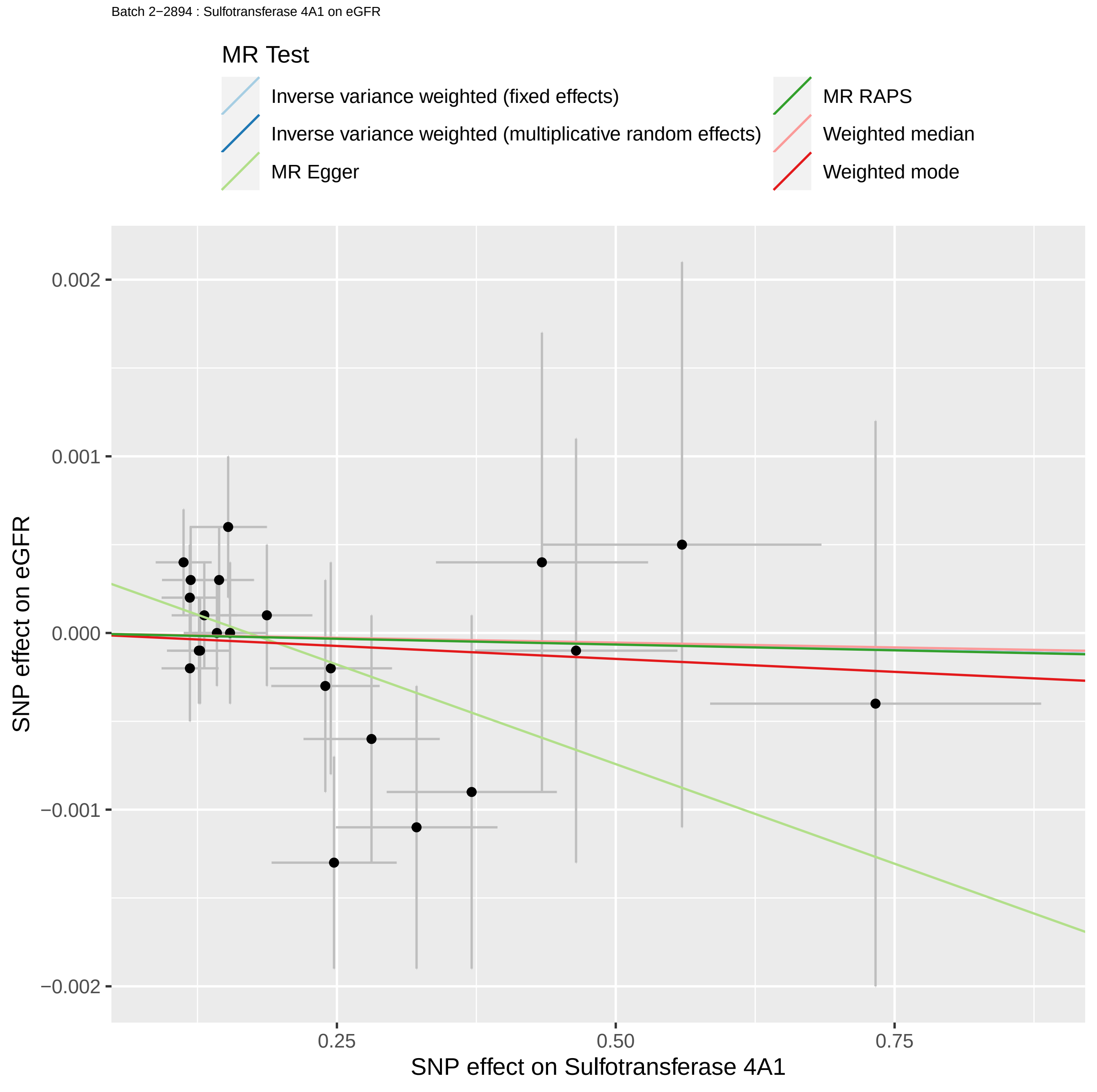
Batch 2−2894 : Sulfotransferase 4A1 on eGFR
MR Test
Inverse variance weighted (fixed effects)
Inverse variance weighted (multiplicative random effects)
MR Egger
MR RAPS
Weighted median
Weighted mode
0.002
0.001
0.000
−0.001
−0.002
SNP effect on eGFR
0.25
0.50
0.75
SNP effect on Sulfotransferase 4A1

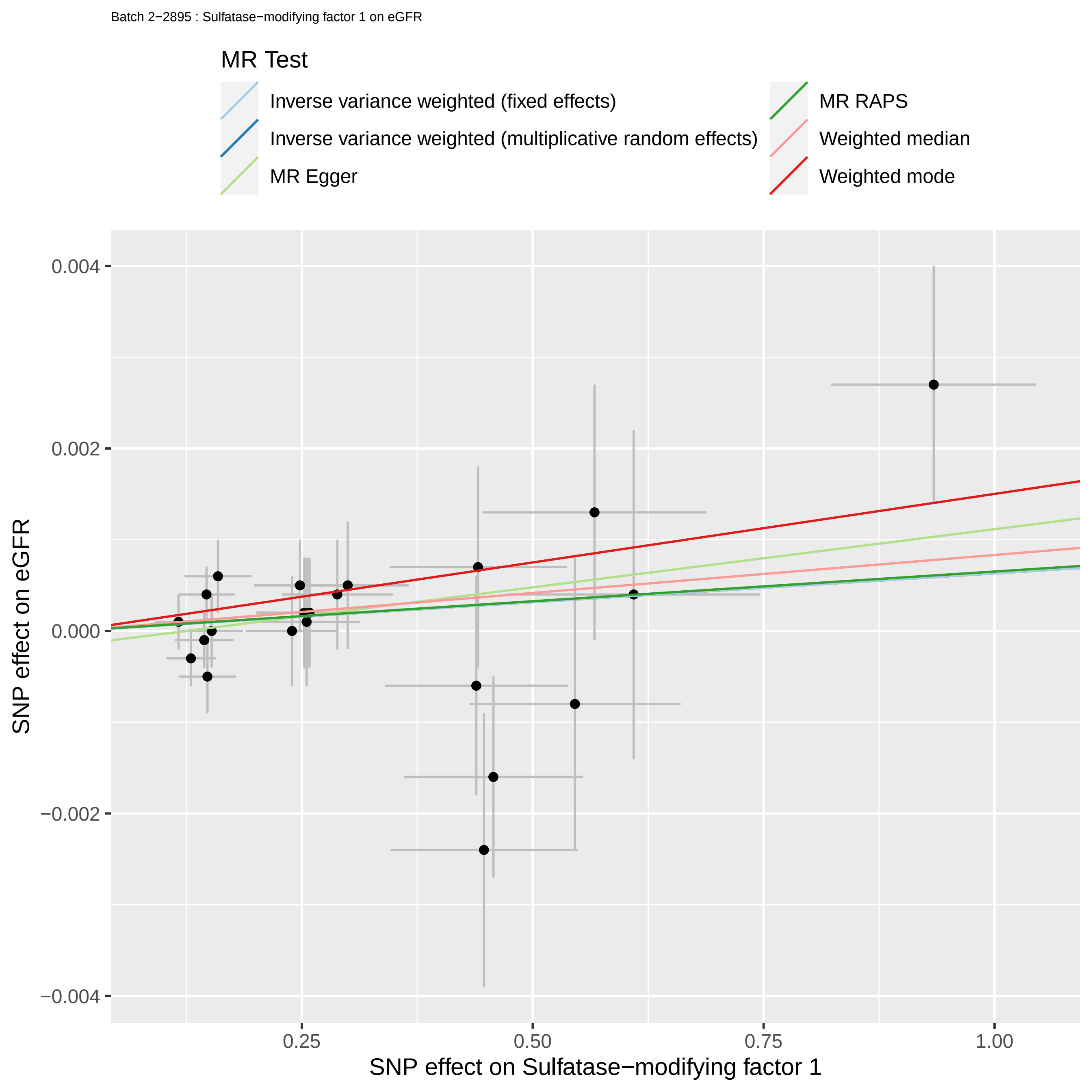

Batch 2−2895 : Sulfatase−modifying factor 1 on eGFR
MR Test
Inverse variance weighted (fixed effects)
Inverse variance weighted (multiplicative random effects)
MR Egger
MR RAPS
Weighted median
Weighted mode
0.004
0.002
0.000
−0.002
−0.004
0.25
0.50
0.75
1.00
SNP effect on eGFR
SNP effect on Sulfatase−modifying factor 1

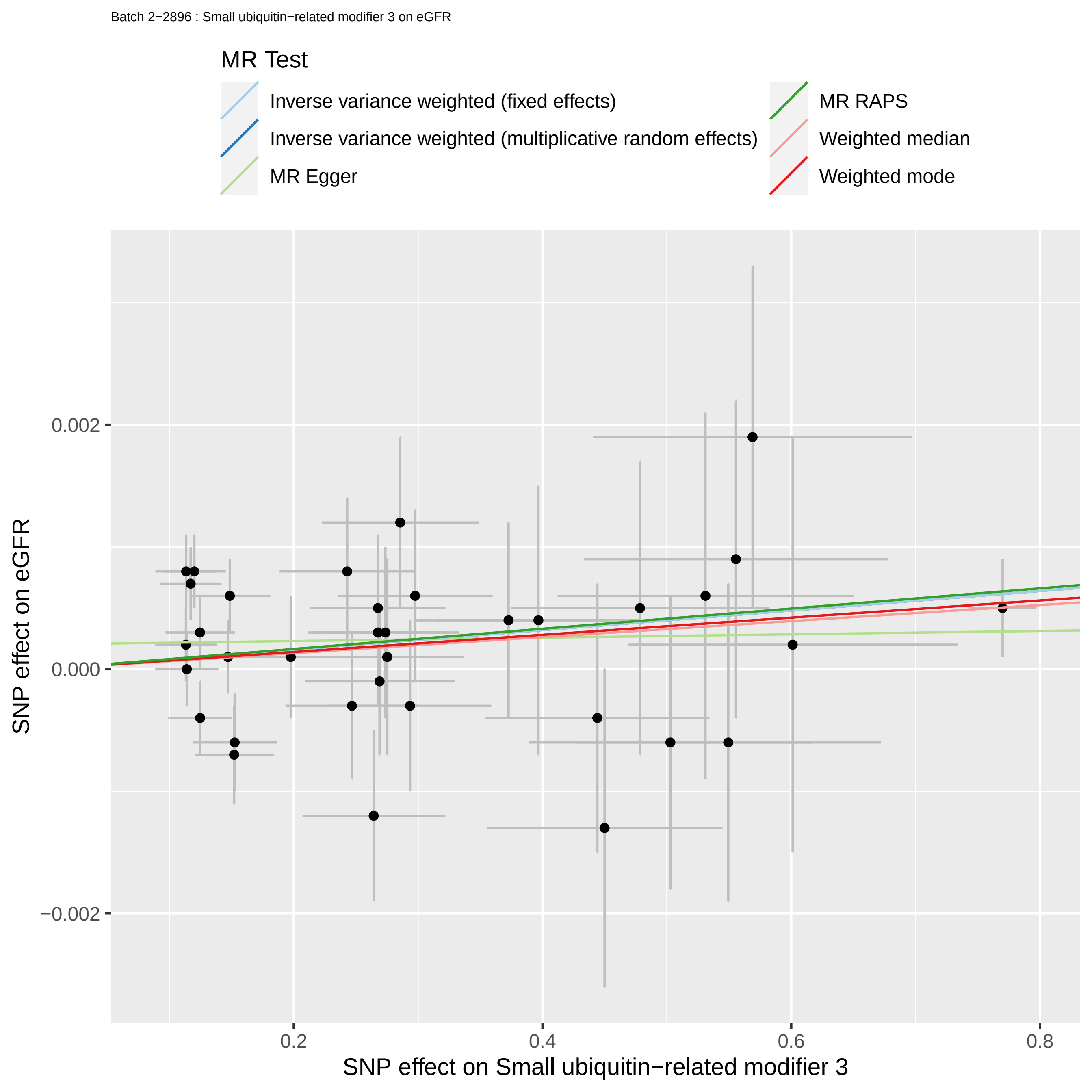

Batch 2−2896 : Small ubiquitin−related modifier 3 on eGFR
MR Test
Inverse variance weighted (fixed effects)
Inverse variance weighted (multiplicative random effects)
MR Egger
MR RAPS
Weighted median
Weighted mode
SNP effect on eGFR
0.002
0.000
−0.002
0.2
0.4
0.6
0.8
SNP effect on Small ubiquitin−related modifier 3

Batch 2−2897 : Small ubiquitin−related modifier 3 on eGFR

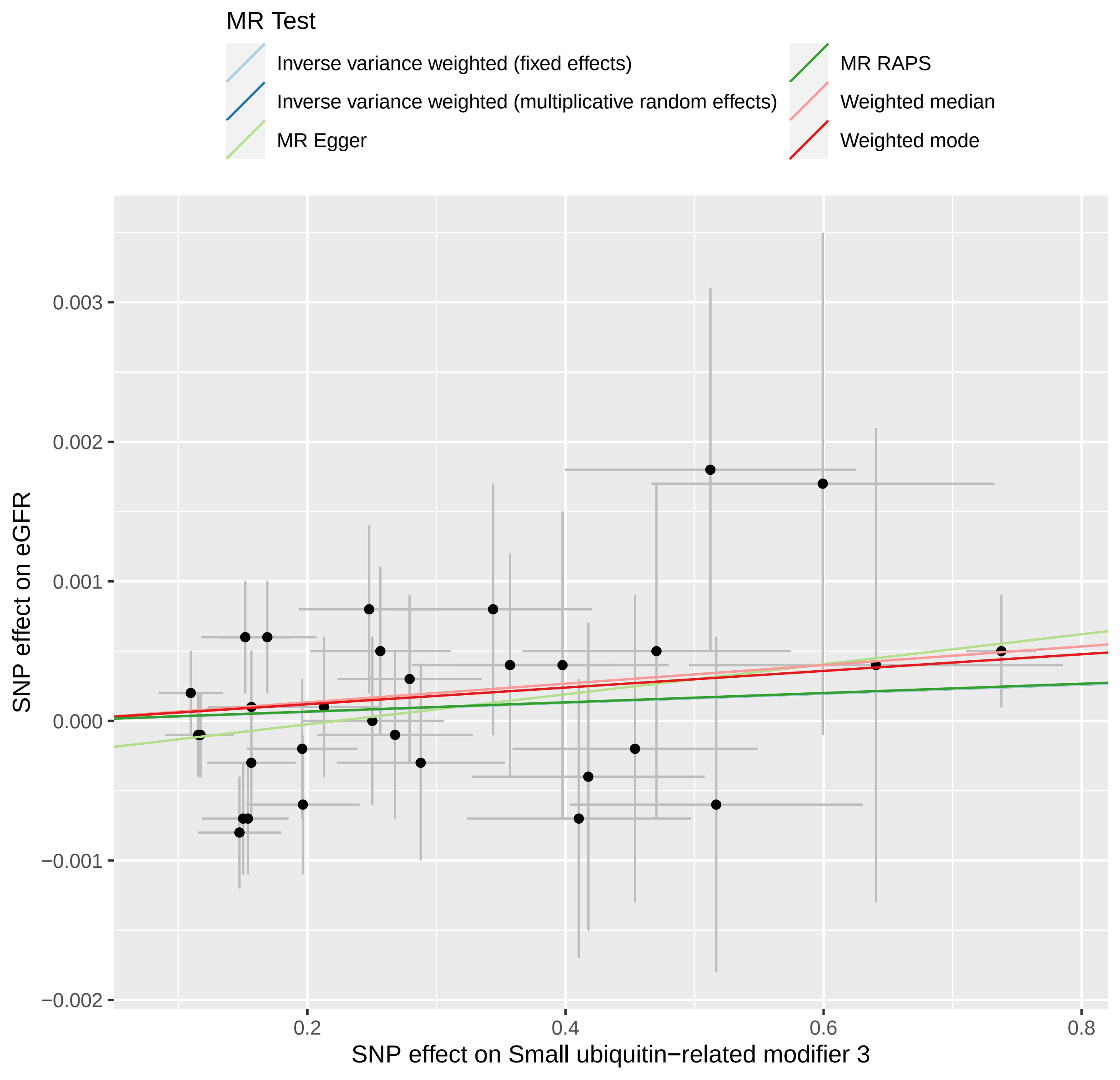

Batch 2−2898 : SUN domain−containing protein 3 on eGFR

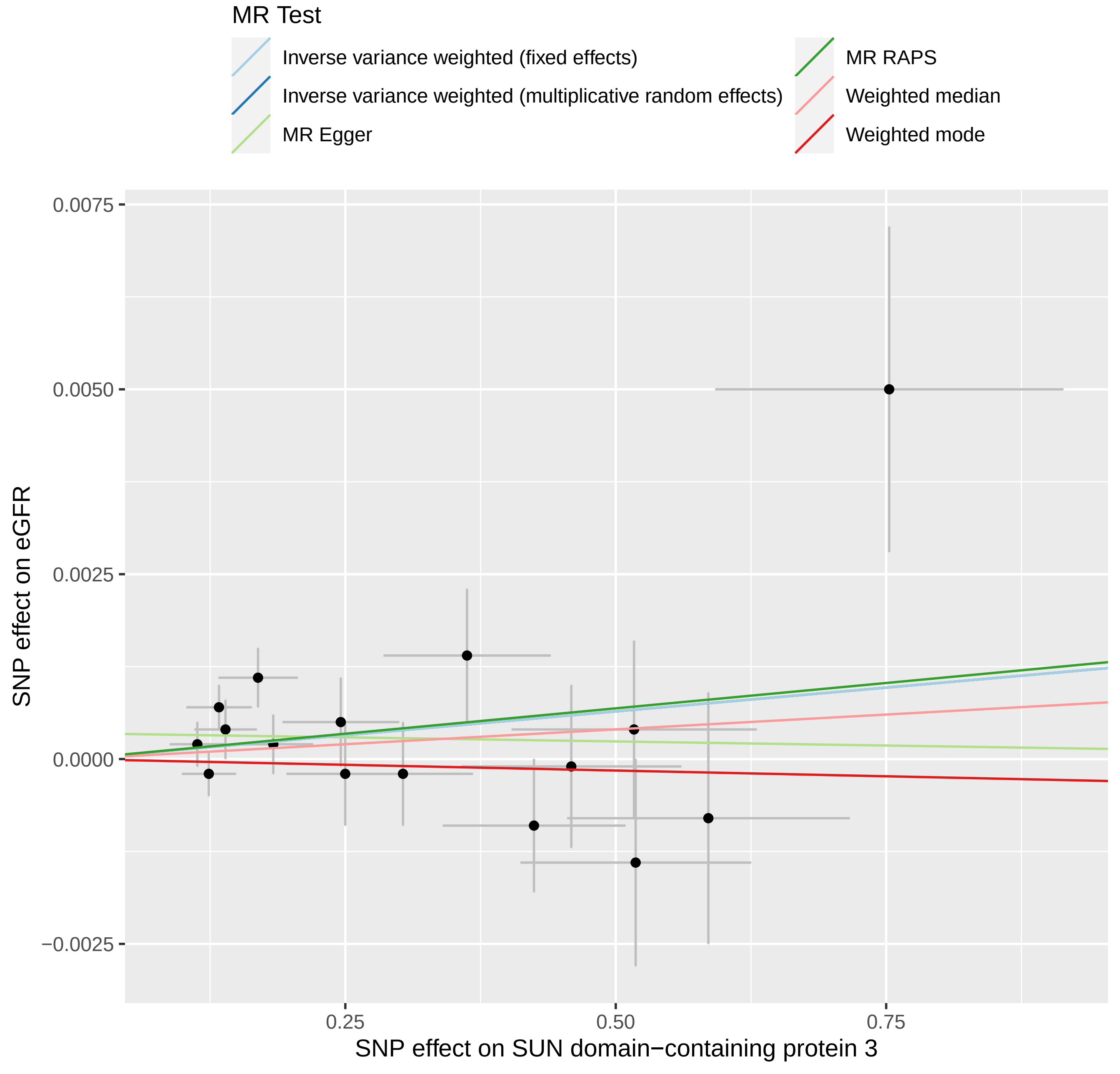

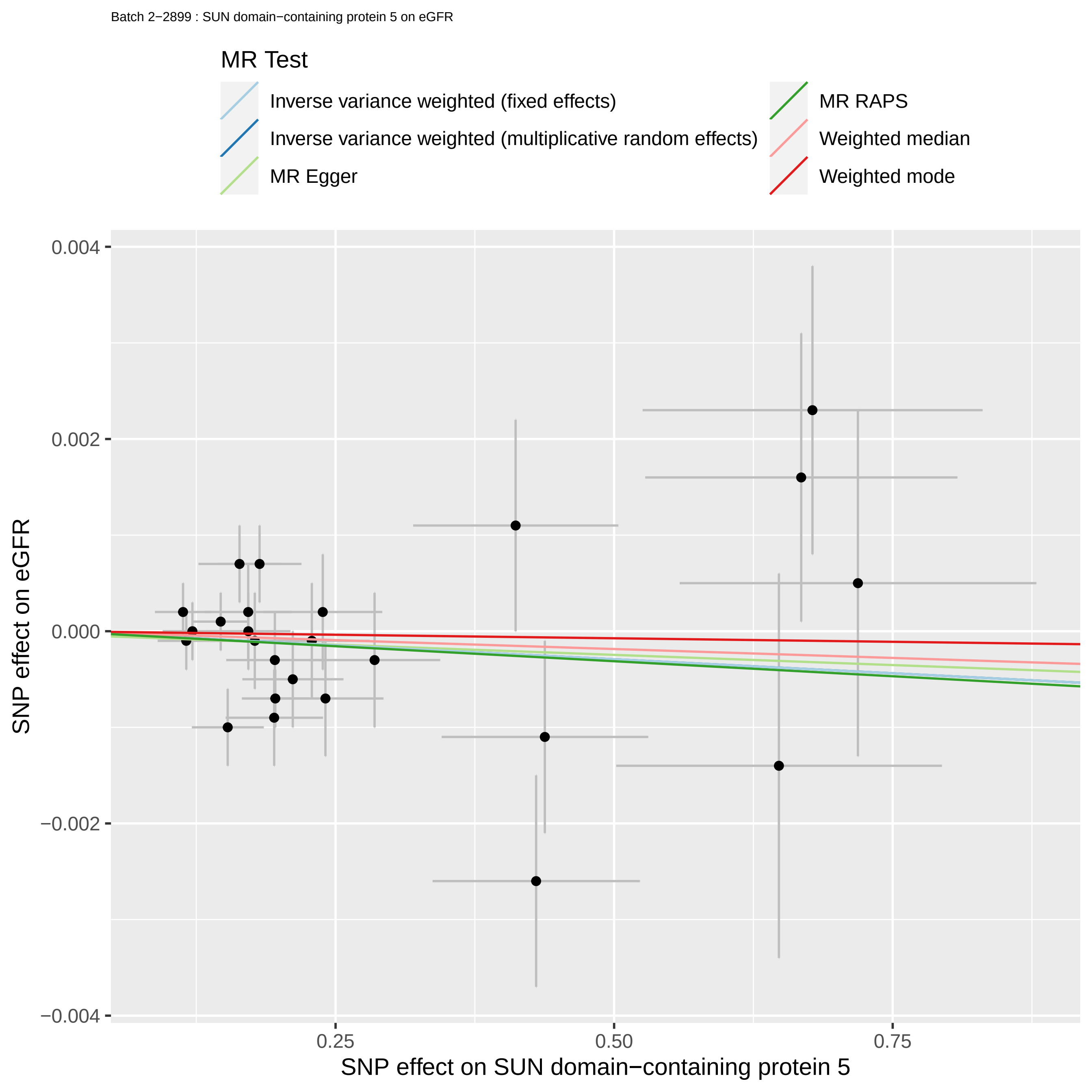
Batch 2−2899 : SUN domain−containing protein 5 on eGFR
MR Test
Inverse variance weighted (fixed effects)
Inverse variance weighted (multiplicative random effects)
MR Egger
MR RAPS
Weighted median
Weighted mode
0.004
0.002
0.000
−0.002
−0.004
0.25
0.50
0.75
SNP effect on eGFR
SNP effect on SUN domain−containing protein 5

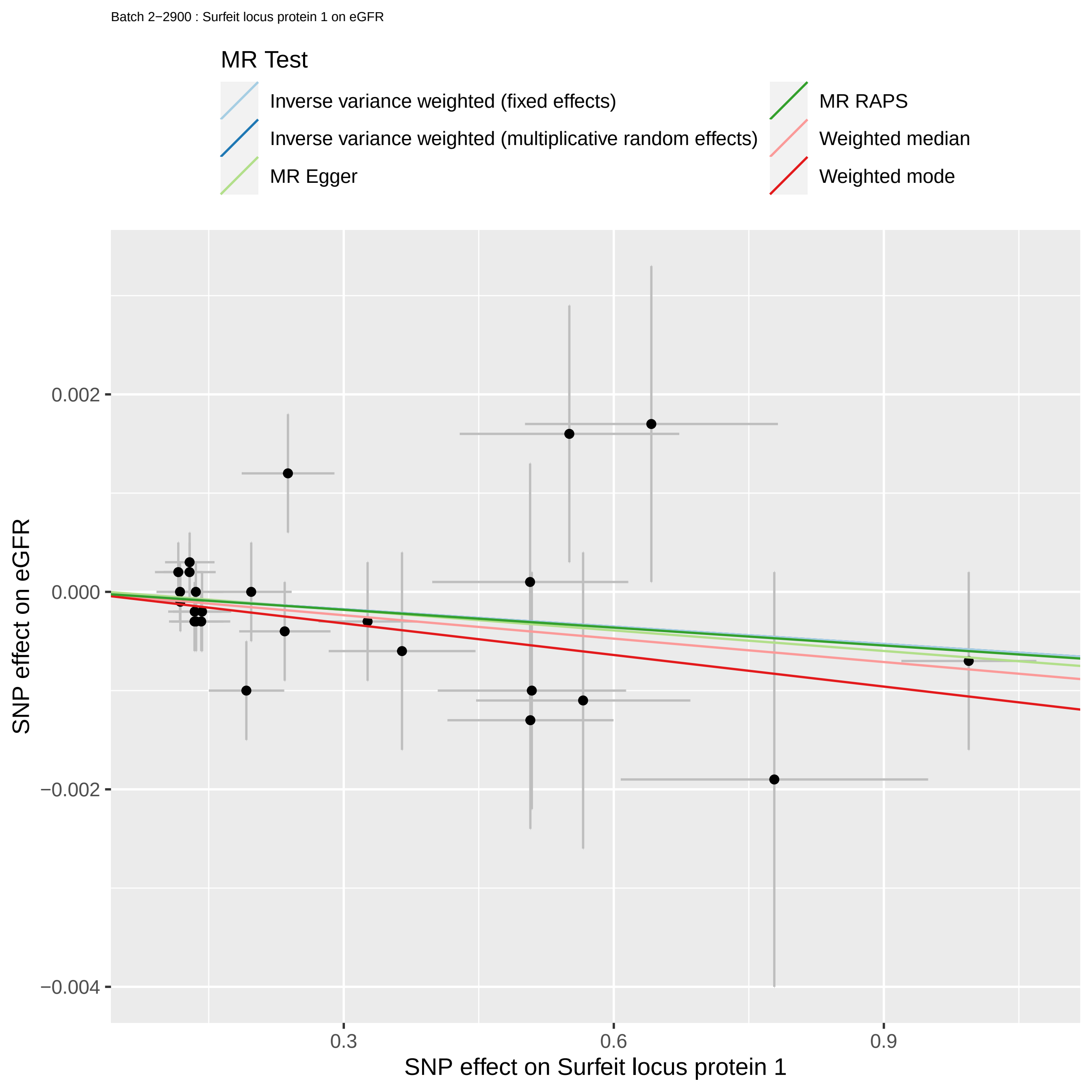

Batch 2−2900 : Surfeit locus protein 1 on eGFR
MR Test
Inverse variance weighted (fixed effects)
Inverse variance weighted (multiplicative random effects)
MR Egger
MR RAPS
Weighted median
Weighted mode
0.002
0.000
−0.002
−0.004
SNP effect on eGFR
0.3
0.6
0.9
SNP effect on Surfeit locus protein 1

Batch 2−2901 : Sushi domain−containing protein 1 on eGFR

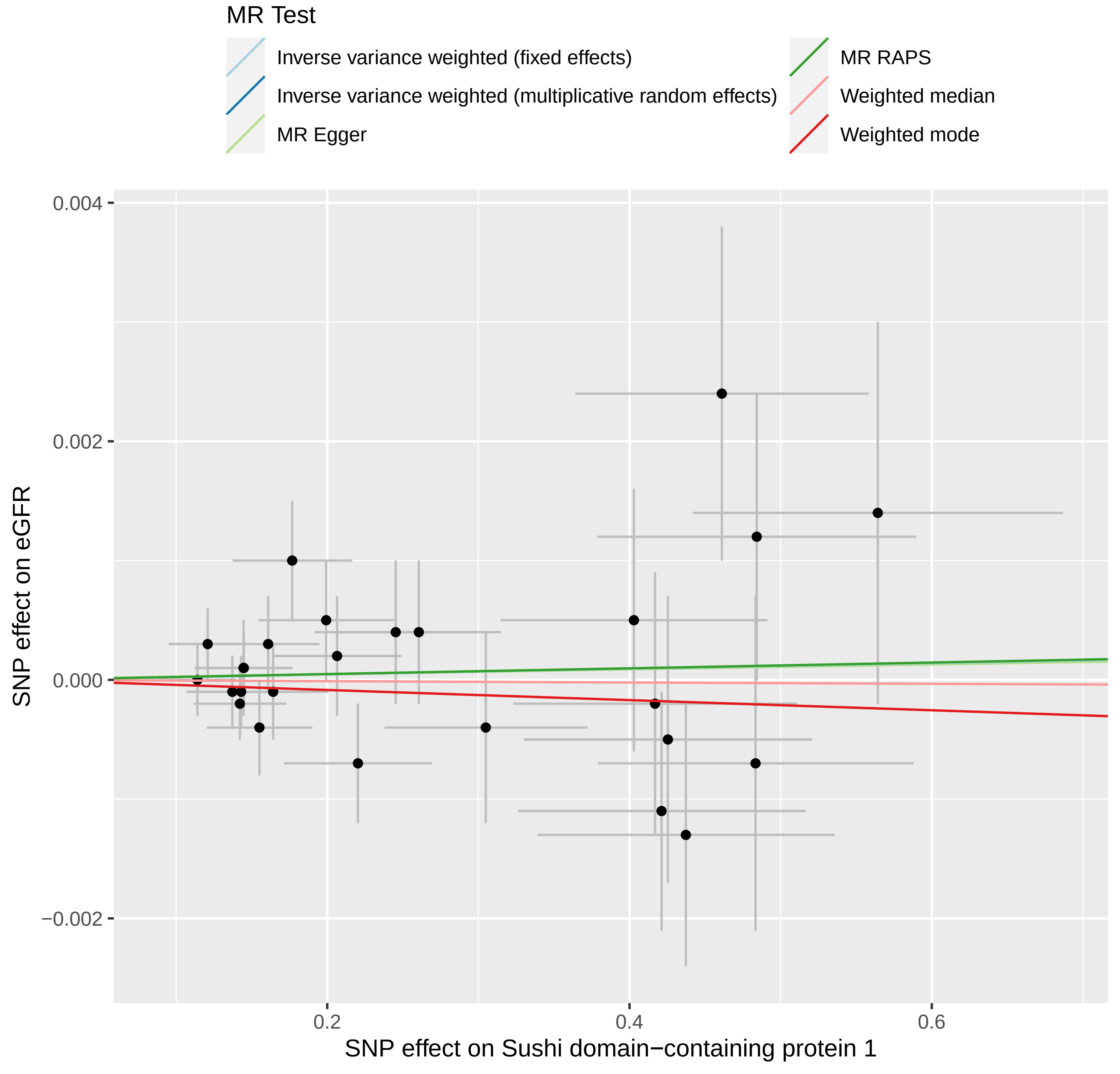

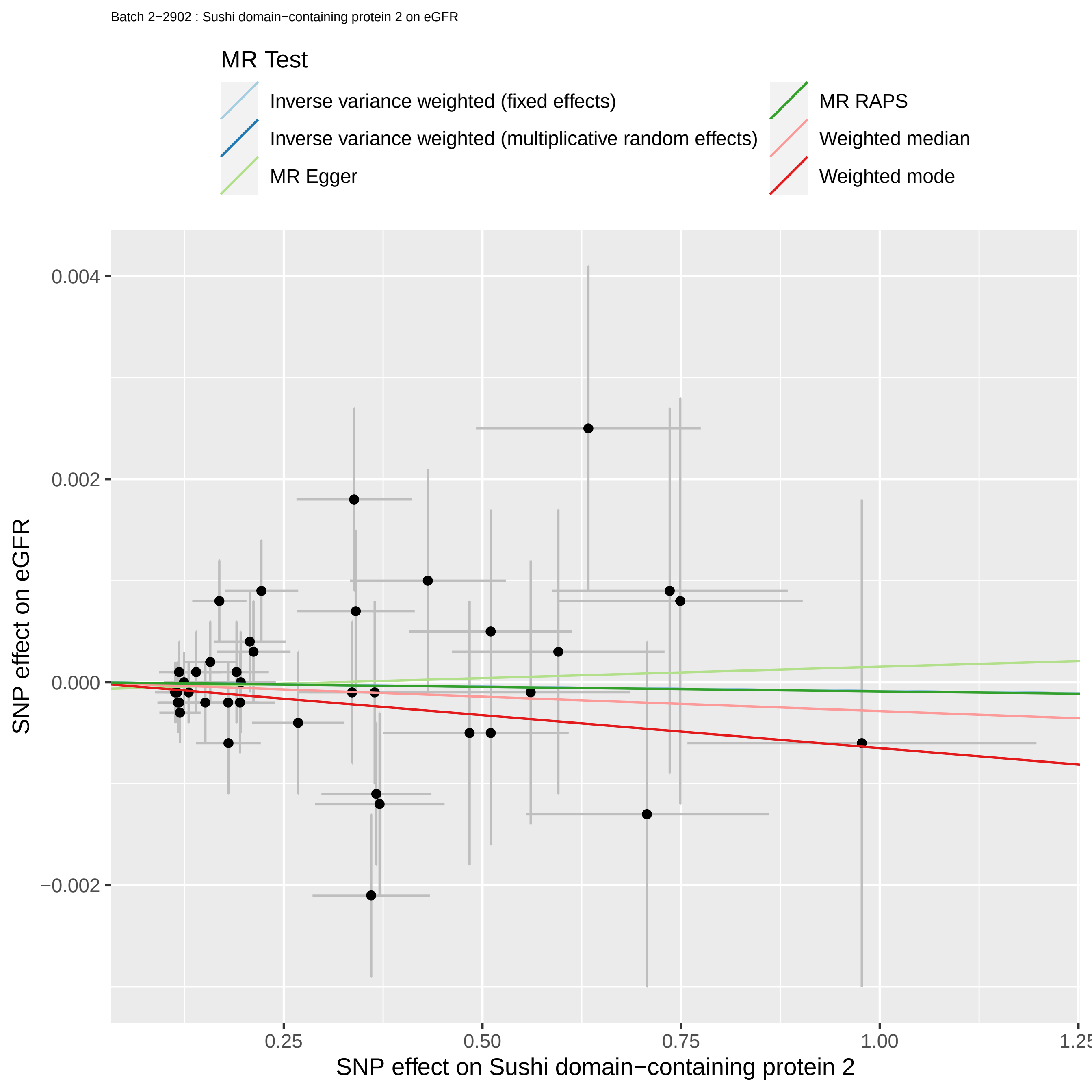

Batch 2−2902 : Sushi domain−containing protein 2 on eGFR
MR Test
Inverse variance weighted (fixed effects)
Inverse variance weighted (multiplicative random effects)
MR Egger
MR RAPS
Weighted median
Weighted mode
0.004
0.002
0.000
−0.002
SNP effect on eGFR
0.25
0.50
0.75
1.00
1.25
SNP effect on Sushi domain−containing protein 2

Batch 2−2903 : Sushi domain−containing protein 3 on eGFR

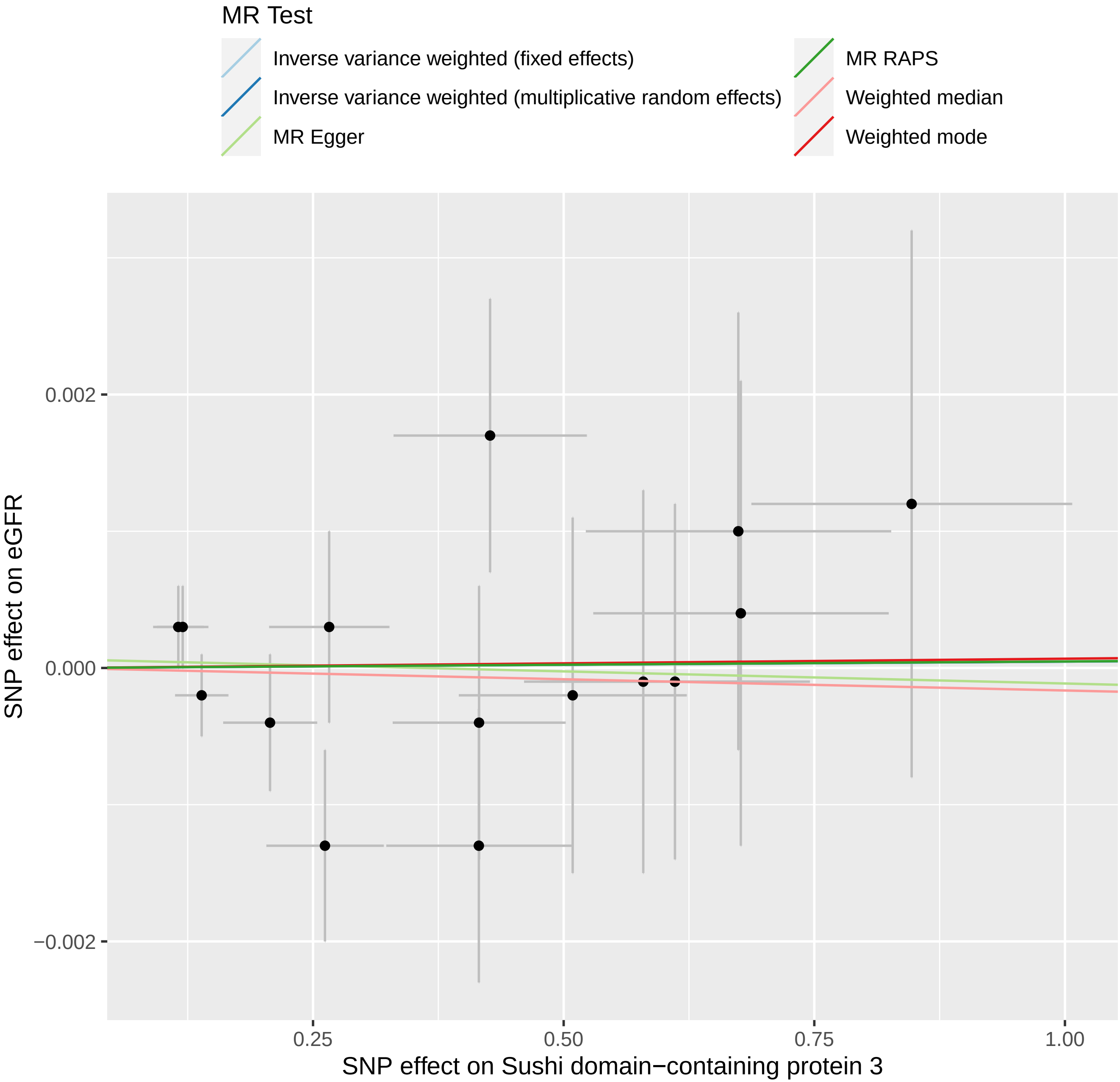

Batch 2−2904 : Histone−lysine N−methyltransferase SUV420H2 on eGFR

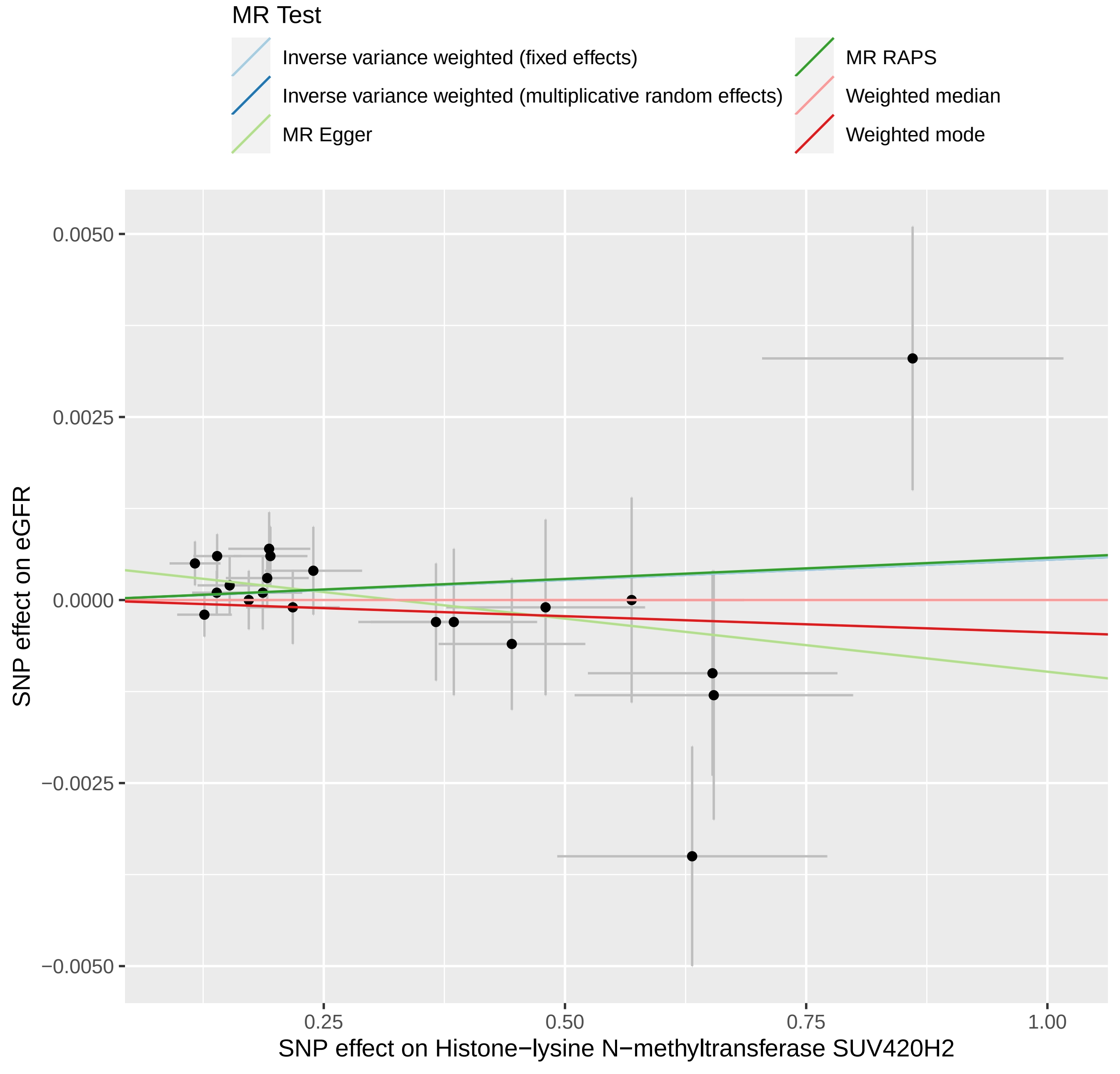

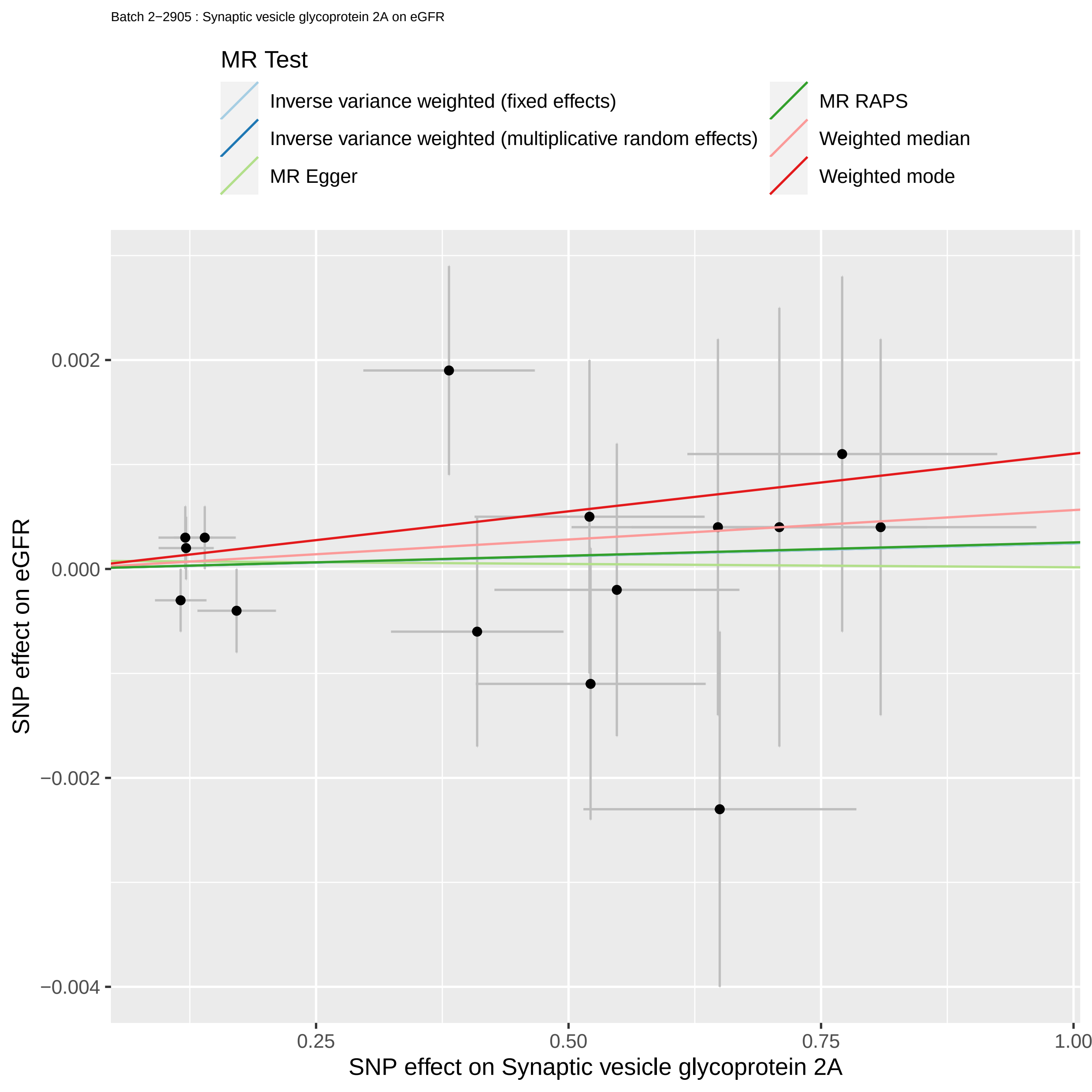

Batch 2−2905 : Synaptic vesicle glycoprotein 2A on eGFR
MR Test
Inverse variance weighted (fixed effects)
Inverse variance weighted (multiplicative random effects)
MR Egger
MR RAPS
Weighted median
Weighted mode
0.002
0.000
−0.002
−0.004
0.25
0.50
0.75
1.00
SNP effect on eGFR
SNP effect on Synaptic vesicle glycoprotein 2A

Batch 2−2906 : Sushi, von Willebrand factor type A, EGF and pentraxin domain−containing protein 1 on eGFR

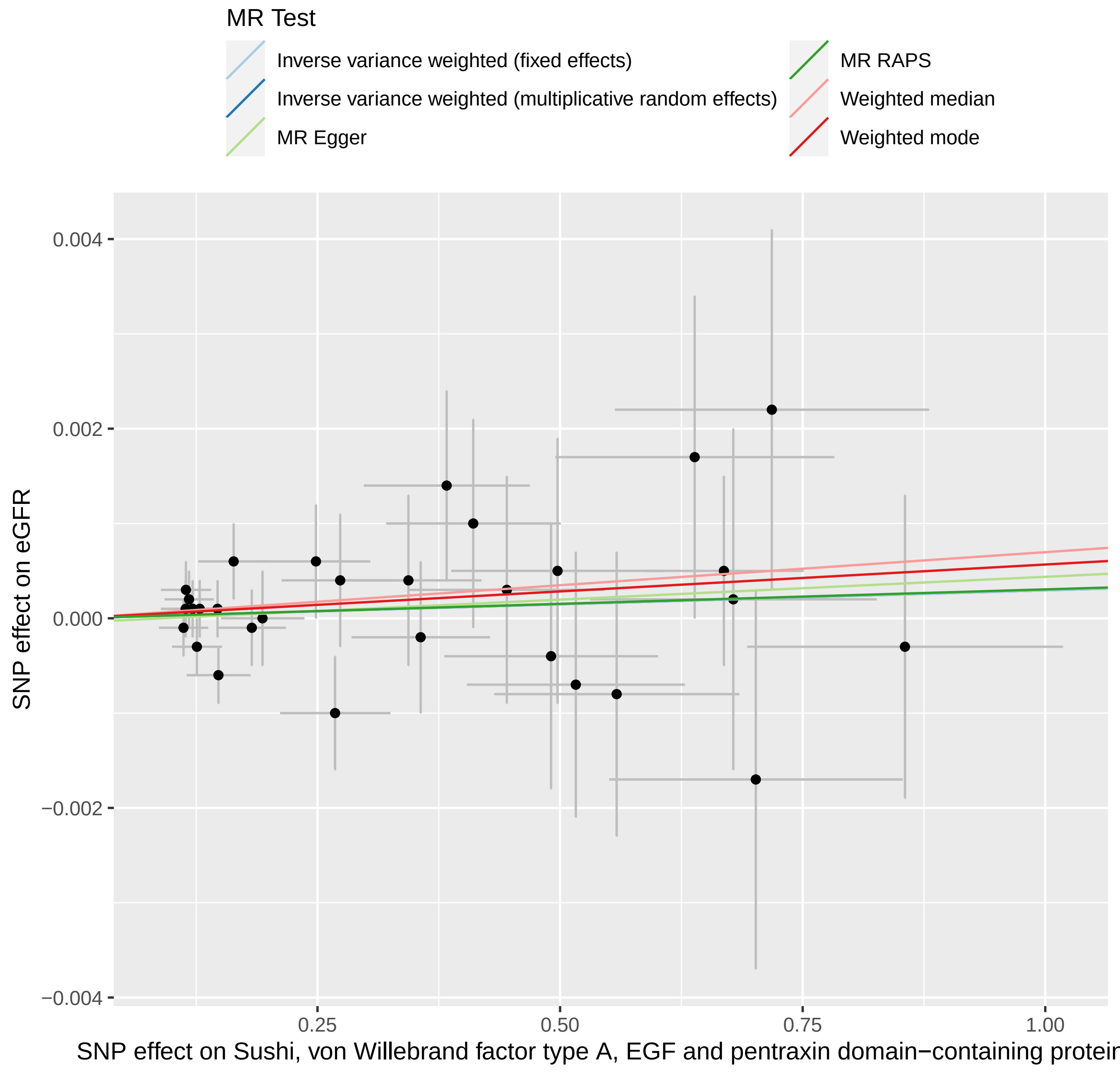

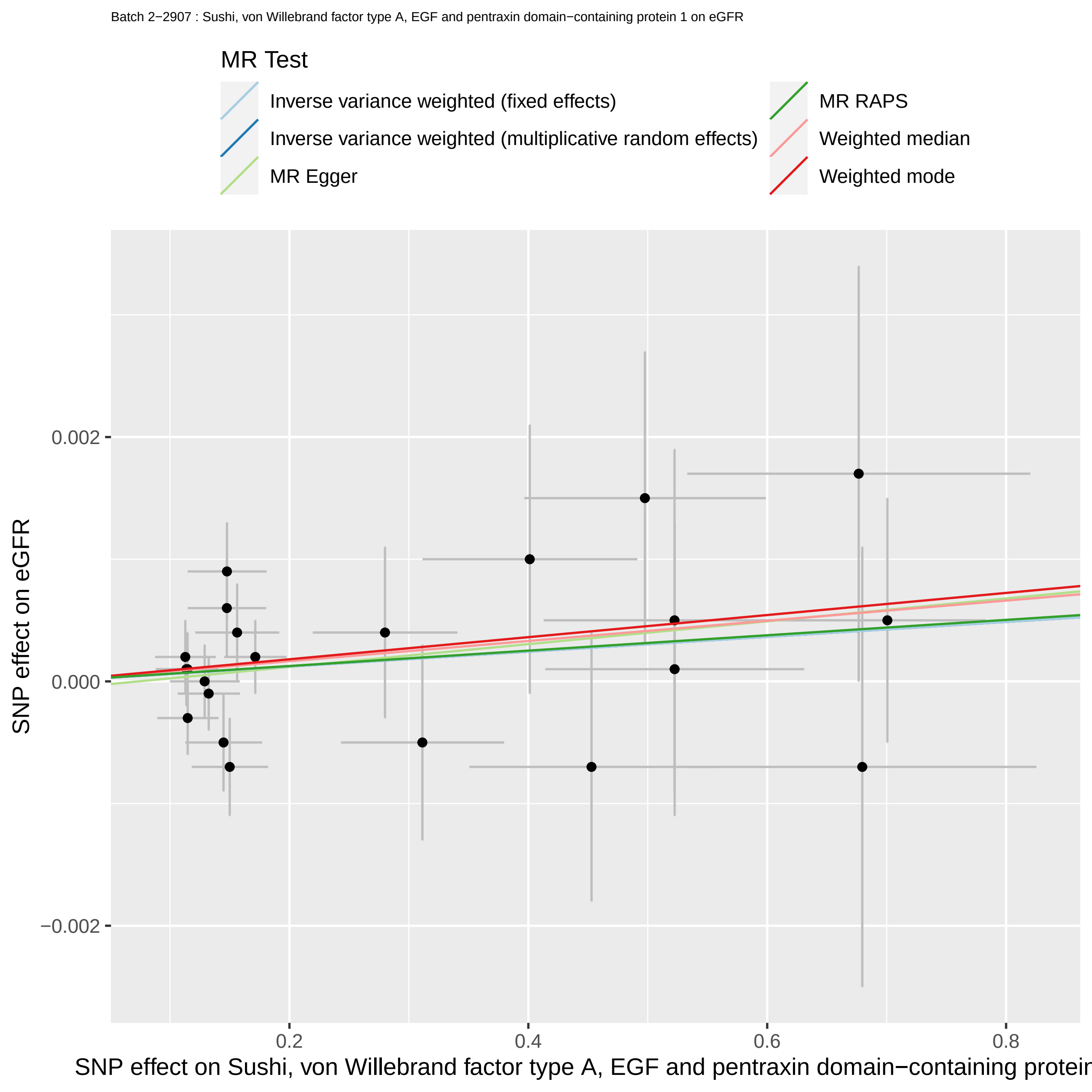
Batch 2−2907 : Sushi, von Willebrand factor type A, EGF and pentraxin domain−containing protein 1 on eGFR
MR Test
Inverse variance weighted (fixed effects)
Inverse variance weighted (multiplicative random effects)
MR Egger
MR RAPS
Weighted median
Weighted mode
0.002
0.000
−0.002
0.2
0.4
0.6
0.8
SNP effect on eGFR
SNP effect on Sushi, von Willebrand factor type A, EGF and pentraxin domain−containing protein

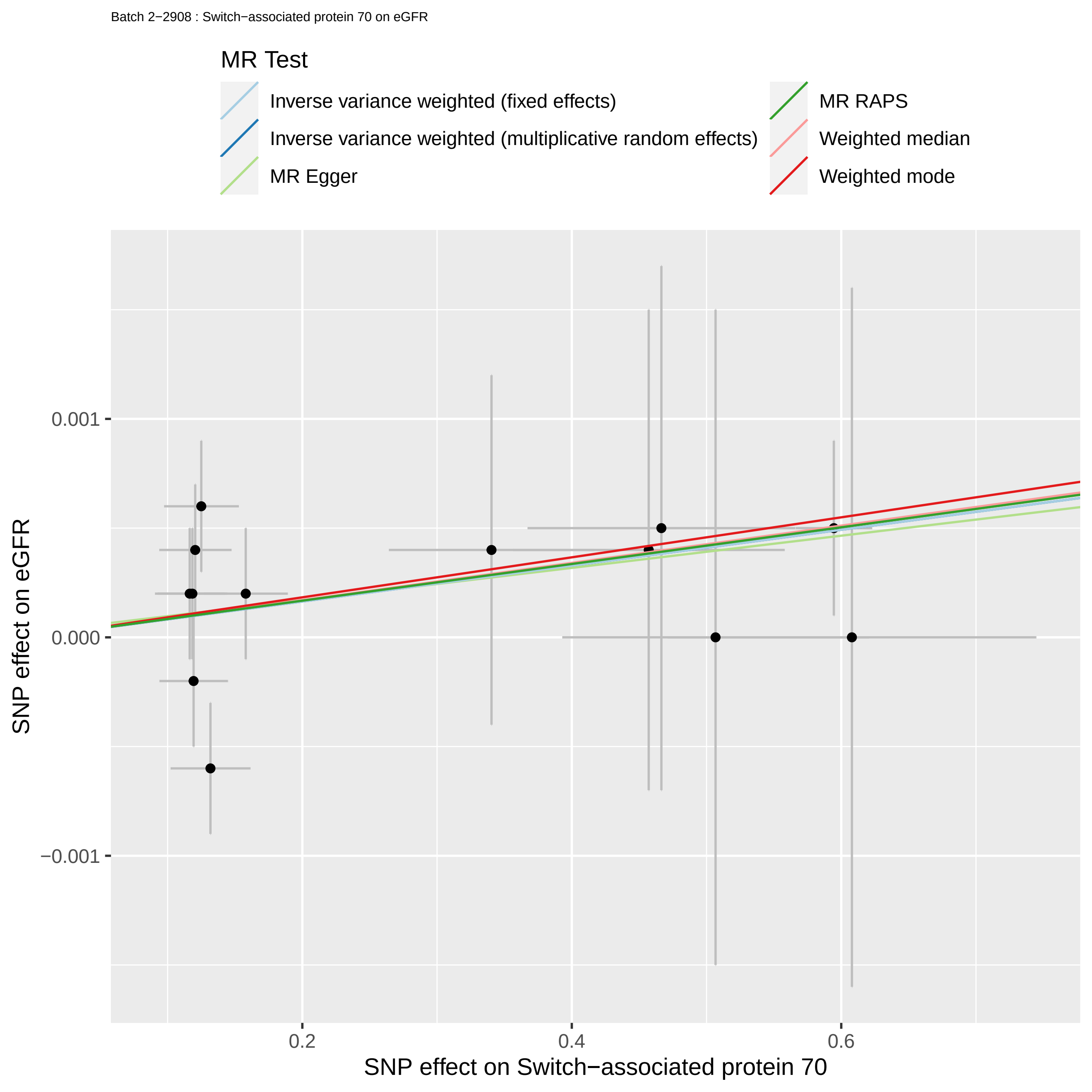
Batch 2−2908 : Switch−associated protein 70 on eGFR
MR Test
Inverse variance weighted (fixed effects)
Inverse variance weighted (multiplicative random effects)
MR Egger
MR RAPS
Weighted median
Weighted mode
0.001
0.000
−0.001
0.2
0.4
0.6
SNP effect on eGFR
SNP effect on Switch−associated protein 70

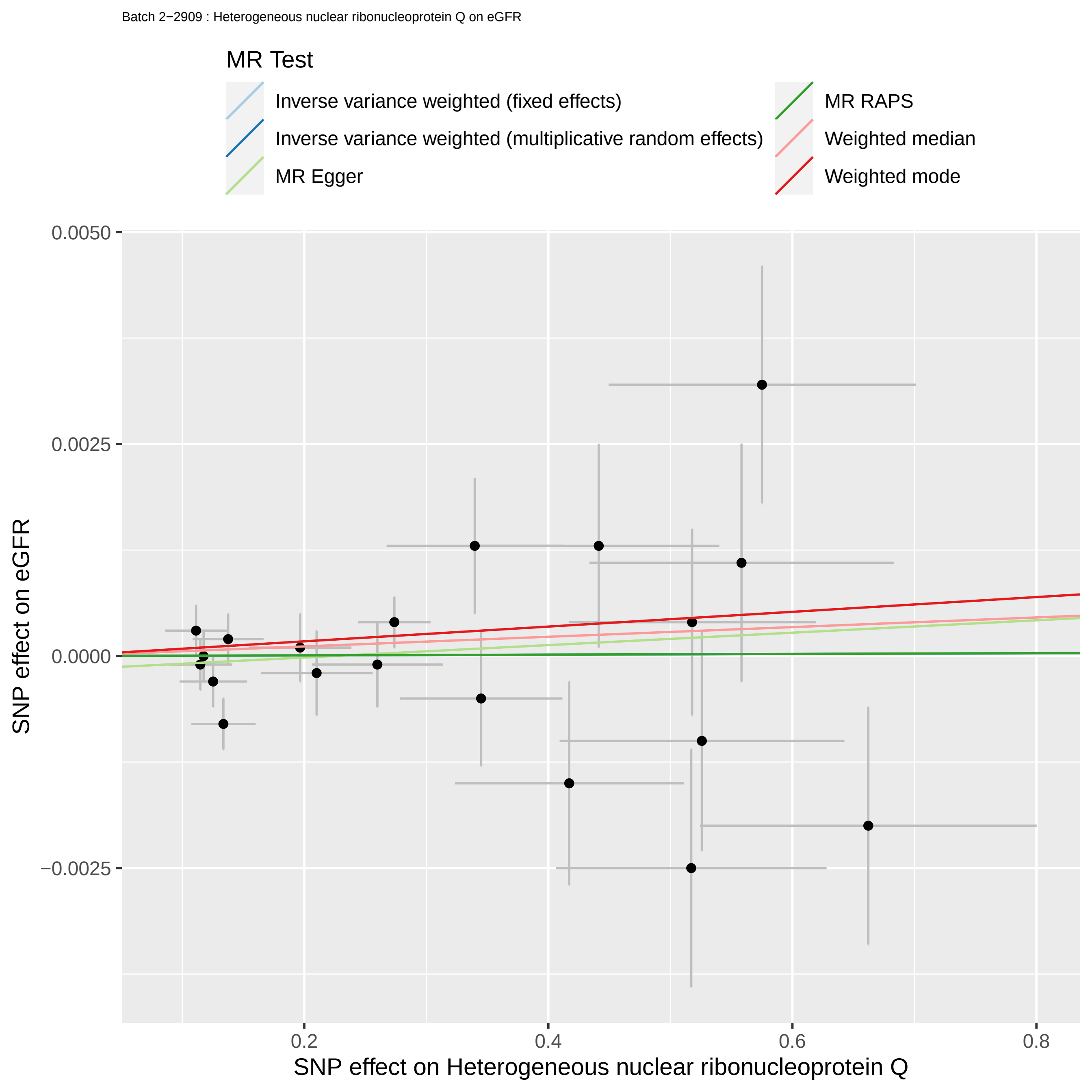

Batch 2−2909 : Heterogeneous nuclear ribonucleoprotein Q on eGFR
MR Test
Inverse variance weighted (fixed effects)
Inverse variance weighted (multiplicative random effects)
MR Egger
MR RAPS
Weighted median
Weighted mode
0.0050
0.0025
0.0000
−0.0025
SNP effect on eGFR
0.2
0.4
0.6
0.8
SNP effect on Heterogeneous nuclear ribonucleoprotein Q

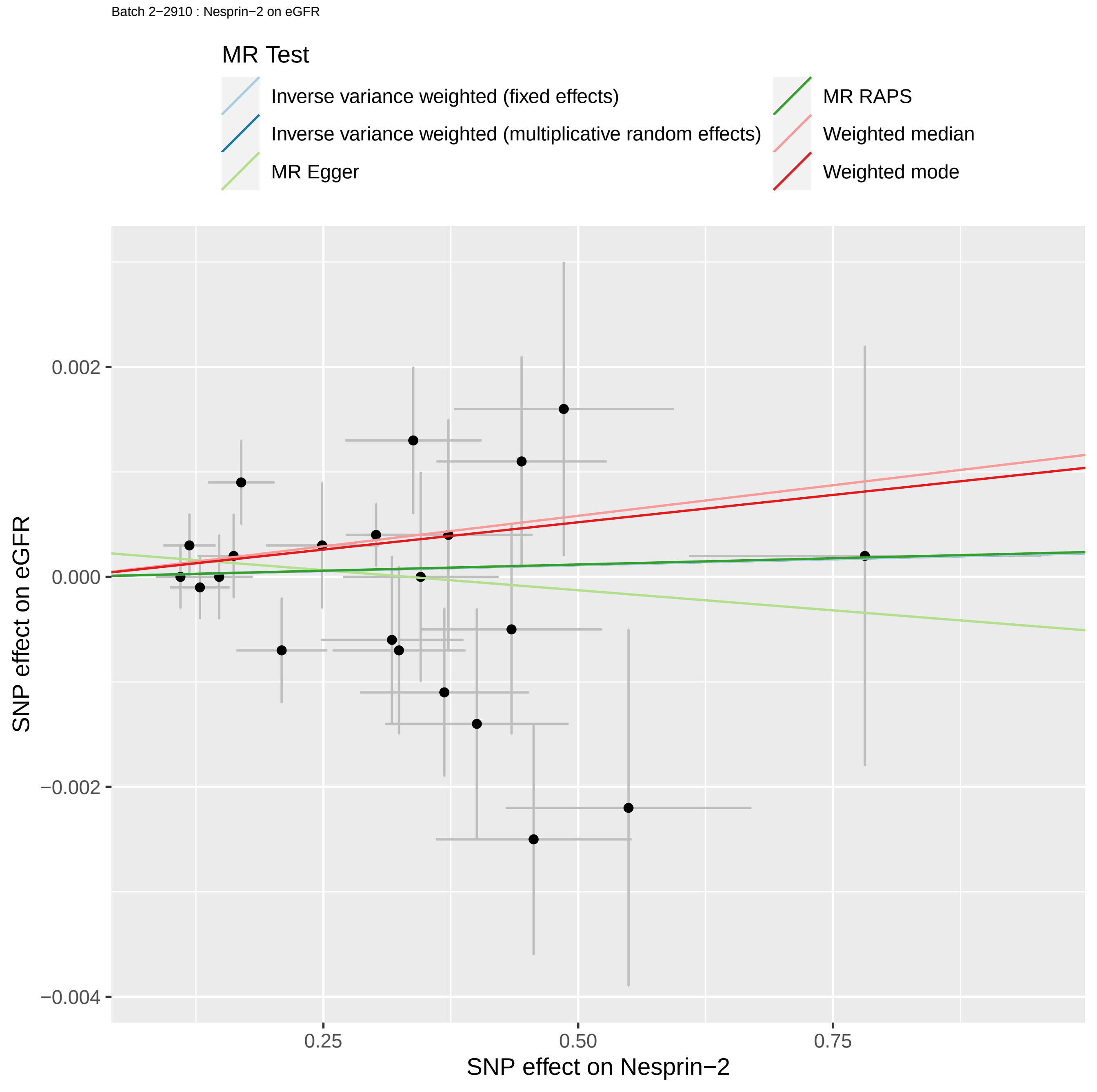
Batch 2−2910 : Nesprin−2 on eGFR
MR Test
Inverse variance weighted (fixed effects)
Inverse variance weighted (multiplicative random effects)
MR Egger
MR RAPS
Weighted median
Weighted mode
0.002
0.000
−0.002
−0.004
0.25
0.50
0.75
SNP effect on eGFR
SNP effect on Nesprin−2

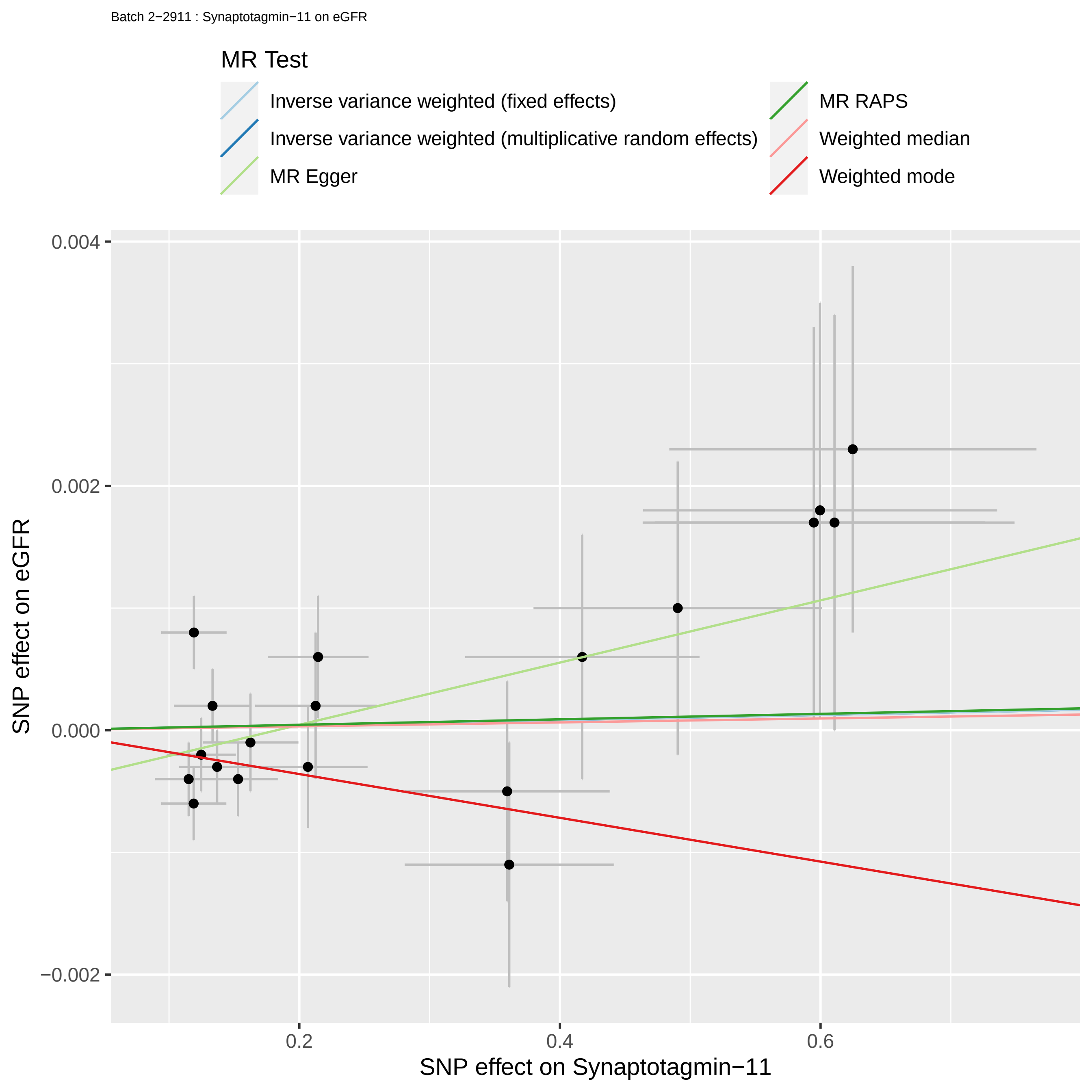
Batch 2−2911 : Synaptotagmin−11 on eGFR
MR Test
Inverse variance weighted (fixed effects)
Inverse variance weighted (multiplicative random effects)
MR Egger
MR RAPS
Weighted median
Weighted mode
0.004
0.002
0.000
−0.002
0.2
0.4
0.6
SNP effect on eGFR
SNP effect on Synaptotagmin−11

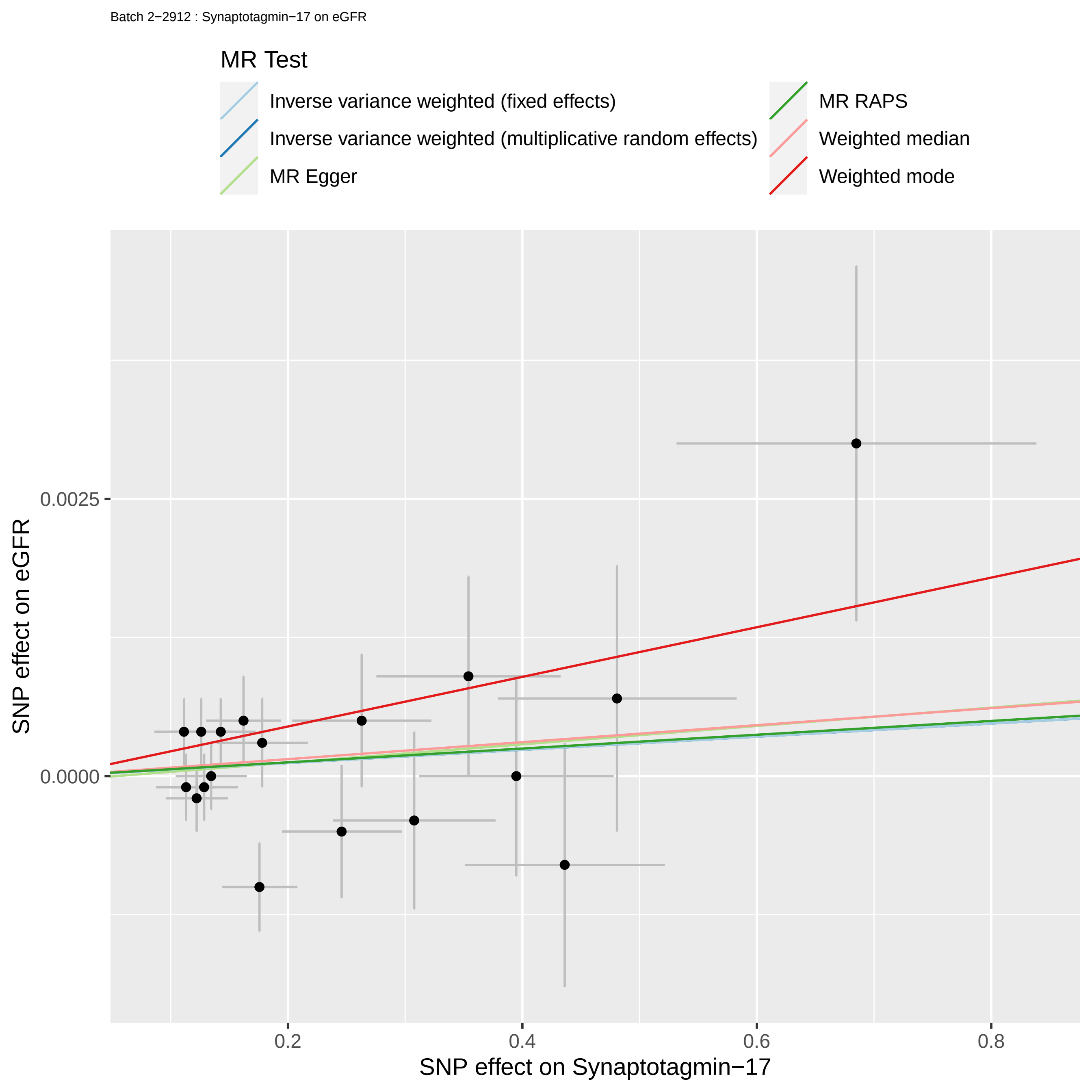

Batch 2−2912 : Synaptotagmin−17 on eGFR
MR Test
Inverse variance weighted (fixed effects)
Inverse variance weighted (multiplicative random effects)
MR Egger
MR RAPS
Weighted median
Weighted mode
0.0025
0.0000
0.2
0.4
0.6
0.8
SNP effect on eGFR
SNP effect on Synaptotagmin−17

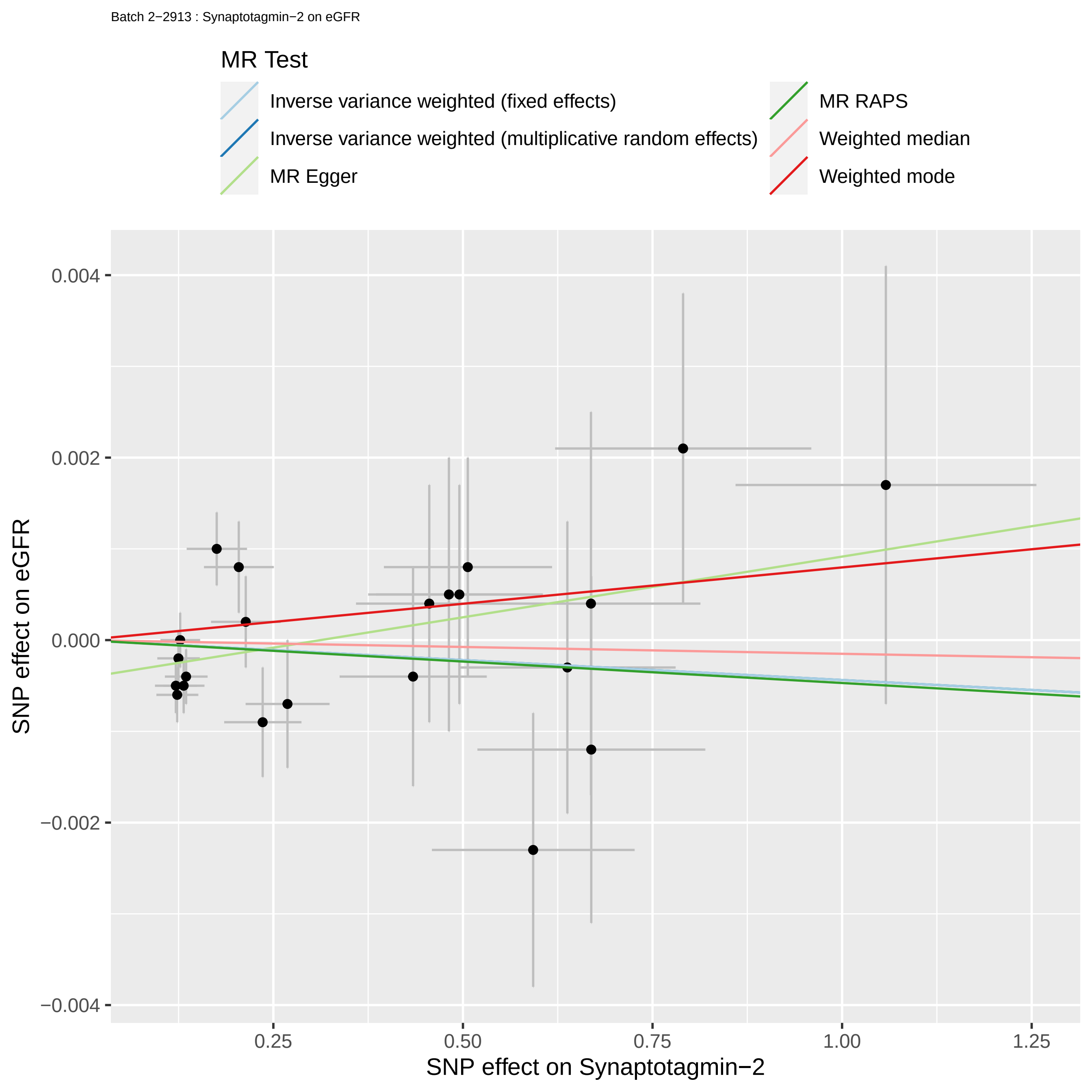
Batch 2−2913 : Synaptotagmin−2 on eGFR
MR Test
Inverse variance weighted (fixed effects)
Inverse variance weighted (multiplicative random effects)
MR Egger
MR RAPS
Weighted median
Weighted mode
0.004
0.002
0.000
−0.002
−0.004
SNP effect on eGFR
0.25
0.50
0.75
1.00
1.25
SNP effect on Synaptotagmin−2

Batch 2−2914 : Synaptotagmin−3 on eGFR

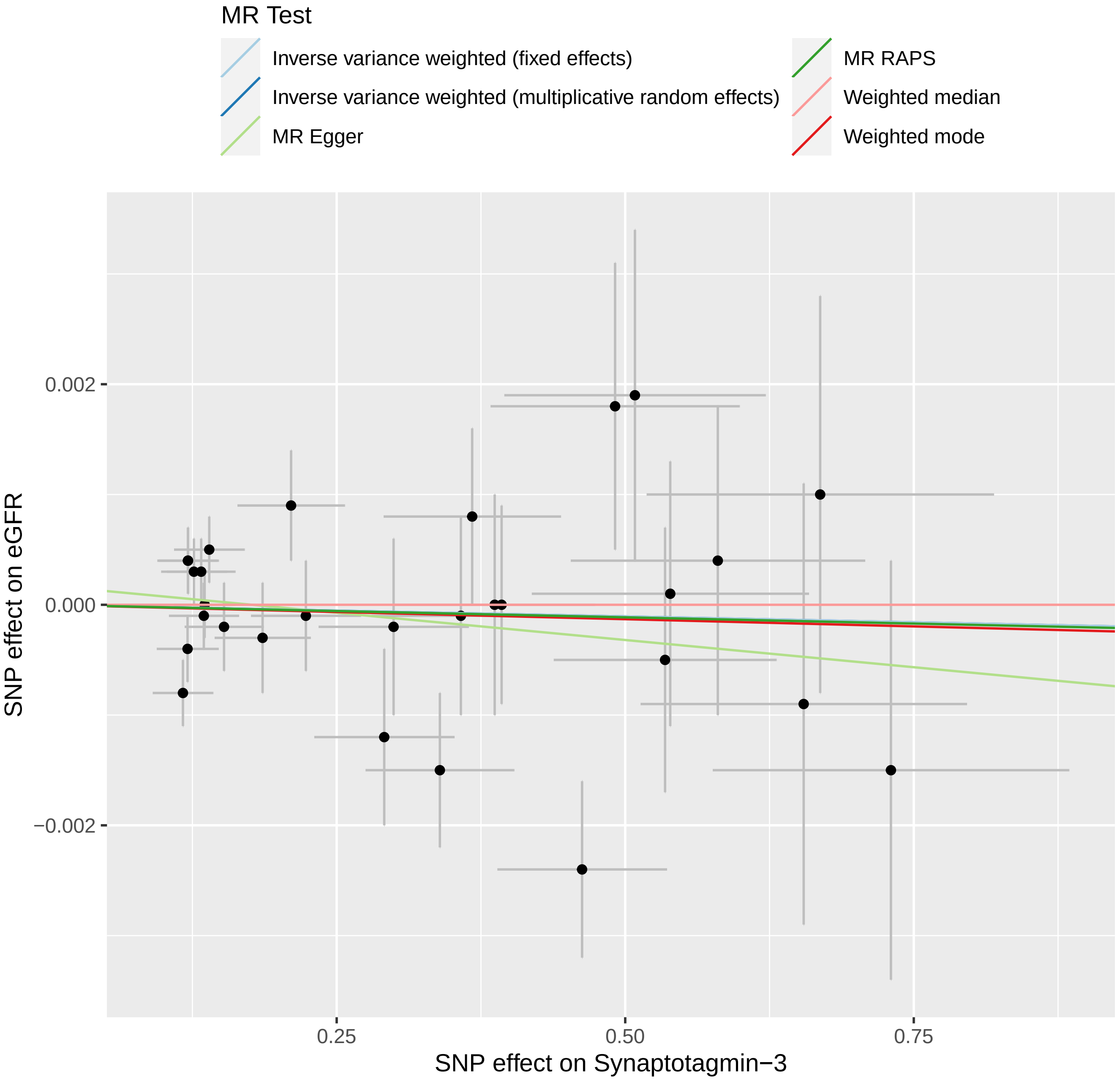

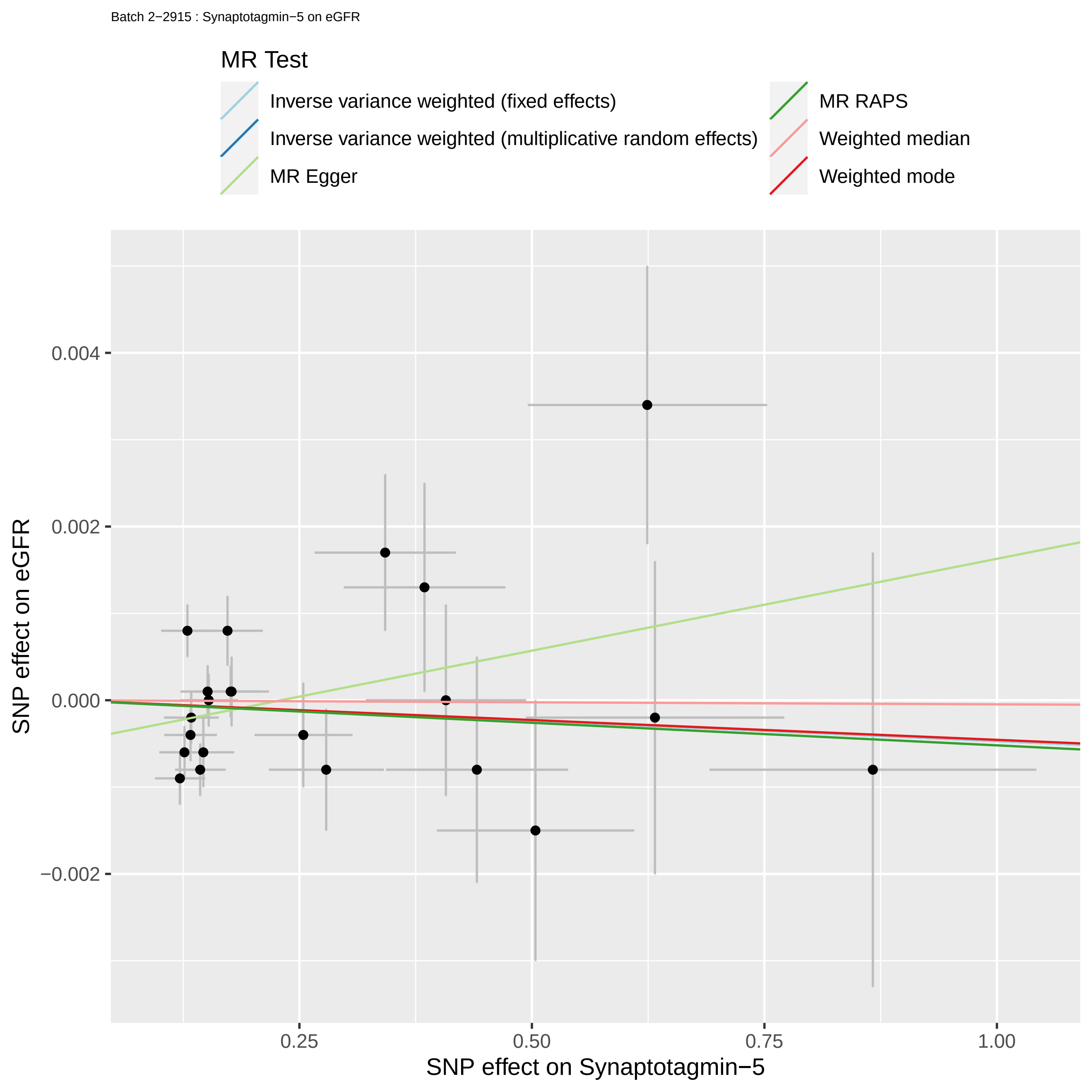
Batch 2−2915 : Synaptotagmin−5 on eGFR
MR Test
Inverse variance weighted (fixed effects)
Inverse variance weighted (multiplicative random effects)
MR Egger
MR RAPS
Weighted median
Weighted mode
0.004
0.002
0.000
−0.002
SNP effect on eGFR
0.25
0.50
0.75
1.00
SNP effect on Synaptotagmin−5

Batch 2−2916 : Synaptotagmin−7 on eGFR

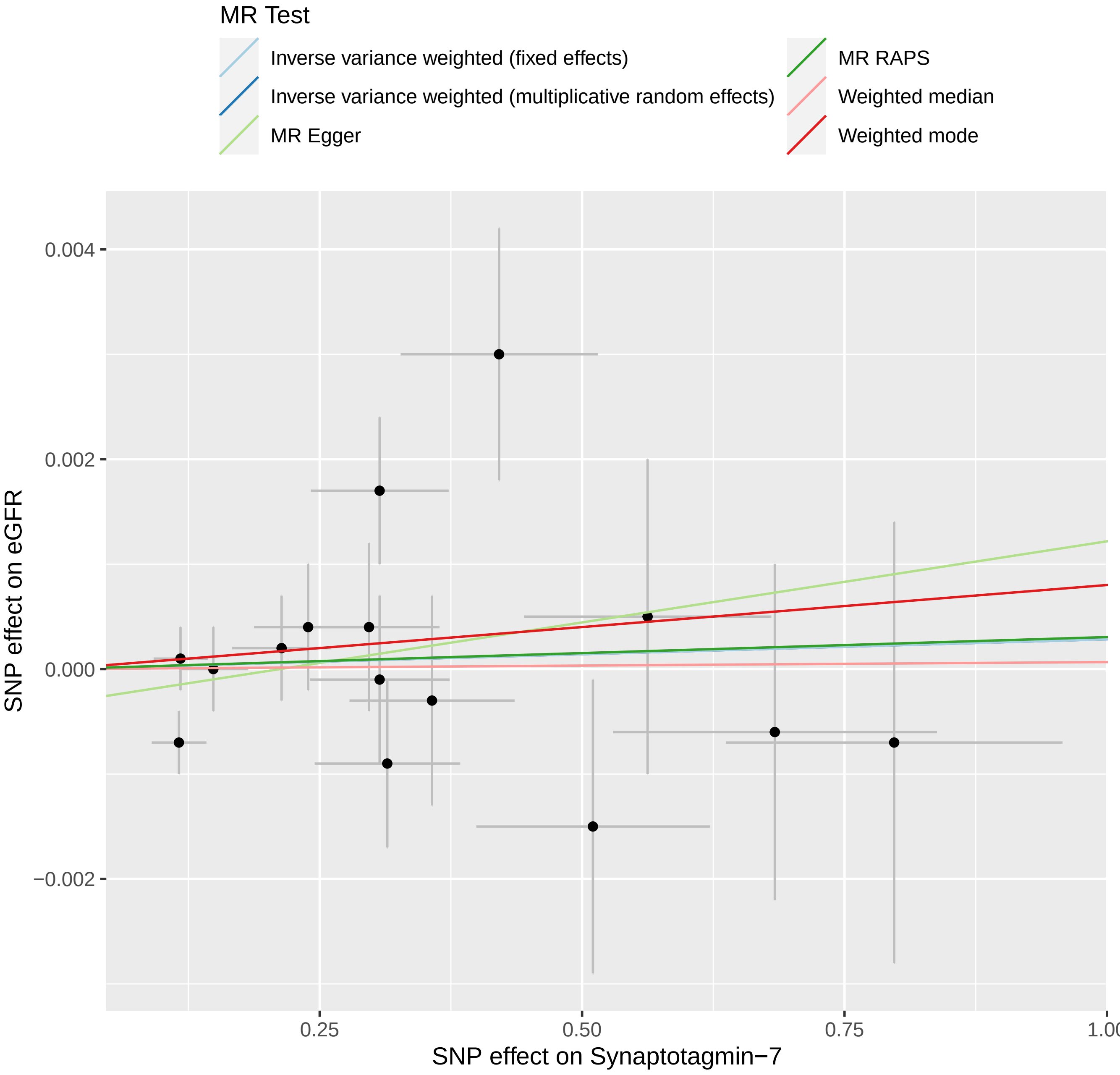

Batch 2−2917 : Synaptotagmin−8 on eGFR

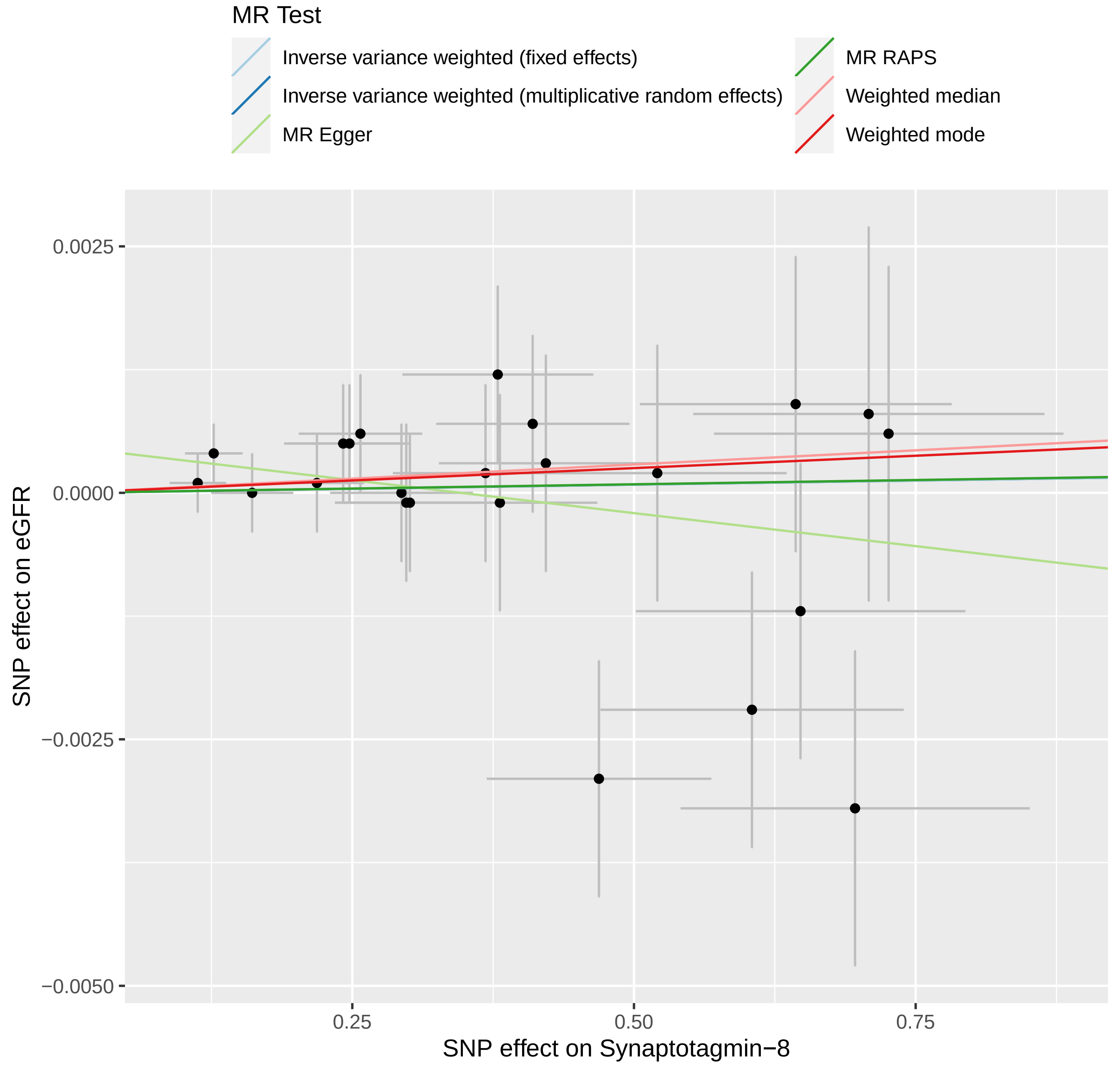

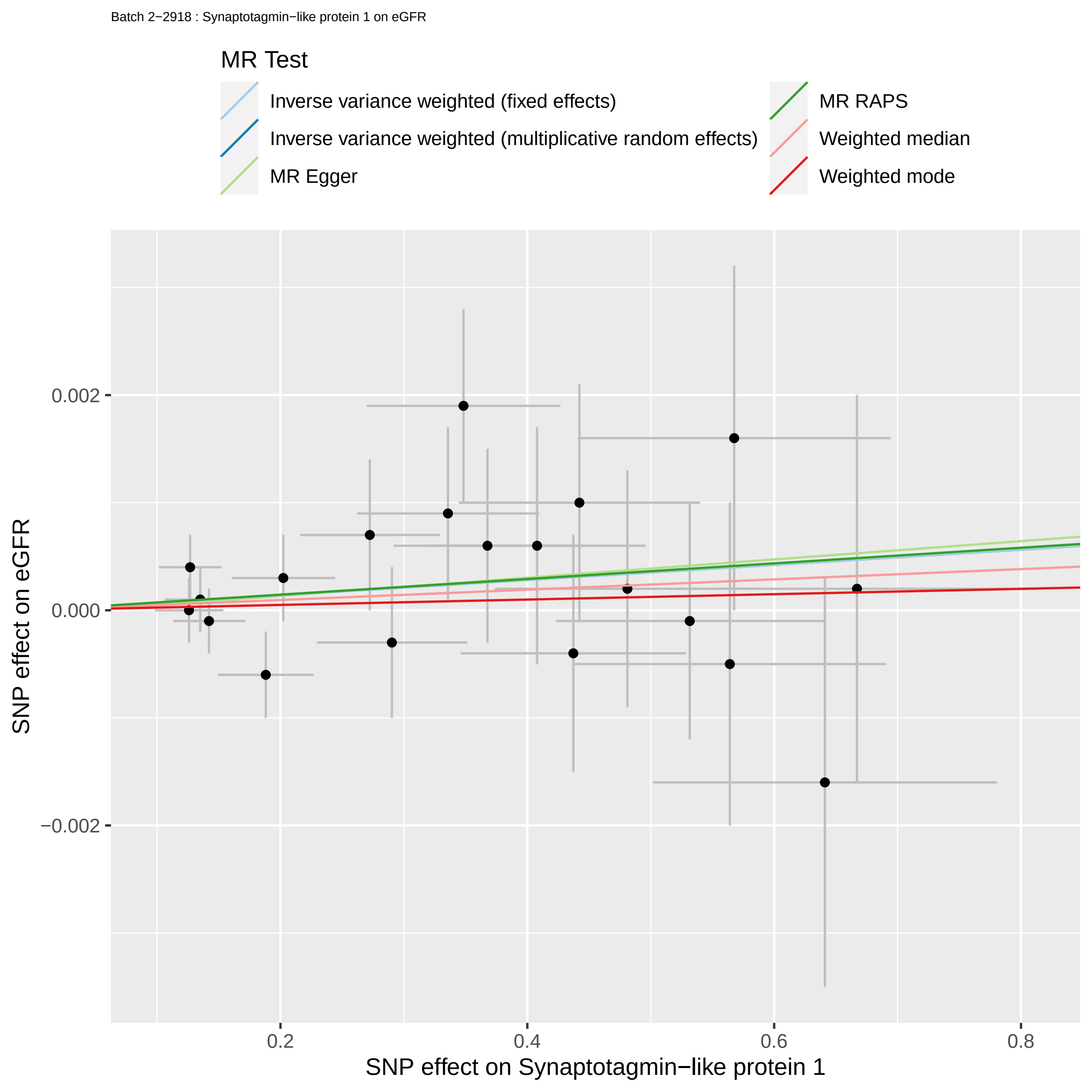

Batch 2−2918 : Synaptotagmin−like protein 1 on eGFR
MR Test
Inverse variance weighted (fixed effects)
Inverse variance weighted (multiplicative random effects)
MR Egger
MR RAPS
Weighted median
Weighted mode
0.002
0.000
−0.002
0.2
0.4
0.6
0.8
SNP effect on eGFR
SNP effect on Synaptotagmin−like protein 1

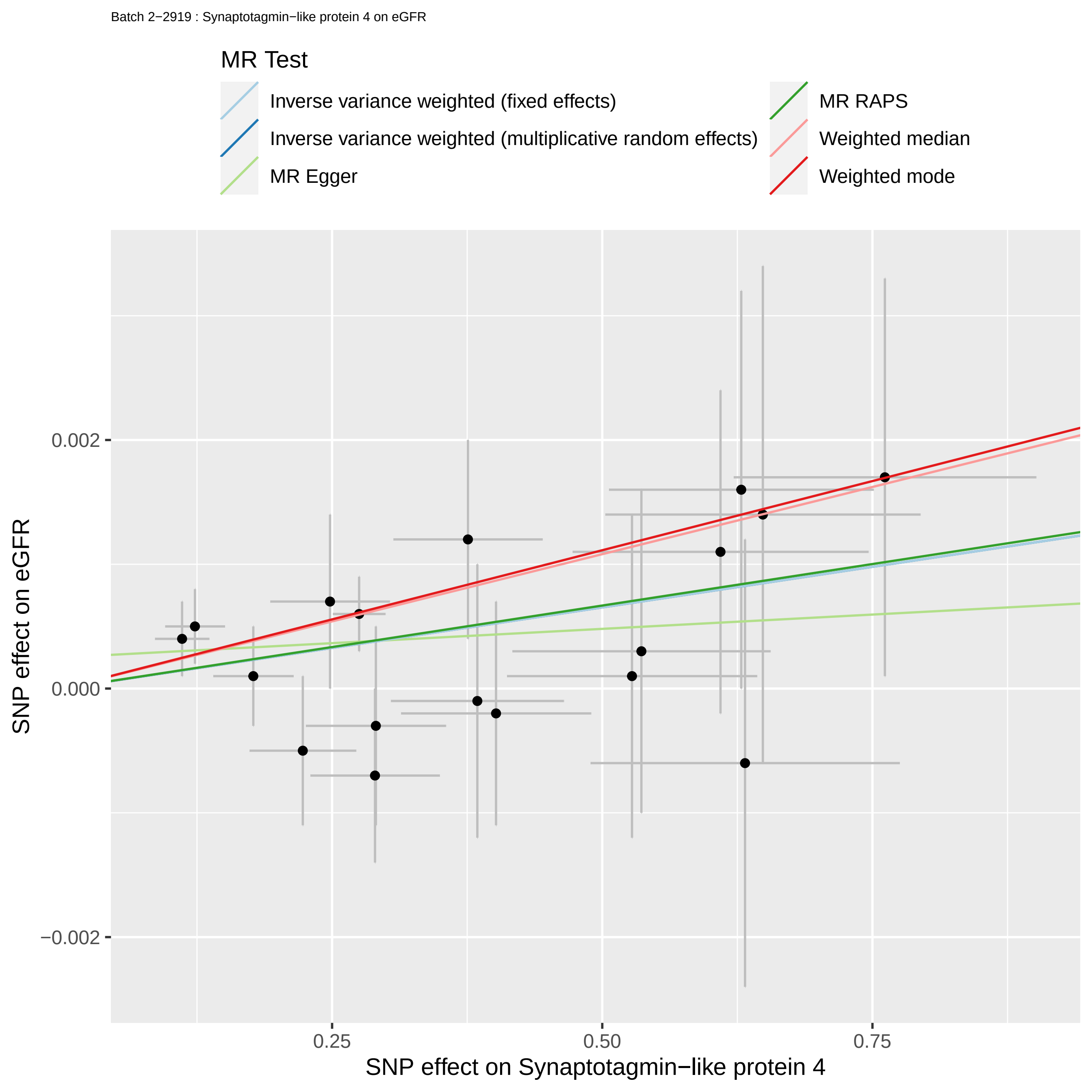
Batch 2−2919 : Synaptotagmin−like protein 4 on eGFR
MR Test
Inverse variance weighted (fixed effects)
Inverse variance weighted (multiplicative random effects)
MR Egger
MR RAPS
Weighted median
Weighted mode
SNP effect on eGFR
0.002
0.000
−0.002
0.25
0.50
0.75
SNP effect on Synaptotagmin−like protein 4

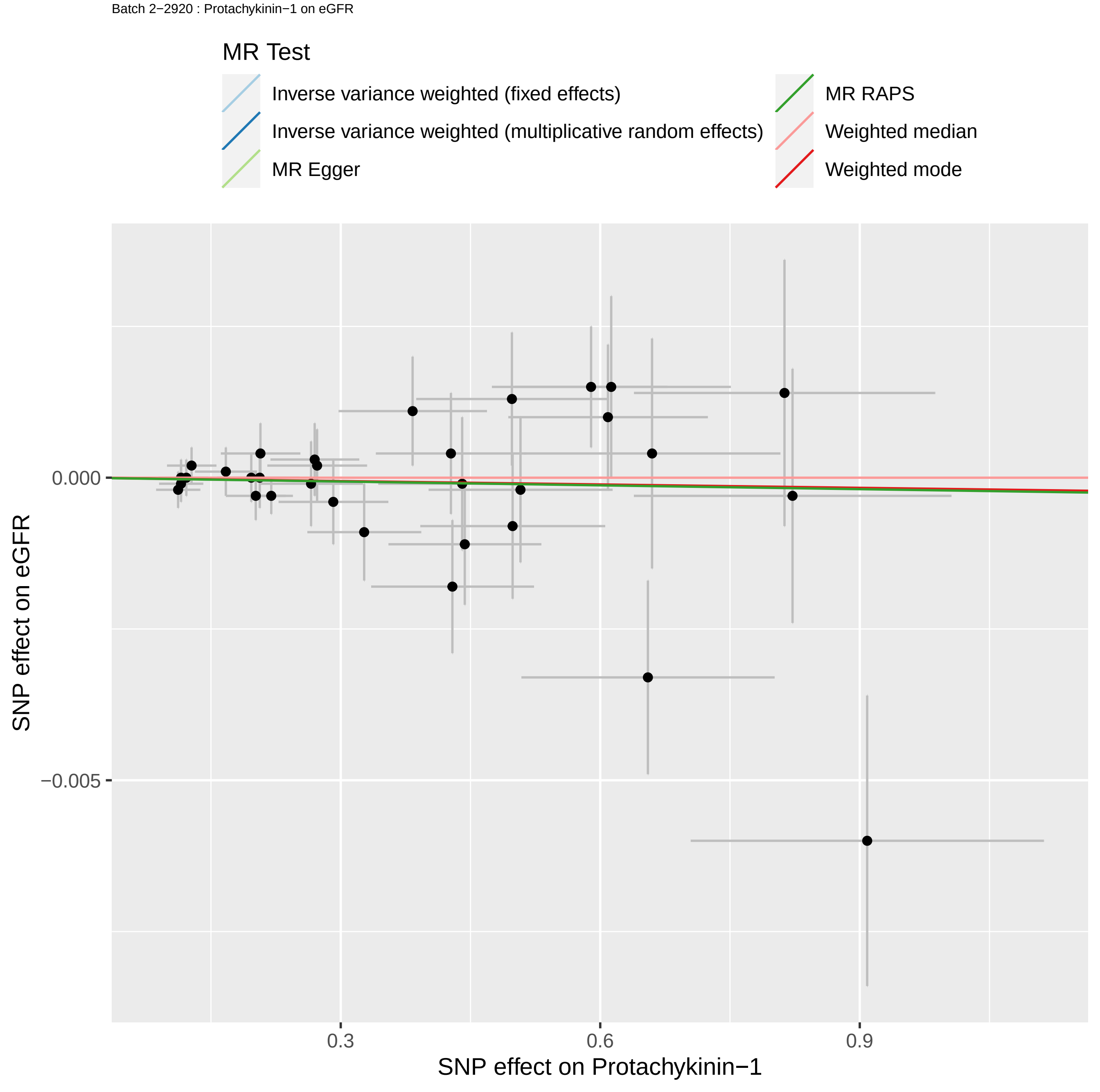
Batch 2−2920 : Protachykinin−1 on eGFR
MR Test
Inverse variance weighted (fixed effects)
Inverse variance weighted (multiplicative random effects)
MR Egger
MR RAPS
Weighted median
Weighted mode
0.000
−0.005
0.3
0.6
0.9
SNP effect on eGFR
SNP effect on Protachykinin−1

Batch 2−2921 : Tachykinin−3 on eGFR

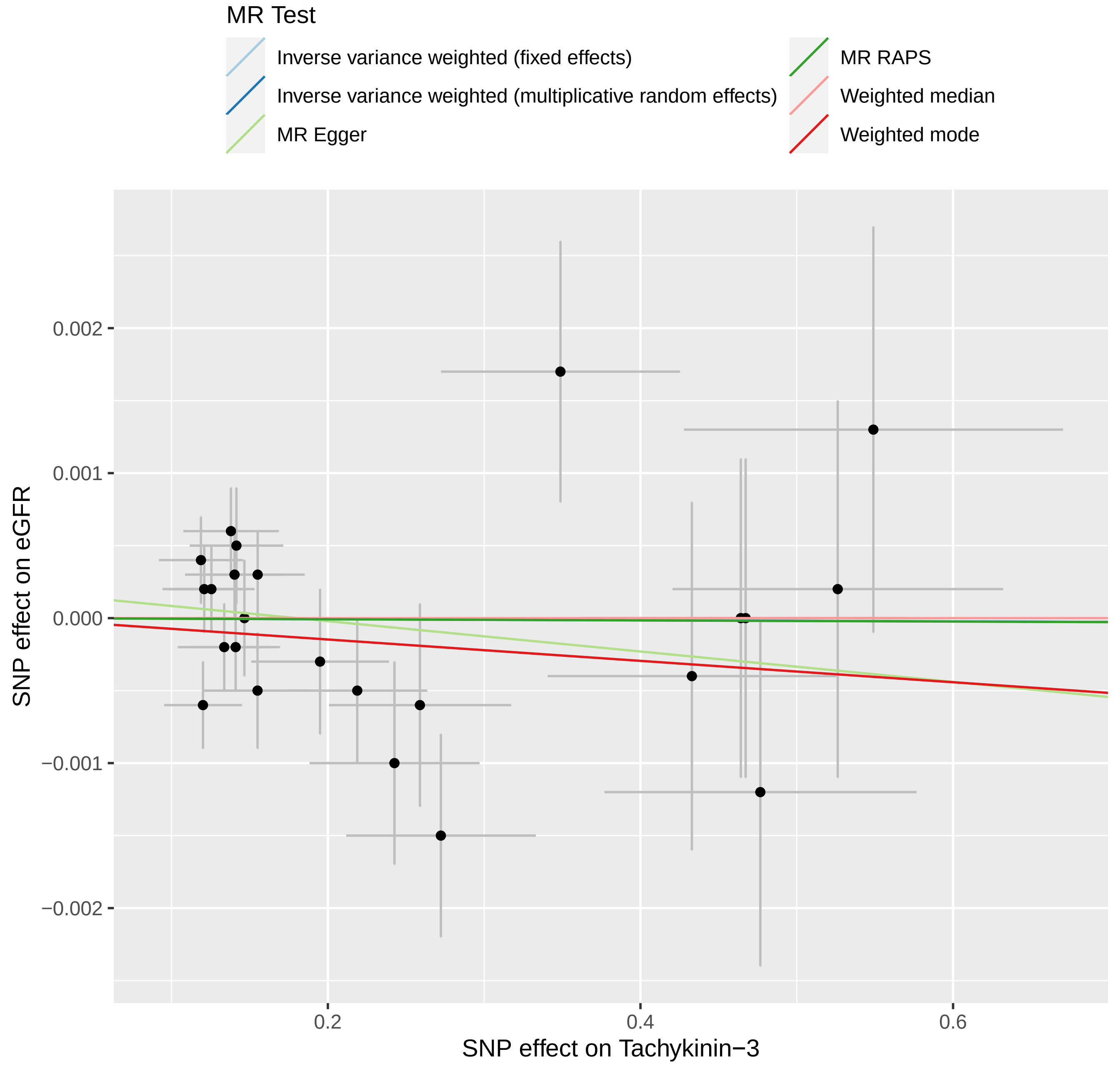

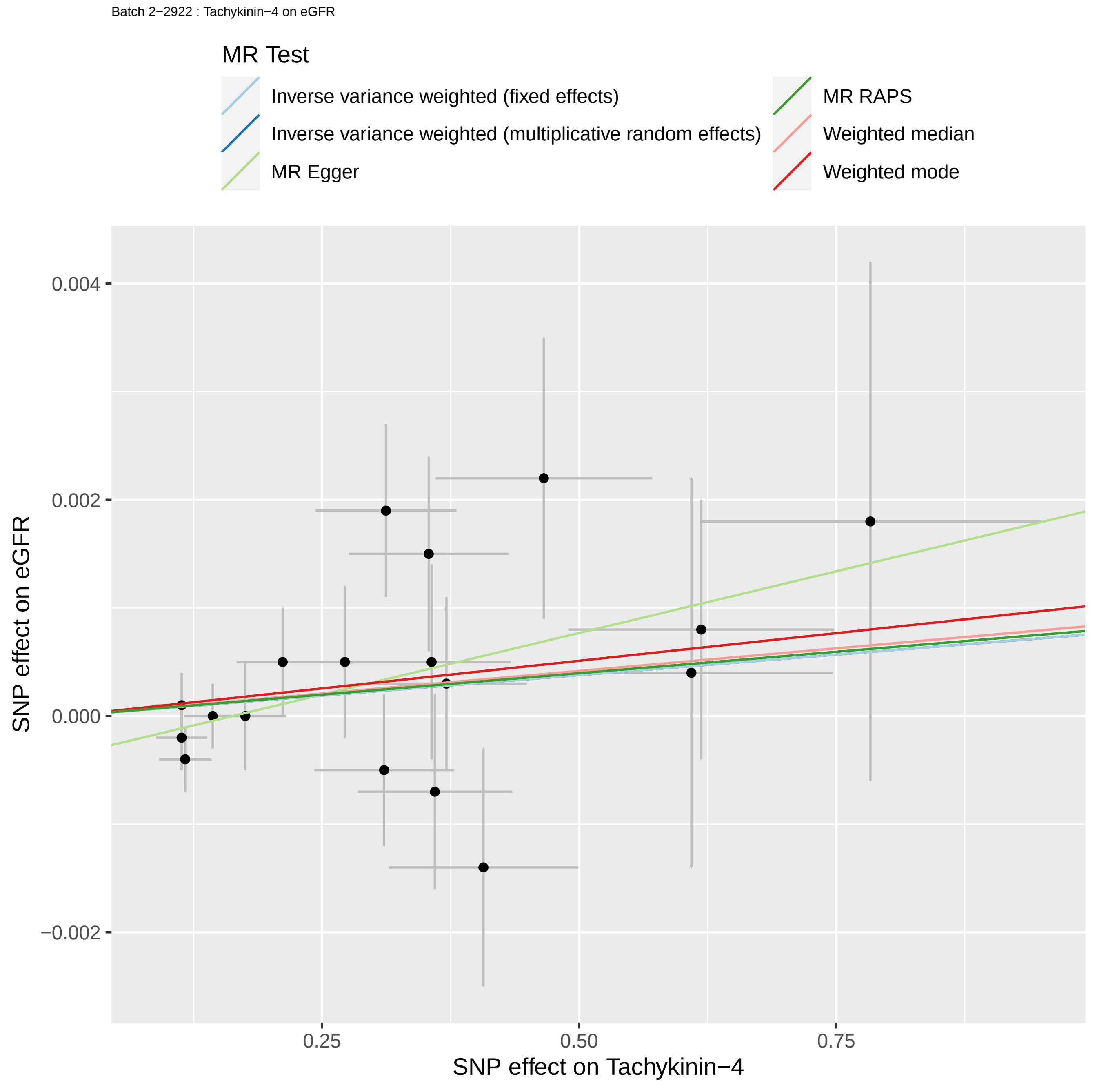
Batch 2−2922 : Tachykinin−4 on eGFR
MR Test
Inverse variance weighted (fixed effects)
Inverse variance weighted (multiplicative random effects)
MR Egger
MR RAPS
Weighted median
Weighted mode
0.004
0.002
0.000
−0.002
SNP effect on eGFR
0.25
0.50
0.75
SNP effect on Tachykinin−4

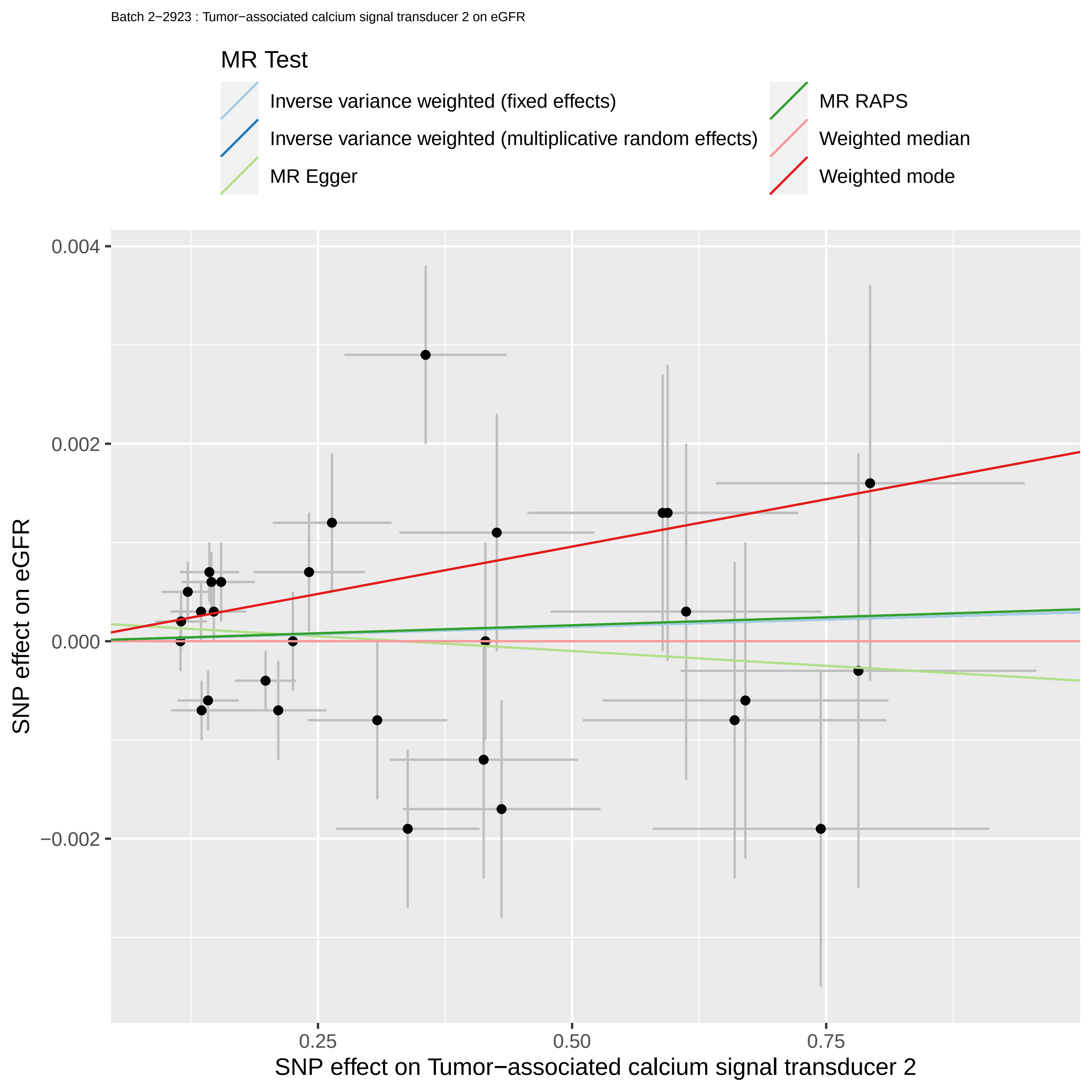

Batch 2−2923 : Tumor−associated calcium signal transducer 2 on eGFR
MR Test
Inverse variance weighted (fixed effects)
Inverse variance weighted (multiplicative random effects)
MR Egger
MR RAPS
Weighted median
Weighted mode
0.004
0.002
0.000
−0.002
0.25
0.50
0.75
SNP effect on eGFR
SNP effect on Tumor−associated calcium signal transducer 2

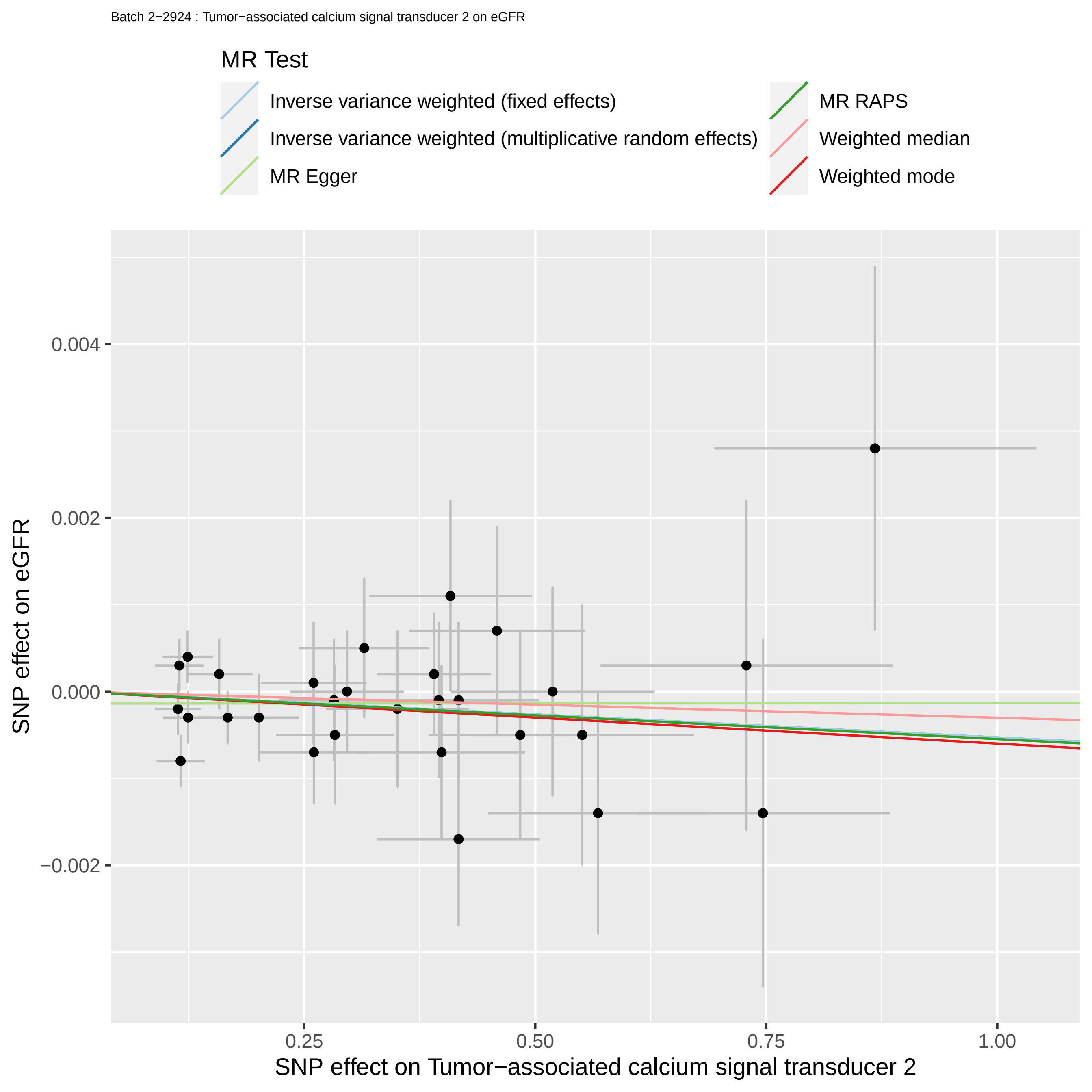
Batch 2−2924 : Tumor−associated calcium signal transducer 2 on eGFR
MR Test
Inverse variance weighted (fixed effects)
Inverse variance weighted (multiplicative random effects)
MR Egger
MR RAPS
Weighted median
Weighted mode
0.004
0.002
0.000
−0.002
0.25
0.50
0.75
1.00
SNP effect on eGFR
SNP effect on Tumor−associated calcium signal transducer 2

Batch 2−2925 : Transgelin on eGFR

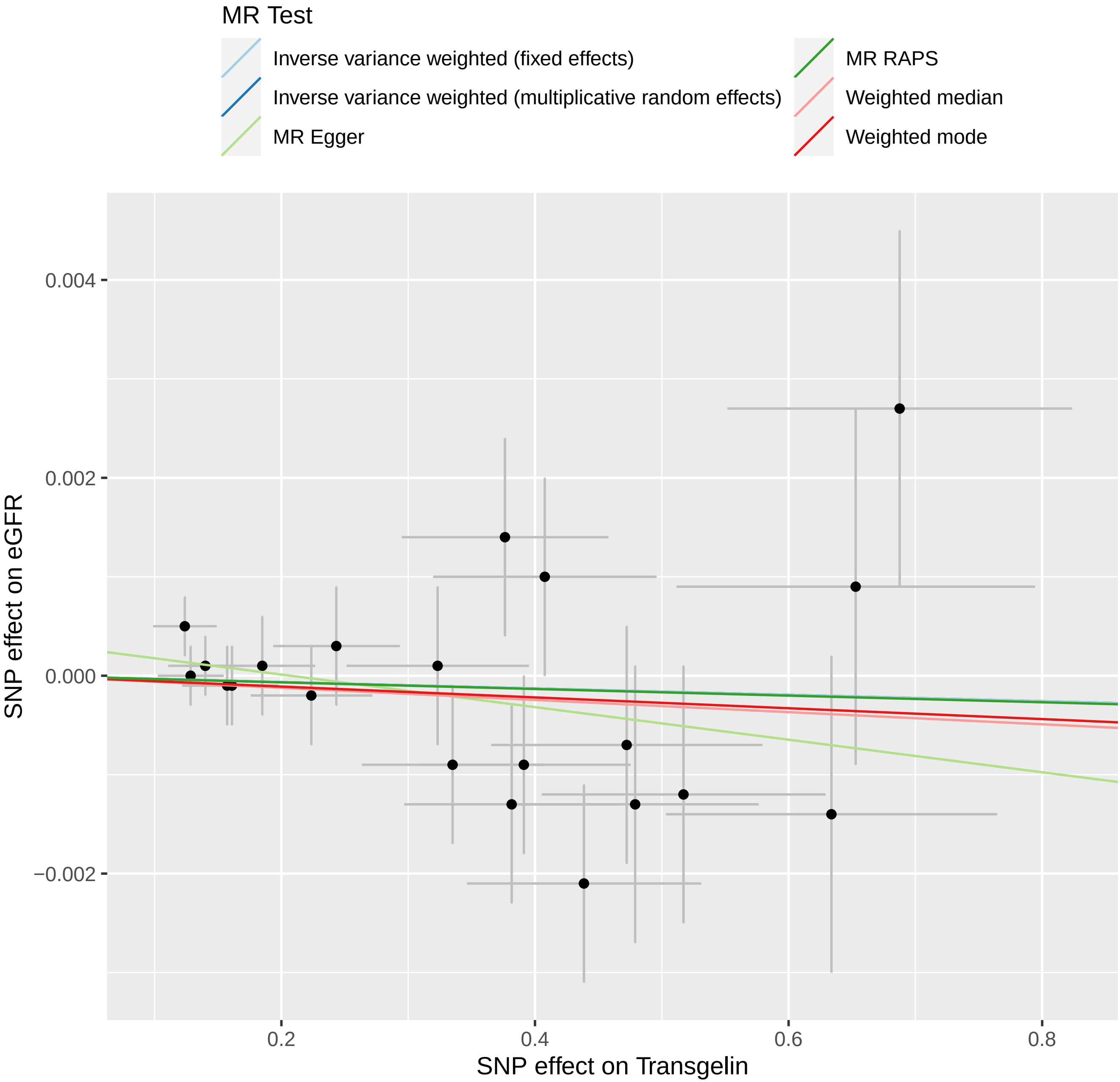

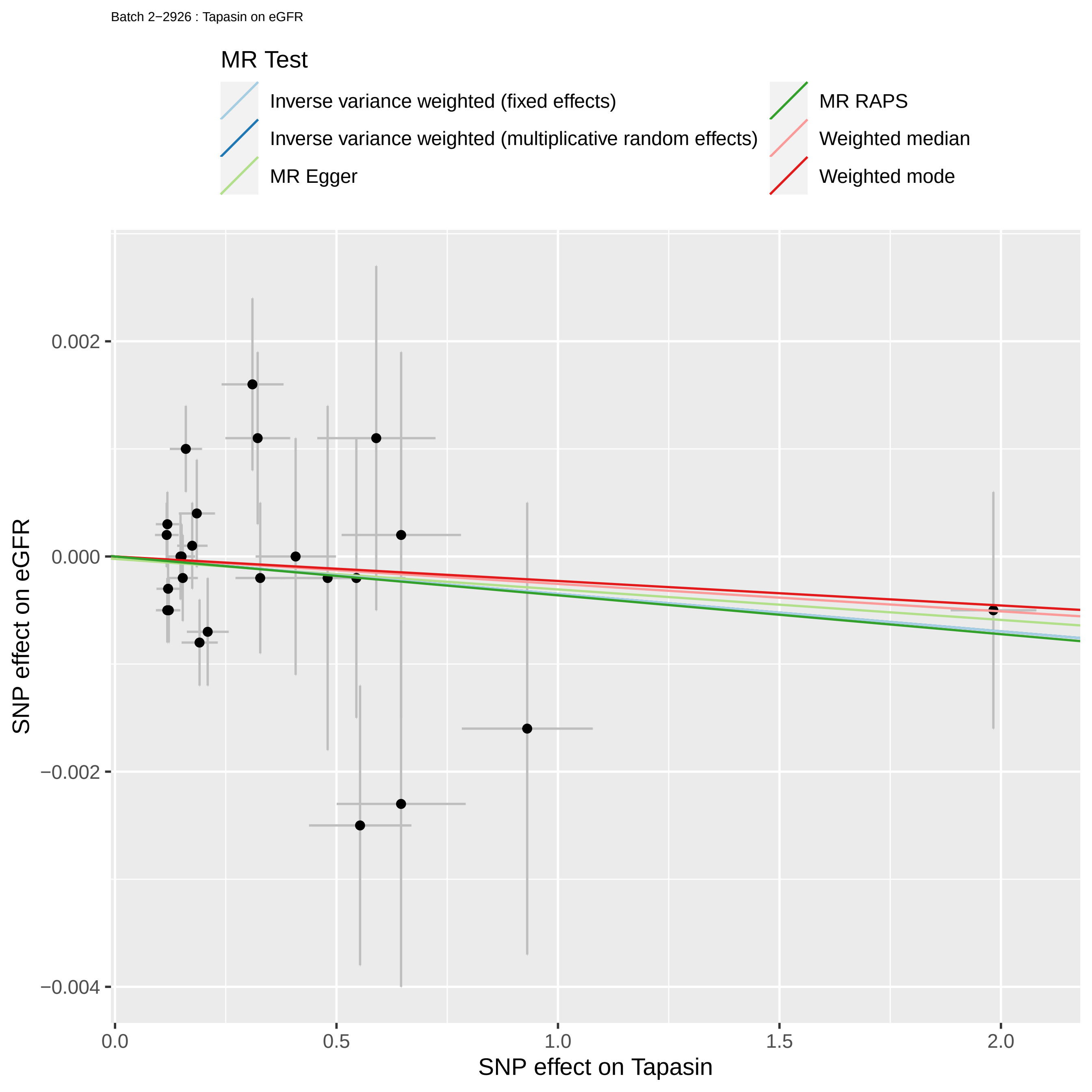
Batch 2−2926 : Tapasin on eGFR
MR Test
Inverse variance weighted (fixed effects)
Inverse variance weighted (multiplicative random effects)
MR Egger
MR RAPS
Weighted median
Weighted mode
0.002
0.000
−0.002
−0.004
0.0
0.5
1.0
1.5
2.0
SNP effect on Tapasin
SNP effect on eGFR

Batch 2−2927 : Tapasin−related protein on eGFR

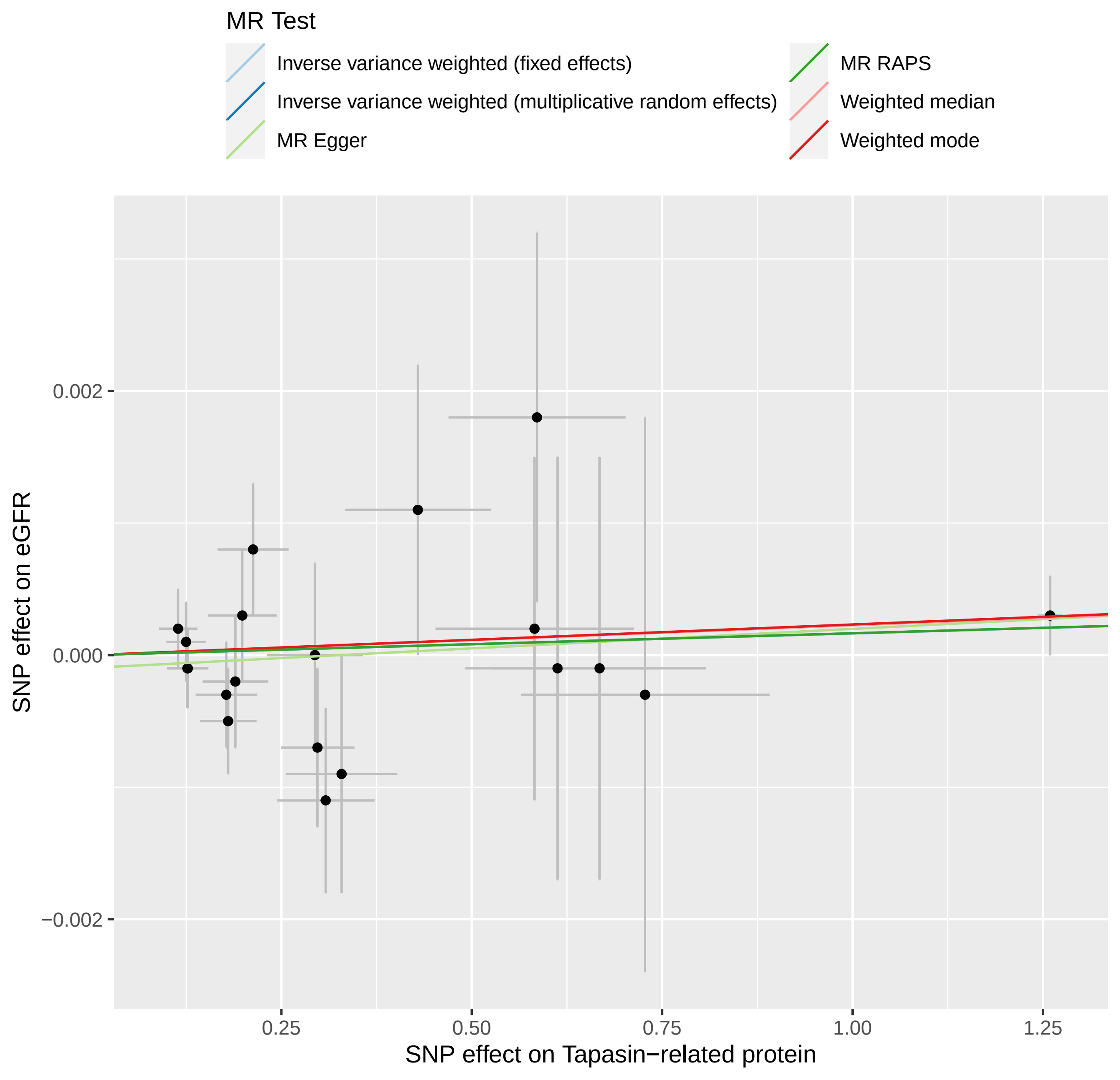

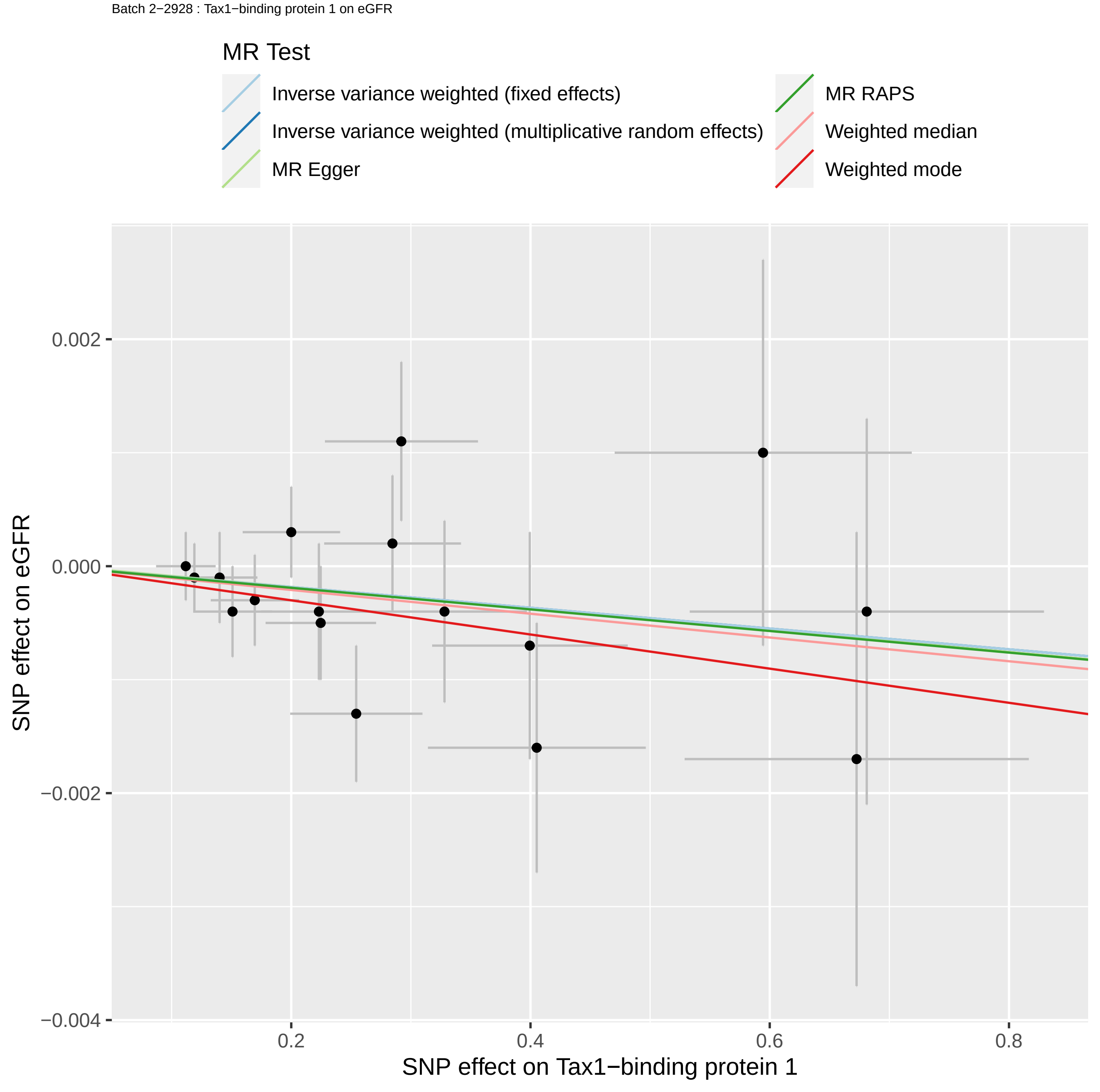

Batch 2−2928 : Tax1−binding protein 1 on eGFR
MR Test
Inverse variance weighted (fixed effects)
Inverse variance weighted (multiplicative random effects)
MR Egger
MR RAPS
Weighted median
Weighted mode
0.002
0.000
−0.002
−0.004
0.2
0.4
0.6
0.8
SNP effect on Tax1−binding protein 1
SNP effect on eGFR

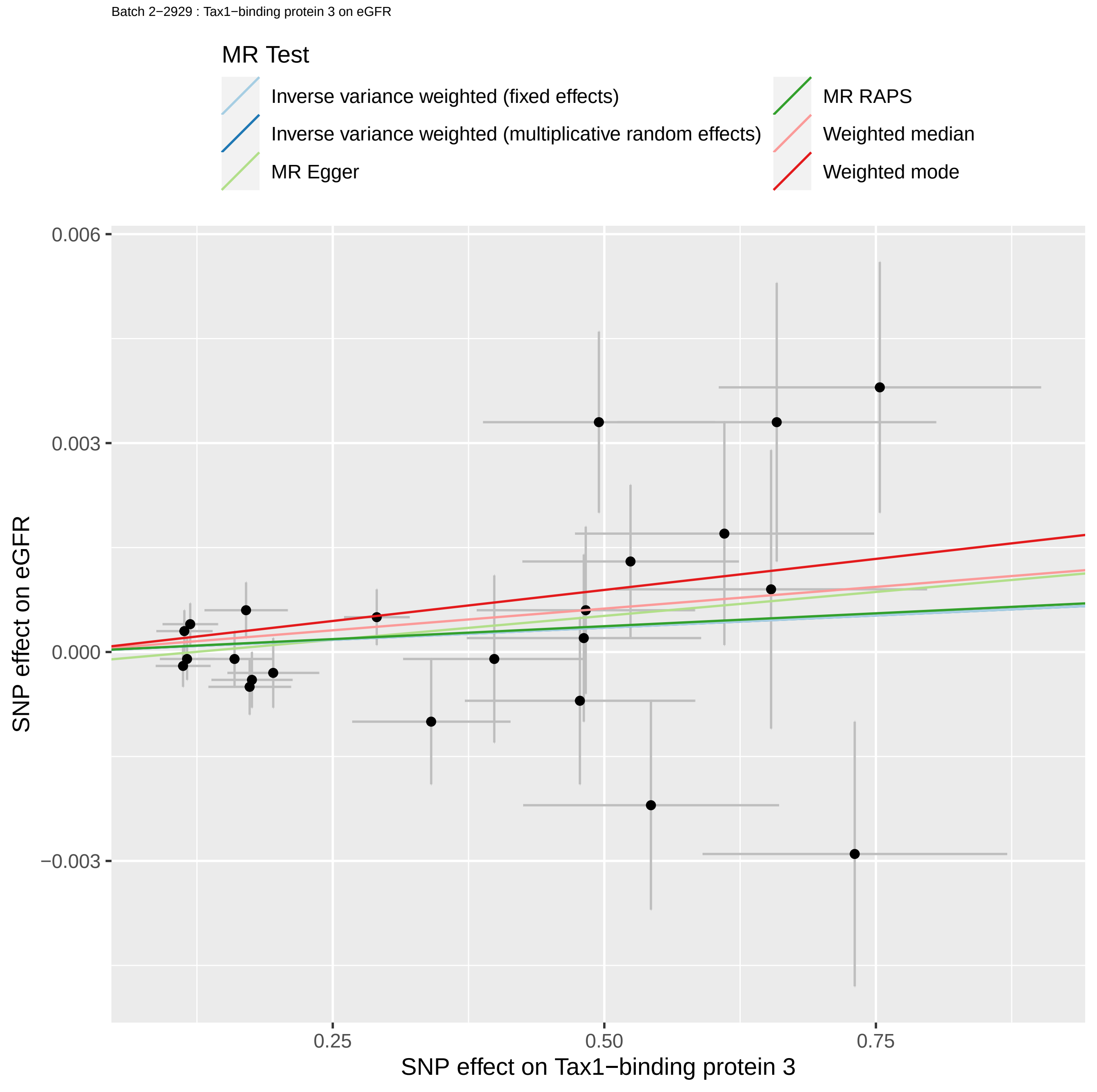
Batch 2−2929 : Tax1−binding protein 3 on eGFR
MR Test
Inverse variance weighted (fixed effects)
Inverse variance weighted (multiplicative random effects)
MR Egger
MR RAPS
Weighted median
Weighted mode
0.006
0.003
0.000
−0.003
SNP effect on eGFR
0.25
0.50
0.75
SNP effect on Tax1−binding protein 3

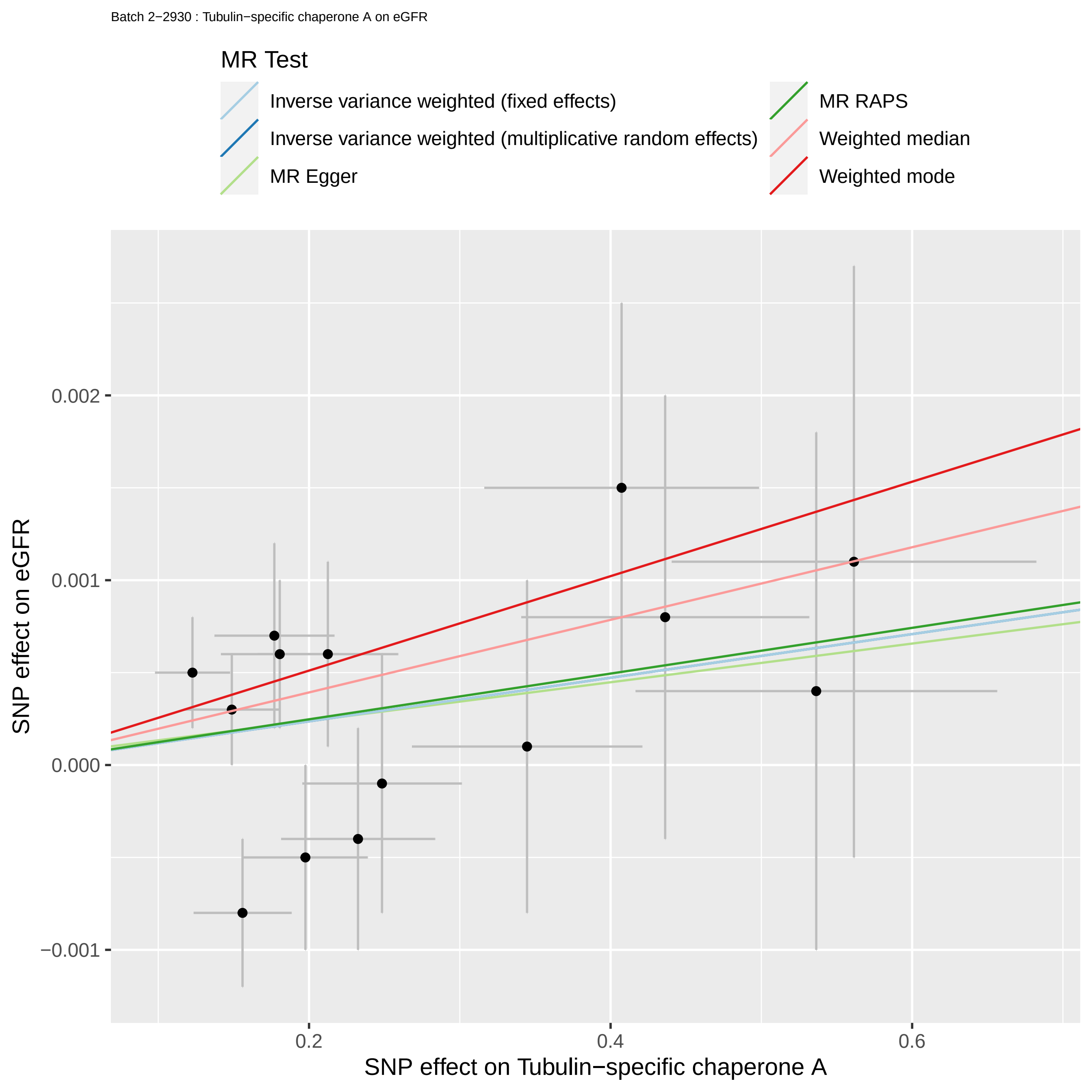
Batch 2−2930 : Tubulin−specific chaperone A on eGFR
MR Test
Inverse variance weighted (fixed effects)
Inverse variance weighted (multiplicative random effects)
MR Egger
MR RAPS
Weighted median
Weighted mode
0.002
0.001
0.000
−0.001
SNP effect on eGFR
0.2
0.4
0.6
SNP effect on Tubulin−specific chaperone A

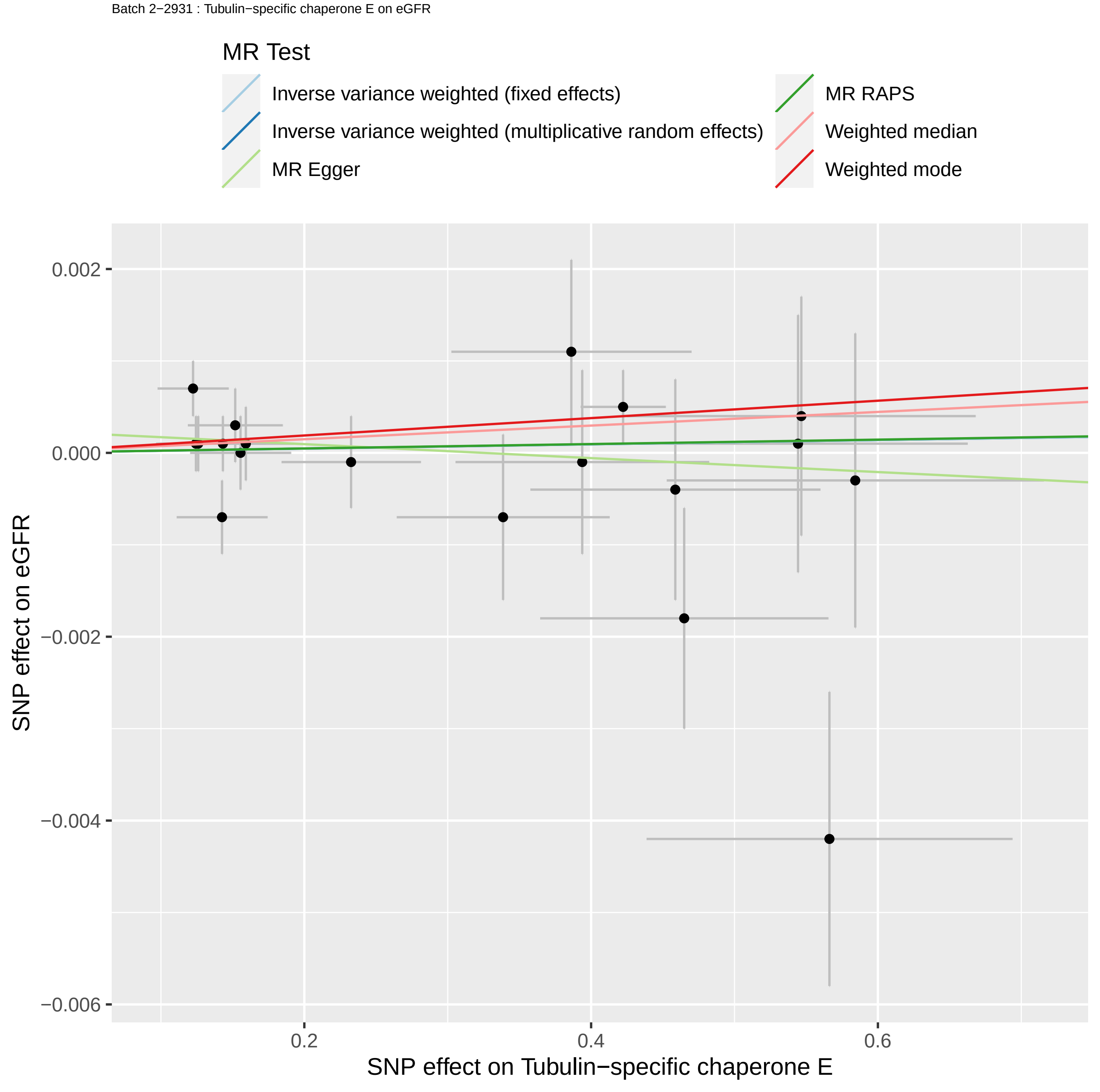

Batch 2−2931 : Tubulin−specific chaperone E on eGFR
MR Test
Inverse variance weighted (fixed effects)
Inverse variance weighted (multiplicative random effects)
MR Egger
MR RAPS
Weighted median
Weighted mode
0.002
0.000
−0.002
−0.004
−0.006
0.2
0.4
0.6
SNP effect on eGFR
SNP effect on Tubulin−specific chaperone E

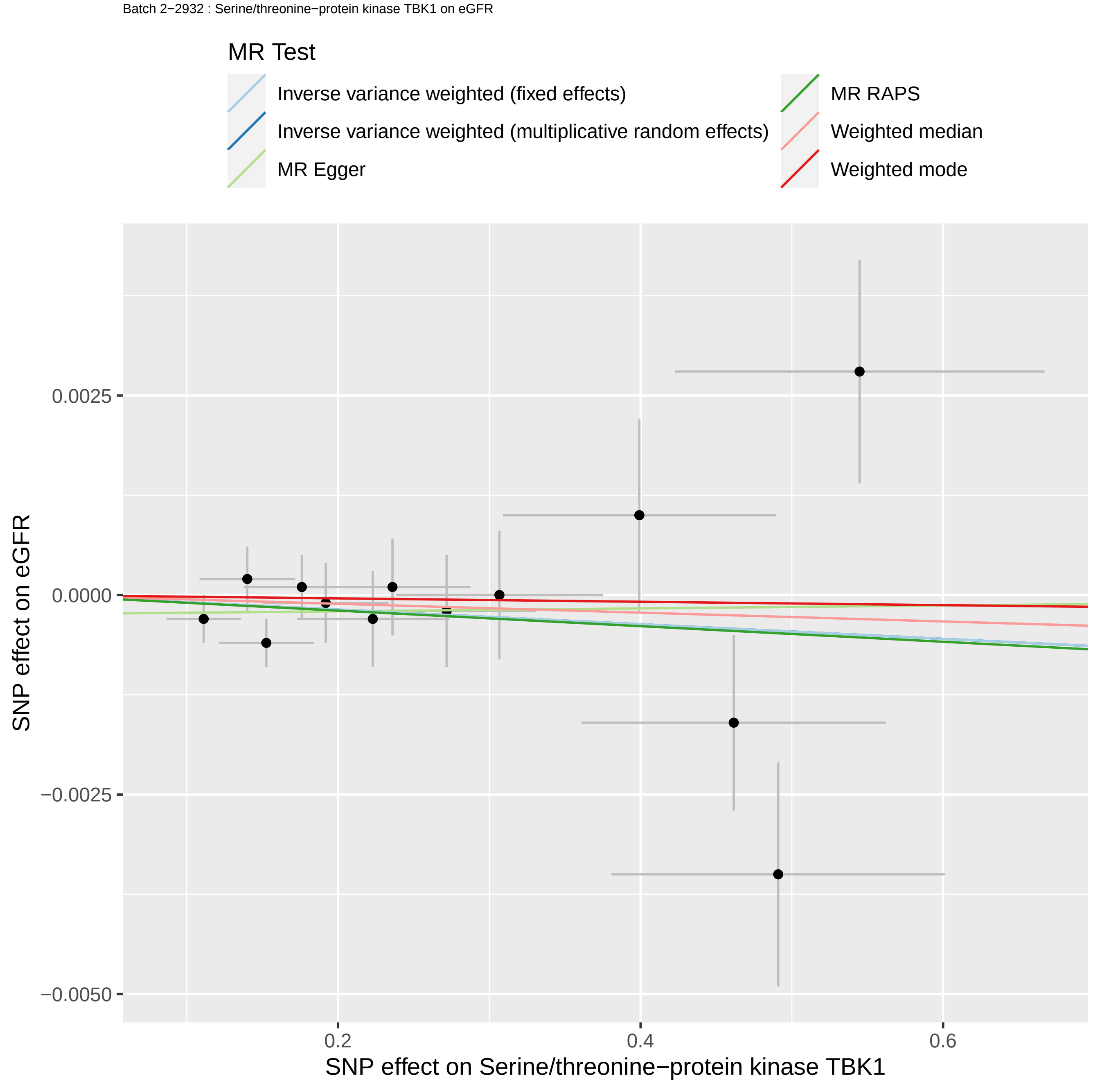

Batch 2−2932 : Serine/threonine−protein kinase TBK1 on eGFR
MR Test
Inverse variance weighted (fixed effects)
Inverse variance weighted (multiplicative random effects)
MR Egger
MR RAPS
Weighted median
Weighted mode
SNP effect on eGFR
0.0025
0.0000
−0.0025
−0.0050
0.2
0.4
0.6
SNP effect on Serine/threonine−protein kinase TBK1

Batch 2−2933 : TATA−box−binding protein on eGFR

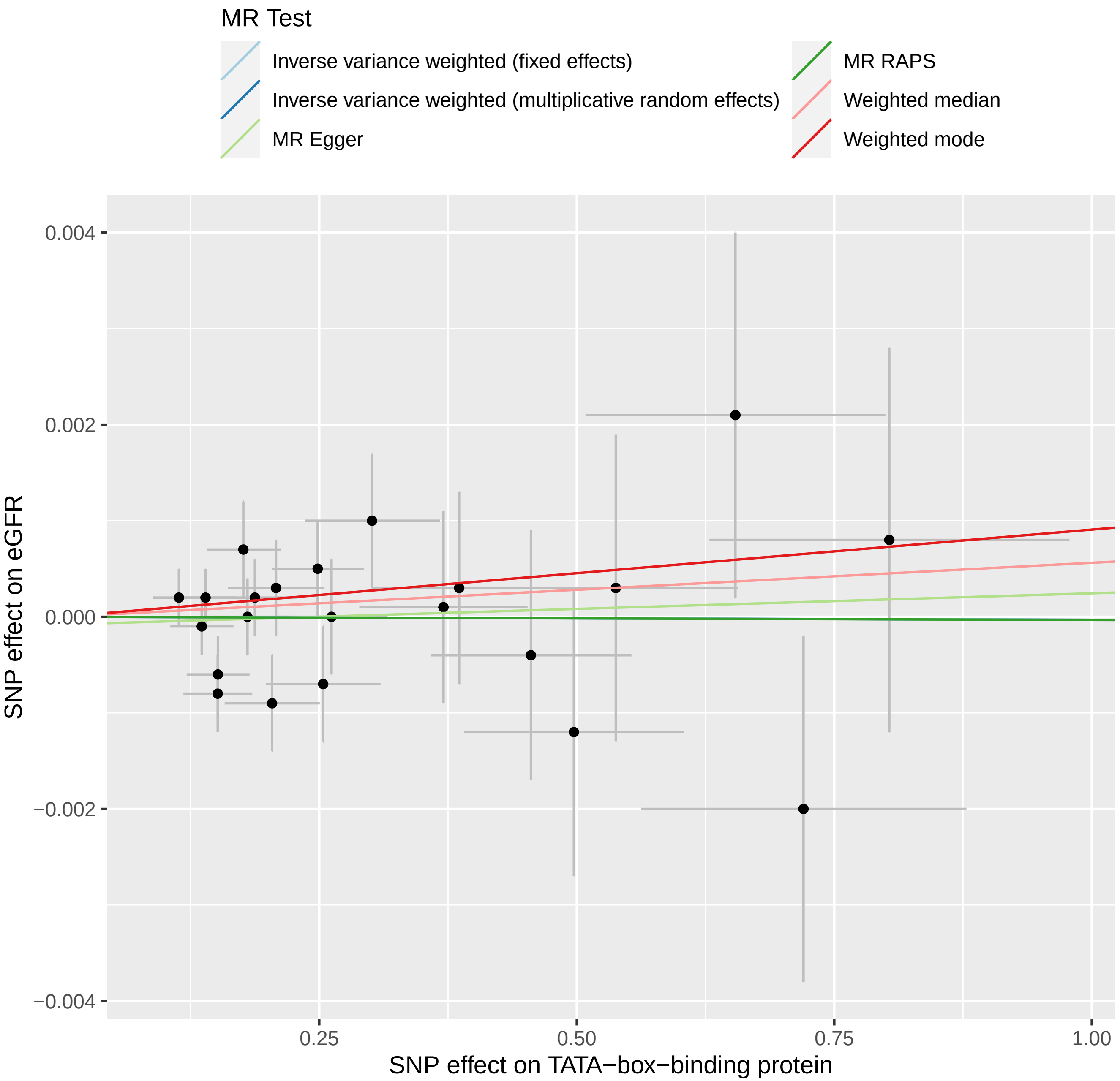

Batch 2−2934 : T−box transcription factor TBX22 on eGFR

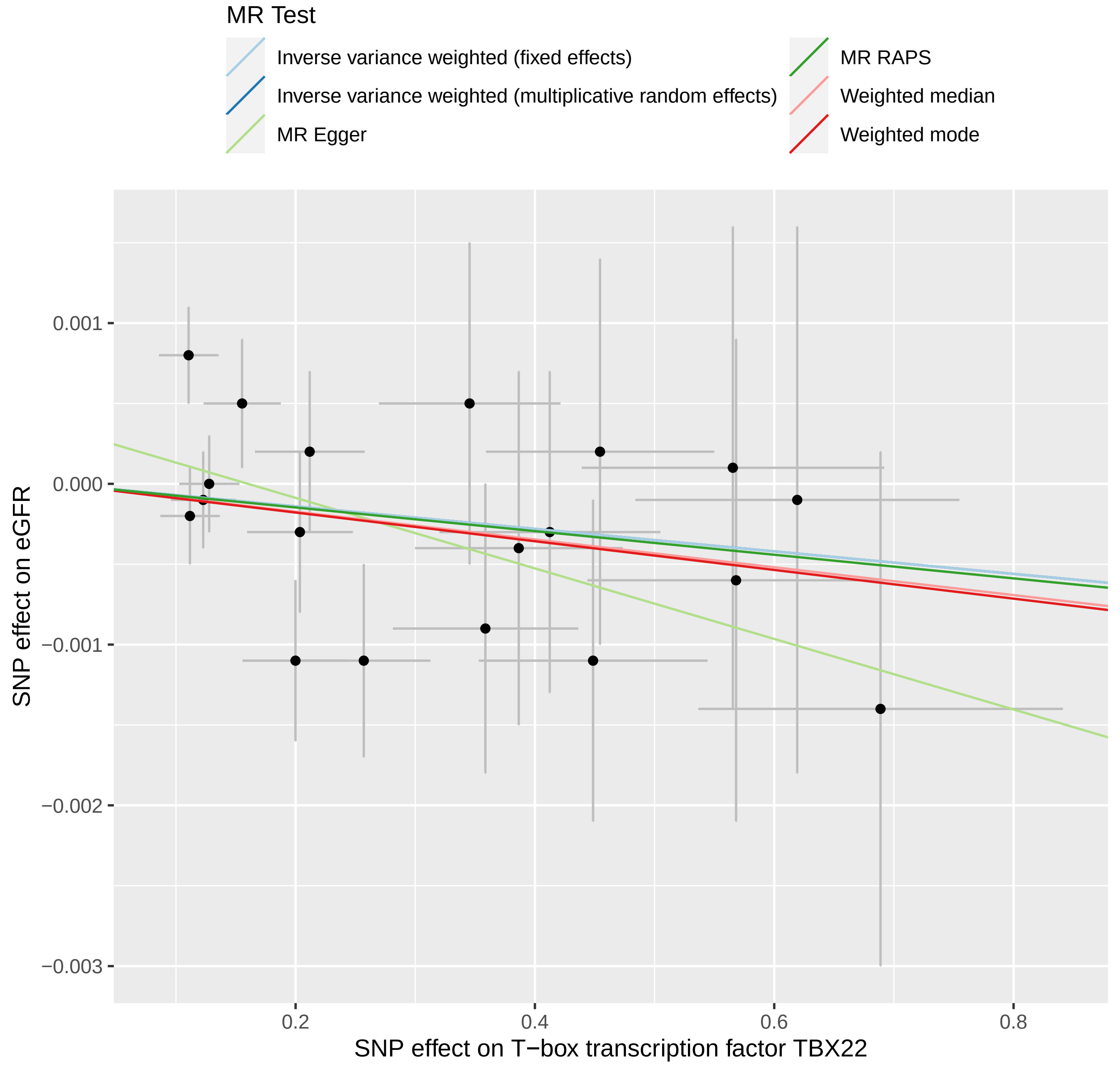

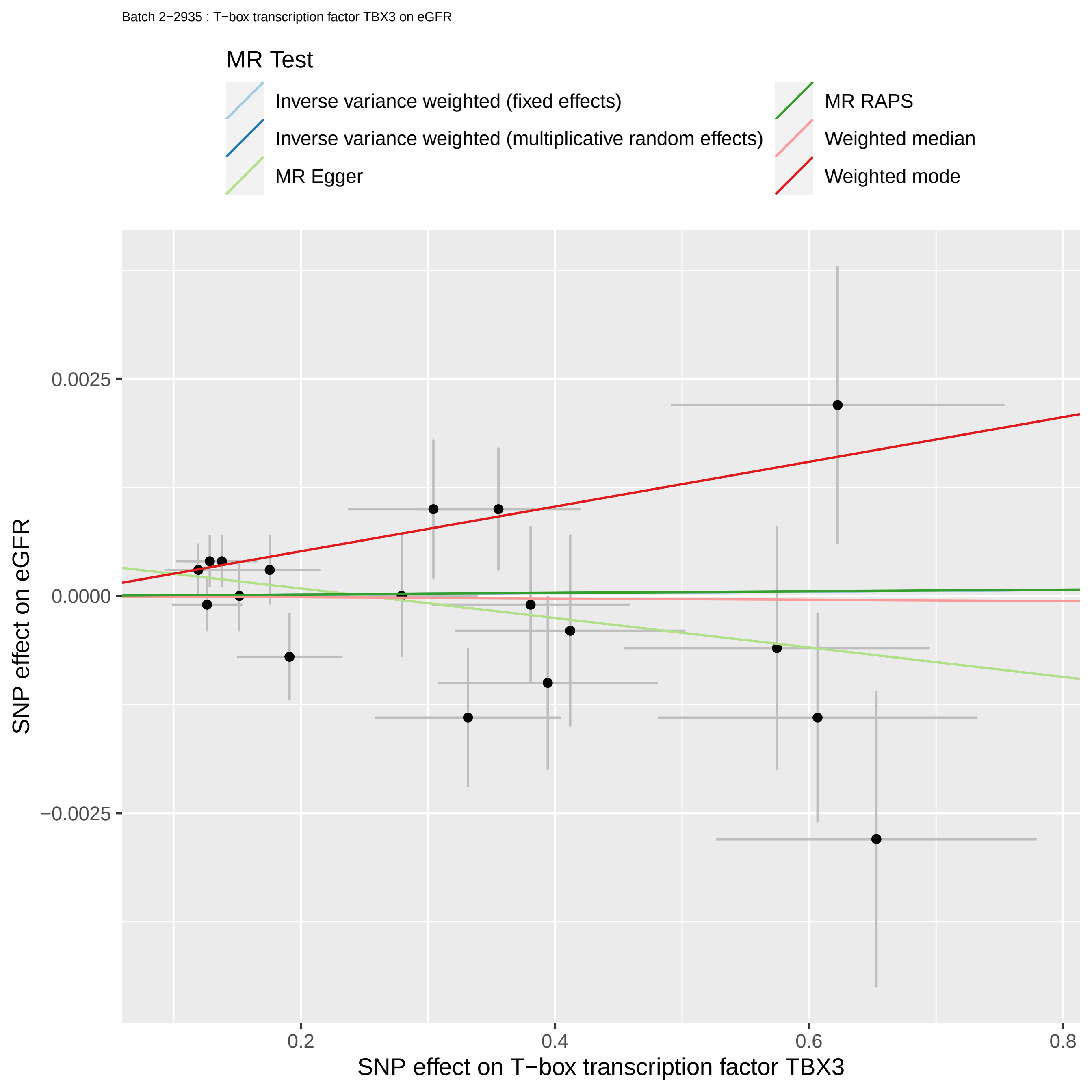

Batch 2−2935 : T−box transcription factor TBX3 on eGFR
MR Test
Inverse variance weighted (fixed effects)
Inverse variance weighted (multiplicative random effects)
MR Egger
MR RAPS
Weighted median
Weighted mode
0.0025
0.0000
−0.0025
SNP effect on eGFR
0.2
0.4
0.6
0.8
SNP effect on T−box transcription factor TBX3

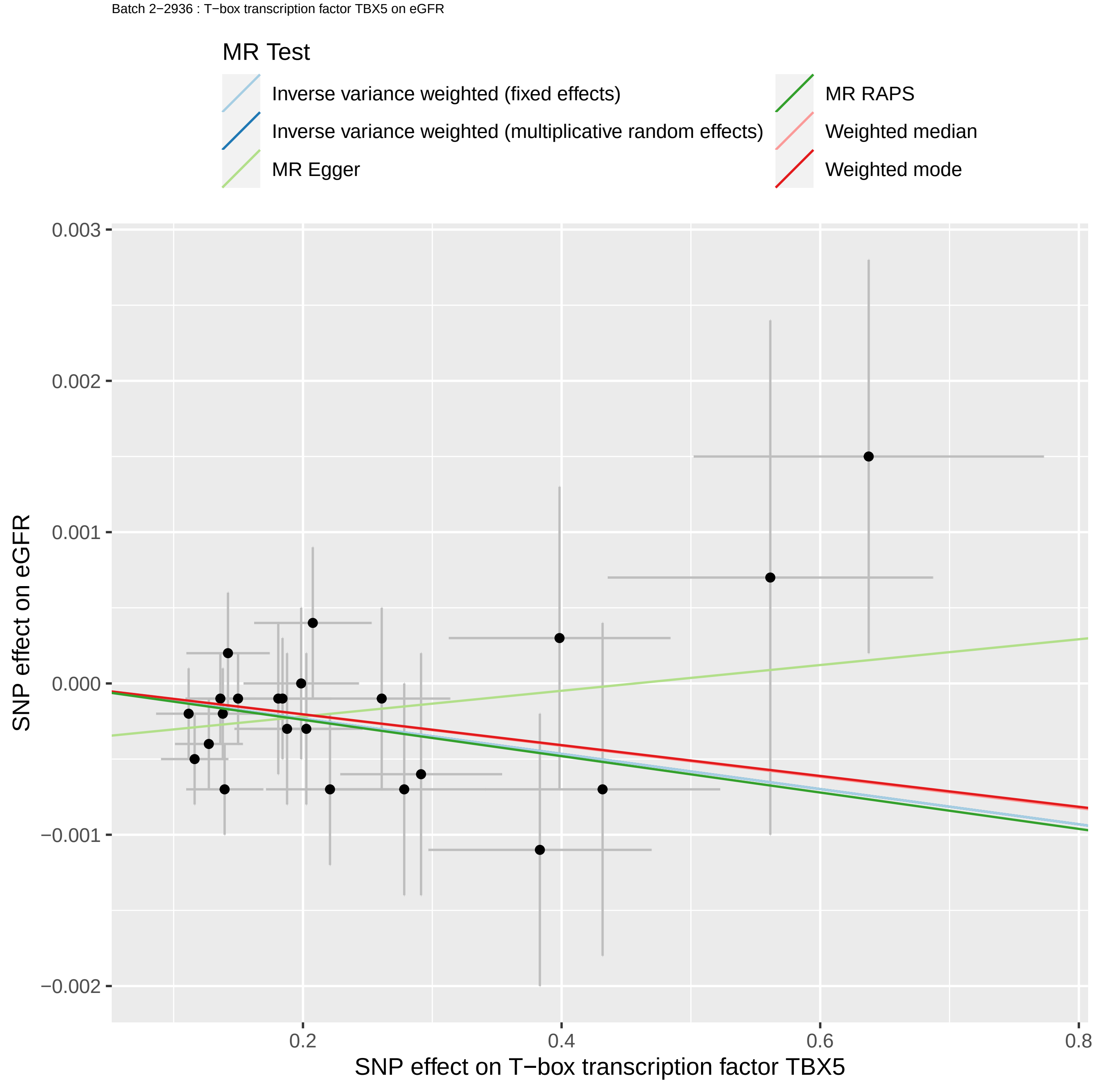

Batch 2−2936 : T−box transcription factor TBX5 on eGFR
MR Test
Inverse variance weighted (fixed effects)
Inverse variance weighted (multiplicative random effects)
MR Egger
MR RAPS
Weighted median
Weighted mode
0.003
0.002
0.001
0.000
−0.001
−0.002
0.2
0.4
0.6
0.8
SNP effect on eGFR
SNP effect on T−box transcription factor TBX5

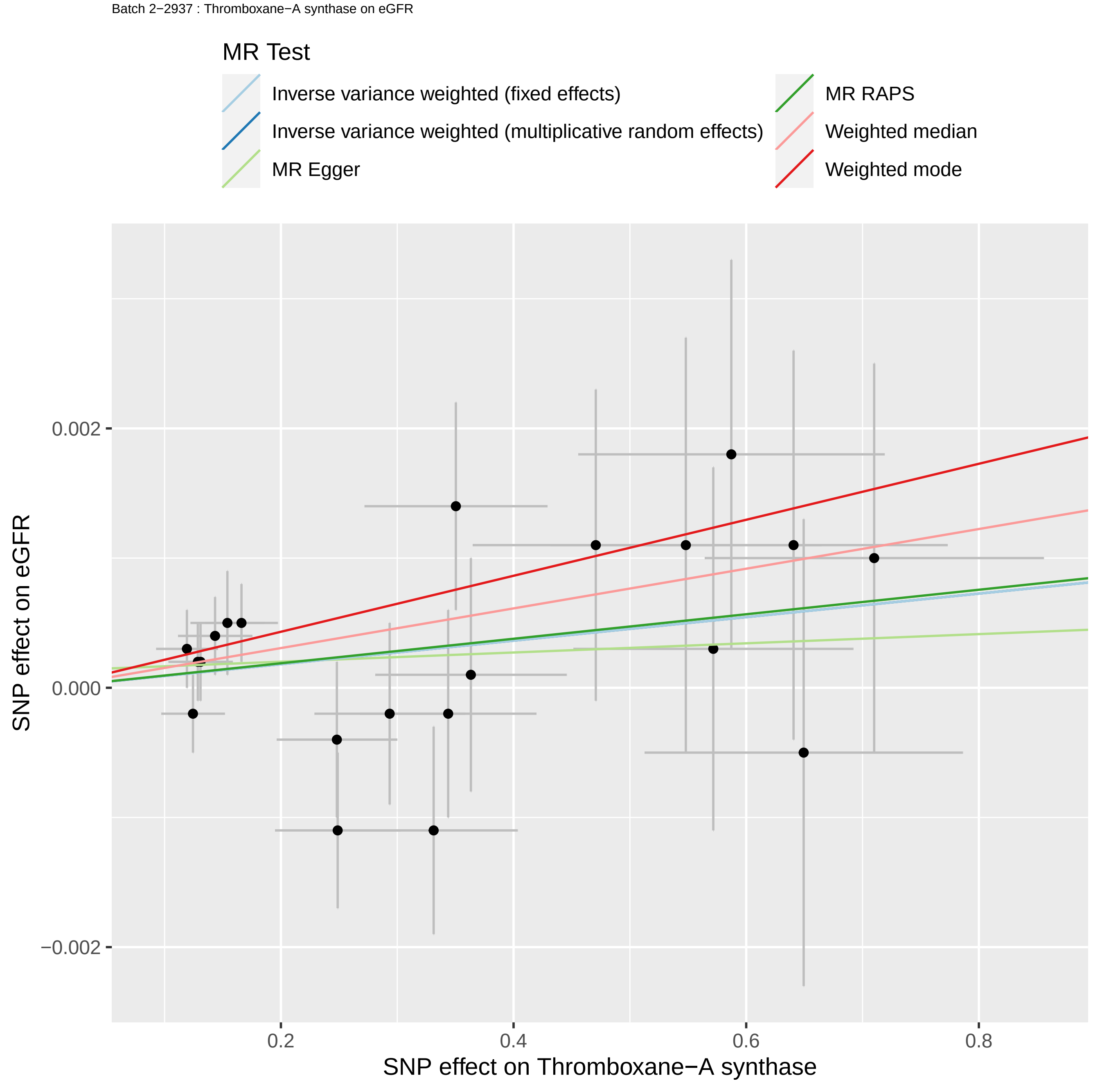

Batch 2−2937 : Thromboxane−A synthase on eGFR
MR Test
Inverse variance weighted (fixed effects)
Inverse variance weighted (multiplicative random effects)
MR Egger
MR RAPS
Weighted median
Weighted mode
0.002
0.000
−0.002
0.2
0.4
0.6
0.8
SNP effect on eGFR
SNP effect on Thromboxane−A synthase

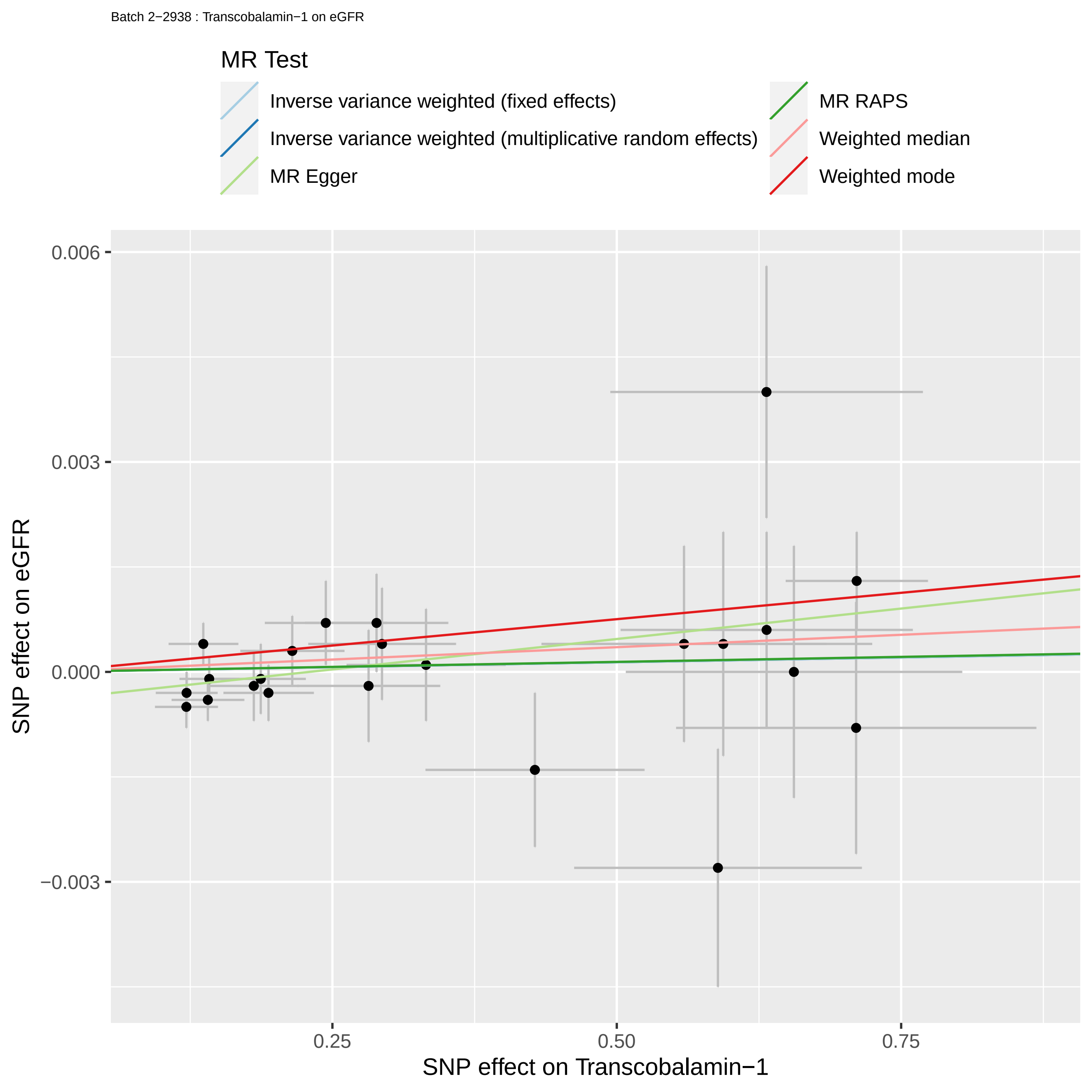
Batch 2−2938 : Transcobalamin−1 on eGFR
MR Test
Inverse variance weighted (fixed effects)
Inverse variance weighted (multiplicative random effects)
MR Egger
MR RAPS
Weighted median
Weighted mode
0.006
0.003
0.000
−0.003
0.25
0.50
0.75
SNP effect on eGFR
SNP effect on Transcobalamin−1

Batch 2−2939 : Holo−Transcobalamin−2 on eGFR

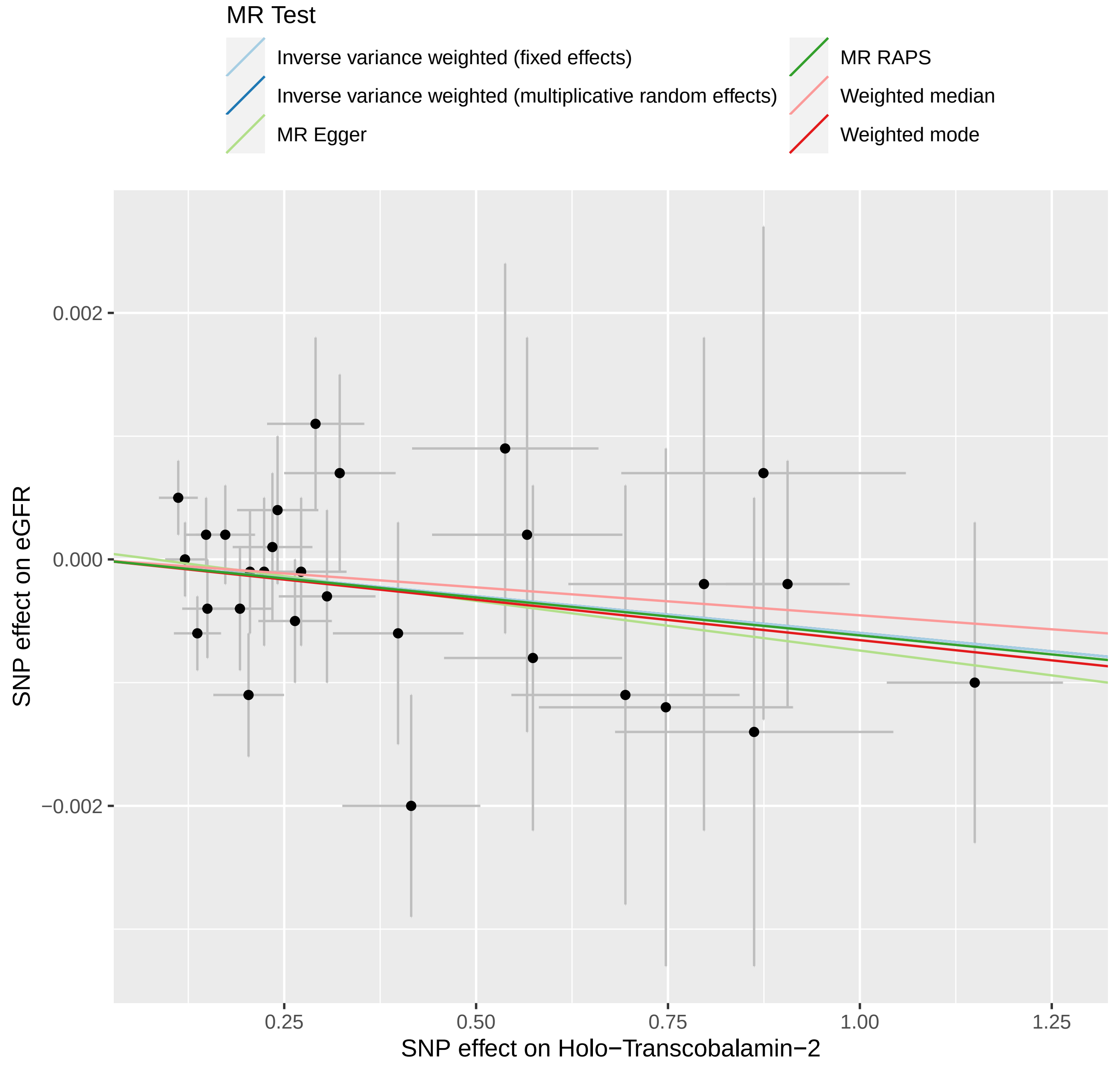

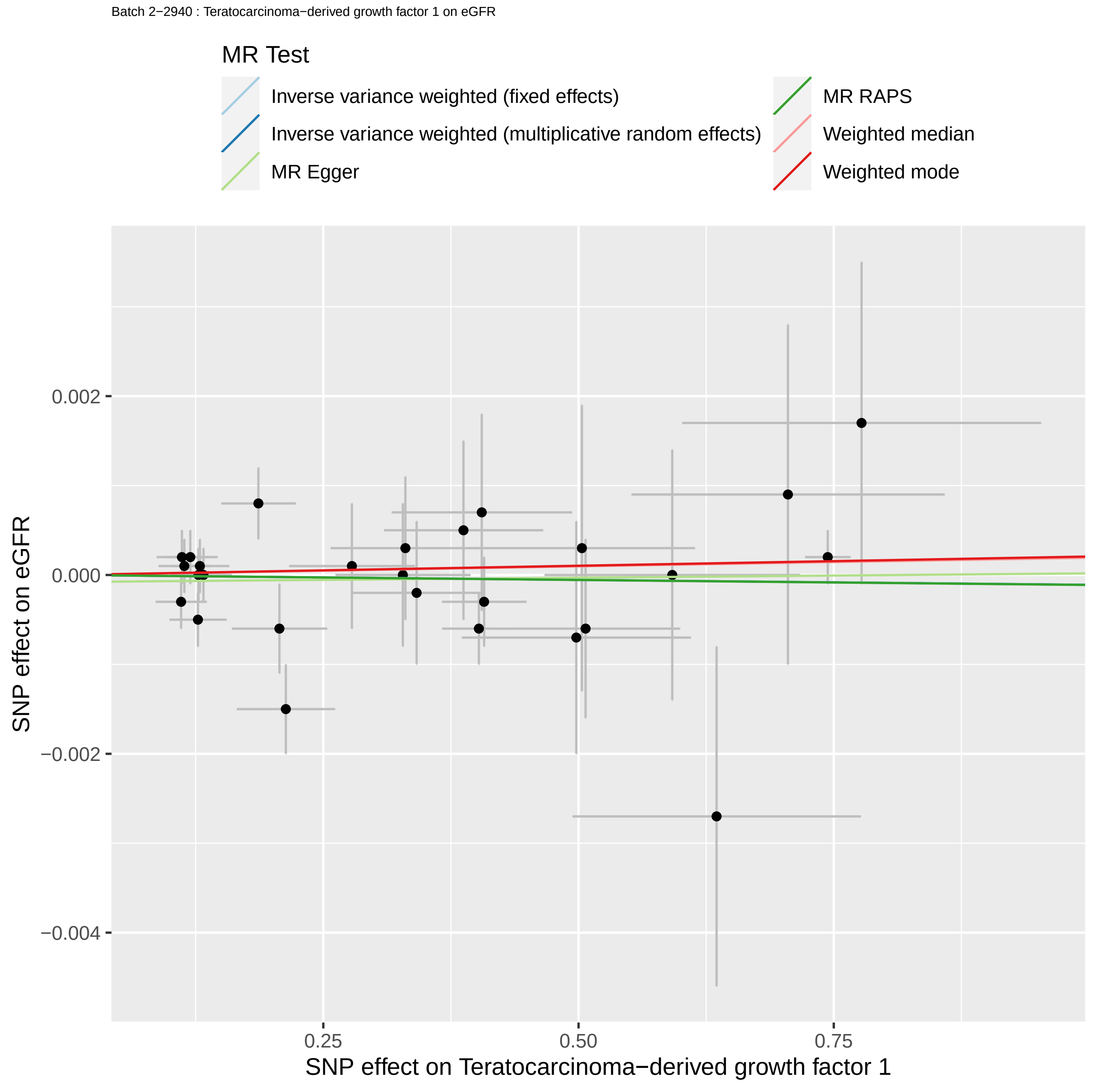
Batch 2−2940 : Teratocarcinoma−derived growth factor 1 on eGFR
MR Test
Inverse variance weighted (fixed effects)
Inverse variance weighted (multiplicative random effects)
MR Egger
MR RAPS
Weighted median
Weighted mode
0.002
0.000
−0.002
−0.004
SNP effect on eGFR
0.25
0.50
0.75
SNP effect on Teratocarcinoma−derived growth factor 1

Batch 2−2941 : Tyrosyl−DNA phosphodiesterase 1 on eGFR

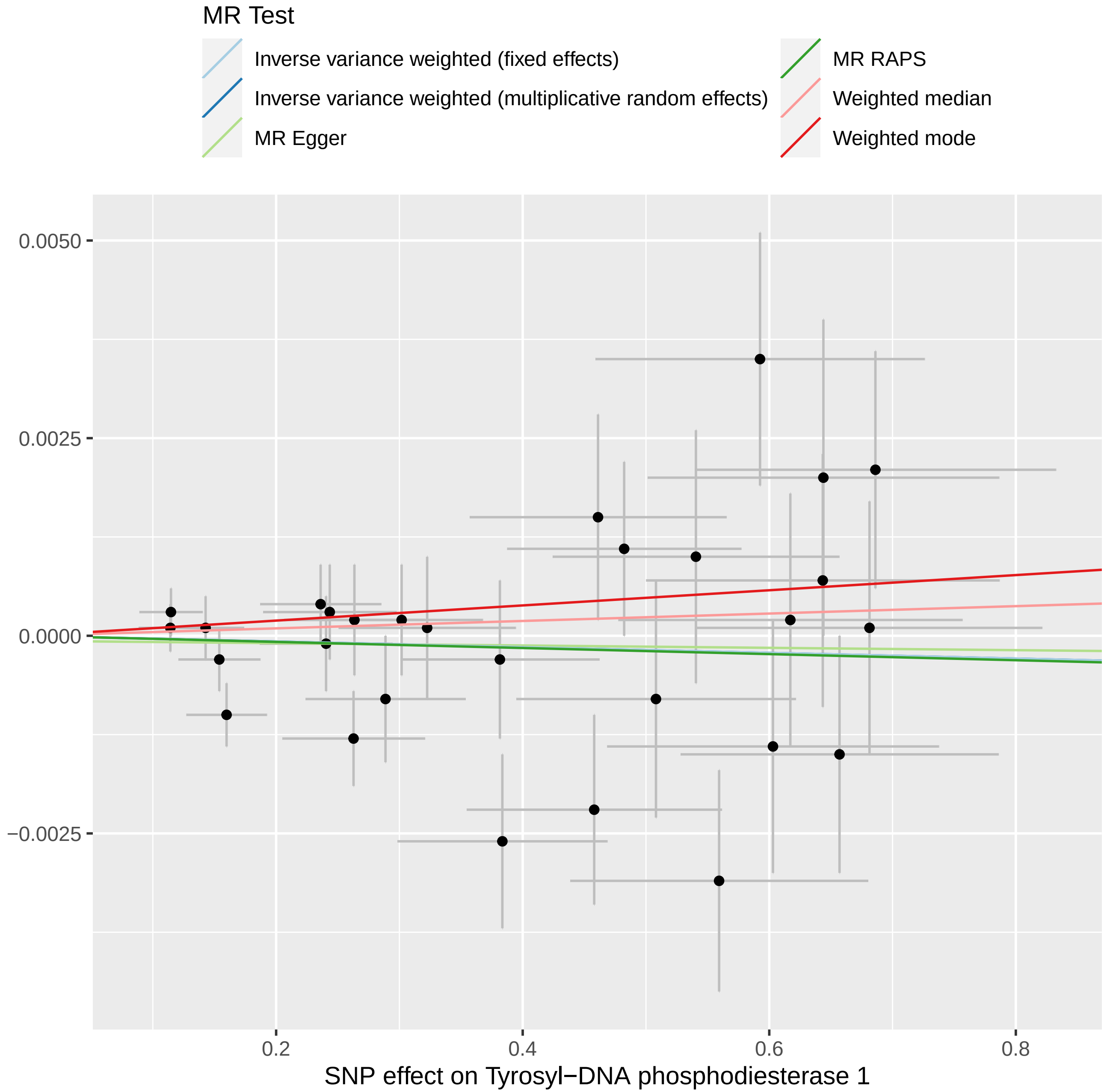

Batch 2−2942 : Transcriptional enhancer factor TEF−5 on eGFR

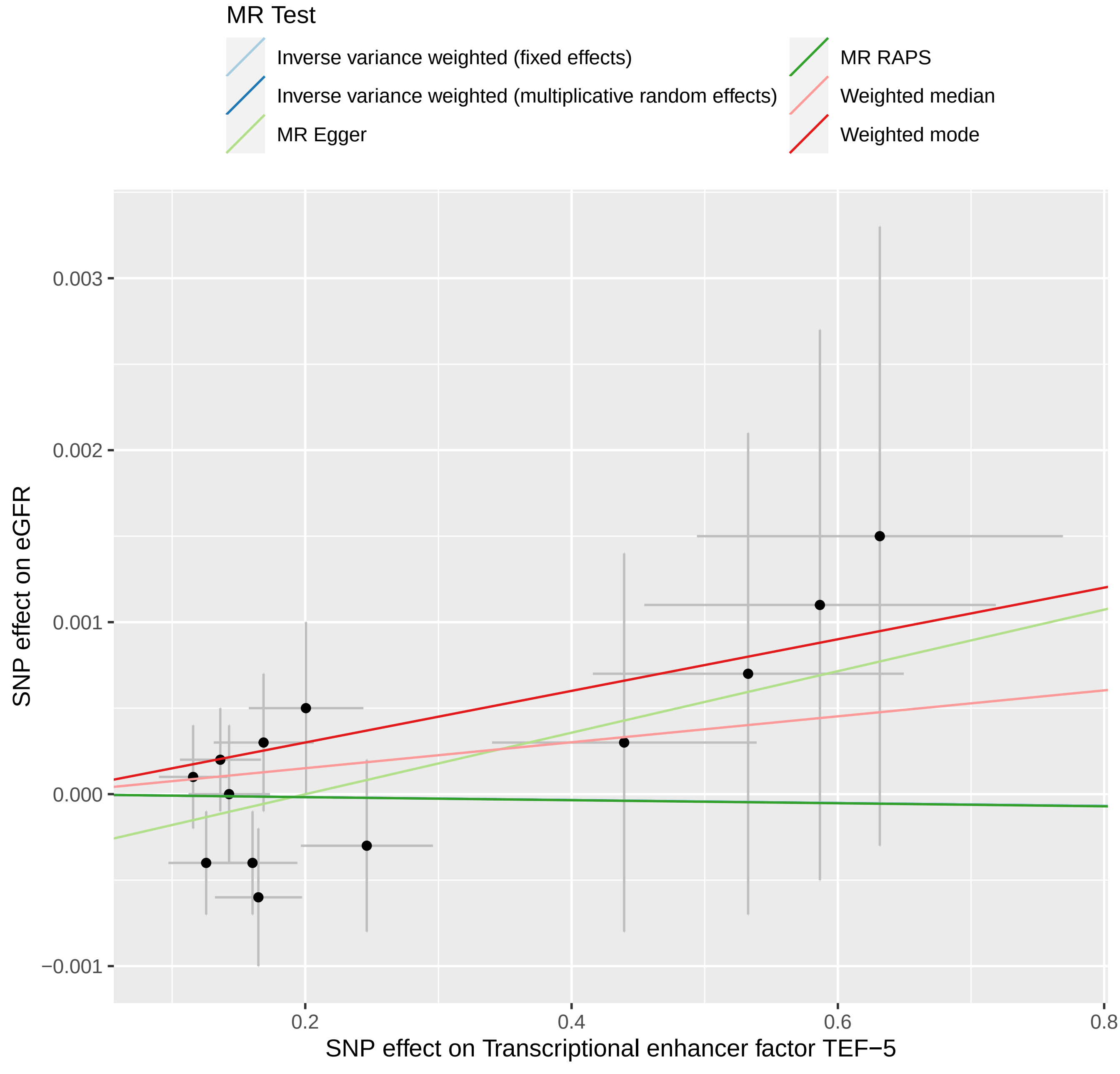

Batch 2−2943 : Transcriptional enhancer factor TEF−3 on eGFR

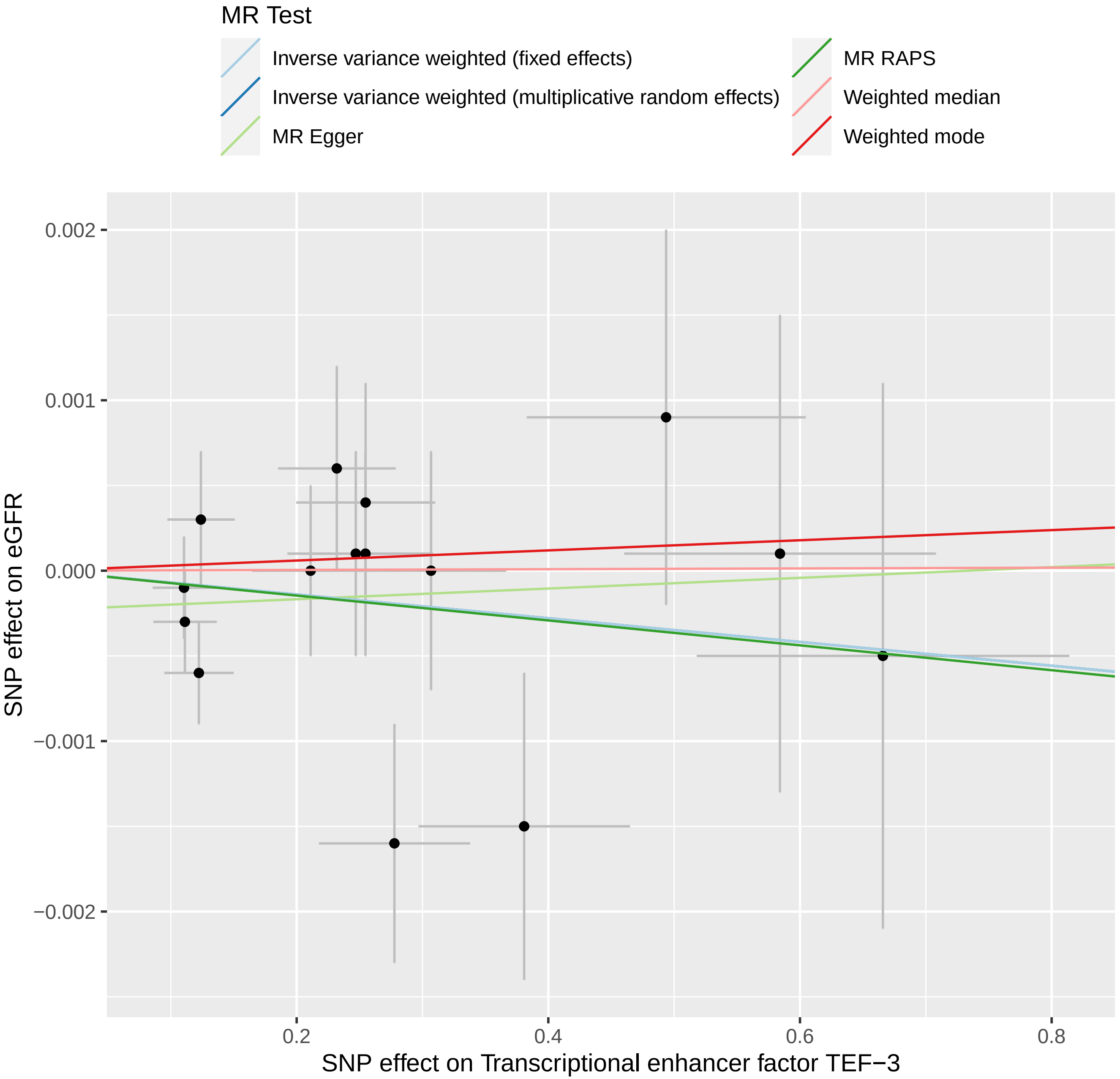

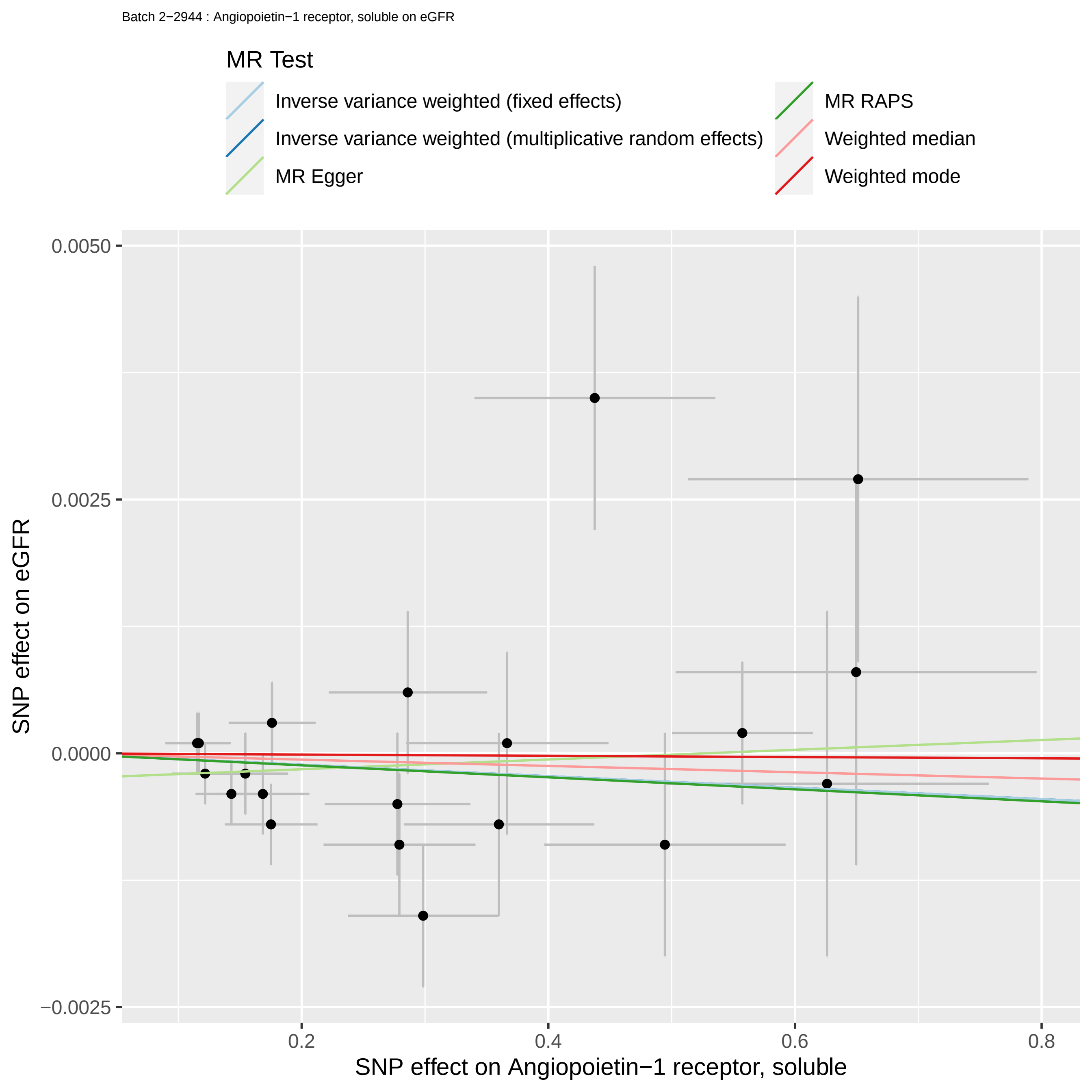

Batch 2−2944 : Angiopoietin−1 receptor, soluble on eGFR
MR Test
Inverse variance weighted (fixed effects)
Inverse variance weighted (multiplicative random effects)
MR Egger
MR RAPS
Weighted median
Weighted mode
0.0050
0.0025
0.0000
−0.0025
SNP effect on eGFR
0.2
0.4
0.6
0.8
SNP effect on Angiopoietin−1 receptor, soluble

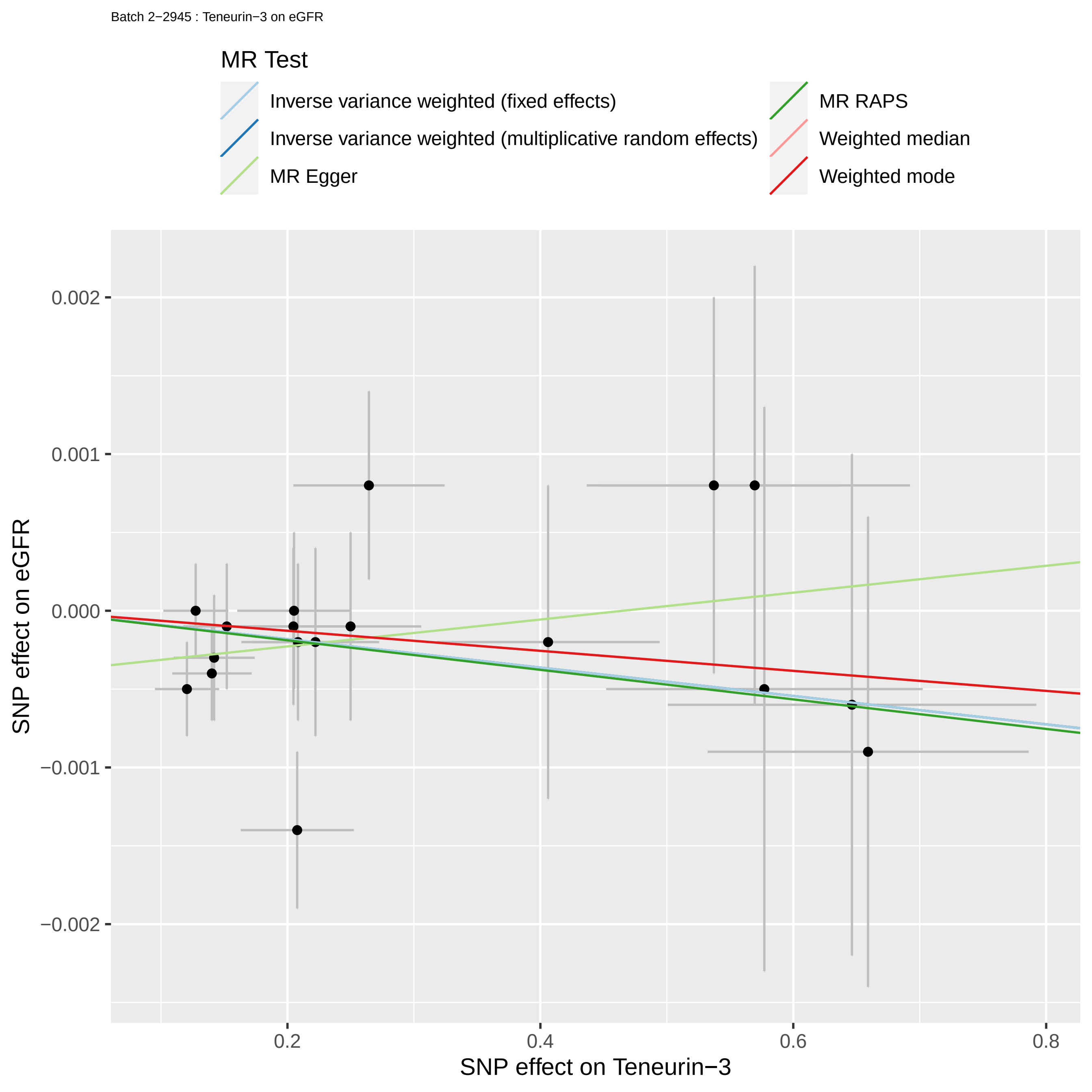

Batch 2−2945 : Teneurin−3 on eGFR
MR Test
Inverse variance weighted (fixed effects)
Inverse variance weighted (multiplicative random effects)
MR Egger
MR RAPS
Weighted median
Weighted mode
0.002
0.001
0.000
−0.001
−0.002
SNP effect on eGFR
0.2
0.4
0.6
0.8
SNP effect on Teneurin−3

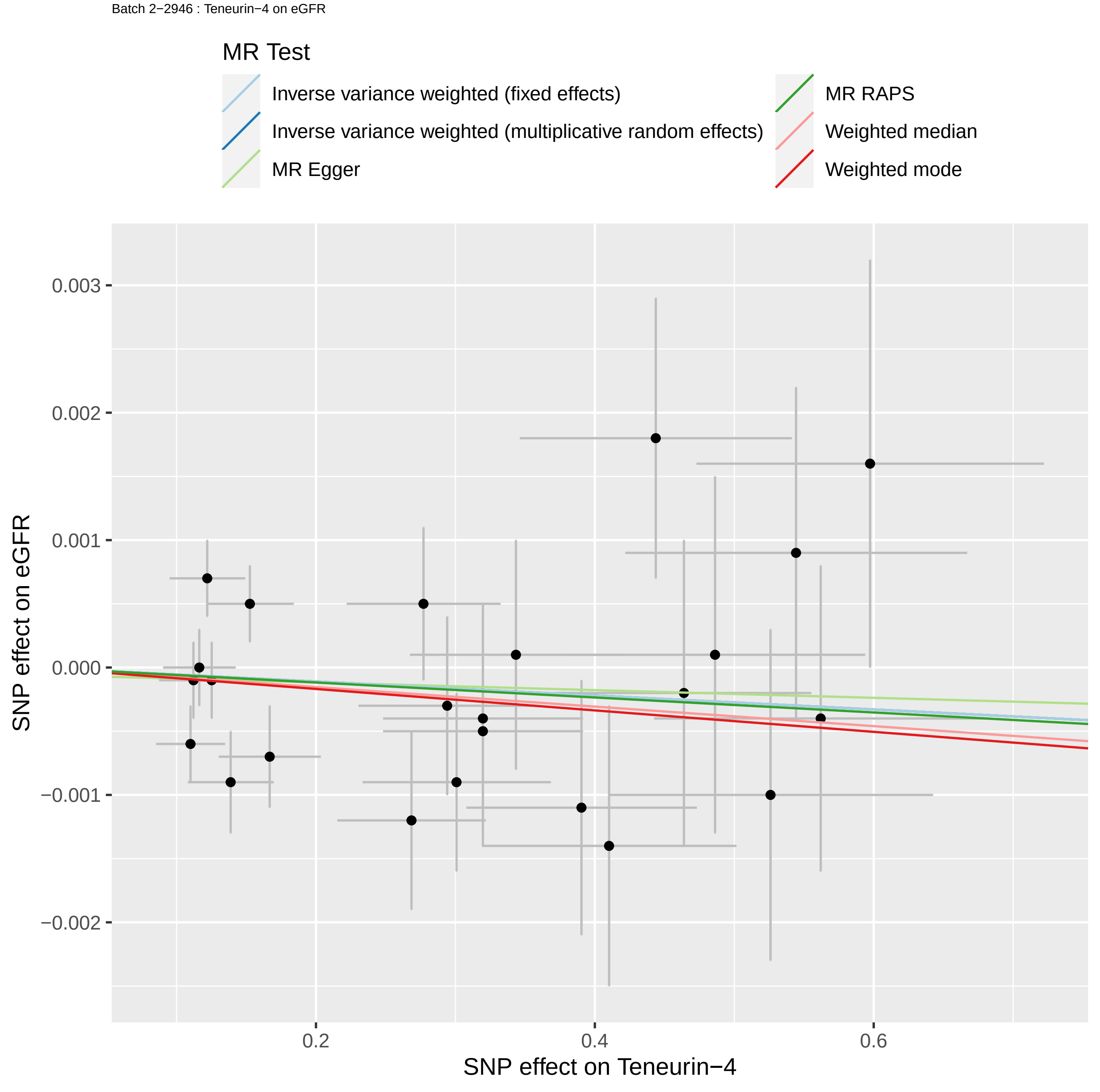
Batch 2−2946 : Teneurin−4 on eGFR
MR Test
Inverse variance weighted (fixed effects)
Inverse variance weighted (multiplicative random effects)
MR Egger
MR RAPS
Weighted median
Weighted mode
0.003
0.002
0.001
0.000
−0.001
−0.002
SNP effect on eGFR
0.2
0.4
0.6
SNP effect on Teneurin−4

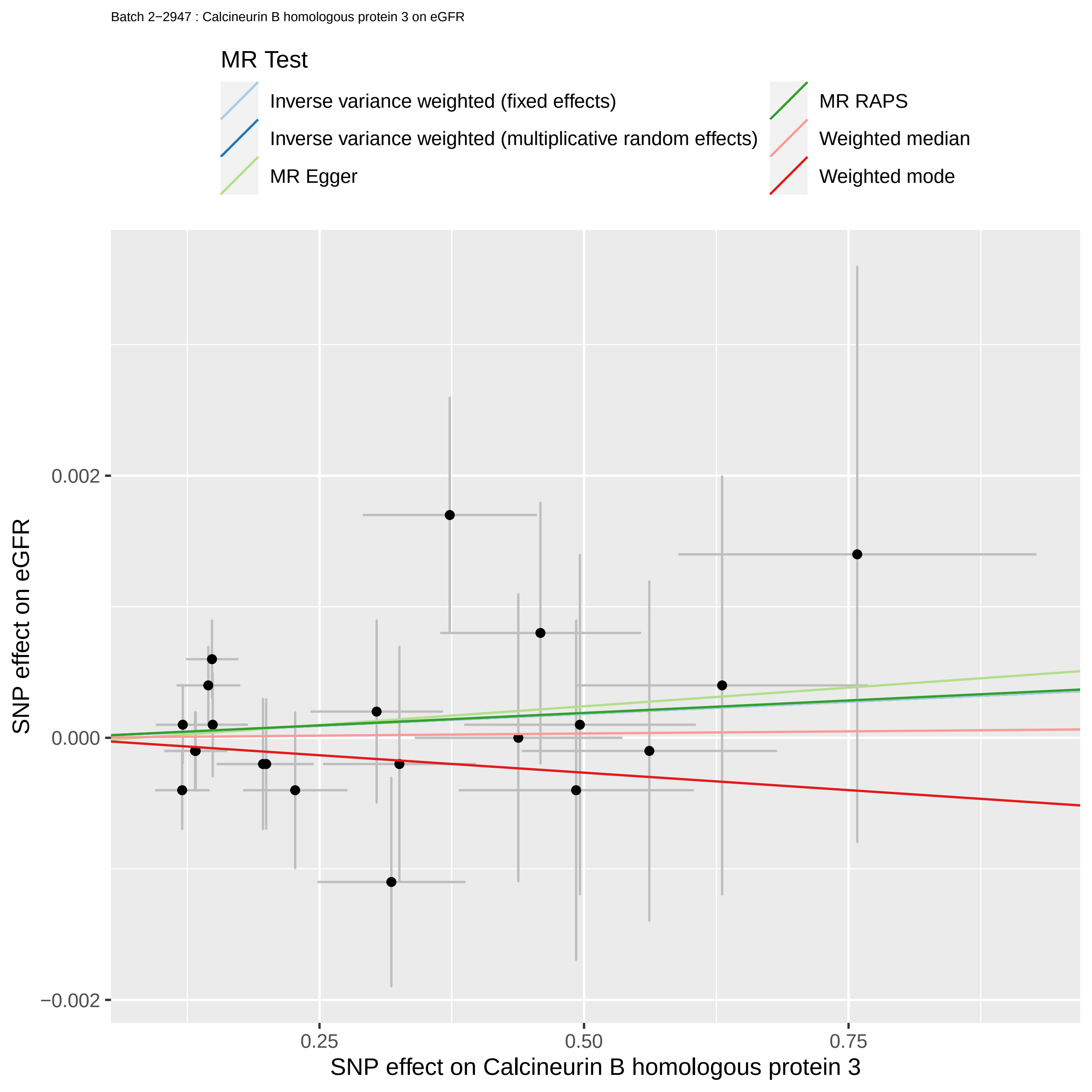
Batch 2−2947 : Calcineurin B homologous protein 3 on eGFR
MR Test
Inverse variance weighted (fixed effects)
Inverse variance weighted (multiplicative random effects)
MR Egger
MR RAPS
Weighted median
Weighted mode
0.002
0.000
−0.002
0.25
0.50
0.75
SNP effect on eGFR
SNP effect on Calcineurin B homologous protein 3

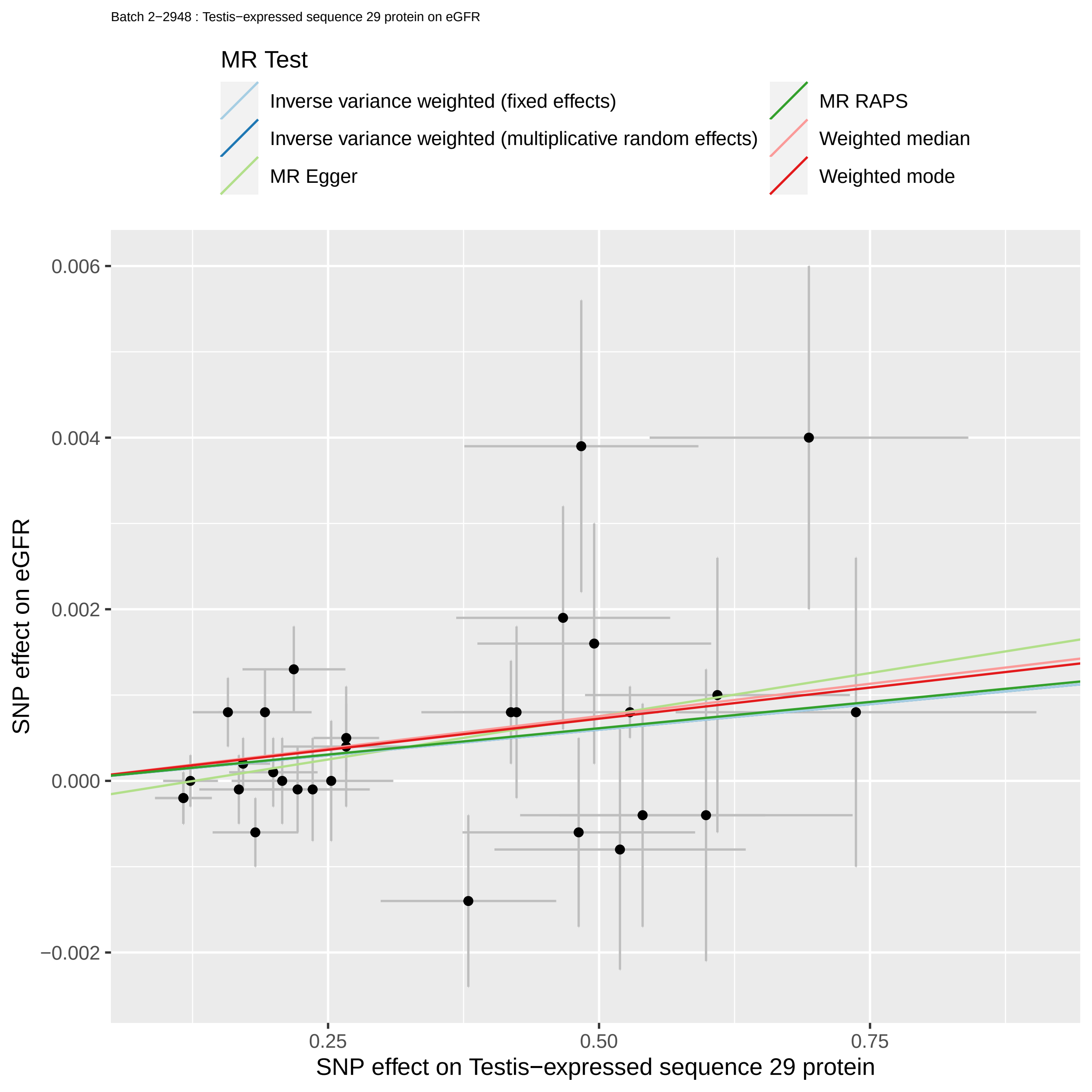
Batch 2−2948 : Testis−expressed sequence 29 protein on eGFR
MR Test
Inverse variance weighted (fixed effects)
Inverse variance weighted (multiplicative random effects)
MR Egger
MR RAPS
Weighted median
Weighted mode
SNP effect on eGFR
0.006
0.004
0.002
0.000
−0.002
0.25
0.50
0.75
SNP effect on Testis−expressed sequence 29 protein

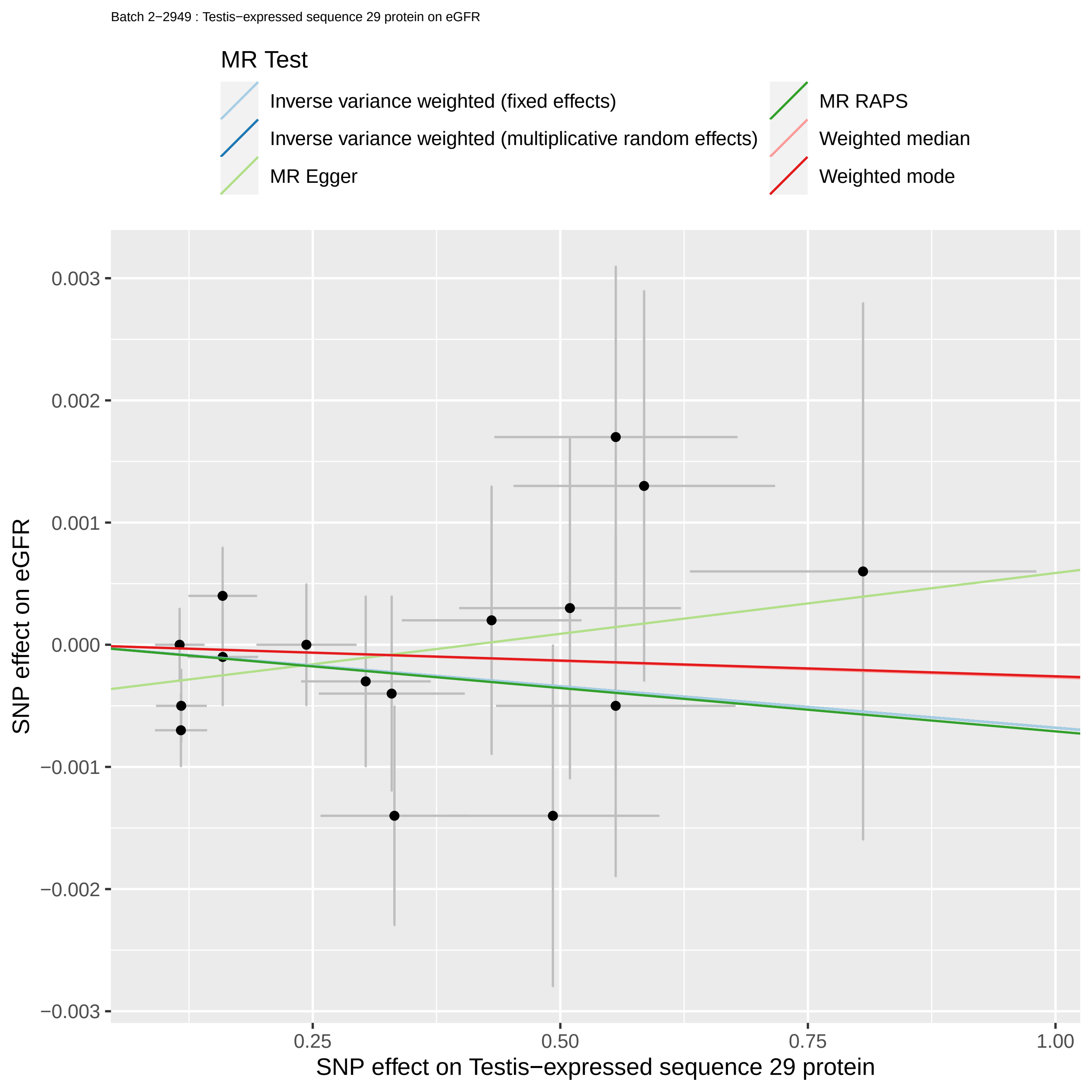

Batch 2−2949 : Testis−expressed sequence 29 protein on eGFR
MR Test
Inverse variance weighted (fixed effects)
Inverse variance weighted (multiplicative random effects)
MR Egger
MR RAPS
Weighted median
Weighted mode
0.003
0.002
0.001
0.000
−0.001
−0.002
−0.003
0.25
0.50
0.75
1.00
SNP effect on eGFR
SNP effect on Testis−expressed sequence 29 protein

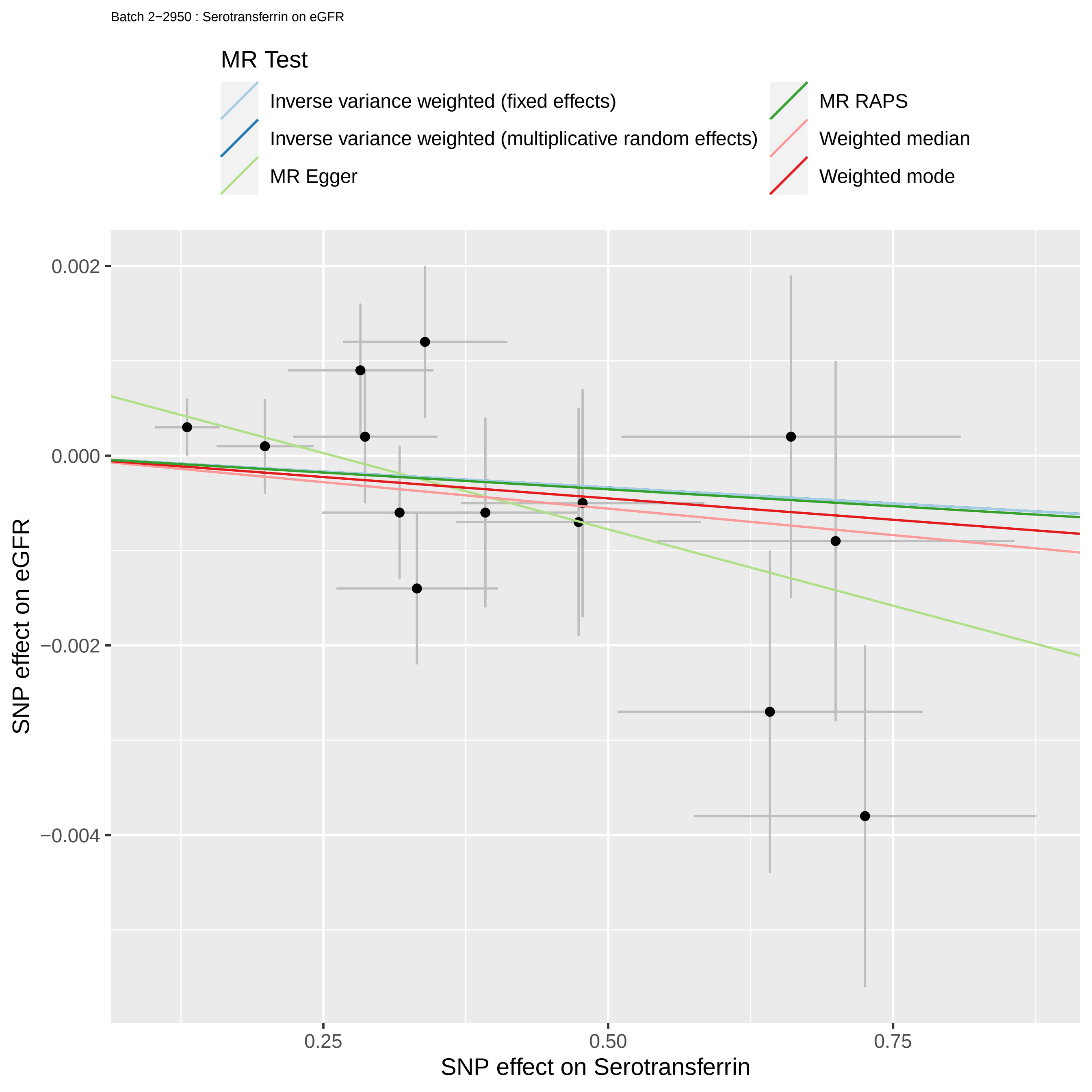
Batch 2−2950 : Serotransferrin on eGFR
MR Test
Inverse variance weighted (fixed effects)
Inverse variance weighted (multiplicative random effects)
MR Egger
MR RAPS
Weighted median
Weighted mode
0.002
0.000
−0.002
−0.004
0.25
0.50
0.75
SNP effect on eGFR
SNP effect on Serotransferrin

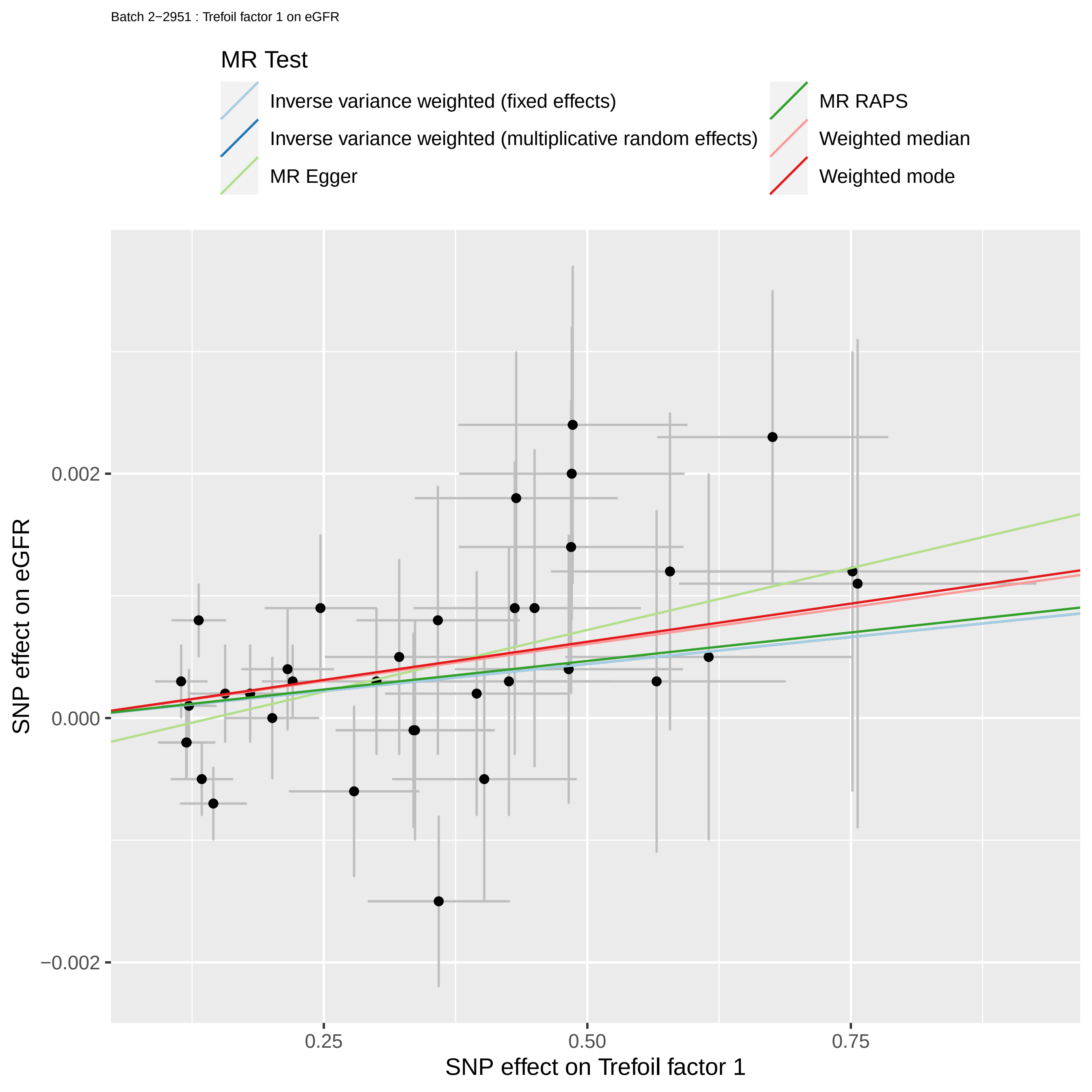
Batch 2−2951 : Trefoil factor 1 on eGFR
MR Test
Inverse variance weighted (fixed effects)
Inverse variance weighted (multiplicative random effects)
MR Egger
MR RAPS
Weighted median
Weighted mode
0.002
0.000
−0.002
0.25
0.50
0.75
SNP effect on eGFR
SNP effect on Trefoil factor 1

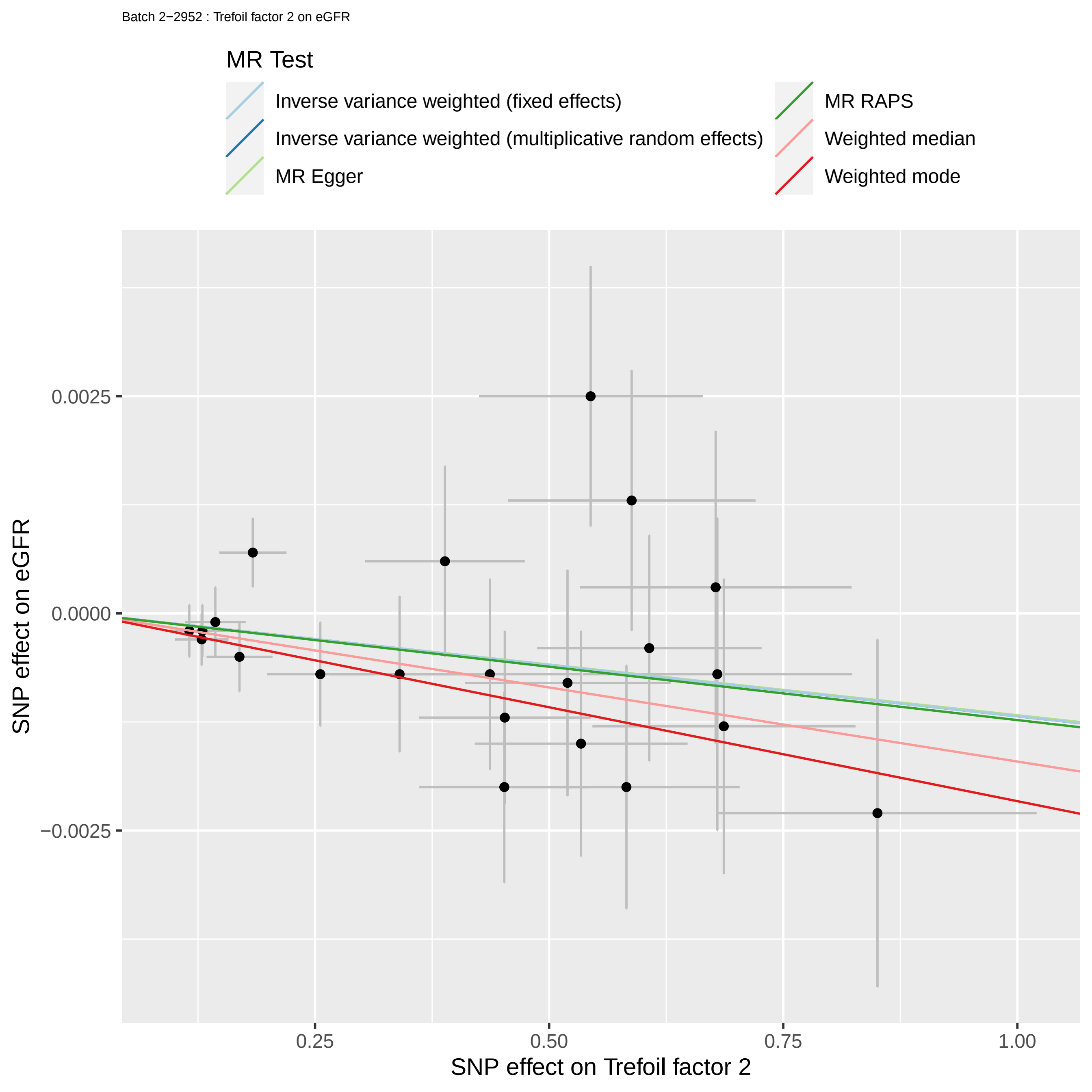

Batch 2−2952 : Trefoil factor 2 on eGFR
MR Test
Inverse variance weighted (fixed effects)
Inverse variance weighted (multiplicative random effects)
MR Egger
MR RAPS
Weighted median
Weighted mode
0.0025
0.0000
−0.0025
0.25
0.50
0.75
1.00
SNP effect on eGFR
SNP effect on Trefoil factor 2

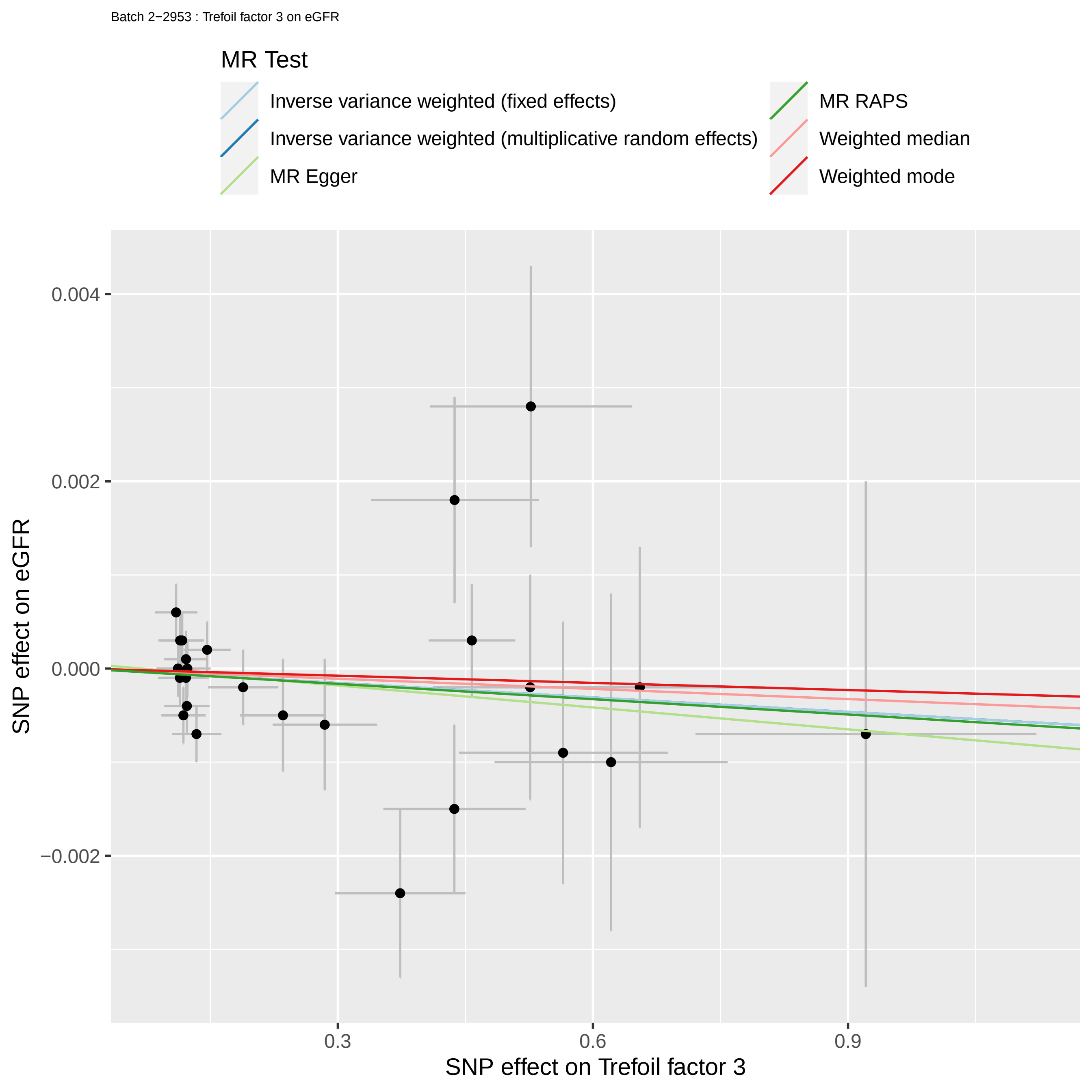

Batch 2−2953 : Trefoil factor 3 on eGFR
MR Test
Inverse variance weighted (fixed effects)
Inverse variance weighted (multiplicative random effects)
MR Egger
MR RAPS
Weighted median
Weighted mode
0.004
0.002
0.000
−0.002
0.3
0.6
0.9
SNP effect on eGFR
SNP effect on Trefoil factor 3

Batch 2−2954 : Trefoil factor 3 on eGFR

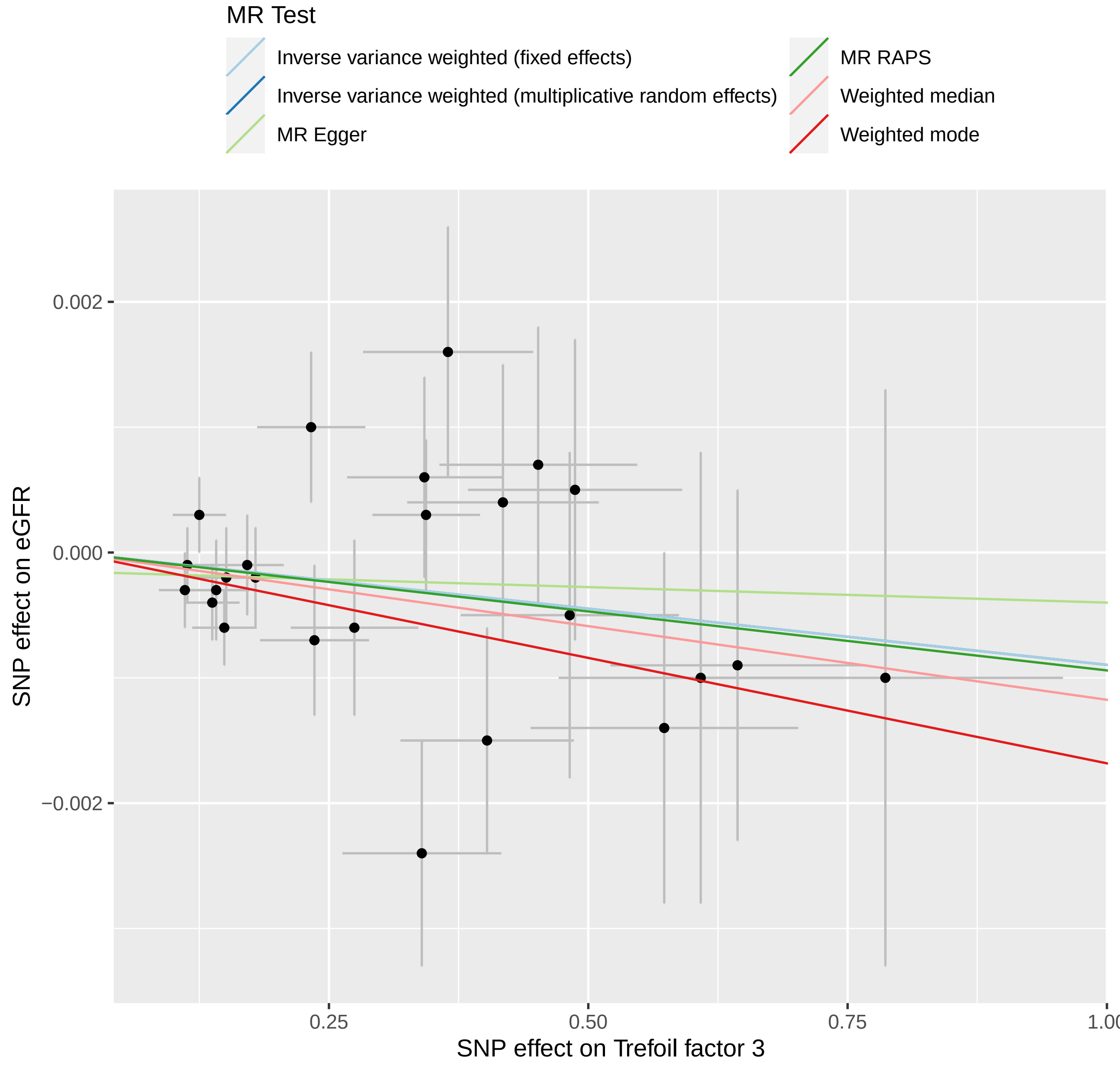

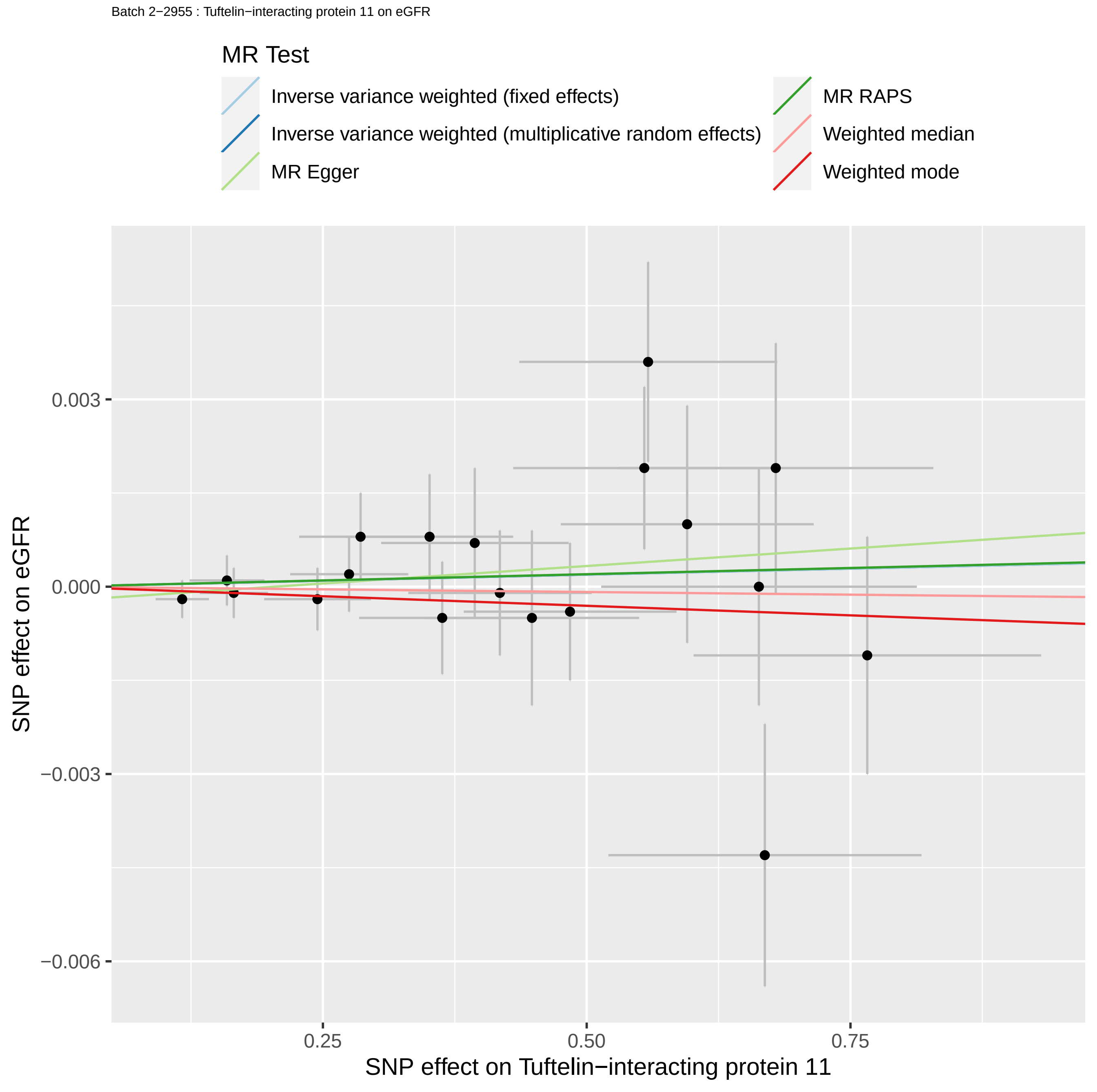

Batch 2−2955 : Tuftelin−interacting protein 11 on eGFR
MR Test
Inverse variance weighted (fixed effects)
Inverse variance weighted (multiplicative random effects)
MR Egger
MR RAPS
Weighted median
Weighted mode
0.003
0.000
−0.003
−0.006
SNP effect on eGFR
0.25
0.50
0.75
SNP effect on Tuftelin−interacting protein 11

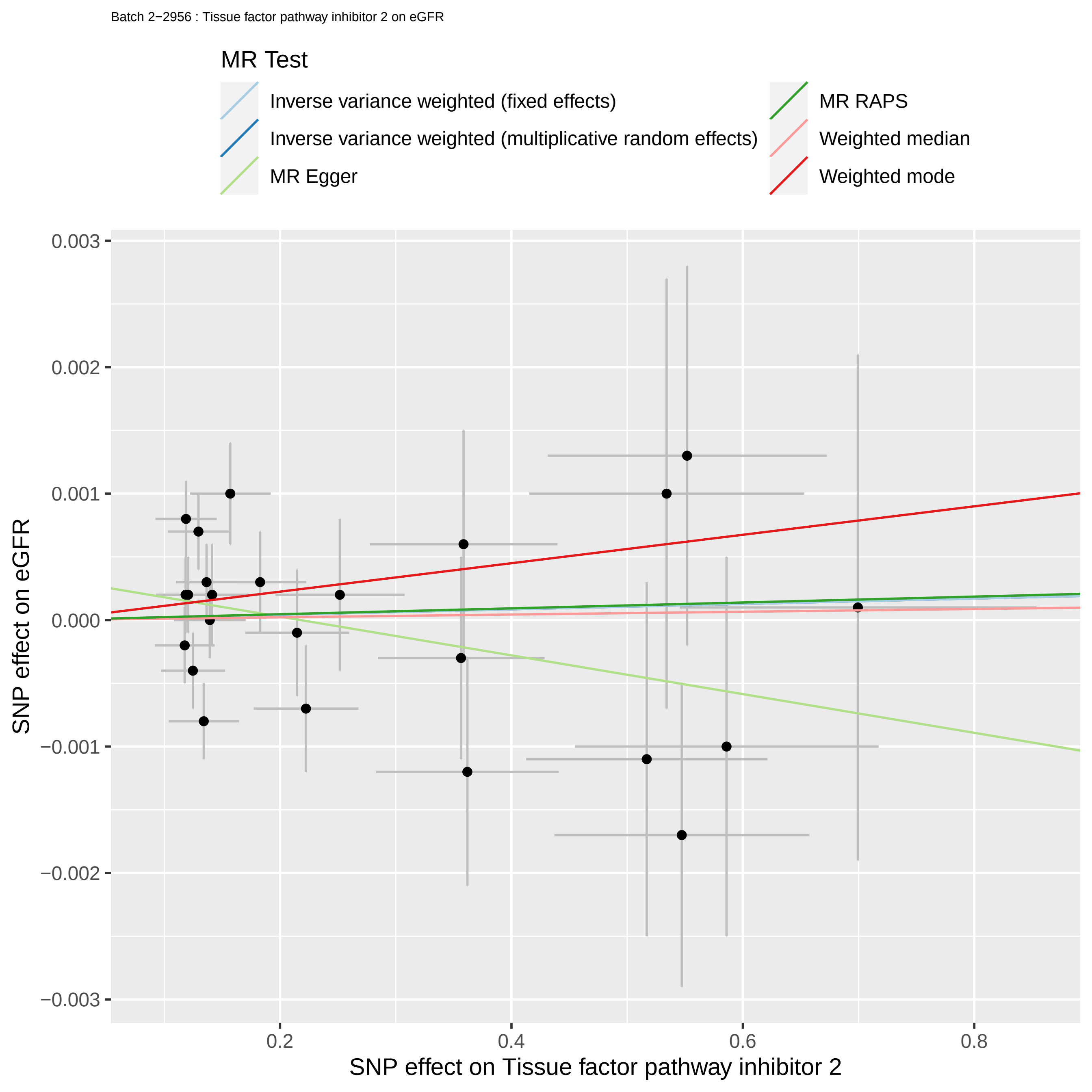

Batch 2−2956 : Tissue factor pathway inhibitor 2 on eGFR
MR Test
Inverse variance weighted (fixed effects)
Inverse variance weighted (multiplicative random effects)
MR Egger
MR RAPS
Weighted median
Weighted mode
0.003
0.002
0.001
0.000
−0.001
−0.002
−0.003
0.2
0.4
0.6
0.8
SNP effect on eGFR
SNP effect on Tissue factor pathway inhibitor 2

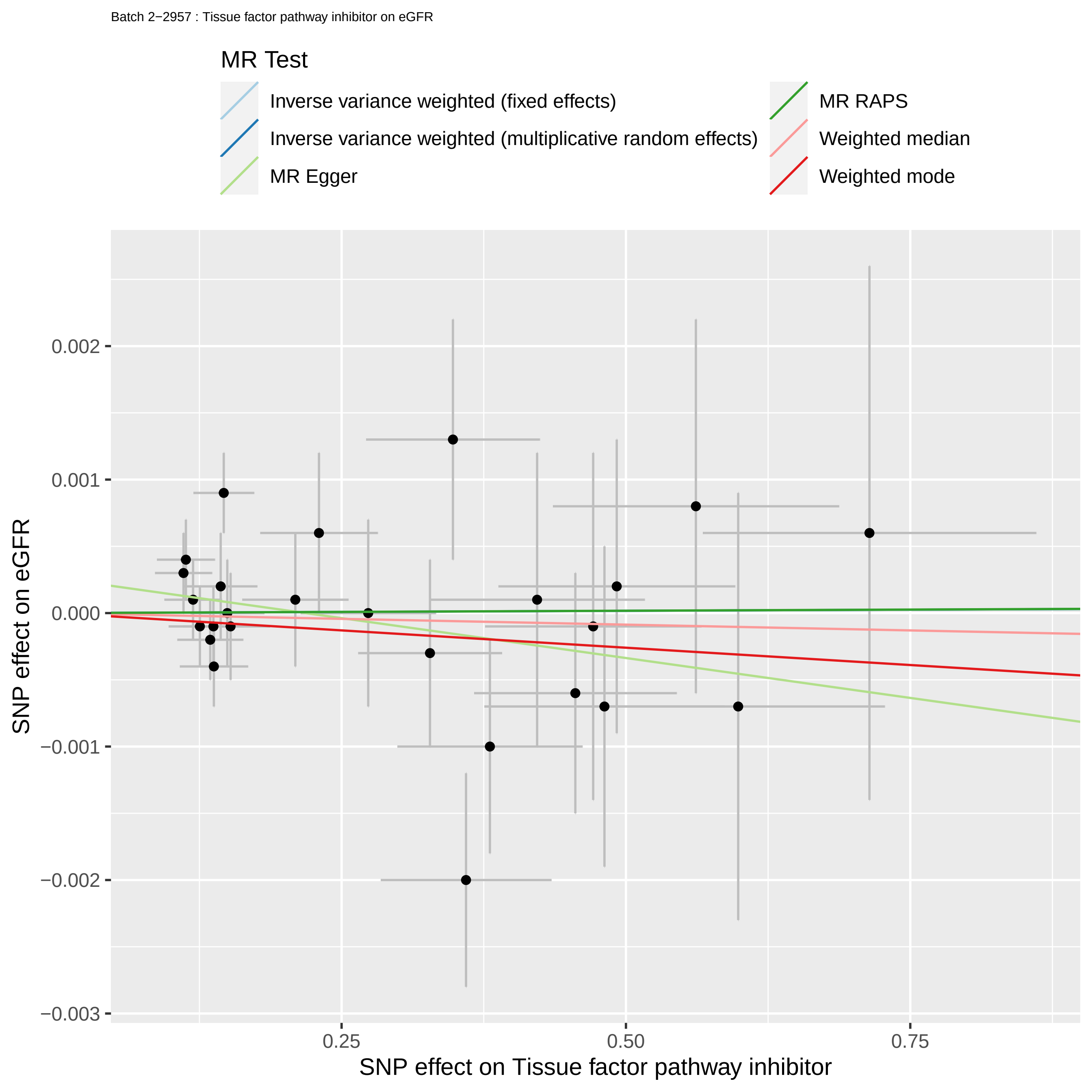
Batch 2−2957 : Tissue factor pathway inhibitor on eGFR
MR Test
Inverse variance weighted (fixed effects)
Inverse variance weighted (multiplicative random effects)
MR Egger
MR RAPS
Weighted median
Weighted mode
0.002
0.001
0.000
−0.001
−0.002
−0.003
SNP effect on eGFR
0.25
0.50
0.75
SNP effect on Tissue factor pathway inhibitor

Batch 2−2958 : Transferrin receptor protein 1 on eGFR

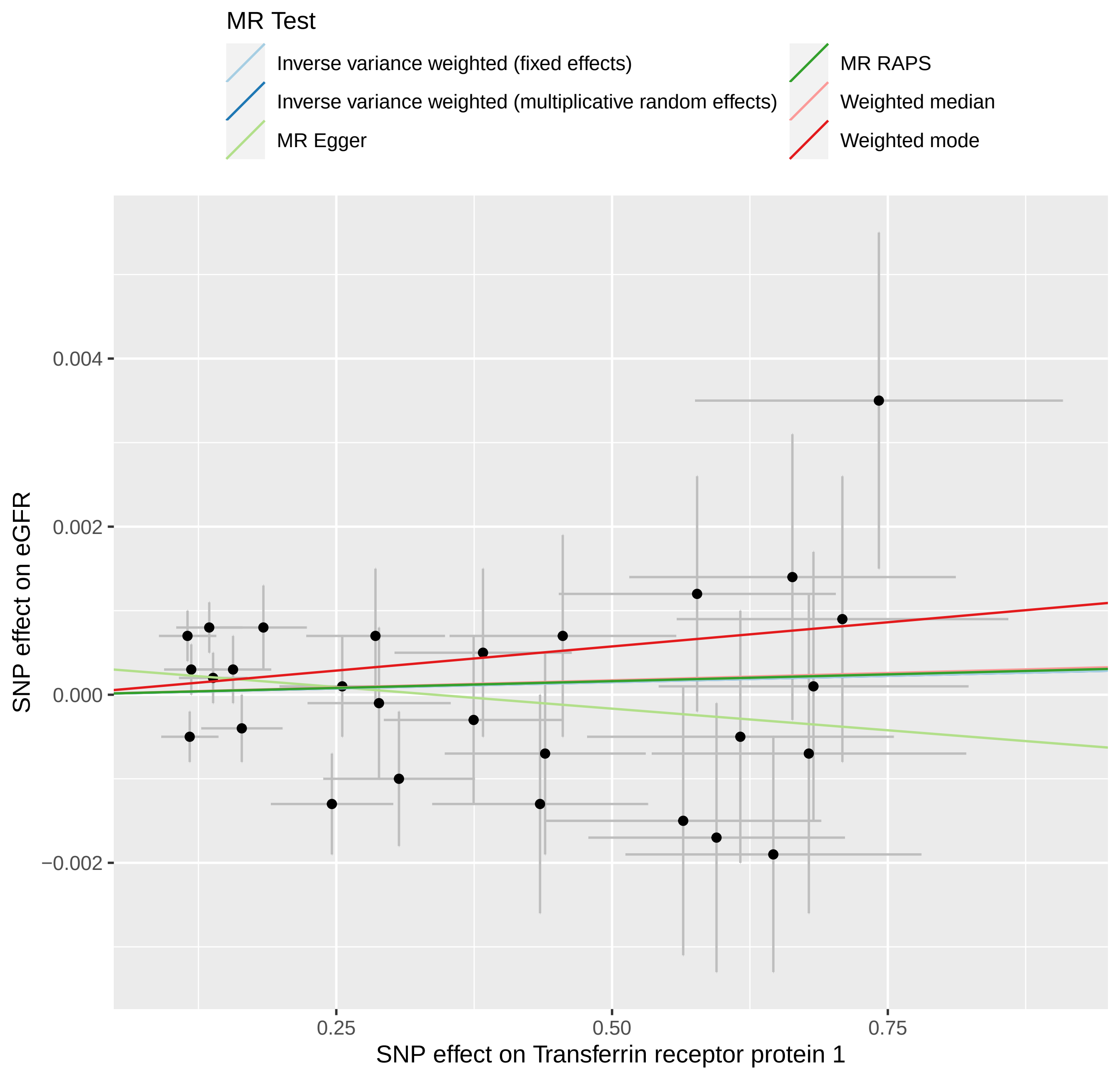

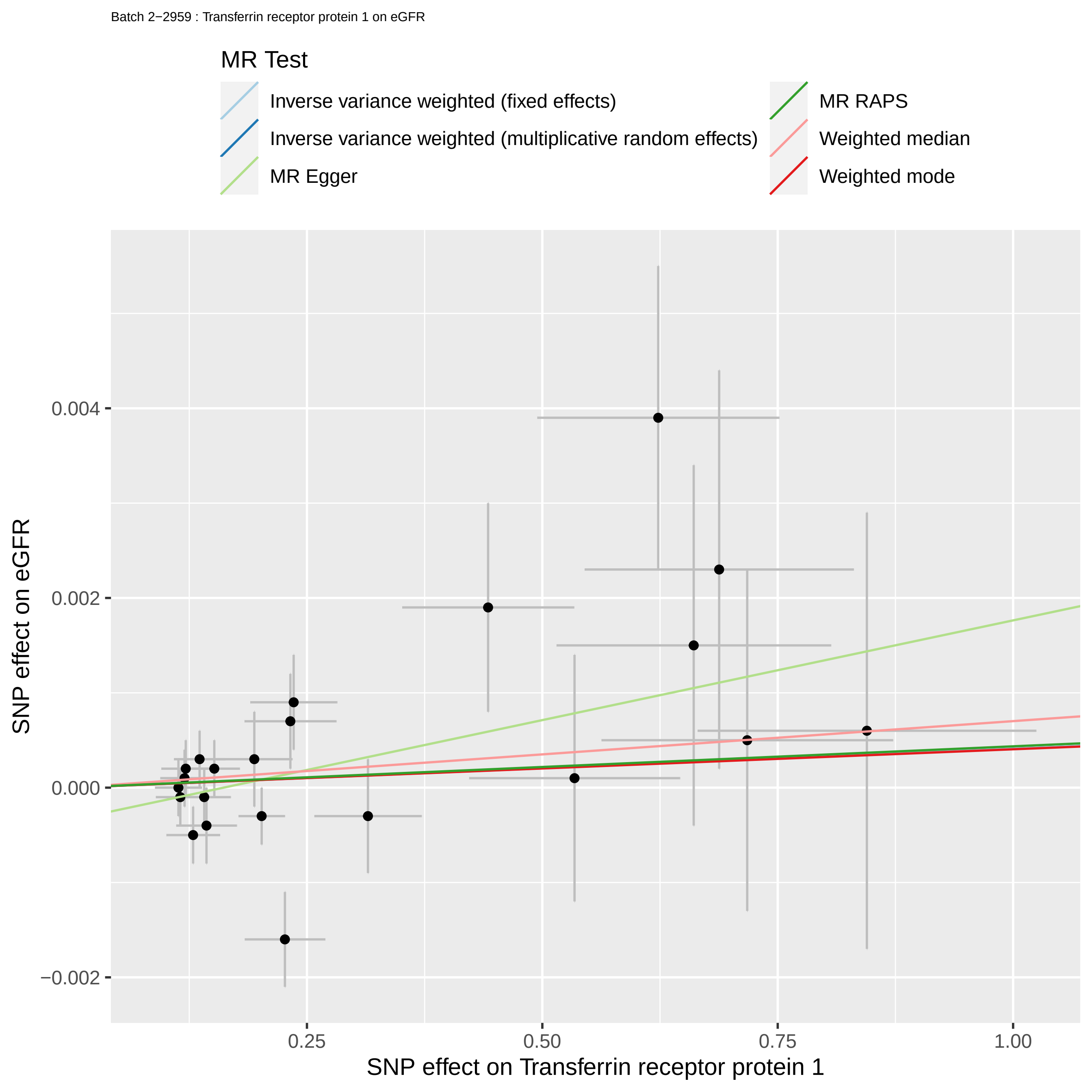

Batch 2−2959 : Transferrin receptor protein 1 on eGFR
MR Test
Inverse variance weighted (fixed effects)
Inverse variance weighted (multiplicative random effects)
MR Egger
MR RAPS
Weighted median
Weighted mode
0.004
0.002
0.000
−0.002
0.25
0.50
0.75
1.00
SNP effect on eGFR
SNP effect on Transferrin receptor protein 1

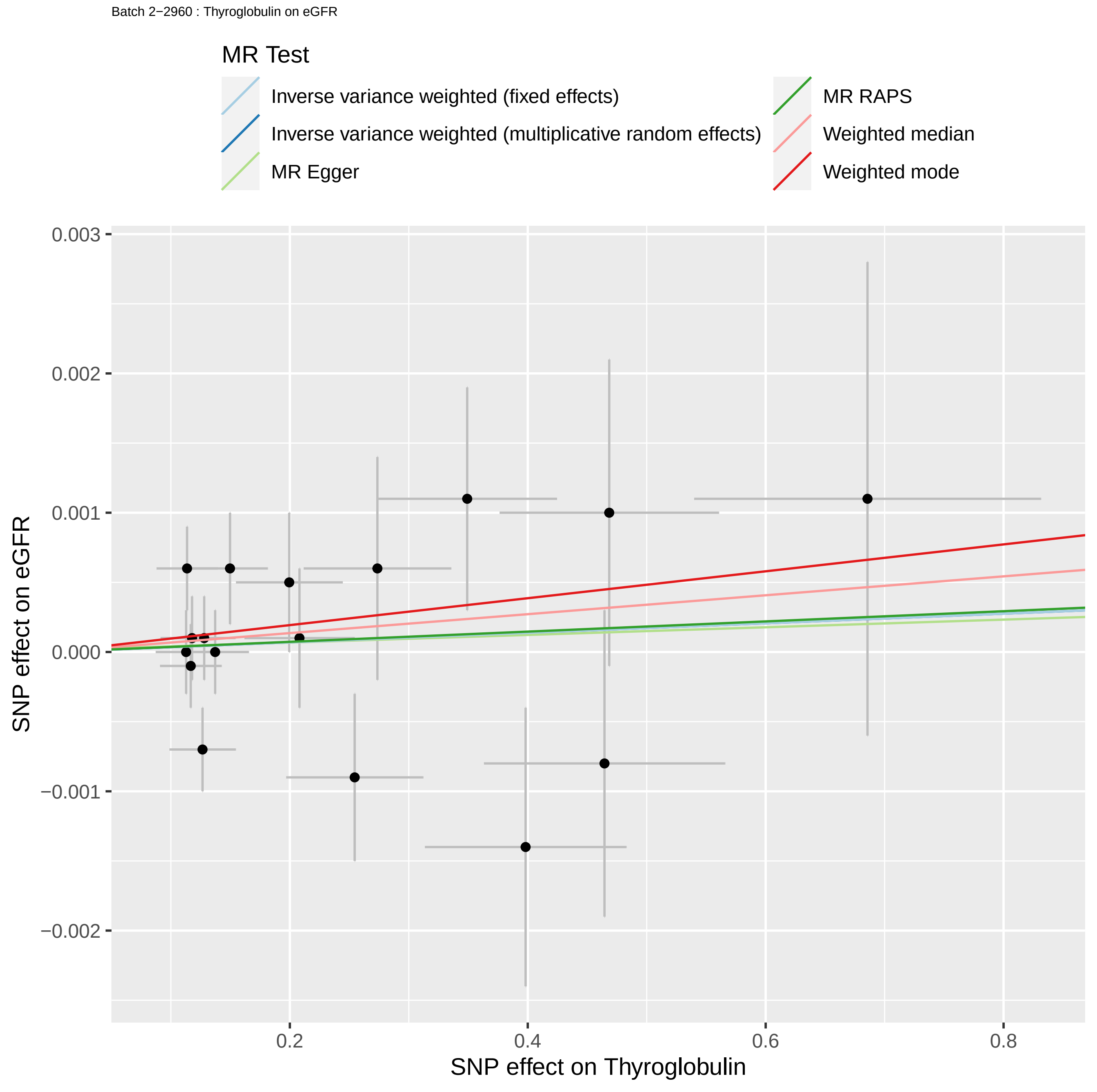
Batch 2−2960 : Thyroglobulin on eGFR
MR Test
Inverse variance weighted (fixed effects)
Inverse variance weighted (multiplicative random effects)
MR Egger
MR RAPS
Weighted median
Weighted mode
0.003
0.002
0.001
0.000
−0.001
−0.002
0.2
0.4
0.6
0.8
SNP effect on eGFR
SNP effect on Thyroglobulin

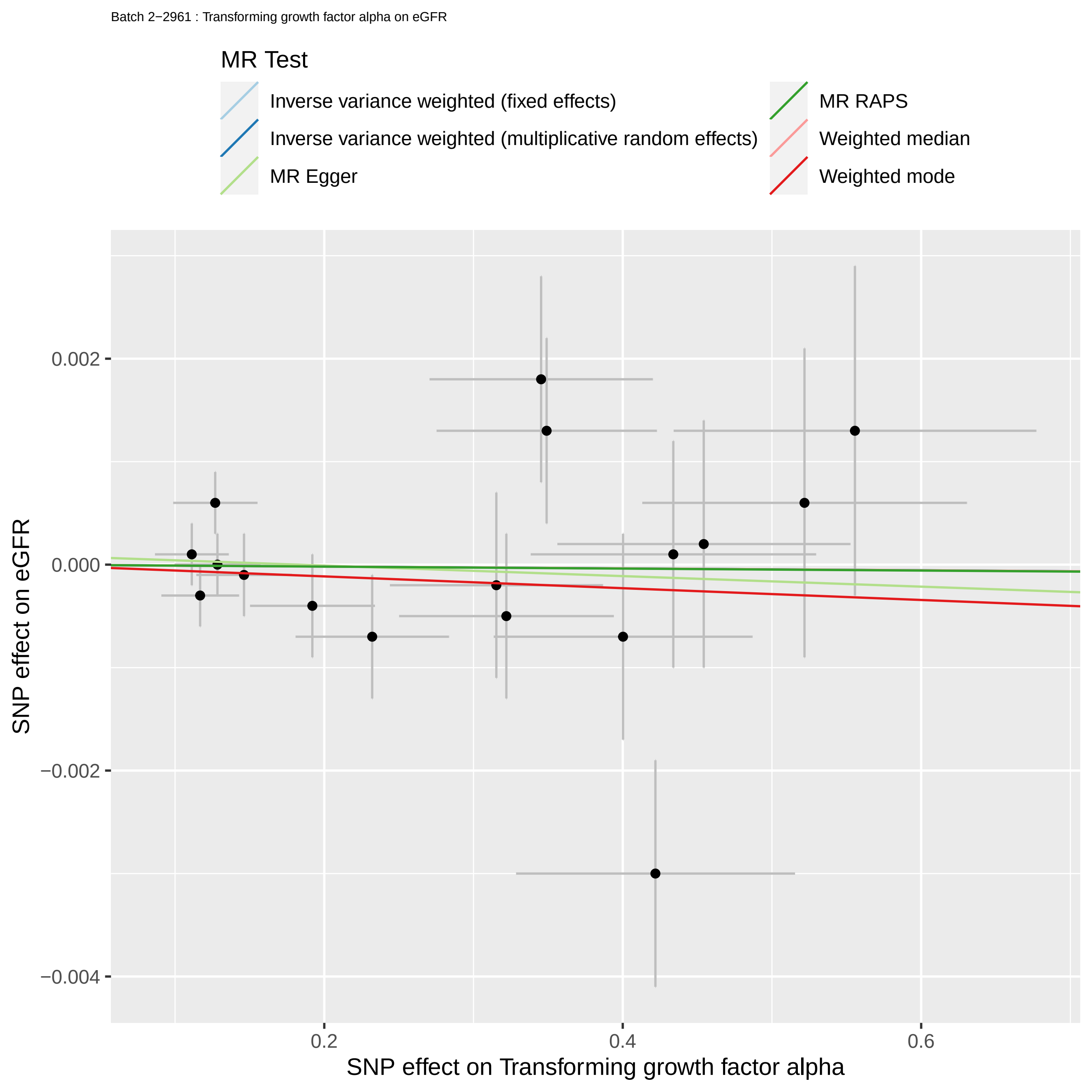
Batch 2−2961 : Transforming growth factor alpha on eGFR
MR Test
Inverse variance weighted (fixed effects)
Inverse variance weighted (multiplicative random effects)
MR Egger
MR RAPS
Weighted median
Weighted mode
0.002
0.000
−0.002
−0.004
0.2
0.4
0.6
SNP effect on eGFR
SNP effect on Transforming growth factor alpha

Batch 2−2962 : Transforming growth factor beta−1 on eGFR

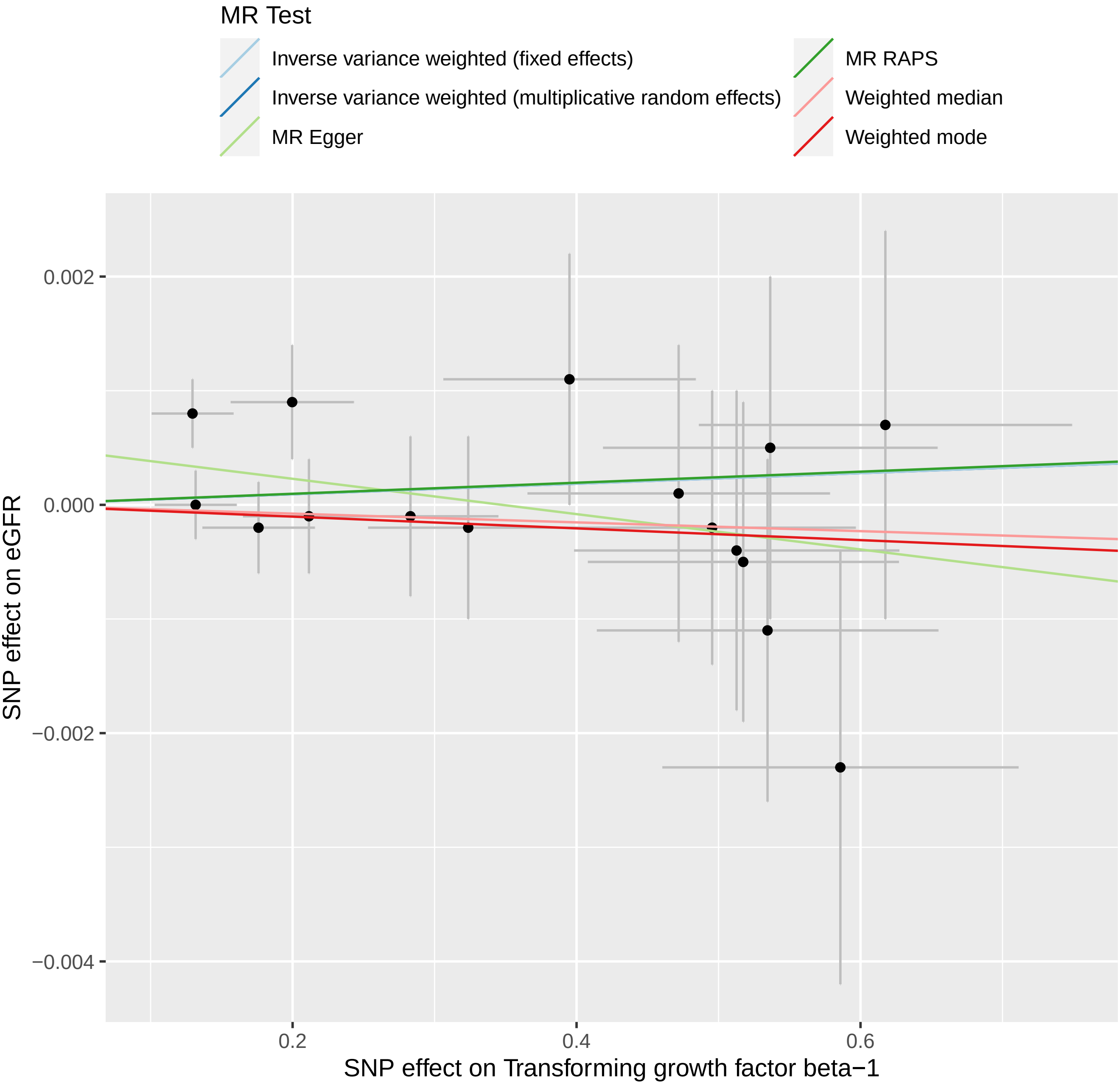

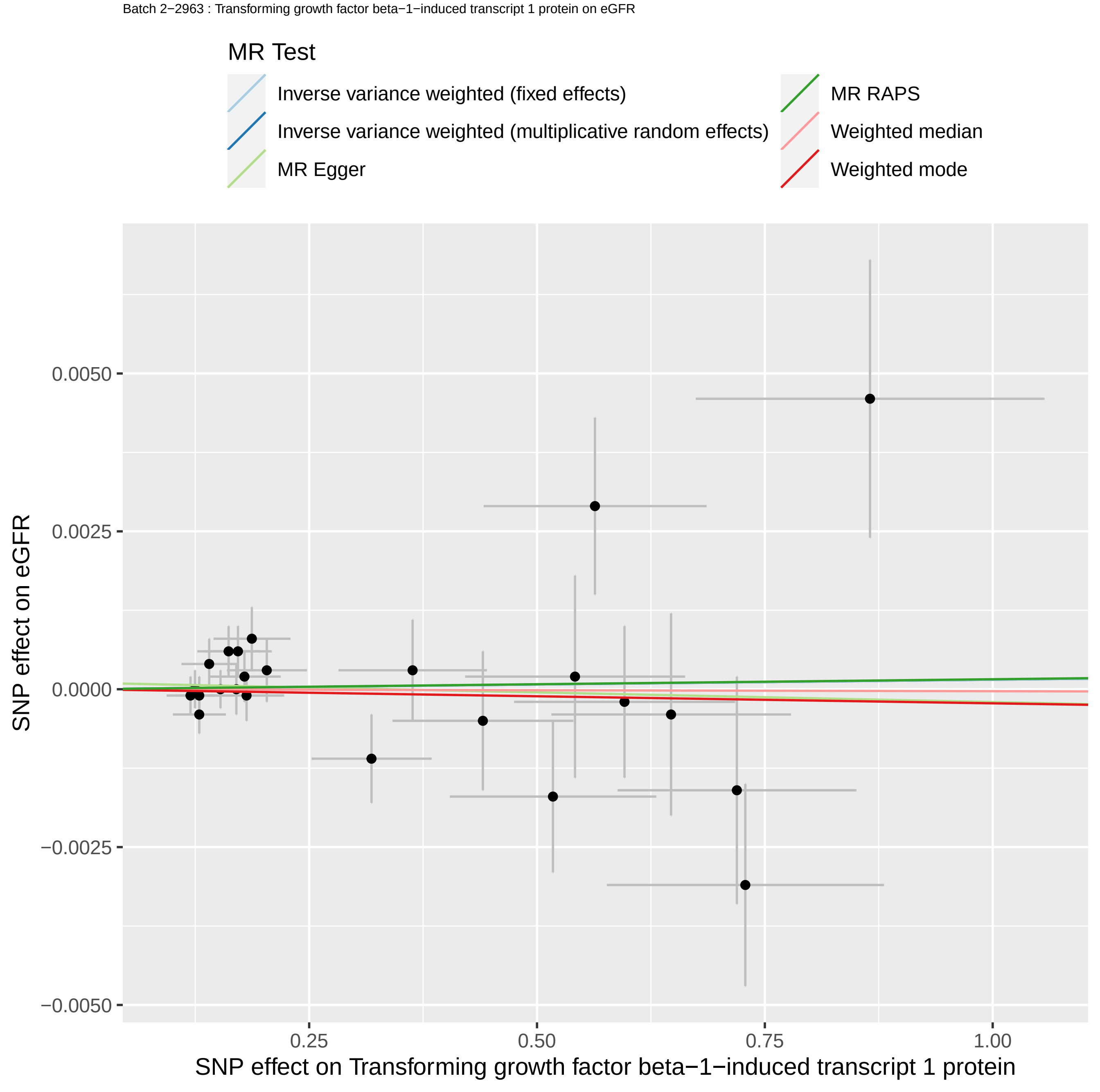
Batch 2−2963 : Transforming growth factor beta−1−induced transcript 1 protein on eGFR
MR Test
Inverse variance weighted (fixed effects)
Inverse variance weighted (multiplicative random effects)
MR Egger
MR RAPS
Weighted median
Weighted mode
0.0050
0.0025
0.0000
−0.0025
−0.0050
0.25
0.50
0.75
1.00
SNP effect on eGFR
SNP effect on Transforming growth factor beta−1−induced transcript 1 protein

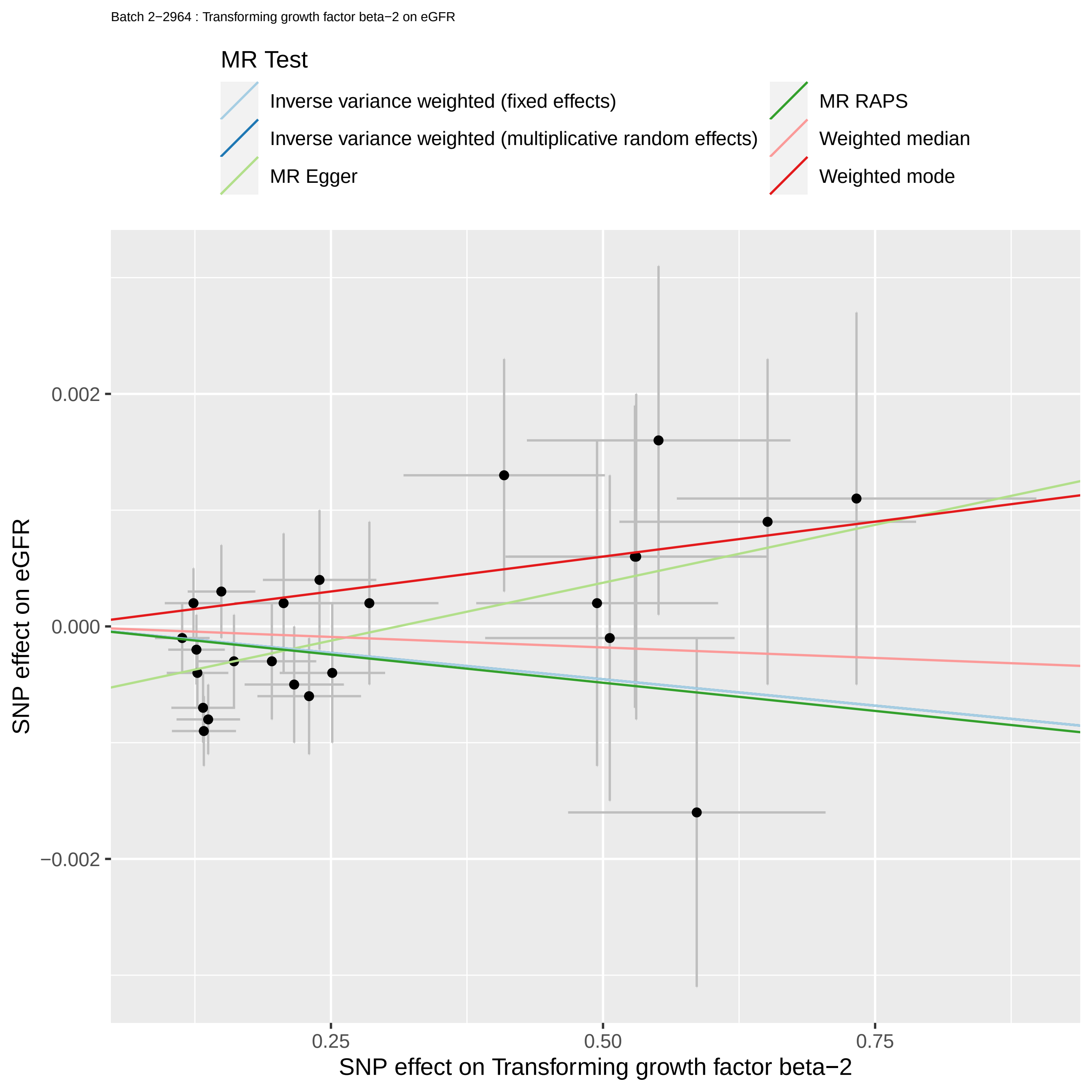
Batch 2−2964 : Transforming growth factor beta−2 on eGFR
MR Test
Inverse variance weighted (fixed effects)
Inverse variance weighted (multiplicative random effects)
MR Egger
MR RAPS
Weighted median
Weighted mode
0.002
0.000
−0.002
0.25
0.50
0.75
SNP effect on eGFR
SNP effect on Transforming growth factor beta−2

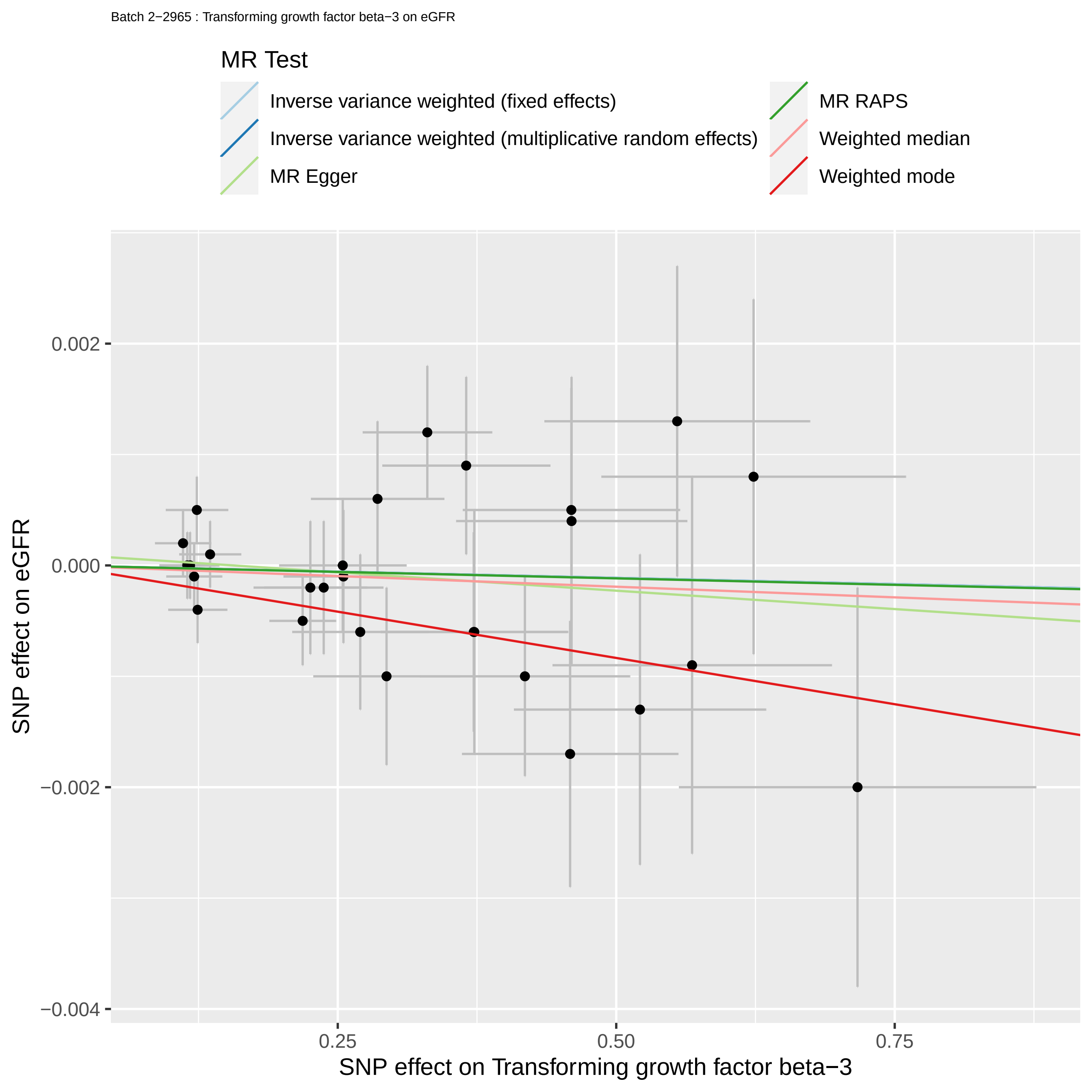
Batch 2−2965 : Transforming growth factor beta−3 on eGFR
MR Test
Inverse variance weighted (fixed effects)
Inverse variance weighted (multiplicative random effects)
MR Egger
MR RAPS
Weighted median
Weighted mode
0.002
0.000
−0.002
−0.004
0.25
0.50
0.75
SNP effect on eGFR
SNP effect on Transforming growth factor beta−3

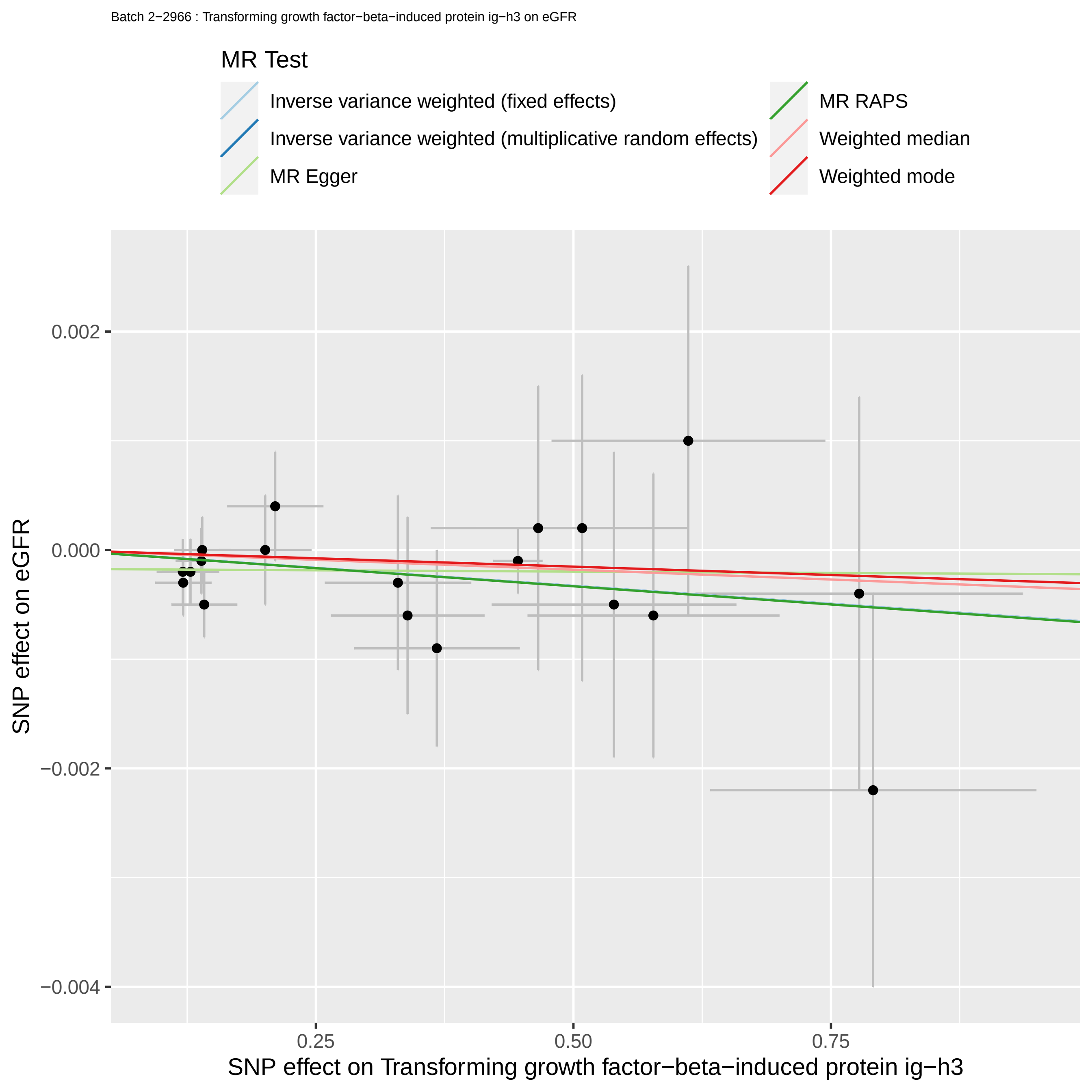
Batch 2−2966 : Transforming growth factor−beta−induced protein ig−h3 on eGFR
MR Test
Inverse variance weighted (fixed effects)
Inverse variance weighted (multiplicative random effects)
MR Egger
MR RAPS
Weighted median
Weighted mode
SNP effect on eGFR
0.002
0.000
−0.002
−0.004
0.25
0.50
0.75
SNP effect on Transforming growth factor−beta−induced protein ig−h3

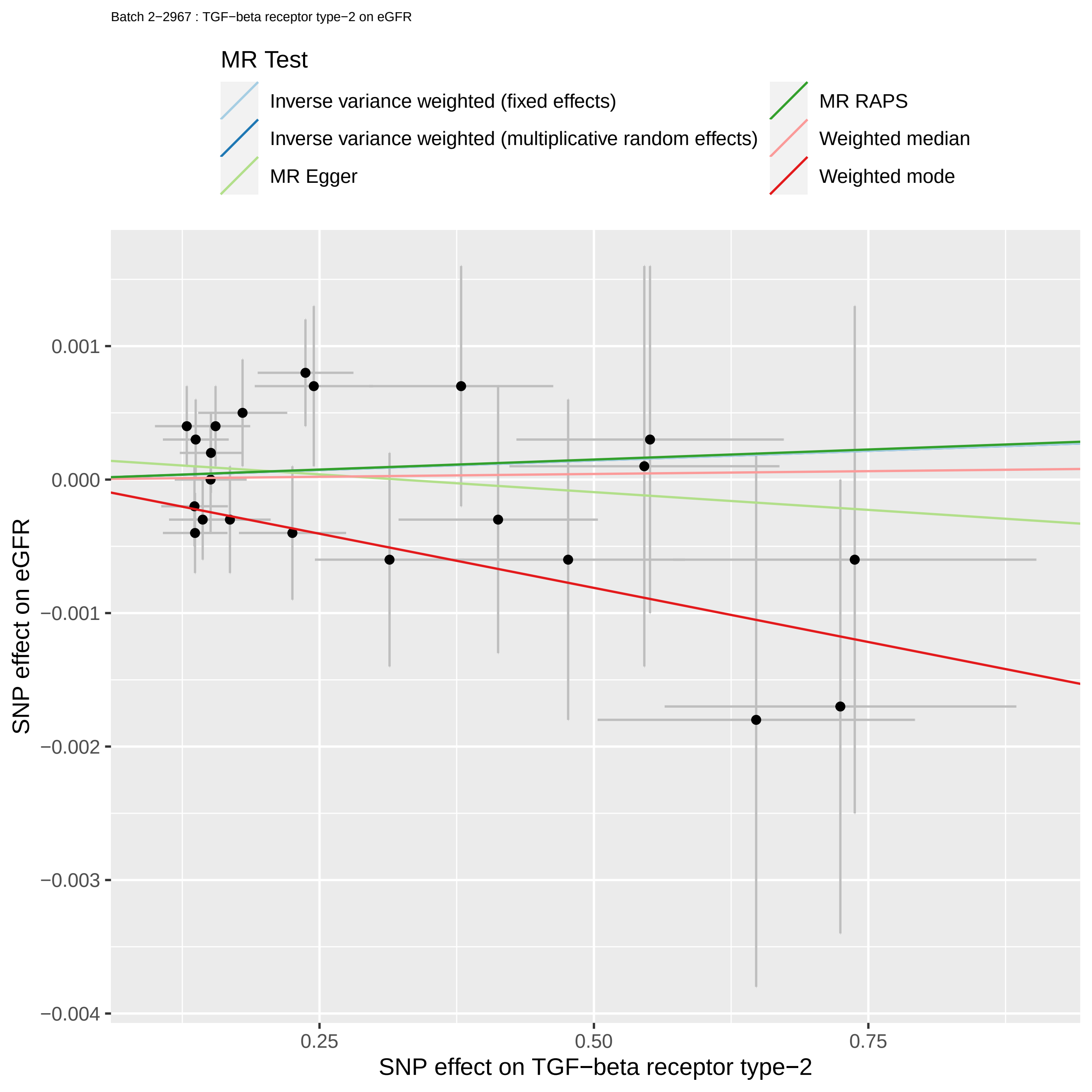

Batch 2−2967 : TGF−beta receptor type−2 on eGFR
MR Test
Inverse variance weighted (fixed effects)
Inverse variance weighted (multiplicative random effects)
MR Egger
MR RAPS
Weighted median
Weighted mode
0.001
0.000
−0.001
−0.002
−0.003
−0.004
0.25
0.50
0.75
SNP effect on eGFR
SNP effect on TGF−beta receptor type−2

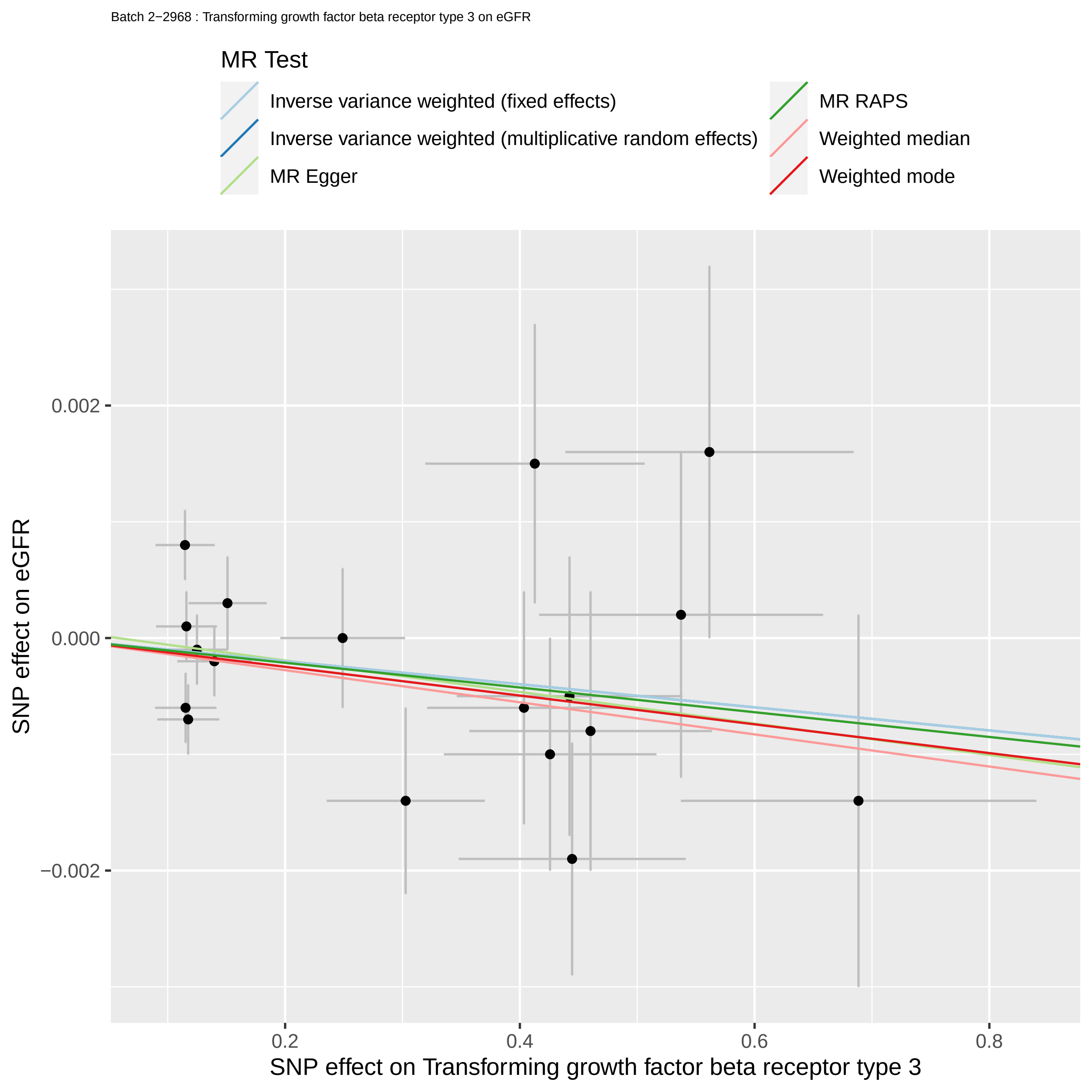
Batch 2−2968 : Transforming growth factor beta receptor type 3 on eGFR
MR Test
Inverse variance weighted (fixed effects)
Inverse variance weighted (multiplicative random effects)
MR Egger
MR RAPS
Weighted median
Weighted mode
0.002
0.000
−0.002
0.2
0.4
0.6
0.8
SNP effect on eGFR
SNP effect on Transforming growth factor beta receptor type 3

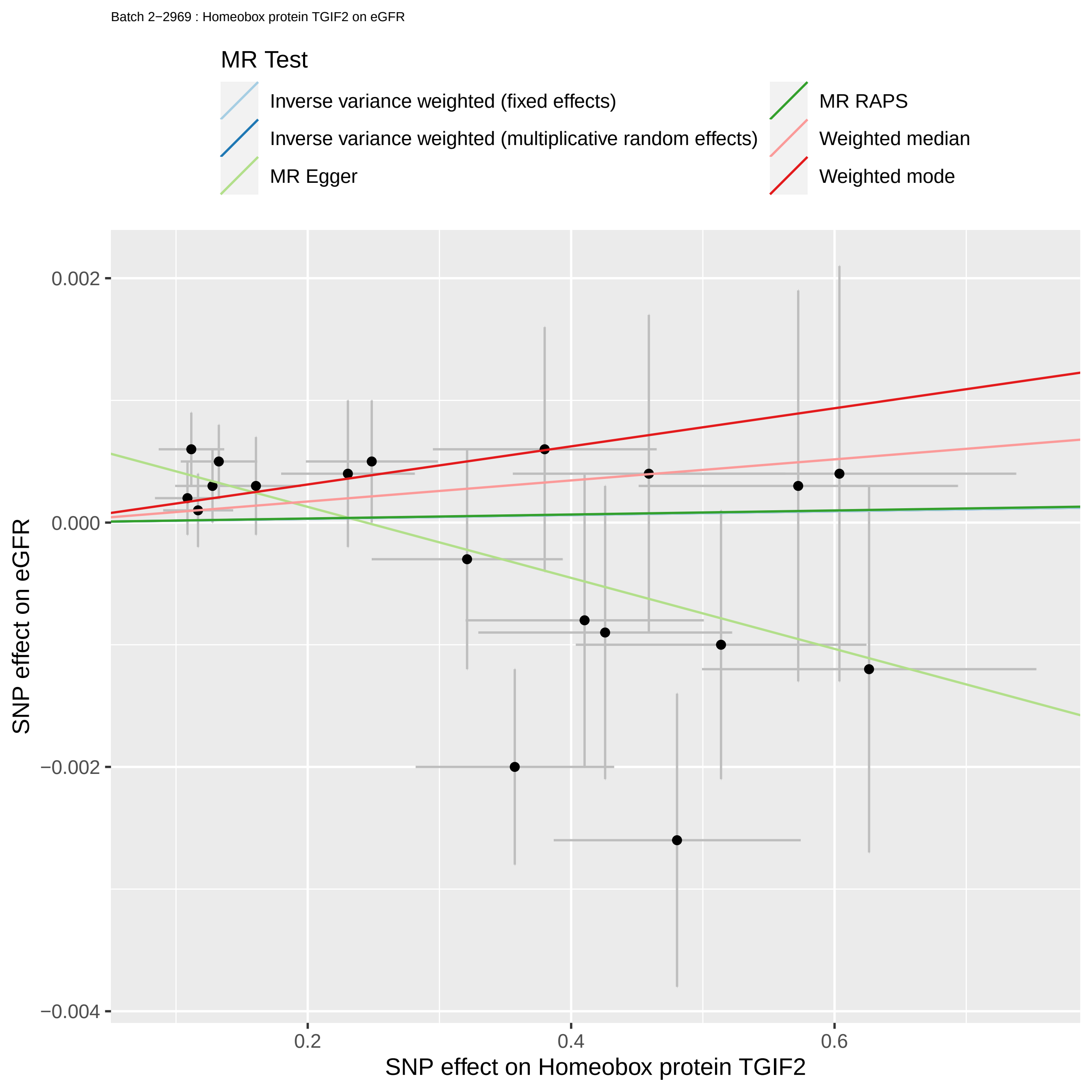
Batch 2−2969 : Homeobox protein TGIF2 on eGFR
MR Test
Inverse variance weighted (fixed effects)
Inverse variance weighted (multiplicative random effects)
MR Egger
MR RAPS
Weighted median
Weighted mode
0.002
0.000
−0.002
−0.004
SNP effect on eGFR
0.2
0.4
0.6
SNP effect on Homeobox protein TGIF2

Batch 2−2970 : Thrombospondin−2 on eGFR

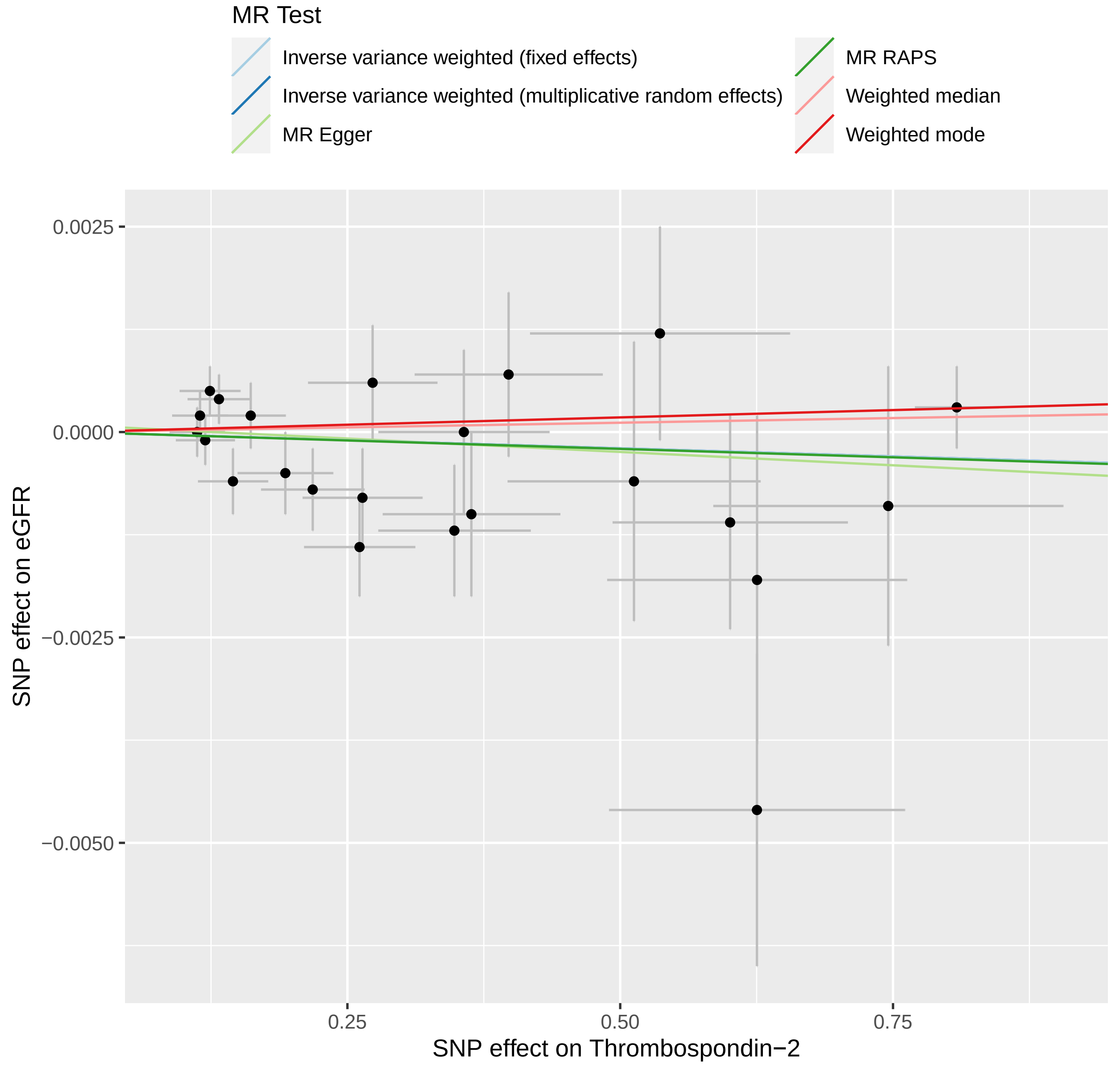

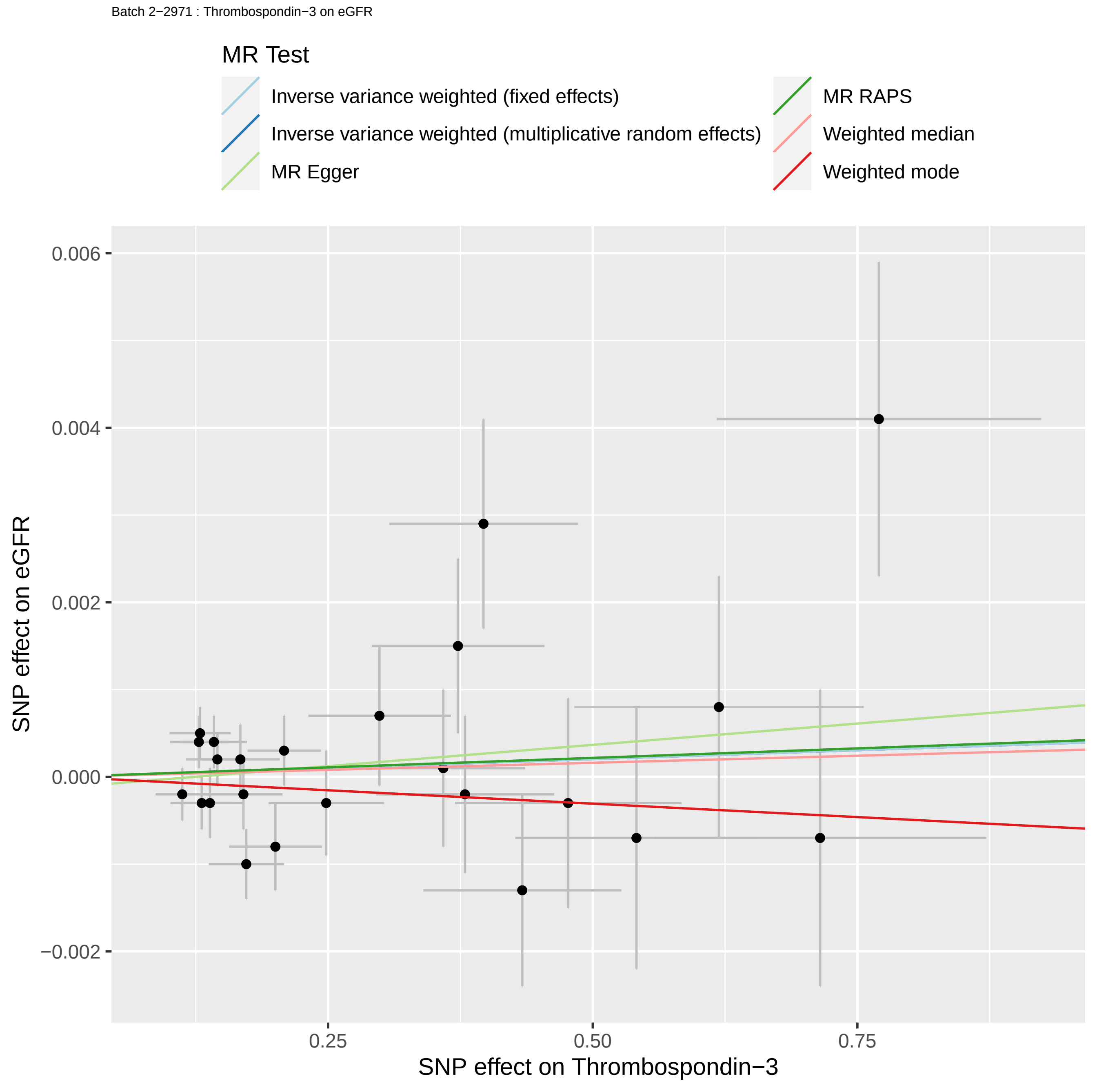
Batch 2−2971 : Thrombospondin−3 on eGFR
MR Test
Inverse variance weighted (fixed effects)
Inverse variance weighted (multiplicative random effects)
MR Egger
MR RAPS
Weighted median
Weighted mode
0.006
0.004
0.002
0.000
−0.002
0.25
0.50
0.75
SNP effect on eGFR
SNP effect on Thrombospondin−3

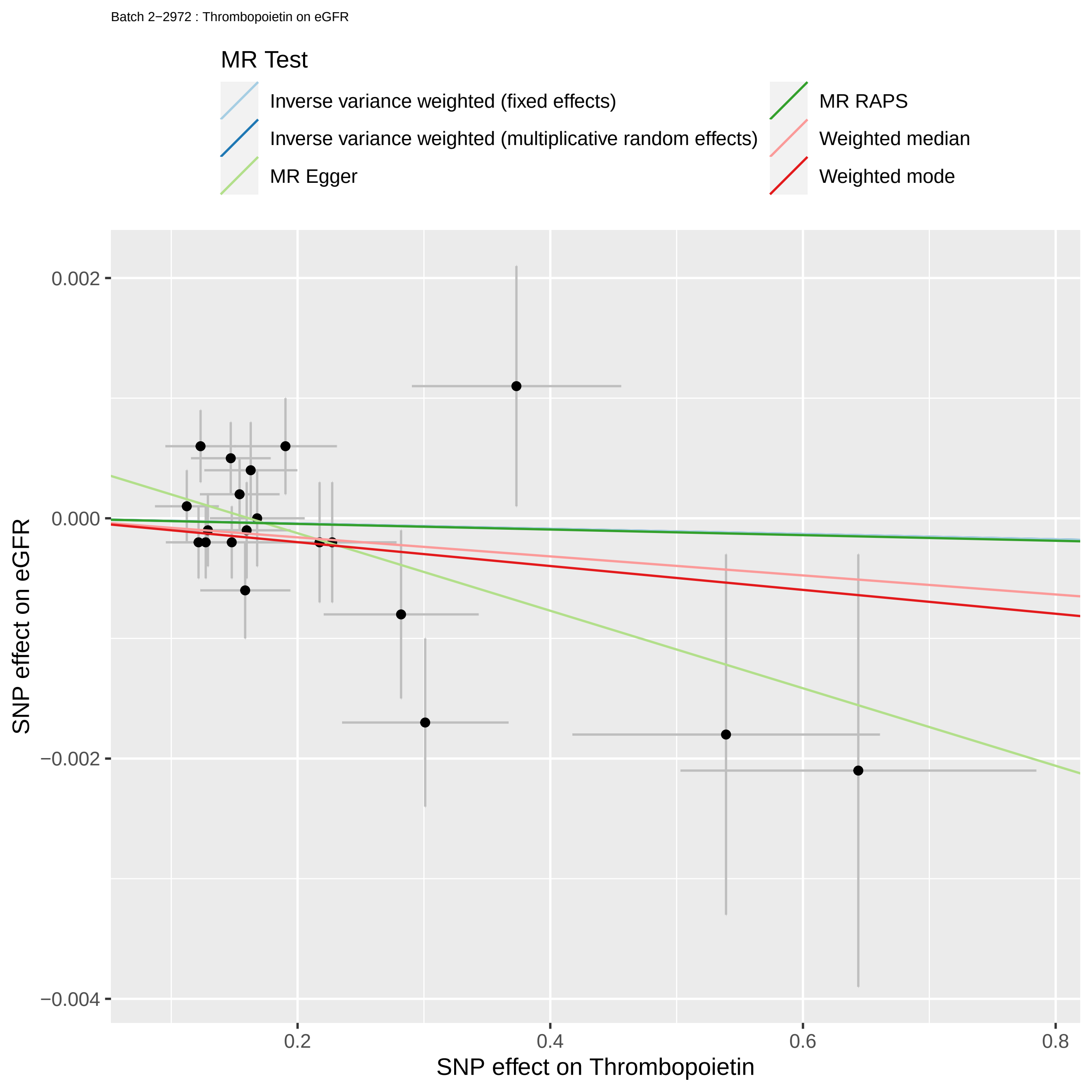
Batch 2−2972 : Thrombopoietin on eGFR
MR Test
Inverse variance weighted (fixed effects)
Inverse variance weighted (multiplicative random effects)
MR Egger
MR RAPS
Weighted median
Weighted mode
0.002
0.000
−0.002
−0.004
0.2
0.4
0.6
0.8
SNP effect on eGFR
SNP effect on Thrombopoietin

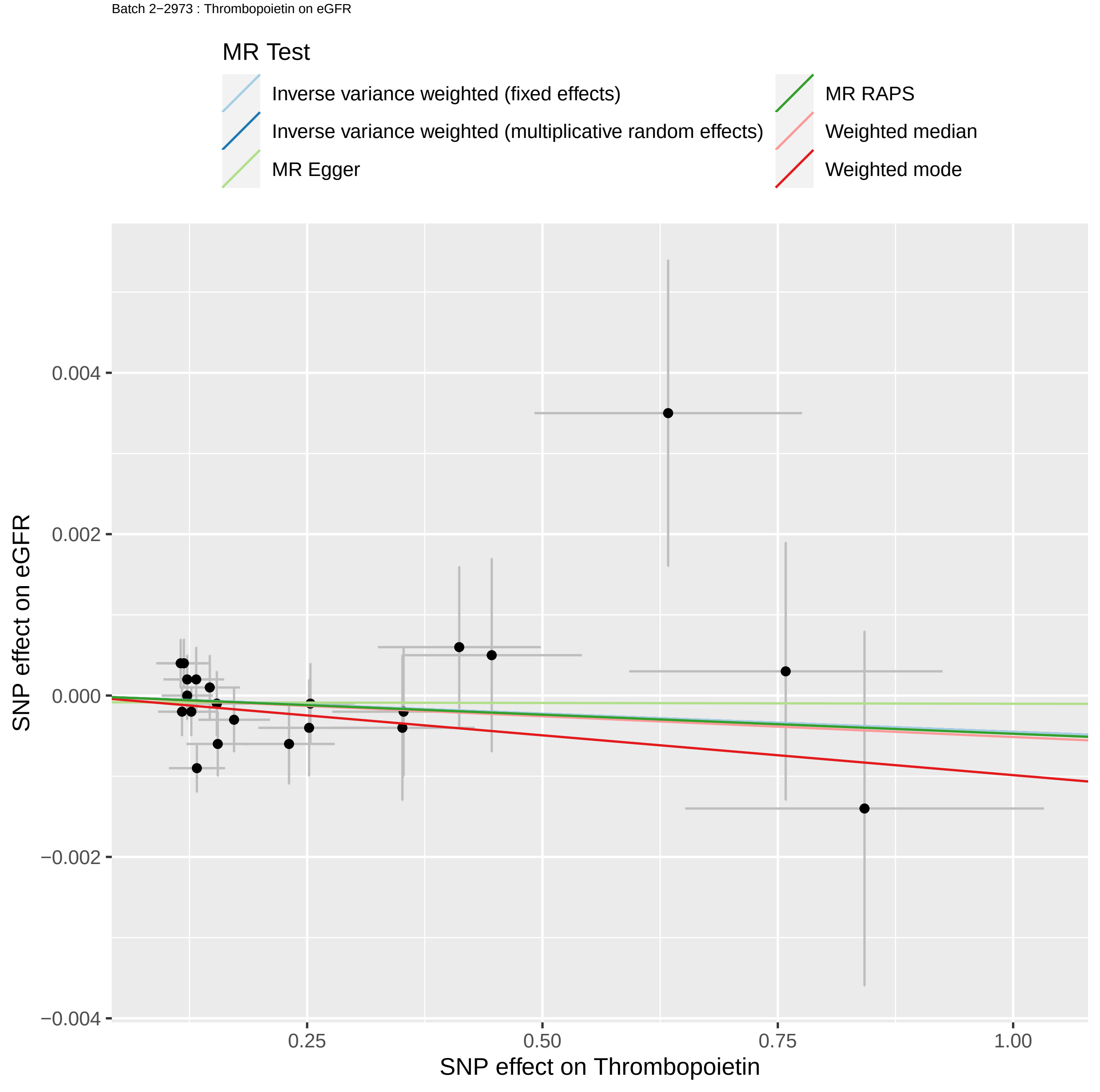

Batch 2−2973 : Thrombopoietin on eGFR
MR Test
Inverse variance weighted (fixed effects)
Inverse variance weighted (multiplicative random effects)
MR Egger
MR RAPS
Weighted median
Weighted mode
0.004
0.002
0.000
−0.002
−0.004
0.25
0.50
0.75
1.00
SNP effect on eGFR
SNP effect on Thrombopoietin

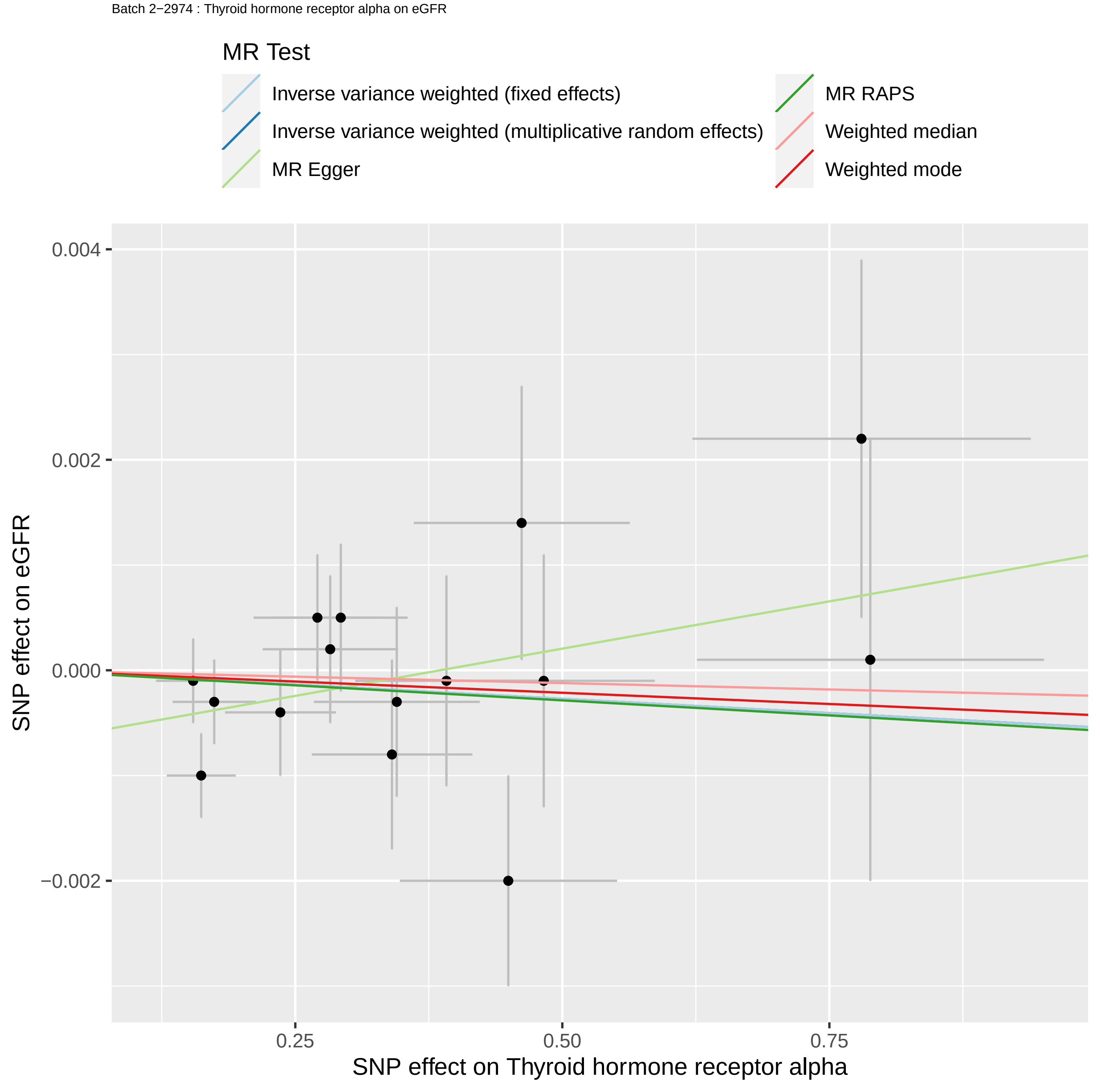
Batch 2−2974 : Thyroid hormone receptor alpha on eGFR
MR Test
Inverse variance weighted (fixed effects)
Inverse variance weighted (multiplicative random effects)
MR Egger
MR RAPS
Weighted median
Weighted mode
0.004
0.002
0.000
−0.002
0.25
0.50
0.75
SNP effect on eGFR
SNP effect on Thyroid hormone receptor alpha

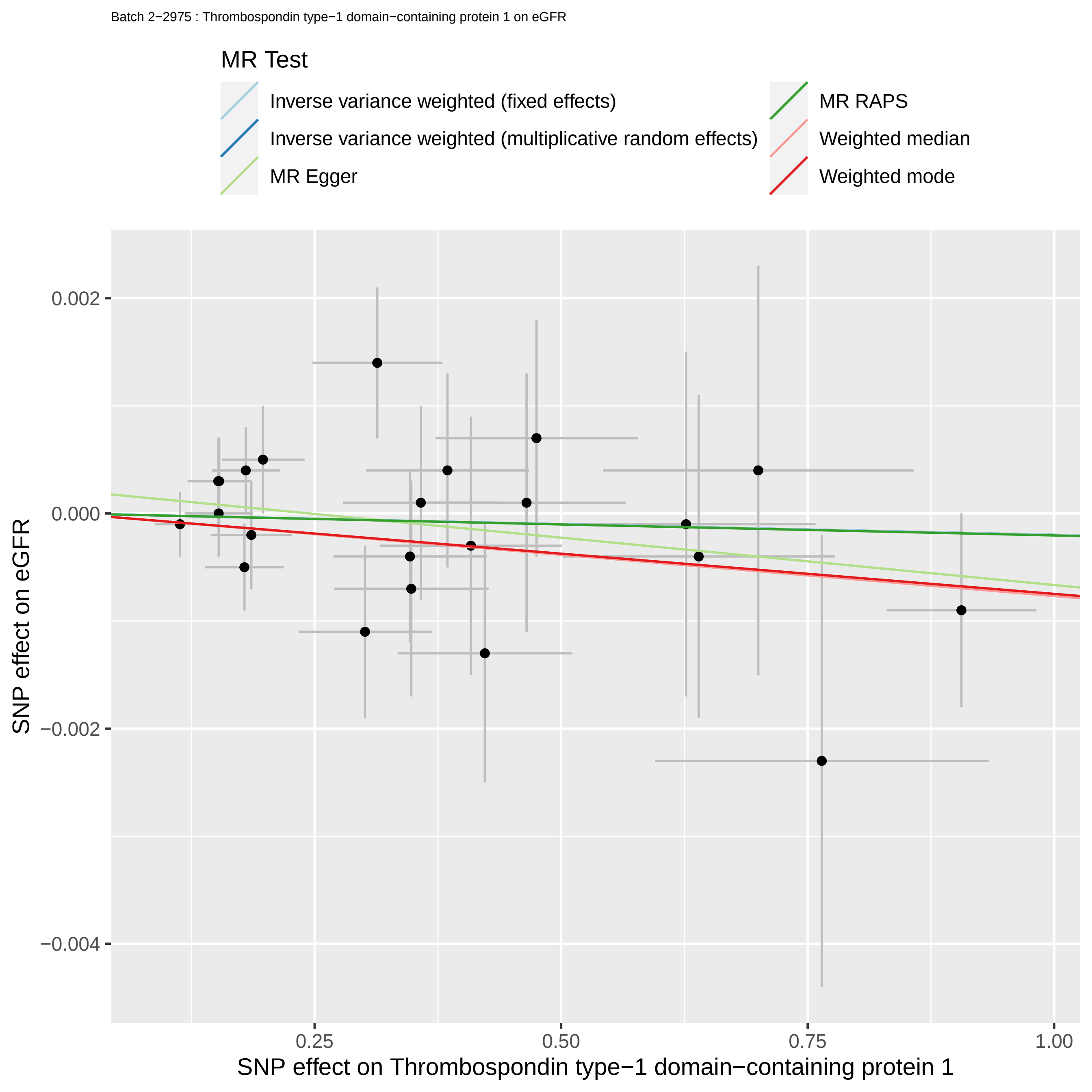

Batch 2−2975 : Thrombospondin type−1 domain−containing protein 1 on eGFR
MR Test
Inverse variance weighted (fixed effects)
Inverse variance weighted (multiplicative random effects)
MR Egger
MR RAPS
Weighted median
Weighted mode
0.002
0.000
−0.002
−0.004
0.25
0.50
0.75
1.00
SNP effect on eGFR
SNP effect on Thrombospondin type−1 domain−containing protein 1

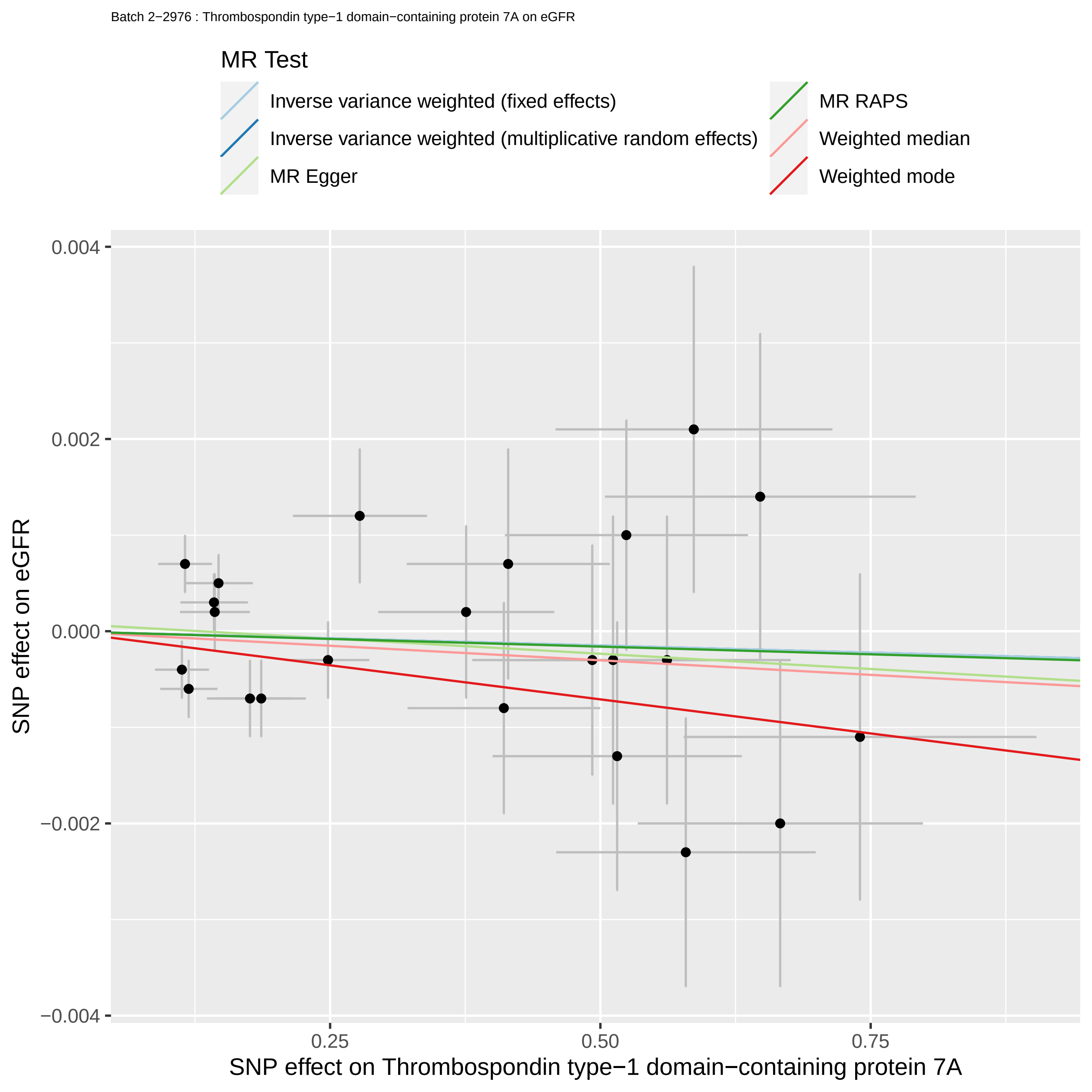

Batch 2−2976 : Thrombospondin type−1 domain−containing protein 7A on eGFR
MR Test
Inverse variance weighted (fixed effects)
Inverse variance weighted (multiplicative random effects)
MR Egger
MR RAPS
Weighted median
Weighted mode
0.004
0.002
0.000
−0.002
−0.004
SNP effect on eGFR
0.25
0.50
0.75
SNP effect on Thrombospondin type−1 domain−containing protein 7A

Batch 2−2977 : Thymocyte nuclear protein 1 on eGFR

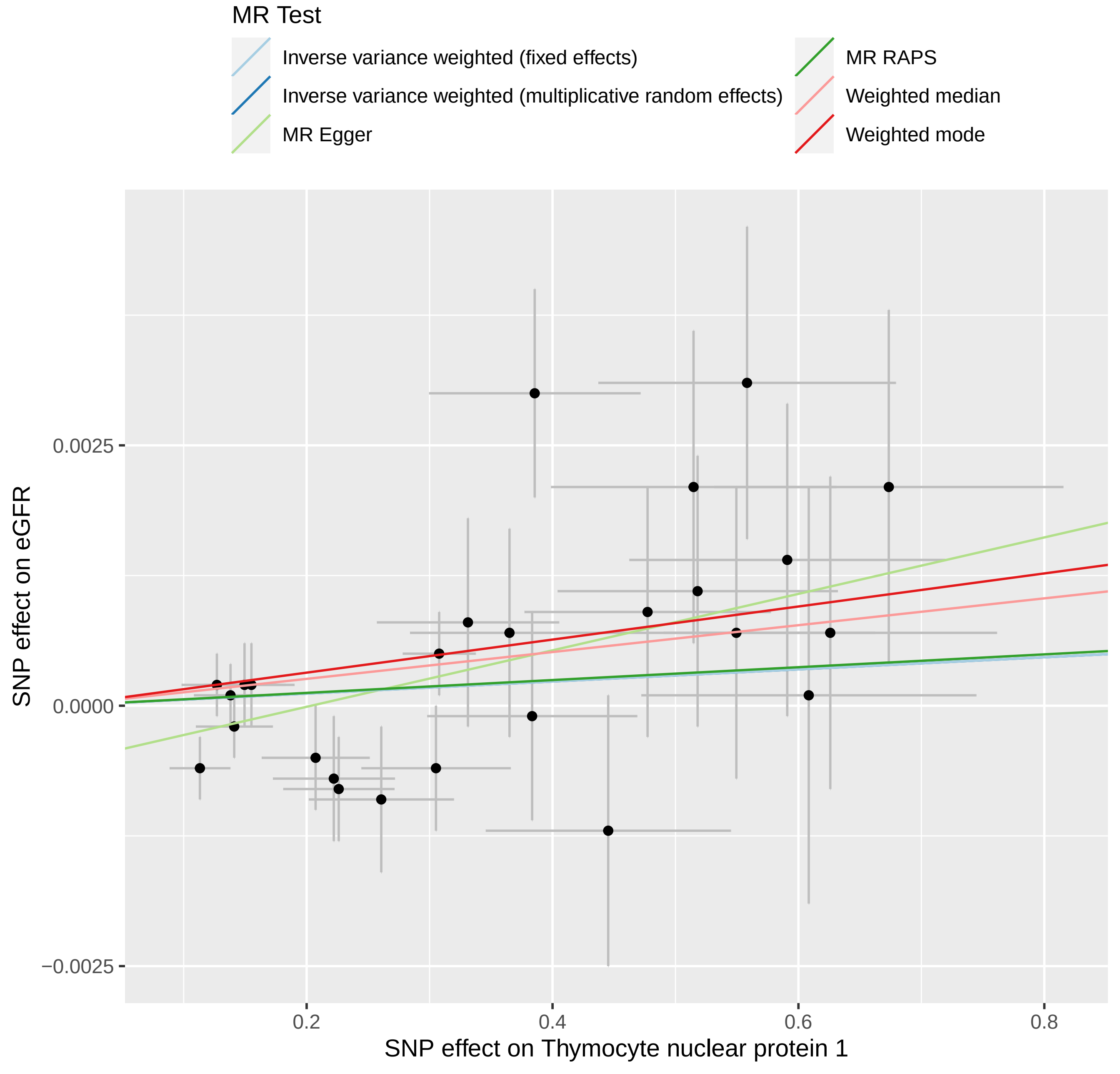

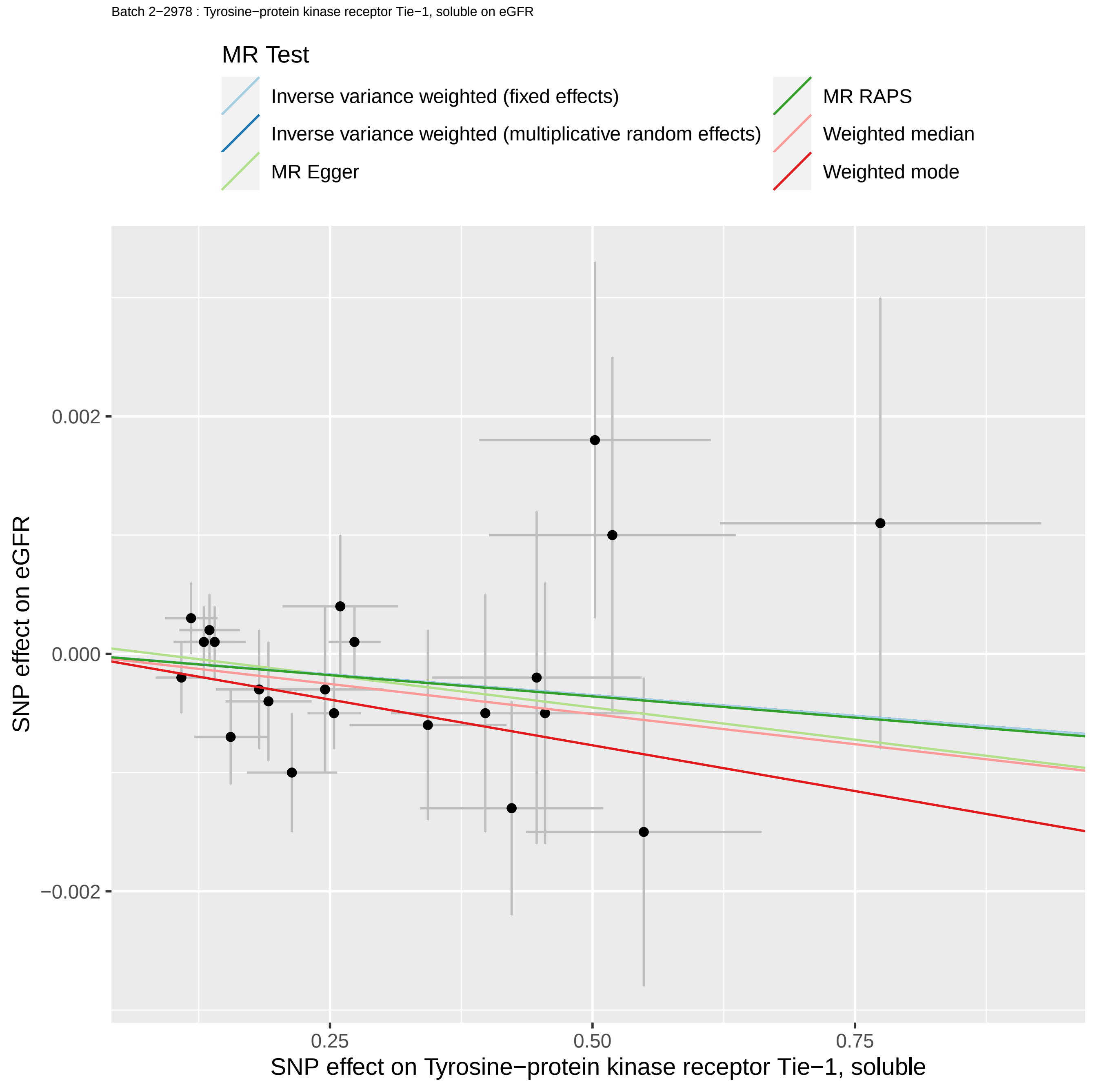

Batch 2−2978 : Tyrosine−protein kinase receptor Tie−1, soluble on eGFR
MR Test
Inverse variance weighted (fixed effects)
Inverse variance weighted (multiplicative random effects)
MR Egger
MR RAPS
Weighted median
Weighted mode
SNP effect on eGFR
0.002
0.000
−0.002
0.25
0.50
0.75
SNP effect on Tyrosine−protein kinase receptor Tie−1, soluble

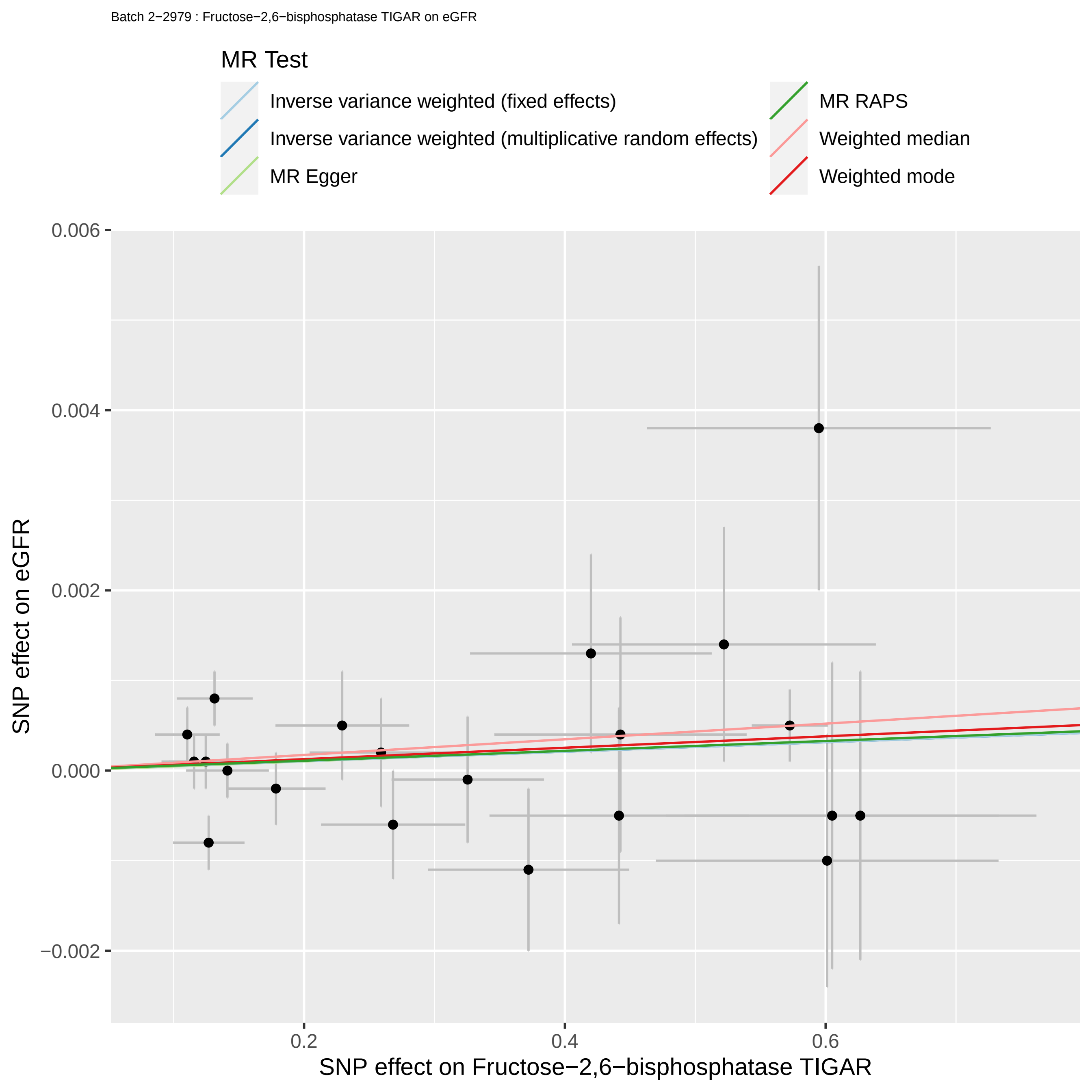
Batch 2−2979 : Fructose−2,6−bisphosphatase TIGAR on eGFR
MR Test
Inverse variance weighted (fixed effects)
Inverse variance weighted (multiplicative random effects)
MR Egger
MR RAPS
Weighted median
Weighted mode
0.006
0.004
0.002
0.000
−0.002
0.2
0.4
0.6
SNP effect on eGFR
SNP effect on Fructose−2,6−bisphosphatase TIGAR

Batch 2−2980 : Metalloproteinase inhibitor 4 on eGFR

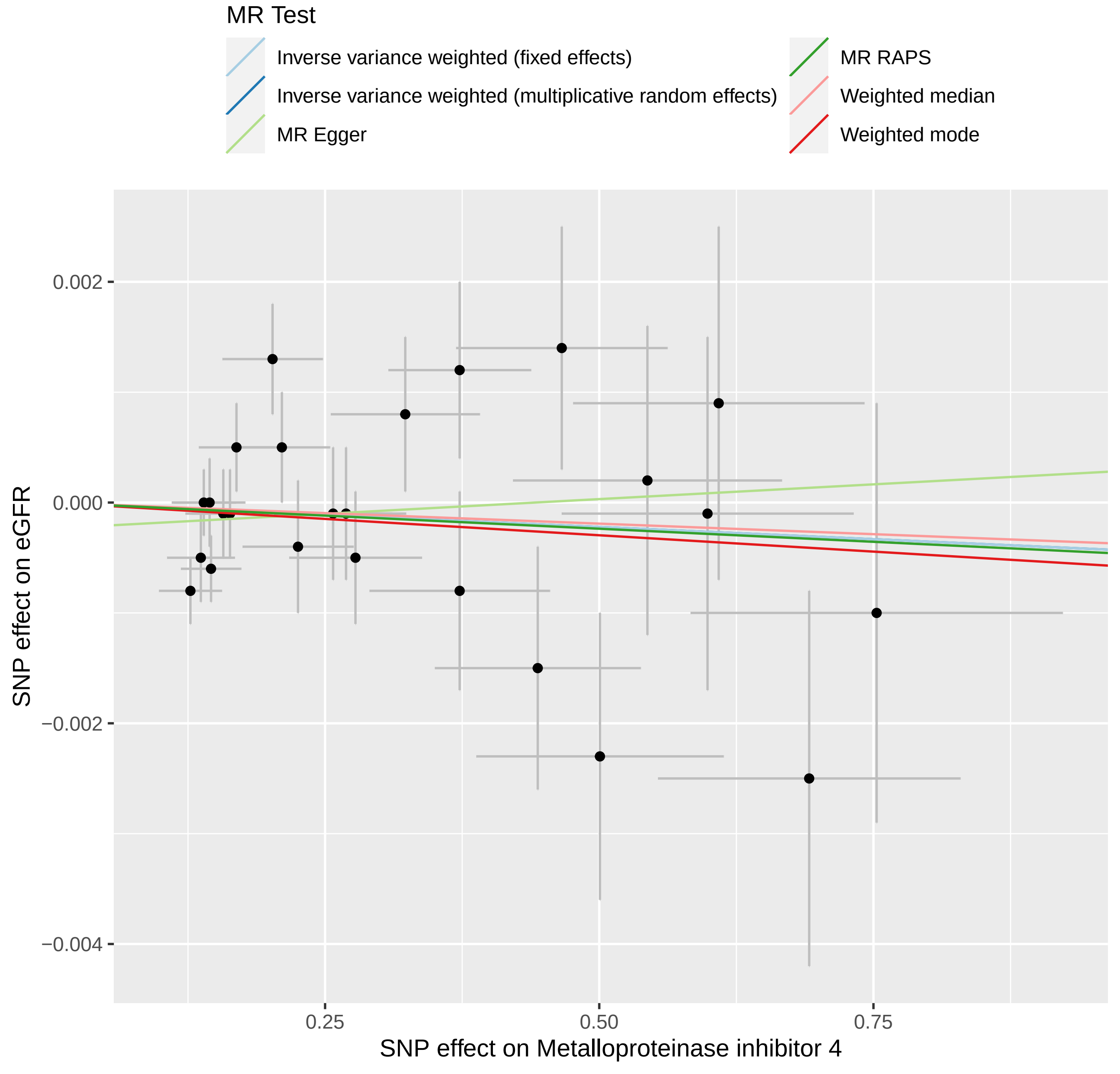

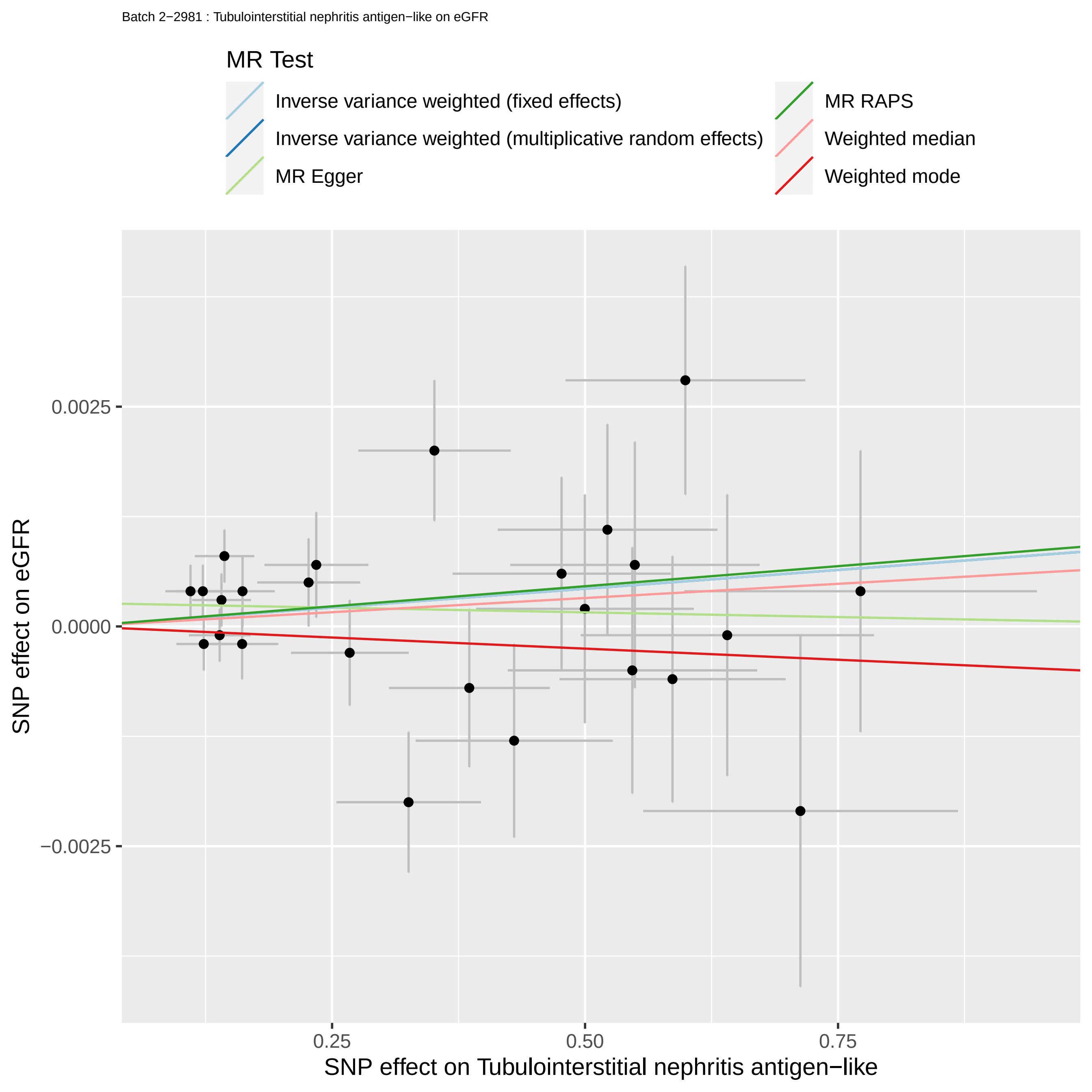
Batch 2−2981 : Tubulointerstitial nephritis antigen−like on eGFR
MR Test
Inverse variance weighted (fixed effects)
Inverse variance weighted (multiplicative random effects)
MR Egger
MR RAPS
Weighted median
Weighted mode
0.0025
0.0000
−0.0025
0.25
0.50
0.75
SNP effect on eGFR
SNP effect on Tubulointerstitial nephritis antigen−like

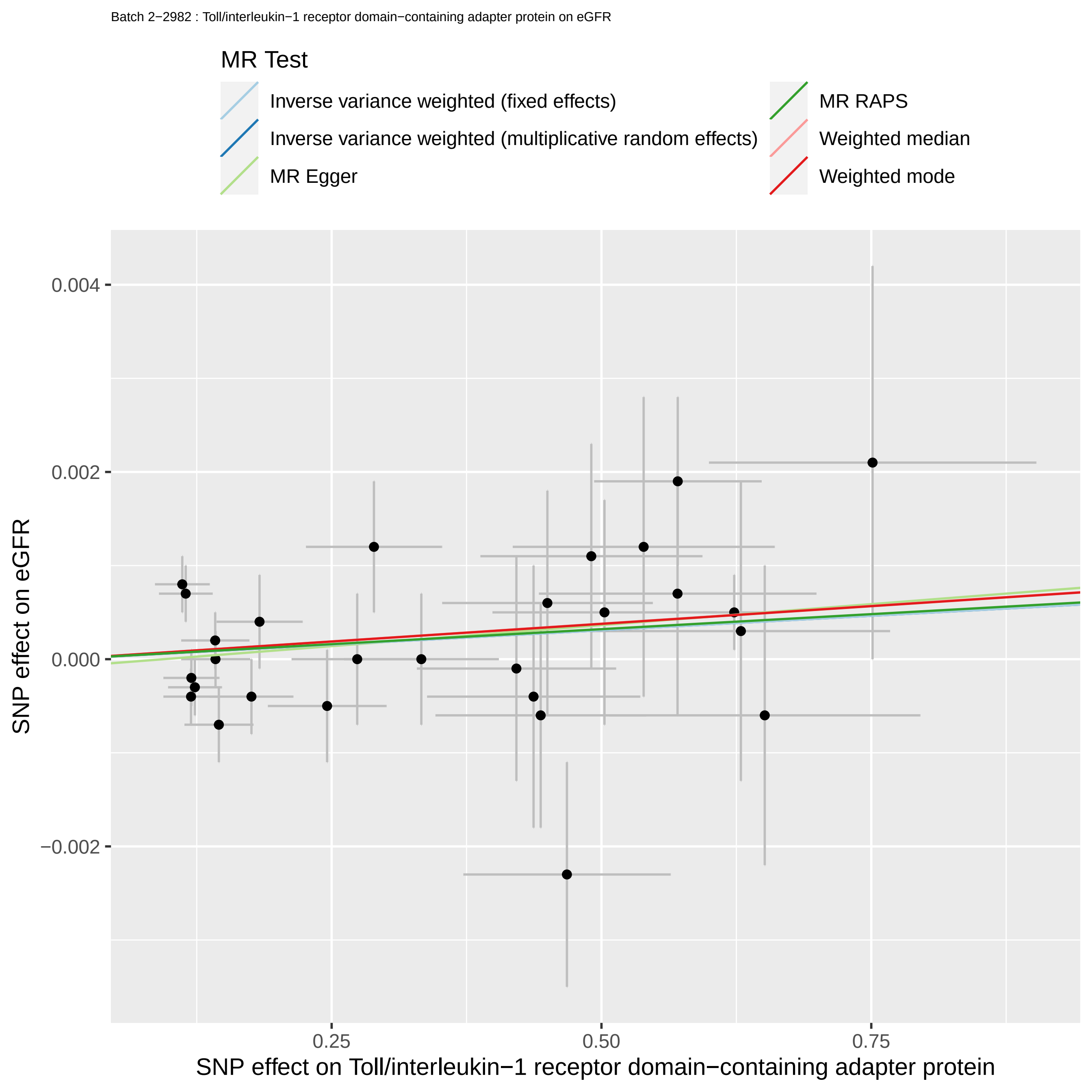

Batch 2−2982 : Toll/interleukin−1 receptor domain−containing adapter protein on eGFR
MR Test
Inverse variance weighted (fixed effects)
Inverse variance weighted (multiplicative random effects)
MR Egger
MR RAPS
Weighted median
Weighted mode
0.004
0.002
0.000
−0.002
0.25
0.50
0.75
SNP effect on eGFR
SNP effect on Toll/interleukin−1 receptor domain−containing adapter protein

Batch 2−2983 : Tight junction protein ZO−1 on eGFR

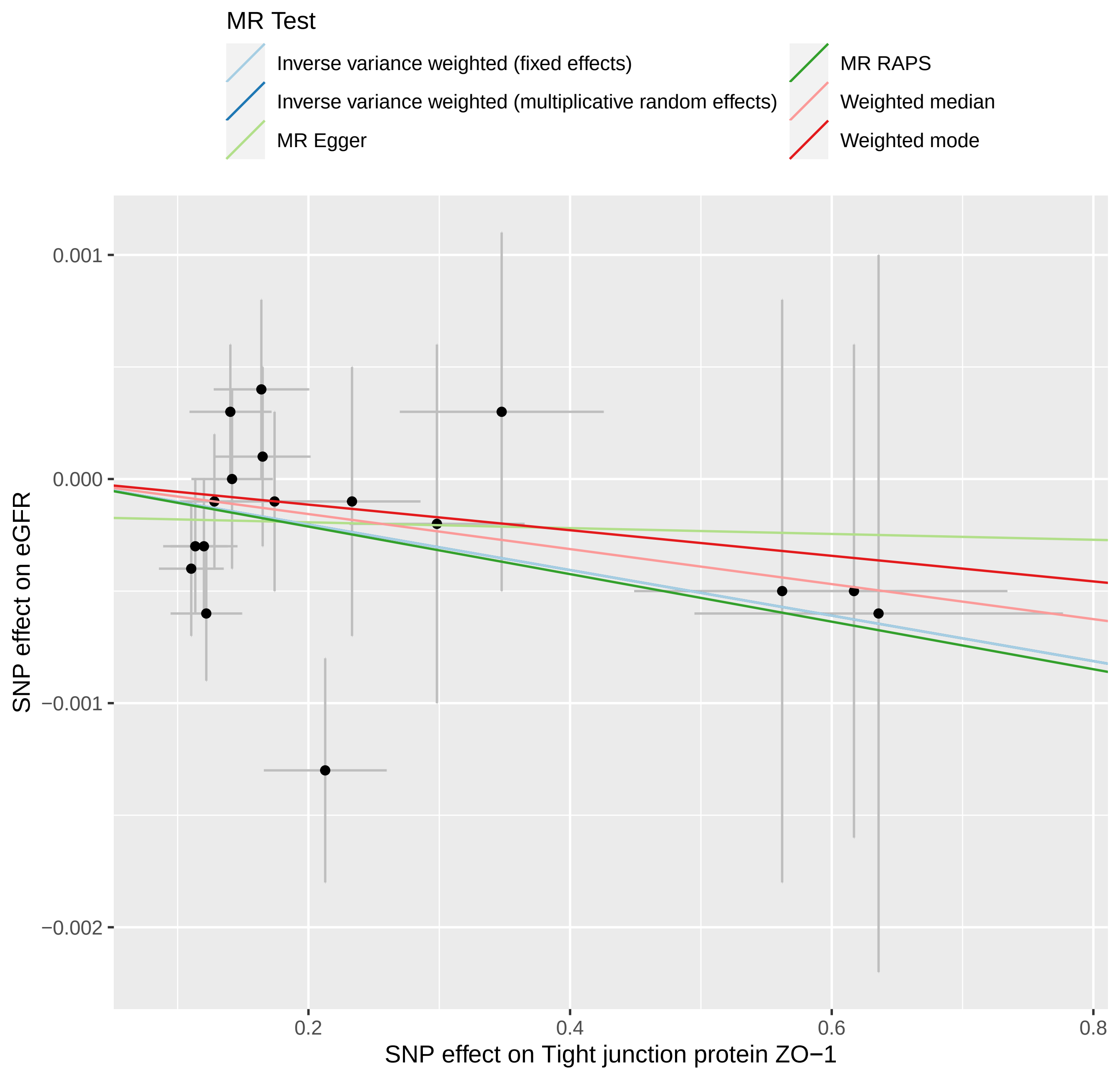

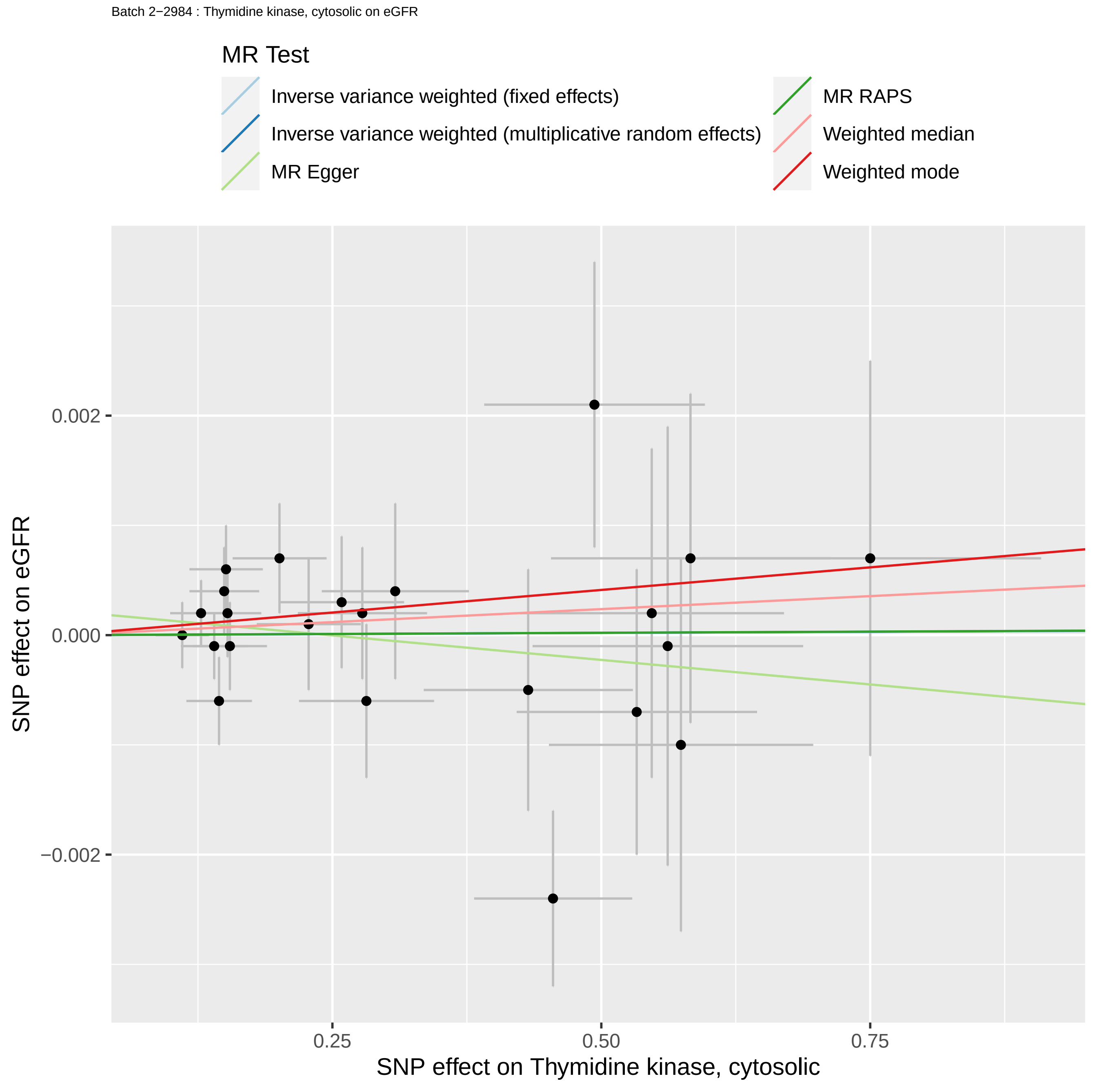
Batch 2−2984 : Thymidine kinase, cytosolic on eGFR
MR Test
Inverse variance weighted (fixed effects)
Inverse variance weighted (multiplicative random effects)
MR Egger
MR RAPS
Weighted median
Weighted mode
SNP effect on eGFR
0.002
0.000
−0.002
0.25
0.50
0.75
SNP effect on Thymidine kinase, cytosolic

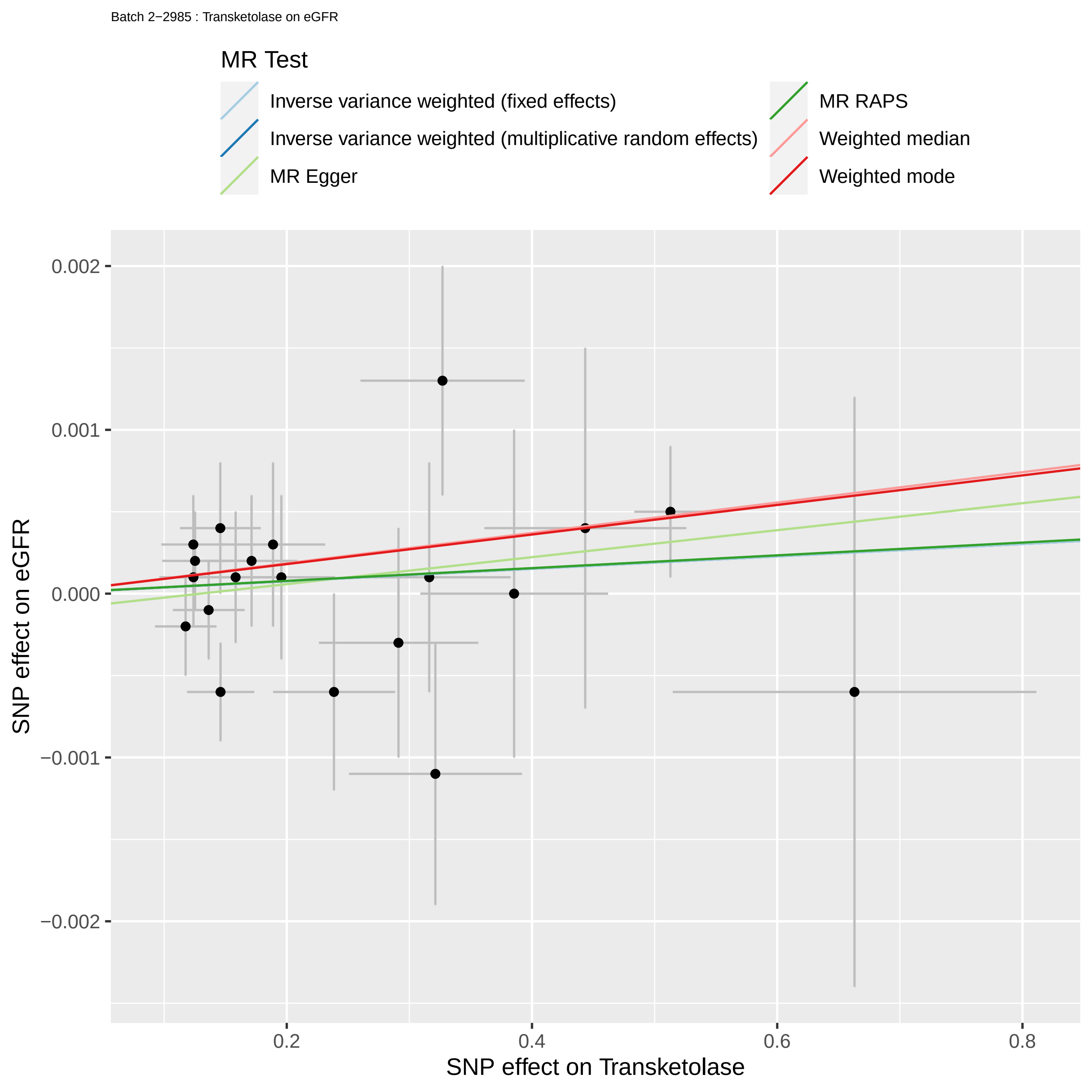

Batch 2−2985 : Transketolase on eGFR
MR Test
Inverse variance weighted (fixed effects)
Inverse variance weighted (multiplicative random effects)
MR Egger
MR RAPS
Weighted median
Weighted mode
0.002
0.001
0.000
−0.001
−0.002
0.2
0.4
0.6
0.8
SNP effect on eGFR
SNP effect on Transketolase

Batch 2−2986 : Tolloid−like protein 1 on eGFR

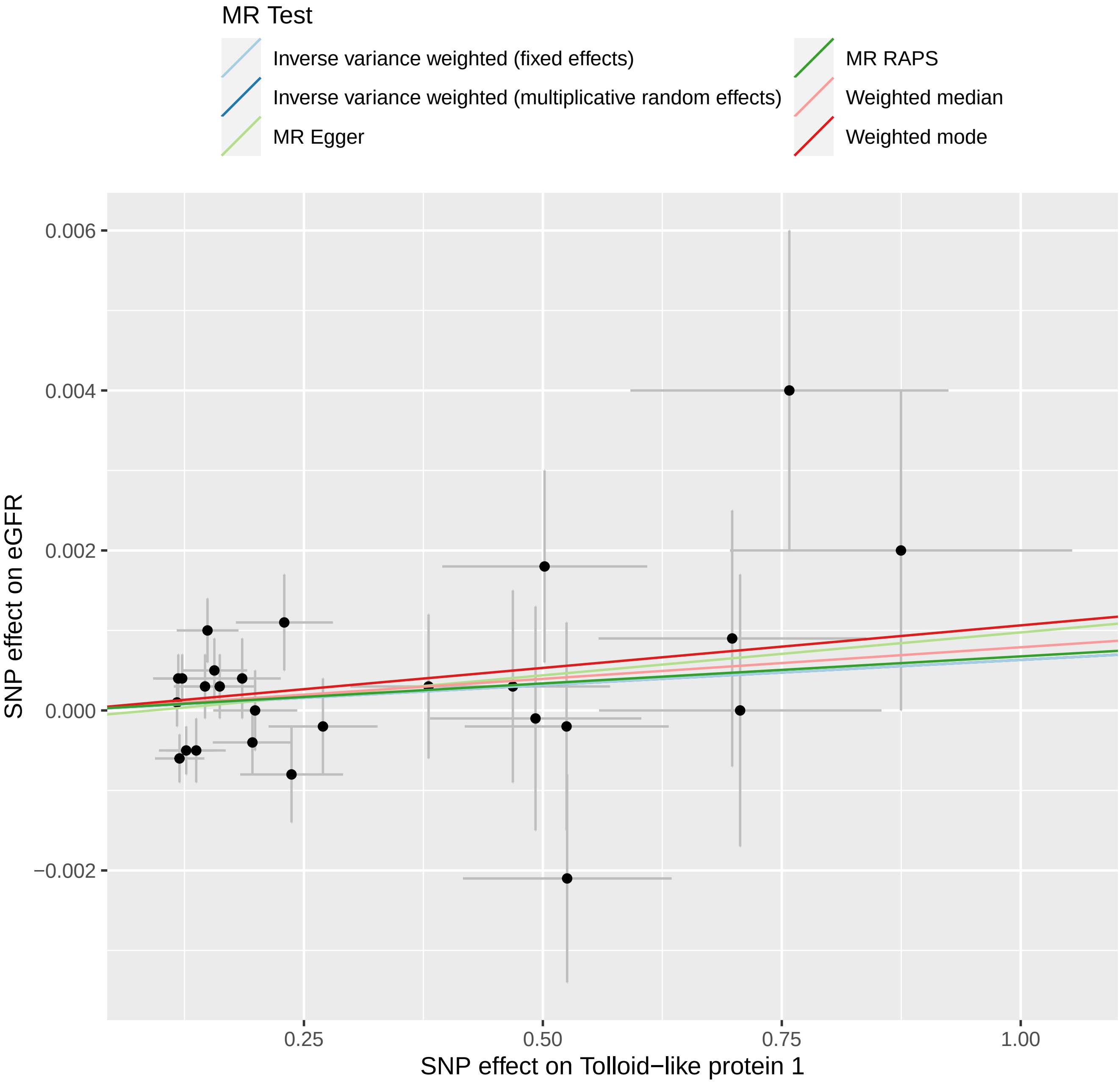

Batch 2−2987 : Talin−2 on eGFR

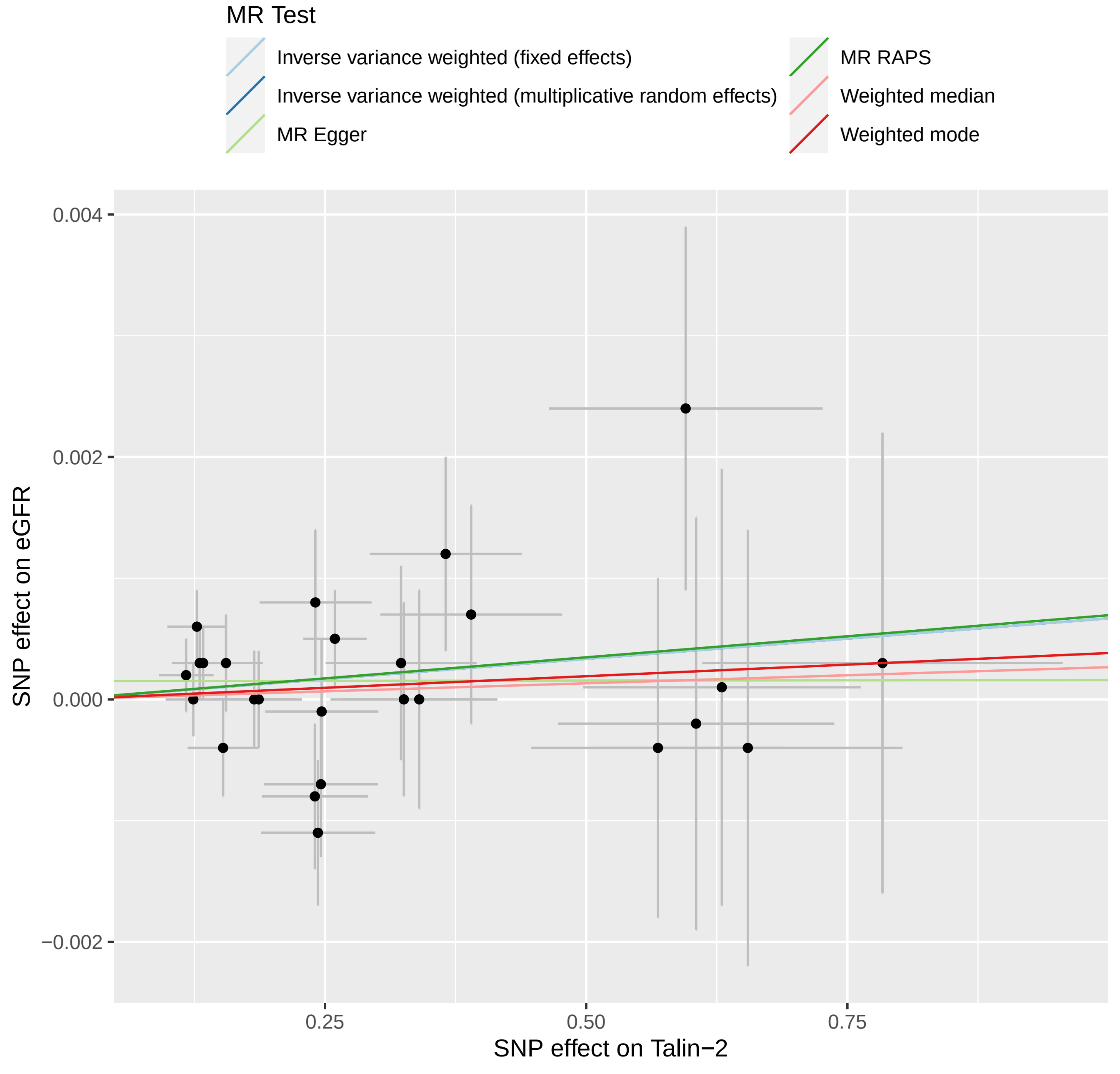

Batch 2−2988 : Toll−like receptor 1 on eGFR

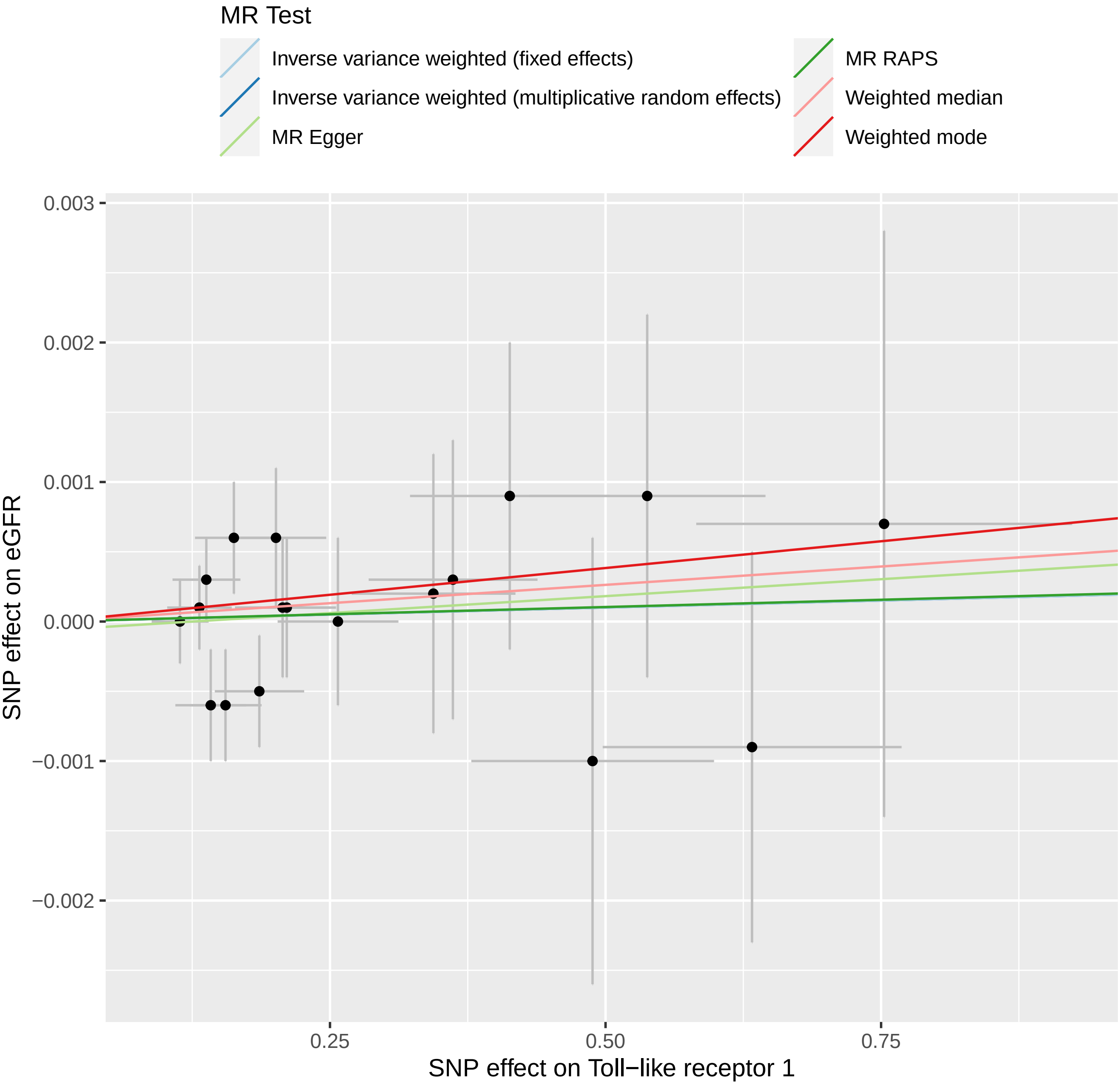

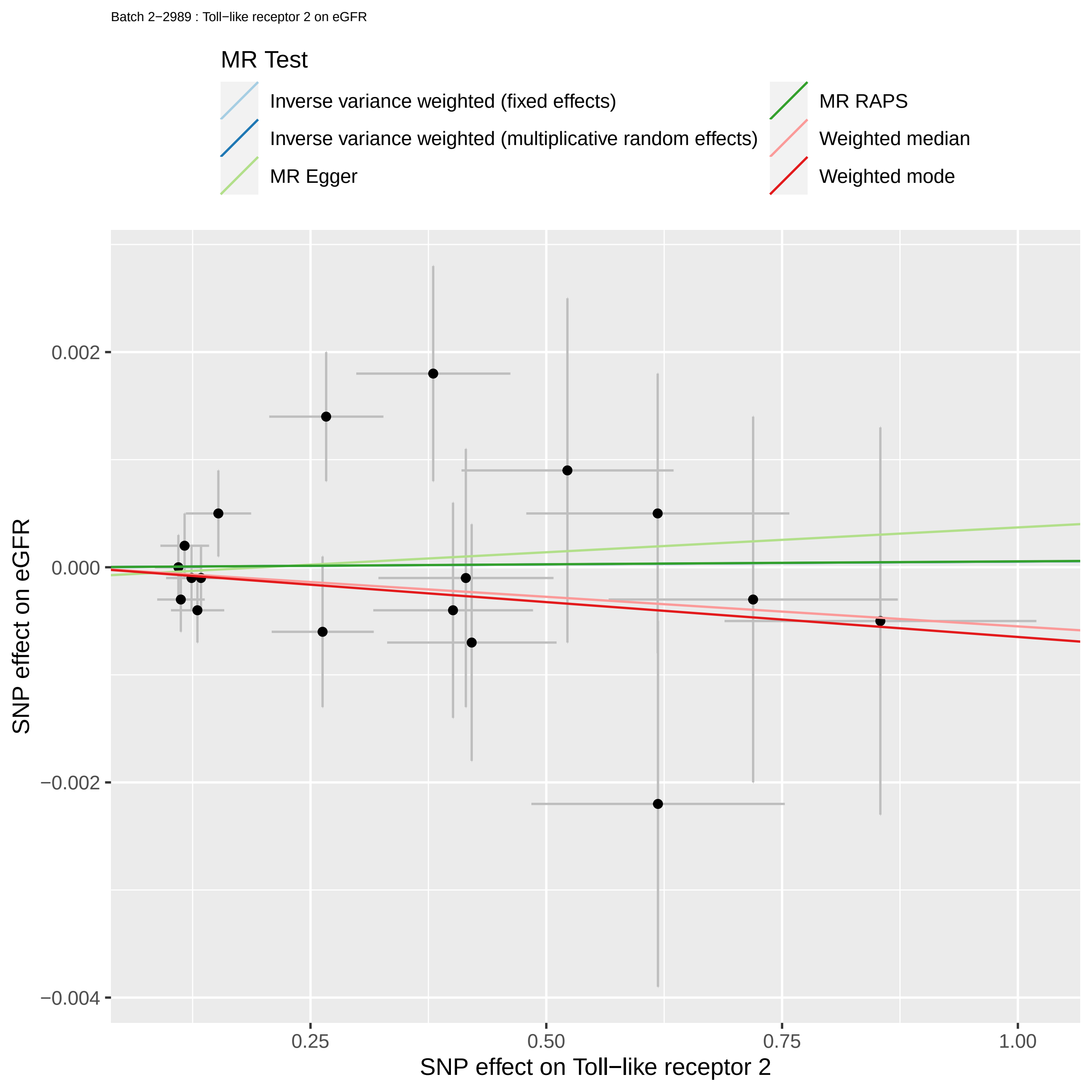
Batch 2−2989 : Toll−like receptor 2 on eGFR
MR Test
Inverse variance weighted (fixed effects)
Inverse variance weighted (multiplicative random effects)
MR Egger
MR RAPS
Weighted median
Weighted mode
0.002
0.000
−0.002
−0.004
SNP effect on eGFR
0.25
0.50
0.75
1.00
SNP effect on Toll−like receptor 2

Batch 2−2990 : Toll−like receptor 4 on eGFR

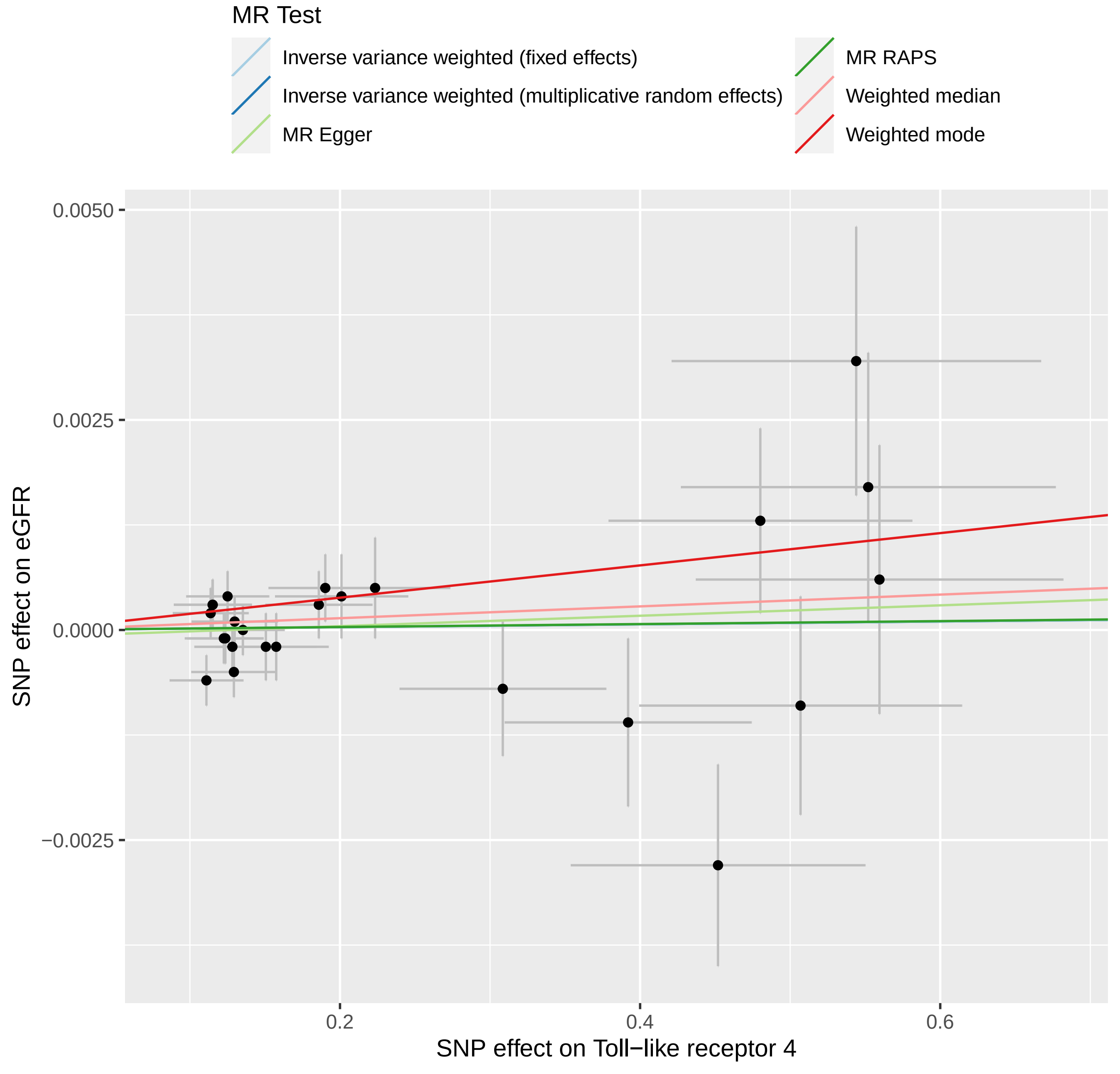

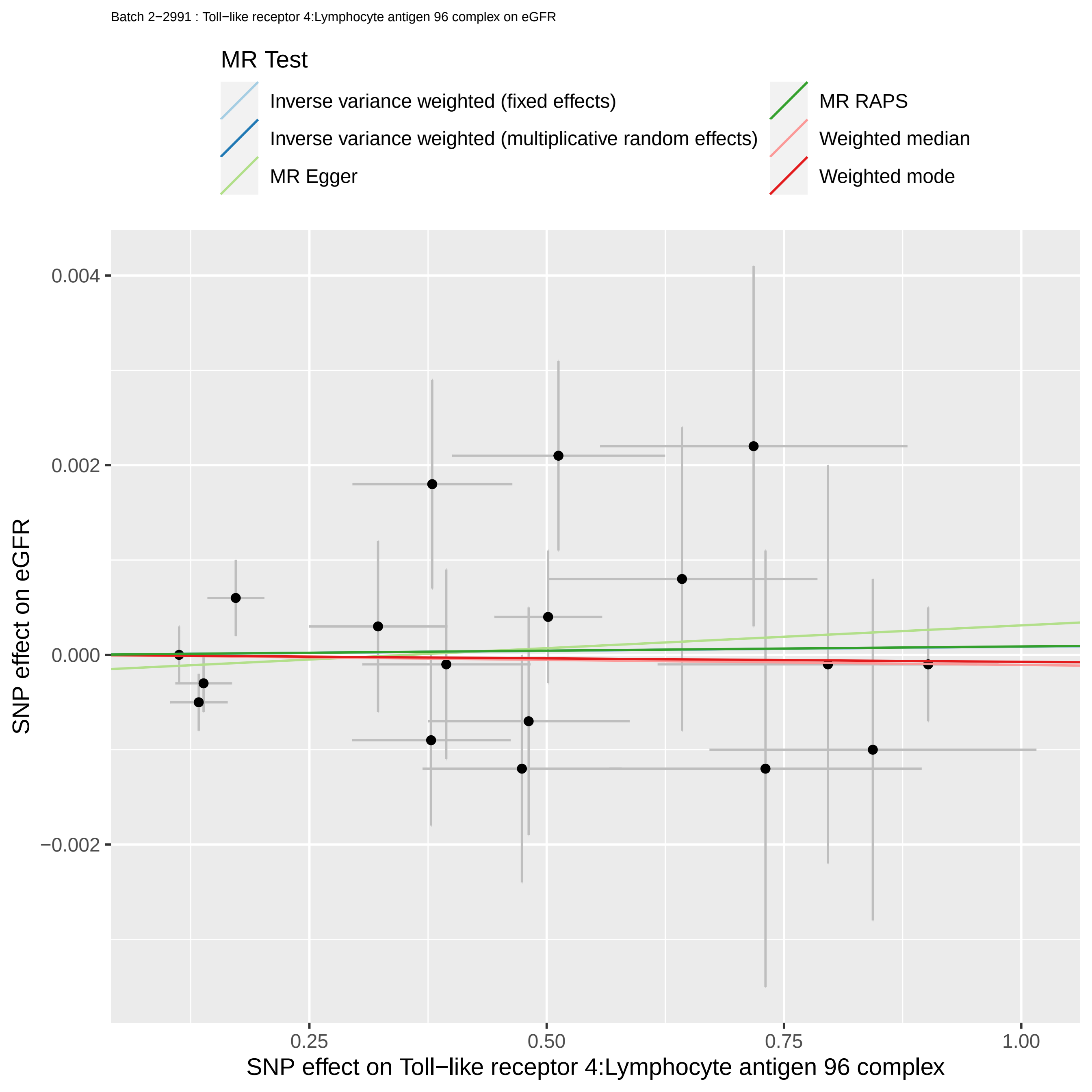
Batch 2−2991 : Toll−like receptor 4:Lymphocyte antigen 96 complex on eGFR
MR Test
Inverse variance weighted (fixed effects)
Inverse variance weighted (multiplicative random effects)
MR Egger
MR RAPS
Weighted median
Weighted mode
0.004
0.002
0.000
−0.002
0.25
0.50
0.75
1.00
SNP effect on eGFR
SNP effect on Toll−like receptor 4:Lymphocyte antigen 96 complex

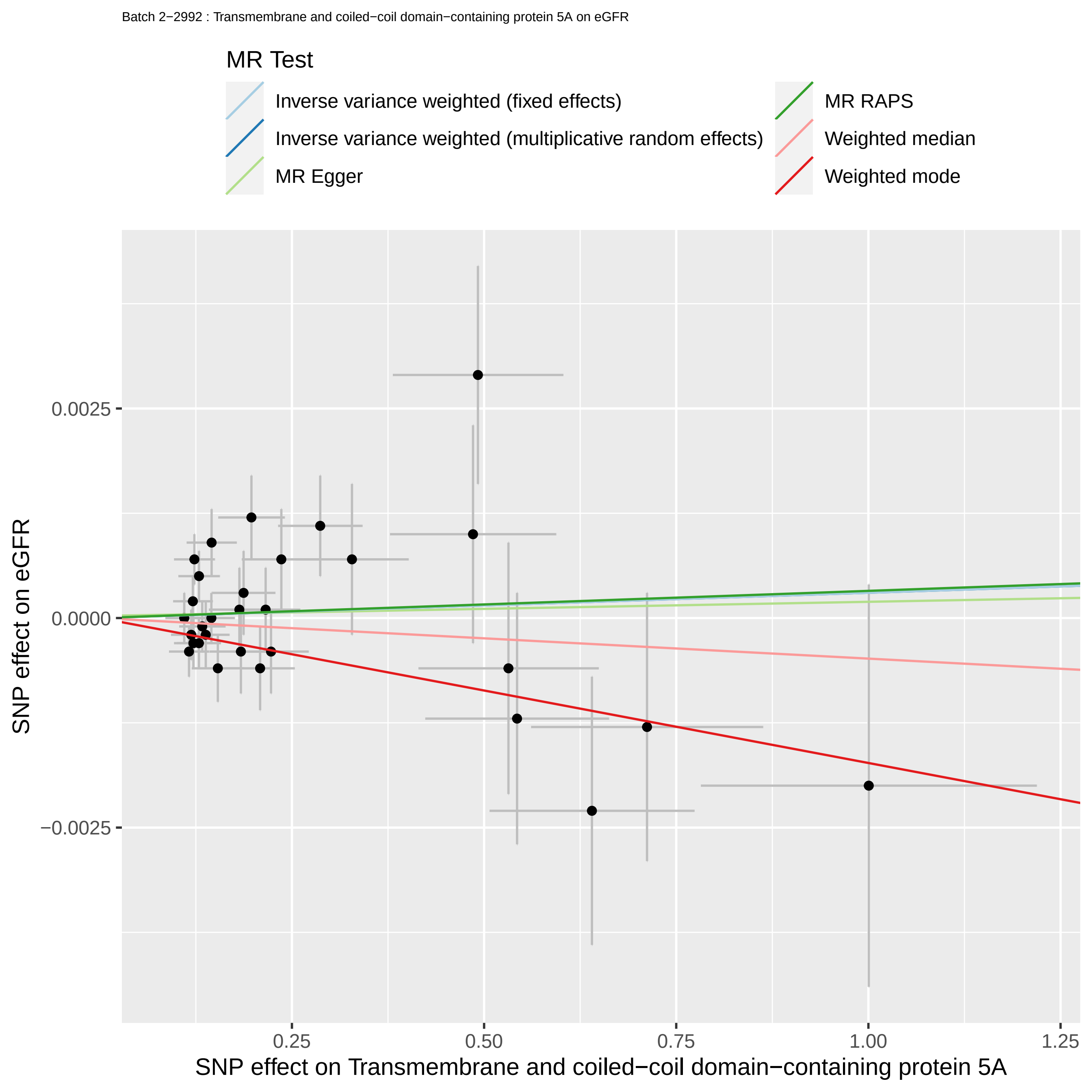

Batch 2−2992 : Transmembrane and coiled−coil domain−containing protein 5A on eGFR
MR Test
Inverse variance weighted (fixed effects)
Inverse variance weighted (multiplicative random effects)
MR Egger
MR RAPS
Weighted median
Weighted mode
0.0025
0.0000
−0.0025
0.25
0.50
0.75
1.00
1.25
SNP effect on eGFR
SNP effect on Transmembrane and coiled−coil domain−containing protein 5A

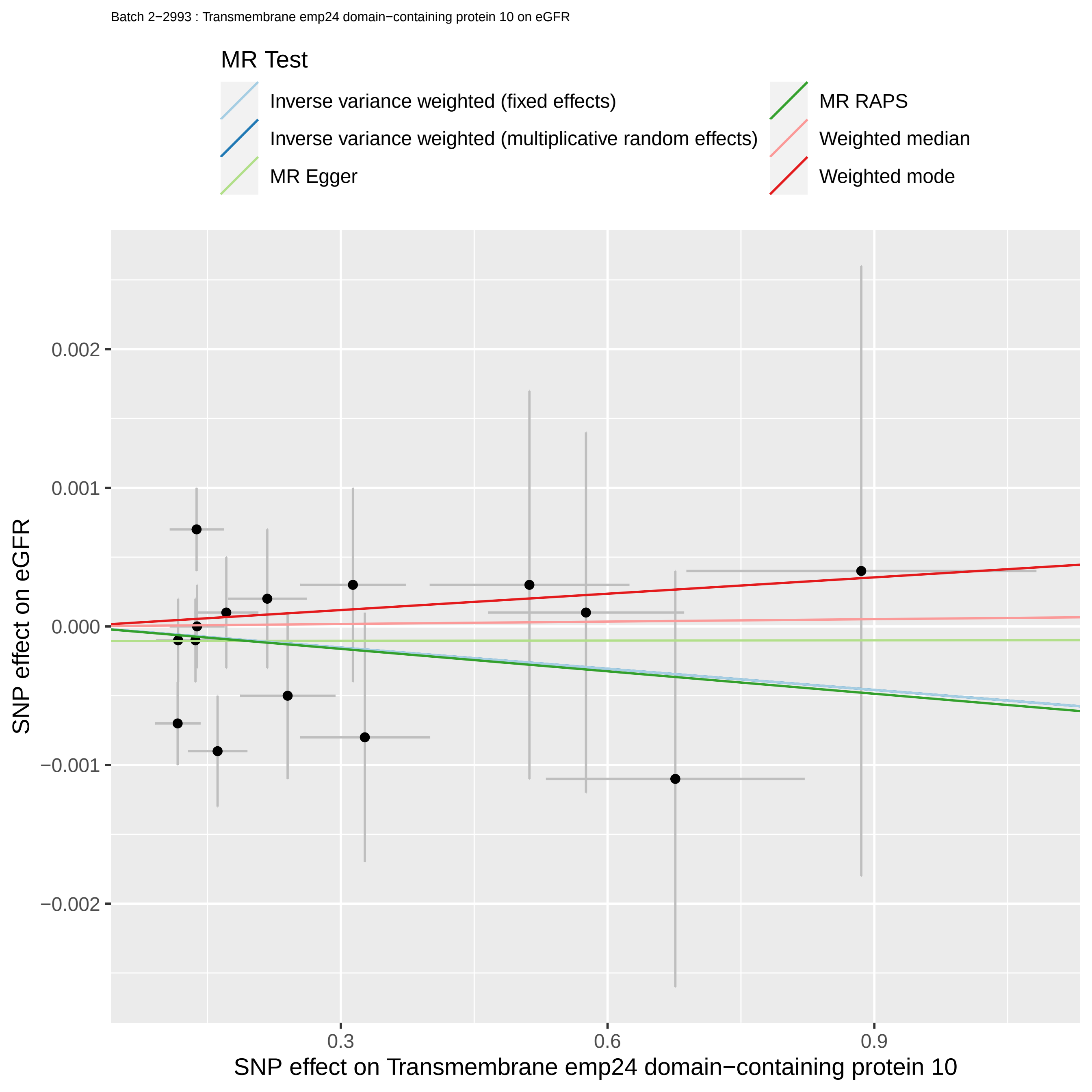
Batch 2−2993 : Transmembrane emp24 domain−containing protein 10 on eGFR
MR Test
Inverse variance weighted (fixed effects)
Inverse variance weighted (multiplicative random effects)
MR Egger
MR RAPS
Weighted median
Weighted mode
0.002
0.001
0.000
−0.001
−0.002
0.3
0.6
0.9
SNP effect on eGFR
SNP effect on Transmembrane emp24 domain−containing protein 10

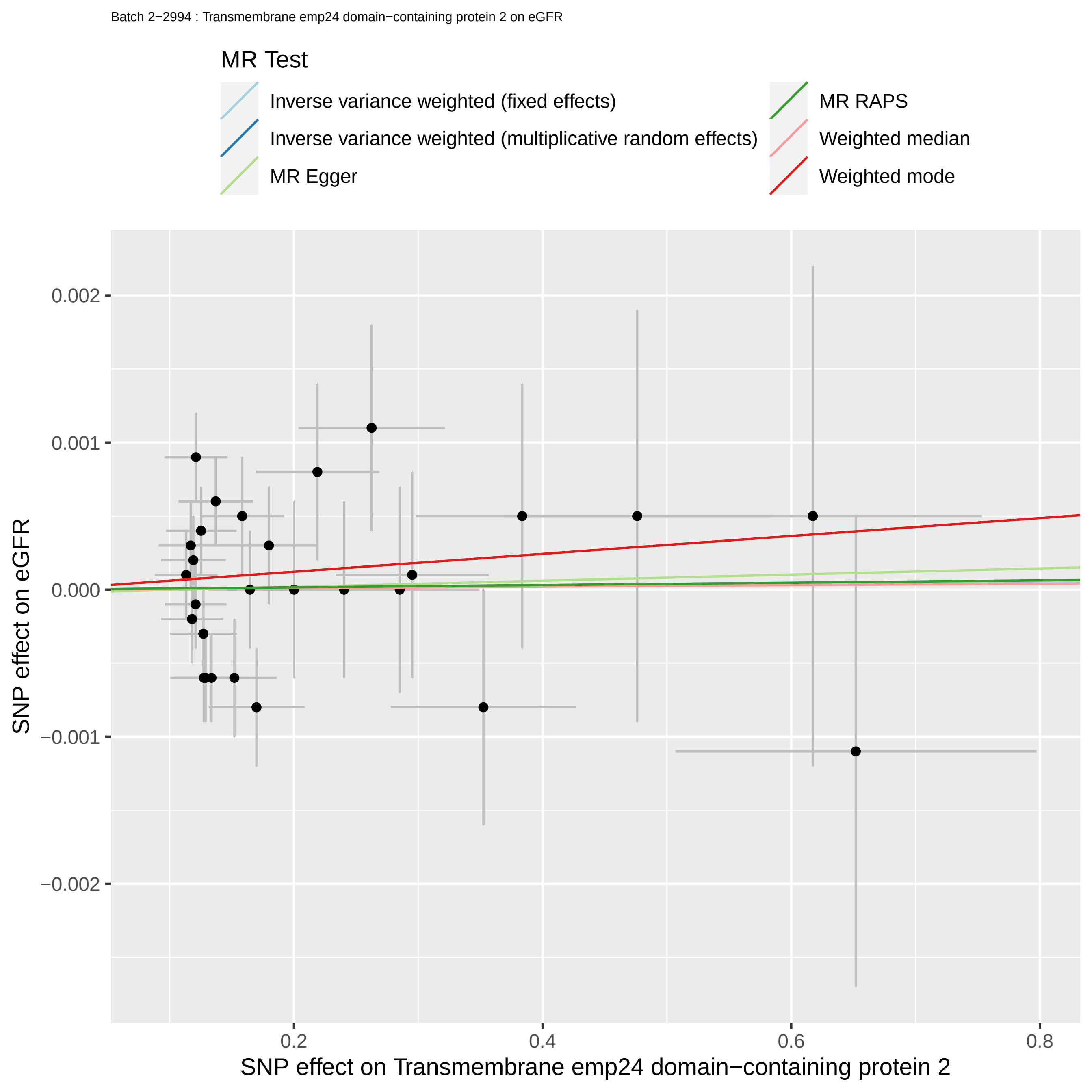
Batch 2−2994 : Transmembrane emp24 domain−containing protein 2 on eGFR
MR Test
Inverse variance weighted (fixed effects)
Inverse variance weighted (multiplicative random effects)
MR Egger
MR RAPS
Weighted median
Weighted mode
0.002
0.001
0.000
−0.001
−0.002
0.2
0.4
0.6
0.8
SNP effect on eGFR
SNP effect on Transmembrane emp24 domain−containing protein 2

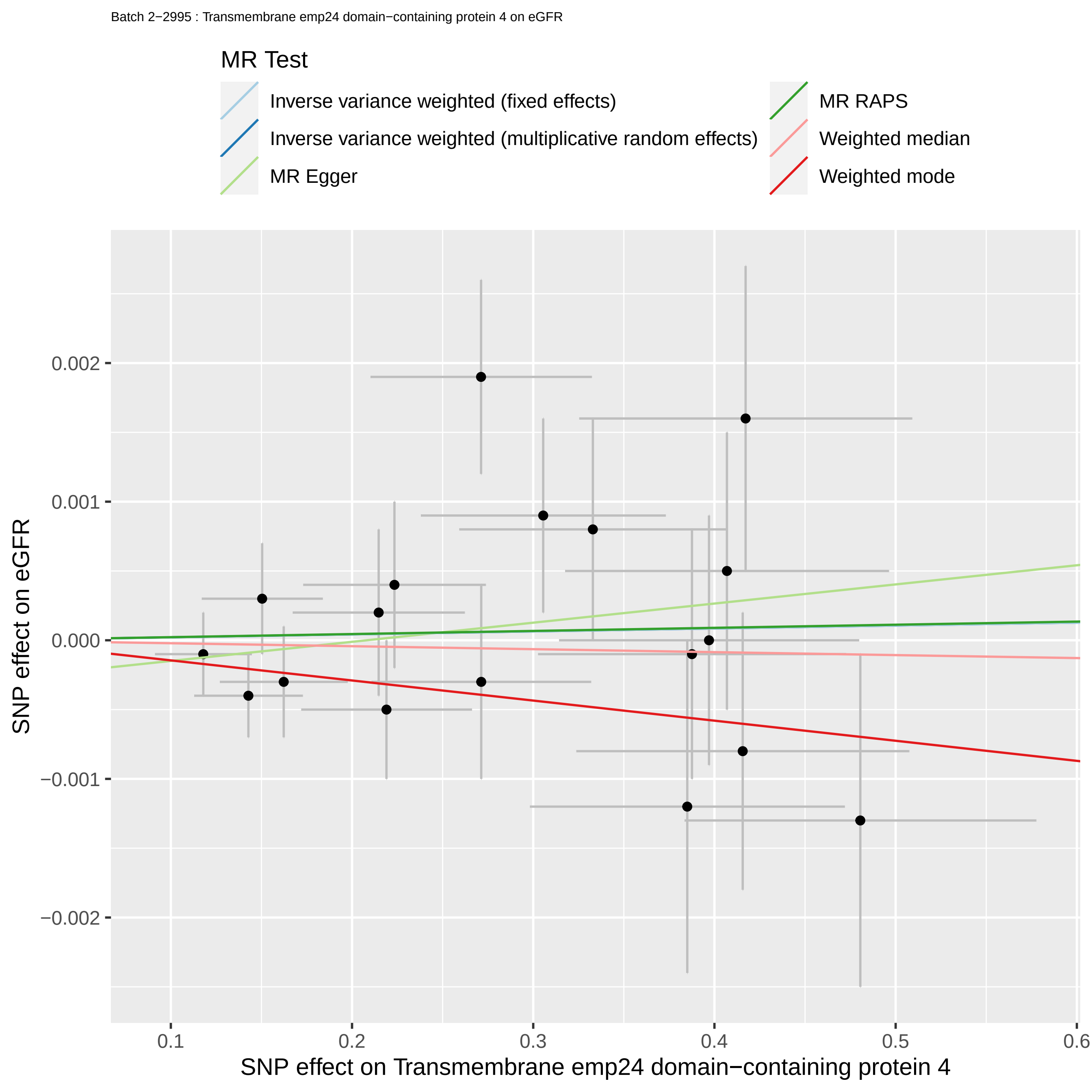

Batch 2−2995 : Transmembrane emp24 domain−containing protein 4 on eGFR
MR Test
Inverse variance weighted (fixed effects)
Inverse variance weighted (multiplicative random effects)
MR Egger
MR RAPS
Weighted median
Weighted mode
0.002
0.001
0.000
−0.001
−0.002
SNP effect on eGFR
0.1
0.2
0.3
0.4
0.5
0.6
SNP effect on Transmembrane emp24 domain−containing protein 4

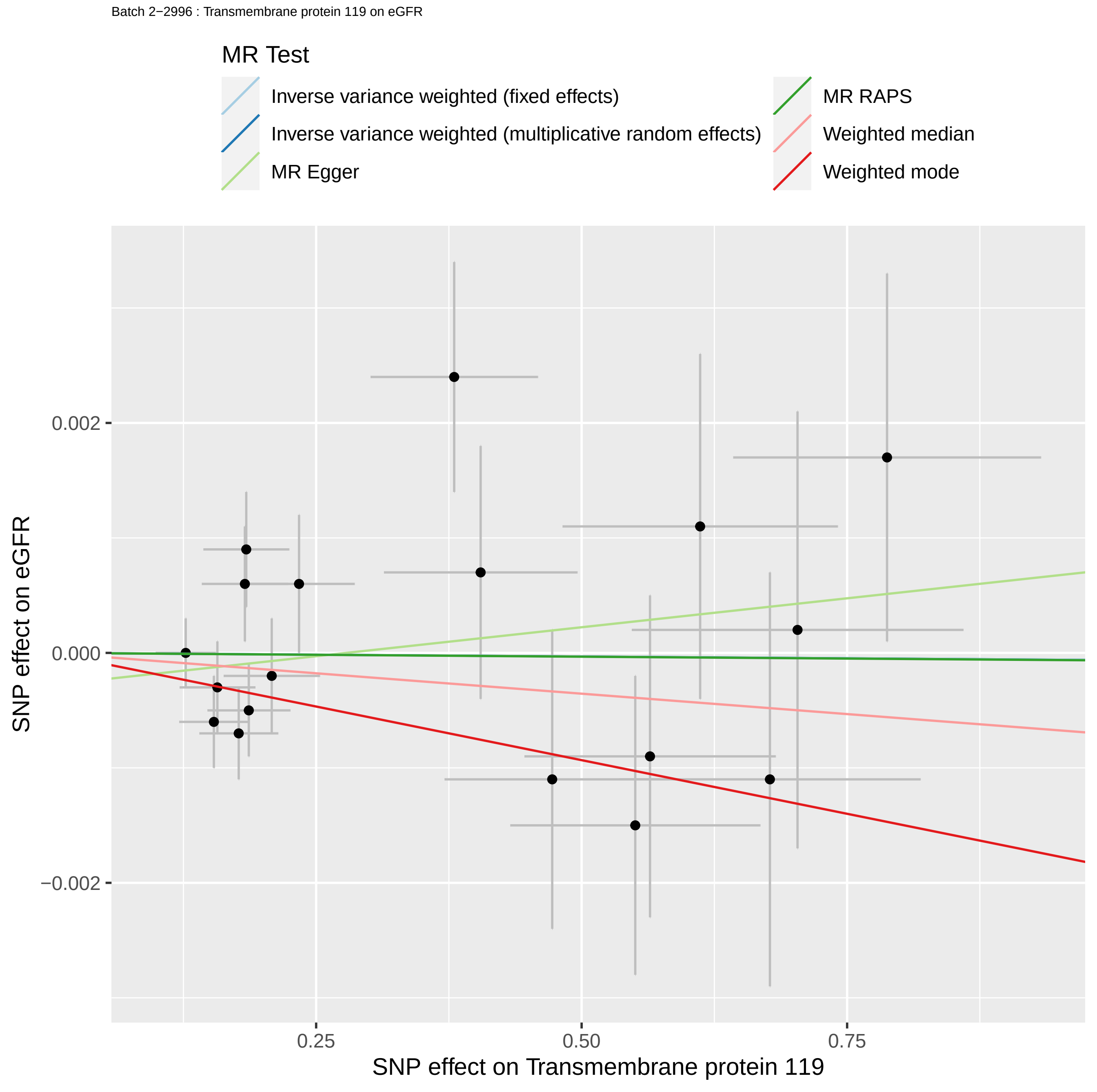

Batch 2−2996 : Transmembrane protein 119 on eGFR
MR Test
Inverse variance weighted (fixed effects)
Inverse variance weighted (multiplicative random effects)
MR Egger
MR RAPS
Weighted median
Weighted mode
0.002
0.000
−0.002
0.25
0.50
0.75
SNP effect on eGFR
SNP effect on Transmembrane protein 119

Batch 2−2997 : Transmembrane protein 132A on eGFR

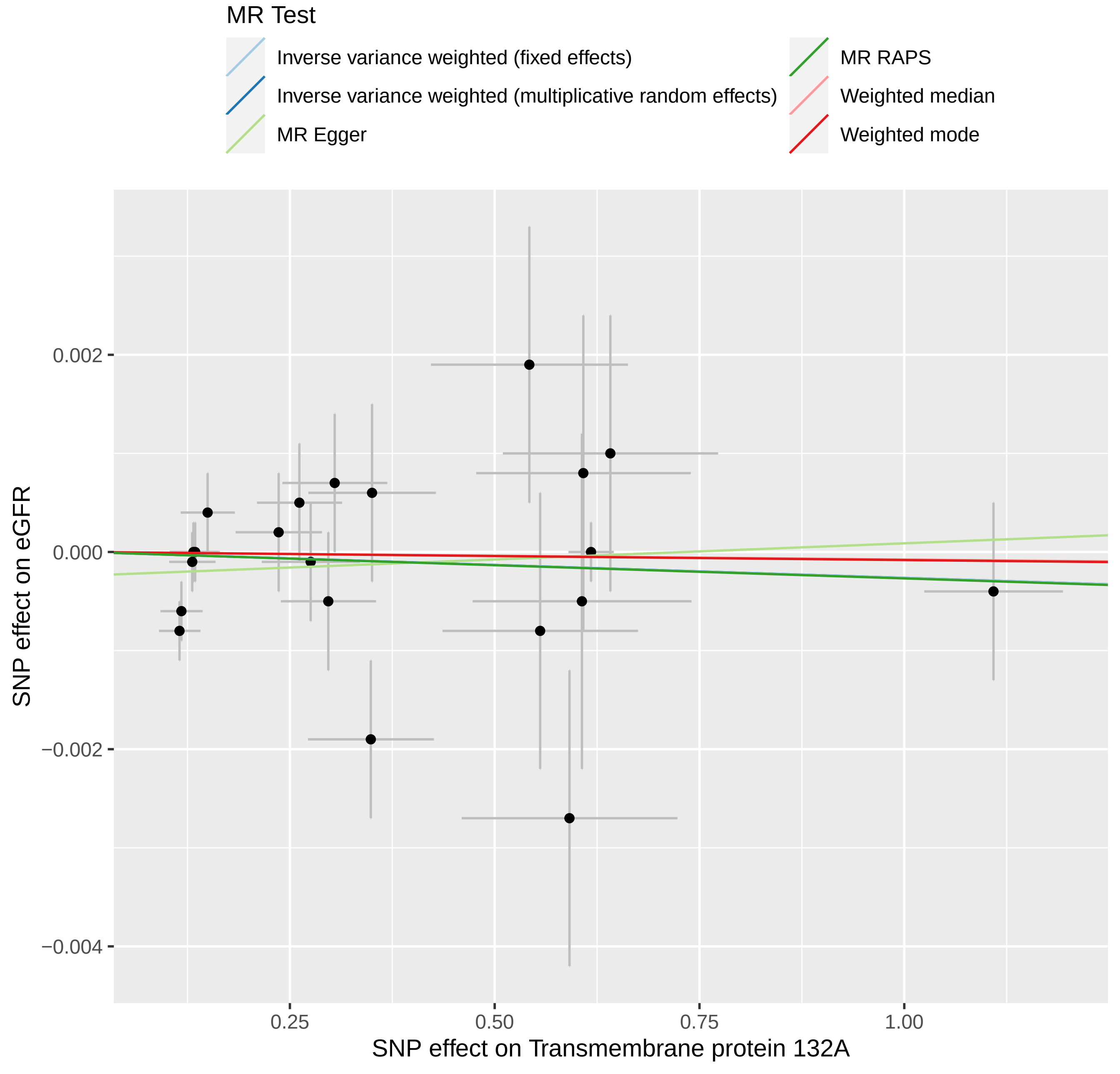

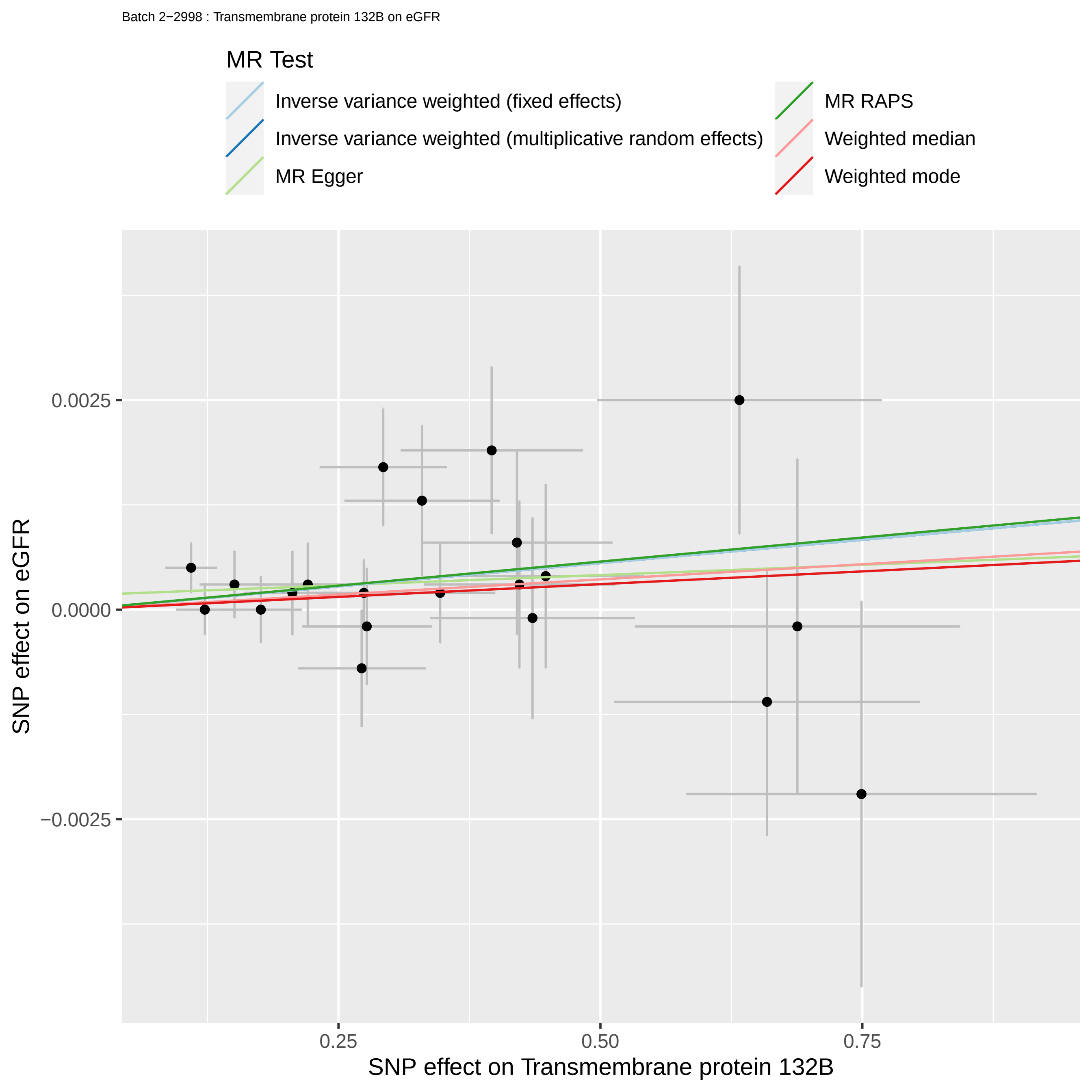
Batch 2−2998 : Transmembrane protein 132B on eGFR
MR Test
Inverse variance weighted (fixed effects)
Inverse variance weighted (multiplicative random effects)
MR Egger
MR RAPS
Weighted median
Weighted mode
0.0025
0.0000
−0.0025
0.25
0.50
0.75
SNP effect on eGFR
SNP effect on Transmembrane protein 132B

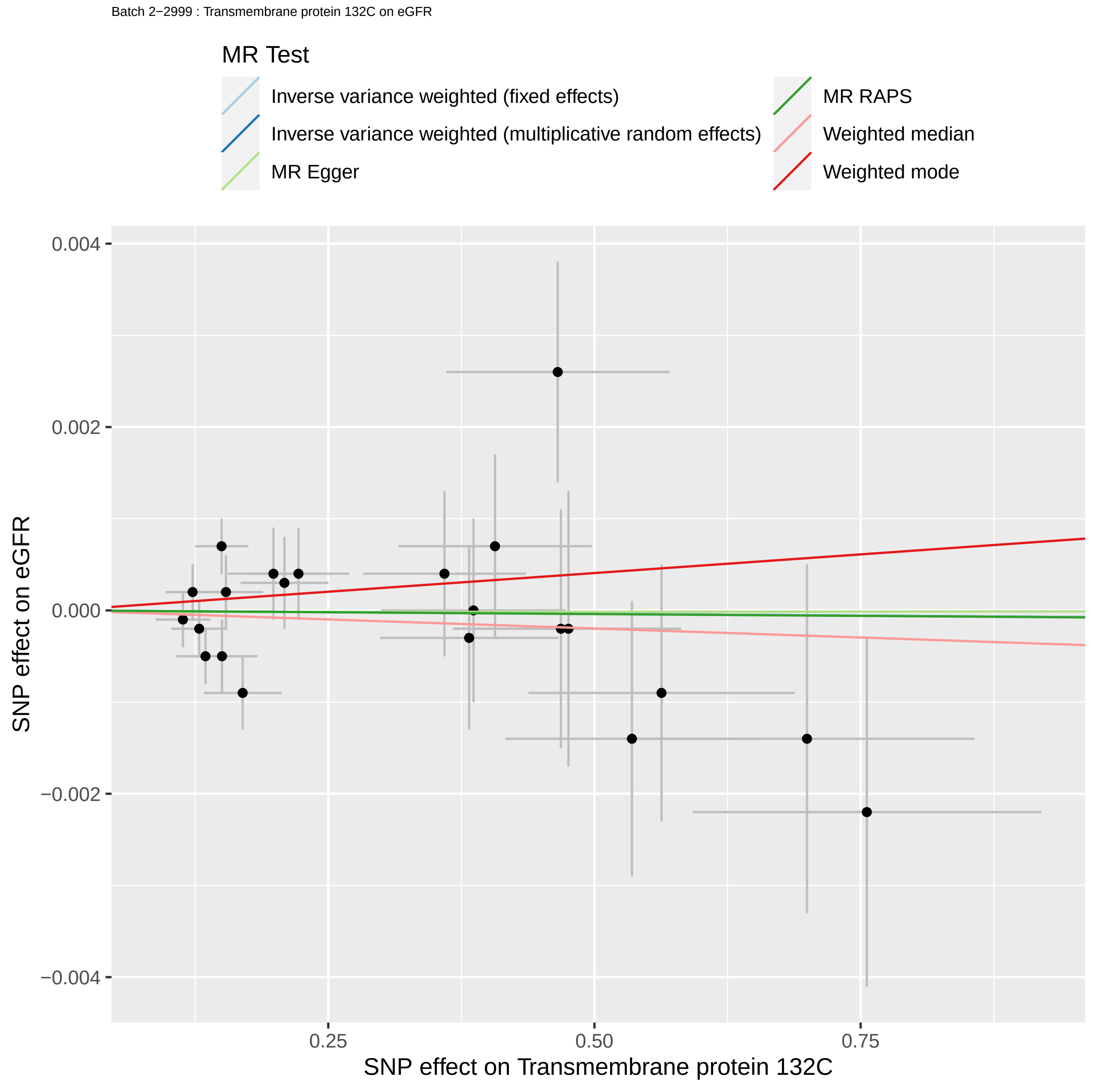

Batch 2−2999 : Transmembrane protein 132C on eGFR
MR Test
Inverse variance weighted (fixed effects)
Inverse variance weighted (multiplicative random effects)
MR Egger
MR RAPS
Weighted median
Weighted mode
0.004
0.002
0.000
−0.002
−0.004
SNP effect on eGFR
0.25
0.50
0.75
SNP effect on Transmembrane protein 132C

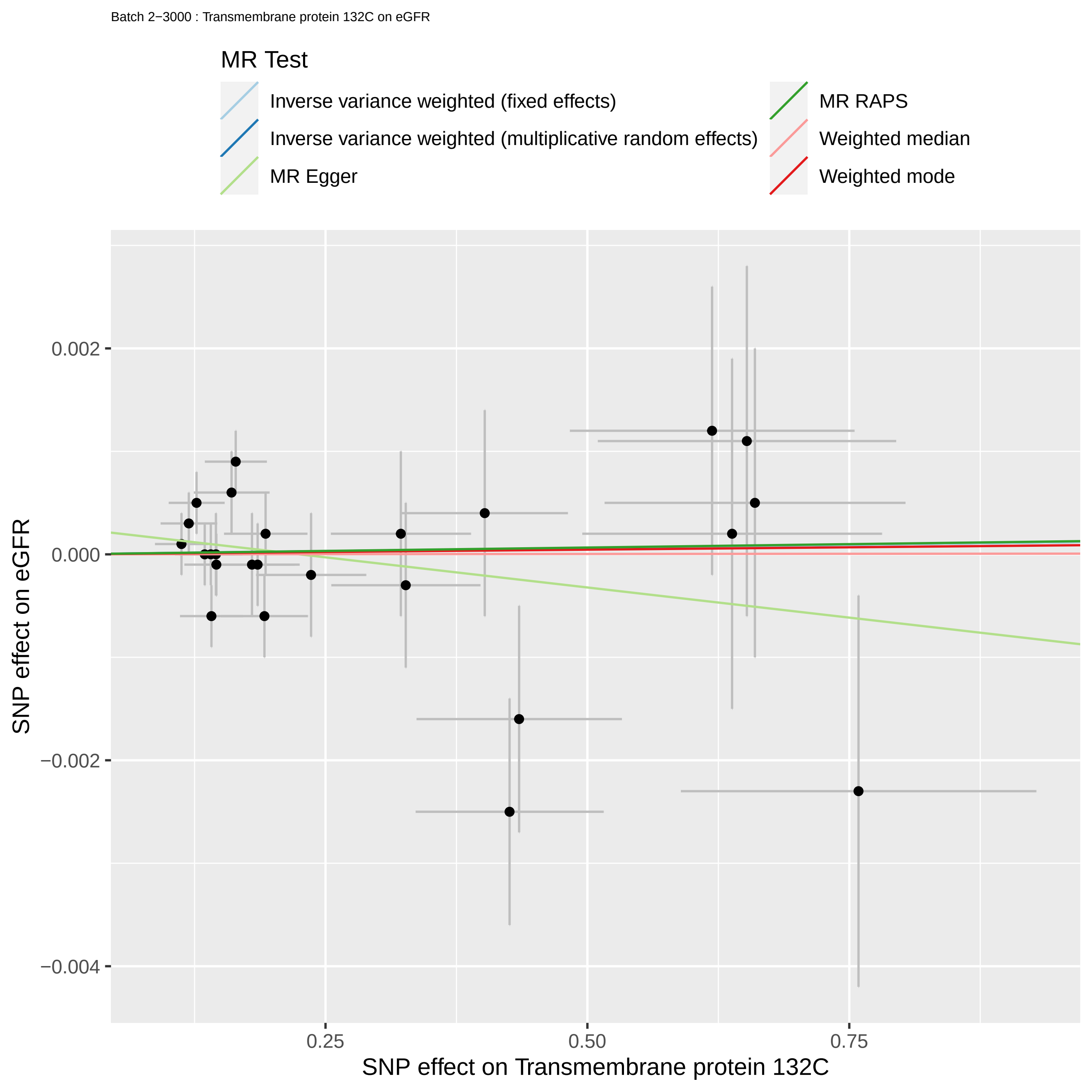

Batch 2−3000 : Transmembrane protein 132C on eGFR
MR Test
Inverse variance weighted (fixed effects)
Inverse variance weighted (multiplicative random effects)
MR Egger
MR RAPS
Weighted median
Weighted mode
0.002
0.000
−0.002
−0.004
0.25
0.50
0.75
SNP effect on eGFR
SNP effect on Transmembrane protein 132C

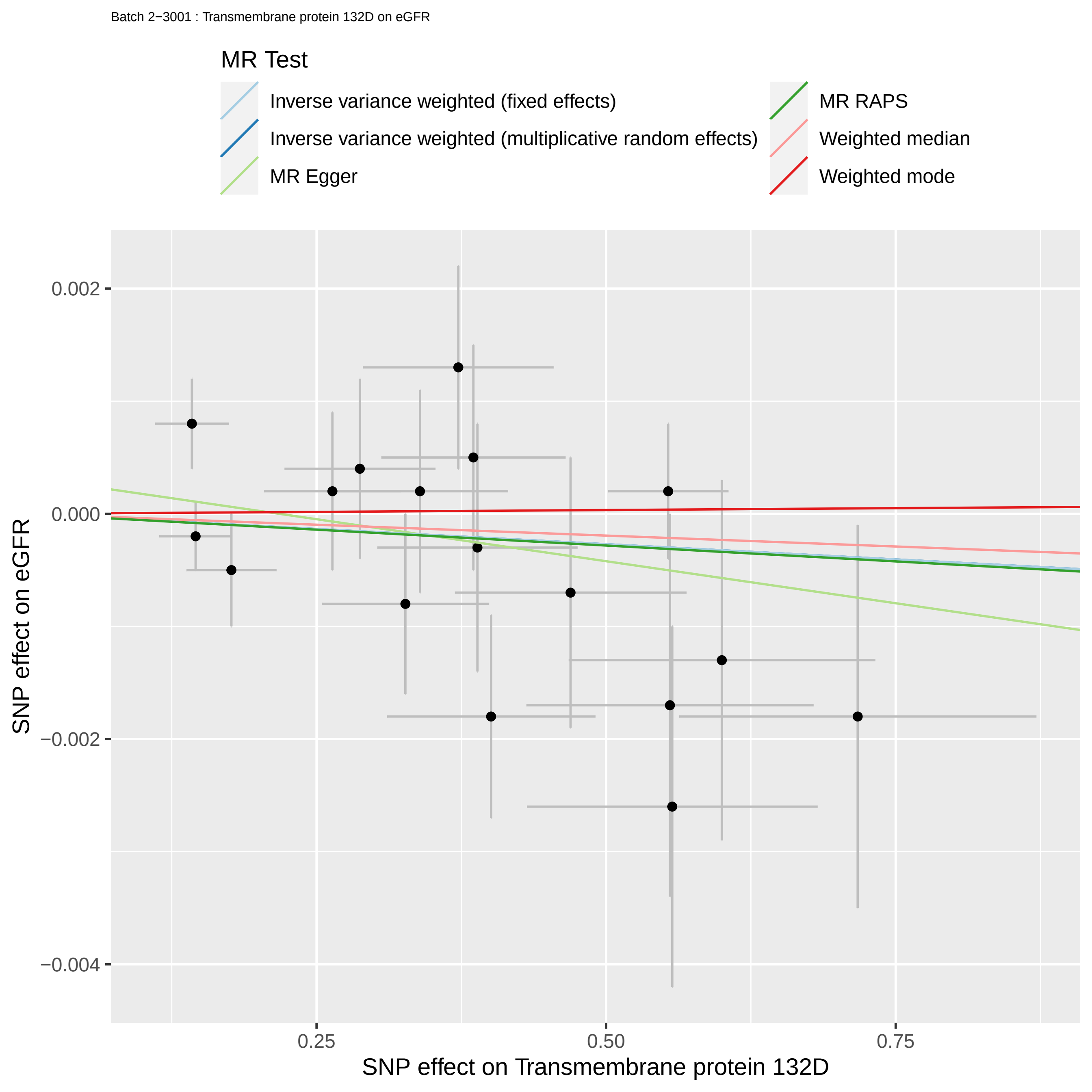

Batch 2−3001 : Transmembrane protein 132D on eGFR
MR Test
Inverse variance weighted (fixed effects)
Inverse variance weighted (multiplicative random effects)
MR Egger
MR RAPS
Weighted median
Weighted mode
0.002
0.000
−0.002
−0.004
0.25
0.50
0.75
SNP effect on eGFR
SNP effect on Transmembrane protein 132D

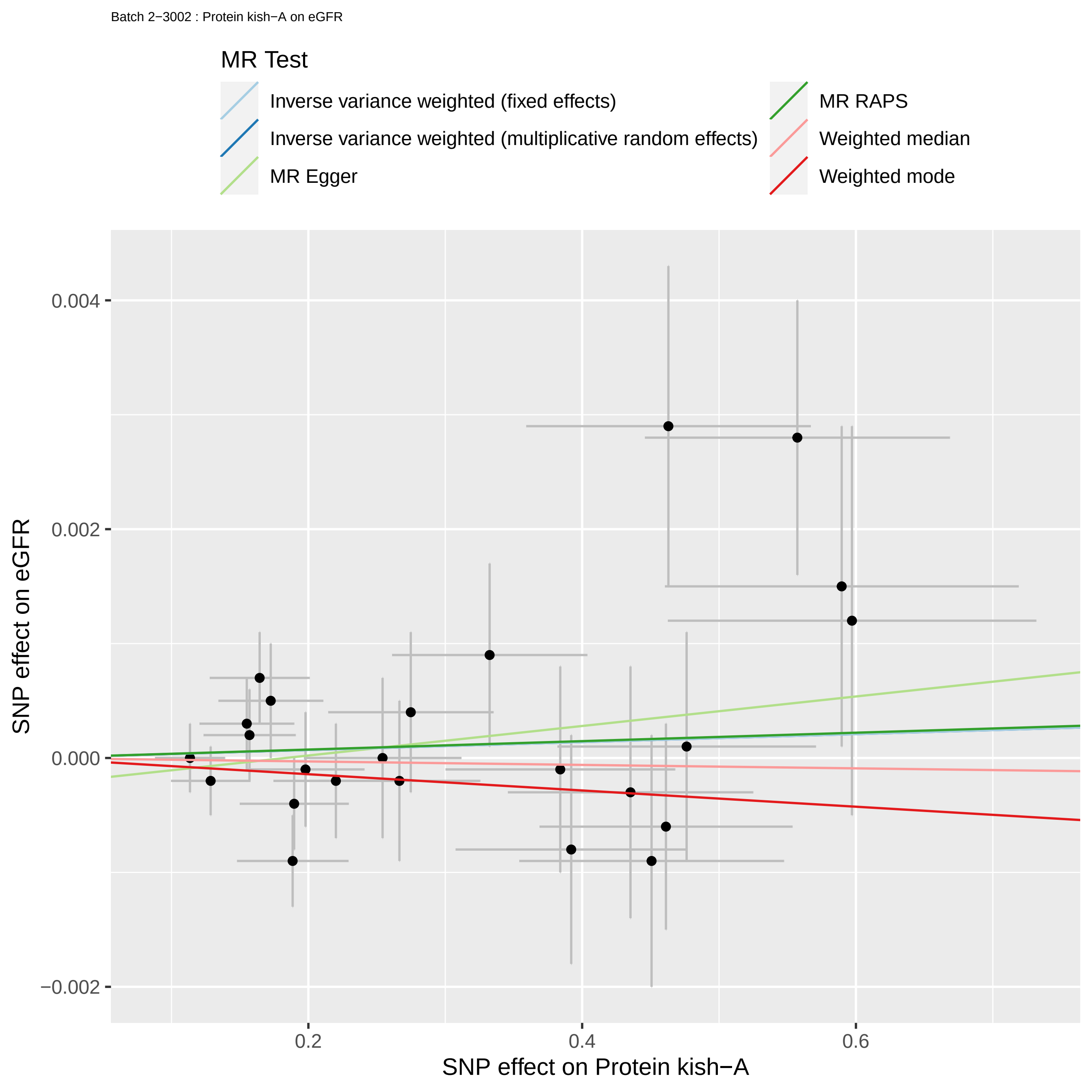

Batch 2−3002 : Protein kish−A on eGFR
MR Test
Inverse variance weighted (fixed effects)
Inverse variance weighted (multiplicative random effects)
MR Egger
MR RAPS
Weighted median
Weighted mode
0.004
0.002
0.000
−0.002
0.2
0.4
0.6
SNP effect on eGFR
SNP effect on Protein kish−A

Batch 2−3003 : Protein kish−B on eGFR

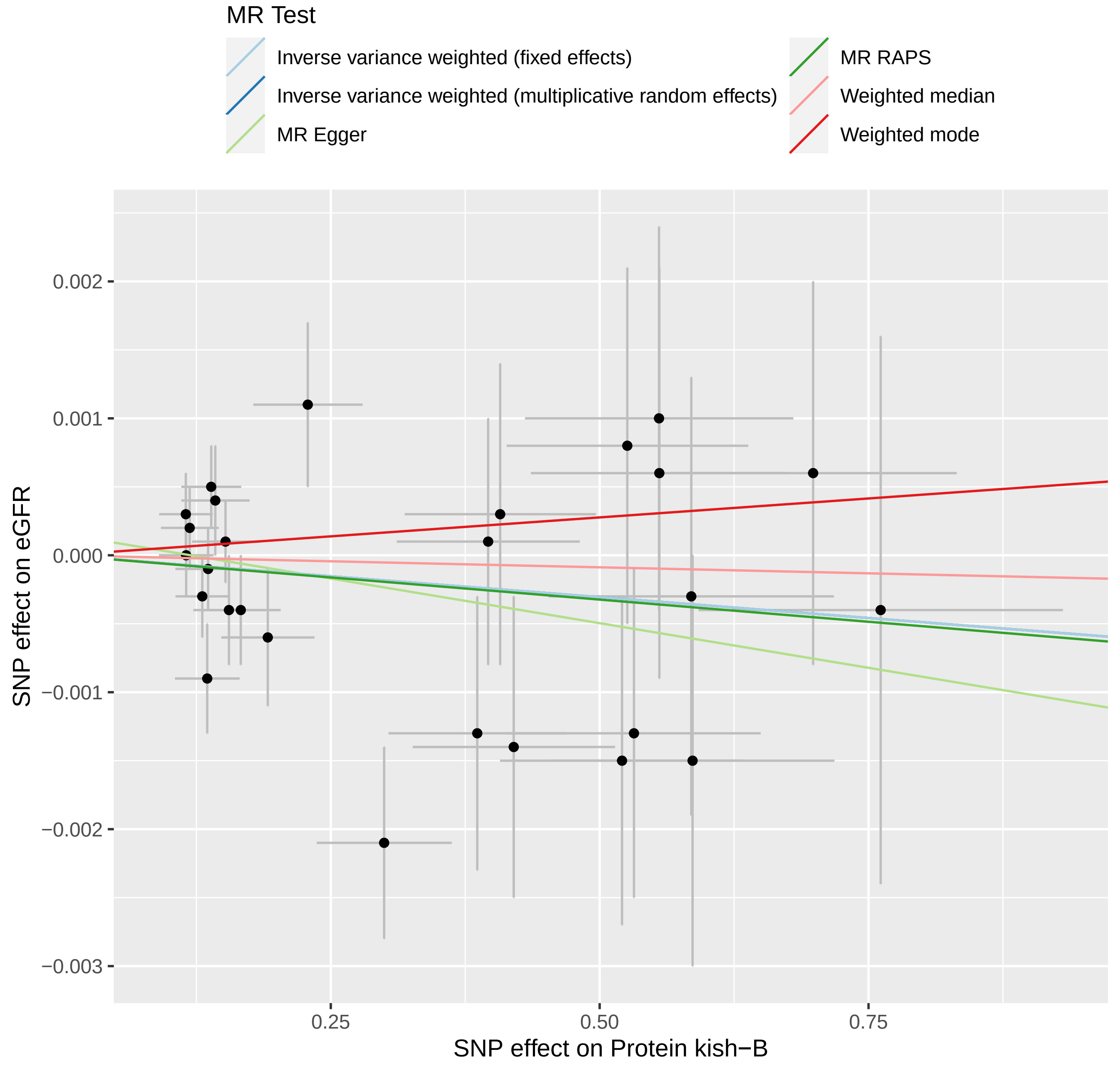

Batch 2−3004 : Transmembrane protein 190 on eGFR

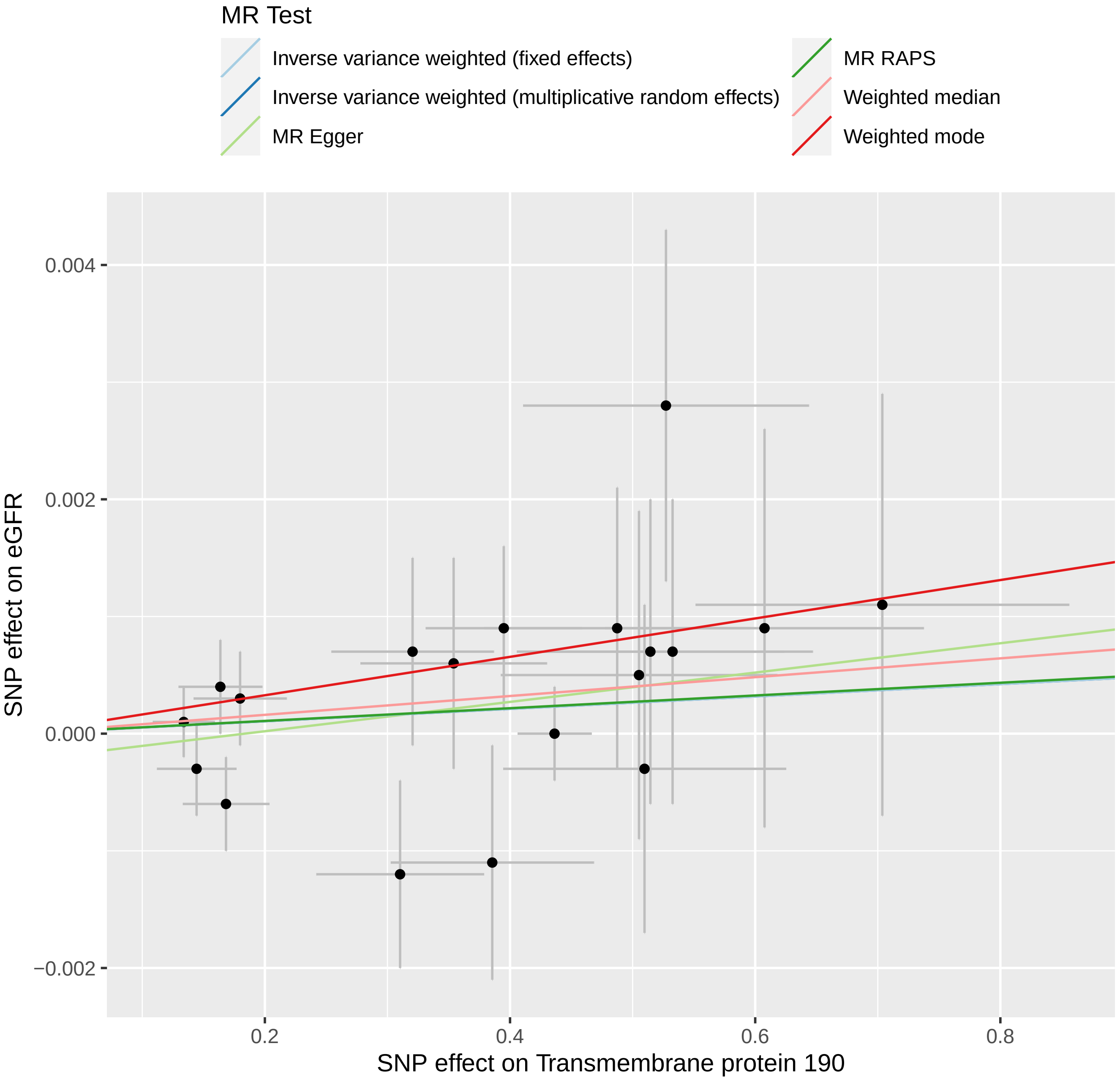

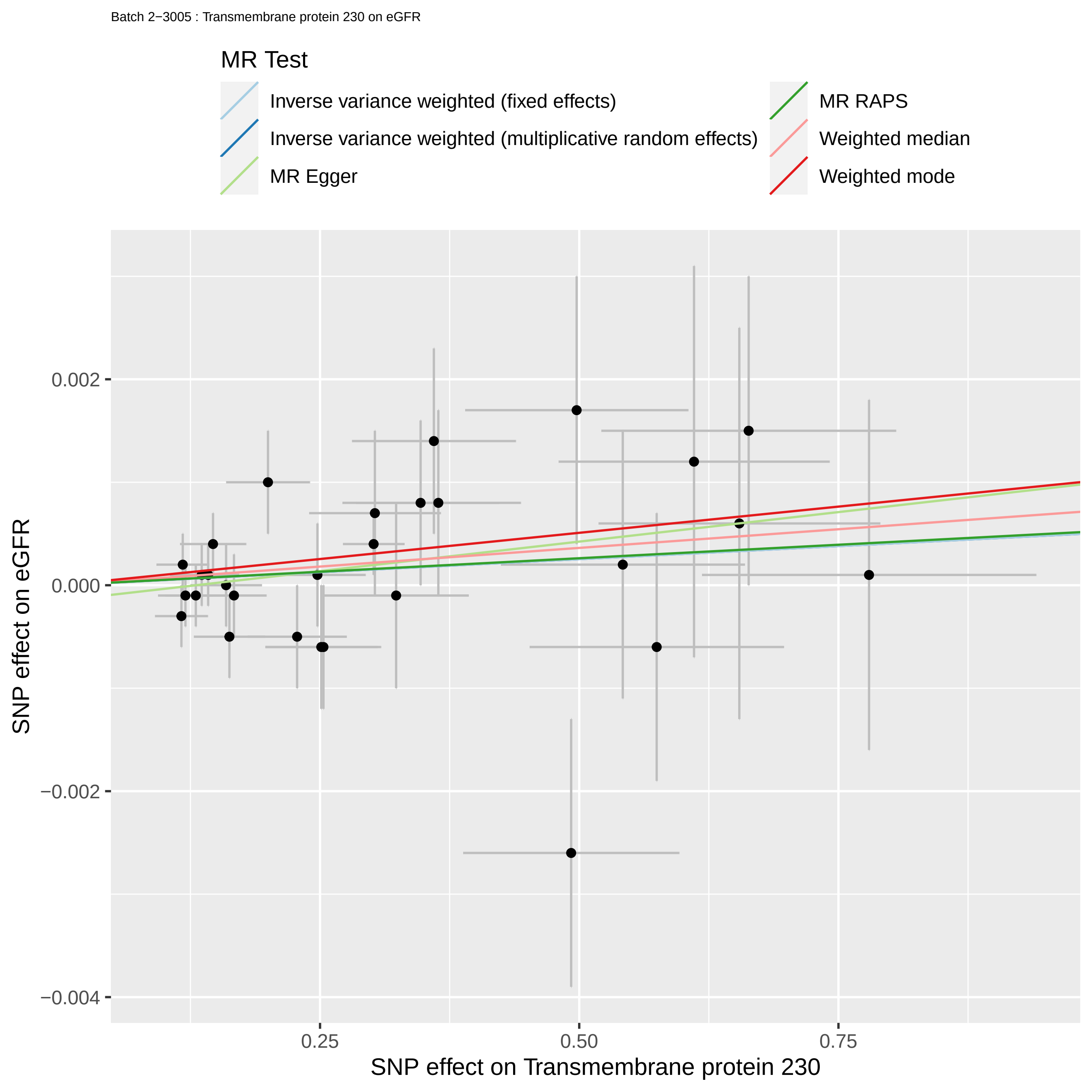
Batch 2−3005 : Transmembrane protein 230 on eGFR
MR Test
Inverse variance weighted (fixed effects)
Inverse variance weighted (multiplicative random effects)
MR Egger
MR RAPS
Weighted median
Weighted mode
0.002
0.000
−0.002
−0.004
0.25
0.50
0.75
SNP effect on eGFR
SNP effect on Transmembrane protein 230

Batch 2−3006 : Transmembrane protein 234 on eGFR

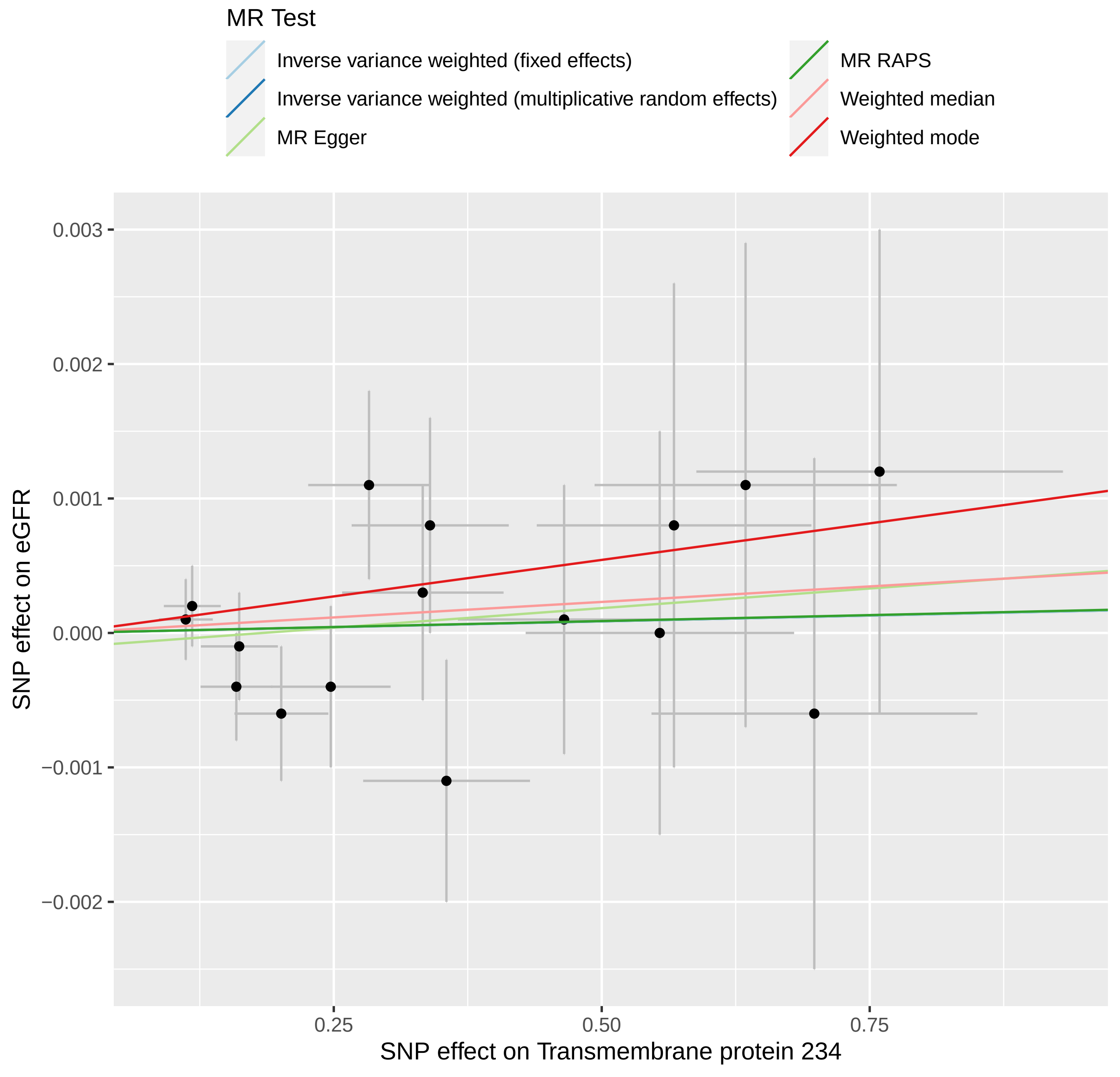

Batch 2−3007 : Transmembrane protein 237 on eGFR

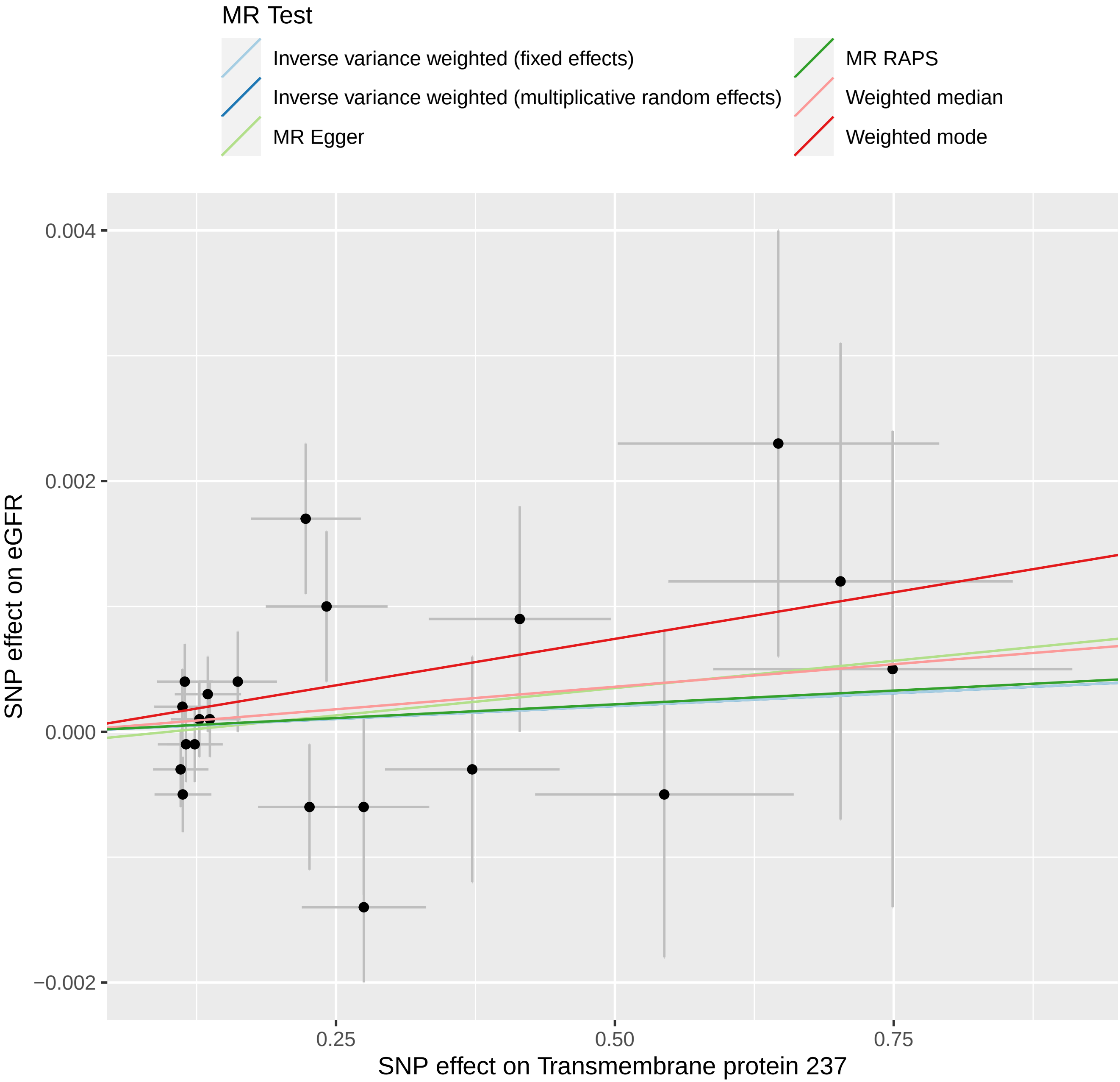

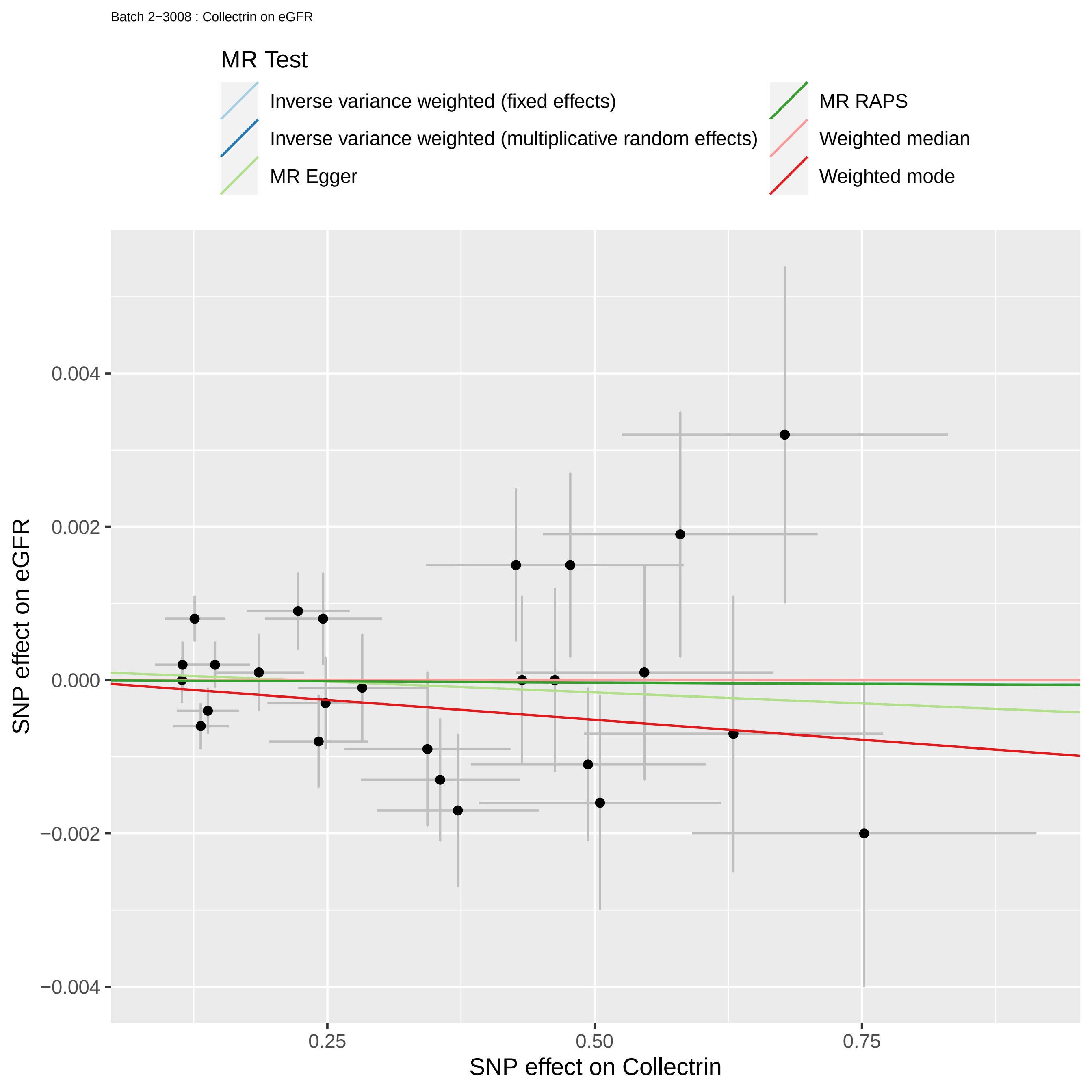

Batch 2−3008 : Collectrin on eGFR
MR Test
Inverse variance weighted (fixed effects)
Inverse variance weighted (multiplicative random effects)
MR Egger
MR RAPS
Weighted median
Weighted mode
0.004
0.002
0.000
−0.002
−0.004
SNP effect on eGFR
0.25
0.50
0.75
SNP effect on Collectrin

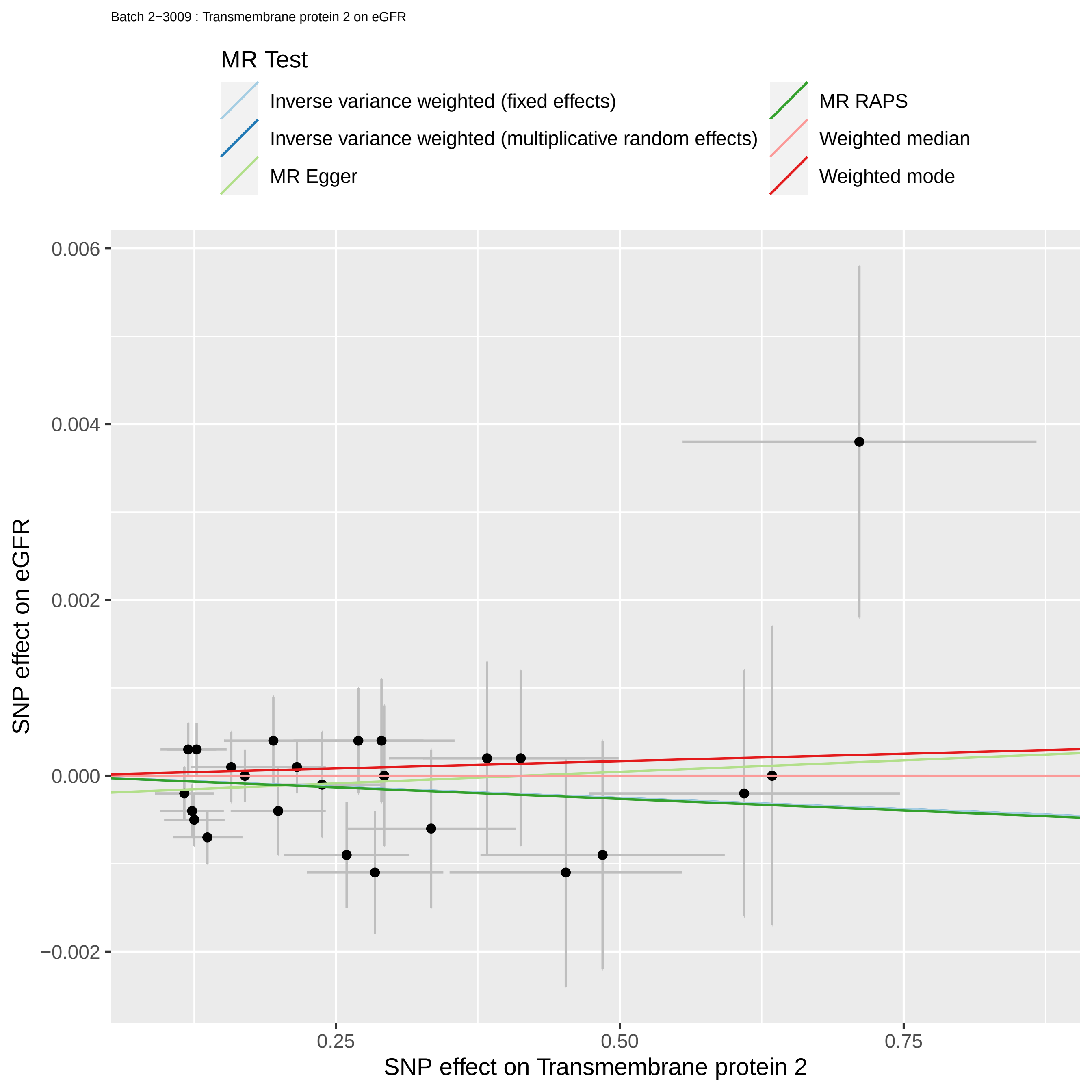

Batch 2−3009 : Transmembrane protein 2 on eGFR
MR Test
Inverse variance weighted (fixed effects)
Inverse variance weighted (multiplicative random effects)
MR Egger
MR RAPS
Weighted median
Weighted mode
0.006
0.004
0.002
0.000
−0.002
SNP effect on eGFR
0.25
0.50
0.75
SNP effect on Transmembrane protein 2

Batch 2−3010 : Transmembrane protein 40 on eGFR

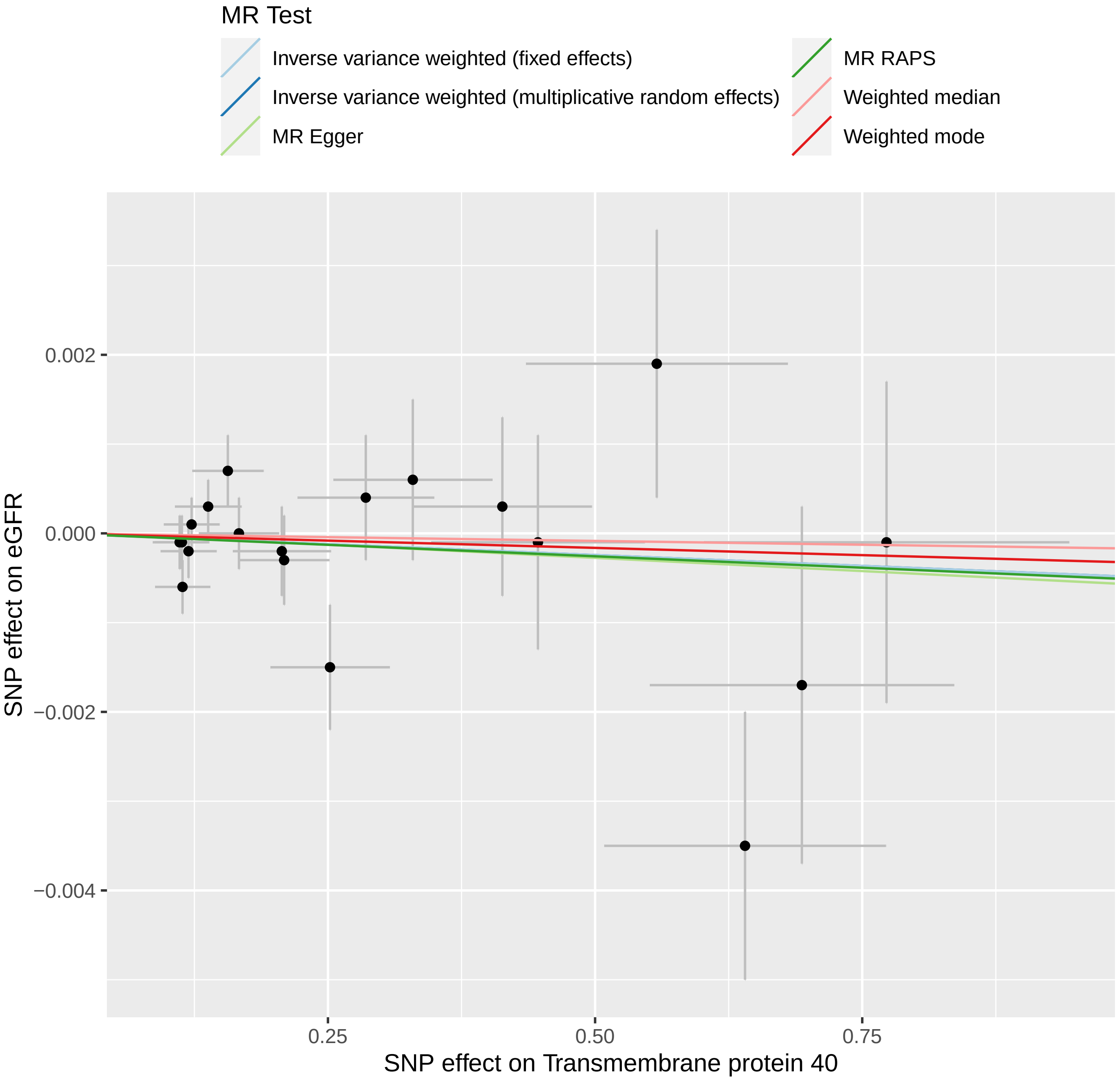

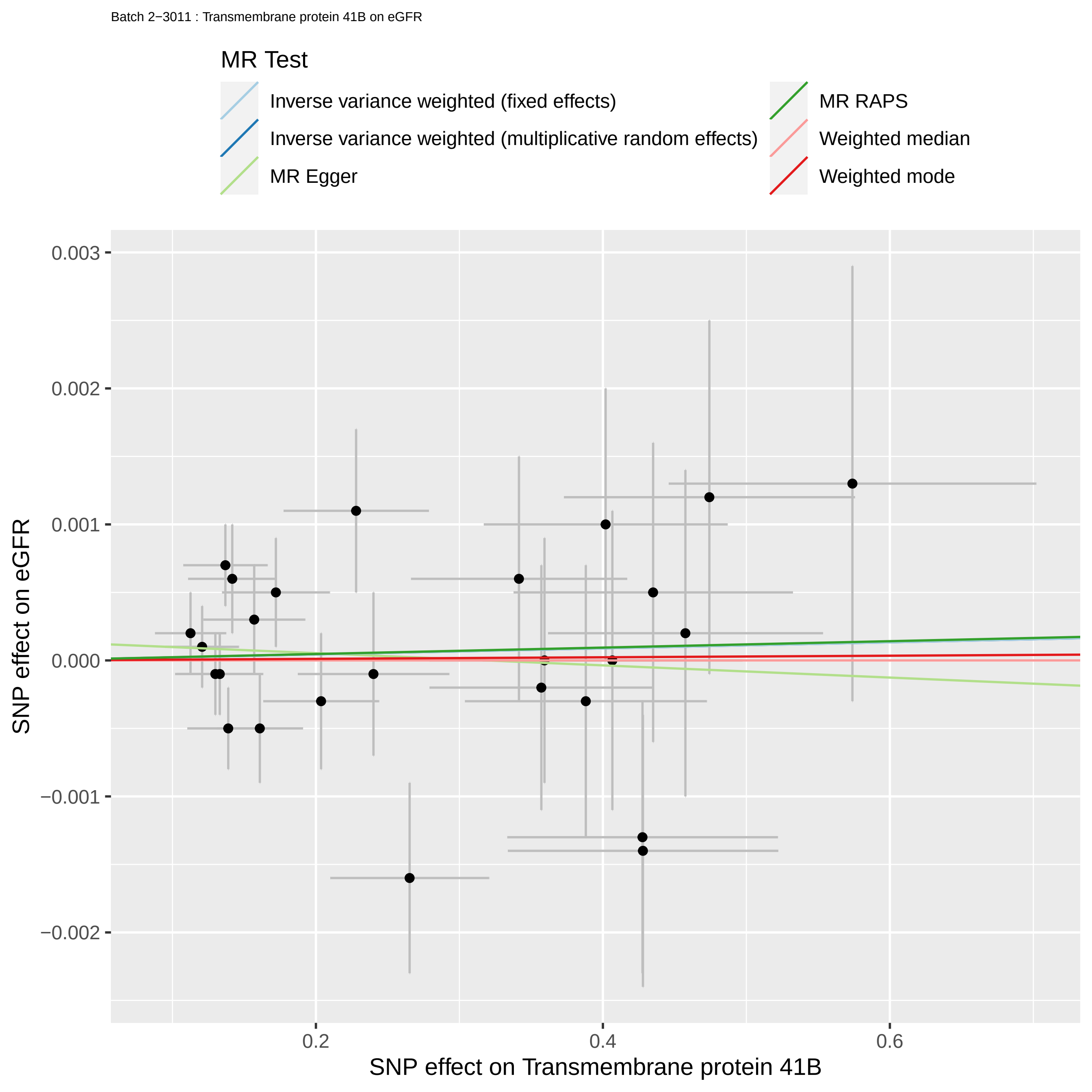

Batch 2−3011 : Transmembrane protein 41B on eGFR
MR Test
Inverse variance weighted (fixed effects)
Inverse variance weighted (multiplicative random effects)
MR Egger
MR RAPS
Weighted median
Weighted mode
0.003
0.002
0.001
0.000
−0.001
−0.002
0.2
0.4
0.6
SNP effect on eGFR
SNP effect on Transmembrane protein 41B

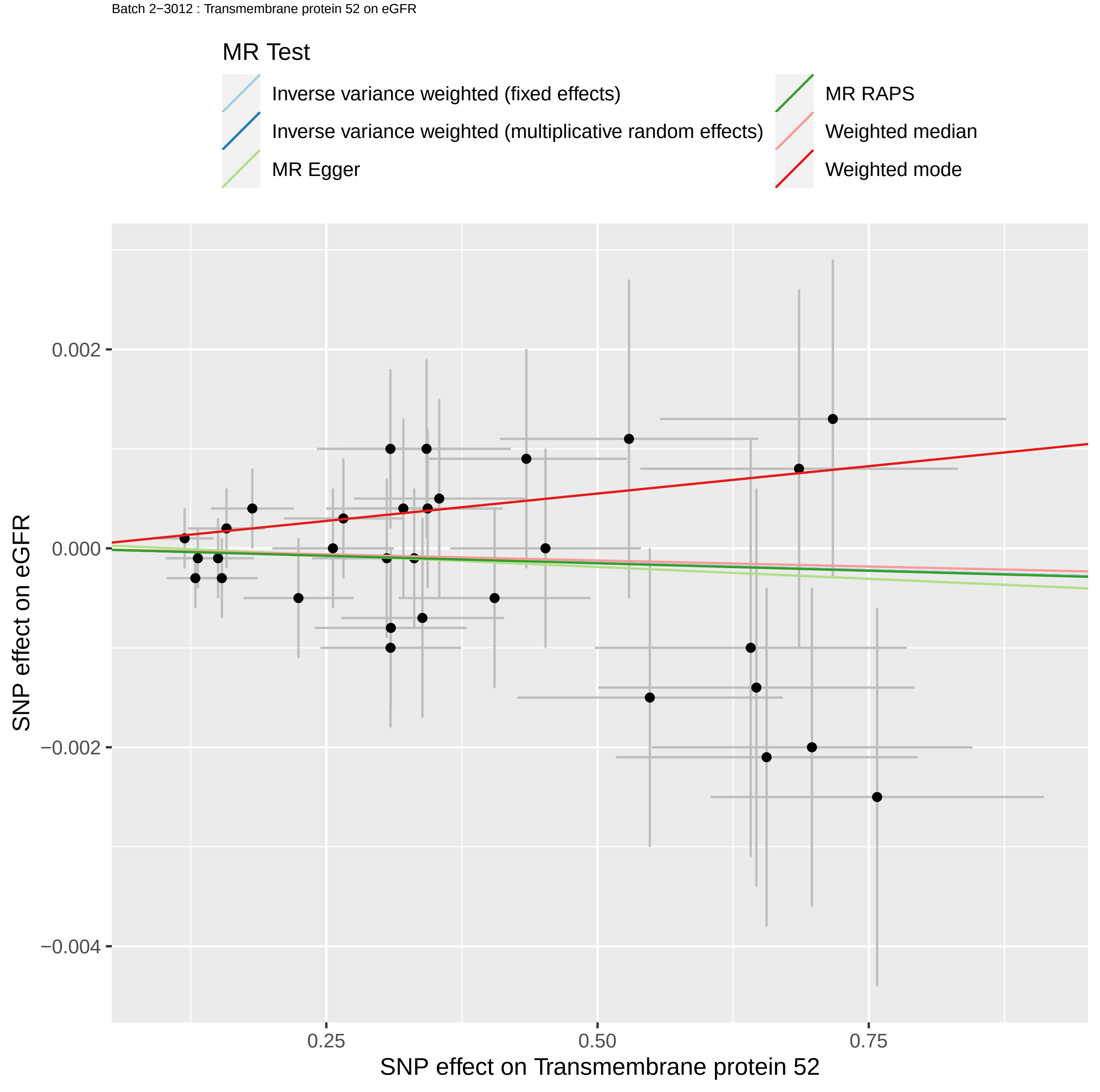

Batch 2−3012 : Transmembrane protein 52 on eGFR
MR Test
Inverse variance weighted (fixed effects)
Inverse variance weighted (multiplicative random effects)
MR Egger
MR RAPS
Weighted median
Weighted mode
0.002
0.000
−0.002
−0.004
SNP effect on eGFR
0.25
0.50
0.75
SNP effect on Transmembrane protein 52

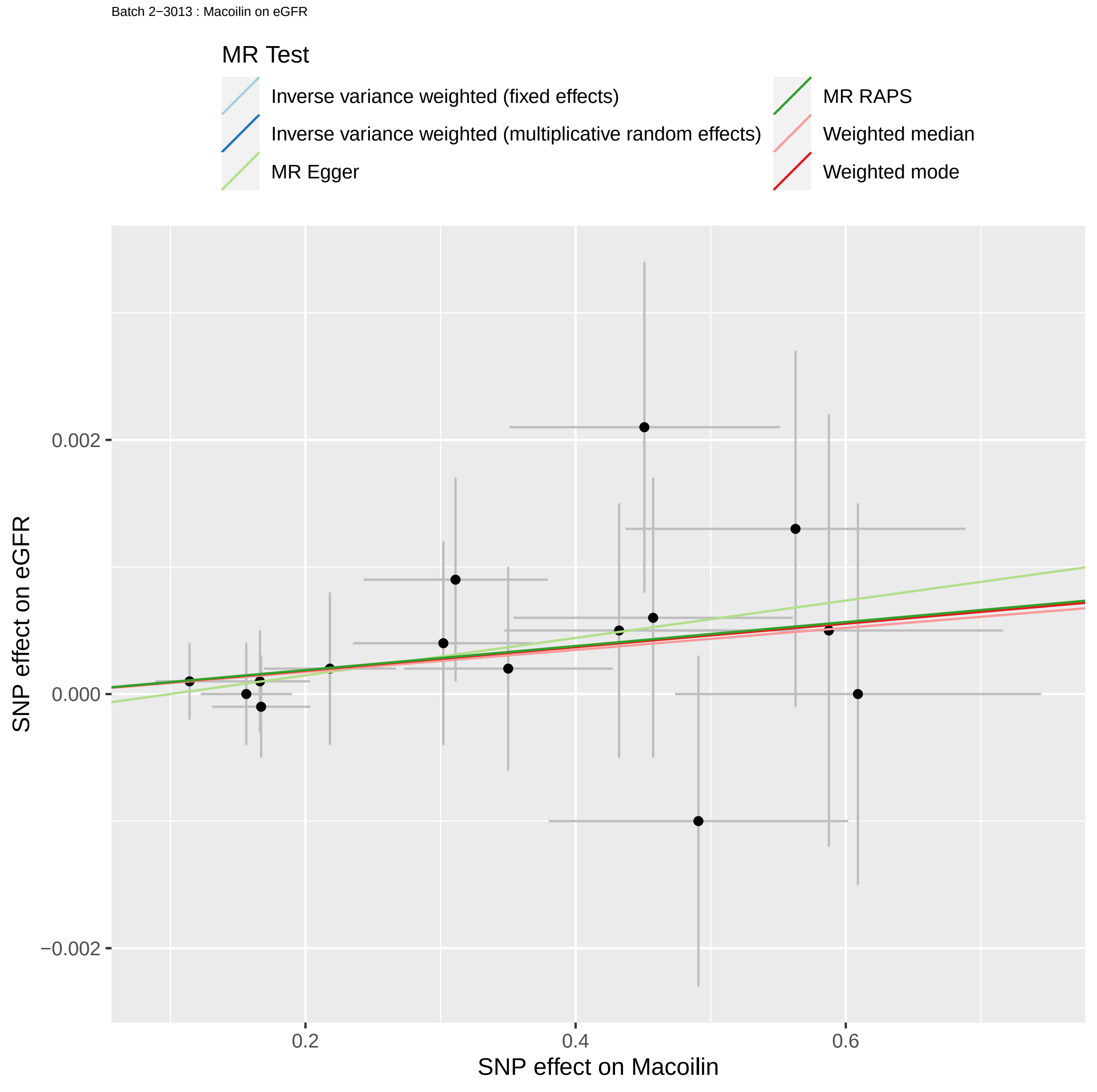

Batch 2−3013 : Macoilin on eGFR
MR Test
Inverse variance weighted (fixed effects)
Inverse variance weighted (multiplicative random effects)
MR Egger
MR RAPS
Weighted median
Weighted mode
0.002
0.000
−0.002
0.2
0.4
0.6
SNP effect on Macoilin
SNP effect on eGFR

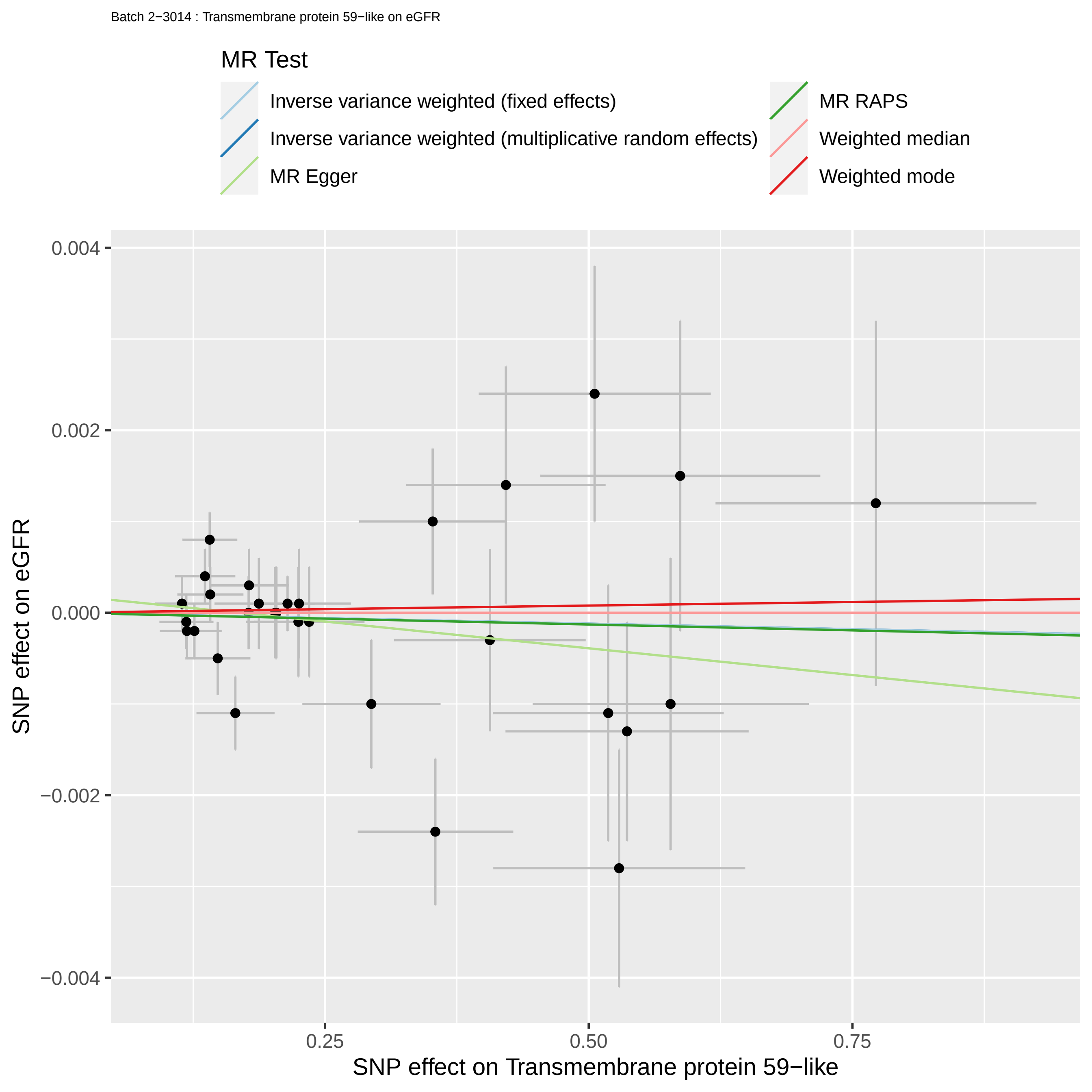

Batch 2−3014 : Transmembrane protein 59−like on eGFR
MR Test
Inverse variance weighted (fixed effects)
Inverse variance weighted (multiplicative random effects)
MR Egger
MR RAPS
Weighted median
Weighted mode
0.004
0.002
0.000
−0.002
−0.004
0.25
0.50
0.75
SNP effect on eGFR
SNP effect on Transmembrane protein 59−like

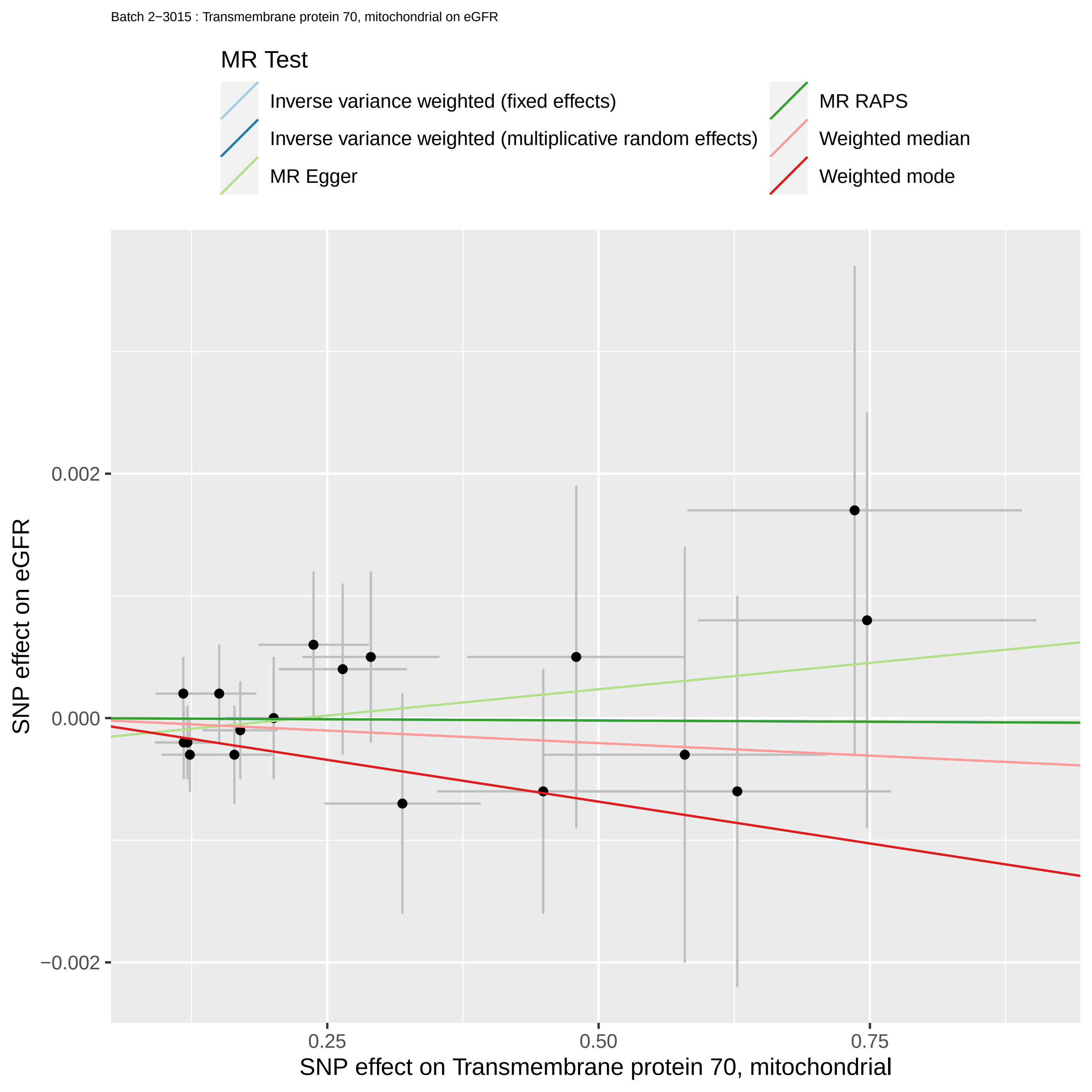
Batch 2−3015 : Transmembrane protein 70, mitochondrial on eGFR
MR Test
Inverse variance weighted (fixed effects)
Inverse variance weighted (multiplicative random effects)
MR Egger
MR RAPS
Weighted median
Weighted mode
0.002
0.000
−0.002
0.25
0.50
0.75
SNP effect on eGFR
SNP effect on Transmembrane protein 70, mitochondrial

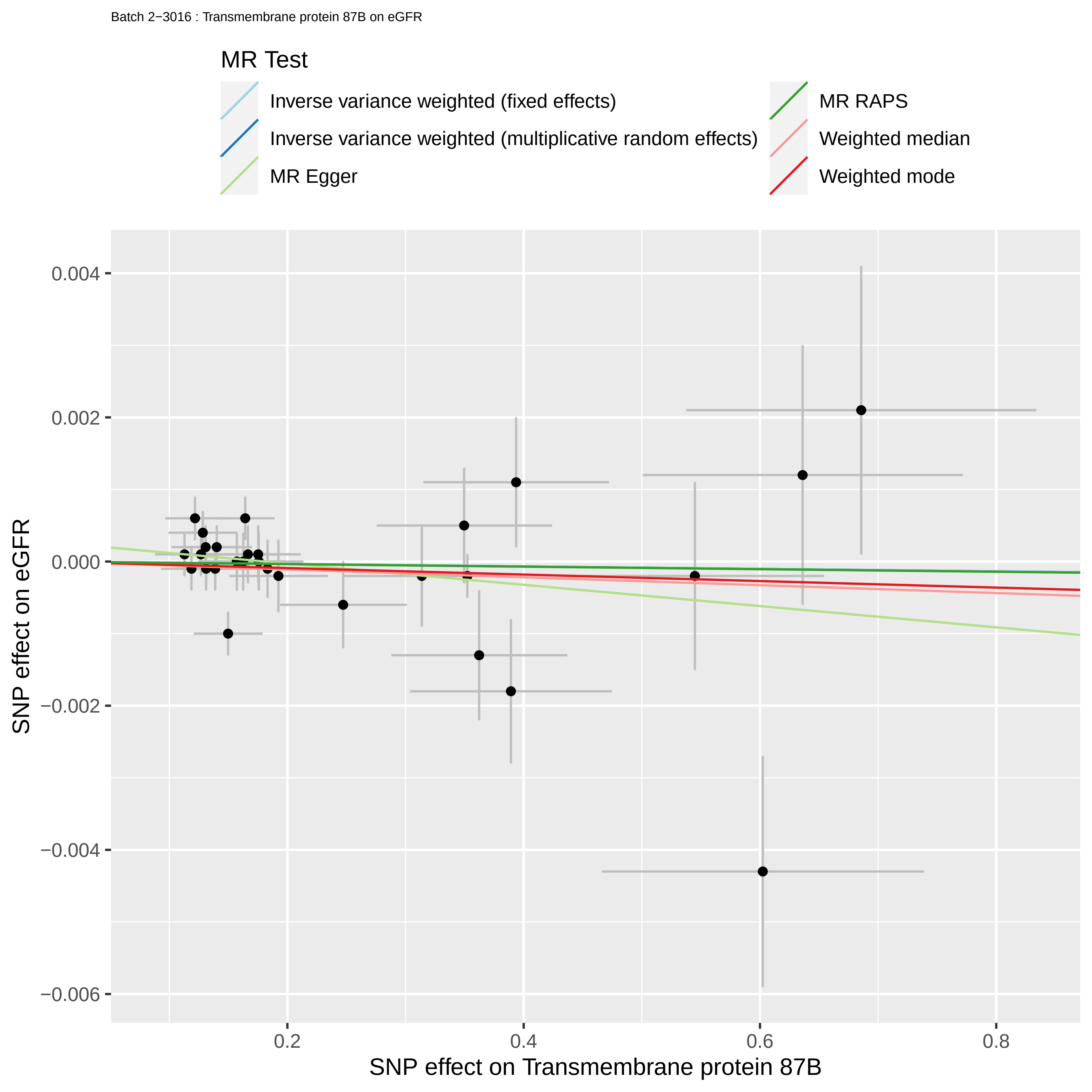

Batch 2−3016 : Transmembrane protein 87B on eGFR
MR Test
Inverse variance weighted (fixed effects)
Inverse variance weighted (multiplicative random effects)
MR Egger
MR RAPS
Weighted median
Weighted mode
0.004
0.002
0.000
−0.002
−0.004
−0.006
SNP effect on eGFR
0.2
0.4
0.6
0.8
SNP effect on Transmembrane protein 87B

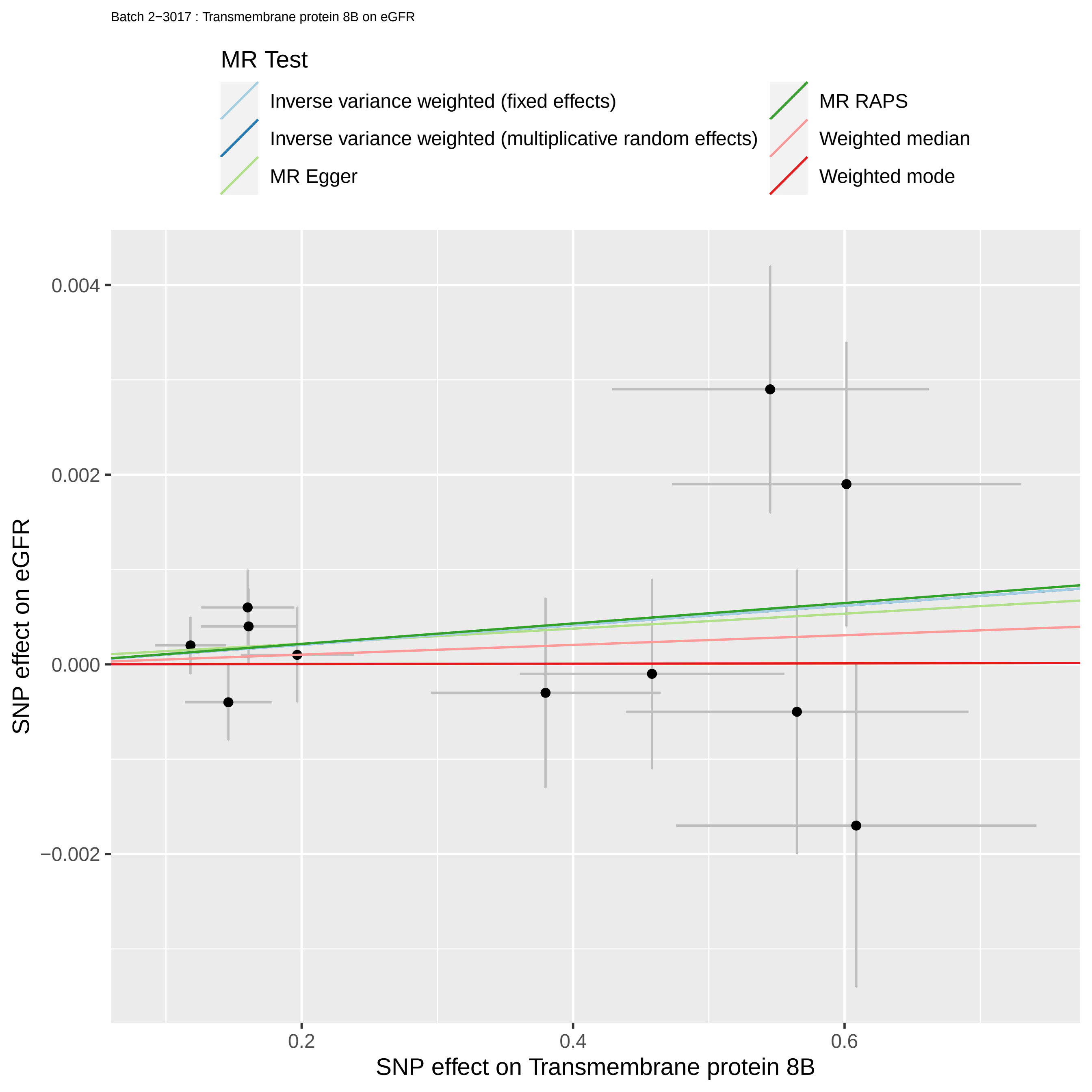
Batch 2−3017 : Transmembrane protein 8B on eGFR
MR Test
Inverse variance weighted (fixed effects)
Inverse variance weighted (multiplicative random effects)
MR Egger
MR RAPS
Weighted median
Weighted mode
0.004
0.002
0.000
−0.002
0.2
0.4
0.6
SNP effect on eGFR
SNP effect on Transmembrane protein 8B

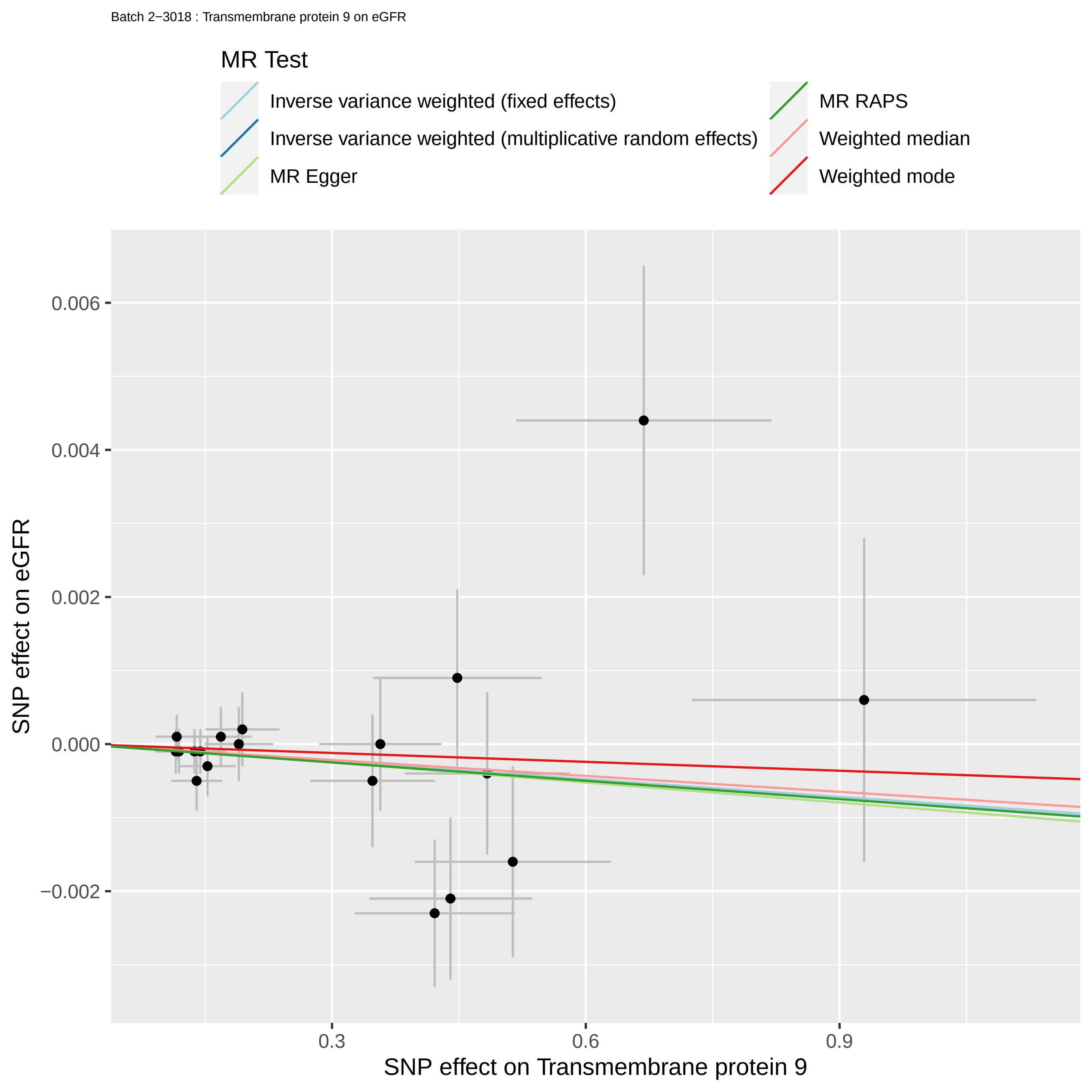
Batch 2−3018 : Transmembrane protein 9 on eGFR
MR Test
Inverse variance weighted (fixed effects)
Inverse variance weighted (multiplicative random effects)
MR Egger
MR RAPS
Weighted median
Weighted mode
0.006
0.004
0.002
0.000
−0.002
0.3
0.6
0.9
SNP effect on eGFR
SNP effect on Transmembrane protein 9

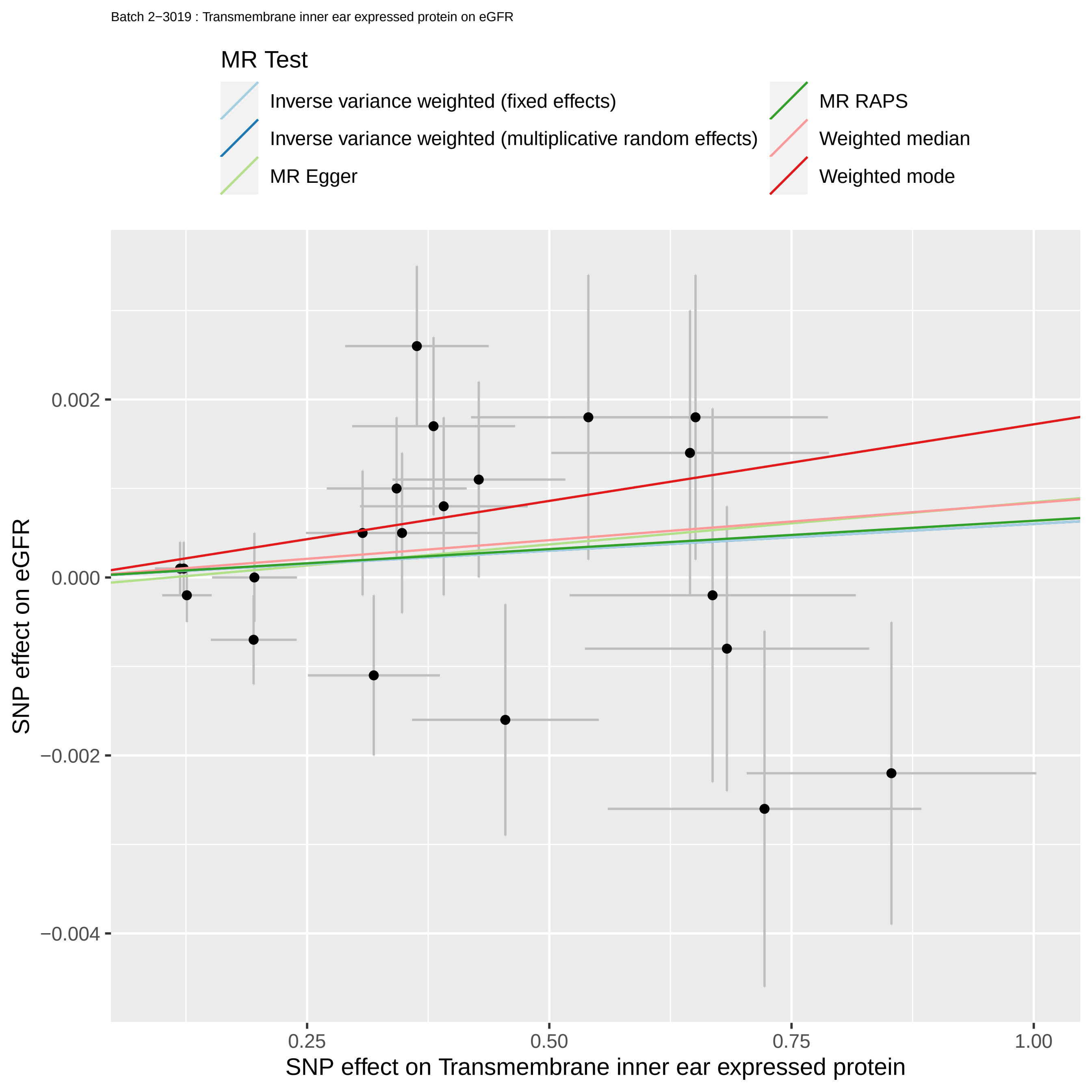

Batch 2−3019 : Transmembrane inner ear expressed protein on eGFR
MR Test
Inverse variance weighted (fixed effects)
Inverse variance weighted (multiplicative random effects)
MR Egger
MR RAPS
Weighted median
Weighted mode
0.002
0.000
−0.002
−0.004
SNP effect on eGFR
0.25
0.50
0.75
1.00
SNP effect on Transmembrane inner ear expressed protein

Batch 2−3020 : Tropomodulin−1 on eGFR

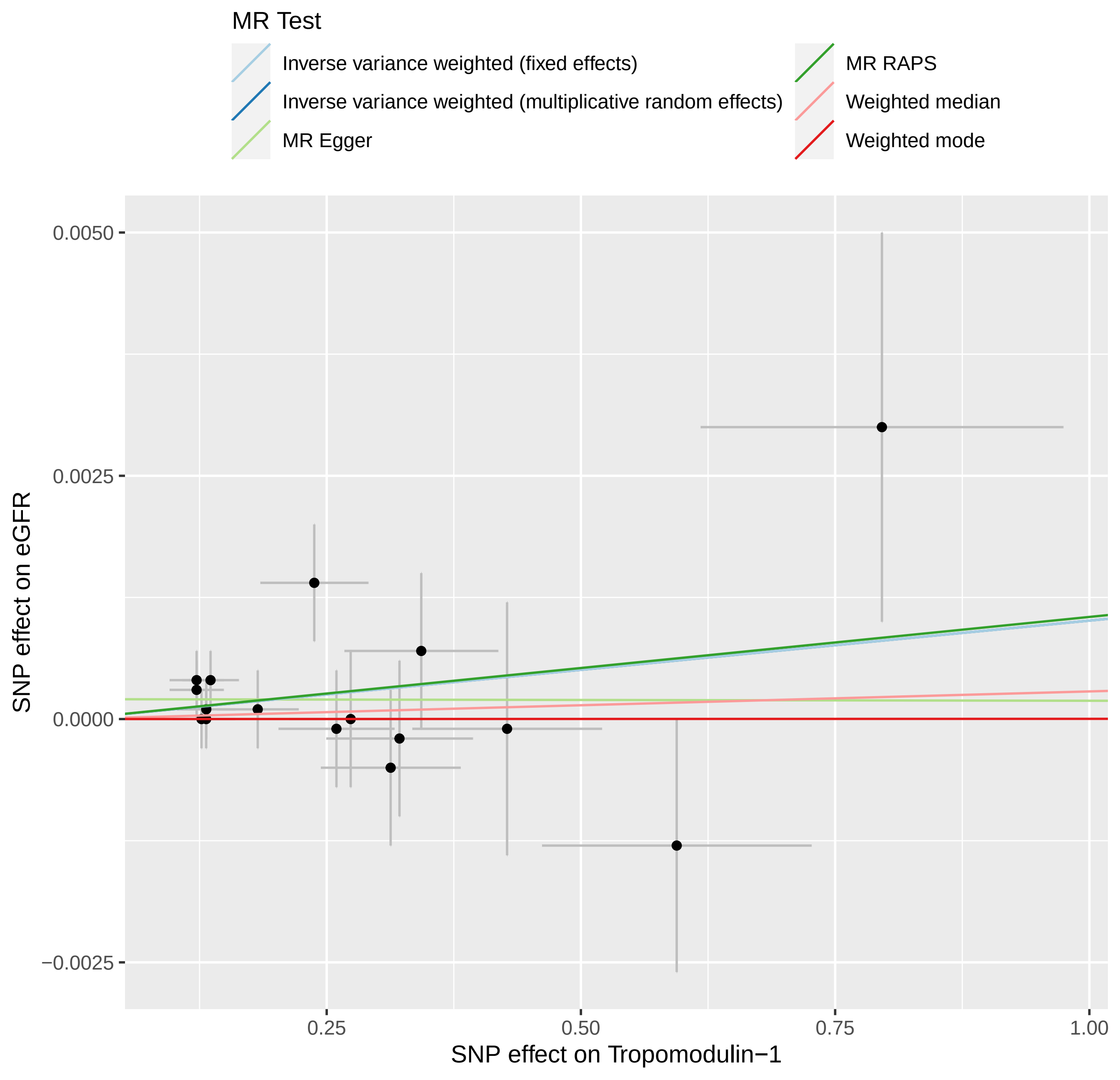

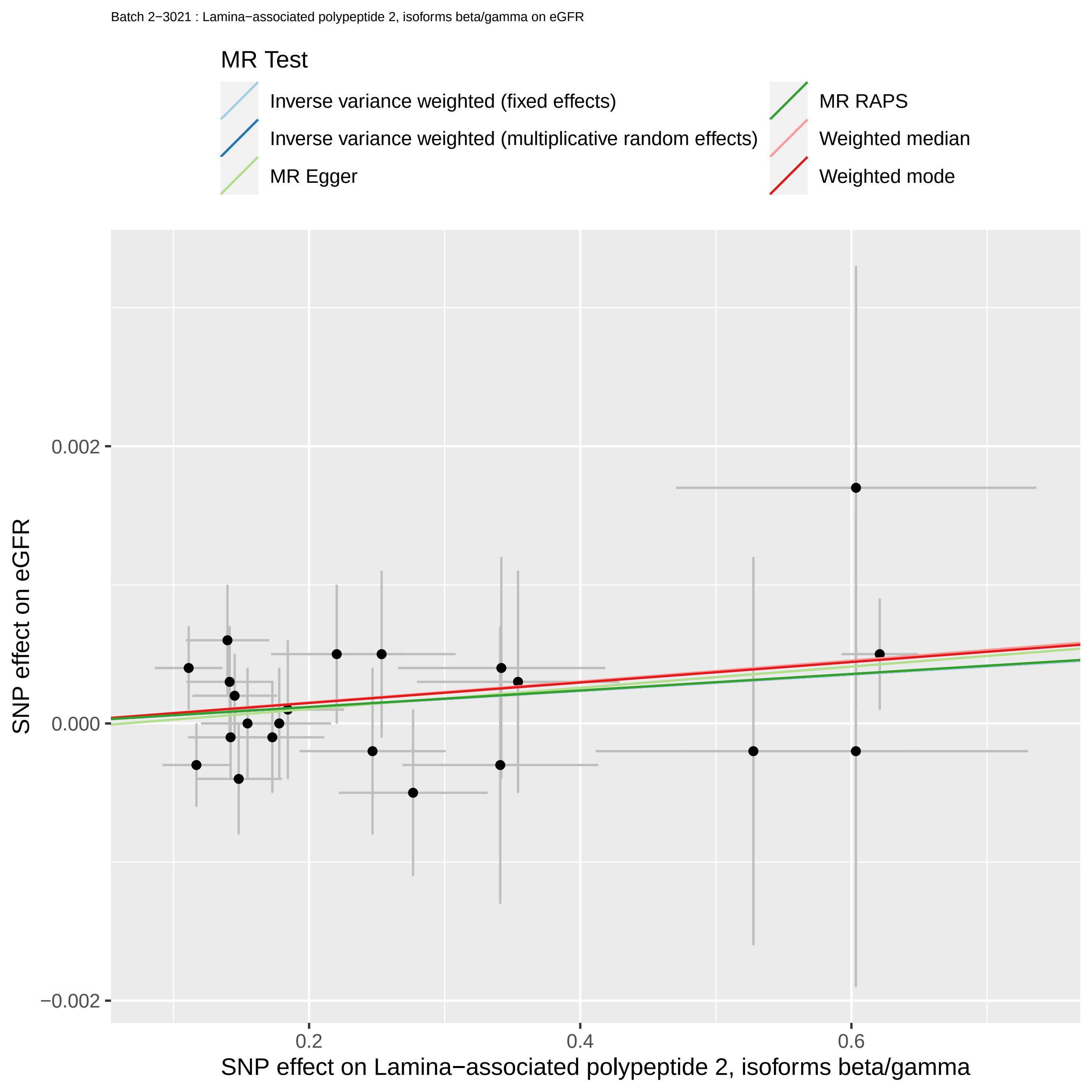

Batch 2−3021 : Lamina−associated polypeptide 2, isoforms beta/gamma on eGFR
MR Test
Inverse variance weighted (fixed effects)
Inverse variance weighted (multiplicative random effects)
MR Egger
MR RAPS
Weighted median
Weighted mode
0.002
0.000
−0.002
SNP effect on eGFR
0.2
0.4
0.6
SNP effect on Lamina−associated polypeptide 2, isoforms beta/gamma

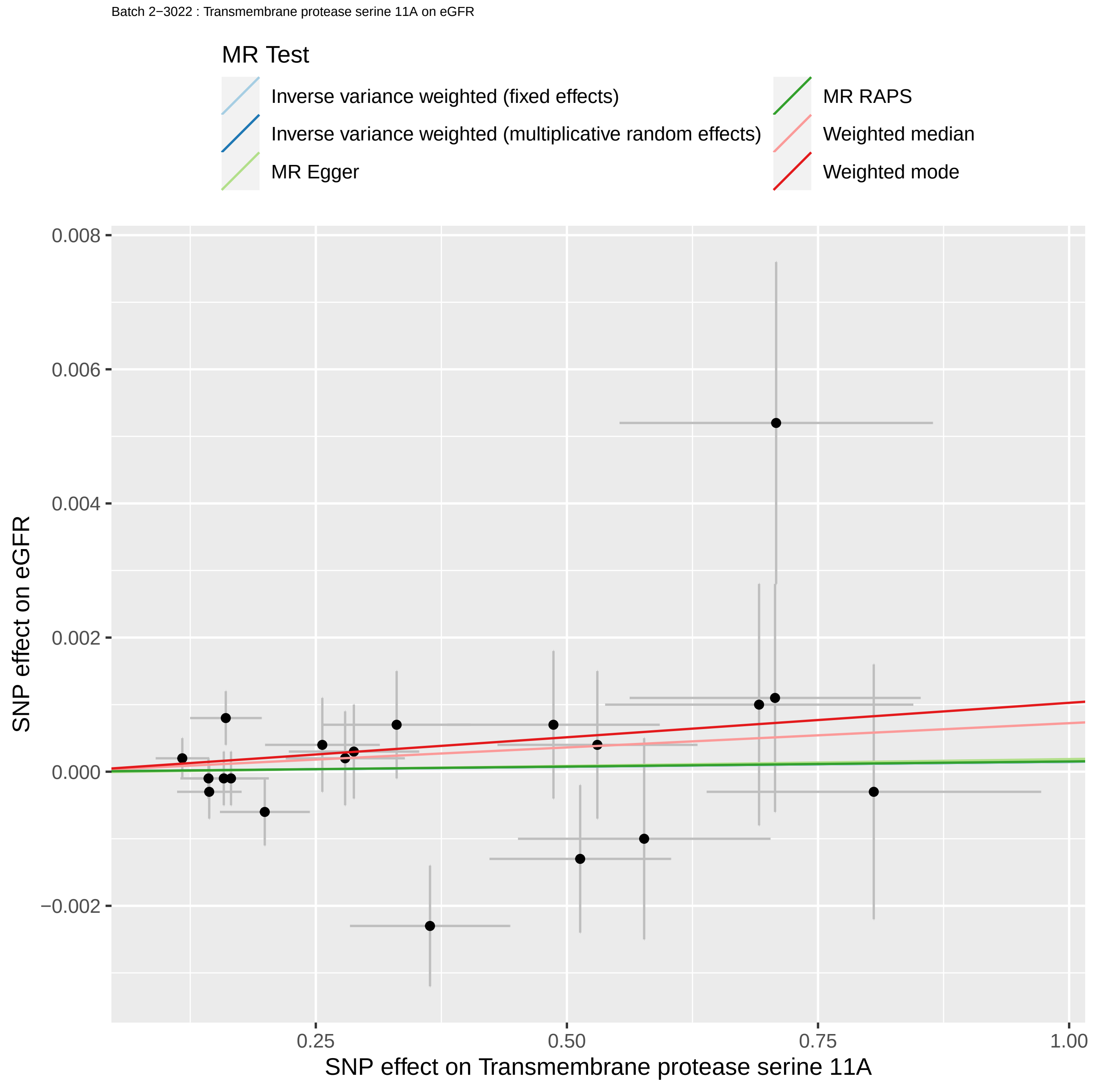

Batch 2−3022 : Transmembrane protease serine 11A on eGFR
MR Test
Inverse variance weighted (fixed effects)
Inverse variance weighted (multiplicative random effects)
MR Egger
MR RAPS
Weighted median
Weighted mode
0.008
0.006
0.004
0.002
0.000
−0.002
SNP effect on eGFR
0.25
0.50
0.75
1.00
SNP effect on Transmembrane protease serine 11A

Batch 2−3023 : Transmembrane protease serine 11B on eGFR

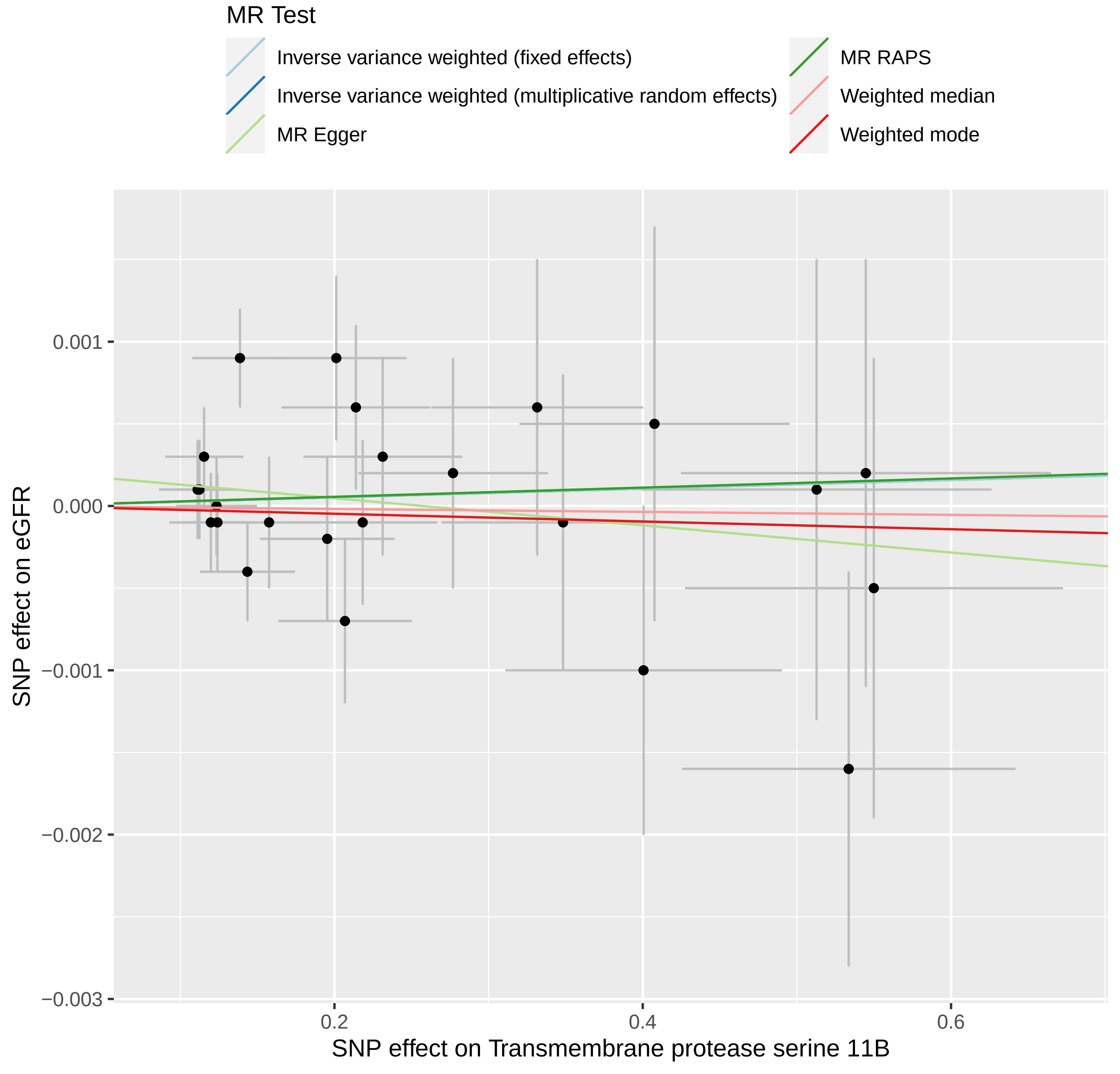

Batch 2−3024 : Transmembrane protease serine 11D on eGFR

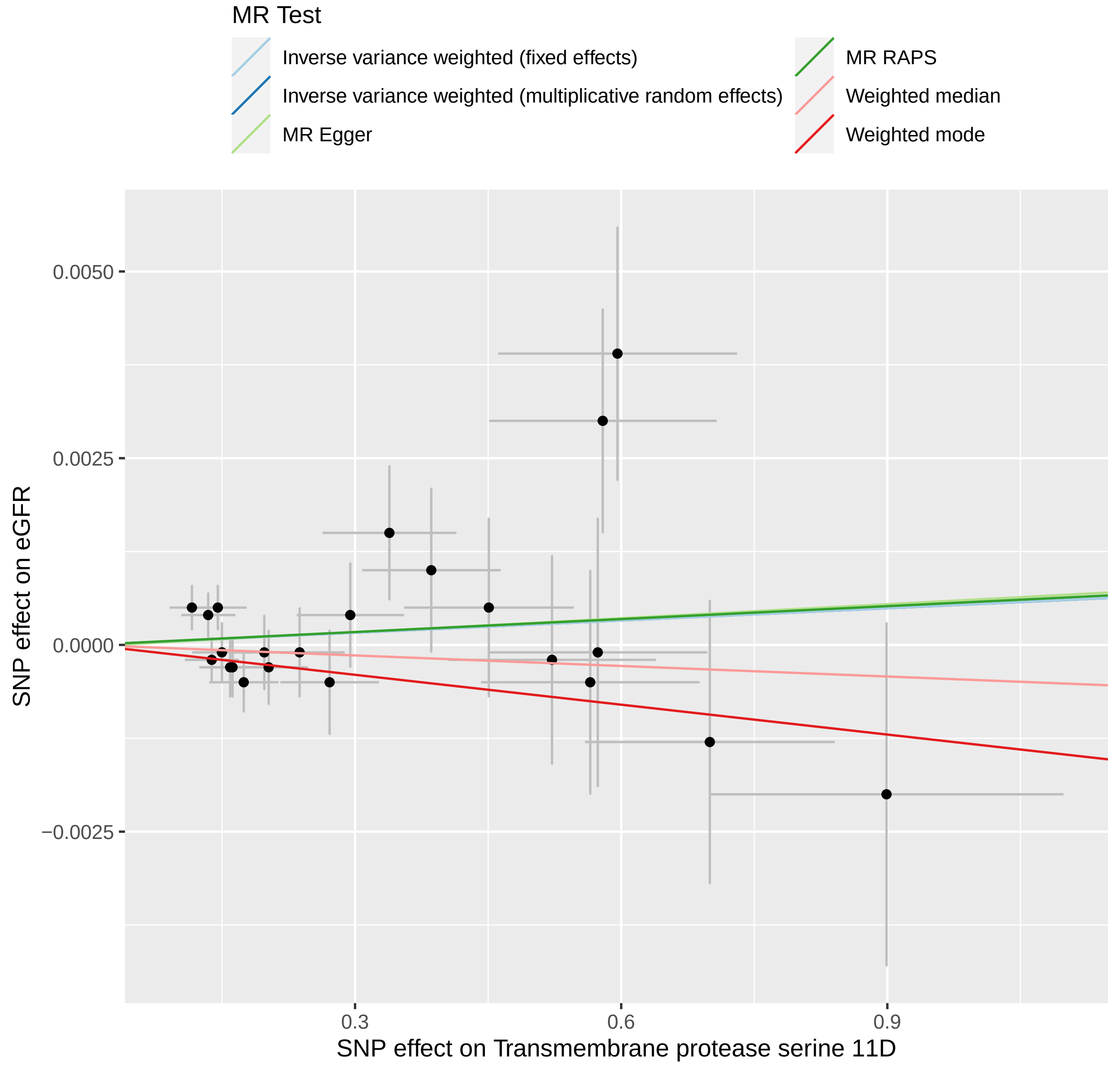

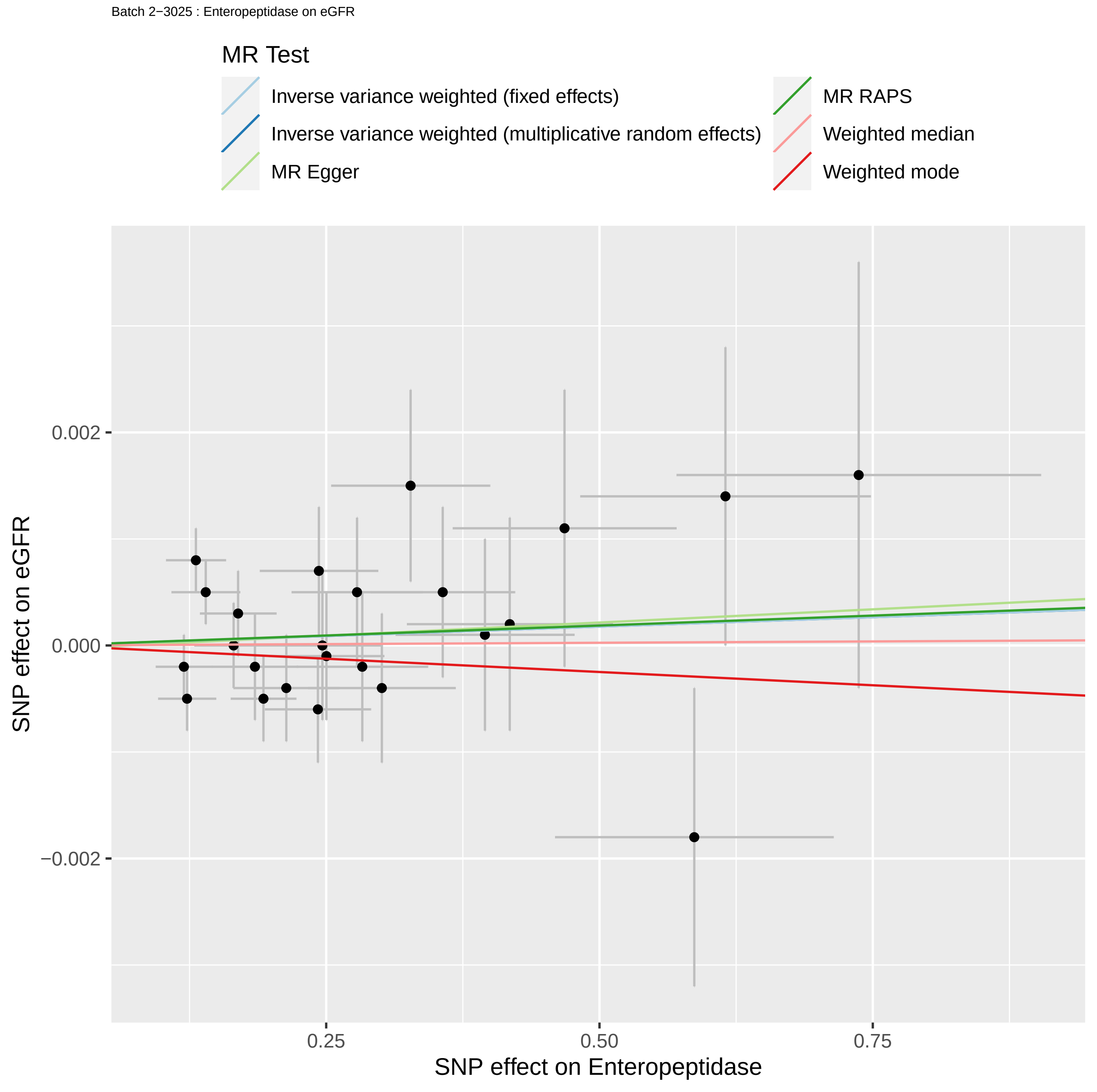

Batch 2−3025 : Enteropeptidase on eGFR
MR Test
Inverse variance weighted (fixed effects)
Inverse variance weighted (multiplicative random effects)
MR Egger
MR RAPS
Weighted median
Weighted mode
0.002
0.000
−0.002
0.25
0.50
0.75
SNP effect on eGFR
SNP effect on Enteropeptidase

Batch 2−3026 : Transmembrane protease serine 5 on eGFR

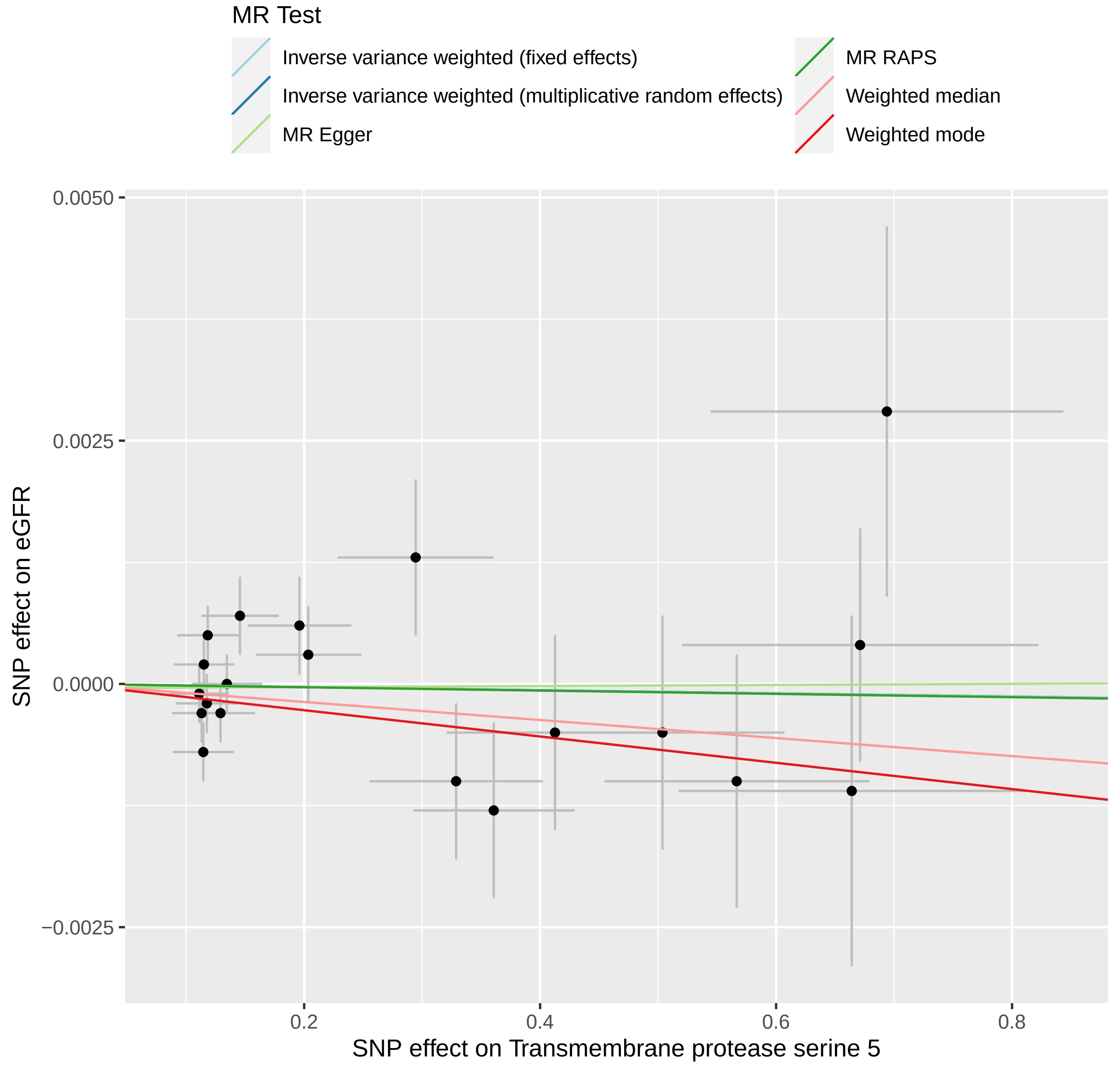

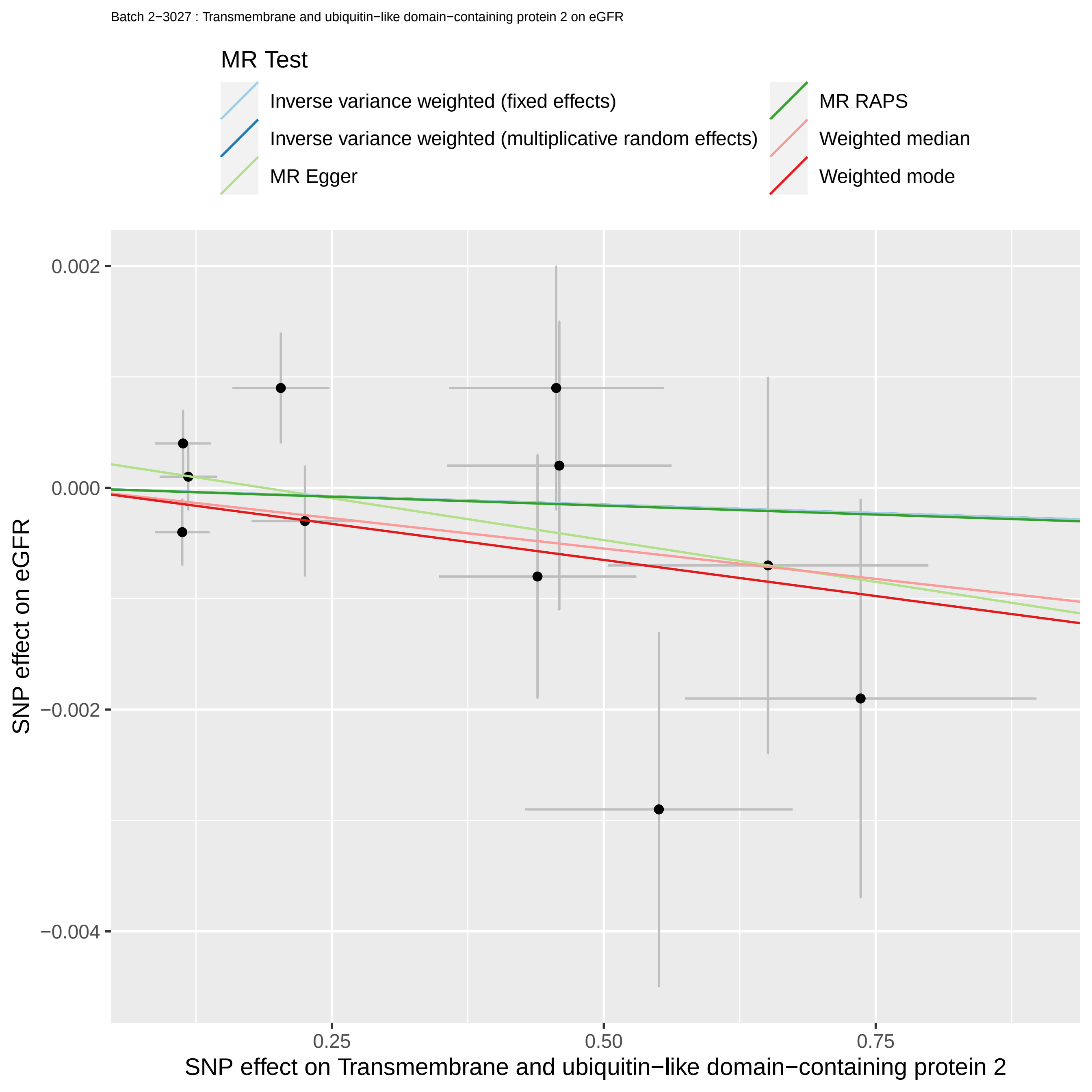

Batch 2−3027 : Transmembrane and ubiquitin−like domain−containing protein 2 on eGFR
MR Test
Inverse variance weighted (fixed effects)
Inverse variance weighted (multiplicative random effects)
MR Egger
MR RAPS
Weighted median
Weighted mode
0.002
0.000
−0.002
−0.004
0.25
0.50
0.75
SNP effect on eGFR
SNP effect on Transmembrane and ubiquitin−like domain−containing protein 2

Batch 2−3028 : Tenascin on eGFR

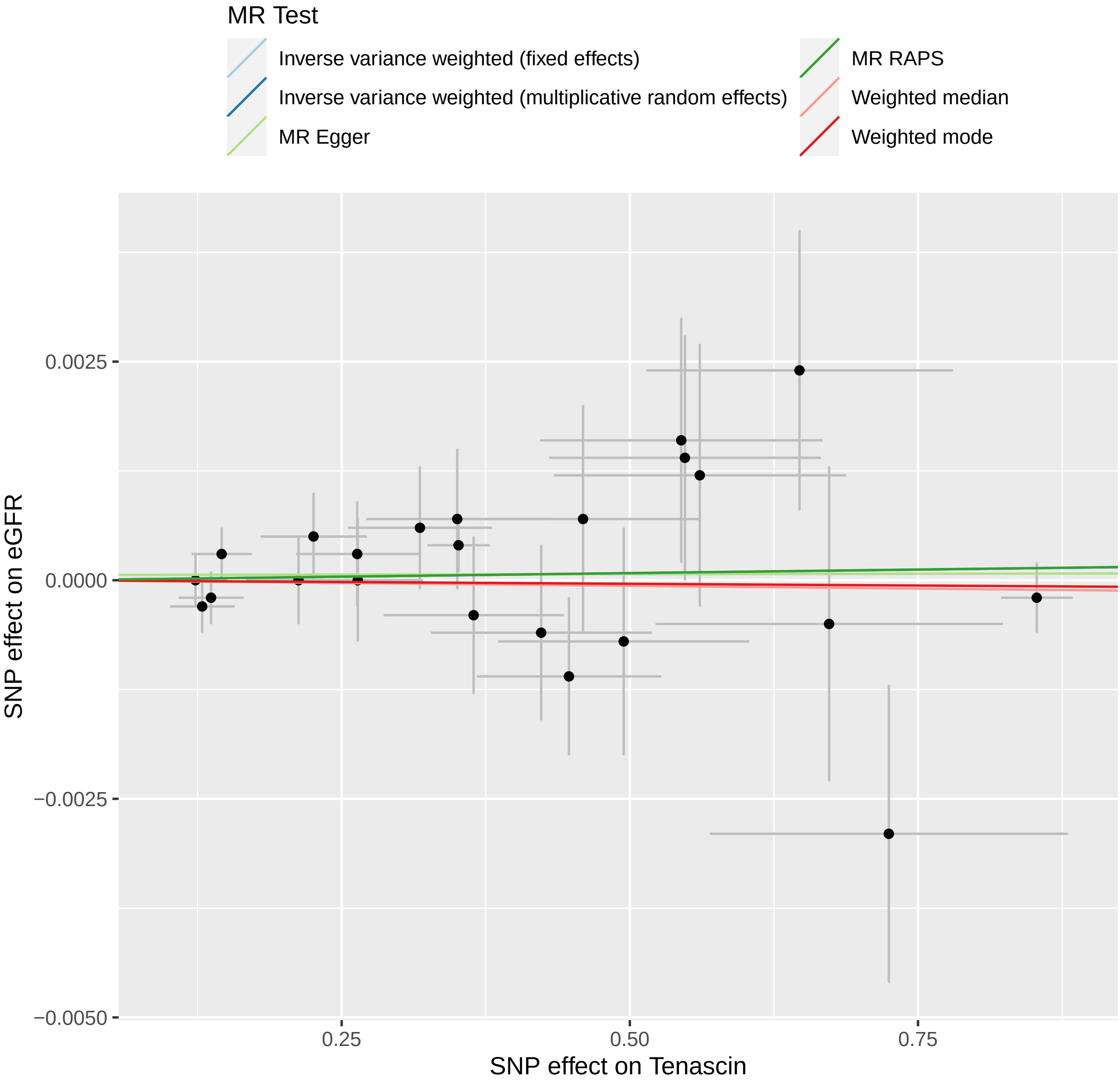

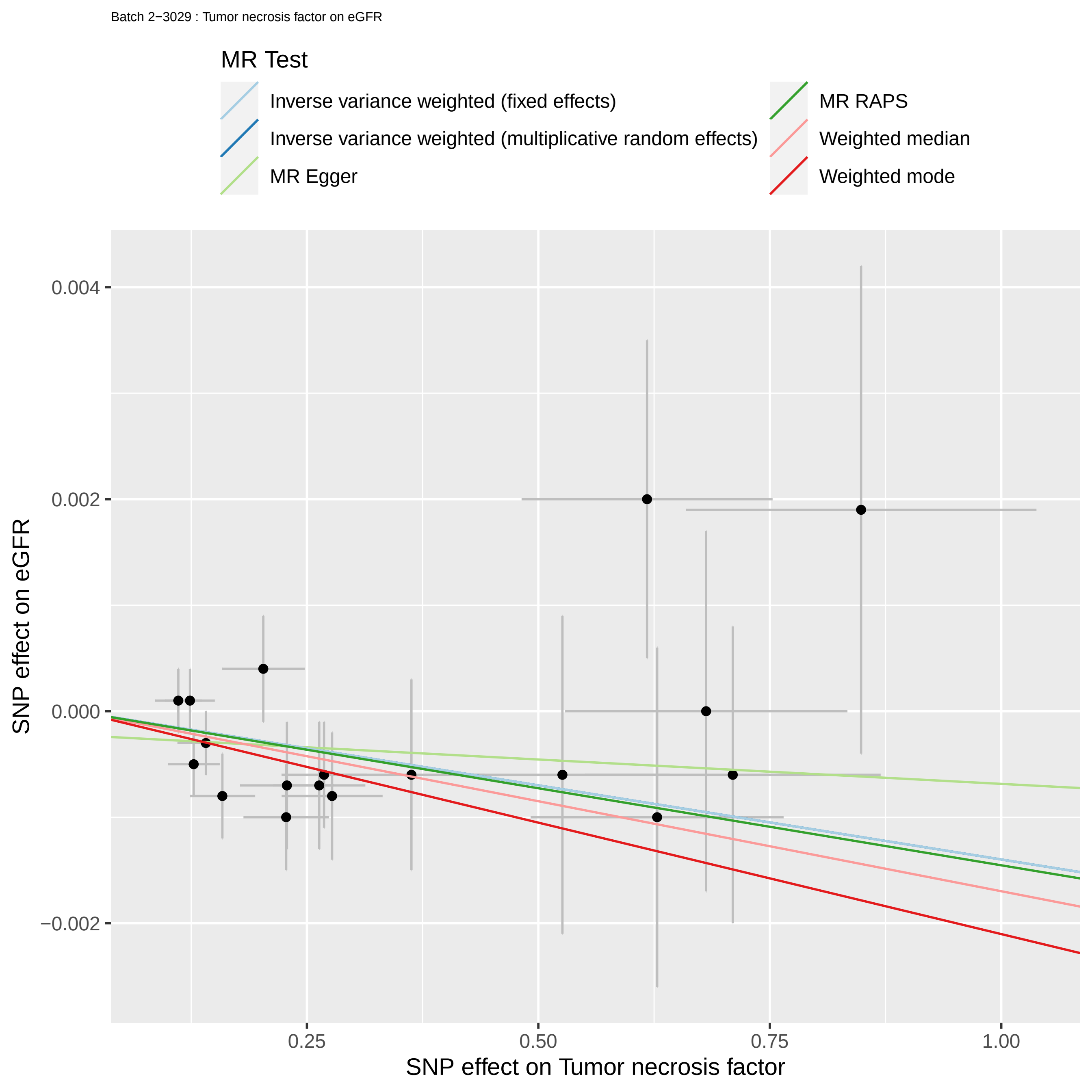

Batch 2−3029 : Tumor necrosis factor on eGFR
MR Test
Inverse variance weighted (fixed effects)
Inverse variance weighted (multiplicative random effects)
MR Egger
MR RAPS
Weighted median
Weighted mode
0.004
0.002
0.000
−0.002
SNP effect on eGFR
0.25
0.50
0.75
1.00
SNP effect on Tumor necrosis factor

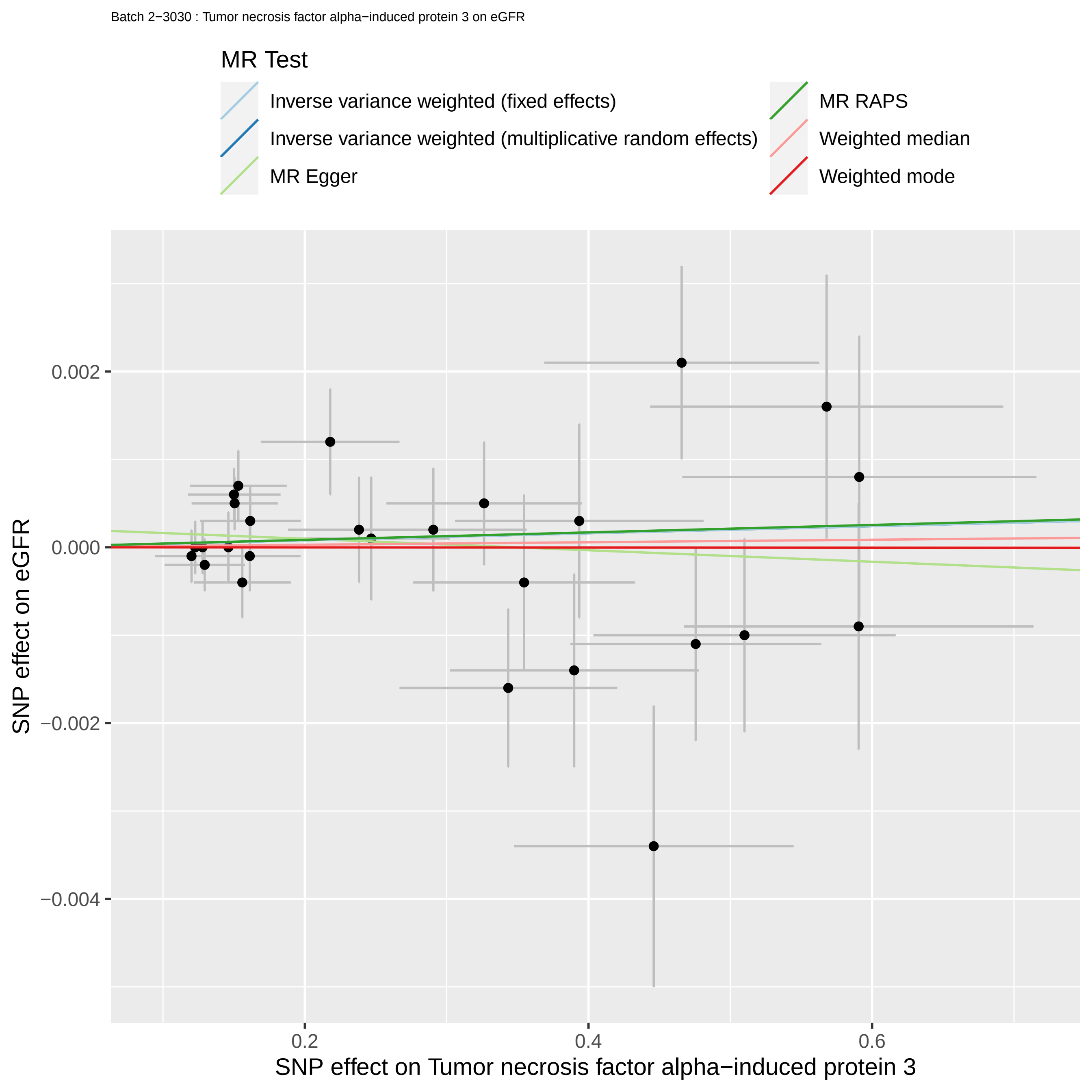
Batch 2−3030 : Tumor necrosis factor alpha−induced protein 3 on eGFR
MR Test
Inverse variance weighted (fixed effects)
Inverse variance weighted (multiplicative random effects)
MR Egger
MR RAPS
Weighted median
Weighted mode
0.002
0.000
−0.002
−0.004
SNP effect on eGFR
0.2
0.4
0.6
SNP effect on Tumor necrosis factor alpha−induced protein 3

Batch 2−3031 : Tumor necrosis factor−inducible gene 6 protein on eGFR

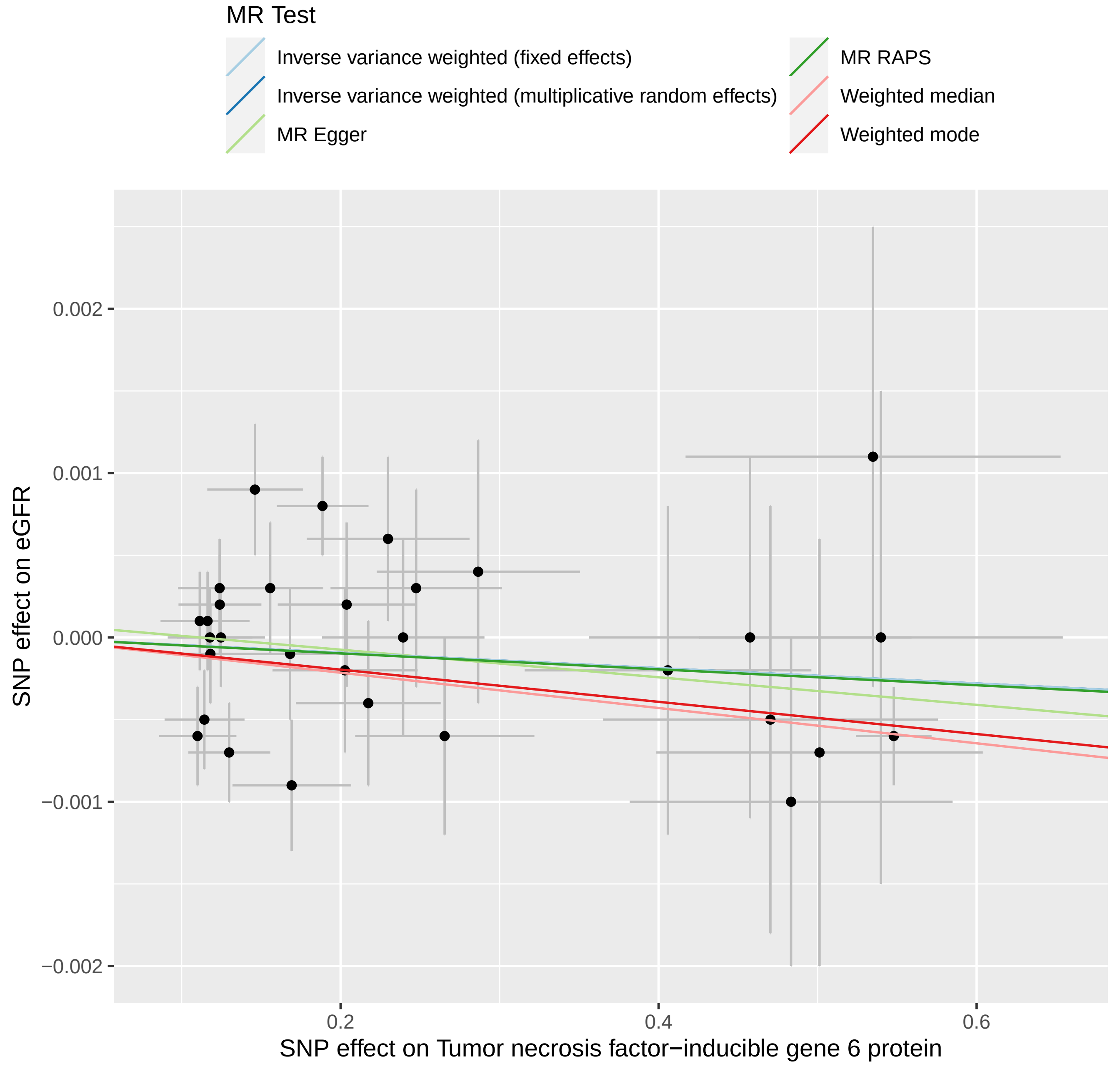

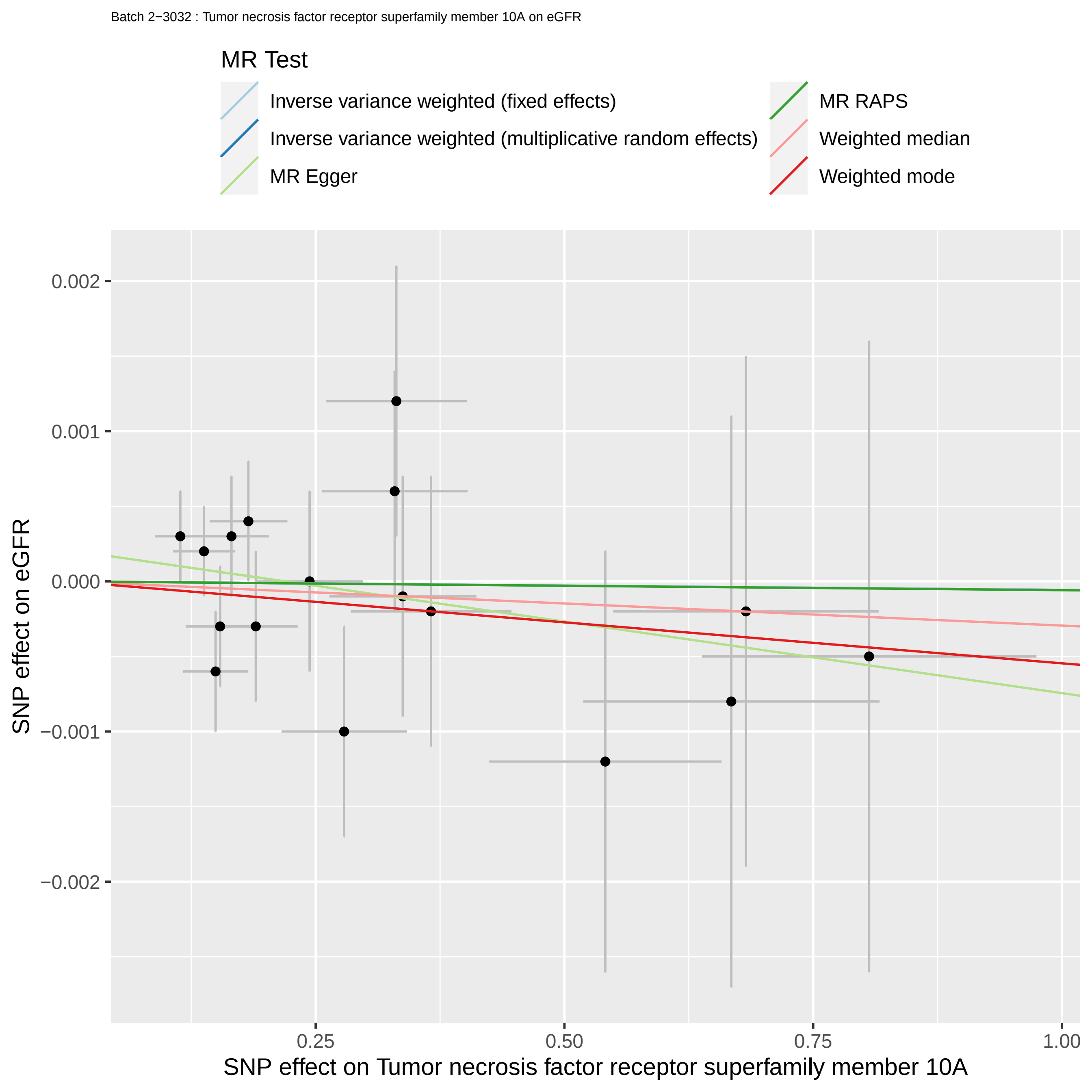

Batch 2−3032 : Tumor necrosis factor receptor superfamily member 10A on eGFR
MR Test
Inverse variance weighted (fixed effects)
Inverse variance weighted (multiplicative random effects)
MR Egger
MR RAPS
Weighted median
Weighted mode
0.002
0.001
0.000
−0.001
−0.002
0.25
0.50
0.75
1.00
SNP effect on eGFR
SNP effect on Tumor necrosis factor receptor superfamily member 10A

Batch 2−3033 : Tumor necrosis factor receptor superfamily member 10B on eGFR

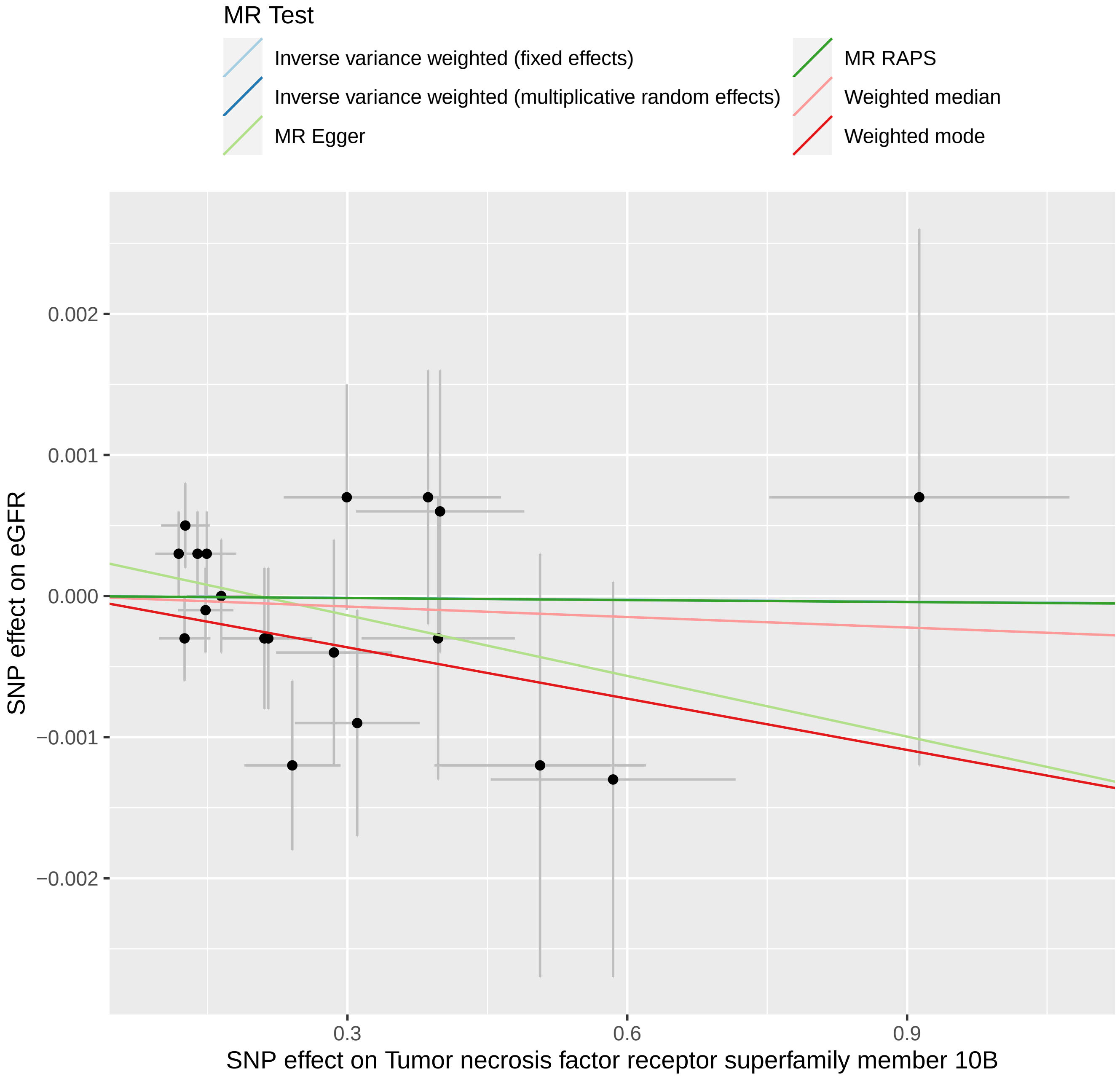

Batch 2−3034 : Tumor necrosis factor receptor superfamily member 10D on eGFR

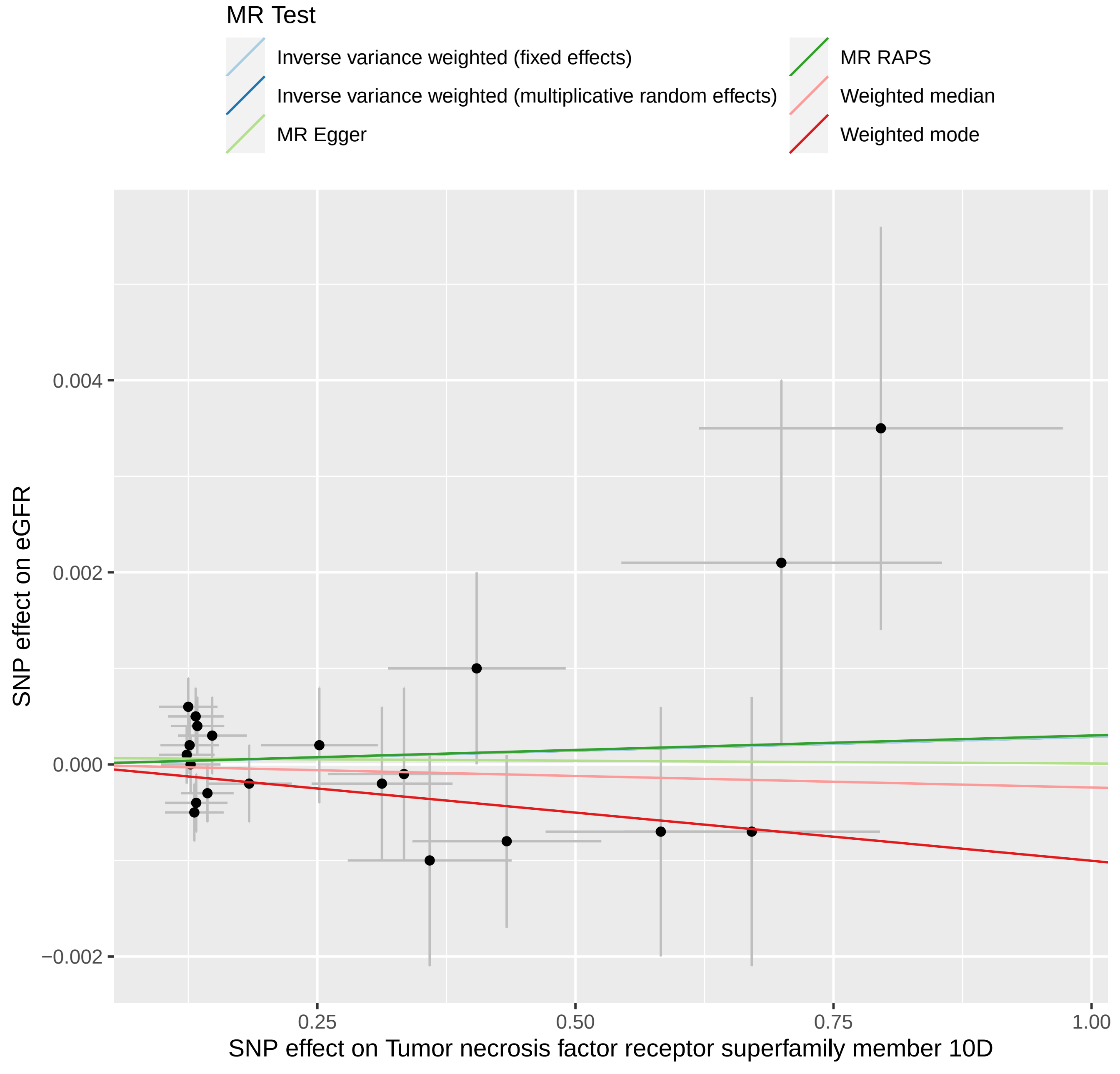

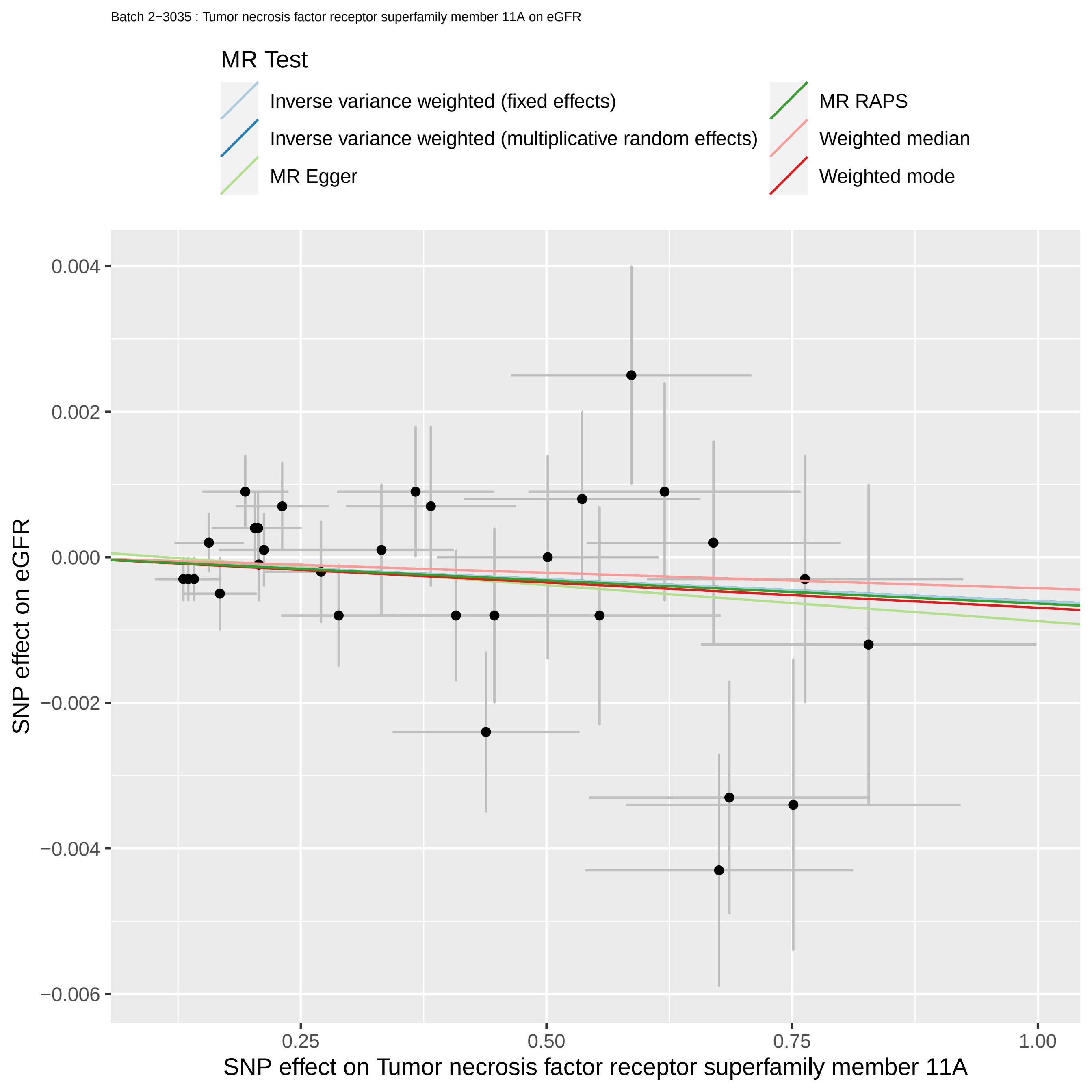
Batch 2−3035 : Tumor necrosis factor receptor superfamily member 11A on eGFR
MR Test
Inverse variance weighted (fixed effects)
Inverse variance weighted (multiplicative random effects)
MR Egger
MR RAPS
Weighted median
Weighted mode
0.004
0.002
0.000
−0.002
−0.004
−0.006
0.25
0.50
0.75
1.00
SNP effect on eGFR
SNP effect on Tumor necrosis factor receptor superfamily member 11A

Batch 2−3036 : Tumor necrosis factor receptor superfamily member 11A on eGFR

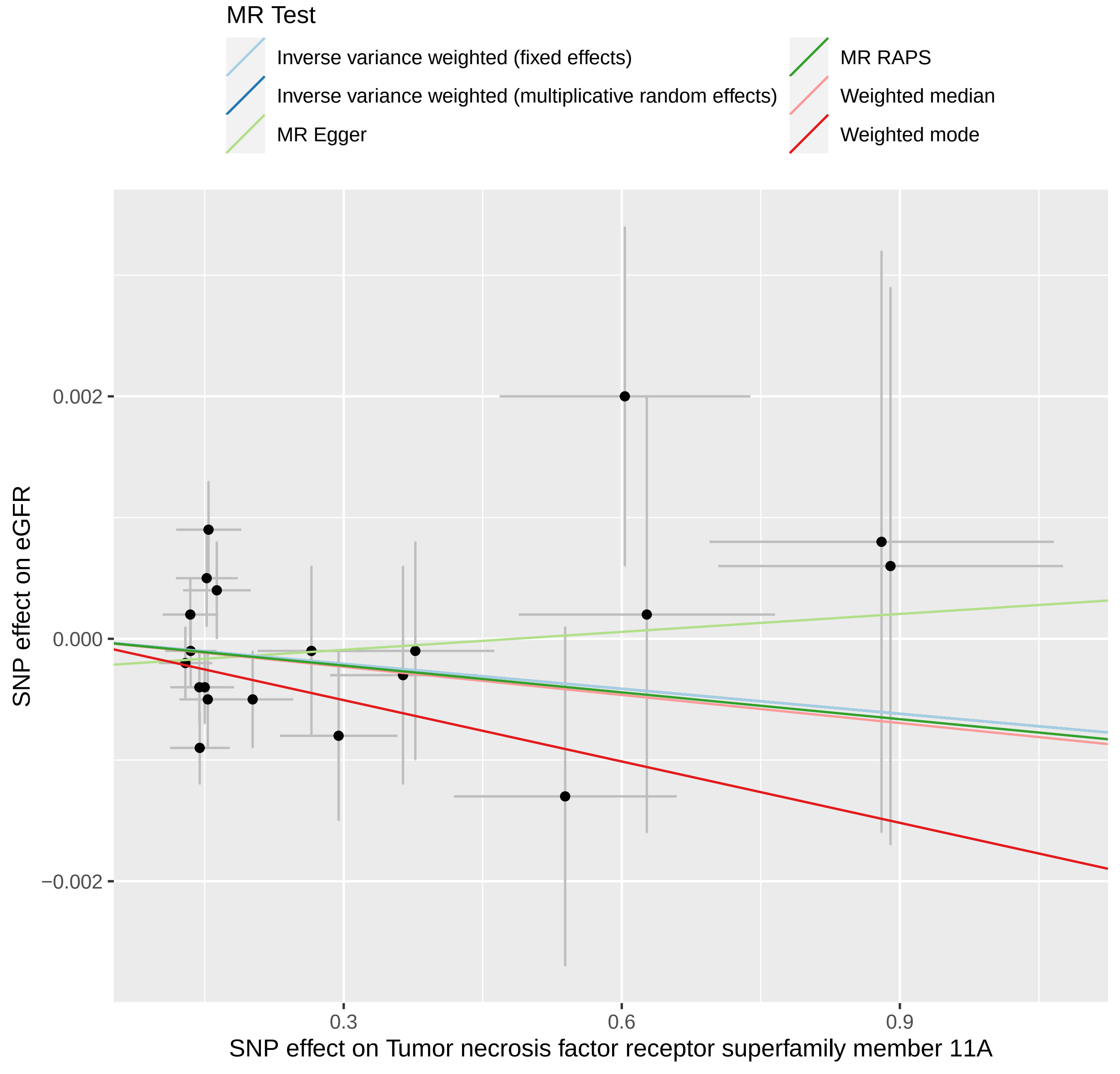

Batch 2−3037 : Tumor necrosis factor receptor superfamily member 11A on eGFR

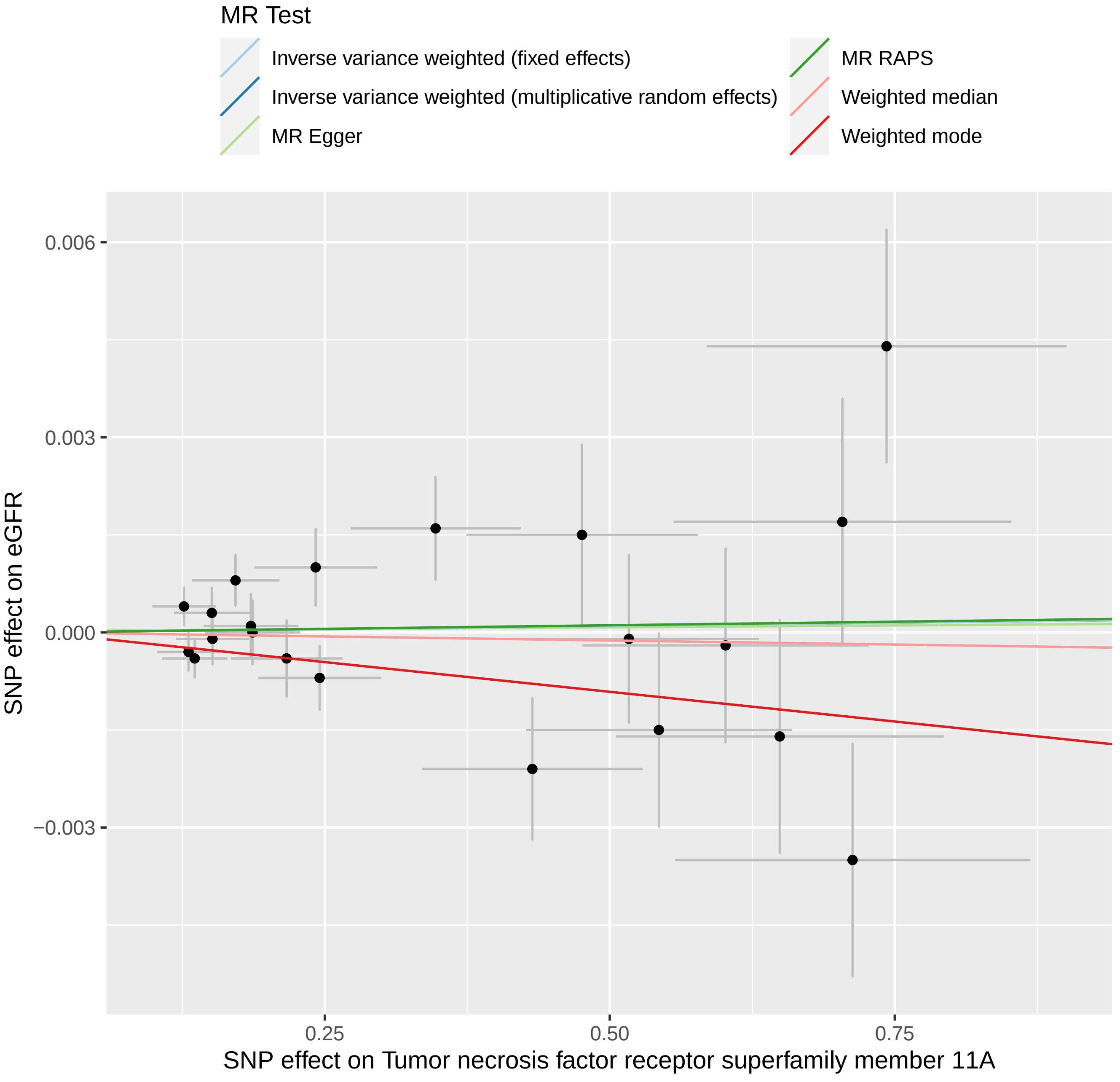

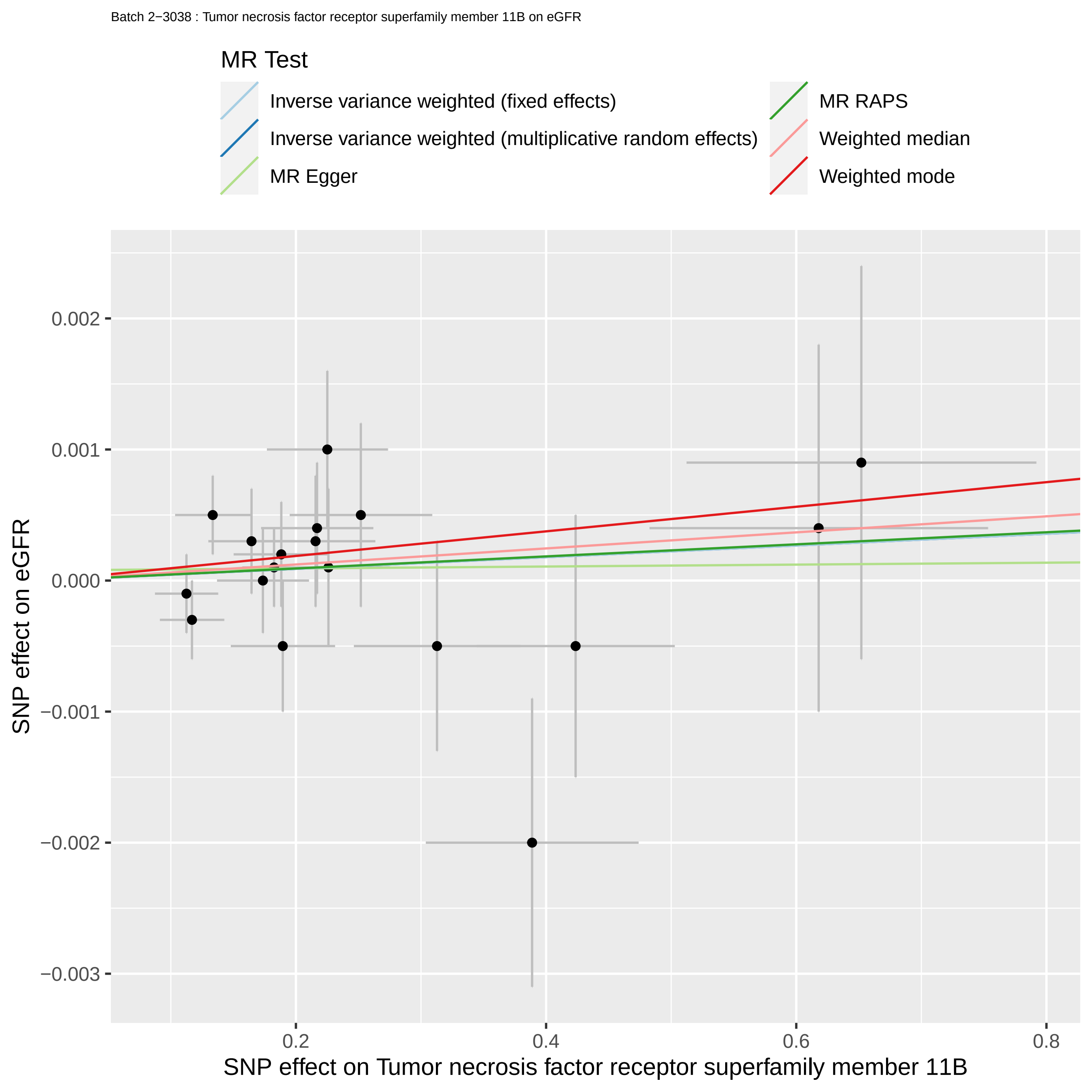
Batch 2−3038 : Tumor necrosis factor receptor superfamily member 11B on eGFR
MR Test
Inverse variance weighted (fixed effects)
Inverse variance weighted (multiplicative random effects)
MR Egger
MR RAPS
Weighted median
Weighted mode
0.002
0.001
0.000
−0.001
−0.002
−0.003
SNP effect on eGFR
0.2
0.4
0.6
0.8
SNP effect on Tumor necrosis factor receptor superfamily member 11B

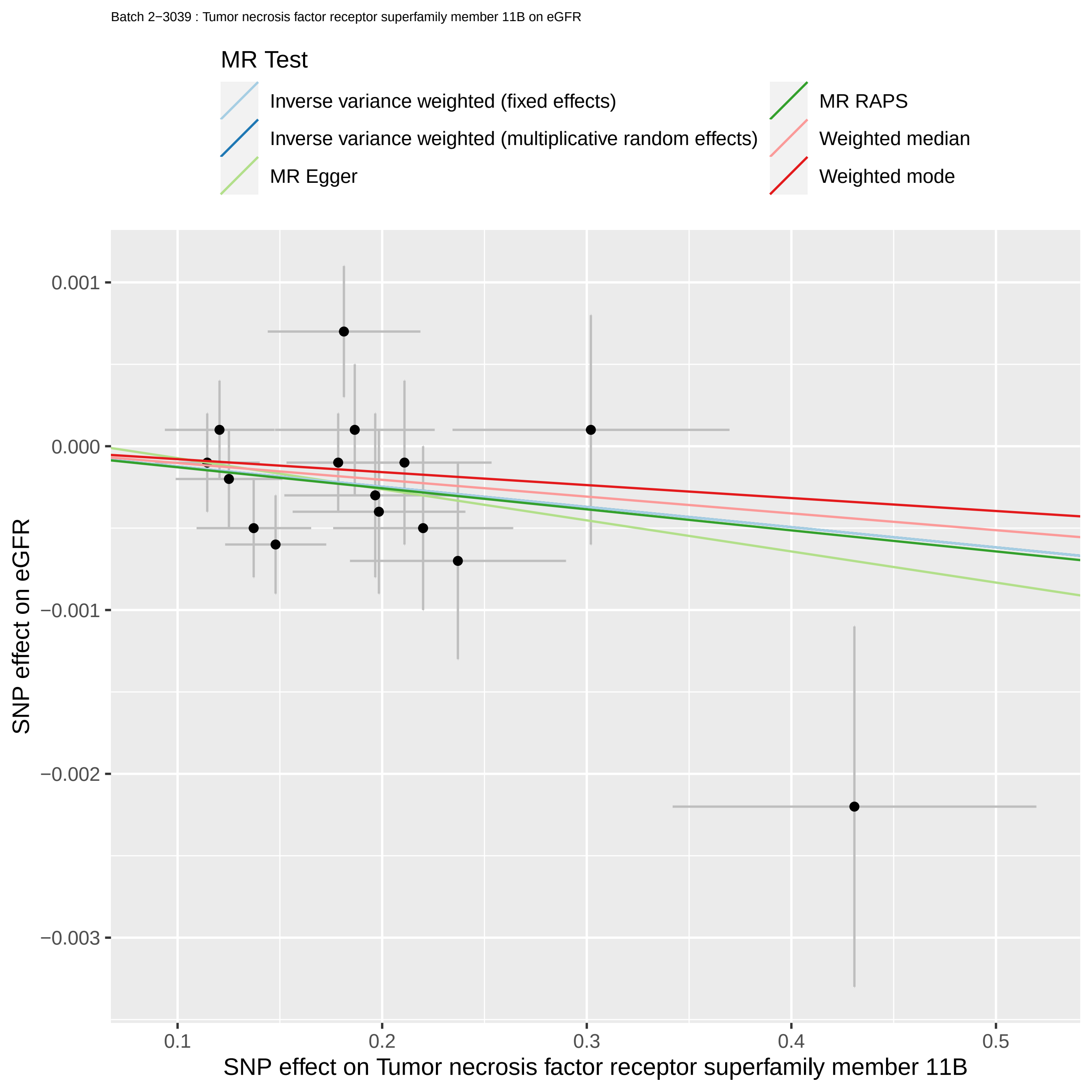

Batch 2−3039 : Tumor necrosis factor receptor superfamily member 11B on eGFR
MR Test
Inverse variance weighted (fixed effects)
Inverse variance weighted (multiplicative random effects)
MR Egger
MR RAPS
Weighted median
Weighted mode
0.001
0.000
−0.001
−0.002
−0.003
SNP effect on eGFR
0.1
0.2
0.3
0.4
0.5
SNP effect on Tumor necrosis factor receptor superfamily member 11B

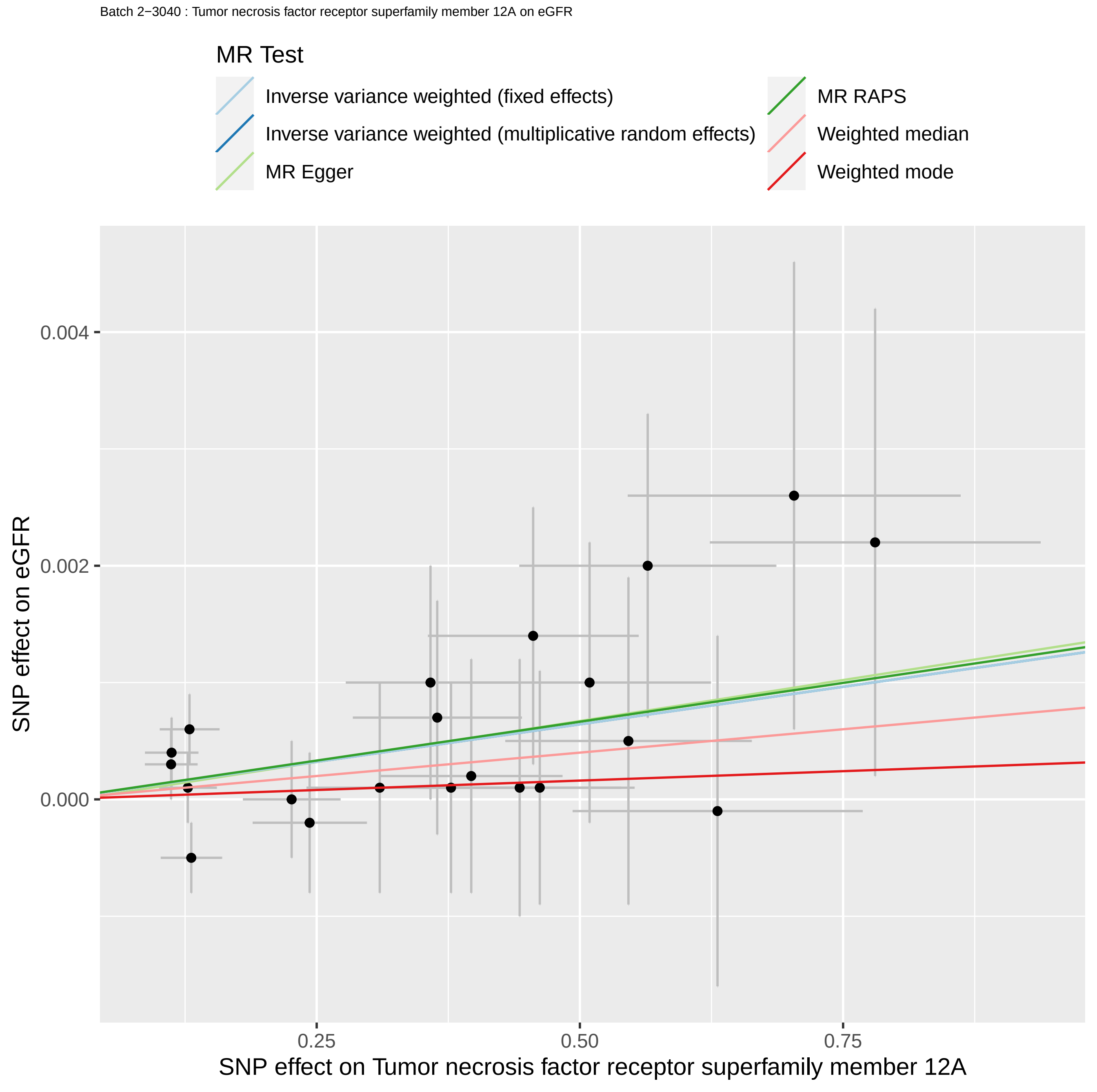

Batch 2−3040 : Tumor necrosis factor receptor superfamily member 12A on eGFR
MR Test
Inverse variance weighted (fixed effects)
Inverse variance weighted (multiplicative random effects)
MR Egger
MR RAPS
Weighted median
Weighted mode
0.004
0.002
0.000
0.25
0.50
0.75
SNP effect on eGFR
SNP effect on Tumor necrosis factor receptor superfamily member 12A

Batch 2−3041 : Tumor necrosis factor receptor superfamily member 13B on eGFR

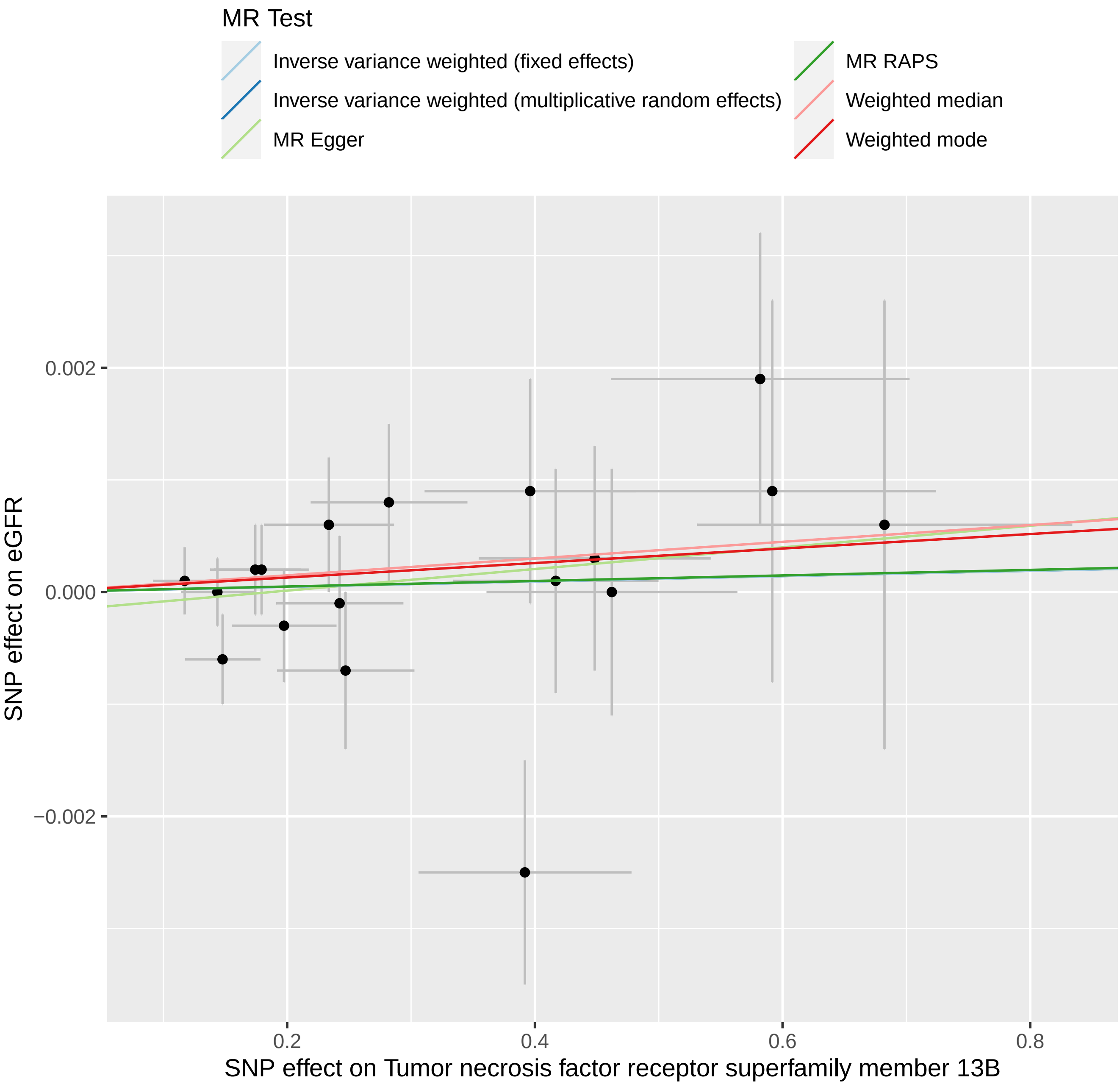

Batch 2−3042 : Tumor necrosis factor receptor superfamily member 14 on eGFR

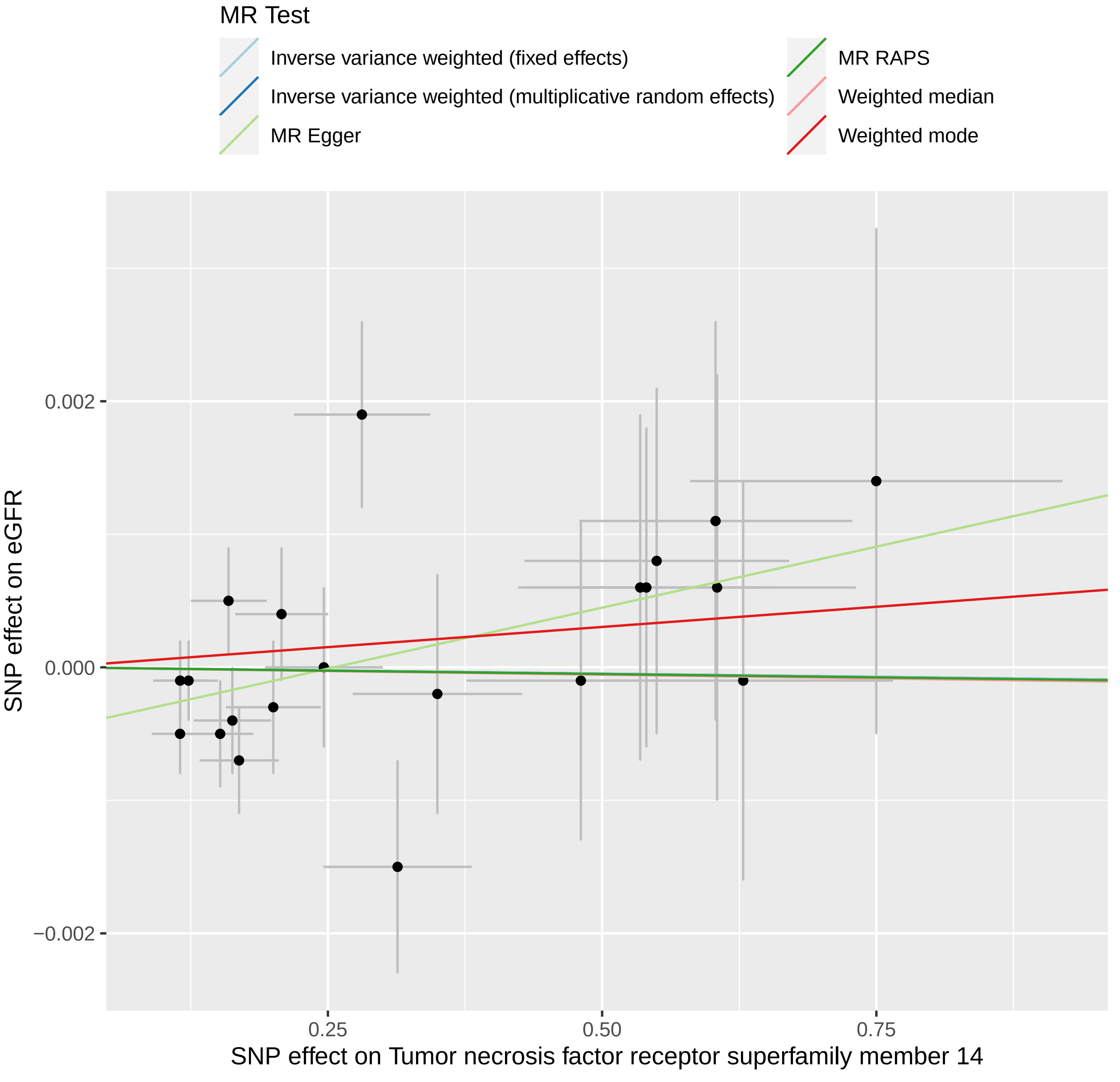

Batch 2−3043 : Tumor necrosis factor receptor superfamily member 17 on eGFR

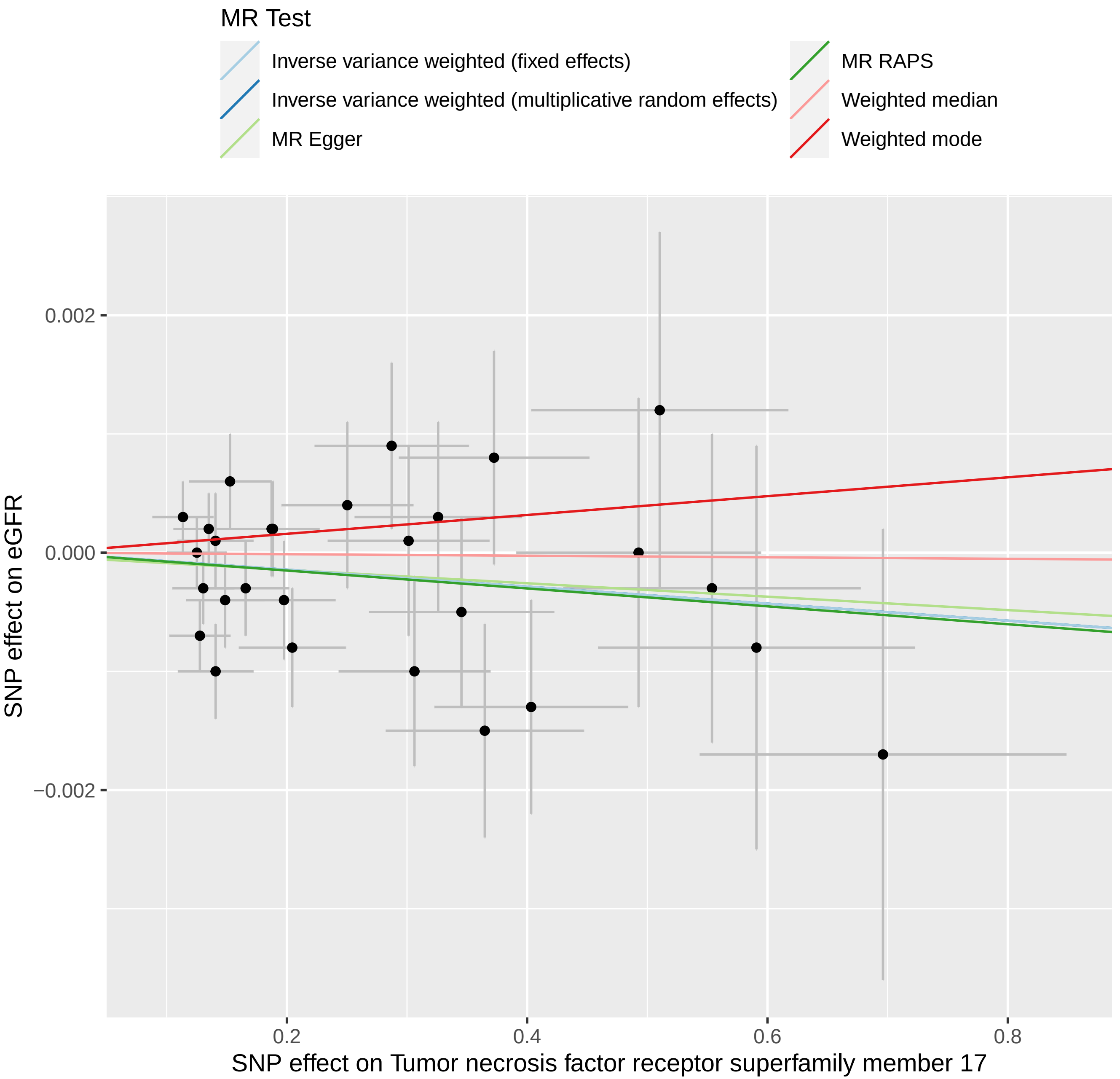

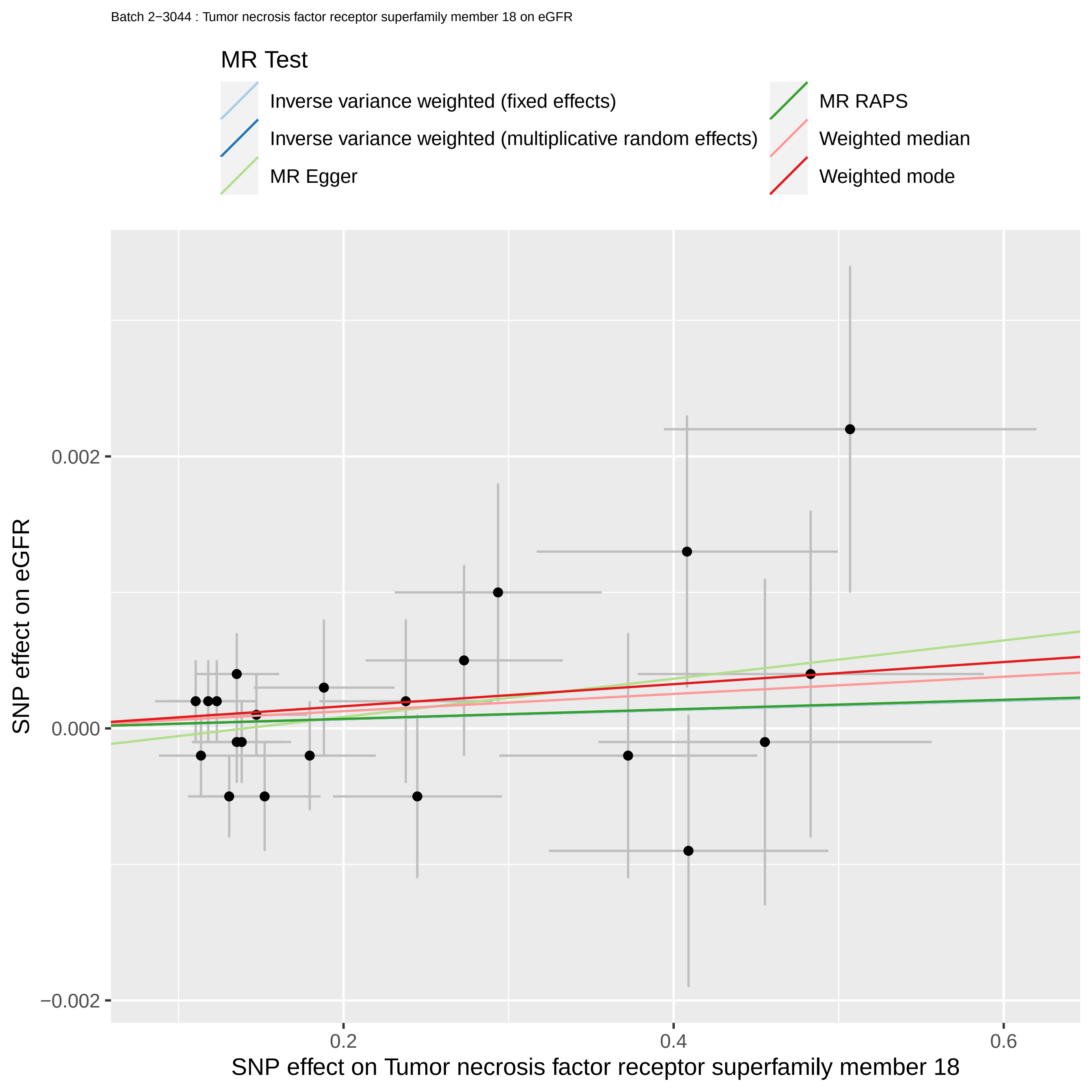

Batch 2−3044 : Tumor necrosis factor receptor superfamily member 18 on eGFR
MR Test
Inverse variance weighted (fixed effects)
Inverse variance weighted (multiplicative random effects)
MR Egger
MR RAPS
Weighted median
Weighted mode
SNP effect on eGFR
0.002
0.000
−0.002
0.2
0.4
0.6
SNP effect on Tumor necrosis factor receptor superfamily member 18

Batch 2−3045 : Tumor necrosis factor receptor superfamily member 19 on eGFR

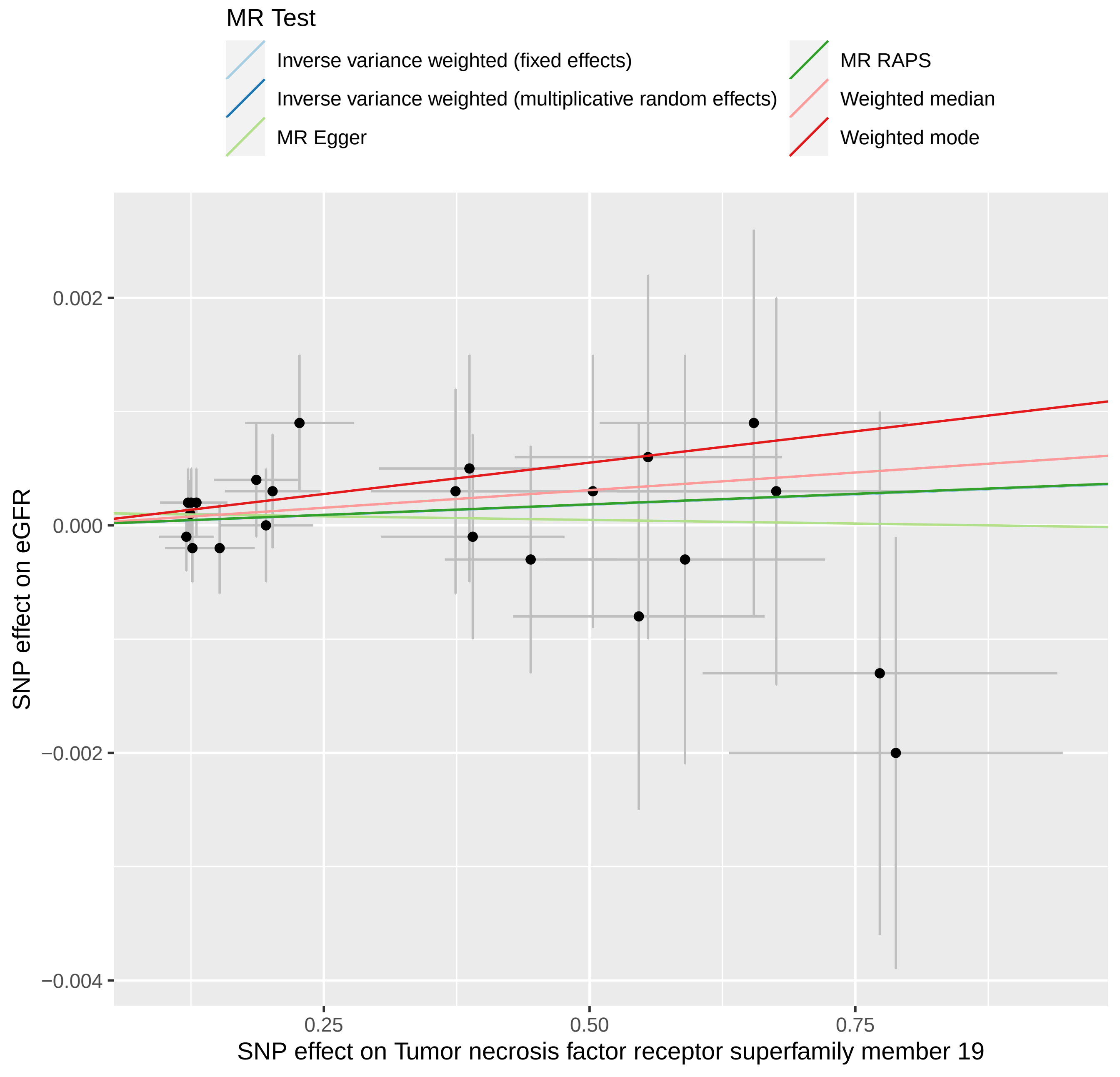

Batch 2−3046 : Tumor necrosis factor receptor superfamily member 1A on eGFR

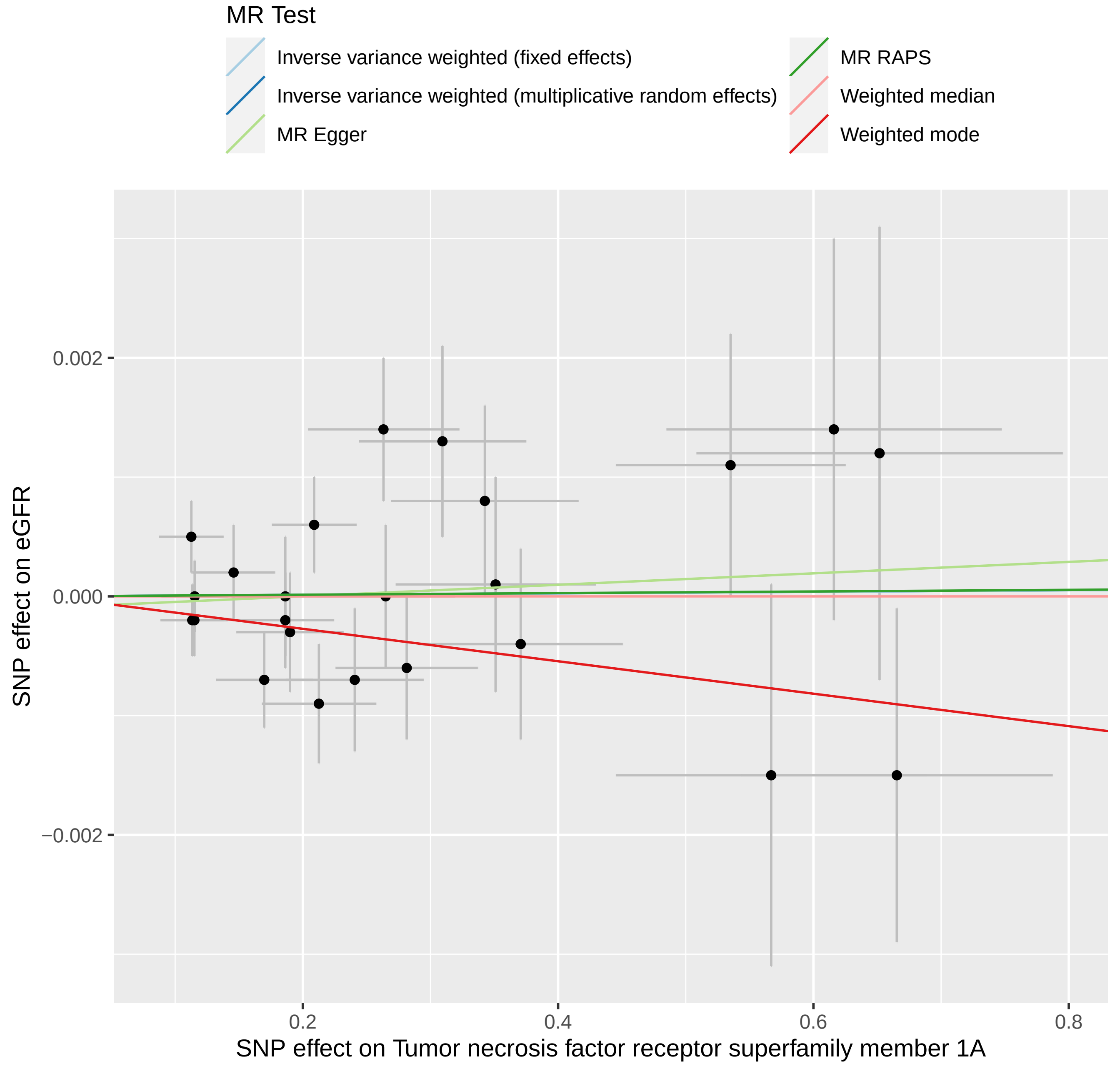

Batch 2−3047 : Tumor necrosis factor receptor superfamily member 1B on eGFR

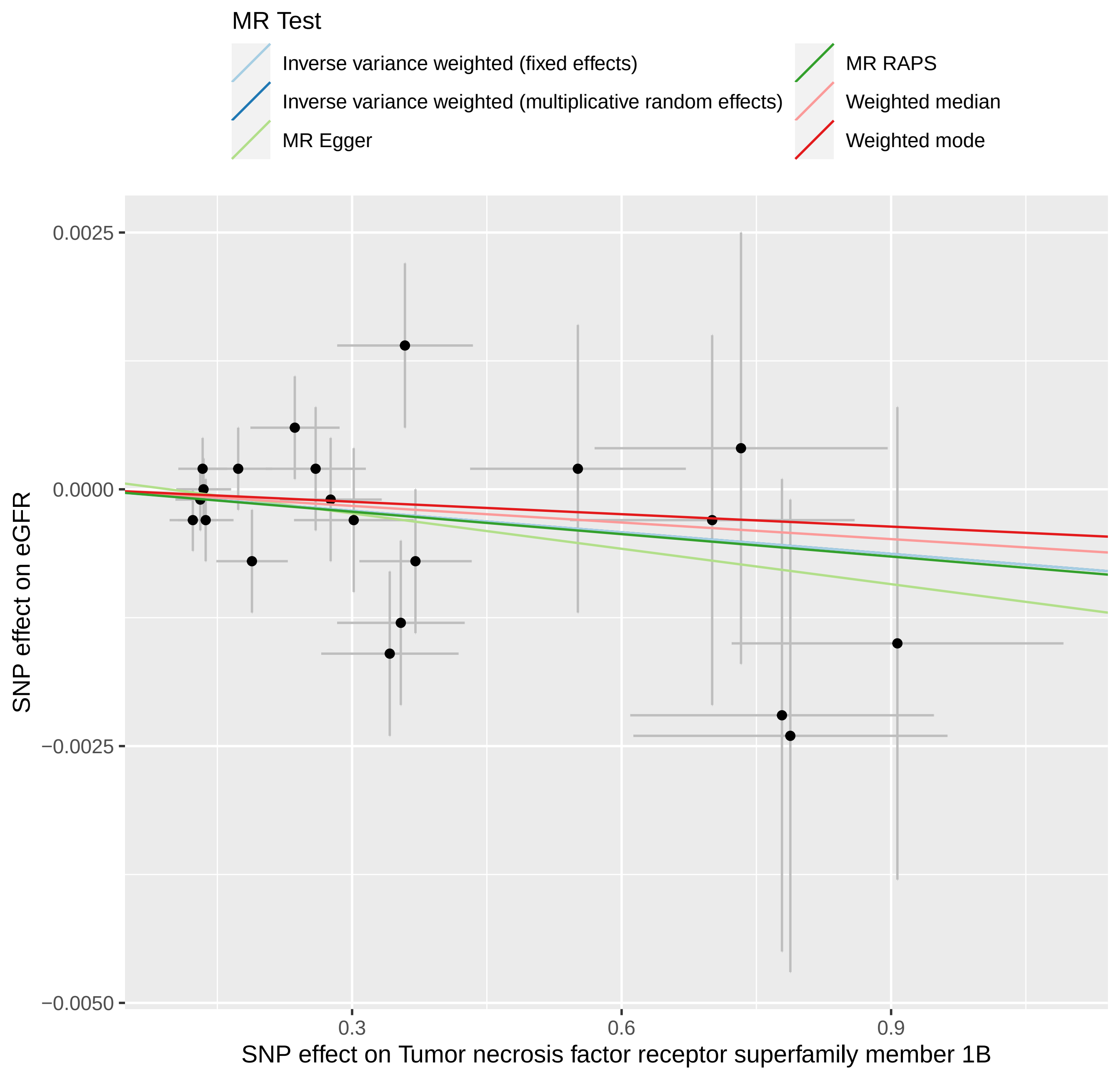

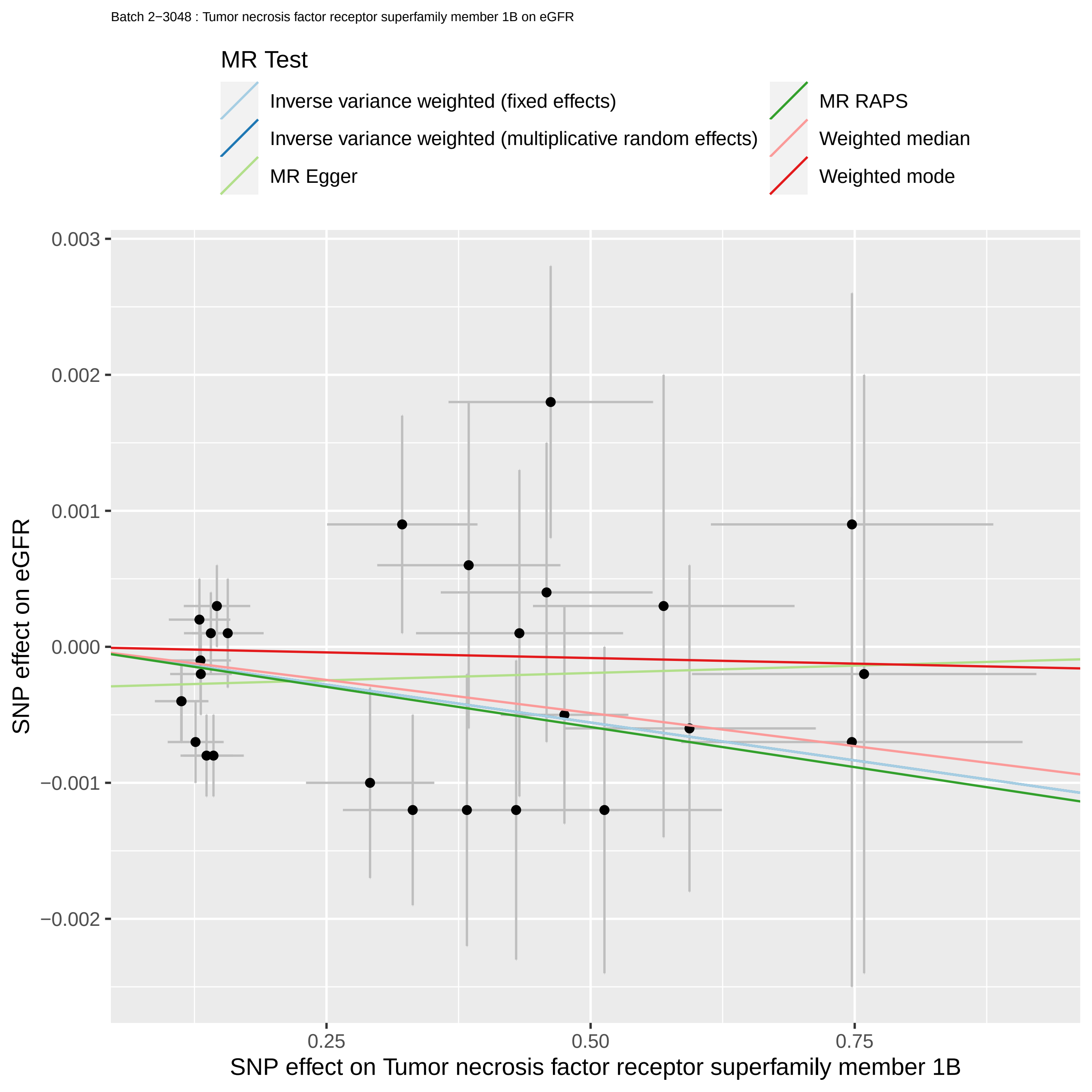

Batch 2−3048 : Tumor necrosis factor receptor superfamily member 1B on eGFR
MR Test
Inverse variance weighted (fixed effects)
Inverse variance weighted (multiplicative random effects)
MR Egger
MR RAPS
Weighted median
Weighted mode
0.003
0.002
0.001
0.000
−0.001
−0.002
SNP effect on eGFR
0.25
0.50
0.75
SNP effect on Tumor necrosis factor receptor superfamily member 1B

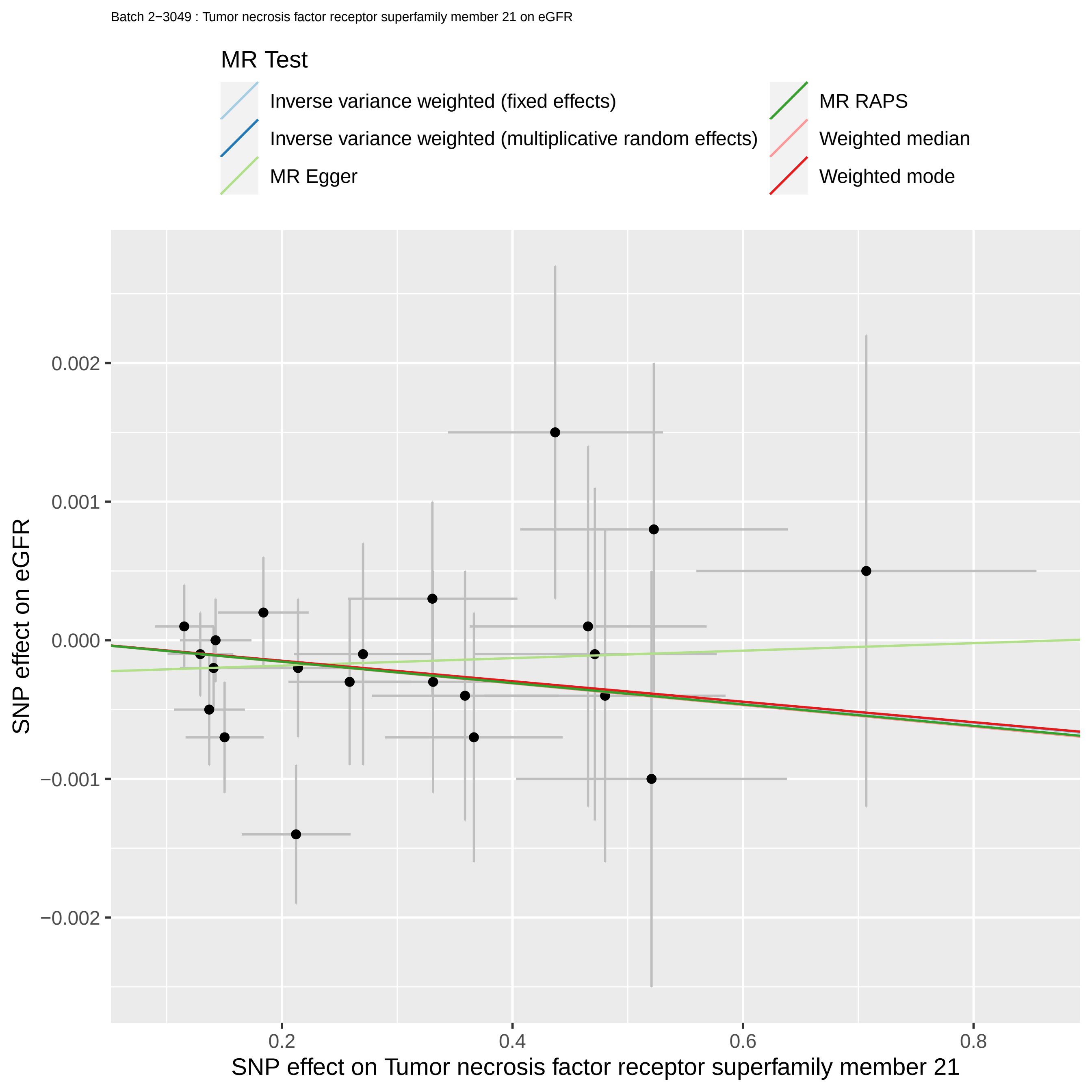
Batch 2−3049 : Tumor necrosis factor receptor superfamily member 21 on eGFR
MR Test
Inverse variance weighted (fixed effects)
Inverse variance weighted (multiplicative random effects)
MR Egger
MR RAPS
Weighted median
Weighted mode
0.002
0.001
0.000
−0.001
−0.002
0.2
0.4
0.6
0.8
SNP effect on eGFR
SNP effect on Tumor necrosis factor receptor superfamily member 21

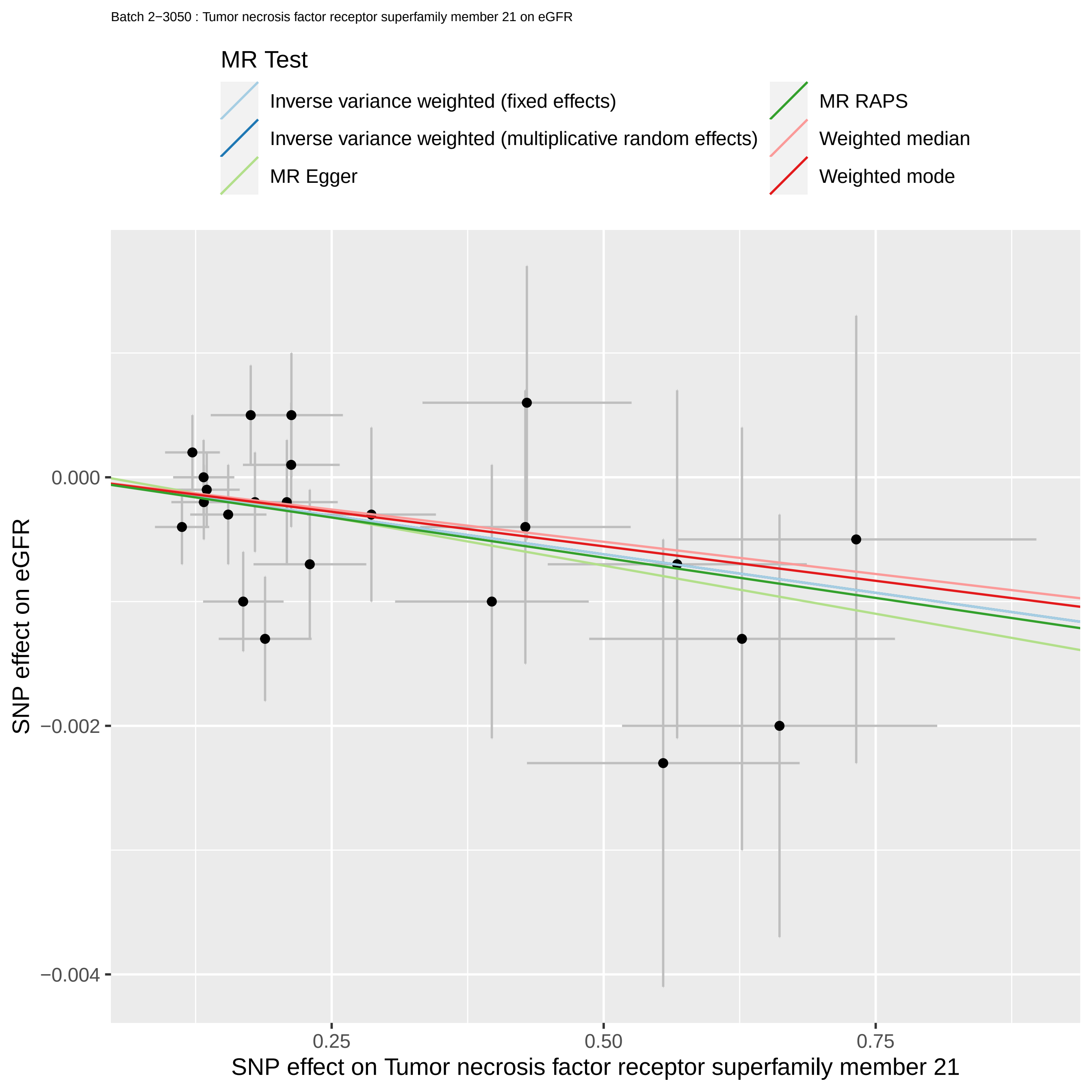
Batch 2−3050 : Tumor necrosis factor receptor superfamily member 21 on eGFR
MR Test
Inverse variance weighted (fixed effects)
Inverse variance weighted (multiplicative random effects)
MR Egger
MR RAPS
Weighted median
Weighted mode
0.000
−0.002
−0.004
0.25
0.50
0.75
SNP effect on eGFR
SNP effect on Tumor necrosis factor receptor superfamily member 21

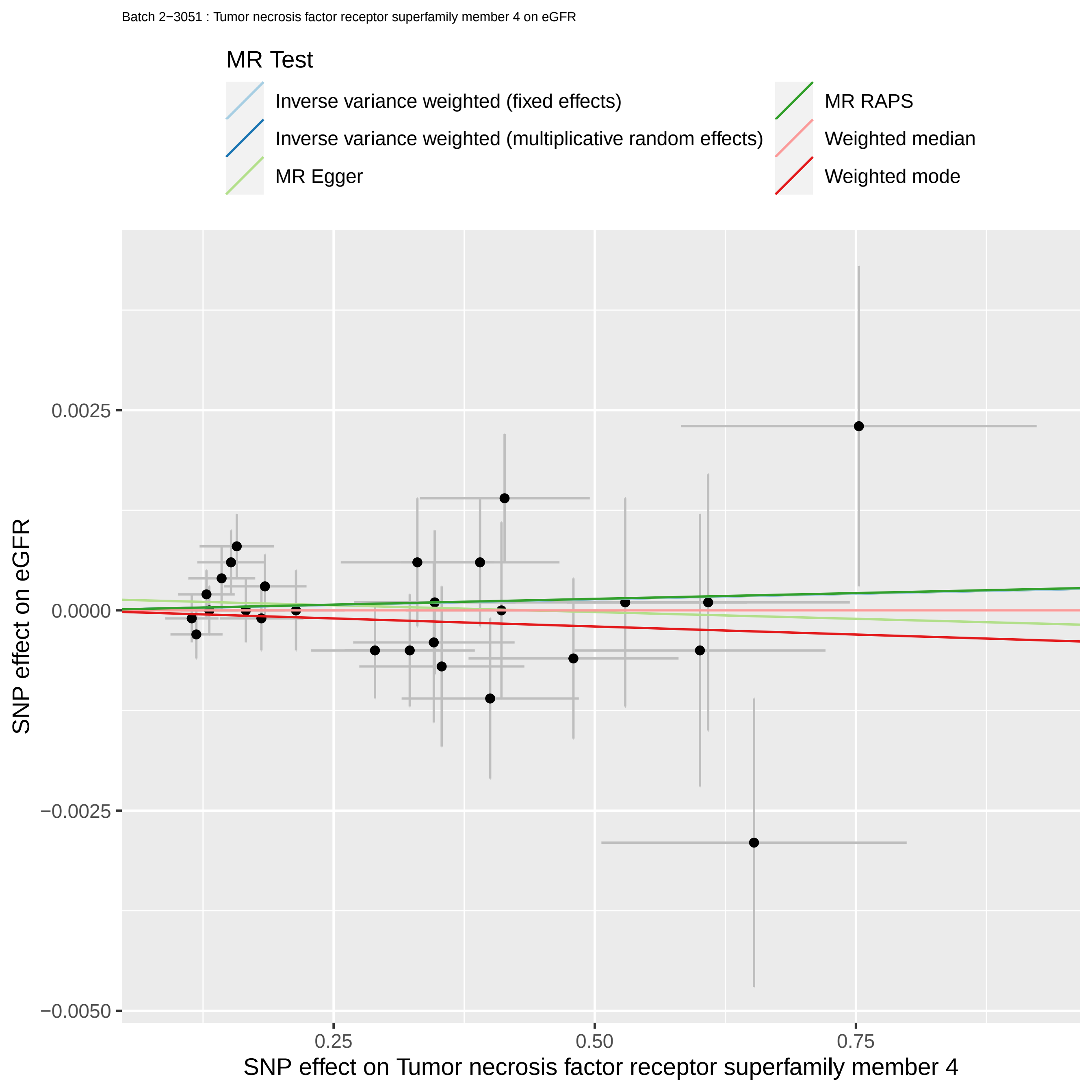

Batch 2−3051 : Tumor necrosis factor receptor superfamily member 4 on eGFR
MR Test
Inverse variance weighted (fixed effects)
Inverse variance weighted (multiplicative random effects)
MR Egger
MR RAPS
Weighted median
Weighted mode
0.0025
0.0000
−0.0025
−0.0050
0.25
0.50
0.75
SNP effect on eGFR
SNP effect on Tumor necrosis factor receptor superfamily member 4

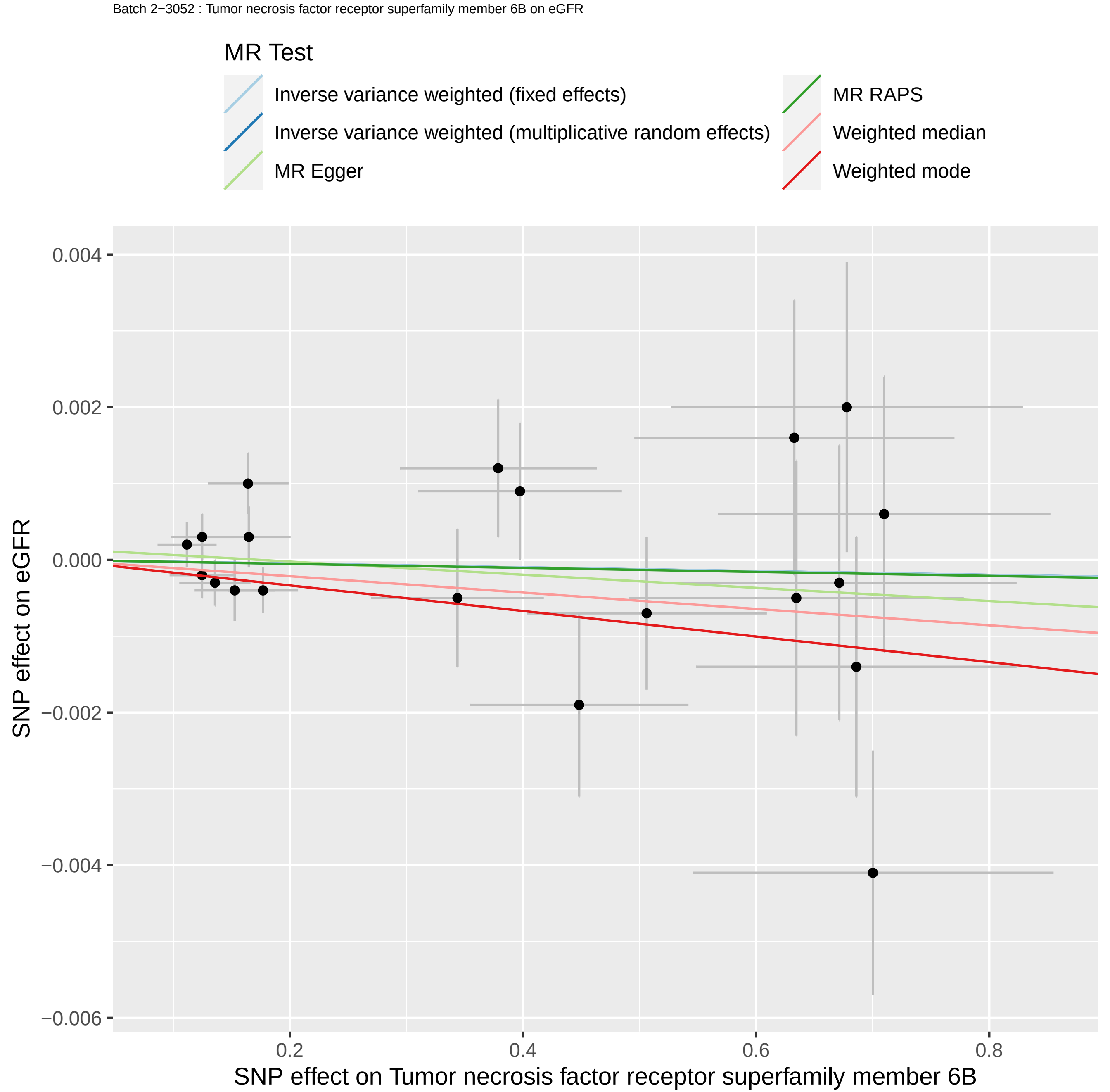
Batch 2−3052 : Tumor necrosis factor receptor superfamily member 6B on eGFR
MR Test
Inverse variance weighted (fixed effects)
Inverse variance weighted (multiplicative random effects)
MR Egger
MR RAPS
Weighted median
Weighted mode
0.004
0.002
0.000
−0.002
−0.004
−0.006
0.2
0.4
0.6
0.8
SNP effect on eGFR
SNP effect on Tumor necrosis factor receptor superfamily member 6B

Batch 2−3053 : Tumor necrosis factor receptor superfamily member 8 on eGFR

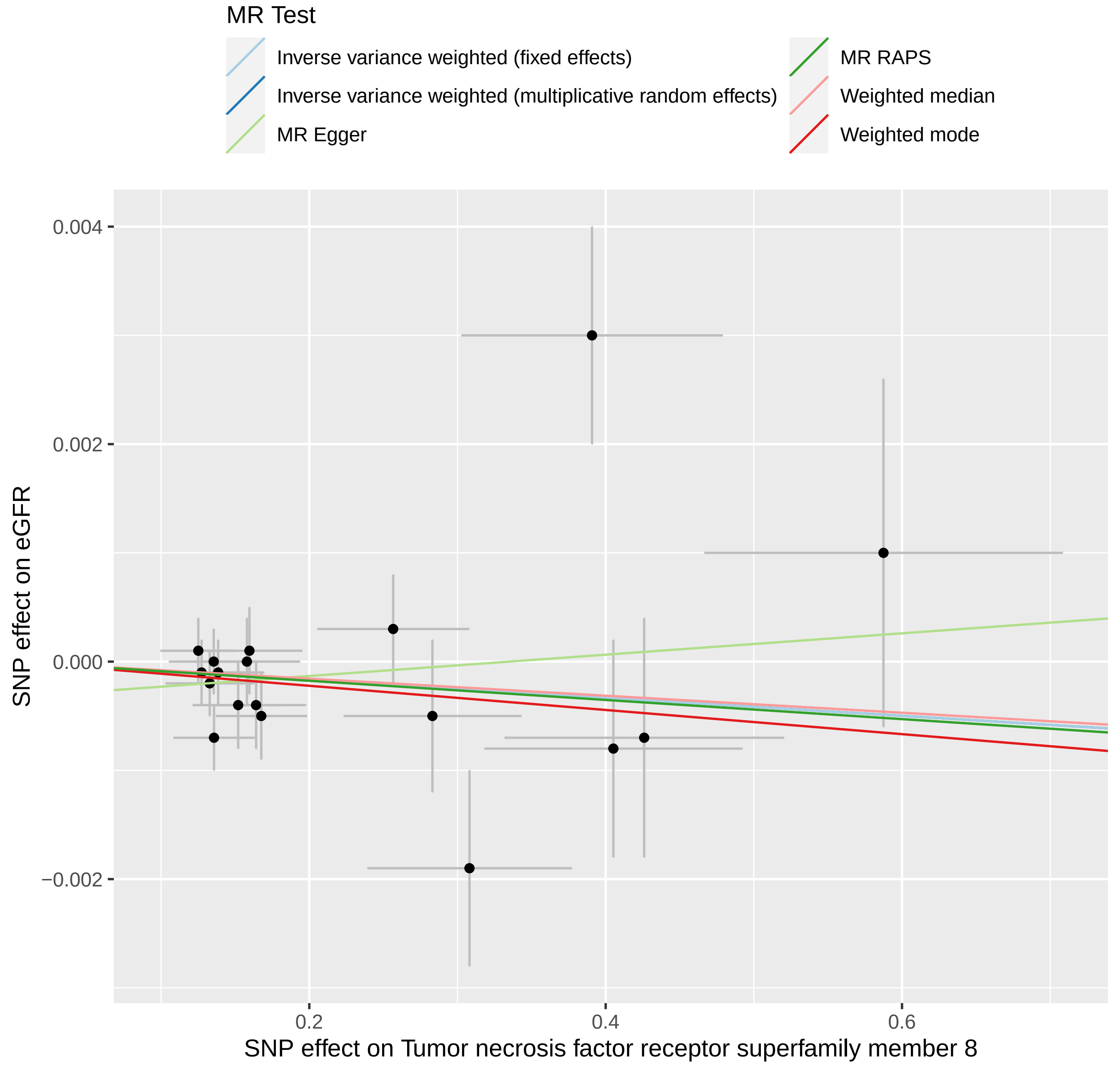

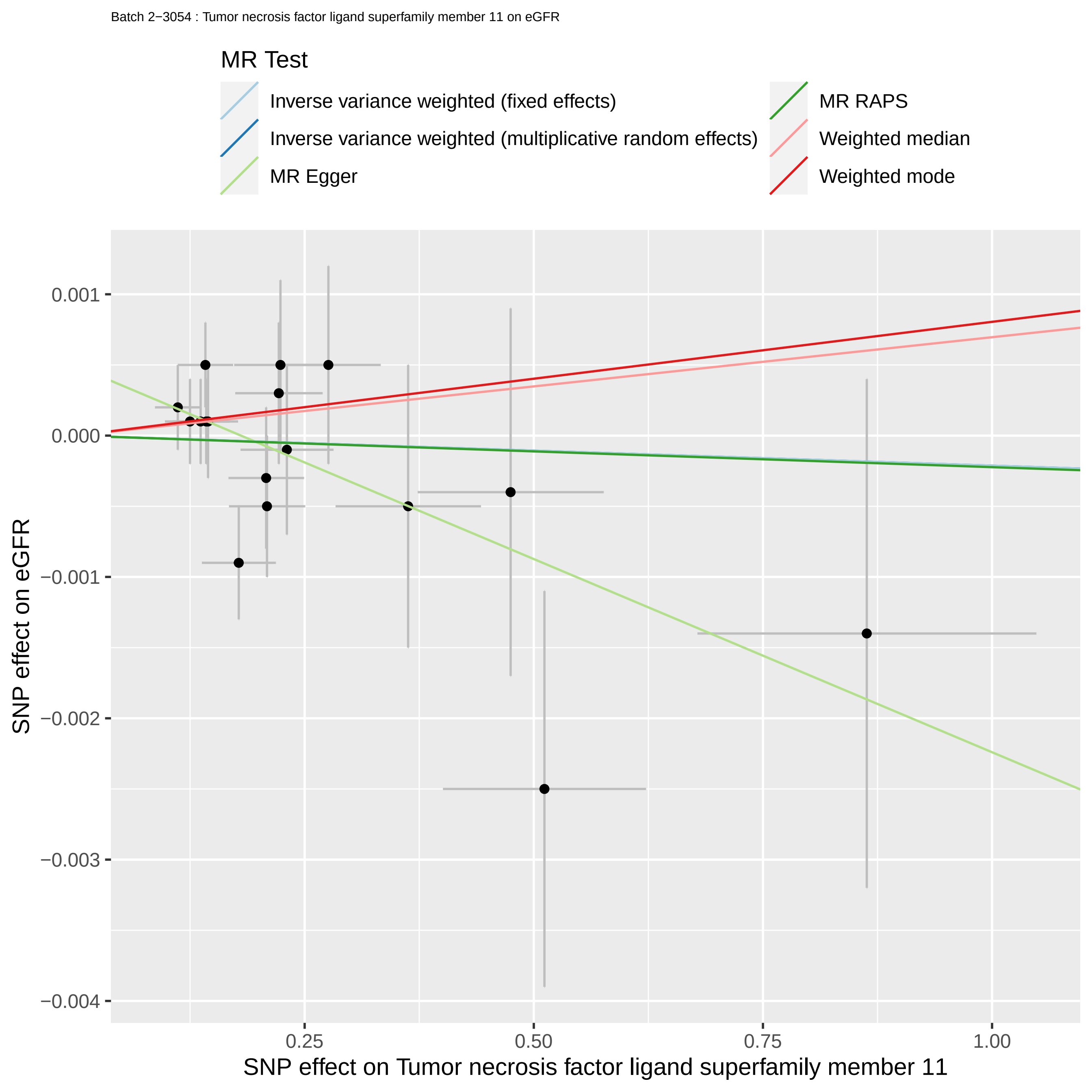
Batch 2−3054 : Tumor necrosis factor ligand superfamily member 11 on eGFR
MR Test
Inverse variance weighted (fixed effects)
Inverse variance weighted (multiplicative random effects)
MR Egger
MR RAPS
Weighted median
Weighted mode
0.001
0.000
−0.001
−0.002
−0.003
−0.004
SNP effect on eGFR
0.25
0.50
0.75
1.00
SNP effect on Tumor necrosis factor ligand superfamily member 11

Batch 2−3055 : Tumor necrosis factor ligand superfamily member 12 on eGFR

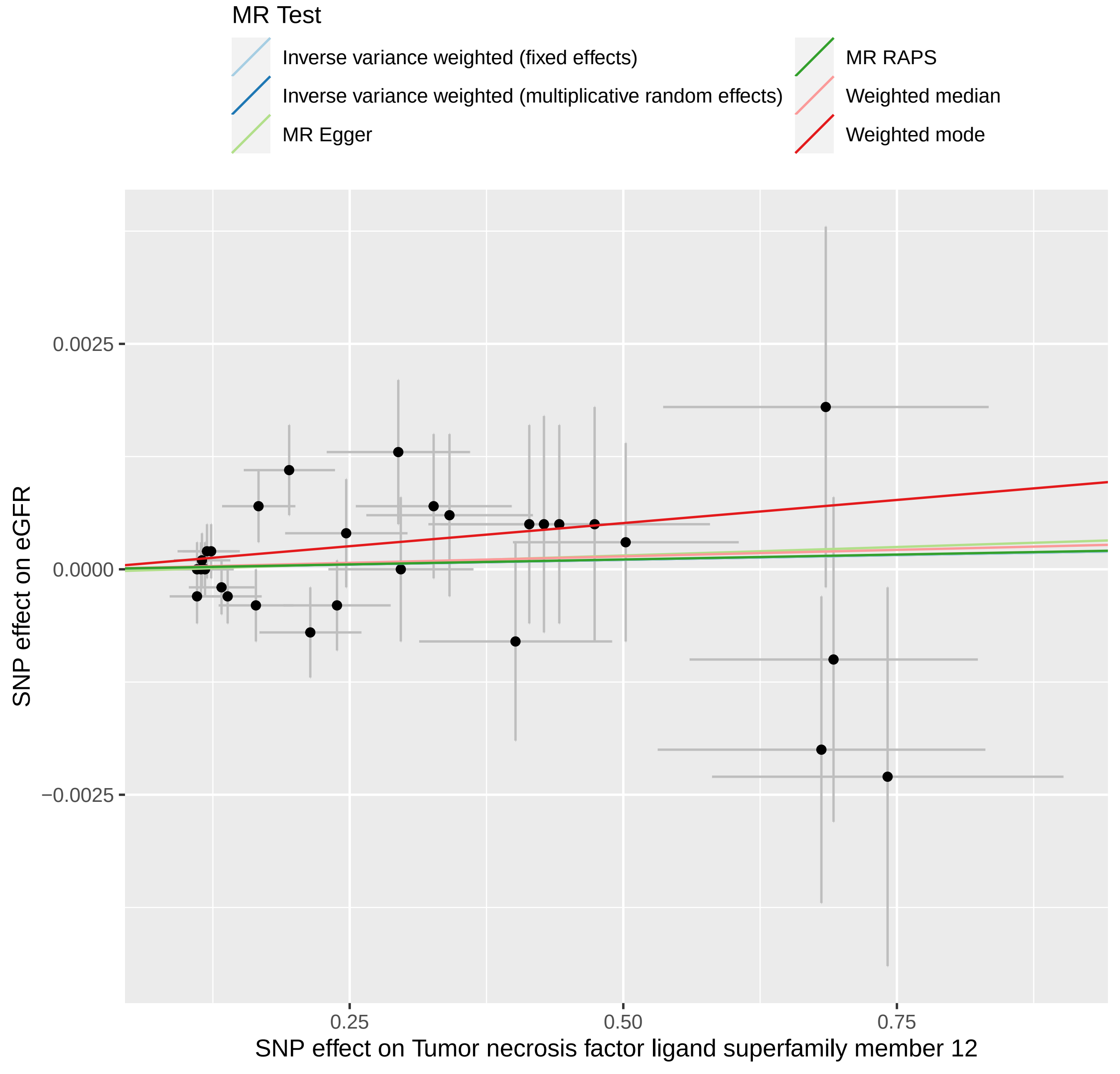

Batch 2−3056 : Tumor necrosis factor ligand superfamily member 13B on eGFR

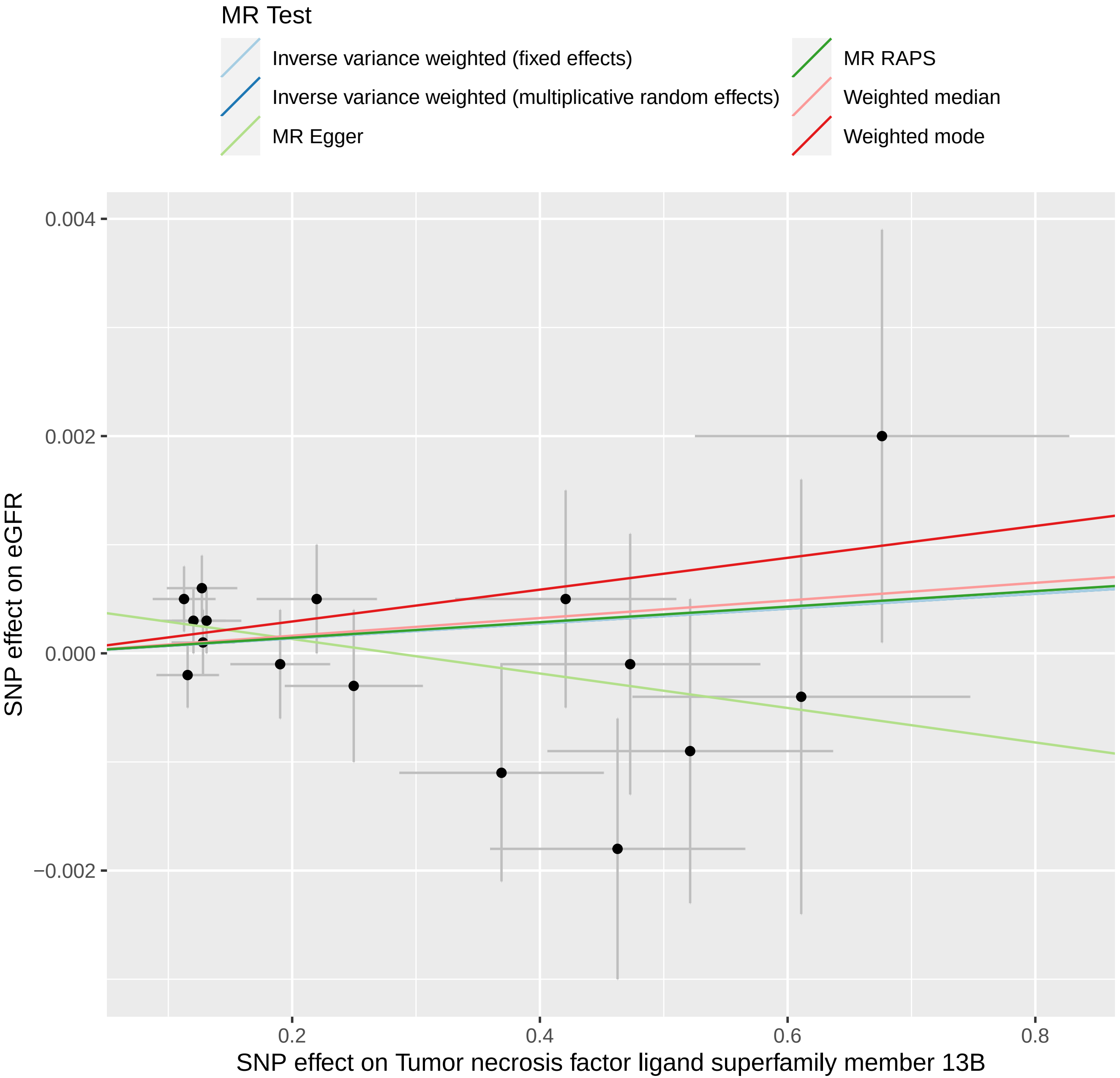

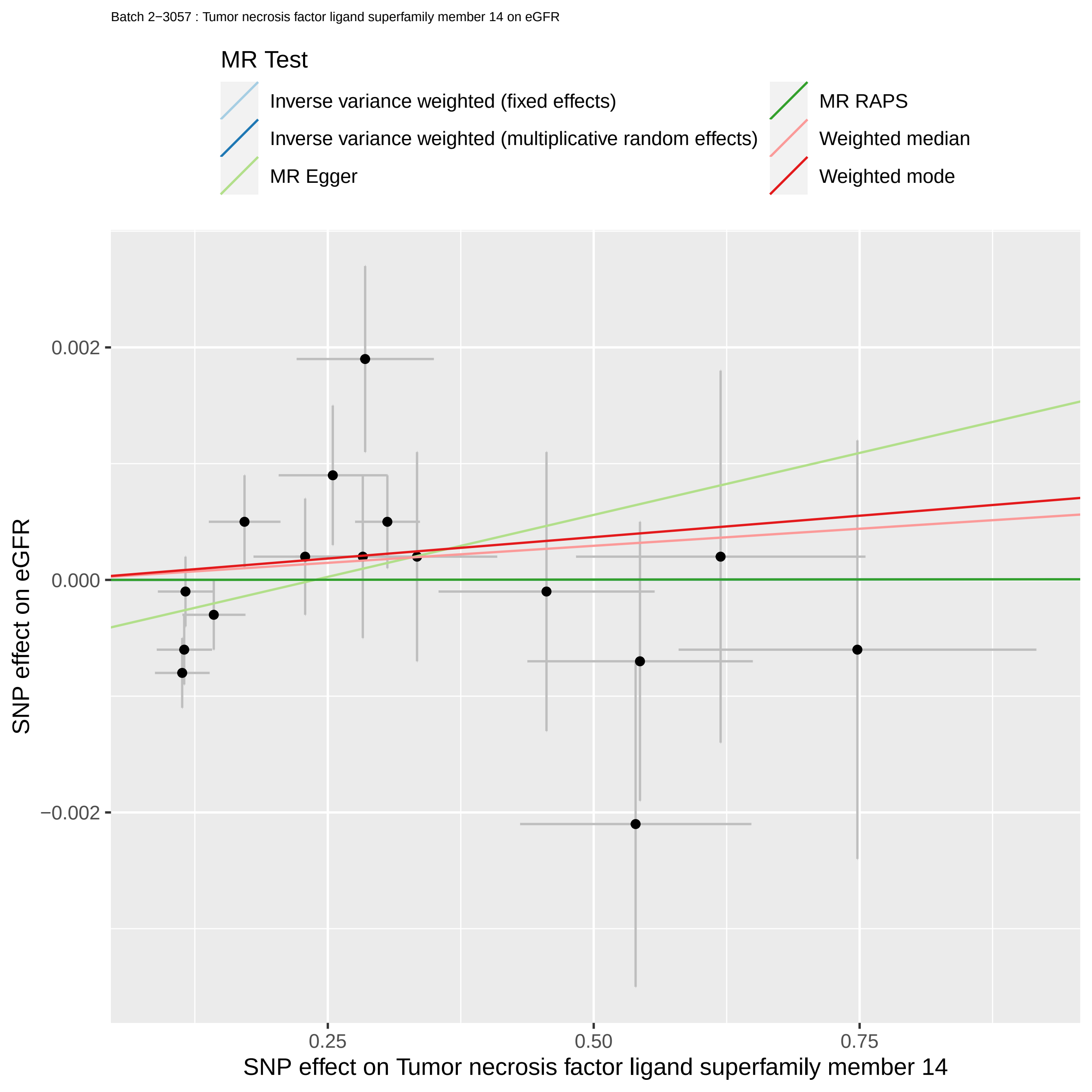
Batch 2−3057 : Tumor necrosis factor ligand superfamily member 14 on eGFR
MR Test
Inverse variance weighted (fixed effects)
Inverse variance weighted (multiplicative random effects)
MR Egger
MR RAPS
Weighted median
Weighted mode
0.002
0.000
−0.002
SNP effect on eGFR
0.25
0.50
0.75
SNP effect on Tumor necrosis factor ligand superfamily member 14

Batch 2−3058 : Tumor necrosis factor ligand superfamily member 15 on eGFR

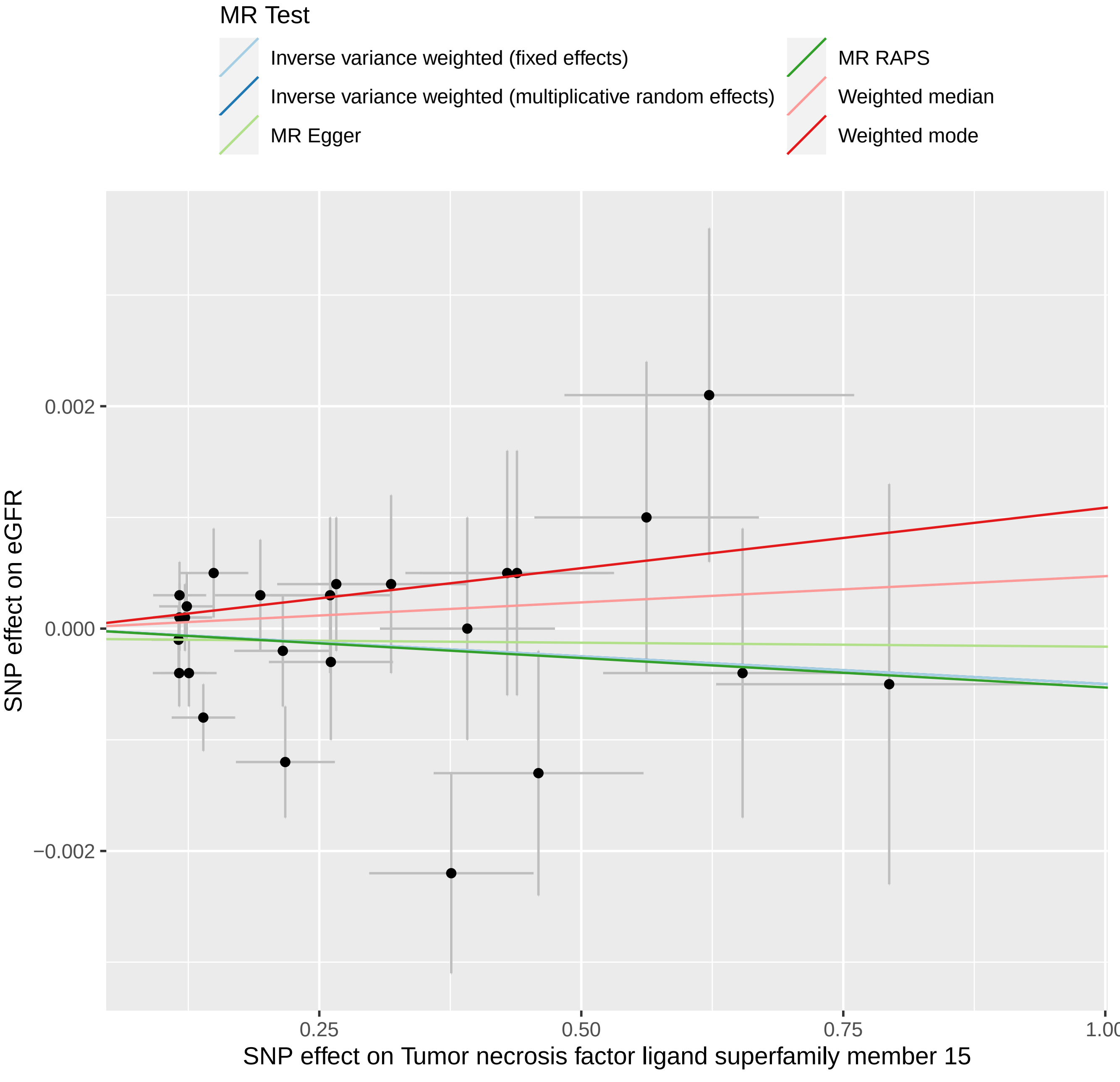

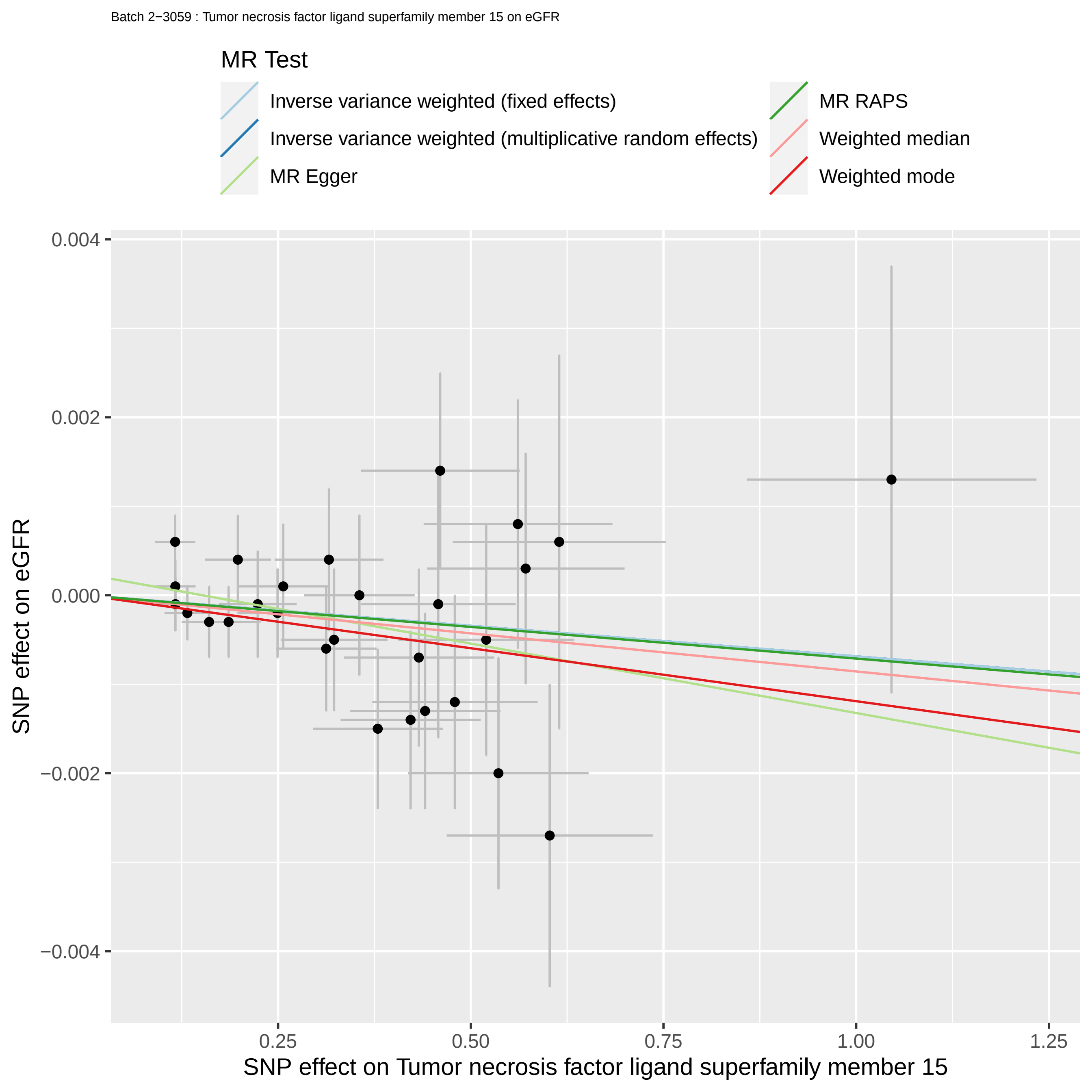

Batch 2−3059 : Tumor necrosis factor ligand superfamily member 15 on eGFR
MR Test
Inverse variance weighted (fixed effects)
Inverse variance weighted (multiplicative random effects)
MR Egger
MR RAPS
Weighted median
Weighted mode
0.004
0.002
0.000
−0.002
−0.004
SNP effect on eGFR
0.25
0.50
0.75
1.00
1.25
SNP effect on Tumor necrosis factor ligand superfamily member 15

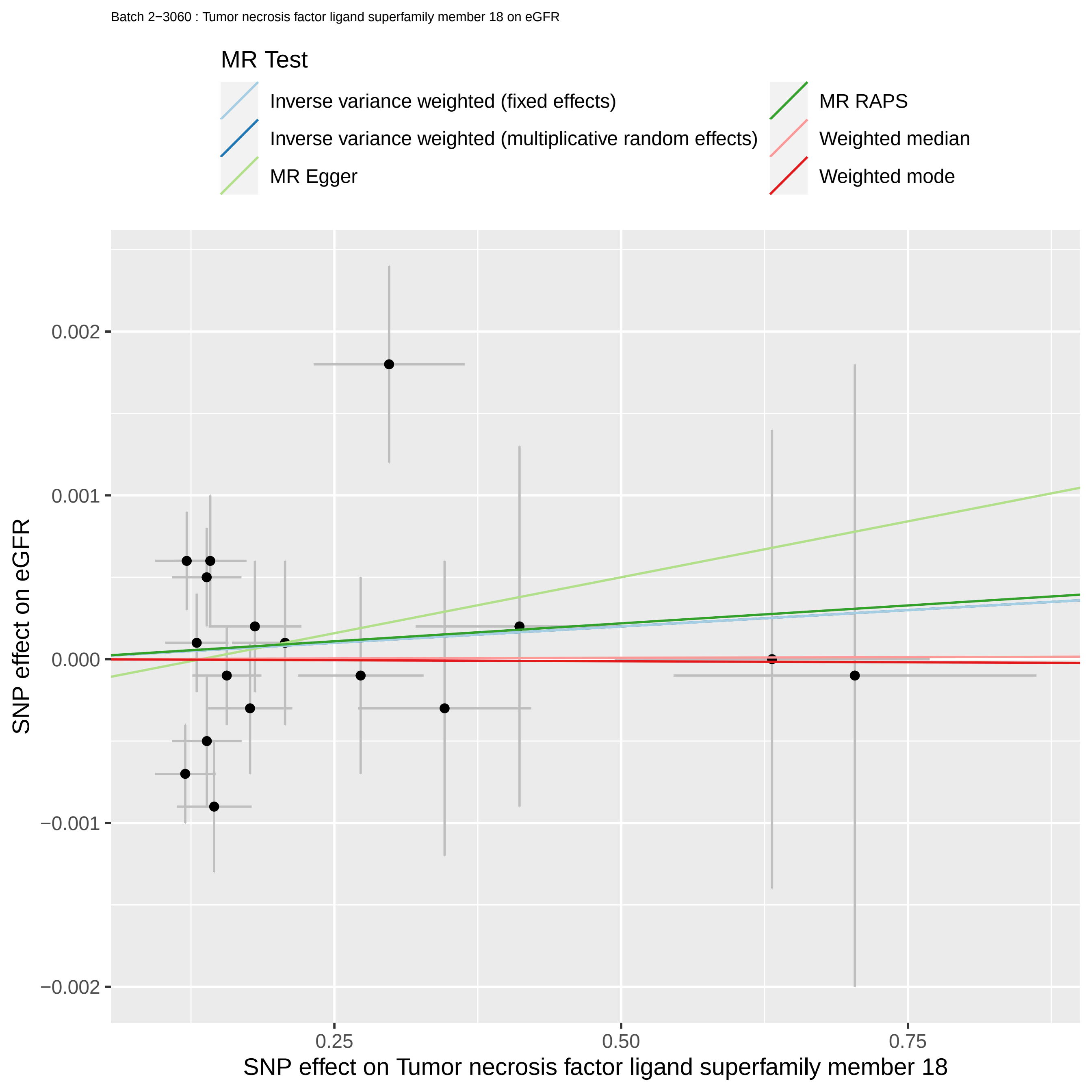

Batch 2−3060 : Tumor necrosis factor ligand superfamily member 18 on eGFR
MR Test
Inverse variance weighted (fixed effects)
Inverse variance weighted (multiplicative random effects)
MR Egger
MR RAPS
Weighted median
Weighted mode
0.002
0.001
0.000
−0.001
−0.002
0.25
0.50
0.75
SNP effect on eGFR
SNP effect on Tumor necrosis factor ligand superfamily member 18

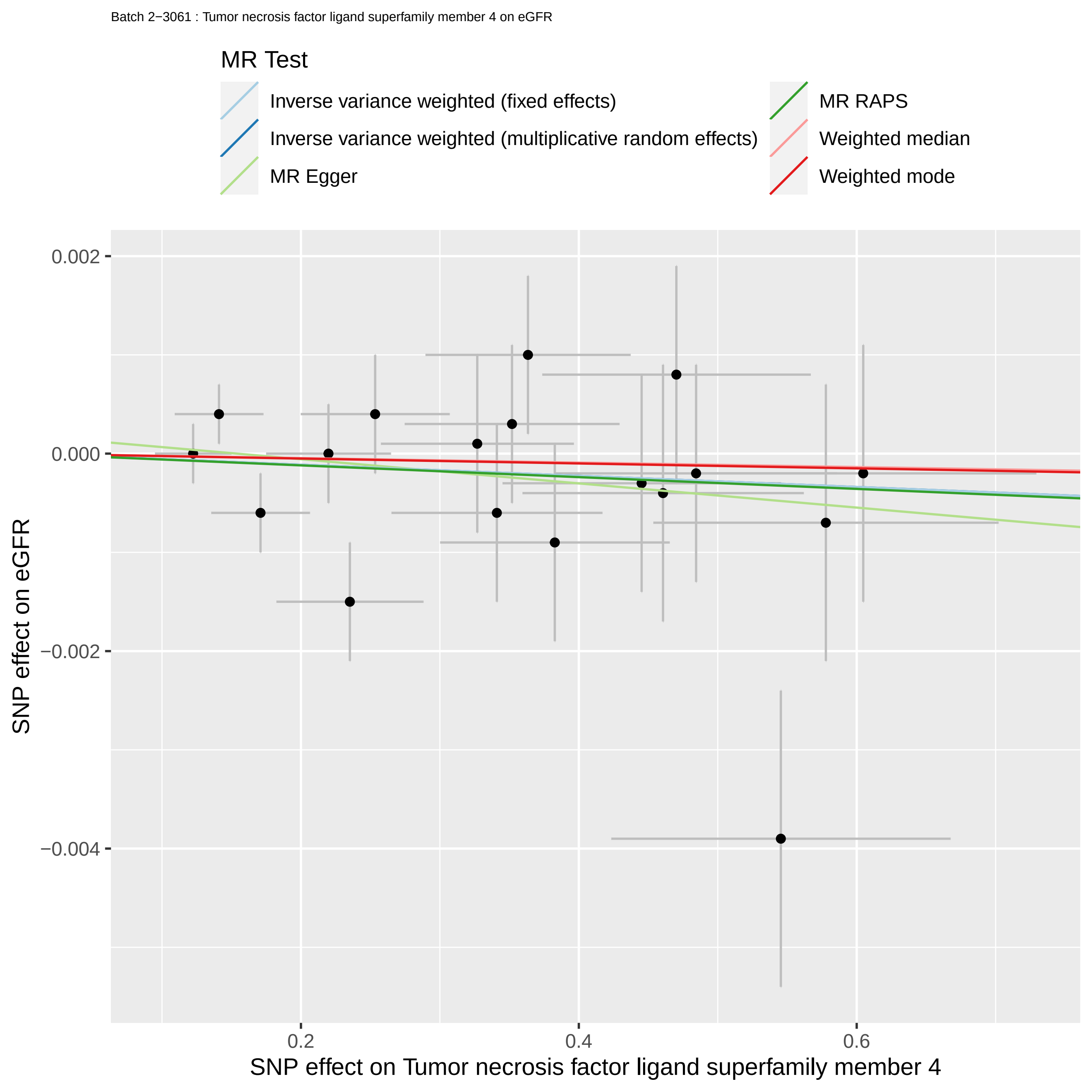

Batch 2−3061 : Tumor necrosis factor ligand superfamily member 4 on eGFR
MR Test
Inverse variance weighted (fixed effects)
Inverse variance weighted (multiplicative random effects)
MR Egger
MR RAPS
Weighted median
Weighted mode
0.002
0.000
−0.002
−0.004
0.2
0.4
0.6
SNP effect on eGFR
SNP effect on Tumor necrosis factor ligand superfamily member 4

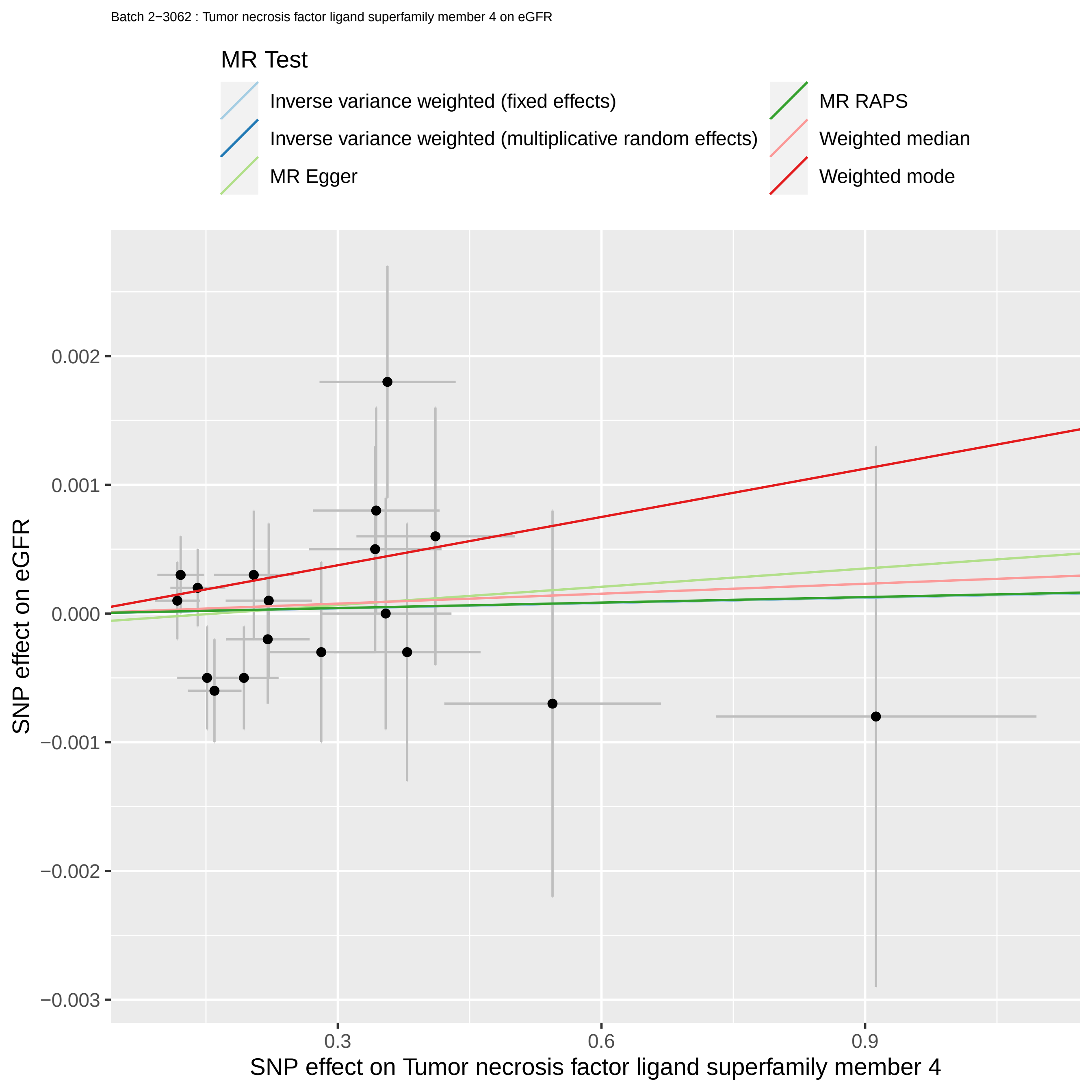
Batch 2−3062 : Tumor necrosis factor ligand superfamily member 4 on eGFR
MR Test
Inverse variance weighted (fixed effects)
Inverse variance weighted (multiplicative random effects)
MR Egger
MR RAPS
Weighted median
Weighted mode
0.002
0.001
0.000
−0.001
−0.002
−0.003
0.3
0.6
0.9
SNP effect on eGFR
SNP effect on Tumor necrosis factor ligand superfamily member 4

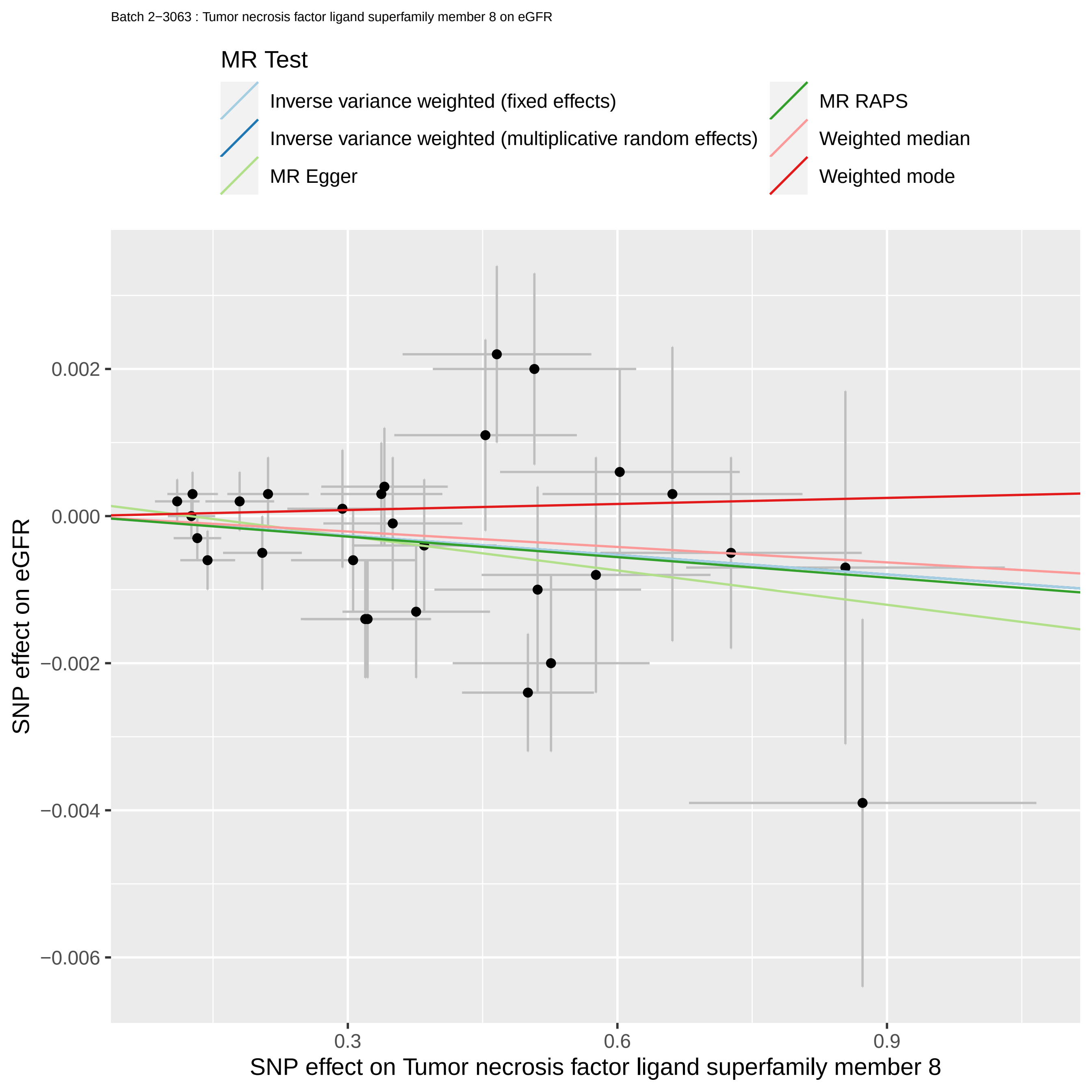

Batch 2−3063 : Tumor necrosis factor ligand superfamily member 8 on eGFR
MR Test
Inverse variance weighted (fixed effects)
Inverse variance weighted (multiplicative random effects)
MR Egger
MR RAPS
Weighted median
Weighted mode
0.002
0.000
−0.002
−0.004
−0.006
0.3
0.6
0.9
SNP effect on eGFR
SNP effect on Tumor necrosis factor ligand superfamily member 8

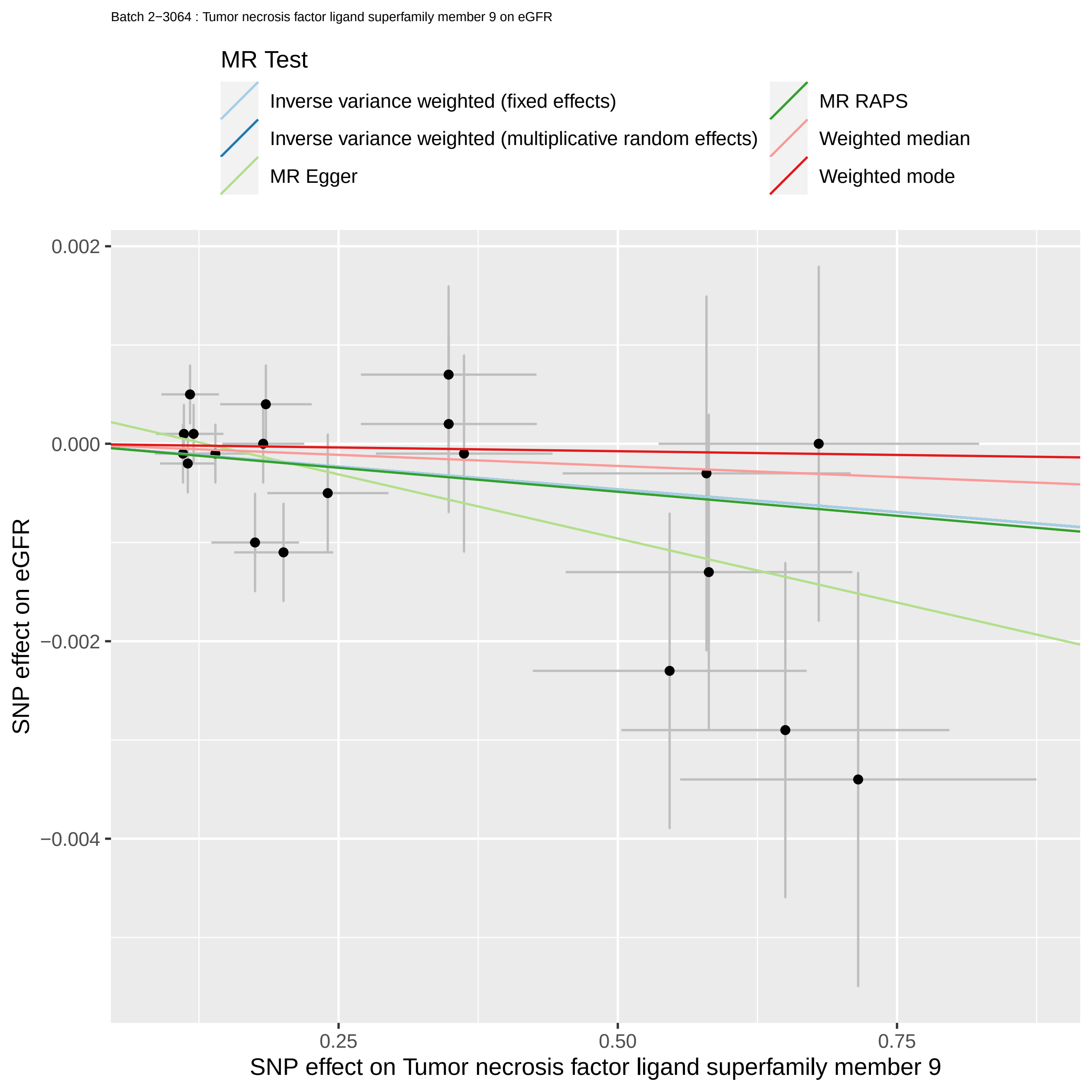
Batch 2−3064 : Tumor necrosis factor ligand superfamily member 9 on eGFR
MR Test
Inverse variance weighted (fixed effects)
Inverse variance weighted (multiplicative random effects)
MR Egger
MR RAPS
Weighted median
Weighted mode
0.002
0.000
−0.002
−0.004
SNP effect on eGFR
0.25
0.50
0.75
SNP effect on Tumor necrosis factor ligand superfamily member 9

Batch 2−3065 : Tenomodulin on eGFR

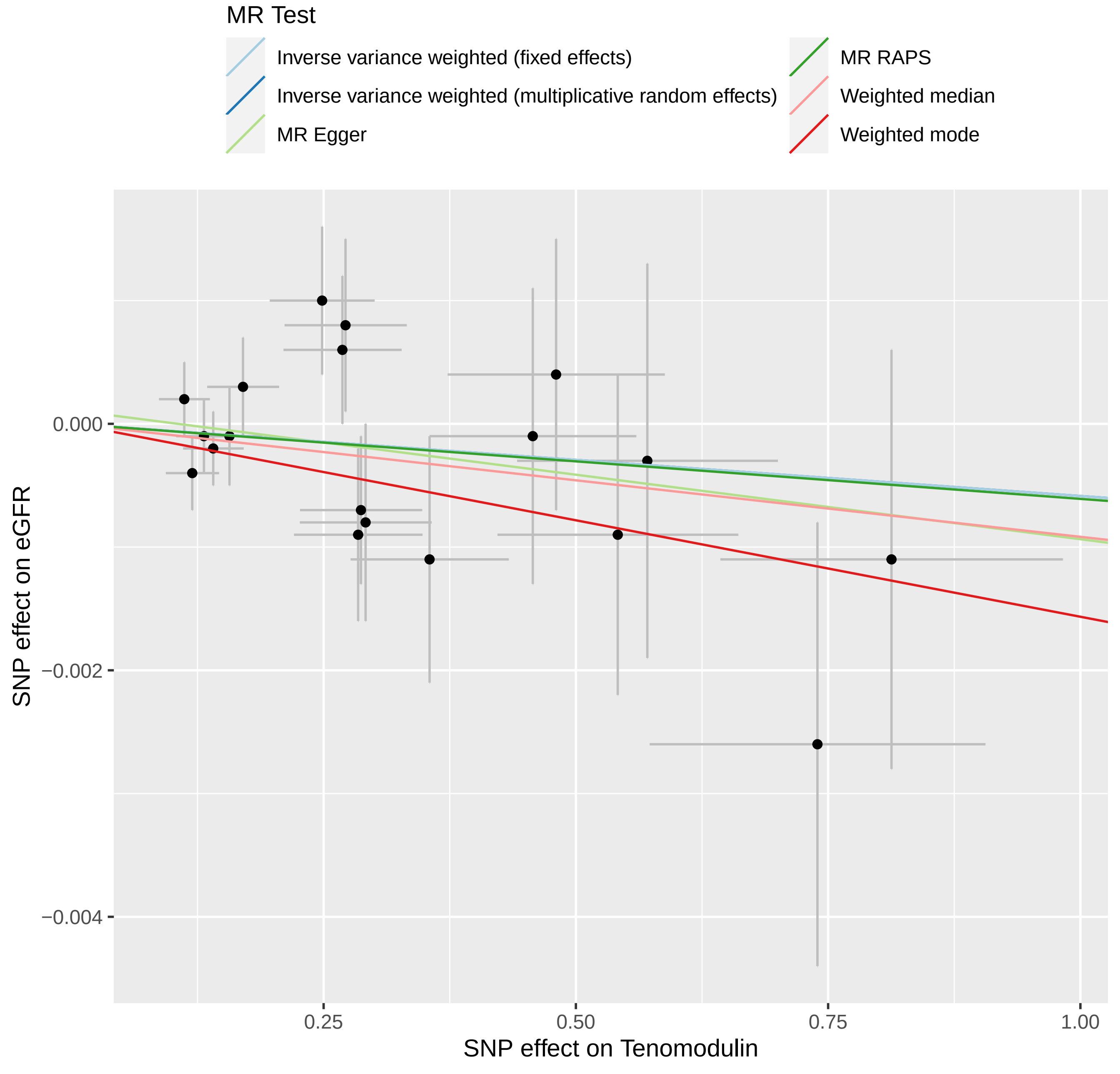

Batch 2−3066 : Troponin I, cardiac muscle on eGFR

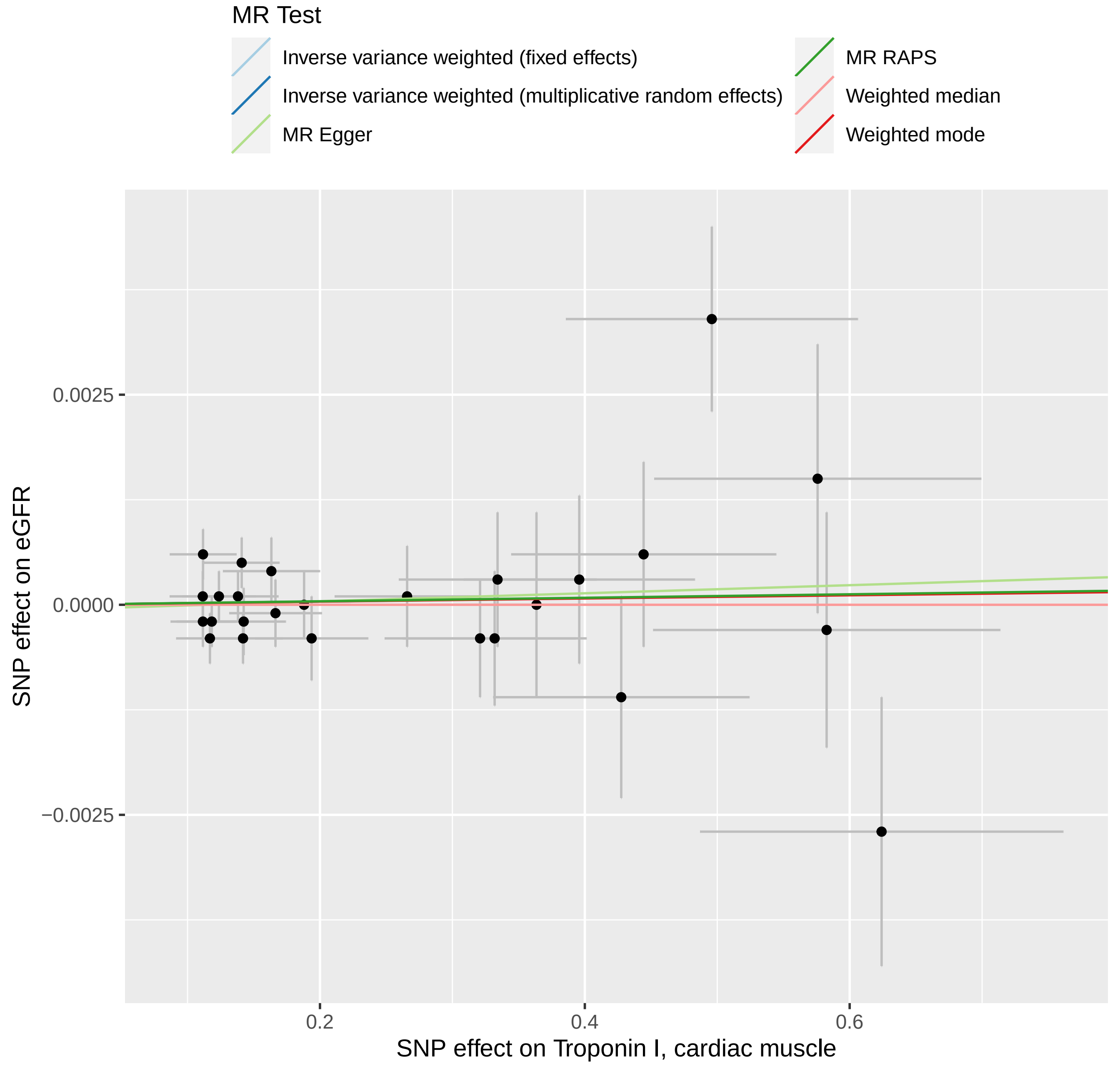

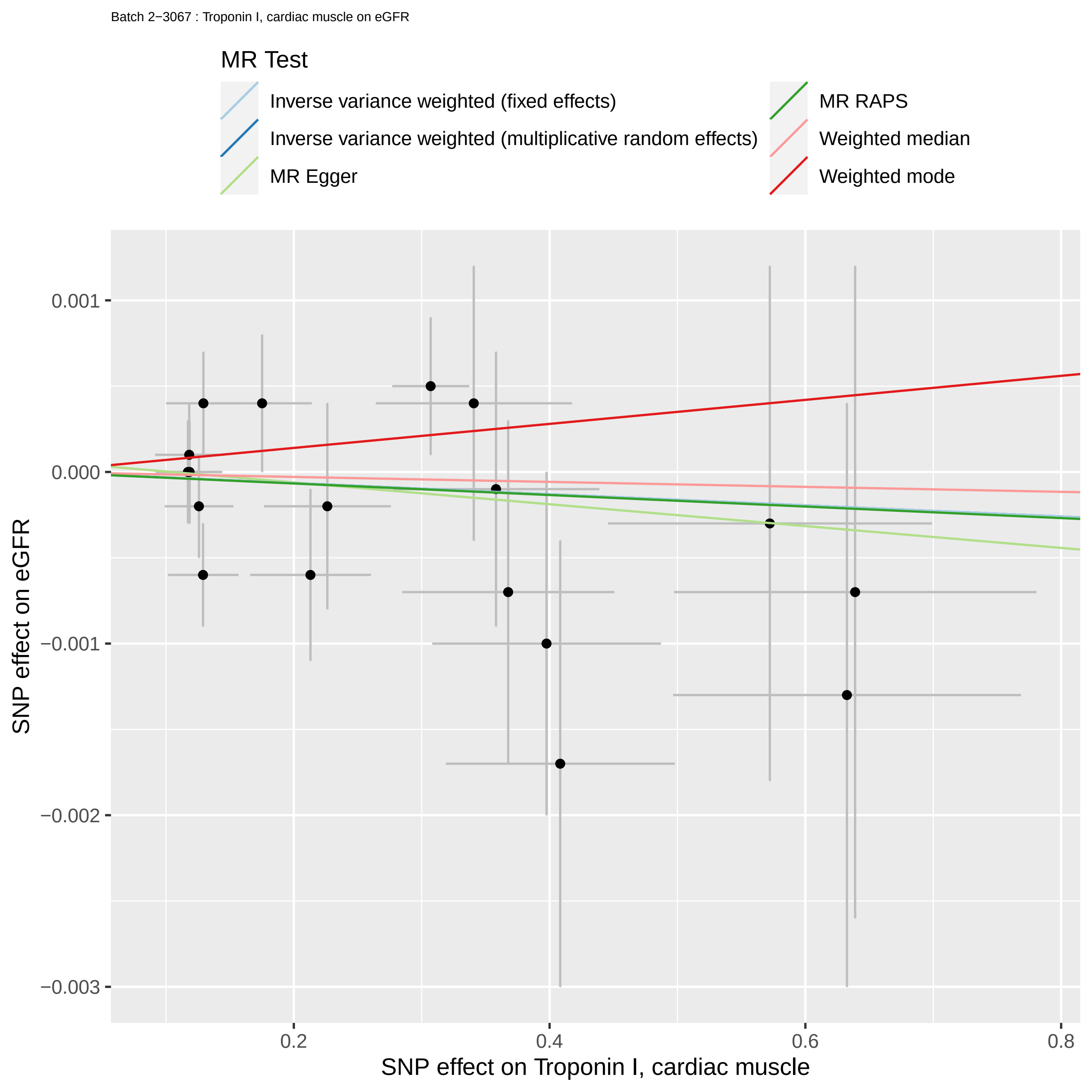

Batch 2−3067 : Troponin I, cardiac muscle on eGFR
MR Test
Inverse variance weighted (fixed effects)
Inverse variance weighted (multiplicative random effects)
MR Egger
MR RAPS
Weighted median
Weighted mode
0.001
0.000
−0.001
−0.002
−0.003
0.2
0.4
0.6
0.8
SNP effect on eGFR
SNP effect on Troponin I, cardiac muscle

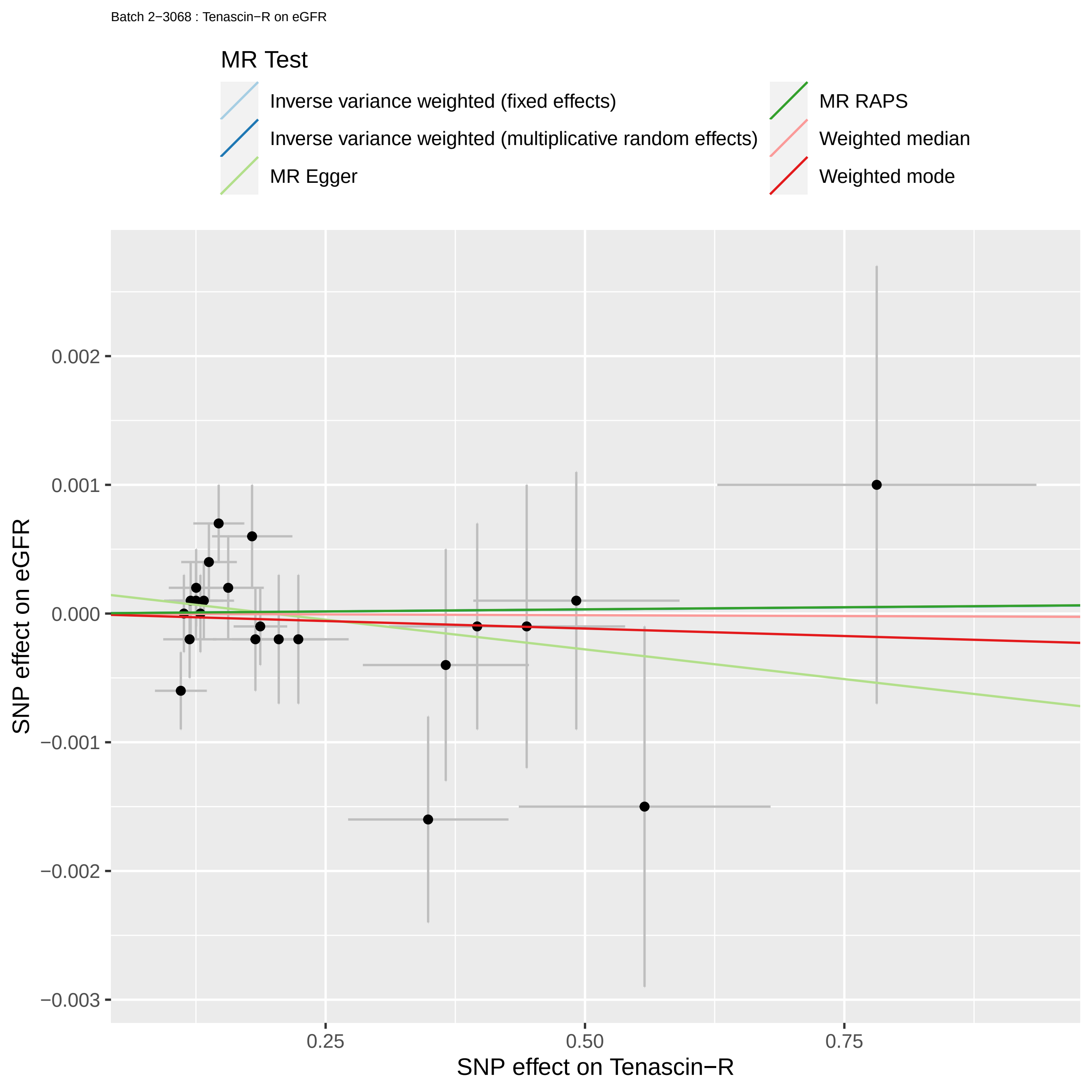
Batch 2−3068 : Tenascin−R on eGFR
MR Test
Inverse variance weighted (fixed effects)
Inverse variance weighted (multiplicative random effects)
MR Egger
MR RAPS
Weighted median
Weighted mode
0.002
0.001
0.000
−0.001
−0.002
−0.003
SNP effect on eGFR
0.25
0.50
0.75
SNP effect on Tenascin−R

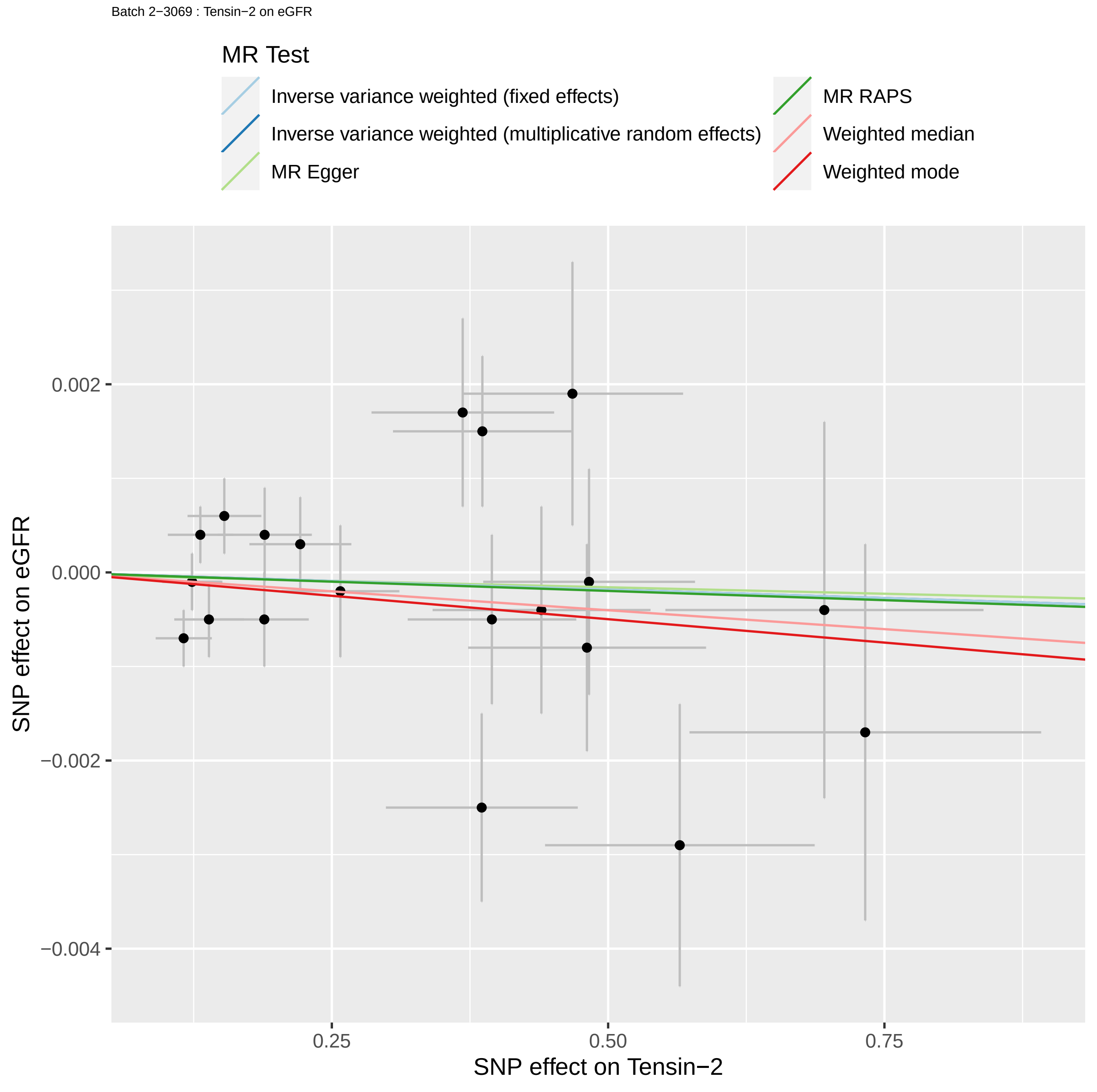

Batch 2−3069 : Tensin−2 on eGFR
MR Test
Inverse variance weighted (fixed effects)
Inverse variance weighted (multiplicative random effects)
MR Egger
MR RAPS
Weighted median
Weighted mode
0.002
0.000
−0.002
−0.004
0.25
0.50
0.75
SNP effect on eGFR
SNP effect on Tensin−2

Batch 2−3070 : Tensin−4 on eGFR

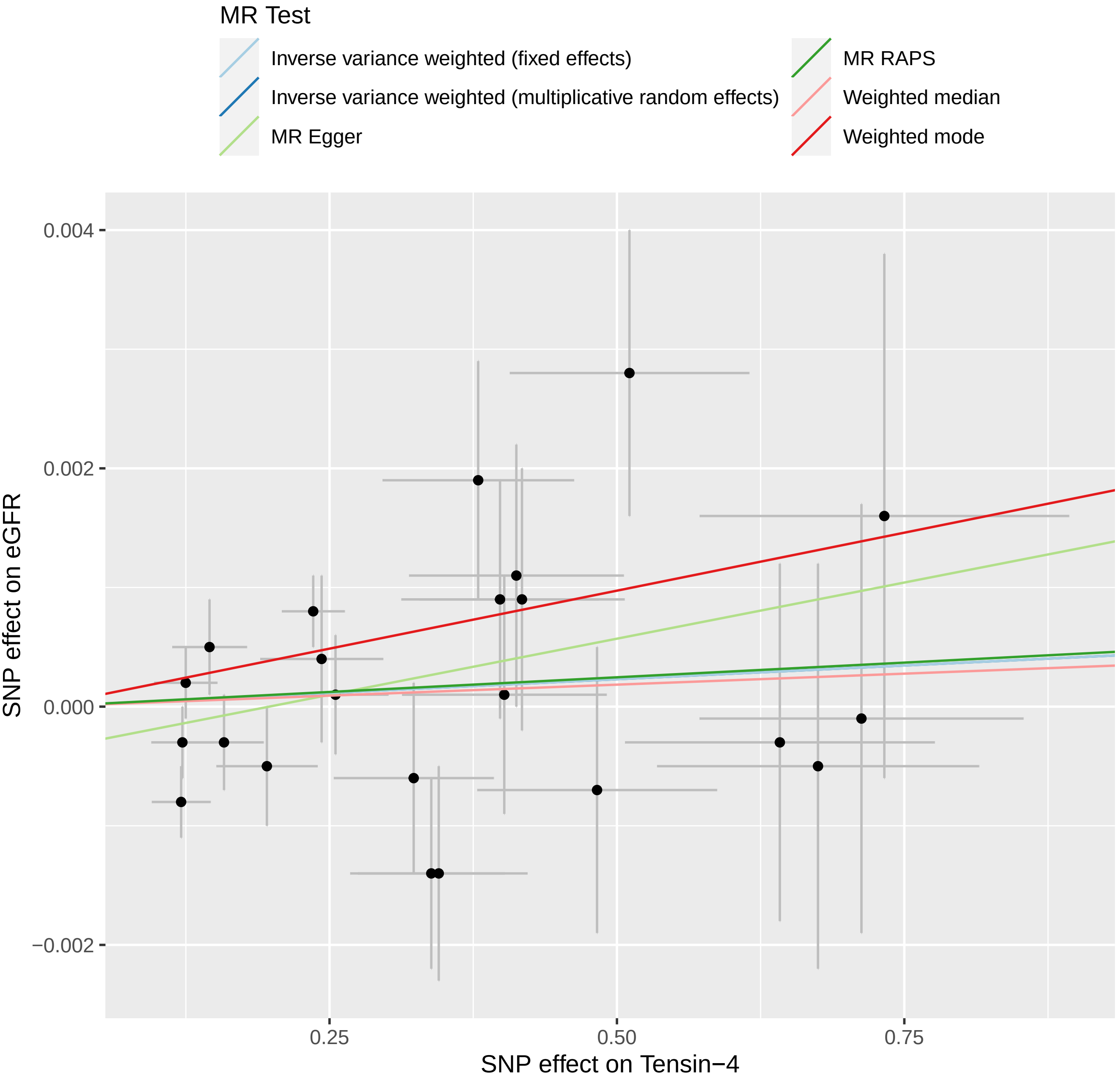

Batch 2−3071 : Tenascin−X on eGFR

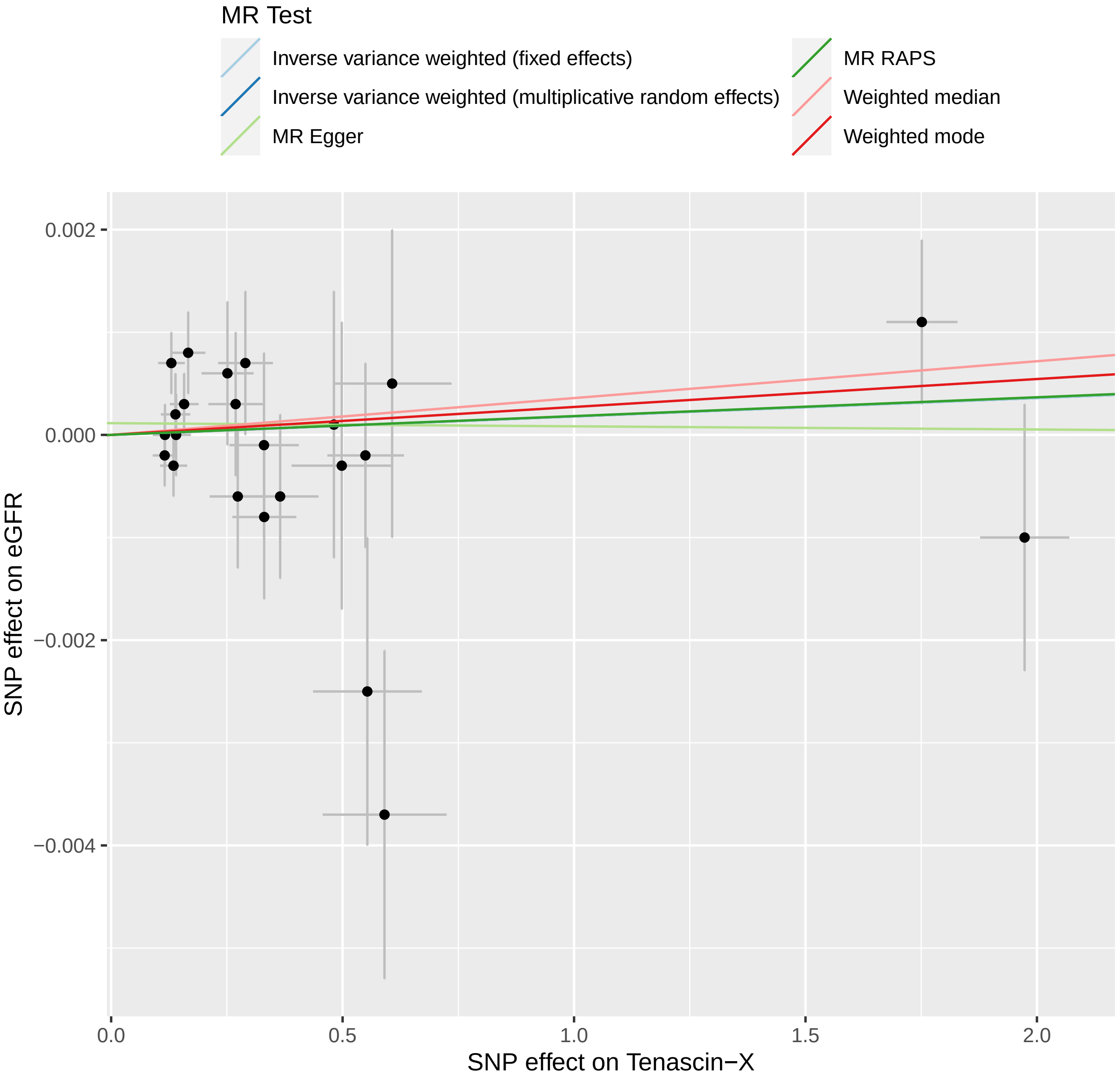

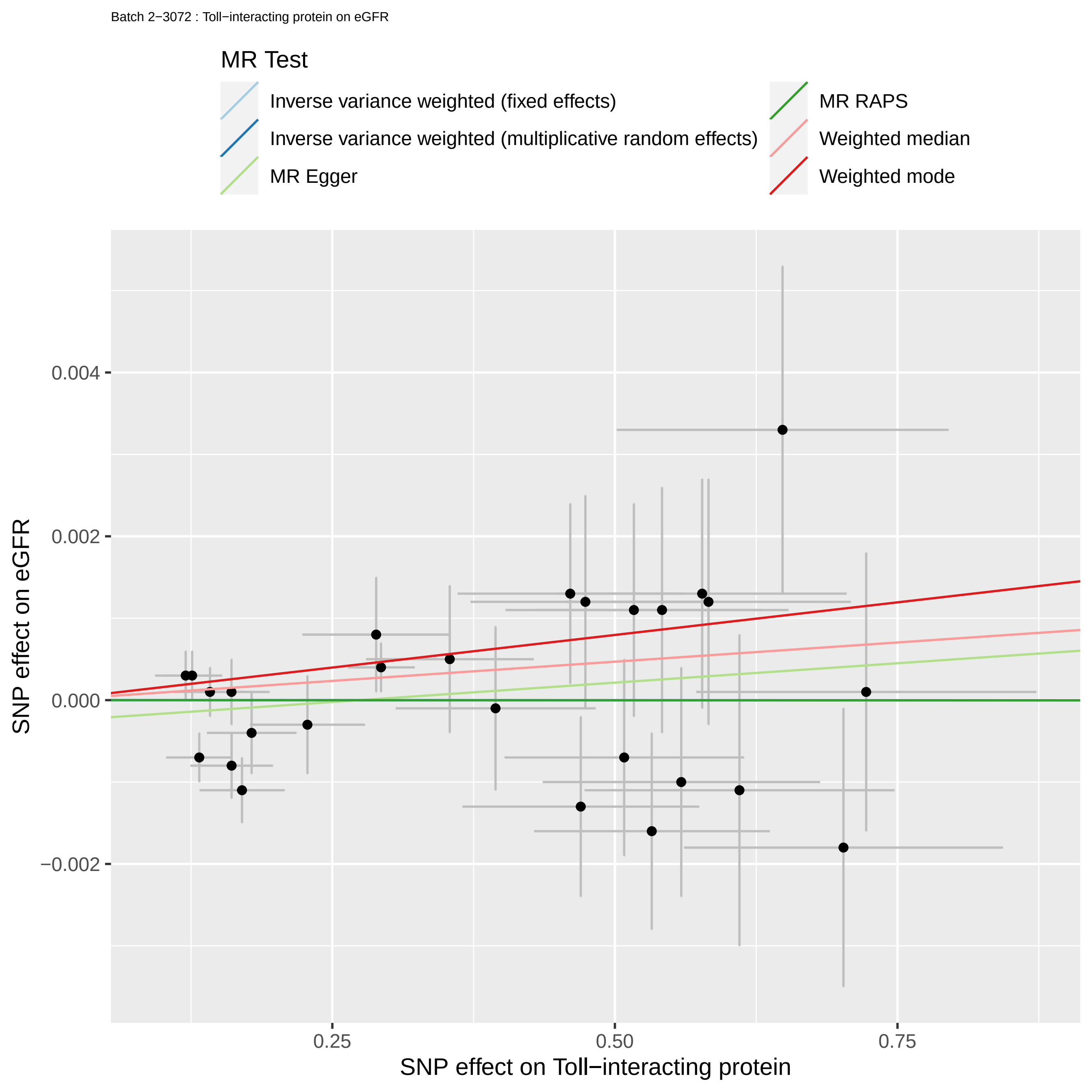
Batch 2−3072 : Toll−interacting protein on eGFR
MR Test
Inverse variance weighted (fixed effects)
Inverse variance weighted (multiplicative random effects)
MR Egger
MR RAPS
Weighted median
Weighted mode
0.004
0.002
0.000
−0.002
0.25
0.50
0.75
SNP effect on eGFR
SNP effect on Toll−interacting protein

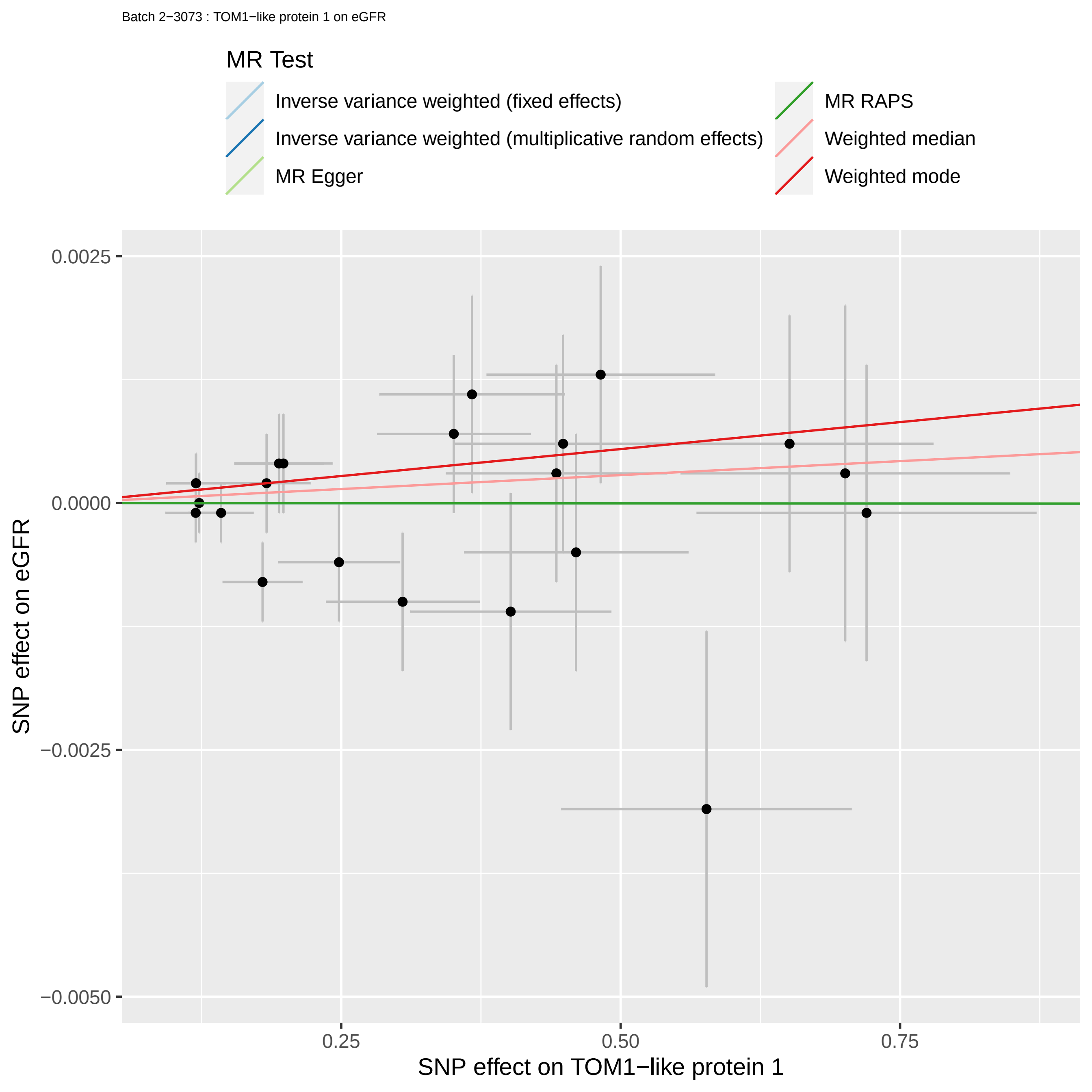

Batch 2−3073 : TOM1−like protein 1 on eGFR
MR Test
Inverse variance weighted (fixed effects)
Inverse variance weighted (multiplicative random effects)
MR Egger
MR RAPS
Weighted median
Weighted mode
0.0025
0.0000
−0.0025
−0.0050
SNP effect on eGFR
0.25
0.50
0.75
SNP effect on TOM1−like protein 1

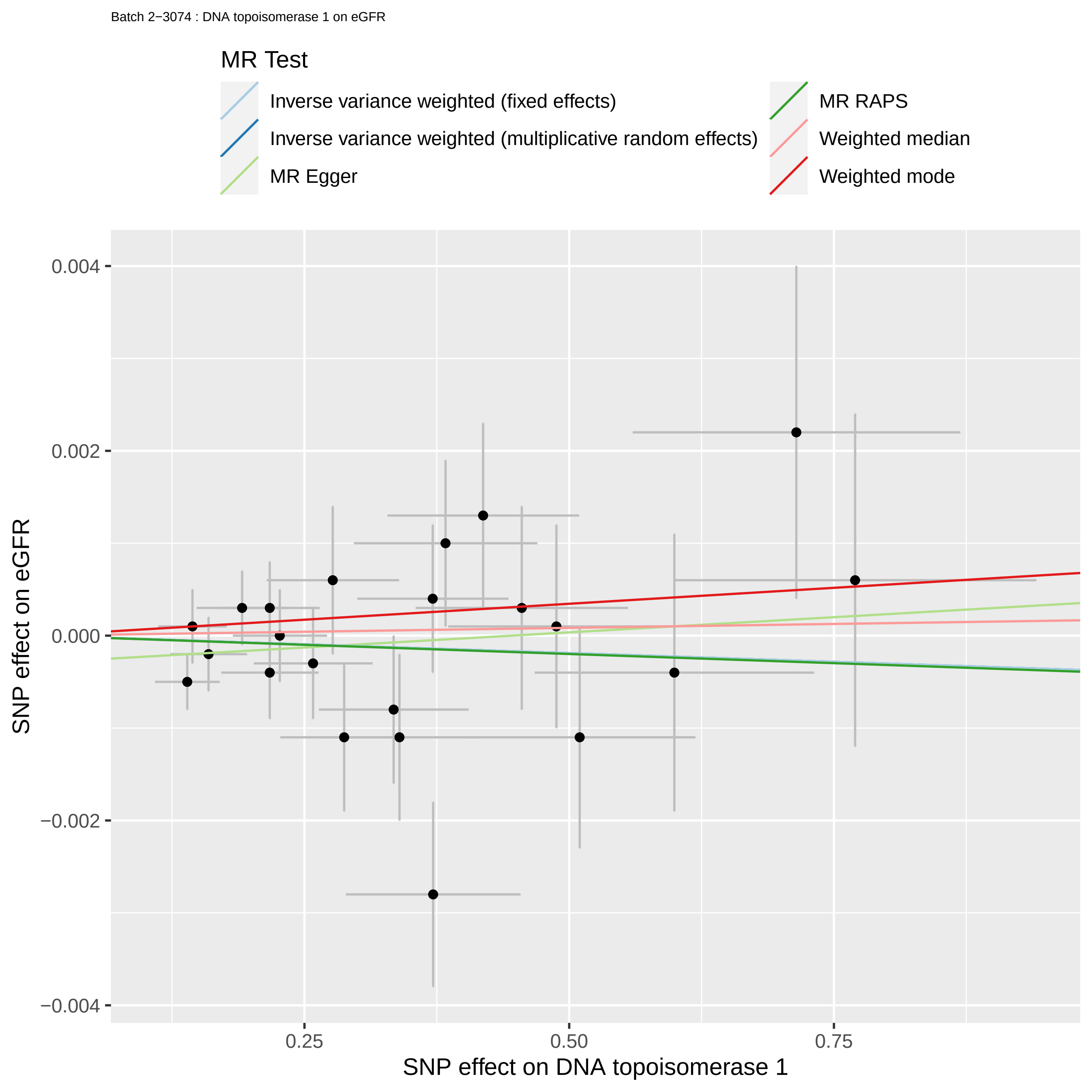

Batch 2−3074 : DNA topoisomerase 1 on eGFR
MR Test
Inverse variance weighted (fixed effects)
Inverse variance weighted (multiplicative random effects)
MR Egger
MR RAPS
Weighted median
Weighted mode
0.004
0.002
0.000
−0.002
−0.004
0.25
0.50
0.75
SNP effect on eGFR
SNP effect on DNA topoisomerase 1

Batch 2−3075 : DNA topoisomerase 2−binding protein 1 on eGFR

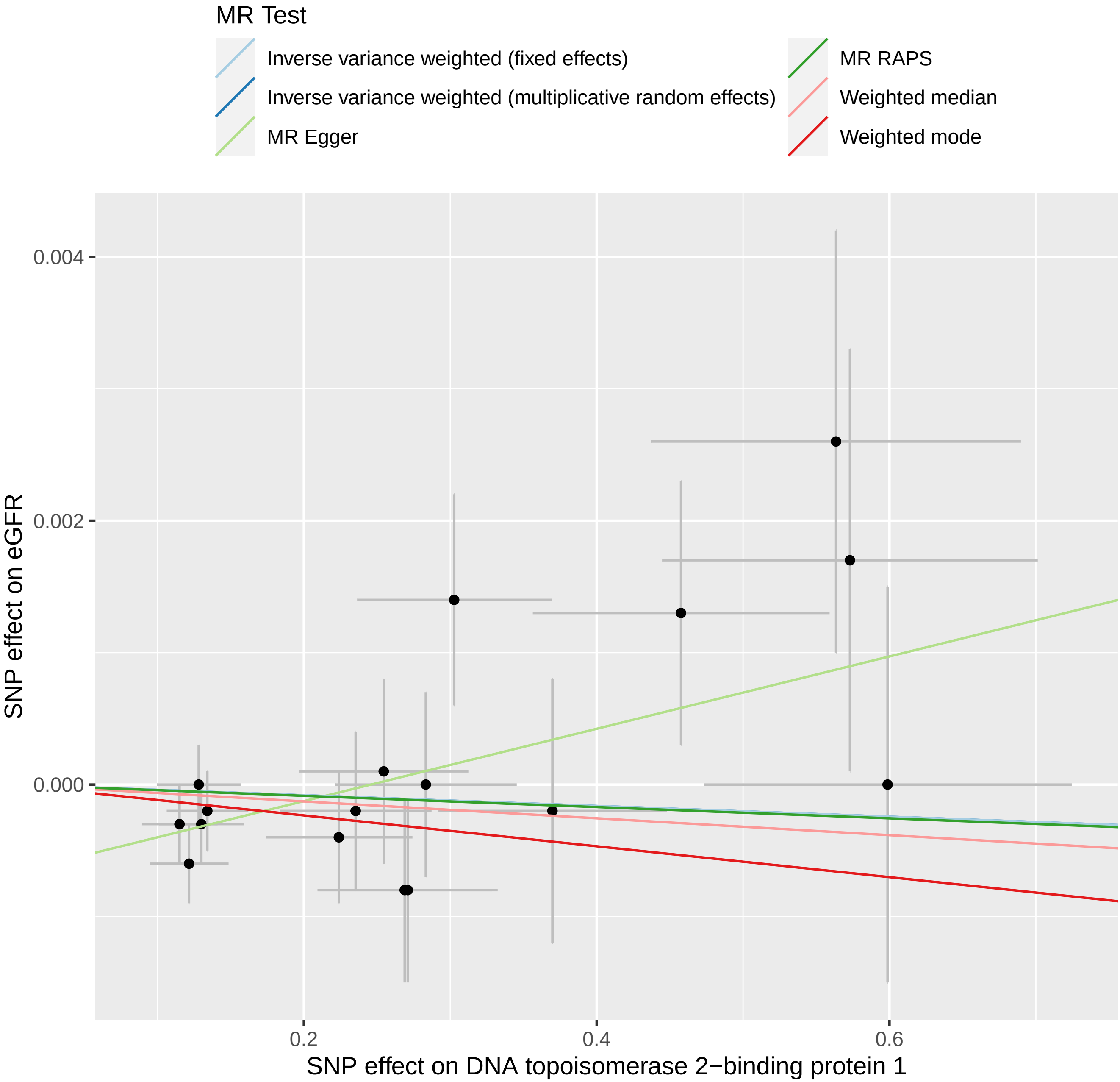

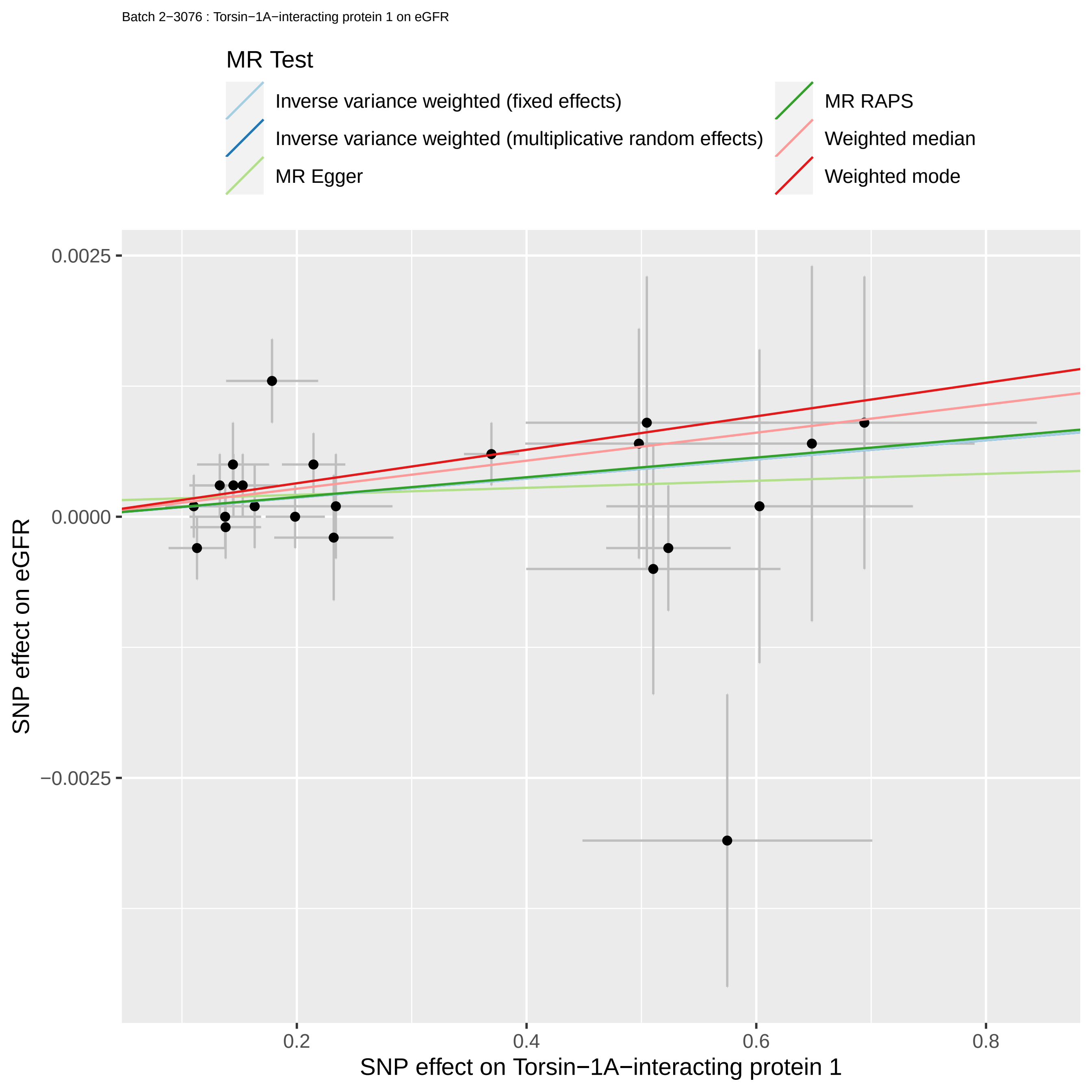

Batch 2−3076 : Torsin−1A−interacting protein 1 on eGFR
MR Test
Inverse variance weighted (fixed effects)
Inverse variance weighted (multiplicative random effects)
MR Egger
MR RAPS
Weighted median
Weighted mode
0.0025
0.0000
−0.0025
SNP effect on eGFR
0.2
0.4
0.6
0.8
SNP effect on Torsin−1A−interacting protein 1

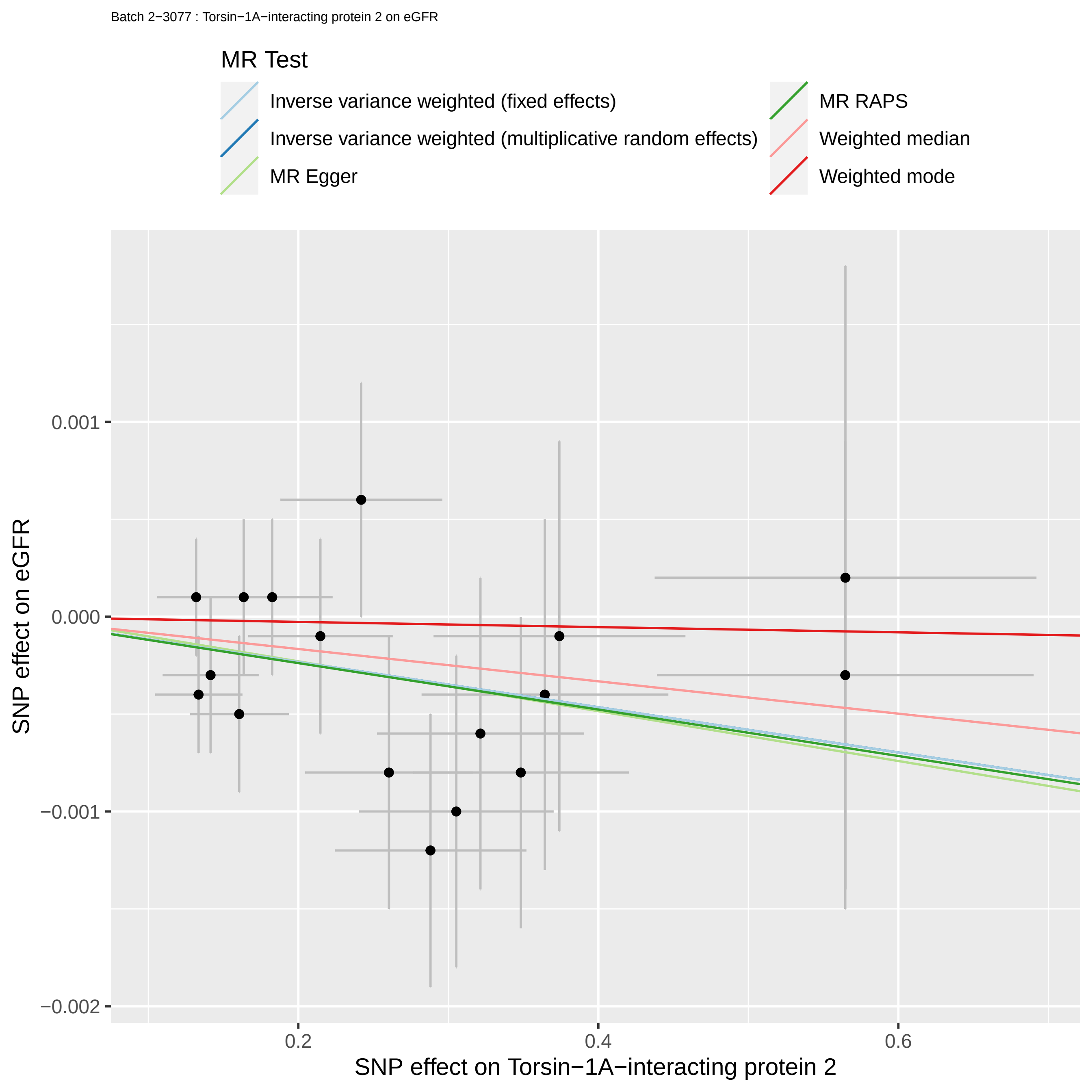

Batch 2−3077 : Torsin−1A−interacting protein 2 on eGFR
MR Test
Inverse variance weighted (fixed effects)
Inverse variance weighted (multiplicative random effects)
MR Egger
MR RAPS
Weighted median
Weighted mode
0.001
0.000
−0.001
−0.002
0.2
0.4
0.6
SNP effect on eGFR
SNP effect on Torsin−1A−interacting protein 2

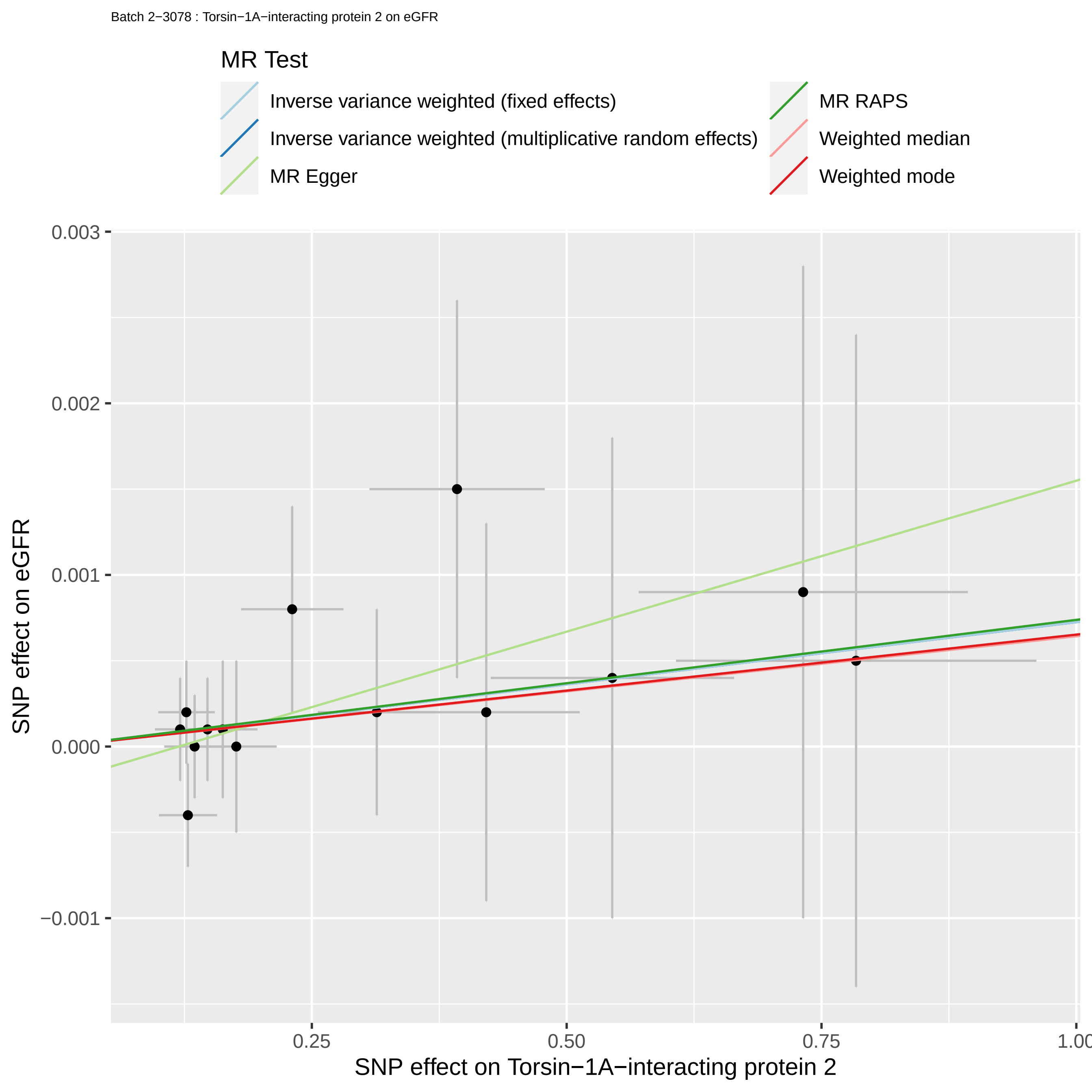
Batch 2−3078 : Torsin−1A−interacting protein 2 on eGFR
MR Test
Inverse variance weighted (fixed effects)
Inverse variance weighted (multiplicative random effects)
MR Egger
MR RAPS
Weighted median
Weighted mode
0.003
0.002
0.001
0.000
−0.001
0.25
0.50
0.75
1.00
SNP effect on eGFR
SNP effect on Torsin−1A−interacting protein 2

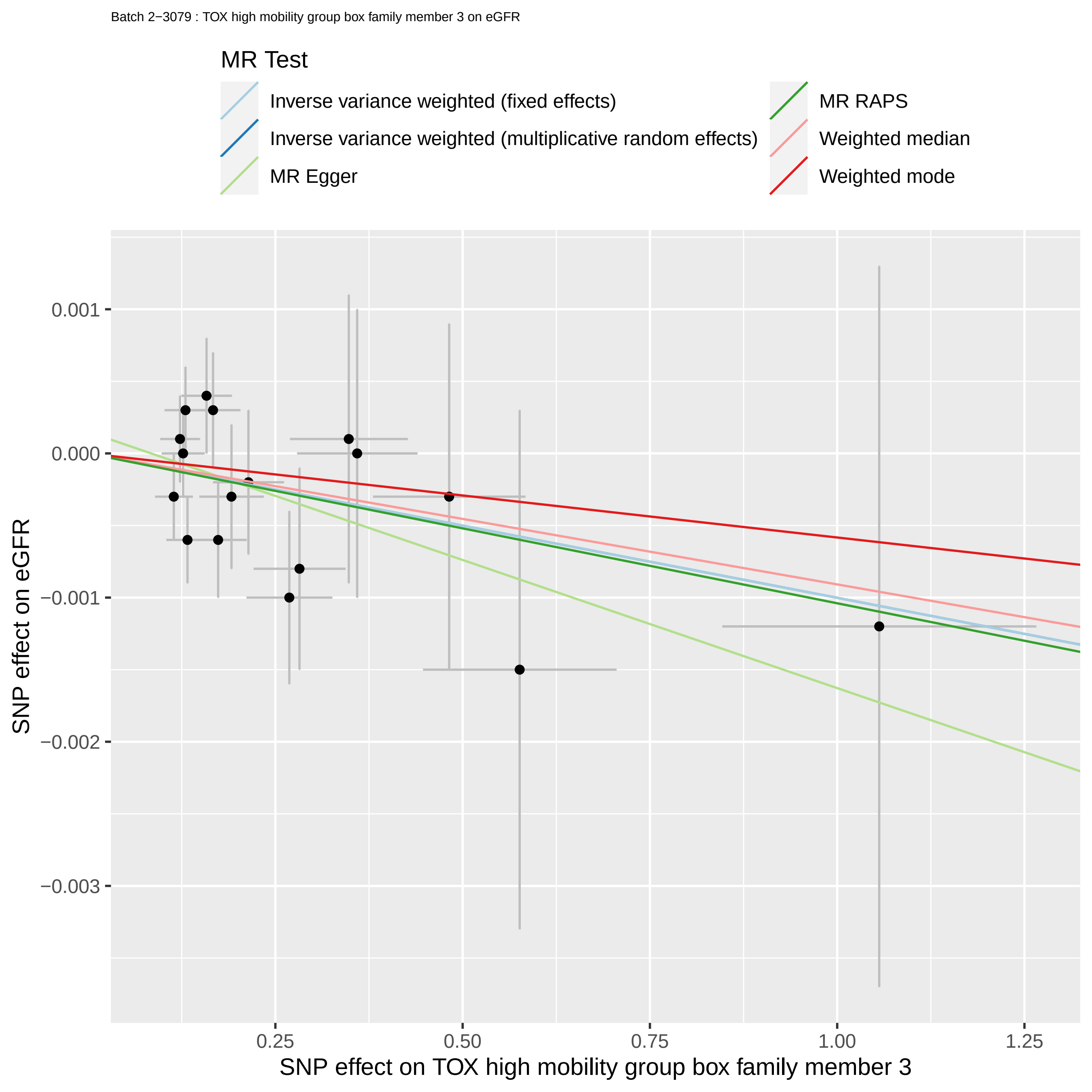
Batch 2−3079 : TOX high mobility group box family member 3 on eGFR
MR Test
Inverse variance weighted (fixed effects)
Inverse variance weighted (multiplicative random effects)
MR Egger
MR RAPS
Weighted median
Weighted mode
0.001
0.000
−0.001
−0.002
−0.003
0.25
0.50
0.75
1.00
1.25
SNP effect on eGFR
SNP effect on TOX high mobility group box family member 3

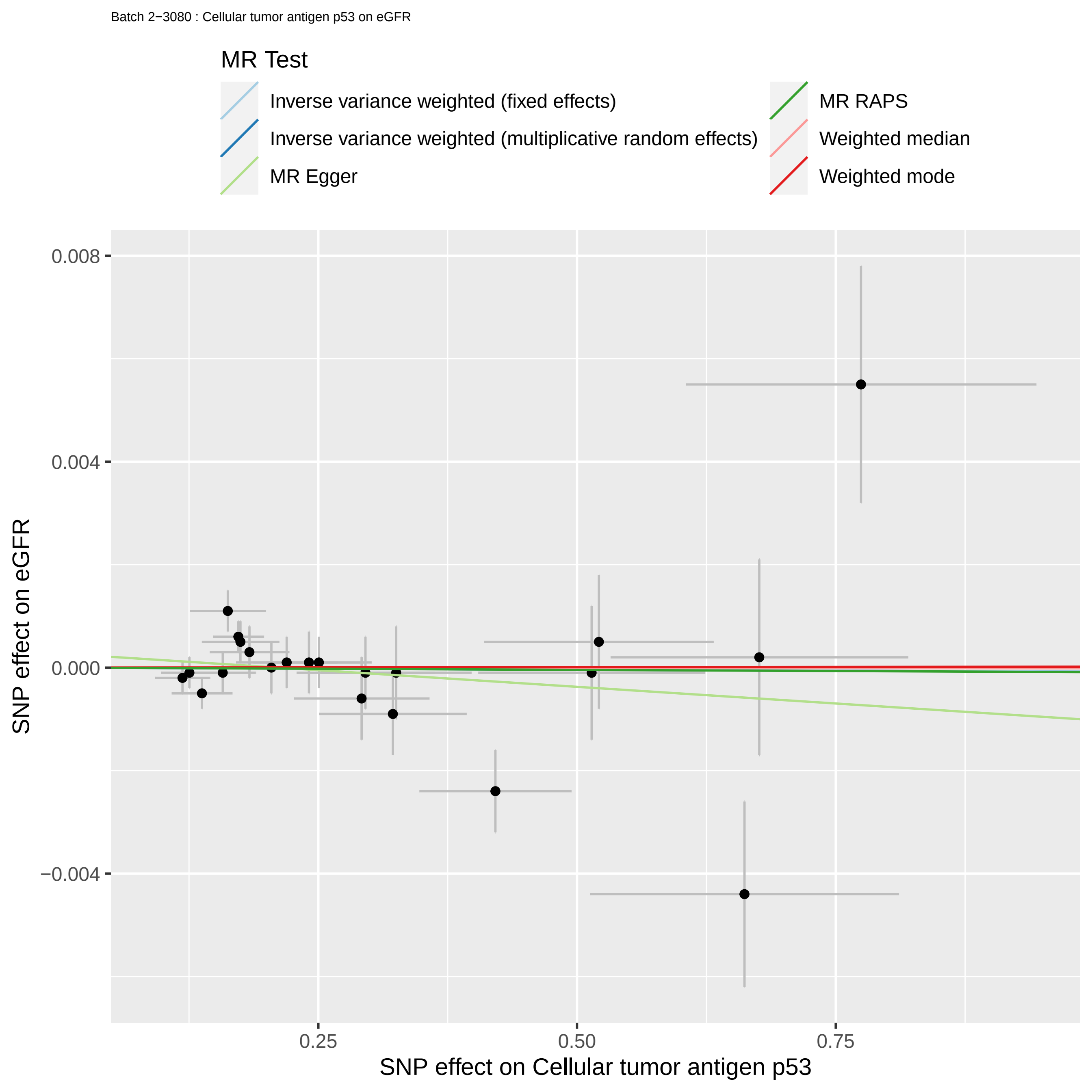

Batch 2−3080 : Cellular tumor antigen p53 on eGFR
MR Test
Inverse variance weighted (fixed effects)
Inverse variance weighted (multiplicative random effects)
MR Egger
MR RAPS
Weighted median
Weighted mode
0.008
0.004
0.000
−0.004
0.25
0.50
0.75
SNP effect on eGFR
SNP effect on Cellular tumor antigen p53

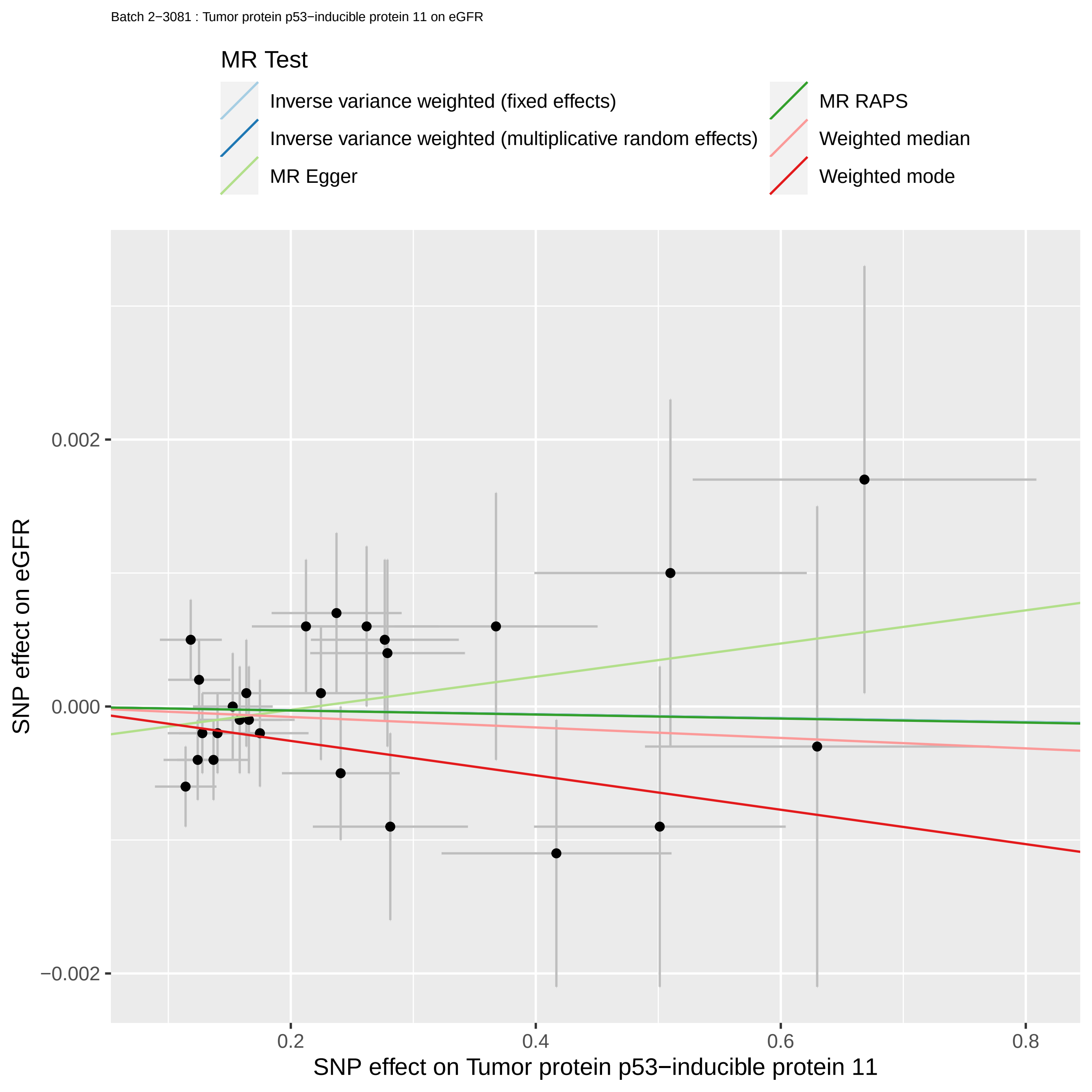

Batch 2−3081 : Tumor protein p53−inducible protein 11 on eGFR
MR Test
Inverse variance weighted (fixed effects)
Inverse variance weighted (multiplicative random effects)
MR Egger
MR RAPS
Weighted median
Weighted mode
0.002
0.000
−0.002
0.2
0.4
0.6
0.8
SNP effect on eGFR
SNP effect on Tumor protein p53−inducible protein 11

Batch 2−3082 : Tumor protein 63 on eGFR

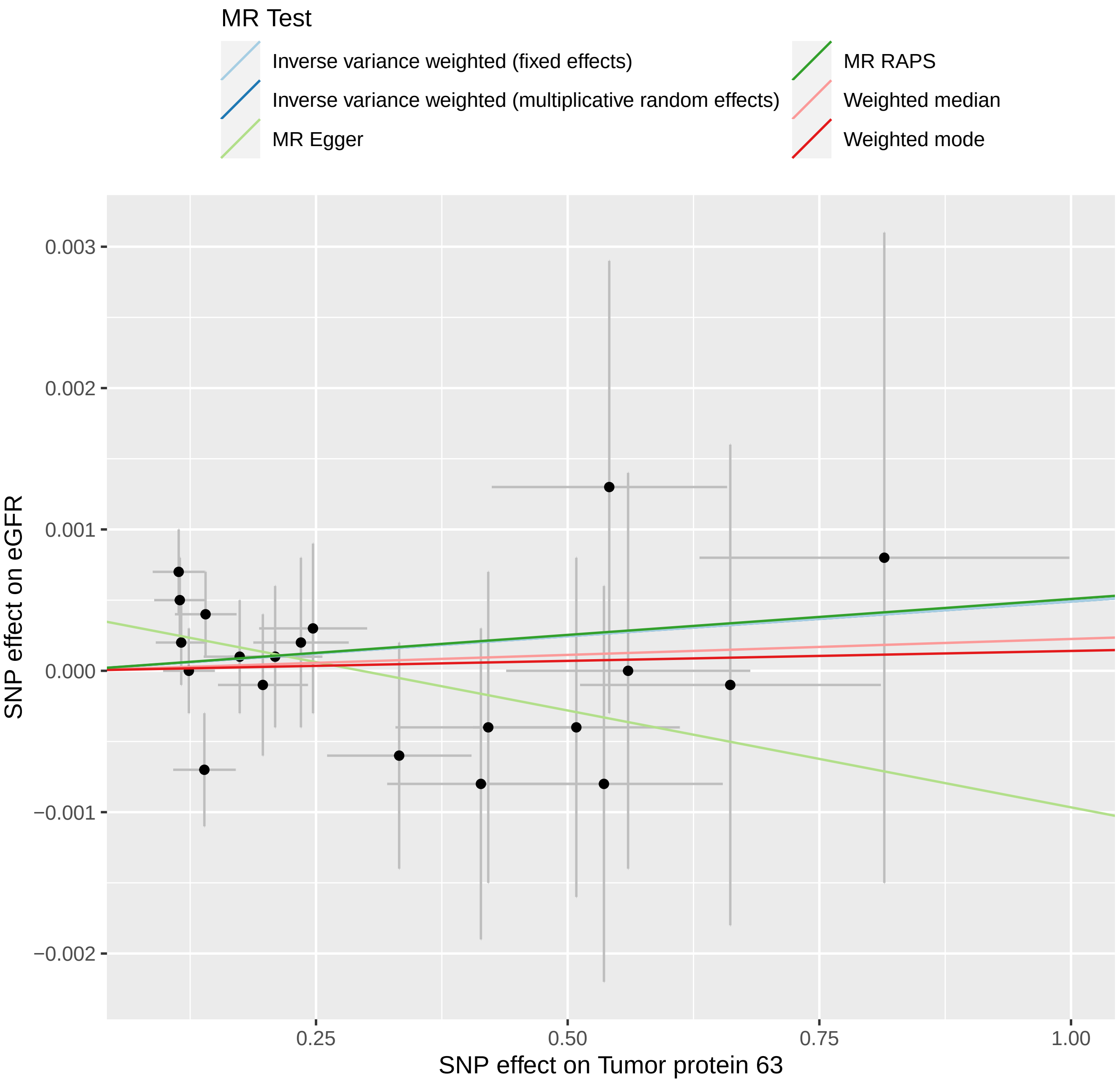

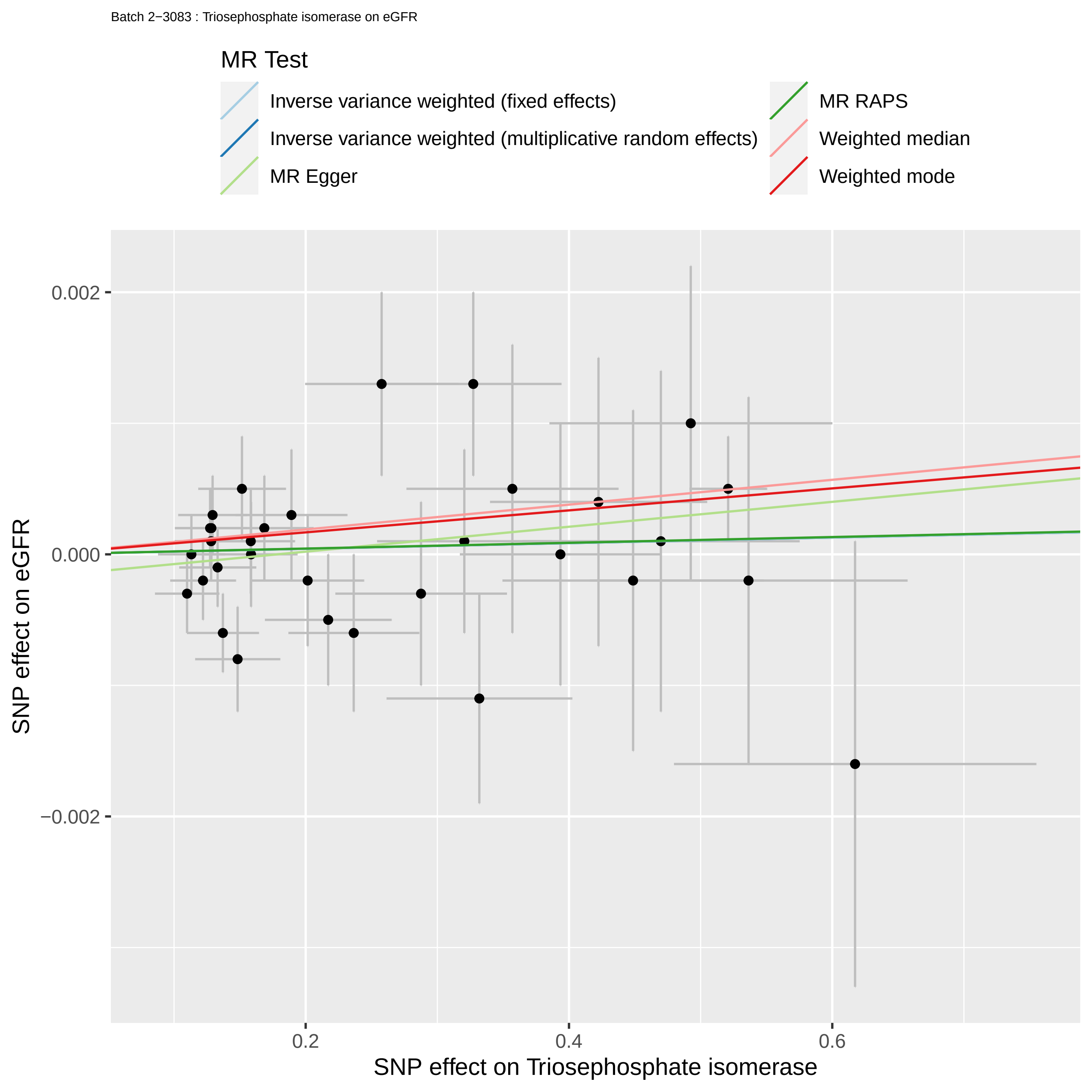

Batch 2−3083 : Triosephosphate isomerase on eGFR
MR Test
Inverse variance weighted (fixed effects)
Inverse variance weighted (multiplicative random effects)
MR Egger
MR RAPS
Weighted median
Weighted mode
0.002
0.000
−0.002
0.2
0.4
0.6
SNP effect on eGFR
SNP effect on Triosephosphate isomerase

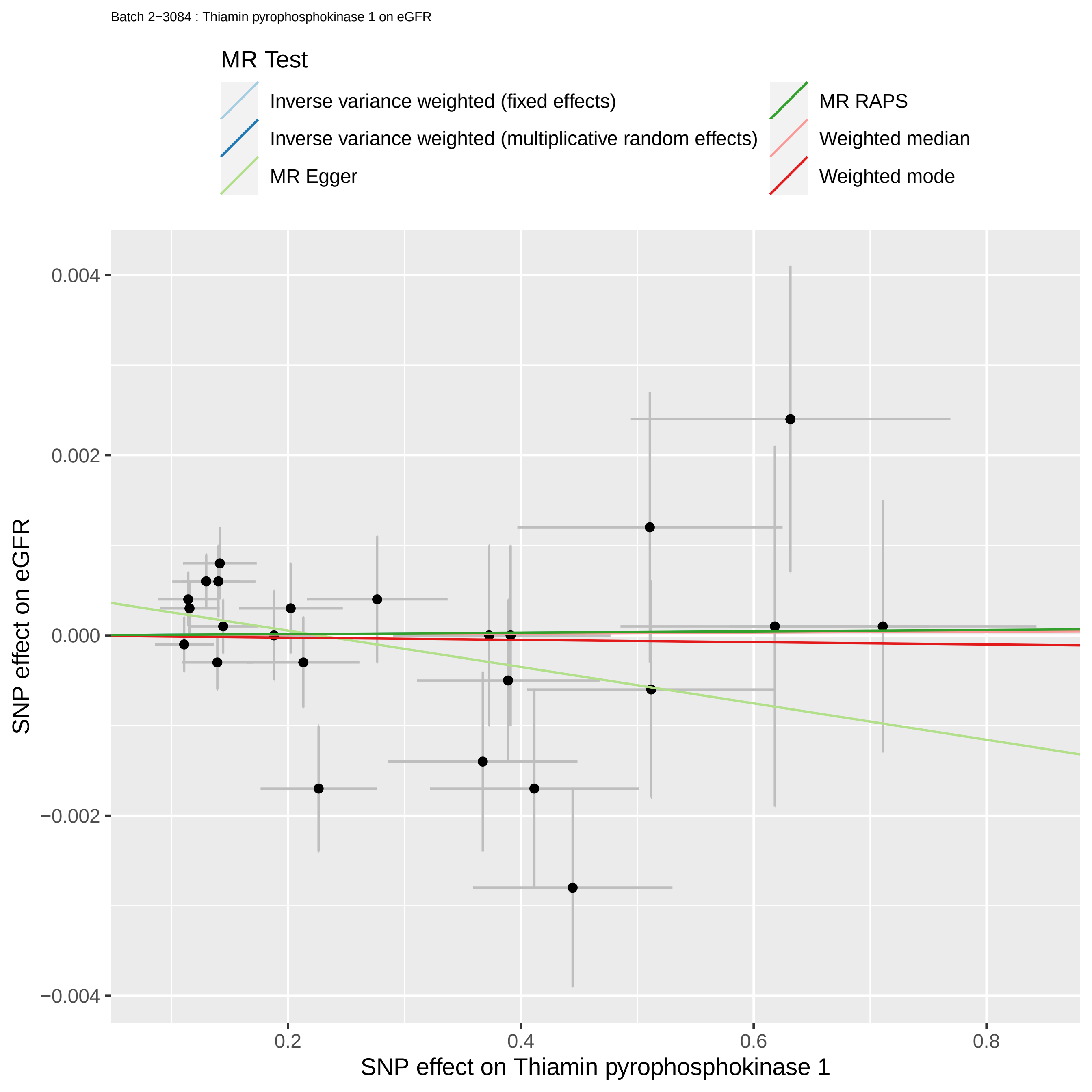

Batch 2−3084 : Thiamin pyrophosphokinase 1 on eGFR
MR Test
Inverse variance weighted (fixed effects)
Inverse variance weighted (multiplicative random effects)
MR Egger
MR RAPS
Weighted median
Weighted mode
0.004
0.002
0.000
−0.002
−0.004
0.2
0.4
0.6
0.8
SNP effect on eGFR
SNP effect on Thiamin pyrophosphokinase 1

Batch 2−3085 : Tropomyosin alpha−1 chain on eGFR

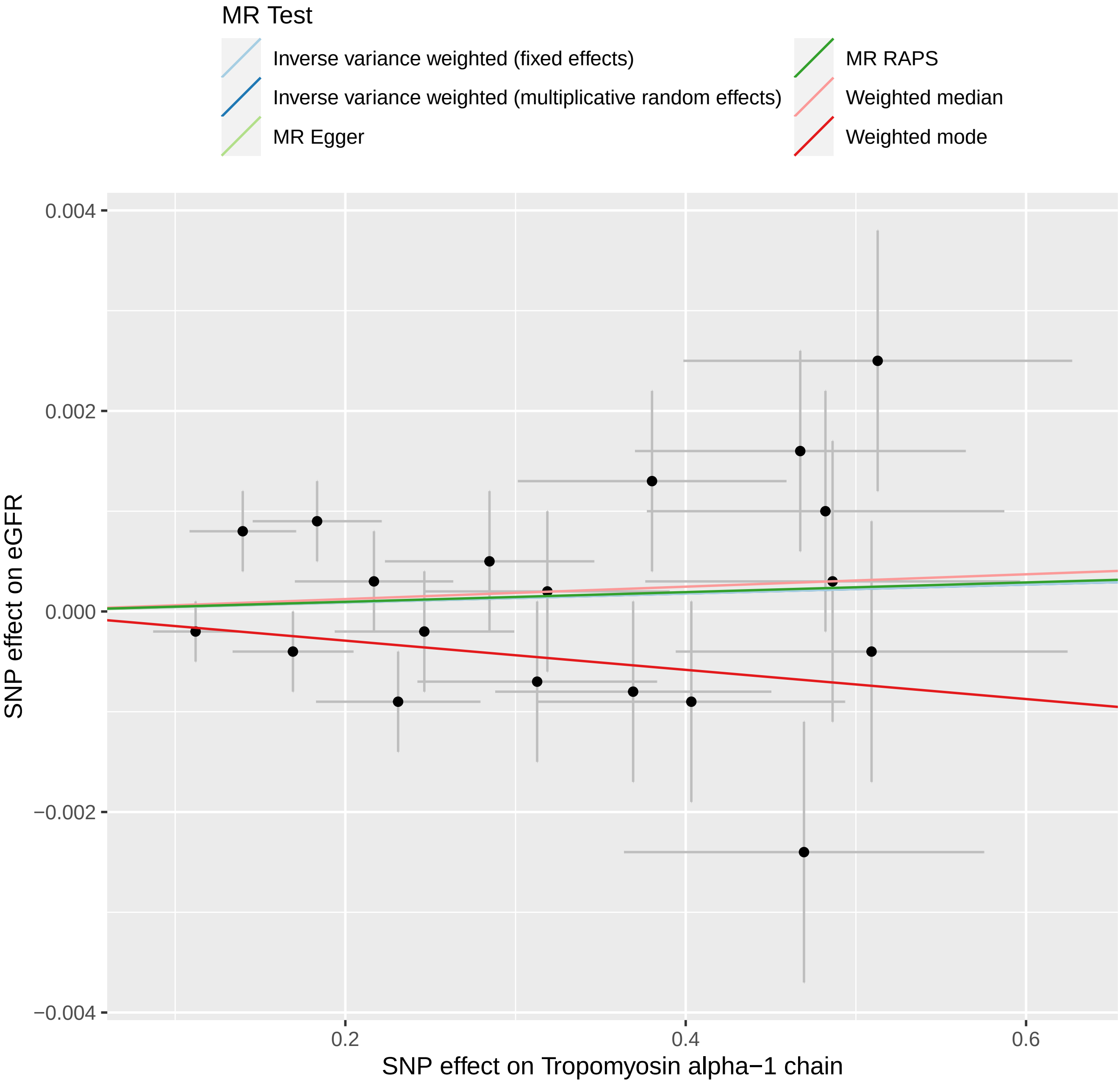

Batch 2−3086 : Tropomyosin beta chain on eGFR

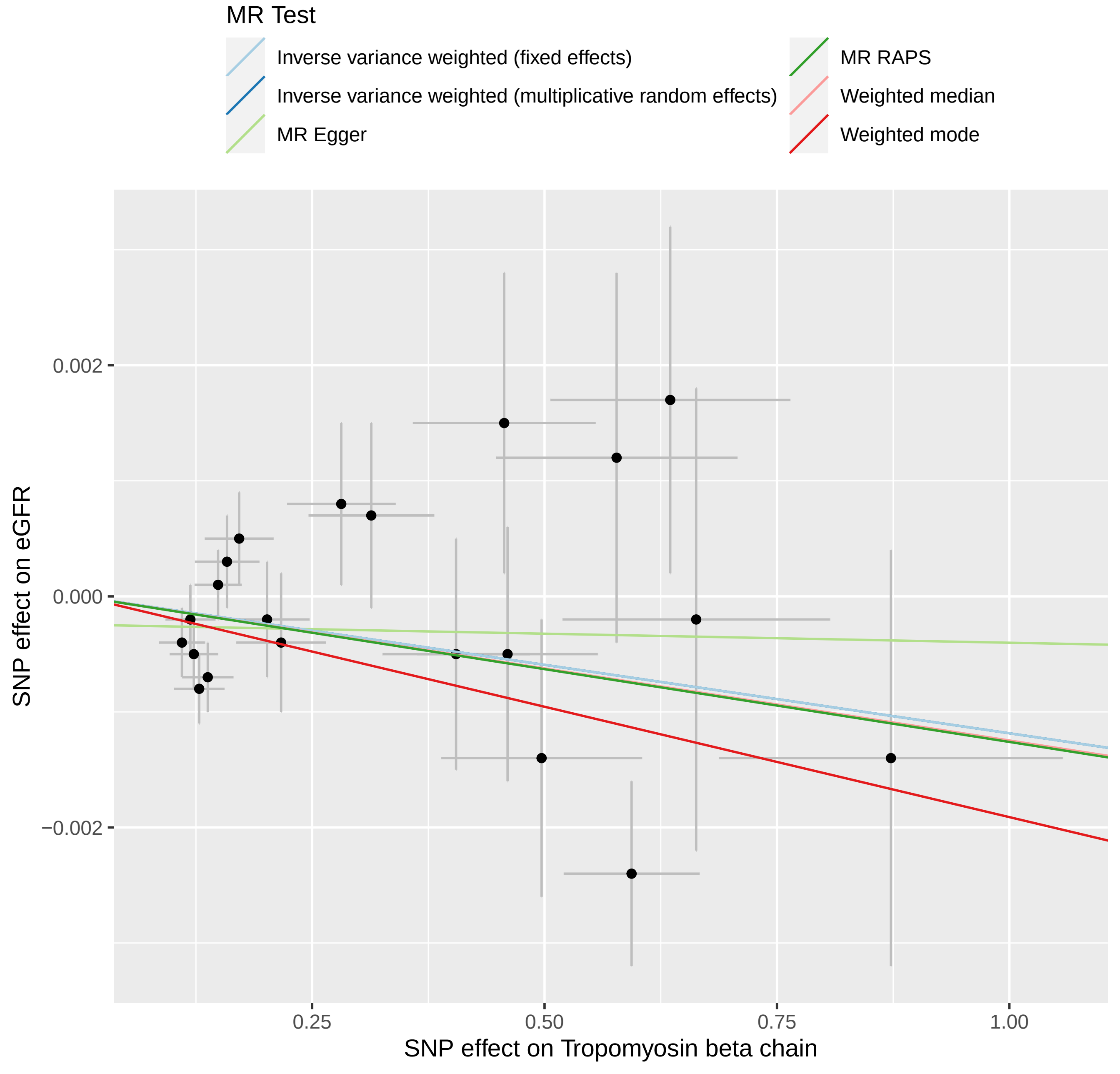

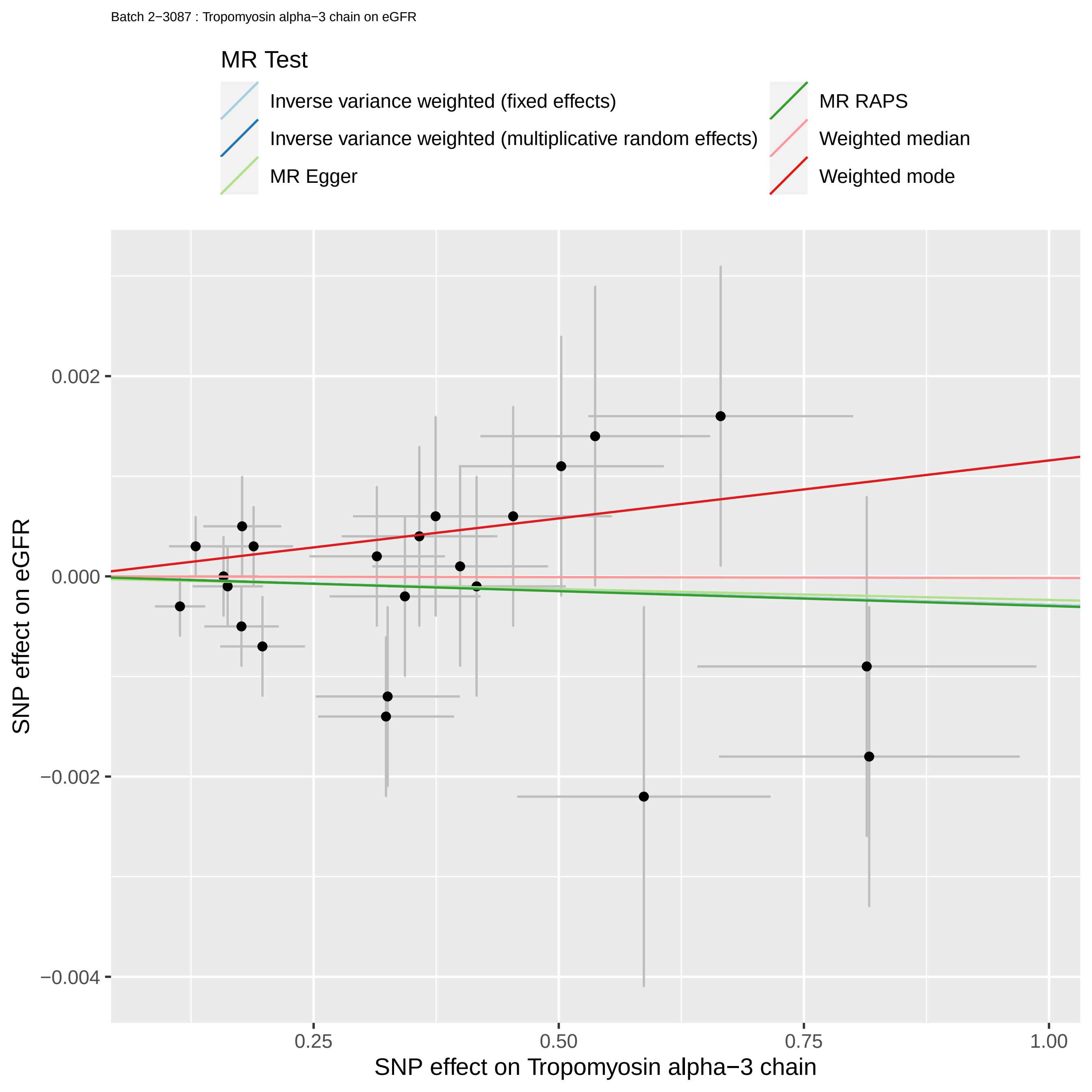
Batch 2−3087 : Tropomyosin alpha−3 chain on eGFR
MR Test
Inverse variance weighted (fixed effects)
Inverse variance weighted (multiplicative random effects)
MR Egger
MR RAPS
Weighted median
Weighted mode
0.002
0.000
−0.002
−0.004
SNP effect on eGFR
0.25
0.50
0.75
1.00
SNP effect on Tropomyosin alpha−3 chain

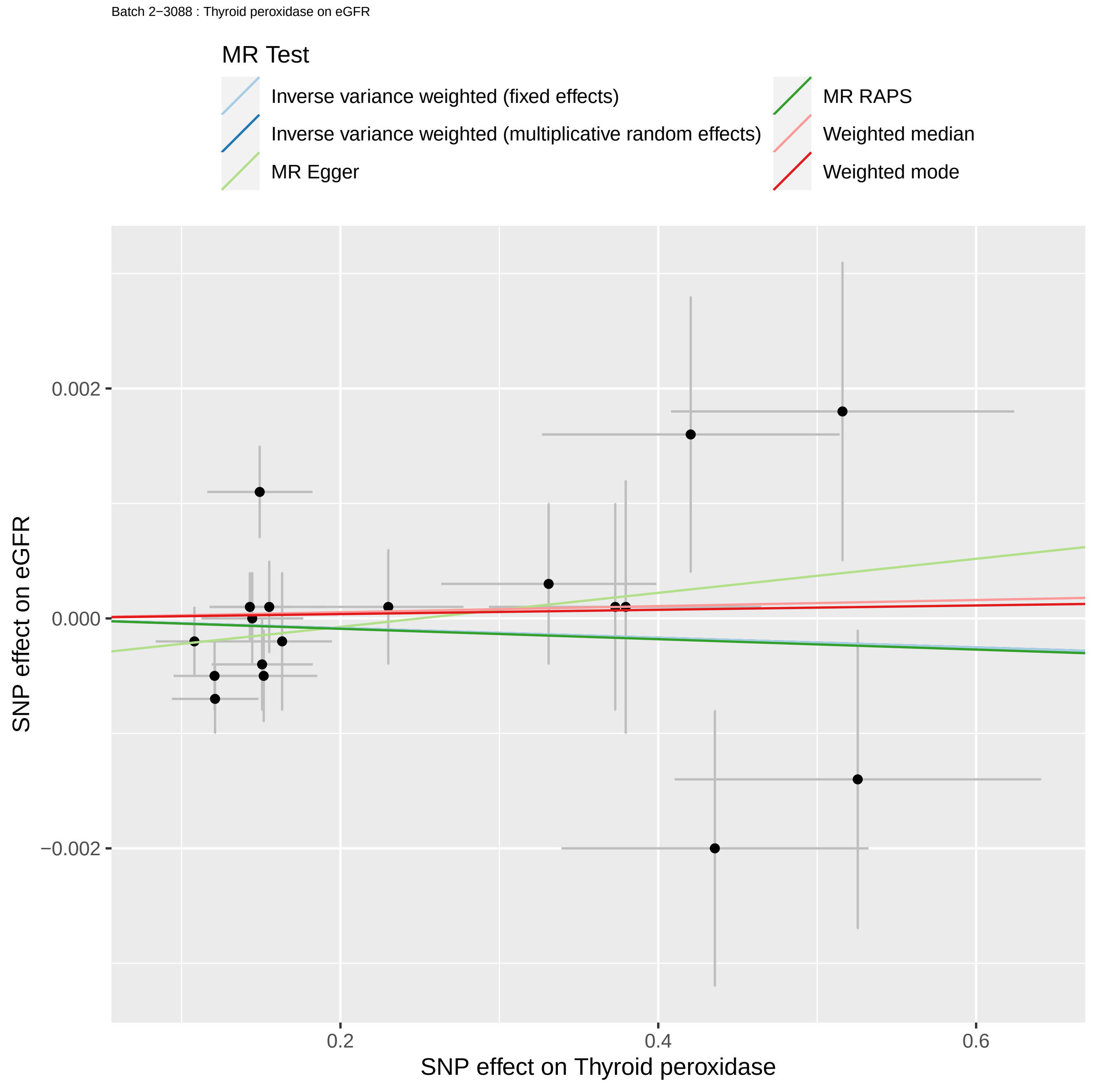

Batch 2−3088 : Thyroid peroxidase on eGFR
MR Test
Inverse variance weighted (fixed effects)
Inverse variance weighted (multiplicative random effects)
MR Egger
MR RAPS
Weighted median
Weighted mode
0.002
0.000
−0.002
0.2
0.4
0.6
SNP effect on eGFR
SNP effect on Thyroid peroxidase

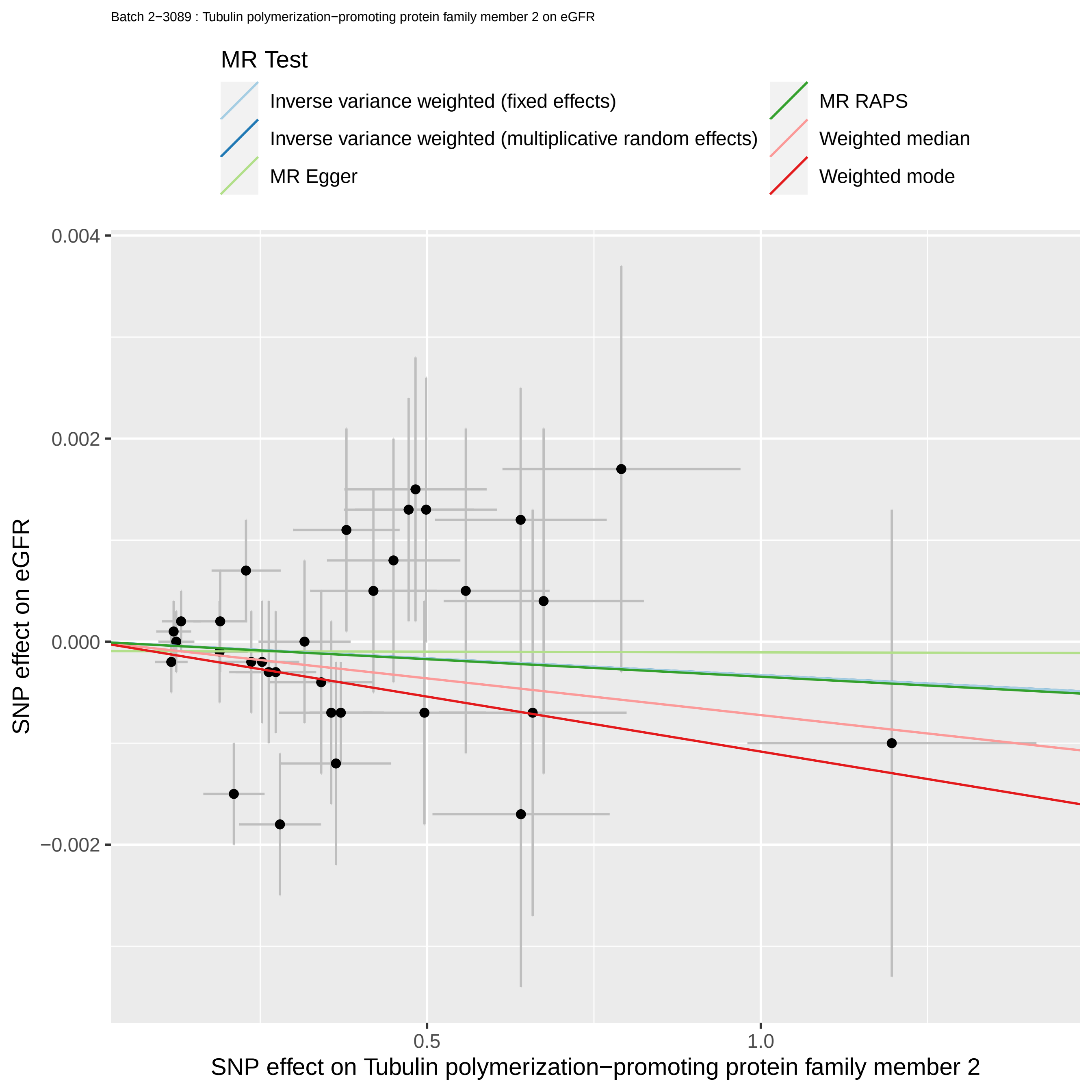

Batch 2−3089 : Tubulin polymerization−promoting protein family member 2 on eGFR
MR Test
Inverse variance weighted (fixed effects)
Inverse variance weighted (multiplicative random effects)
MR Egger
MR RAPS
Weighted median
Weighted mode
0.004
0.002
0.000
−0.002
0.5
1.0
SNP effect on eGFR
SNP effect on Tubulin polymerization−promoting protein family member 2

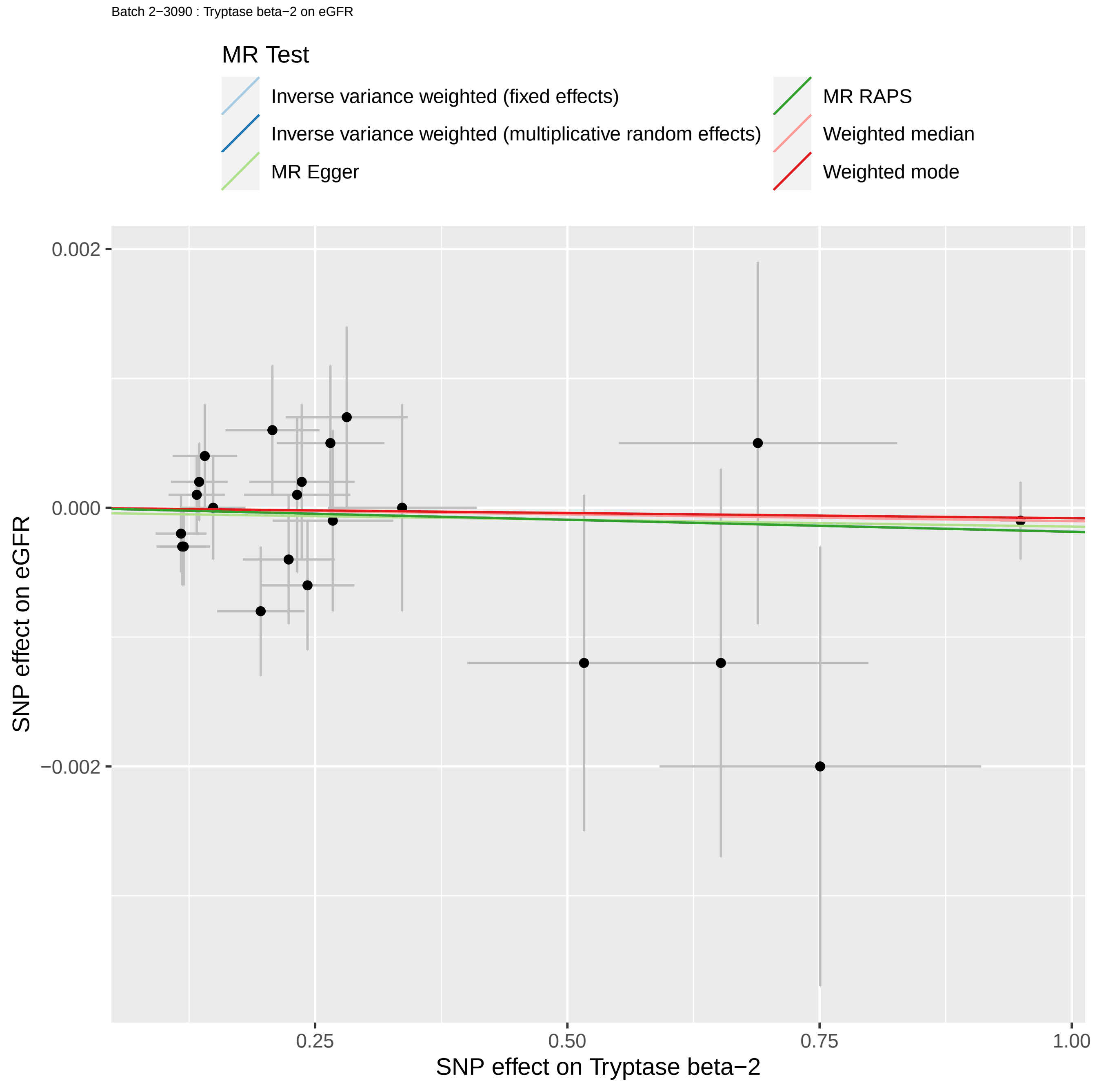
Batch 2−3090 : Tryptase beta−2 on eGFR
MR Test
Inverse variance weighted (fixed effects)
Inverse variance weighted (multiplicative random effects)
MR Egger
MR RAPS
Weighted median
Weighted mode
0.002
0.000
−0.002
SNP effect on eGFR
0.25
0.50
0.75
1.00
SNP effect on Tryptase beta−2

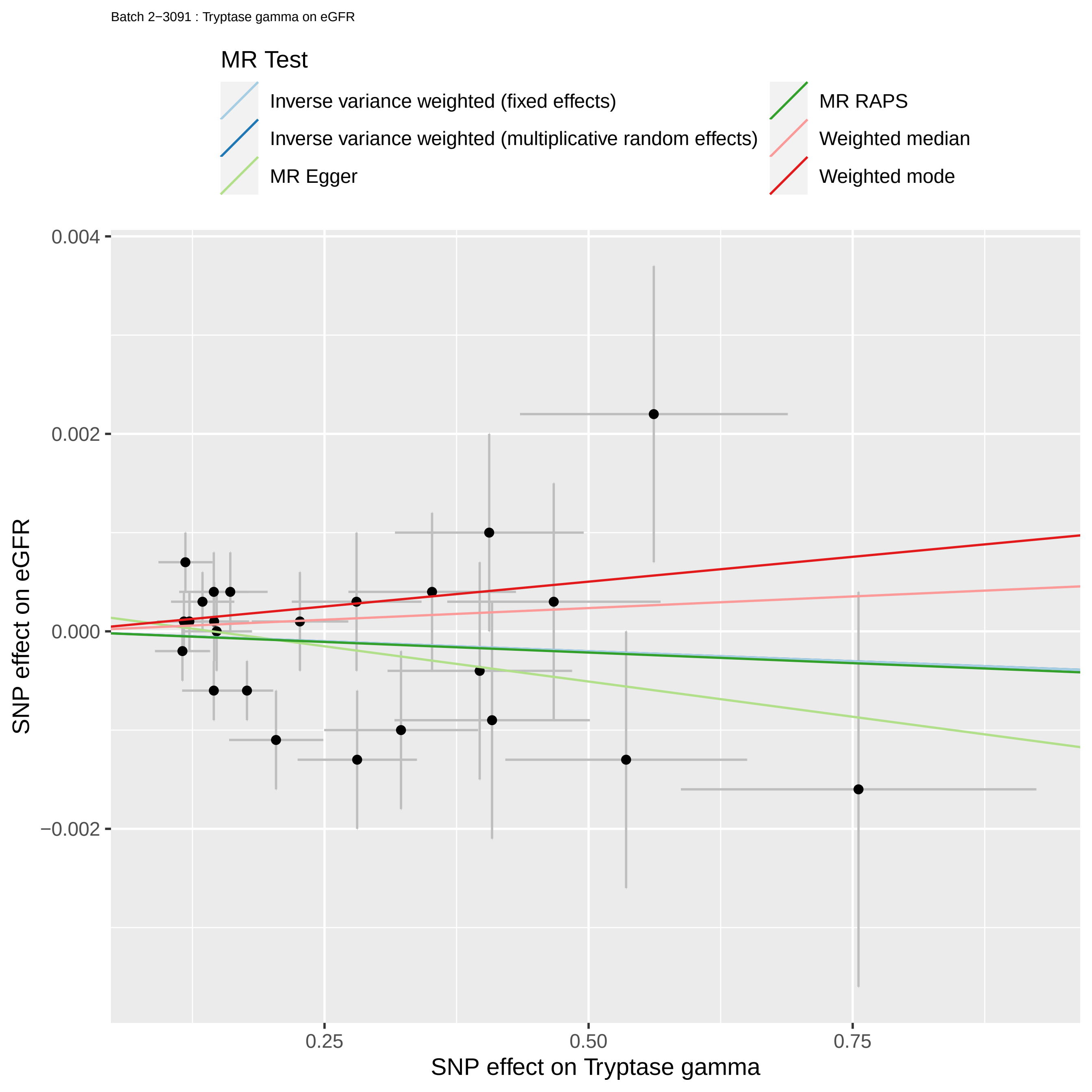

Batch 2−3091 : Tryptase gamma on eGFR
MR Test
Inverse variance weighted (fixed effects)
Inverse variance weighted (multiplicative random effects)
MR Egger
MR RAPS
Weighted median
Weighted mode
0.004
0.002
0.000
−0.002
SNP effect on eGFR
0.25
0.50
0.75
SNP effect on Tryptase gamma

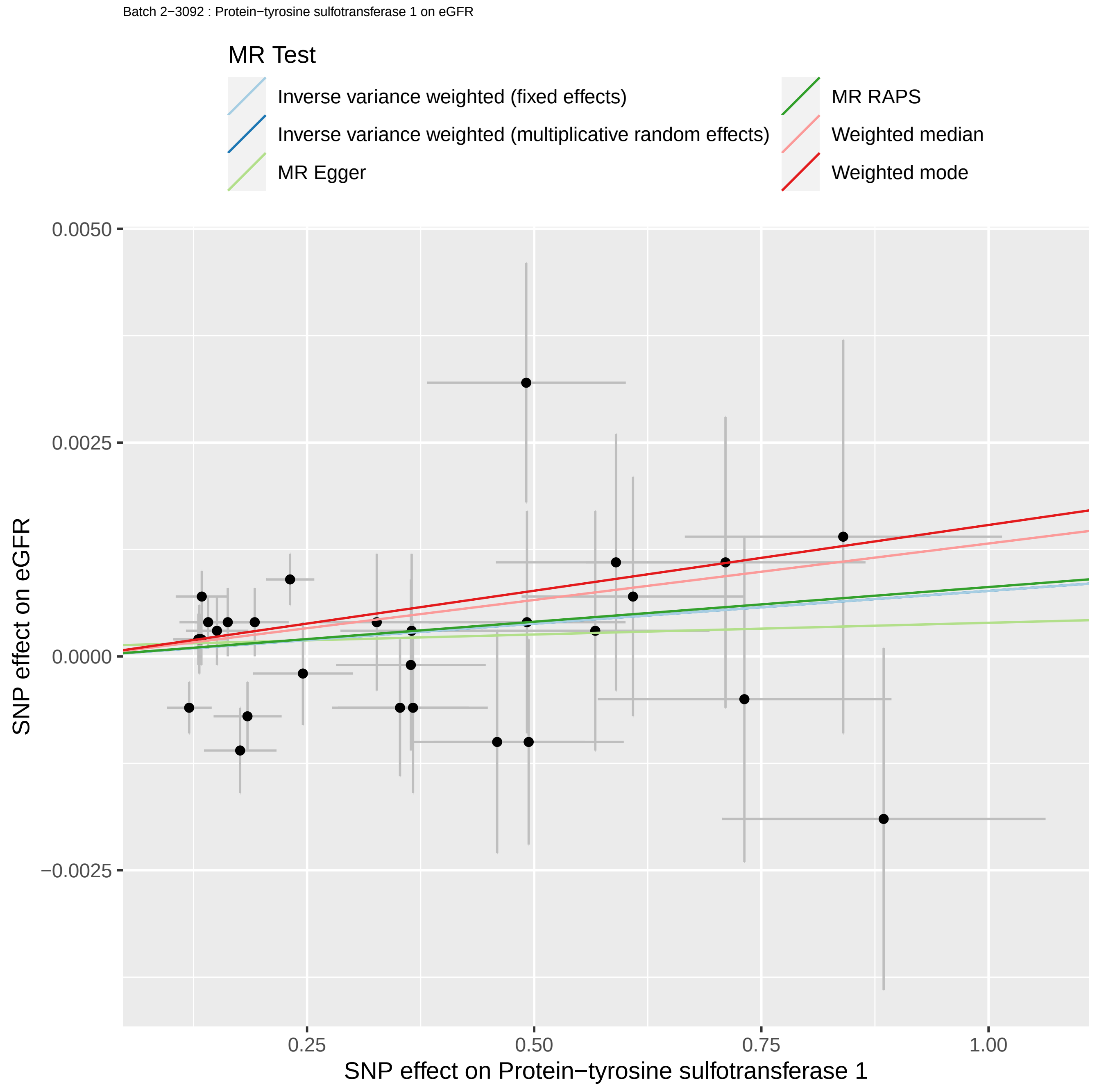
Batch 2−3092 : Protein−tyrosine sulfotransferase 1 on eGFR
MR Test
Inverse variance weighted (fixed effects)
Inverse variance weighted (multiplicative random effects)
MR Egger
MR RAPS
Weighted median
Weighted mode
0.0050
0.0025
0.0000
−0.0025
SNP effect on eGFR
0.25
0.50
0.75
1.00
SNP effect on Protein−tyrosine sulfotransferase 1

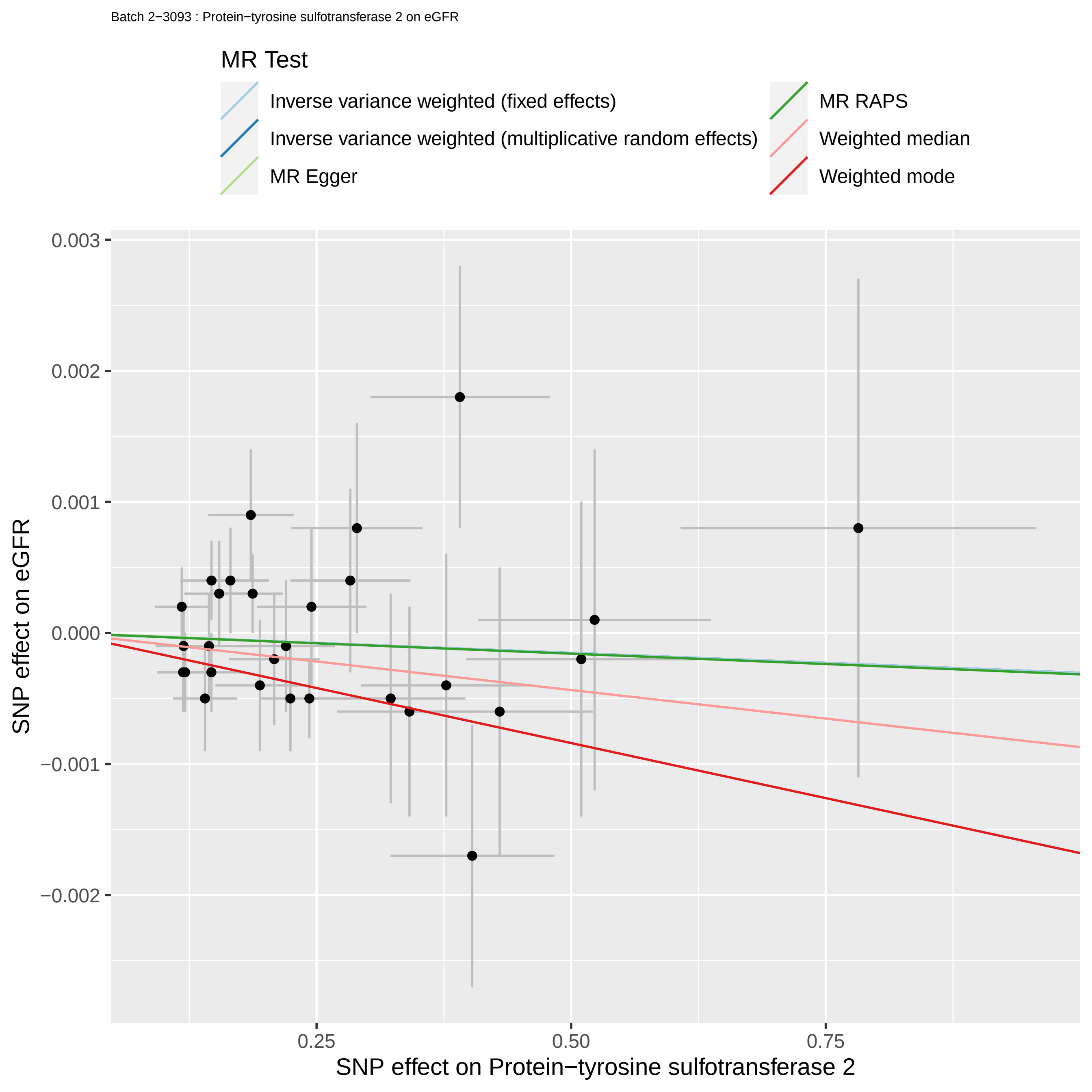

Batch 2−3093 : Protein−tyrosine sulfotransferase 2 on eGFR
MR Test
Inverse variance weighted (fixed effects)
Inverse variance weighted (multiplicative random effects)
MR Egger
MR RAPS
Weighted median
Weighted mode
0.003
0.002
0.001
0.000
−0.001
−0.002
SNP effect on eGFR
0.25
0.50
0.75
SNP effect on Protein−tyrosine sulfotransferase 2

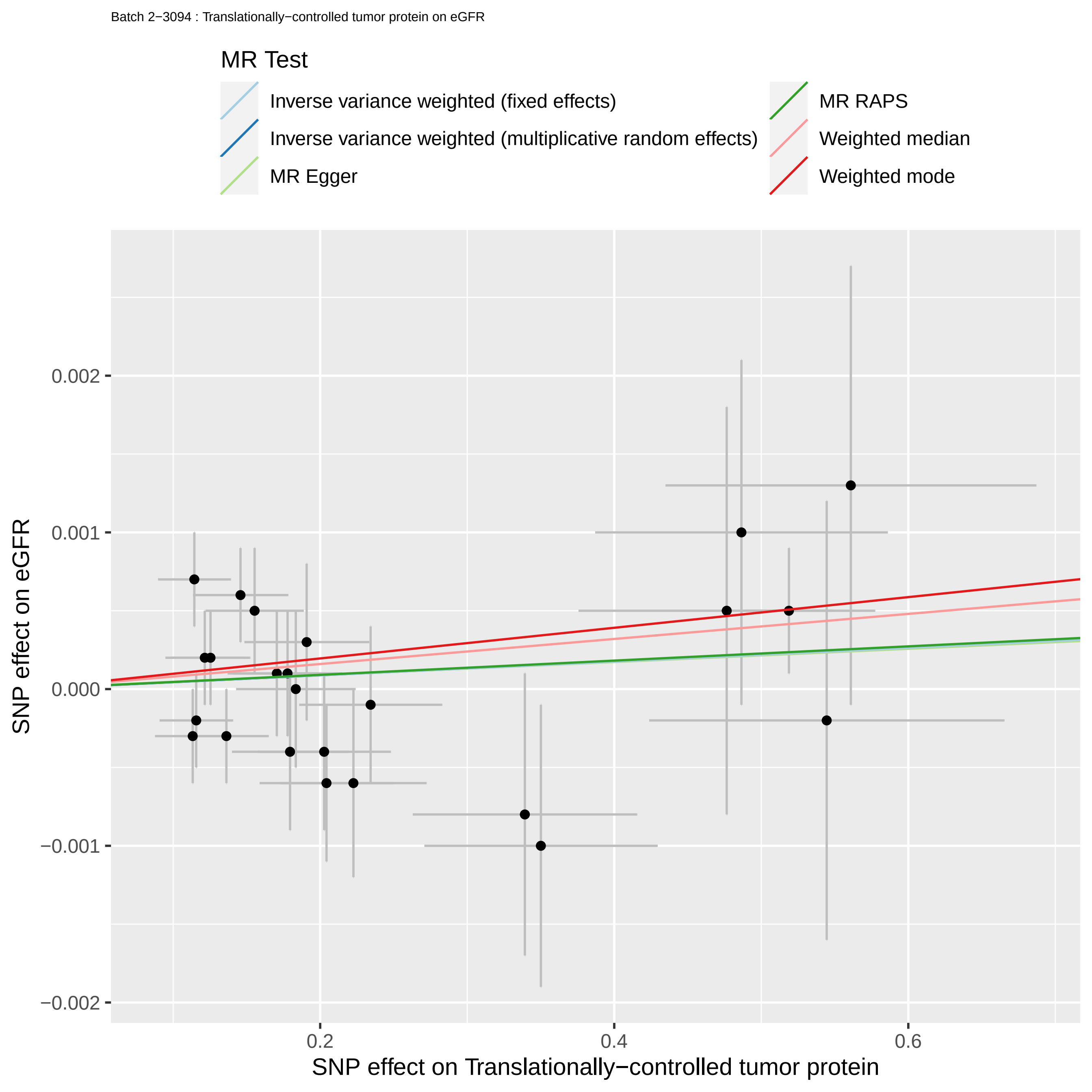

Batch 2−3094 : Translationally−controlled tumor protein on eGFR
MR Test
Inverse variance weighted (fixed effects)
Inverse variance weighted (multiplicative random effects)
MR Egger
MR RAPS
Weighted median
Weighted mode
0.002
0.001
0.000
−0.001
−0.002
0.2
0.4
0.6
SNP effect on eGFR
SNP effect on Translationally−controlled tumor protein

Batch 2−3095 : Transformer−2 protein homolog beta on eGFR

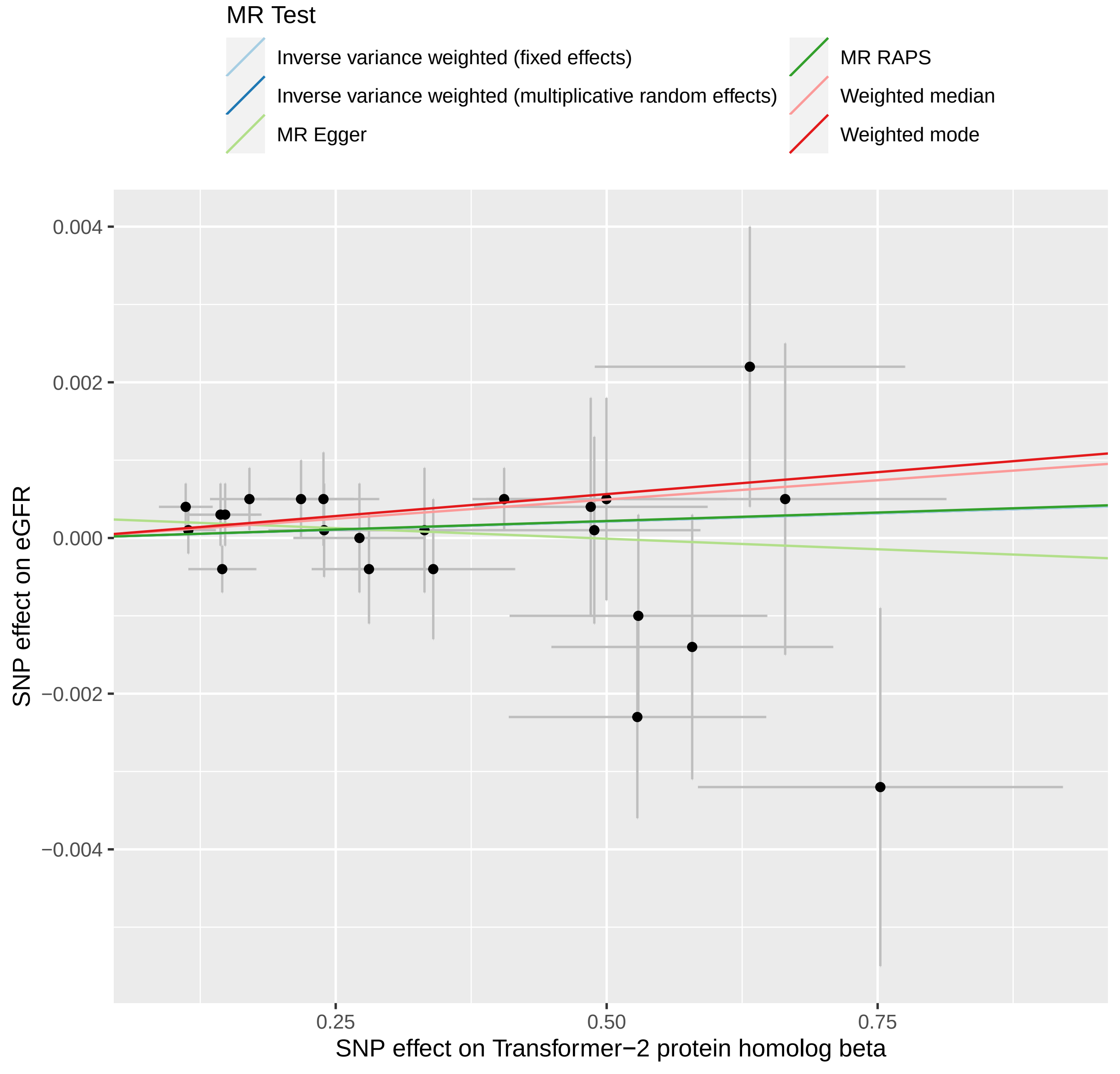

Batch 2−3096 : TNF receptor−associated factor 4 on eGFR

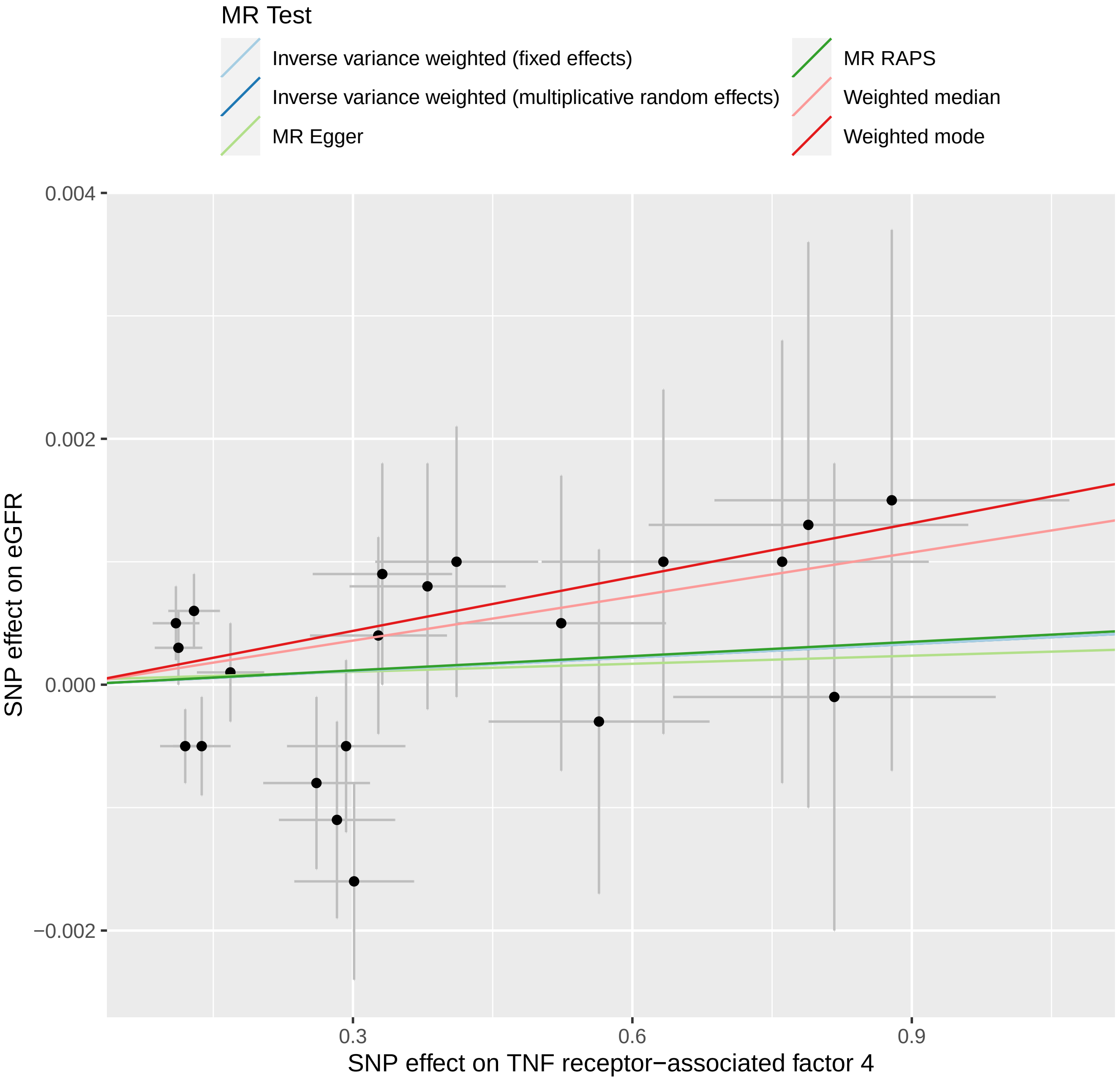

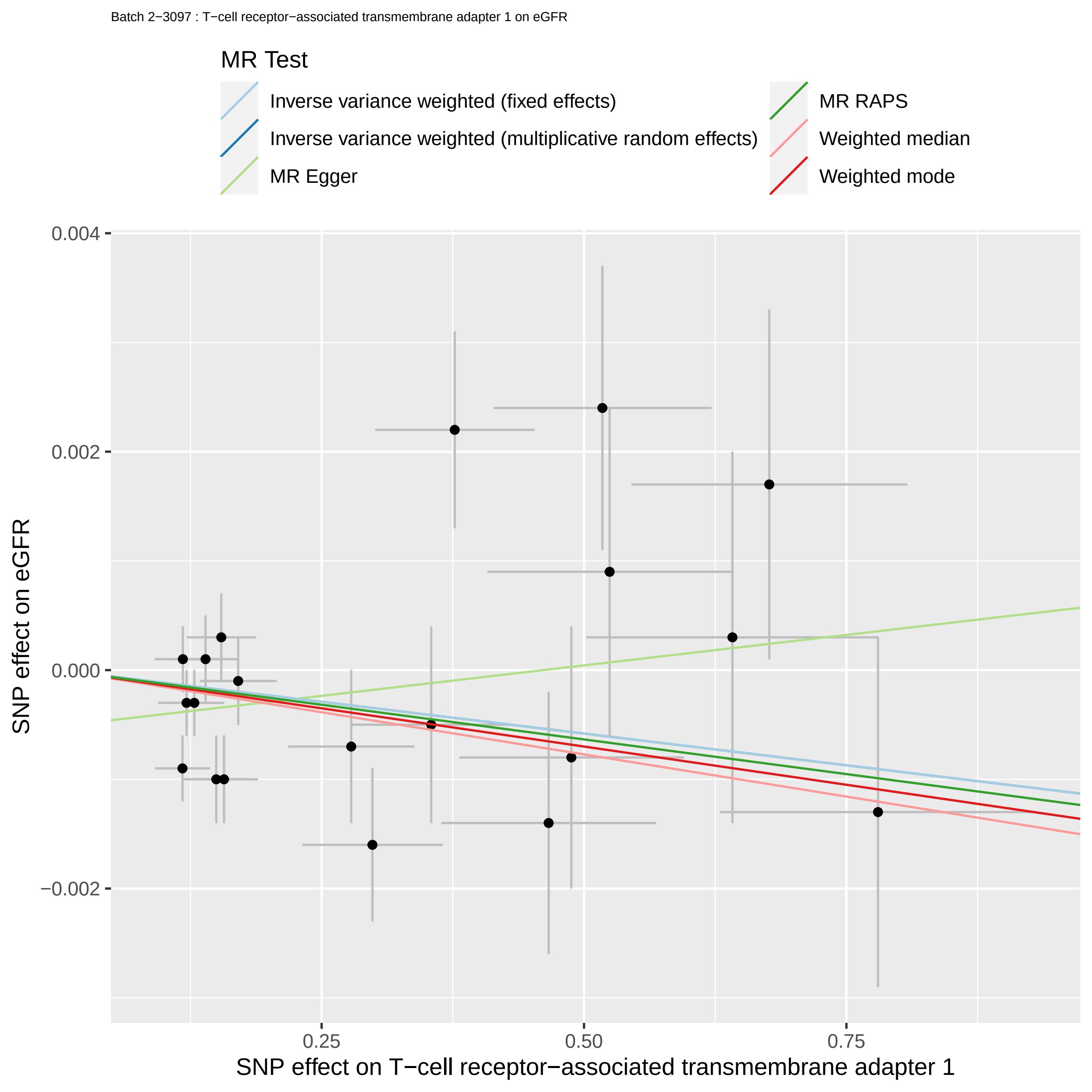
Batch 2−3097 : T−cell receptor−associated transmembrane adapter 1 on eGFR
MR Test
Inverse variance weighted (fixed effects)
Inverse variance weighted (multiplicative random effects)
MR Egger
MR RAPS
Weighted median
Weighted mode
0.004
0.002
0.000
−0.002
0.25
0.50
0.75
SNP effect on eGFR
SNP effect on T−cell receptor−associated transmembrane adapter 1

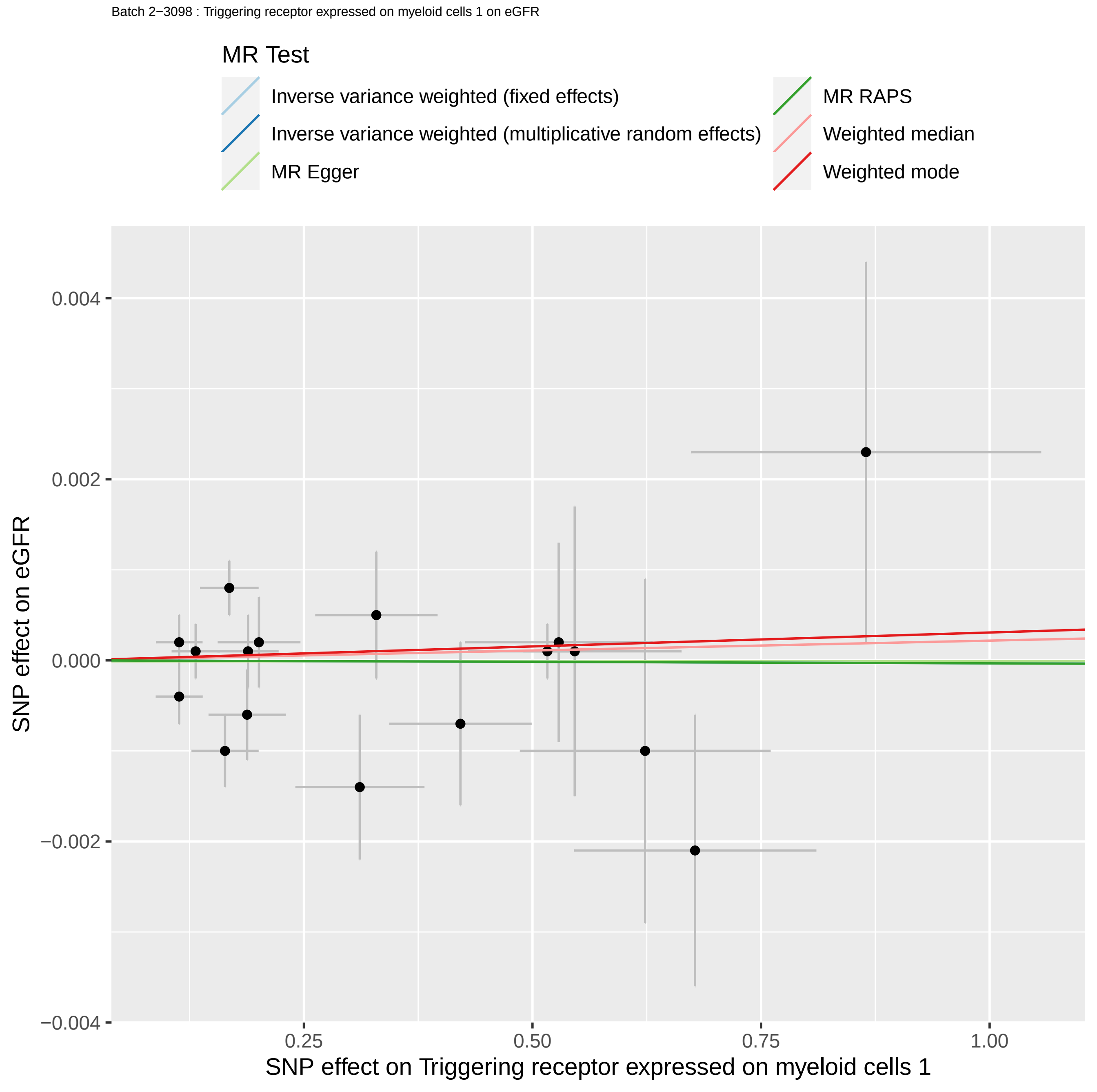

Batch 2−3098 : Triggering receptor expressed on myeloid cells 1 on eGFR
MR Test
Inverse variance weighted (fixed effects)
Inverse variance weighted (multiplicative random effects)
MR Egger
MR RAPS
Weighted median
Weighted mode
0.004
0.002
0.000
−0.002
−0.004
0.25
0.50
0.75
1.00
SNP effect on eGFR
SNP effect on Triggering receptor expressed on myeloid cells 1

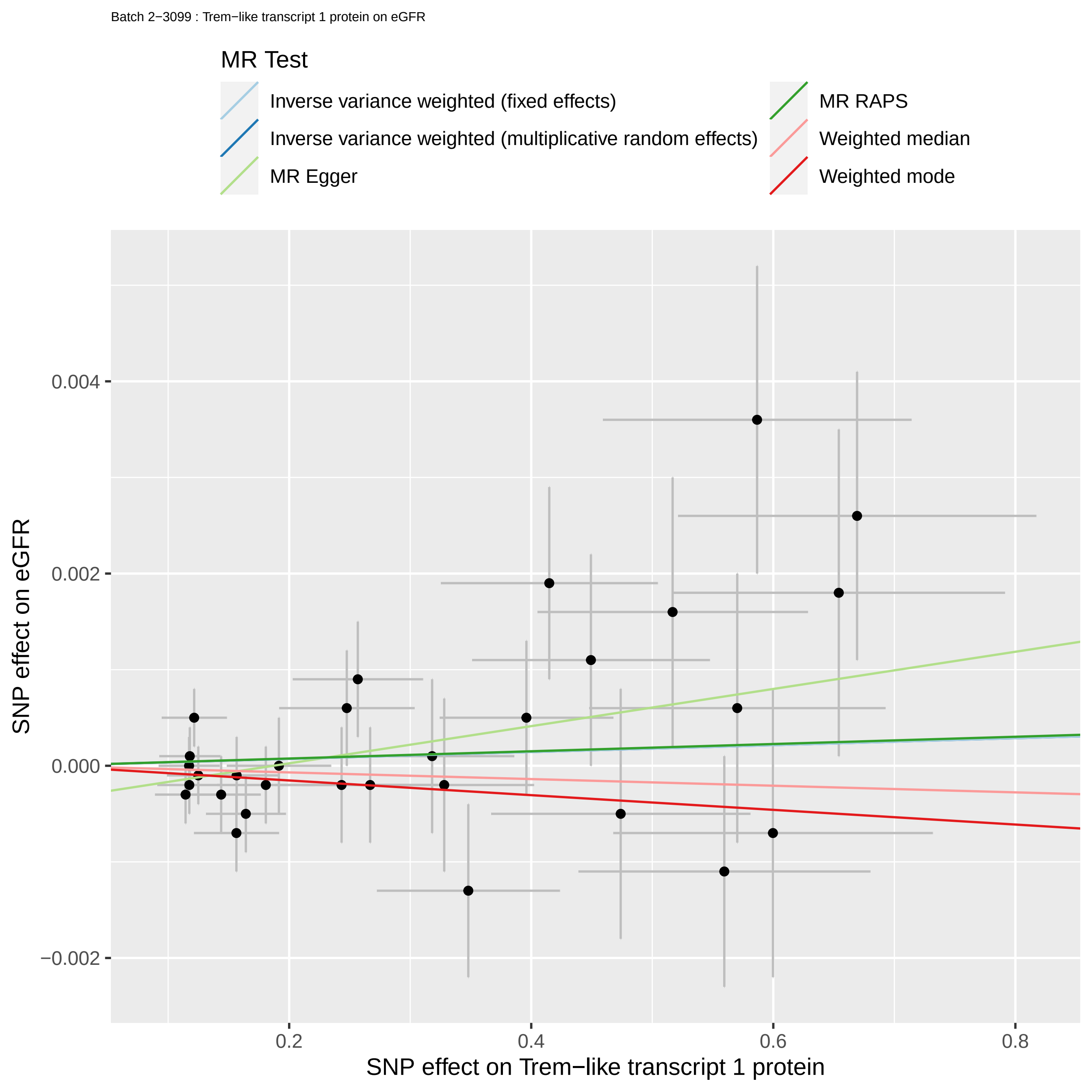

Batch 2−3099 : Trem−like transcript 1 protein on eGFR
MR Test
Inverse variance weighted (fixed effects)
Inverse variance weighted (multiplicative random effects)
MR Egger
MR RAPS
Weighted median
Weighted mode
0.004
0.002
0.000
−0.002
0.2
0.4
0.6
0.8
SNP effect on eGFR
SNP effect on Trem−like transcript 1 protein

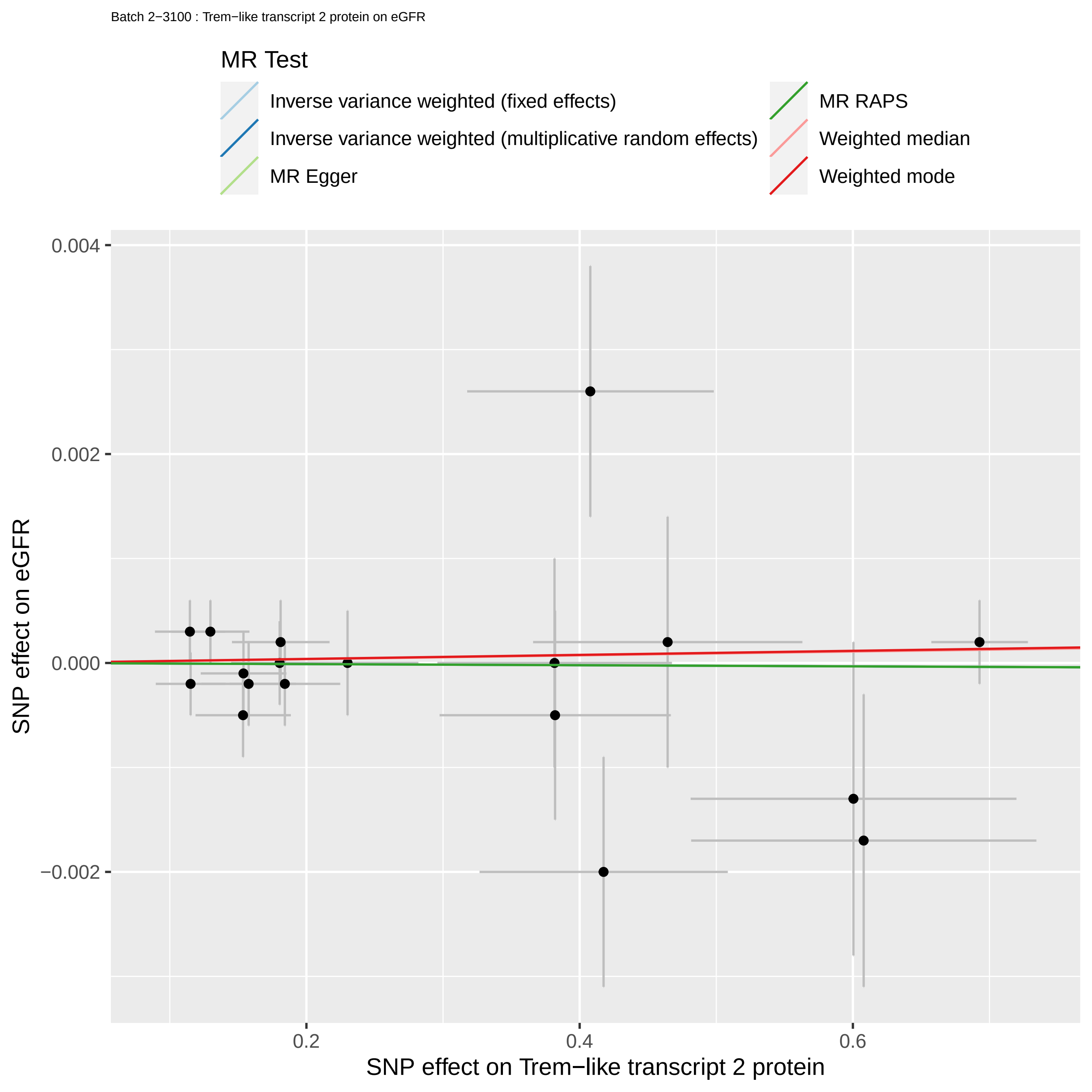

Batch 2−3100 : Trem−like transcript 2 protein on eGFR
MR Test
Inverse variance weighted (fixed effects)
Inverse variance weighted (multiplicative random effects)
MR Egger
MR RAPS
Weighted median
Weighted mode
0.004
0.002
0.000
−0.002
0.2
0.4
0.6
SNP effect on eGFR
SNP effect on Trem−like transcript 2 protein

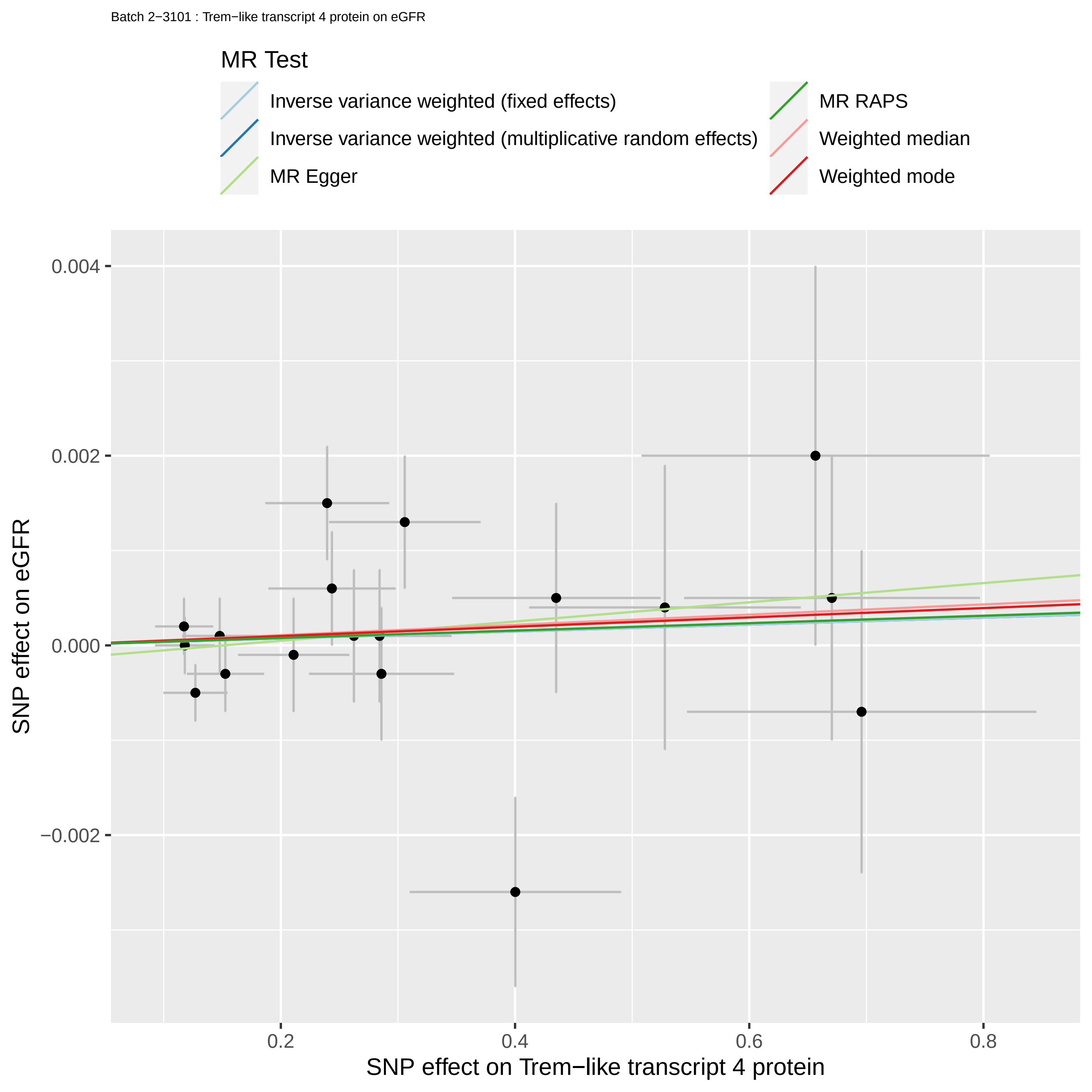

Batch 2−3101 : Trem−like transcript 4 protein on eGFR
MR Test
Inverse variance weighted (fixed effects)
Inverse variance weighted (multiplicative random effects)
MR Egger
MR RAPS
Weighted median
Weighted mode
0.004
0.002
0.000
−0.002
0.2
0.4
0.6
0.8
SNP effect on eGFR
SNP effect on Trem−like transcript 4 protein

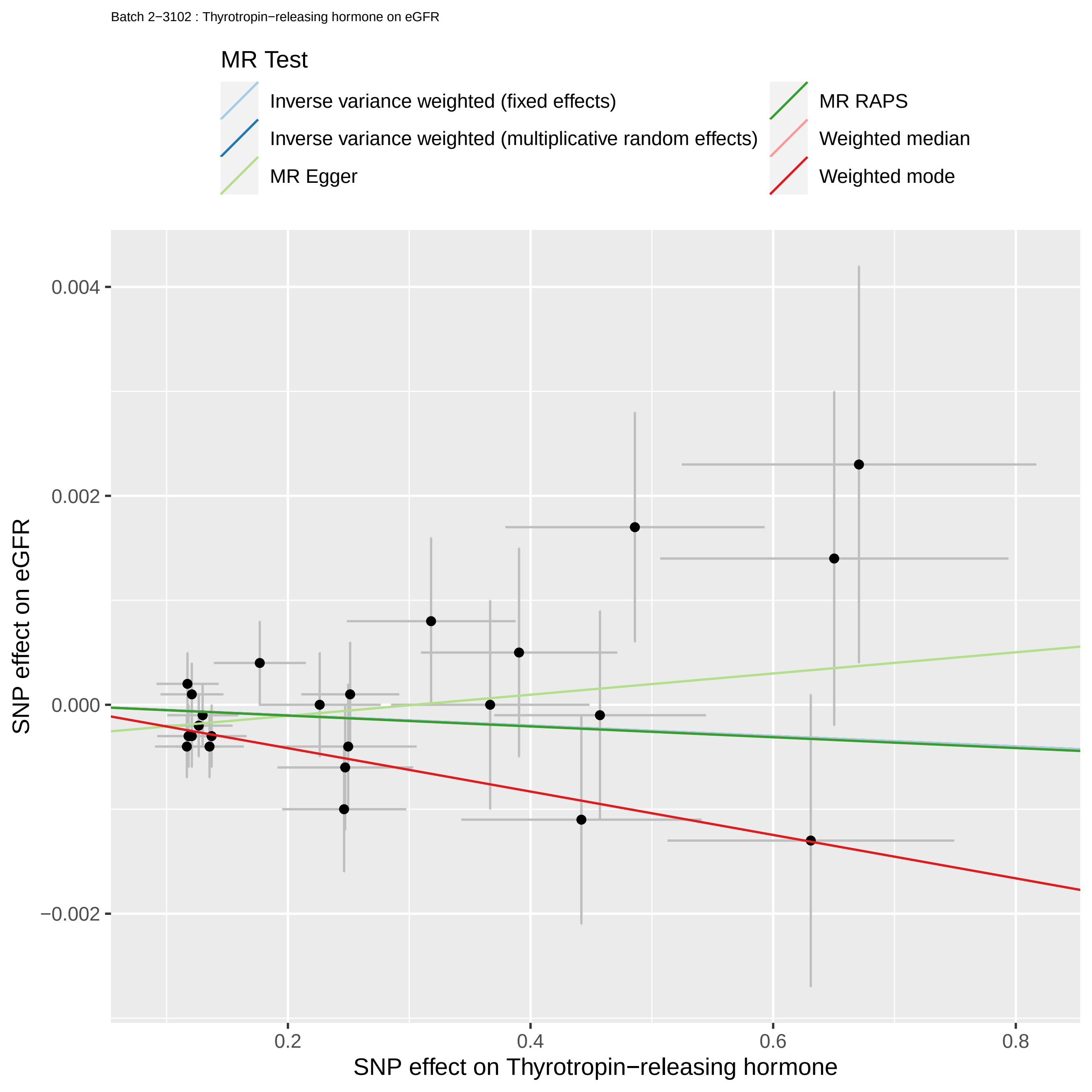

Batch 2−3102 : Thyrotropin−releasing hormone on eGFR
MR Test
Inverse variance weighted (fixed effects)
Inverse variance weighted (multiplicative random effects)
MR Egger
MR RAPS
Weighted median
Weighted mode
0.004
0.002
0.000
−0.002
0.2
0.4
0.6
0.8
SNP effect on eGFR
SNP effect on Thyrotropin−releasing hormone

Batch 2−3103 : Tumor susceptibility gene 101 protein on eGFR

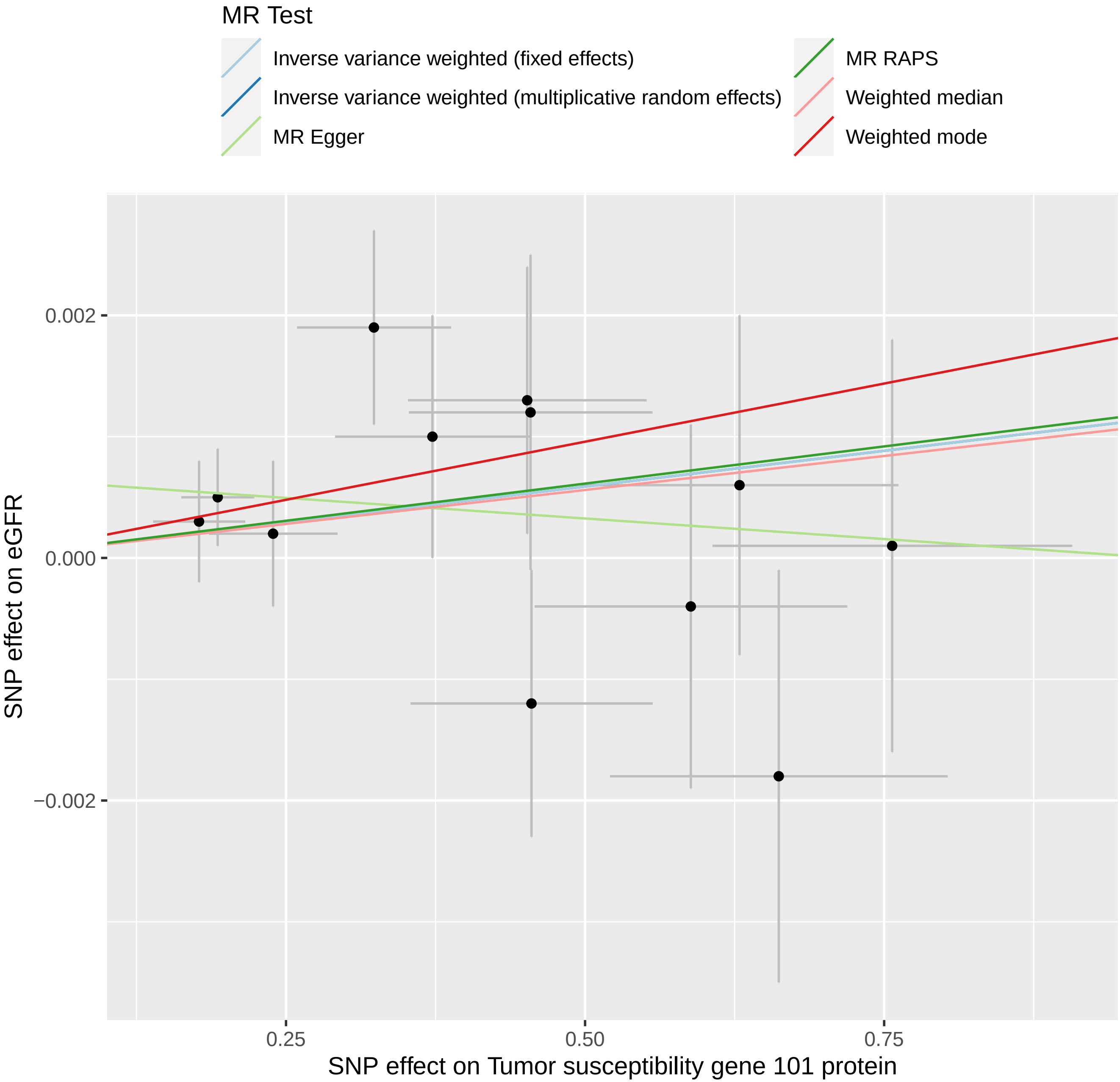

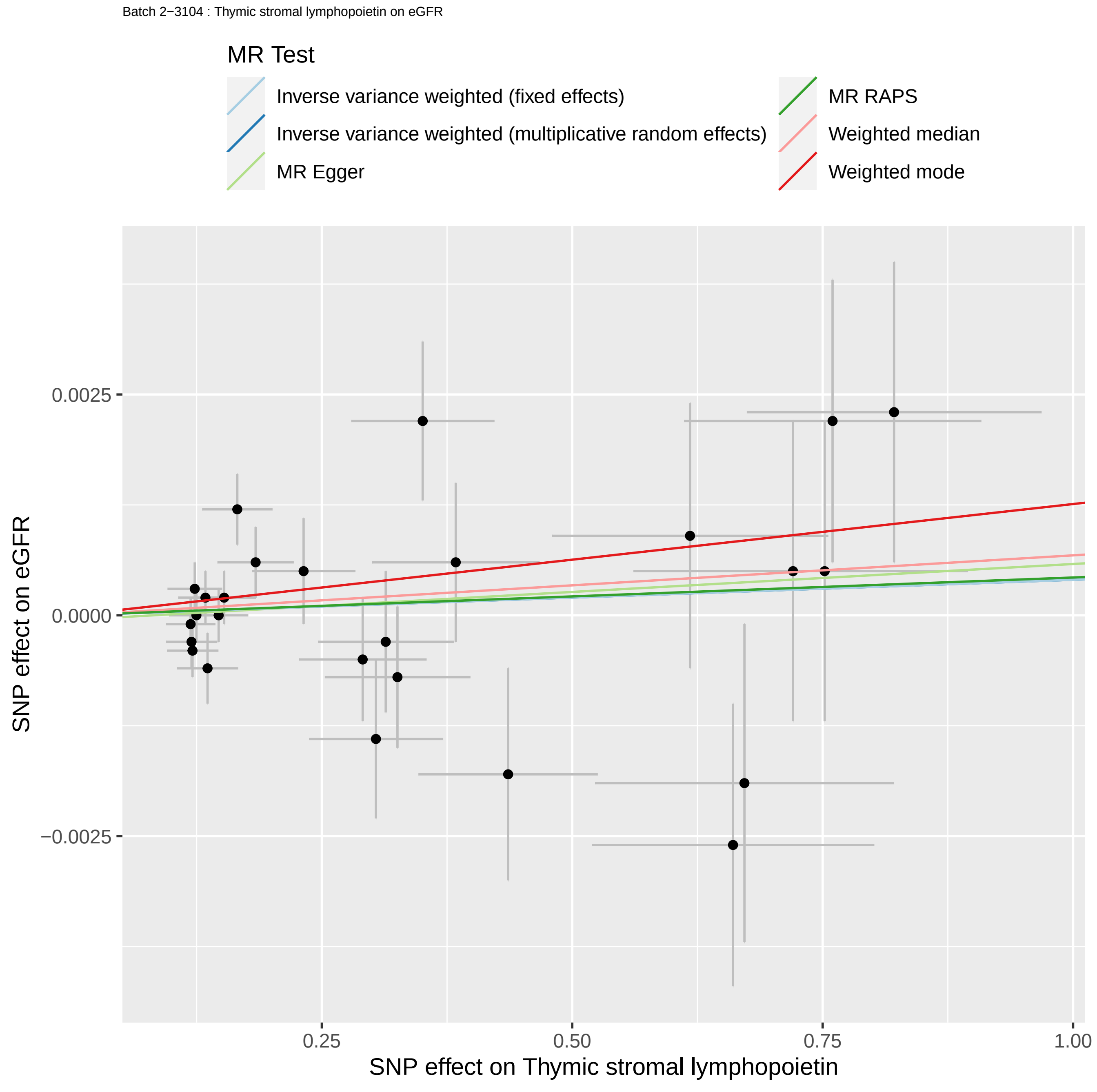

Batch 2−3104 : Thymic stromal lymphopoietin on eGFR
MR Test
Inverse variance weighted (fixed effects)
Inverse variance weighted (multiplicative random effects)
MR Egger
MR RAPS
Weighted median
Weighted mode
0.0025
0.0000
−0.0025
0.25
0.50
0.75
1.00
SNP effect on eGFR
SNP effect on Thymic stromal lymphopoietin

Batch 2−3105 : Translin on eGFR

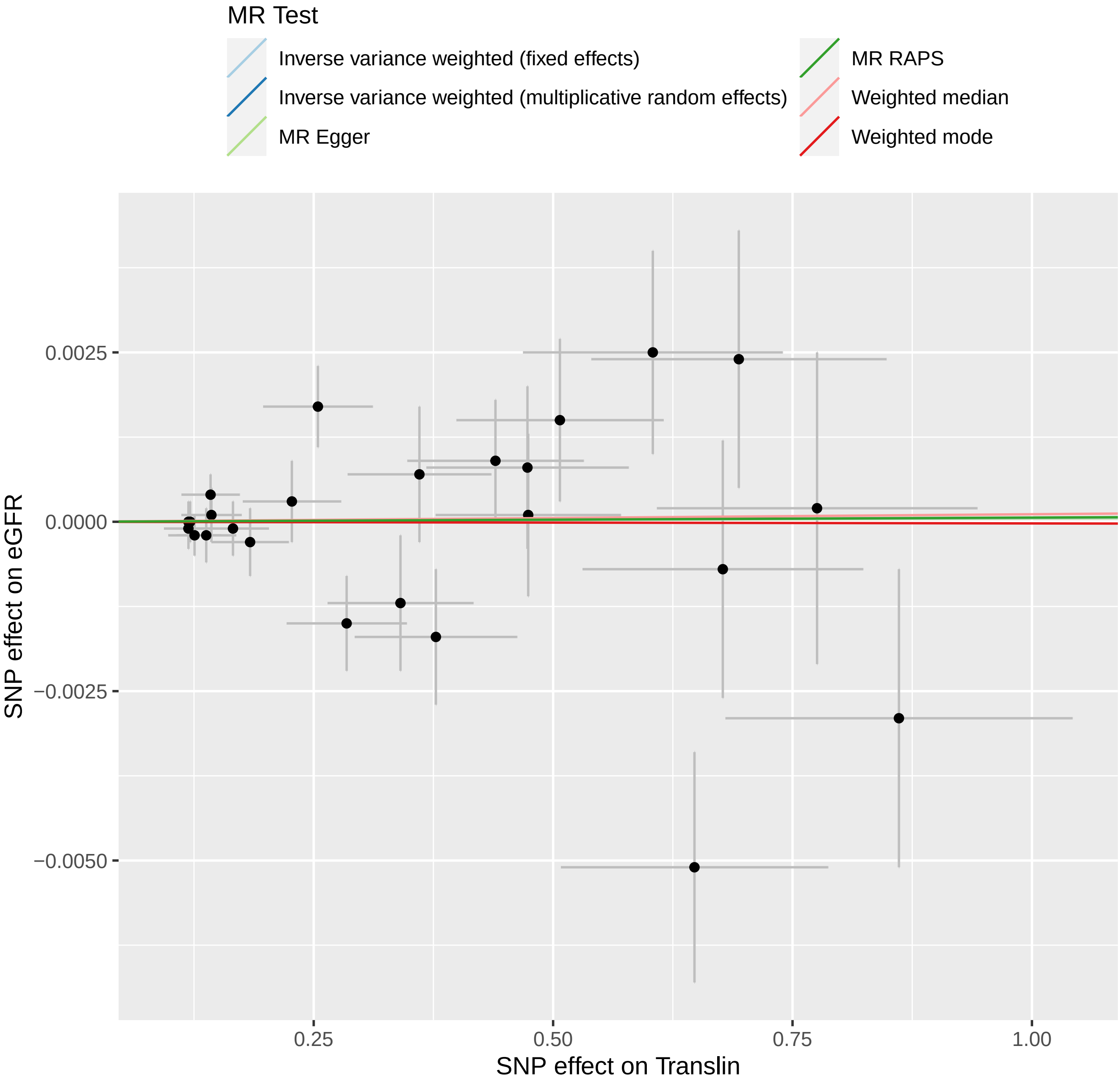

Batch 2−3106 : t−SNARE domain−containing protein 1 on eGFR

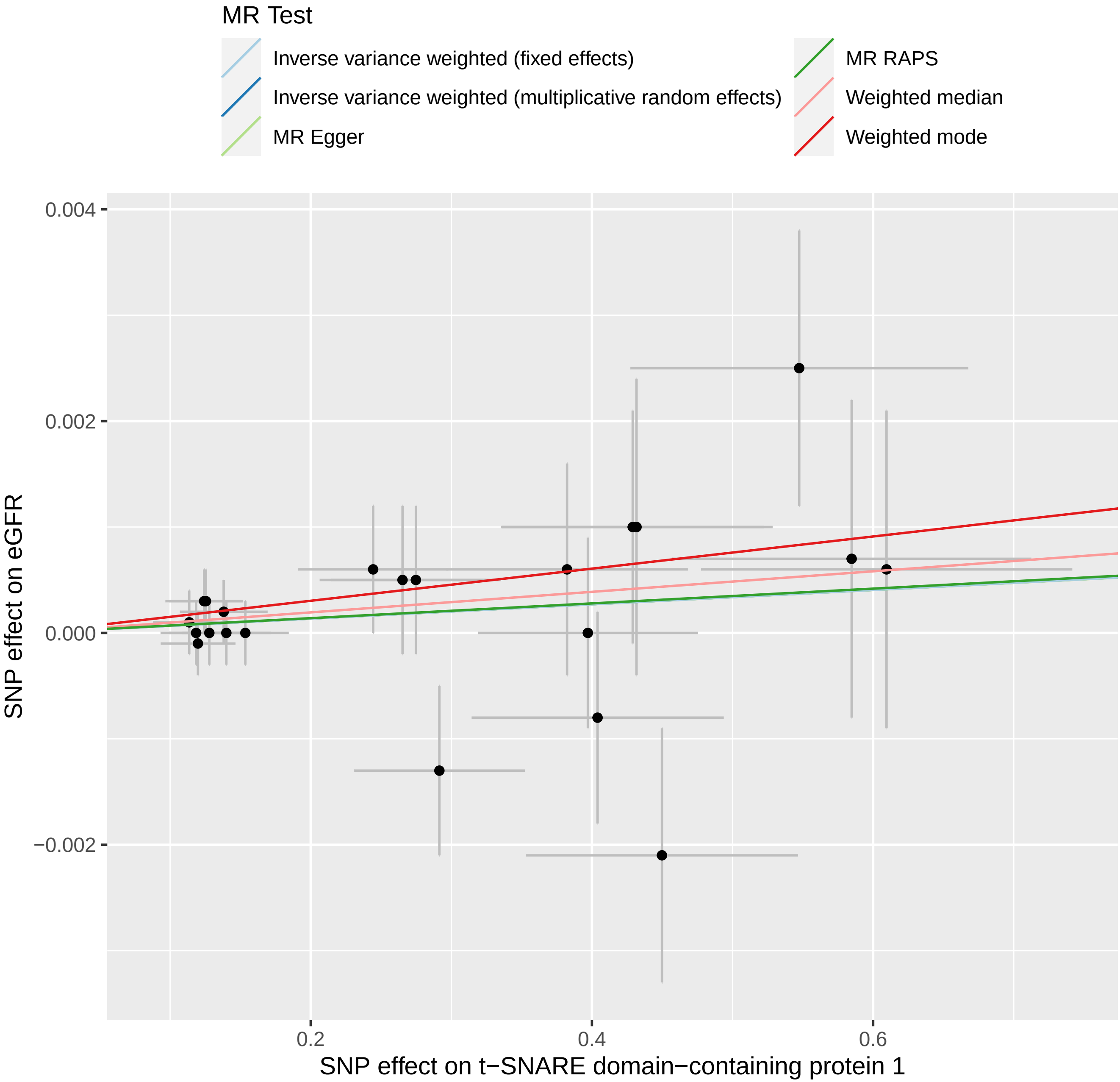

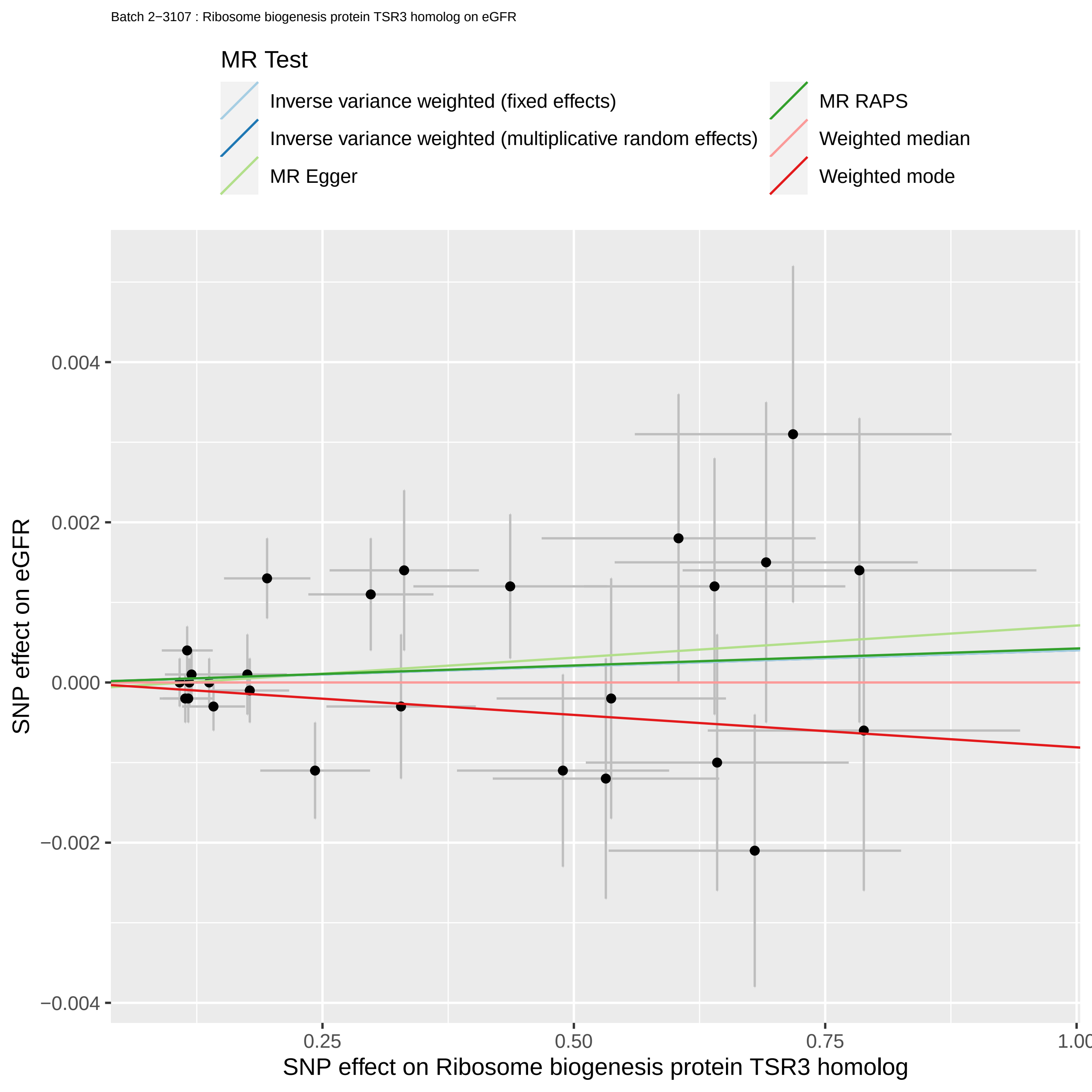

Batch 2−3107 : Ribosome biogenesis protein TSR3 homolog on eGFR
MR Test
Inverse variance weighted (fixed effects)
Inverse variance weighted (multiplicative random effects)
MR Egger
MR RAPS
Weighted median
Weighted mode
0.004
0.002
0.000
−0.002
−0.004
0.25
0.50
0.75
1.00
SNP effect on eGFR
SNP effect on Ribosome biogenesis protein TSR3 homolog

Batch 2−3108 : Testis−specific serine/threonine−protein kinase 2 on eGFR

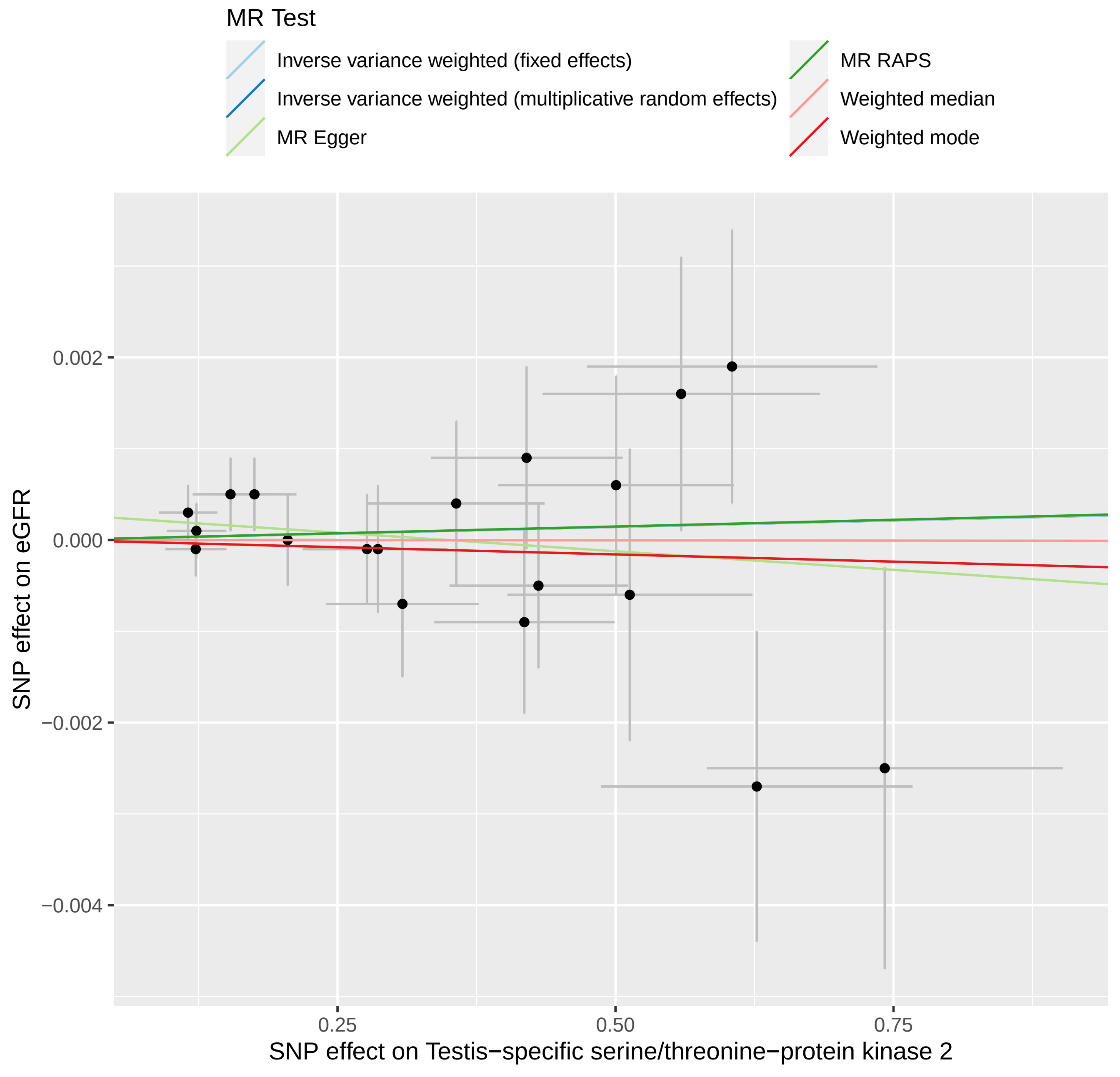

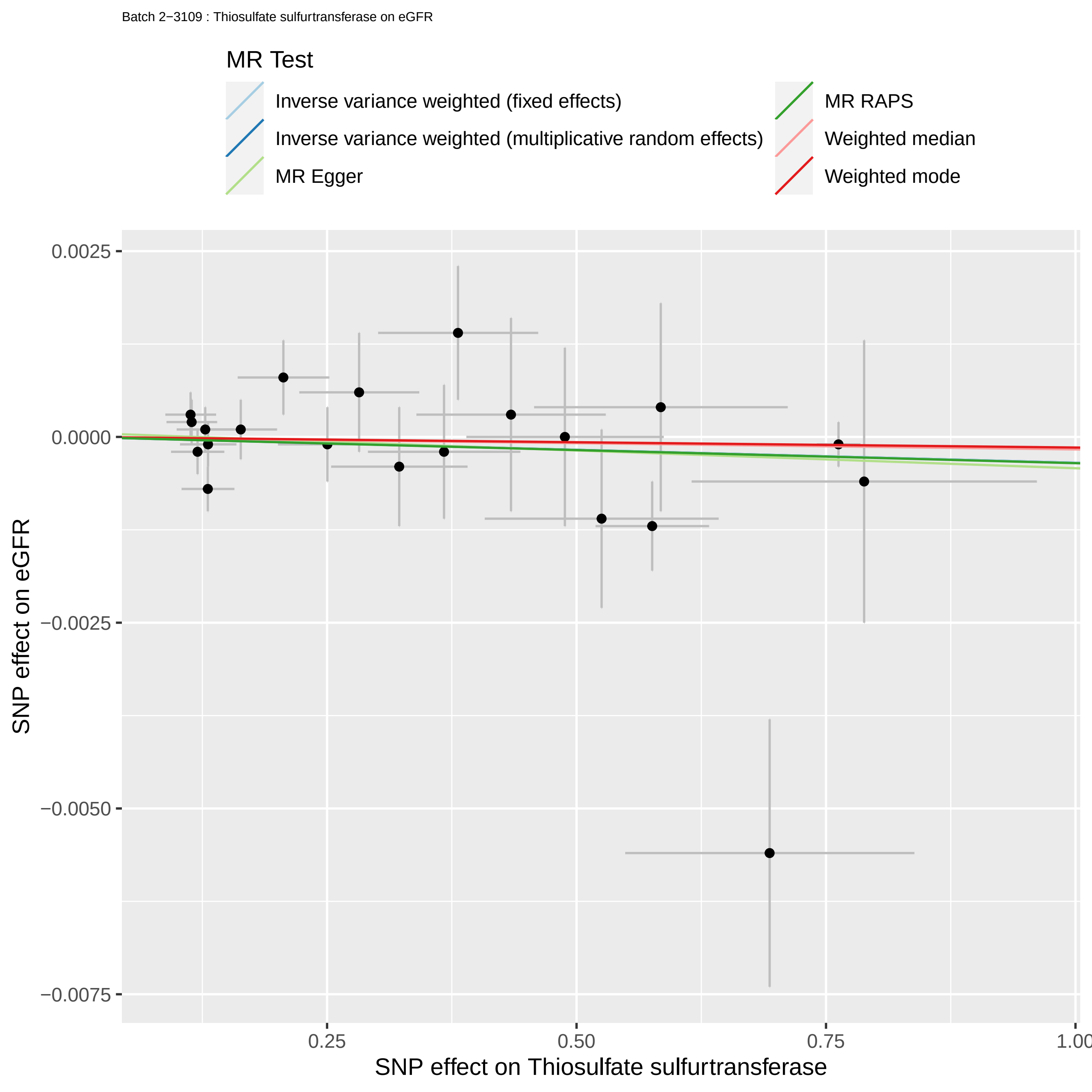

Batch 2−3109 : Thiosulfate sulfurtransferase on eGFR
MR Test
Inverse variance weighted (fixed effects)
Inverse variance weighted (multiplicative random effects)
MR Egger
MR RAPS
Weighted median
Weighted mode
0.0025
0.0000
−0.0025
−0.0050
−0.0075
SNP effect on eGFR
0.25
0.50
0.75
1.00
SNP effect on Thiosulfate sulfurtransferase

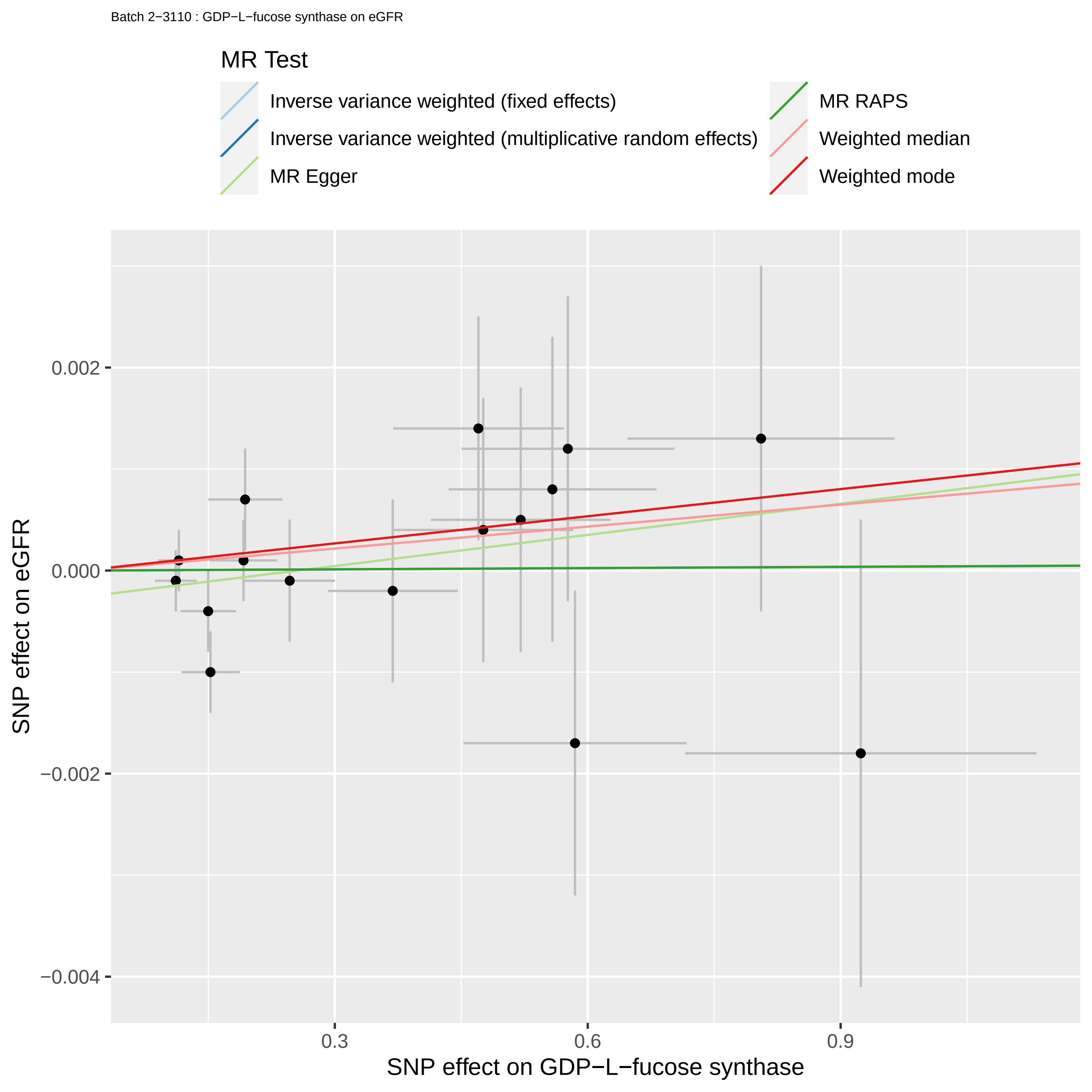

Batch 2−3110 : GDP−L−fucose synthase on eGFR
MR Test
Inverse variance weighted (fixed effects)
Inverse variance weighted (multiplicative random effects)
MR Egger
MR RAPS
Weighted median
Weighted mode
0.002
0.000
−0.002
−0.004
SNP effect on eGFR
0.3
0.6
0.9
SNP effect on GDP−L−fucose synthase

Batch 2−3111 : Tetratricopeptide repeat protein 17 on eGFR

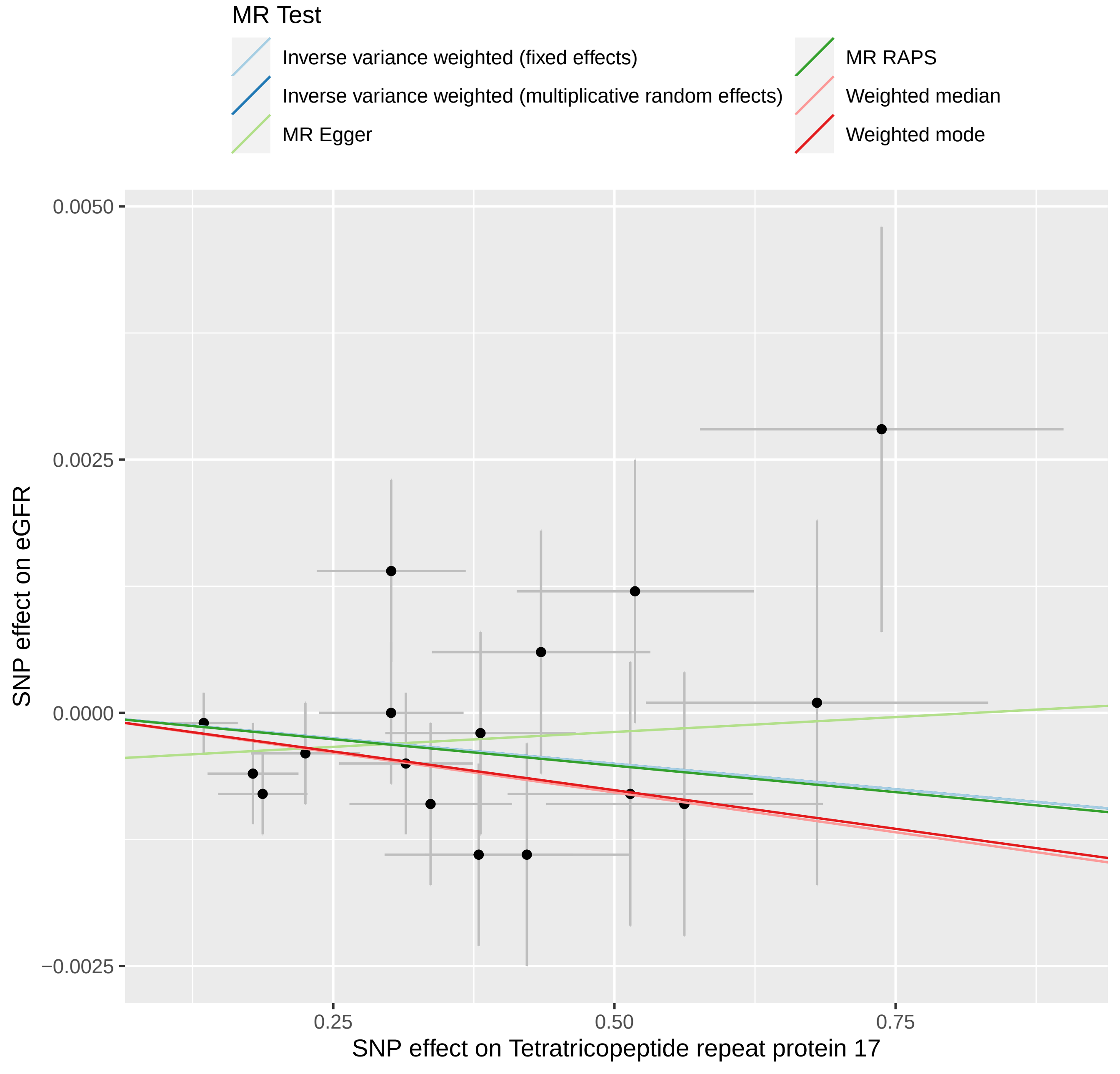

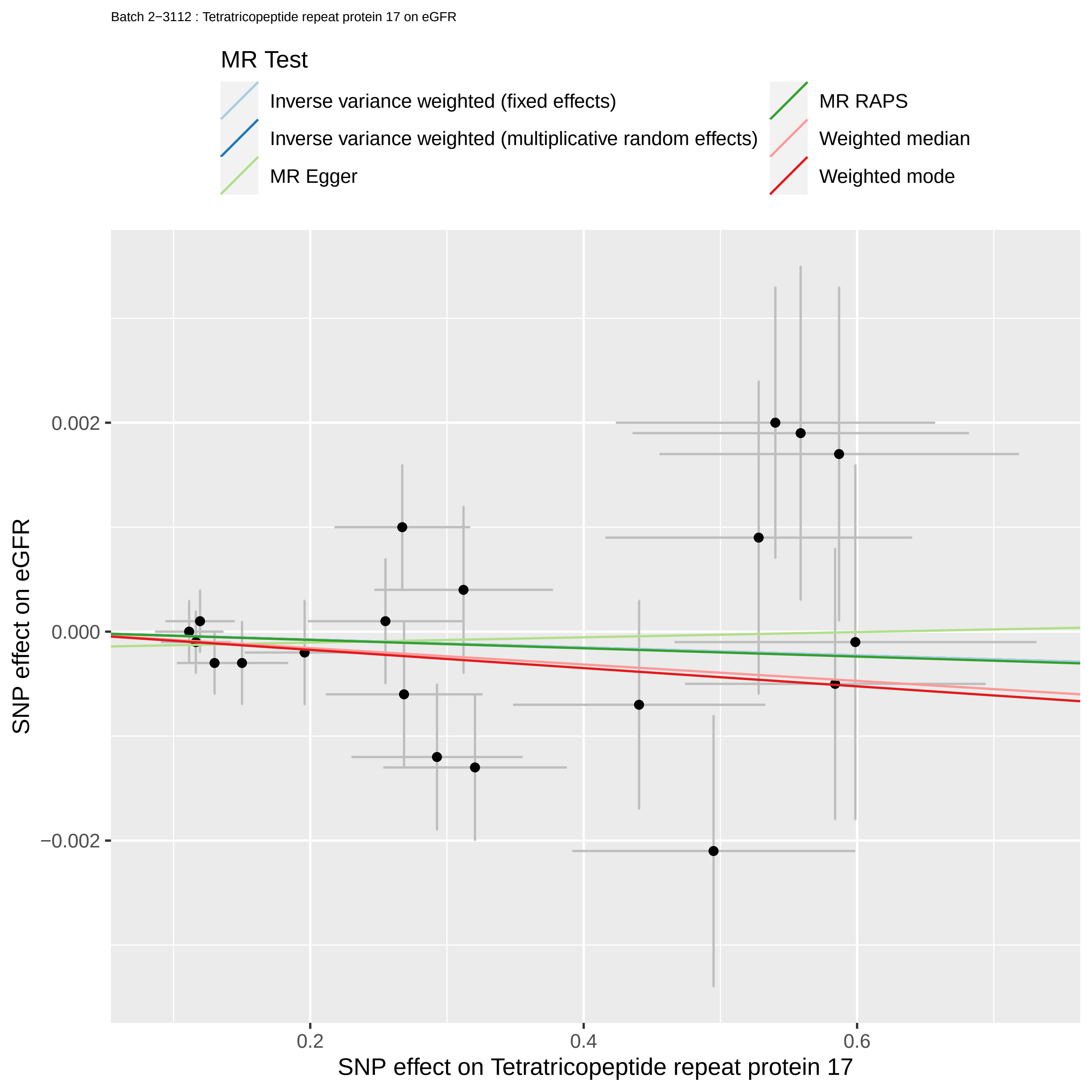

Batch 2−3112 : Tetratricopeptide repeat protein 17 on eGFR
MR Test
Inverse variance weighted (fixed effects)
Inverse variance weighted (multiplicative random effects)
MR Egger
MR RAPS
Weighted median
Weighted mode
0.002
0.000
−0.002
0.2
0.4
0.6
SNP effect on eGFR
SNP effect on Tetratricopeptide repeat protein 17

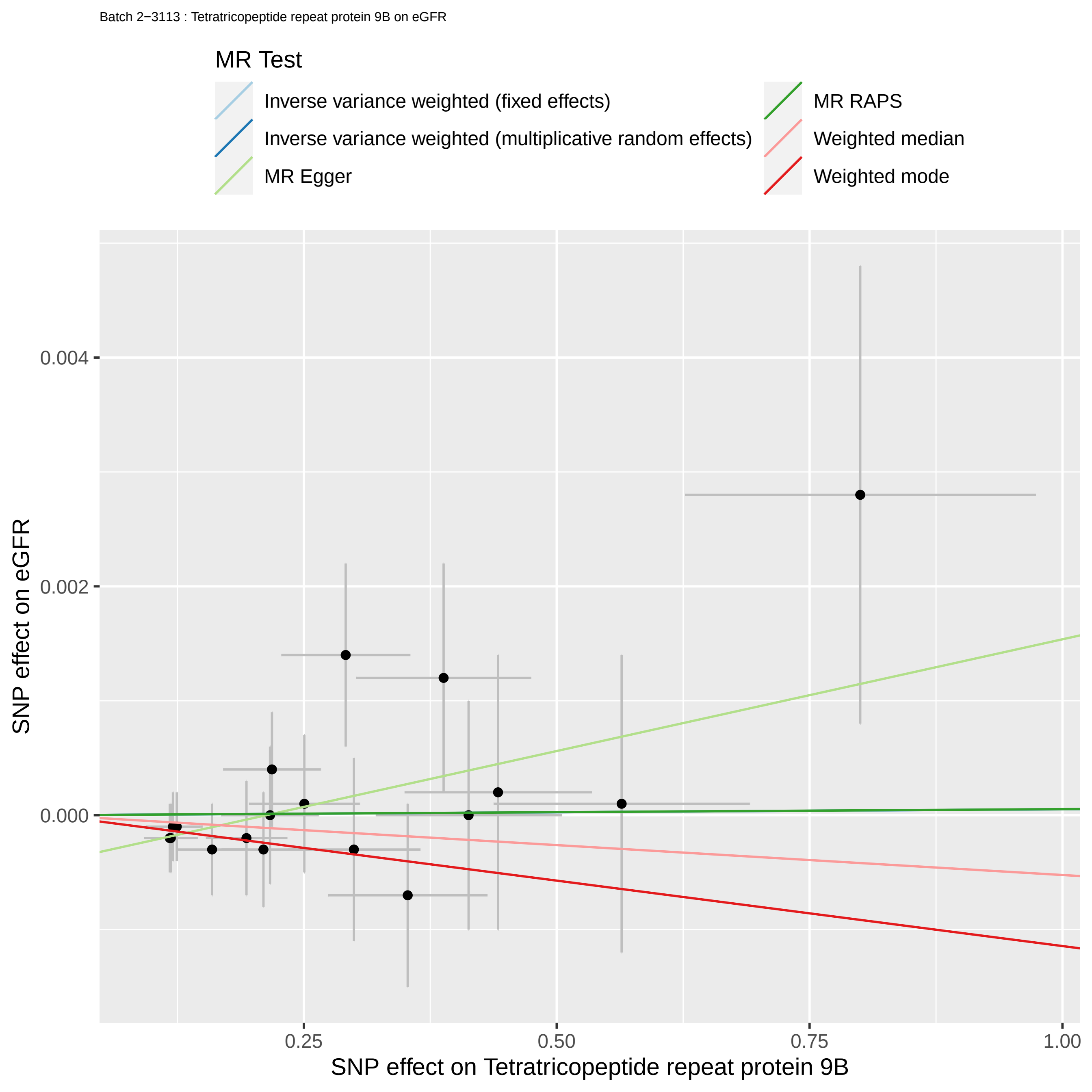

Batch 2−3113 : Tetratricopeptide repeat protein 9B on eGFR
MR Test
Inverse variance weighted (fixed effects)
Inverse variance weighted (multiplicative random effects)
MR Egger
MR RAPS
Weighted median
Weighted mode
0.004
0.002
0.000
SNP effect on eGFR
0.25
0.50
0.75
1.00
SNP effect on Tetratricopeptide repeat protein 9B

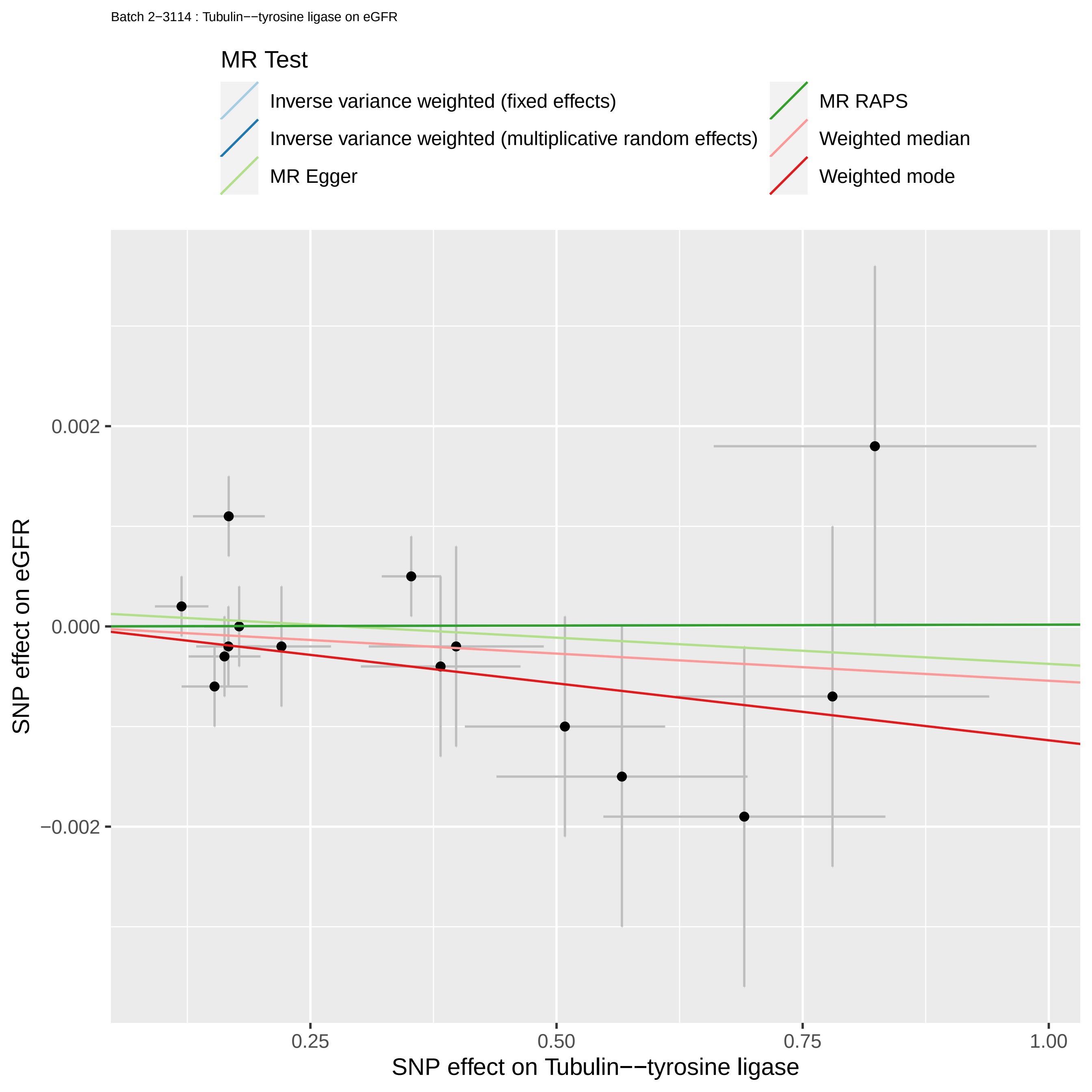
Batch 2−3114 : Tubulin−−tyrosine ligase on eGFR
MR Test
Inverse variance weighted (fixed effects)
Inverse variance weighted (multiplicative random effects)
MR Egger
MR RAPS
Weighted median
Weighted mode
0.002
0.000
−0.002
SNP effect on eGFR
0.25
0.50
0.75
1.00
SNP effect on Tubulin−−tyrosine ligase

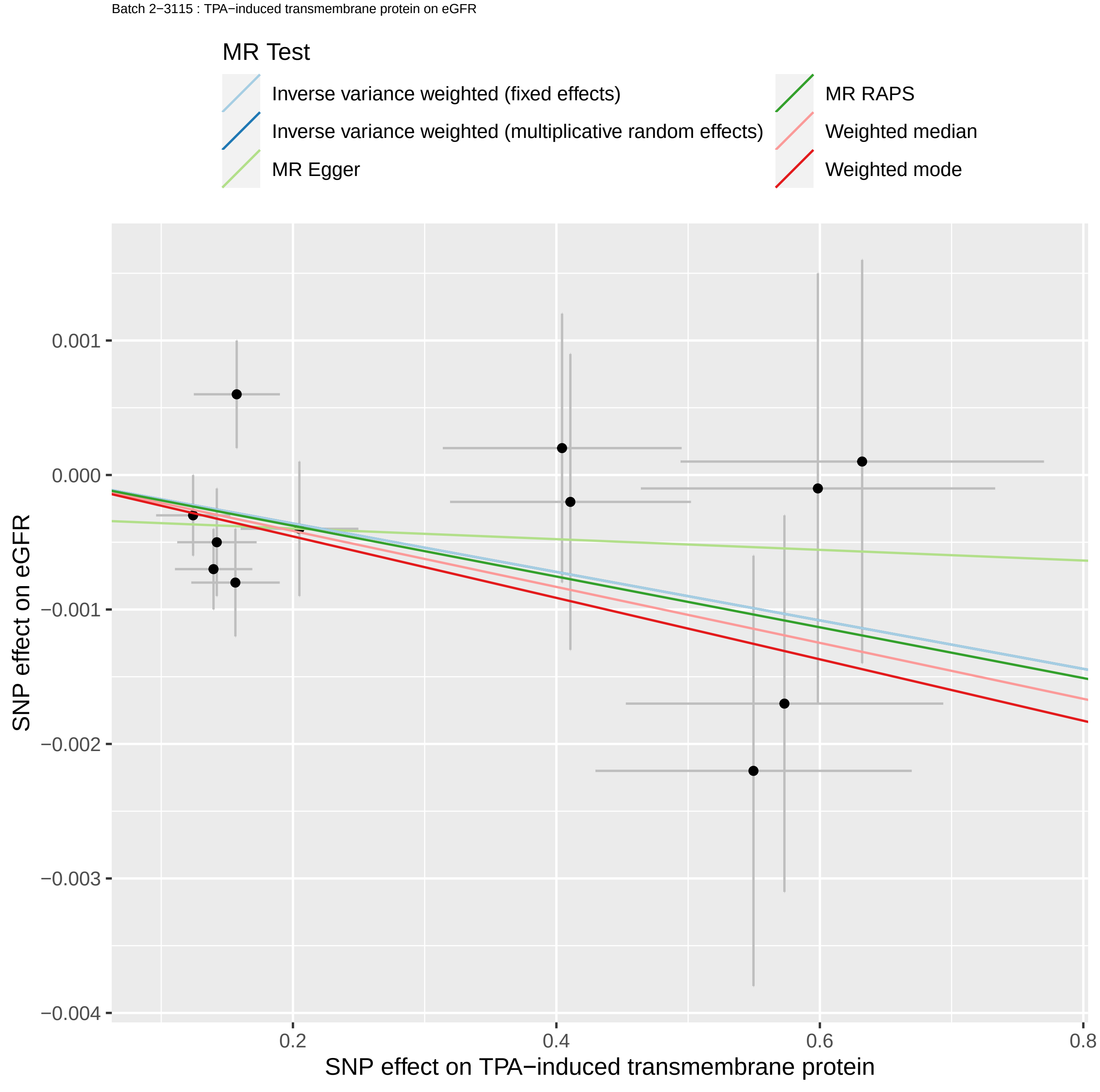
Batch 2−3115 : TPA−induced transmembrane protein on eGFR
MR Test
Inverse variance weighted (fixed effects)
Inverse variance weighted (multiplicative random effects)
MR Egger
MR RAPS
Weighted median
Weighted mode
0.001
0.000
−0.001
−0.002
−0.003
−0.004
SNP effect on eGFR
0.2
0.4
0.6
0.8
SNP effect on TPA−induced transmembrane protein

Batch 2−3116 : Titin on eGFR

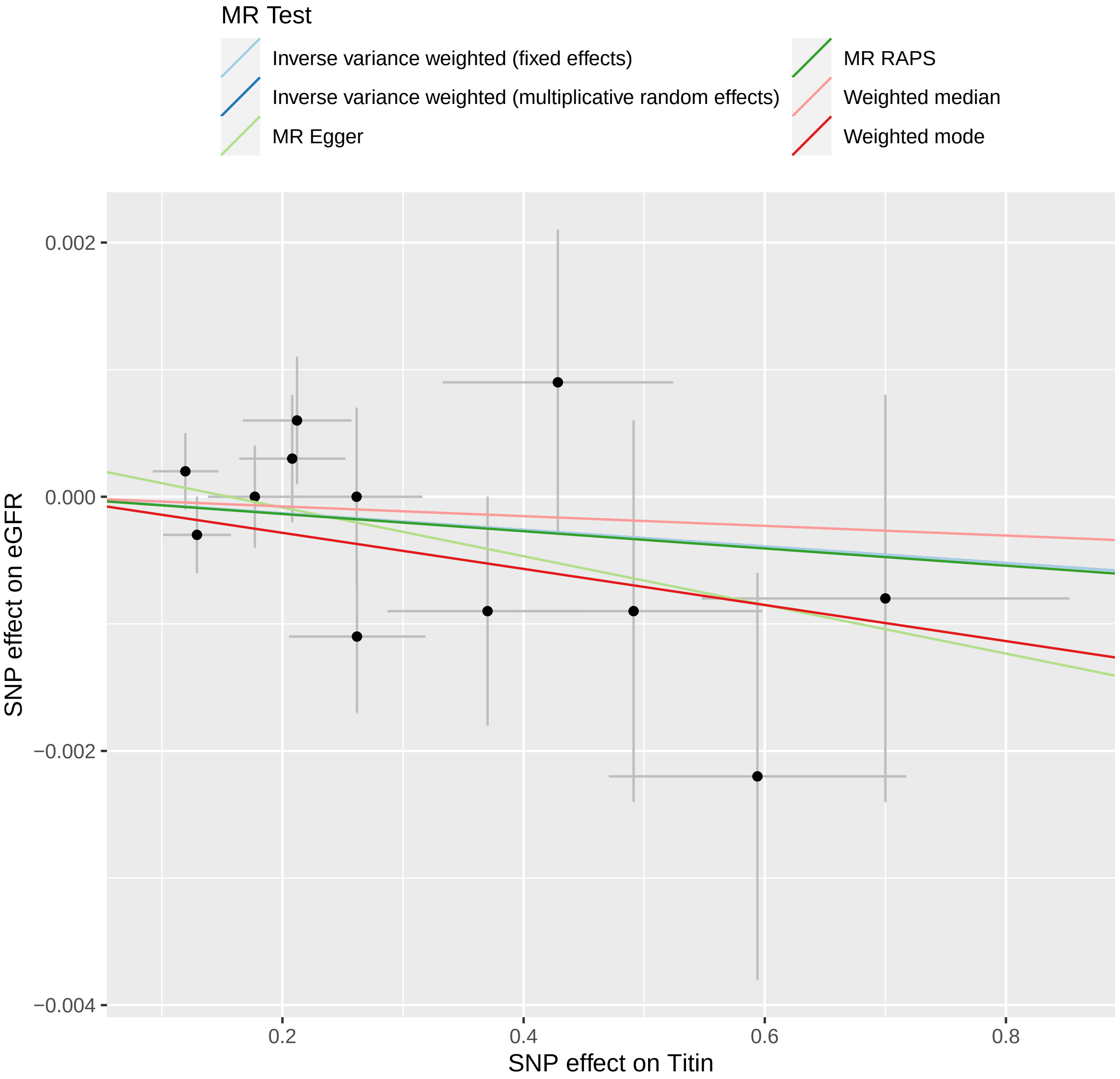

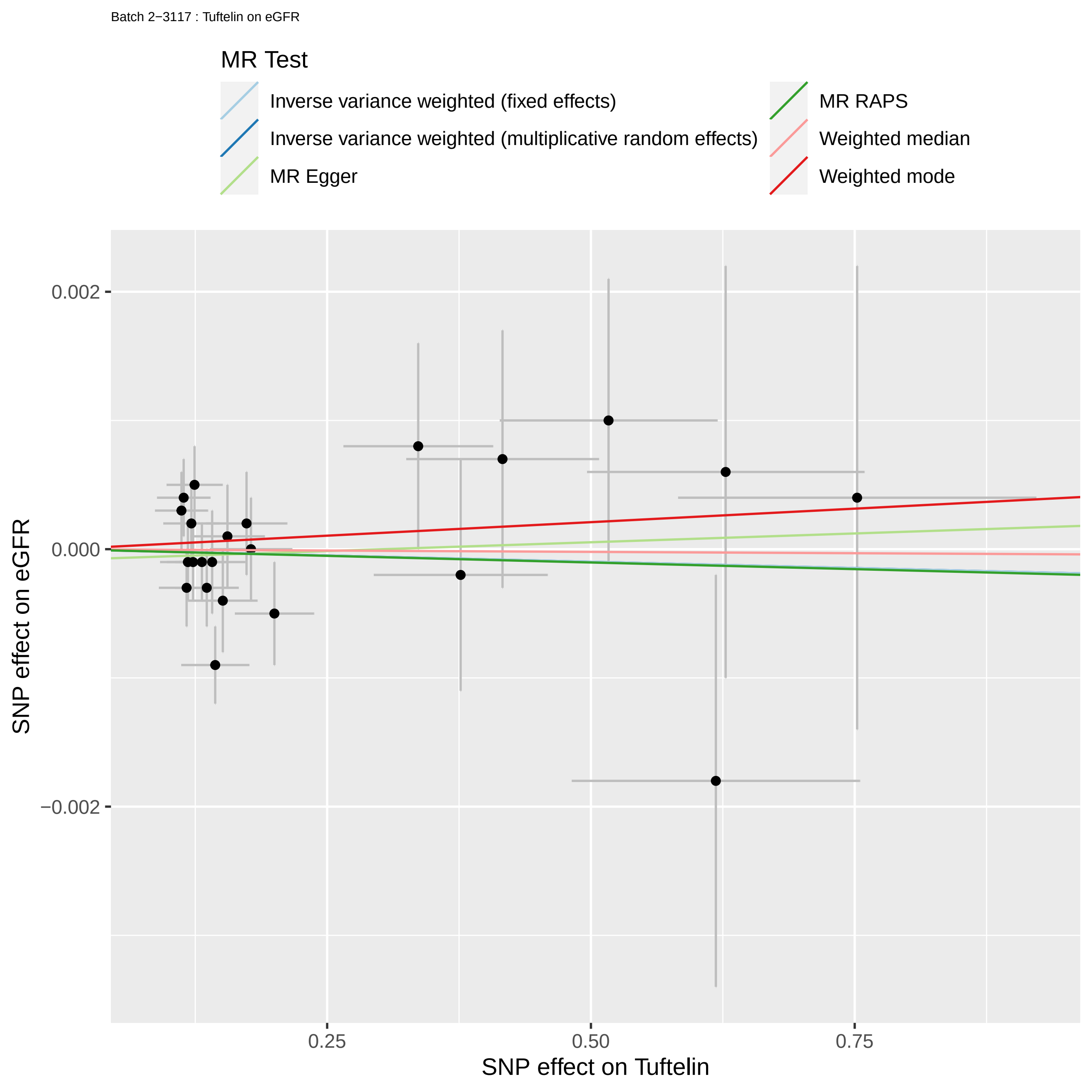
Batch 2−3117 : Tuftelin on eGFR
MR Test
Inverse variance weighted (fixed effects)
Inverse variance weighted (multiplicative random effects)
MR Egger
MR RAPS
Weighted median
Weighted mode
0.002
0.000
−0.002
0.25
0.50
0.75
SNP effect on eGFR
SNP effect on Tuftelin

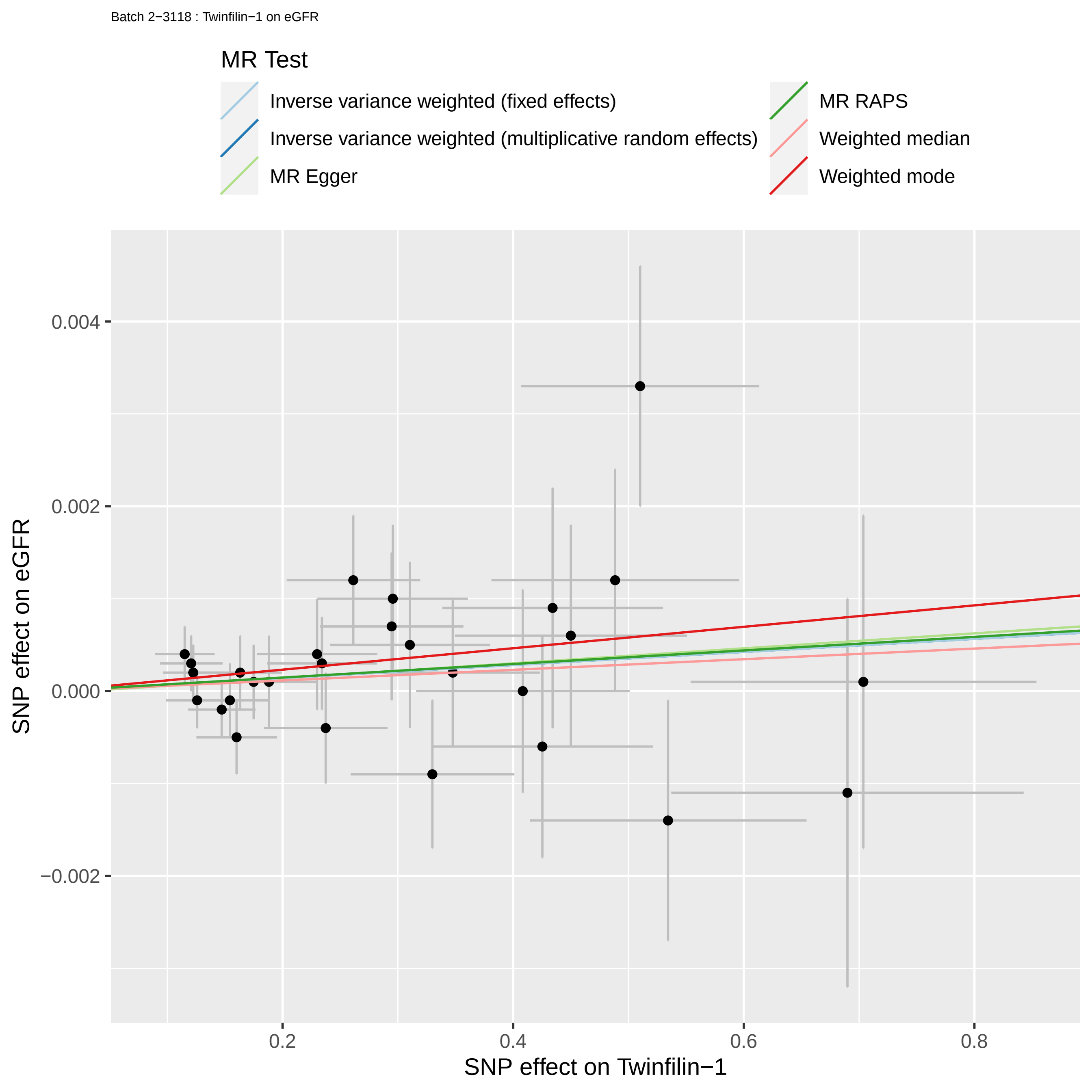

Batch 2−3118 : Twinfilin−1 on eGFR
MR Test
Inverse variance weighted (fixed effects)
Inverse variance weighted (multiplicative random effects)
MR Egger
MR RAPS
Weighted median
Weighted mode
0.004
0.002
0.000
−0.002
0.2
0.4
0.6
0.8
SNP effect on eGFR
SNP effect on Twinfilin−1

Batch 2−3119 : Twisted gastrulation protein homolog 1 on eGFR

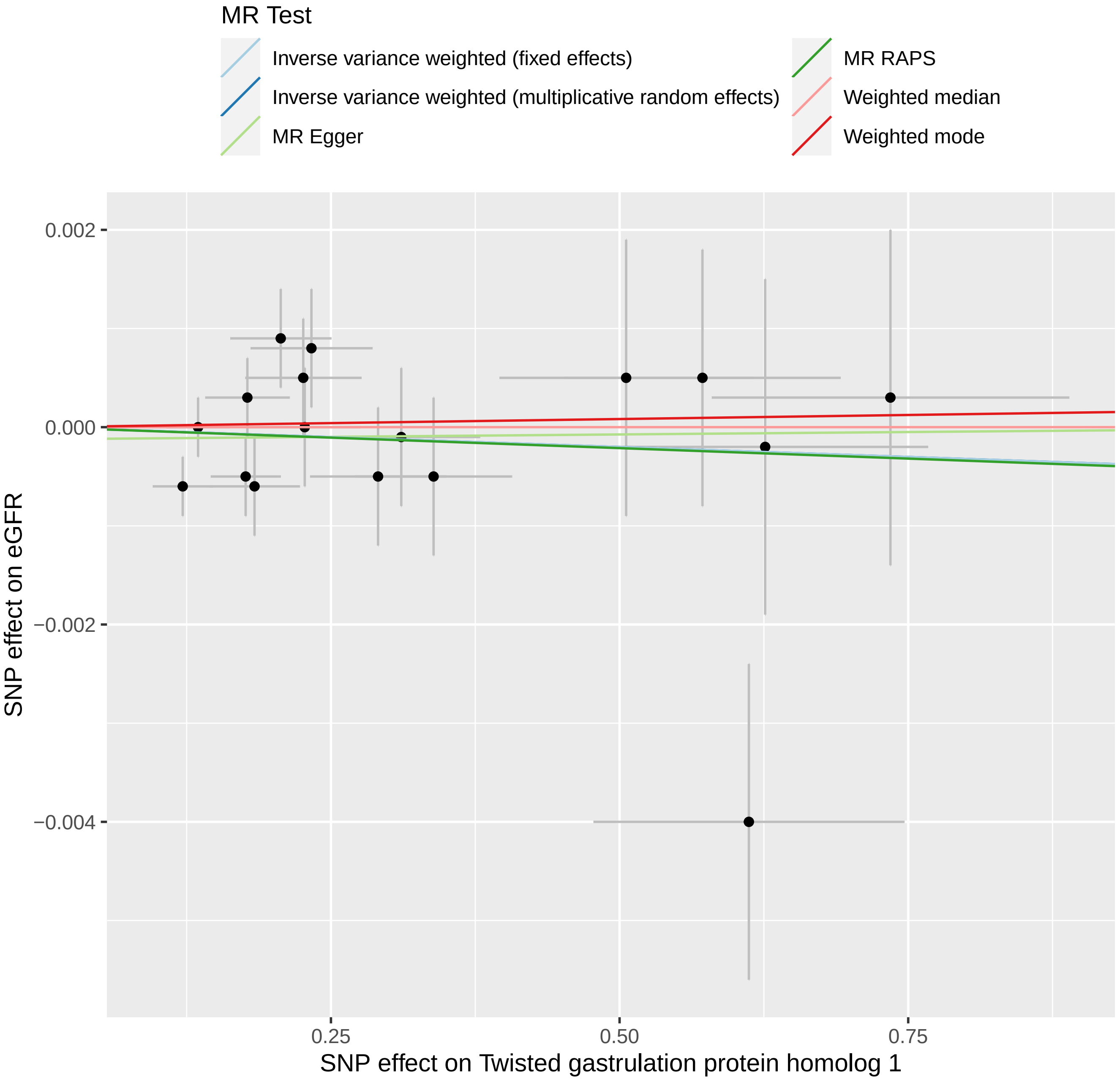

Batch 2−3120 : Alpha−taxilin on eGFR

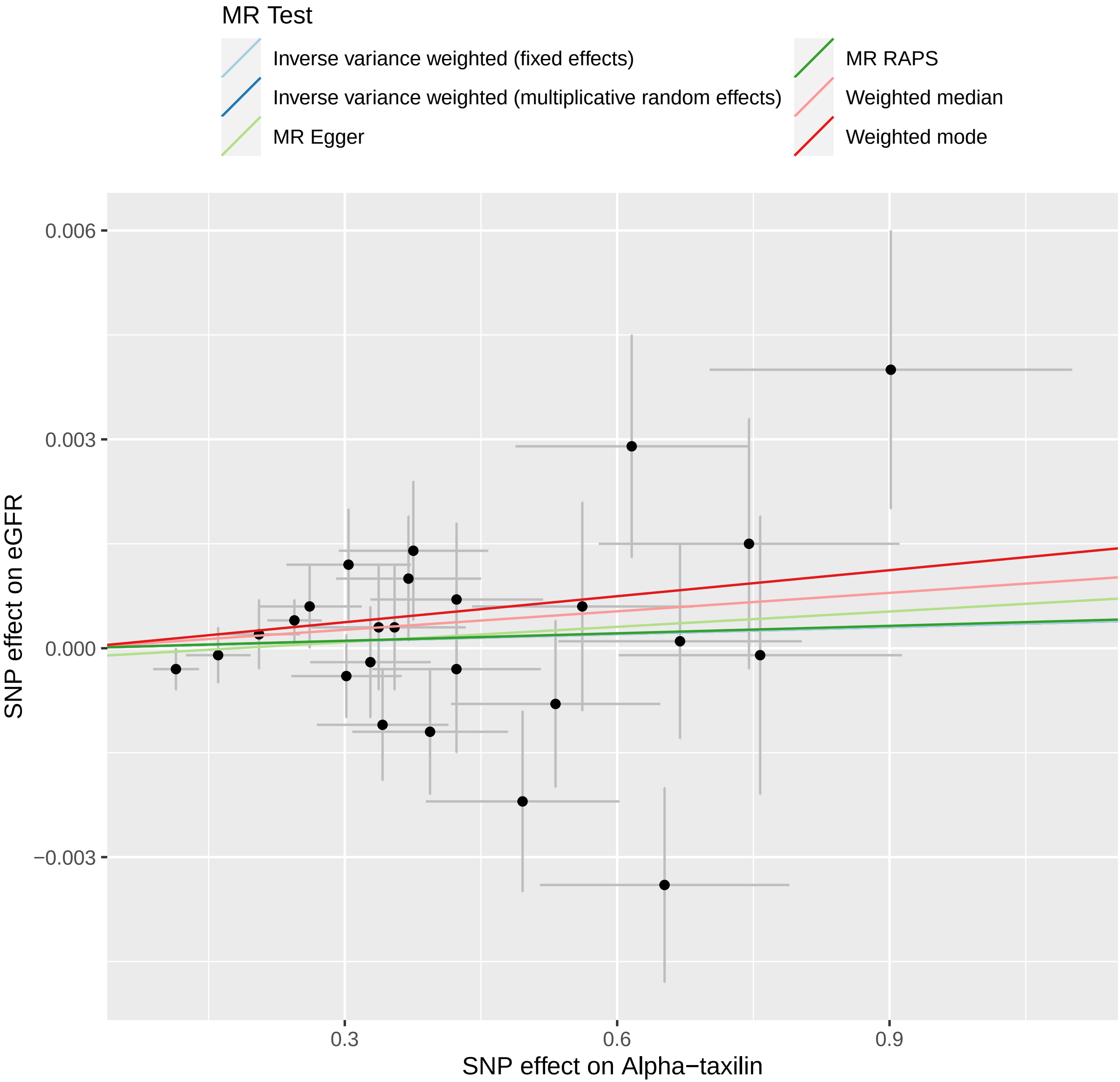

Batch 2−3121 : Thioredoxin on eGFR

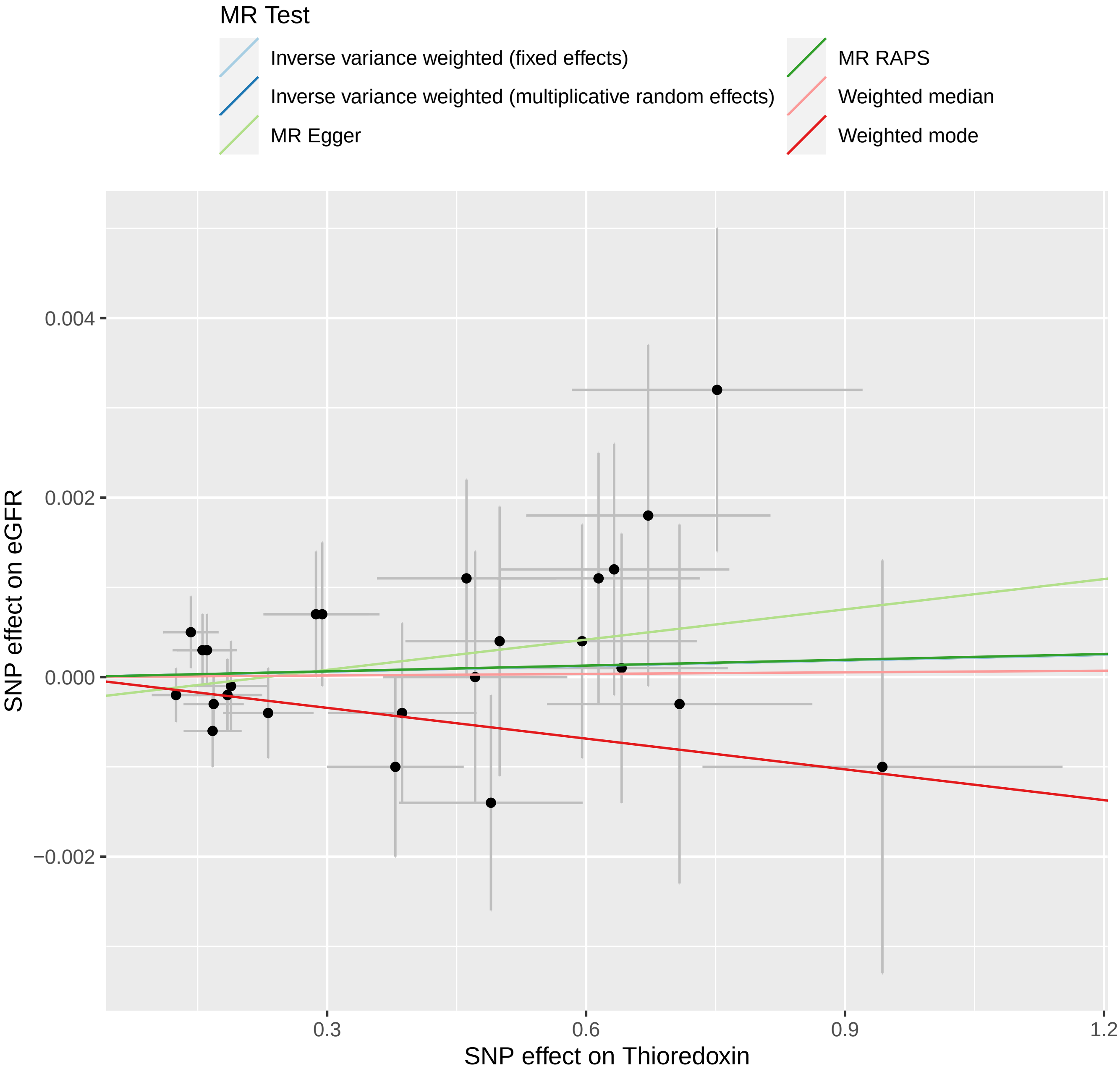

Batch 2−3122 : Thioredoxin domain−containing protein 11 on eGFR

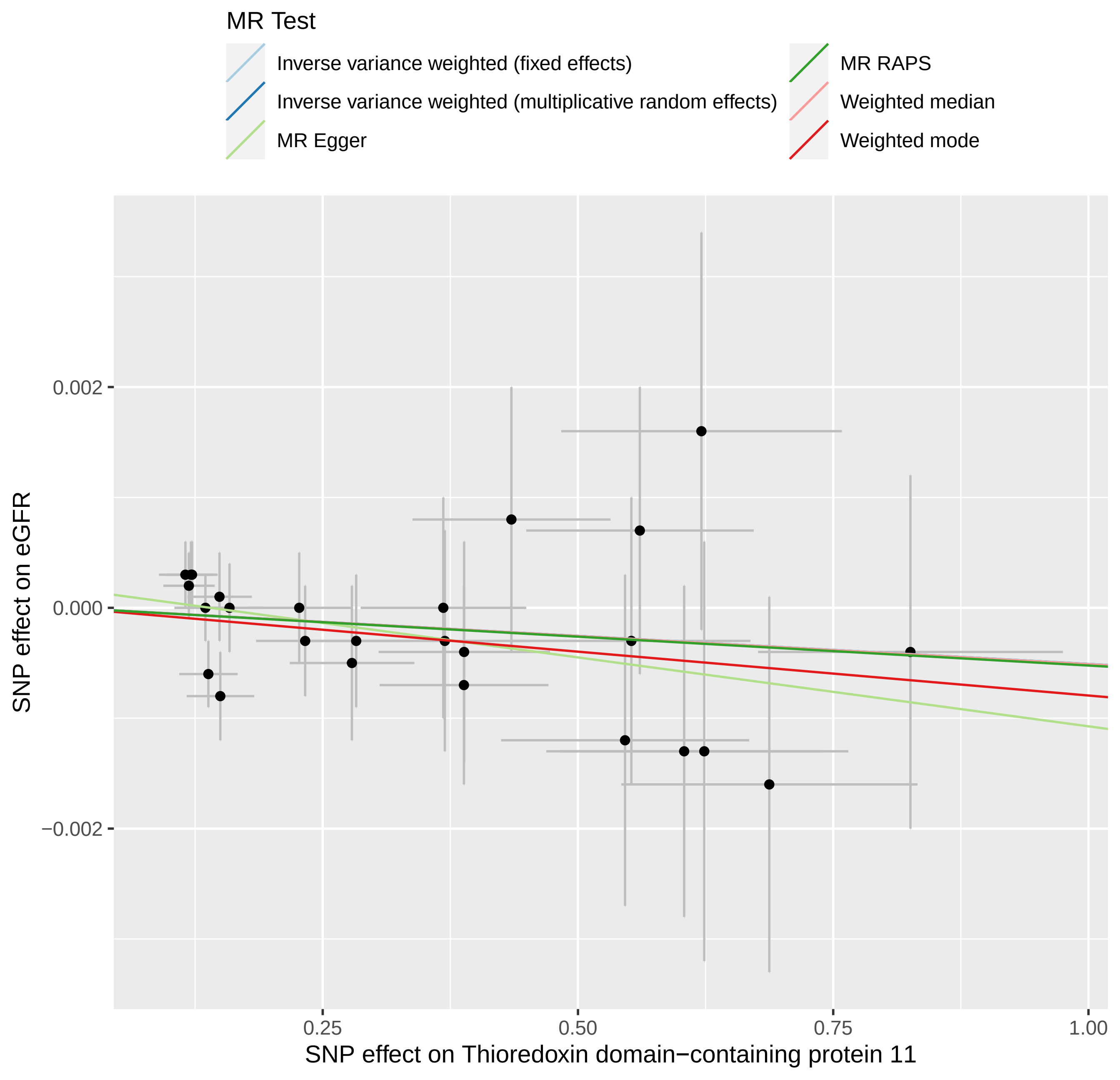

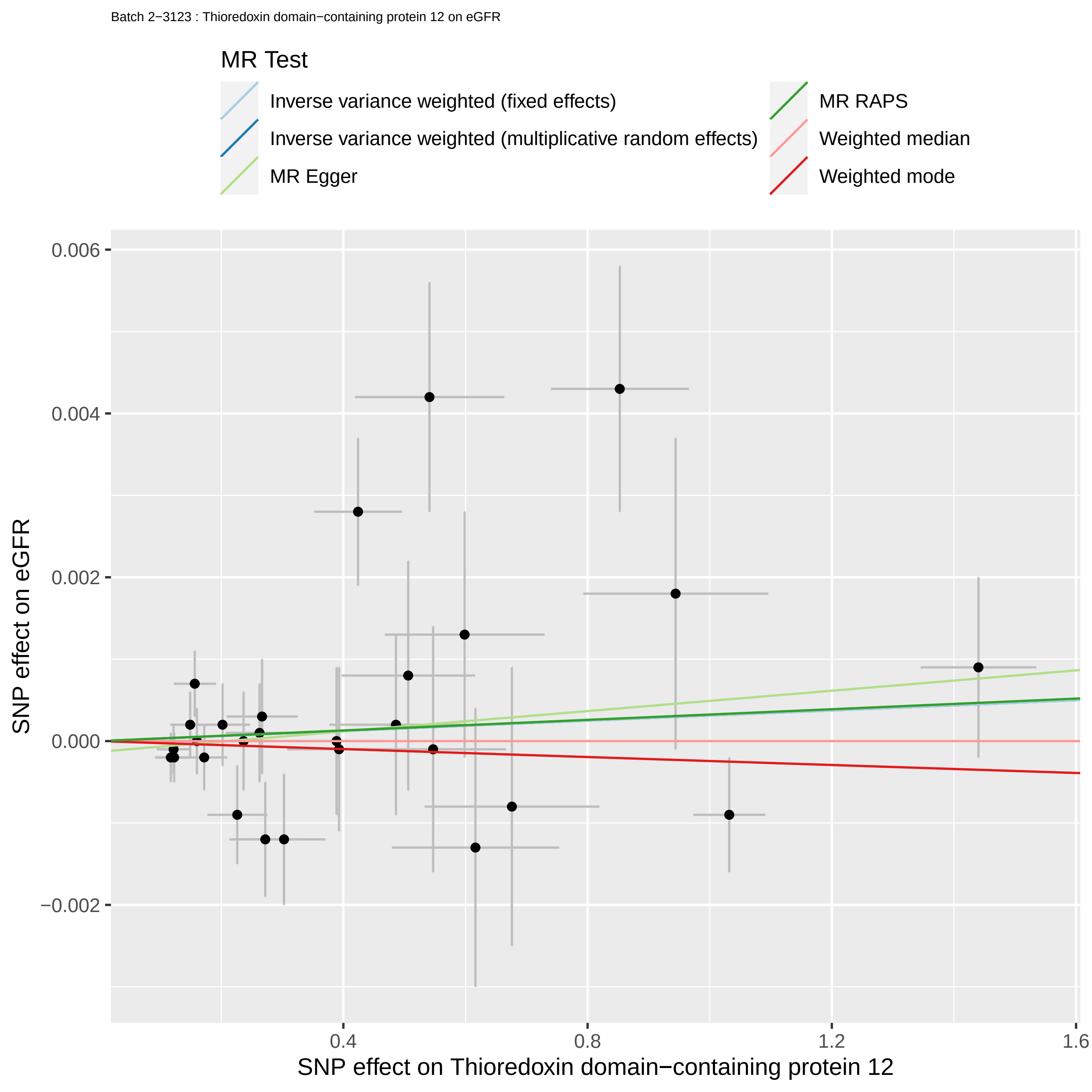
Batch 2−3123 : Thioredoxin domain−containing protein 12 on eGFR
MR Test
Inverse variance weighted (fixed effects)
Inverse variance weighted (multiplicative random effects)
MR Egger
MR RAPS
Weighted median
Weighted mode
0.006
0.004
0.002
0.000
−0.002
0.4
0.8
1.2
1.6
SNP effect on eGFR
SNP effect on Thioredoxin domain−containing protein 12

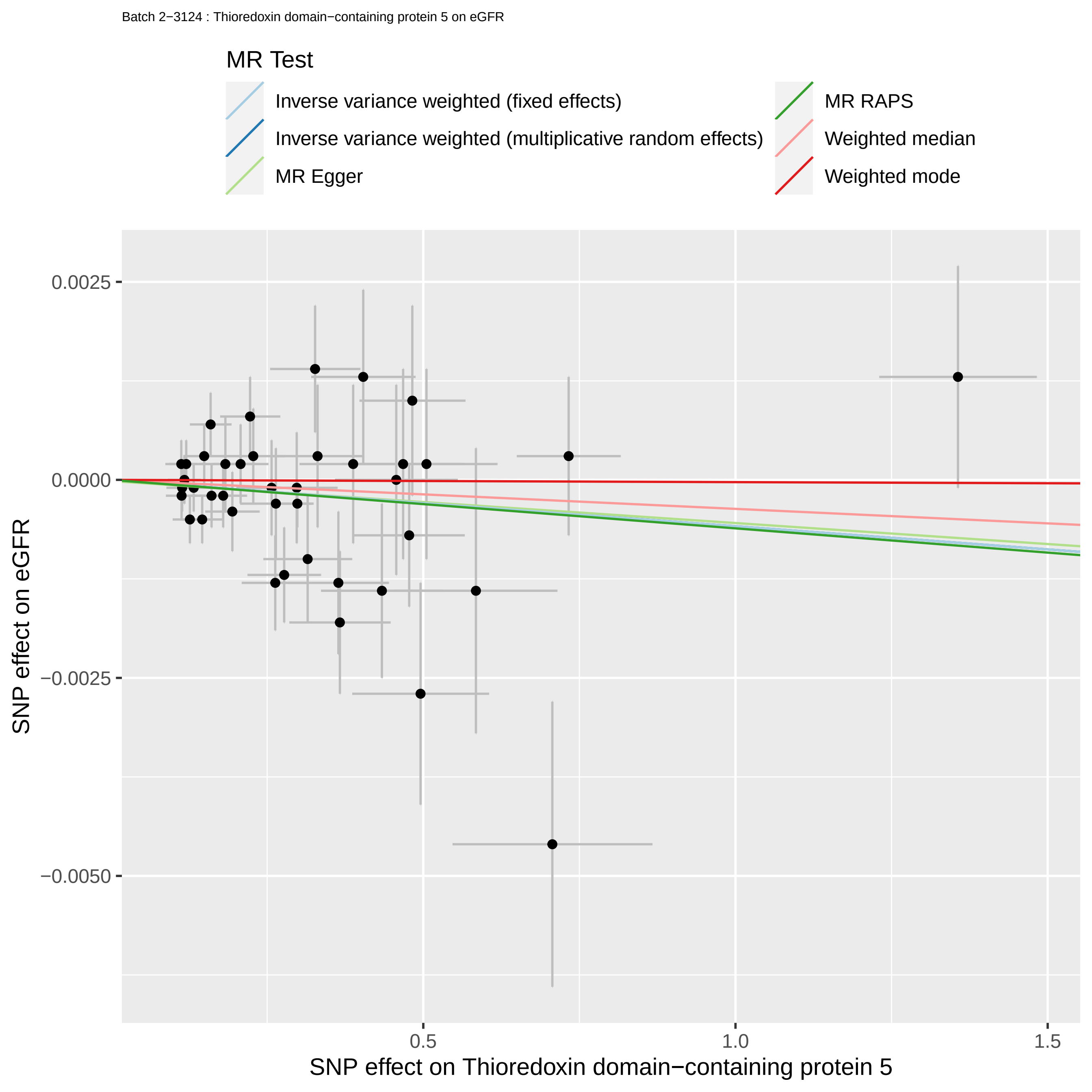

Batch 2−3124 : Thioredoxin domain−containing protein 5 on eGFR
MR Test
Inverse variance weighted (fixed effects)
Inverse variance weighted (multiplicative random effects)
MR Egger
MR RAPS
Weighted median
Weighted mode
0.0025
0.0000
−0.0025
−0.0050
0.5
1.0
1.5
SNP effect on eGFR
SNP effect on Thioredoxin domain−containing protein 5

Batch 2−3125 : Thioredoxin−interacting protein on eGFR

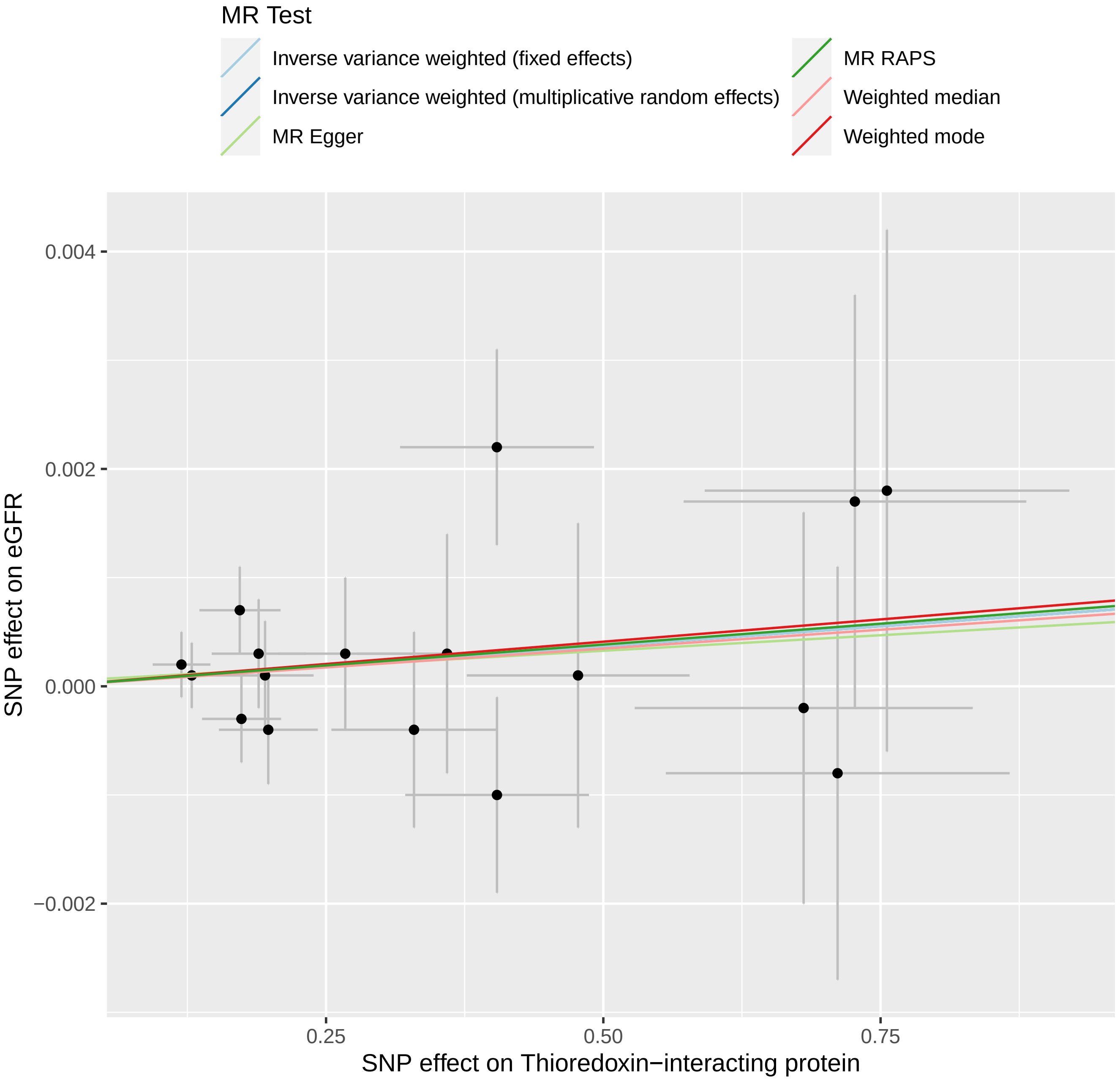

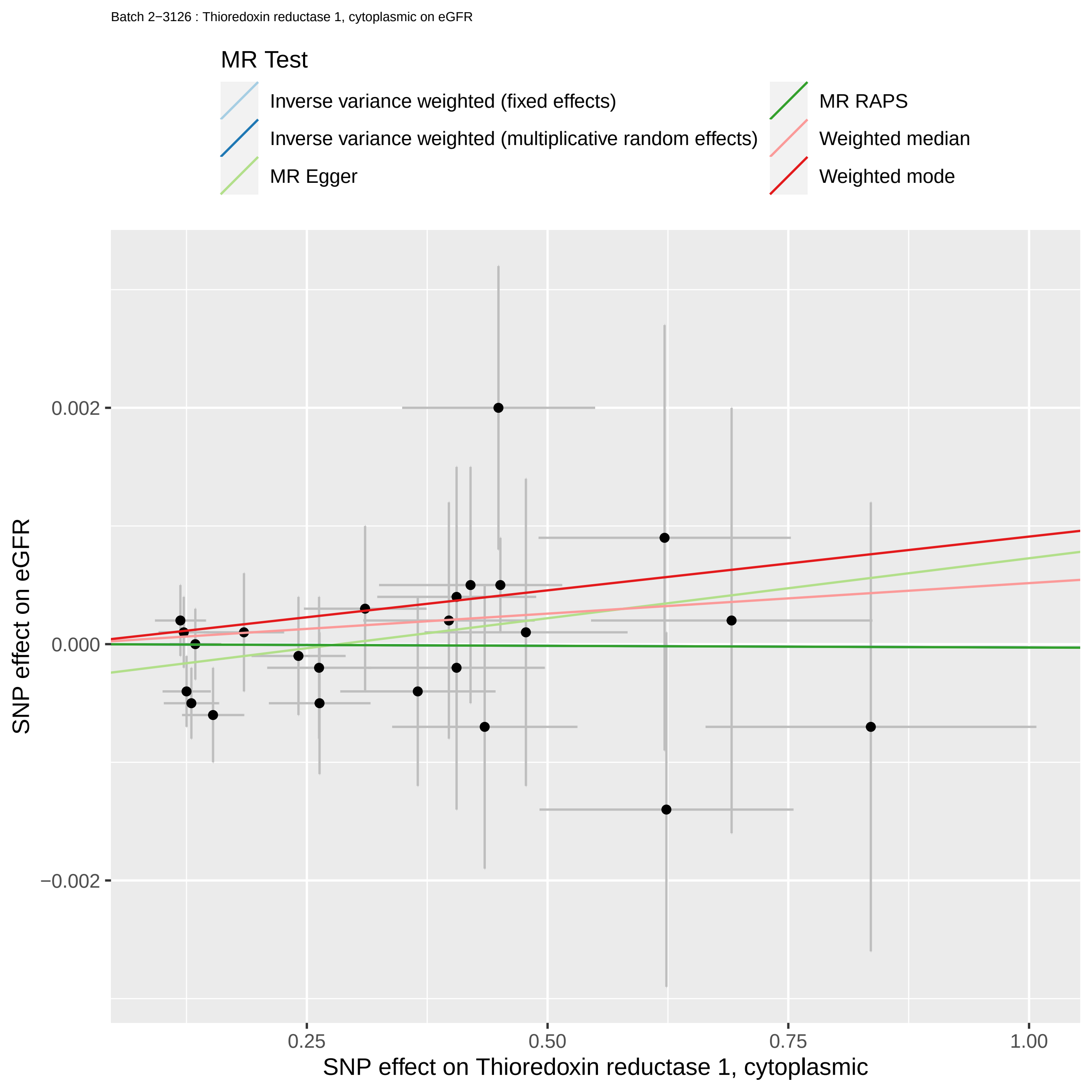

Batch 2−3126 : Thioredoxin reductase 1, cytoplasmic on eGFR
MR Test
Inverse variance weighted (fixed effects)
Inverse variance weighted (multiplicative random effects)
MR Egger
MR RAPS
Weighted median
Weighted mode
0.002
0.000
−0.002
0.25
0.50
0.75
1.00
SNP effect on eGFR
SNP effect on Thioredoxin reductase 1, cytoplasmic

Batch 2−3127 : Non−receptor tyrosine−protein kinase TYK2 on eGFR

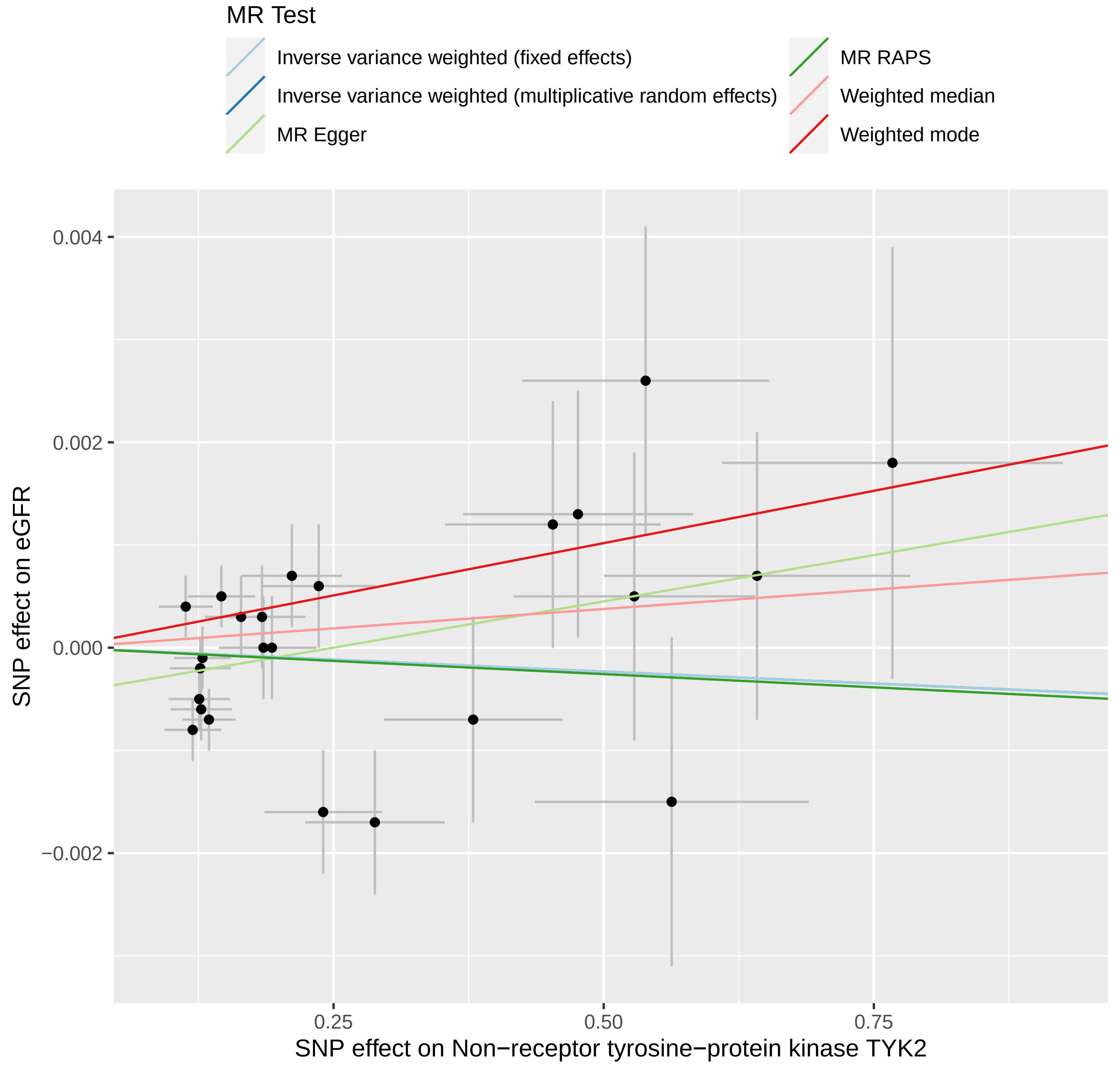

Batch 2−3128 : TYMS opposite strand protein on eGFR

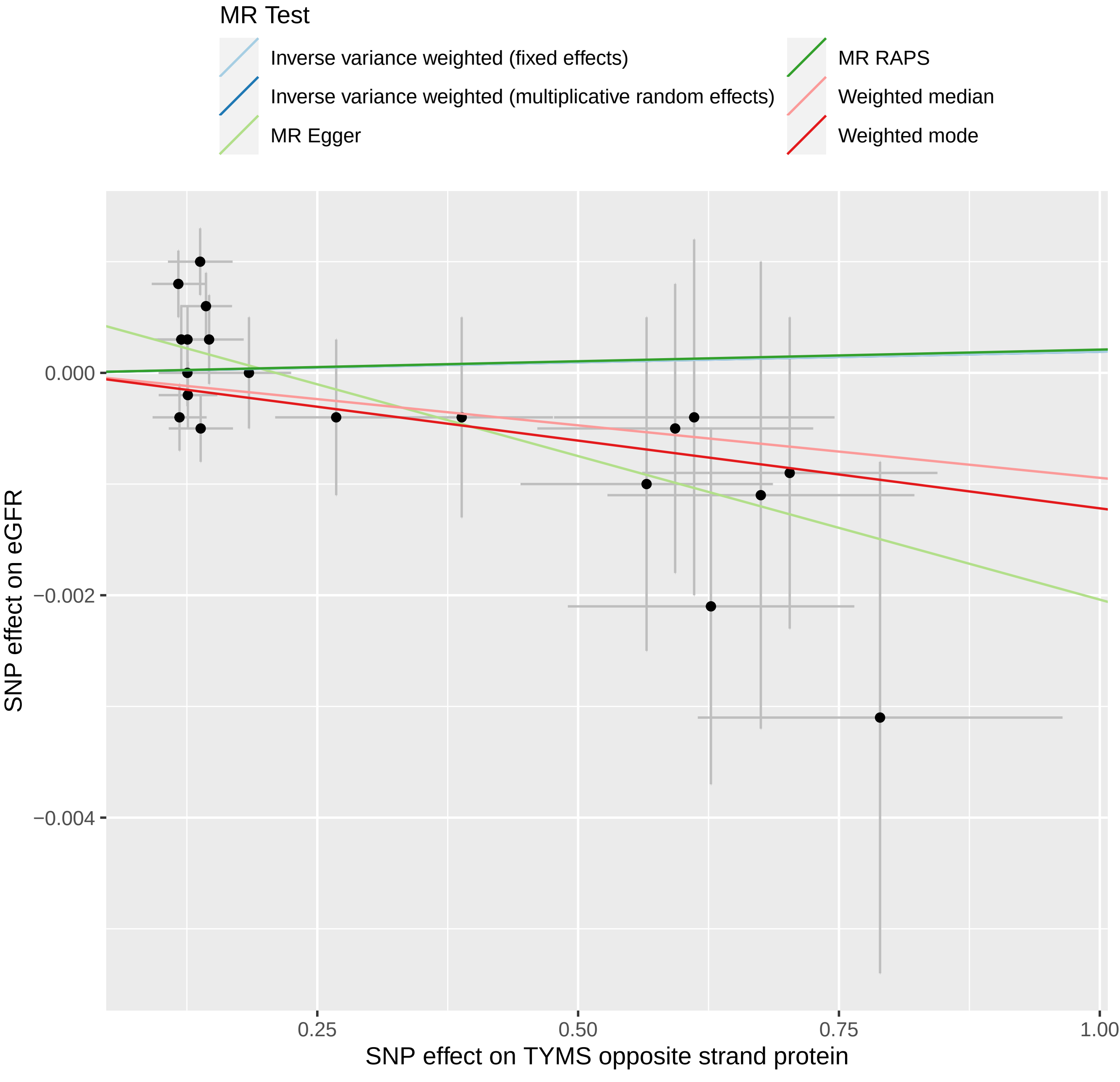

Batch 2−3129 : Tyrosine−protein kinase receptor TYRO3 on eGFR

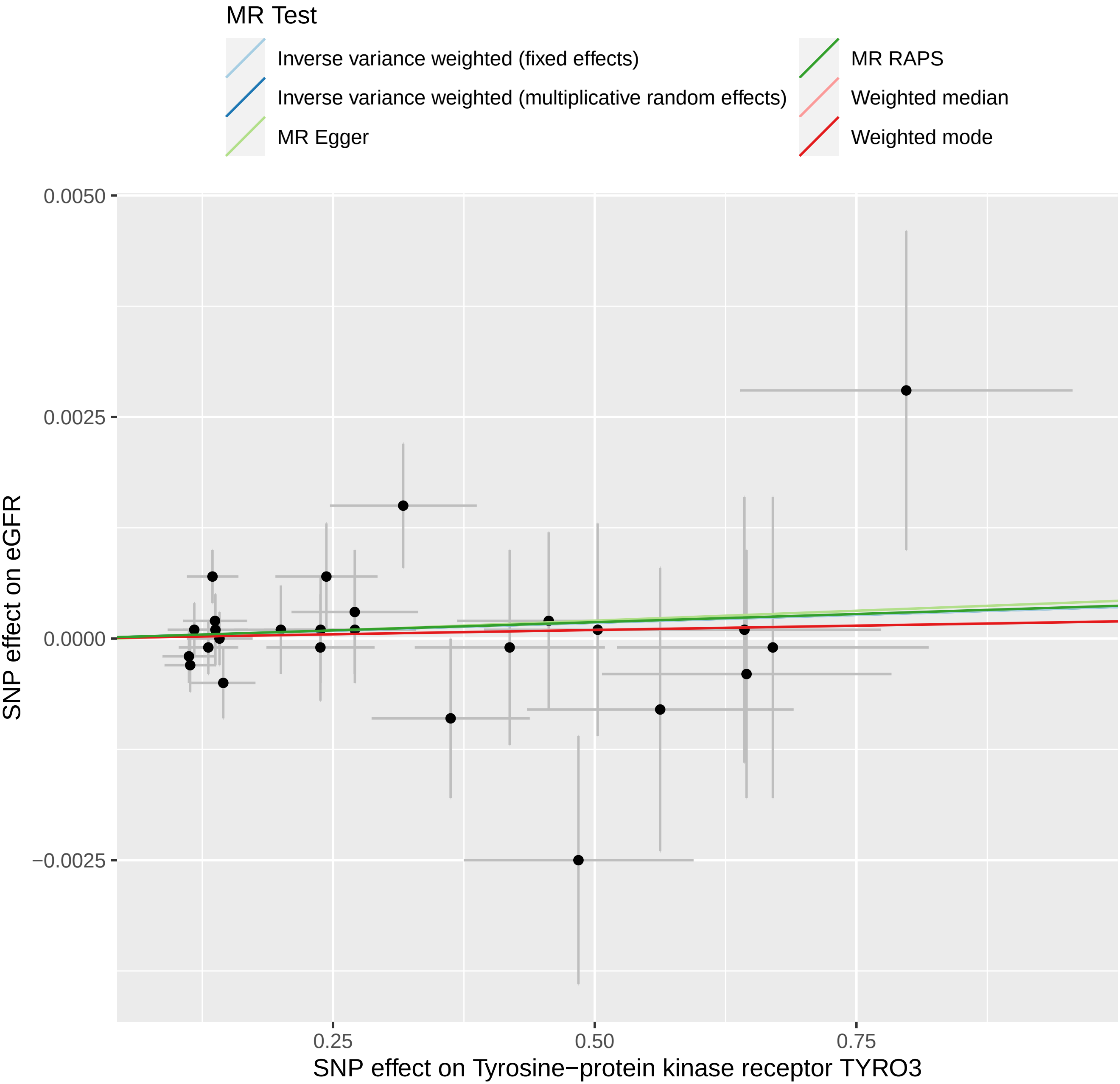

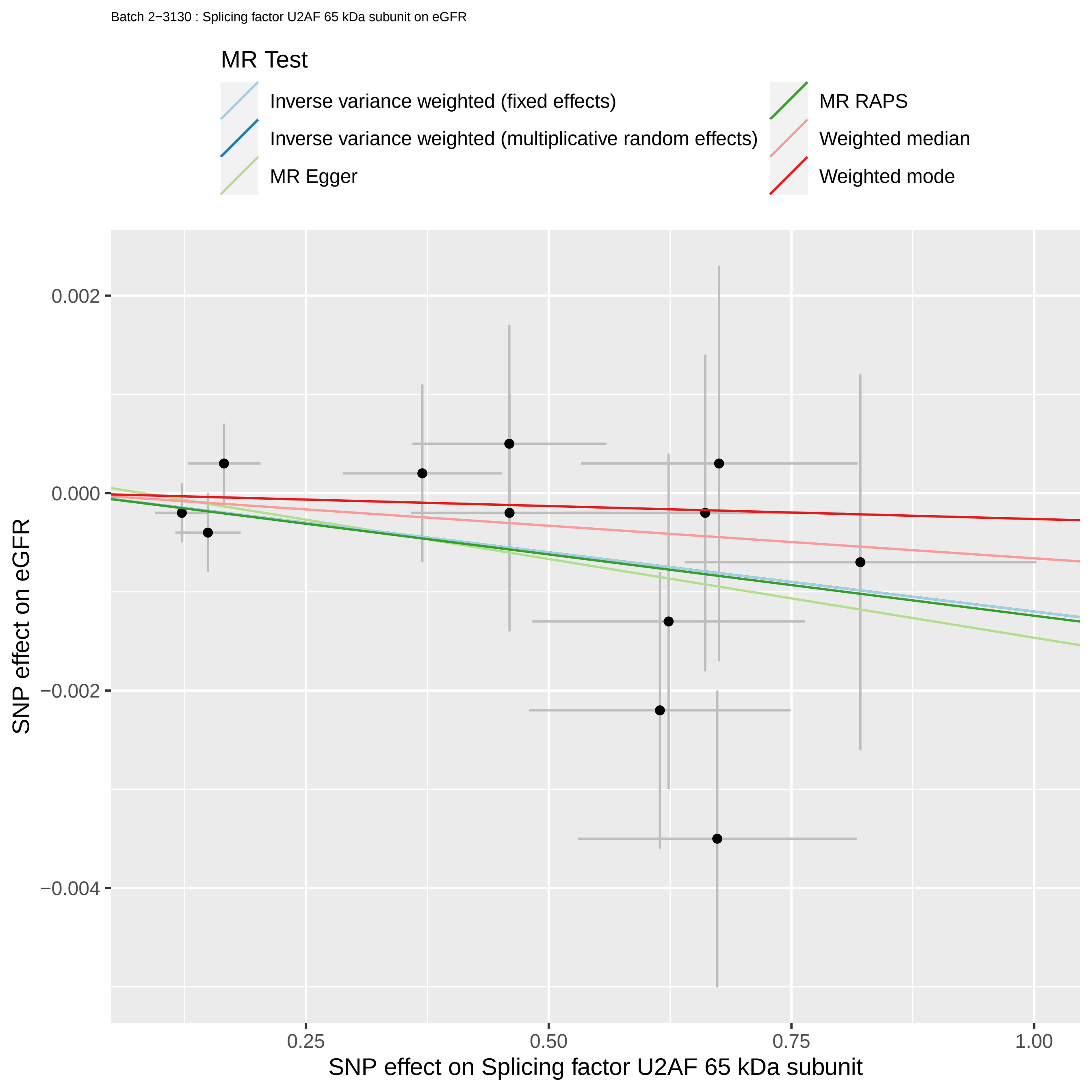

Batch 2−3130 : Splicing factor U2AF 65 kDa subunit on eGFR
MR Test
Inverse variance weighted (fixed effects)
Inverse variance weighted (multiplicative random effects)
MR Egger
MR RAPS
Weighted median
Weighted mode
0.002
0.000
−0.002
−0.004
0.25
0.50
0.75
1.00
SNP effect on eGFR
SNP effect on Splicing factor U2AF 65 kDa subunit

Batch 2−3131 : UDP−N−acetylhexosamine pyrophosphorylase on eGFR

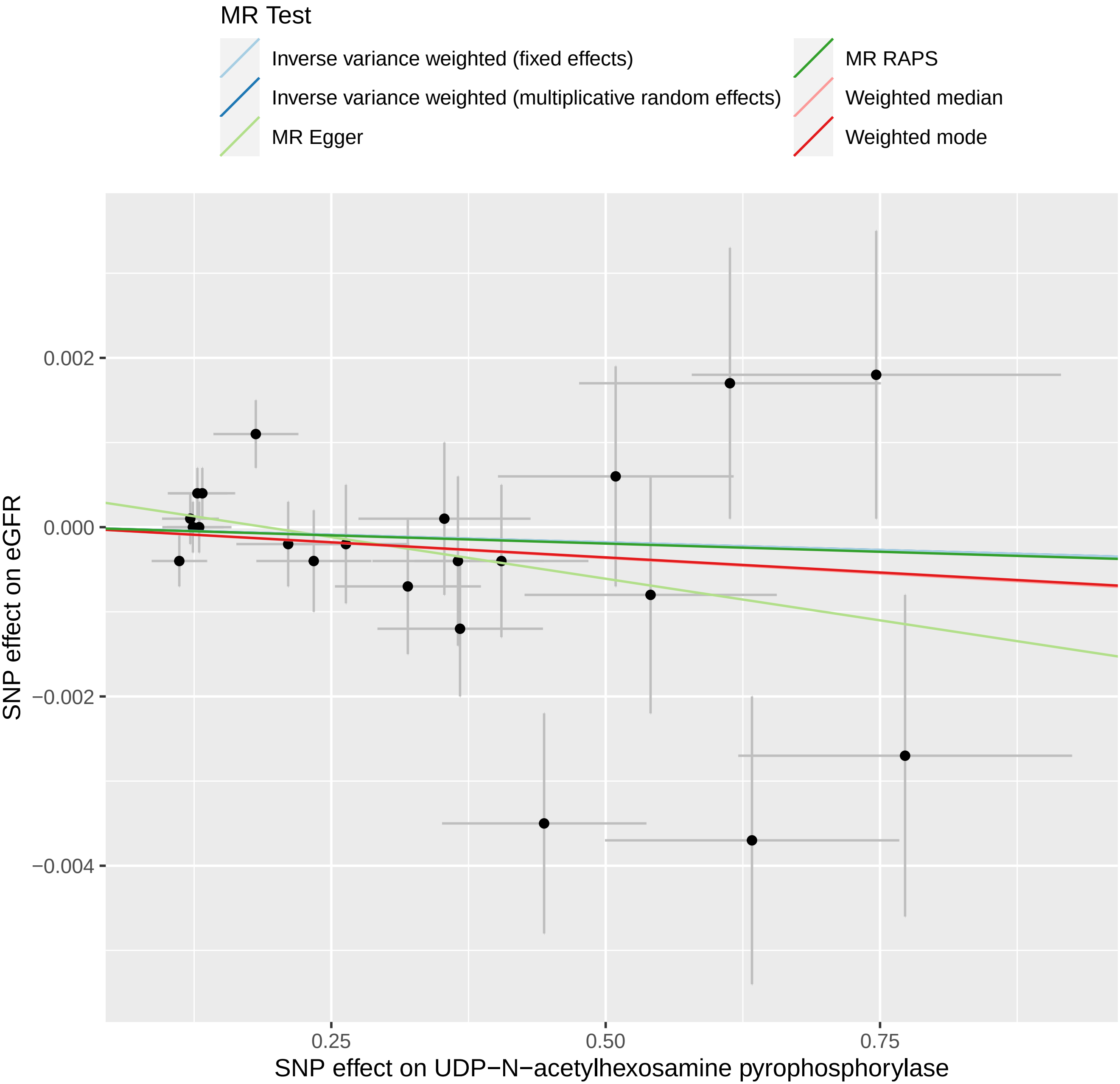

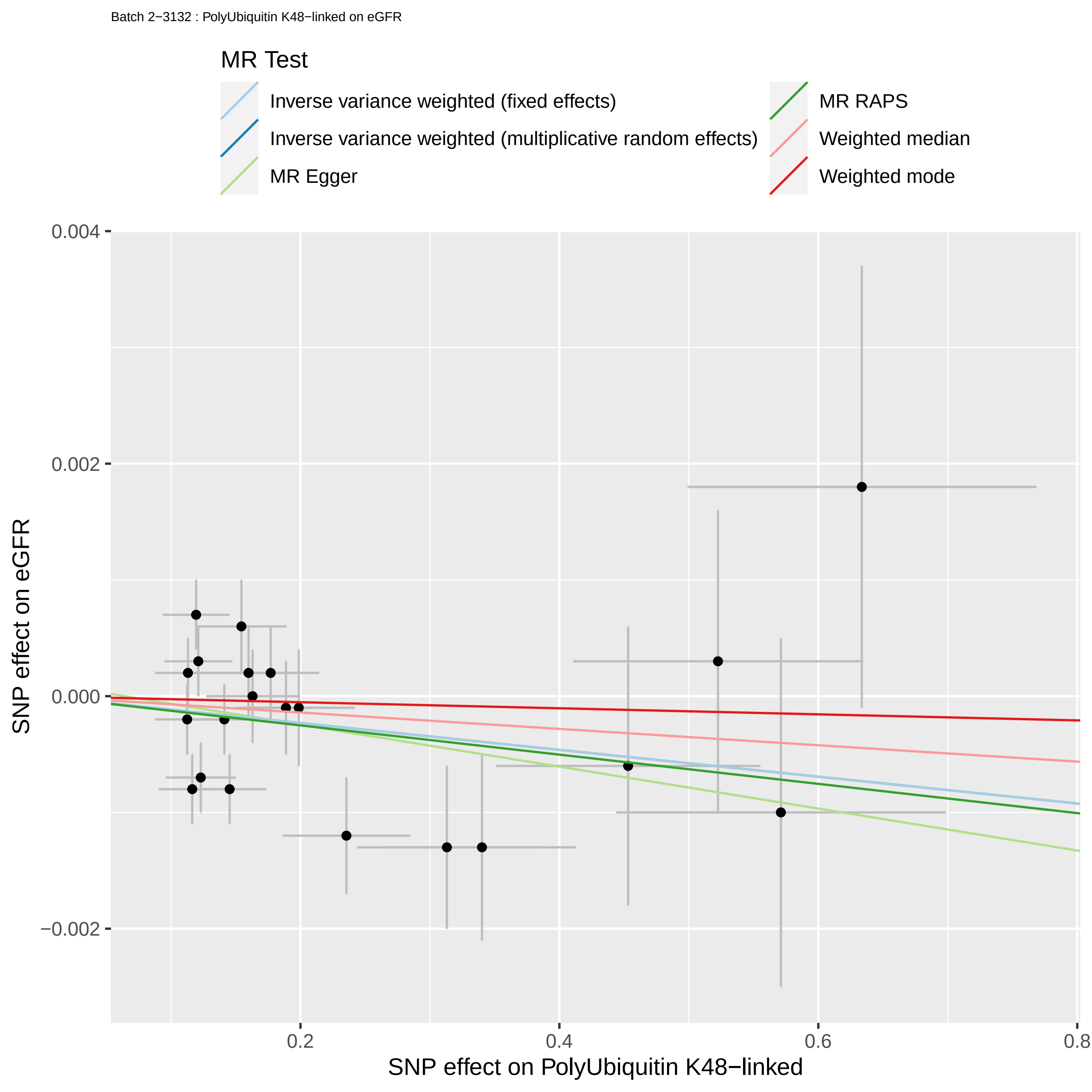
Batch 2−3132 : PolyUbiquitin K48−linked on eGFR
MR Test
Inverse variance weighted (fixed effects)
Inverse variance weighted (multiplicative random effects)
MR Egger
MR RAPS
Weighted median
Weighted mode
0.004
0.002
0.000
−0.002
0.2
0.4
0.6
0.8
SNP effect on eGFR
SNP effect on PolyUbiquitin K48−linked

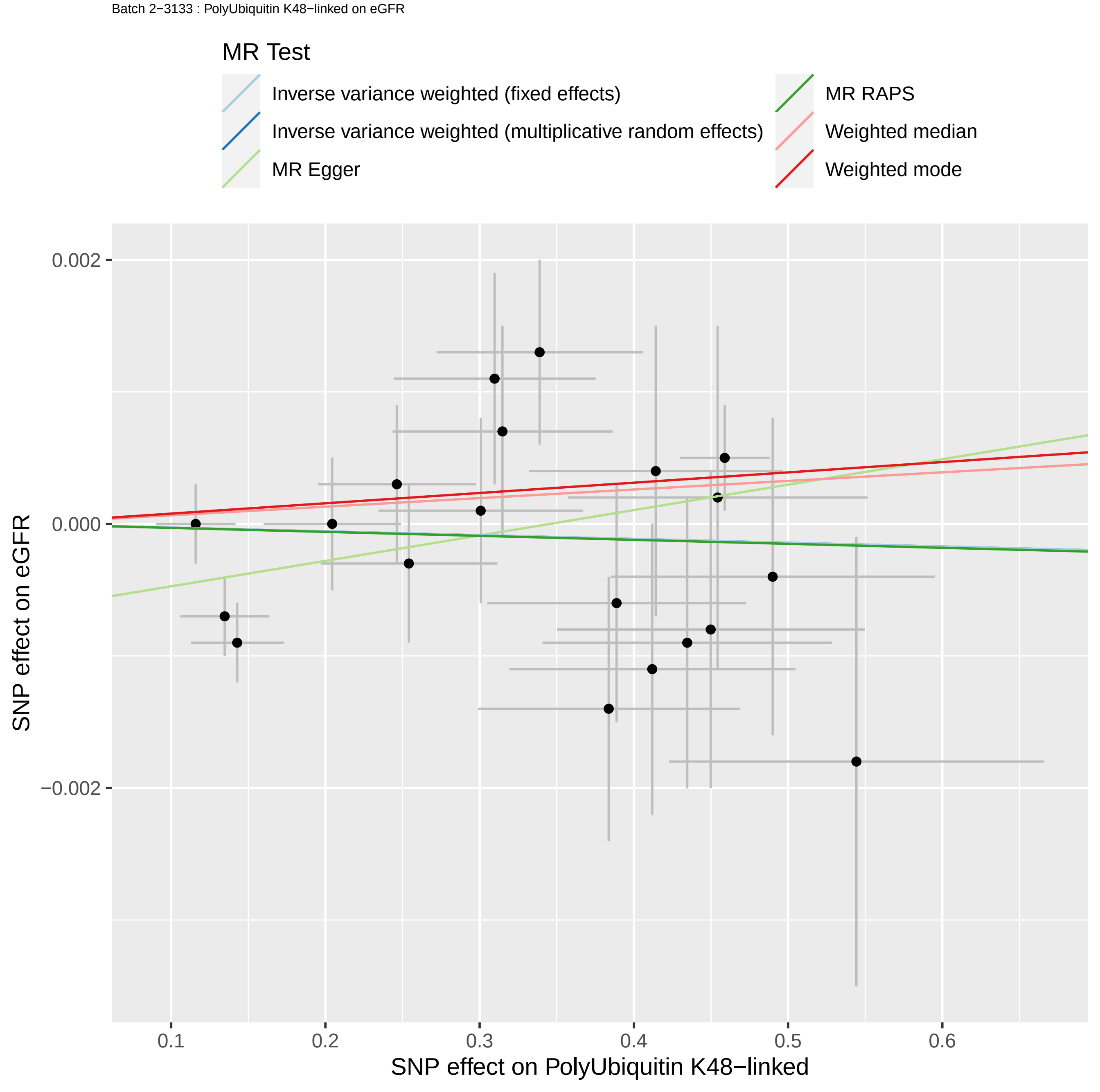
Batch 2−3133 : PolyUbiquitin K48−linked on eGFR
MR Test
Inverse variance weighted (fixed effects)
Inverse variance weighted (multiplicative random effects)
MR Egger
MR RAPS
Weighted median
Weighted mode
0.002
0.000
−0.002
0.1
0.2
0.3
0.4
0.5
0.6
SNP effect on eGFR
SNP effect on PolyUbiquitin K48−linked

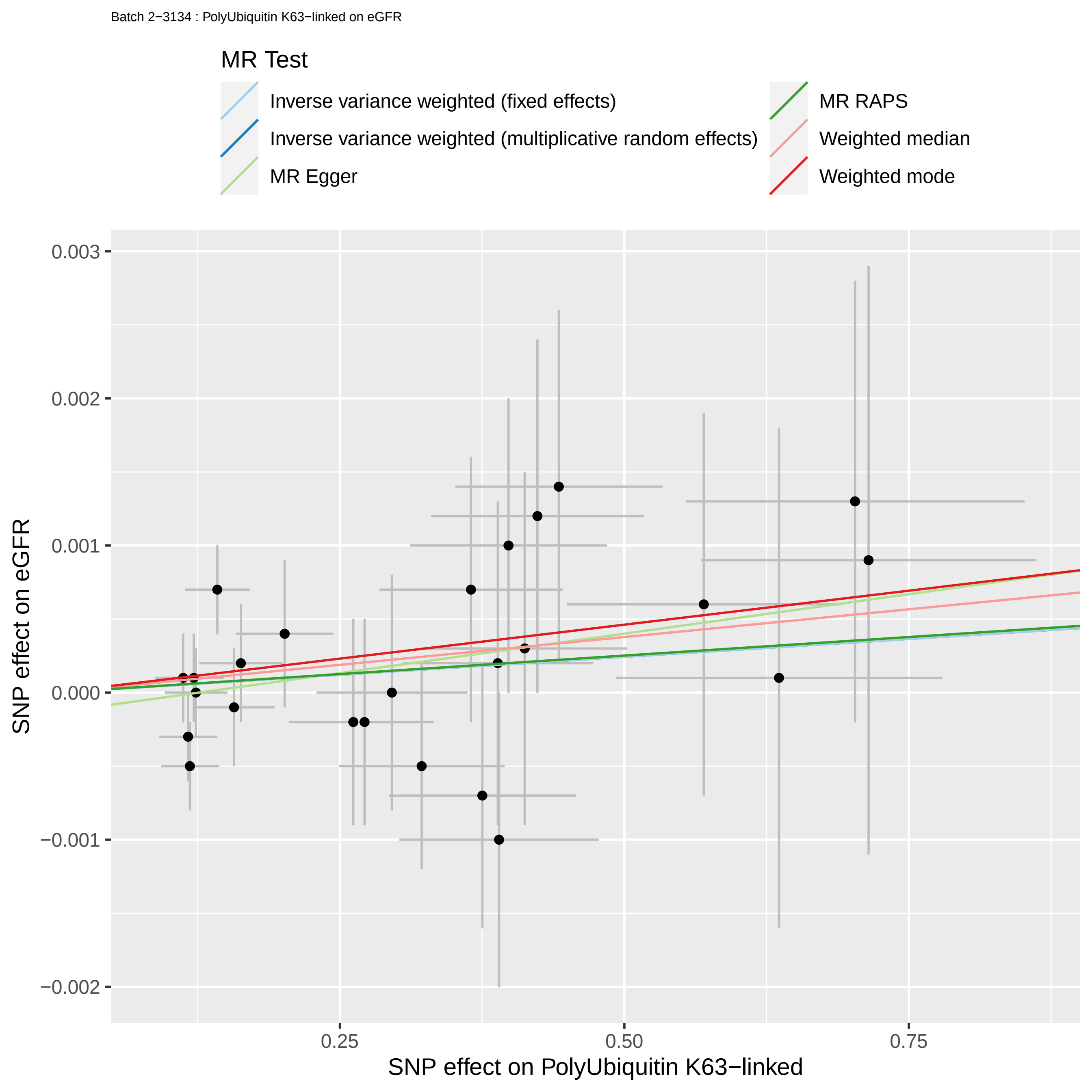

Batch 2−3134 : PolyUbiquitin K63−linked on eGFR
MR Test
Inverse variance weighted (fixed effects)
Inverse variance weighted (multiplicative random effects)
MR Egger
MR RAPS
Weighted median
Weighted mode
0.003
0.002
0.001
0.000
−0.001
−0.002
SNP effect on eGFR
0.25
0.50
0.75
SNP effect on PolyUbiquitin K63−linked

Batch 2−3135 : Ubiquitin−conjugating enzyme E2 B on eGFR

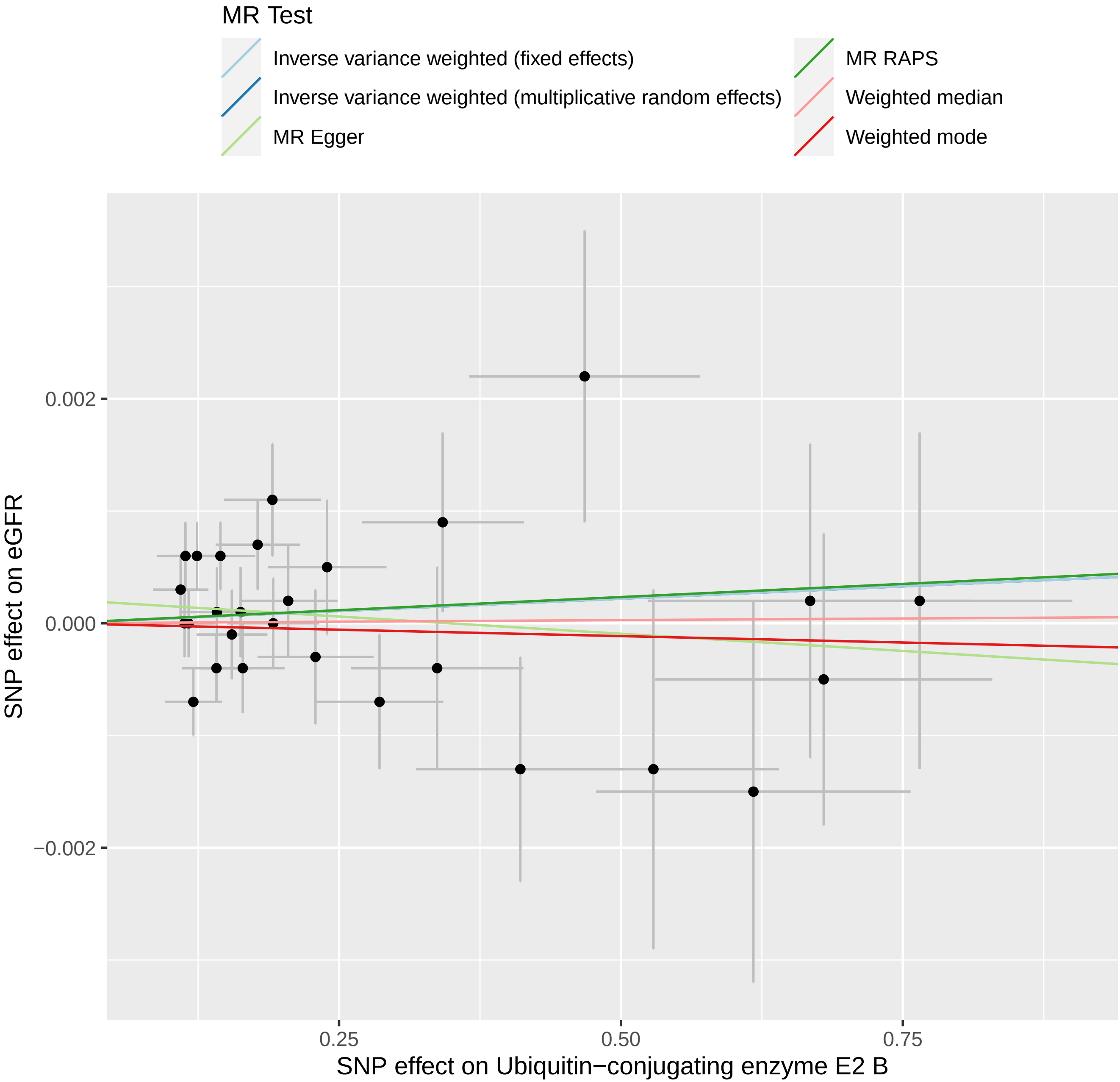

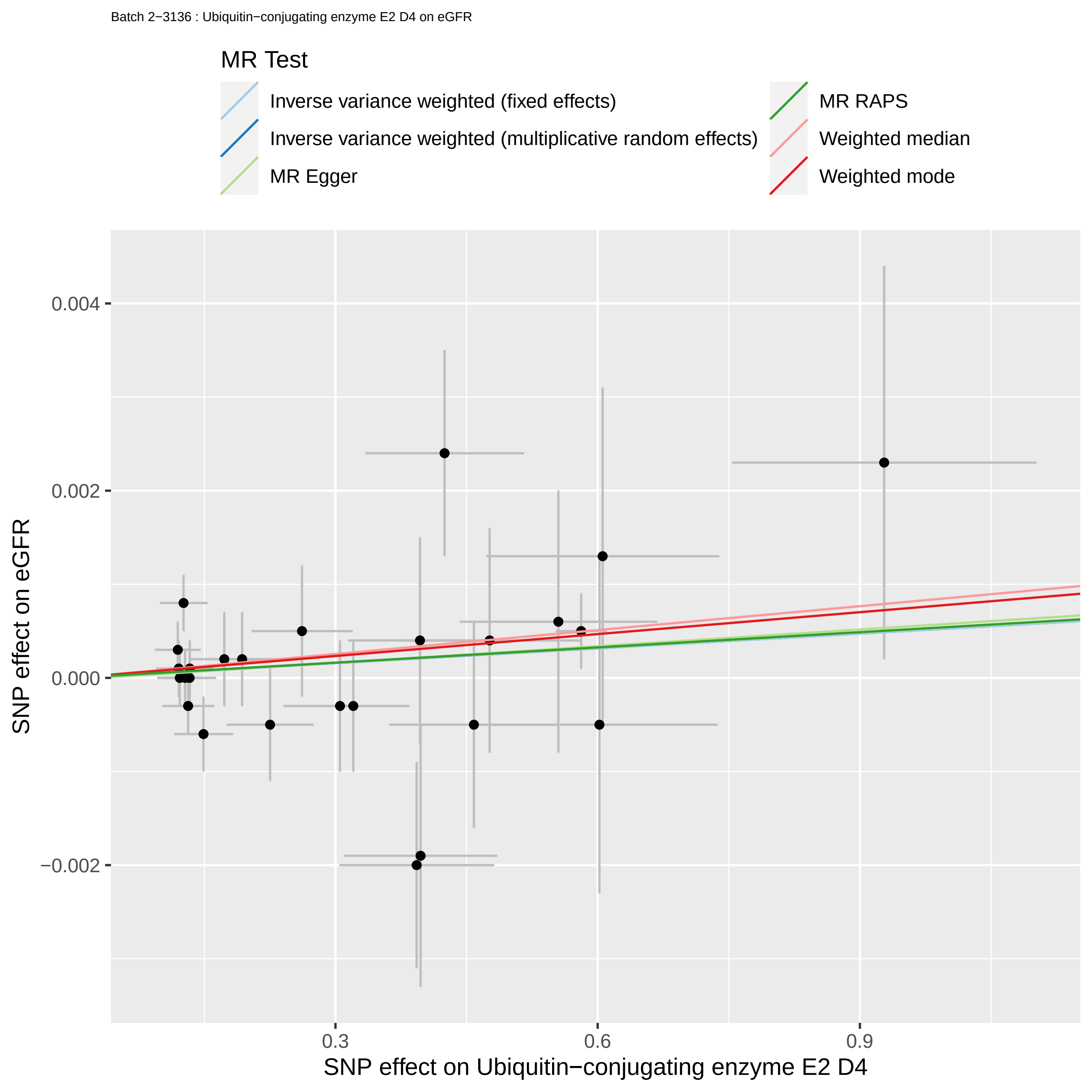

Batch 2−3136 : Ubiquitin−conjugating enzyme E2 D4 on eGFR
MR Test
Inverse variance weighted (fixed effects)
Inverse variance weighted (multiplicative random effects)
MR Egger
MR RAPS
Weighted median
Weighted mode
0.004
0.002
0.000
−0.002
0.3
0.6
0.9
SNP effect on eGFR
SNP effect on Ubiquitin−conjugating enzyme E2 D4

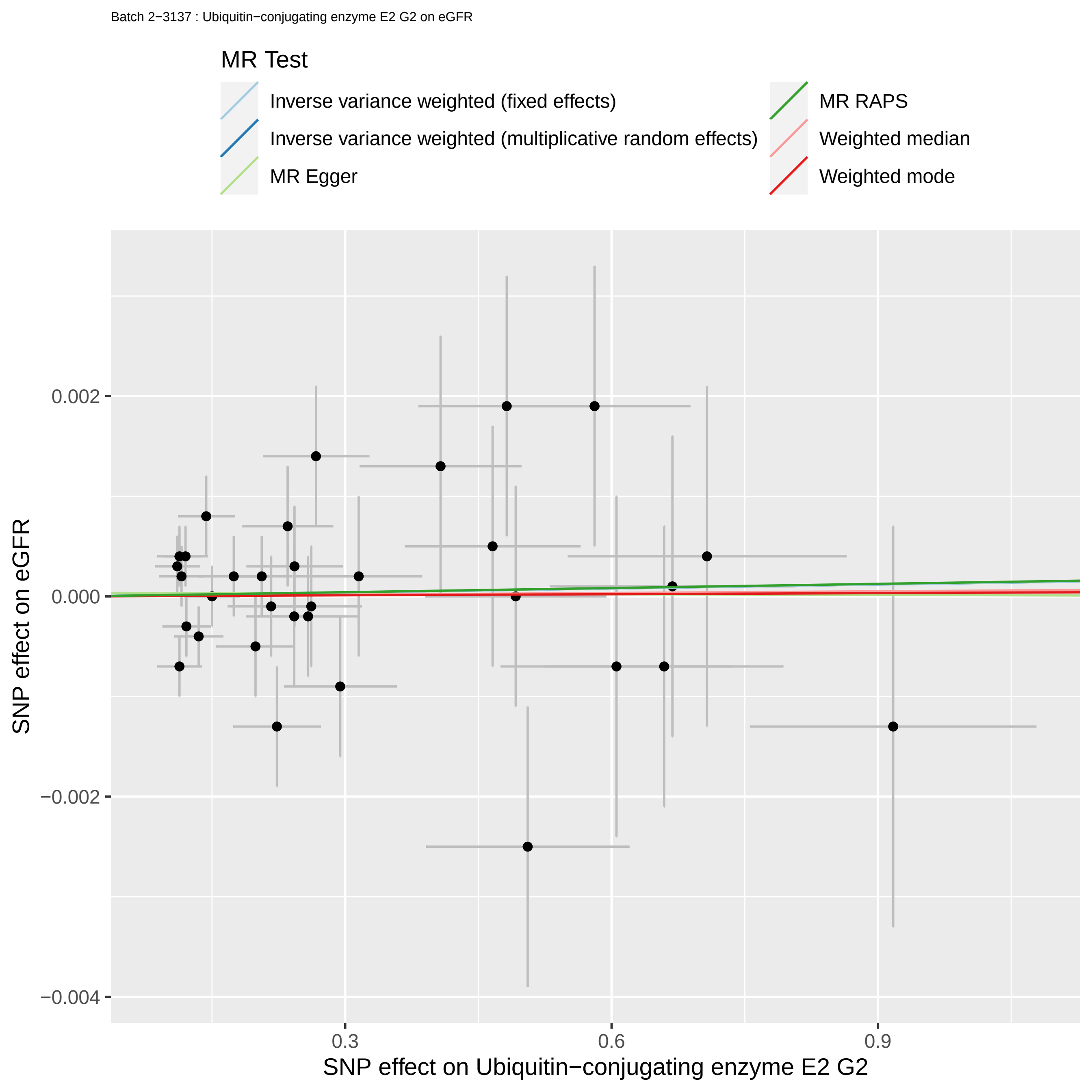
Batch 2−3137 : Ubiquitin−conjugating enzyme E2 G2 on eGFR
MR Test
Inverse variance weighted (fixed effects)
Inverse variance weighted (multiplicative random effects)
MR Egger
MR RAPS
Weighted median
Weighted mode
0.002
0.000
−0.002
−0.004
SNP effect on eGFR
0.3
0.6
0.9
SNP effect on Ubiquitin−conjugating enzyme E2 G2

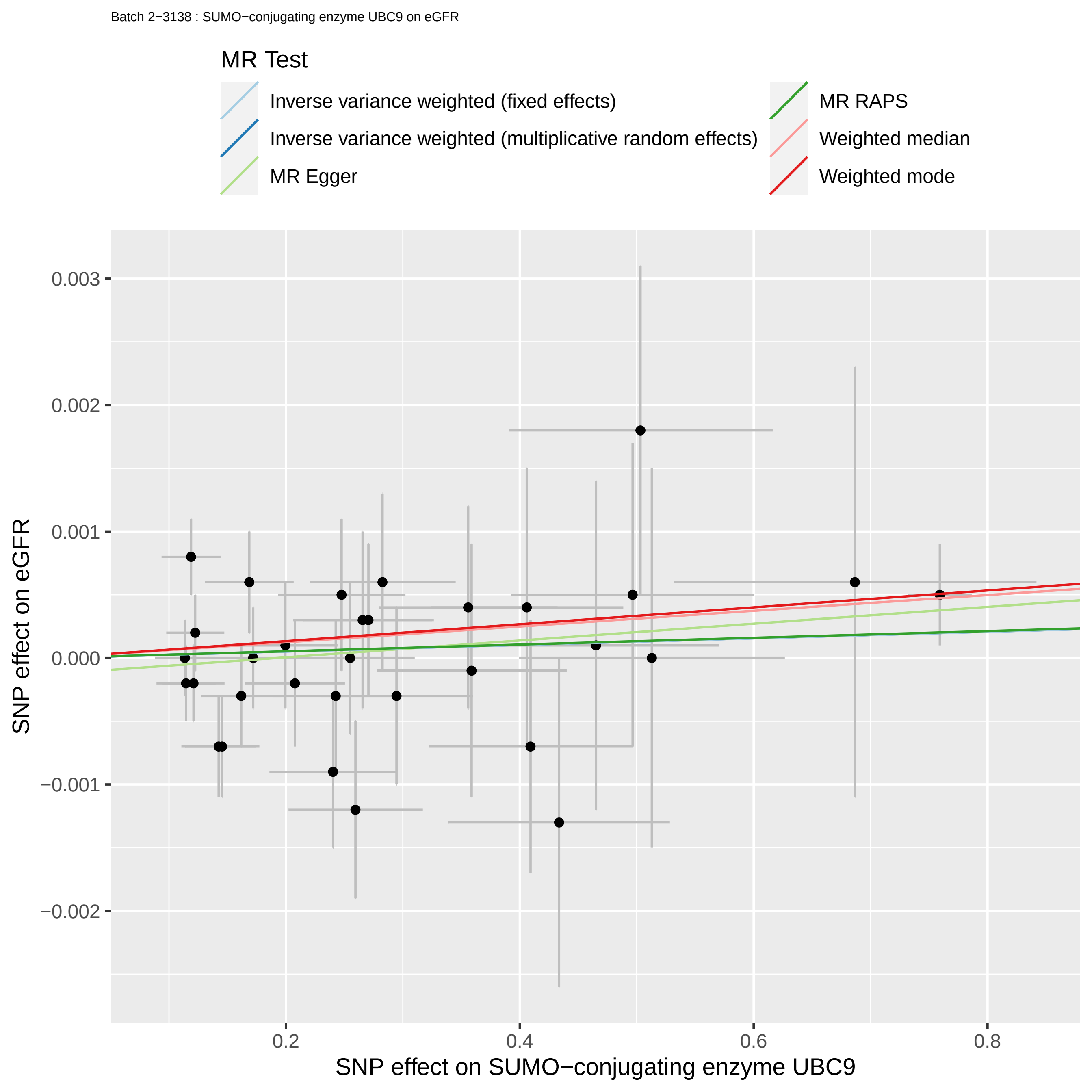

Batch 2−3138 : SUMO−conjugating enzyme UBC9 on eGFR
MR Test
Inverse variance weighted (fixed effects)
Inverse variance weighted (multiplicative random effects)
MR Egger
MR RAPS
Weighted median
Weighted mode
0.003
0.002
0.001
0.000
−0.001
−0.002
SNP effect on eGFR
0.2
0.4
0.6
0.8
SNP effect on SUMO−conjugating enzyme UBC9

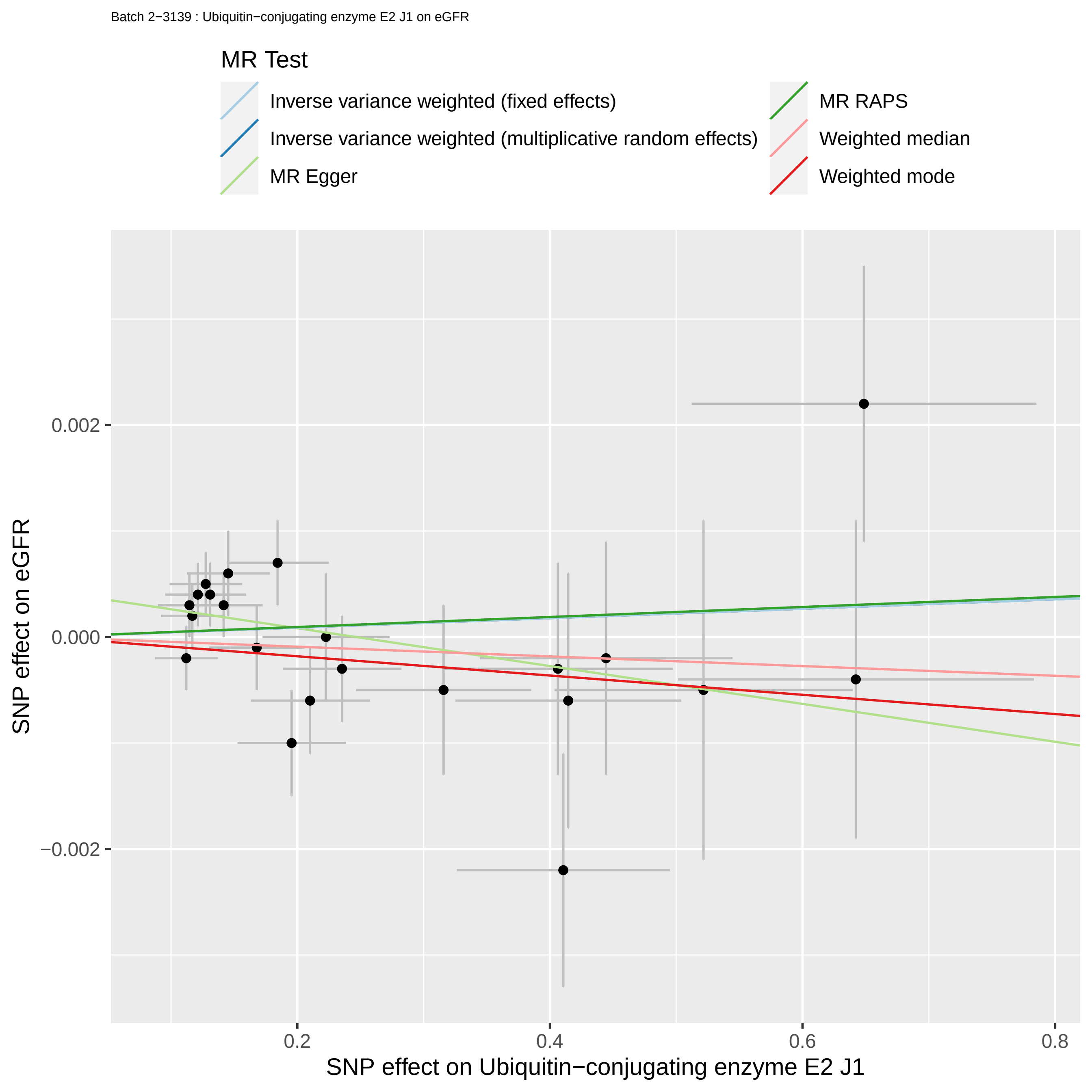
Batch 2−3139 : Ubiquitin−conjugating enzyme E2 J1 on eGFR
MR Test
Inverse variance weighted (fixed effects)
Inverse variance weighted (multiplicative random effects)
MR Egger
MR RAPS
Weighted median
Weighted mode
0.002
0.000
−0.002
0.2
0.4
0.6
0.8
SNP effect on eGFR
SNP effect on Ubiquitin−conjugating enzyme E2 J1

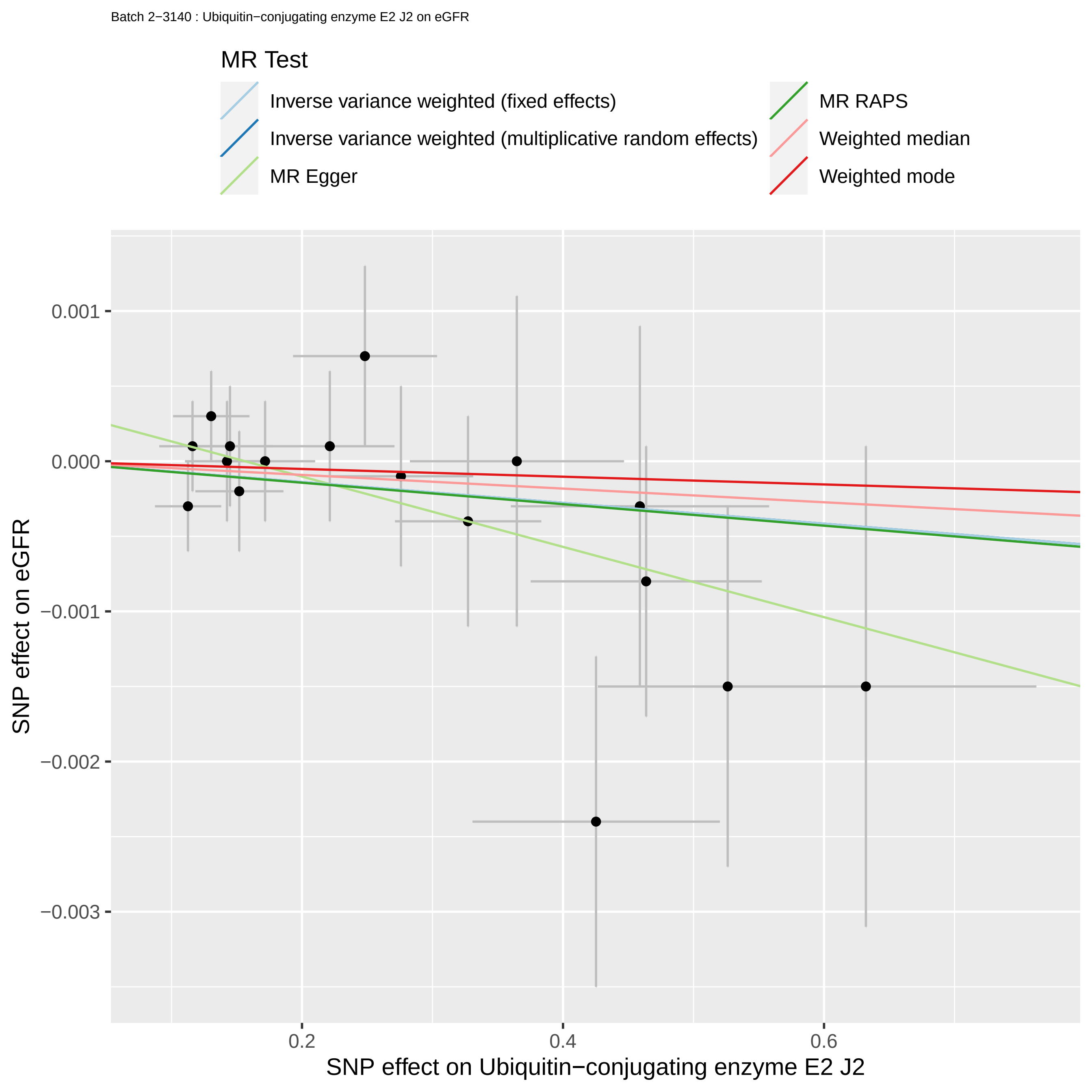

Batch 2−3140 : Ubiquitin−conjugating enzyme E2 J2 on eGFR
MR Test
Inverse variance weighted (fixed effects)
Inverse variance weighted (multiplicative random effects)
MR Egger
MR RAPS
Weighted median
Weighted mode
0.001
0.000
−0.001
−0.002
−0.003
0.2
0.4
0.6
SNP effect on eGFR
SNP effect on Ubiquitin−conjugating enzyme E2 J2

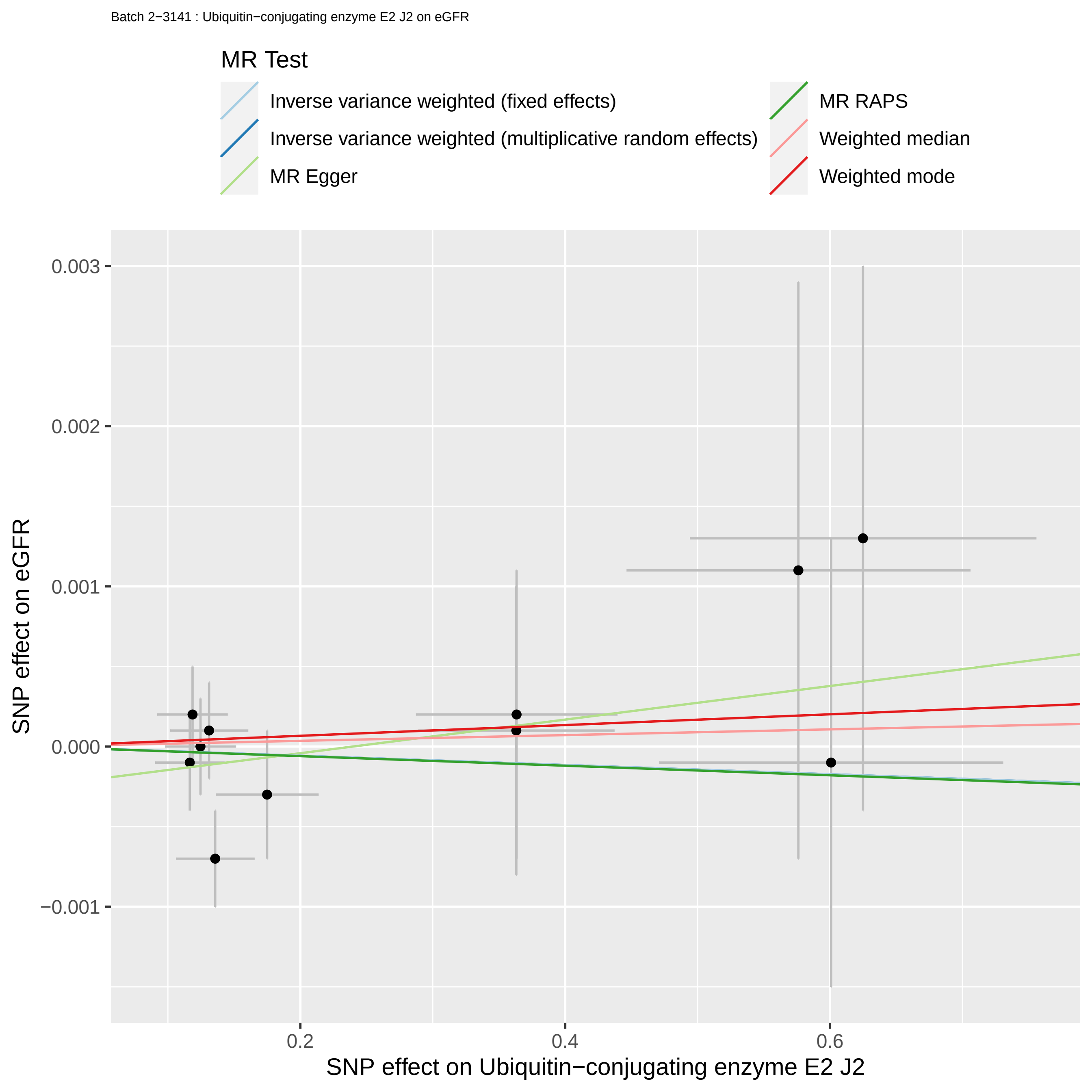

Batch 2−3141 : Ubiquitin−conjugating enzyme E2 J2 on eGFR
MR Test
Inverse variance weighted (fixed effects)
Inverse variance weighted (multiplicative random effects)
MR Egger
MR RAPS
Weighted median
Weighted mode
0.003
0.002
0.001
0.000
−0.001
0.2
0.4
0.6
SNP effect on eGFR
SNP effect on Ubiquitin−conjugating enzyme E2 J2

Batch 2−3142 : Ubiquitin−conjugating enzyme E2 L3 on eGFR

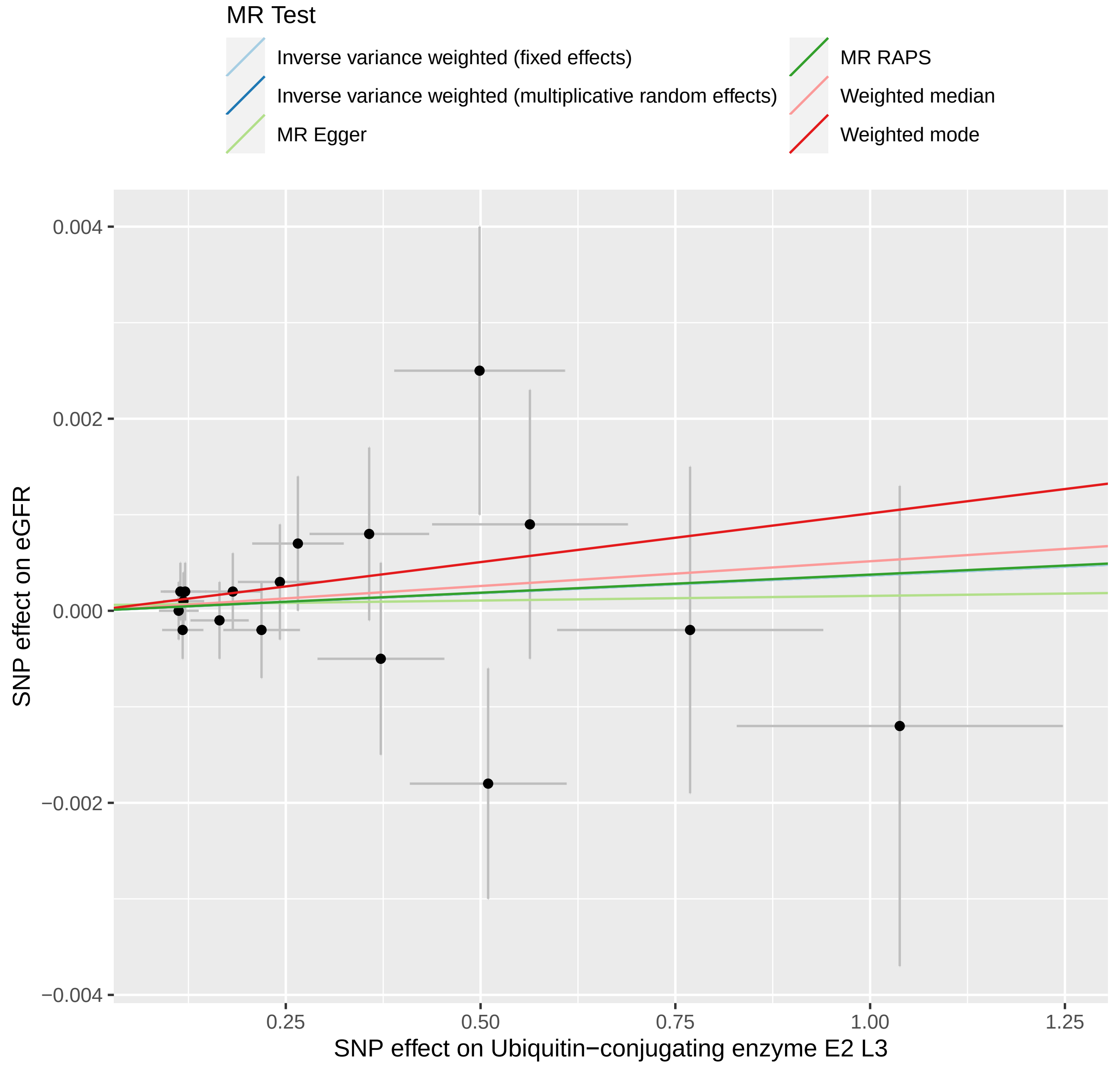

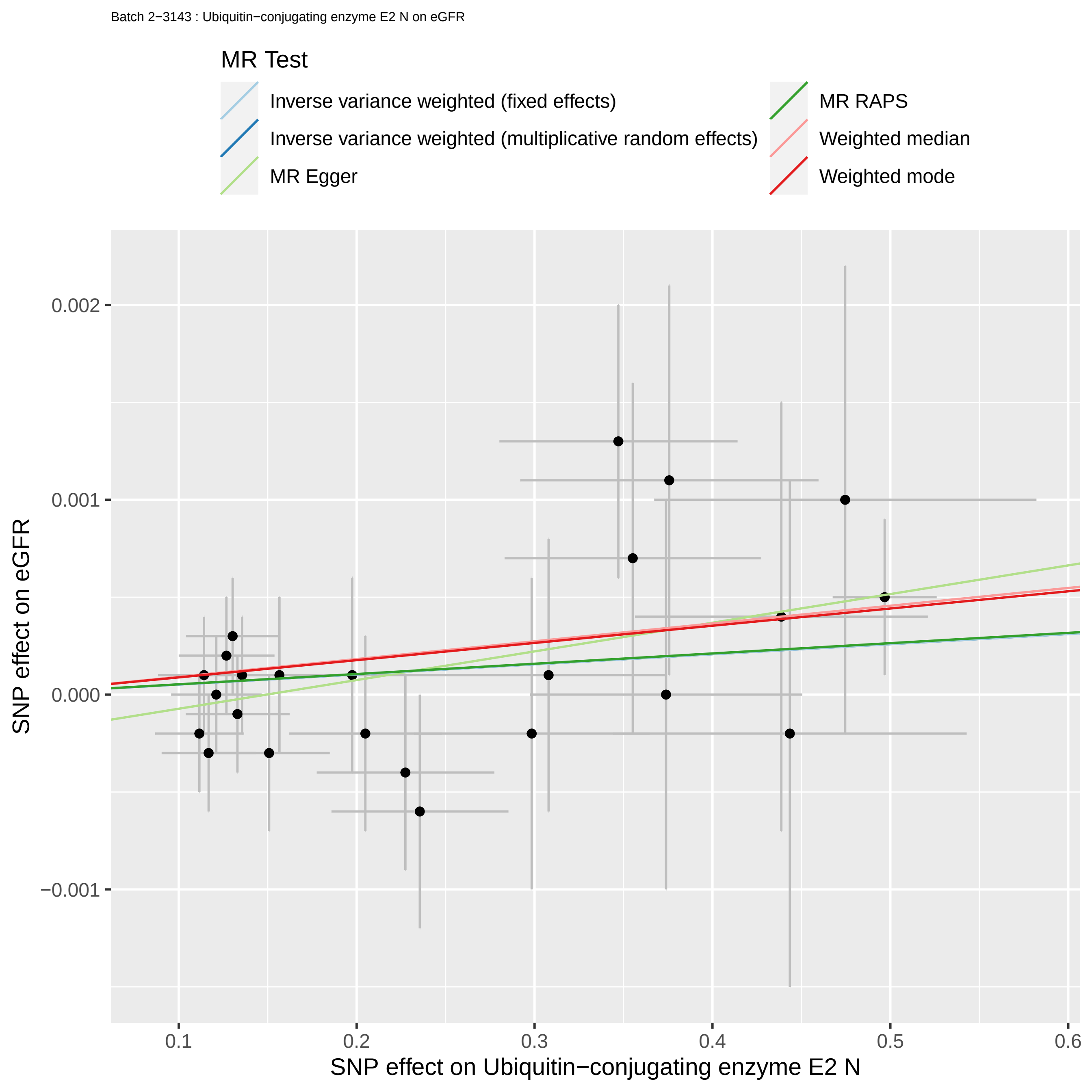
Batch 2−3143 : Ubiquitin−conjugating enzyme E2 N on eGFR
MR Test
Inverse variance weighted (fixed effects)
Inverse variance weighted (multiplicative random effects)
MR Egger
MR RAPS
Weighted median
Weighted mode
0.002
0.001
0.000
−0.001
SNP effect on eGFR
0.1
0.2
0.3
0.4
0.5
0.6
SNP effect on Ubiquitin−conjugating enzyme E2 N

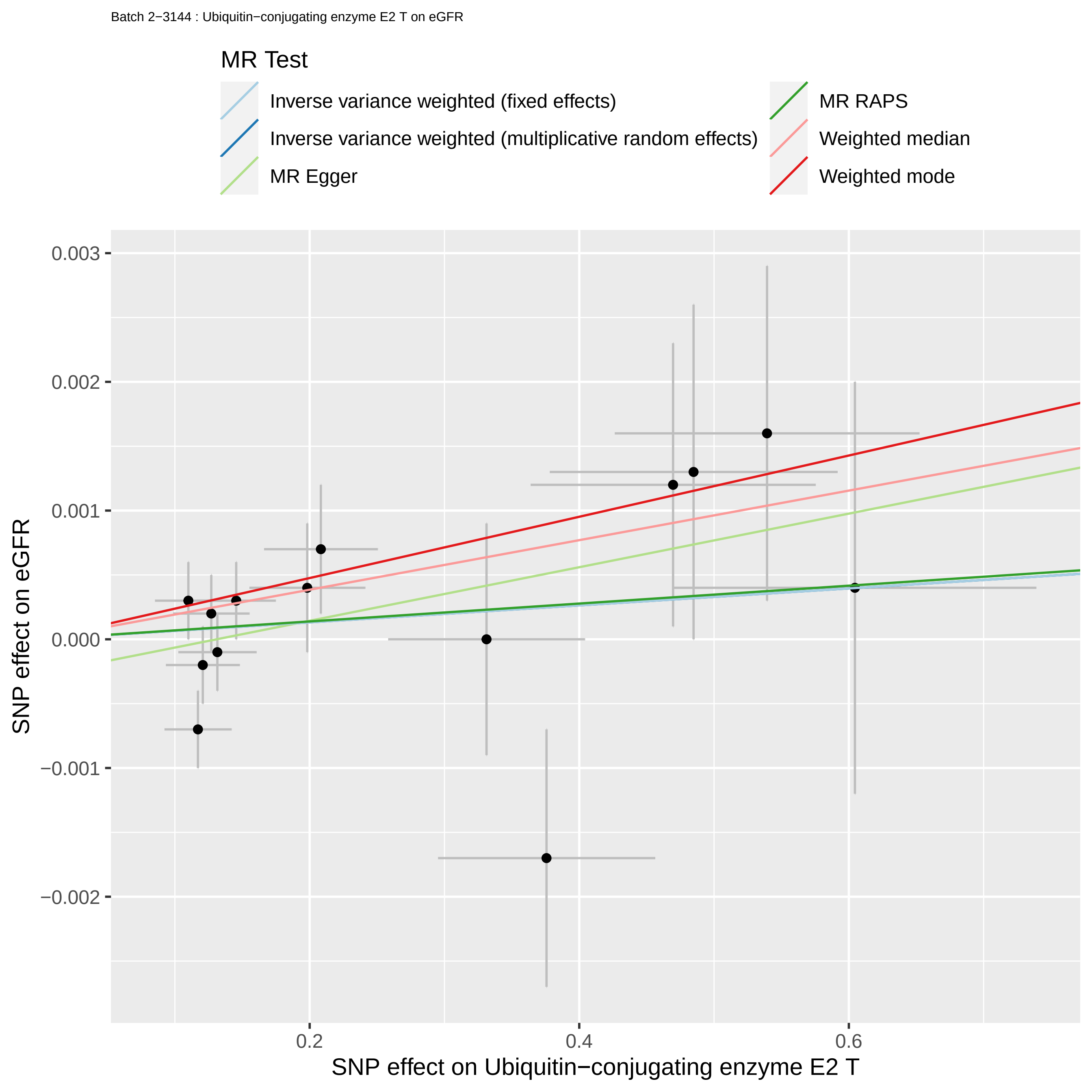

Batch 2−3144 : Ubiquitin−conjugating enzyme E2 T on eGFR
MR Test
Inverse variance weighted (fixed effects)
Inverse variance weighted (multiplicative random effects)
MR Egger
MR RAPS
Weighted median
Weighted mode
0.003
0.002
0.001
0.000
−0.001
−0.002
SNP effect on eGFR
0.2
0.4
0.6
SNP effect on Ubiquitin−conjugating enzyme E2 T

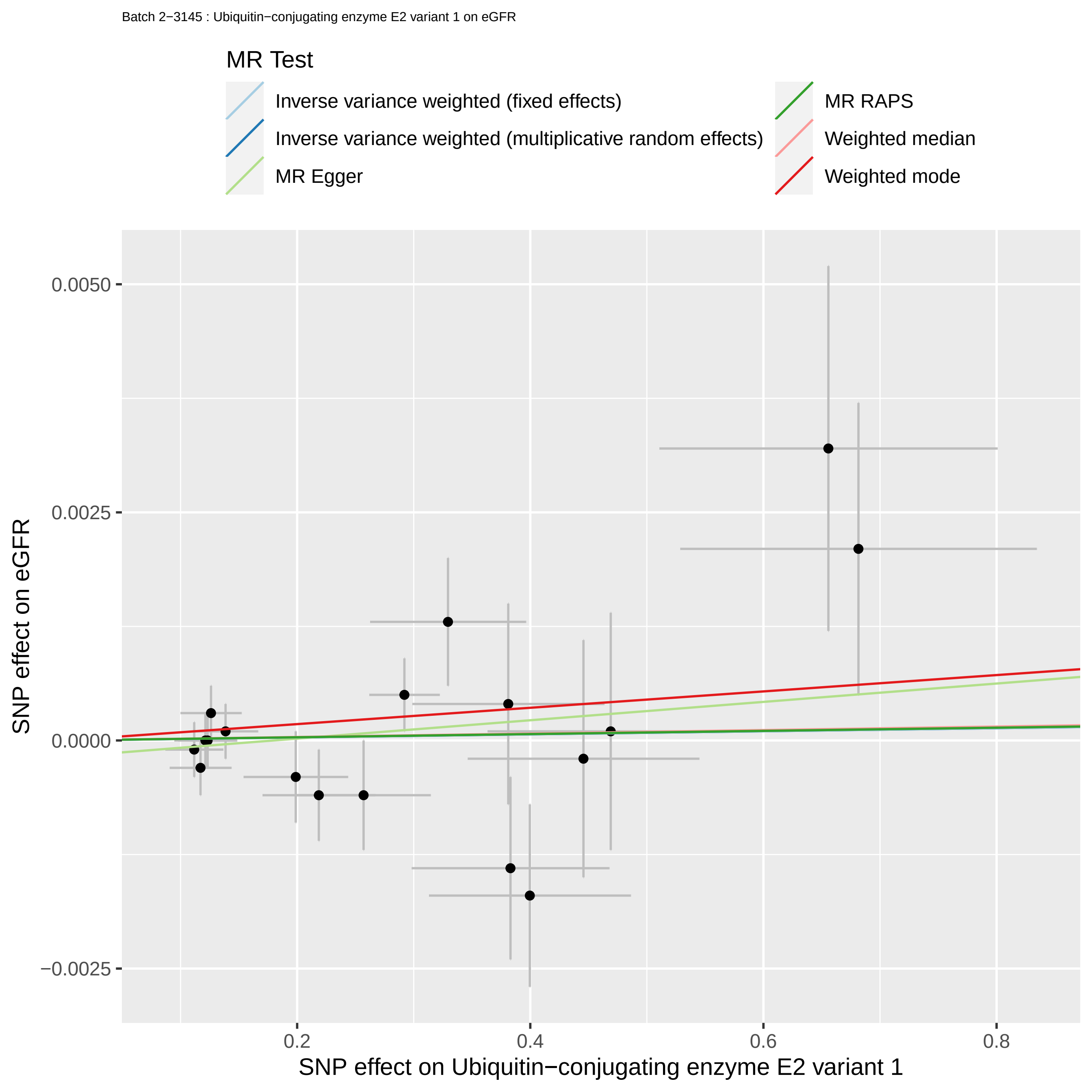
Batch 2−3145 : Ubiquitin−conjugating enzyme E2 variant 1 on eGFR
MR Test
Inverse variance weighted (fixed effects)
Inverse variance weighted (multiplicative random effects)
MR Egger
MR RAPS
Weighted median
Weighted mode
0.0050
0.0025
0.0000
−0.0025
0.2
0.4
0.6
0.8
SNP effect on eGFR
SNP effect on Ubiquitin−conjugating enzyme E2 variant 1

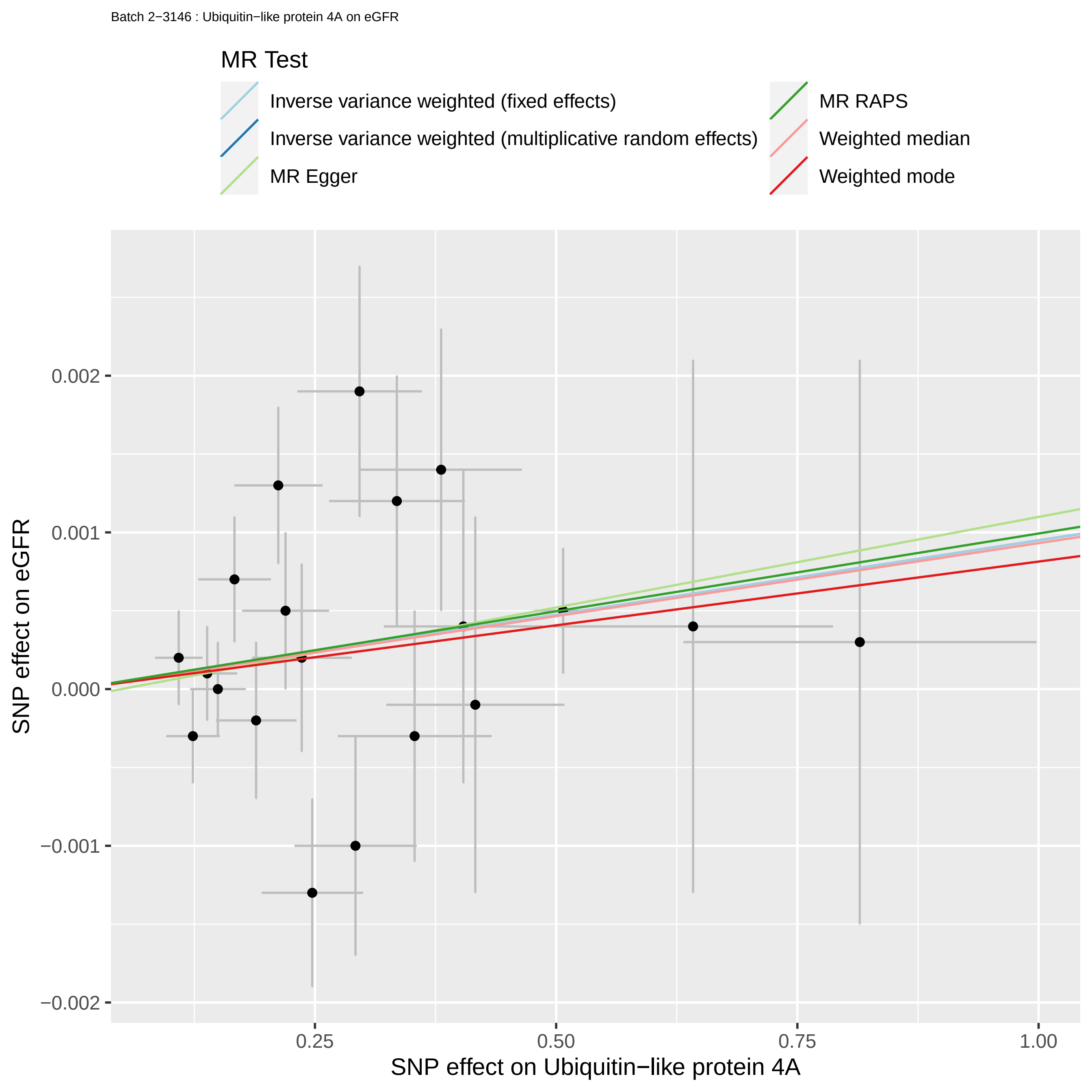
Batch 2−3146 : Ubiquitin−like protein 4A on eGFR
MR Test
Inverse variance weighted (fixed effects)
Inverse variance weighted (multiplicative random effects)
MR Egger
MR RAPS
Weighted median
Weighted mode
0.002
0.001
0.000
−0.001
−0.002
SNP effect on eGFR
0.25
0.50
0.75
1.00
SNP effect on Ubiquitin−like protein 4A

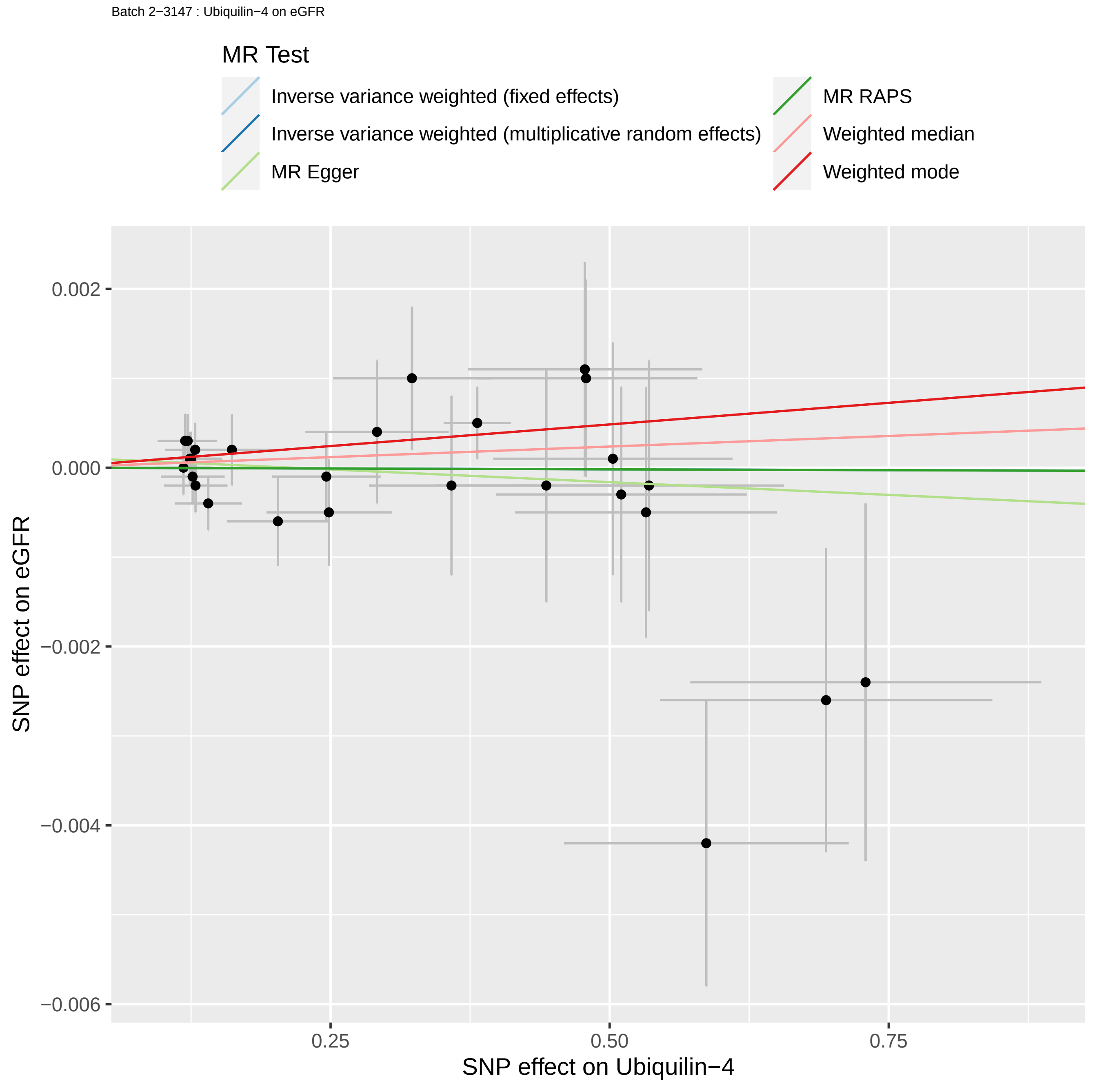
Batch 2−3147 : Ubiquilin−4 on eGFR
MR Test
Inverse variance weighted (fixed effects)
Inverse variance weighted (multiplicative random effects)
MR Egger
MR RAPS
Weighted median
Weighted mode
0.002
0.000
−0.002
−0.004
−0.006
SNP effect on eGFR
0.25
0.50
0.75
SNP effect on Ubiquilin−4

Batch 2−3148 : Ubiquitin domain−containing protein 2 on eGFR

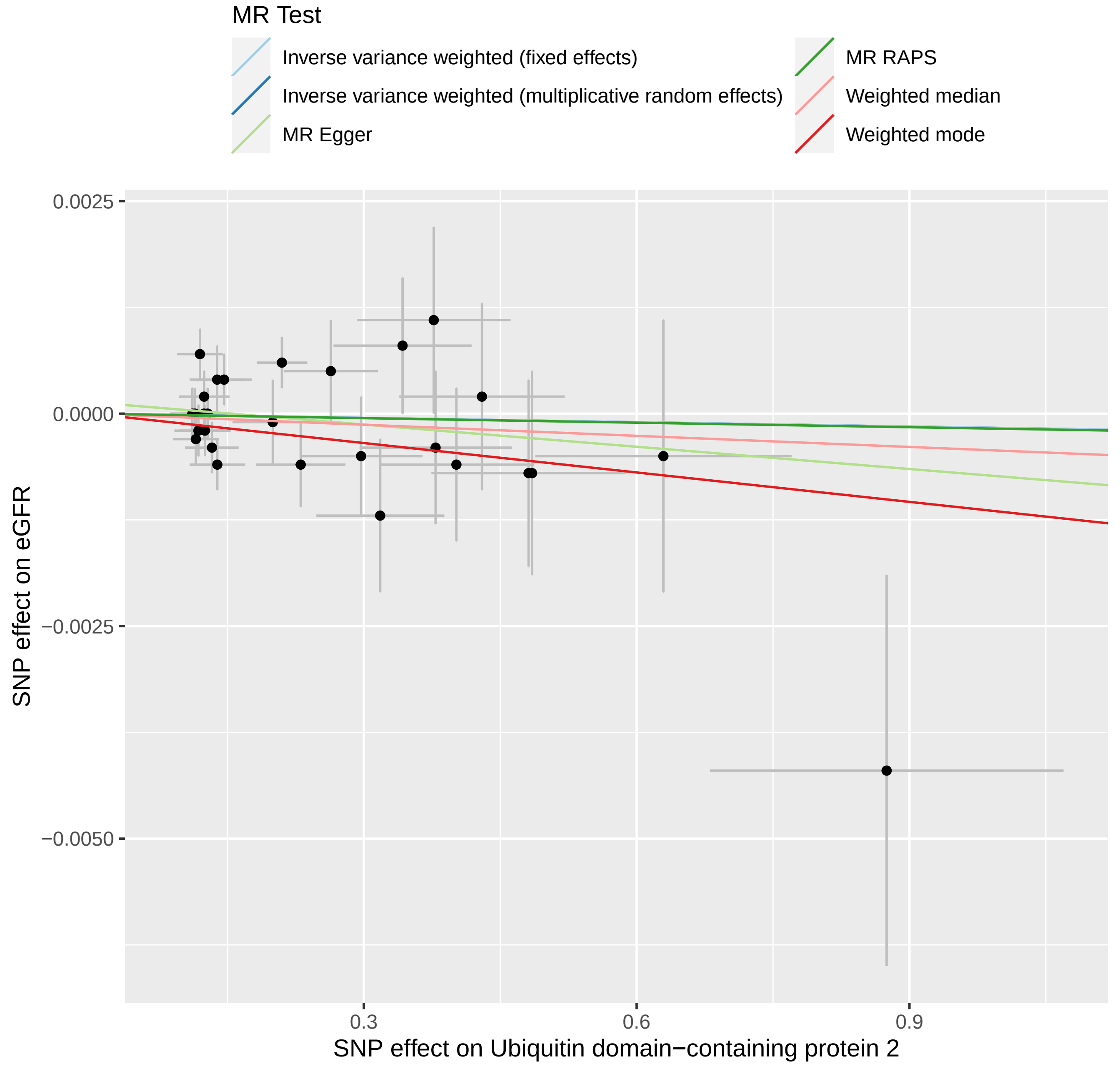

Batch 2−3149 : UBX domain−containing protein 4 on eGFR

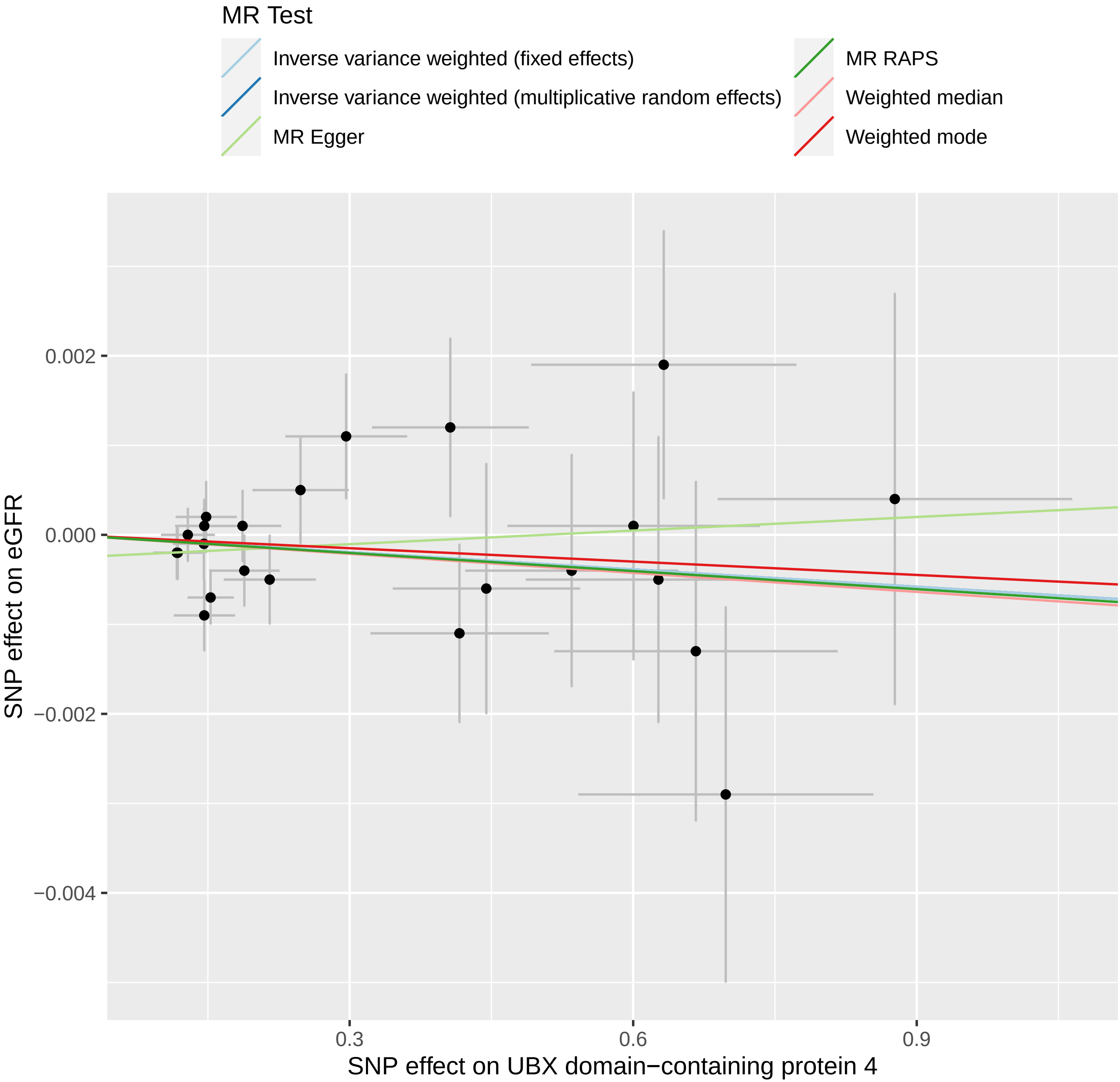

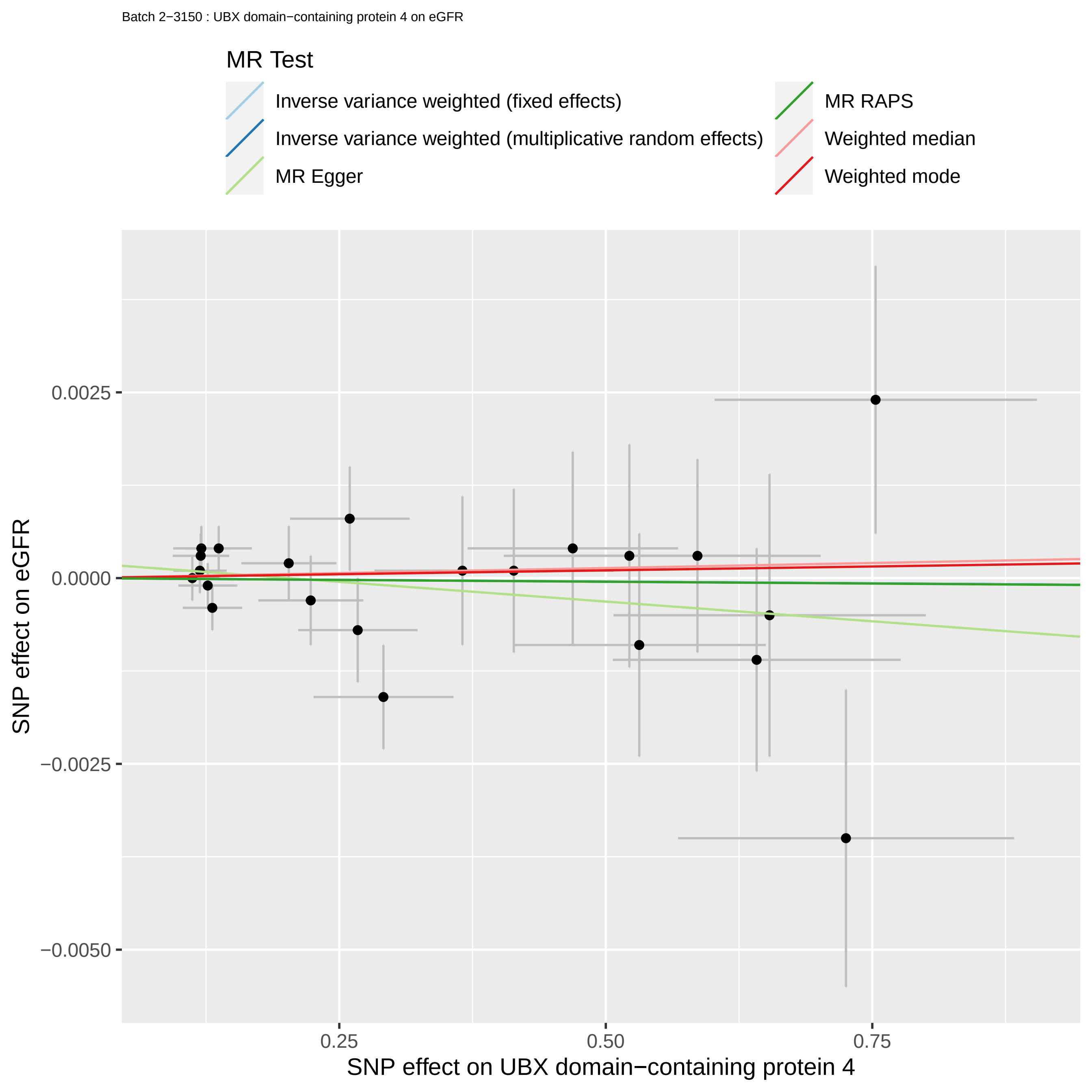
Batch 2−3150 : UBX domain−containing protein 4 on eGFR
MR Test
Inverse variance weighted (fixed effects)
Inverse variance weighted (multiplicative random effects)
MR Egger
MR RAPS
Weighted median
Weighted mode
0.0025
0.0000
−0.0025
−0.0050
0.25
0.50
0.75
SNP effect on eGFR
SNP effect on UBX domain−containing protein 4

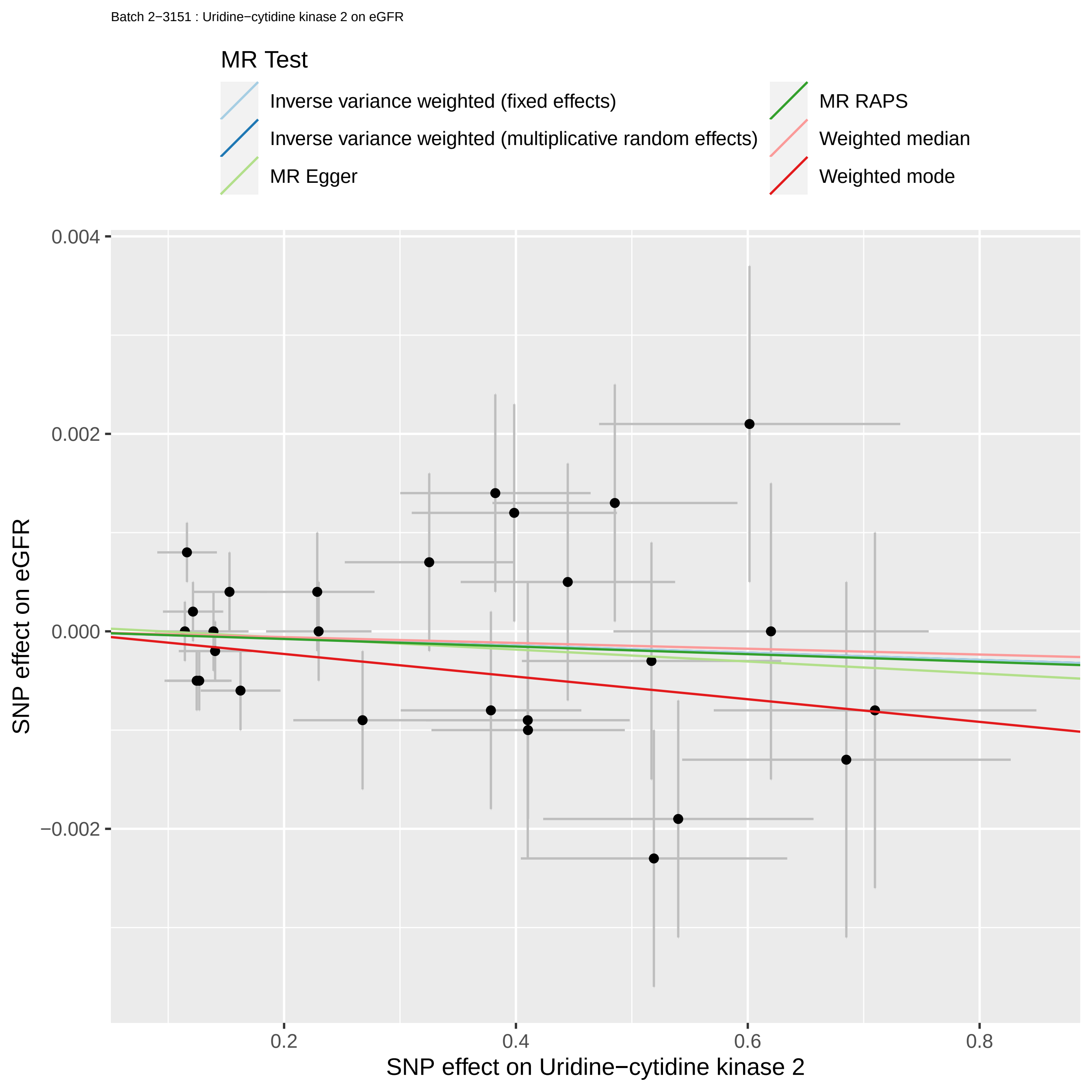
Batch 2−3151 : Uridine−cytidine kinase 2 on eGFR
MR Test
Inverse variance weighted (fixed effects)
Inverse variance weighted (multiplicative random effects)
MR Egger
MR RAPS
Weighted median
Weighted mode
0.004
0.002
0.000
−0.002
SNP effect on eGFR
0.2
0.4
0.6
0.8
SNP effect on Uridine−cytidine kinase 2

Batch 2−3152 : Unique cartilage matrix−associated protein on eGFR

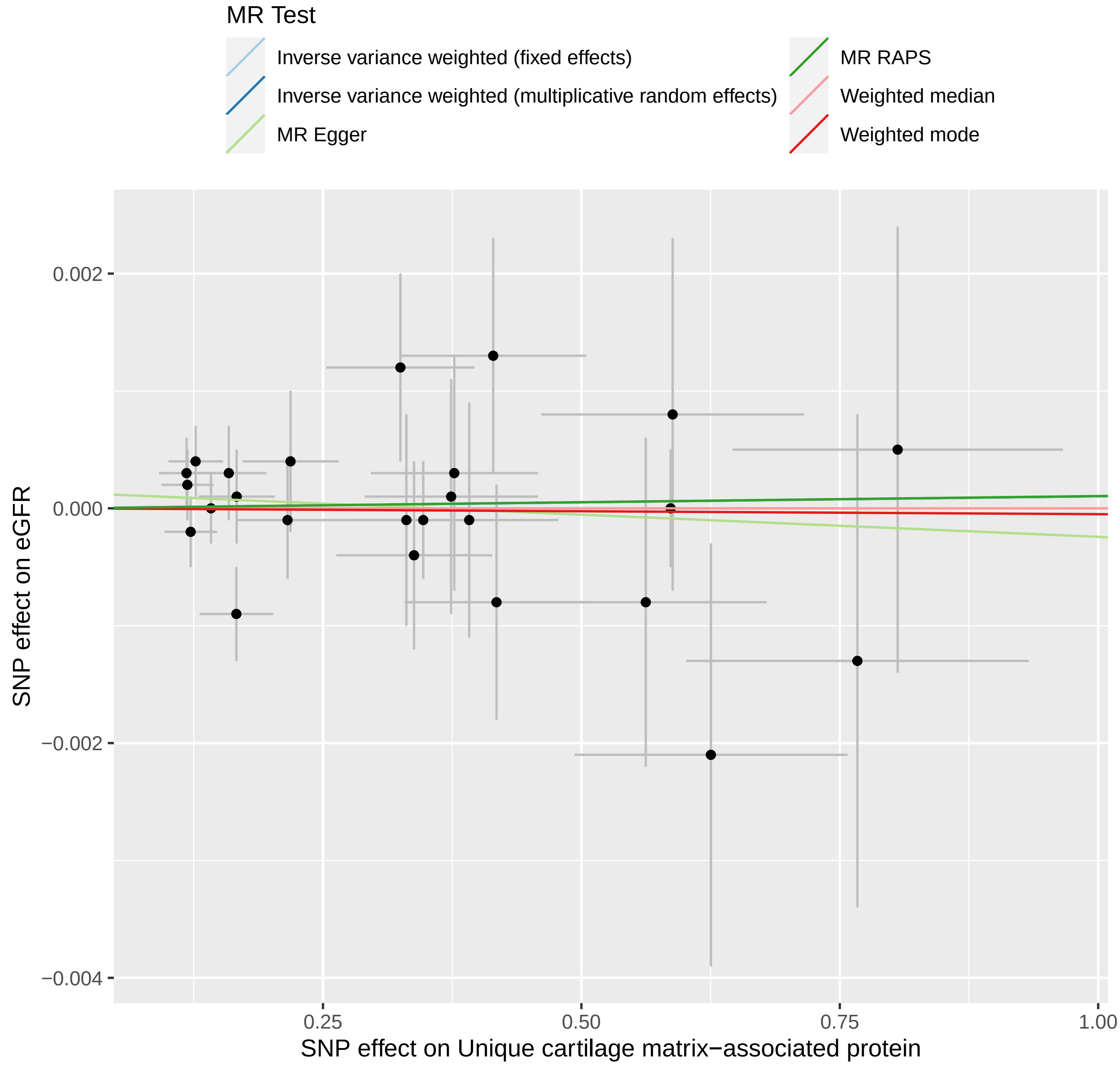

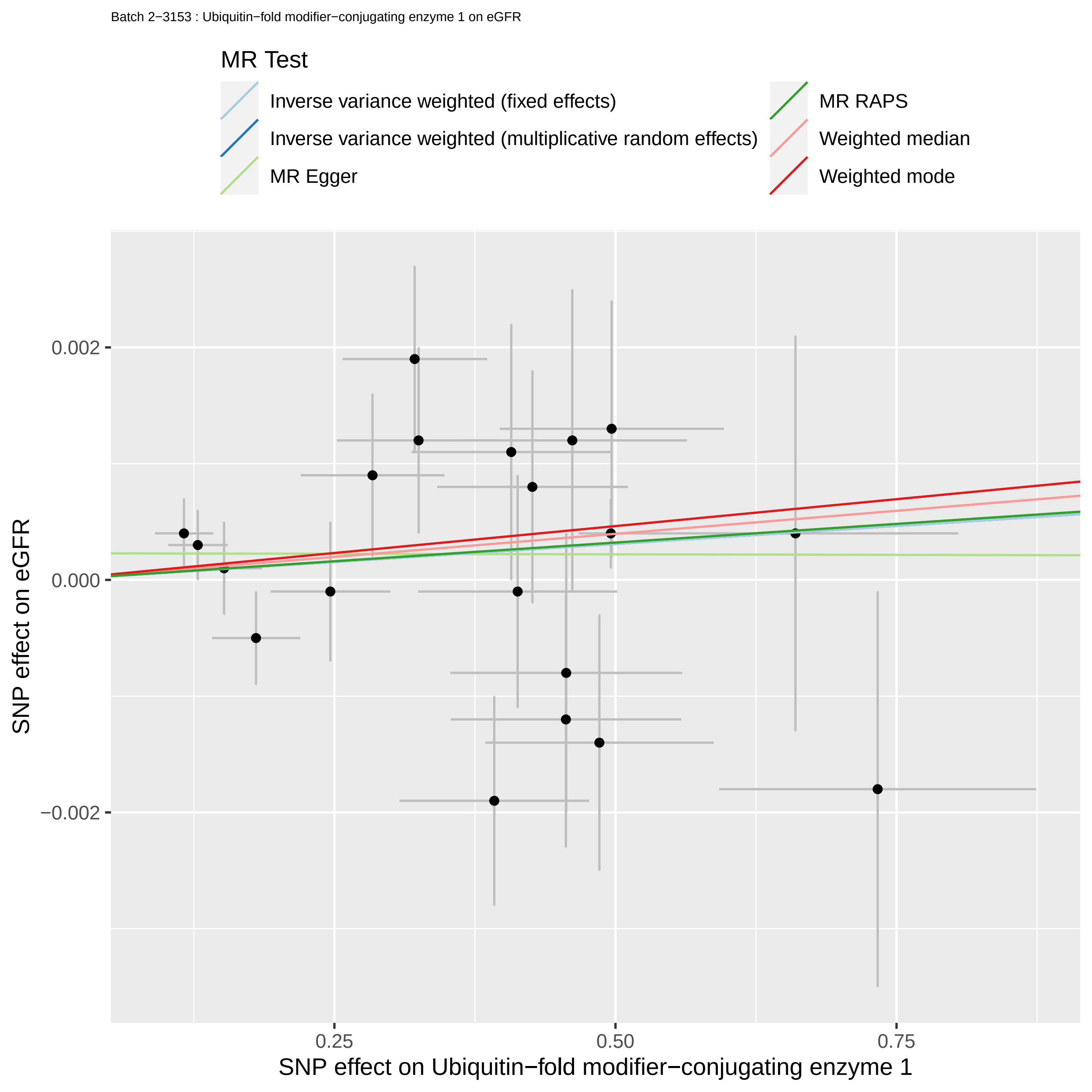
Batch 2−3153 : Ubiquitin−fold modifier−conjugating enzyme 1 on eGFR
MR Test
Inverse variance weighted (fixed effects)
Inverse variance weighted (multiplicative random effects)
MR Egger
MR RAPS
Weighted median
Weighted mode
0.002
0.000
−0.002
0.25
0.50
0.75
SNP effect on eGFR
SNP effect on Ubiquitin−fold modifier−conjugating enzyme 1

Batch 2−3154 : Ubiquitin−fold modifier 1 on eGFR

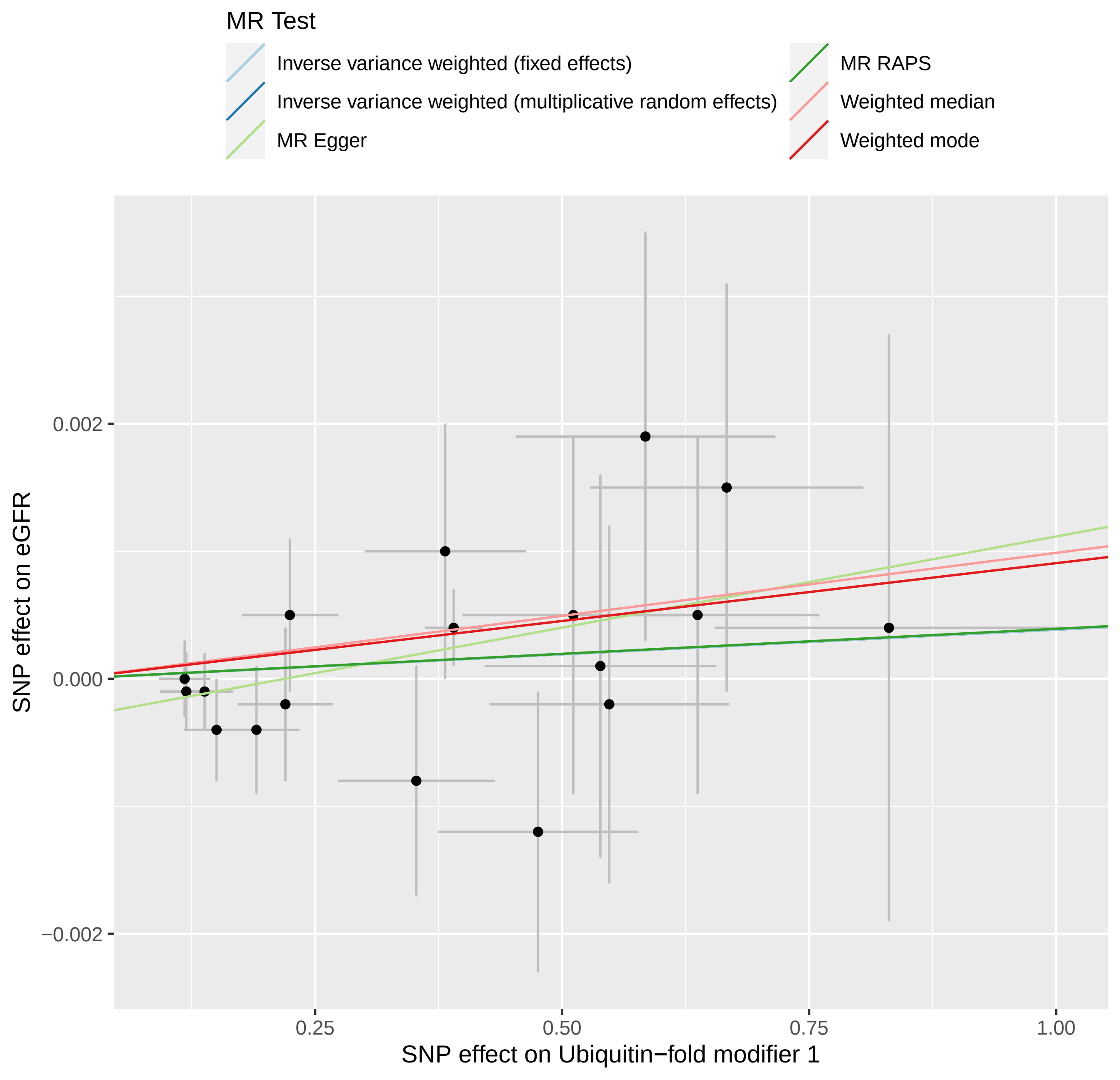

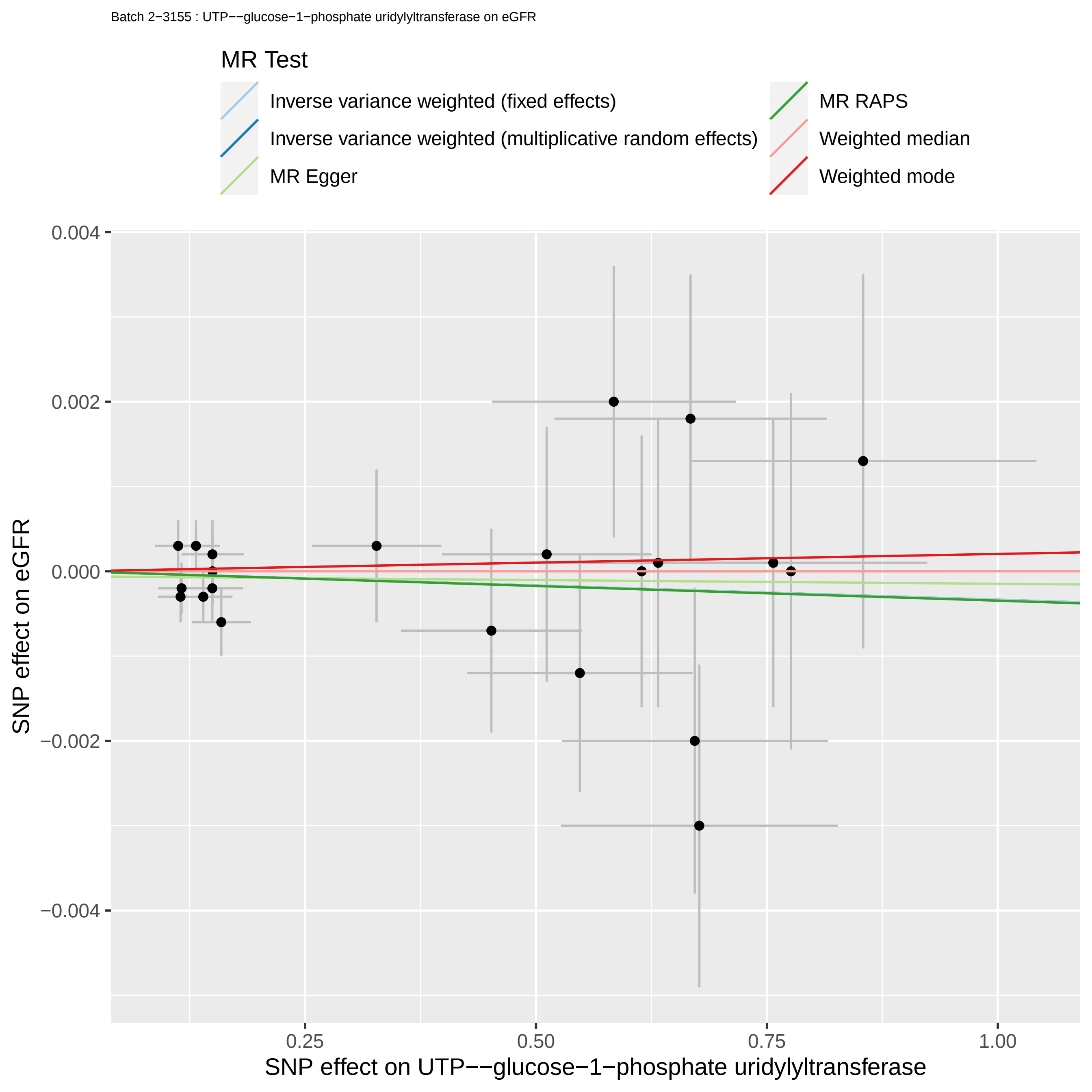
Batch 2−3155 : UTP−−glucose−1−phosphate uridylyltransferase on eGFR
MR Test
Inverse variance weighted (fixed effects)
Inverse variance weighted (multiplicative random effects)
MR Egger
MR RAPS
Weighted median
Weighted mode
0.004
0.002
0.000
−0.002
−0.004
SNP effect on eGFR
0.25
0.50
0.75
1.00
SNP effect on UTP−−glucose−1−phosphate uridylyltransferase

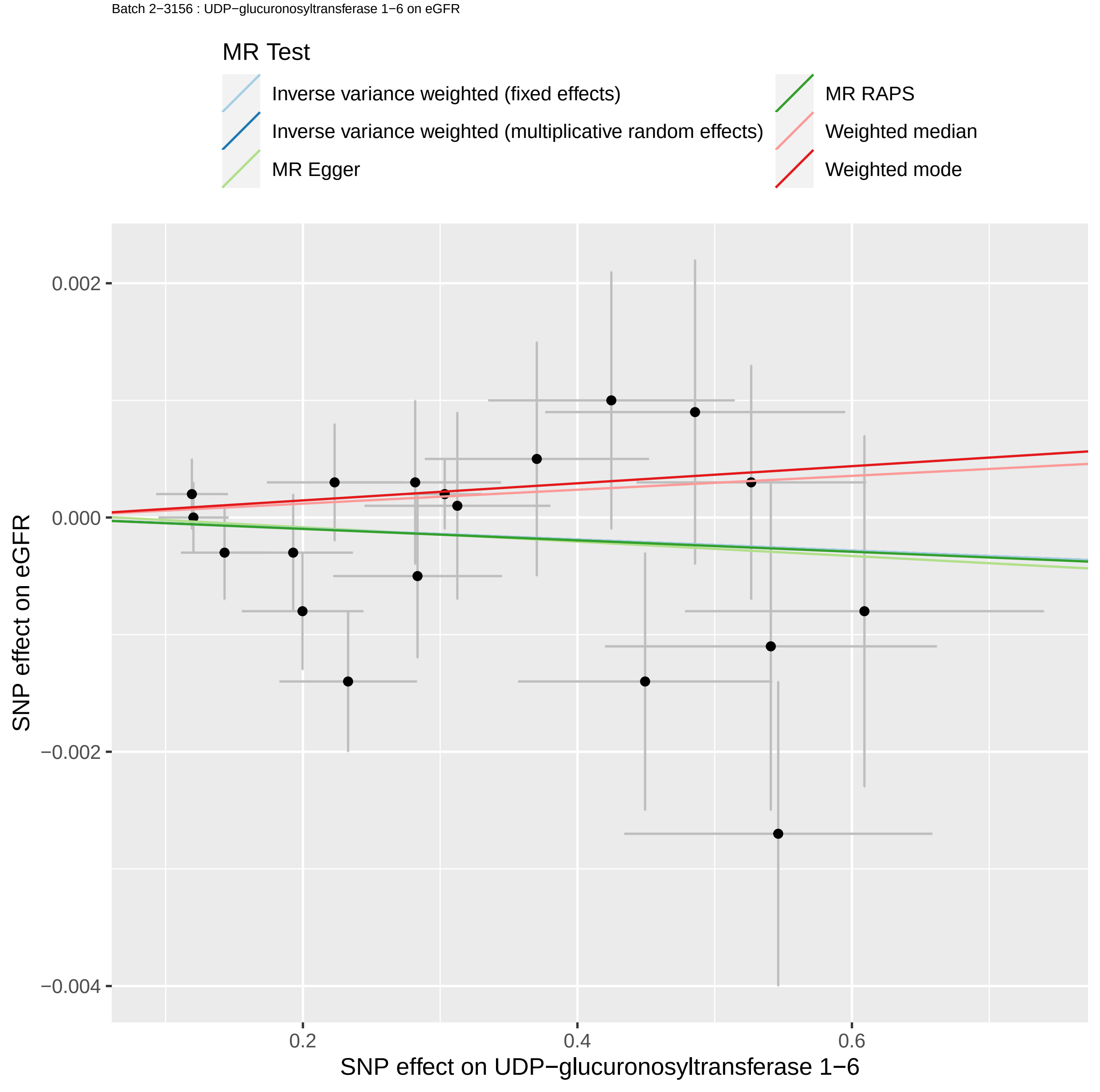
Batch 2−3156 : UDP−glucuronosyltransferase 1−6 on eGFR
MR Test
Inverse variance weighted (fixed effects)
Inverse variance weighted (multiplicative random effects)
MR Egger
MR RAPS
Weighted median
Weighted mode
0.002
0.000
−0.002
−0.004
0.2
0.4
0.6
SNP effect on eGFR
SNP effect on UDP−glucuronosyltransferase 1−6

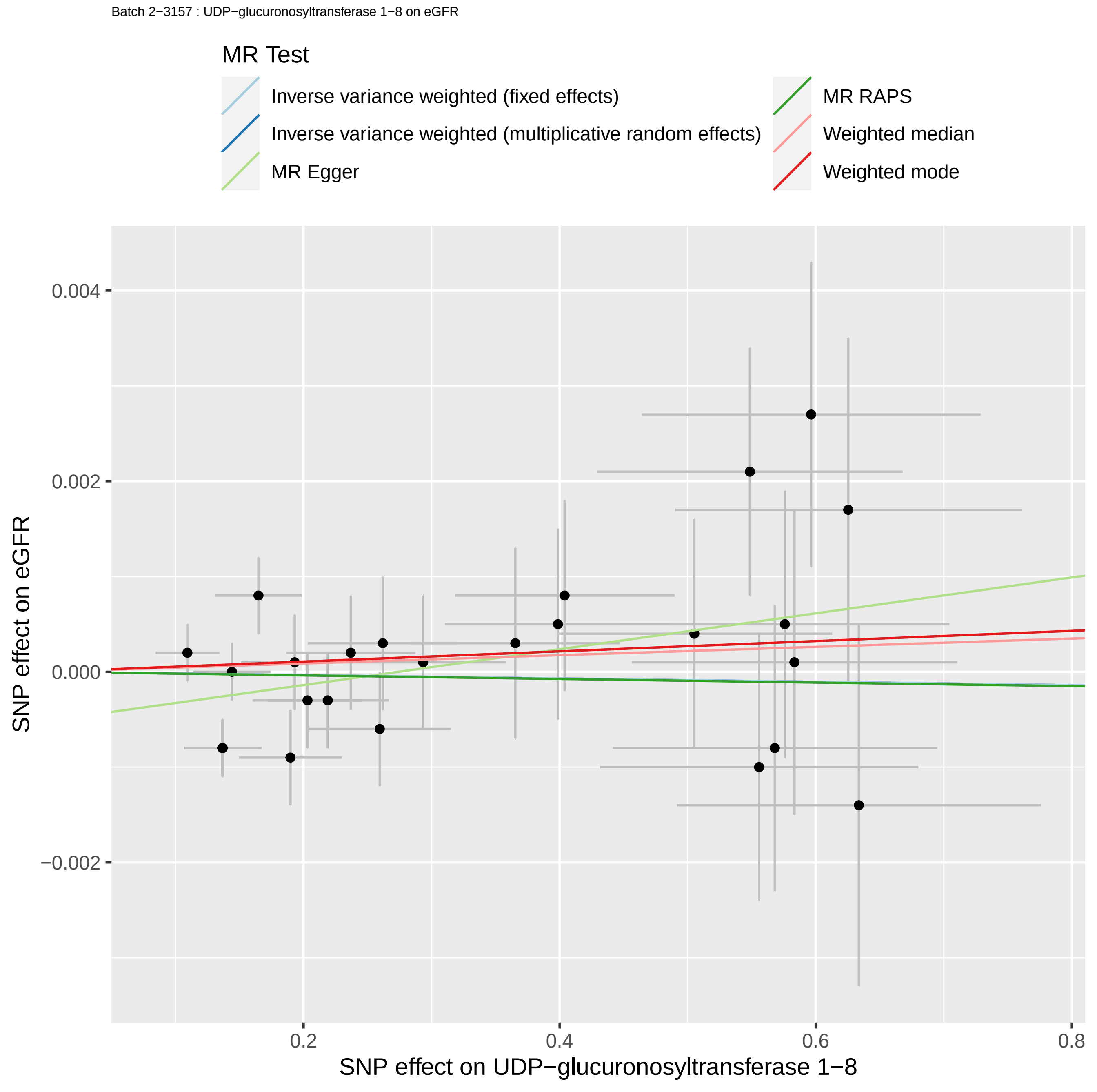

Batch 2−3157 : UDP−glucuronosyltransferase 1−8 on eGFR
MR Test
Inverse variance weighted (fixed effects)
Inverse variance weighted (multiplicative random effects)
MR Egger
MR RAPS
Weighted median
Weighted mode
0.004
0.002
0.000
−0.002
0.2
0.4
0.6
0.8
SNP effect on eGFR
SNP effect on UDP−glucuronosyltransferase 1−8

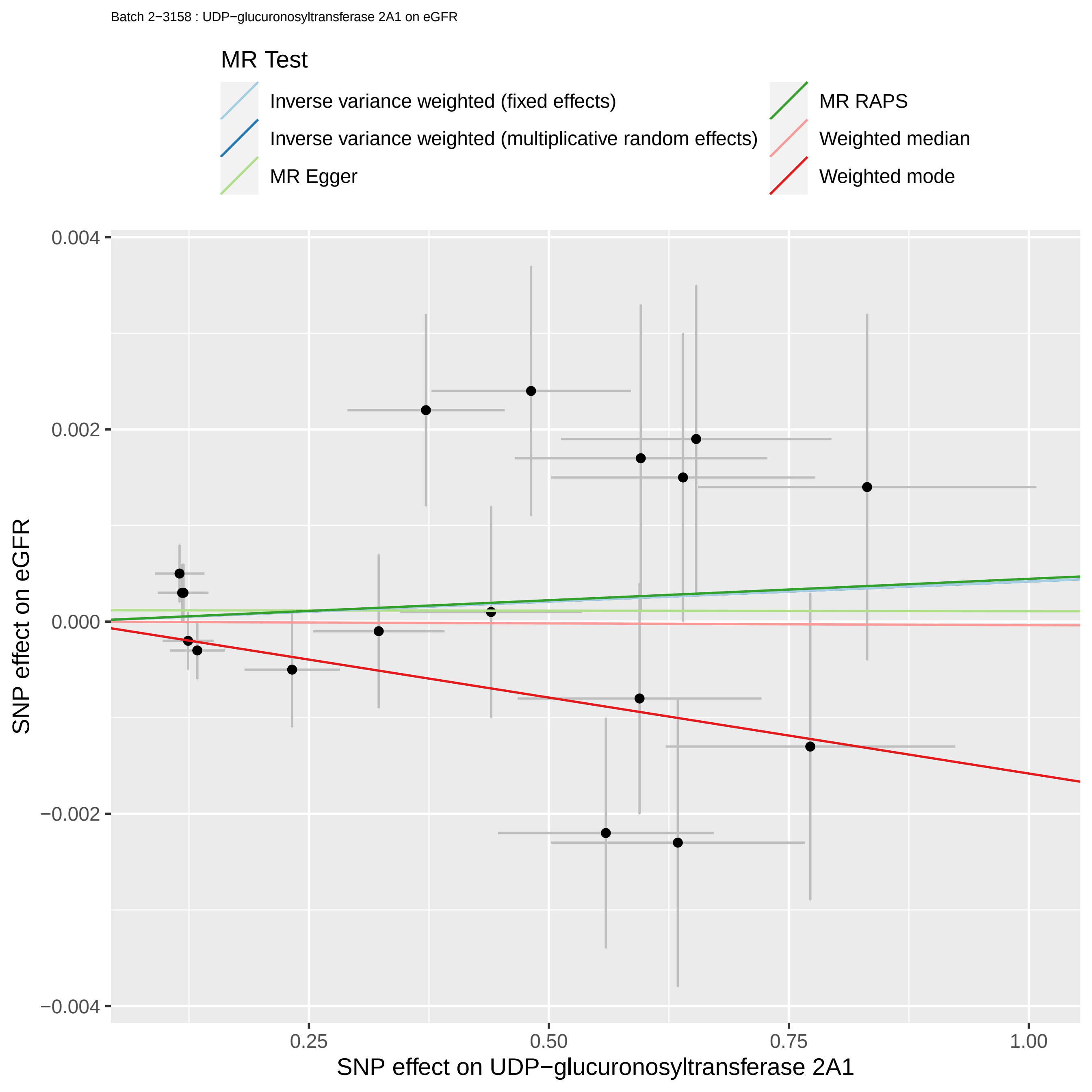

Batch 2−3158 : UDP−glucuronosyltransferase 2A1 on eGFR
MR Test
Inverse variance weighted (fixed effects)
Inverse variance weighted (multiplicative random effects)
MR Egger
MR RAPS
Weighted median
Weighted mode
0.004
0.002
0.000
−0.002
−0.004
0.25
0.50
0.75
1.00
SNP effect on eGFR
SNP effect on UDP−glucuronosyltransferase 2A1

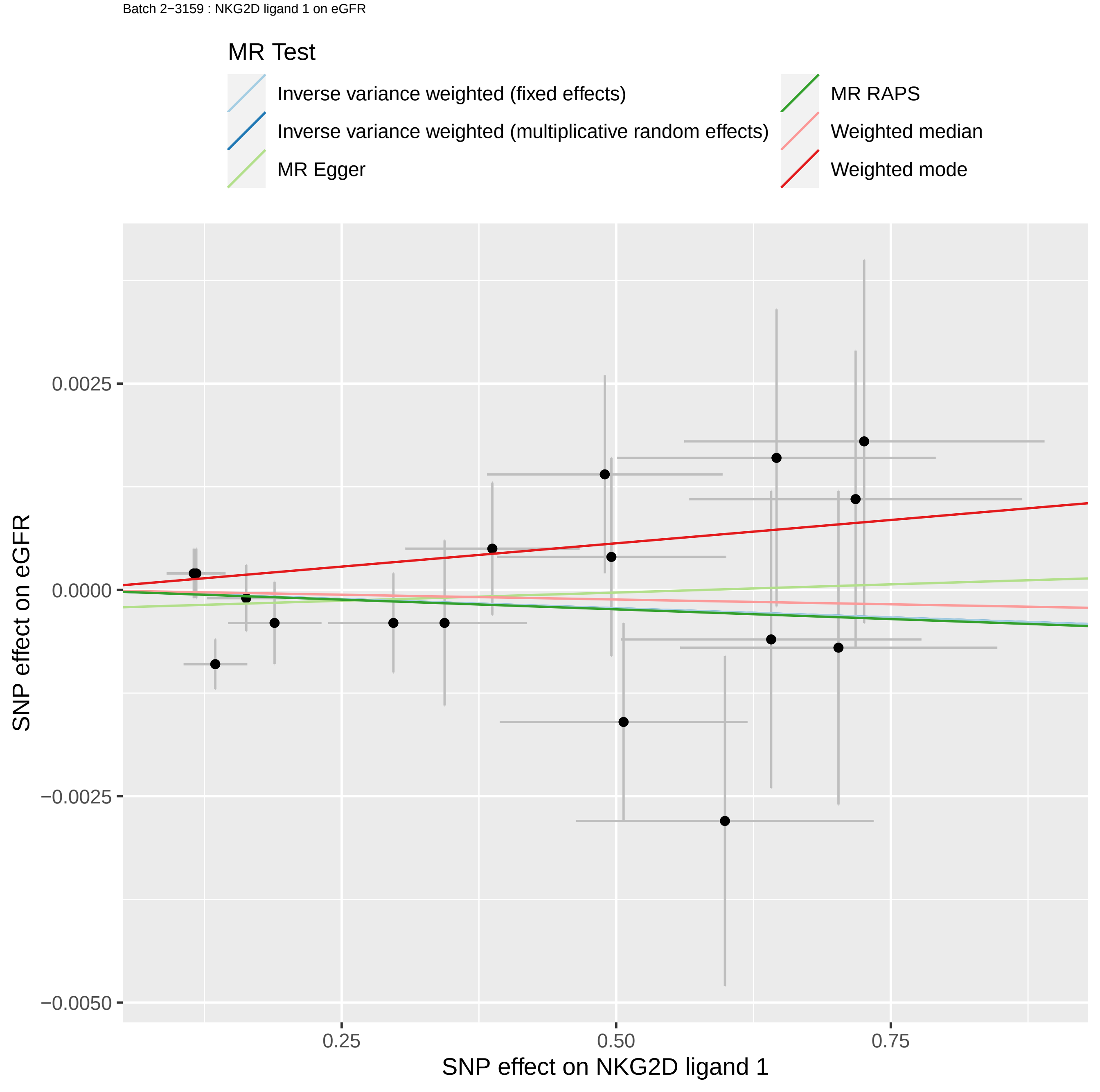
Batch 2−3159 : NKG2D ligand 1 on eGFR
MR Test
Inverse variance weighted (fixed effects)
Inverse variance weighted (multiplicative random effects)
MR Egger
MR RAPS
Weighted median
Weighted mode
0.0025
0.0000
−0.0025
−0.0050
0.25
0.50
0.75
SNP effect on eGFR
SNP effect on NKG2D ligand 1

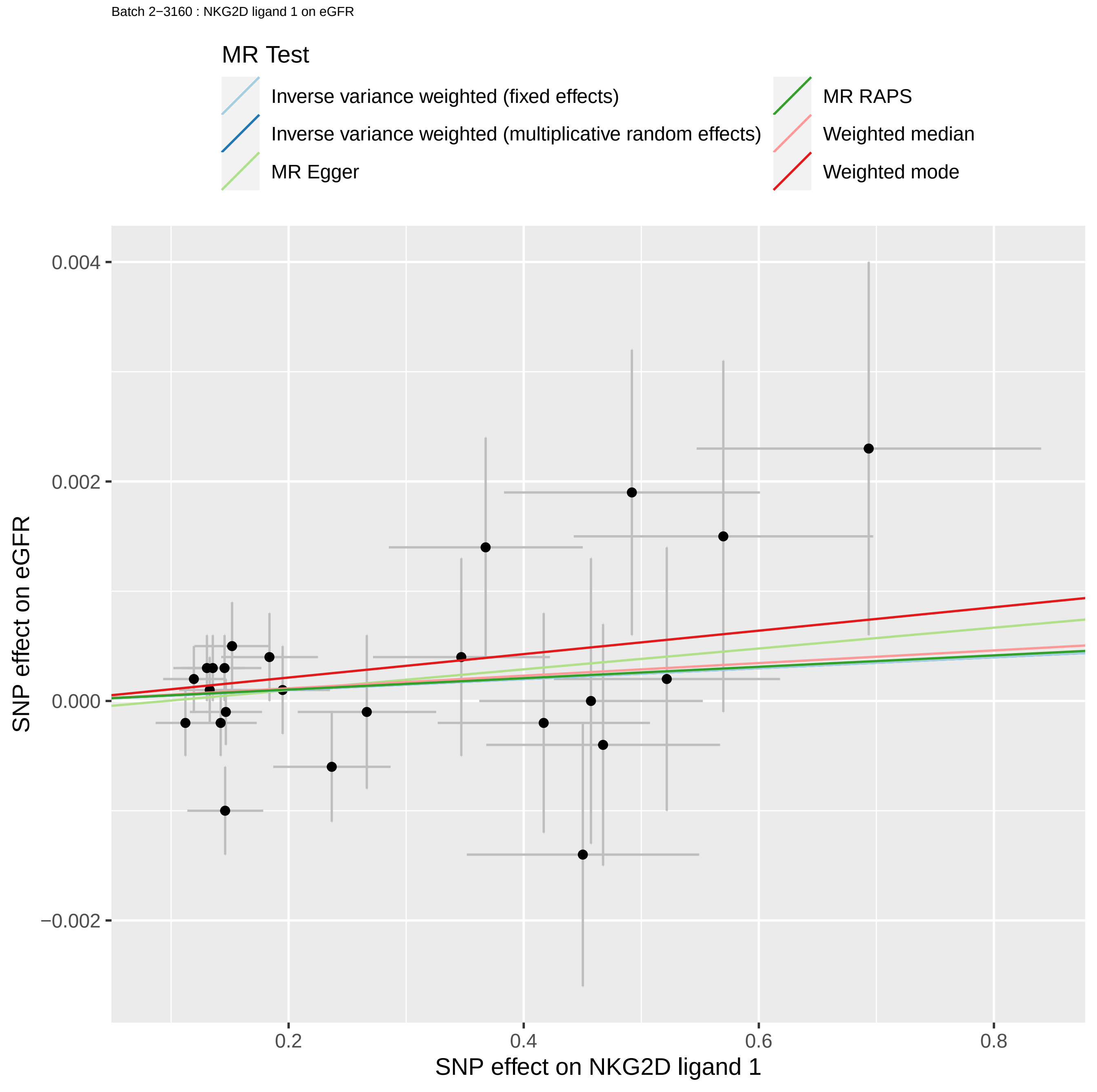
Batch 2−3160 : NKG2D ligand 1 on eGFR
MR Test
Inverse variance weighted (fixed effects)
Inverse variance weighted (multiplicative random effects)
MR Egger
MR RAPS
Weighted median
Weighted mode
0.004
0.002
0.000
−0.002
0.2
0.4
0.6
0.8
SNP effect on eGFR
SNP effect on NKG2D ligand 1

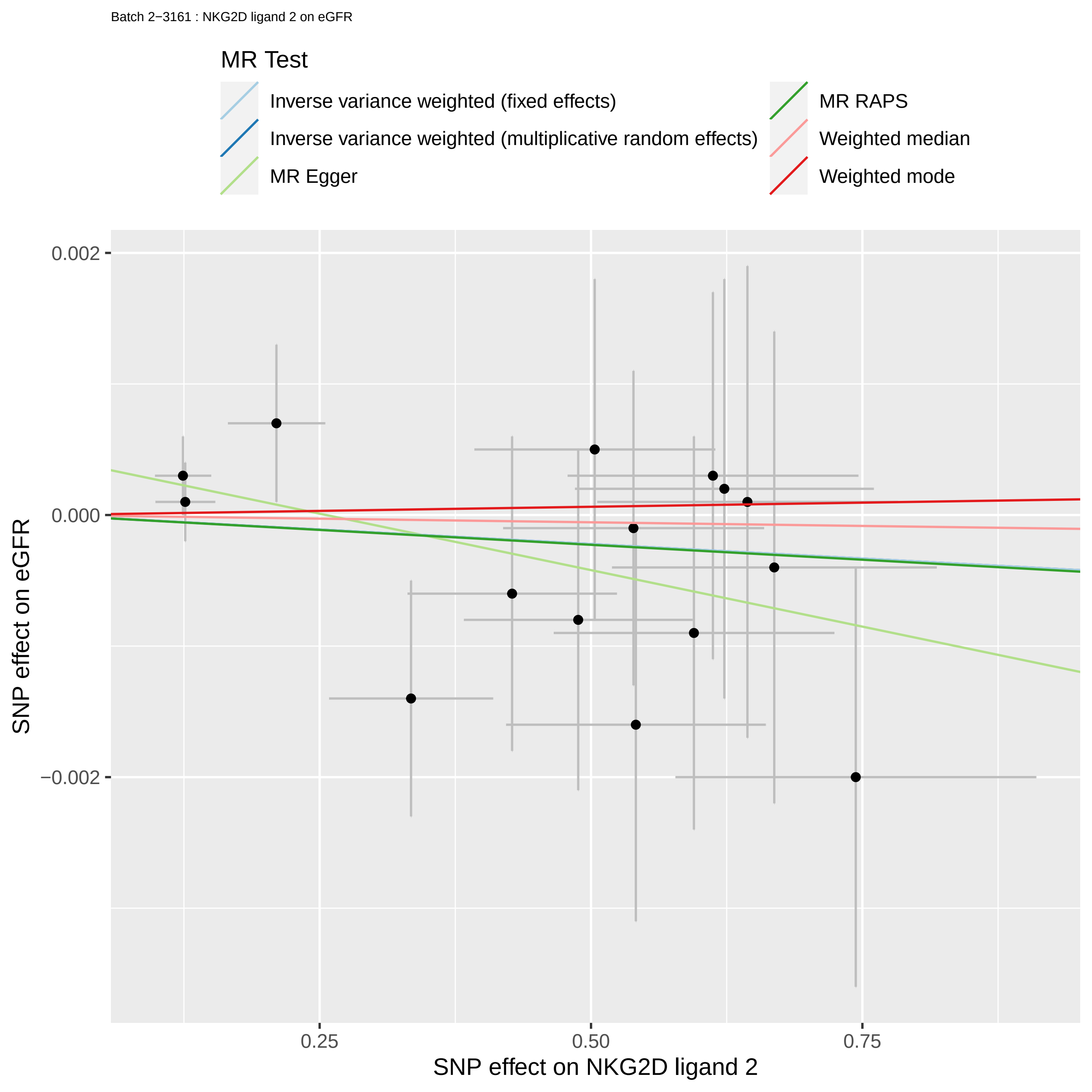
Batch 2−3161 : NKG2D ligand 2 on eGFR
MR Test
Inverse variance weighted (fixed effects)
Inverse variance weighted (multiplicative random effects)
MR Egger
MR RAPS
Weighted median
Weighted mode
0.002
0.000
−0.002
0.25
0.50
0.75
SNP effect on eGFR
SNP effect on NKG2D ligand 2

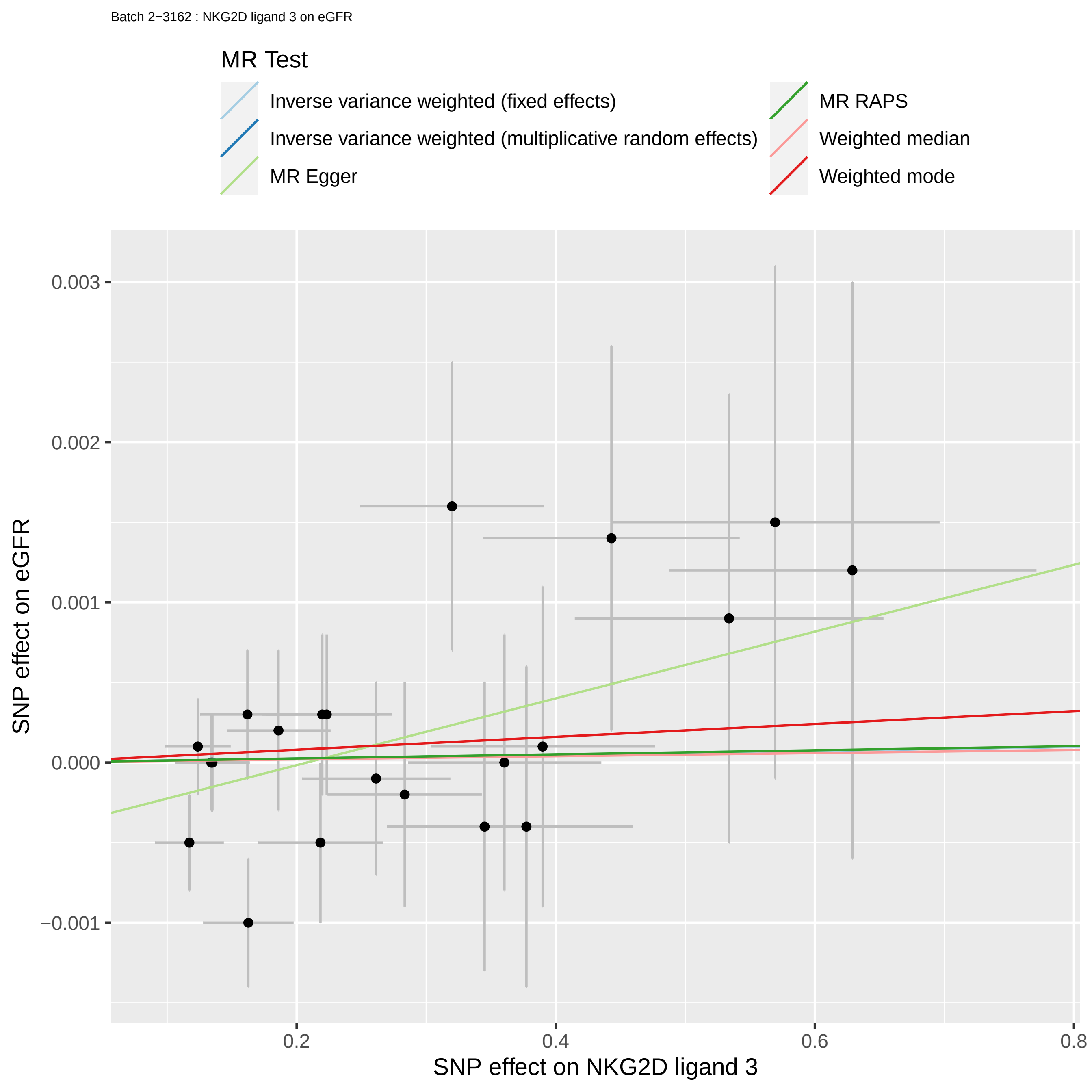

Batch 2−3162 : NKG2D ligand 3 on eGFR
MR Test
Inverse variance weighted (fixed effects)
Inverse variance weighted (multiplicative random effects)
MR Egger
MR RAPS
Weighted median
Weighted mode
0.003
0.002
0.001
0.000
−0.001
0.2
0.4
0.6
0.8
SNP effect on eGFR
SNP effect on NKG2D ligand 3

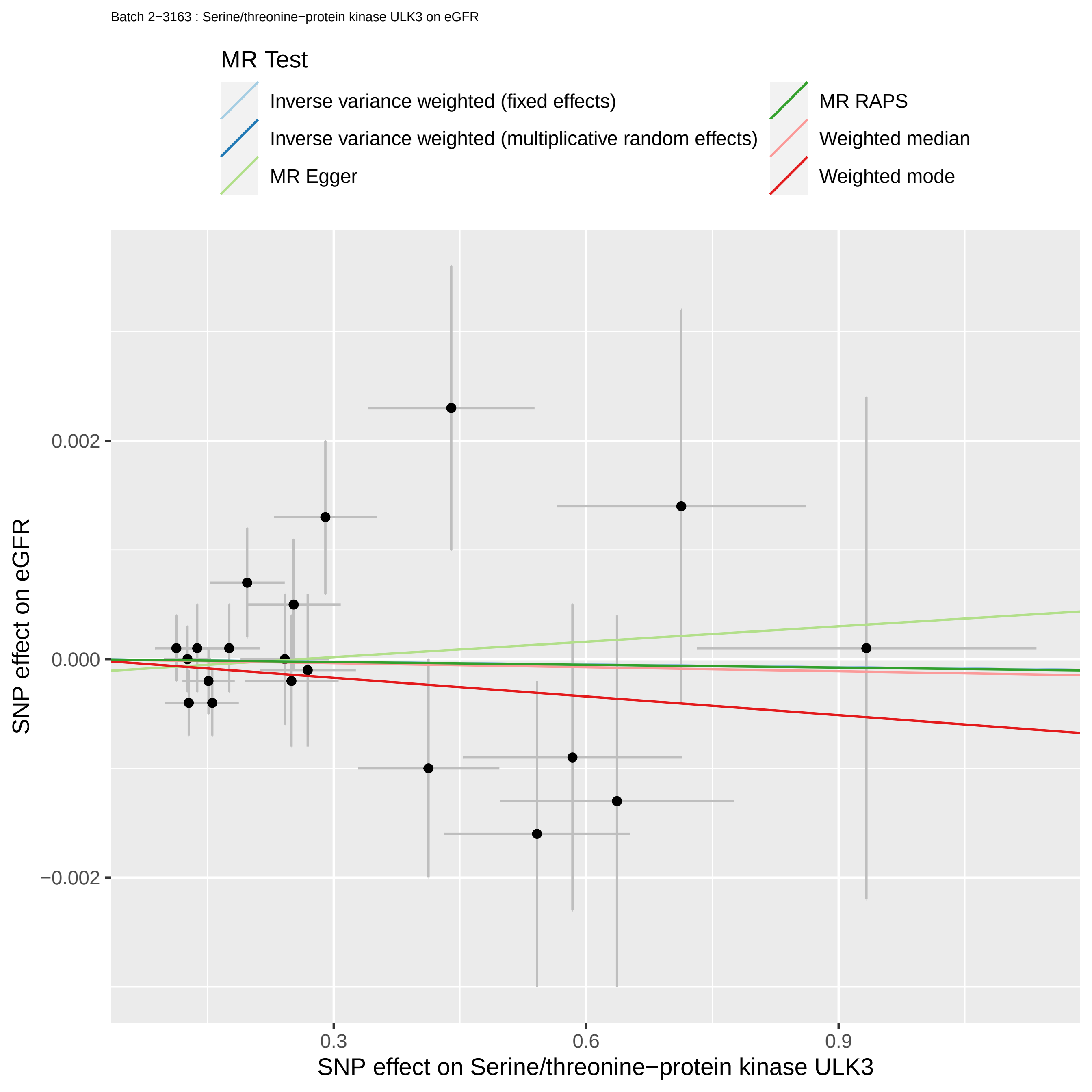
Batch 2−3163 : Serine/threonine−protein kinase ULK3 on eGFR
MR Test
Inverse variance weighted (fixed effects)
Inverse variance weighted (multiplicative random effects)
MR Egger
MR RAPS
Weighted median
Weighted mode
0.002
0.000
−0.002
0.3
0.6
0.9
SNP effect on eGFR
SNP effect on Serine/threonine−protein kinase ULK3

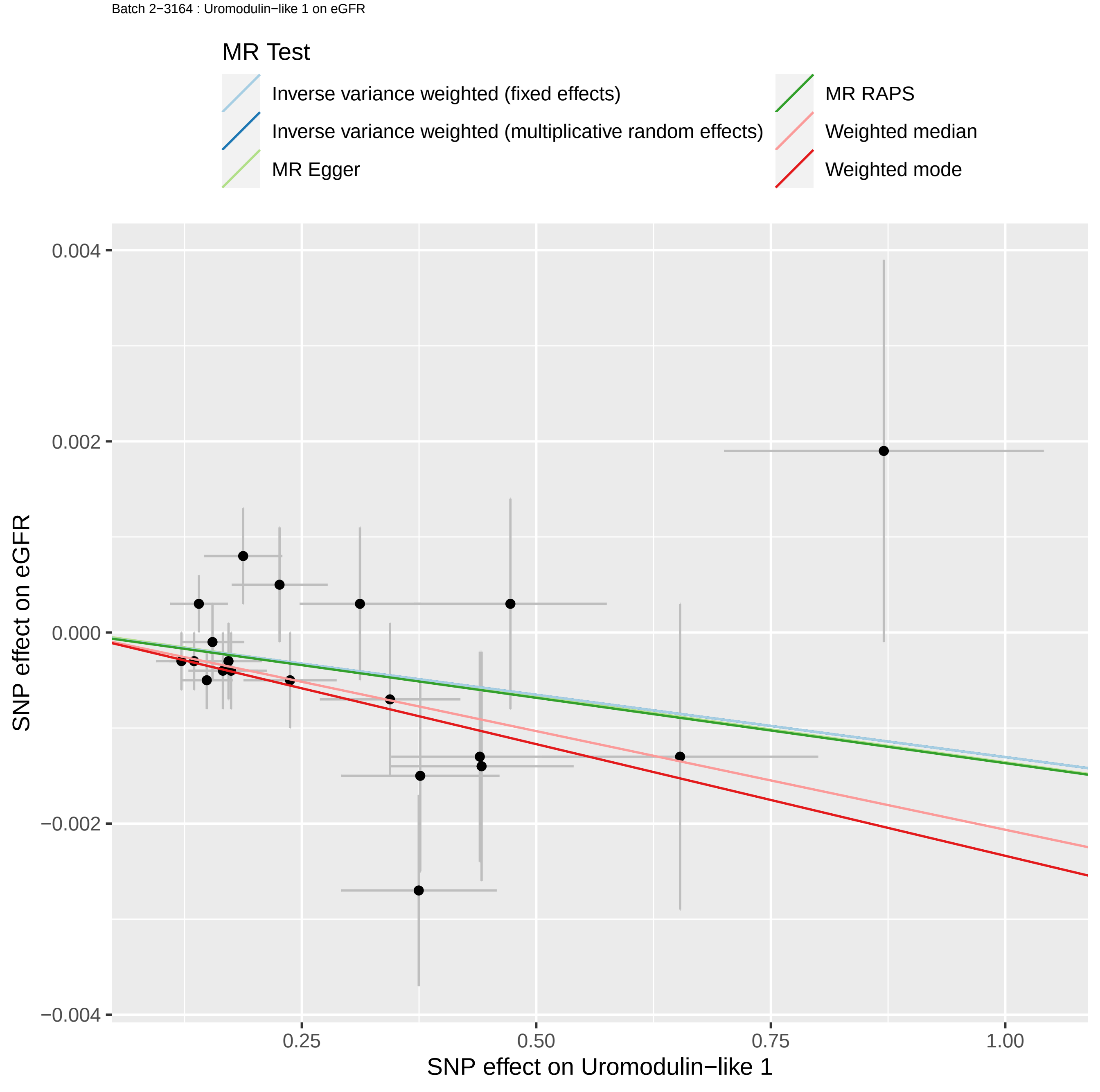

Batch 2−3164 : Uromodulin−like 1 on eGFR
MR Test
Inverse variance weighted (fixed effects)
Inverse variance weighted (multiplicative random effects)
MR Egger
MR RAPS
Weighted median
Weighted mode
0.004
0.002
0.000
−0.002
−0.004
SNP effect on eGFR
0.25
0.50
0.75
1.00
SNP effect on Uromodulin−like 1

Batch 2−3165 : Protein unc−13 homolog A on eGFR

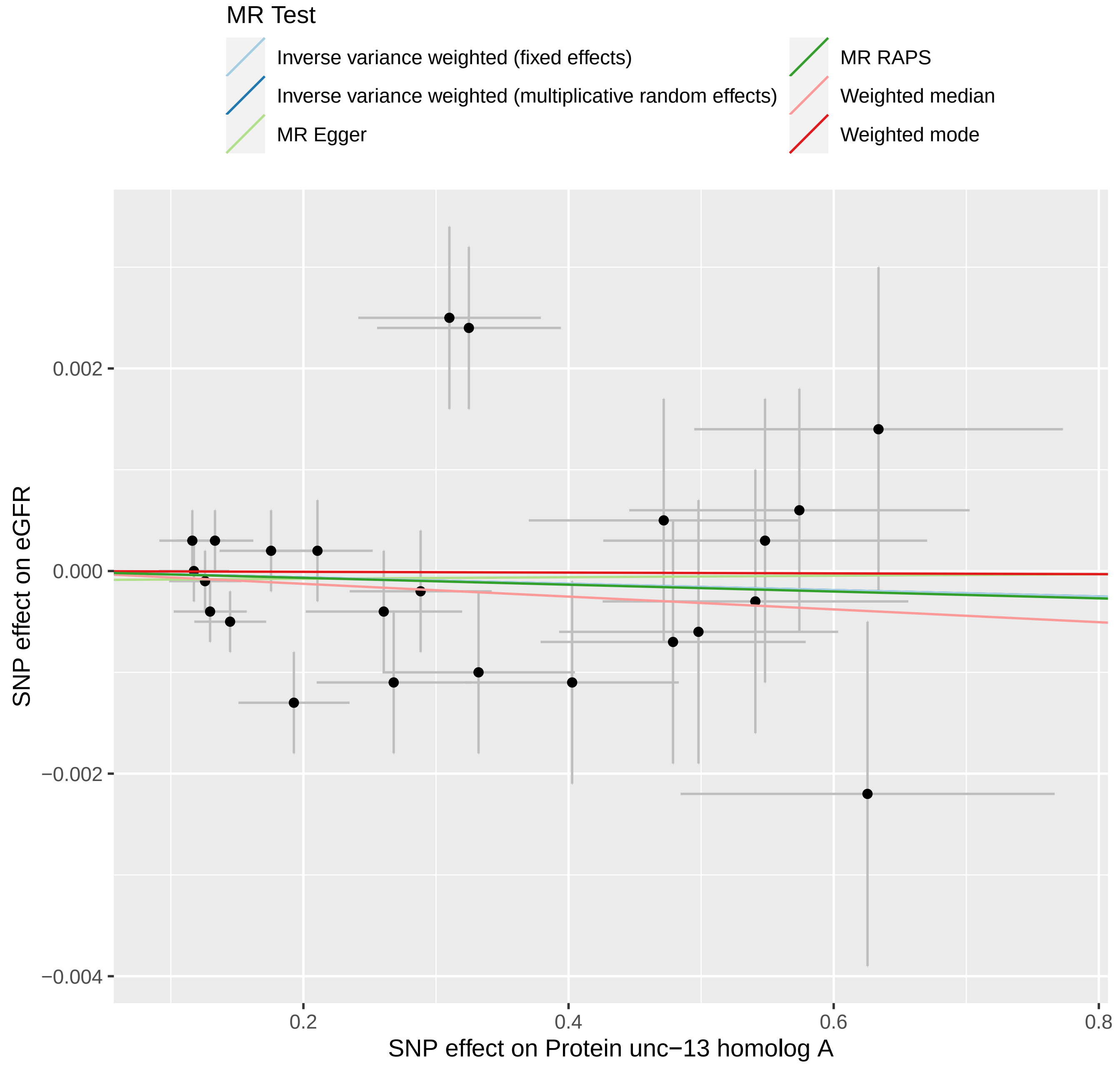

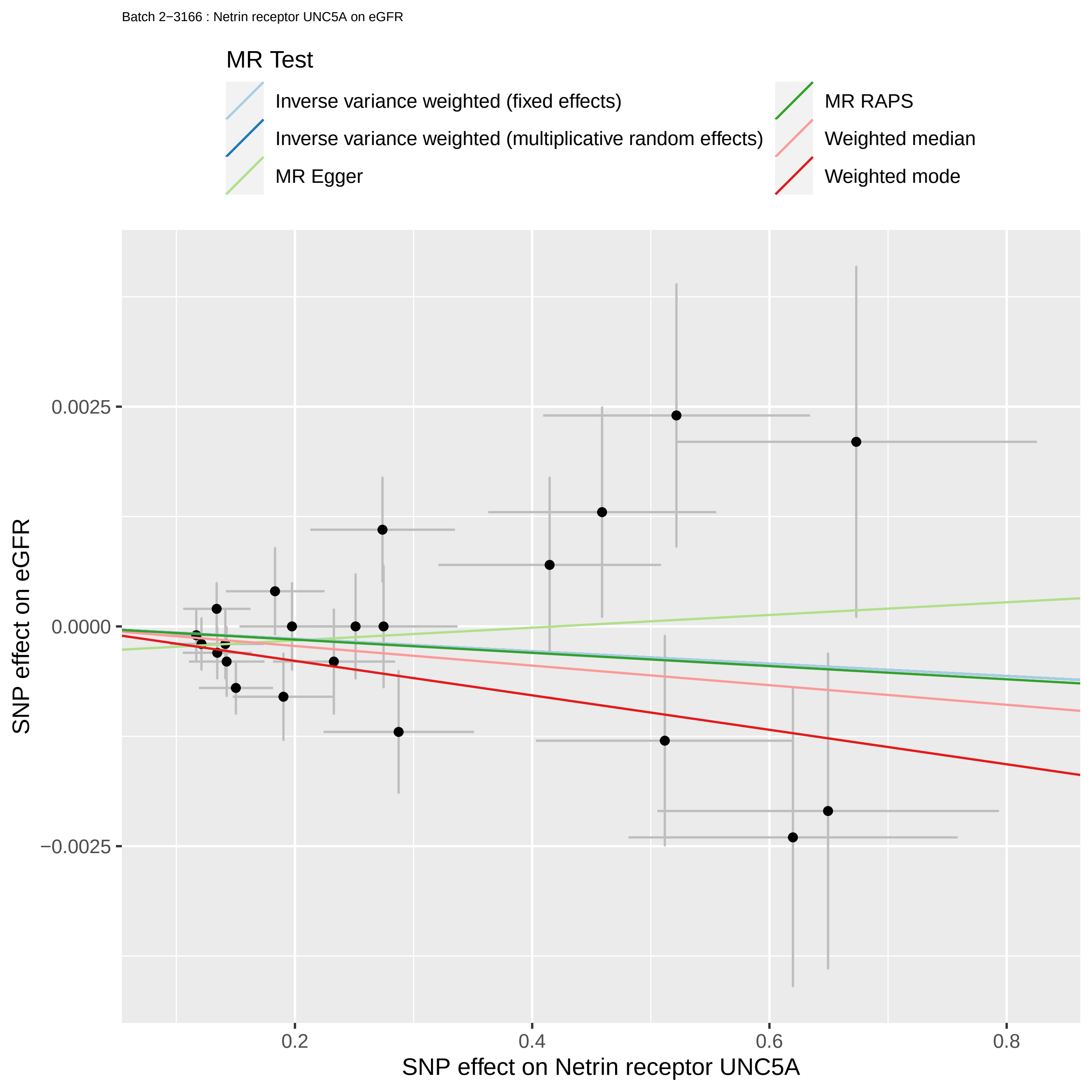

Batch 2−3166 : Netrin receptor UNC5A on eGFR
MR Test
Inverse variance weighted (fixed effects)
Inverse variance weighted (multiplicative random effects)
MR Egger
MR RAPS
Weighted median
Weighted mode
0.0025
0.0000
−0.0025
0.2
0.4
0.6
0.8
SNP effect on eGFR
SNP effect on Netrin receptor UNC5A

Batch 2−3167 : Netrin receptor UNC5B on eGFR

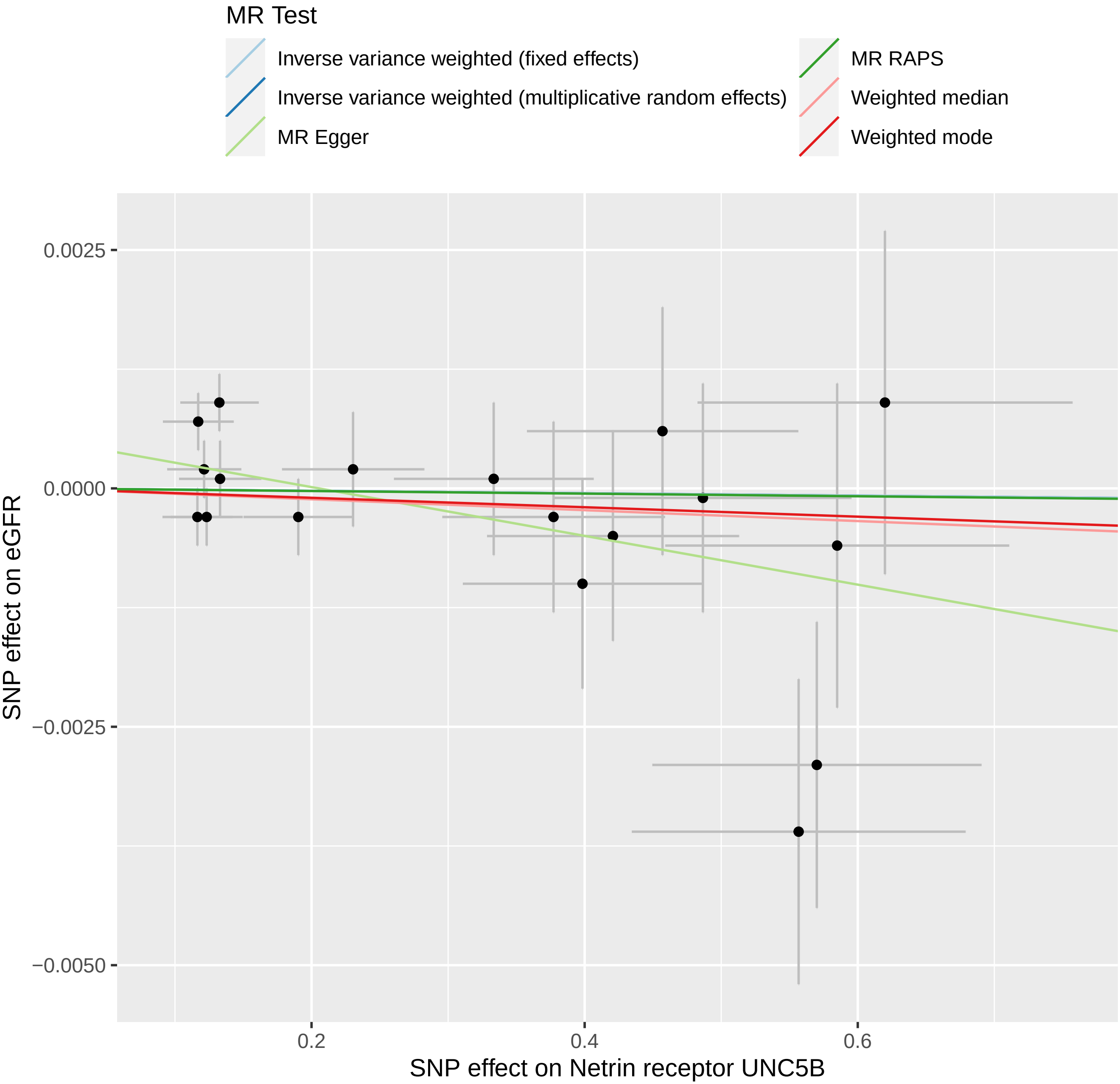

Batch 2−3168 : Netrin receptor UNC5C on eGFR

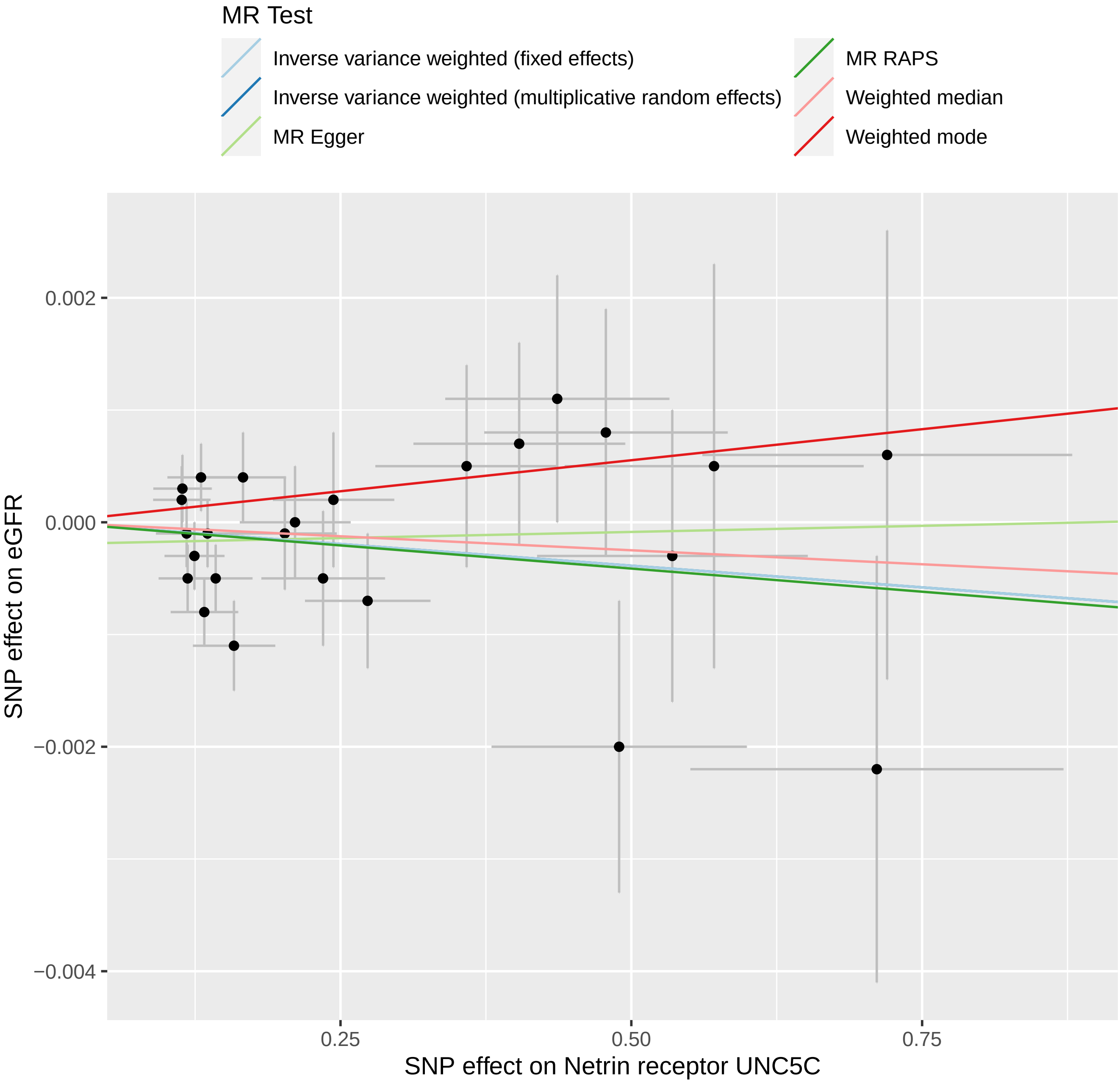

Batch 2−3169 : Netrin receptor UNC5D on eGFR

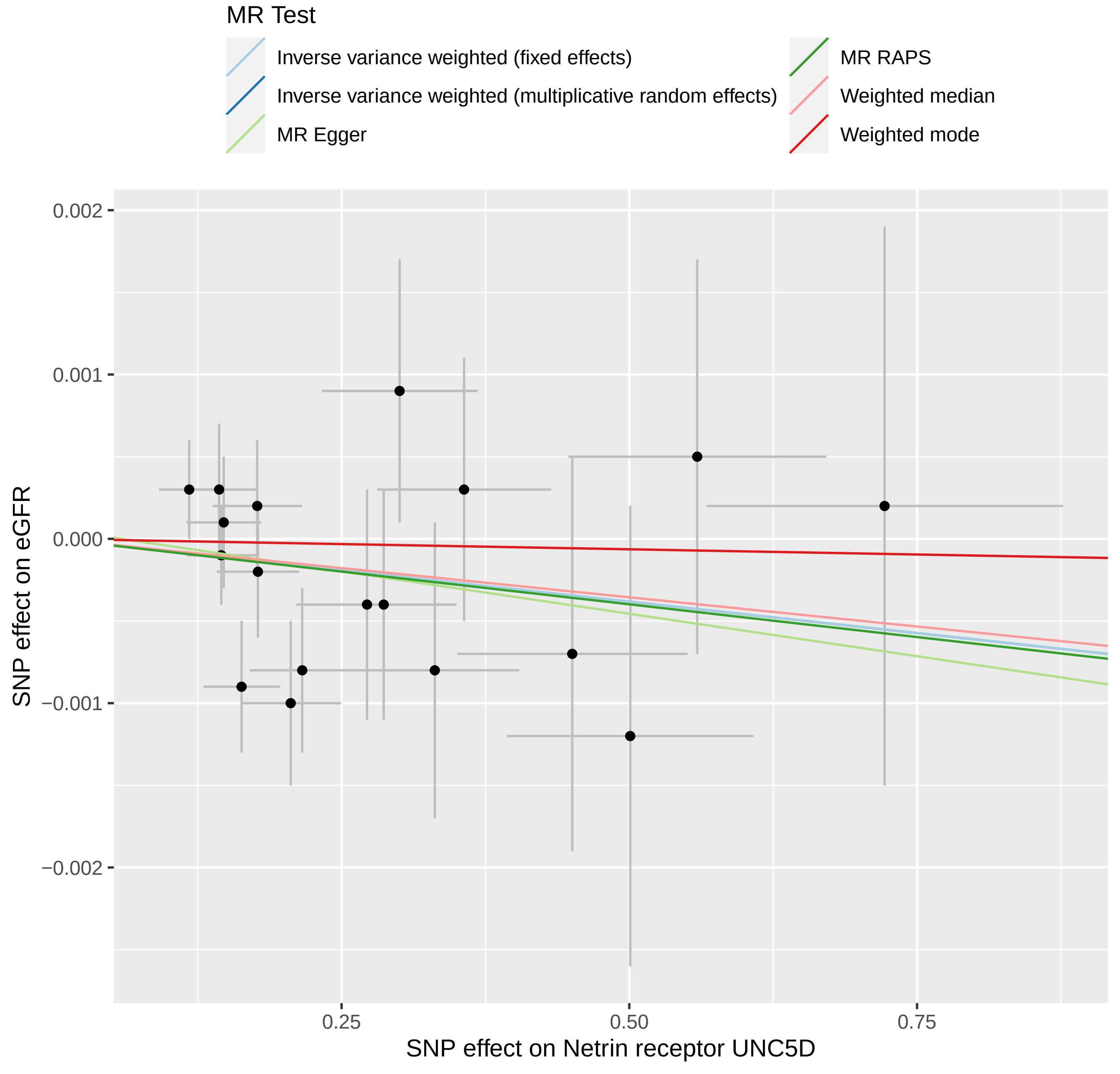

Batch 2−3170 : Netrin receptor UNC5D on eGFR

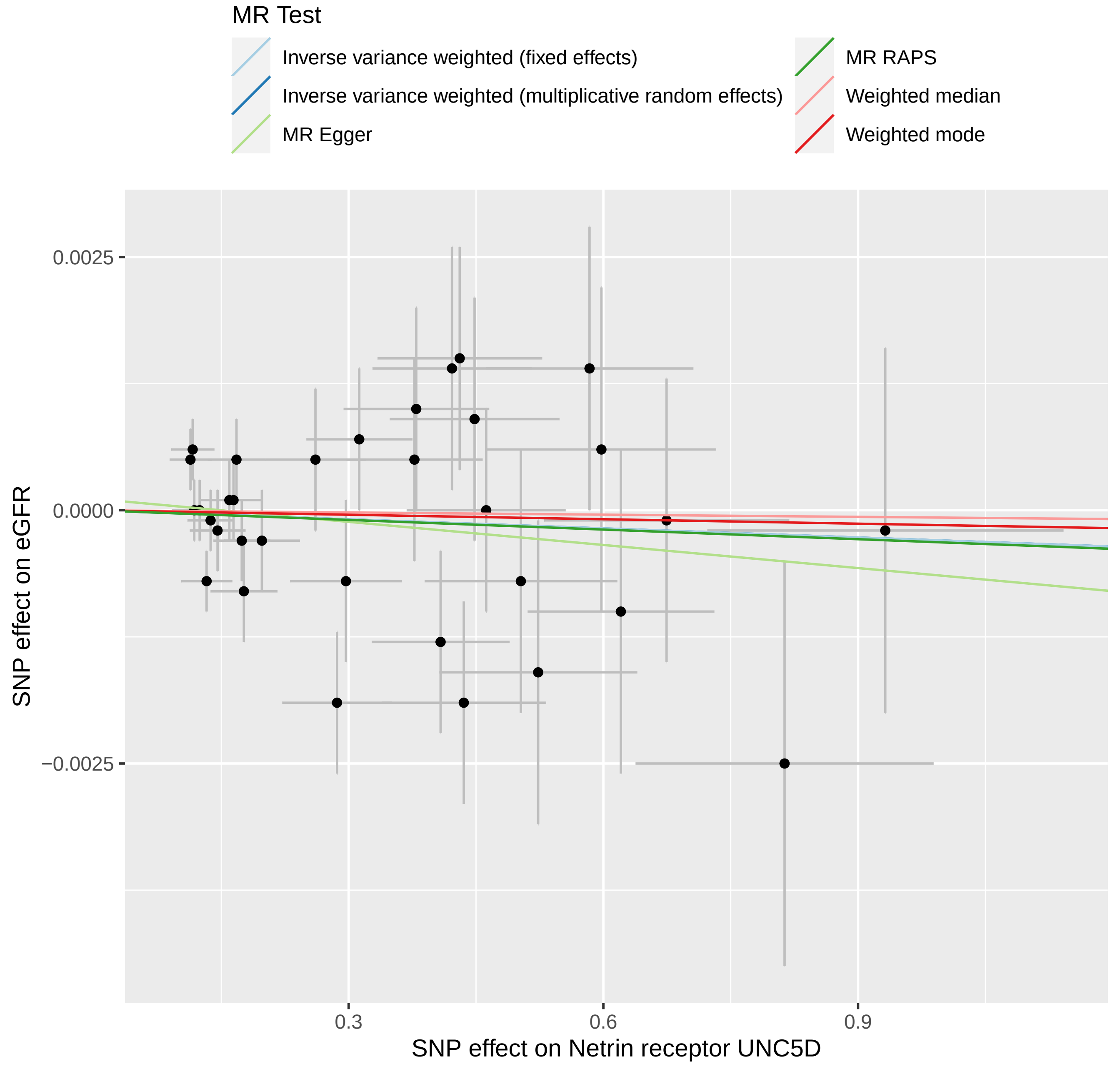

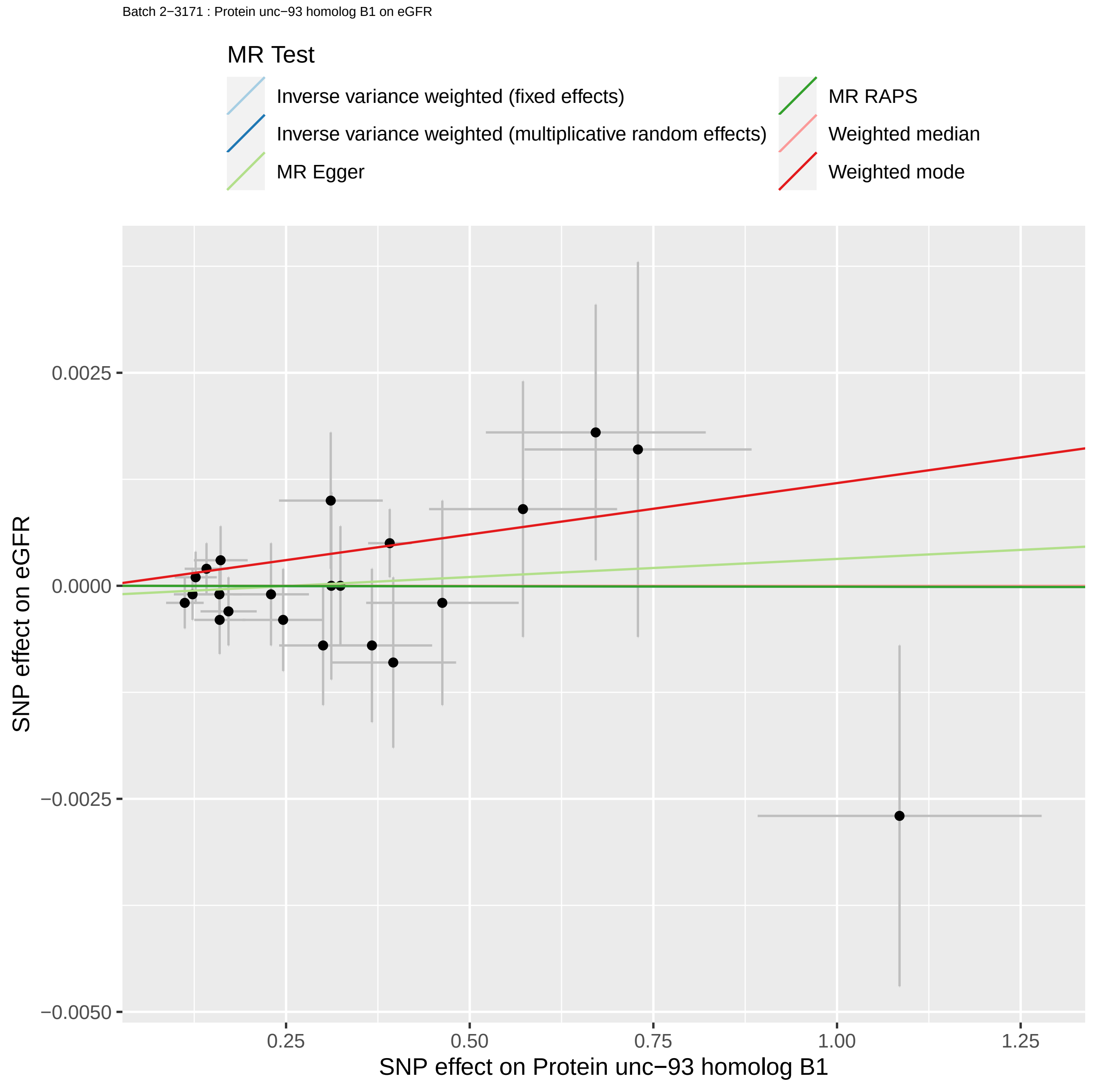
Batch 2−3171 : Protein unc−93 homolog B1 on eGFR
MR Test
Inverse variance weighted (fixed effects)
Inverse variance weighted (multiplicative random effects)
MR Egger
MR RAPS
Weighted median
Weighted mode
0.0025
0.0000
−0.0025
−0.0050
0.25
0.50
0.75
1.00
1.25
SNP effect on eGFR
SNP effect on Protein unc−93 homolog B1

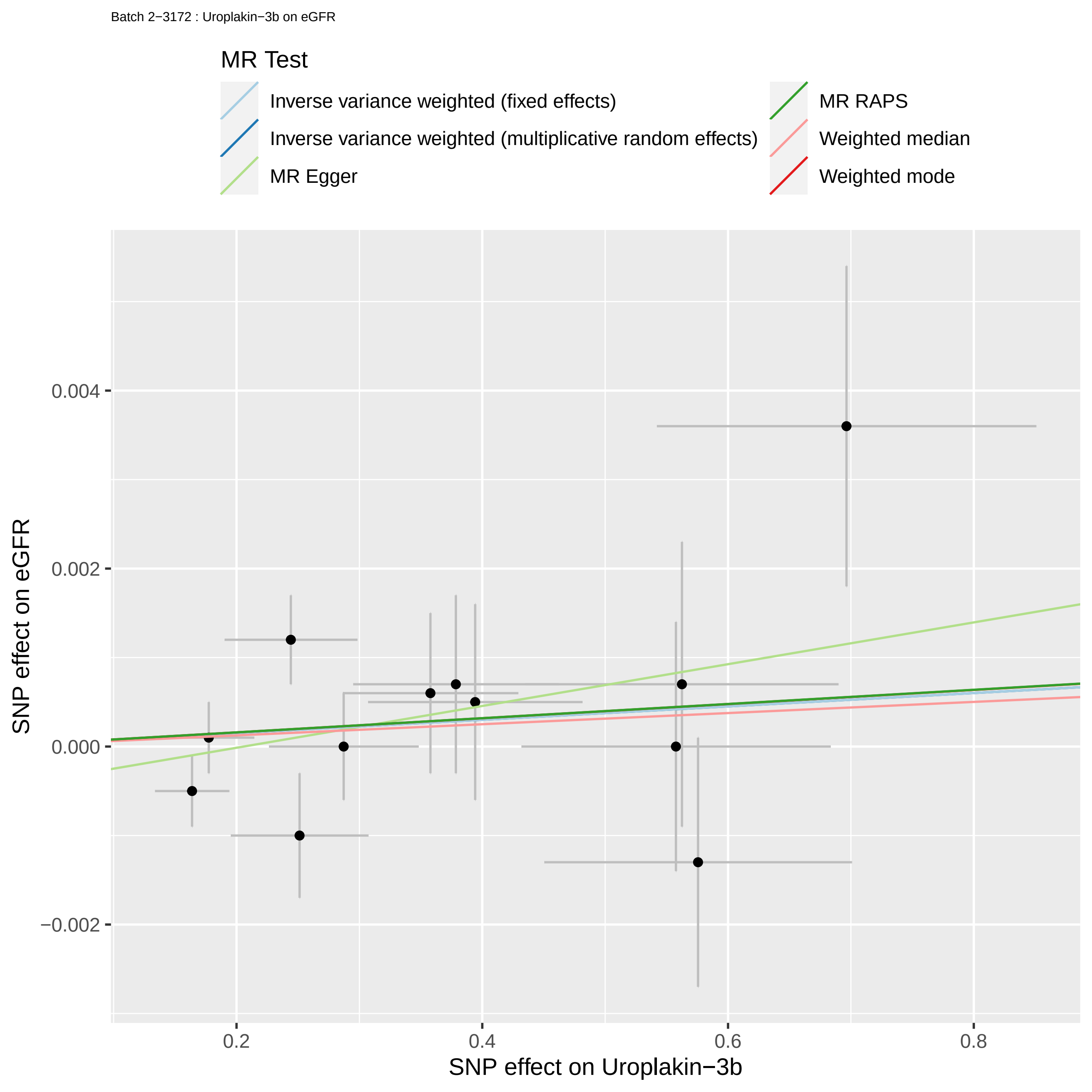
Batch 2−3172 : Uroplakin−3b on eGFR
MR Test
Inverse variance weighted (fixed effects)
Inverse variance weighted (multiplicative random effects)
MR Egger
MR RAPS
Weighted median
Weighted mode
SNP effect on eGFR
0.004
0.002
0.000
−0.002
0.2
0.4
0.6
0.8
SNP effect on Uroplakin−3b

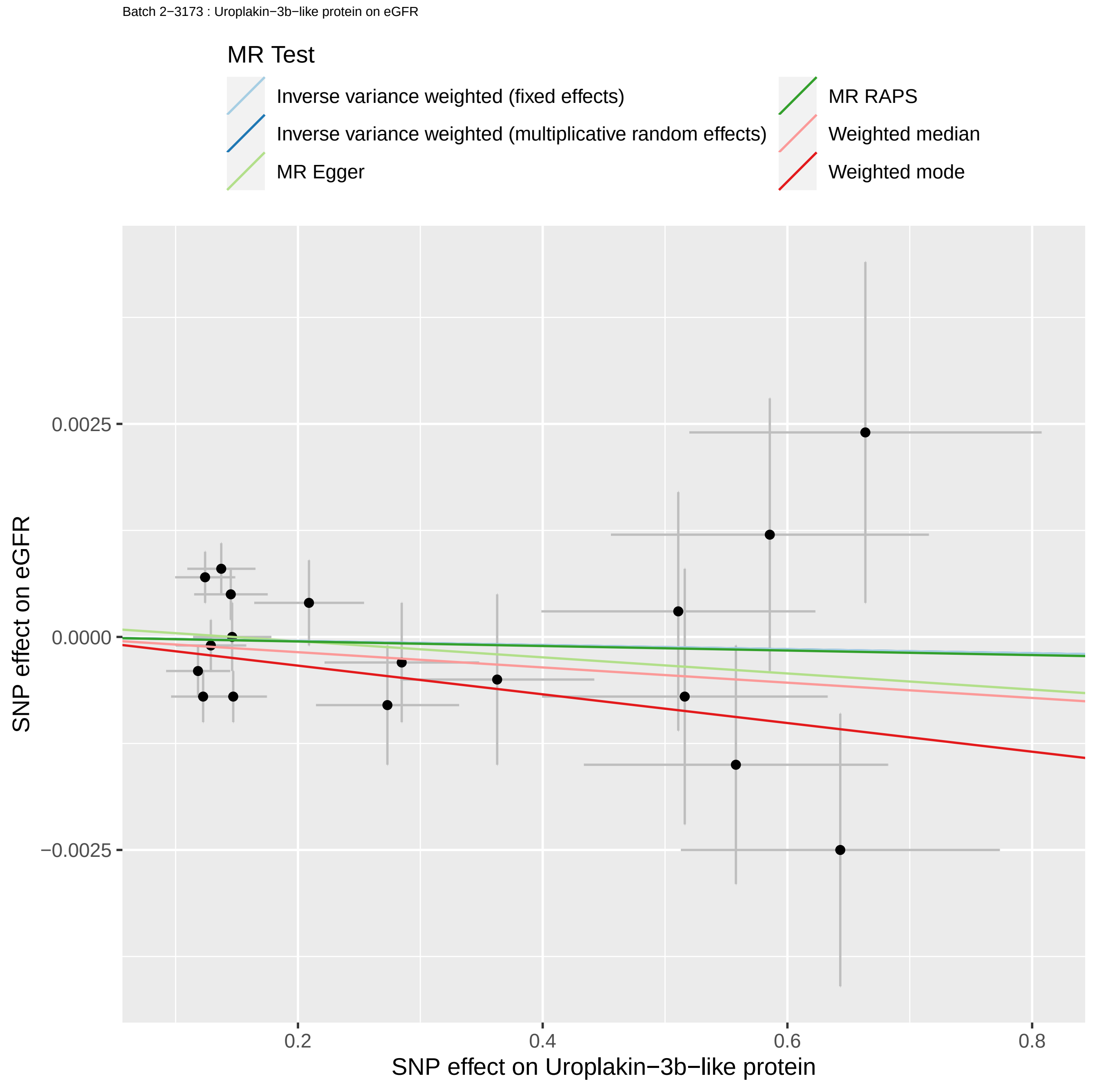
Batch 2−3173 : Uroplakin−3b−like protein on eGFR
MR Test
Inverse variance weighted (fixed effects)
Inverse variance weighted (multiplicative random effects)
MR Egger
MR RAPS
Weighted median
Weighted mode
0.0025
0.0000
−0.0025
0.2
0.4
0.6
0.8
SNP effect on eGFR
SNP effect on Uroplakin−3b−like protein

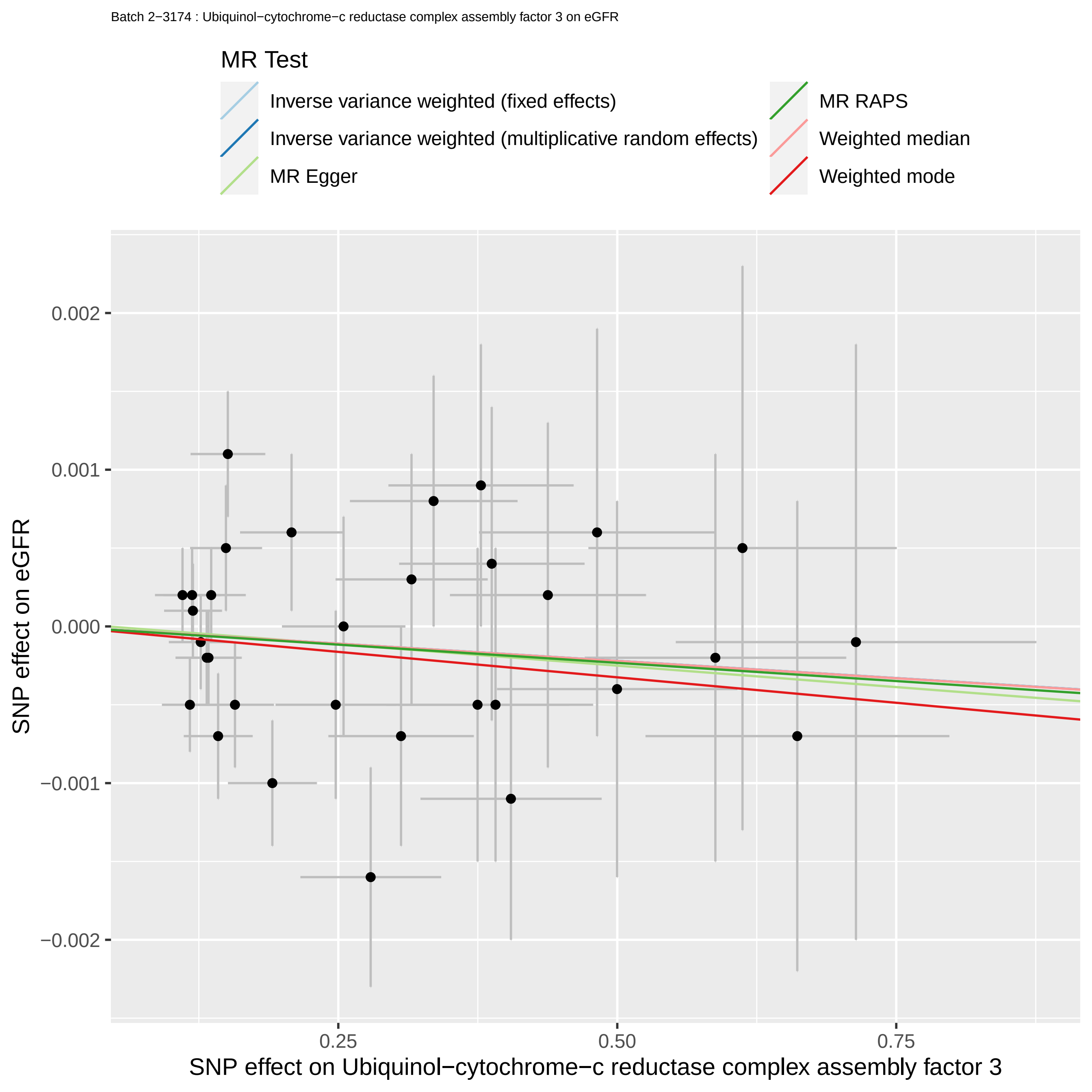
Batch 2−3174 : Ubiquinol−cytochrome−c reductase complex assembly factor 3 on eGFR
MR Test
Inverse variance weighted (fixed effects)
Inverse variance weighted (multiplicative random effects)
MR Egger
MR RAPS
Weighted median
Weighted mode
0.002
0.001
0.000
−0.001
−0.002
0.25
0.50
0.75
SNP effect on eGFR
SNP effect on Ubiquinol−cytochrome−c reductase complex assembly factor 3

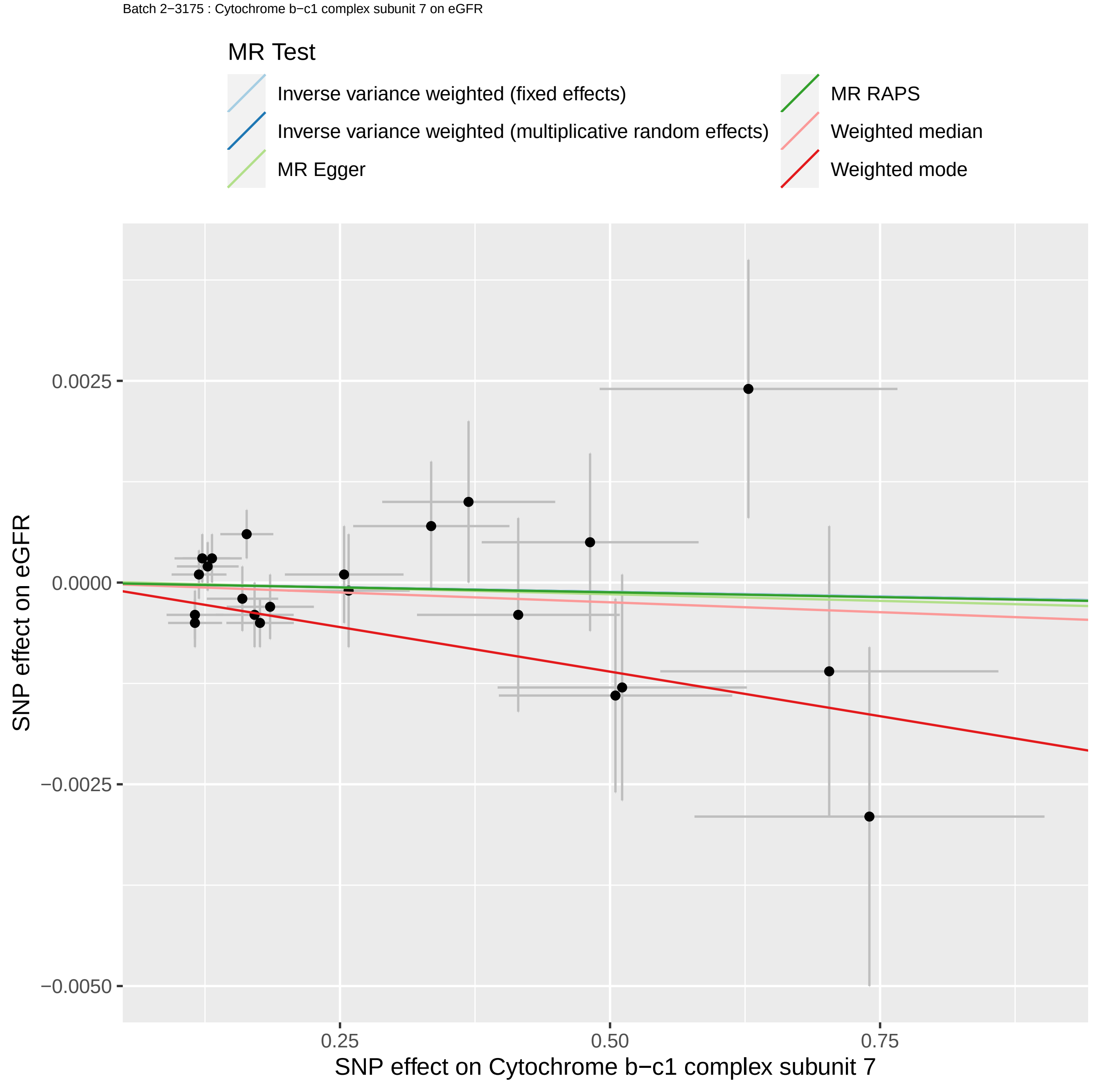
Batch 2−3175 : Cytochrome b−c1 complex subunit 7 on eGFR
MR Test
Inverse variance weighted (fixed effects)
Inverse variance weighted (multiplicative random effects)
MR Egger
MR RAPS
Weighted median
Weighted mode
0.0025
0.0000
−0.0025
−0.0050
0.25
0.50
0.75
SNP effect on eGFR
SNP effect on Cytochrome b−c1 complex subunit 7

Batch 2−3176 : Uroporphyrinogen−III synthase on eGFR

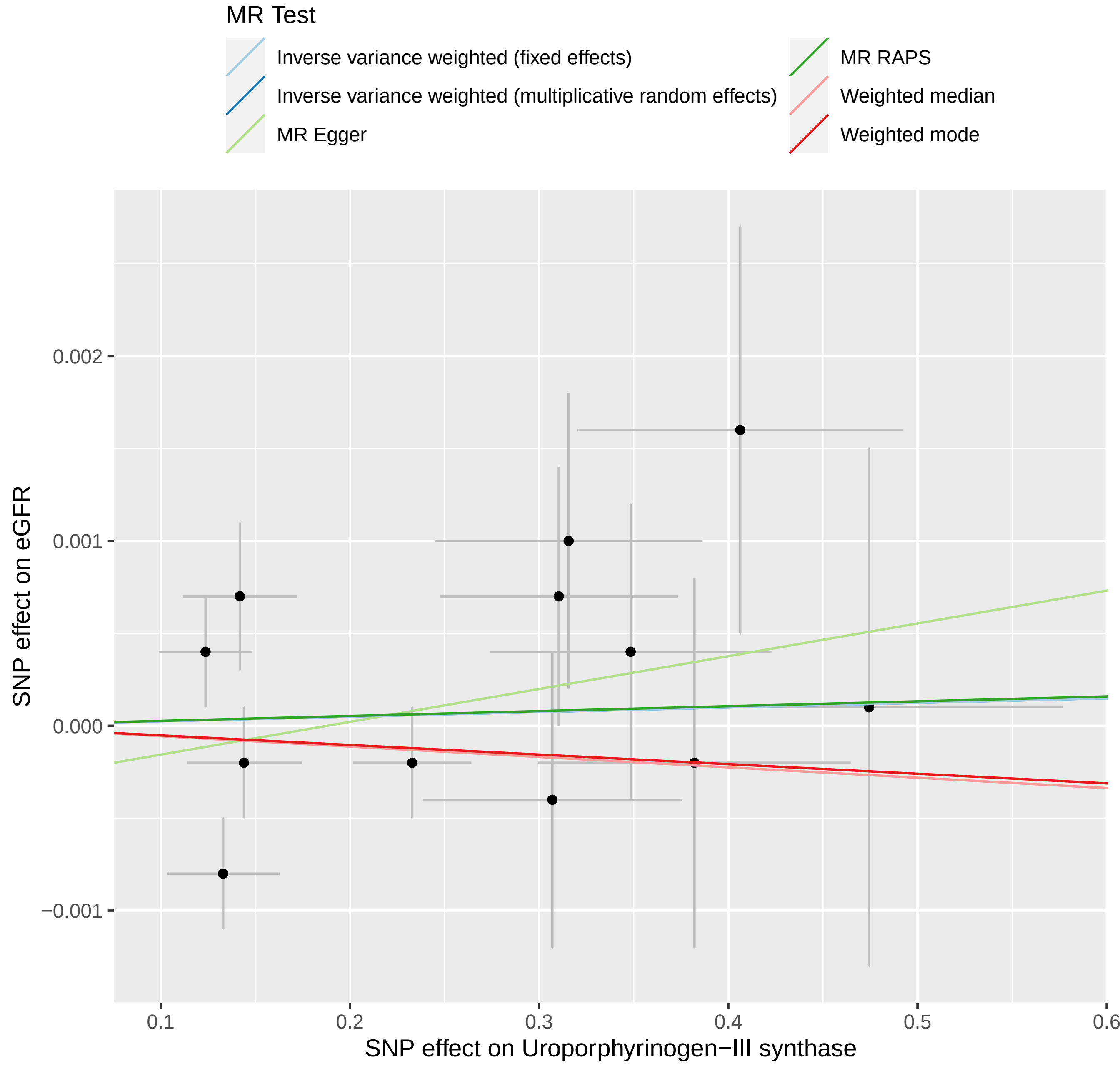

Batch 2−3177 : U6 snRNA phosphodiesterase on eGFR

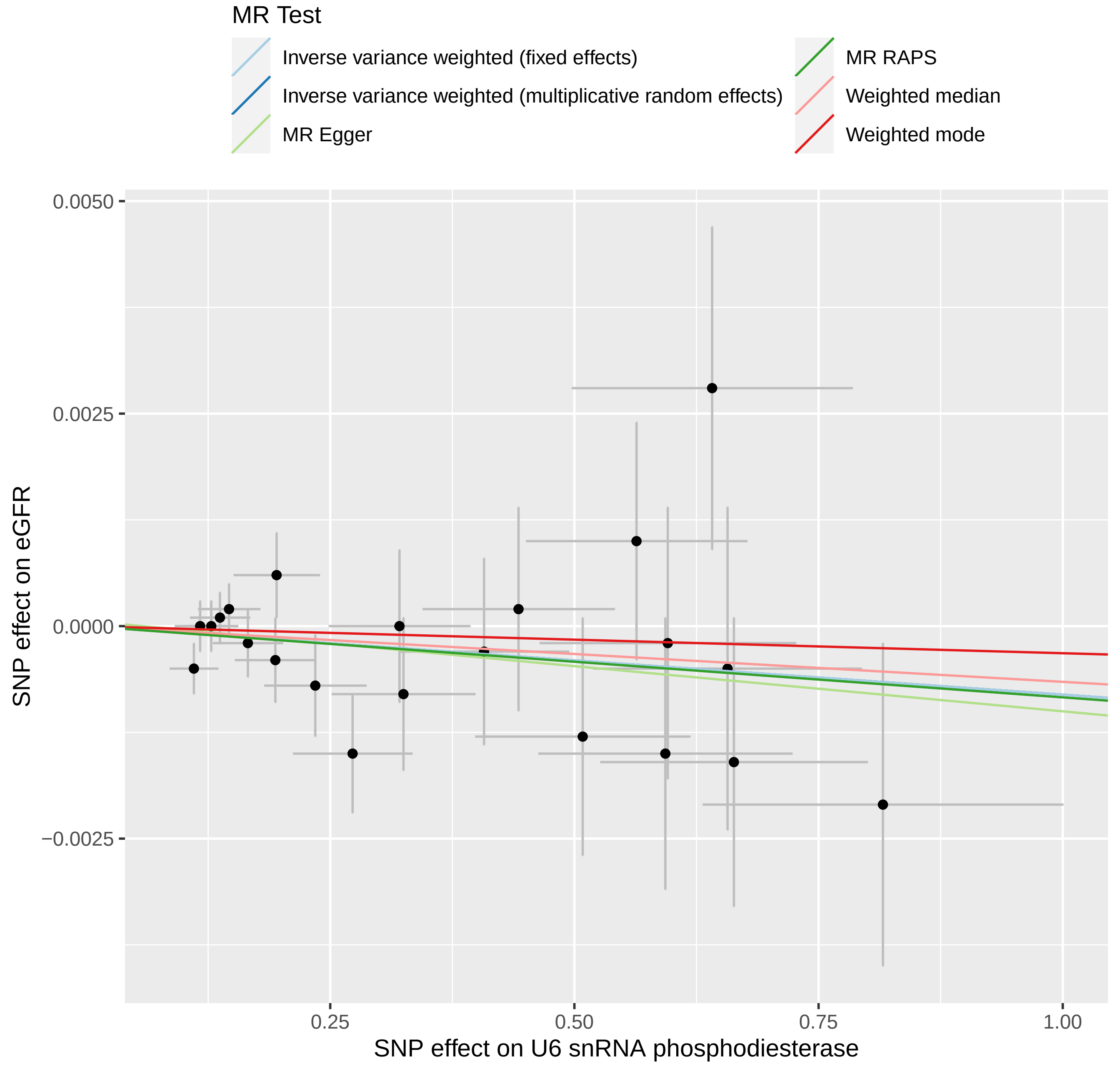

Batch 2−3178 : Vesicle transport protein USE1 on eGFR

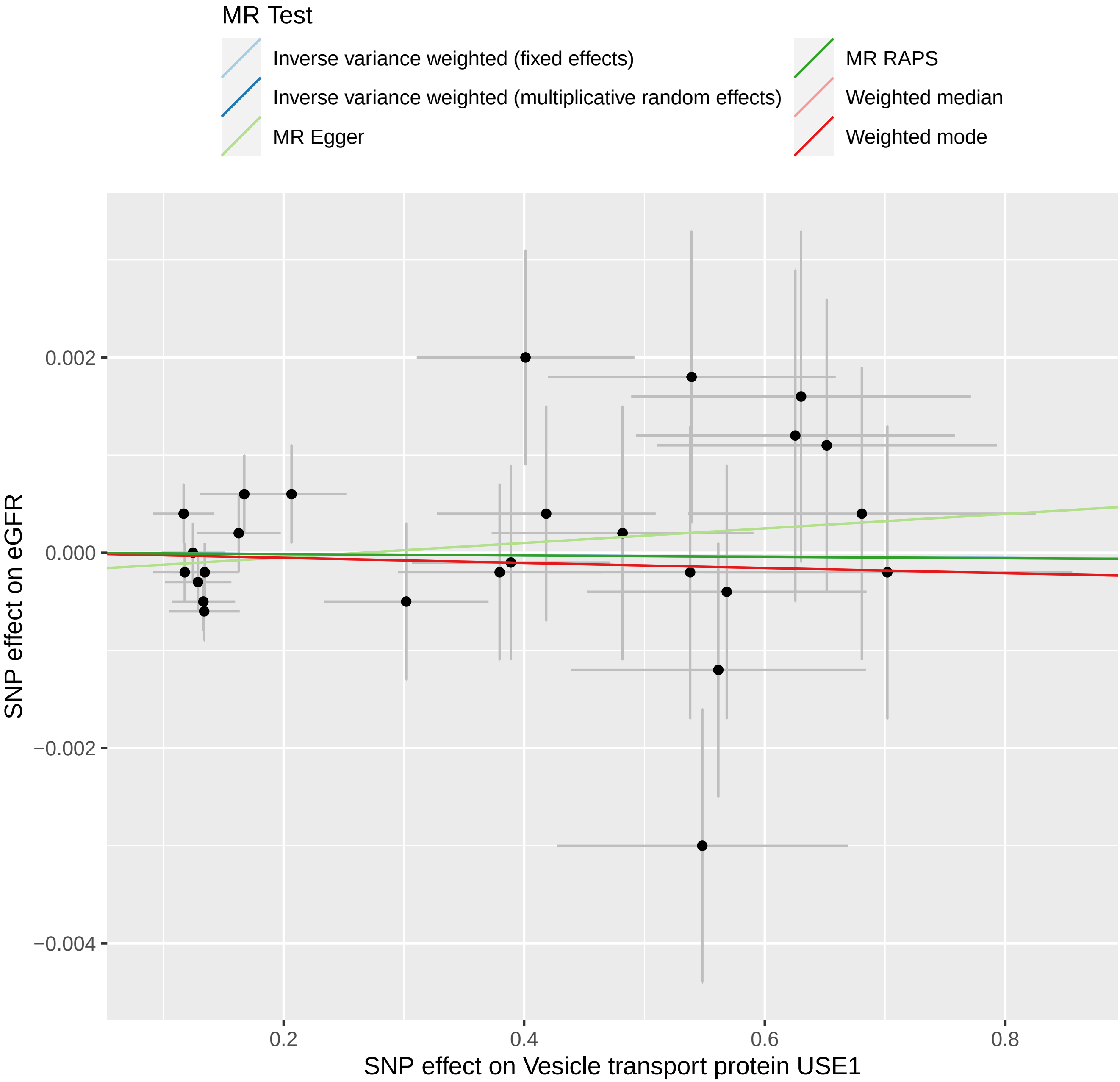

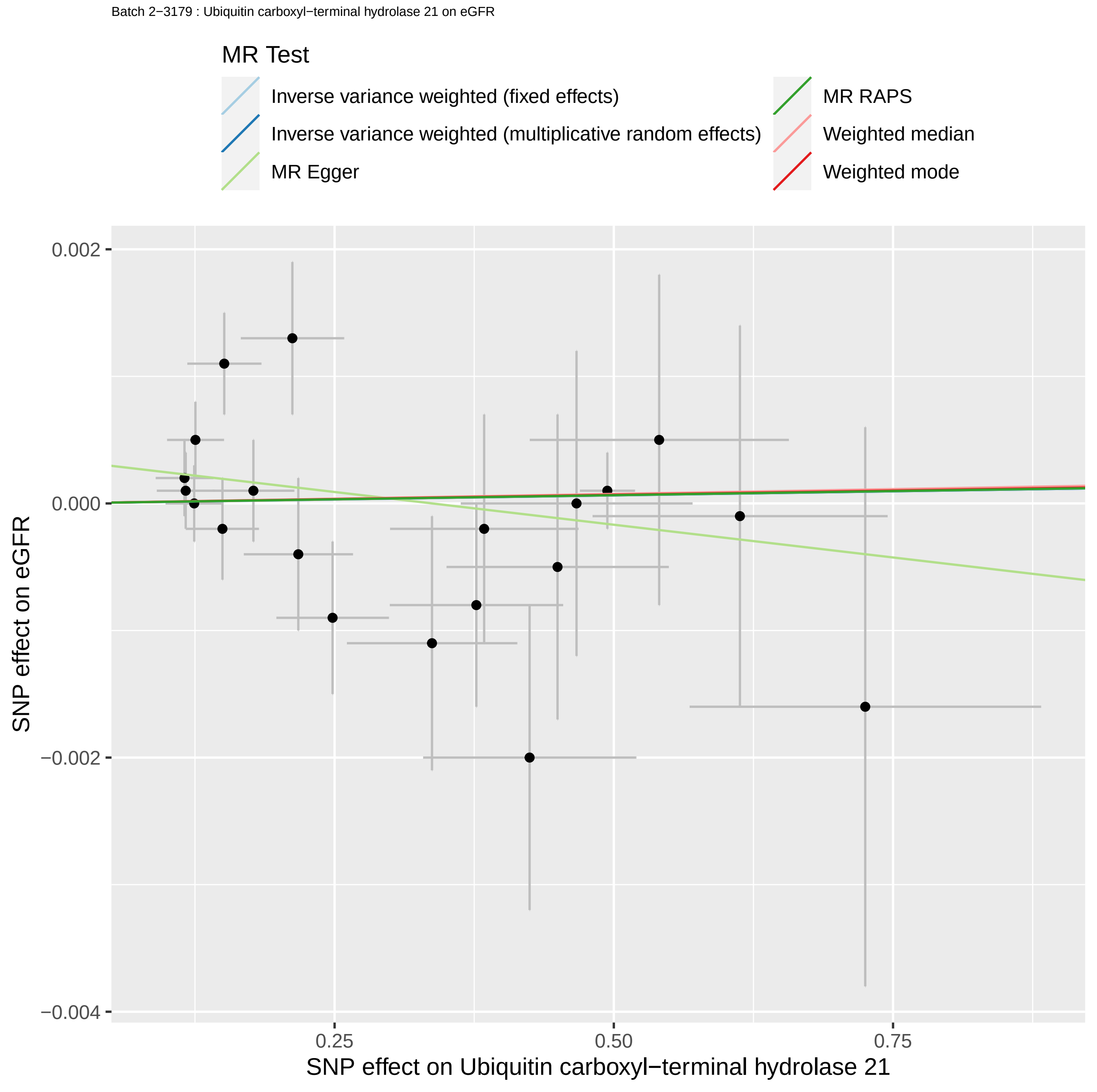
Batch 2−3179 : Ubiquitin carboxyl−terminal hydrolase 21 on eGFR
MR Test
Inverse variance weighted (fixed effects)
Inverse variance weighted (multiplicative random effects)
MR Egger
MR RAPS
Weighted median
Weighted mode
0.002
0.000
−0.002
−0.004
SNP effect on eGFR
0.25
0.50
0.75
SNP effect on Ubiquitin carboxyl−terminal hydrolase 21

Batch 2−3180 : Ubiquitin carboxyl−terminal hydrolase 25 on eGFR

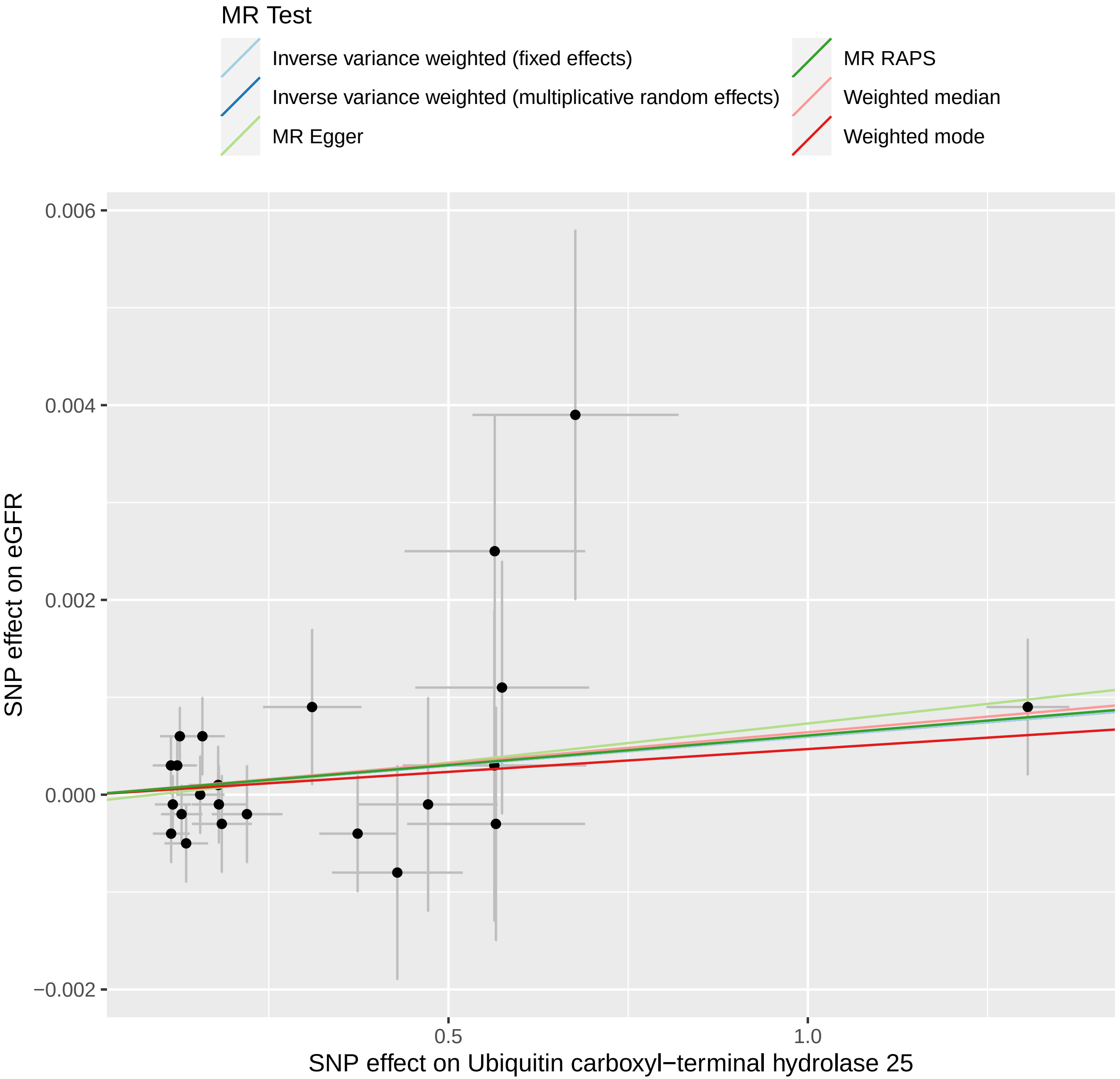

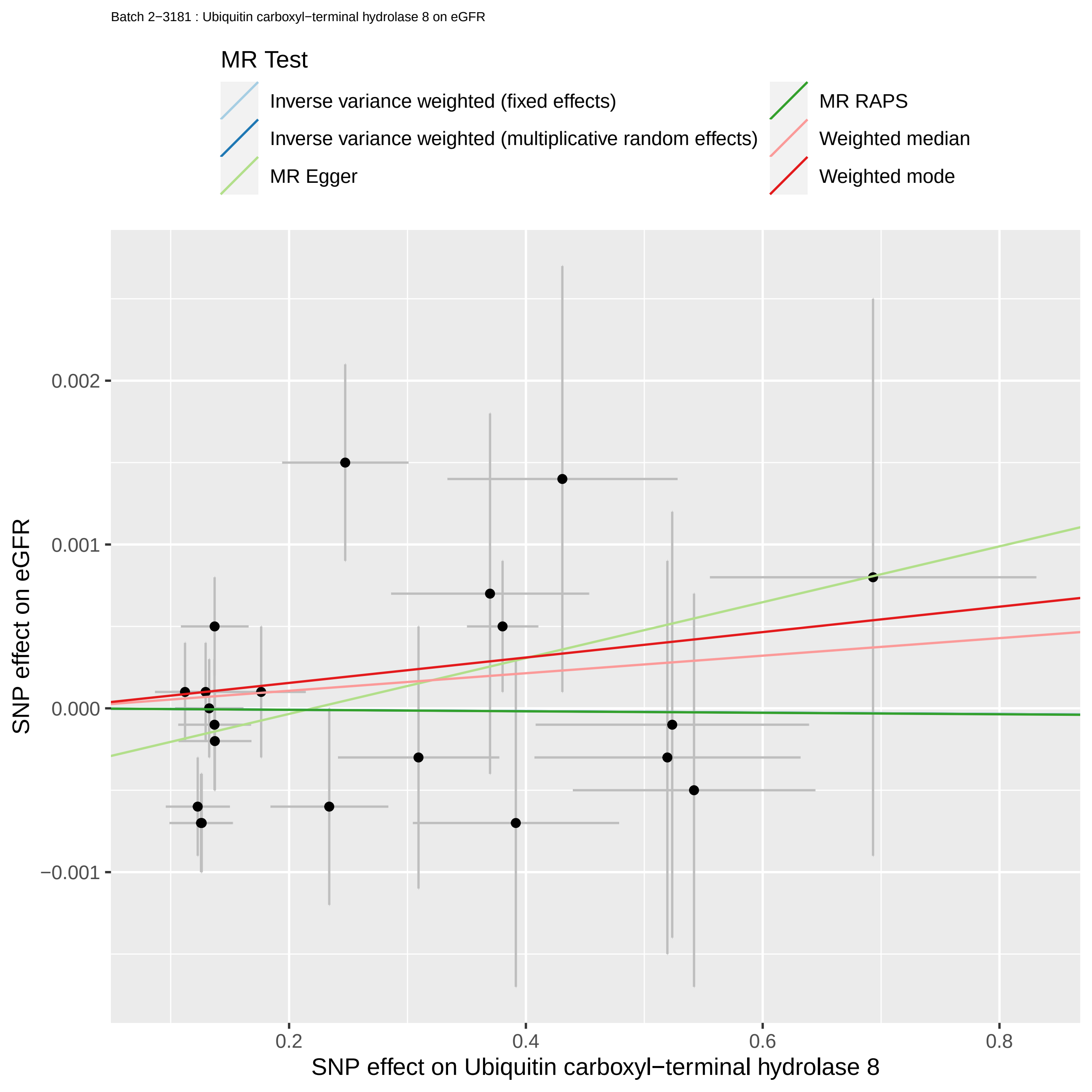
Batch 2−3181 : Ubiquitin carboxyl−terminal hydrolase 8 on eGFR
MR Test
Inverse variance weighted (fixed effects)
Inverse variance weighted (multiplicative random effects)
MR Egger
MR RAPS
Weighted median
Weighted mode
0.002
0.001
0.000
−0.001
0.2
0.4
0.6
0.8
SNP effect on eGFR
SNP effect on Ubiquitin carboxyl−terminal hydrolase 8

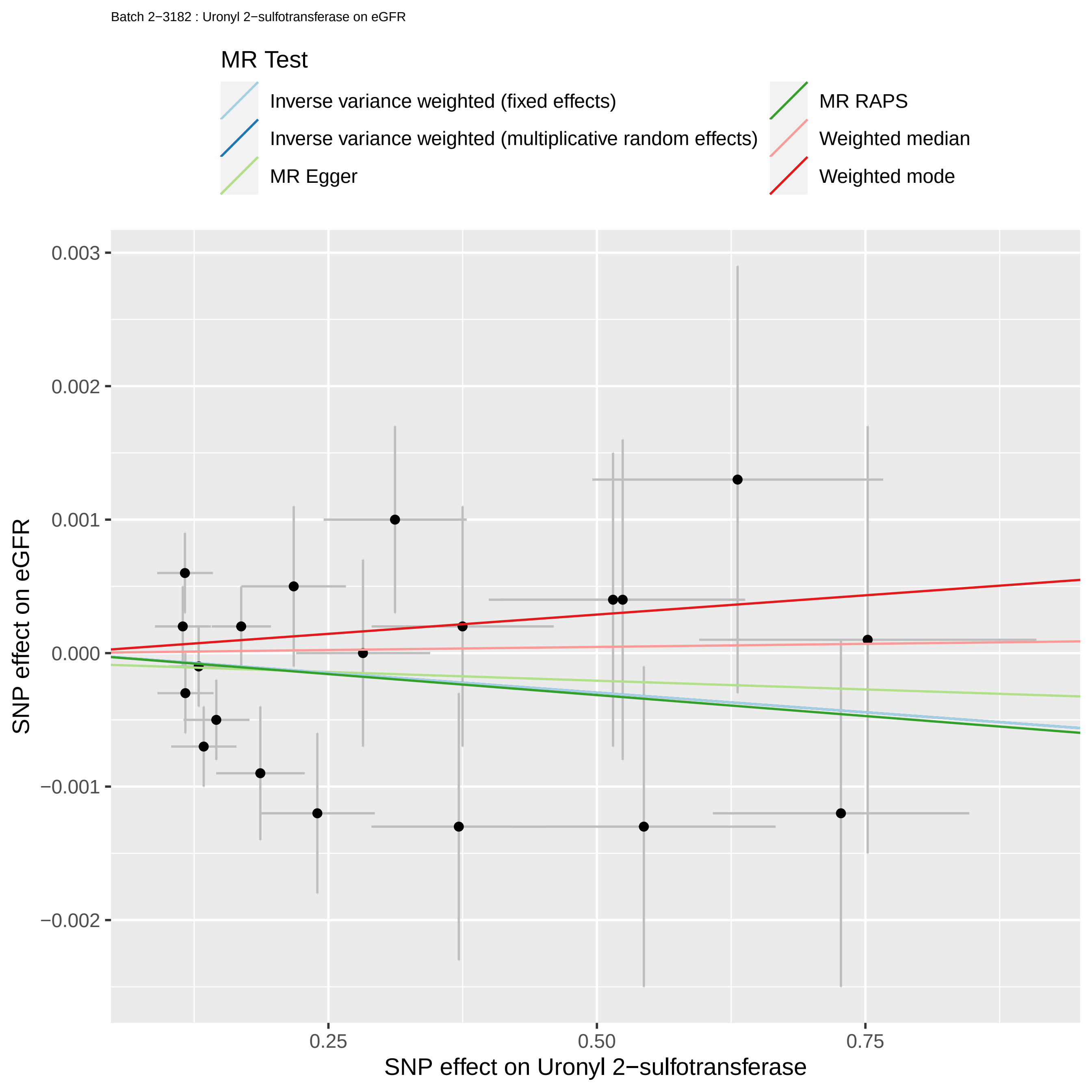

Batch 2−3182 : Uronyl 2−sulfotransferase on eGFR
MR Test
Inverse variance weighted (fixed effects)
Inverse variance weighted (multiplicative random effects)
MR Egger
MR RAPS
Weighted median
Weighted mode
0.003
0.002
0.001
0.000
−0.001
−0.002
SNP effect on eGFR
0.25
0.50
0.75
SNP effect on Uronyl 2−sulfotransferase

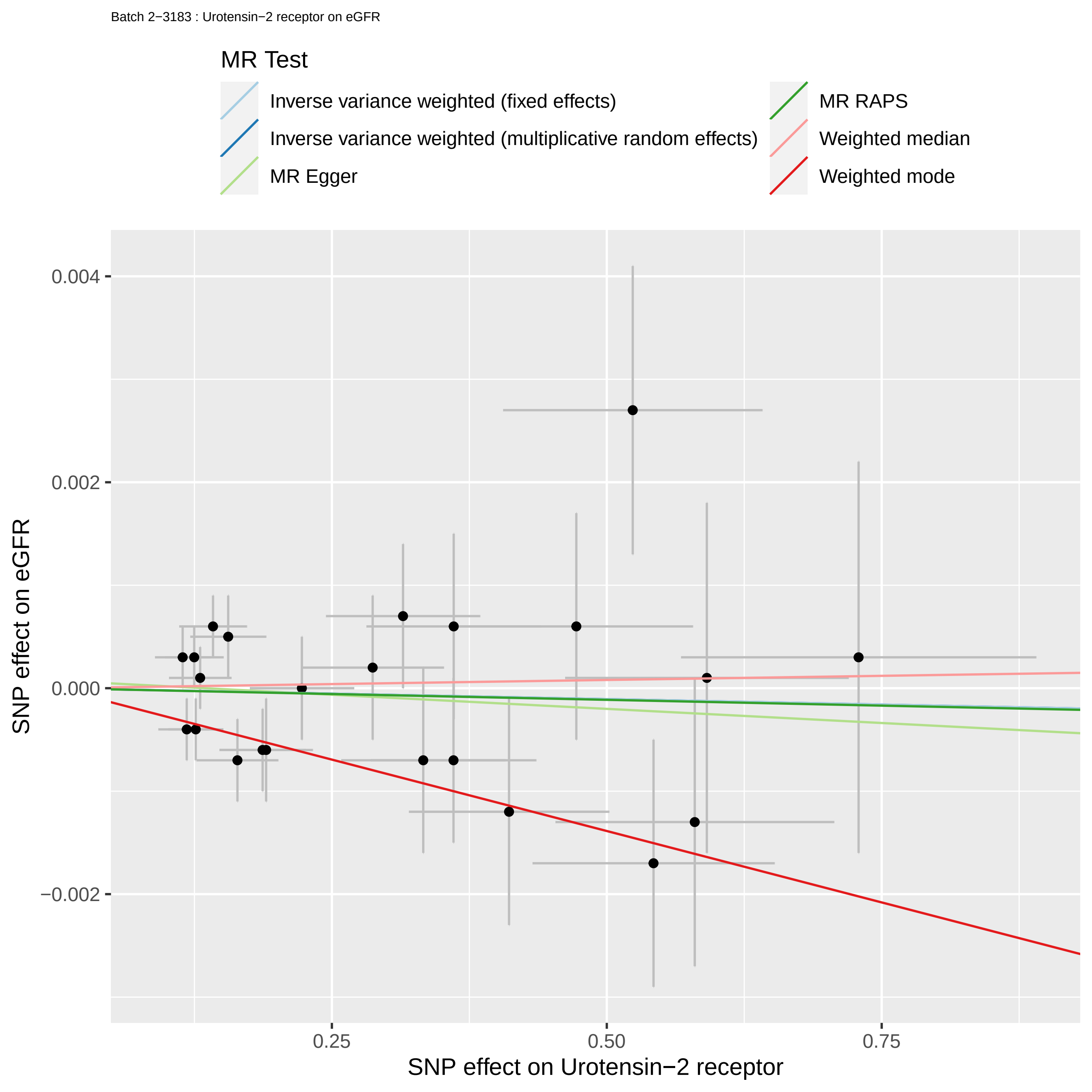
Batch 2−3183 : Urotensin−2 receptor on eGFR
MR Test
Inverse variance weighted (fixed effects)
Inverse variance weighted (multiplicative random effects)
MR Egger
MR RAPS
Weighted median
Weighted mode
0.004
0.002
0.000
−0.002
0.25
0.50
0.75
SNP effect on eGFR
SNP effect on Urotensin−2 receptor

Batch 2−3184 : UDP−glucuronic acid decarboxylase 1 on eGFR

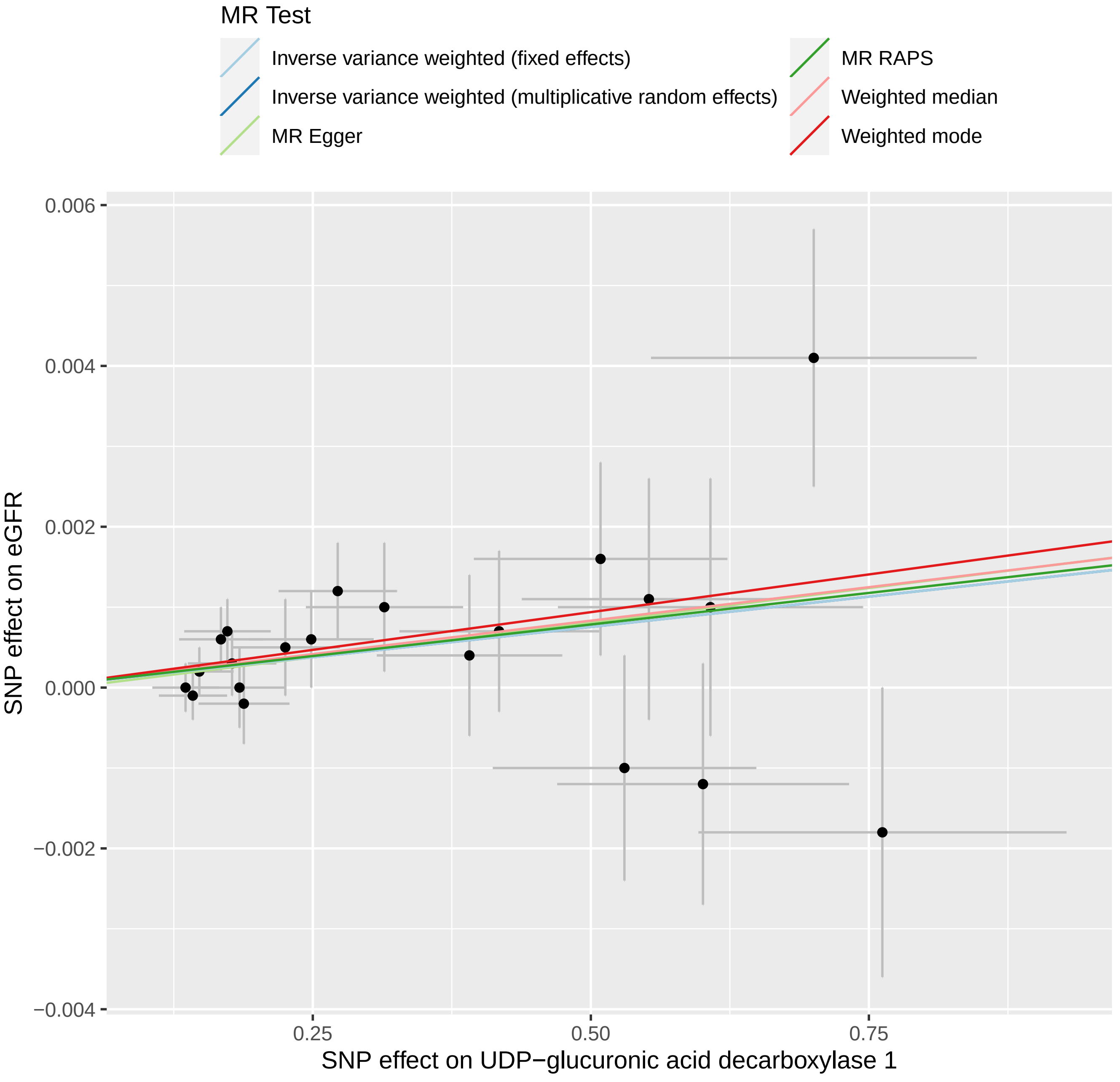

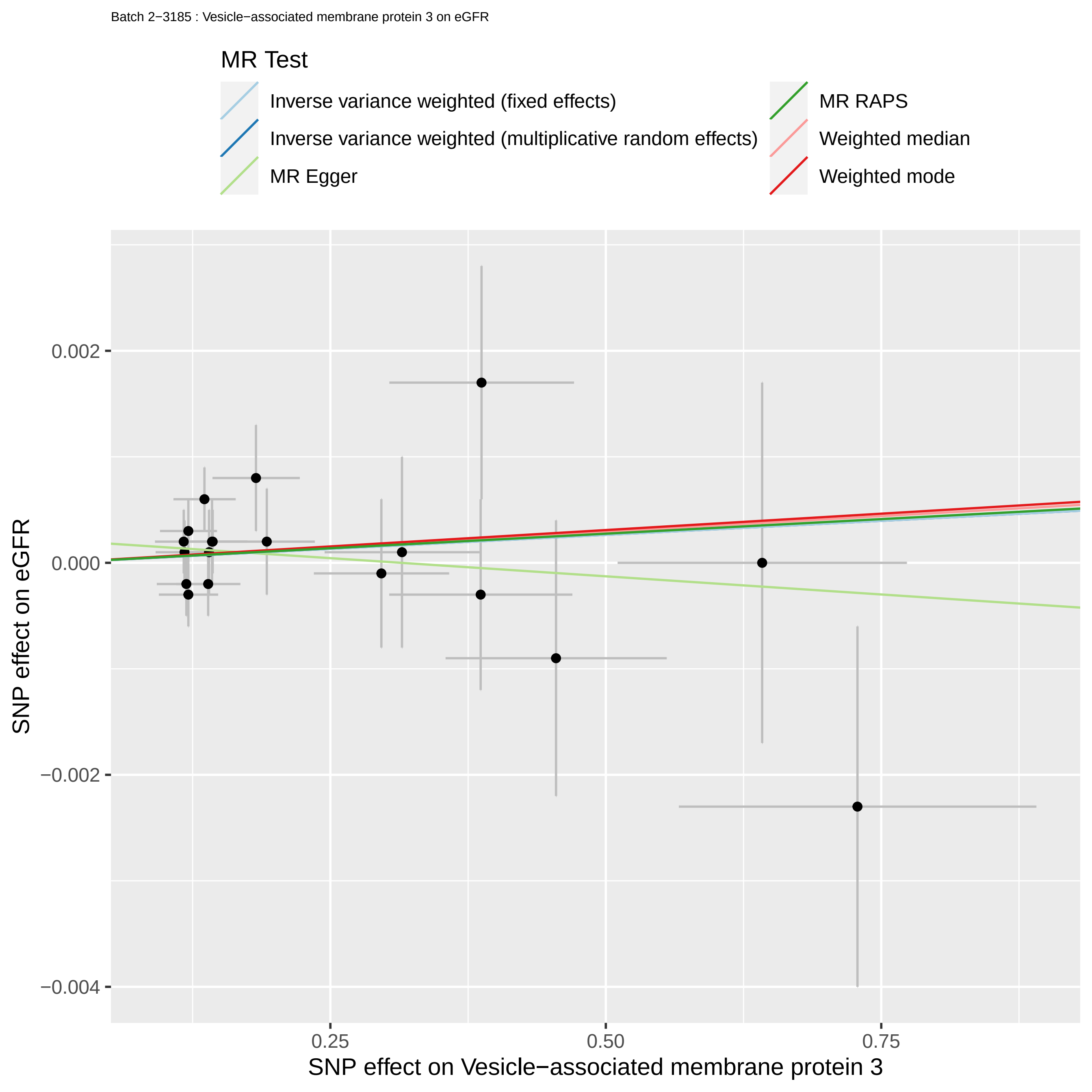
Batch 2−3185 : Vesicle−associated membrane protein 3 on eGFR
MR Test
Inverse variance weighted (fixed effects)
Inverse variance weighted (multiplicative random effects)
MR Egger
MR RAPS
Weighted median
Weighted mode
0.002
0.000
−0.002
−0.004
0.25
0.50
0.75
SNP effect on eGFR
SNP effect on Vesicle−associated membrane protein 3

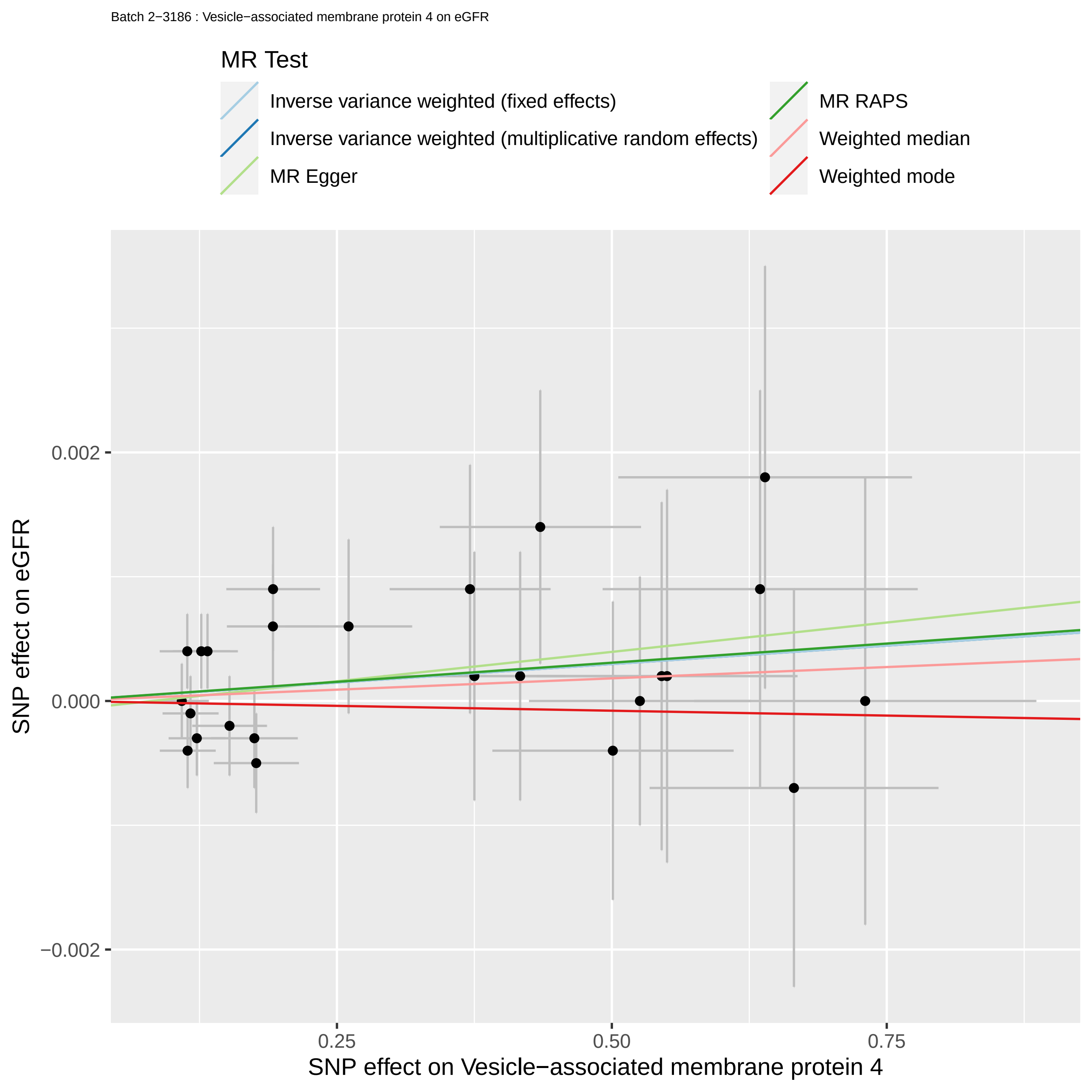
Batch 2−3186 : Vesicle−associated membrane protein 4 on eGFR
MR Test
Inverse variance weighted (fixed effects)
Inverse variance weighted (multiplicative random effects)
MR Egger
MR RAPS
Weighted median
Weighted mode
0.002
0.000
−0.002
SNP effect on eGFR
0.25
0.50
0.75
SNP effect on Vesicle−associated membrane protein 4

Batch 2−3187 : Vesicle−associated membrane protein 5 on eGFR

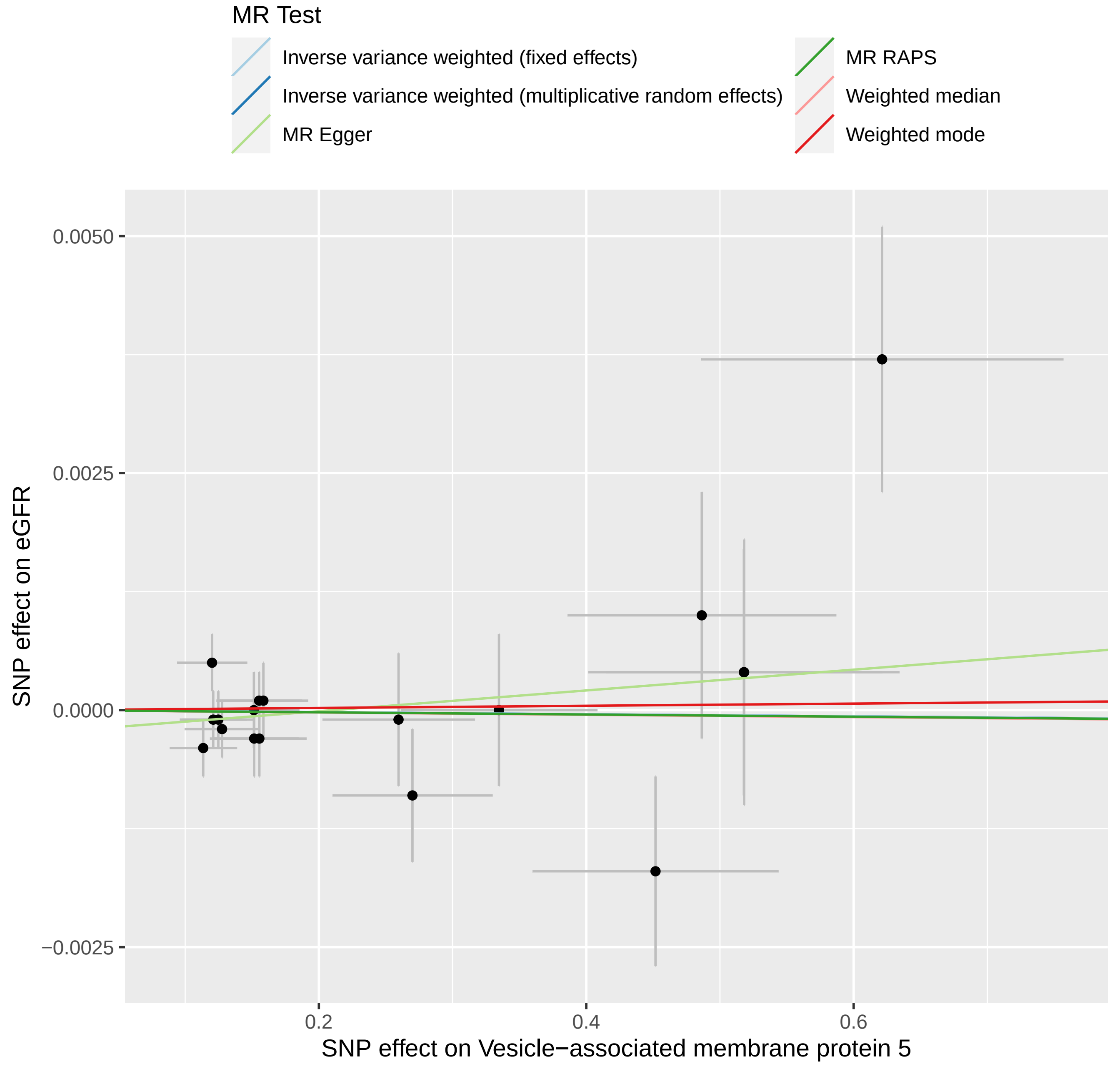

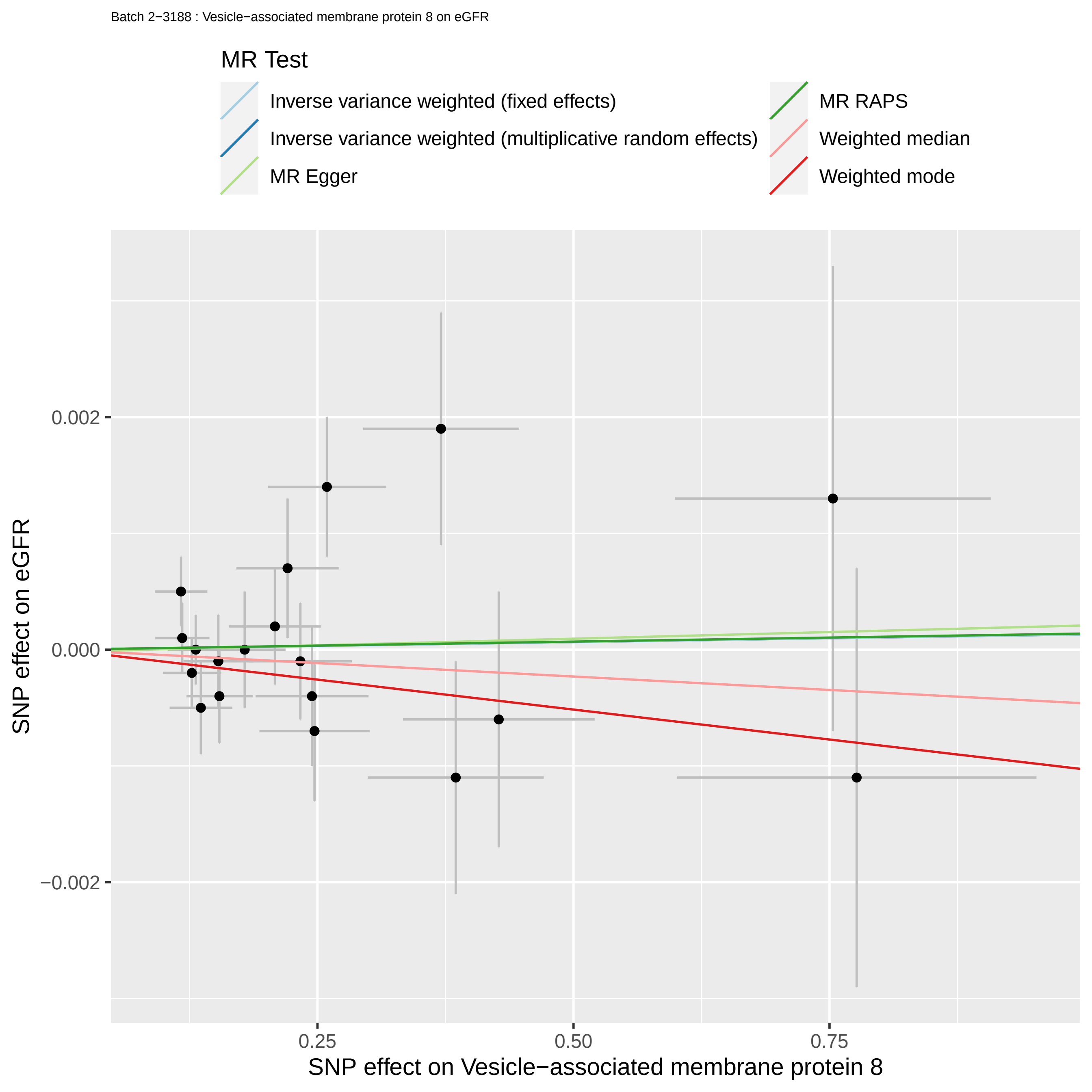

Batch 2−3188 : Vesicle−associated membrane protein 8 on eGFR
MR Test
Inverse variance weighted (fixed effects)
Inverse variance weighted (multiplicative random effects)
MR Egger
MR RAPS
Weighted median
Weighted mode
0.002
0.000
−0.002
0.25
0.50
0.75
SNP effect on eGFR
SNP effect on Vesicle−associated membrane protein 8

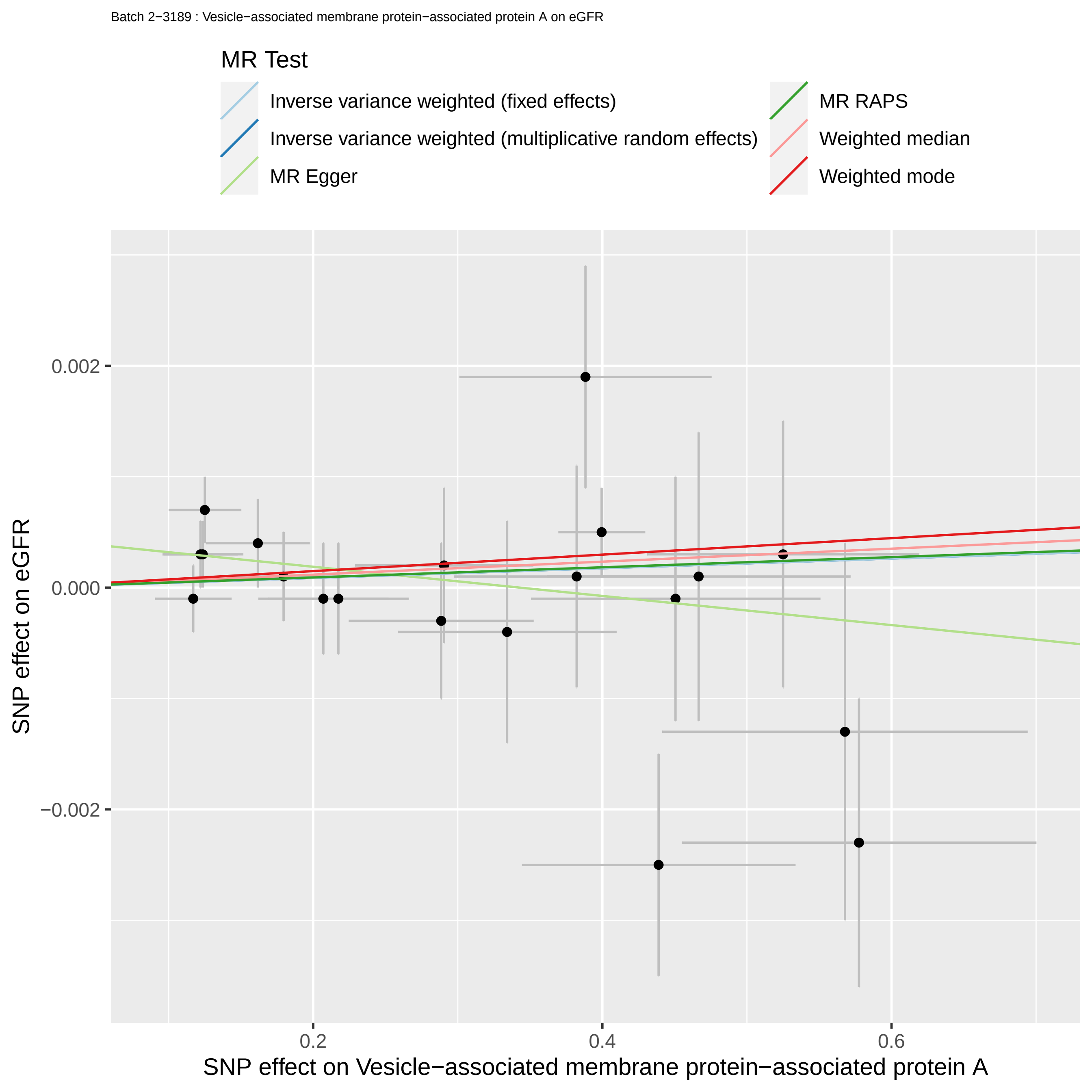

Batch 2−3189 : Vesicle−associated membrane protein−associated protein A on eGFR
MR Test
Inverse variance weighted (fixed effects)
Inverse variance weighted (multiplicative random effects)
MR Egger
MR RAPS
Weighted median
Weighted mode
0.002
0.000
−0.002
SNP effect on eGFR
0.2
0.4
0.6
SNP effect on Vesicle−associated membrane protein−associated protein A

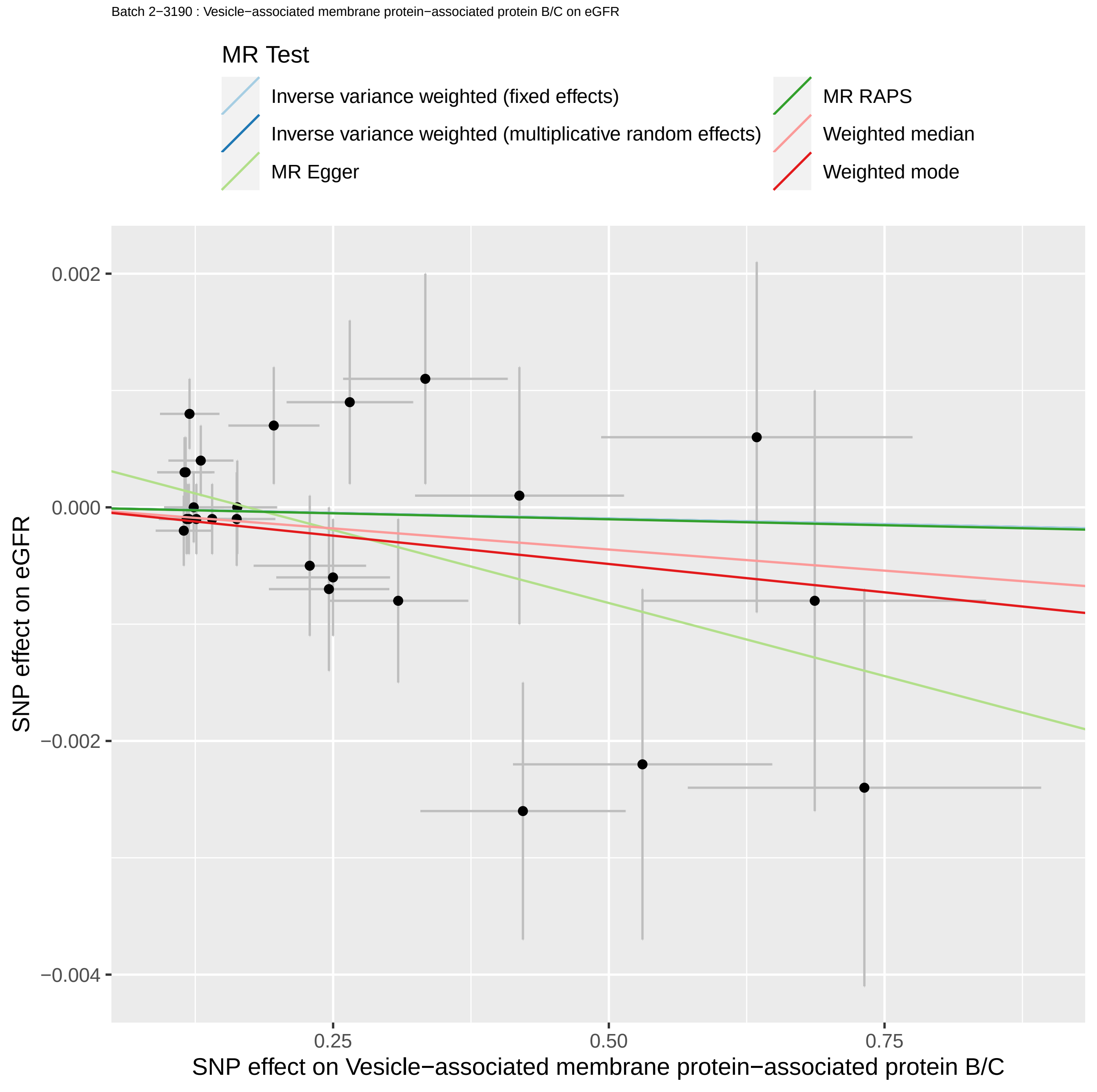

Batch 2−3190 : Vesicle−associated membrane protein−associated protein B/C on eGFR
MR Test
Inverse variance weighted (fixed effects)
Inverse variance weighted (multiplicative random effects)
MR Egger
MR RAPS
Weighted median
Weighted mode
0.002
0.000
−0.002
−0.004
SNP effect on eGFR
0.25
0.50
0.75
SNP effect on Vesicle−associated membrane protein−associated protein B/C

Batch 2−3191 : Valine−−tRNA ligase on eGFR

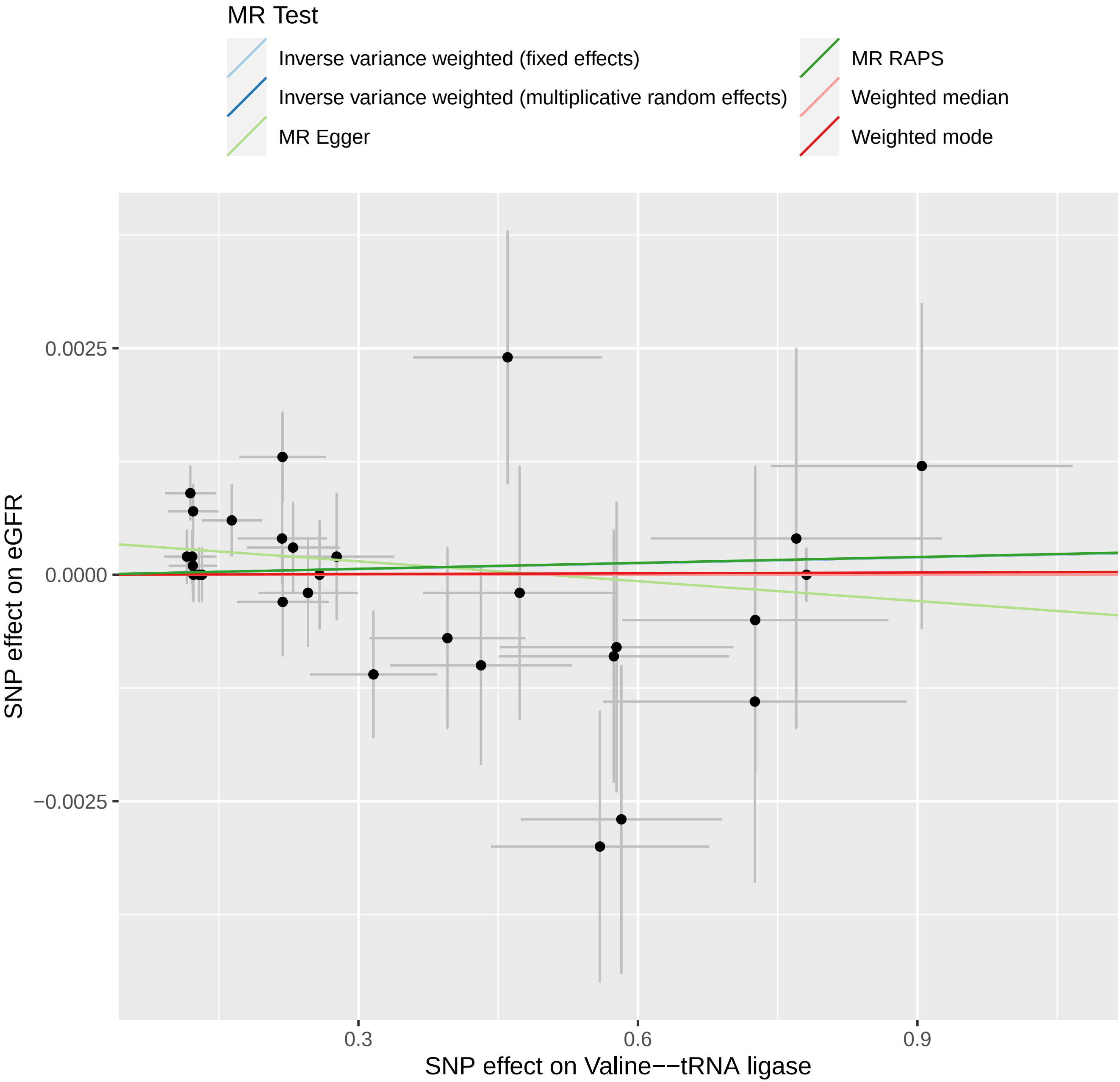

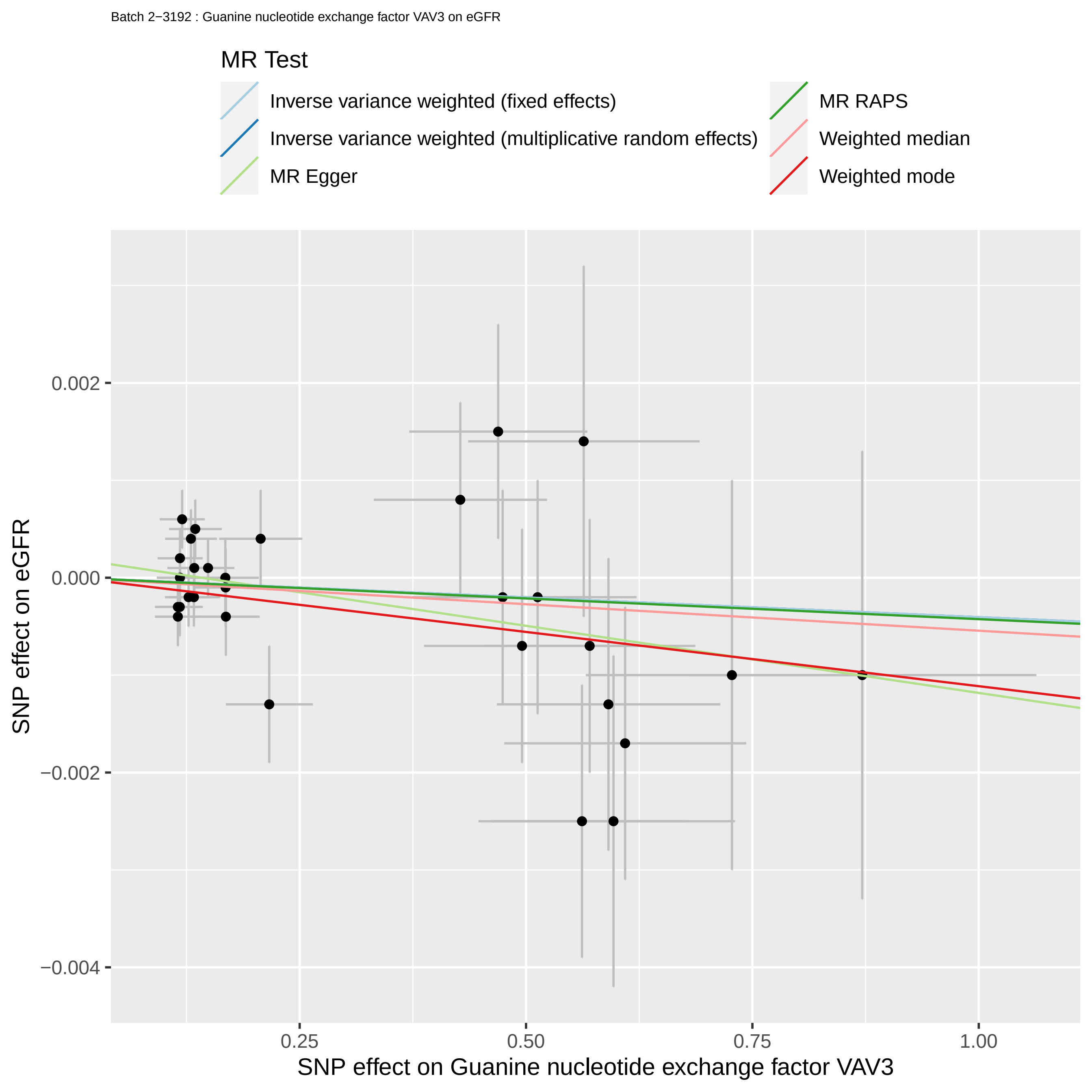
Batch 2−3192 : Guanine nucleotide exchange factor VAV3 on eGFR
MR Test
Inverse variance weighted (fixed effects)
Inverse variance weighted (multiplicative random effects)
MR Egger
MR RAPS
Weighted median
Weighted mode
0.002
0.000
−0.002
−0.004
SNP effect on eGFR
0.25
0.50
0.75
1.00
SNP effect on Guanine nucleotide exchange factor VAV3

Batch 2−3193 : Vascular cell adhesion protein 1 on eGFR

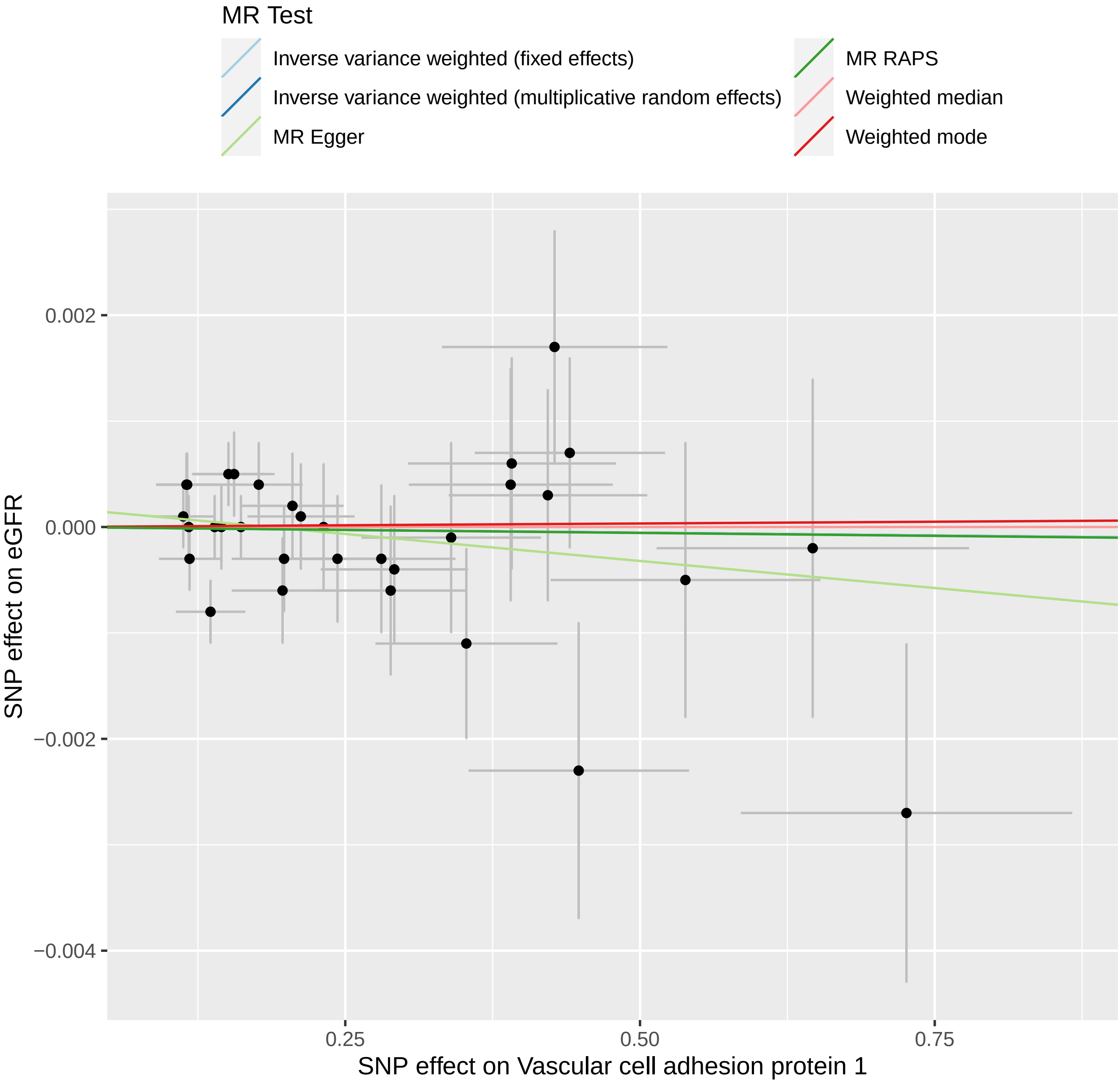

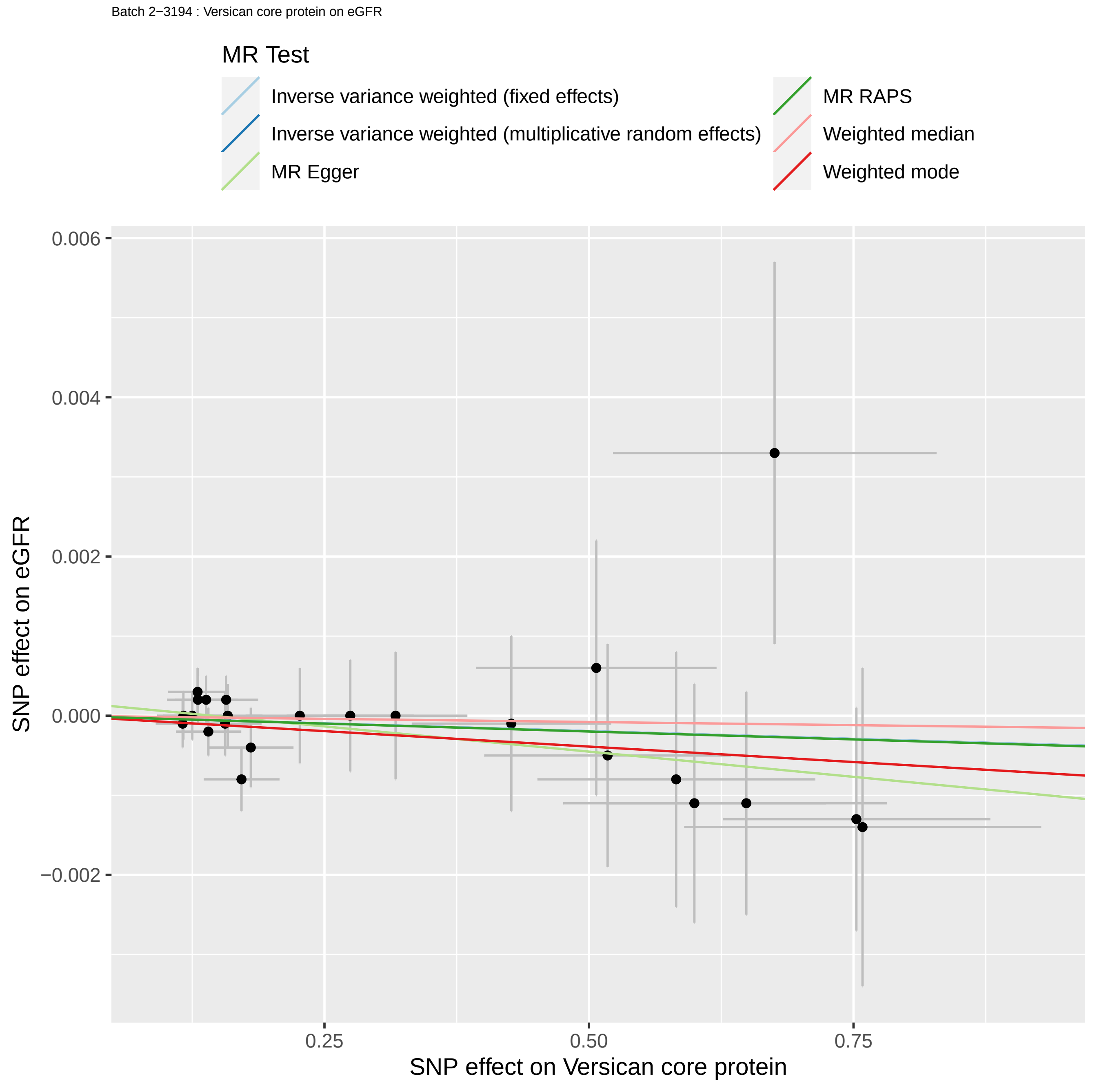
Batch 2−3194 : Versican core protein on eGFR
MR Test
Inverse variance weighted (fixed effects)
Inverse variance weighted (multiplicative random effects)
MR Egger
MR RAPS
Weighted median
Weighted mode
0.006
0.004
0.002
0.000
−0.002
0.25
0.50
0.75
SNP effect on eGFR
SNP effect on Versican core protein

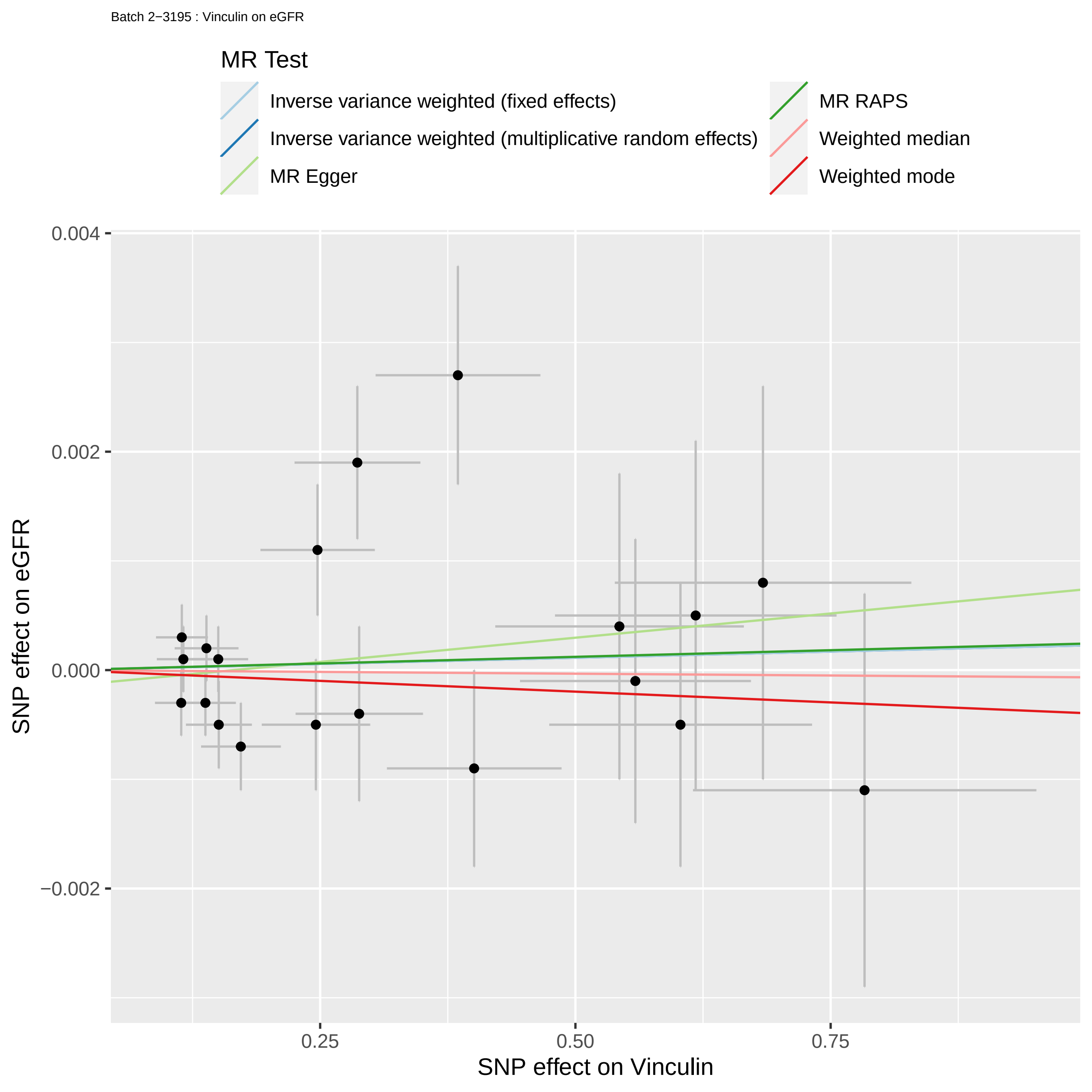

Batch 2−3195 : Vinculin on eGFR
MR Test
Inverse variance weighted (fixed effects)
Inverse variance weighted (multiplicative random effects)
MR Egger
MR RAPS
Weighted median
Weighted mode
0.004
0.002
0.000
−0.002
0.25
0.50
0.75
SNP effect on Vinculin
SNP effect on eGFR

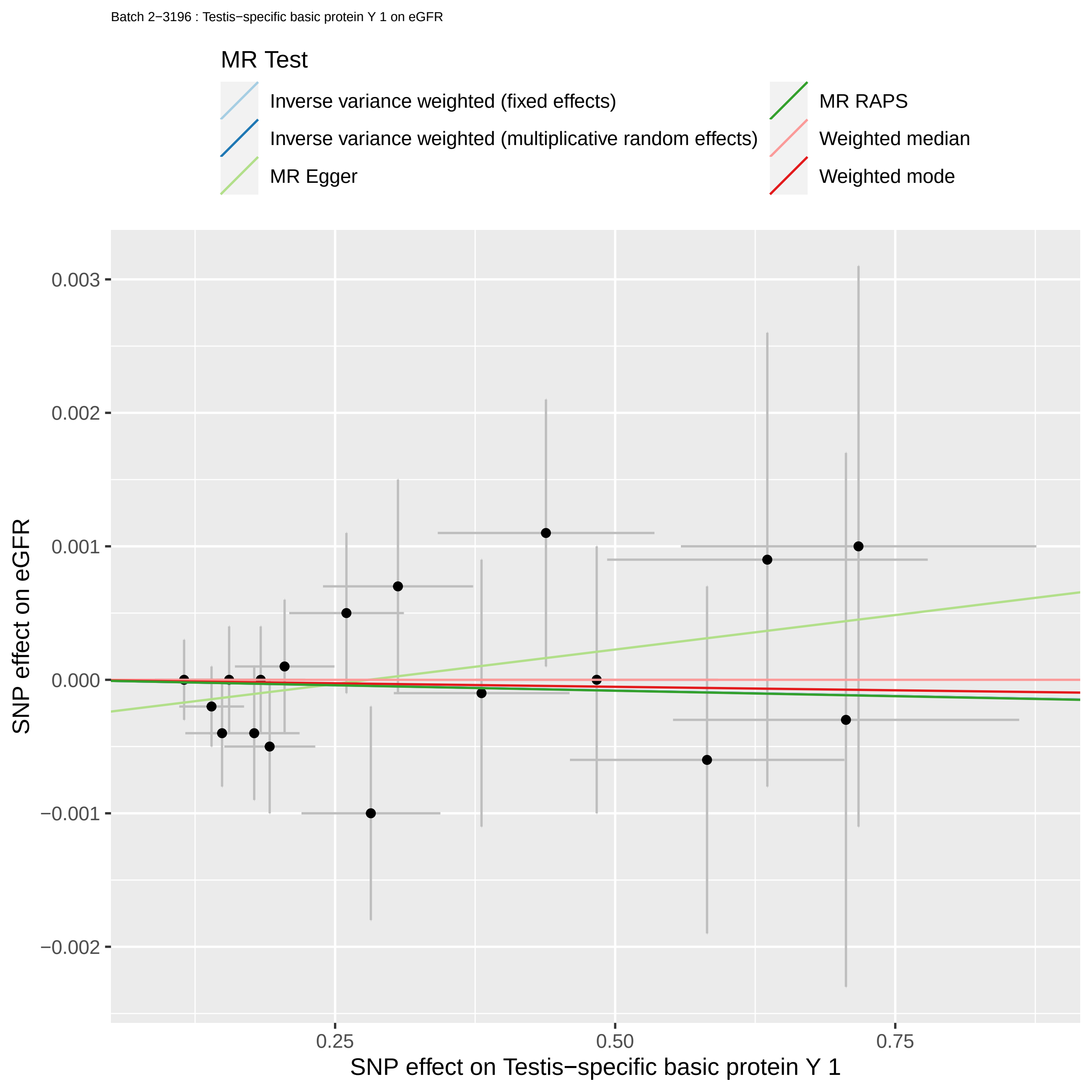

Batch 2−3196 : Testis−specific basic protein Y 1 on eGFR
MR Test
Inverse variance weighted (fixed effects)
Inverse variance weighted (multiplicative random effects)
MR Egger
MR RAPS
Weighted median
Weighted mode
0.003
0.002
0.001
0.000
−0.001
−0.002
0.25
0.50
0.75
SNP effect on eGFR
SNP effect on Testis−specific basic protein Y 1

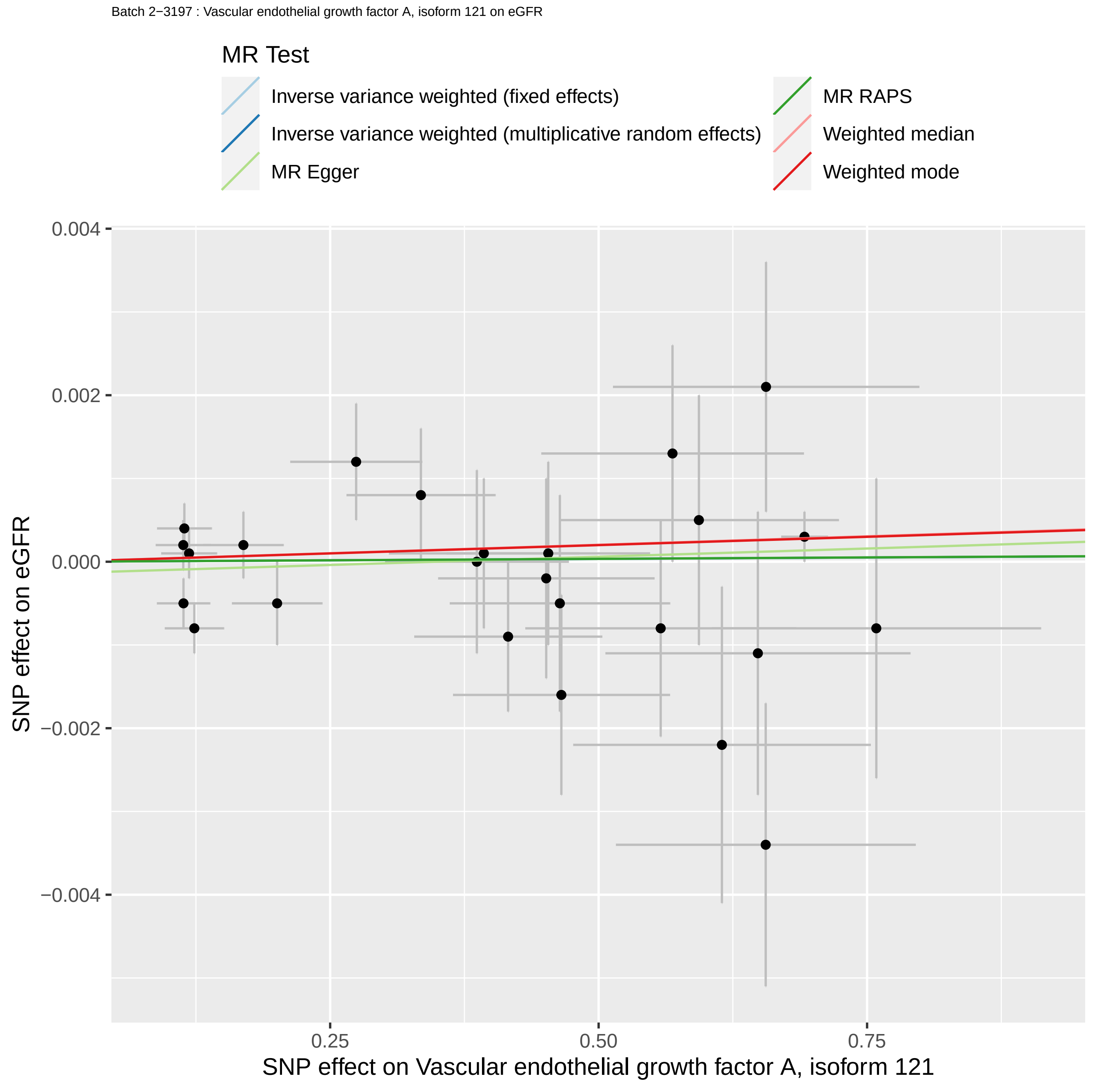

Batch 2−3197 : Vascular endothelial growth factor A, isoform 121 on eGFR
MR Test
Inverse variance weighted (fixed effects)
Inverse variance weighted (multiplicative random effects)
MR Egger
MR RAPS
Weighted median
Weighted mode
0.004
0.002
0.000
−0.002
−0.004
0.25
0.50
0.75
SNP effect on eGFR
SNP effect on Vascular endothelial growth factor A, isoform 121

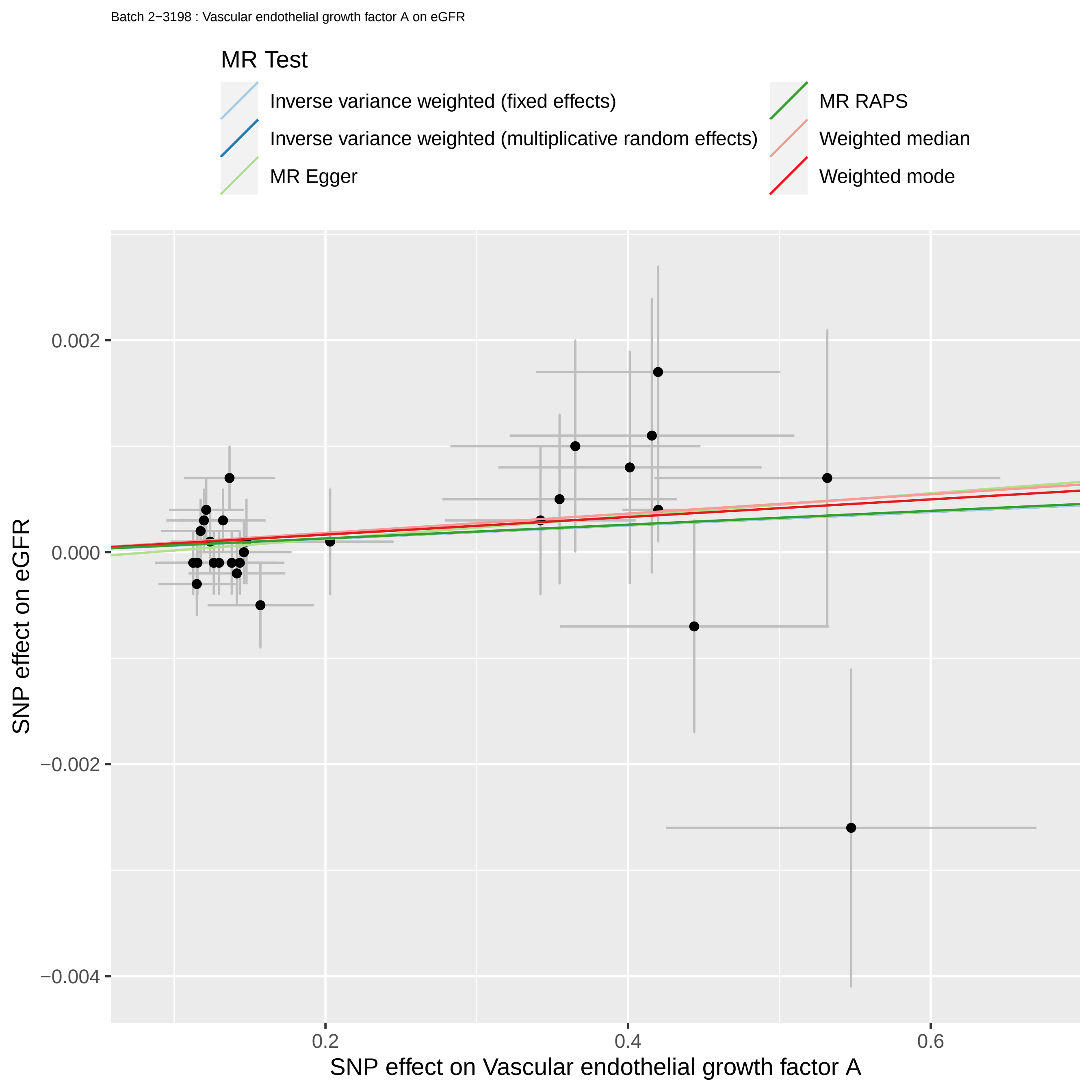

Batch 2−3198 : Vascular endothelial growth factor A on eGFR
MR Test
Inverse variance weighted (fixed effects)
Inverse variance weighted (multiplicative random effects)
MR Egger
MR RAPS
Weighted median
Weighted mode
0.002
0.000
−0.002
−0.004
0.2
0.4
0.6
SNP effect on eGFR
SNP effect on Vascular endothelial growth factor A

Batch 2−3199 : Vascular endothelial growth factor C on eGFR

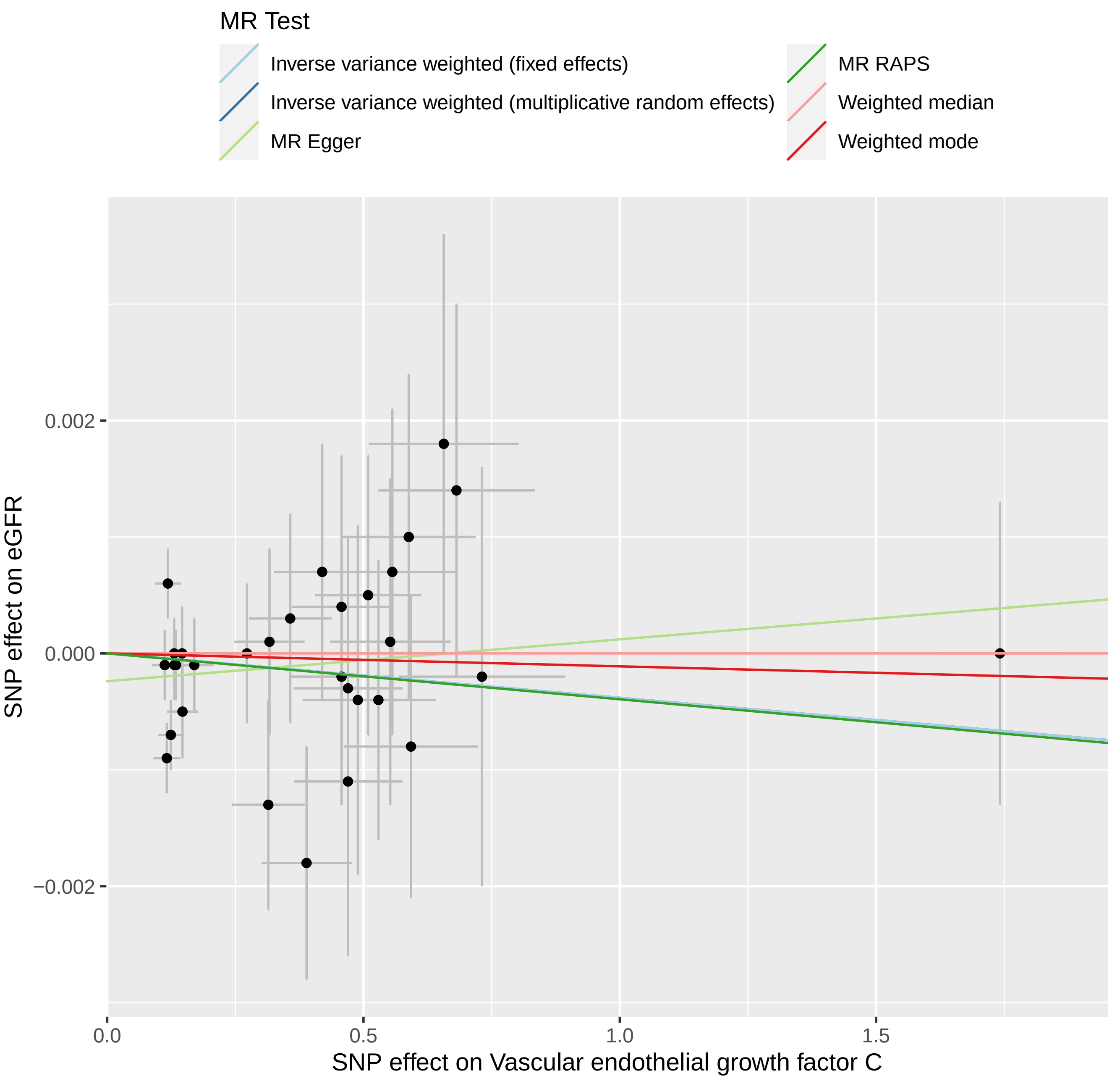

Batch 2−3200 : Selenoprotein S on eGFR

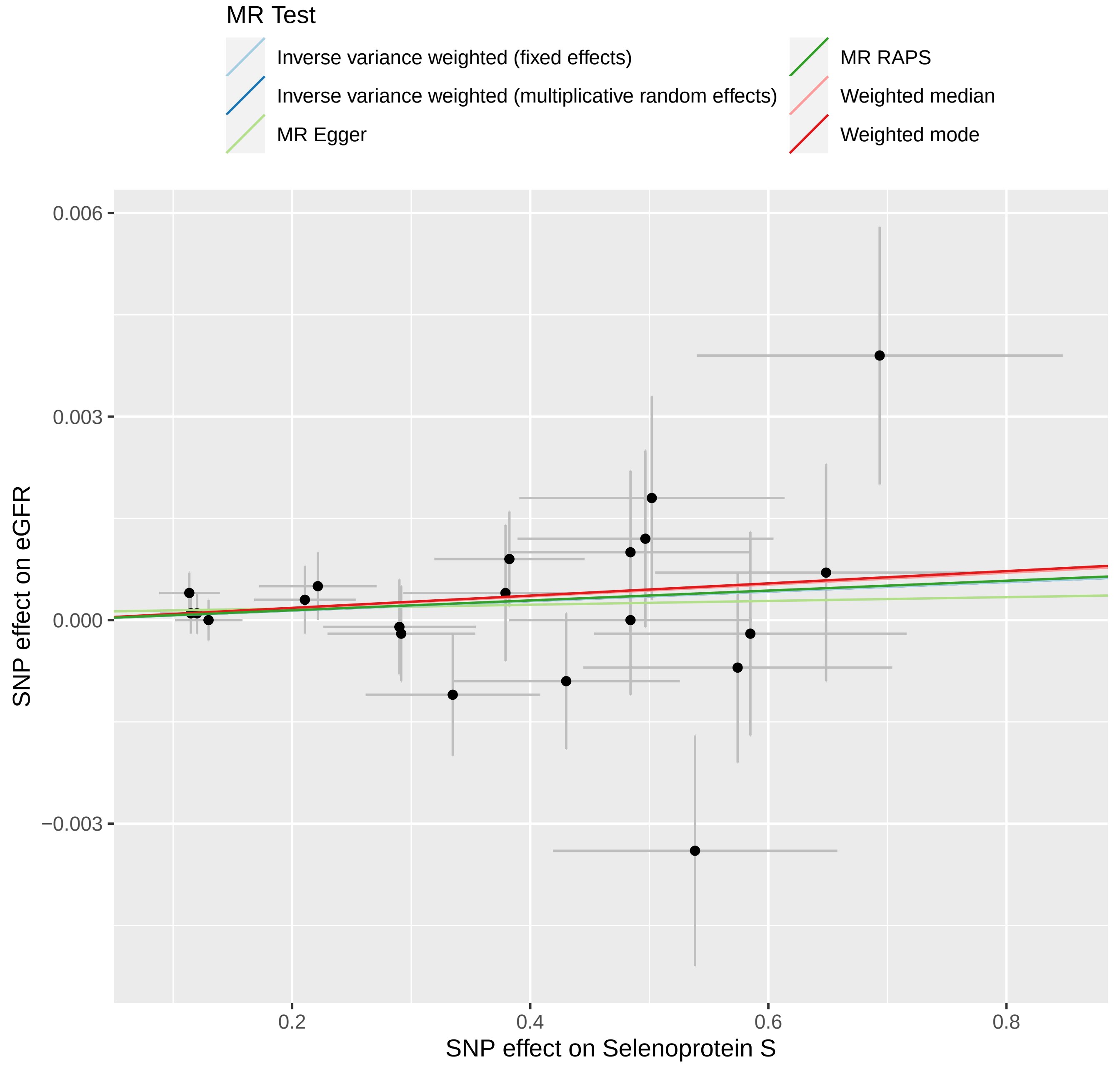

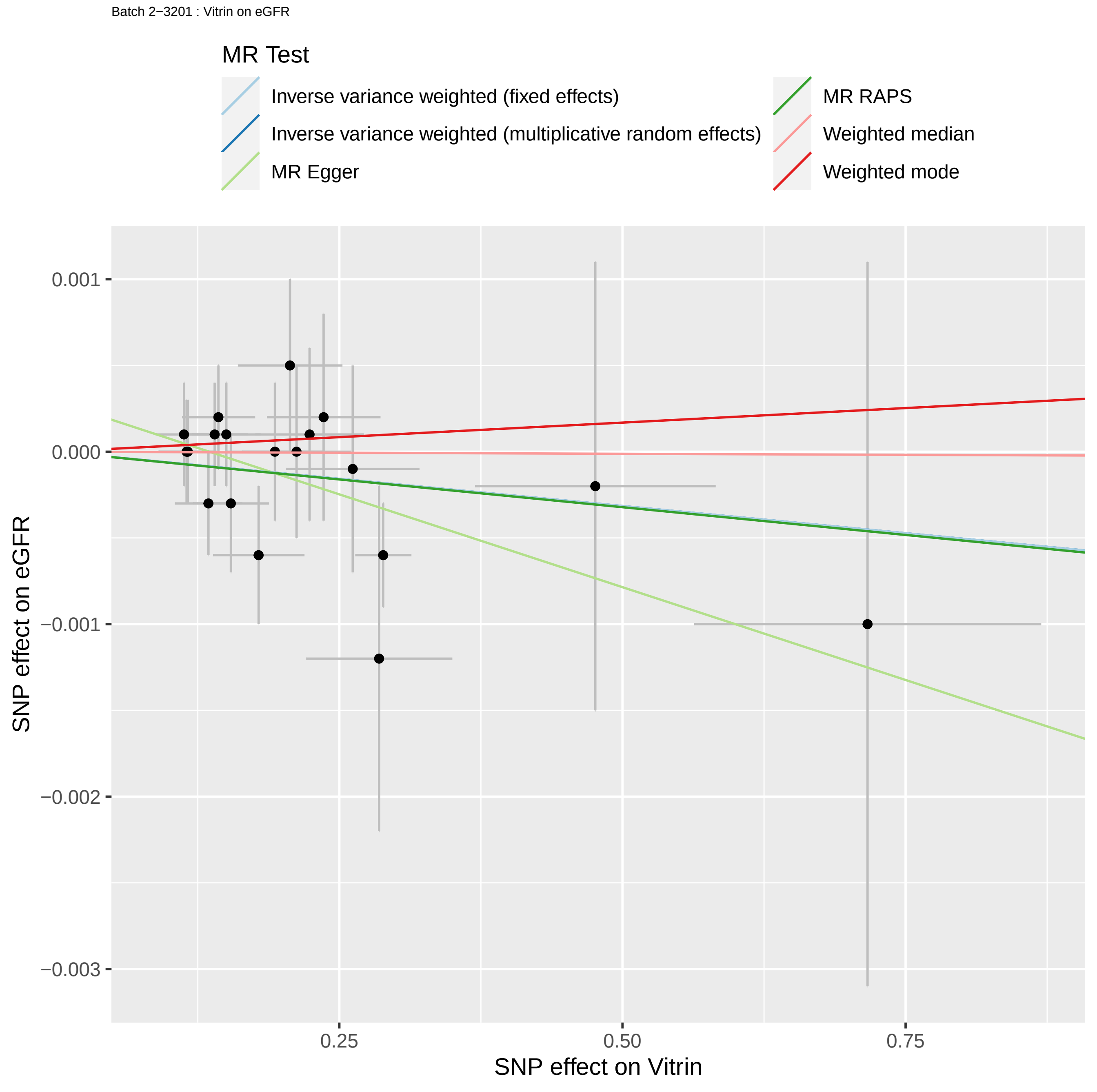
Batch 2−3201 : Vitrin on eGFR
MR Test
Inverse variance weighted (fixed effects)
Inverse variance weighted (multiplicative random effects)
MR Egger
MR RAPS
Weighted median
Weighted mode
0.001
0.000
−0.001
−0.002
−0.003
0.25
0.50
0.75
SNP effect on eGFR
SNP effect on Vitrin

Batch 2−3202 : Charged multivesicular body protein 3 on eGFR

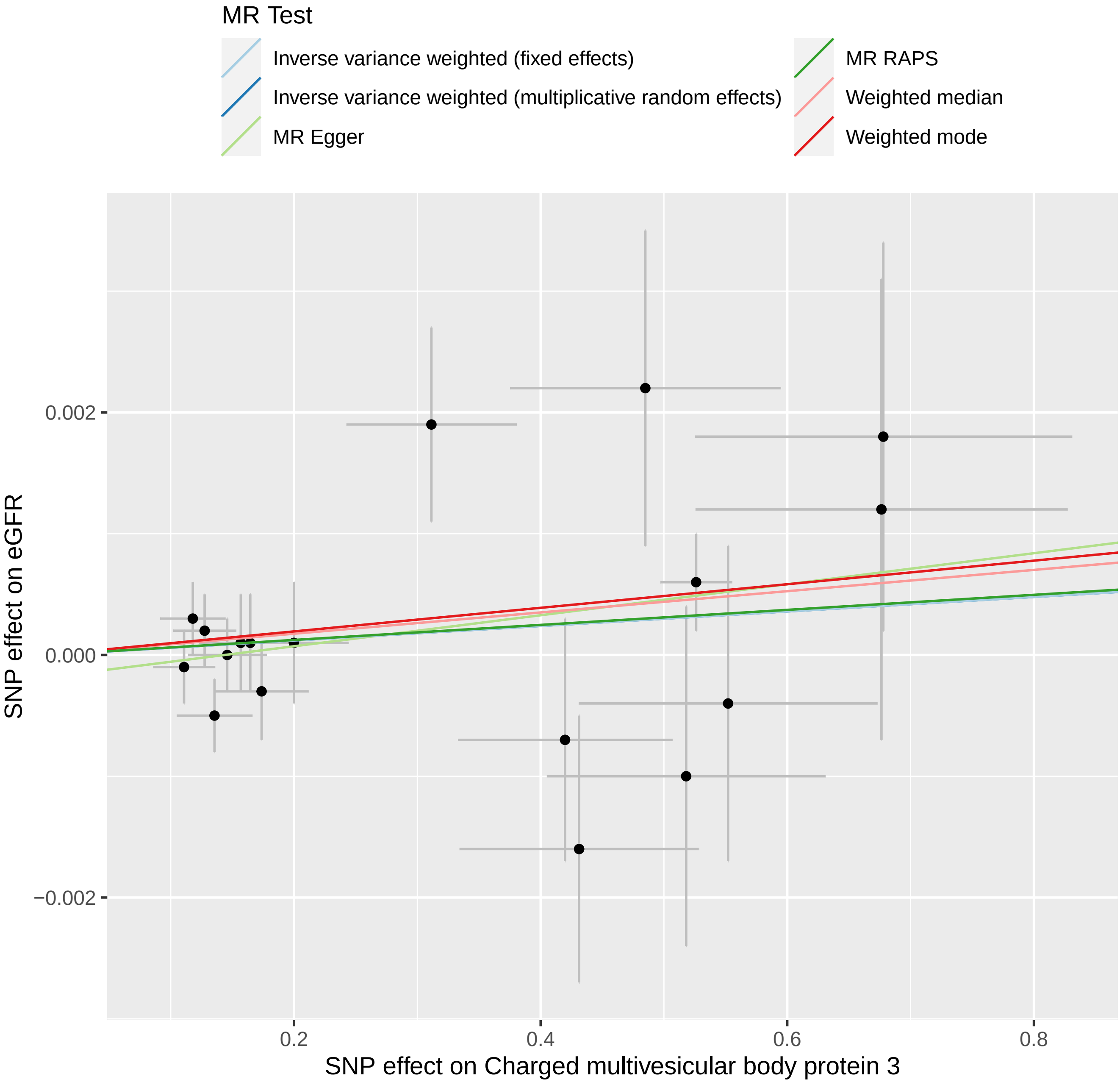

Batch 2−3203 : Vacuolar protein sorting−associated protein 29 on eGFR

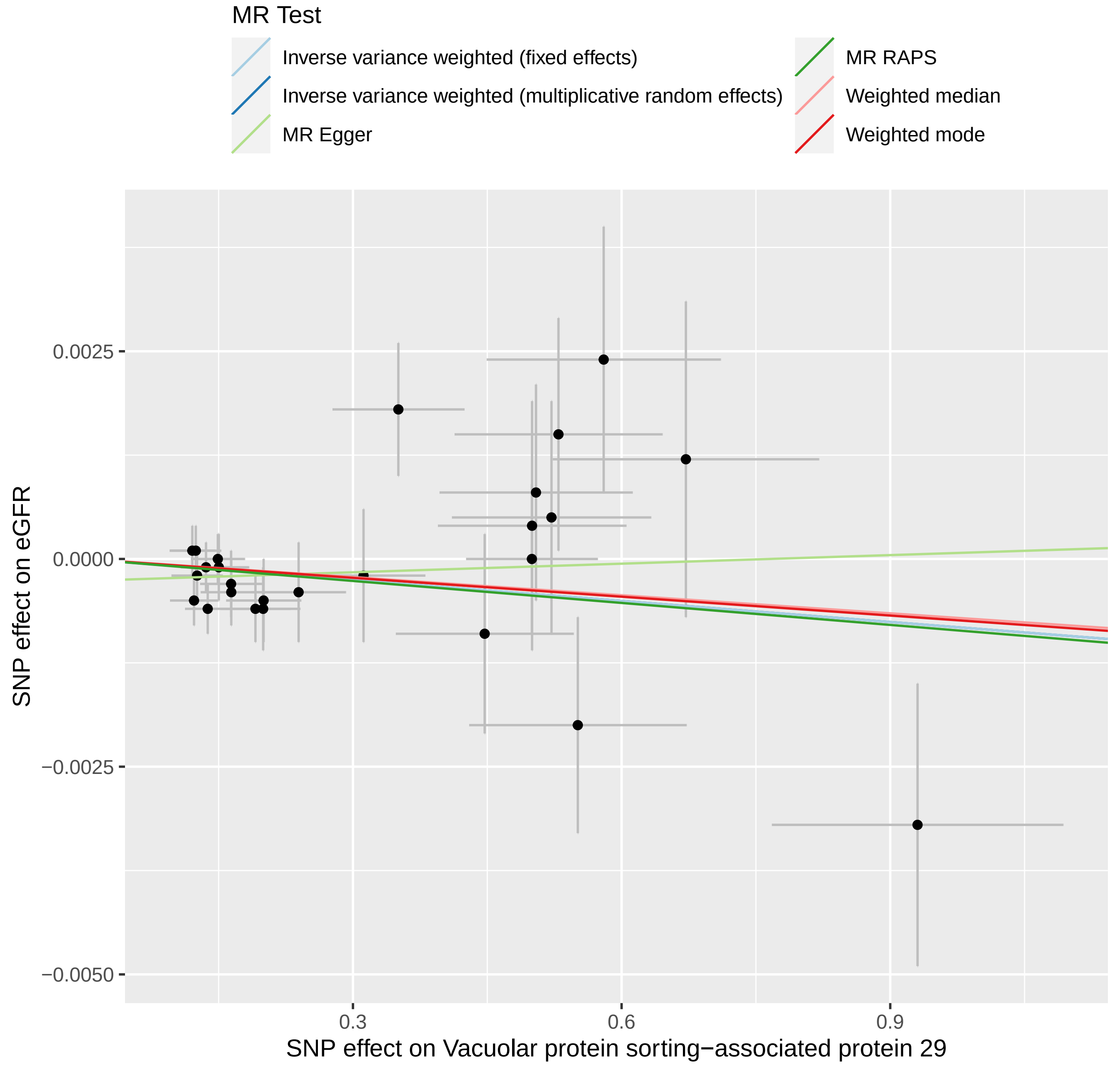

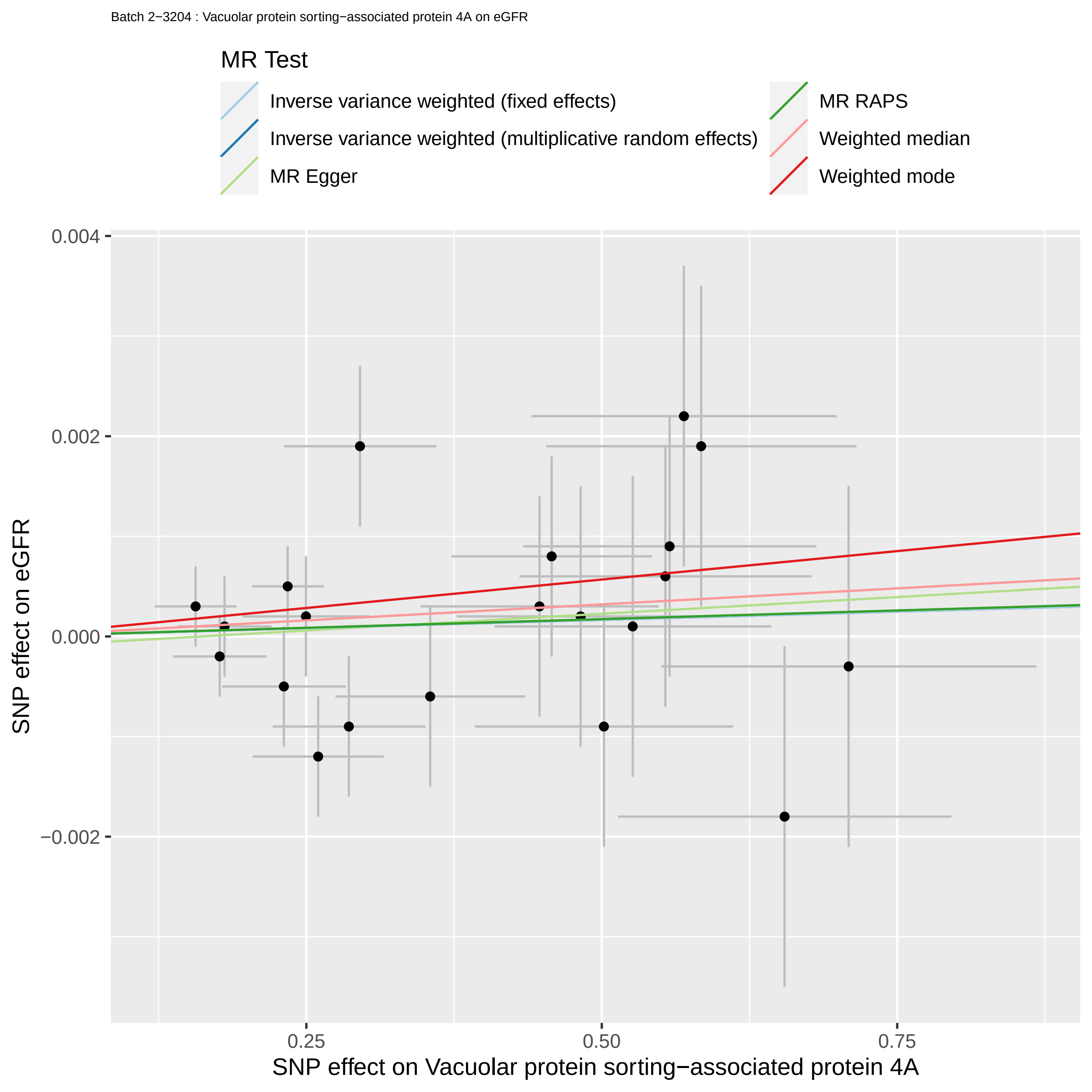

Batch 2−3204 : Vacuolar protein sorting−associated protein 4A on eGFR
MR Test
Inverse variance weighted (fixed effects)
Inverse variance weighted (multiplicative random effects)
MR Egger
MR RAPS
Weighted median
Weighted mode
0.004
0.002
0.000
−0.002
SNP effect on eGFR
0.25
0.50
0.75
SNP effect on Vacuolar protein sorting−associated protein 4A

Batch 2−3205 : Vacuolar protein sorting−associated protein 4B on eGFR

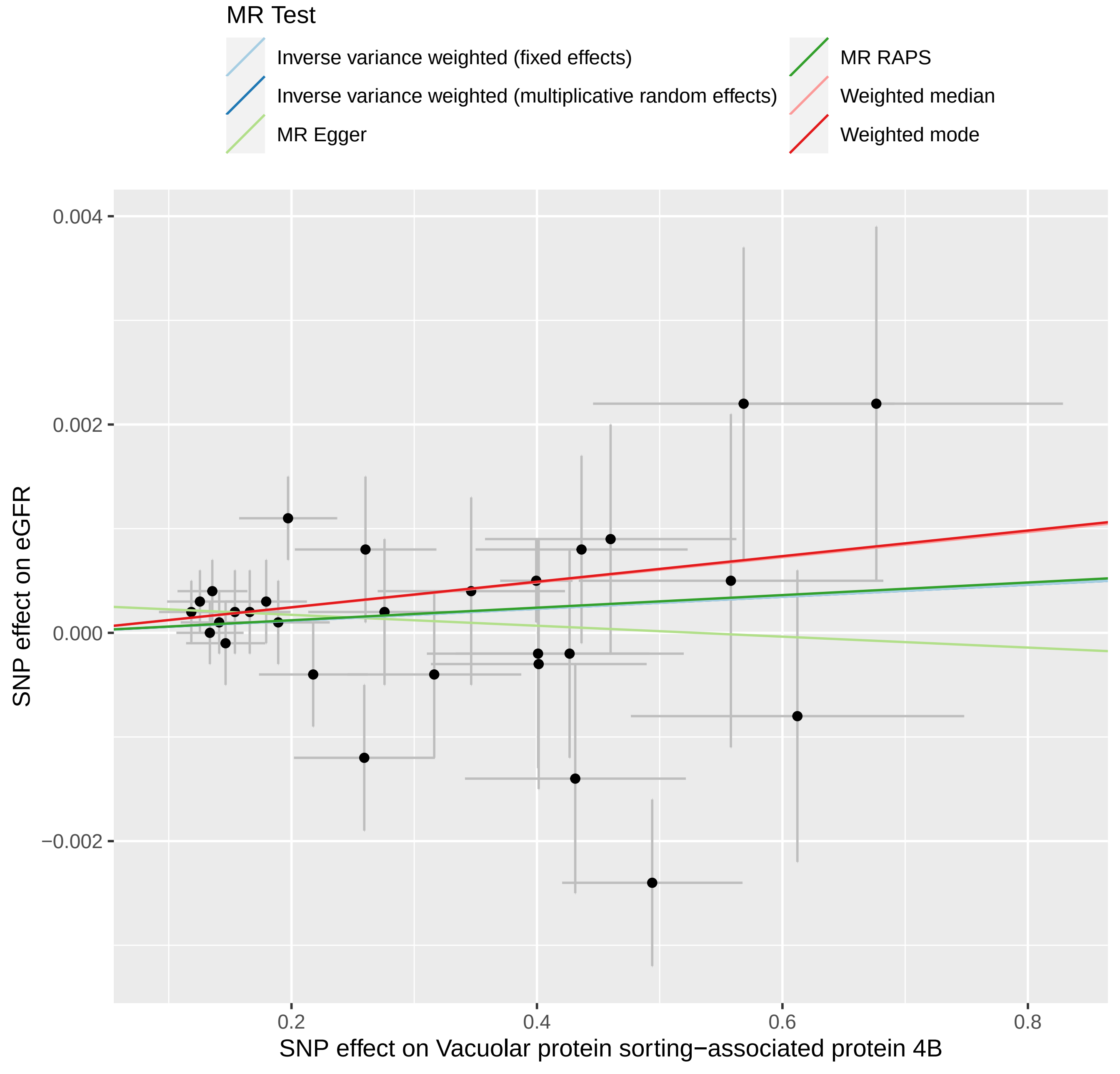

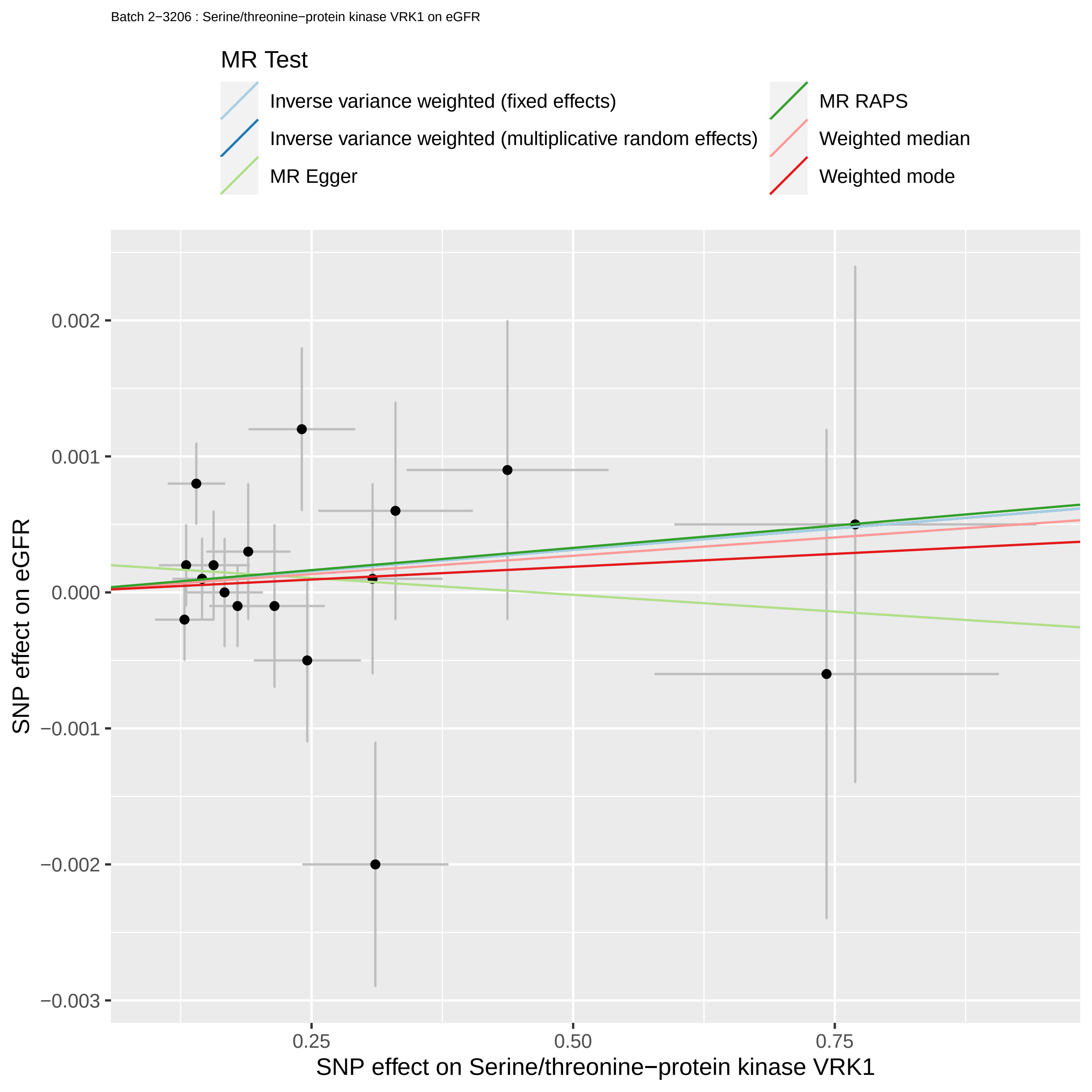

Batch 2−3206 : Serine/threonine−protein kinase VRK1 on eGFR
MR Test
Inverse variance weighted (fixed effects)
Inverse variance weighted (multiplicative random effects)
MR Egger
MR RAPS
Weighted median
Weighted mode
0.002
0.001
0.000
−0.001
−0.002
−0.003
0.25
0.50
0.75
SNP effect on eGFR
SNP effect on Serine/threonine−protein kinase VRK1

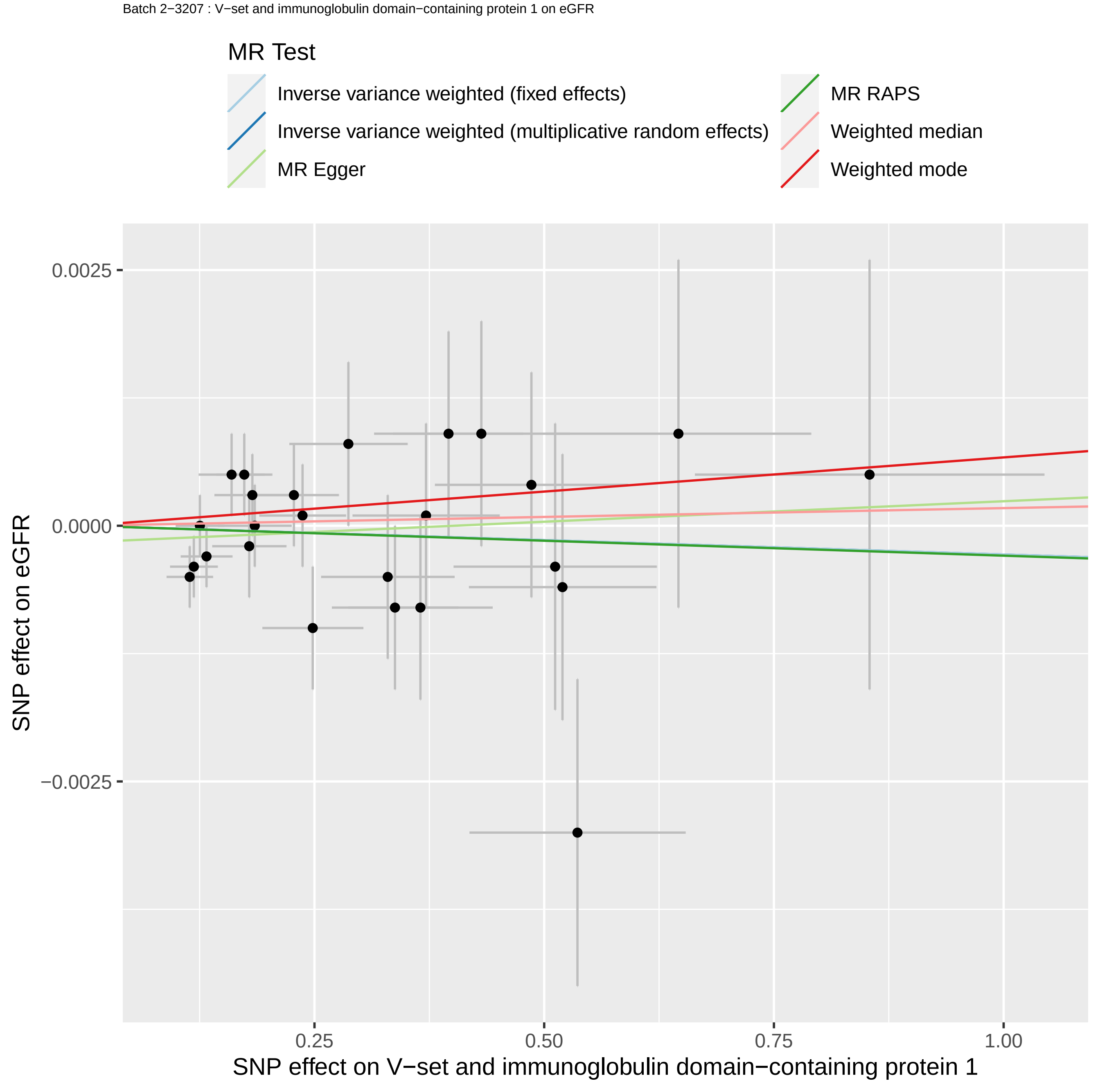
Batch 2−3207 : V−set and immunoglobulin domain−containing protein 1 on eGFR
MR Test
Inverse variance weighted (fixed effects)
Inverse variance weighted (multiplicative random effects)
MR Egger
MR RAPS
Weighted median
Weighted mode
0.0025
0.0000
−0.0025
0.25
0.50
0.75
1.00
SNP effect on eGFR
SNP effect on V−set and immunoglobulin domain−containing protein 1

Batch 2−3208 : V−set and immunoglobulin domain−containing protein 2 on eGFR

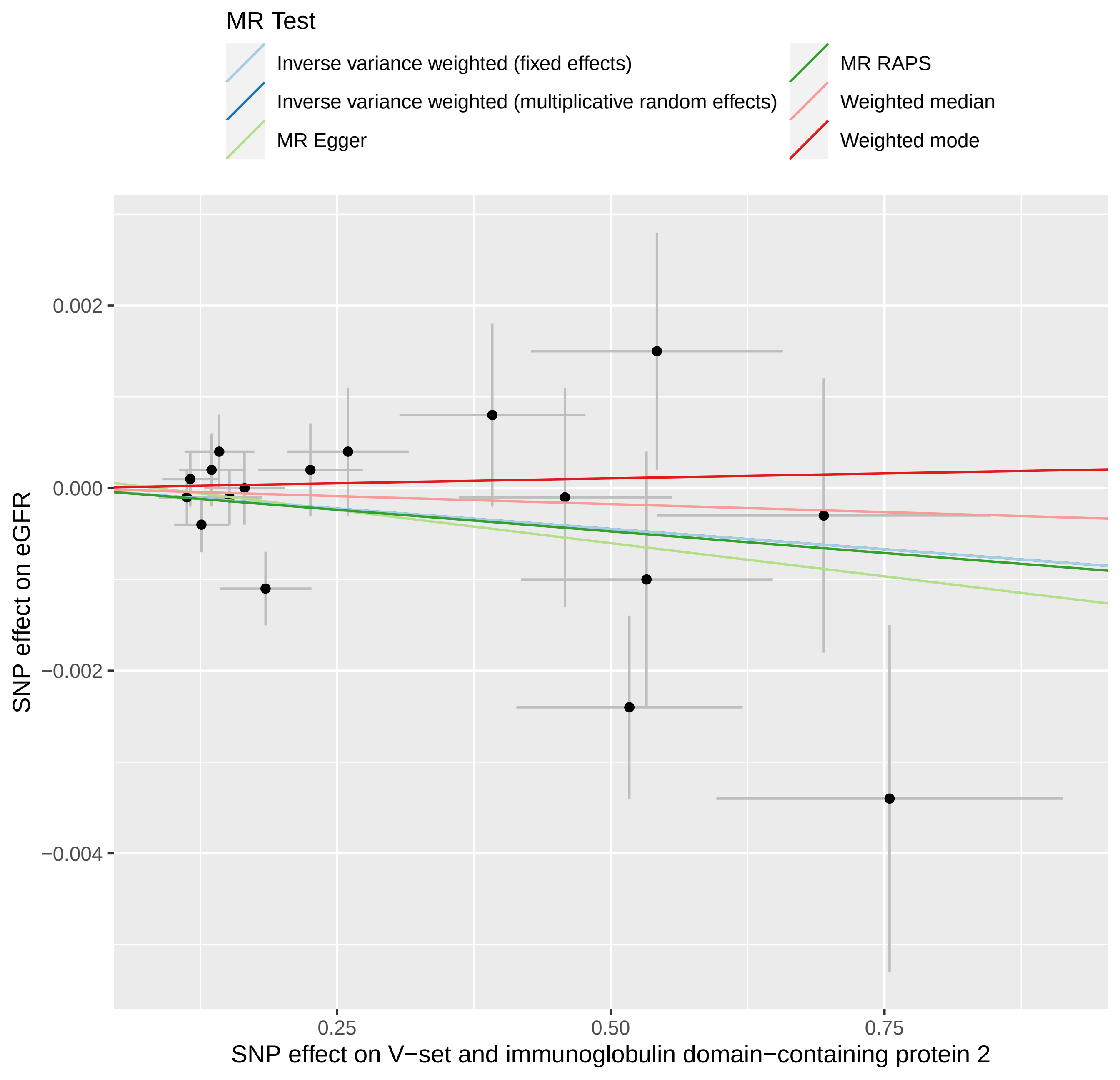

Batch 2−3209 : V−set and transmembrane domain−containing protein 2−like protein on eGFR

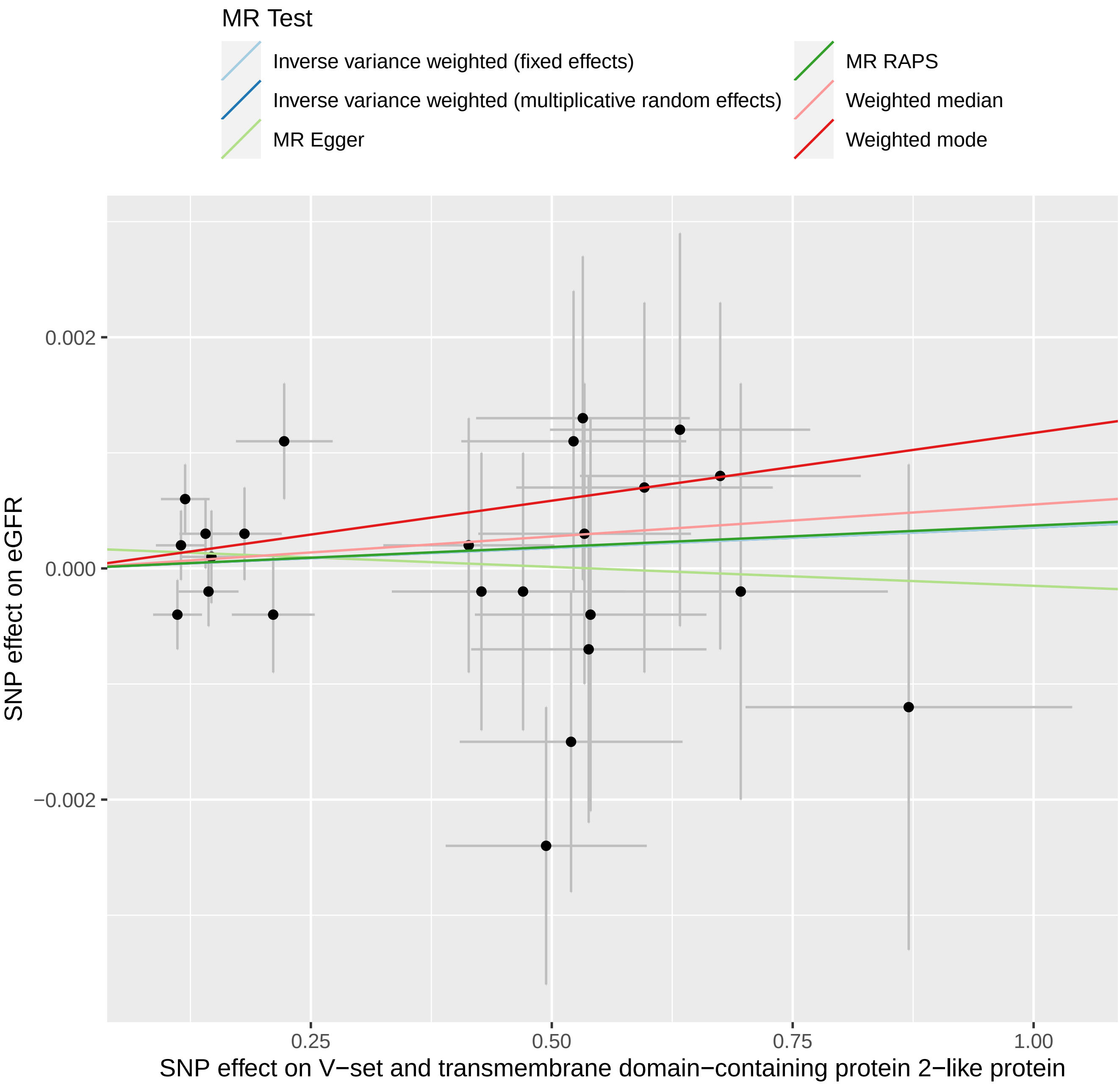

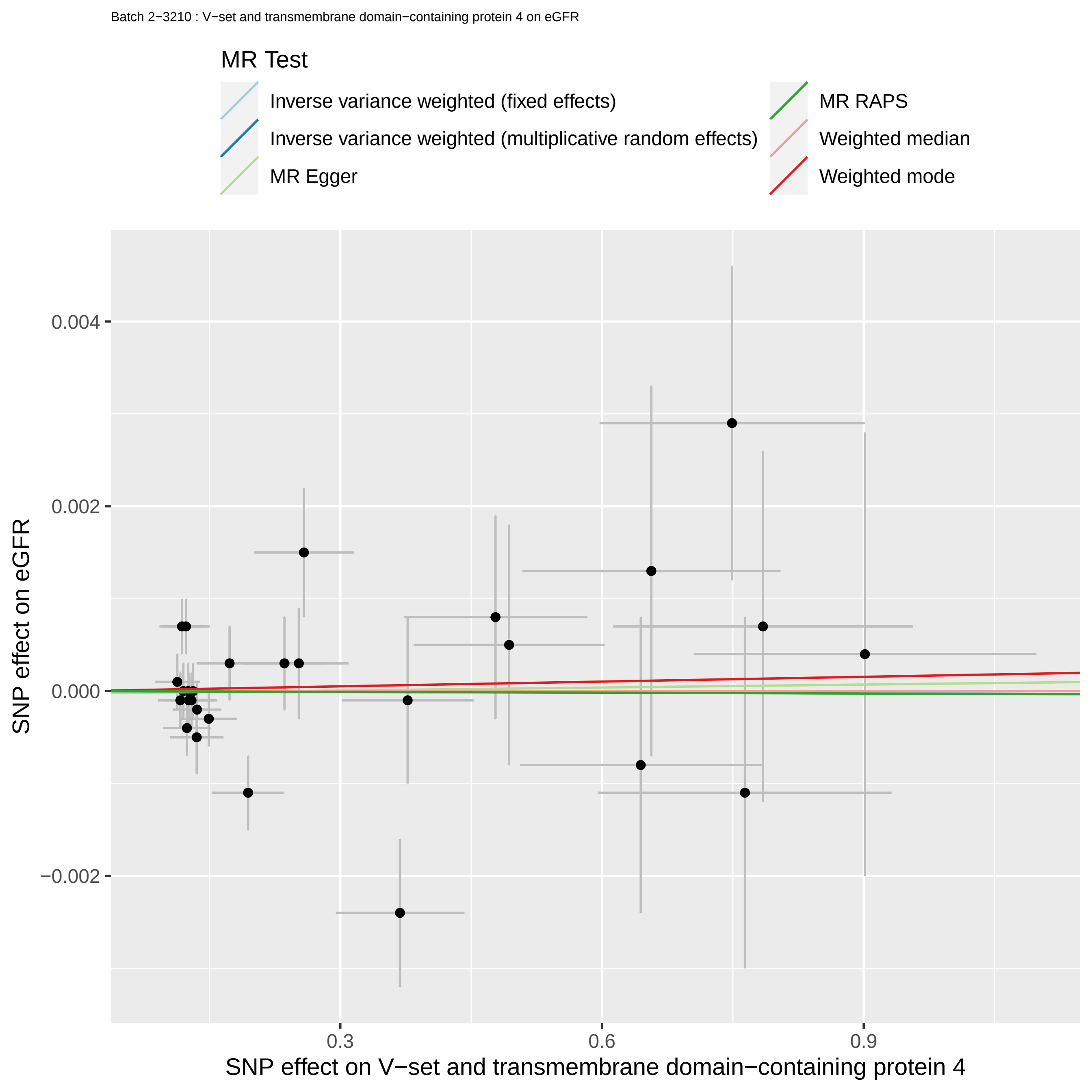

Batch 2−3210 : V−set and transmembrane domain−containing protein 4 on eGFR
MR Test
Inverse variance weighted (fixed effects)
Inverse variance weighted (multiplicative random effects)
MR Egger
MR RAPS
Weighted median
Weighted mode
0.004
0.002
0.000
−0.002
0.3
0.6
0.9
SNP effect on eGFR
SNP effect on V−set and transmembrane domain−containing protein 4

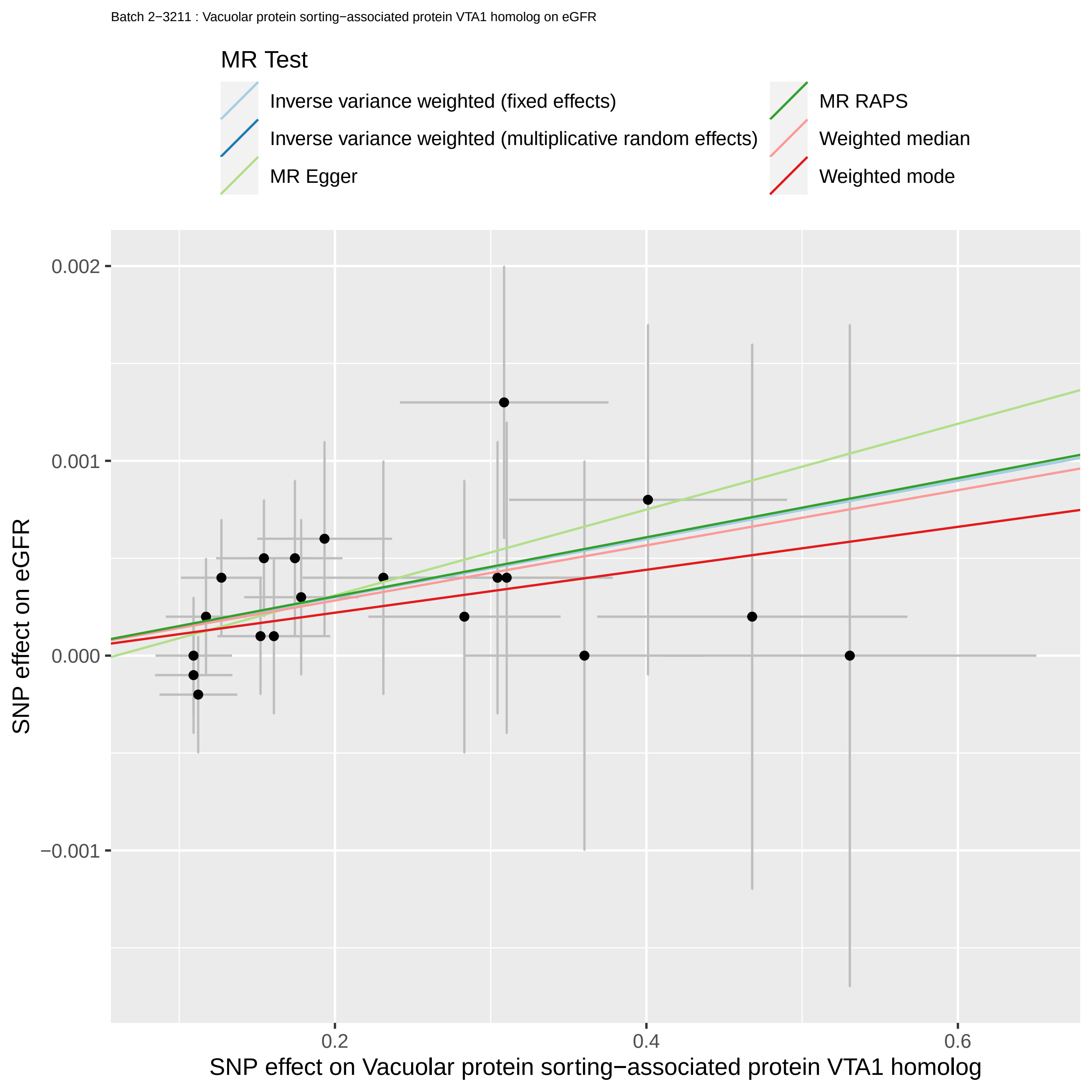

Batch 2−3211 : Vacuolar protein sorting−associated protein VTA1 homolog on eGFR
MR Test
Inverse variance weighted (fixed effects)
Inverse variance weighted (multiplicative random effects)
MR Egger
MR RAPS
Weighted median
Weighted mode
0.002
0.001
0.000
−0.001
0.2
0.4
0.6
SNP effect on eGFR
SNP effect on Vacuolar protein sorting−associated protein VTA1 homolog

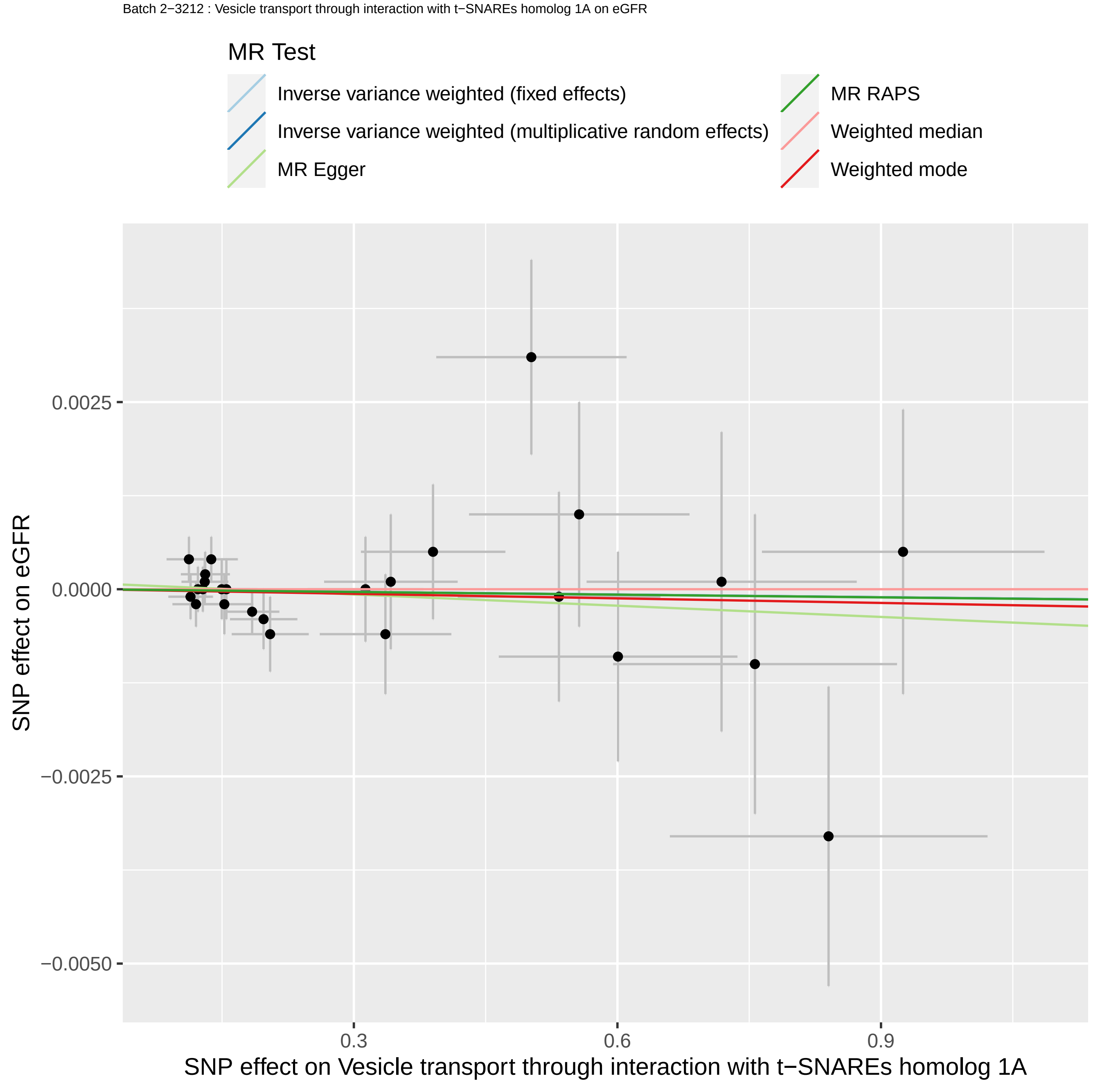
Batch 2−3212 : Vesicle transport through interaction with t−SNAREs homolog 1A on eGFR
MR Test
Inverse variance weighted (fixed effects)
Inverse variance weighted (multiplicative random effects)
MR Egger
MR RAPS
Weighted median
Weighted mode
0.0025
0.0000
−0.0025
−0.0050
SNP effect on eGFR
0.3
0.6
0.9
SNP effect on Vesicle transport through interaction with t−SNAREs homolog 1A

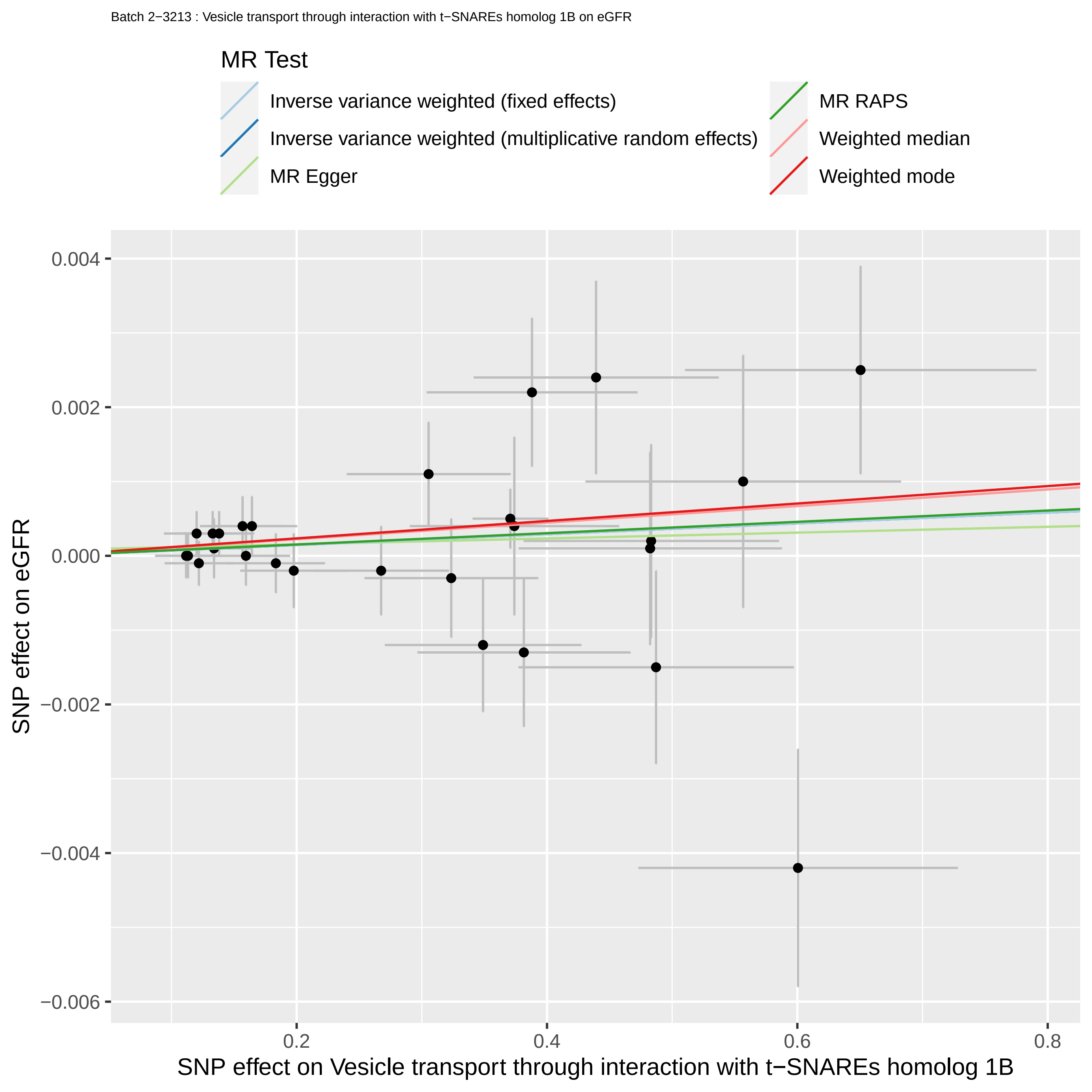
Batch 2−3213 : Vesicle transport through interaction with t−SNAREs homolog 1B on eGFR
MR Test
Inverse variance weighted (fixed effects)
Inverse variance weighted (multiplicative random effects)
MR Egger
MR RAPS
Weighted median
Weighted mode
0.004
0.002
0.000
−0.002
−0.004
−0.006
0.2
0.4
0.6
0.8
SNP effect on eGFR
SNP effect on Vesicle transport through interaction with t−SNAREs homolog 1B

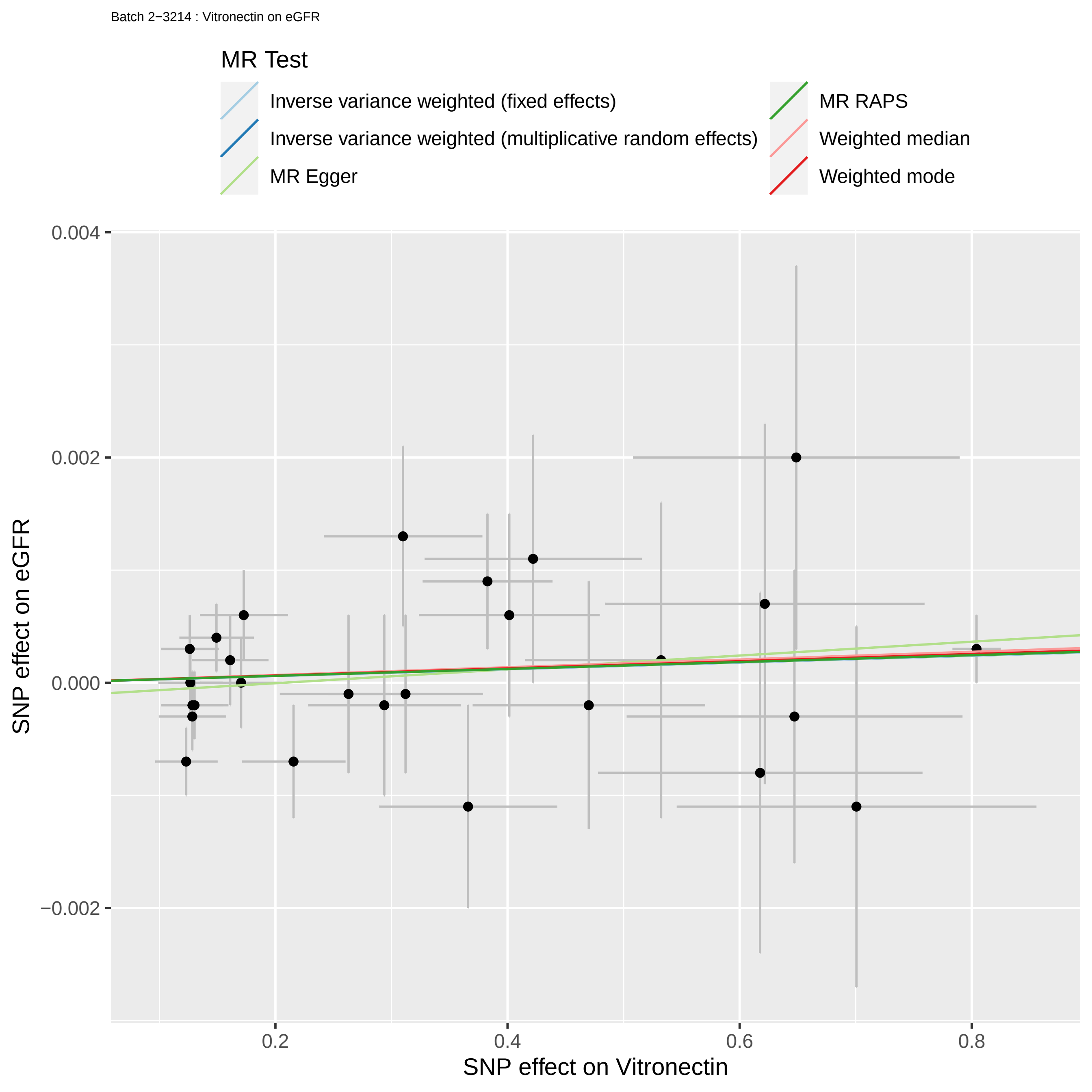

Batch 2−3214 : Vitronectin on eGFR
MR Test
Inverse variance weighted (fixed effects)
Inverse variance weighted (multiplicative random effects)
MR Egger
MR RAPS
Weighted median
Weighted mode
0.004
0.002
0.000
−0.002
SNP effect on eGFR
0.2
0.4
0.6
0.8
SNP effect on Vitronectin

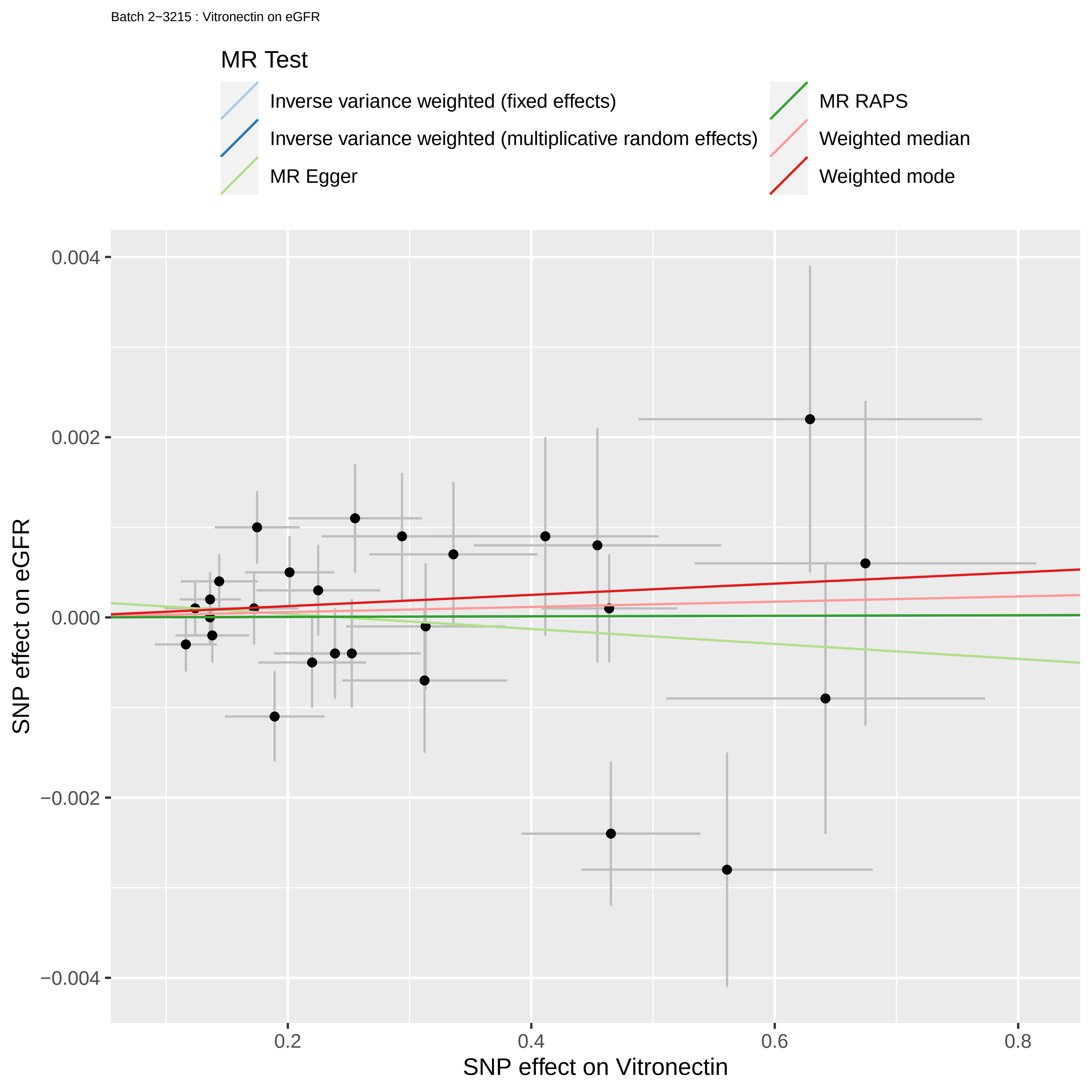

Batch 2−3215 : Vitronectin on eGFR
MR Test
Inverse variance weighted (fixed effects)
Inverse variance weighted (multiplicative random effects)
MR Egger
MR RAPS
Weighted median
Weighted mode
0.004
0.002
0.000
−0.002
−0.004
SNP effect on eGFR
0.2
0.4
0.6
0.8
SNP effect on Vitronectin

Batch 2−3216 : Brorin on eGFR

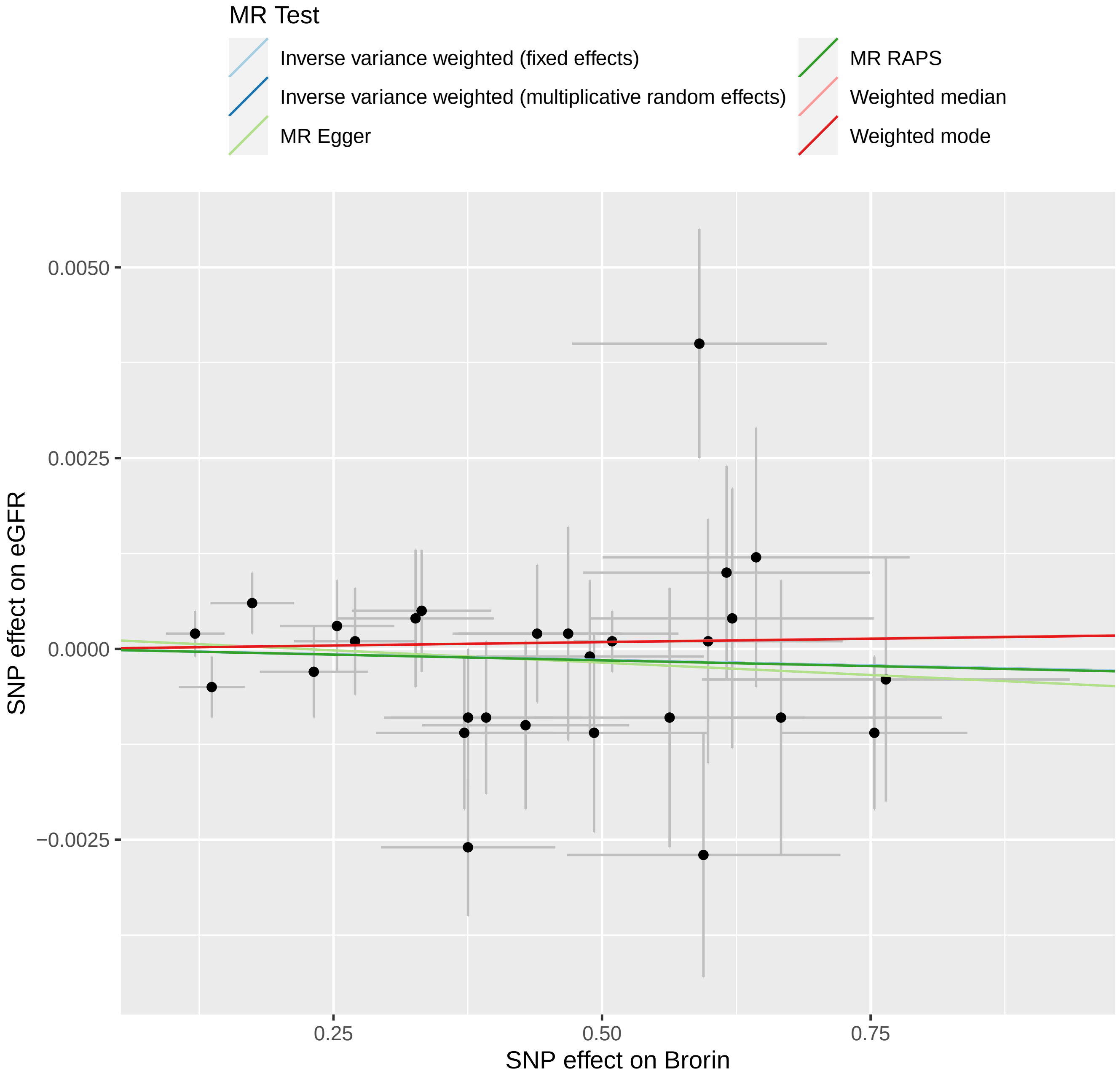

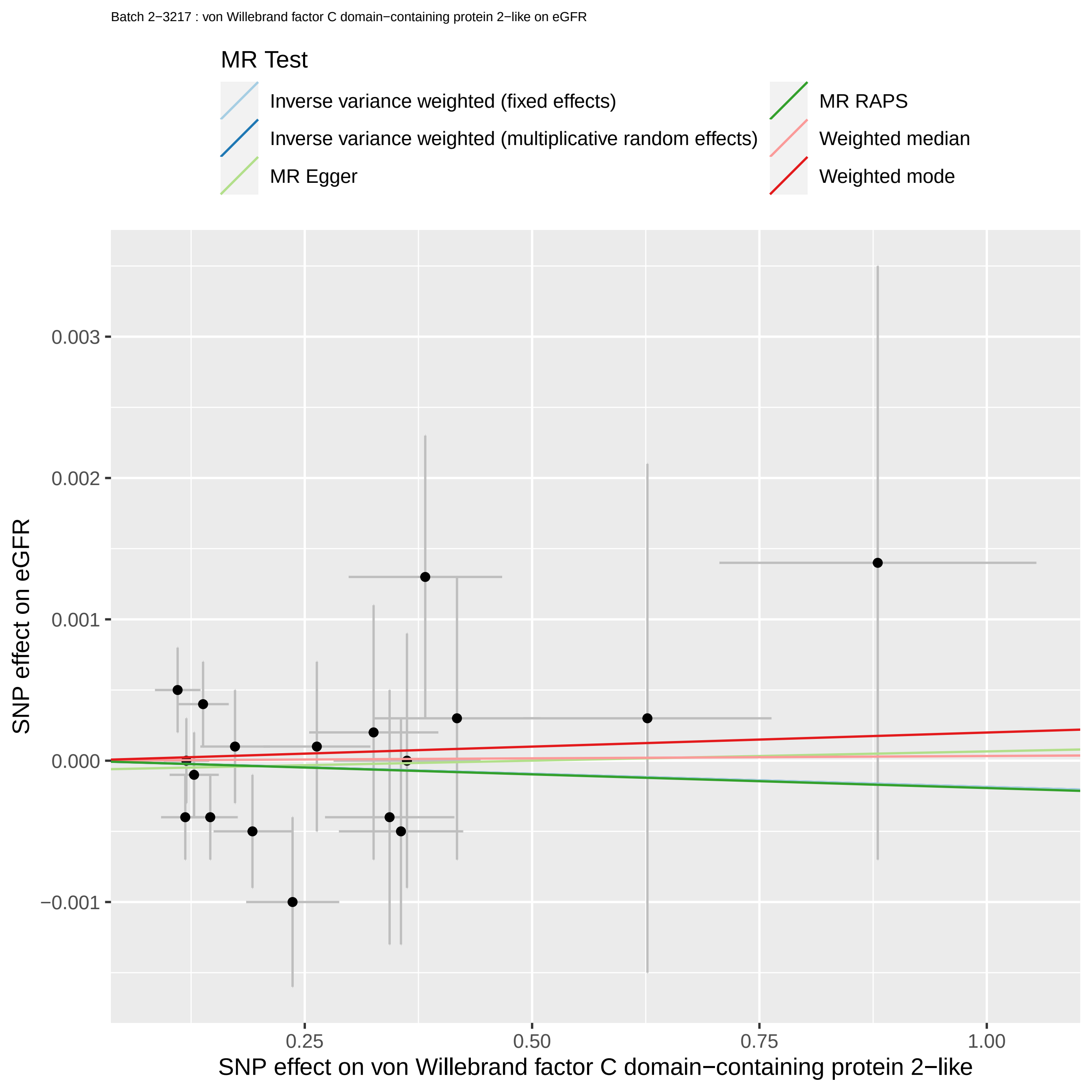
Batch 2−3217 : von Willebrand factor C domain−containing protein 2−like on eGFR
MR Test
Inverse variance weighted (fixed effects)
Inverse variance weighted (multiplicative random effects)
MR Egger
MR RAPS
Weighted median
Weighted mode
0.003
0.002
0.001
0.000
−0.001
0.25
0.50
0.75
1.00
SNP effect on eGFR
SNP effect on von Willebrand factor C domain−containing protein 2−like

Batch 2−3218 : WW domain−binding protein 1 on eGFR

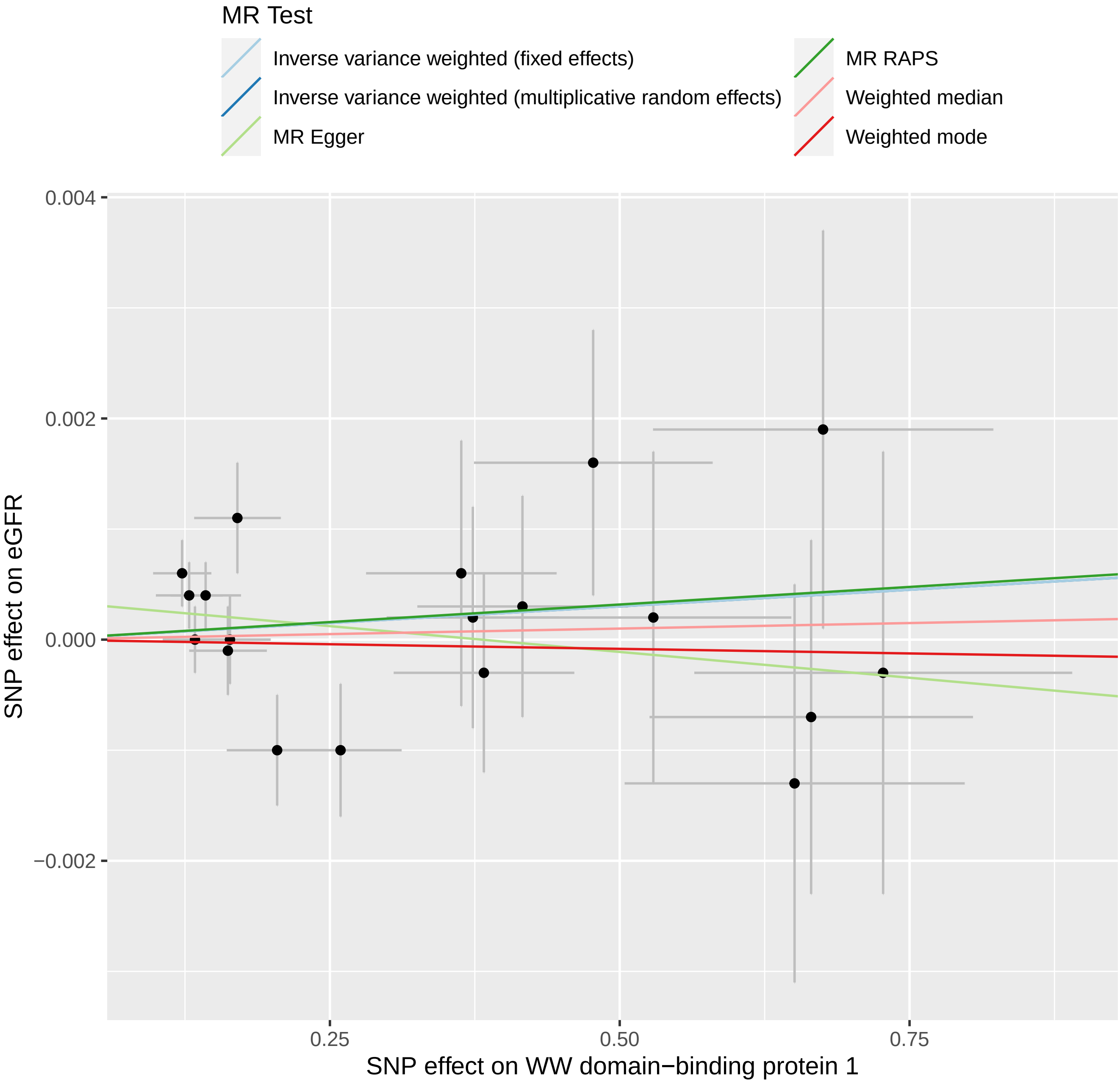

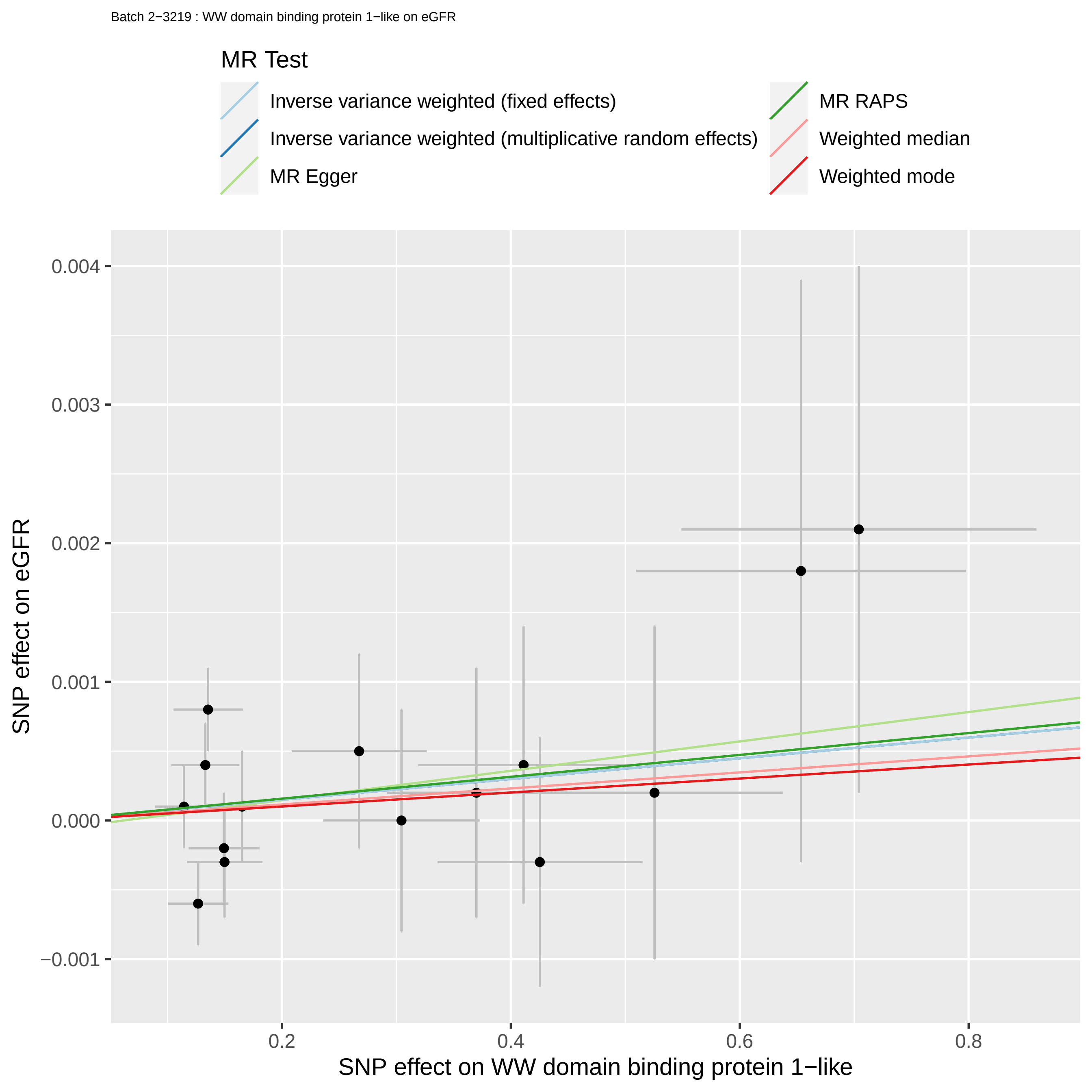

Batch 2−3219 : WW domain binding protein 1−like on eGFR
MR Test
Inverse variance weighted (fixed effects)
Inverse variance weighted (multiplicative random effects)
MR Egger
MR RAPS
Weighted median
Weighted mode
0.004
0.003
0.002
0.001
0.000
−0.001
0.2
0.4
0.6
0.8
SNP effect on eGFR
SNP effect on WW domain binding protein 1−like

Batch 2−3220 : WD repeat−containing protein 1 on eGFR

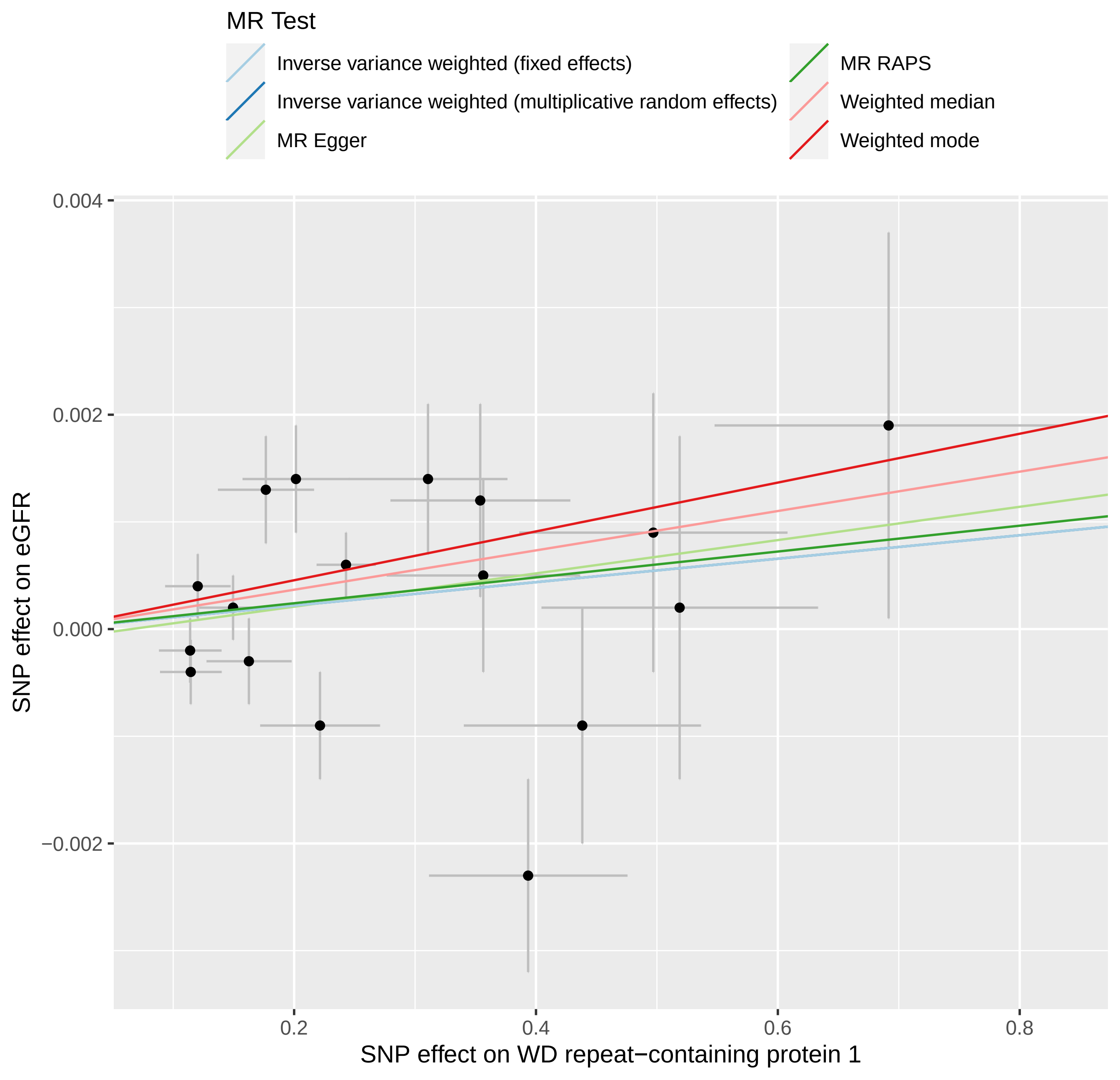

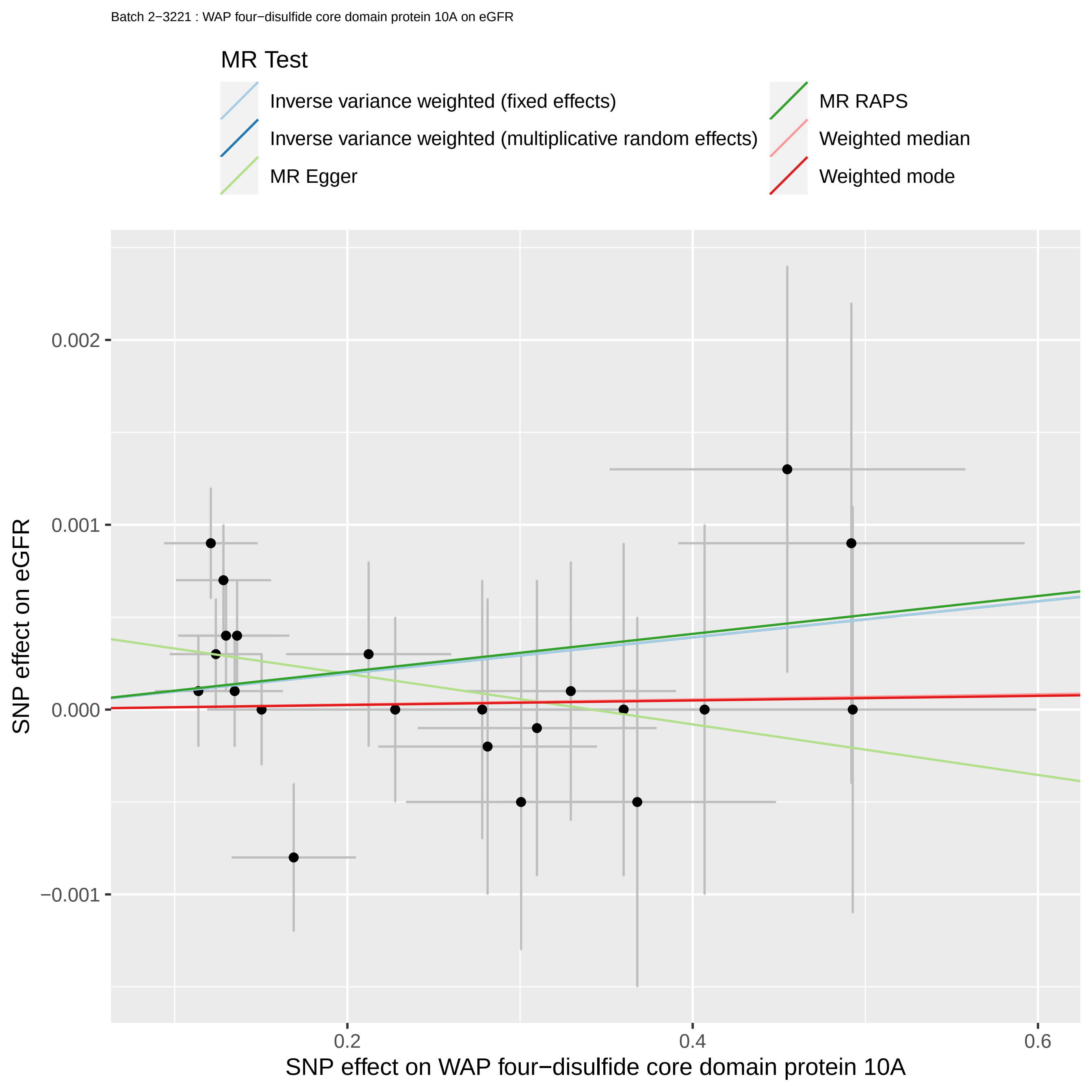

Batch 2−3221 : WAP four−disulfide core domain protein 10A on eGFR
MR Test
Inverse variance weighted (fixed effects)
Inverse variance weighted (multiplicative random effects)
MR Egger
MR RAPS
Weighted median
Weighted mode
0.002
0.001
0.000
−0.001
SNP effect on eGFR
0.2
0.4
0.6
SNP effect on WAP four−disulfide core domain protein 10A

Batch 2−3222 : WAP four−disulfide core domain protein 12 on eGFR

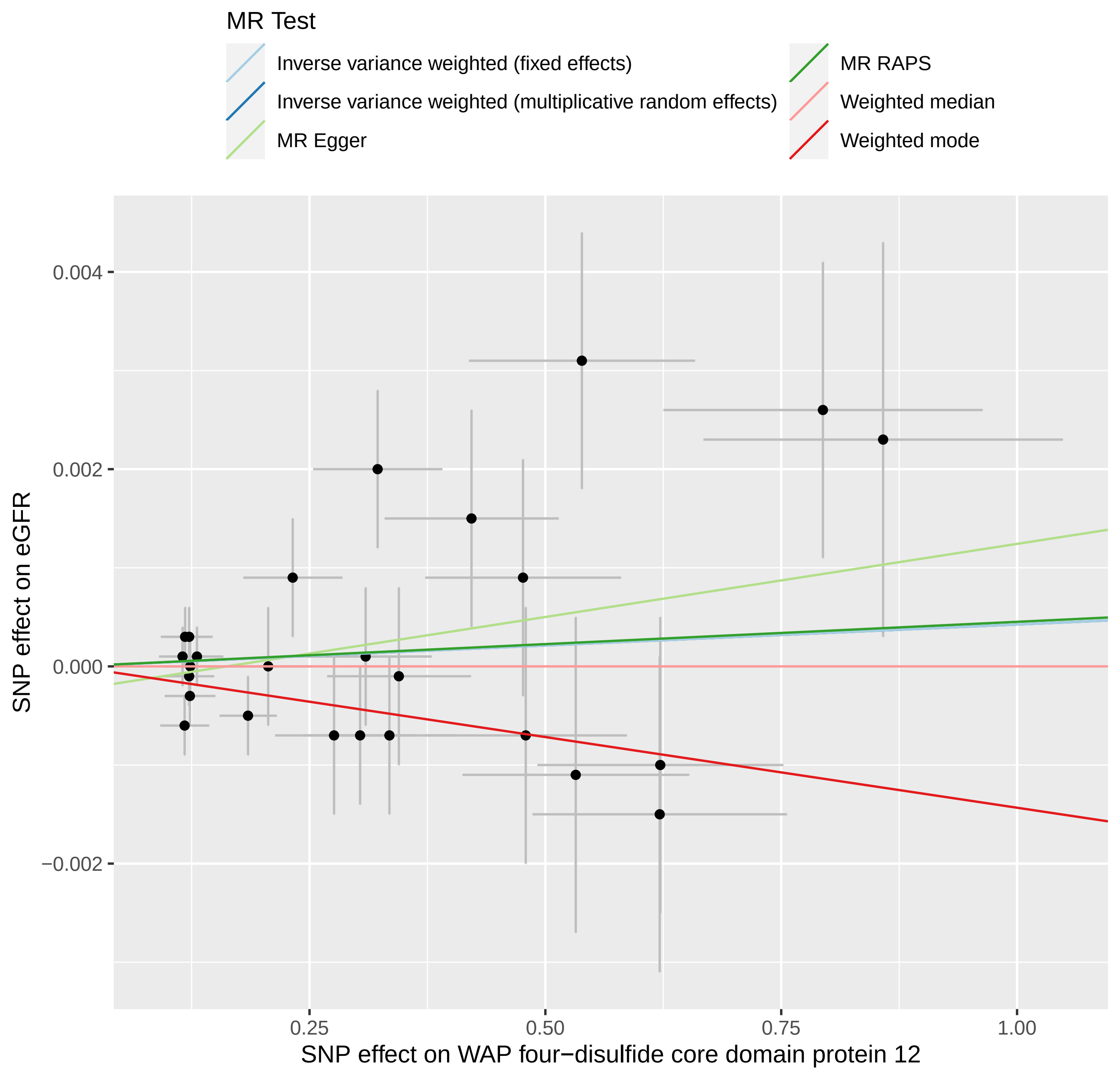

Batch 2−3223 : WAP four−disulfide core domain protein 13 on eGFR

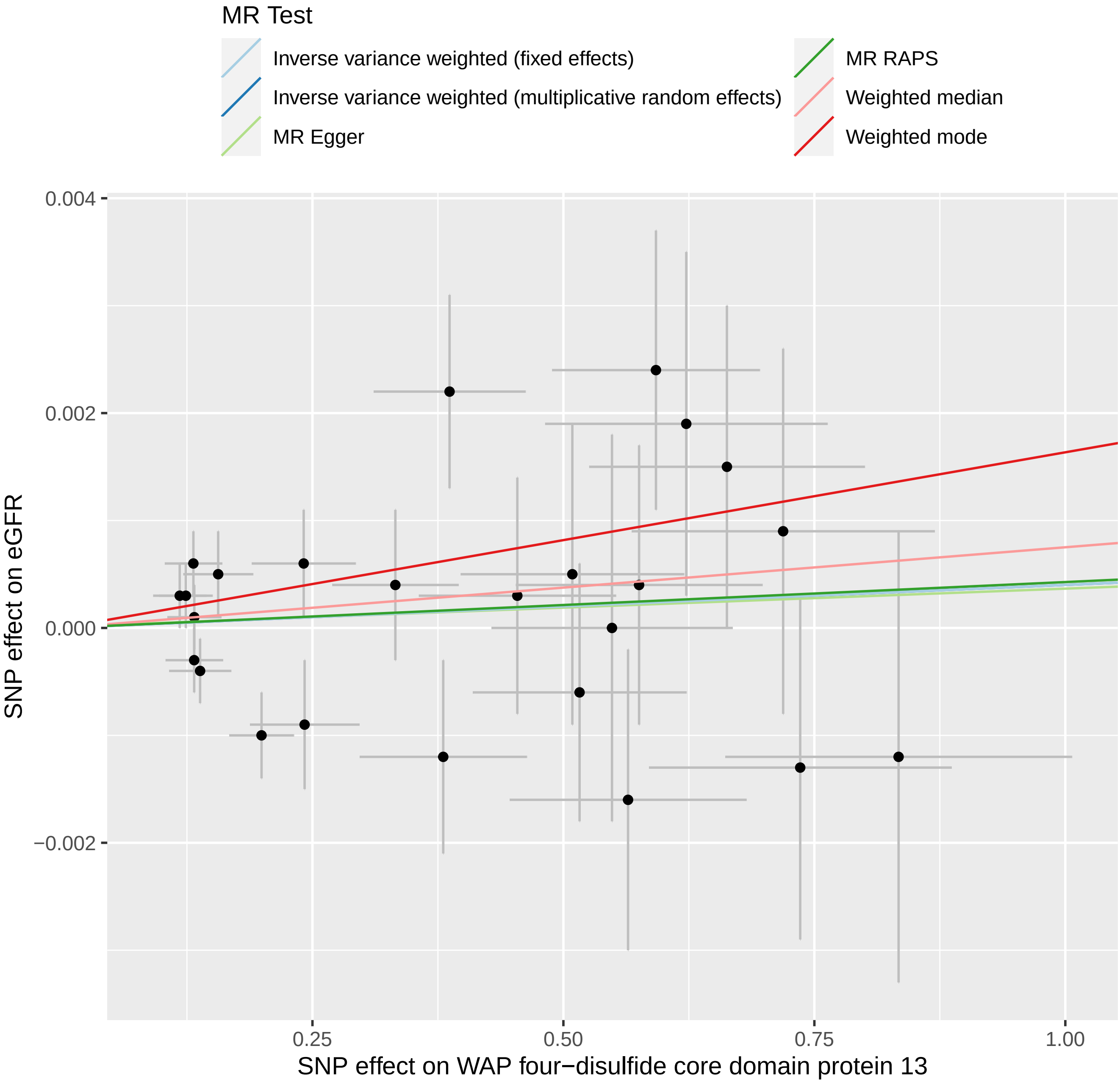

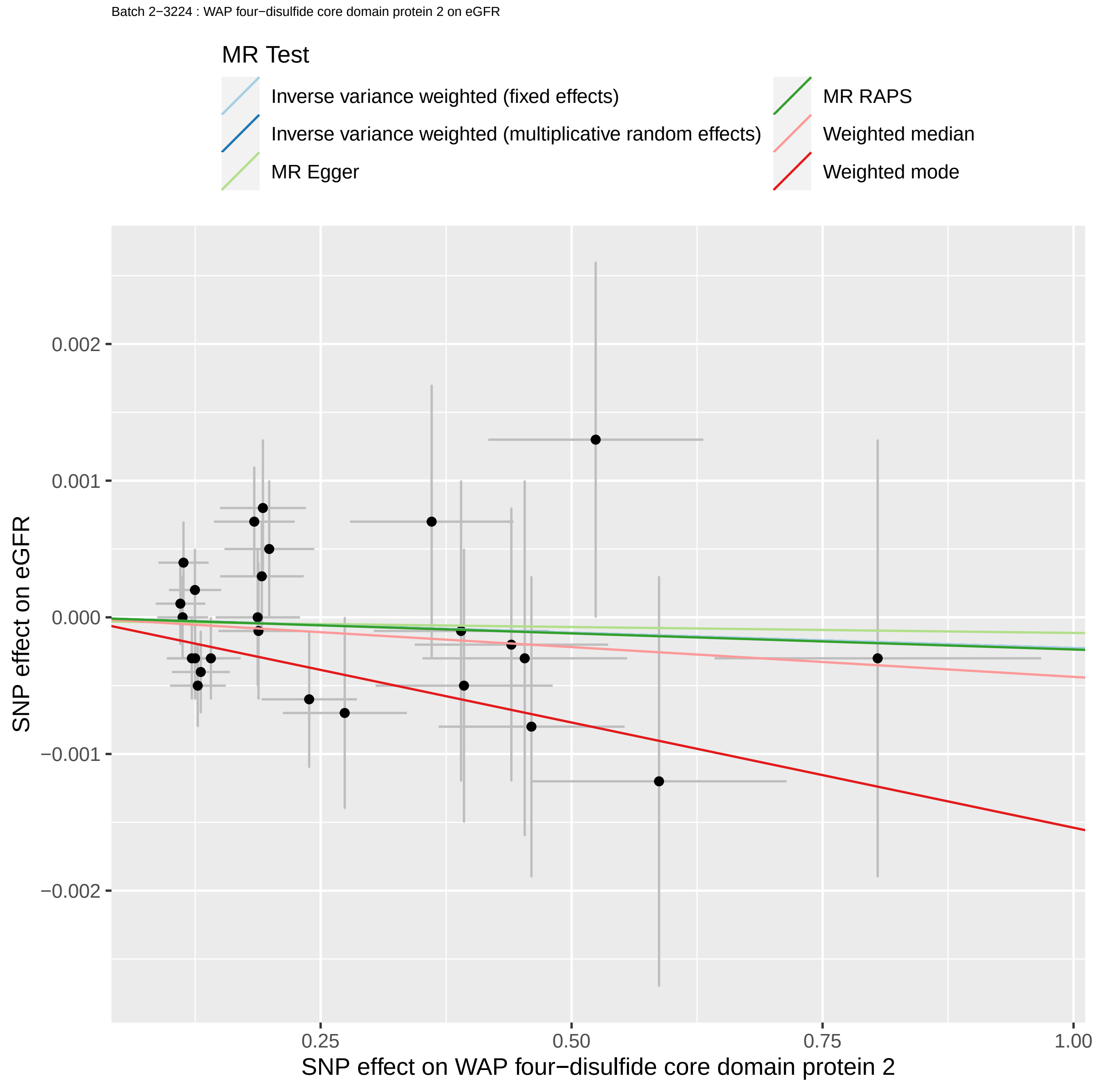

Batch 2−3224 : WAP four−disulfide core domain protein 2 on eGFR
MR Test
Inverse variance weighted (fixed effects)
Inverse variance weighted (multiplicative random effects)
MR Egger
MR RAPS
Weighted median
Weighted mode
0.002
0.001
0.000
−0.001
−0.002
SNP effect on eGFR
0.25
0.50
0.75
1.00
SNP effect on WAP four−disulfide core domain protein 2

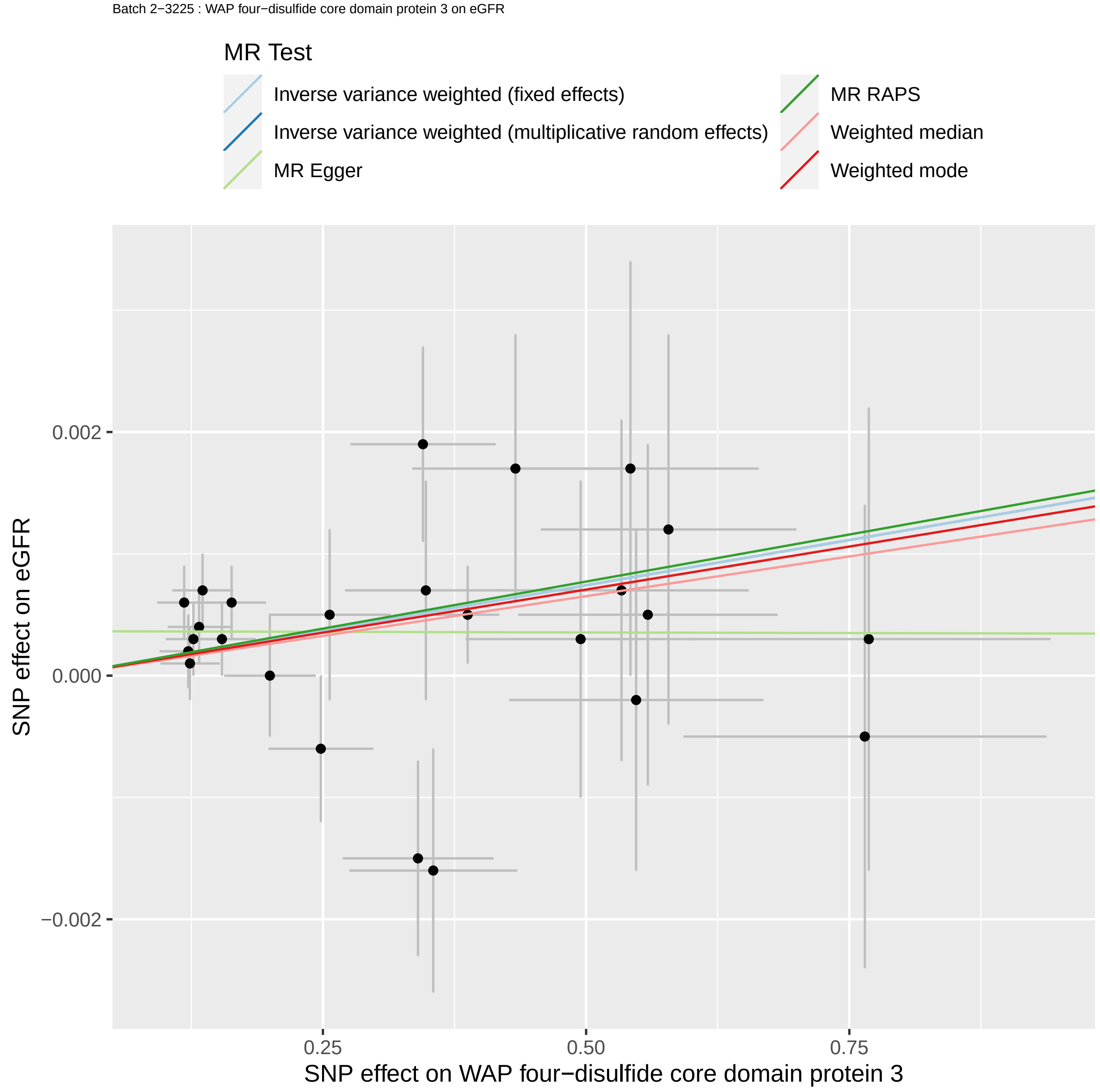

Batch 2−3225 : WAP four−disulfide core domain protein 3 on eGFR
MR Test
Inverse variance weighted (fixed effects)
Inverse variance weighted (multiplicative random effects)
MR Egger
MR RAPS
Weighted median
Weighted mode
SNP effect on eGFR
0.002
0.000
−0.002
0.25
0.50
0.75
SNP effect on WAP four−disulfide core domain protein 3

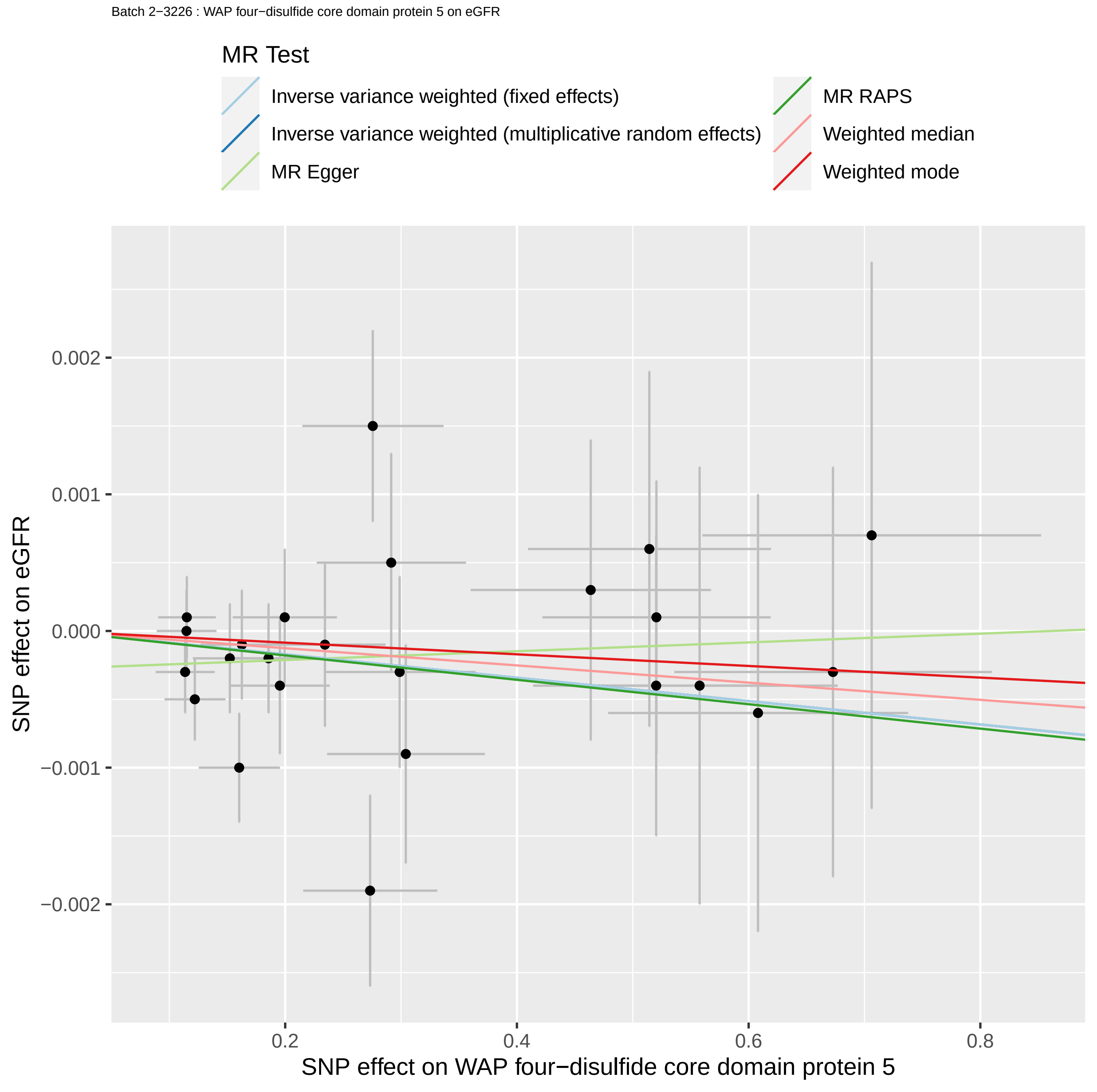

Batch 2−3226 : WAP four−disulfide core domain protein 5 on eGFR
MR Test
Inverse variance weighted (fixed effects)
Inverse variance weighted (multiplicative random effects)
MR Egger
MR RAPS
Weighted median
Weighted mode
0.002
0.001
0.000
−0.001
−0.002
0.2
0.4
0.6
0.8
SNP effect on eGFR
SNP effect on WAP four−disulfide core domain protein 5

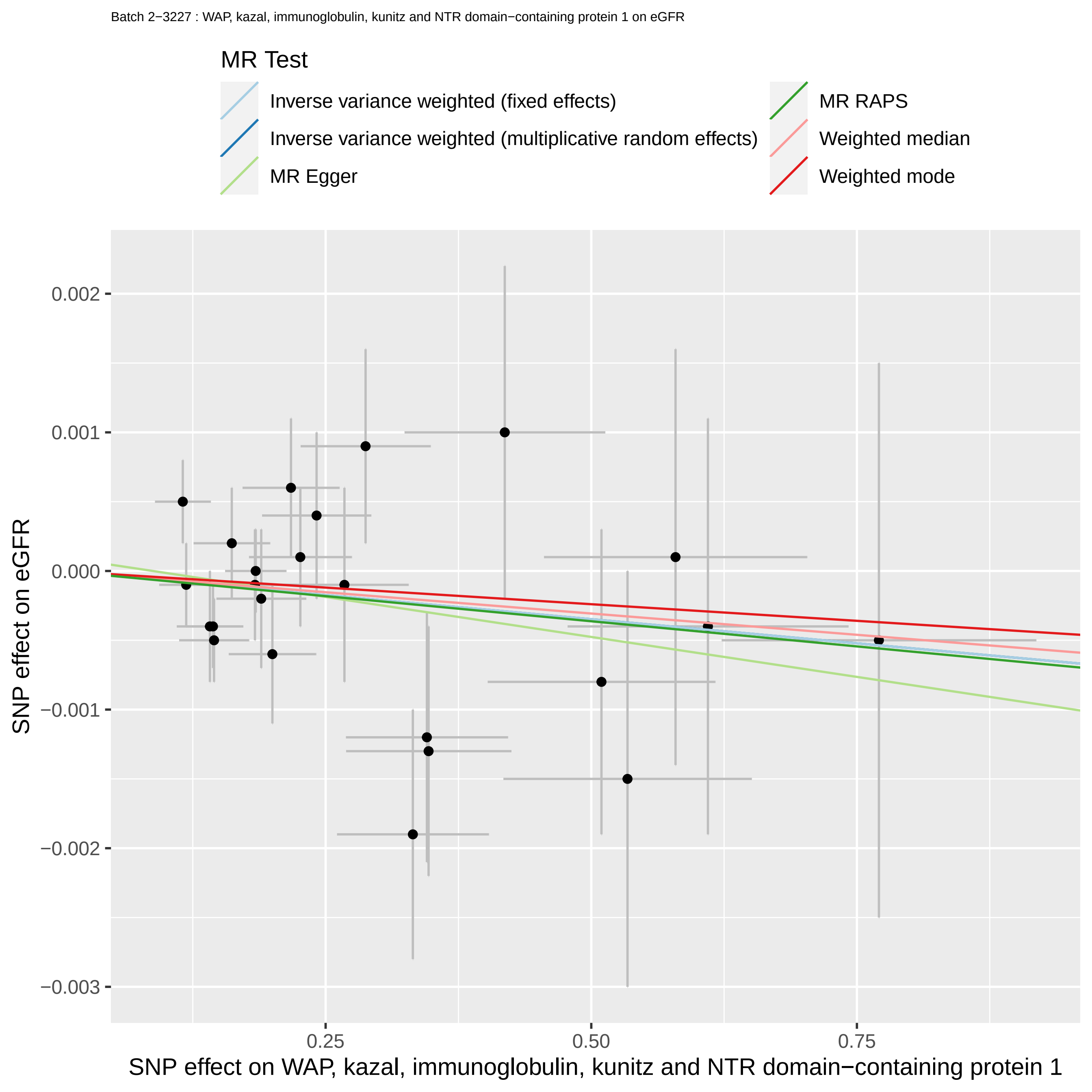
Batch 2−3227 : WAP, kazal, immunoglobulin, kunitz and NTR domain−containing protein 1 on eGFR
MR Test
Inverse variance weighted (fixed effects)
Inverse variance weighted (multiplicative random effects)
MR Egger
MR RAPS
Weighted median
Weighted mode
0.002
0.001
0.000
−0.001
−0.002
−0.003
SNP effect on eGFR
0.25
0.50
0.75
SNP effect on WAP, kazal, immunoglobulin, kunitz and NTR domain−containing protein 1

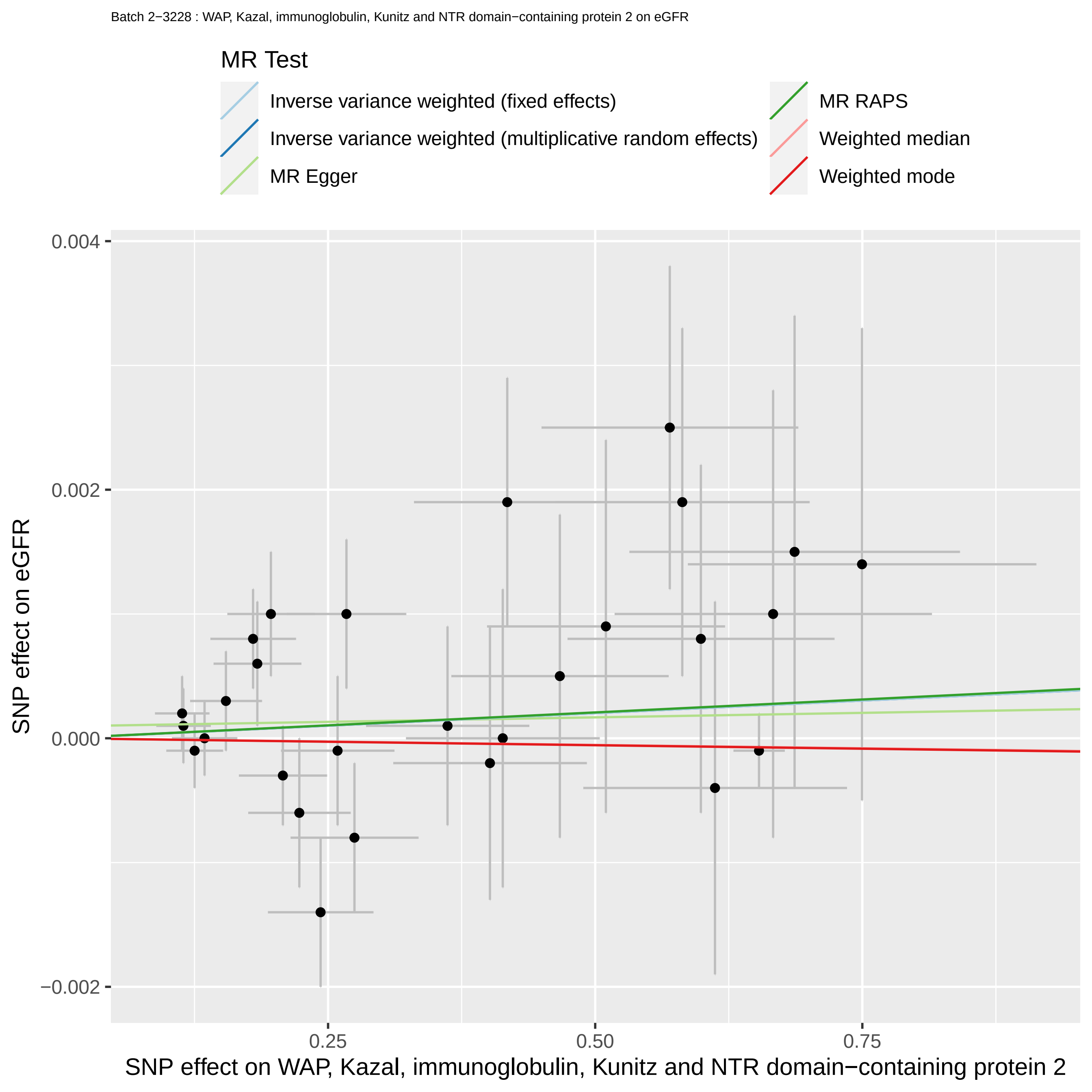

Batch 2−3228 : WAP, Kazal, immunoglobulin, Kunitz and NTR domain−containing protein 2 on eGFR
MR Test
Inverse variance weighted (fixed effects)
Inverse variance weighted (multiplicative random effects)
MR Egger
MR RAPS
Weighted median
Weighted mode
0.004
0.002
0.000
−0.002
0.25
0.50
0.75
SNP effect on eGFR
SNP effect on WAP, Kazal, immunoglobulin, Kunitz and NTR domain−containing protein 2

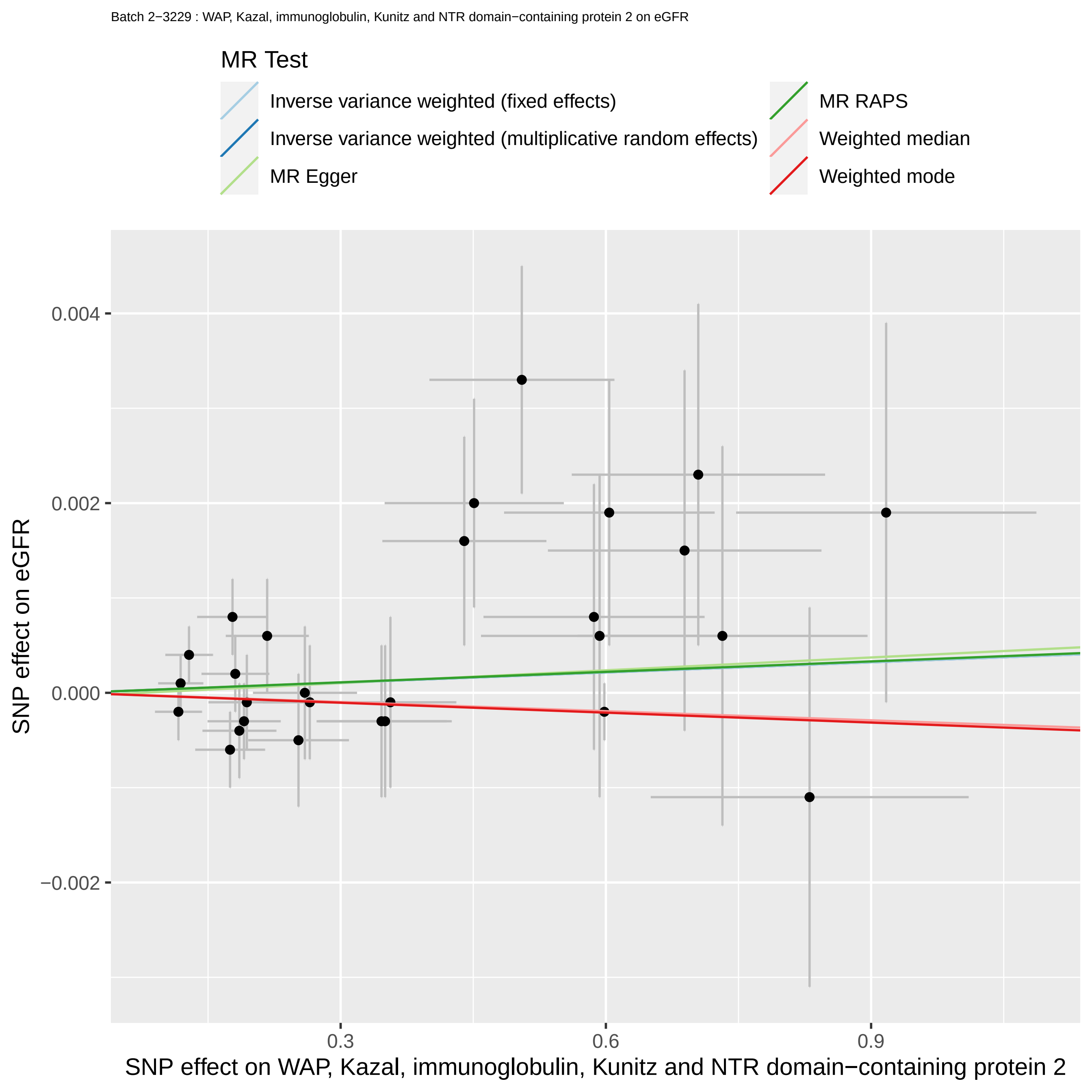

Batch 2−3229 : WAP, Kazal, immunoglobulin, Kunitz and NTR domain−containing protein 2 on eGFR
MR Test
Inverse variance weighted (fixed effects)
Inverse variance weighted (multiplicative random effects)
MR Egger
MR RAPS
Weighted median
Weighted mode
0.004
0.002
0.000
−0.002
0.3
0.6
0.9
SNP effect on eGFR
SNP effect on WAP, Kazal, immunoglobulin, Kunitz and NTR domain−containing protein 2

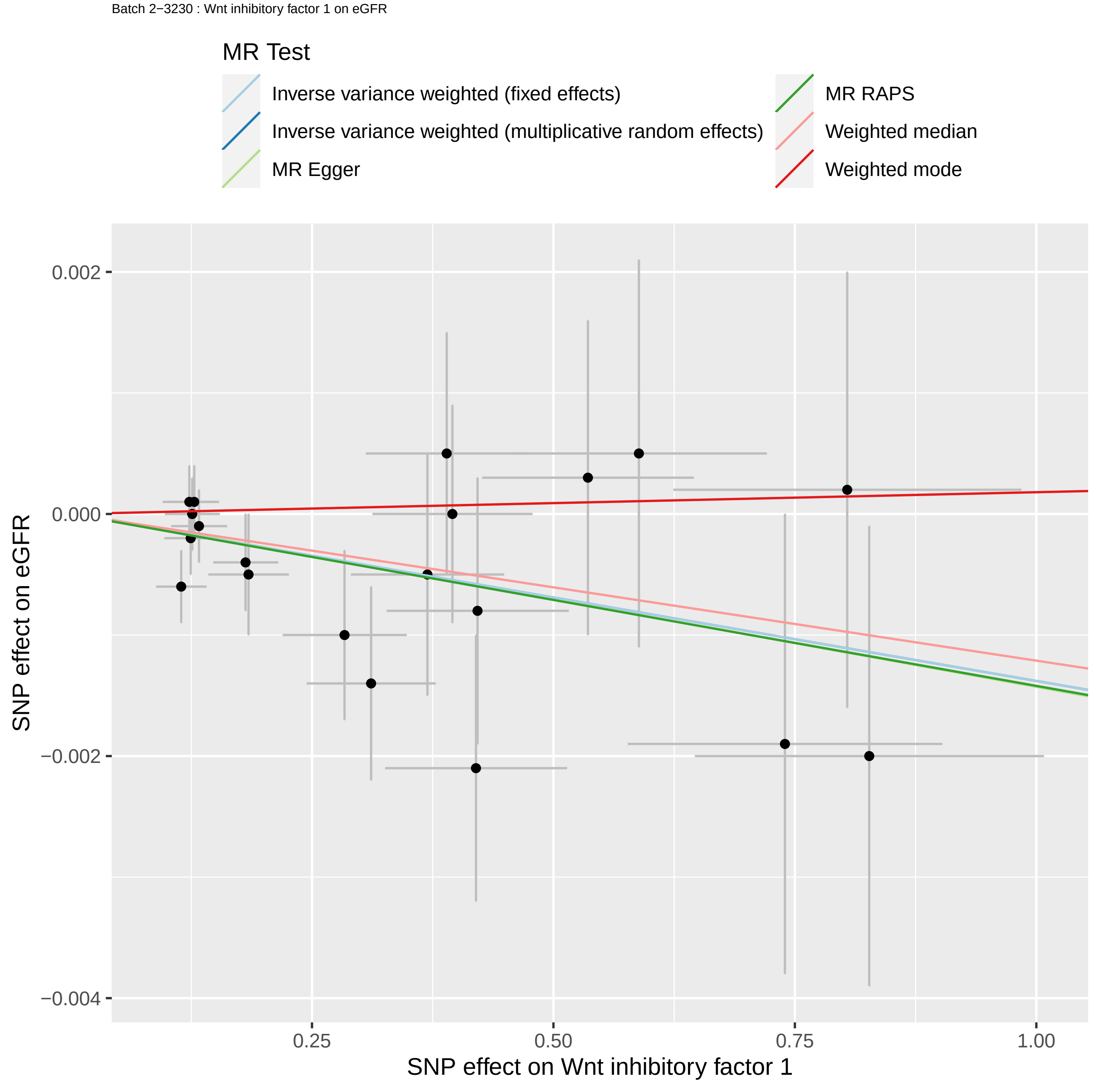

Batch 2−3230 : Wnt inhibitory factor 1 on eGFR
MR Test
Inverse variance weighted (fixed effects)
Inverse variance weighted (multiplicative random effects)
MR Egger
MR RAPS
Weighted median
Weighted mode
0.002
0.000
−0.002
−0.004
0.25
0.50
0.75
1.00
SNP effect on eGFR
SNP effect on Wnt inhibitory factor 1

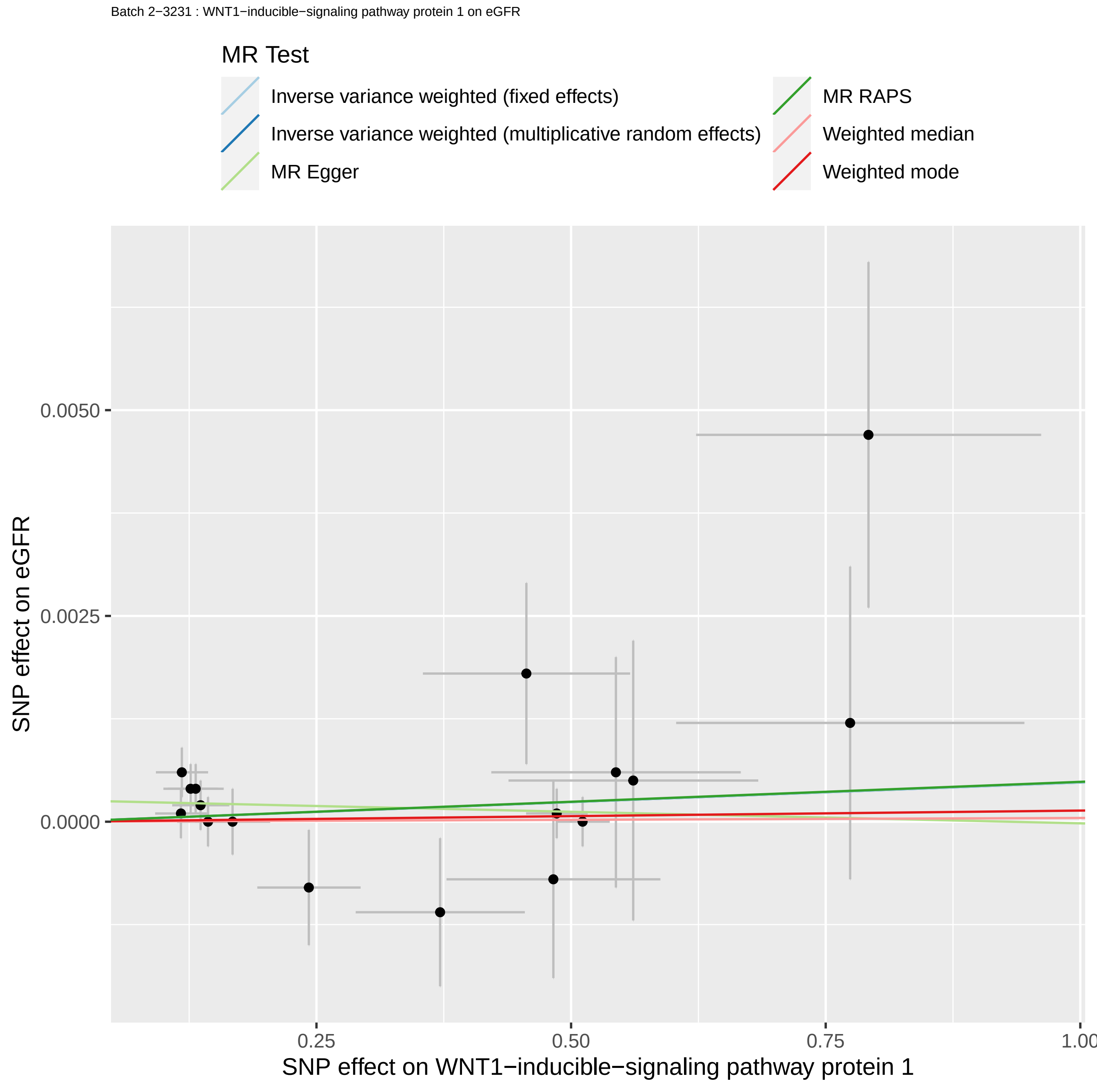
Batch 2−3231 : WNT1−inducible−signaling pathway protein 1 on eGFR
MR Test
Inverse variance weighted (fixed effects)
Inverse variance weighted (multiplicative random effects)
MR Egger
MR RAPS
Weighted median
Weighted mode
0.0050
0.0025
0.0000
SNP effect on eGFR
0.25
0.50
0.75
1.00
SNP effect on WNT1−inducible−signaling pathway protein 1

Batch 2−3232 : WNT1−inducible−signaling pathway protein 3 on eGFR

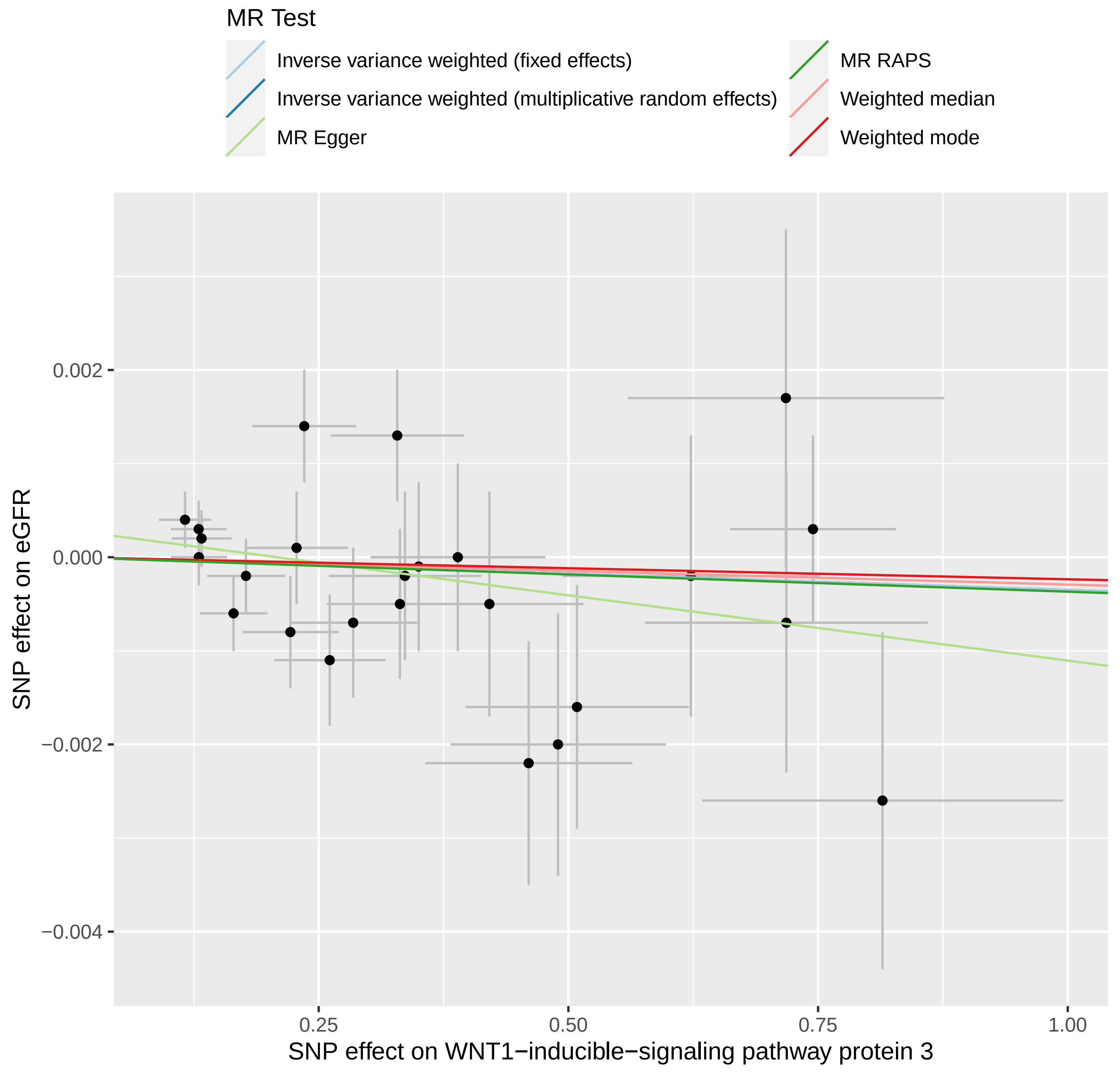

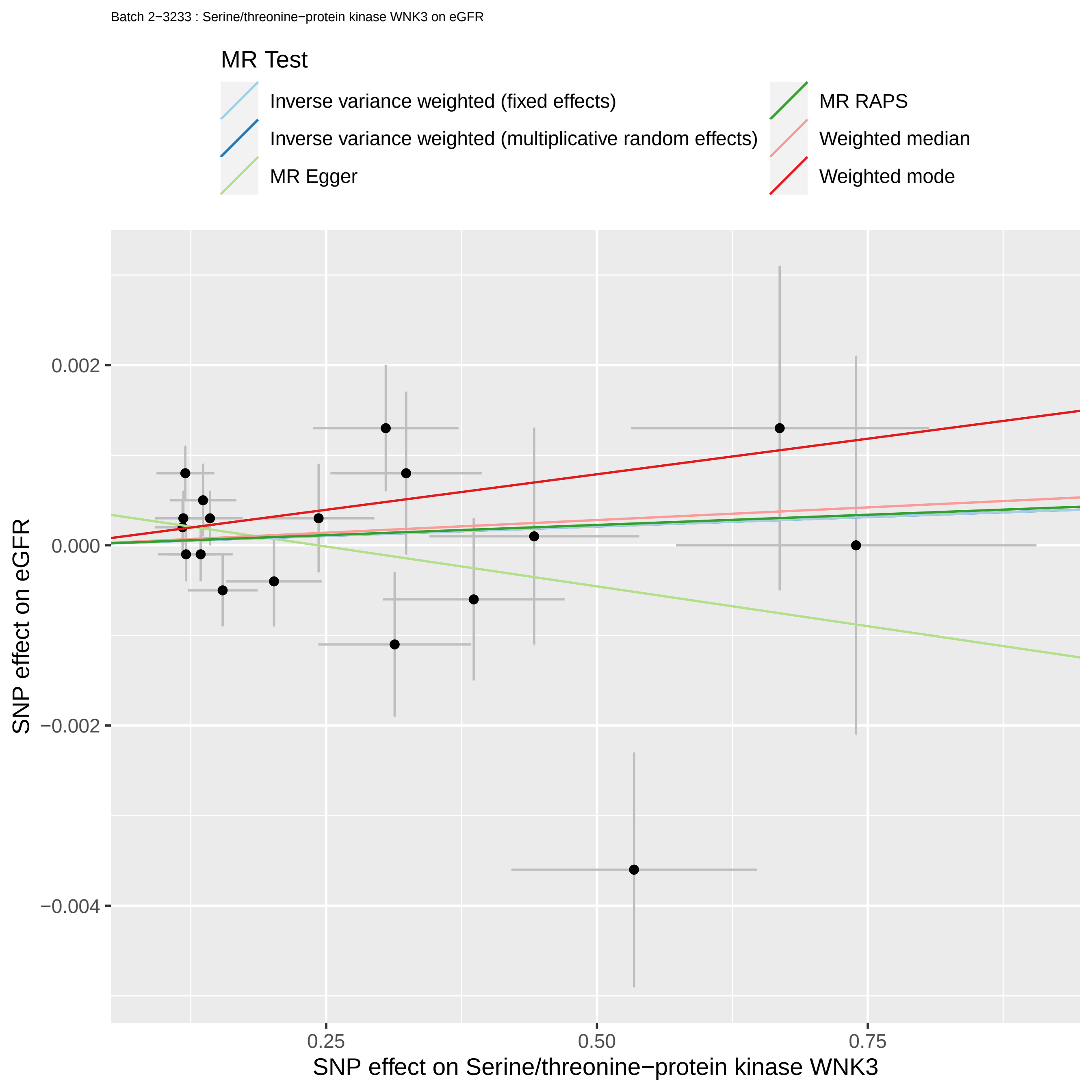
Batch 2−3233 : Serine/threonine−protein kinase WNK3 on eGFR
MR Test
Inverse variance weighted (fixed effects)
Inverse variance weighted (multiplicative random effects)
MR Egger
MR RAPS
Weighted median
Weighted mode
0.002
0.000
−0.002
−0.004
0.25
0.50
0.75
SNP effect on eGFR
SNP effect on Serine/threonine−protein kinase WNK3

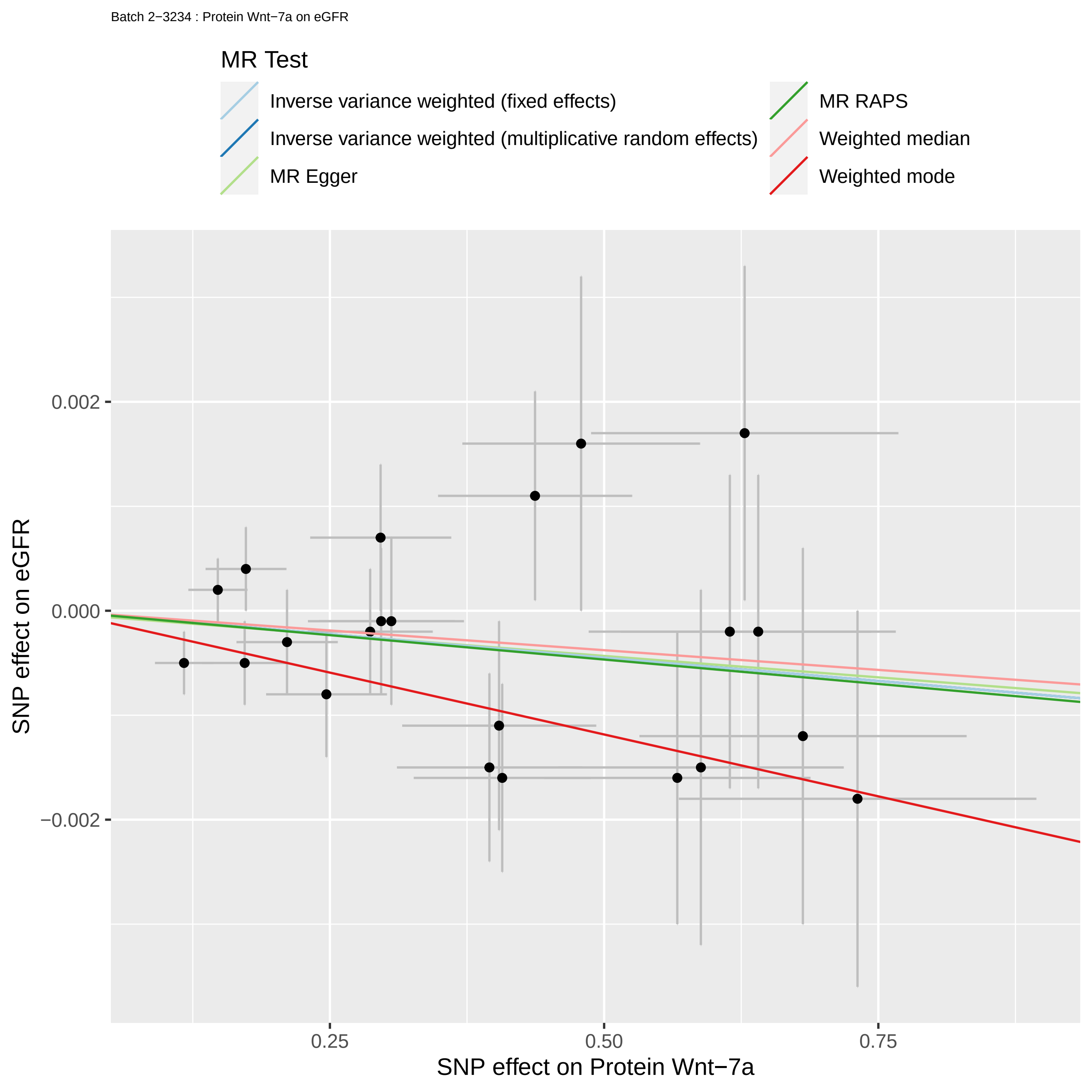

Batch 2−3234 : Protein Wnt−7a on eGFR
MR Test
Inverse variance weighted (fixed effects)
Inverse variance weighted (multiplicative random effects)
MR Egger
MR RAPS
Weighted median
Weighted mode
0.002
0.000
−0.002
0.25
0.50
0.75
SNP effect on eGFR
SNP effect on Protein Wnt−7a

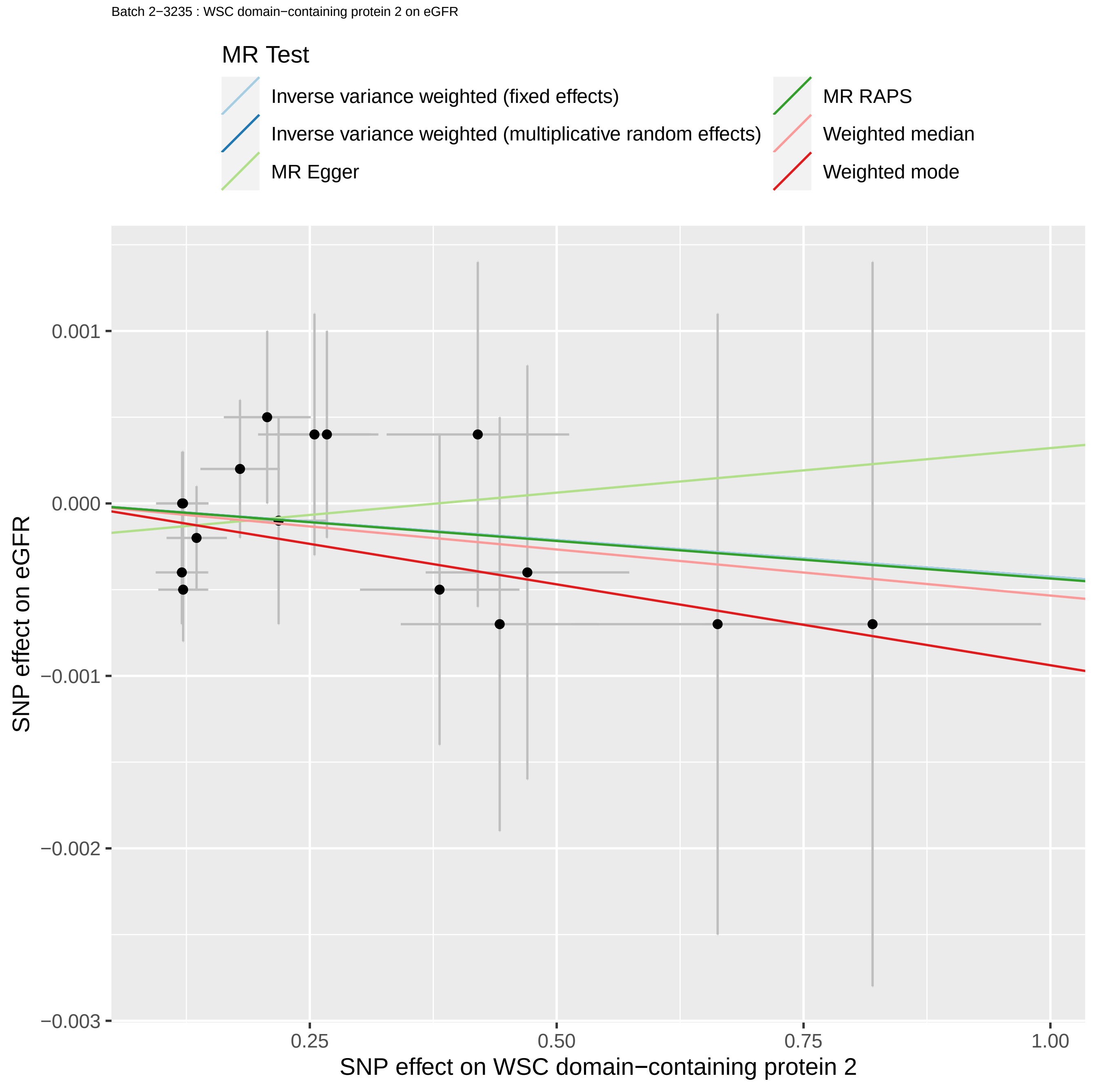

Batch 2−3235 : WSC domain−containing protein 2 on eGFR
MR Test
Inverse variance weighted (fixed effects)
Inverse variance weighted (multiplicative random effects)
MR Egger
MR RAPS
Weighted median
Weighted mode
0.001
0.000
−0.001
−0.002
−0.003
0.25
0.50
0.75
1.00
SNP effect on eGFR
SNP effect on WSC domain−containing protein 2

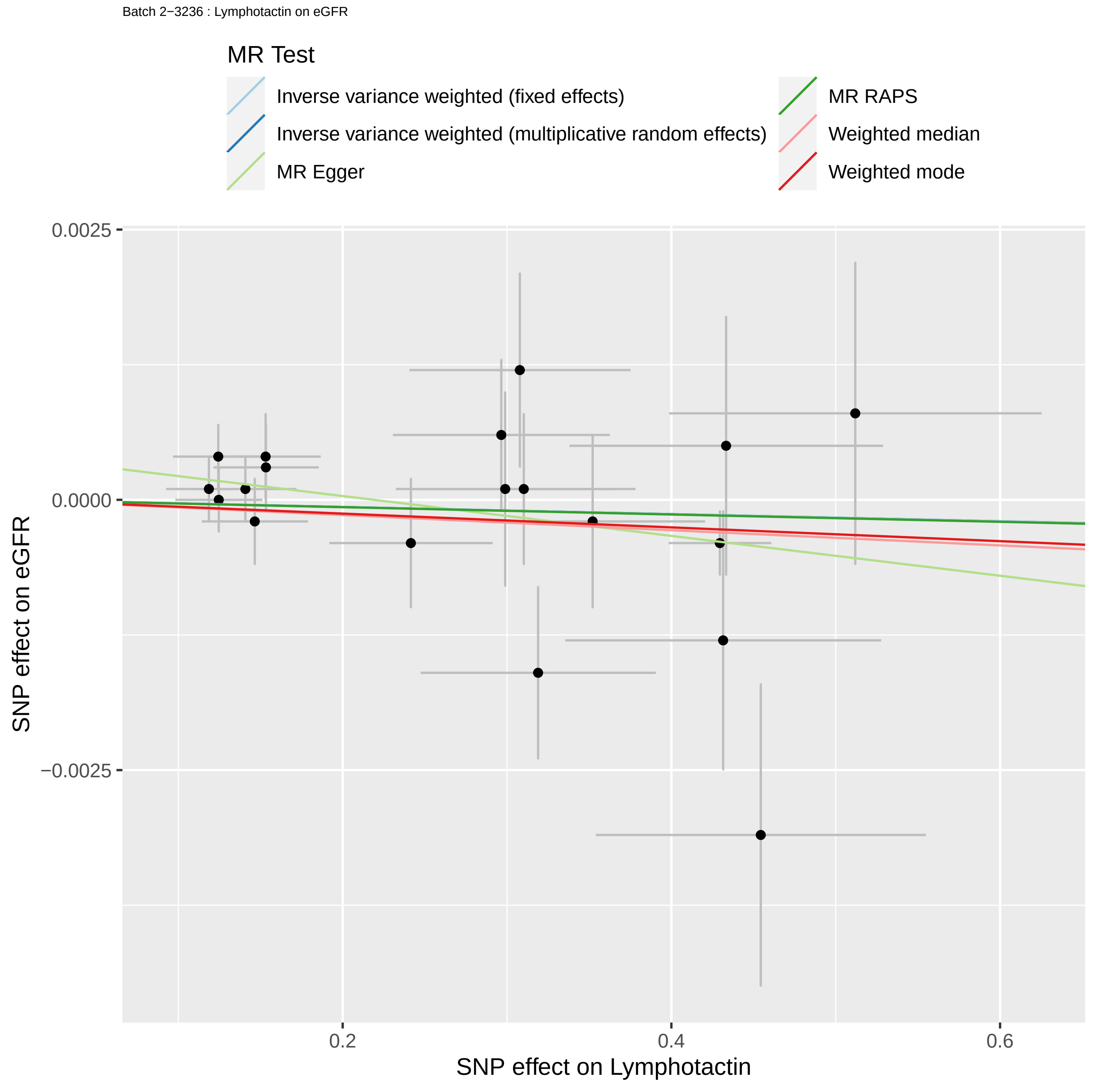

Batch 2−3236 : Lymphotactin on eGFR
MR Test
Inverse variance weighted (fixed effects)
Inverse variance weighted (multiplicative random effects)
MR Egger
MR RAPS
Weighted median
Weighted mode
0.0025
0.0000
−0.0025
SNP effect on eGFR
0.2
0.4
0.6
SNP effect on Lymphotactin

Batch 2−3237 : Lymphotactin on eGFR

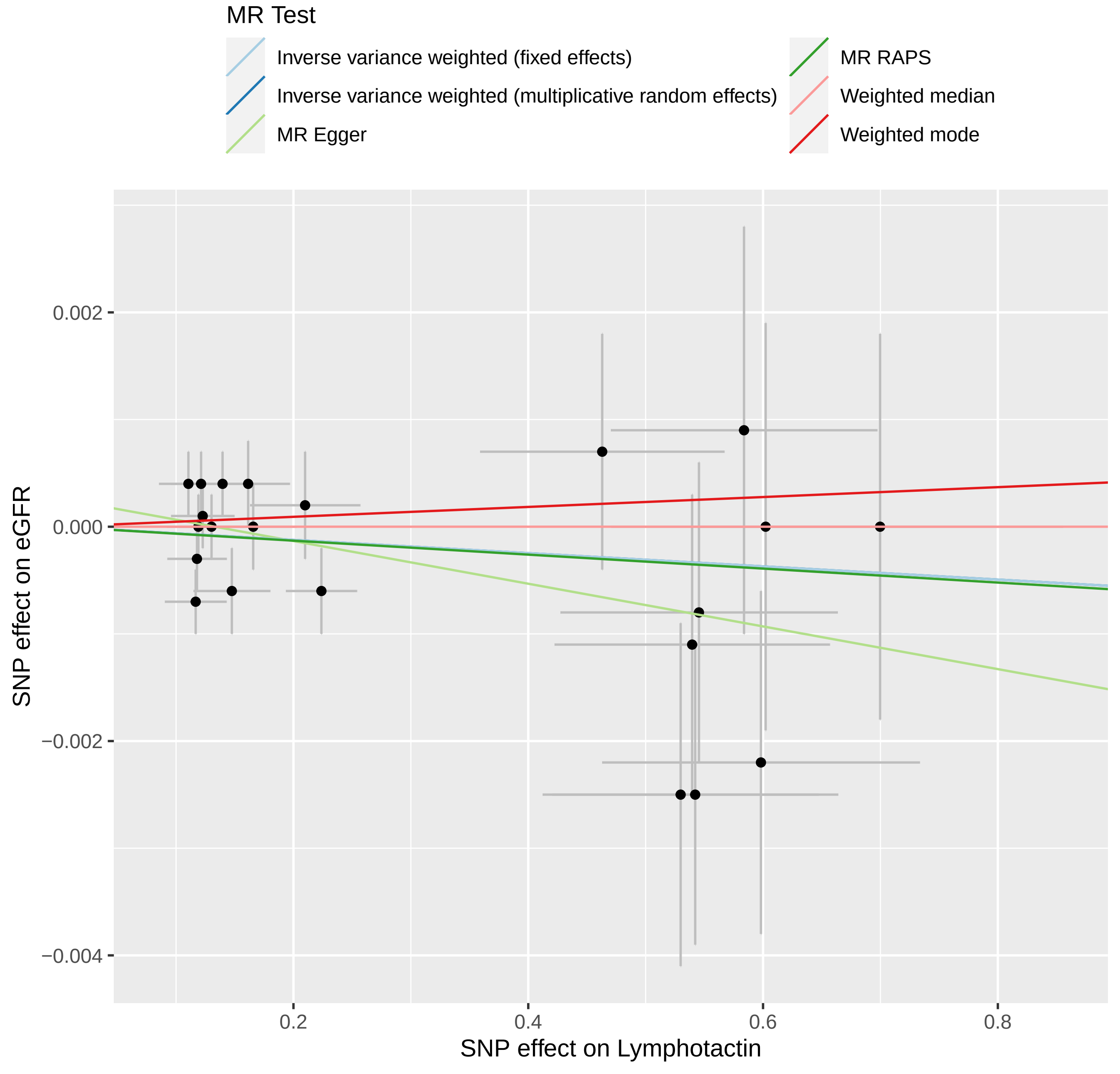

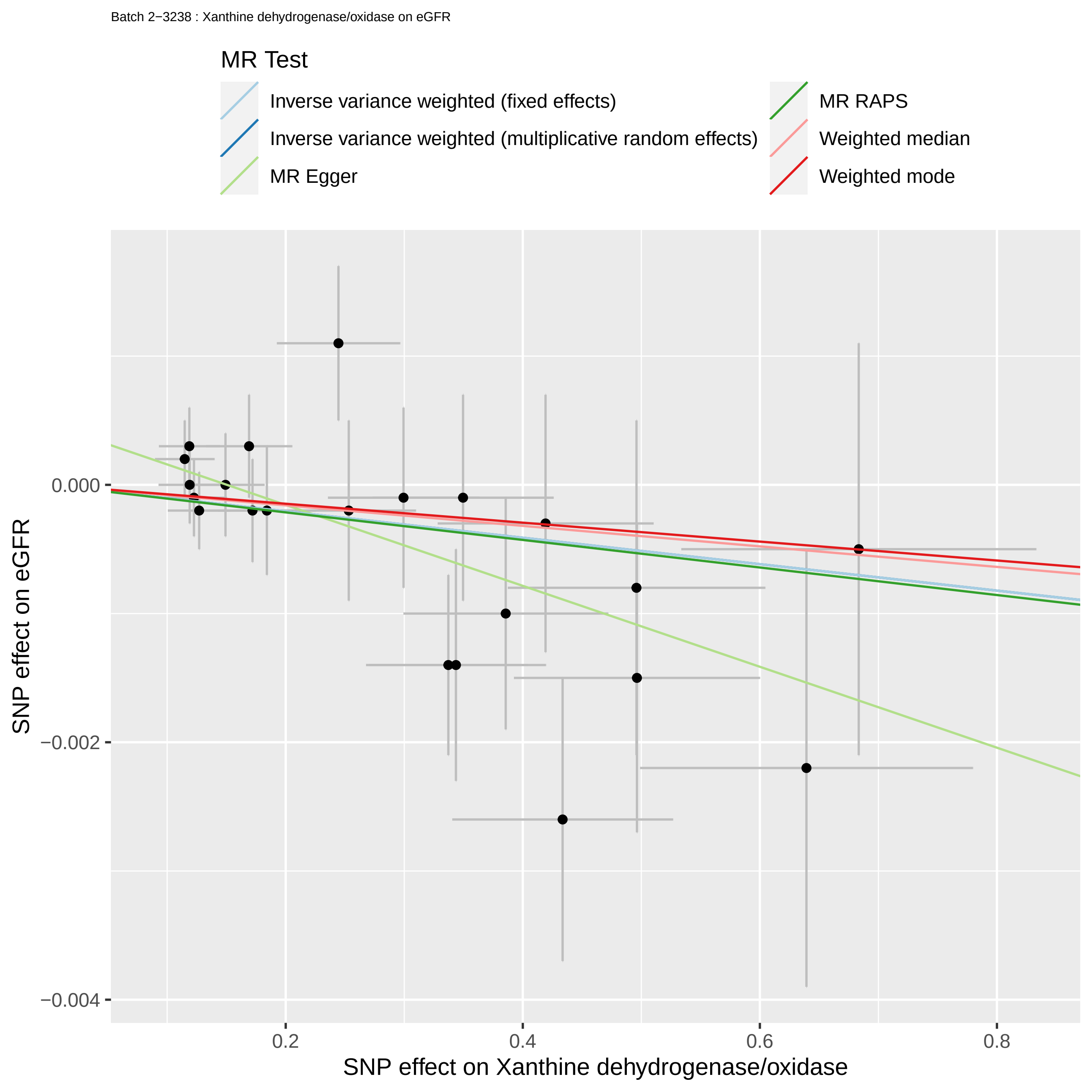

Batch 2−3238 : Xanthine dehydrogenase/oxidase on eGFR
MR Test
Inverse variance weighted (fixed effects)
Inverse variance weighted (multiplicative random effects)
MR Egger
MR RAPS
Weighted median
Weighted mode
0.000
−0.002
−0.004
0.2
0.4
0.6
0.8
SNP effect on eGFR
SNP effect on Xanthine dehydrogenase/oxidase

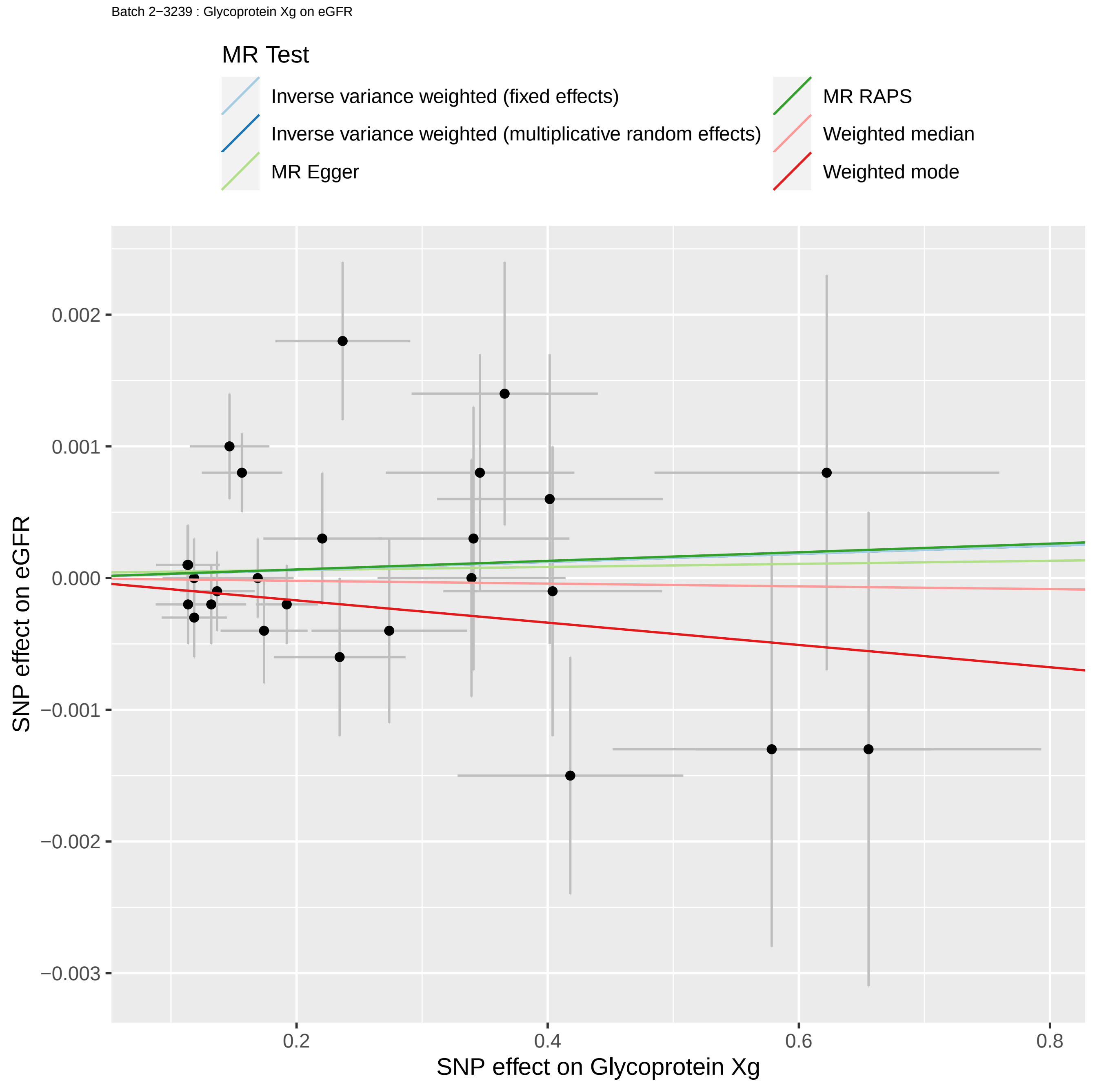

Batch 2−3239 : Glycoprotein Xg on eGFR
MR Test
Inverse variance weighted (fixed effects)
Inverse variance weighted (multiplicative random effects)
MR Egger
MR RAPS
Weighted median
Weighted mode
0.002
0.001
0.000
−0.001
−0.002
−0.003
SNP effect on eGFR
0.2
0.4
0.6
0.8
SNP effect on Glycoprotein Xg

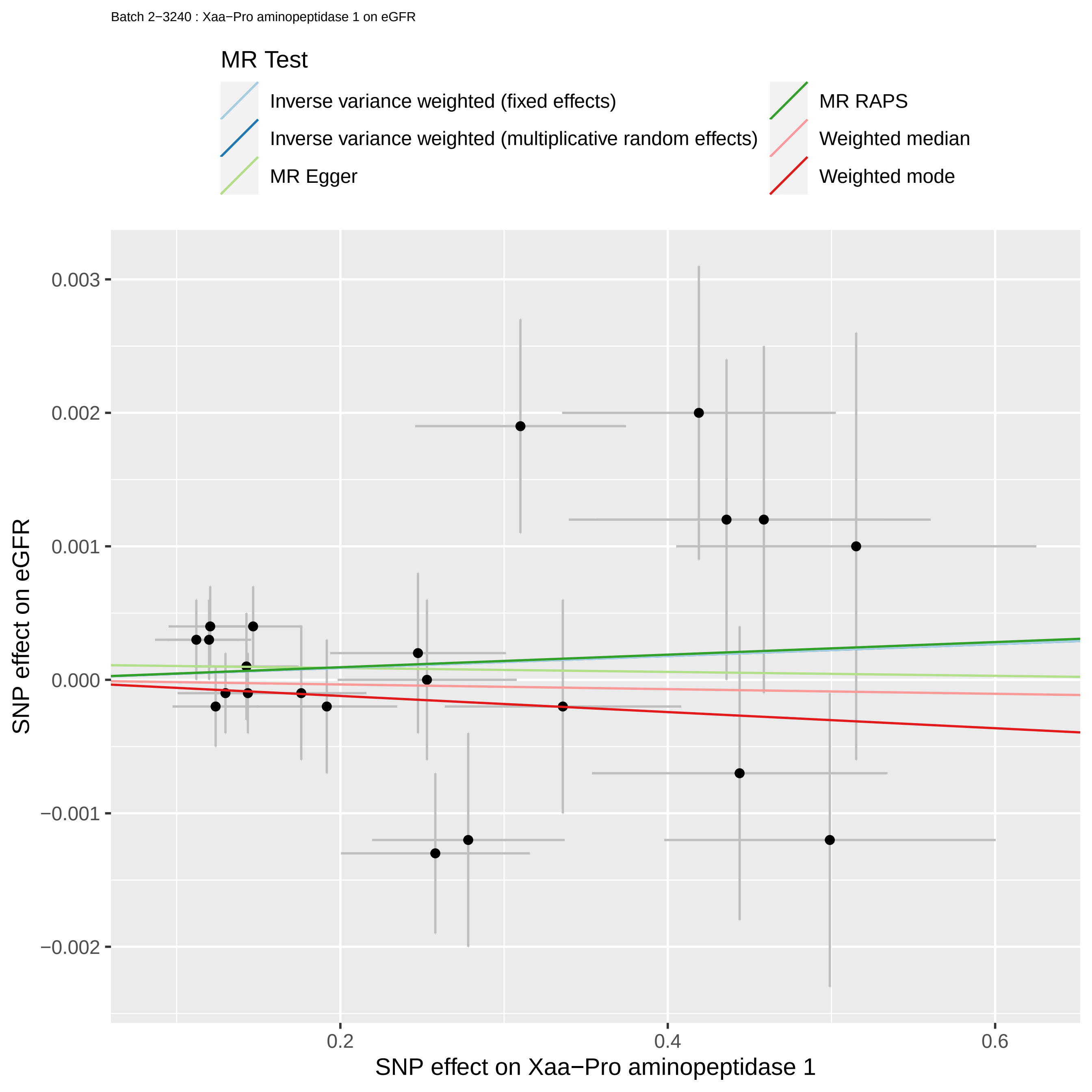

Batch 2−3240 : Xaa−Pro aminopeptidase 1 on eGFR
MR Test
Inverse variance weighted (fixed effects)
Inverse variance weighted (multiplicative random effects)
MR Egger
MR RAPS
Weighted median
Weighted mode
0.003
0.002
0.001
0.000
−0.001
−0.002
SNP effect on eGFR
0.2
0.4
0.6
SNP effect on Xaa−Pro aminopeptidase 1

Batch 2−3241 : DNA repair protein XRCC1 on eGFR

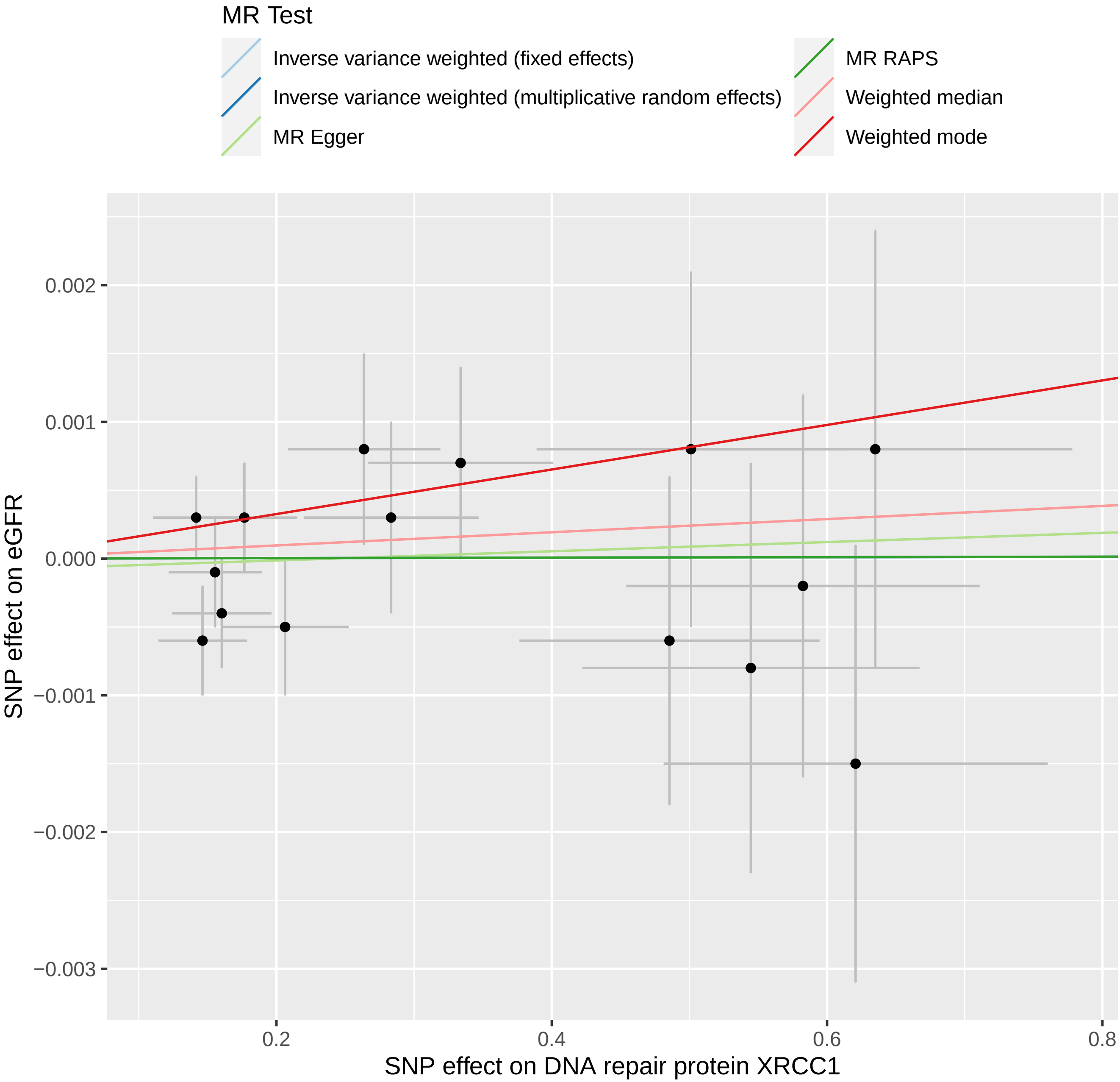

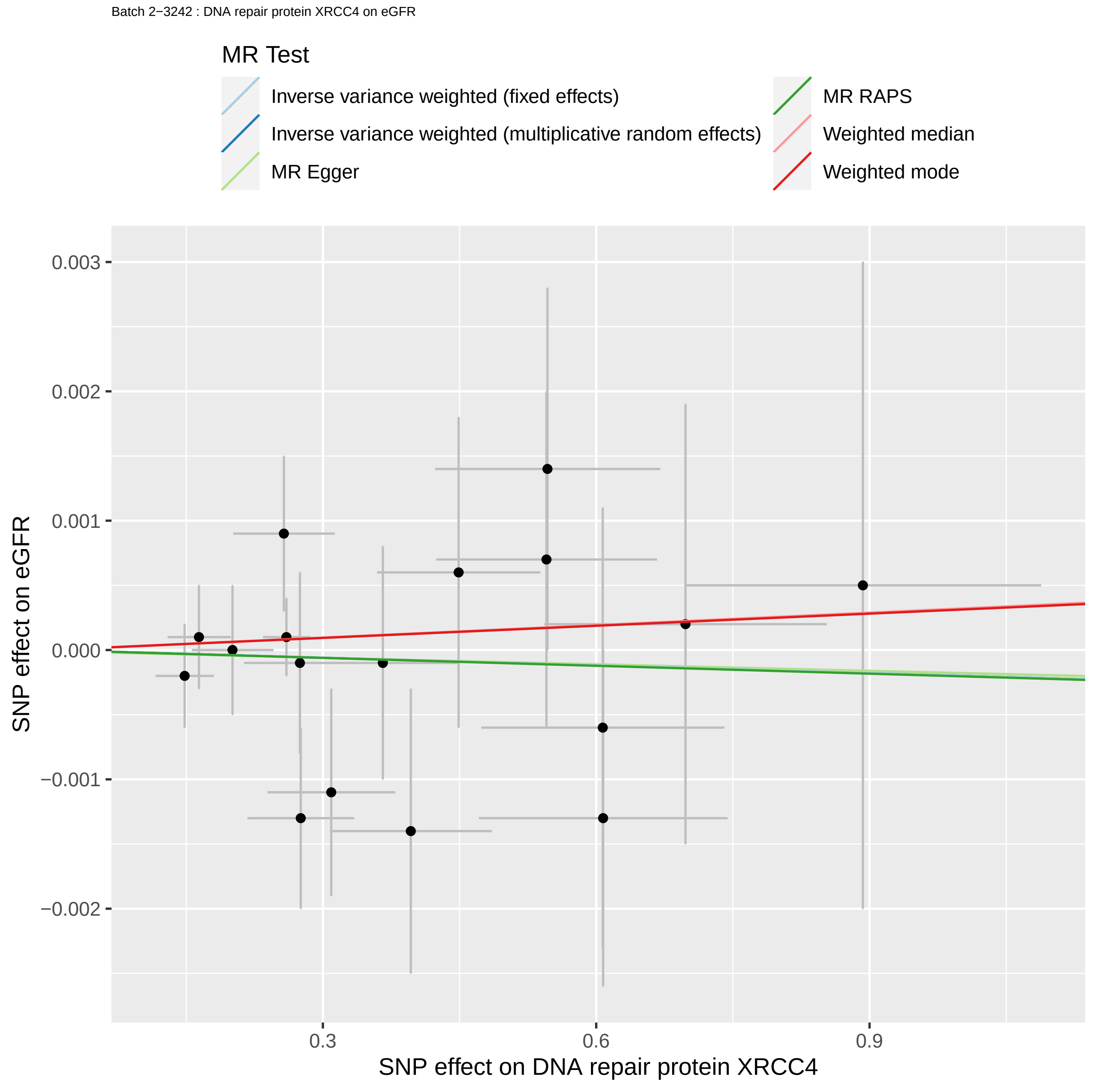
Batch 2−3242 : DNA repair protein XRCC4 on eGFR
MR Test
Inverse variance weighted (fixed effects)
Inverse variance weighted (multiplicative random effects)
MR Egger
MR RAPS
Weighted median
Weighted mode
0.003
0.002
0.001
0.000
−0.001
−0.002
SNP effect on eGFR
0.3
0.6
0.9
SNP effect on DNA repair protein XRCC4

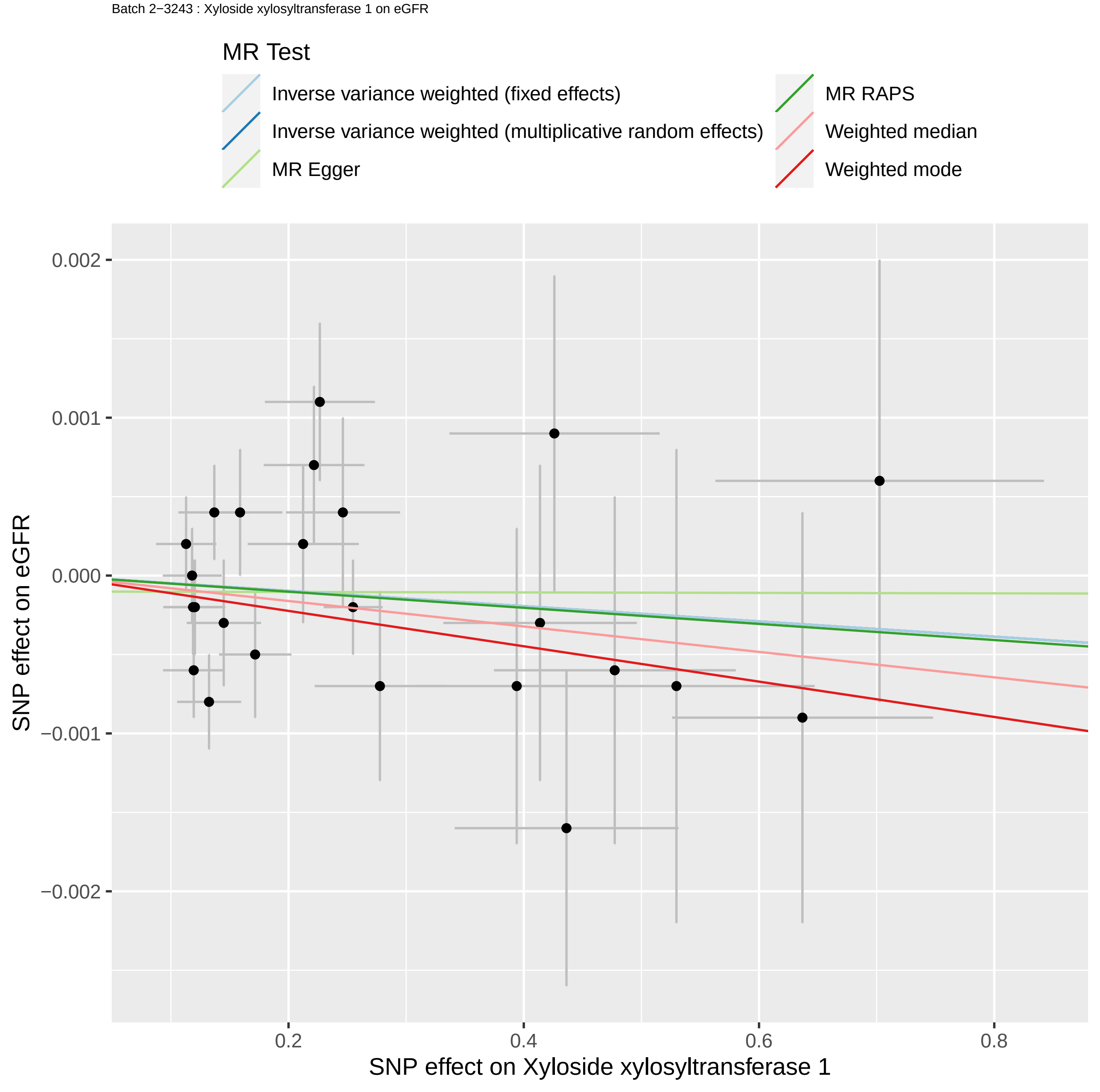

Batch 2−3243 : Xyloside xylosyltransferase 1 on eGFR
MR Test
Inverse variance weighted (fixed effects)
Inverse variance weighted (multiplicative random effects)
MR Egger
MR RAPS
Weighted median
Weighted mode
0.002
0.001
0.000
−0.001
−0.002
0.2
0.4
0.6
0.8
SNP effect on eGFR
SNP effect on Xyloside xylosyltransferase 1

Batch 2−3244 : Y−box−binding protein 2 on eGFR

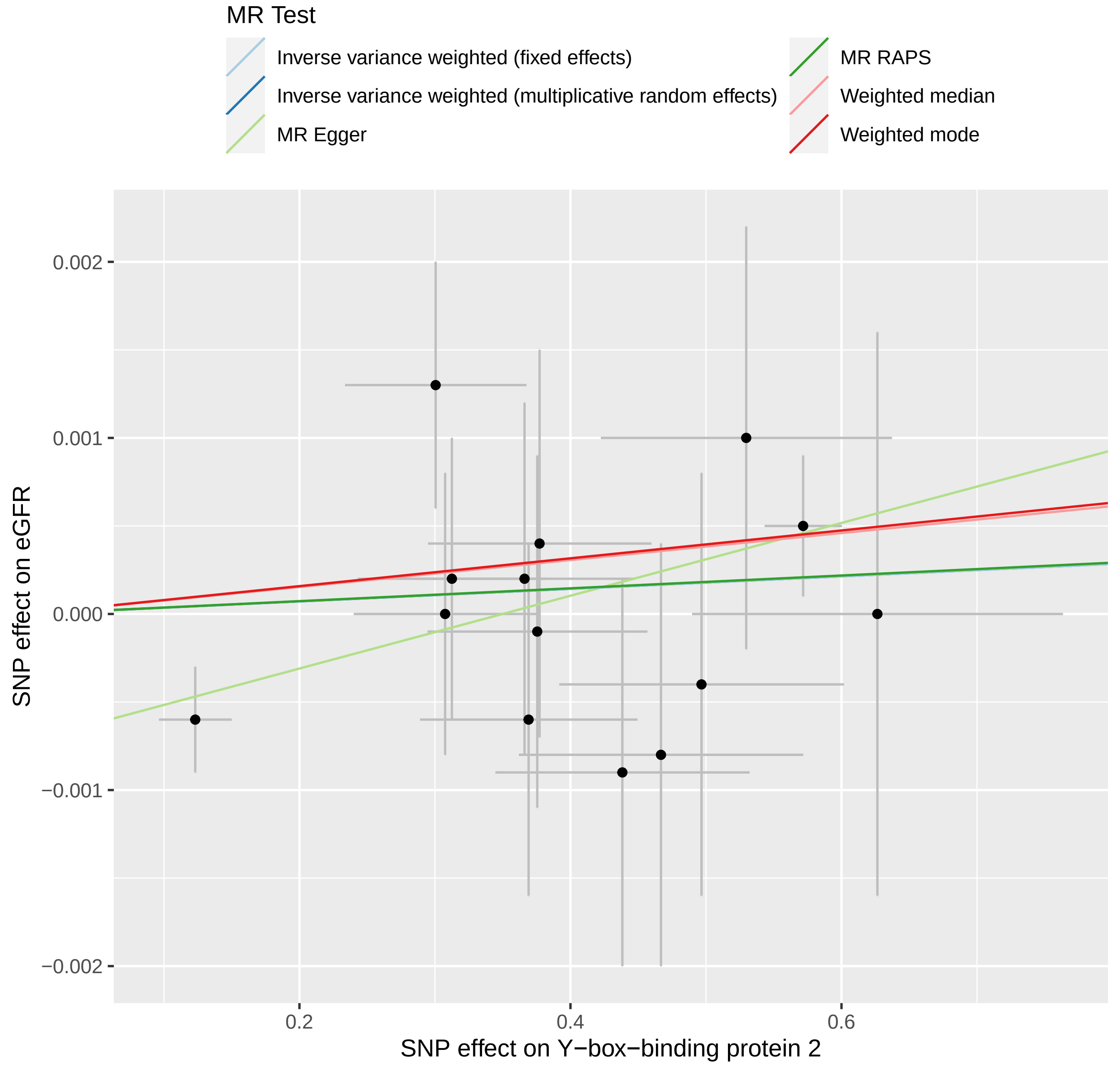

Batch 2−3245 : Tyrosine−protein kinase Yes on eGFR

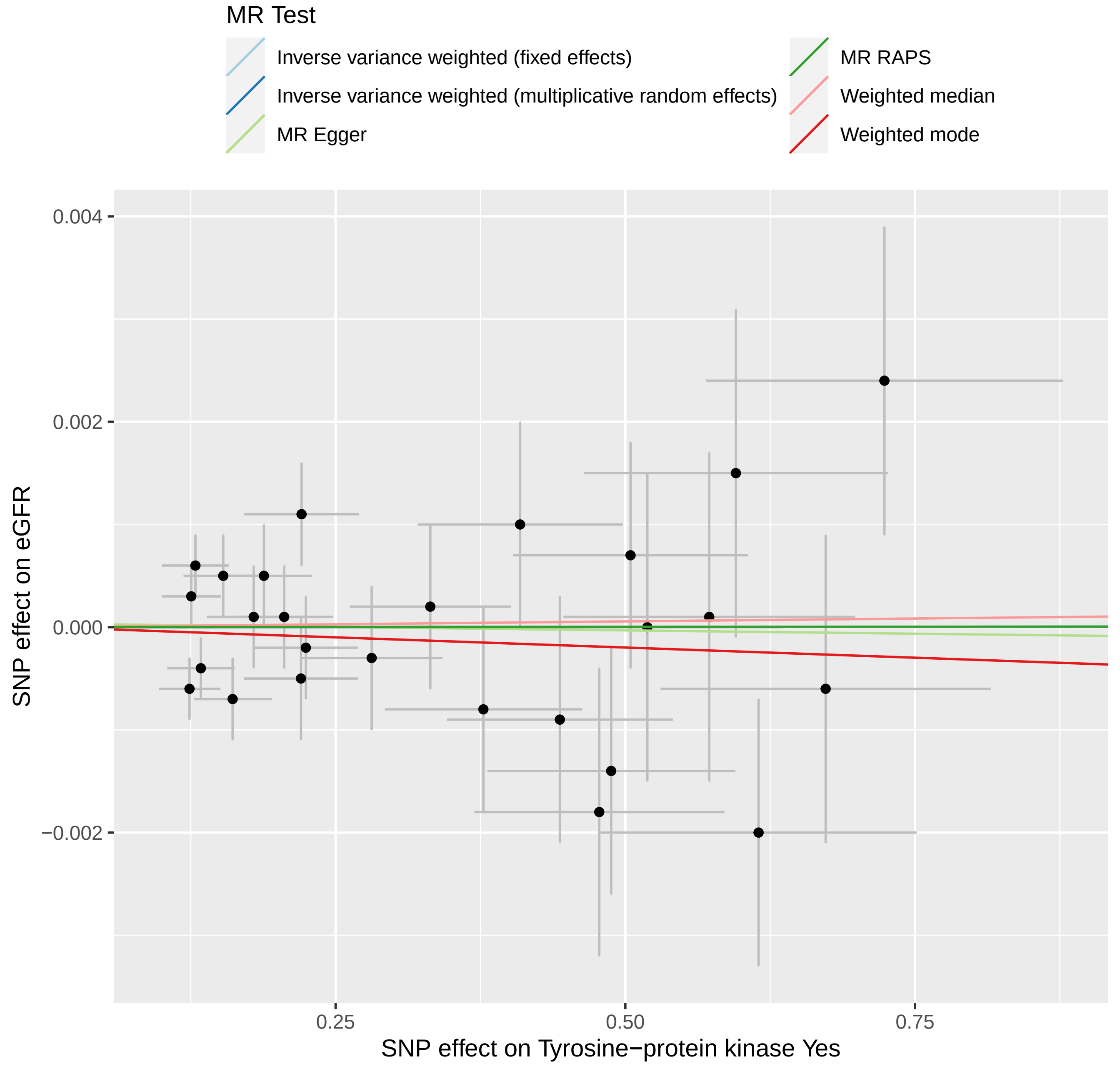

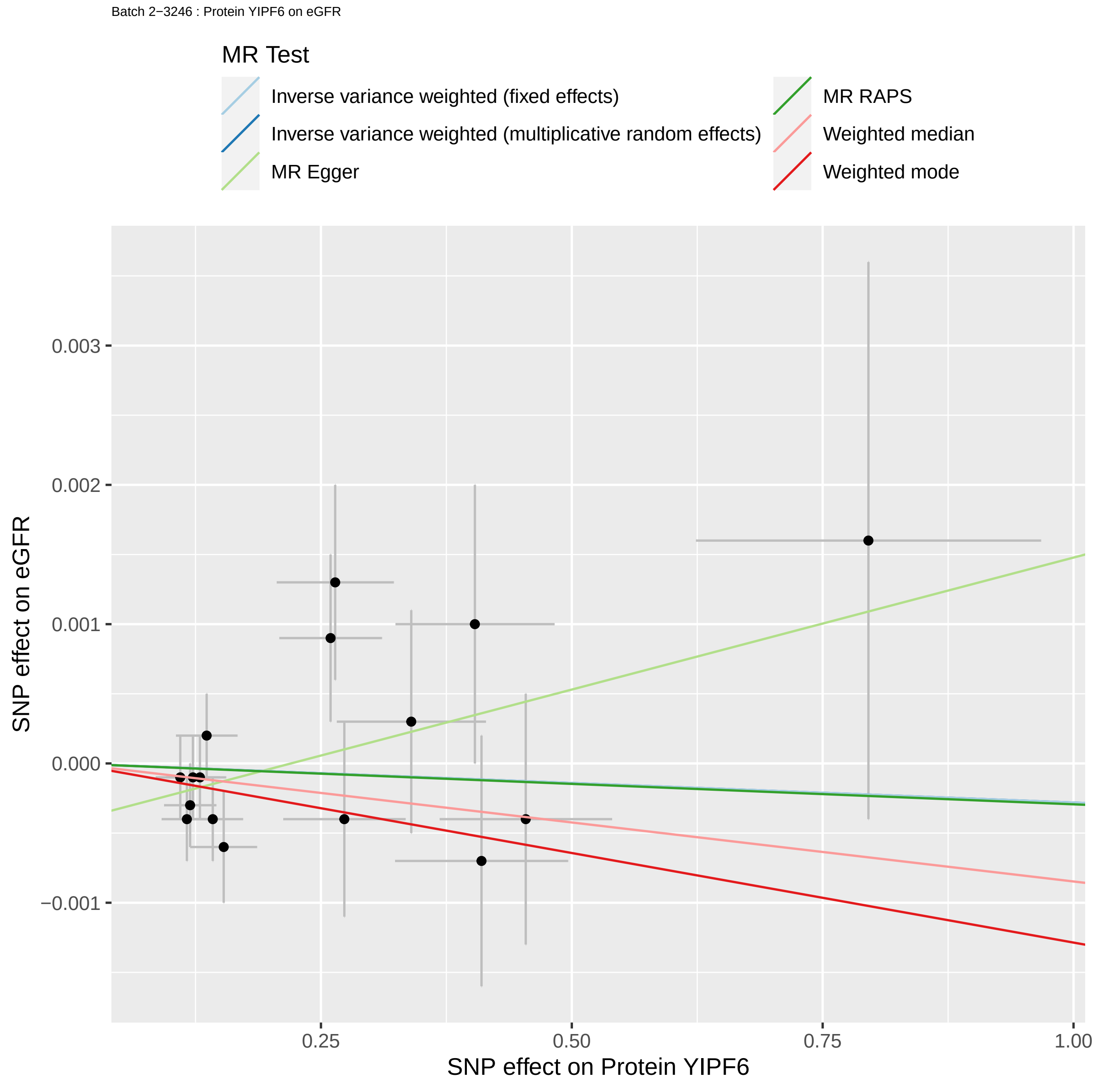
Batch 2−3246 : Protein YIPF6 on eGFR
MR Test
Inverse variance weighted (fixed effects)
Inverse variance weighted (multiplicative random effects)
MR Egger
MR RAPS
Weighted median
Weighted mode
0.003
0.002
0.001
0.000
−0.001
SNP effect on eGFR
0.25
0.50
0.75
1.00
SNP effect on Protein YIPF6

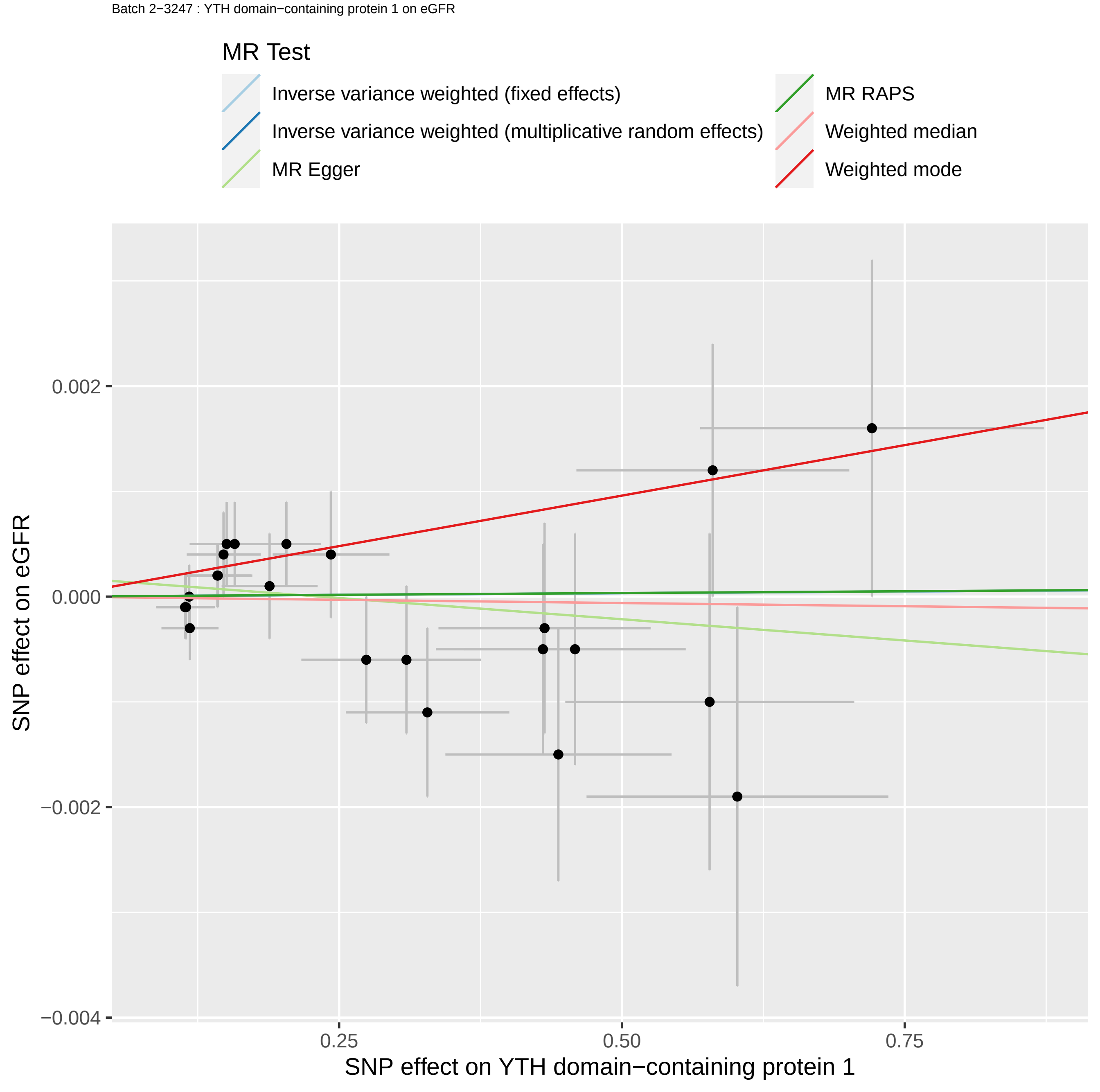

Batch 2−3247 : YTH domain−containing protein 1 on eGFR
MR Test
Inverse variance weighted (fixed effects)
Inverse variance weighted (multiplicative random effects)
MR Egger
MR RAPS
Weighted median
Weighted mode
0.002
0.000
−0.002
−0.004
0.25
0.50
0.75
SNP effect on eGFR
SNP effect on YTH domain−containing protein 1

Batch 2−3248 : 14−3−3 protein family on eGFR

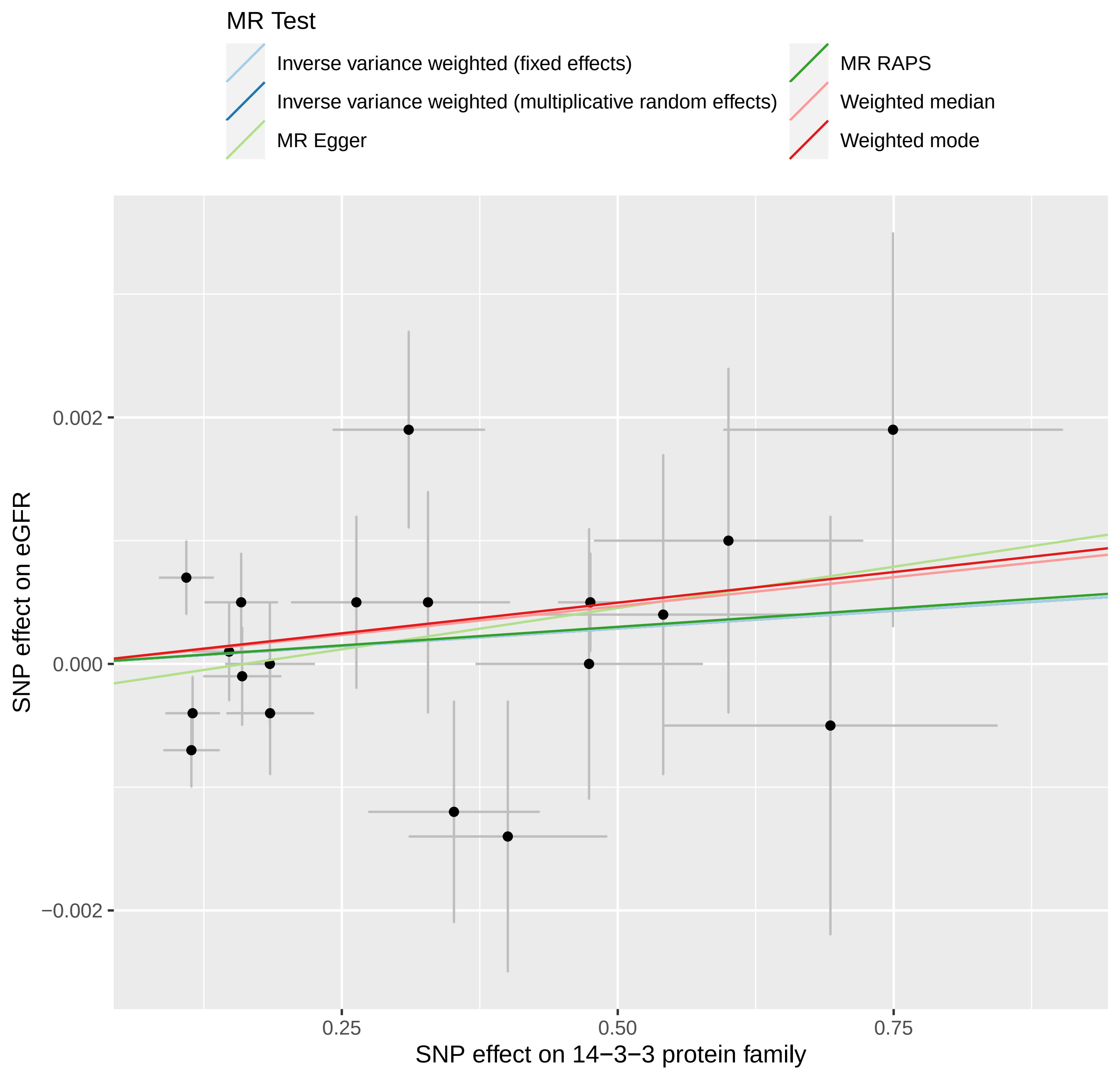

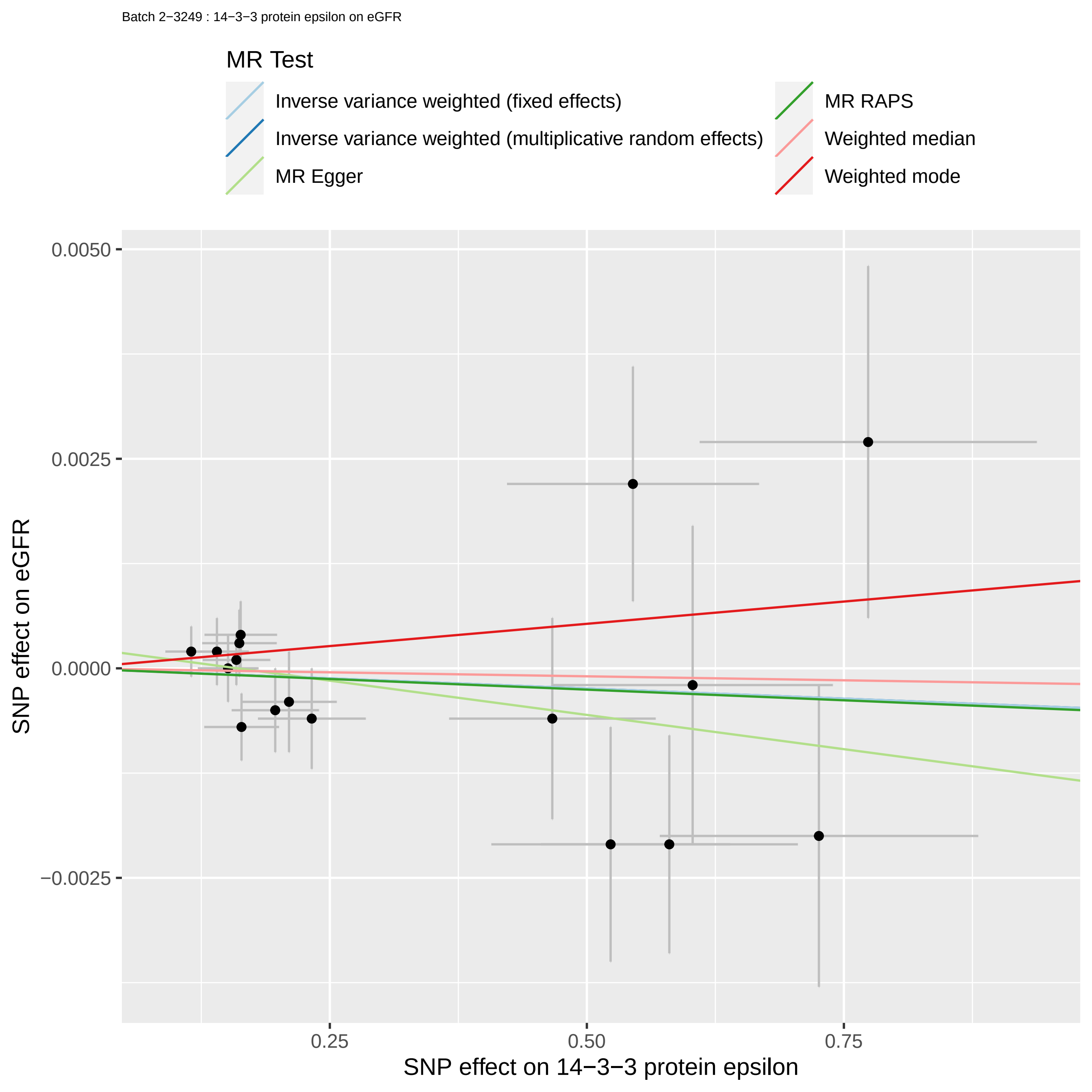
Batch 2−3249 : 14−3−3 protein epsilon on eGFR
MR Test
Inverse variance weighted (fixed effects)
Inverse variance weighted (multiplicative random effects)
MR Egger
MR RAPS
Weighted median
Weighted mode
0.0050
0.0025
0.0000
−0.0025
SNP effect on eGFR
0.25
0.50
0.75
SNP effect on 14−3−3 protein epsilon

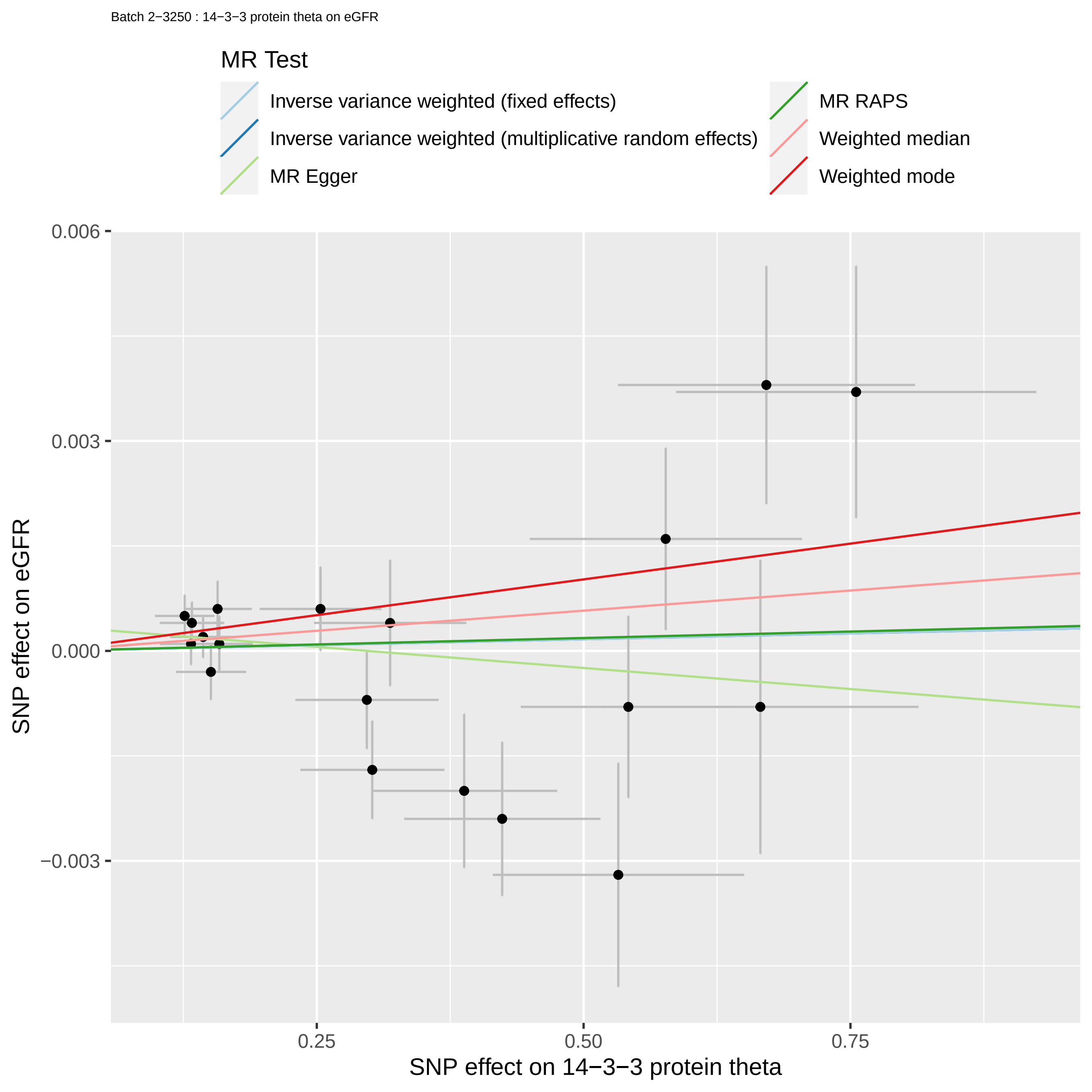

Batch 2−3250 : 14−3−3 protein theta on eGFR
MR Test
Inverse variance weighted (fixed effects)
Inverse variance weighted (multiplicative random effects)
MR Egger
MR RAPS
Weighted median
Weighted mode
0.006
0.003
0.000
−0.003
0.25
0.50
0.75
SNP effect on eGFR
SNP effect on 14−3−3 protein theta

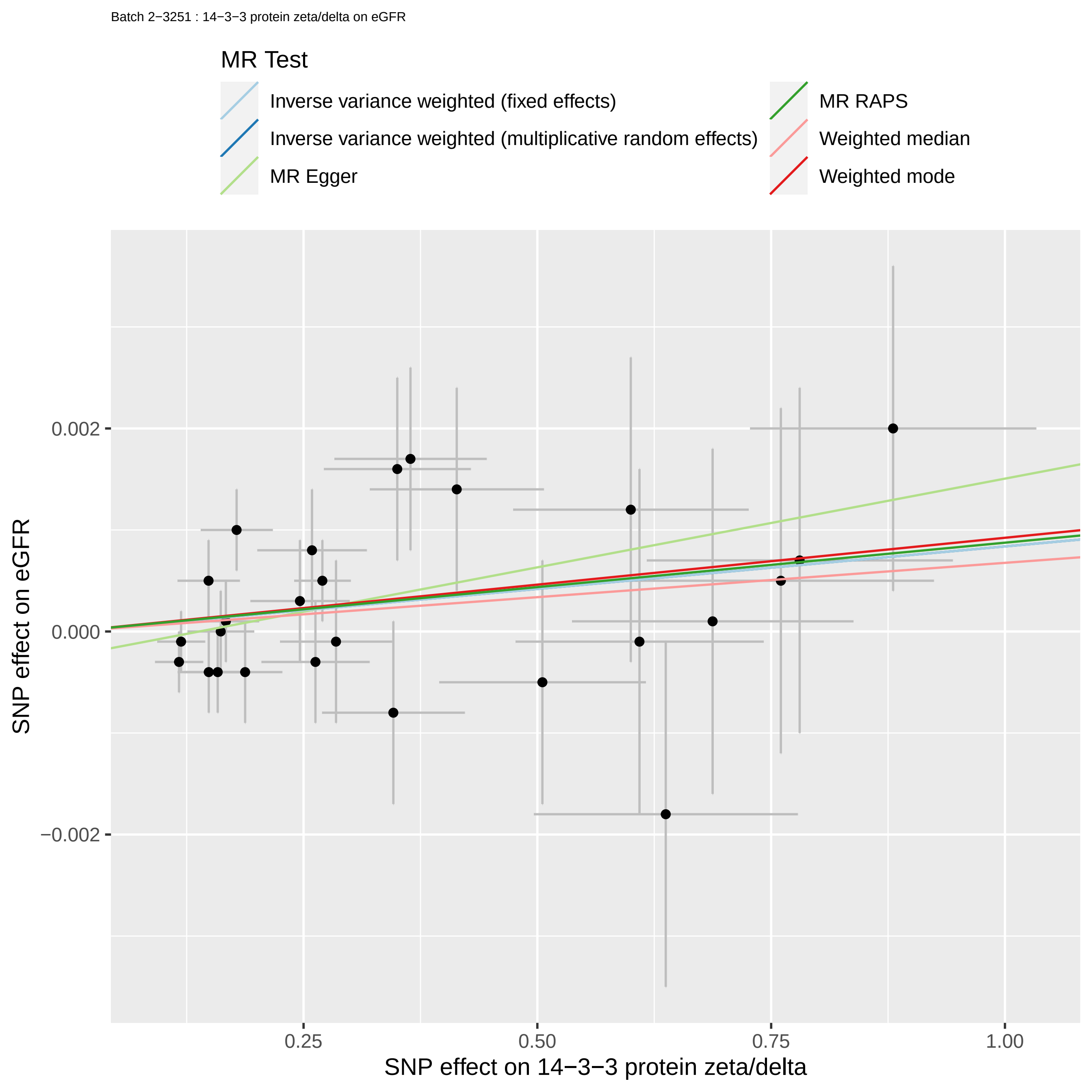

Batch 2−3251 : 14−3−3 protein zeta/delta on eGFR
MR Test
Inverse variance weighted (fixed effects)
Inverse variance weighted (multiplicative random effects)
MR Egger
MR RAPS
Weighted median
Weighted mode
0.002
0.000
−0.002
0.25
0.50
0.75
1.00
SNP effect on eGFR
SNP effect on 14−3−3 protein zeta/delta

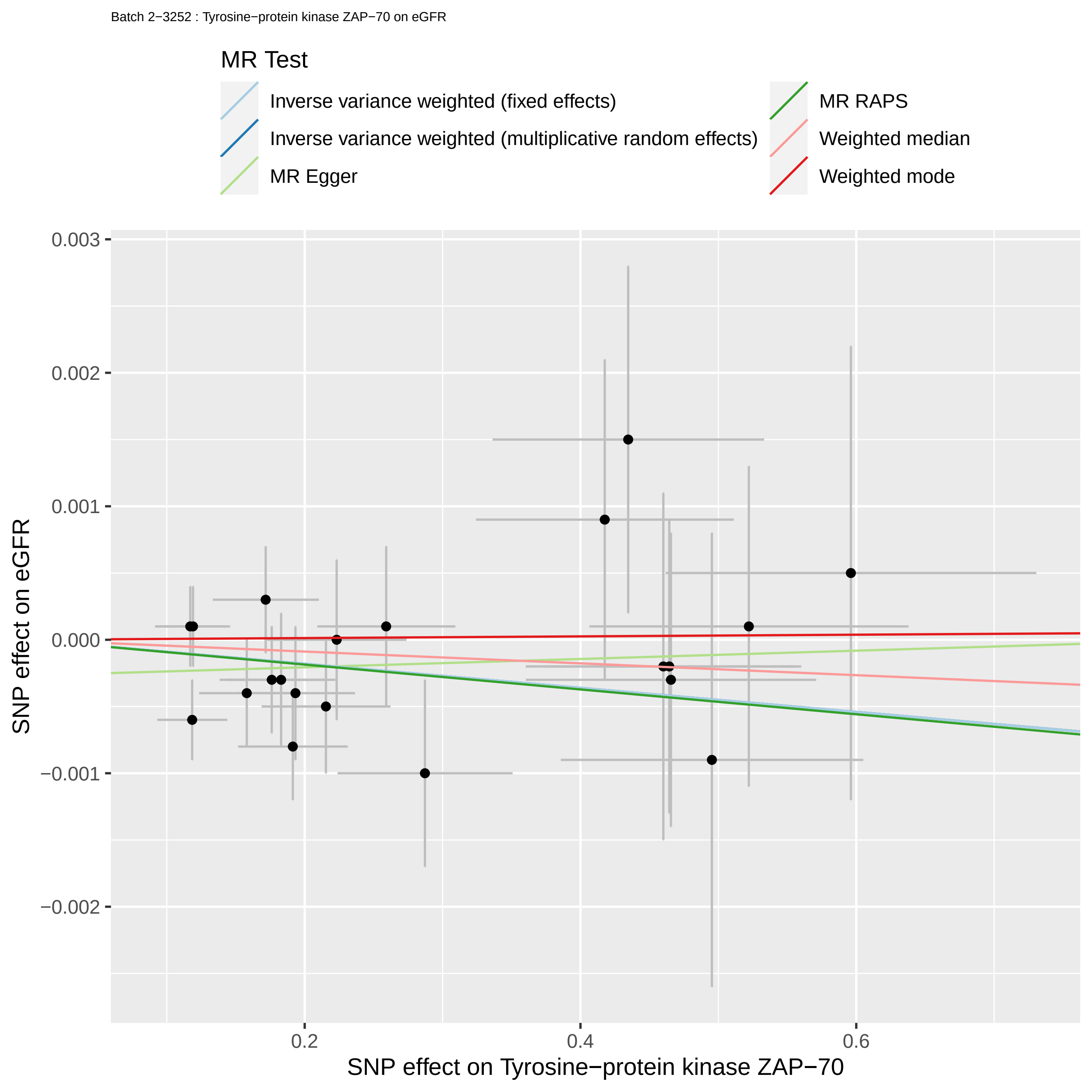
Batch 2−3252 : Tyrosine−protein kinase ZAP−70 on eGFR
MR Test
Inverse variance weighted (fixed effects)
Inverse variance weighted (multiplicative random effects)
MR Egger
MR RAPS
Weighted median
Weighted mode
0.003
0.002
0.001
0.000
−0.001
−0.002
SNP effect on eGFR
0.2
0.4
0.6
SNP effect on Tyrosine−protein kinase ZAP−70

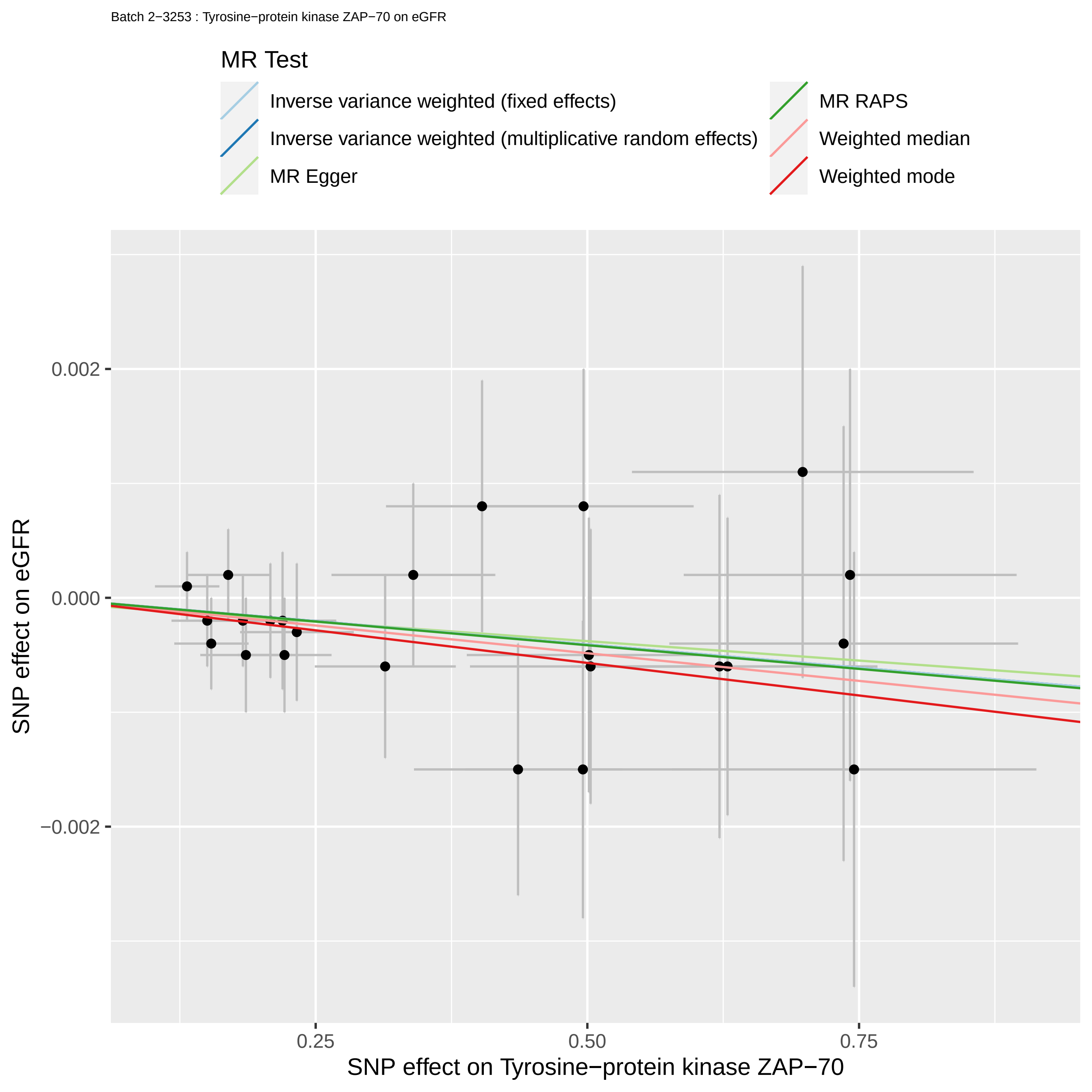
Batch 2−3253 : Tyrosine−protein kinase ZAP−70 on eGFR
MR Test
Inverse variance weighted (fixed effects)
Inverse variance weighted (multiplicative random effects)
MR Egger
MR RAPS
Weighted median
Weighted mode
0.002
0.000
−0.002
0.25
0.50
0.75
SNP effect on eGFR
SNP effect on Tyrosine−protein kinase ZAP−70

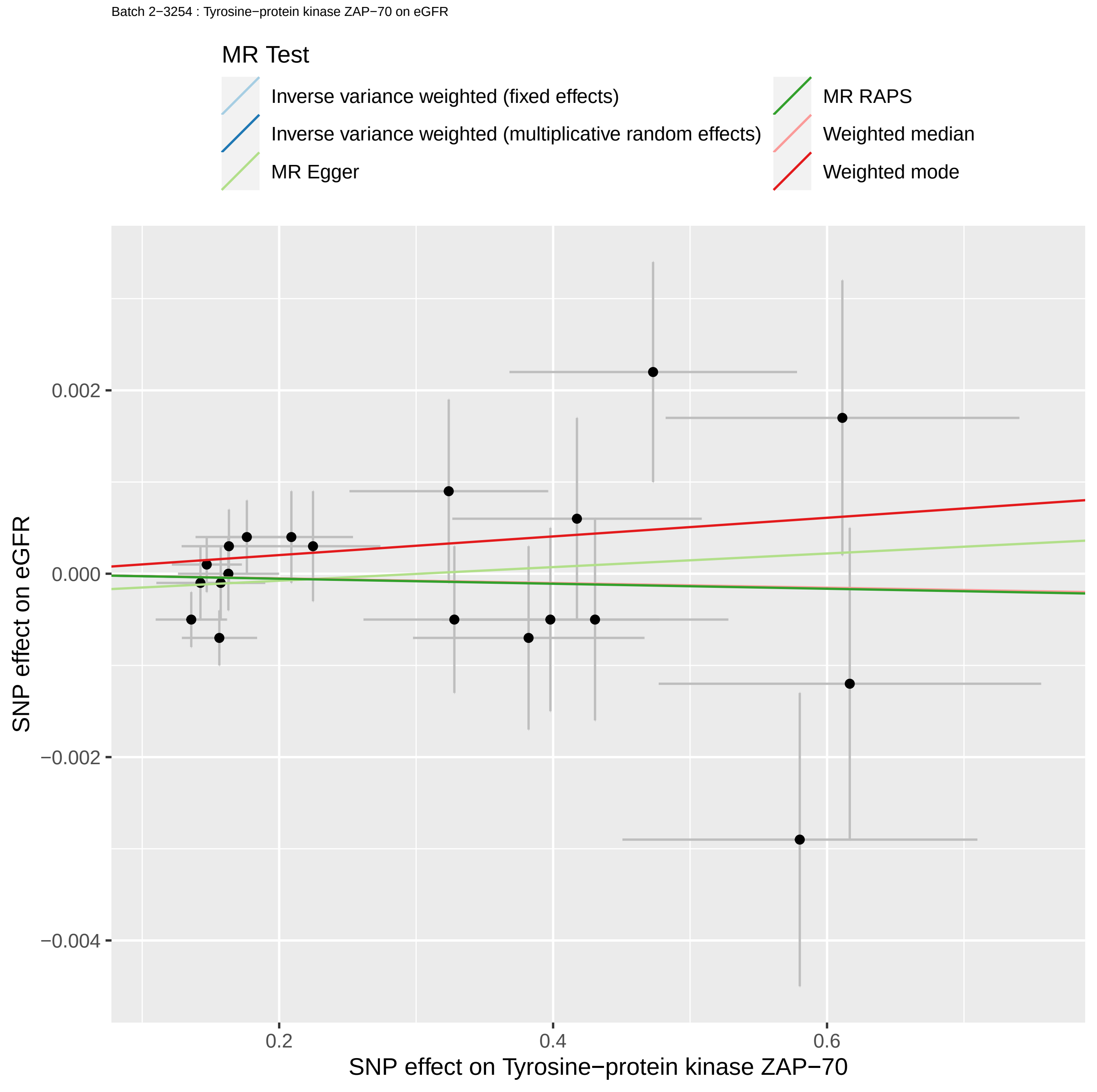

Batch 2−3254 : Tyrosine−protein kinase ZAP−70 on eGFR
MR Test
Inverse variance weighted (fixed effects)
Inverse variance weighted (multiplicative random effects)
MR Egger
MR RAPS
Weighted median
Weighted mode
0.002
0.000
−0.002
−0.004
0.2
0.4
0.6
SNP effect on eGFR
SNP effect on Tyrosine−protein kinase ZAP−70

Batch 2−3255 : Transcriptional regulator Kaiso on eGFR

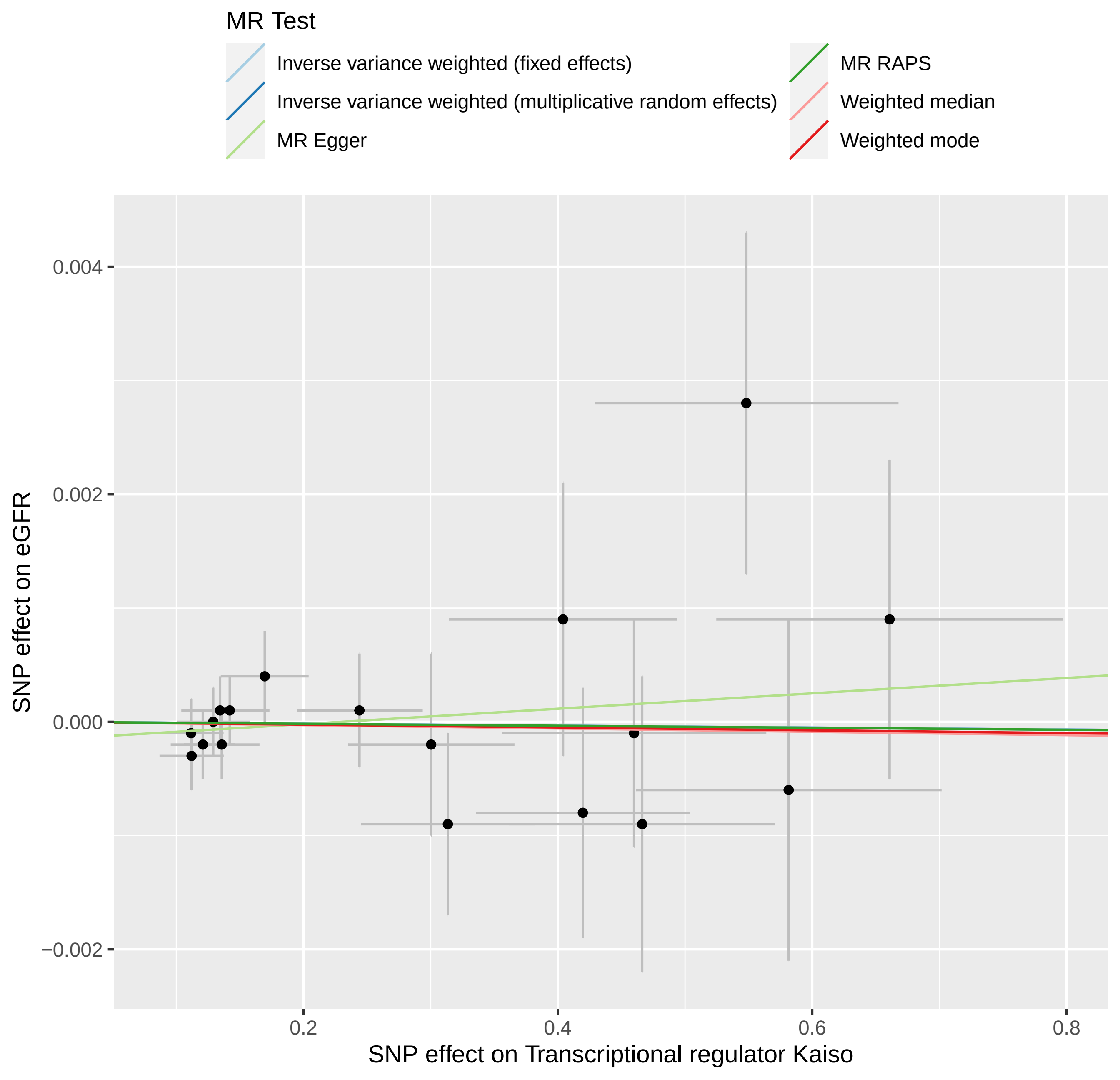

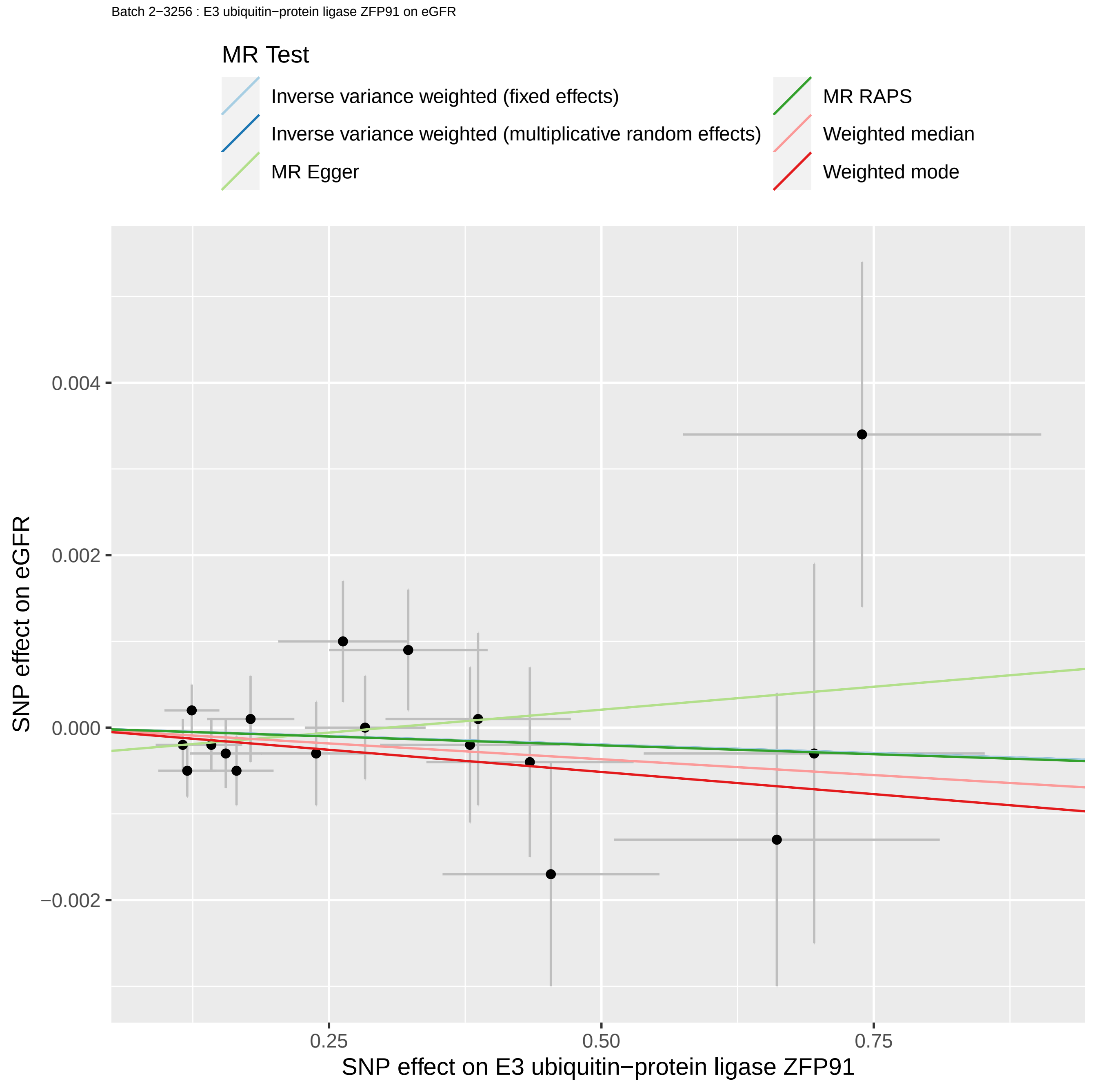

Batch 2−3256 : E3 ubiquitin−protein ligase ZFP91 on eGFR
MR Test
Inverse variance weighted (fixed effects)
Inverse variance weighted (multiplicative random effects)
MR Egger
MR RAPS
Weighted median
Weighted mode
0.004
0.002
0.000
−0.002
0.25
0.50
0.75
SNP effect on eGFR
SNP effect on E3 ubiquitin−protein ligase ZFP91

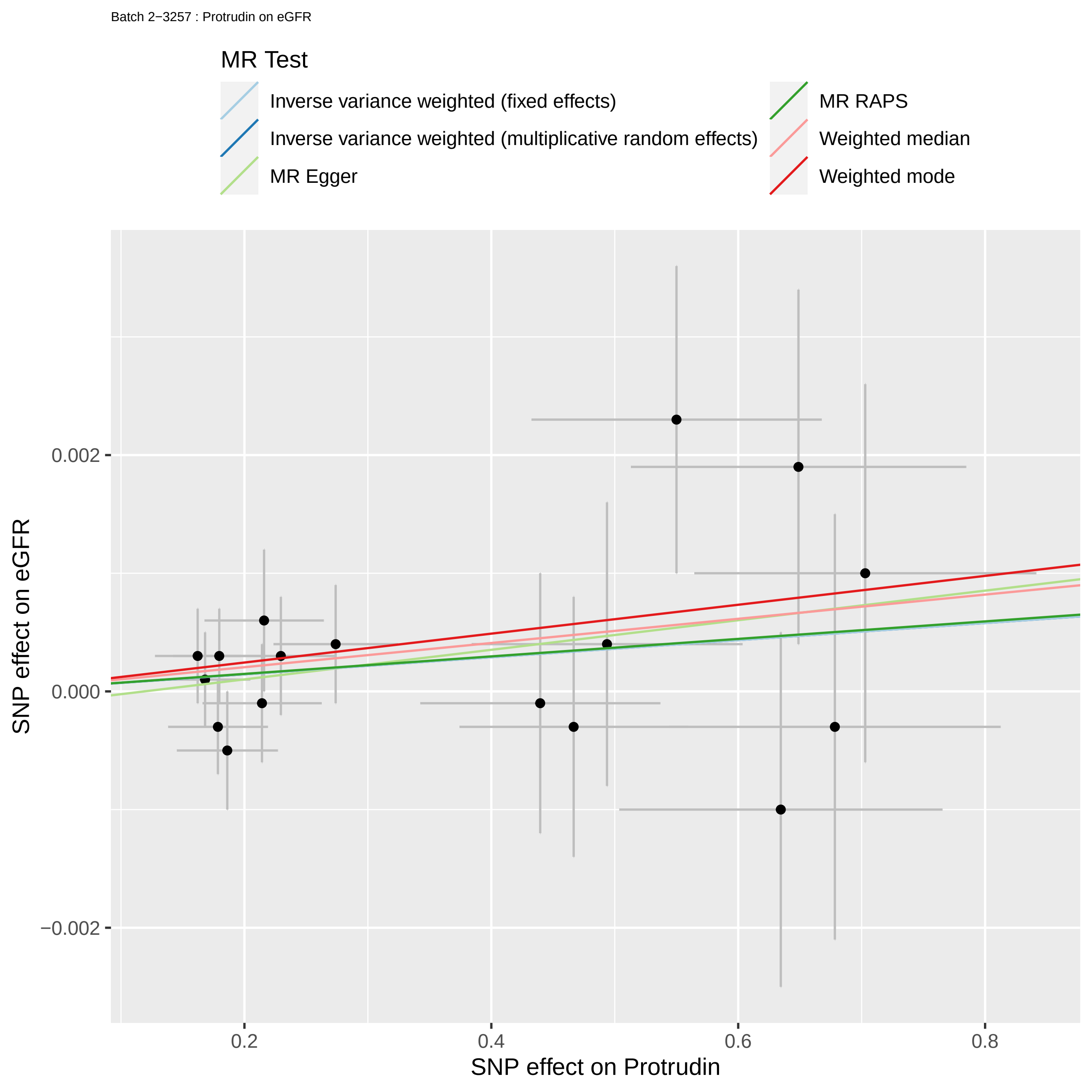
Batch 2−3257 : Protrudin on eGFR
MR Test
Inverse variance weighted (fixed effects)
Inverse variance weighted (multiplicative random effects)
MR Egger
MR RAPS
Weighted median
Weighted mode
SNP effect on eGFR
0.002
0.000
−0.002
SNP effect on Protrudin
0.2
0.4
0.6
0.8

Batch 2−3258 : Zinc fingers and homeoboxes protein 3 on eGFR

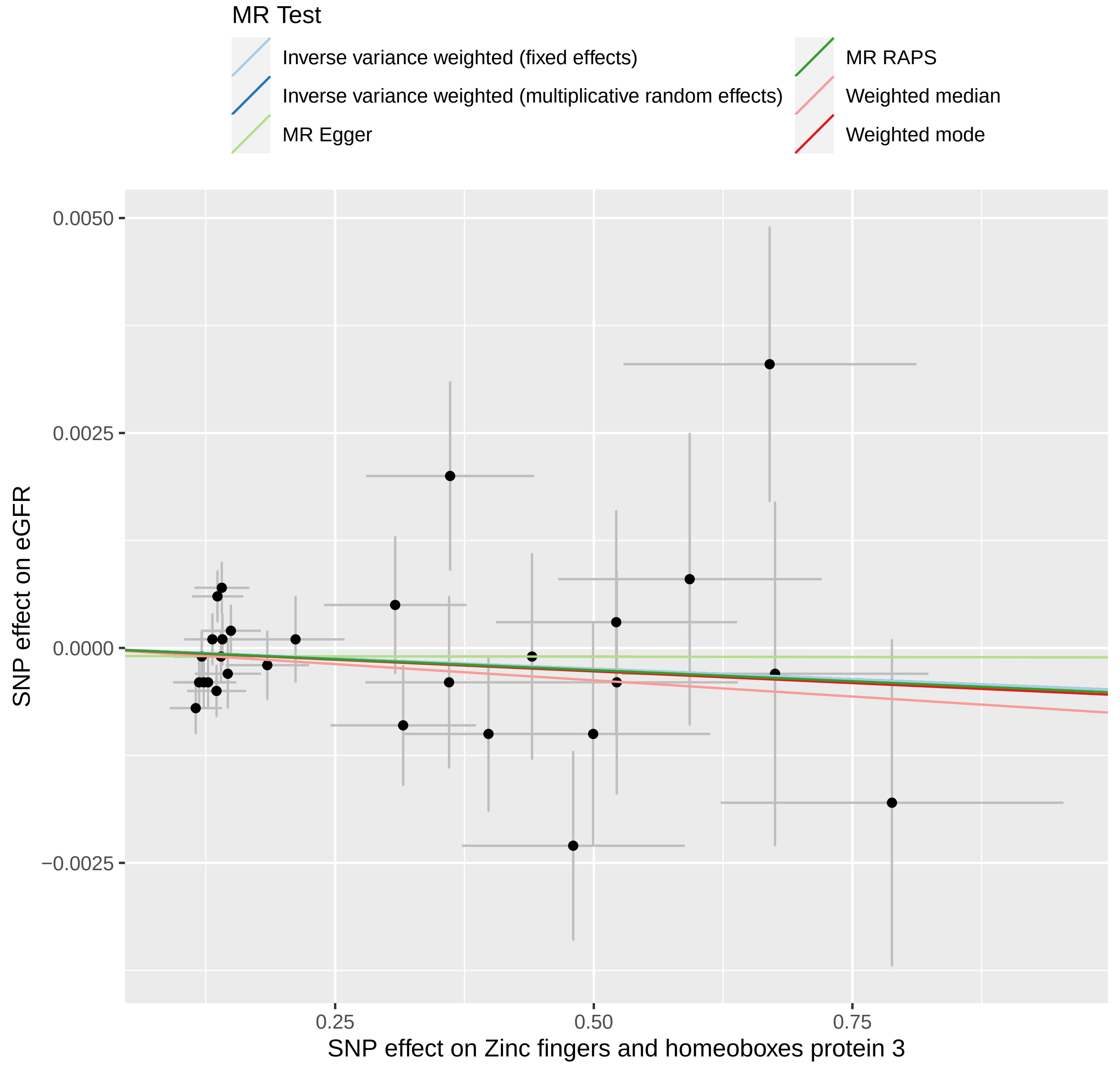

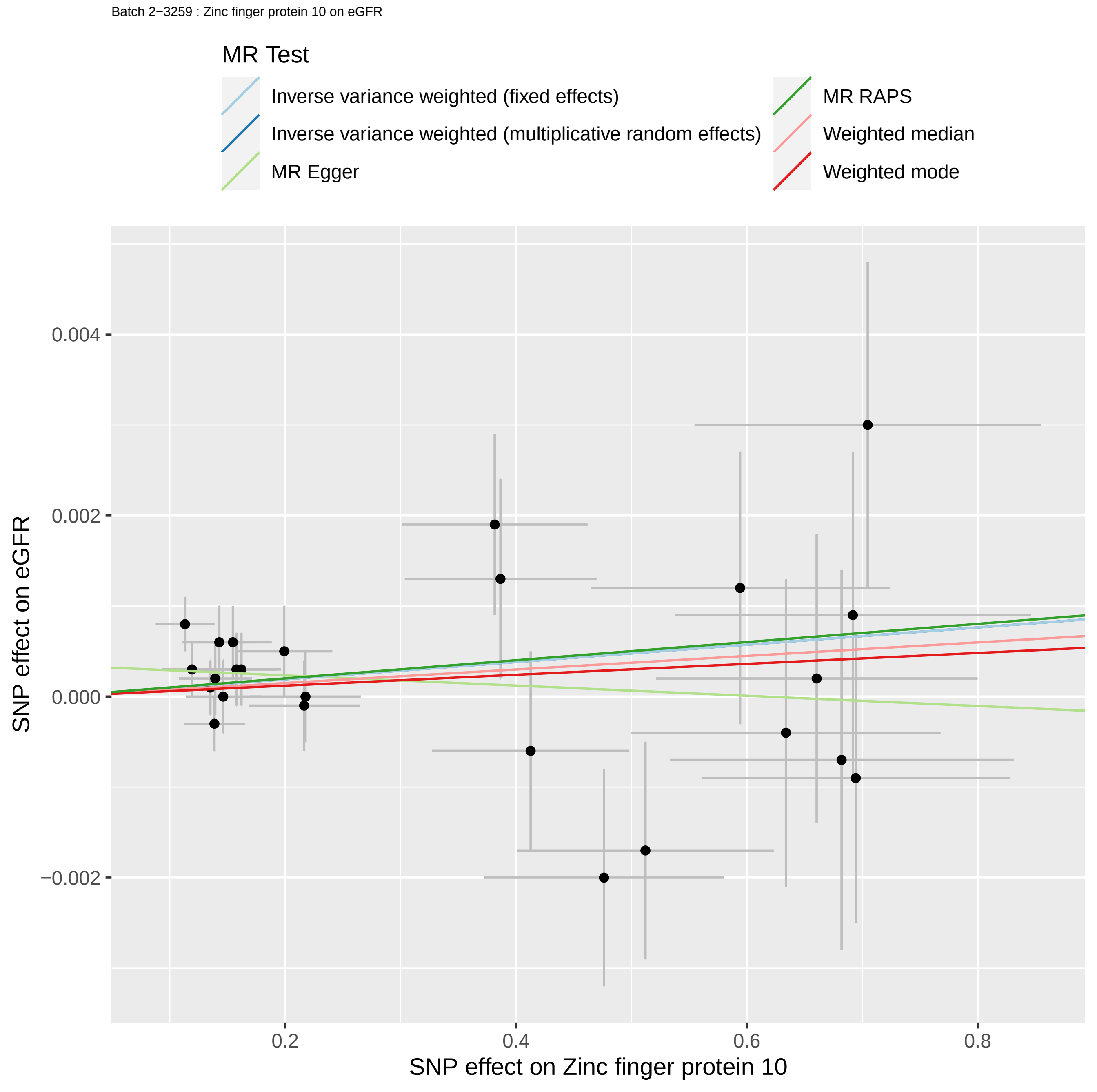
Batch 2−3259 : Zinc finger protein 10 on eGFR
MR Test
Inverse variance weighted (fixed effects)
Inverse variance weighted (multiplicative random effects)
MR Egger
MR RAPS
Weighted median
Weighted mode
0.004
0.002
0.000
−0.002
0.2
0.4
0.6
0.8
SNP effect on eGFR
SNP effect on Zinc finger protein 10

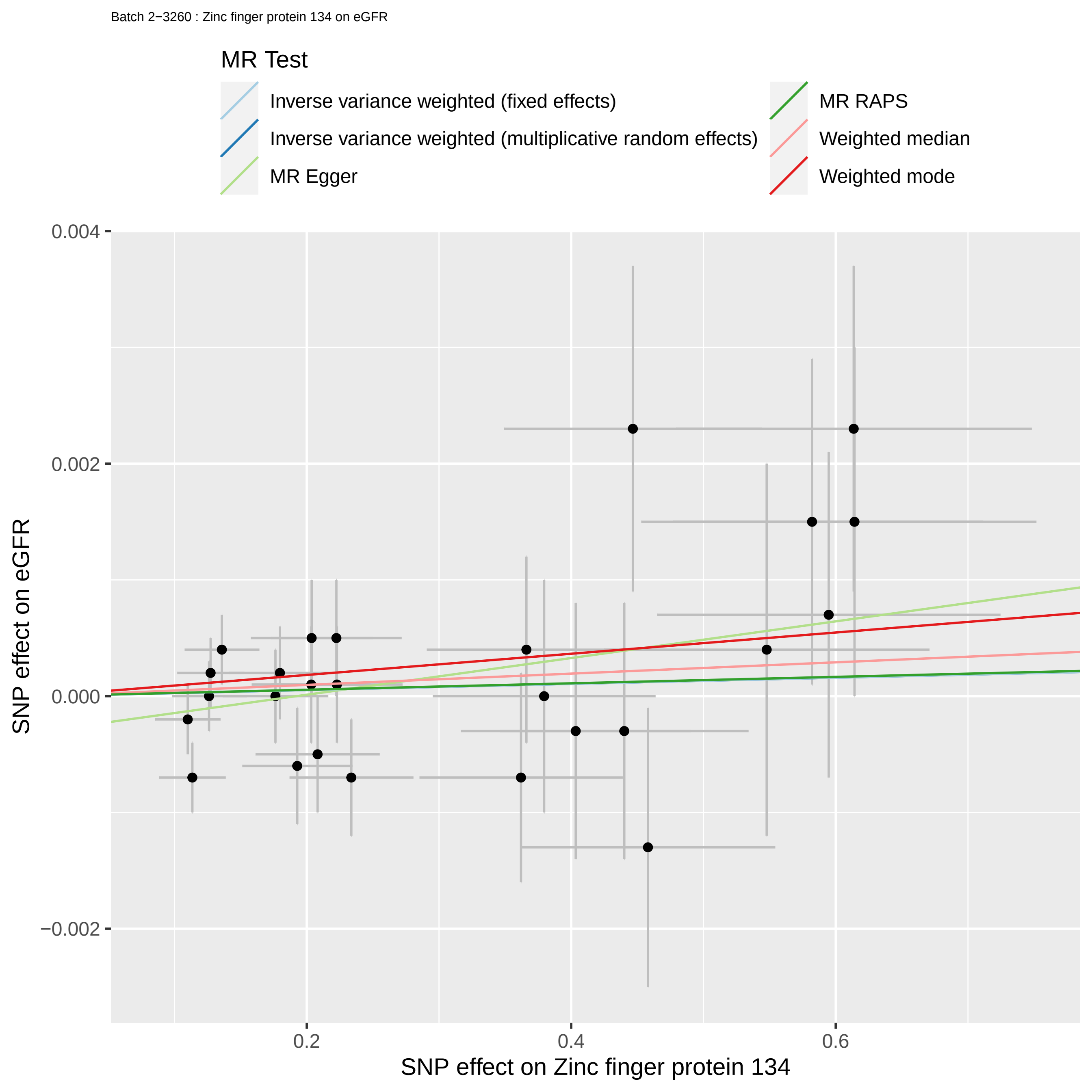

Batch 2−3260 : Zinc finger protein 134 on eGFR
MR Test
Inverse variance weighted (fixed effects)
Inverse variance weighted (multiplicative random effects)
MR Egger
MR RAPS
Weighted median
Weighted mode
0.004
0.002
0.000
−0.002
0.2
0.4
0.6
SNP effect on eGFR
SNP effect on Zinc finger protein 134

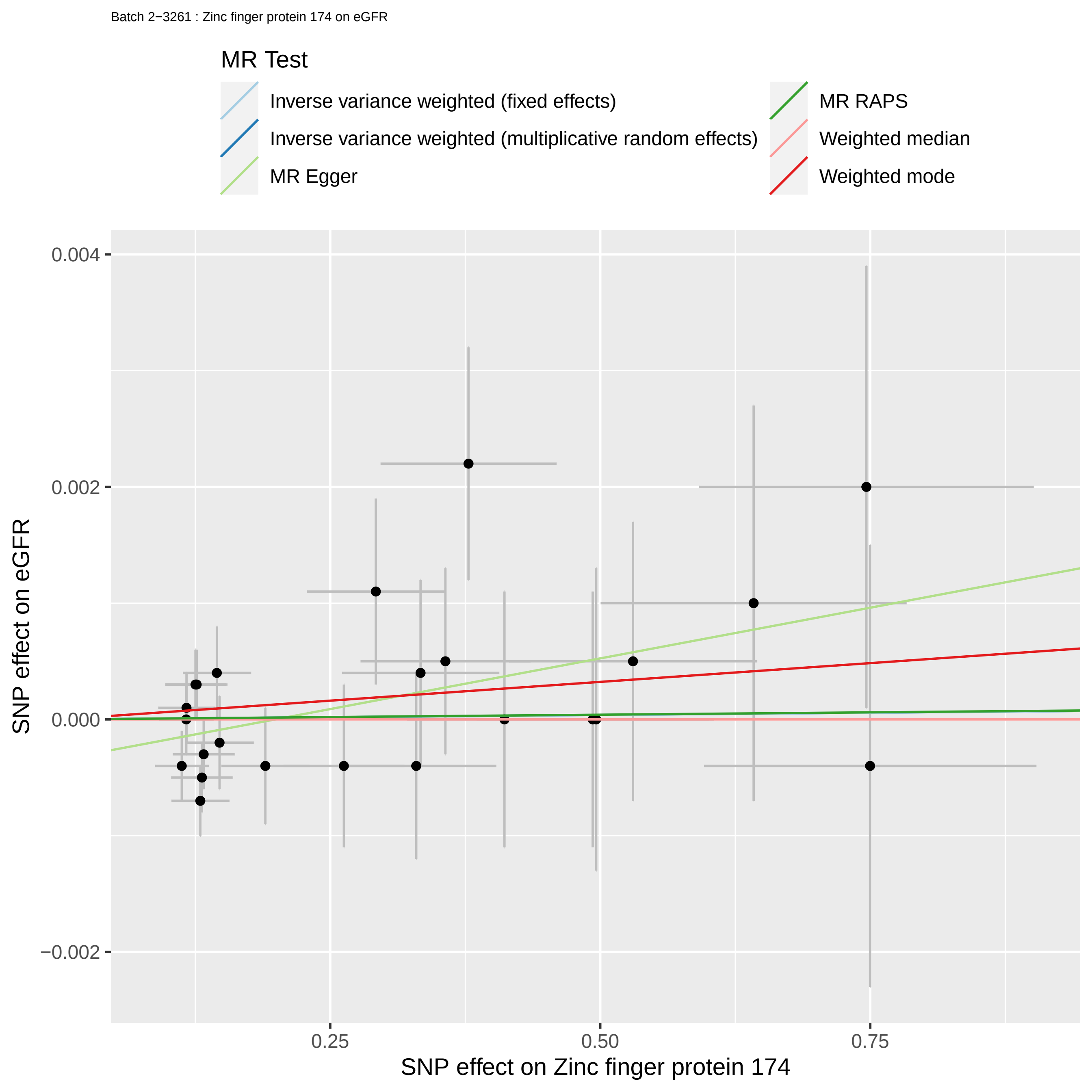
Batch 2−3261 : Zinc finger protein 174 on eGFR
MR Test
Inverse variance weighted (fixed effects)
Inverse variance weighted (multiplicative random effects)
MR Egger
MR RAPS
Weighted median
Weighted mode
0.004
0.002
0.000
−0.002
0.25
0.50
0.75
SNP effect on eGFR
SNP effect on Zinc finger protein 174

Batch 2−3262 : Zinc finger protein 175 on eGFR

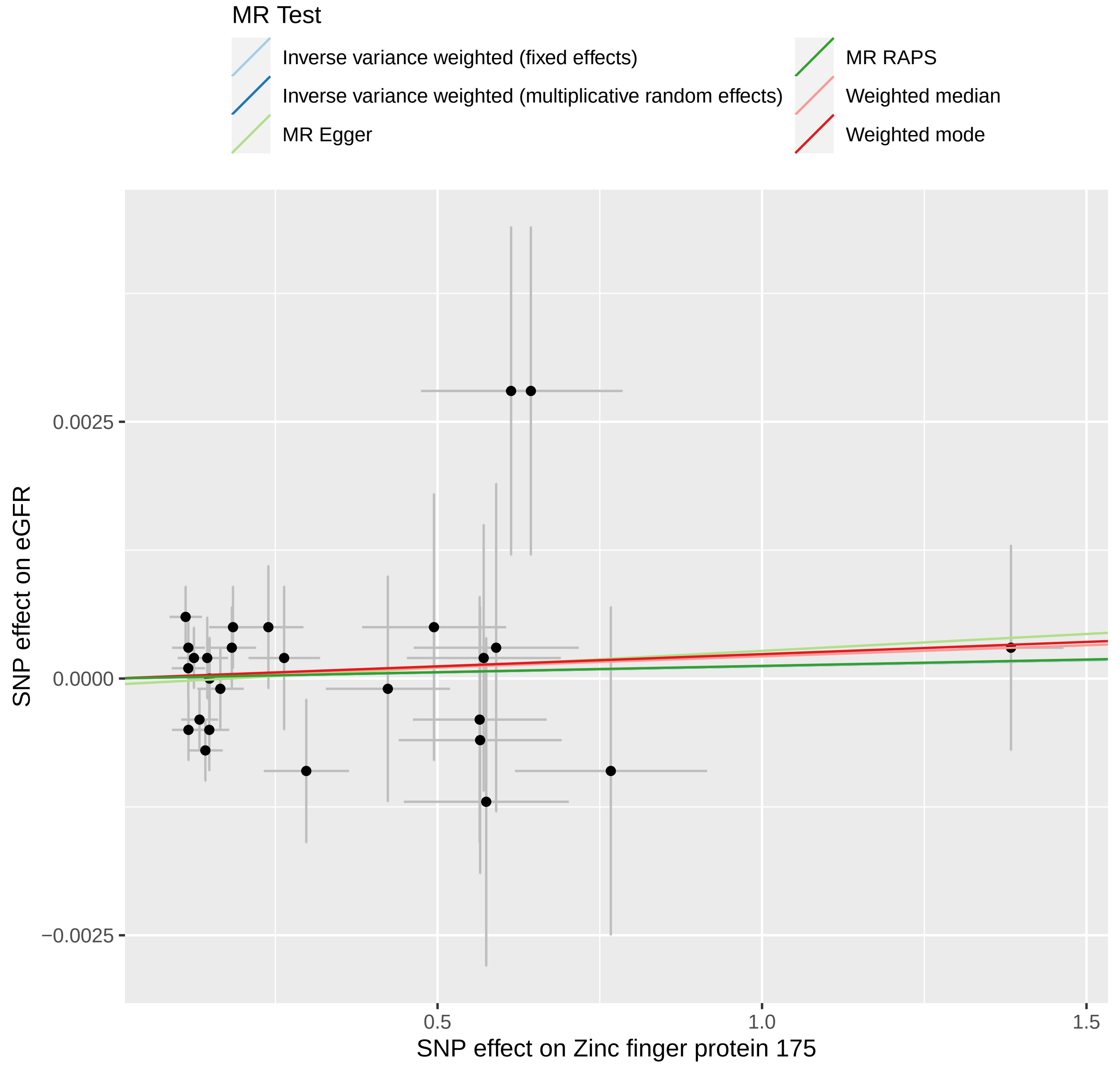

Batch 2−3263 : Zinc finger protein 180 on eGFR

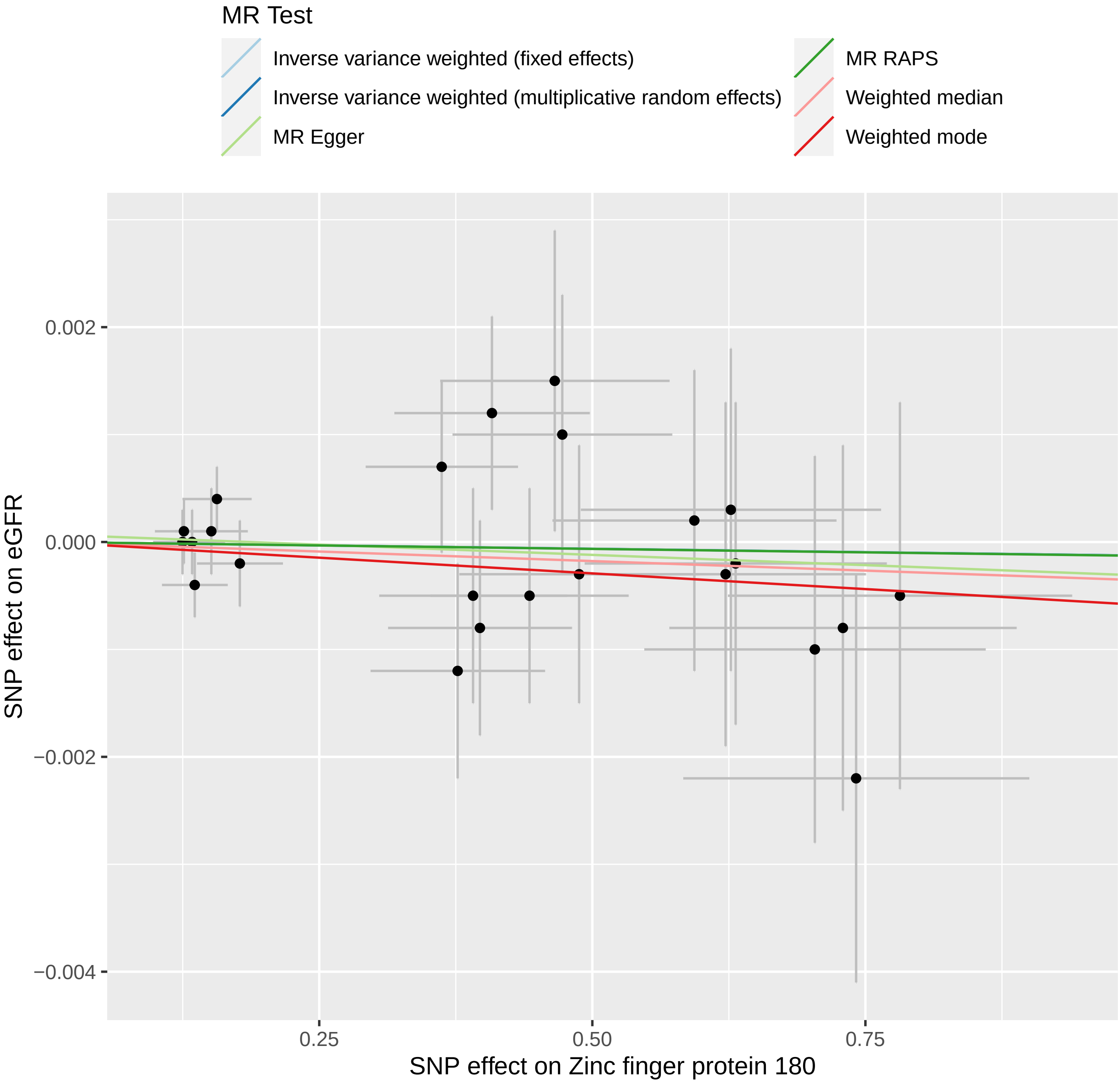

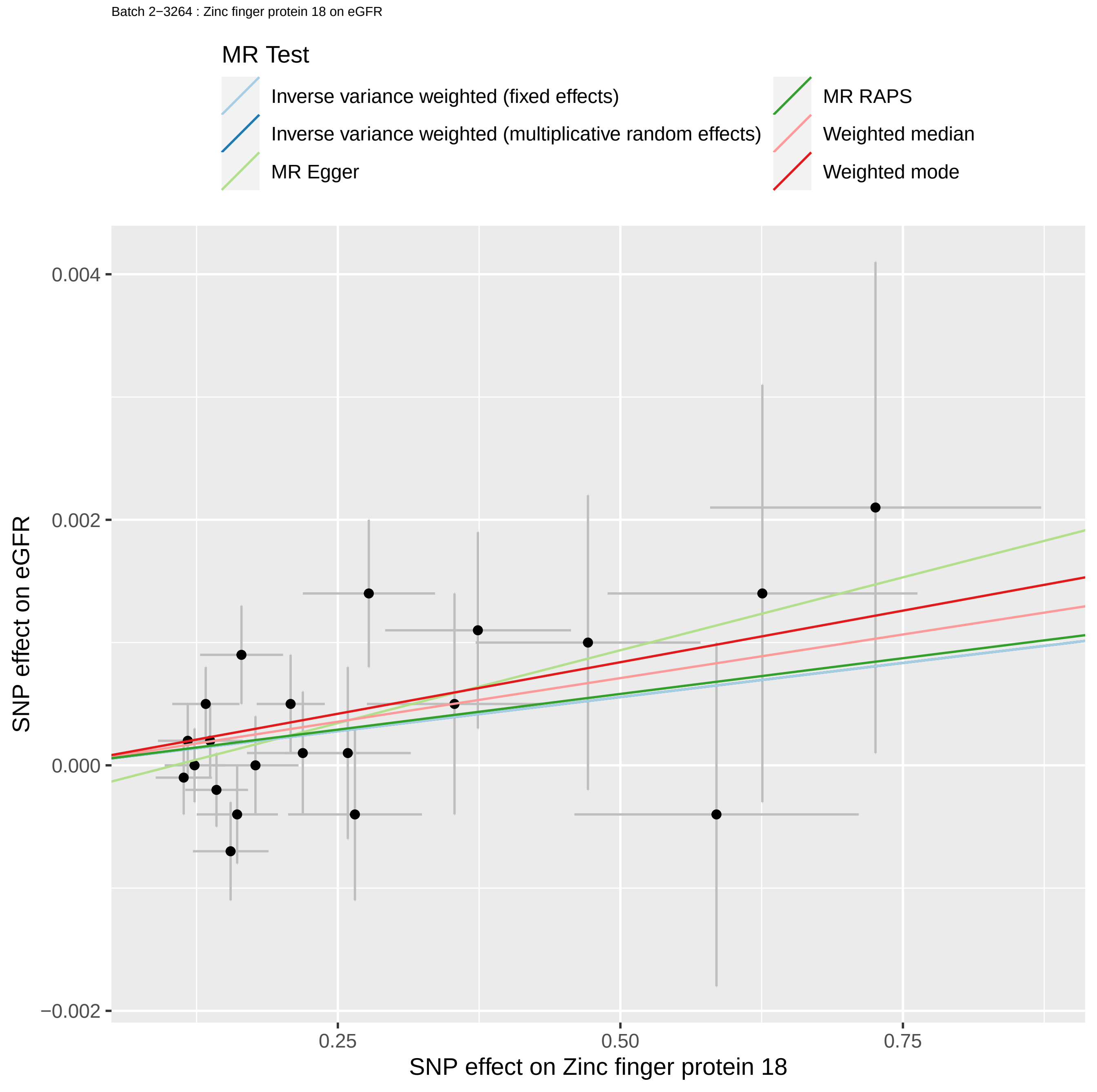
Batch 2−3264 : Zinc finger protein 18 on eGFR
MR Test
Inverse variance weighted (fixed effects)
Inverse variance weighted (multiplicative random effects)
MR Egger
MR RAPS
Weighted median
Weighted mode
0.004
0.002
0.000
−0.002
SNP effect on eGFR
0.25
0.50
0.75
SNP effect on Zinc finger protein 18

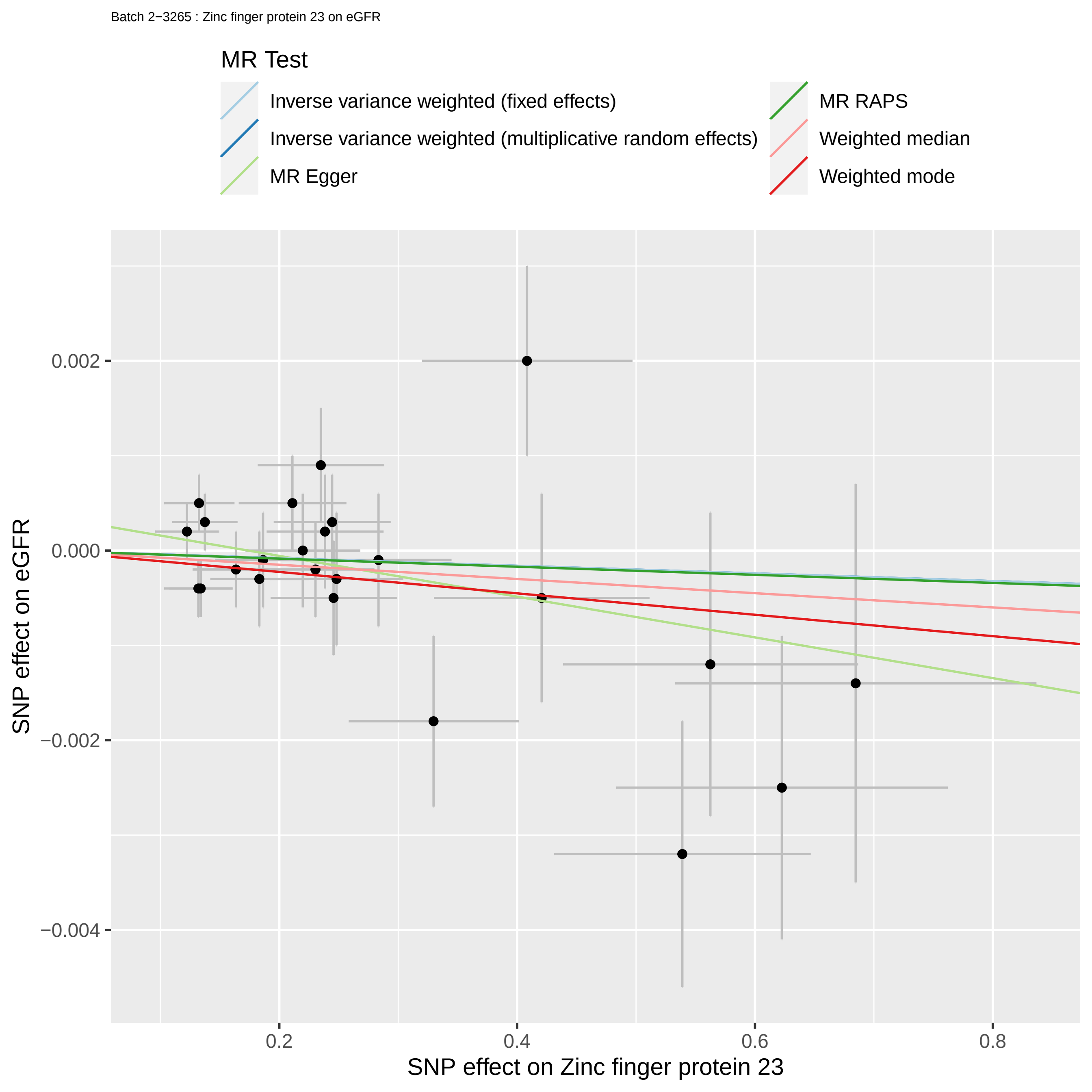

Batch 2−3265 : Zinc finger protein 23 on eGFR
MR Test
Inverse variance weighted (fixed effects)
Inverse variance weighted (multiplicative random effects)
MR Egger
MR RAPS
Weighted median
Weighted mode
0.002
0.000
−0.002
−0.004
SNP effect on eGFR
0.2
0.4
0.6
0.8
SNP effect on Zinc finger protein 23

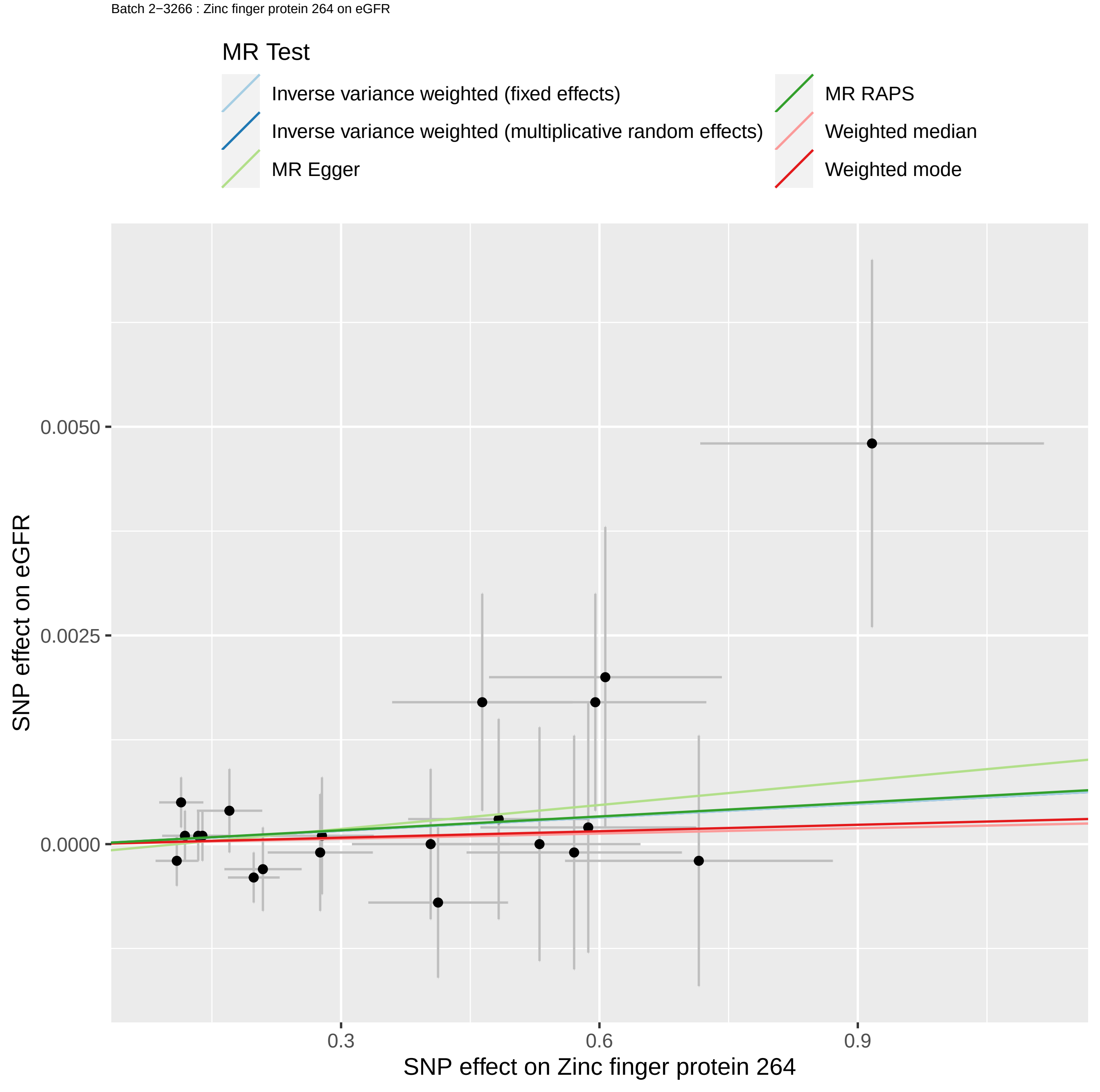

Batch 2−3266 : Zinc finger protein 264 on eGFR
MR Test
Inverse variance weighted (fixed effects)
Inverse variance weighted (multiplicative random effects)
MR Egger
MR RAPS
Weighted median
Weighted mode
0.0050
0.0025
0.0000
0.3
0.6
0.9
SNP effect on eGFR
SNP effect on Zinc finger protein 264

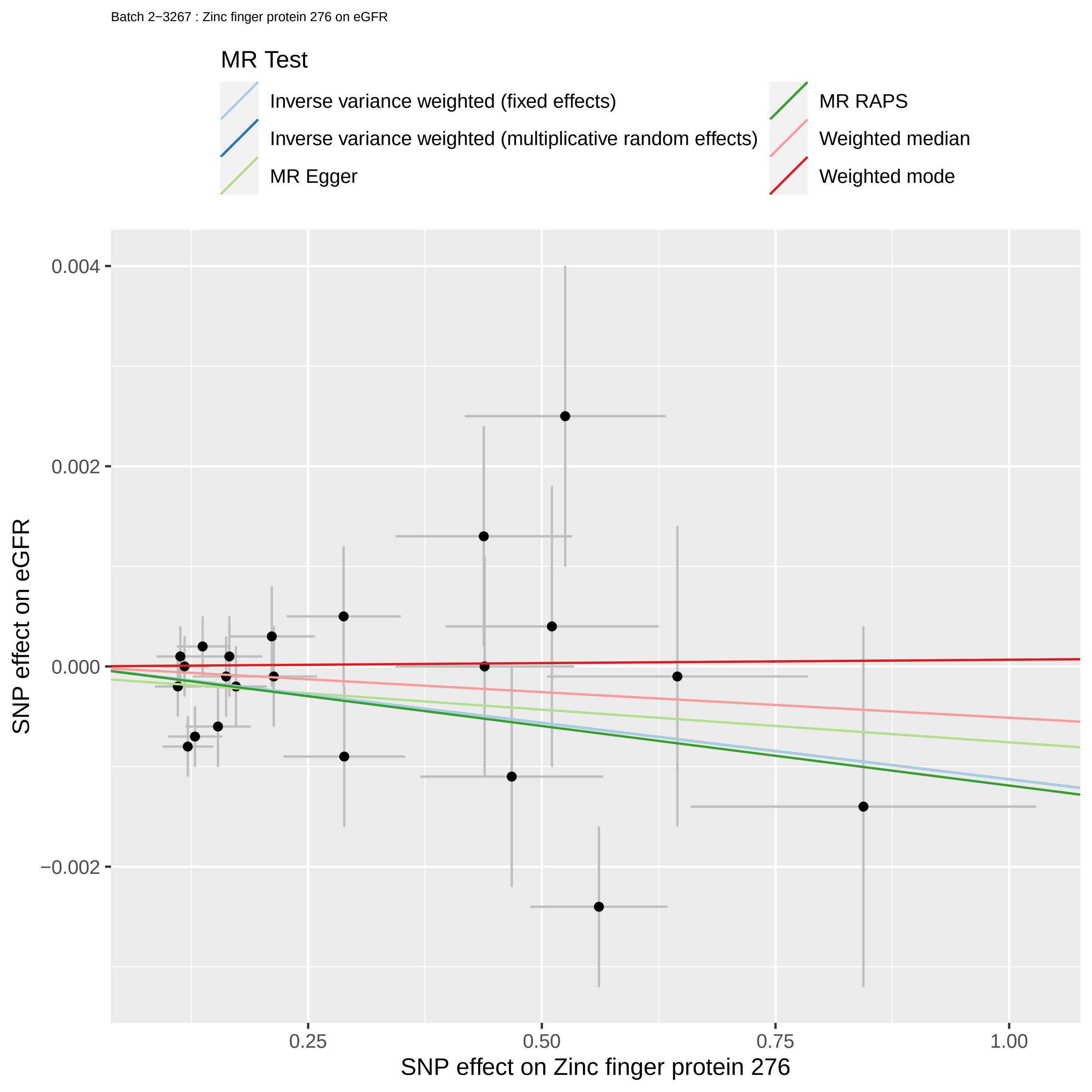
Batch 2−3267 : Zinc finger protein 276 on eGFR
MR Test
Inverse variance weighted (fixed effects)
Inverse variance weighted (multiplicative random effects)
MR Egger
MR RAPS
Weighted median
Weighted mode
0.004
0.002
0.000
−0.002
SNP effect on eGFR
0.25
0.50
0.75
1.00
SNP effect on Zinc finger protein 276

Batch 2−3268 : Zinc finger protein 329 on eGFR

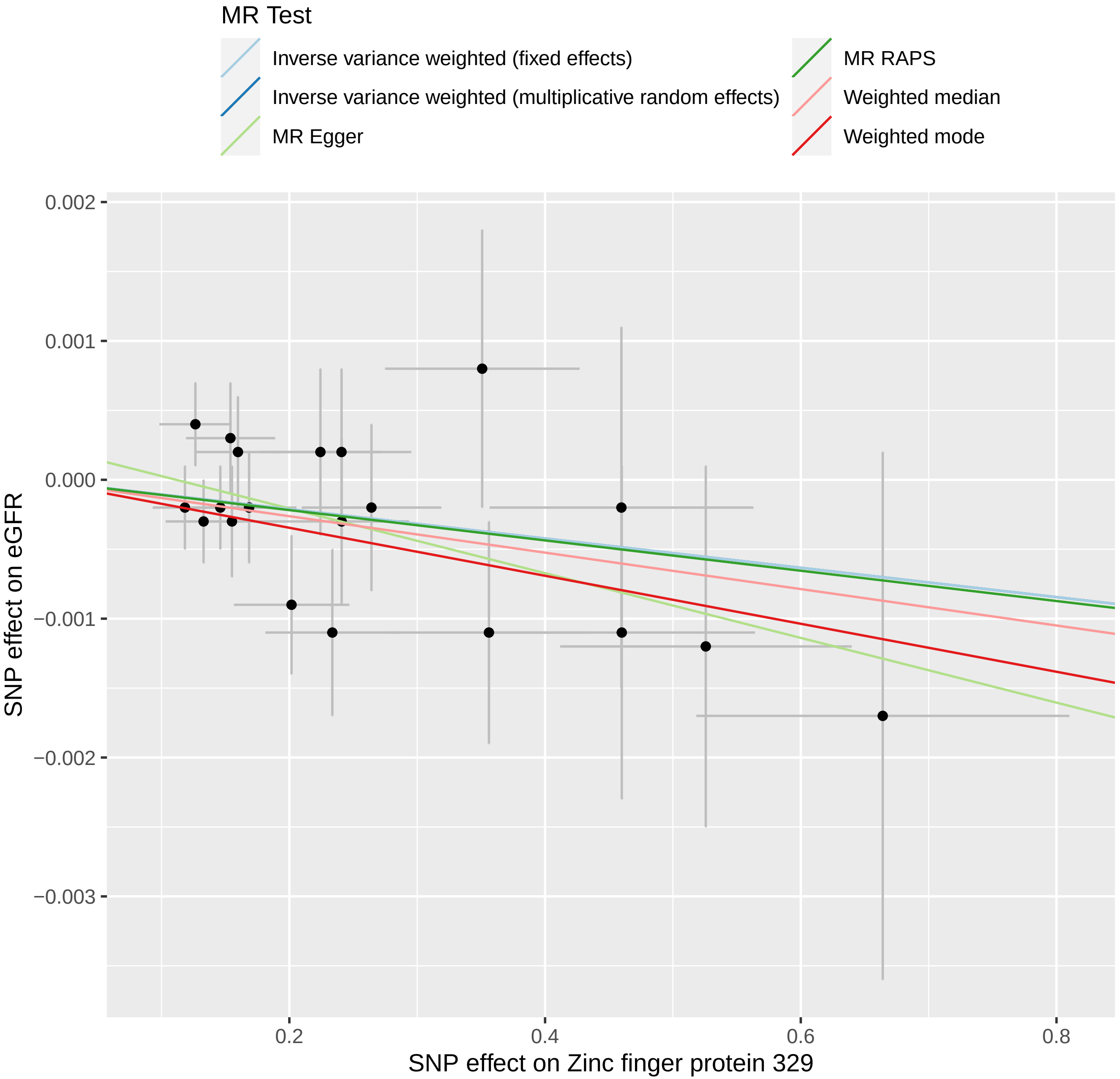

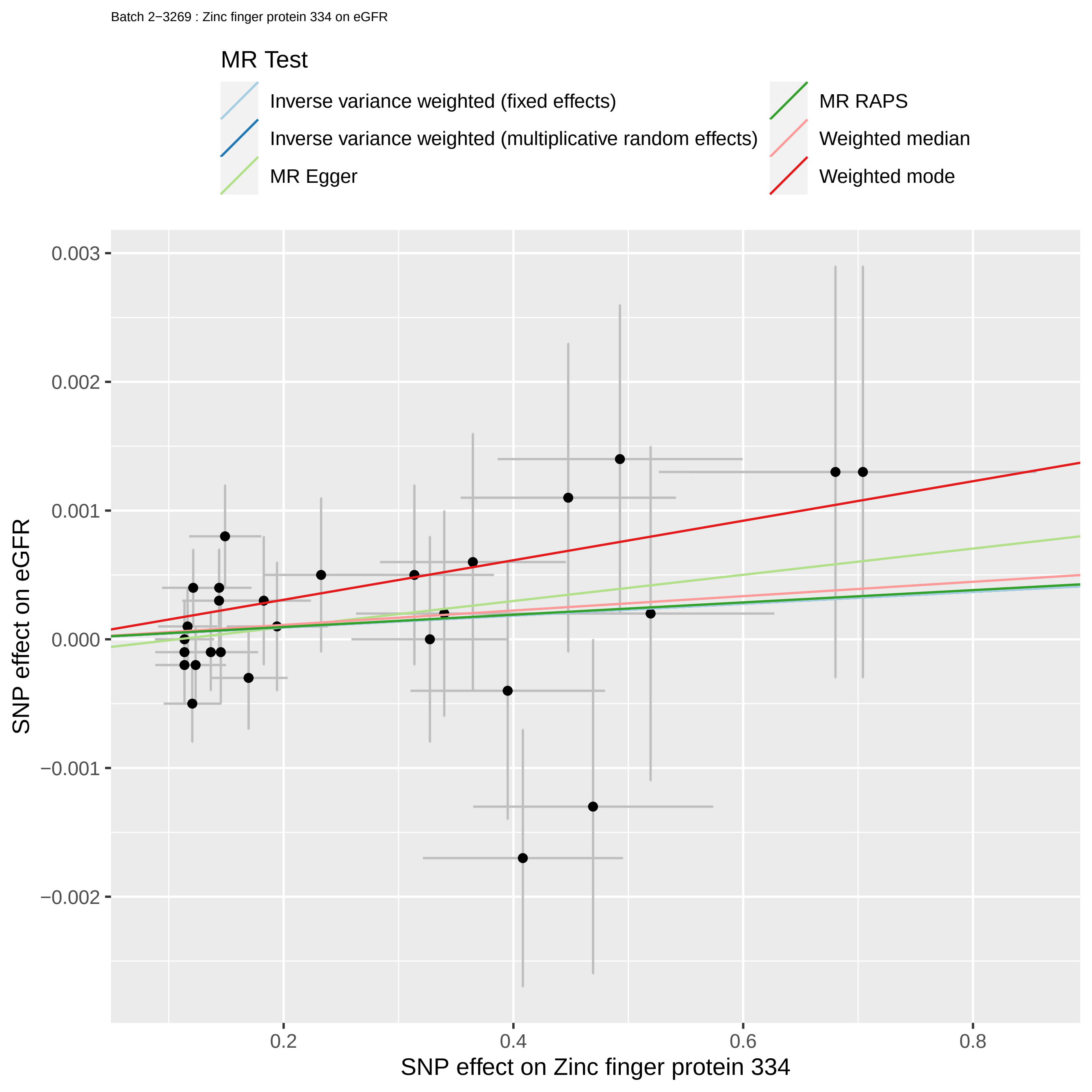

Batch 2−3269 : Zinc finger protein 334 on eGFR
MR Test
Inverse variance weighted (fixed effects)
Inverse variance weighted (multiplicative random effects)
MR Egger
MR RAPS
Weighted median
Weighted mode
0.003
0.002
0.001
0.000
−0.001
−0.002
SNP effect on eGFR
0.2
0.4
0.6
0.8
SNP effect on Zinc finger protein 334

Batch 2−3270 : Zinc finger protein 358 on eGFR

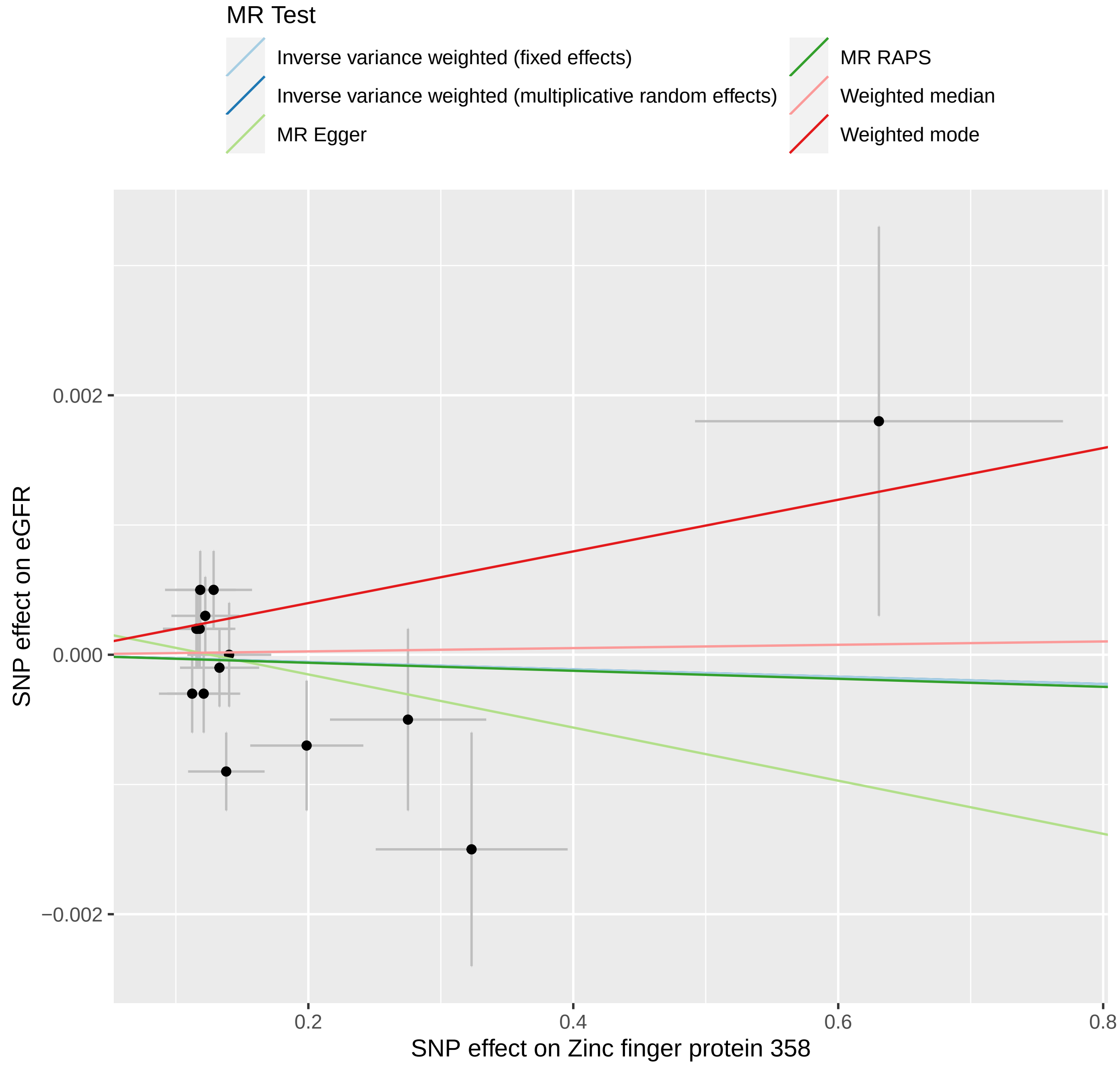

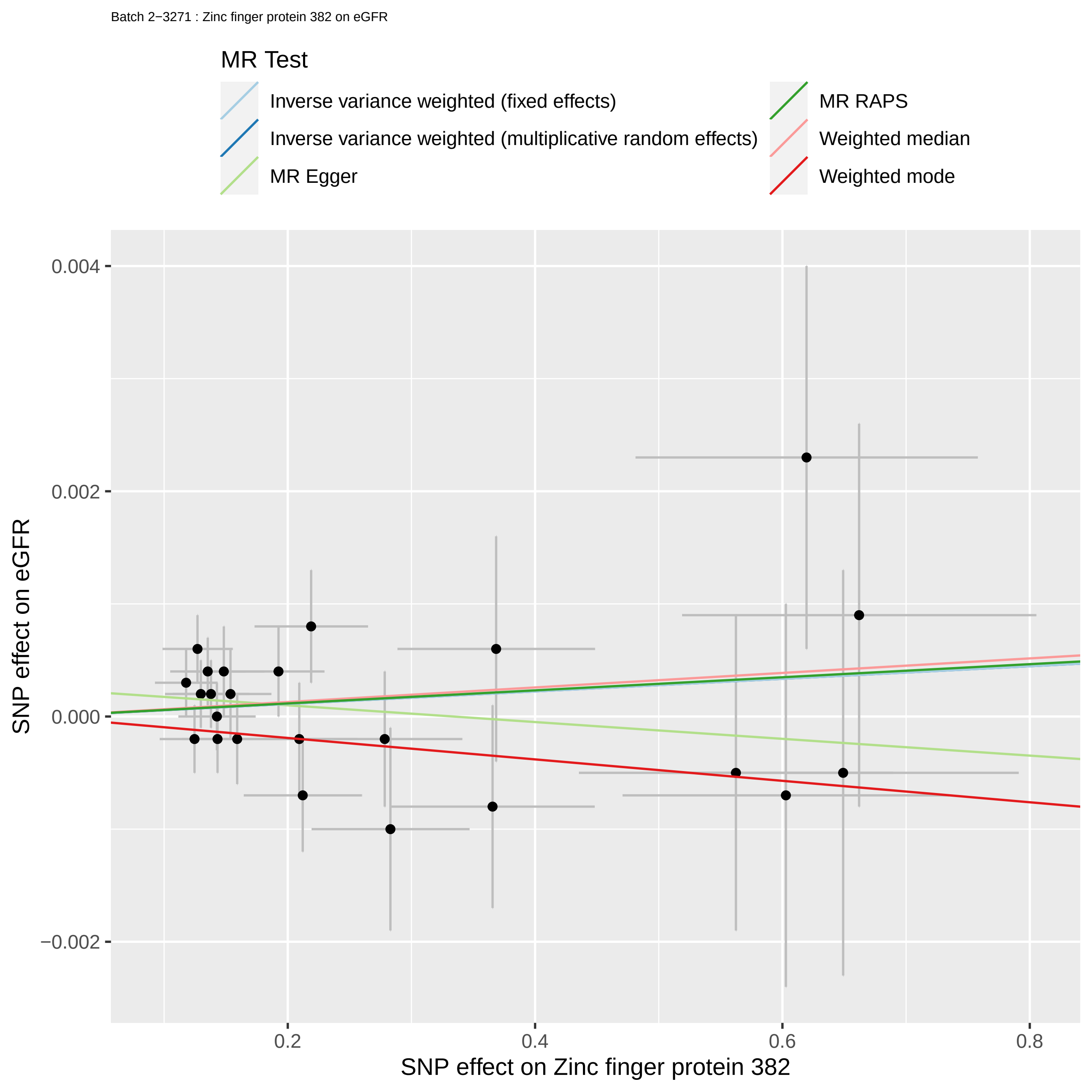
Batch 2−3271 : Zinc finger protein 382 on eGFR
MR Test
Inverse variance weighted (fixed effects)
Inverse variance weighted (multiplicative random effects)
MR Egger
MR RAPS
Weighted median
Weighted mode
0.004
0.002
0.000
−0.002
0.2
0.4
0.6
0.8
SNP effect on eGFR
SNP effect on Zinc finger protein 382

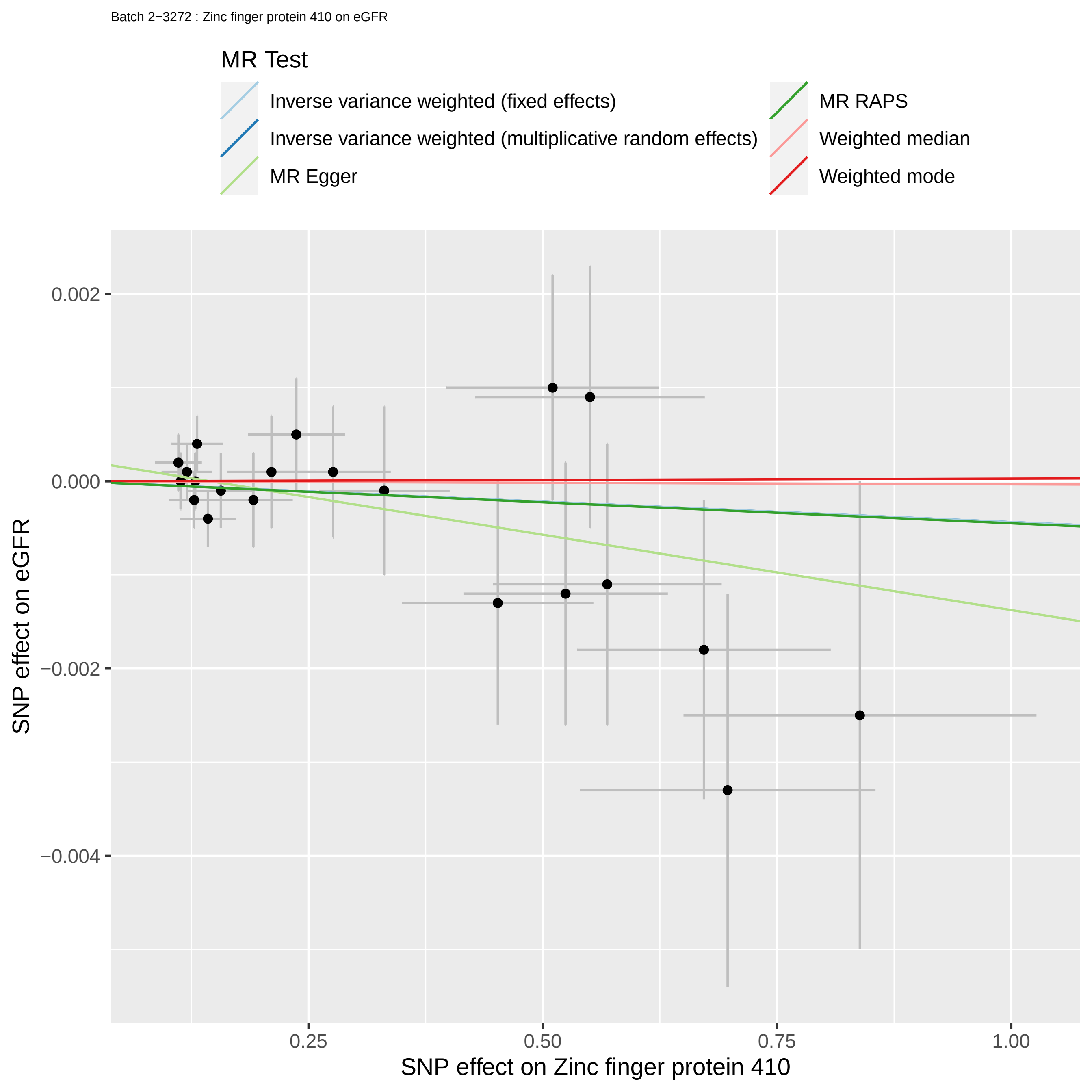

Batch 2−3272 : Zinc finger protein 410 on eGFR
MR Test
Inverse variance weighted (fixed effects)
Inverse variance weighted (multiplicative random effects)
MR Egger
MR RAPS
Weighted median
Weighted mode
0.002
0.000
−0.002
−0.004
0.25
0.50
0.75
1.00
SNP effect on eGFR
SNP effect on Zinc finger protein 410

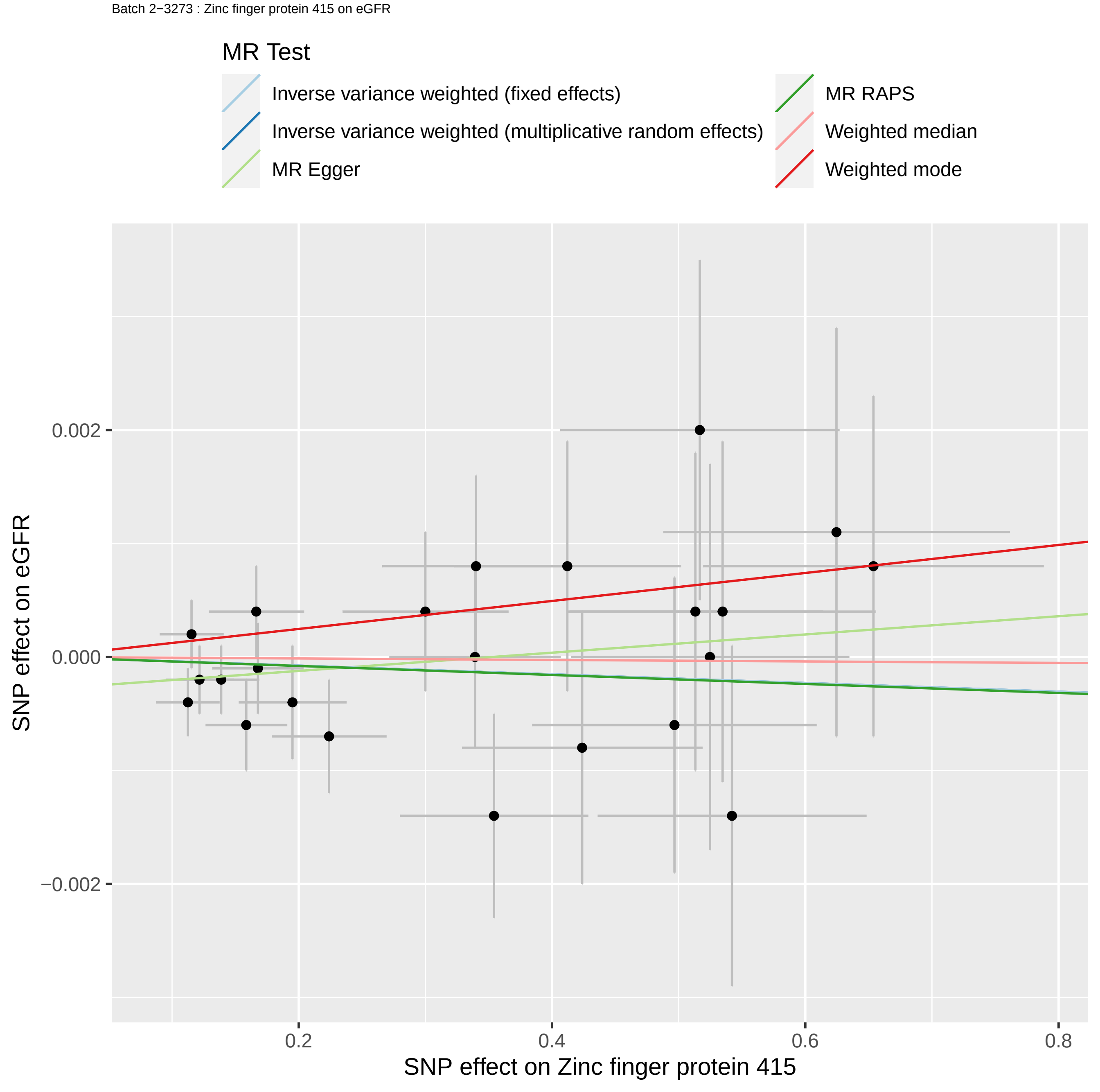
Batch 2−3273 : Zinc finger protein 415 on eGFR
MR Test
Inverse variance weighted (fixed effects)
Inverse variance weighted (multiplicative random effects)
MR Egger
MR RAPS
Weighted median
Weighted mode
0.002
0.000
−0.002
0.2
0.4
0.6
0.8
SNP effect on eGFR
SNP effect on Zinc finger protein 415

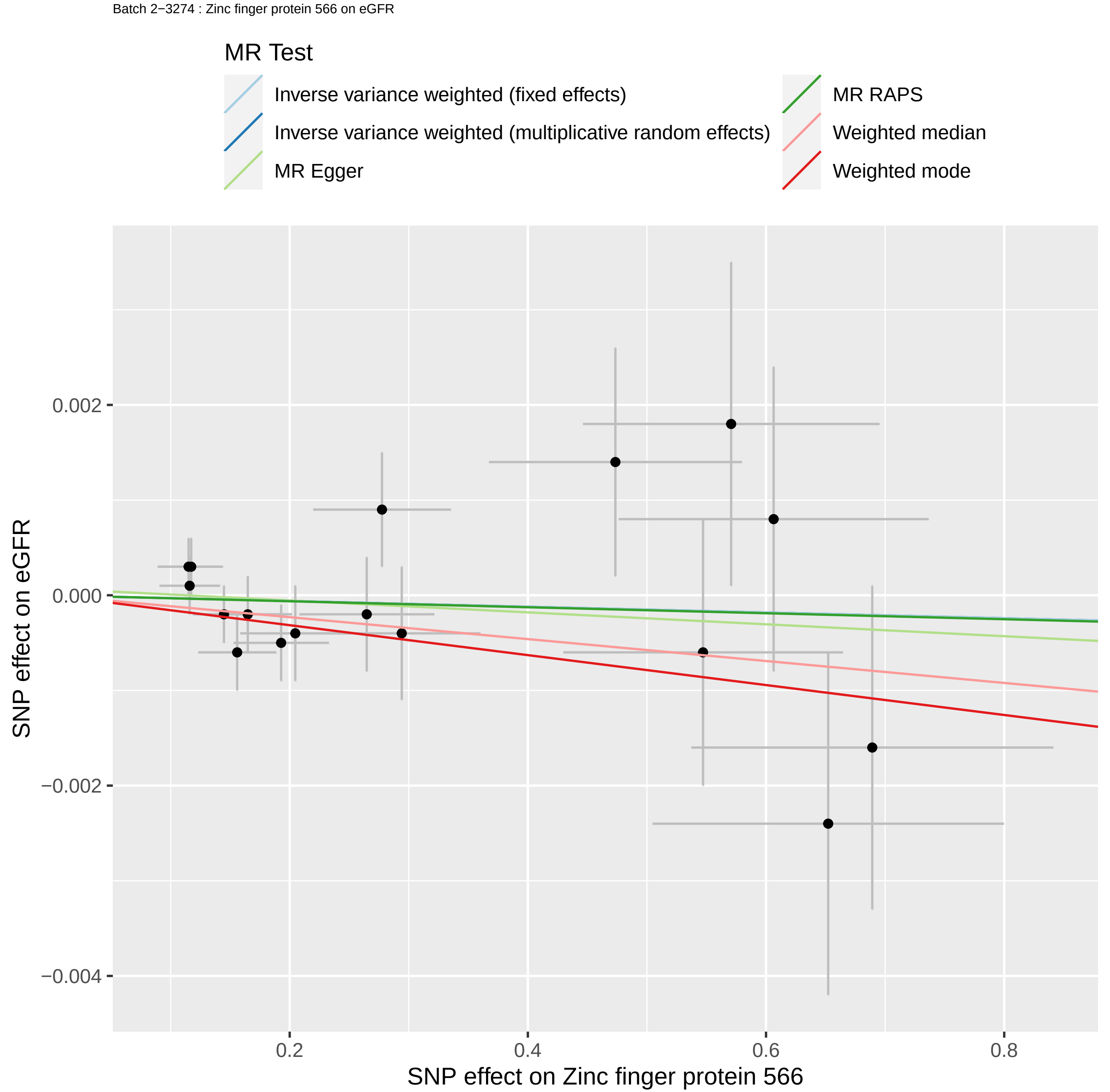

Batch 2−3274 : Zinc finger protein 566 on eGFR
MR Test
Inverse variance weighted (fixed effects)
Inverse variance weighted (multiplicative random effects)
MR Egger
MR RAPS
Weighted median
Weighted mode
0.002
0.000
−0.002
−0.004
0.2
0.4
0.6
0.8
SNP effect on eGFR
SNP effect on Zinc finger protein 566

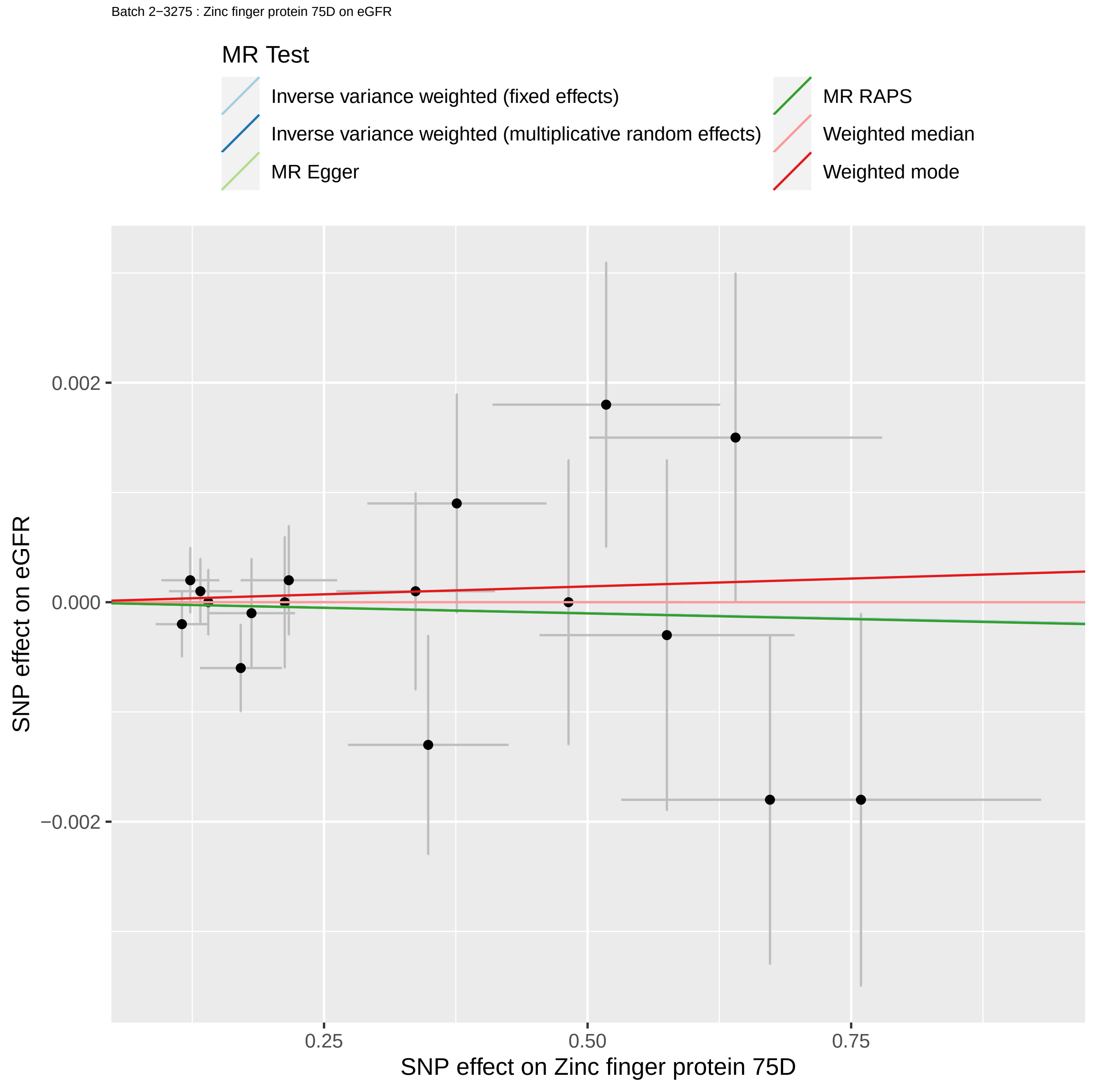
Batch 2−3275 : Zinc finger protein 75D on eGFR
MR Test
Inverse variance weighted (fixed effects)
Inverse variance weighted (multiplicative random effects)
MR Egger
MR RAPS
Weighted median
Weighted mode
0.002
0.000
−0.002
0.25
0.50
0.75
SNP effect on eGFR
SNP effect on Zinc finger protein 75D

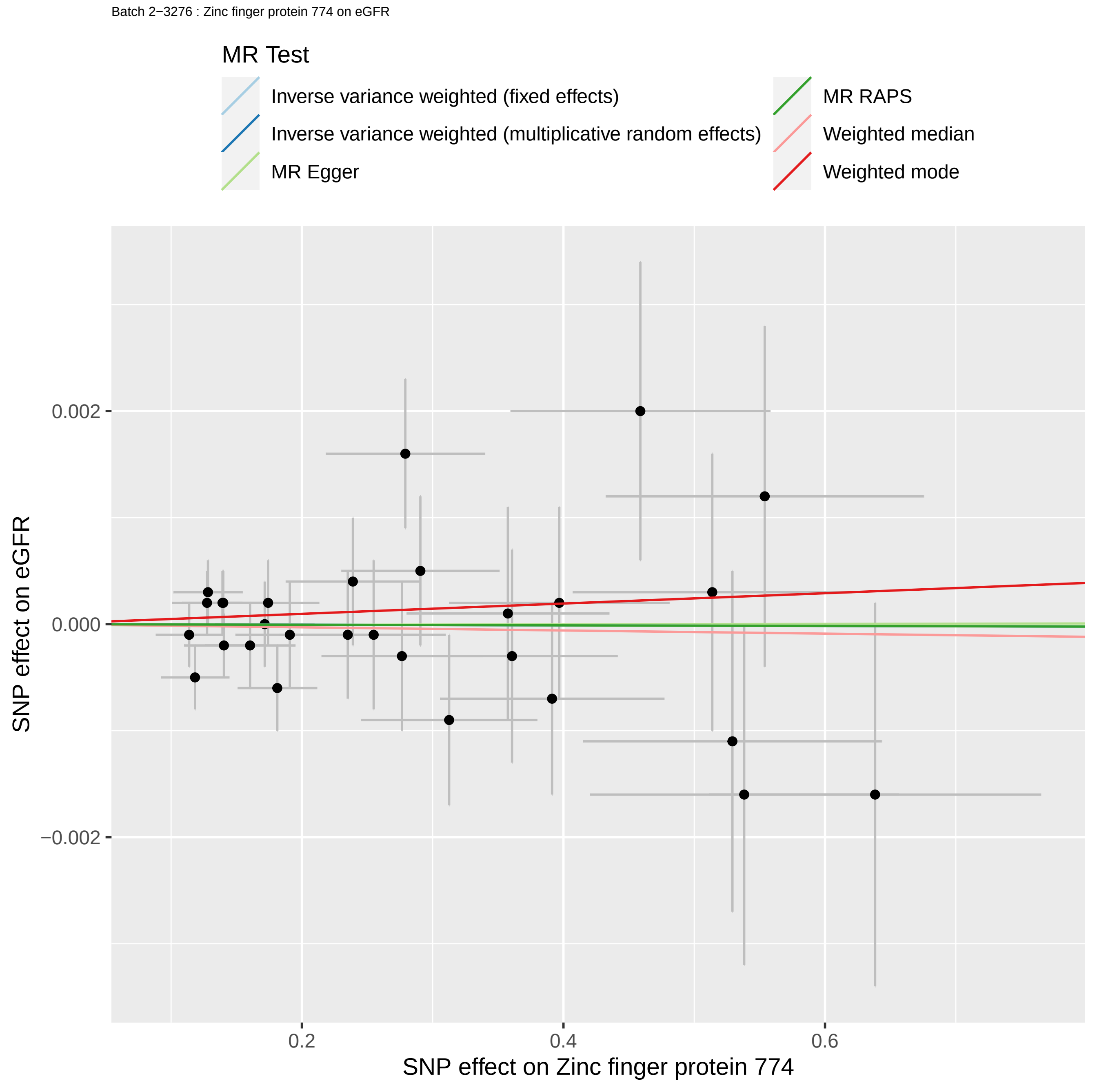

Batch 2−3276 : Zinc finger protein 774 on eGFR
MR Test
Inverse variance weighted (fixed effects)
Inverse variance weighted (multiplicative random effects)
MR Egger
MR RAPS
Weighted median
Weighted mode
0.002
0.000
−0.002
0.2
0.4
0.6
SNP effect on eGFR
SNP effect on Zinc finger protein 774

Batch 2−3277 : Zinc finger protein 843 on eGFR

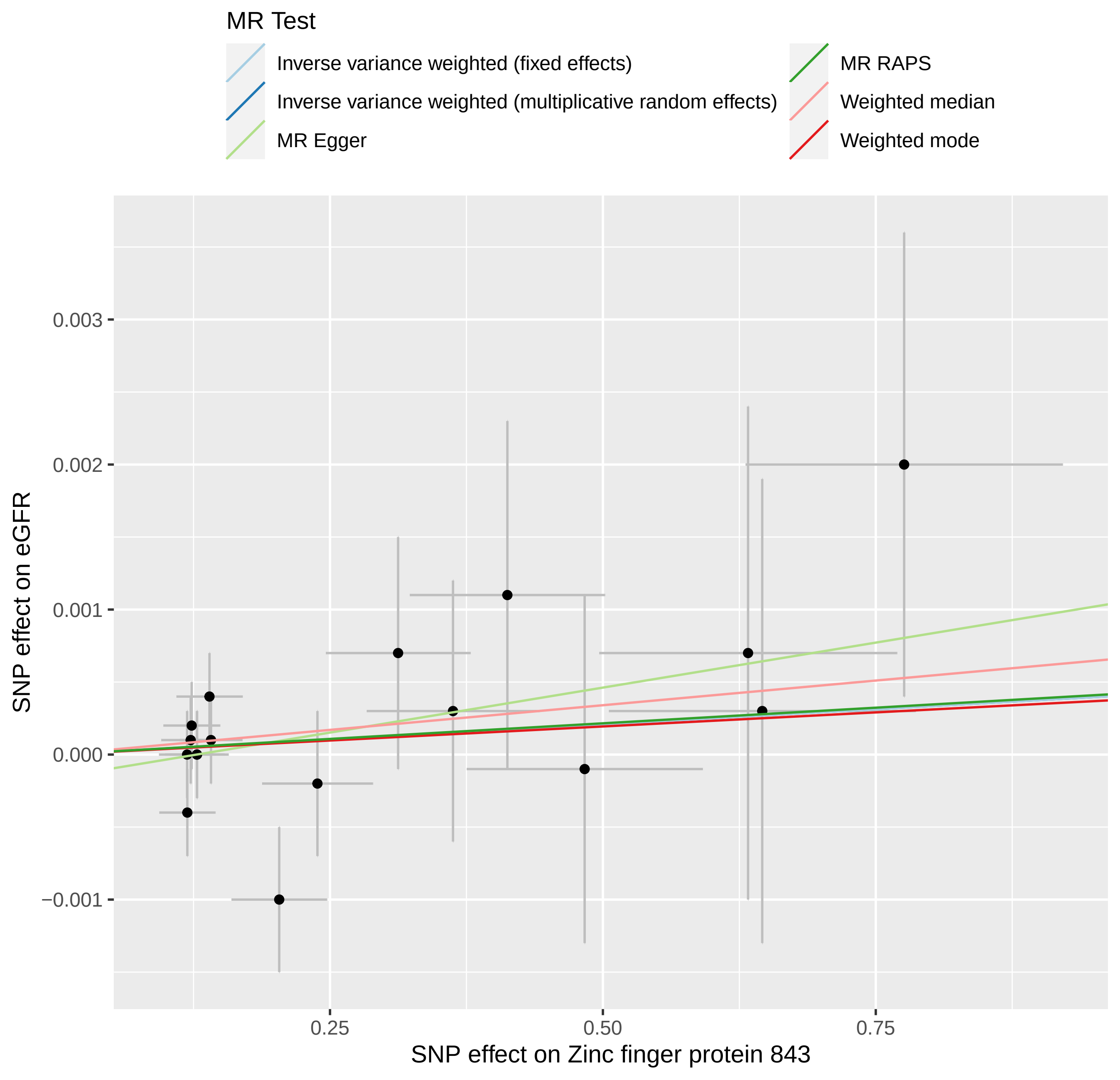

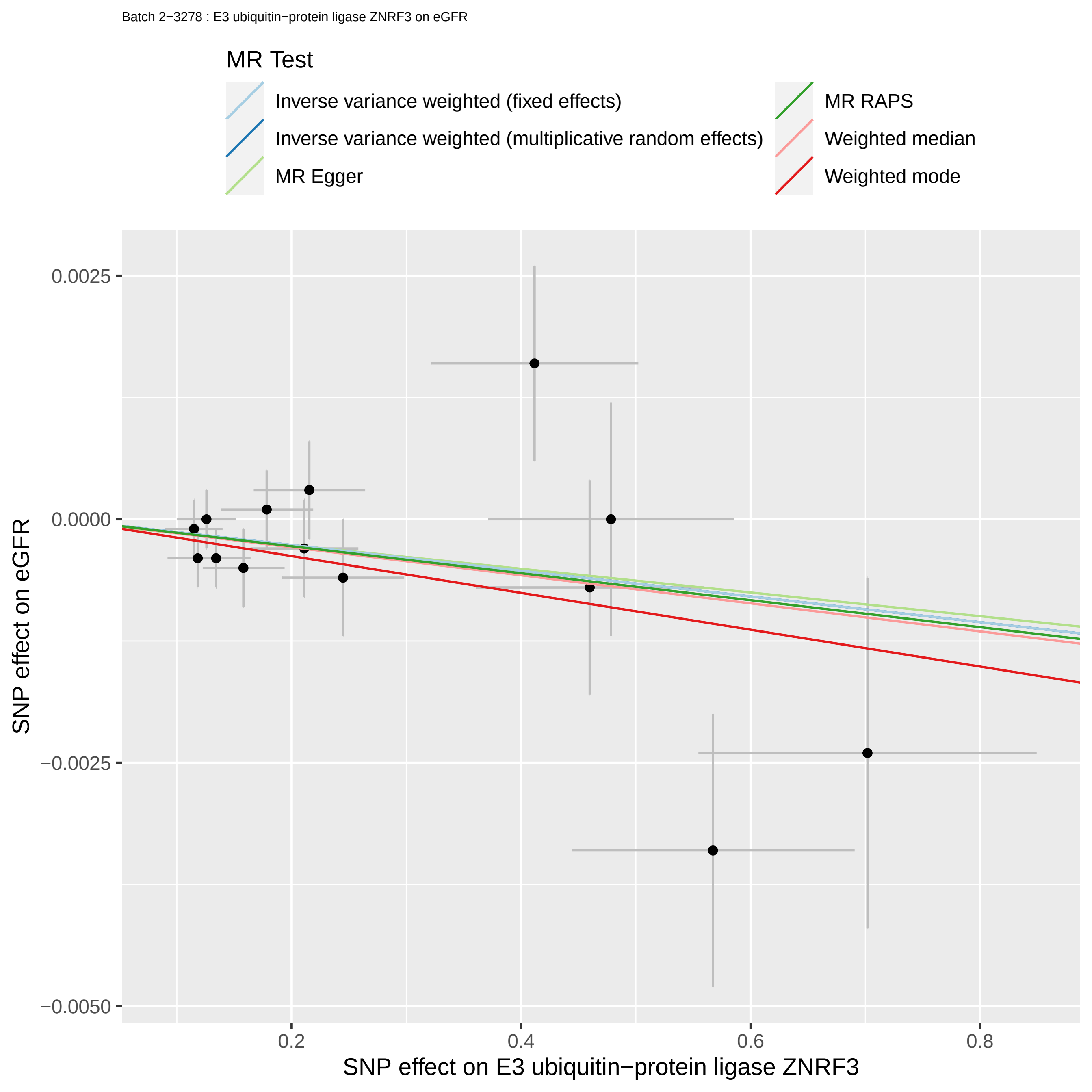
Batch 2−3278 : E3 ubiquitin−protein ligase ZNRF3 on eGFR
MR Test
Inverse variance weighted (fixed effects)
Inverse variance weighted (multiplicative random effects)
MR Egger
MR RAPS
Weighted median
Weighted mode
0.0025
0.0000
−0.0025
−0.0050
SNP effect on eGFR
0.2
0.4
0.6
0.8
SNP effect on E3 ubiquitin−protein ligase ZNRF3

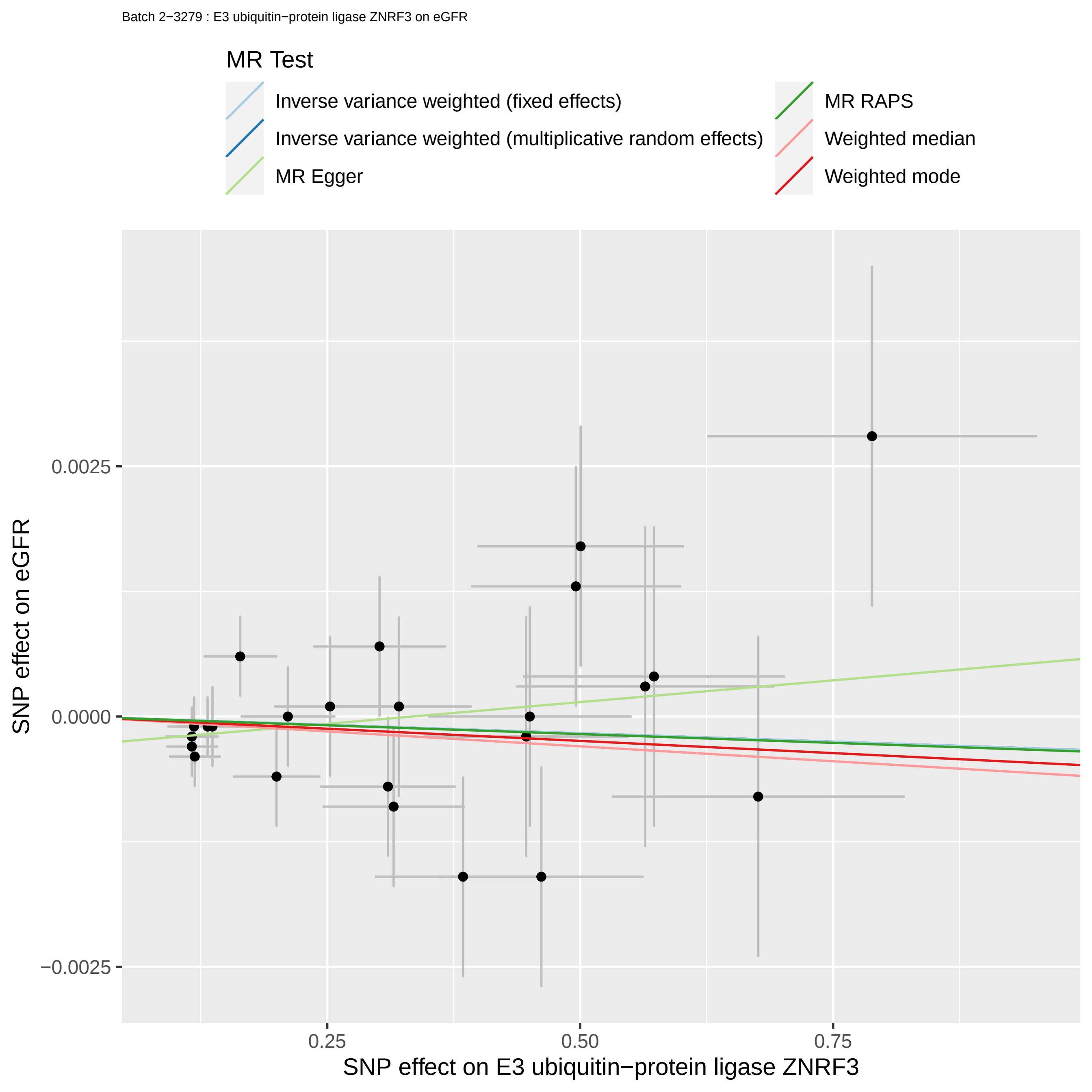

Batch 2−3279 : E3 ubiquitin−protein ligase ZNRF3 on eGFR
MR Test
Inverse variance weighted (fixed effects)
Inverse variance weighted (multiplicative random effects)
MR Egger
MR RAPS
Weighted median
Weighted mode
0.0025
0.0000
−0.0025
SNP effect on eGFR
0.25
0.50
0.75
SNP effect on E3 ubiquitin−protein ligase ZNRF3

Batch 2−3280 : Zinc/RING finger protein 4 on eGFR

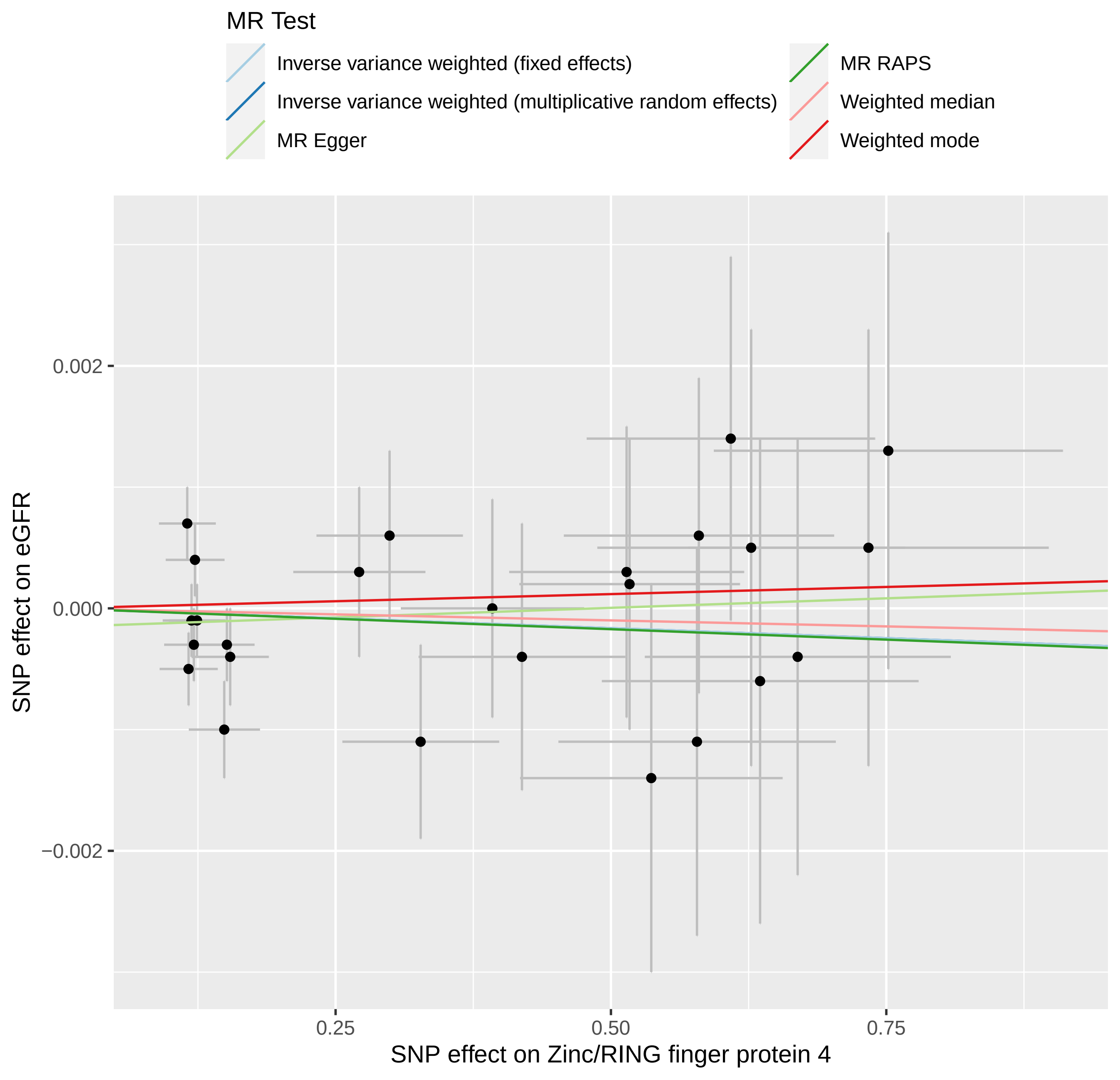

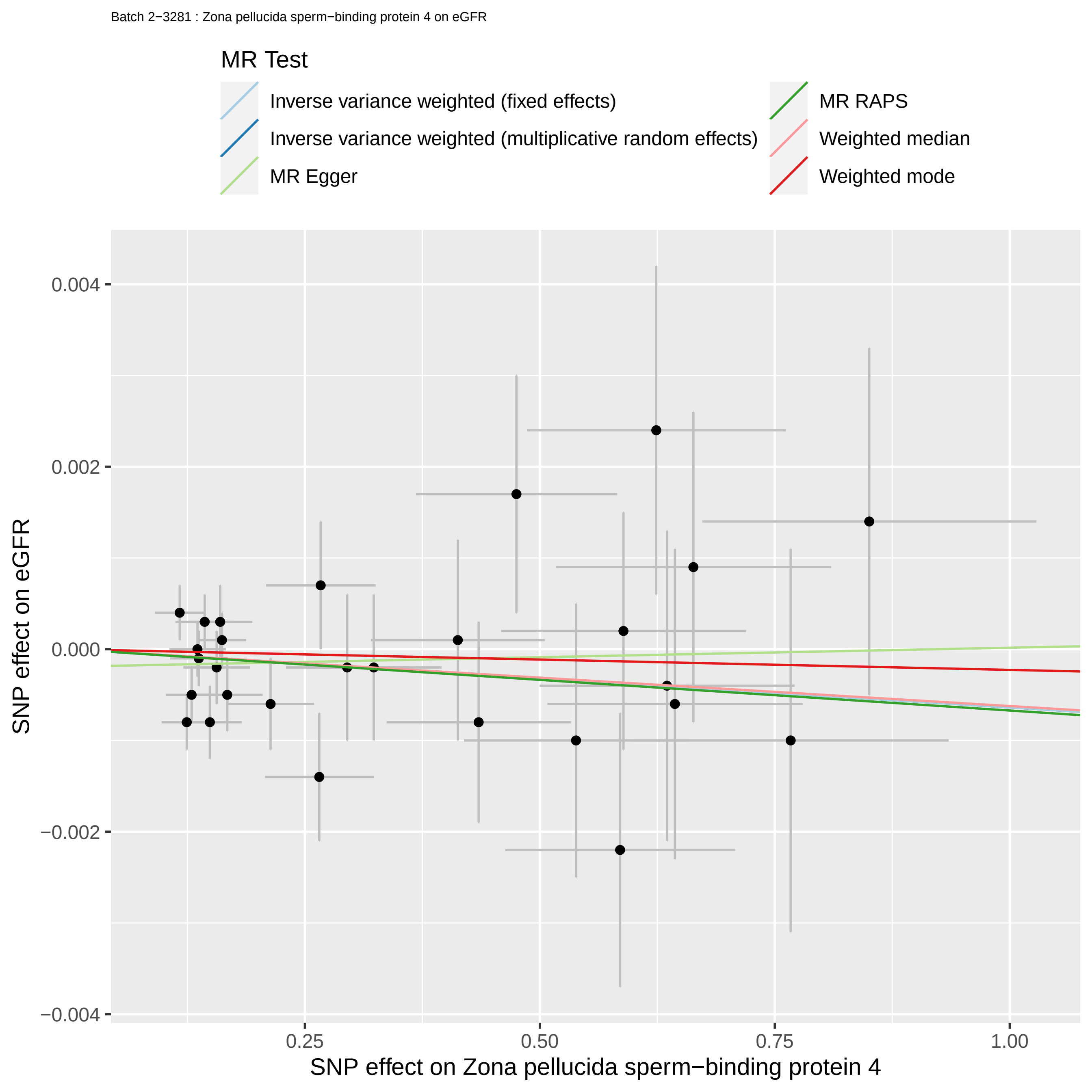

Batch 2−3281 : Zona pellucida sperm−binding protein 4 on eGFR
MR Test
Inverse variance weighted (fixed effects)
Inverse variance weighted (multiplicative random effects)
MR Egger
MR RAPS
Weighted median
Weighted mode
0.004
0.002
0.000
−0.002
−0.004
0.25
0.50
0.75
1.00
SNP effect on eGFR
SNP effect on Zona pellucida sperm−binding protein 4

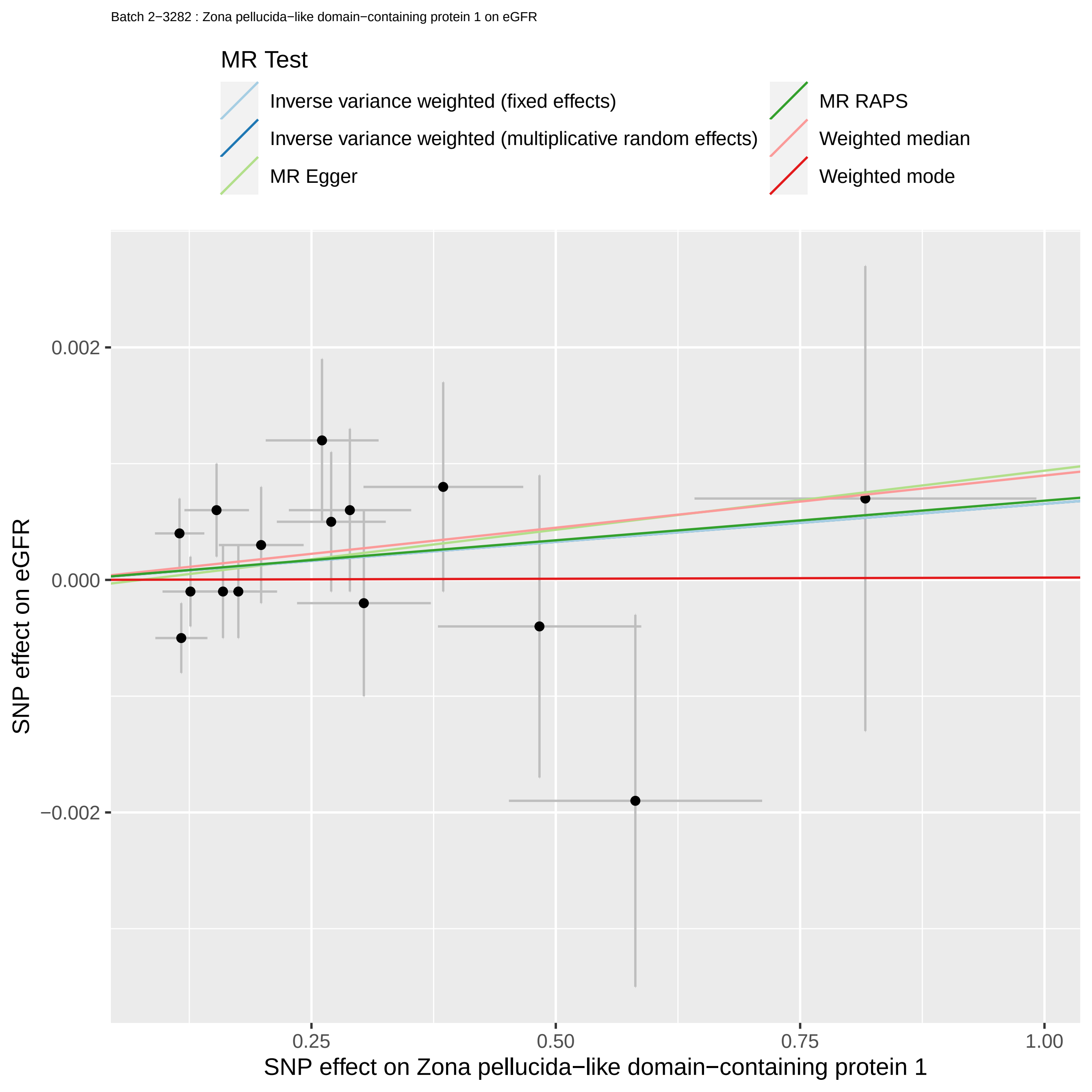

Batch 2−3282 : Zona pellucida−like domain−containing protein 1 on eGFR
MR Test
Inverse variance weighted (fixed effects)
Inverse variance weighted (multiplicative random effects)
MR Egger
MR RAPS
Weighted median
Weighted mode
0.002
0.000
−0.002
0.25
0.50
0.75
1.00
SNP effect on eGFR
SNP effect on Zona pellucida−like domain−containing protein 1
